# Causal Pathways Linking Gut Microbiota, Serum Metabolites, and Meningioma Risk: A Mendelian Randomization Analysis

**DOI:** 10.1002/brb3.71220

**Published:** 2026-02-09

**Authors:** Xuanli Gong, Jinxiang Zhang, Mengjiao He, Xiaochen Zhang, Kelan Wang, Yulu Yang, Qingyun Zhao, Xin Zhao, Wei Zou

**Affiliations:** ^1^ Yunnan Provincial Key Laboratory of Public Health and Biosafety & School of Public Health Kunming Medical University Kunming Yunnan People's Republic of China; ^2^ Basic Medical College Kunming Medical University Kunming Yunnan People's Republic of China; ^3^ The Second Clinical Medical College Kunming Medical University Kunming Yunnan People's Republic of China; ^4^ Infection Control Office Xi'an Public Health Center (Xi'an Emergency Medical Center) Xi'an Shaanxi People's Republic of China

**Keywords:** gut–brain axis, Mendelian randomization, meningioma, serum metabolites

## Abstract

**Objectives:**

Meningioma is a common tumor of the adult central nervous system (CNS), but its origin remains unclear. Increasing evidence suggests that the gut microbiota affects CNS disorders through the “microbiota–gut–brain axis,” yet its link to meningioma is still uncertain. This study used Mendelian randomization (MR) to explore causal relationships between gut microbiota, serum metabolites, and meningioma, and to investigate potential mediation by serum metabolites.

**Methods:**

A two‐sample MR framework was applied using publicly available genome‐wide association study data on 473 gut microbial taxa, 1400 serum metabolites, and meningioma. Primary estimates were obtained using the inverse variance weighted method, with MR‐Egger and weighted median methods as complementary approaches. A two‐step MR was used to assess mediation. Sensitivity analyses were performed to confirm robustness.

**Results:**

Nineteen gut microbial taxa were causally associated with meningioma. A reverse causal association was identified only for Lachnospirales. A total of 49 serum metabolites showed potential causal associations, involving inflammatory, hormonal, and lipid pathways. Arachidonate (20:4n6) may mediate the effect of CAG−873 sp001701165 on meningioma.

**Conclusion:**

This study provides new insights into the causal roles of gut microbiota and metabolites in meningioma, suggesting novel prevention and treatment strategies.

## Graphical abstract

This Mendelian randomization analysis elucidates causal pathways linking gut microbiota and serum metabolites to meningioma. We identified specific risk factors and found that arachidonic acid potentially mediates the effect of CAG−873 sp001701165 on meningioma risk.

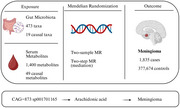

## 1 Introduction

Meningioma is one of the most prevalent intracranial neoplasms, accounting for 40%–56% of primary central nervous system tumors, mostly affecting women aged 50–70 years (Degeneffe et al. 2023; CBTRUS 2024). The WHO categorization indicates that 80%–81% of meningiomas are benign (Grade I), 17%–18% are atypical (Grade II), and roughly 1.7% are malignant (Grade III) (Yarabarla et al. 2023). Grades II–III meningiomas have elevated recurrence rates, increased invasiveness, and worse prognosis (Sescu et al. 2023). Benign tumors may need surgical excision if situated in functionally essential regions or if they are of considerable size, which poses hazards including brain harm. Prevalent symptoms include headache (32.3%–50%), neurological impairments (e.g., hemiplegia, sensory abnormalities, aphasia, affecting around 17.3%), and visual or cranial nerve damage (13%–42.5%). Meningiomas located at the skull base or superior orbital fissure are more prone to impact cranial nerves (Mederer et al. 2022). Exploring the etiology of meningioma is essential for identifying therapeutic targets.

The exact etiology of meningioma remains unclear but may involve multiple factors. Recognized high‐risk factors include exposure to ionizing radiation, especially head and neck radiotherapy during childhood (Banerjee et al. 2009). Hormonal aspects are crucial; over 70% of meningiomas exhibit progesterone receptors (PR), with some also displaying estrogen receptors. The prolonged use of certain progestin medicines further elevates the risk (Roland et al. 2024; Wiemels et al. 2010). Inflammation and immunological responses contribute to the formation and pathological progression of meningioma. This is evidenced by irregularities in pro‐inflammatory metabolic pathways, including heightened arachidonic acid concentrations and enhanced activity of its enzymes COX and lipoxygenase, resulting in the production of tumor‐promoting eicosanoids and the infiltration of immune cells such as tumor‐associated macrophages (Nathoo et al. 2004; Roland et al. 2024). Metabolic disorders, including altered plasma levels of choline, indole, tryptophan, and lipids, together with family history and genetic diseases such as neurofibromatosis type 2, are related to the formation of meningiomas (Goutagny et al. 2012; Kökoğlu et al. 1998).

The brain, gastrointestinal system, and microbiome interact via the gut–brain axis to affect neurological health and disorders (Morais et al. 2021). The gut microbiota is involved not only in nutrient absorption but also in systemic regulation of immunity, inflammation, and metabolism (Staby et al. 2017). The gut microbiota composition in individuals with brain tumors, including meningioma, differs from that of healthy individuals, indicating that bacteria may promote brain tumor growth via immunological and inflammatory mechanisms (Jiang et al. 2022; Mehrian‐Shai et al. 2019). We posited a possible correlation between gut microbiota and meningioma risk and evaluated this notion by Mendelian randomization (MR) analysis. MR used genetic variations as instrumental factors to deduce causation in evaluating this hypothesis. This research seeks to investigate the pathogenic causes and possible treatment modalities for meningioma via the perspective of the gut–brain axis. A two‐step MR analysis was performed: first, to assess the causal relationships between the abundance of 473 fecal gut microbial taxa and 1,400 serum metabolites with meningioma; second, to examine the causal associations between gut microbiota and serum metabolites; and finally, to evaluate the mediating effects of serum metabolites in the relationship between gut microbiota and meningioma.

## 2 Methods

### 2.1 MR Assumptions and Study Design

**FIGURE 1:**
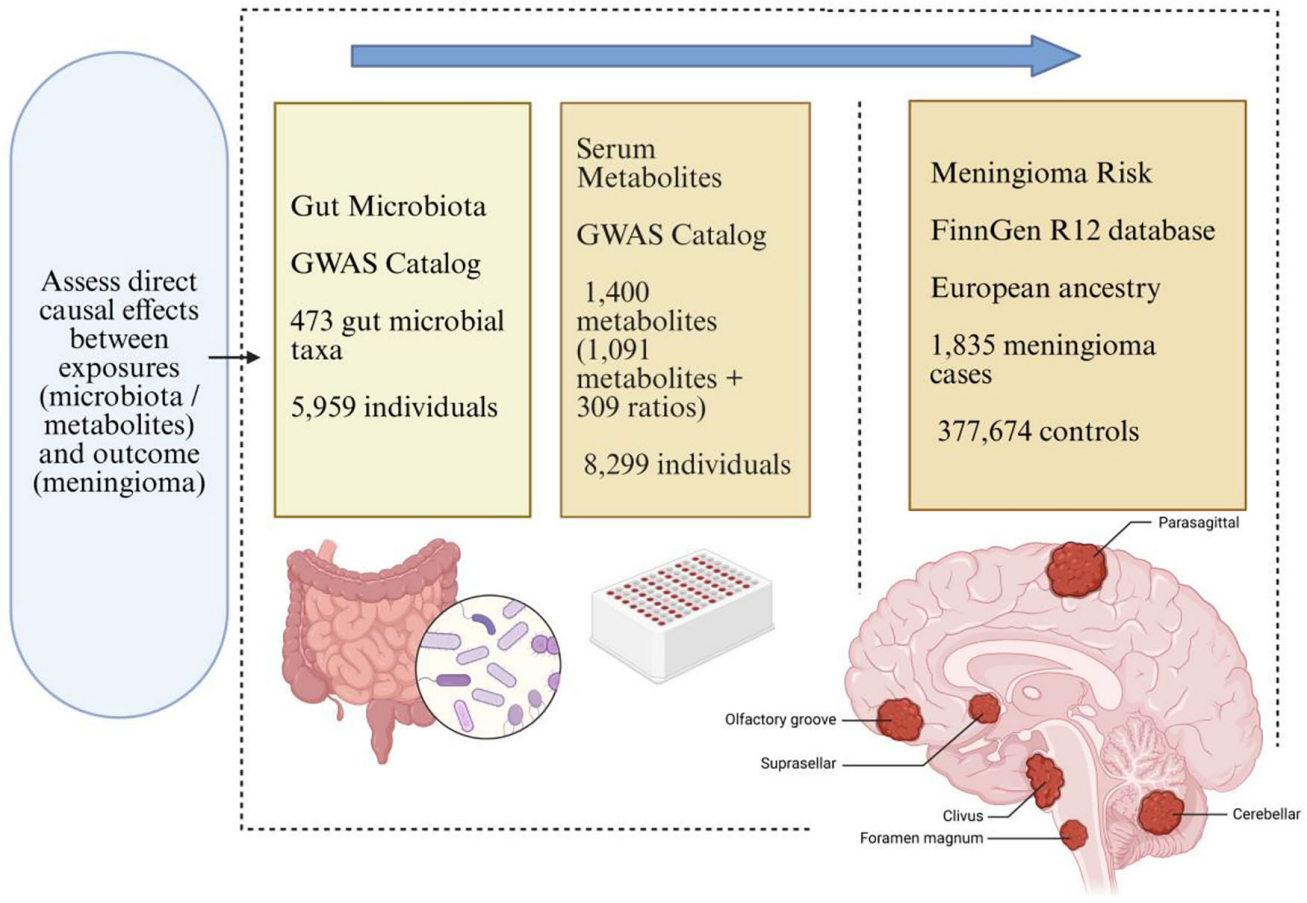
Overall analysis framework for research. The study aims to assess direct causal effects between exposures (gut microbiota and serum metabolites) and meningioma risk (outcome). Data sources for Mendelian randomization analysis are shown, detailing the summary statistics utilized for exposures from the GWAS Catalog (gut microbiota, *n* = 5959; serum metabolites, *n *= 8299) and for the outcome from the FinnGen R12 database (1835 meningioma cases and 377,674 controls).

The study used a two‐sample MR strategy to examine possible causal associations among 473 gut microbiota species, 1400 blood metabolites, and meningioma. The MR approach employs genetic variations as instrumental variables (IVs) to deduce causality between exposures and health outcomes, successfully reducing biases seen in observational research, such as confounding and reverse causation (Lawlor et al. 2008; L. N. Wang and Zhang 2017). Valid MR inference requires adherence to three essential assumptions (Allman et al. 2021; Zhu et al. 2018): relevance assumption (a strong association between the IV and the exposure), the independence assumption (no correlation between the IV and confounders), and the exclusion restriction (IV influence outcomes exclusively through the exposure). This research used MR analysis in a three‐step process: (i) evaluated the affirmative causal relationships of 473 gut microbiota with meningioma and performed reverse MR analysis to eliminate reverse causality; (ii) examined the causal relationships of 1400 serum metabolites with meningioma; (iii) investigated the causal interactions between gut microbiota and serum metabolites, and quantified the mediated impact of serum metabolites between specific gut microbiota and meningioma via mediation analysis. Figure 1 illustrates the analytical framework of the MR analysis.

### 2.2 Data Sources

The publicly accessible genome‐wide association study (GWAS) summary results for gut microbiota were acquired from the GWAS Catalog (https://www.ebi.ac.uk/gwas), with accession numbers spanning from GCST90032172 to GCST90032644. This dataset includes 473 gut microbial taxa identified from 5959 people, which includes 11 phyla, 18 classes, 24 orders, 62 families, 145 genera, and 213 species. The GWAS data on serum metabolites were obtained from the GWAS Catalog (https://www.ebi.ac.uk/gwas), with accession numbers spanning from GCST90199621 to GCST90201020. This dataset comprises information from 8,299 persons, including genome‐wide relationships of 1091 serum metabolites and 309 metabolite ratios. The GWAS summary data for meningioma were sourced from the FinnGen R12 database, including a European population‐based cohort of 1835 meningioma cases and 377,674 controls. Comprehensive information may be found at https://www.finngen.fi/en/access_results. All summary‐level data from GWAS used in this study are publicly available, and signed informed permission was acquired from all individuals by the relevant institutional review boards. This research, being a secondary examination of already published data, did not need further ethical clearance. The complete list of gut microbiota GWAS accession IDs used in this study is provided in Appendix S1. The complete list of serum metabolite GWAS accession IDs used in this study is provided in Appendix S2.

### 2.3 Selection of IVs

The same IVs selection strategy was applied for both gut microbiota and serum metabolite exposures. Single nucleotide polymorphisms (SNPs) strongly associated with the exposures were chosen for IVs. Drawing from previous research, overly stringent thresholds often result in too few IVs and reduced statistical efficiency. Therefore, we adopted a relatively lenient significance threshold of *p* < 1.0 × 10^−5^ to select SNPs (Fei et al. 2024). To guarantee the independence of SNPs and regulate linkage disequilibrium (LD), we established an LD threshold of *r* ^2^ < 0.001 and a window size of 10,000 kb. Data harmonization was performed to guarantee uniform effect directions and allele coding for all SNPs. The *F*‐statistic was calculated for every qualifying SNP to assess the strength of IVs, ensuring that all instruments had an *F*‐statistic over 10 to avoid weak instrument bias. All statistical analyses were performed using R software (version 4.4.2) in conjunction with the MRPRESSO (version 1.0) and TwoSampleMR (version 0.6.8) packages.

### 2.4 Statistical Analysis

This research used several MR techniques, including MR‐Egger, weighted median, inverse variance weighted (IVW), simple mode, and weighted mode. MR‐Egger regression allows for directional pleiotropy by including an intercept term and estimates the causal effect from the slope. Weighted Median estimator takes the weighted median of the individual effect estimates as the causal‐effect estimate. The IVW method pools effect estimates from the individual IVs to infer the causal effect, weighting each estimate by the inverse of its variance. Simple Mode estimator selects the most frequently occurring value in the distribution of effect estimates as the point estimate. Weighted Mode estimator first weights the individual effect estimates by the inverse of their variances before computing the mode. Among these, the IVW method, extensively used and notably robust (Larsson and Burgess 2022; Use of Progestogens, and the Risk of Intracranial Meningioma: National Case‐Control Study 2024; Yavorska and Burgess 2017), functioned as the principal analytical technique. A *p* value less than 0.05 was deemed statistically significant. The variability of SNPs was evaluated using Cochran's *Q* statistic and funnel plots. Horizontal pleiotropy was assessed via MR‐Egger and MR‐PRESSO, accompanied by a leave‐one‐out sensitivity analysis to determine the impact of individual SNPs. To determine the directionality of causal relationships and exclude reverse causality, we used reverse MR analysis, with meningioma as the exposure and gut microbiota as the outcome. In this reverse MR investigation, IVs for meningioma were chosen based on the criteria of *p* < 5 × 10^−6^, *r* ^2^ < 0.001, kb = 10,000, and *F* > 10. A two‐step MR analysis using the coefficient product method was conducted to investigate whether the gut microbiota can influence the risk of meningioma by affecting serum metabolites, and to examine the potential mediating role of serum metabolites in the causal chain between gut microbiota and meningioma. A two‐step MR research was conducted to examine the influence of gut microbiota on meningioma risk via blood metabolites and to evaluate the potential mediating role of serum metabolites in the causal link between gut microbiota and meningioma. The mediation analysis included the overall impact of gut microbiota on meningioma (*β*), the influence of gut microbiota on serum metabolites (*β*_1_), and the impact of serum metabolites on meningioma (*β*_2_). The mediation effect was calculated as *β*_1_ × *β*_2_, the mediation percentage as (*β*_1_ × *β*_2_)/*β*, and the direct effect as the total impact minus the mediation effect. This manuscript is reported in accordance with the STROBE‐MR reporting guideline; the completed checklist is provided in Appendix S3.

## 3 Results

### 3.1 Investigation of the Causal Impacts of Gut Microbiota on Meningioma

**FIGURE 2:**
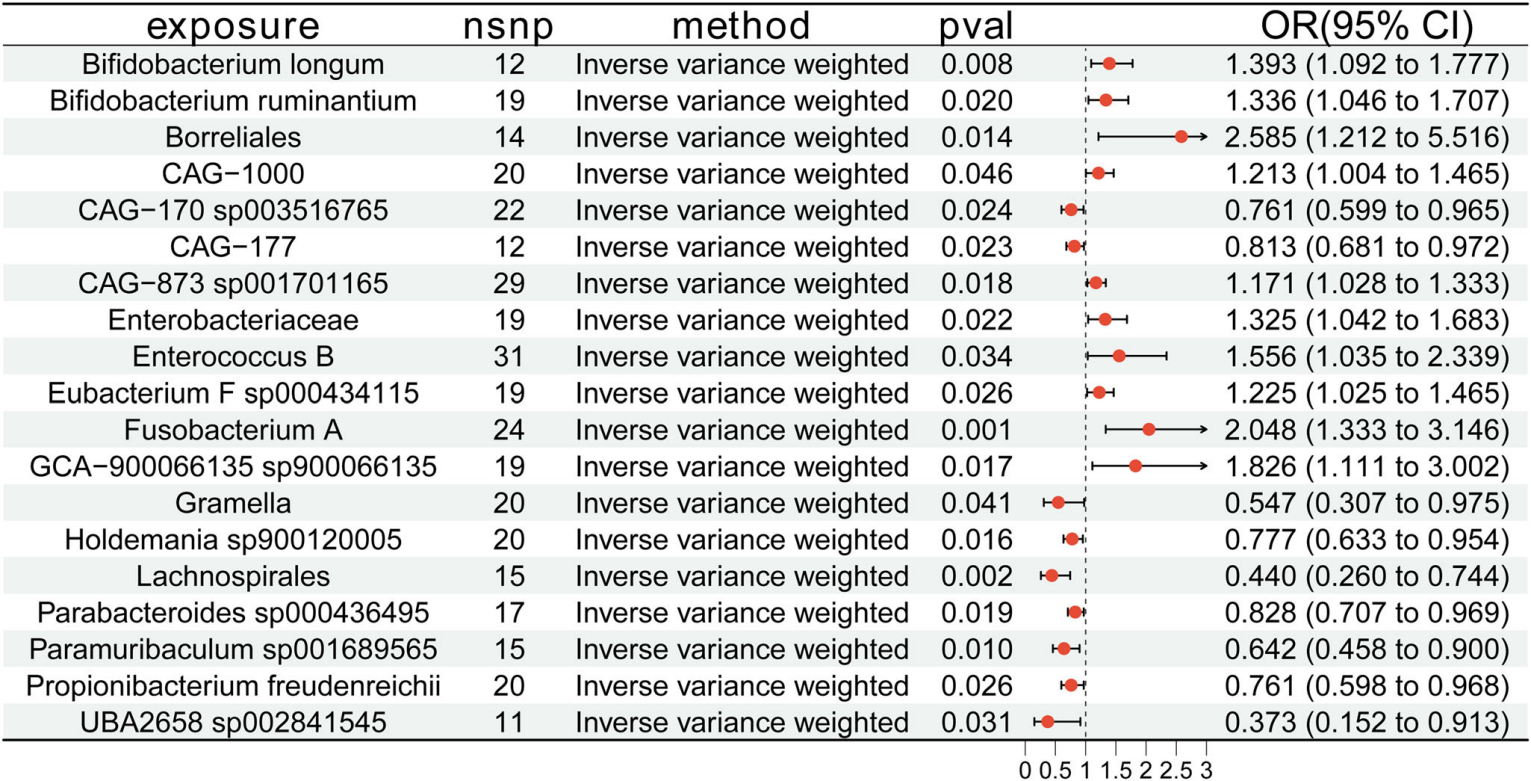
Forest plots illustrate the causative impact of the prevalence of 19 gut bacteria on meningioma. Estimates were calculated using the inverse variance weighted (IVW) method. Red dots indicate odds ratios (ORs), and horizontal lines represent 95% confidence intervals (CIs). nsnp, number of single‐nucleotide polymorphisms utilized as instrumental variables.

Using publicly available GWAS datasets, we performed two‐sample MR experiments to examine the potential causative effects of 473 gut microbial taxa (as exposures) on the occurrence of meningioma (as the outcome). The main approach used was the IVW method. Nineteen gut microbial species were found as causally related with meningioma after extensive testing for horizontal pleiotropy and heterogeneity. Of these, 10 taxa had odds ratios (ORs) over 1, suggesting they might be risk factors for meningioma. The results included CAG−873 sp001701165 (OR = 1.171, 95% CI = 1.028–1.333, *p *= 0.018), Enterobacteriaceae (OR = 1.325, 95% CI = 1.042–1.683, *p* = 0.022), and also *Fusobacterium* A (OR = 2.048, 95% CI = 1.333–3.146, *p =* 0.001). Nine taxa had ORs below 1, indicating a possible preventive effect against meningioma. The findings included *Propionibacterium freudenreichii* (OR = 0.761, 95% CI = 0.598–0.968, *p* = 0.026), CAG−170 sp003516765 (OR = 0.761, 95% CI = 0.599–0.965, *p* = 0.024), CAG−177 (OR = 0.813, 95% CI = 0.681–0.972, *p* = 0.023), and also UBA2658 sp002841545 (OR = 0.373, 95% CI = 0.152–0.913, *p* = 0.031). Figure 2 displays the MR results with the 19 microbial taxa as exposures and meningioma as the outcome; none of these associations showed evidence of horizontal pleiotropy. Detailed sensitivity analyses and results from additional MR methods are provided in Appendices S4 and S5.

### 3.2 Investigation of the Causal Impacts of Meningioma on Gut Microbiota

**FIGURE 3:**
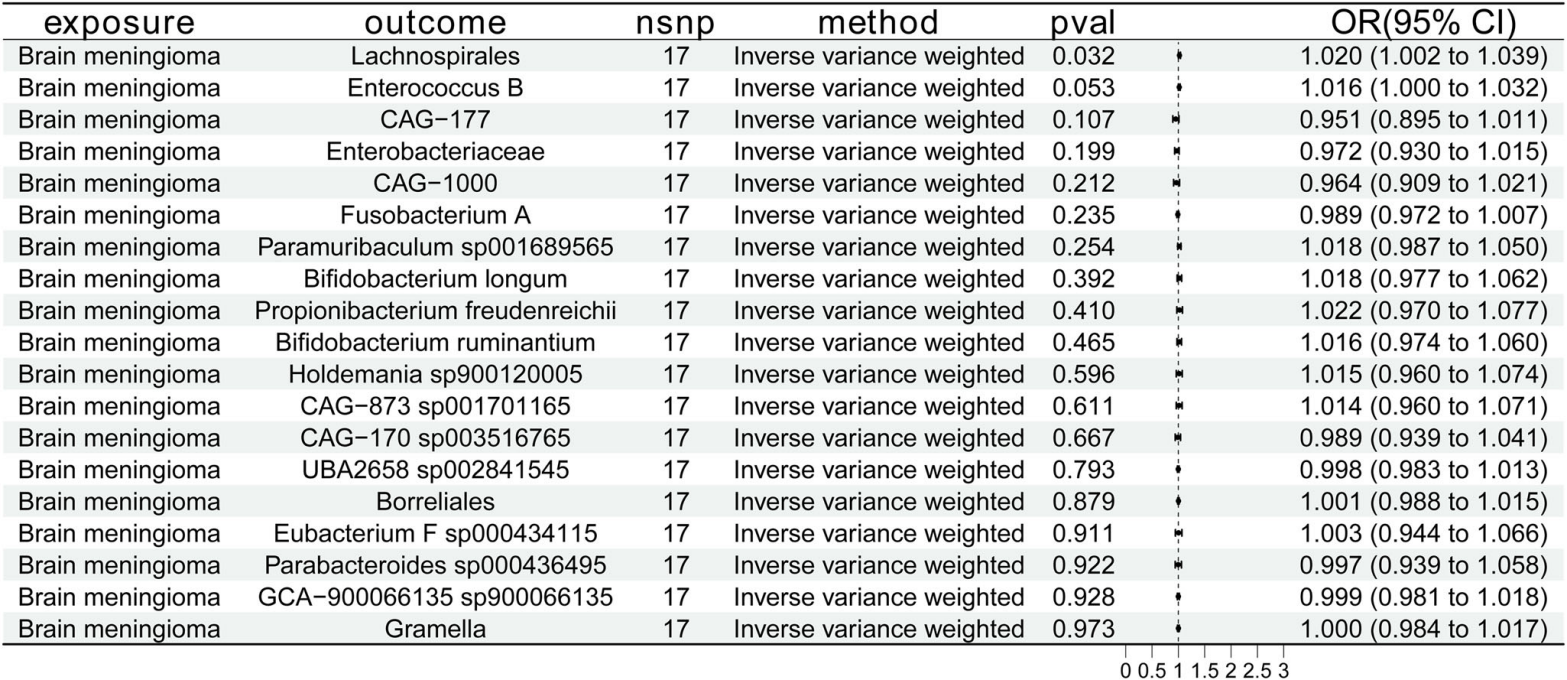
Forest plots illustrate the causative impact of meningioma on the prevalence of 19 gut bacteria. Estimates were based on the inverse variance weighted (IVW) method. OR, odds ratio; CI, 95% confidence interval; nsnp, number of single‐nucleotide polymorphisms.

This study treated meningioma as the exposure factor and the aforementioned 19 gut microbiota as outcomes. Using IVW as the primary method for reverse MR analysis, it revealed a reverse causal relationship between meningioma and the gut bacterium Lachnospirales, which will be excluded in subsequent analyses. No inverse causal correlations were identified between meningioma and the other 18 gut microbial species. Figure 3 depicts the findings of the MR study, with meningioma serving as the exposure and the 19 gut microbiota taxa as outcomes.

### 3.3 Investigation of the Causal Impacts of Serum Metabolites on Meningioma

**FIGURE 4:**
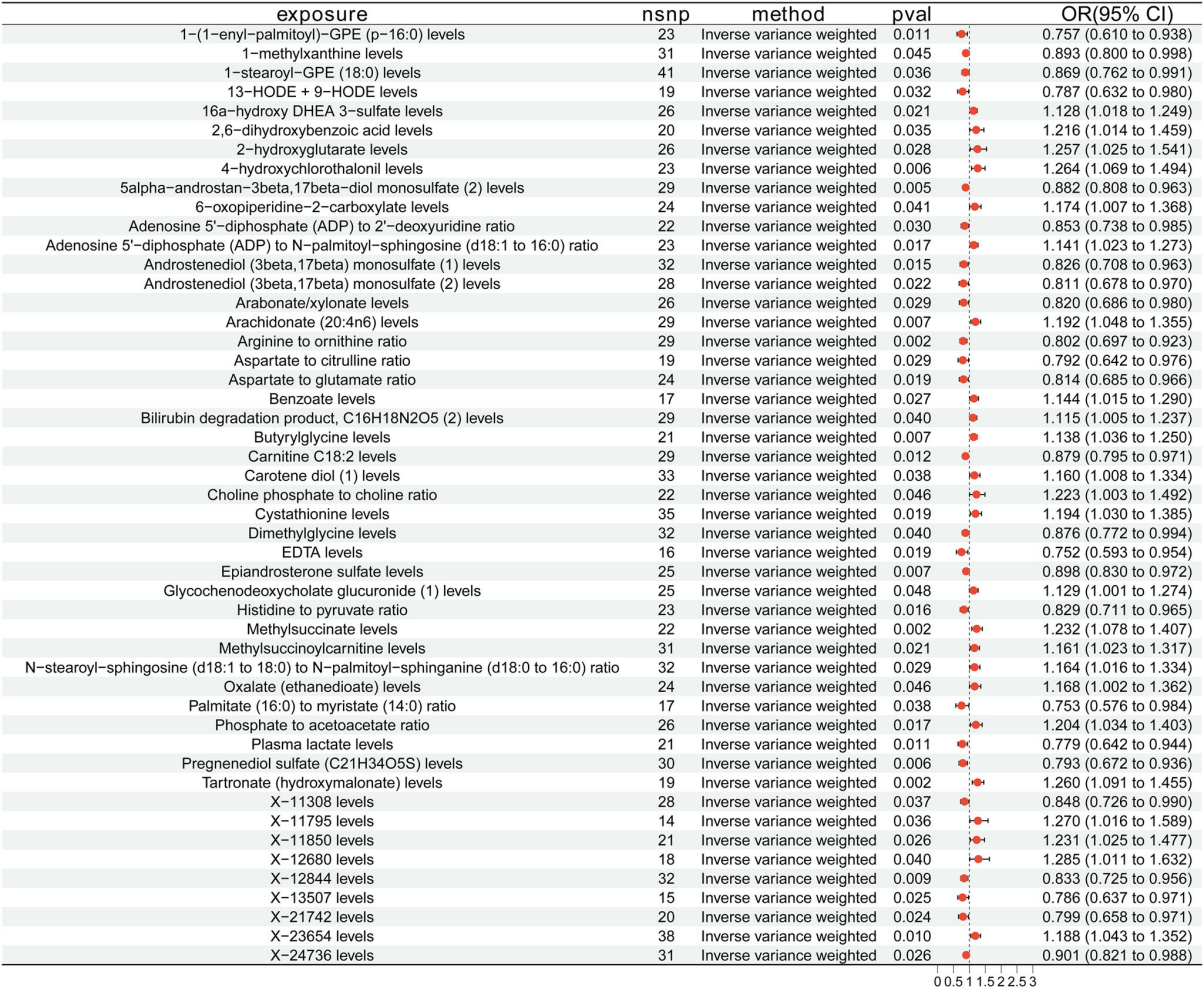
Forest plots illustrate the causative impact of 49 metabolite levels on meningioma. Estimates were calculated using the inverse variance weighted (IVW) method. OR, odds ratio; CI, 95% confidence interval; nsnp, number of single‐nucleotide polymorphisms.

Consequently, we conducted a two‐sample MR research to investigate the potential causal influence of 1400 serum metabolites on meningioma risk. Among them, 24 metabolites were found as significantly associated with an increased risk of meningioma. Prominent instances encompass arachidonate (20:4n6) (OR = 1.192; 95% CI: 1.048–1.355; *p* = 0.007), 2‐hydroxyglutarate (OR = 1.257; 95% CI: 1.025–1.541; *p* = 0.028), 16α‐hydroxy DHEA 3‐sulfate (OR = 1.128; 95% CI: 1.018–1.249; *p* = 0.021), and the phosphate‐to‐acetoacetate ratio (OR = 1.204; 95% CI: 1.034–1.403; *p* = 0.017). In contrast, 25 metabolites were linked to a reduced incidence of meningioma. The substances include 5alpha‐androstan‐3beta,17beta‐diol monosulfate(2) (OR = 0.882; 95% CI: 0.808–0.963; *p* = 0.005), epiandrosterone sulfate (OR = 0.898; 95% CI: 0.830–0.972; *p* = 0.007), 1‐(1‐enyl‐palmitoyl)‐GPE (p‐16:0) (OR = 0.757; 95% CI: 0.610–0.938; *p* = 0.011), and 1‐stearoyl‐GPE (18:0) (OR = 0.869; 95% CI: 0.762–0.991; *p* = 0.036). Figure 4 displays a forest plot that encapsulates the MR estimates for these 49 metabolites. No indication of horizontal pleiotropy was seen among the included instruments. Comprehensive results from sensitivity analyses and supplementary MR methodologies are included in Appendices S6 and S7.

### 3.4 Investigation of the Causal Impact of Gut Microbiota on Serum Metabolites

**FIGURE 5:**
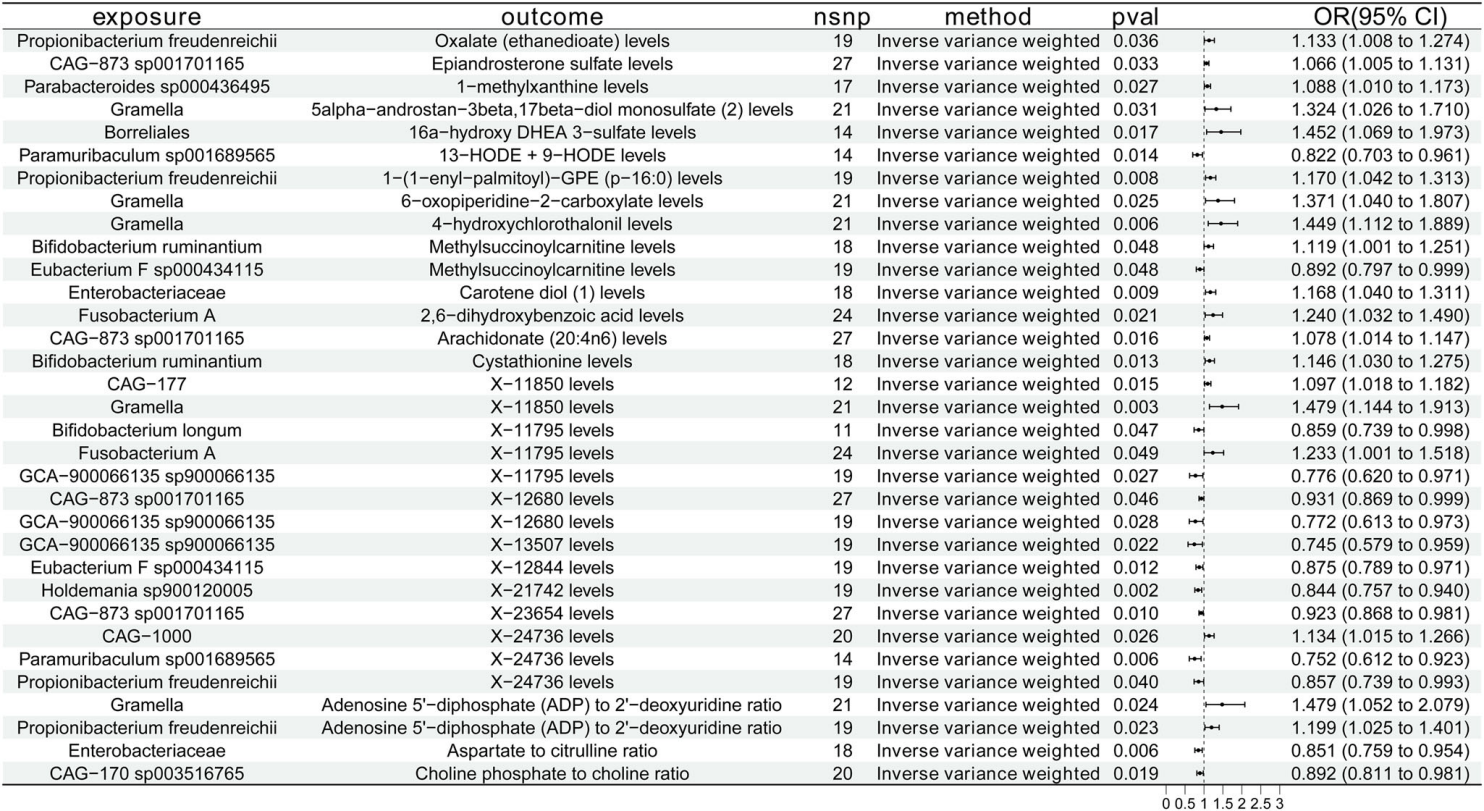
Forest plots illustrate the causative influence of 16 gut bacteria on metabolite concentrations. Estimates were calculated using the inverse variance weighted (IVW) method. OR, odds ratio; CI, 95% confidence interval; nsnp, number of single‐nucleotide polymorphisms.

Subsequent to the finding of gut microbiota and blood metabolites with causal links to meningioma, a two‐sample MR research was done between these two variables. A reverse causal link was identified between meningioma and the gut microbiota Lachnospirales in the second phase of the MR analysis, leading to the exclusion of this taxon from the present study. Figure 5 presents a forest plot of the IVW method results using 25 serum metabolites as outcomes and 16 gut microbiota taxa as exposures. Detailed sensitivity analyses and results from other MR methods are provided in Appendices S8 and S9.

### 3.5 Genetically Predicted Serum Metabolites Mediate the Complex Causal Relationship Between Gut Microbiota and Meningioma

**FIGURE 6:**
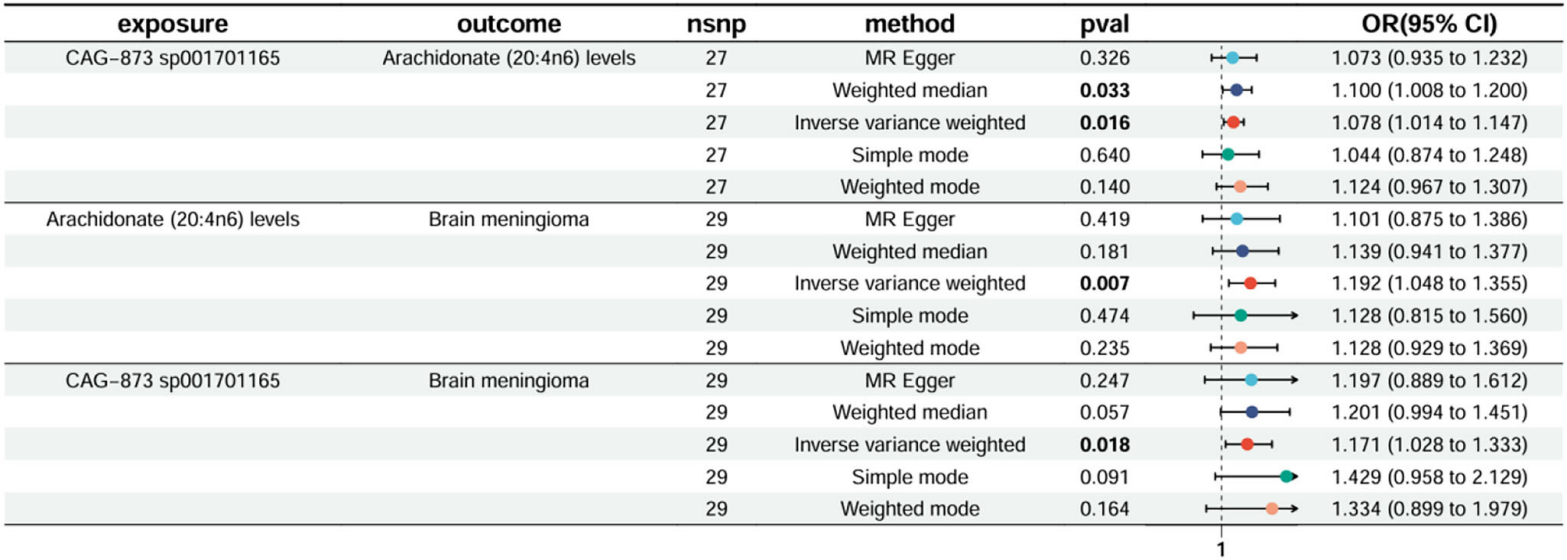
Forest plot illustrating the mediation Mendelian randomization analysis of arachidonate (20:4n6) levels in the association between *CAG‐873 sp001701165* and meningioma. Causal estimates are shown for the three steps of mediation analysis: (1) effect of *CAG‐873* on arachidonate levels, (2) effect of arachidonate levels on meningioma, and (3) total effect of *CAG‐873* on meningioma. Multiple MR methods were employed (MR Egger, weighted median, IVW, simple mode, weighted mode). CI, 95% confidence interval; nsnp, number of single‐nucleotide polymorphisms; OR, odds ratio.

**FIGURE 7:**
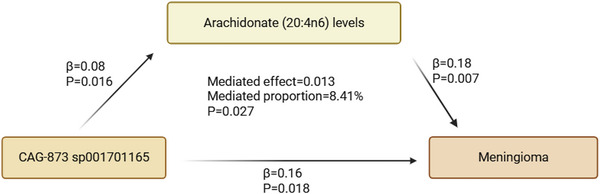
Schematic representation of the mediation Mendelian randomization analysis. Arrows indicate causal pathways, with *β* values representing causal effect estimates and *p* values indicating statistical significance. The analysis reveals that arachidonate (20:4n6) levels partially mediate the effect of *CAG‐873 sp001701165* on meningioma risk (mediated effect = 0.013, $P = 0.027), with a mediation proportion of 8.41%.

Through mediation analysis, we further clarified that Arachidonate (20:4n6) levels mediate the relationship between CAG‐873 sp001701165 and meningioma (*p* = 0.027), with a mediation effect of 0.0132 (95% CI: 0.00148, 0.025), accounting for 8.41% (95% CI: 0.938%, 15.9%) of the total effect. CAG‐873 sp001701165 increases the risk of meningioma by upregulating arachidonic acid levels. No significant pleiotropy or heterogeneity was detected, supporting the robustness of the findings. Figure 6 displays the outcomes of the MR analysis, while Figure 7 illustrates the findings of the mediation study. Sensitivity analyses and additional MR analysis results can be found in Appendices S10 and S11.

## 4 Discussion

This research, using two‐sample MR analysis, revealed 19 gut microbial species that are causally linked to the risk of meningioma. Among them, ten taxa—including CAG‐873 sp001701165 from the order Bacteroidales, Enterobacteriaceae, *Fusobacterium* A from the family *Fusobacteriaceae*, *Bifidobacterium*, *Enterococcus*, *Eubacterium* F sp000434115, and CAG‐1000 from the order Bacteroidales—were linked to an increased risk of meningioma. The tumor‐promoting effects of these gut microbes on meningioma may involve mechanisms including the activation of inflammatory signaling pathways (e.g., TLR4/NF‐κB pathway), the release of pro‐inflammatory cytokines (IL‐6, TNF‐α), dysregulation of polyunsaturated fatty acid metabolism (notably the accumulation of arachidonic acid), and the reprogramming of the immune microenvironment.

CAG‐873 sp001701165 is a gut microbe belonging to the order Bacteroidales, whose members possess complex metabolic networks capable of regulating host fatty acid metabolism (Park et al. 2022; H. Zhang et al. 2024). The expression of proteins associated with fatty acid metabolism is significantly linked to meningioma grading and prognosis, and aberrant expression of these proteins may enhance tumor cell proliferation and survival (Shibuya 2015). Arachidonic acid, an ω‐6 polyunsaturated fatty acid, may be processed via the cyclooxygenase‐2 (COX‐2) route to generate prostaglandin E2 (PGE2), which facilitates tumor angiogenesis and immunological regulation (Cui et al. 2017; Jin et al. 2023). This study found a significant association between CAG‐873 sp001701165 and elevated Arachidonate (20:4n6) levels, with part of the tumor‐promoting mechanism mediated by Arachidonate (20:4n6) (mediation effect accounted for 8.41%, *p* = 0.027), suggesting that this microbe may participate in fatty acid metabolism and regulation through multiple pathways. Additionally, the cell walls of Bacteroidales bacteria typically contain components such as lipopolysaccharides (LPS), some of which exhibit pro‐inflammatory effects. The pro‐inflammatory activity of the LPS from CAG‐873 sp001701165 remains ambiguous; however, drawing from mechanisms observed in similar taxa, it may activate the Toll‐like Receptor 4 (TLR4)/NF‐κB pathway, thereby facilitating the release of pro‐inflammatory cytokines (e.g., IL‐6, TNF‐α) (Chen et al. 2023), which could amplify inflammatory signaling cascades associated with meningioma. Likewise, *Fusobacterium* A, a member of the *Fusobacteriaceae* family, may affect meningioma risk via the modulation of inflammation. Enterobacteriaceae may induce chronic intestinal mucosal inflammation and the production of reactive oxygen species (ROS), leading to increased genomic instability. (Jiang et al. 2024; Zhou et al. 2024). Consequently, Enterobacteriaceae may affect meningioma risk through similar mechanisms.

Conversely, nine microbial taxa—including *Propionibacterium freudenreichii* from the genus *Propionibacterium*, CAG‐170 sp003516765 and CAG‐177 from the genus *Ruminococcus*, UBA2658 sp002841545 from the phylum Firmicutes, Lachnospirales from the order Clostridiales, *Holdemania* sp900120005, and *Parabacteroides* sp000436495 from the genus *Parabacteroides*—were associated with a decreased risk of meningioma. The protective effects of these microbes may involve mechanisms such as short‐chain fatty acid (SCFA)‐mediated inhibition of histone deacetylases (HDAC), enhanced differentiation of regulatory T cells (Tregs), antioxidative stress, and modulation of secondary bile acid metabolism. Specifically, *Propionibacterium freudenreichii*, a member of the *Propionibacterium* genus, exhibits antitumor properties that are likely related to its secretion of SCFAs, such as propionate. SCFAs can inhibit HDAC activity, upregulate tumor suppressor genes (e.g., p21), and promote Treg differentiation, thereby suppressing tumor immune evasion (Luu et al. 2021; Ryu et al. 2022).

This study further identified 34 serum metabolites significantly associated with meningioma risk. Among them, Arachidonate (20:4n6) showed a potential causal relationship with meningioma (OR = 1.192, 95% CI: 1.048–1.355, *p* = 0.007). Arachidonic acid and its metabolites are known to regulate multiple mammalian processes, including inflammation, aging, ion channel function, and neuropsychiatric disorders (Chopra et al. 2012). Arachidonic acid is closely linked to various inflammatory mechanisms; as a precursor of prostaglandins, Arachidonic acid can be metabolized via the COX‐2 pathway to produce PGE2. PGE2 promotes tumor cell proliferation, angiogenesis, and the formation of an immunosuppressive microenvironment by activating EP2/EP4 receptors (Jin et al. 2023). Overexpression of COX‐2 and elevated PGE2 levels in meningioma tissues have been confirmed to correlate with poor prognosis (Aung et al. 2024; Kulesza et al. 2023), suggesting that this pathway may represent a therapeutic target. A MR research indicated that heightened levels of TNF‐*β* and CXCL1, together with diminished levels of IL‐9, correlate with an increased risk of meningioma (Z. Zhang et al. 2023). Arachidonic acid can amplify inflammatory responses and modulate other inflammatory mediators (Gorica and Calderone 2022; Regulska et al. 2021; Z. Zhang et al. 2023). Thus, the association between arachidonic acid and meningioma is closely related to inflammation regulation.

Levels of 16α‐hydroxy DHEA 3‐sulfate, 2,6‐dihydroxybenzoic acid, 2‐hydroxyglutarate, 4‐hydroxychlorothalonil, methylsuccinate, methylsuccinoylcarnitine, along with X‐11795, X‐11850, X‐12600, X‐23654, and tartronate (hydroxymalonate) are correlated with an elevated risk of meningioma. 2‐hydroxyglutarate, functioning as an oncometabolite, can competitively inhibit α‐ketoglutarate‐dependent dioxygenases such as TET2 and JMJD3, leading to genome‐wide DNA hypermethylation and stabilization of hypoxia‐inducible factor‐1α (HIF‐1α), thereby mimicking the “metabolic reprogramming” phenotype seen in gliomas (Kadiyala et al. 2021; Reiter‐Brennan et al. 2018; Tong et al. 2024). Furthermore, 2‐hydroxyglutarate may exacerbate oxidative stress and promote the accumulation of genomic mutations by disrupting the tricarboxylic acid (TCA) cycle and mitochondrial electron transport chain.

Conversely, serum metabolites—1‐(1‐enyl‐palmitoyl)‐GPE, 1‐stearoyl‐GPE, the arginine to ornithine ratio, plasma lactate levels, pregnenediol sulfate levels, and levels of X‐11308, X‐12484, X‐13507, and X‐21742—correlate with a reduced risk of meningioma. These metabolites may affect the incidence and advancement of meningioma by altering inflammatory responses, the immunological microenvironment, or hormone‐dependent mechanisms. 1‐stearoyl‐GPE may inhibit pro‐inflammatory signaling by altering membrane phospholipid metabolism; plasma lactate levels might reflect reduced glycolytic activity in the tumor microenvironment, correlated with benign tumor morphologies. Moreover, pregnenediol sulfate may impede the phosphorylation of PR via feedback mechanisms, hence hindering tumor proliferation associated with the MAPK/ERK pathway, consistent with the hormone‐dependent nature of meningiomas.

Our research demonstrated that CAG‐873 sp001701165 influences meningioma risk via the upregulation of Arachidonate (20:4n6) levels (mediation effect *p* = 0.027), indicating that gut microbiota might remotely modulate the central tumor microenvironment by altering metabolite levels. Previous research has shown that circulating PGE2 may disrupt blood‐brain barrier integrity, infiltrate meningioma tissue, and activate the STAT3 pathway to drive tumor stem cell self‐renewal (H. Q. Wang et al. 2022). These results support the “microbiota–gut–brain axis” paradigm, which posits that chemicals originating from gut microbiota affect central tumors via systemic inflammation and neuroendocrine mechanisms. This research first evaluates the causal relationship between fecal gut microbiota abundance, blood metabolites, and the risk of meningioma by MR analysis. However, the GWAS data used are mostly from North American and European populations, so further validation is needed for better generalizability to other populations. While larger sample sizes can boost MR analysis accuracy, obtaining large—scale datasets is challenging in practice. As MR analysis depends on genetic instruments to infer causality, additional research is required to enhance the reliability of the results. From the brain—gut axis perspective, the study explores arachidonate (20:4n6)'s mediating role between gut bacterium CAG ‐ 873 sp001701165 and meningioma. The remaining associated signals also deserve more exploration. Future studies should dissect the brain—gut axis metabolic regulatory network and employ multi—omics or complementary analytical approaches to elucidate its role in meningioma pathogenesis.

This study has several limitations. In this study, MR‐Egger and MR‐PRESSO were employed to assess horizontal pleiotropy; however, both methods rely on the InSIDE (Instrument Strength Independent of Direct Effect) assumption, which requires that the strength of the instruments for the exposure be independent of their direct effects on the outcome. This assumption cannot be empirically verified and may be violated due to factors such as pathway enrichment, LD, or the use of a relatively relaxed instrument selection threshold (*p* < 1 × 10^−5^), potentially leading to bias. Considering the complex, multi‐pathway biological characteristics of gut microbiota and metabolites, we interpret our findings with caution and recommend that future studies confirm these results using multi‐ancestry replication, stronger IVs, more robust MR approaches, and complementary experimental validation. Moreover, because the genetic architecture, allele frequencies, and LD patterns of gut microbiota and metabolites differ across populations—which may affect instrument strength and direction—the generalizability of our results is mainly limited to individuals of European ancestry. Future studies should therefore perform cross‐population validation and homogeneity tests to further strengthen the robustness of causal inferences.

## 5 Conclusion

This study rigorously assessed the causal relationships among gut microbiota, blood metabolites, and meningioma by MR analysis. MR mitigated the influence of confounding variables prevalent in conventional observational research, hence increasing the credibility of the results. Mediation analysis was used to elucidate the mediating function of serum metabolites in the gut microbiota‐meningioma relationship. This study offers novel insights into the pathophysiology and possible therapeutic targets of meningioma via the lens of the brain–gut axis. However, the intricate interactions between gut microbiota, serum metabolites, and meningioma need more exploration.

## Author Contributions

The project was managed by X.G., J.Z., and W.Z. Statistical analyses were conducted by J.Z., K.W., and X.G. Data extraction and literature review were performed by M.H. Figures and visualizations were created by X.Z. Methodological framework and sensitivity analyses were contributed by K.W. Manuscript drafting and writing were undertaken by X.G. and Y.Y. The study was supervised and critically revised by X.Z., Q.Z., and W.Z., who also served as the corresponding authors. The final manuscript was reviewed and approved by all authors.

## Funding

The authors have nothing to report.

## Ethics Statement

This research used publicly accessible summary‐level GWAS data. The first investigations obtained ethical clearance and informed permission from subjects. This secondary analysis does not need further ethical clearance.

## Conflicts of Interest

The authors declare no conflicts of interests.

## Supporting information




**Supplementary Appendix S1**: brb371220‐sup‐0001‐Appendix1.xlsx

**Supplementary Appendix S2**: brb371220‐sup‐0002‐Appendix2.xlsx

**Supplementary Appendix S3**: brb371220‐sup‐0003‐Appendix3.docx

**Supplementary Appendix S4**: brb371220‐sup‐0004‐Appendix4.pdf

**Supplementary Appendix S5**: brb371220‐sup‐0005‐Appendix5.xlsx

**Supplementary Appendix S6**: brb371220‐sup‐0006‐Appendix6.pdf

**Supplementary Appendix S7**: brb371220‐sup‐0007‐Appendix7.xlsx

**Supplementary Appendix S8**: brb371220‐sup‐0008‐Appendix8.pdf

**Supplementary Appendix S9**: brb371220‐sup‐0009‐Appendix9.xls

**Supplementary Appendix S10**: brb371220‐sup‐00010‐Appendix10.pdf

**Supplementary Appendix S11**: brb371220‐sup‐00011‐Appendix11.xlsx

## Data Availability

All data used in this study are publicly available from GWAS repositories. Details and access links are provided in the Supporting Information.

## References

[brb371220-bib-0001] “Use of Progestogens and the Risk of Intracranial Meningioma: National Case‐Control Study.” 2024. *Bmj* 384: q776. 10.1136/bmj.q776. PMC10977609 38548284

[brb371220-bib-0002] Allman, P. H. , I. Aban , D. M. Long , et al. 2021. “A Novel Mendelian Randomization Method With Binary Risk Factor and Outcome.” Genetic Epidemiology 45, no. 5: 549–560. 10.1002/gepi.22387. 33998053

[brb371220-bib-0003] Aung, T. M. , C. Ngamjarus , T. Proungvitaya , C. Saengboonmee , and S. Proungvitaya . 2024. “Biomarkers for Prognosis of Meningioma Patients: A Systematic Review and Meta‐Analysis.” PLoS ONE 19, no. 5: e0303337. 10.1371/journal.pone.0303337. 38758750 PMC11101050

[brb371220-bib-0004] Banerjee, J. , E. Pääkkö , M. Harila , et al. 2009. “Radiation‐Induced Meningiomas: A Shadow in the Success Story of Childhood Leukemia.” Neuro‐Oncology 11, no. 5: 543–549. 10.1215/15228517-2008-122. 19179425 PMC2765343

[brb371220-bib-0005] CBTRUS . 2024. “CBTRUS Fact Sheet 2024.” https://cbtrus.org/.

[brb371220-bib-0006] Chen, X. , J. Wu , X. Fu , P. Wang , and C. Chen . 2023. “Fructus Mori Polysaccharide Alleviates Diabetic Symptoms by Regulating Intestinal Microbiota and Intestinal Barrier Against TLR4/NF‐κB Pathway.” International Journal of Biological Macromolecules 249: 126038. 10.1016/j.ijbiomac.2023.126038. 37516223

[brb371220-bib-0007] Chopra, A. , L. Shan , W. C. Eckelman , et al. 2012. “Molecular Imaging and Contrast Agent Database (MICAD): Evolution and Progress.” Molecular Imaging and Biology 14, no. 1: 4–13. 10.1007/s11307-011-0521-3. 21989943 PMC3259264

[brb371220-bib-0008] Cui, Y. , X. O. Shu , H. L. Li , et al. 2017. “Prospective Study of Urinary Prostaglandin E2 Metabolite and Pancreatic Cancer Risk.” International Journal of Cancer 141, no. 12: 2423–2429. 10.1002/ijc.31007. 28815606 PMC5749224

[brb371220-bib-0009] Degeneffe, A. , V. De Maertelaer , O. De Witte , and F. Lefranc . 2023. “The Association Between Meningioma and Breast Cancer: A Systematic Review and Meta‐Analysis.” JAMA Network Open 6, no. 6: e2318620. 10.1001/jamanetworkopen.2023.18620. 37326990 PMC10276307

[brb371220-bib-0010] Fei, Y. , H. Yu , Y. Wu , and S. Gong . 2024. “The Causal Relationship Between Immune Cells and Ankylosing Spondylitis: A Bidirectional Mendelian Randomization Study.” Arthritis Research & Therapy 26, no. 1: 24. 10.1186/s13075-024-03266-0. 38229175 PMC10790477

[brb371220-bib-0011] Gorica, E. , and V. Calderone . 2022. “Arachidonic Acid Derivatives and Neuroinflammation.” CNS & Neurological Disorders—Drug Targets 21, no. 2: 118–129. 10.2174/1871527320666210208130412. 33557740

[brb371220-bib-0012] Goutagny, S. , A. B. Bah , D. Henin , et al. 2012. “Long‐Term Follow‐Up of 287 Meningiomas in Neurofibromatosis Type 2 Patients: Clinical, Radiological, and Molecular Features.” Neuro‐Oncology 14, no. 8: 1090–1096. 10.1093/neuonc/nos129. 22711605 PMC3408259

[brb371220-bib-0013] Jiang, Y. F. , Y. Q. Huang , Y. E. Hu , et al. 2024. “Banxia Xiexin Decoction Inhibiting Colitis‐Associated Colorectal Cancer Infected With *Fusobacterium nucleatum* by Regulating Wnt/β‐Catenin Pathway.” [In Chinese] Zhongguo Zhong Yao Za Zhi = Zhongguo Zhongyao Zazhi = China Journal of Chinese Materia Medica 49, no. 5: 1266–1274. 10.19540/j.cnki.cjcmm.20231114.701. 38621974

[brb371220-bib-0014] Jiang, H. , W. Zeng , X. Zhang , Y. Pei , H. Zhang , and Y. Li . 2022. “The Role of Gut Microbiota in Patients With Benign and Malignant Brain Tumors: A Pilot Study.” Bioengineered 13, no. 3: 7847–7859. 10.1080/21655979.2022.2049959. 35291914 PMC9208447

[brb371220-bib-0015] Jin, K. , C. Qian , J. Lin , and B. Liu . 2023. “Cyclooxygenase‐2‐Prostaglandin E2 Pathway: A Key Player in Tumor‐Associated Immune Cells.” Frontiers in Oncology 13: 1099811. 10.3389/fonc.2023.1099811. 36776289 PMC9911818

[brb371220-bib-0016] Kadiyala, P. , S. V. Carney , J. C. Gauss , et al. 2021. “Inhibition of 2‐Hydroxyglutarate Elicits Metabolic Reprogramming and Mutant IDH1 Glioma Immunity in Mice.” Journal of Clinical Investigation 131, no. 4: e139542. 10.1172/jci139542. 33332283 PMC7880418

[brb371220-bib-0017] Kökoğlu, E. , Y. Tüter , K. S. Sandikçi , et al. 1998. “Prostaglandin E2 Levels in Human Brain Tumor Tissues and Arachidonic Acid Levels in the Plasma Membrane of Human Brain Tumors.” Cancer Letters 132, no. 1–2: 17–21. 10.1016/s0304-3835(98)00127-x. 10397448

[brb371220-bib-0018] Kulesza, A. , L. Paczek , and A. Burdzinska . 2023. “The Role of COX‐2 and PGE2 in the Regulation of Immunomodulation and Other Functions of Mesenchymal Stromal Cells.” Biomedicines 11, no. 2: 445. 10.3390/biomedicines11020445. 36830980 PMC9952951

[brb371220-bib-0019] Larsson, S. C. , and S. Burgess . 2022. “Appraising the Causal Role of Smoking in Multiple Diseases: A Systematic Review and Meta‐Analysis of Mendelian Randomization Studies.” eBioMedicine 82: 104154. 10.1016/j.ebiom.2022.104154. 35816897 PMC9278068

[brb371220-bib-0020] Lawlor, D. A. , R. M. Harbord , J. A. Sterne , N. Timpson , and G. Davey Smith . 2008. “Mendelian Randomization: Using Genes as Instruments for Making Causal Inferences in Epidemiology.” Statistics in Medicine 27, no. 8: 1133–1163. 10.1002/sim.3034. 17886233

[brb371220-bib-0021] Luu, M. , Z. Riester , A. Baldrich , et al. 2021. “Microbial Short‐Chain Fatty Acids Modulate CD8^+^ T Cell Responses and Improve Adoptive Immunotherapy for Cancer.” Nature Communications 12, no. 1: 4077. 10.1038/s41467-021-24331-1. PMC8249424 34210970

[brb371220-bib-0022] Mederer, T. , S. Schachinger , K. Rosengarth , et al. 2022. “Symptom Burden and Surgical Outcome in Non‐Skull Base Meningiomas.” Frontiers in Oncology 12: 967420. 10.3389/fonc.2022.967420. 36212448 PMC9532974

[brb371220-bib-0023] Mehrian‐Shai, R. , J. K. V. Reichardt , C. C. Harris , and A. Toren . 2019. “The Gut–Brain Axis, Paving the Way to Brain Cancer.” Trends in Cancer 5, no. 4: 200–207. 10.1016/j.trecan.2019.02.008. 30961828 PMC6734924

[brb371220-bib-0024] Morais, L. H. , H. L. t. Schreiber , and S. K. Mazmanian . 2021. “The Gut Microbiota‐Brain Axis in Behaviour and Brain Disorders.” Nature Reviews Microbiology 19, no. 4: 241–255. 10.1038/s41579-020-00460-0. 33093662

[brb371220-bib-0025] Nathoo, N. , G. H. Barnett , and M. Golubic . 2004. “The Eicosanoid Cascade: Possible Role in Gliomas and Meningiomas.” Journal of Clinical Pathology 57, no. 1: 6–13. 10.1136/jcp.57.1.6. 14693827 PMC1770171

[brb371220-bib-0026] Park, S. Y. , C. Rao , K. Z. Coyte , et al. 2022. “Strain‐Level Fitness in the Gut Microbiome Is an Emergent Property of Glycans and a Single Metabolite.” Cell 185, no. 3: 513–529.e521. 10.1016/j.cell.2022.01.002. 35120663 PMC8896310

[brb371220-bib-0027] Regulska, M. , M. Szuster‐Głuszczak , E. Trojan , M. Leśkiewicz , and A. Basta‐Kaim . 2021. “The Emerging Role of the Double‐Edged Impact of Arachidonic Acid‐Derived Eicosanoids in the Neuroinflammatory Background of Depression.” Current Neuropharmacology 19, no. 2: 278–293. 10.2174/1570159x18666200807144530. 32851950 PMC8033972

[brb371220-bib-0028] Reiter‐Brennan, C. , L. Semmler , and A. Klein . 2018. “The Effects of 2‐Hydroxyglutarate on the Tumorigenesis of Gliomas.” Contemporary Oncology 22, no. 4: 215–222. 10.5114/wo.2018.82642. 30783384 PMC6377424

[brb371220-bib-0029] Roland, N. , A. Neumann , L. Hoisnard , et al. 2024. “Use of Progestogens and the Risk of Intracranial Meningioma: National Case‐Control Study.” Bmj 384: e078078. 10.1136/bmj-2023-078078. 38537944 PMC10966896

[brb371220-bib-0030] Ryu, T. Y. , K. Kim , T. S. Han , et al. 2022. “Human Gut‐Microbiome‐Derived Propionate Coordinates Proteasomal Degradation via HECTD2 Upregulation to Target EHMT2 in Colorectal Cancer.” ISME Journal 16, no. 5: 1205–1221. 10.1038/s41396-021-01119-1. 34972816 PMC9038766

[brb371220-bib-0031] Sescu, D. , A. Chansiriwongs , K. J. Minta , J. Vasudevan , and C. Kaliaperumal . 2023. “Early Preventive Strategies and CNS Meningioma—Is This Feasible? A Comprehensive Review of the Literature.” World Neurosurgery 180: 123–133. 10.1016/j.wneu.2023.09.075. 37774783

[brb371220-bib-0032] Shibuya, M. 2015. “Pathology and Molecular Genetics of Meningioma: Recent Advances Supplement.” Neurologia Medico‐Chirurgica 55, no. S1: 14–27. 10.2176/nmc.ra.2014-0233 PMC4533397 25744347

[brb371220-bib-0033] Staby, L. , C. O'Shea , M. Willemoës , F. Theisen , B. B. Kragelund , and K. Skriver . 2017. “Eukaryotic Transcription Factors: Paradigms of Protein Intrinsic Disorder.” Biochemical Journal 474, no. 15: 2509–2532. 10.1042/bcj20160631. 28701416

[brb371220-bib-0034] Tong, S. , J. Wu , Y. Song , et al. 2024. “IDH1‐Mutant Metabolite D‐2‐Hydroxyglutarate Inhibits Proliferation and Sensitizes Glioma to Temozolomide via Down‐Regulating ITGB4/PI3K/AKT.” Cell Death Discovery 10, no. 1: 317. 10.1038/s41420-024-02088-y. 38982076 PMC11233597

[brb371220-bib-0035] Wang, H. Q. , Q. W. Man , F. Y. Huo , et al. 2022. “STAT3 Pathway in Cancers: Past, Present, and Future.” MedComm 3, no. 2: e124. 10.1002/mco2.124. 35356799 PMC8942302

[brb371220-bib-0036] Wang, L. N. , and Z. Zhang . 2017. “Mendelian Randomization Approach, Used for Causal Inferences.” [In Chinese] Zhonghua Liu Xing Bing Xue Za Zhi = Zhonghua Liuxingbingxue Zazhi 38, no. 4: 547–552. 10.3760/cma.j.issn.0254-6450.2017.04.027. 28468080

[brb371220-bib-0037] Wiemels, J. , M. Wrensch , and E. B. Claus . 2010. “Epidemiology and Etiology of Meningioma.” Journal of Neuro‐Oncology 99, no. 3: 307–314. 10.1007/s11060-010-0386-3. 20821343 PMC2945461

[brb371220-bib-0038] Yarabarla, V. , A. Mylarapu , T. J. Han , S. L. McGovern , S. M. Raza , and T. H. Beckham . 2023. “Intracranial Meningiomas: An Update of the 2021 World Health Organization Classifications and Review of Management With a Focus on Radiation Therapy.” Frontiers in Oncology 13: 1137849. 10.3389/fonc.2023.1137849. 37675219 PMC10477988

[brb371220-bib-0039] Yavorska, O. O. , and S. Burgess . 2017. “Mendelian Randomization: An R Package for Performing Mendelian Randomization Analyses Using Summarized Data.” International Journal of Epidemiology 46, no. 6: 1734–1739. 10.1093/ije/dyx034. 28398548 PMC5510723

[brb371220-bib-0040] Zhang, Z. , S. Wang , F. Ren , et al. 2023. “Inflammatory Factors and Risk of Meningiomas: A Bidirectional Mendelian‐Randomization Study.” Frontiers in Neuroscience 17: 1186312. 10.3389/fnins.2023.1186312. 37425011 PMC10325787

[brb371220-bib-0041] Zhang, H. , Y. Xie , F. Cao , and X. Song . 2024. “Gut Microbiota‐Derived Fatty Acid and Sterol Metabolites: Biotransformation and Immunomodulatory Functions.” Gut Microbes 16, no. 1: 2382336. 10.1080/19490976.2024.2382336. 39046079 PMC11271093

[brb371220-bib-0042] Zhou, J. Q. , H. J. Li , Y. H. Zeng , et al. 2024. “Baicalin Induces Ferroptosis in HepG2 Cells by Inhibiting ROS‐Mediated PI3K/Akt/FoxO3a Signaling Pathway.” [In Chinese] Zhongguo Zhong Yao Za Zhi = Zhongguo Zhongyao Zazhi = China Journal of Chinese Materia Medica 49, no. 5: 1327–1334. 10.19540/j.cnki.cjcmm.20231010.403. 38621980

[brb371220-bib-0043] Zhu, Z. , Z. Zheng , F. Zhang , et al. 2018. “Causal Associations Between Risk Factors and Common Diseases Inferred From GWAS Summary Data.” Nature Communications 9, no. 1: 224. 10.1038/s41467-017-02317-2. PMC5768719 29335400

