# Supplementary material for: Causal Pathways Linking Gut Microbiota, Serum Metabolites, and Meningioma Risk: A Mendelian Randomization Analysis
Source: Brain Behav. 2026 Feb 9;16(2):e71220. doi: 10.1002/brb3.71220 (PMC12887442; doi:10.1002/brb3.71220)
Supplement: Supplementary file 3 — Supplementary Appendix S3: brb371220‐sup‐0003‐Appendix3.docx [file BRB3-16-e71220-s006.docx]

**STROBE-MR checklist of recommended items to address in reports of Mendelian randomization studies**^1^ ^2^

| **Item No.** | **Section** | **Checklist item** | **Page No.** | **Relevant text from manuscript** |
| --- | --- | --- | --- | --- |
| 1 | **TITLE and ABSTRACT** | Indicate Mendelian randomization (MR) as the study’s design in the title and/or the abstract if that is a main purpose of the study | Pages 1-2 | Title, Abstract |
|  | **INTRODUCTION** |  |  |  |
| 2 | **Background** | Explain the scientific background and rationale for the reported study. What is the exposure? Is a potential causal relationship between exposure and outcome plausible? Justify why MR is a helpful method to address the study question | Pages 2-4 | Abstract, Introduction: paragraphs 1-3 |
| 3 | **Objectives** | State specific objectives clearly, including pre-specified causal hypotheses (if any). State that MR is a method that, under specific assumptions, intends to estimate causal effects | Pages 4 | Abstract, Introduction: paragraphs 3 |
|  | **METHODS** |  |  |  |
| 4 | **Study design and data sources** | Present key elements of the study design early in the article. Consider including a table listing sources of data for all phases of the study. For each data source contributing to the analysis, describe the following: |  |  |
|  | a) | Setting: Describe the study design and the underlying population, if possible. Describe the setting, locations, and relevant dates, including periods of recruitment, exposure, follow-up, and data collection, when available. | Pages 5-8 | Materials and methods: Data Sources |
|  | b) | Participants: Give the eligibility criteria, and the sources and methods of selection of participants. Report the sample size, and whether any power or sample size calculations were carried out prior to the main analysis | Pages 5-8 | Materials and methods: Data Sources |
|  | c) | Describe measurement, quality control and selection of genetic variants | Pages 6 | Materials and Methods: Selection of IVs |
|  | d) | For each exposure, outcome, and other relevant variables, describe methods of assessment and diagnostic criteria for diseases | Pages 5-8 | Materials and methods: Data Sources |
|  | e) | Provide details of ethics committee approval and participant informed consent, if relevant | Pages 5-8 | Materials and methods: Data Sources |
| 5 | **Assumptions** | Explicitly state the three core IV assumptions for the main analysis (relevance, independence and exclusion restriction) as well assumptions for any additional or sensitivity analysis | Pages 4-5 | Materials and Methods: MR Assumptions and Study Design |
| 6 | **Statistical methods: main analysis** | Describe statistical methods and statistics used |  |  |
|  | a) | Describe how quantitative variables were handled in the analyses (i.e., scale, units, model) | Pages 5-8 | Materials and Methods: Data Sources; Selection of IVs; Statistical Analysis |
|  | b) | Describe how genetic variants were handled in the analyses and, if applicable, how their weights were selected |  |  |
|  | c) | Describe the MR estimator (e.g. two-stage least squares, Wald ratio) and related statistics. Detail the included covariates and, in case of two-sample MR, whether the same covariate set was used for adjustment in the two samples |  |  |
|  | d) | Explain how missing data were addressed |  |  |
|  | e) | If applicable, indicate how multiple testing was addressed |  |  |
| 7 | **Assessment of assumptions** | Describe any methods or prior knowledge used to assess the assumptions or justify their validity | Pages 4-5 | Materials and Methods: MR Assumptions and Study Design |
| 8 | **Sensitivity analyses and additional analyses** | Describe any sensitivity analyses or additional analyses performed (e.g. comparison of effect estimates from different approaches, independent replication, bias analytic techniques, validation of instruments, simulations) | Pages 6-8 | Materials and Methods: Statistical Analysis |
| 9 | **Software and pre-registration** |  |  |  |
|  | a) | Name statistical software and package(s), including version and settings used | Pages 6-8 | Materials and Methods: Statistical Analysis |
|  | b) | State whether the study protocol and details were pre-registered (as well as when and where) |  | N/A |
|  | **RESULTS** |  |  |  |
| 10 | **Descriptive data** |  |  |  |
|  | a) | Report the numbers of individuals at each stage of included studies and reasons for exclusion. Consider use of a flow diagram | Pages 5-8 | Materials and methods: Data Sources |
|  | b) | Report summary statistics for phenotypic exposure(s), outcome(s), and other relevant variables (e.g. means, SDs, proportions) | Pages 5-8 | Materials and methods: Data Sources |
|  | c) | If the data sources include meta-analyses of previous studies, provide the assessments of heterogeneity across these studies |  | N/A |
|  | d) | For two-sample MR:  i. Provide justification of the similarity of the genetic variant-exposure associations between the exposure and outcome samples  ii. Provide information on the number of individuals who overlap between the exposure and outcome studies | Pages 5-8 | Materials and methods: Data Sources |
| 11 | **Main results** |  |  |  |
|  | a) | Report the associations between genetic variant and exposure, and between genetic variant and outcome, preferably on an interpretable scale | Pages 8 | "Single nucleotide polymorphisms (SNPs) strongly associated with the exposures were chosen for IVs."; "The main approach used was the IVW method. Nineteen gut microbial species were found as causally related with meningioma... Forty-nine serum metabolites showed potential causal associations..." |
|  | b) | Report MR estimates of the relationship between exposure and outcome, and the measures of uncertainty from the MR analysis, on an interpretable scale, such as odds ratio or relative risk per SD difference | Pages 8-9 | "Nineteen gut microbial species were found as causally related with meningioma... Of these, 10 taxa had odds ratios (ORs) over 1... The results included CAG−873 sp001701165 (OR = 1.171, 95% CI = 1.028–1.333, P = 0.018), Enterobacteriaceae (OR = 1.325, 95% CI = 1.042–1.683, P = 0.022), and also Fusobacterium A (OR = 2.048, 95% CI = 1.333–3.146, P = 0.001)."; "24 metabolites were found as significantly associated with an increased risk of meningioma. Prominent instances encompass arachidonate (20:4n6) (OR = 1.192; 95% CI: 1.048–1.355; P = 0.007)... In contrast, 25 metabolites were linked to a reduced incidence of meningioma. The substances include 5alpha-androstan-3beta,17beta-diol monosulfate (OR = 0.882; 95% CI: 0.808–0.963; P = 0.005)..." |
|  | c) | If relevant, consider translating estimates of relative risk into absolute risk for a meaningful time period |  | N/A |
|  | d) | Consider plots to visualize results (e.g. forest plot, scatterplot of associations between genetic variants and outcome versus between genetic variants and exposure) | Pages 9-10 | Figure 2-7 |
| 12 | **Assessment of assumptions** |  |  |  |
|  | a) | Report the assessment of the validity of the assumptions | Pages 10 | "none of these associations showed evidence of horizontal pleiotropy."; "No indication of horizontal pleiotropy was seen among the included instruments."; "The results showed no evidence of pleiotropy and heterogeneity." |
|  | b) | Report any additional statistics (e.g., assessments of heterogeneity across genetic variants, such as *I^2^*, Q statistic or E-value) | Pages 4-7 | The variability of SNPs was evaluated using Cochran's Q statistic and funnel plots. |
| 13 | **Sensitivity analyses and additional analyses** |  |  |  |
|  | a) | Report any sensitivity analyses to assess the robustness of the main results to violations of the assumptions | Pages 8-9 | "Detailed sensitivity analyses and results from additional MR methods are provided in Appendices S4 and S5."; "Comprehensive results from sensitivity analyses and supplementary MR methodologies are included in Appendices S6 and S7."; "Detailed sensitivity analyses and results from other MR methods are provided in Appendices S8 and S9."; "Sensitivity analyses and additional MR analysis results can be found in Appendices S10 and S11." |
|  | b) | Report results from other sensitivity analyses or additional analyses | Pages 8-9 | "Detailed sensitivity analyses and results from additional MR methods are provided in Appendices S4 and S5."; "Comprehensive results from sensitivity analyses and supplementary MR methodologies are included in Appendices S6 and S7."; "Detailed sensitivity analyses and results from other MR methods are provided in Appendices S8 and S9."; "Sensitivity analyses and additional MR analysis results can be found in Appendices S10 and S11." |
|  | c) | Report any assessment of direction of causal relationship (e.g., bidirectional MR) |  |  |
|  | d) | When relevant, report and compare with estimates from non-MR analyses |  | N/A |
|  | e) | Consider additional plots to visualize results (e.g., leave-one-out analyses) | Page 8-10 | Appendices S4-S11. |
|  | **DISCUSSION** |  |  |  |
| 14 | **Key results** | Summarize key results with reference to study objectives | Page 10-11 | Discussion:paragraph 1 |
| 15 | **Limitations** | Discuss limitations of the study, taking into account the validity of the IV assumptions, other sources of potential bias, and imprecision. Discuss both direction and magnitude of any potential bias and any efforts to address them | Page 15 | Discussion:paragraph 8 |
| 16 | **Interpretation** |  |  |  |
|  | a) | Meaning: Give a cautious overall interpretation of results in the context of their limitations and in comparison with other studies | Pages 11-15 | Discussion:paragraph 2-7 |
|  | b) | Mechanism: Discuss underlying biological mechanisms that could drive a potential causal relationship between the investigated exposure and the outcome, and whether the gene-environment equivalence assumption is reasonable. Use causal language carefully, clarifying that IV estimates may provide causal effects only under certain assumptions | Pages 11-15 | Discussion:paragraph 2-7 |
|  | c) | Clinical relevance: Discuss whether the results have clinical or public policy relevance, and to what extent they inform effect sizes of possible interventions | Page 10-11 | Discussion:paragraph 1 |
| 17 | **Generalizability** | Discuss the generalizability of the study results (a) to other populations, (b) across other exposure periods/timings, and (c) across other levels of exposure | Page 15 | Discussion:paragraph 8 |
|  | **OTHER INFORMATION** |  |  |  |
| 18 | **Funding** | Describe sources of funding and the role of funders in the present study and, if applicable, sources of funding for the databases and original study or studies on which the present study is based | Page 16 | Acknowledgments |
| 19 | **Data and data sharing** | Provide the data used to perform all analyses or report where and how the data can be accessed, and reference these sources in the article. Provide the statistical code needed to reproduce the results in the article, or report whether the code is publicly accessible and if so, where | Page 16 | Data Availability Statement |
| 20 | **Conflicts of Interest** | All authors should declare all potential conflicts of interest | Page 16-17 | Conflicts of Interest |

This checklist is copyrighted by the Equator Network under the Creative Commons Attribution 3.0 Unported (CC BY 3.0) license.

1. Skrivankova VW, Richmond RC, Woolf BAR, Yarmolinsky J, Davies NM, Swanson SA, et al. Strengthening the Reporting of Observational Studies in Epidemiology using Mendelian Randomization (STROBE-MR) Statement. JAMA. 2021;under review.

2. Skrivankova VW, Richmond RC, Woolf BAR, Davies NM, Swanson SA, VanderWeele TJ, et al. Strengthening the Reporting of Observational Studies in Epidemiology using Mendelian Randomisation (STROBE-MR): Explanation and Elaboration. BMJ. 2021;375:n2233.
