# Supplementary material for: Causal Pathways Linking Gut Microbiota, Serum Metabolites, and Meningioma Risk: A Mendelian Randomization Analysis
Source: Brain Behav. 2026 Feb 9;16(2):e71220. doi: 10.1002/brb3.71220 (PMC12887442; doi:10.1002/brb3.71220)
Supplement: Supplementary file 4 — Supplementary Appendix S4: brb371220‐sup‐0004‐Appendix4.pdf [file BRB3-16-e71220-s005.pdf]

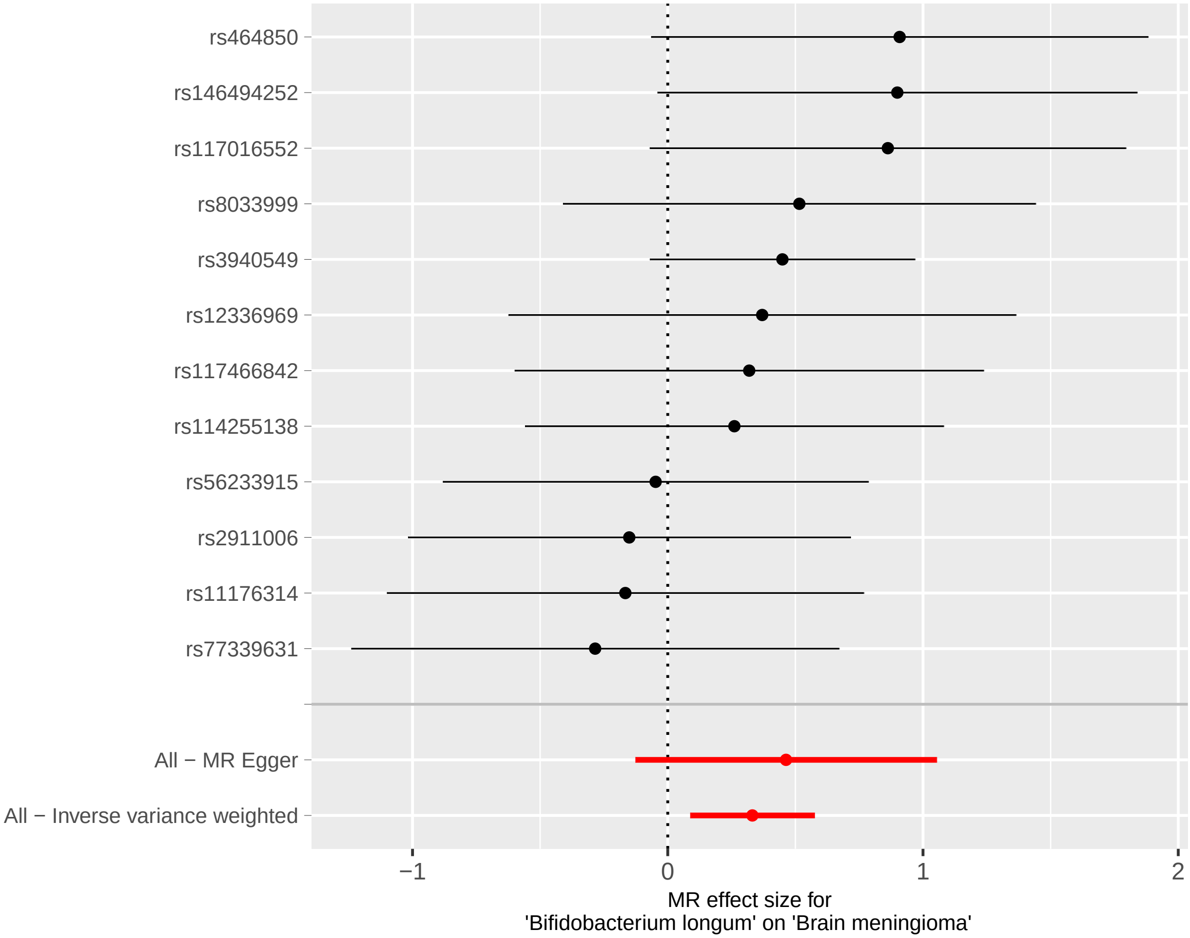

rs464850
rs146494252
rs117016552
rs8033999
rs3940549
rs12336969
rs117466842
rs114255138
rs56233915
rs2911006
rs11176314
rs77339631
All − MR Egger
All − Inverse variance weighted
−1
0
1
2
MR effect size for
'Bifidobacterium longum' on 'Brain meningioma'

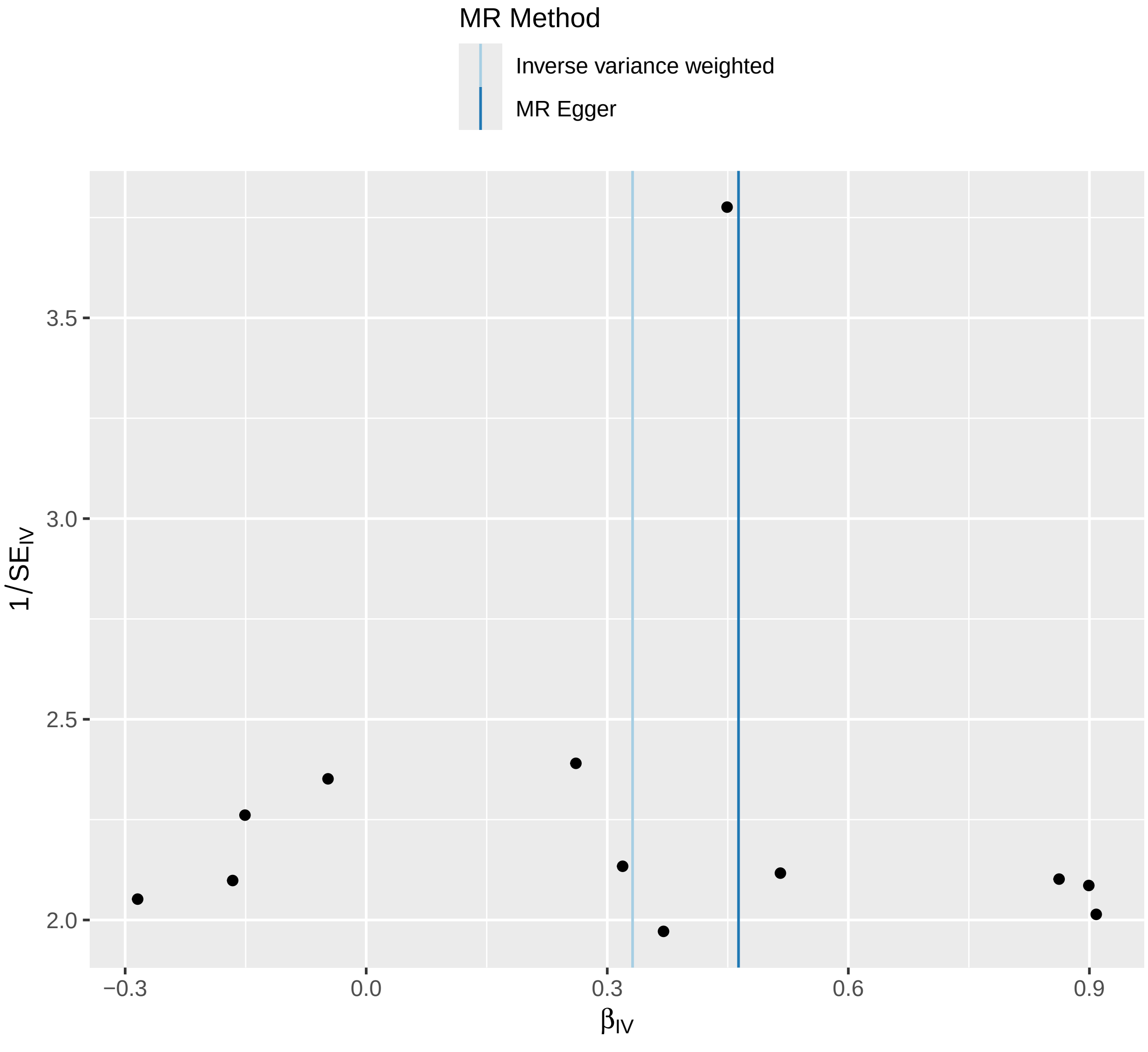

MR Method
Inverse variance weighted
MR Egger
3.5
3.0
2.5
2.0
1/SE_IV
−0.3
0.0
0.3
0.6
0.9
β_IV

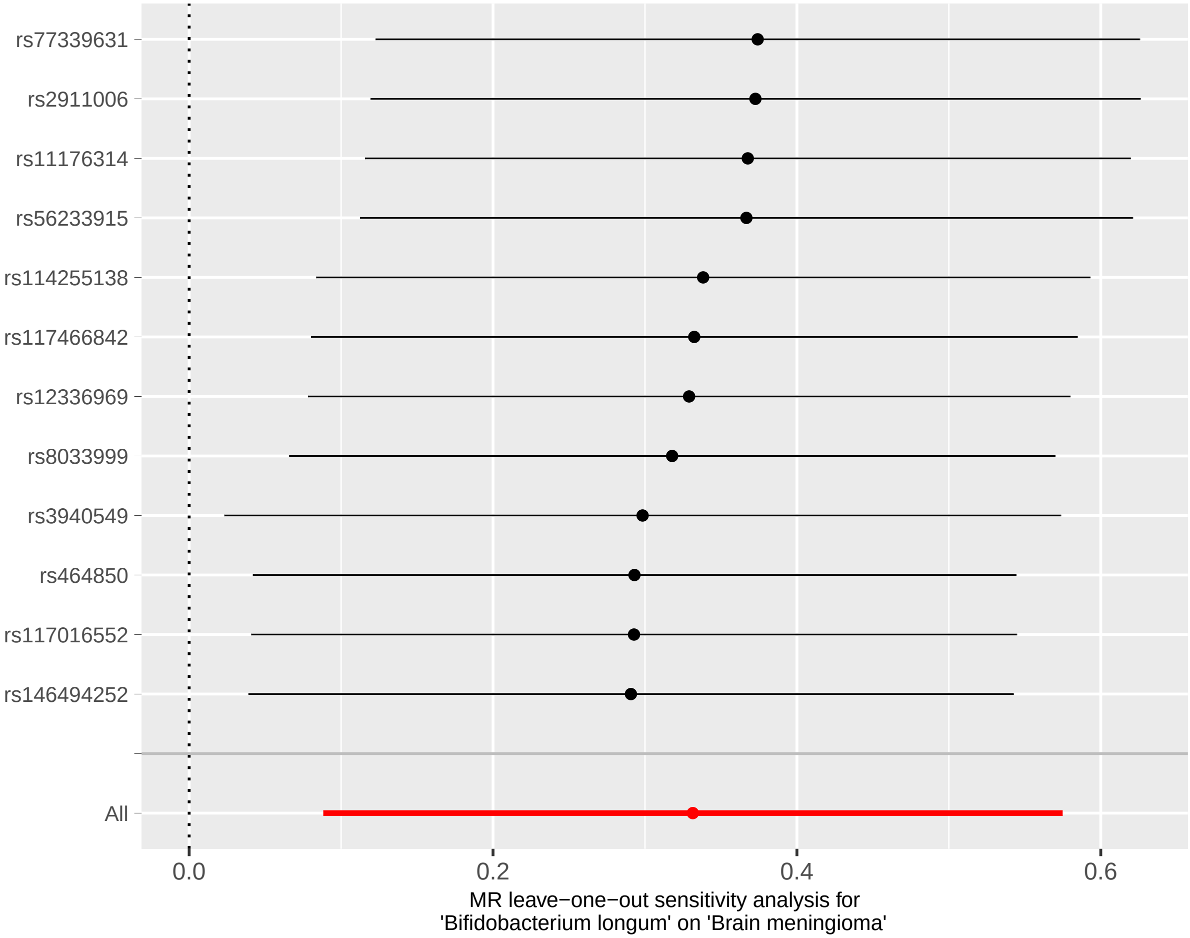
rs77339631
rs2911006
rs11176314
rs56233915
rs114255138
rs117466842
rs12336969
rs8033999
rs3940549
rs464850
rs117016552
rs146494252
All
0.0
0.2
0.4
0.6
MR leave−one−out sensitivity analysis for
'Bifidobacterium longum' on 'Brain meningioma'

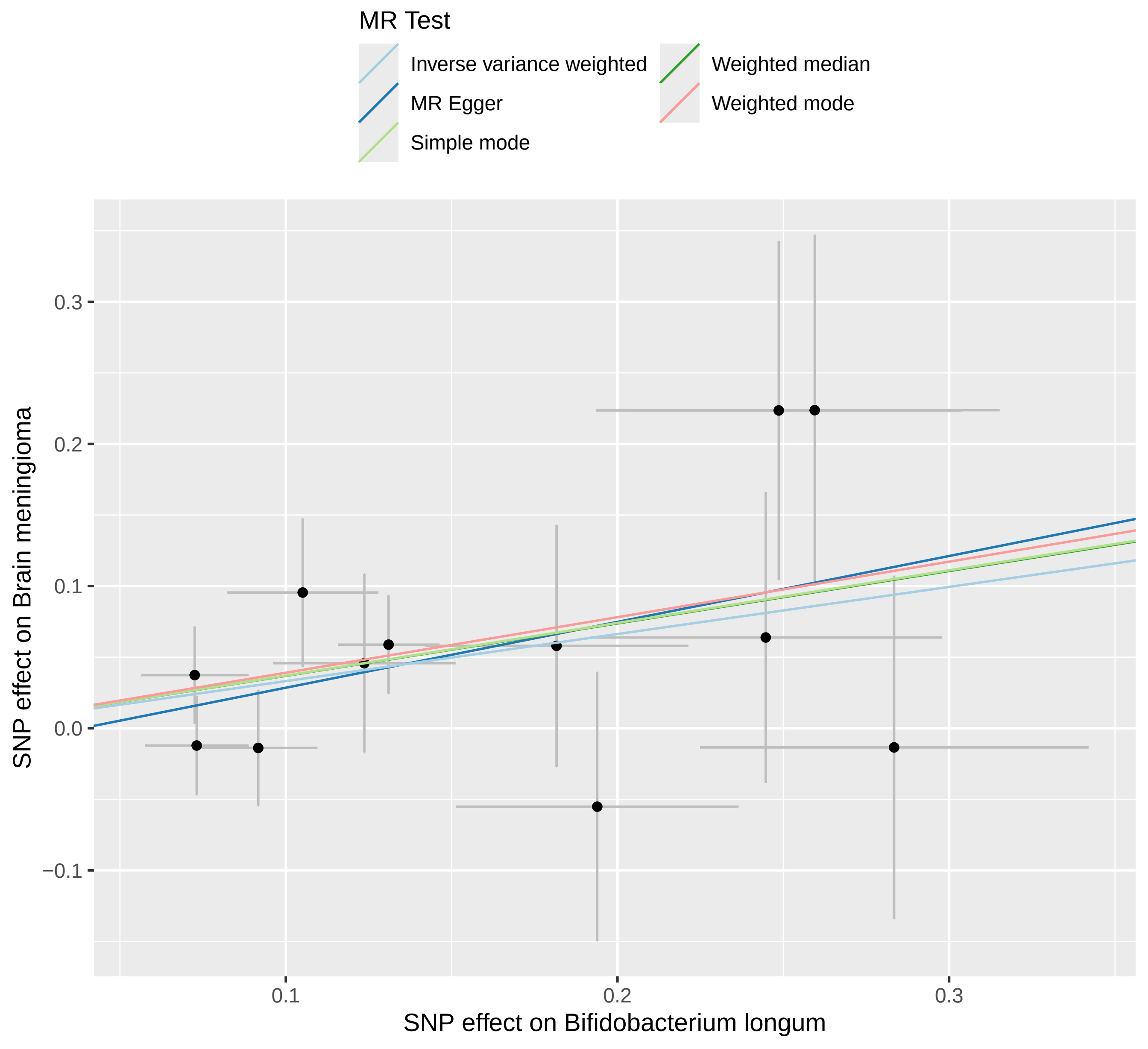
MR Test
Inverse variance weighted
MR Egger
Simple mode
Weighted median
Weighted mode
SNP effect on Brain meningioma
0.3
0.2
0.1
0.0
−0.1
0.1
0.2
0.3
SNP effect on Bifidobacterium longum

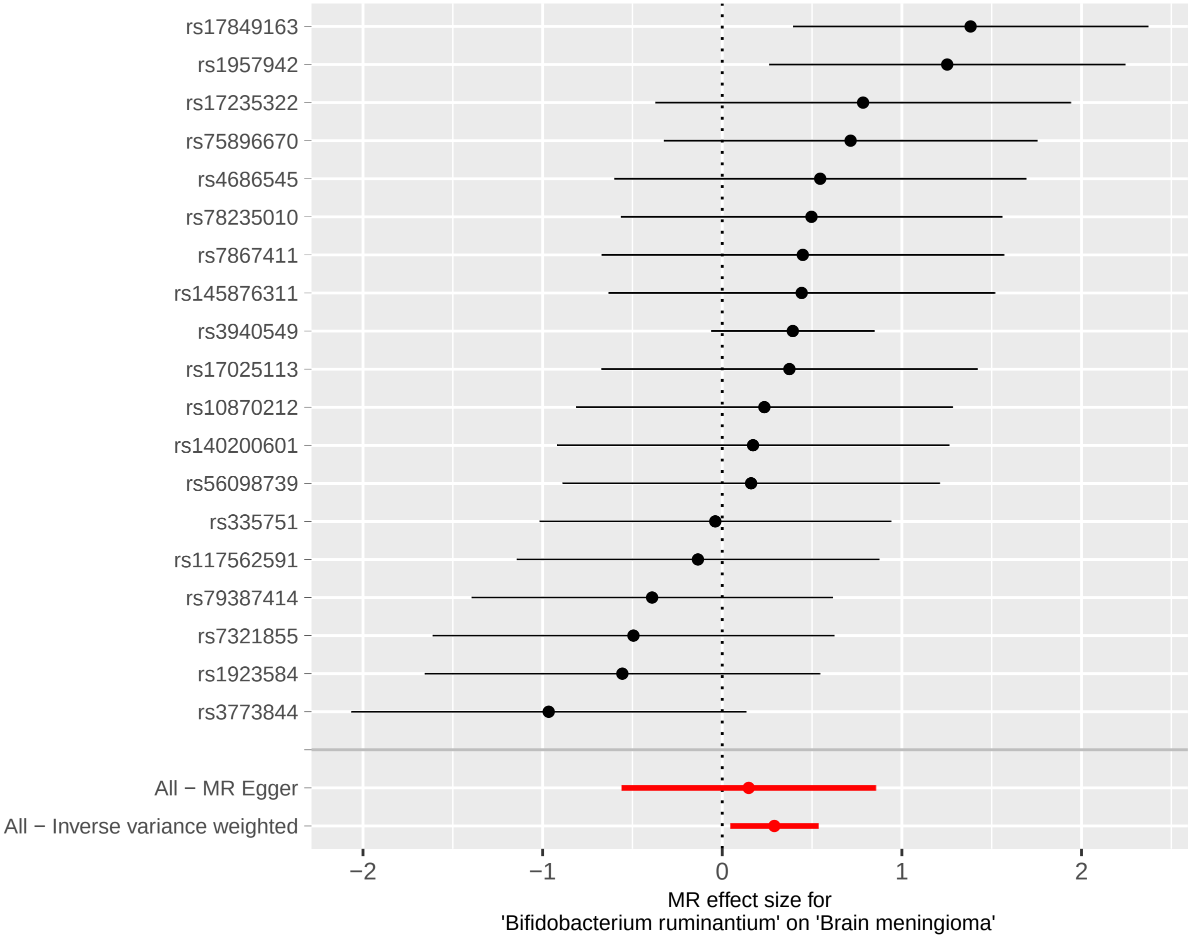
rs17849163
rs1957942
rs17235322
rs75896670
rs4686545
rs78235010
rs7867411
rs145876311
rs3940549
rs17025113
rs10870212
rs140200601
rs56098739
rs335751
rs117562591
rs79387414
rs7321855
rs1923584
rs3773844
All − MR Egger
All − Inverse variance weighted
−2
−1
0
1
2
MR effect size for
'Bifidobacterium ruminantium' on 'Brain meningioma'

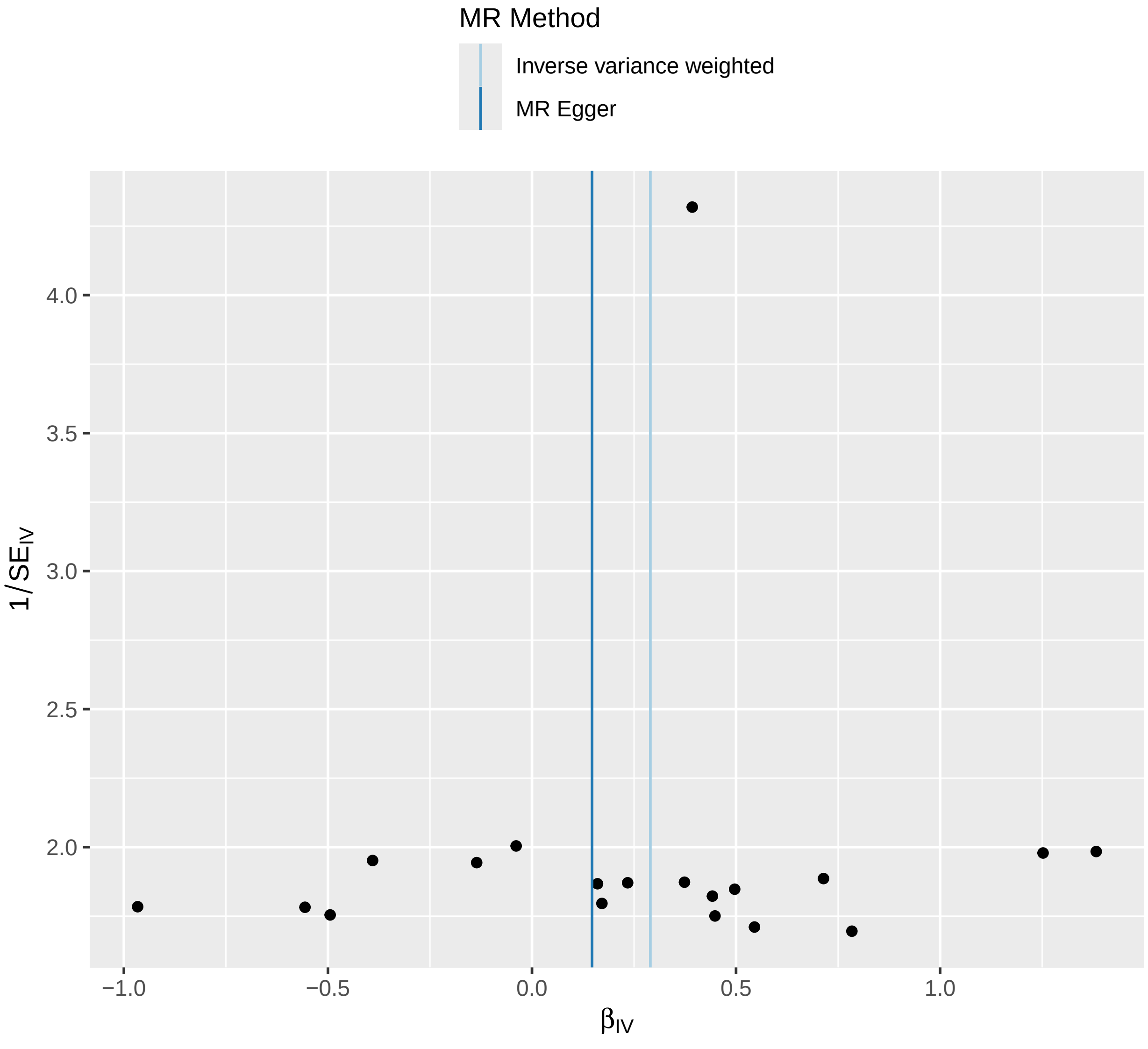
MR Method
Inverse variance weighted
MR Egger
4.0
3.5
3.0
2.5
2.0
$1/SE_{IV}$
−1.0
−0.5
0.0
0.5
1.0
β_{IV}

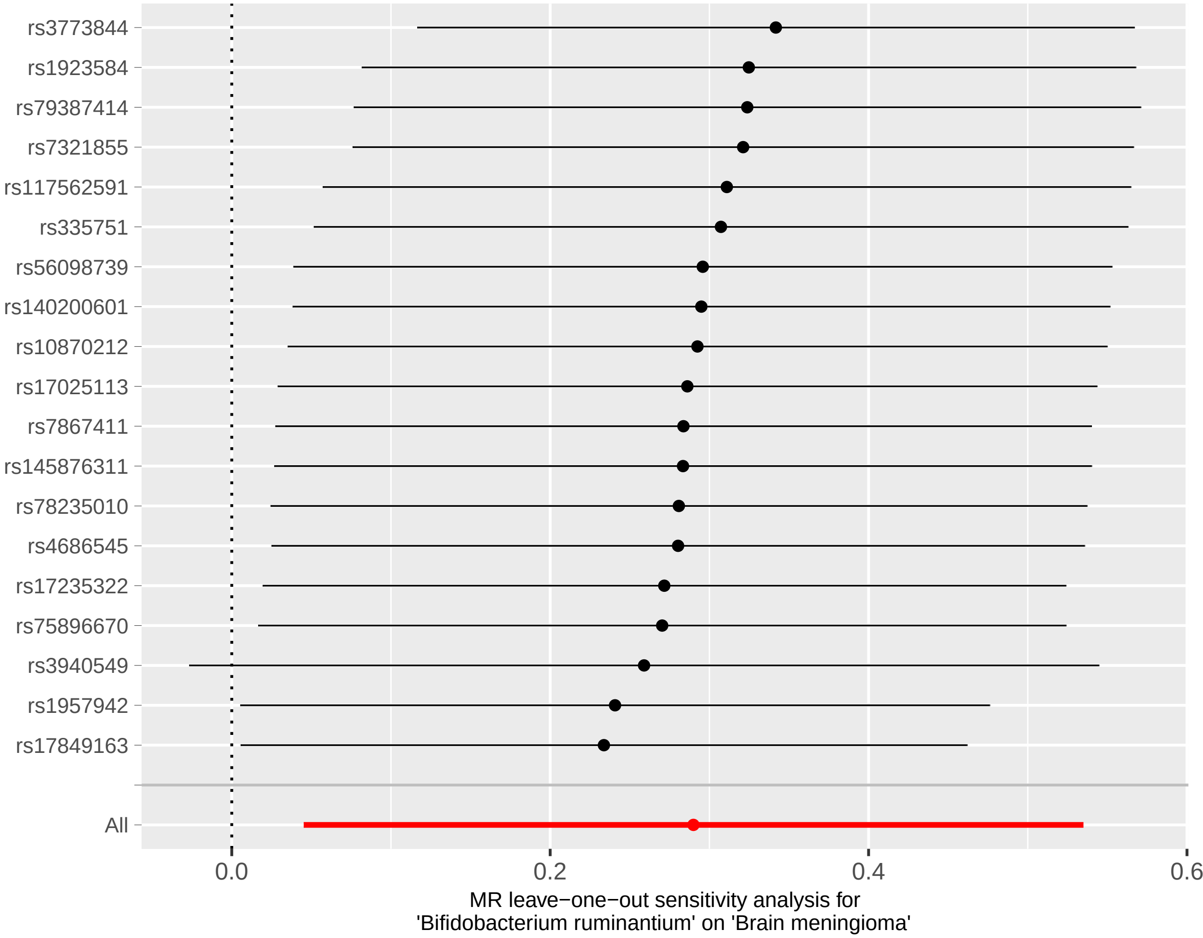
rs3773844
rs1923584
rs79387414
rs7321855
rs117562591
rs335751
rs56098739
rs140200601
rs10870212
rs17025113
rs7867411
rs145876311
rs78235010
rs4686545
rs17235322
rs75896670
rs3940549
rs1957942
rs17849163
All
0.0
0.2
0.4
0.6
MR leave−one−out sensitivity analysis for
'Bifidobacterium ruminantium' on 'Brain meningioma'

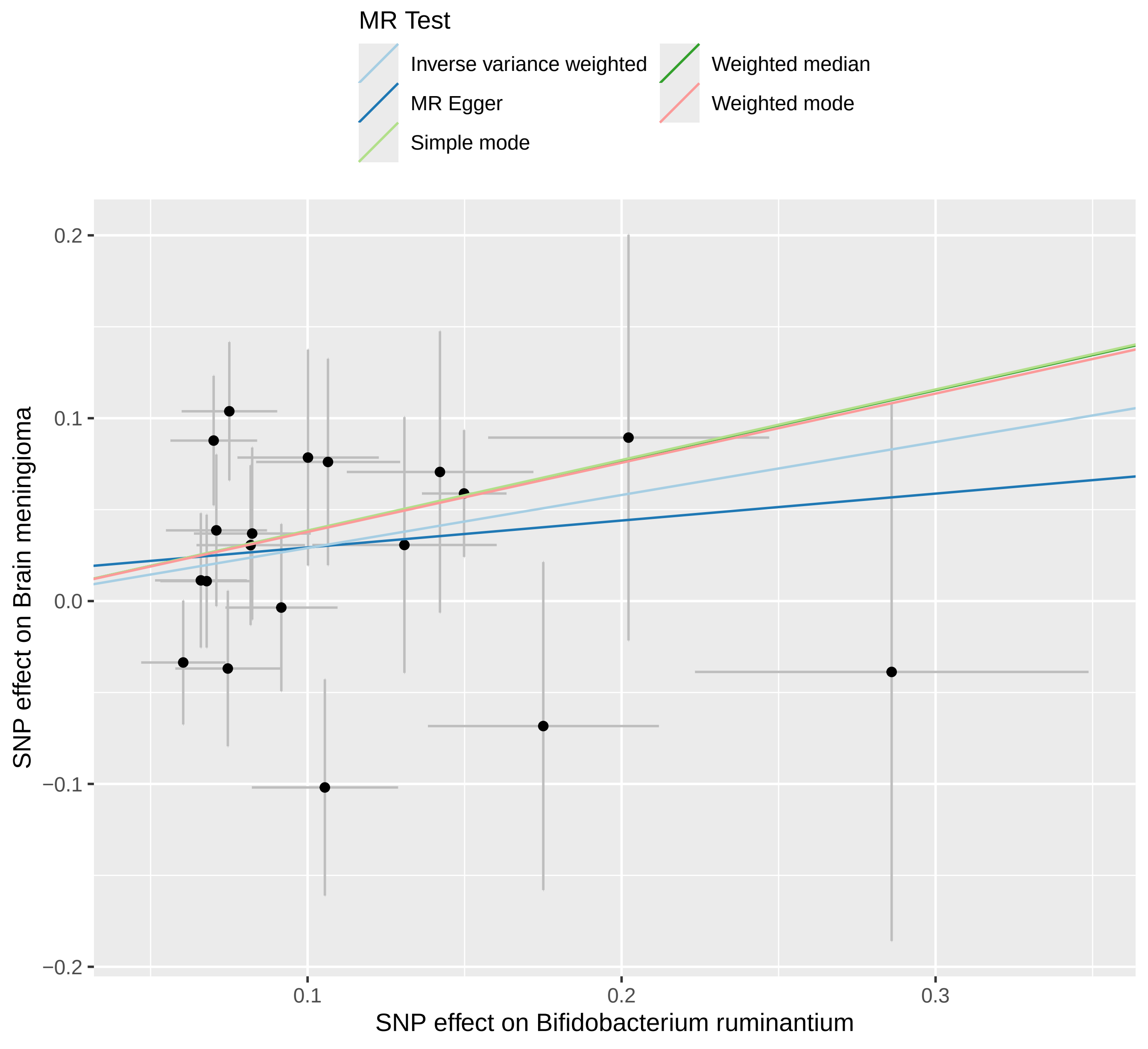
MR Test
Inverse variance weighted
MR Egger
Simple mode
Weighted median
Weighted mode
0.2
0.1
0.0
−0.1
−0.2
SNP effect on Brain meningioma
0.1
0.2
0.3
SNP effect on Bifidobacterium ruminantium

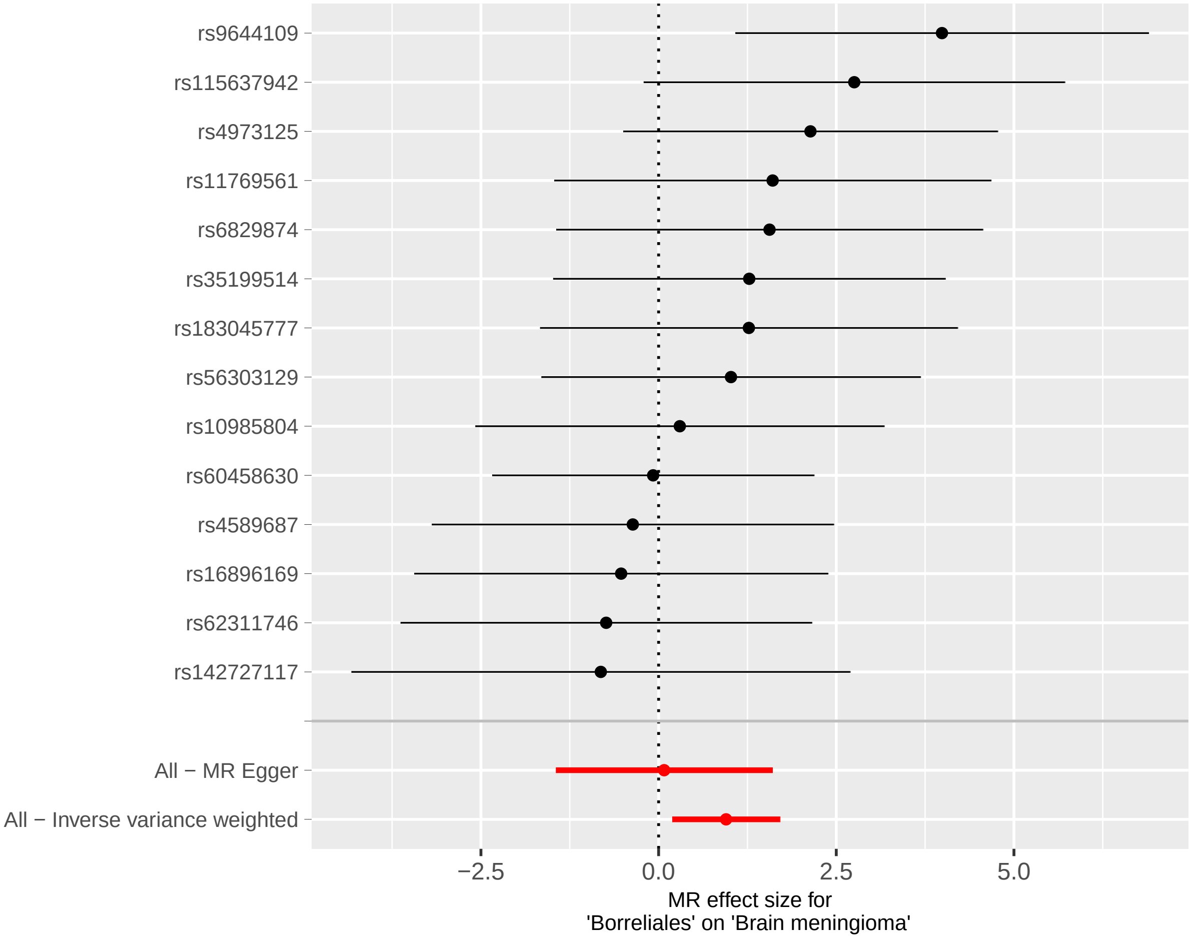

rs9644109
rs115637942
rs4973125
rs11769561
rs6829874
rs35199514
rs183045777
rs56303129
rs10985804
rs60458630
rs4589687
rs16896169
rs62311746
rs142727117
All − MR Egger
All − Inverse variance weighted
−2.5
0.0
2.5
5.0
MR effect size for
'Borreliales' on 'Brain meningioma'

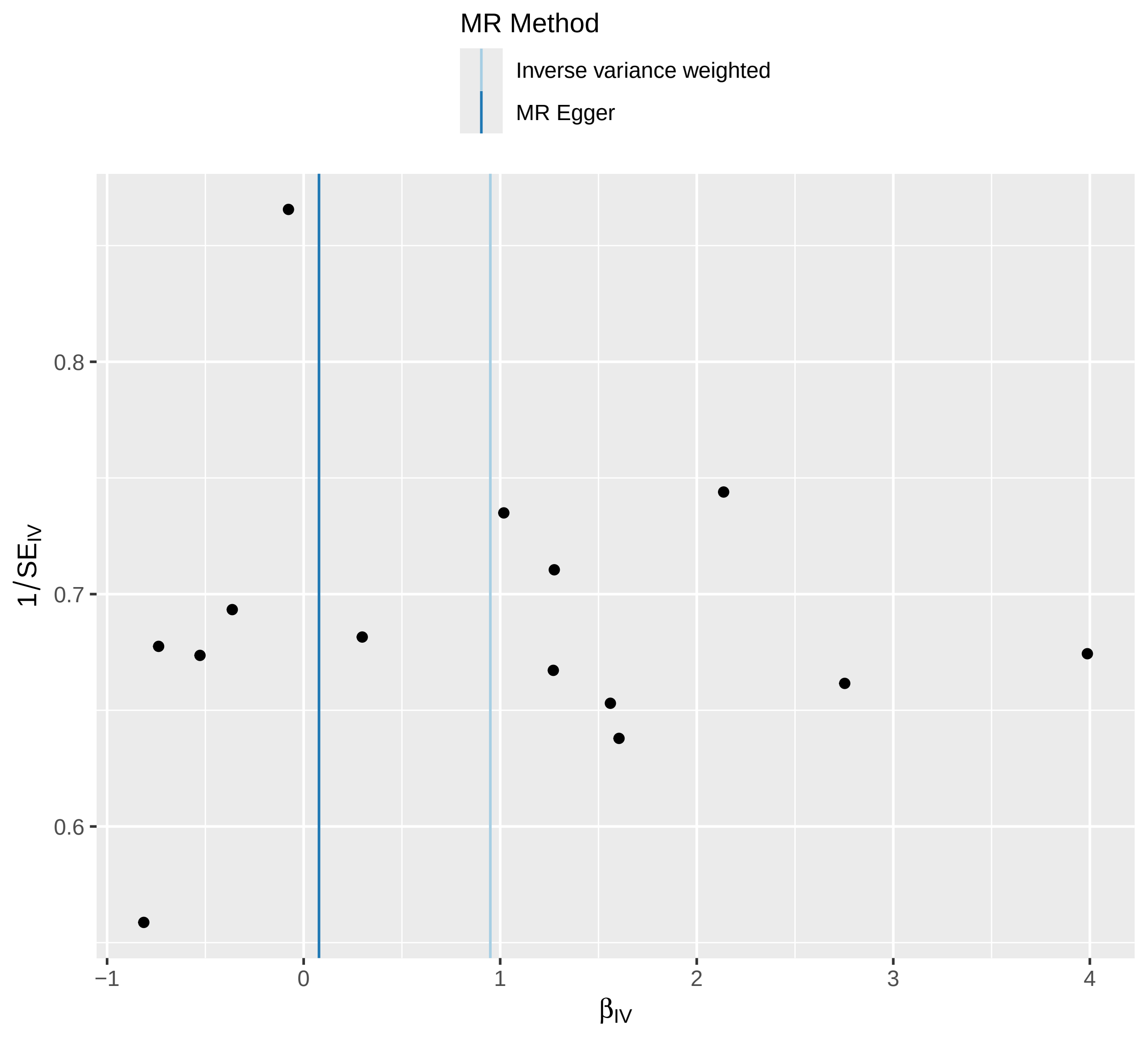
MR Method
Inverse variance weighted
MR Egger
1/SE_IV
0.6
0.7
0.8
β_IV
−1
0
1
2
3
4

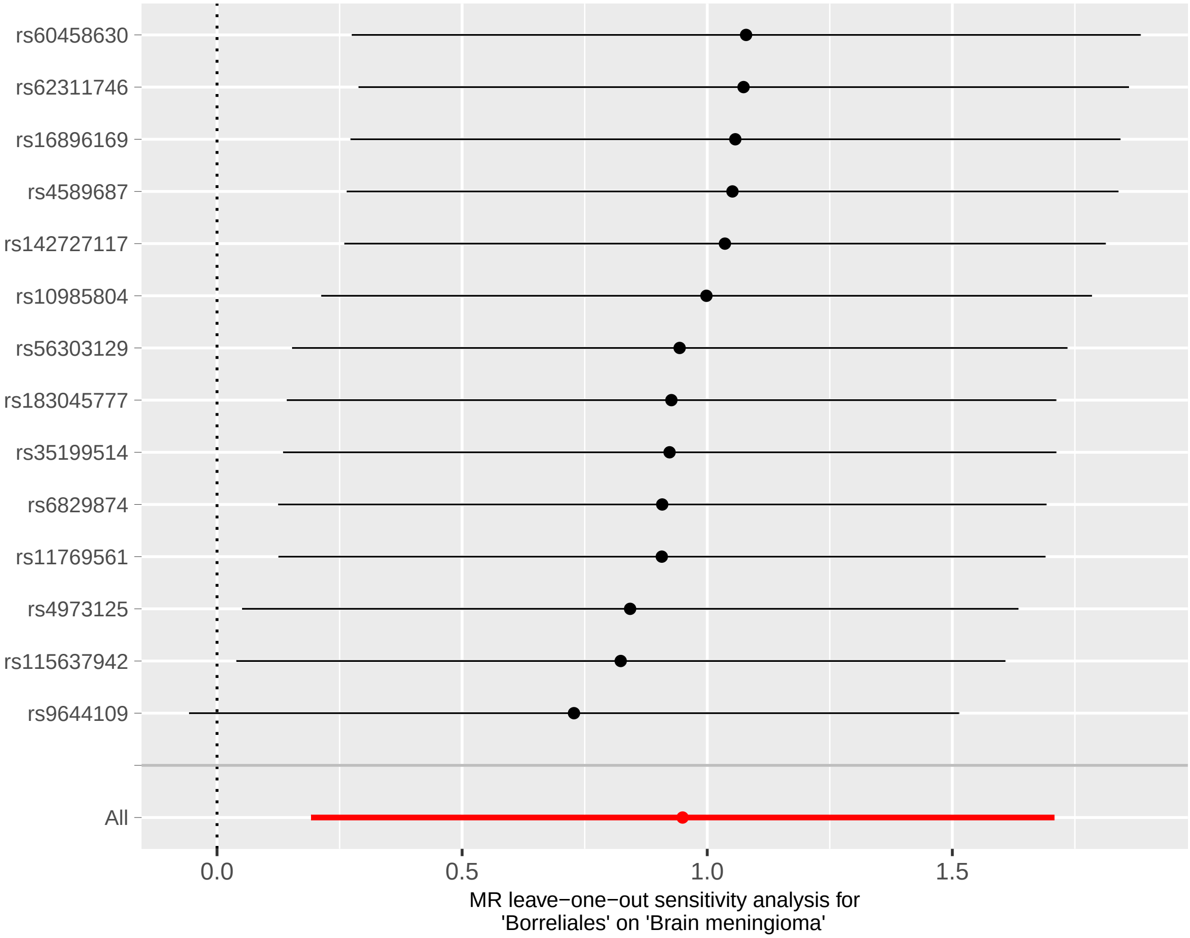

rs60458630
rs62311746
rs16896169
rs4589687
rs142727117
rs10985804
rs56303129
rs183045777
rs35199514
rs6829874
rs11769561
rs4973125
rs115637942
rs9644109
All
0.0
0.5
1.0
1.5
MR leave−one−out sensitivity analysis for
'Borreliales' on 'Brain meningioma'

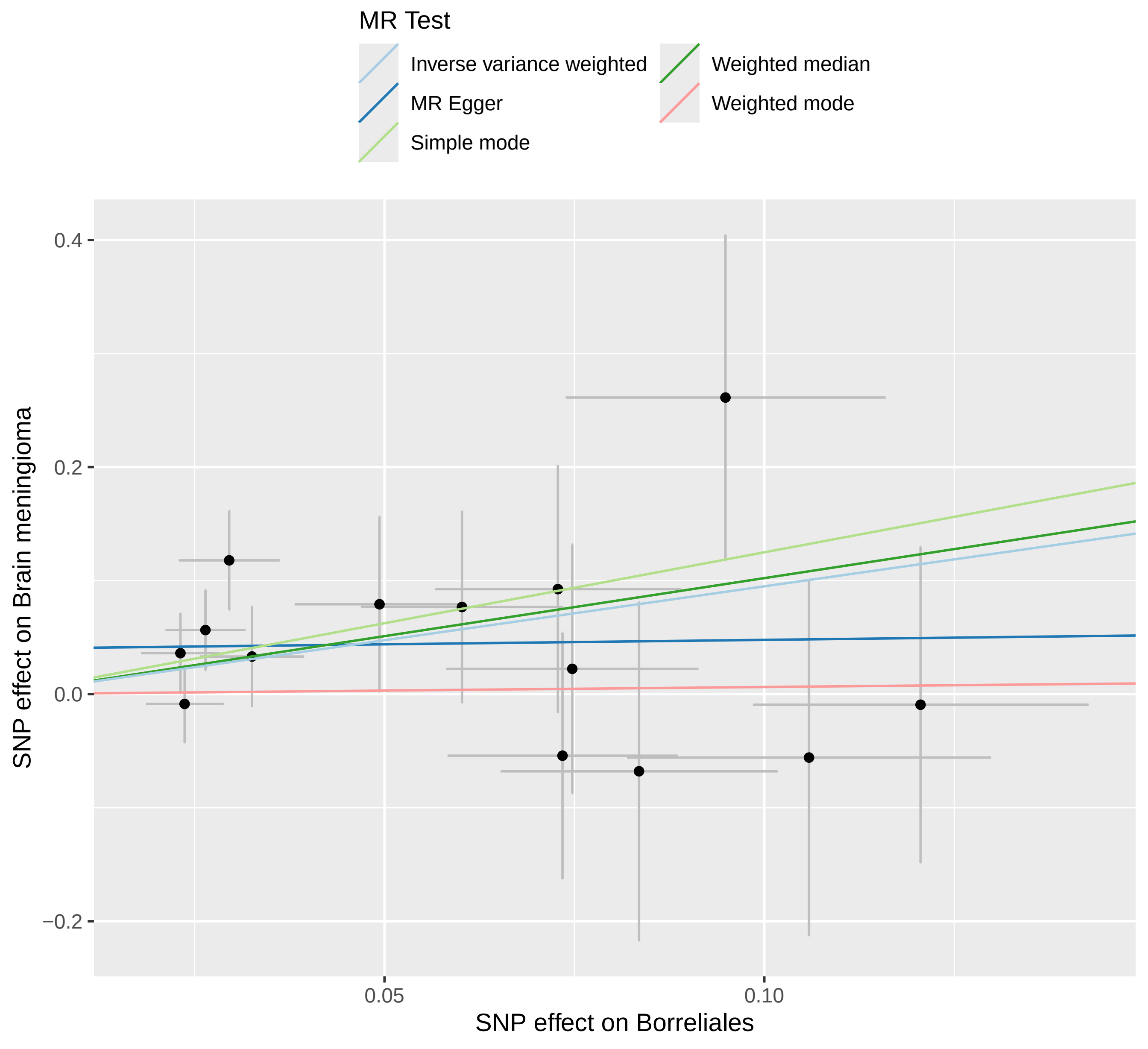
MR Test
Inverse variance weighted
MR Egger
Simple mode
Weighted median
Weighted mode
0.4
0.2
0.0
−0.2
0.05
0.10
SNP effect on Brain meningioma
SNP effect on Borreliales

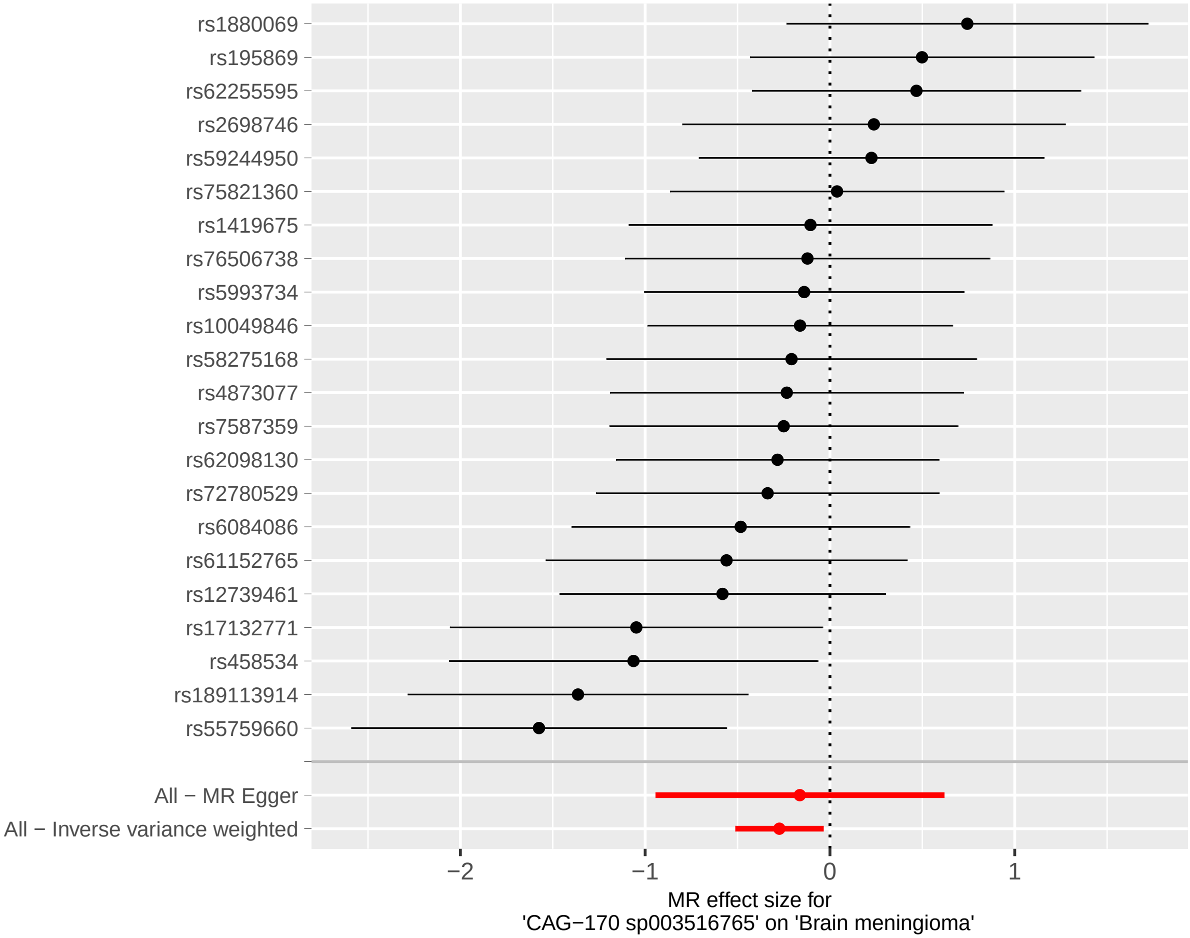
rs1880069
rs195869
rs62255595
rs2698746
rs59244950
rs75821360
rs1419675
rs76506738
rs5993734
rs10049846
rs58275168
rs4873077
rs7587359
rs62098130
rs72780529
rs6084086
rs61152765
rs12739461
rs17132771
rs458534
rs189113914
rs55759660
All − MR Egger
All − Inverse variance weighted
−2
−1
0
1
MR effect size for
'CAG−170 sp003516765' on 'Brain meningioma'

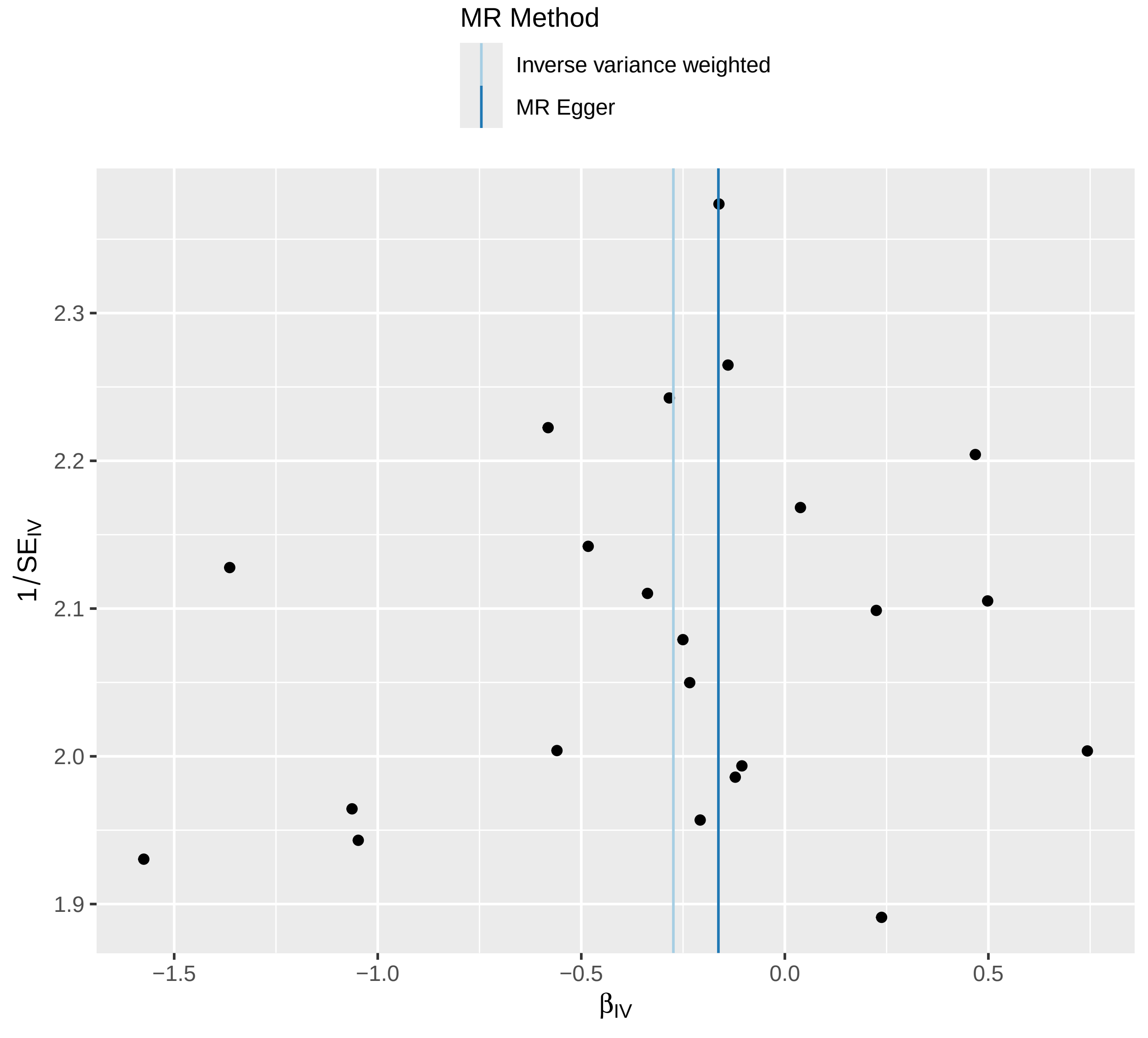

MR Method
Inverse variance weighted
MR Egger
1/SE$_{IV}$
2.3
2.2
2.1
2.0
1.9
−1.5
−1.0
−0.5
0.0
0.5
β$_{IV}$

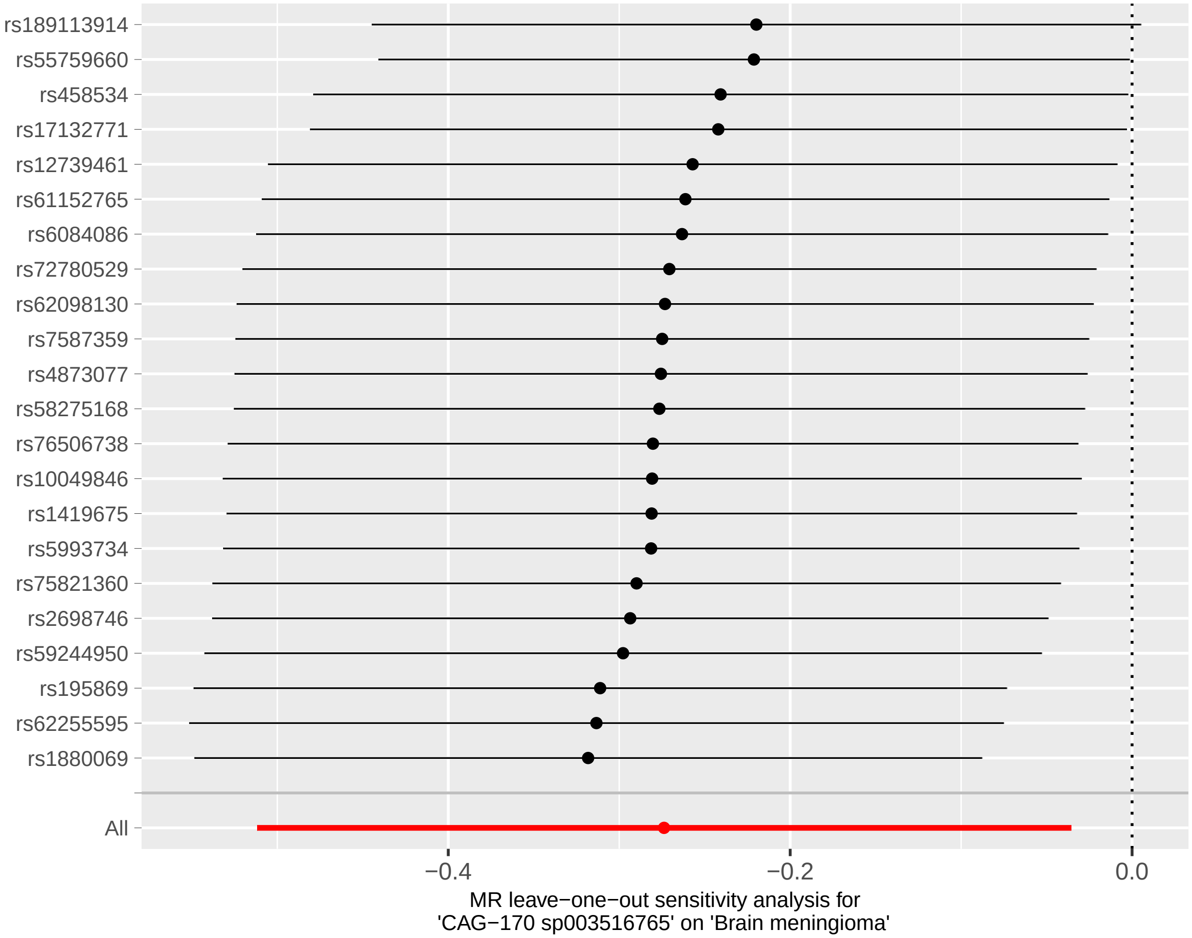
rs189113914
rs55759660
rs458534
rs17132771
rs12739461
rs61152765
rs6084086
rs72780529
rs62098130
rs7587359
rs4873077
rs58275168
rs76506738
rs10049846
rs1419675
rs5993734
rs75821360
rs2698746
rs59244950
rs195869
rs62255595
rs1880069
All
−0.4
−0.2
0.0
MR leave−one−out sensitivity analysis for
'CAG−170 sp003516765' on 'Brain meningioma'

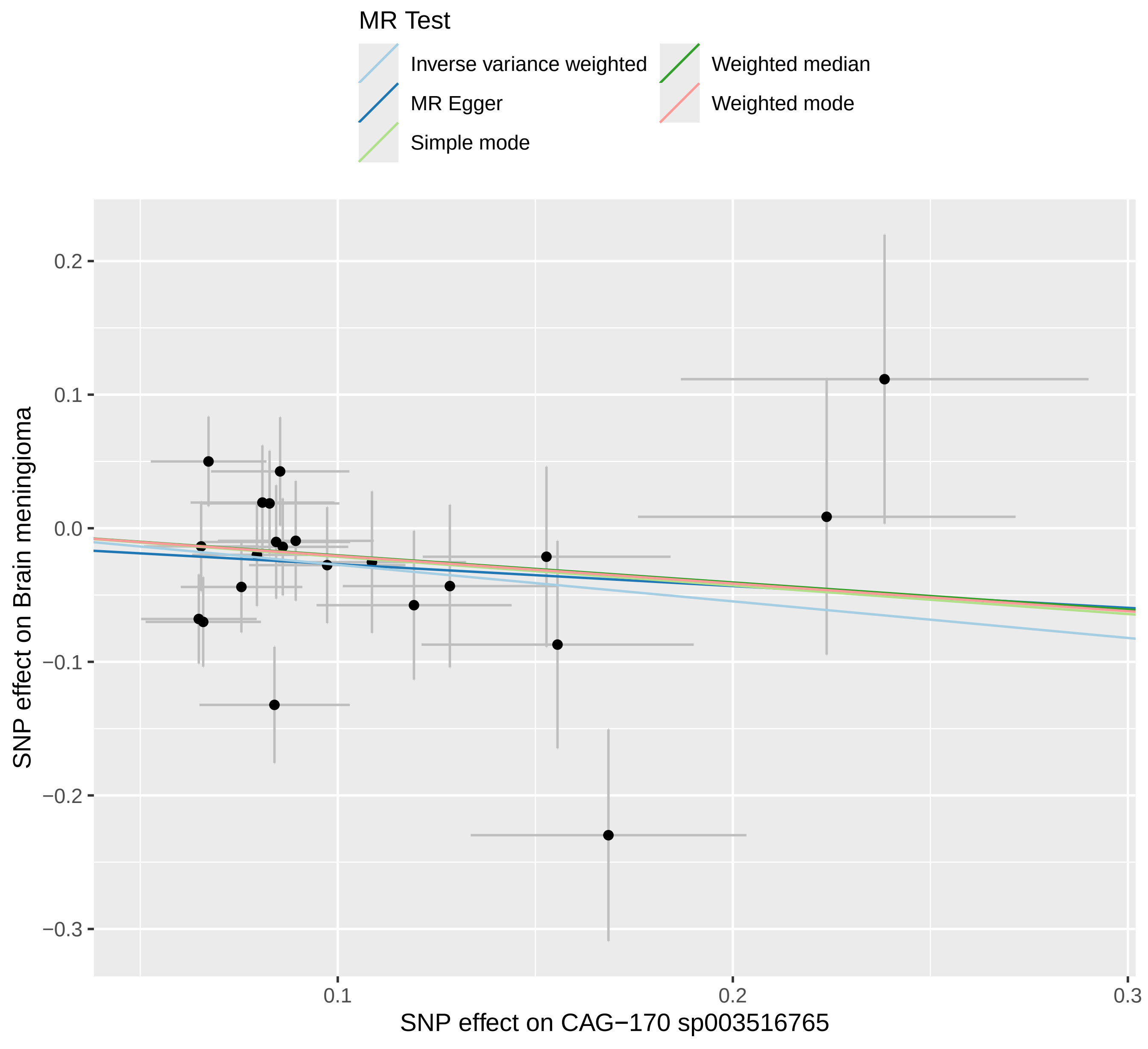
MR Test
Inverse variance weighted
MR Egger
Simple mode
Weighted median
Weighted mode
SNP effect on Brain meningioma
0.2
0.1
0.0
−0.1
−0.2
−0.3
0.1
0.2
0.3
SNP effect on CAG−170 sp003516765

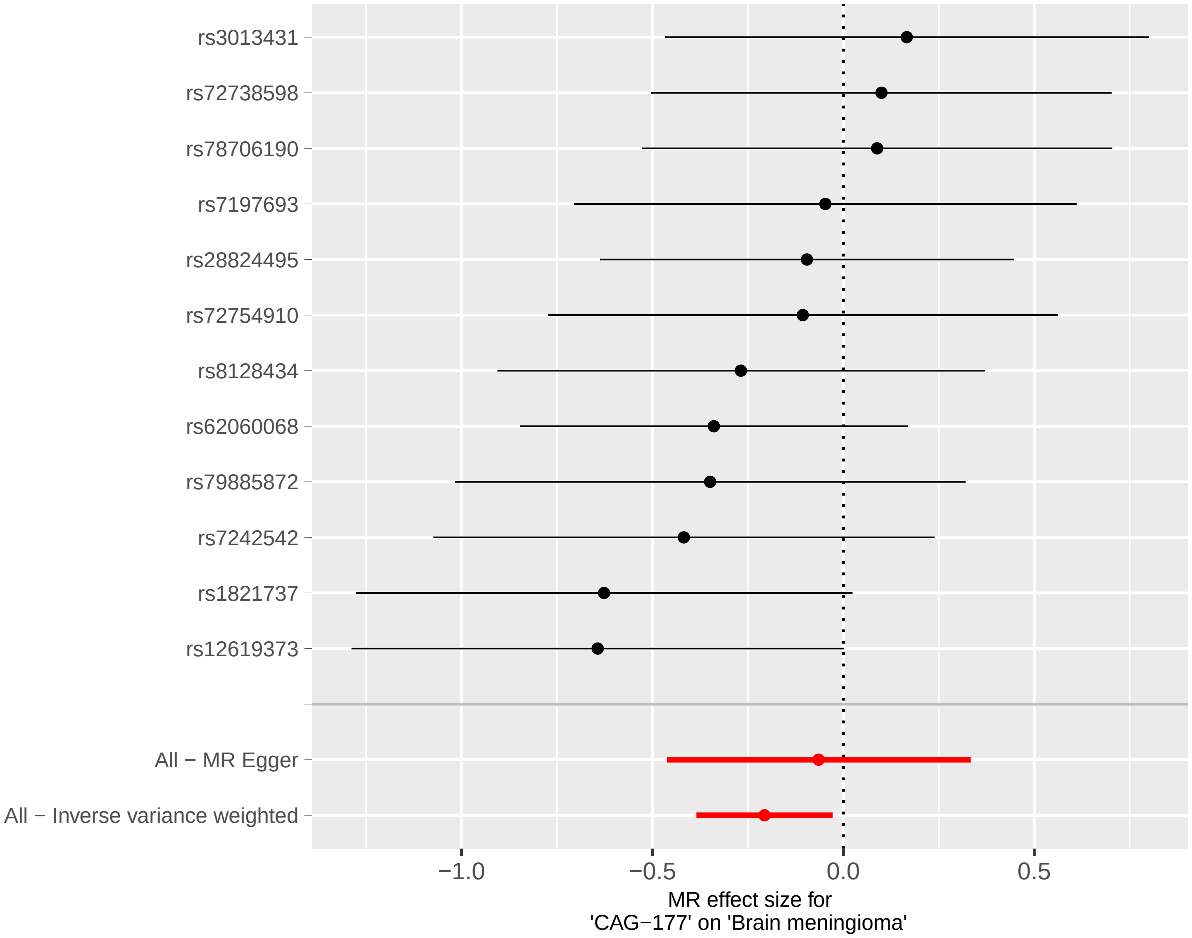

rs3013431
rs72738598
rs78706190
rs7197693
rs28824495
rs72754910
rs8128434
rs62060068
rs79885872
rs7242542
rs1821737
rs12619373
All − MR Egger
All − Inverse variance weighted
−1.0
−0.5
0.0
0.5
MR effect size for
'CAG−177' on 'Brain meningioma'

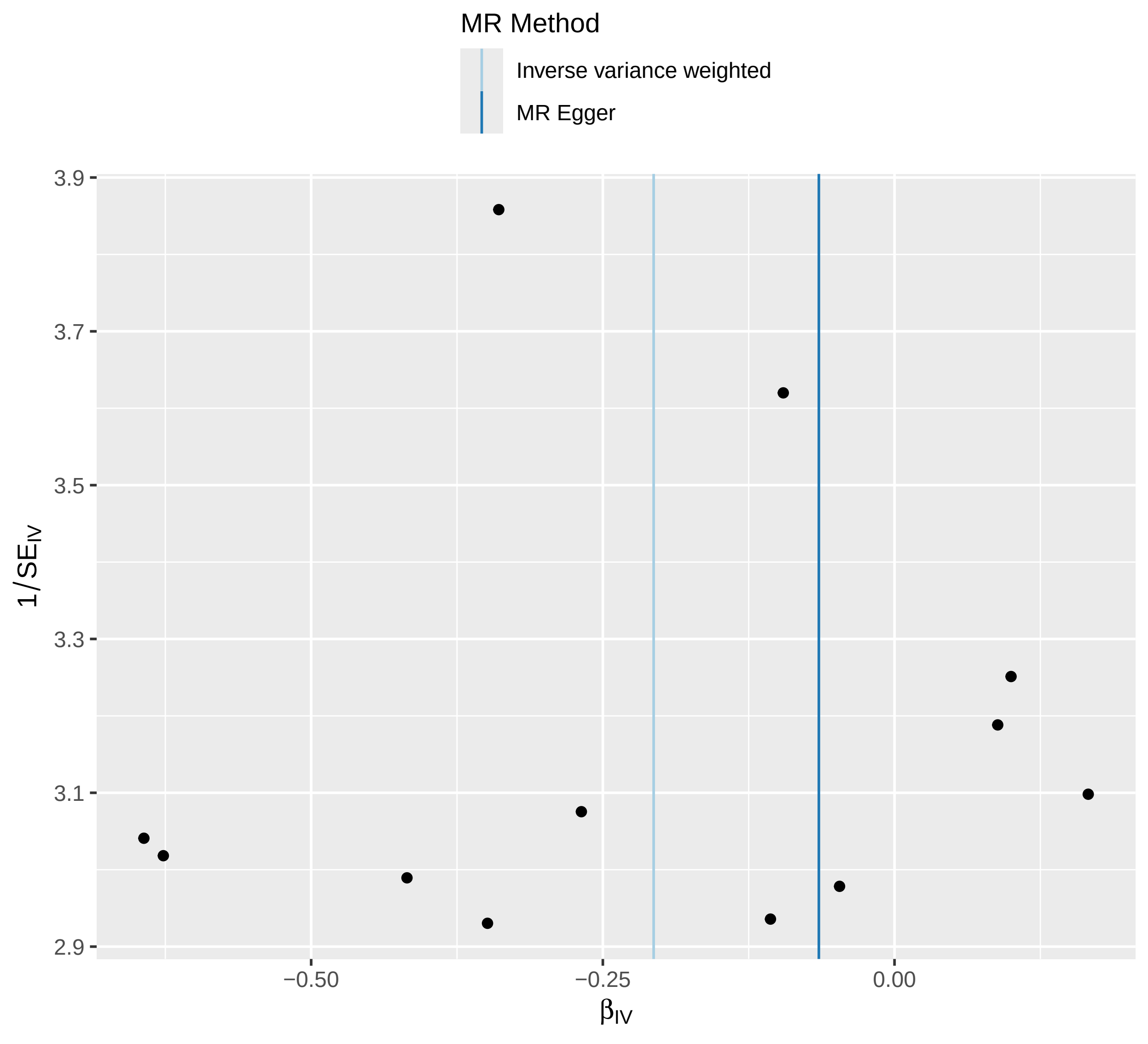
MR Method
Inverse variance weighted
MR Egger
3.9
3.7
3.5
3.3
3.1
2.9
1/SE_IV
−0.50
−0.25
0.00
β_IV

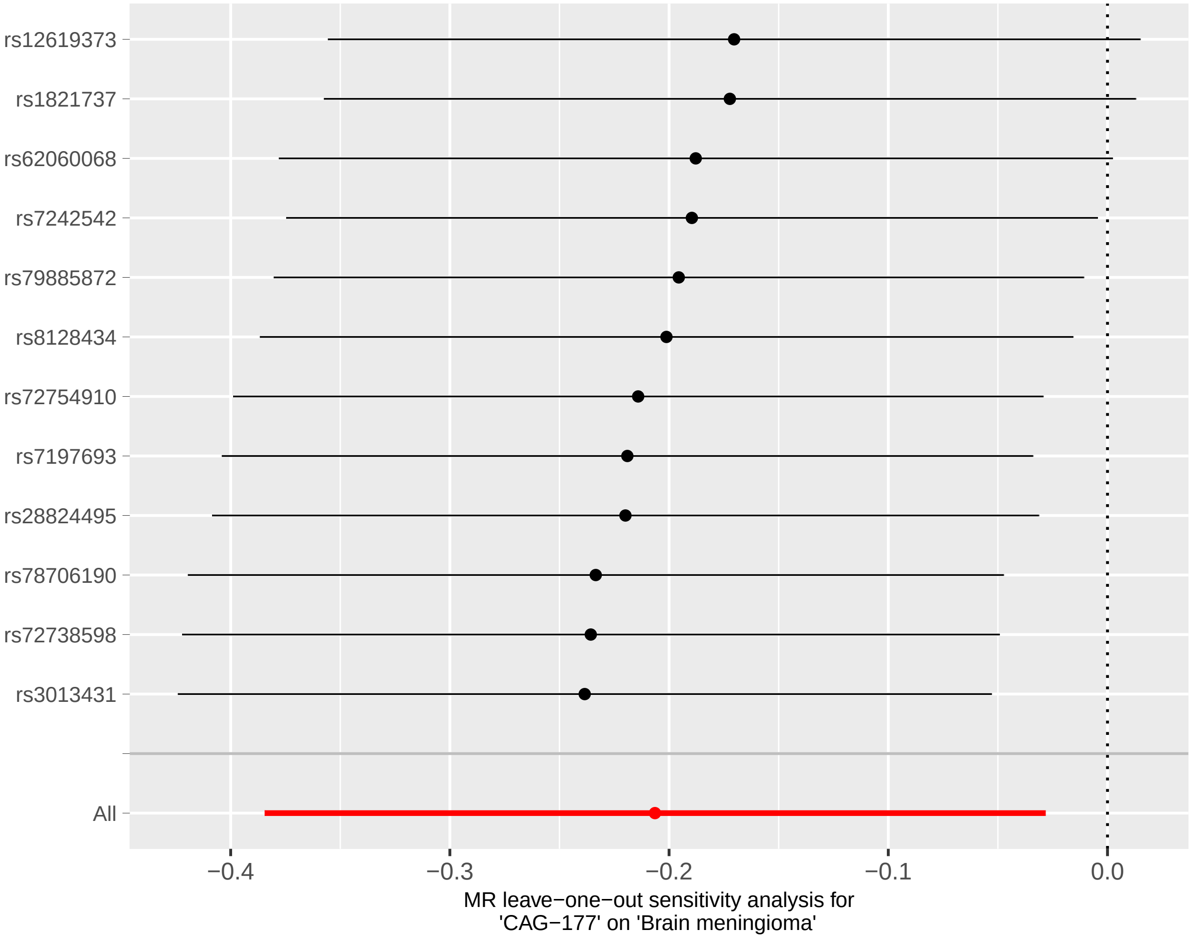
rs12619373
rs1821737
rs62060068
rs7242542
rs79885872
rs8128434
rs72754910
rs7197693
rs28824495
rs78706190
rs72738598
rs3013431
All
−0.4
−0.3
−0.2
−0.1
0.0
MR leave−one−out sensitivity analysis for
'CAG−177' on 'Brain meningioma'

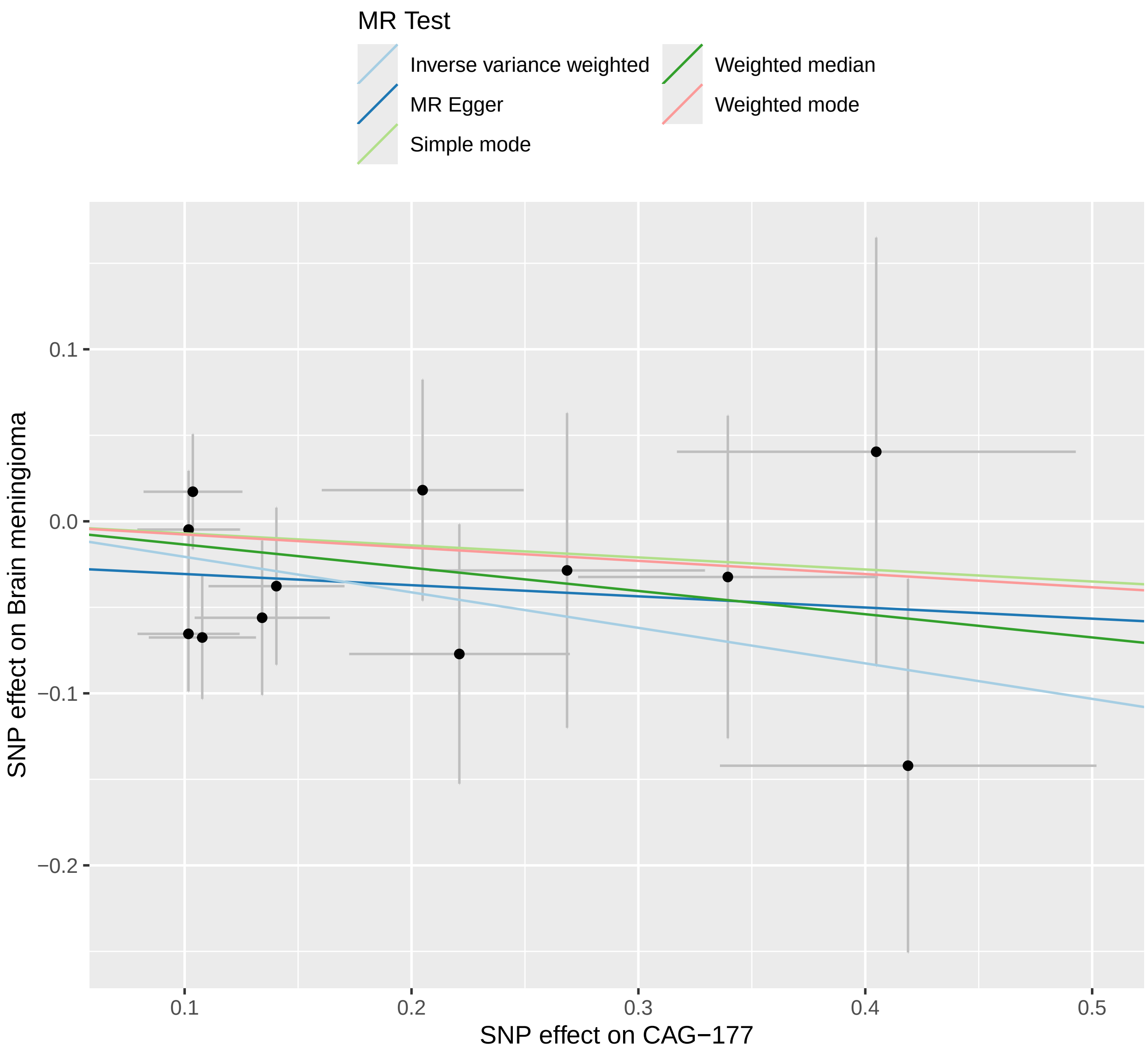
MR Test
Inverse variance weighted
MR Egger
Simple mode
Weighted median
Weighted mode
0.1
0.0
−0.1
−0.2
SNP effect on Brain meningioma
0.1
0.2
0.3
0.4
0.5
SNP effect on CAG−177

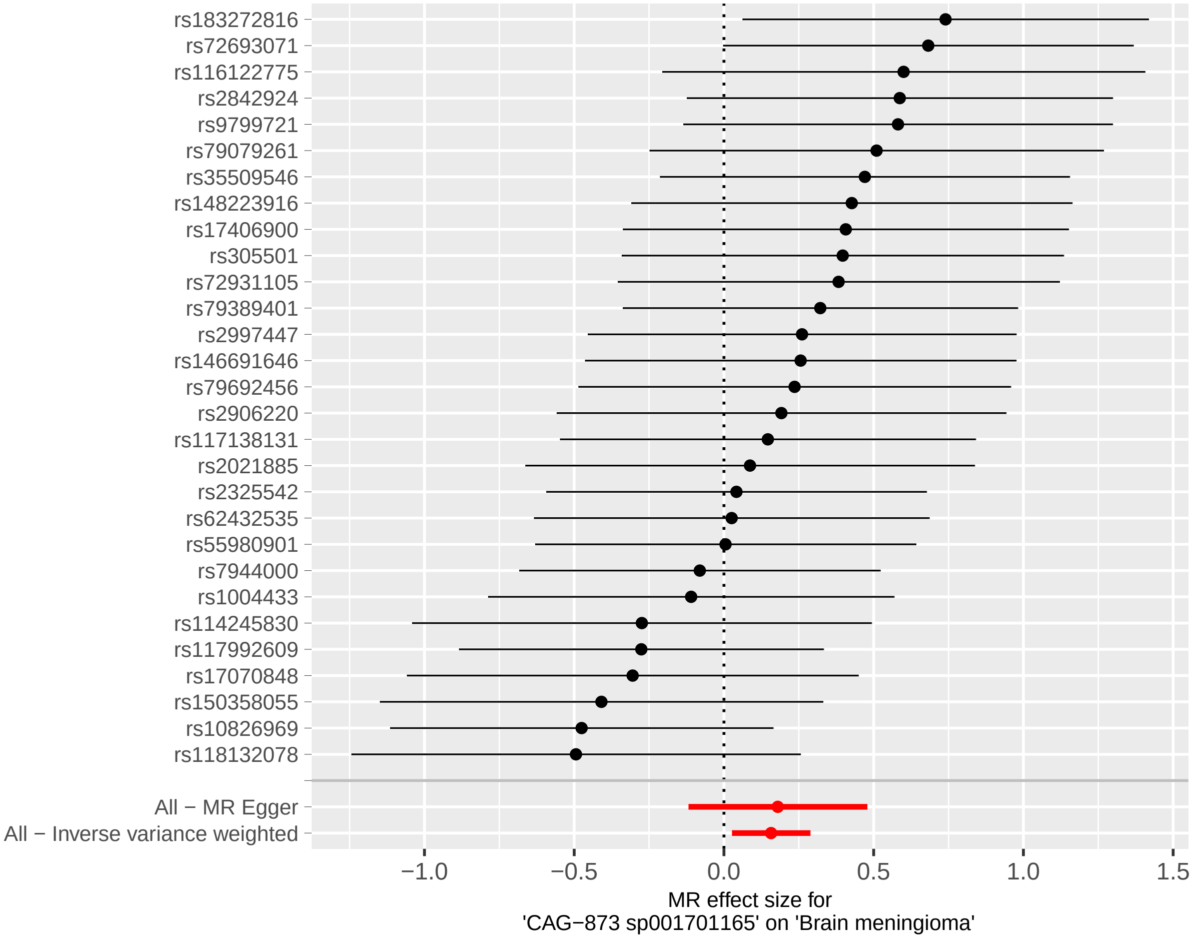

rs183272816
rs72693071
rs116122775
rs2842924
rs9799721
rs79079261
rs35509546
rs148223916
rs17406900
rs305501
rs72931105
rs79389401
rs2997447
rs146691646
rs79692456
rs2906220
rs117138131
rs2021885
rs2325542
rs62432535
rs55980901
rs7944000
rs1004433
rs114245830
rs117992609
rs17070848
rs150358055
rs10826969
rs118132078
All − MR Egger
All − Inverse variance weighted
−1.0
−0.5
0.0
0.5
1.0
1.5
MR effect size for
'CAG−873 sp001701165' on 'Brain meningioma'

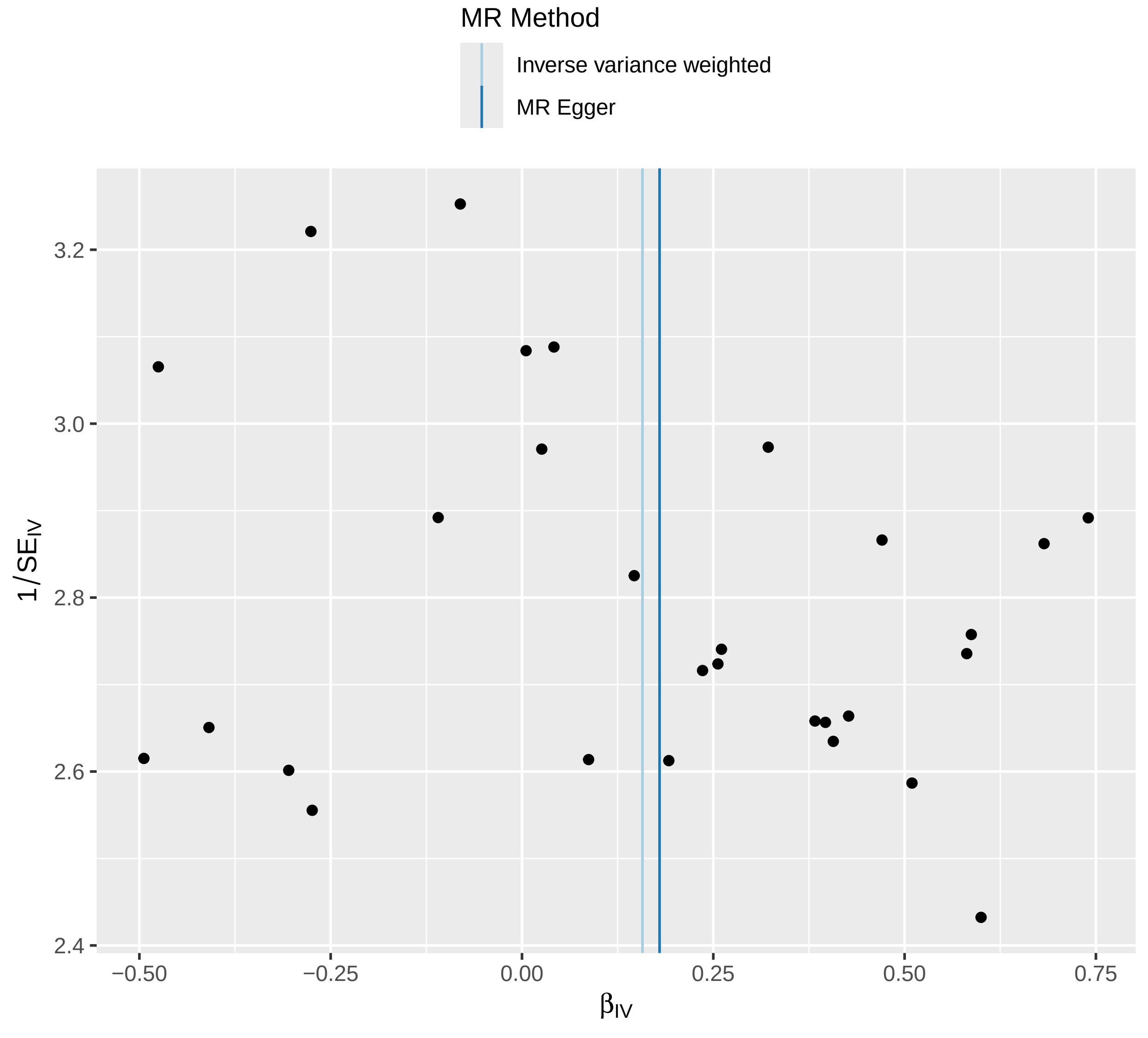

MR Method
Inverse variance weighted
MR Egger
3.2
3.0
2.8
2.6
2.4
1/SE_IV
−0.50
−0.25
0.00
0.25
0.50
0.75
β_IV

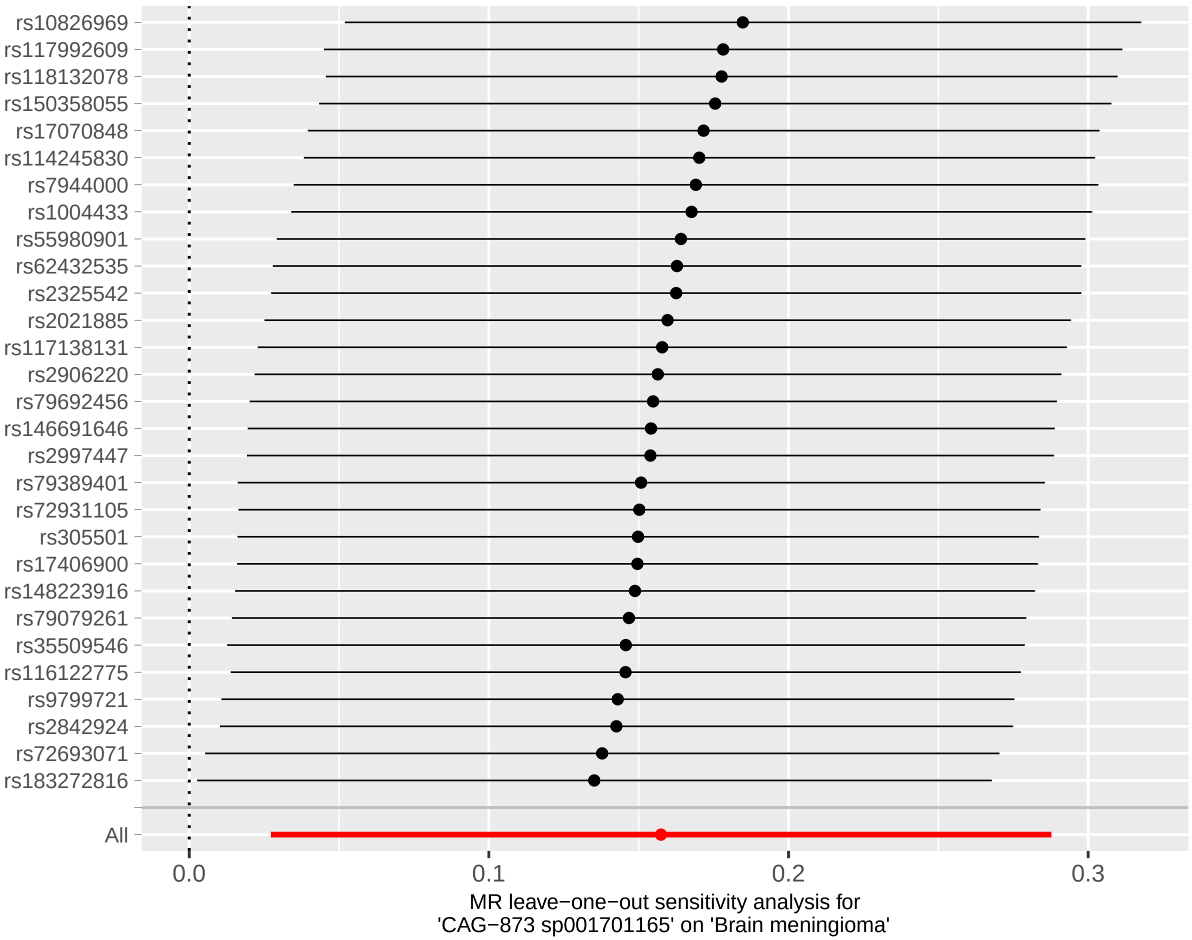

rs10826969
rs117992609
rs118132078
rs150358055
rs17070848
rs114245830
rs7944000
rs1004433
rs55980901
rs62432535
rs2325542
rs2021885
rs117138131
rs2906220
rs79692456
rs146691646
rs2997447
rs79389401
rs72931105
rs305501
rs17406900
rs148223916
rs79079261
rs35509546
rs116122775
rs9799721
rs2842924
rs72693071
rs183272816
All
0.0
0.1
0.2
0.3
MR leave−one−out sensitivity analysis for
'CAG−873 sp001701165' on 'Brain meningioma'

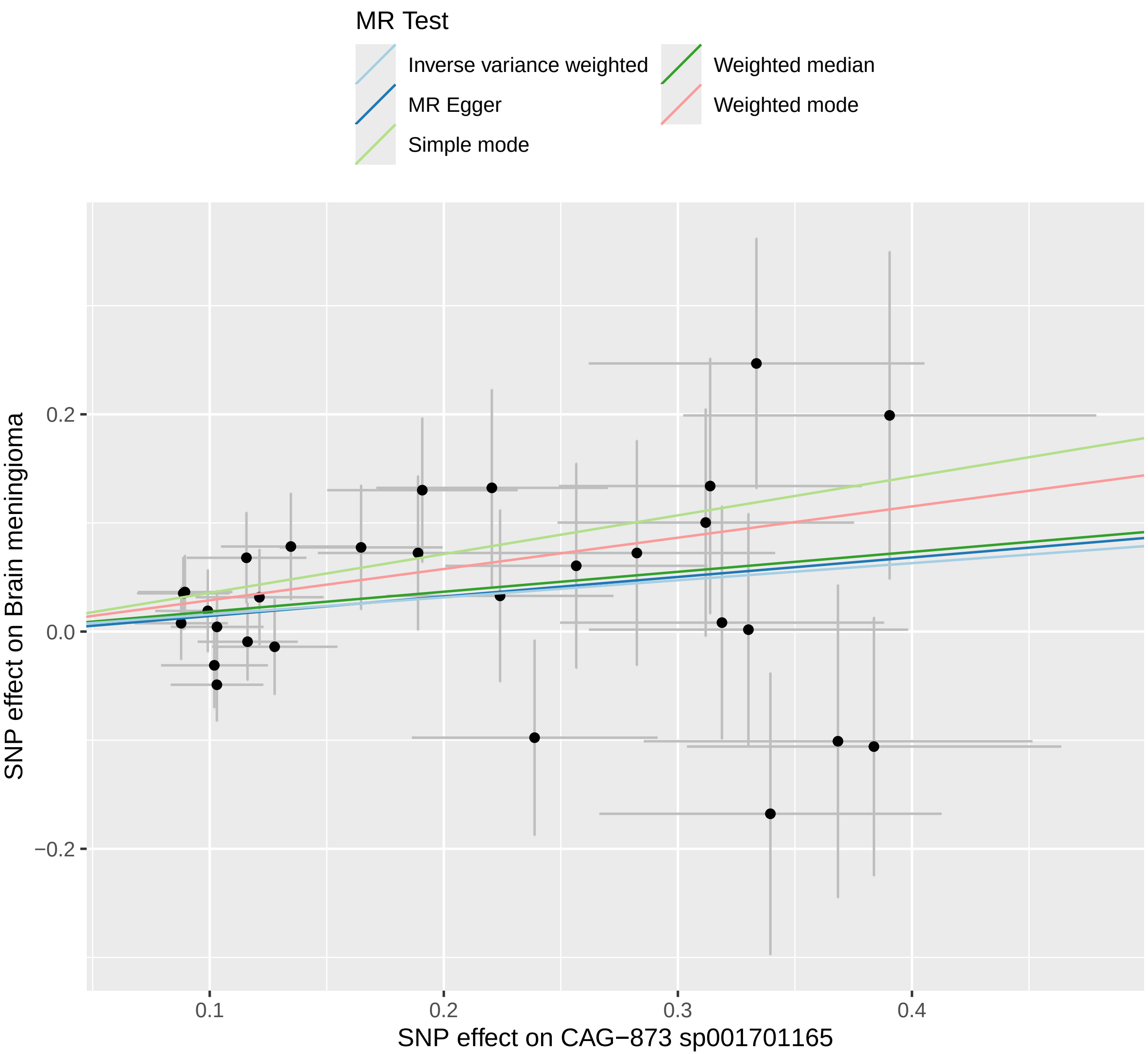
MR Test
Inverse variance weighted
MR Egger
Simple mode
Weighted median
Weighted mode
0.2
0.0
−0.2
0.1
0.2
0.3
0.4
SNP effect on Brain meningioma
SNP effect on CAG−873 sp001701165

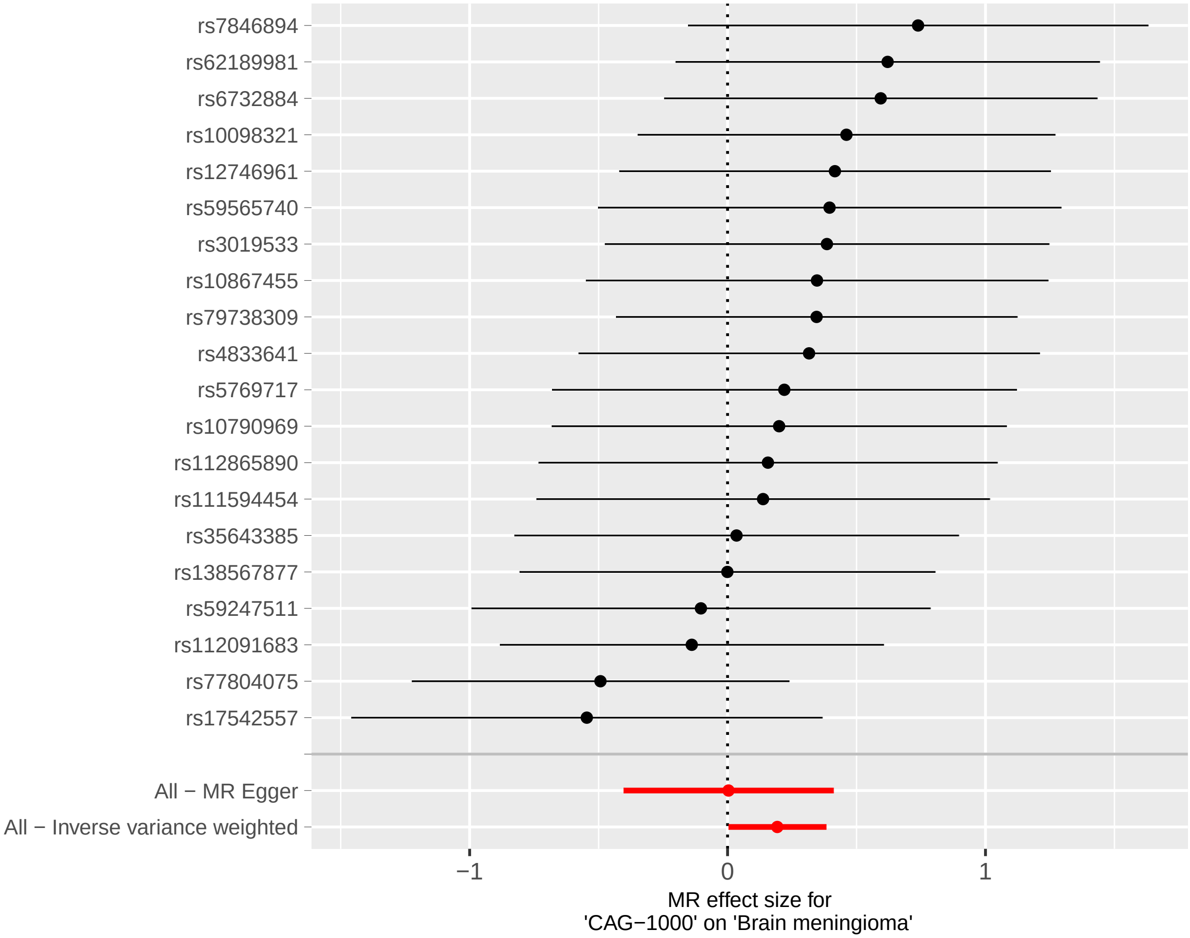
rs7846894
rs62189981
rs6732884
rs10098321
rs12746961
rs59565740
rs3019533
rs10867455
rs79738309
rs4833641
rs5769717
rs10790969
rs112865890
rs111594454
rs35643385
rs138567877
rs59247511
rs112091683
rs77804075
rs17542557
All − MR Egger
All − Inverse variance weighted
−1
0
1
MR effect size for
'CAG−1000' on 'Brain meningioma'

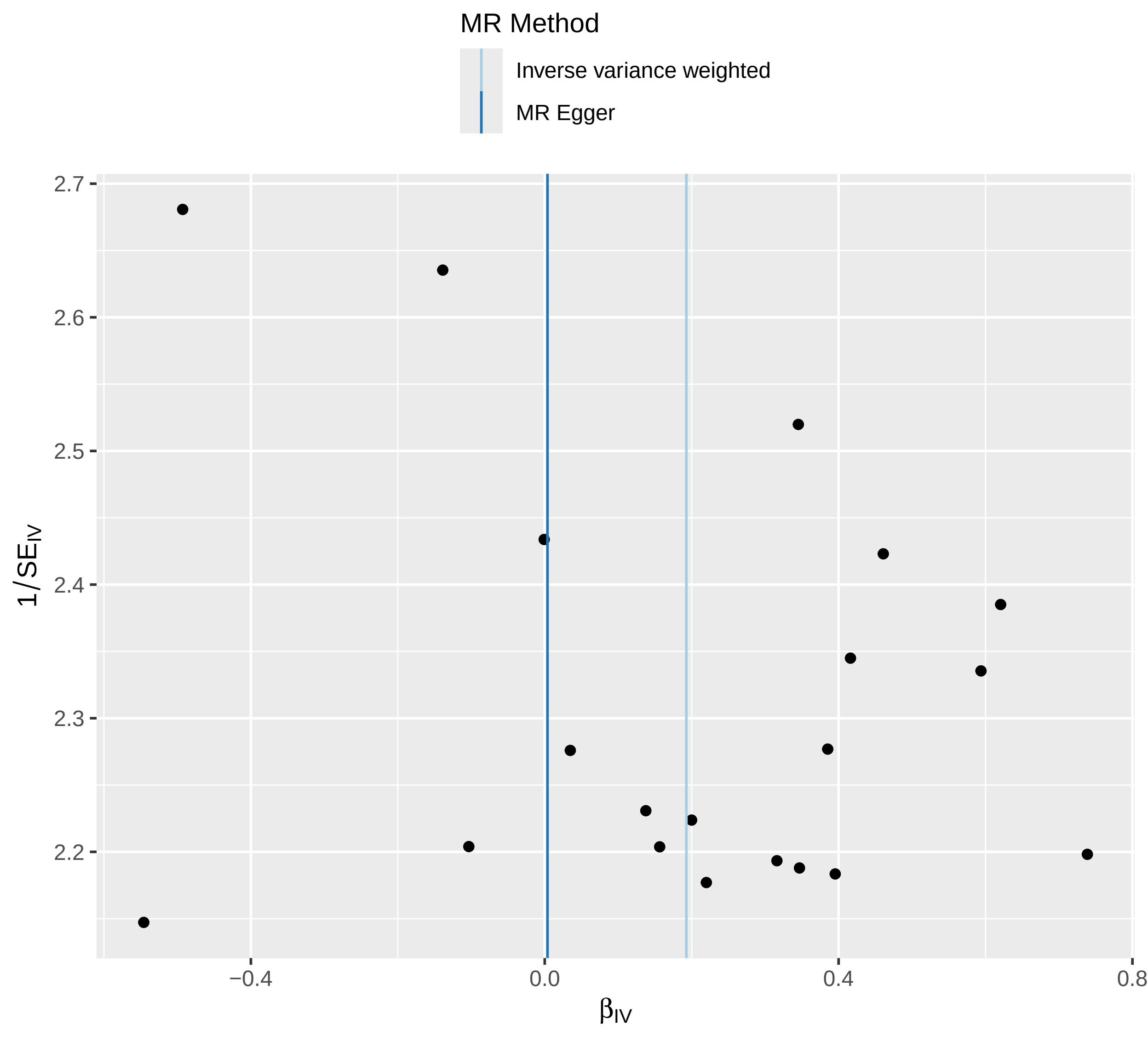
MR Method
Inverse variance weighted
MR Egger
1/SE_IV
2.7
2.6
2.5
2.4
2.3
2.2
β_IV
−0.4
0.0
0.4
0.8

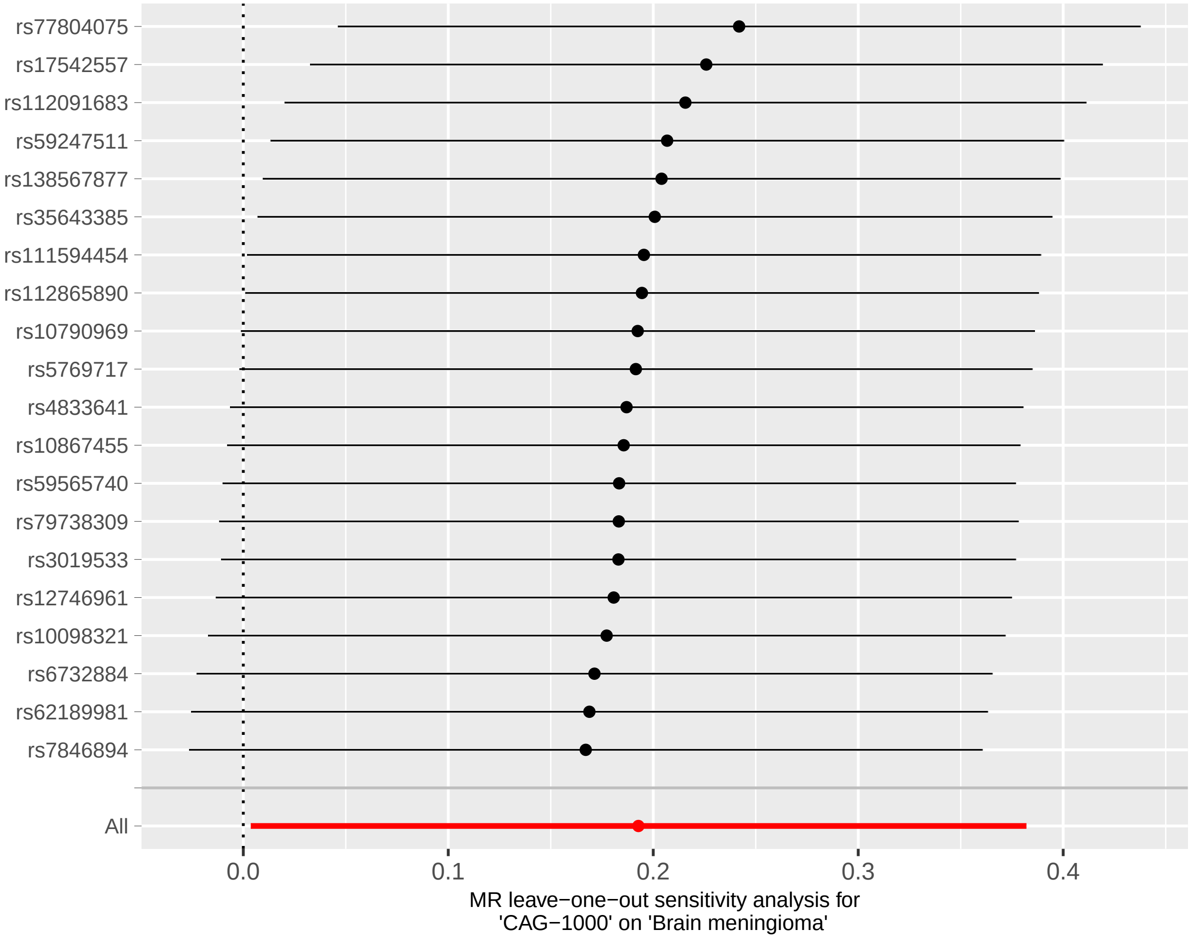

rs77804075
rs17542557
rs112091683
rs59247511
rs138567877
rs35643385
rs111594454
rs112865890
rs10790969
rs5769717
rs4833641
rs10867455
rs59565740
rs79738309
rs3019533
rs12746961
rs10098321
rs6732884
rs62189981
rs7846894
All
0.0
0.1
0.2
0.3
0.4
MR leave−one−out sensitivity analysis for
'CAG−1000' on 'Brain meningioma'

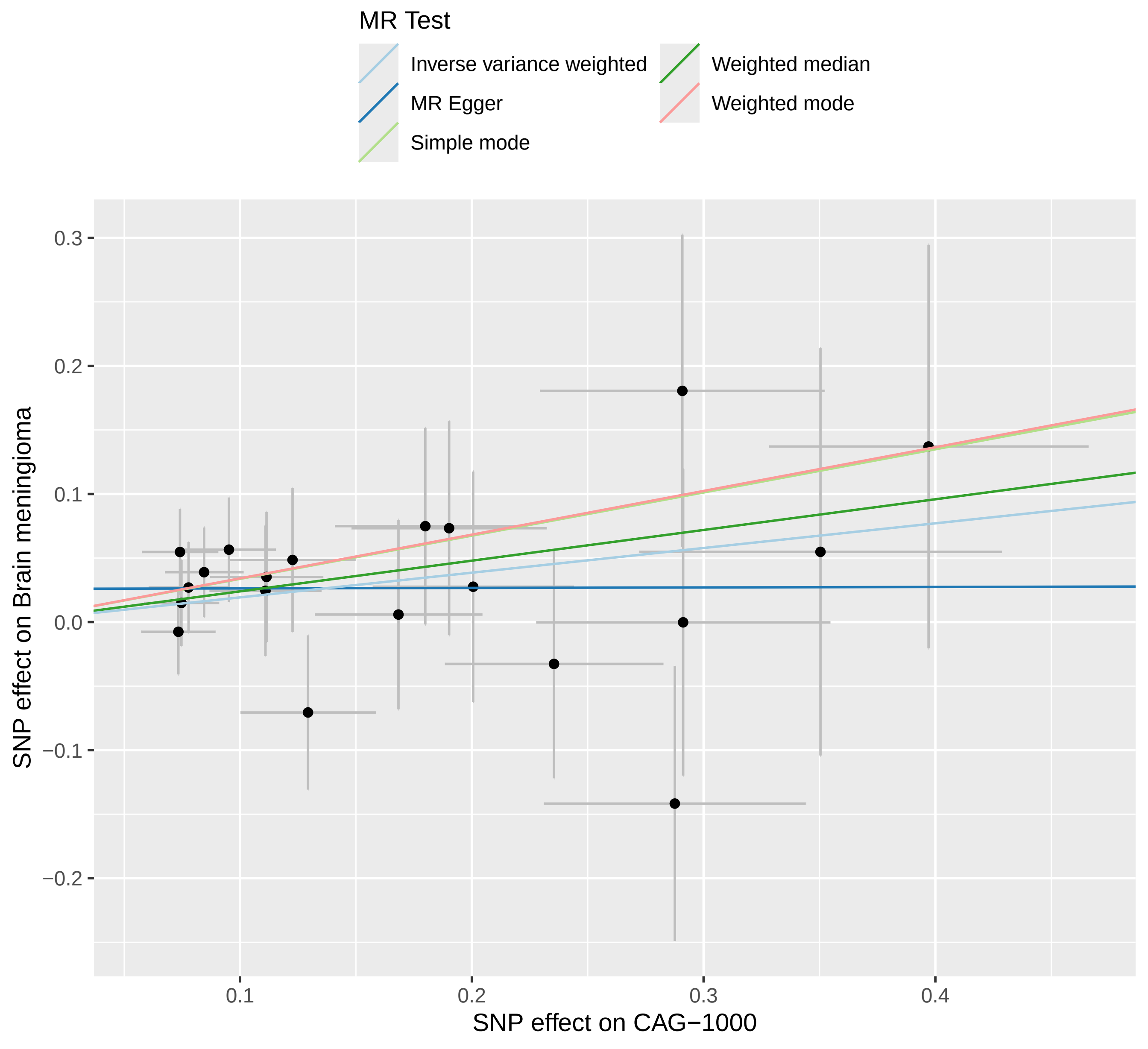
MR Test
Inverse variance weighted
MR Egger
Simple mode
Weighted median
Weighted mode
SNP effect on Brain meningioma
SNP effect on CAG−1000
0.3
0.2
0.1
0.0
−0.1
−0.2
0.1
0.2
0.3
0.4

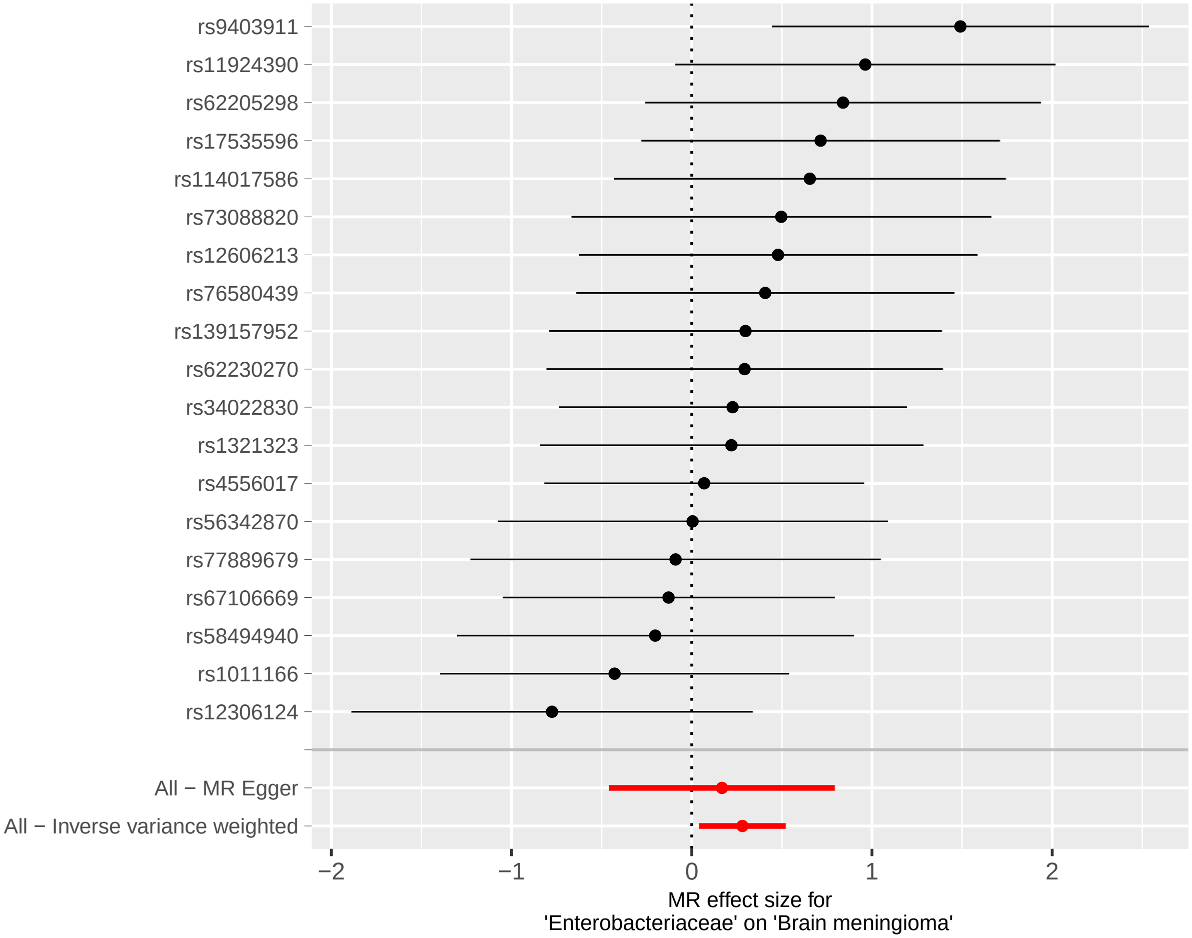
rs9403911
rs11924390
rs62205298
rs17535596
rs114017586
rs73088820
rs12606213
rs76580439
rs139157952
rs62230270
rs34022830
rs1321323
rs4556017
rs56342870
rs77889679
rs67106669
rs58494940
rs1011166
rs12306124
All − MR Egger
All − Inverse variance weighted
−2
−1
0
1
2
MR effect size for
'Enterobacteriaceae' on 'Brain meningioma'

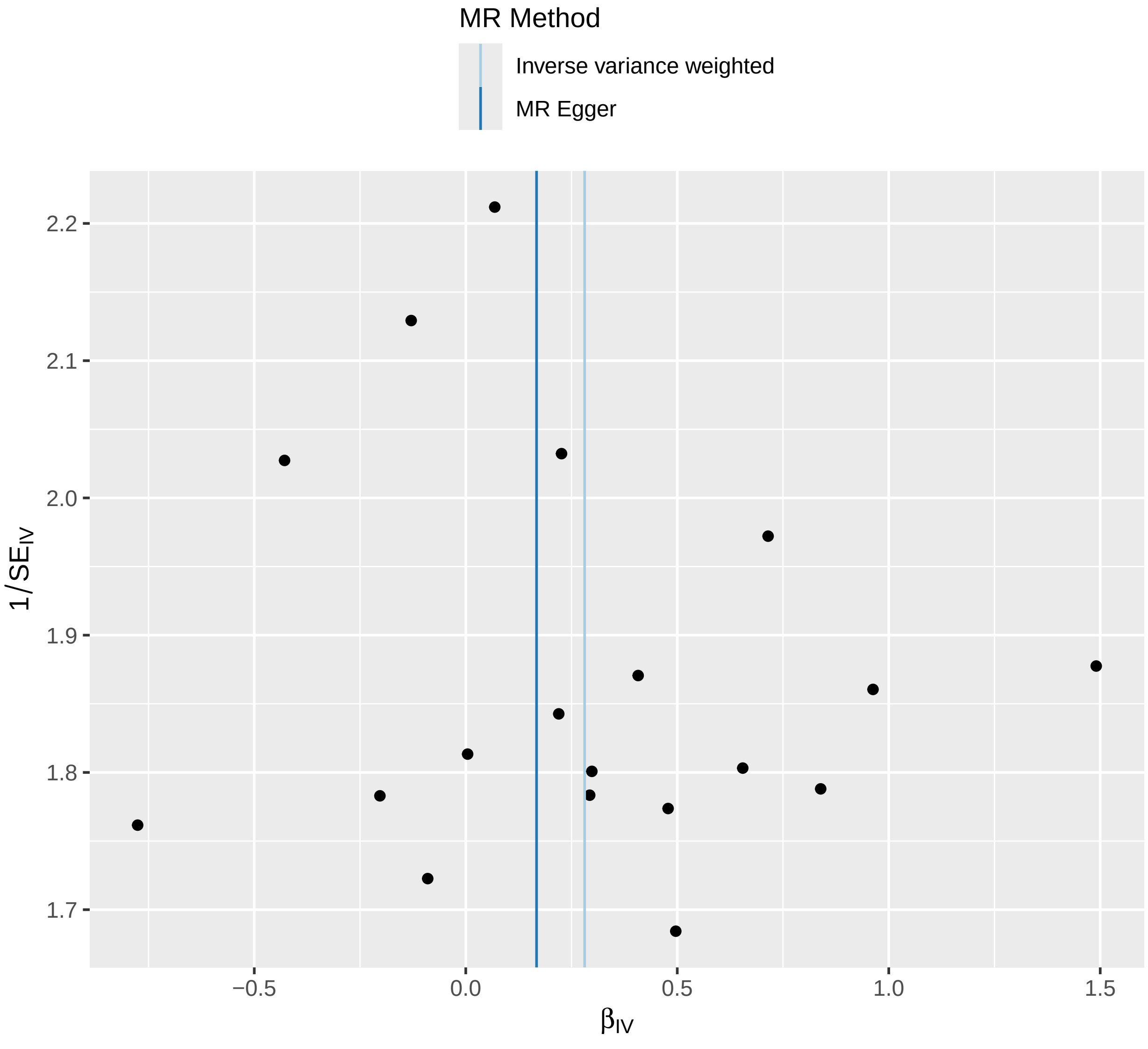
MR Method
Inverse variance weighted
MR Egger
2.2
2.1
2.0
1.9
1.8
1.7
$1/SE_{IV}$
−0.5
0.0
0.5
1.0
1.5
β_{IV}

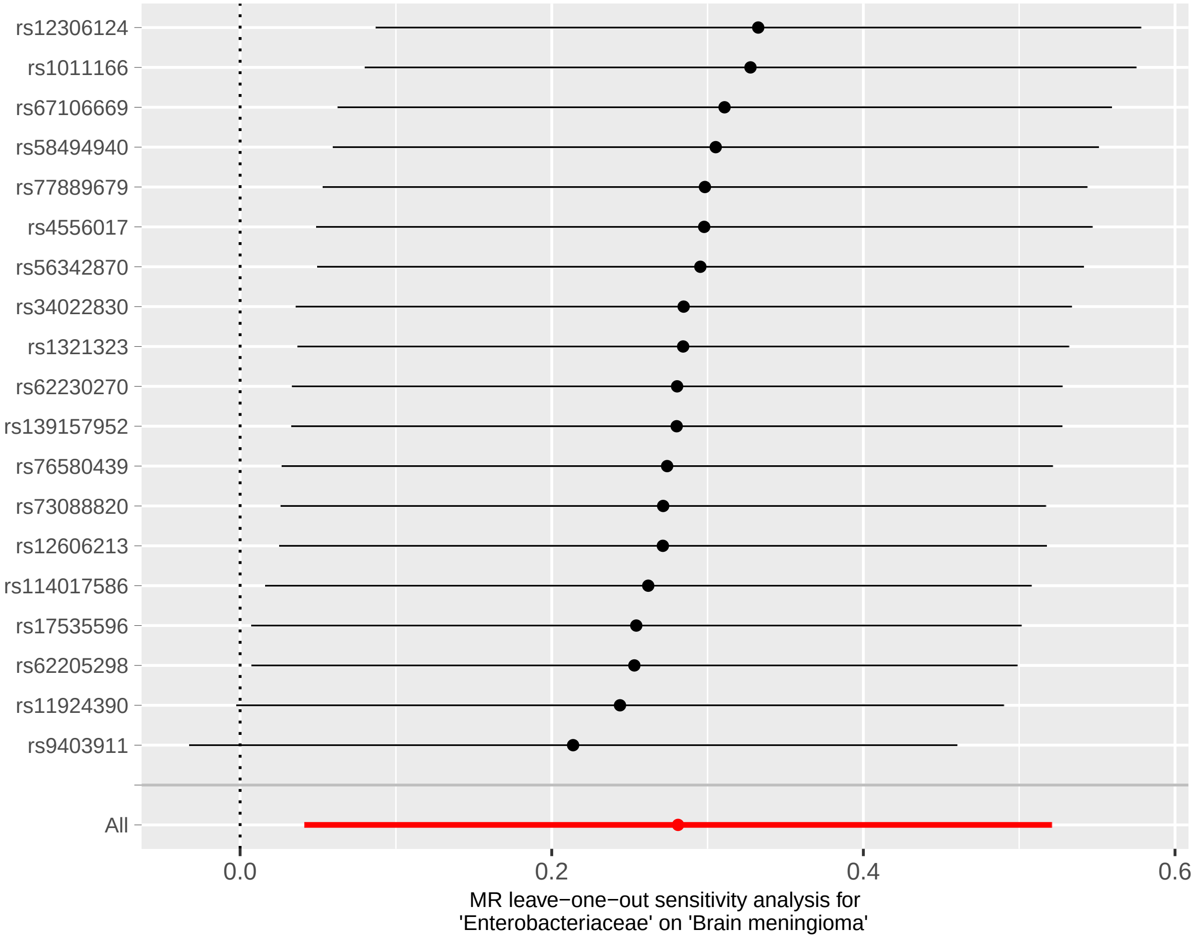
rs12306124
rs1011166
rs67106669
rs58494940
rs77889679
rs4556017
rs56342870
rs34022830
rs1321323
rs62230270
rs139157952
rs76580439
rs73088820
rs12606213
rs114017586
rs17535596
rs62205298
rs11924390
rs9403911
All
0.0
0.2
0.4
0.6
MR leave−one−out sensitivity analysis for
'Enterobacteriaceae' on 'Brain meningioma'

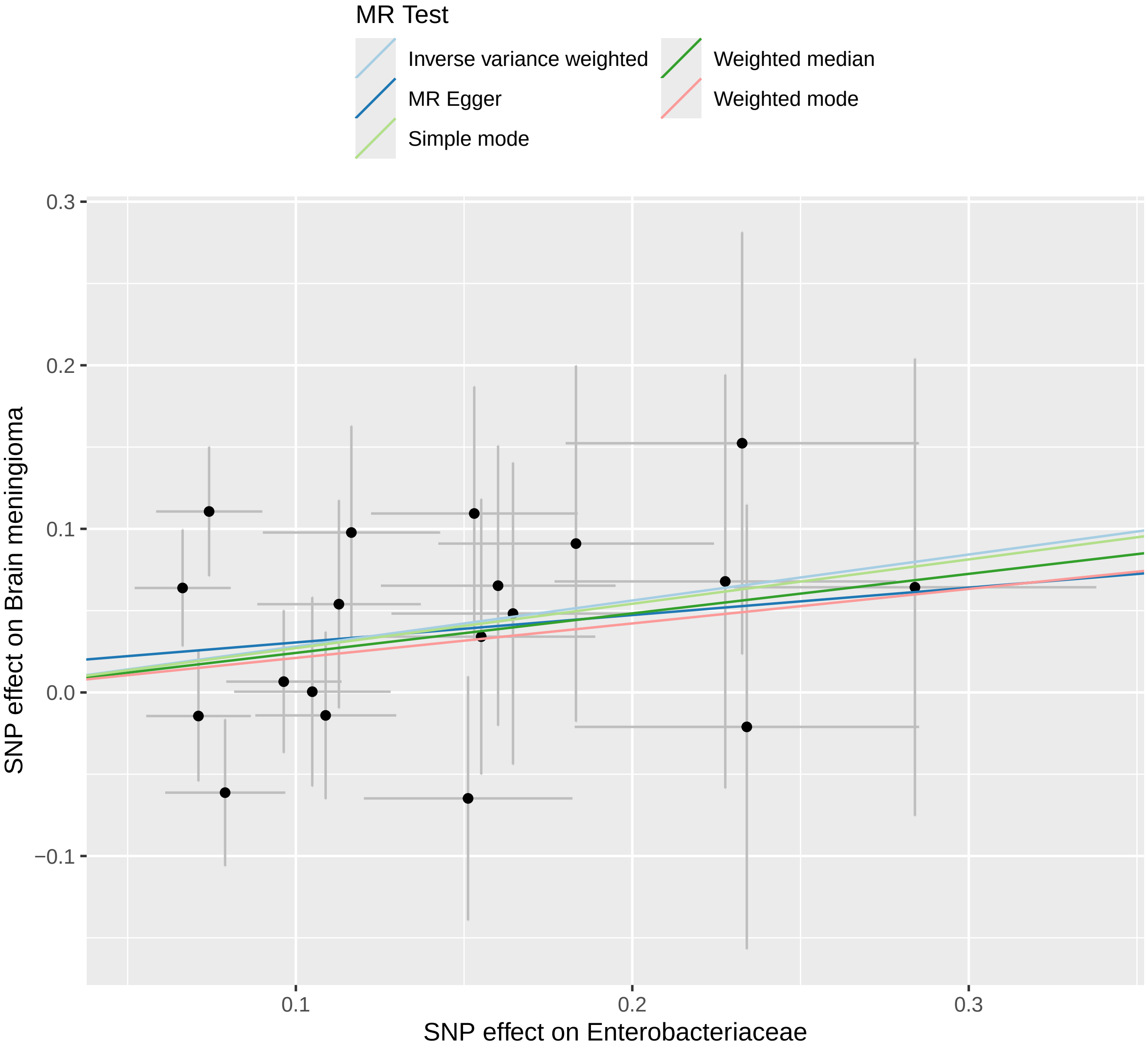

MR Test
Inverse variance weighted
MR Egger
Simple mode
Weighted median
Weighted mode
0.3
0.2
0.1
0.0
−0.1
SNP effect on Brain meningioma
0.1
0.2
0.3
SNP effect on Enterobacteriaceae

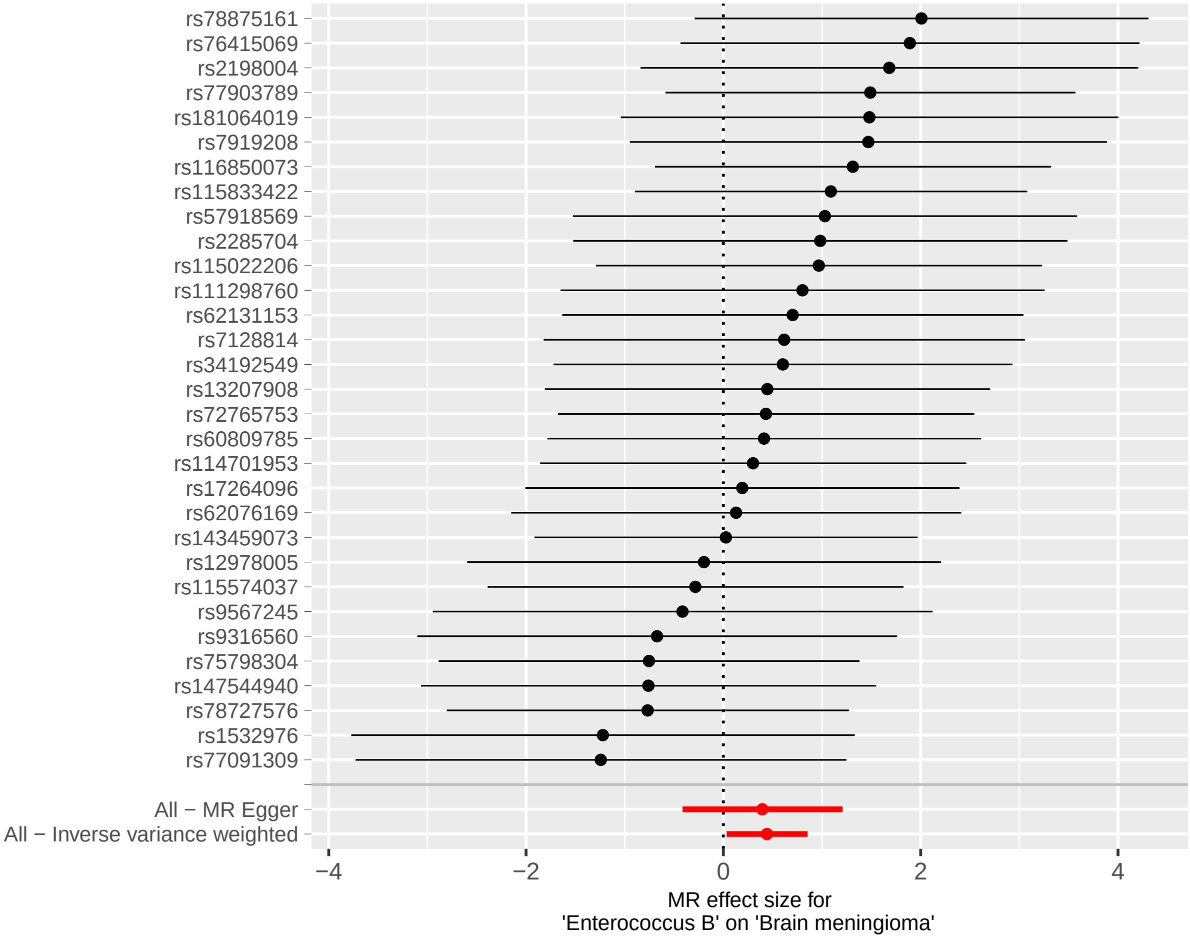

rs78875161
rs76415069
rs2198004
rs77903789
rs181064019
rs7919208
rs116850073
rs115833422
rs57918569
rs2285704
rs115022206
rs111298760
rs62131153
rs7128814
rs34192549
rs13207908
rs72765753
rs60809785
rs114701953
rs17264096
rs62076169
rs143459073
rs12978005
rs115574037
rs9567245
rs9316560
rs75798304
rs147544940
rs78727576
rs1532976
rs77091309
All − MR Egger
All − Inverse variance weighted
−4
−2
0
2
4
MR effect size for
'Enterococcus B' on 'Brain meningioma'

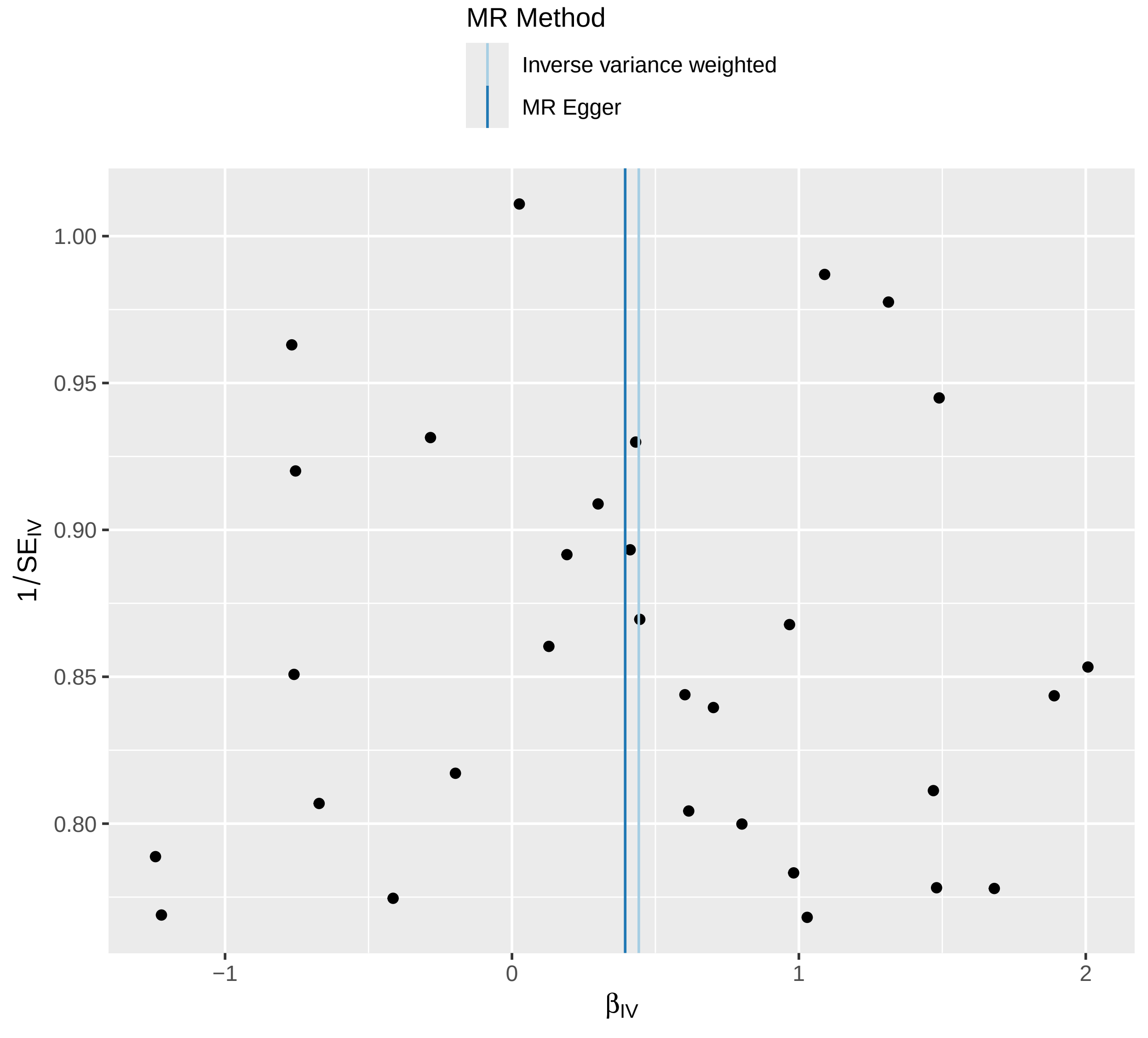
MR Method
Inverse variance weighted
MR Egger
1.00
0.95
0.90
0.85
0.80
1/SE_IV
−1
0
1
2
β_IV

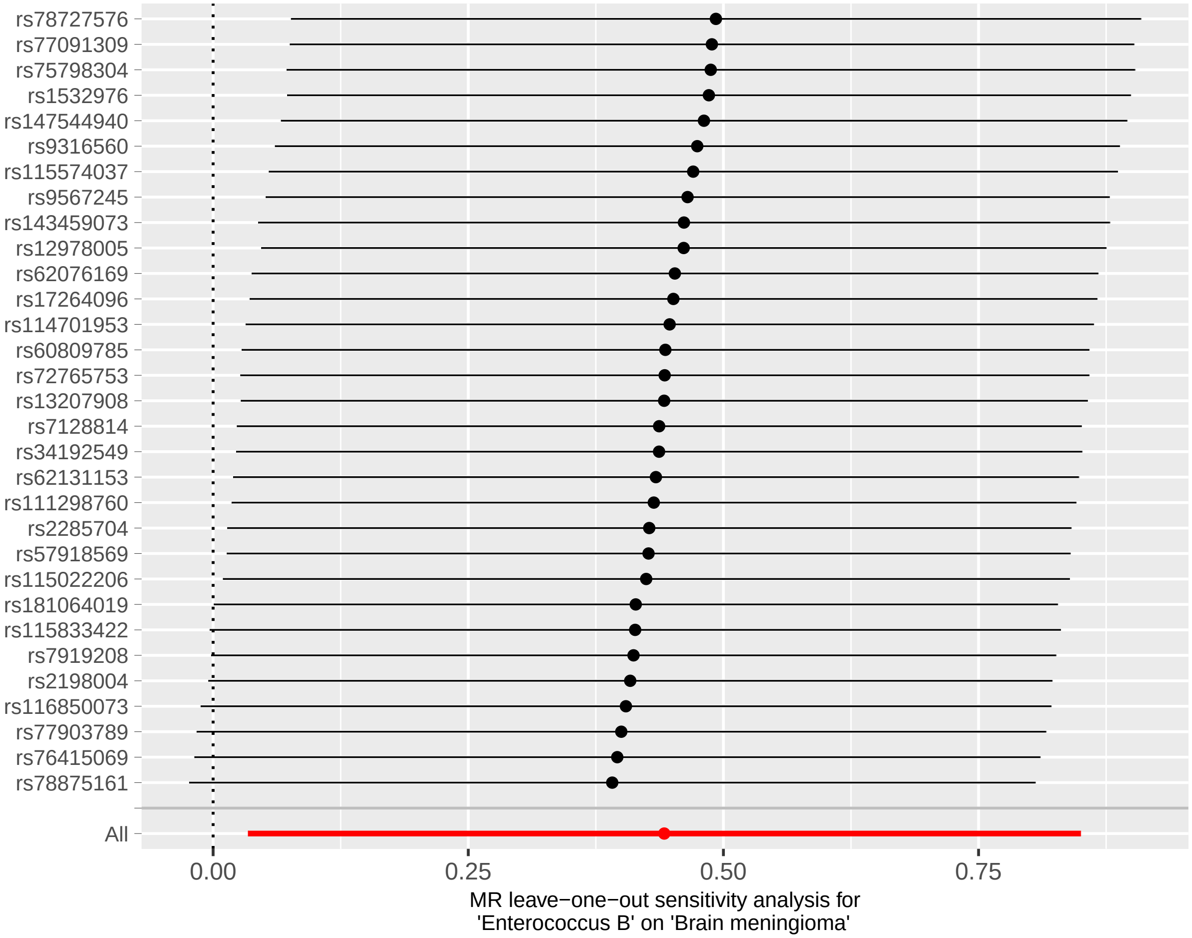

rs78727576
rs77091309
rs75798304
rs1532976
rs147544940
rs9316560
rs115574037
rs9567245
rs143459073
rs12978005
rs62076169
rs17264096
rs114701953
rs60809785
rs72765753
rs13207908
rs7128814
rs34192549
rs62131153
rs111298760
rs2285704
rs57918569
rs115022206
rs181064019
rs115833422
rs7919208
rs2198004
rs116850073
rs77903789
rs76415069
rs78875161
All
0.00
0.25
0.50
0.75
MR leave−one−out sensitivity analysis for
'Enterococcus B' on 'Brain meningioma'

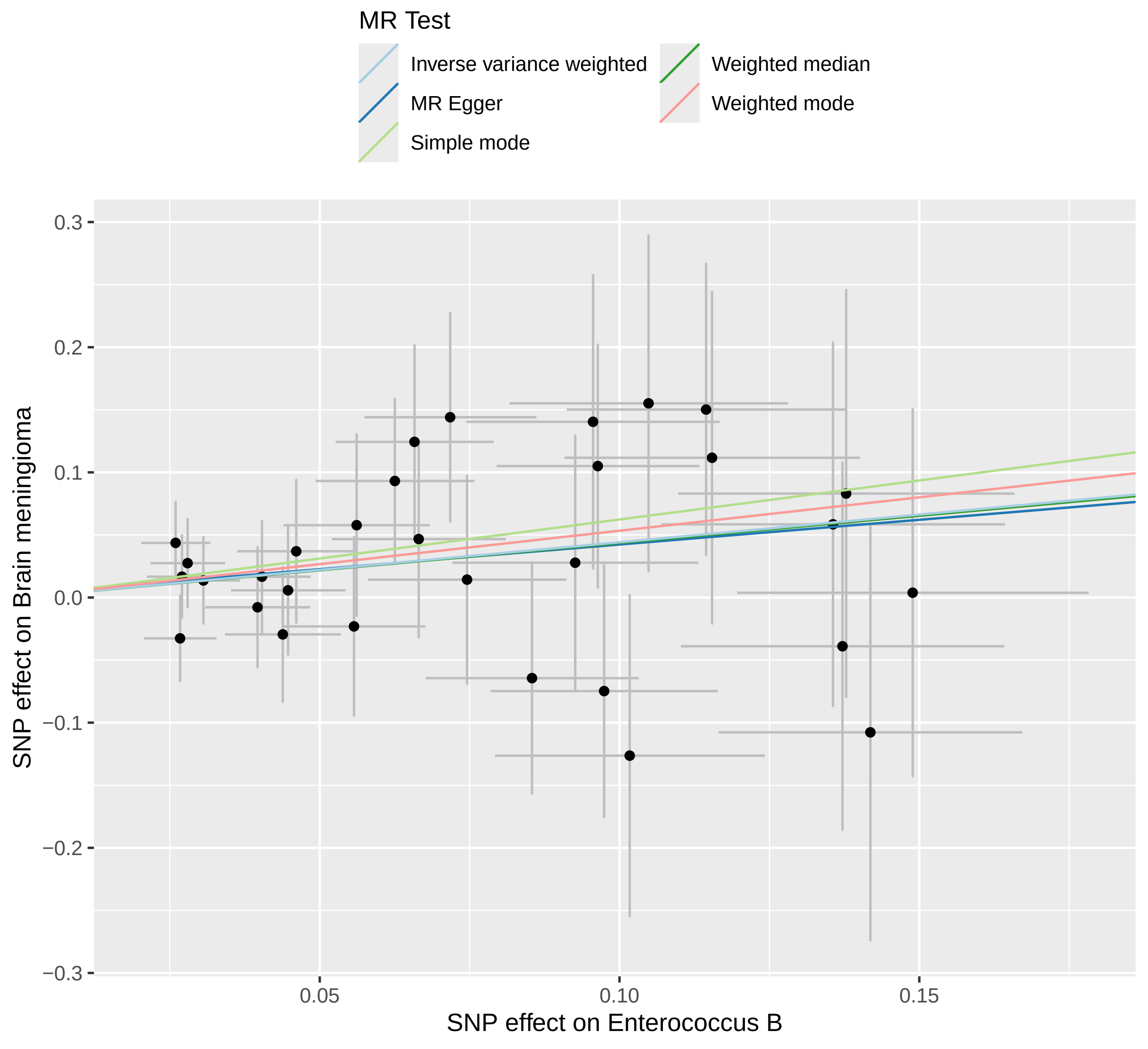
MR Test
Inverse variance weighted
MR Egger
Simple mode
Weighted median
Weighted mode
0.3
0.2
0.1
0.0
−0.1
−0.2
−0.3
0.05
0.10
0.15
SNP effect on Brain meningioma
SNP effect on Enterococcus B

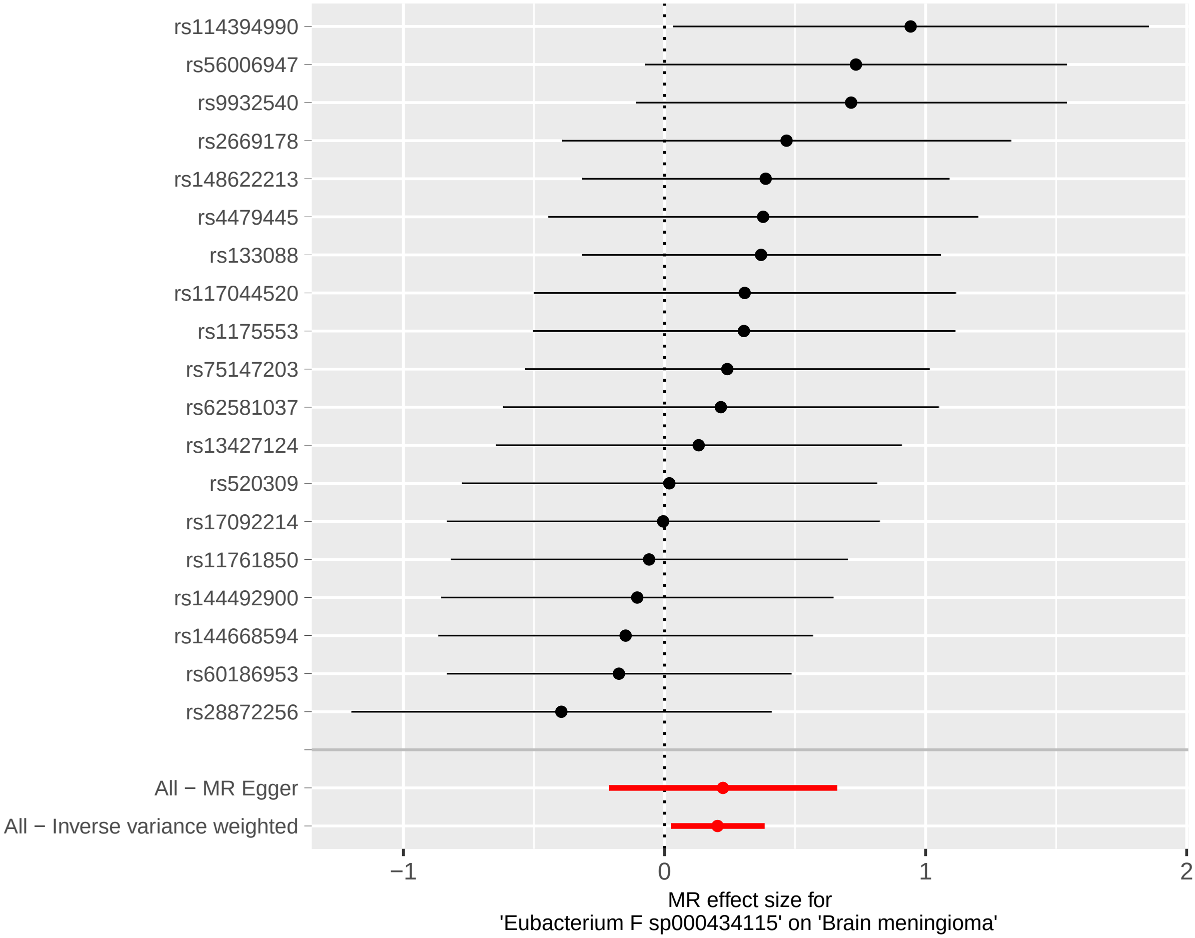
rs114394990
rs56006947
rs9932540
rs2669178
rs148622213
rs4479445
rs133088
rs117044520
rs1175553
rs75147203
rs62581037
rs13427124
rs520309
rs17092214
rs11761850
rs144492900
rs144668594
rs60186953
rs28872256
All − MR Egger
All − Inverse variance weighted
−1
0
1
2
MR effect size for
'Eubacterium F sp000434115' on 'Brain meningioma'

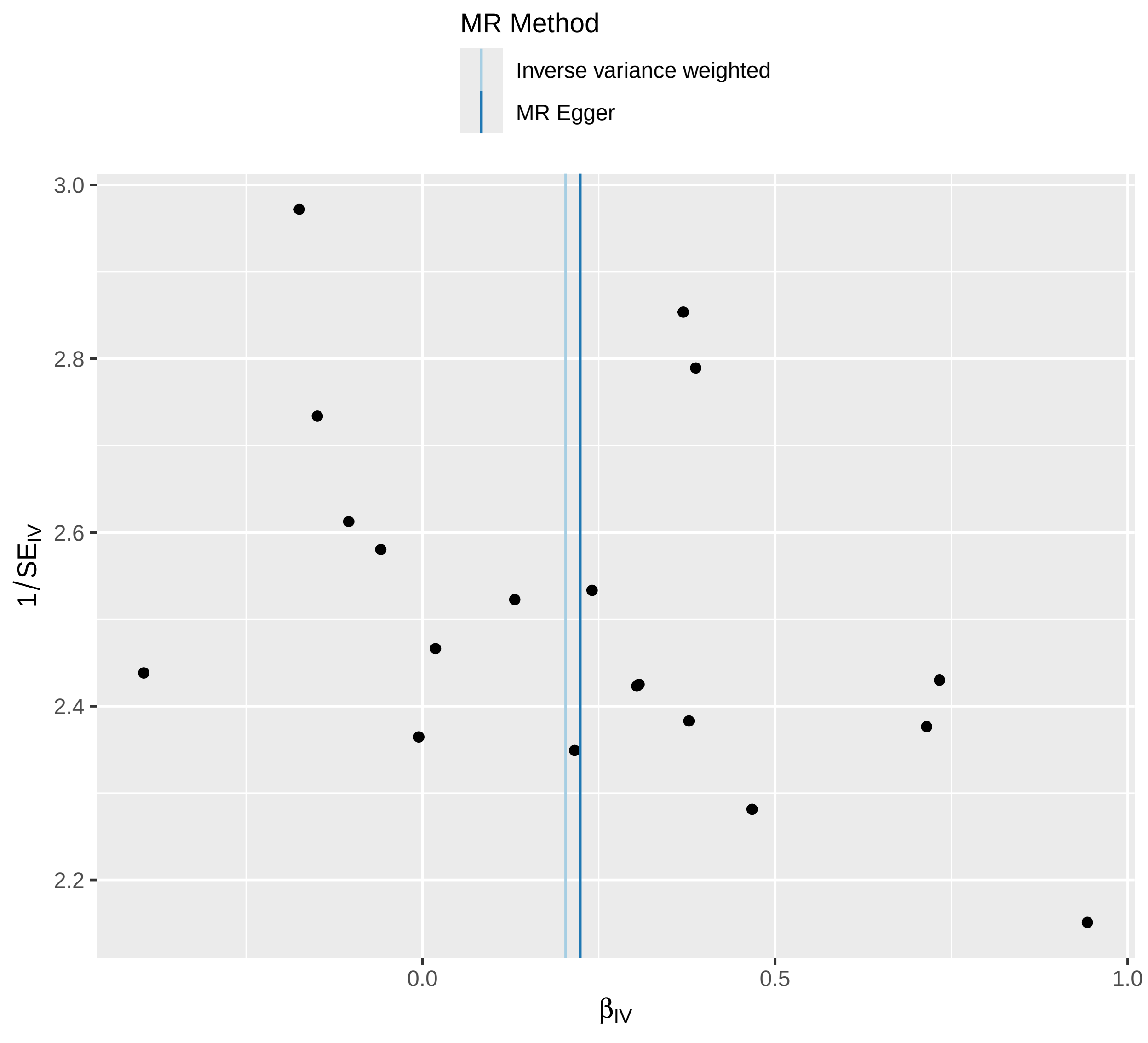

MR Method
Inverse variance weighted
MR Egger
3.0
2.8
2.6
2.4
2.2
1/SE_IV
0.0
0.5
1.0
β_IV

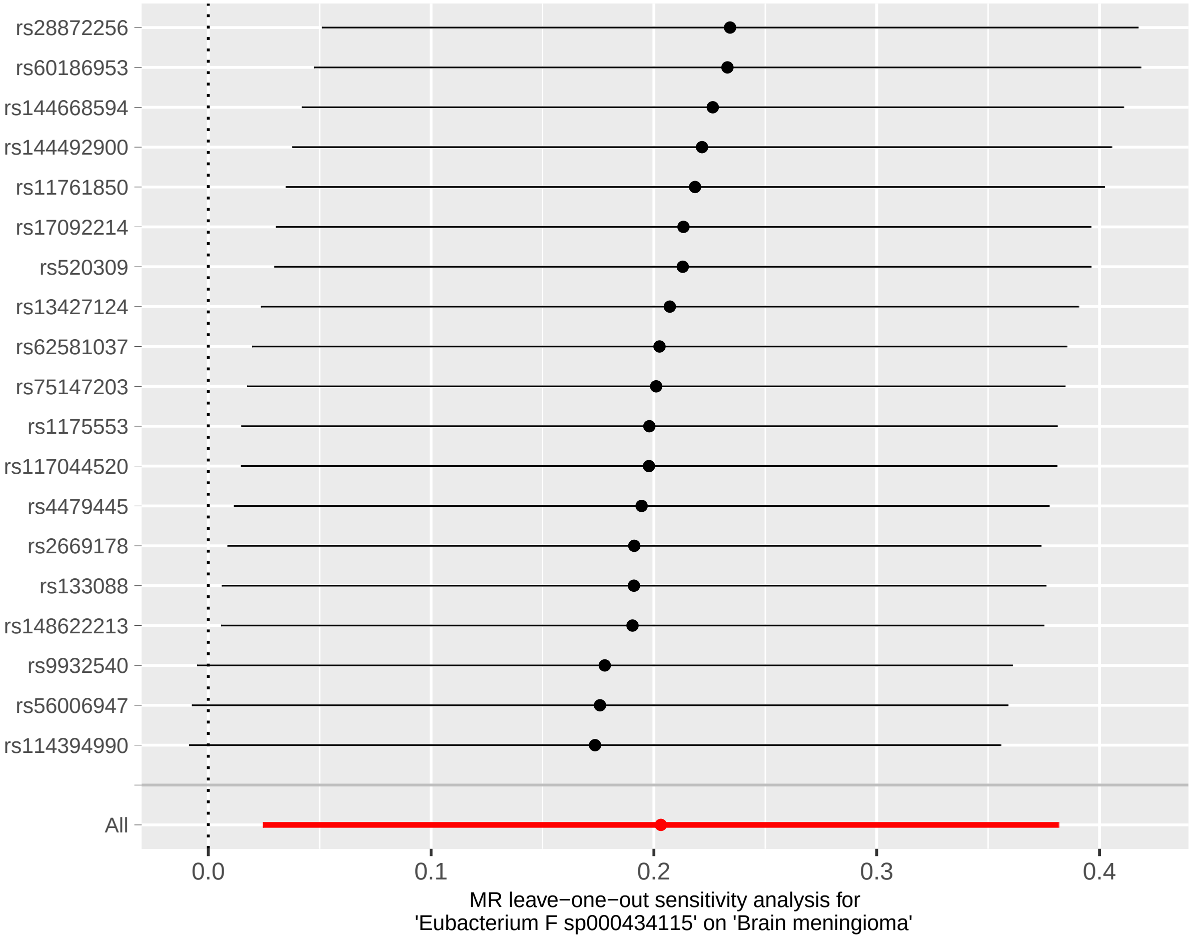
rs28872256
rs60186953
rs144668594
rs144492900
rs11761850
rs17092214
rs520309
rs13427124
rs62581037
rs75147203
rs1175553
rs117044520
rs4479445
rs2669178
rs133088
rs148622213
rs9932540
rs56006947
rs114394990
All
0.0
0.1
0.2
0.3
0.4
MR leave−one−out sensitivity analysis for
'Eubacterium F sp000434115' on 'Brain meningioma'

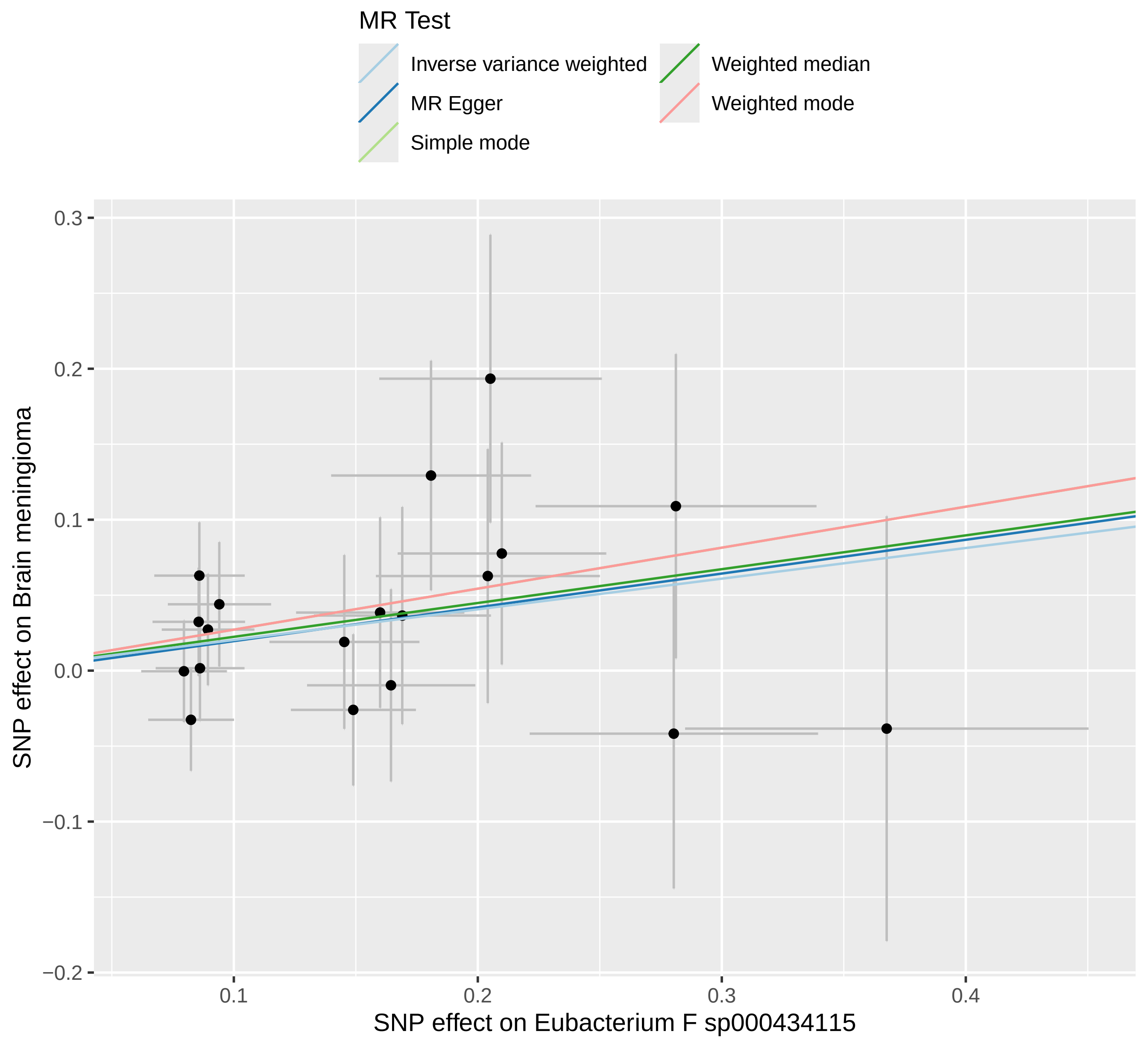

MR Test
Inverse variance weighted
MR Egger
Simple mode
Weighted median
Weighted mode
SNP effect on Brain meningioma
SNP effect on Eubacterium F sp000434115
0.3
0.2
0.1
0.0
−0.1
−0.2
0.1
0.2
0.3
0.4

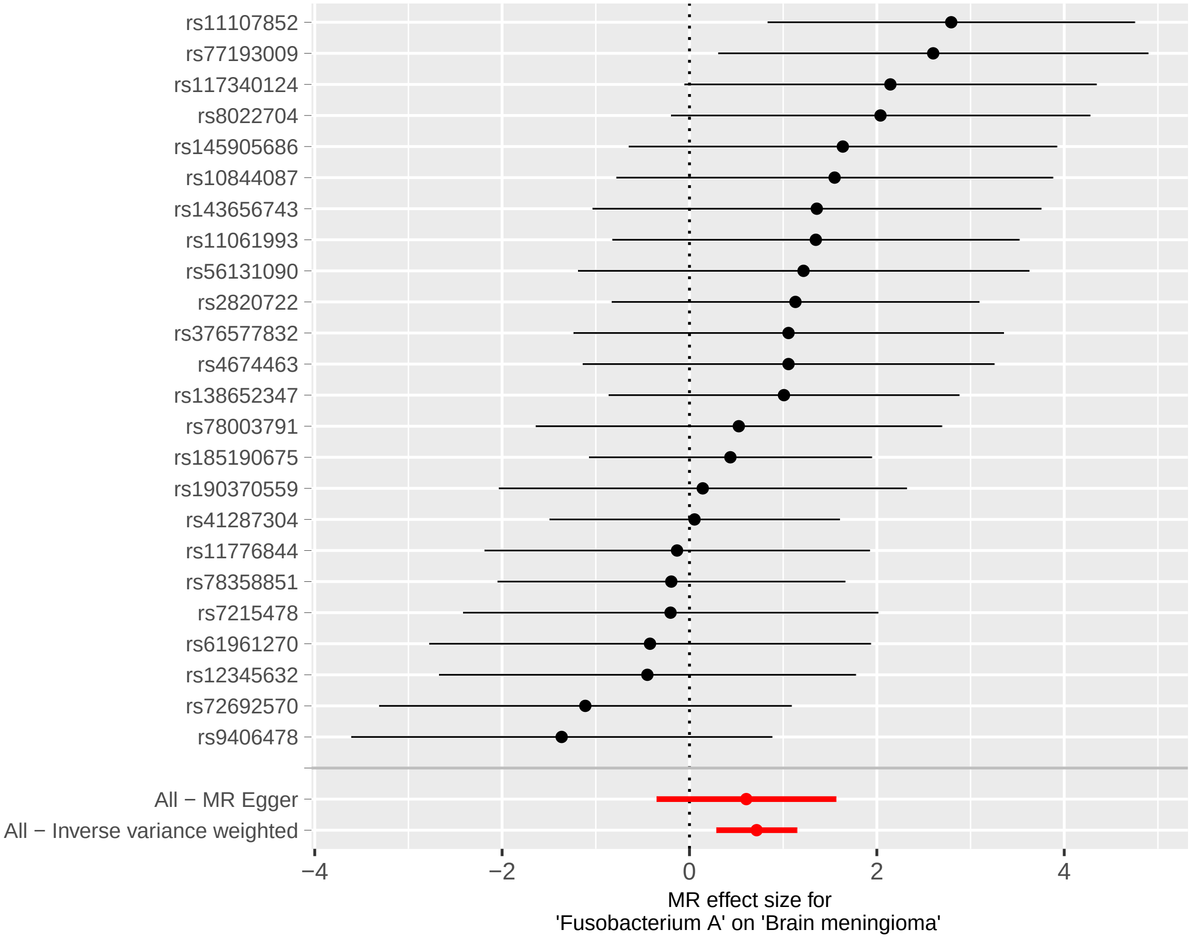

rs11107852
rs77193009
rs117340124
rs8022704
rs145905686
rs10844087
rs143656743
rs11061993
rs56131090
rs2820722
rs376577832
rs4674463
rs138652347
rs78003791
rs185190675
rs190370559
rs41287304
rs11776844
rs78358851
rs7215478
rs61961270
rs12345632
rs72692570
rs9406478
All − MR Egger
All − Inverse variance weighted
−4
−2
0
2
4
MR effect size for
'Fusobacterium A' on 'Brain meningioma'

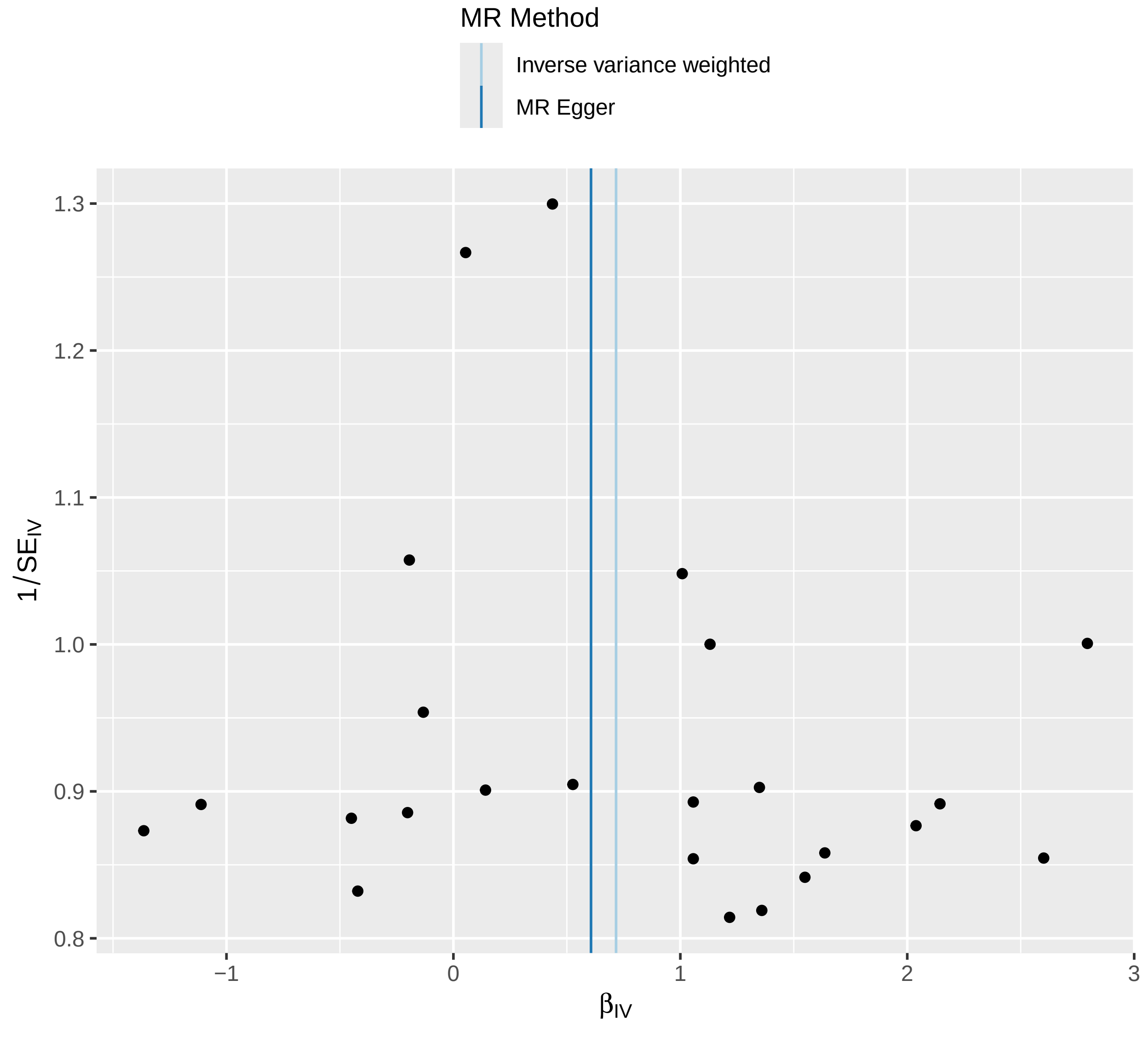
MR Method
Inverse variance weighted
MR Egger
1.3
1.2
1.1
1.0
0.9
0.8
1/SE$_{IV}$
−1
0
1
2
3
β$_{IV}$

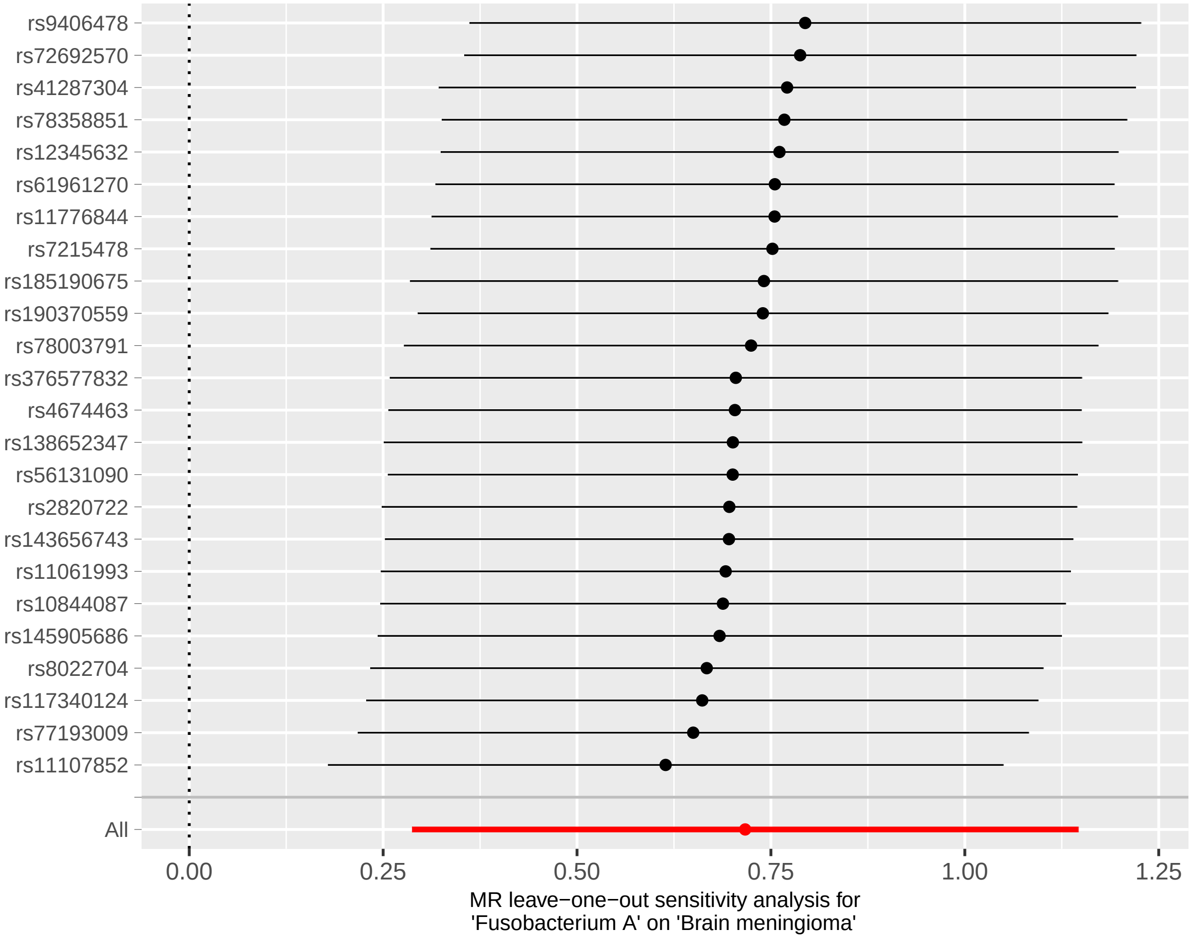
rs9406478
rs72692570
rs41287304
rs78358851
rs12345632
rs61961270
rs11776844
rs7215478
rs185190675
rs190370559
rs78003791
rs376577832
rs4674463
rs138652347
rs56131090
rs2820722
rs143656743
rs11061993
rs10844087
rs145905686
rs8022704
rs117340124
rs77193009
rs11107852
All
0.00
0.25
0.50
0.75
1.00
1.25
MR leave−one−out sensitivity analysis for
'Fusobacterium A' on 'Brain meningioma'

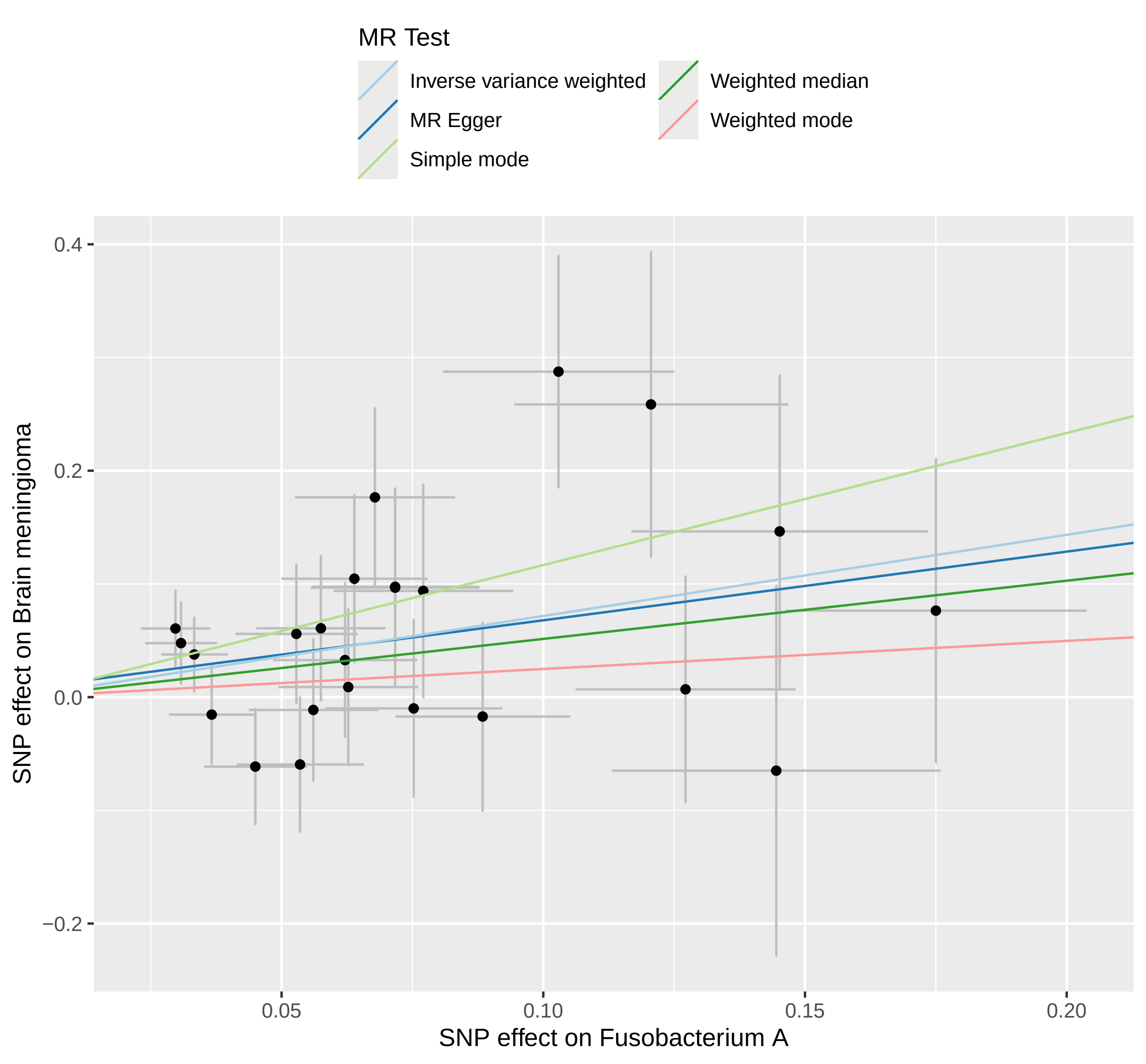
MR Test
Inverse variance weighted
MR Egger
Simple mode
Weighted median
Weighted mode
SNP effect on Brain meningioma
SNP effect on Fusobacterium A
0.4
0.2
0.0
−0.2
0.05
0.10
0.15
0.20

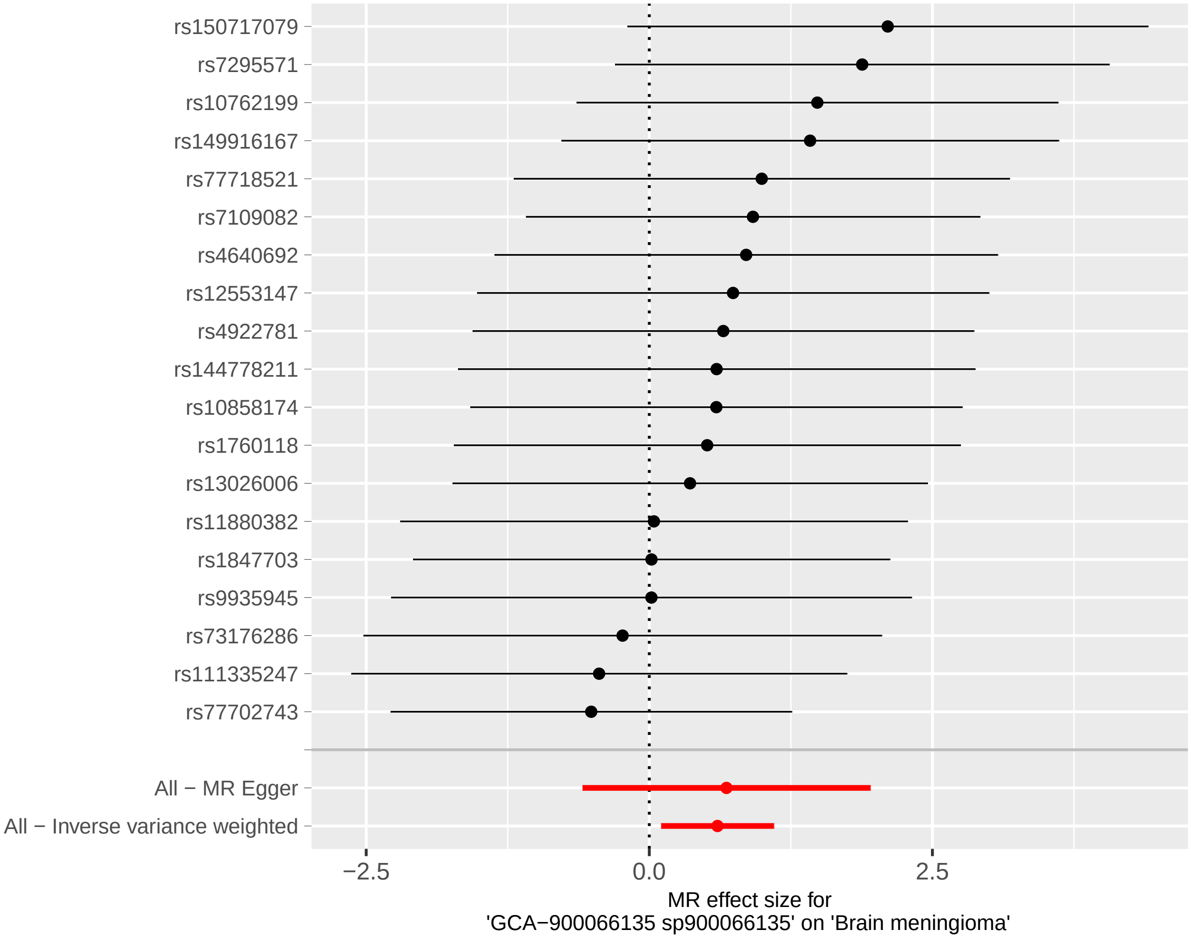
rs150717079
rs7295571
rs10762199
rs149916167
rs77718521
rs7109082
rs4640692
rs12553147
rs4922781
rs144778211
rs10858174
rs1760118
rs13026006
rs11880382
rs1847703
rs9935945
rs73176286
rs111335247
rs77702743
All − MR Egger
All − Inverse variance weighted
−2.5
0.0
2.5
MR effect size for
'GCA−900066135 sp900066135' on 'Brain meningioma'

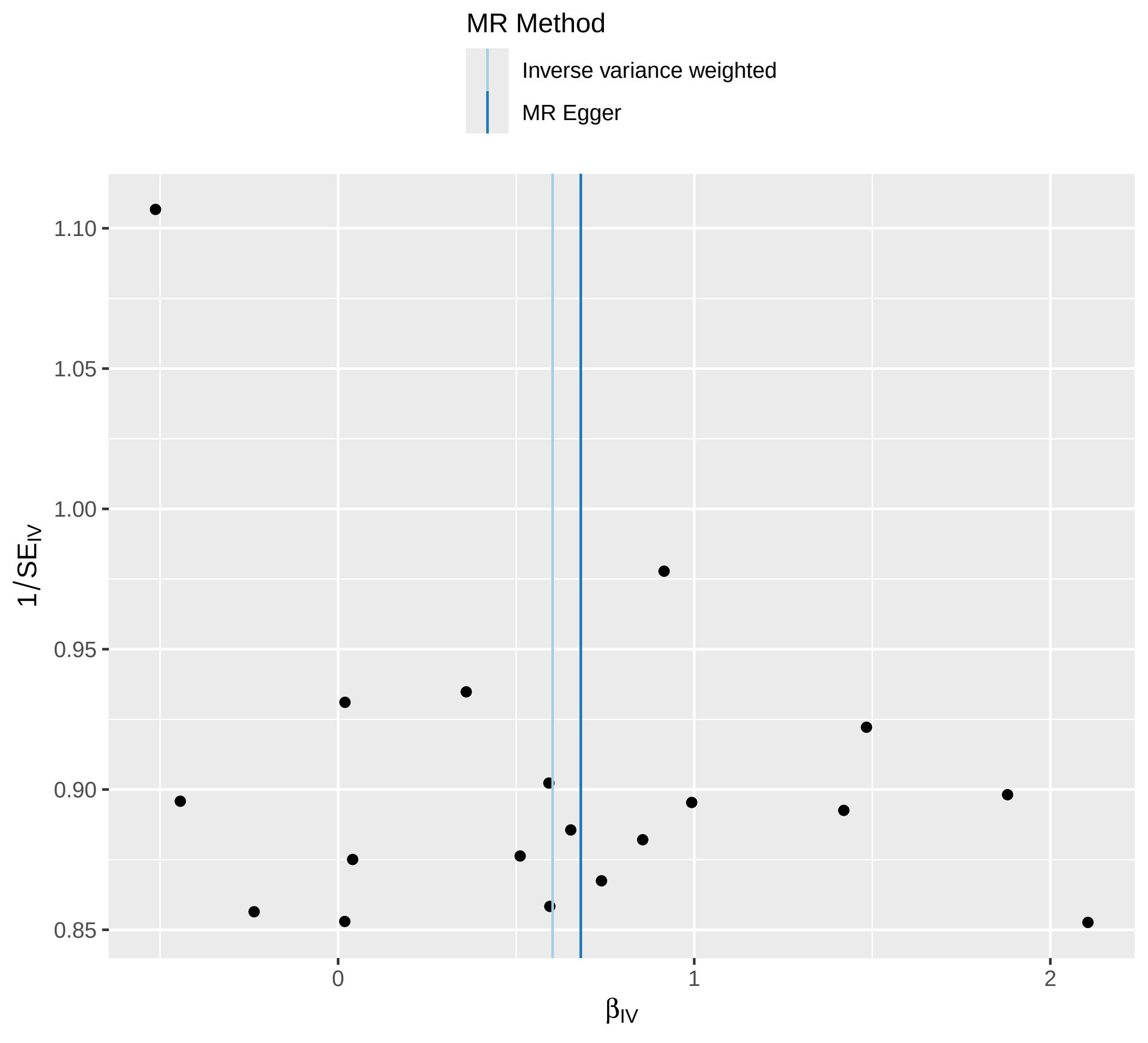
MR Method
Inverse variance weighted
MR Egger
1.10
1.05
1.00
0.95
0.90
0.85
$1/SE_{IV}$
0
1
2
β_{IV}

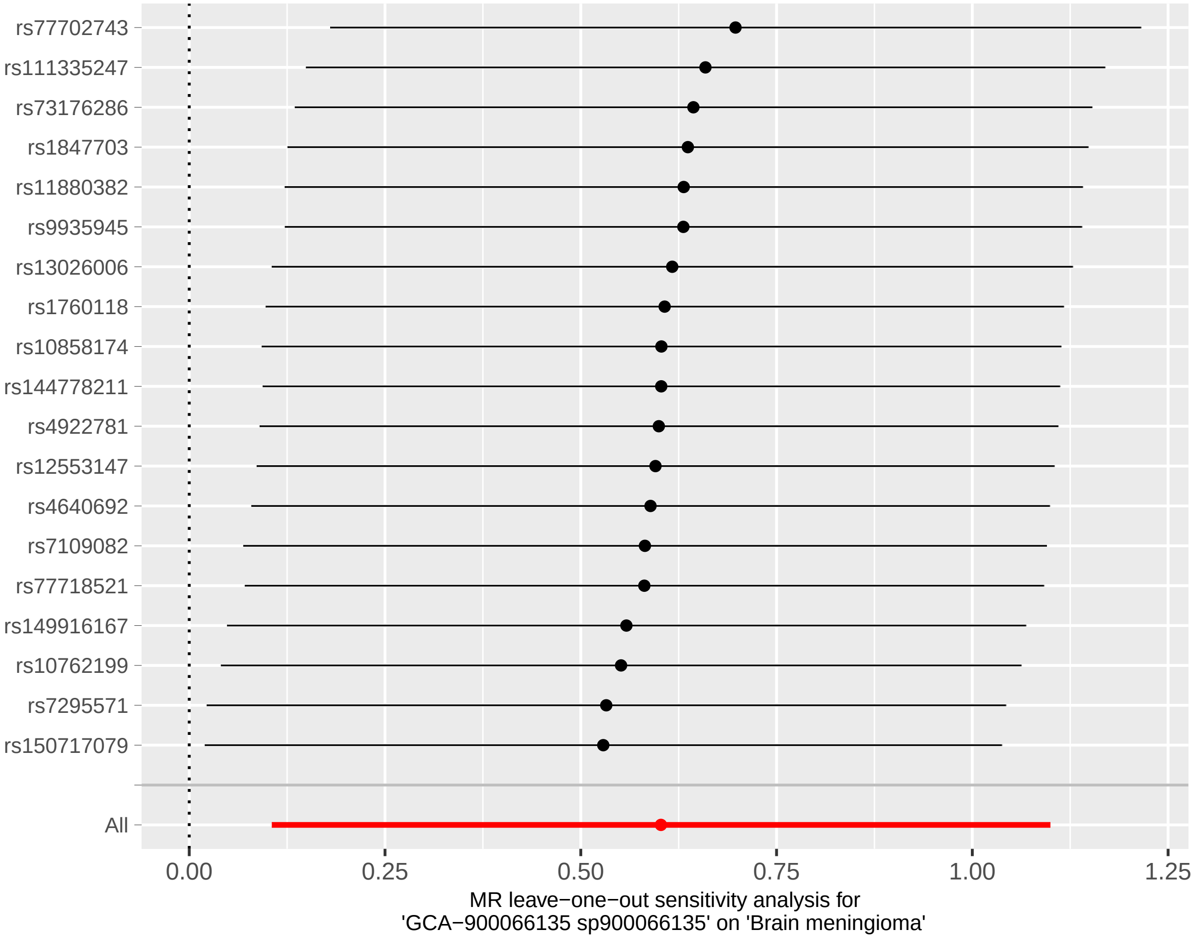
rs77702743
rs111335247
rs73176286
rs1847703
rs11880382
rs9935945
rs13026006
rs1760118
rs10858174
rs144778211
rs4922781
rs12553147
rs4640692
rs7109082
rs77718521
rs149916167
rs10762199
rs7295571
rs150717079
All
0.00
0.25
0.50
0.75
1.00
1.25
MR leave−one−out sensitivity analysis for
'GCA−900066135 sp900066135' on 'Brain meningioma'

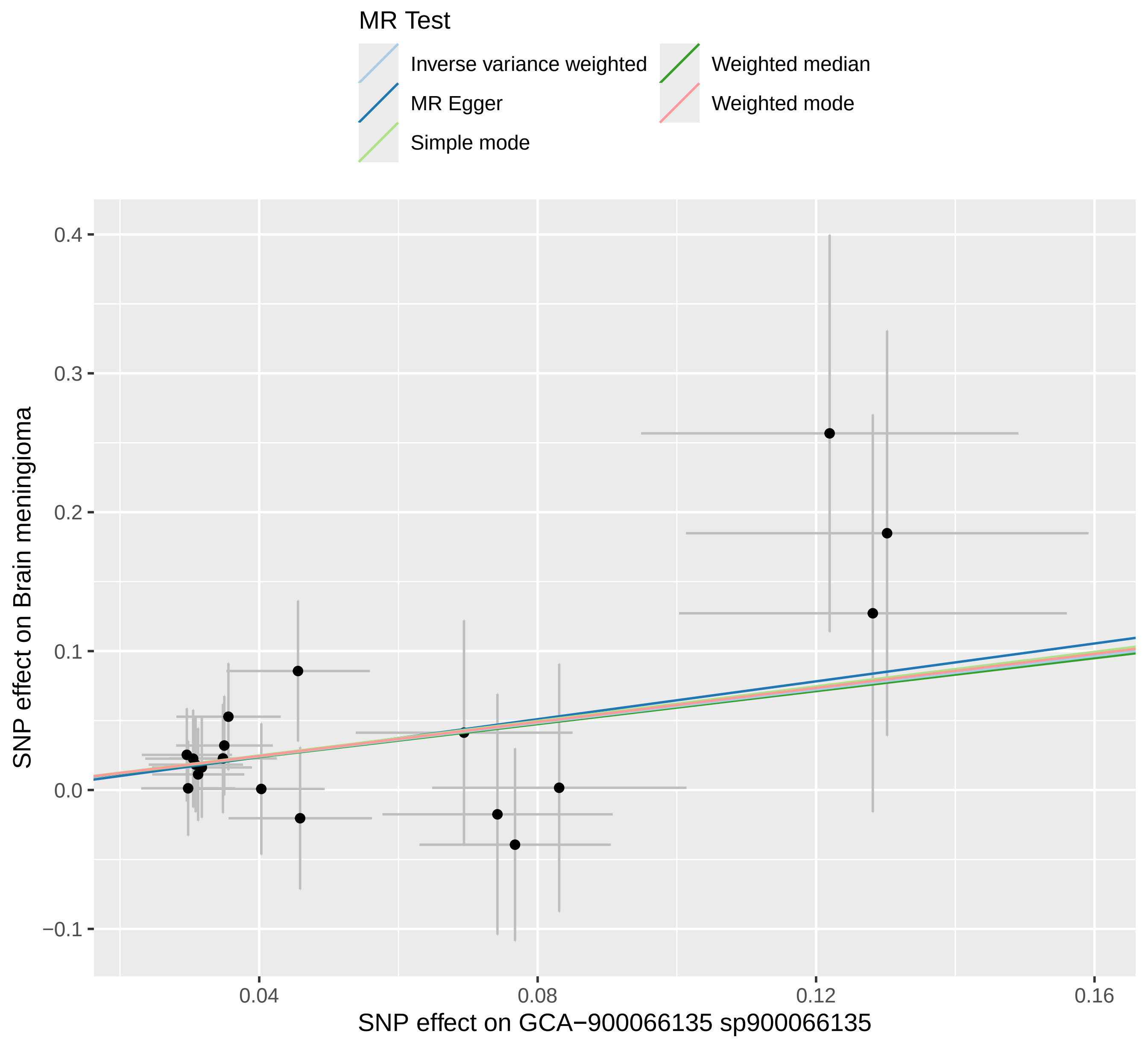

MR Test
Inverse variance weighted
MR Egger
Simple mode
Weighted median
Weighted mode
0.4
0.3
0.2
0.1
0.0
−0.1
0.04
0.08
0.12
0.16
SNP effect on Brain meningioma
SNP effect on GCA−900066135 sp900066135

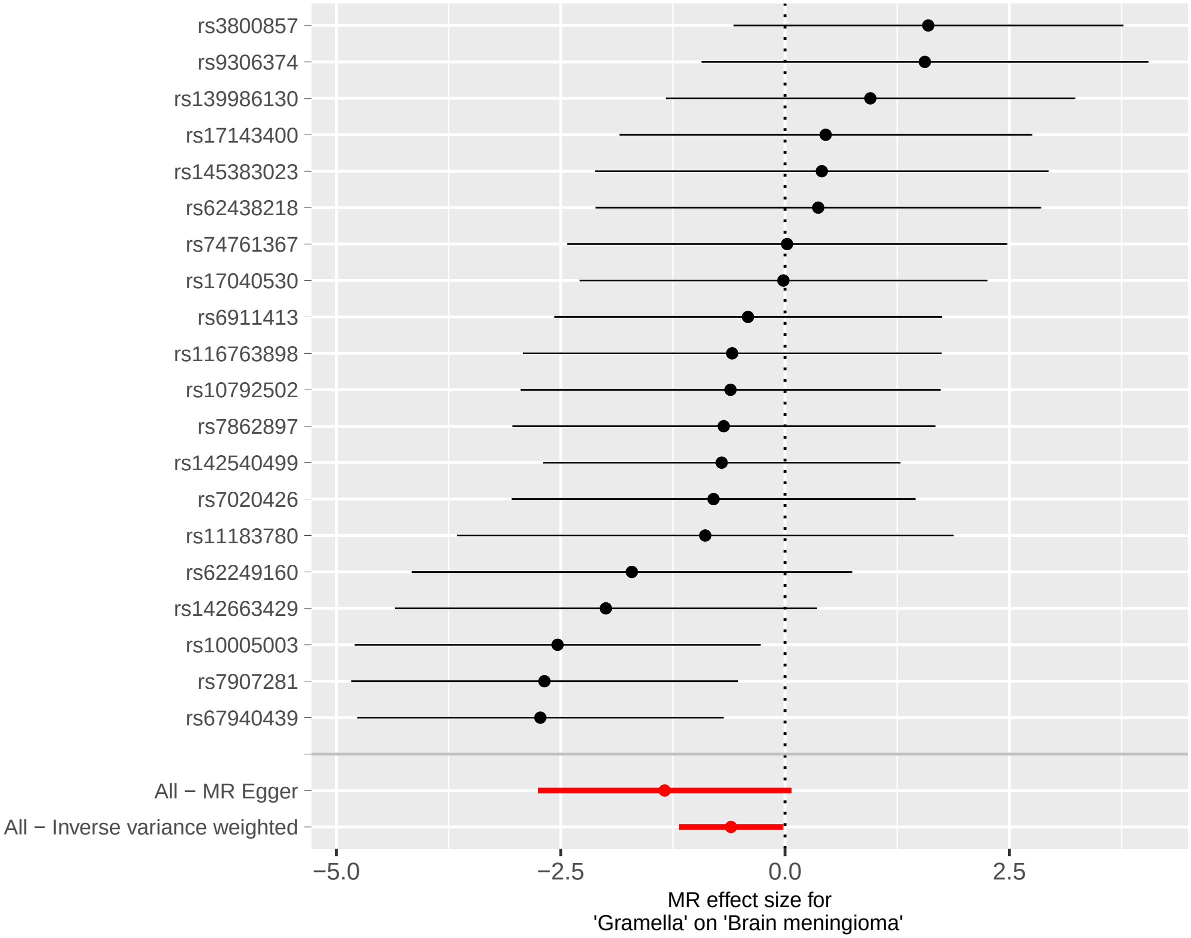

rs3800857
rs9306374
rs139986130
rs17143400
rs145383023
rs62438218
rs74761367
rs17040530
rs6911413
rs116763898
rs10792502
rs7862897
rs142540499
rs7020426
rs11183780
rs62249160
rs142663429
rs10005003
rs7907281
rs67940439
All − MR Egger
All − Inverse variance weighted
−5.0
−2.5
0.0
2.5
MR effect size for
'Gramella' on 'Brain meningioma'

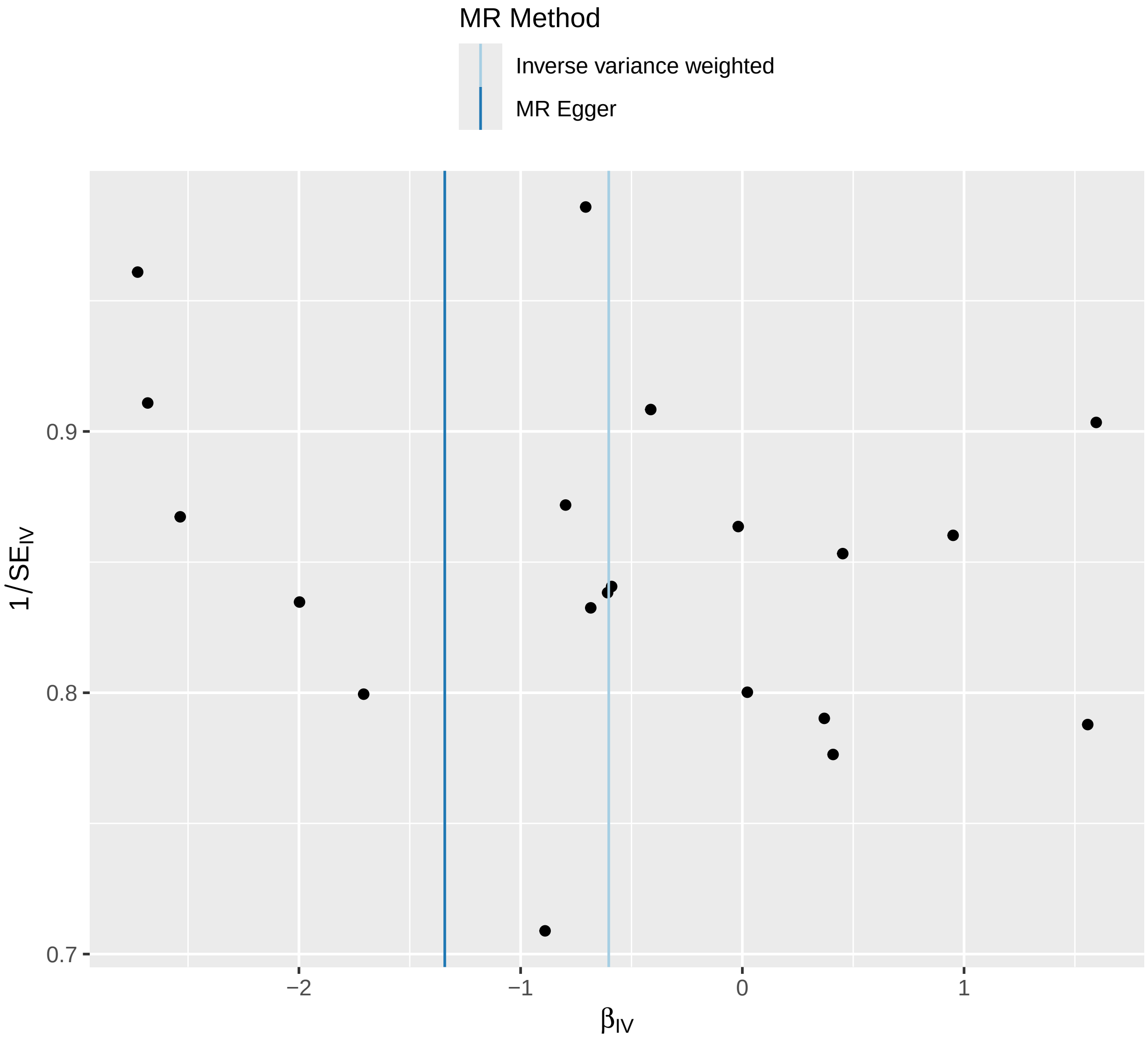

MR Method
Inverse variance weighted
MR Egger
0.9
0.8
0.7
−2
−1
0
1
1/SE_IV
β_IV

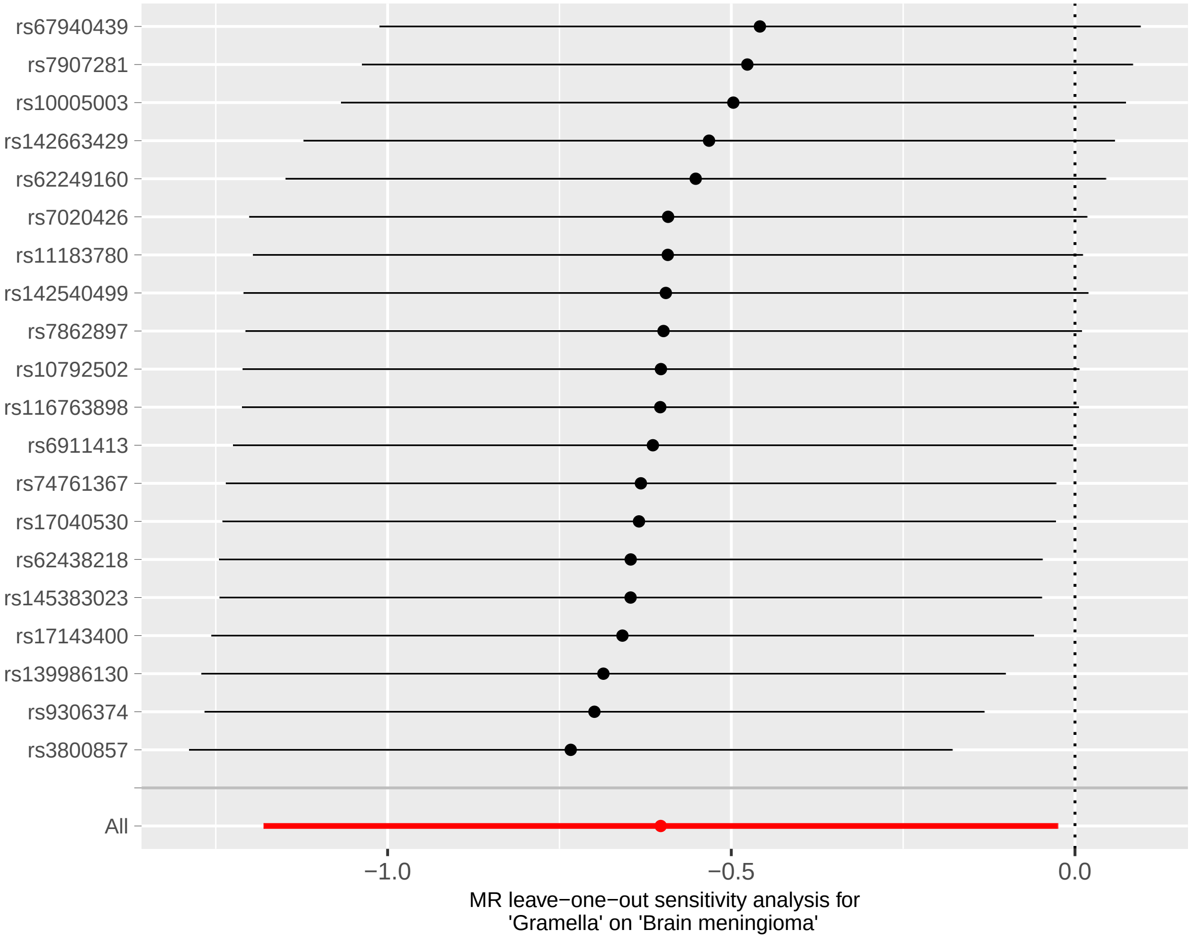
rs67940439
rs7907281
rs10005003
rs142663429
rs62249160
rs7020426
rs11183780
rs142540499
rs7862897
rs10792502
rs116763898
rs6911413
rs74761367
rs17040530
rs62438218
rs145383023
rs17143400
rs139986130
rs9306374
rs3800857
All
−1.0
−0.5
0.0
MR leave−one−out sensitivity analysis for
'Gramella' on 'Brain meningioma'

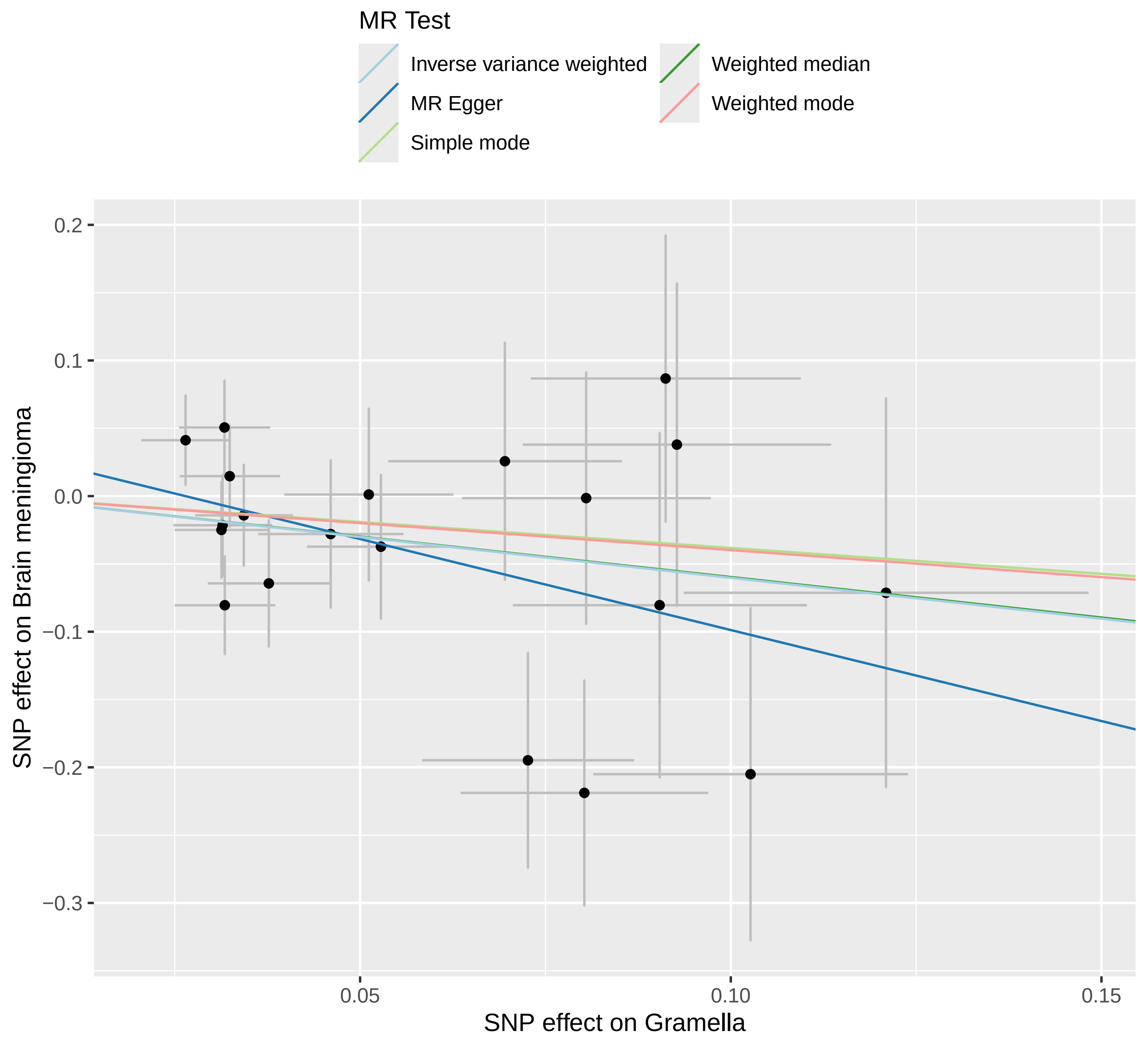
MR Test
Inverse variance weighted
MR Egger
Simple mode
Weighted median
Weighted mode
SNP effect on Brain meningioma
0.2
0.1
0.0
−0.1
−0.2
−0.3
0.05
0.10
0.15
SNP effect on Gramella

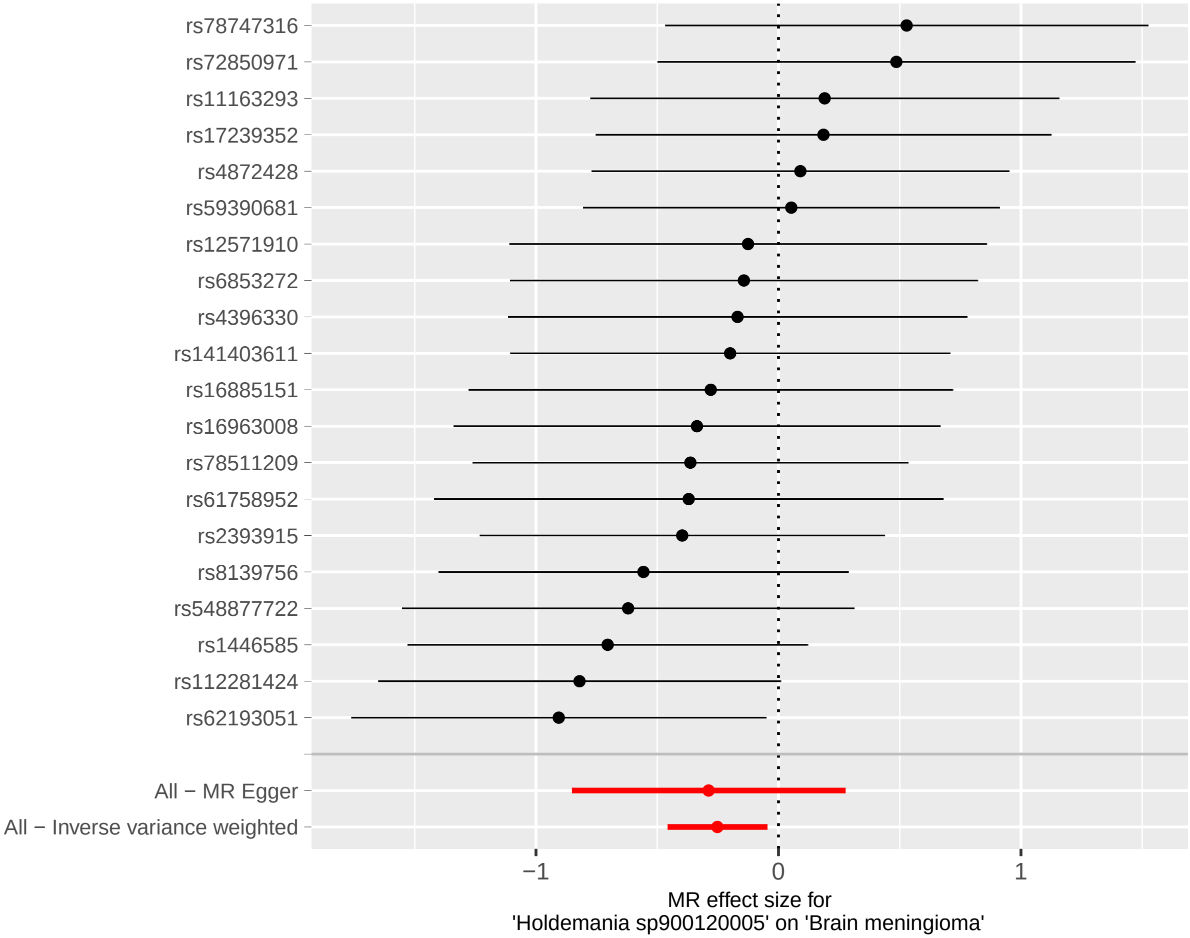
rs78747316
rs72850971
rs11163293
rs17239352
rs4872428
rs59390681
rs12571910
rs6853272
rs4396330
rs141403611
rs16885151
rs16963008
rs78511209
rs61758952
rs2393915
rs8139756
rs548877722
rs1446585
rs112281424
rs62193051
All − MR Egger
All − Inverse variance weighted
−1
0
1
MR effect size for
'Holdemania sp900120005' on 'Brain meningioma'

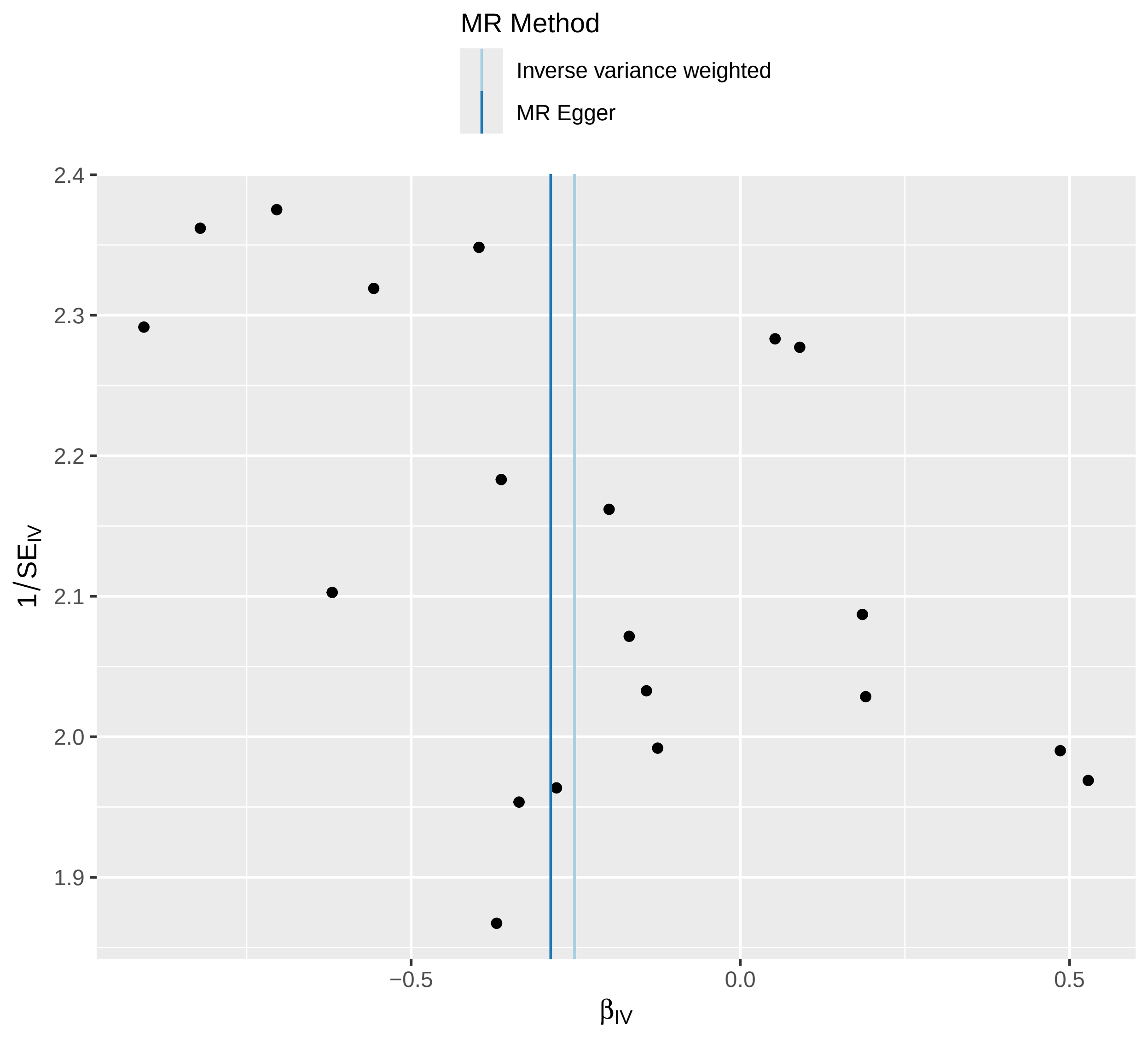
MR Method
Inverse variance weighted
MR Egger
2.4
2.3
2.2
2.1
2.0
1.9
1/SE$_{IV}$
−0.5
0.0
0.5
β$_{IV}$

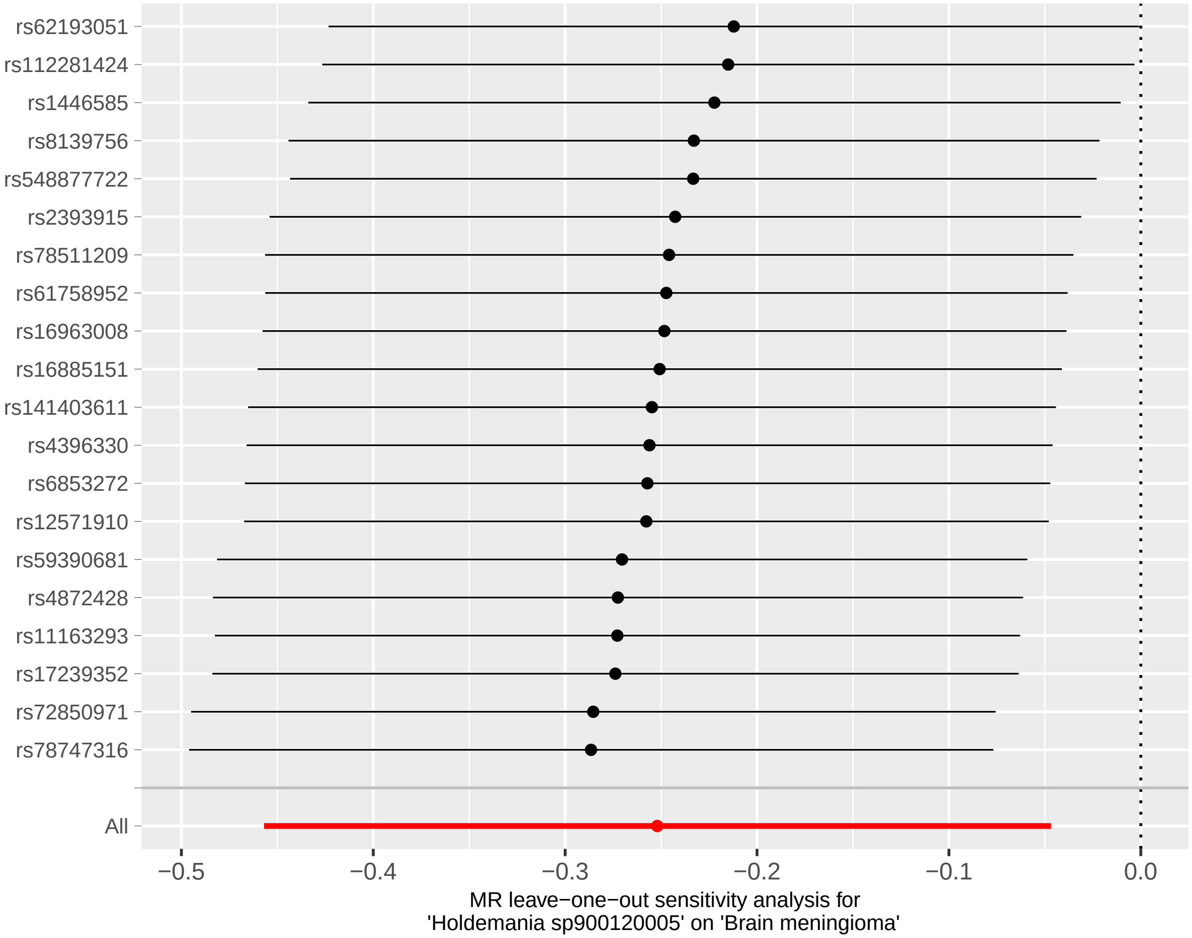
rs62193051
rs112281424
rs1446585
rs8139756
rs548877722
rs2393915
rs78511209
rs61758952
rs16963008
rs16885151
rs141403611
rs4396330
rs6853272
rs12571910
rs59390681
rs4872428
rs11163293
rs17239352
rs72850971
rs78747316
All
−0.5
−0.4
−0.3
−0.2
−0.1
0.0
MR leave−one−out sensitivity analysis for
'Holdemania sp900120005' on 'Brain meningioma'

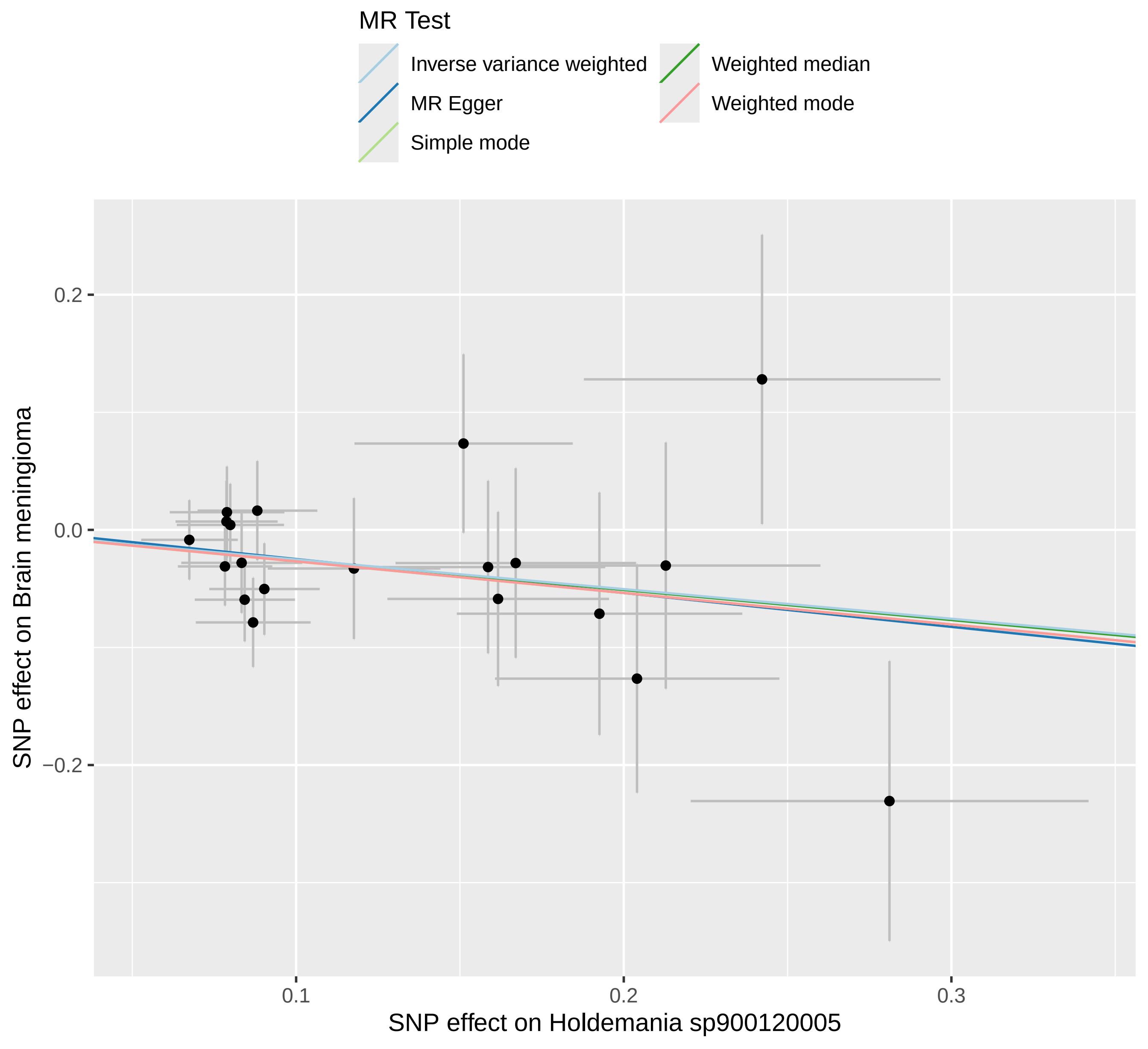
MR Test
Inverse variance weighted
MR Egger
Simple mode
Weighted median
Weighted mode
0.2
0.0
−0.2
0.1
0.2
0.3
SNP effect on Brain meningioma
SNP effect on Holdemania sp900120005

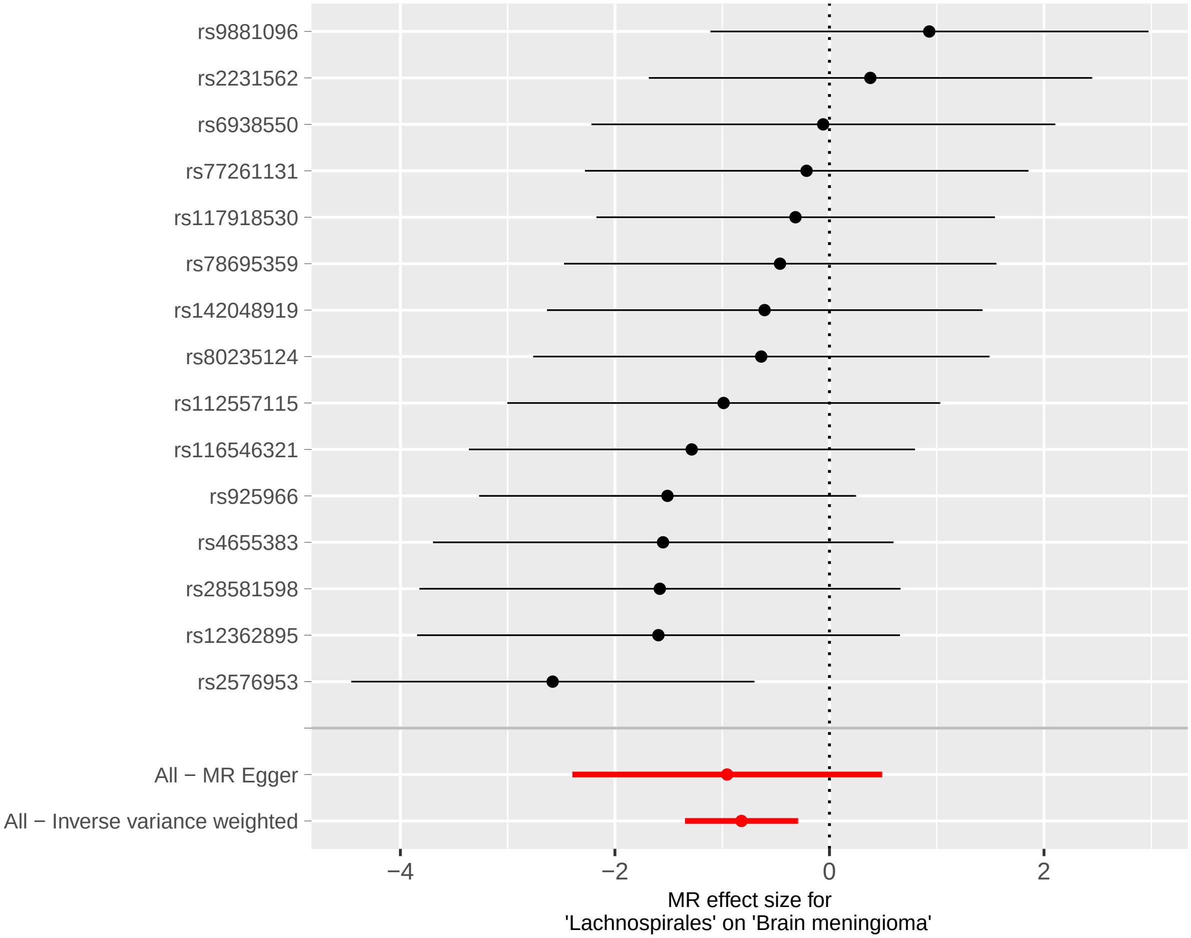
rs9881096
rs2231562
rs6938550
rs77261131
rs117918530
rs78695359
rs142048919
rs80235124
rs112557115
rs116546321
rs925966
rs4655383
rs28581598
rs12362895
rs2576953
All − MR Egger
All − Inverse variance weighted
−4
−2
0
2
MR effect size for
'Lachnospirales' on 'Brain meningioma'

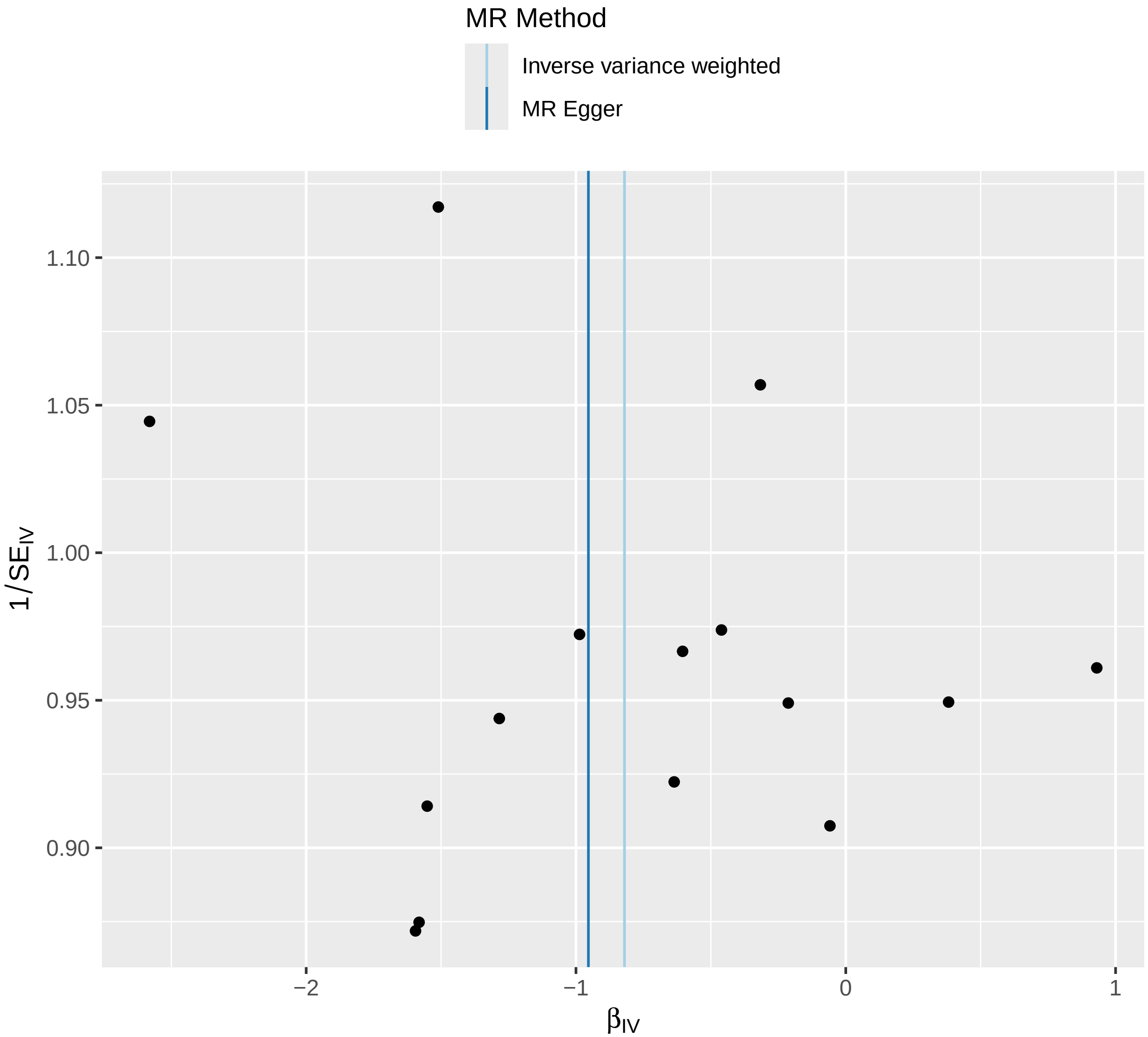

MR Method
Inverse variance weighted
MR Egger
1.10
1.05
1.00
0.95
0.90
$1/SE_{IV}$
−2
−1
0
1
β_{IV}

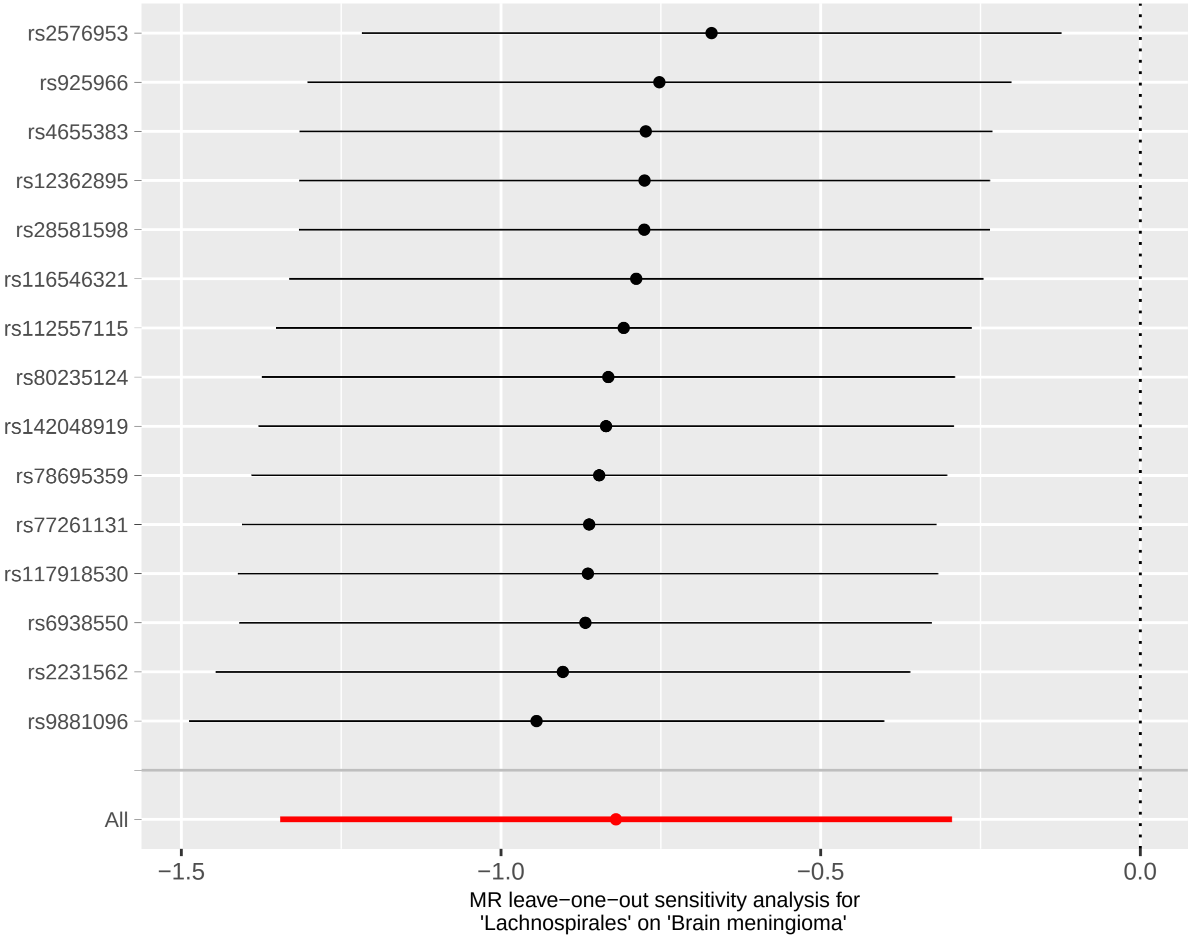
rs2576953
rs925966
rs4655383
rs12362895
rs28581598
rs116546321
rs112557115
rs80235124
rs142048919
rs78695359
rs77261131
rs117918530
rs6938550
rs2231562
rs9881096
All
−1.5
−1.0
−0.5
0.0
MR leave−one−out sensitivity analysis for
'Lachnospirales' on 'Brain meningioma'

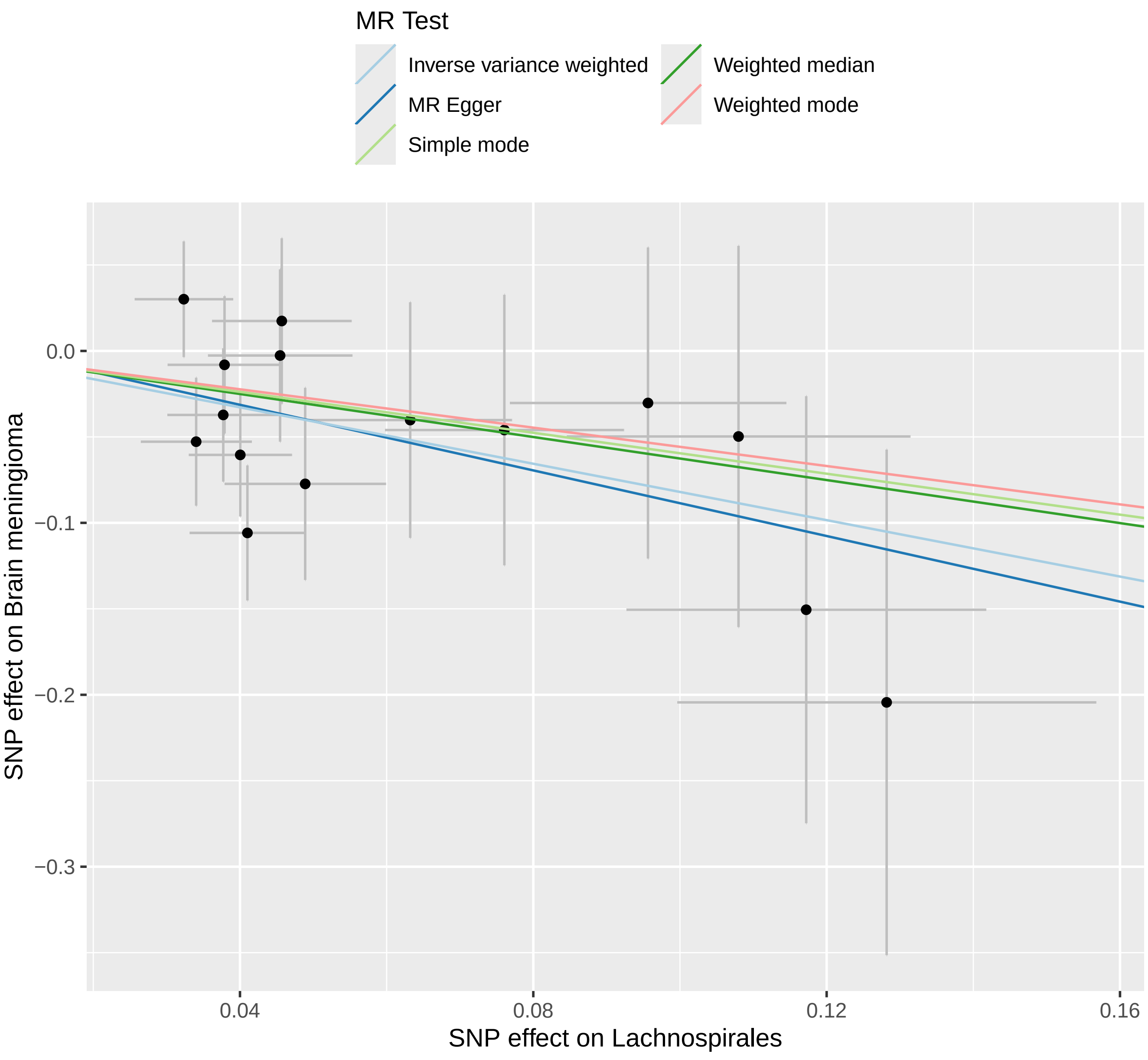

MR Test
Inverse variance weighted
MR Egger
Simple mode
Weighted median
Weighted mode
SNP effect on Brain meningioma
0.0
−0.1
−0.2
−0.3
0.04
0.08
0.12
0.16
SNP effect on Lachnospirales

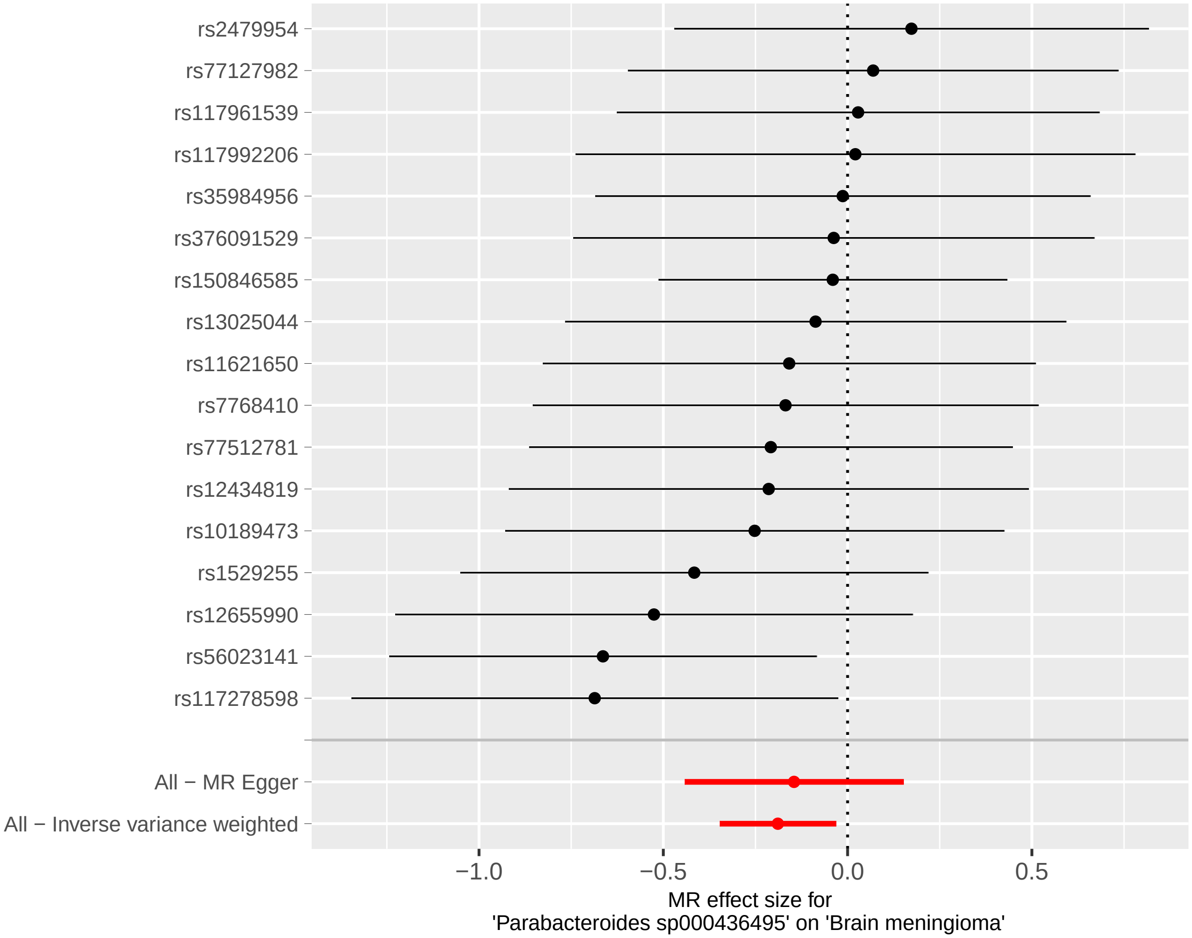

rs2479954
rs77127982
rs117961539
rs117992206
rs35984956
rs376091529
rs150846585
rs13025044
rs11621650
rs7768410
rs77512781
rs12434819
rs10189473
rs1529255
rs12655990
rs56023141
rs117278598
All − MR Egger
All − Inverse variance weighted
−1.0
−0.5
0.0
0.5
MR effect size for
'Parabacteroides sp000436495' on 'Brain meningioma'

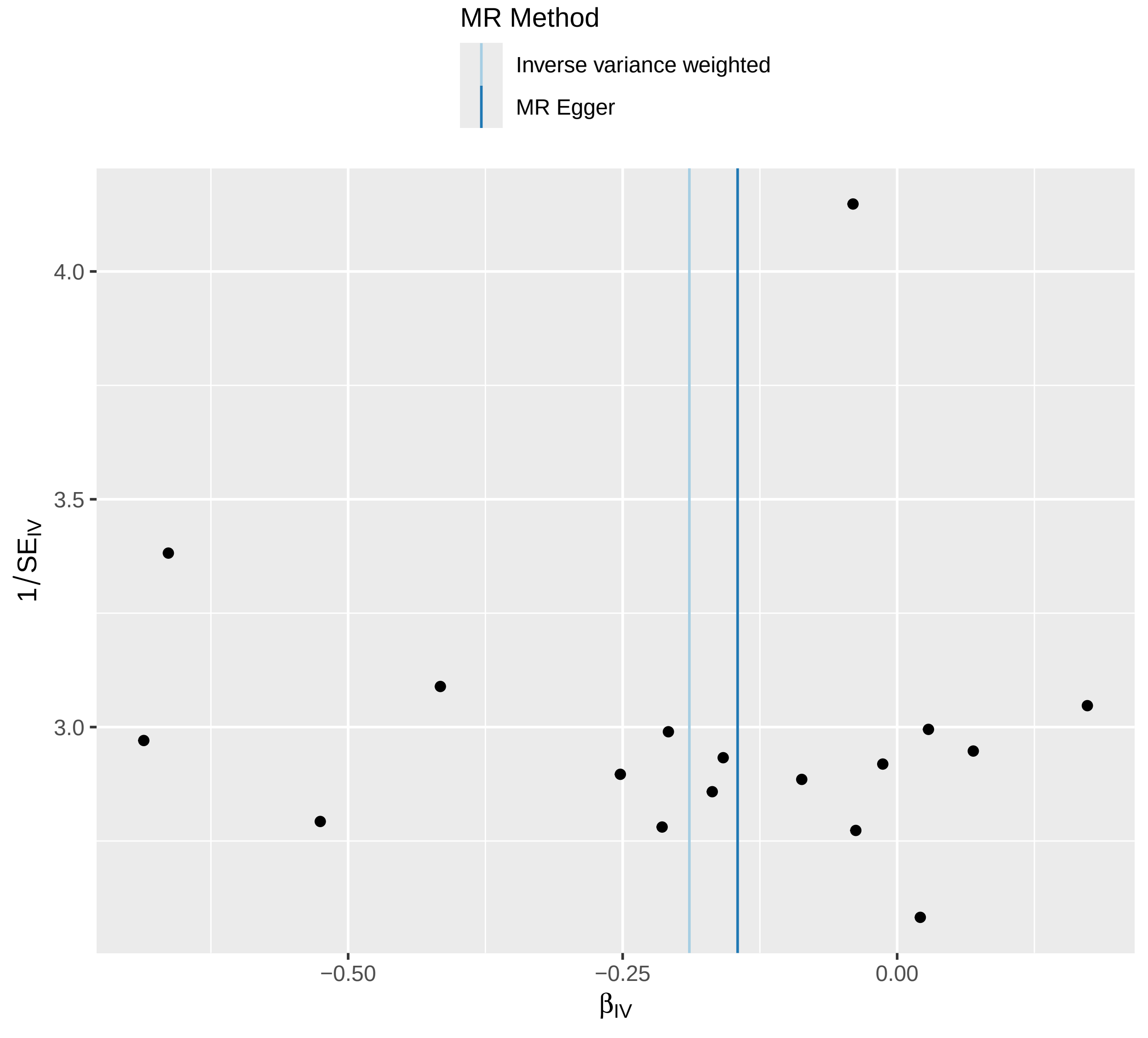
MR Method
Inverse variance weighted
MR Egger
4.0
3.5
3.0
1/SE_IV
−0.50
−0.25
0.00
β_IV

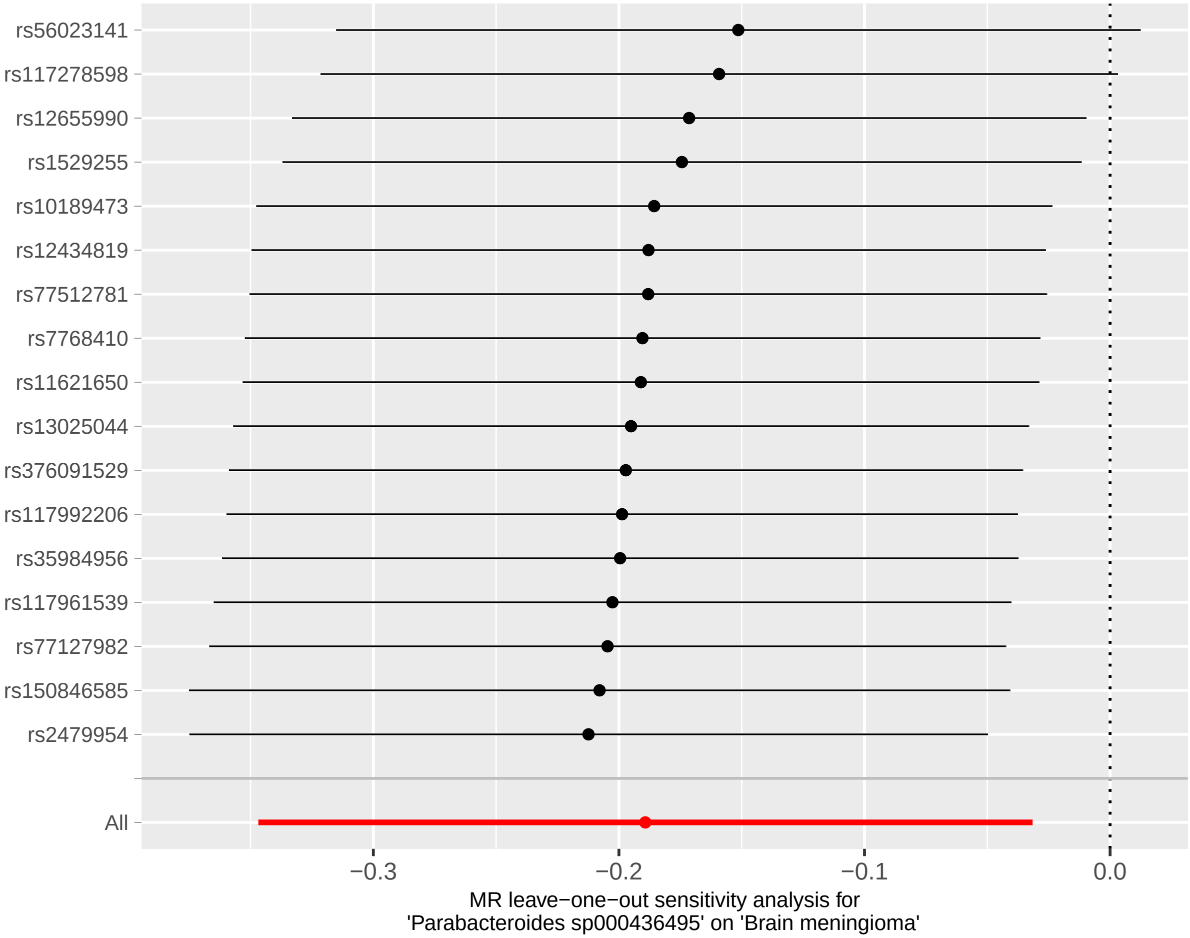

rs56023141
rs117278598
rs12655990
rs1529255
rs10189473
rs12434819
rs77512781
rs7768410
rs11621650
rs13025044
rs376091529
rs117992206
rs35984956
rs117961539
rs77127982
rs150846585
rs2479954
All
−0.3
−0.2
−0.1
0.0
MR leave−one−out sensitivity analysis for
'Parabacteroides sp000436495' on 'Brain meningioma'

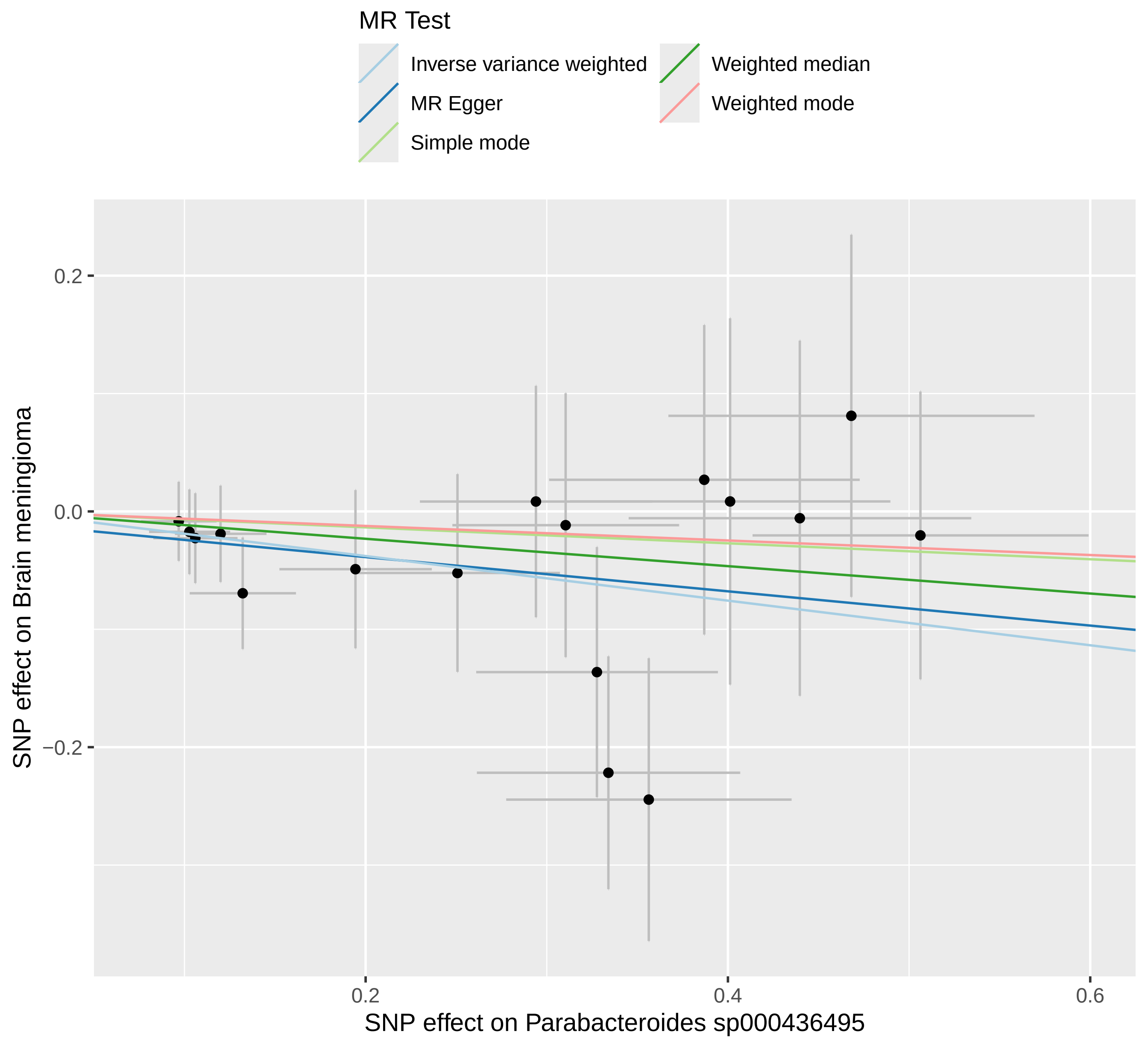
MR Test
Inverse variance weighted
MR Egger
Simple mode
Weighted median
Weighted mode
0.2
0.0
−0.2
SNP effect on Brain meningioma
0.2
0.4
0.6
SNP effect on Parabacteroides sp000436495

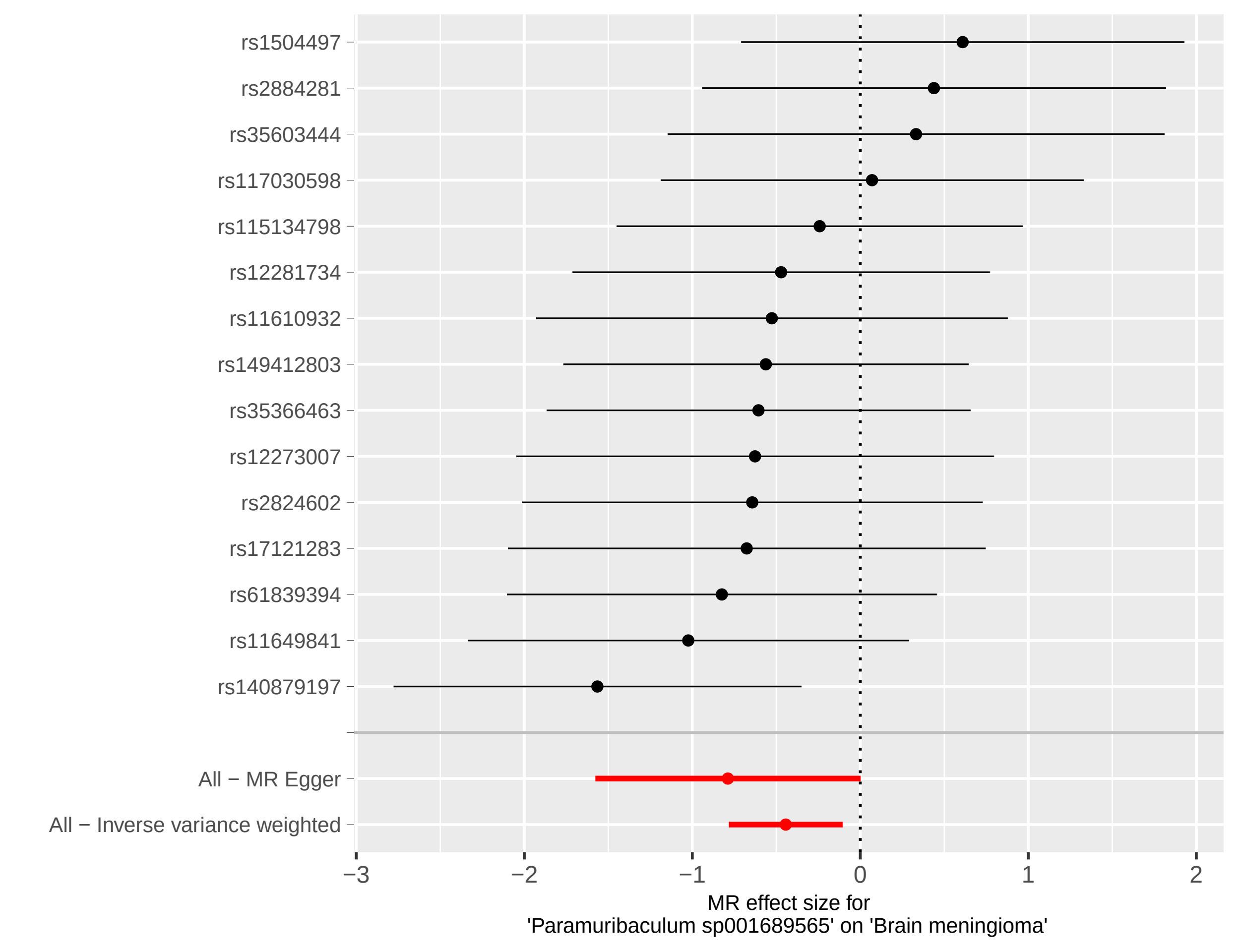

rs1504497
rs2884281
rs35603444
rs117030598
rs115134798
rs12281734
rs11610932
rs149412803
rs35366463
rs12273007
rs2824602
rs17121283
rs61839394
rs11649841
rs140879197
All − MR Egger
All − Inverse variance weighted
−3
−2
−1
0
1
2
MR effect size for
'Paramuribaculum sp001689565' on 'Brain meningioma'

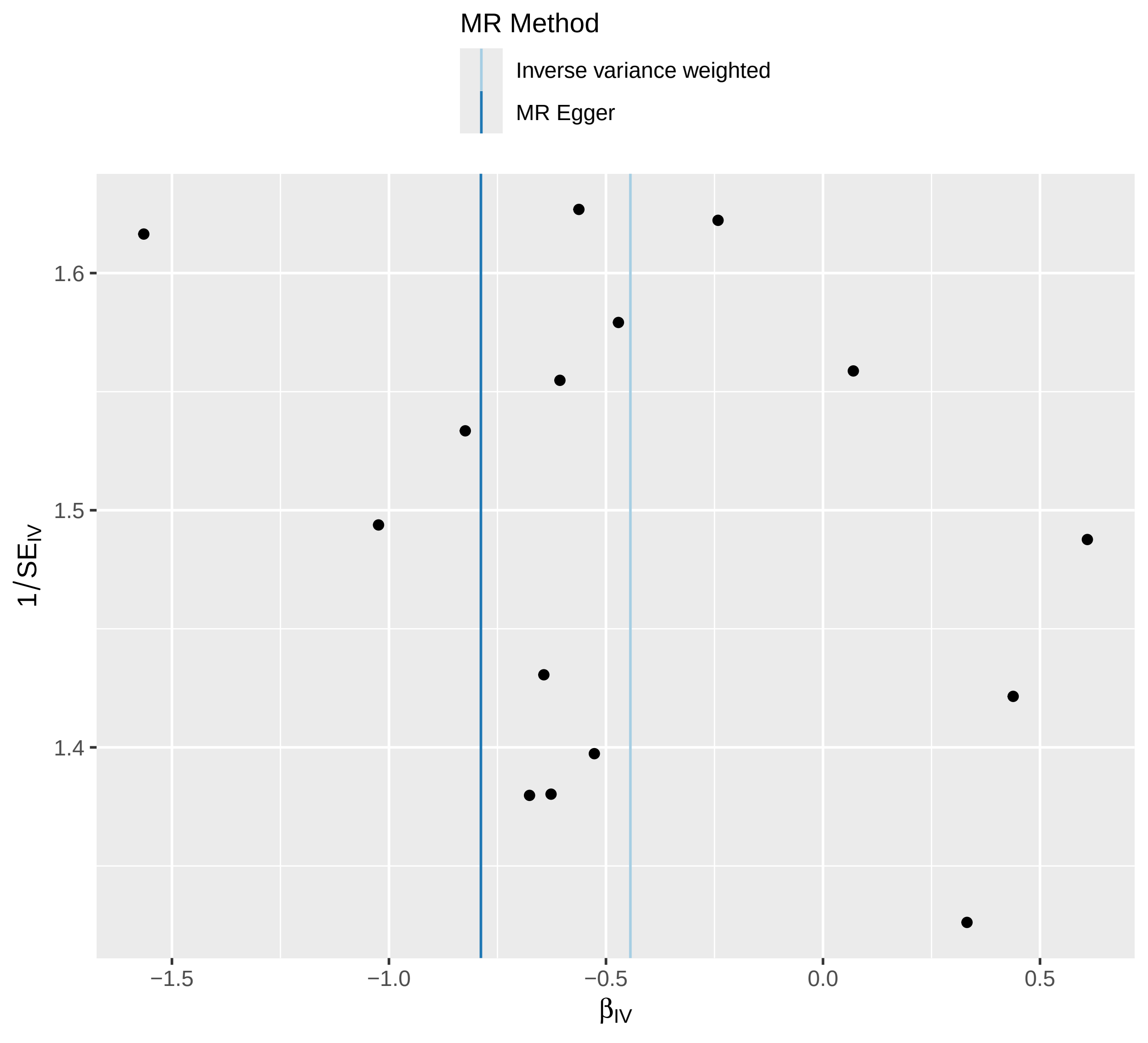
MR Method
Inverse variance weighted
MR Egger
1.6
1.5
1.4
1/SE_IV
−1.5
−1.0
−0.5
0.0
0.5
β_IV

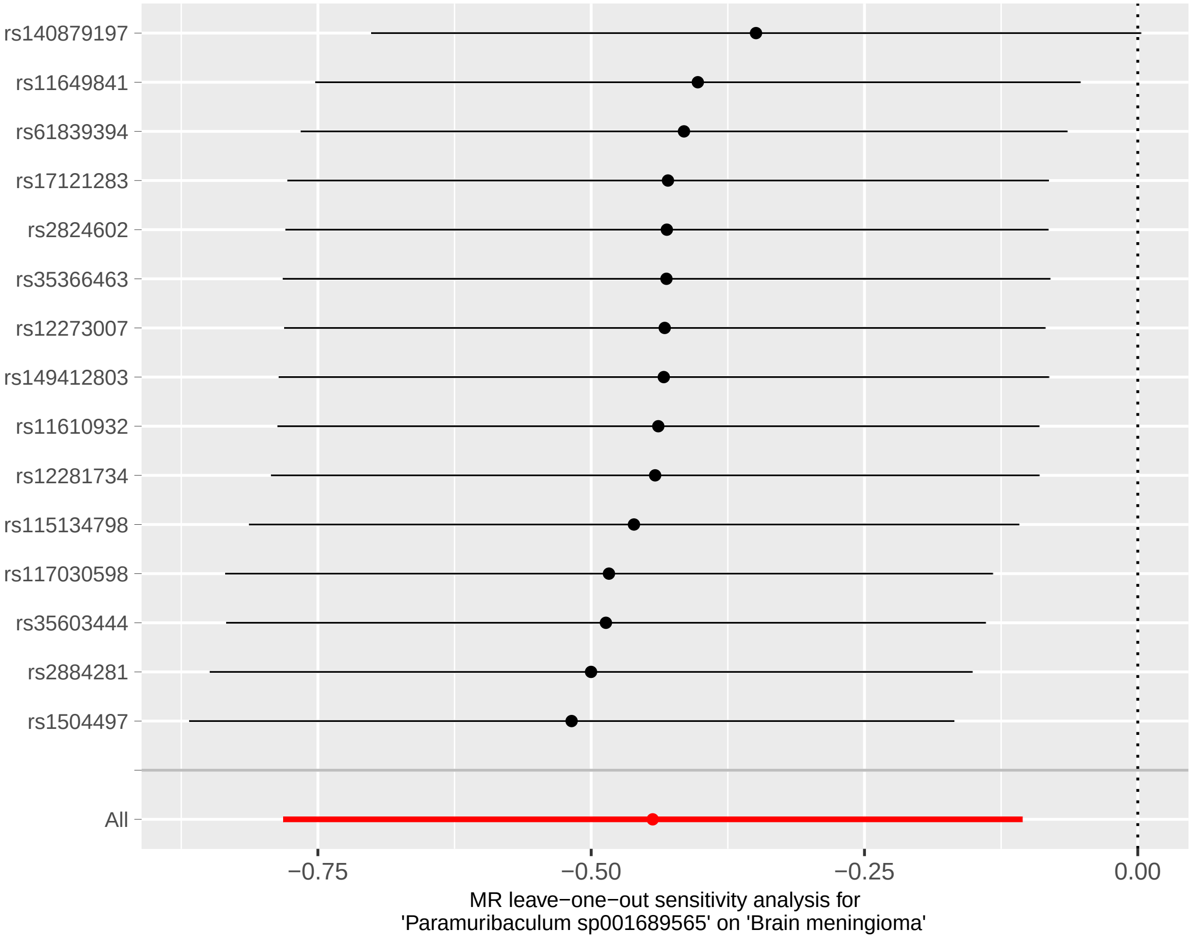
rs140879197
rs11649841
rs61839394
rs17121283
rs2824602
rs35366463
rs12273007
rs149412803
rs11610932
rs12281734
rs115134798
rs117030598
rs35603444
rs2884281
rs1504497
All
−0.75
−0.50
−0.25
0.00
MR leave−one−out sensitivity analysis for
'Paramuribaculum sp001689565' on 'Brain meningioma'

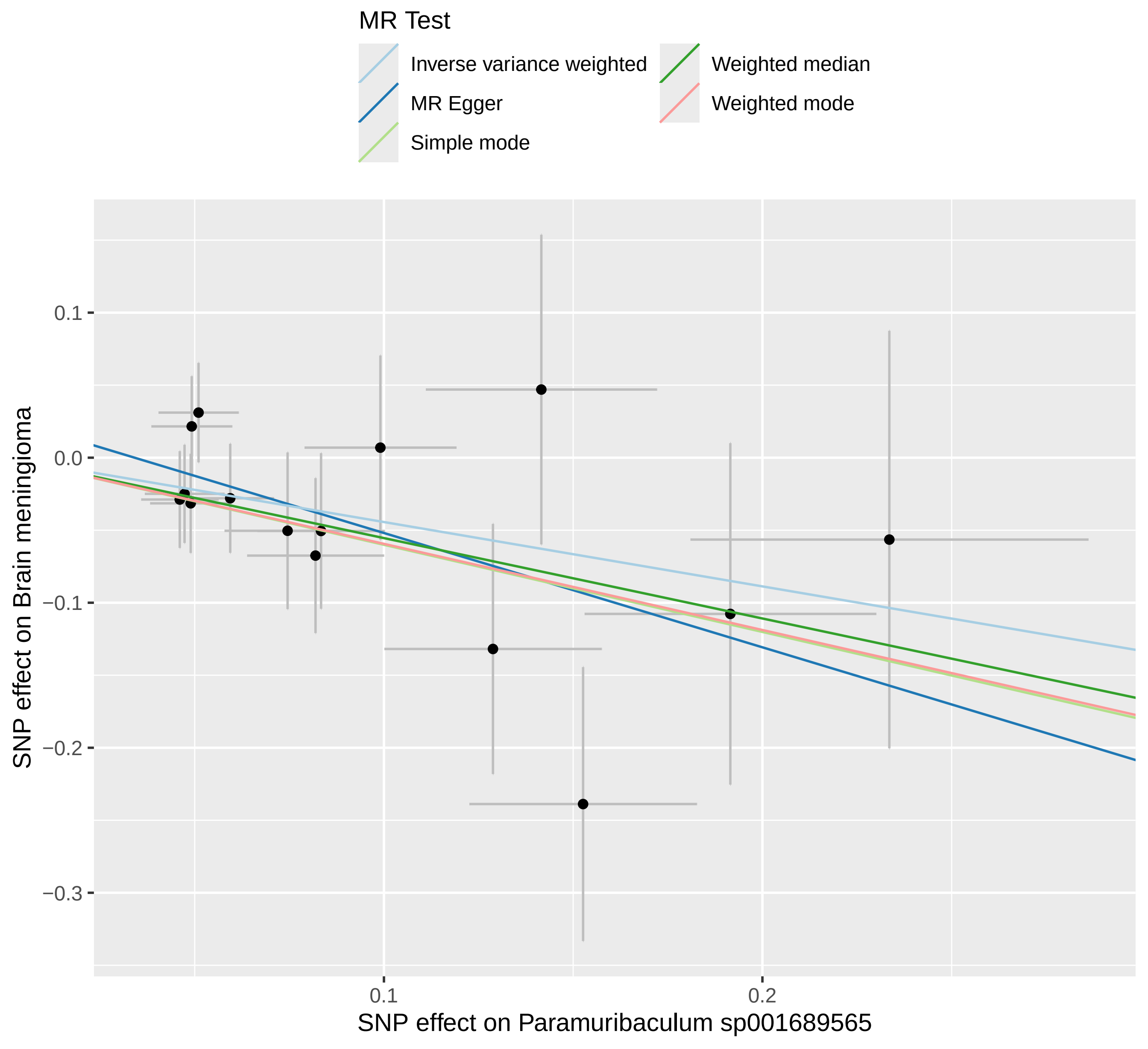
MR Test
Inverse variance weighted
MR Egger
Simple mode
Weighted median
Weighted mode
0.1
0.0
−0.1
−0.2
−0.3
0.1
0.2
SNP effect on Brain meningioma
SNP effect on Paramuribaculum sp001689565

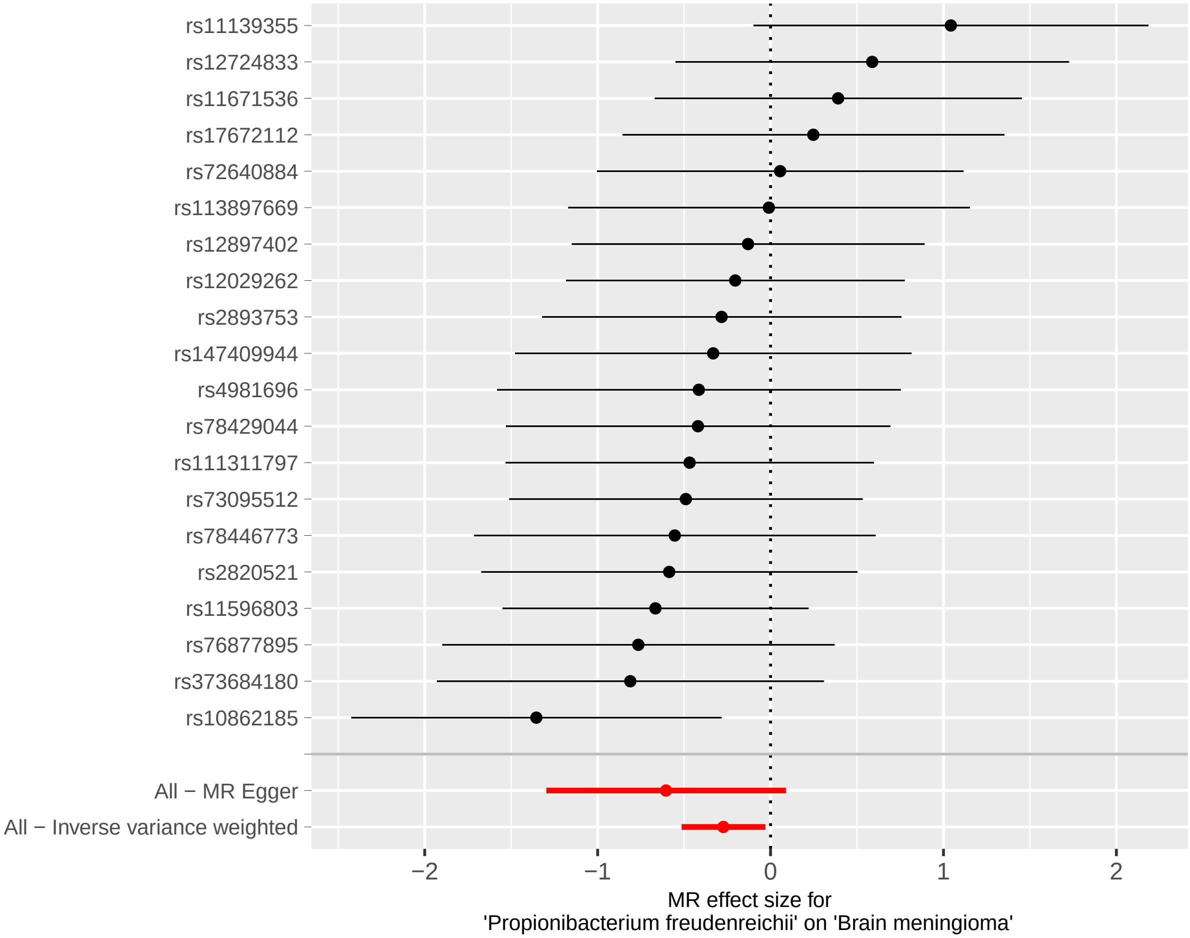

rs11139355
rs12724833
rs11671536
rs17672112
rs72640884
rs113897669
rs12897402
rs12029262
rs2893753
rs147409944
rs4981696
rs78429044
rs111311797
rs73095512
rs78446773
rs2820521
rs11596803
rs76877895
rs373684180
rs10862185
All − MR Egger
All − Inverse variance weighted
−2
−1
0
1
2
MR effect size for
'Propionibacterium freudenreichii' on 'Brain meningioma'

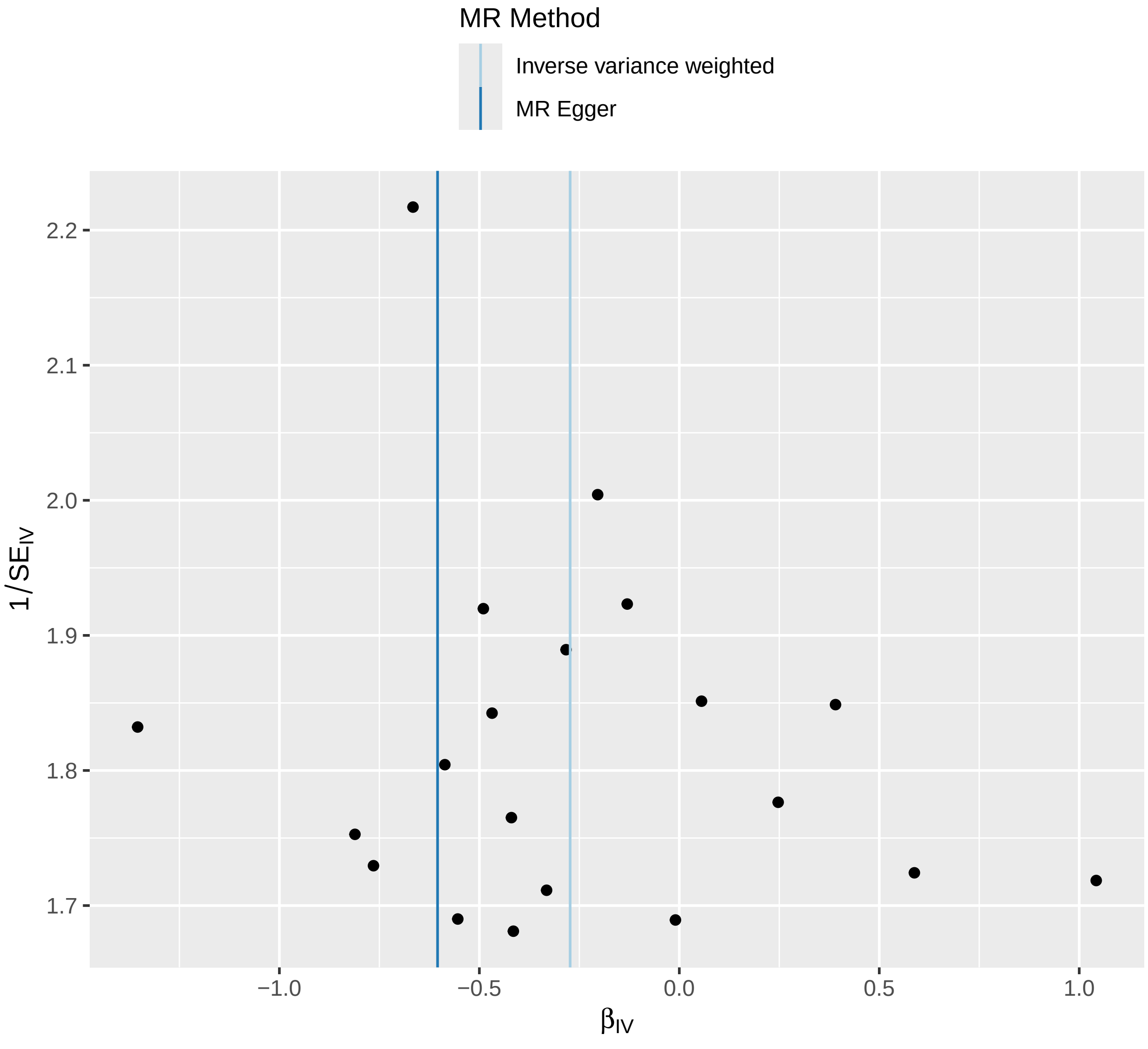
MR Method
Inverse variance weighted
MR Egger
1/SE_IV
2.2
2.1
2.0
1.9
1.8
1.7
−1.0
−0.5
0.0
0.5
1.0
β_IV

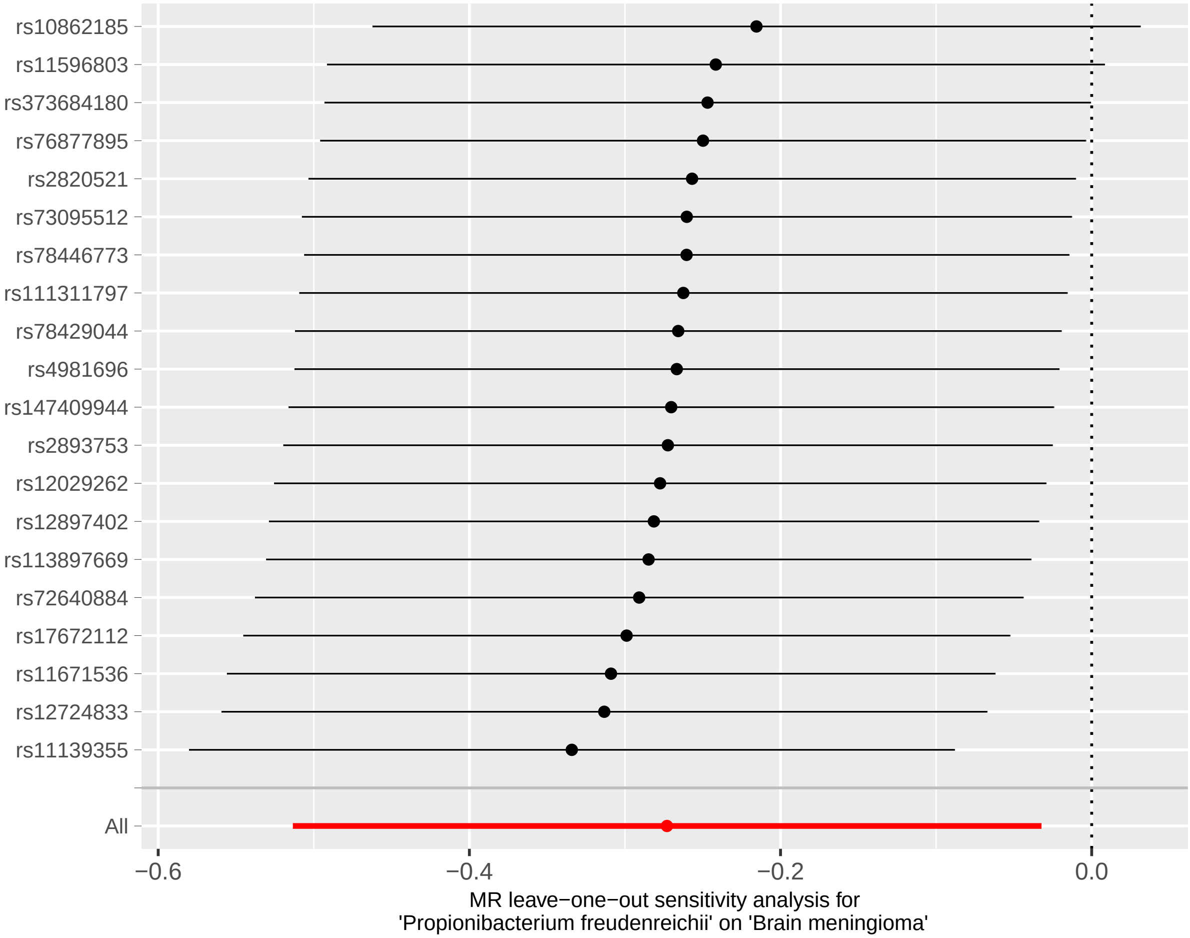
rs10862185
rs11596803
rs373684180
rs76877895
rs2820521
rs73095512
rs78446773
rs111311797
rs78429044
rs4981696
rs147409944
rs2893753
rs12029262
rs12897402
rs113897669
rs72640884
rs17672112
rs11671536
rs12724833
rs11139355
All
−0.6
−0.4
−0.2
0.0
MR leave−one−out sensitivity analysis for
'Propionibacterium freudenreichii' on 'Brain meningioma'

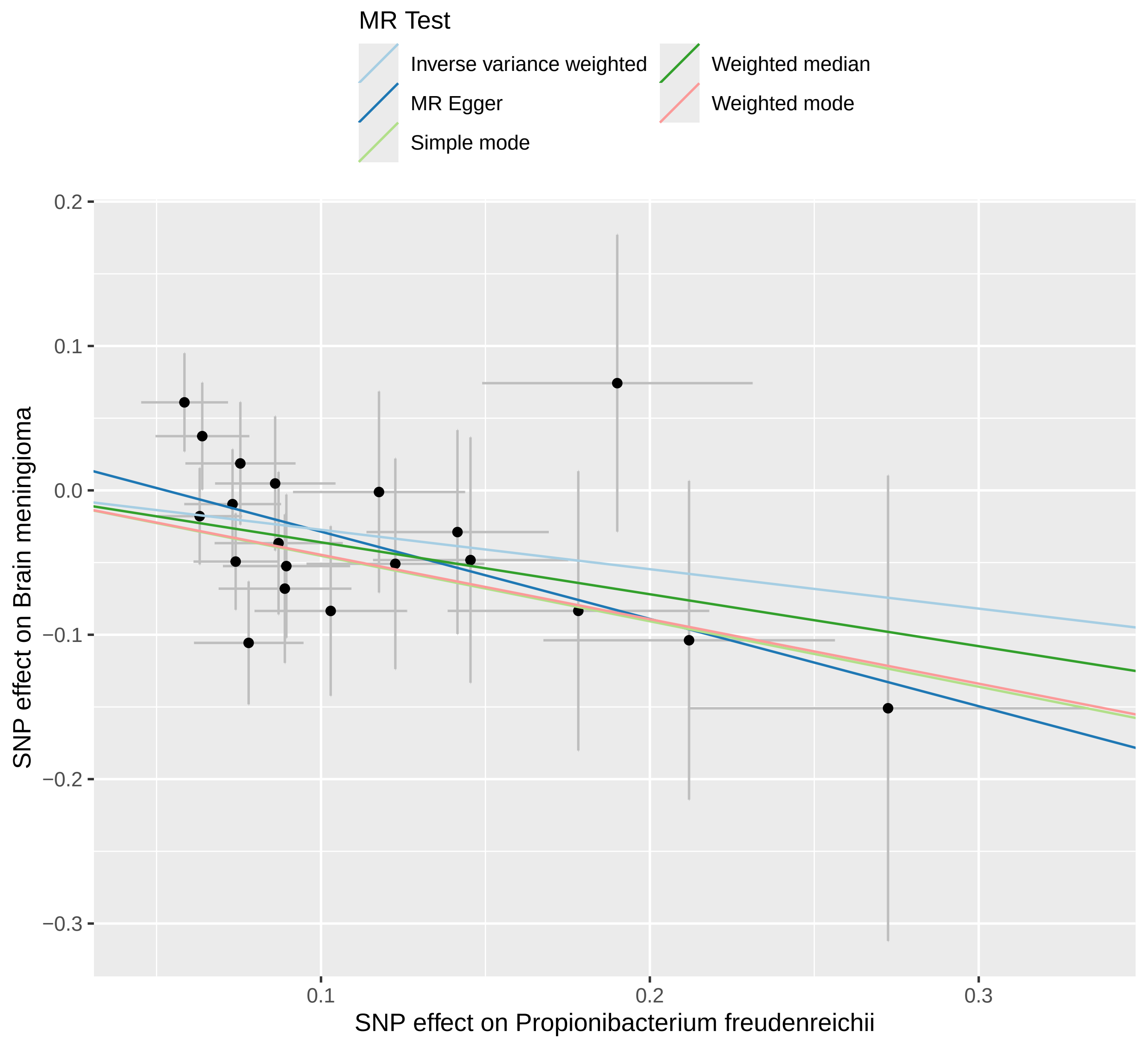

MR Test
Inverse variance weighted
MR Egger
Simple mode
Weighted median
Weighted mode
0.2
0.1
0.0
−0.1
−0.2
−0.3
0.1
0.2
0.3
SNP effect on Brain meningioma
SNP effect on Propionibacterium freudenreichii

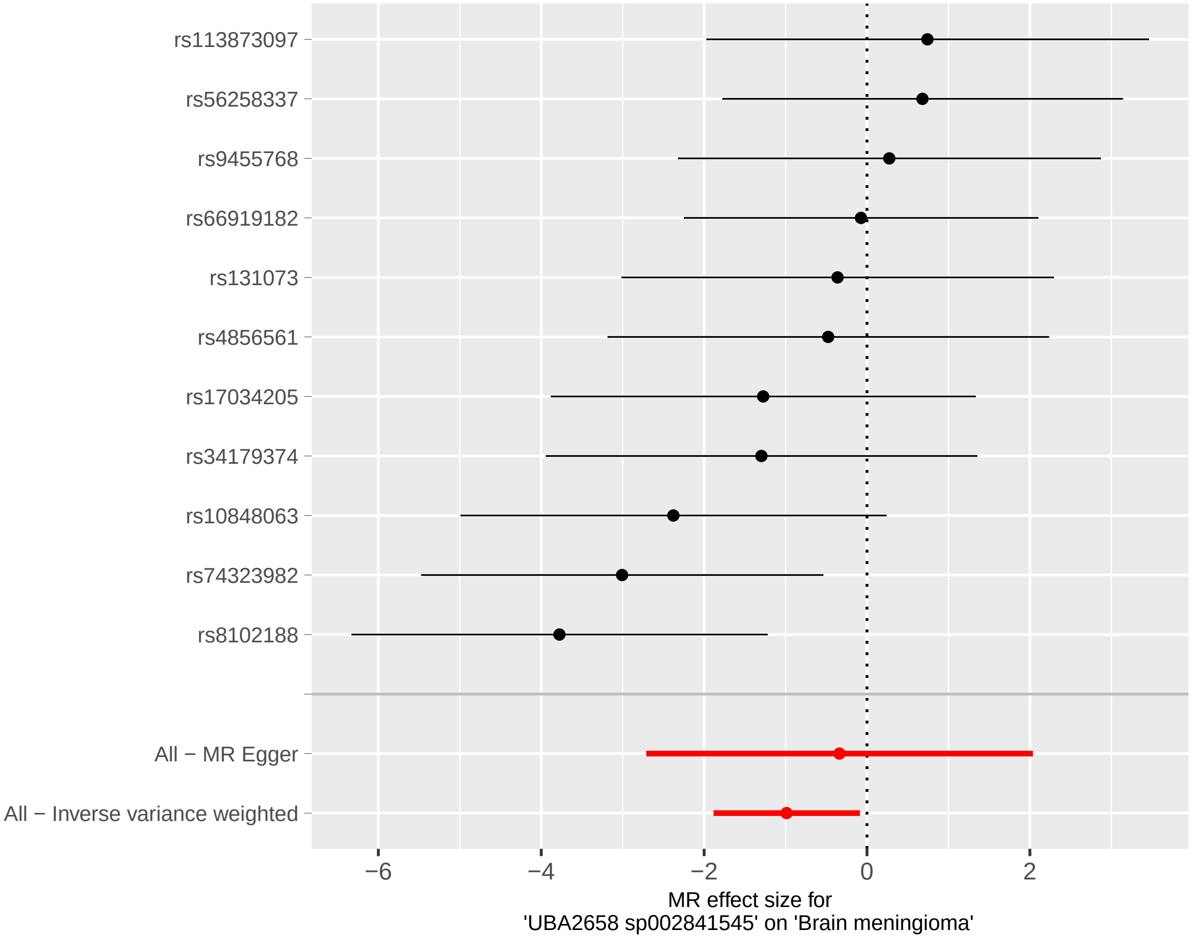
rs113873097
rs56258337
rs9455768
rs66919182
rs131073
rs4856561
rs17034205
rs34179374
rs10848063
rs74323982
rs8102188
All − MR Egger
All − Inverse variance weighted
−6
−4
−2
0
2
MR effect size for
'UBA2658 sp002841545' on 'Brain meningioma'

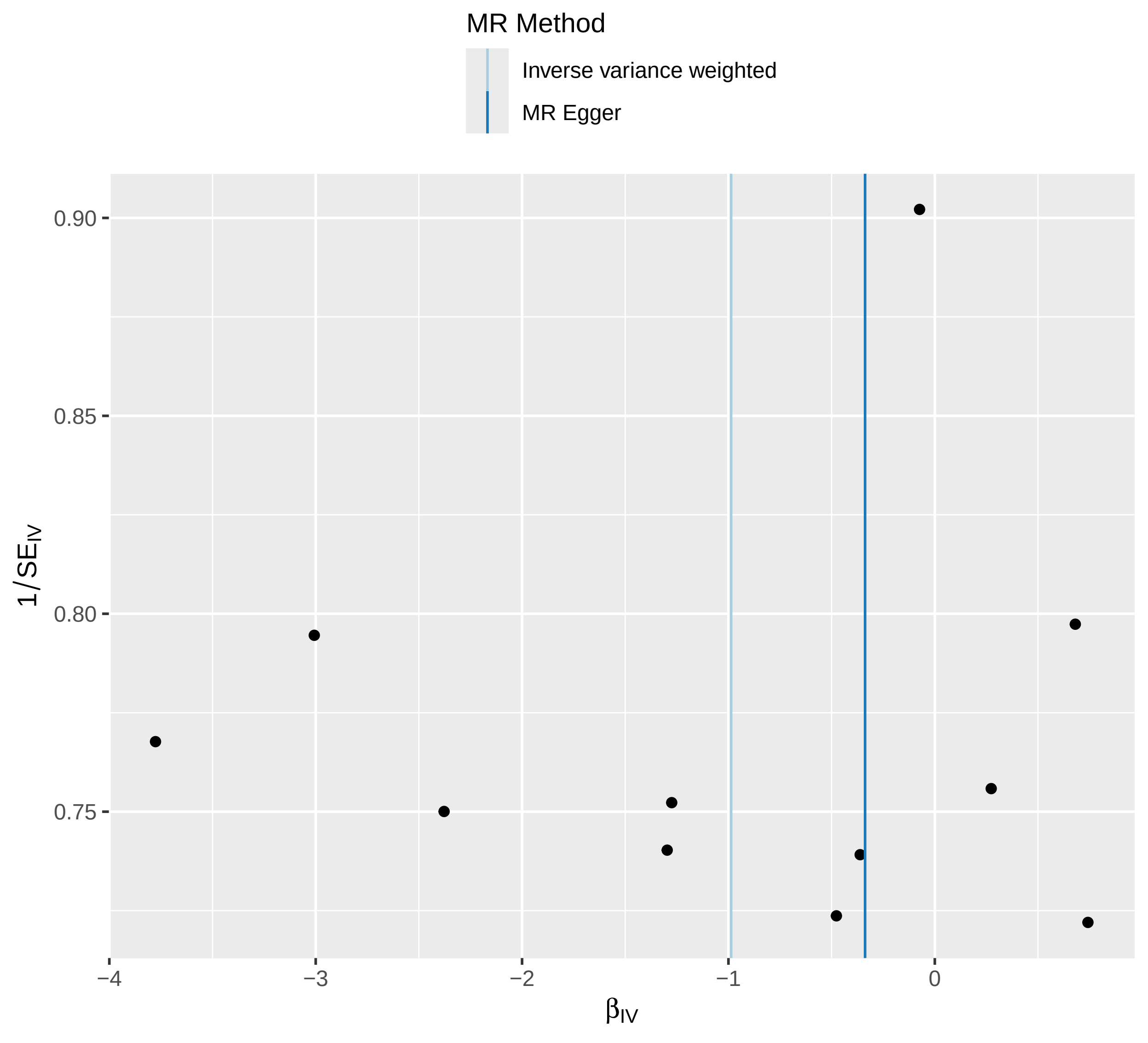

MR Method
Inverse variance weighted
MR Egger
0.90
0.85
0.80
0.75
1/SE_IV
−4
−3
−2
−1
0
β_IV

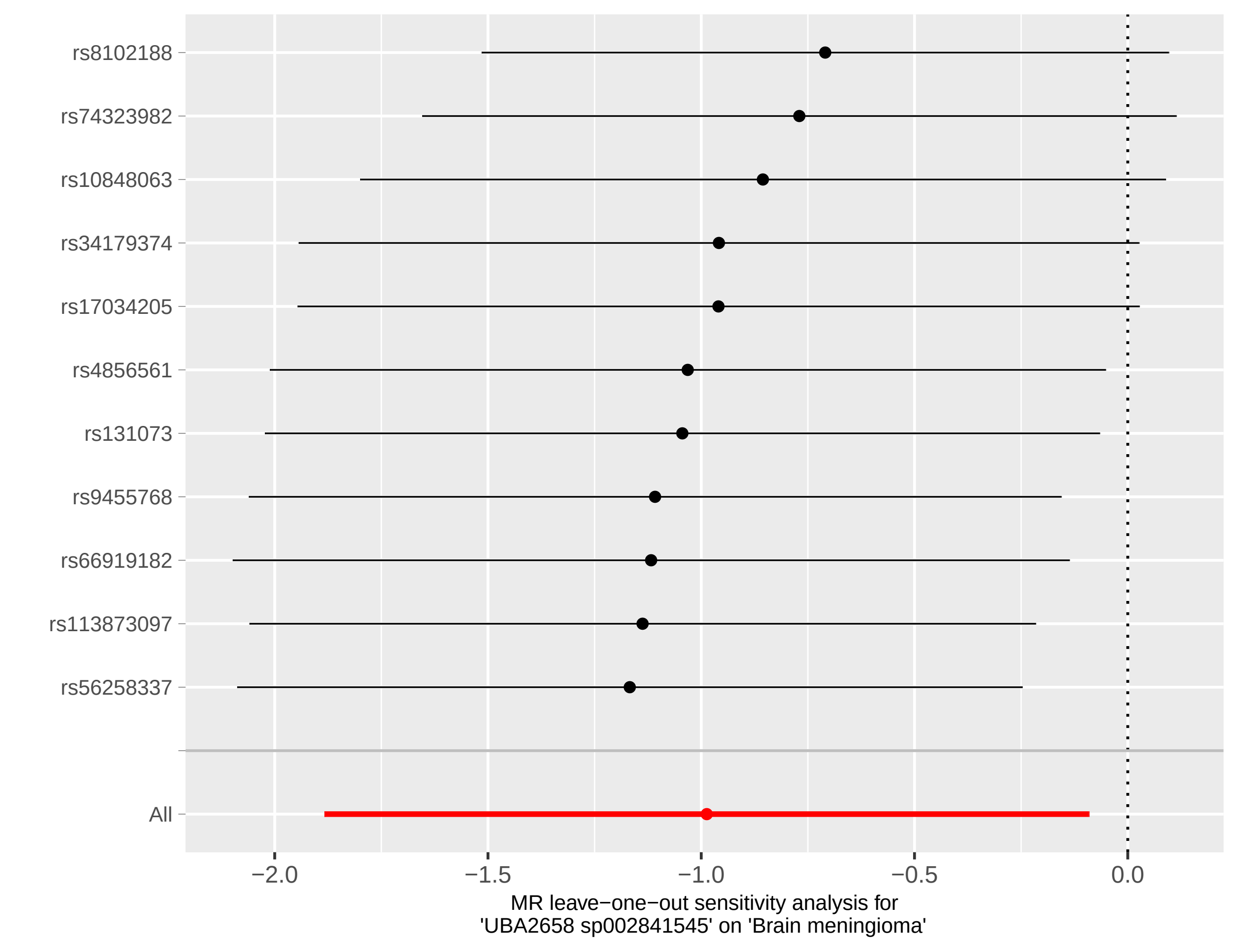

rs8102188
rs74323982
rs10848063
rs34179374
rs17034205
rs4856561
rs131073
rs9455768
rs66919182
rs113873097
rs56258337
All
−2.0
−1.5
−1.0
−0.5
0.0
MR leave−one−out sensitivity analysis for
'UBA2658 sp002841545' on 'Brain meningioma'

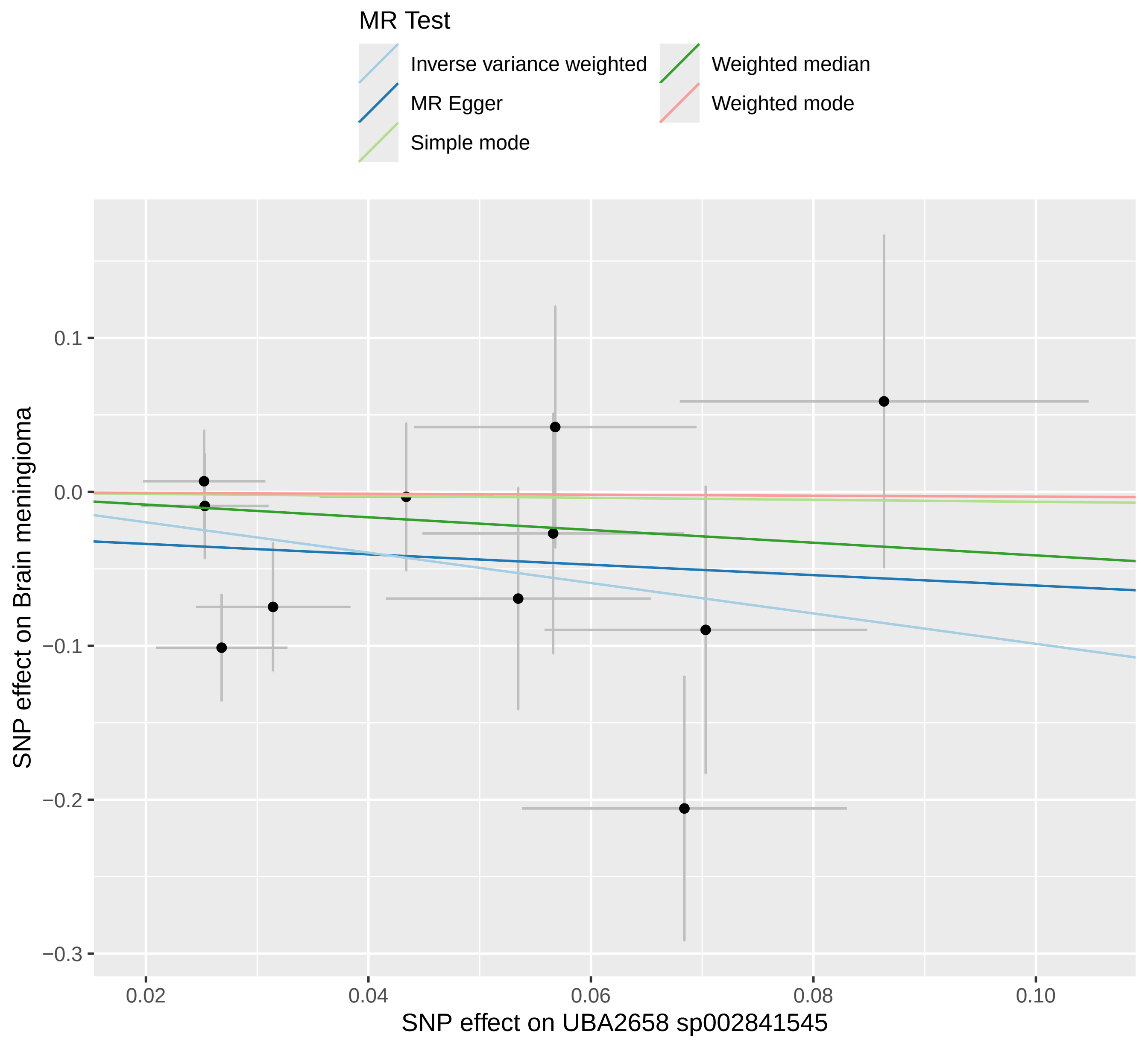
MR Test
Inverse variance weighted
MR Egger
Simple mode
Weighted median
Weighted mode
SNP effect on Brain meningioma
0.1
0.0
−0.1
−0.2
−0.3
0.02
0.04
0.06
0.08
0.10
SNP effect on UBA2658 sp002841545
