# Supplementary material for: Causal Pathways Linking Gut Microbiota, Serum Metabolites, and Meningioma Risk: A Mendelian Randomization Analysis
Source: Brain Behav. 2026 Feb 9;16(2):e71220. doi: 10.1002/brb3.71220 (PMC12887442; doi:10.1002/brb3.71220)
Supplement: Supplementary file 6 — Supplementary Appendix S6: brb371220‐sup‐0006‐Appendix6.pdf [file BRB3-16-e71220-s008.pdf]

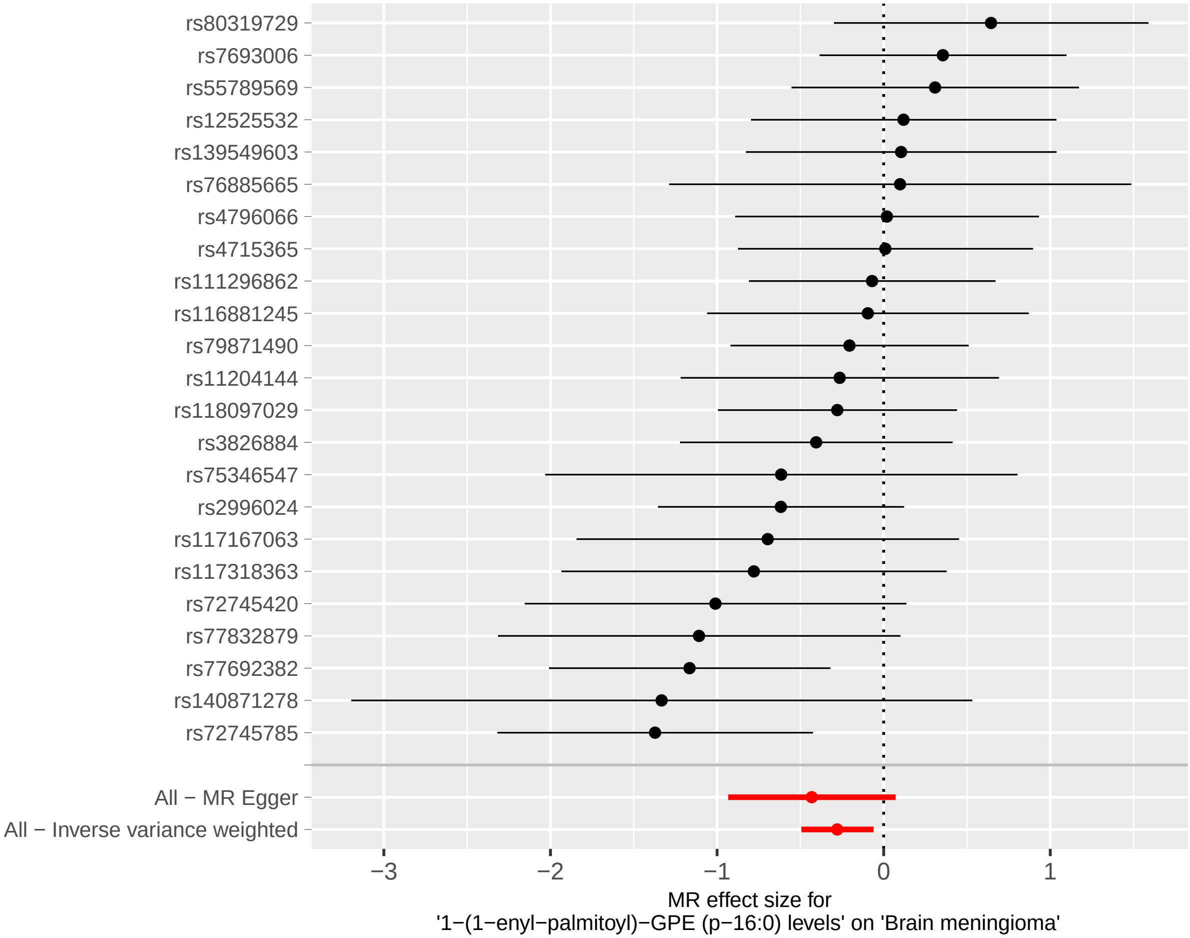
rs80319729
rs7693006
rs55789569
rs12525532
rs139549603
rs76885665
rs4796066
rs4715365
rs111296862
rs116881245
rs79871490
rs11204144
rs118097029
rs3826884
rs75346547
rs2996024
rs117167063
rs117318363
rs72745420
rs77832879
rs77692382
rs140871278
rs72745785
All − MR Egger
All − Inverse variance weighted
−3
−2
−1
0
1
MR effect size for
'1−(1−enyl−palmitoyl)−GPE (p−16:0) levels' on 'Brain meningioma'

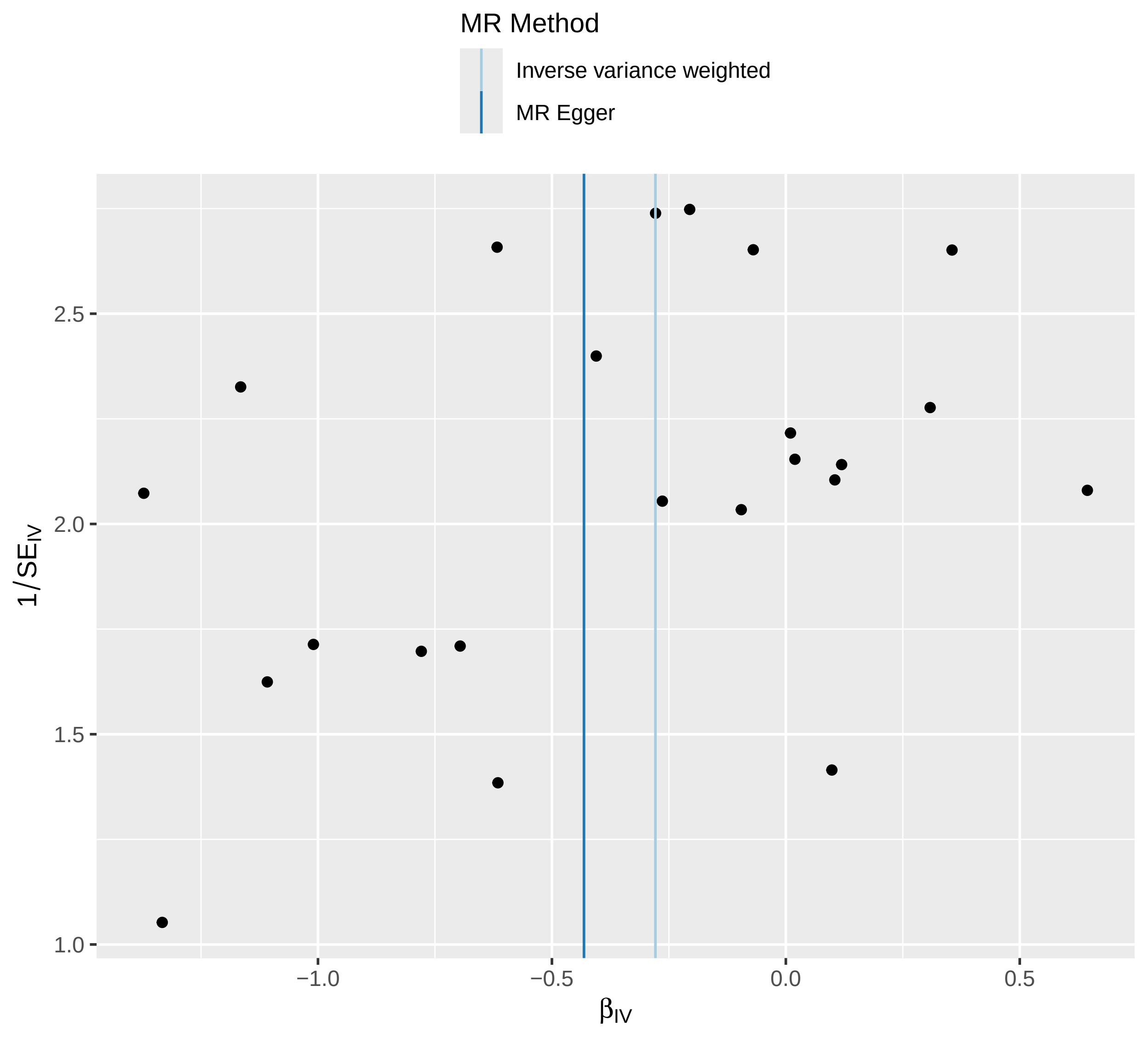

MR Method
Inverse variance weighted
MR Egger
2.5
2.0
1.5
1.0
1/SE_IV
−1.0
−0.5
0.0
0.5
β_IV

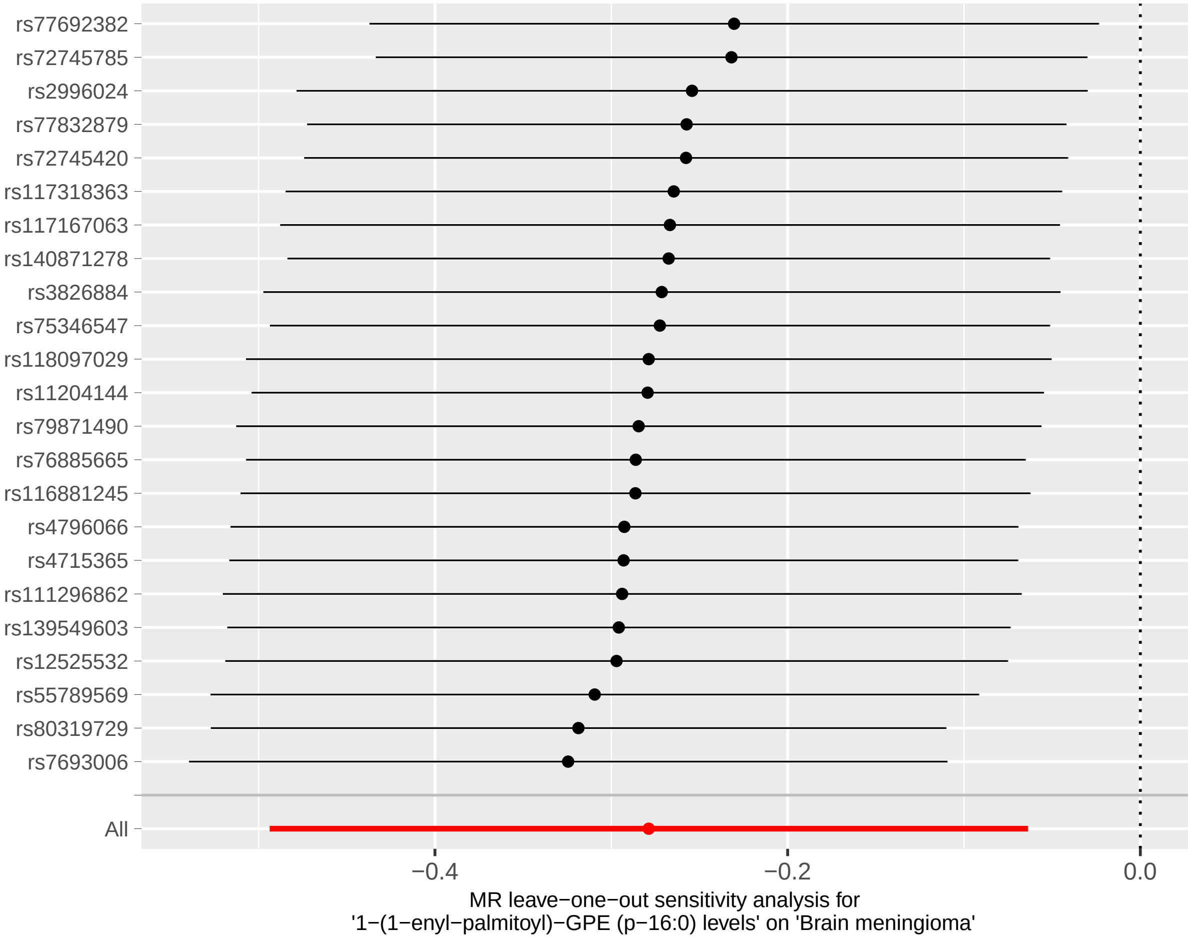
rs77692382
rs72745785
rs2996024
rs77832879
rs72745420
rs117318363
rs117167063
rs140871278
rs3826884
rs75346547
rs118097029
rs11204144
rs79871490
rs76885665
rs116881245
rs4796066
rs4715365
rs111296862
rs139549603
rs12525532
rs55789569
rs80319729
rs7693006
All
−0.4
−0.2
0.0
MR leave−one−out sensitivity analysis for
'1−(1−enyl−palmitoyl)−GPE (p−16:0) levels' on 'Brain meningioma'

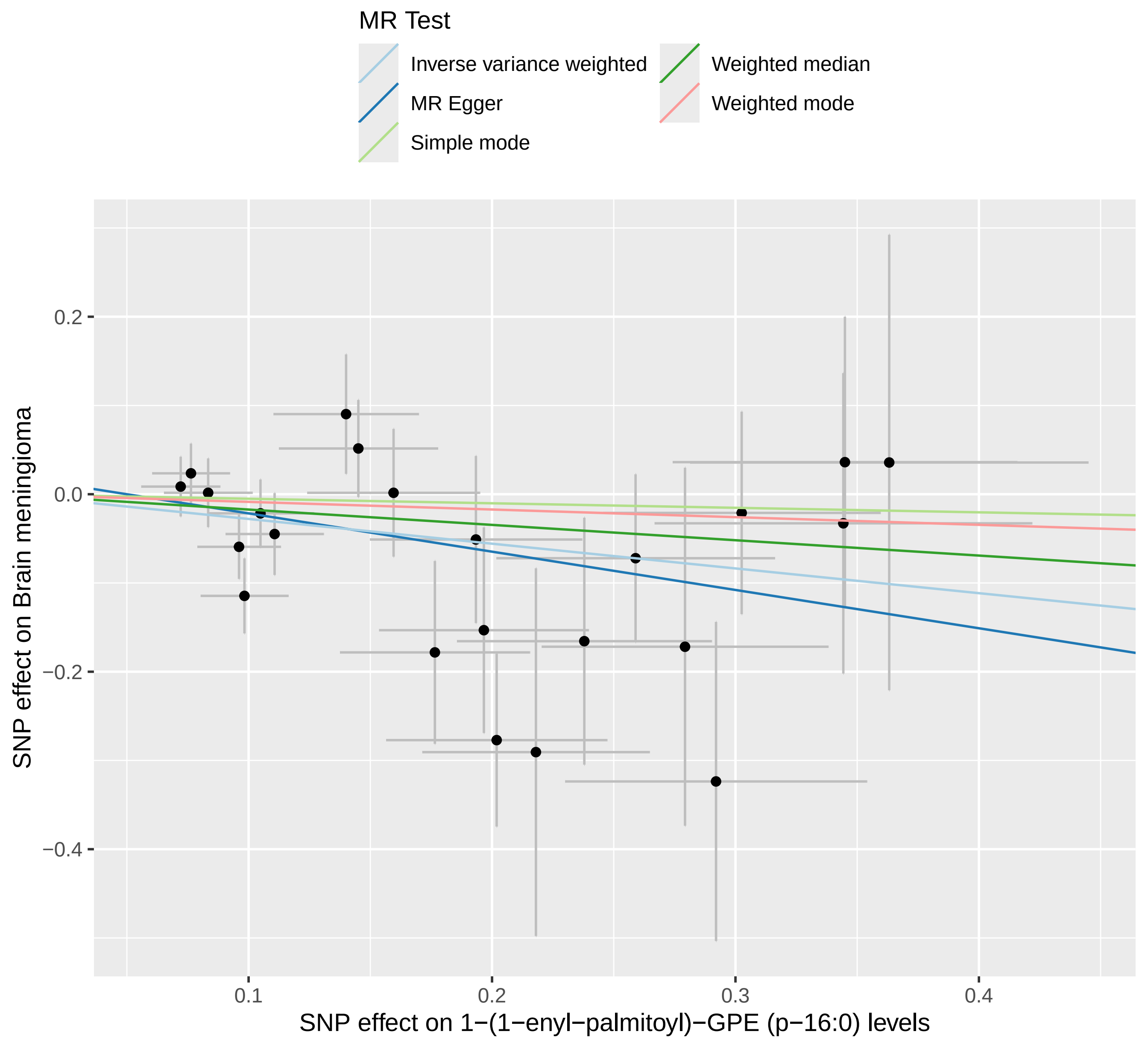
MR Test
Inverse variance weighted
MR Egger
Simple mode
Weighted median
Weighted mode
SNP effect on Brain meningioma
0.2
0.0
−0.2
−0.4
0.1
0.2
0.3
0.4
SNP effect on 1−(1−enyl−palmitoyl)−GPE (p−16:0) levels

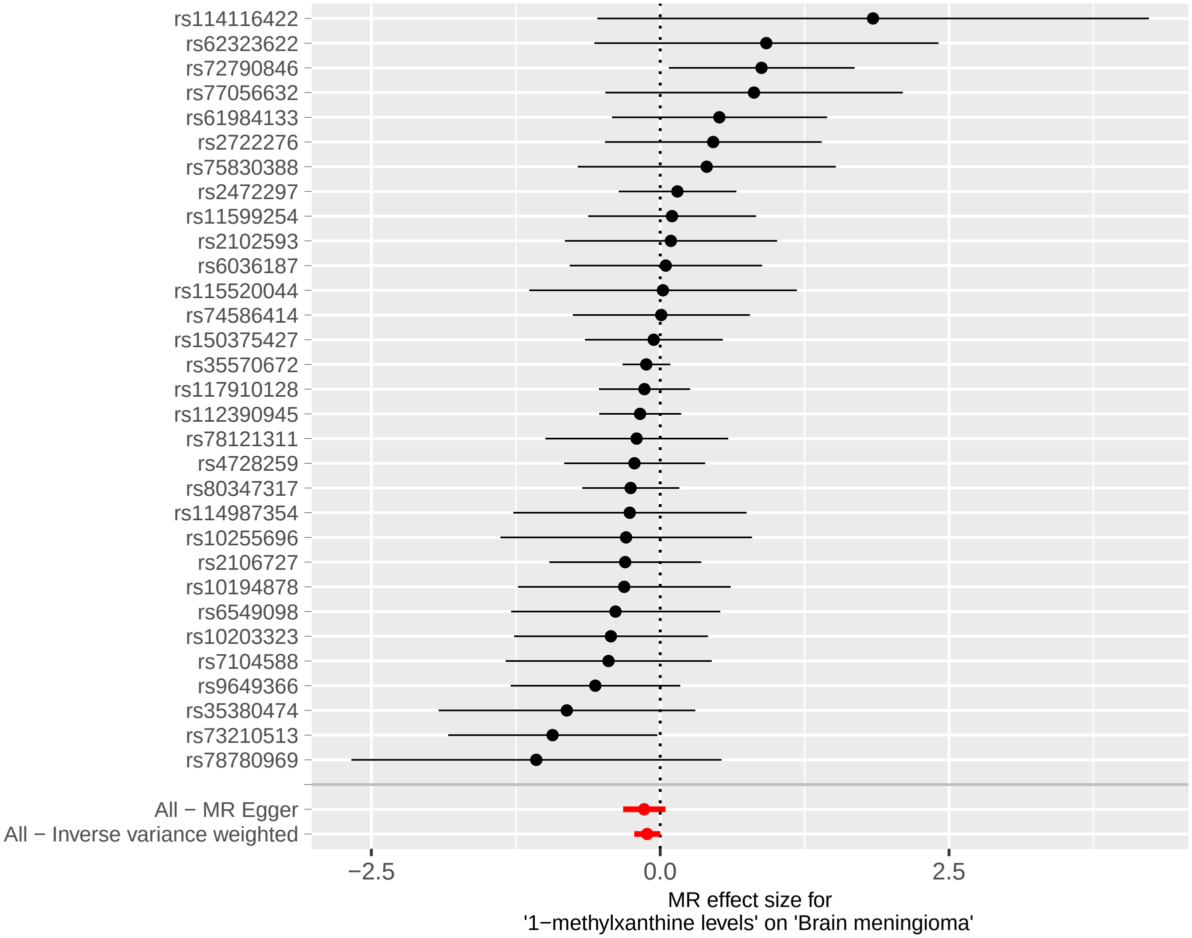
rs114116422
rs62323622
rs72790846
rs77056632
rs61984133
rs2722276
rs75830388
rs2472297
rs11599254
rs2102593
rs6036187
rs115520044
rs74586414
rs150375427
rs35570672
rs117910128
rs112390945
rs78121311
rs4728259
rs80347317
rs114987354
rs10255696
rs2106727
rs10194878
rs6549098
rs10203323
rs7104588
rs9649366
rs35380474
rs73210513
rs78780969
All − MR Egger
All − Inverse variance weighted
−2.5
0.0
2.5
MR effect size for
'1−methylxanthine levels' on 'Brain meningioma'

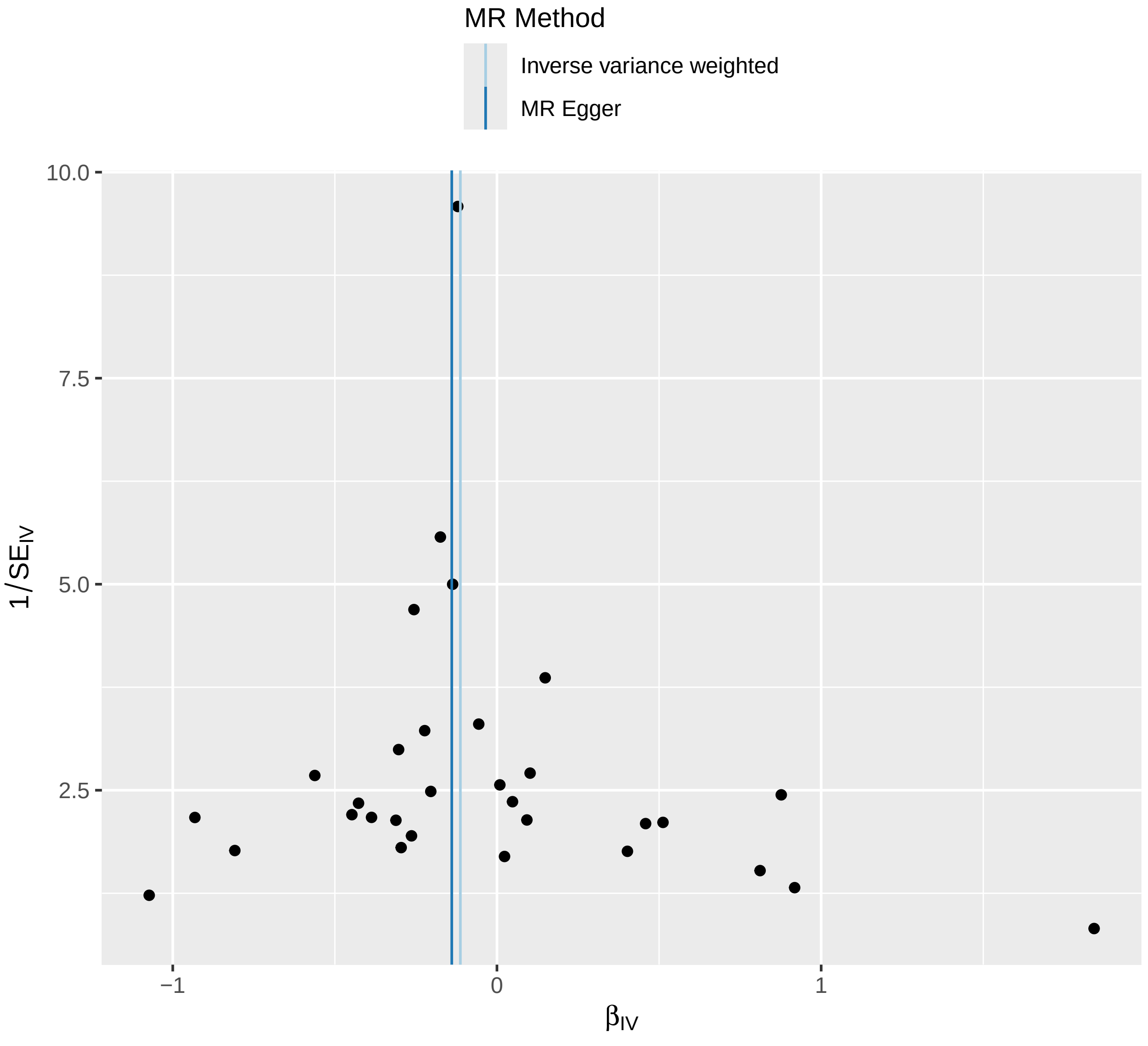

MR Method
Inverse variance weighted
MR Egger
10.0
7.5
5.0
2.5
$1/SE_{IV}$
−1
0
1
β_{IV}

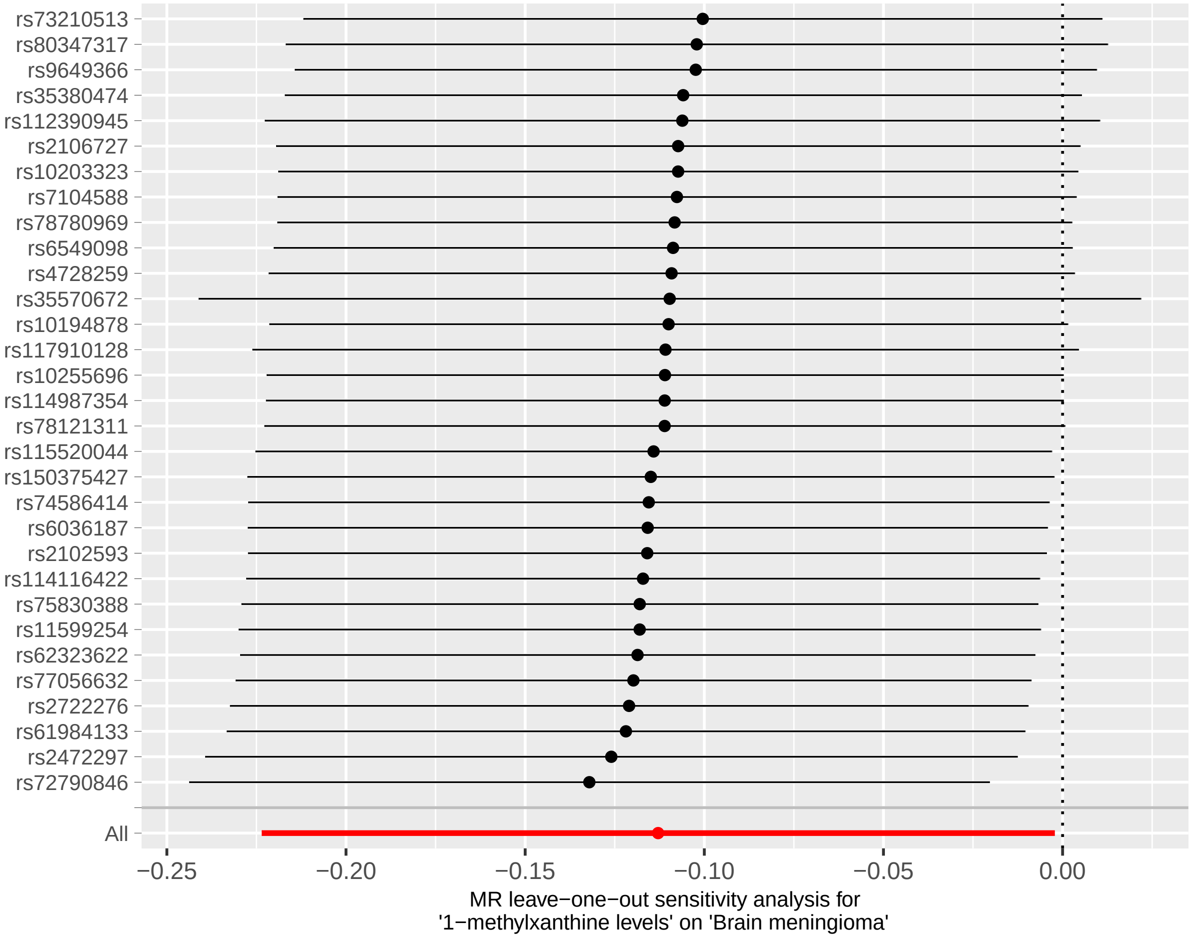
rs73210513
rs80347317
rs9649366
rs35380474
rs112390945
rs2106727
rs10203323
rs7104588
rs78780969
rs6549098
rs4728259
rs35570672
rs10194878
rs117910128
rs10255696
rs114987354
rs78121311
rs115520044
rs150375427
rs74586414
rs6036187
rs2102593
rs114116422
rs75830388
rs11599254
rs62323622
rs77056632
rs2722276
rs61984133
rs2472297
rs72790846
All
−0.25
−0.20
−0.15
−0.10
−0.05
0.00
MR leave−one−out sensitivity analysis for
'1−methylxanthine levels' on 'Brain meningioma'

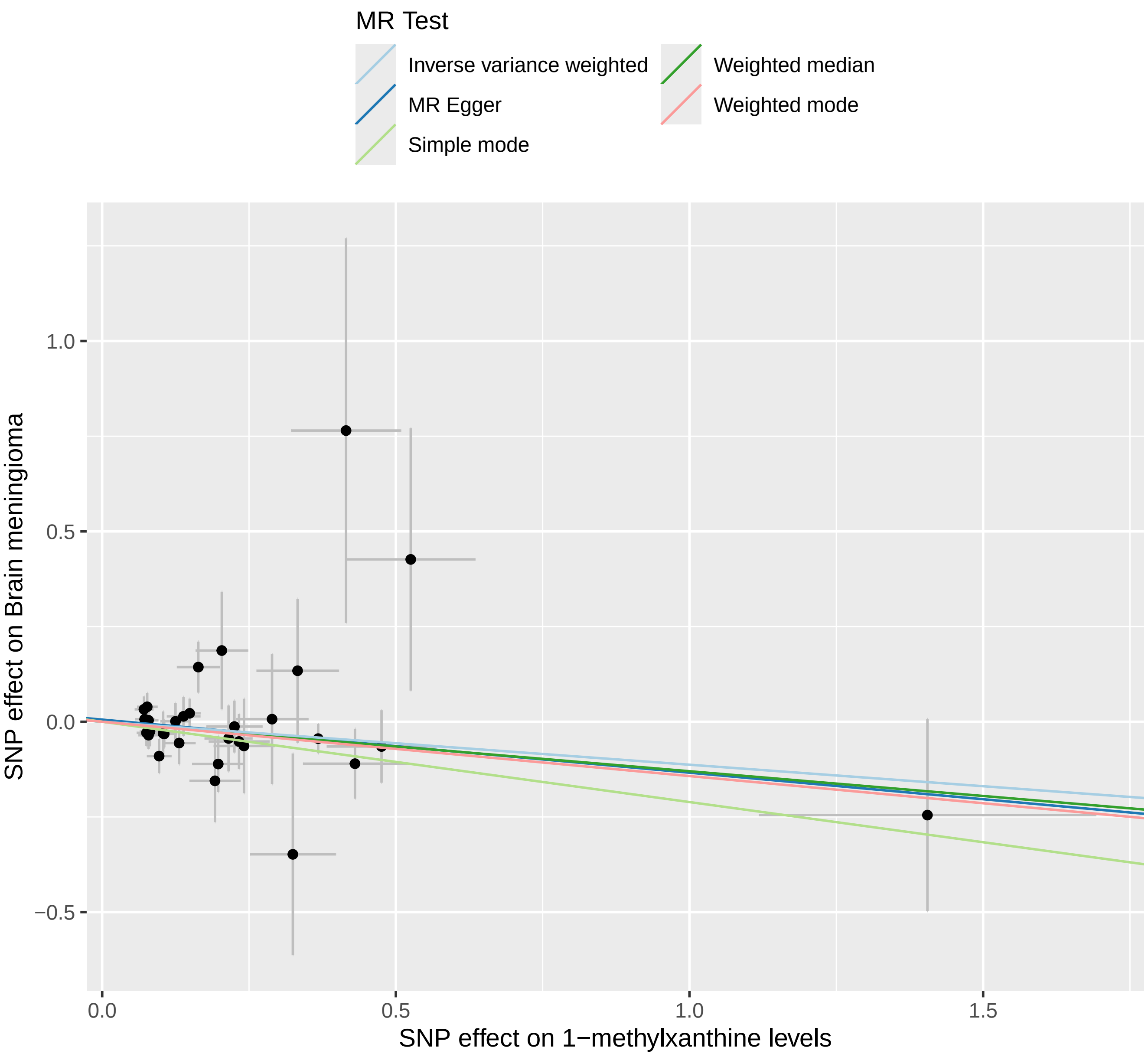

MR Test
Inverse variance weighted
MR Egger
Simple mode
Weighted median
Weighted mode
1.0
0.5
0.0
−0.5
0.0
0.5
1.0
1.5
SNP effect on Brain meningioma
SNP effect on 1−methylxanthine levels

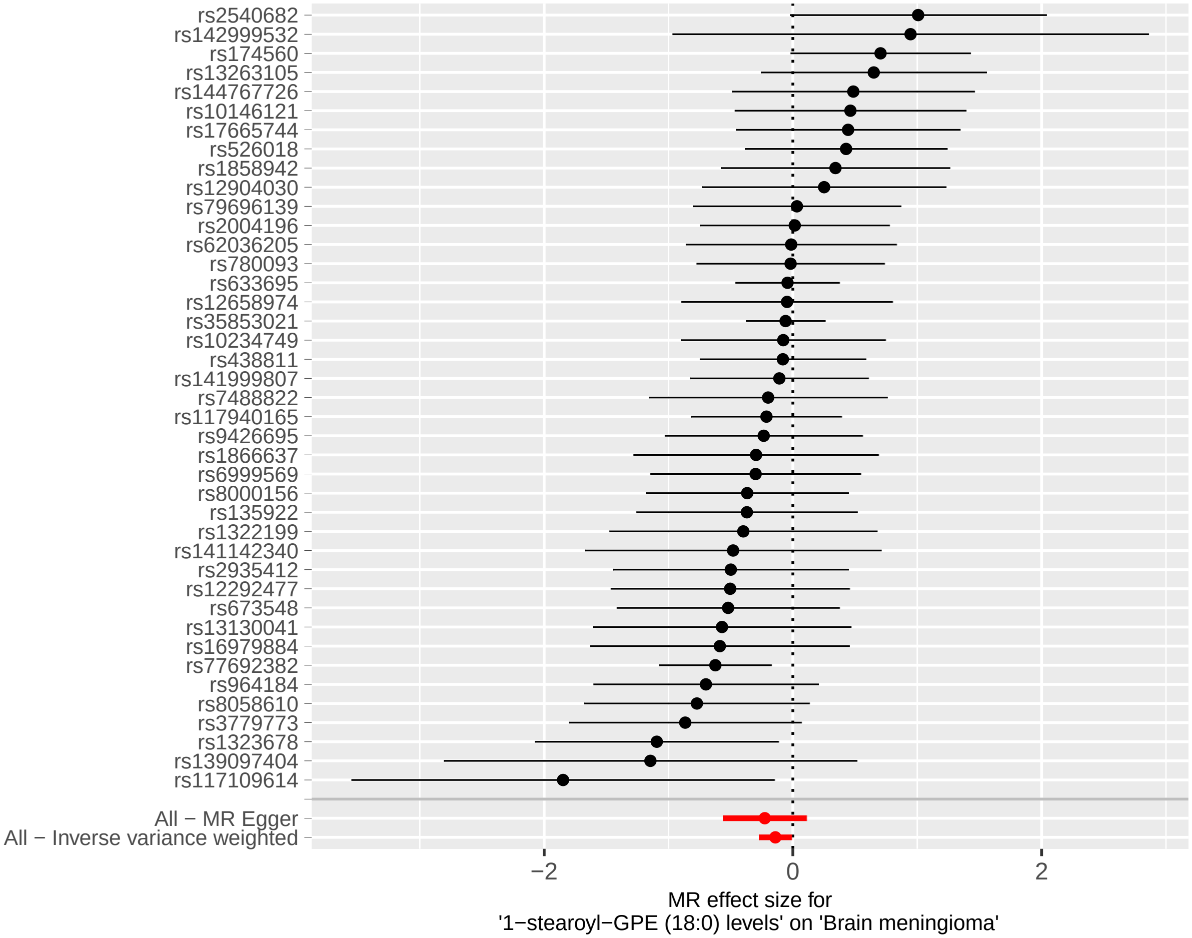
rs2540682
rs142999532
rs174560
rs13263105
rs144767726
rs10146121
rs17665744
rs526018
rs1858942
rs12904030
rs79696139
rs2004196
rs62036205
rs780093
rs633695
rs12658974
rs35853021
rs10234749
rs438811
rs141999807
rs7488822
rs117940165
rs9426695
rs1866637
rs6999569
rs8000156
rs135922
rs1322199
rs141142340
rs2935412
rs12292477
rs673548
rs13130041
rs16979884
rs77692382
rs964184
rs8058610
rs3779773
rs1323678
rs139097404
rs117109614
All − MR Egger
All − Inverse variance weighted
−2
0
2
MR effect size for
'1−stearoyl−GPE (18:0) levels' on 'Brain meningioma'

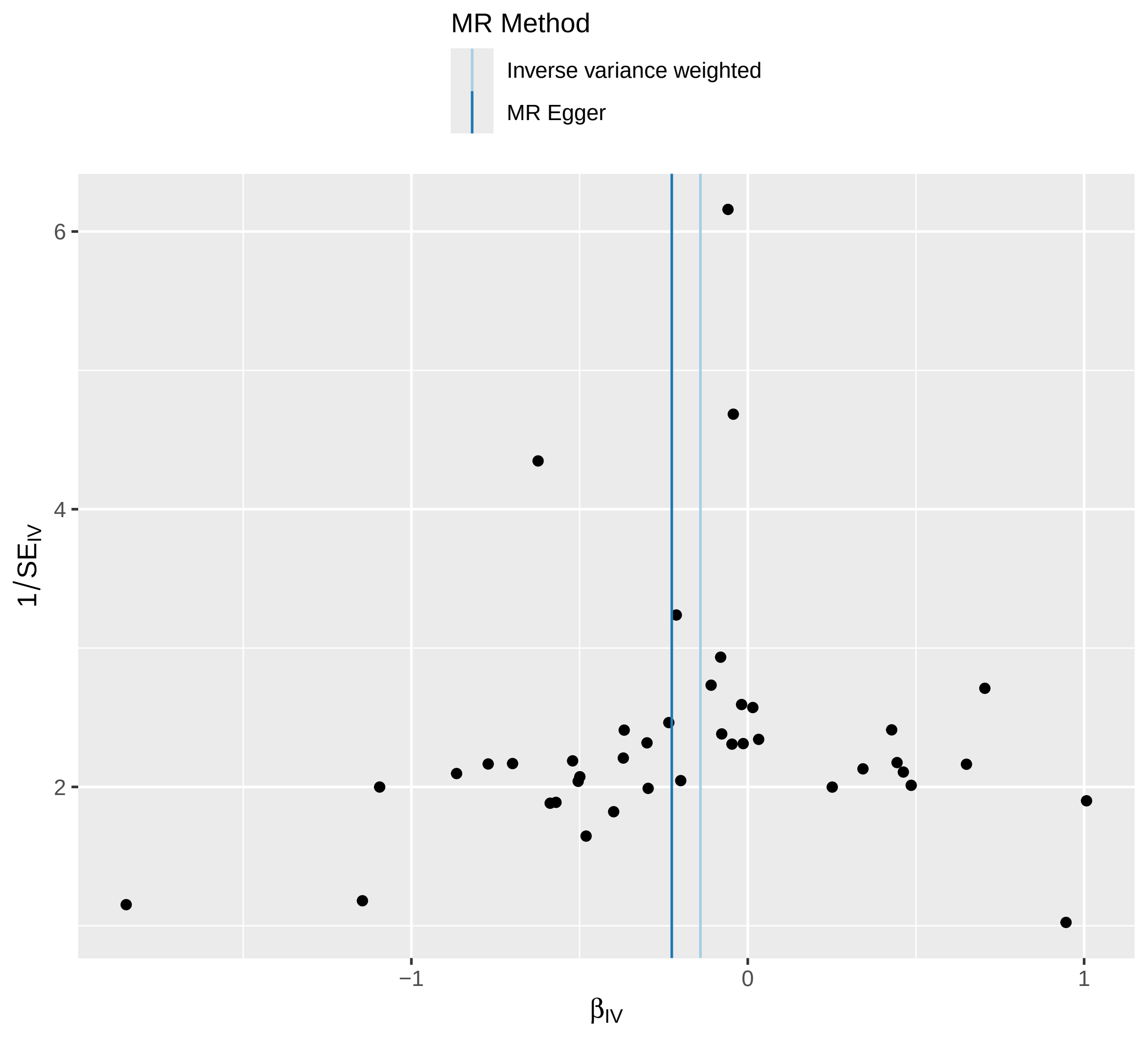
MR Method
Inverse variance weighted
MR Egger
6
4
2
1/SE_IV
−1
0
1
β_IV

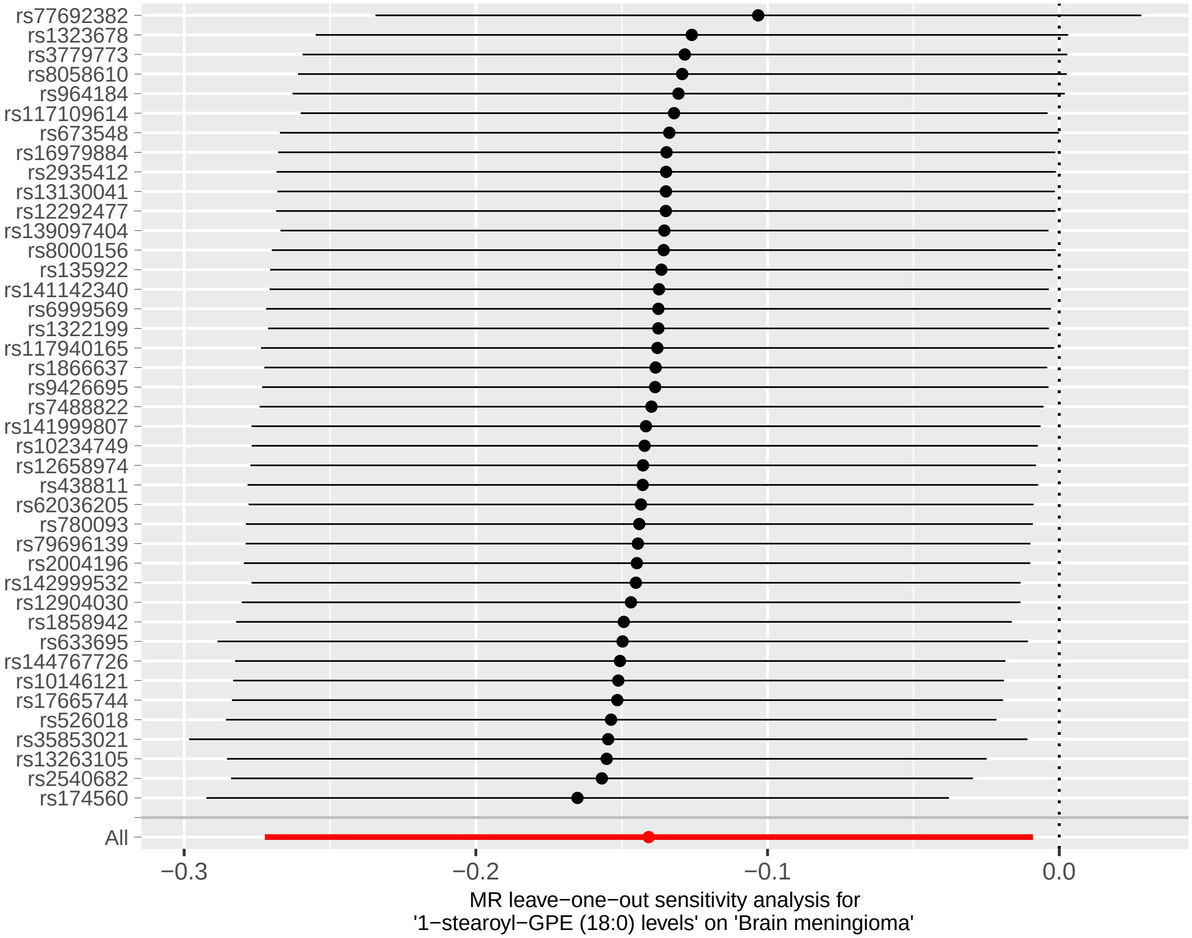
rs77692382
rs1323678
rs3779773
rs8058610
rs964184
rs117109614
rs673548
rs16979884
rs2935412
rs13130041
rs12292477
rs139097404
rs8000156
rs135922
rs141142340
rs6999569
rs1322199
rs117940165
rs1866637
rs9426695
rs7488822
rs141999807
rs10234749
rs12658974
rs438811
rs62036205
rs780093
rs79696139
rs2004196
rs142999532
rs12904030
rs1858942
rs633695
rs144767726
rs10146121
rs17665744
rs526018
rs35853021
rs13263105
rs2540682
rs174560
All
−0.3
−0.2
−0.1
0.0
MR leave−one−out sensitivity analysis for
'1−stearoyl−GPE (18:0) levels' on 'Brain meningioma'

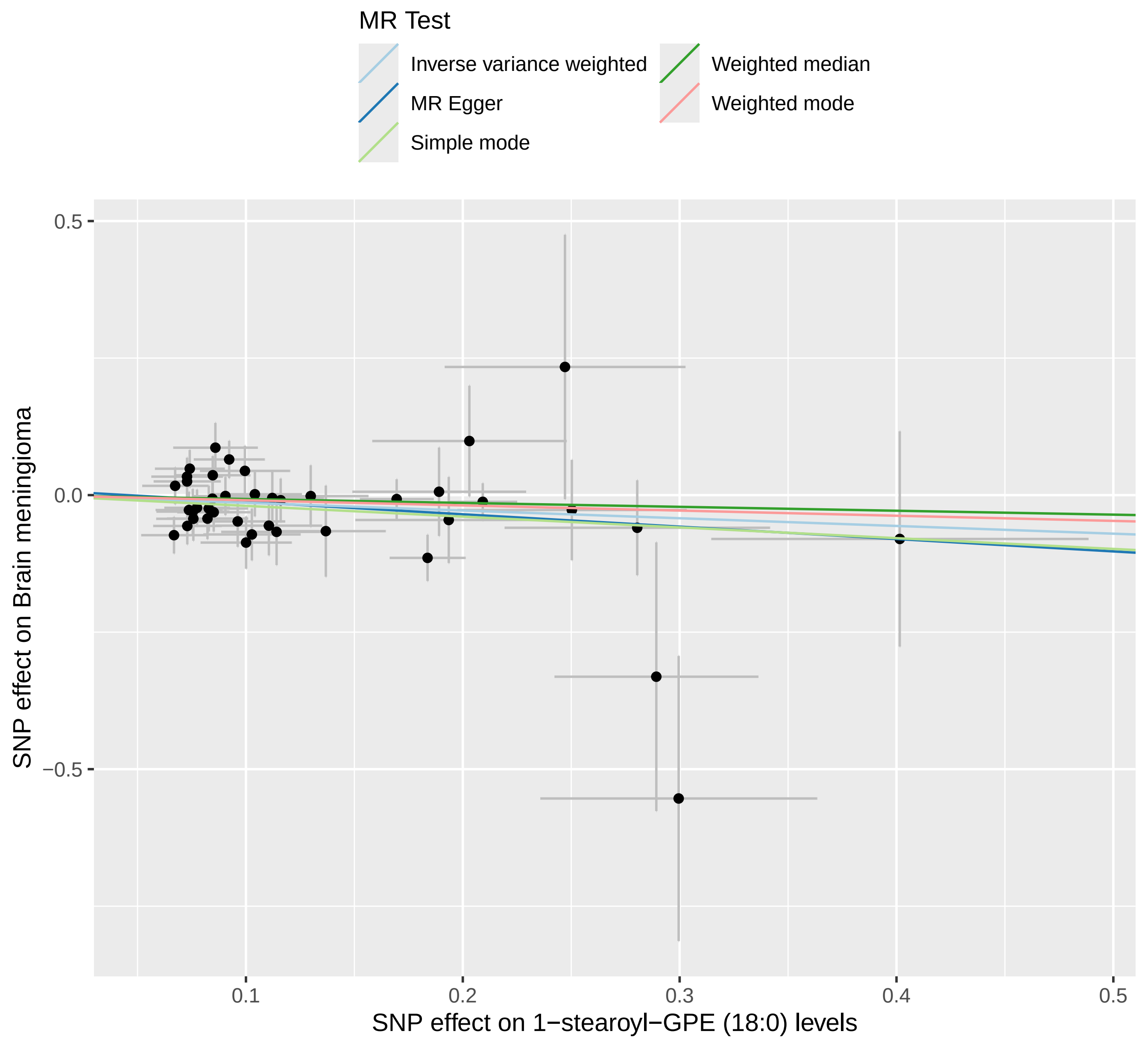
MR Test
Inverse variance weighted
MR Egger
Simple mode
Weighted median
Weighted mode
SNP effect on Brain meningioma
0.5
0.0
−0.5
0.1
0.2
0.3
0.4
0.5
SNP effect on 1−stearoyl−GPE (18:0) levels

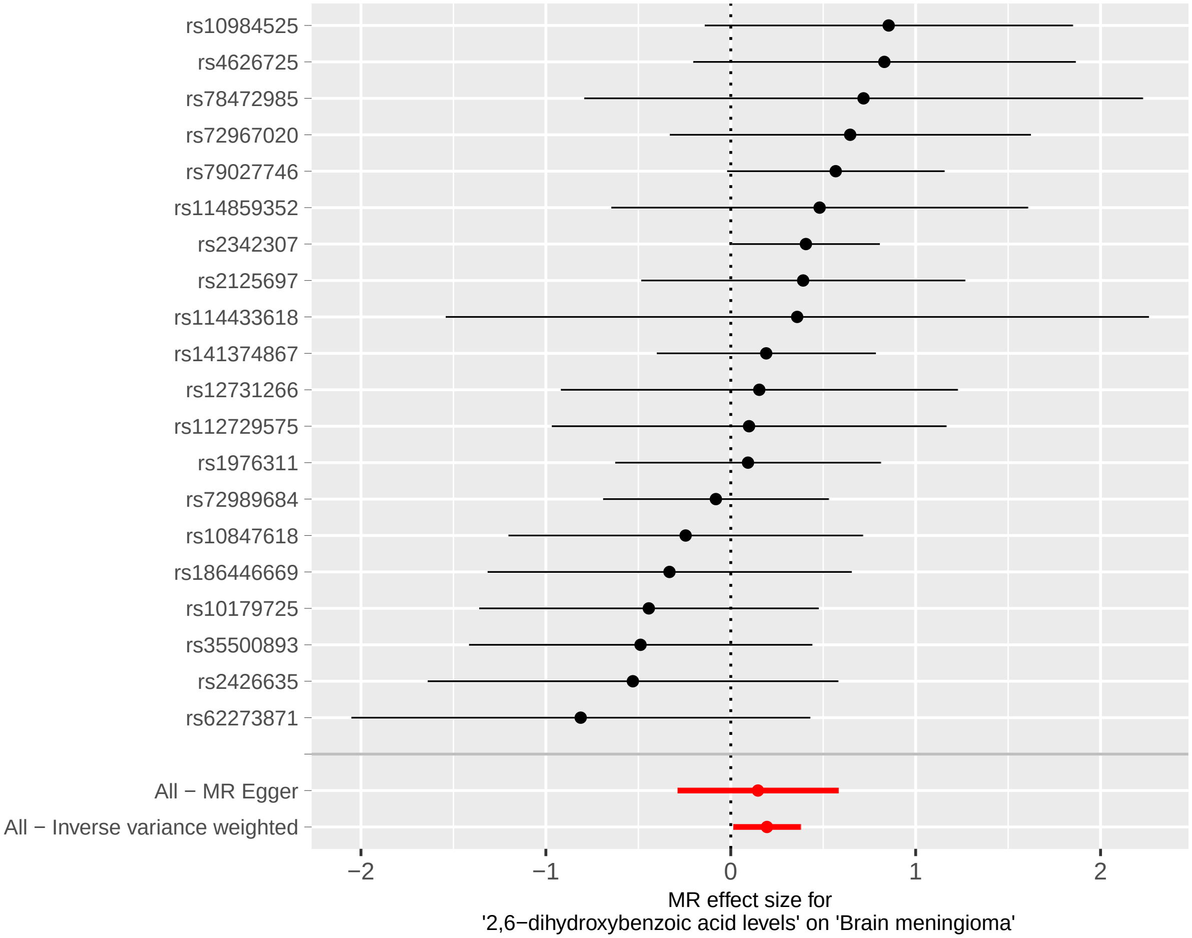

rs10984525
rs4626725
rs78472985
rs72967020
rs79027746
rs114859352
rs2342307
rs2125697
rs114433618
rs141374867
rs12731266
rs112729575
rs1976311
rs72989684
rs10847618
rs186446669
rs10179725
rs35500893
rs2426635
rs62273871
All − MR Egger
All − Inverse variance weighted
−2
−1
0
1
2
MR effect size for
'2,6−dihydroxybenzoic acid levels' on 'Brain meningioma'

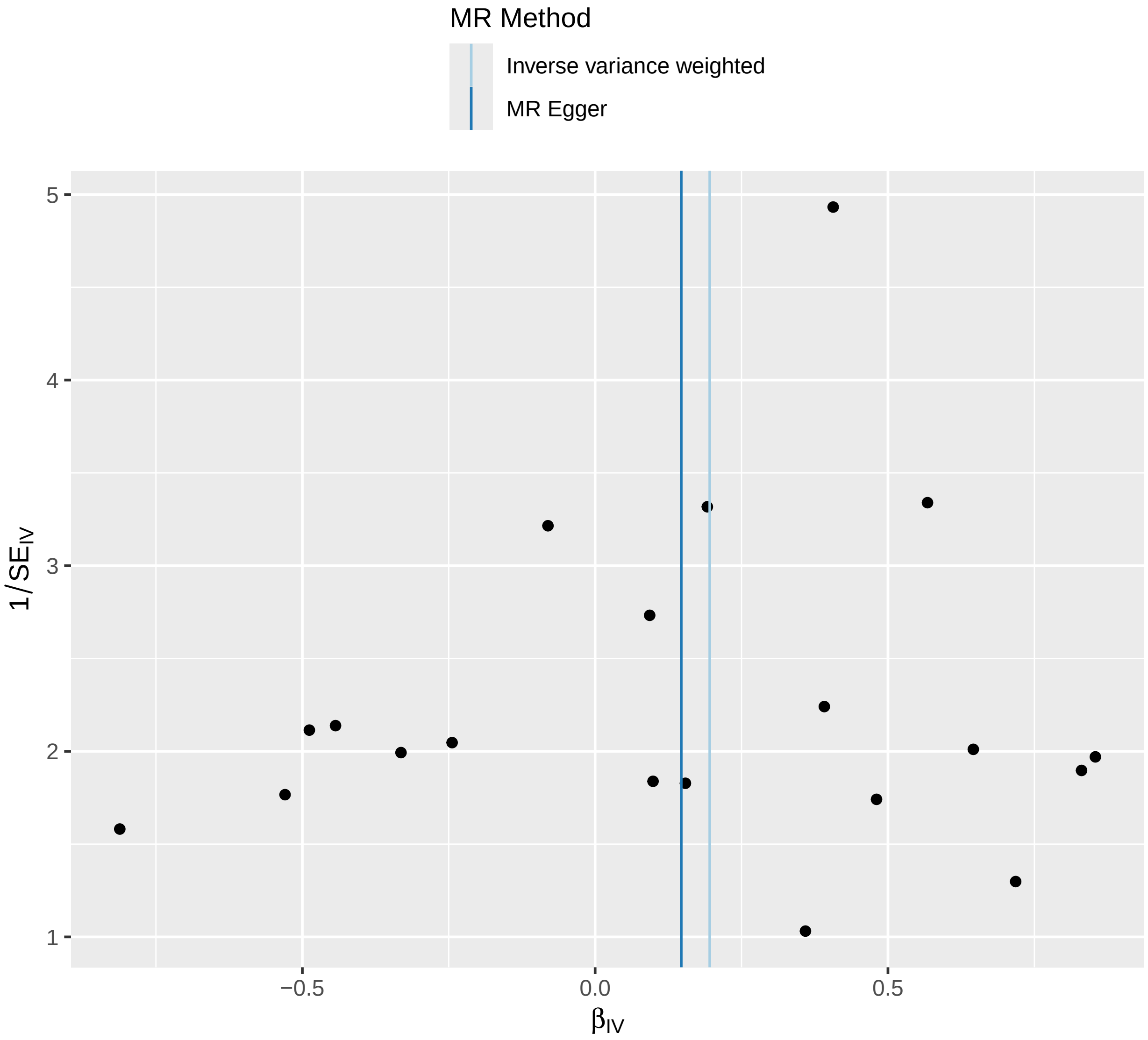

MR Method
Inverse variance weighted
MR Egger
1/SE_IV
β_IV
5
4
3
2
1
−0.5
0.0
0.5

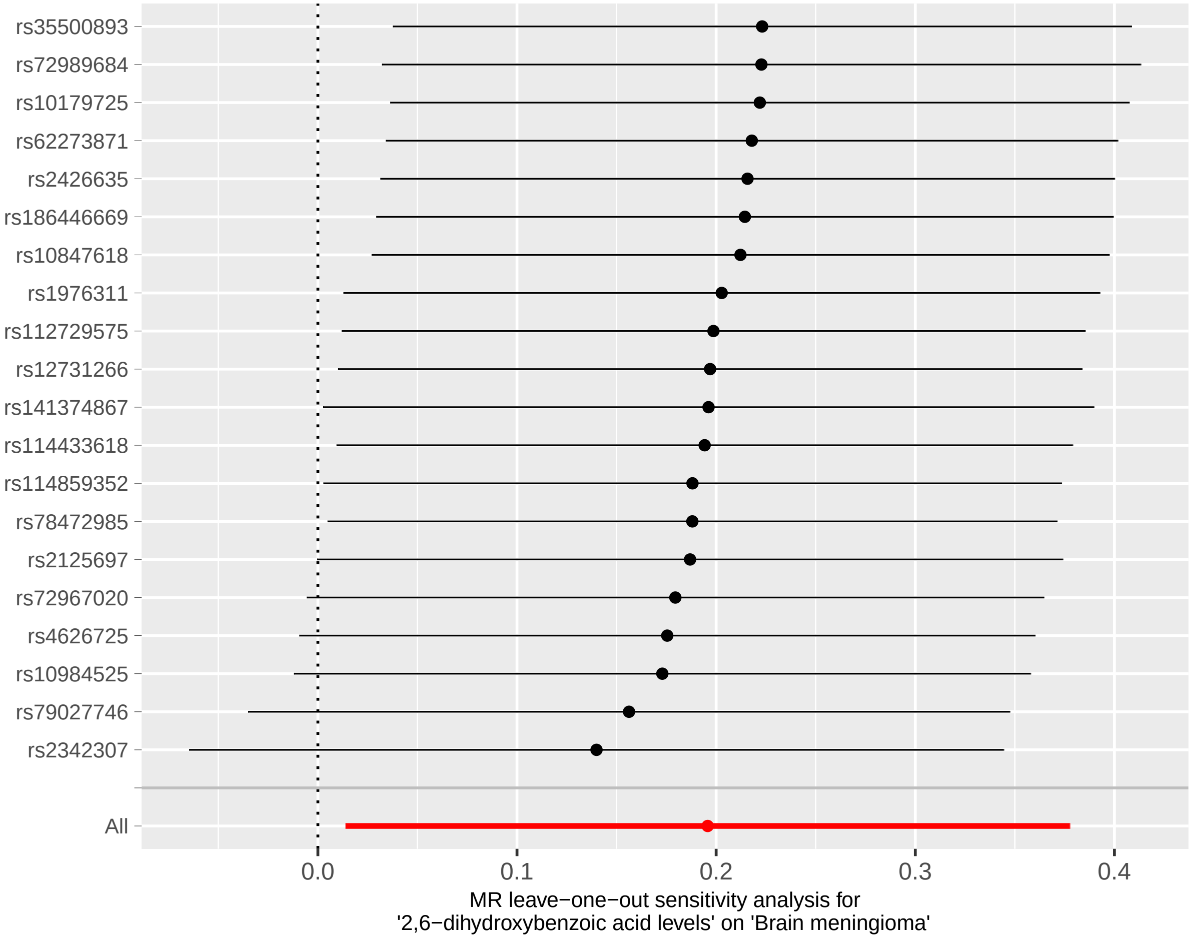

rs35500893
rs72989684
rs10179725
rs62273871
rs2426635
rs186446669
rs10847618
rs1976311
rs112729575
rs12731266
rs141374867
rs114433618
rs114859352
rs78472985
rs2125697
rs72967020
rs4626725
rs10984525
rs79027746
rs2342307
All
0.0
0.1
0.2
0.3
0.4
MR leave−one−out sensitivity analysis for
'2,6−dihydroxybenzoic acid levels' on 'Brain meningioma'

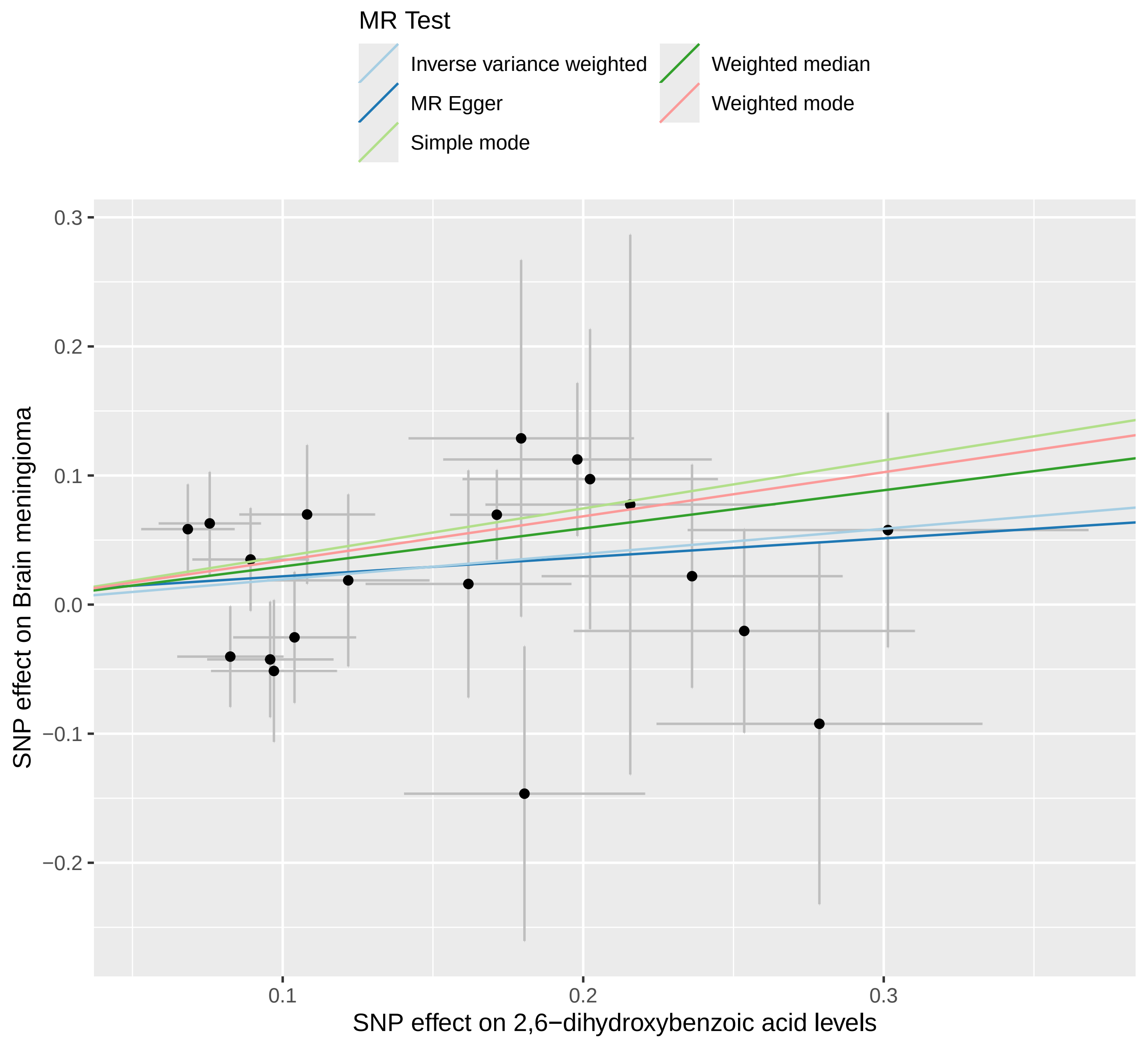
MR Test
Inverse variance weighted
MR Egger
Simple mode
Weighted median
Weighted mode
0.3
0.2
0.1
0.0
−0.1
−0.2
SNP effect on Brain meningioma
0.1
0.2
0.3
SNP effect on 2,6−dihydroxybenzoic acid levels

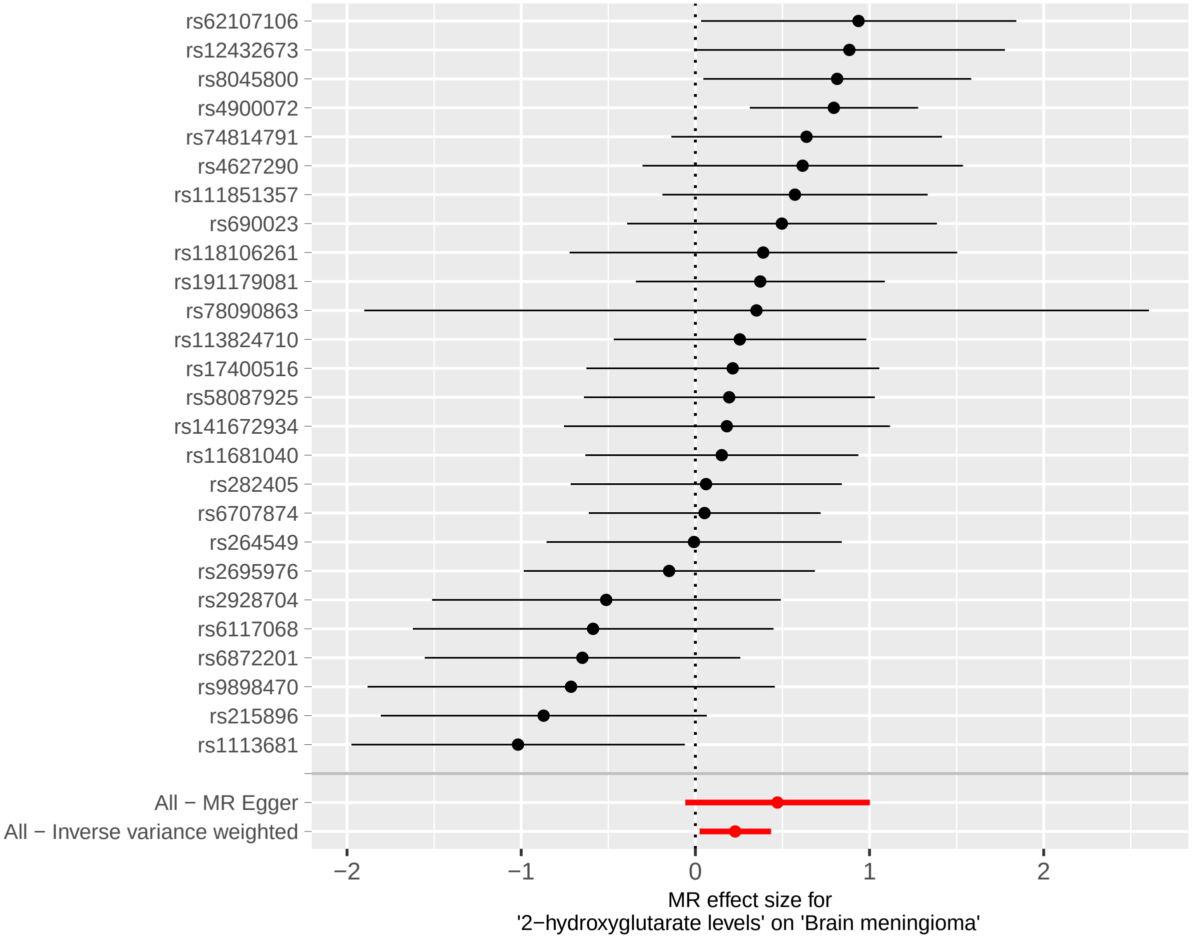
rs62107106
rs12432673
rs8045800
rs4900072
rs74814791
rs4627290
rs111851357
rs690023
rs118106261
rs191179081
rs78090863
rs113824710
rs17400516
rs58087925
rs141672934
rs11681040
rs282405
rs6707874
rs264549
rs2695976
rs2928704
rs6117068
rs6872201
rs9898470
rs215896
rs1113681
All − MR Egger
All − Inverse variance weighted
−2
−1
0
1
2
MR effect size for
'2−hydroxyglutarate levels' on 'Brain meningioma'

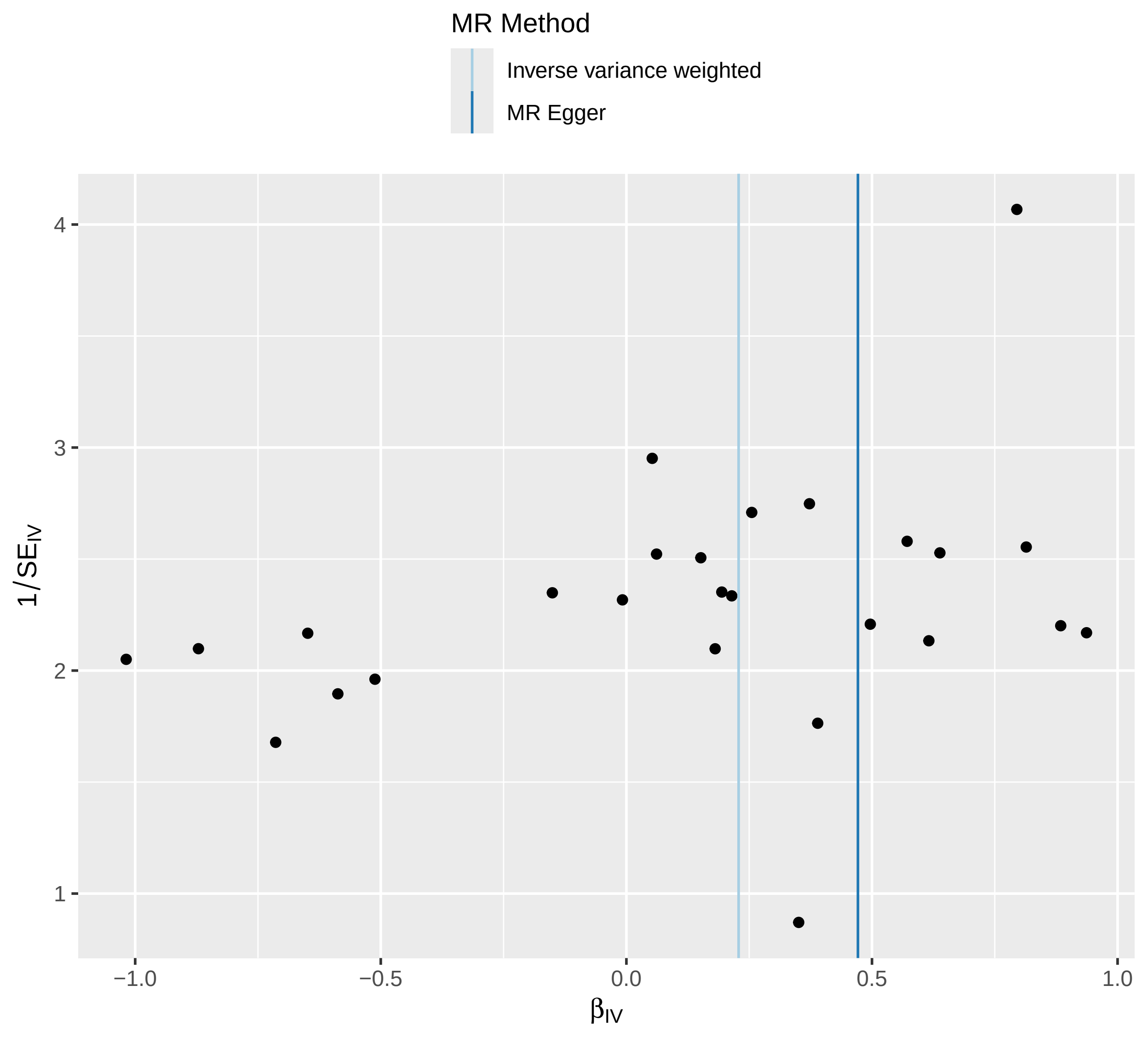

MR Method
Inverse variance weighted
MR Egger
4
3
2
1
1/SE$_{IV}$
−1.0
−0.5
0.0
0.5
1.0
β$_{IV}$

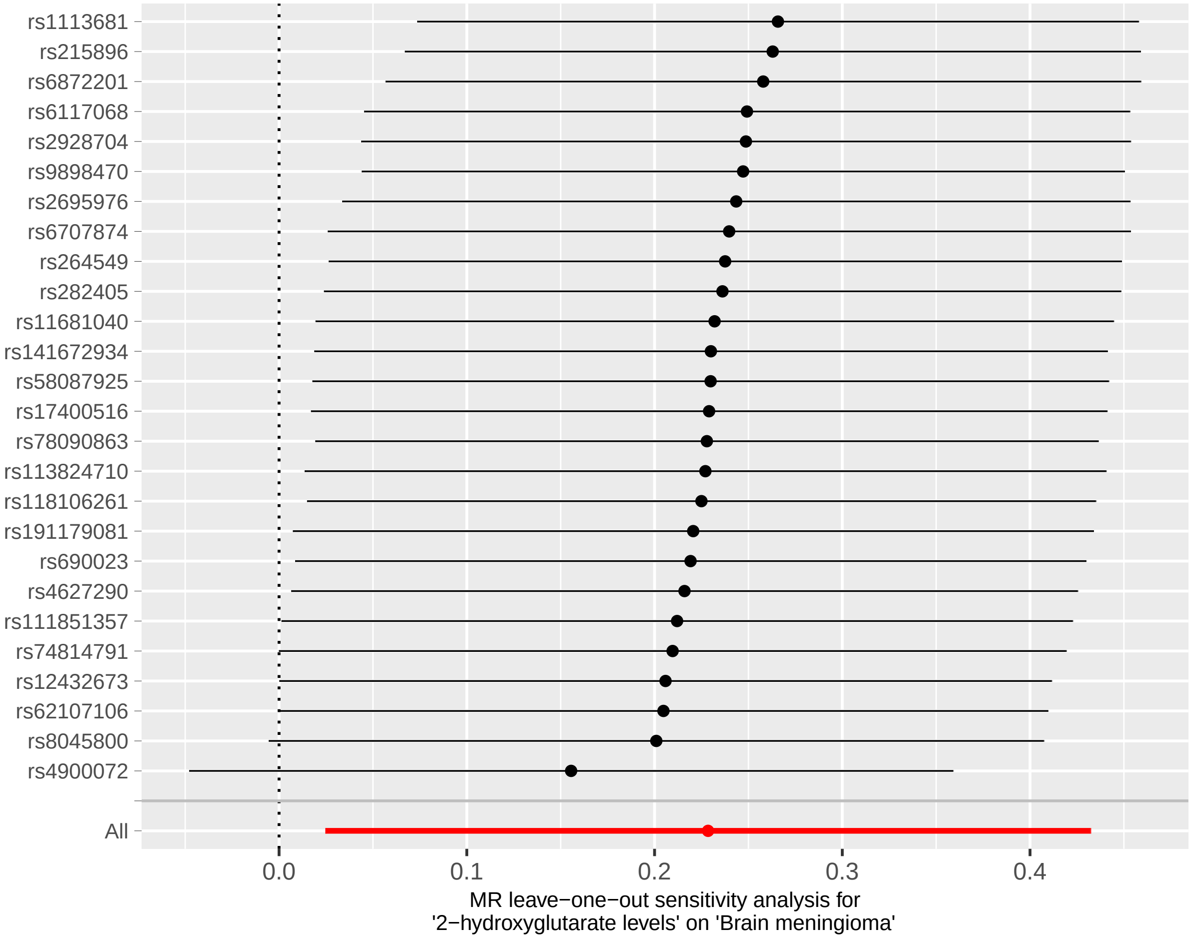
rs1113681
rs215896
rs6872201
rs6117068
rs2928704
rs9898470
rs2695976
rs6707874
rs264549
rs282405
rs11681040
rs141672934
rs58087925
rs17400516
rs78090863
rs113824710
rs118106261
rs191179081
rs690023
rs4627290
rs111851357
rs74814791
rs12432673
rs62107106
rs8045800
rs4900072
All
0.0
0.1
0.2
0.3
0.4
MR leave−one−out sensitivity analysis for
'2−hydroxyglutarate levels' on 'Brain meningioma'

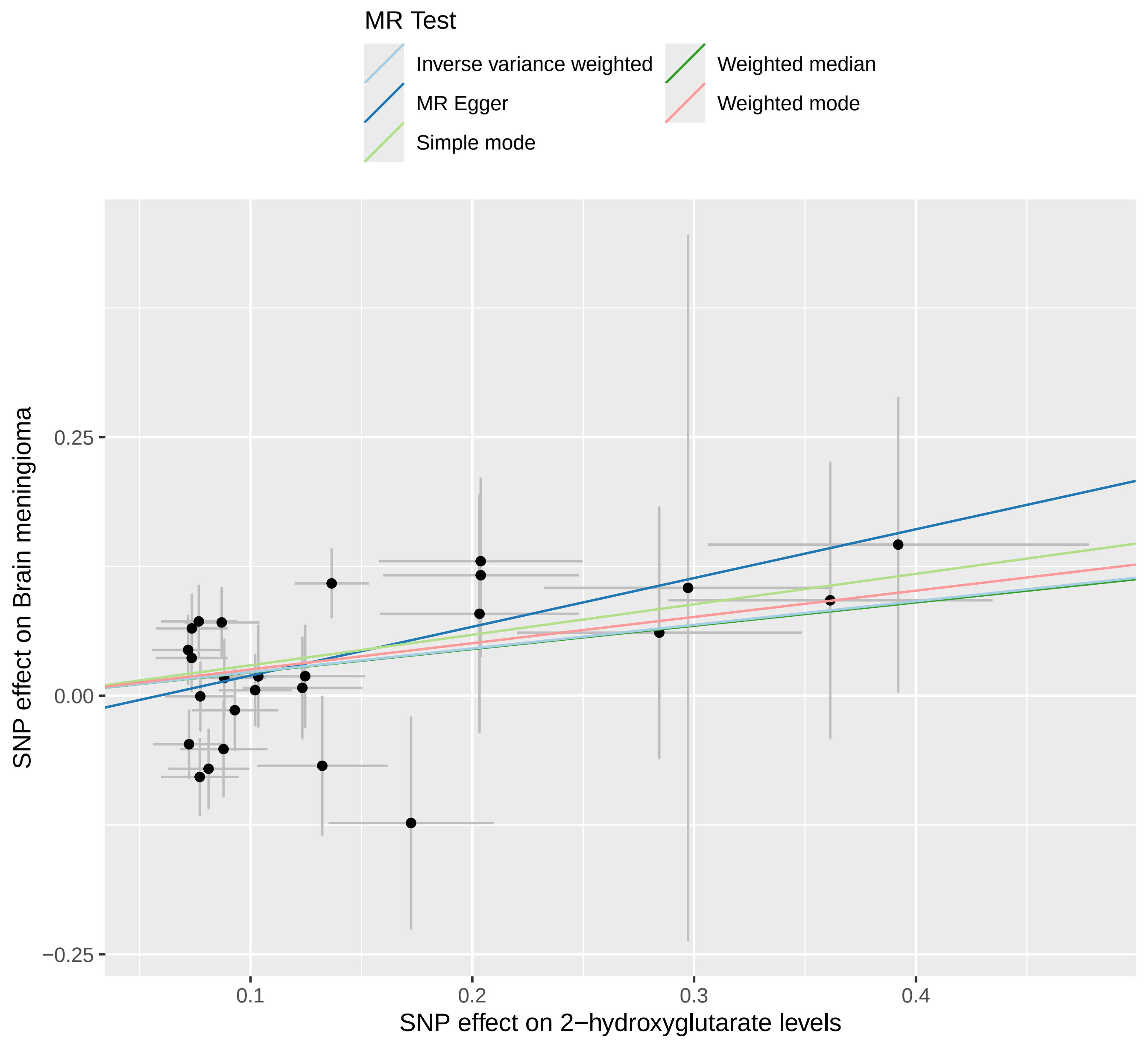

MR Test
Inverse variance weighted
MR Egger
Simple mode
Weighted median
Weighted mode
0.25
0.00
−0.25
0.1
0.2
0.3
0.4
SNP effect on Brain meningioma
SNP effect on 2−hydroxyglutarate levels

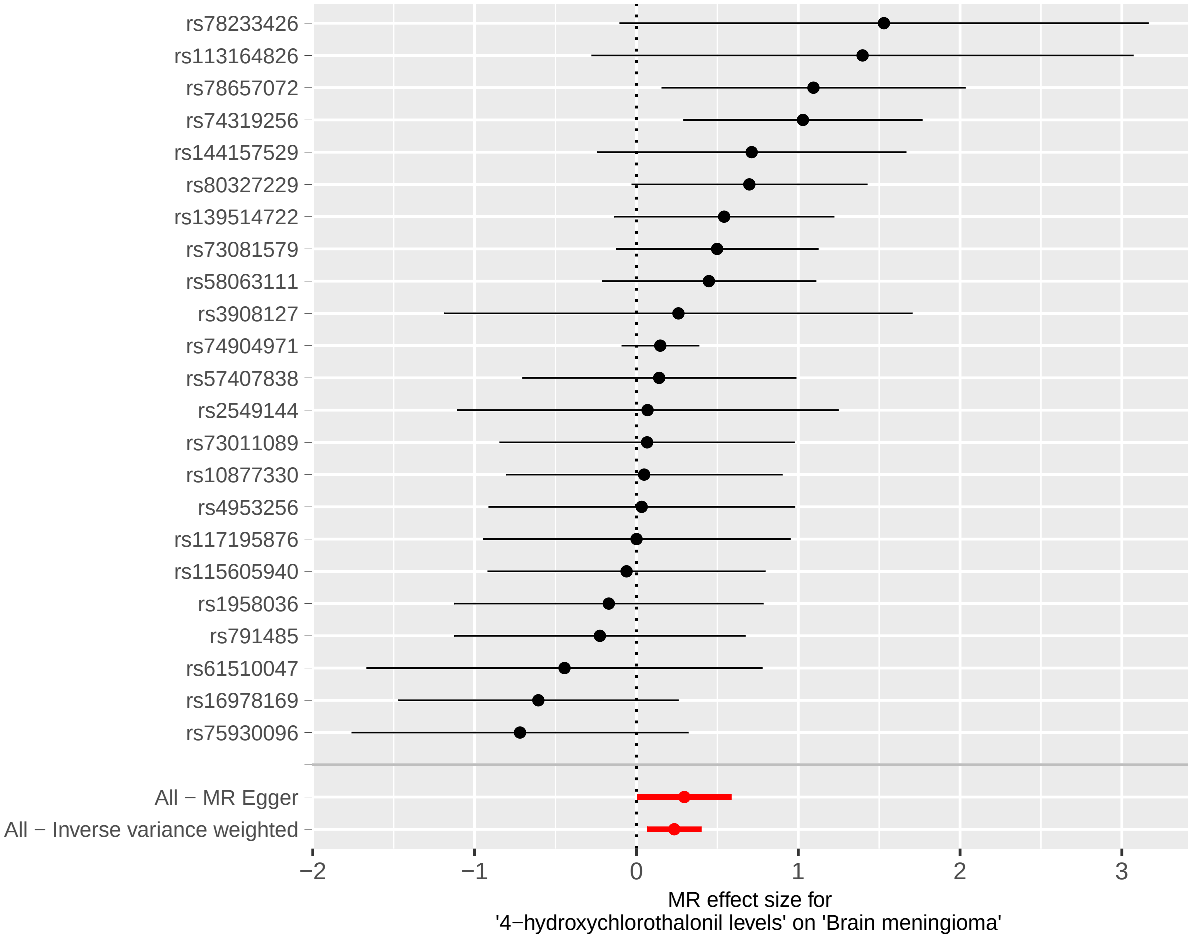
rs78233426
rs113164826
rs78657072
rs74319256
rs144157529
rs80327229
rs139514722
rs73081579
rs58063111
rs3908127
rs74904971
rs57407838
rs2549144
rs73011089
rs10877330
rs4953256
rs117195876
rs115605940
rs1958036
rs791485
rs61510047
rs16978169
rs75930096
All − MR Egger
All − Inverse variance weighted
−2
−1
0
1
2
3
MR effect size for
'4−hydroxychlorothalonil levels' on 'Brain meningioma'

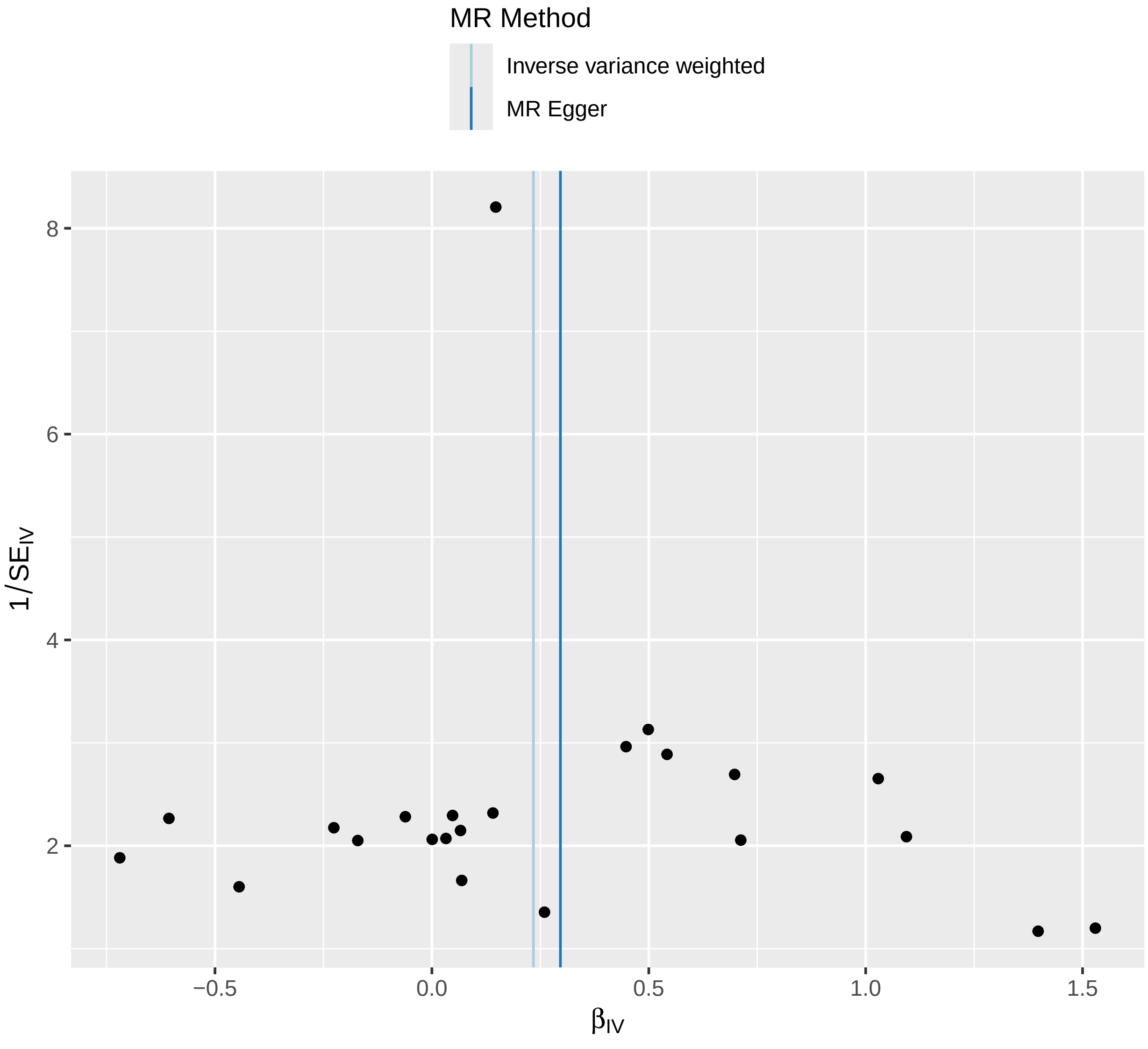

MR Method
Inverse variance weighted
MR Egger
8
6
4
2
1/SE_IV
−0.5
0.0
0.5
1.0
1.5
β_IV

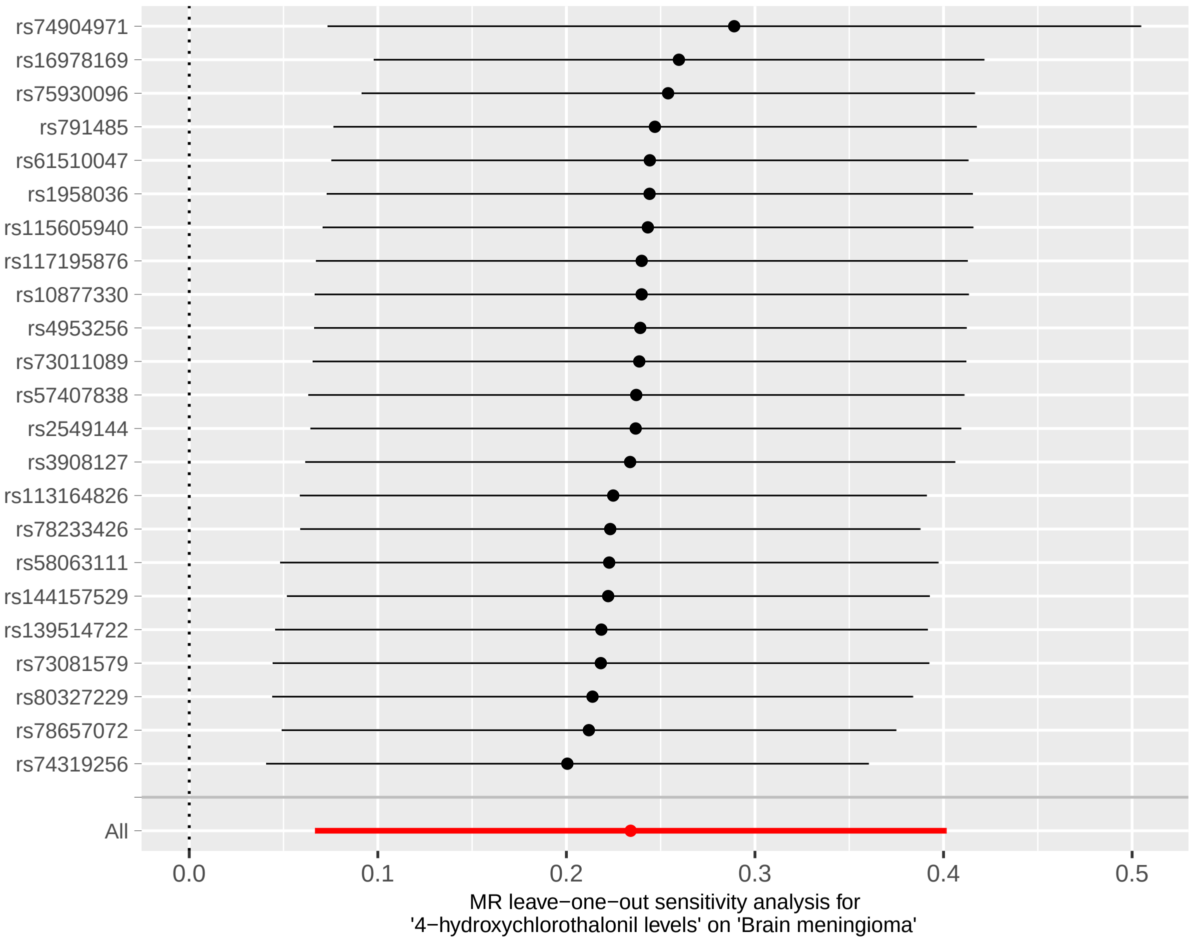

rs74904971
rs16978169
rs75930096
rs791485
rs61510047
rs1958036
rs115605940
rs117195876
rs10877330
rs4953256
rs73011089
rs57407838
rs2549144
rs3908127
rs113164826
rs78233426
rs58063111
rs144157529
rs139514722
rs73081579
rs80327229
rs78657072
rs74319256
All
0.0
0.1
0.2
0.3
0.4
0.5
MR leave−one−out sensitivity analysis for
'4−hydroxychlorothalonil levels' on 'Brain meningioma'

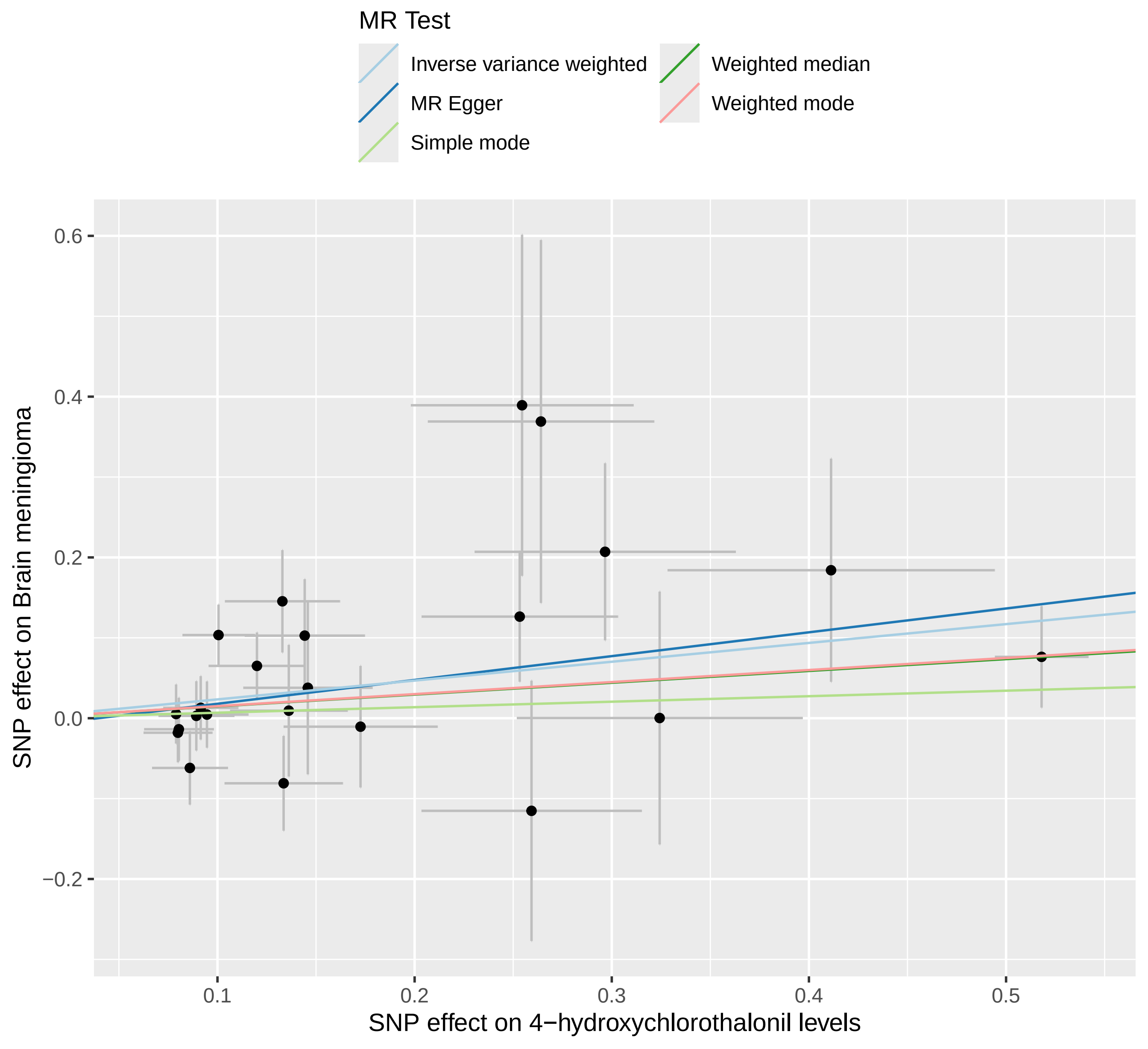

MR Test
Inverse variance weighted
MR Egger
Simple mode
Weighted median
Weighted mode
SNP effect on Brain meningioma
0.6
0.4
0.2
0.0
−0.2
0.1
0.2
0.3
0.4
0.5
SNP effect on 4−hydroxychlorothalonil levels

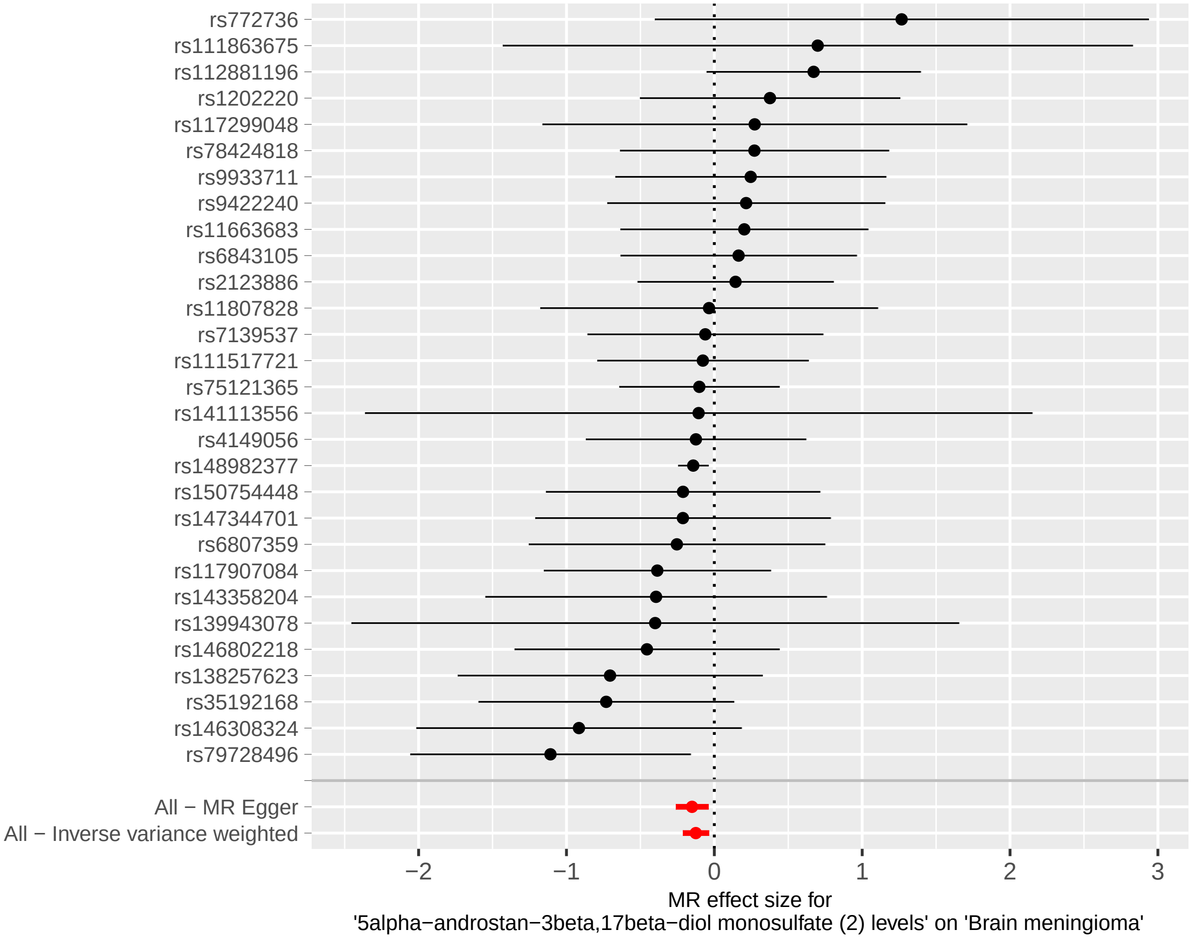
rs772736
rs111863675
rs112881196
rs1202220
rs117299048
rs78424818
rs9933711
rs9422240
rs11663683
rs6843105
rs2123886
rs11807828
rs7139537
rs111517721
rs75121365
rs141113556
rs4149056
rs148982377
rs150754448
rs147344701
rs6807359
rs117907084
rs143358204
rs139943078
rs146802218
rs138257623
rs35192168
rs146308324
rs79728496
All − MR Egger
All − Inverse variance weighted
−2
−1
0
1
2
3
MR effect size for
'5alpha−androstan−3beta,17beta−diol monosulfate (2) levels' on 'Brain meningioma'

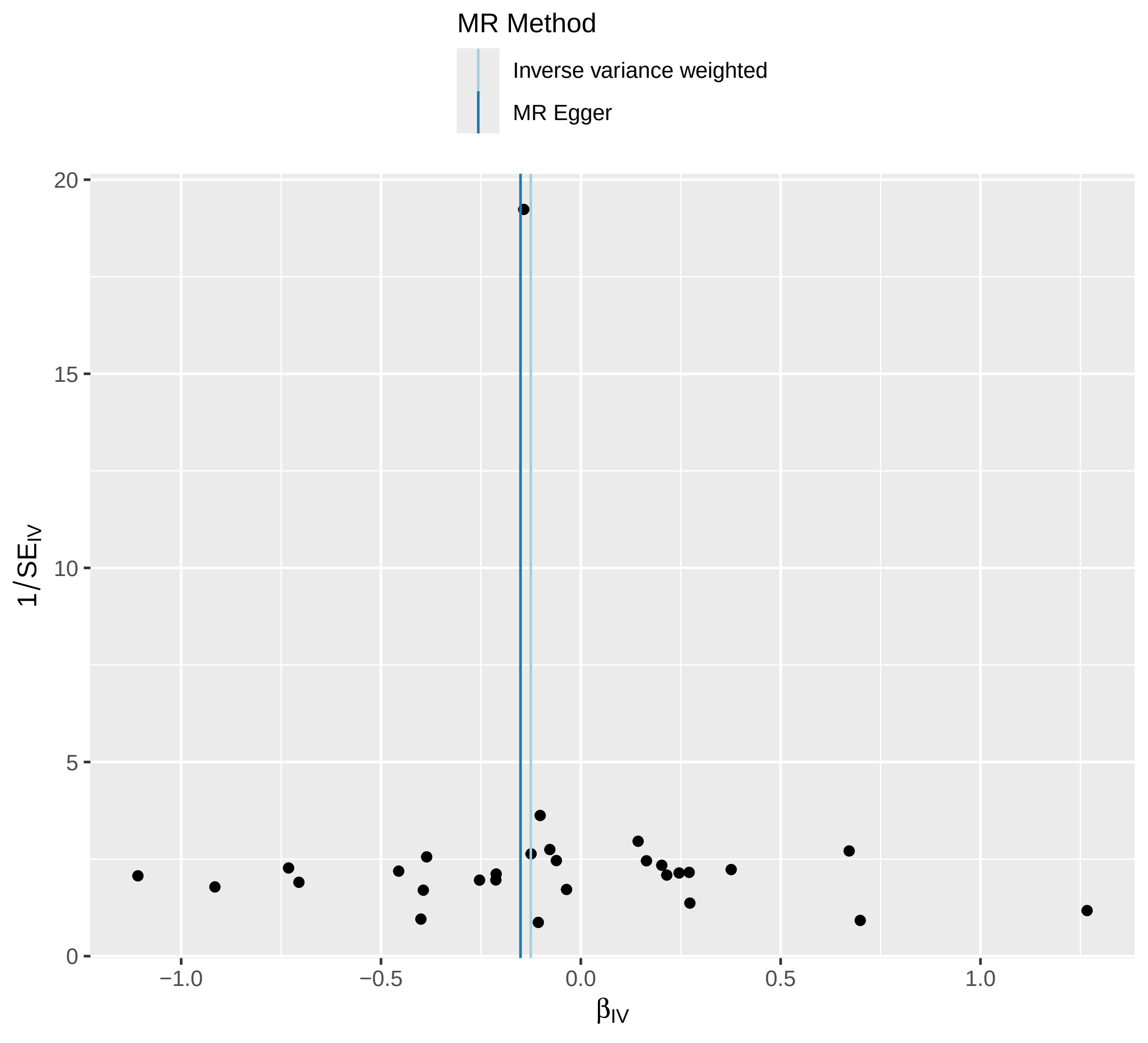

MR Method
Inverse variance weighted
MR Egger
20
15
10
5
0
1/SE_IV
−1.0
−0.5
0.0
0.5
1.0
β_IV

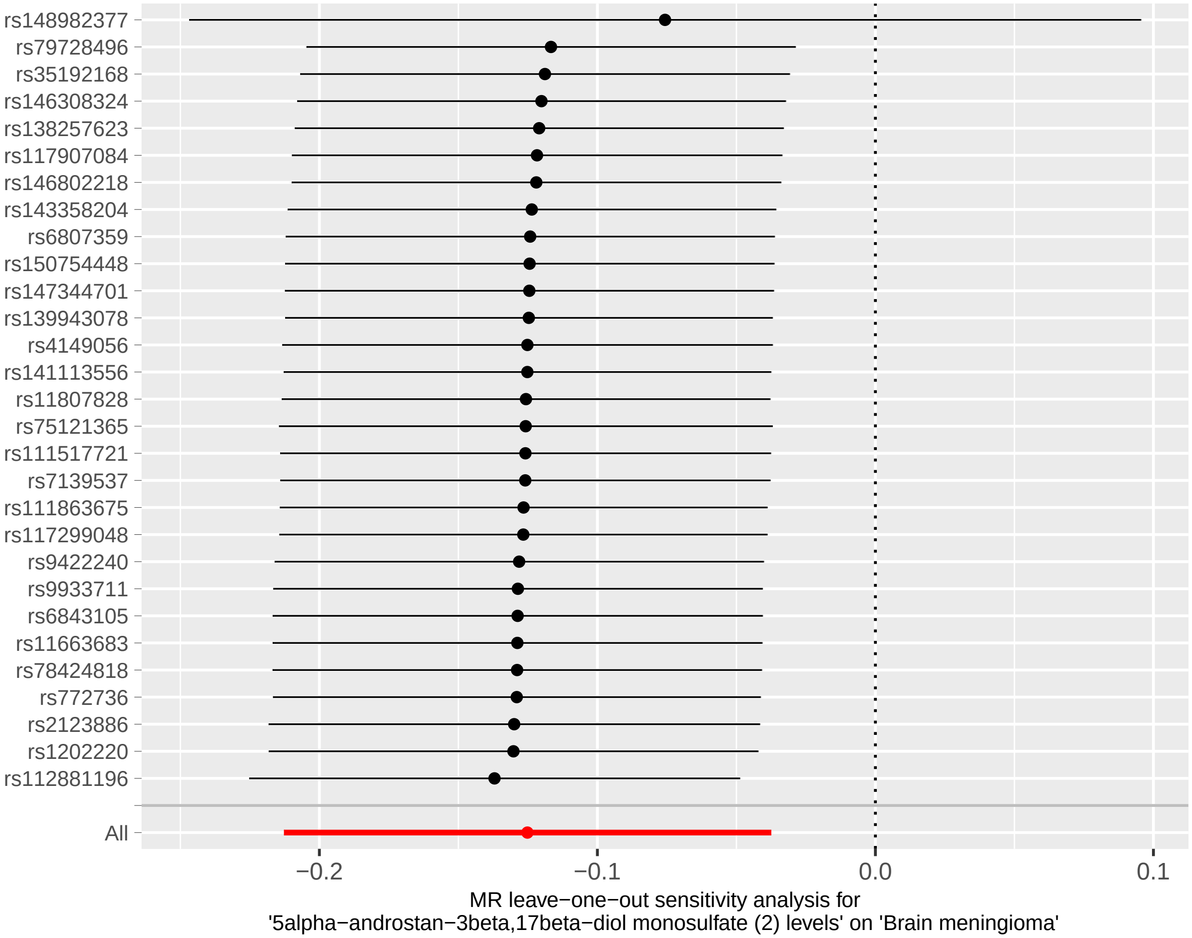

rs148982377
rs79728496
rs35192168
rs146308324
rs138257623
rs117907084
rs146802218
rs143358204
rs6807359
rs150754448
rs147344701
rs139943078
rs4149056
rs141113556
rs11807828
rs75121365
rs111517721
rs7139537
rs111863675
rs117299048
rs9422240
rs9933711
rs6843105
rs11663683
rs78424818
rs772736
rs2123886
rs1202220
rs112881196
All
−0.2
−0.1
0.0
0.1
MR leave−one−out sensitivity analysis for
'5alpha−androstan−3beta,17beta−diol monosulfate (2) levels' on 'Brain meningioma'

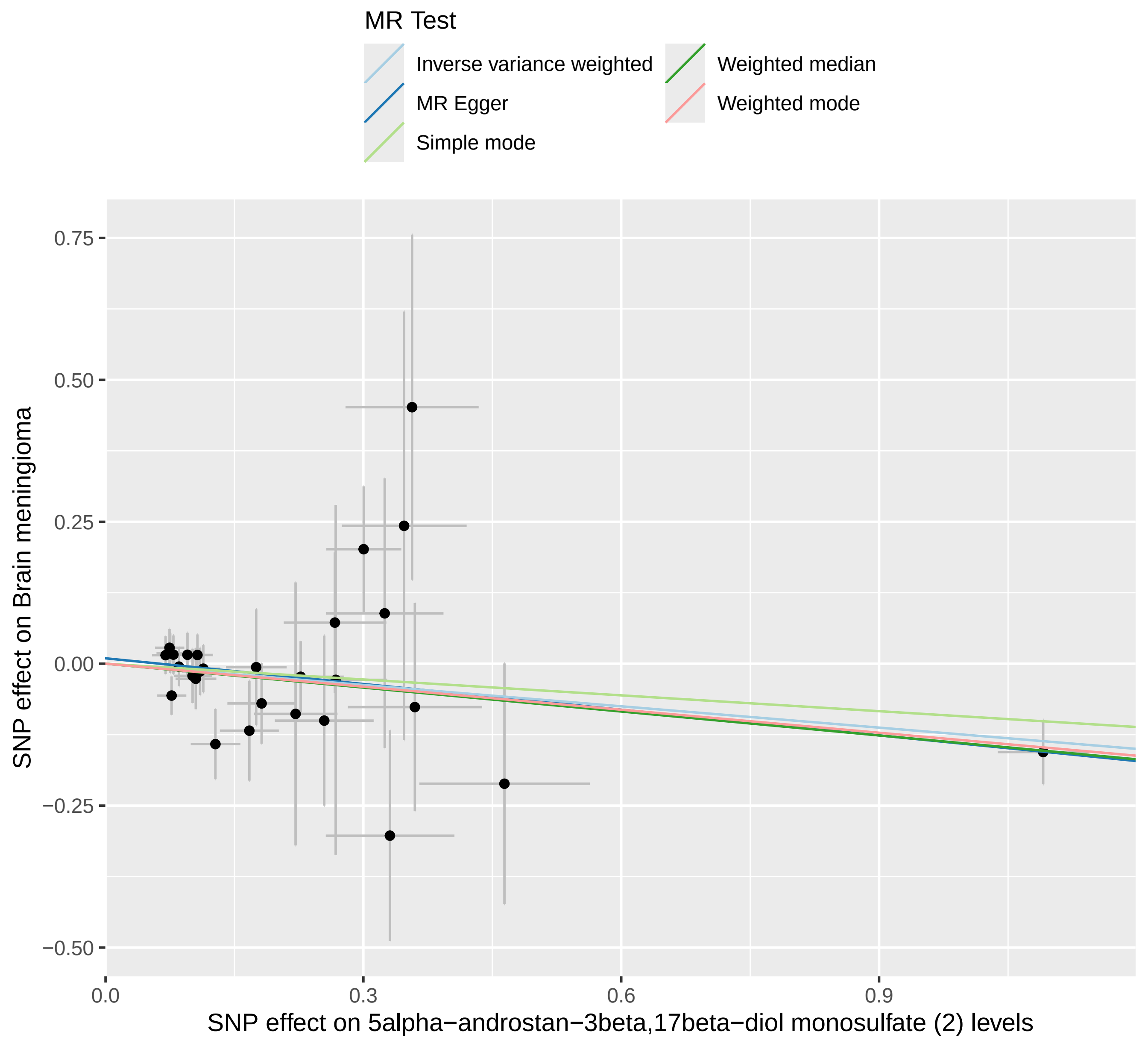

MR Test
Inverse variance weighted
MR Egger
Simple mode
Weighted median
Weighted mode
0.75
0.50
0.25
0.00
−0.25
−0.50
SNP effect on Brain meningioma
0.0
0.3
0.6
0.9
SNP effect on 5alpha−androstan−3beta,17beta−diol monosulfate (2) levels

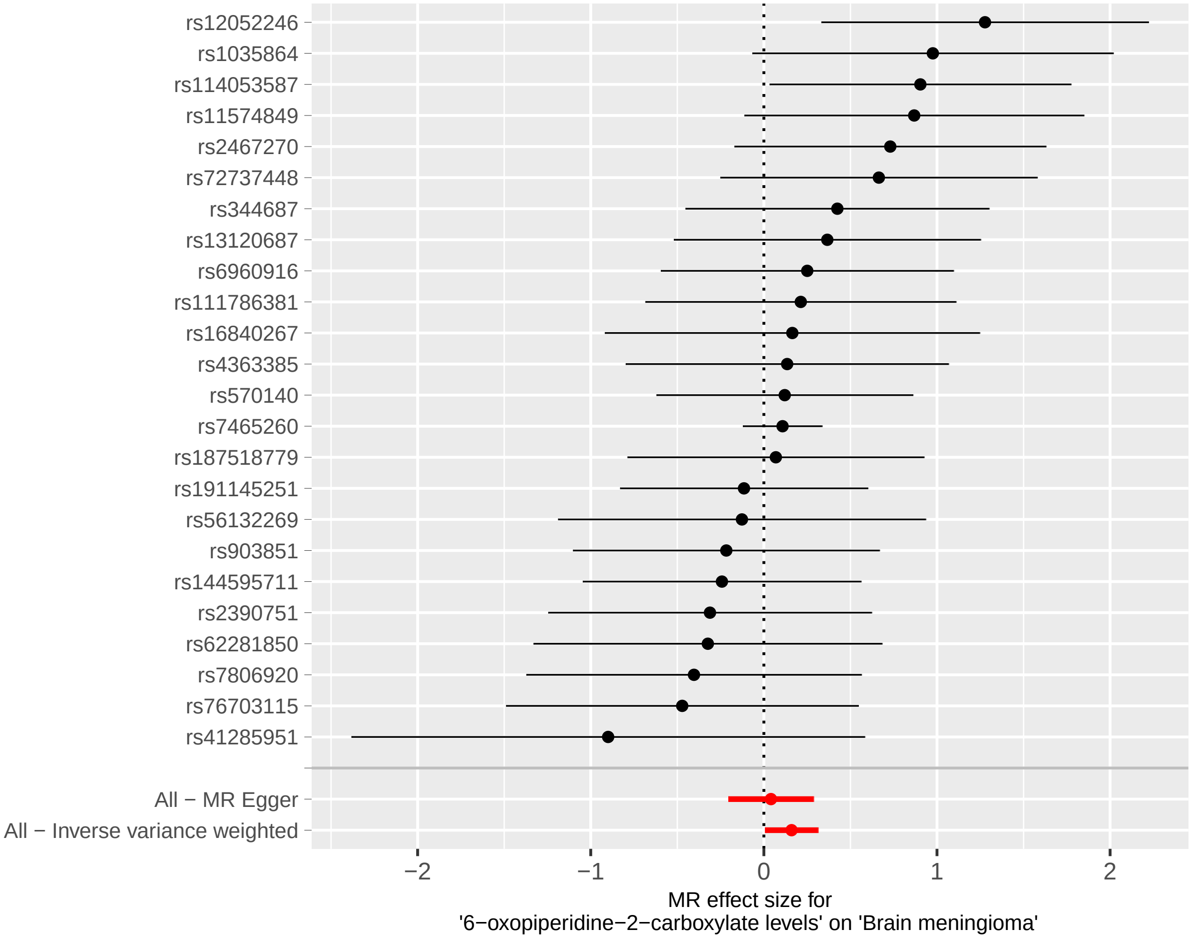

rs12052246
rs1035864
rs114053587
rs11574849
rs2467270
rs72737448
rs344687
rs13120687
rs6960916
rs111786381
rs16840267
rs4363385
rs570140
rs7465260
rs187518779
rs191145251
rs56132269
rs903851
rs144595711
rs2390751
rs62281850
rs7806920
rs76703115
rs41285951
All − MR Egger
All − Inverse variance weighted
−2
−1
0
1
2
MR effect size for
'6−oxopiperidine−2−carboxylate levels' on 'Brain meningioma'

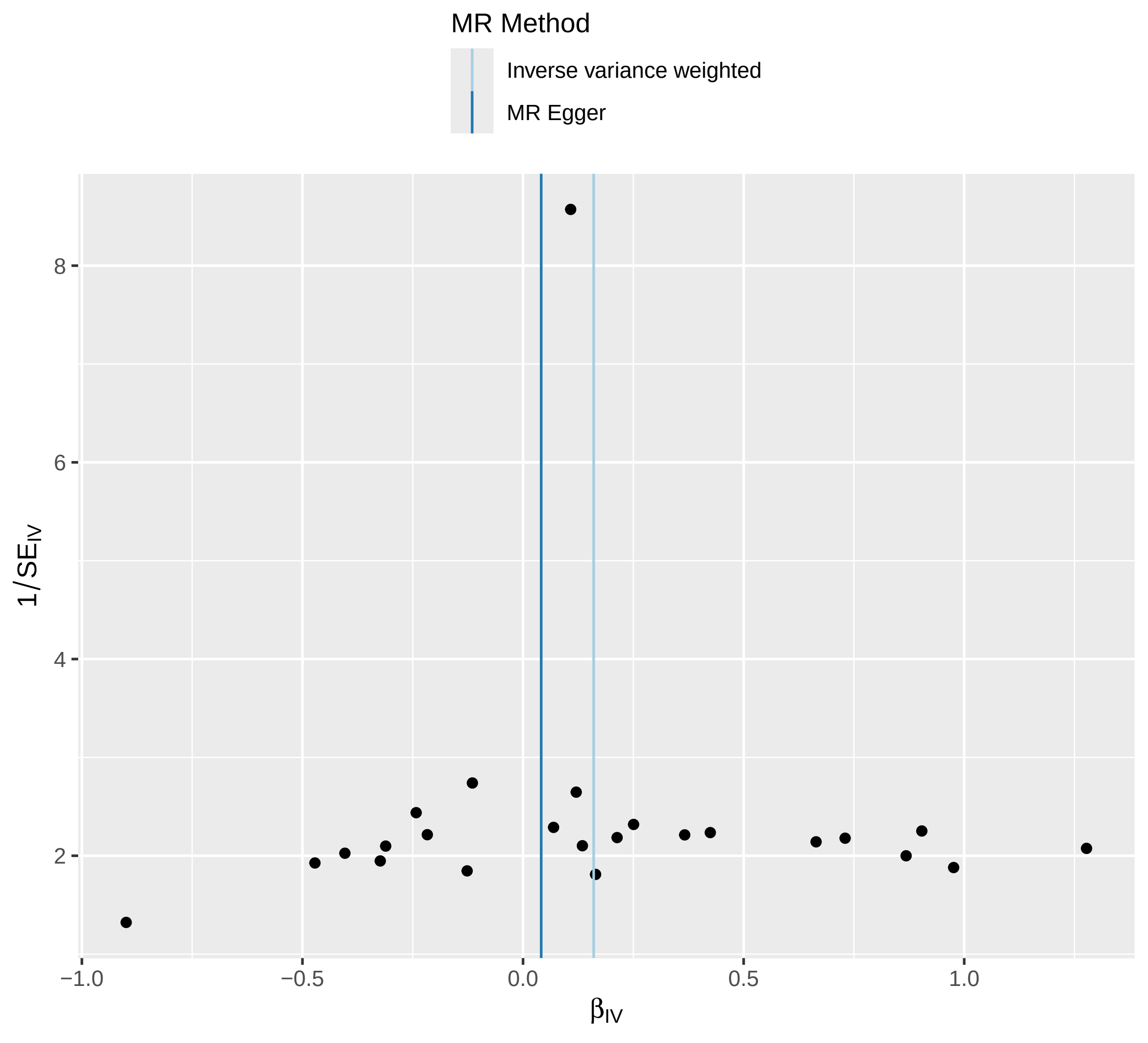
MR Method
Inverse variance weighted
MR Egger
1/SE_IV
8
6
4
2
−1.0
−0.5
0.0
0.5
1.0
β_IV

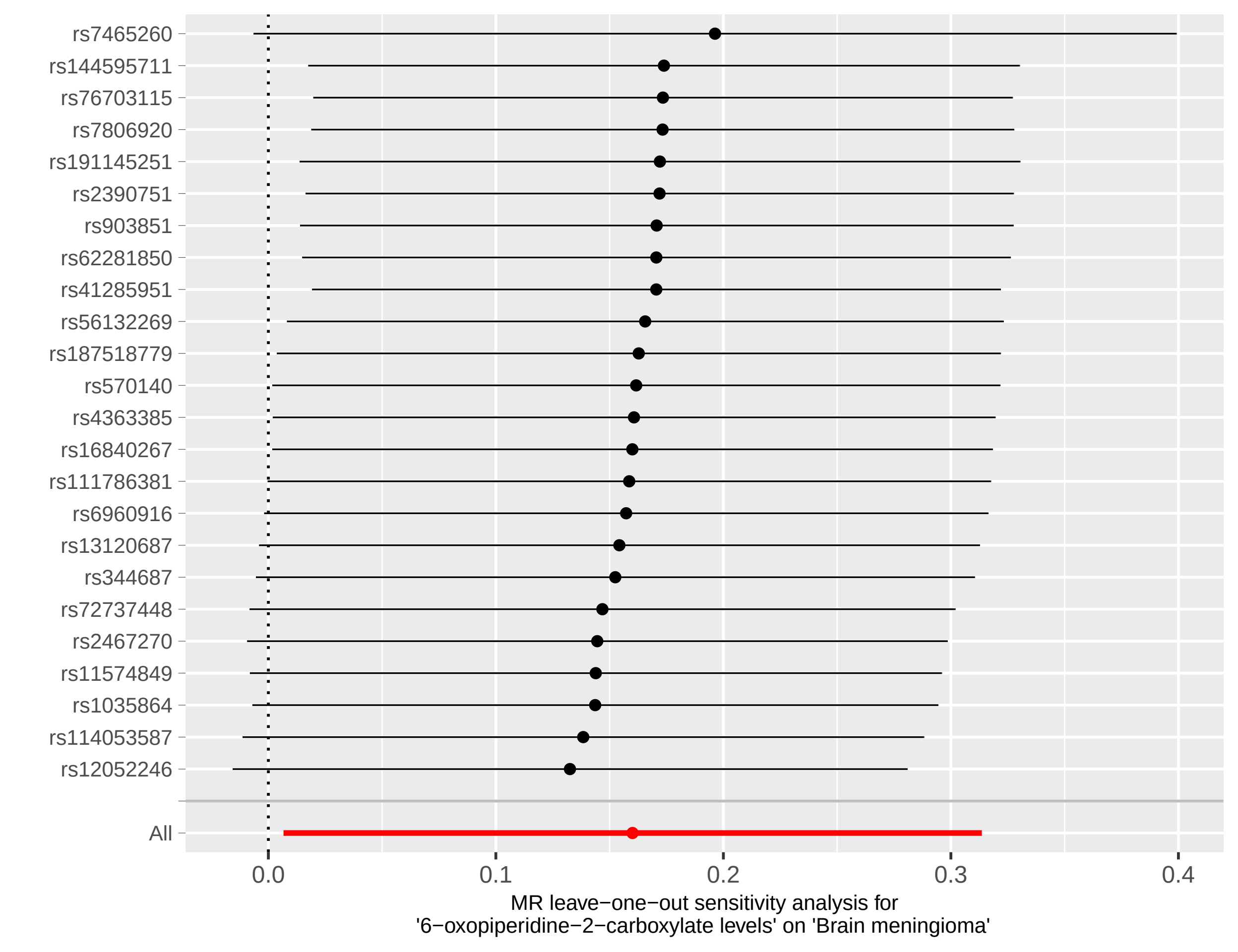
rs7465260
rs144595711
rs76703115
rs7806920
rs191145251
rs2390751
rs903851
rs62281850
rs41285951
rs56132269
rs187518779
rs570140
rs4363385
rs16840267
rs111786381
rs6960916
rs13120687
rs344687
rs72737448
rs2467270
rs11574849
rs1035864
rs114053587
rs12052246
All
0.0
0.1
0.2
0.3
0.4
MR leave−one−out sensitivity analysis for
'6−oxopiperidine−2−carboxylate levels' on 'Brain meningioma'

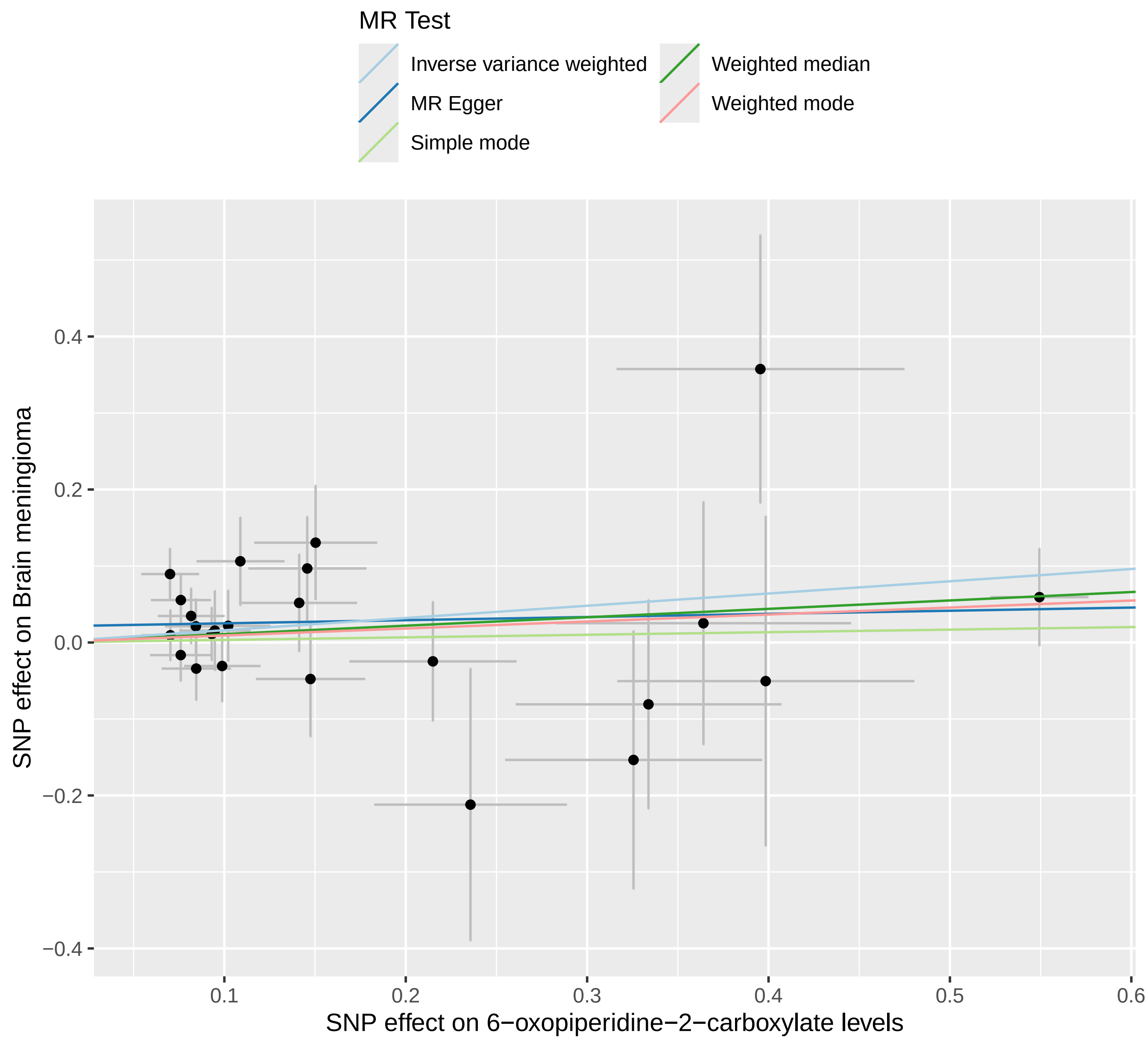

MR Test
Inverse variance weighted
MR Egger
Simple mode
Weighted median
Weighted mode
SNP effect on Brain meningioma
0.4
0.2
0.0
−0.2
−0.4
0.1
0.2
0.3
0.4
0.5
0.6
SNP effect on 6−oxopiperidine−2−carboxylate levels

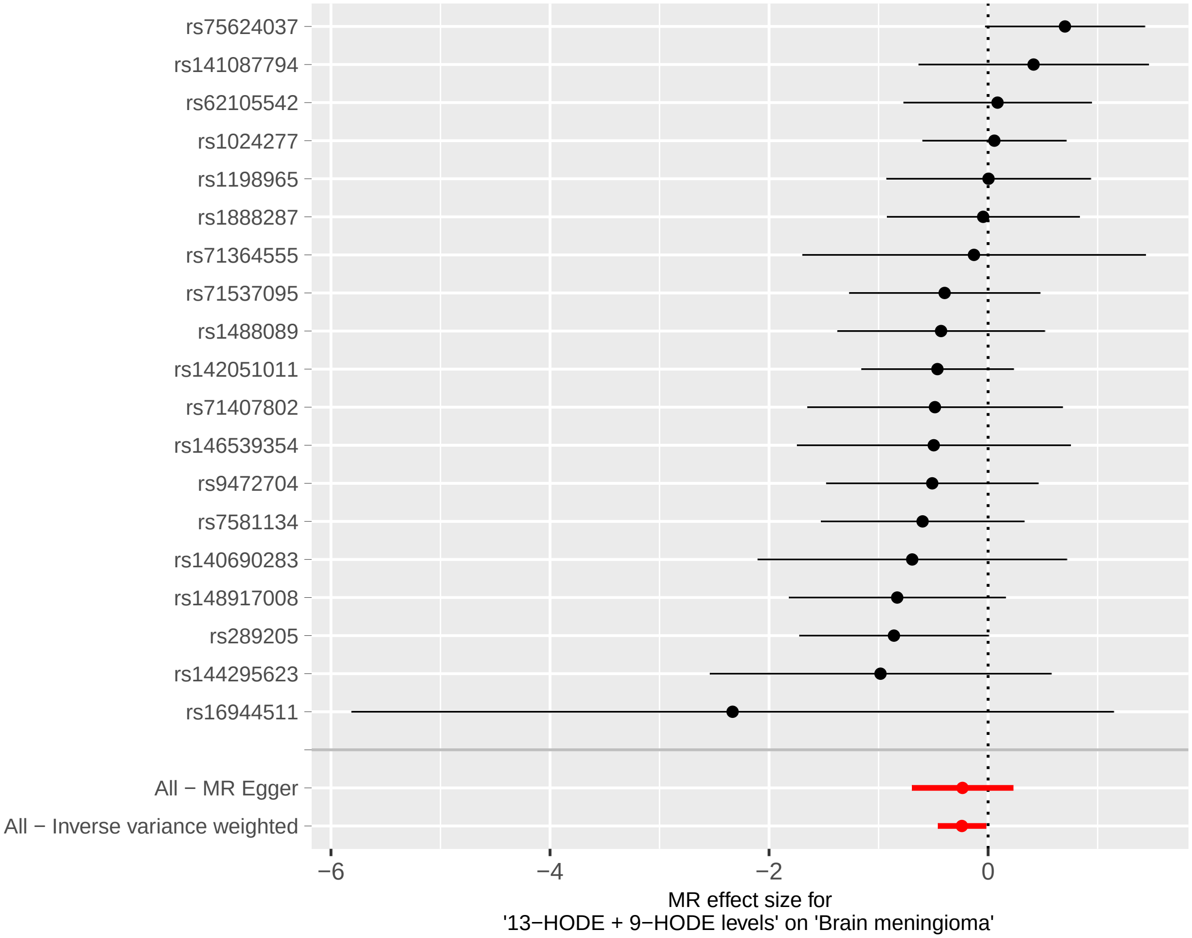
rs75624037
rs141087794
rs62105542
rs1024277
rs1198965
rs1888287
rs71364555
rs71537095
rs1488089
rs142051011
rs71407802
rs146539354
rs9472704
rs7581134
rs140690283
rs148917008
rs289205
rs144295623
rs16944511
All − MR Egger
All − Inverse variance weighted
−6
−4
−2
0
MR effect size for
'13−HODE + 9−HODE levels' on 'Brain meningioma'

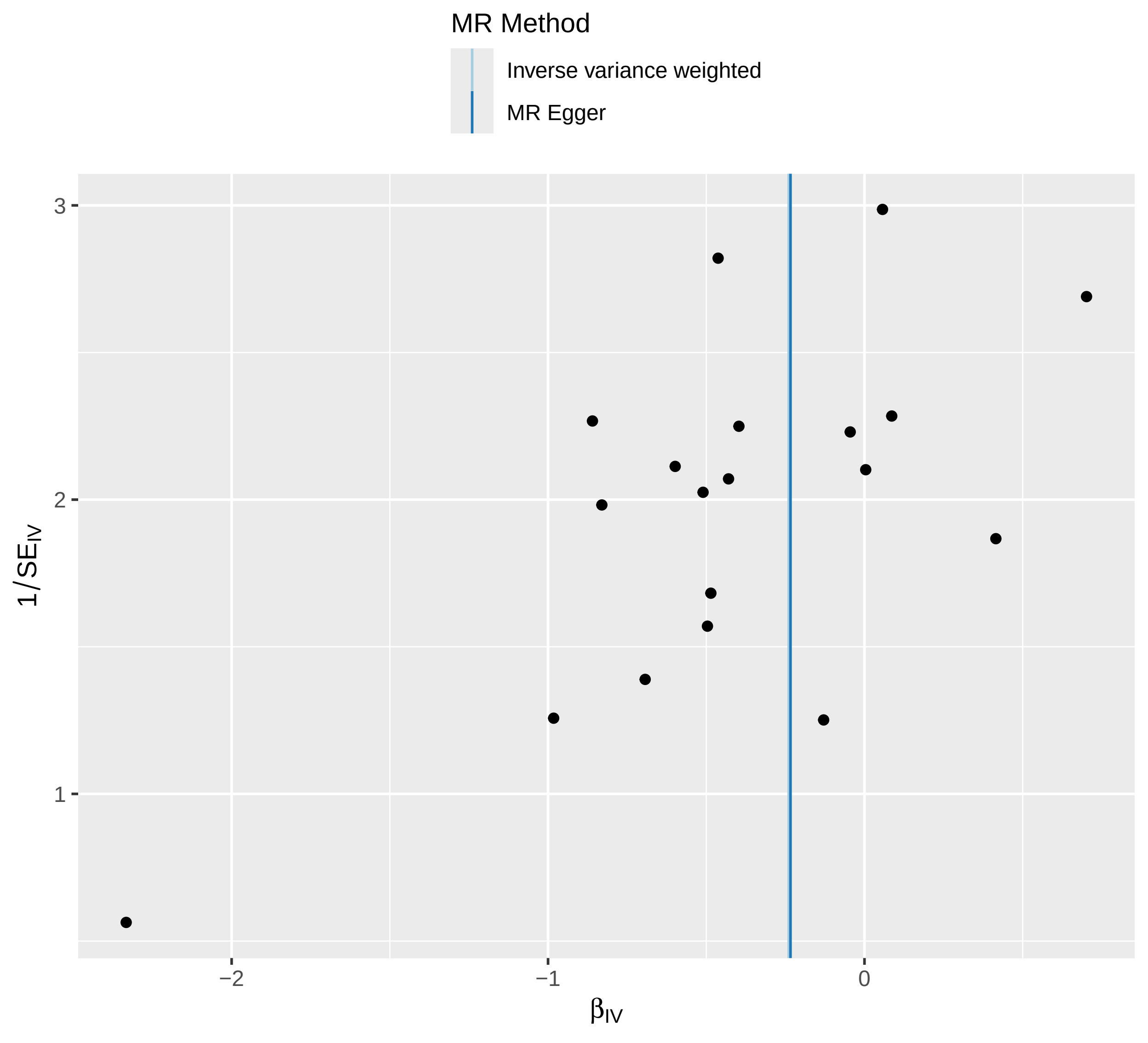

MR Method
Inverse variance weighted
MR Egger
3
2
1
1/SE_IV
−2
−1
0
β_IV

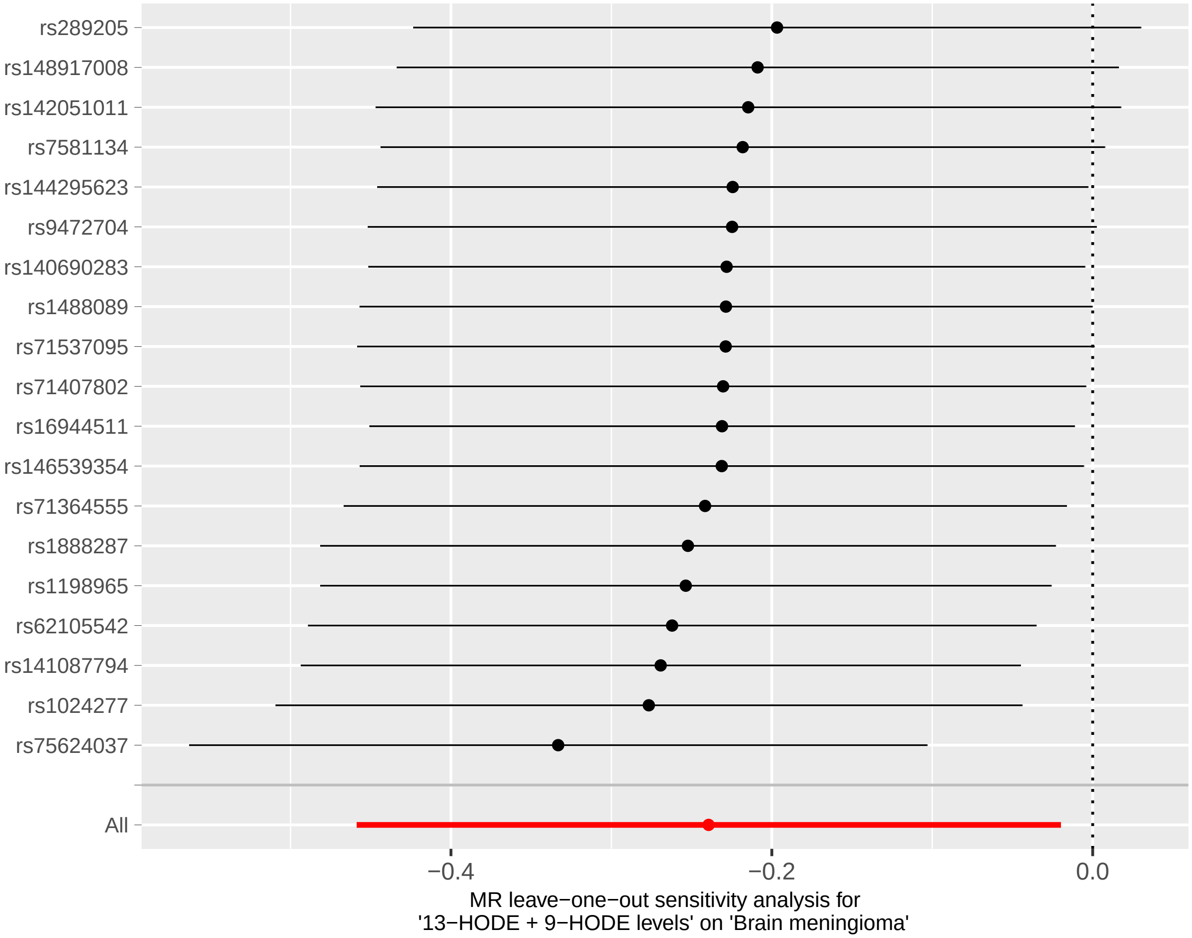
rs289205
rs148917008
rs142051011
rs7581134
rs144295623
rs9472704
rs140690283
rs1488089
rs71537095
rs71407802
rs16944511
rs146539354
rs71364555
rs1888287
rs1198965
rs62105542
rs141087794
rs1024277
rs75624037
All
−0.4
−0.2
0.0
MR leave−one−out sensitivity analysis for
'13−HODE + 9−HODE levels' on 'Brain meningioma'

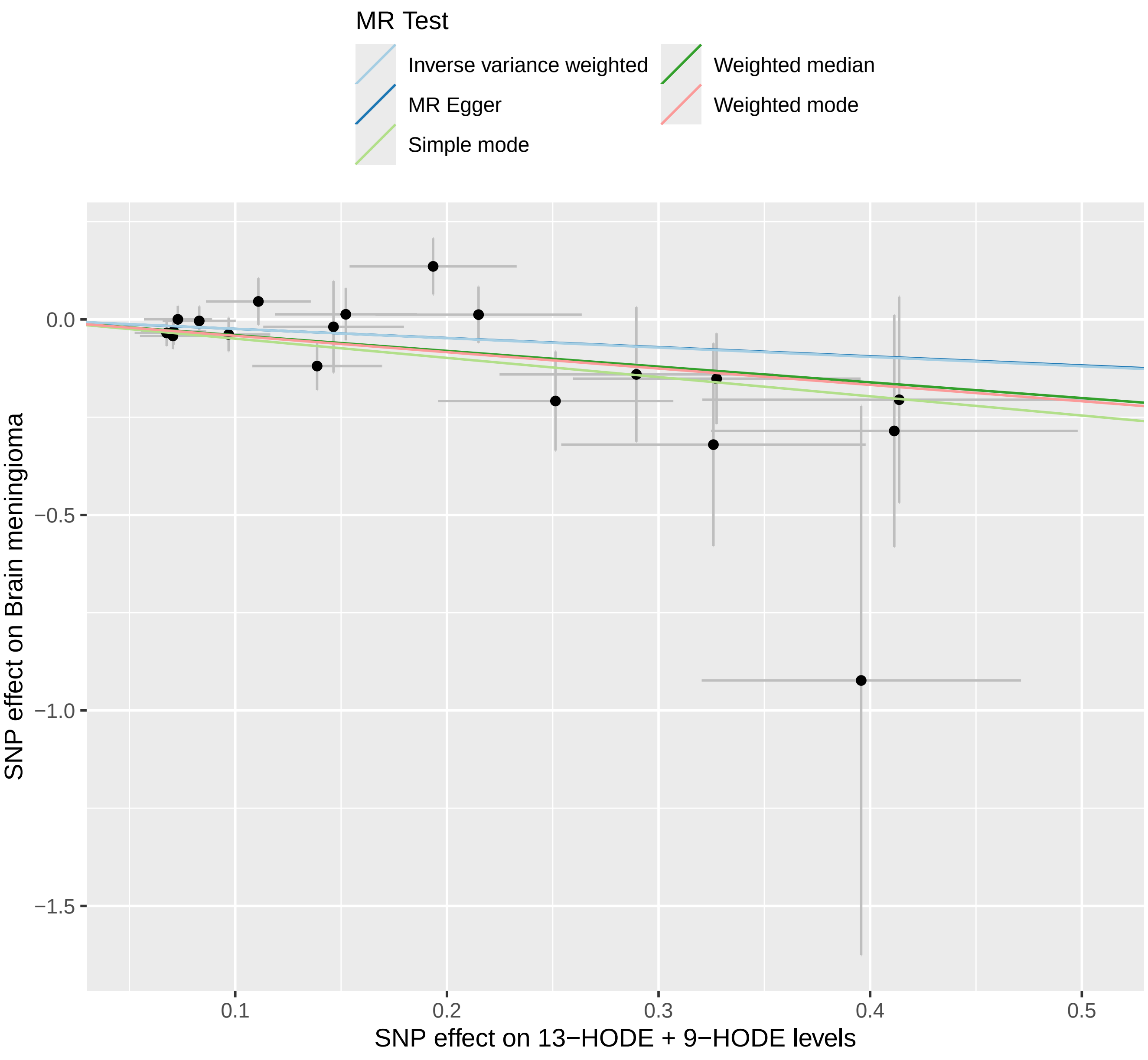
MR Test
Inverse variance weighted
MR Egger
Simple mode
Weighted median
Weighted mode
0.0
−0.5
−1.0
−1.5
SNP effect on Brain meningioma
0.1
0.2
0.3
0.4
0.5
SNP effect on 13−HODE + 9−HODE levels

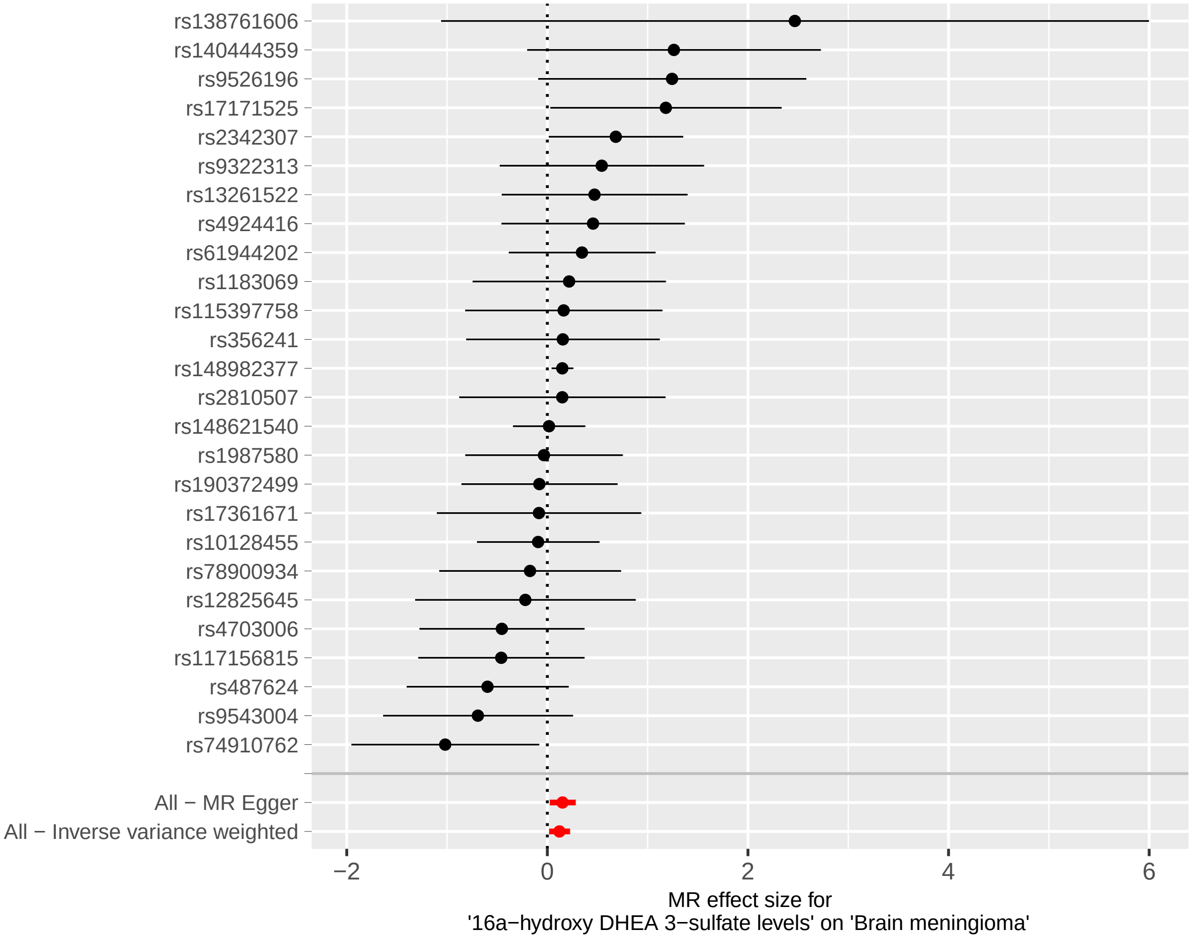
rs138761606
rs140444359
rs9526196
rs17171525
rs2342307
rs9322313
rs13261522
rs4924416
rs61944202
rs1183069
rs115397758
rs356241
rs148982377
rs2810507
rs148621540
rs1987580
rs190372499
rs17361671
rs10128455
rs78900934
rs12825645
rs4703006
rs117156815
rs487624
rs9543004
rs74910762
All − MR Egger
All − Inverse variance weighted
−2
0
2
4
6
MR effect size for
'16a−hydroxy DHEA 3−sulfate levels' on 'Brain meningioma'

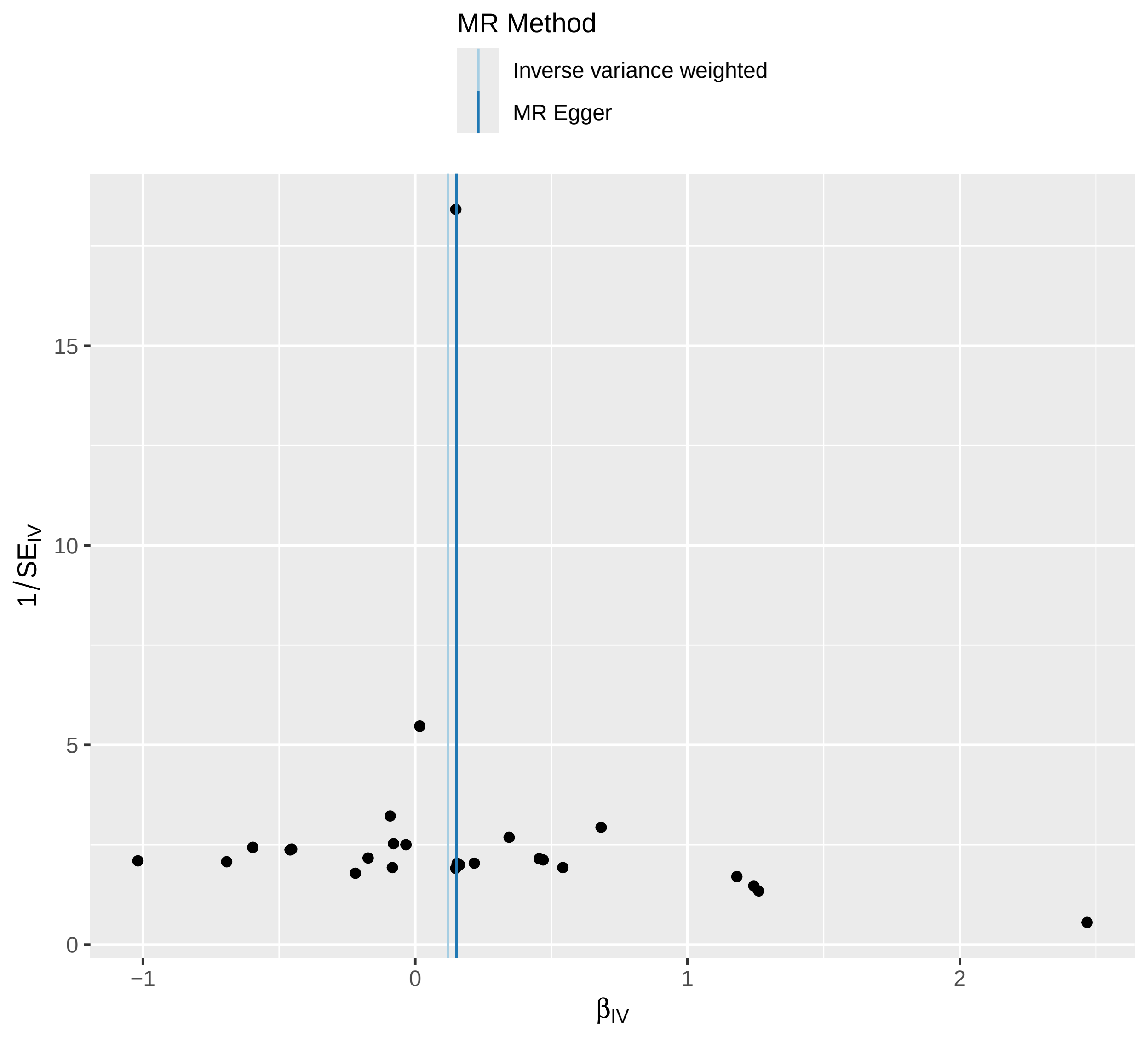

MR Method
Inverse variance weighted
MR Egger
1/SE_IV
15
10
5
0
−1
0
1
2
β_IV

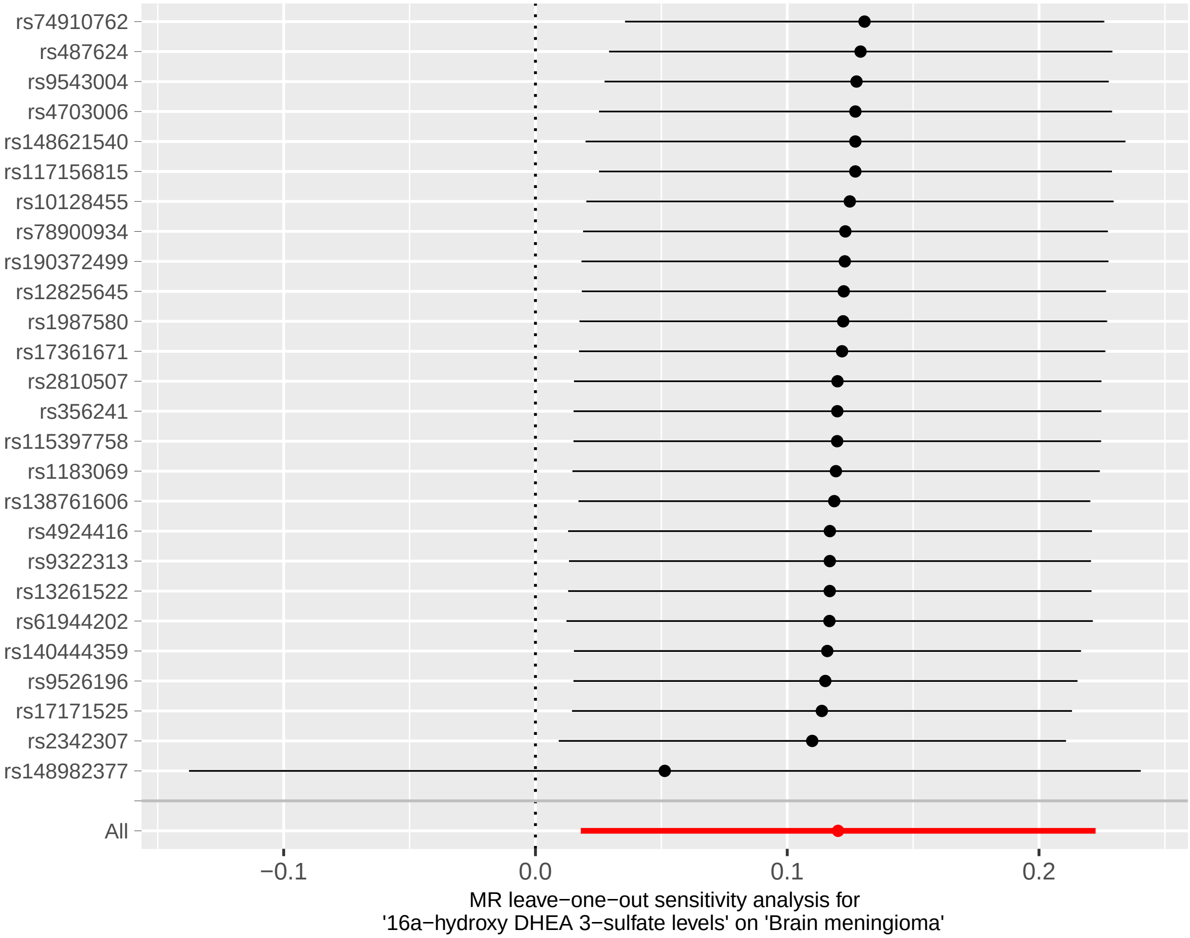
rs74910762
rs487624
rs9543004
rs4703006
rs148621540
rs117156815
rs10128455
rs78900934
rs190372499
rs12825645
rs1987580
rs17361671
rs2810507
rs356241
rs115397758
rs1183069
rs138761606
rs4924416
rs9322313
rs13261522
rs61944202
rs140444359
rs9526196
rs17171525
rs2342307
rs148982377
All
−0.1
0.0
0.1
0.2
MR leave−one−out sensitivity analysis for
'16a−hydroxy DHEA 3−sulfate levels' on 'Brain meningioma'

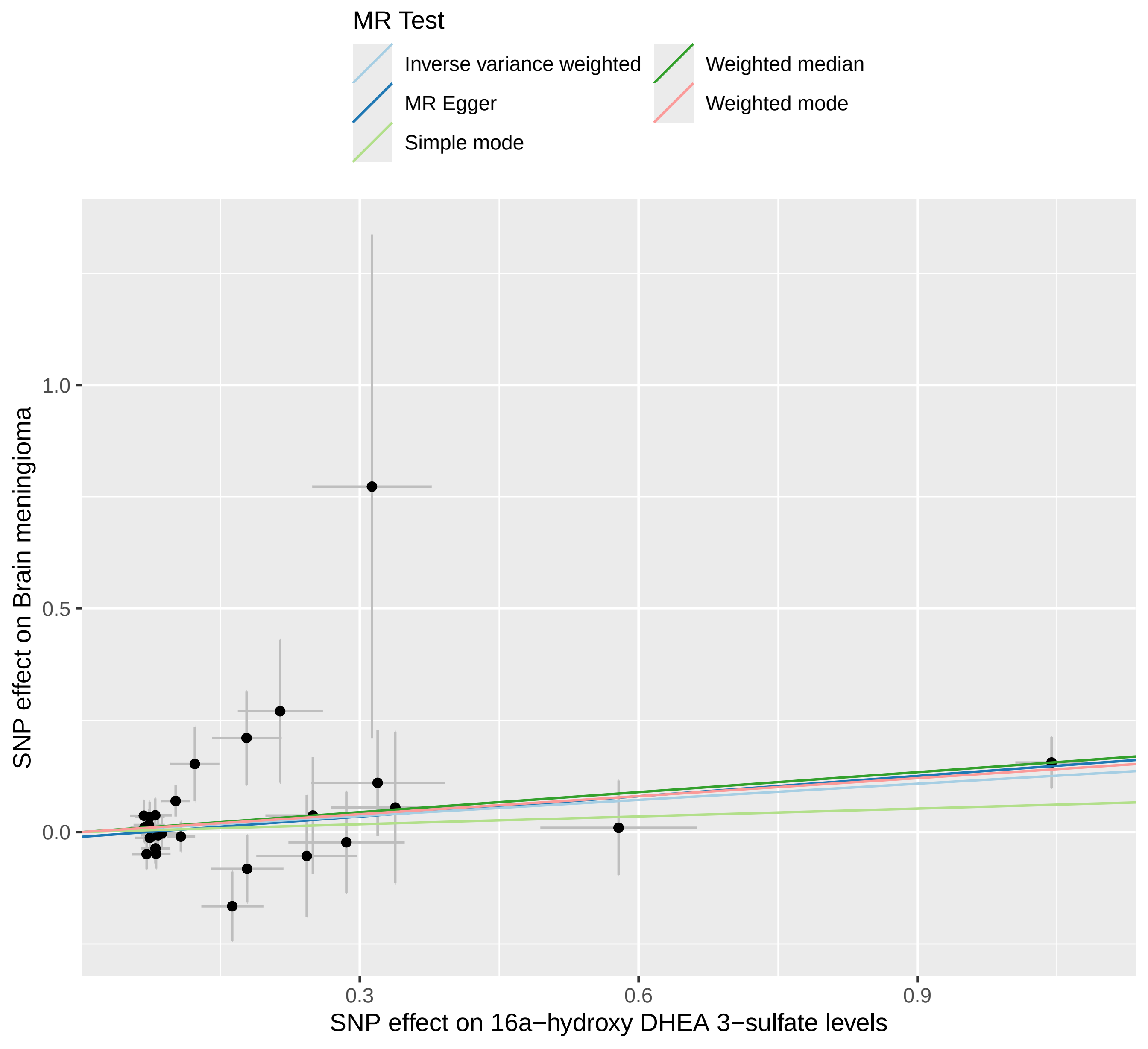
MR Test
Inverse variance weighted
MR Egger
Simple mode
Weighted median
Weighted mode
1.0
0.5
0.0
0.3
0.6
0.9
SNP effect on Brain meningioma
SNP effect on 16a−hydroxy DHEA 3−sulfate levels

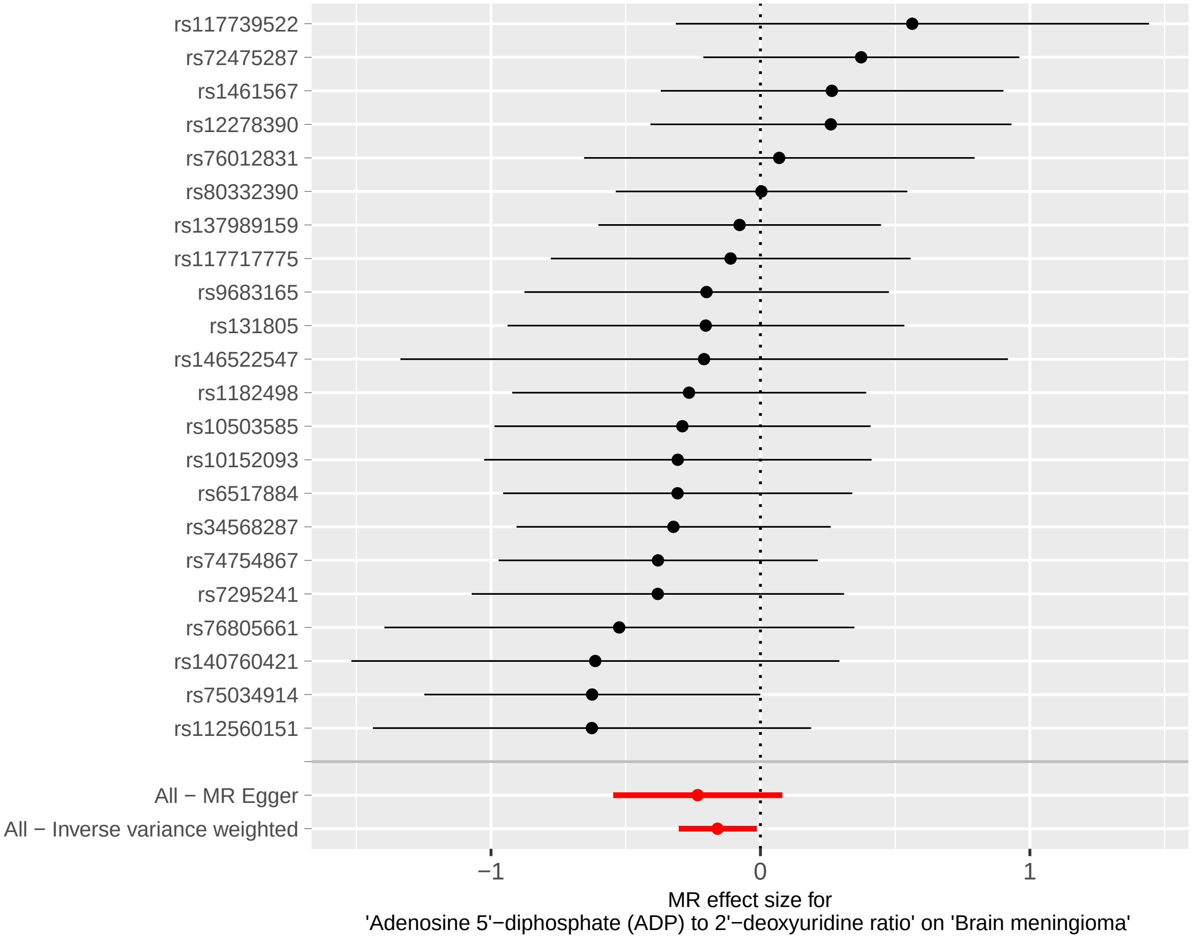
rs117739522
rs72475287
rs1461567
rs12278390
rs76012831
rs80332390
rs137989159
rs117717775
rs9683165
rs131805
rs146522547
rs1182498
rs10503585
rs10152093
rs6517884
rs34568287
rs74754867
rs7295241
rs76805661
rs140760421
rs75034914
rs112560151
All − MR Egger
All − Inverse variance weighted
−1
0
1
MR effect size for
'Adenosine 5'−diphosphate (ADP) to 2'−deoxyuridine ratio' on 'Brain meningioma'

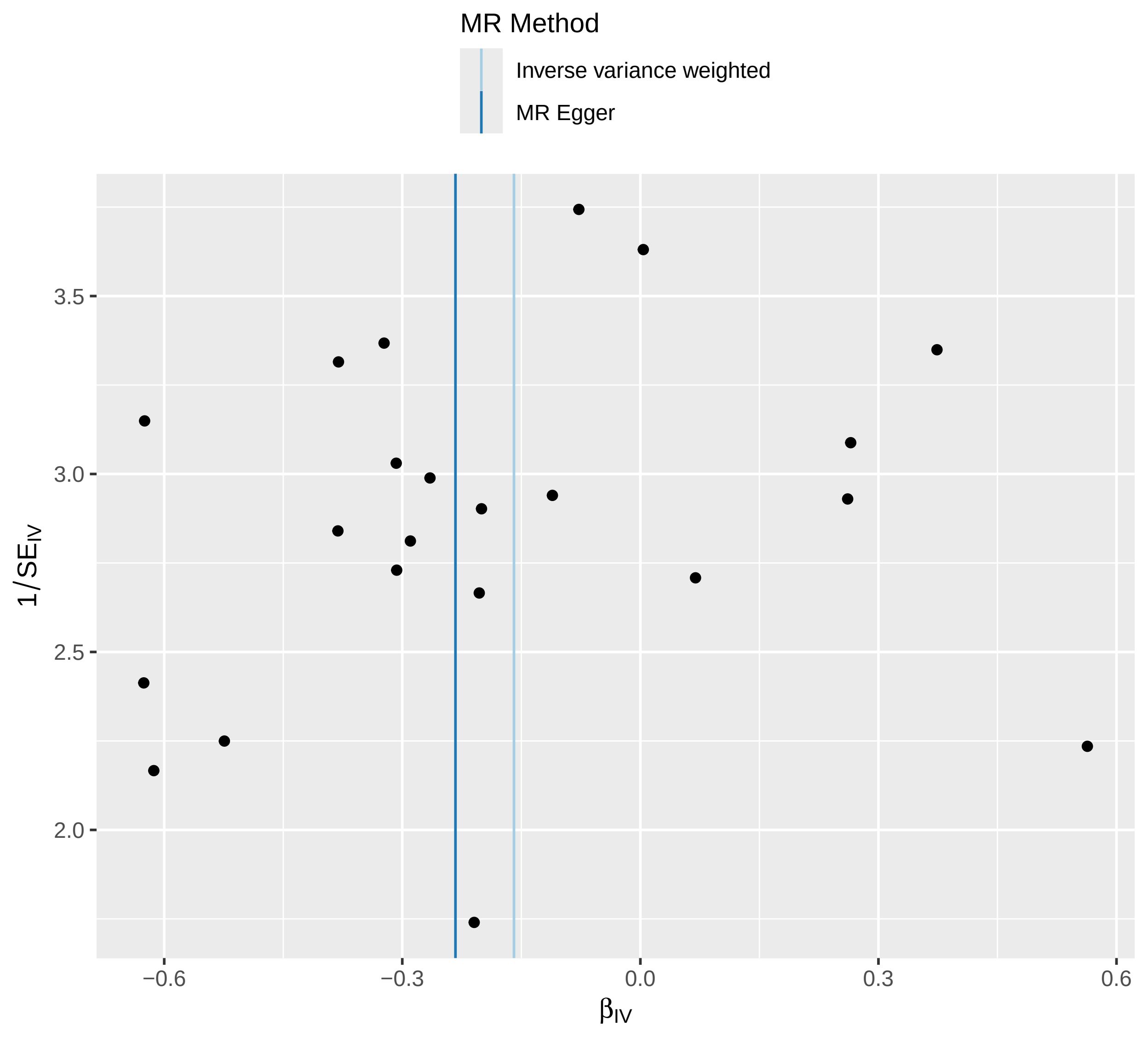
MR Method
Inverse variance weighted
MR Egger
3.5
3.0
2.5
2.0
1/SE_IV
−0.6
−0.3
0.0
0.3
0.6
β_IV

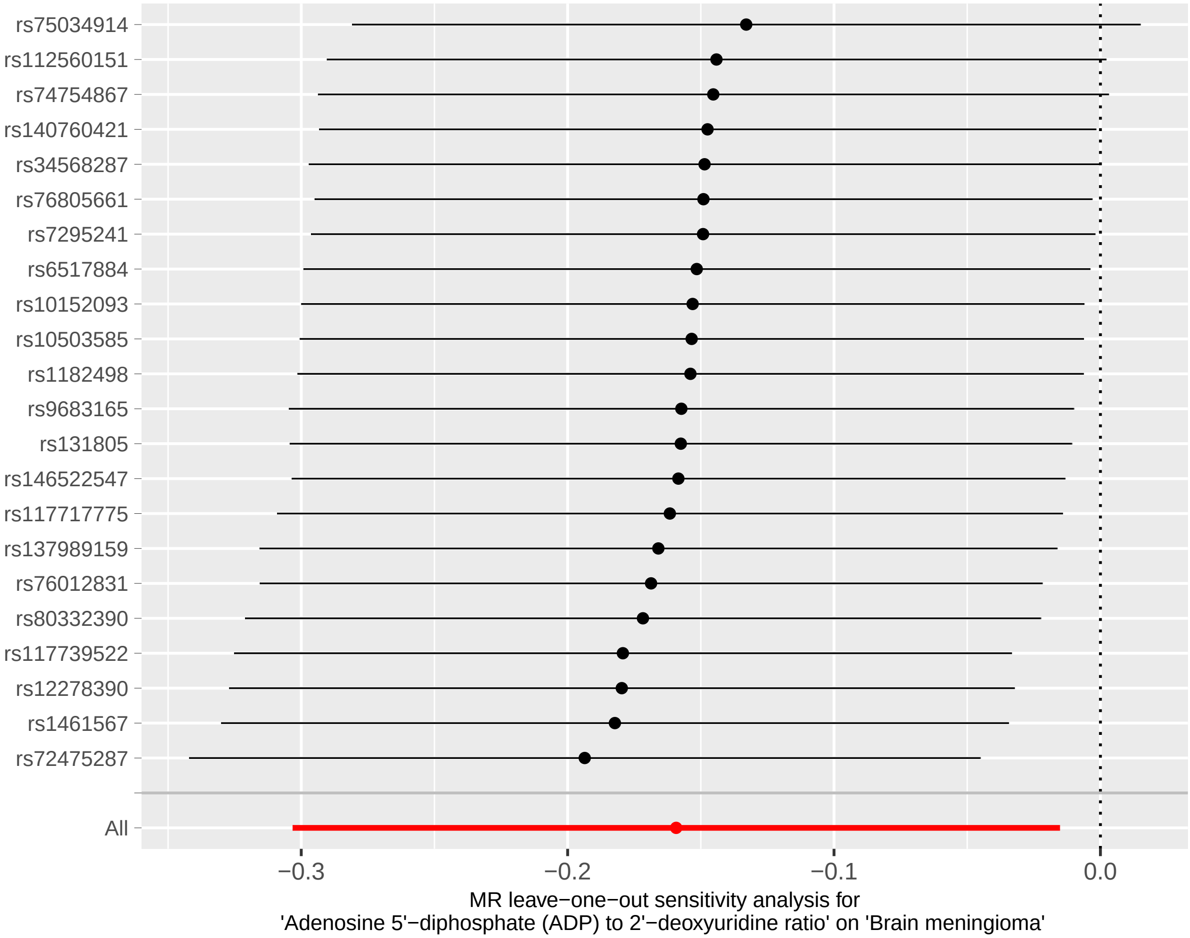

rs75034914
rs112560151
rs74754867
rs140760421
rs34568287
rs76805661
rs7295241
rs6517884
rs10152093
rs10503585
rs1182498
rs9683165
rs131805
rs146522547
rs117717775
rs137989159
rs76012831
rs80332390
rs117739522
rs12278390
rs1461567
rs72475287
All
−0.3
−0.2
−0.1
0.0
MR leave−one−out sensitivity analysis for
'Adenosine 5'−diphosphate (ADP) to 2'−deoxyuridine ratio' on 'Brain meningioma'

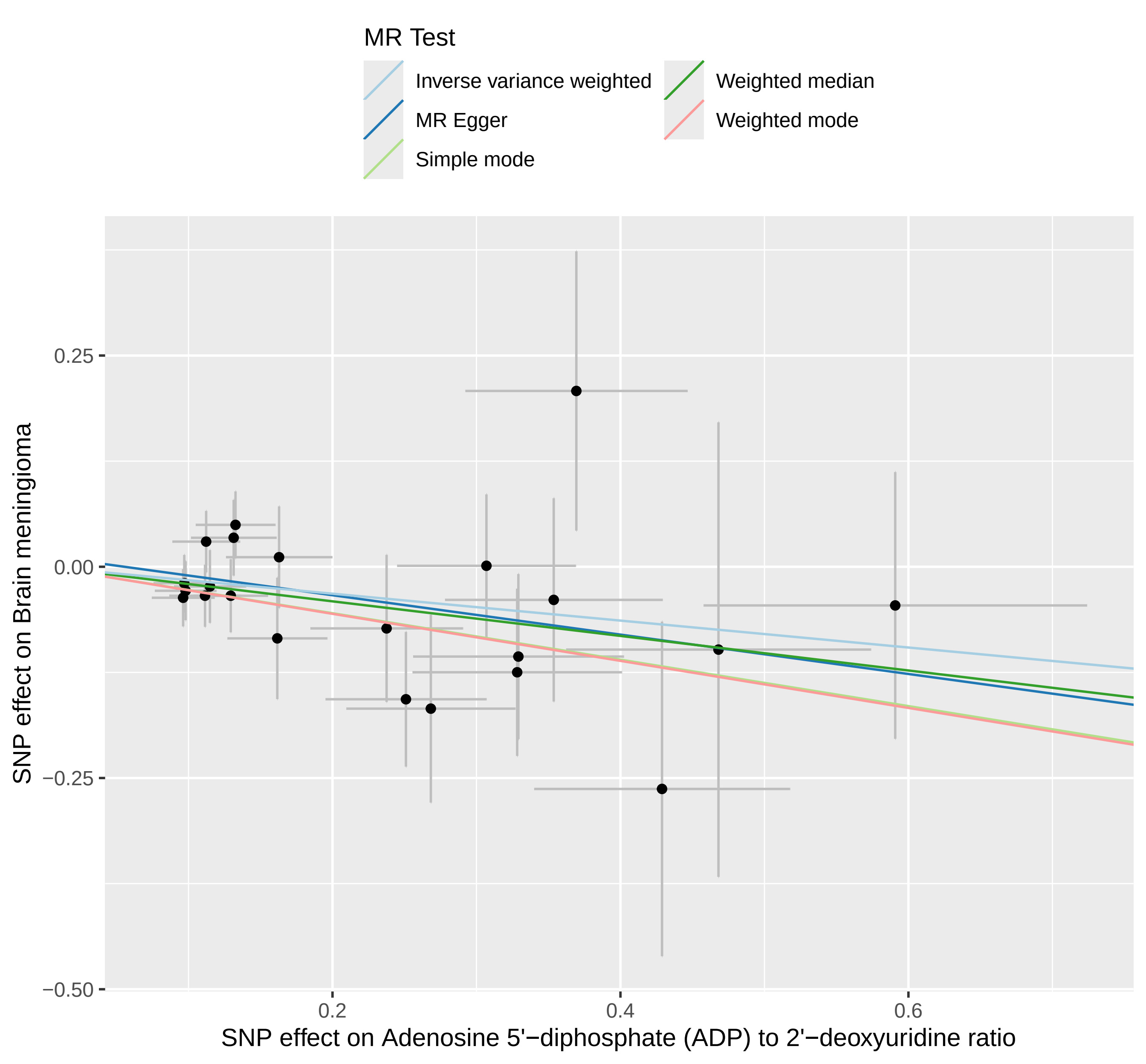
MR Test
Inverse variance weighted
MR Egger
Simple mode
Weighted median
Weighted mode
SNP effect on Brain meningioma
0.25
0.00
−0.25
−0.50
0.2
0.4
0.6
SNP effect on Adenosine 5'−diphosphate (ADP) to 2'−deoxyuridine ratio

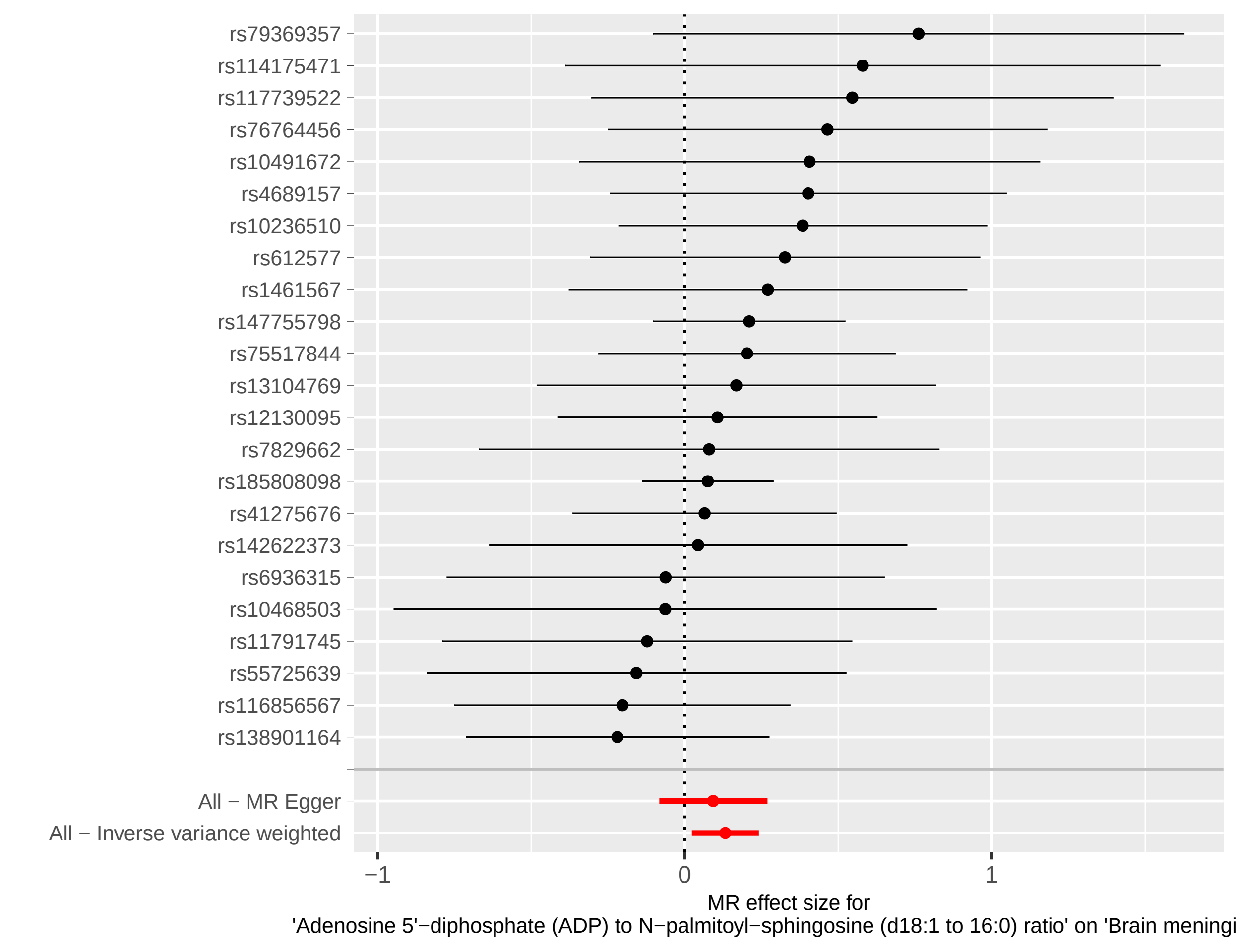
rs79369357
rs114175471
rs117739522
rs76764456
rs10491672
rs4689157
rs10236510
rs612577
rs1461567
rs147755798
rs75517844
rs13104769
rs12130095
rs7829662
rs185808098
rs41275676
rs142622373
rs6936315
rs10468503
rs11791745
rs55725639
rs116856567
rs138901164
All − MR Egger
All − Inverse variance weighted
−1
0
1
MR effect size for
'Adenosine 5'−diphosphate (ADP) to N−palmitoyl−sphingosine (d18:1 to 16:0) ratio' on 'Brain meningi

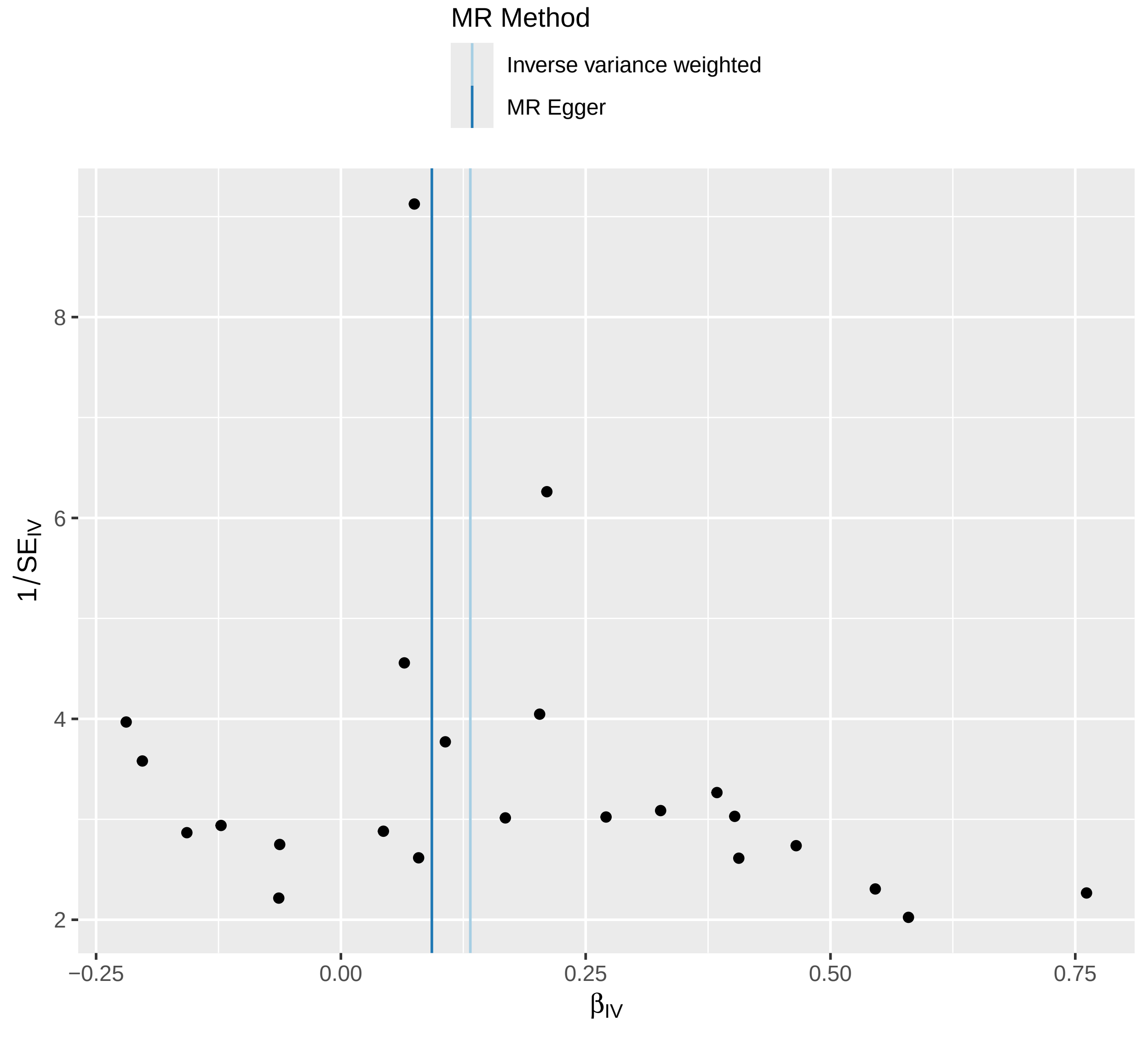
MR Method
Inverse variance weighted
MR Egger
1/SE_IV
8
6
4
2
−0.25
0.00
0.25
0.50
0.75
β_IV

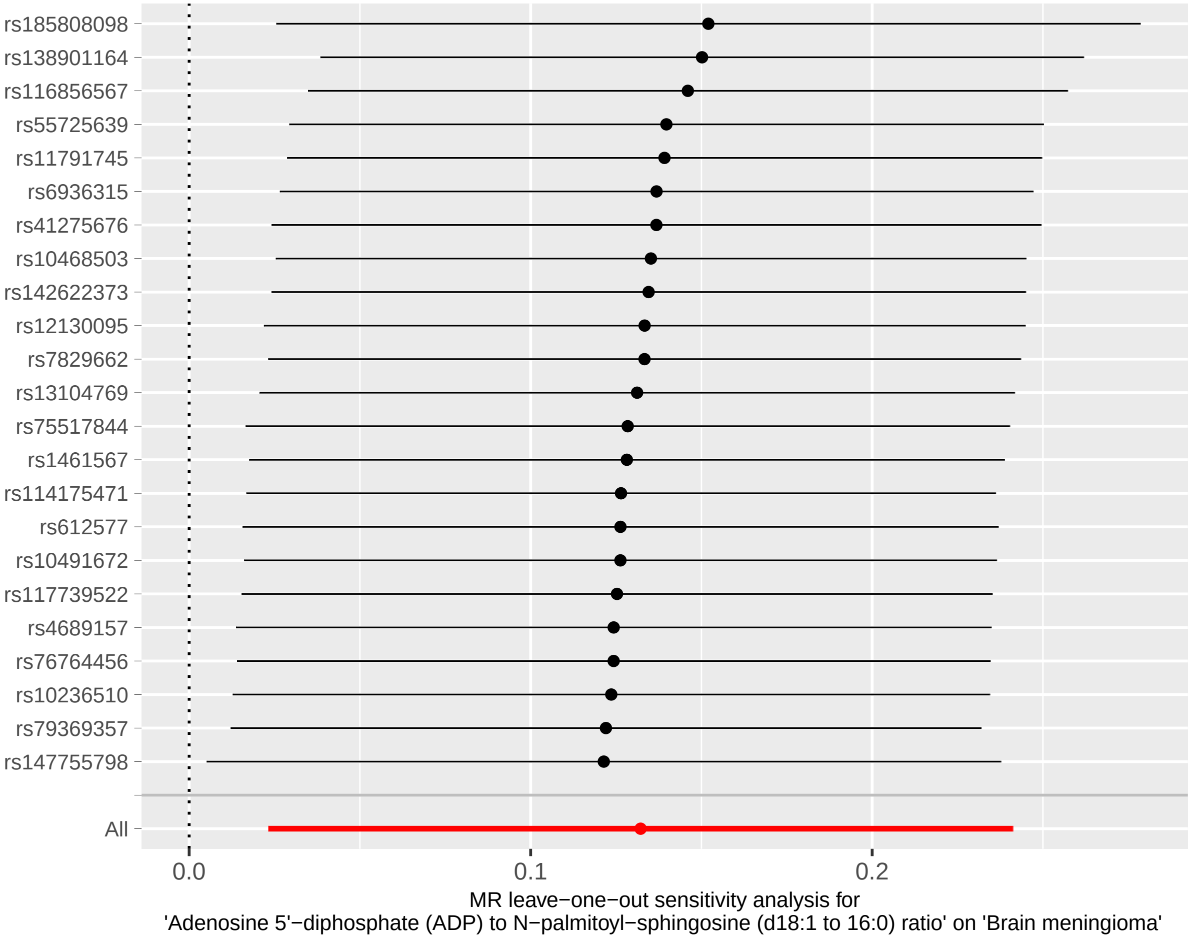
rs185808098
rs138901164
rs116856567
rs55725639
rs11791745
rs6936315
rs41275676
rs10468503
rs142622373
rs12130095
rs7829662
rs13104769
rs75517844
rs1461567
rs114175471
rs612577
rs10491672
rs117739522
rs4689157
rs76764456
rs10236510
rs79369357
rs147755798
All
0.0
0.1
0.2
MR leave−one−out sensitivity analysis for
'Adenosine 5'−diphosphate (ADP) to N−palmitoyl−sphingosine (d18:1 to 16:0) ratio' on 'Brain meningioma'

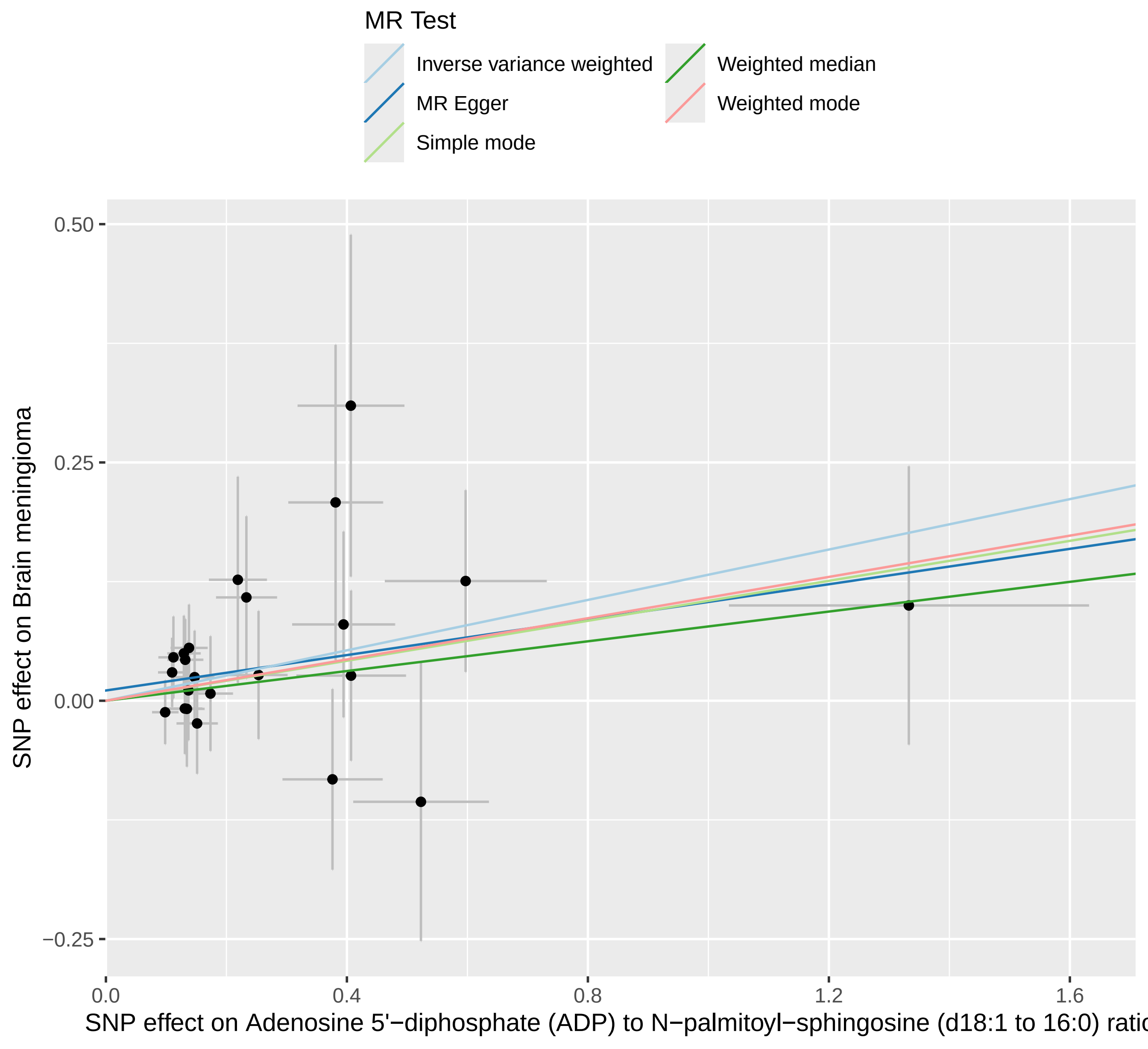
MR Test
Inverse variance weighted
MR Egger
Simple mode
Weighted median
Weighted mode
SNP effect on Brain meningioma
0.50
0.25
0.00
−0.25
0.0
0.4
0.8
1.2
1.6
SNP effect on Adenosine 5'−diphosphate (ADP) to N−palmitoyl−sphingosine (d18:1 to 16:0) ratio

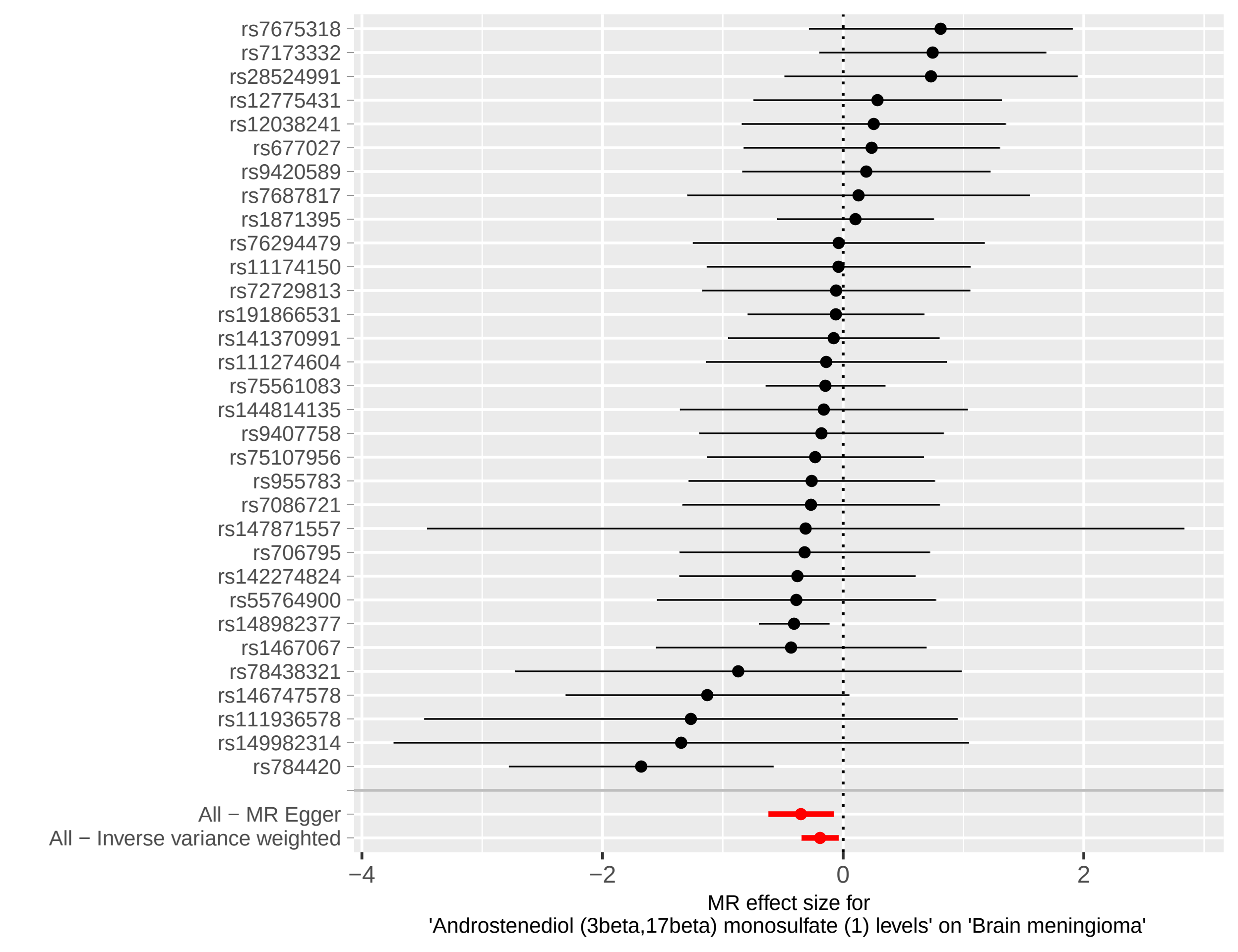
rs7675318
rs7173332
rs28524991
rs12775431
rs12038241
rs677027
rs9420589
rs7687817
rs1871395
rs76294479
rs11174150
rs72729813
rs191866531
rs141370991
rs111274604
rs75561083
rs144814135
rs9407758
rs75107956
rs955783
rs7086721
rs147871557
rs706795
rs142274824
rs55764900
rs148982377
rs1467067
rs78438321
rs146747578
rs111936578
rs149982314
rs784420
All − MR Egger
All − Inverse variance weighted
−4
−2
0
2
MR effect size for
'Androstenediol (3beta,17beta) monosulfate (1) levels' on 'Brain meningioma'

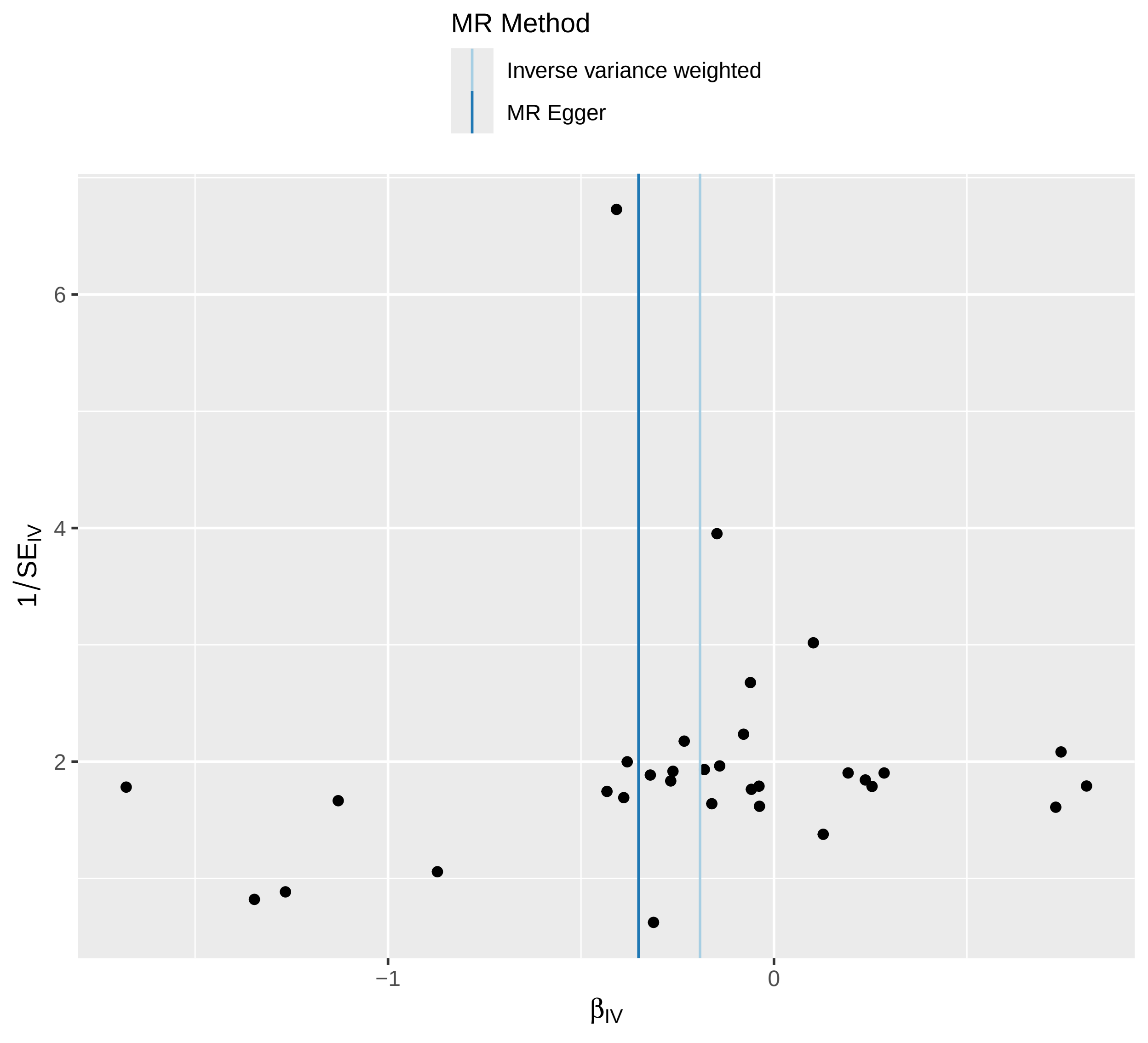

MR Method
Inverse variance weighted
MR Egger
6
4
2
1/SE_IV
−1
0
β_IV

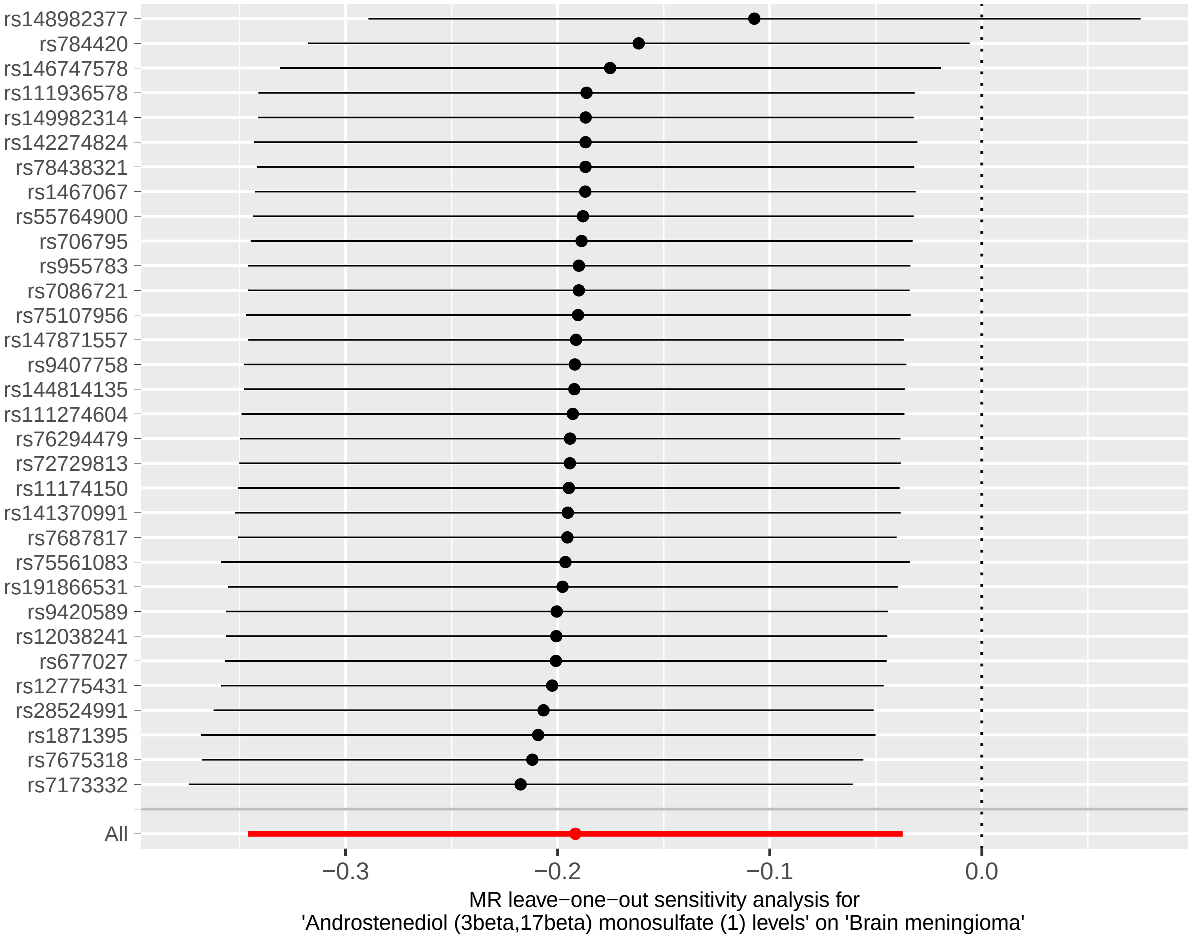
rs148982377
rs784420
rs146747578
rs111936578
rs149982314
rs142274824
rs78438321
rs1467067
rs55764900
rs706795
rs955783
rs7086721
rs75107956
rs147871557
rs9407758
rs144814135
rs111274604
rs76294479
rs72729813
rs11174150
rs141370991
rs7687817
rs75561083
rs191866531
rs9420589
rs12038241
rs677027
rs12775431
rs28524991
rs1871395
rs7675318
rs7173332
All
−0.3
−0.2
−0.1
0.0
MR leave−one−out sensitivity analysis for
'Androstenediol (3beta,17beta) monosulfate (1) levels' on 'Brain meningioma'

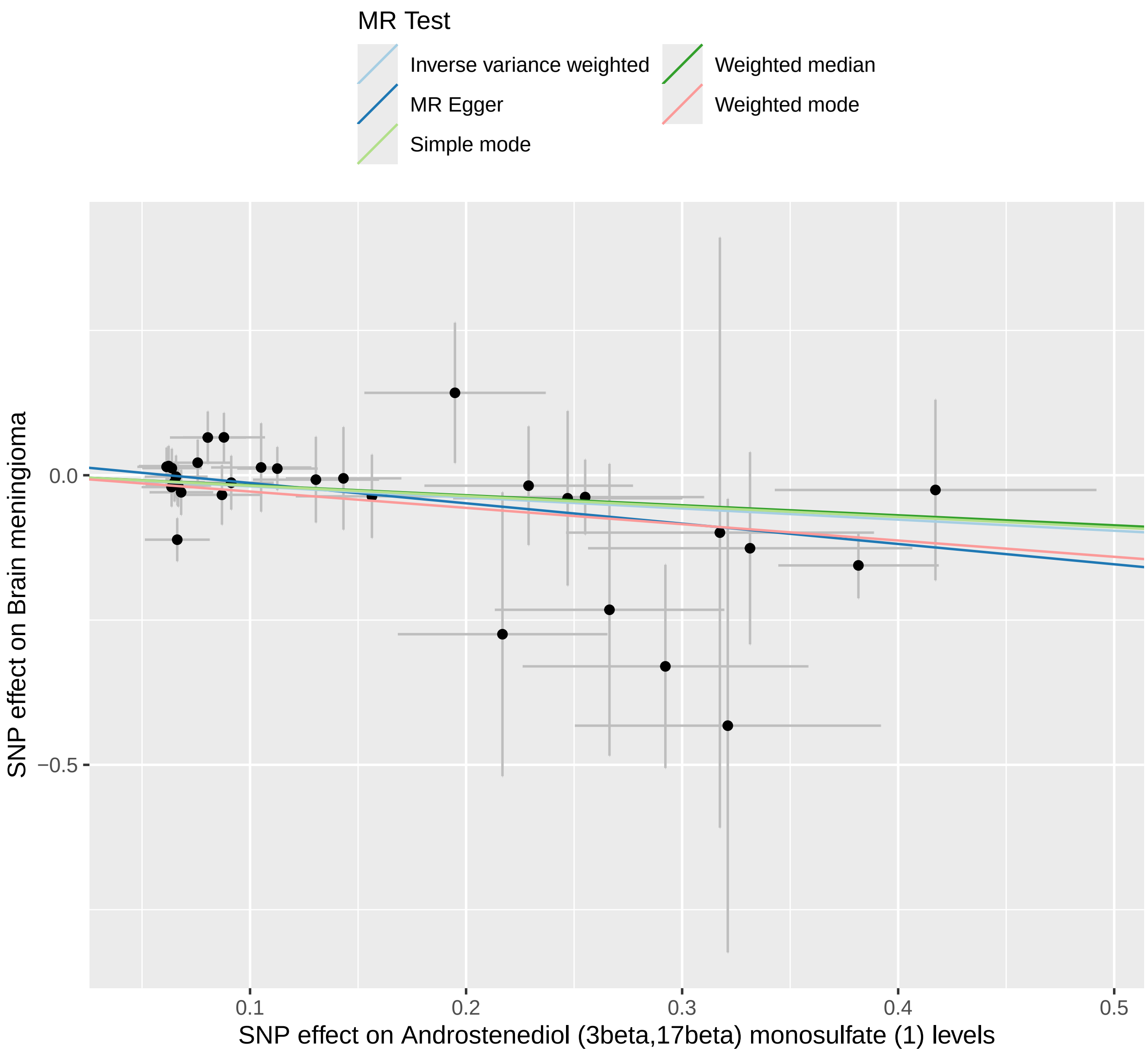
MR Test
Inverse variance weighted
MR Egger
Simple mode
Weighted median
Weighted mode
SNP effect on Brain meningioma
0.0
−0.5
0.1
0.2
0.3
0.4
0.5
SNP effect on Androstenediol (3beta,17beta) monosulfate (1) levels

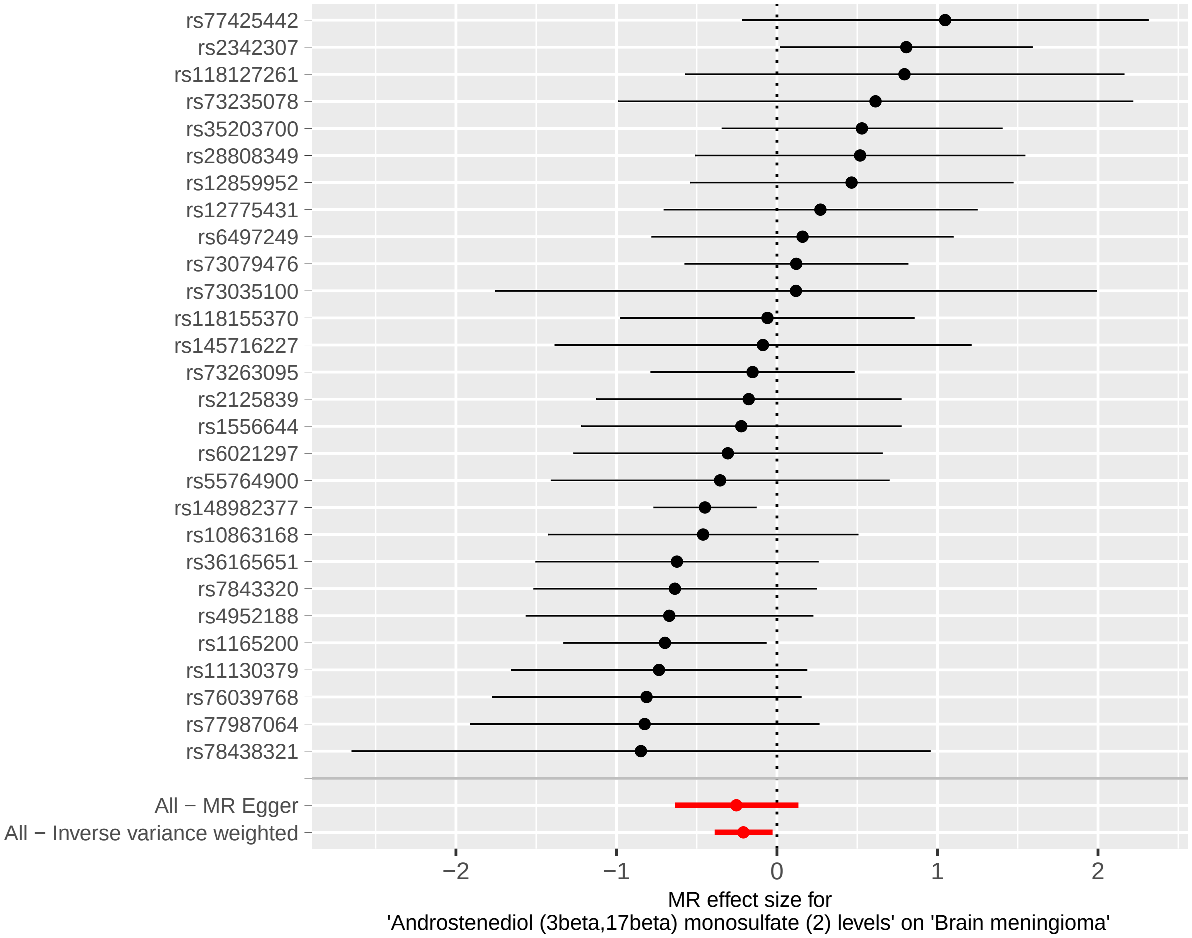
rs77425442
rs2342307
rs118127261
rs73235078
rs35203700
rs28808349
rs12859952
rs12775431
rs6497249
rs73079476
rs73035100
rs118155370
rs145716227
rs73263095
rs2125839
rs1556644
rs6021297
rs55764900
rs148982377
rs10863168
rs36165651
rs7843320
rs4952188
rs1165200
rs11130379
rs76039768
rs77987064
rs78438321
All − MR Egger
All − Inverse variance weighted
−2
−1
0
1
2
MR effect size for
'Androstenediol (3beta,17beta) monosulfate (2) levels' on 'Brain meningioma'

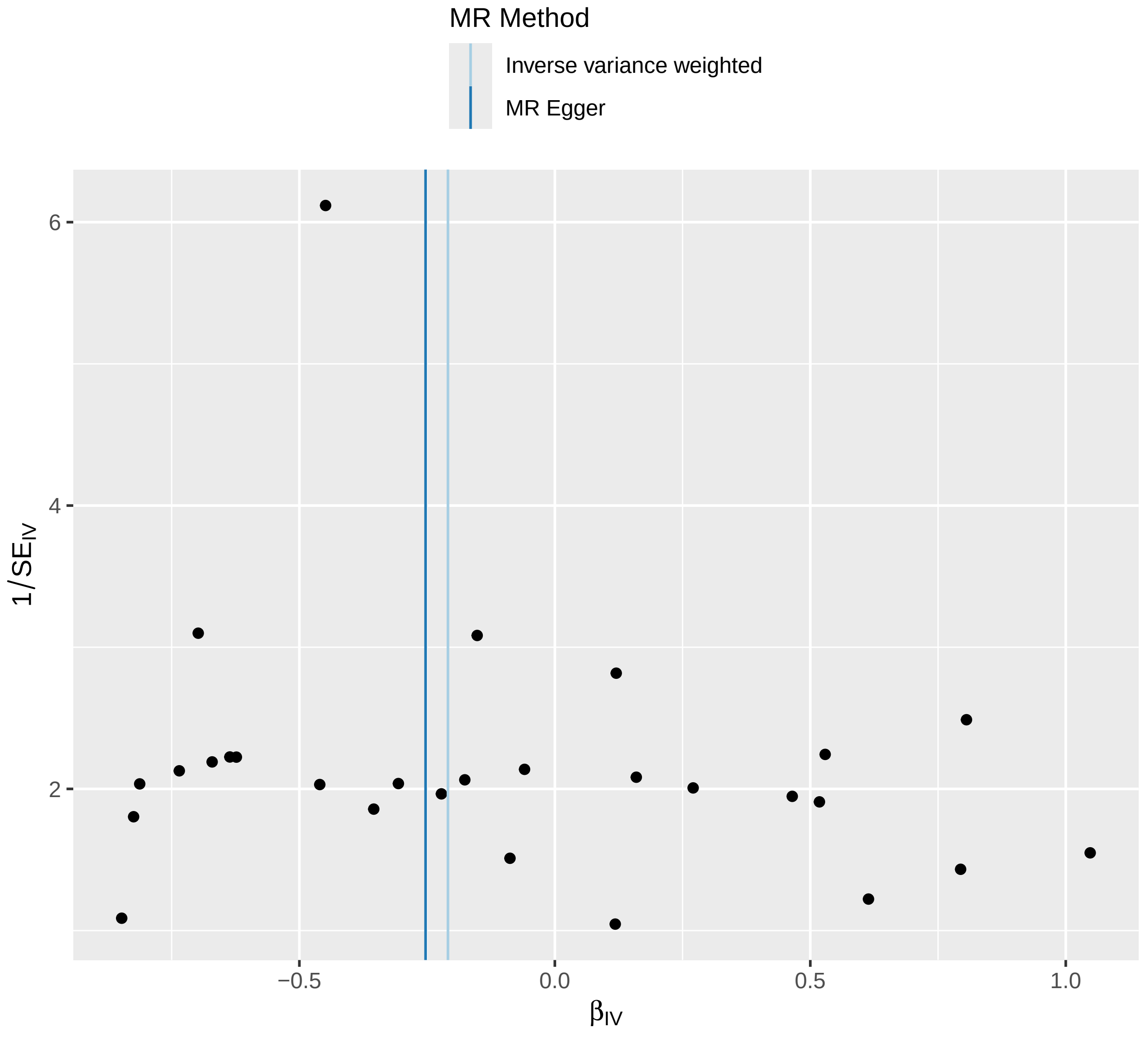
MR Method
Inverse variance weighted
MR Egger
6
4
2
$1/SE_{IV}$
−0.5
0.0
0.5
1.0
β_{IV}

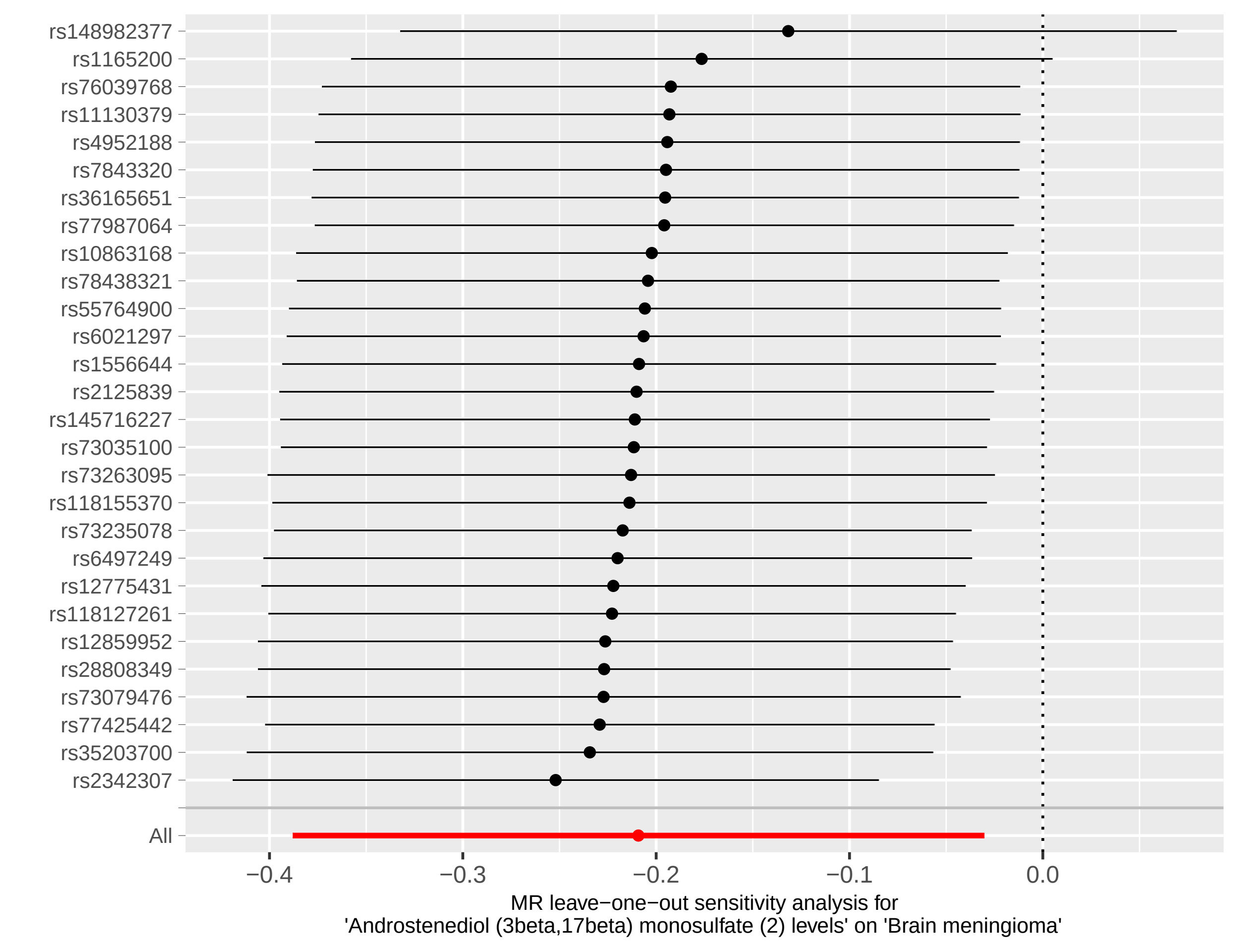
rs148982377
rs1165200
rs76039768
rs11130379
rs4952188
rs7843320
rs36165651
rs77987064
rs10863168
rs78438321
rs55764900
rs6021297
rs1556644
rs2125839
rs145716227
rs73035100
rs73263095
rs118155370
rs73235078
rs6497249
rs12775431
rs118127261
rs12859952
rs28808349
rs73079476
rs77425442
rs35203700
rs2342307
All
−0.4
−0.3
−0.2
−0.1
0.0
MR leave−one−out sensitivity analysis for
'Androstenediol (3beta,17beta) monosulfate (2) levels' on 'Brain meningioma'

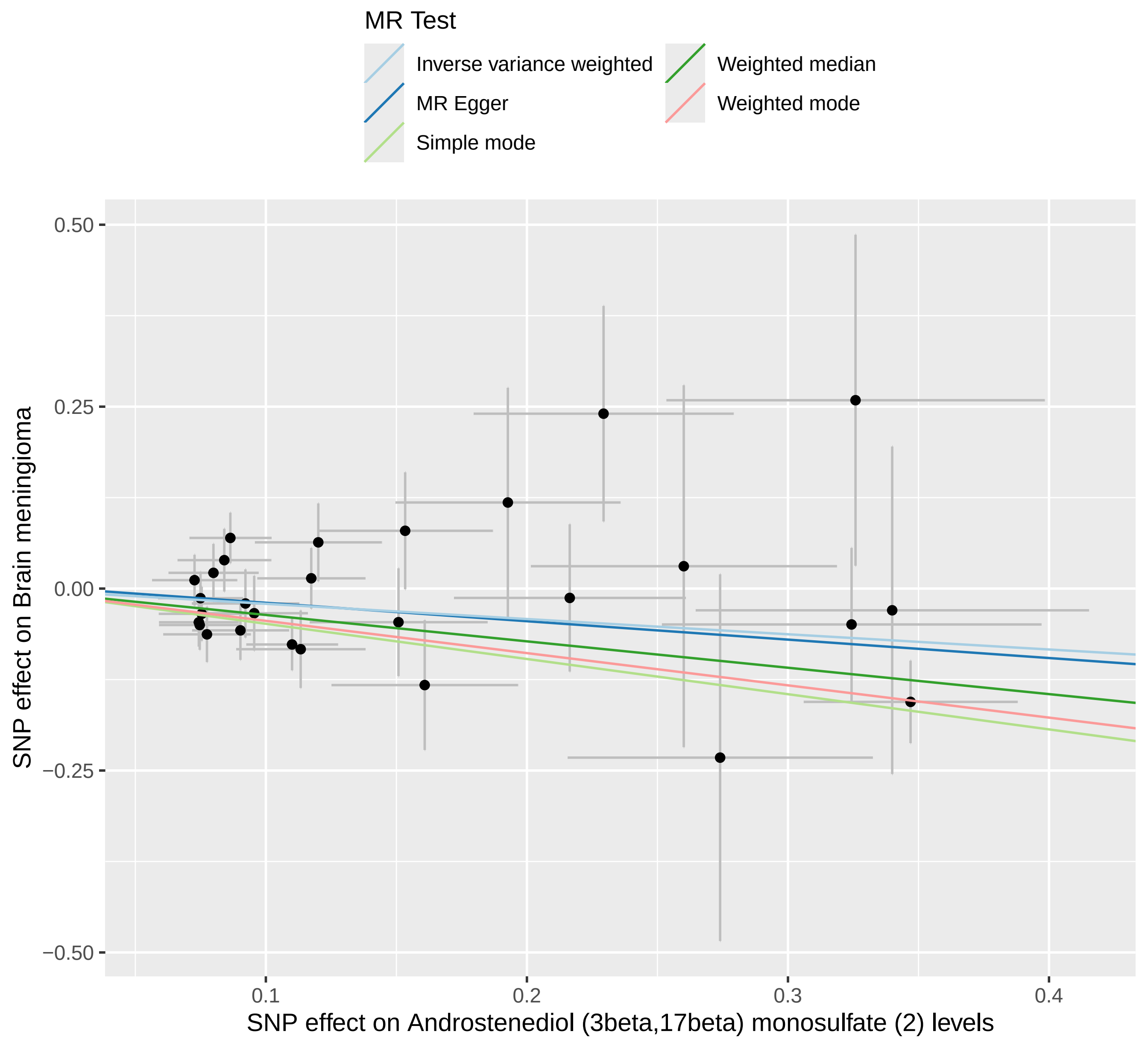

MR Test
Inverse variance weighted
MR Egger
Simple mode
Weighted median
Weighted mode
0.50
0.25
0.00
−0.25
−0.50
0.1
0.2
0.3
0.4
SNP effect on Brain meningioma
SNP effect on Androstenediol (3beta,17beta) monosulfate (2) levels

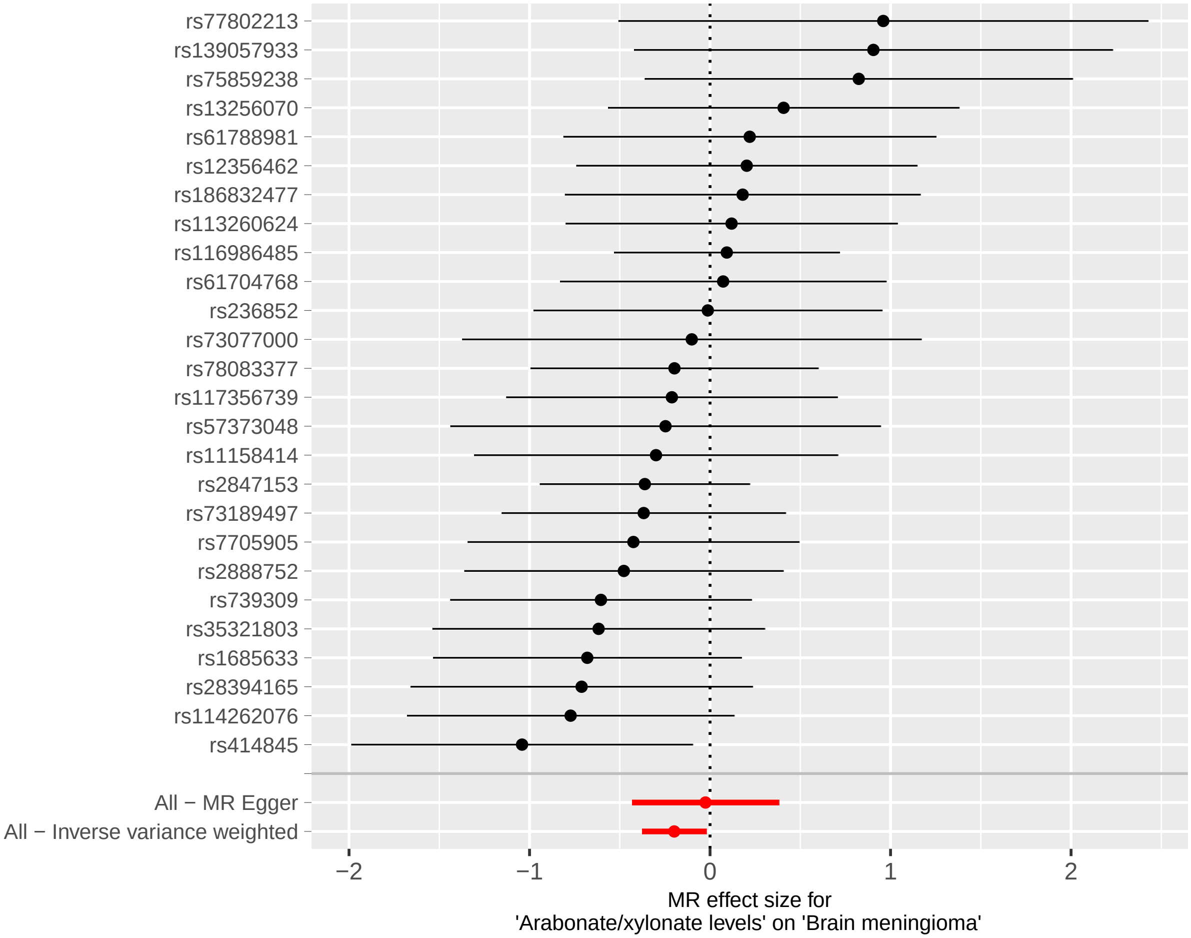

rs77802213
rs139057933
rs75859238
rs13256070
rs61788981
rs12356462
rs186832477
rs113260624
rs116986485
rs61704768
rs236852
rs73077000
rs78083377
rs117356739
rs57373048
rs11158414
rs2847153
rs73189497
rs7705905
rs2888752
rs739309
rs35321803
rs1685633
rs28394165
rs114262076
rs414845
All − MR Egger
All − Inverse variance weighted
−2
−1
0
1
2
MR effect size for
'Arabonate/xylonate levels' on 'Brain meningioma'

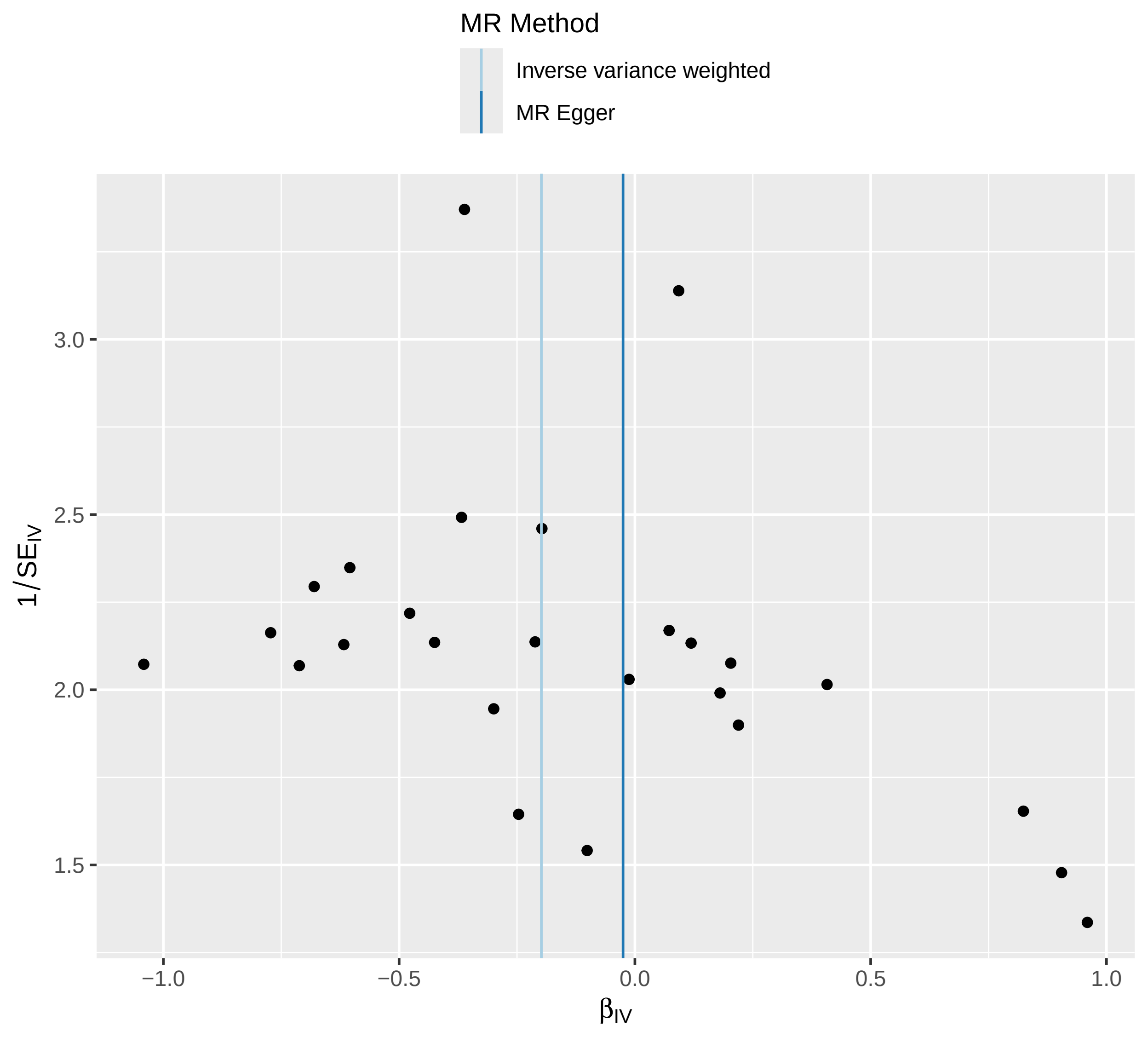
MR Method
Inverse variance weighted
MR Egger
3.0
2.5
2.0
1.5
1/SE_IV
−1.0
−0.5
0.0
0.5
1.0
β_IV

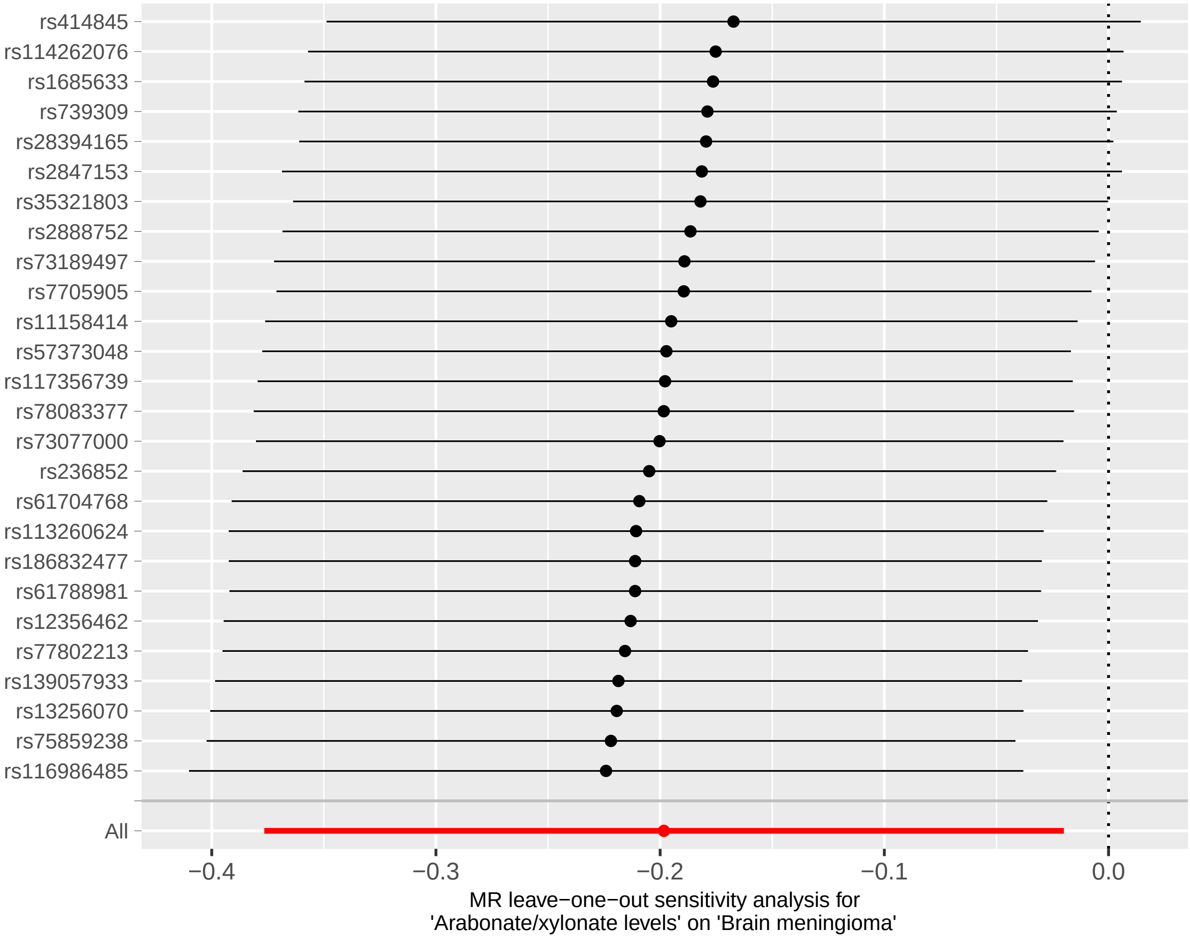

rs414845
rs114262076
rs1685633
rs739309
rs28394165
rs2847153
rs35321803
rs2888752
rs73189497
rs7705905
rs11158414
rs57373048
rs117356739
rs78083377
rs73077000
rs236852
rs61704768
rs113260624
rs186832477
rs61788981
rs12356462
rs77802213
rs139057933
rs13256070
rs75859238
rs116986485
All
−0.4
−0.3
−0.2
−0.1
0.0
MR leave−one−out sensitivity analysis for
'Arabonate/xylonate levels' on 'Brain meningioma'

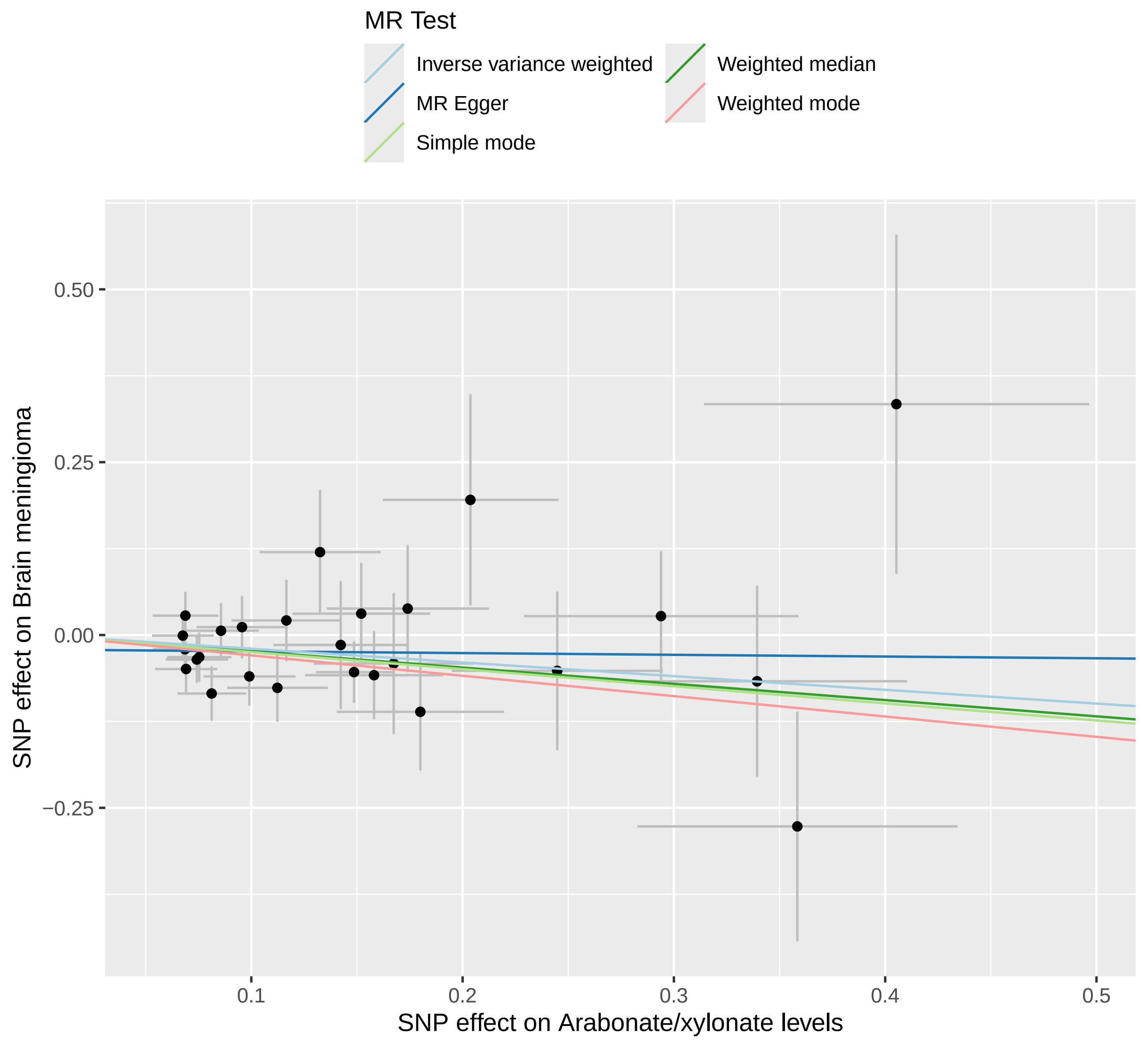
MR Test
Inverse variance weighted
MR Egger
Simple mode
Weighted median
Weighted mode
SNP effect on Brain meningioma
0.50
0.25
0.00
−0.25
0.1
0.2
0.3
0.4
0.5
SNP effect on Arabonate/xylonate levels

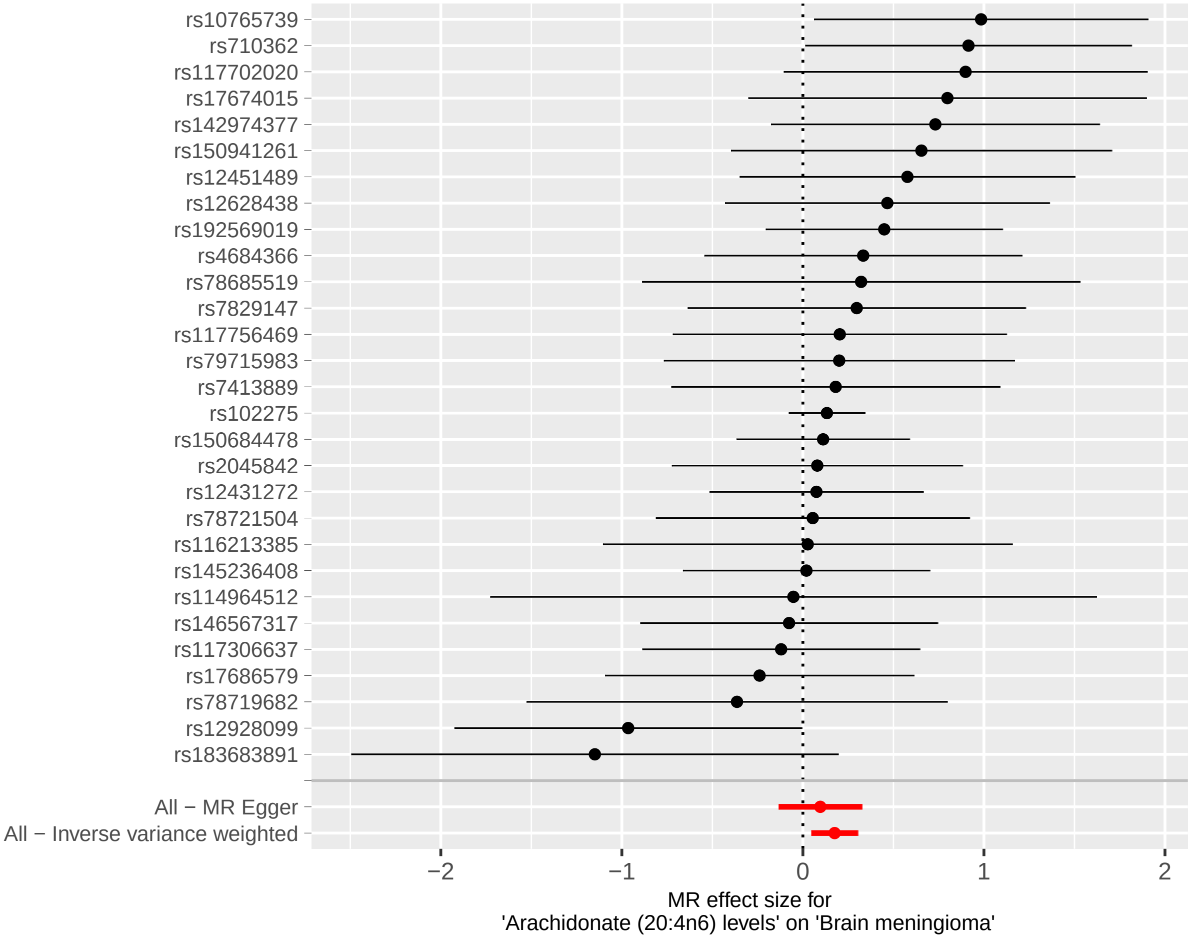

rs10765739
rs710362
rs117702020
rs17674015
rs142974377
rs150941261
rs12451489
rs12628438
rs192569019
rs4684366
rs78685519
rs7829147
rs117756469
rs79715983
rs7413889
rs102275
rs150684478
rs2045842
rs12431272
rs78721504
rs116213385
rs145236408
rs114964512
rs146567317
rs117306637
rs17686579
rs78719682
rs12928099
rs183683891
All − MR Egger
All − Inverse variance weighted
−2
−1
0
1
2
MR effect size for
'Arachidonate (20:4n6) levels' on 'Brain meningioma'

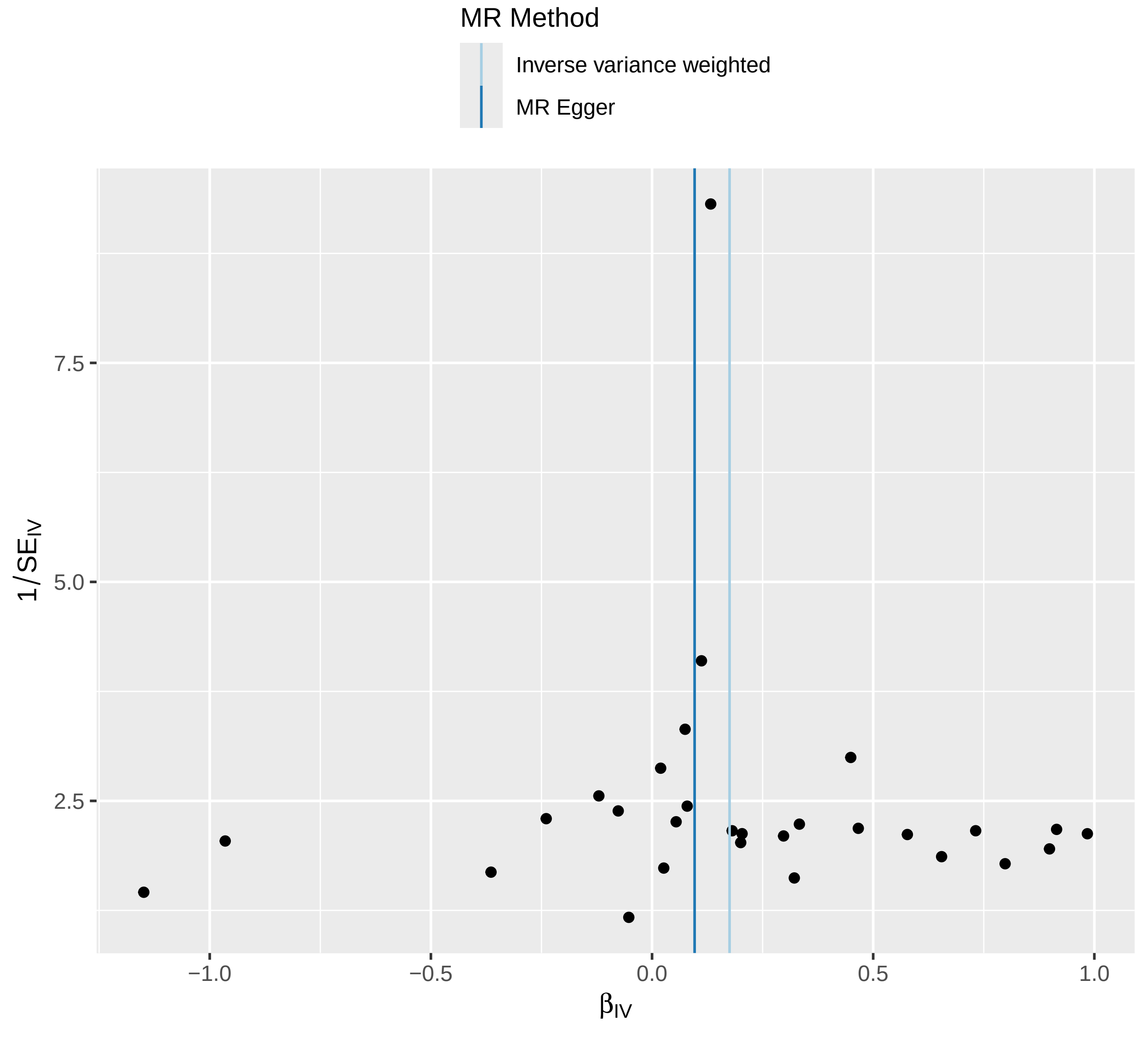

MR Method
Inverse variance weighted
MR Egger
7.5
5.0
2.5
1/SE_IV
−1.0
−0.5
0.0
0.5
1.0
β_IV

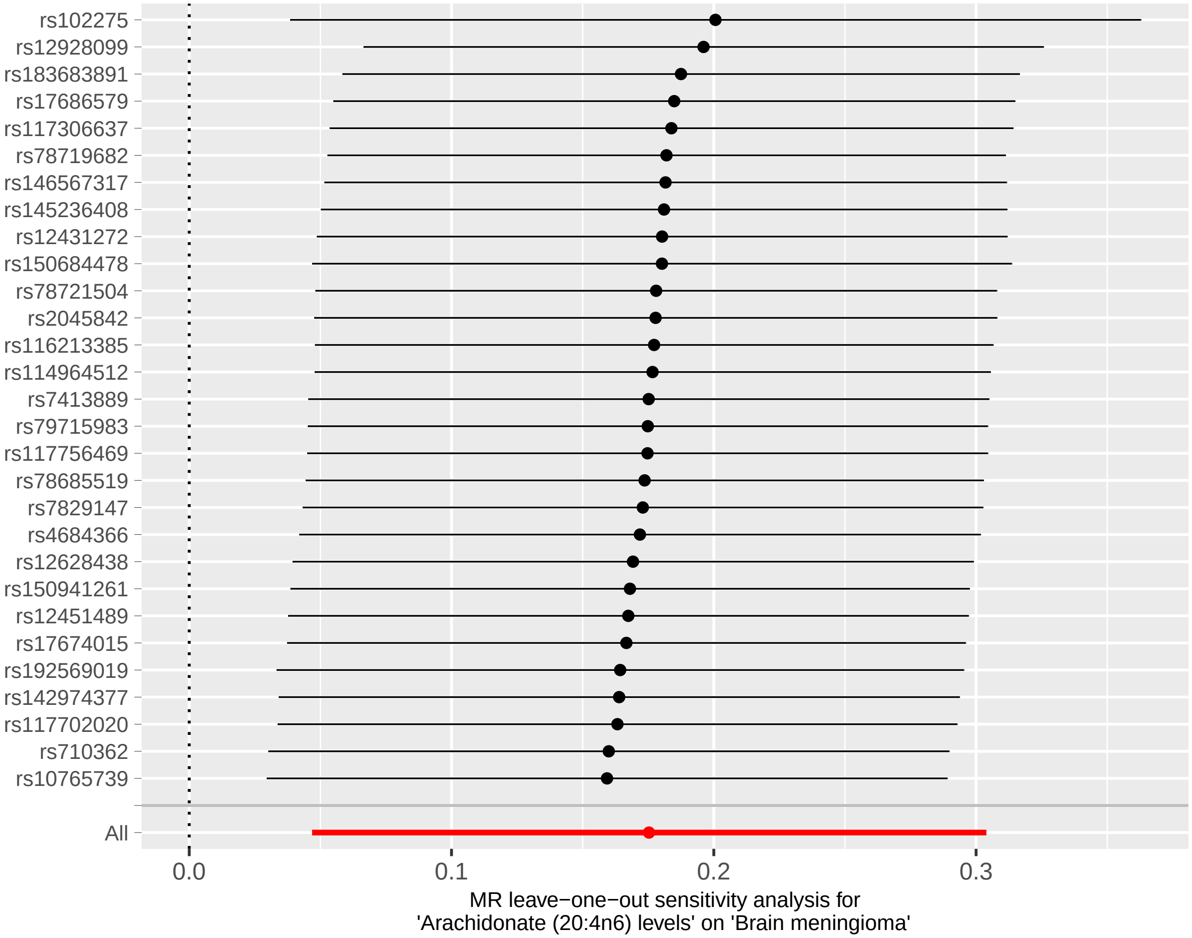
rs102275
rs12928099
rs183683891
rs17686579
rs117306637
rs78719682
rs146567317
rs145236408
rs12431272
rs150684478
rs78721504
rs2045842
rs116213385
rs114964512
rs7413889
rs79715983
rs117756469
rs78685519
rs7829147
rs4684366
rs12628438
rs150941261
rs12451489
rs17674015
rs192569019
rs142974377
rs117702020
rs710362
rs10765739
All
0.0
0.1
0.2
0.3
MR leave−one−out sensitivity analysis for
'Arachidonate (20:4n6) levels' on 'Brain meningioma'

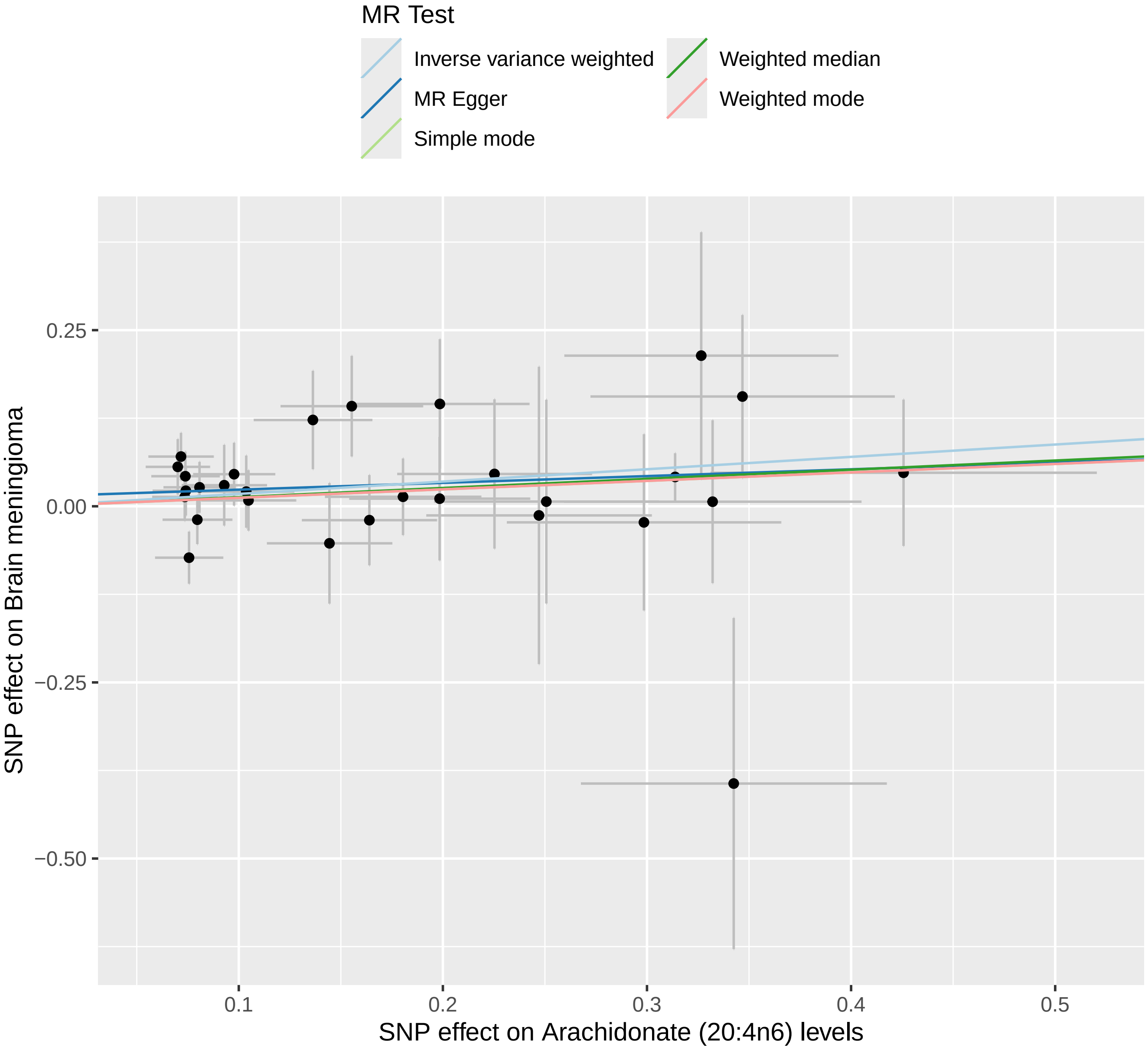
MR Test
Inverse variance weighted
MR Egger
Simple mode
Weighted median
Weighted mode
0.25
0.00
−0.25
−0.50
0.1
0.2
0.3
0.4
0.5
SNP effect on Brain meningioma
SNP effect on Arachidonate (20:4n6) levels

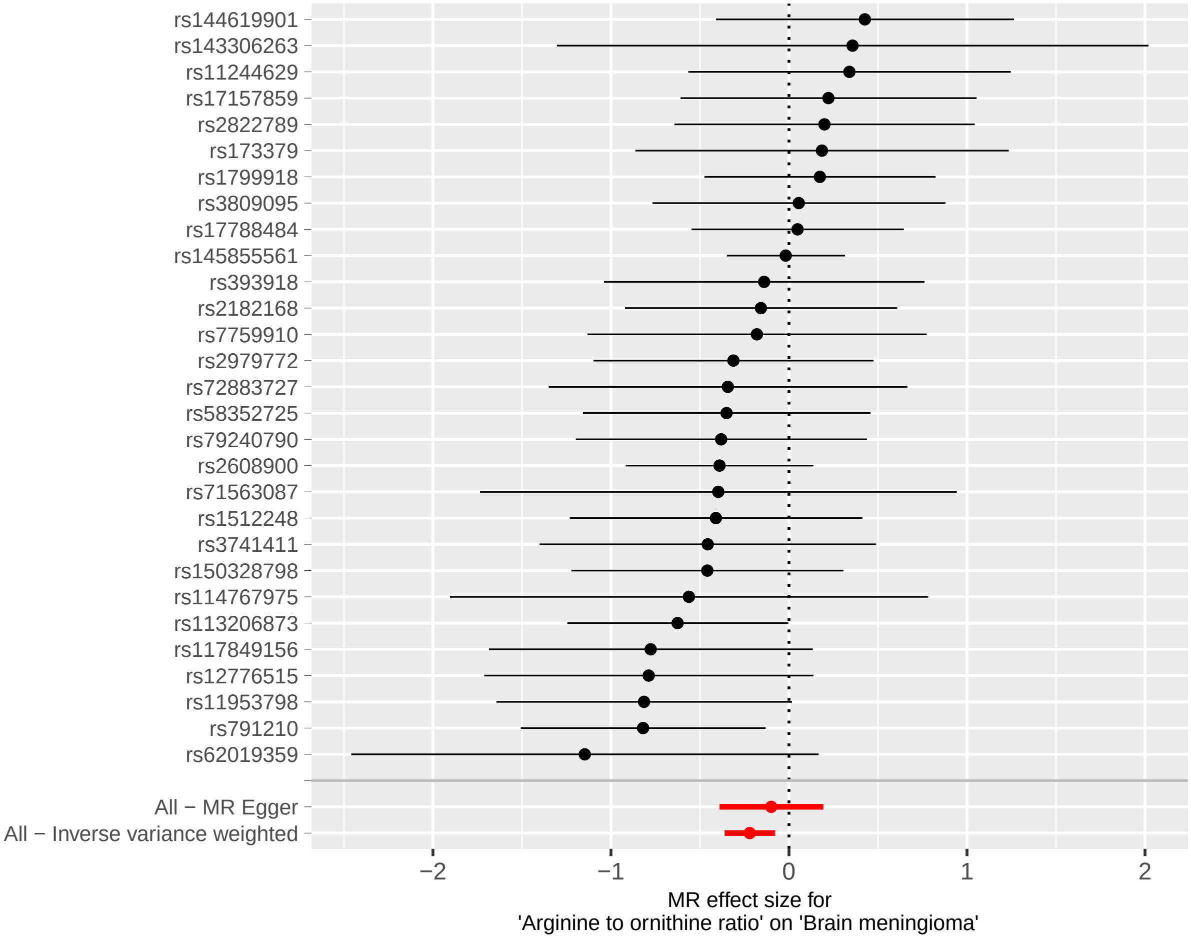
rs144619901
rs143306263
rs11244629
rs17157859
rs2822789
rs173379
rs1799918
rs3809095
rs17788484
rs145855561
rs393918
rs2182168
rs7759910
rs2979772
rs72883727
rs58352725
rs79240790
rs2608900
rs71563087
rs1512248
rs3741411
rs150328798
rs114767975
rs113206873
rs117849156
rs12776515
rs11953798
rs791210
rs62019359
All − MR Egger
All − Inverse variance weighted
−2
−1
0
1
2
MR effect size for
'Arginine to ornithine ratio' on 'Brain meningioma'

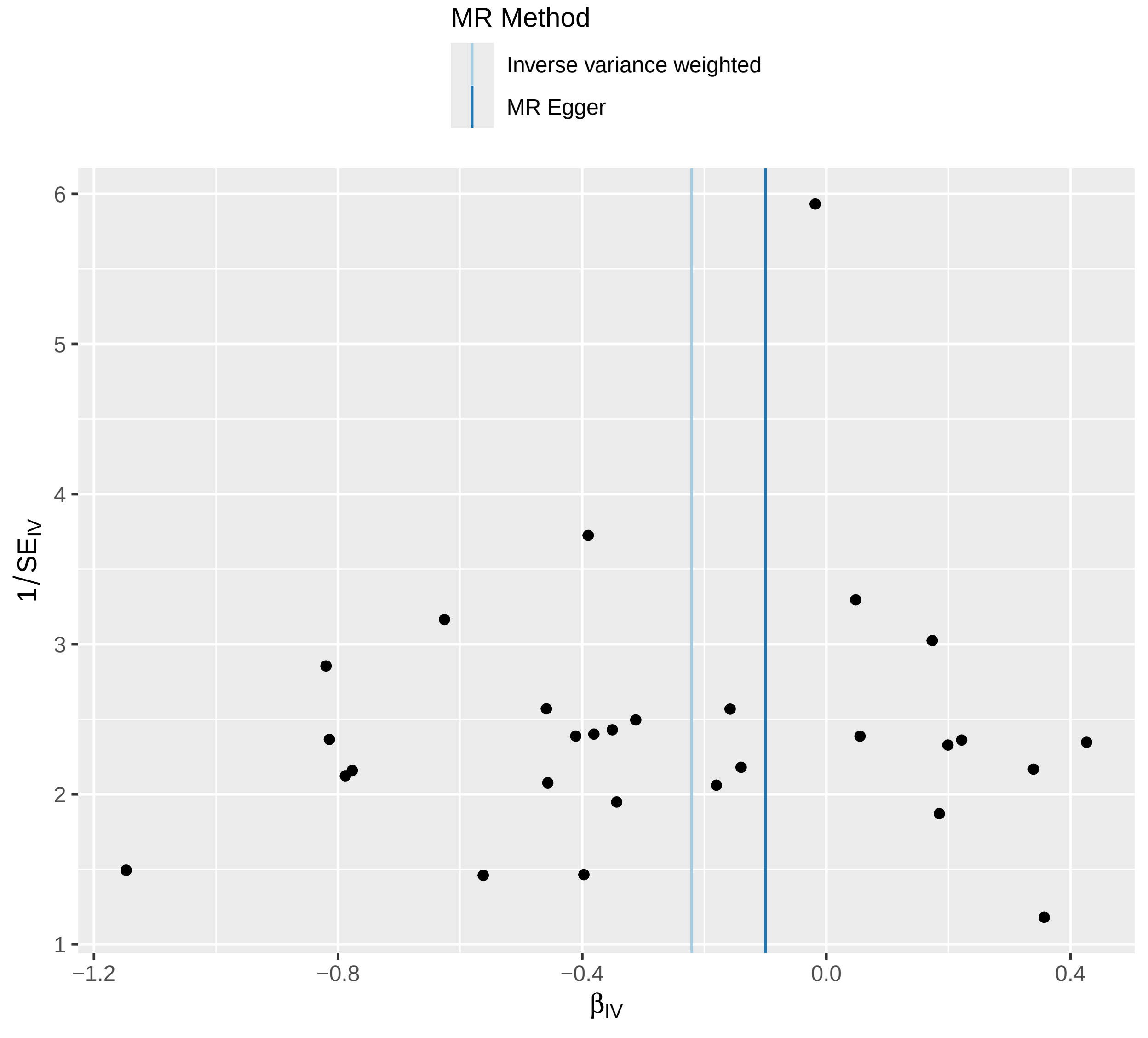

MR Method
Inverse variance weighted
MR Egger
6
5
4
3
2
1
1/SE$_{IV}$
−1.2
−0.8
−0.4
0.0
0.4
β$_{IV}$

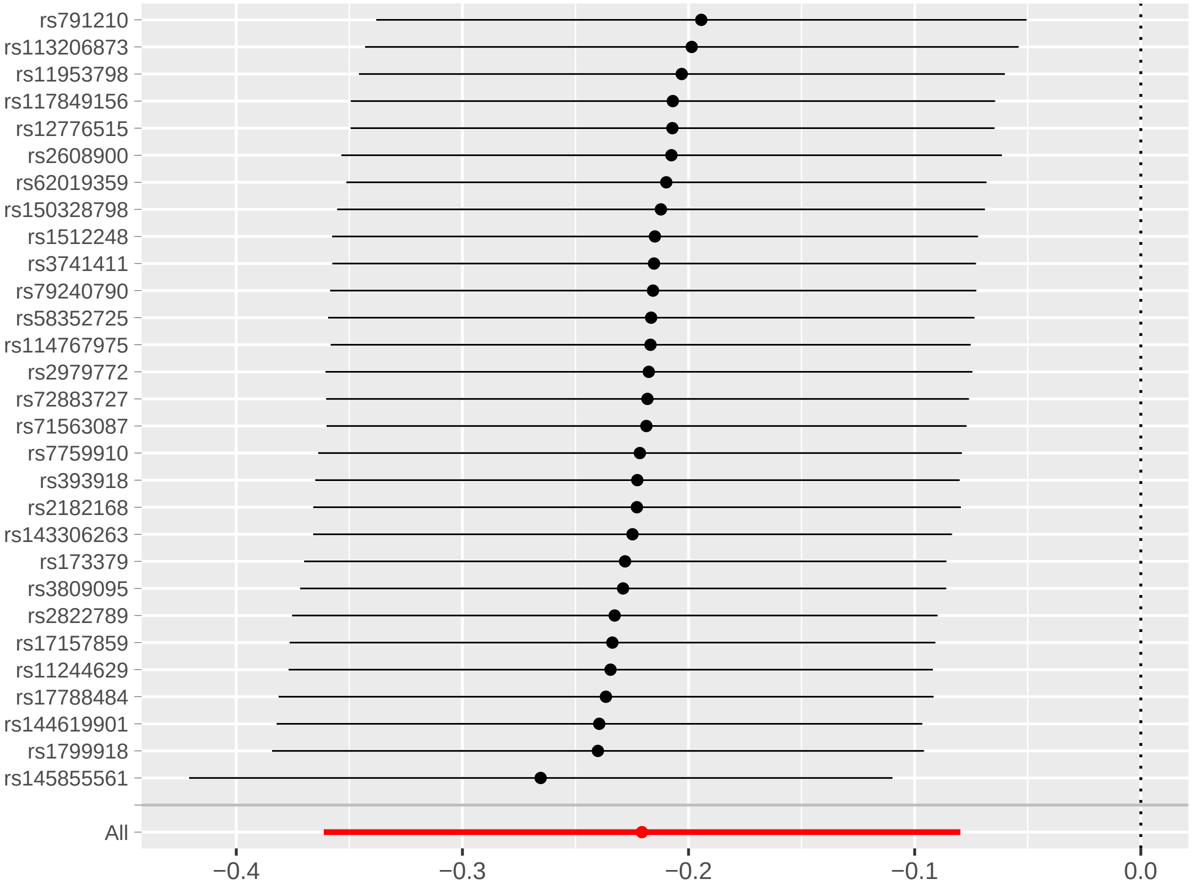

MR leave−one−out sensitivity analysis for
'Arginine to ornithine ratio' on 'Brain meningioma'

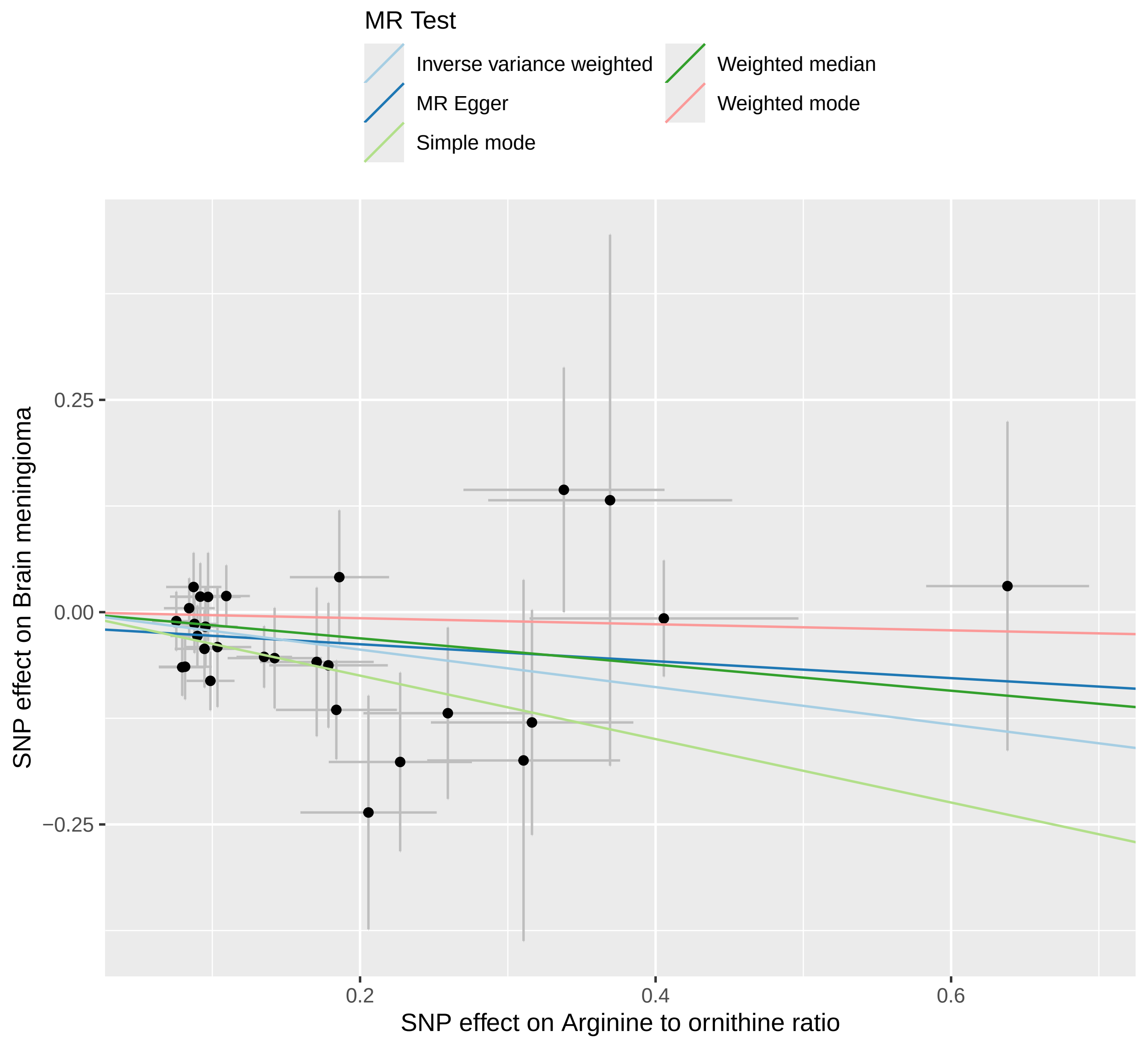

MR Test
Inverse variance weighted
MR Egger
Simple mode
Weighted median
Weighted mode
0.25
0.00
−0.25
0.2
0.4
0.6
SNP effect on Brain meningioma
SNP effect on Arginine to ornithine ratio

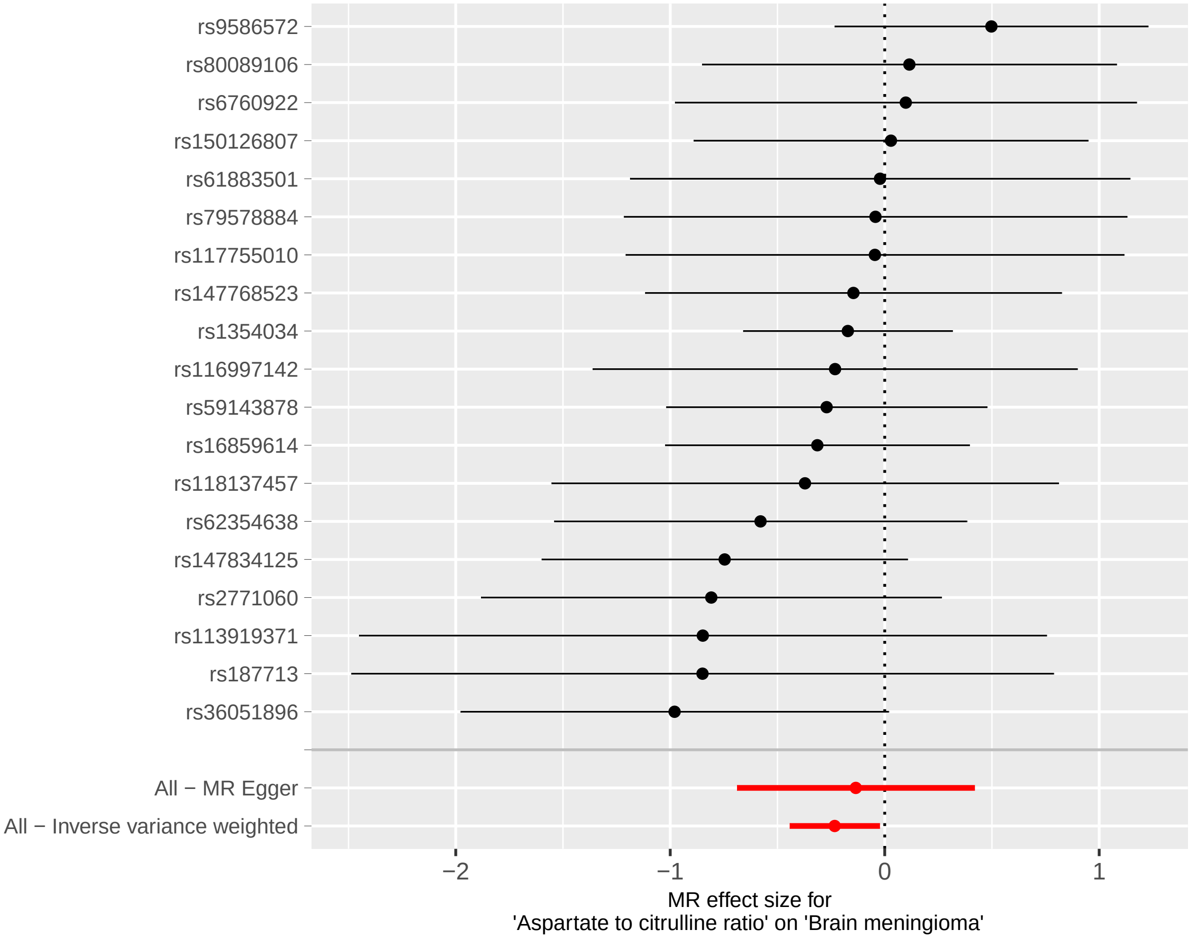
rs9586572
rs80089106
rs6760922
rs150126807
rs61883501
rs79578884
rs117755010
rs147768523
rs1354034
rs116997142
rs59143878
rs16859614
rs118137457
rs62354638
rs147834125
rs2771060
rs113919371
rs187713
rs36051896
All − MR Egger
All − Inverse variance weighted
−2
−1
0
1
MR effect size for
'Aspartate to citrulline ratio' on 'Brain meningioma'

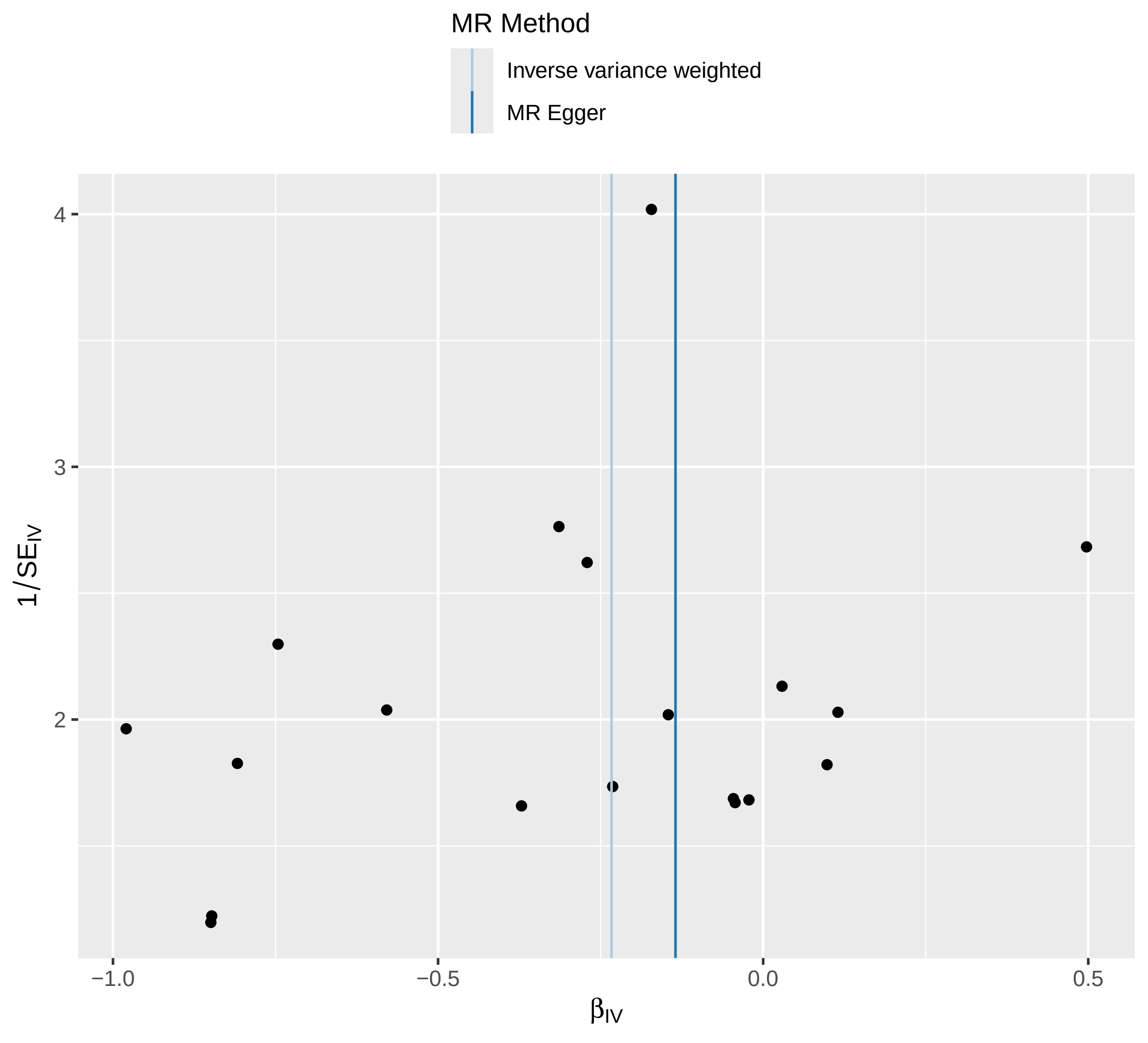

MR Method
Inverse variance weighted
MR Egger
4
3
2
1/SE_IV
−1.0
−0.5
0.0
0.5
β_IV

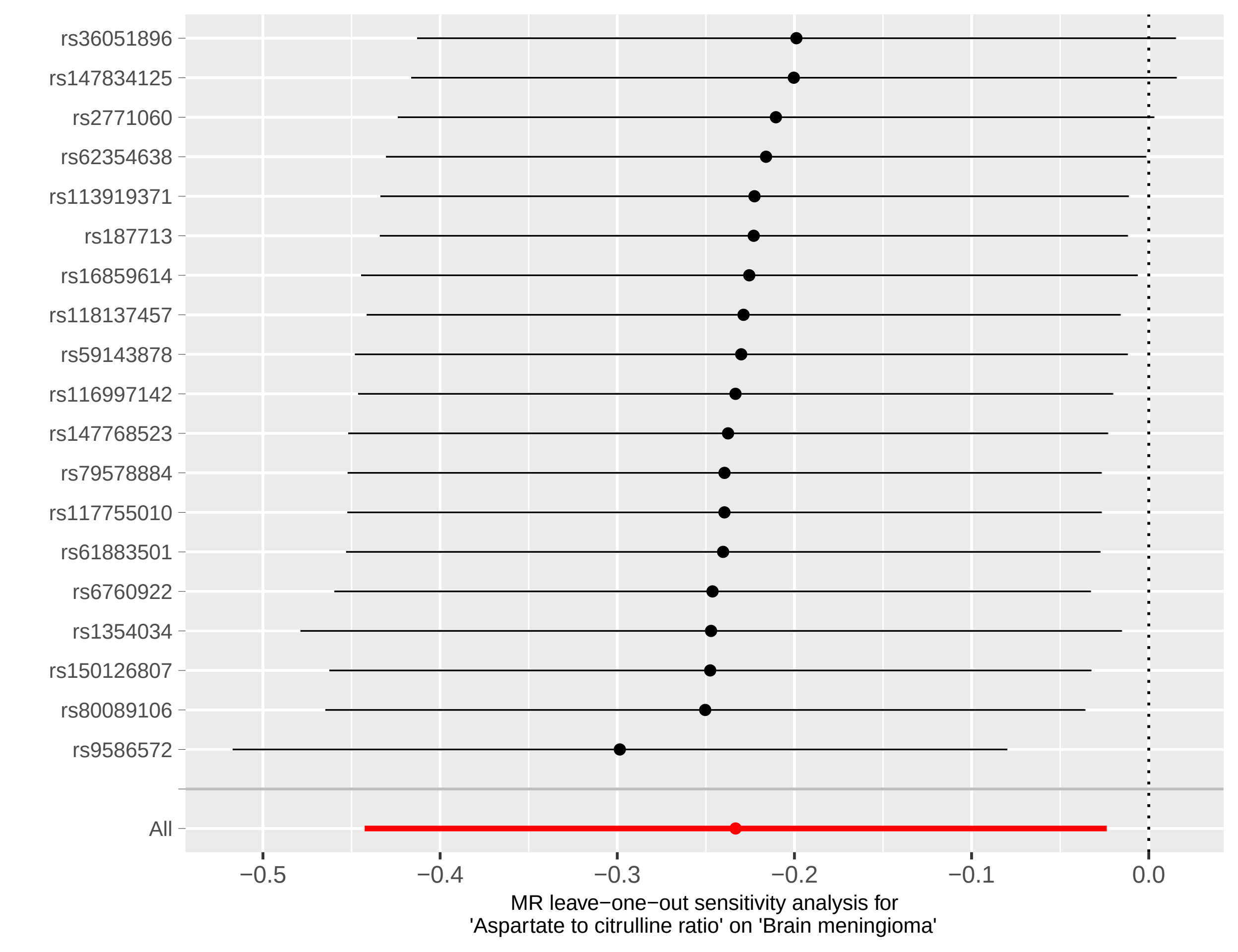
rs36051896
rs147834125
rs2771060
rs62354638
rs113919371
rs187713
rs16859614
rs118137457
rs59143878
rs116997142
rs147768523
rs79578884
rs117755010
rs61883501
rs6760922
rs1354034
rs150126807
rs80089106
rs9586572
All
−0.5
−0.4
−0.3
−0.2
−0.1
0.0
MR leave−one−out sensitivity analysis for
'Aspartate to citrulline ratio' on 'Brain meningioma'

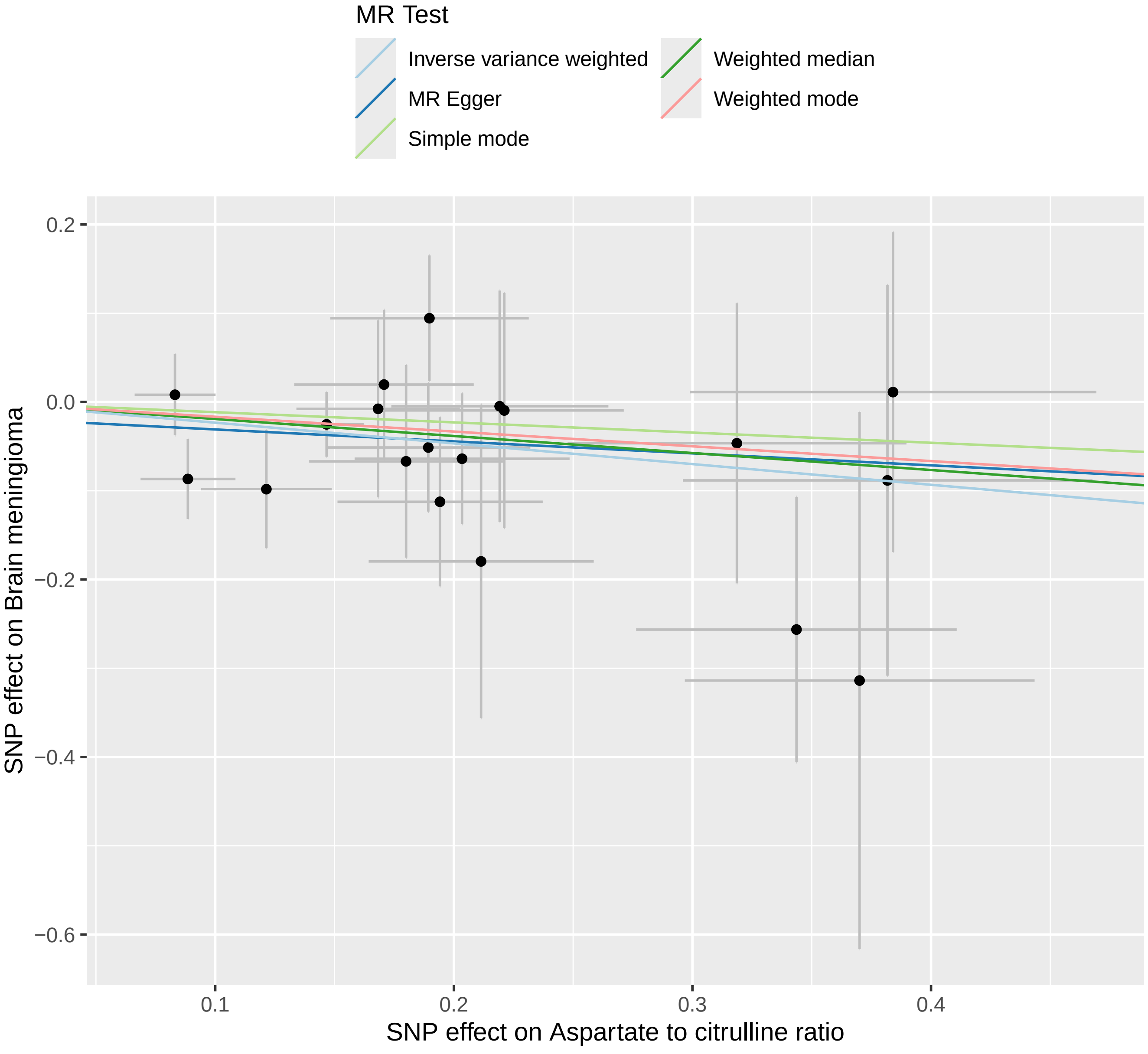
MR Test
Inverse variance weighted
MR Egger
Simple mode
Weighted median
Weighted mode
0.2
0.0
−0.2
−0.4
−0.6
SNP effect on Brain meningioma
0.1
0.2
0.3
0.4
SNP effect on Aspartate to citrulline ratio

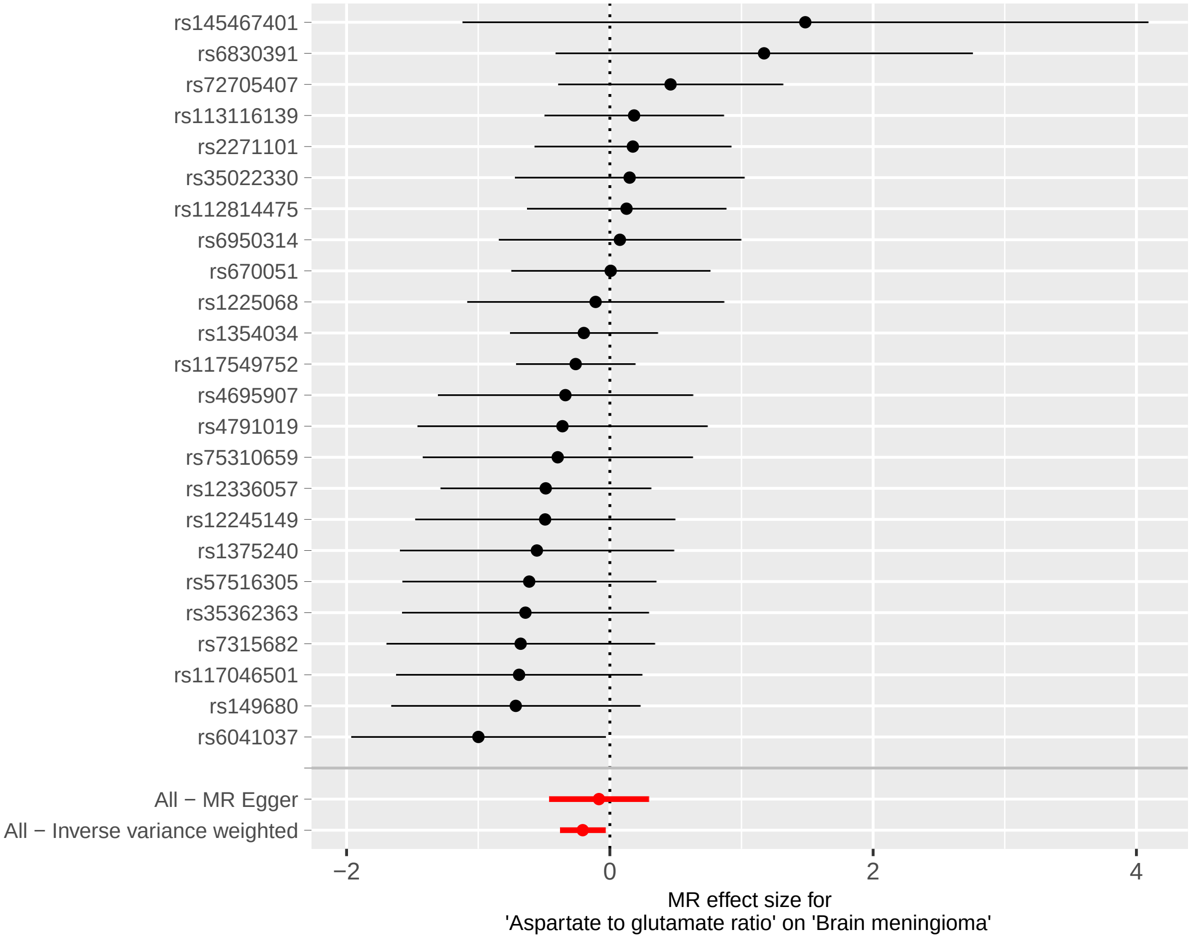
rs145467401
rs6830391
rs72705407
rs113116139
rs2271101
rs35022330
rs112814475
rs6950314
rs670051
rs1225068
rs1354034
rs117549752
rs4695907
rs4791019
rs75310659
rs12336057
rs12245149
rs1375240
rs57516305
rs35362363
rs7315682
rs117046501
rs149680
rs6041037
All − MR Egger
All − Inverse variance weighted
−2
0
2
4
MR effect size for
'Aspartate to glutamate ratio' on 'Brain meningioma'

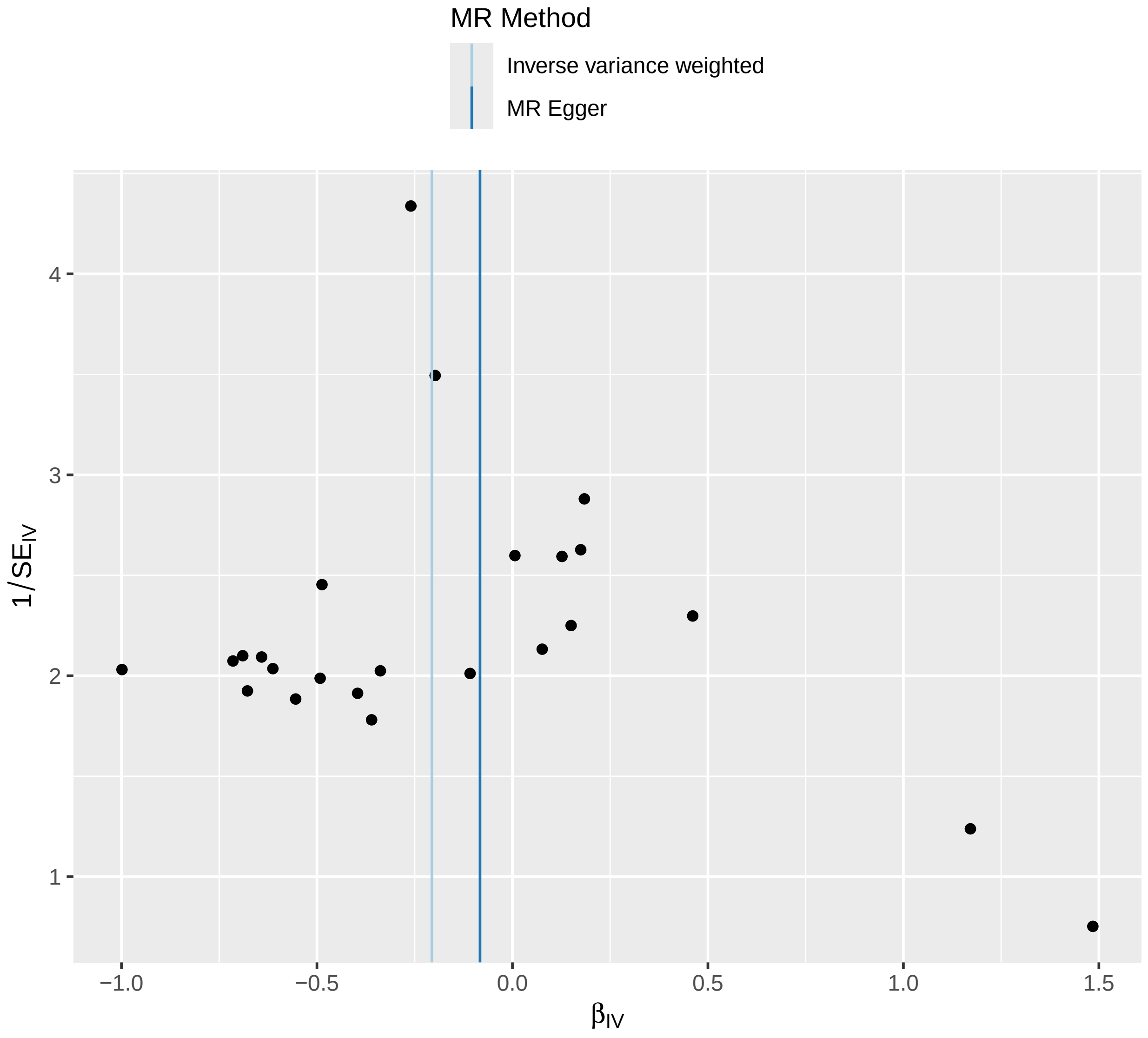
MR Method
Inverse variance weighted
MR Egger
1/SE_IV
β_IV
−1.0
−0.5
0.0
0.5
1.0
1.5
1
2
3
4

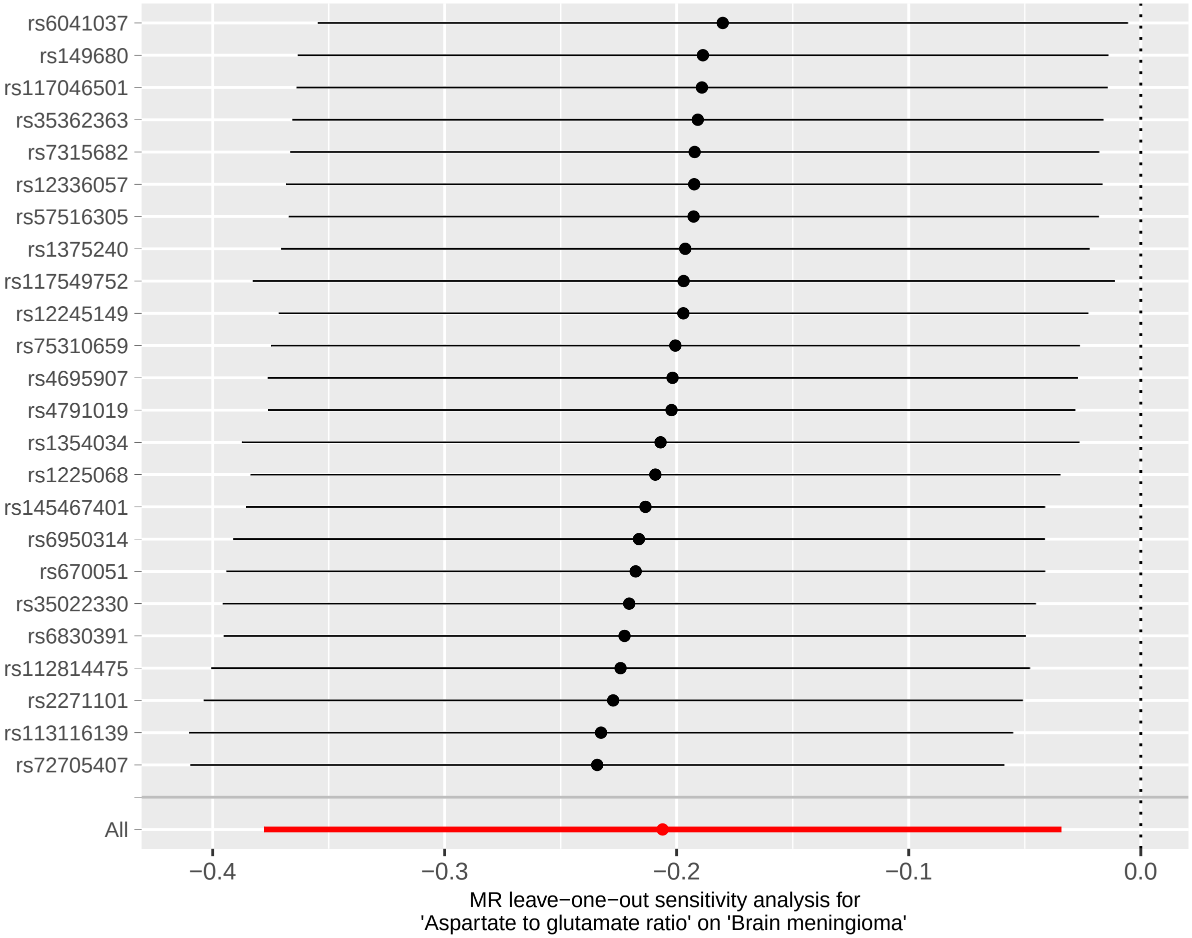
rs6041037
rs149680
rs117046501
rs35362363
rs7315682
rs12336057
rs57516305
rs1375240
rs117549752
rs12245149
rs75310659
rs4695907
rs4791019
rs1354034
rs1225068
rs145467401
rs6950314
rs670051
rs35022330
rs6830391
rs112814475
rs2271101
rs113116139
rs72705407
All
−0.4
−0.3
−0.2
−0.1
0.0
MR leave−one−out sensitivity analysis for
'Aspartate to glutamate ratio' on 'Brain meningioma'

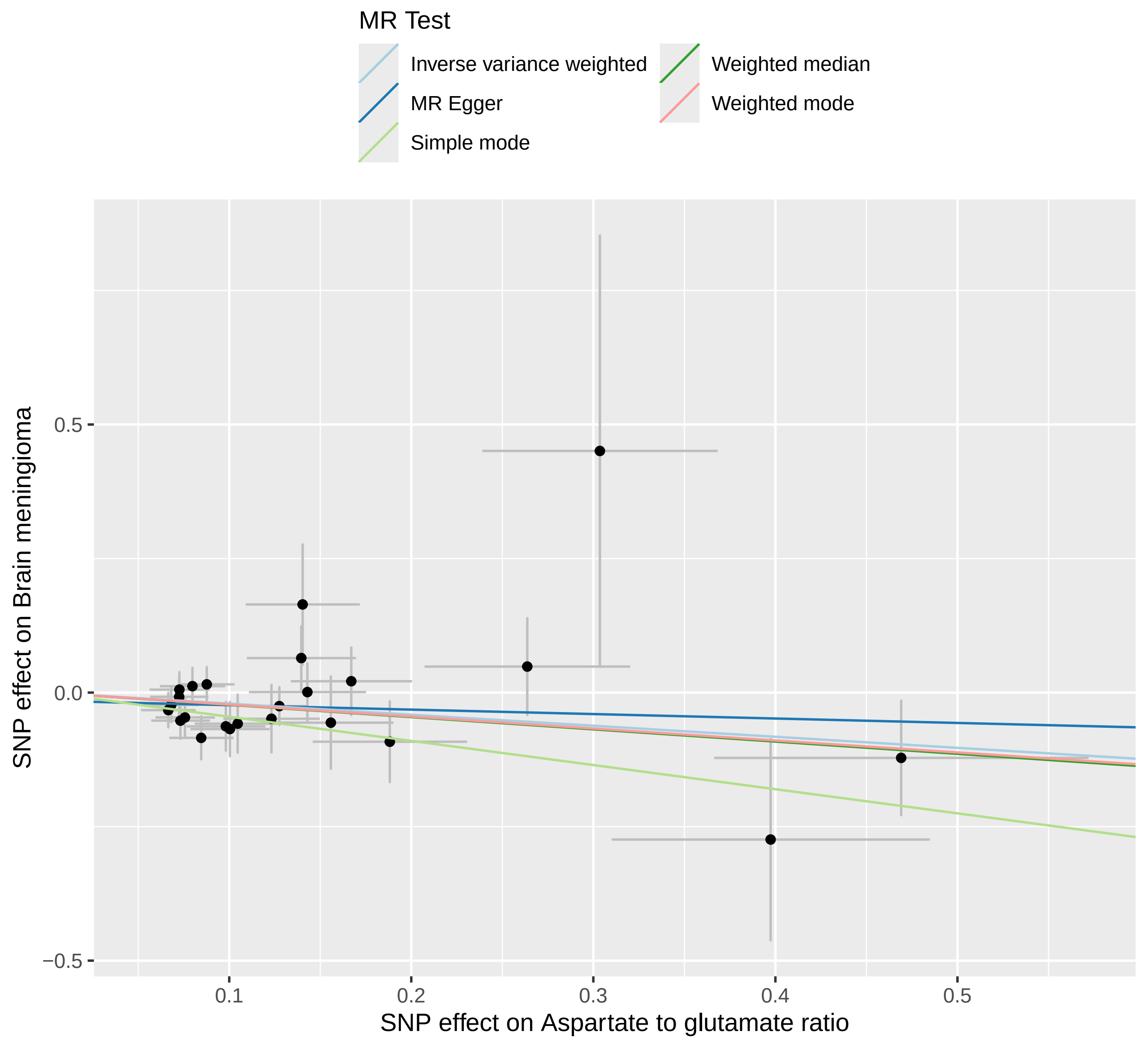
MR Test
Inverse variance weighted
MR Egger
Simple mode
Weighted median
Weighted mode
SNP effect on Brain meningioma
0.5
0.0
−0.5
0.1
0.2
0.3
0.4
0.5
SNP effect on Aspartate to glutamate ratio

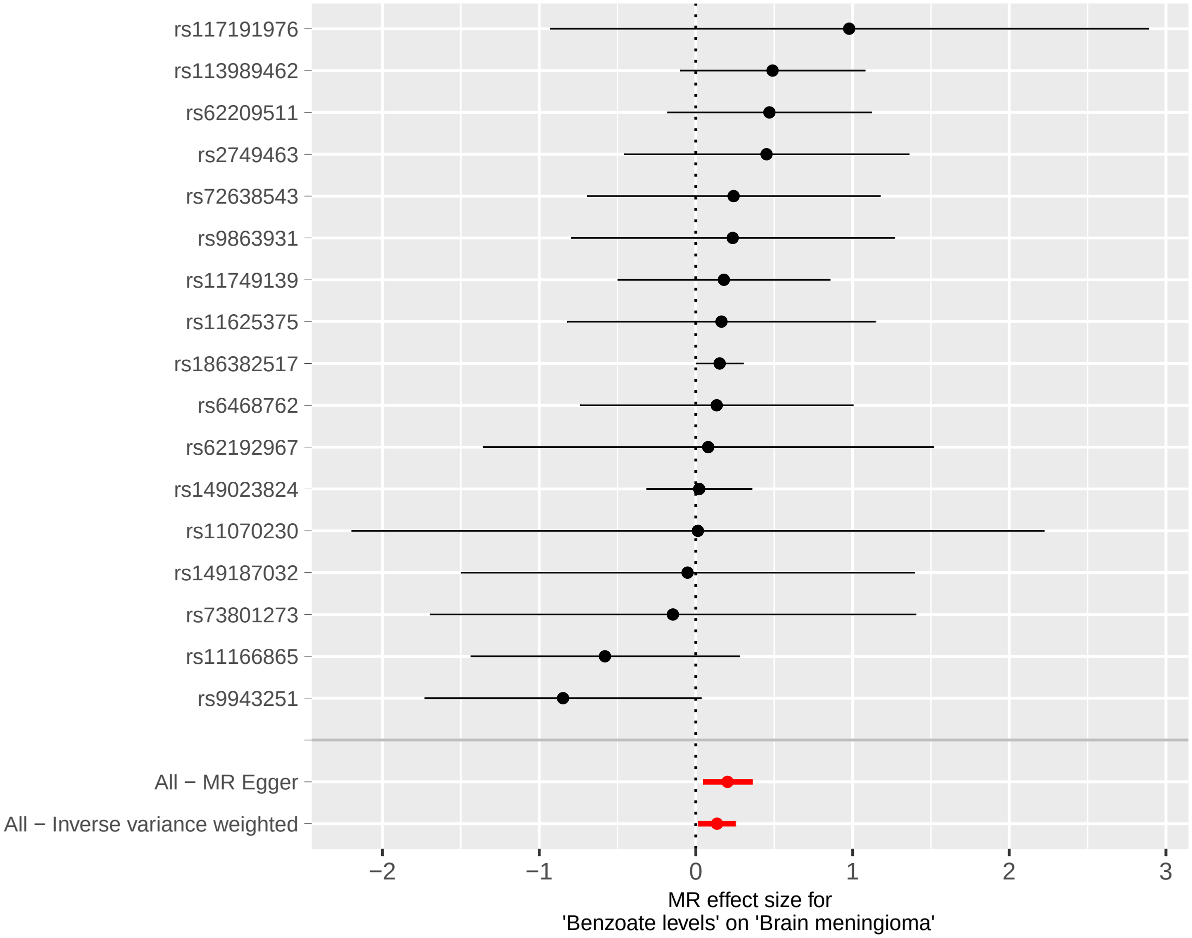
rs117191976
rs113989462
rs62209511
rs2749463
rs72638543
rs9863931
rs11749139
rs11625375
rs186382517
rs6468762
rs62192967
rs149023824
rs11070230
rs149187032
rs73801273
rs11166865
rs9943251
All − MR Egger
All − Inverse variance weighted
−2
−1
0
1
2
3
MR effect size for
'Benzoate levels' on 'Brain meningioma'

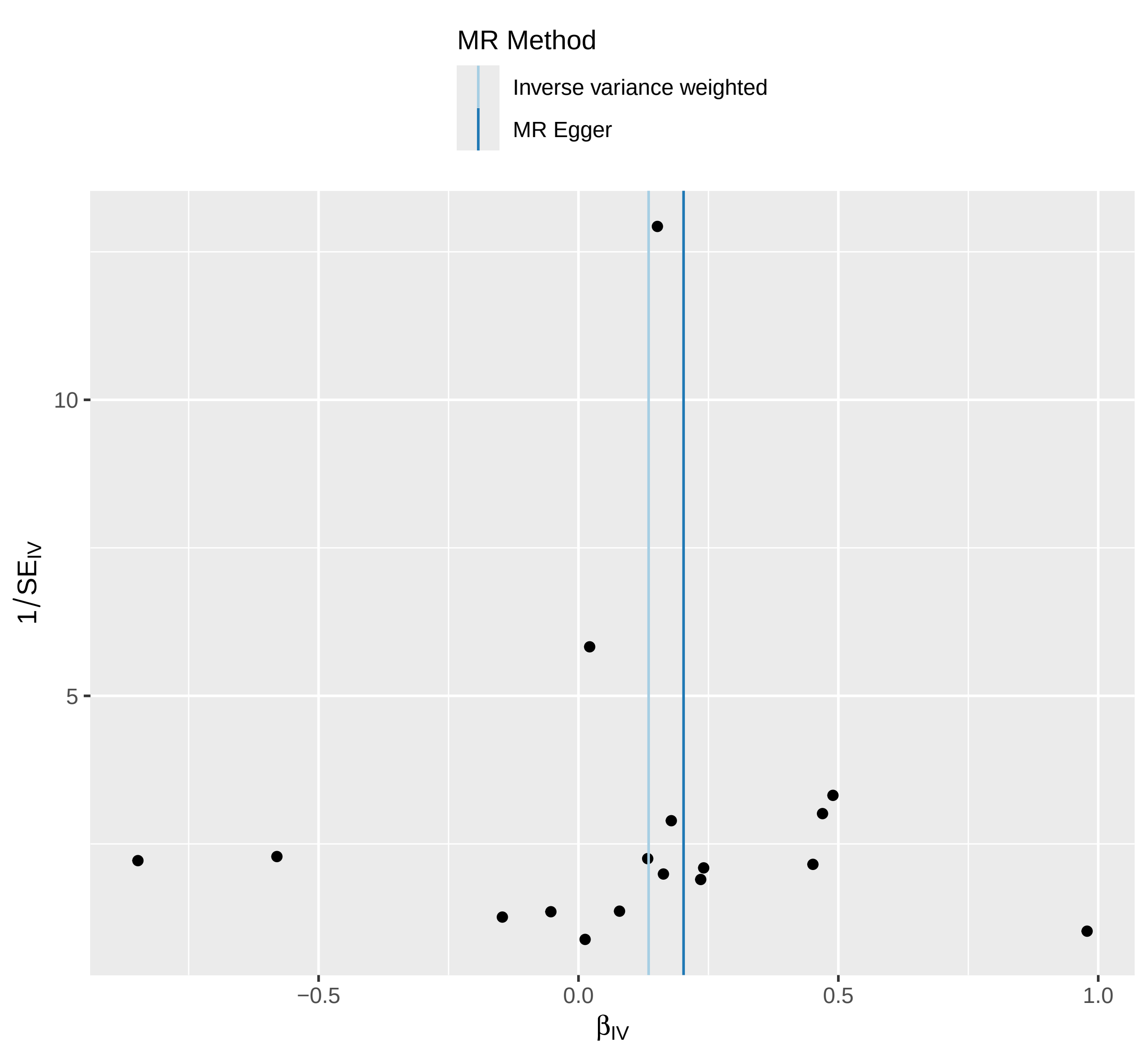
MR Method
Inverse variance weighted
MR Egger
10
5
1/SE_IV
−0.5
0.0
0.5
1.0
β_IV

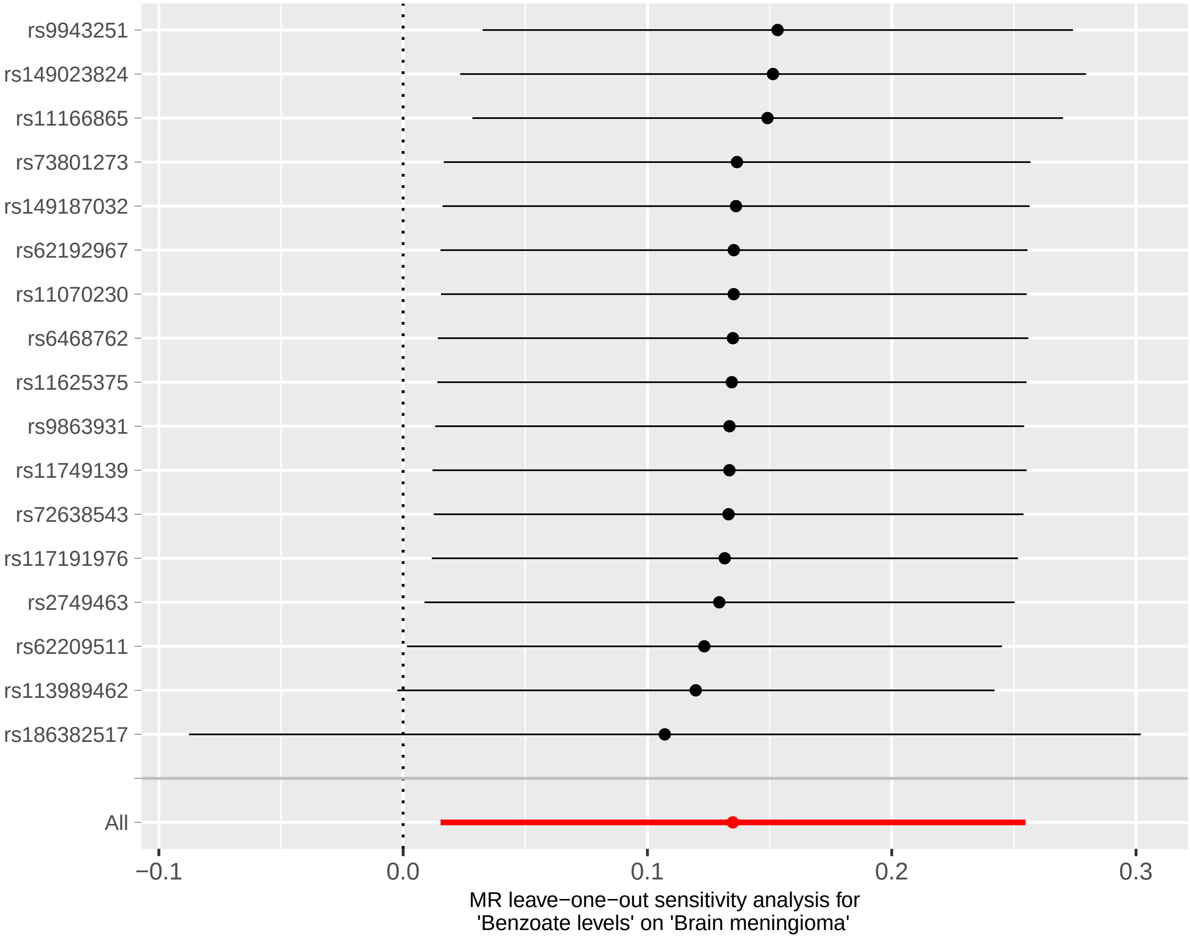
rs9943251
rs149023824
rs11166865
rs73801273
rs149187032
rs62192967
rs11070230
rs6468762
rs11625375
rs9863931
rs11749139
rs72638543
rs117191976
rs2749463
rs62209511
rs113989462
rs186382517
All
−0.1
0.0
0.1
0.2
0.3
MR leave−one−out sensitivity analysis for
'Benzoate levels' on 'Brain meningioma'

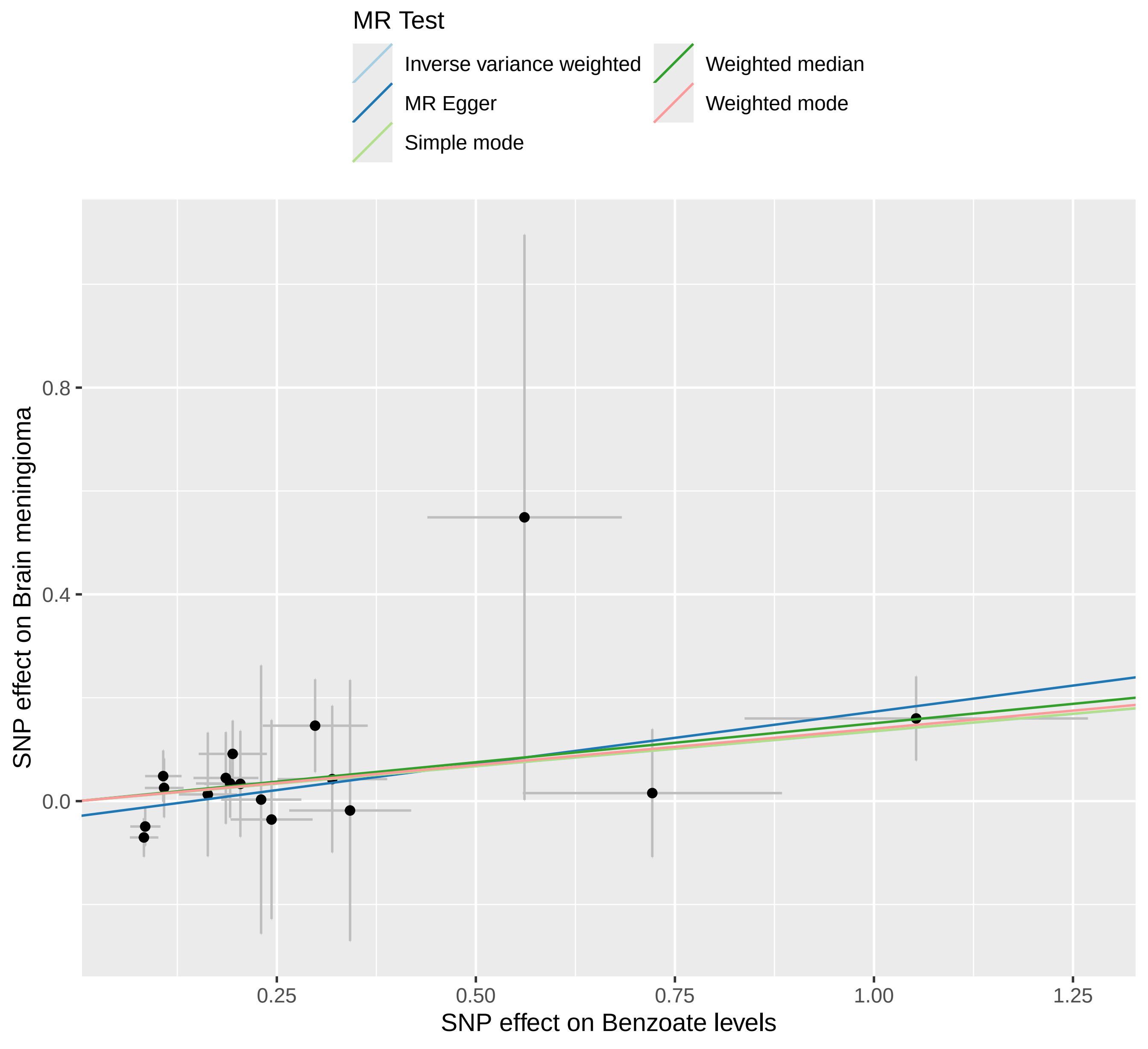
MR Test
Inverse variance weighted
MR Egger
Simple mode
Weighted median
Weighted mode
0.8
0.4
0.0
0.25
0.50
0.75
1.00
1.25
SNP effect on Brain meningioma
SNP effect on Benzoate levels

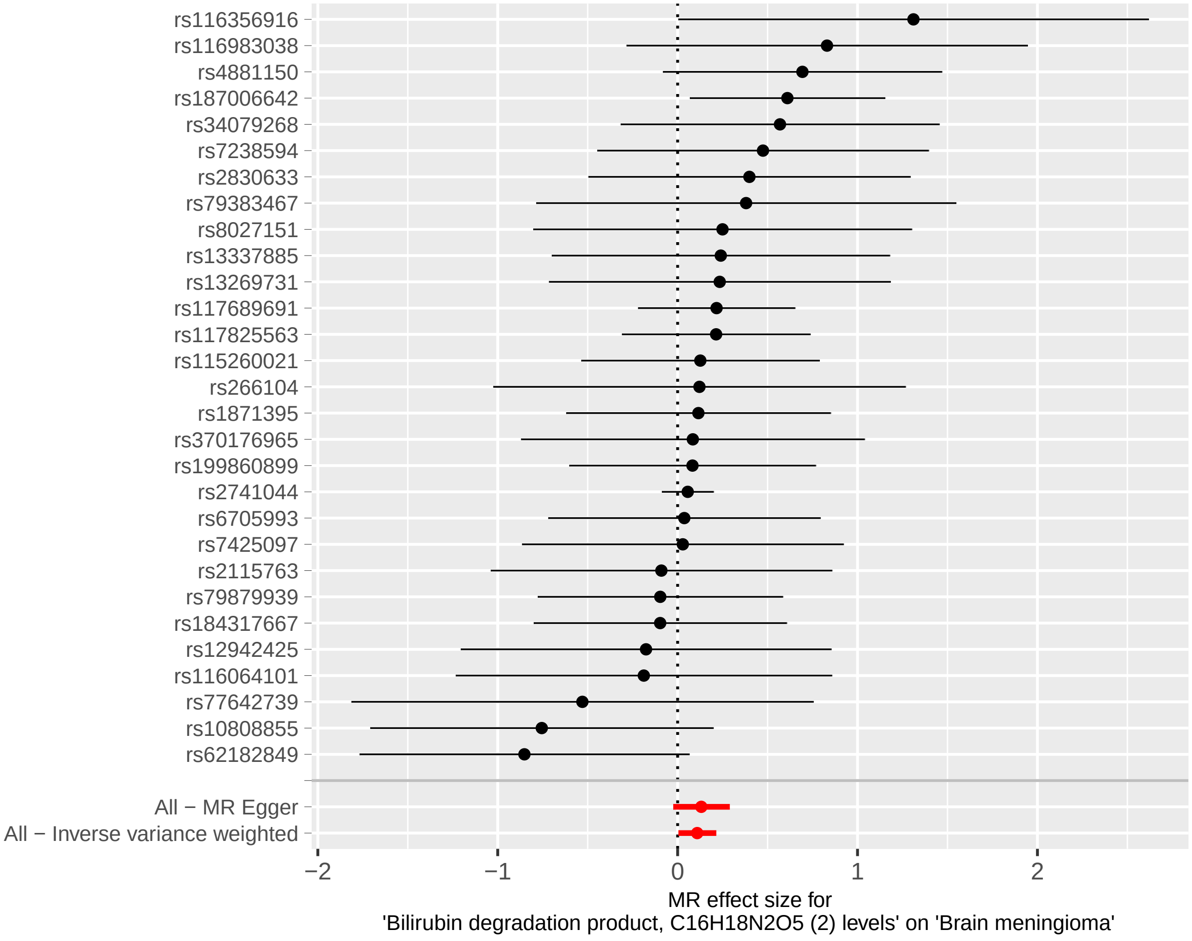

rs116356916
rs116983038
rs4881150
rs187006642
rs34079268
rs7238594
rs2830633
rs79383467
rs8027151
rs13337885
rs13269731
rs117689691
rs117825563
rs115260021
rs266104
rs1871395
rs370176965
rs199860899
rs2741044
rs6705993
rs7425097
rs2115763
rs79879939
rs184317667
rs12942425
rs116064101
rs77642739
rs10808855
rs62182849
All − MR Egger
All − Inverse variance weighted
−2
−1
0
1
2
MR effect size for
'Bilirubin degradation product, C16H18N2O5 (2) levels' on 'Brain meningioma'

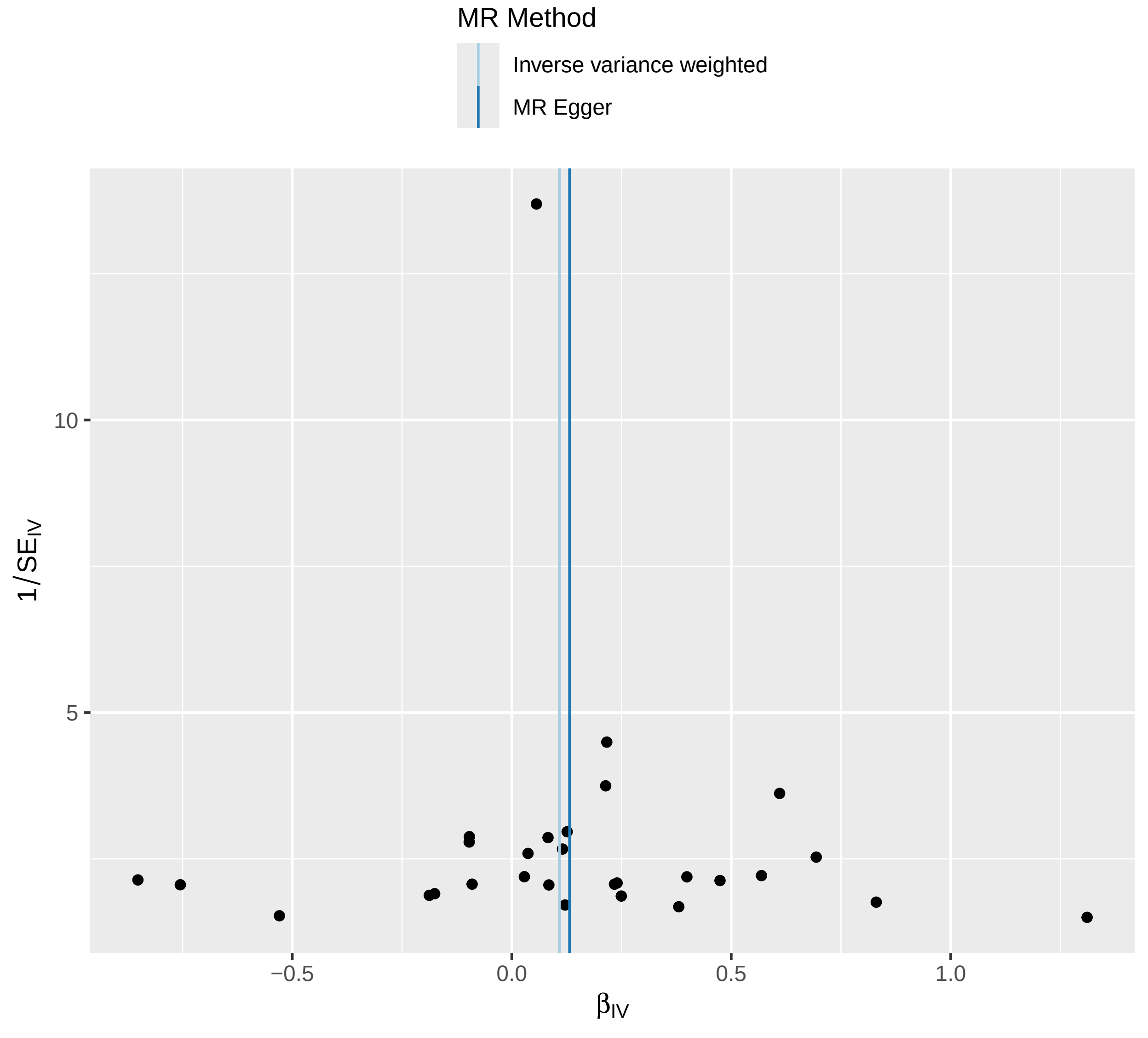
MR Method
Inverse variance weighted
MR Egger
10
5
1/SE_IV
−0.5
0.0
0.5
1.0
β_IV

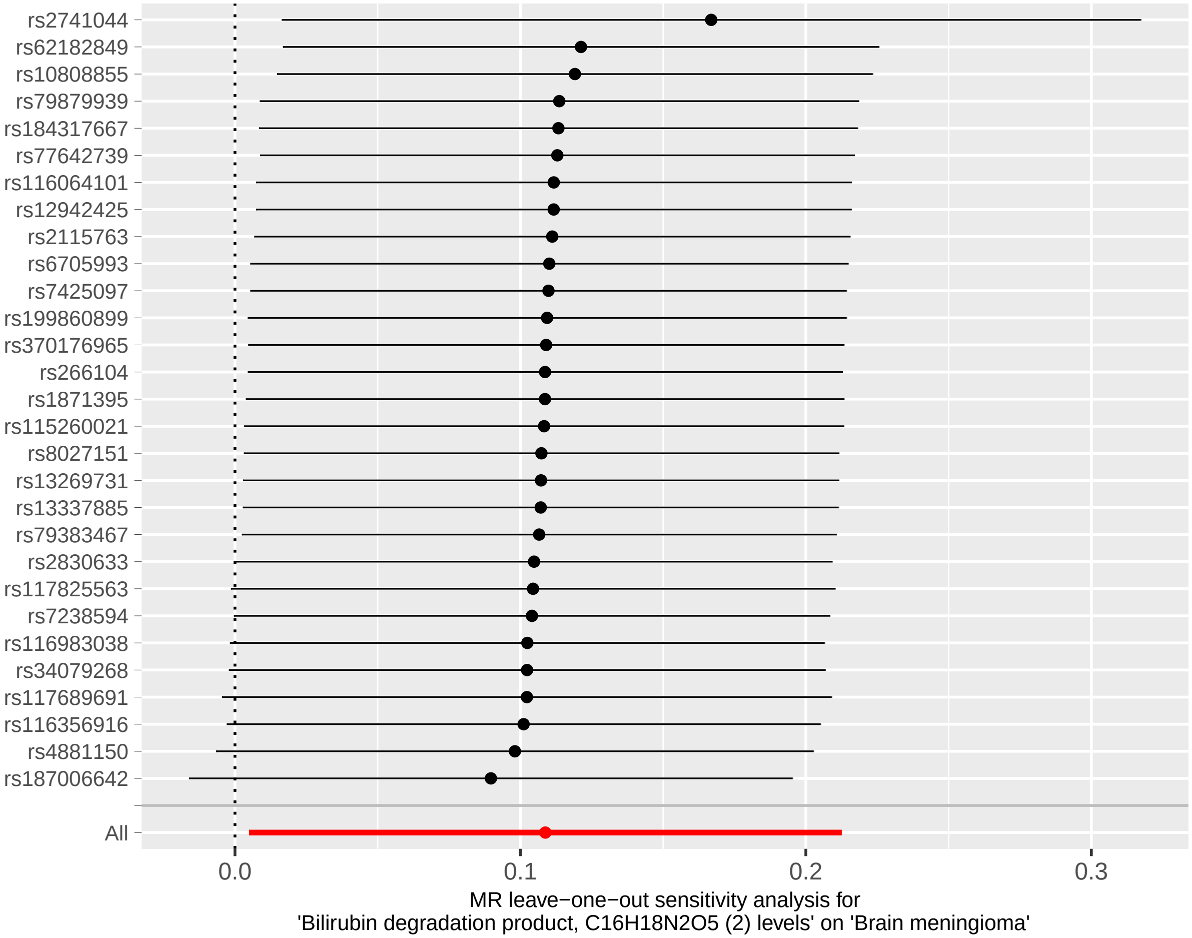
rs2741044
rs62182849
rs10808855
rs79879939
rs184317667
rs77642739
rs116064101
rs12942425
rs2115763
rs6705993
rs7425097
rs199860899
rs370176965
rs266104
rs1871395
rs115260021
rs8027151
rs13269731
rs13337885
rs79383467
rs2830633
rs117825563
rs7238594
rs116983038
rs34079268
rs117689691
rs116356916
rs4881150
rs187006642
All
0.0
0.1
0.2
0.3
MR leave−one−out sensitivity analysis for
'Bilirubin degradation product, C16H18N2O5 (2) levels' on 'Brain meningioma'

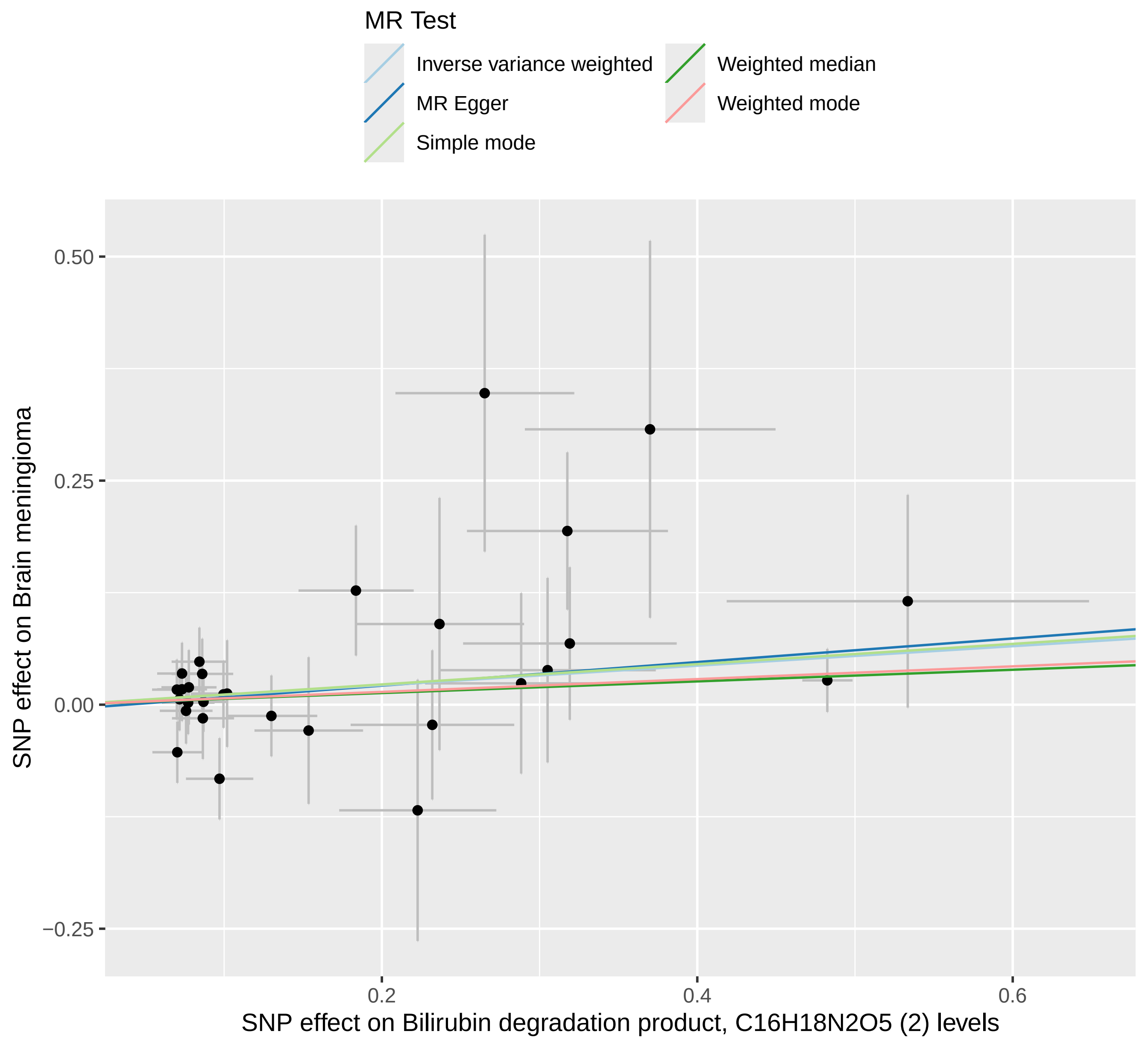
MR Test
Inverse variance weighted
MR Egger
Simple mode
Weighted median
Weighted mode
0.50
0.25
0.00
−0.25
0.2
0.4
0.6
SNP effect on Brain meningioma
SNP effect on Bilirubin degradation product, C16H18N2O5 (2) levels

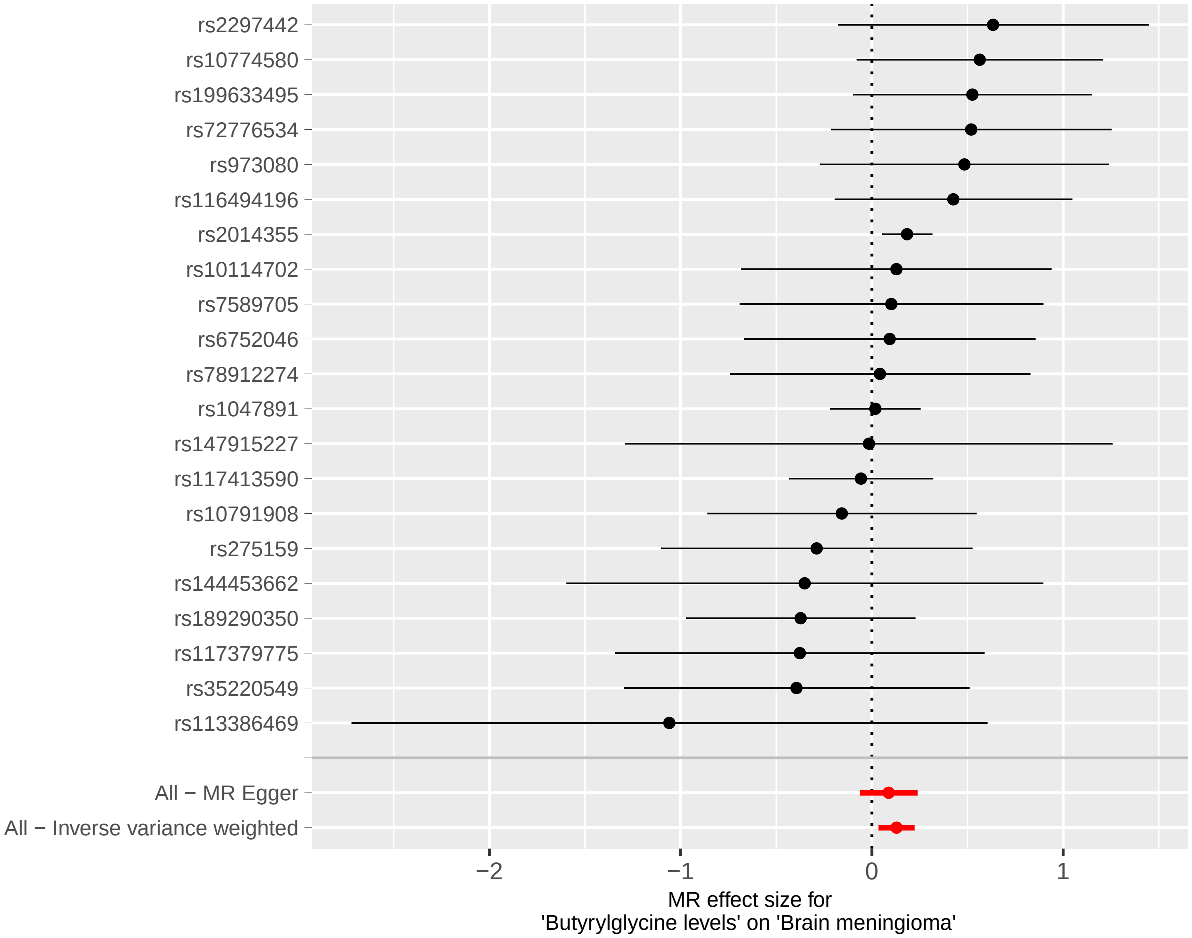
rs2297442
rs10774580
rs199633495
rs72776534
rs973080
rs116494196
rs2014355
rs10114702
rs7589705
rs6752046
rs78912274
rs1047891
rs147915227
rs117413590
rs10791908
rs275159
rs144453662
rs189290350
rs117379775
rs35220549
rs113386469
All − MR Egger
All − Inverse variance weighted
−2
−1
0
1
MR effect size for
'Butyrylglycine levels' on 'Brain meningioma'

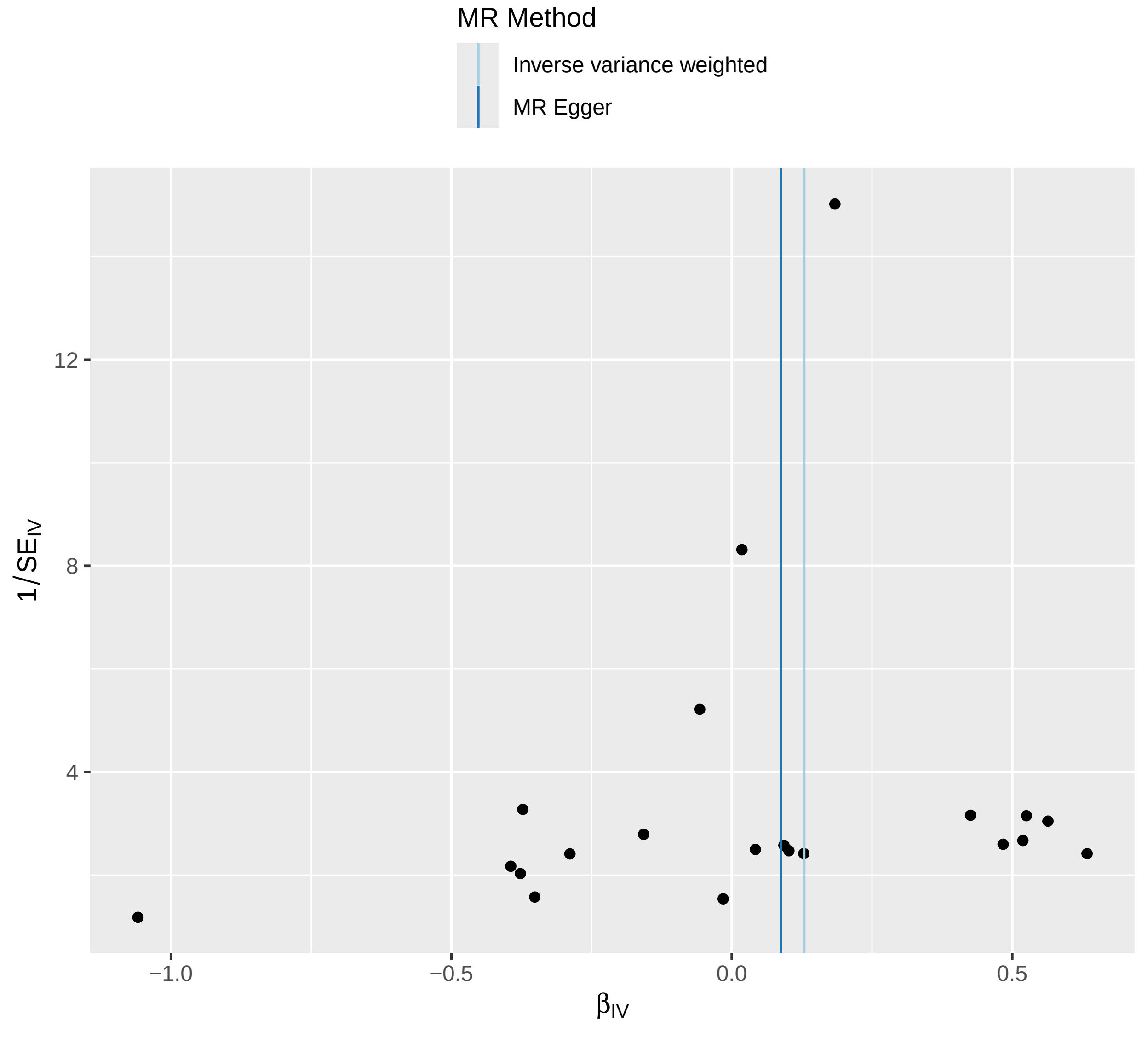
MR Method
Inverse variance weighted
MR Egger
12
8
4
$1/SE_{IV}$
−1.0
−0.5
0.0
0.5
β_{IV}

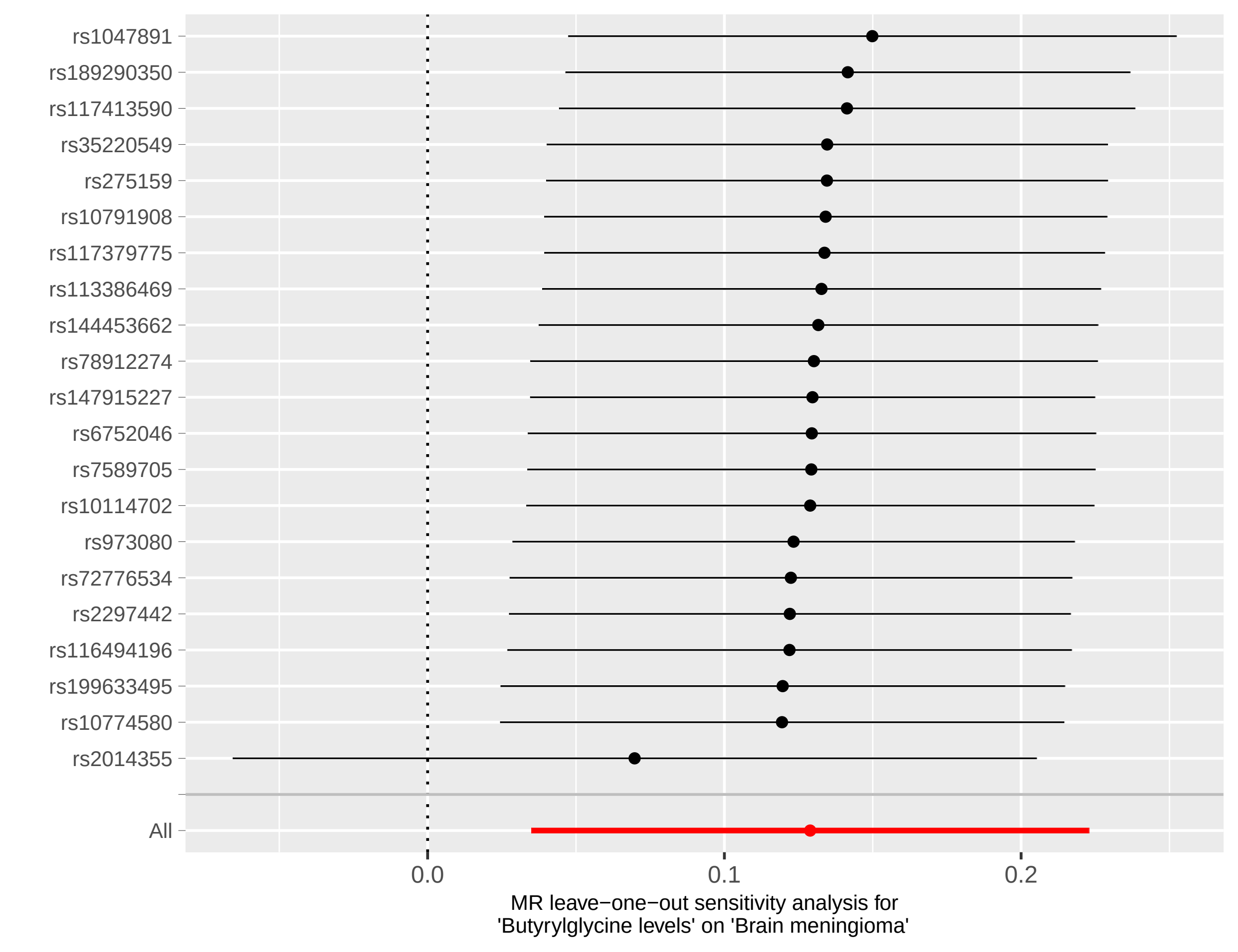
rs1047891
rs189290350
rs117413590
rs35220549
rs275159
rs10791908
rs117379775
rs113386469
rs144453662
rs78912274
rs147915227
rs6752046
rs7589705
rs10114702
rs973080
rs72776534
rs2297442
rs116494196
rs199633495
rs10774580
rs2014355
All
0.0
0.1
0.2
MR leave−one−out sensitivity analysis for
'Butyrylglycine levels' on 'Brain meningioma'

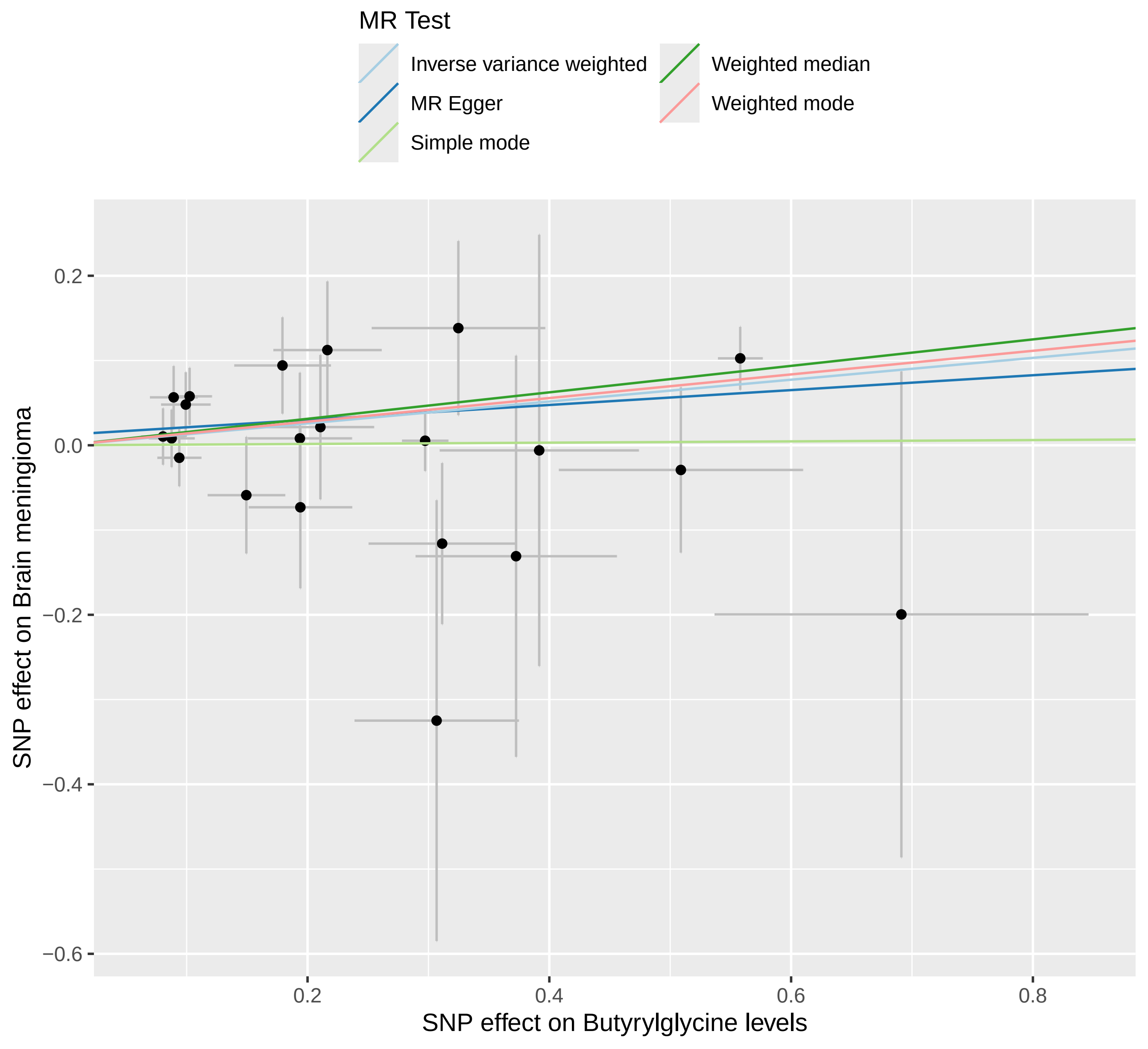

MR Test
Inverse variance weighted
MR Egger
Simple mode
Weighted median
Weighted mode
0.2
0.0
−0.2
−0.4
−0.6
SNP effect on Brain meningioma
0.2
0.4
0.6
0.8
SNP effect on Butyrylglycine levels

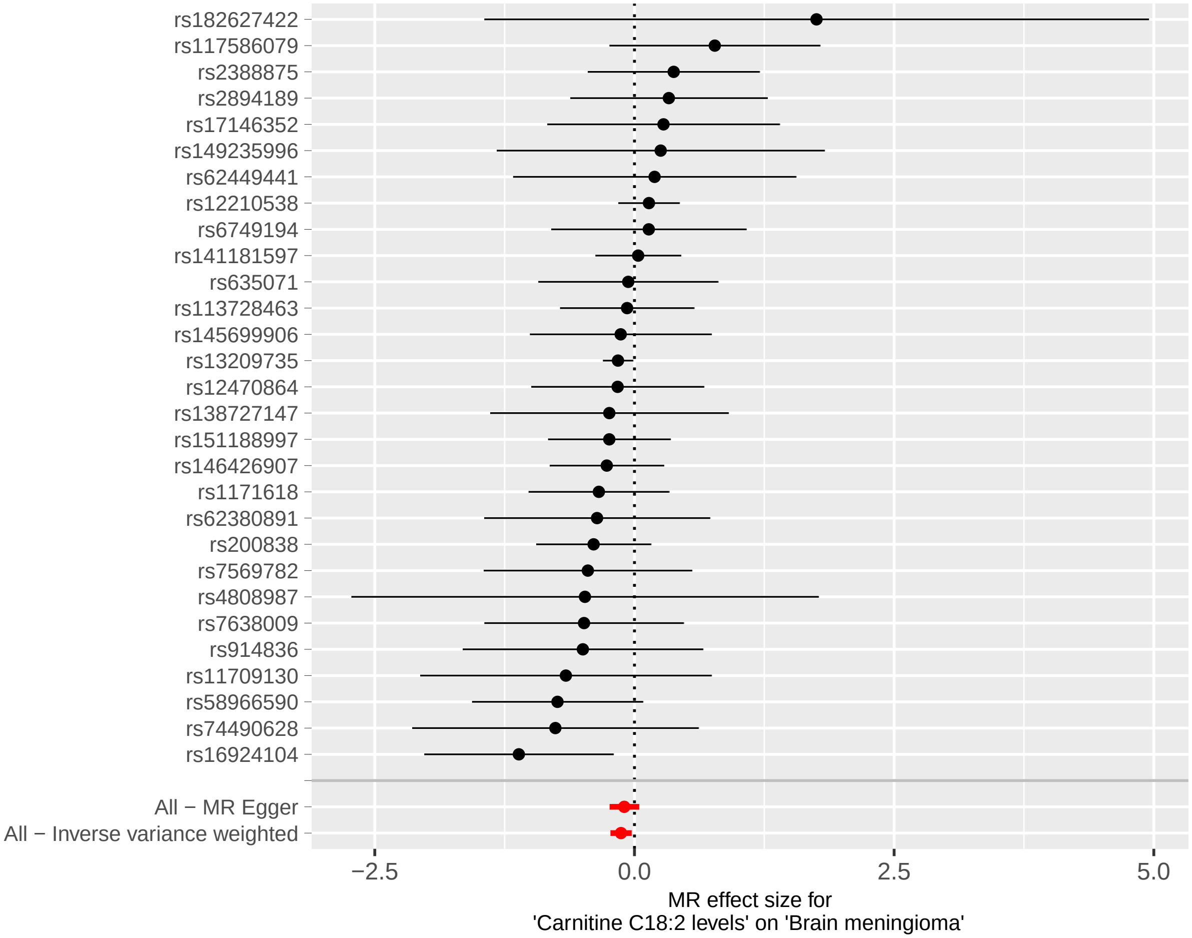

rs182627422
rs117586079
rs2388875
rs2894189
rs17146352
rs149235996
rs62449441
rs12210538
rs6749194
rs141181597
rs635071
rs113728463
rs145699906
rs13209735
rs12470864
rs138727147
rs151188997
rs146426907
rs1171618
rs62380891
rs200838
rs7569782
rs4808987
rs7638009
rs914836
rs11709130
rs58966590
rs74490628
rs16924104
All − MR Egger
All − Inverse variance weighted
−2.5
0.0
2.5
5.0
MR effect size for
'Carnitine C18:2 levels' on 'Brain meningioma'

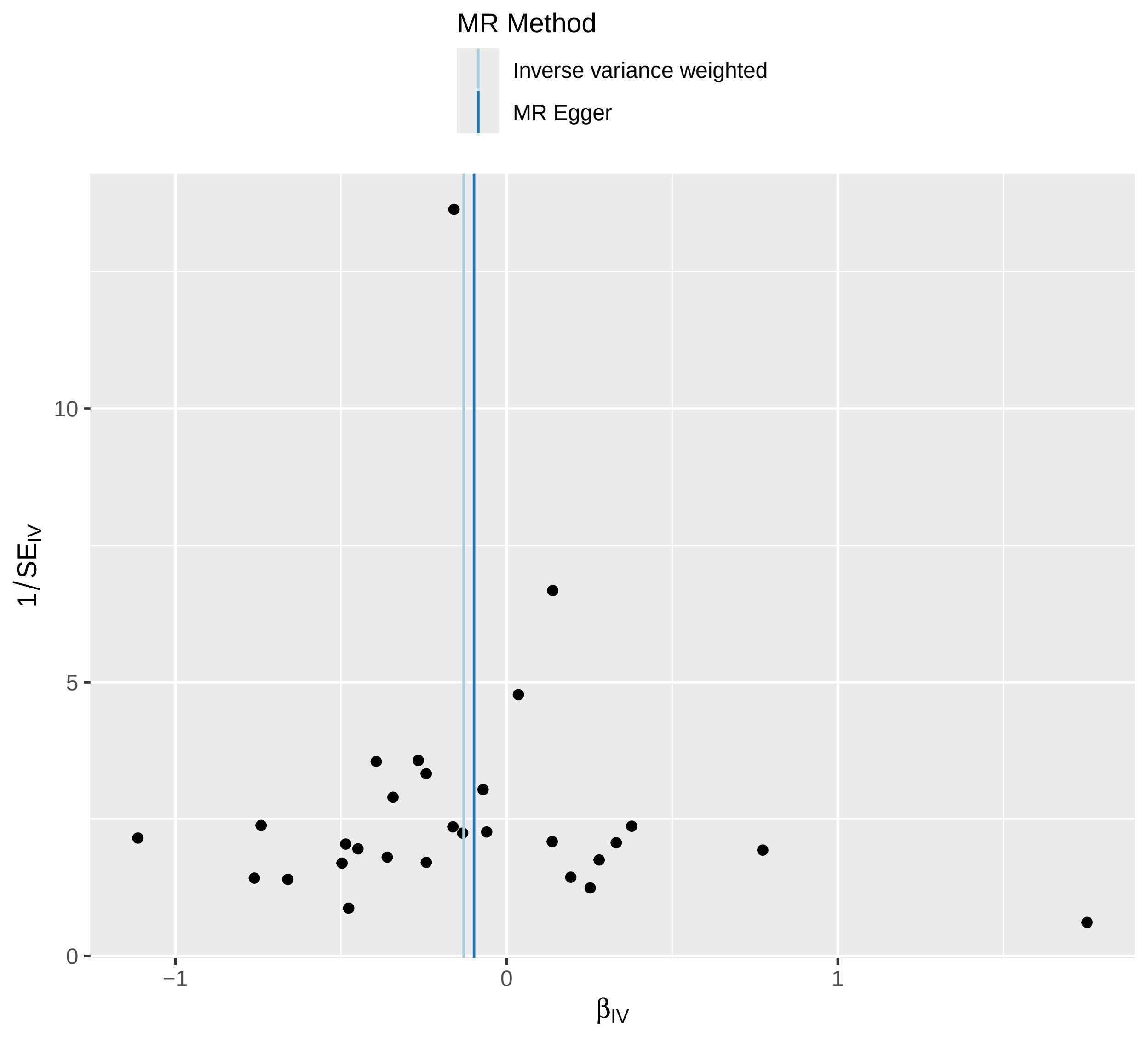

MR Method
Inverse variance weighted
MR Egger
1/SE_IV
10
5
0
−1
0
1
β_IV

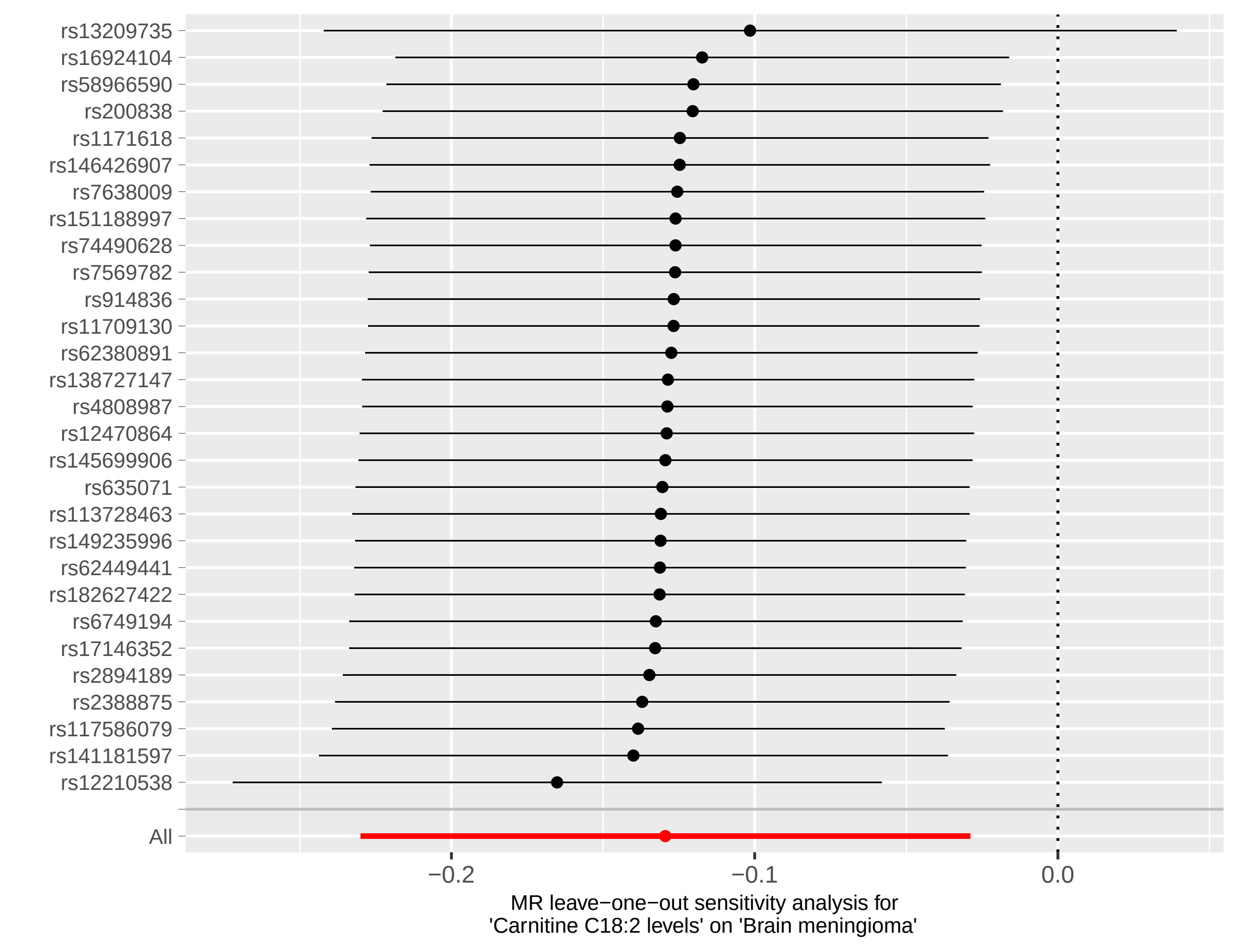
rs13209735
rs16924104
rs58966590
rs200838
rs1171618
rs146426907
rs7638009
rs151188997
rs74490628
rs7569782
rs914836
rs11709130
rs62380891
rs138727147
rs4808987
rs12470864
rs145699906
rs635071
rs113728463
rs149235996
rs62449441
rs182627422
rs6749194
rs17146352
rs2894189
rs2388875
rs117586079
rs141181597
rs12210538
All
−0.2
−0.1
0.0
MR leave−one−out sensitivity analysis for
'Carnitine C18:2 levels' on 'Brain meningioma'

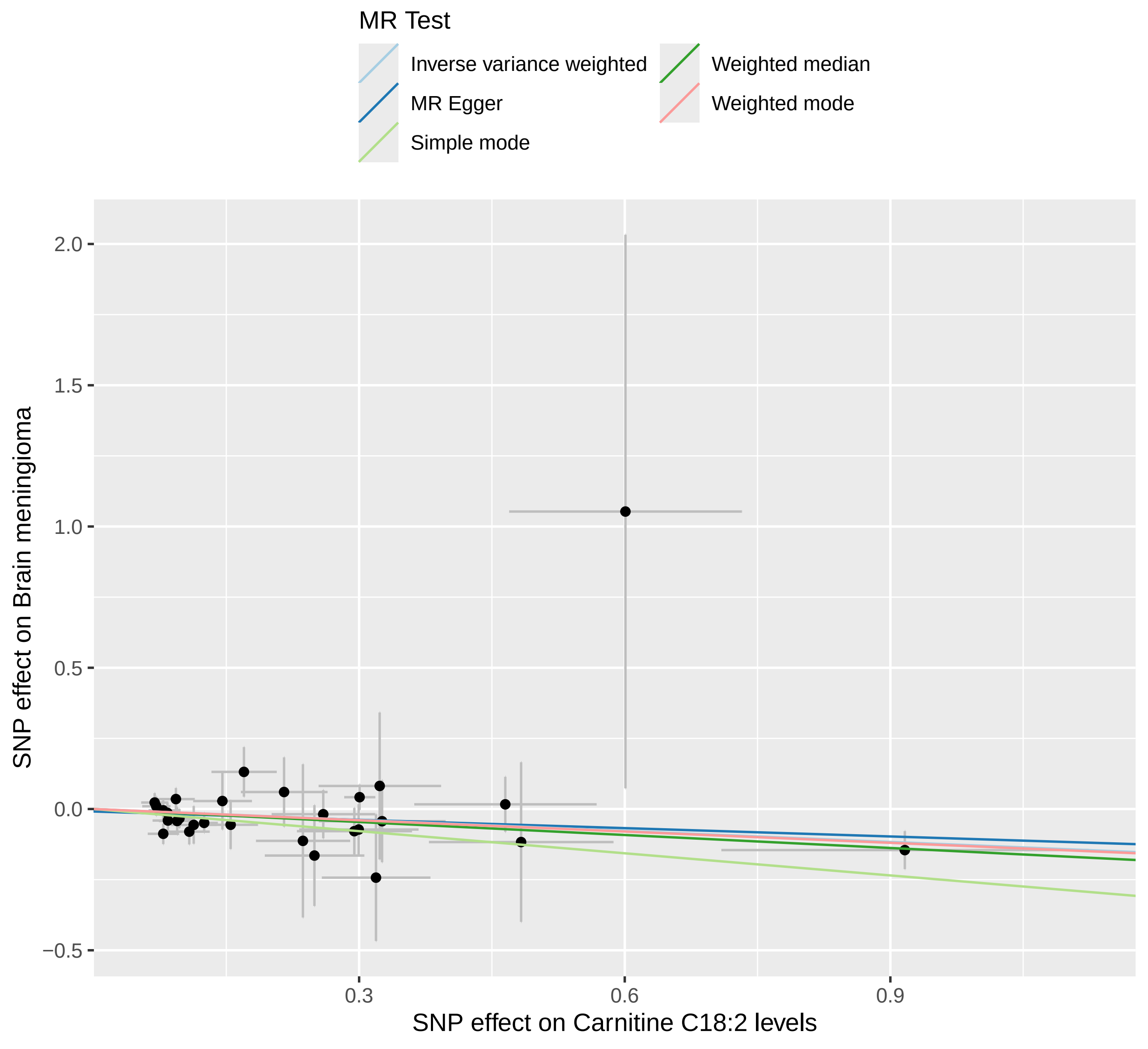
MR Test
Inverse variance weighted
MR Egger
Simple mode
Weighted median
Weighted mode
2.0
1.5
1.0
0.5
0.0
−0.5
0.3
0.6
0.9
SNP effect on Brain meningioma
SNP effect on Carnitine C18:2 levels

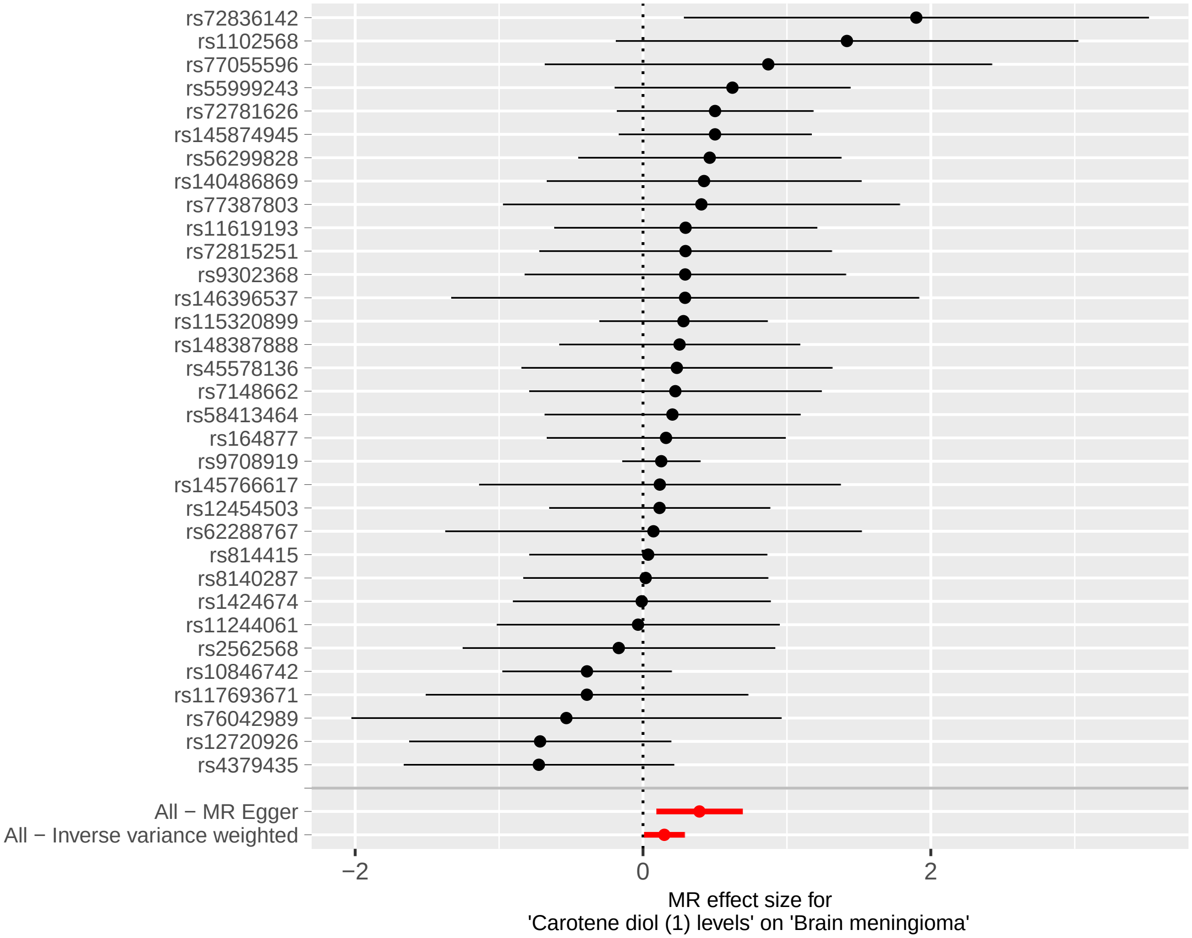
rs72836142
rs1102568
rs77055596
rs55999243
rs72781626
rs145874945
rs56299828
rs140486869
rs77387803
rs11619193
rs72815251
rs9302368
rs146396537
rs115320899
rs148387888
rs45578136
rs7148662
rs58413464
rs164877
rs9708919
rs145766617
rs12454503
rs62288767
rs814415
rs8140287
rs1424674
rs11244061
rs2562568
rs10846742
rs117693671
rs76042989
rs12720926
rs4379435
All − MR Egger
All − Inverse variance weighted
−2
0
2
MR effect size for
'Carotene diol (1) levels' on 'Brain meningioma'

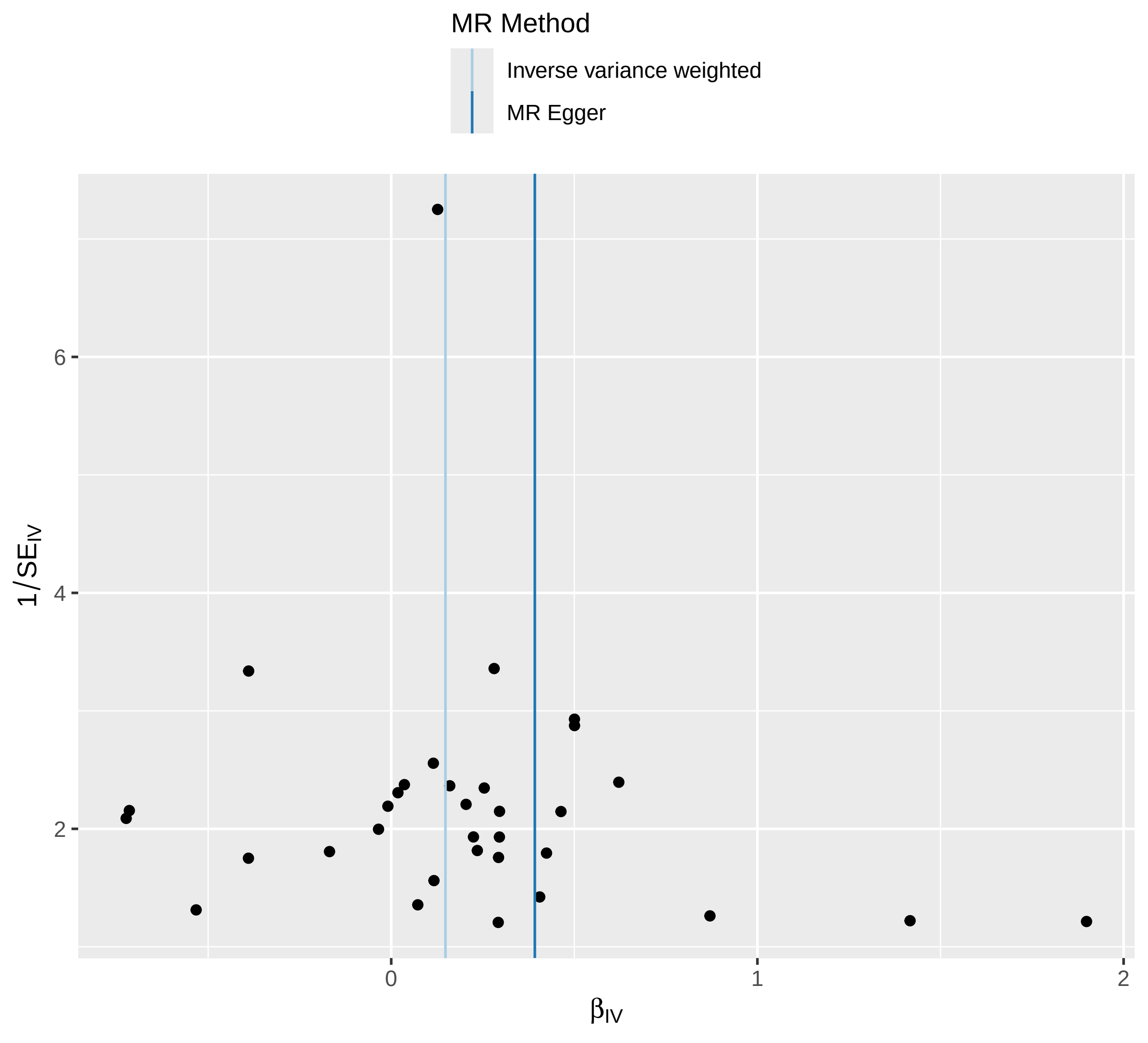

MR Method
Inverse variance weighted
MR Egger
1/SE_IV
6
4
2
0
1
2
β_IV

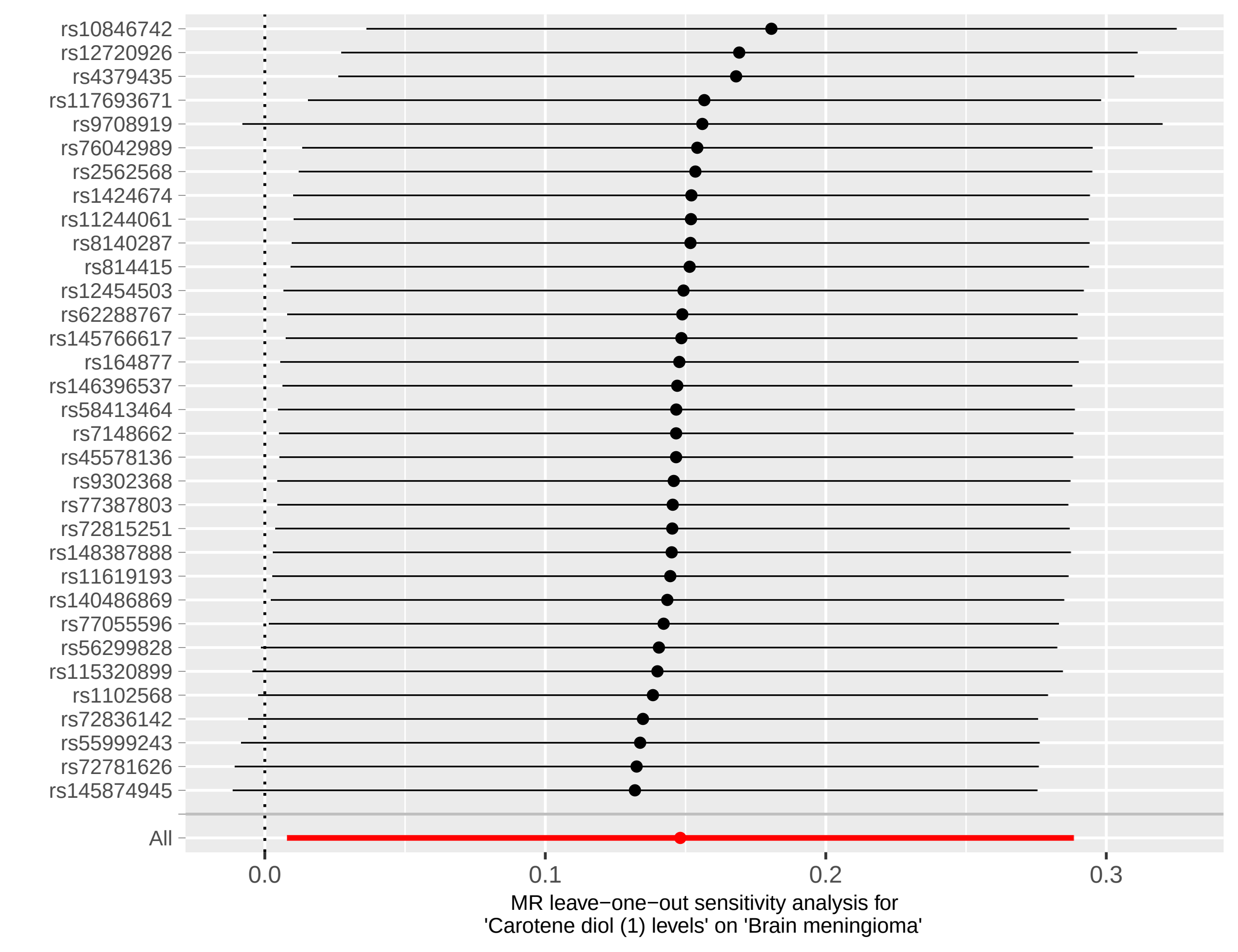

rs10846742
rs12720926
rs4379435
rs117693671
rs9708919
rs76042989
rs2562568
rs1424674
rs11244061
rs8140287
rs814415
rs12454503
rs62288767
rs145766617
rs164877
rs146396537
rs58413464
rs7148662
rs45578136
rs9302368
rs77387803
rs72815251
rs148387888
rs11619193
rs140486869
rs77055596
rs56299828
rs115320899
rs1102568
rs72836142
rs55999243
rs72781626
rs145874945
All
0.0
0.1
0.2
0.3
MR leave−one−out sensitivity analysis for
'Carotene diol (1) levels' on 'Brain meningioma'

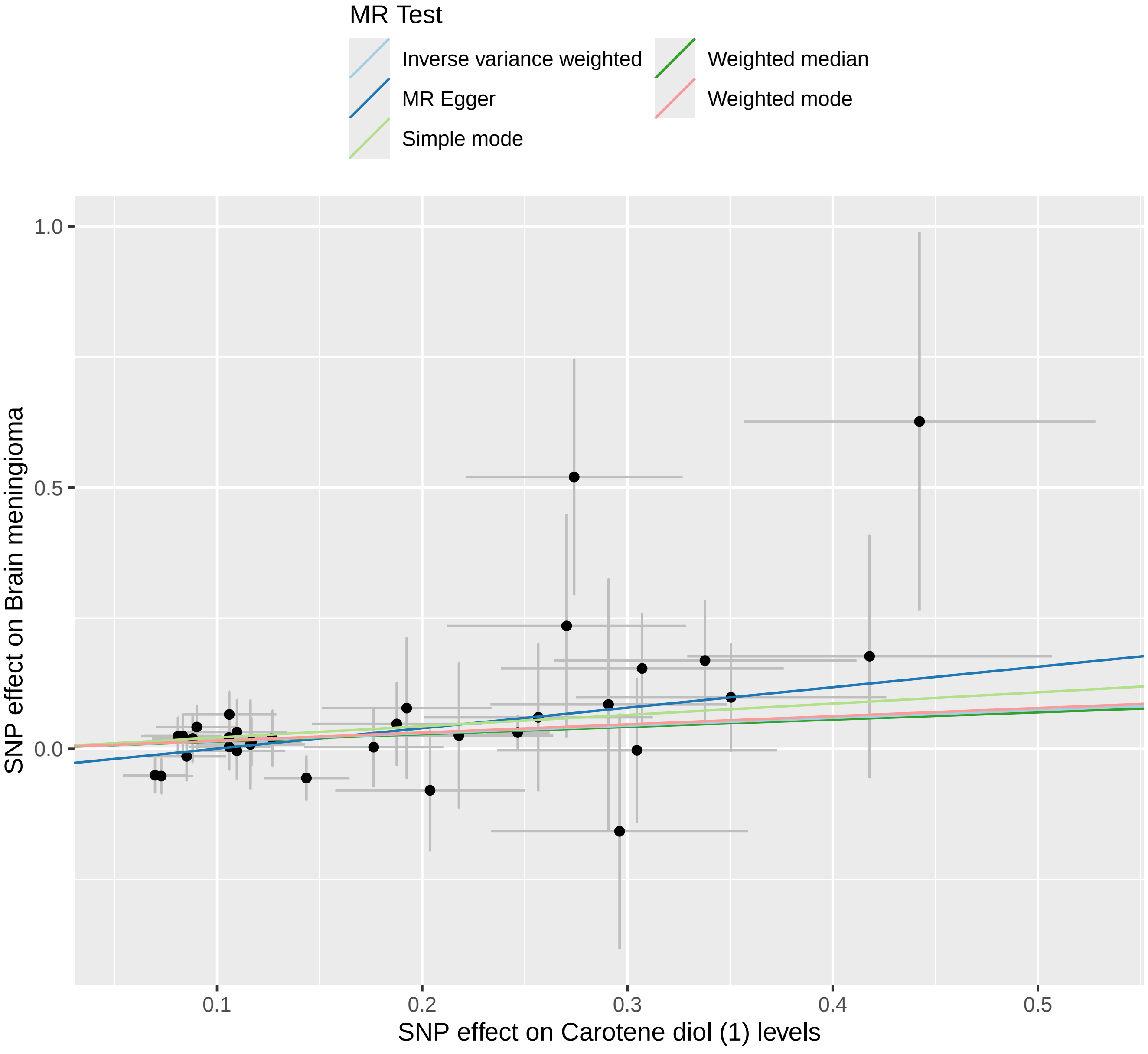
MR Test
Inverse variance weighted
MR Egger
Simple mode
Weighted median
Weighted mode
1.0
0.5
0.0
0.1
0.2
0.3
0.4
0.5
SNP effect on Brain meningioma
SNP effect on Carotene diol (1) levels

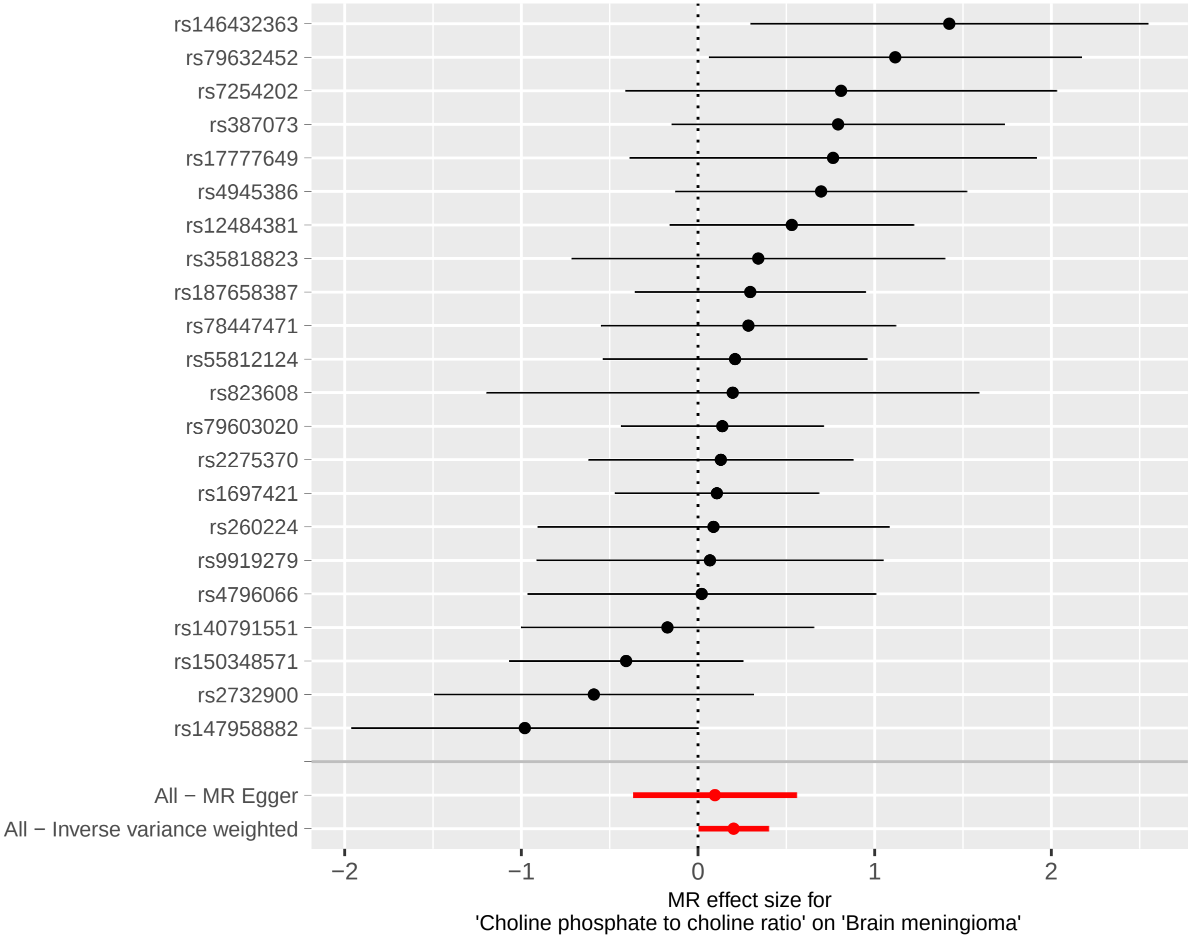
rs146432363
rs79632452
rs7254202
rs387073
rs17777649
rs4945386
rs12484381
rs35818823
rs187658387
rs78447471
rs55812124
rs823608
rs79603020
rs2275370
rs1697421
rs260224
rs9919279
rs4796066
rs140791551
rs150348571
rs2732900
rs147958882
All − MR Egger
All − Inverse variance weighted
−2
−1
0
1
2
MR effect size for
'Choline phosphate to choline ratio' on 'Brain meningioma'

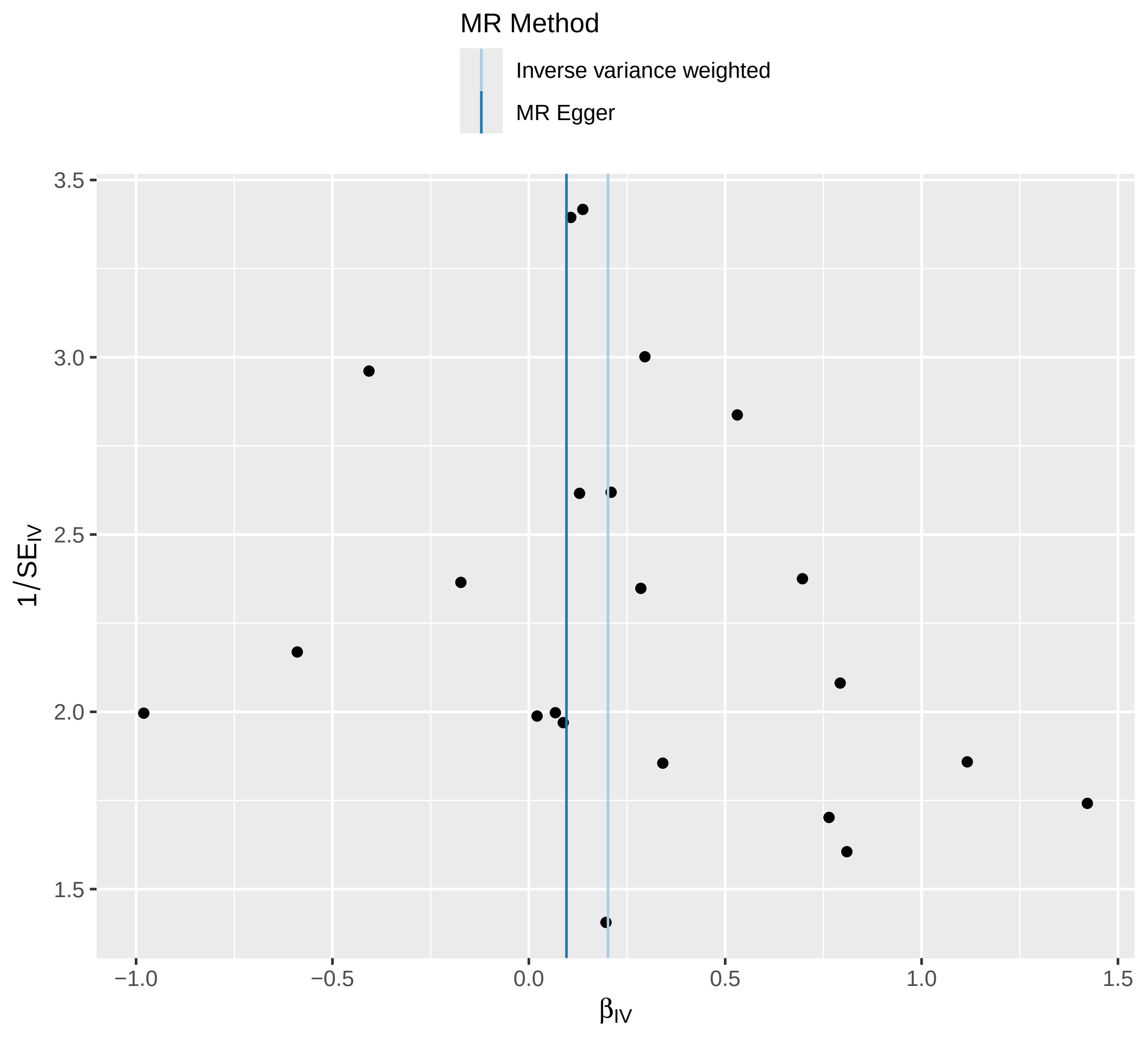
MR Method
Inverse variance weighted
MR Egger
3.5
3.0
2.5
2.0
1.5
1/SE_IV
−1.0
−0.5
0.0
0.5
1.0
1.5
β_IV

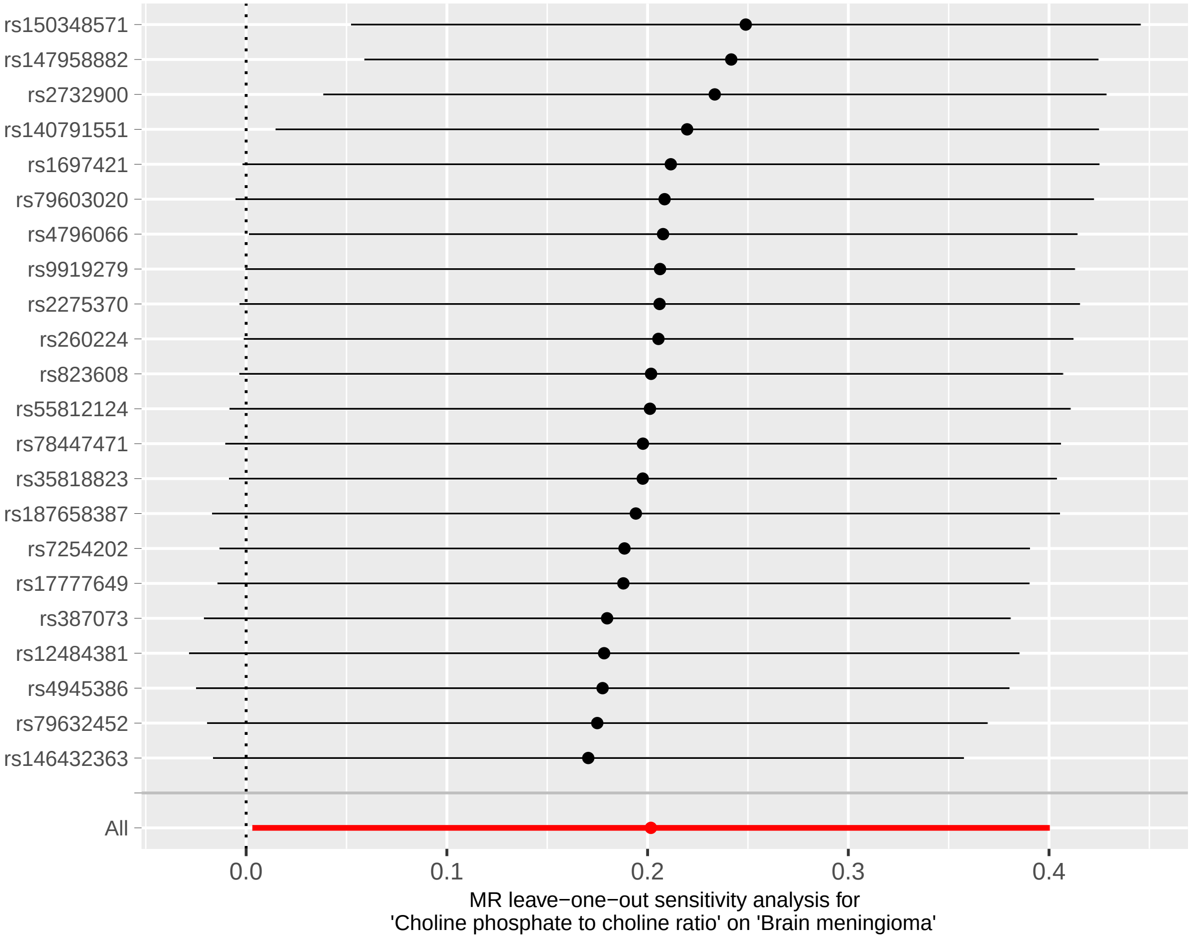
rs150348571
rs147958882
rs2732900
rs140791551
rs1697421
rs79603020
rs4796066
rs9919279
rs2275370
rs260224
rs823608
rs55812124
rs78447471
rs35818823
rs187658387
rs7254202
rs17777649
rs387073
rs12484381
rs4945386
rs79632452
rs146432363
All
0.0
0.1
0.2
0.3
0.4
MR leave−one−out sensitivity analysis for
'Choline phosphate to choline ratio' on 'Brain meningioma'

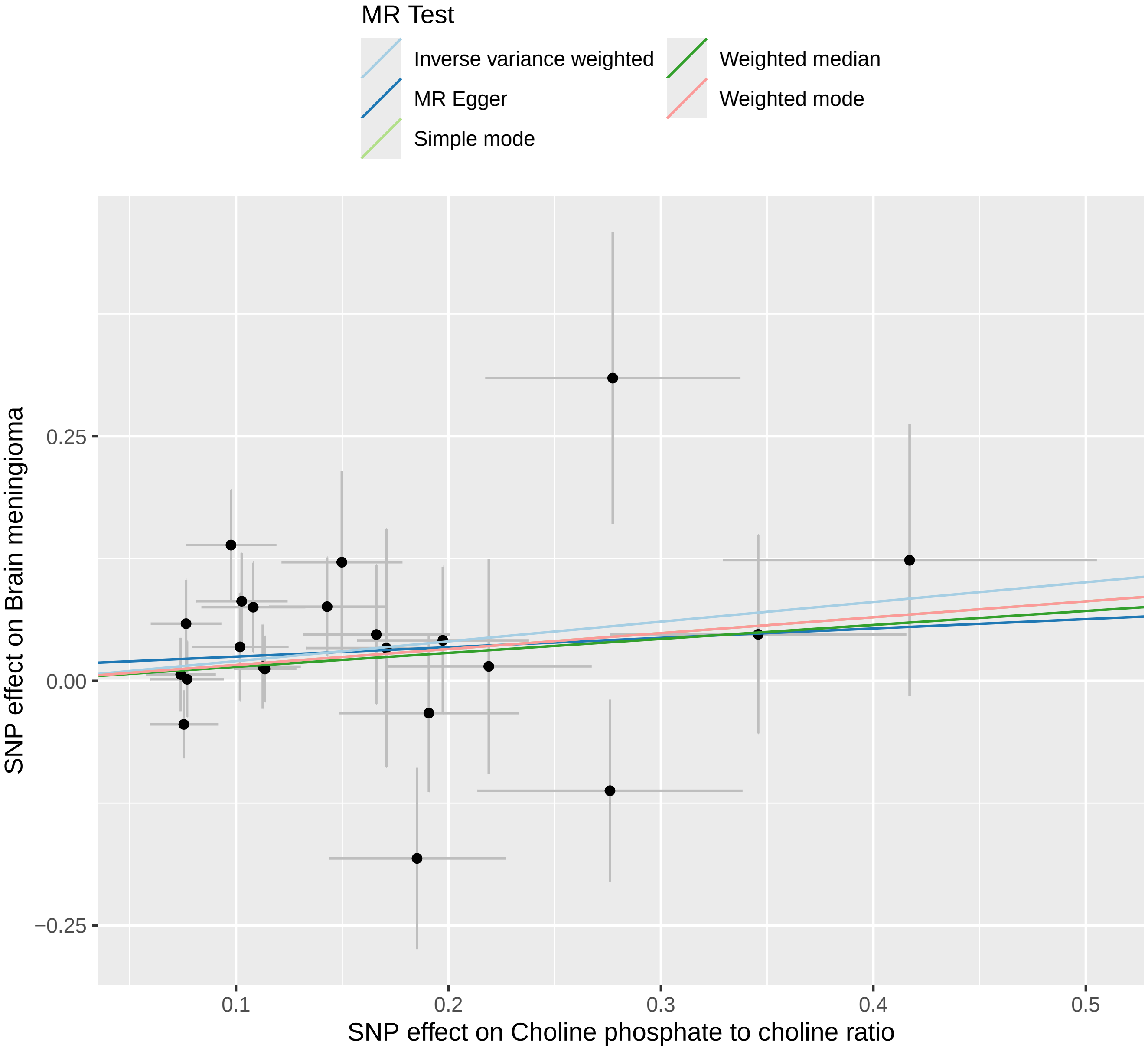
MR Test
Inverse variance weighted
MR Egger
Simple mode
Weighted median
Weighted mode
0.25
0.00
−0.25
0.1
0.2
0.3
0.4
0.5
SNP effect on Brain meningioma
SNP effect on Choline phosphate to choline ratio

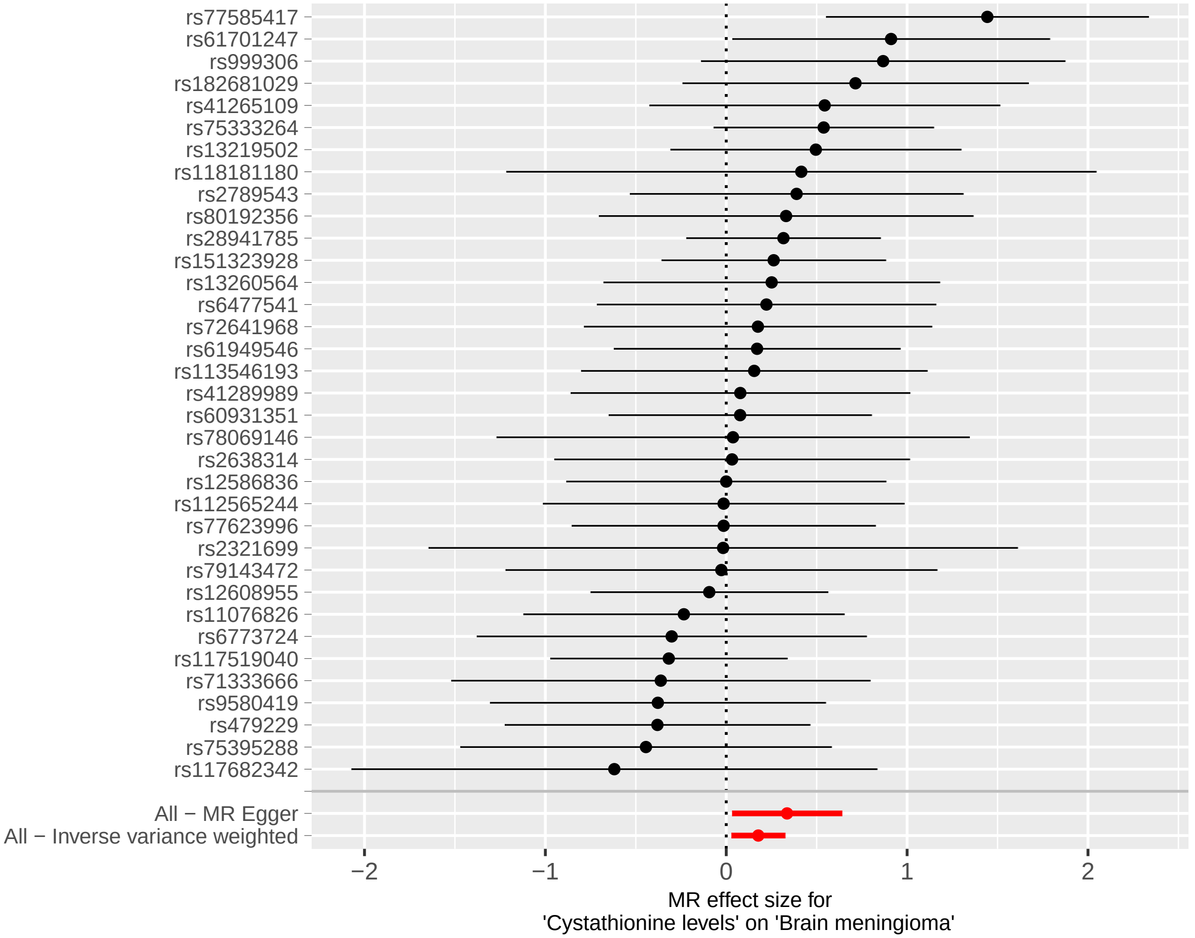
rs77585417
rs61701247
rs999306
rs182681029
rs41265109
rs75333264
rs13219502
rs118181180
rs2789543
rs80192356
rs28941785
rs151323928
rs13260564
rs6477541
rs72641968
rs61949546
rs113546193
rs41289989
rs60931351
rs78069146
rs2638314
rs12586836
rs112565244
rs77623996
rs2321699
rs79143472
rs12608955
rs11076826
rs6773724
rs117519040
rs71333666
rs9580419
rs479229
rs75395288
rs117682342
All − MR Egger
All − Inverse variance weighted
−2
−1
0
1
2
MR effect size for
'Cystathionine levels' on 'Brain meningioma'

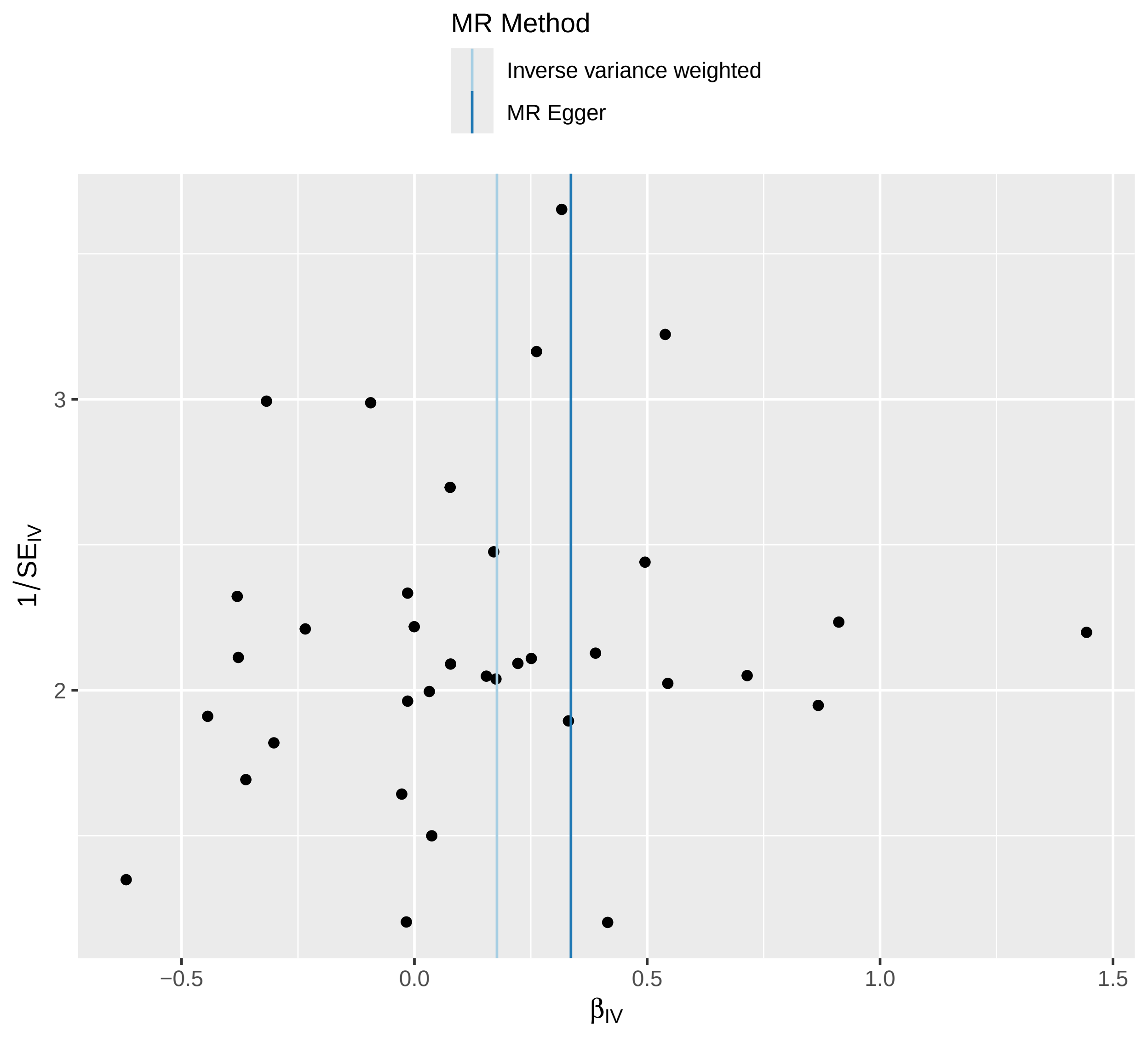
MR Method
Inverse variance weighted
MR Egger
3
2
1/SE$_{IV}$
−0.5
0.0
0.5
1.0
1.5
β$_{IV}$

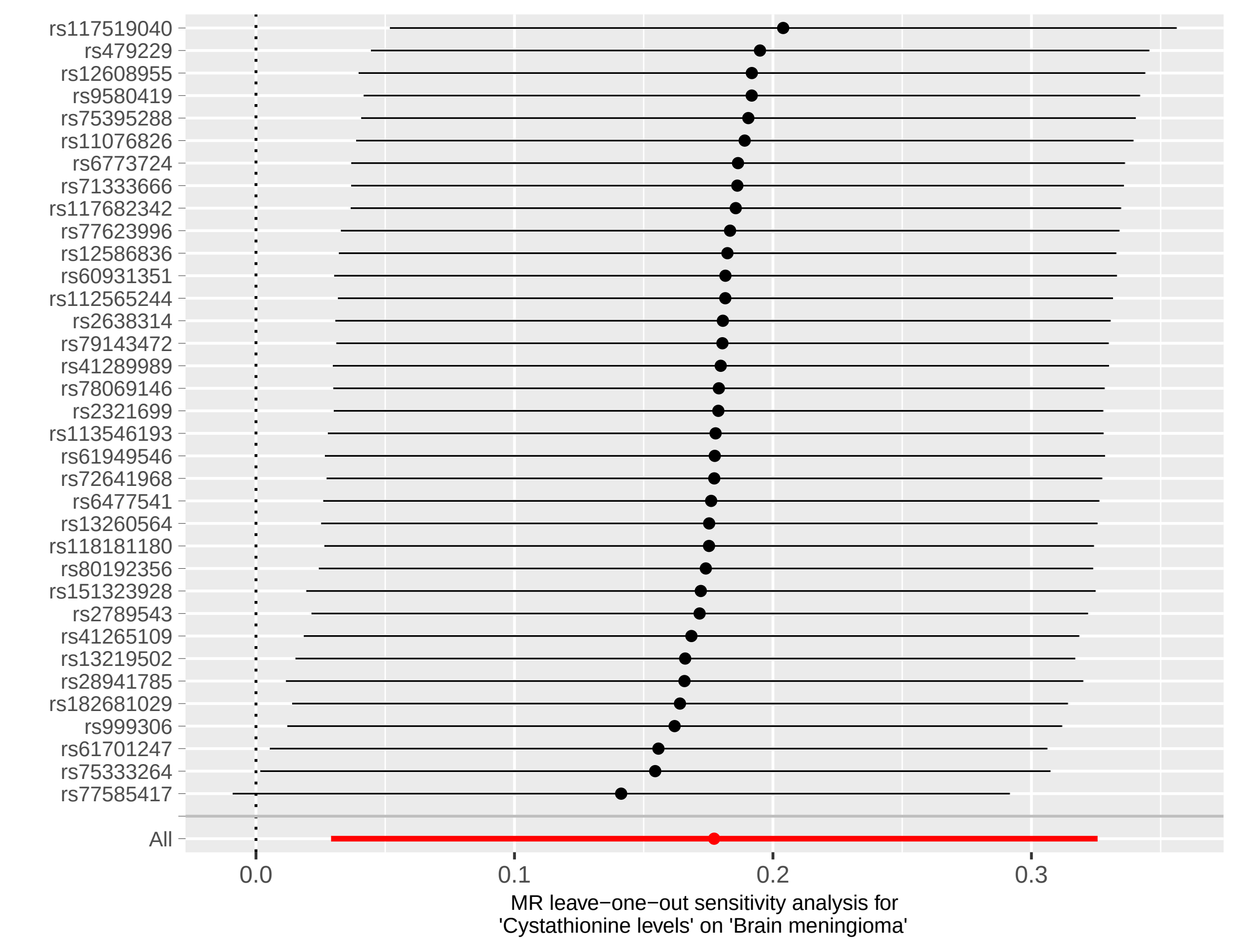
rs117519040
rs479229
rs12608955
rs9580419
rs75395288
rs11076826
rs6773724
rs71333666
rs117682342
rs77623996
rs12586836
rs60931351
rs112565244
rs2638314
rs79143472
rs41289989
rs78069146
rs2321699
rs113546193
rs61949546
rs72641968
rs6477541
rs13260564
rs118181180
rs80192356
rs151323928
rs2789543
rs41265109
rs13219502
rs28941785
rs182681029
rs999306
rs61701247
rs75333264
rs77585417
All
0.0
0.1
0.2
0.3
MR leave−one−out sensitivity analysis for
'Cystathionine levels' on 'Brain meningioma'

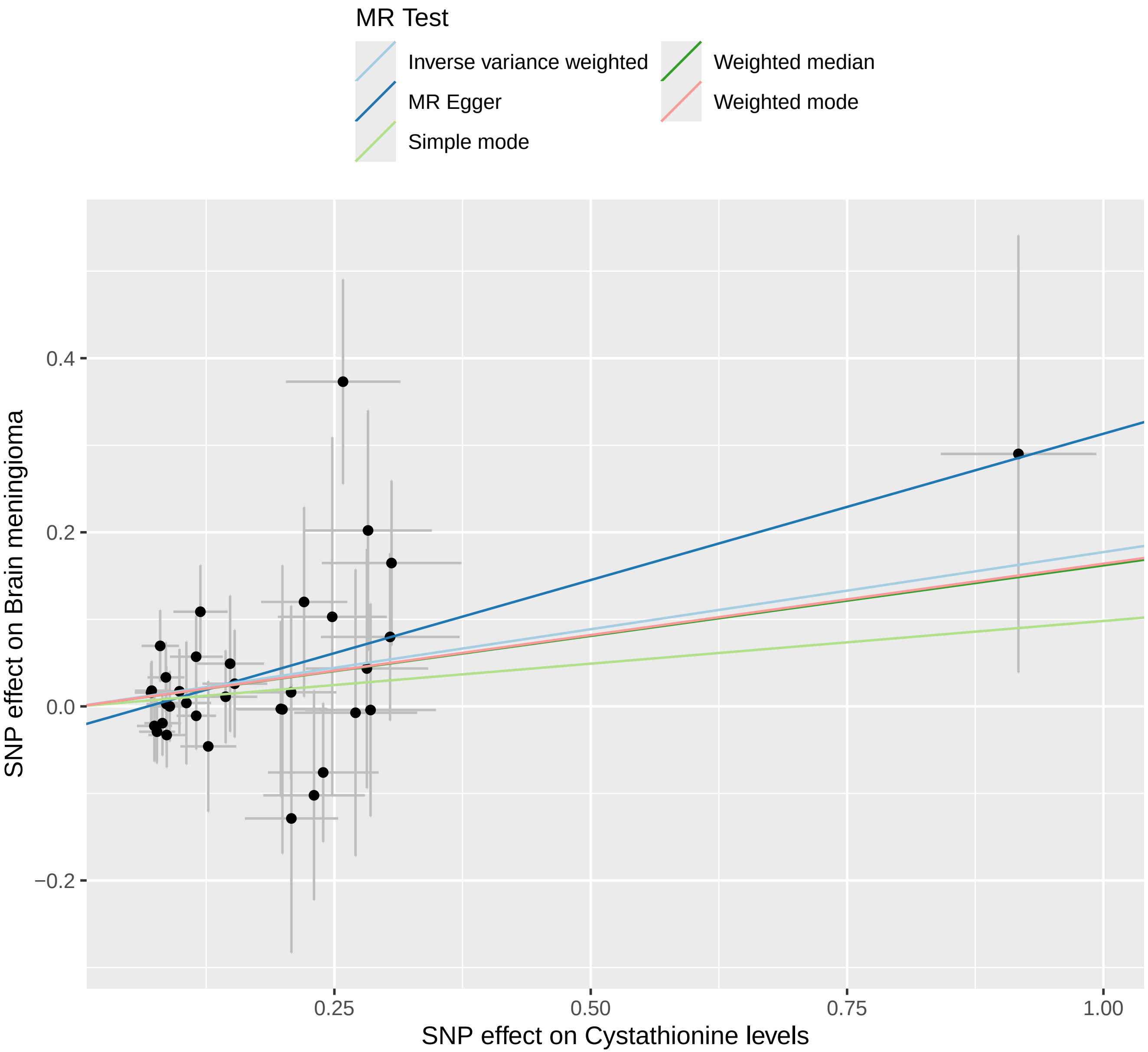
MR Test
Inverse variance weighted
MR Egger
Simple mode
Weighted median
Weighted mode
0.4
0.2
0.0
−0.2
0.25
0.50
0.75
1.00
SNP effect on Brain meningioma
SNP effect on Cystathionine levels

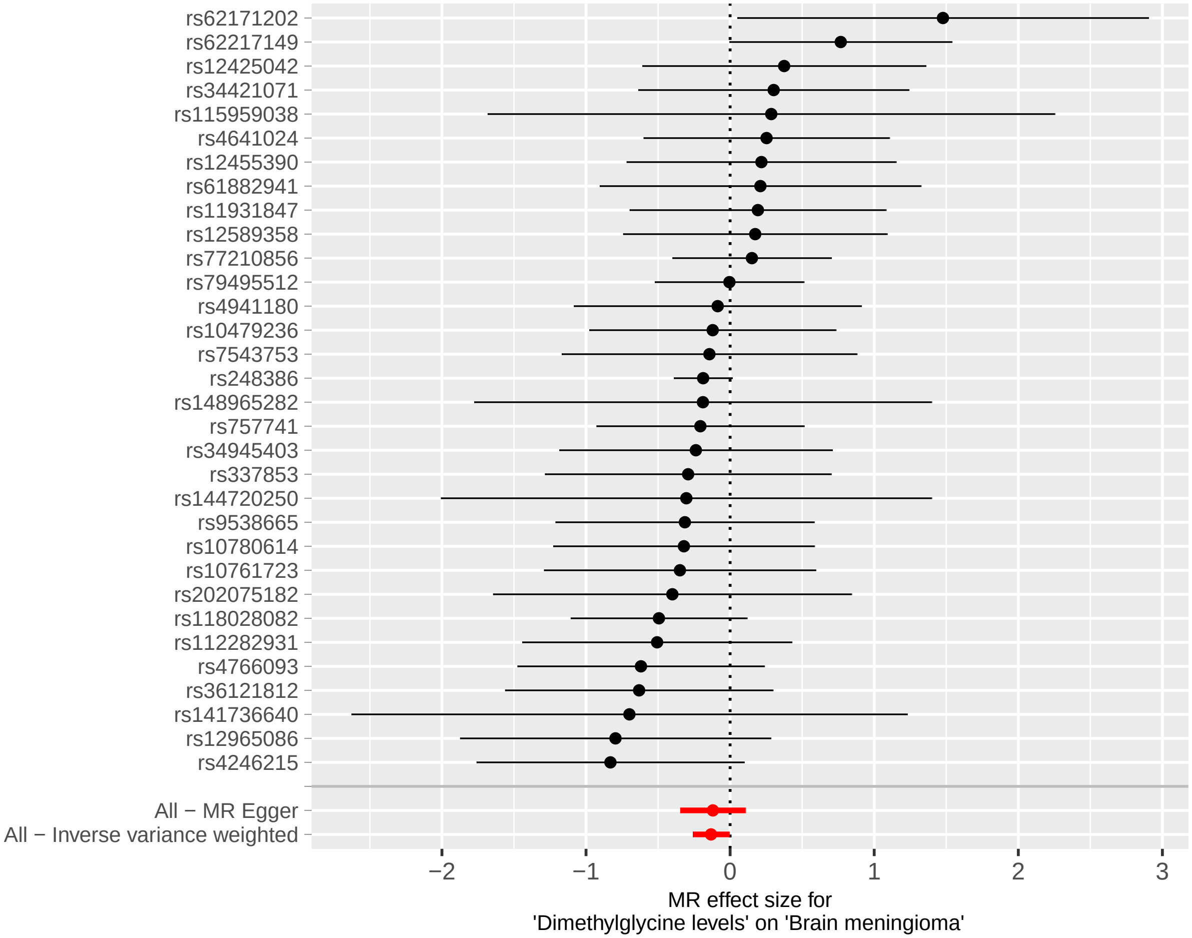
rs62171202
rs62217149
rs12425042
rs34421071
rs115959038
rs4641024
rs12455390
rs61882941
rs11931847
rs12589358
rs77210856
rs79495512
rs4941180
rs10479236
rs7543753
rs248386
rs148965282
rs757741
rs34945403
rs337853
rs144720250
rs9538665
rs10780614
rs10761723
rs202075182
rs118028082
rs112282931
rs4766093
rs36121812
rs141736640
rs12965086
rs4246215
All − MR Egger
All − Inverse variance weighted
−2
−1
0
1
2
3
MR effect size for
'Dimethylglycine levels' on 'Brain meningioma'

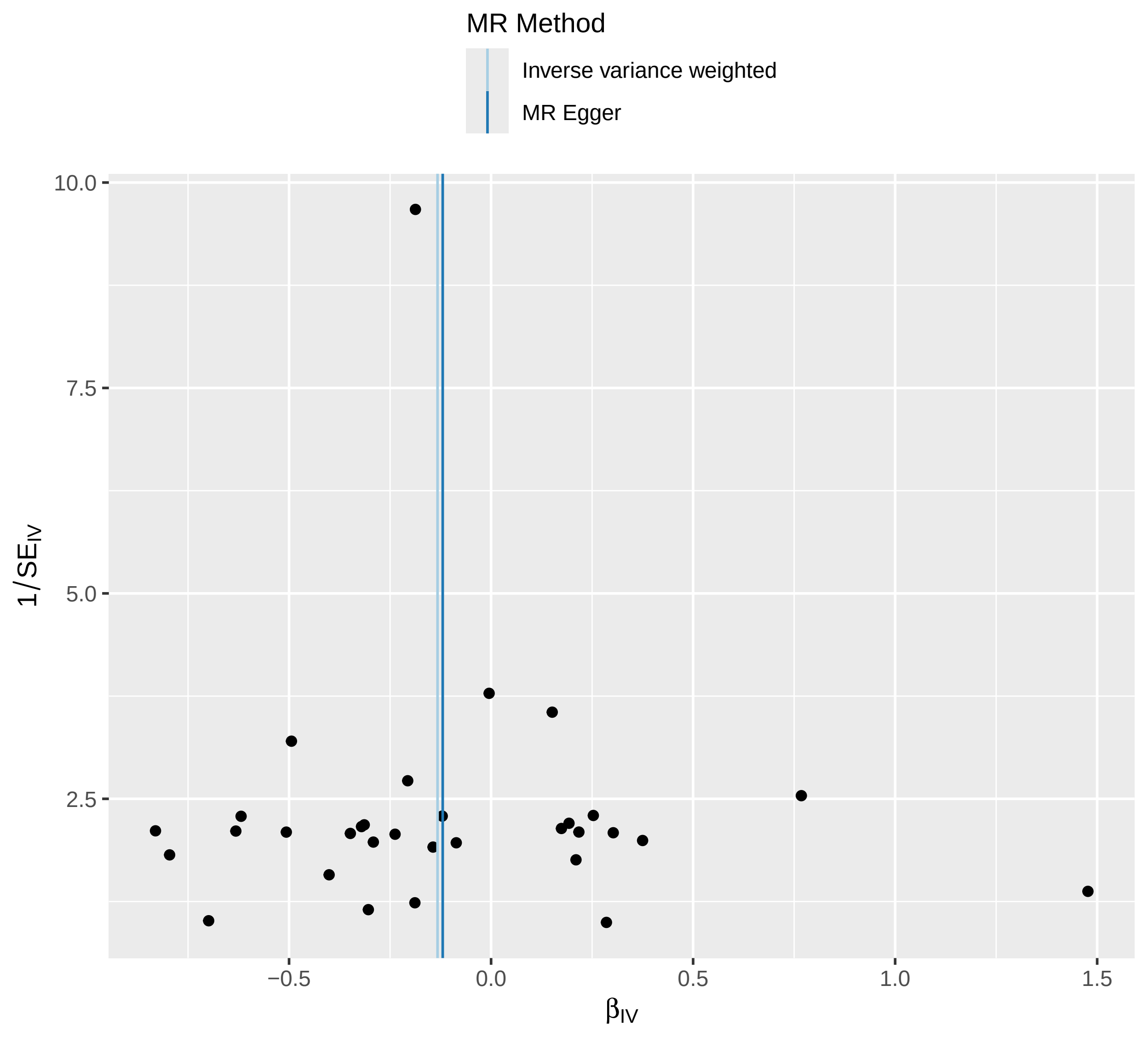

MR Method
Inverse variance weighted
MR Egger
10.0
7.5
5.0
2.5
1/SE_IV
−0.5
0.0
0.5
1.0
1.5
β_IV

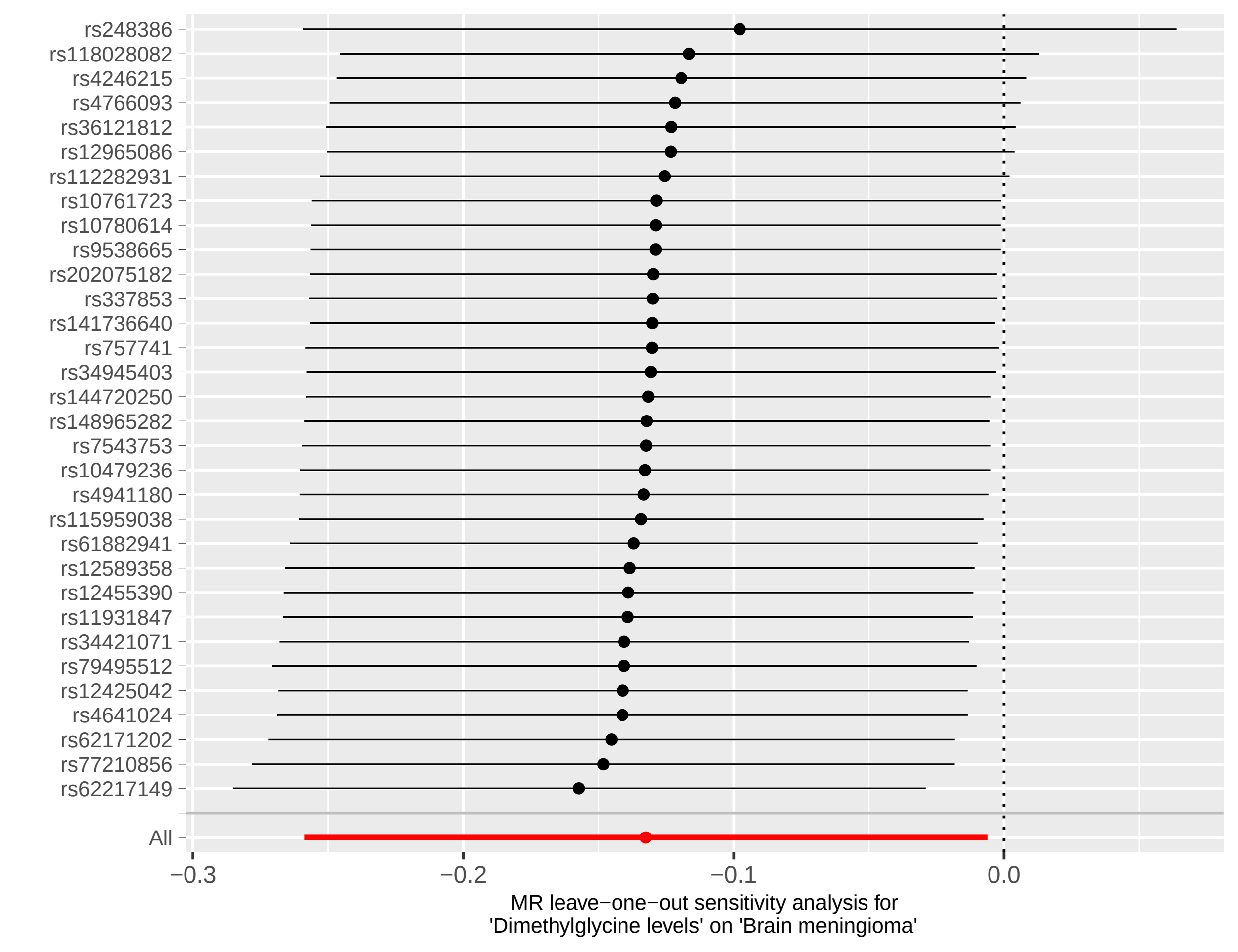
rs248386
rs118028082
rs4246215
rs4766093
rs36121812
rs12965086
rs112282931
rs10761723
rs10780614
rs9538665
rs202075182
rs337853
rs141736640
rs757741
rs34945403
rs144720250
rs148965282
rs7543753
rs10479236
rs4941180
rs115959038
rs61882941
rs12589358
rs12455390
rs11931847
rs34421071
rs79495512
rs12425042
rs4641024
rs62171202
rs77210856
rs62217149
All
−0.3
−0.2
−0.1
0.0
MR leave−one−out sensitivity analysis for
'Dimethylglycine levels' on 'Brain meningioma'

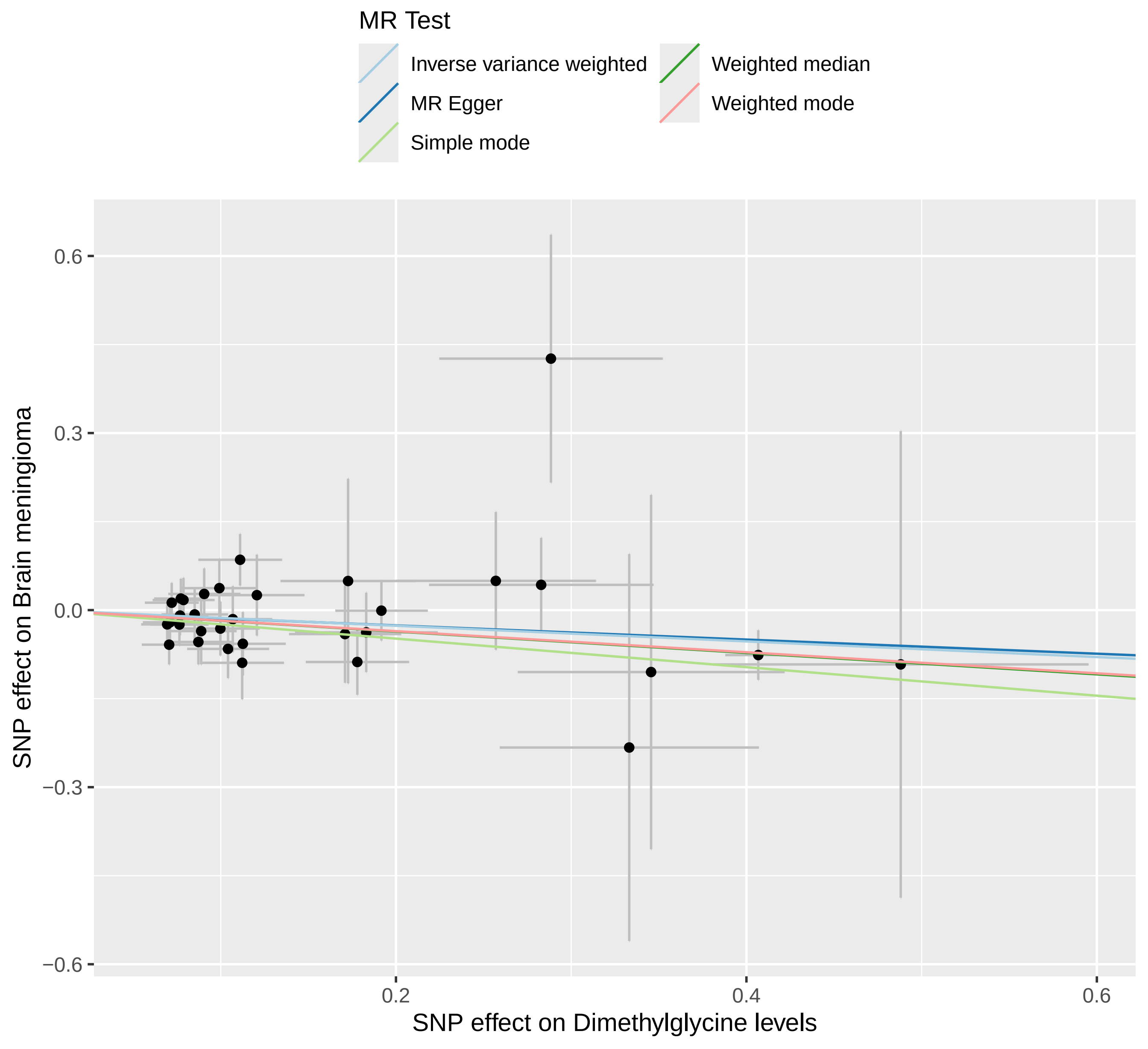
MR Test
Inverse variance weighted
MR Egger
Simple mode
Weighted median
Weighted mode
0.6
0.3
0.0
−0.3
−0.6
SNP effect on Brain meningioma
0.2
0.4
0.6
SNP effect on Dimethylglycine levels

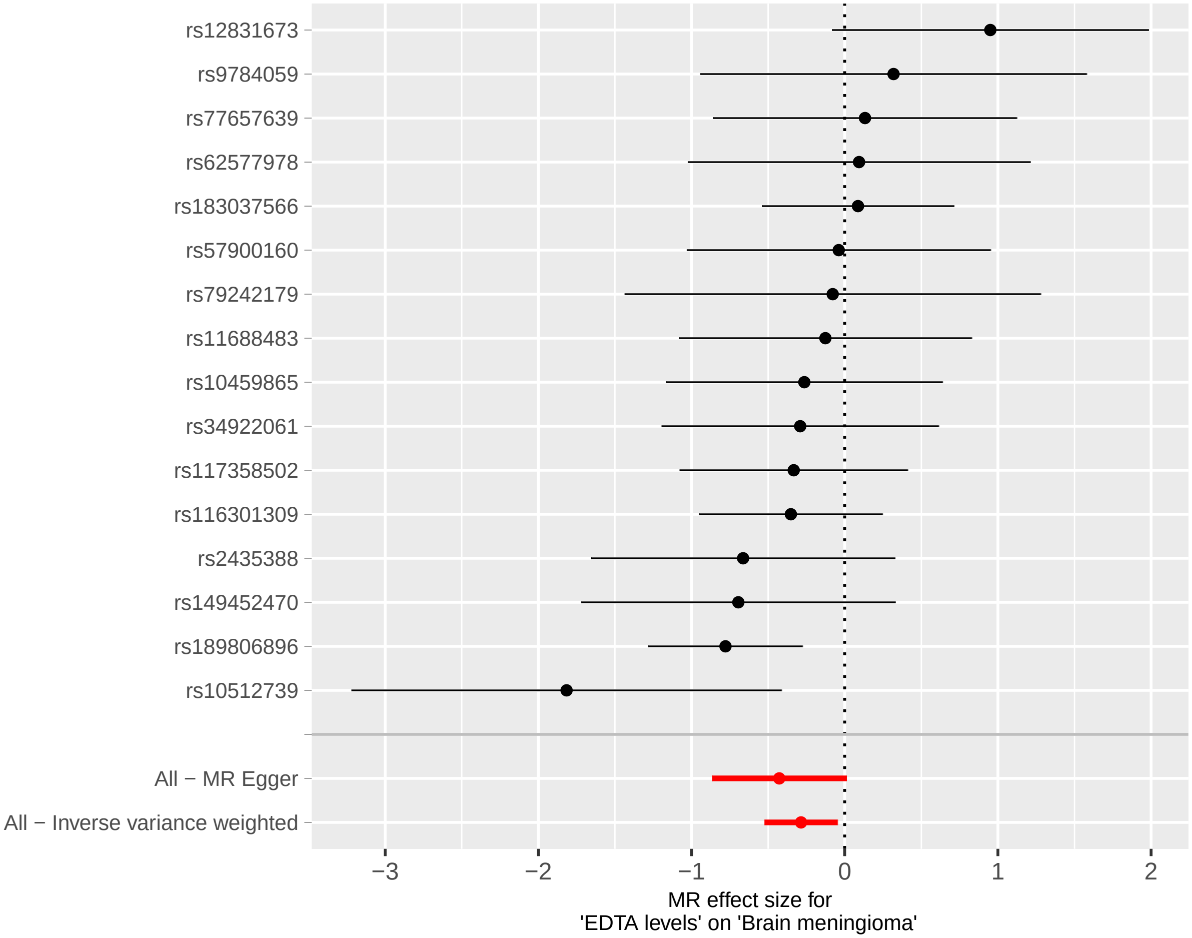
rs12831673
rs9784059
rs77657639
rs62577978
rs183037566
rs57900160
rs79242179
rs11688483
rs10459865
rs34922061
rs117358502
rs116301309
rs2435388
rs149452470
rs189806896
rs10512739
All − MR Egger
All − Inverse variance weighted
−3
−2
−1
0
1
2
MR effect size for
'EDTA levels' on 'Brain meningioma'

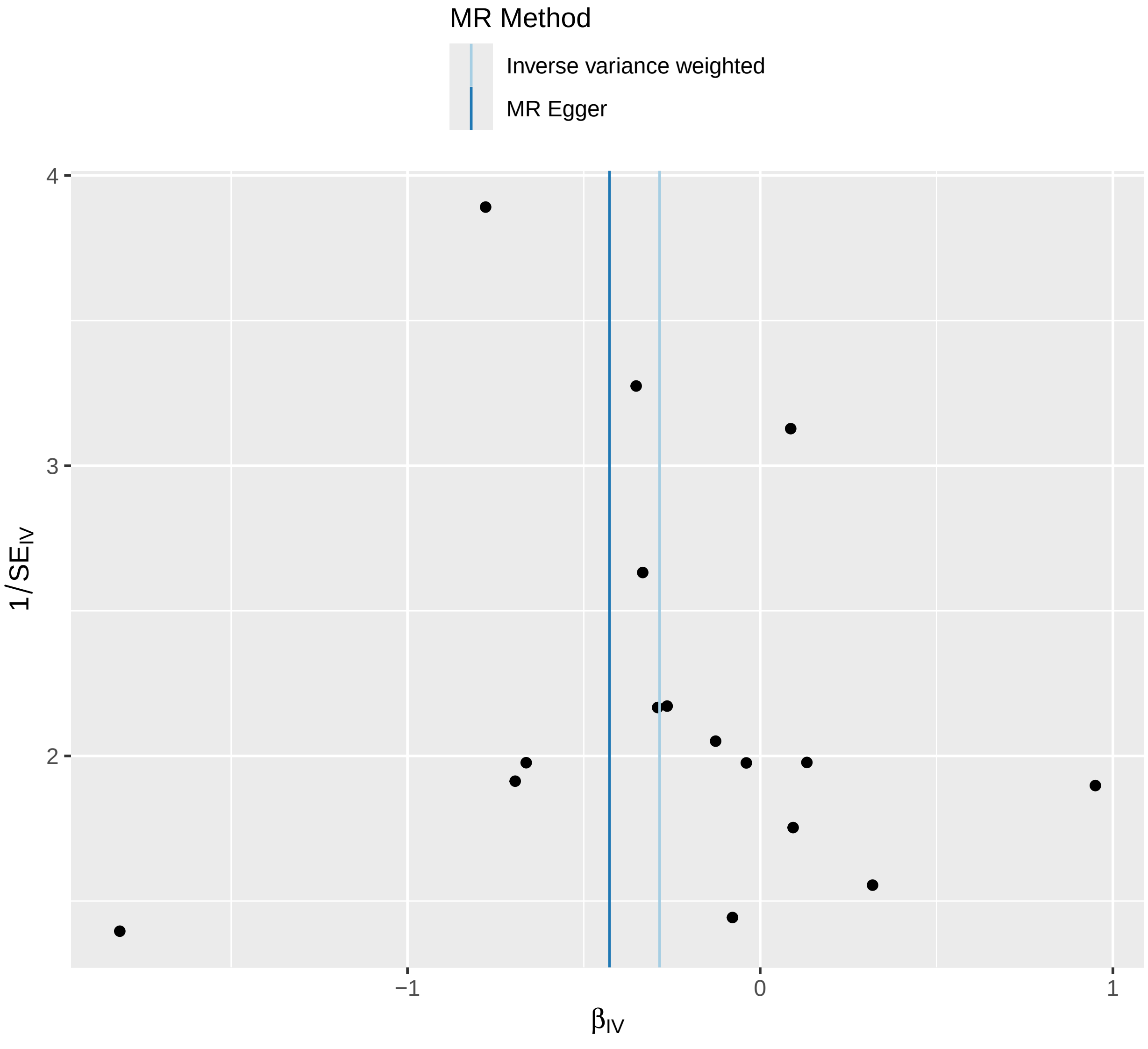
MR Method
Inverse variance weighted
MR Egger
4
3
2
1/SE$_{IV}$
−1
0
1
β$_{IV}$

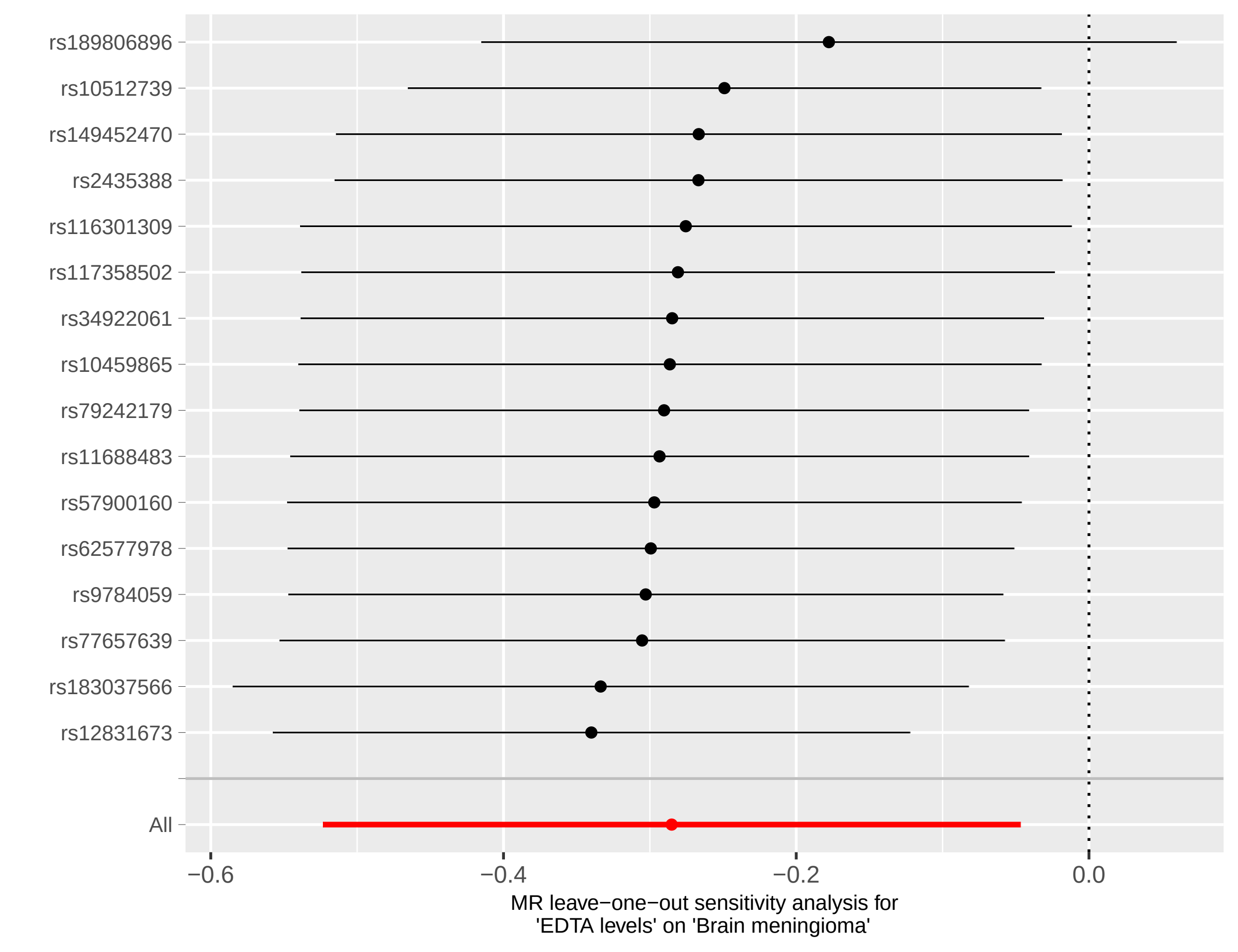
rs189806896
rs10512739
rs149452470
rs2435388
rs116301309
rs117358502
rs34922061
rs10459865
rs79242179
rs11688483
rs57900160
rs62577978
rs9784059
rs77657639
rs183037566
rs12831673
All
−0.6
−0.4
−0.2
0.0
MR leave−one−out sensitivity analysis for
'EDTA levels' on 'Brain meningioma'

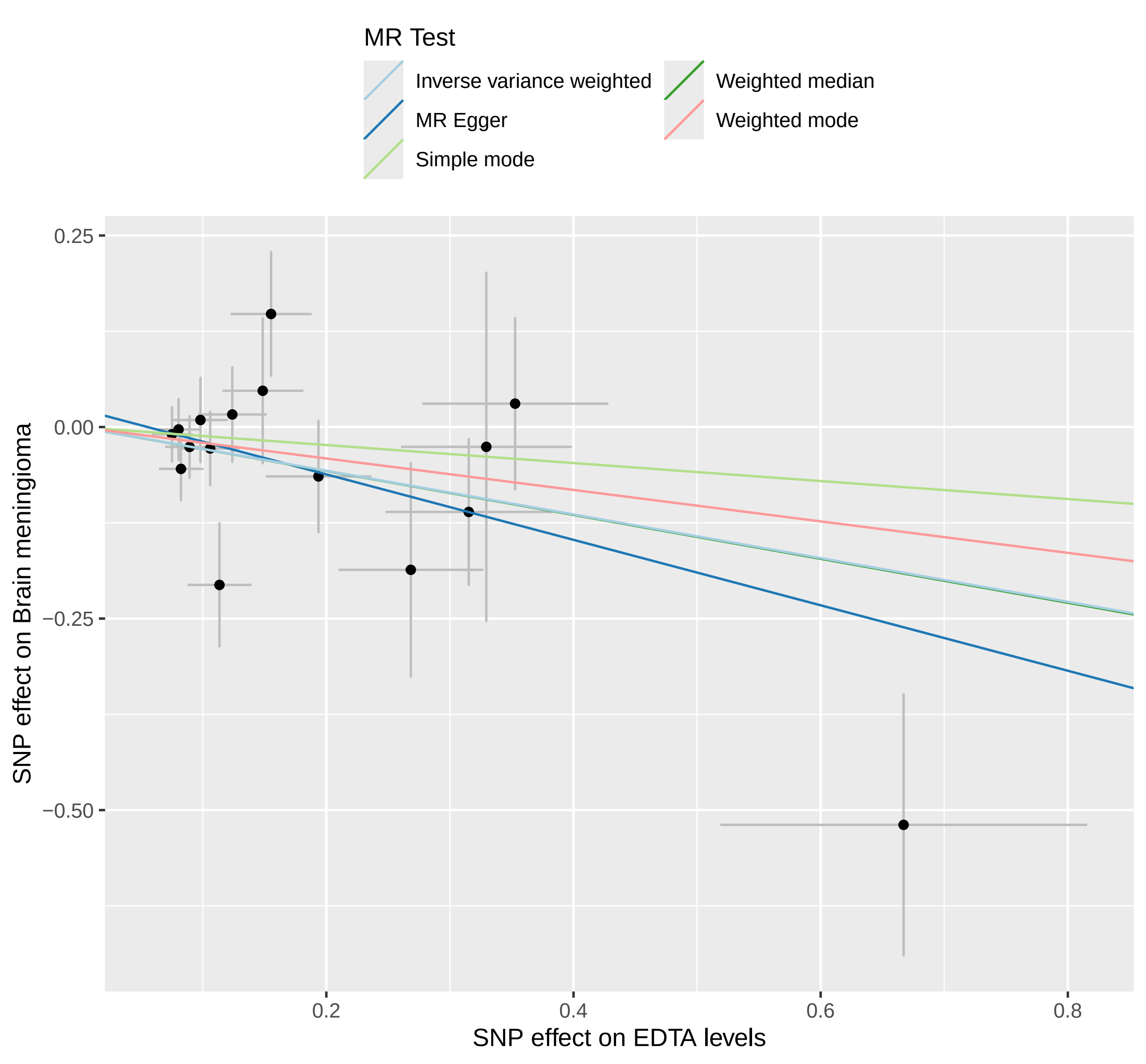
MR Test
Inverse variance weighted
MR Egger
Simple mode
Weighted median
Weighted mode
0.25
0.00
−0.25
−0.50
SNP effect on Brain meningioma
0.2
0.4
0.6
0.8
SNP effect on EDTA levels

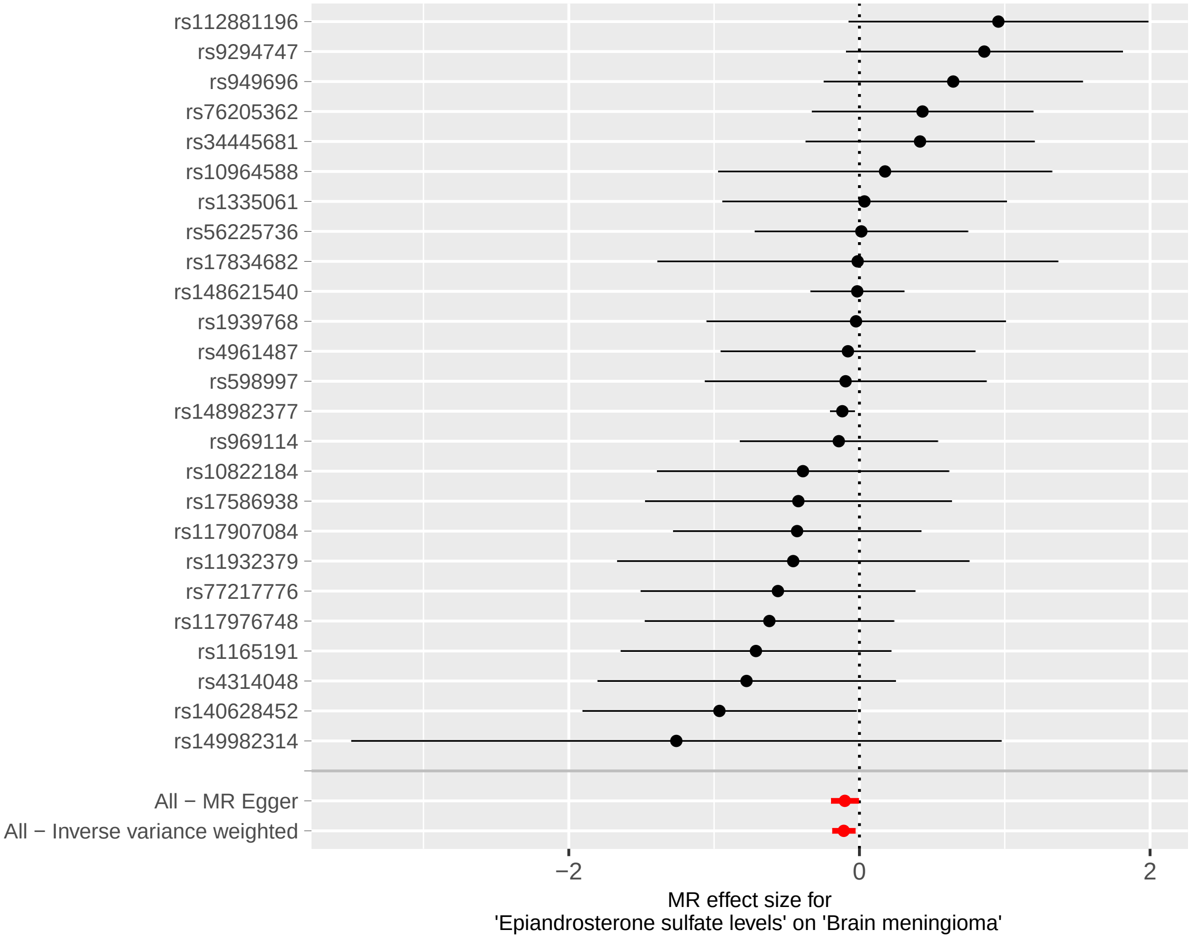
rs112881196
rs9294747
rs949696
rs76205362
rs34445681
rs10964588
rs1335061
rs56225736
rs17834682
rs148621540
rs1939768
rs4961487
rs598997
rs148982377
rs969114
rs10822184
rs17586938
rs117907084
rs11932379
rs77217776
rs117976748
rs1165191
rs4314048
rs140628452
rs149982314
All − MR Egger
All − Inverse variance weighted
−2
0
2
MR effect size for
'Epiandrosterone sulfate levels' on 'Brain meningioma'

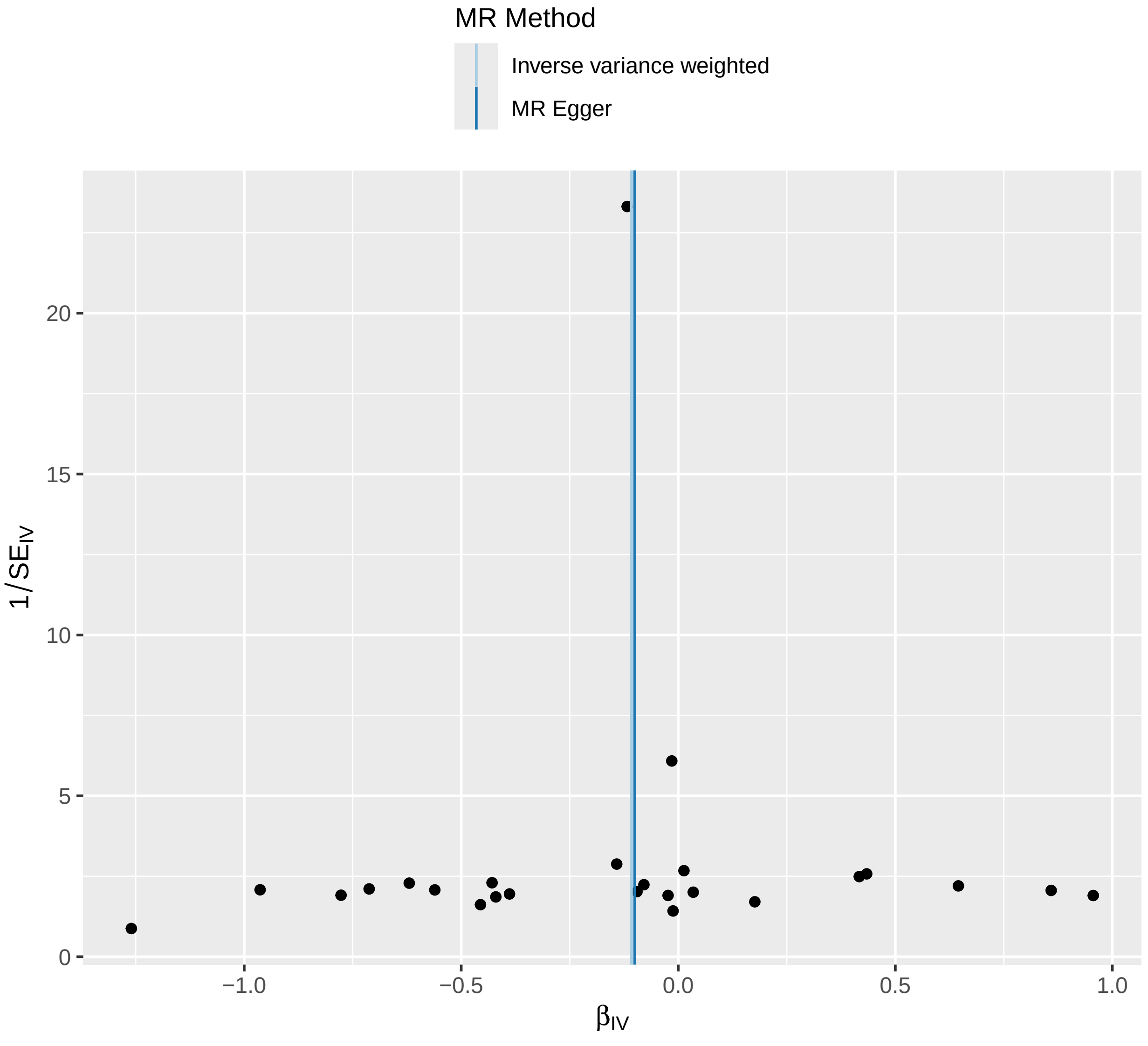
MR Method
Inverse variance weighted
MR Egger
20
15
10
5
0
1/SE_IV
−1.0
−0.5
0.0
0.5
1.0
β_IV

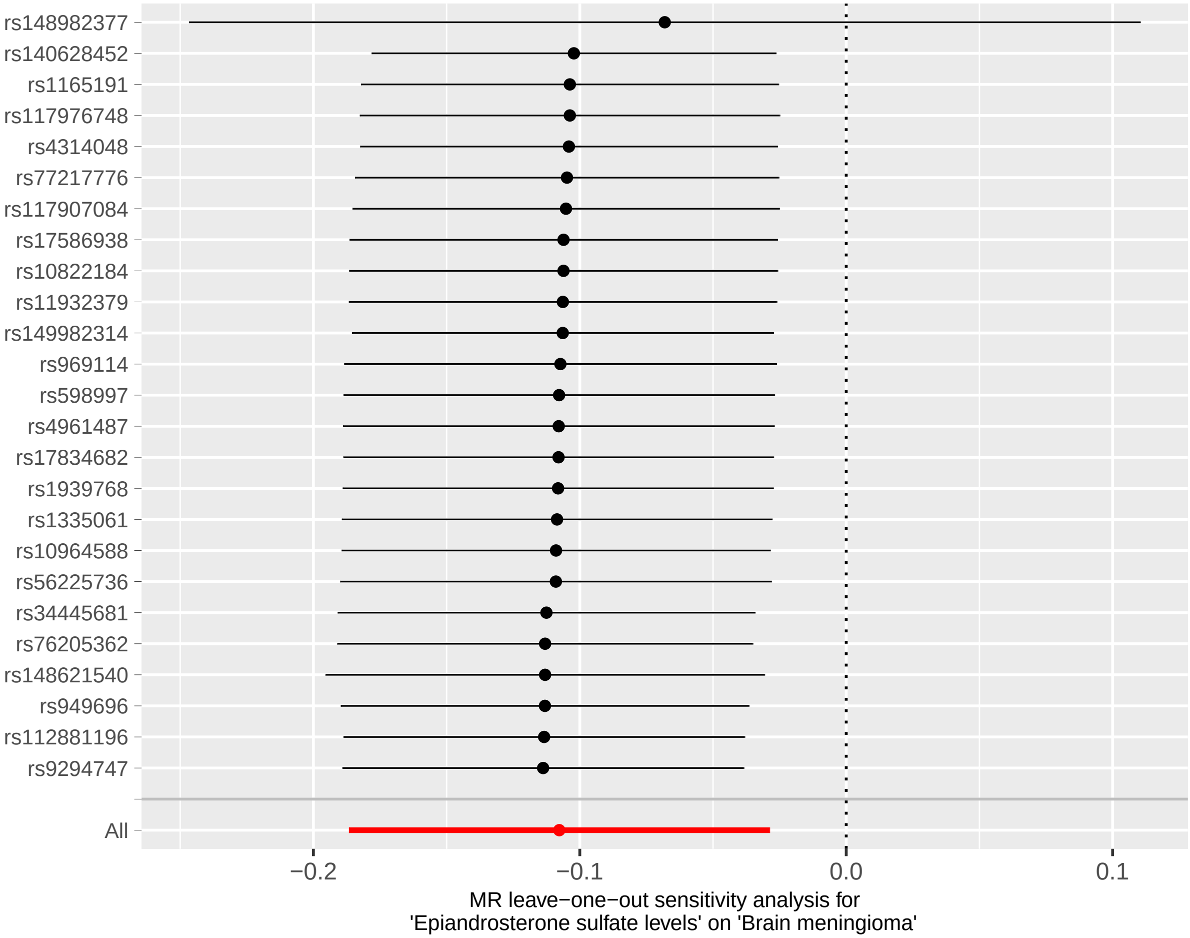
rs148982377
rs140628452
rs1165191
rs117976748
rs4314048
rs77217776
rs117907084
rs17586938
rs10822184
rs11932379
rs149982314
rs969114
rs598997
rs4961487
rs17834682
rs1939768
rs1335061
rs10964588
rs56225736
rs34445681
rs76205362
rs148621540
rs949696
rs112881196
rs9294747
All
−0.2
−0.1
0.0
0.1
MR leave−one−out sensitivity analysis for
'Epiandrosterone sulfate levels' on 'Brain meningioma'

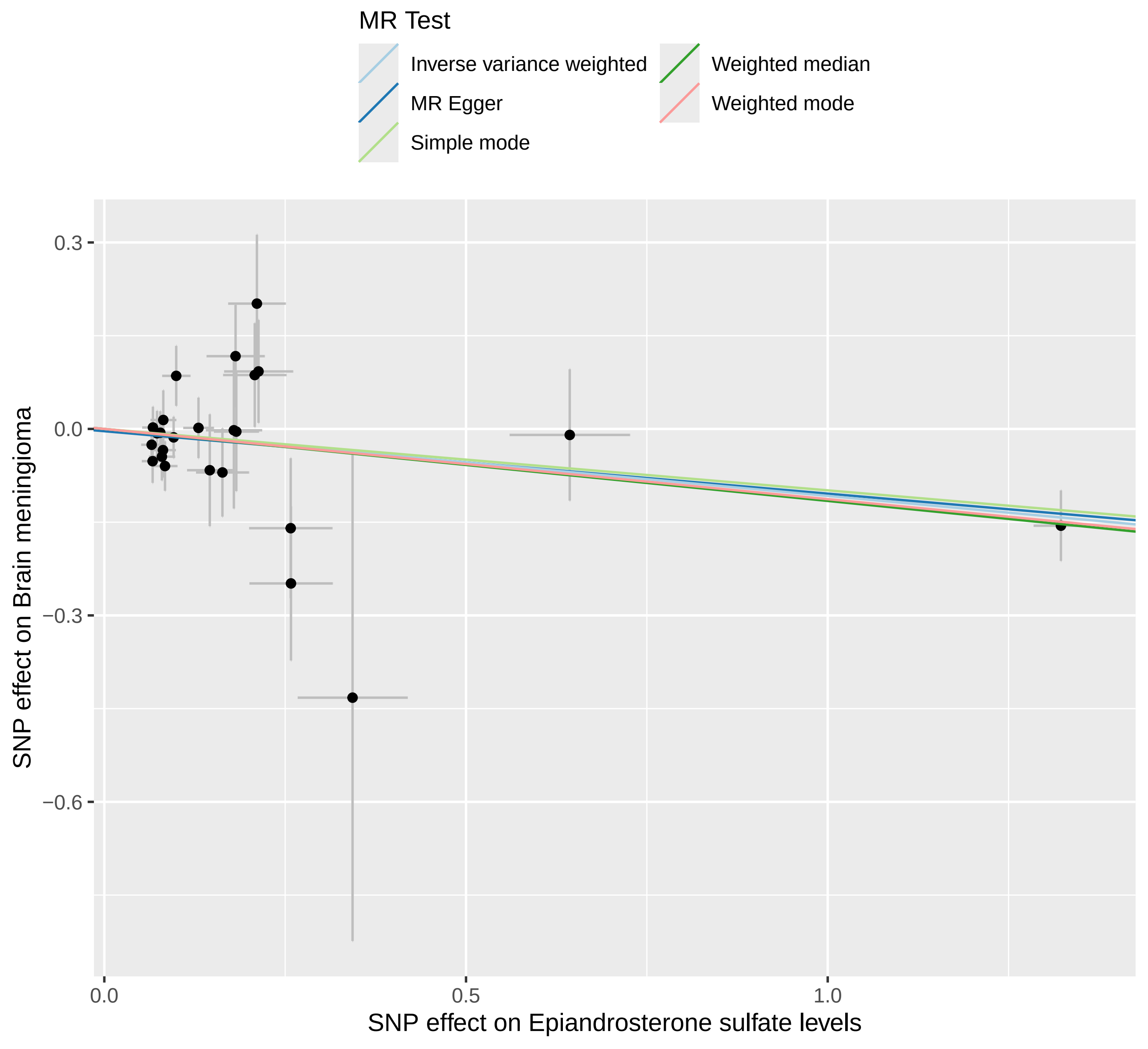

MR Test
Inverse variance weighted
MR Egger
Simple mode
Weighted median
Weighted mode
0.3
0.0
−0.3
−0.6
0.0
0.5
1.0
SNP effect on Brain meningioma
SNP effect on Epiandrosterone sulfate levels

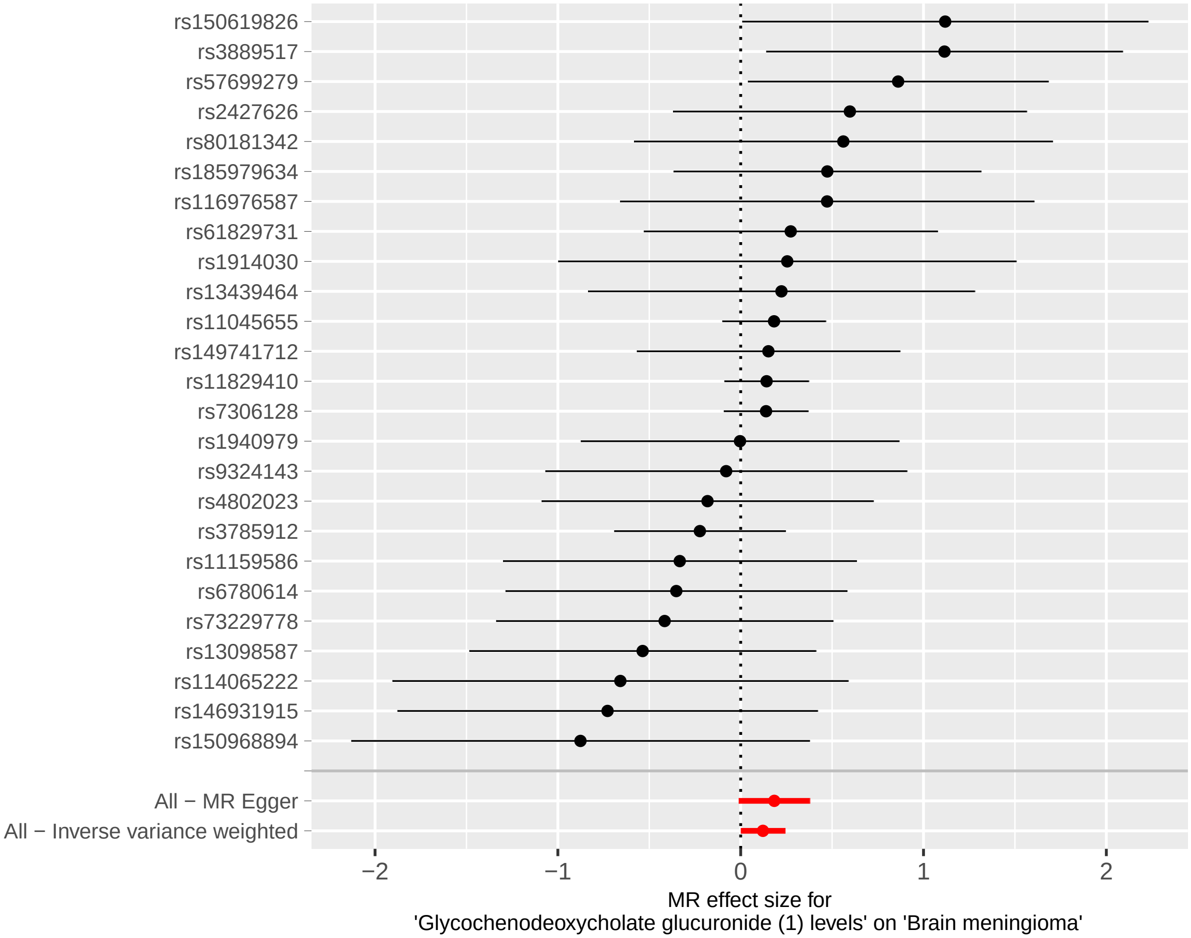
rs150619826
rs3889517
rs57699279
rs2427626
rs80181342
rs185979634
rs116976587
rs61829731
rs1914030
rs13439464
rs11045655
rs149741712
rs11829410
rs7306128
rs1940979
rs9324143
rs4802023
rs3785912
rs11159586
rs6780614
rs73229778
rs13098587
rs114065222
rs146931915
rs150968894
All − MR Egger
All − Inverse variance weighted
−2
−1
0
1
2
MR effect size for
'Glycochenodeoxycholate glucuronide (1) levels' on 'Brain meningioma'

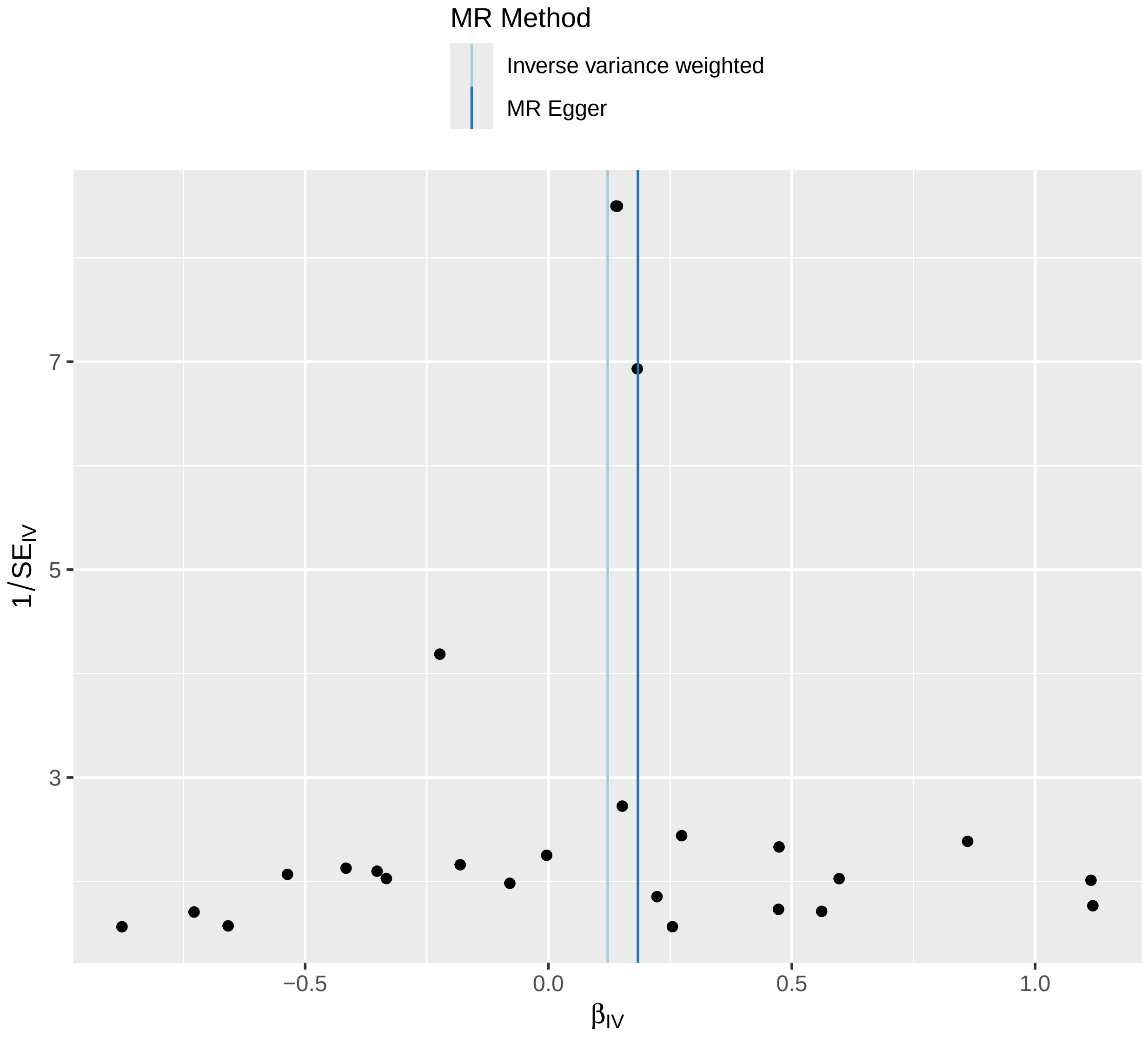

MR Method
Inverse variance weighted
MR Egger
7
5
3
−0.5
0.0
0.5
1.0
$1/SE_{IV}$
β_{IV}

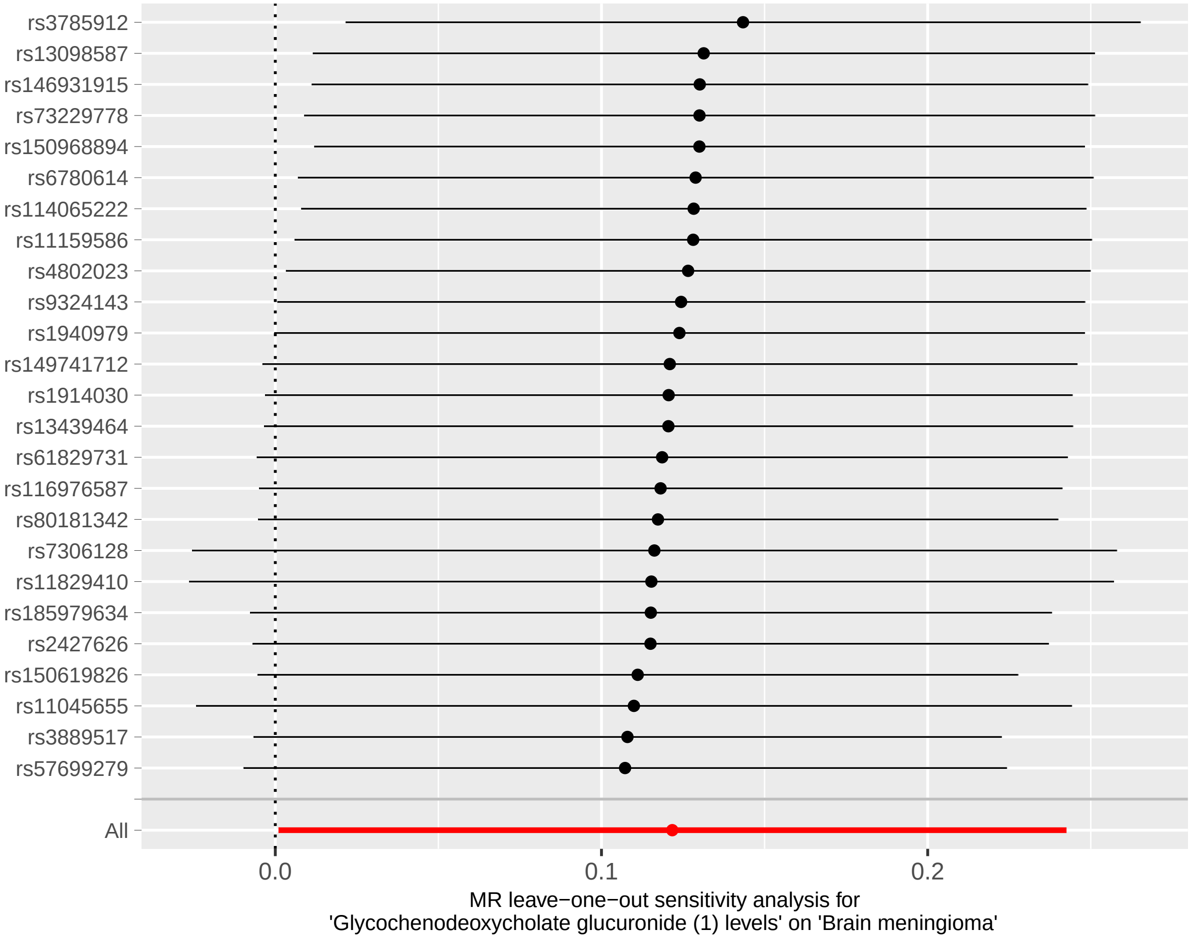

rs3785912
rs13098587
rs146931915
rs73229778
rs150968894
rs6780614
rs114065222
rs11159586
rs4802023
rs9324143
rs1940979
rs149741712
rs1914030
rs13439464
rs61829731
rs116976587
rs80181342
rs7306128
rs11829410
rs185979634
rs2427626
rs150619826
rs11045655
rs3889517
rs57699279
All
0.0
0.1
0.2
MR leave−one−out sensitivity analysis for
'Glycochenodeoxycholate glucuronide (1) levels' on 'Brain meningioma'

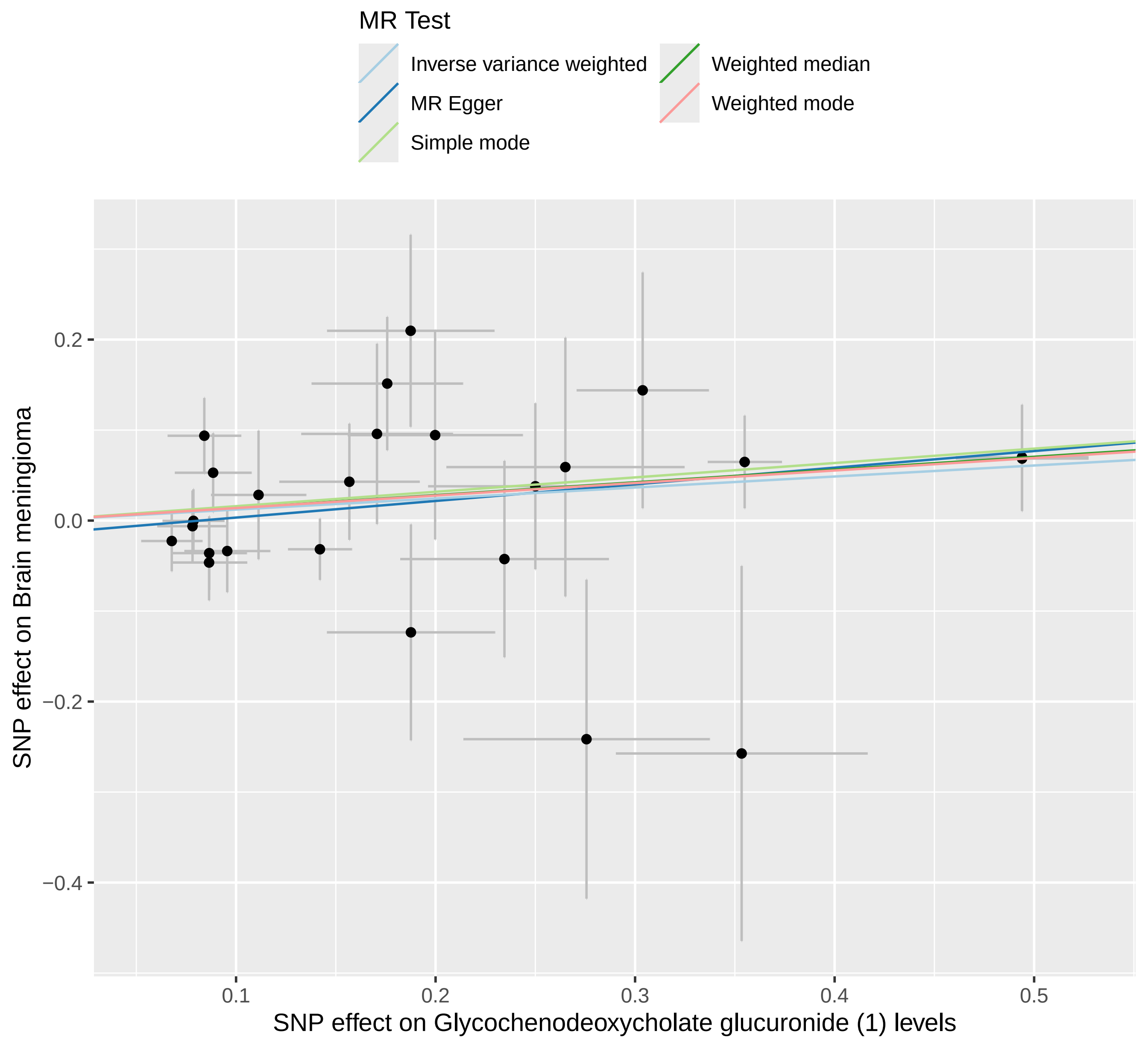
MR Test
Inverse variance weighted
MR Egger
Simple mode
Weighted median
Weighted mode
0.2
0.0
−0.2
−0.4
0.1
0.2
0.3
0.4
0.5
SNP effect on Brain meningioma
SNP effect on Glycochenodeoxycholate glucuronide (1) levels

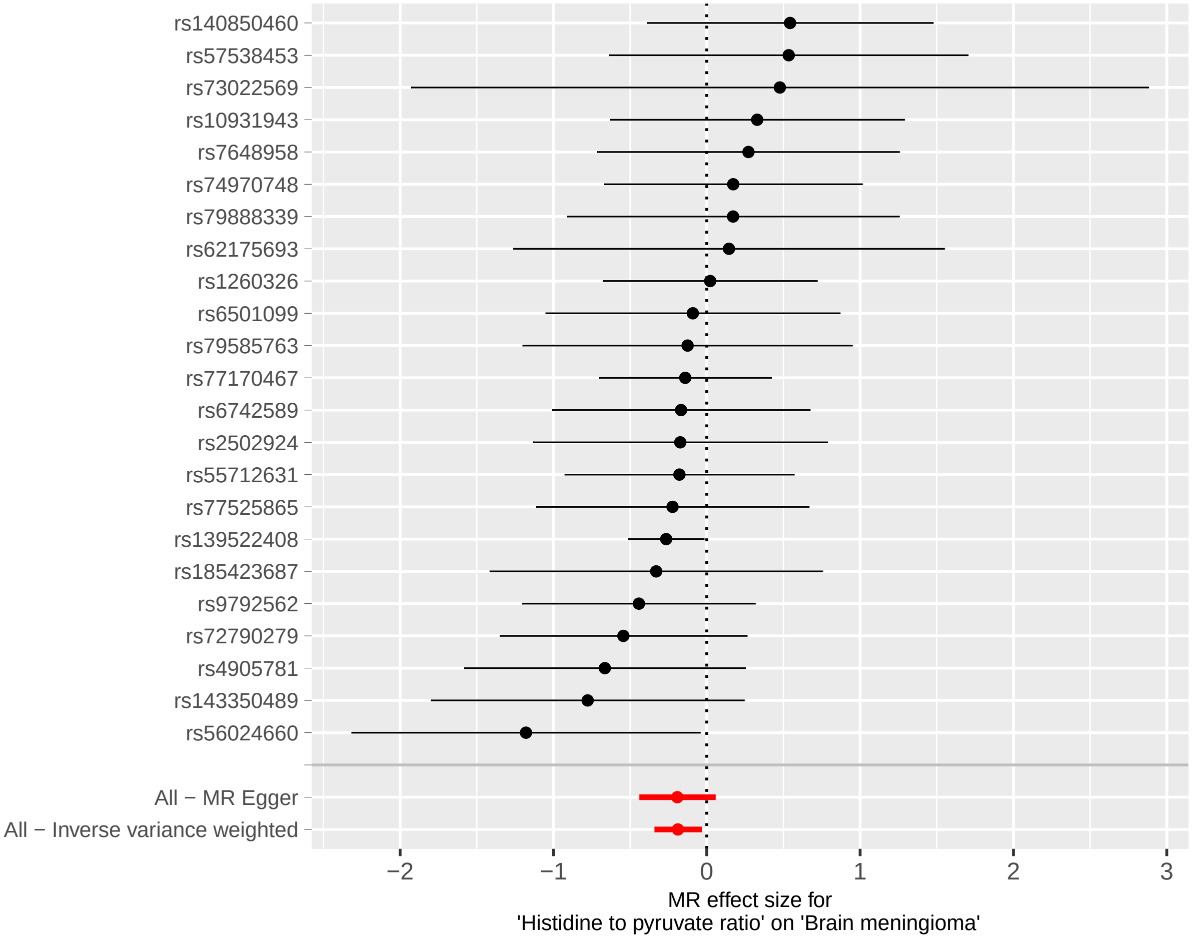
rs140850460
rs57538453
rs73022569
rs10931943
rs7648958
rs74970748
rs79888339
rs62175693
rs1260326
rs6501099
rs79585763
rs77170467
rs6742589
rs2502924
rs55712631
rs77525865
rs139522408
rs185423687
rs9792562
rs72790279
rs4905781
rs143350489
rs56024660
All − MR Egger
All − Inverse variance weighted
−2
−1
0
1
2
3
MR effect size for
'Histidine to pyruvate ratio' on 'Brain meningioma'

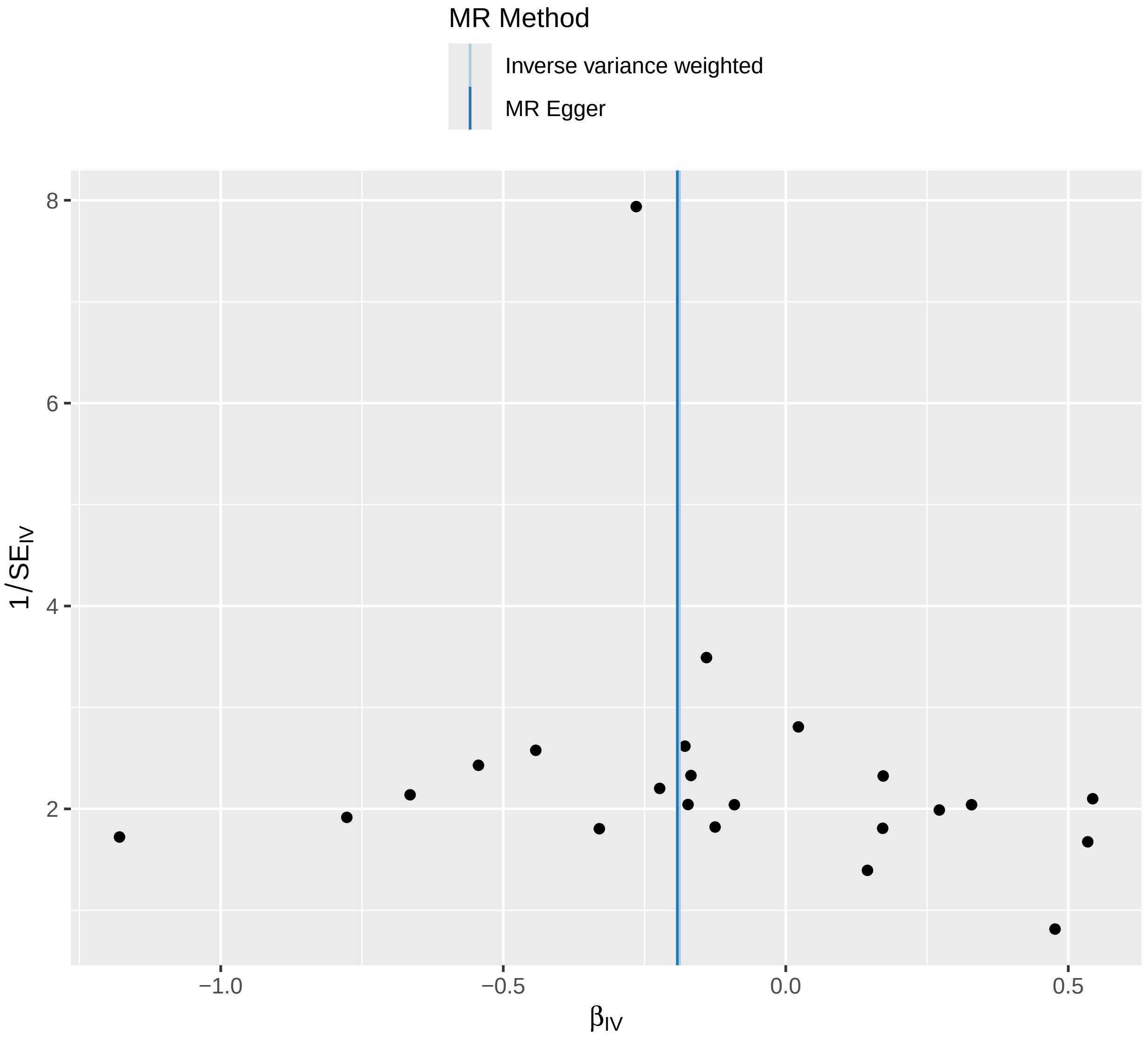
MR Method
Inverse variance weighted
MR Egger
8
6
4
2
1/SE_IV
−1.0
−0.5
0.0
0.5
β_IV

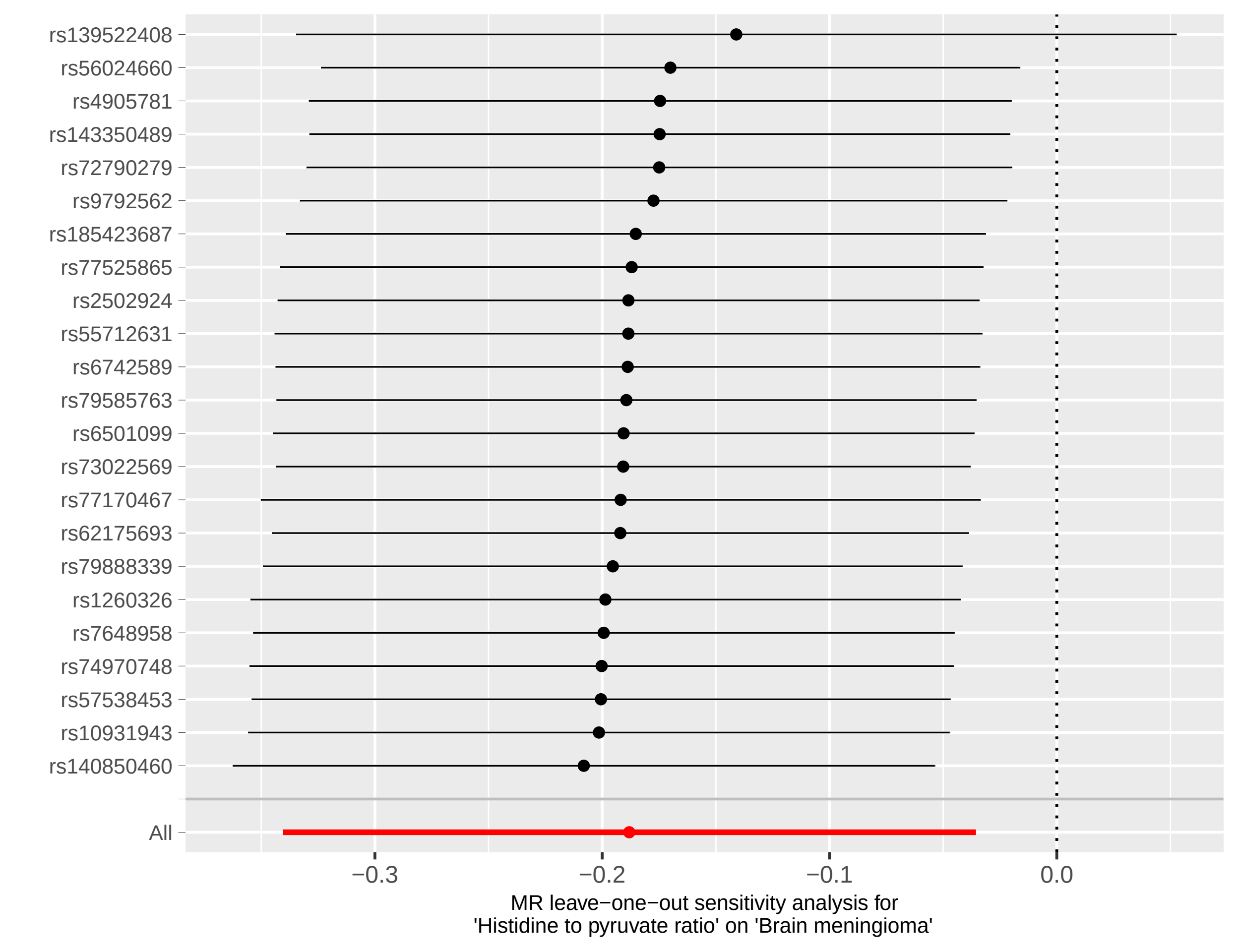
rs139522408
rs56024660
rs4905781
rs143350489
rs72790279
rs9792562
rs185423687
rs77525865
rs2502924
rs55712631
rs6742589
rs79585763
rs6501099
rs73022569
rs77170467
rs62175693
rs79888339
rs1260326
rs7648958
rs74970748
rs57538453
rs10931943
rs140850460
All
−0.3
−0.2
−0.1
0.0
MR leave−one−out sensitivity analysis for
'Histidine to pyruvate ratio' on 'Brain meningioma'

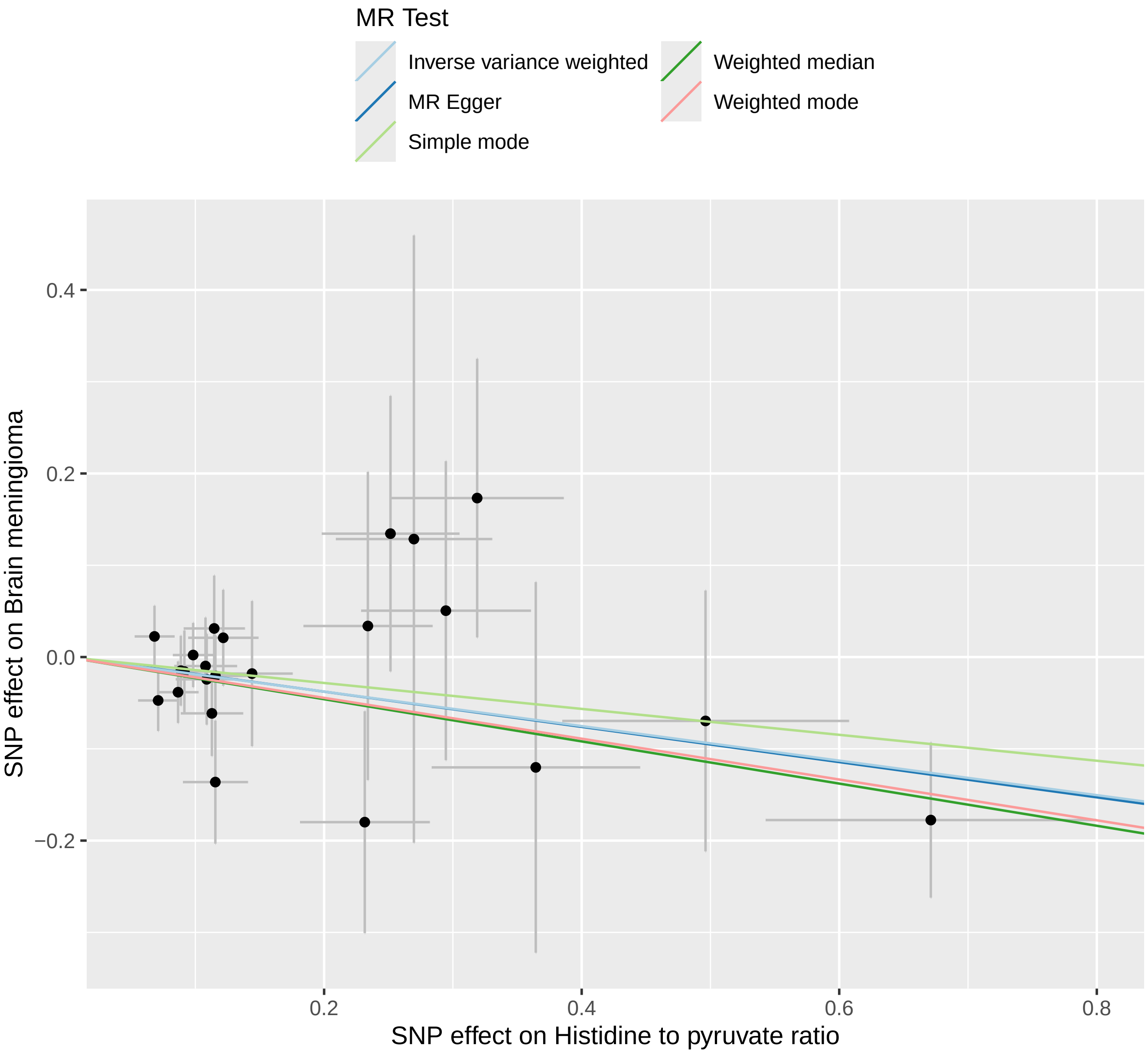
MR Test
Inverse variance weighted
MR Egger
Simple mode
Weighted median
Weighted mode
0.4
0.2
0.0
−0.2
SNP effect on Brain meningioma
0.2
0.4
0.6
0.8
SNP effect on Histidine to pyruvate ratio

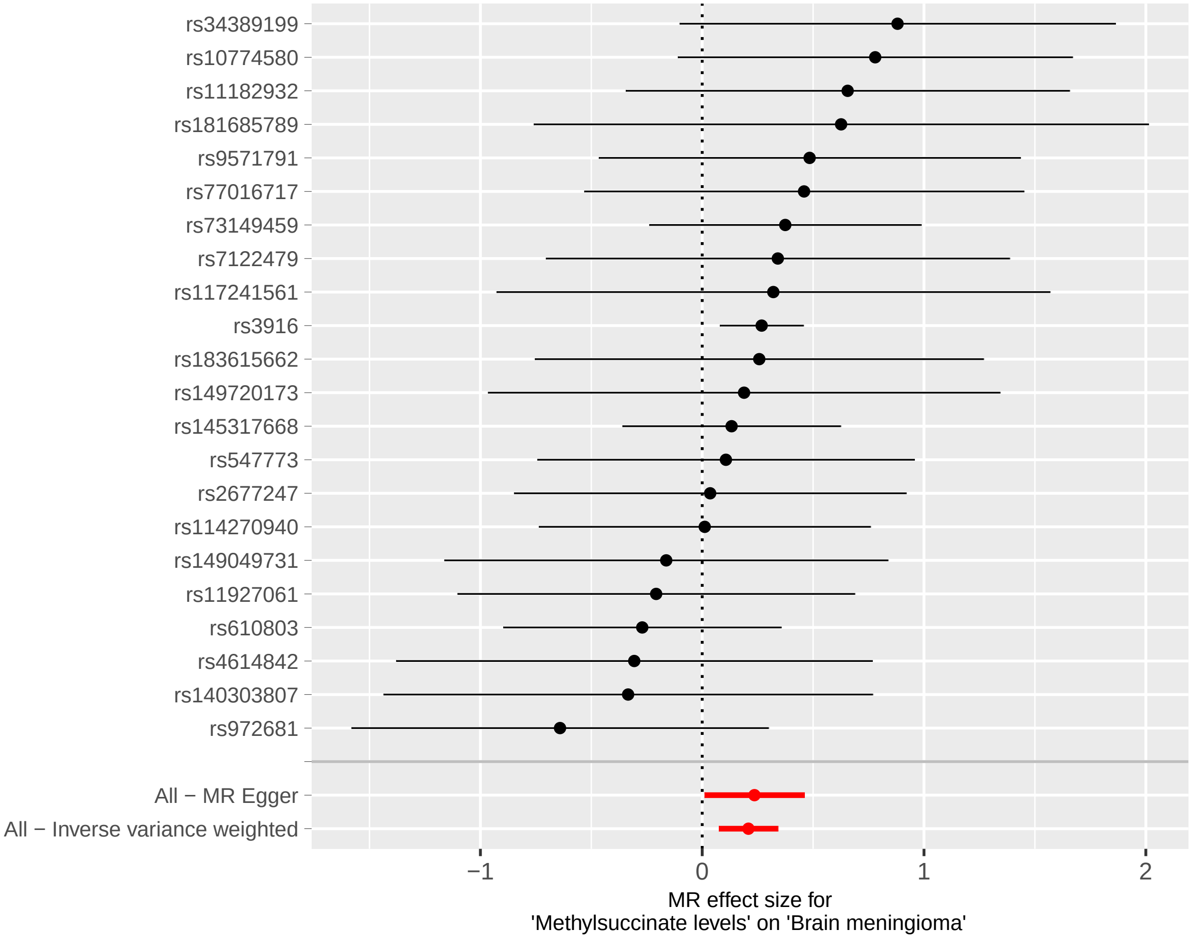
rs34389199
rs10774580
rs11182932
rs181685789
rs9571791
rs77016717
rs73149459
rs7122479
rs117241561
rs3916
rs183615662
rs149720173
rs145317668
rs547773
rs2677247
rs114270940
rs149049731
rs11927061
rs610803
rs4614842
rs140303807
rs972681
All − MR Egger
All − Inverse variance weighted
−1
0
1
2
MR effect size for
'Methylsuccinate levels' on 'Brain meningioma'

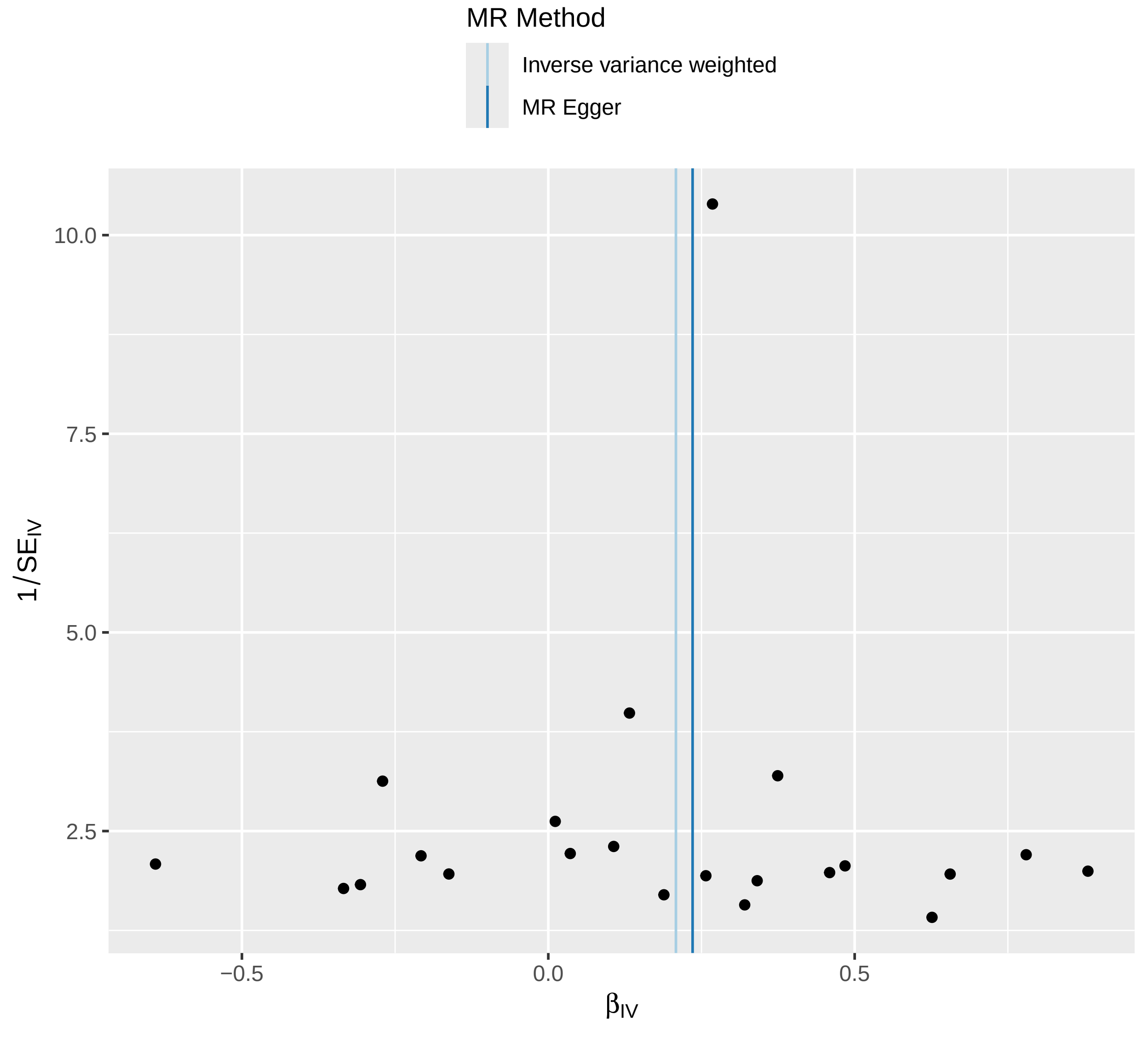
MR Method
Inverse variance weighted
MR Egger
10.0
7.5
5.0
2.5
$1/SE_{IV}$
−0.5
0.0
0.5
β_{IV}

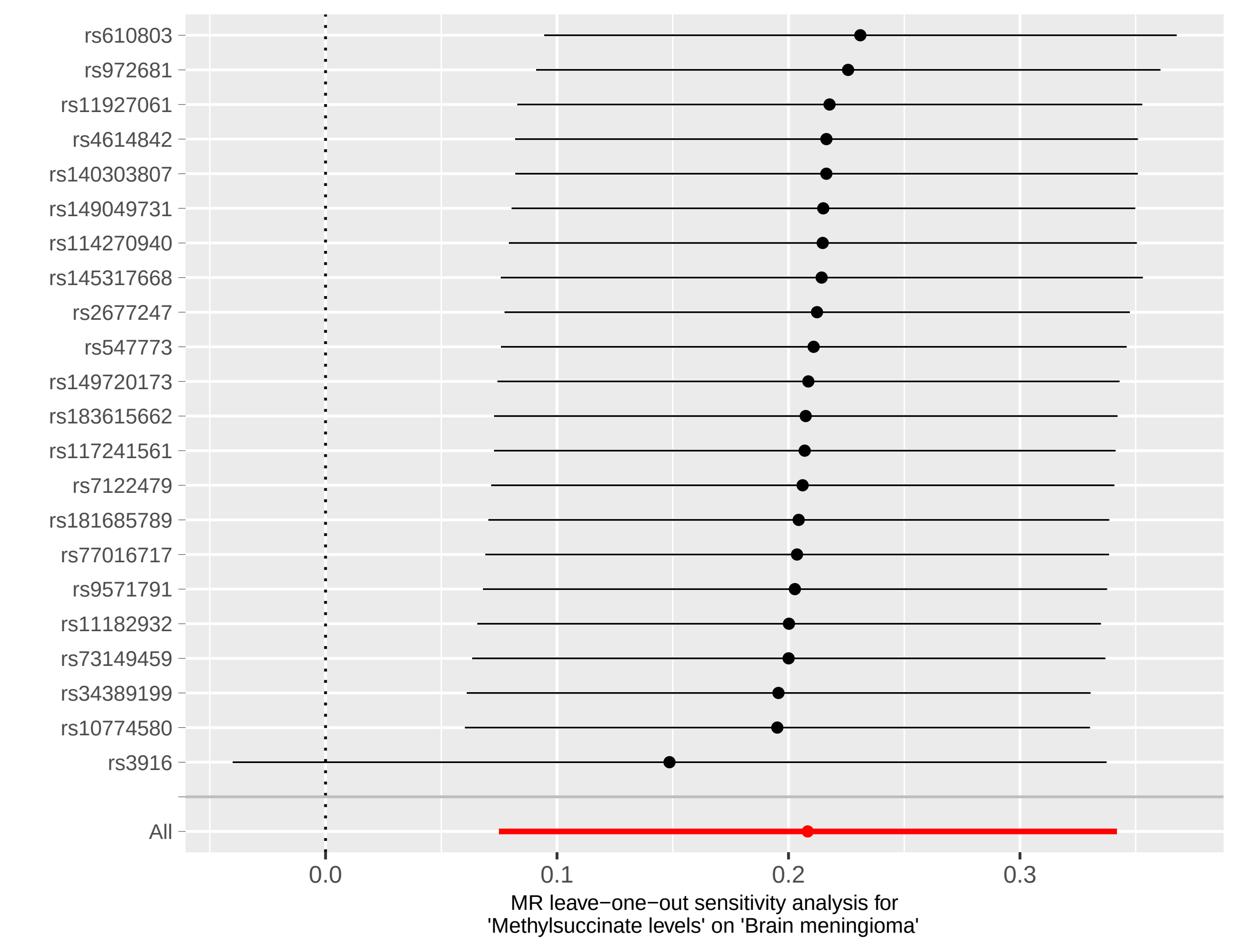
rs610803
rs972681
rs11927061
rs4614842
rs140303807
rs149049731
rs114270940
rs145317668
rs2677247
rs547773
rs149720173
rs183615662
rs117241561
rs7122479
rs181685789
rs77016717
rs9571791
rs11182932
rs73149459
rs34389199
rs10774580
rs3916
All
0.0
0.1
0.2
0.3
MR leave−one−out sensitivity analysis for
'Methylsuccinate levels' on 'Brain meningioma'

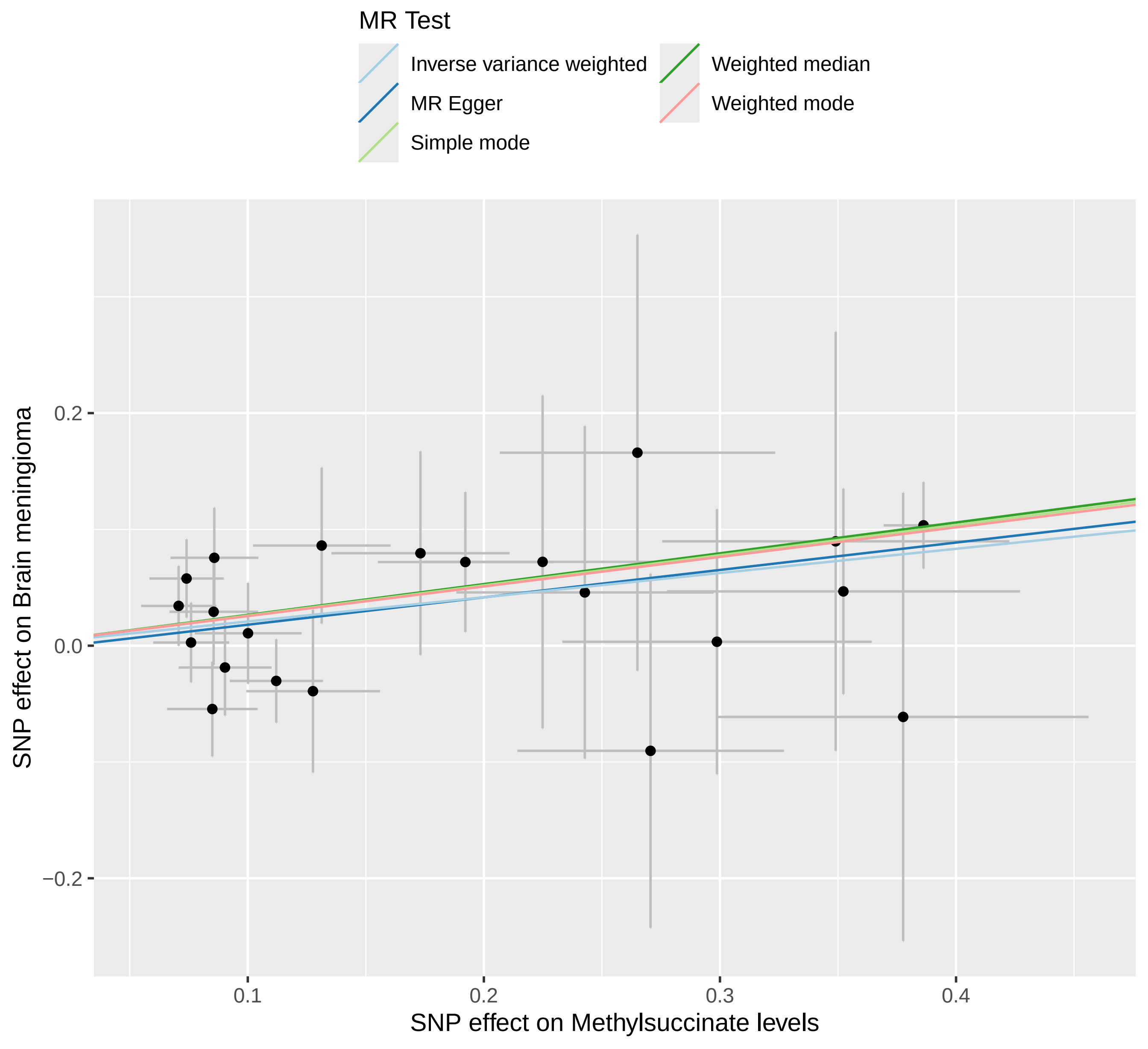
MR Test
Inverse variance weighted
MR Egger
Simple mode
Weighted median
Weighted mode
SNP effect on Brain meningioma
0.2
0.0
−0.2
0.1
0.2
0.3
0.4
SNP effect on Methylsuccinate levels

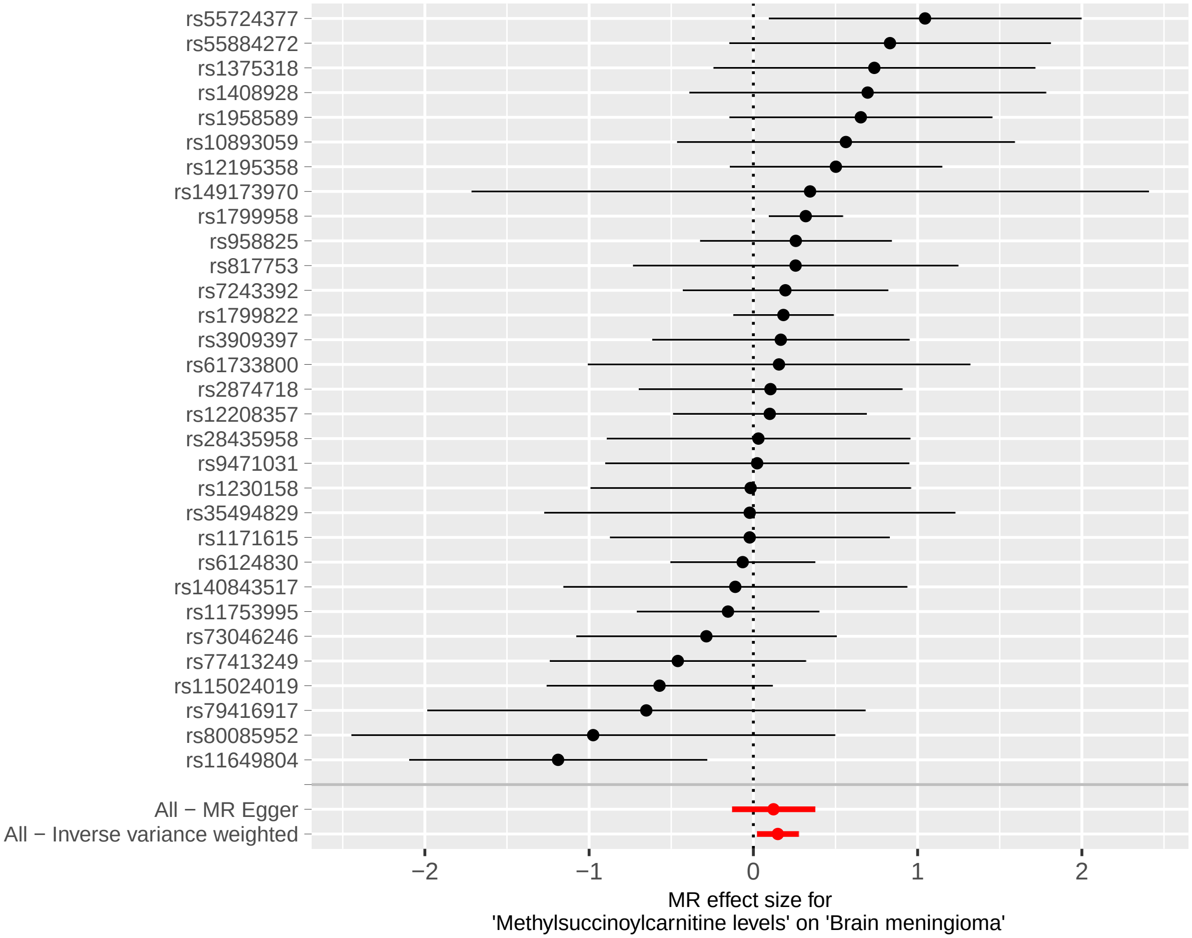
rs55724377
rs55884272
rs1375318
rs1408928
rs1958589
rs10893059
rs12195358
rs149173970
rs1799958
rs958825
rs817753
rs7243392
rs1799822
rs3909397
rs61733800
rs2874718
rs12208357
rs28435958
rs9471031
rs1230158
rs35494829
rs1171615
rs6124830
rs140843517
rs11753995
rs73046246
rs77413249
rs115024019
rs79416917
rs80085952
rs11649804
All − MR Egger
All − Inverse variance weighted
−2
−1
0
1
2
MR effect size for
'Methylsuccinoylcarnitine levels' on 'Brain meningioma'

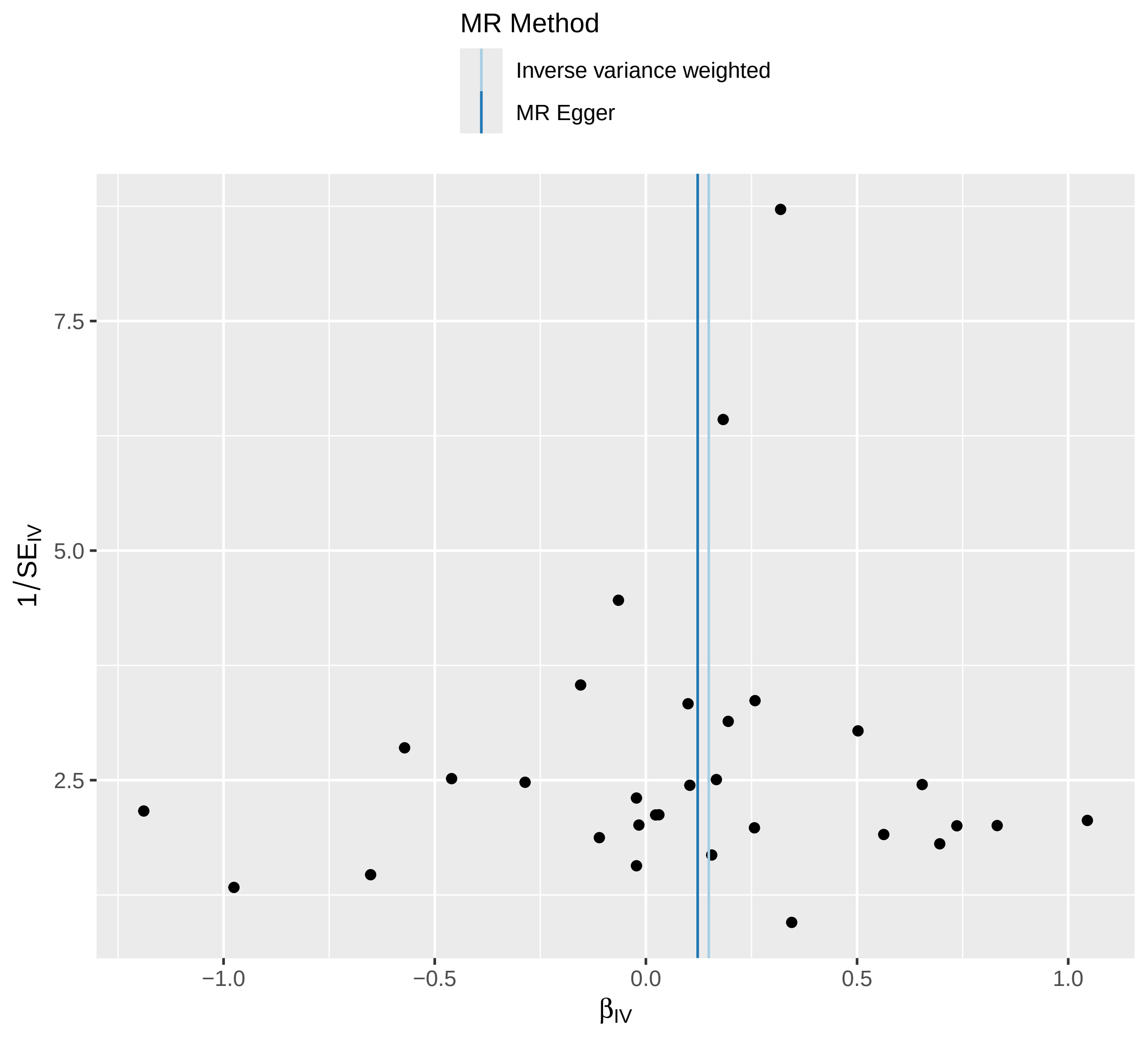
MR Method
Inverse variance weighted
MR Egger
7.5
5.0
2.5
1/SE_IV
−1.0
−0.5
0.0
0.5
1.0
β_IV

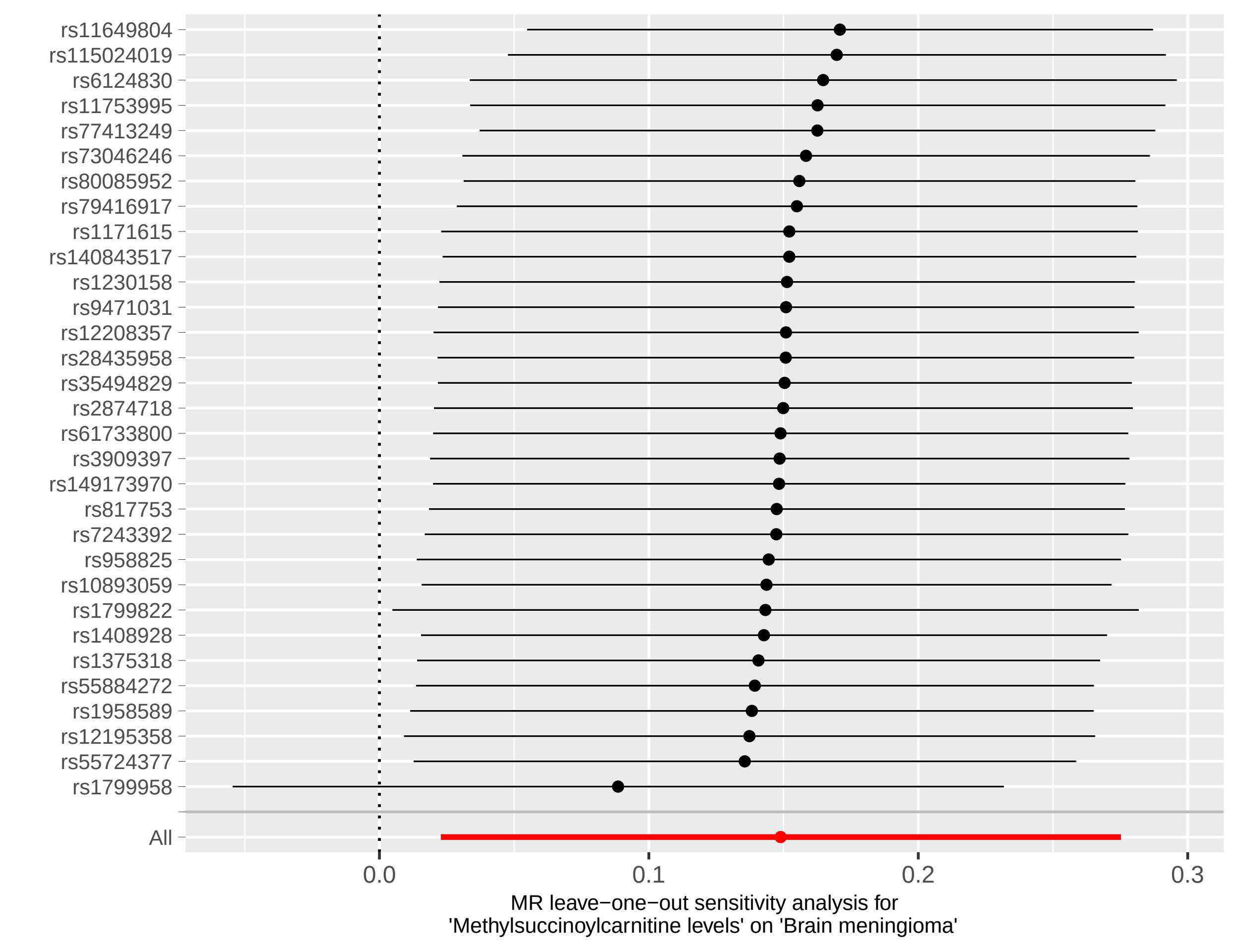
rs11649804
rs115024019
rs6124830
rs11753995
rs77413249
rs73046246
rs80085952
rs79416917
rs1171615
rs140843517
rs1230158
rs9471031
rs12208357
rs28435958
rs35494829
rs2874718
rs61733800
rs3909397
rs149173970
rs817753
rs7243392
rs958825
rs10893059
rs1799822
rs1408928
rs1375318
rs55884272
rs1958589
rs12195358
rs55724377
rs1799958
All
0.0
0.1
0.2
0.3
MR leave−one−out sensitivity analysis for
'Methylsuccinoylcarnitine levels' on 'Brain meningioma'

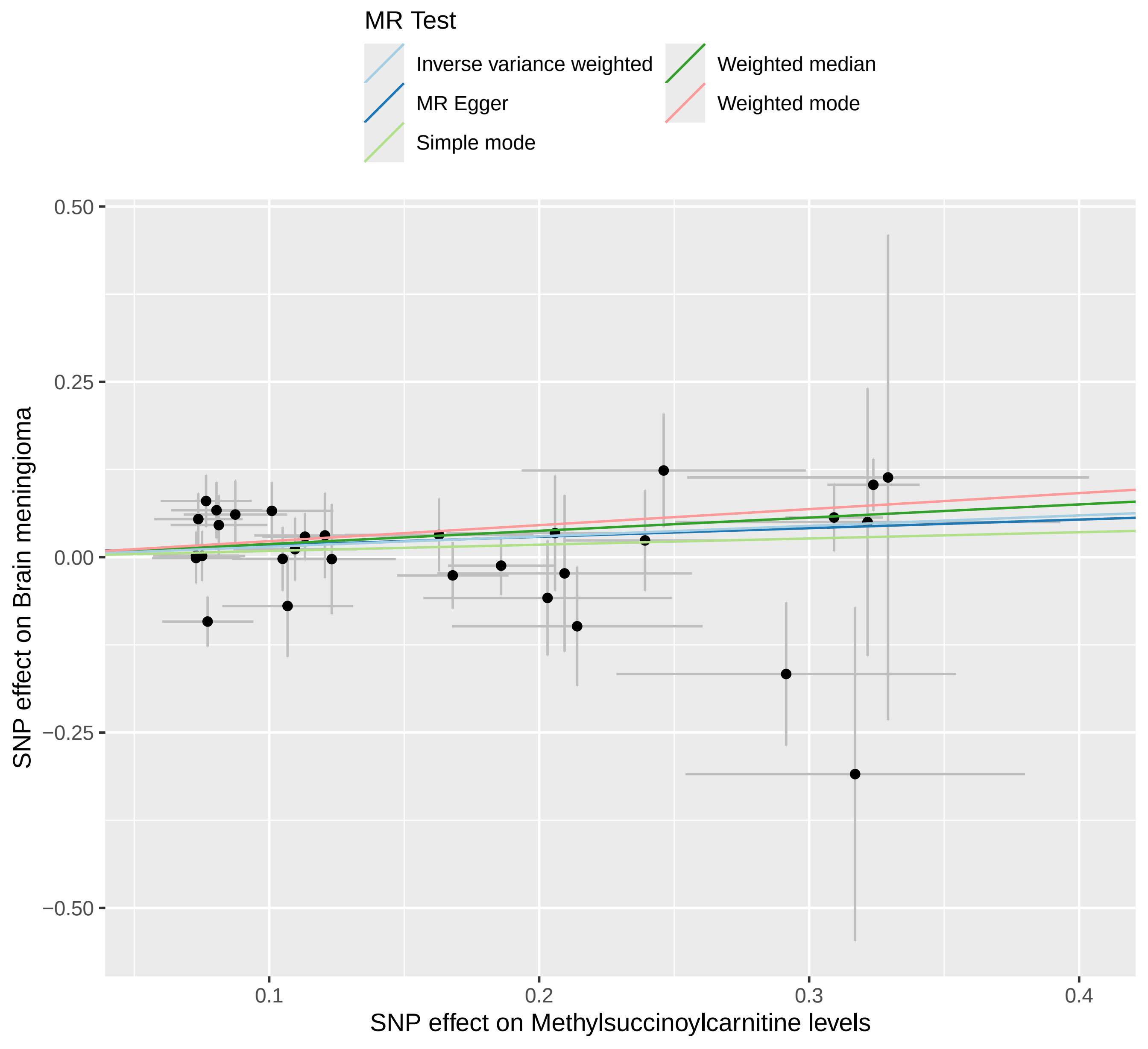
MR Test
Inverse variance weighted
MR Egger
Simple mode
Weighted median
Weighted mode
0.50
0.25
0.00
−0.25
−0.50
SNP effect on Brain meningioma
0.1
0.2
0.3
0.4
SNP effect on Methylsuccinoylcarnitine levels

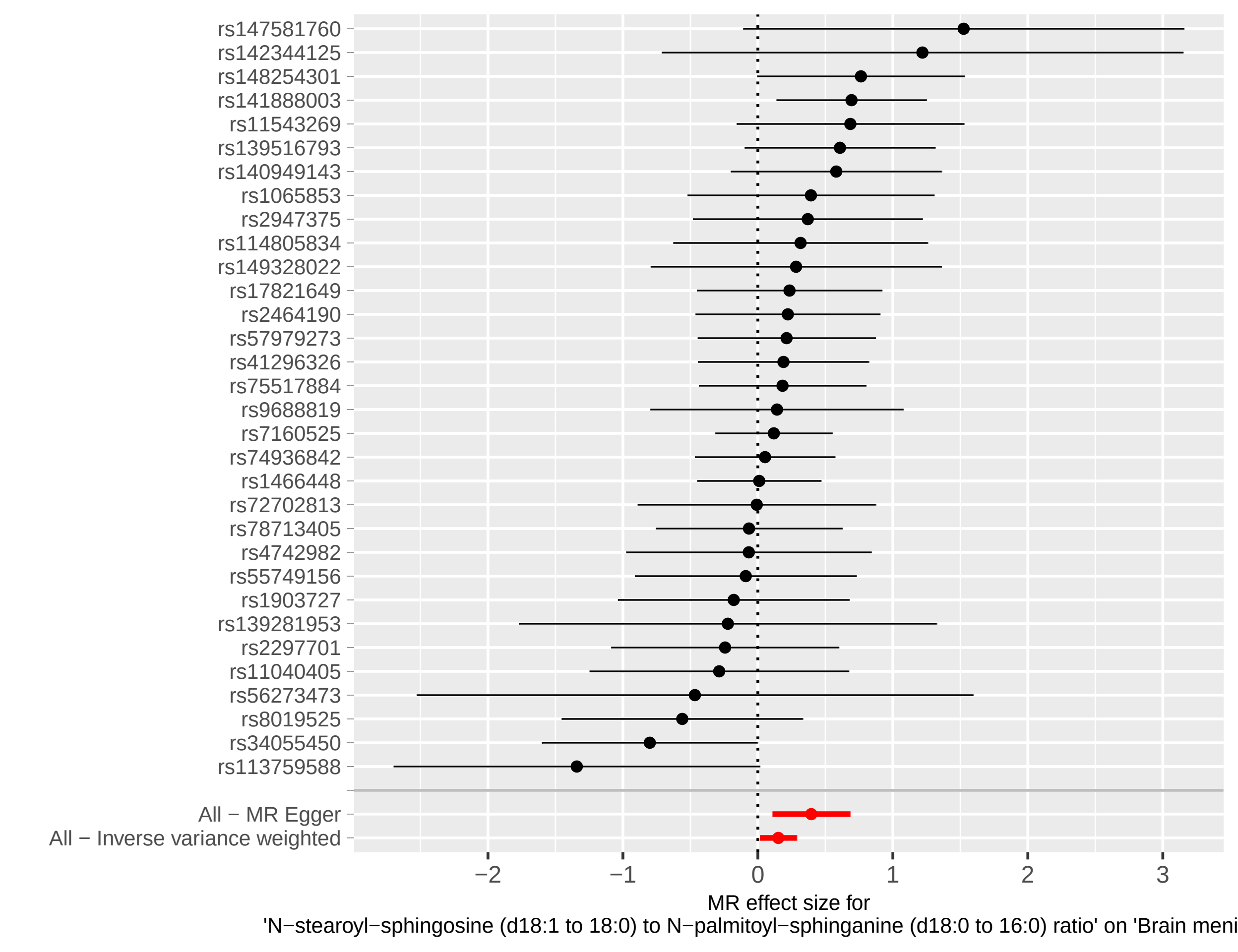

rs147581760
rs142344125
rs148254301
rs141888003
rs11543269
rs139516793
rs140949143
rs1065853
rs2947375
rs114805834
rs149328022
rs17821649
rs2464190
rs57979273
rs41296326
rs75517884
rs9688819
rs7160525
rs74936842
rs1466448
rs72702813
rs78713405
rs4742982
rs55749156
rs1903727
rs139281953
rs2297701
rs11040405
rs56273473
rs8019525
rs34055450
rs113759588
All − MR Egger
All − Inverse variance weighted
−2
−1
0
1
2
3
MR effect size for
'N−stearoyl−sphingosine (d18:1 to 18:0) to N−palmitoyl−sphinganine (d18:0 to 16:0) ratio' on 'Brain meni

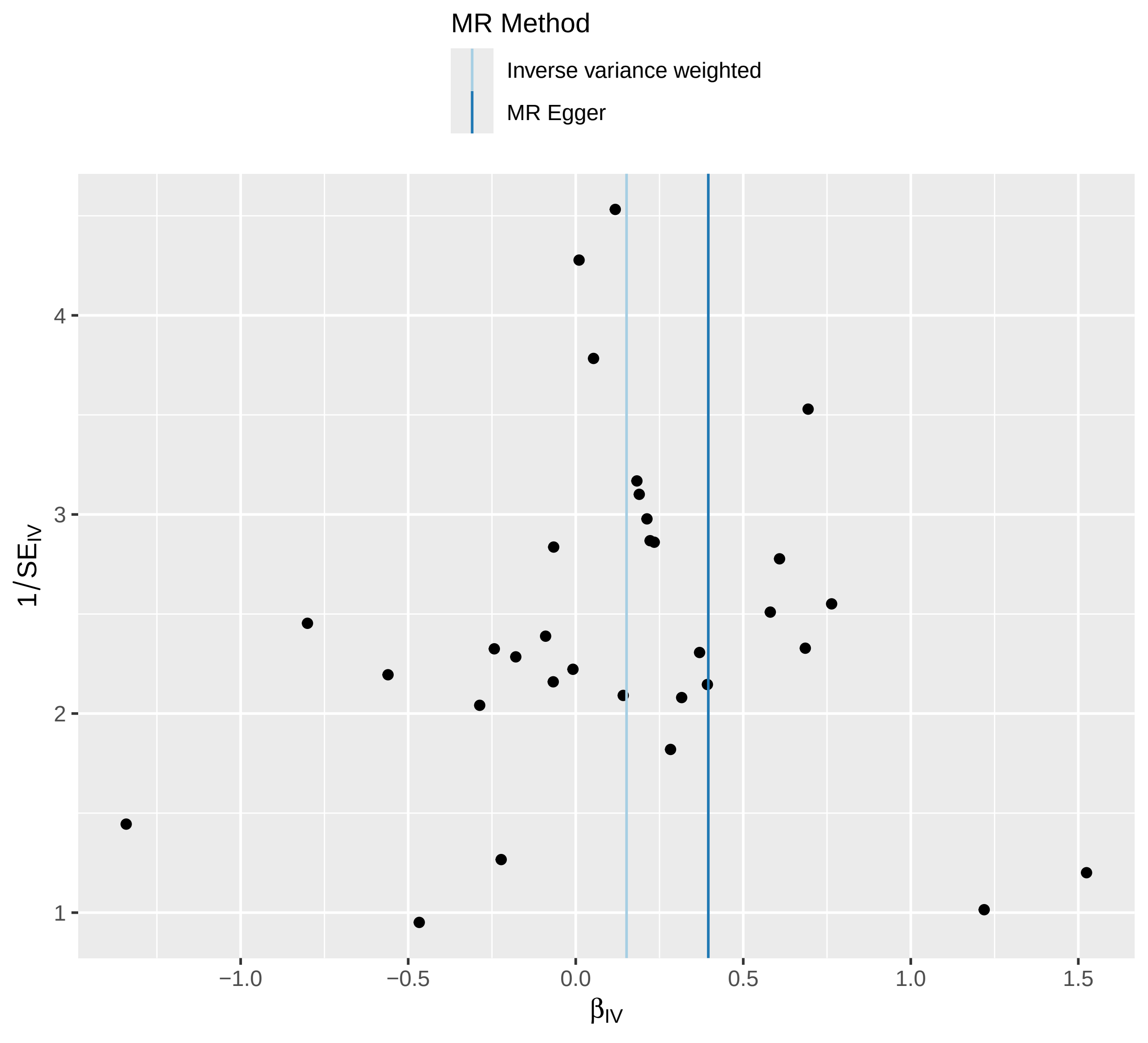
MR Method
Inverse variance weighted
MR Egger
1/SE_IV
β_IV
4
3
2
1
−1.0
−0.5
0.0
0.5
1.0
1.5

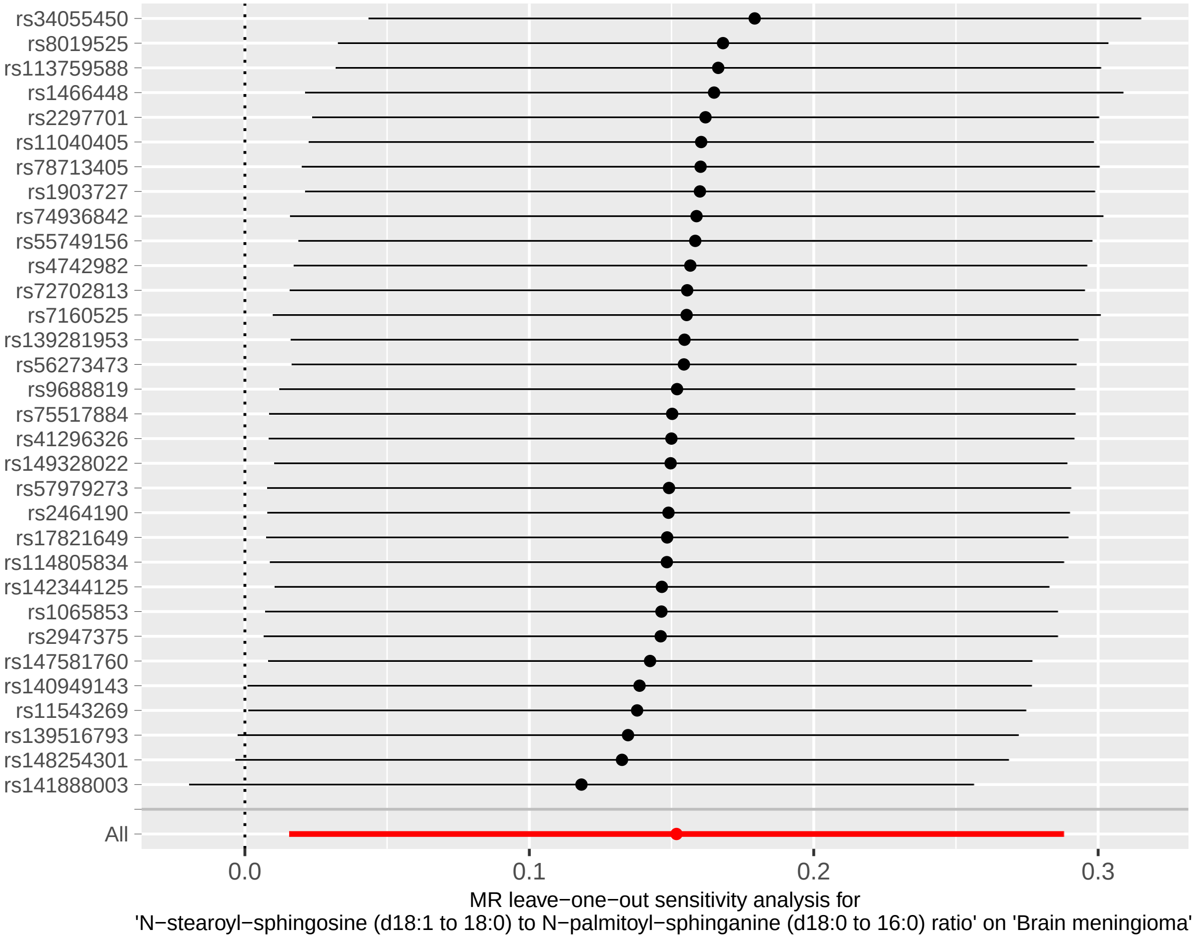
rs34055450
rs8019525
rs113759588
rs1466448
rs2297701
rs11040405
rs78713405
rs1903727
rs74936842
rs55749156
rs4742982
rs72702813
rs7160525
rs139281953
rs56273473
rs9688819
rs75517884
rs41296326
rs149328022
rs57979273
rs2464190
rs17821649
rs114805834
rs142344125
rs1065853
rs2947375
rs147581760
rs140949143
rs11543269
rs139516793
rs148254301
rs141888003
All
0.0
0.1
0.2
0.3
MR leave−one−out sensitivity analysis for
'N−stearoyl−sphingosine (d18:1 to 18:0) to N−palmitoyl−sphinganine (d18:0 to 16:0) ratio' on 'Brain meningioma'

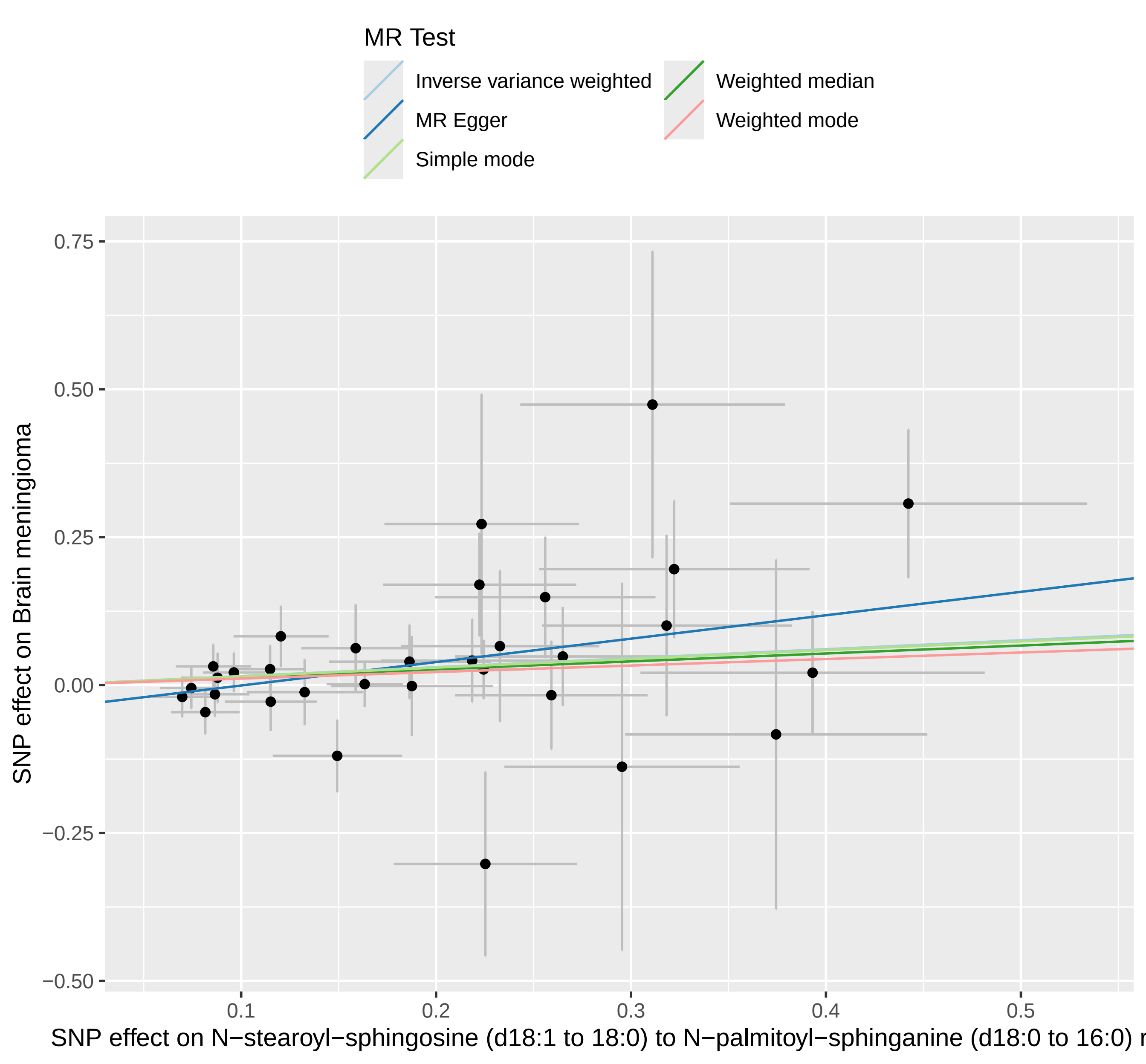
MR Test
Inverse variance weighted
MR Egger
Simple mode
Weighted median
Weighted mode
0.75
0.50
0.25
0.00
−0.25
−0.50
0.1
0.2
0.3
0.4
0.5
SNP effect on Brain meningioma
SNP effect on N−stearoyl−sphingosine (d18:1 to 18:0) to N−palmitoyl−sphinganine (d18:0 to 16:0) r

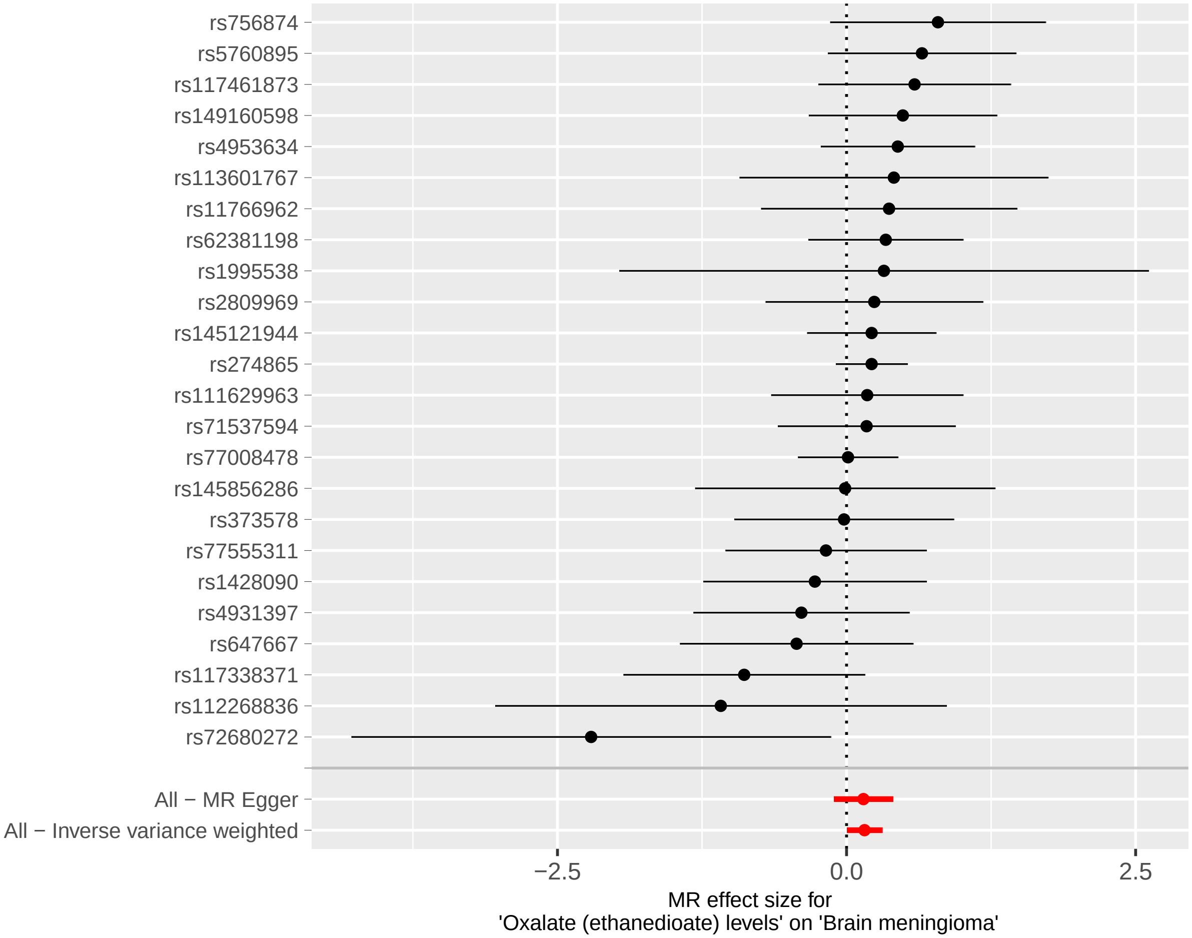
rs756874
rs5760895
rs117461873
rs149160598
rs4953634
rs113601767
rs11766962
rs62381198
rs1995538
rs2809969
rs145121944
rs274865
rs111629963
rs71537594
rs77008478
rs145856286
rs373578
rs77555311
rs1428090
rs4931397
rs647667
rs117338371
rs112268836
rs72680272
All − MR Egger
All − Inverse variance weighted
−2.5
0.0
2.5
MR effect size for
'Oxalate (ethanedioate) levels' on 'Brain meningioma'

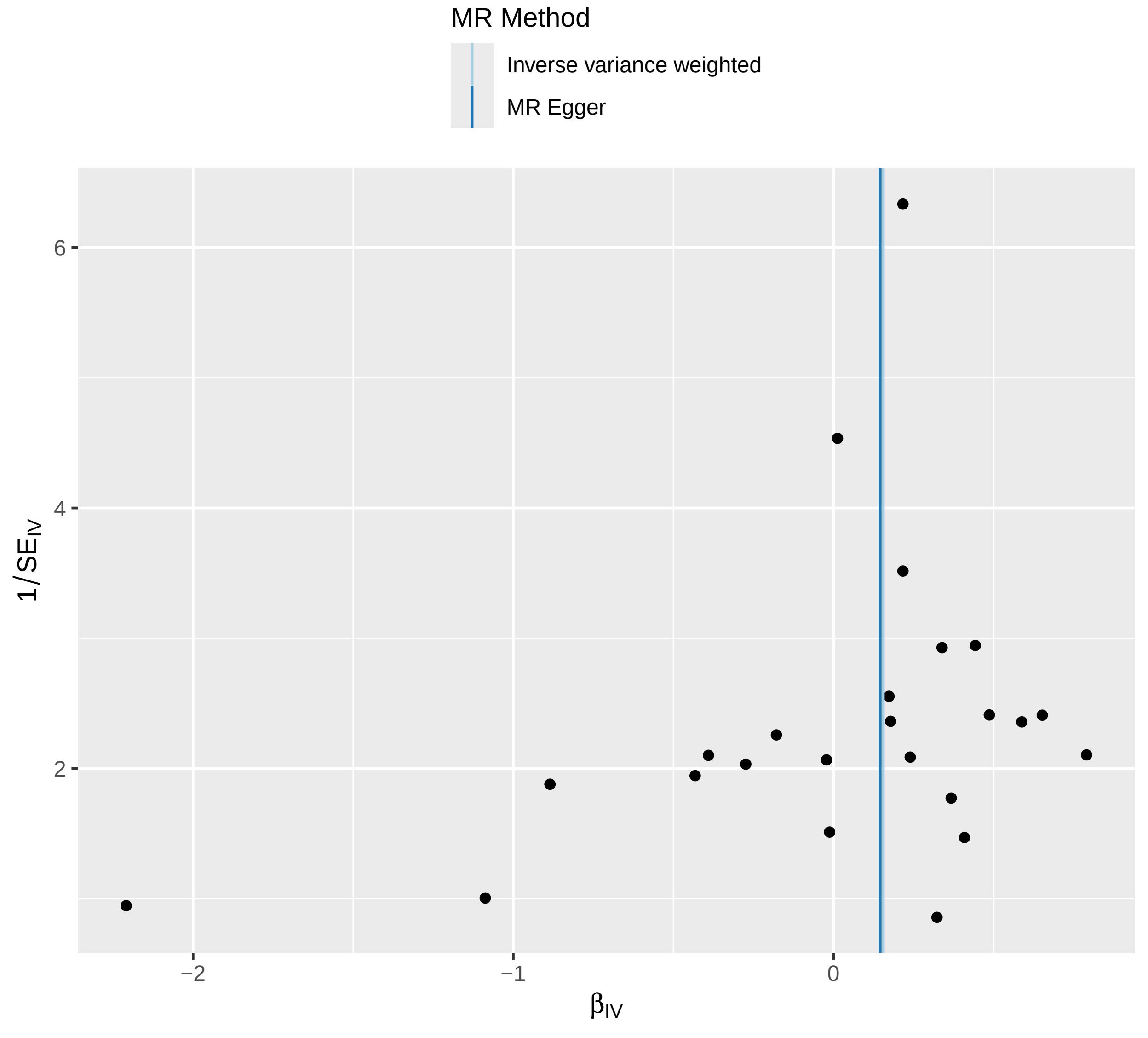
MR Method
Inverse variance weighted
MR Egger
6
4
2
1/SE_IV
−2
−1
0
β_IV

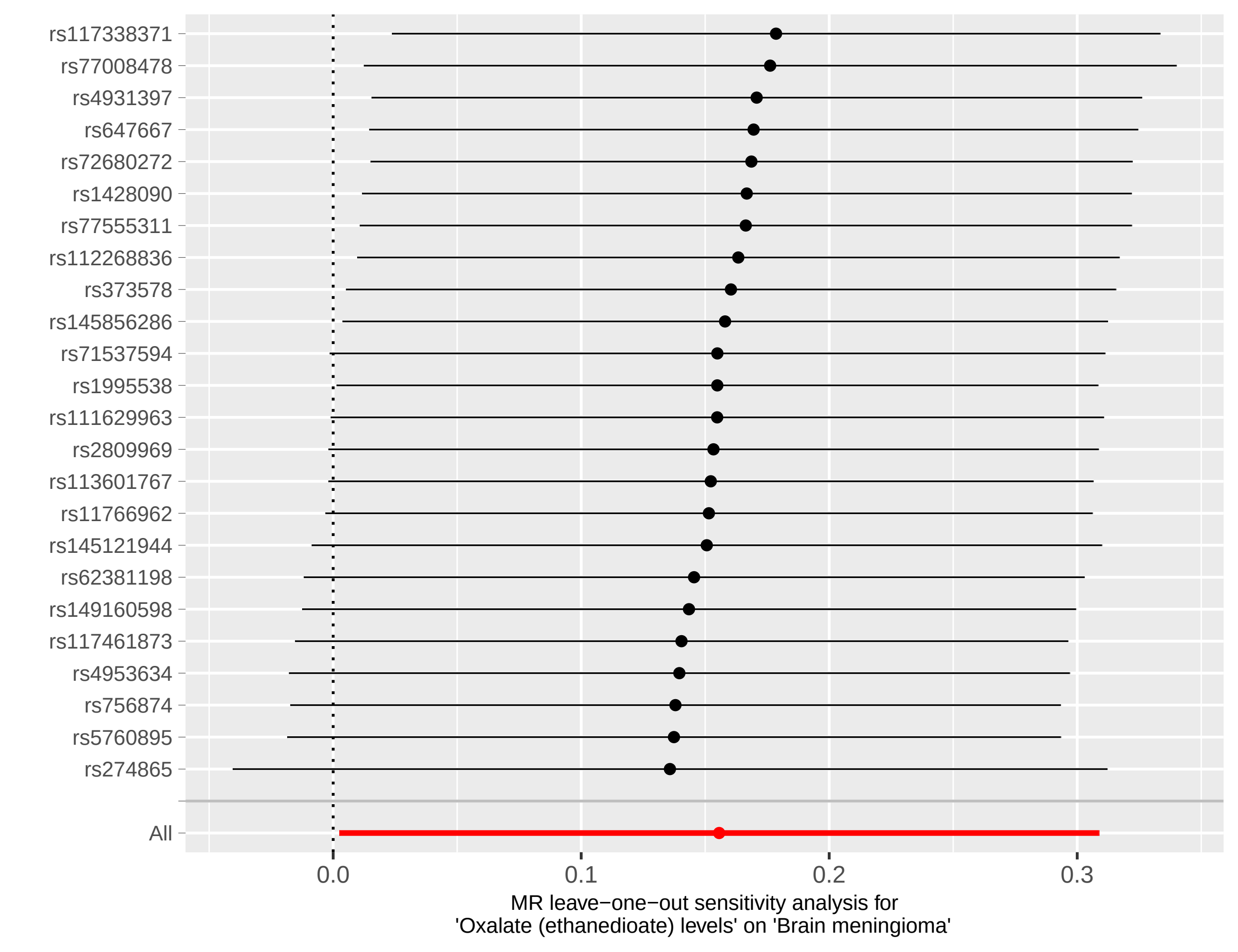
rs117338371
rs77008478
rs4931397
rs647667
rs72680272
rs1428090
rs77555311
rs112268836
rs373578
rs145856286
rs71537594
rs1995538
rs111629963
rs2809969
rs113601767
rs11766962
rs145121944
rs62381198
rs149160598
rs117461873
rs4953634
rs756874
rs5760895
rs274865
All
0.0
0.1
0.2
0.3
MR leave−one−out sensitivity analysis for
'Oxalate (ethanedioate) levels' on 'Brain meningioma'

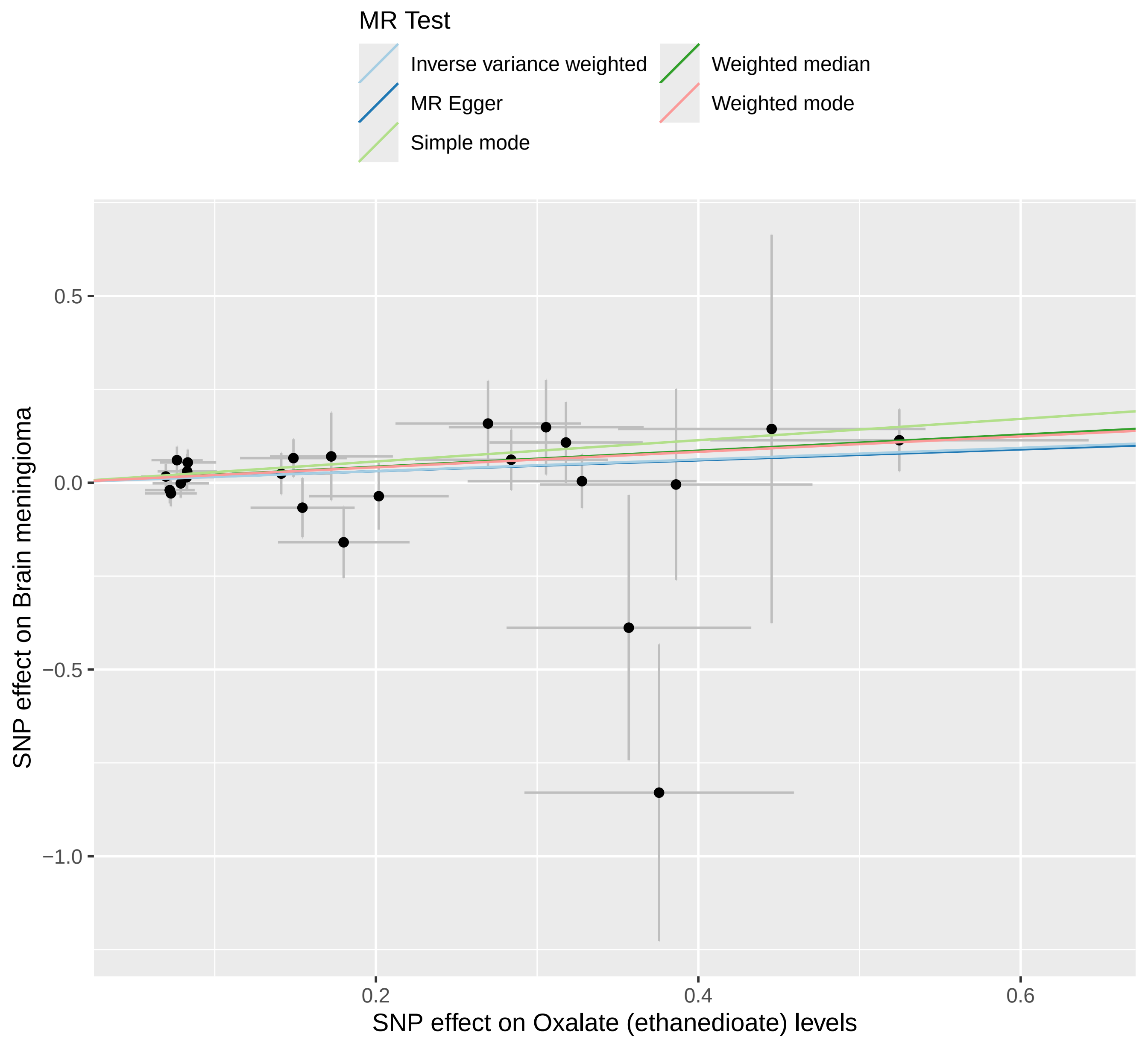
MR Test
Inverse variance weighted
MR Egger
Simple mode
Weighted median
Weighted mode
0.5
0.0
−0.5
−1.0
0.2
0.4
0.6
SNP effect on Brain meningioma
SNP effect on Oxalate (ethanedioate) levels

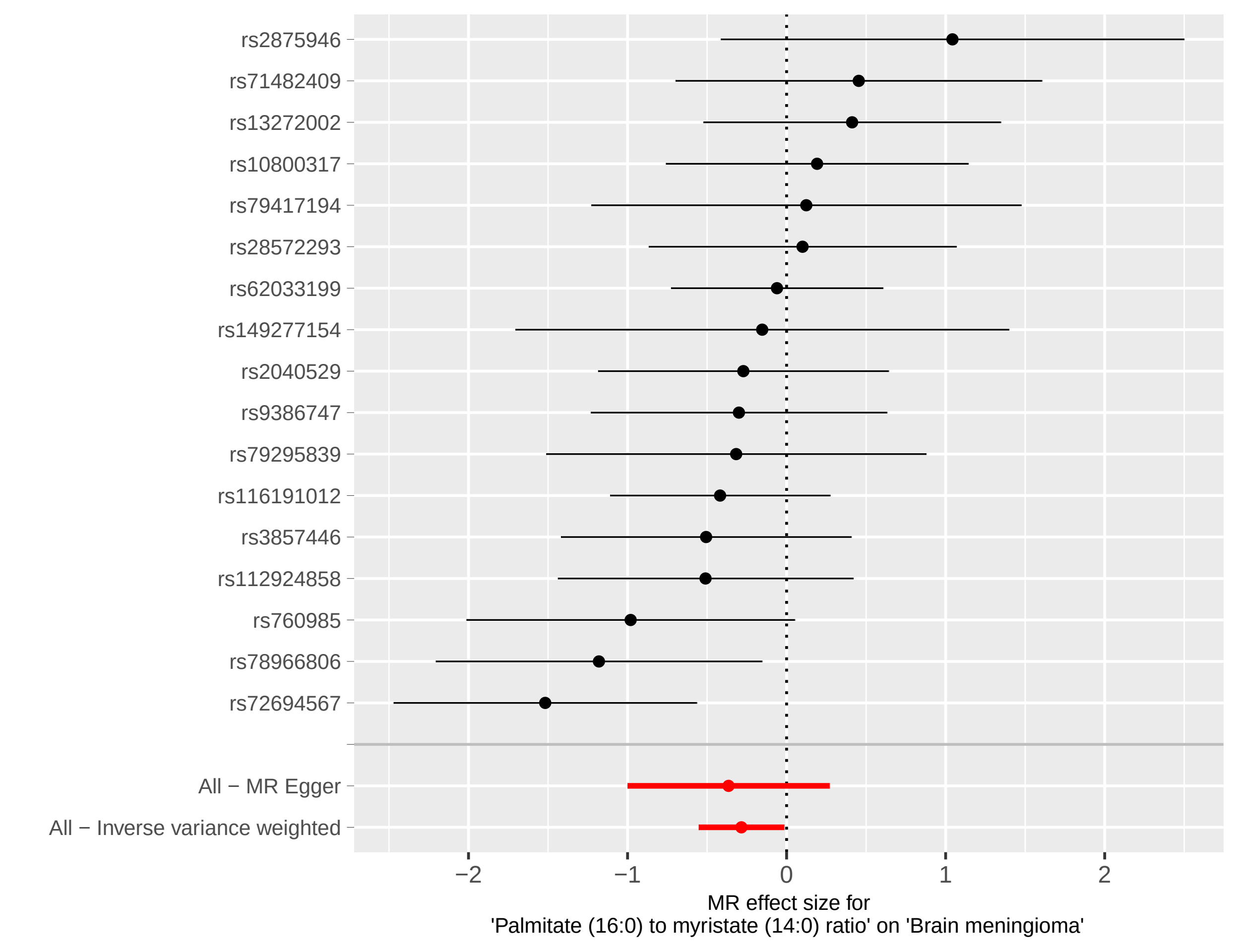
rs2875946
rs71482409
rs13272002
rs10800317
rs79417194
rs28572293
rs62033199
rs149277154
rs2040529
rs9386747
rs79295839
rs116191012
rs3857446
rs112924858
rs760985
rs78966806
rs72694567
All − MR Egger
All − Inverse variance weighted
−2
−1
0
1
2
MR effect size for
'Palmitate (16:0) to myristate (14:0) ratio' on 'Brain meningioma'

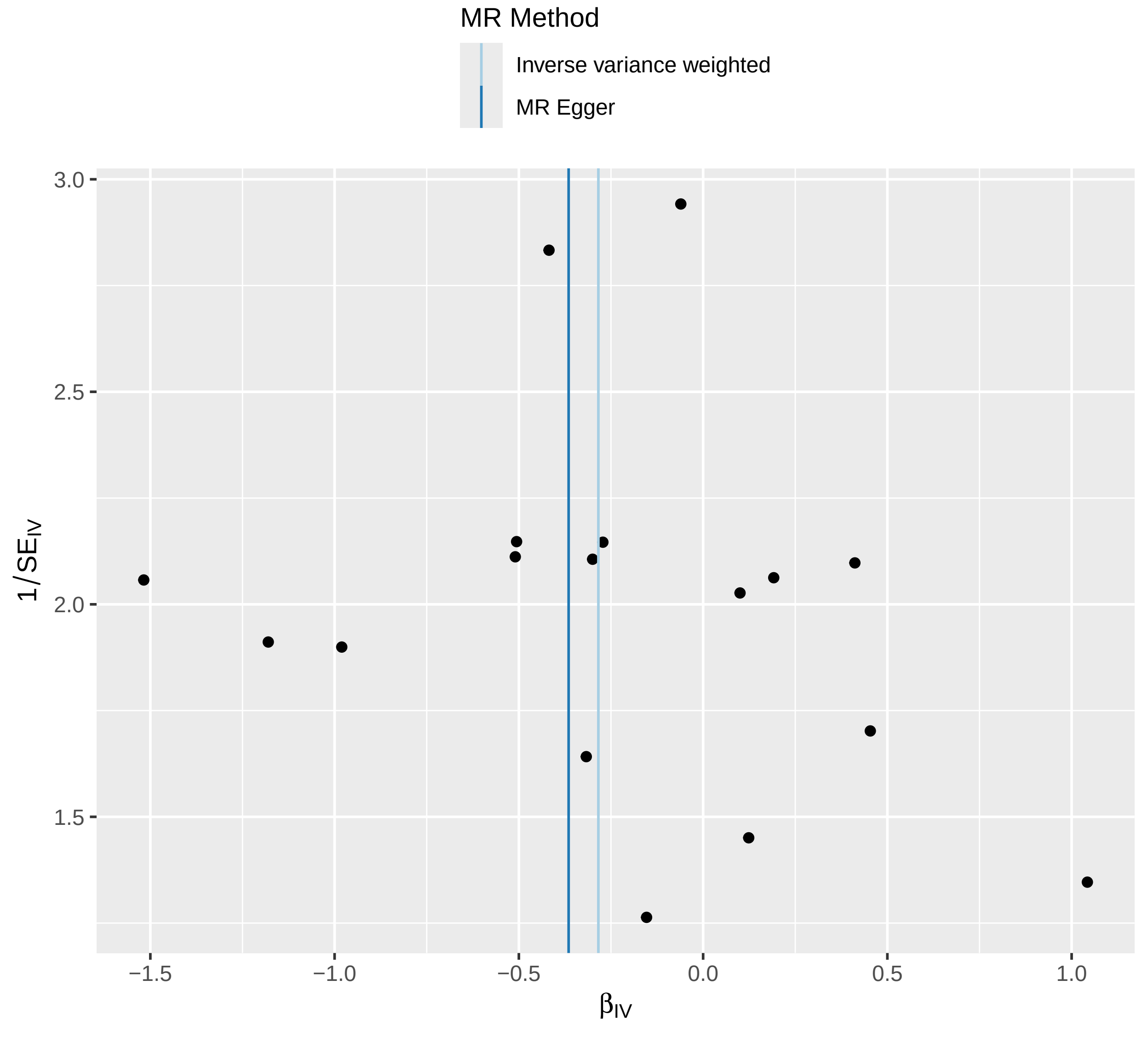

MR Method
Inverse variance weighted
MR Egger
3.0
2.5
2.0
1.5
1/SE_IV
−1.5
−1.0
−0.5
0.0
0.5
1.0
β_IV

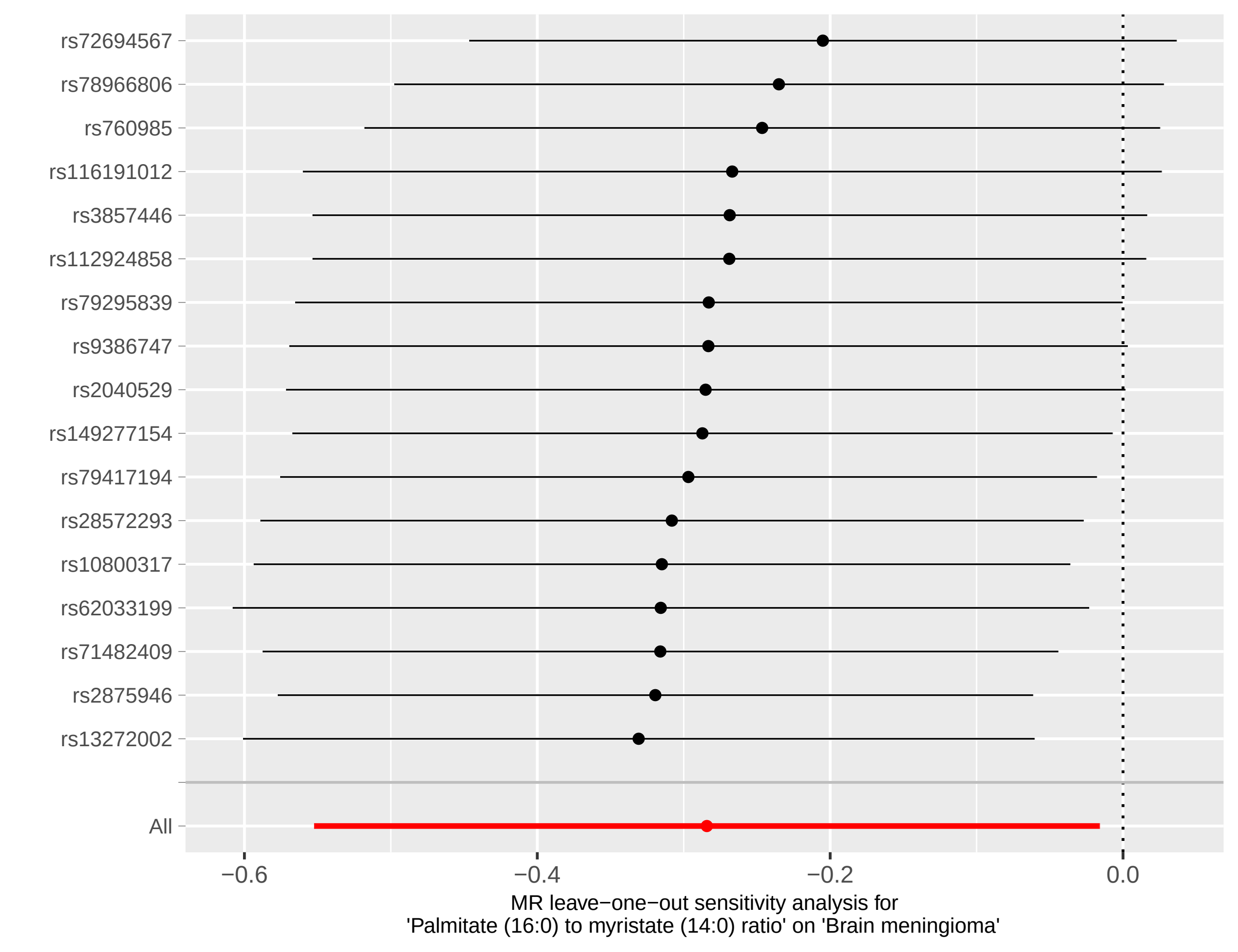

rs72694567
rs78966806
rs760985
rs116191012
rs3857446
rs112924858
rs79295839
rs9386747
rs2040529
rs149277154
rs79417194
rs28572293
rs10800317
rs62033199
rs71482409
rs2875946
rs13272002
All
−0.6
−0.4
−0.2
0.0
MR leave−one−out sensitivity analysis for
'Palmitate (16:0) to myristate (14:0) ratio' on 'Brain meningioma'

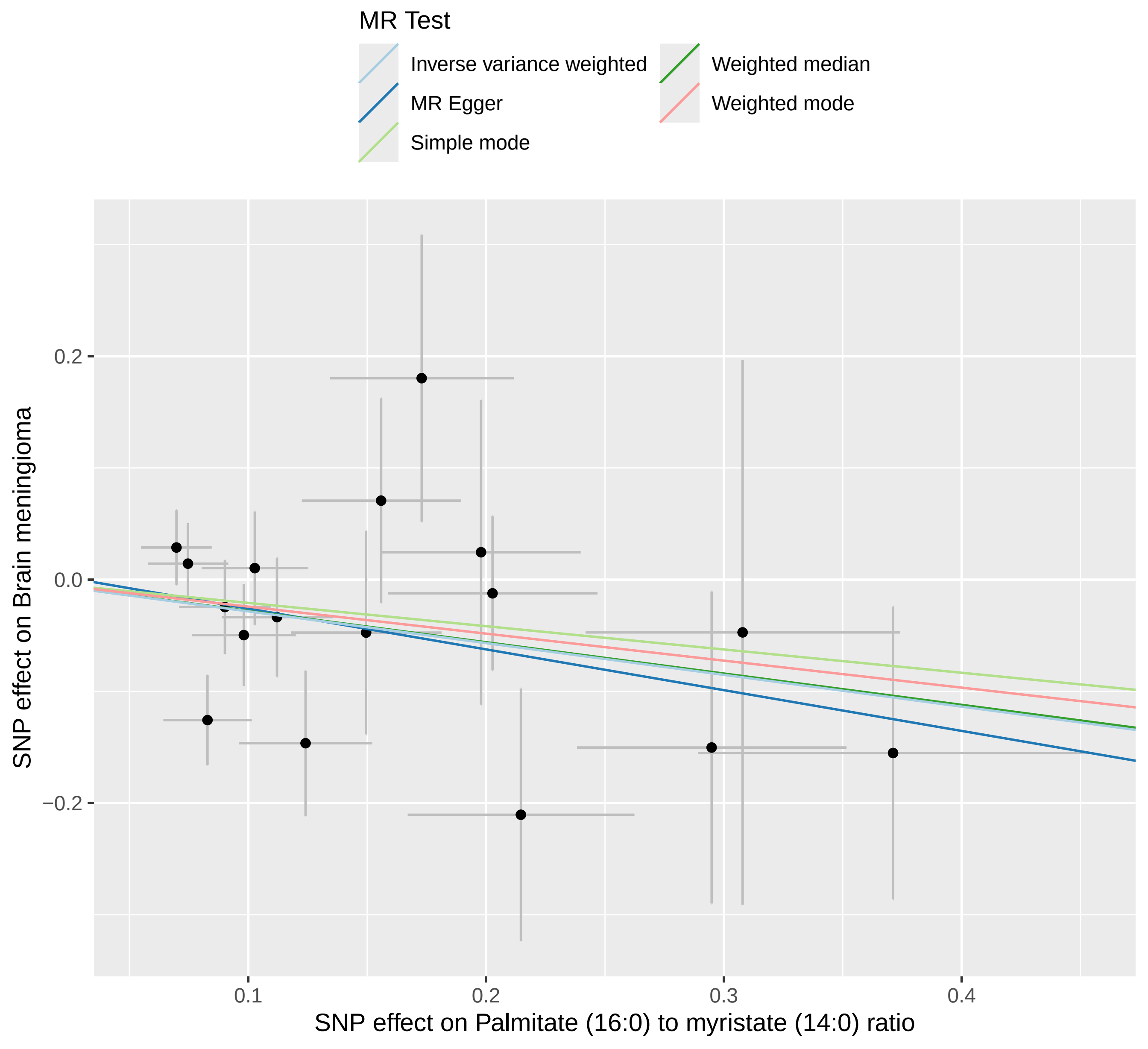
MR Test
Inverse variance weighted
MR Egger
Simple mode
Weighted median
Weighted mode
SNP effect on Brain meningioma
0.2
0.0
−0.2
0.1
0.2
0.3
0.4
SNP effect on Palmitate (16:0) to myristate (14:0) ratio

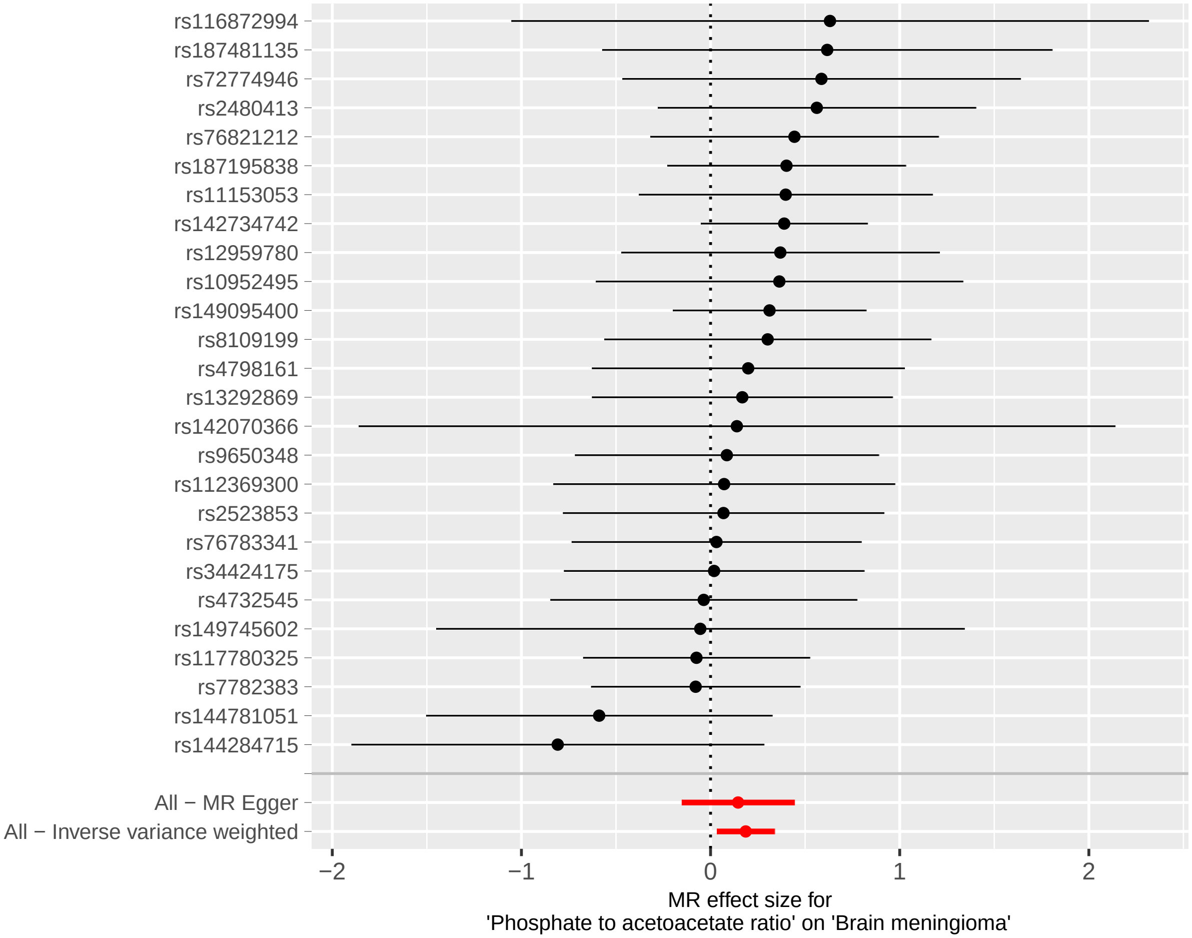
rs116872994
rs187481135
rs72774946
rs2480413
rs76821212
rs187195838
rs11153053
rs142734742
rs12959780
rs10952495
rs149095400
rs8109199
rs4798161
rs13292869
rs142070366
rs9650348
rs112369300
rs2523853
rs76783341
rs34424175
rs4732545
rs149745602
rs117780325
rs7782383
rs144781051
rs144284715
All − MR Egger
All − Inverse variance weighted
−2
−1
0
1
2
MR effect size for
'Phosphate to acetoacetate ratio' on 'Brain meningioma'

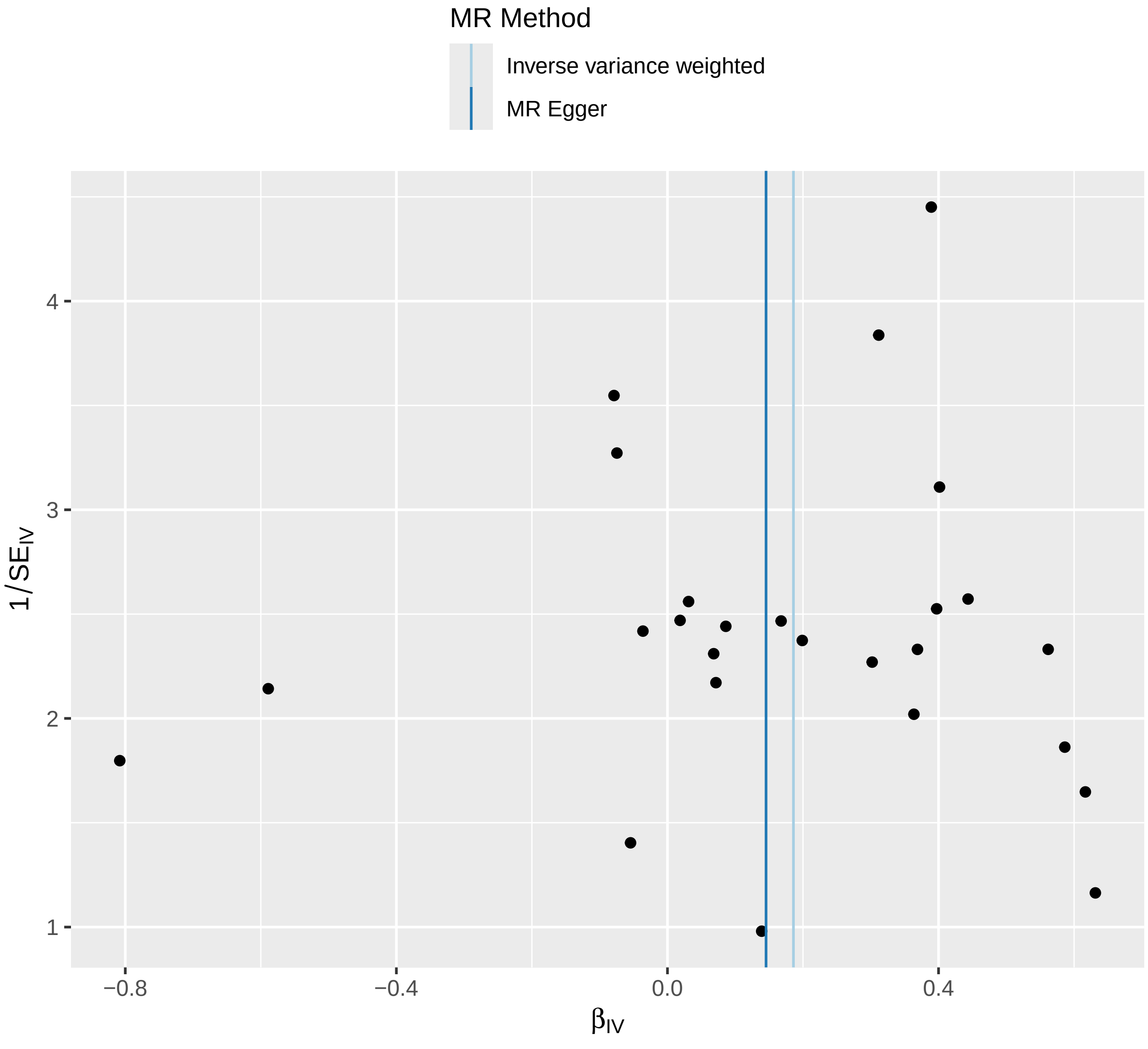
MR Method
Inverse variance weighted
MR Egger
1/SE_IV
4
3
2
1
−0.8
−0.4
0.0
0.4
β_IV

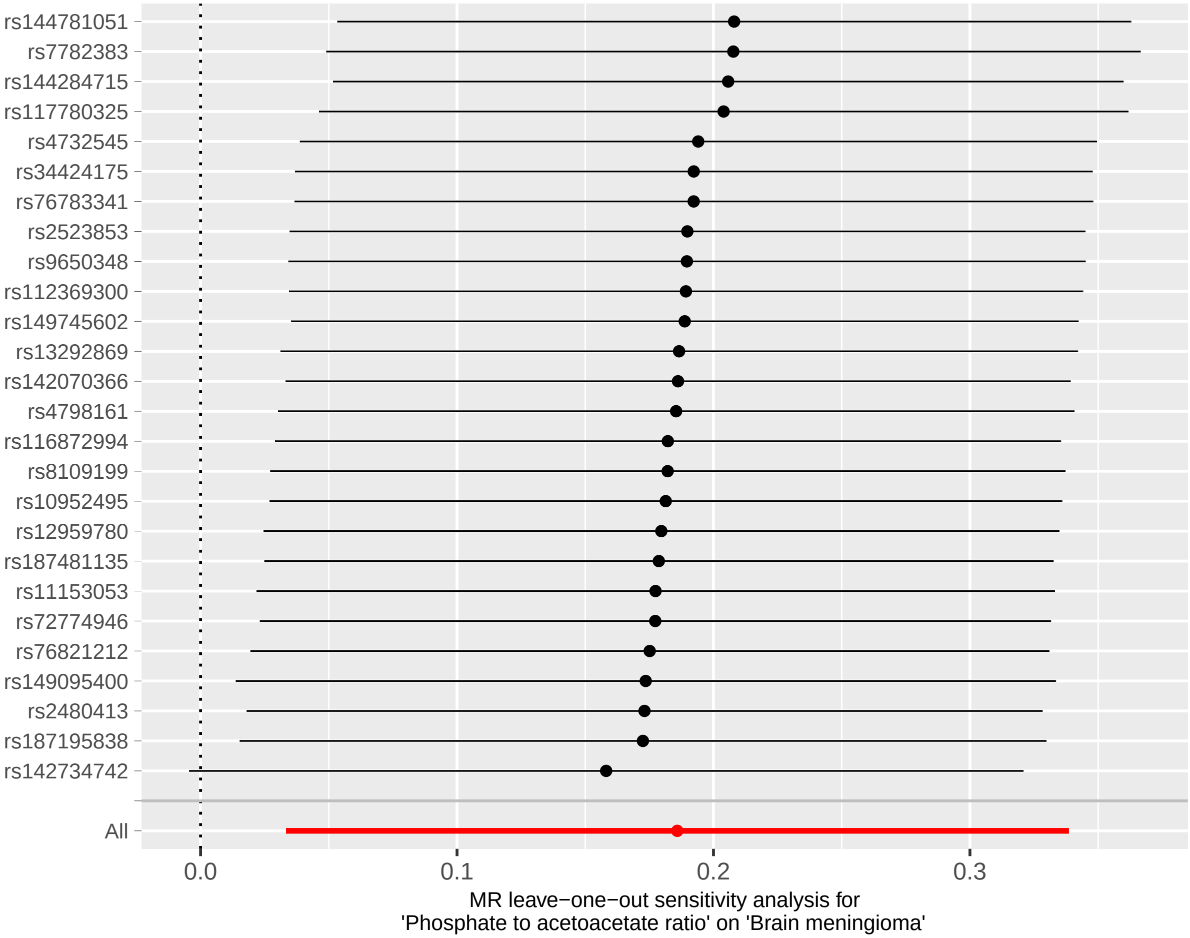
rs144781051
rs7782383
rs144284715
rs117780325
rs4732545
rs34424175
rs76783341
rs2523853
rs9650348
rs112369300
rs149745602
rs13292869
rs142070366
rs4798161
rs116872994
rs8109199
rs10952495
rs12959780
rs187481135
rs11153053
rs72774946
rs76821212
rs149095400
rs2480413
rs187195838
rs142734742
All
0.0
0.1
0.2
0.3
MR leave−one−out sensitivity analysis for
'Phosphate to acetoacetate ratio' on 'Brain meningioma'

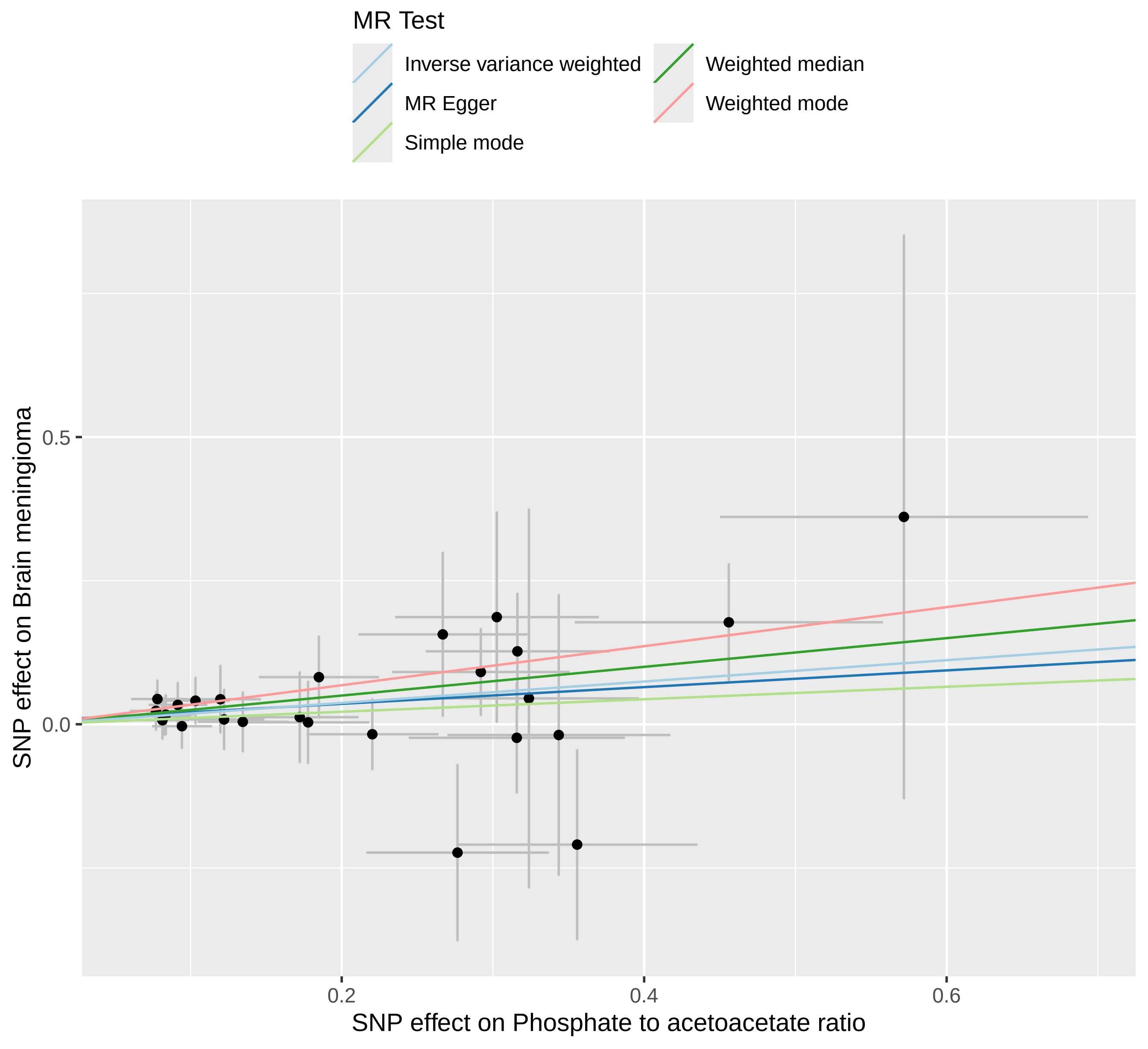

MR Test
Inverse variance weighted
MR Egger
Simple mode
Weighted median
Weighted mode
0.5
0.0
0.2
0.4
0.6
SNP effect on Brain meningioma
SNP effect on Phosphate to acetoacetate ratio

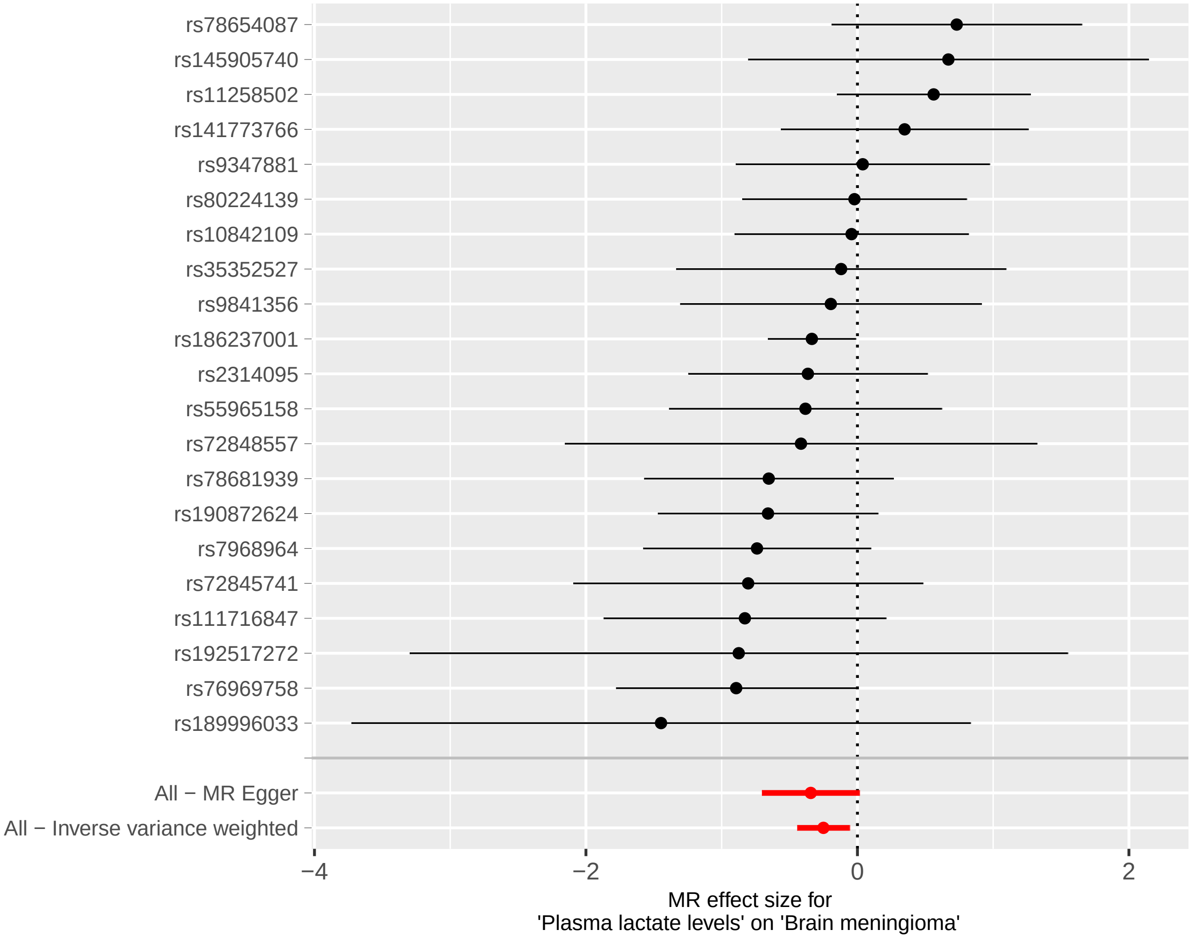
rs78654087
rs145905740
rs11258502
rs141773766
rs9347881
rs80224139
rs10842109
rs35352527
rs9841356
rs186237001
rs2314095
rs55965158
rs72848557
rs78681939
rs190872624
rs7968964
rs72845741
rs111716847
rs192517272
rs76969758
rs189996033
All − MR Egger
All − Inverse variance weighted
−4
−2
0
2
MR effect size for
'Plasma lactate levels' on 'Brain meningioma'

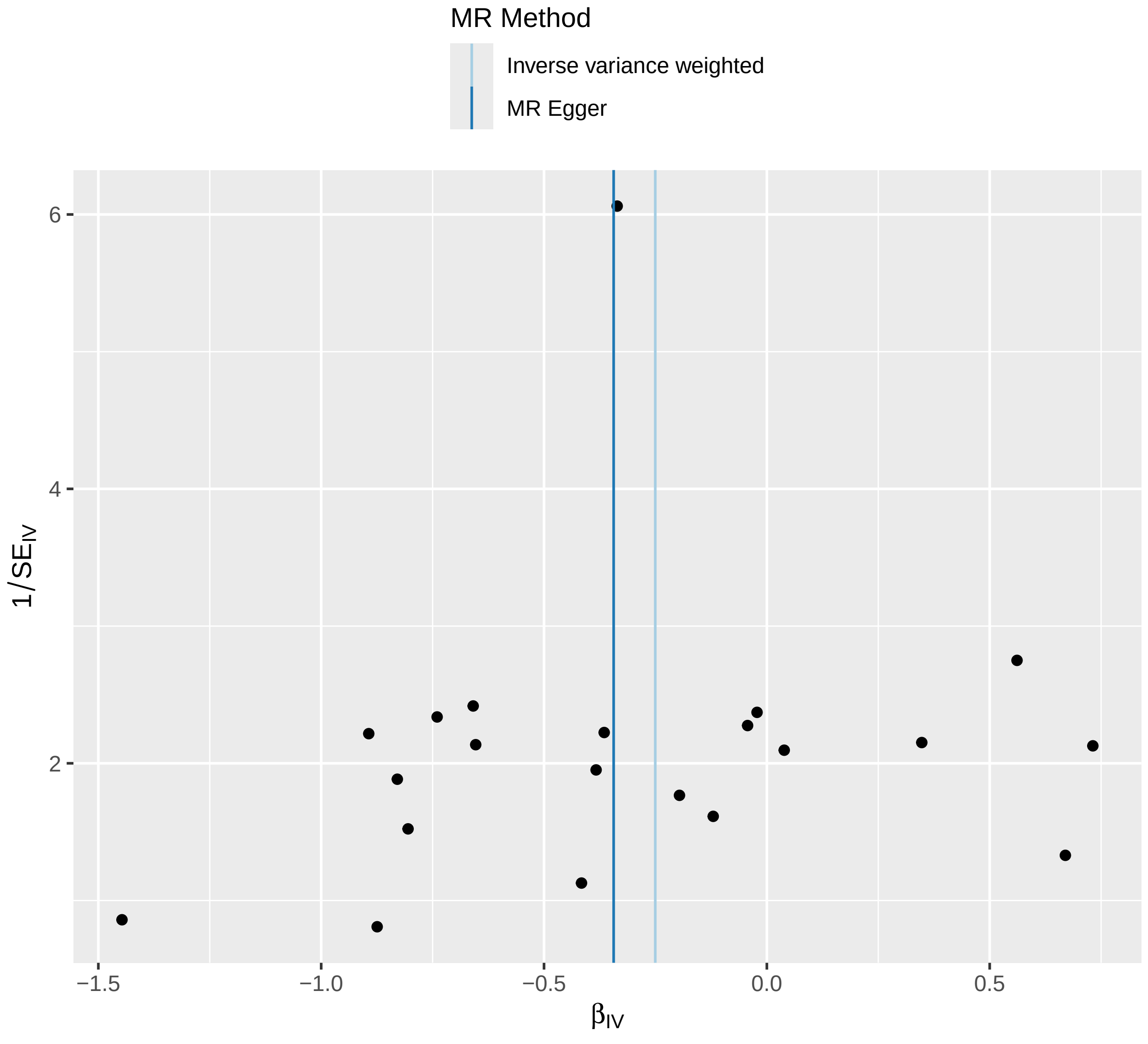
MR Method
Inverse variance weighted
MR Egger
6
4
2
1/SE_IV
−1.5
−1.0
−0.5
0.0
0.5
β_IV

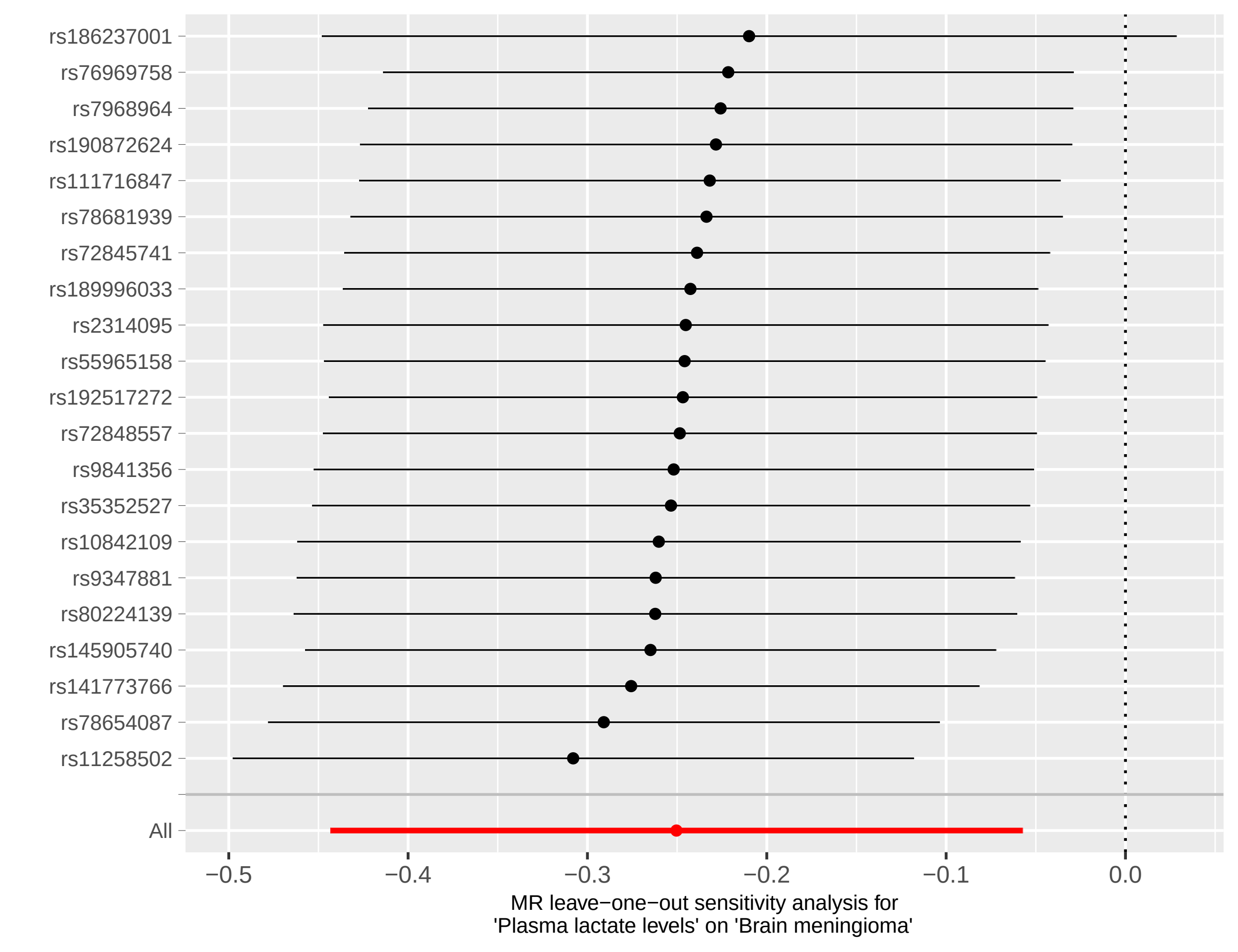
rs186237001
rs76969758
rs7968964
rs190872624
rs111716847
rs78681939
rs72845741
rs189996033
rs2314095
rs55965158
rs192517272
rs72848557
rs9841356
rs35352527
rs10842109
rs9347881
rs80224139
rs145905740
rs141773766
rs78654087
rs11258502
All
−0.5
−0.4
−0.3
−0.2
−0.1
0.0
MR leave−one−out sensitivity analysis for
'Plasma lactate levels' on 'Brain meningioma'

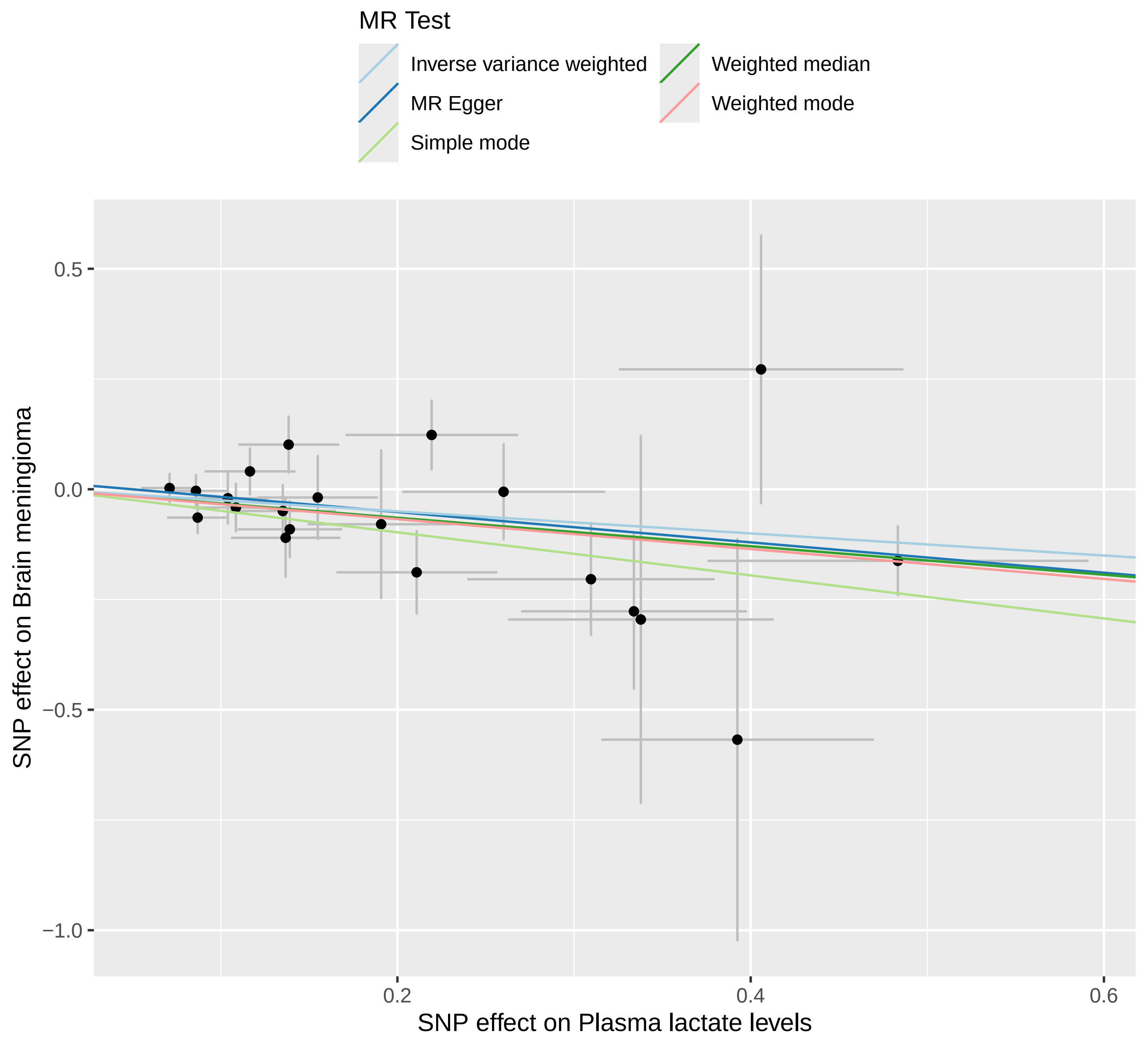
MR Test
Inverse variance weighted
MR Egger
Simple mode
Weighted median
Weighted mode
0.5
0.0
−0.5
−1.0
0.2
0.4
0.6
SNP effect on Brain meningioma
SNP effect on Plasma lactate levels

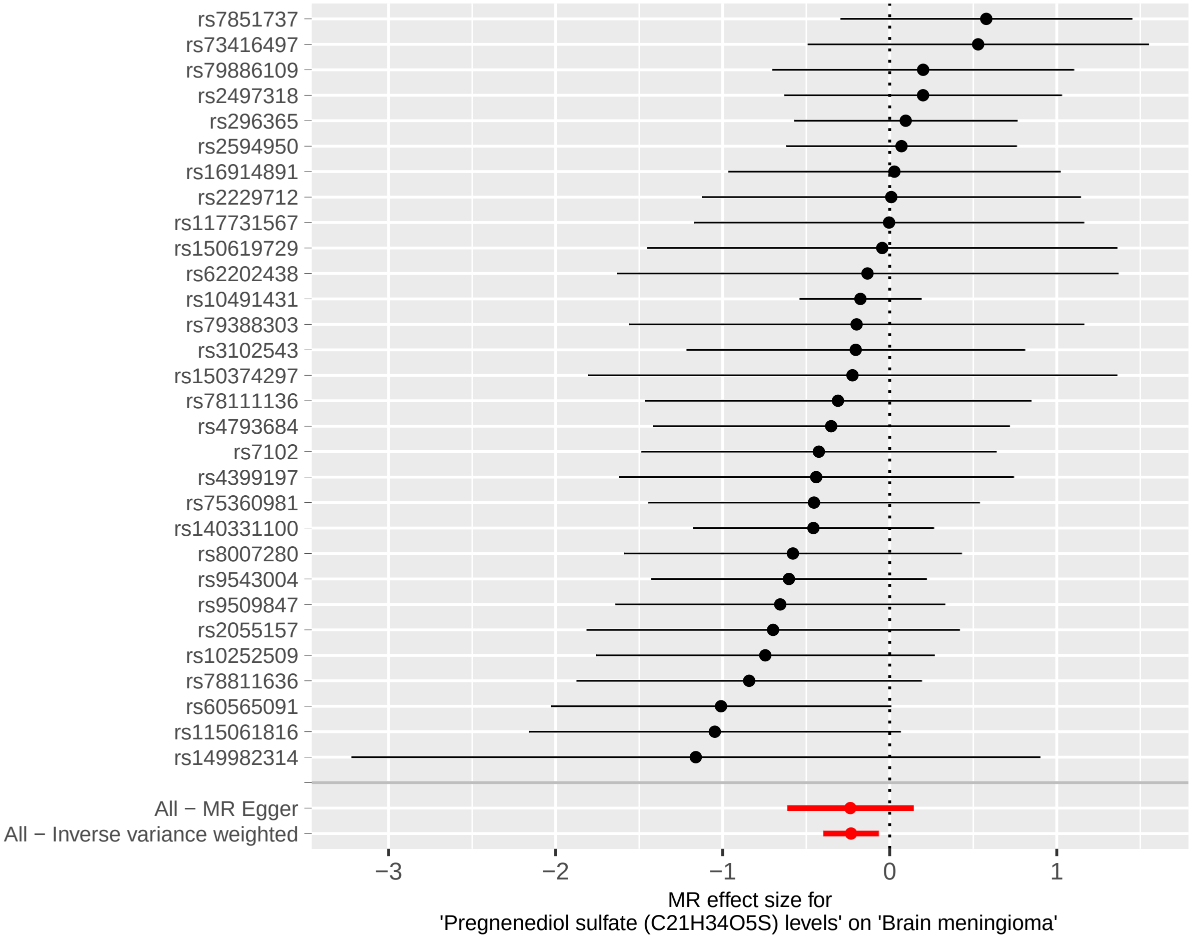
rs7851737
rs73416497
rs79886109
rs2497318
rs296365
rs2594950
rs16914891
rs2229712
rs117731567
rs150619729
rs62202438
rs10491431
rs79388303
rs3102543
rs150374297
rs78111136
rs4793684
rs7102
rs4399197
rs75360981
rs140331100
rs8007280
rs9543004
rs9509847
rs2055157
rs10252509
rs78811636
rs60565091
rs115061816
rs149982314
All − MR Egger
All − Inverse variance weighted
−3
−2
−1
0
1
MR effect size for
'Pregnenediol sulfate (C21H34O5S) levels' on 'Brain meningioma'

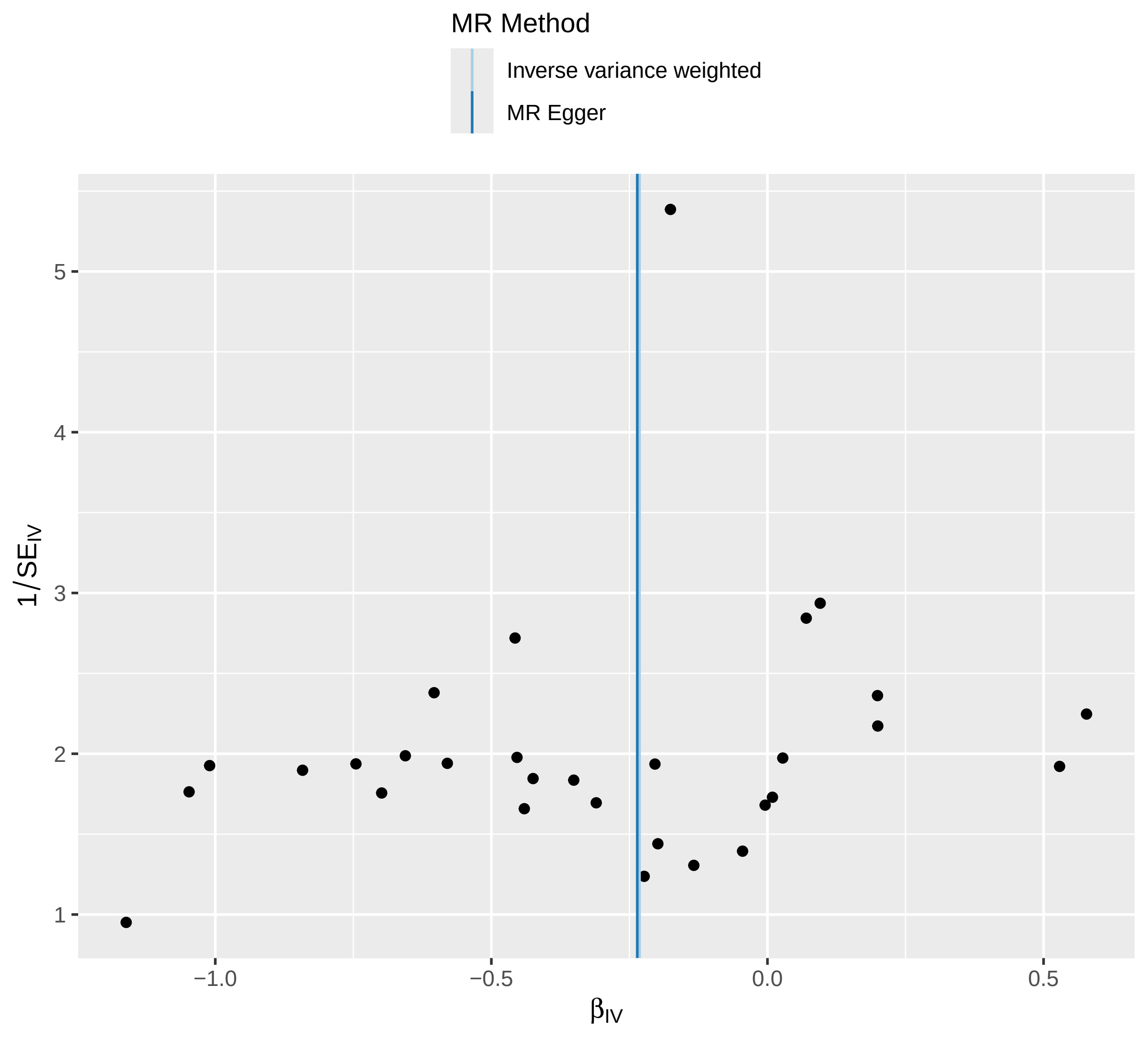

MR Method
Inverse variance weighted
MR Egger
1/SE_IV
β_IV
5
4
3
2
1
−1.0
−0.5
0.0
0.5

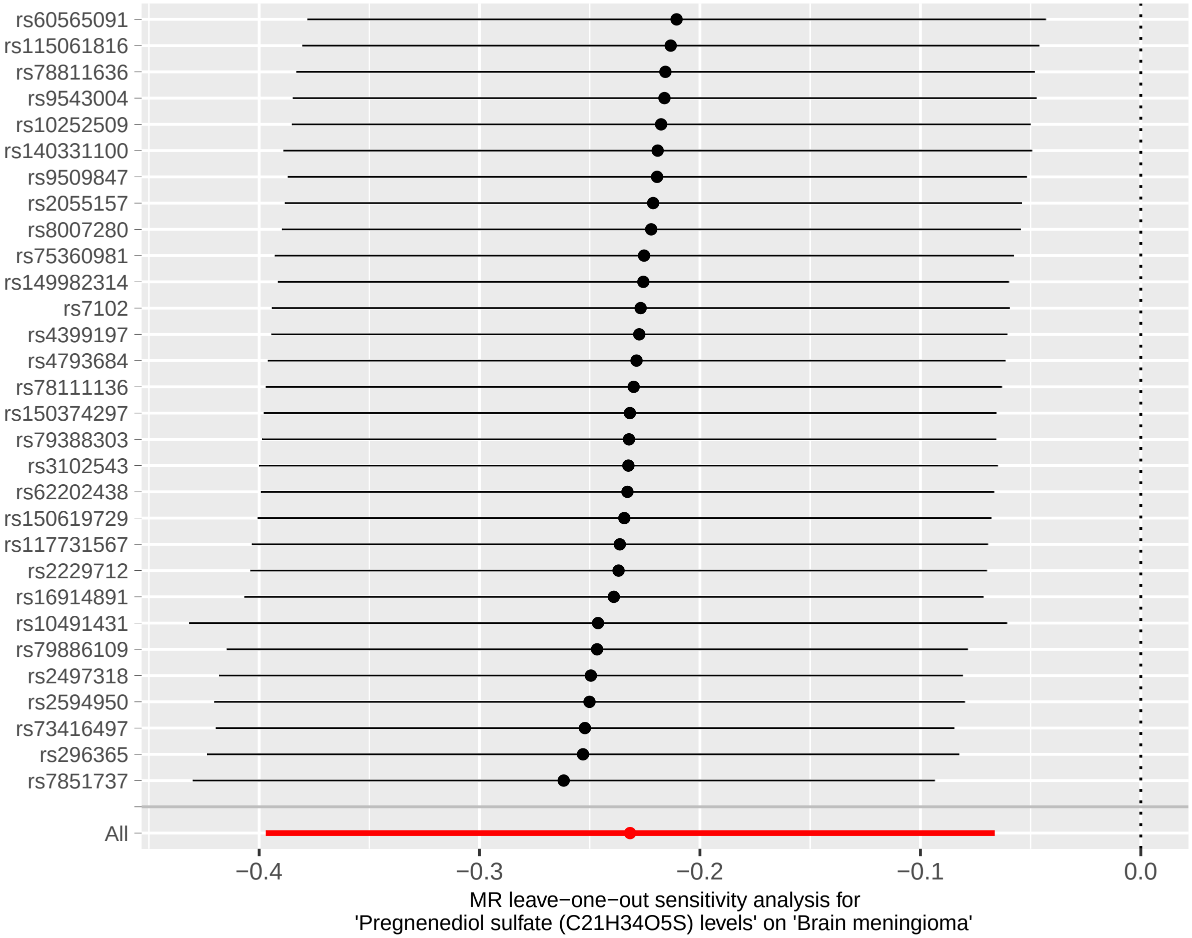
rs60565091
rs115061816
rs78811636
rs9543004
rs10252509
rs140331100
rs9509847
rs2055157
rs8007280
rs75360981
rs149982314
rs7102
rs4399197
rs4793684
rs78111136
rs150374297
rs79388303
rs3102543
rs62202438
rs150619729
rs117731567
rs2229712
rs16914891
rs10491431
rs79886109
rs2497318
rs2594950
rs73416497
rs296365
rs7851737
All
−0.4
−0.3
−0.2
−0.1
0.0
MR leave−one−out sensitivity analysis for
'Pregnenediol sulfate (C21H34O5S) levels' on 'Brain meningioma'

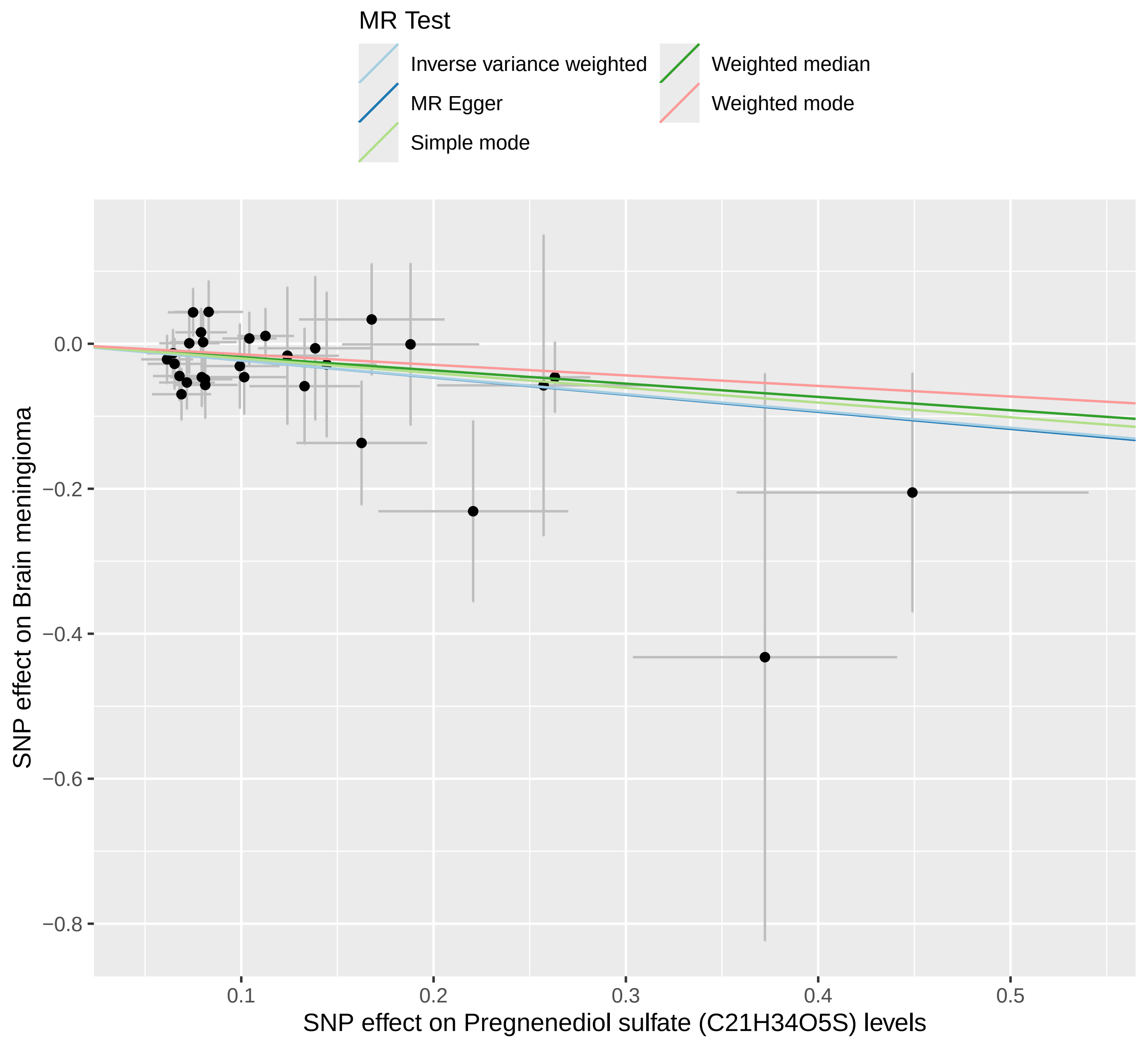
MR Test
Inverse variance weighted
MR Egger
Simple mode
Weighted median
Weighted mode
0.0
−0.2
−0.4
−0.6
−0.8
0.1
0.2
0.3
0.4
0.5
SNP effect on Brain meningioma
SNP effect on Pregnenediol sulfate (C21H34O5S) levels

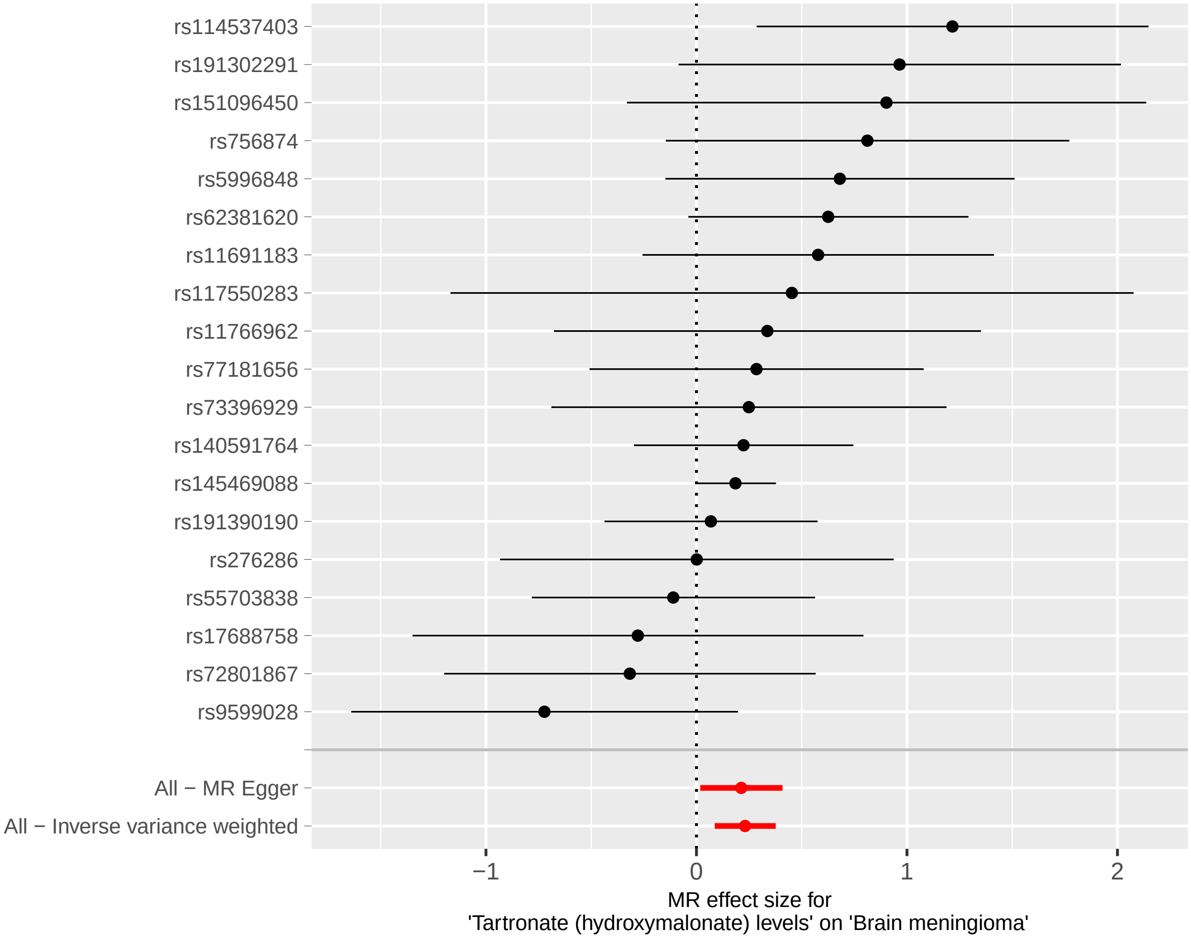
rs114537403
rs191302291
rs151096450
rs756874
rs5996848
rs62381620
rs11691183
rs117550283
rs11766962
rs77181656
rs73396929
rs140591764
rs145469088
rs191390190
rs276286
rs55703838
rs17688758
rs72801867
rs9599028
All − MR Egger
All − Inverse variance weighted
−1
0
1
2
MR effect size for
'Tartronate (hydroxymalonate) levels' on 'Brain meningioma'

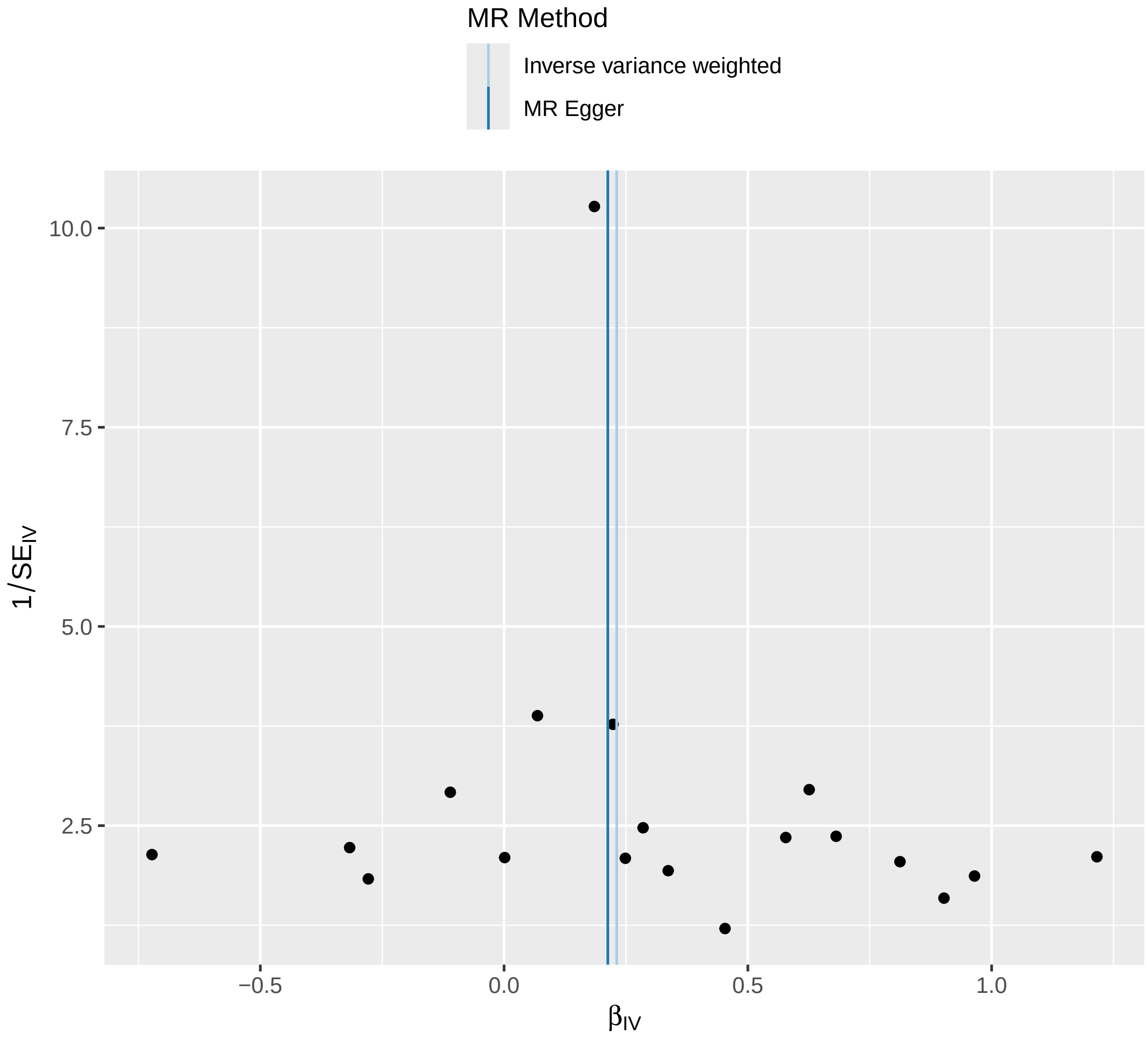
MR Method
Inverse variance weighted
MR Egger
10.0
7.5
5.0
2.5
1/SE$_{IV}$
−0.5
0.0
0.5
1.0
β$_{IV}$

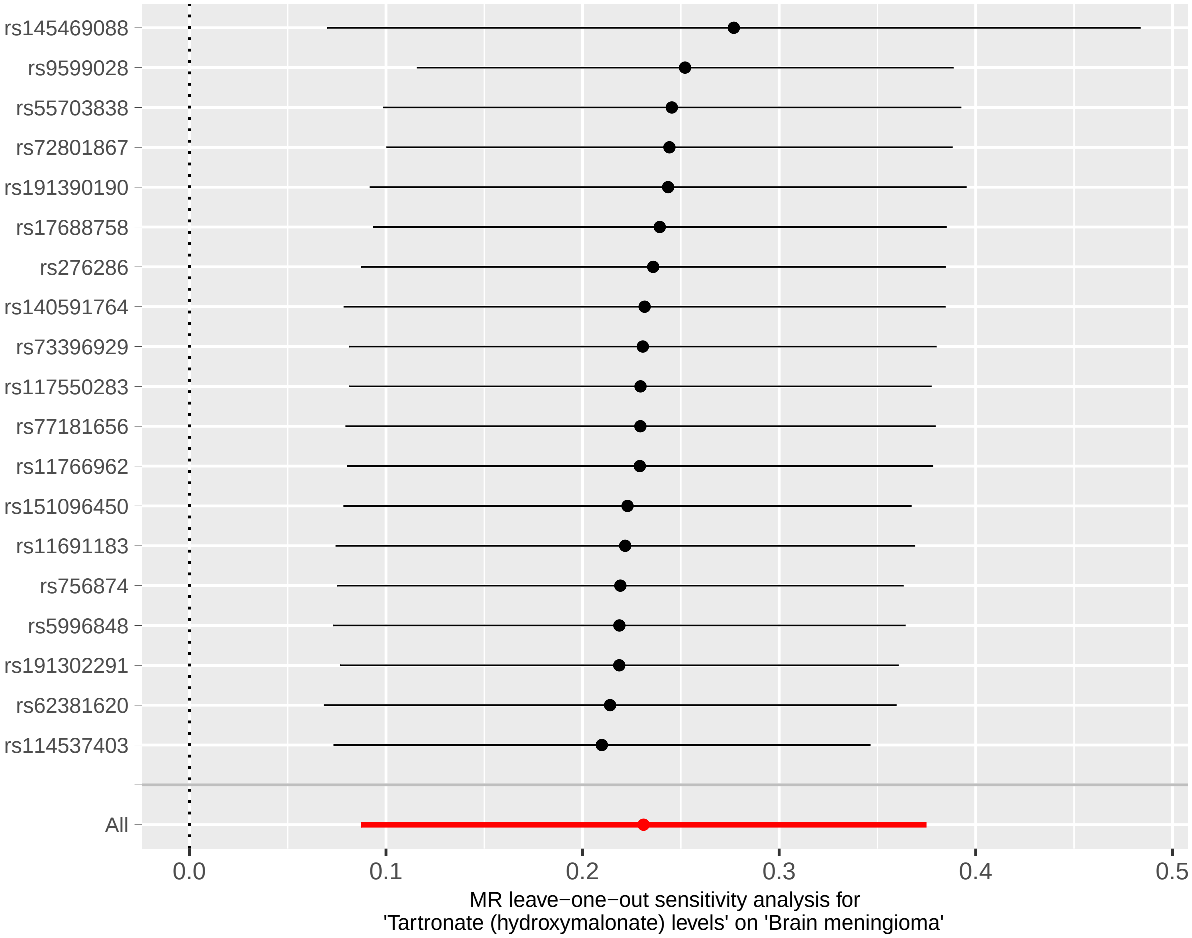
rs145469088
rs9599028
rs55703838
rs72801867
rs191390190
rs17688758
rs276286
rs140591764
rs73396929
rs117550283
rs77181656
rs11766962
rs151096450
rs11691183
rs756874
rs5996848
rs191302291
rs62381620
rs114537403
All
0.0
0.1
0.2
0.3
0.4
0.5
MR leave−one−out sensitivity analysis for
'Tartronate (hydroxymalonate) levels' on 'Brain meningioma'

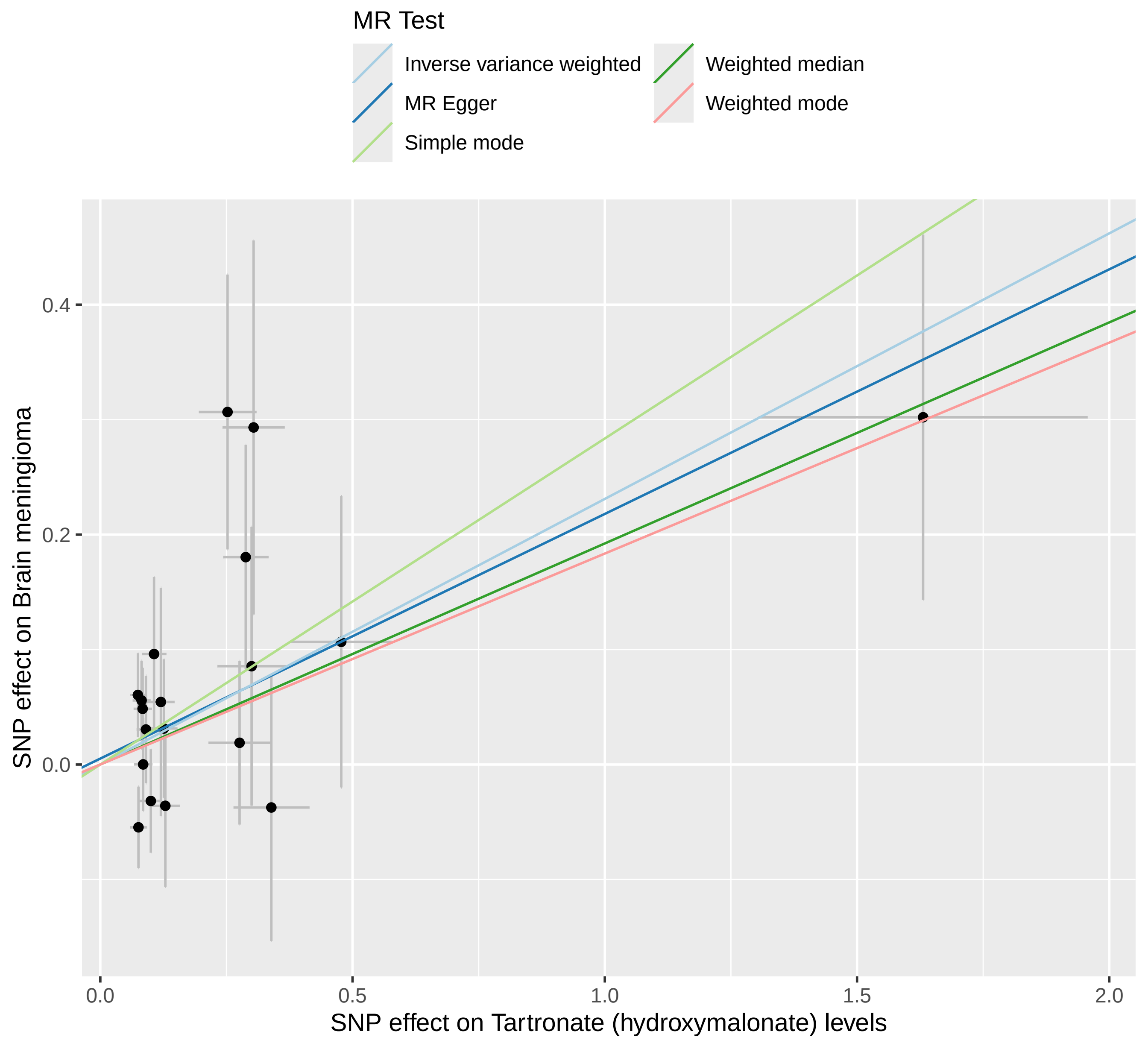
MR Test
Inverse variance weighted
MR Egger
Simple mode
Weighted median
Weighted mode
0.4
0.2
0.0
0.0
0.5
1.0
1.5
2.0
SNP effect on Brain meningioma
SNP effect on Tartronate (hydroxymalonate) levels

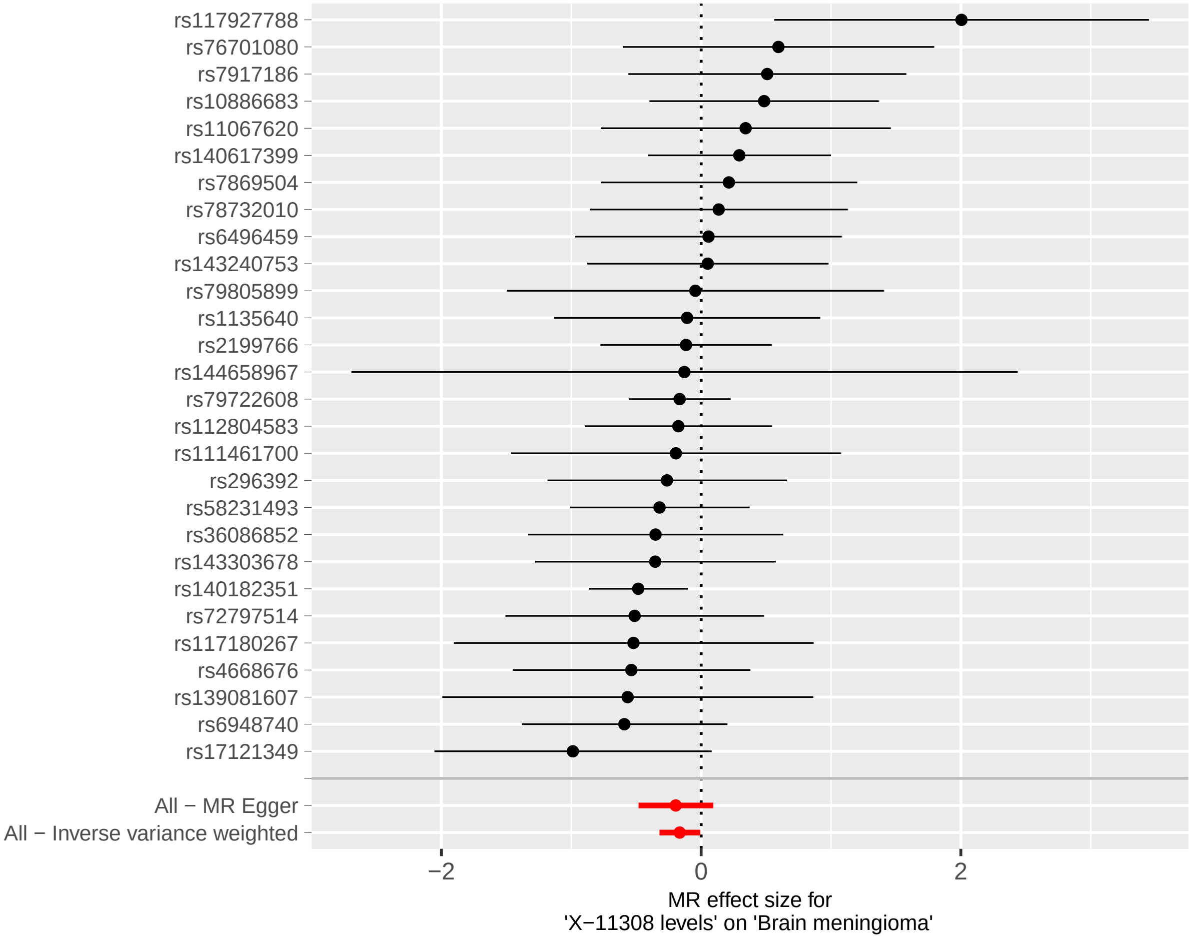
rs117927788
rs76701080
rs7917186
rs10886683
rs11067620
rs140617399
rs7869504
rs78732010
rs6496459
rs143240753
rs79805899
rs1135640
rs2199766
rs144658967
rs79722608
rs112804583
rs111461700
rs296392
rs58231493
rs36086852
rs143303678
rs140182351
rs72797514
rs117180267
rs4668676
rs139081607
rs6948740
rs17121349
All − MR Egger
All − Inverse variance weighted
−2
0
2
MR effect size for
'X−11308 levels' on 'Brain meningioma'

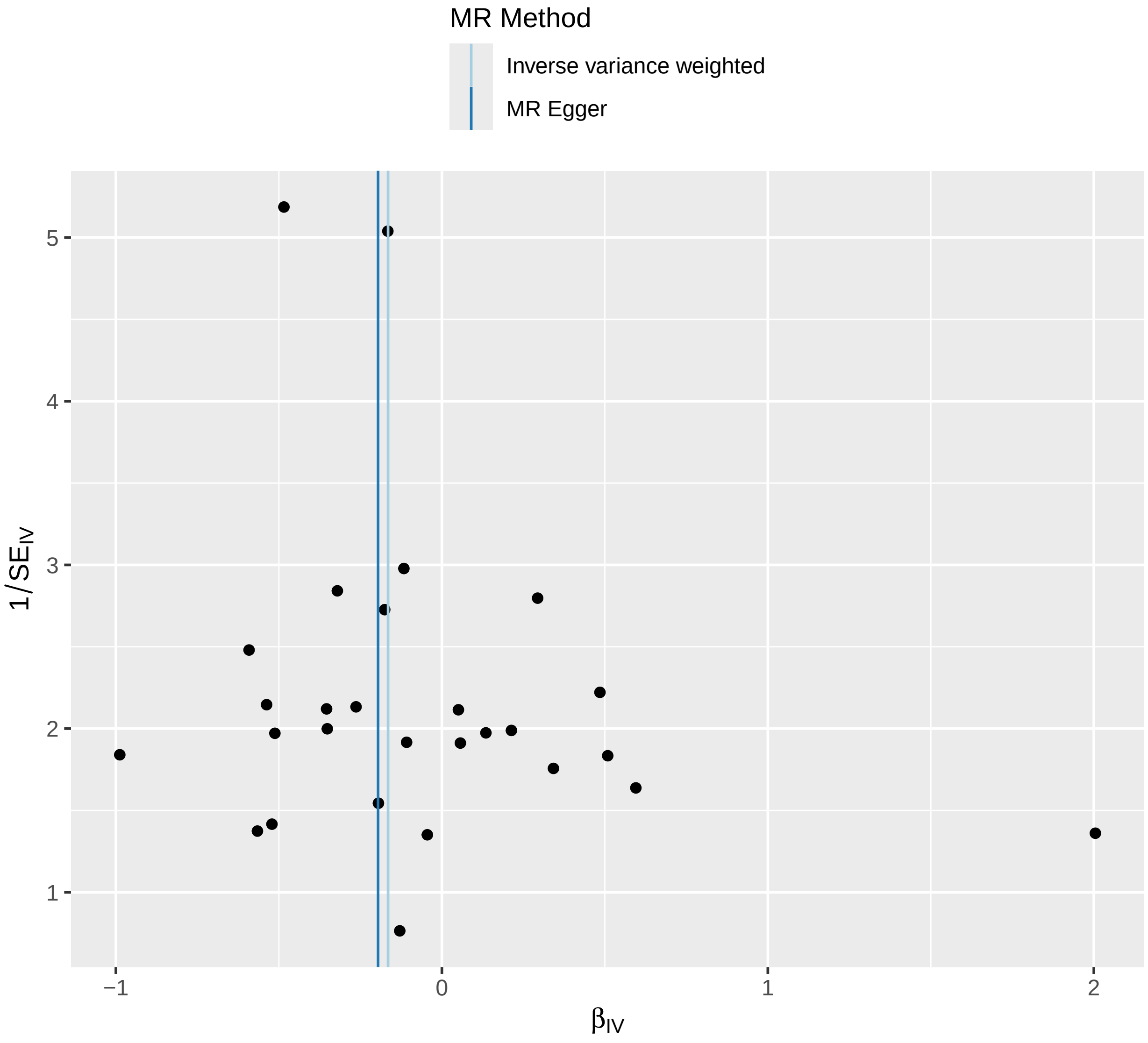

MR Method
Inverse variance weighted
MR Egger
1/SE_IV
β_IV
5
4
3
2
1
−1
0
1
2

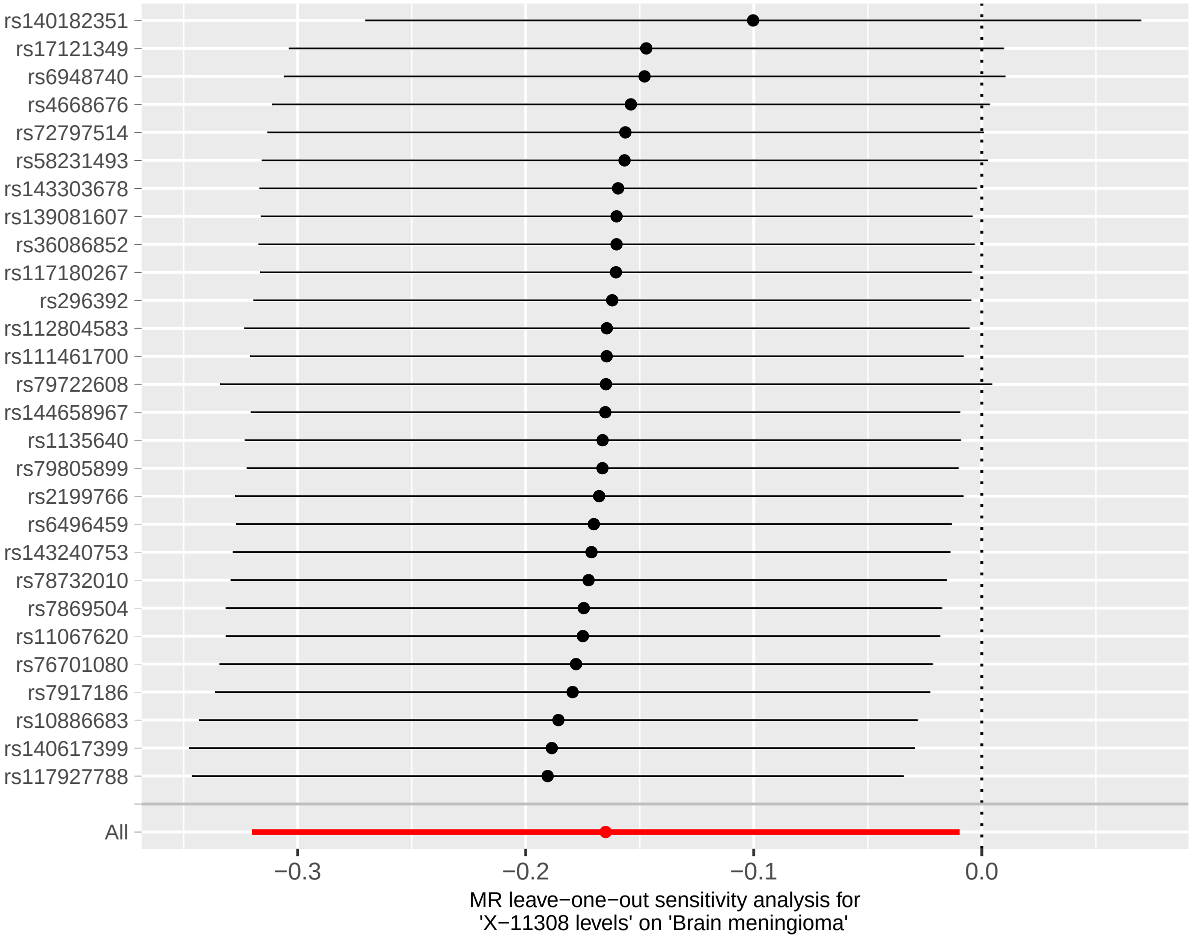
rs140182351
rs17121349
rs6948740
rs4668676
rs72797514
rs58231493
rs143303678
rs139081607
rs36086852
rs117180267
rs296392
rs112804583
rs111461700
rs79722608
rs144658967
rs1135640
rs79805899
rs2199766
rs6496459
rs143240753
rs78732010
rs7869504
rs11067620
rs76701080
rs7917186
rs10886683
rs140617399
rs117927788
All
−0.3
−0.2
−0.1
0.0
MR leave−one−out sensitivity analysis for
'X−11308 levels' on 'Brain meningioma'

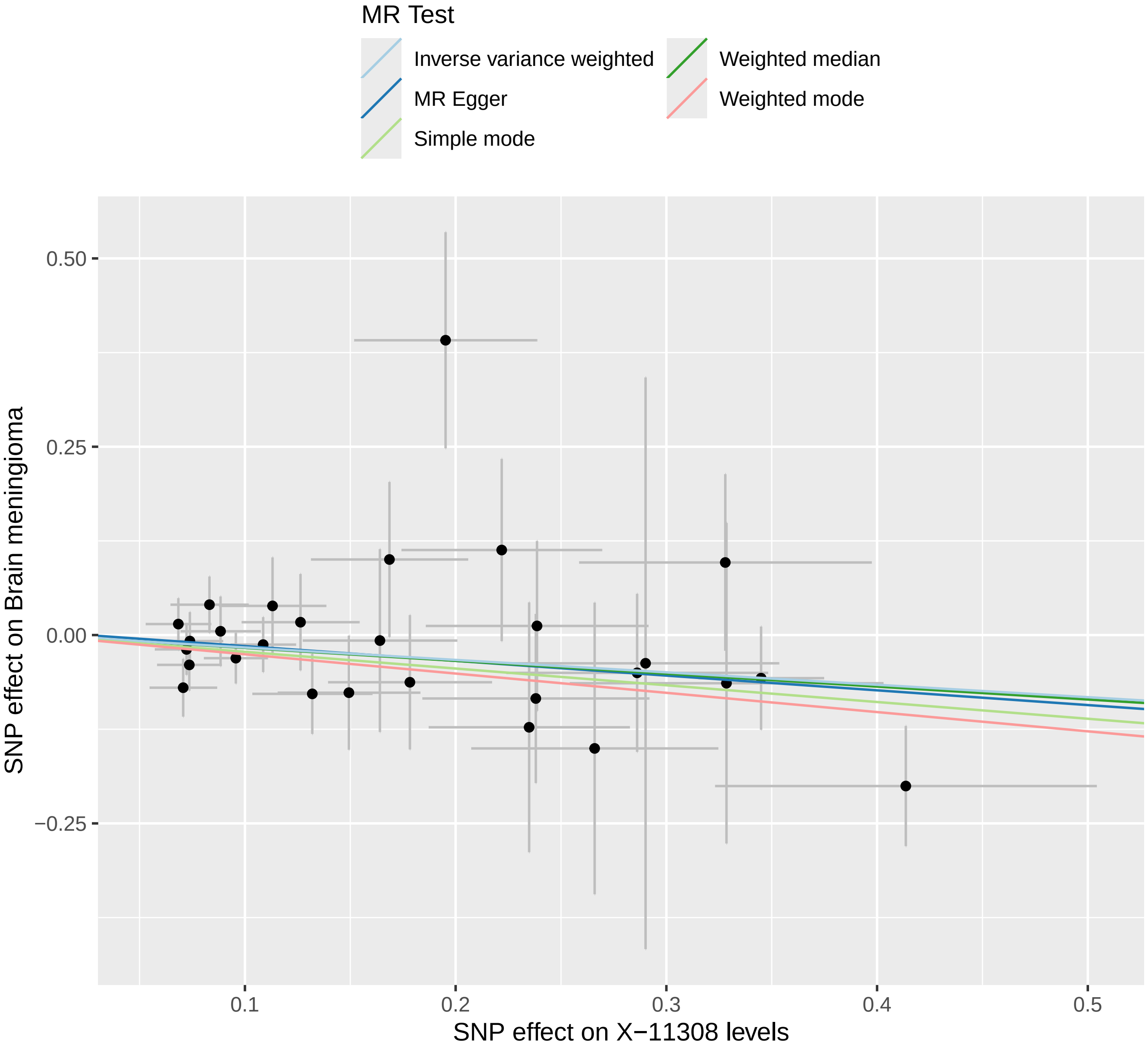
MR Test
Inverse variance weighted
MR Egger
Simple mode
Weighted median
Weighted mode
0.50
0.25
0.00
−0.25
0.1
0.2
0.3
0.4
0.5
SNP effect on Brain meningioma
SNP effect on X−11308 levels

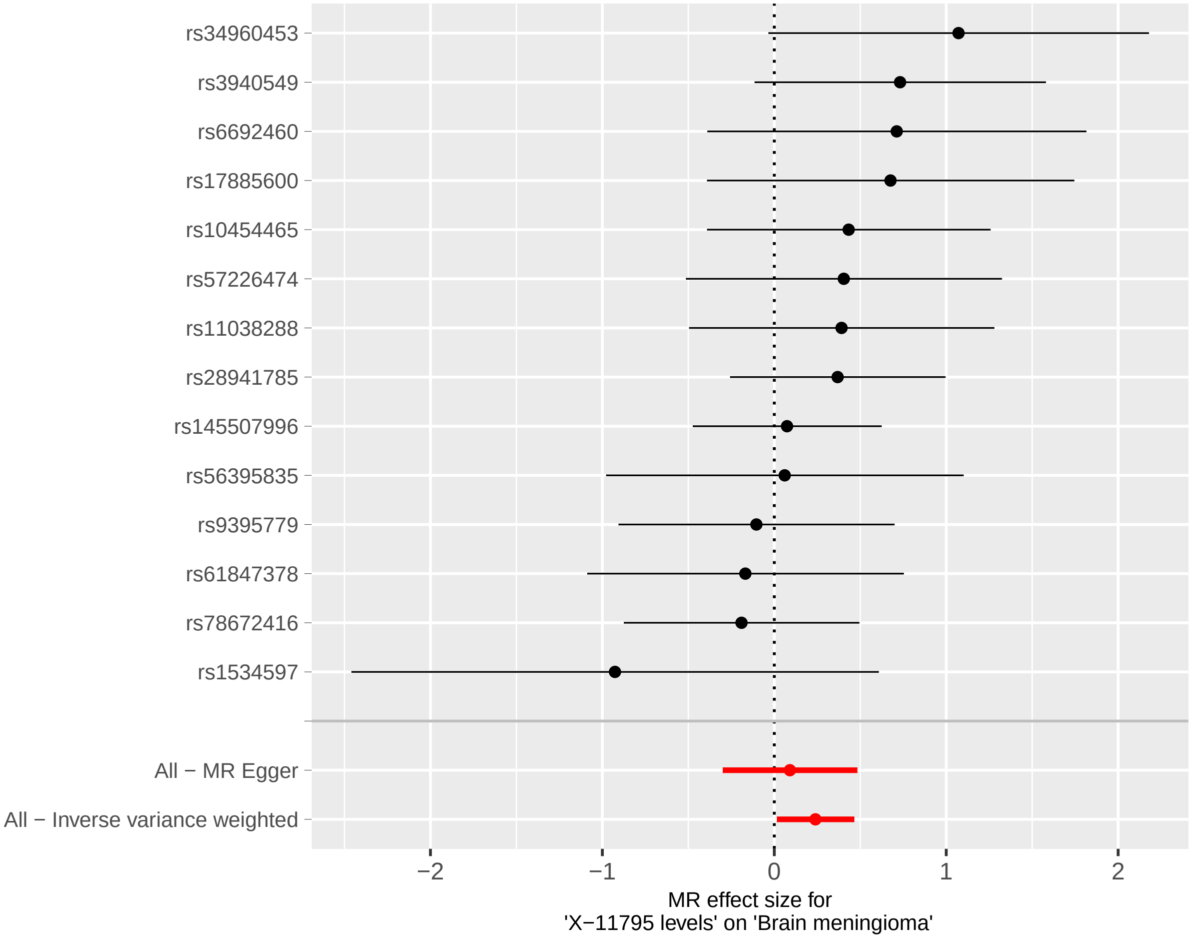

rs34960453
rs3940549
rs6692460
rs17885600
rs10454465
rs57226474
rs11038288
rs28941785
rs145507996
rs56395835
rs9395779
rs61847378
rs78672416
rs1534597
All − MR Egger
All − Inverse variance weighted
−2
−1
0
1
2
MR effect size for
'X−11795 levels' on 'Brain meningioma'

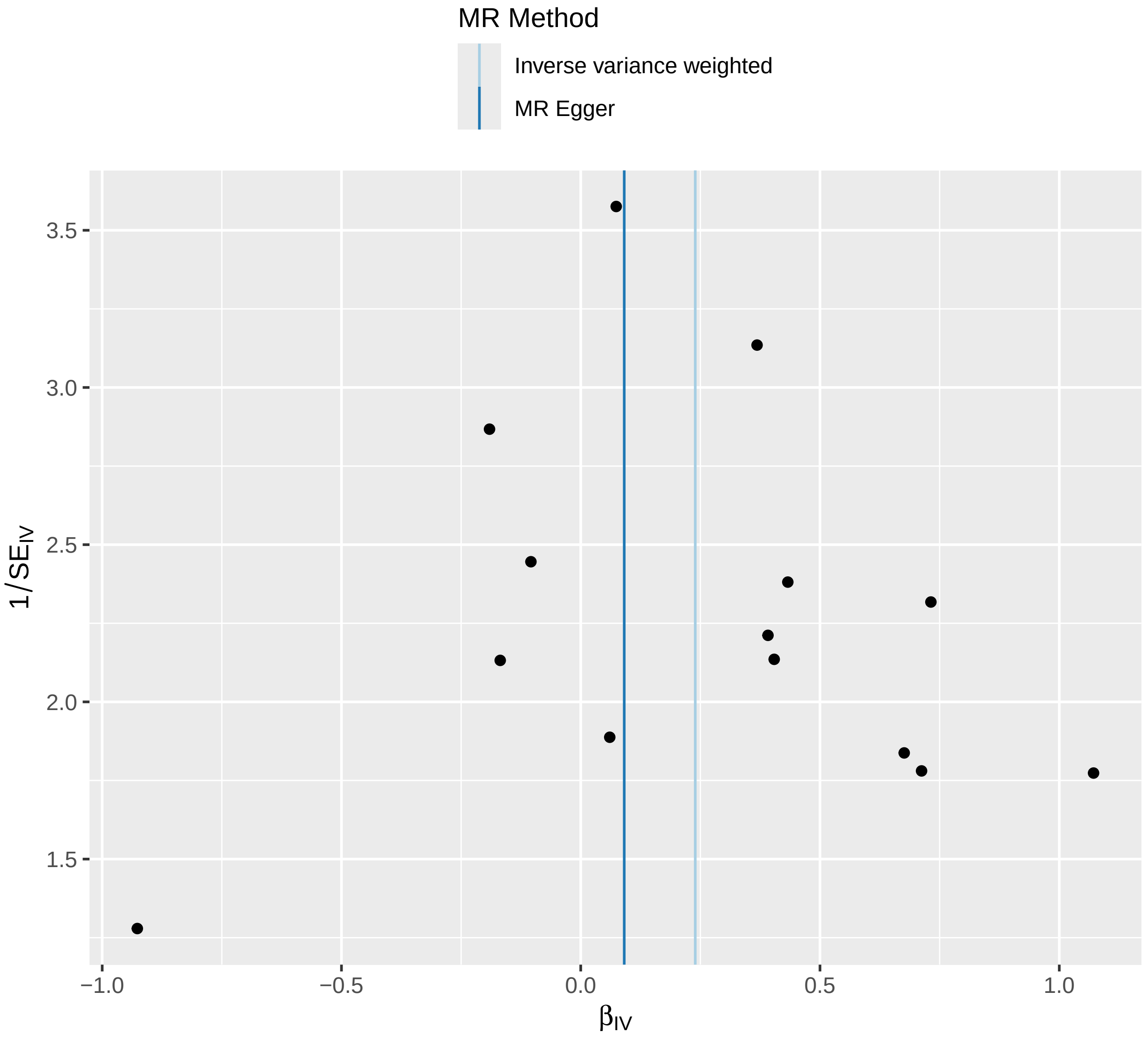

MR Method
Inverse variance weighted
MR Egger
3.5
3.0
2.5
2.0
1.5
1/SE_IV
−1.0
−0.5
0.0
0.5
1.0
β_IV

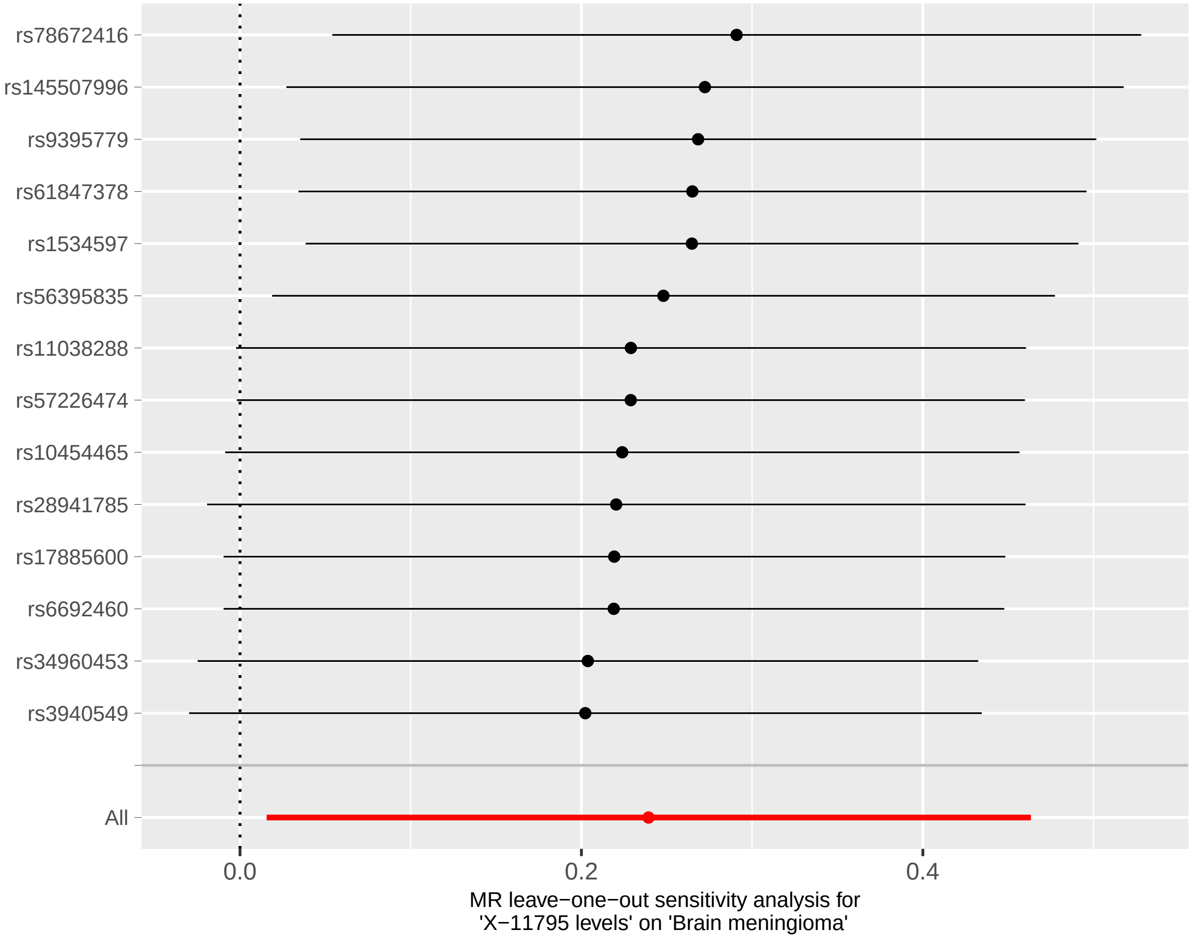
rs78672416
rs145507996
rs9395779
rs61847378
rs1534597
rs56395835
rs11038288
rs57226474
rs10454465
rs28941785
rs17885600
rs6692460
rs34960453
rs3940549
All
0.0
0.2
0.4
MR leave−one−out sensitivity analysis for
'X−11795 levels' on 'Brain meningioma'

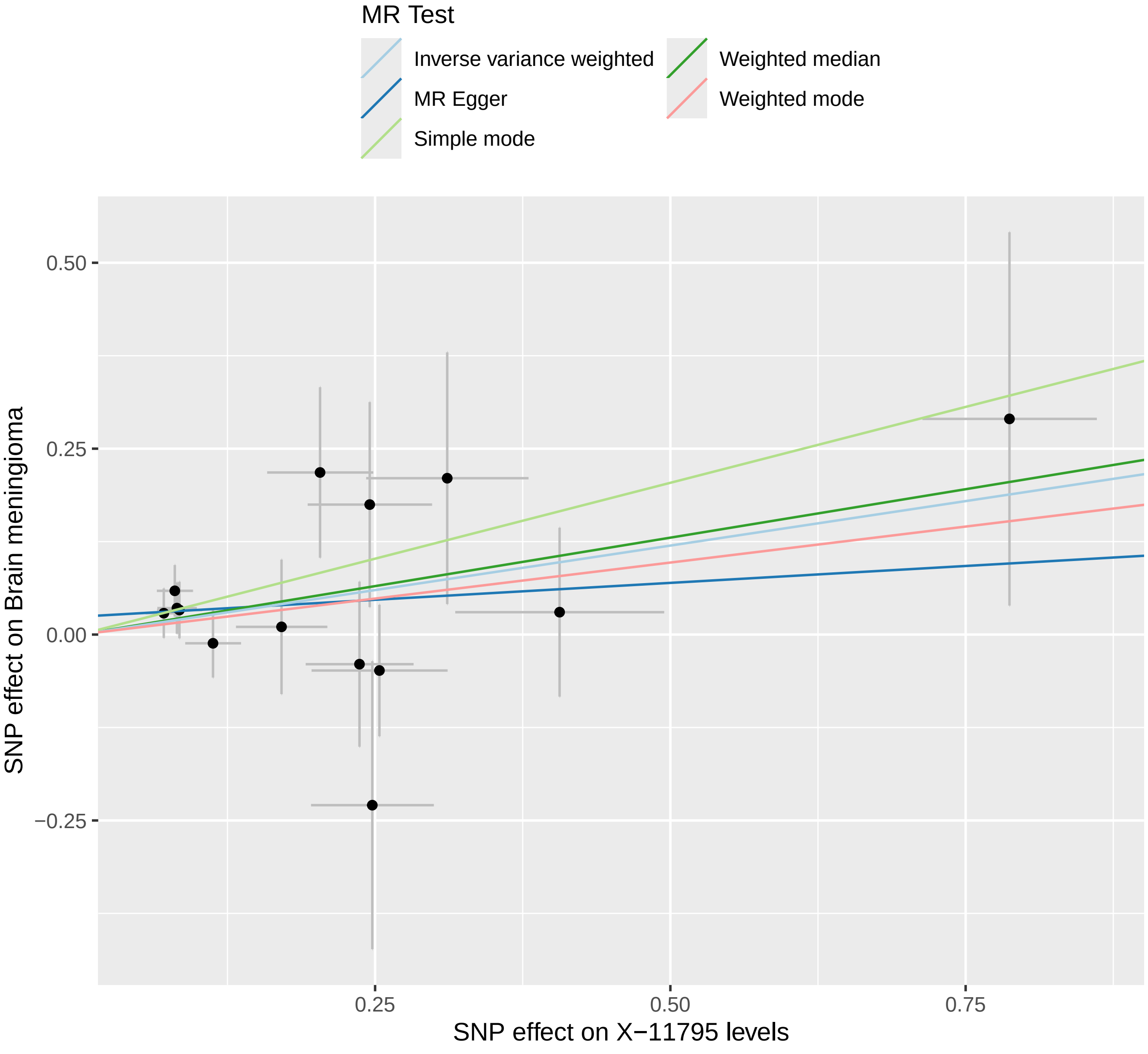
MR Test
Inverse variance weighted
MR Egger
Simple mode
Weighted median
Weighted mode
0.50
0.25
0.00
−0.25
SNP effect on Brain meningioma
0.25
0.50
0.75
SNP effect on X−11795 levels

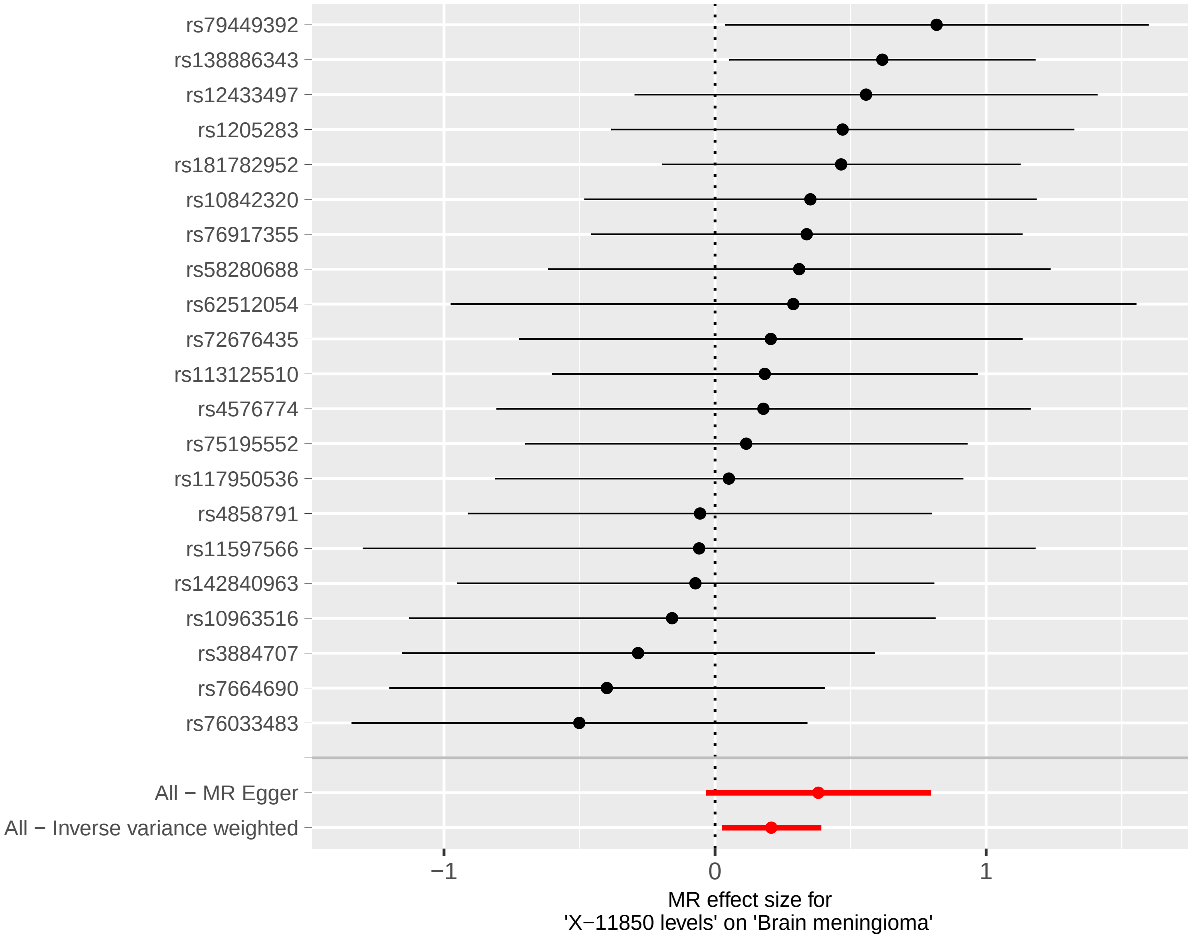
rs79449392
rs138886343
rs12433497
rs1205283
rs181782952
rs10842320
rs76917355
rs58280688
rs62512054
rs72676435
rs113125510
rs4576774
rs75195552
rs117950536
rs4858791
rs11597566
rs142840963
rs10963516
rs3884707
rs7664690
rs76033483
All − MR Egger
All − Inverse variance weighted
−1
0
1
MR effect size for
'X−11850 levels' on 'Brain meningioma'

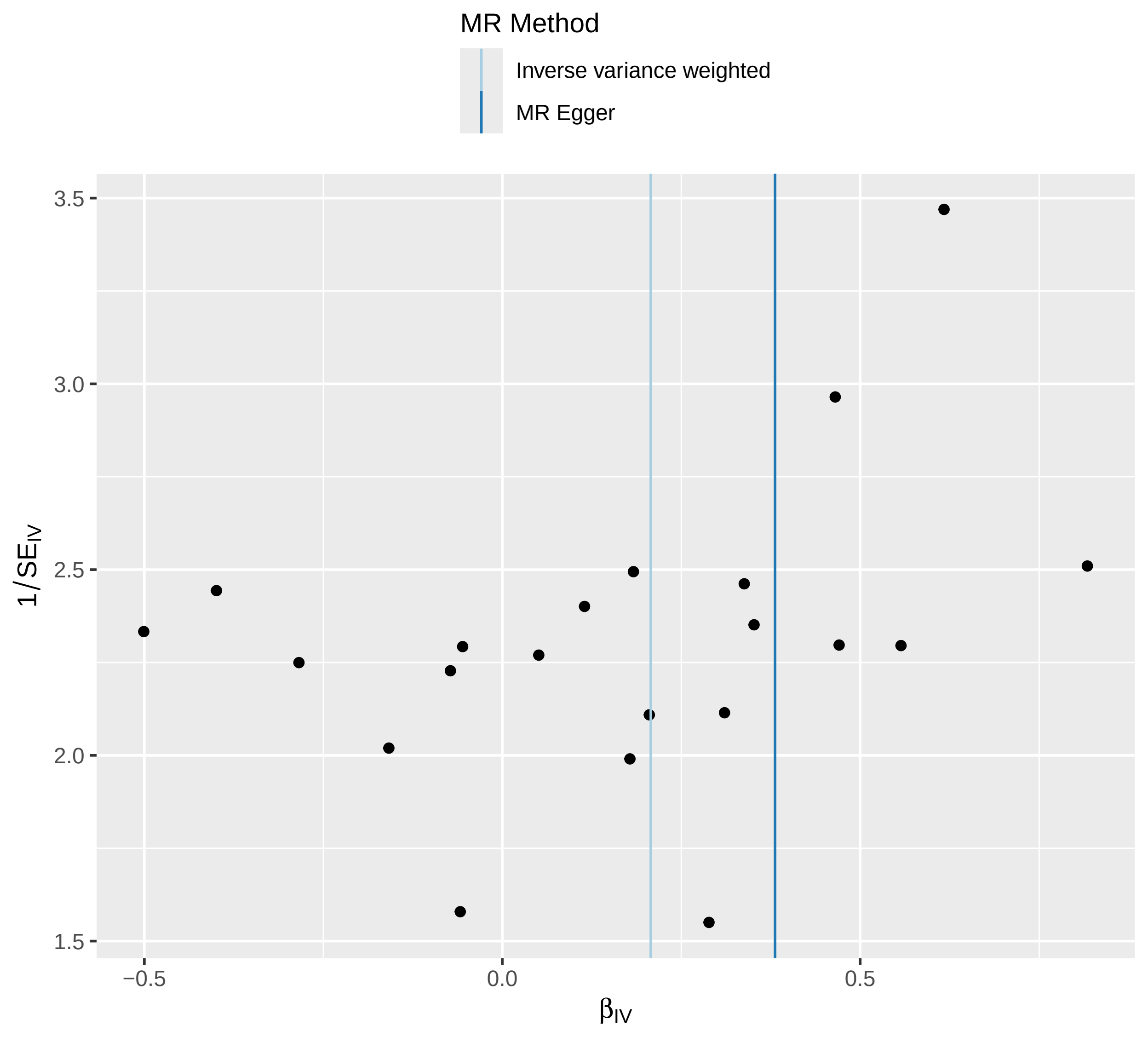

MR Method
Inverse variance weighted
MR Egger
3.5
3.0
2.5
2.0
1.5
$1/SE_{IV}$
−0.5
0.0
0.5
β_{IV}

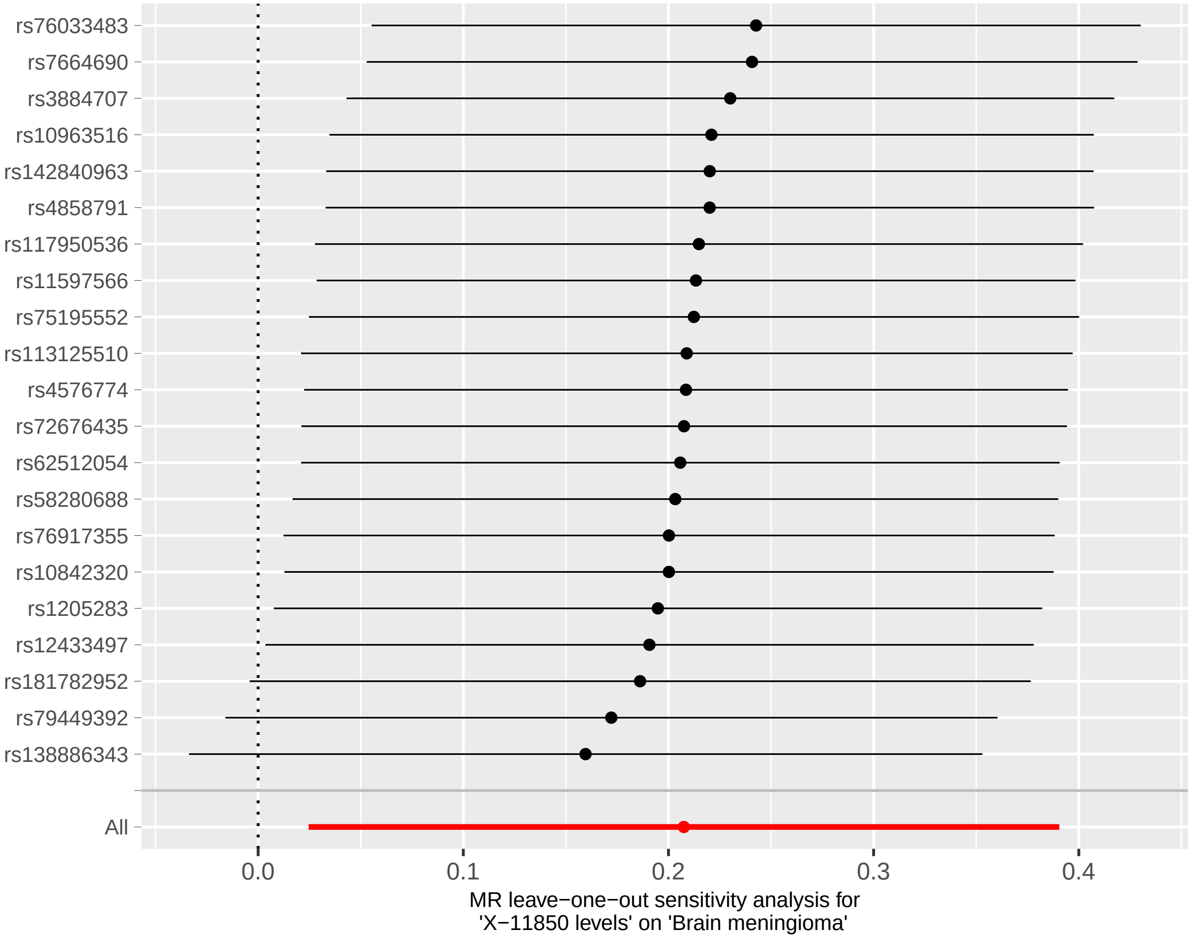
rs76033483
rs7664690
rs3884707
rs10963516
rs142840963
rs4858791
rs117950536
rs11597566
rs75195552
rs113125510
rs4576774
rs72676435
rs62512054
rs58280688
rs76917355
rs10842320
rs1205283
rs12433497
rs181782952
rs79449392
rs138886343
All
0.0
0.1
0.2
0.3
0.4
MR leave−one−out sensitivity analysis for
'X−11850 levels' on 'Brain meningioma'

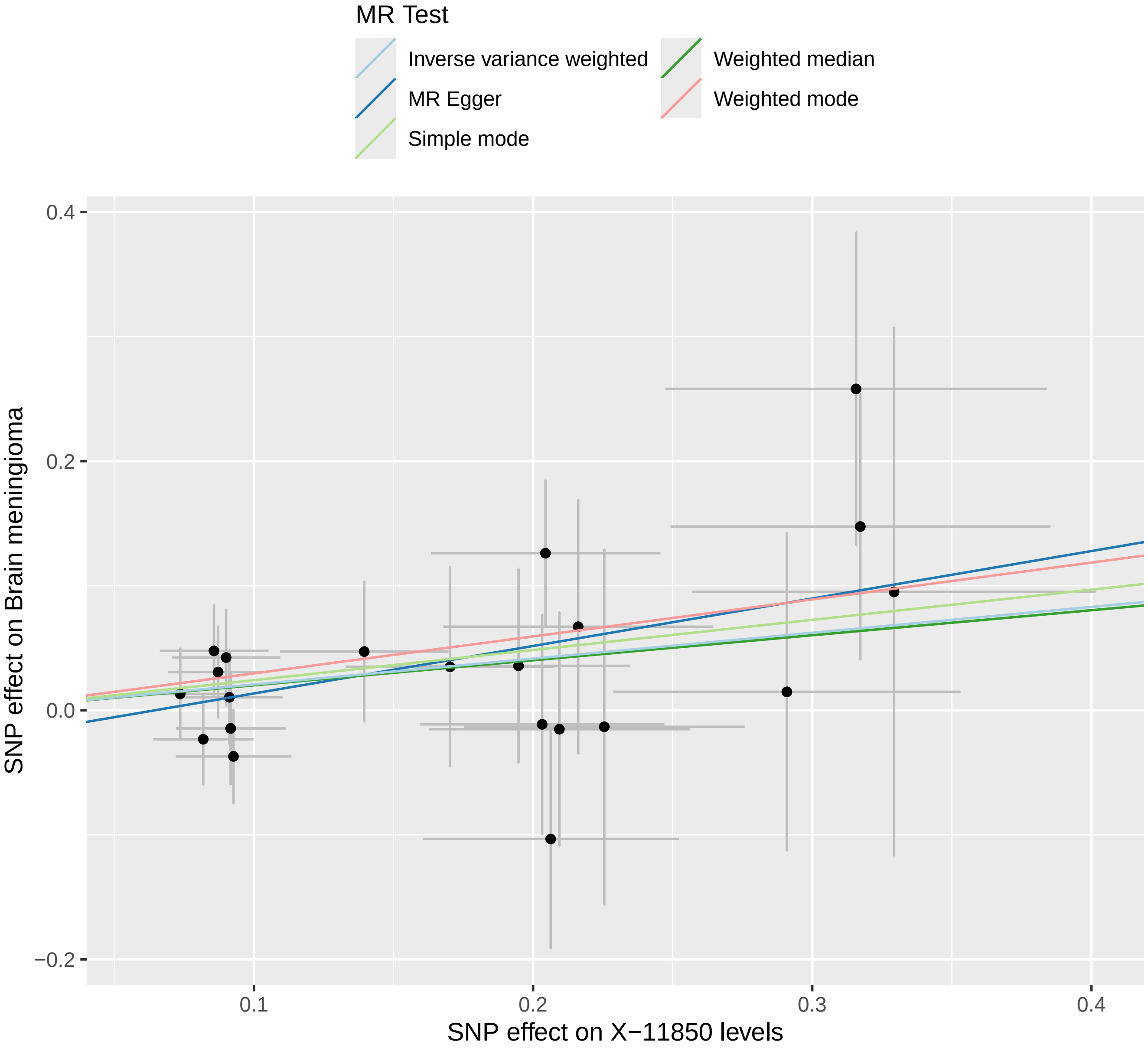

MR Test
Inverse variance weighted
MR Egger
Simple mode
Weighted median
Weighted mode
SNP effect on Brain meningioma
SNP effect on X−11850 levels
0.4
0.2
0.0
−0.2
0.1
0.2
0.3
0.4

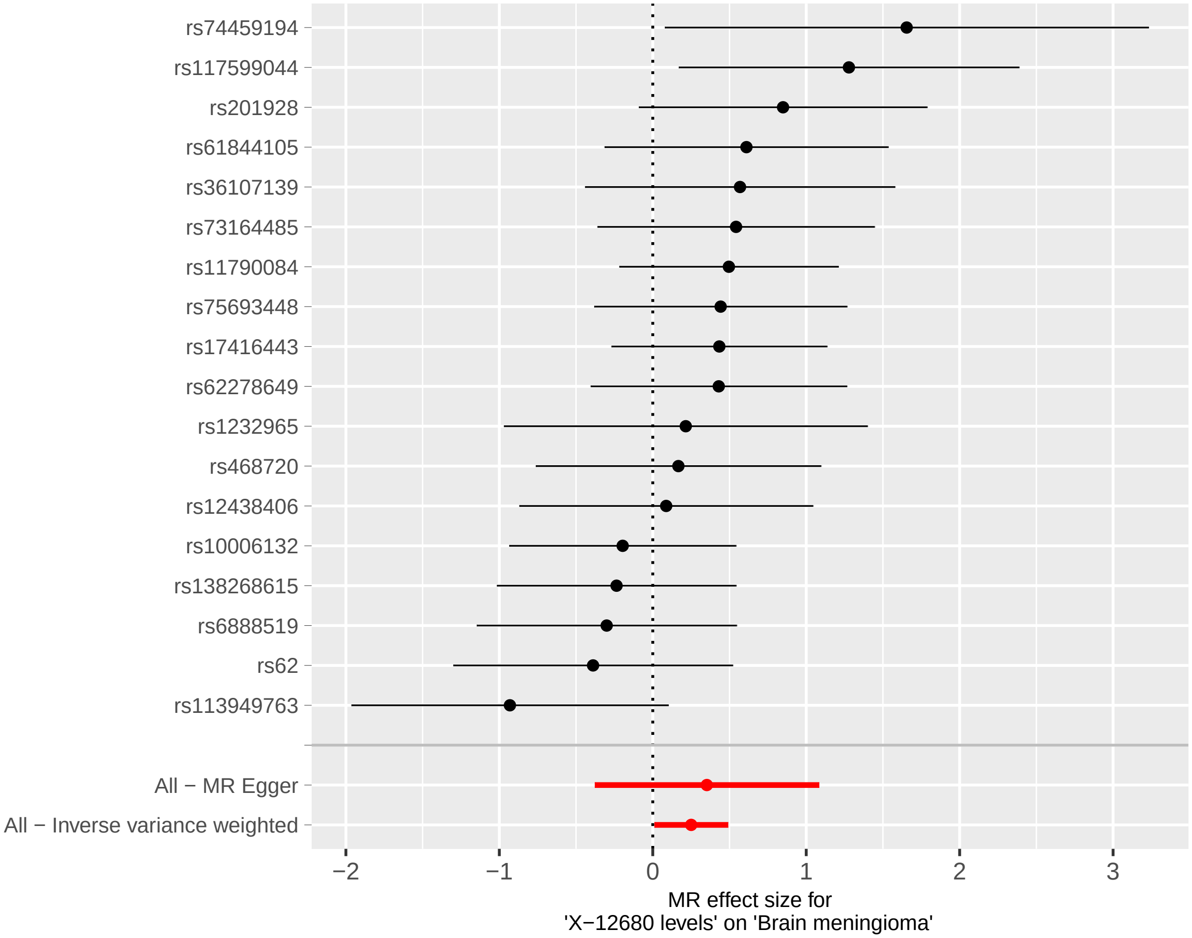
rs74459194
rs117599044
rs201928
rs61844105
rs36107139
rs73164485
rs11790084
rs75693448
rs17416443
rs62278649
rs1232965
rs468720
rs12438406
rs10006132
rs138268615
rs6888519
rs62
rs113949763
All − MR Egger
All − Inverse variance weighted
−2
−1
0
1
2
3
MR effect size for
'X−12680 levels' on 'Brain meningioma'

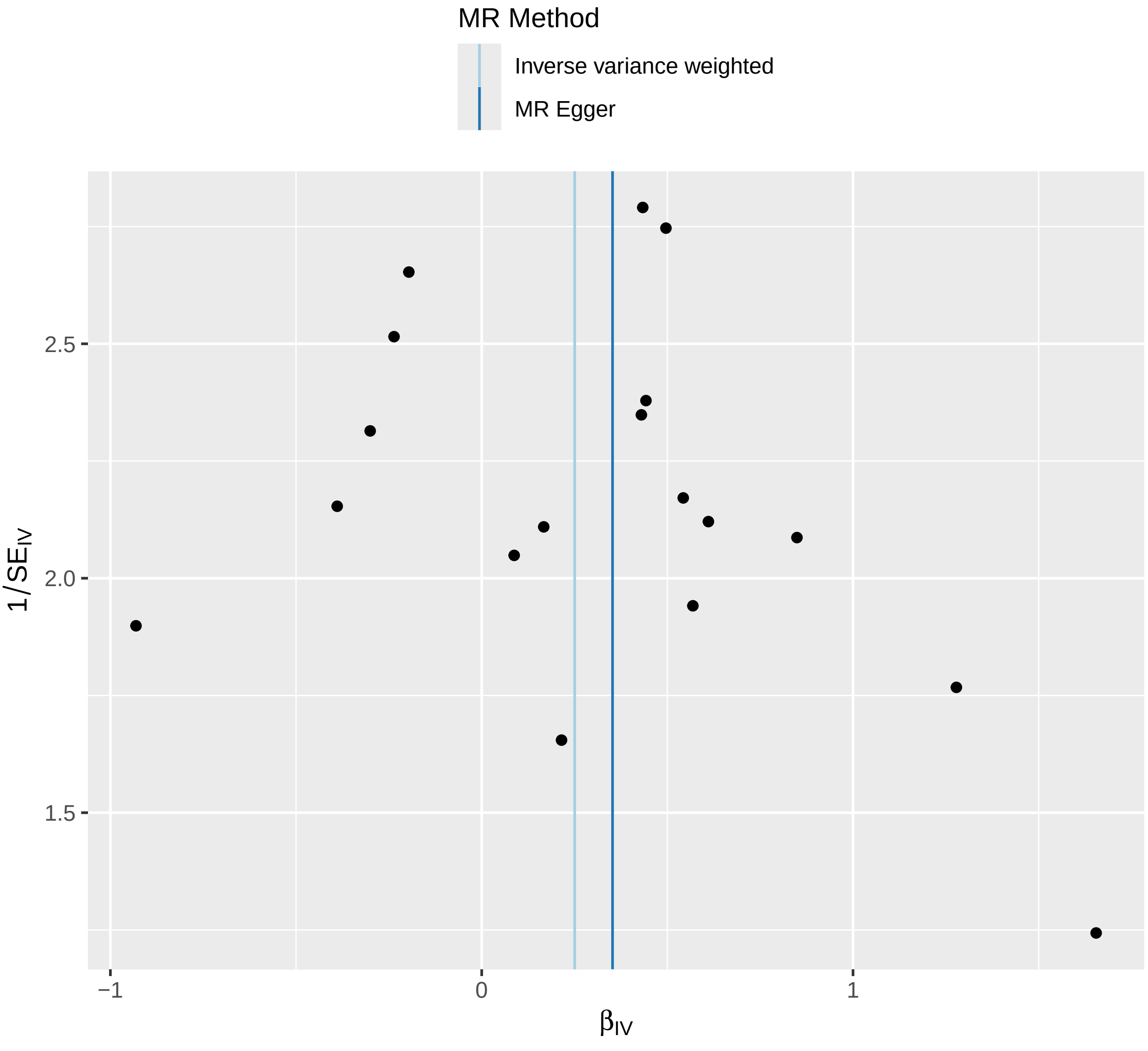

MR Method
Inverse variance weighted
MR Egger
2.5
2.0
1.5
$1/SE_{IV}$
−1
0
1
β_{IV}

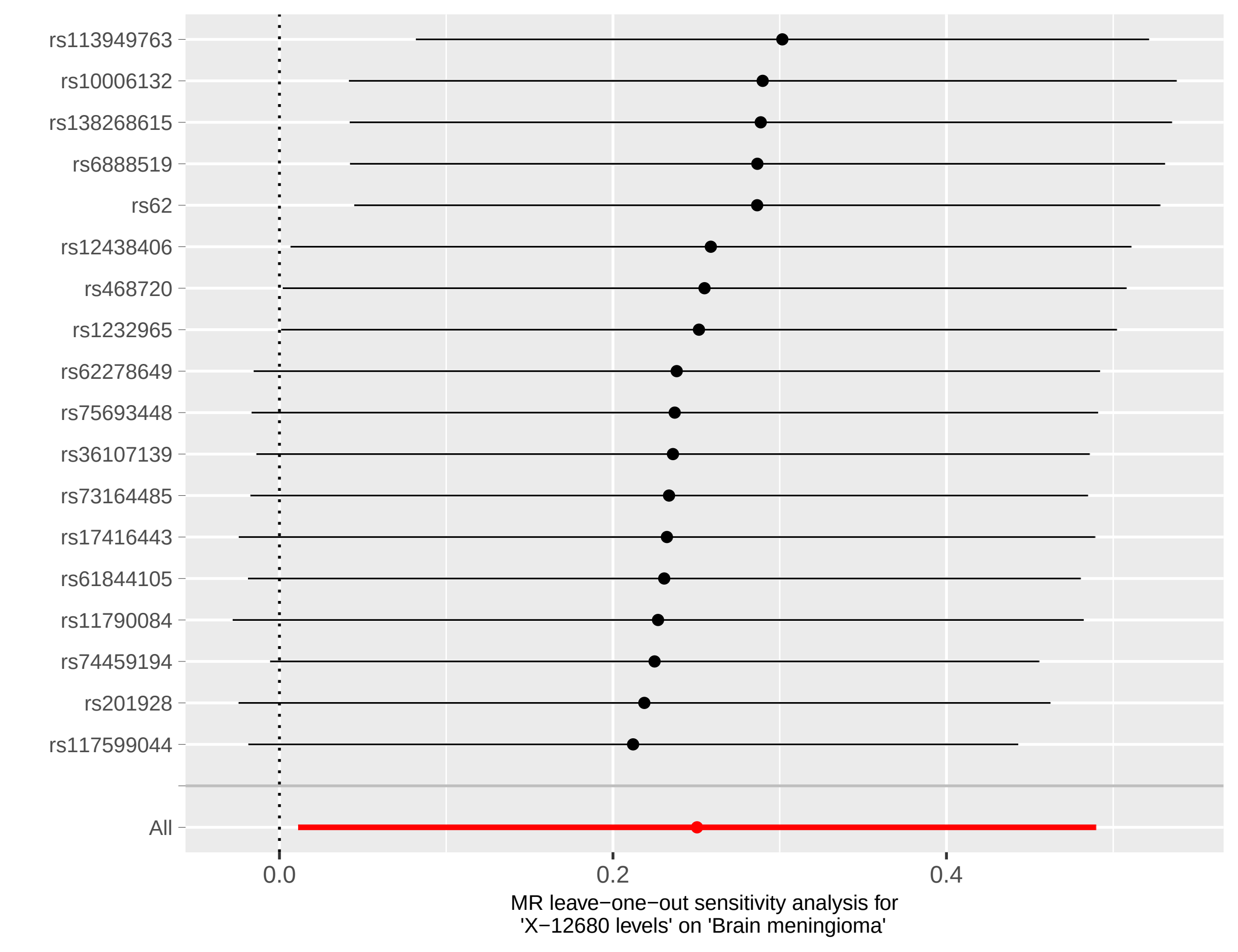

rs113949763
rs10006132
rs138268615
rs6888519
rs62
rs12438406
rs468720
rs1232965
rs62278649
rs75693448
rs36107139
rs73164485
rs17416443
rs61844105
rs11790084
rs74459194
rs201928
rs117599044
All
0.0
0.2
0.4
MR leave−one−out sensitivity analysis for
'X−12680 levels' on 'Brain meningioma'

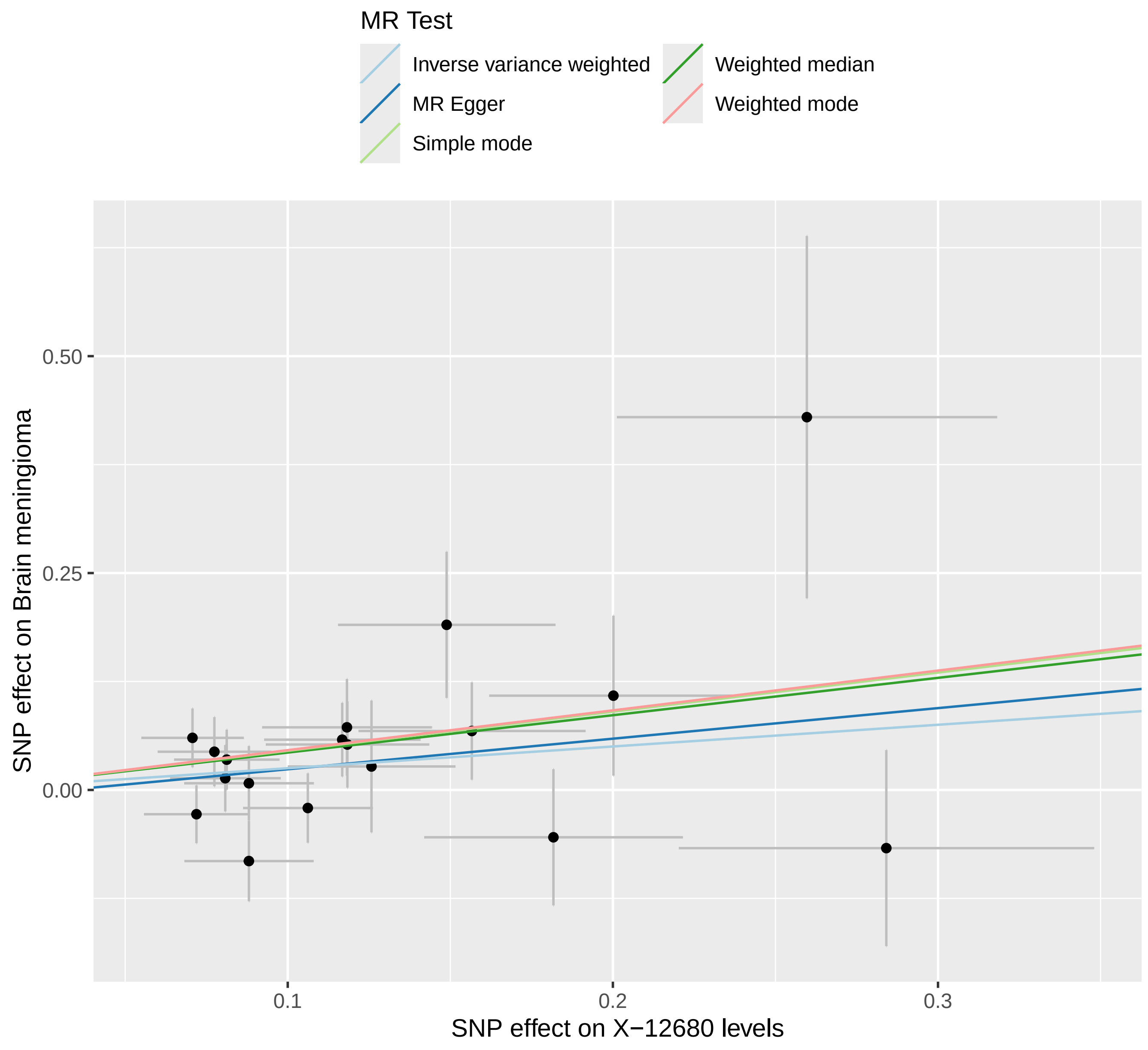
MR Test
Inverse variance weighted
MR Egger
Simple mode
Weighted median
Weighted mode
SNP effect on Brain meningioma
0.50
0.25
0.00
0.1
0.2
0.3
SNP effect on X−12680 levels

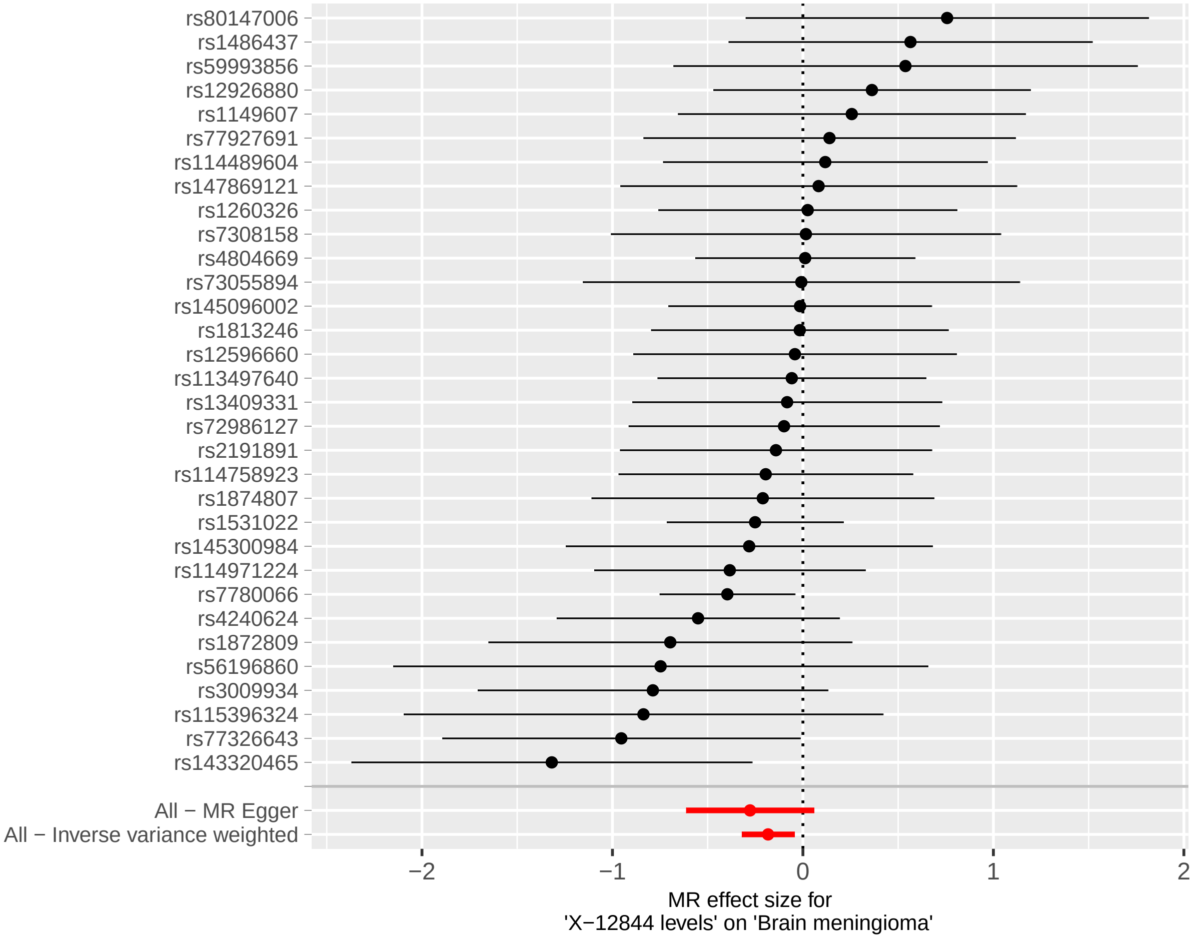
rs80147006
rs1486437
rs59993856
rs12926880
rs1149607
rs77927691
rs114489604
rs147869121
rs1260326
rs7308158
rs4804669
rs73055894
rs145096002
rs1813246
rs12596660
rs113497640
rs13409331
rs72986127
rs2191891
rs114758923
rs1874807
rs1531022
rs145300984
rs114971224
rs7780066
rs4240624
rs1872809
rs56196860
rs3009934
rs115396324
rs77326643
rs143320465
All − MR Egger
All − Inverse variance weighted
−2
−1
0
1
2
MR effect size for
'X−12844 levels' on 'Brain meningioma'

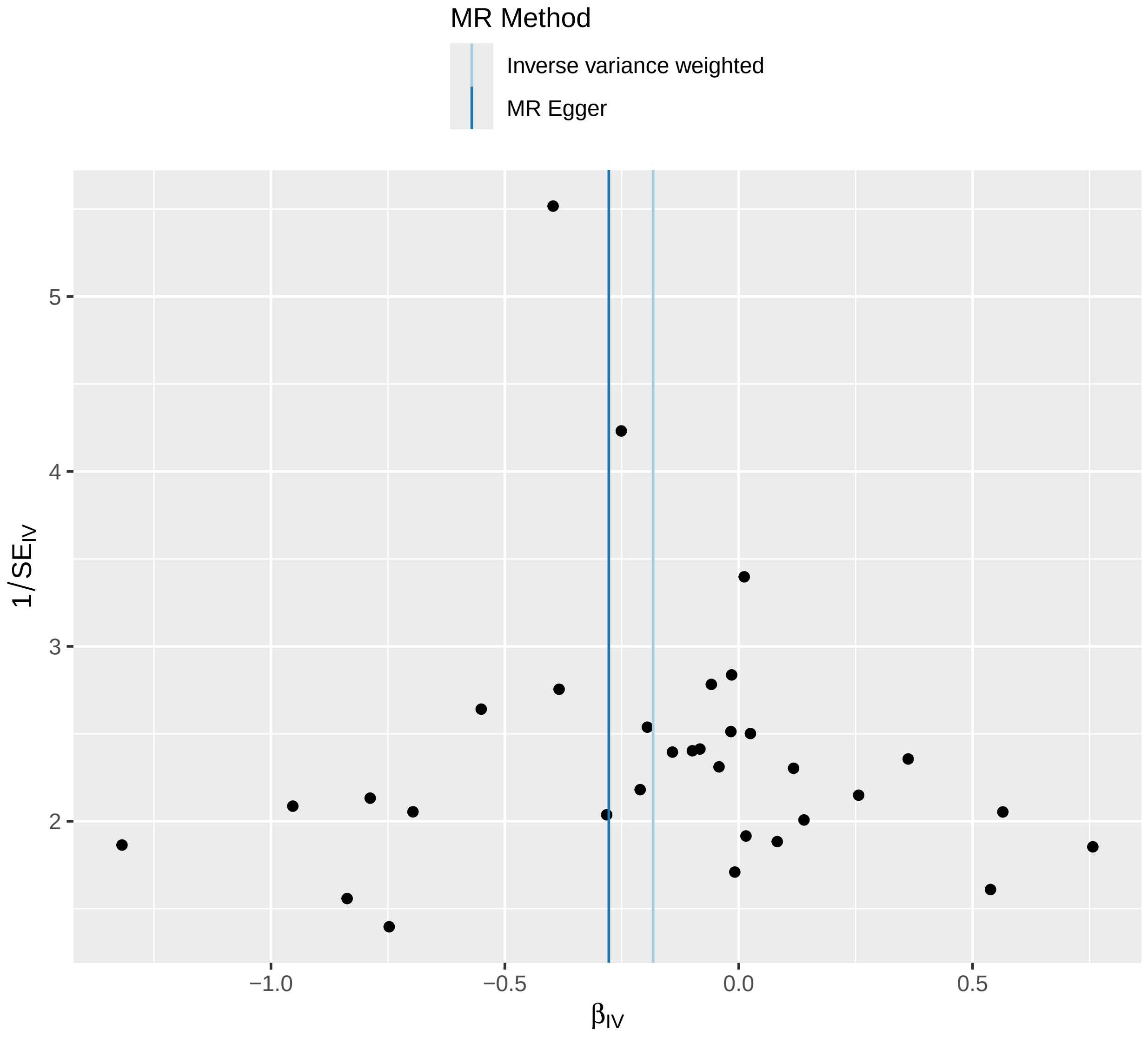
MR Method
Inverse variance weighted
MR Egger
5
4
3
2
−1.0
−0.5
0.0
0.5
$1/SE_{IV}$
β_{IV}

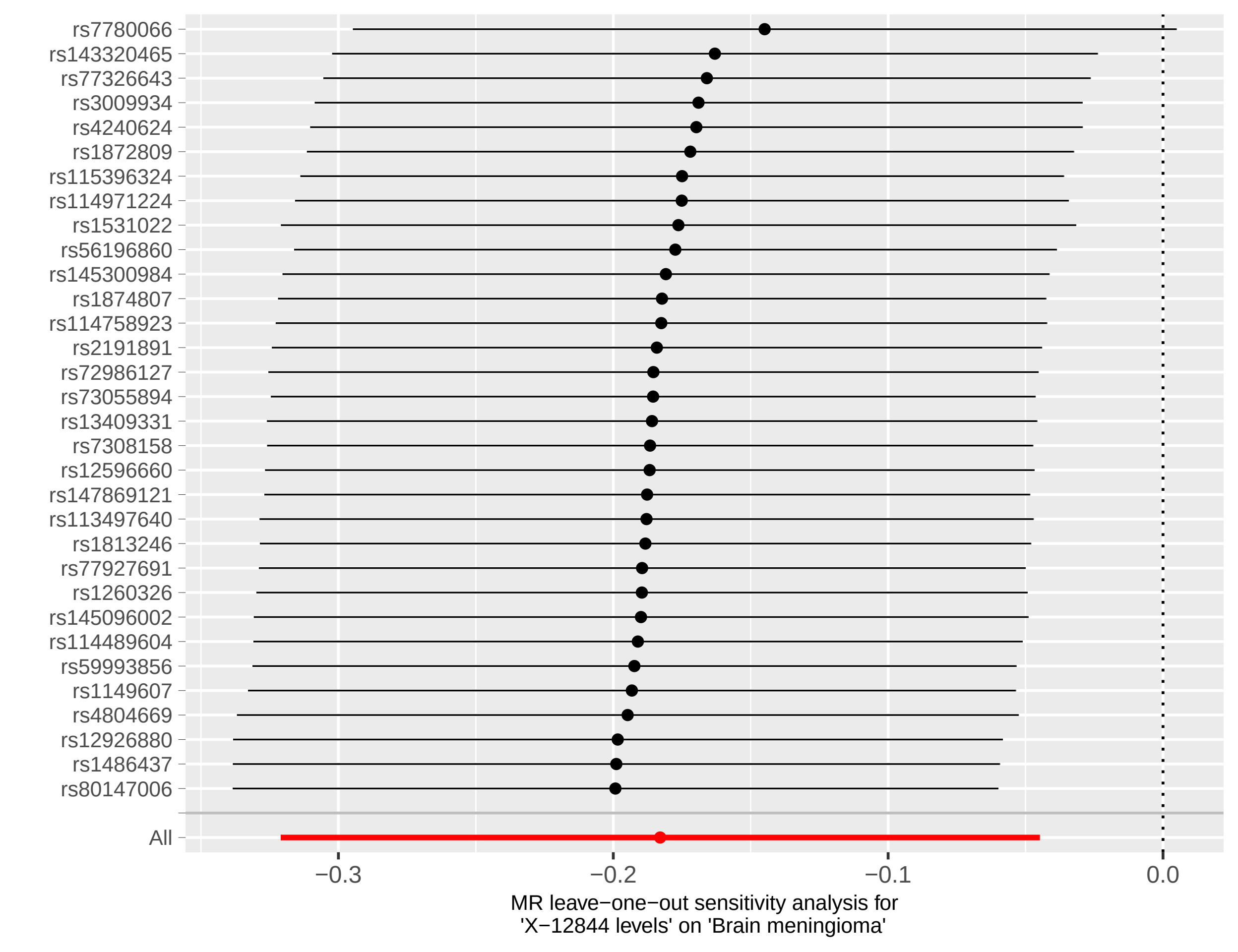
rs7780066
rs143320465
rs77326643
rs3009934
rs4240624
rs1872809
rs115396324
rs114971224
rs1531022
rs56196860
rs145300984
rs1874807
rs114758923
rs2191891
rs72986127
rs73055894
rs13409331
rs7308158
rs12596660
rs147869121
rs113497640
rs1813246
rs77927691
rs1260326
rs145096002
rs114489604
rs59993856
rs1149607
rs4804669
rs12926880
rs1486437
rs80147006
All
−0.3
−0.2
−0.1
0.0
MR leave−one−out sensitivity analysis for
'X−12844 levels' on 'Brain meningioma'

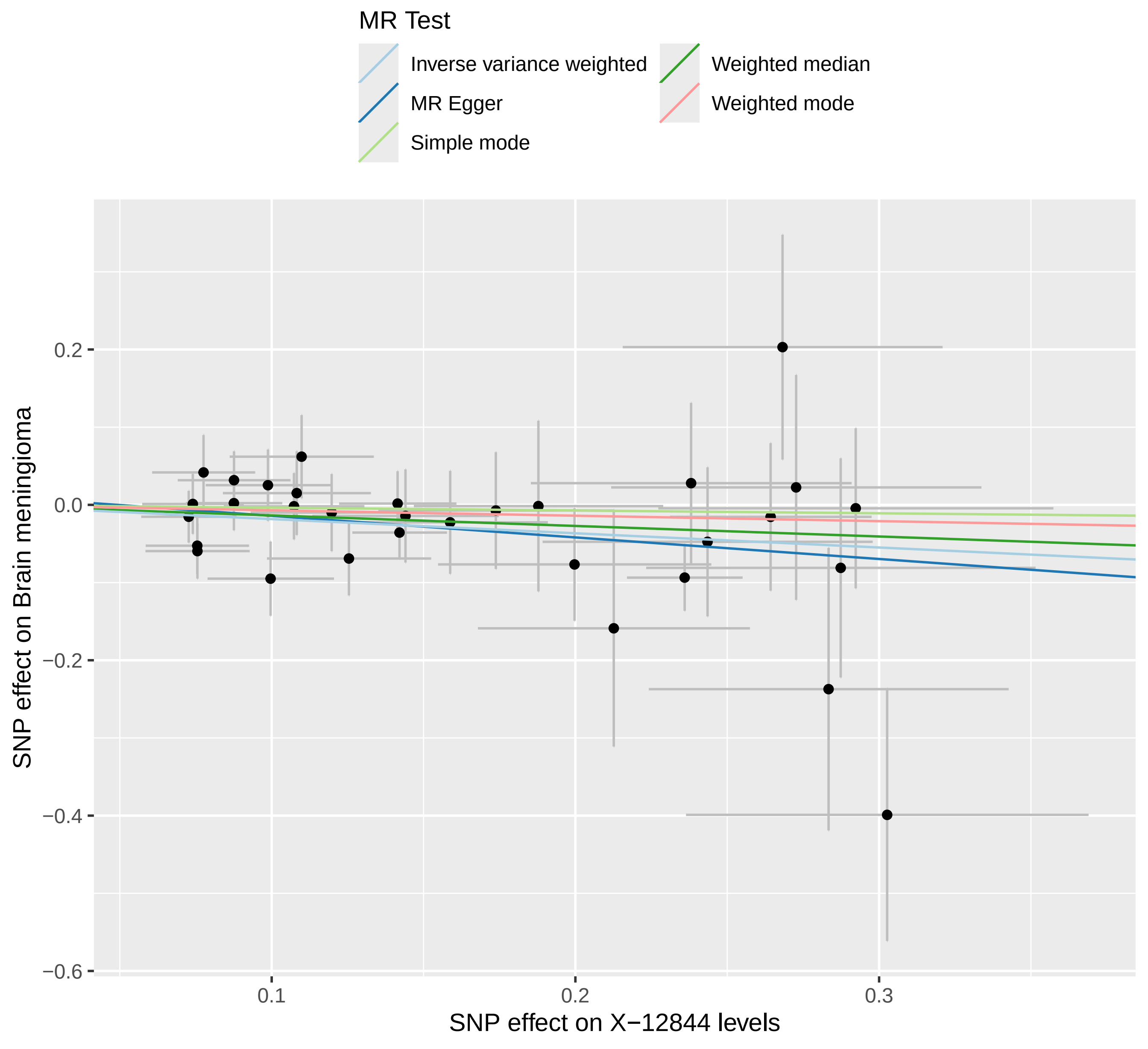
MR Test
Inverse variance weighted
MR Egger
Simple mode
Weighted median
Weighted mode
SNP effect on Brain meningioma
0.2
0.0
−0.2
−0.4
−0.6
0.1
0.2
0.3
SNP effect on X−12844 levels

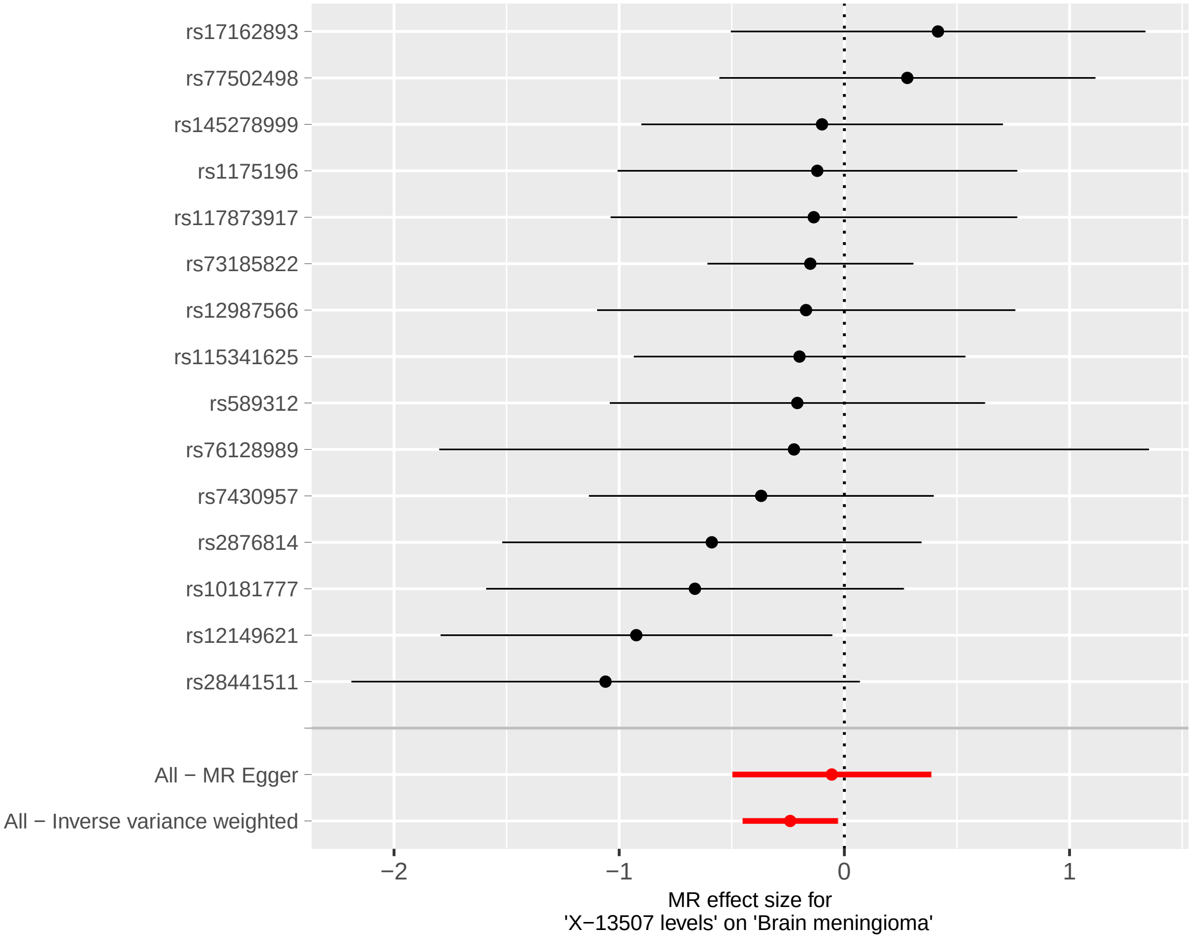

rs17162893
rs77502498
rs145278999
rs1175196
rs117873917
rs73185822
rs12987566
rs115341625
rs589312
rs76128989
rs7430957
rs2876814
rs10181777
rs12149621
rs28441511
All − MR Egger
All − Inverse variance weighted
−2
−1
0
1
MR effect size for
'X−13507 levels' on 'Brain meningioma'

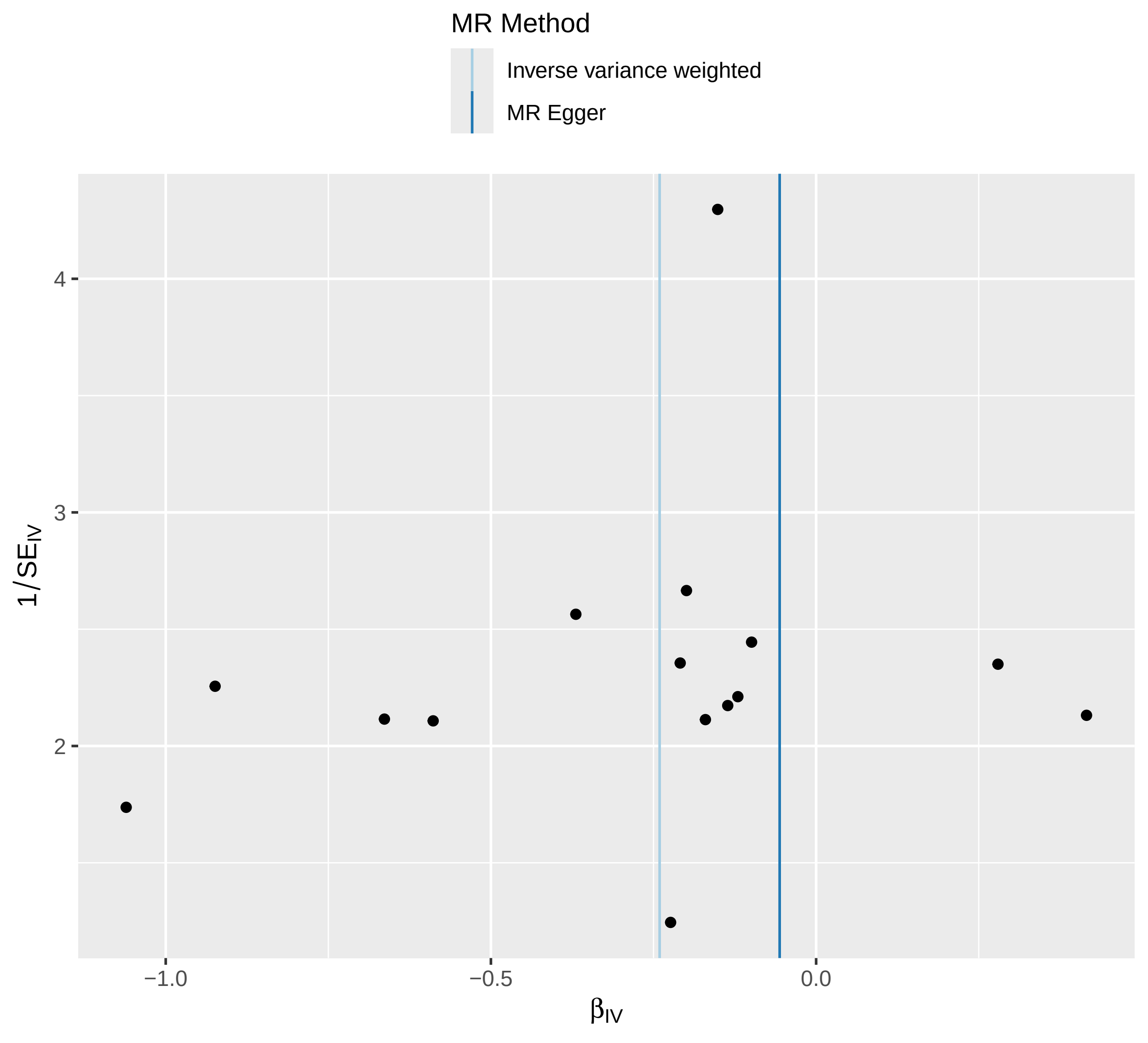

MR Method
Inverse variance weighted
MR Egger
4
3
2
1/SE_IV
−1.0
−0.5
0.0
β_IV

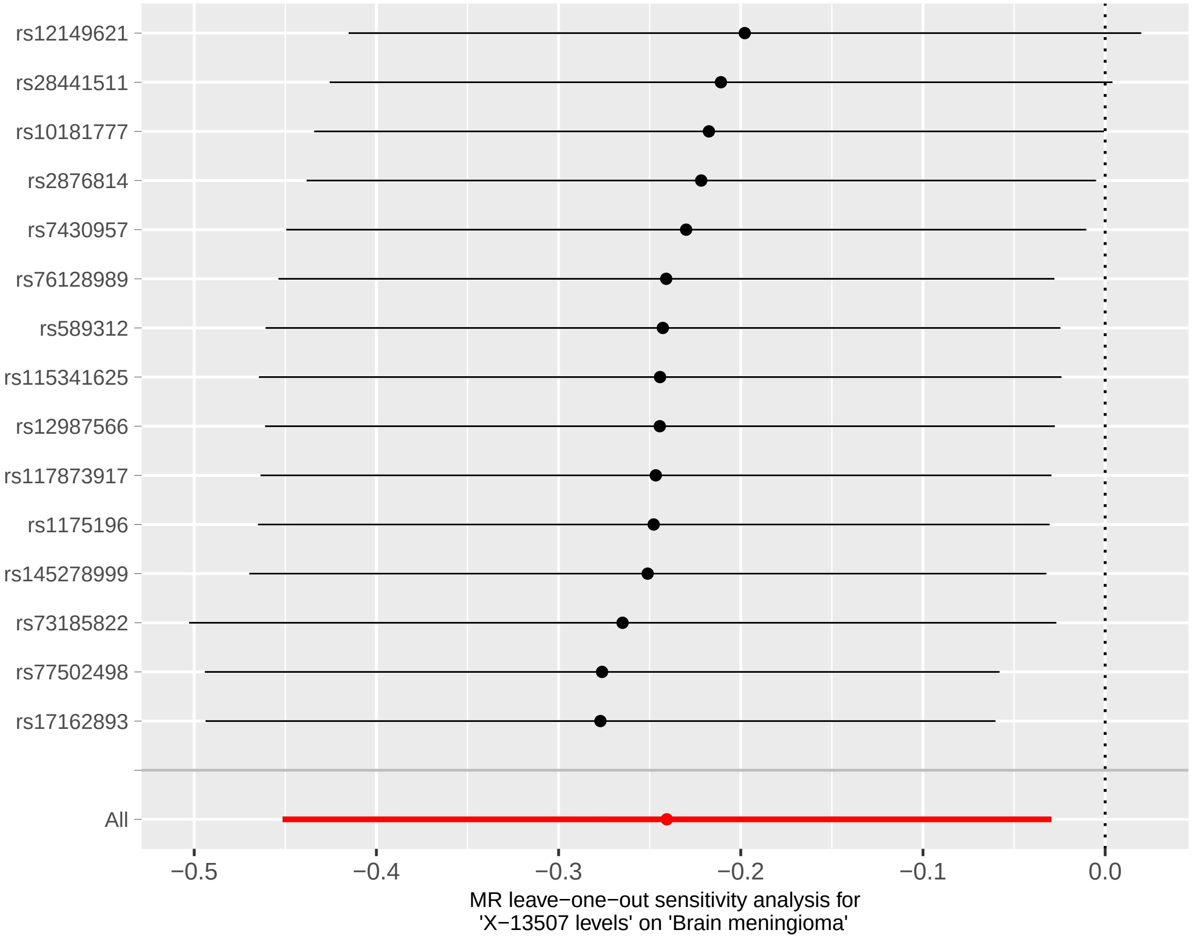

rs12149621
rs28441511
rs10181777
rs2876814
rs7430957
rs76128989
rs589312
rs115341625
rs12987566
rs117873917
rs1175196
rs145278999
rs73185822
rs77502498
rs17162893
All
−0.5
−0.4
−0.3
−0.2
−0.1
0.0
MR leave−one−out sensitivity analysis for
'X−13507 levels' on 'Brain meningioma'

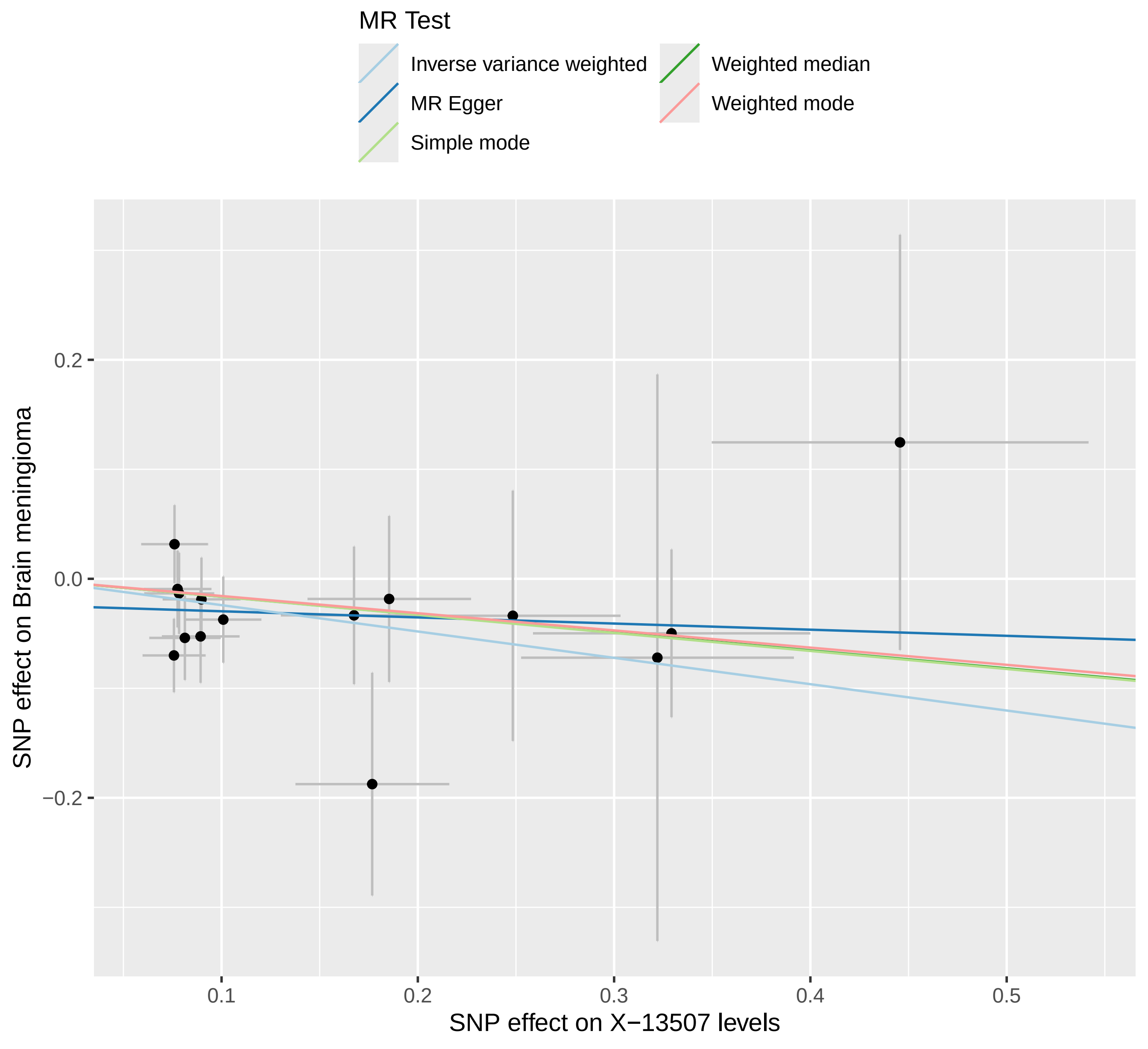

MR Test
Inverse variance weighted
MR Egger
Simple mode
Weighted median
Weighted mode
0.2
0.0
−0.2
0.1
0.2
0.3
0.4
0.5
SNP effect on Brain meningioma
SNP effect on X−13507 levels

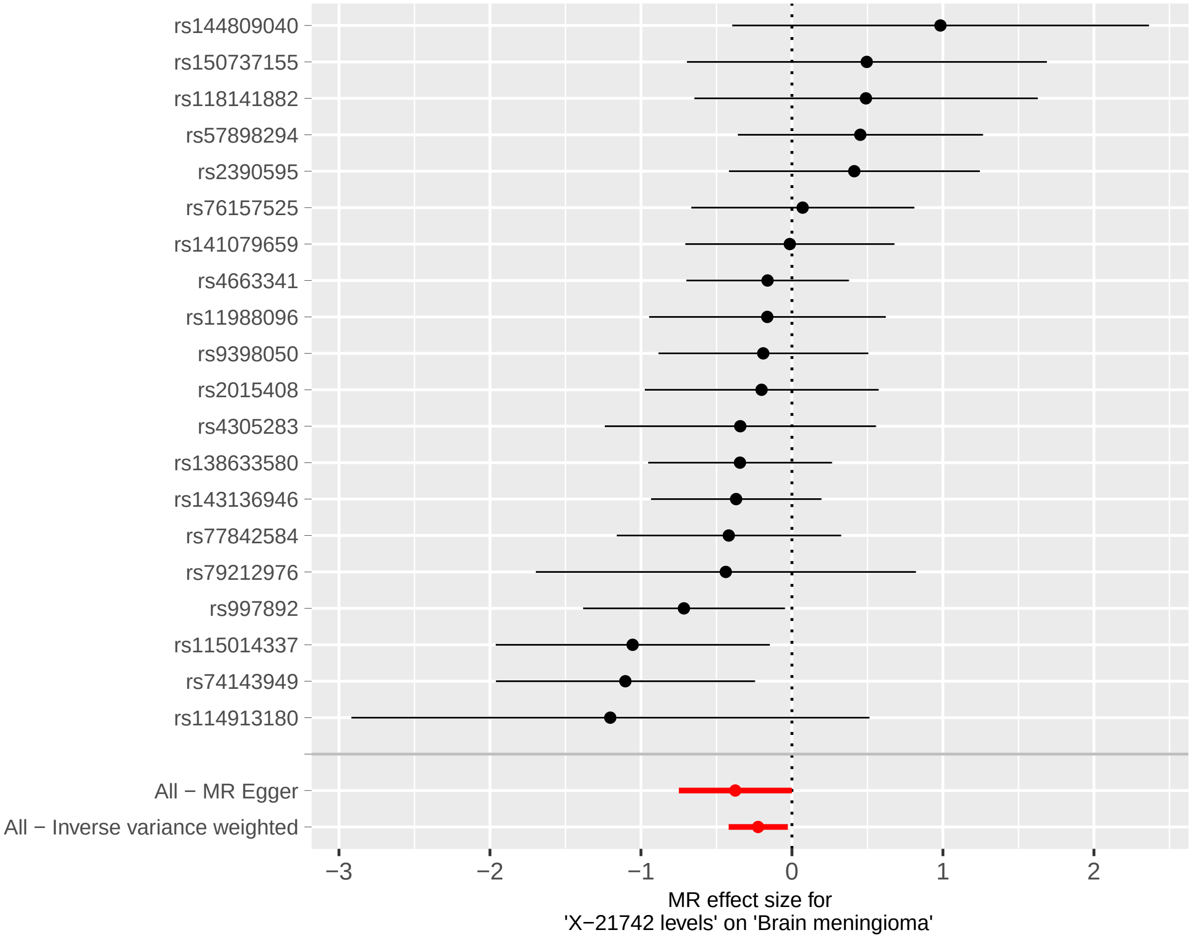
rs144809040
rs150737155
rs118141882
rs57898294
rs2390595
rs76157525
rs141079659
rs4663341
rs11988096
rs9398050
rs2015408
rs4305283
rs138633580
rs143136946
rs77842584
rs79212976
rs997892
rs115014337
rs74143949
rs114913180
All − MR Egger
All − Inverse variance weighted
−3
−2
−1
0
1
2
MR effect size for
'X−21742 levels' on 'Brain meningioma'

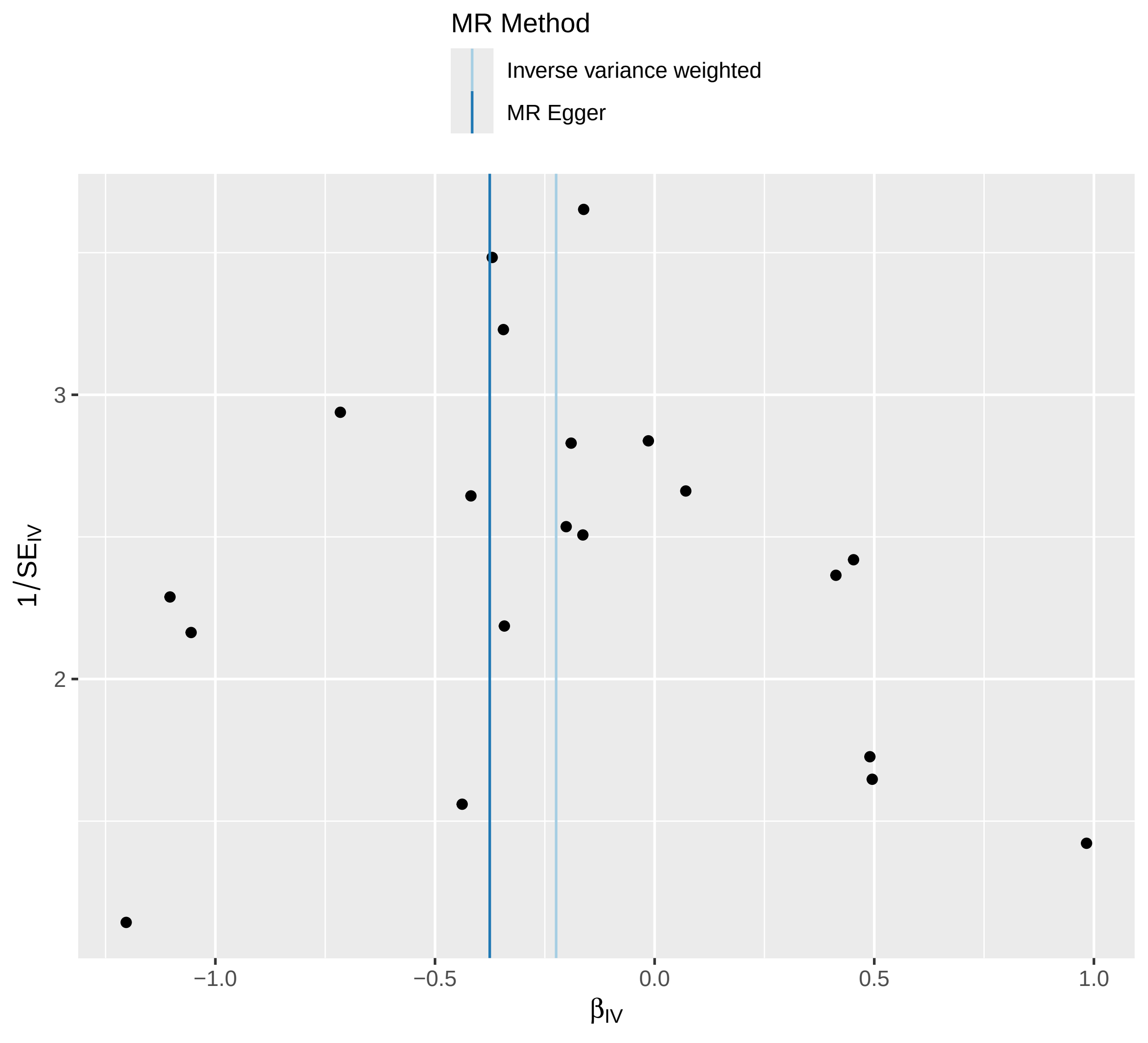

MR Method
Inverse variance weighted
MR Egger
3
2
1/SE_IV
−1.0
−0.5
0.0
0.5
1.0
β_IV

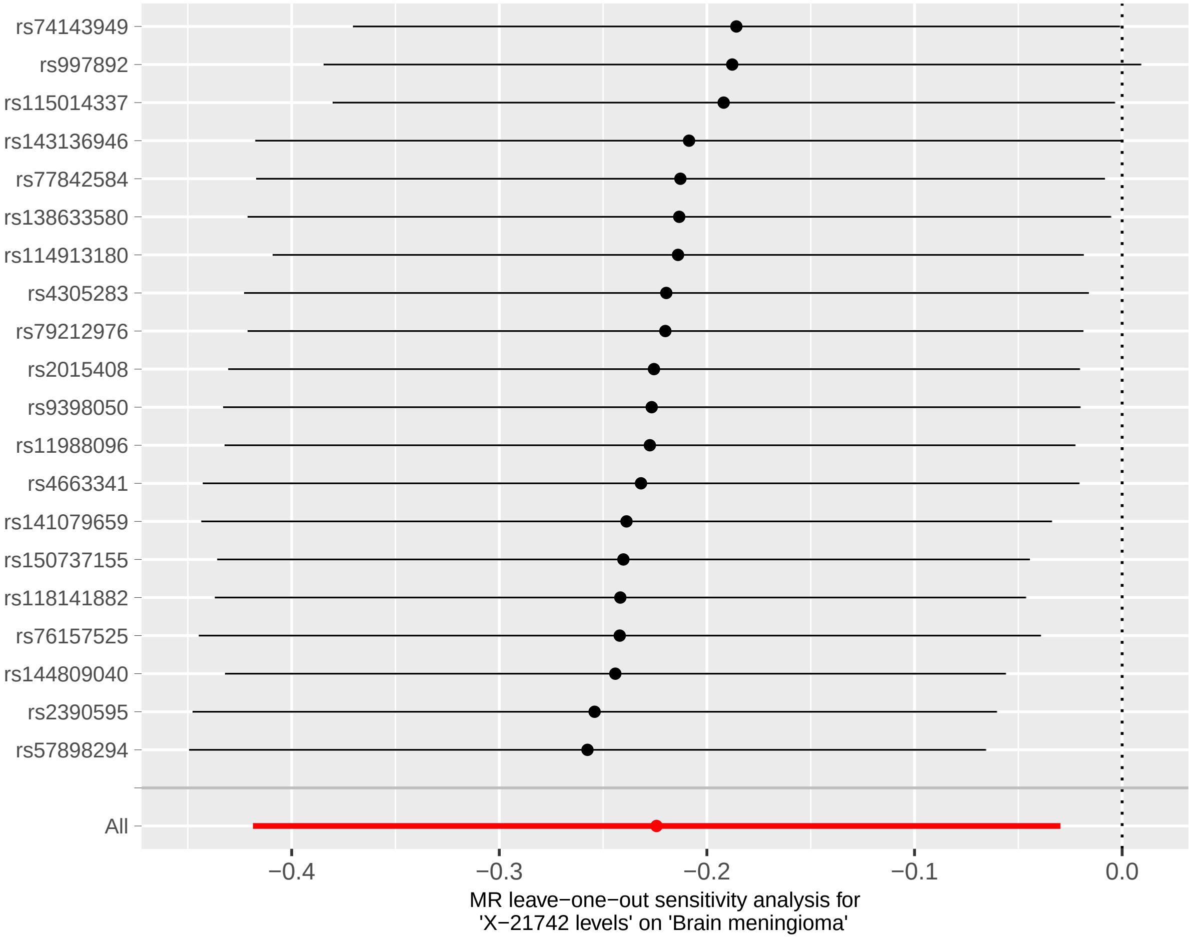
rs74143949
rs997892
rs115014337
rs143136946
rs77842584
rs138633580
rs114913180
rs4305283
rs79212976
rs2015408
rs9398050
rs11988096
rs4663341
rs141079659
rs150737155
rs118141882
rs76157525
rs144809040
rs2390595
rs57898294
All
−0.4
−0.3
−0.2
−0.1
0.0
MR leave−one−out sensitivity analysis for
'X−21742 levels' on 'Brain meningioma'

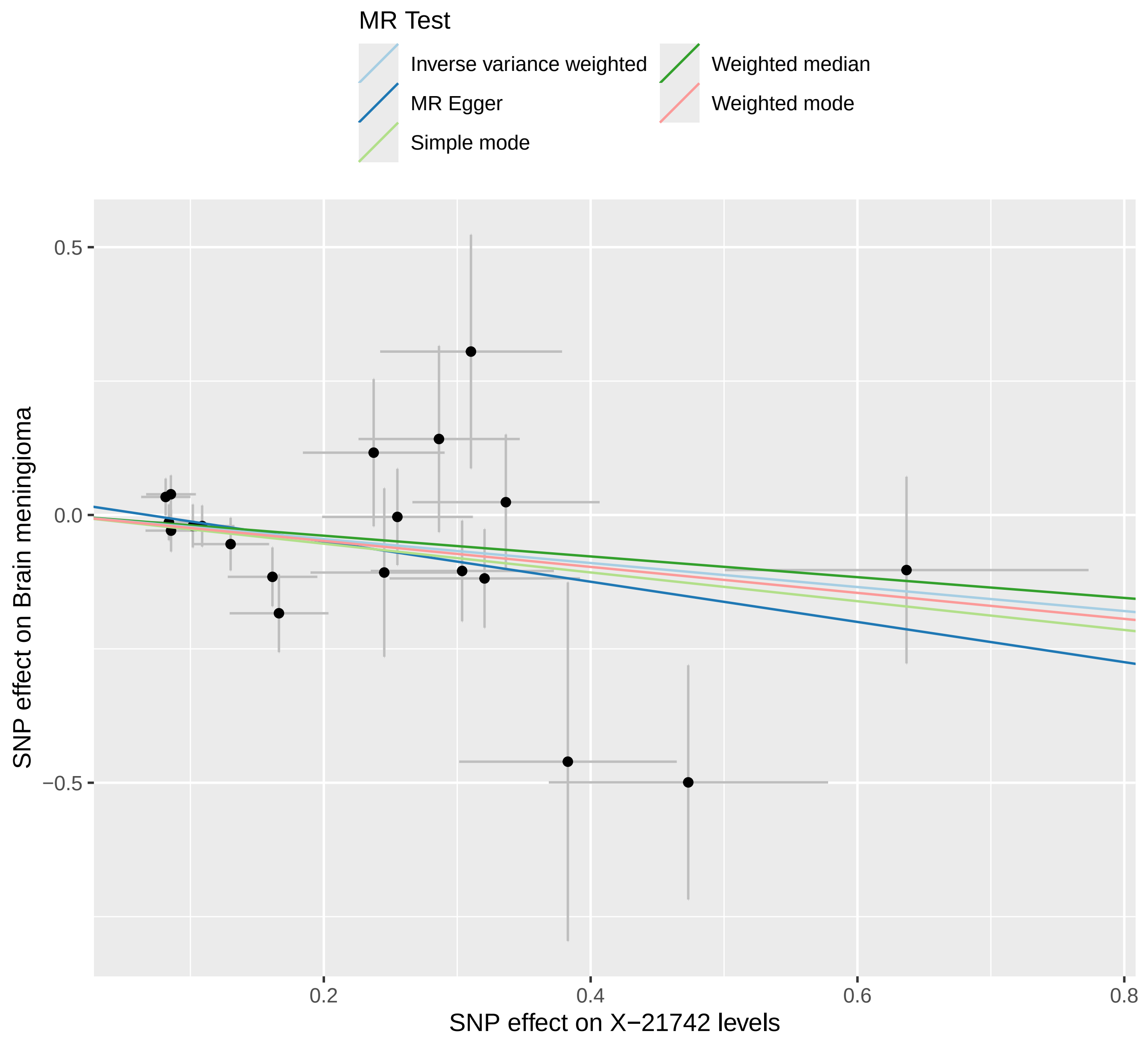

MR Test
Inverse variance weighted
MR Egger
Simple mode
Weighted median
Weighted mode
0.5
0.0
−0.5
0.2
0.4
0.6
0.8
SNP effect on Brain meningioma
SNP effect on X−21742 levels

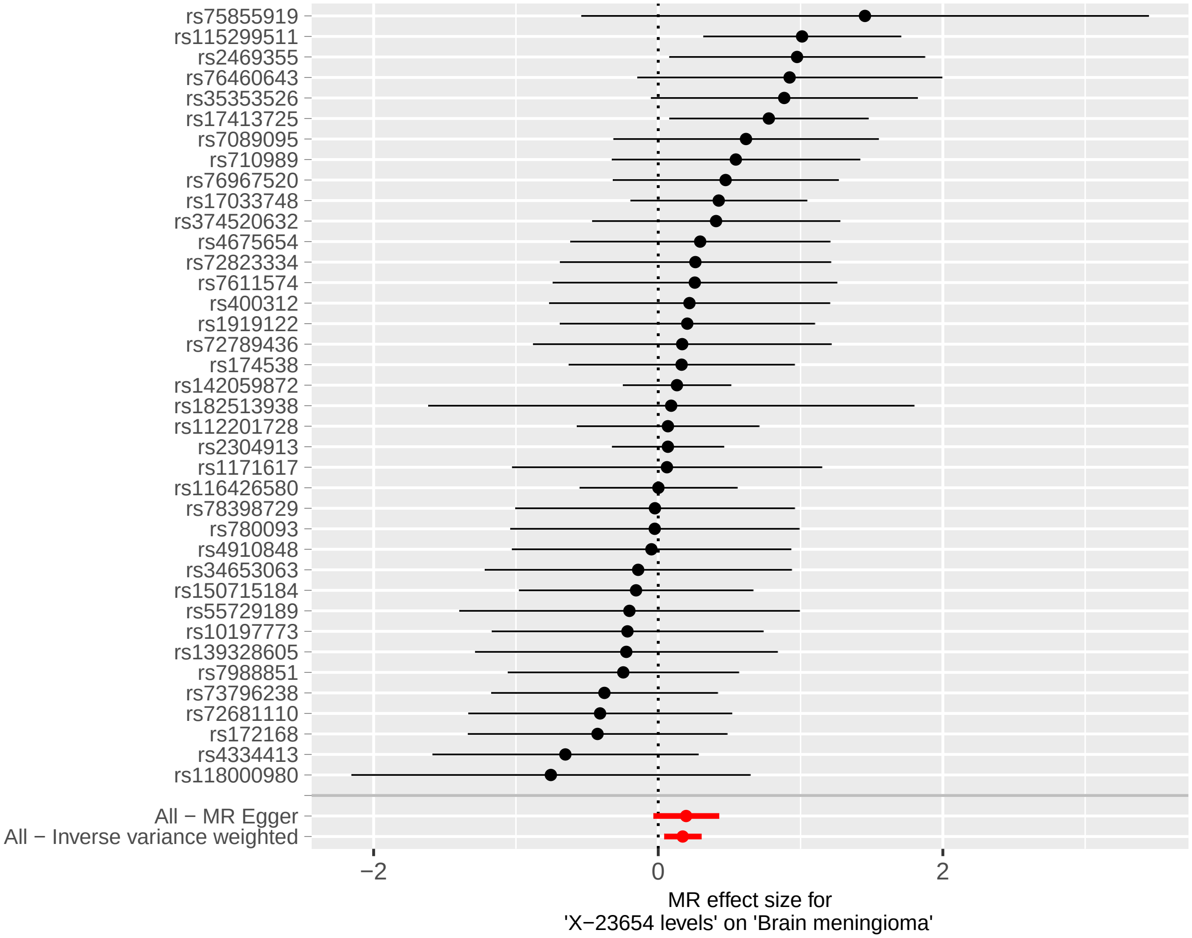
rs75855919
rs115299511
rs2469355
rs76460643
rs35353526
rs17413725
rs7089095
rs710989
rs76967520
rs17033748
rs374520632
rs4675654
rs72823334
rs7611574
rs400312
rs1919122
rs72789436
rs174538
rs142059872
rs182513938
rs112201728
rs2304913
rs1171617
rs116426580
rs78398729
rs780093
rs4910848
rs34653063
rs150715184
rs55729189
rs10197773
rs139328605
rs7988851
rs73796238
rs72681110
rs172168
rs4334413
rs118000980
All − MR Egger
All − Inverse variance weighted
−2
0
2
MR effect size for
'X−23654 levels' on 'Brain meningioma'

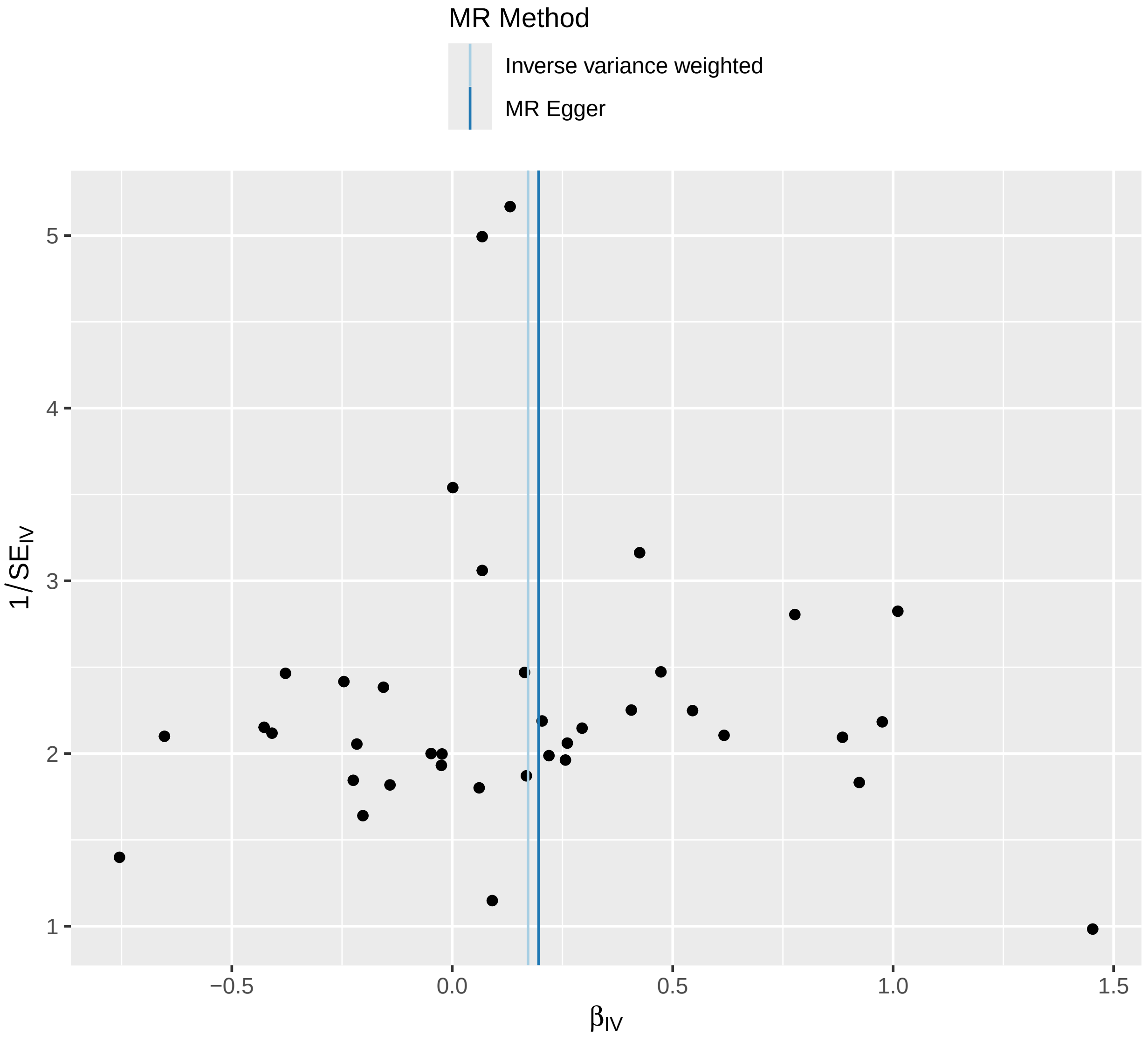
MR Method
Inverse variance weighted
MR Egger
1/SE_IV
β_IV
5
4
3
2
1
−0.5
0.0
0.5
1.0
1.5

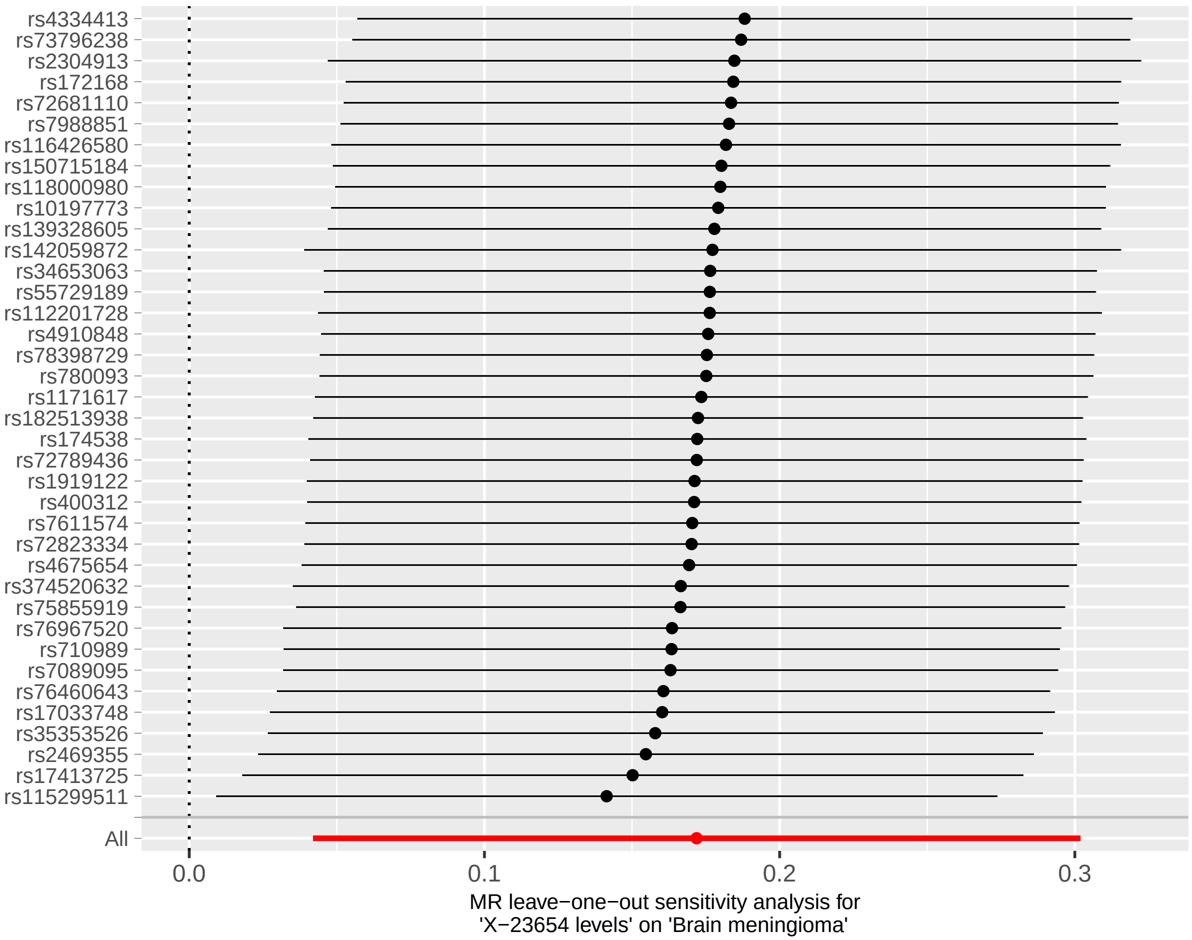

rs4334413
rs73796238
rs2304913
rs172168
rs72681110
rs7988851
rs116426580
rs150715184
rs118000980
rs10197773
rs139328605
rs142059872
rs34653063
rs55729189
rs112201728
rs4910848
rs78398729
rs780093
rs1171617
rs182513938
rs174538
rs72789436
rs1919122
rs400312
rs7611574
rs72823334
rs4675654
rs374520632
rs75855919
rs76967520
rs710989
rs7089095
rs76460643
rs17033748
rs35353526
rs2469355
rs17413725
rs115299511
All
0.0
0.1
0.2
0.3
MR leave−one−out sensitivity analysis for
'X−23654 levels' on 'Brain meningioma'

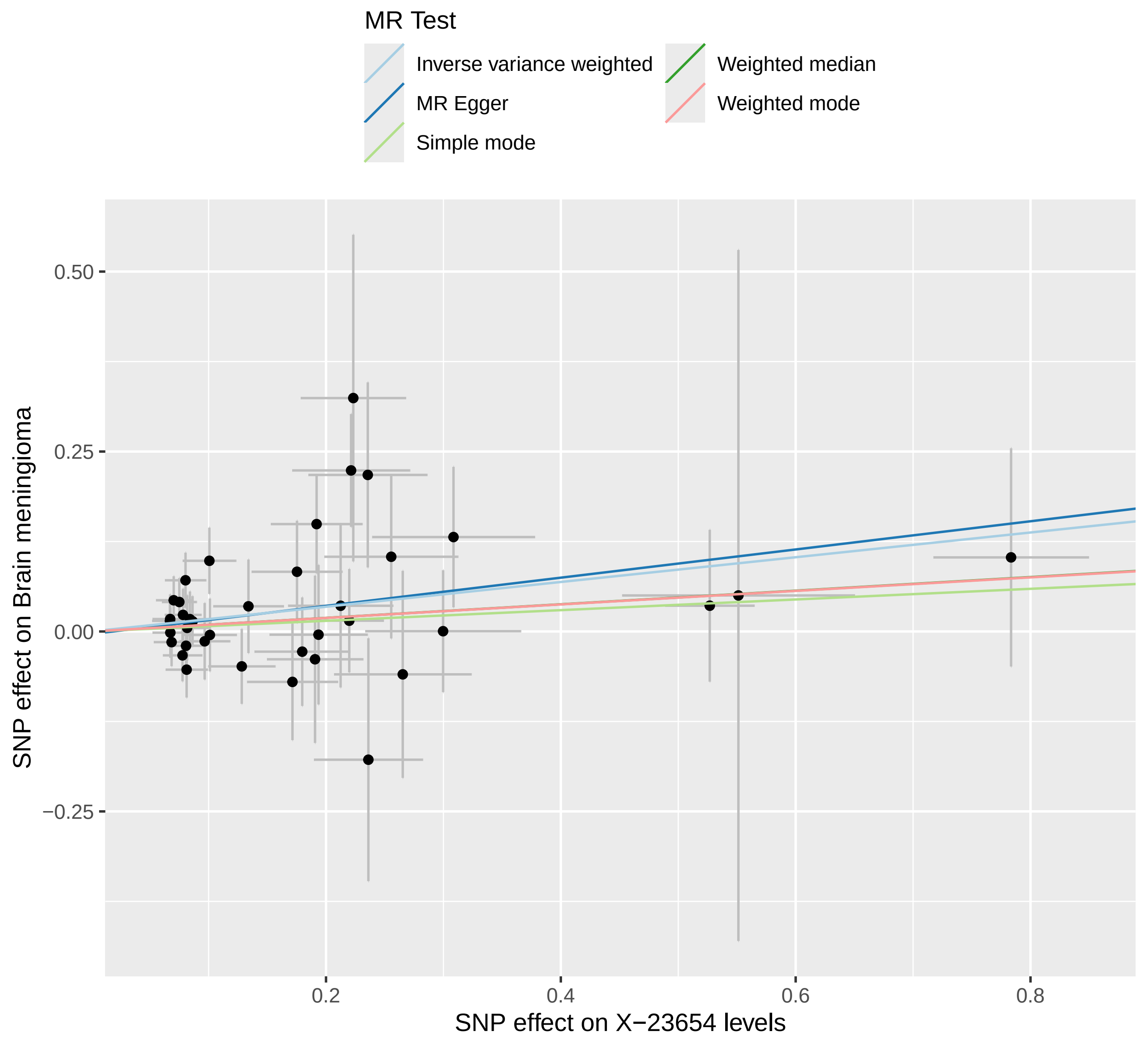

MR Test
Inverse variance weighted
MR Egger
Simple mode
Weighted median
Weighted mode
SNP effect on Brain meningioma
0.50
0.25
0.00
−0.25
0.2
0.4
0.6
0.8
SNP effect on X−23654 levels

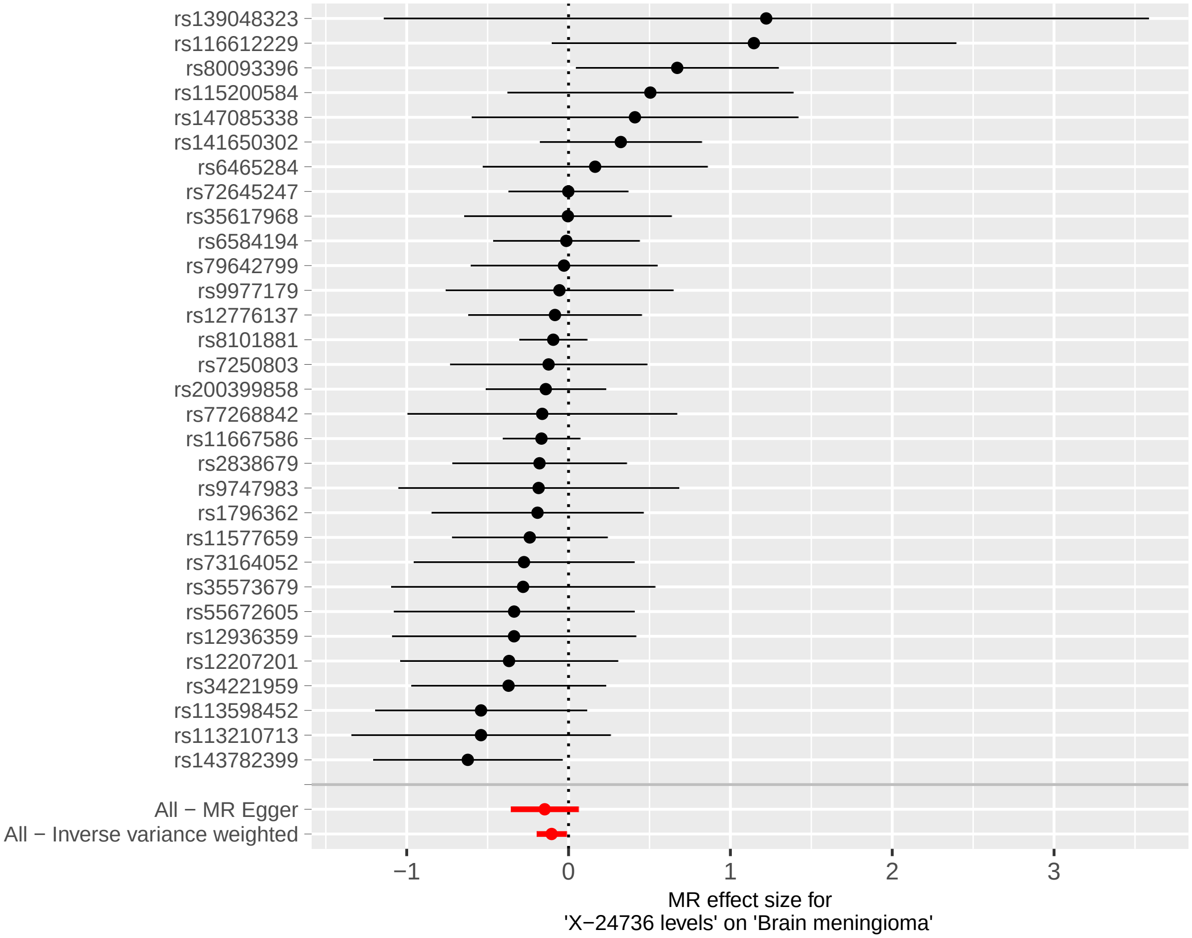

rs139048323
rs116612229
rs80093396
rs115200584
rs147085338
rs141650302
rs6465284
rs72645247
rs35617968
rs6584194
rs79642799
rs9977179
rs12776137
rs8101881
rs7250803
rs200399858
rs77268842
rs11667586
rs2838679
rs9747983
rs1796362
rs11577659
rs73164052
rs35573679
rs55672605
rs12936359
rs12207201
rs34221959
rs113598452
rs113210713
rs143782399
All − MR Egger
All − Inverse variance weighted
−1
0
1
2
3
MR effect size for
'X−24736 levels' on 'Brain meningioma'

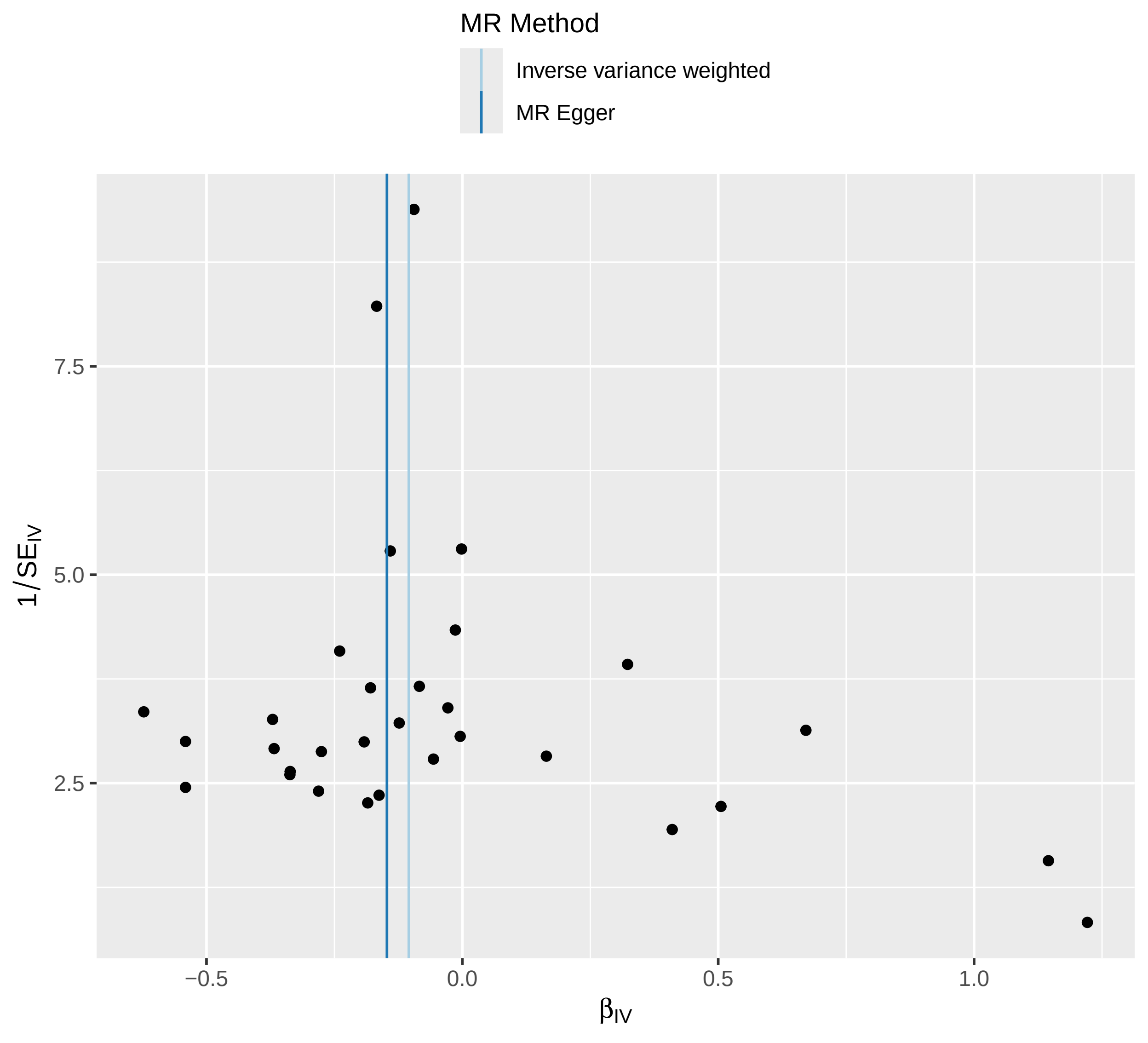
MR Method
Inverse variance weighted
MR Egger
7.5
5.0
2.5
1/SE_IV
−0.5
0.0
0.5
1.0
β_IV

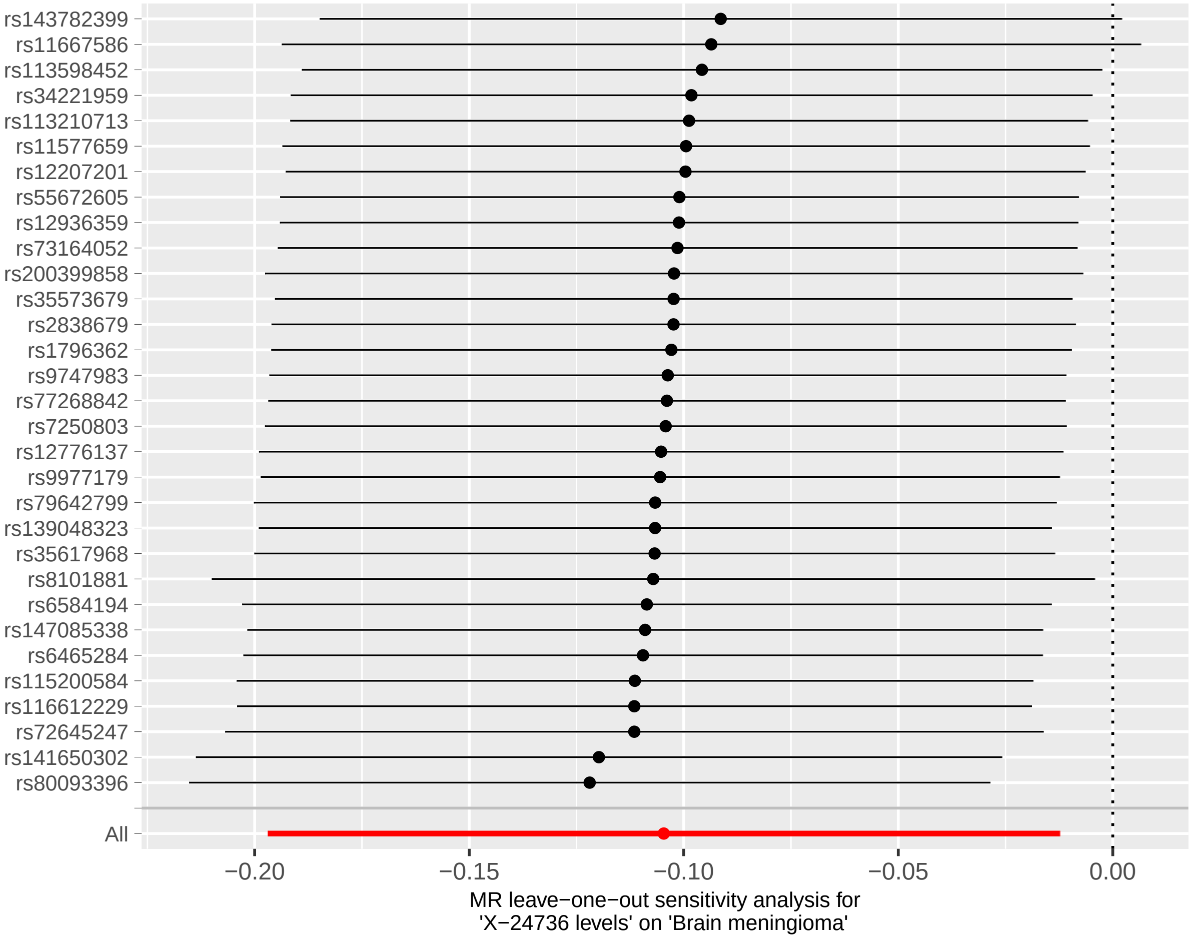
rs143782399
rs11667586
rs113598452
rs34221959
rs113210713
rs11577659
rs12207201
rs55672605
rs12936359
rs73164052
rs200399858
rs35573679
rs2838679
rs1796362
rs9747983
rs77268842
rs7250803
rs12776137
rs9977179
rs79642799
rs139048323
rs35617968
rs8101881
rs6584194
rs147085338
rs6465284
rs115200584
rs116612229
rs72645247
rs141650302
rs80093396
All
−0.20
−0.15
−0.10
−0.05
0.00
MR leave−one−out sensitivity analysis for
'X−24736 levels' on 'Brain meningioma'

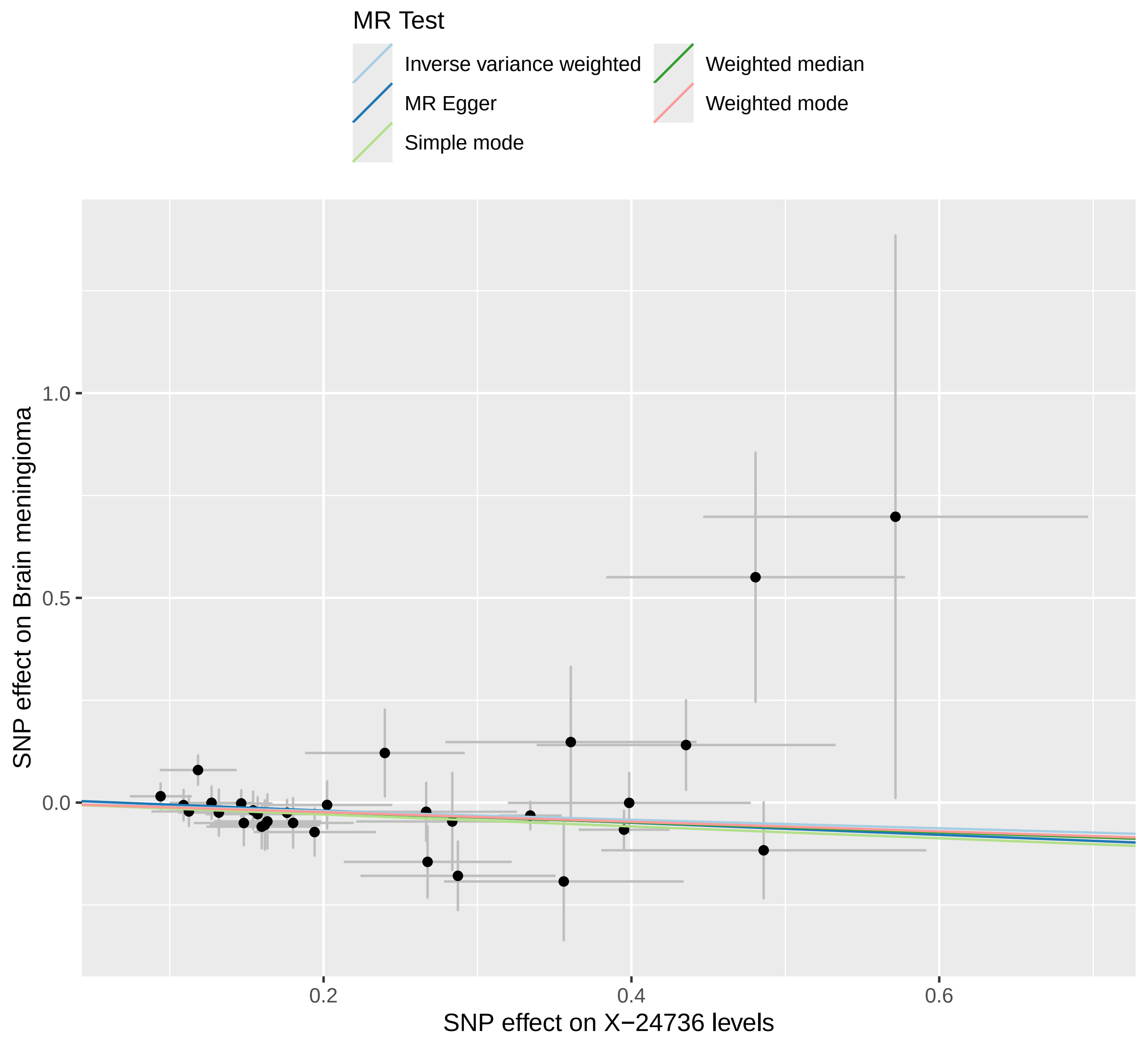
MR Test
Inverse variance weighted
MR Egger
Simple mode
Weighted median
Weighted mode
SNP effect on Brain meningioma
1.0
0.5
0.0
0.2
0.4
0.6
SNP effect on X−24736 levels
