# Supplementary material for: Causal Pathways Linking Gut Microbiota, Serum Metabolites, and Meningioma Risk: A Mendelian Randomization Analysis
Source: Brain Behav. 2026 Feb 9;16(2):e71220. doi: 10.1002/brb3.71220 (PMC12887442; doi:10.1002/brb3.71220)
Supplement: Supplementary file 10 — Supplementary Appendix S10: brb371220‐sup‐00010‐Appendix10.pdf [file BRB3-16-e71220-s010.pdf]

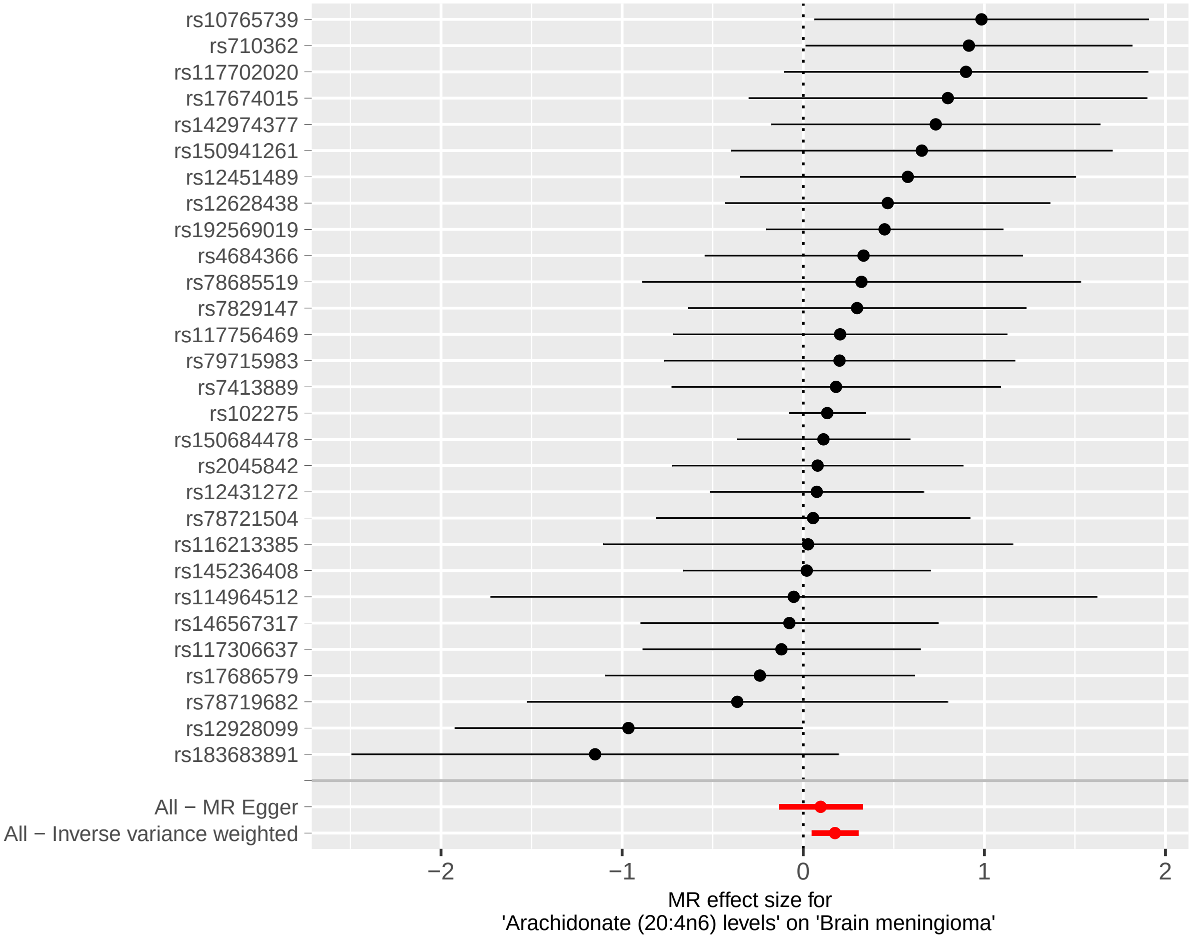
rs10765739
rs710362
rs117702020
rs17674015
rs142974377
rs150941261
rs12451489
rs12628438
rs192569019
rs4684366
rs78685519
rs7829147
rs117756469
rs79715983
rs7413889
rs102275
rs150684478
rs2045842
rs12431272
rs78721504
rs116213385
rs145236408
rs114964512
rs146567317
rs117306637
rs17686579
rs78719682
rs12928099
rs183683891
All − MR Egger
All − Inverse variance weighted
−2
−1
0
1
2
MR effect size for
'Arachidonate (20:4n6) levels' on 'Brain meningioma'

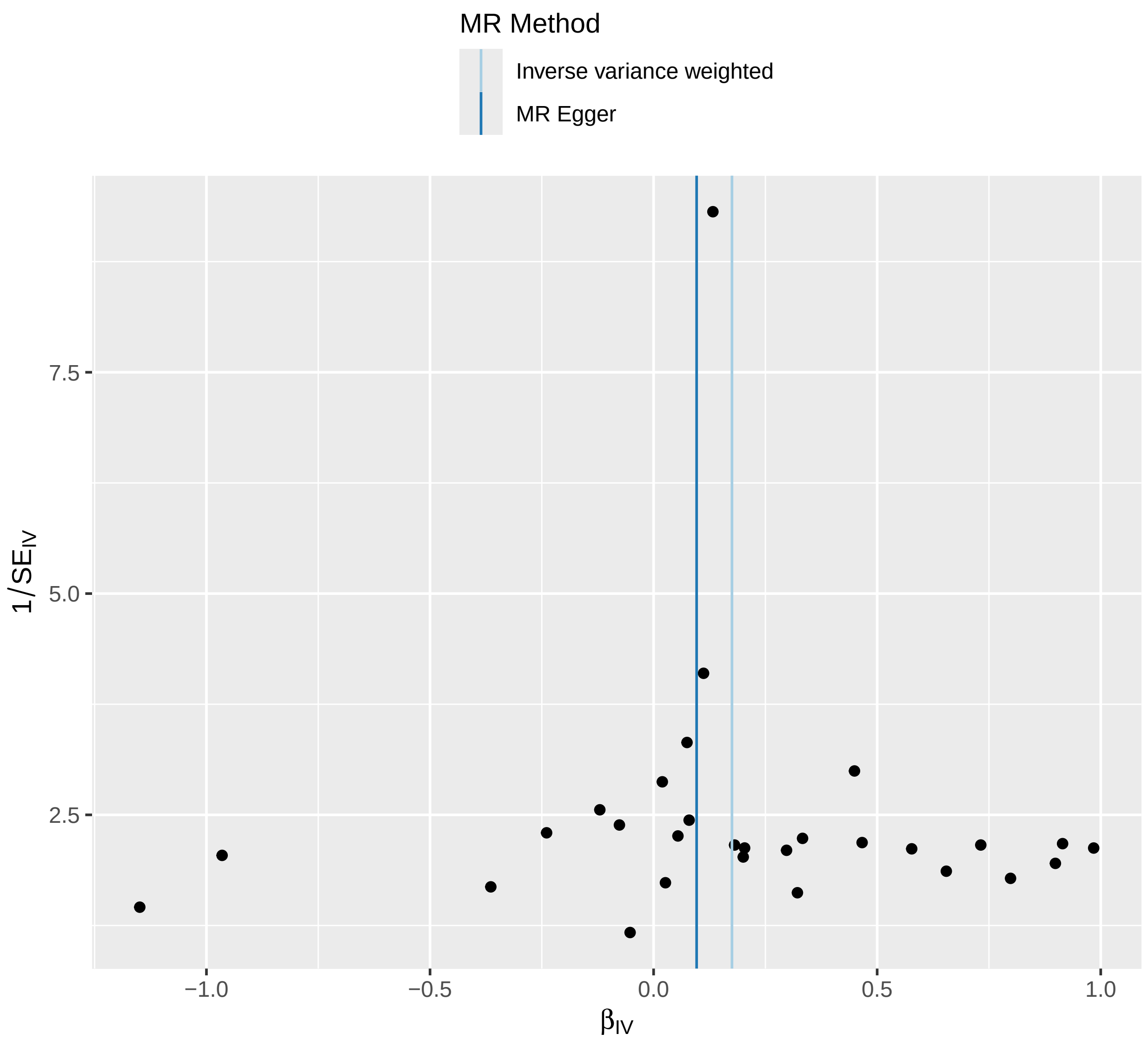

MR Method
Inverse variance weighted
MR Egger
7.5
5.0
2.5
1/SE_IV
−1.0
−0.5
0.0
0.5
1.0
β_IV

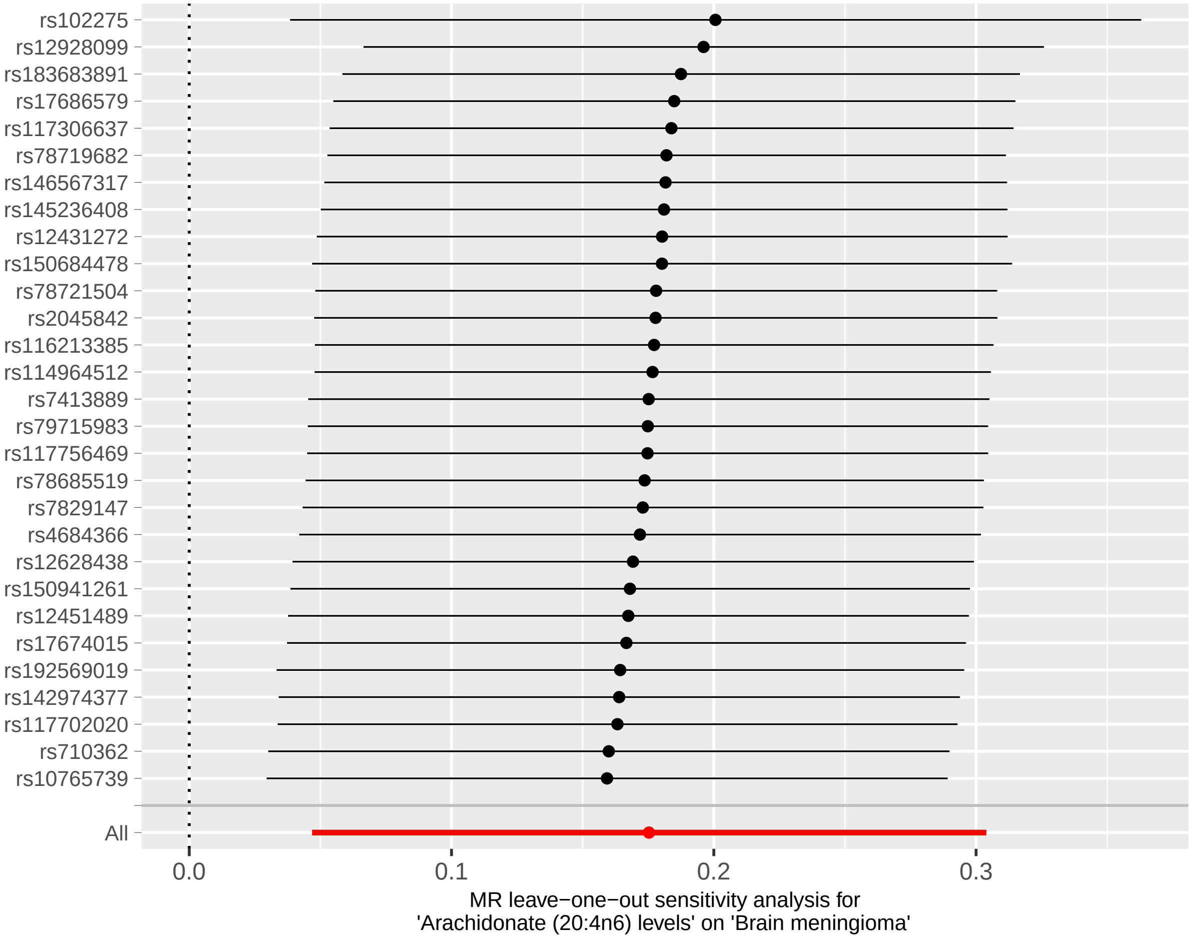

rs102275
rs12928099
rs183683891
rs17686579
rs117306637
rs78719682
rs146567317
rs145236408
rs12431272
rs150684478
rs78721504
rs2045842
rs116213385
rs114964512
rs7413889
rs79715983
rs117756469
rs78685519
rs7829147
rs4684366
rs12628438
rs150941261
rs12451489
rs17674015
rs192569019
rs142974377
rs117702020
rs710362
rs10765739
All
0.0
0.1
0.2
0.3
MR leave−one−out sensitivity analysis for
'Arachidonate (20:4n6) levels' on 'Brain meningioma'

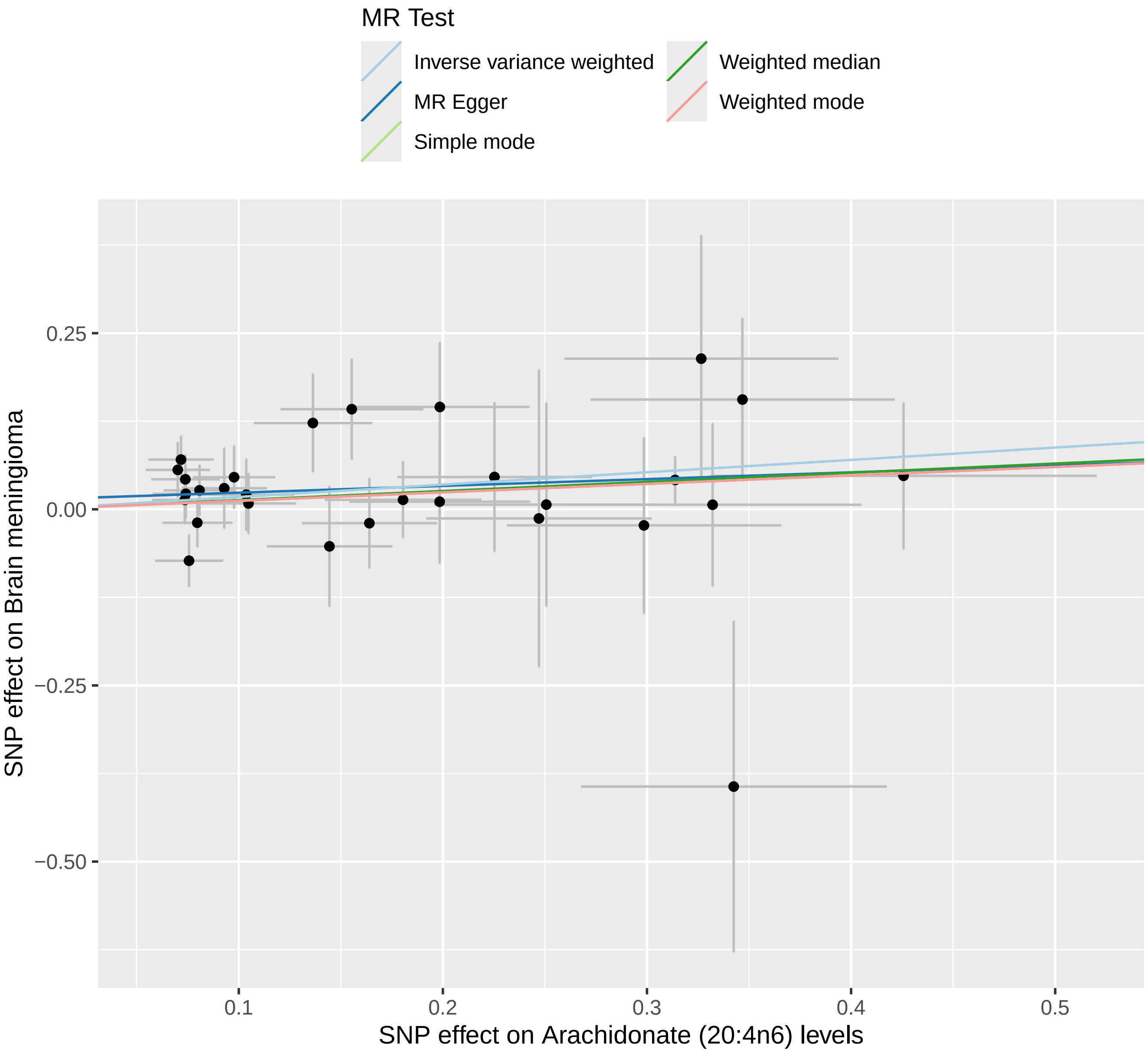
MR Test
Inverse variance weighted
MR Egger
Simple mode
Weighted median
Weighted mode
0.25
0.00
−0.25
−0.50
0.1
0.2
0.3
0.4
0.5
SNP effect on Brain meningioma
SNP effect on Arachidonate (20:4n6) levels

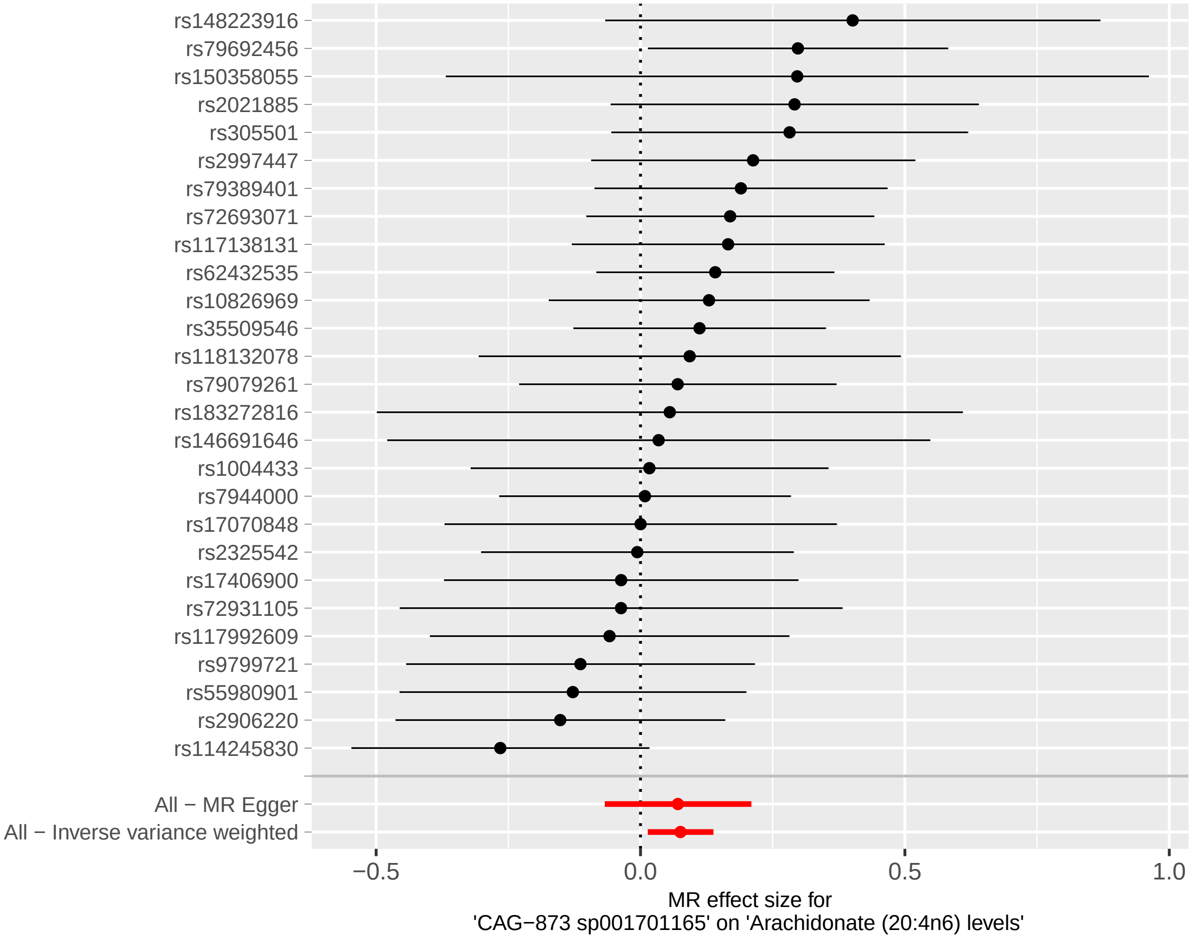

rs148223916
rs79692456
rs150358055
rs2021885
rs305501
rs2997447
rs79389401
rs72693071
rs117138131
rs62432535
rs10826969
rs35509546
rs118132078
rs79079261
rs183272816
rs146691646
rs1004433
rs7944000
rs17070848
rs2325542
rs17406900
rs72931105
rs117992609
rs9799721
rs55980901
rs2906220
rs114245830
All − MR Egger
All − Inverse variance weighted
−0.5
0.0
0.5
1.0
MR effect size for
'CAG−873 sp001701165' on 'Arachidonate (20:4n6) levels'

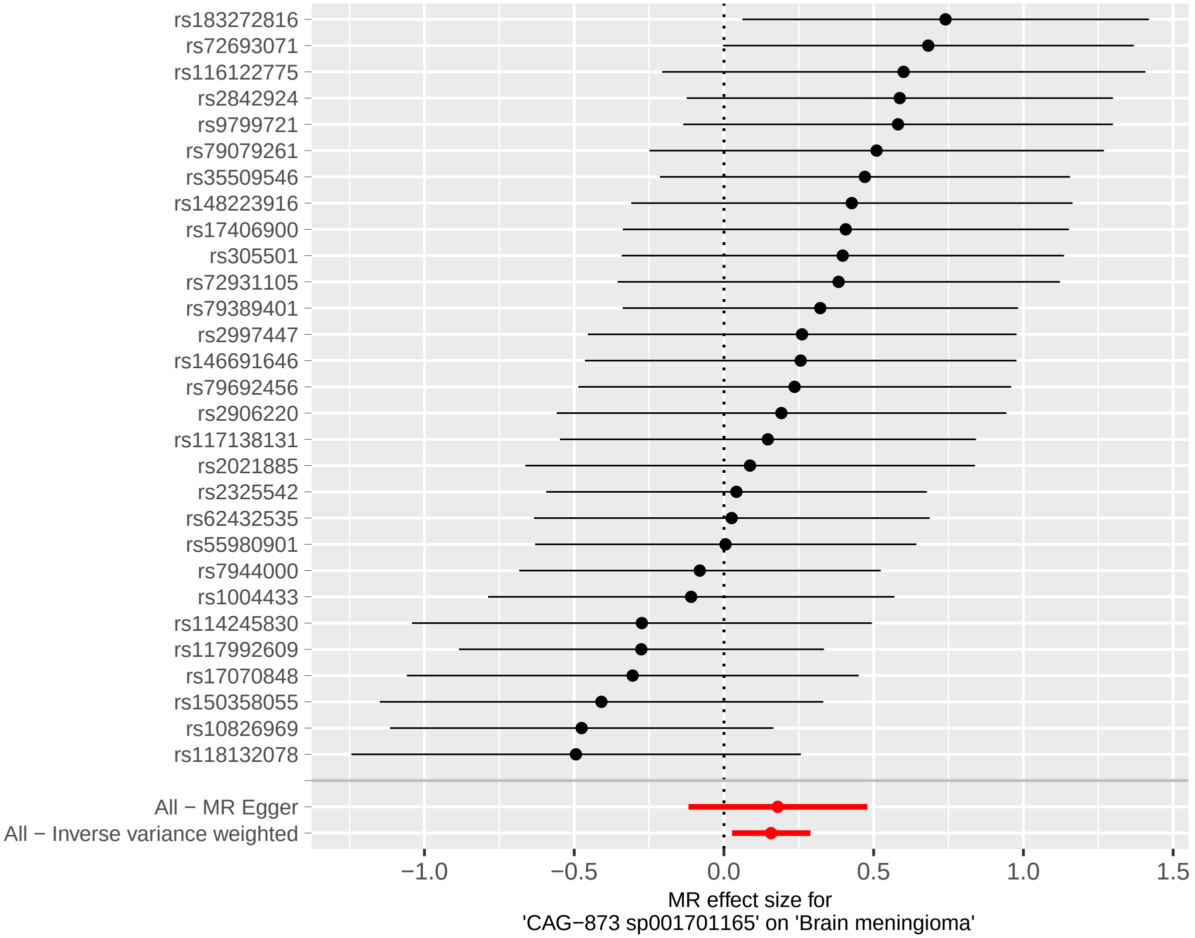
rs183272816
rs72693071
rs116122775
rs2842924
rs9799721
rs79079261
rs35509546
rs148223916
rs17406900
rs305501
rs72931105
rs79389401
rs2997447
rs146691646
rs79692456
rs2906220
rs117138131
rs2021885
rs2325542
rs62432535
rs55980901
rs7944000
rs1004433
rs114245830
rs117992609
rs17070848
rs150358055
rs10826969
rs118132078
All − MR Egger
All − Inverse variance weighted
−1.0
−0.5
0.0
0.5
1.0
1.5
MR effect size for
'CAG−873 sp001701165' on 'Brain meningioma'

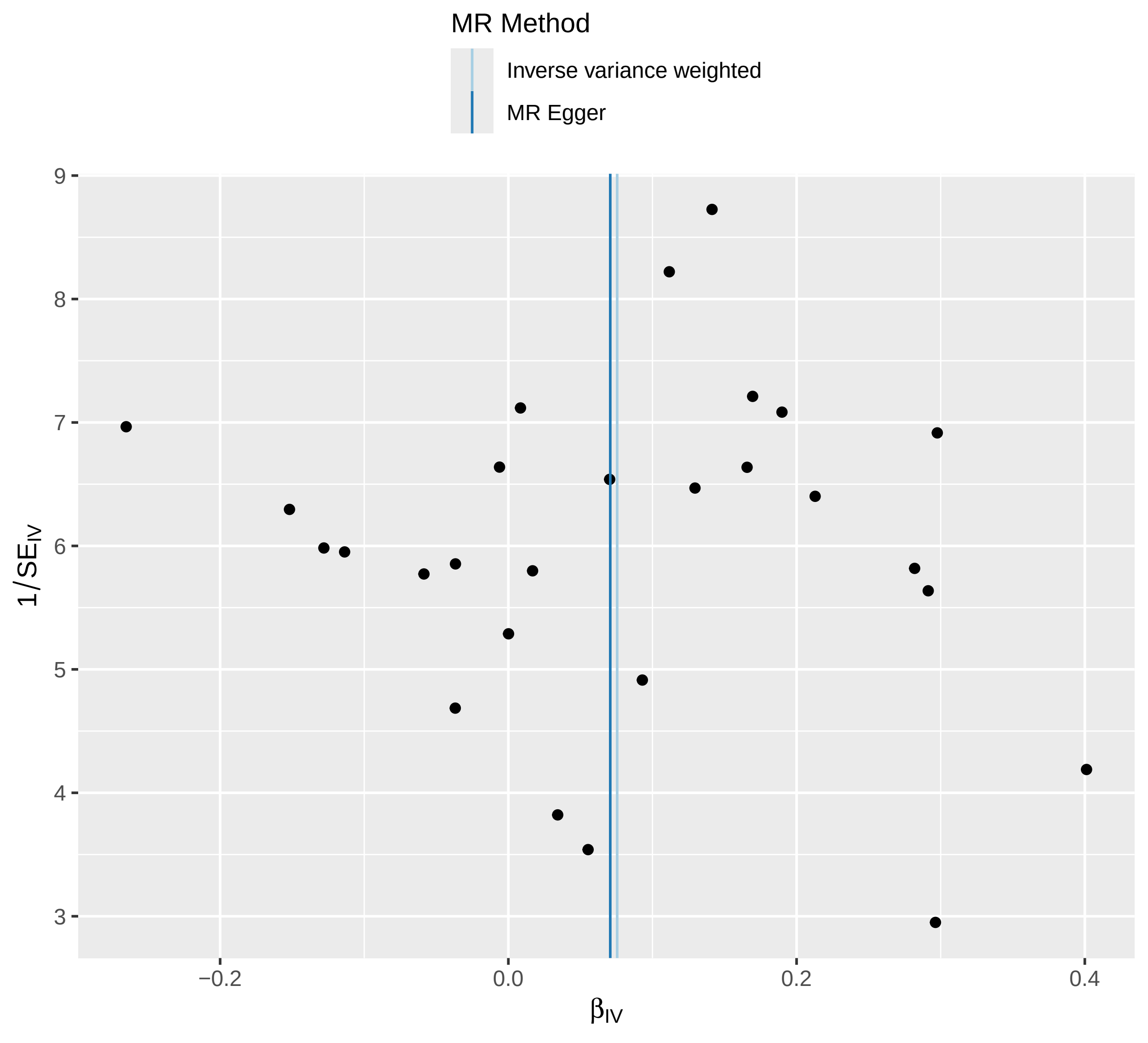
MR Method
Inverse variance weighted
MR Egger
9
8
7
6
5
4
3
1/SE_IV
−0.2
0.0
0.2
0.4
β_IV

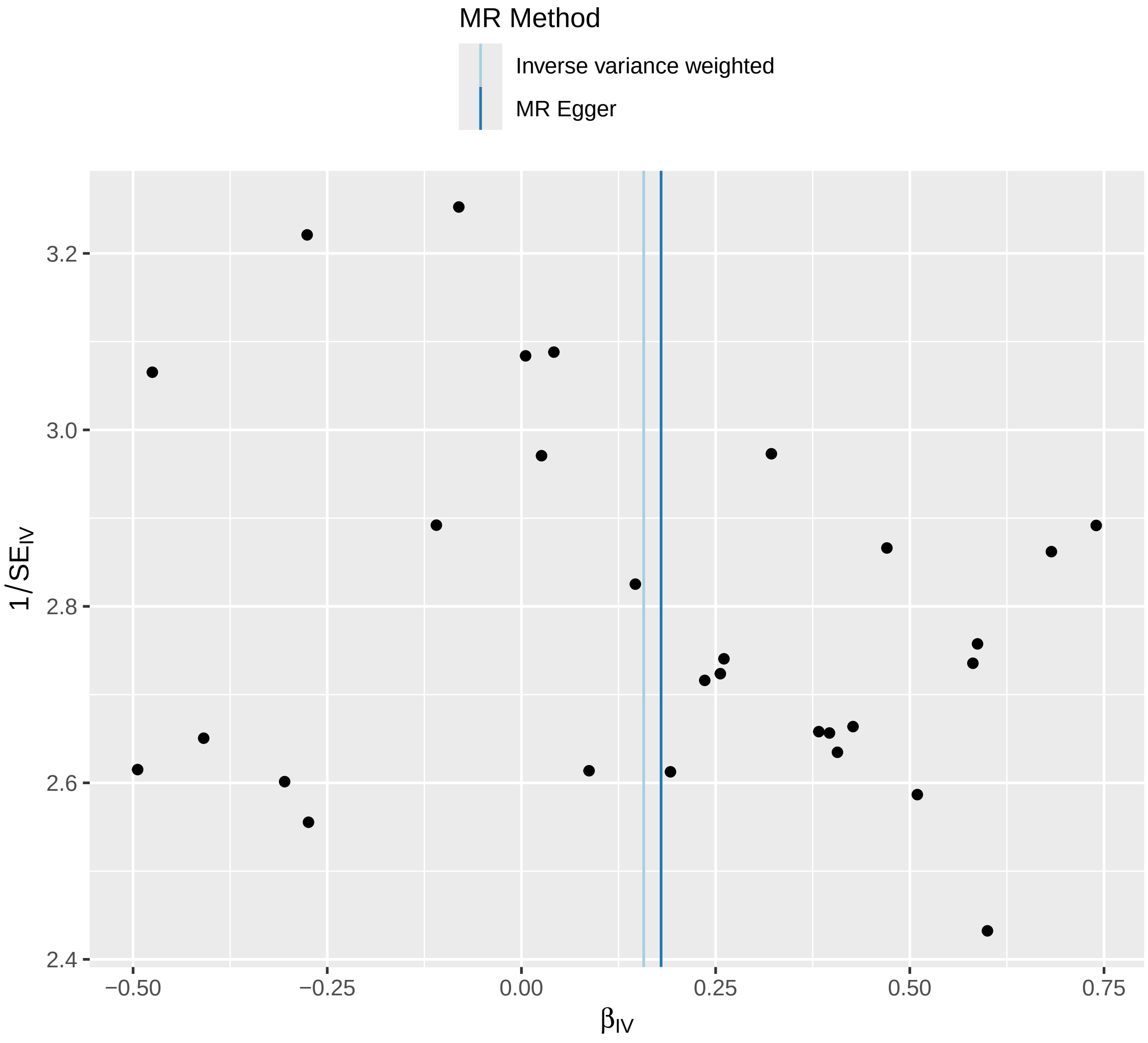
MR Method
Inverse variance weighted
MR Egger
3.2
3.0
2.8
2.6
2.4
1/SE_IV
−0.50
−0.25
0.00
0.25
0.50
0.75
β_IV

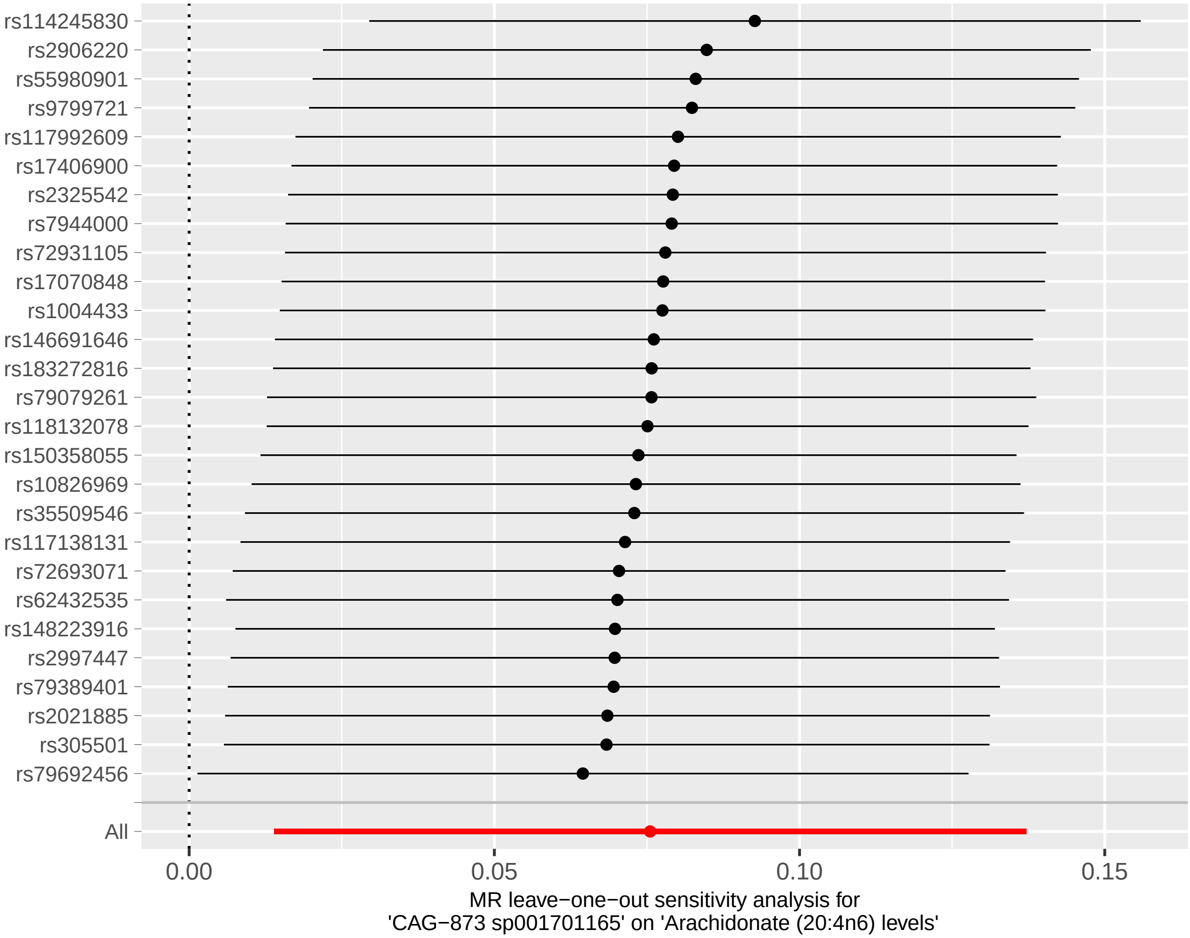
rs114245830
rs2906220
rs55980901
rs9799721
rs117992609
rs17406900
rs2325542
rs7944000
rs72931105
rs17070848
rs1004433
rs146691646
rs183272816
rs79079261
rs118132078
rs150358055
rs10826969
rs35509546
rs117138131
rs72693071
rs62432535
rs148223916
rs2997447
rs79389401
rs2021885
rs305501
rs79692456
All
0.00
0.05
0.10
0.15
MR leave−one−out sensitivity analysis for
'CAG−873 sp001701165' on 'Arachidonate (20:4n6) levels'

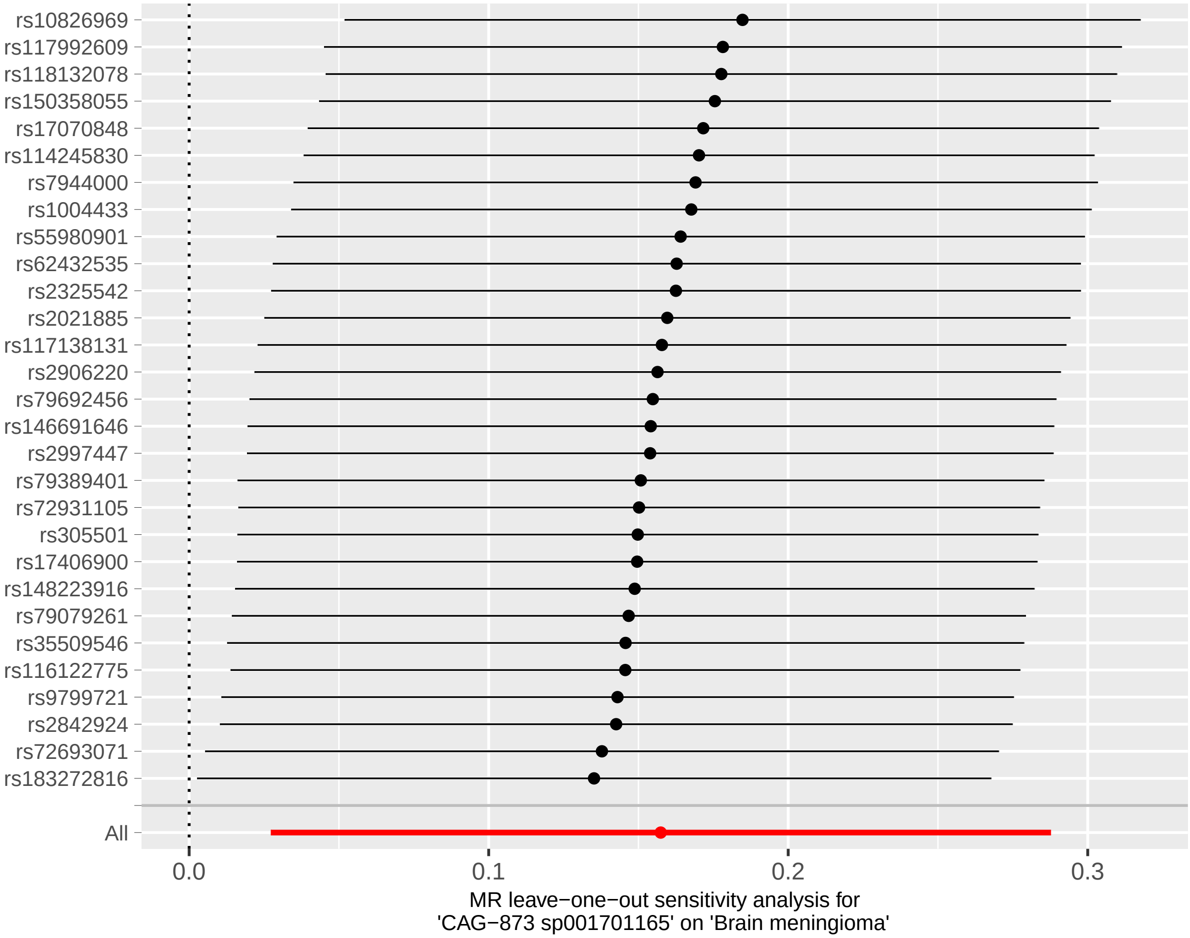
rs10826969
rs117992609
rs118132078
rs150358055
rs17070848
rs114245830
rs7944000
rs1004433
rs55980901
rs62432535
rs2325542
rs2021885
rs117138131
rs2906220
rs79692456
rs146691646
rs2997447
rs79389401
rs72931105
rs305501
rs17406900
rs148223916
rs79079261
rs35509546
rs116122775
rs9799721
rs2842924
rs72693071
rs183272816
All
0.0
0.1
0.2
0.3
MR leave−one−out sensitivity analysis for
'CAG−873 sp001701165' on 'Brain meningioma'

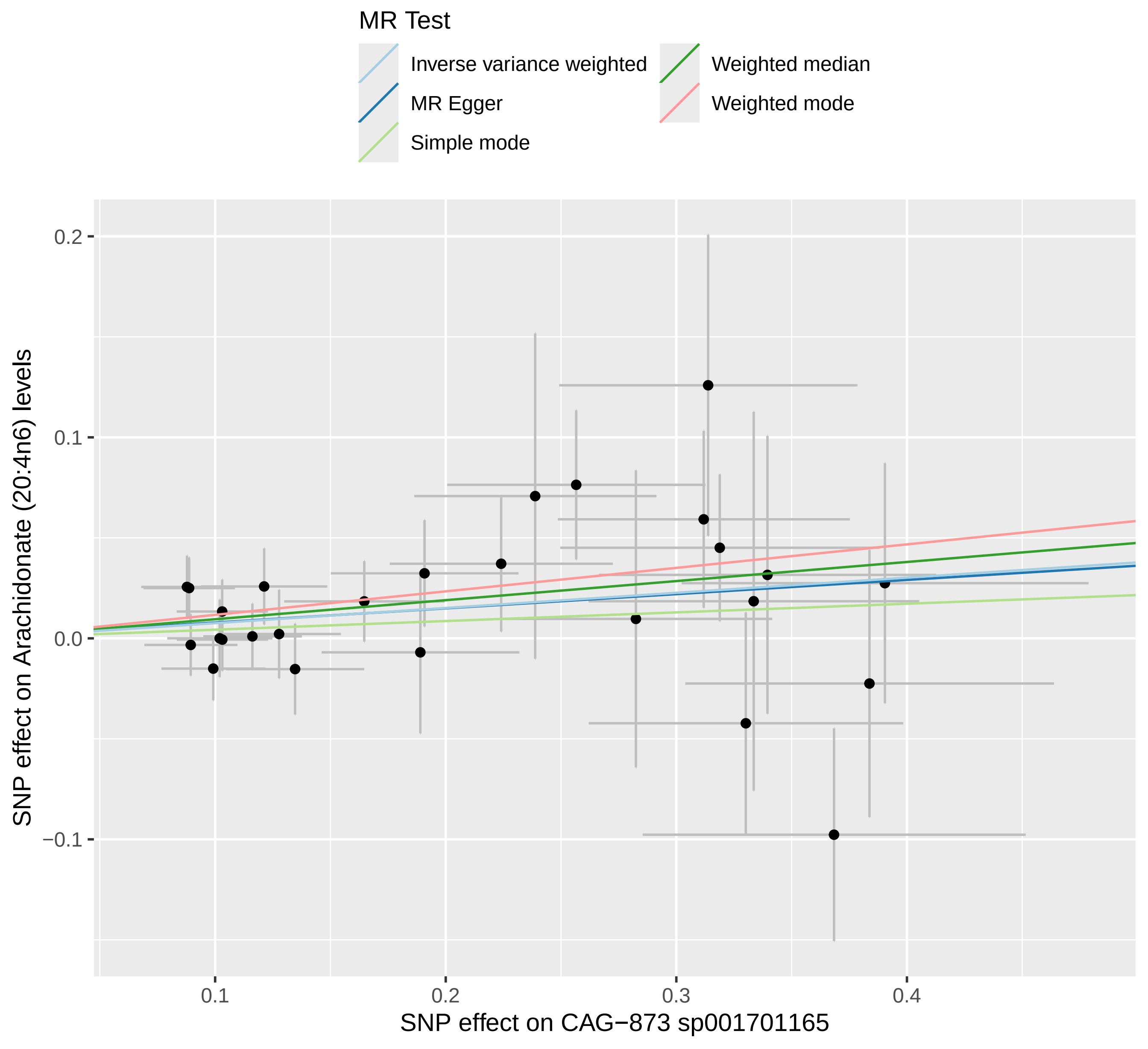

MR Test
Inverse variance weighted
MR Egger
Simple mode
Weighted median
Weighted mode
0.2
0.1
0.0
−0.1
0.1
0.2
0.3
0.4
SNP effect on Arachidonate (20:4n6) levels
SNP effect on CAG−873 sp001701165

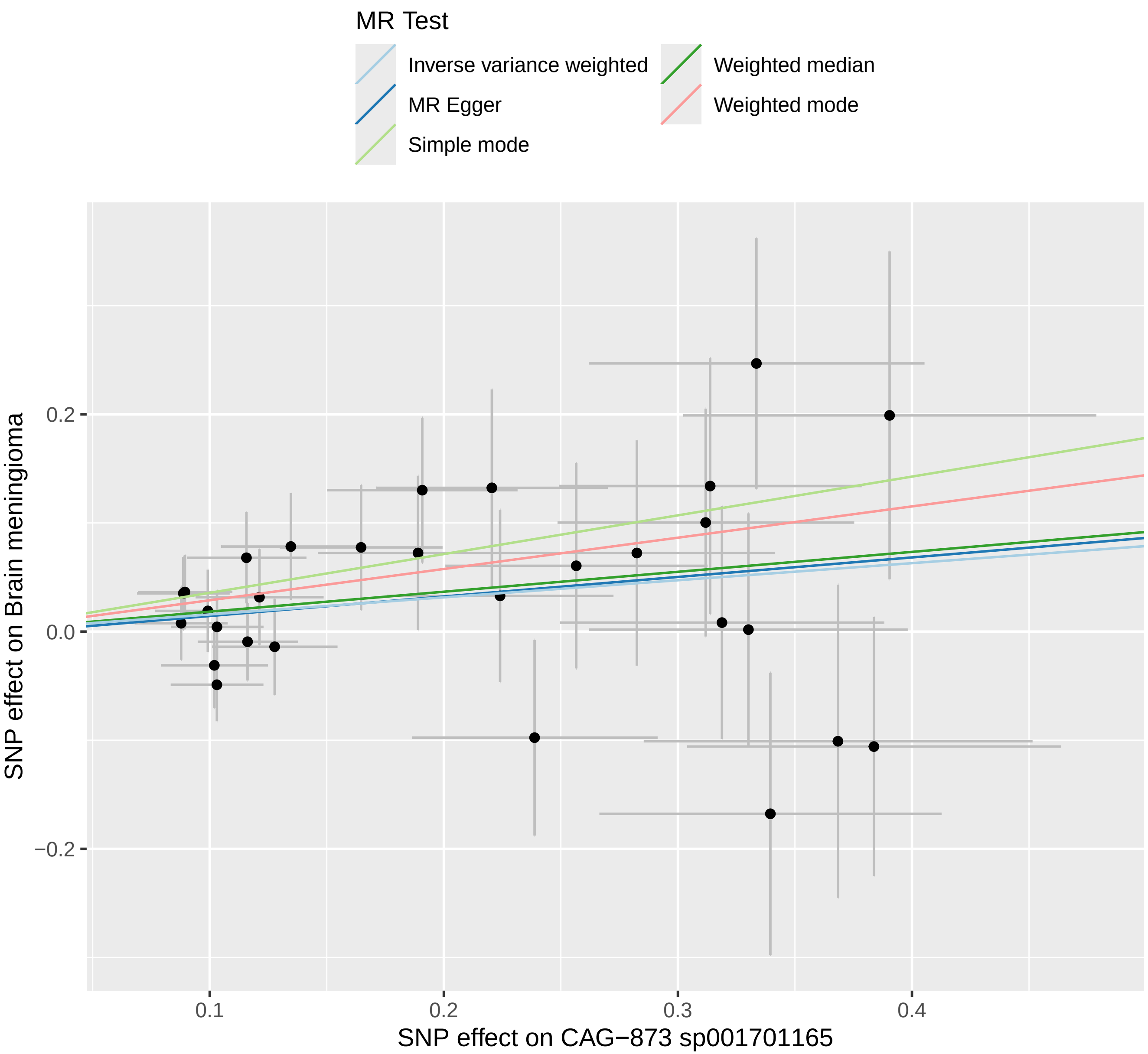
MR Test
Inverse variance weighted
MR Egger
Simple mode
Weighted median
Weighted mode
0.2
0.0
−0.2
0.1
0.2
0.3
0.4
SNP effect on Brain meningioma
SNP effect on CAG−873 sp001701165
