# Supplementary material for: Causal Pathways Linking Gut Microbiota, Serum Metabolites, and Meningioma Risk: A Mendelian Randomization Analysis
Source: Brain Behav. 2026 Feb 9;16(2):e71220. doi: 10.1002/brb3.71220 (PMC12887442; doi:10.1002/brb3.71220)
Supplement: Supplementary file 8 — Supplementary Appendix S8: brb371220‐sup‐0008‐Appendix8.pdf [file BRB3-16-e71220-s001.pdf]

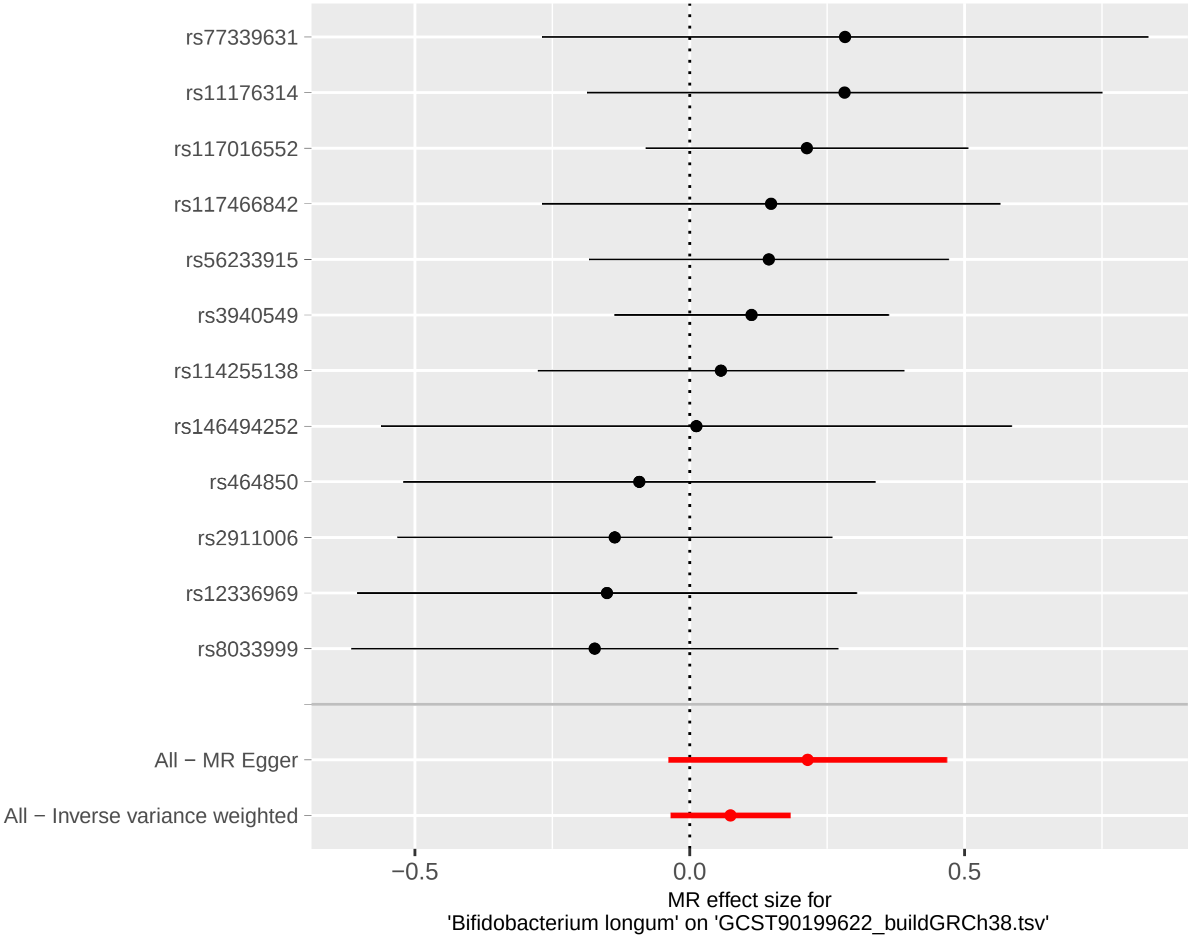
rs77339631
rs11176314
rs117016552
rs117466842
rs56233915
rs3940549
rs114255138
rs146494252
rs464850
rs2911006
rs12336969
rs8033999
All − MR Egger
All − Inverse variance weighted
−0.5
0.0
0.5
MR effect size for
'Bifidobacterium longum' on 'GCST90199622_buildGRCh38.tsv'

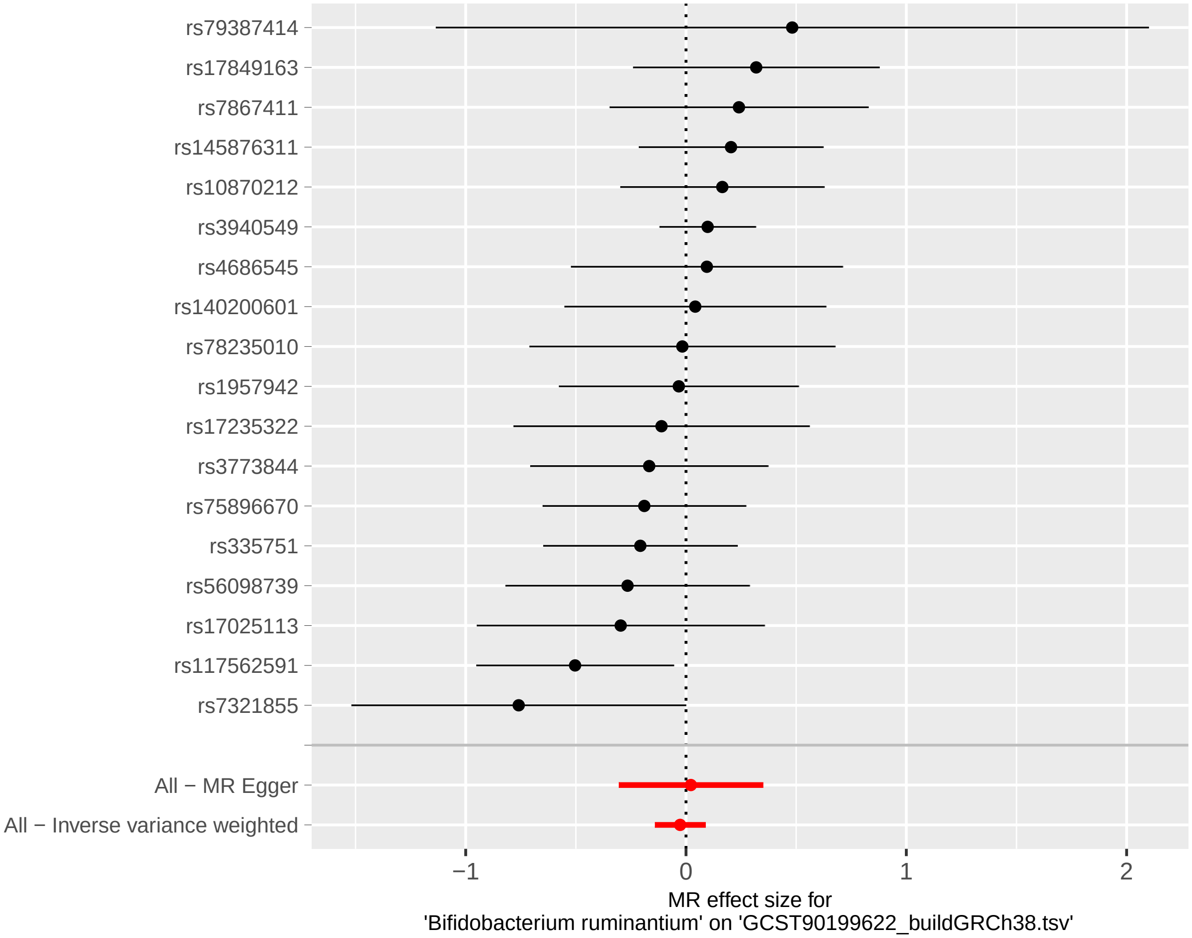
rs79387414
rs17849163
rs7867411
rs145876311
rs10870212
rs3940549
rs4686545
rs140200601
rs78235010
rs1957942
rs17235322
rs3773844
rs75896670
rs335751
rs56098739
rs17025113
rs117562591
rs7321855
All − MR Egger
All − Inverse variance weighted
−1
0
1
2
MR effect size for
'Bifidobacterium ruminantium' on 'GCST90199622_buildGRCh38.tsv'

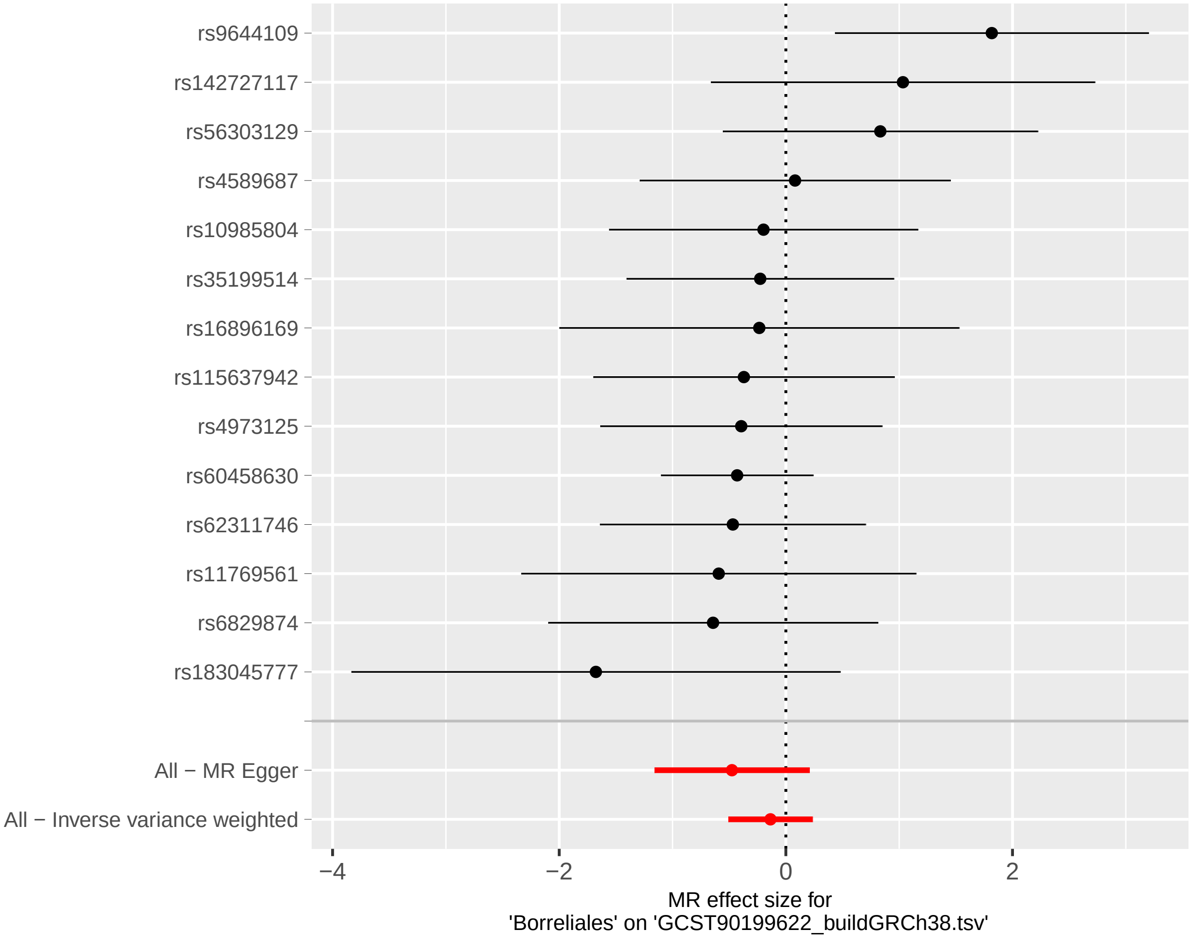
rs9644109
rs142727117
rs56303129
rs4589687
rs10985804
rs35199514
rs16896169
rs115637942
rs4973125
rs60458630
rs62311746
rs11769561
rs6829874
rs183045777
All − MR Egger
All − Inverse variance weighted
−4
−2
0
2
MR effect size for
'Borreliales' on 'GCST90199622_buildGRCh38.tsv'

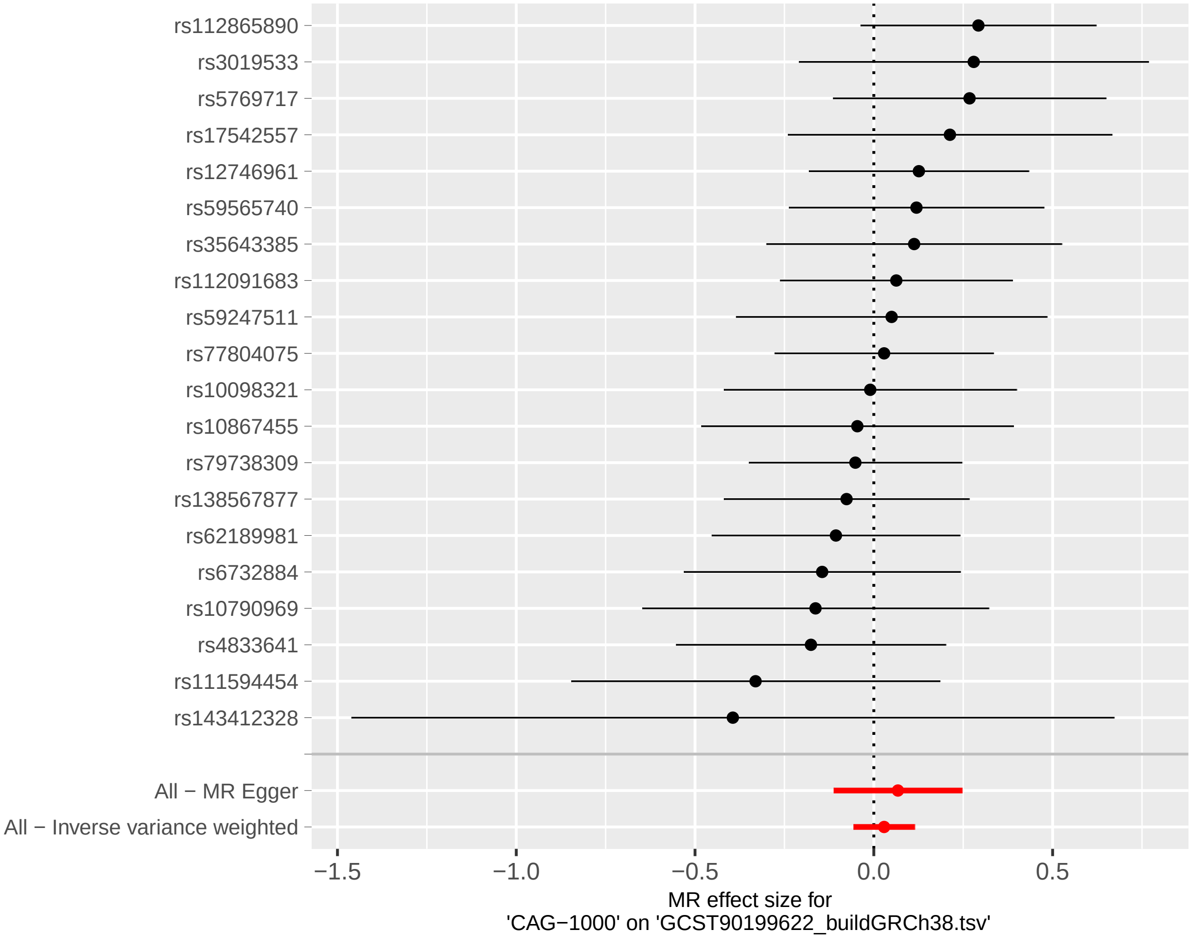

rs112865890
rs3019533
rs5769717
rs17542557
rs12746961
rs59565740
rs35643385
rs112091683
rs59247511
rs77804075
rs10098321
rs10867455
rs79738309
rs138567877
rs62189981
rs6732884
rs10790969
rs4833641
rs111594454
rs143412328
All − MR Egger
All − Inverse variance weighted
−1.5
−1.0
−0.5
0.0
0.5
MR effect size for
'CAG−1000' on 'GCST90199622_buildGRCh38.tsv'

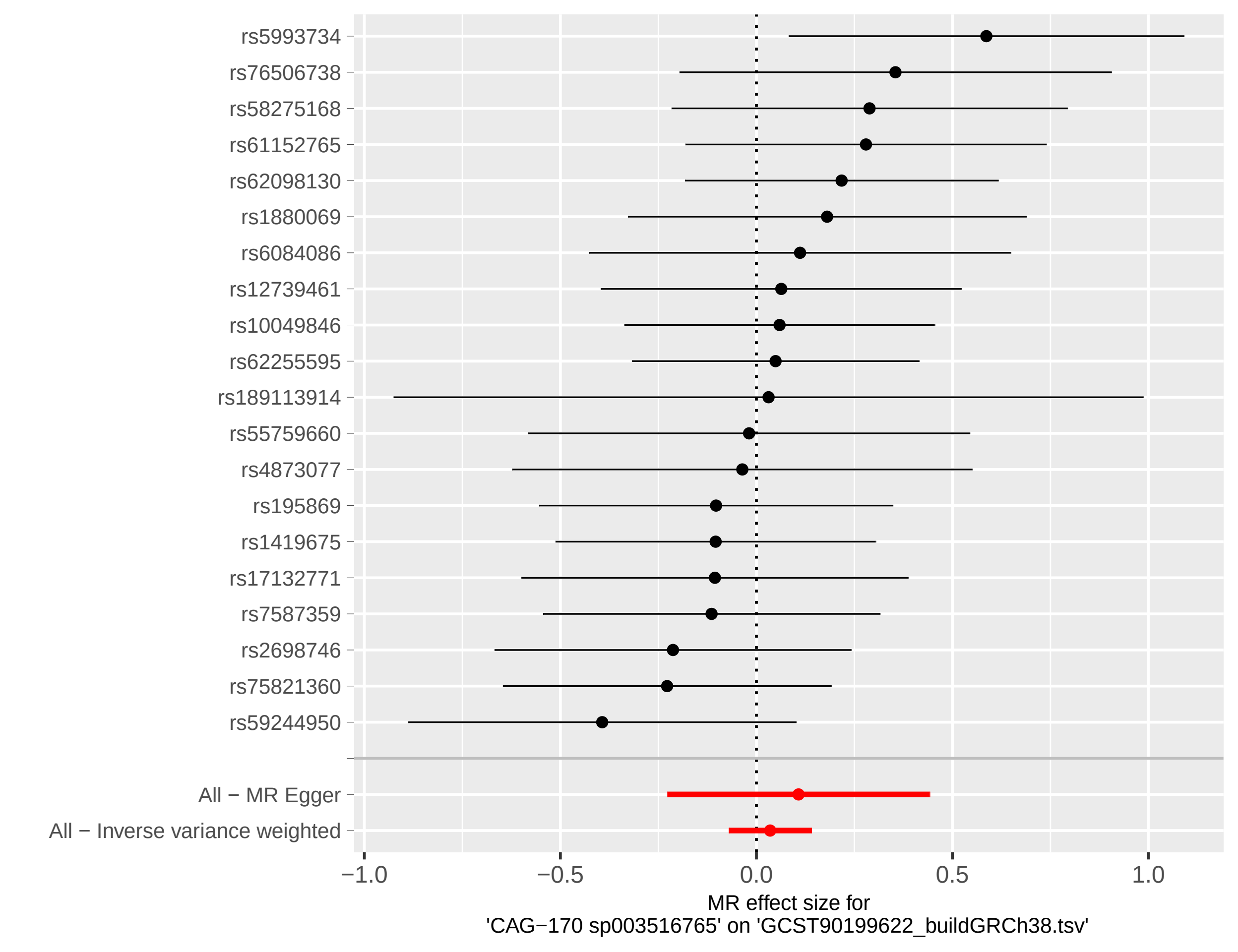

rs5993734
rs76506738
rs58275168
rs61152765
rs62098130
rs1880069
rs6084086
rs12739461
rs10049846
rs62255595
rs189113914
rs55759660
rs4873077
rs195869
rs1419675
rs17132771
rs7587359
rs2698746
rs75821360
rs59244950
All − MR Egger
All − Inverse variance weighted
−1.0
−0.5
0.0
0.5
1.0
MR effect size for
'CAG−170 sp003516765' on 'GCST90199622_buildGRCh38.tsv'

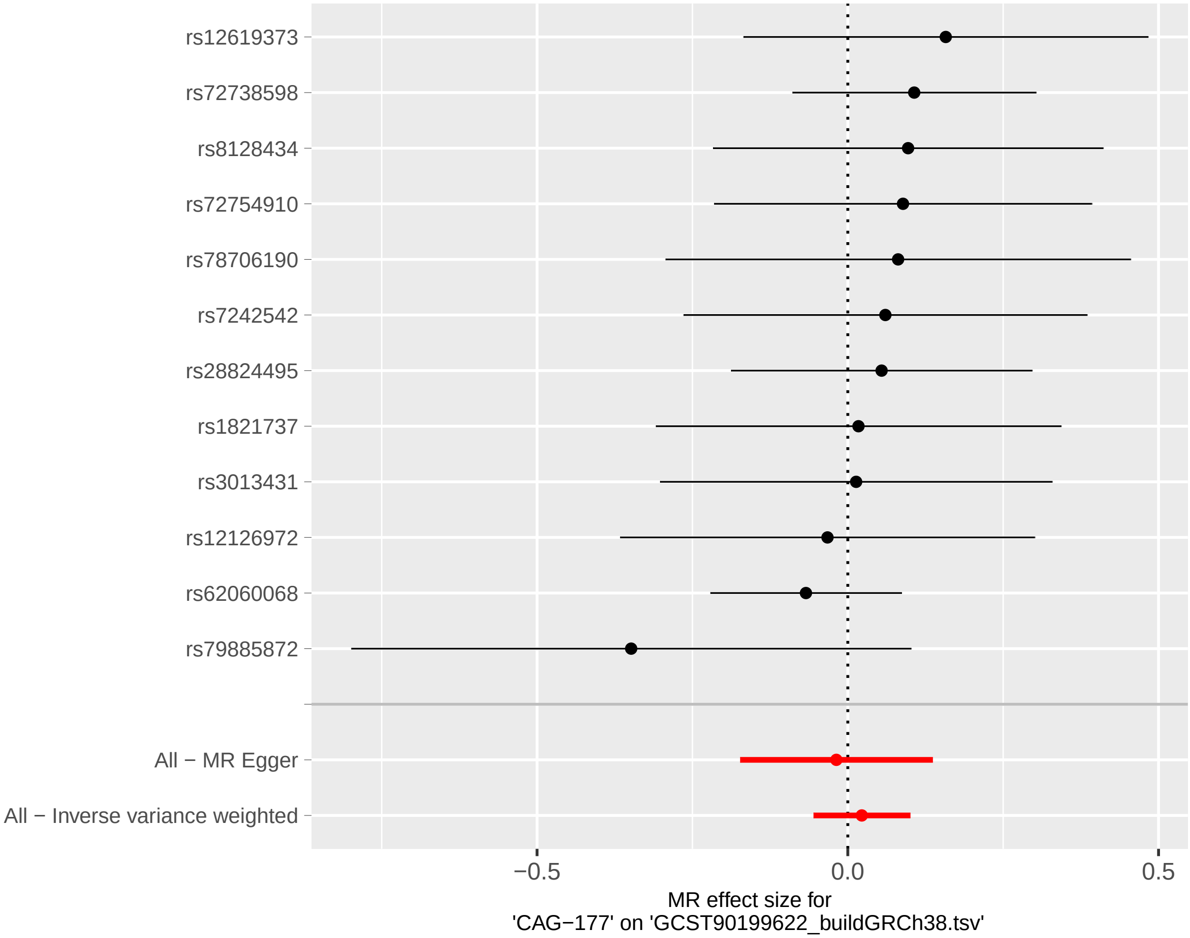

rs12619373
rs72738598
rs8128434
rs72754910
rs78706190
rs7242542
rs28824495
rs1821737
rs3013431
rs12126972
rs62060068
rs79885872
All − MR Egger
All − Inverse variance weighted
−0.5
0.0
0.5
MR effect size for
'CAG−177' on 'GCST90199622_buildGRCh38.tsv'

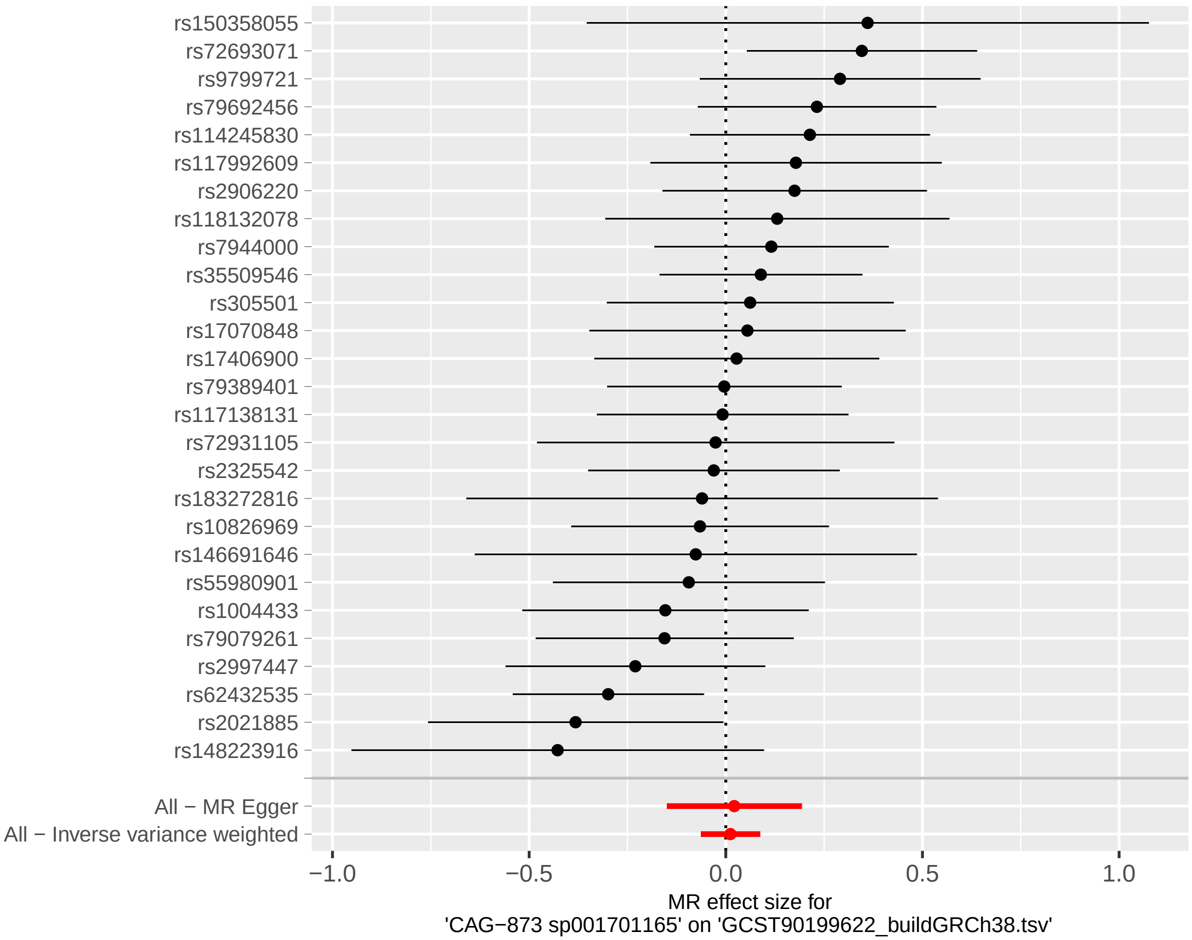
rs150358055
rs72693071
rs9799721
rs79692456
rs114245830
rs117992609
rs2906220
rs118132078
rs7944000
rs35509546
rs305501
rs17070848
rs17406900
rs79389401
rs117138131
rs72931105
rs2325542
rs183272816
rs10826969
rs146691646
rs55980901
rs1004433
rs79079261
rs2997447
rs62432535
rs2021885
rs148223916
All − MR Egger
All − Inverse variance weighted
−1.0
−0.5
0.0
0.5
1.0
MR effect size for
'CAG−873 sp001701165' on 'GCST90199622_buildGRCh38.tsv'

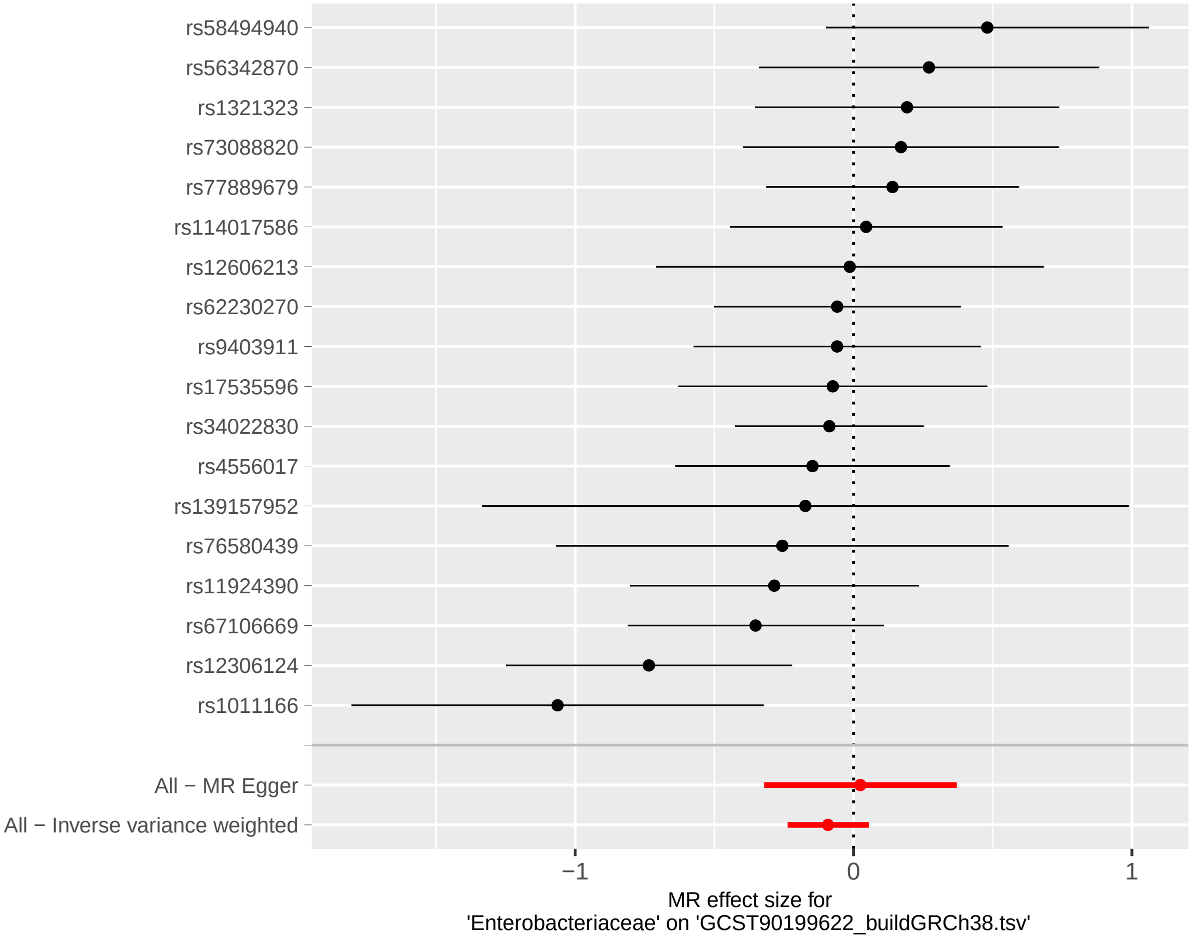

rs58494940
rs56342870
rs1321323
rs73088820
rs77889679
rs114017586
rs12606213
rs62230270
rs9403911
rs17535596
rs34022830
rs4556017
rs139157952
rs76580439
rs11924390
rs67106669
rs12306124
rs1011166
All − MR Egger
All − Inverse variance weighted
−1
0
1
MR effect size for
'Enterobacteriaceae' on 'GCST90199622_buildGRCh38.tsv'

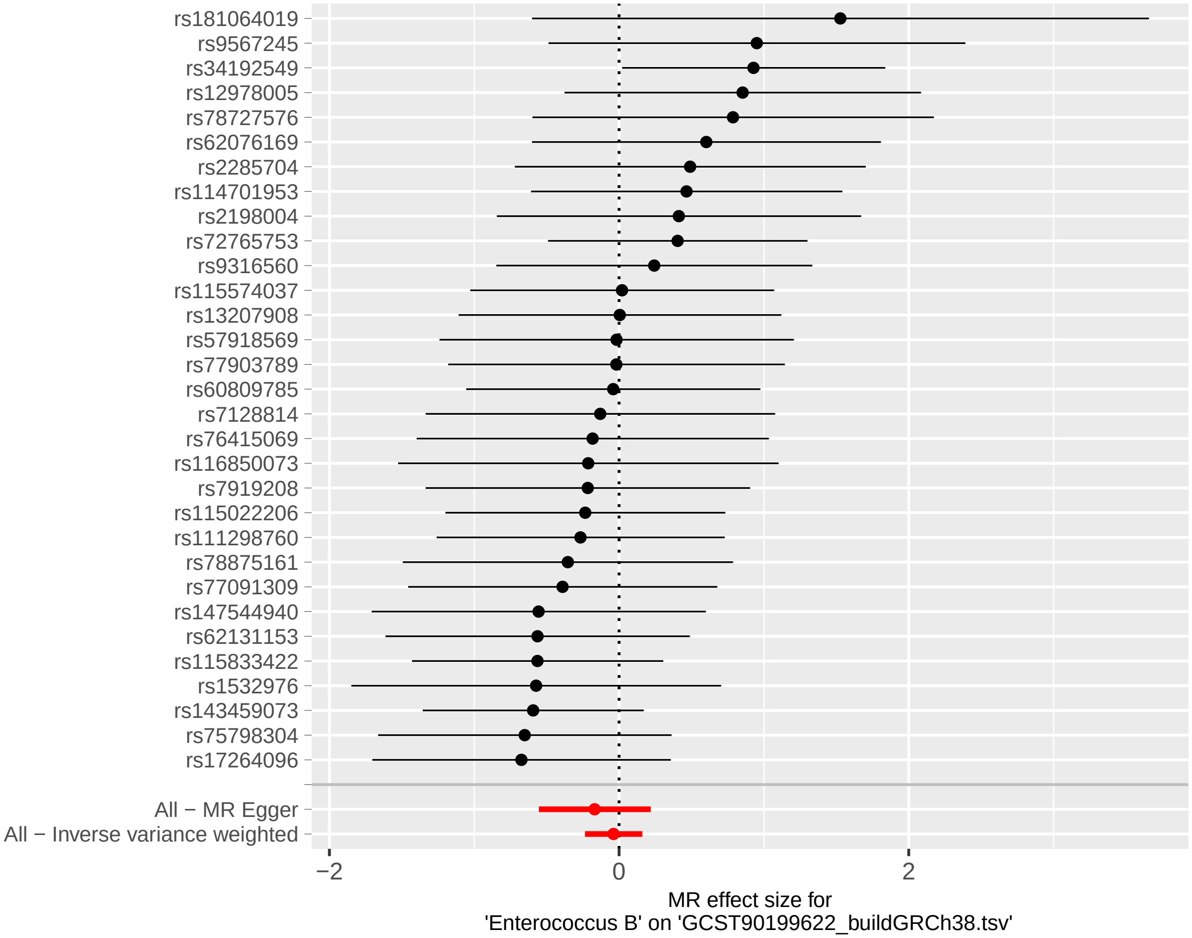

rs181064019
rs9567245
rs34192549
rs12978005
rs78727576
rs62076169
rs2285704
rs114701953
rs2198004
rs72765753
rs9316560
rs115574037
rs13207908
rs57918569
rs77903789
rs60809785
rs7128814
rs76415069
rs116850073
rs7919208
rs115022206
rs111298760
rs78875161
rs77091309
rs147544940
rs62131153
rs115833422
rs1532976
rs143459073
rs75798304
rs17264096
All − MR Egger
All − Inverse variance weighted
−2
0
2
MR effect size for
'Enterococcus B' on 'GCST90199622_buildGRCh38.tsv'

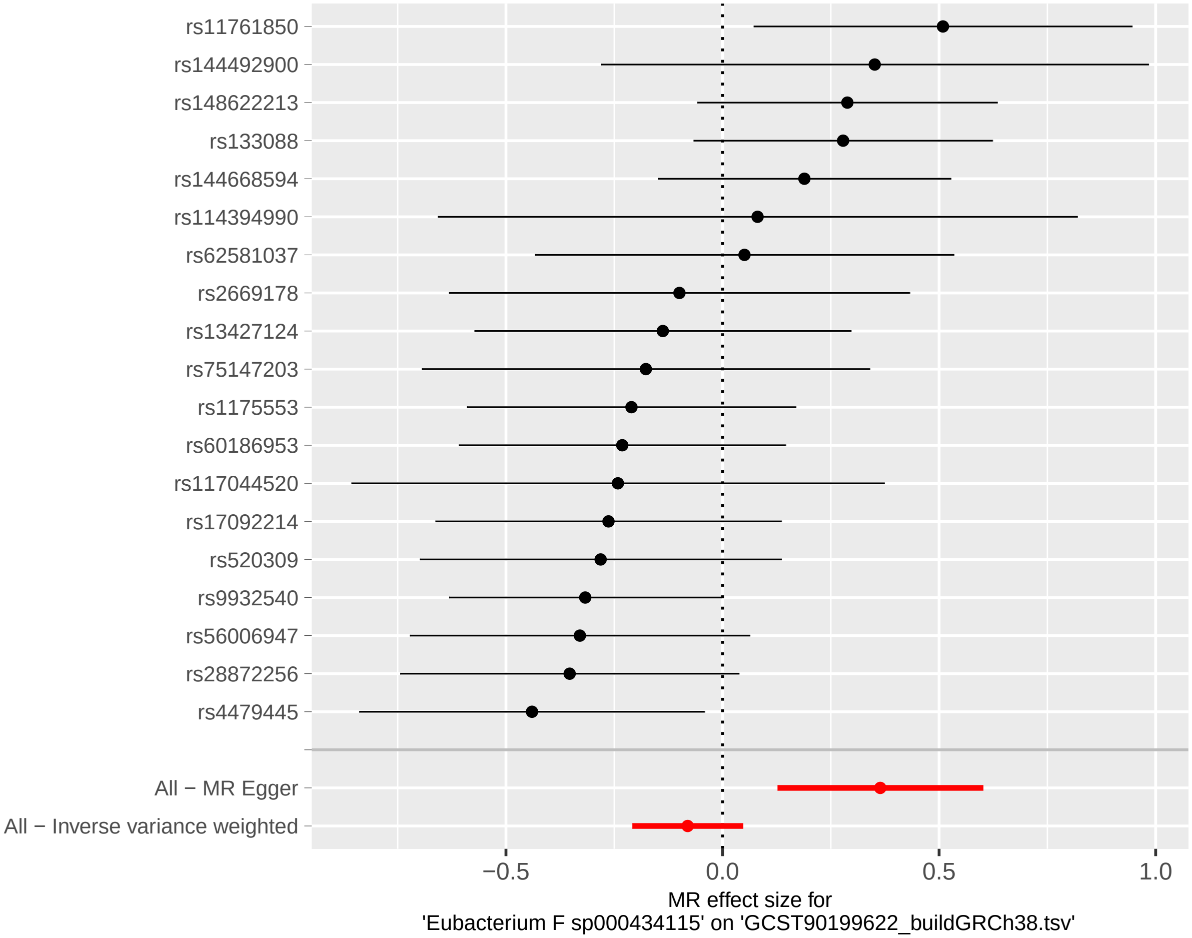
rs11761850
rs144492900
rs148622213
rs133088
rs144668594
rs114394990
rs62581037
rs2669178
rs13427124
rs75147203
rs1175553
rs60186953
rs117044520
rs17092214
rs520309
rs9932540
rs56006947
rs28872256
rs4479445
All − MR Egger
All − Inverse variance weighted
−0.5
0.0
0.5
1.0
MR effect size for
'Eubacterium F sp000434115' on 'GCST90199622_buildGRCh38.tsv'

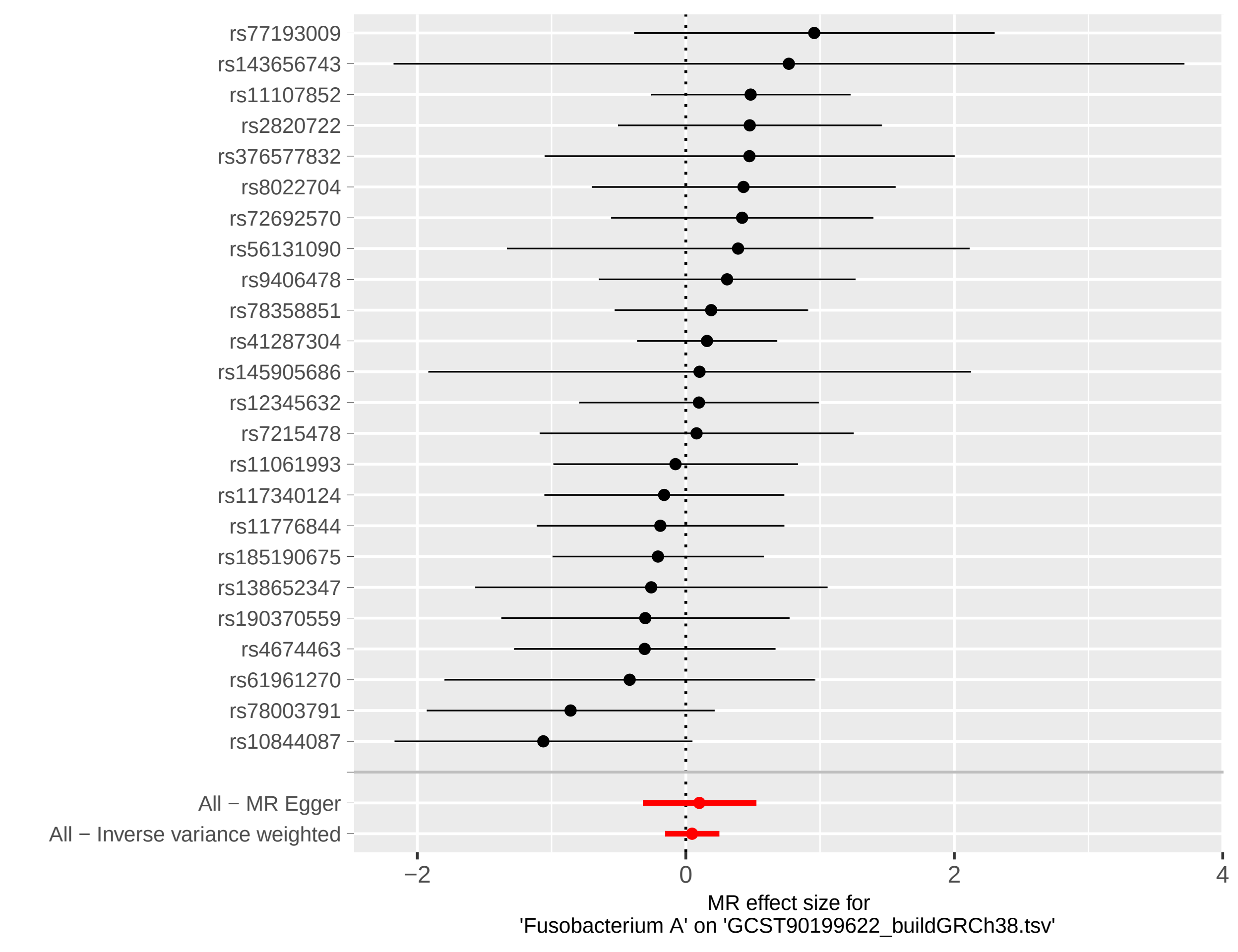
rs77193009
rs143656743
rs11107852
rs2820722
rs376577832
rs8022704
rs72692570
rs56131090
rs9406478
rs78358851
rs41287304
rs145905686
rs12345632
rs7215478
rs11061993
rs117340124
rs11776844
rs185190675
rs138652347
rs190370559
rs4674463
rs61961270
rs78003791
rs10844087
All − MR Egger
All − Inverse variance weighted
−2
0
2
4
MR effect size for
'Fusobacterium A' on 'GCST90199622_buildGRCh38.tsv'

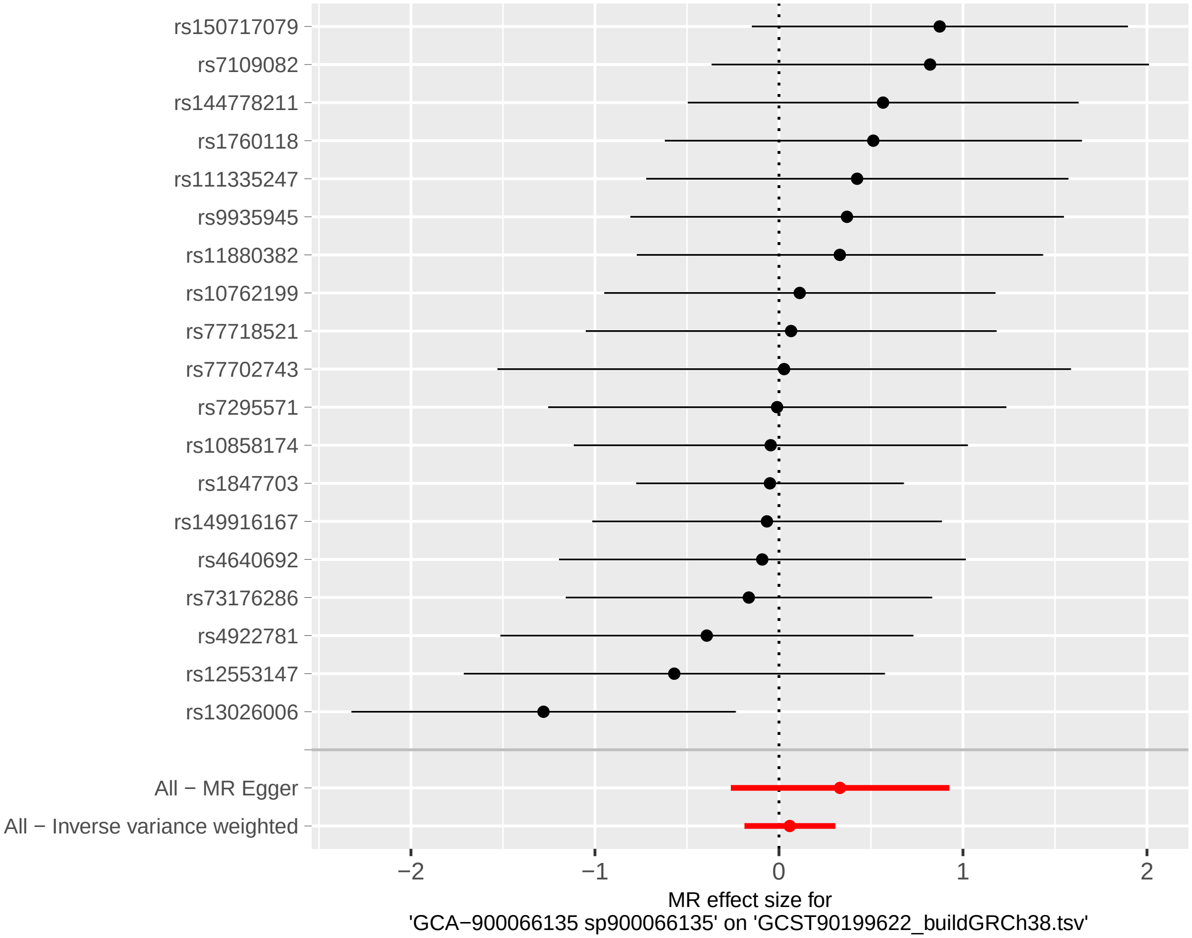

rs150717079
rs7109082
rs144778211
rs1760118
rs111335247
rs9935945
rs11880382
rs10762199
rs77718521
rs77702743
rs7295571
rs10858174
rs1847703
rs149916167
rs4640692
rs73176286
rs4922781
rs12553147
rs13026006
All − MR Egger
All − Inverse variance weighted
−2
−1
0
1
2
MR effect size for
'GCA−900066135 sp900066135' on 'GCST90199622_buildGRCh38.tsv'

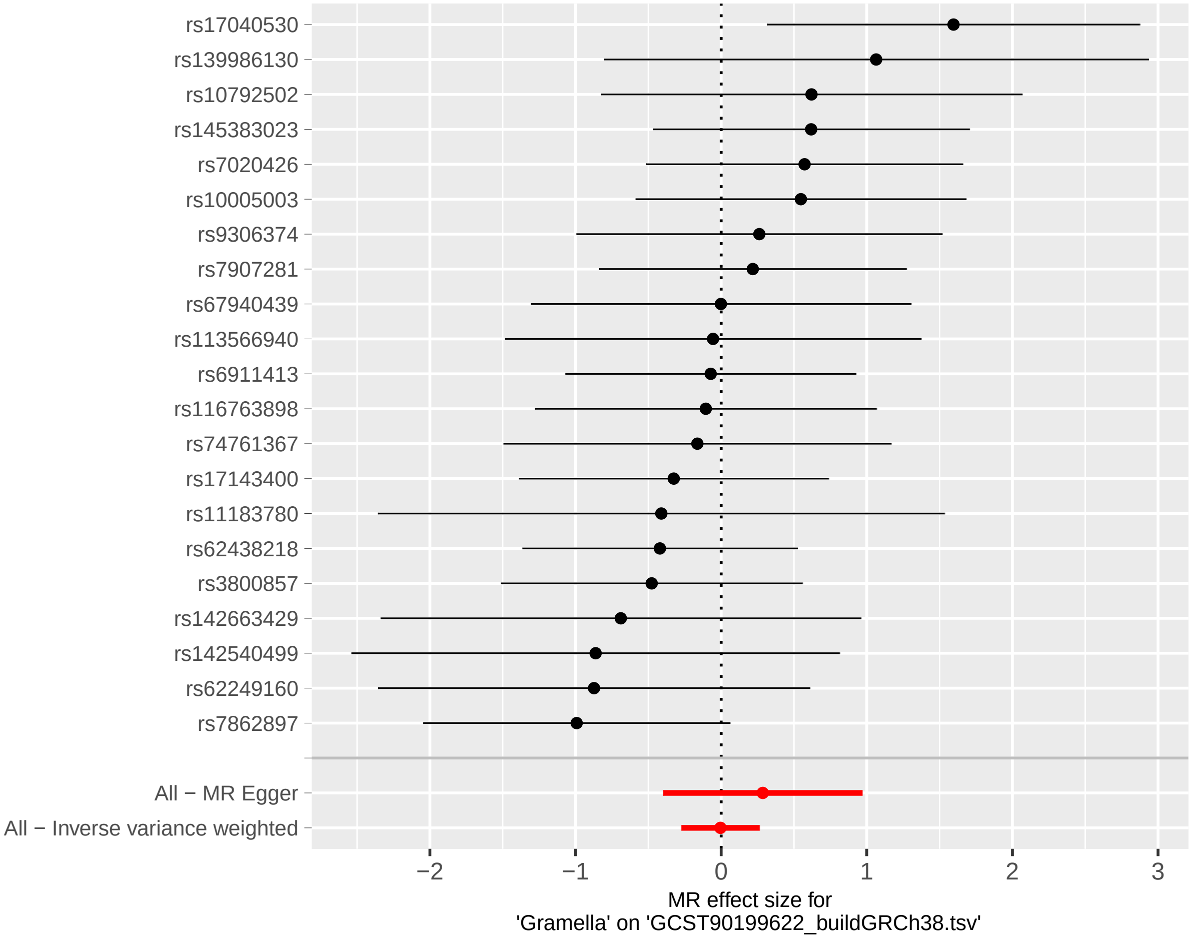

rs17040530
rs139986130
rs10792502
rs145383023
rs7020426
rs10005003
rs9306374
rs7907281
rs67940439
rs113566940
rs6911413
rs116763898
rs74761367
rs17143400
rs11183780
rs62438218
rs3800857
rs142663429
rs142540499
rs62249160
rs7862897
All − MR Egger
All − Inverse variance weighted
−2
−1
0
1
2
3
MR effect size for
'Gramella' on 'GCST90199622_buildGRCh38.tsv'

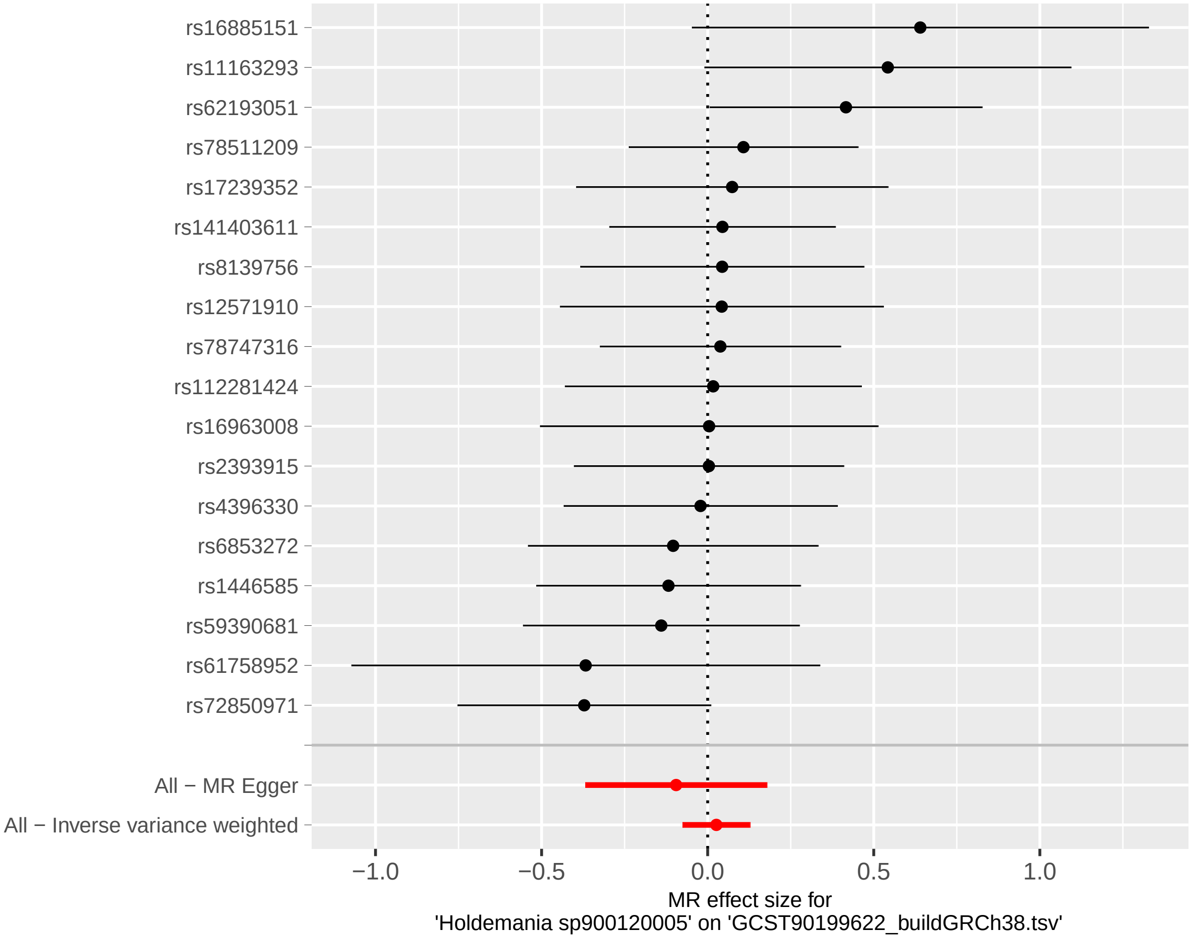

rs16885151
rs11163293
rs62193051
rs78511209
rs17239352
rs141403611
rs8139756
rs12571910
rs78747316
rs112281424
rs16963008
rs2393915
rs4396330
rs6853272
rs1446585
rs59390681
rs61758952
rs72850971
All − MR Egger
All − Inverse variance weighted
−1.0
−0.5
0.0
0.5
1.0
MR effect size for
'Holdemania sp900120005' on 'GCST90199622_buildGRCh38.tsv'

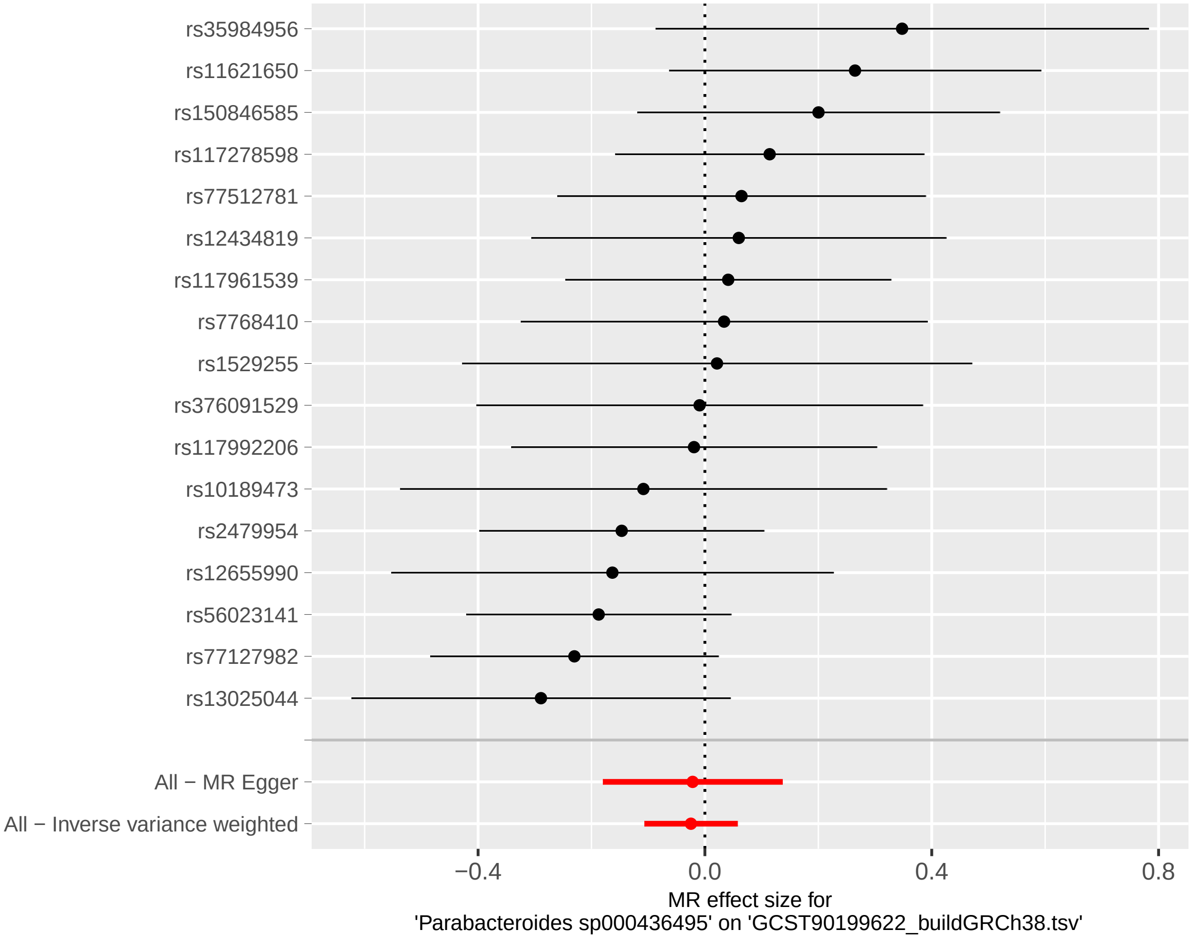
rs35984956
rs11621650
rs150846585
rs117278598
rs77512781
rs12434819
rs117961539
rs7768410
rs1529255
rs376091529
rs117992206
rs10189473
rs2479954
rs12655990
rs56023141
rs77127982
rs13025044
All − MR Egger
All − Inverse variance weighted
−0.4
0.0
0.4
0.8
MR effect size for
'Parabacteroides sp000436495' on 'GCST90199622_buildGRCh38.tsv'

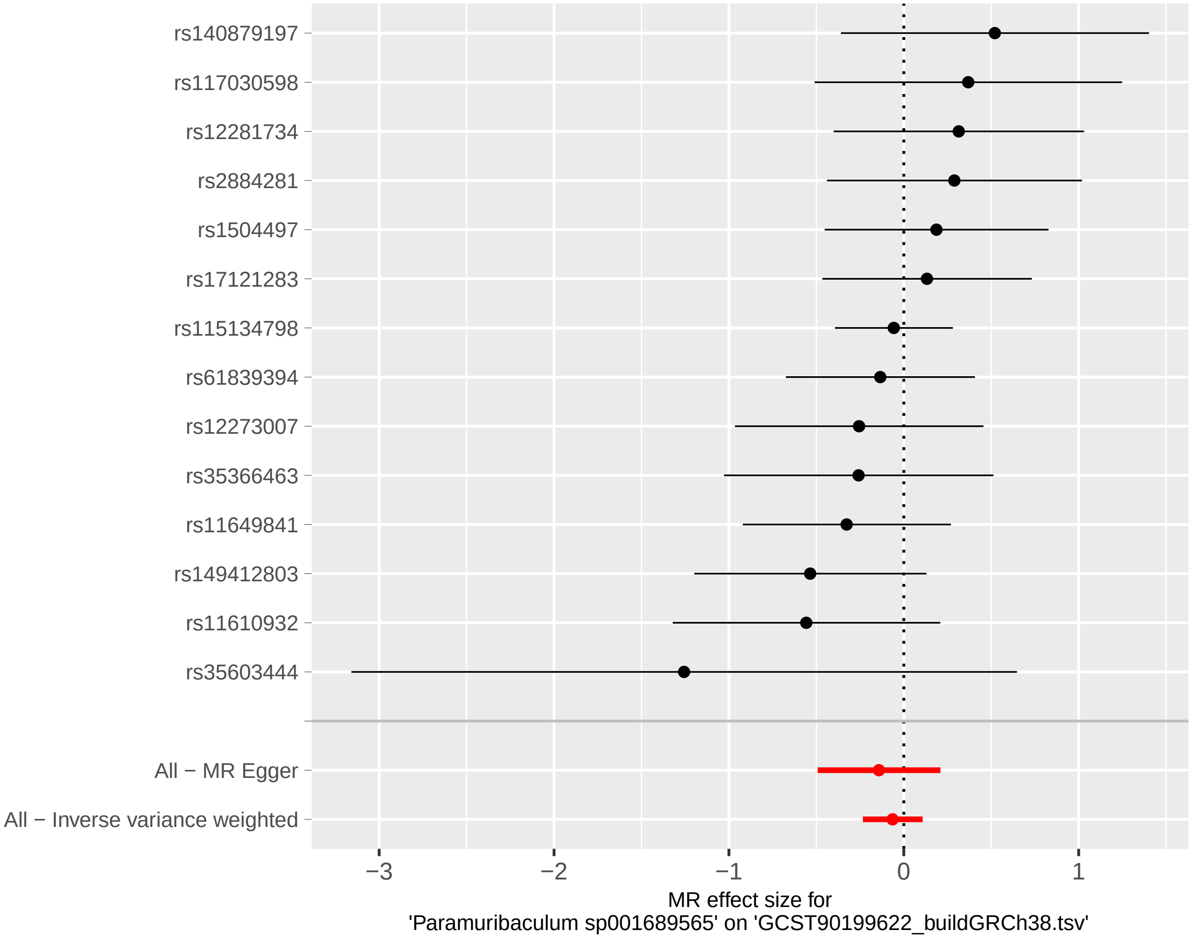
rs140879197
rs117030598
rs12281734
rs2884281
rs1504497
rs17121283
rs115134798
rs61839394
rs12273007
rs35366463
rs11649841
rs149412803
rs11610932
rs35603444
All − MR Egger
All − Inverse variance weighted
−3
−2
−1
0
1
MR effect size for
'Paramuribaculum sp001689565' on 'GCST90199622_buildGRCh38.tsv'

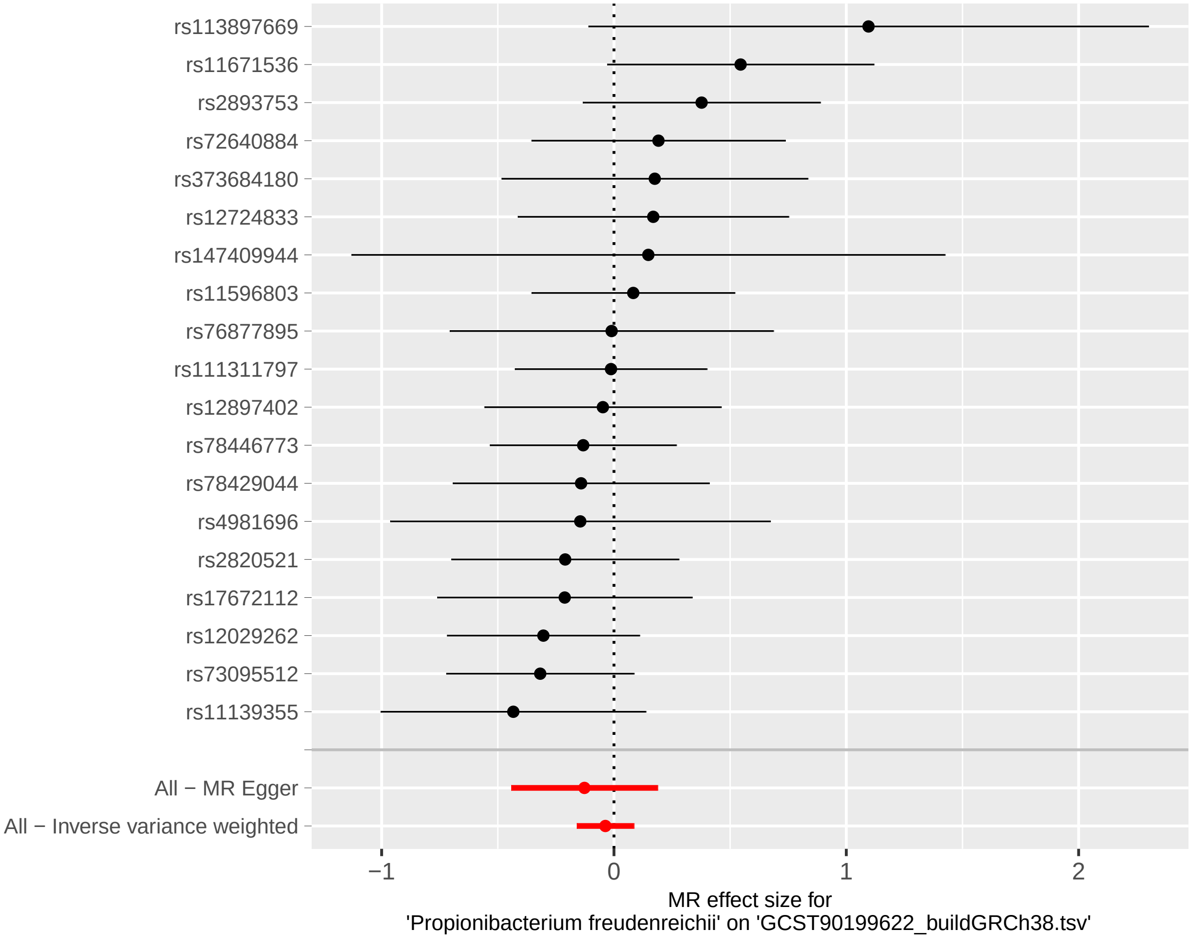

rs113897669
rs11671536
rs2893753
rs72640884
rs373684180
rs12724833
rs147409944
rs11596803
rs76877895
rs111311797
rs12897402
rs78446773
rs78429044
rs4981696
rs2820521
rs17672112
rs12029262
rs73095512
rs11139355
All − MR Egger
All − Inverse variance weighted
−1
0
1
2
MR effect size for
'Propionibacterium freudenreichii' on 'GCST90199622_buildGRCh38.tsv'

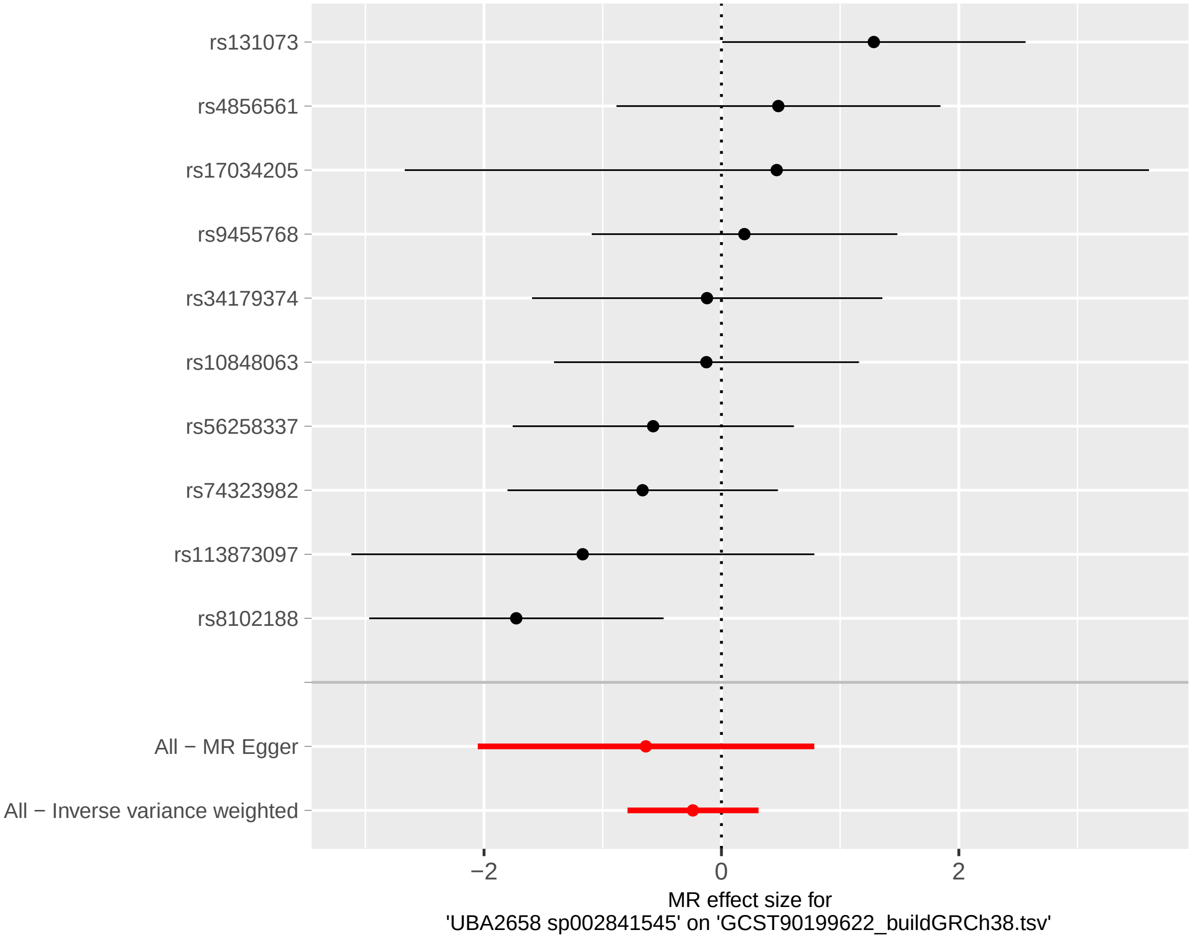
rs131073
rs4856561
rs17034205
rs9455768
rs34179374
rs10848063
rs56258337
rs74323982
rs113873097
rs8102188
All − MR Egger
All − Inverse variance weighted
−2
0
2
MR effect size for
'UBA2658 sp002841545' on 'GCST90199622_buildGRCh38.tsv'

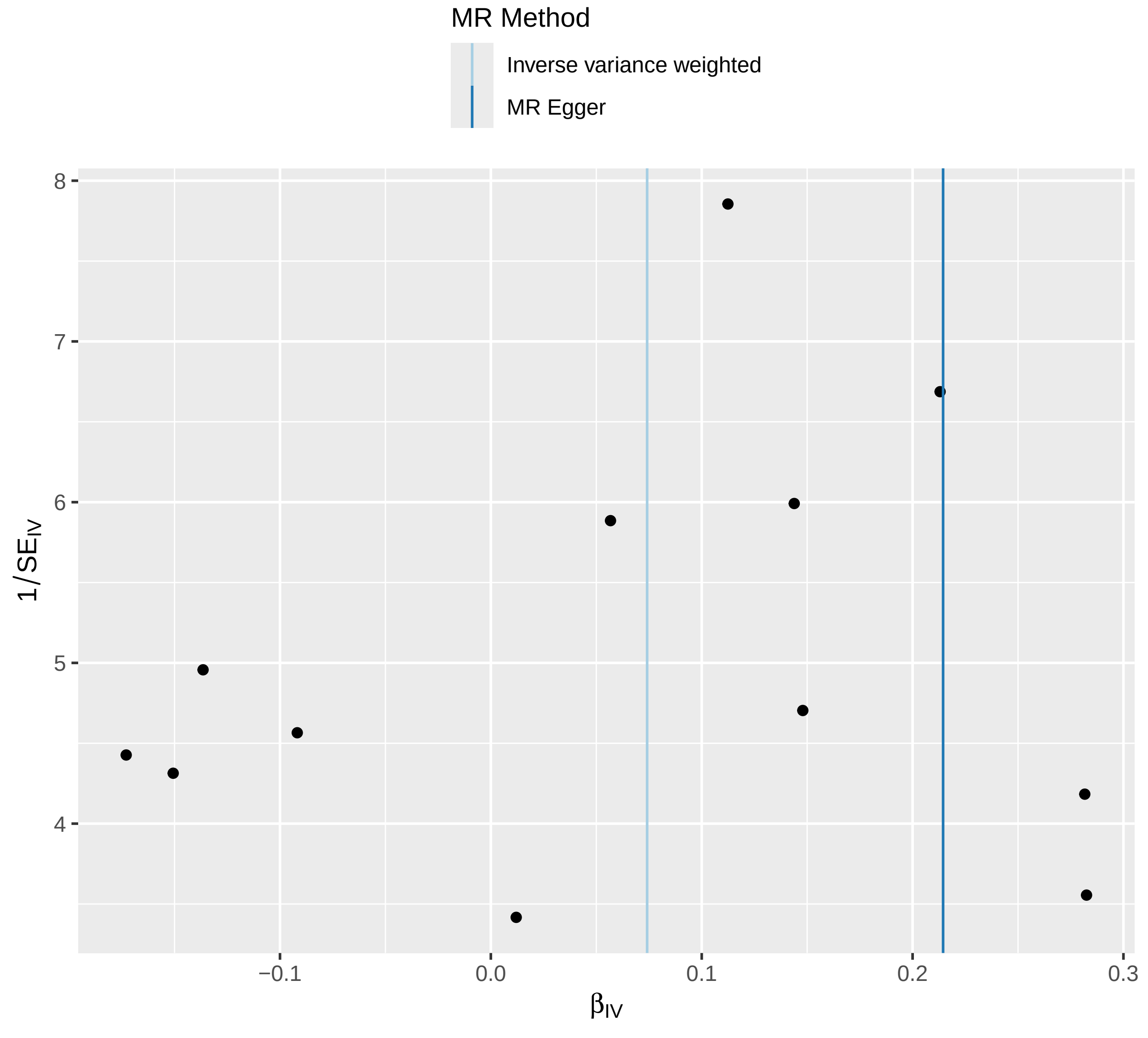

MR Method
Inverse variance weighted
MR Egger
8
7
6
5
4
1/SE_IV
−0.1
0.0
0.1
0.2
0.3
β_IV

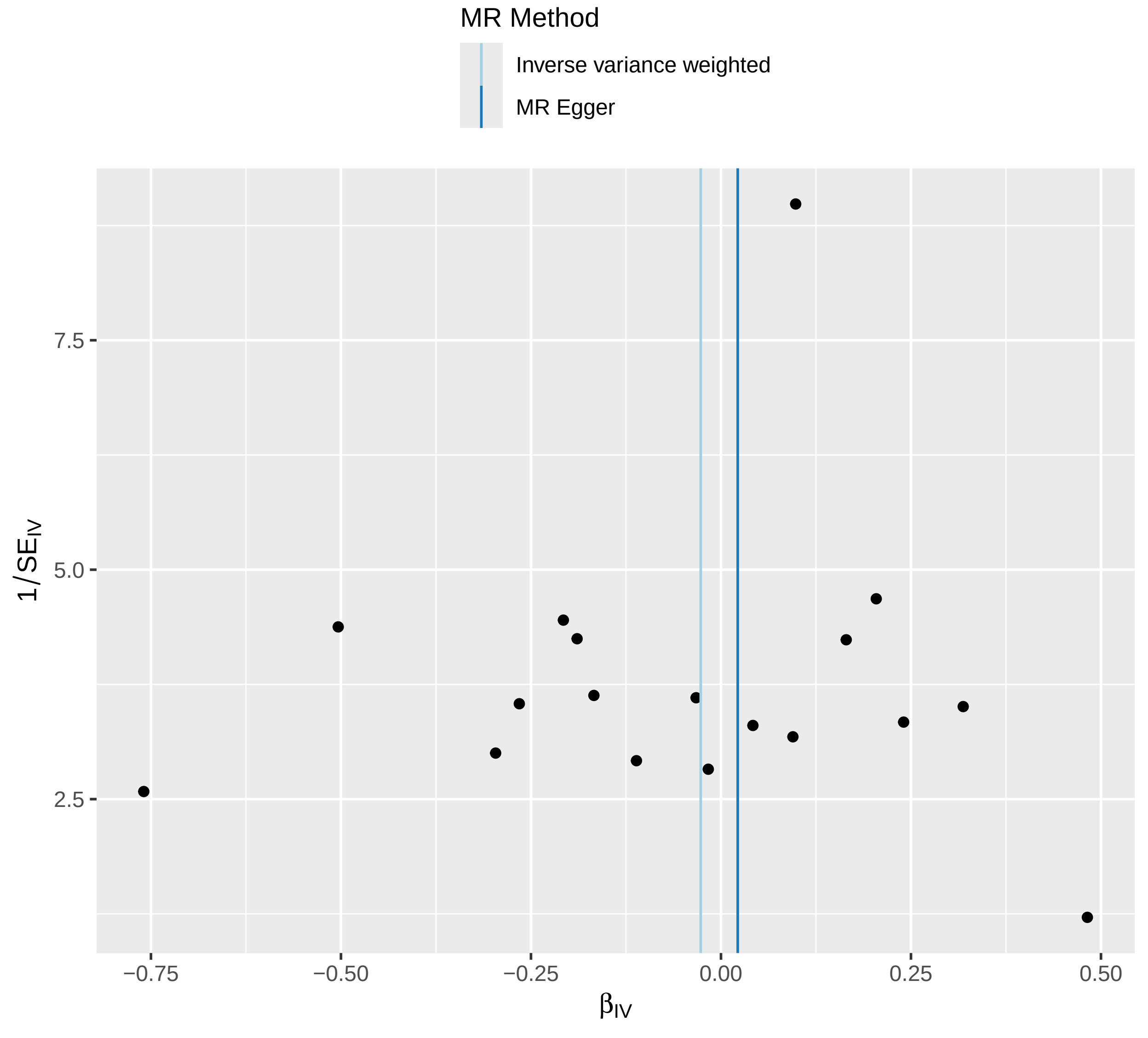

MR Method
Inverse variance weighted
MR Egger
7.5
5.0
2.5
$1/SE_{IV}$
−0.75
−0.50
−0.25
0.00
0.25
0.50
β_{IV}

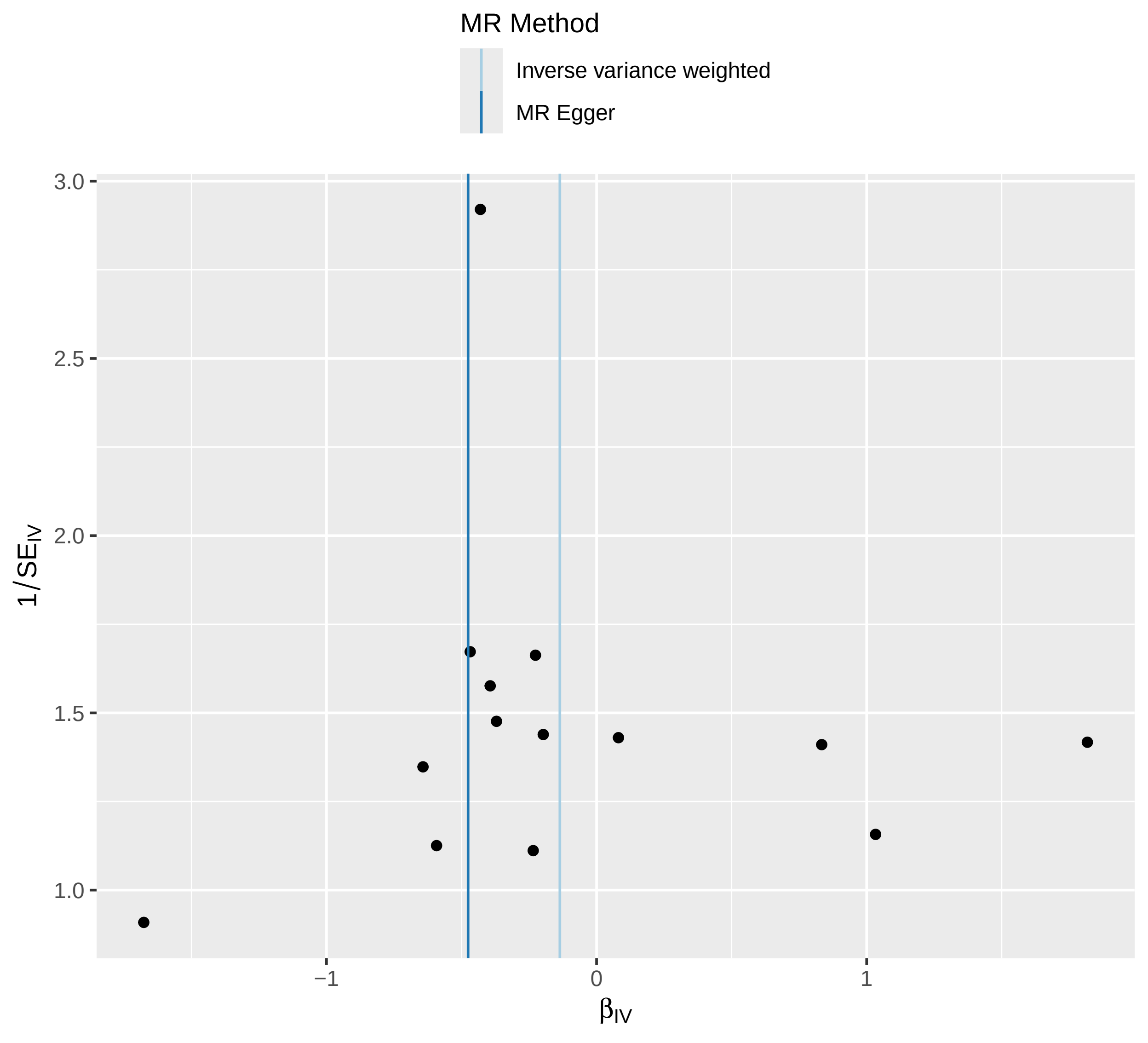
MR Method
Inverse variance weighted
MR Egger
3.0
2.5
2.0
1.5
1.0
$1/SE_{IV}$
−1
0
1
β_{IV}

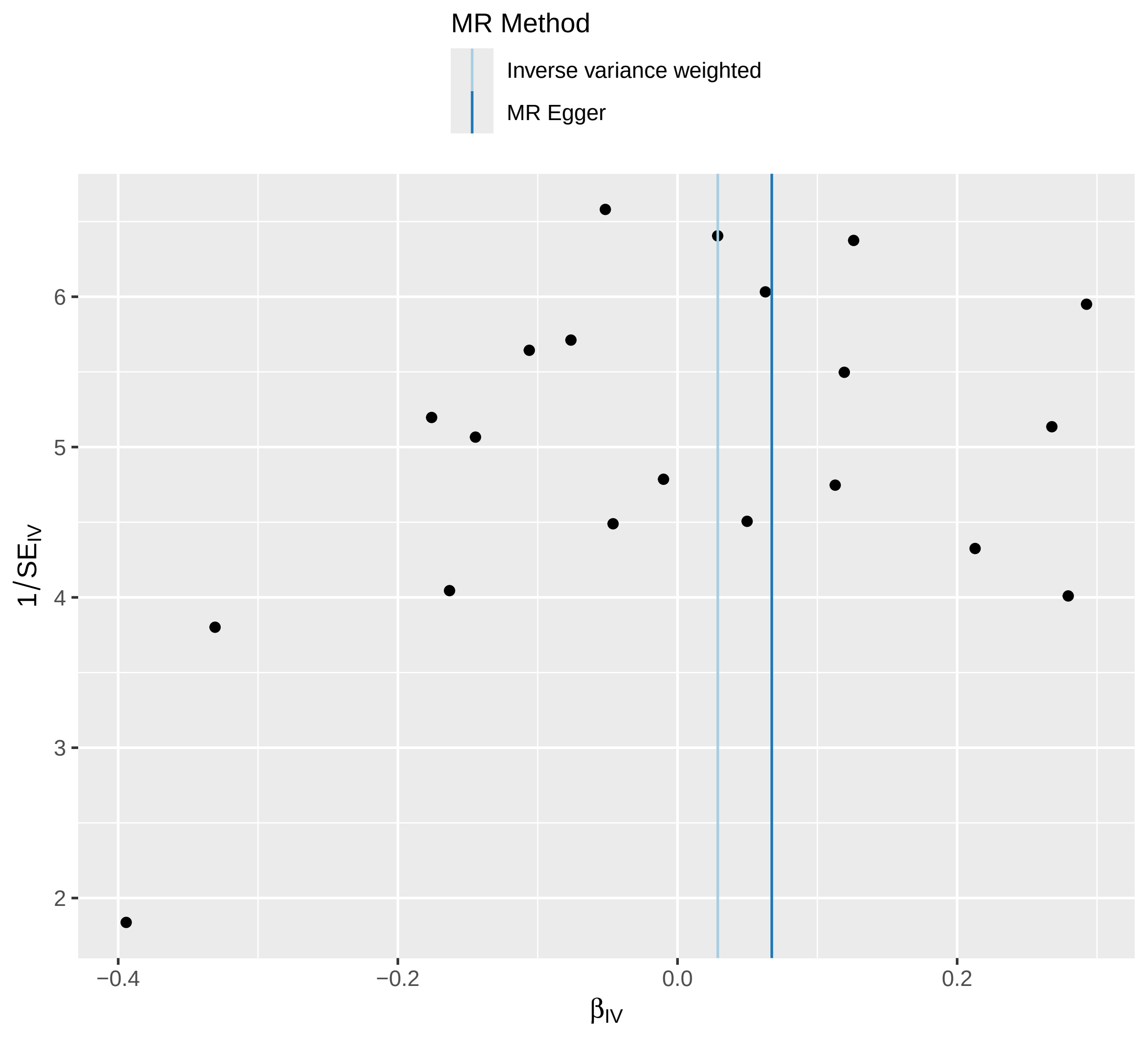
MR Method
Inverse variance weighted
MR Egger
1/SE_IV
β_IV
6
5
4
3
2
−0.4
−0.2
0.0
0.2

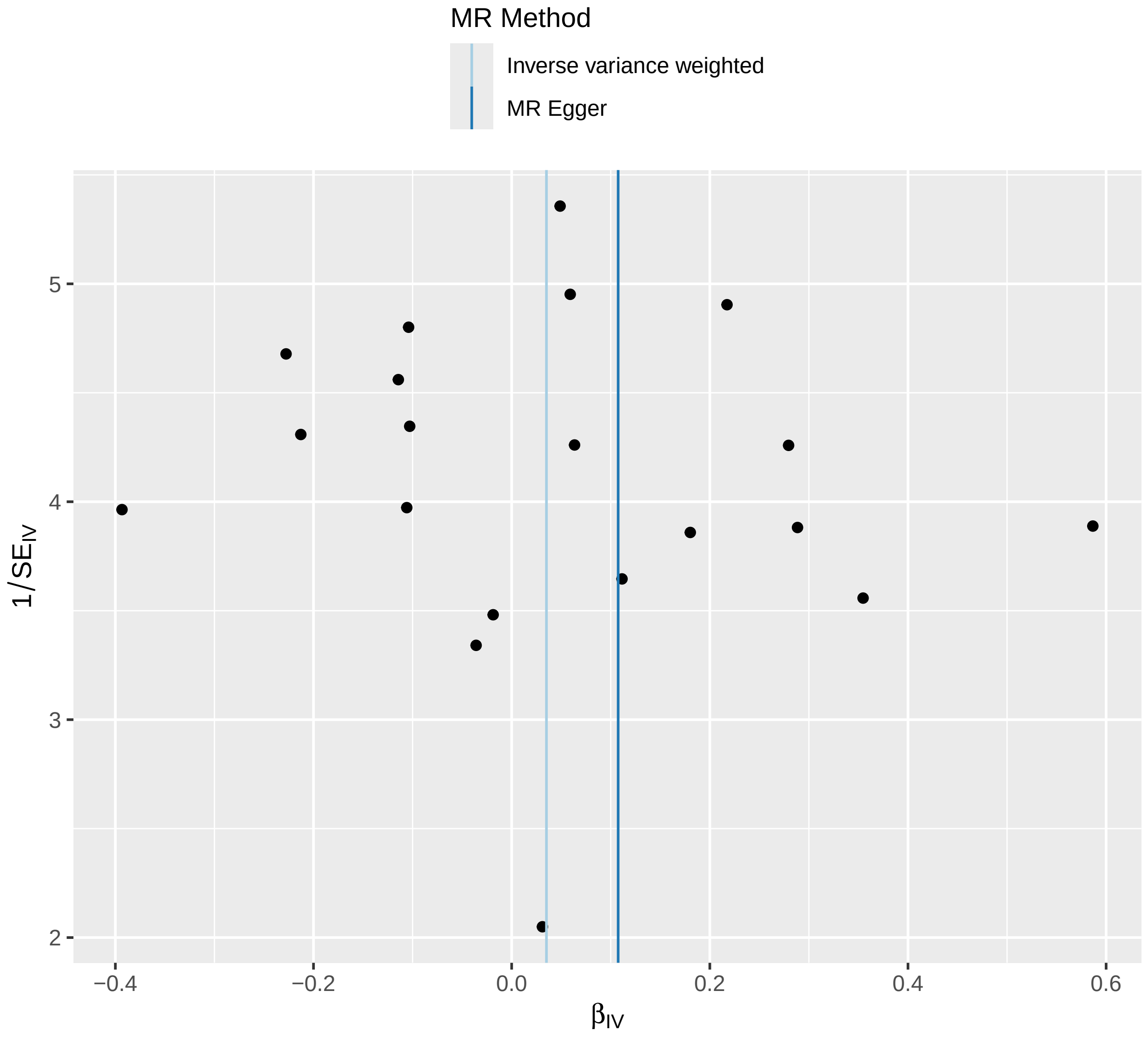
MR Method
Inverse variance weighted
MR Egger
5
4
3
2
1/SE_IV
−0.4
−0.2
0.0
0.2
0.4
0.6
β_IV

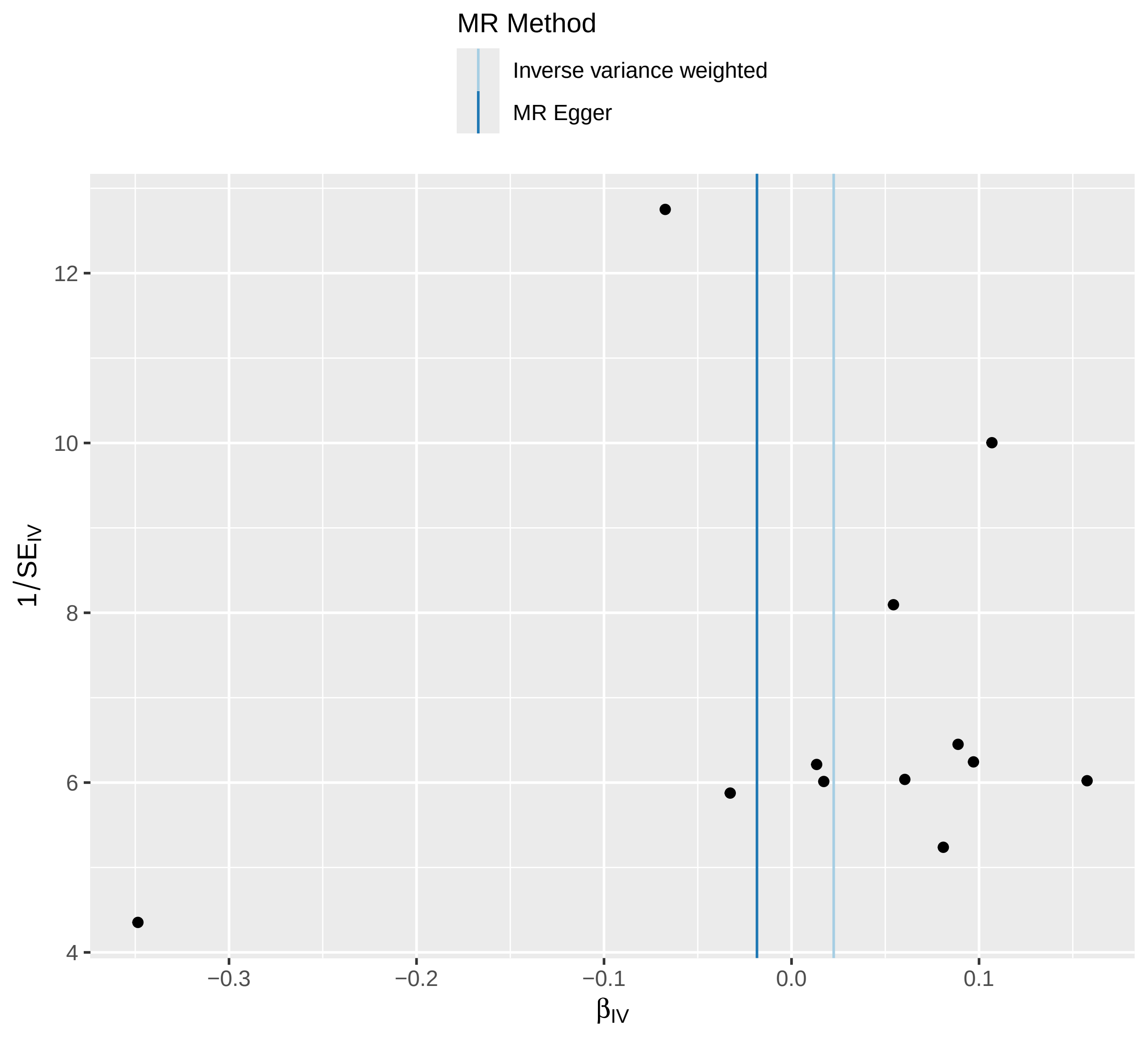

MR Method
Inverse variance weighted
MR Egger
1/SE_IV
12
10
8
6
4
−0.3
−0.2
−0.1
0.0
0.1
β_IV

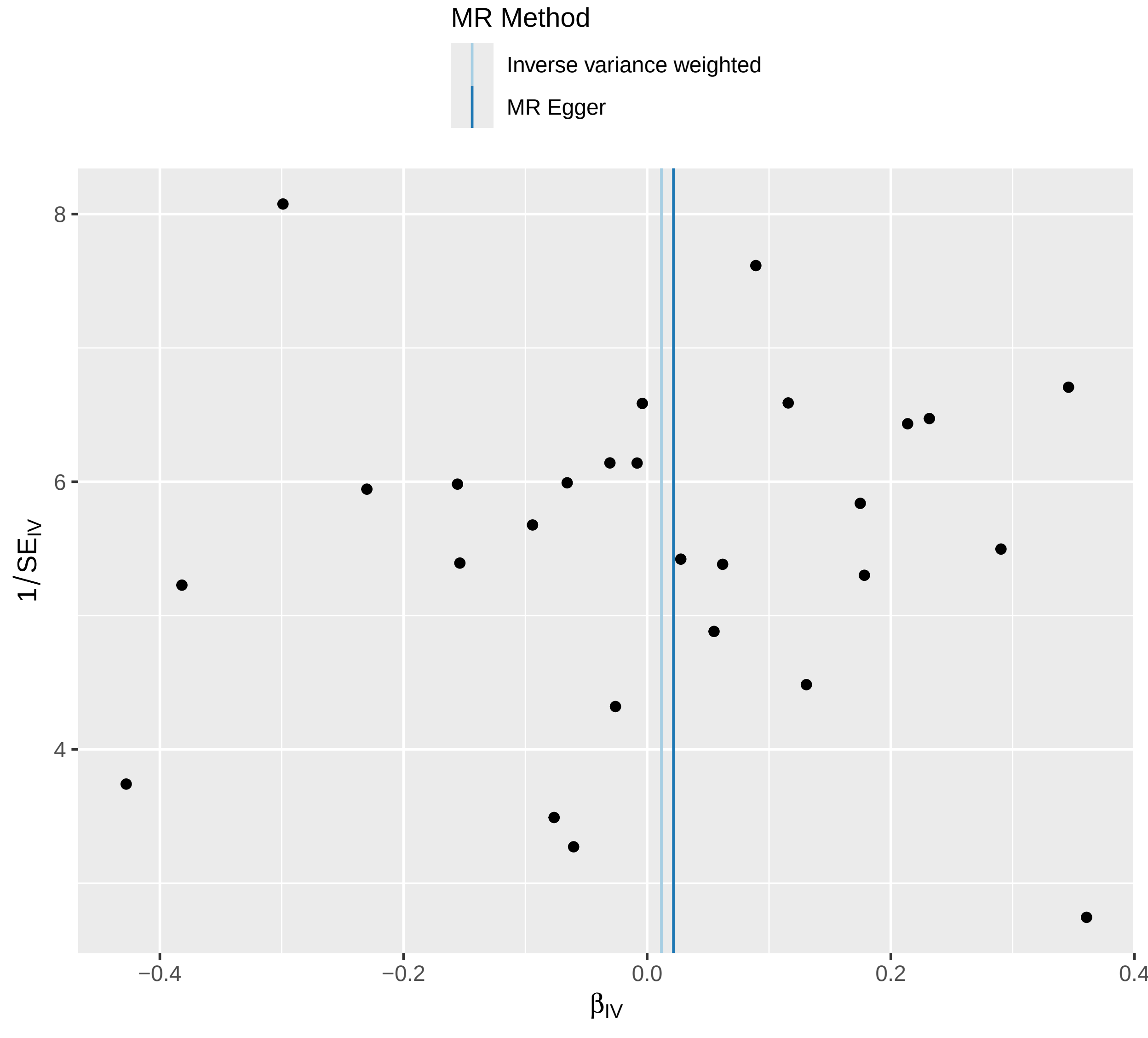

MR Method
Inverse variance weighted
MR Egger
8
6
4
1/SE_IV
−0.4
−0.2
0.0
0.2
0.4
β_IV

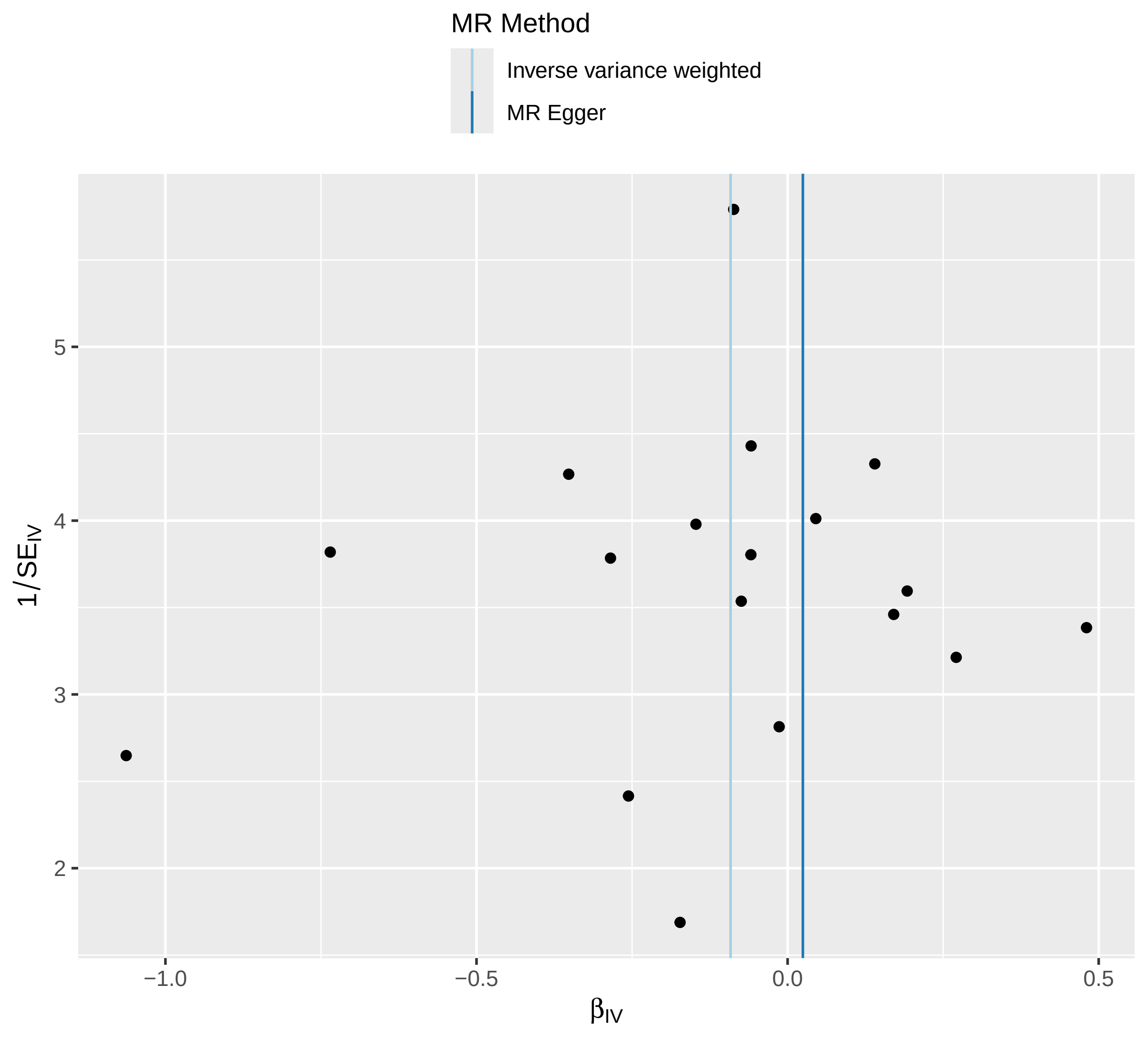
MR Method
Inverse variance weighted
MR Egger
5
4
3
2
1/SE_IV
−1.0
−0.5
0.0
0.5
β_IV

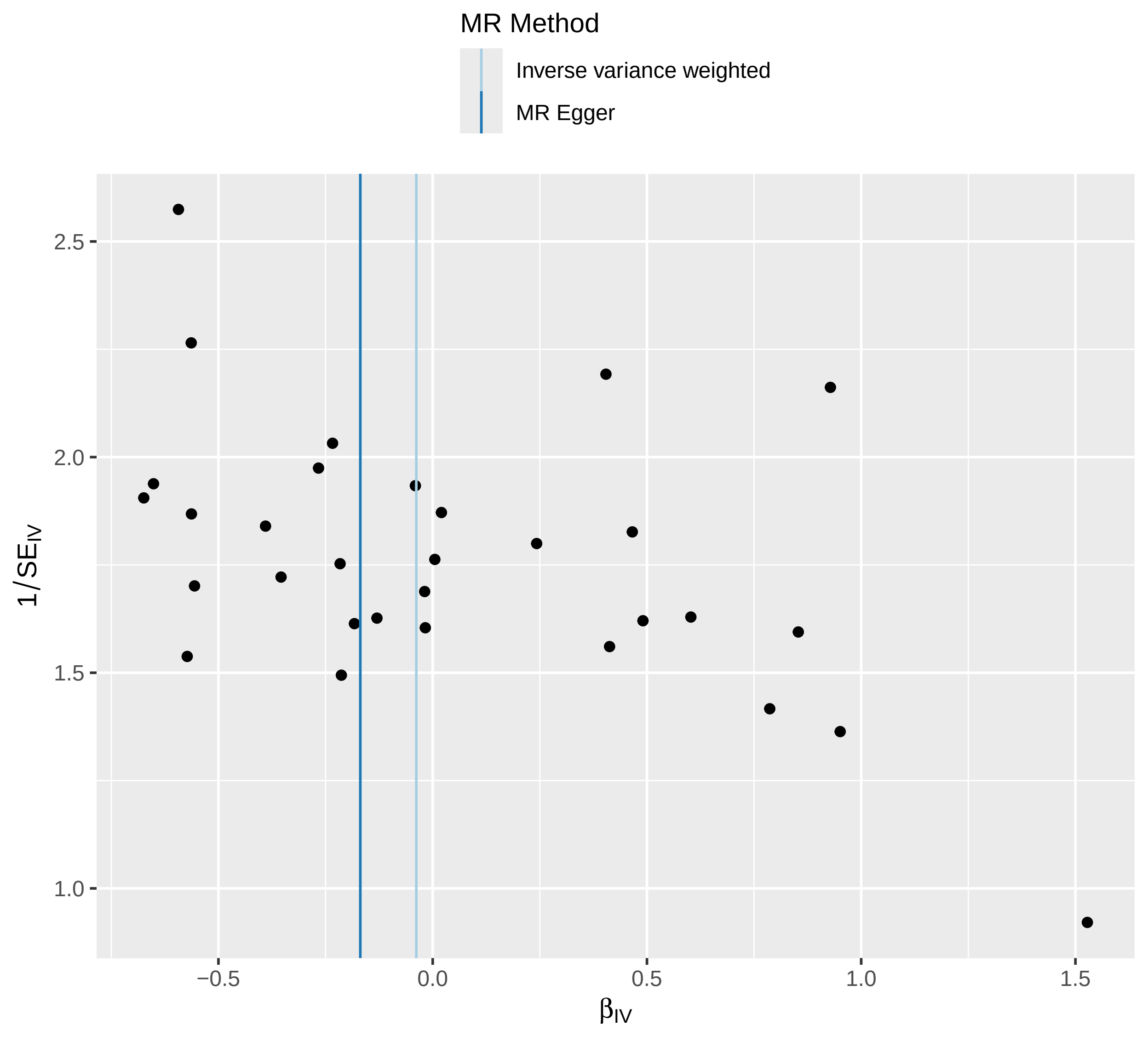
MR Method
Inverse variance weighted
MR Egger
2.5
2.0
1.5
1.0
$1/SE_{IV}$
−0.5
0.0
0.5
1.0
1.5
β_{IV}

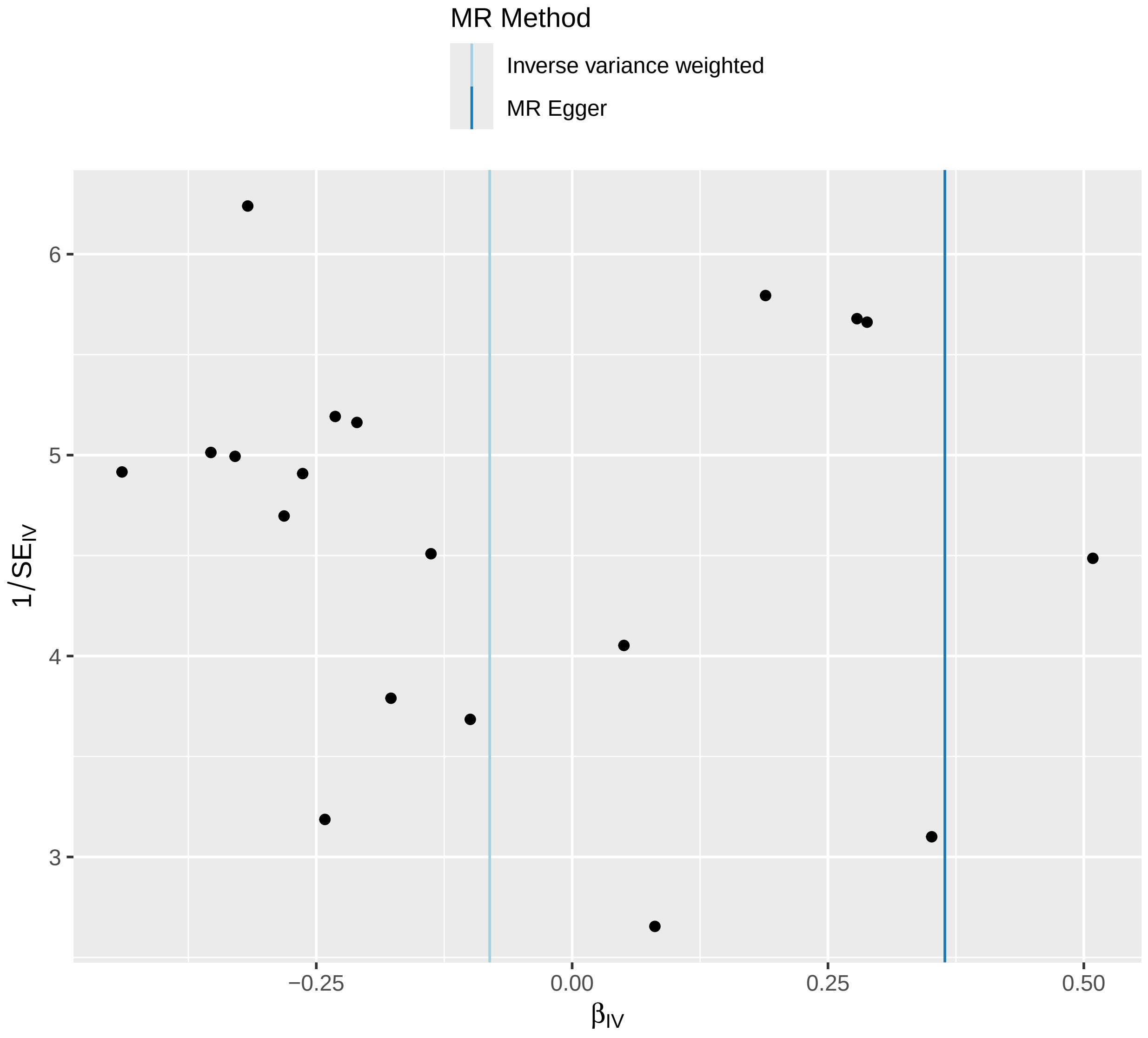

MR Method
Inverse variance weighted
MR Egger
6
5
4
3
$1/SE_{IV}$
−0.25
0.00
0.25
0.50
β_{IV}

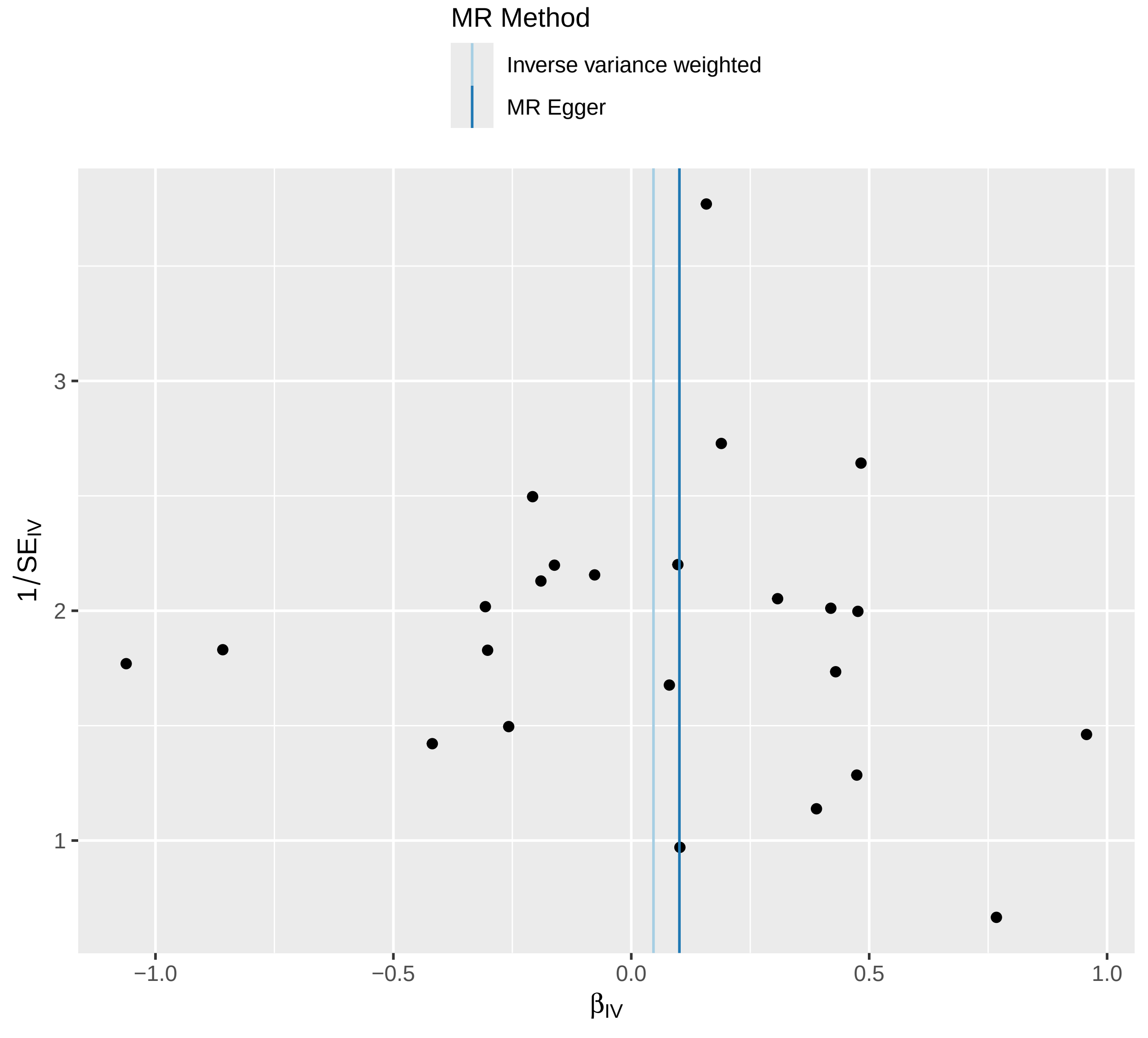

MR Method
Inverse variance weighted
MR Egger
3
2
1
1/SE$_{IV}$
−1.0
−0.5
0.0
0.5
1.0
β$_{IV}$

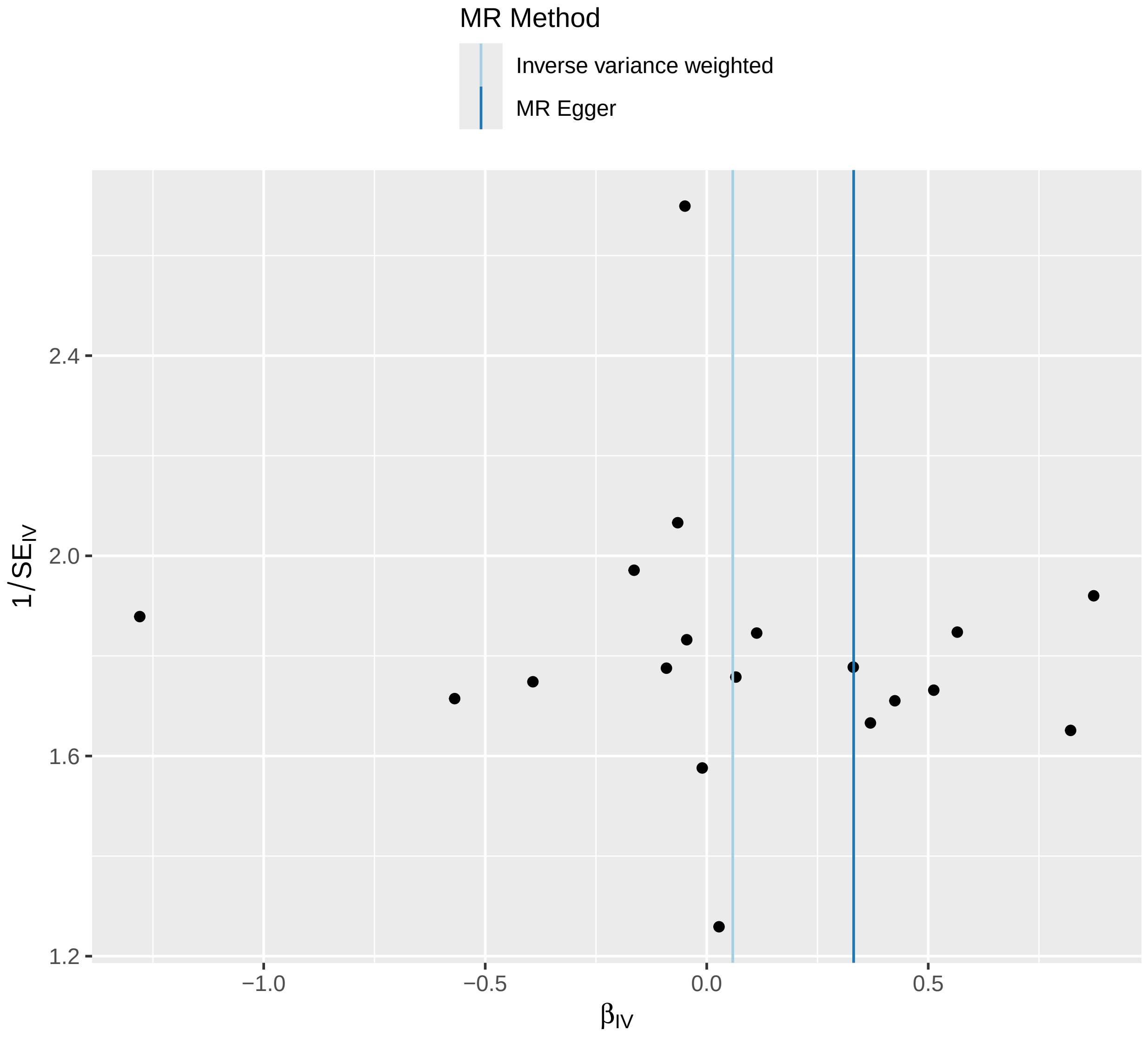
MR Method
Inverse variance weighted
MR Egger
2.4
2.0
1.6
1.2
$1/SE_{IV}$
−1.0
−0.5
0.0
0.5
β_{IV}

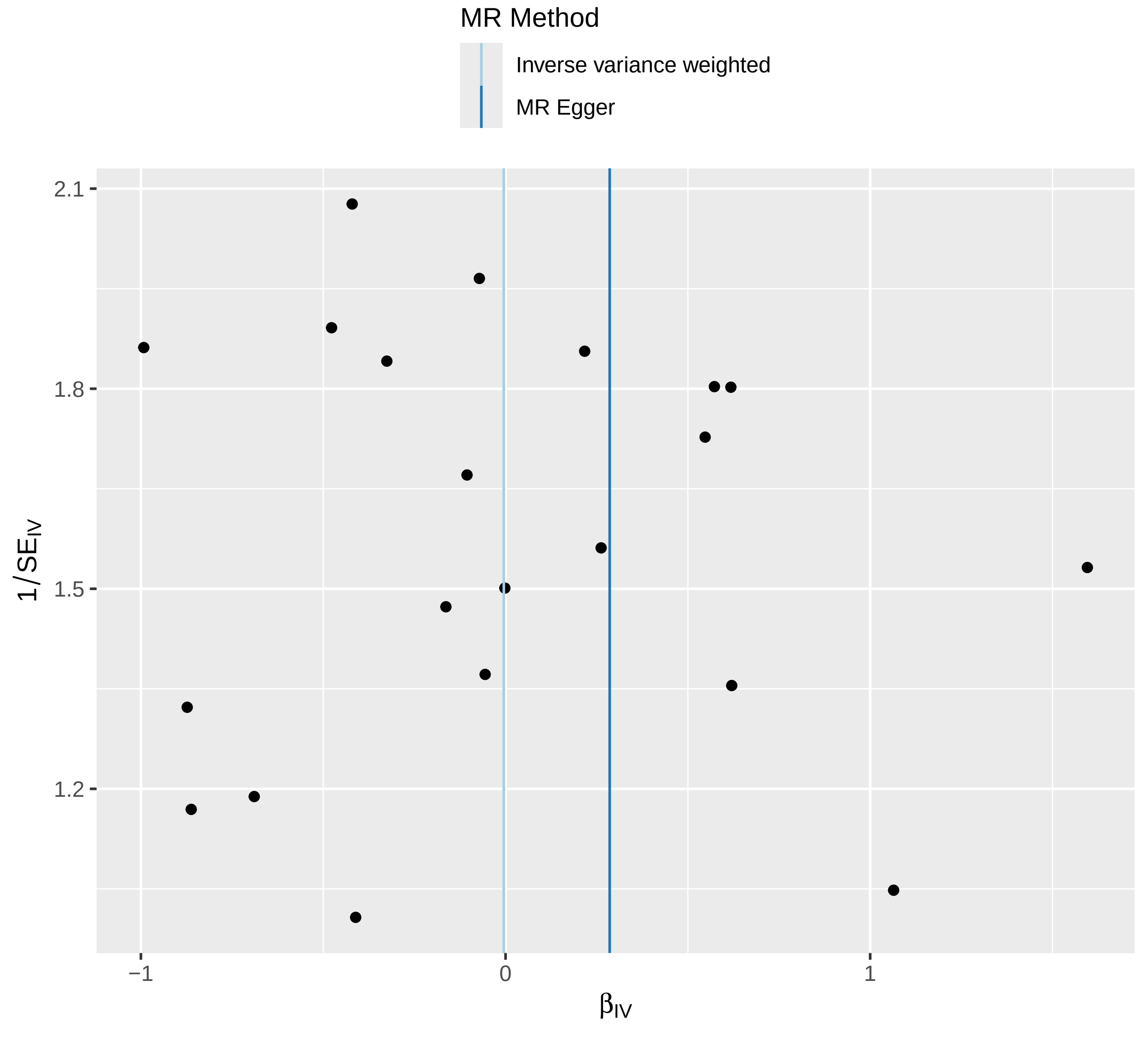
MR Method
Inverse variance weighted
MR Egger
2.1
1.8
1.5
1.2
1/SE$_{IV}$
−1
0
1
β$_{IV}$

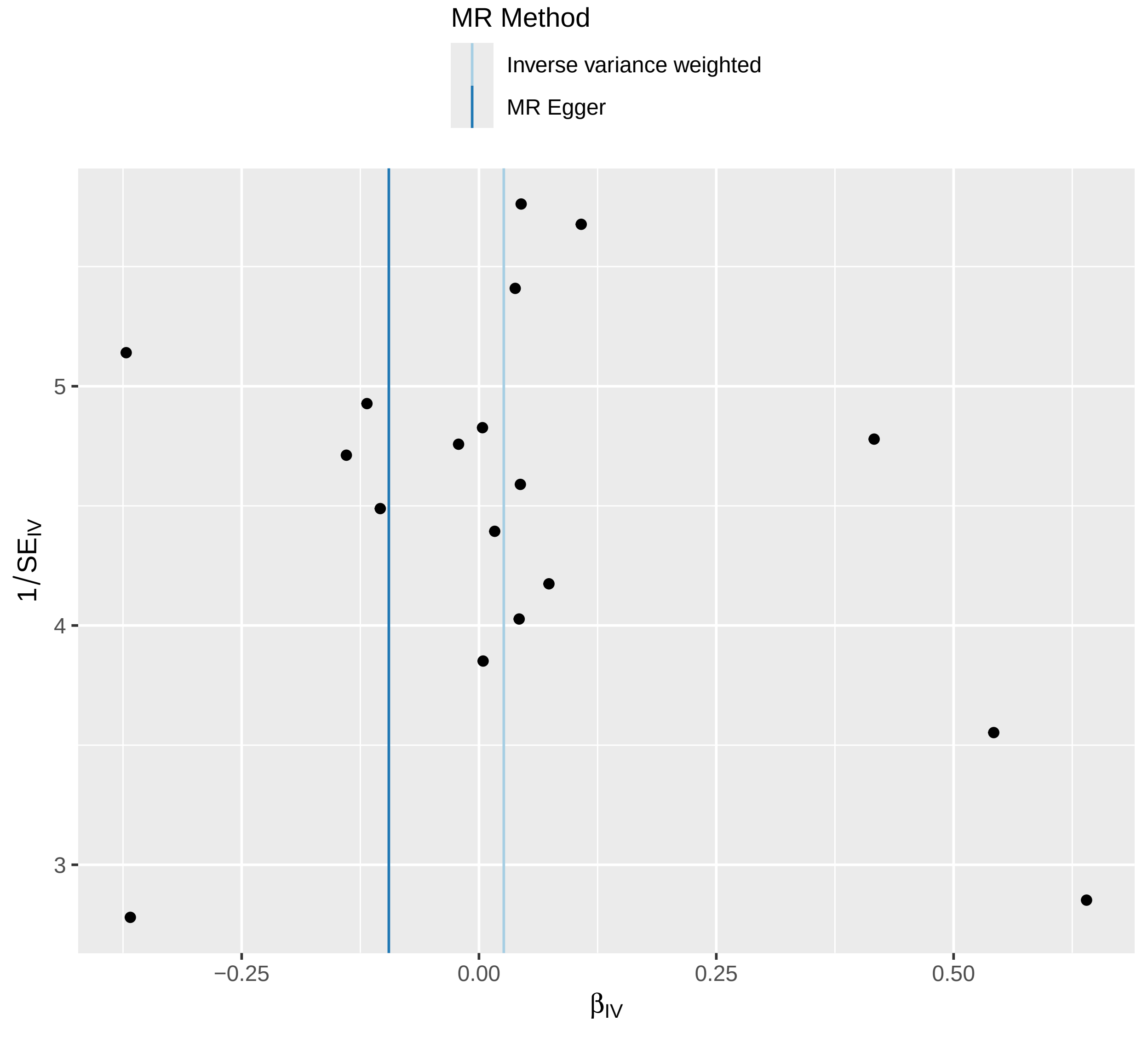

MR Method
Inverse variance weighted
MR Egger
5
4
3
$1/SE_{IV}$
−0.25
0.00
0.25
0.50
β_{IV}

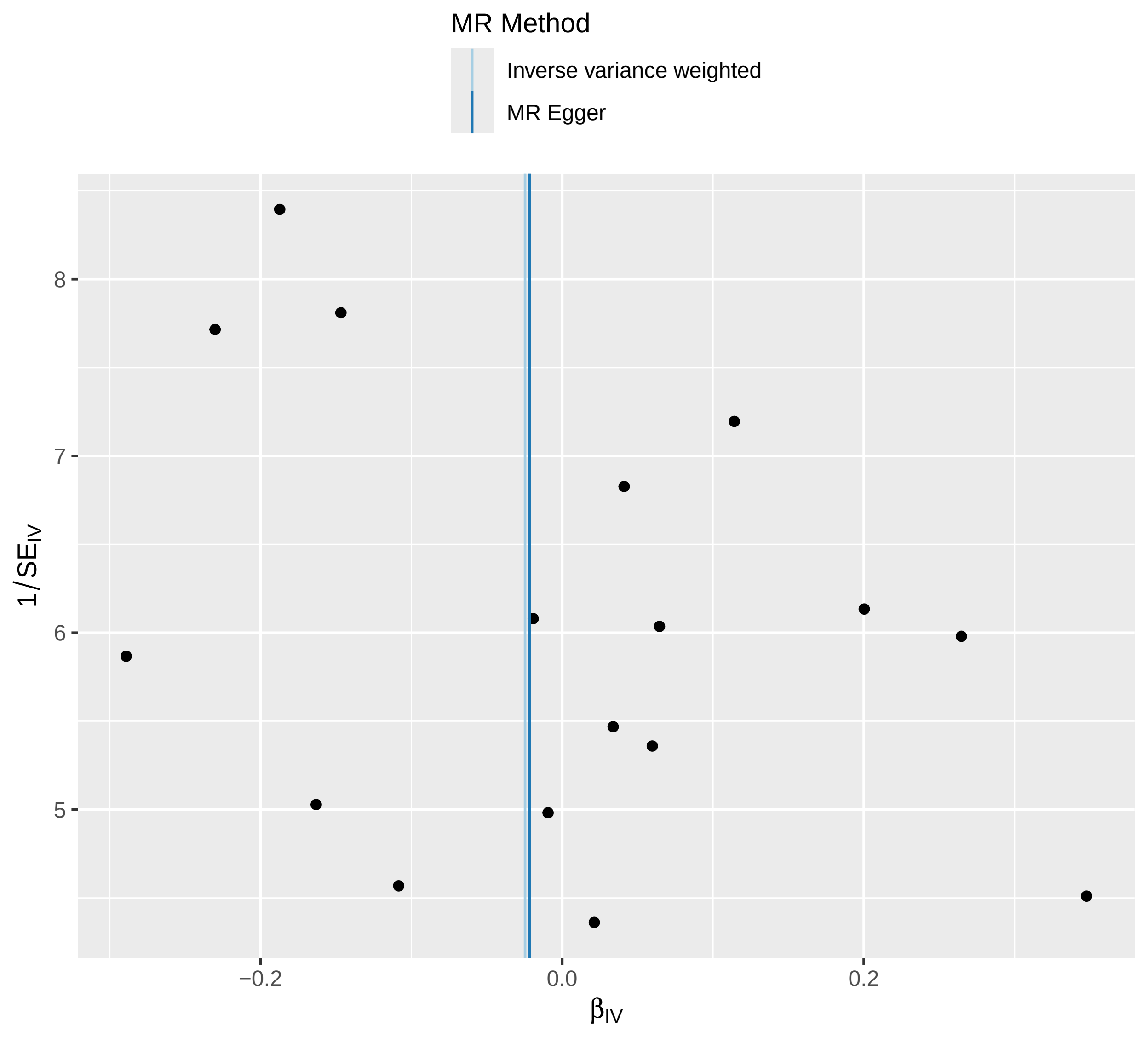
MR Method
Inverse variance weighted
MR Egger
8
7
6
5
1/SE_IV
−0.2
0.0
0.2
β_IV

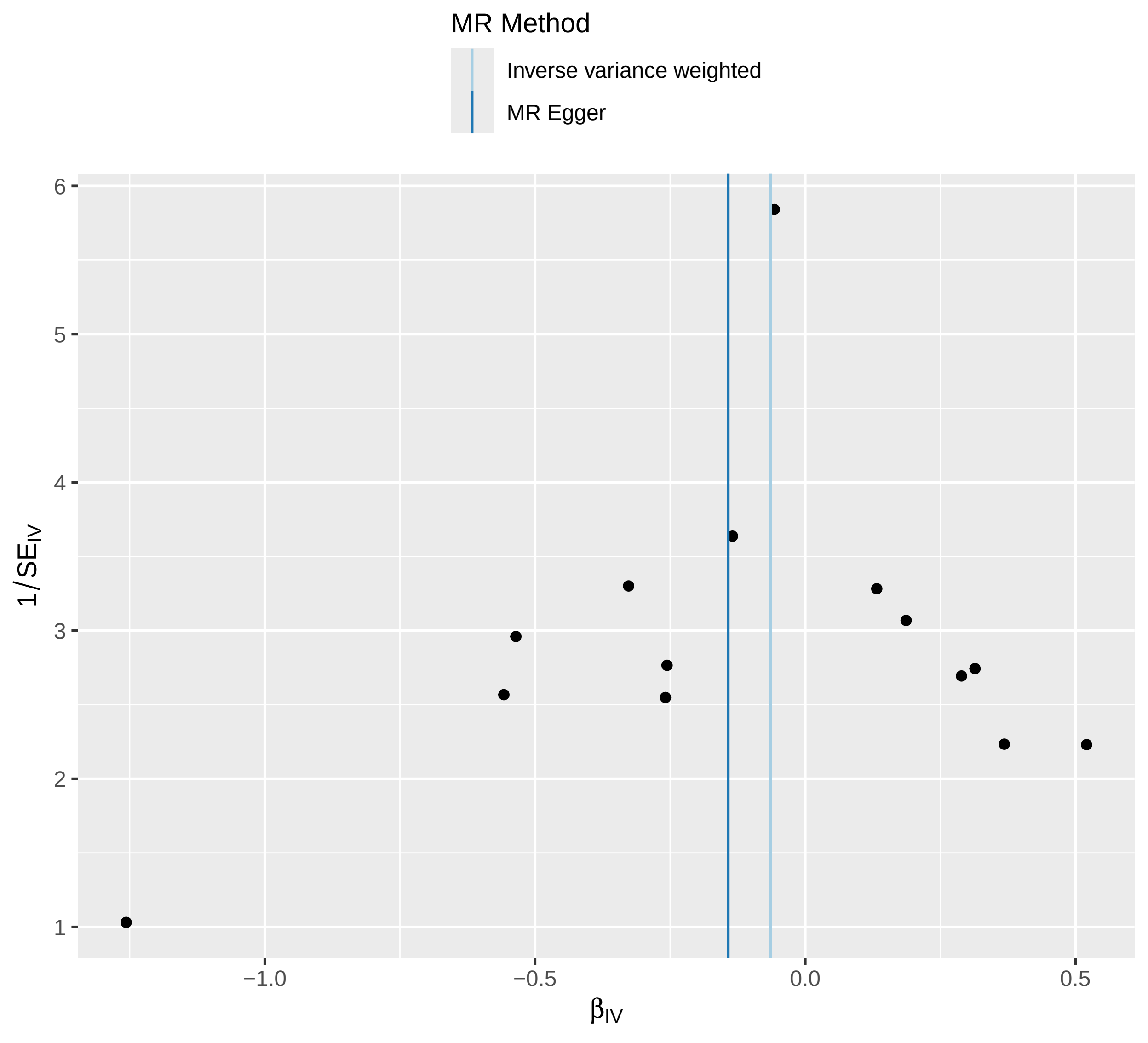
MR Method
Inverse variance weighted
MR Egger
1/SE$_{IV}$
6
5
4
3
2
1
−1.0
−0.5
0.0
0.5
β$_{IV}$

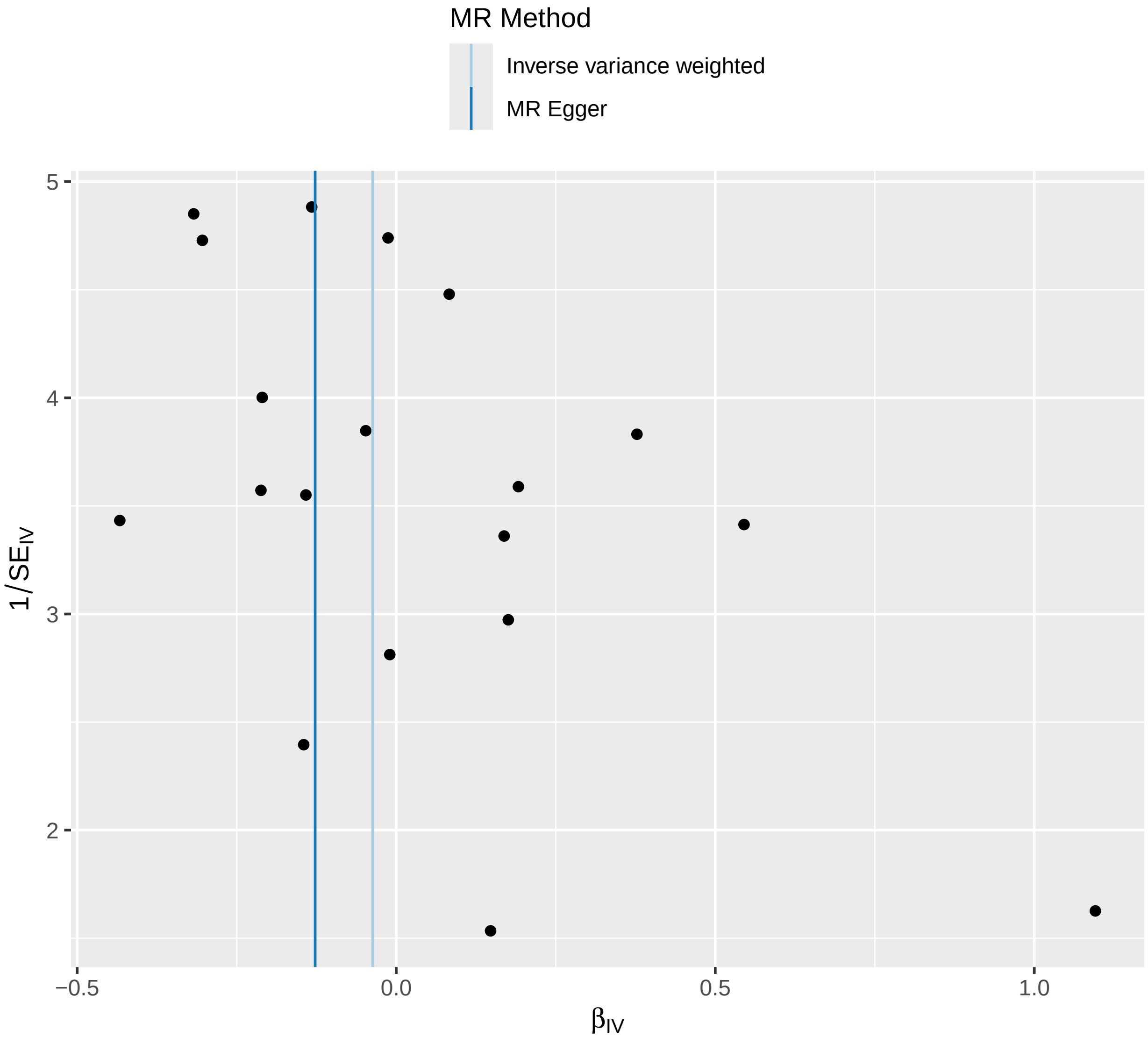
MR Method
Inverse variance weighted
MR Egger
5
4
3
2
$1/SE_{IV}$
−0.5
0.0
0.5
1.0
β_{IV}

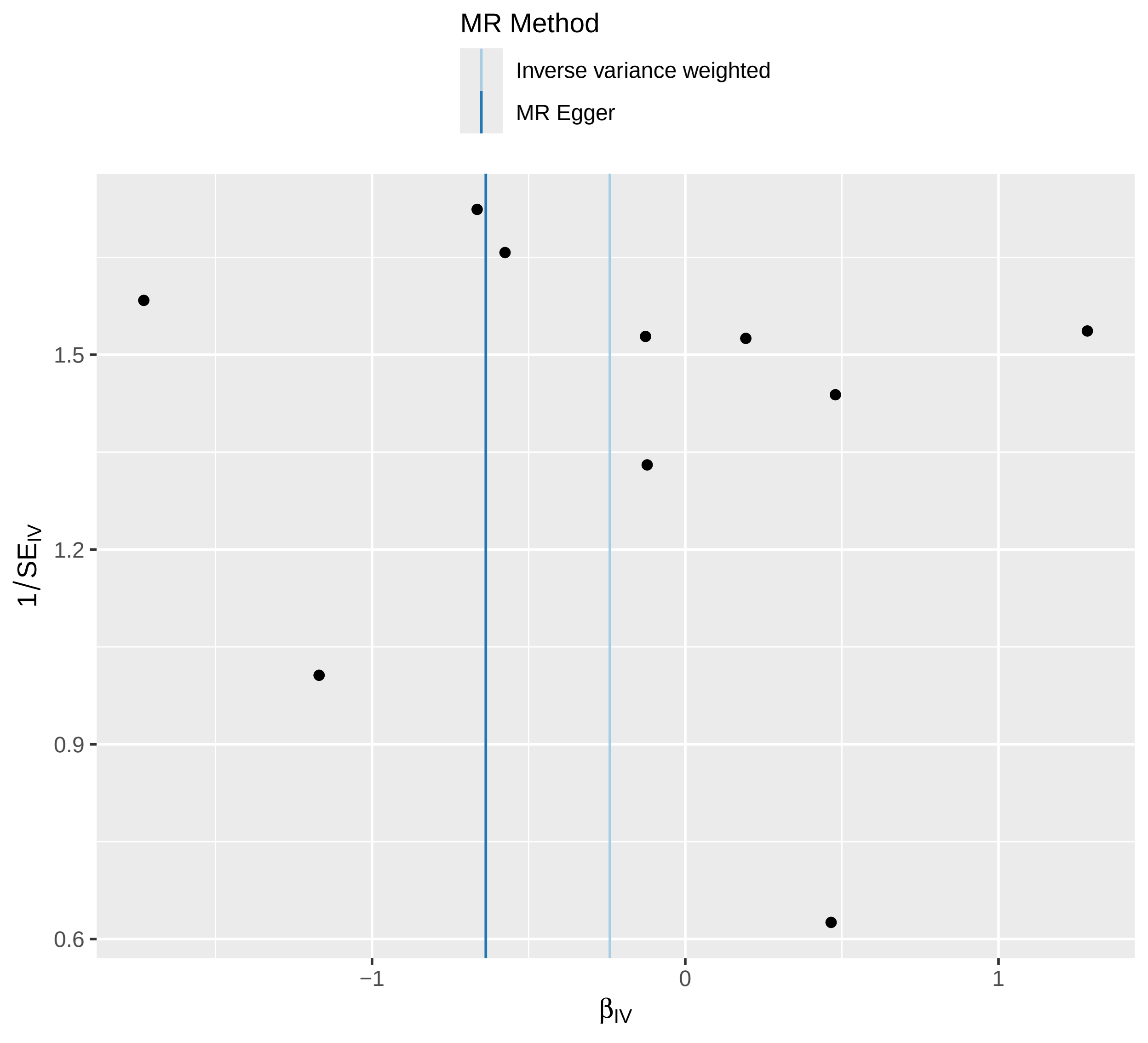
MR Method
Inverse variance weighted
MR Egger
1/SE_IV
1.5
1.2
0.9
0.6
−1
0
1
β_IV

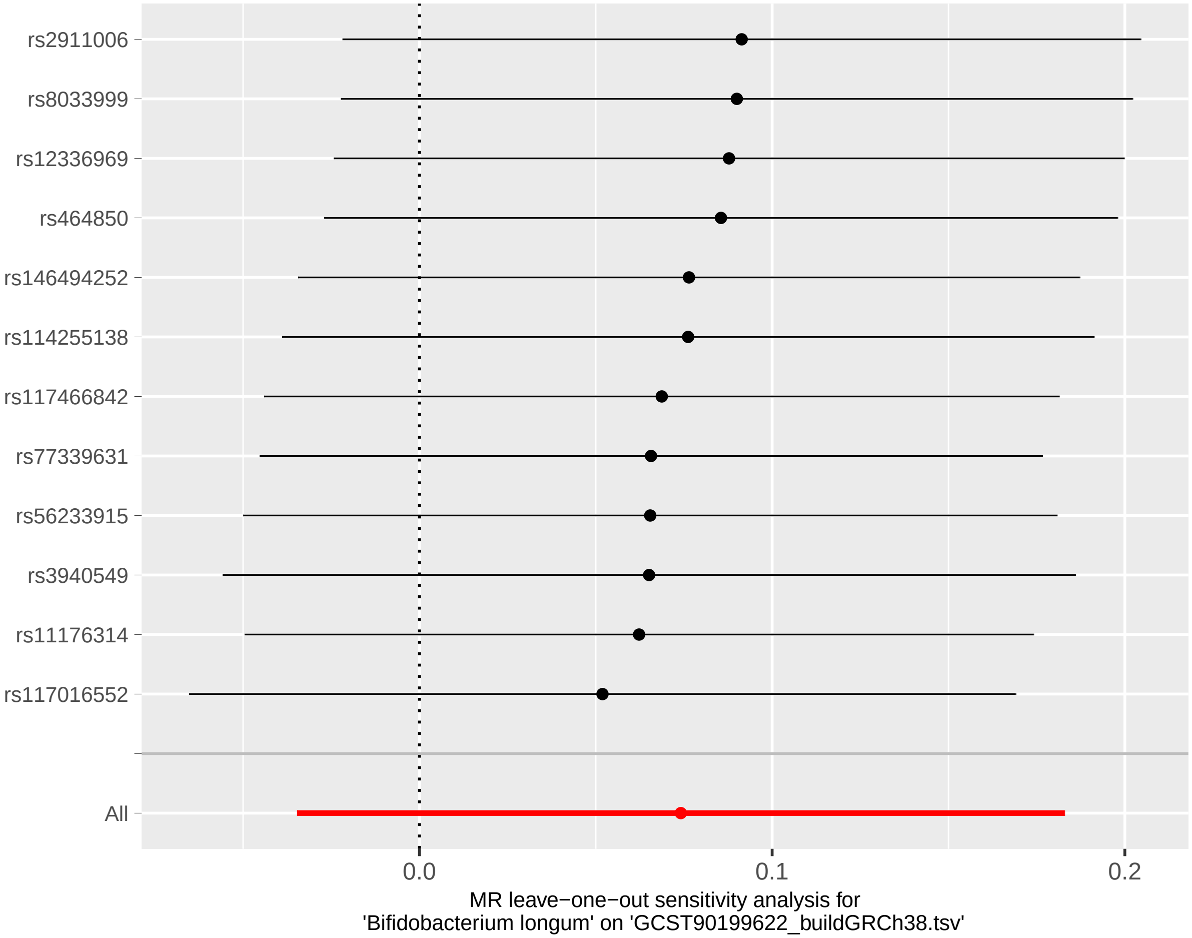
rs2911006
rs8033999
rs12336969
rs464850
rs146494252
rs114255138
rs117466842
rs77339631
rs56233915
rs3940549
rs11176314
rs117016552
All
0.0
0.1
0.2
MR leave−one−out sensitivity analysis for
'Bifidobacterium longum' on 'GCST90199622_buildGRCh38.tsv'

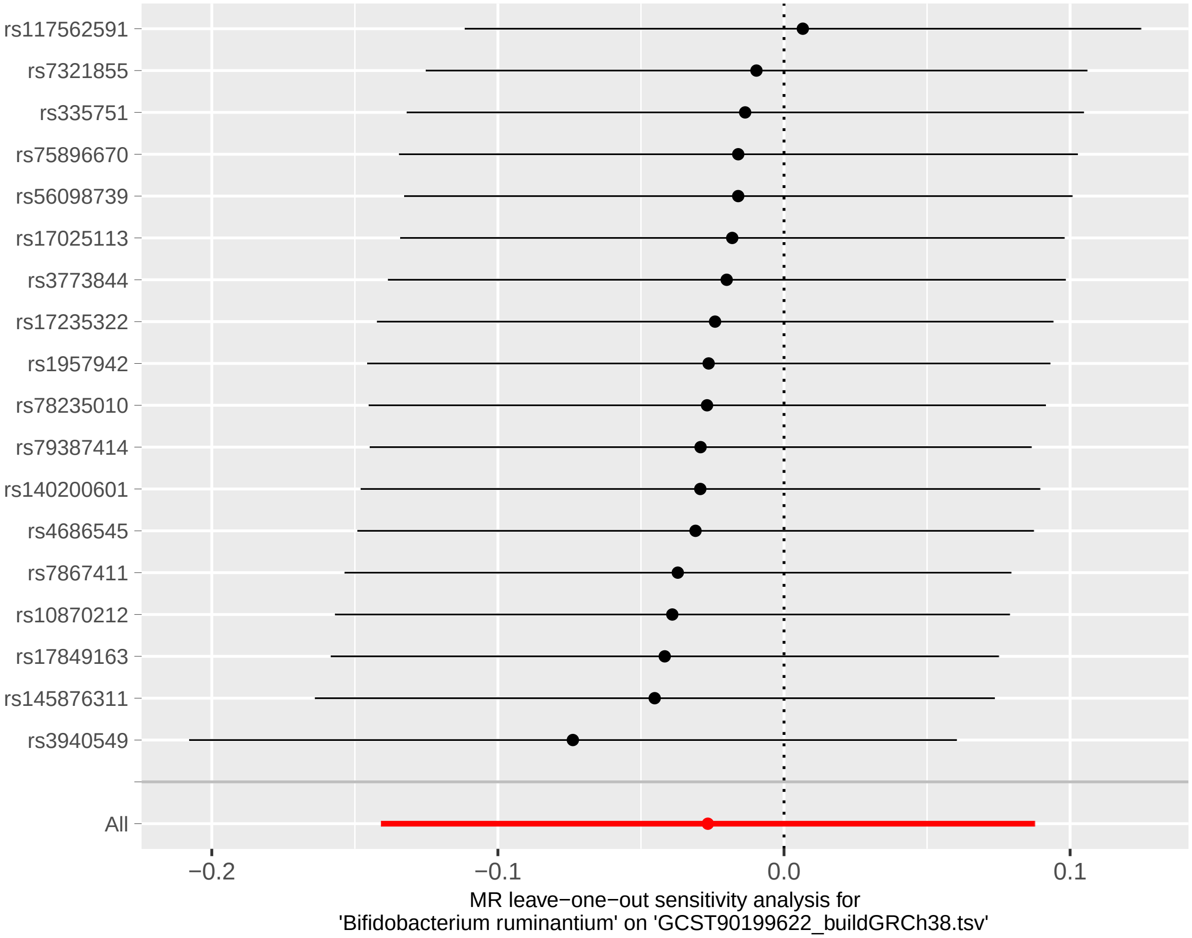
rs117562591
rs7321855
rs335751
rs75896670
rs56098739
rs17025113
rs3773844
rs17235322
rs1957942
rs78235010
rs79387414
rs140200601
rs4686545
rs7867411
rs10870212
rs17849163
rs145876311
rs3940549
All
−0.2
−0.1
0.0
0.1
MR leave−one−out sensitivity analysis for
'Bifidobacterium ruminantium' on 'GCST90199622_buildGRCh38.tsv'

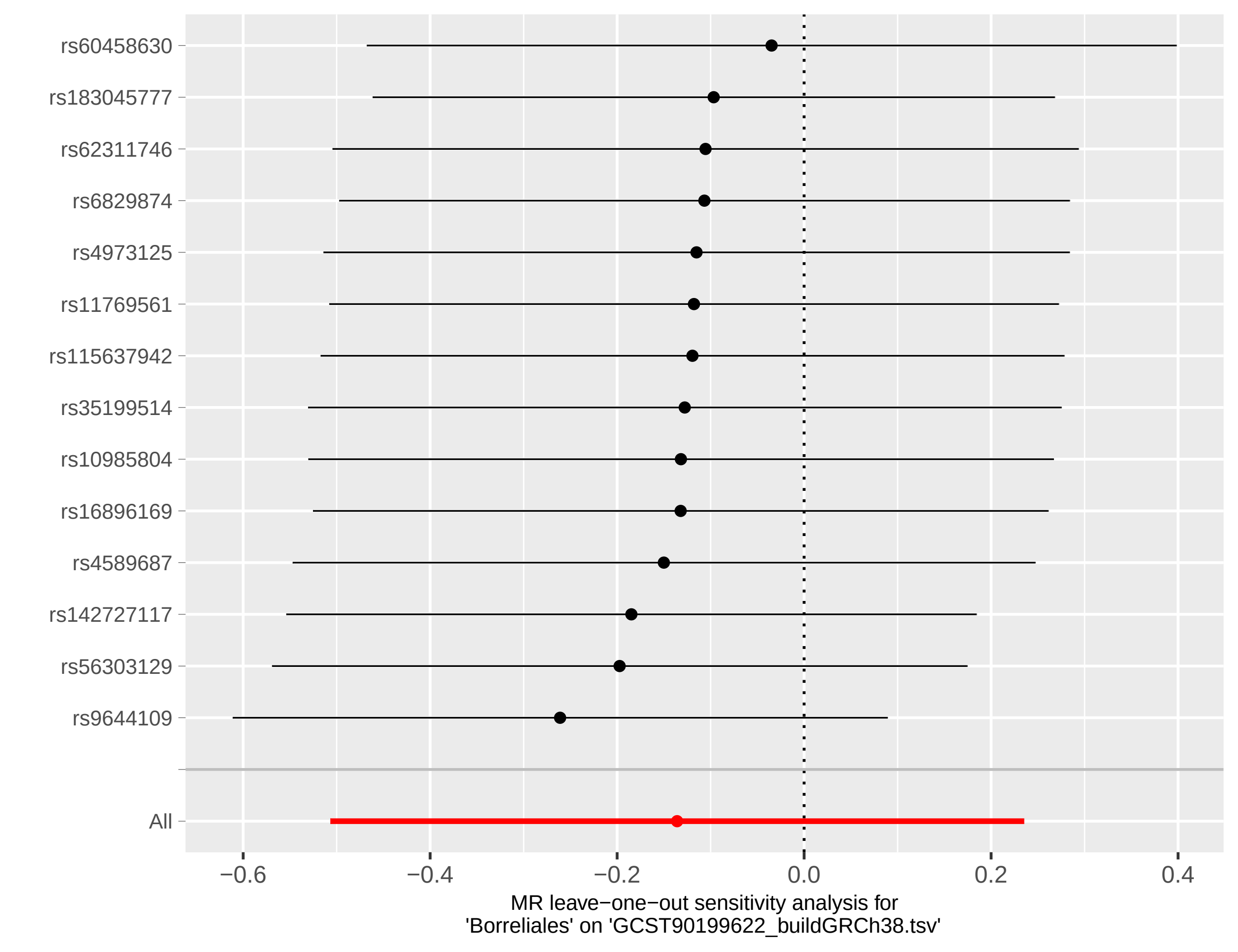
rs60458630
rs183045777
rs62311746
rs6829874
rs4973125
rs11769561
rs115637942
rs35199514
rs10985804
rs16896169
rs4589687
rs142727117
rs56303129
rs9644109
All
−0.6
−0.4
−0.2
0.0
0.2
0.4
MR leave−one−out sensitivity analysis for
'Borreliales' on 'GCST90199622_buildGRCh38.tsv'

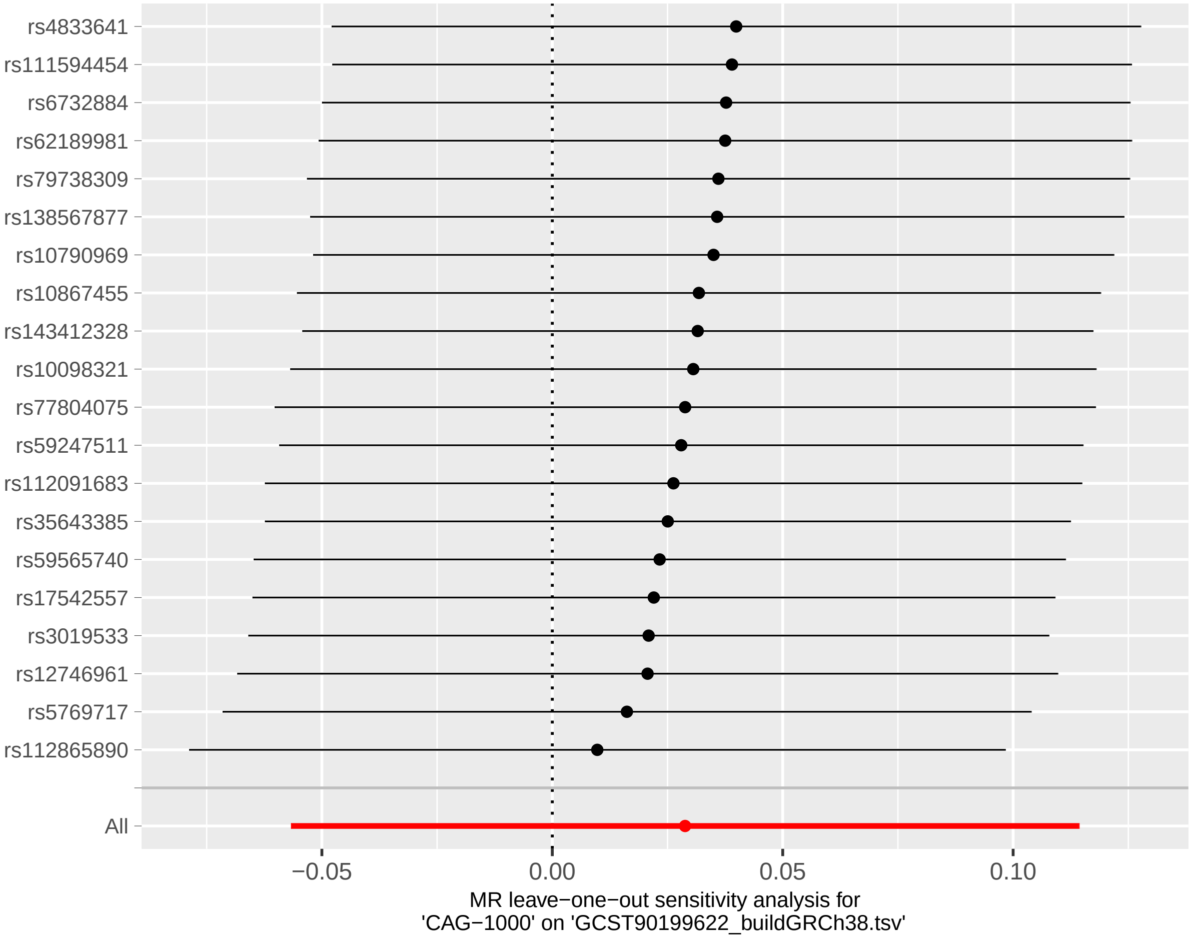
rs4833641
rs111594454
rs6732884
rs62189981
rs79738309
rs138567877
rs10790969
rs10867455
rs143412328
rs10098321
rs77804075
rs59247511
rs112091683
rs35643385
rs59565740
rs17542557
rs3019533
rs12746961
rs5769717
rs112865890
All
−0.05
0.00
0.05
0.10
MR leave−one−out sensitivity analysis for
'CAG−1000' on 'GCST90199622_buildGRCh38.tsv'

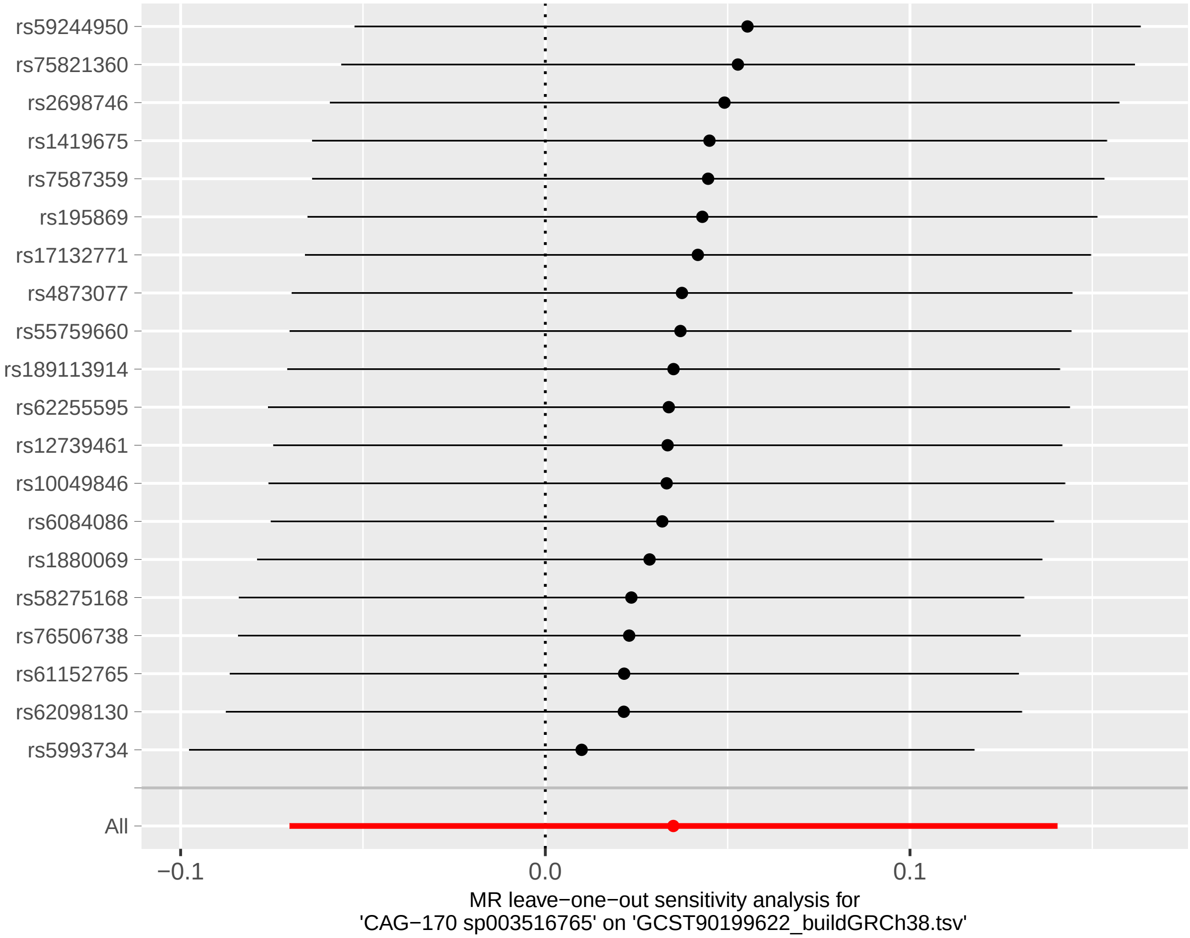
rs59244950
rs75821360
rs2698746
rs1419675
rs7587359
rs195869
rs17132771
rs4873077
rs55759660
rs189113914
rs62255595
rs12739461
rs10049846
rs6084086
rs1880069
rs58275168
rs76506738
rs61152765
rs62098130
rs5993734
All
−0.1
0.0
0.1
MR leave−one−out sensitivity analysis for
'CAG−170 sp003516765' on 'GCST90199622_buildGRCh38.tsv'

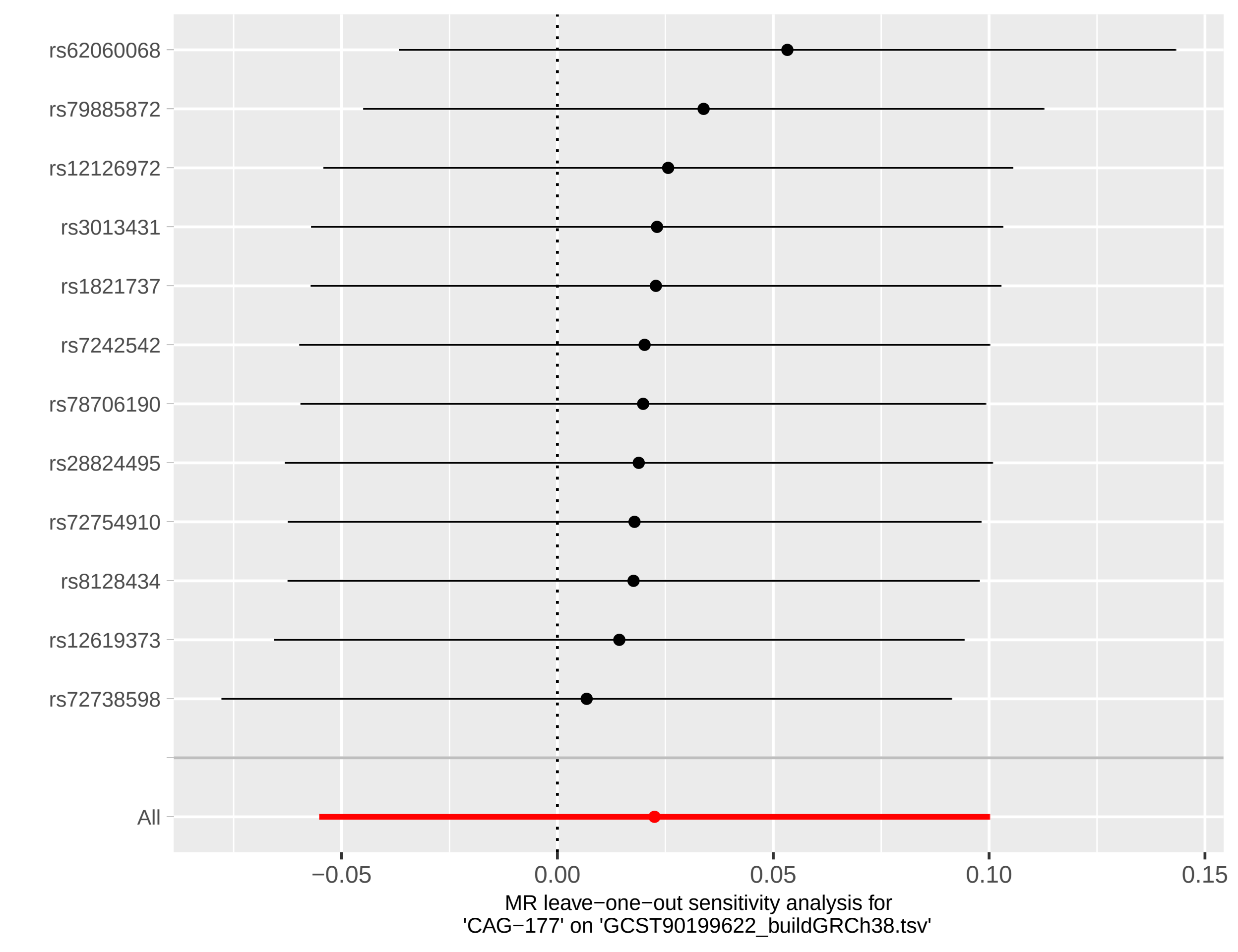
rs62060068
rs79885872
rs12126972
rs3013431
rs1821737
rs7242542
rs78706190
rs28824495
rs72754910
rs8128434
rs12619373
rs72738598
All
−0.05
0.00
0.05
0.10
0.15
MR leave−one−out sensitivity analysis for
'CAG−177' on 'GCST90199622_buildGRCh38.tsv'

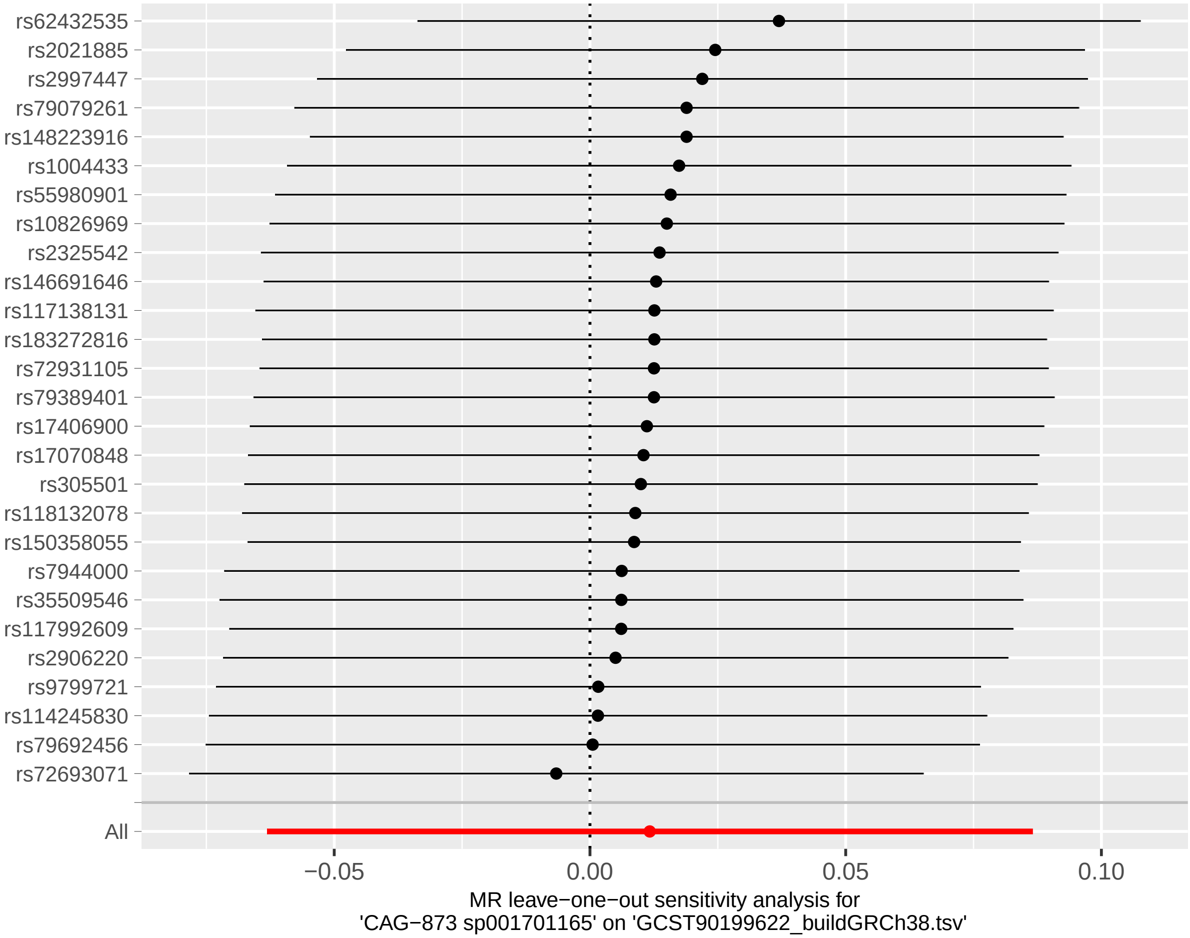
rs62432535
rs2021885
rs2997447
rs79079261
rs148223916
rs1004433
rs55980901
rs10826969
rs2325542
rs146691646
rs117138131
rs183272816
rs72931105
rs79389401
rs17406900
rs17070848
rs305501
rs118132078
rs150358055
rs7944000
rs35509546
rs117992609
rs2906220
rs9799721
rs114245830
rs79692456
rs72693071
All
−0.05
0.00
0.05
0.10
MR leave−one−out sensitivity analysis for
'CAG−873 sp001701165' on 'GCST90199622_buildGRCh38.tsv'

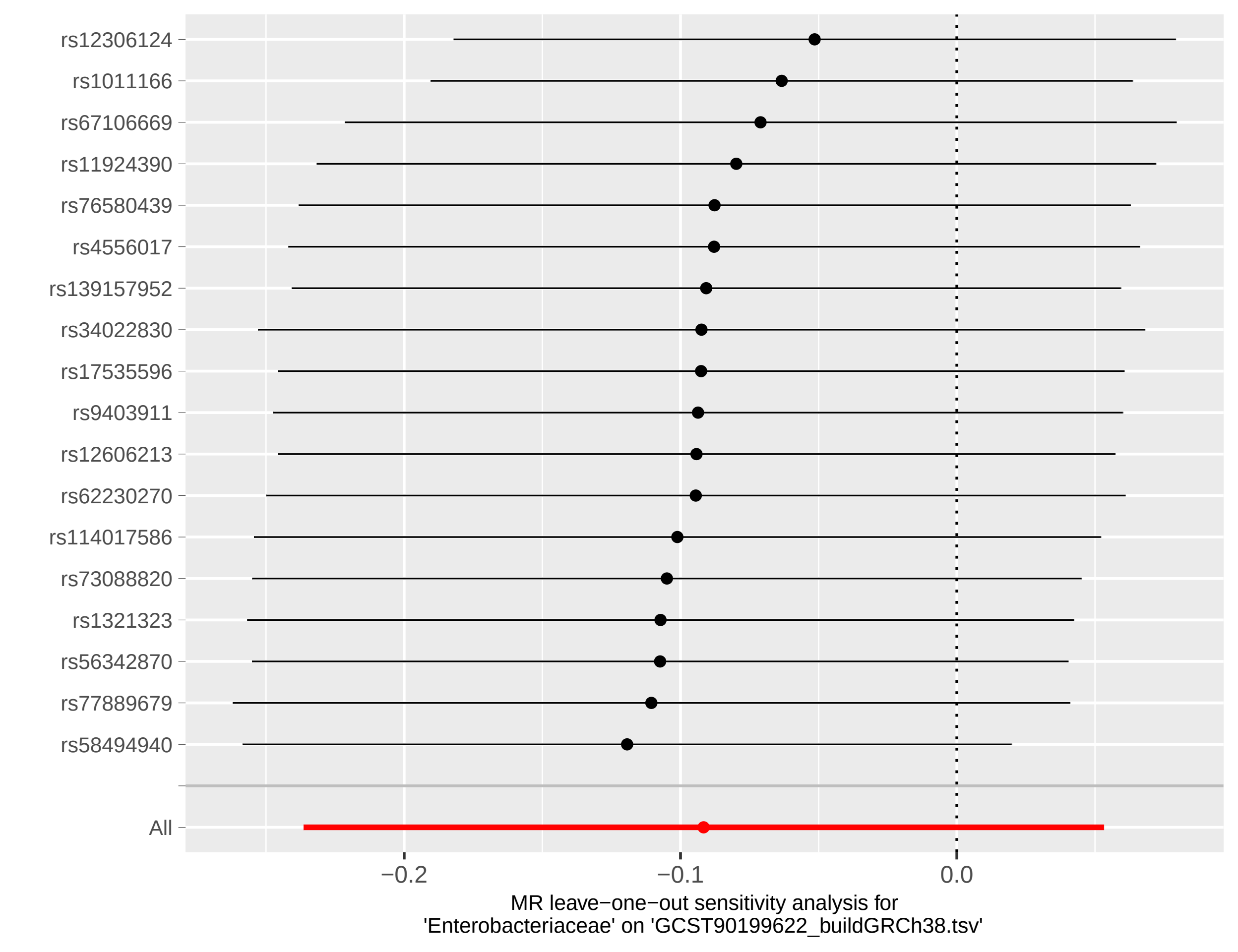
rs12306124
rs1011166
rs67106669
rs11924390
rs76580439
rs4556017
rs139157952
rs34022830
rs17535596
rs9403911
rs12606213
rs62230270
rs114017586
rs73088820
rs1321323
rs56342870
rs77889679
rs58494940
All
−0.2
−0.1
0.0
MR leave−one−out sensitivity analysis for
'Enterobacteriaceae' on 'GCST90199622_buildGRCh38.tsv'

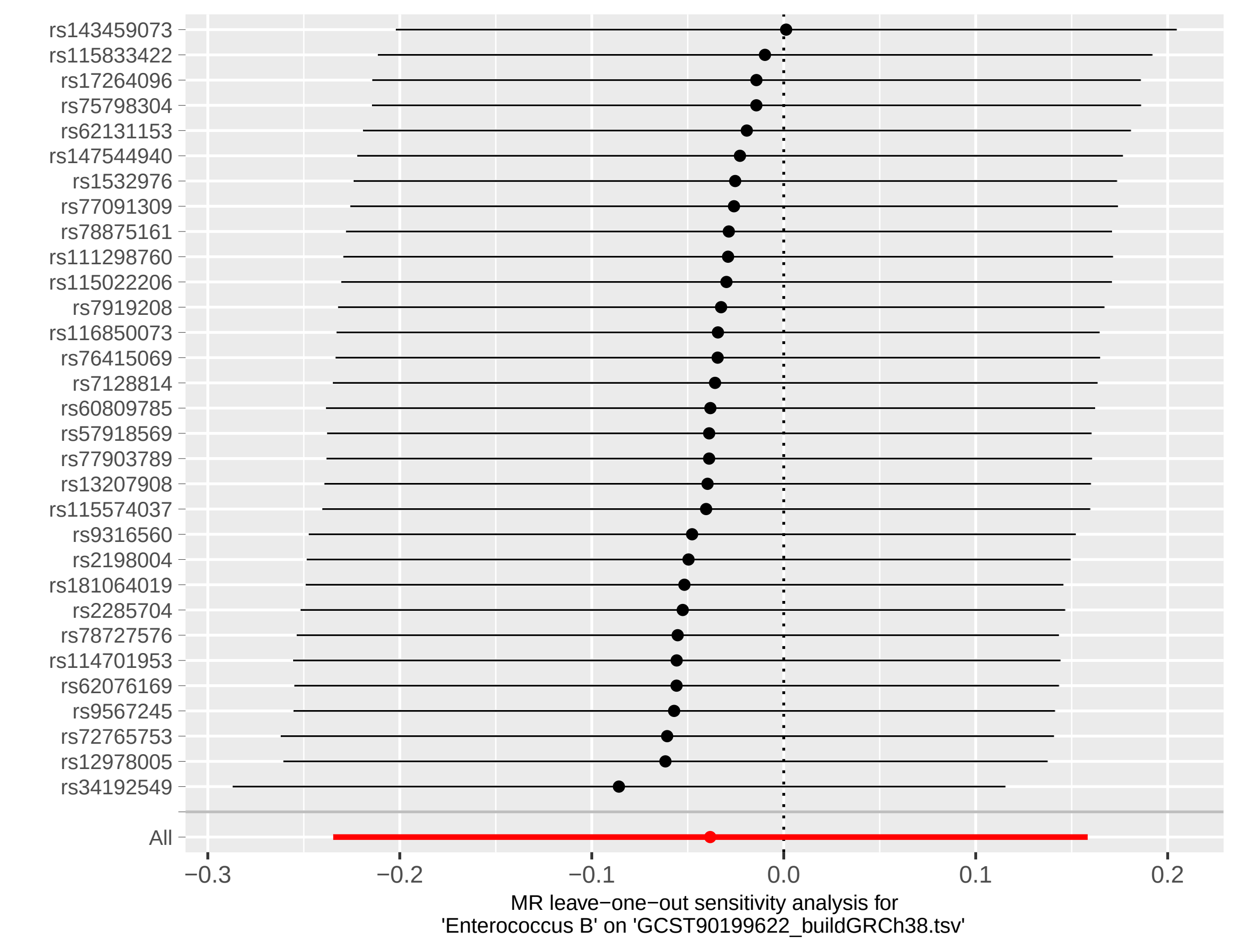
rs143459073
rs115833422
rs17264096
rs75798304
rs62131153
rs147544940
rs1532976
rs77091309
rs78875161
rs111298760
rs115022206
rs7919208
rs116850073
rs76415069
rs7128814
rs60809785
rs57918569
rs77903789
rs13207908
rs115574037
rs9316560
rs2198004
rs181064019
rs2285704
rs78727576
rs114701953
rs62076169
rs9567245
rs72765753
rs12978005
rs34192549
All
−0.3
−0.2
−0.1
0.0
0.1
0.2
MR leave−one−out sensitivity analysis for
'Enterococcus B' on 'GCST90199622_buildGRCh38.tsv'

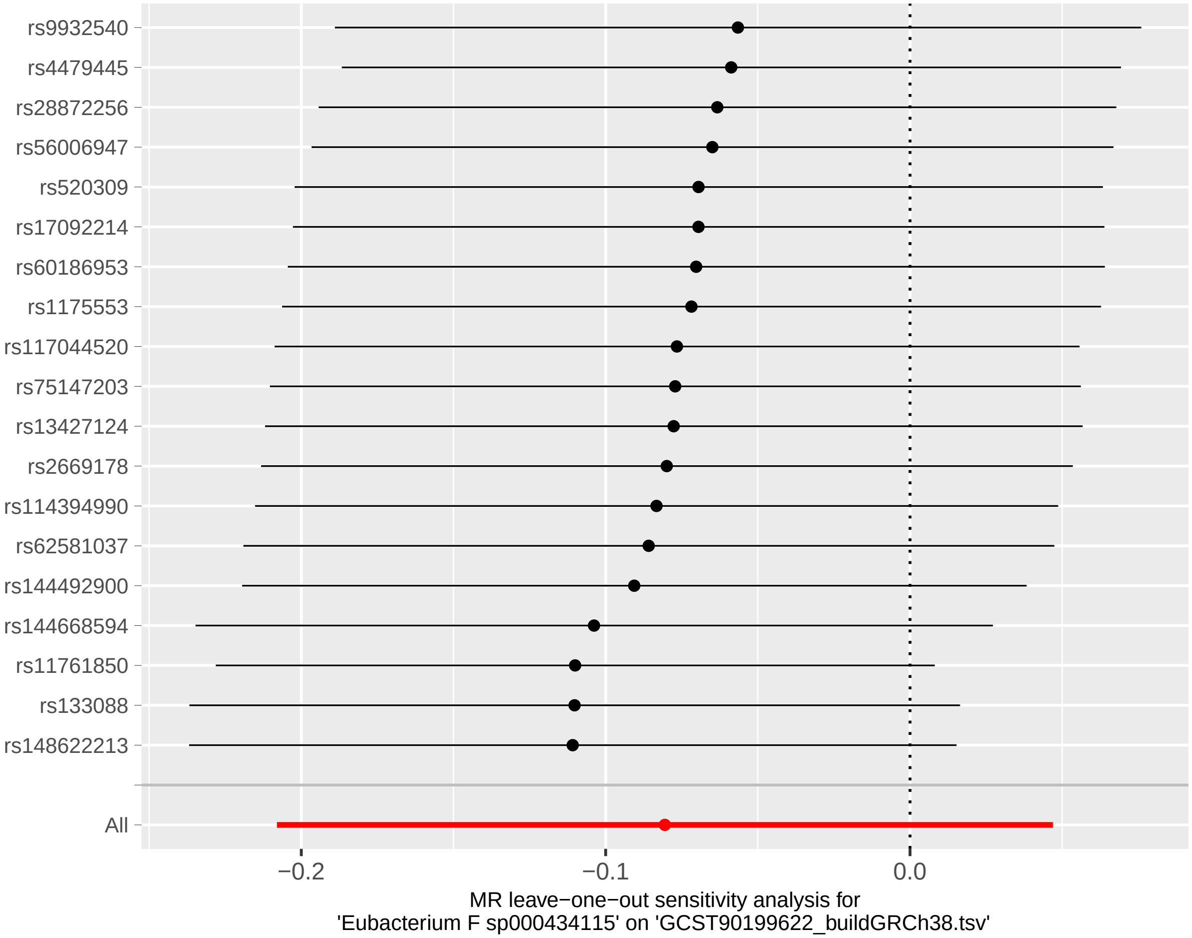

rs9932540
rs4479445
rs28872256
rs56006947
rs520309
rs17092214
rs60186953
rs1175553
rs117044520
rs75147203
rs13427124
rs2669178
rs114394990
rs62581037
rs144492900
rs144668594
rs11761850
rs133088
rs148622213
All
−0.2
−0.1
0.0
MR leave−one−out sensitivity analysis for
'Eubacterium F sp000434115' on 'GCST90199622_buildGRCh38.tsv'

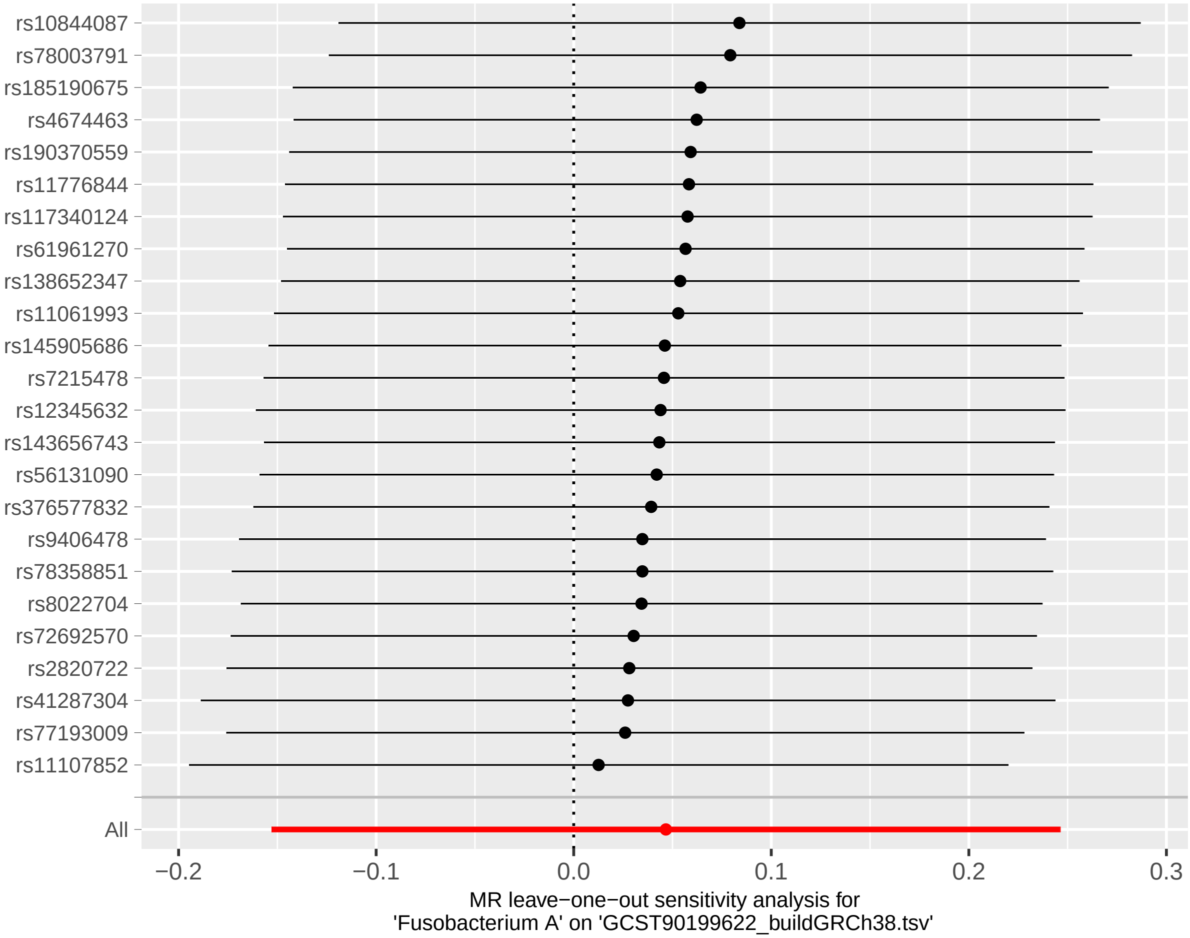
rs10844087
rs78003791
rs185190675
rs4674463
rs190370559
rs11776844
rs117340124
rs61961270
rs138652347
rs11061993
rs145905686
rs7215478
rs12345632
rs143656743
rs56131090
rs376577832
rs9406478
rs78358851
rs8022704
rs72692570
rs2820722
rs41287304
rs77193009
rs11107852
All
−0.2
−0.1
0.0
0.1
0.2
0.3
MR leave−one−out sensitivity analysis for
'Fusobacterium A' on 'GCST90199622_buildGRCh38.tsv'

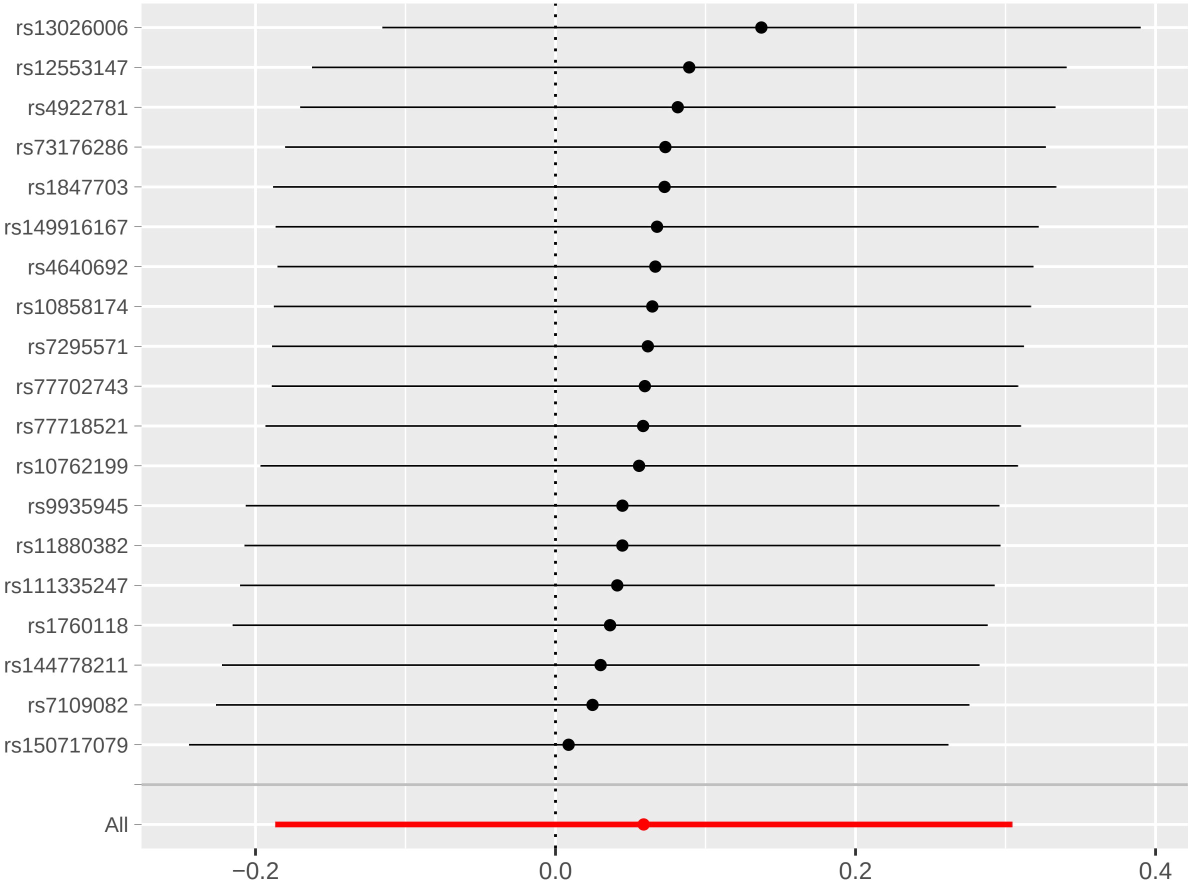

MR leave−one−out sensitivity analysis for
'GCA−900066135 sp900066135' on 'GCST90199622_buildGRCh38.tsv'

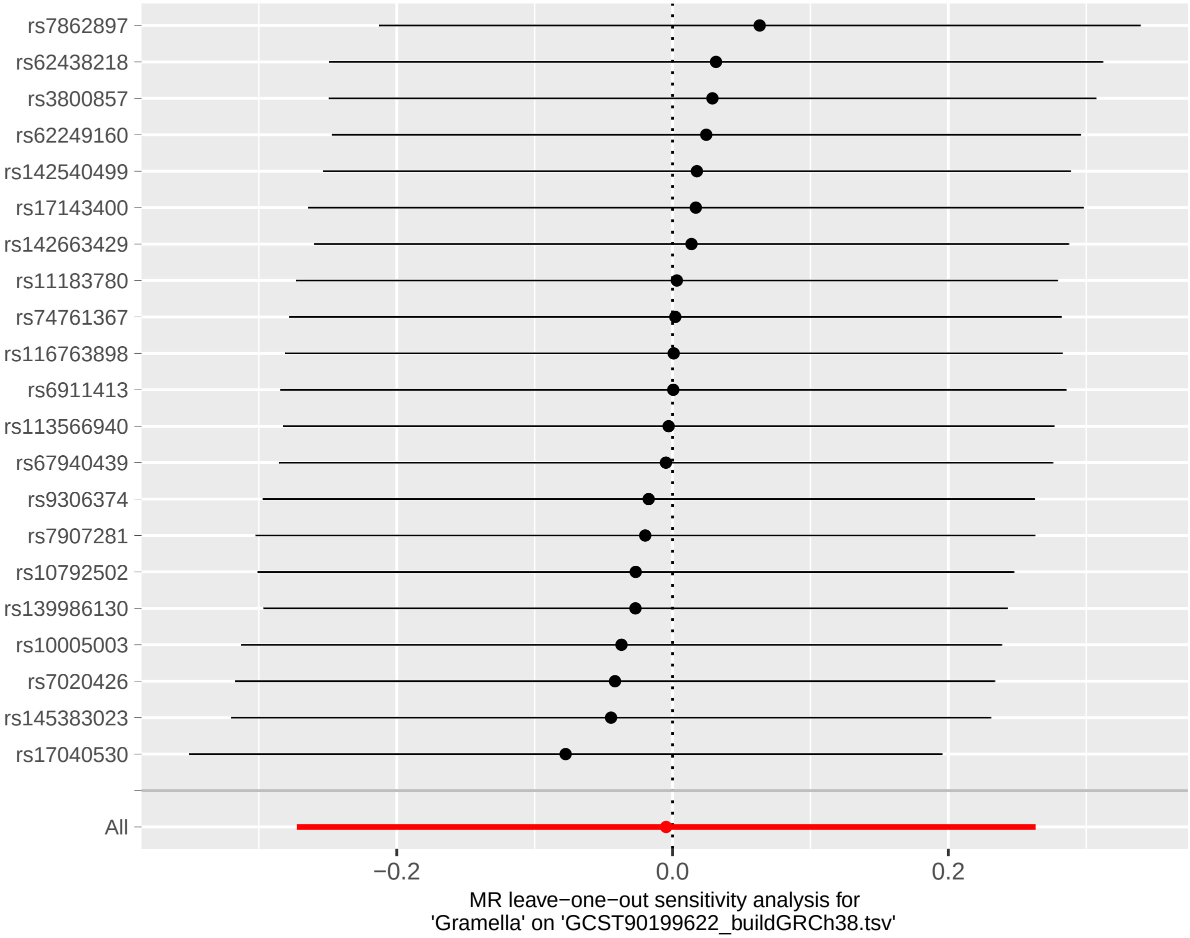

rs7862897
rs62438218
rs3800857
rs62249160
rs142540499
rs17143400
rs142663429
rs11183780
rs74761367
rs116763898
rs6911413
rs113566940
rs67940439
rs9306374
rs7907281
rs10792502
rs139986130
rs10005003
rs7020426
rs145383023
rs17040530
All
−0.2
0.0
0.2
MR leave−one−out sensitivity analysis for
'Gramella' on 'GCST90199622_buildGRCh38.tsv'

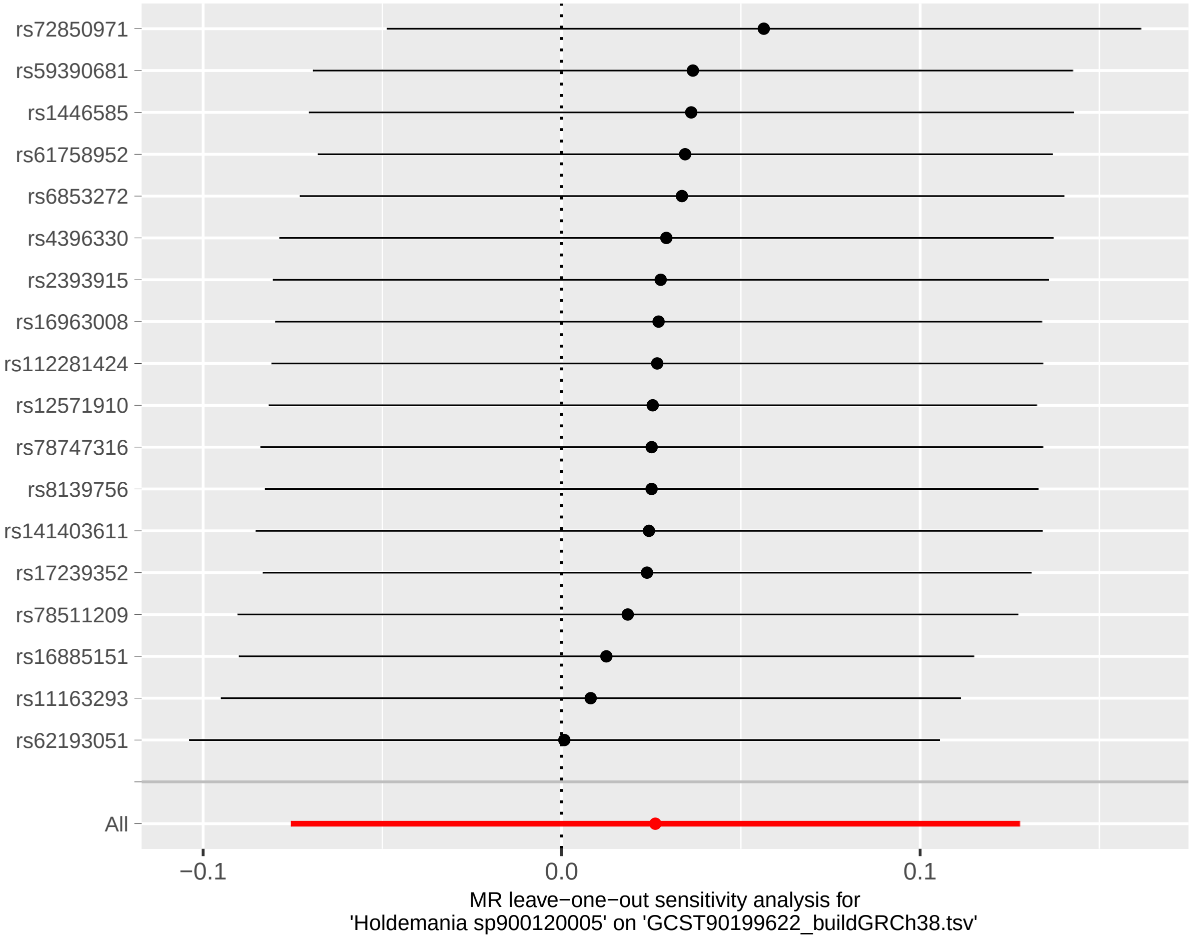
rs72850971
rs59390681
rs1446585
rs61758952
rs6853272
rs4396330
rs2393915
rs16963008
rs112281424
rs12571910
rs78747316
rs8139756
rs141403611
rs17239352
rs78511209
rs16885151
rs11163293
rs62193051
All
−0.1
0.0
0.1
MR leave−one−out sensitivity analysis for
'Holdemania sp900120005' on 'GCST90199622_buildGRCh38.tsv'

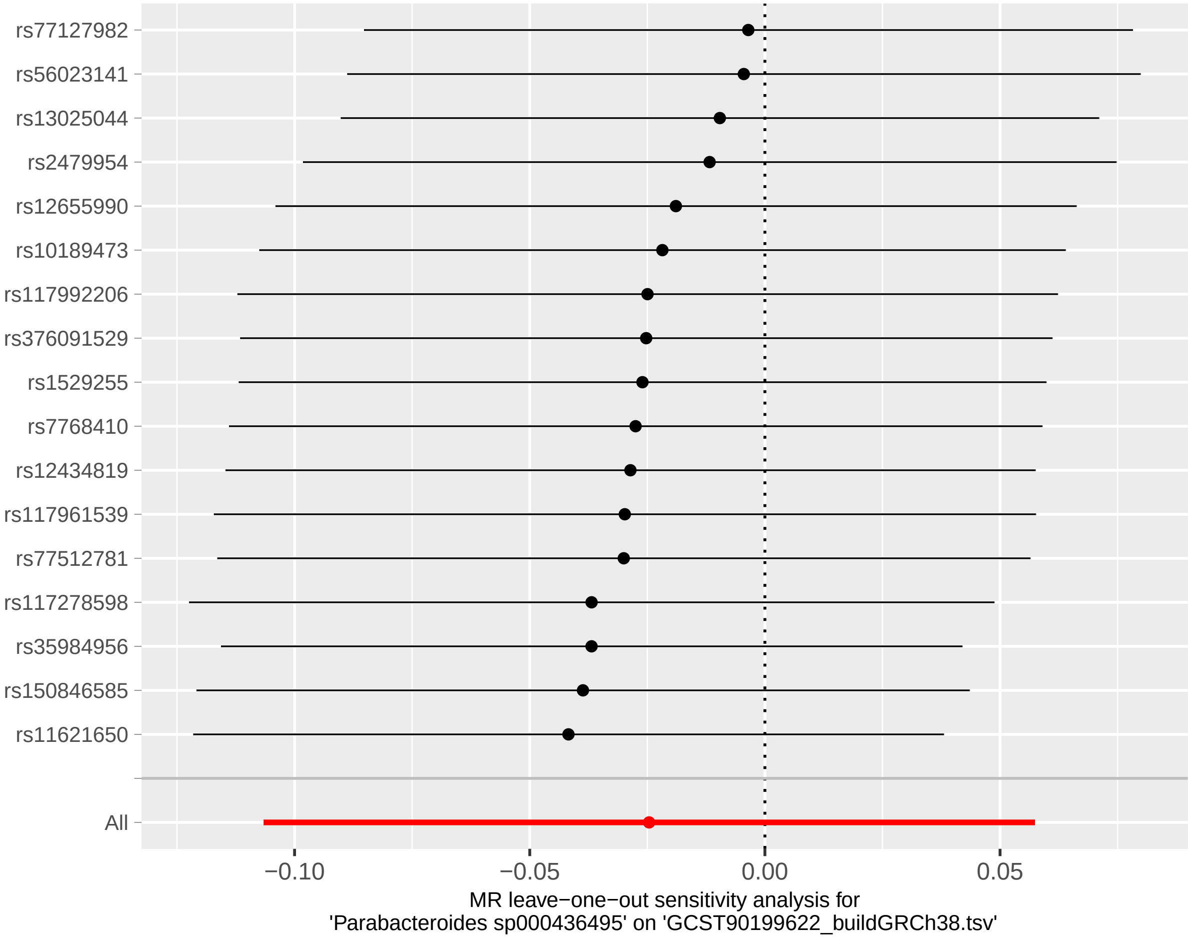
rs77127982
rs56023141
rs13025044
rs2479954
rs12655990
rs10189473
rs117992206
rs376091529
rs1529255
rs7768410
rs12434819
rs117961539
rs77512781
rs117278598
rs35984956
rs150846585
rs11621650
All
−0.10
−0.05
0.00
0.05
MR leave−one−out sensitivity analysis for
'Parabacteroides sp000436495' on 'GCST90199622_buildGRCh38.tsv'

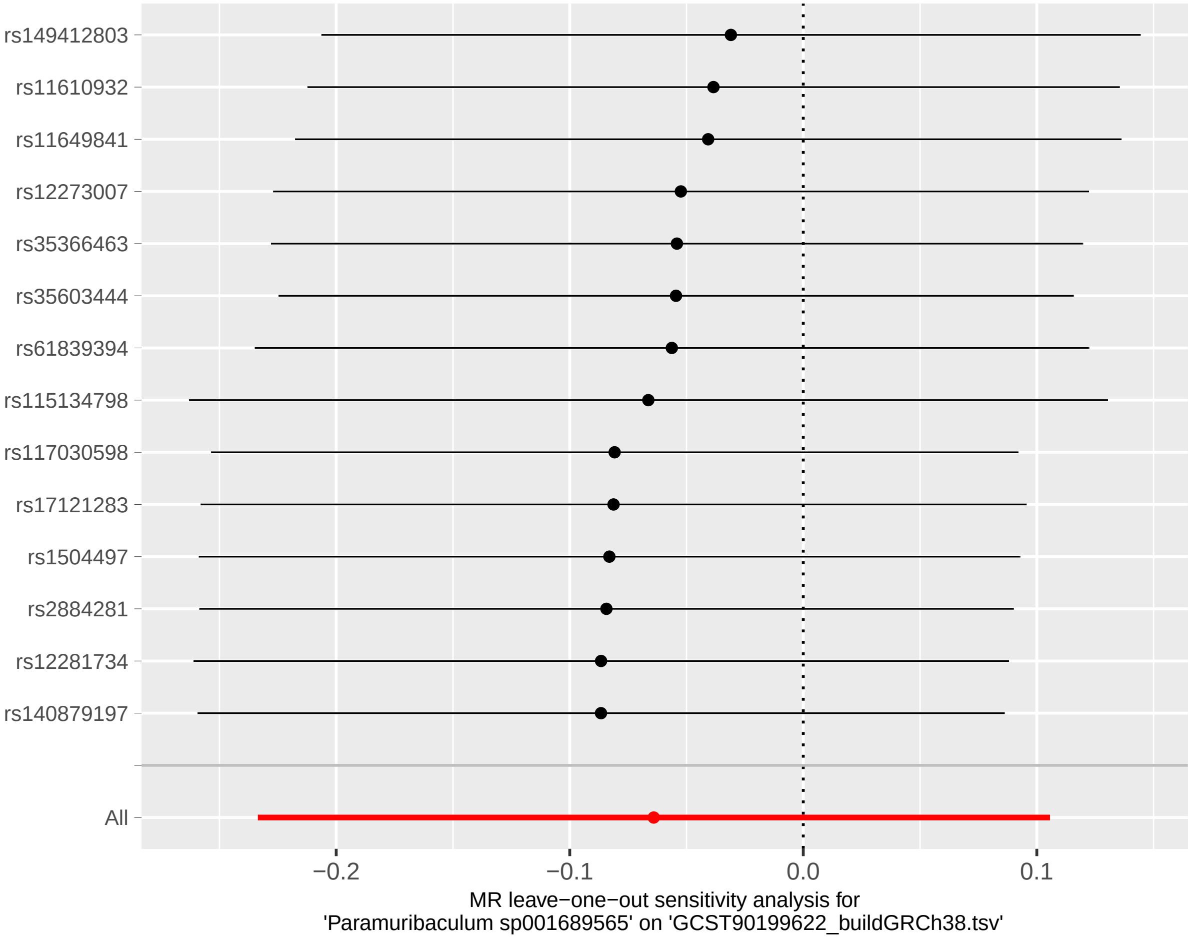
rs149412803
rs11610932
rs11649841
rs12273007
rs35366463
rs35603444
rs61839394
rs115134798
rs117030598
rs17121283
rs1504497
rs2884281
rs12281734
rs140879197
All
−0.2
−0.1
0.0
0.1
MR leave−one−out sensitivity analysis for
'Paramuribaculum sp001689565' on 'GCST90199622_buildGRCh38.tsv'

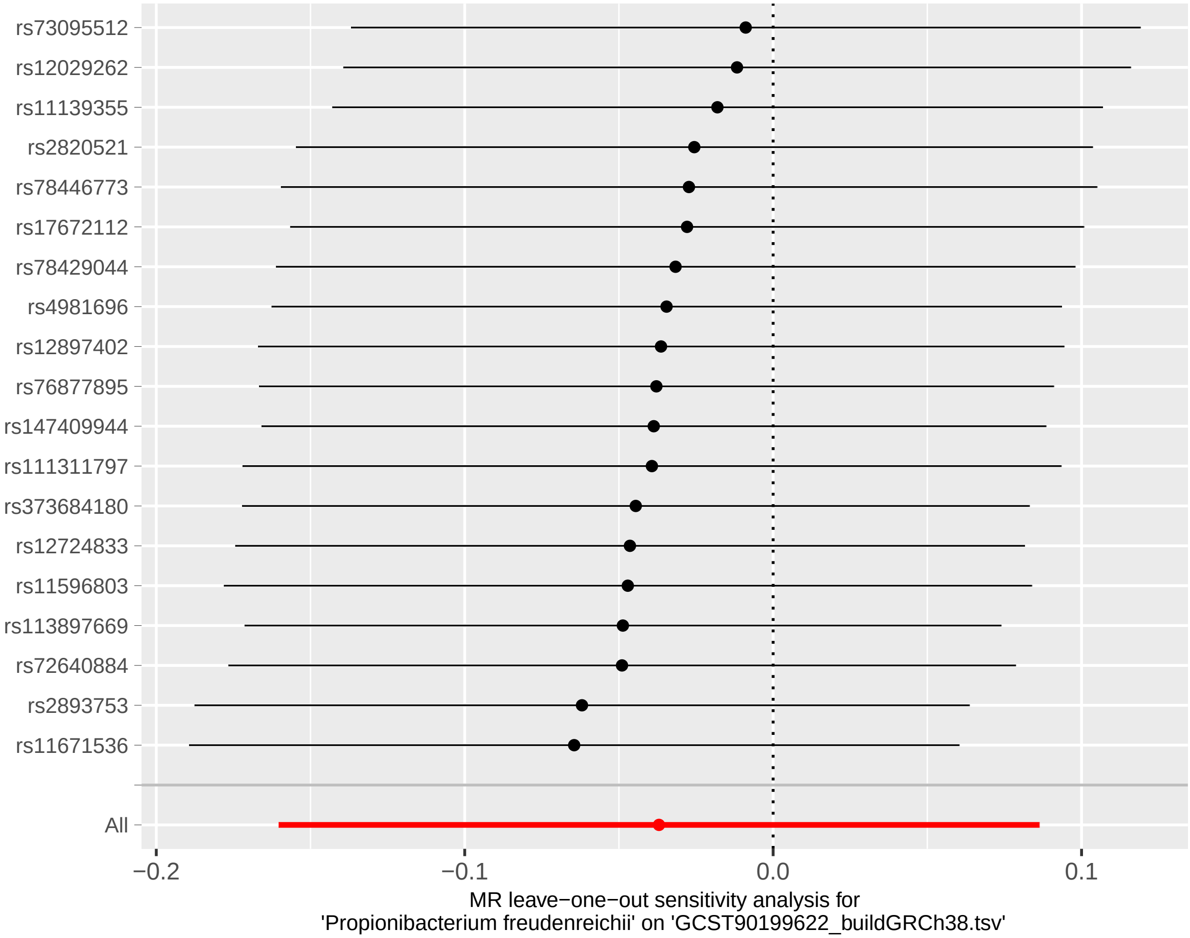
rs73095512
rs12029262
rs11139355
rs2820521
rs78446773
rs17672112
rs78429044
rs4981696
rs12897402
rs76877895
rs147409944
rs111311797
rs373684180
rs12724833
rs11596803
rs113897669
rs72640884
rs2893753
rs11671536
All
−0.2
−0.1
0.0
0.1
MR leave−one−out sensitivity analysis for
'Propionibacterium freudenreichii' on 'GCST90199622_buildGRCh38.tsv'

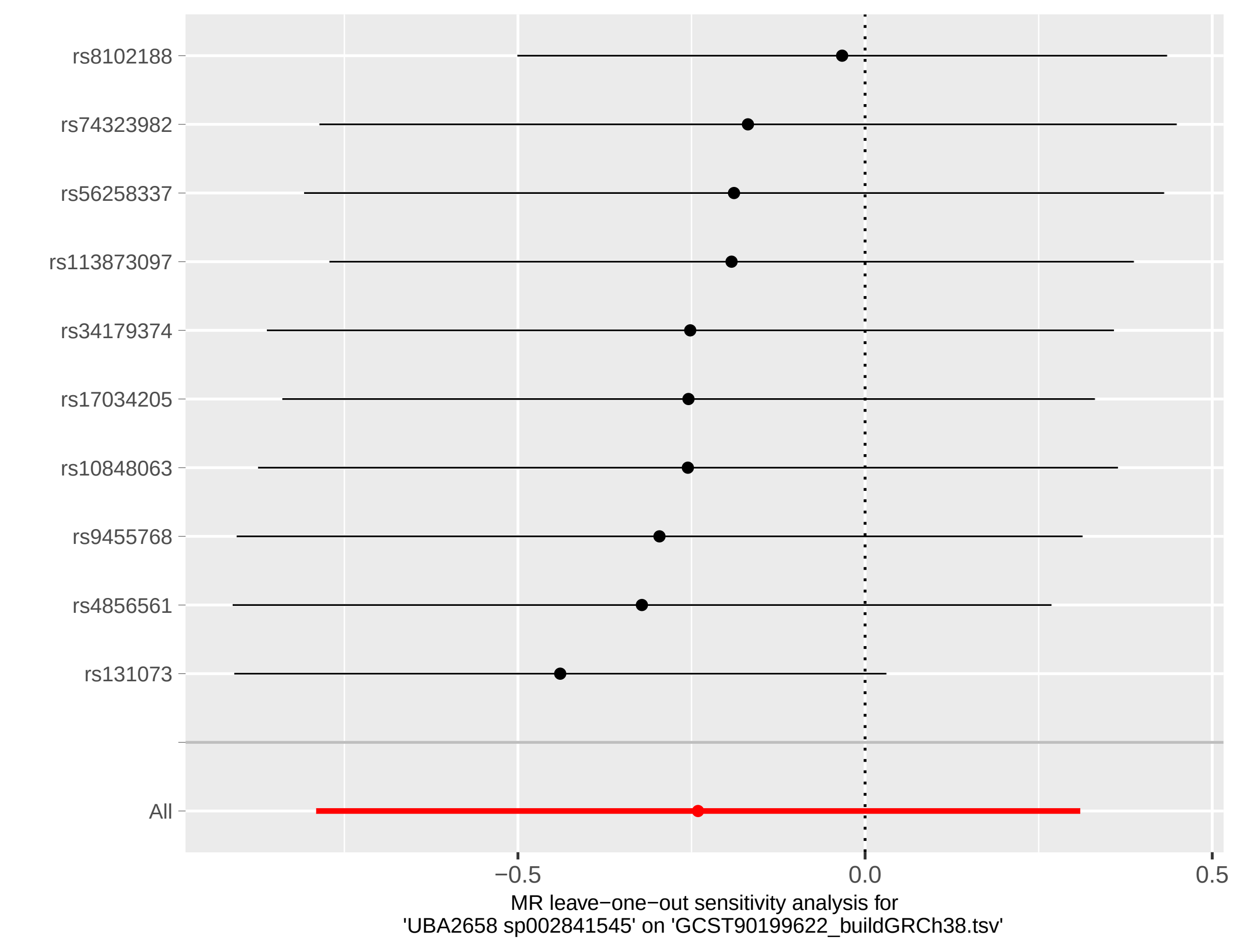

rs8102188
rs74323982
rs56258337
rs113873097
rs34179374
rs17034205
rs10848063
rs9455768
rs4856561
rs131073
All
−0.5
0.0
0.5
MR leave−one−out sensitivity analysis for
'UBA2658 sp002841545' on 'GCST90199622_buildGRCh38.tsv'

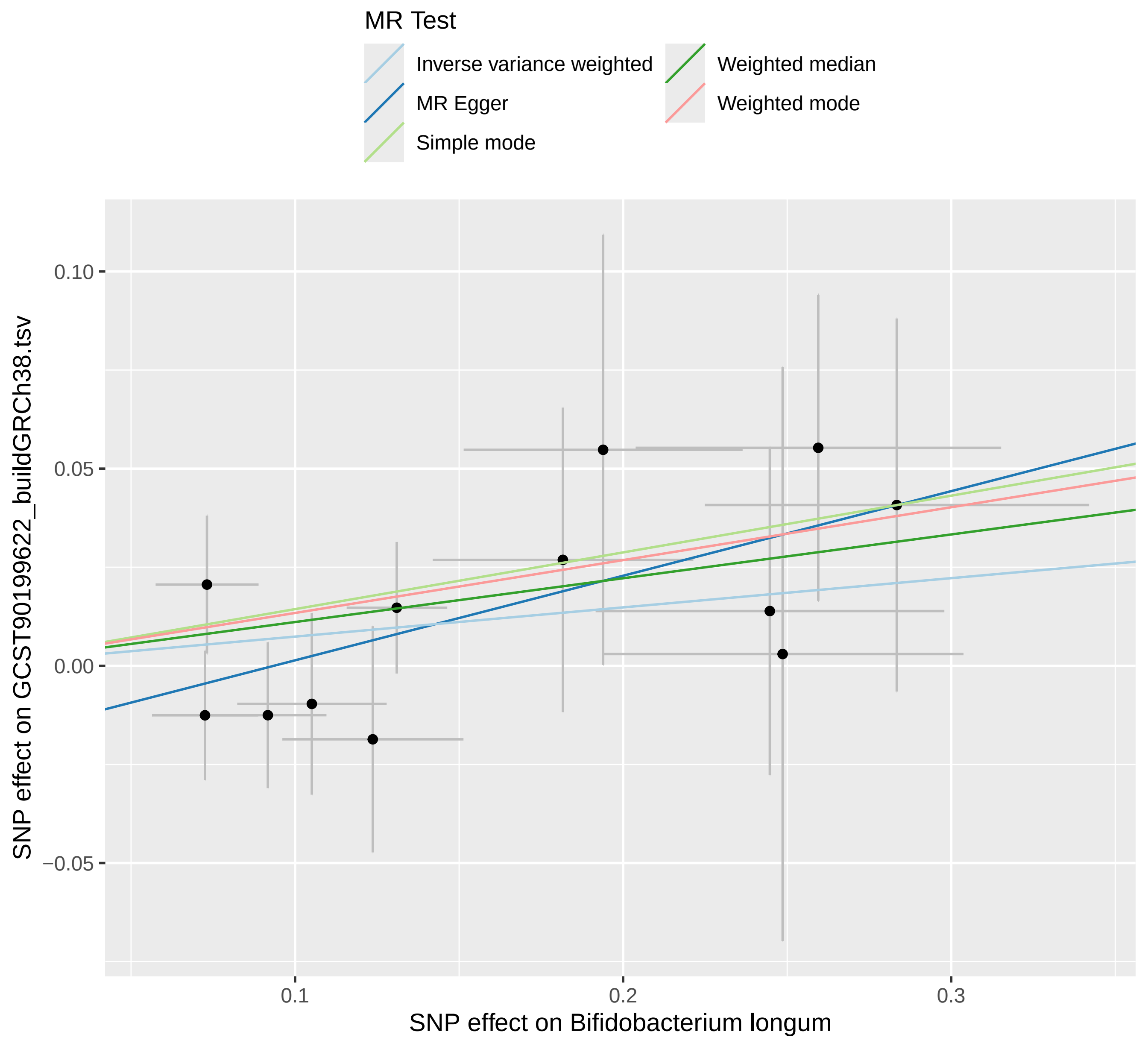
MR Test
Inverse variance weighted
MR Egger
Simple mode
Weighted median
Weighted mode
0.10
0.05
0.00
−0.05
0.1
0.2
0.3
SNP effect on GCST90199622_buildGRCh38.tsv
SNP effect on Bifidobacterium longum

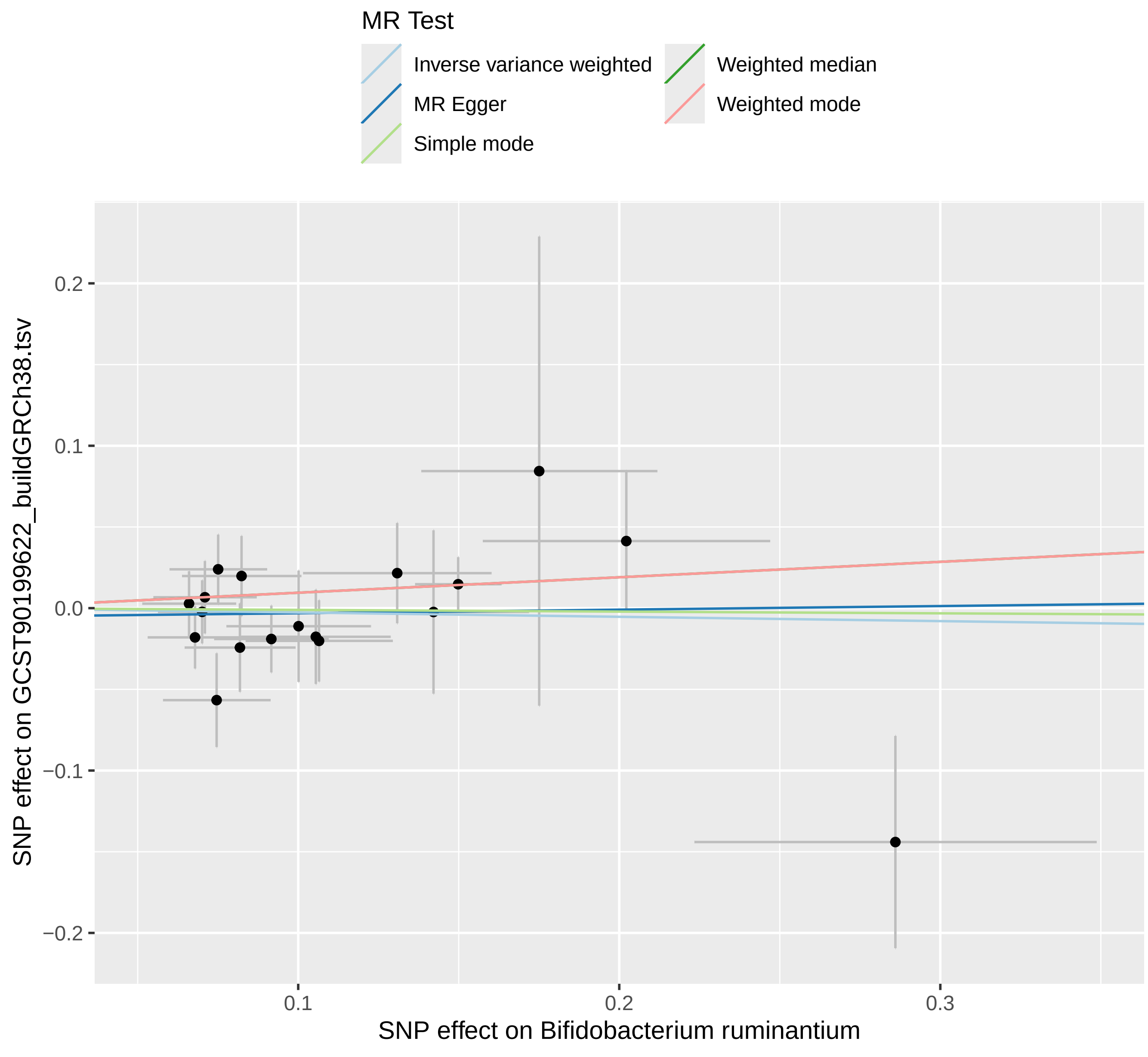

MR Test
Inverse variance weighted
MR Egger
Simple mode
Weighted median
Weighted mode
0.2
0.1
0.0
−0.1
−0.2
0.1
0.2
0.3
SNP effect on GCST90199622_buildGRCh38.tsv
SNP effect on Bifidobacterium ruminantium

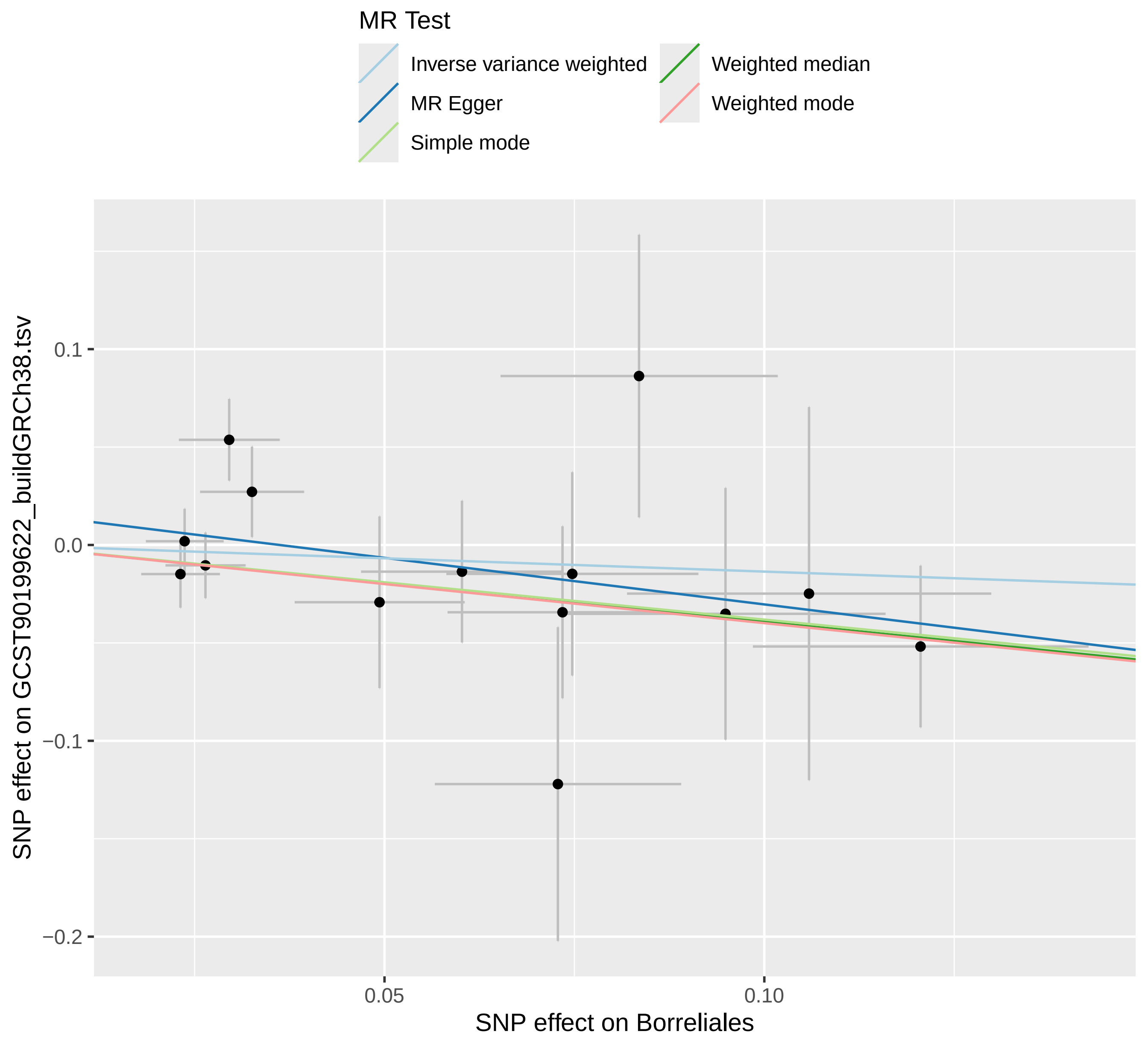

MR Test
Inverse variance weighted
MR Egger
Simple mode
Weighted median
Weighted mode
SNP effect on GCST90199622_buildGRCh38.tsv
0.1
0.0
−0.1
−0.2
0.05
0.10
SNP effect on Borreliales

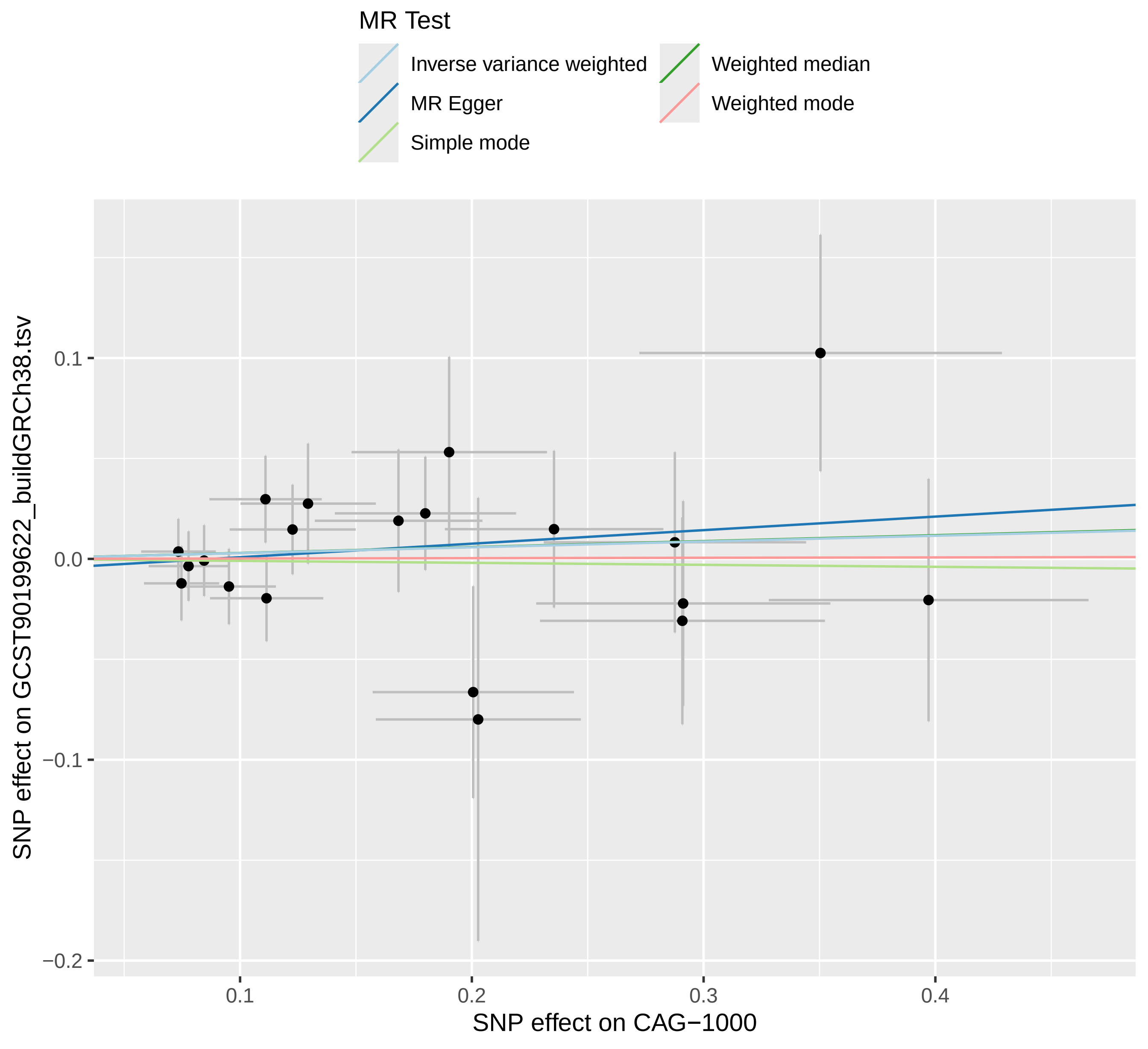

MR Test
Inverse variance weighted
MR Egger
Simple mode
Weighted median
Weighted mode
SNP effect on GCST90199622_buildGRCh38.tsv
0.1
0.0
−0.1
−0.2
0.1
0.2
0.3
0.4
SNP effect on CAG−1000

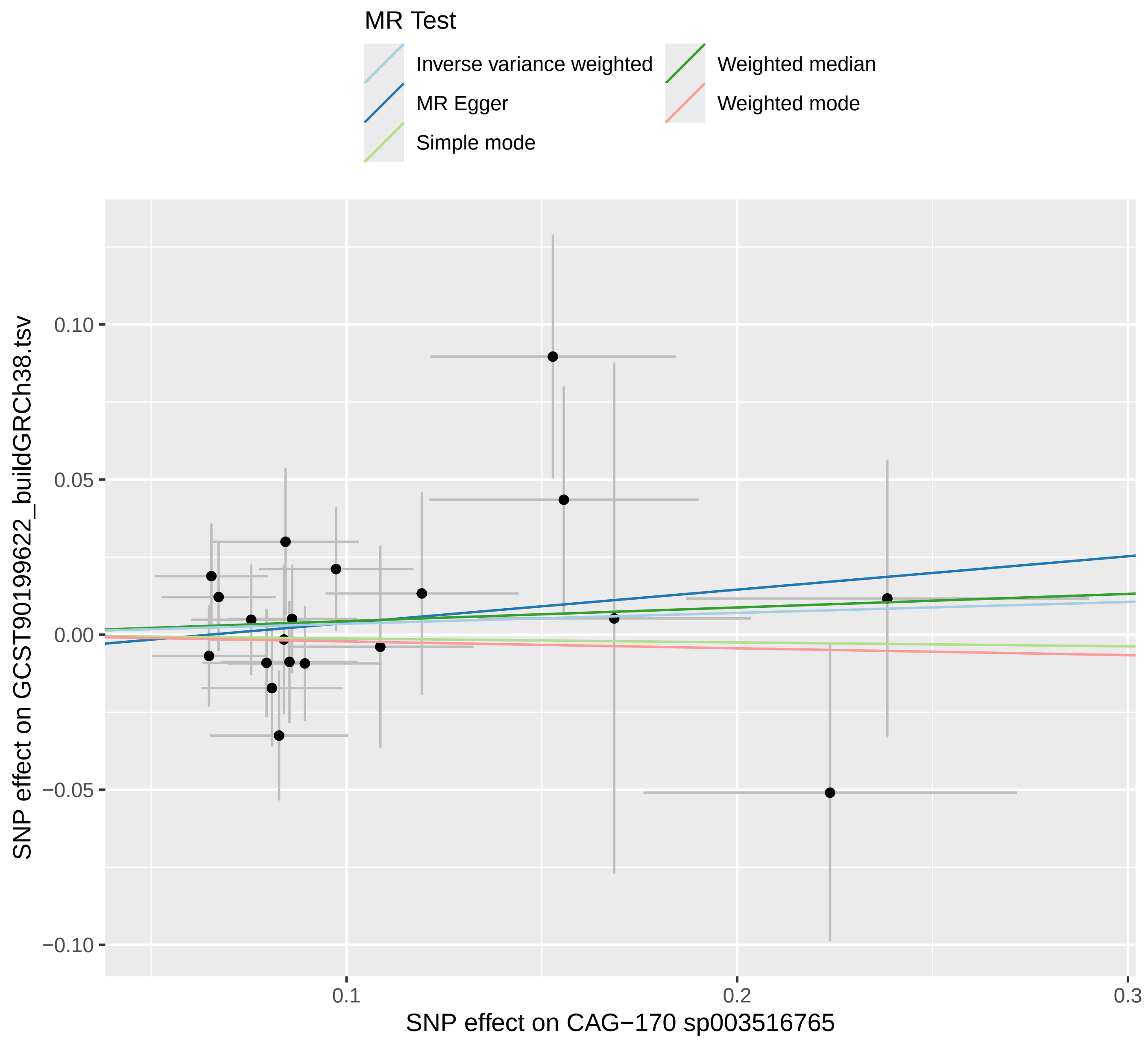
MR Test
Inverse variance weighted
MR Egger
Simple mode
Weighted median
Weighted mode
0.10
0.05
0.00
−0.05
−0.10
0.1
0.2
0.3
SNP effect on GCST90199622_buildGRCh38.tsv
SNP effect on CAG−170 sp003516765

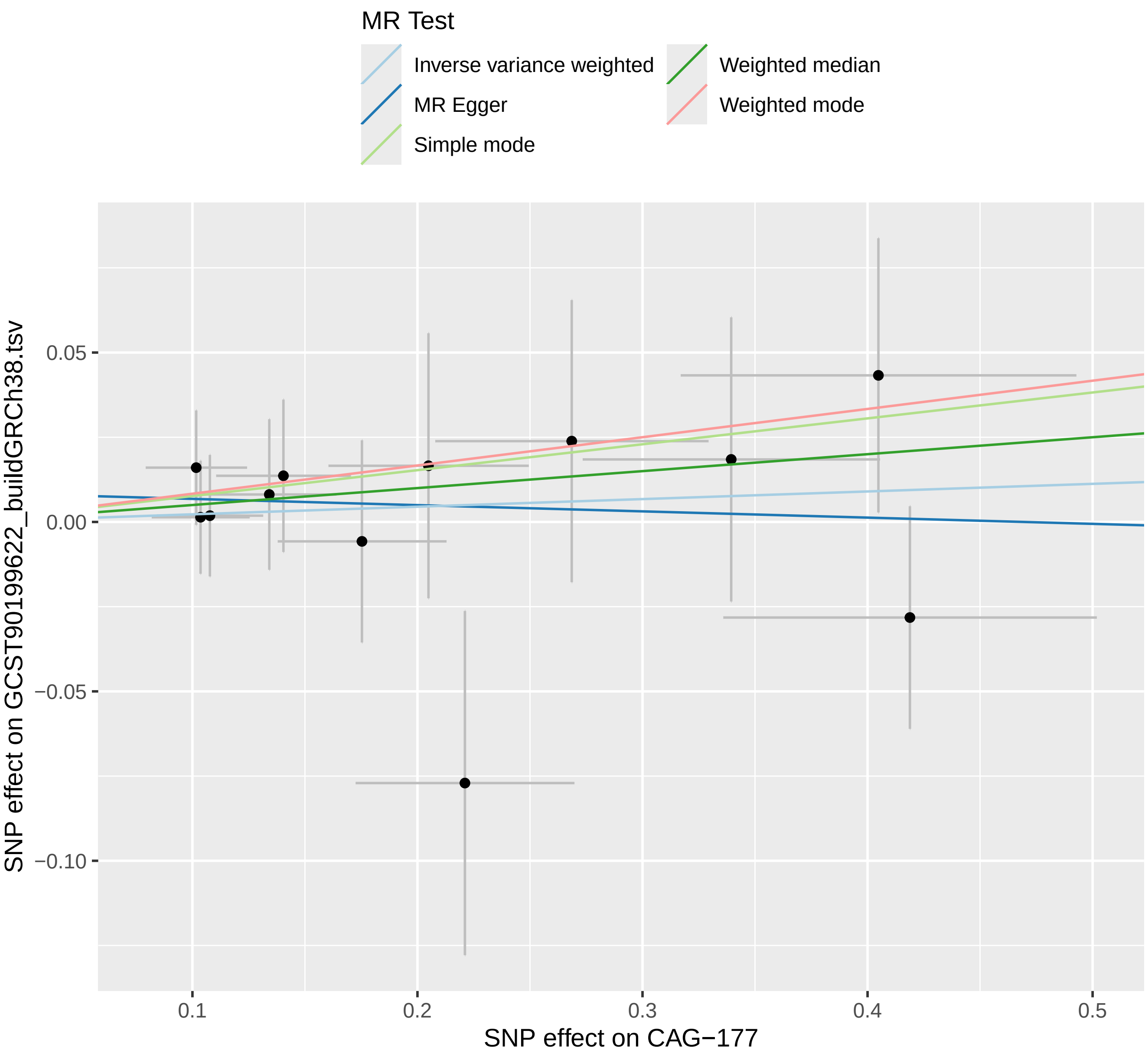

MR Test
Inverse variance weighted
MR Egger
Simple mode
Weighted median
Weighted mode
0.05
0.00
−0.05
−0.10
0.1
0.2
0.3
0.4
0.5
SNP effect on GCST90199622_buildGRCh38.tsv
SNP effect on CAG−177

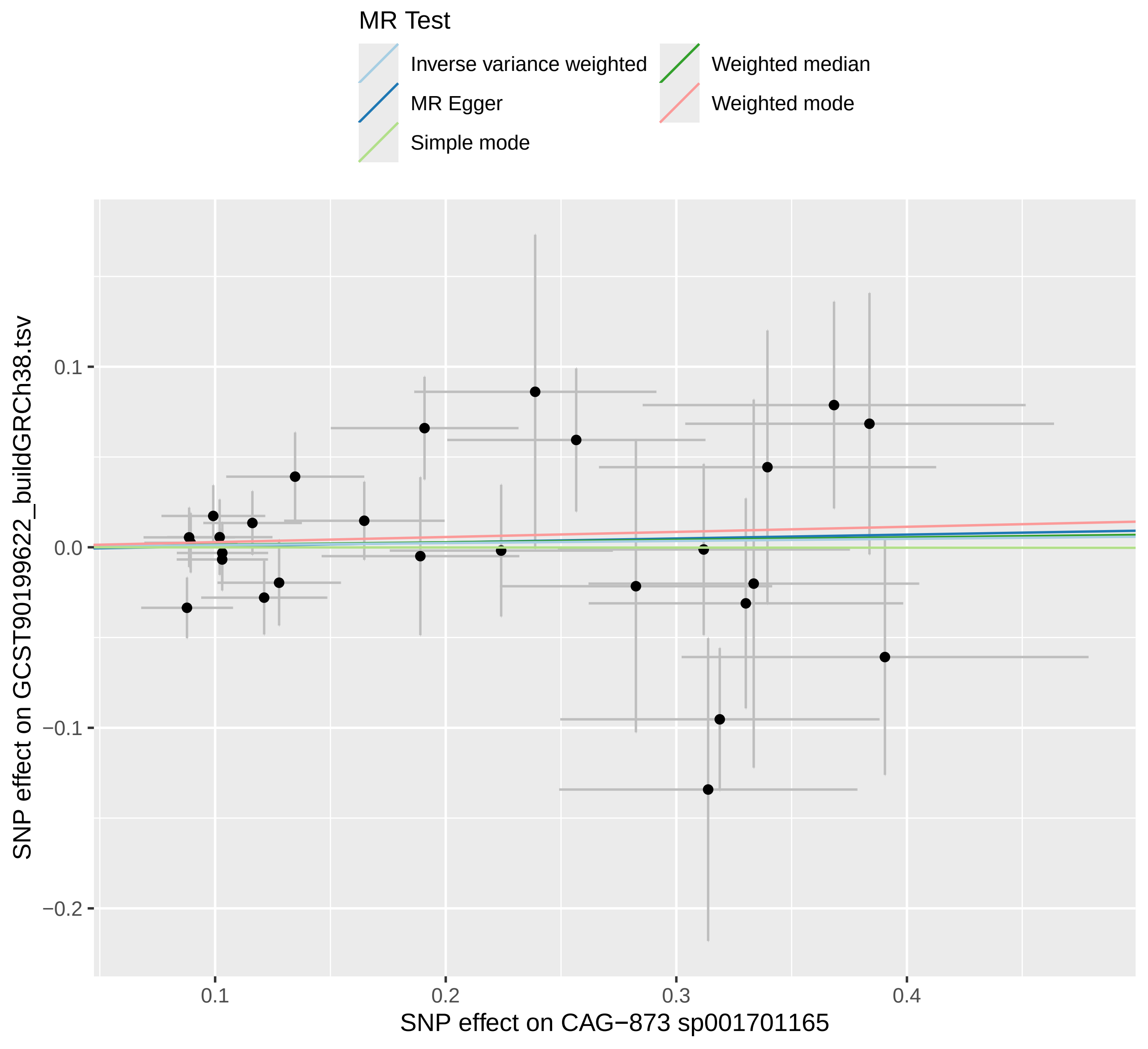

MR Test
Inverse variance weighted
MR Egger
Simple mode
Weighted median
Weighted mode
SNP effect on GCST90199622_buildGRCh38.tsv
0.1
0.0
−0.1
−0.2
0.1
0.2
0.3
0.4
SNP effect on CAG−873 sp001701165

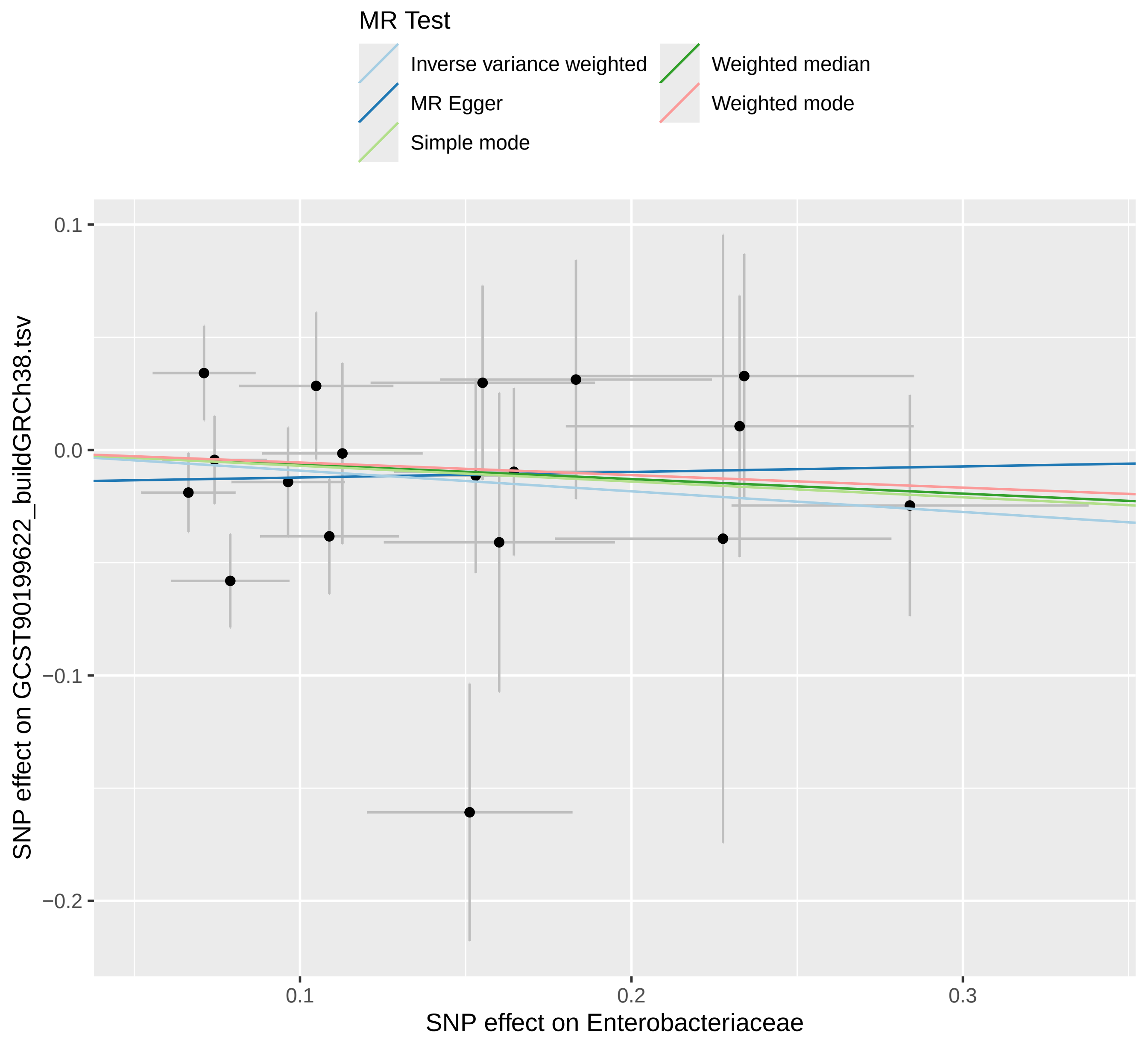

MR Test
Inverse variance weighted
MR Egger
Simple mode
Weighted median
Weighted mode
0.1
0.0
−0.1
−0.2
SNP effect on GCST90199622_buildGRCh38.tsv
0.1
0.2
0.3
SNP effect on Enterobacteriaceae

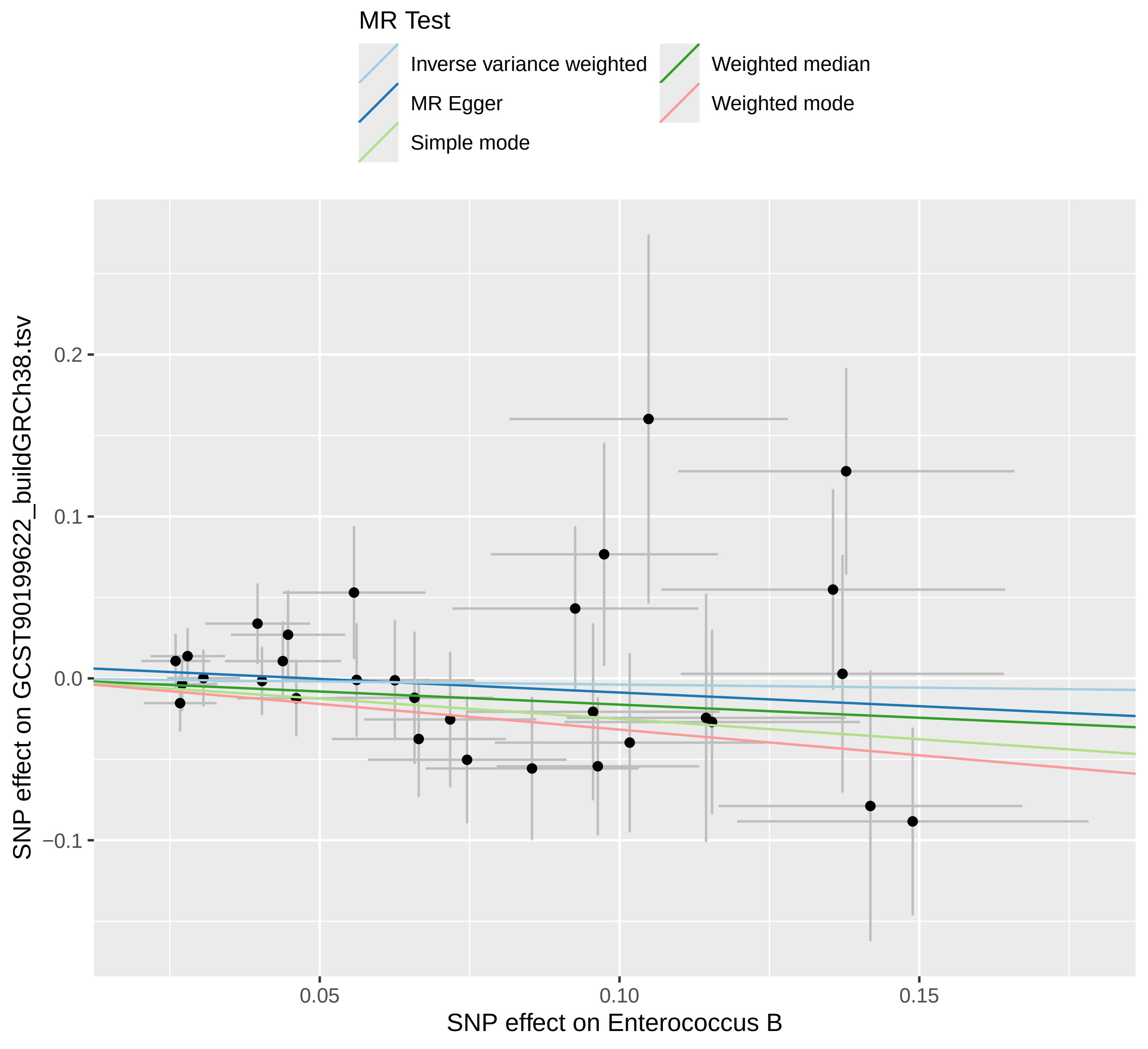

MR Test
Inverse variance weighted
MR Egger
Simple mode
Weighted median
Weighted mode
SNP effect on GCST90199622_buildGRCh38.tsv
0.2
0.1
0.0
−0.1
0.05
0.10
0.15
SNP effect on Enterococcus B

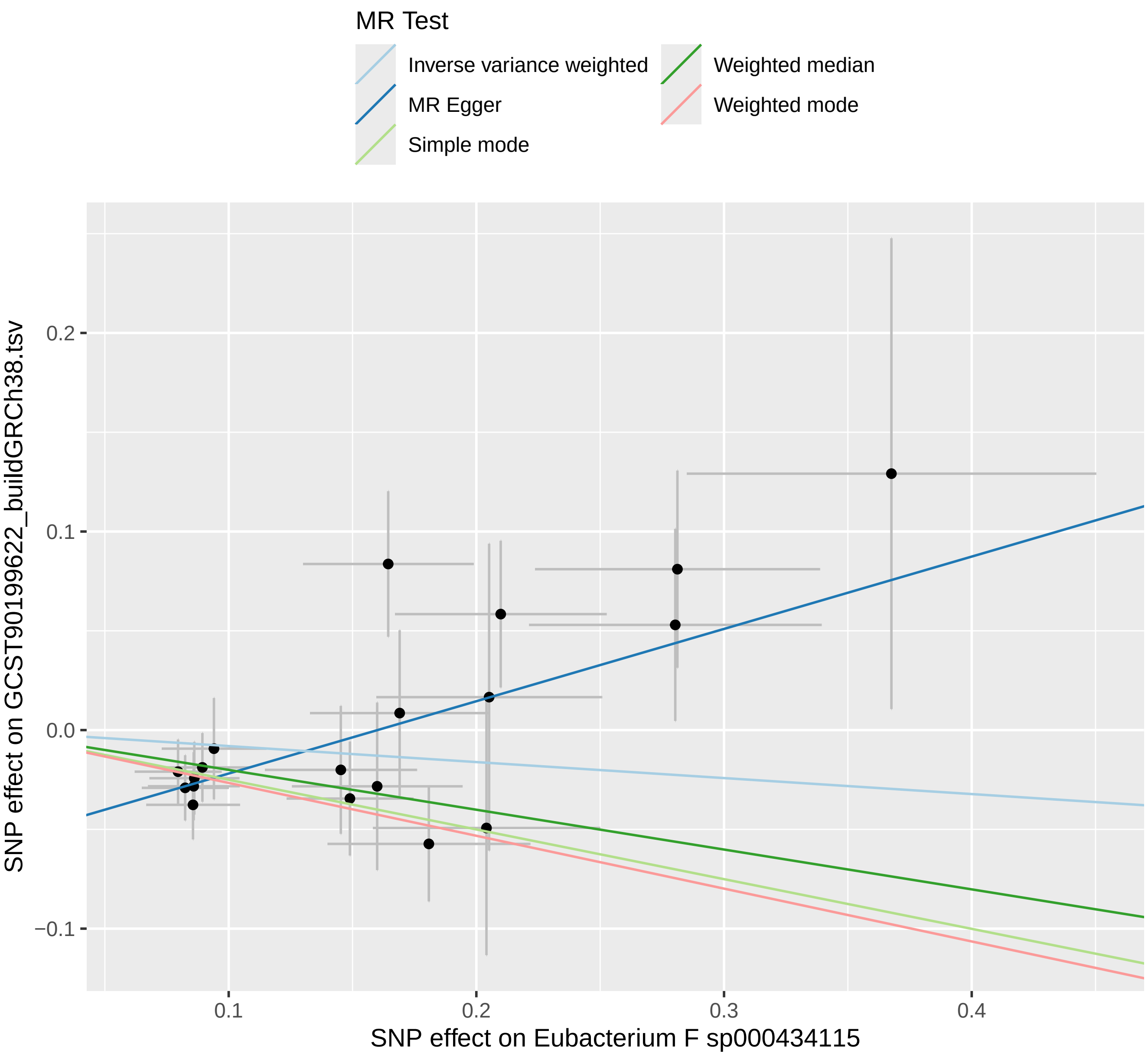
MR Test
Inverse variance weighted
MR Egger
Simple mode
Weighted median
Weighted mode
0.2
0.1
0.0
−0.1
0.1
0.2
0.3
0.4
SNP effect on GCST90199622_buildGRCh38.tsv
SNP effect on Eubacterium F sp000434115

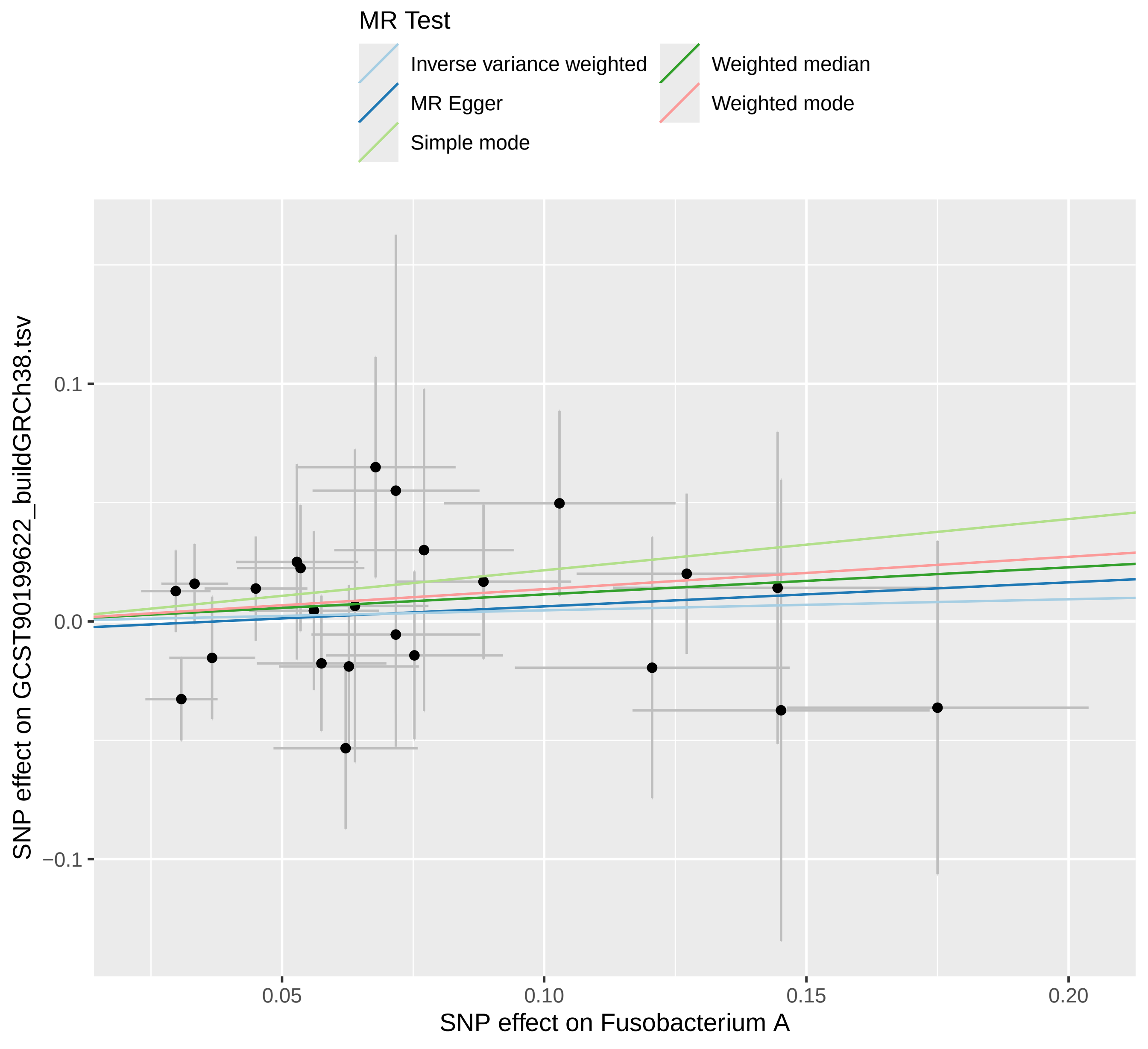
MR Test
Inverse variance weighted
MR Egger
Simple mode
Weighted median
Weighted mode
SNP effect on GCST90199622_buildGRCh38.tsv
0.1
0.0
−0.1
0.05
0.10
0.15
0.20
SNP effect on Fusobacterium A

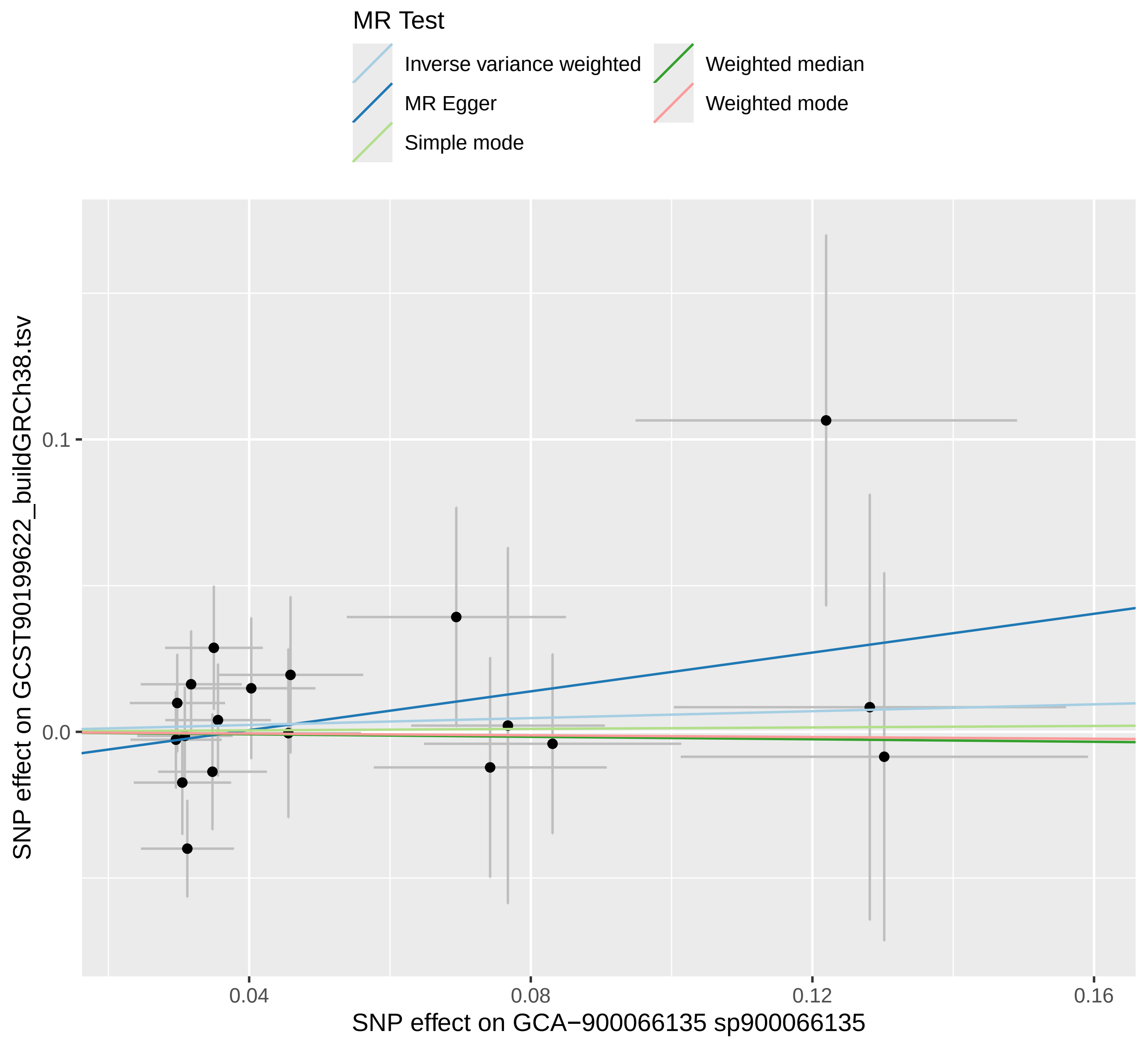
MR Test
Inverse variance weighted
MR Egger
Simple mode
Weighted median
Weighted mode
SNP effect on GCST90199622_buildGRCh38.tsv
0.1
0.0
0.04
0.08
0.12
0.16
SNP effect on GCA−900066135 sp900066135

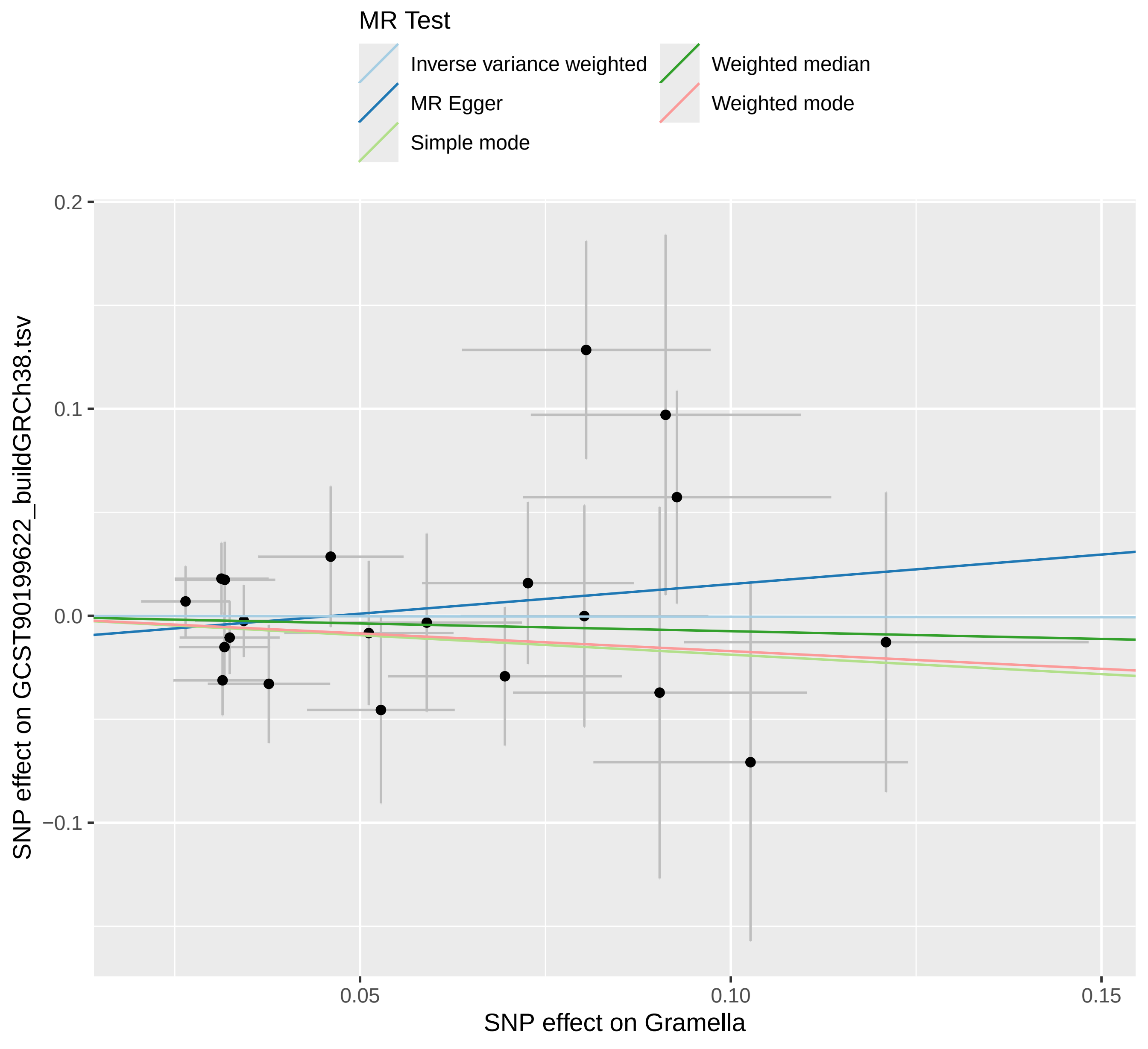
MR Test
Inverse variance weighted
MR Egger
Simple mode
Weighted median
Weighted mode
0.2
0.1
0.0
−0.1
0.05
0.10
0.15
SNP effect on GCST90199622_buildGRCh38.tsv
SNP effect on Gramella

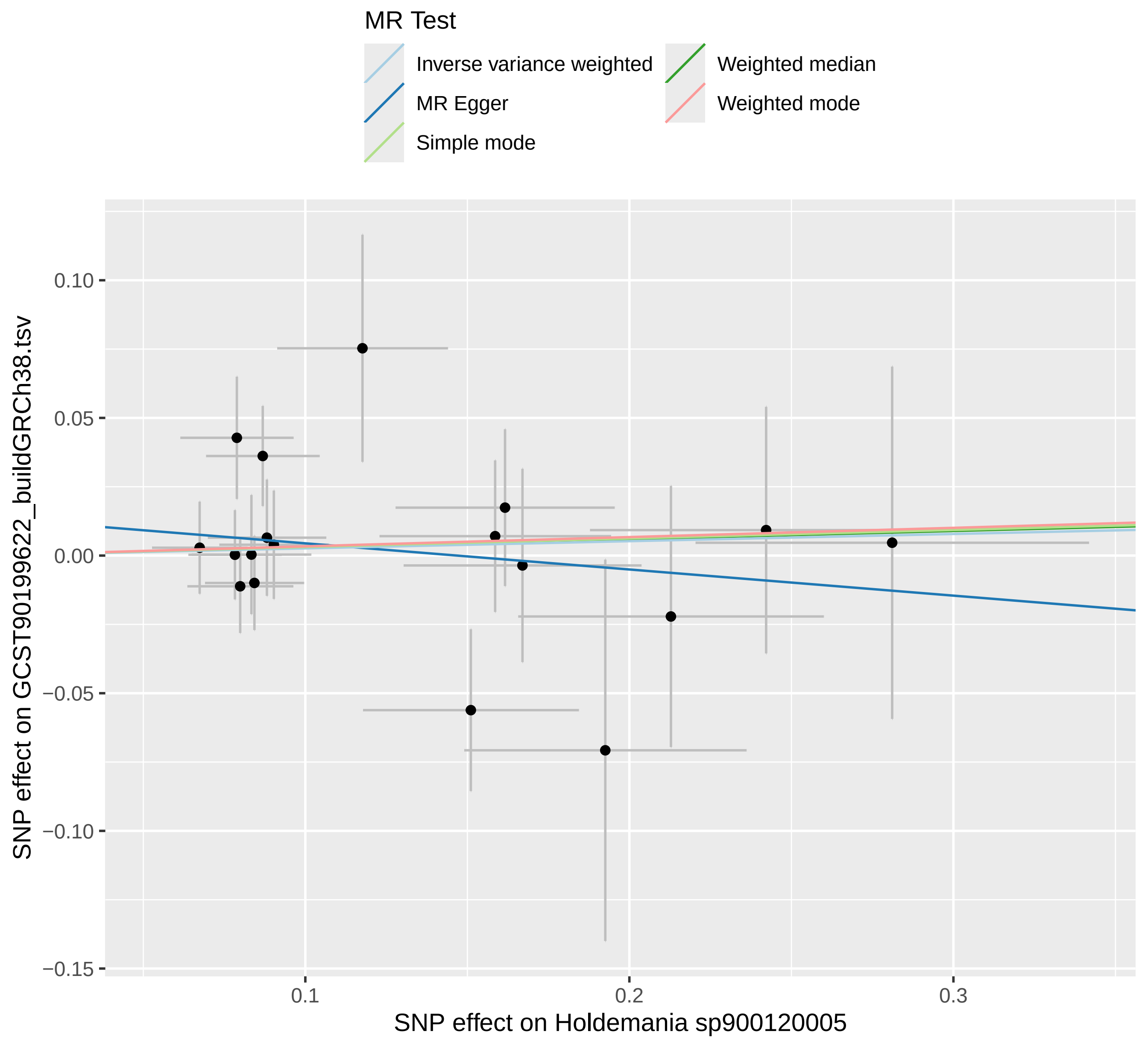

MR Test
Inverse variance weighted
MR Egger
Simple mode
Weighted median
Weighted mode
0.10
0.05
0.00
−0.05
−0.10
−0.15
0.1
0.2
0.3
SNP effect on GCST90199622_buildGRCh38.tsv
SNP effect on Holdemania sp900120005

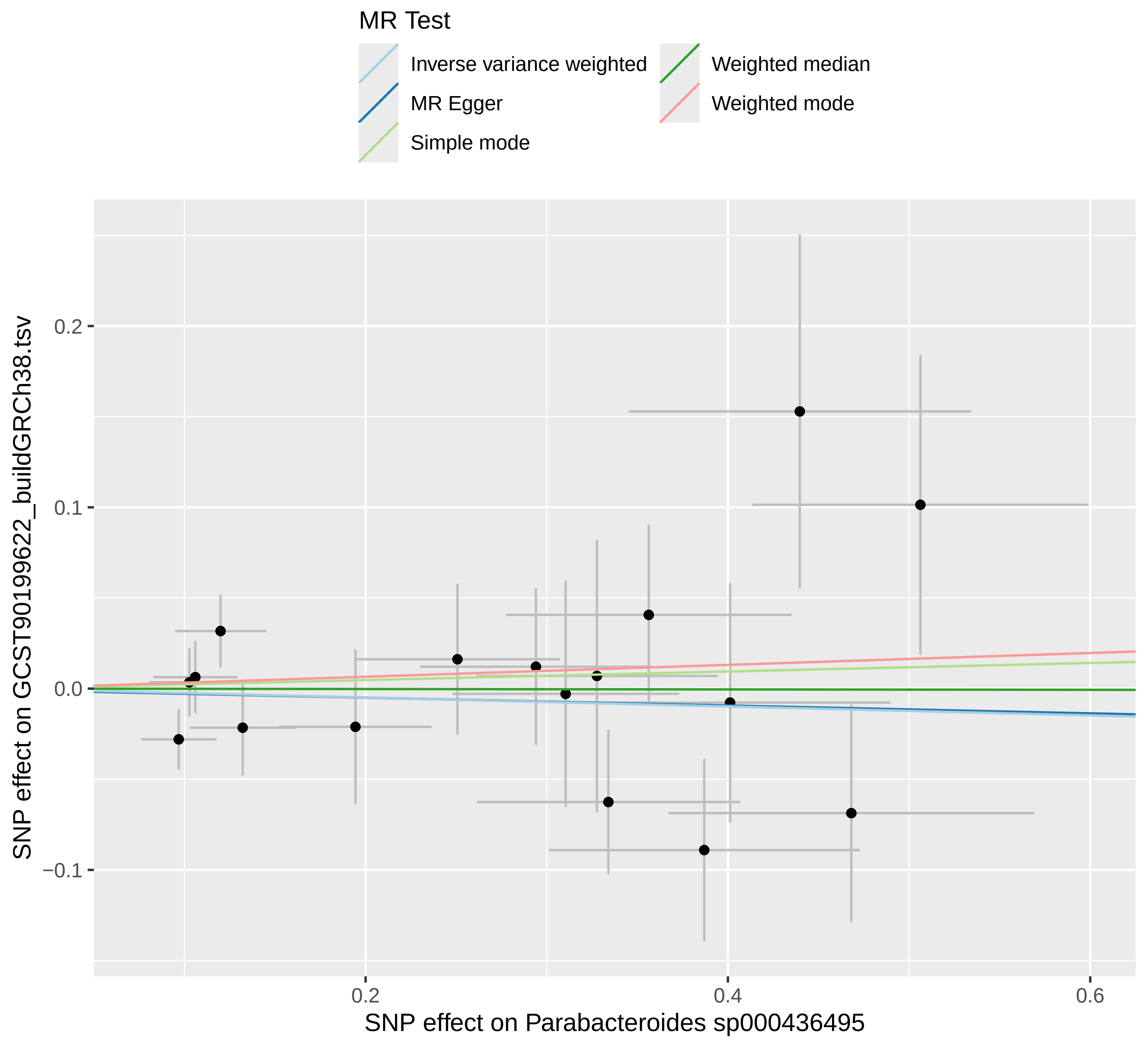

MR Test
Inverse variance weighted
MR Egger
Simple mode
Weighted median
Weighted mode
SNP effect on GCST90199622_buildGRCh38.tsv
0.2
0.1
0.0
−0.1
0.2
0.4
0.6
SNP effect on Parabacteroides sp000436495

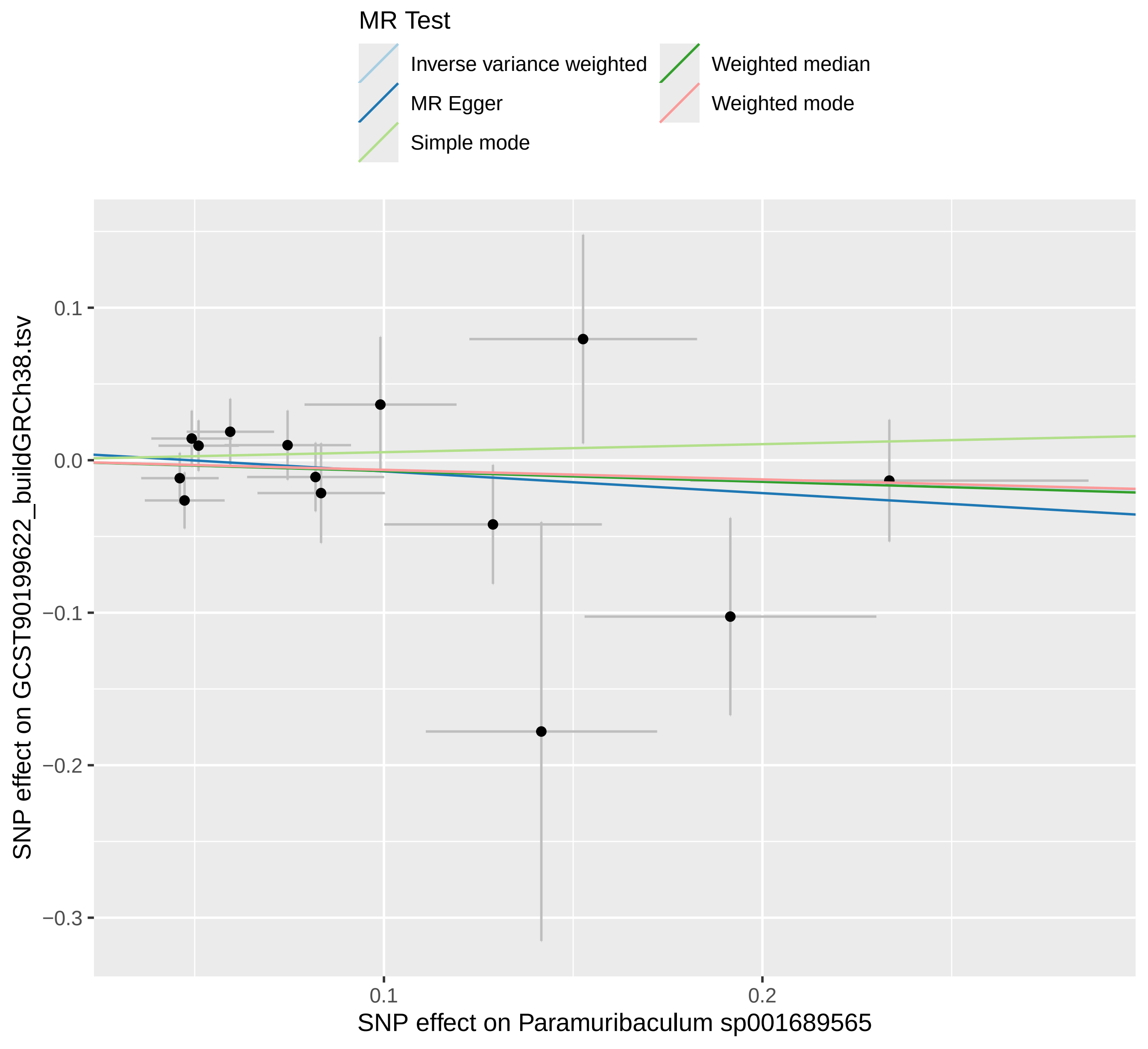

MR Test
Inverse variance weighted
MR Egger
Simple mode
Weighted median
Weighted mode
0.1
0.0
−0.1
−0.2
−0.3
0.1
0.2
SNP effect on GCST90199622_buildGRCh38.tsv
SNP effect on Paramuribaculum sp001689565

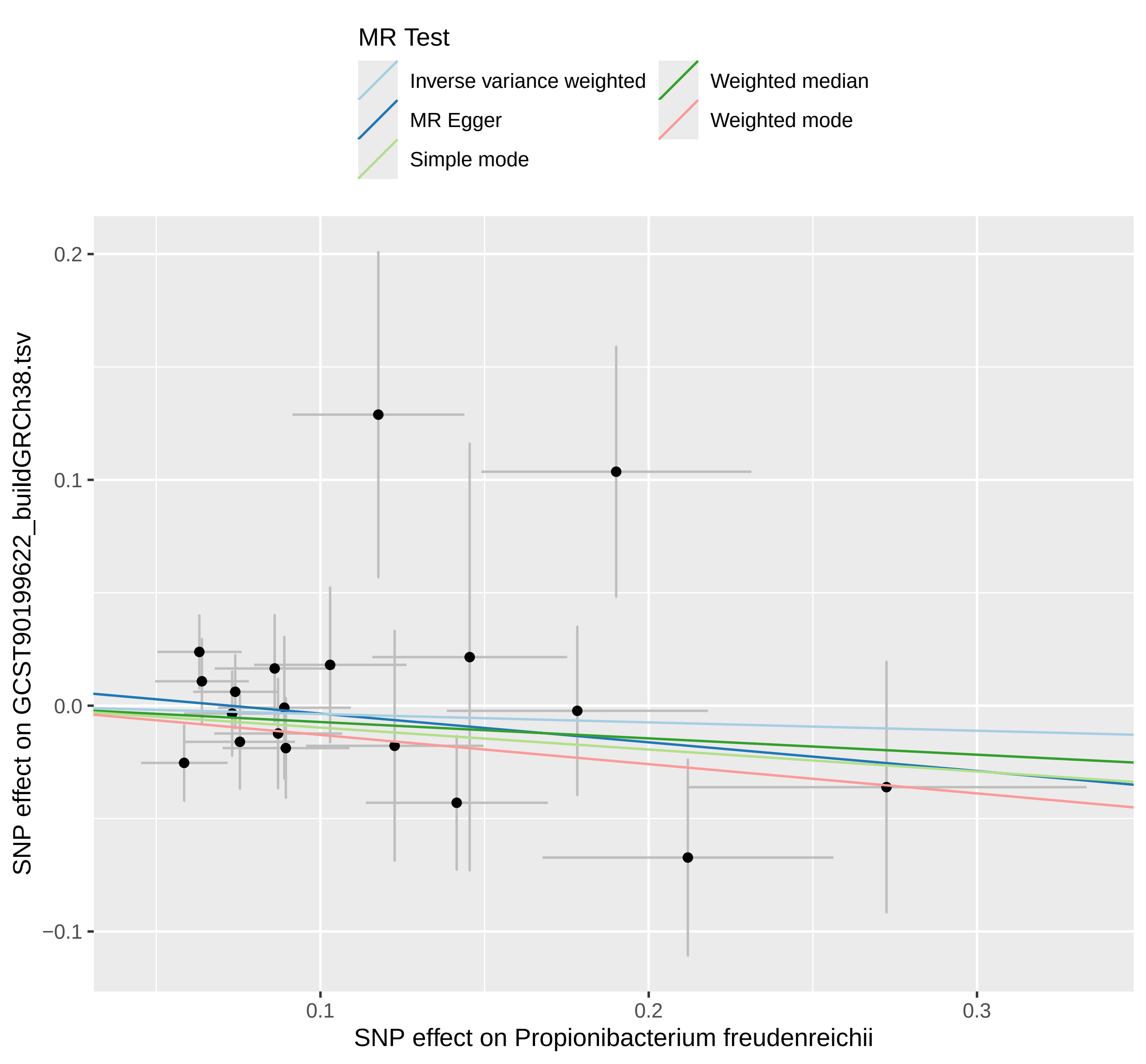
MR Test
Inverse variance weighted
MR Egger
Simple mode
Weighted median
Weighted mode
0.2
0.1
0.0
−0.1
0.1
0.2
0.3
SNP effect on GCST90199622_buildGRCh38.tsv
SNP effect on Propionibacterium freudenreichii

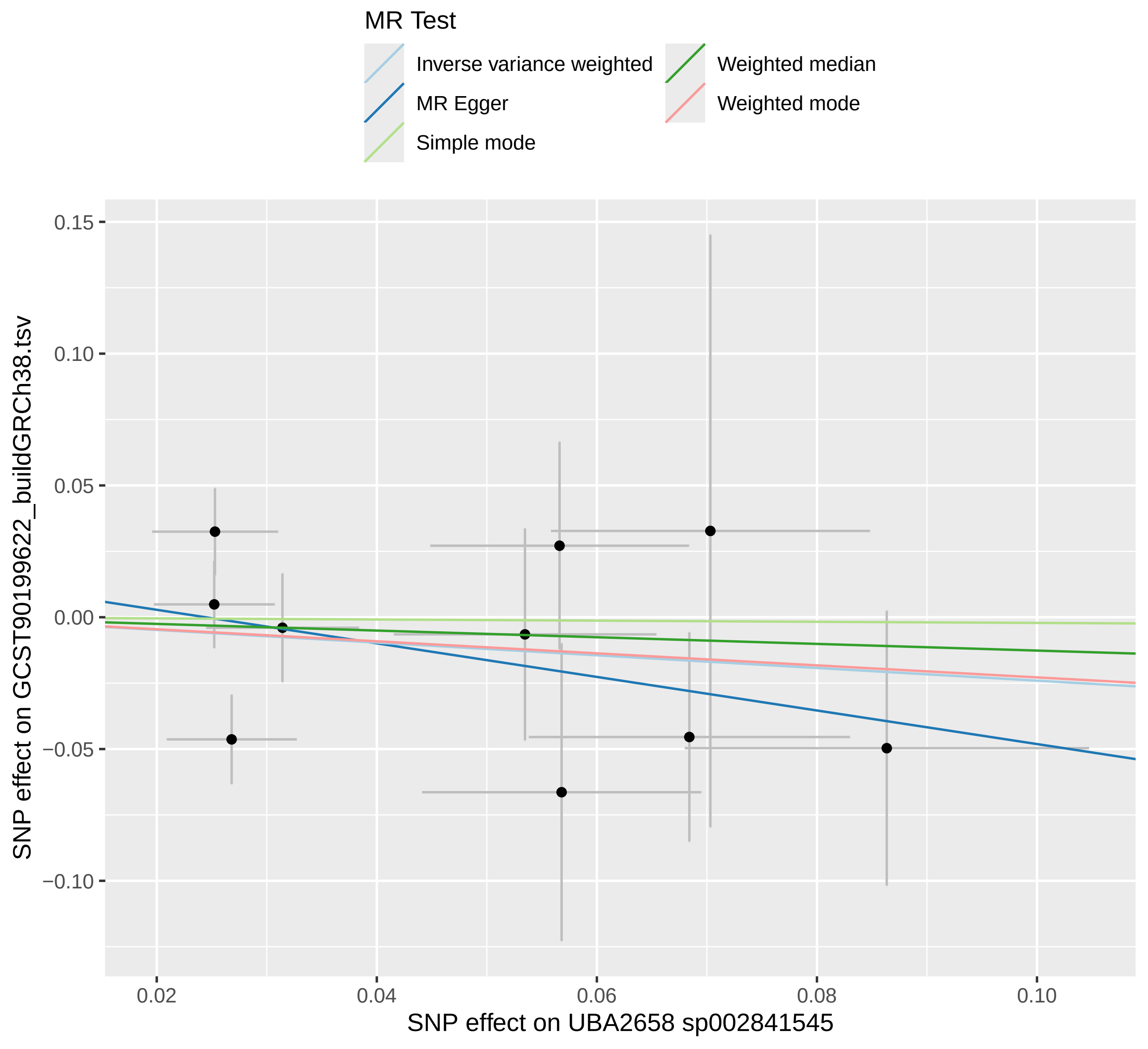
MR Test
Inverse variance weighted
MR Egger
Simple mode
Weighted median
Weighted mode
SNP effect on GCST90199622_buildGRCh38.tsv
0.15
0.10
0.05
0.00
−0.05
−0.10
0.02
0.04
0.06
0.08
0.10
SNP effect on UBA2658 sp002841545

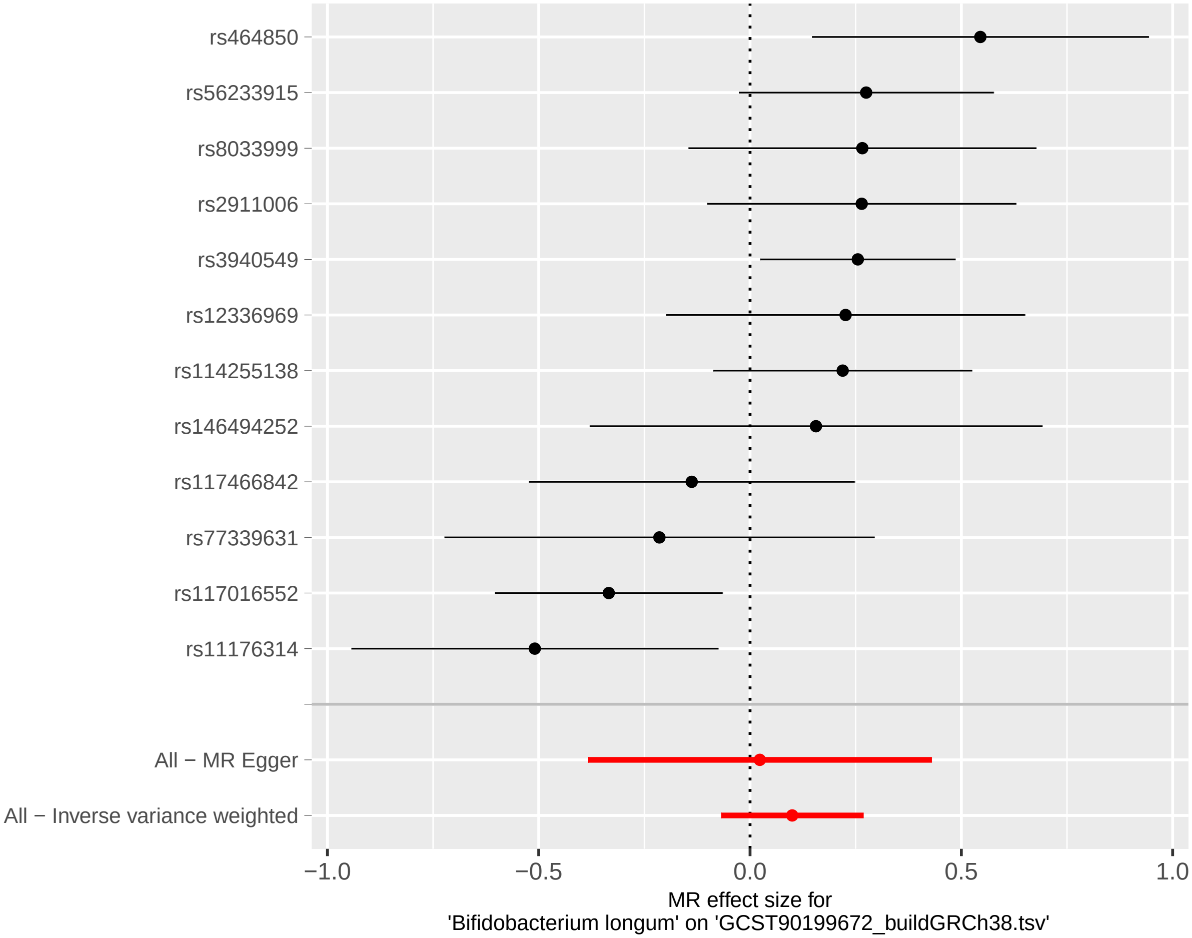
rs464850
rs56233915
rs8033999
rs2911006
rs3940549
rs12336969
rs114255138
rs146494252
rs117466842
rs77339631
rs117016552
rs11176314
All − MR Egger
All − Inverse variance weighted
−1.0
−0.5
0.0
0.5
1.0
MR effect size for
'Bifidobacterium longum' on 'GCST90199672_buildGRCh38.tsv'

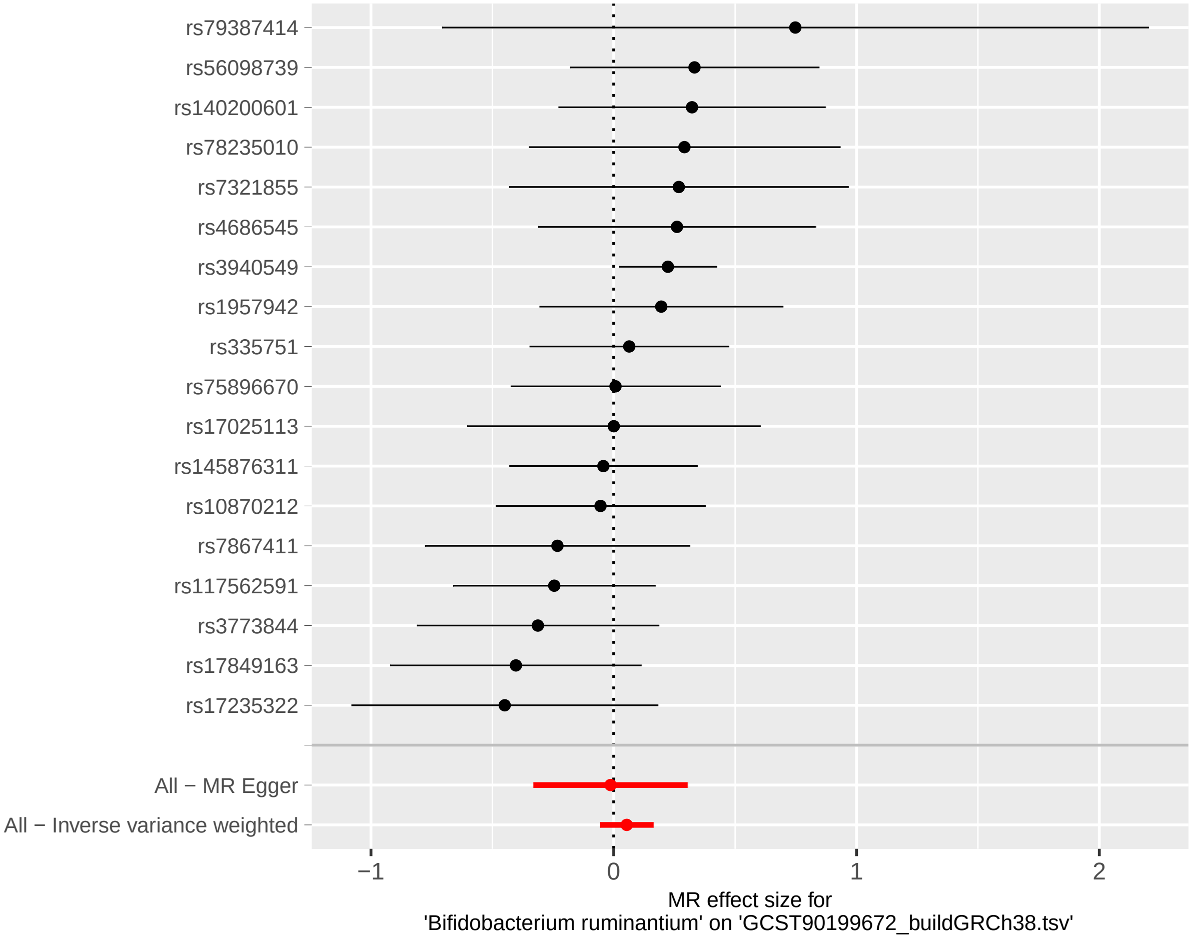
rs79387414
rs56098739
rs140200601
rs78235010
rs7321855
rs4686545
rs3940549
rs1957942
rs335751
rs75896670
rs17025113
rs145876311
rs10870212
rs7867411
rs117562591
rs3773844
rs17849163
rs17235322
All − MR Egger
All − Inverse variance weighted
−1
0
1
2
MR effect size for
'Bifidobacterium ruminantium' on 'GCST90199672_buildGRCh38.tsv'

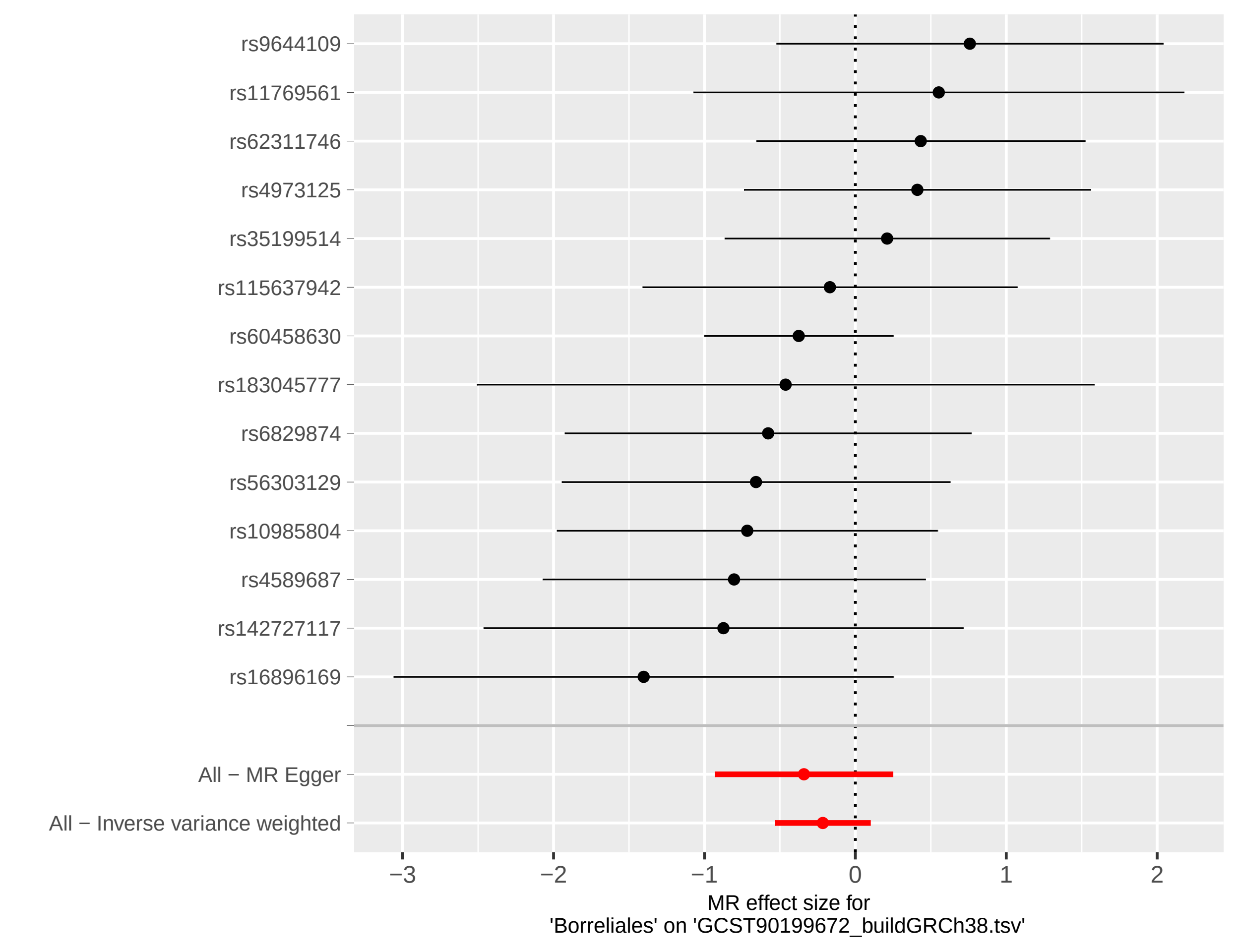

rs9644109
rs11769561
rs62311746
rs4973125
rs35199514
rs115637942
rs60458630
rs183045777
rs6829874
rs56303129
rs10985804
rs4589687
rs142727117
rs16896169
All − MR Egger
All − Inverse variance weighted
−3
−2
−1
0
1
2
MR effect size for
'Borreliales' on 'GCST90199672_buildGRCh38.tsv'

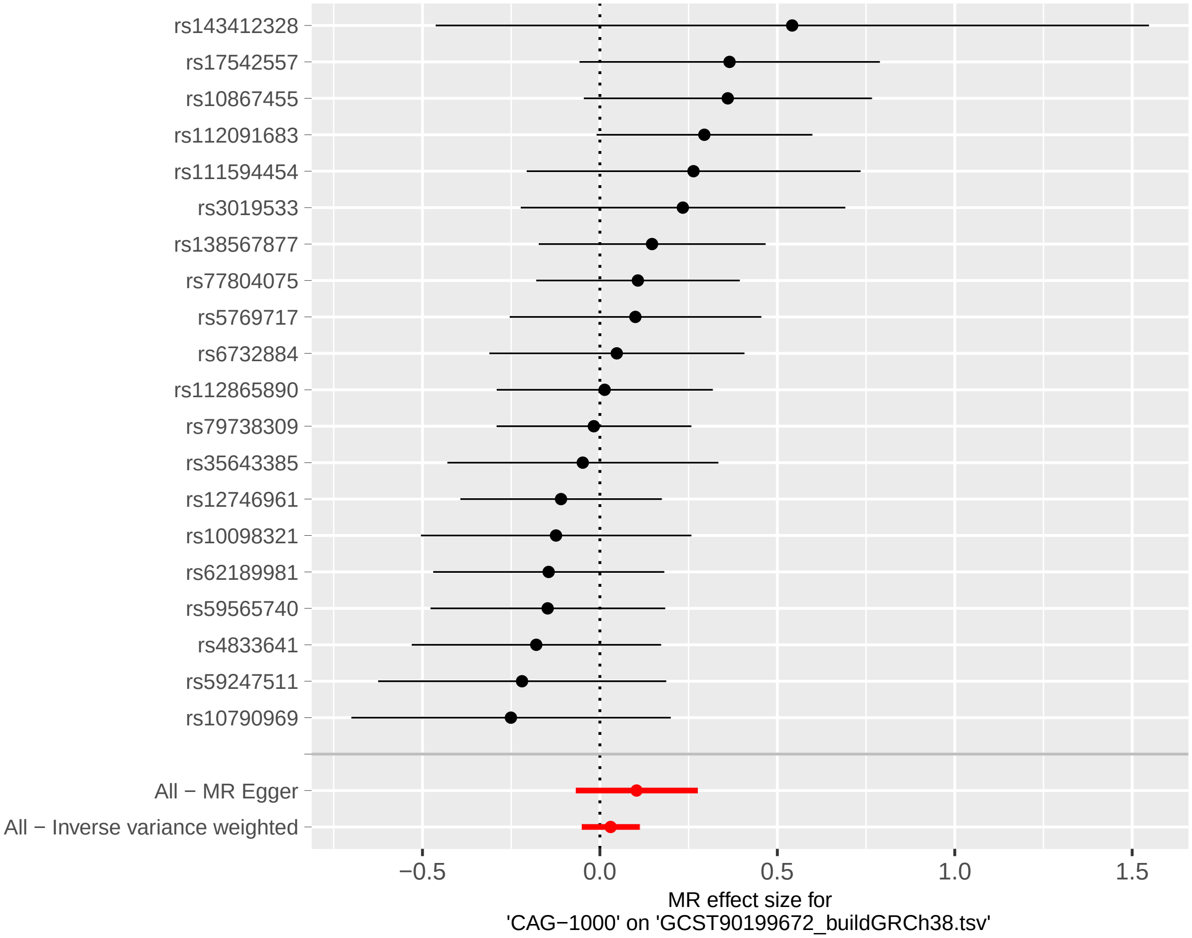
rs143412328
rs17542557
rs10867455
rs112091683
rs111594454
rs3019533
rs138567877
rs77804075
rs5769717
rs6732884
rs112865890
rs79738309
rs35643385
rs12746961
rs10098321
rs62189981
rs59565740
rs4833641
rs59247511
rs10790969
All − MR Egger
All − Inverse variance weighted
−0.5
0.0
0.5
1.0
1.5
MR effect size for
'CAG−1000' on 'GCST90199672_buildGRCh38.tsv'

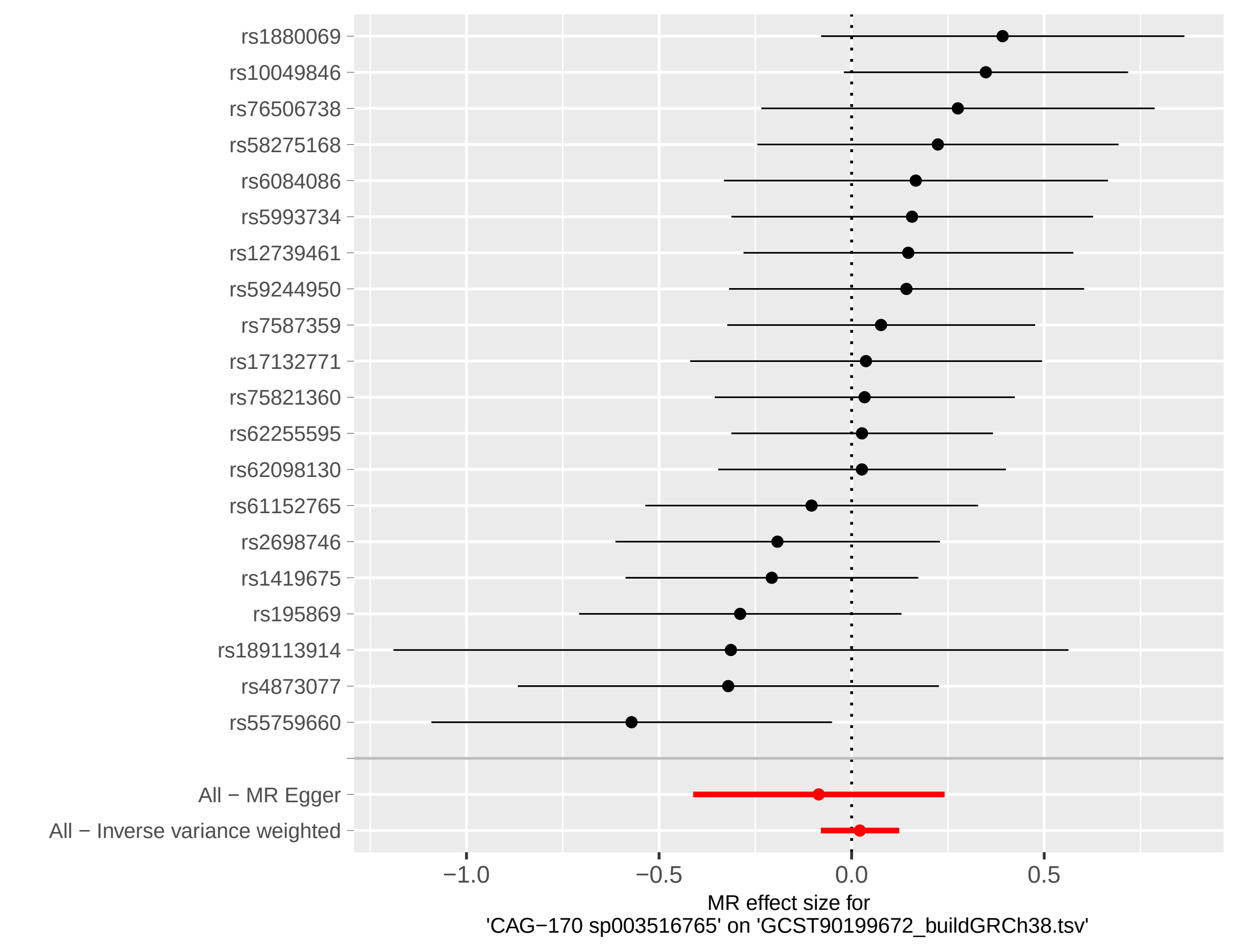

rs1880069
rs10049846
rs76506738
rs58275168
rs6084086
rs5993734
rs12739461
rs59244950
rs7587359
rs17132771
rs75821360
rs62255595
rs62098130
rs61152765
rs2698746
rs1419675
rs195869
rs189113914
rs4873077
rs55759660
All − MR Egger
All − Inverse variance weighted
−1.0
−0.5
0.0
0.5
MR effect size for
'CAG−170 sp003516765' on 'GCST90199672_buildGRCh38.tsv'

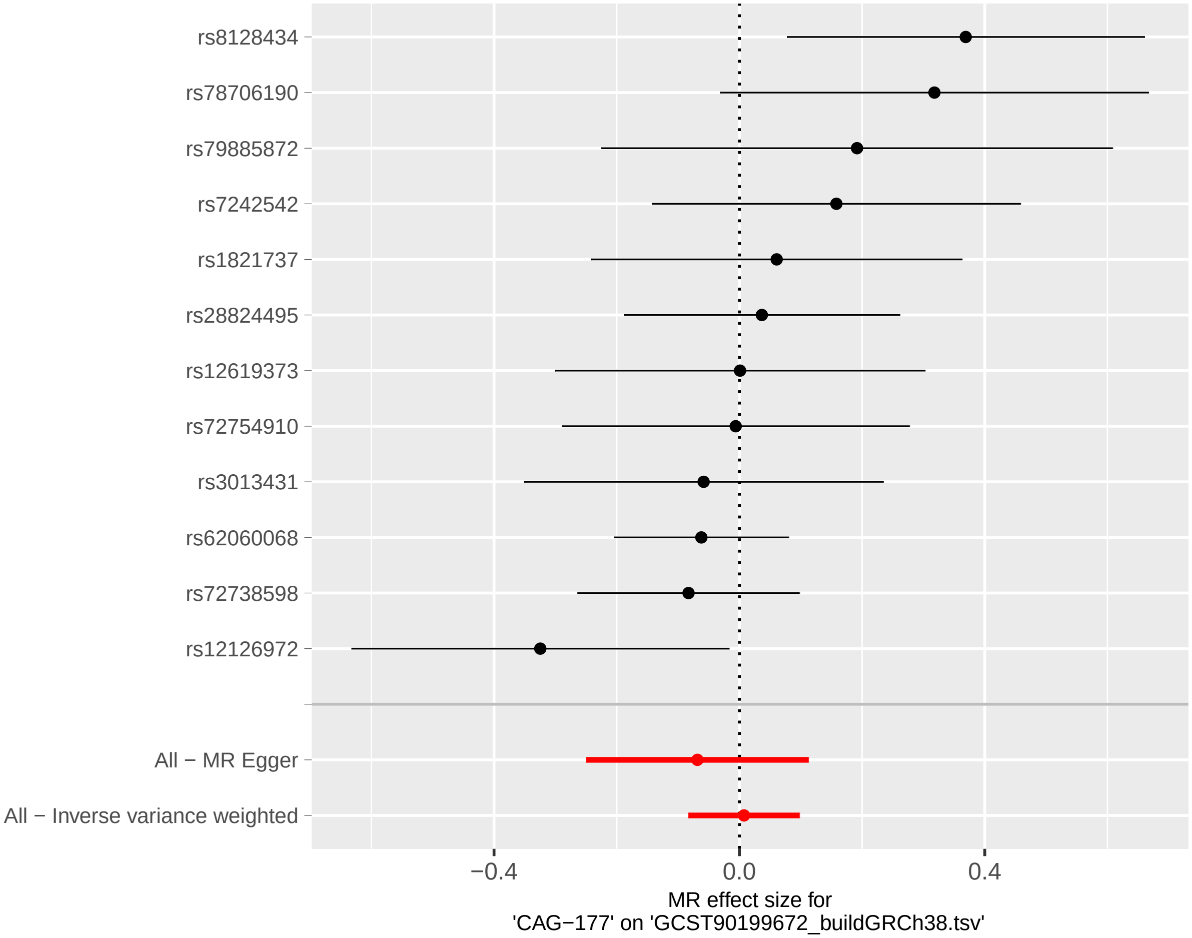
rs8128434
rs78706190
rs79885872
rs7242542
rs1821737
rs28824495
rs12619373
rs72754910
rs3013431
rs62060068
rs72738598
rs12126972
All − MR Egger
All − Inverse variance weighted
−0.4
0.0
0.4
MR effect size for
'CAG−177' on 'GCST90199672_buildGRCh38.tsv'

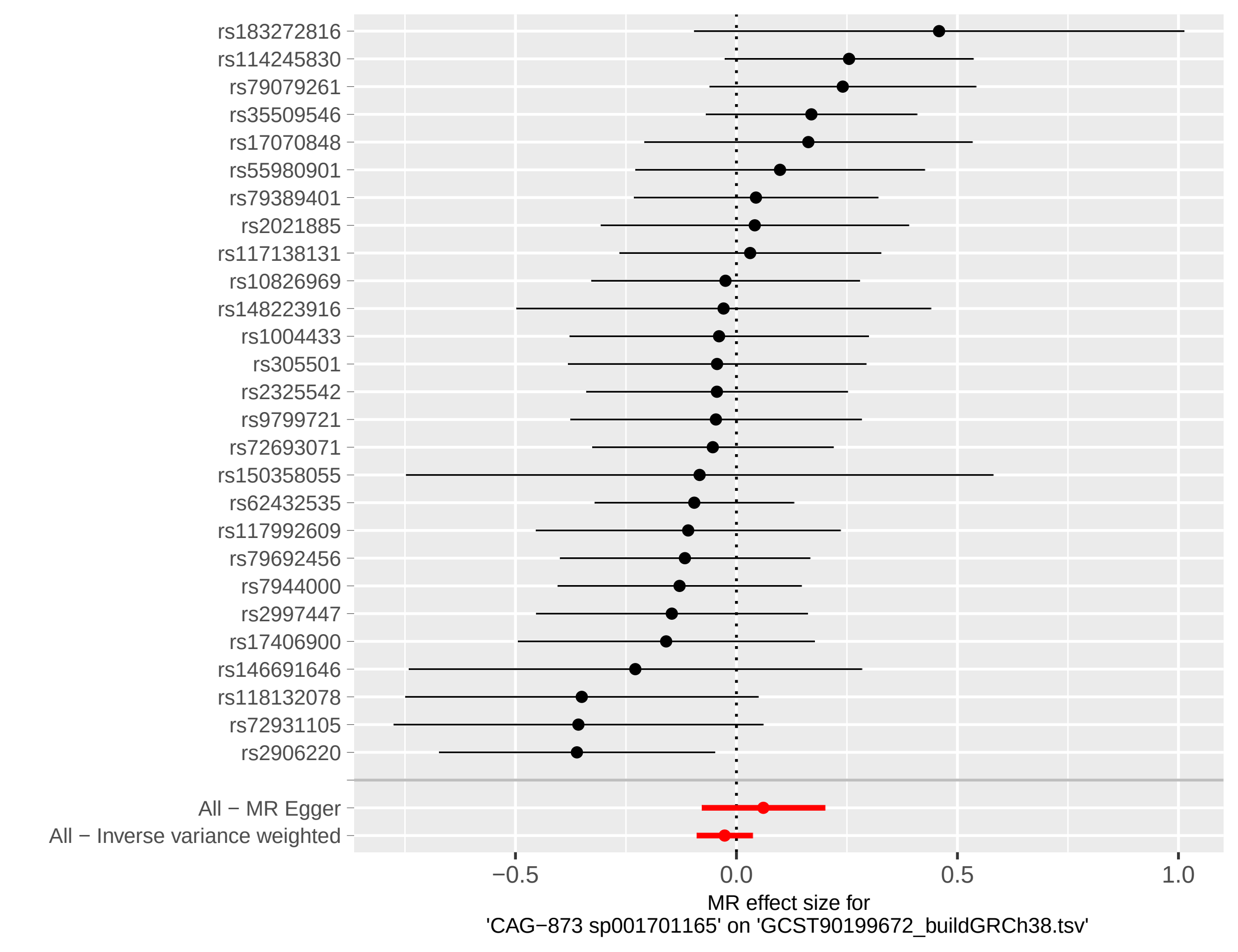

rs183272816
rs114245830
rs79079261
rs35509546
rs17070848
rs55980901
rs79389401
rs2021885
rs117138131
rs10826969
rs148223916
rs1004433
rs305501
rs2325542
rs9799721
rs72693071
rs150358055
rs62432535
rs117992609
rs79692456
rs7944000
rs2997447
rs17406900
rs146691646
rs118132078
rs72931105
rs2906220
All − MR Egger
All − Inverse variance weighted
−0.5
0.0
0.5
1.0
MR effect size for
'CAG−873 sp001701165' on 'GCST90199672_buildGRCh38.tsv'

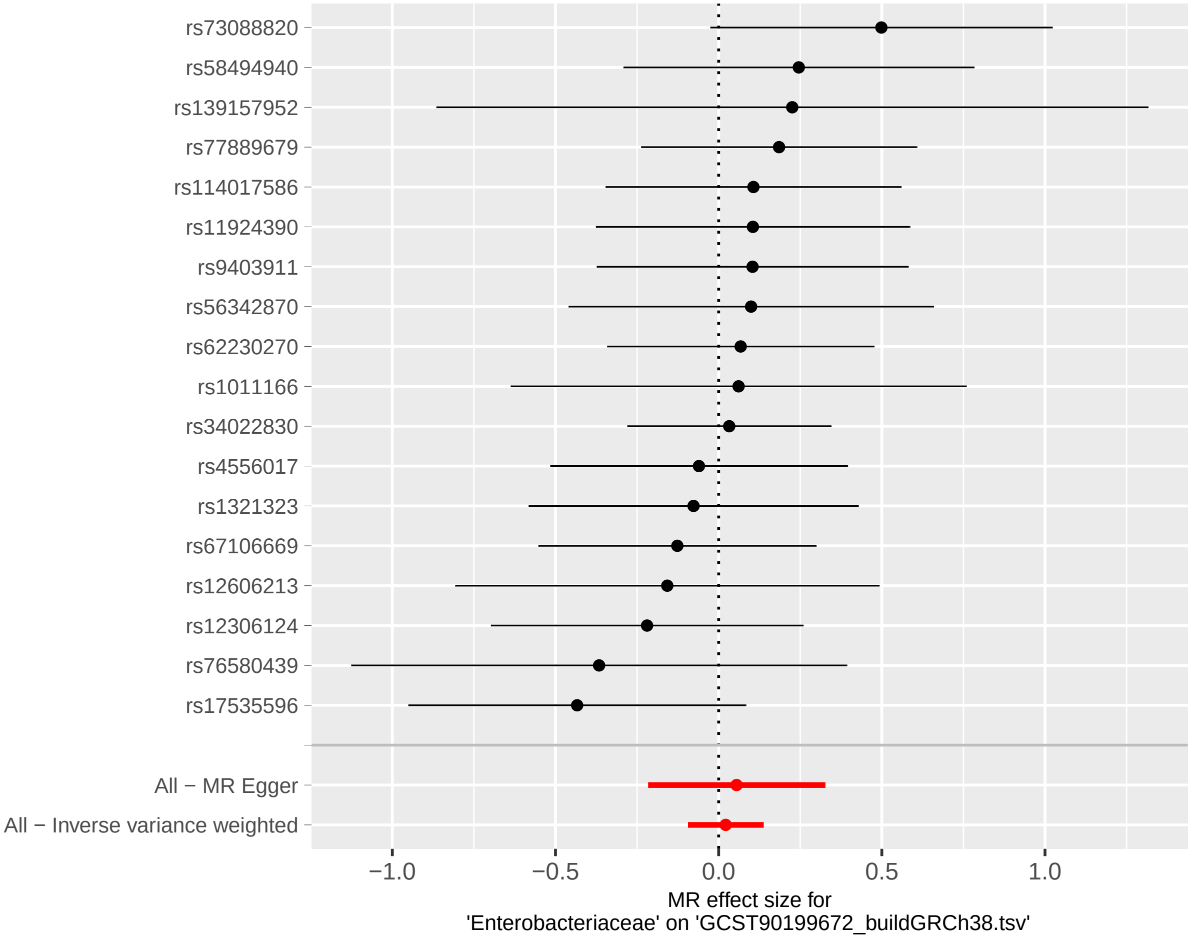
rs73088820
rs58494940
rs139157952
rs77889679
rs114017586
rs11924390
rs9403911
rs56342870
rs62230270
rs1011166
rs34022830
rs4556017
rs1321323
rs67106669
rs12606213
rs12306124
rs76580439
rs17535596
All − MR Egger
All − Inverse variance weighted
−1.0
−0.5
0.0
0.5
1.0
MR effect size for
'Enterobacteriaceae' on 'GCST90199672_buildGRCh38.tsv'

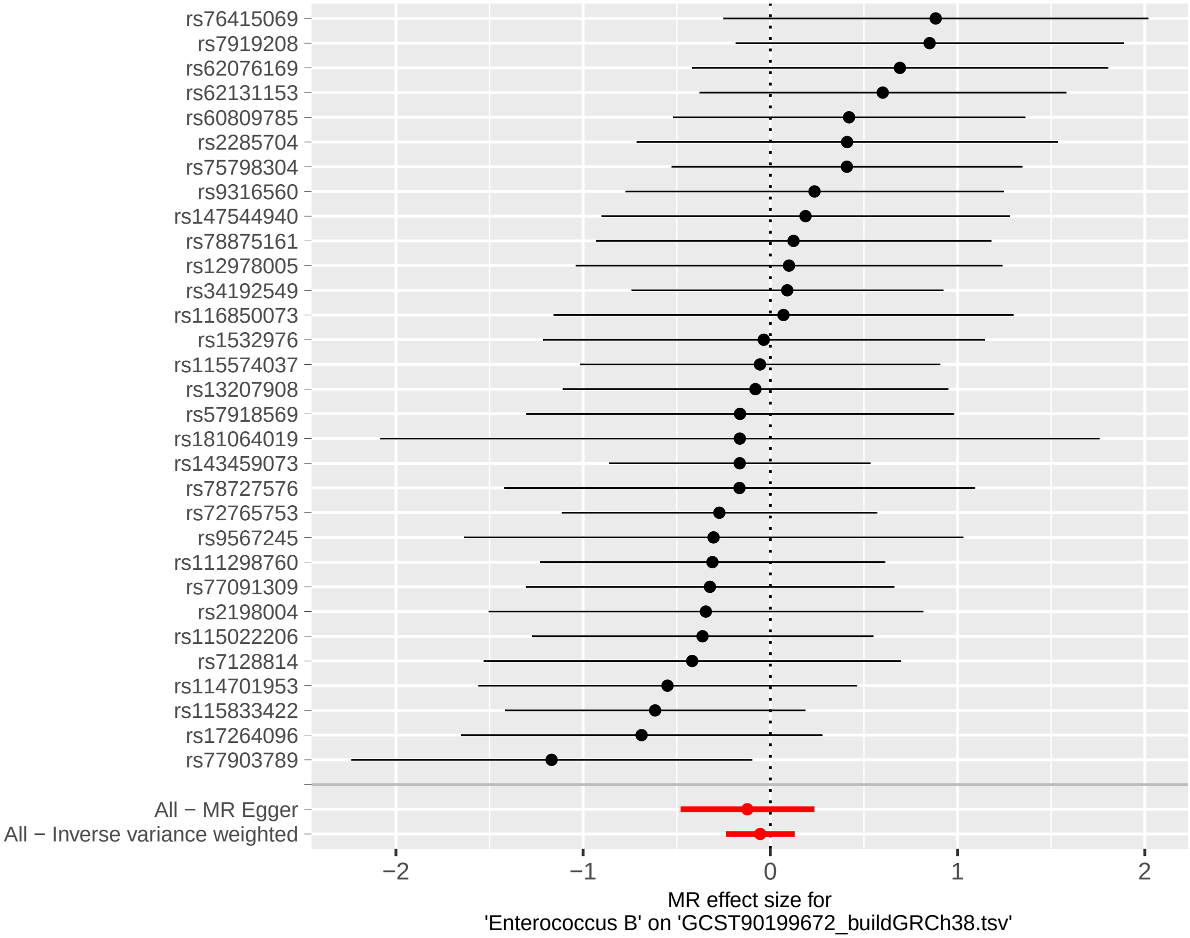
rs76415069
rs7919208
rs62076169
rs62131153
rs60809785
rs2285704
rs75798304
rs9316560
rs147544940
rs78875161
rs12978005
rs34192549
rs116850073
rs1532976
rs115574037
rs13207908
rs57918569
rs181064019
rs143459073
rs78727576
rs72765753
rs9567245
rs111298760
rs77091309
rs2198004
rs115022206
rs7128814
rs114701953
rs115833422
rs17264096
rs77903789
All − MR Egger
All − Inverse variance weighted
−2
−1
0
1
2
MR effect size for
'Enterococcus B' on 'GCST90199672_buildGRCh38.tsv'

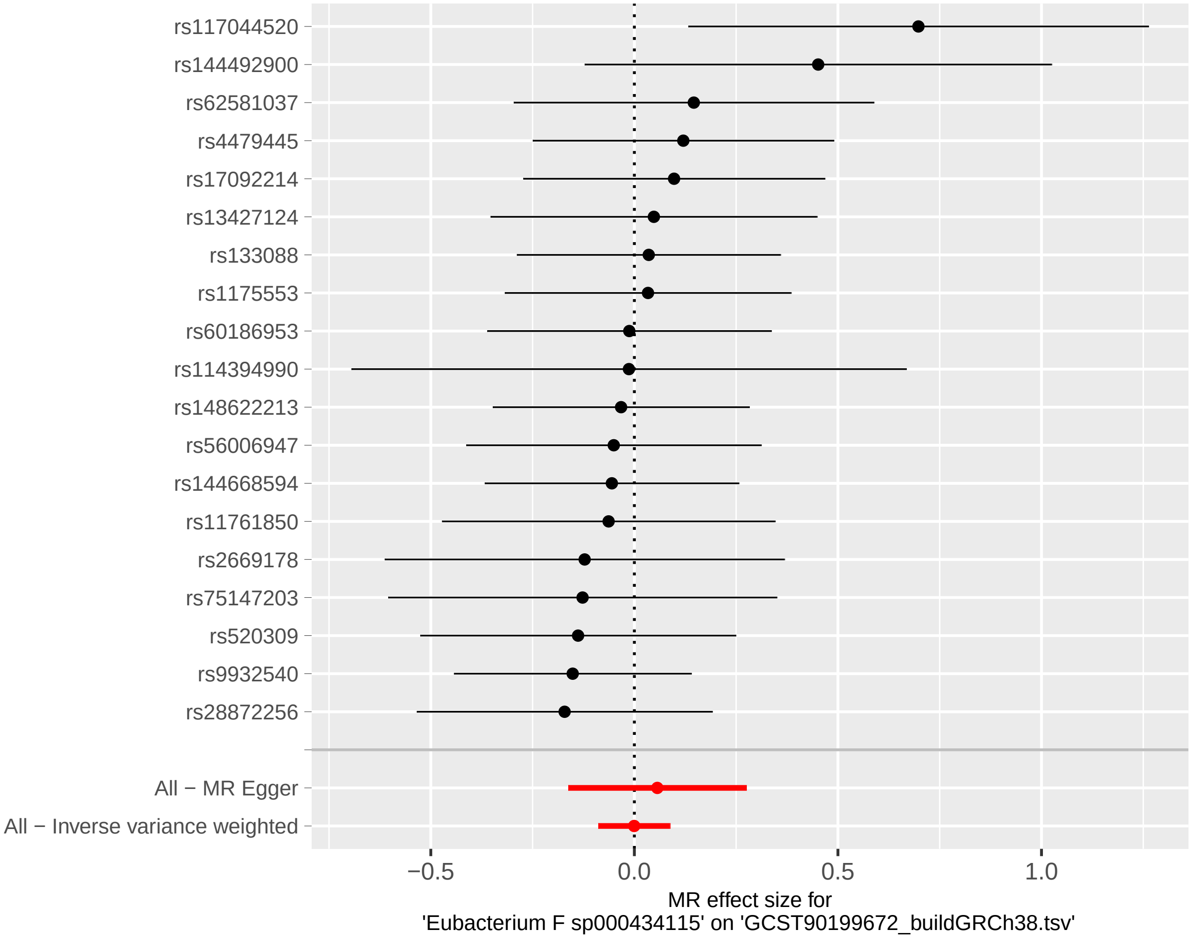
rs117044520
rs144492900
rs62581037
rs4479445
rs17092214
rs13427124
rs133088
rs1175553
rs60186953
rs114394990
rs148622213
rs56006947
rs144668594
rs11761850
rs2669178
rs75147203
rs520309
rs9932540
rs28872256
All − MR Egger
All − Inverse variance weighted
−0.5
0.0
0.5
1.0
MR effect size for
'Eubacterium F sp000434115' on 'GCST90199672_buildGRCh38.tsv'

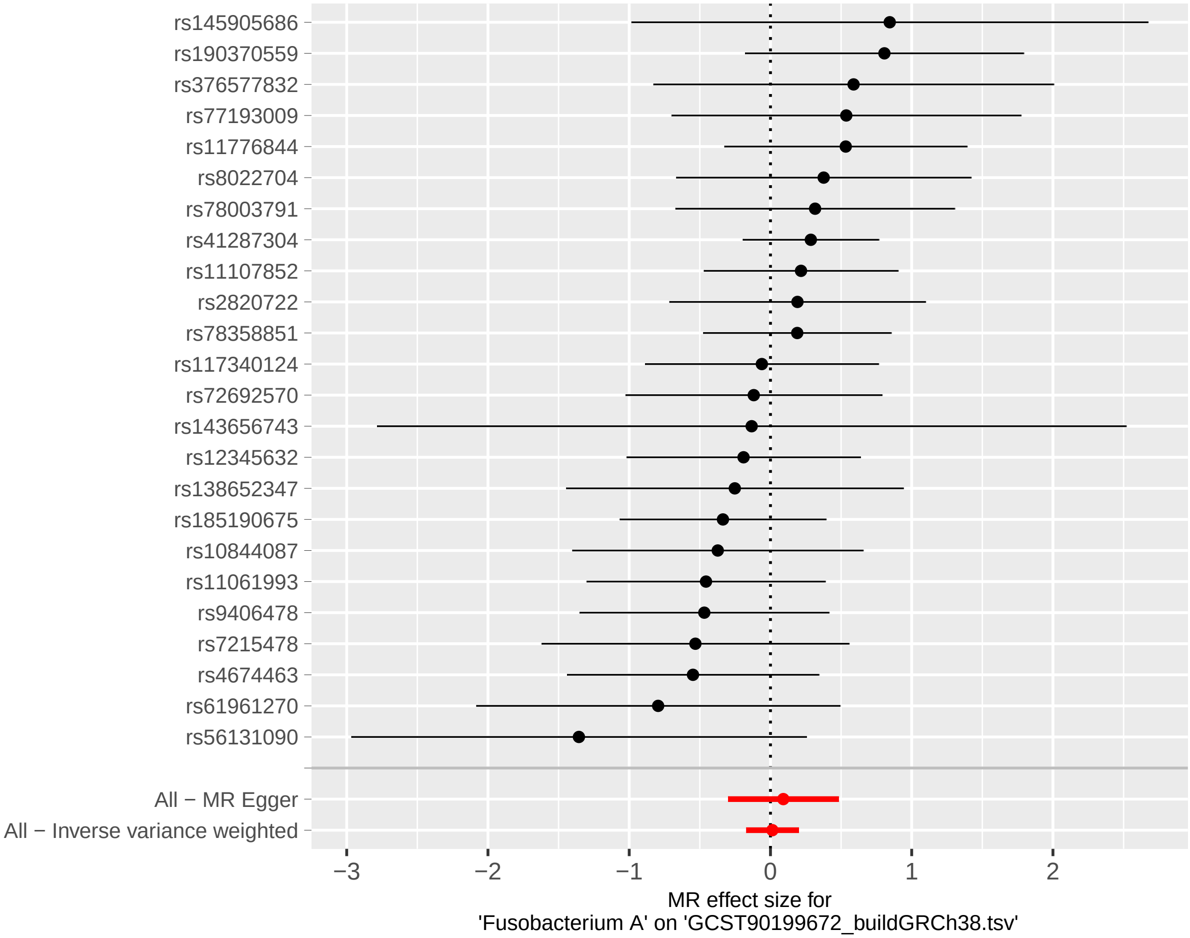
rs145905686
rs190370559
rs376577832
rs77193009
rs11776844
rs8022704
rs78003791
rs41287304
rs11107852
rs2820722
rs78358851
rs117340124
rs72692570
rs143656743
rs12345632
rs138652347
rs185190675
rs10844087
rs11061993
rs9406478
rs7215478
rs4674463
rs61961270
rs56131090
All − MR Egger
All − Inverse variance weighted
−3
−2
−1
0
1
2
MR effect size for
'Fusobacterium A' on 'GCST90199672_buildGRCh38.tsv'

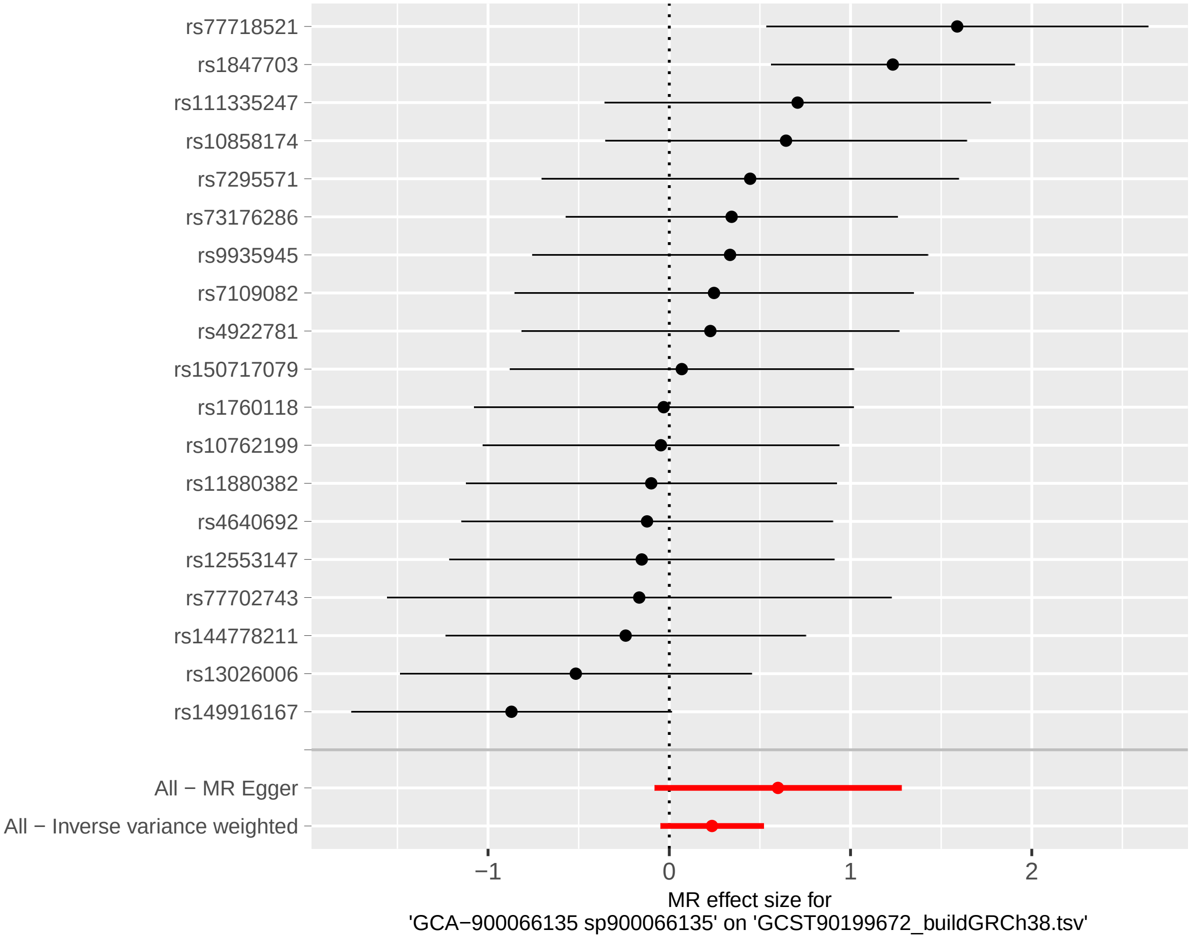
rs77718521
rs1847703
rs111335247
rs10858174
rs7295571
rs73176286
rs9935945
rs7109082
rs4922781
rs150717079
rs1760118
rs10762199
rs11880382
rs4640692
rs12553147
rs77702743
rs144778211
rs13026006
rs149916167
All − MR Egger
All − Inverse variance weighted
−1
0
1
2
MR effect size for
'GCA−900066135 sp900066135' on 'GCST90199672_buildGRCh38.tsv'

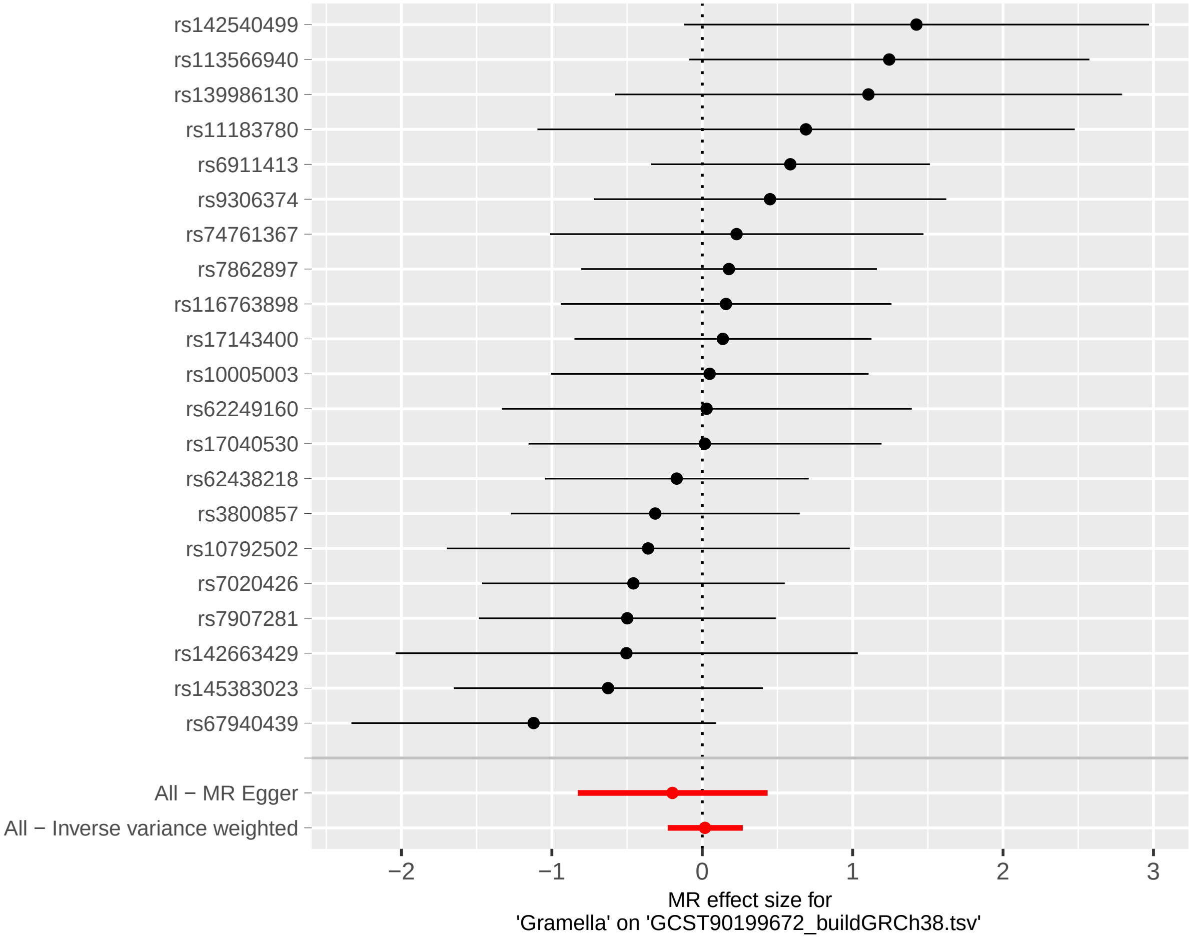

rs142540499
rs113566940
rs139986130
rs11183780
rs6911413
rs9306374
rs74761367
rs7862897
rs116763898
rs17143400
rs10005003
rs62249160
rs17040530
rs62438218
rs3800857
rs10792502
rs7020426
rs7907281
rs142663429
rs145383023
rs67940439
All − MR Egger
All − Inverse variance weighted
−2
−1
0
1
2
3
MR effect size for
'Gramella' on 'GCST90199672_buildGRCh38.tsv'

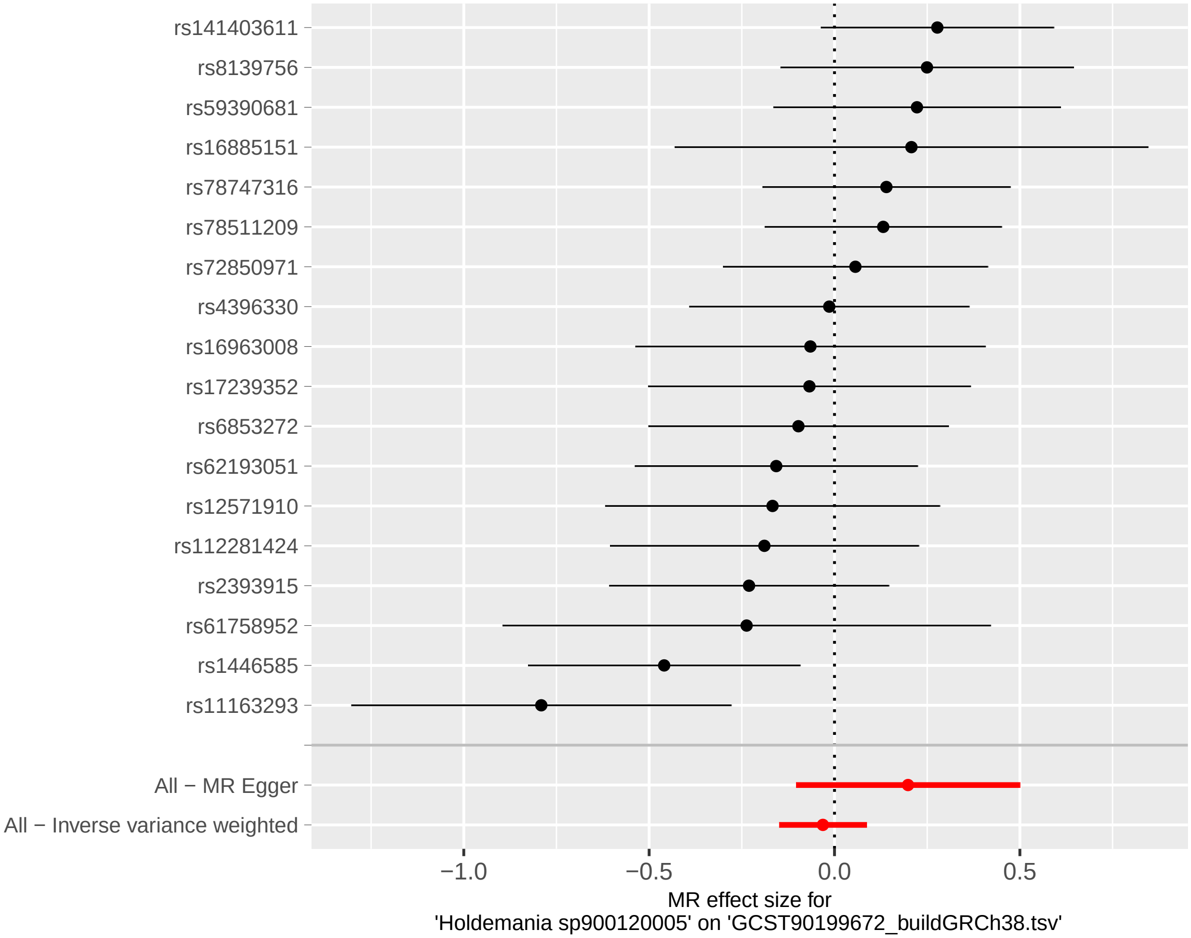

rs141403611
rs8139756
rs59390681
rs16885151
rs78747316
rs78511209
rs72850971
rs4396330
rs16963008
rs17239352
rs6853272
rs62193051
rs12571910
rs112281424
rs2393915
rs61758952
rs1446585
rs11163293
All − MR Egger
All − Inverse variance weighted
−1.0
−0.5
0.0
0.5
MR effect size for
'Holdemania sp900120005' on 'GCST90199672_buildGRCh38.tsv'

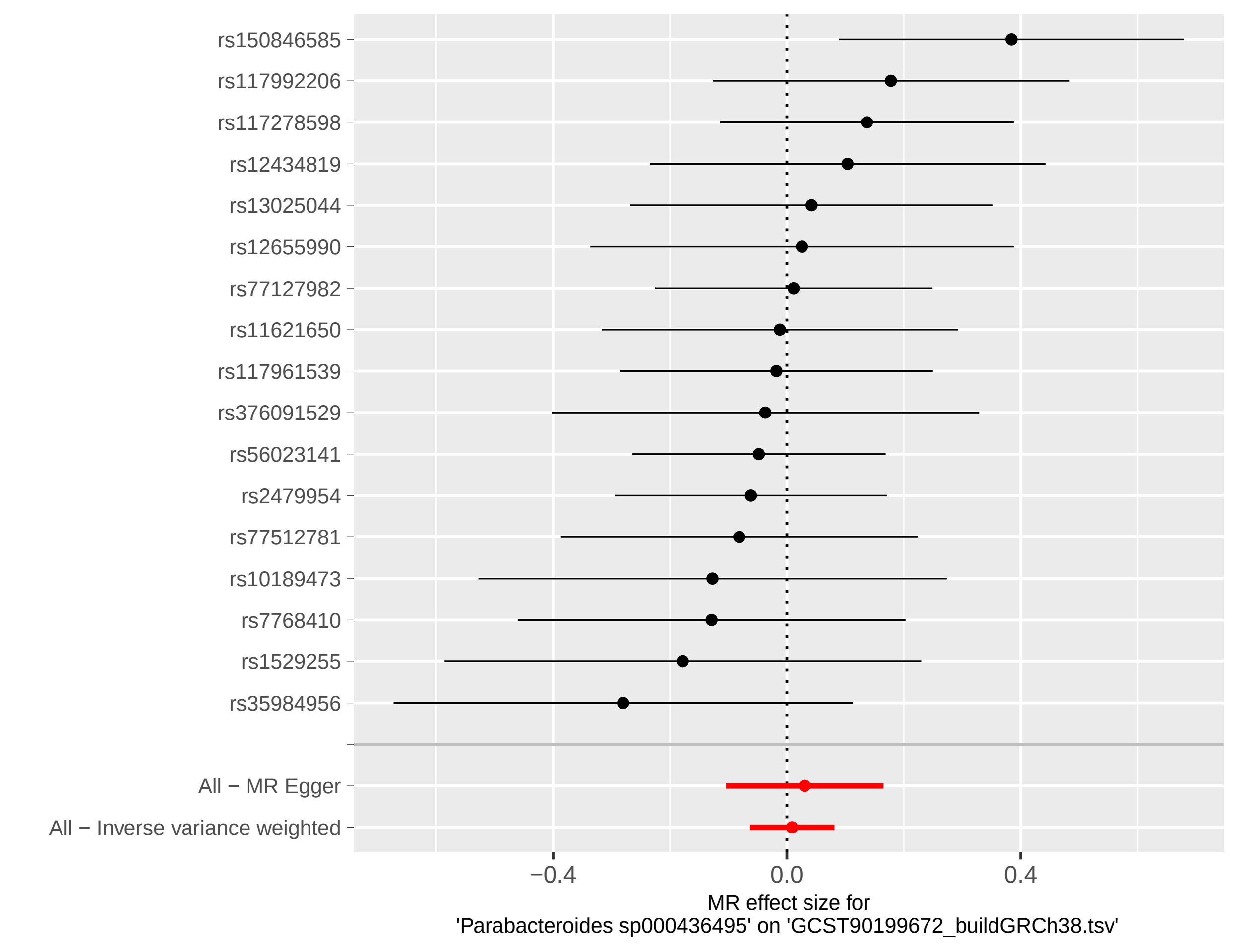
rs150846585
rs117992206
rs117278598
rs12434819
rs13025044
rs12655990
rs77127982
rs11621650
rs117961539
rs376091529
rs56023141
rs2479954
rs77512781
rs10189473
rs7768410
rs1529255
rs35984956
All − MR Egger
All − Inverse variance weighted
−0.4
0.0
0.4
MR effect size for
'Parabacteroides sp000436495' on 'GCST90199672_buildGRCh38.tsv'

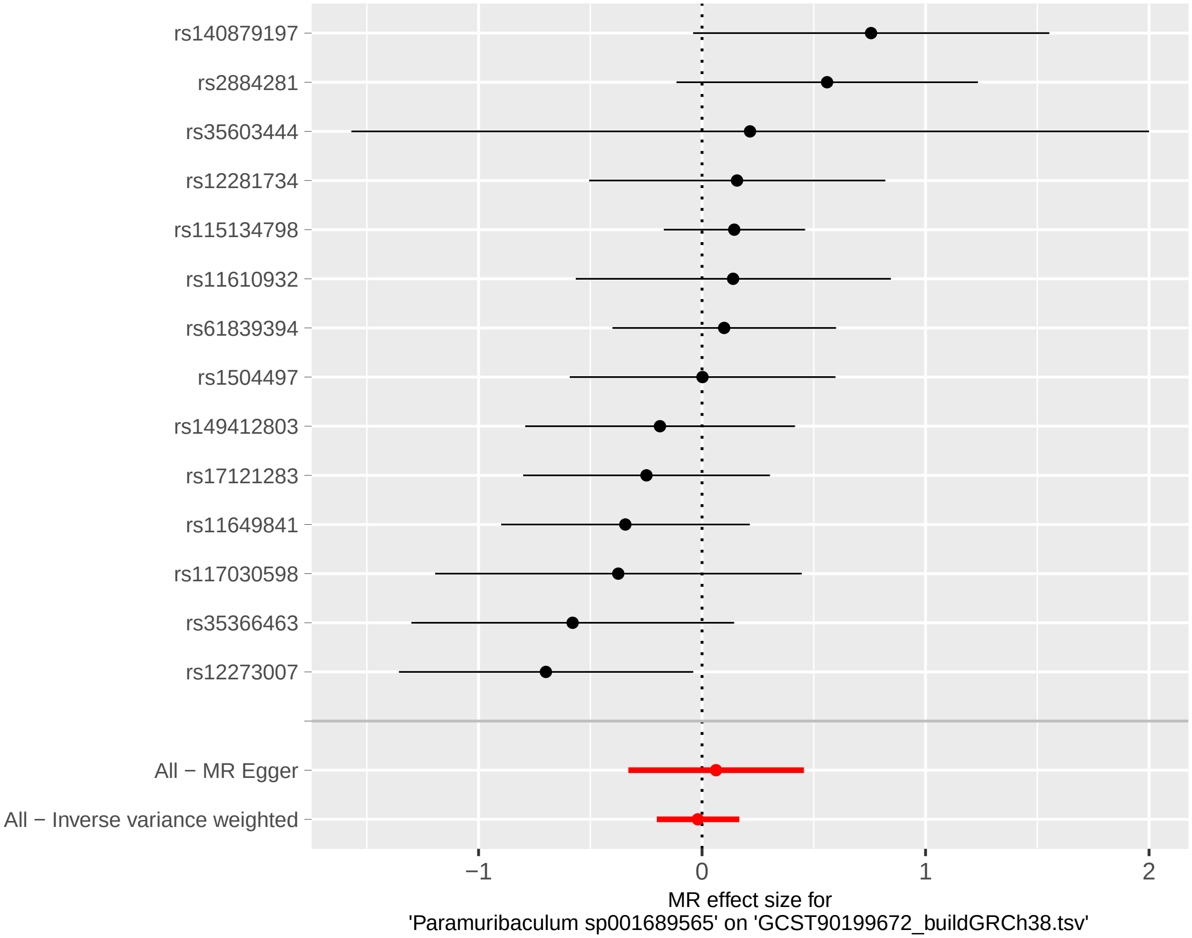
rs140879197
rs2884281
rs35603444
rs12281734
rs115134798
rs11610932
rs61839394
rs1504497
rs149412803
rs17121283
rs11649841
rs117030598
rs35366463
rs12273007
All − MR Egger
All − Inverse variance weighted
−1
0
1
2
MR effect size for
'Paramuribaculum sp001689565' on 'GCST90199672_buildGRCh38.tsv'

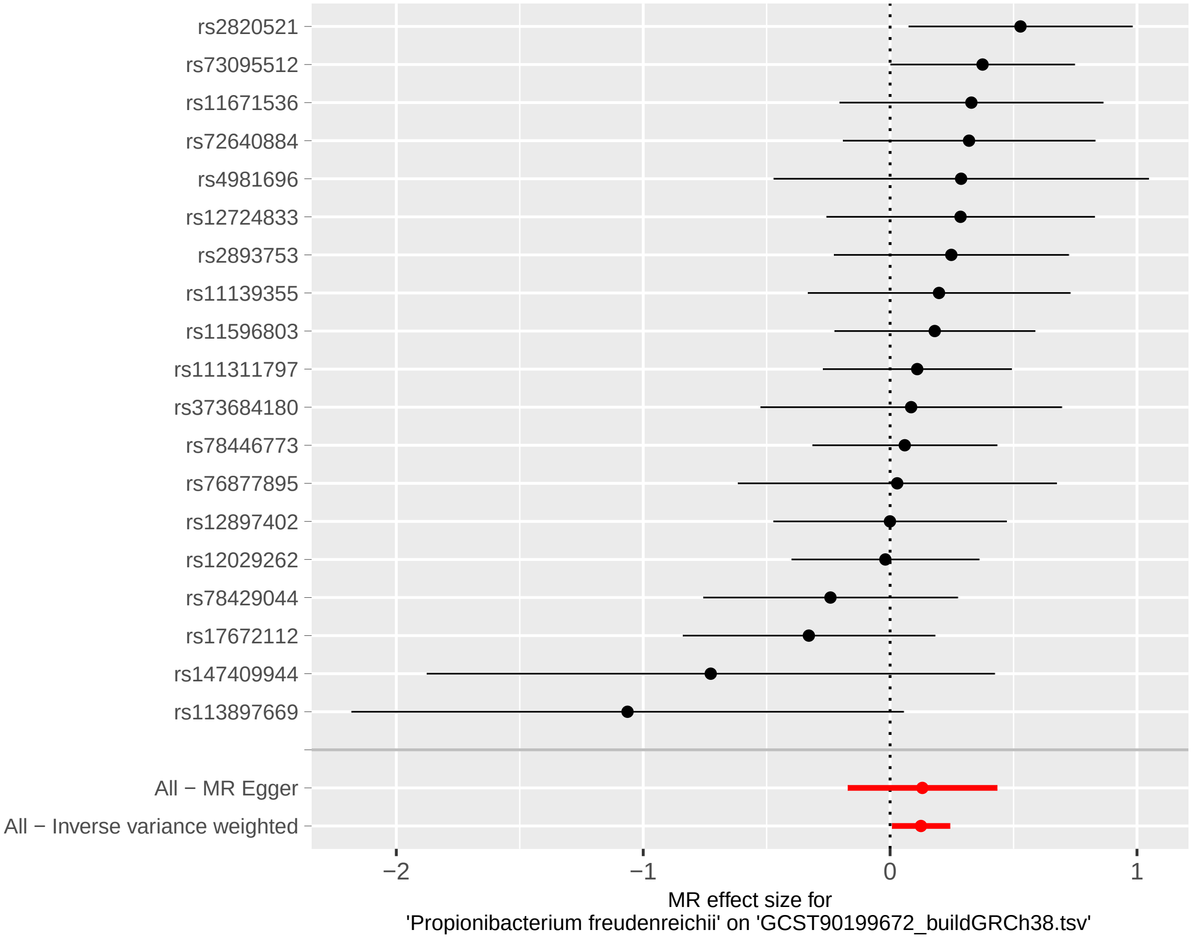
rs2820521
rs73095512
rs11671536
rs72640884
rs4981696
rs12724833
rs2893753
rs11139355
rs11596803
rs111311797
rs373684180
rs78446773
rs76877895
rs12897402
rs12029262
rs78429044
rs17672112
rs147409944
rs113897669
All − MR Egger
All − Inverse variance weighted
−2
−1
0
1
MR effect size for
'Propionibacterium freudenreichii' on 'GCST90199672_buildGRCh38.tsv'

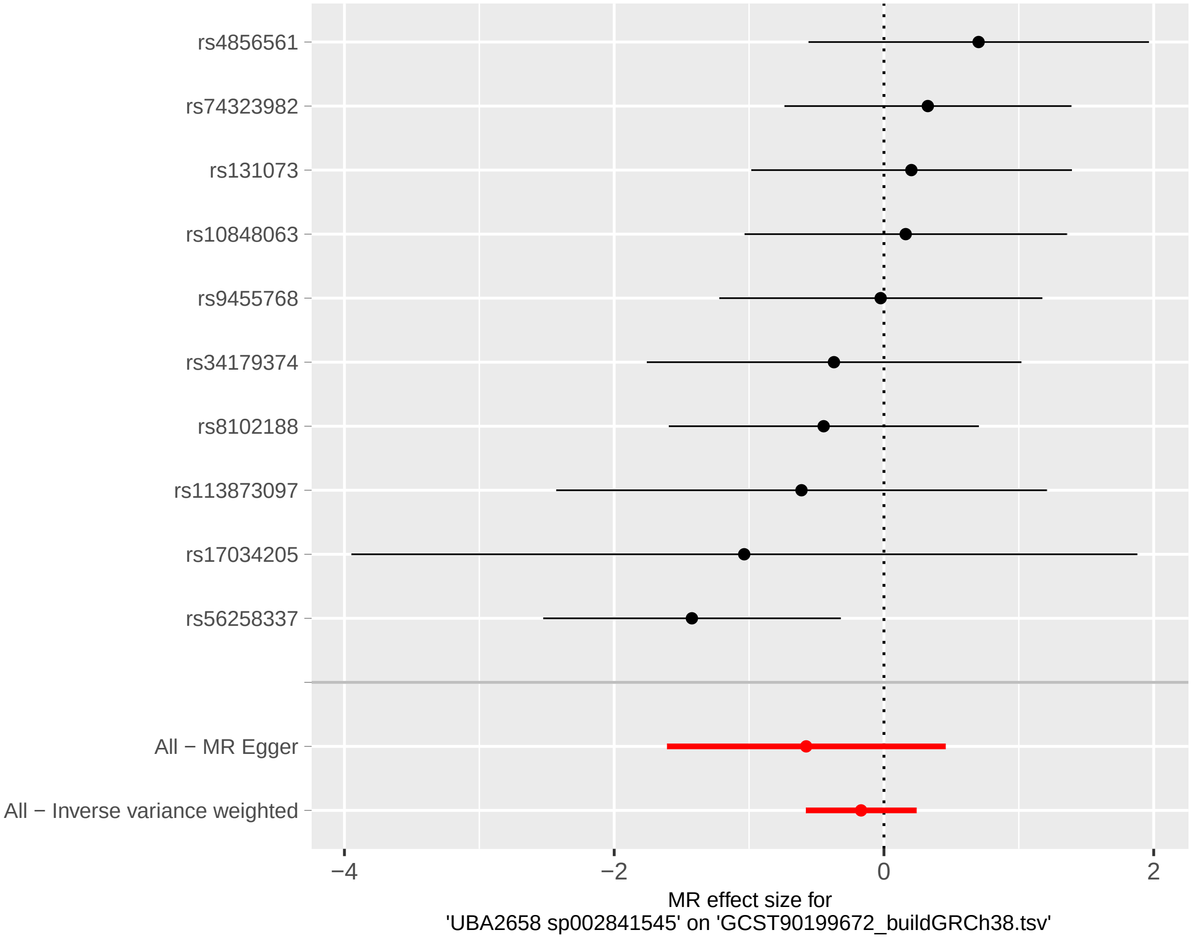

rs4856561
rs74323982
rs131073
rs10848063
rs9455768
rs34179374
rs8102188
rs113873097
rs17034205
rs56258337
All − MR Egger
All − Inverse variance weighted
−4
−2
0
2
MR effect size for
'UBA2658 sp002841545' on 'GCST90199672_buildGRCh38.tsv'

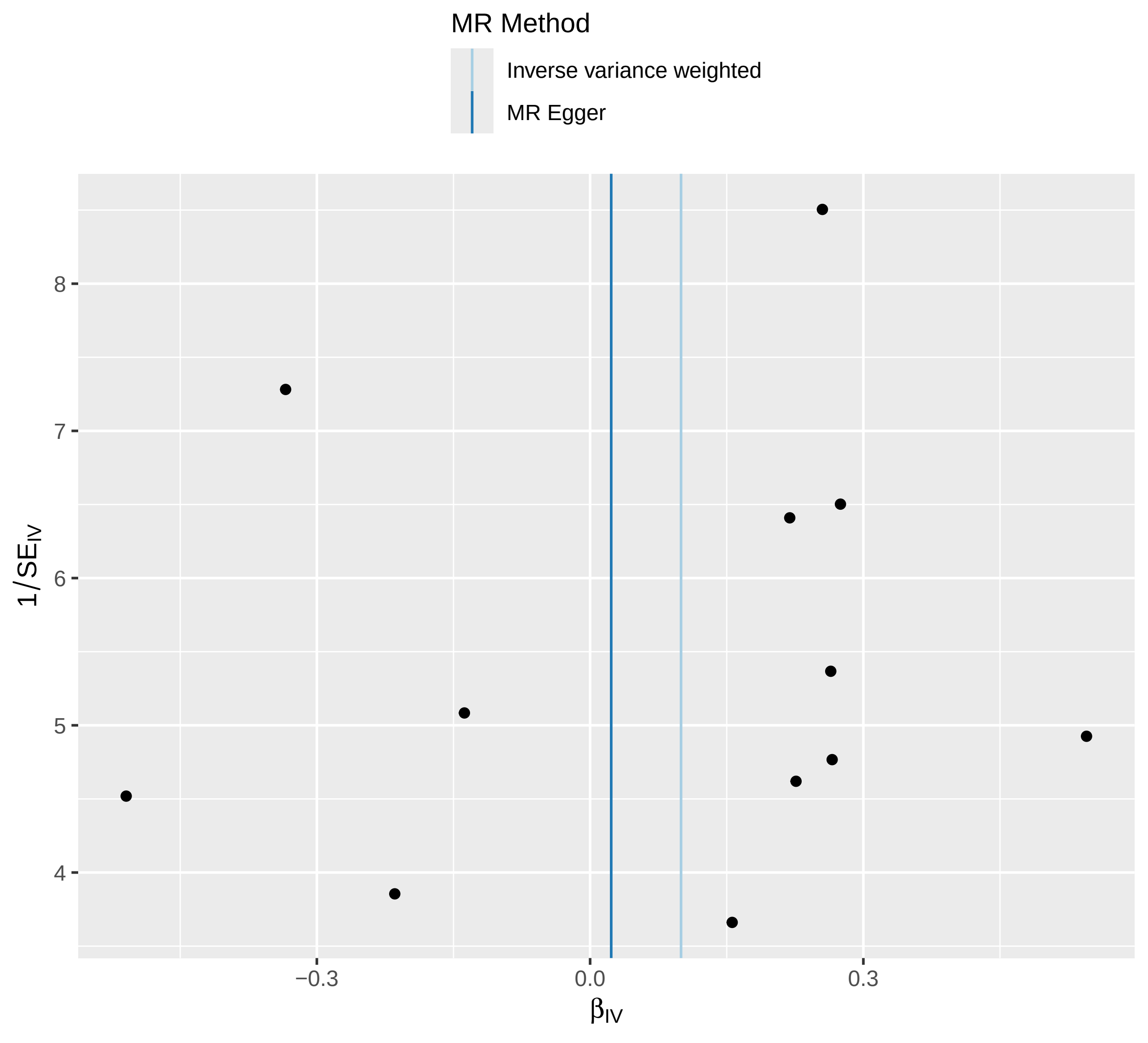
MR Method
Inverse variance weighted
MR Egger
8
7
6
5
4
1/SE_IV
−0.3
0.0
0.3
β_IV

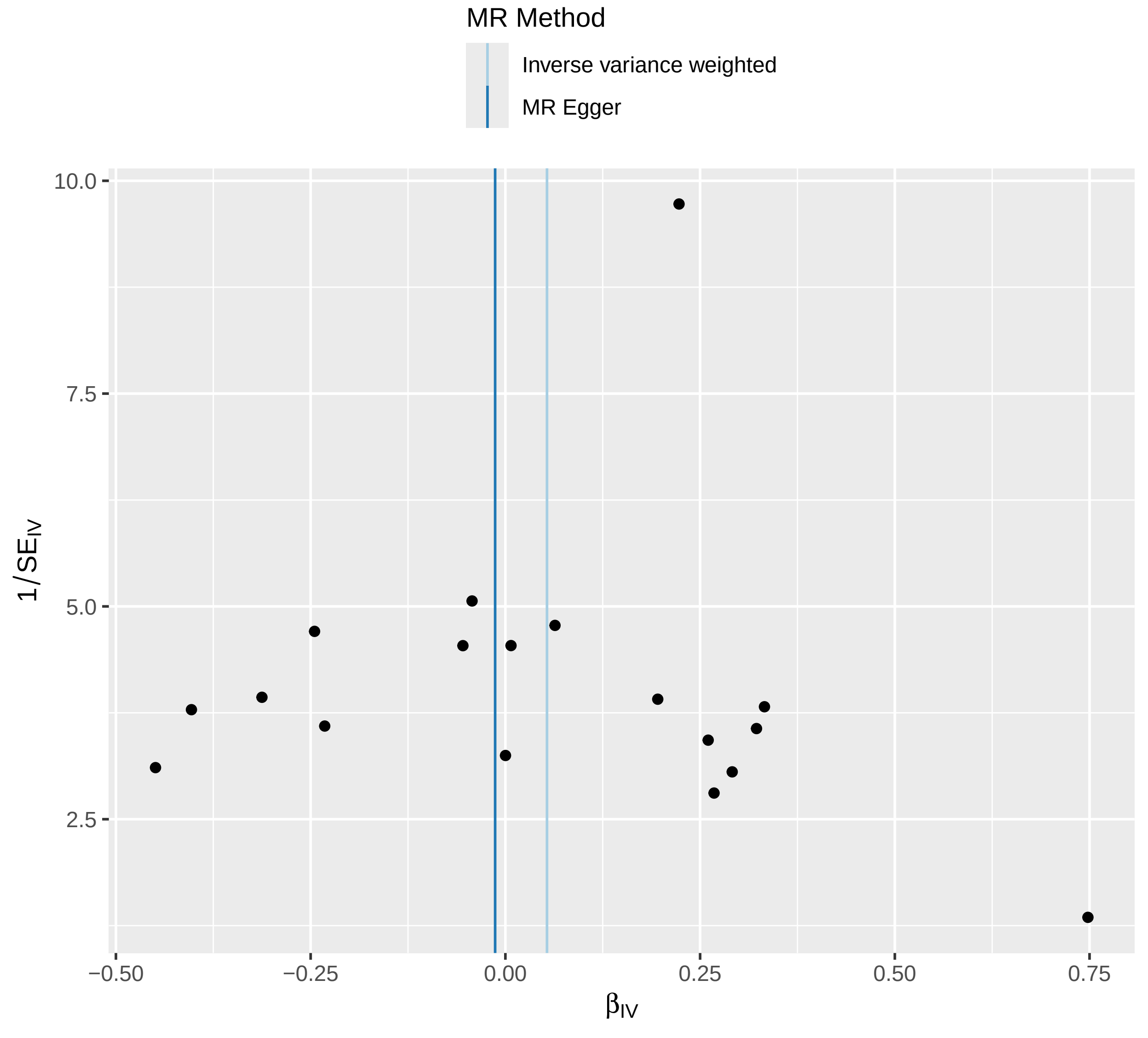
MR Method
Inverse variance weighted
MR Egger
10.0
7.5
5.0
2.5
$1/SE_{IV}$
−0.50
−0.25
0.00
0.25
0.50
0.75
β_{IV}

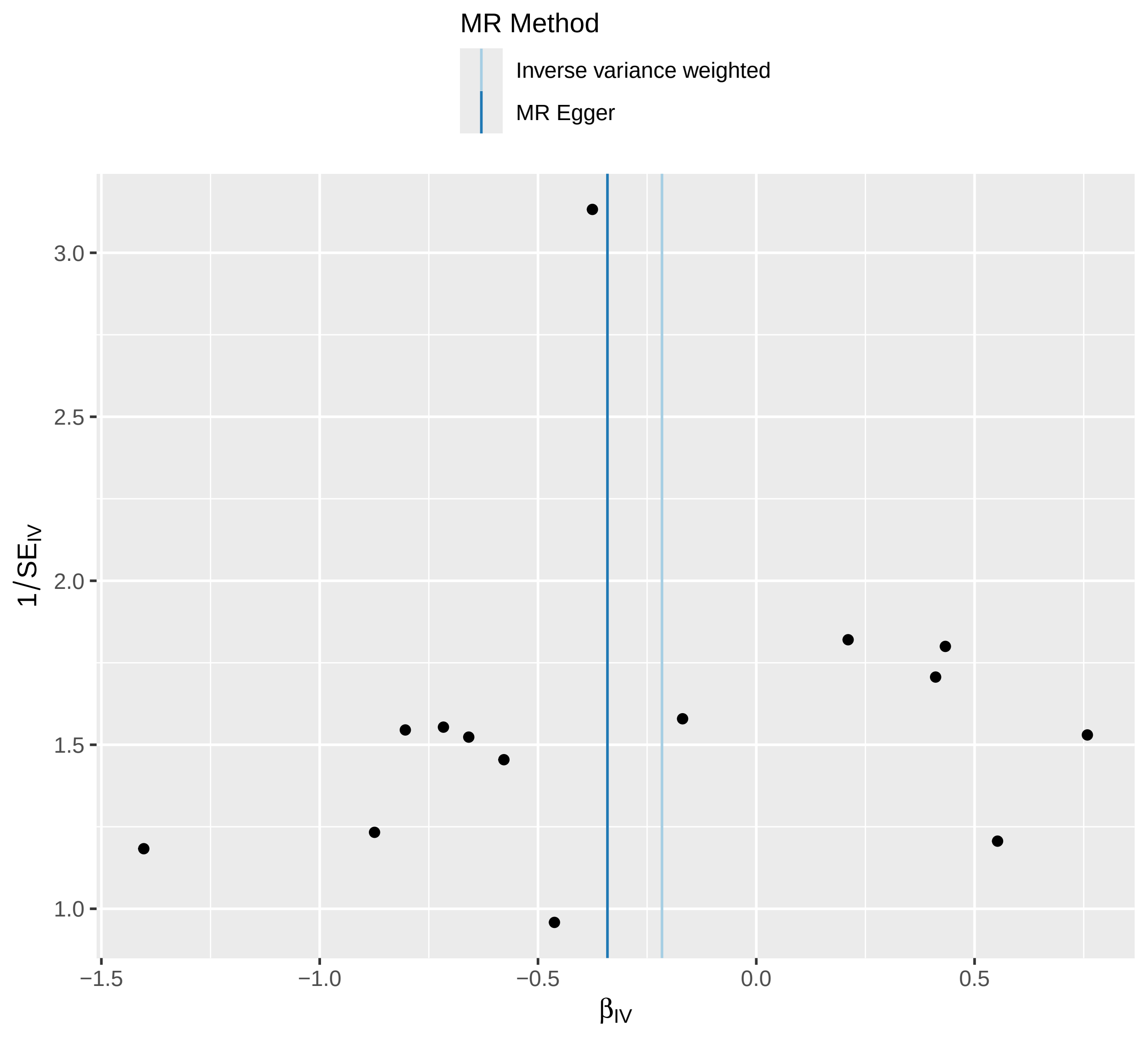
MR Method
Inverse variance weighted
MR Egger
3.0
2.5
2.0
1.5
1.0
$1/SE_{IV}$
−1.5
−1.0
−0.5
0.0
0.5
β_{IV}

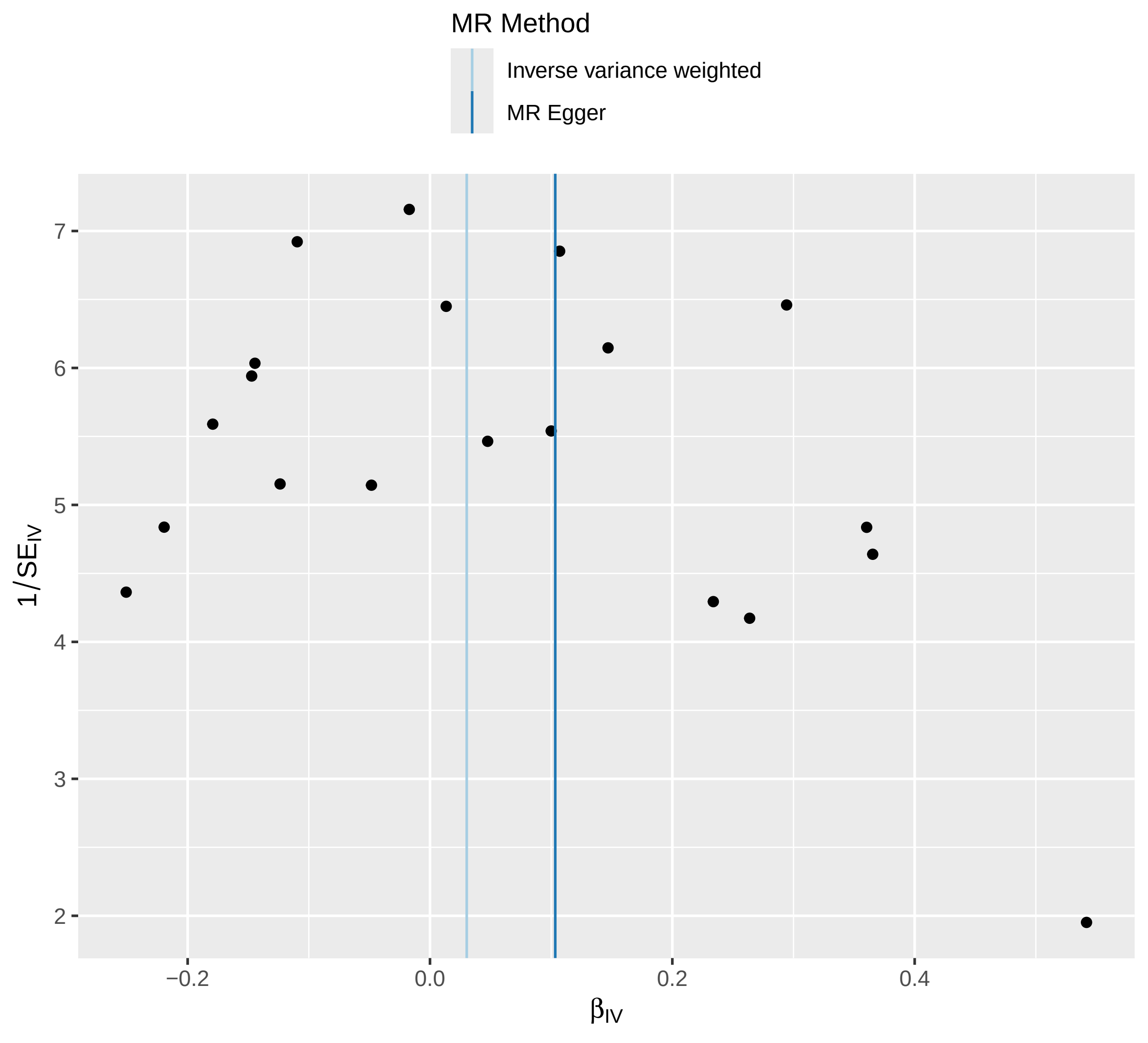
MR Method
Inverse variance weighted
MR Egger
1/SE_IV
β_IV
7
6
5
4
3
2
−0.2
0.0
0.2
0.4

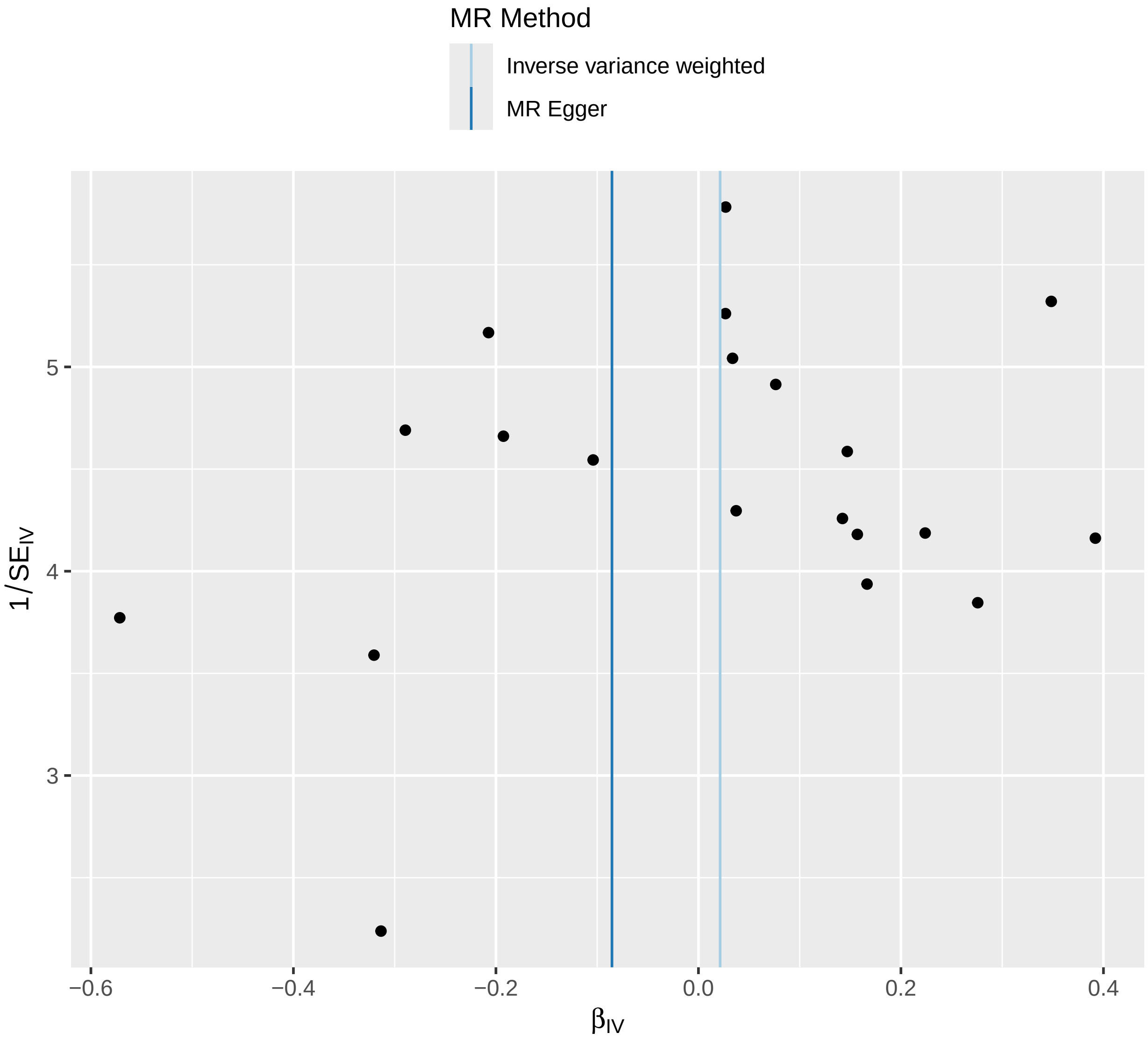
MR Method
Inverse variance weighted
MR Egger
1/SE_IV
5
4
3
−0.6
−0.4
−0.2
0.0
0.2
0.4
β_IV

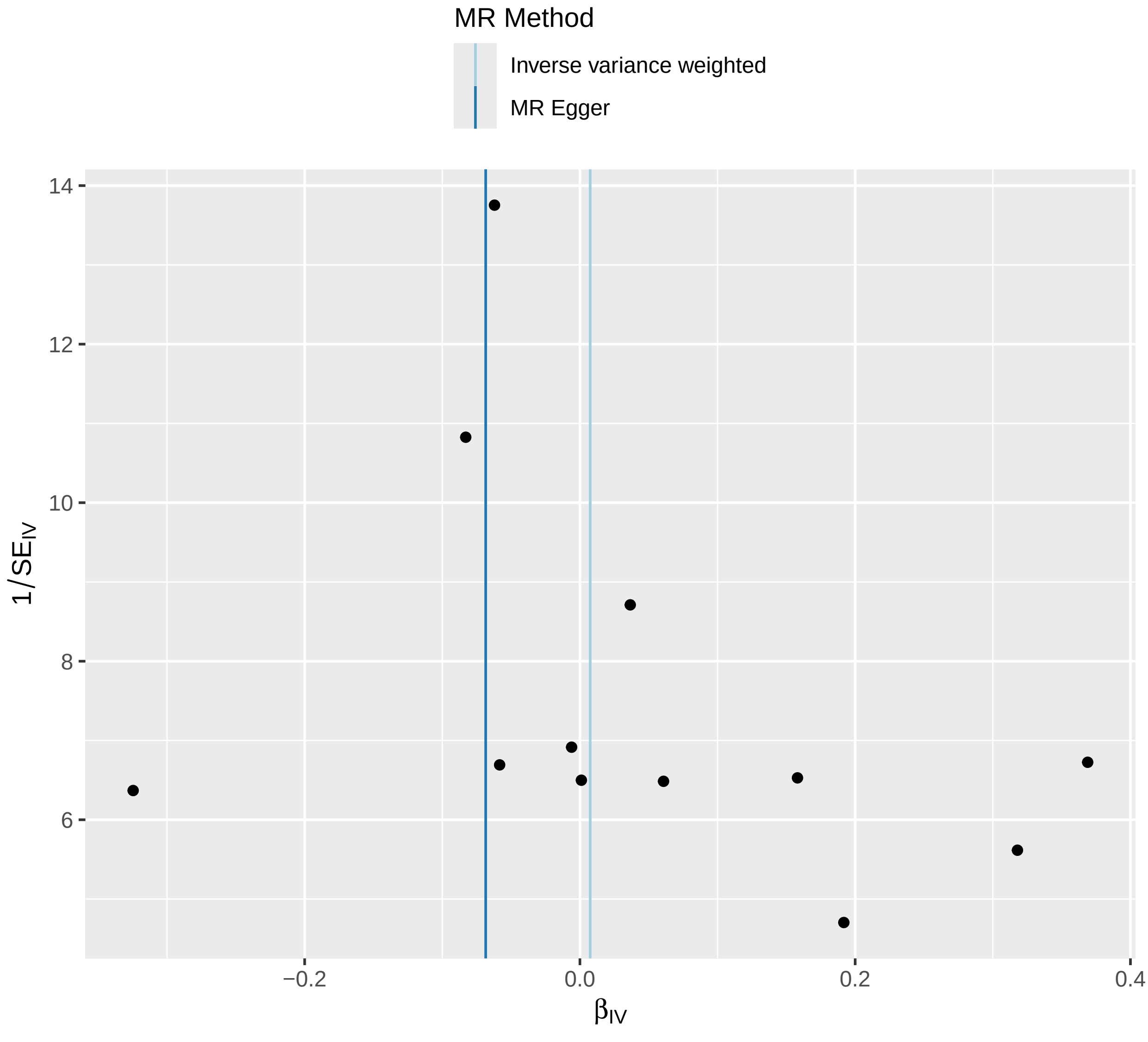

MR Method
Inverse variance weighted
MR Egger
14
12
10
8
6
1/SE$_{IV}$
−0.2
0.0
0.2
0.4
β$_{IV}$

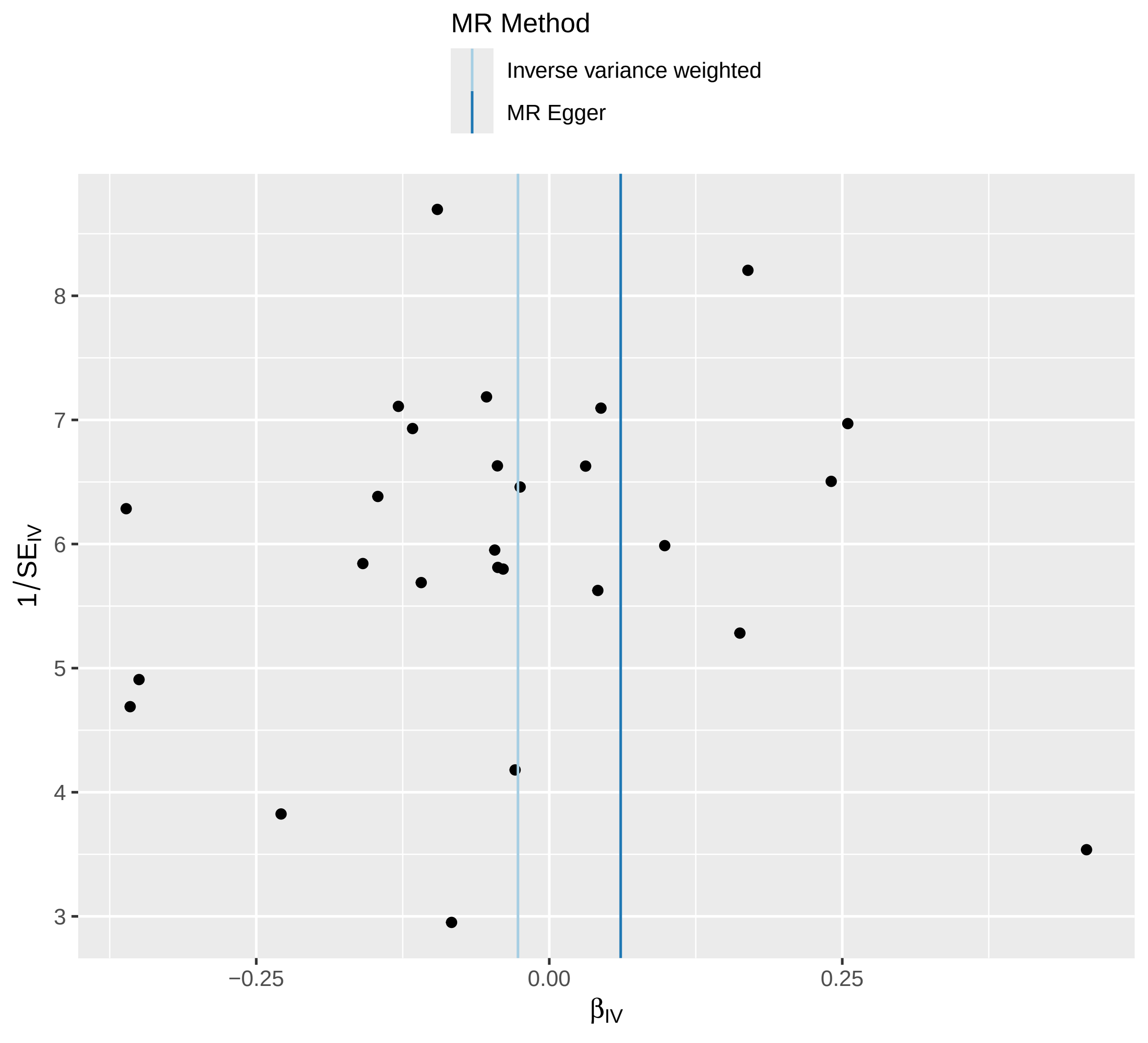
MR Method
Inverse variance weighted
MR Egger
8
7
6
5
4
3
1/SE_IV
−0.25
0.00
0.25
β_IV

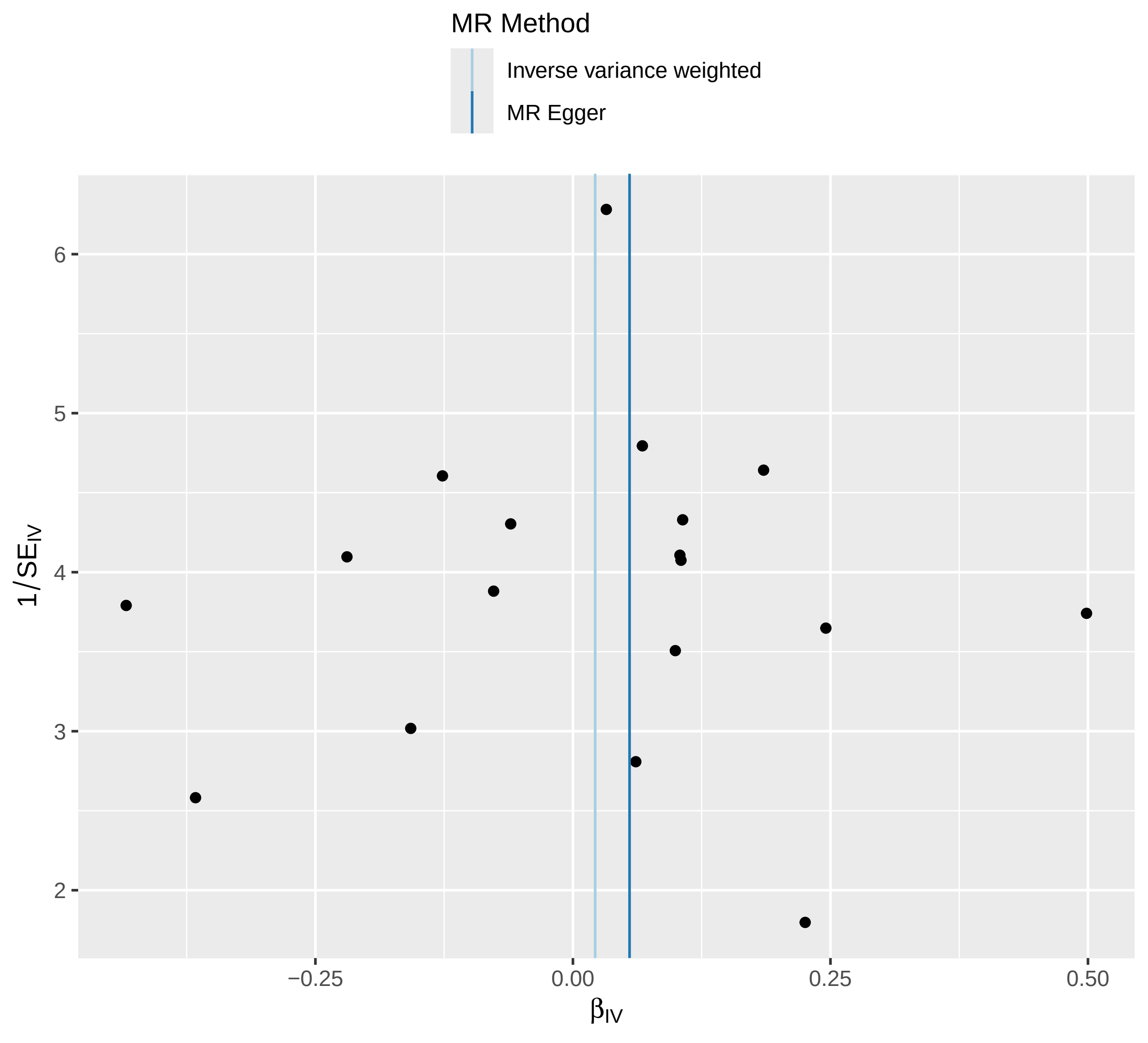

MR Method
Inverse variance weighted
MR Egger
6
5
4
3
2
1/SE_IV
−0.25
0.00
0.25
0.50
β_IV

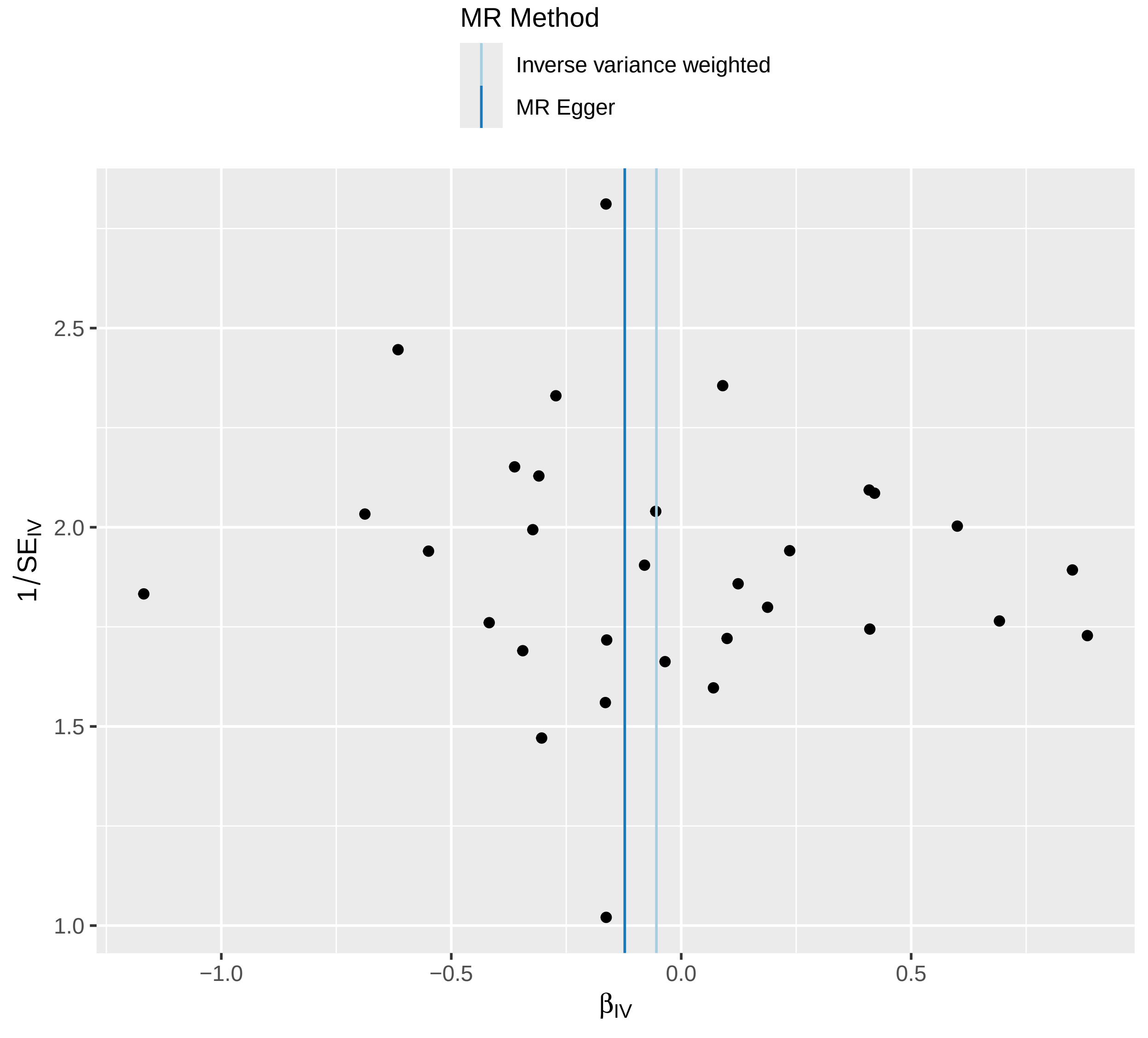
MR Method
Inverse variance weighted
MR Egger
2.5
2.0
1.5
1.0
−1.0
−0.5
0.0
0.5
1/SE_IV
β_IV

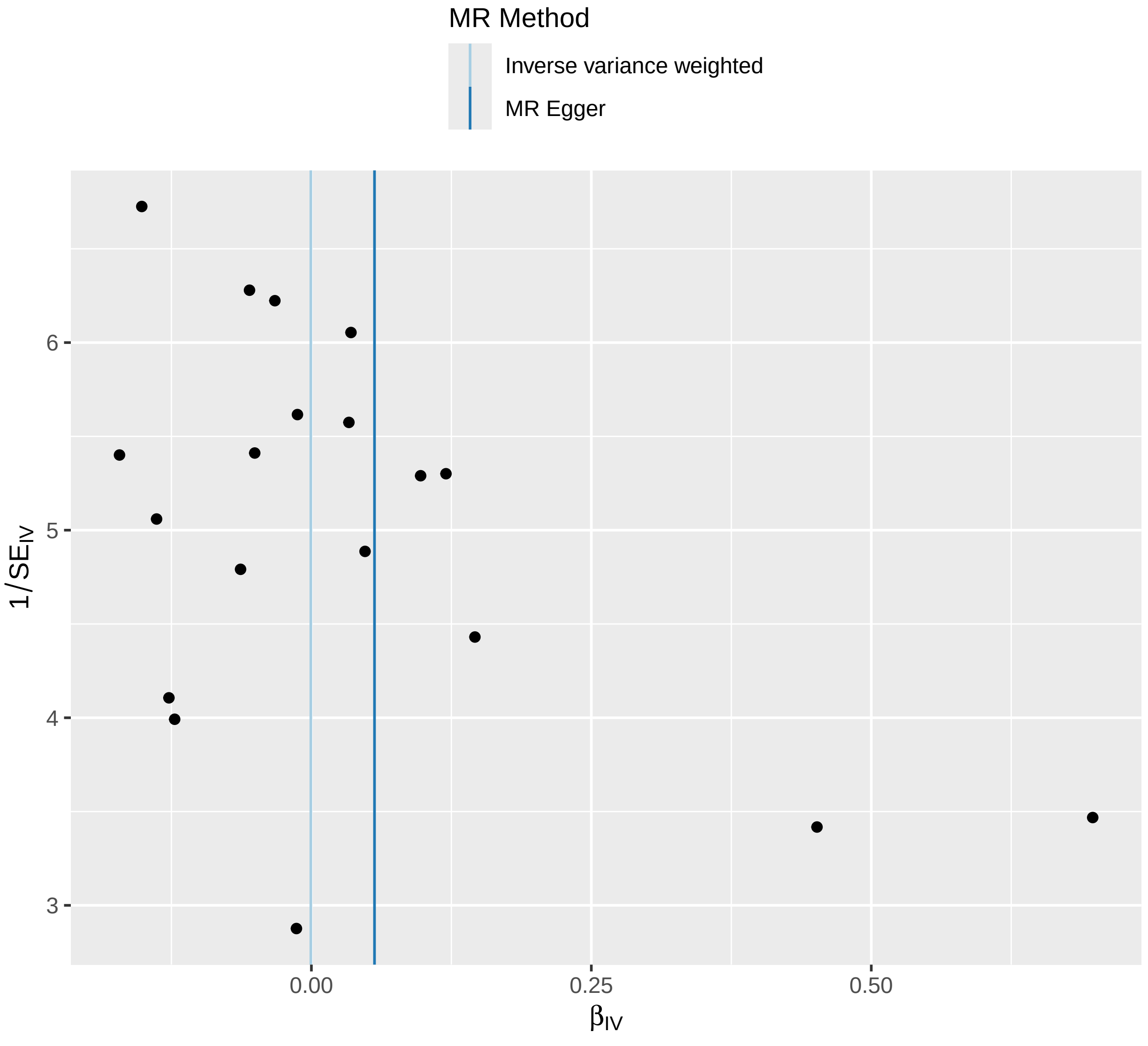

MR Method
Inverse variance weighted
MR Egger
6
5
4
3
1/SE$_{IV}$
0.00
0.25
0.50
β$_{IV}$

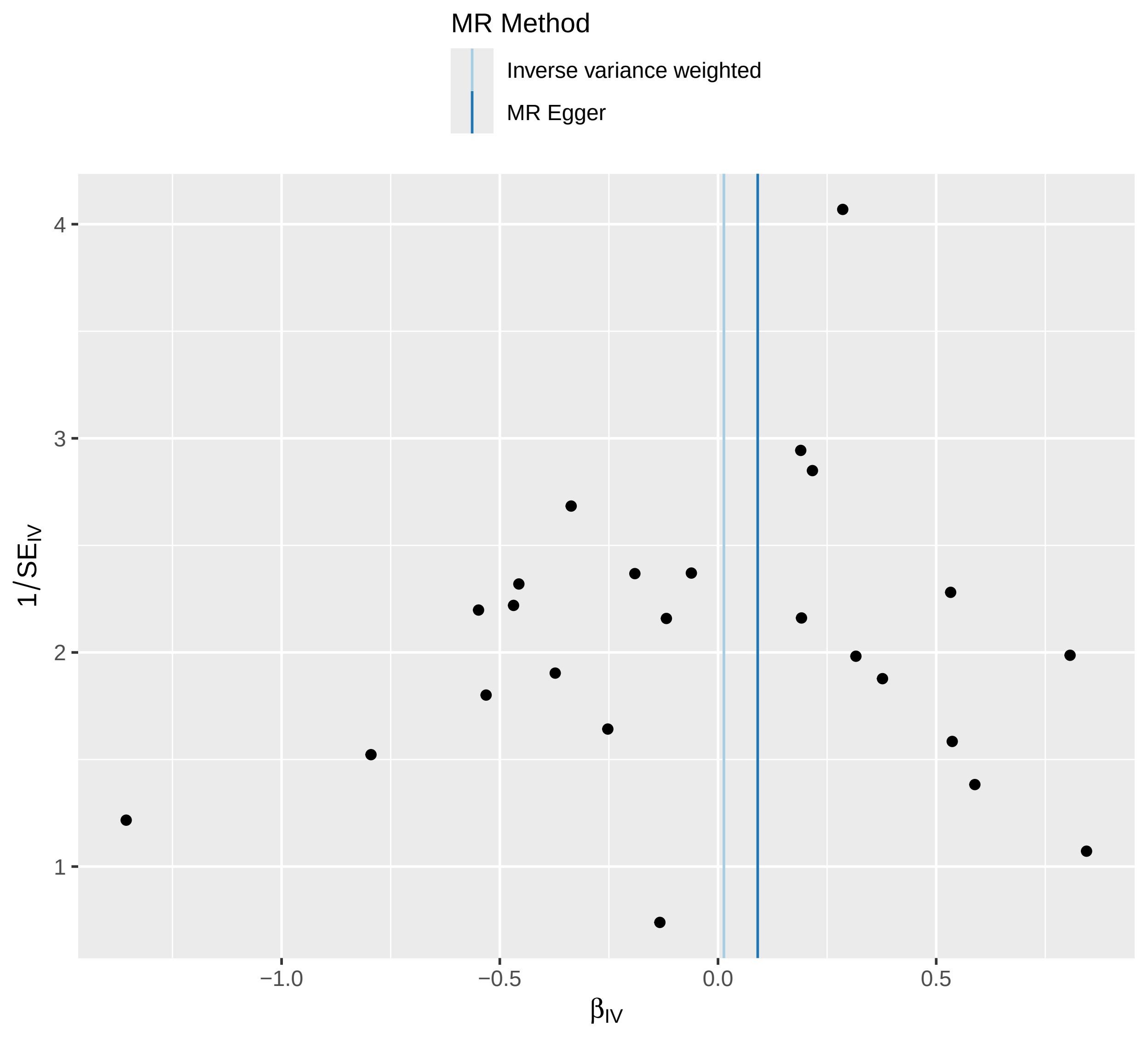

MR Method
Inverse variance weighted
MR Egger
1/SE$_{IV}$
4
3
2
1
−1.0
−0.5
0.0
0.5
β$_{IV}$

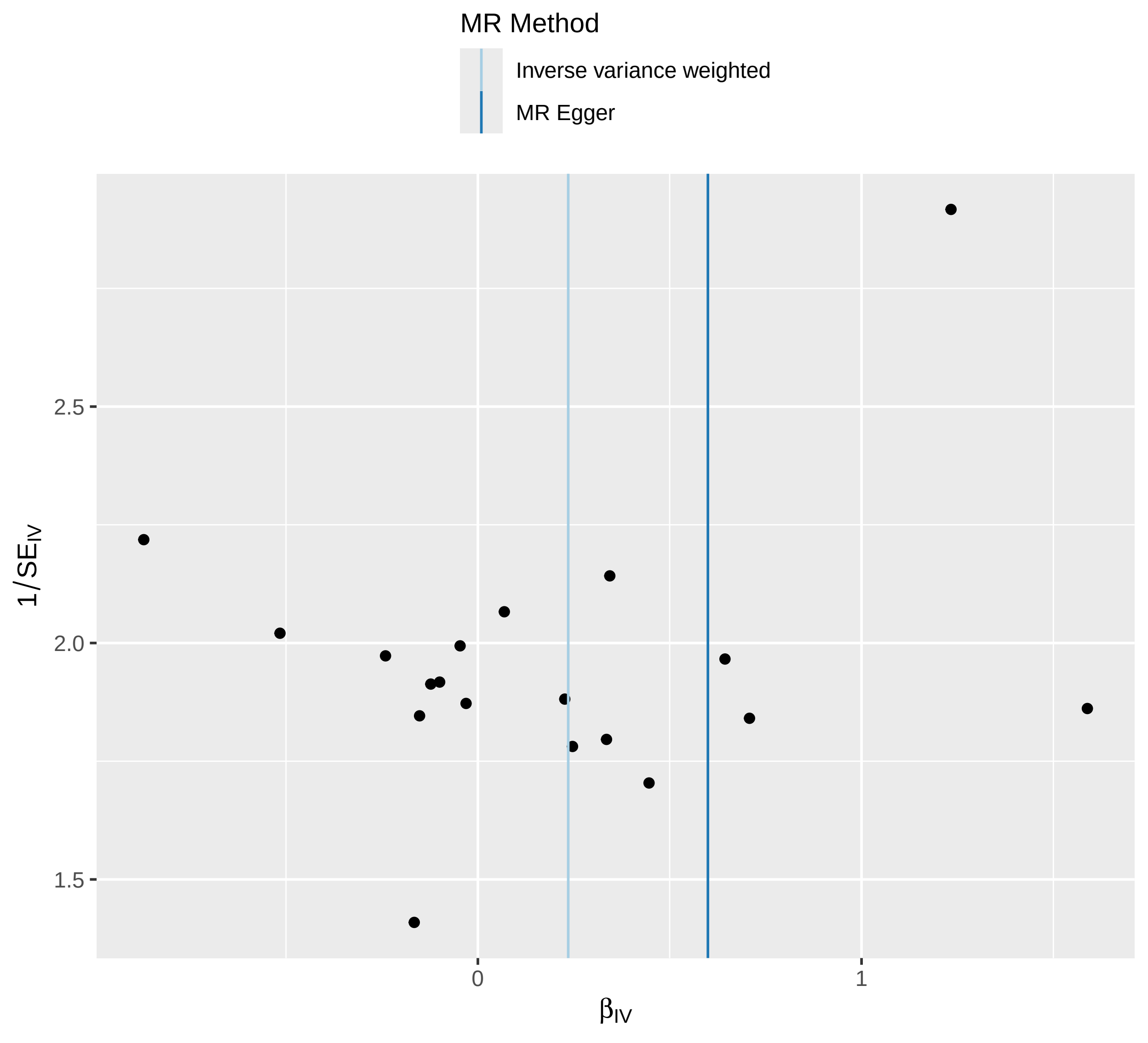

MR Method
Inverse variance weighted
MR Egger
2.5
2.0
1.5
1/SE_IV
0
1
β_IV

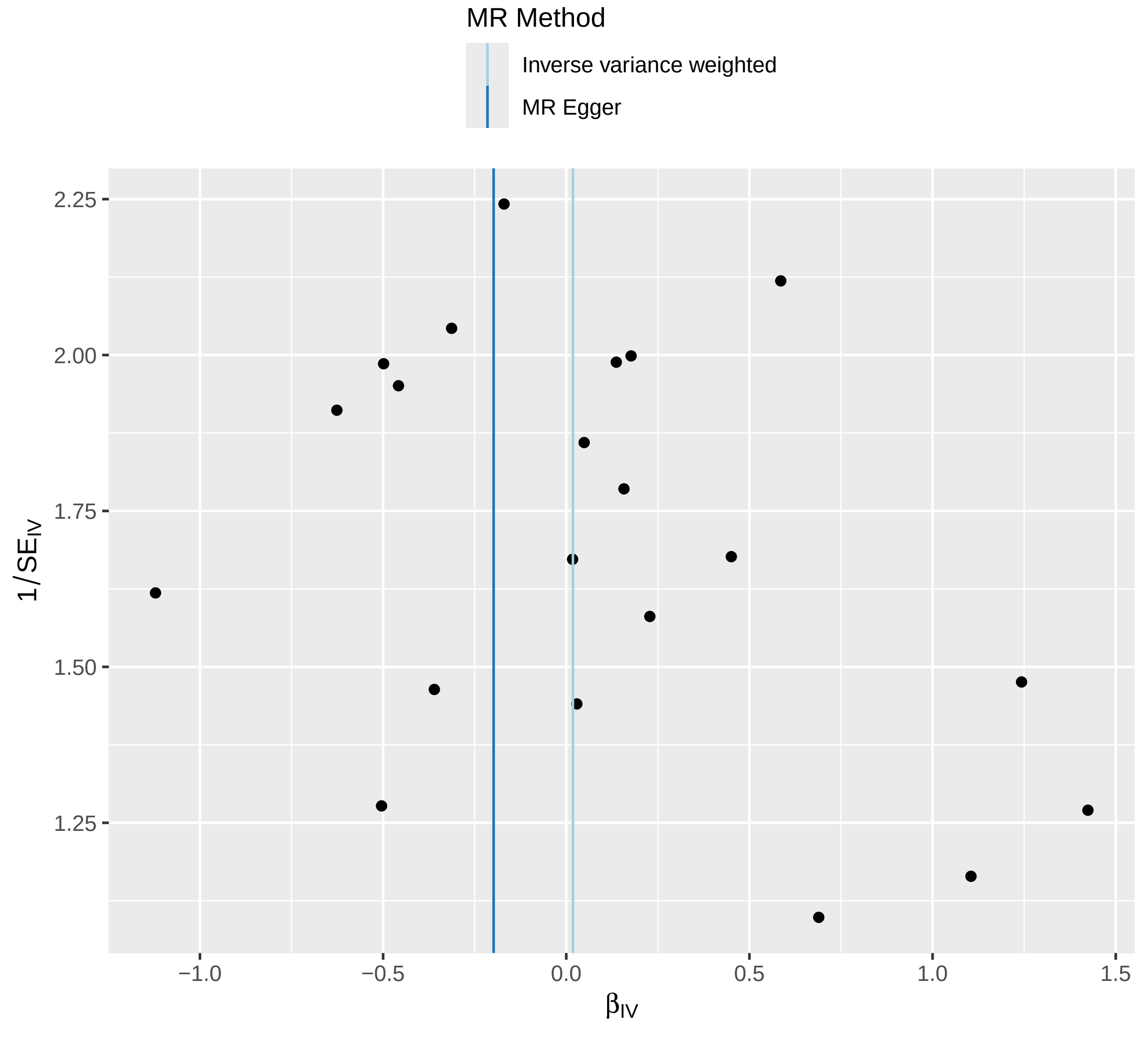

MR Method
Inverse variance weighted
MR Egger
2.25
2.00
1.75
1.50
1.25
1/SE_IV
−1.0
−0.5
0.0
0.5
1.0
1.5
β_IV

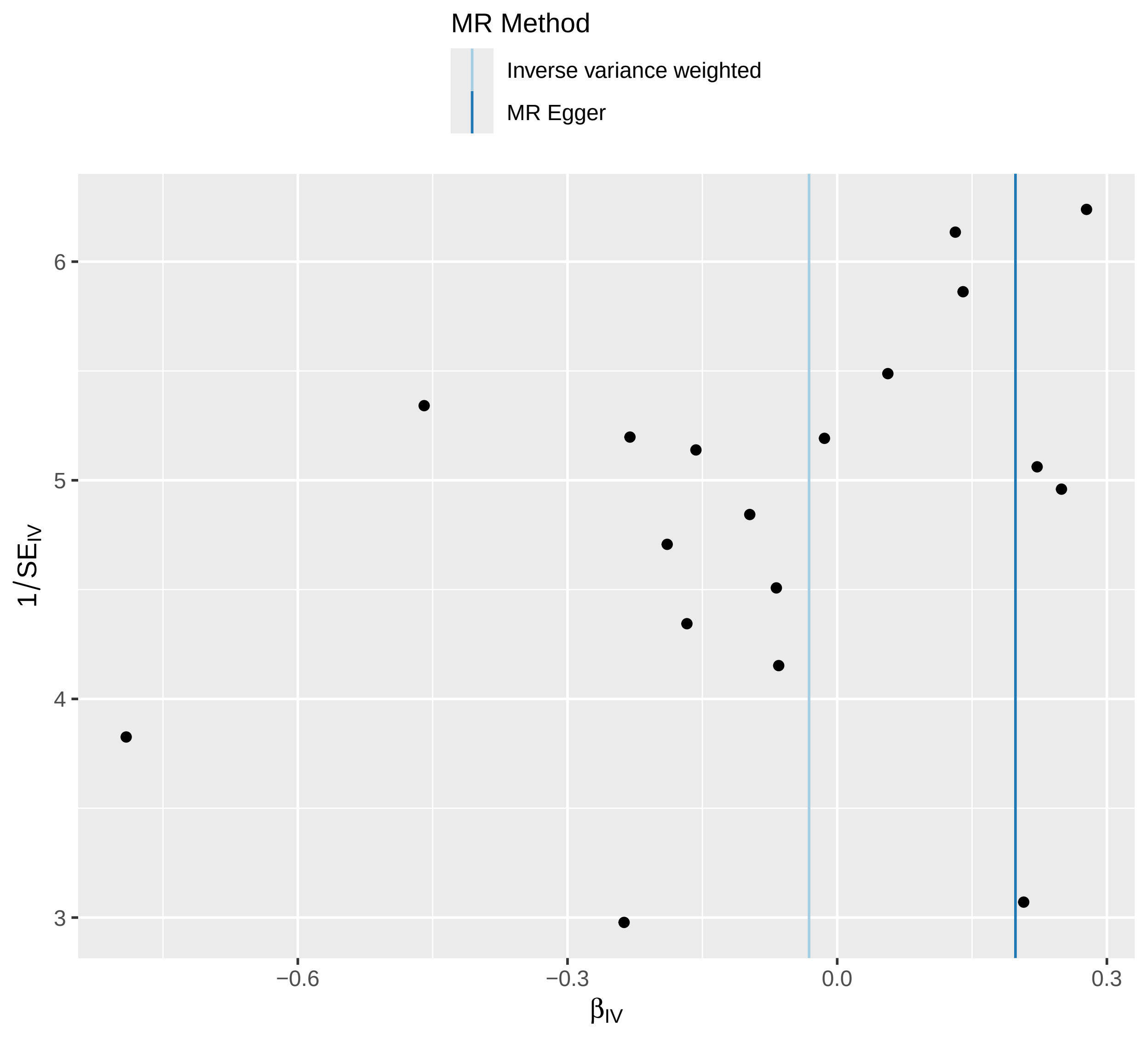
MR Method
Inverse variance weighted
MR Egger
6
5
4
3
$1/SE_{IV}$
−0.6
−0.3
0.0
0.3
β_{IV}

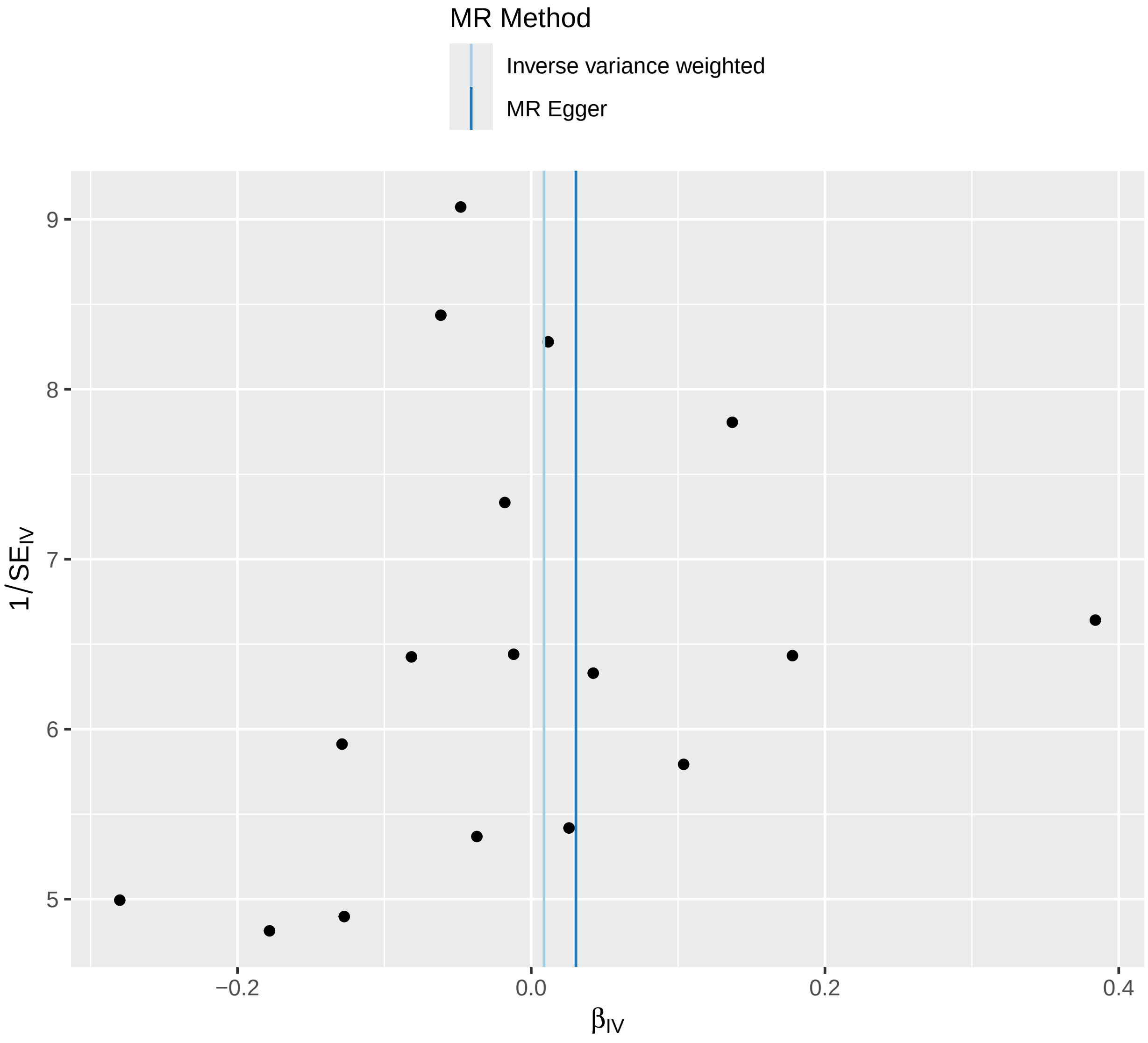
MR Method
Inverse variance weighted
MR Egger
1/SE_IV
9
8
7
6
5
−0.2
0.0
0.2
0.4
β_IV

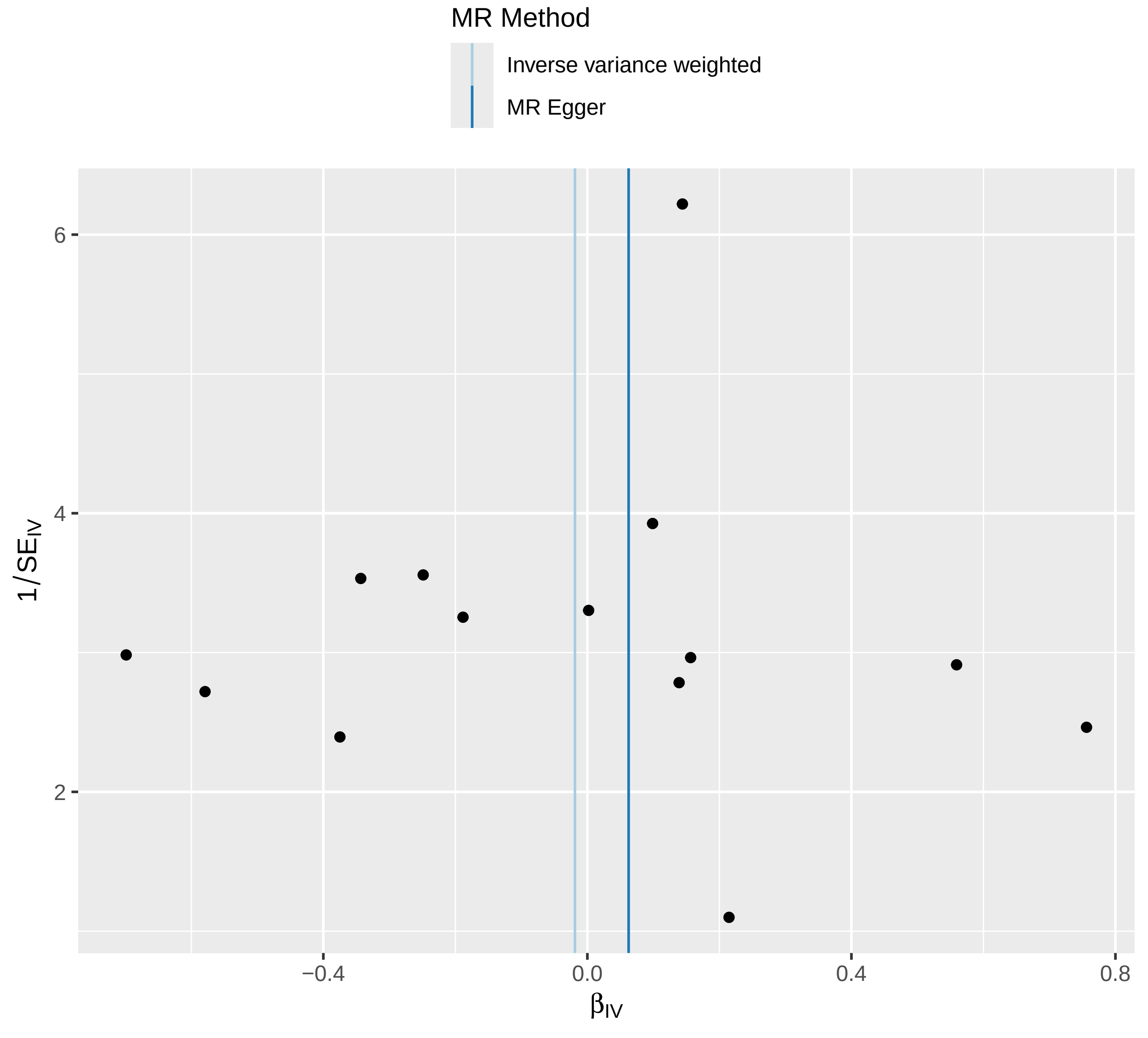
MR Method
Inverse variance weighted
MR Egger
6
4
2
1/SE$_{IV}$
−0.4
0.0
0.4
0.8
β$_{IV}$

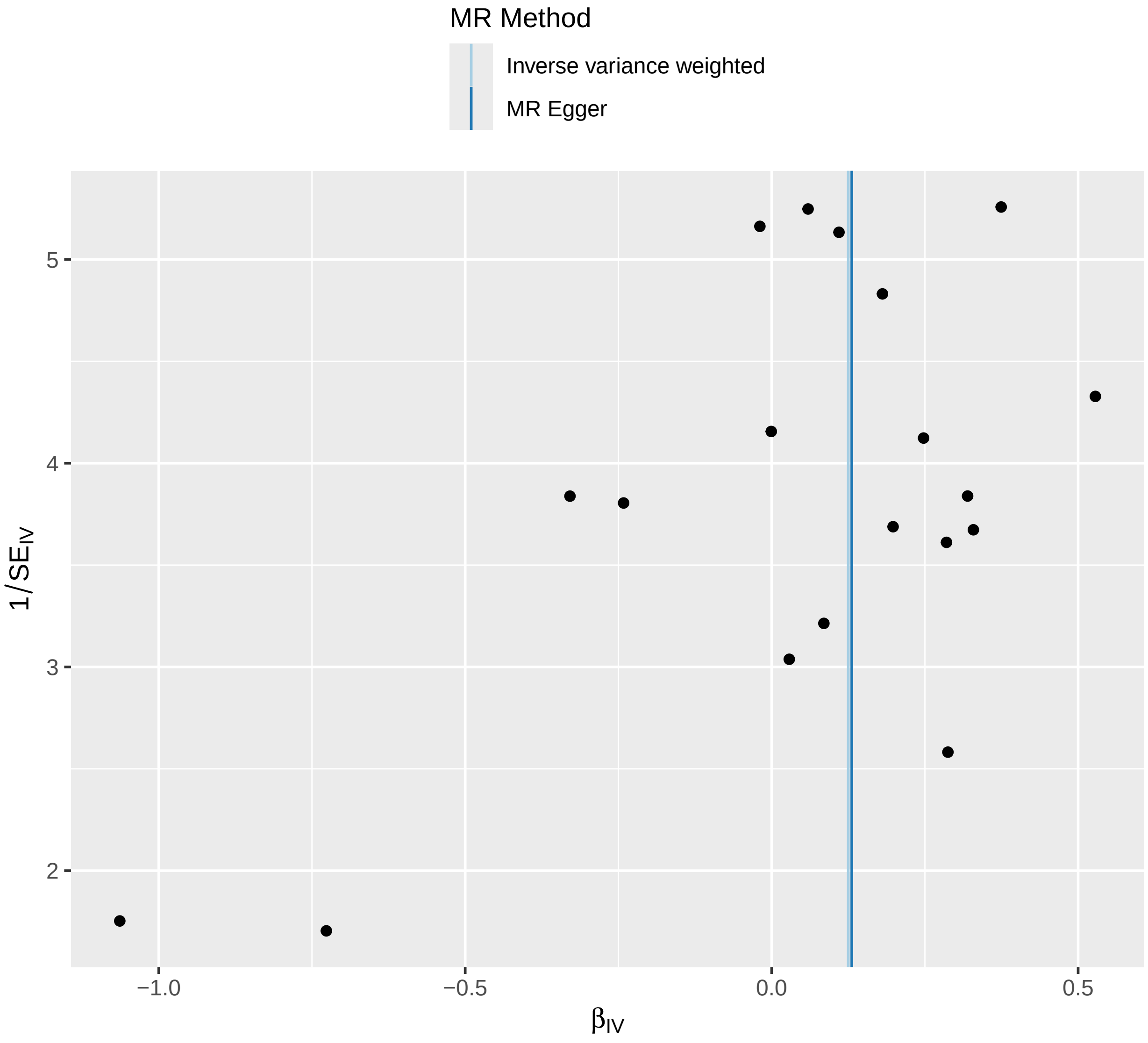

MR Method
Inverse variance weighted
MR Egger
5
4
3
2
1/SE$_{IV}$
−1.0
−0.5
0.0
0.5
β$_{IV}$

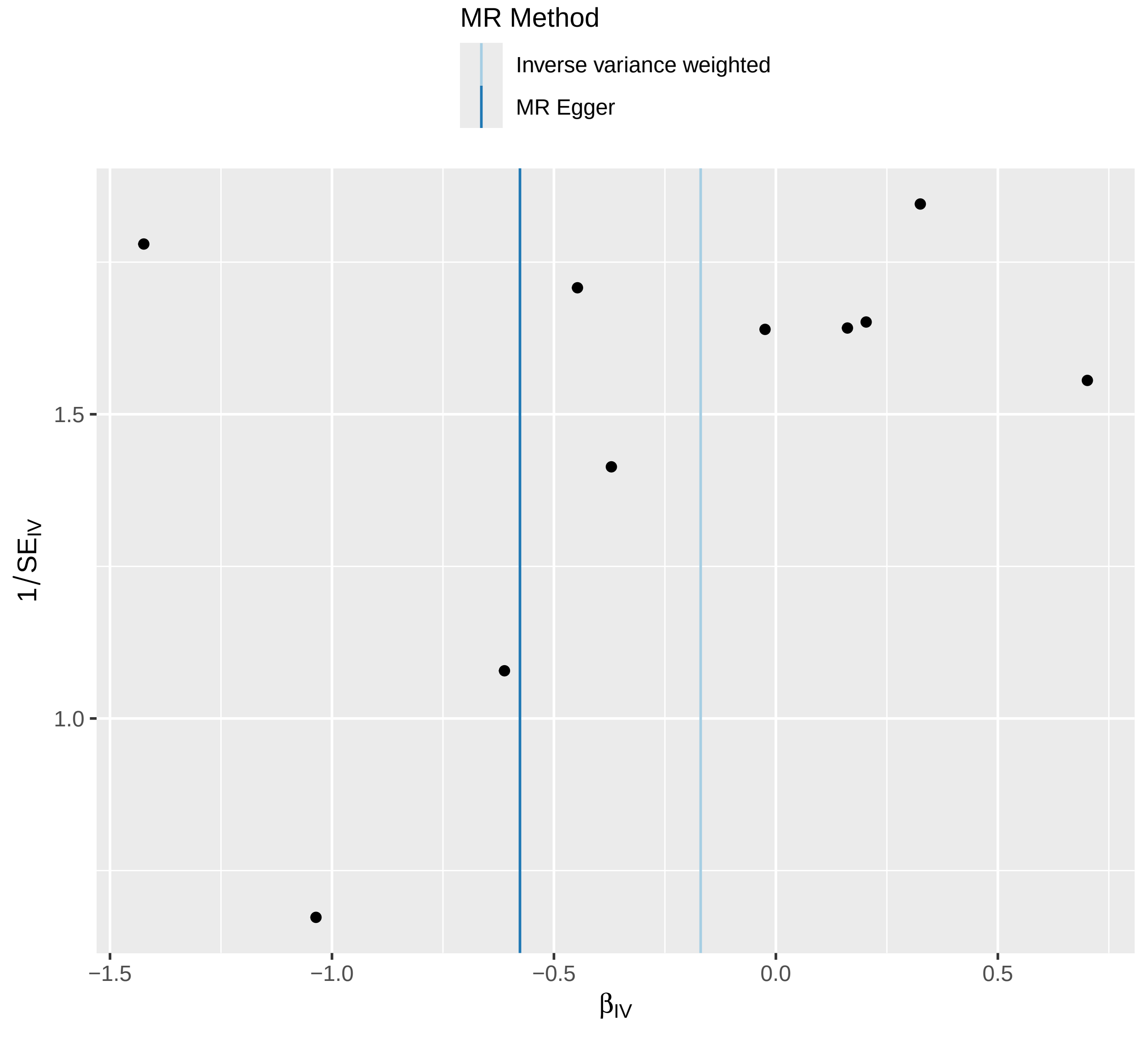
MR Method
Inverse variance weighted
MR Egger
1.5
1.0
−1.5
−1.0
−0.5
0.0
0.5
$1/SE_{IV}$
β_{IV}

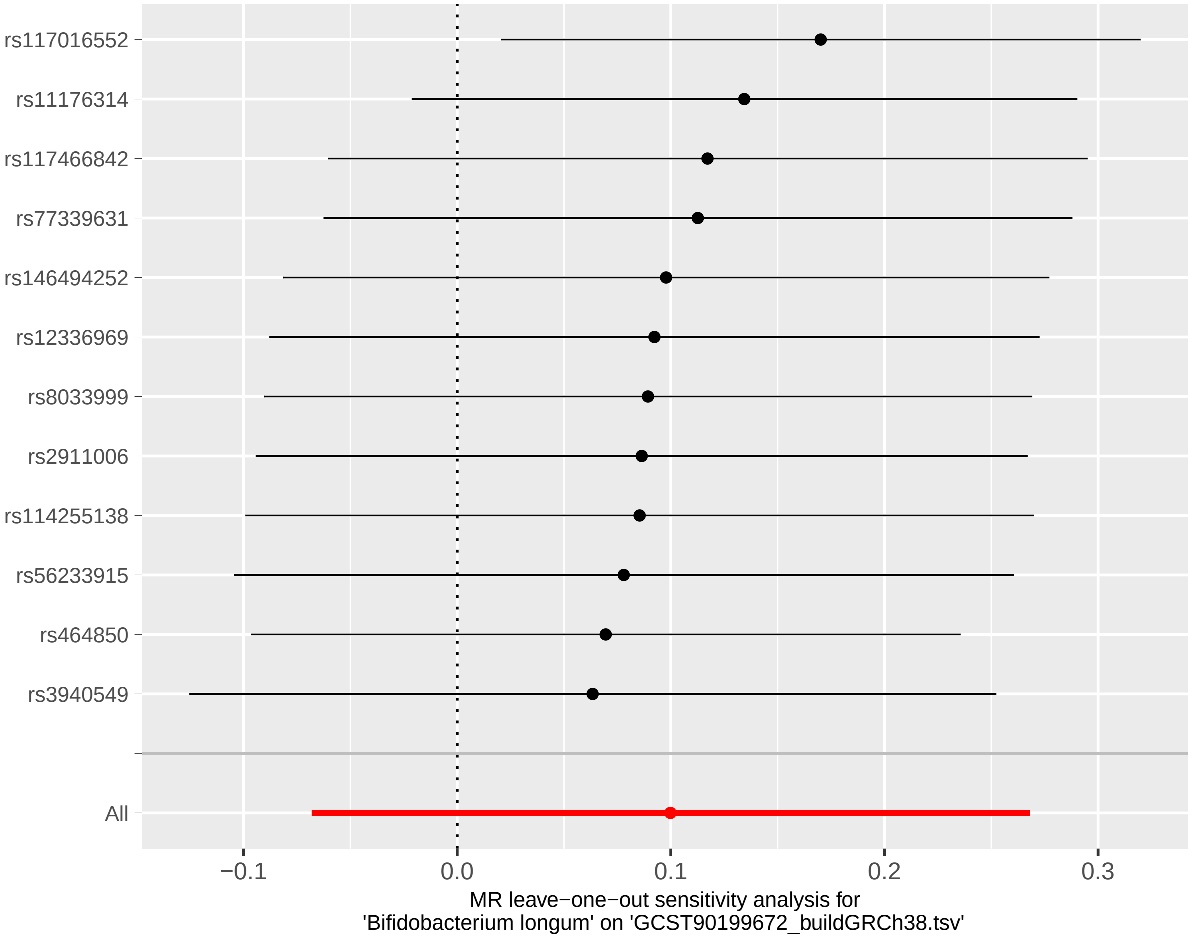
rs117016552
rs11176314
rs117466842
rs77339631
rs146494252
rs12336969
rs8033999
rs2911006
rs114255138
rs56233915
rs464850
rs3940549
All
−0.1
0.0
0.1
0.2
0.3
MR leave−one−out sensitivity analysis for
'Bifidobacterium longum' on 'GCST90199672_buildGRCh38.tsv'

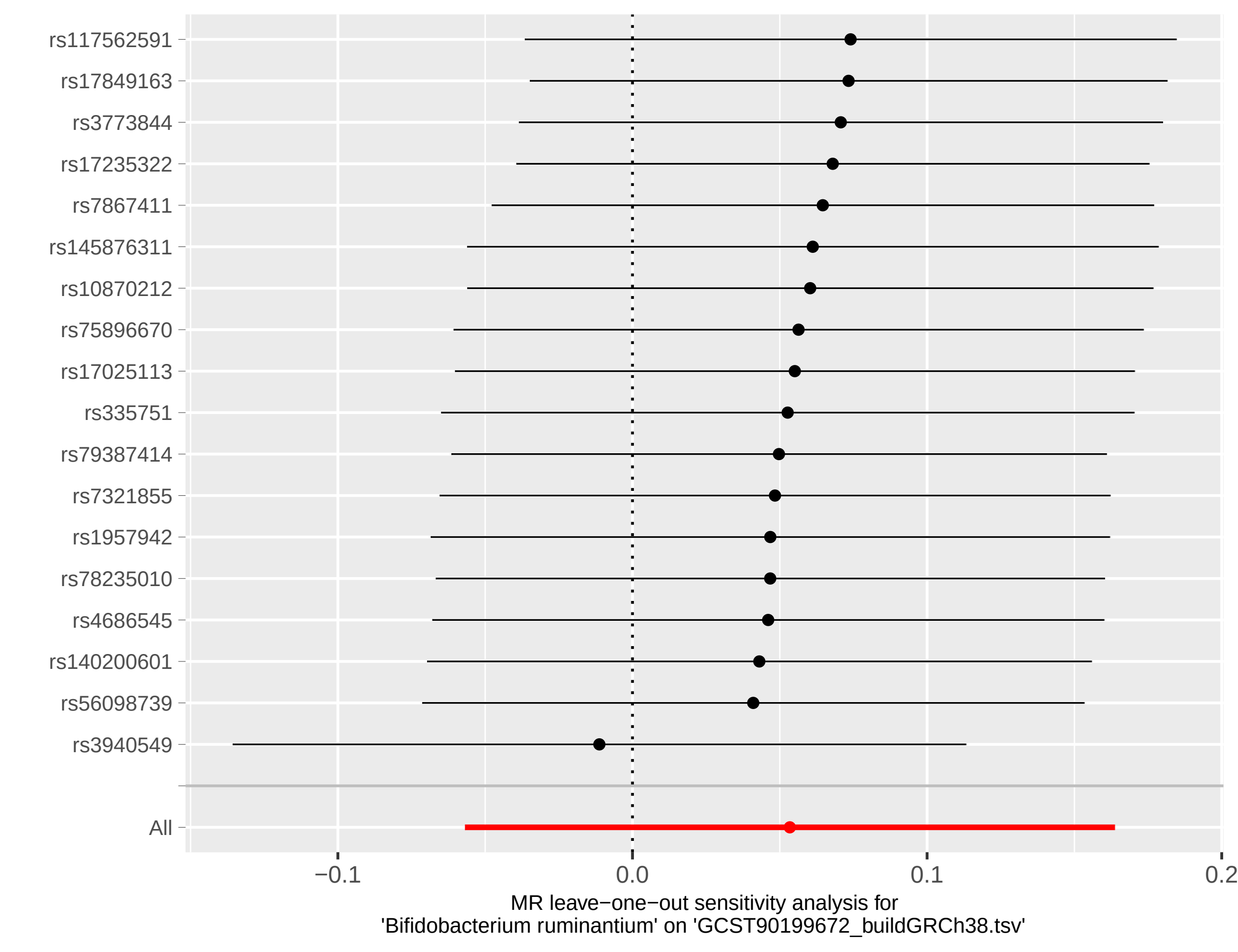

rs117562591
rs17849163
rs3773844
rs17235322
rs7867411
rs145876311
rs10870212
rs75896670
rs17025113
rs335751
rs79387414
rs7321855
rs1957942
rs78235010
rs4686545
rs140200601
rs56098739
rs3940549
All
−0.1
0.0
0.1
0.2
MR leave−one−out sensitivity analysis for
'Bifidobacterium ruminantium' on 'GCST90199672_buildGRCh38.tsv'

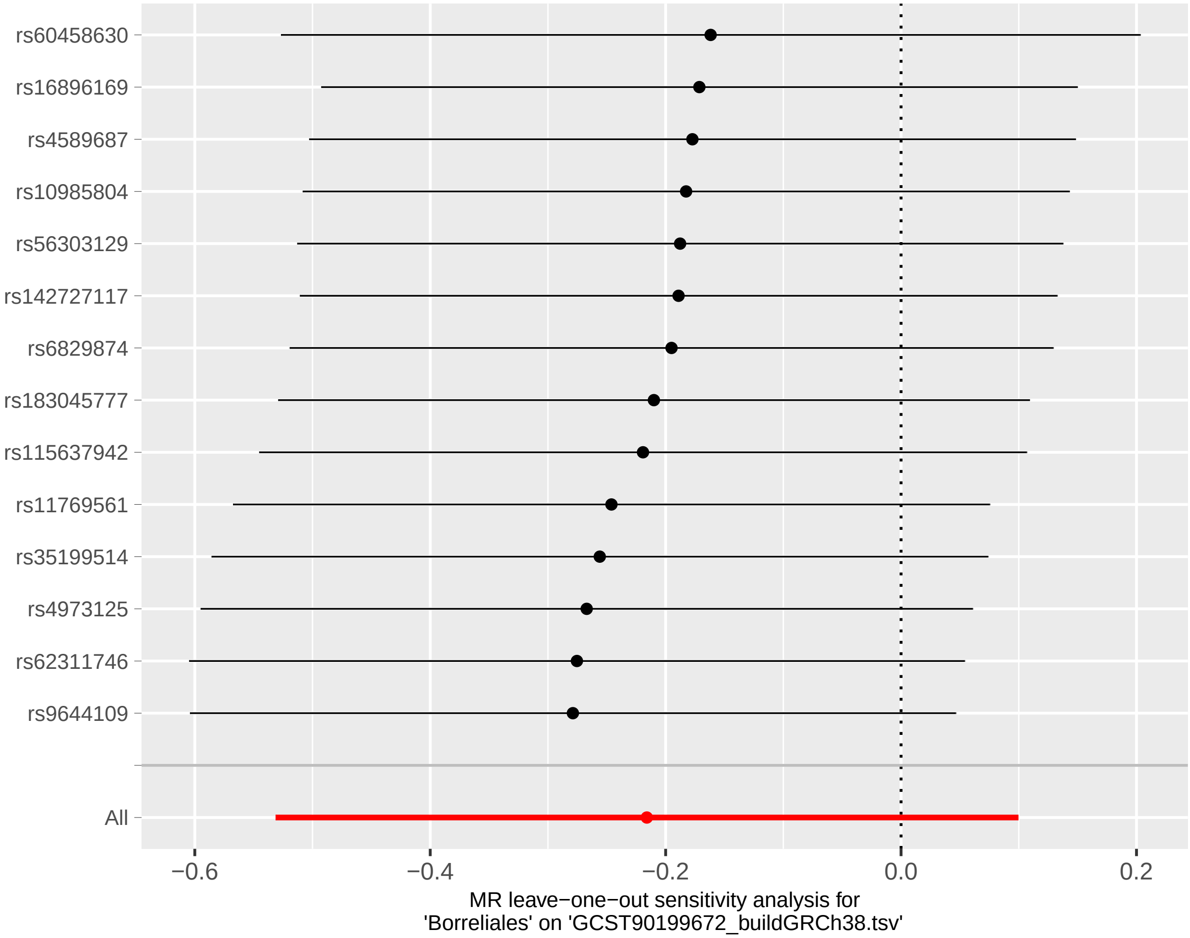
rs60458630
rs16896169
rs4589687
rs10985804
rs56303129
rs142727117
rs6829874
rs183045777
rs115637942
rs11769561
rs35199514
rs4973125
rs62311746
rs9644109
All
−0.6
−0.4
−0.2
0.0
0.2
MR leave−one−out sensitivity analysis for
'Borreliales' on 'GCST90199672_buildGRCh38.tsv'

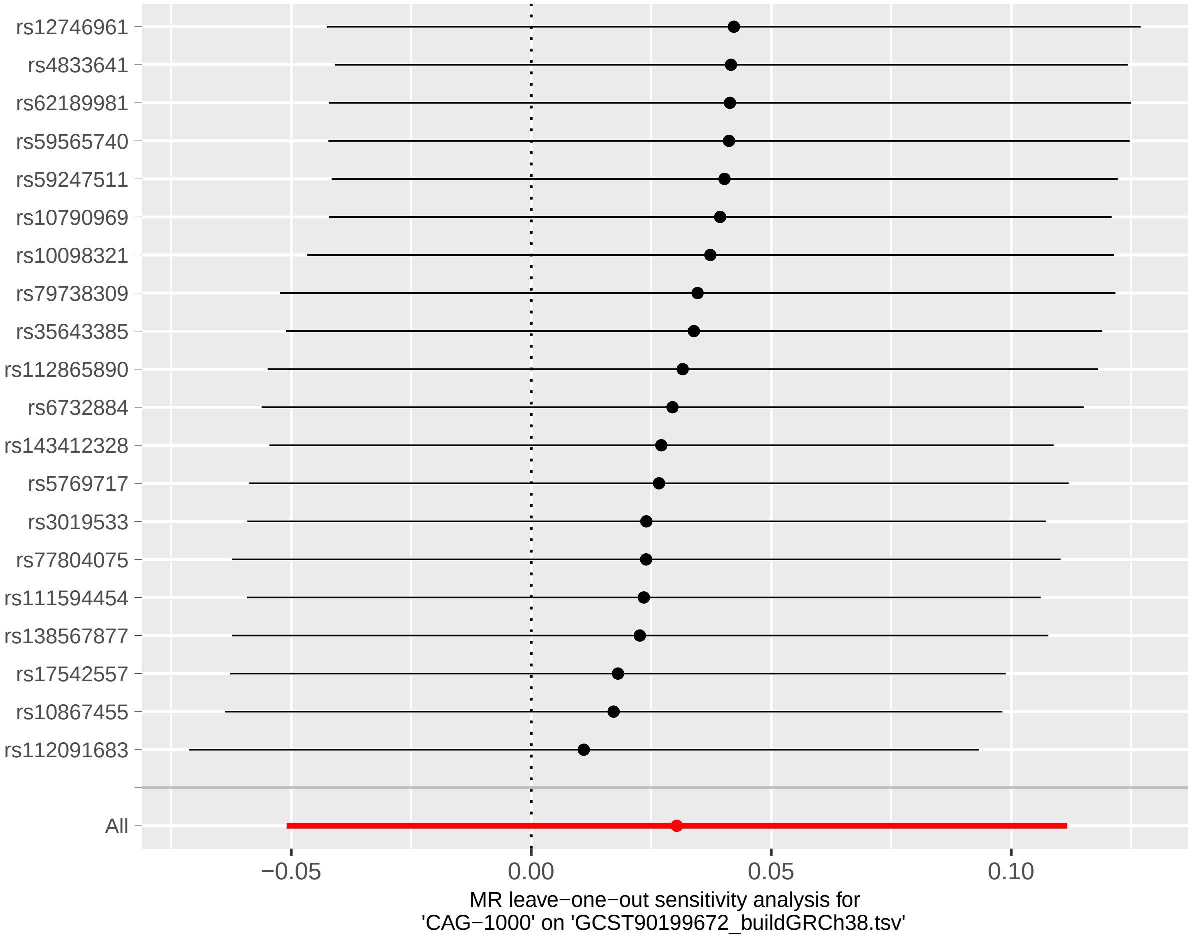
rs12746961
rs4833641
rs62189981
rs59565740
rs59247511
rs10790969
rs10098321
rs79738309
rs35643385
rs112865890
rs6732884
rs143412328
rs5769717
rs3019533
rs77804075
rs111594454
rs138567877
rs17542557
rs10867455
rs112091683
All
−0.05
0.00
0.05
0.10
MR leave−one−out sensitivity analysis for
'CAG−1000' on 'GCST90199672_buildGRCh38.tsv'

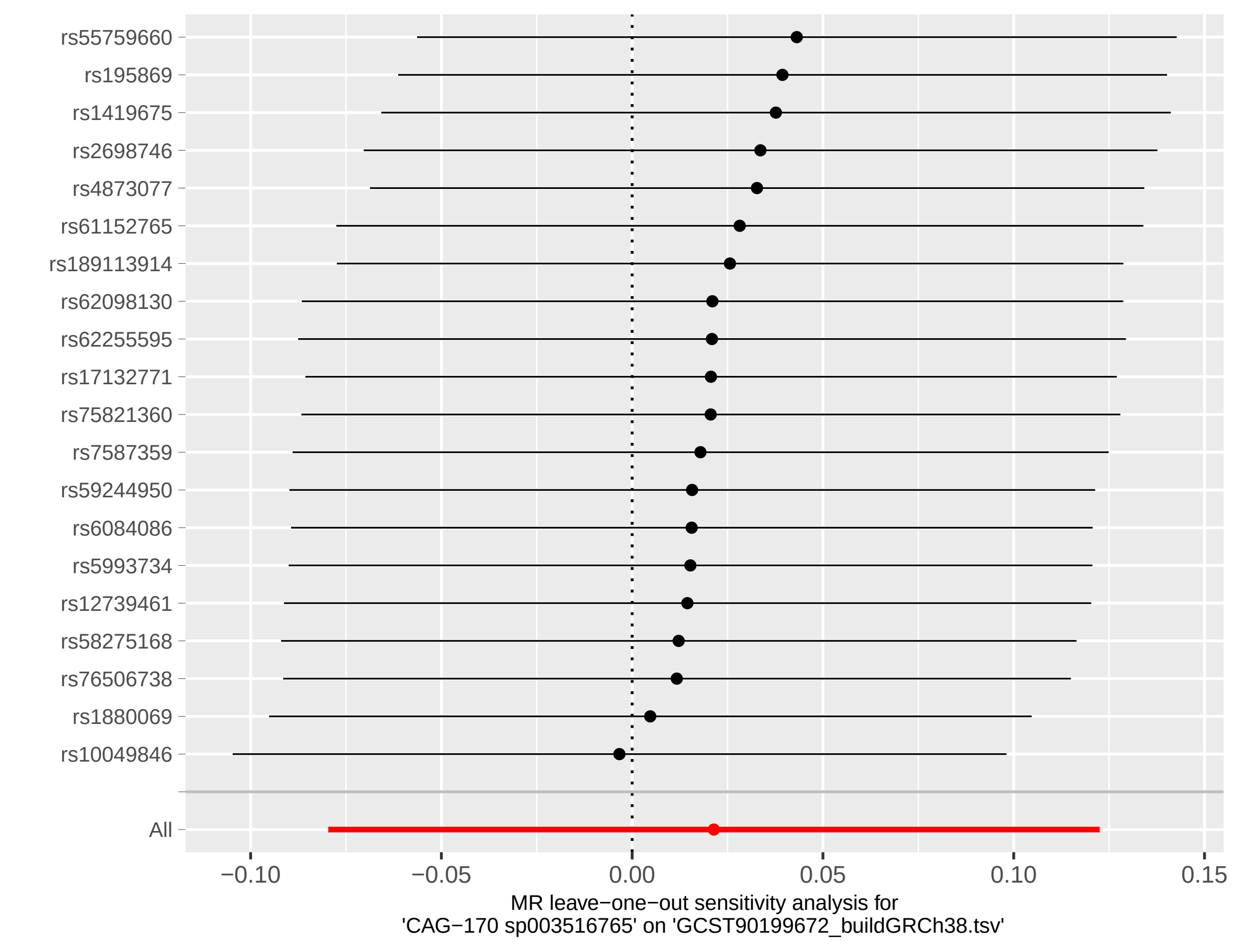

rs55759660
rs195869
rs1419675
rs2698746
rs4873077
rs61152765
rs189113914
rs62098130
rs62255595
rs17132771
rs75821360
rs7587359
rs59244950
rs6084086
rs5993734
rs12739461
rs58275168
rs76506738
rs1880069
rs10049846
All
−0.10
−0.05
0.00
0.05
0.10
0.15
MR leave−one−out sensitivity analysis for
'CAG−170 sp003516765' on 'GCST90199672_buildGRCh38.tsv'

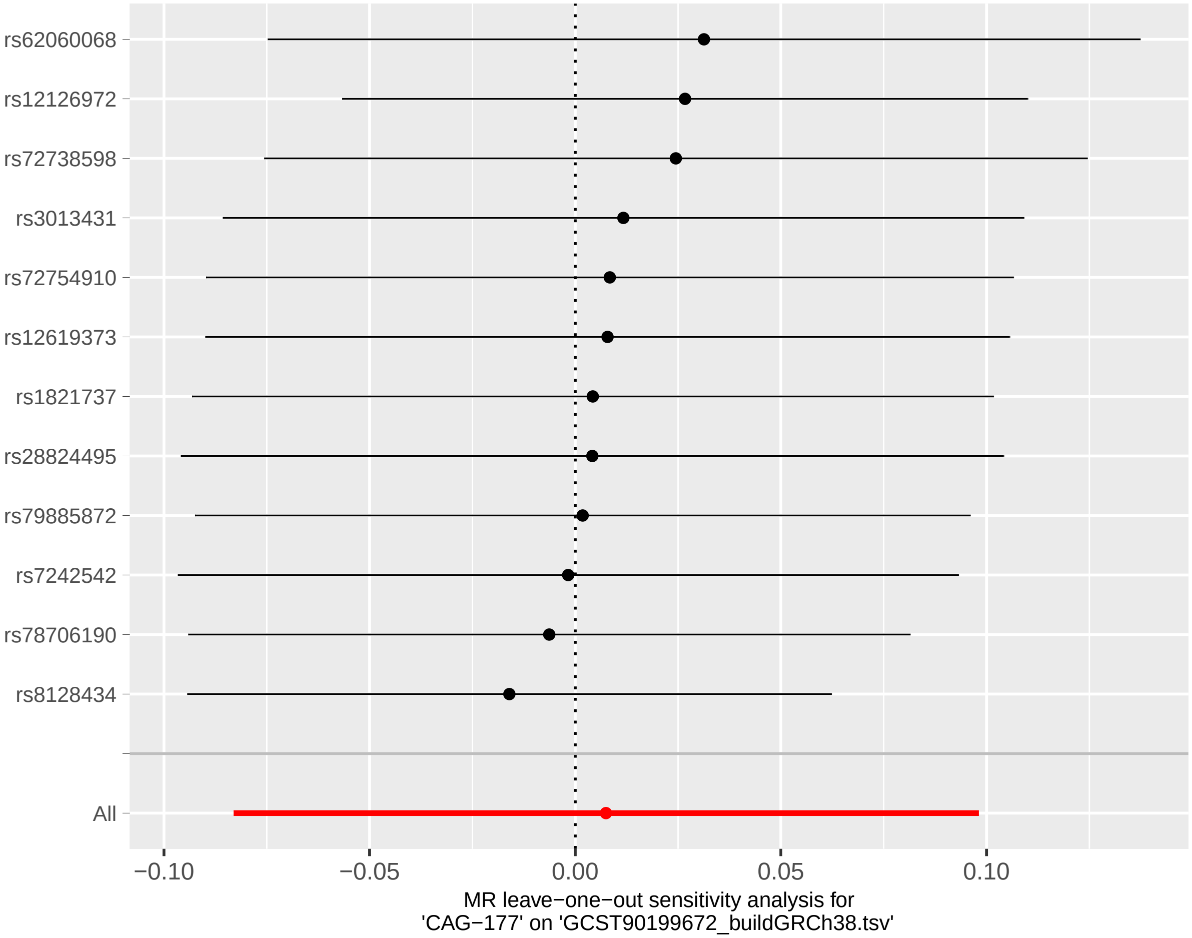

rs62060068
rs12126972
rs72738598
rs3013431
rs72754910
rs12619373
rs1821737
rs28824495
rs79885872
rs7242542
rs78706190
rs8128434
All
−0.10
−0.05
0.00
0.05
0.10
MR leave−one−out sensitivity analysis for
'CAG−177' on 'GCST90199672_buildGRCh38.tsv'

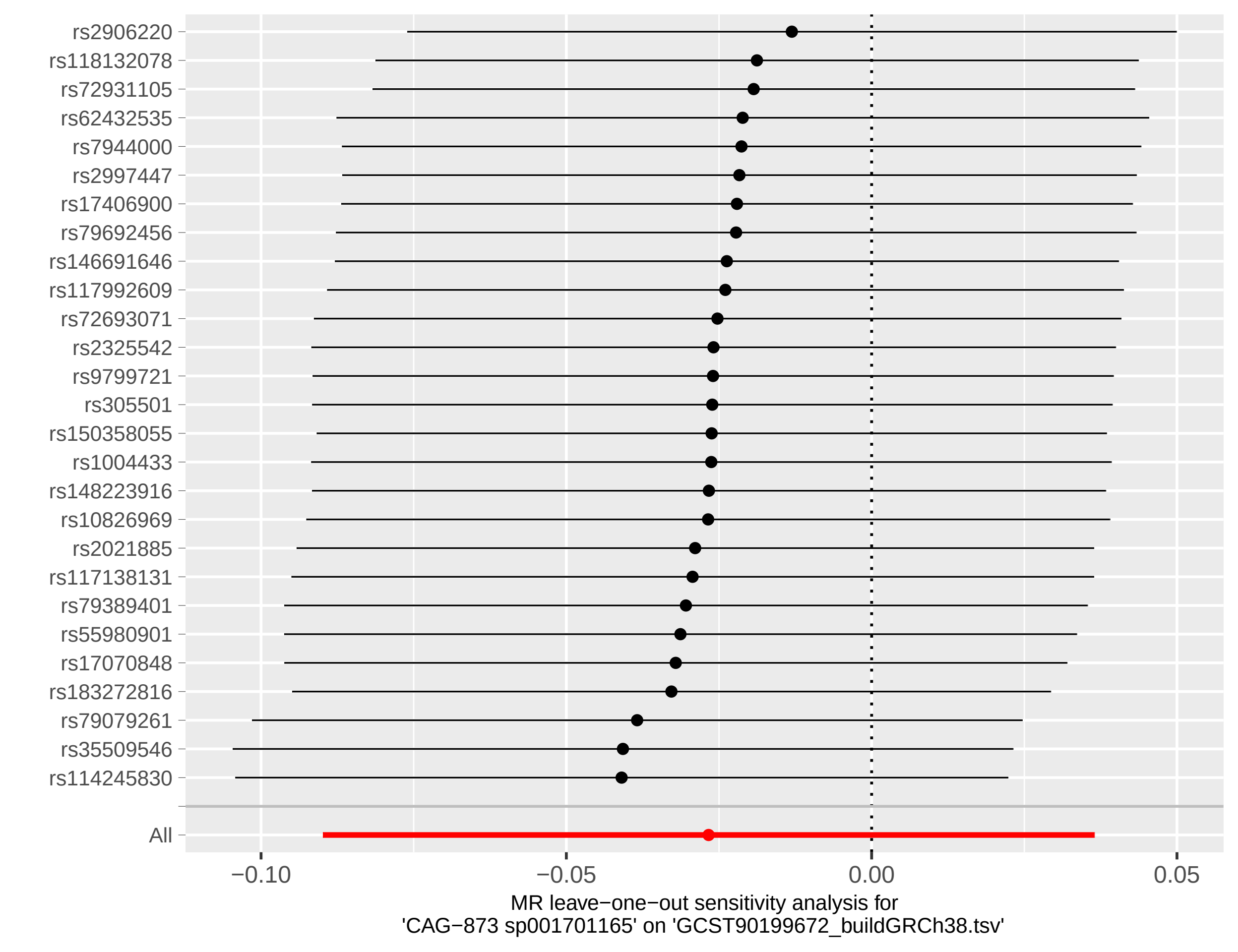

rs2906220
rs118132078
rs72931105
rs62432535
rs7944000
rs2997447
rs17406900
rs79692456
rs146691646
rs117992609
rs72693071
rs2325542
rs9799721
rs305501
rs150358055
rs1004433
rs148223916
rs10826969
rs2021885
rs117138131
rs79389401
rs55980901
rs17070848
rs183272816
rs79079261
rs35509546
rs114245830
All
−0.10
−0.05
0.00
0.05
MR leave−one−out sensitivity analysis for
'CAG−873 sp001701165' on 'GCST90199672_buildGRCh38.tsv'

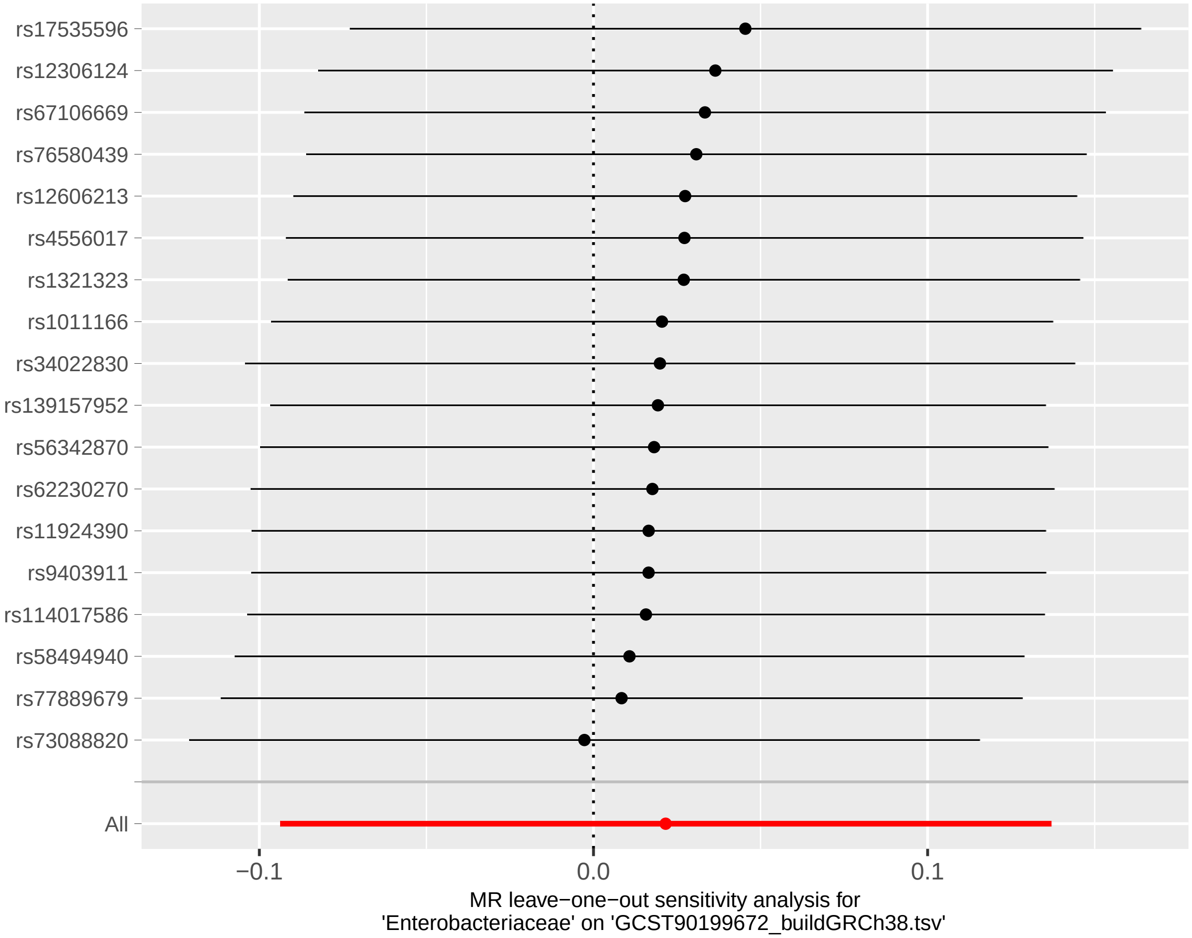
rs17535596
rs12306124
rs67106669
rs76580439
rs12606213
rs4556017
rs1321323
rs1011166
rs34022830
rs139157952
rs56342870
rs62230270
rs11924390
rs9403911
rs114017586
rs58494940
rs77889679
rs73088820
All
−0.1
0.0
0.1
MR leave−one−out sensitivity analysis for
'Enterobacteriaceae' on 'GCST90199672_buildGRCh38.tsv'

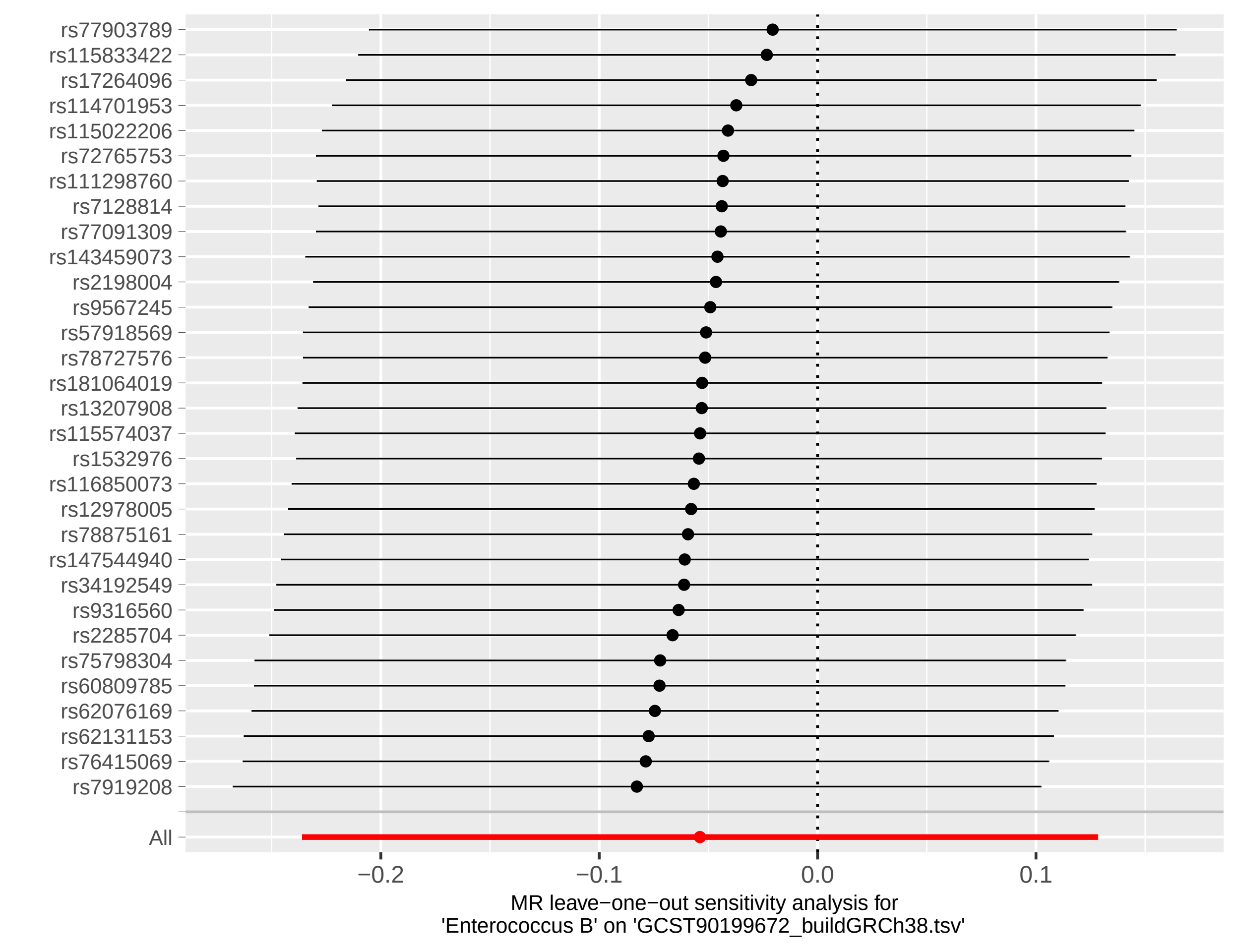

rs77903789
rs115833422
rs17264096
rs114701953
rs115022206
rs72765753
rs111298760
rs7128814
rs77091309
rs143459073
rs2198004
rs9567245
rs57918569
rs78727576
rs181064019
rs13207908
rs115574037
rs1532976
rs116850073
rs12978005
rs78875161
rs147544940
rs34192549
rs9316560
rs2285704
rs75798304
rs60809785
rs62076169
rs62131153
rs76415069
rs7919208
All
−0.2
−0.1
0.0
0.1
MR leave−one−out sensitivity analysis for
'Enterococcus B' on 'GCST90199672_buildGRCh38.tsv'

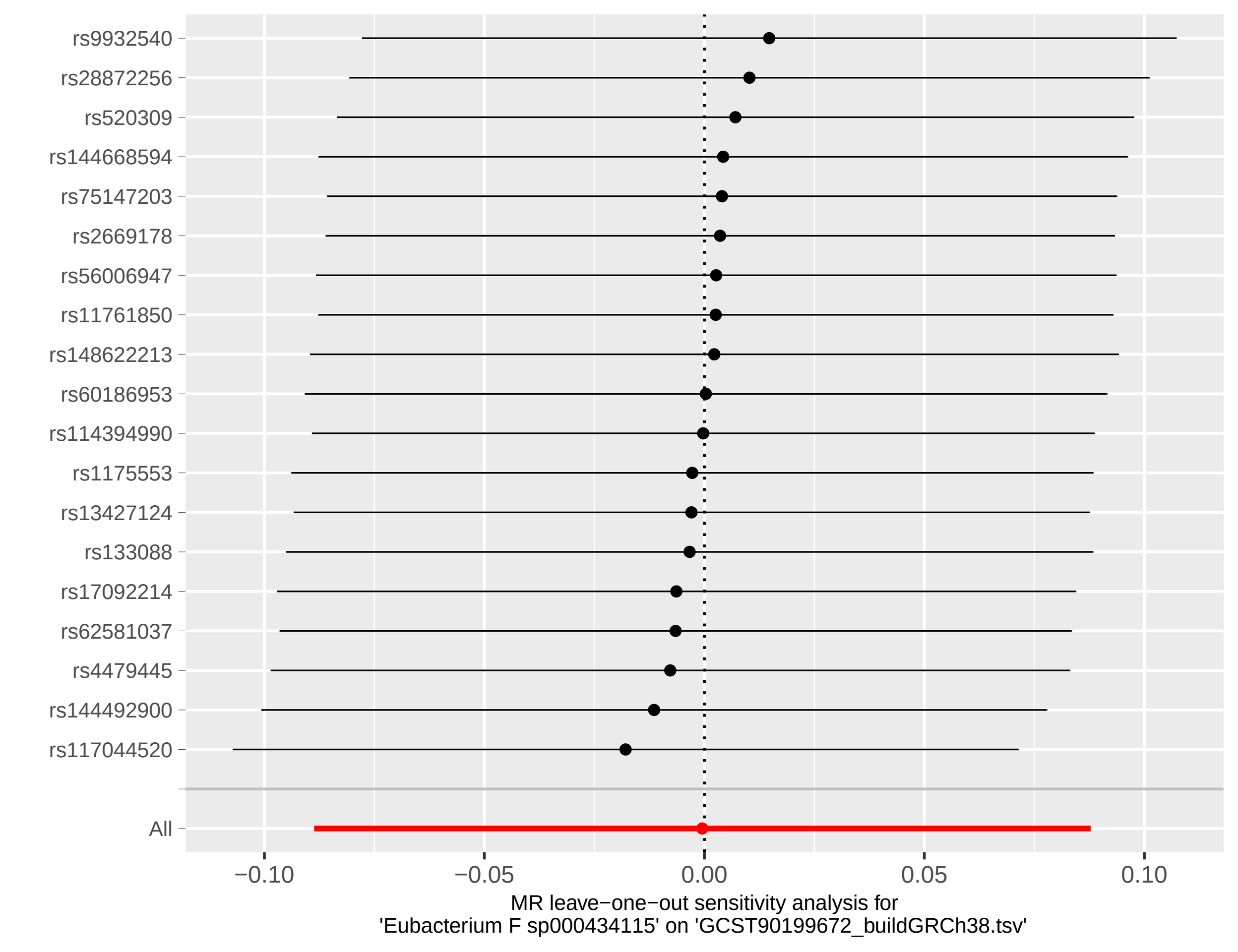
rs9932540
rs28872256
rs520309
rs144668594
rs75147203
rs2669178
rs56006947
rs11761850
rs148622213
rs60186953
rs114394990
rs1175553
rs13427124
rs133088
rs17092214
rs62581037
rs4479445
rs144492900
rs117044520
All
−0.10
−0.05
0.00
0.05
0.10
MR leave−one−out sensitivity analysis for
'Eubacterium F sp000434115' on 'GCST90199672_buildGRCh38.tsv'

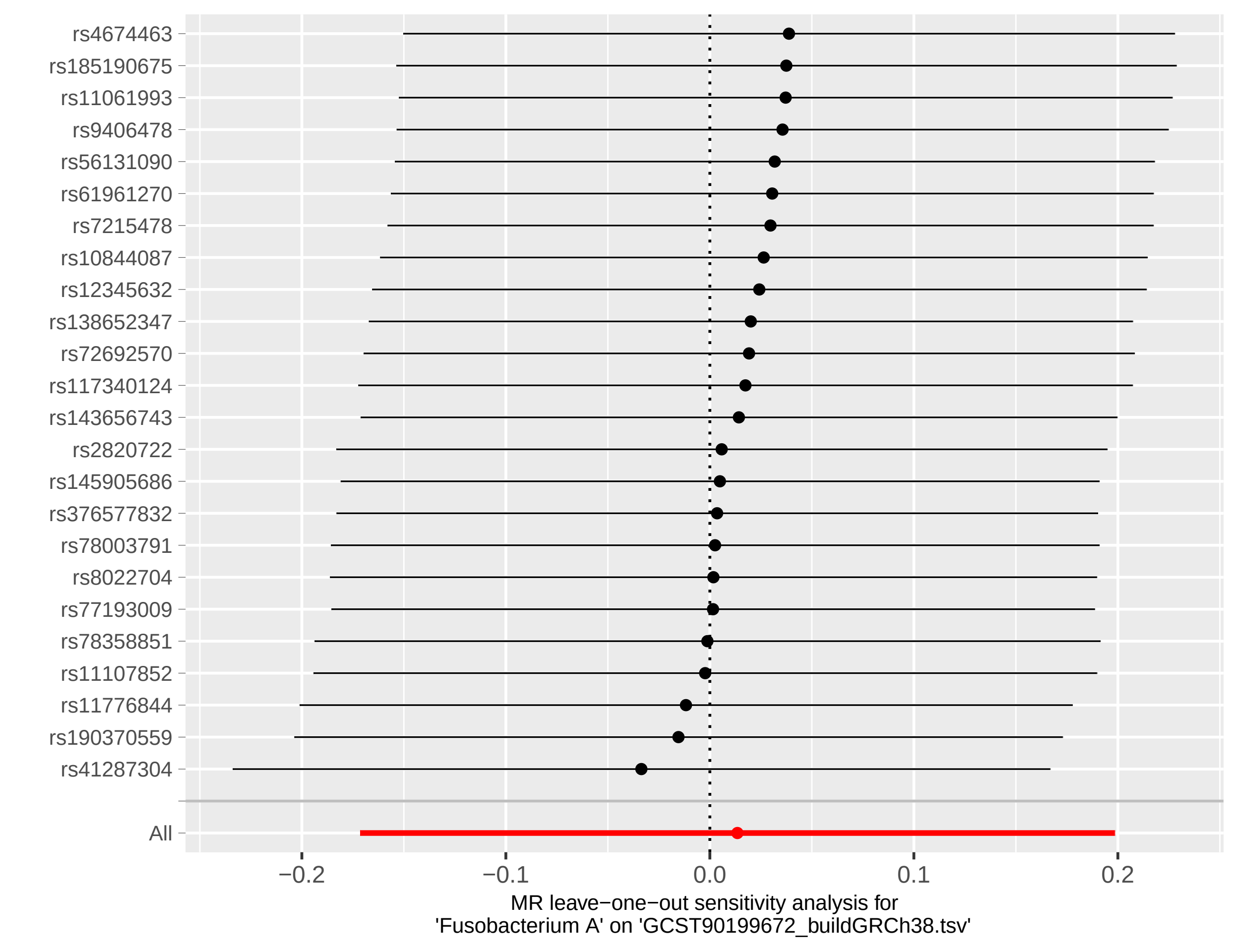
rs4674463
rs185190675
rs11061993
rs9406478
rs56131090
rs61961270
rs7215478
rs10844087
rs12345632
rs138652347
rs72692570
rs117340124
rs143656743
rs2820722
rs145905686
rs376577832
rs78003791
rs8022704
rs77193009
rs78358851
rs11107852
rs11776844
rs190370559
rs41287304
All
−0.2
−0.1
0.0
0.1
0.2
MR leave−one−out sensitivity analysis for
'Fusobacterium A' on 'GCST90199672_buildGRCh38.tsv'

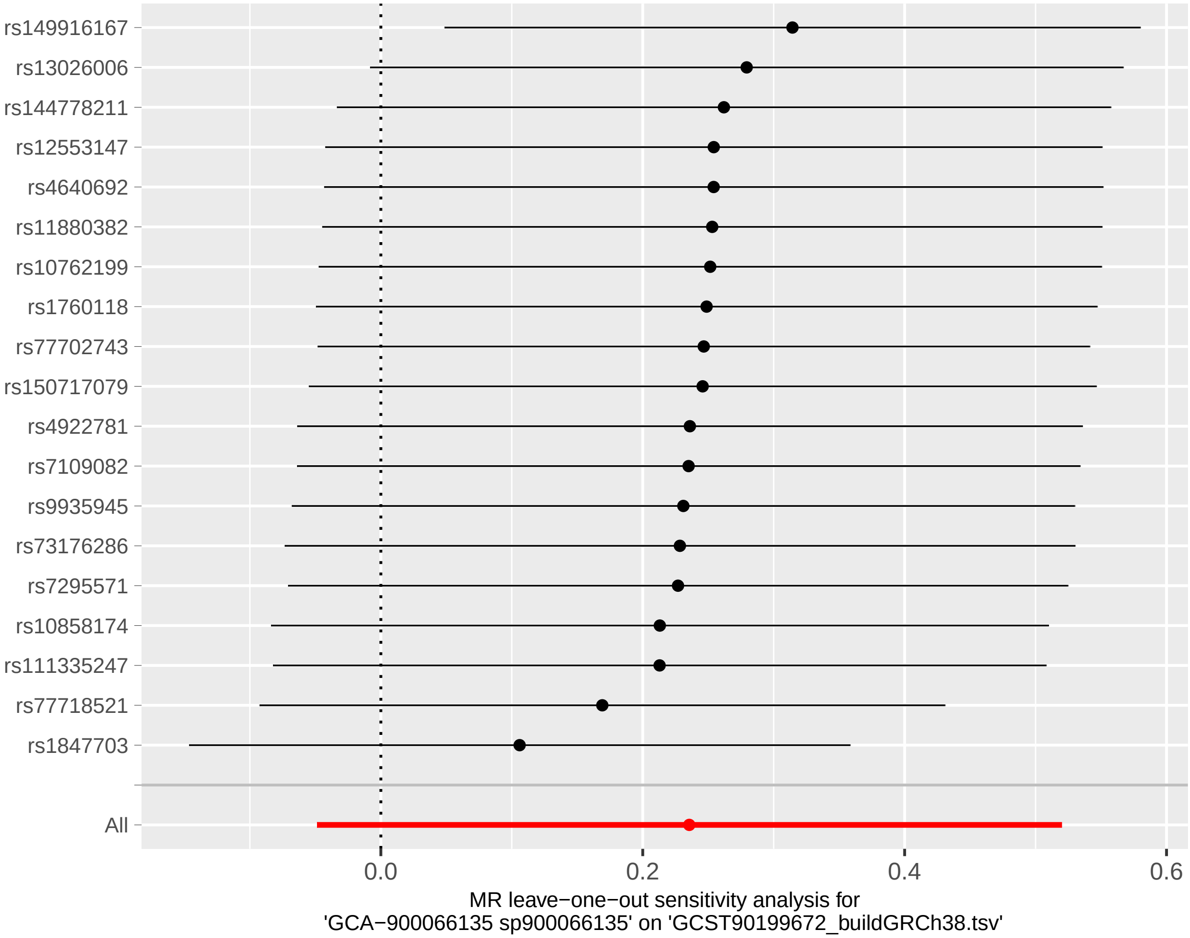

rs149916167
rs13026006
rs144778211
rs12553147
rs4640692
rs11880382
rs10762199
rs1760118
rs77702743
rs150717079
rs4922781
rs7109082
rs9935945
rs73176286
rs7295571
rs10858174
rs111335247
rs77718521
rs1847703
All
0.0
0.2
0.4
0.6
MR leave−one−out sensitivity analysis for
'GCA−900066135 sp900066135' on 'GCST90199672_buildGRCh38.tsv'

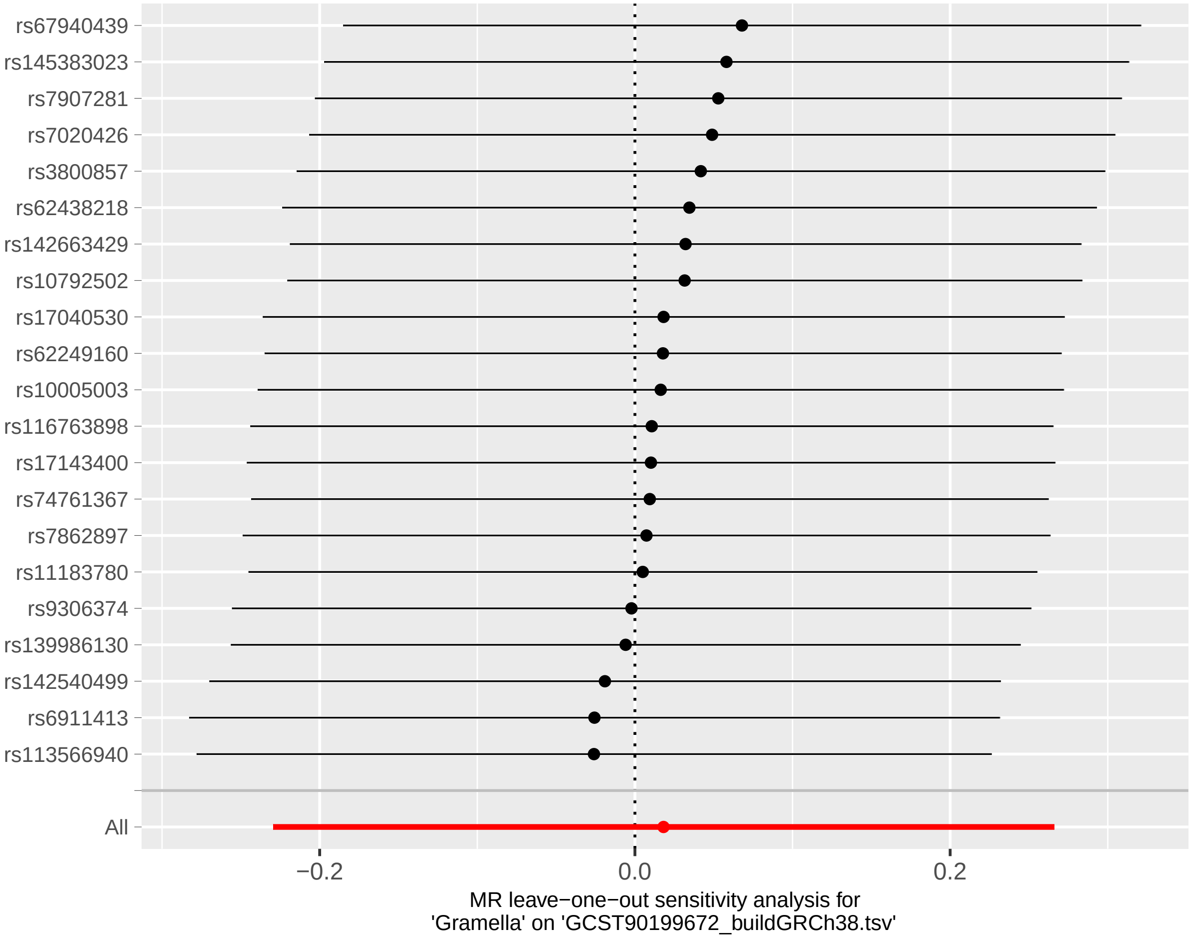
rs67940439
rs145383023
rs7907281
rs7020426
rs3800857
rs62438218
rs142663429
rs10792502
rs17040530
rs62249160
rs10005003
rs116763898
rs17143400
rs74761367
rs7862897
rs11183780
rs9306374
rs139986130
rs142540499
rs6911413
rs113566940
All
−0.2
0.0
0.2
MR leave−one−out sensitivity analysis for
'Gramella' on 'GCST90199672_buildGRCh38.tsv'

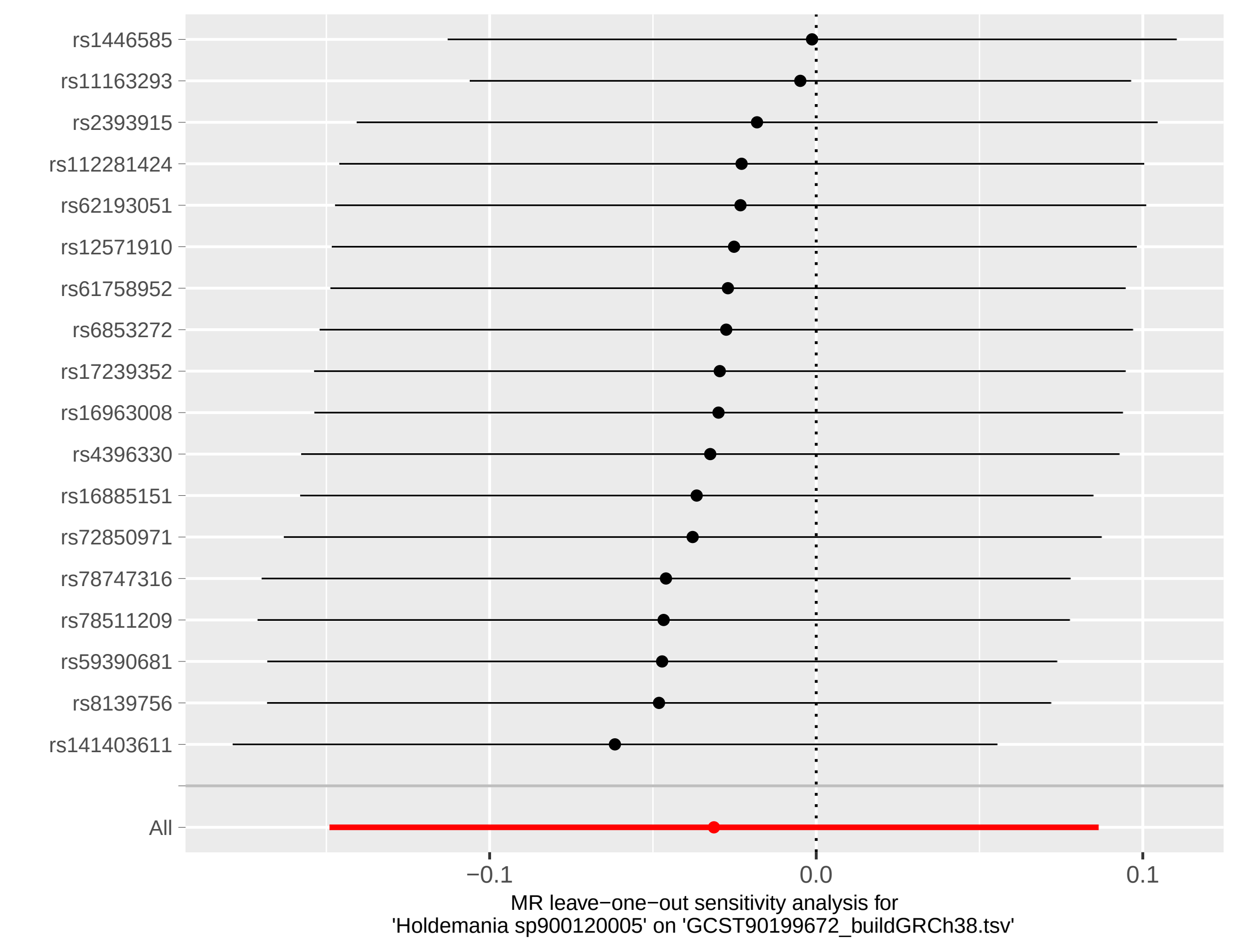

rs1446585
rs11163293
rs2393915
rs112281424
rs62193051
rs12571910
rs61758952
rs6853272
rs17239352
rs16963008
rs4396330
rs16885151
rs72850971
rs78747316
rs78511209
rs59390681
rs8139756
rs141403611
All
−0.1
0.0
0.1
MR leave−one−out sensitivity analysis for
'Holdemania sp900120005' on 'GCST90199672_buildGRCh38.tsv'

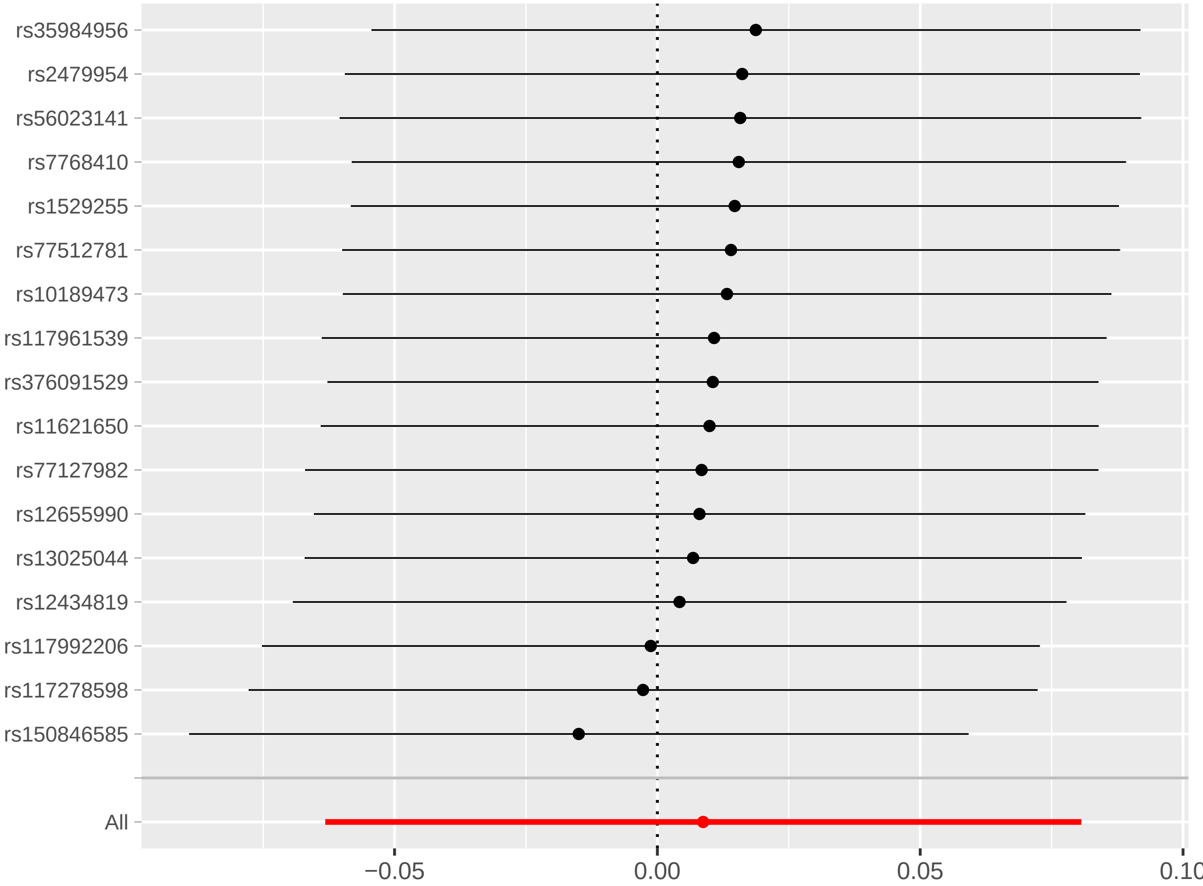

MR leave−one−out sensitivity analysis for
'Parabacteroides sp000436495' on 'GCST90199672_buildGRCh38.tsv'

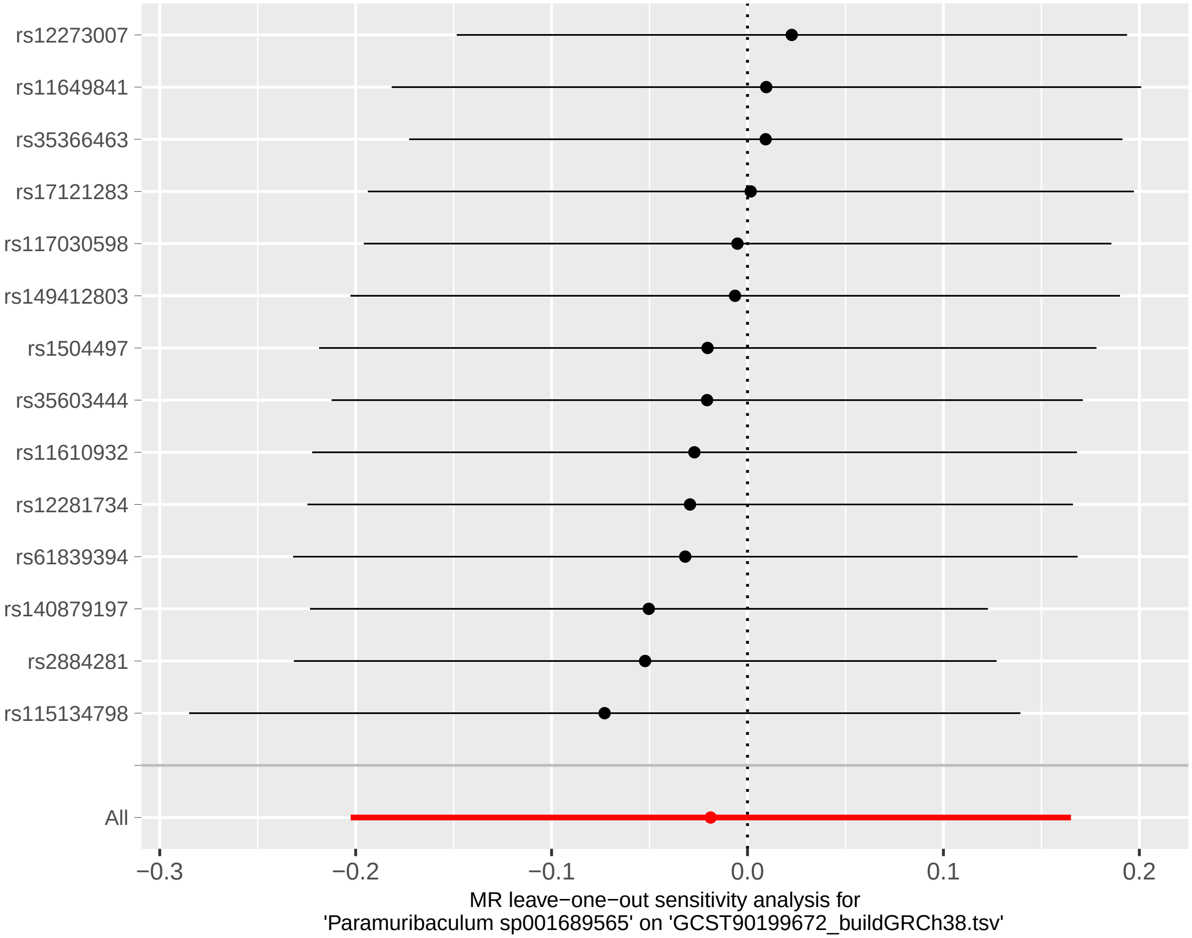

rs12273007
rs11649841
rs35366463
rs17121283
rs117030598
rs149412803
rs1504497
rs35603444
rs11610932
rs12281734
rs61839394
rs140879197
rs2884281
rs115134798
All
−0.3
−0.2
−0.1
0.0
0.1
0.2
MR leave−one−out sensitivity analysis for
'Paramuribaculum sp001689565' on 'GCST90199672_buildGRCh38.tsv'

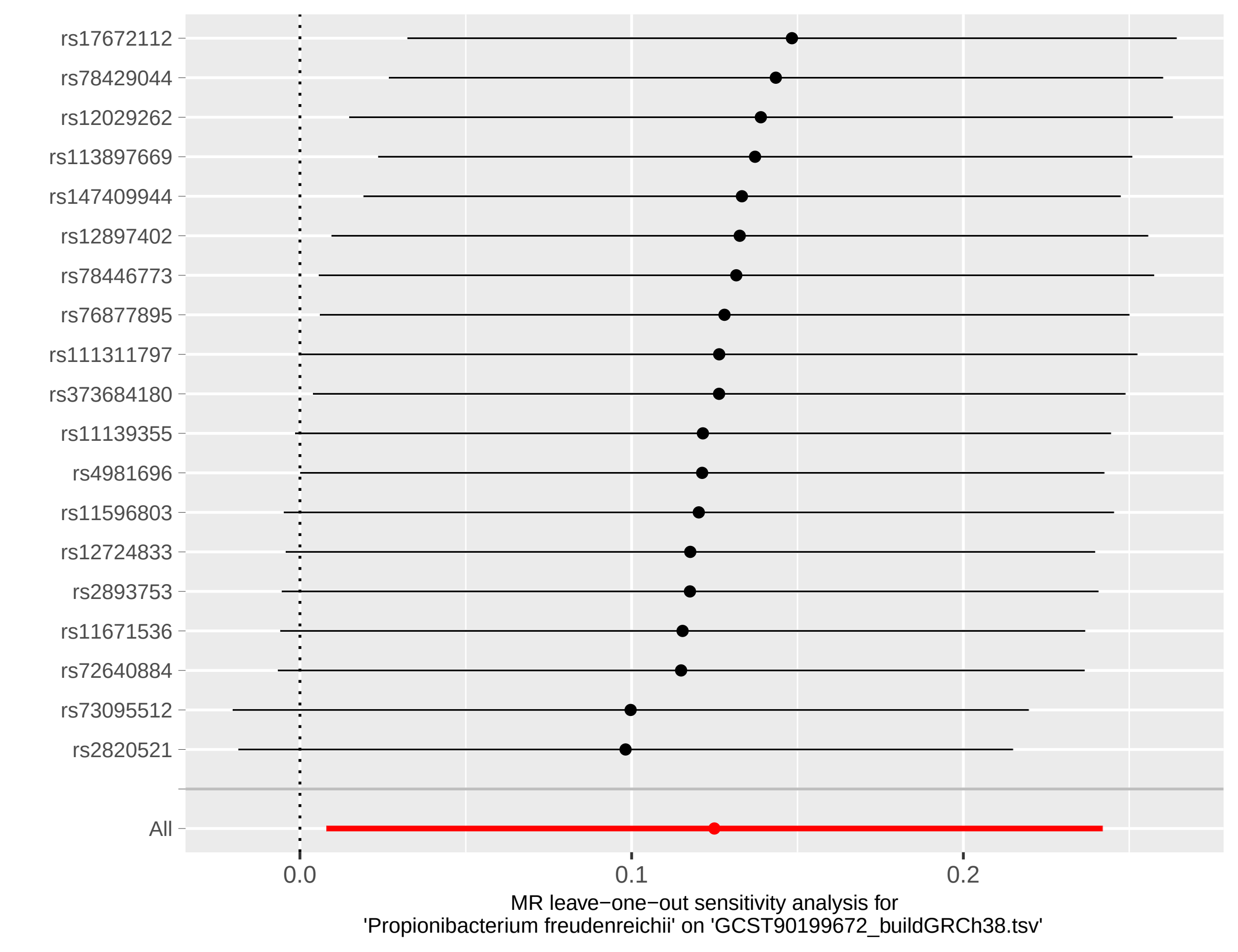

rs17672112
rs78429044
rs12029262
rs113897669
rs147409944
rs12897402
rs78446773
rs76877895
rs111311797
rs373684180
rs11139355
rs4981696
rs11596803
rs12724833
rs2893753
rs11671536
rs72640884
rs73095512
rs2820521
All
0.0
0.1
0.2
MR leave−one−out sensitivity analysis for
'Propionibacterium freudenreichii' on 'GCST90199672_buildGRCh38.tsv'

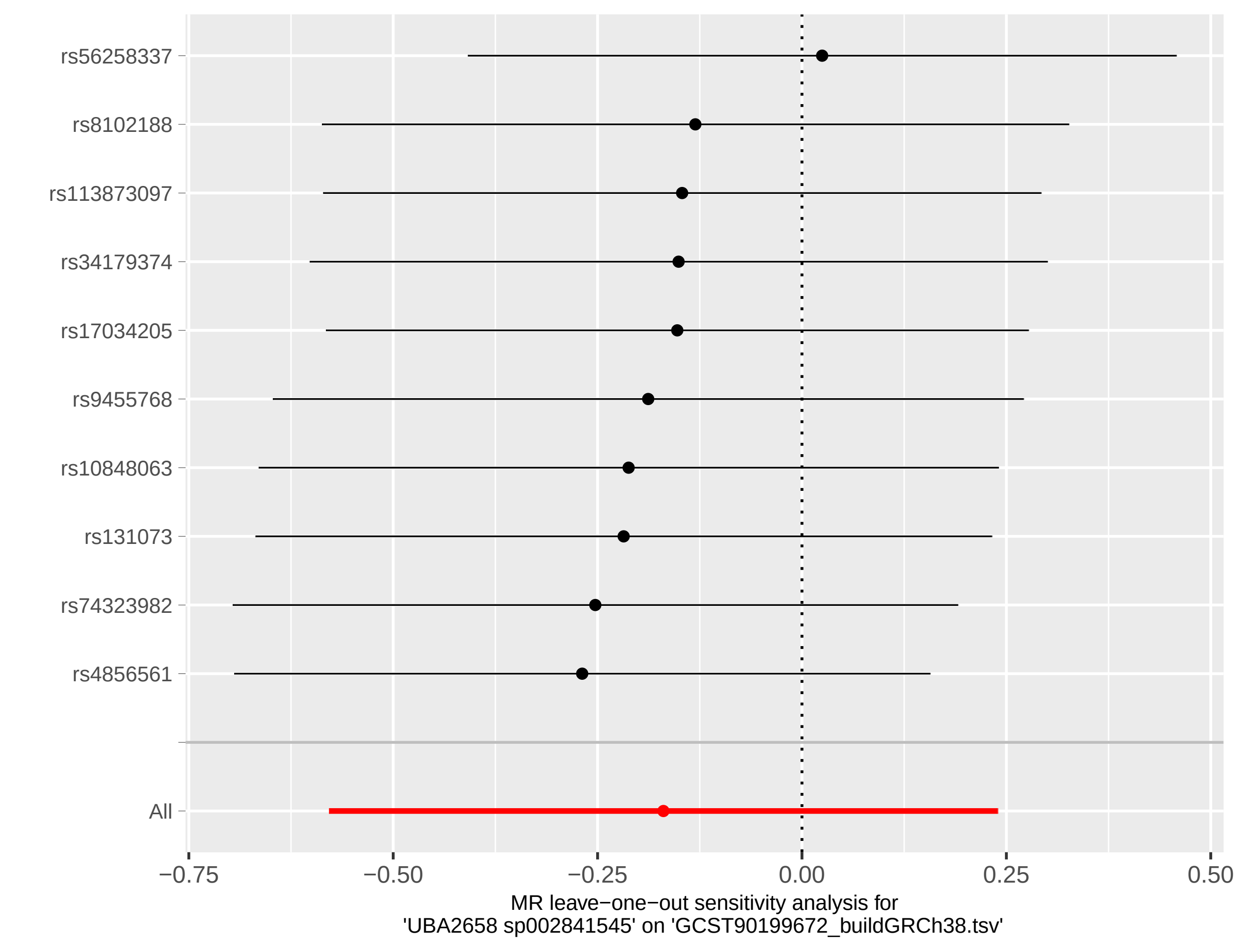

rs56258337
rs8102188
rs113873097
rs34179374
rs17034205
rs9455768
rs10848063
rs131073
rs74323982
rs4856561
All
−0.75
−0.50
−0.25
0.00
0.25
0.50
MR leave−one−out sensitivity analysis for
'UBA2658 sp002841545' on 'GCST90199672_buildGRCh38.tsv'

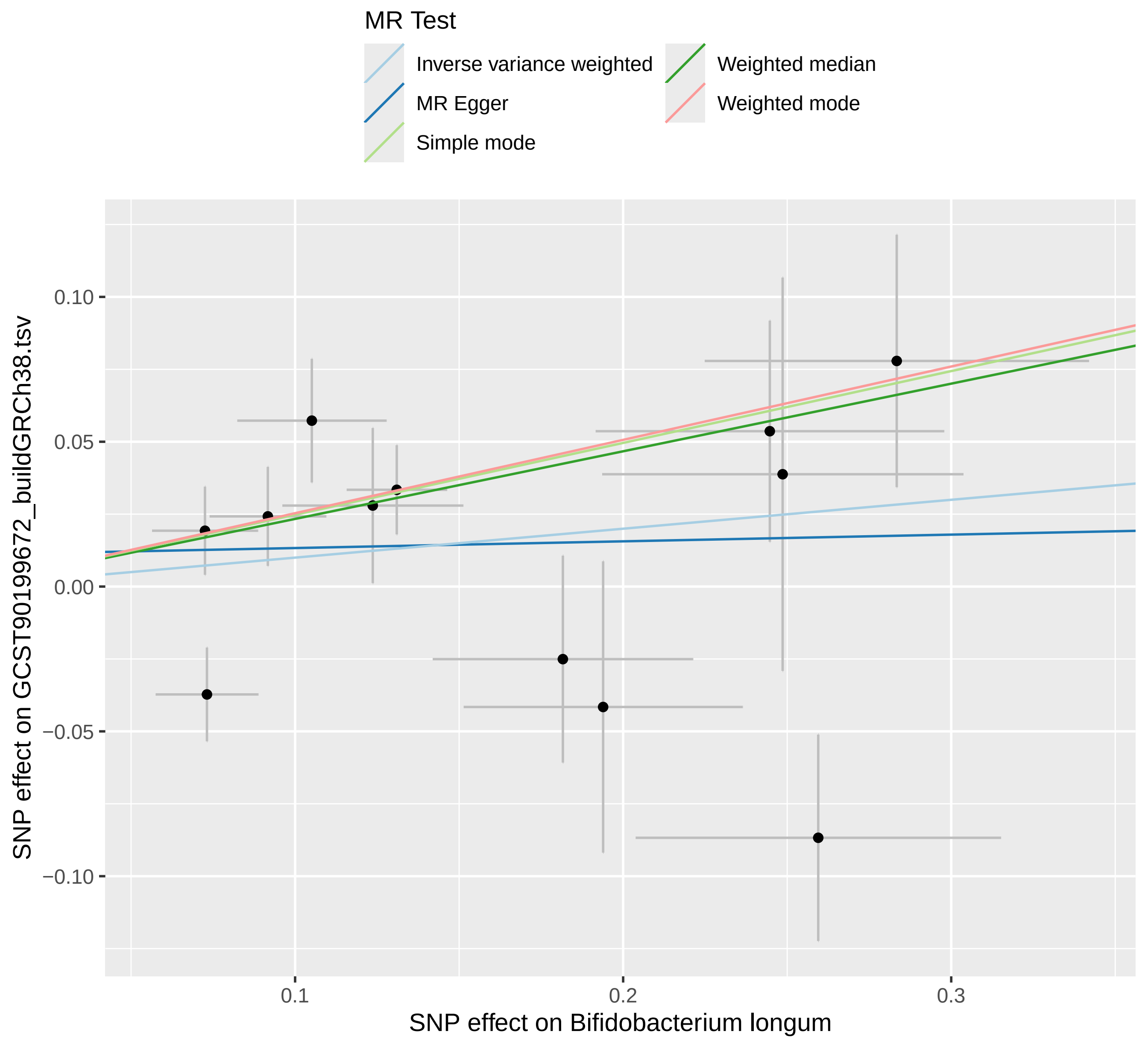

MR Test
Inverse variance weighted
MR Egger
Simple mode
Weighted median
Weighted mode
0.10
0.05
0.00
−0.05
−0.10
SNP effect on GCST90199672_buildGRCh38.tsv
0.1
0.2
0.3
SNP effect on Bifidobacterium longum

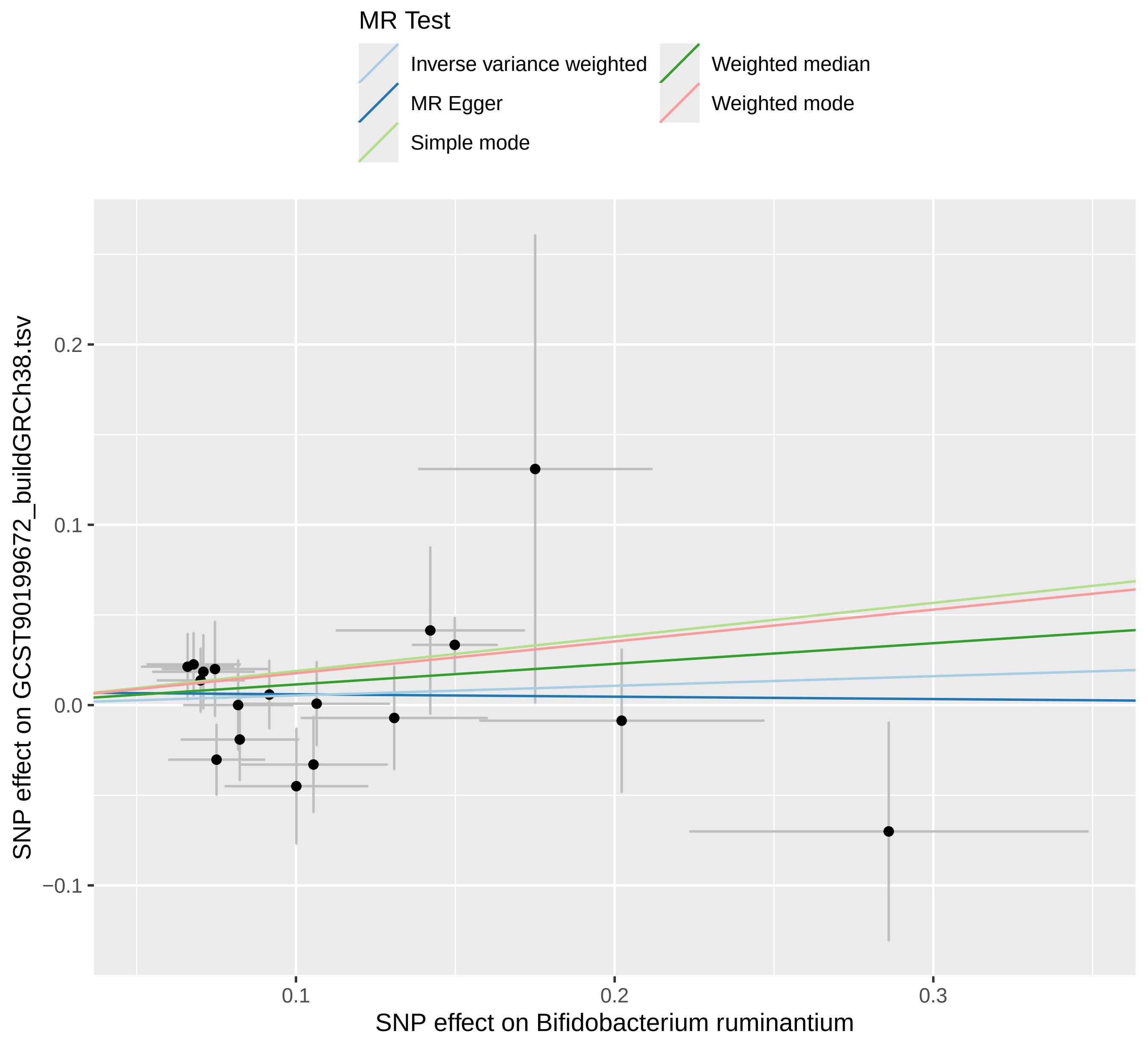
MR Test
Inverse variance weighted
MR Egger
Simple mode
Weighted median
Weighted mode
0.2
0.1
0.0
−0.1
0.1
0.2
0.3
SNP effect on GCST90199672_buildGRCh38.tsv
SNP effect on Bifidobacterium ruminantium

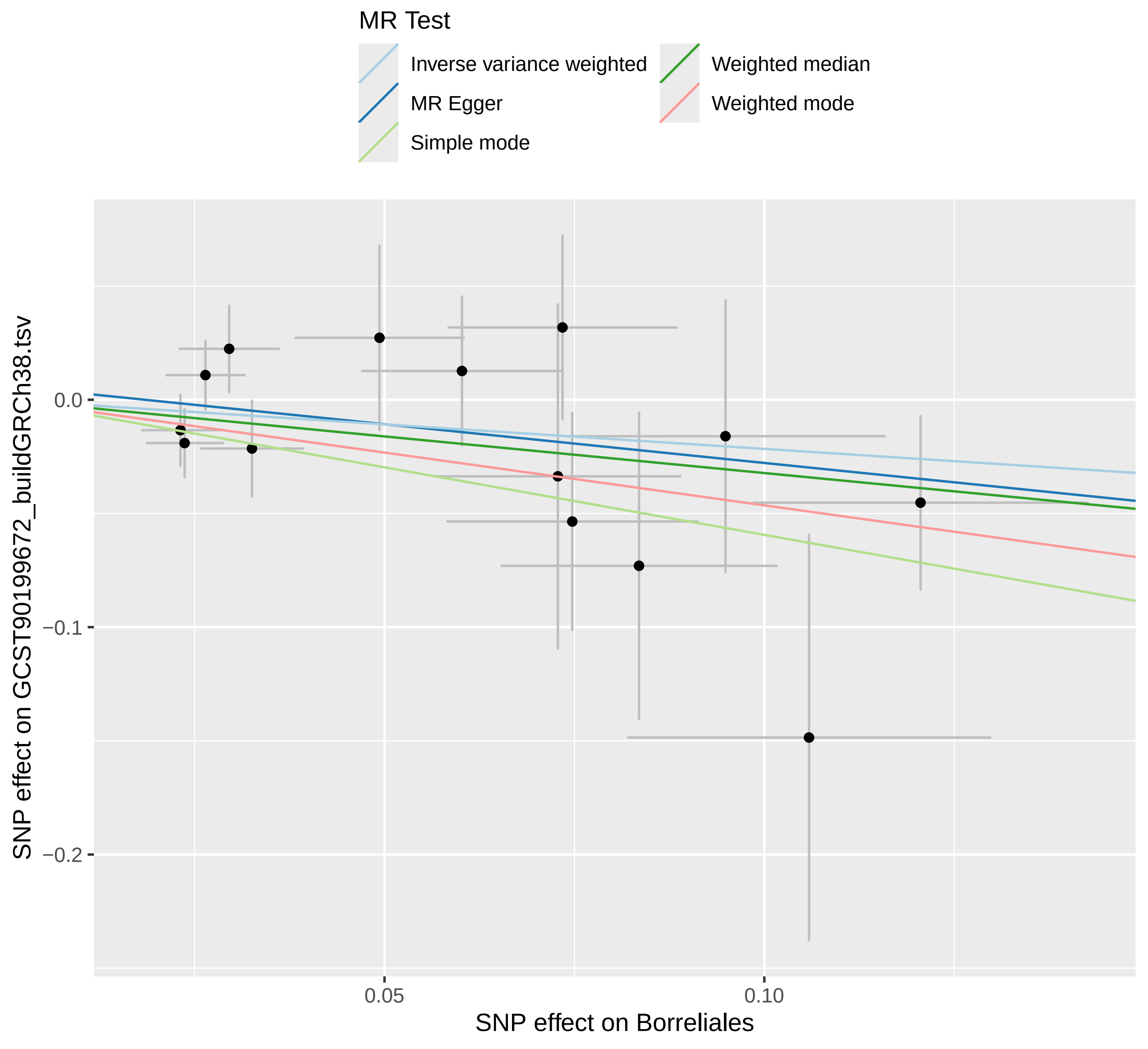
MR Test
Inverse variance weighted
MR Egger
Simple mode
Weighted median
Weighted mode
SNP effect on GCST90199672_buildGRCh38.tsv
0.0
−0.1
−0.2
0.05
0.10
SNP effect on Borreliales

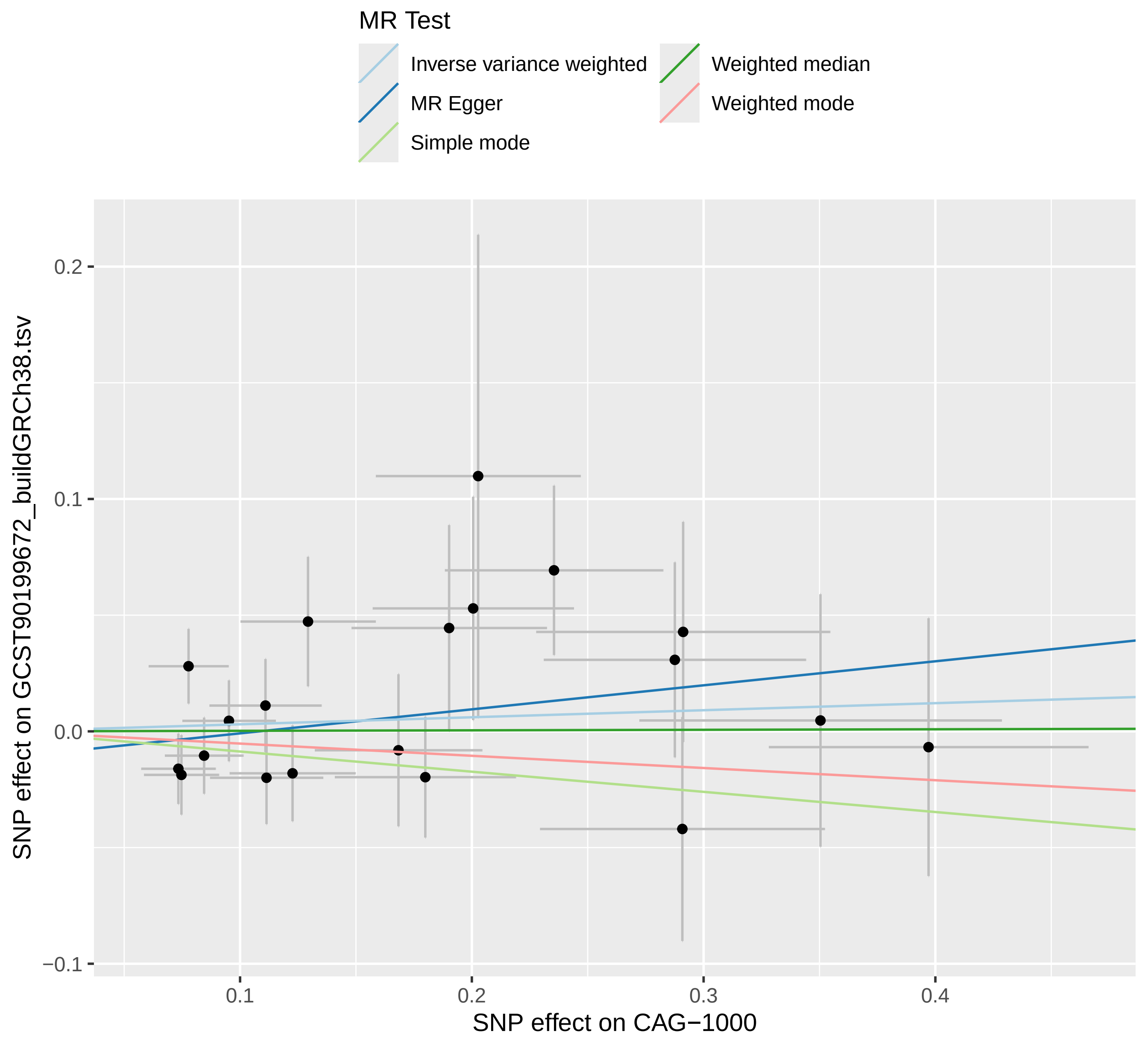
MR Test
Inverse variance weighted
MR Egger
Simple mode
Weighted median
Weighted mode
0.2
0.1
0.0
−0.1
0.1
0.2
0.3
0.4
SNP effect on GCST90199672_buildGRCh38.tsv
SNP effect on CAG−1000

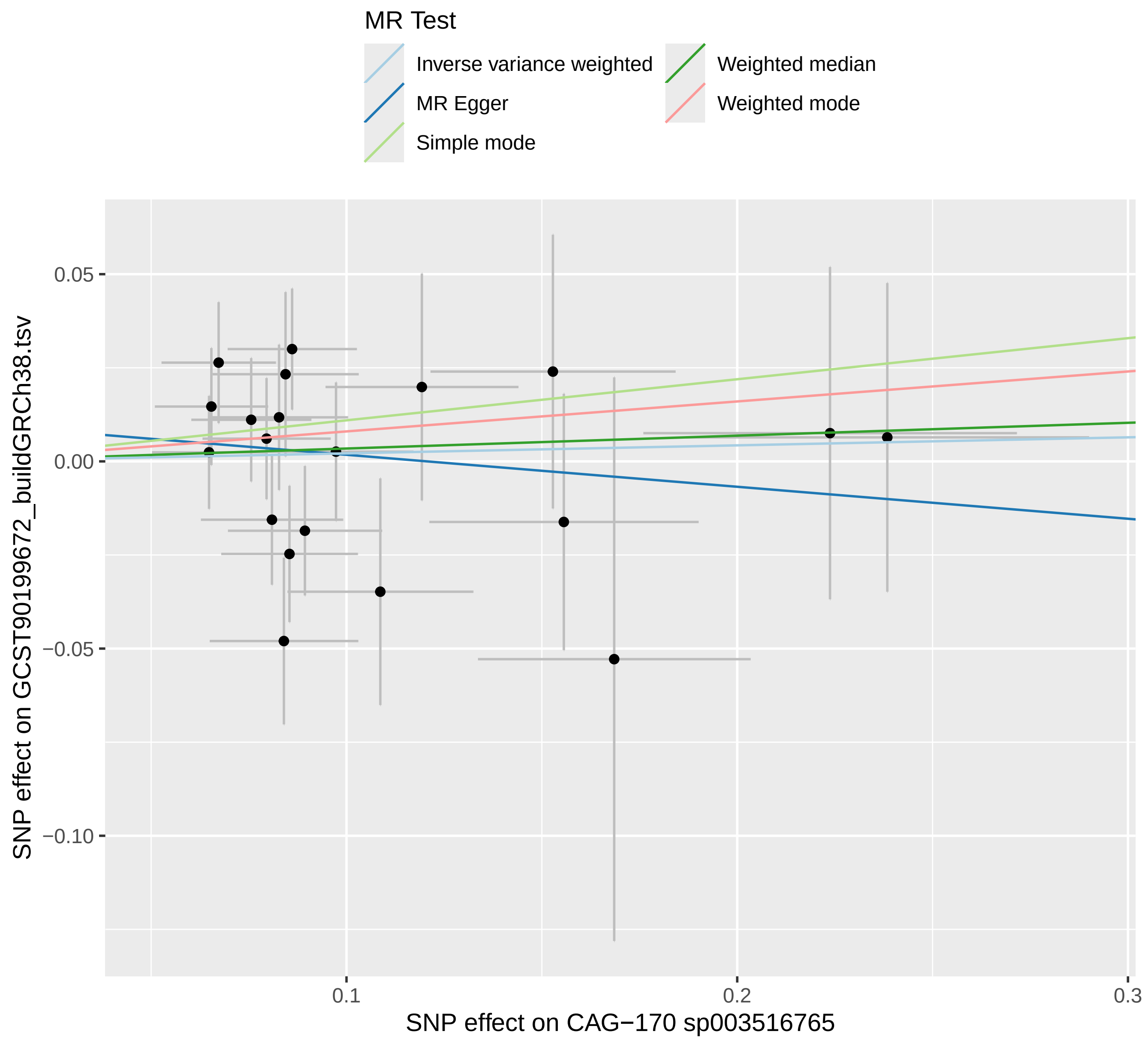
MR Test
Inverse variance weighted
MR Egger
Simple mode
Weighted median
Weighted mode
0.05
0.00
−0.05
−0.10
0.1
0.2
0.3
SNP effect on GCST90199672_buildGRCh38.tsv
SNP effect on CAG−170 sp003516765

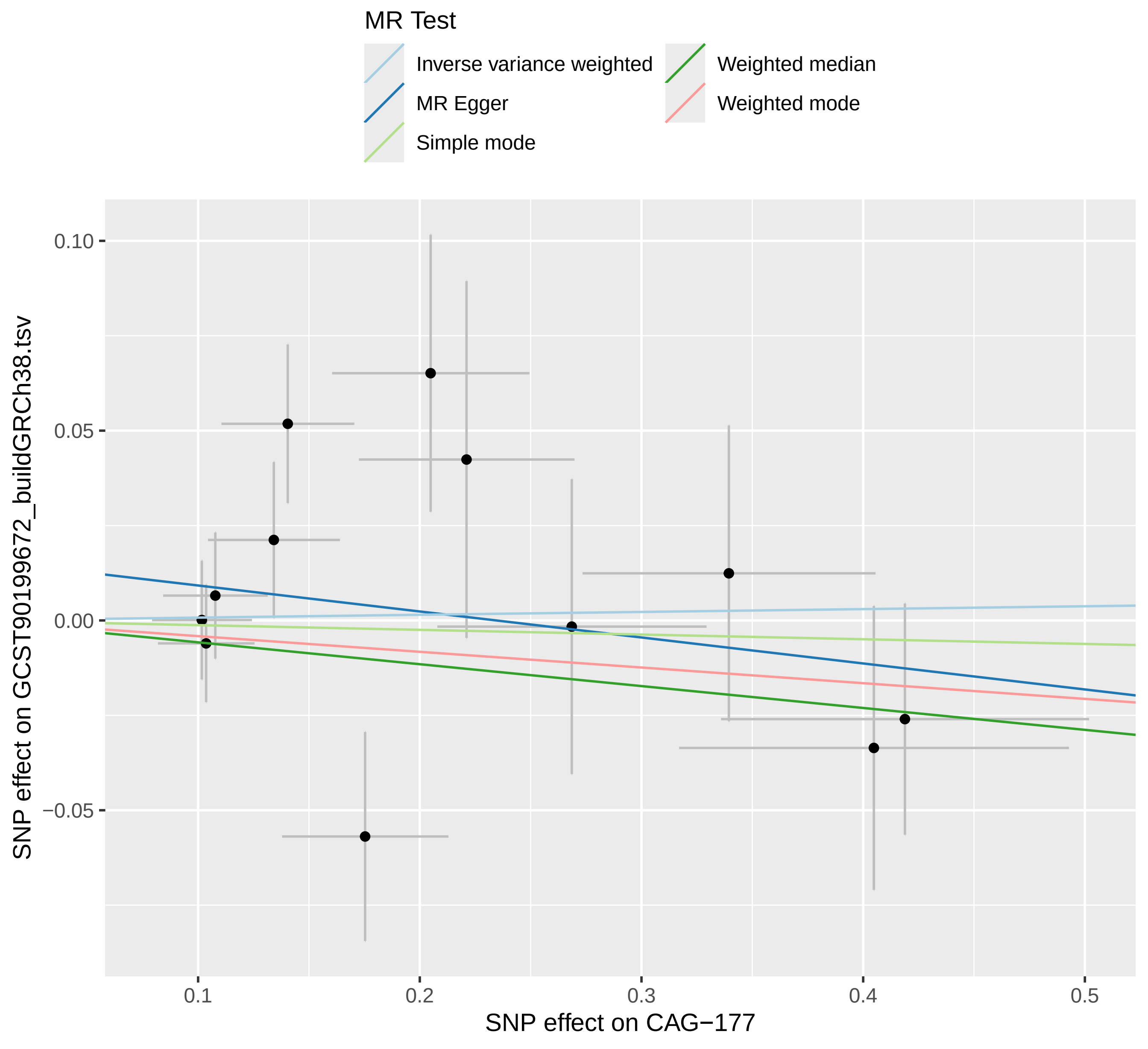

MR Test
Inverse variance weighted
MR Egger
Simple mode
Weighted median
Weighted mode
0.10
0.05
0.00
−0.05
0.1
0.2
0.3
0.4
0.5
SNP effect on GCST90199672_buildGRCh38.tsv
SNP effect on CAG−177

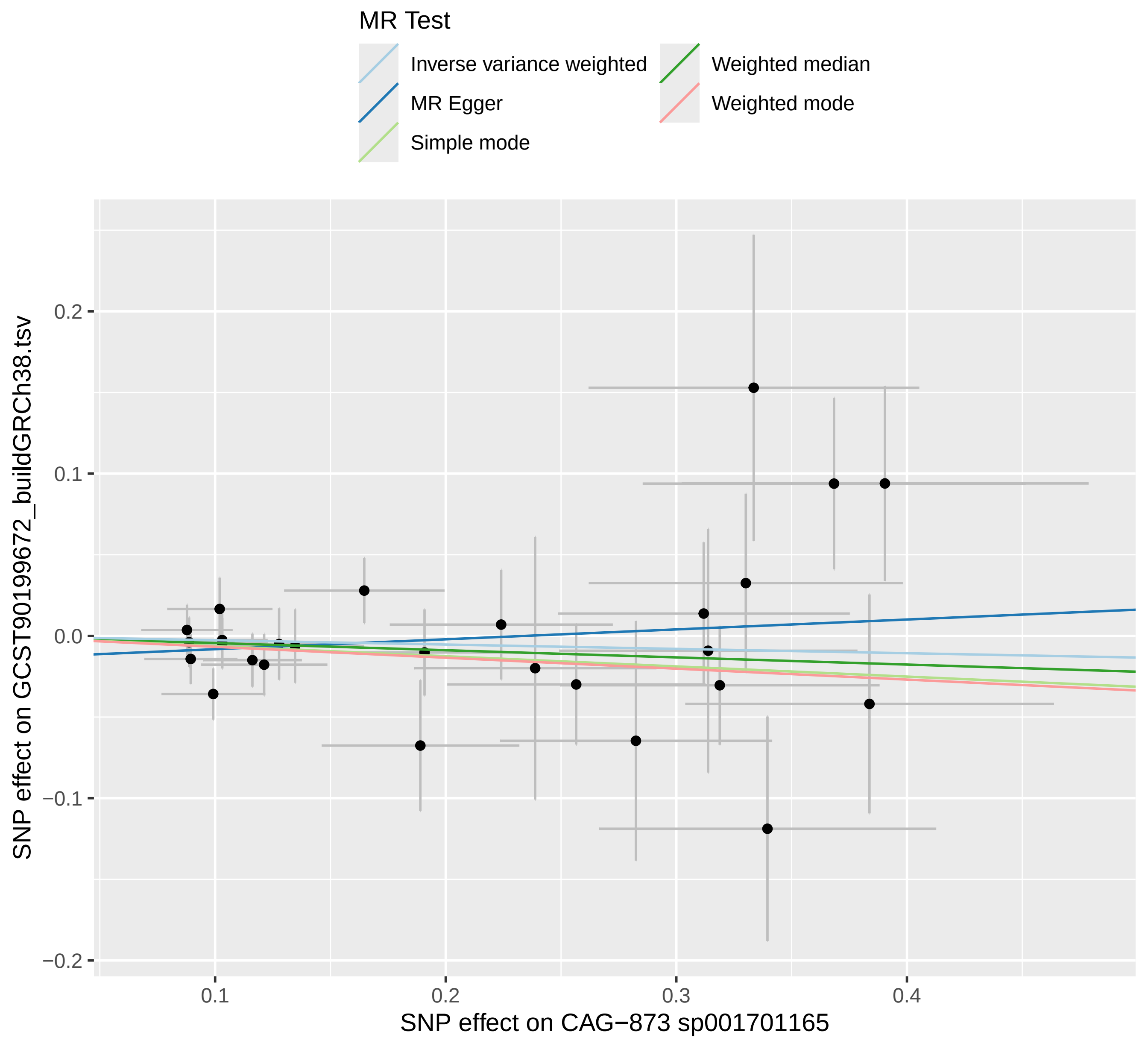
MR Test
Inverse variance weighted
MR Egger
Simple mode
Weighted median
Weighted mode
0.2
0.1
0.0
−0.1
−0.2
0.1
0.2
0.3
0.4
SNP effect on GCST90199672_buildGRCh38.tsv
SNP effect on CAG−873 sp001701165

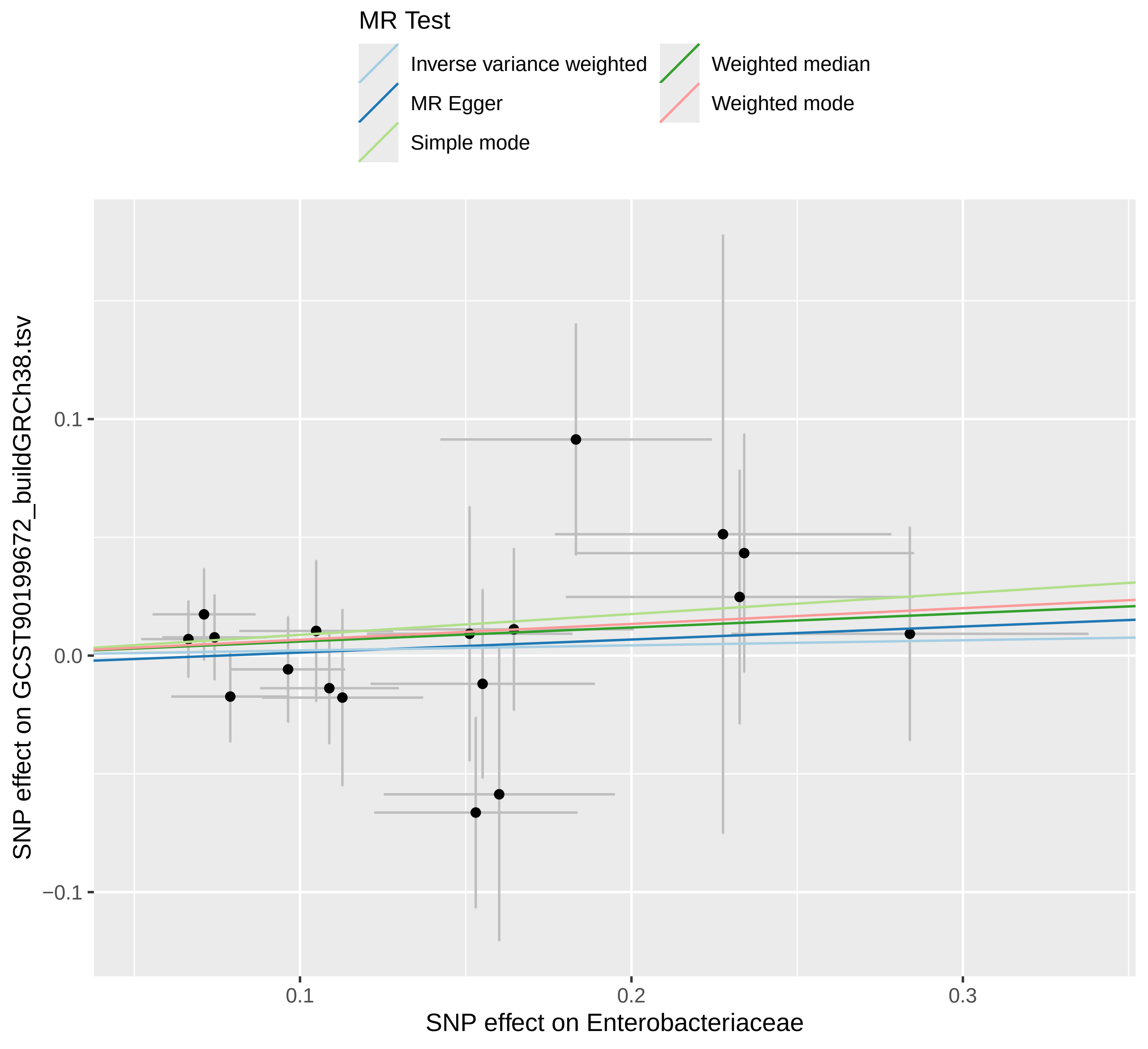
MR Test
Inverse variance weighted
MR Egger
Simple mode
Weighted median
Weighted mode
SNP effect on GCST90199672_buildGRCh38.tsv
0.1
0.0
−0.1
0.1
0.2
0.3
SNP effect on Enterobacteriaceae

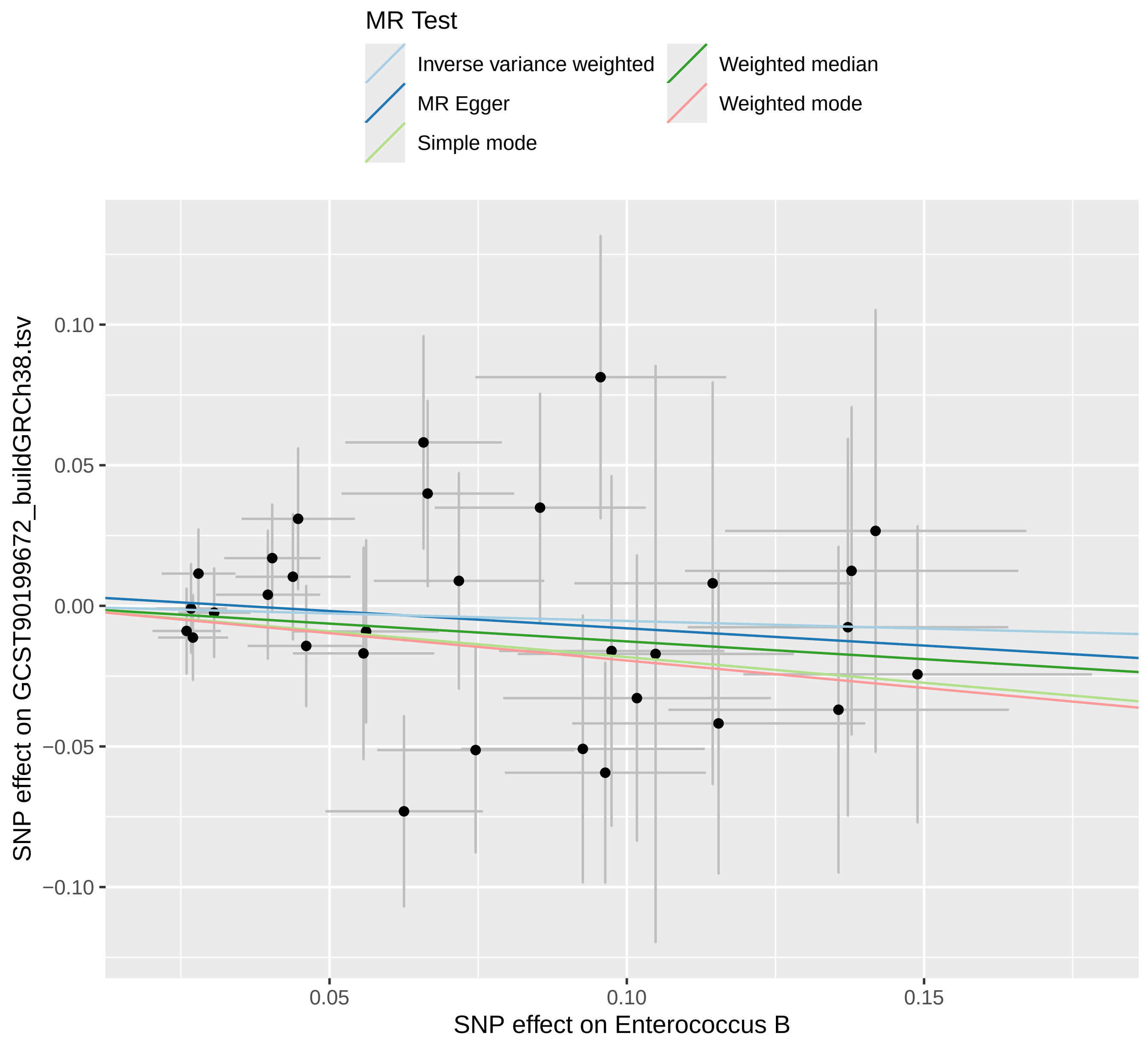
MR Test
Inverse variance weighted
MR Egger
Simple mode
Weighted median
Weighted mode
0.10
0.05
0.00
−0.05
−0.10
0.05
0.10
0.15
SNP effect on GCST90199672_buildGRCh38.tsv
SNP effect on Enterococcus B

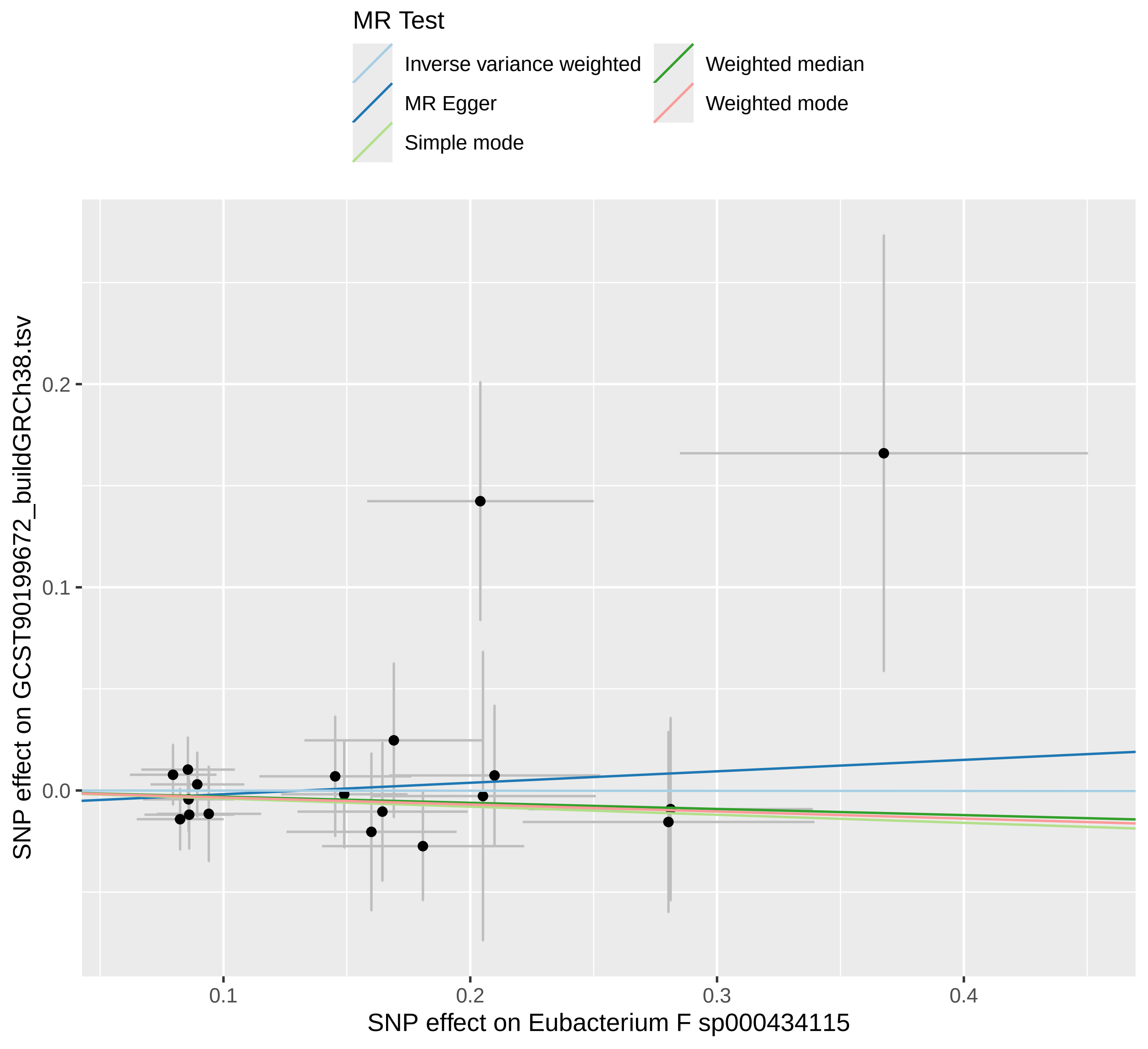
MR Test
Inverse variance weighted
MR Egger
Simple mode
Weighted median
Weighted mode
SNP effect on GCST90199672_buildGRCh38.tsv
0.2
0.1
0.0
0.1
0.2
0.3
0.4
SNP effect on Eubacterium F sp000434115

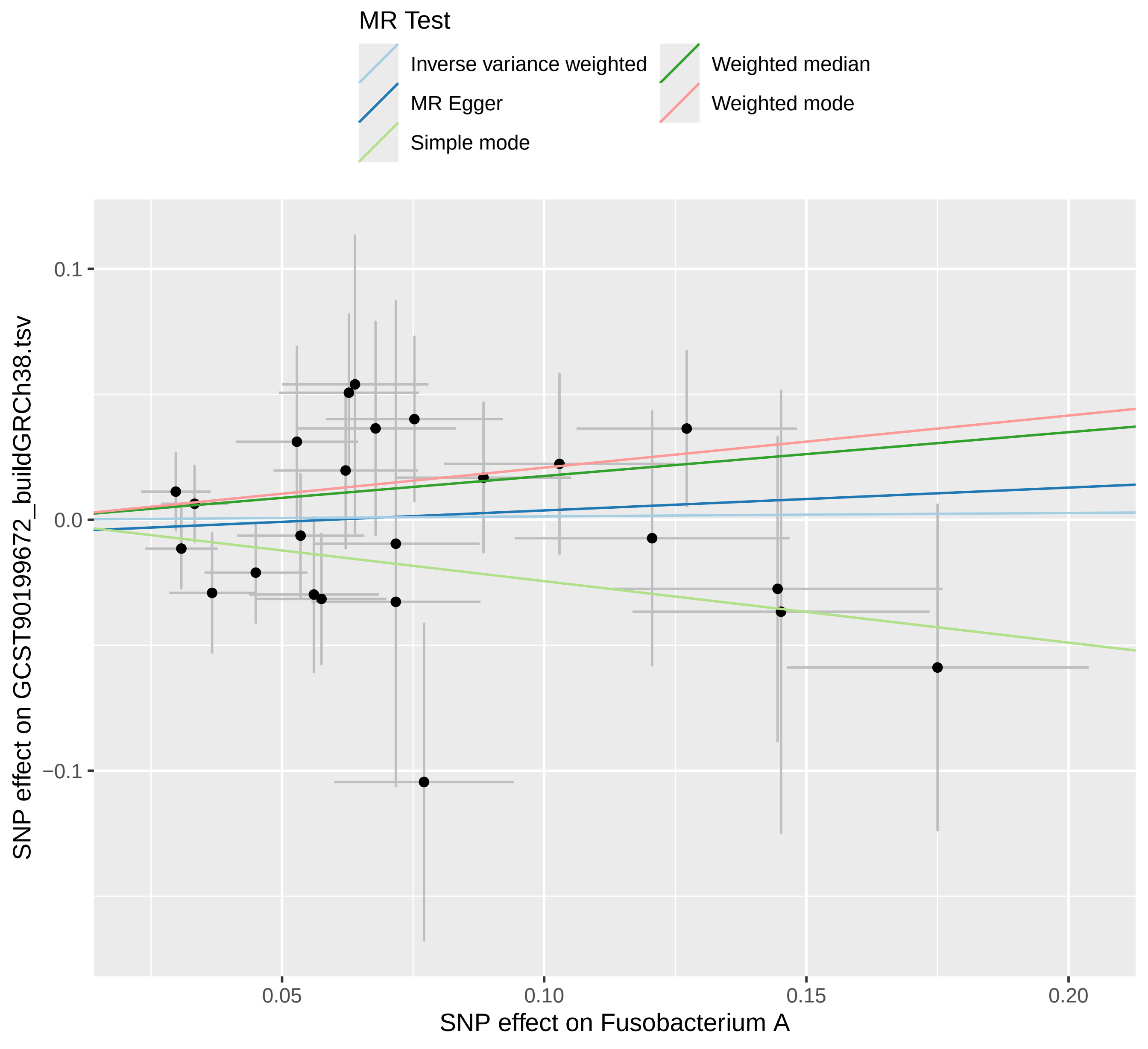

MR Test
Inverse variance weighted
MR Egger
Simple mode
Weighted median
Weighted mode
0.1
0.0
−0.1
0.05
0.10
0.15
0.20
SNP effect on GCST90199672_buildGRCh38.tsv
SNP effect on Fusobacterium A

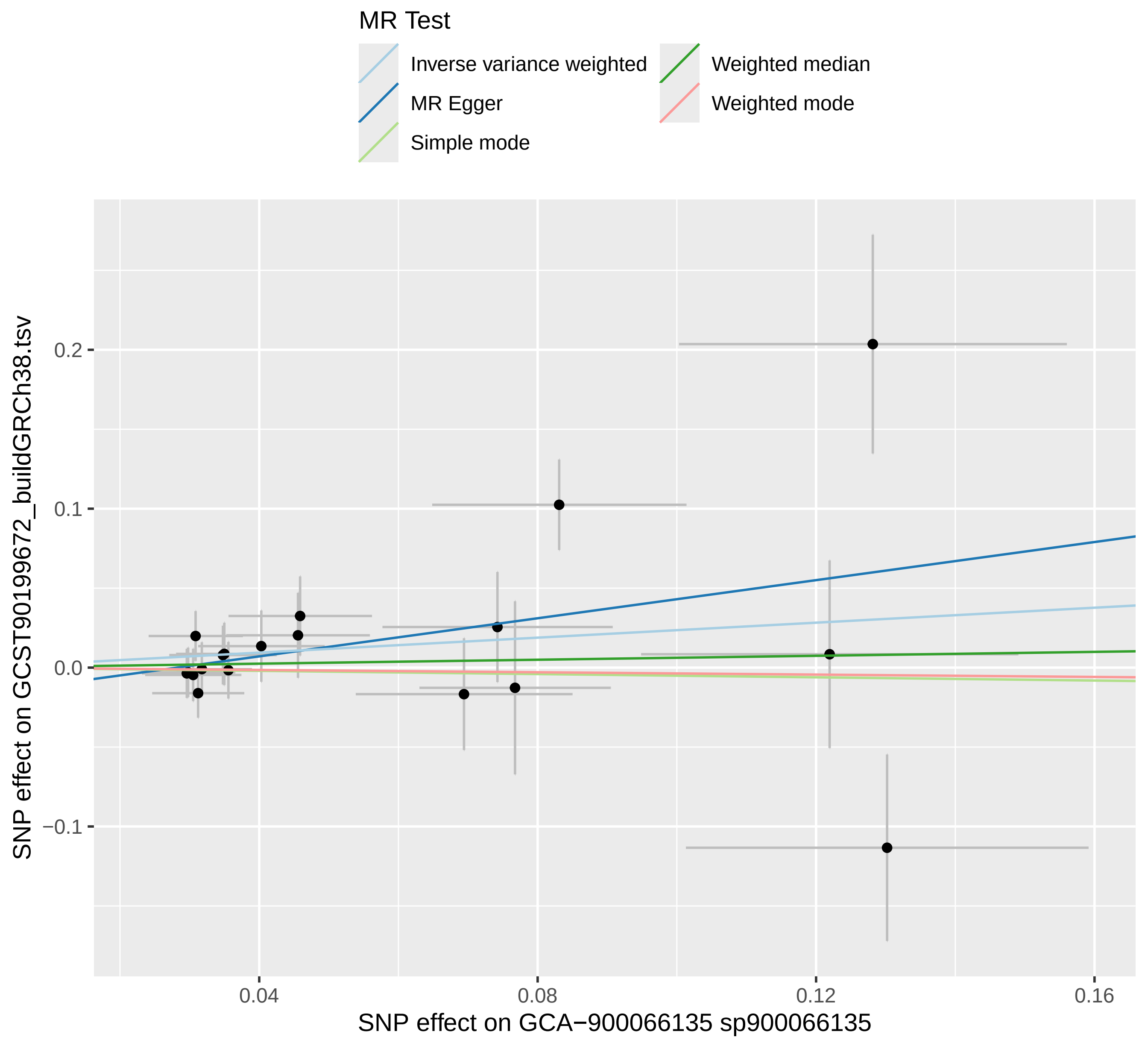
MR Test
Inverse variance weighted
MR Egger
Simple mode
Weighted median
Weighted mode
0.2
0.1
0.0
−0.1
0.04
0.08
0.12
0.16
SNP effect on GCST90199672_buildGRCh38.tsv
SNP effect on GCA−900066135 sp900066135

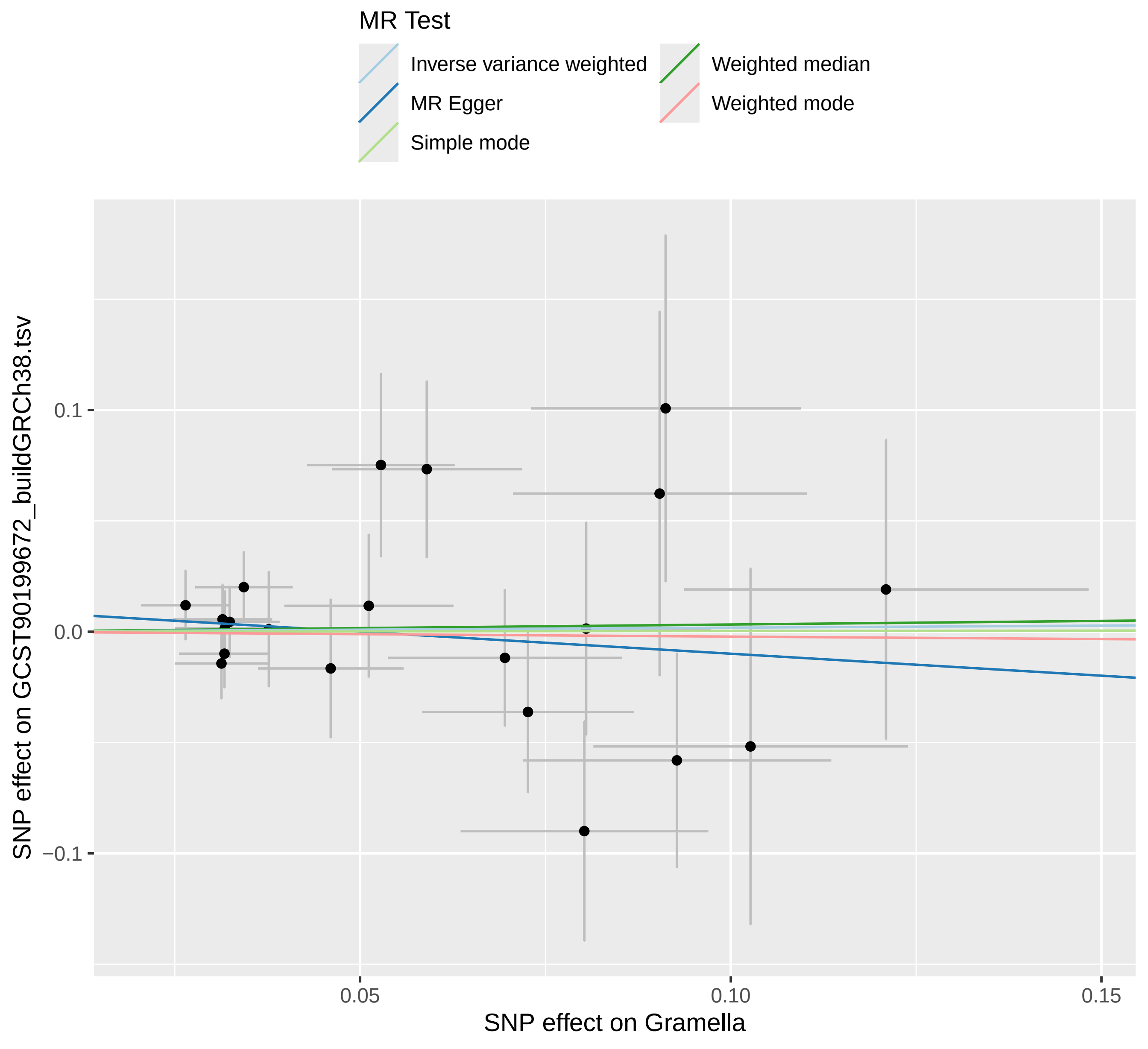

MR Test
Inverse variance weighted
MR Egger
Simple mode
Weighted median
Weighted mode
SNP effect on GCST90199672_buildGRCh38.tsv
0.1
0.0
−0.1
0.05
0.10
0.15
SNP effect on Gramella

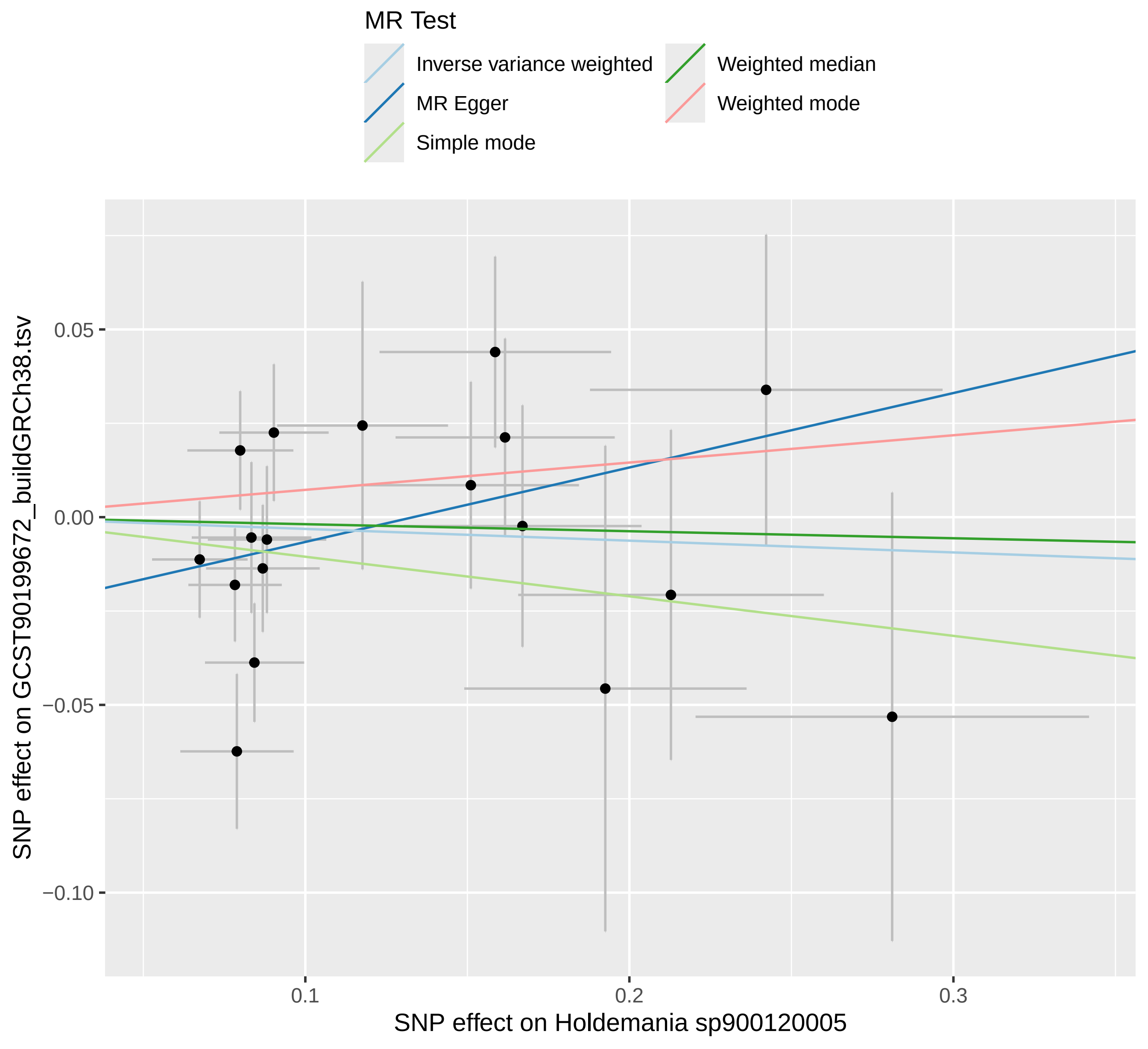

MR Test
Inverse variance weighted
MR Egger
Simple mode
Weighted median
Weighted mode
0.05
0.00
−0.05
−0.10
0.1
0.2
0.3
SNP effect on GCST90199672_buildGRCh38.tsv
SNP effect on Holdemania sp900120005

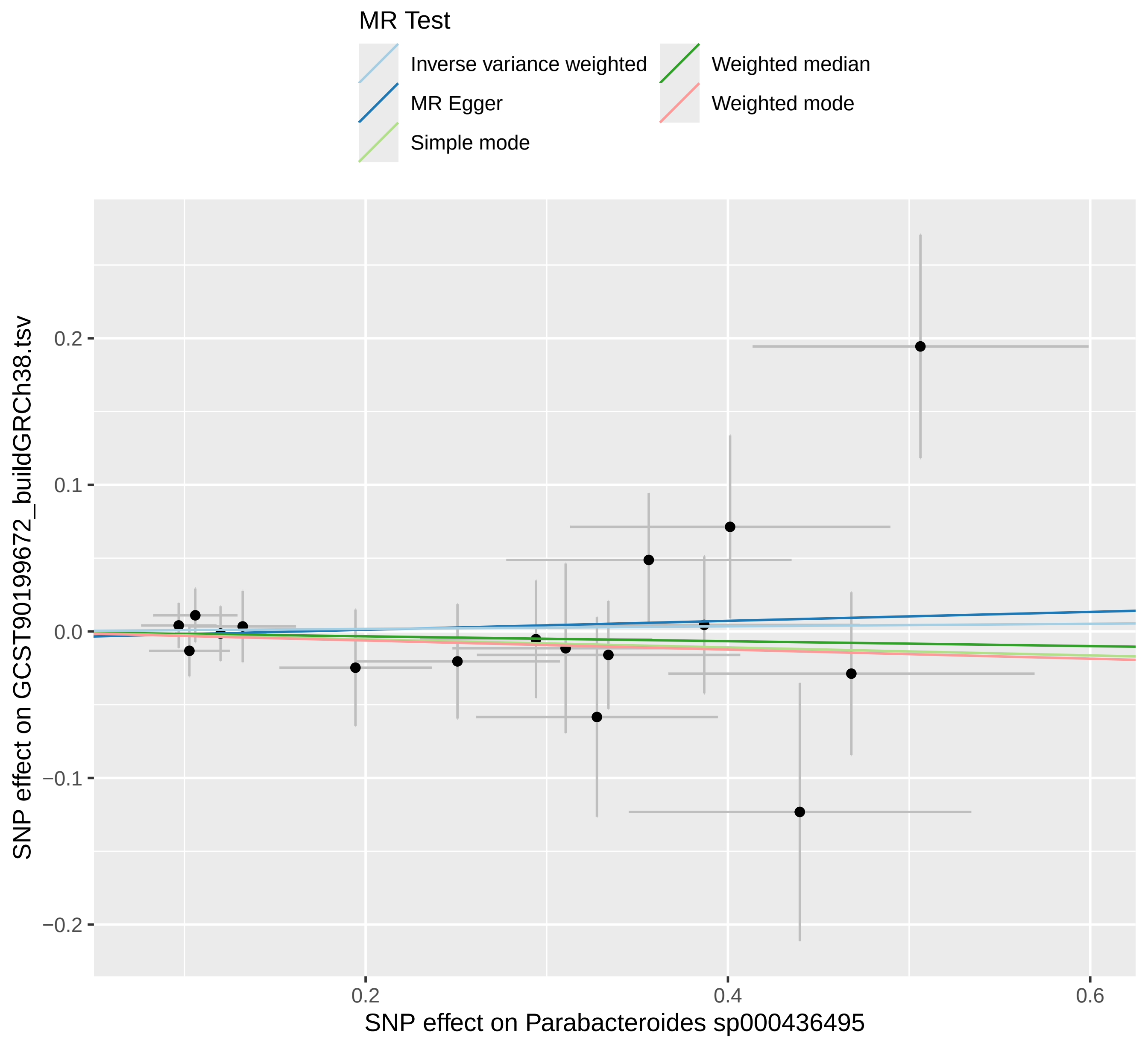
MR Test
Inverse variance weighted
MR Egger
Simple mode
Weighted median
Weighted mode
SNP effect on GCST90199672_buildGRCh38.tsv
0.2
0.1
0.0
−0.1
−0.2
0.2
0.4
0.6
SNP effect on Parabacteroides sp000436495

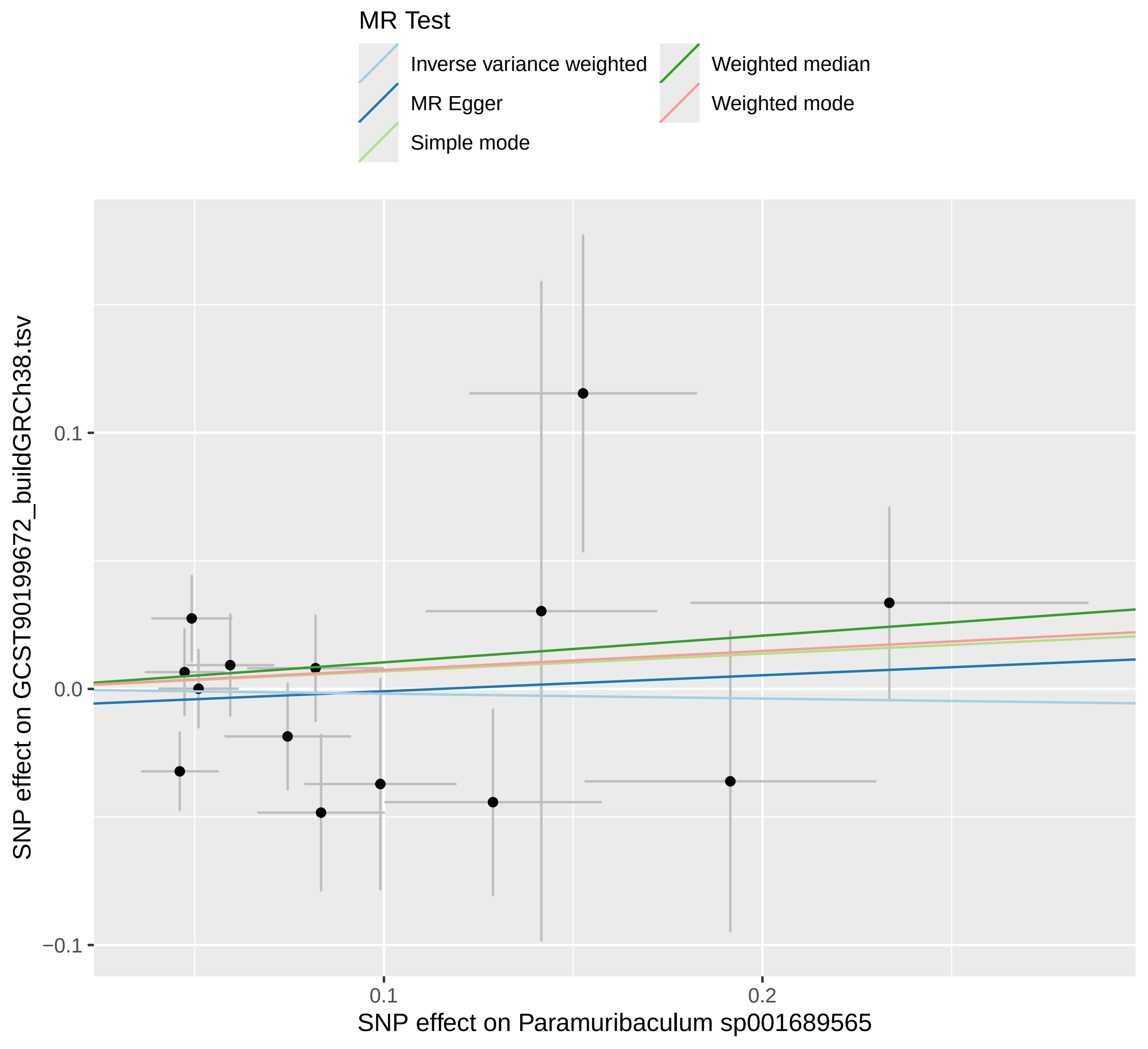
MR Test
Inverse variance weighted
MR Egger
Simple mode
Weighted median
Weighted mode
SNP effect on GCST90199672_buildGRCh38.tsv
0.1
0.0
−0.1
0.1
0.2
SNP effect on Paramuribaculum sp001689565

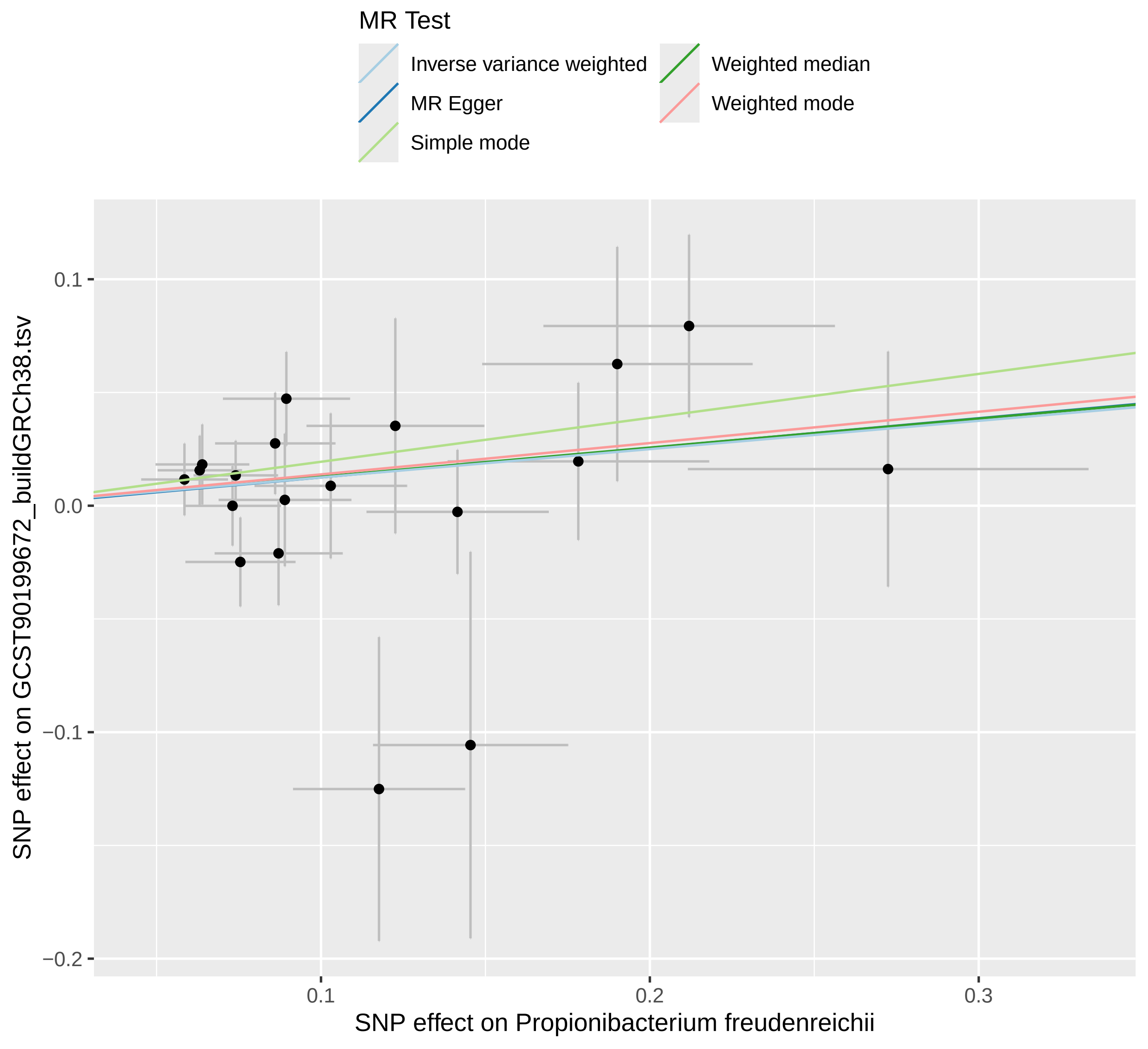

MR Test
Inverse variance weighted
MR Egger
Simple mode
Weighted median
Weighted mode
0.1
0.0
−0.1
−0.2
0.1
0.2
0.3
SNP effect on GCST90199672_buildGRCh38.tsv
SNP effect on Propionibacterium freudenreichii

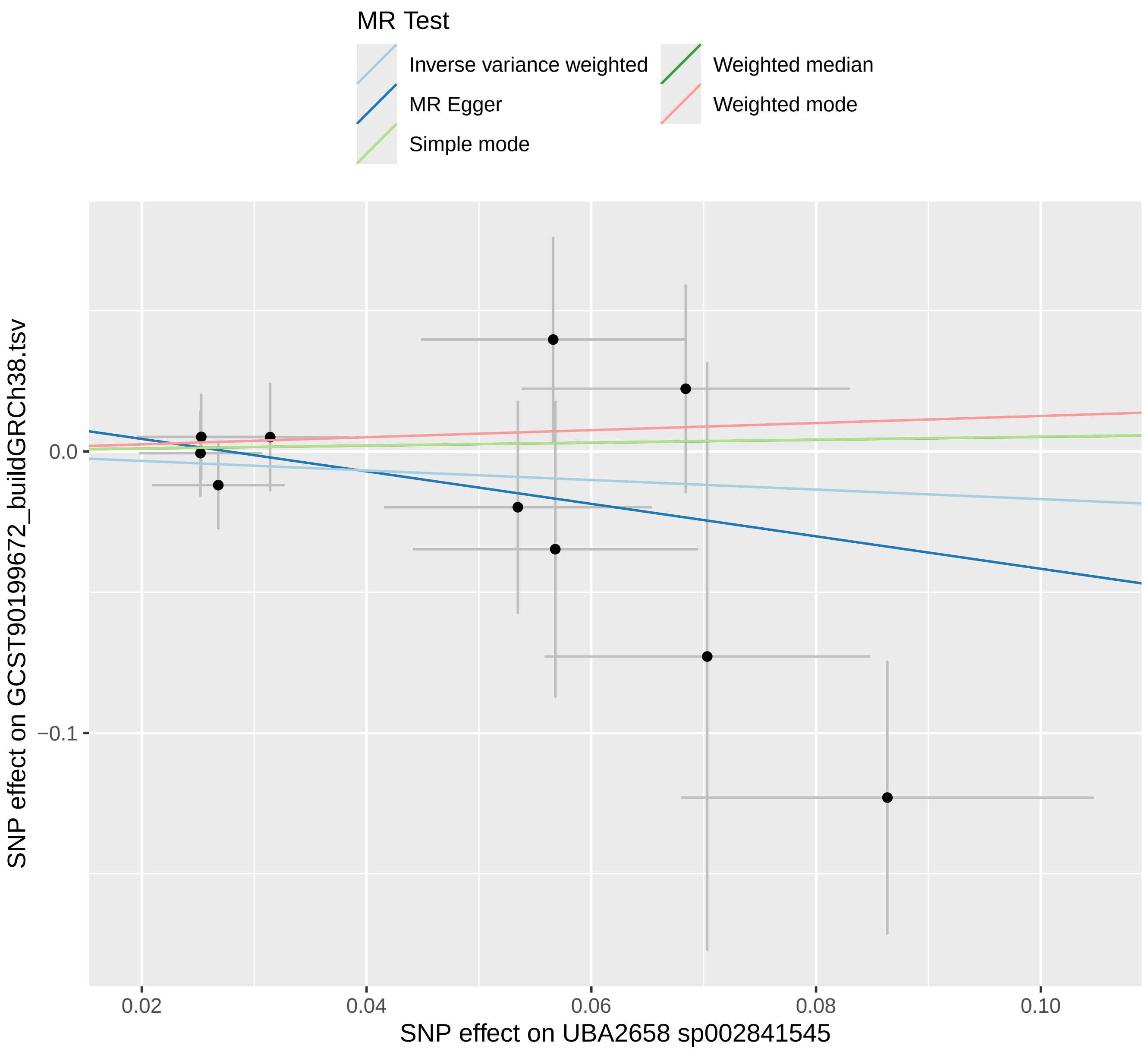

MR Test
Inverse variance weighted
MR Egger
Simple mode
Weighted median
Weighted mode
0.0
−0.1
0.02
0.04
0.06
0.08
0.10
SNP effect on GCST90199672_buildGRCh38.tsv
SNP effect on UBA2658 sp002841545

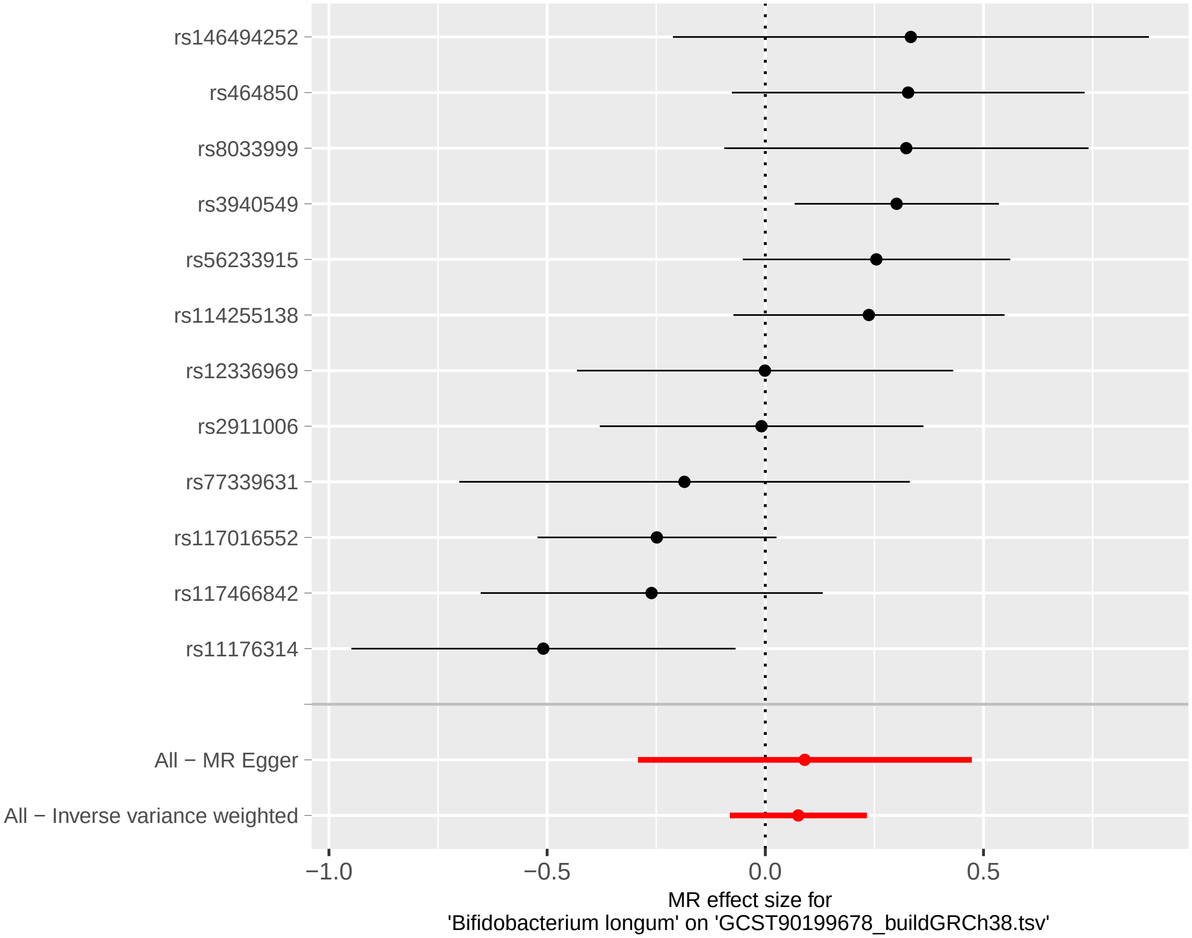
rs146494252
rs464850
rs8033999
rs3940549
rs56233915
rs114255138
rs12336969
rs2911006
rs77339631
rs117016552
rs117466842
rs11176314
All − MR Egger
All − Inverse variance weighted
−1.0
−0.5
0.0
0.5
MR effect size for
'Bifidobacterium longum' on 'GCST90199678_buildGRCh38.tsv'

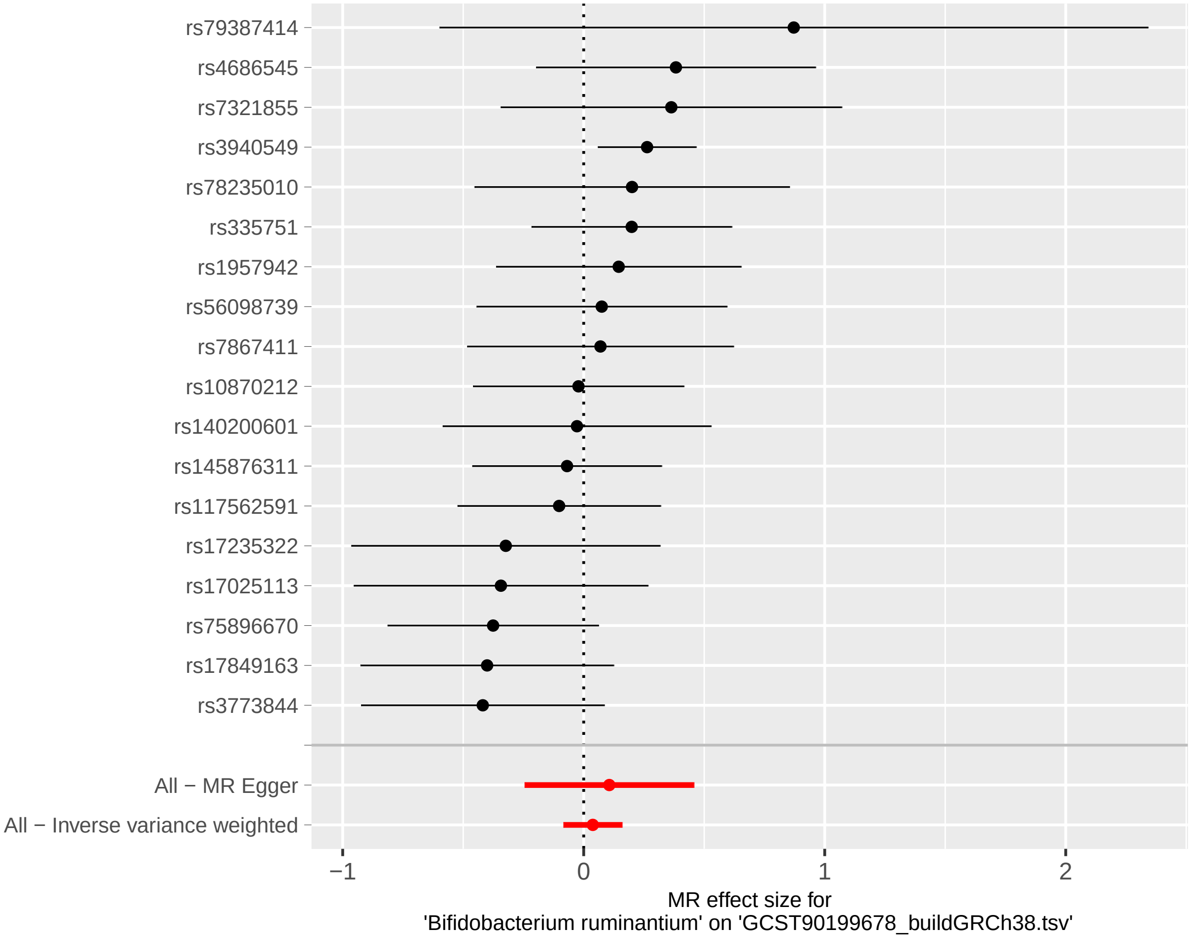
rs79387414
rs4686545
rs7321855
rs3940549
rs78235010
rs335751
rs1957942
rs56098739
rs7867411
rs10870212
rs140200601
rs145876311
rs117562591
rs17235322
rs17025113
rs75896670
rs17849163
rs3773844
All − MR Egger
All − Inverse variance weighted
−1
0
1
2
MR effect size for
'Bifidobacterium ruminantium' on 'GCST90199678_buildGRCh38.tsv'

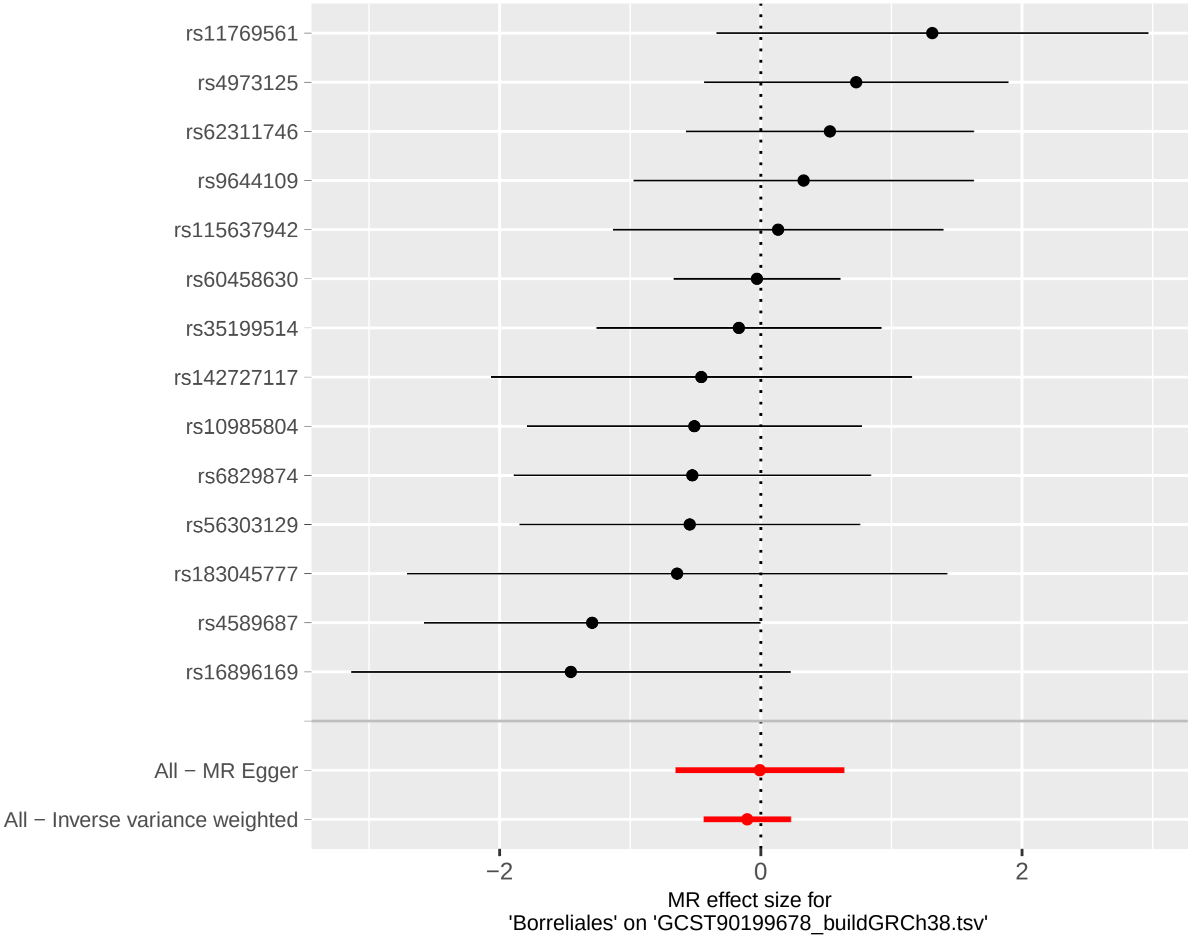

rs11769561
rs4973125
rs62311746
rs9644109
rs115637942
rs60458630
rs35199514
rs142727117
rs10985804
rs6829874
rs56303129
rs183045777
rs4589687
rs16896169
All − MR Egger
All − Inverse variance weighted
−2
0
2
MR effect size for
'Borreliales' on 'GCST90199678_buildGRCh38.tsv'

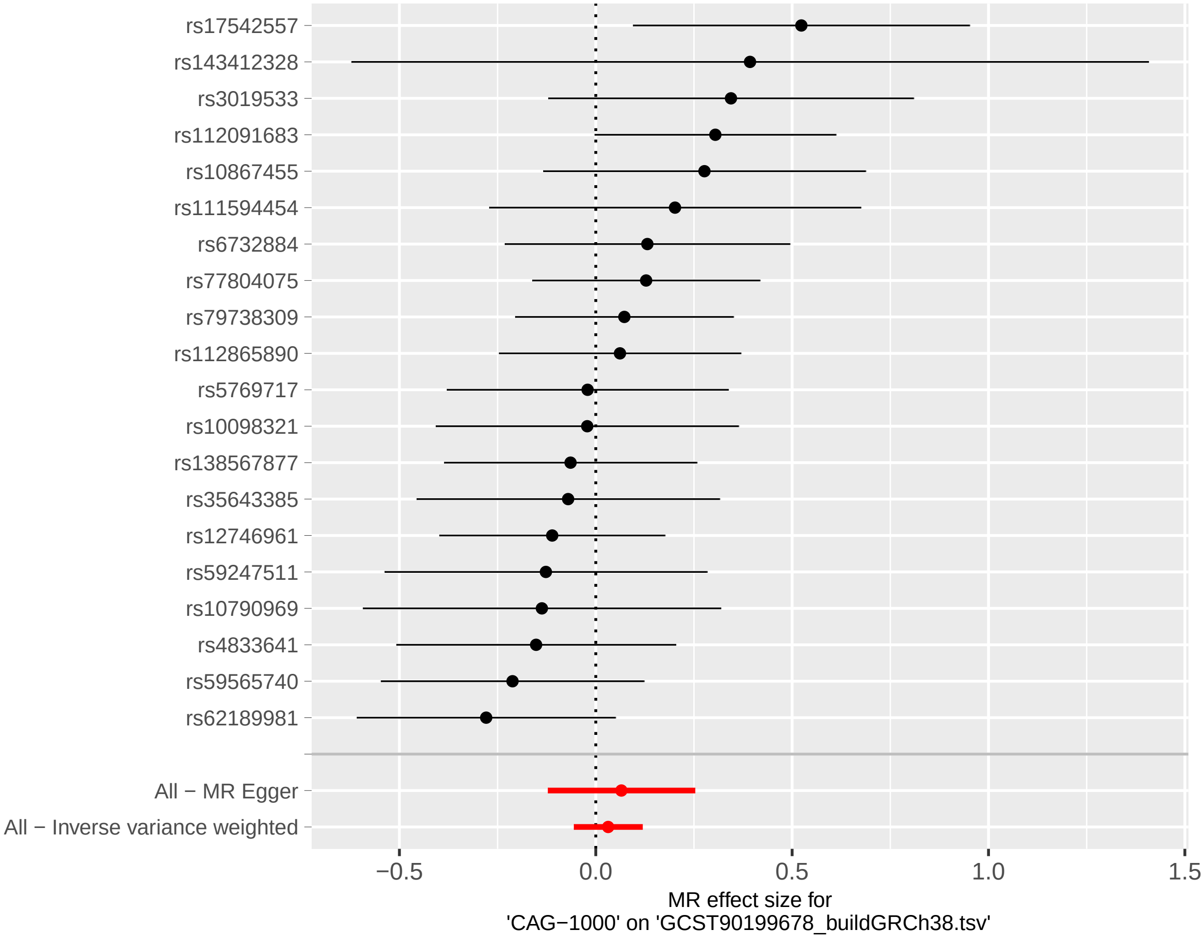
rs17542557
rs143412328
rs3019533
rs112091683
rs10867455
rs111594454
rs6732884
rs77804075
rs79738309
rs112865890
rs5769717
rs10098321
rs138567877
rs35643385
rs12746961
rs59247511
rs10790969
rs4833641
rs59565740
rs62189981
All − MR Egger
All − Inverse variance weighted
−0.5
0.0
0.5
1.0
1.5
MR effect size for
'CAG−1000' on 'GCST90199678_buildGRCh38.tsv'

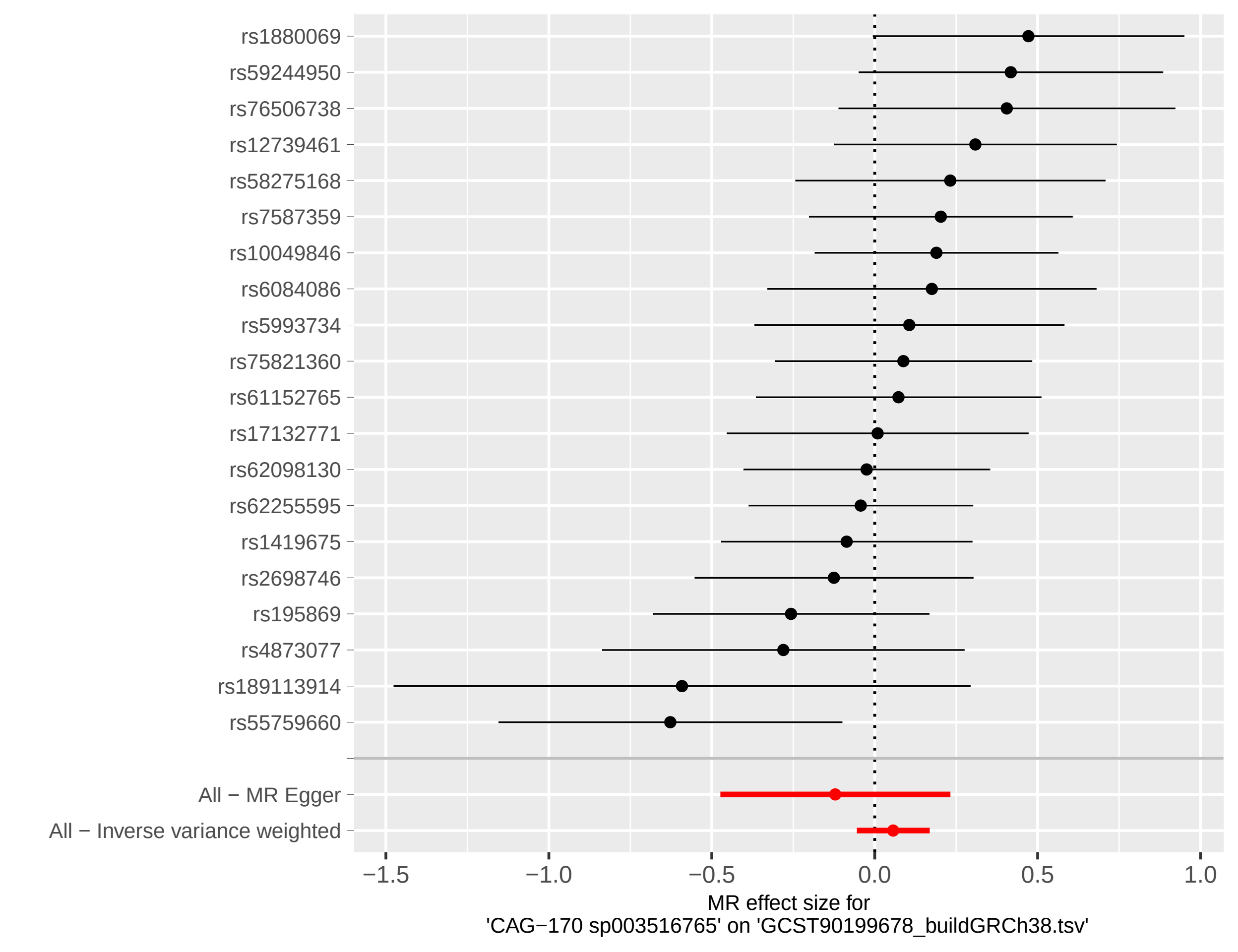
rs1880069
rs59244950
rs76506738
rs12739461
rs58275168
rs7587359
rs10049846
rs6084086
rs5993734
rs75821360
rs61152765
rs17132771
rs62098130
rs62255595
rs1419675
rs2698746
rs195869
rs4873077
rs189113914
rs55759660
All − MR Egger
All − Inverse variance weighted
−1.5
−1.0
−0.5
0.0
0.5
1.0
MR effect size for
'CAG−170 sp003516765' on 'GCST90199678_buildGRCh38.tsv'

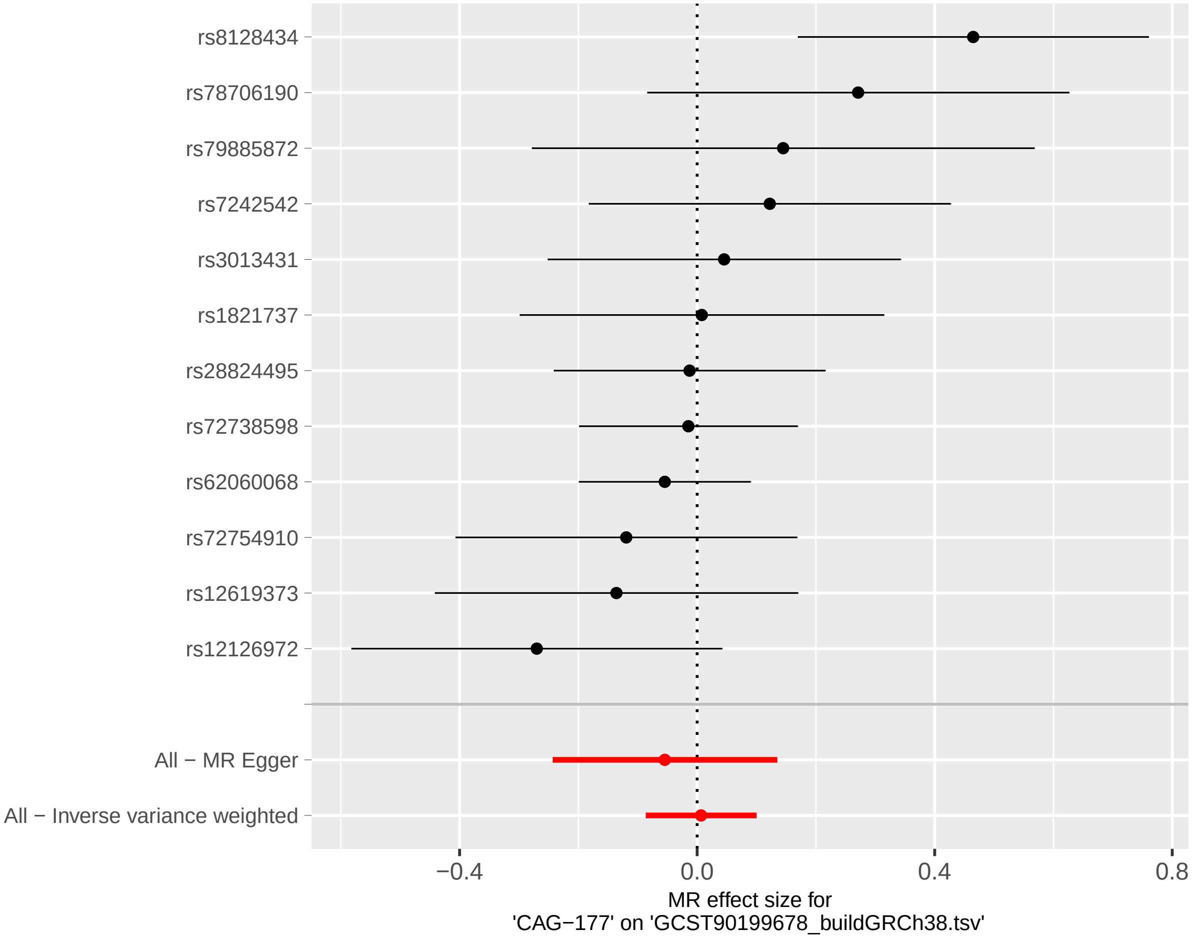
rs8128434
rs78706190
rs79885872
rs7242542
rs3013431
rs1821737
rs28824495
rs72738598
rs62060068
rs72754910
rs12619373
rs12126972
All − MR Egger
All − Inverse variance weighted
−0.4
0.0
0.4
0.8
MR effect size for
'CAG−177' on 'GCST90199678_buildGRCh38.tsv'

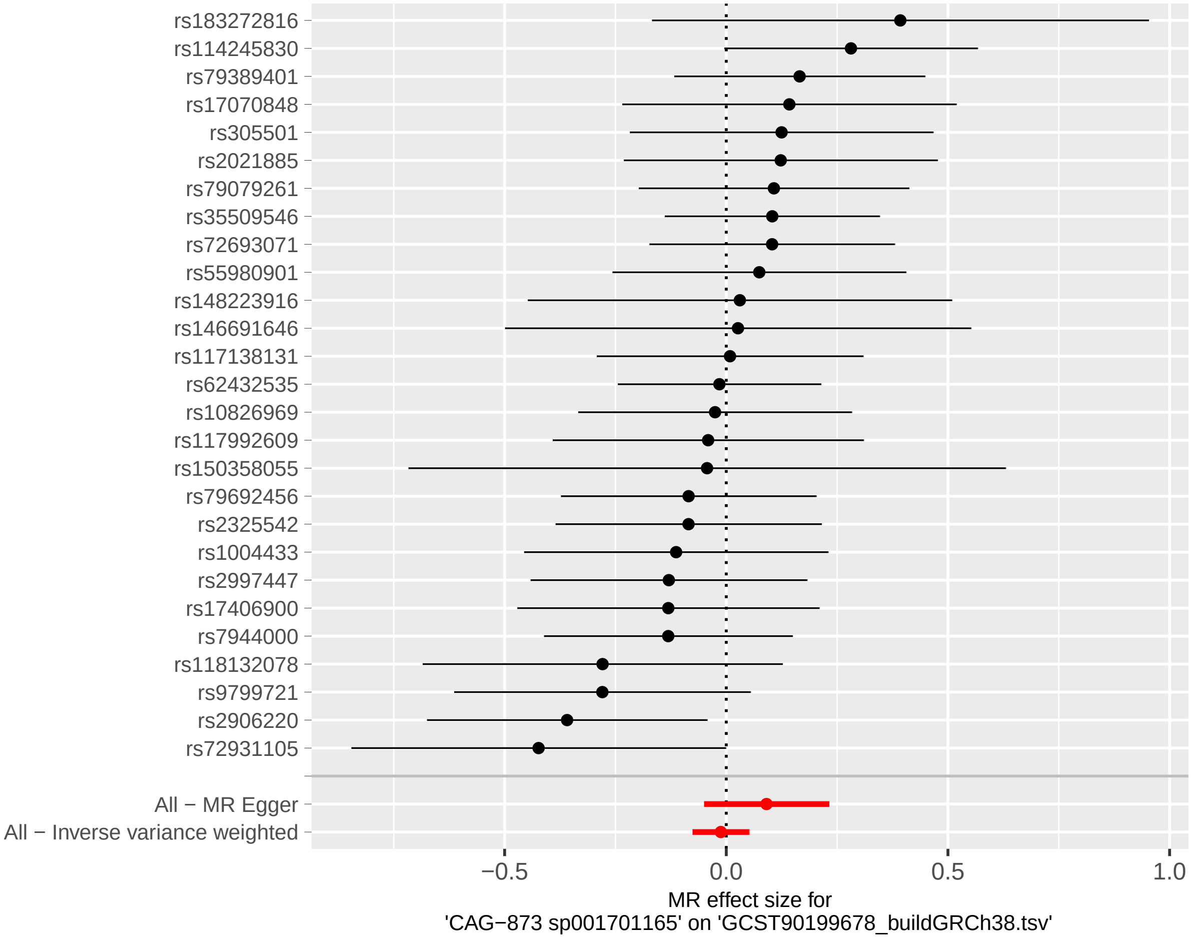

rs183272816
rs114245830
rs79389401
rs17070848
rs305501
rs2021885
rs79079261
rs35509546
rs72693071
rs55980901
rs148223916
rs146691646
rs117138131
rs62432535
rs10826969
rs117992609
rs150358055
rs79692456
rs2325542
rs1004433
rs2997447
rs17406900
rs7944000
rs118132078
rs9799721
rs2906220
rs72931105
All − MR Egger
All − Inverse variance weighted
−0.5
0.0
0.5
1.0
MR effect size for
'CAG−873 sp001701165' on 'GCST90199678_buildGRCh38.tsv'

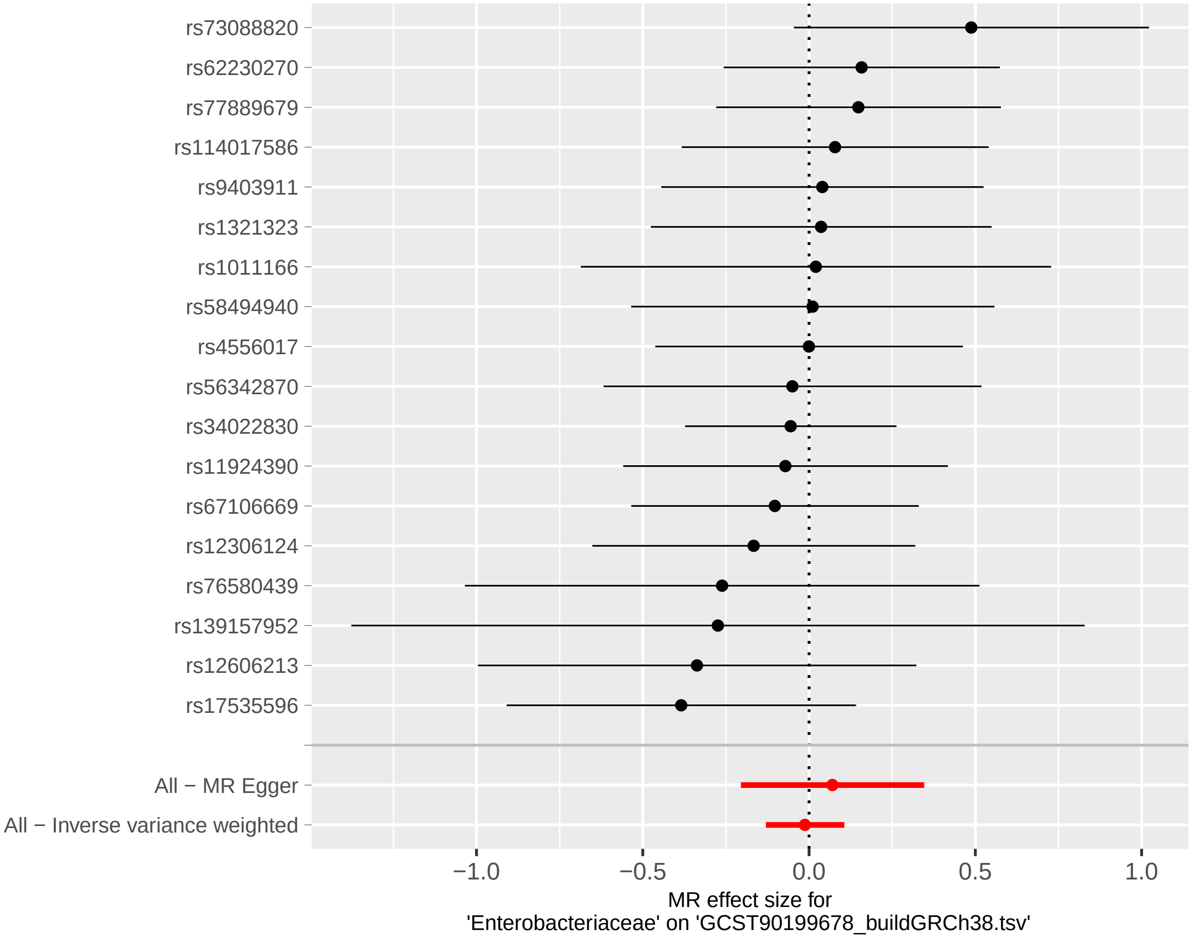

rs73088820
rs62230270
rs77889679
rs114017586
rs9403911
rs1321323
rs1011166
rs58494940
rs4556017
rs56342870
rs34022830
rs11924390
rs67106669
rs12306124
rs76580439
rs139157952
rs12606213
rs17535596
All − MR Egger
All − Inverse variance weighted
−1.0
−0.5
0.0
0.5
1.0
MR effect size for
'Enterobacteriaceae' on 'GCST90199678_buildGRCh38.tsv'

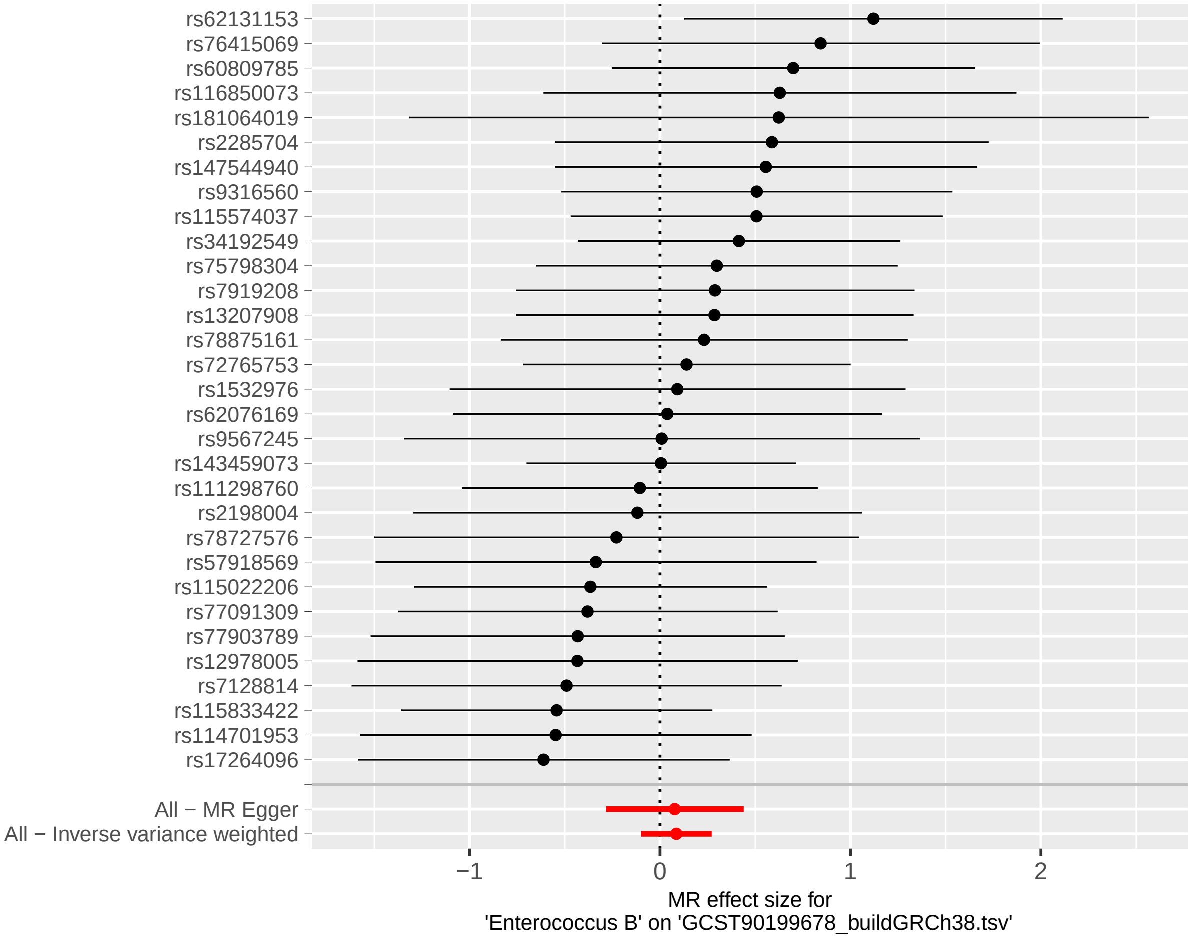
rs62131153
rs76415069
rs60809785
rs116850073
rs181064019
rs2285704
rs147544940
rs9316560
rs115574037
rs34192549
rs75798304
rs7919208
rs13207908
rs78875161
rs72765753
rs1532976
rs62076169
rs9567245
rs143459073
rs111298760
rs2198004
rs78727576
rs57918569
rs115022206
rs77091309
rs77903789
rs12978005
rs7128814
rs115833422
rs114701953
rs17264096
All − MR Egger
All − Inverse variance weighted
−1
0
1
2
MR effect size for
'Enterococcus B' on 'GCST90199678_buildGRCh38.tsv'

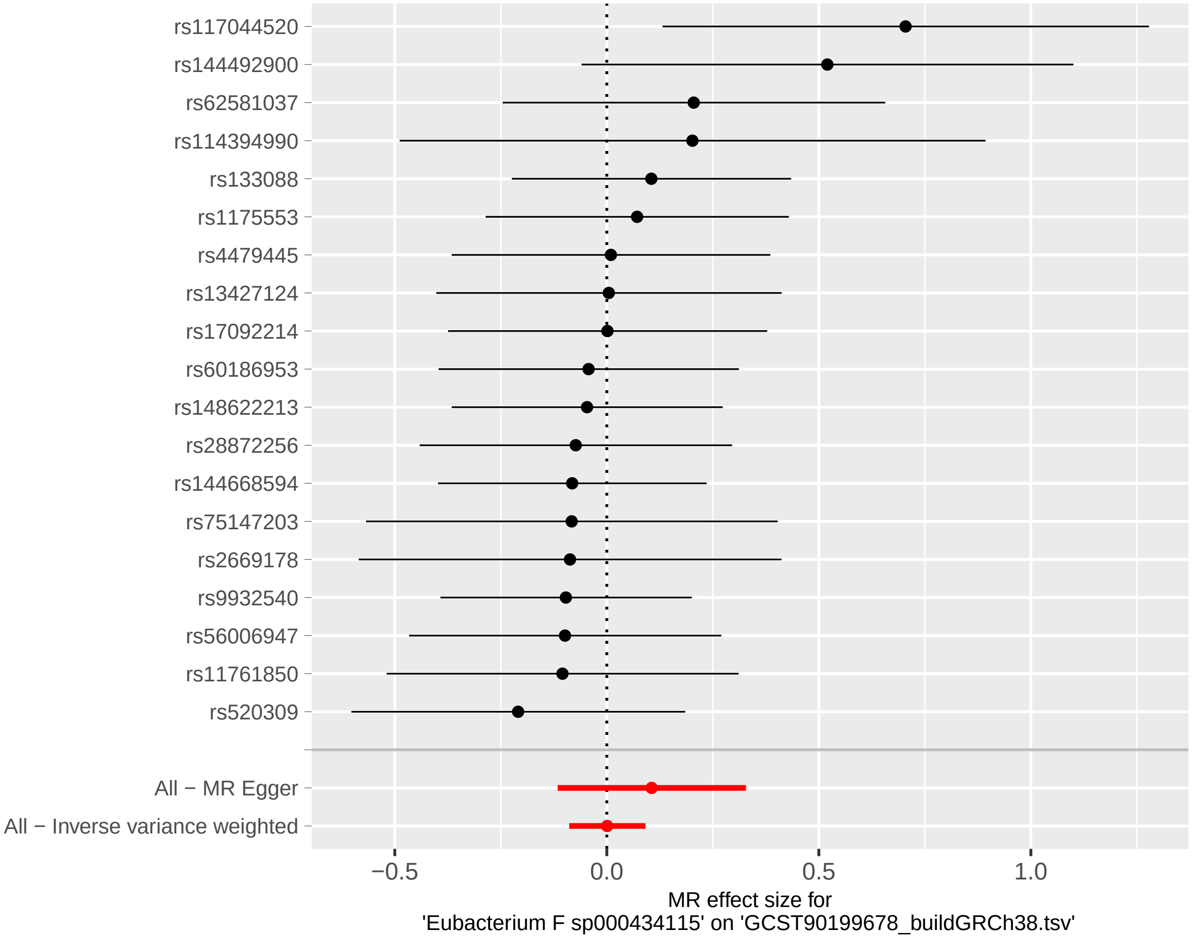
rs117044520
rs144492900
rs62581037
rs114394990
rs133088
rs1175553
rs4479445
rs13427124
rs17092214
rs60186953
rs148622213
rs28872256
rs144668594
rs75147203
rs2669178
rs9932540
rs56006947
rs11761850
rs520309
All − MR Egger
All − Inverse variance weighted
−0.5
0.0
0.5
1.0
MR effect size for
'Eubacterium F sp000434115' on 'GCST90199678_buildGRCh38.tsv'

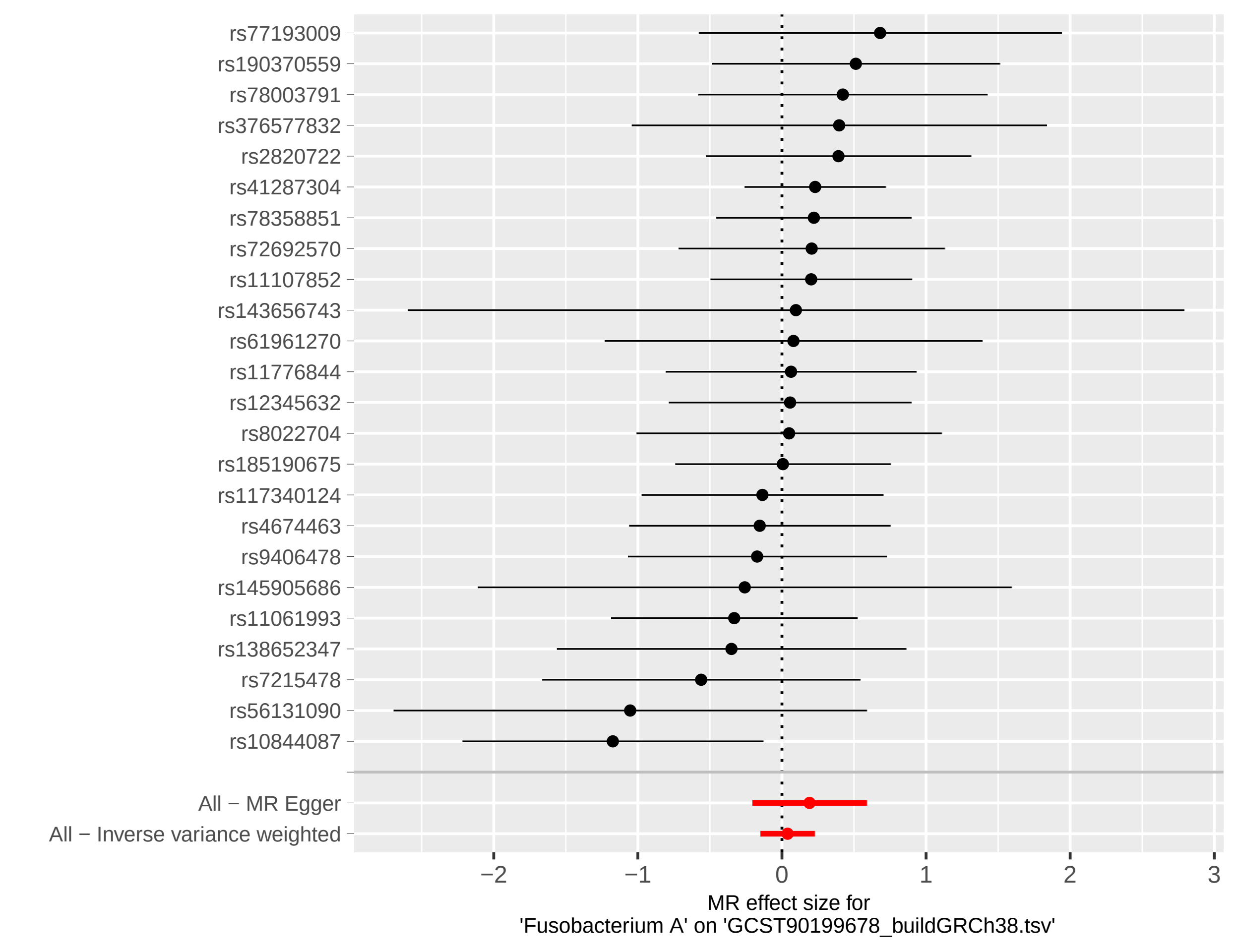
rs77193009
rs190370559
rs78003791
rs376577832
rs2820722
rs41287304
rs78358851
rs72692570
rs11107852
rs143656743
rs61961270
rs11776844
rs12345632
rs8022704
rs185190675
rs117340124
rs4674463
rs9406478
rs145905686
rs11061993
rs138652347
rs7215478
rs56131090
rs10844087
All − MR Egger
All − Inverse variance weighted
−2
−1
0
1
2
3
MR effect size for
'Fusobacterium A' on 'GCST90199678_buildGRCh38.tsv'

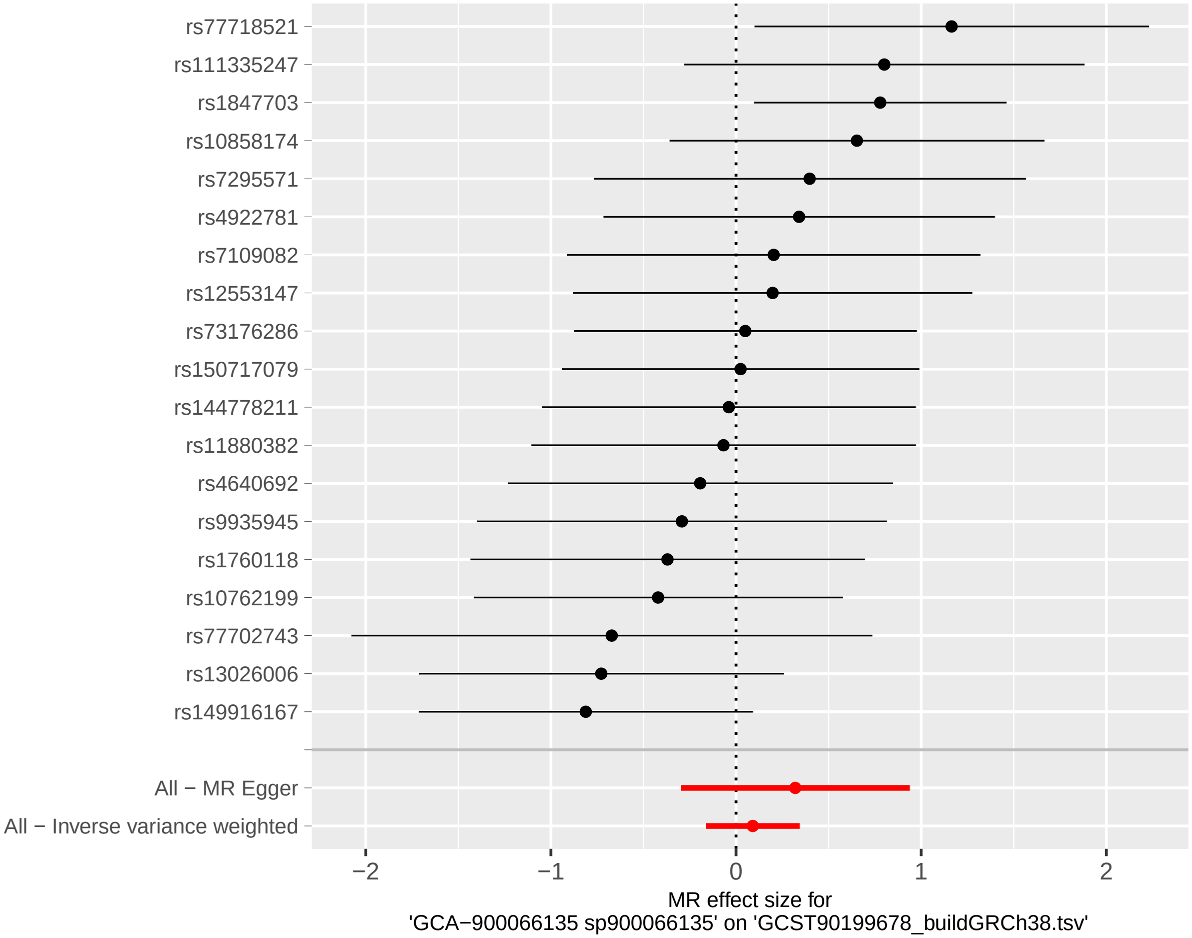

rs77718521
rs111335247
rs1847703
rs10858174
rs7295571
rs4922781
rs7109082
rs12553147
rs73176286
rs150717079
rs144778211
rs11880382
rs4640692
rs9935945
rs1760118
rs10762199
rs77702743
rs13026006
rs149916167
All − MR Egger
All − Inverse variance weighted
−2
−1
0
1
2
MR effect size for
'GCA−900066135 sp900066135' on 'GCST90199678_buildGRCh38.tsv'

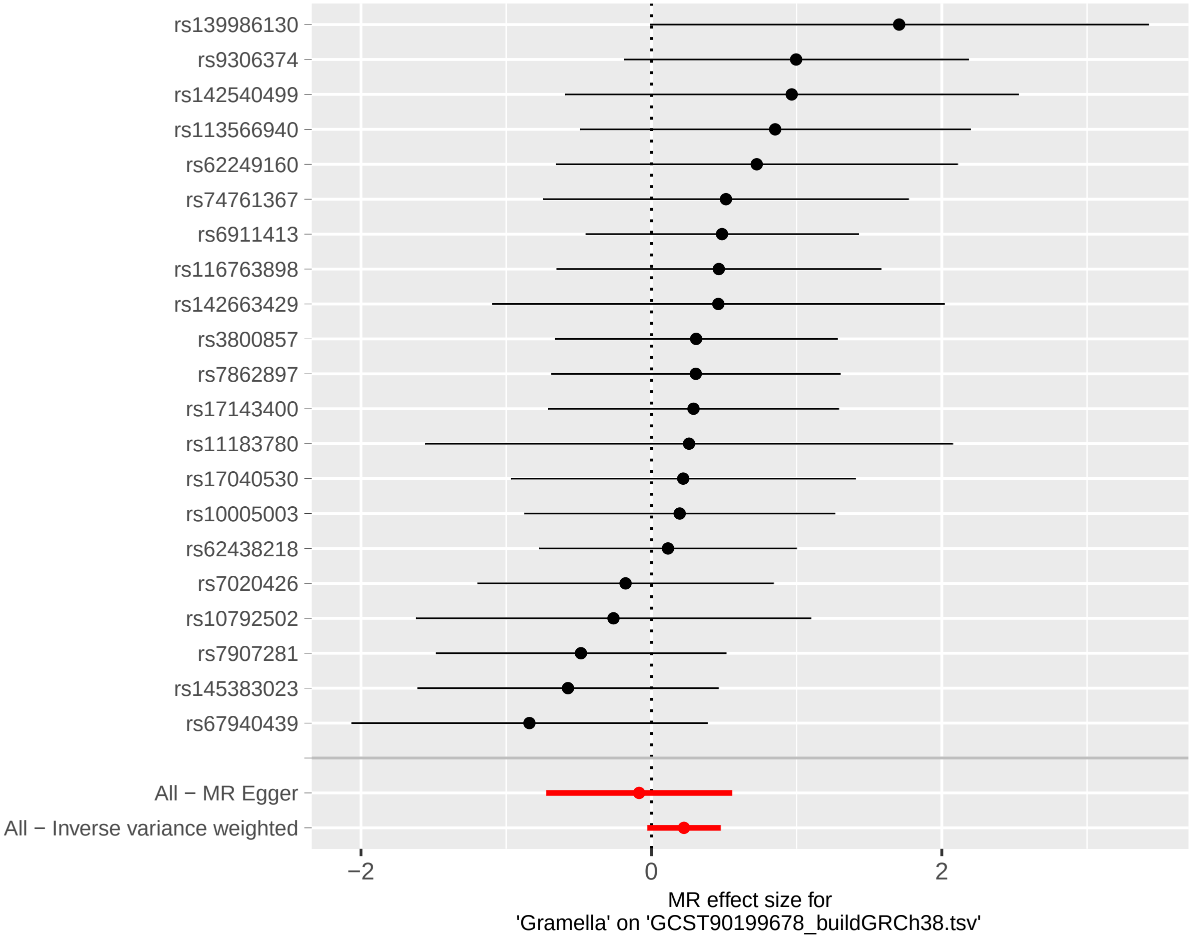

rs139986130
rs9306374
rs142540499
rs113566940
rs62249160
rs74761367
rs6911413
rs116763898
rs142663429
rs3800857
rs7862897
rs17143400
rs11183780
rs17040530
rs10005003
rs62438218
rs7020426
rs10792502
rs7907281
rs145383023
rs67940439
All − MR Egger
All − Inverse variance weighted
−2
0
2
MR effect size for
'Gramella' on 'GCST90199678_buildGRCh38.tsv'

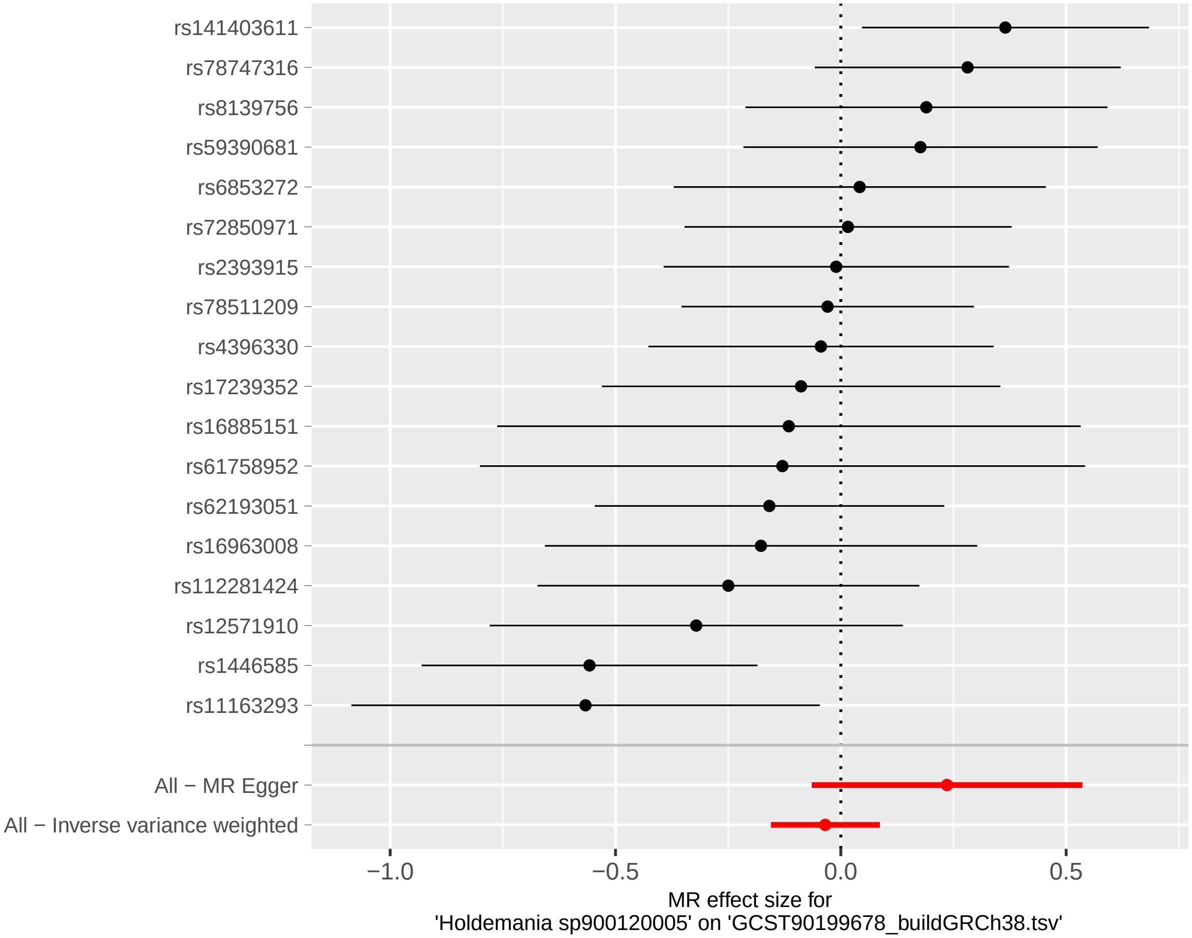

rs141403611
rs78747316
rs8139756
rs59390681
rs6853272
rs72850971
rs2393915
rs78511209
rs4396330
rs17239352
rs16885151
rs61758952
rs62193051
rs16963008
rs112281424
rs12571910
rs1446585
rs11163293
All − MR Egger
All − Inverse variance weighted
−1.0
−0.5
0.0
0.5
MR effect size for
'Holdemania sp900120005' on 'GCST90199678_buildGRCh38.tsv'

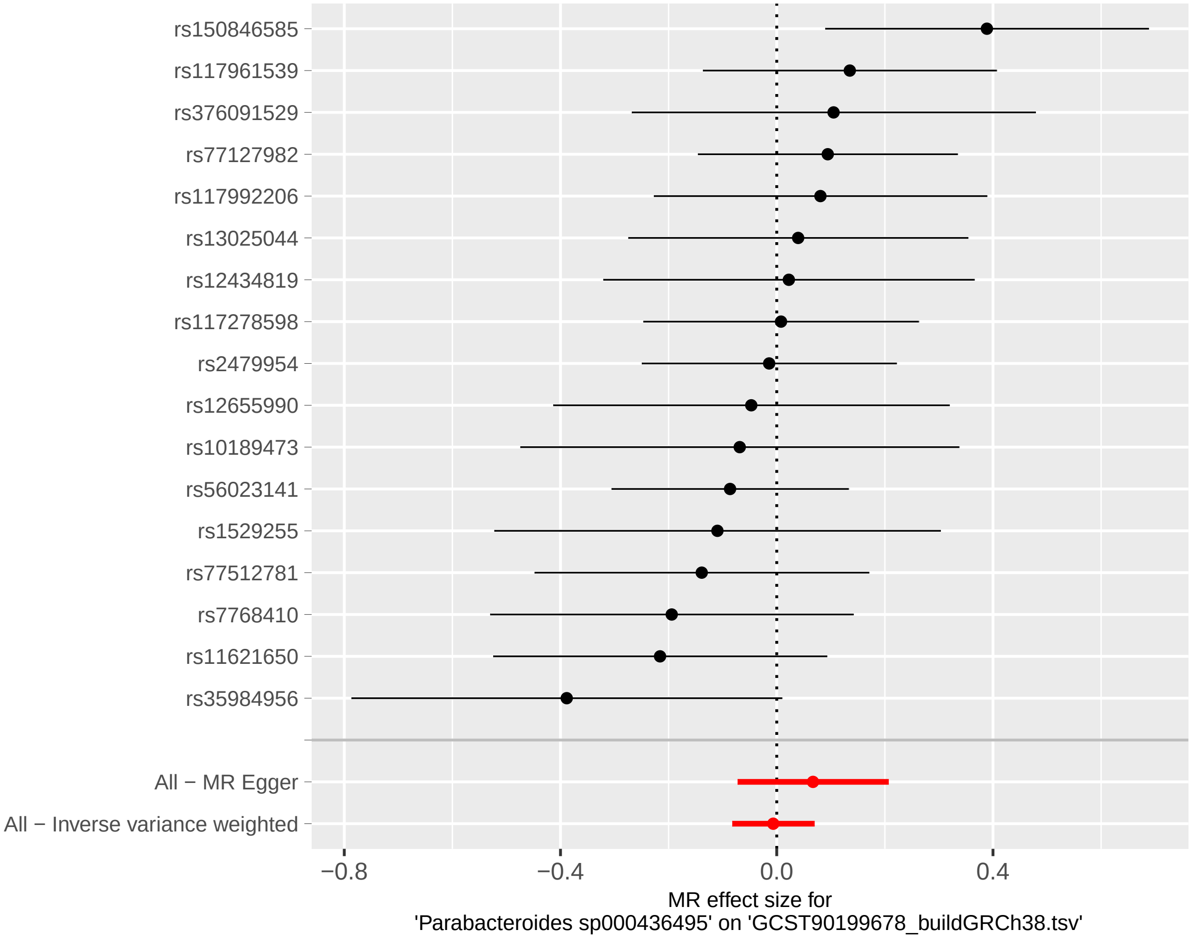
rs150846585
rs117961539
rs376091529
rs77127982
rs117992206
rs13025044
rs12434819
rs117278598
rs2479954
rs12655990
rs10189473
rs56023141
rs1529255
rs77512781
rs7768410
rs11621650
rs35984956
All − MR Egger
All − Inverse variance weighted
−0.8
−0.4
0.0
0.4
MR effect size for
'Parabacteroides sp000436495' on 'GCST90199678_buildGRCh38.tsv'

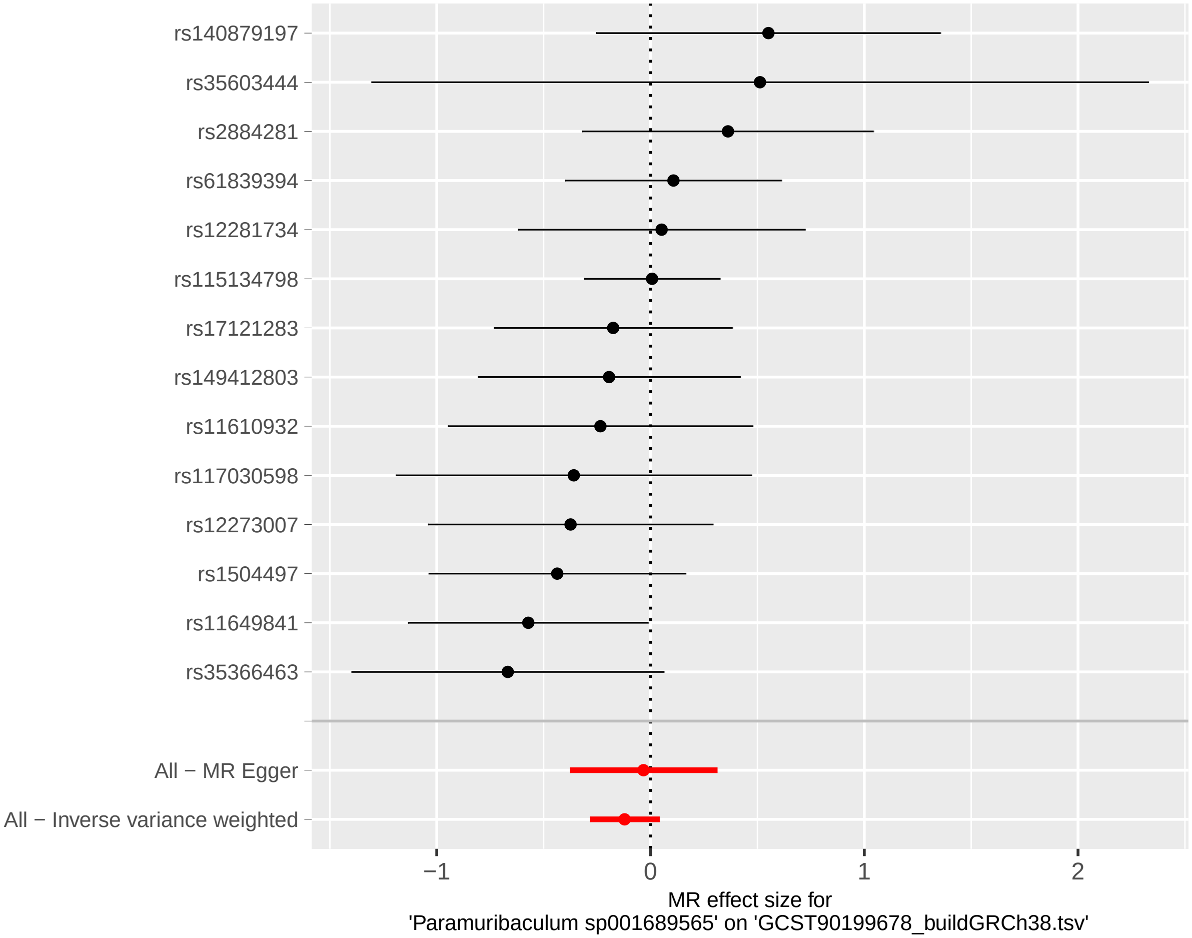

rs140879197
rs35603444
rs2884281
rs61839394
rs12281734
rs115134798
rs17121283
rs149412803
rs11610932
rs117030598
rs12273007
rs1504497
rs11649841
rs35366463
All − MR Egger
All − Inverse variance weighted
−1
0
1
2
MR effect size for
'Paramuribaculum sp001689565' on 'GCST90199678_buildGRCh38.tsv'

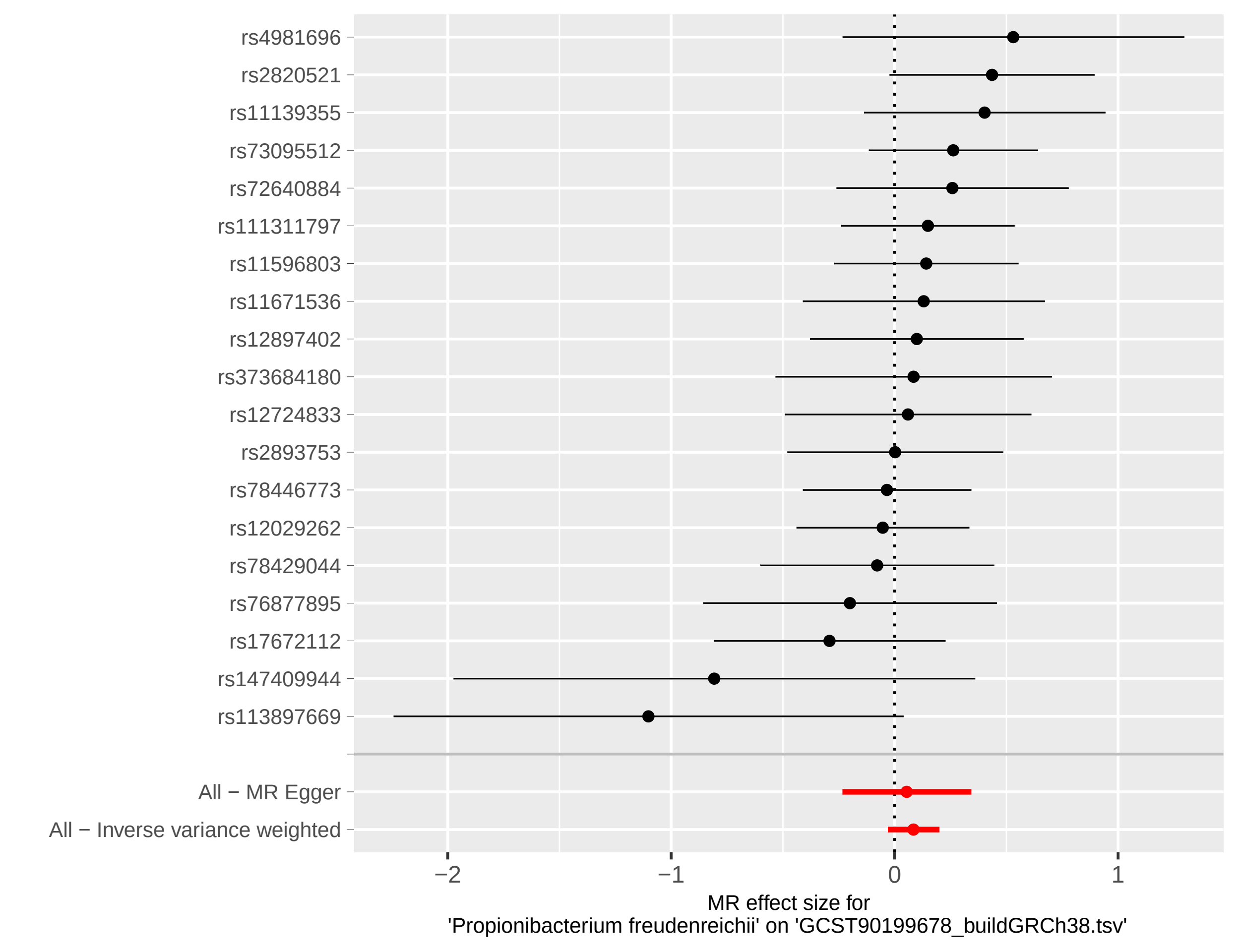
rs4981696
rs2820521
rs11139355
rs73095512
rs72640884
rs111311797
rs11596803
rs11671536
rs12897402
rs373684180
rs12724833
rs2893753
rs78446773
rs12029262
rs78429044
rs76877895
rs17672112
rs147409944
rs113897669
All − MR Egger
All − Inverse variance weighted
−2
−1
0
1
MR effect size for
'Propionibacterium freudenreichii' on 'GCST90199678_buildGRCh38.tsv'

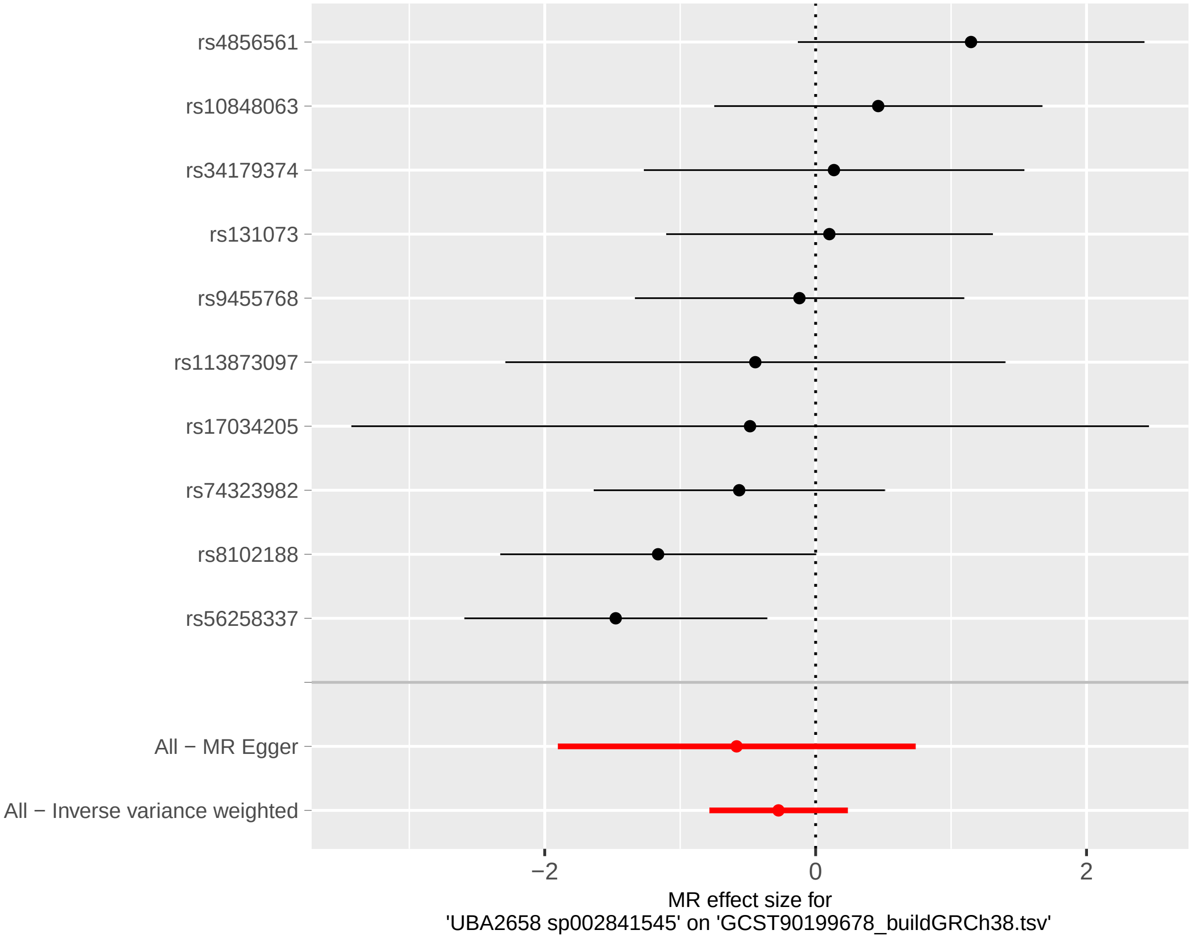

rs4856561
rs10848063
rs34179374
rs131073
rs9455768
rs113873097
rs17034205
rs74323982
rs8102188
rs56258337
All − MR Egger
All − Inverse variance weighted
−2
0
2
MR effect size for
'UBA2658 sp002841545' on 'GCST90199678_buildGRCh38.tsv'

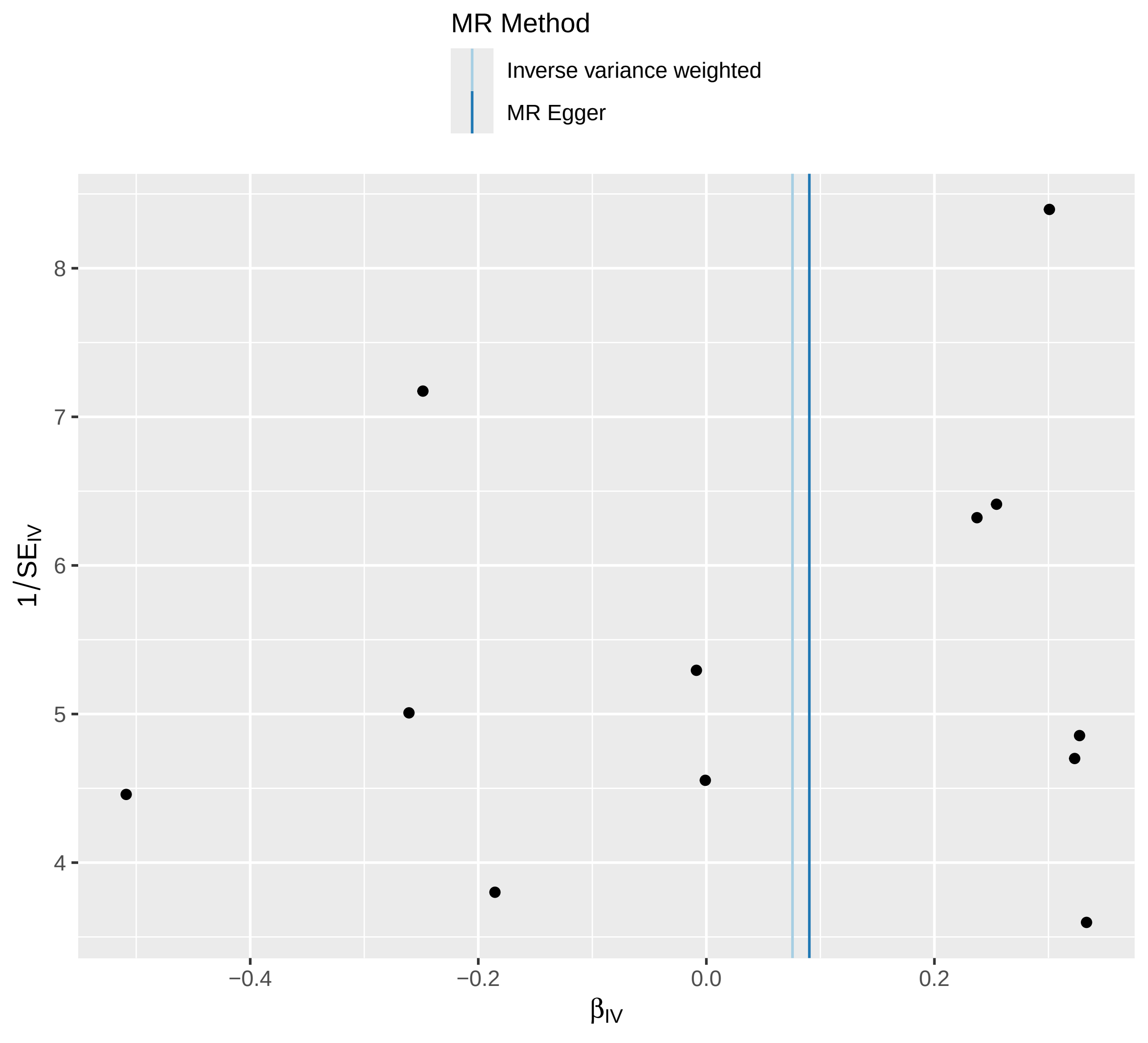

MR Method
Inverse variance weighted
MR Egger
8
7
6
5
4
1/SE_IV
−0.4
−0.2
0.0
0.2
β_IV

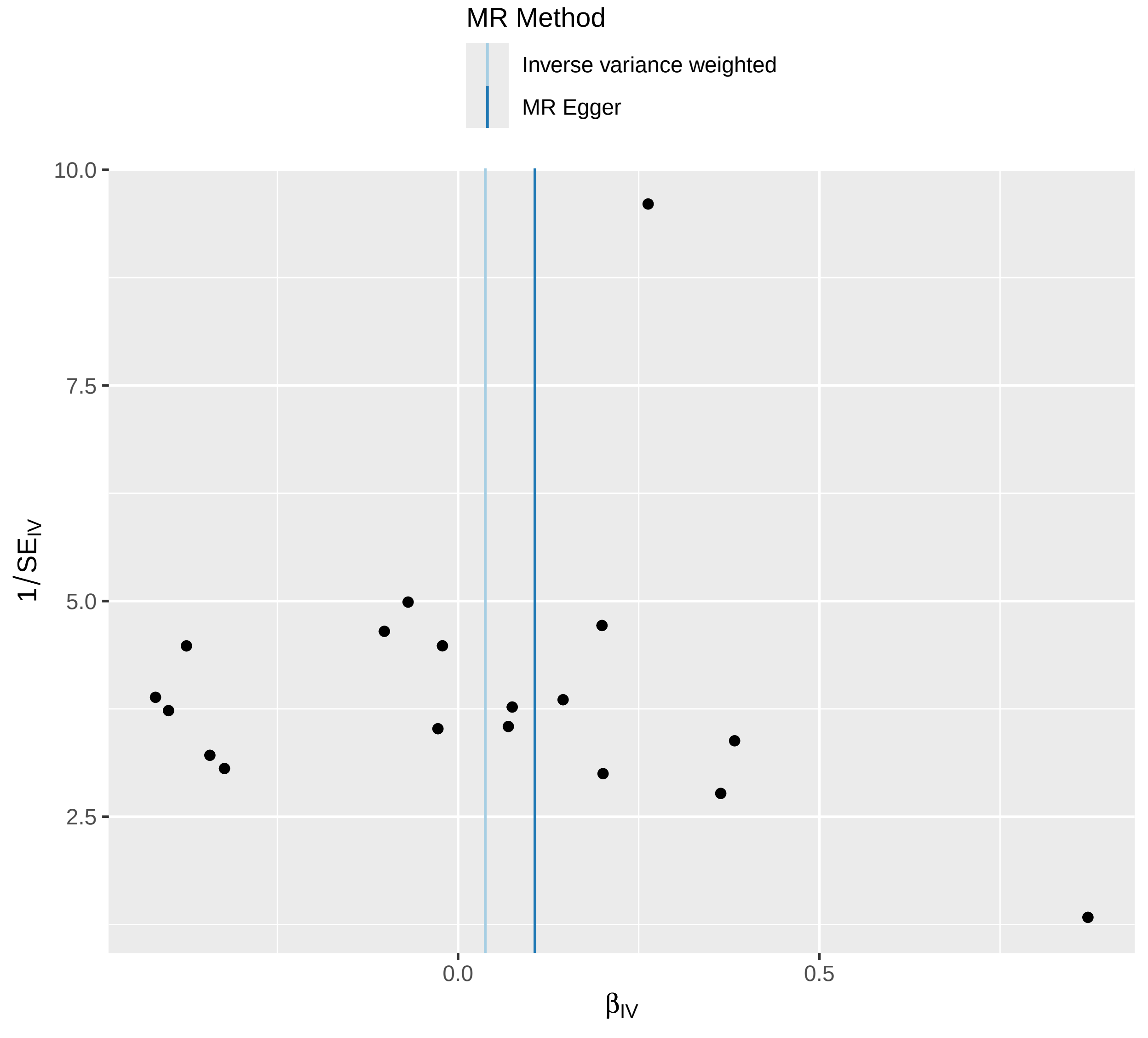
MR Method
Inverse variance weighted
MR Egger
10.0
7.5
5.0
2.5
$1/SE_{IV}$
0.0
0.5
β_{IV}

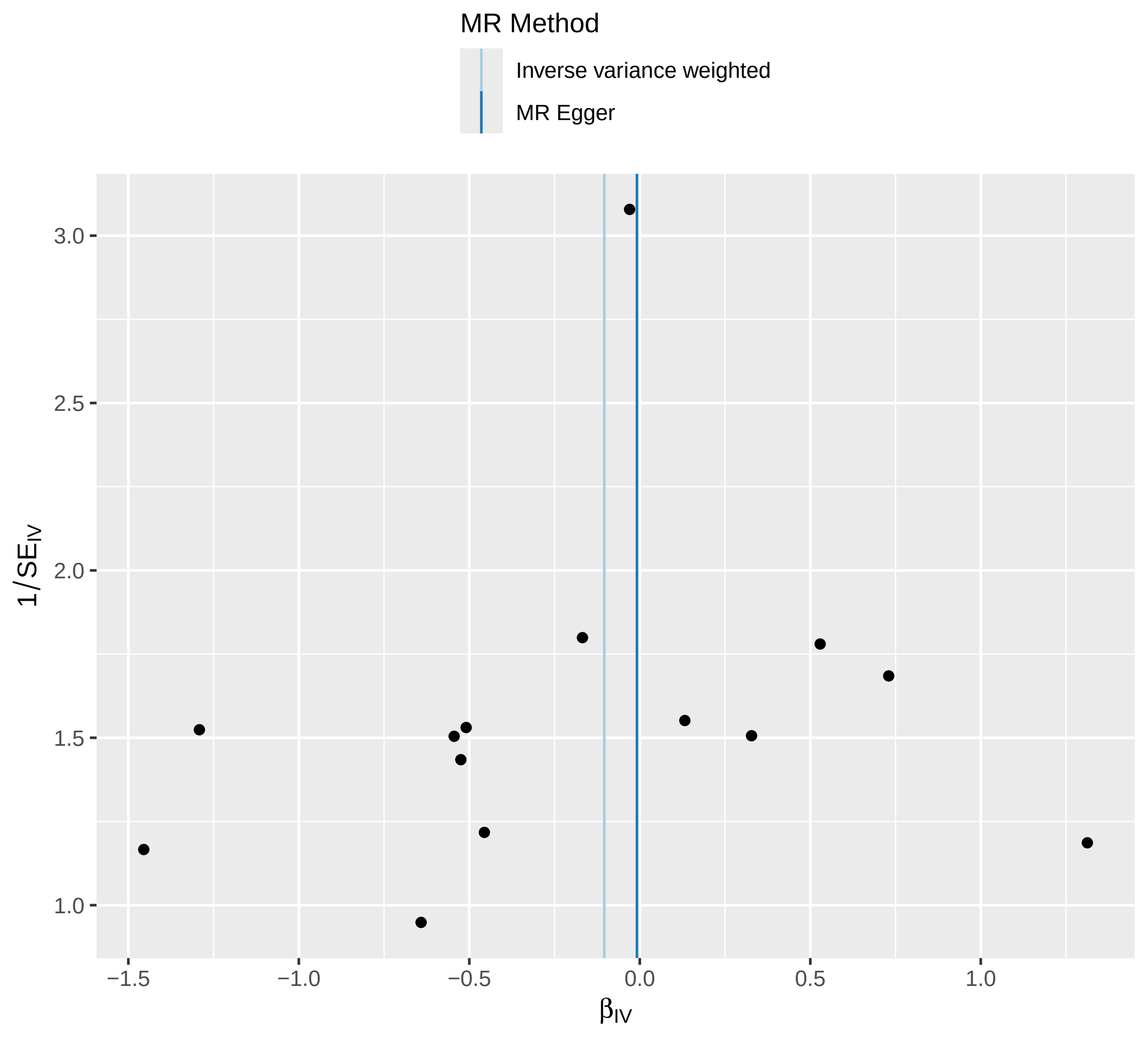
MR Method
Inverse variance weighted
MR Egger
3.0
2.5
2.0
1.5
1.0
1/SE_IV
−1.5
−1.0
−0.5
0.0
0.5
1.0
β_IV

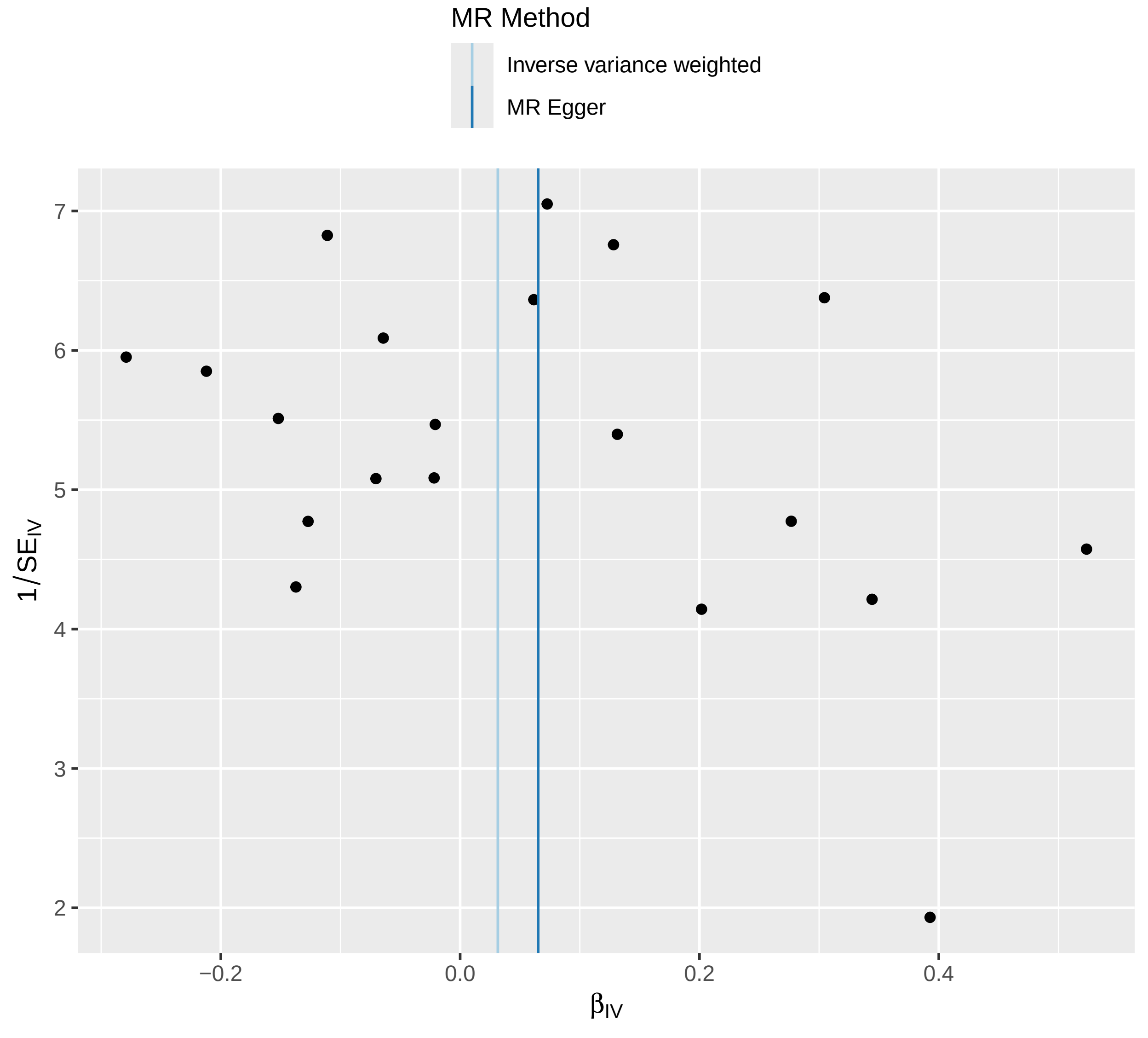

MR Method
Inverse variance weighted
MR Egger
1/SE_IV
β_IV
7
6
5
4
3
2
−0.2
0.0
0.2
0.4

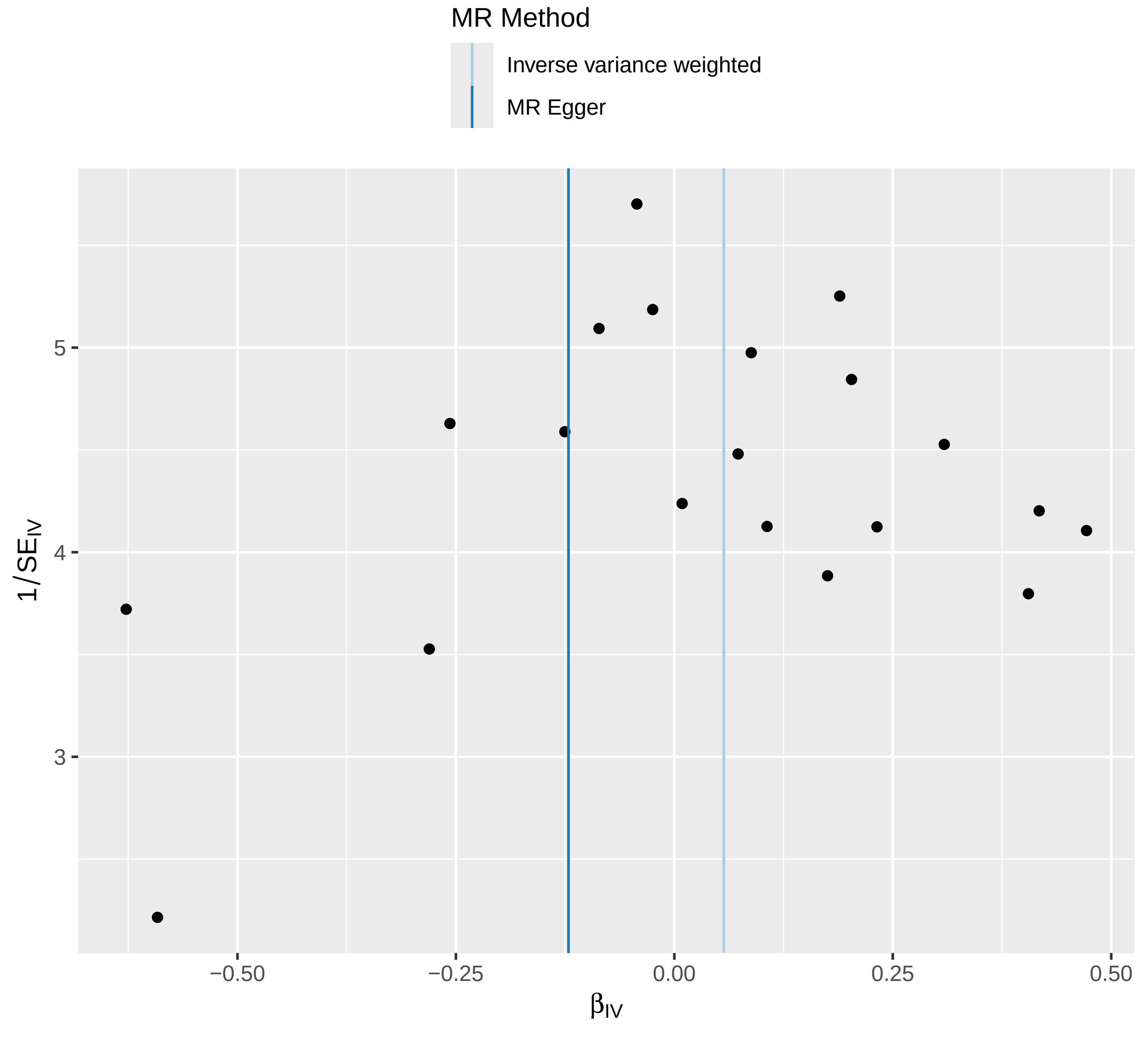

MR Method
Inverse variance weighted
MR Egger
1/SE_IV
5
4
3
−0.50
−0.25
0.00
0.25
0.50
β_IV

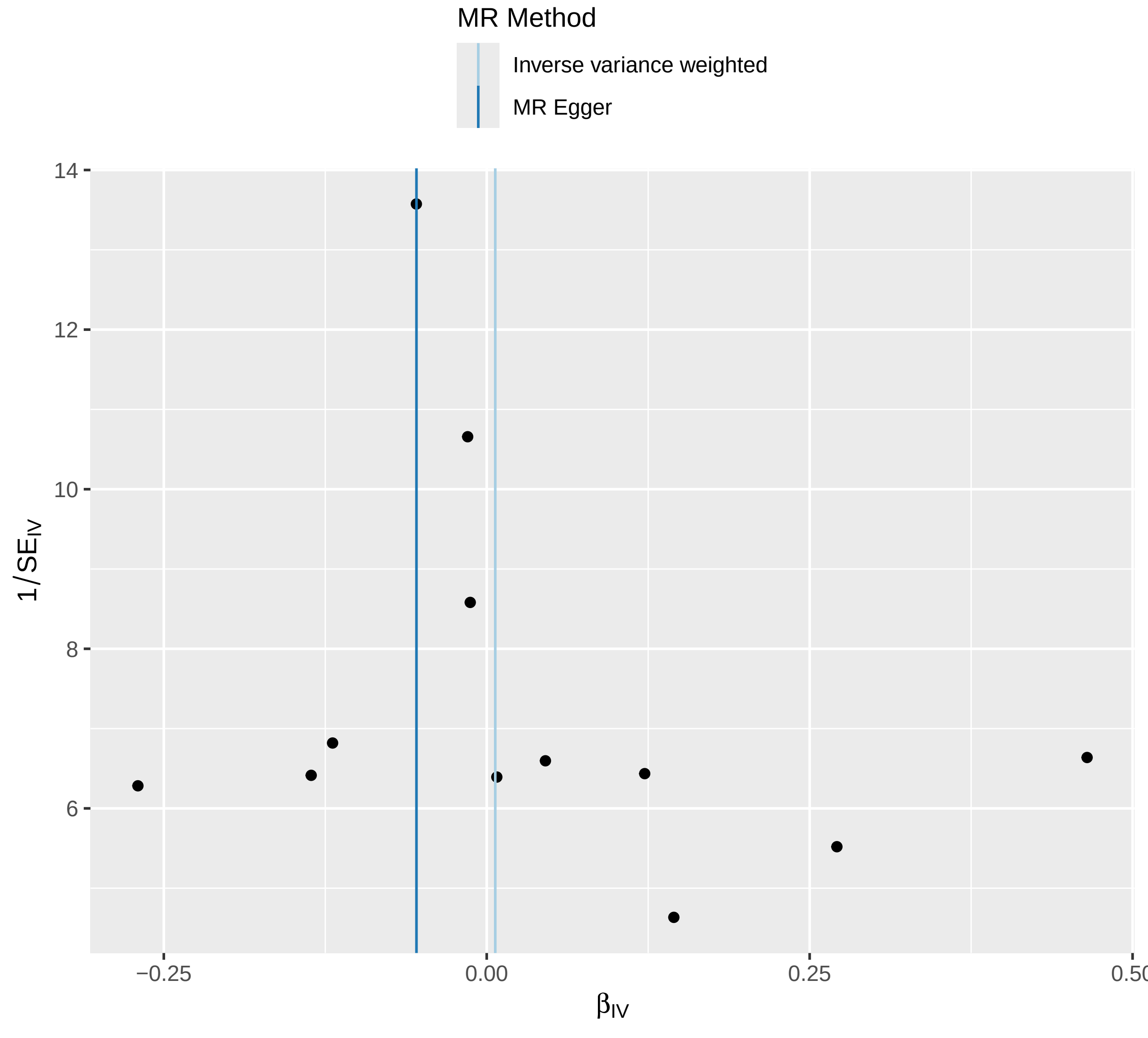
MR Method
Inverse variance weighted
MR Egger
14
12
10
8
6
$1/SE_{IV}$
−0.25
0.00
0.25
0.50
β_{IV}

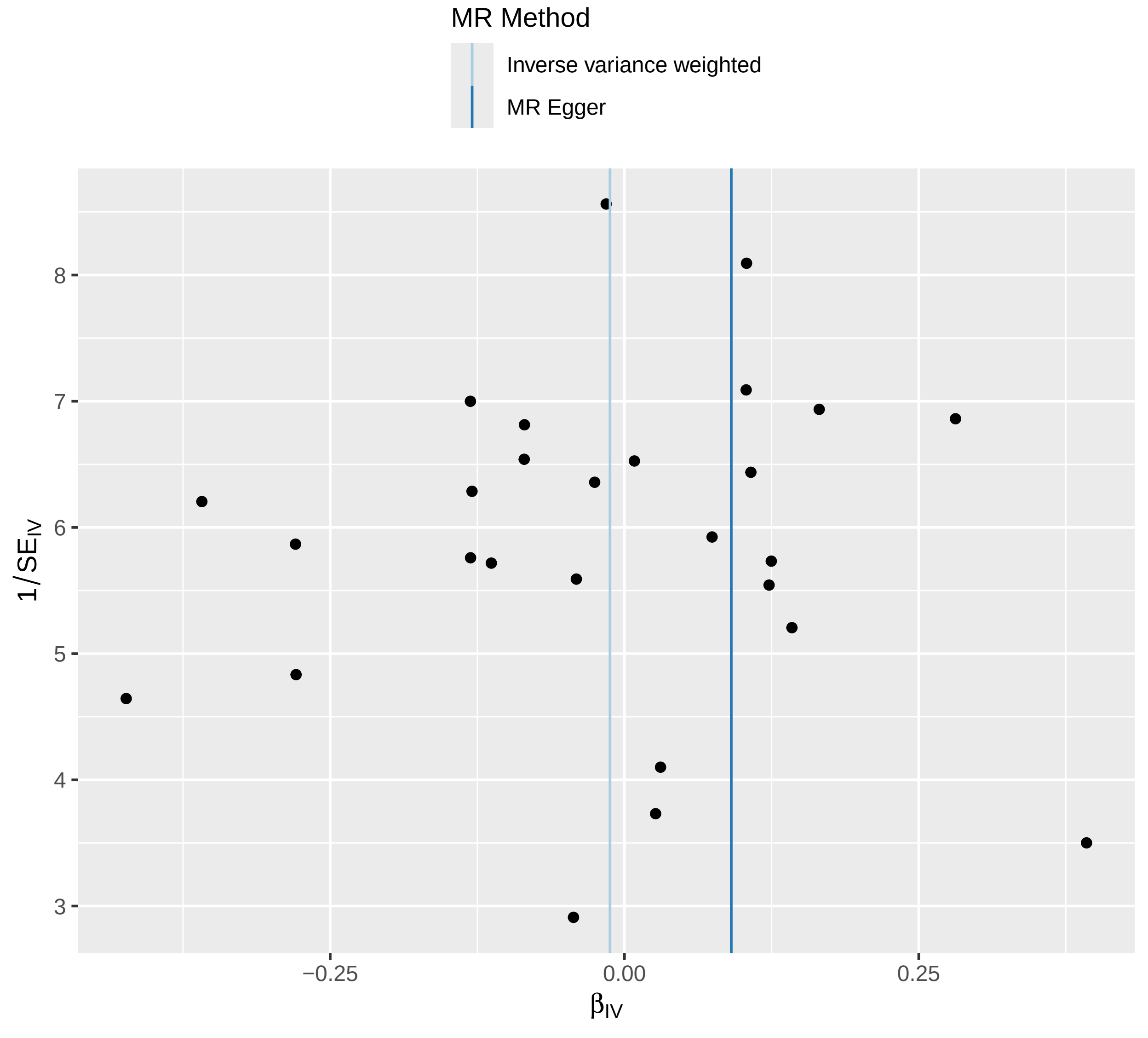

MR Method
Inverse variance weighted
MR Egger
8
7
6
5
4
3
1/SE_IV
−0.25
0.00
0.25
β_IV

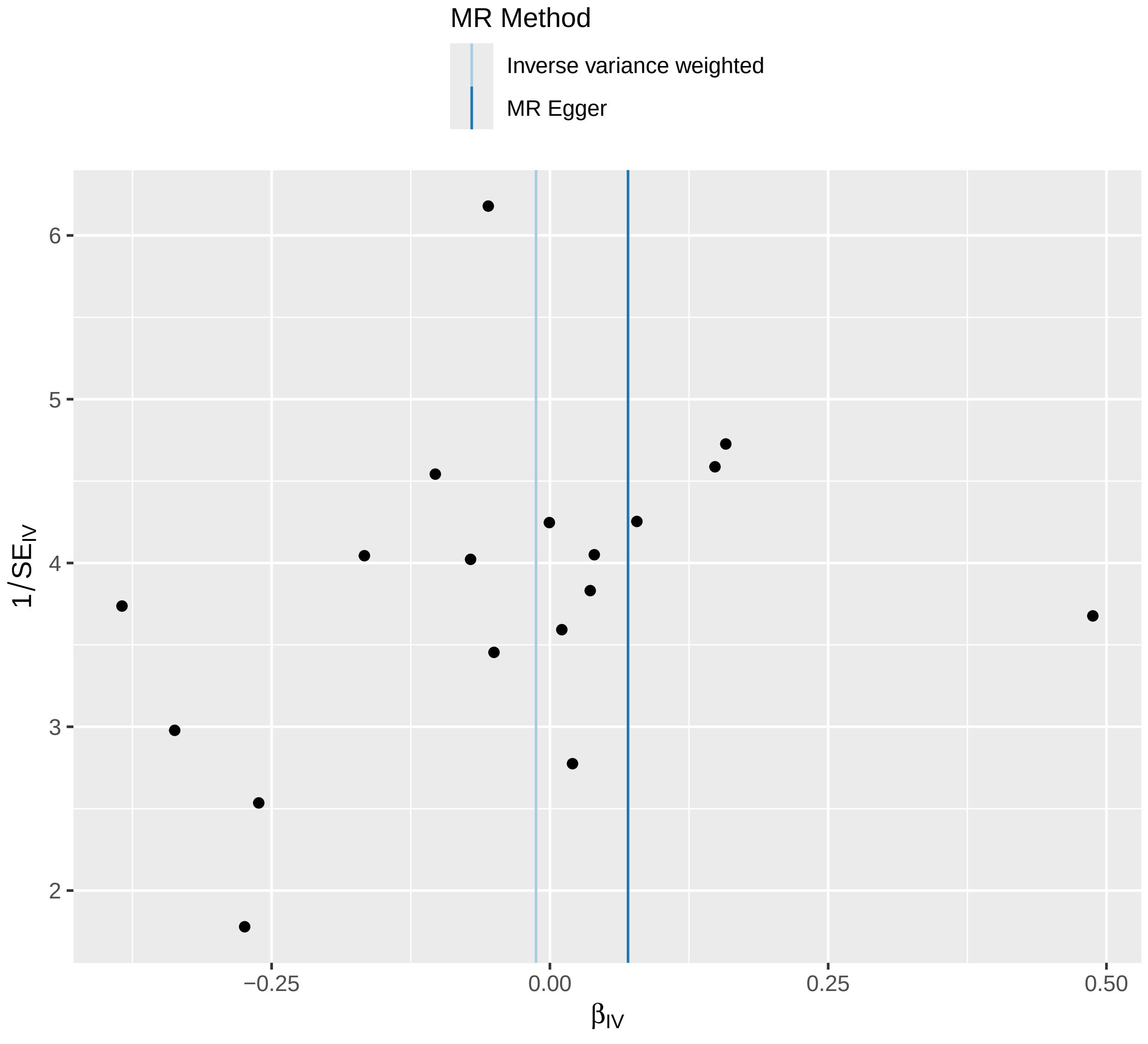
MR Method
Inverse variance weighted
MR Egger
1/SE_IV
6
5
4
3
2
β_IV
−0.25
0.00
0.25
0.50

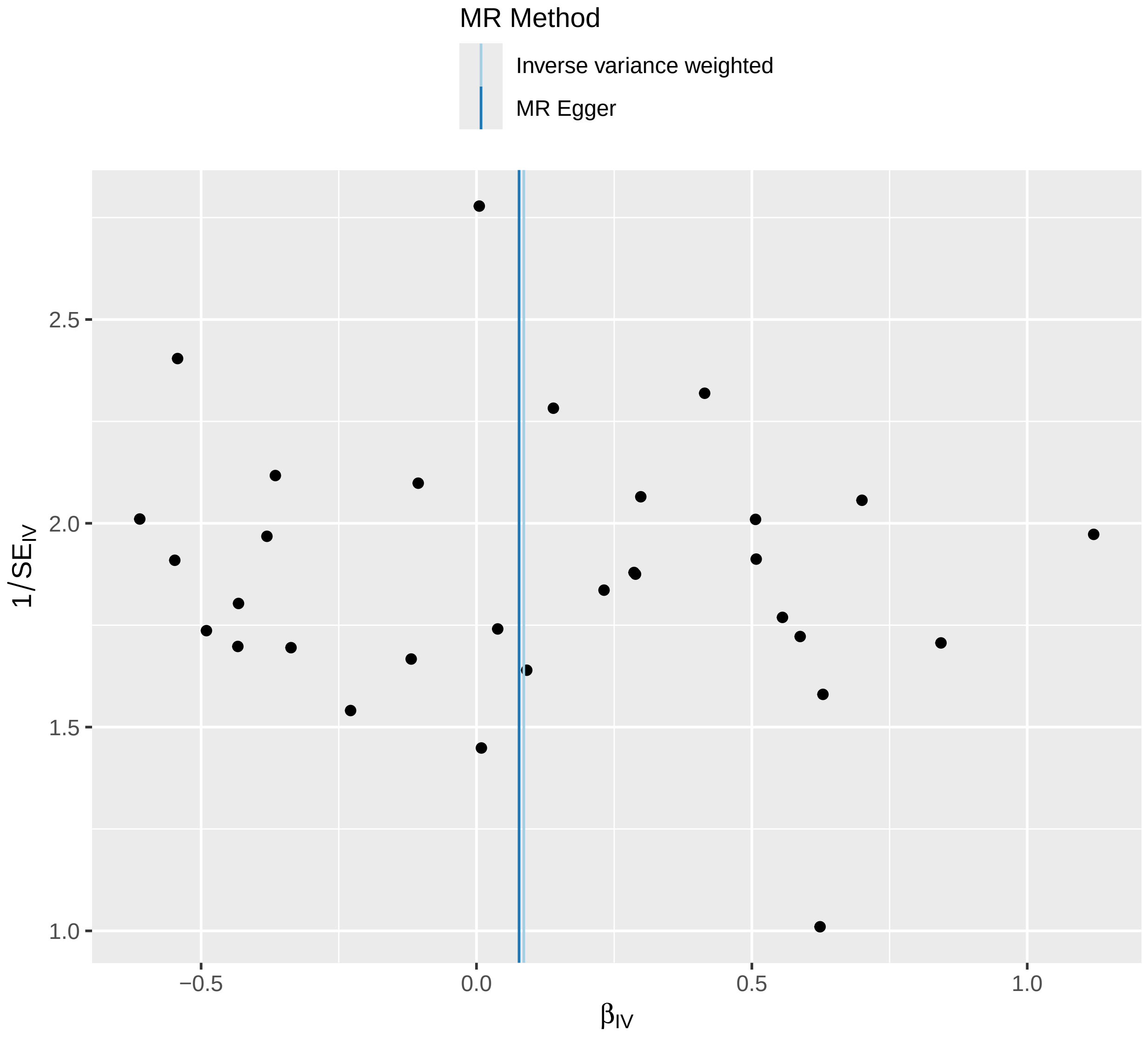

MR Method
Inverse variance weighted
MR Egger
2.5
2.0
1.5
1.0
1/SE_IV
−0.5
0.0
0.5
1.0
β_IV

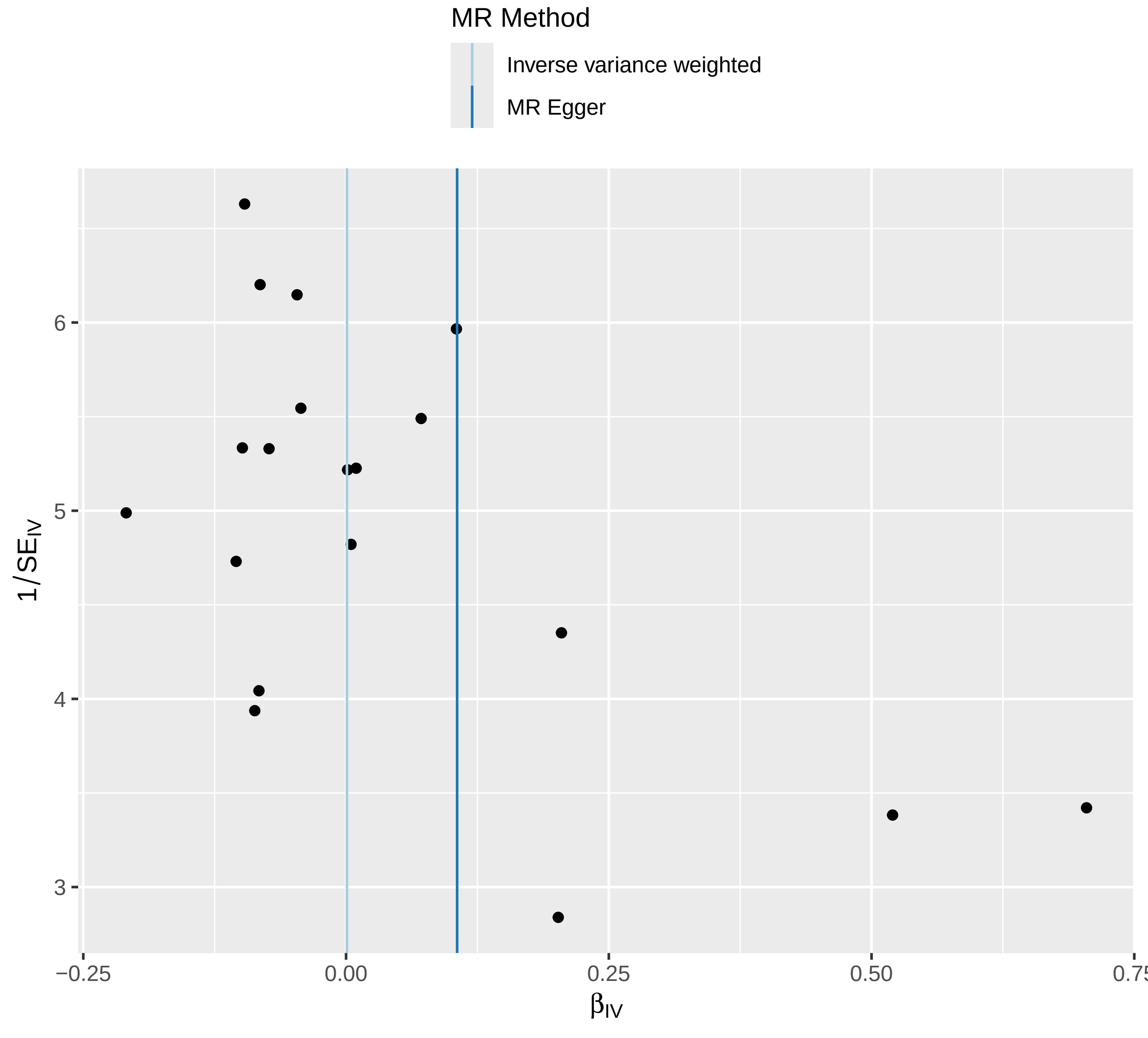
MR Method
Inverse variance weighted
MR Egger
6
5
4
3
1/SE_IV
−0.25
0.00
0.25
0.50
0.75
β_IV

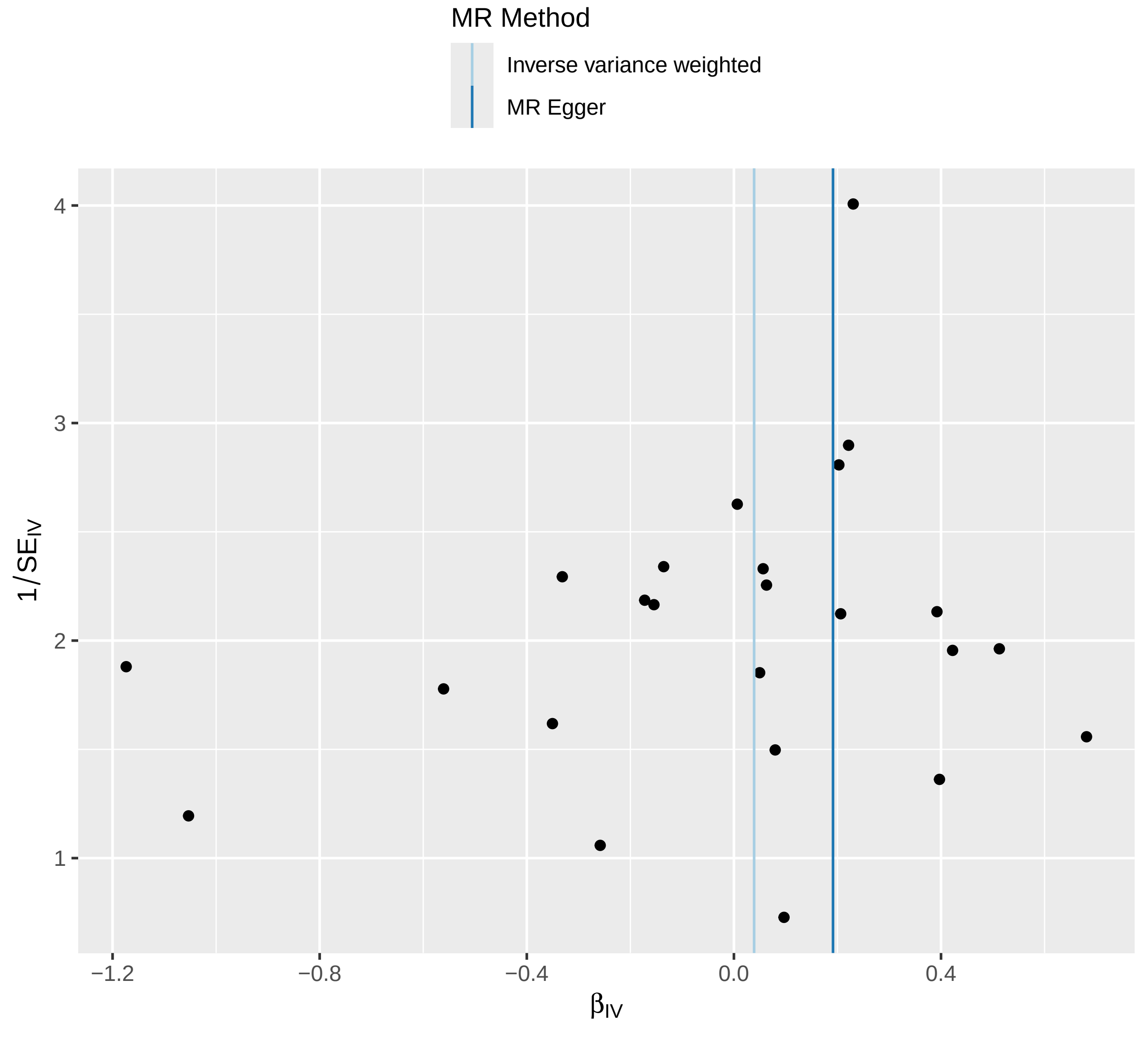
MR Method
Inverse variance weighted
MR Egger
1/SE_IV
4
3
2
1
−1.2
−0.8
−0.4
0.0
0.4
β_IV

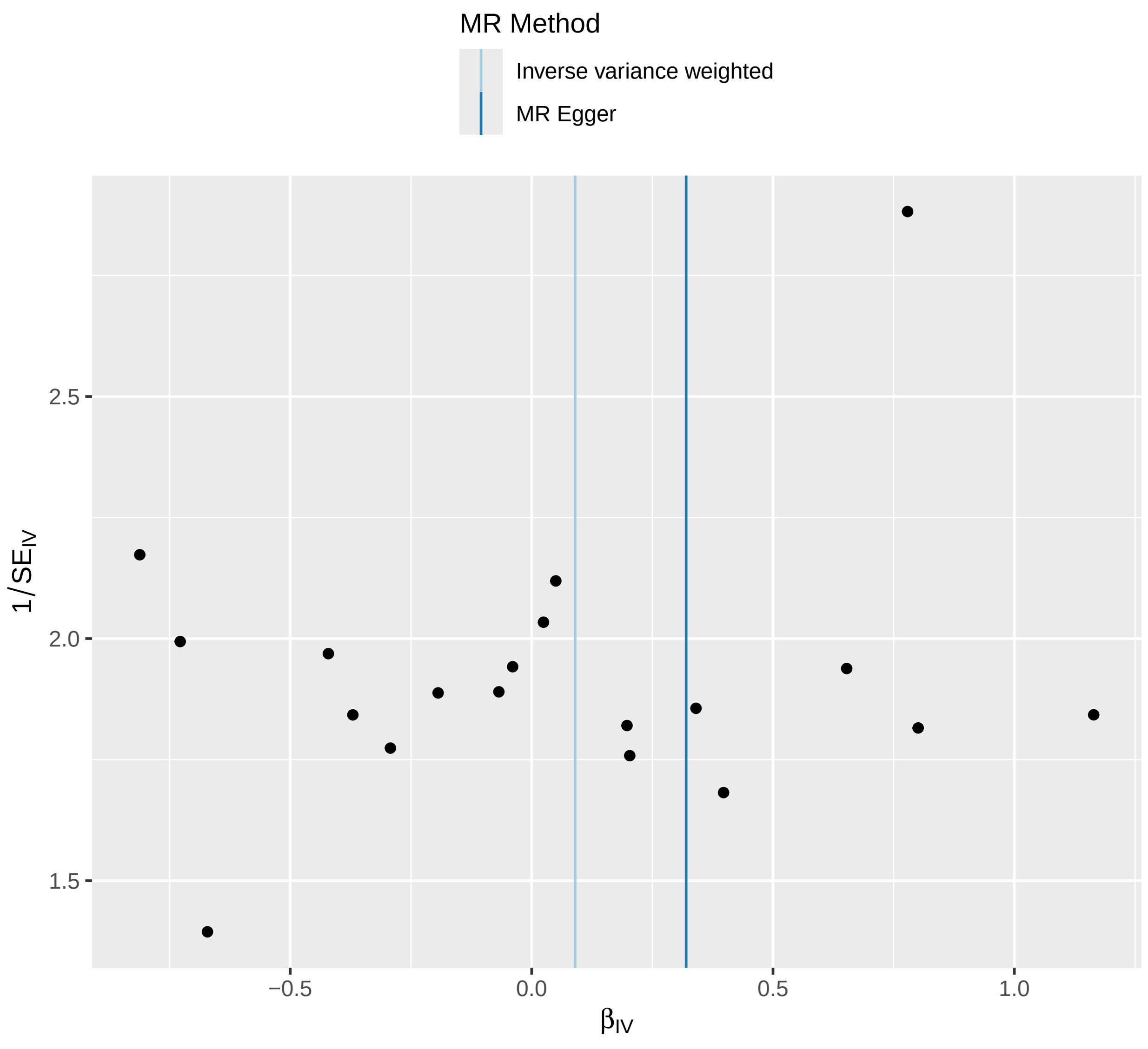

MR Method
Inverse variance weighted
MR Egger
2.5
2.0
1.5
1/SE_IV
−0.5
0.0
0.5
1.0
β_IV

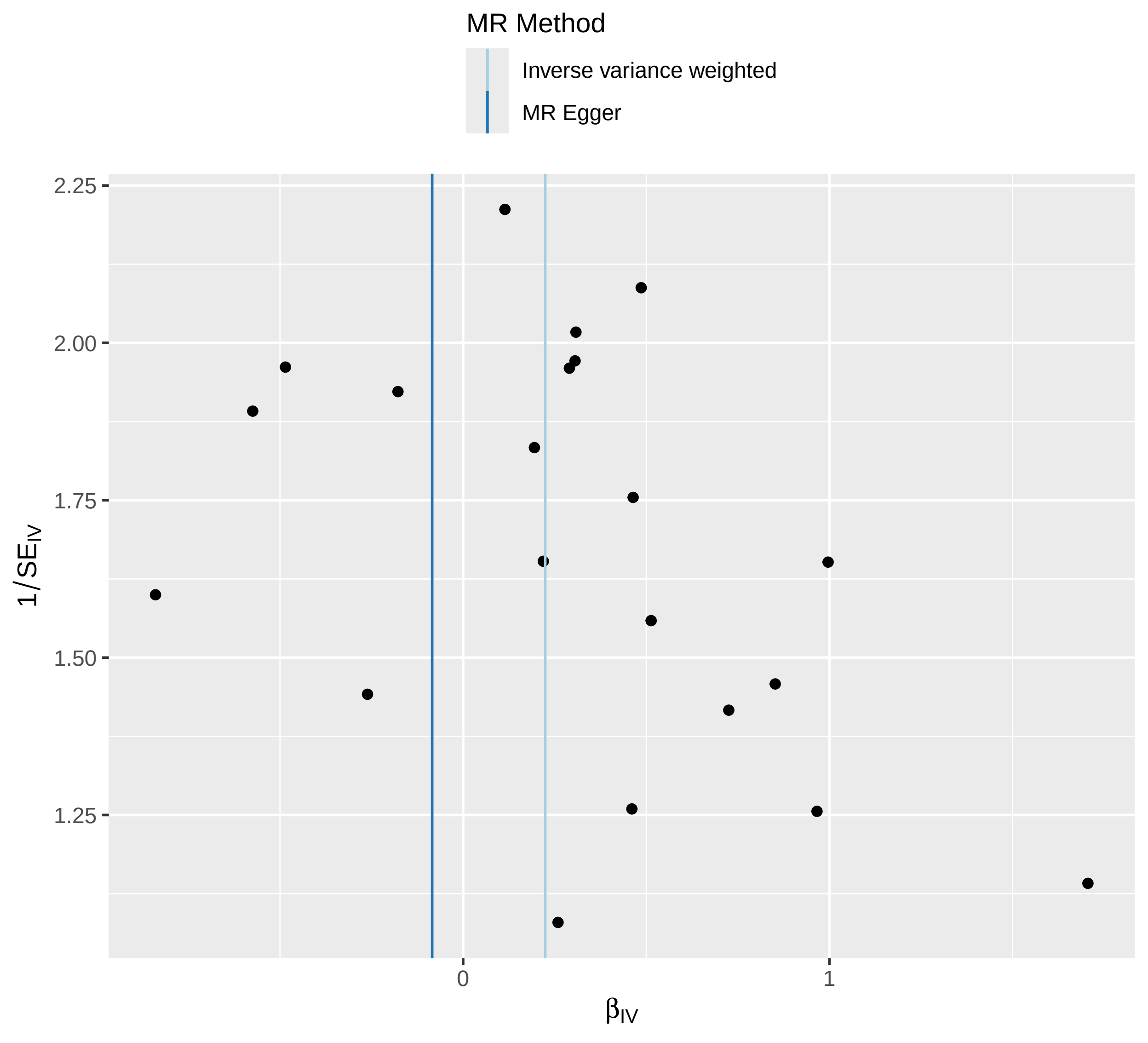
MR Method
Inverse variance weighted
MR Egger
2.25
2.00
1.75
1.50
1.25
$1/SE_{IV}$
0
1
β_{IV}

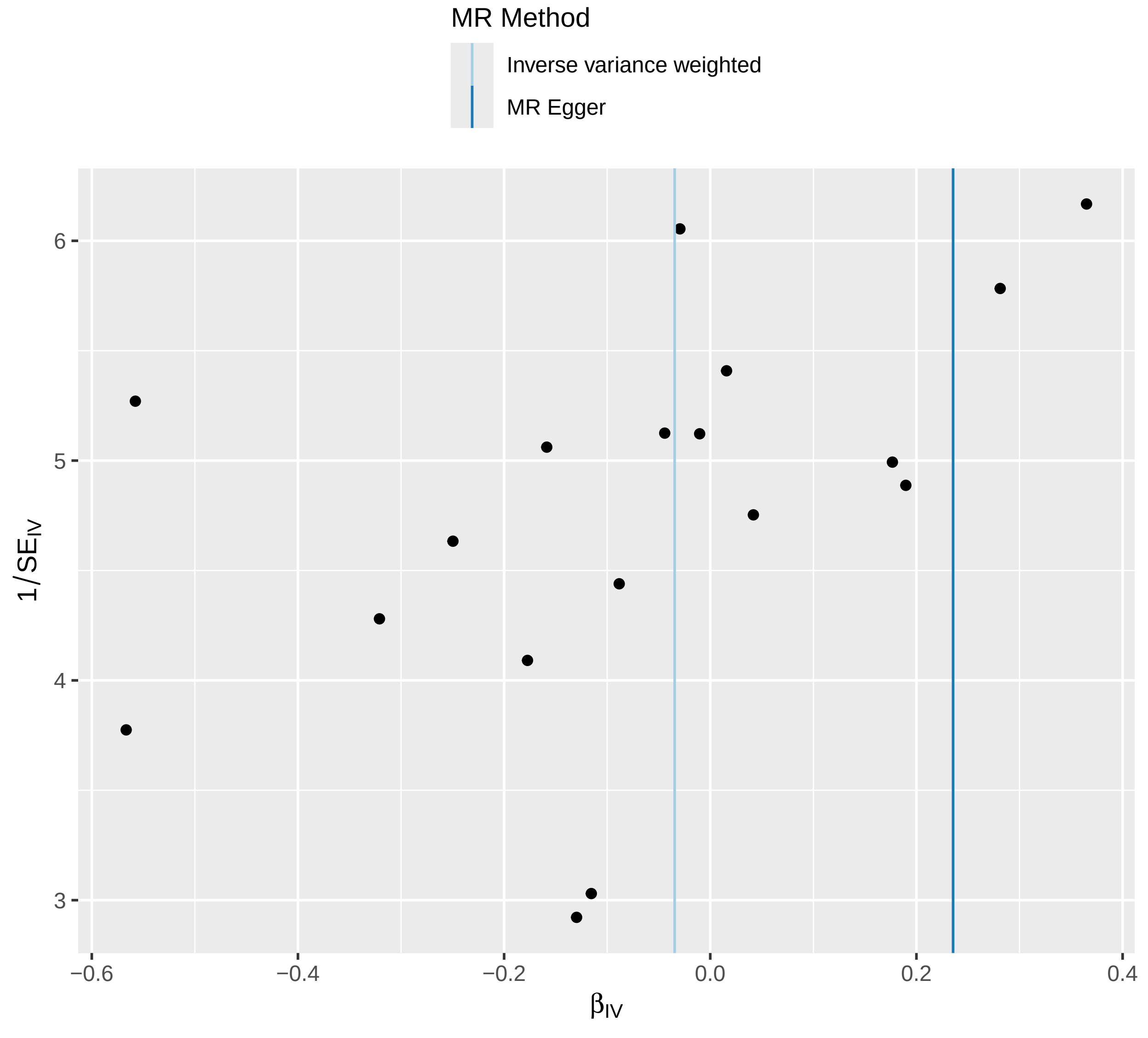

MR Method
Inverse variance weighted
MR Egger
6
5
4
3
1/SE_IV
−0.6
−0.4
−0.2
0.0
0.2
0.4
β_IV

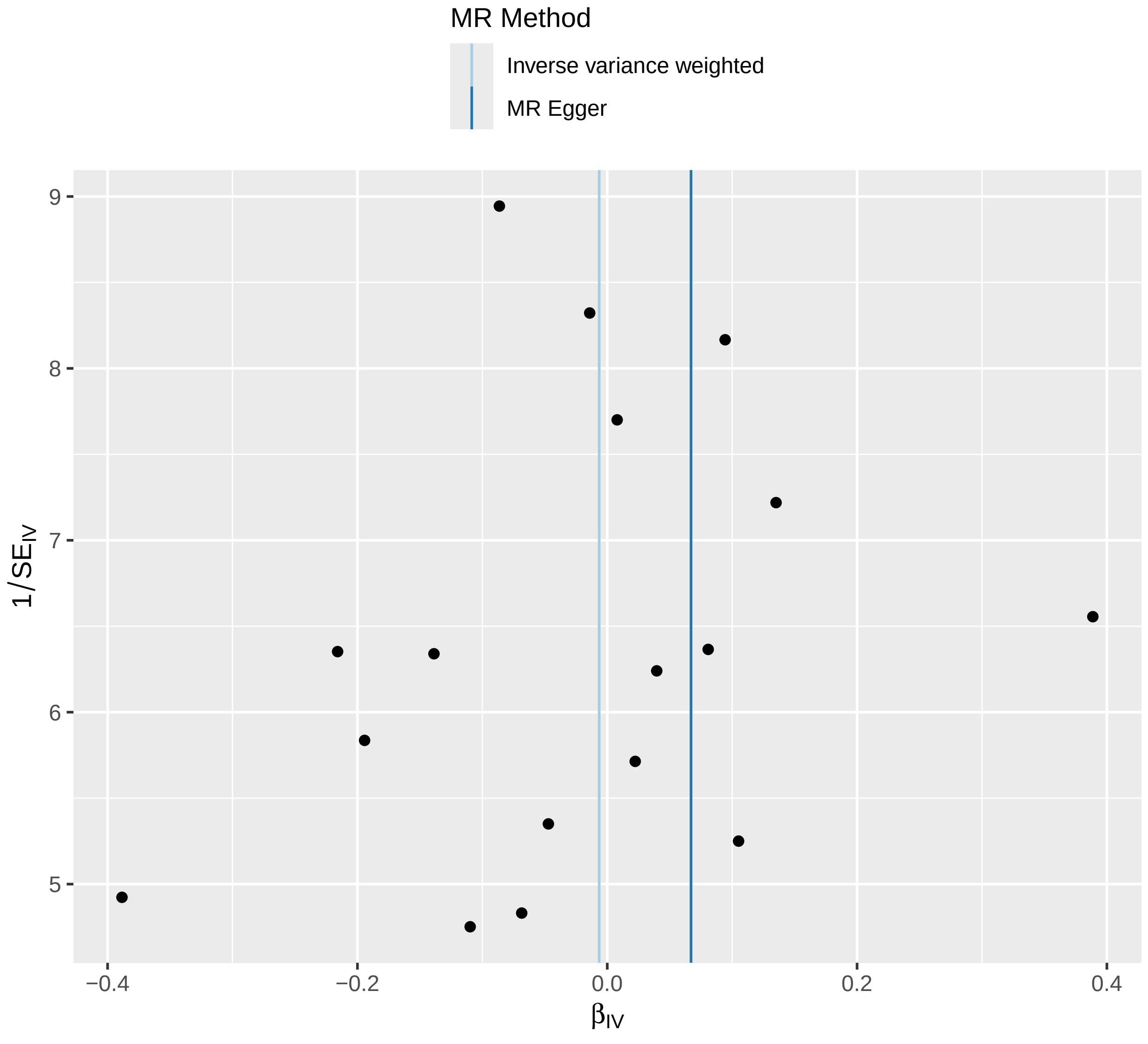
MR Method
Inverse variance weighted
MR Egger
9
8
7
6
5
1/SE_IV
−0.4
−0.2
0.0
0.2
0.4
β_IV

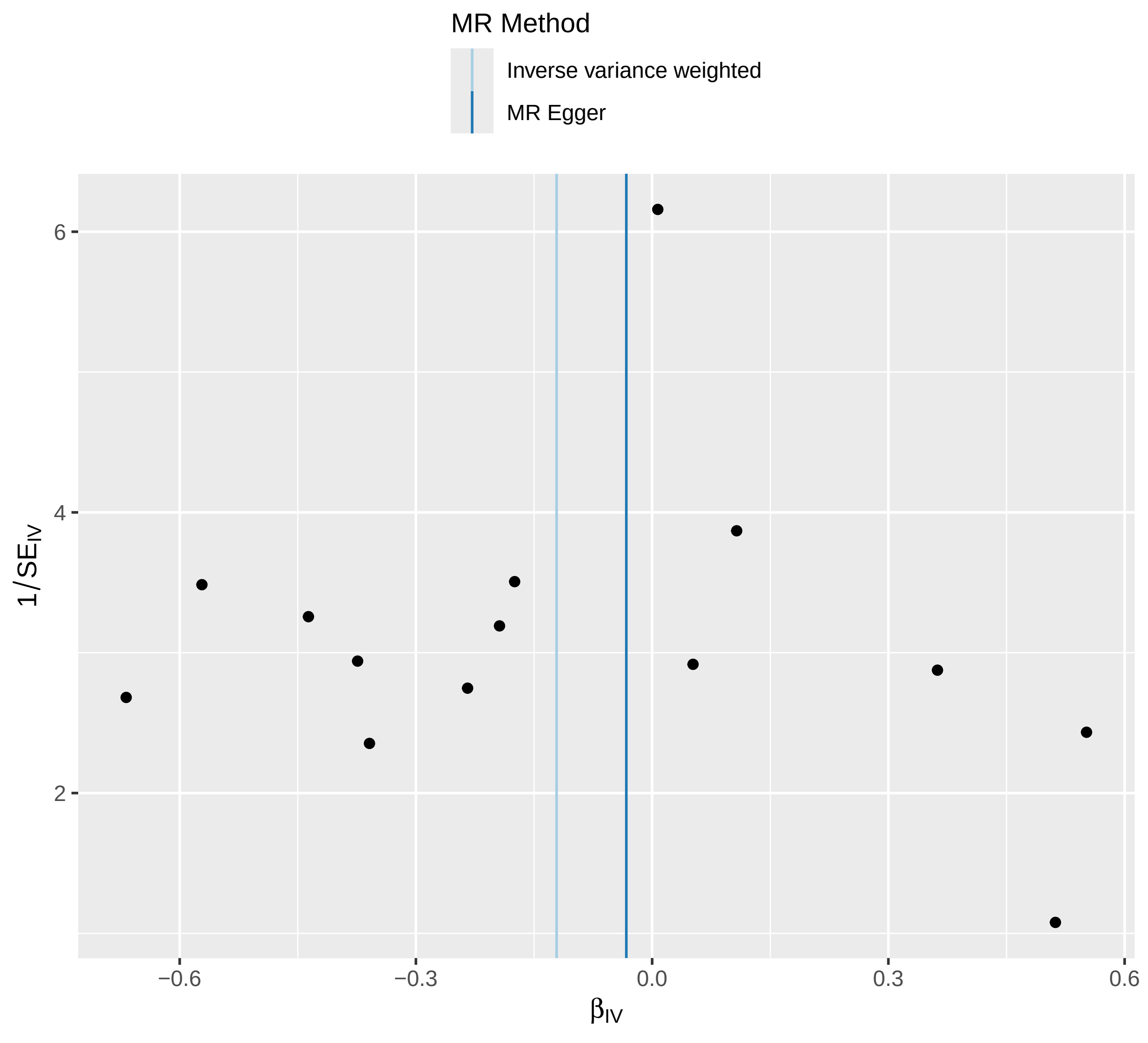
MR Method
Inverse variance weighted
MR Egger
6
4
2
1/SE_IV
−0.6
−0.3
0.0
0.3
0.6
β_IV

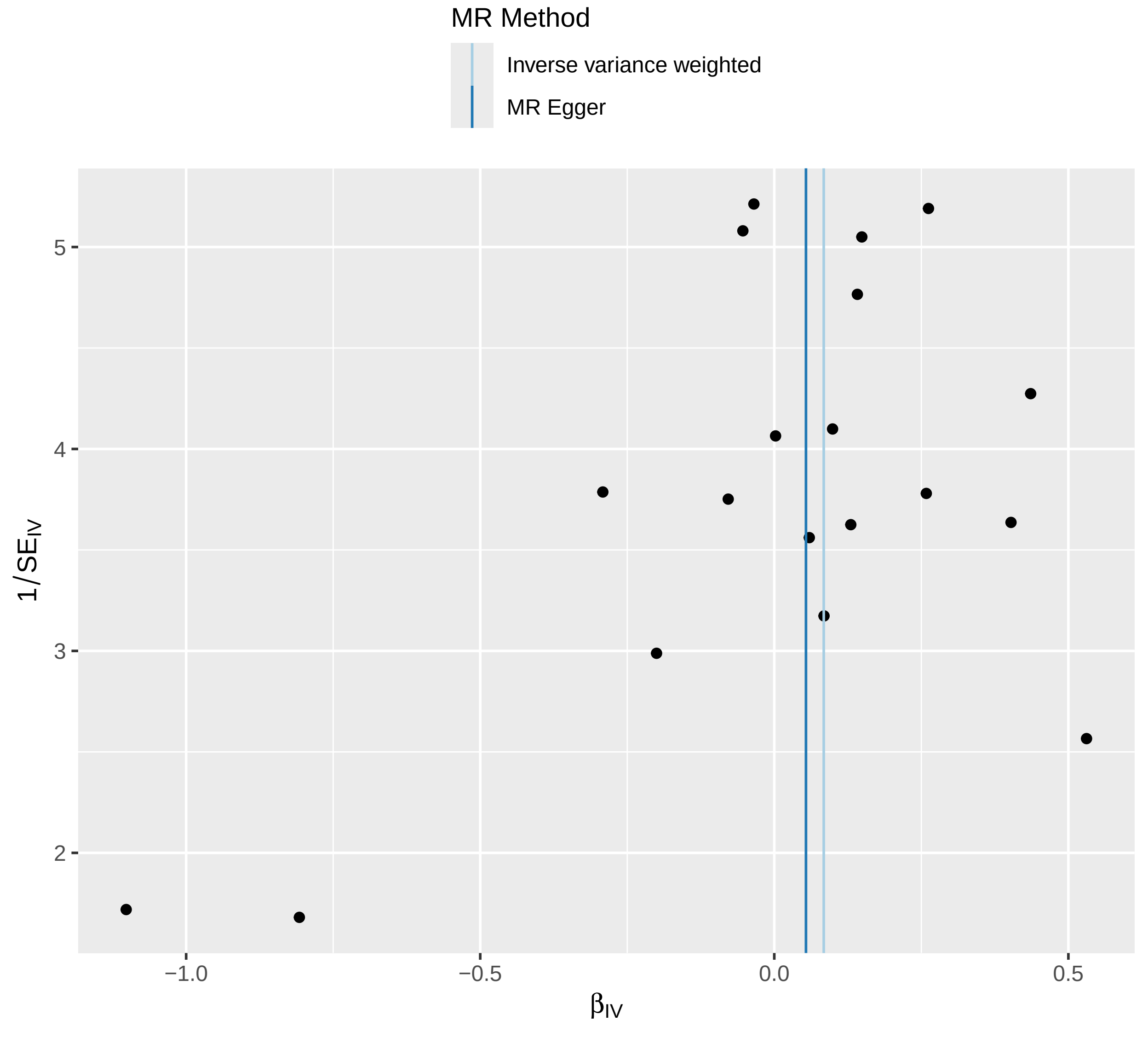
MR Method
Inverse variance weighted
MR Egger
5
4
3
2
1/SE_IV
−1.0
−0.5
0.0
0.5
β_IV

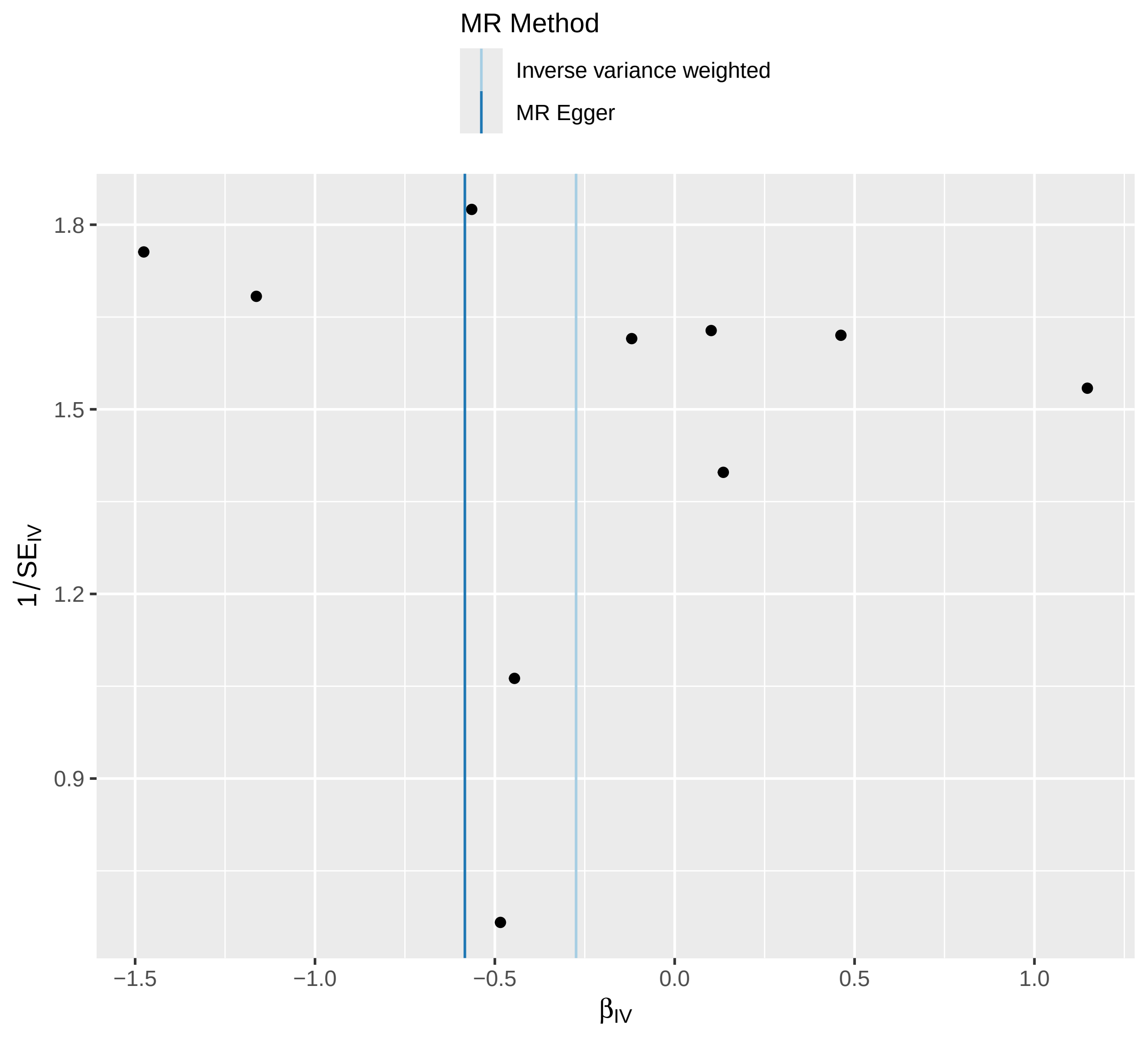
MR Method
Inverse variance weighted
MR Egger
1.8
1.5
1.2
0.9
−1.5
−1.0
−0.5
0.0
0.5
1.0
$1/SE_{IV}$
β_{IV}

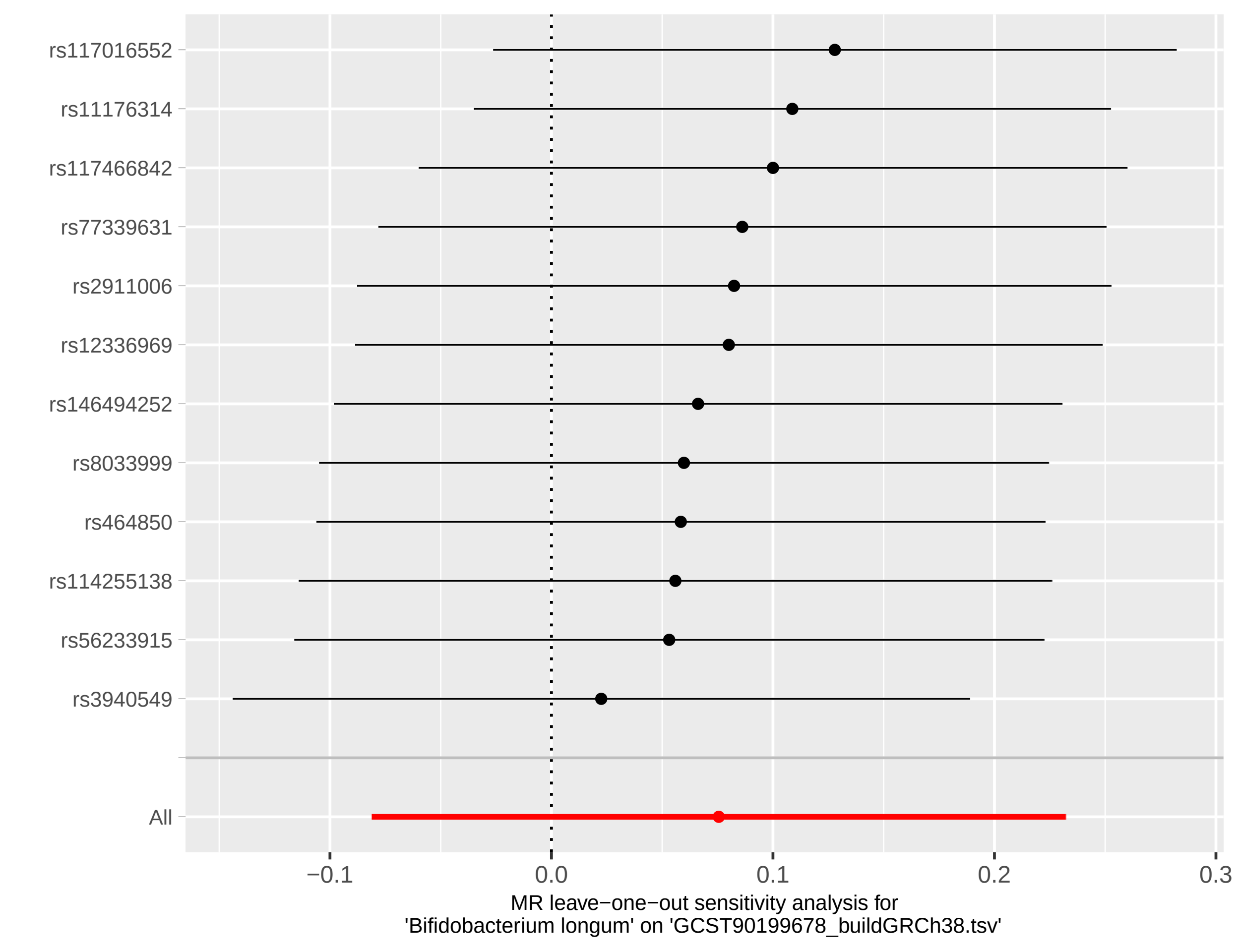
rs117016552
rs11176314
rs117466842
rs77339631
rs2911006
rs12336969
rs146494252
rs8033999
rs464850
rs114255138
rs56233915
rs3940549
All
−0.1
0.0
0.1
0.2
0.3
MR leave−one−out sensitivity analysis for
'Bifidobacterium longum' on 'GCST90199678_buildGRCh38.tsv'

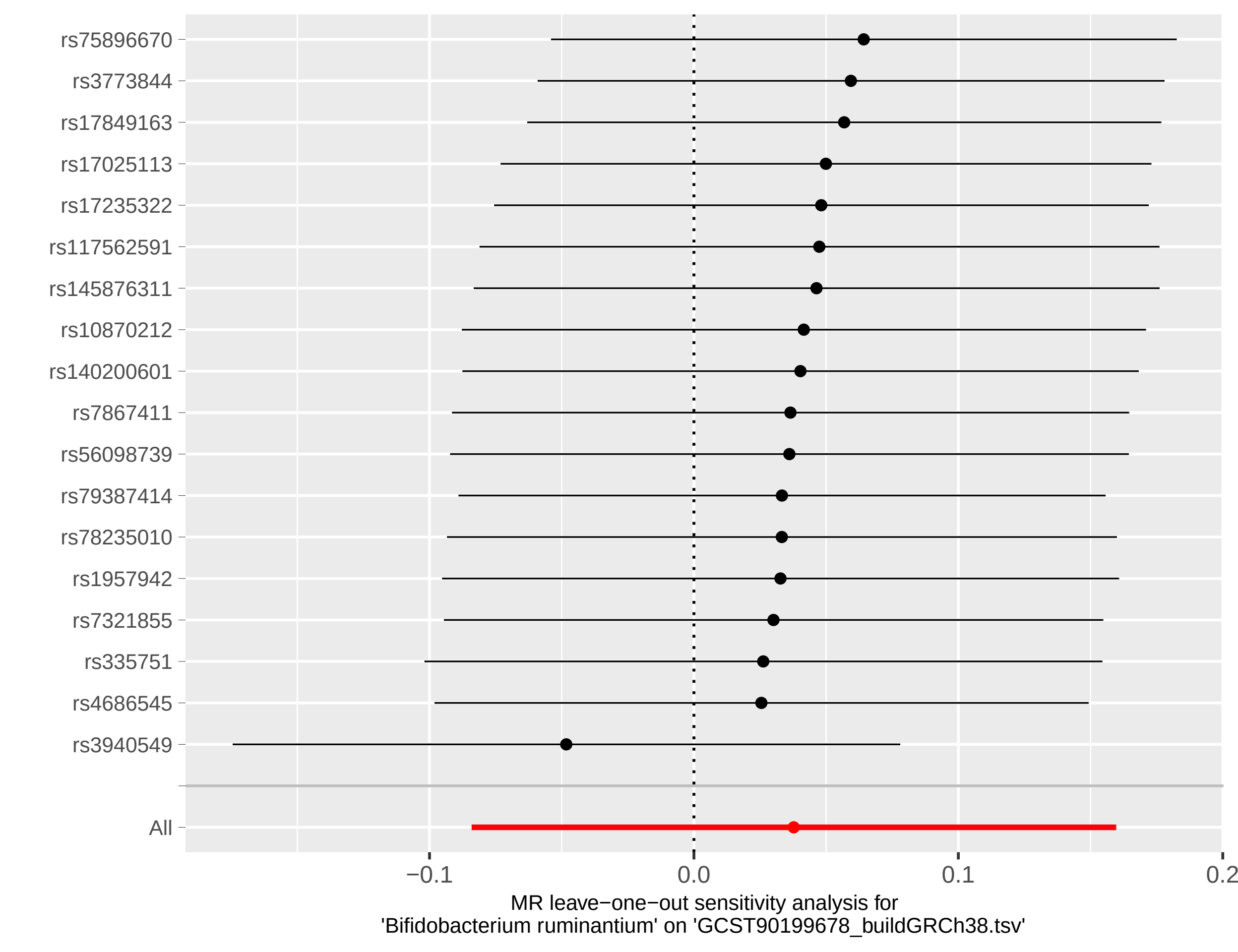
rs75896670
rs3773844
rs17849163
rs17025113
rs17235322
rs117562591
rs145876311
rs10870212
rs140200601
rs7867411
rs56098739
rs79387414
rs78235010
rs1957942
rs7321855
rs335751
rs4686545
rs3940549
All
−0.1
0.0
0.1
0.2
MR leave−one−out sensitivity analysis for
'Bifidobacterium ruminantium' on 'GCST90199678_buildGRCh38.tsv'

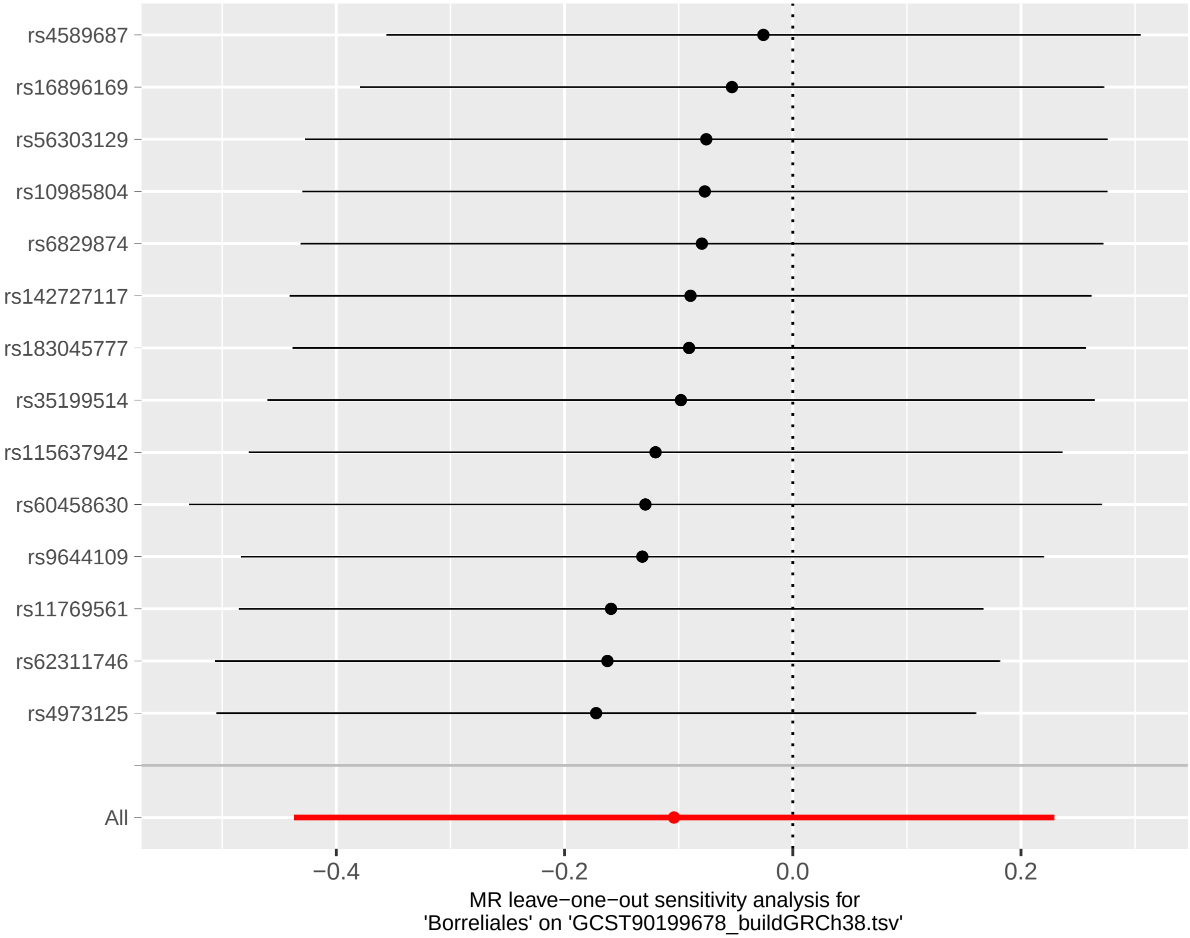
rs4589687
rs16896169
rs56303129
rs10985804
rs6829874
rs142727117
rs183045777
rs35199514
rs115637942
rs60458630
rs9644109
rs11769561
rs62311746
rs4973125
All
−0.4
−0.2
0.0
0.2
MR leave−one−out sensitivity analysis for
'Borreliales' on 'GCST90199678_buildGRCh38.tsv'

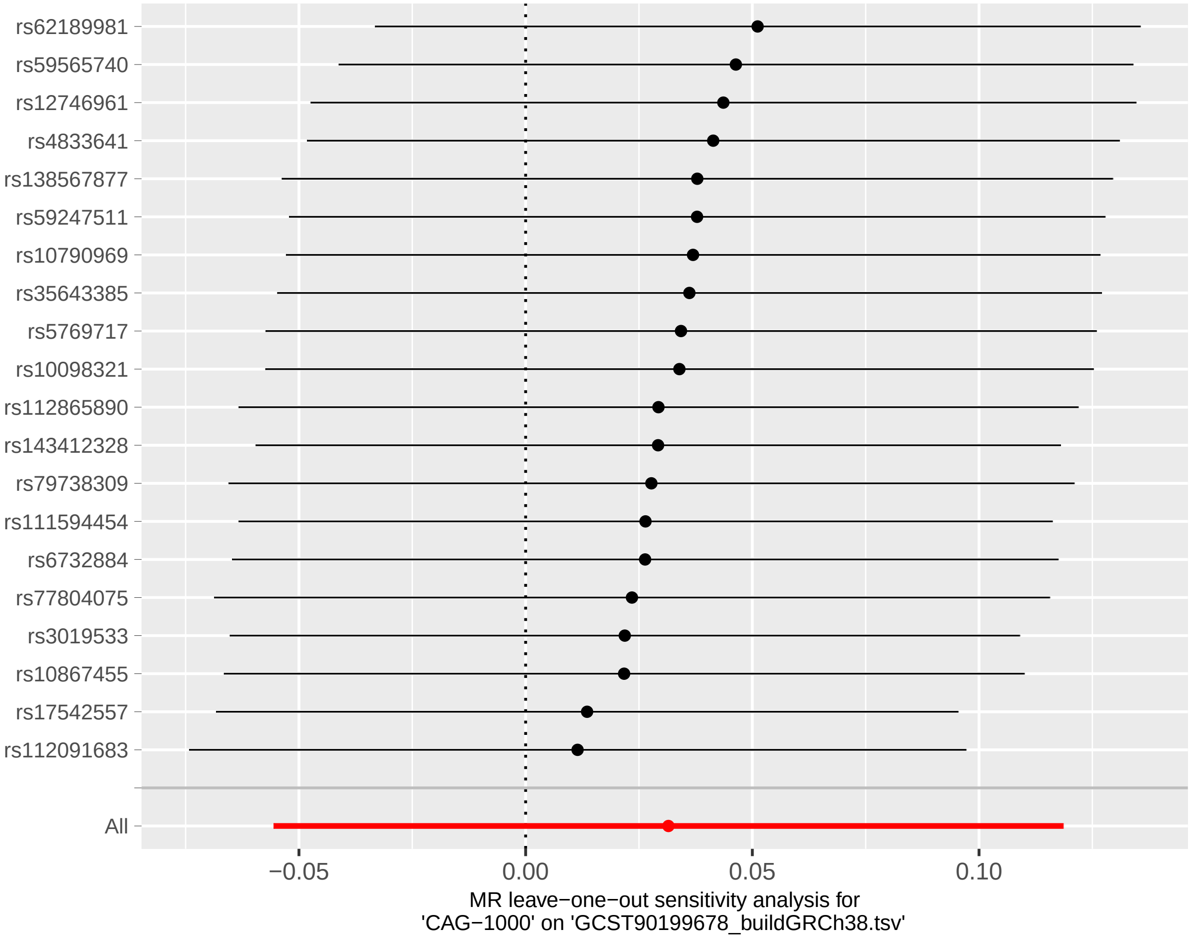
rs62189981
rs59565740
rs12746961
rs4833641
rs138567877
rs59247511
rs10790969
rs35643385
rs5769717
rs10098321
rs112865890
rs143412328
rs79738309
rs111594454
rs6732884
rs77804075
rs3019533
rs10867455
rs17542557
rs112091683
All
−0.05
0.00
0.05
0.10
MR leave−one−out sensitivity analysis for
'CAG−1000' on 'GCST90199678_buildGRCh38.tsv'

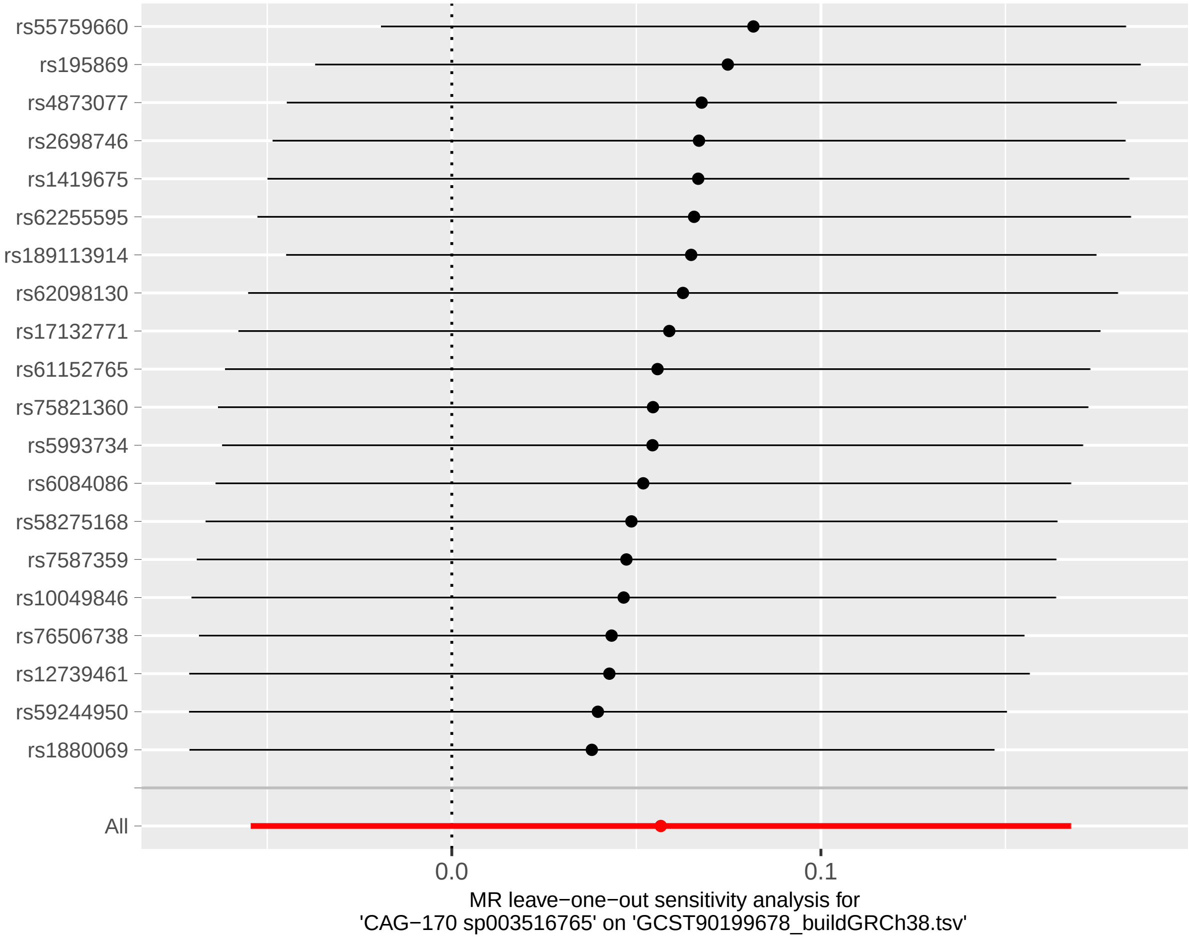
rs55759660
rs195869
rs4873077
rs2698746
rs1419675
rs62255595
rs189113914
rs62098130
rs17132771
rs61152765
rs75821360
rs5993734
rs6084086
rs58275168
rs7587359
rs10049846
rs76506738
rs12739461
rs59244950
rs1880069
All
0.0
0.1
MR leave−one−out sensitivity analysis for
'CAG−170 sp003516765' on 'GCST90199678_buildGRCh38.tsv'

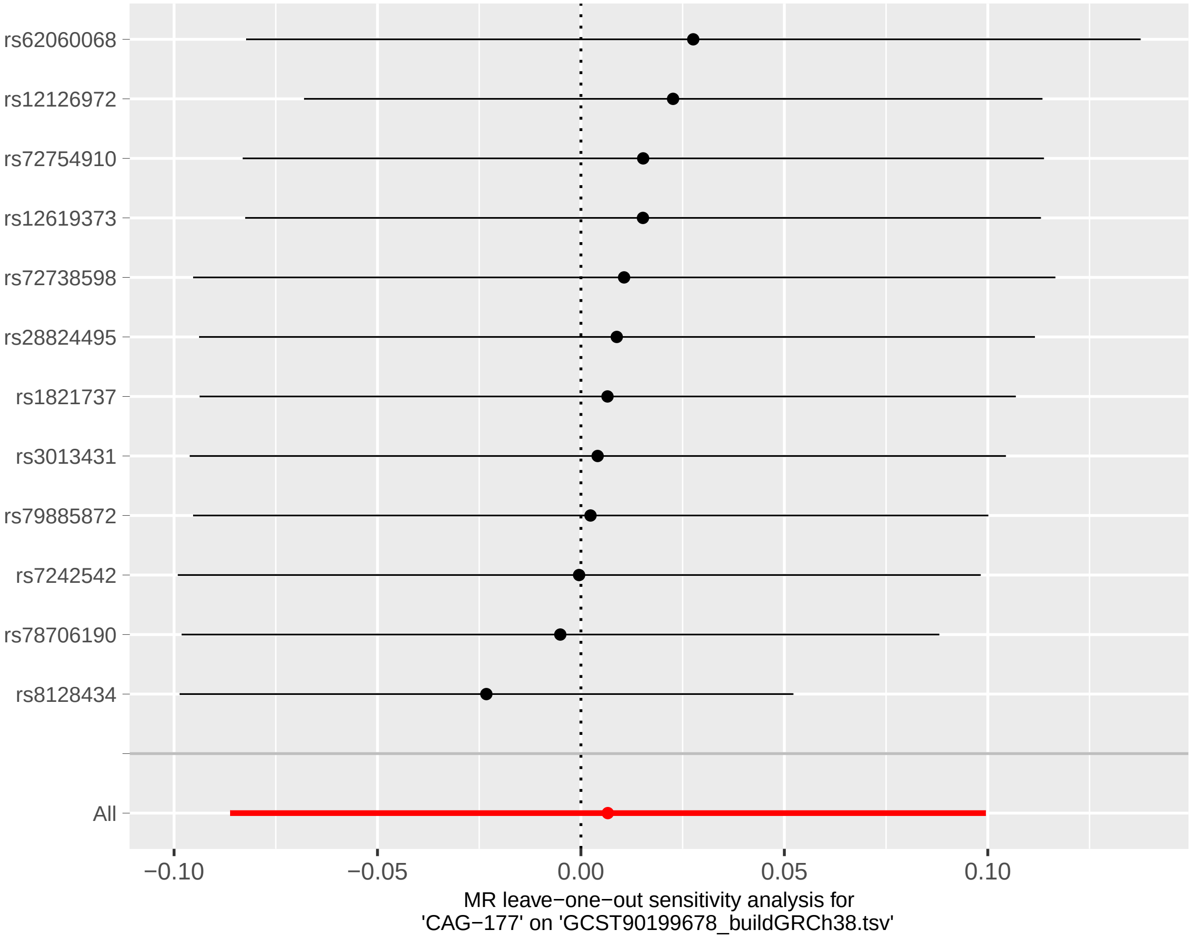
rs62060068
rs12126972
rs72754910
rs12619373
rs72738598
rs28824495
rs1821737
rs3013431
rs79885872
rs7242542
rs78706190
rs8128434
All
−0.10
−0.05
0.00
0.05
0.10
MR leave−one−out sensitivity analysis for
'CAG−177' on 'GCST90199678_buildGRCh38.tsv'

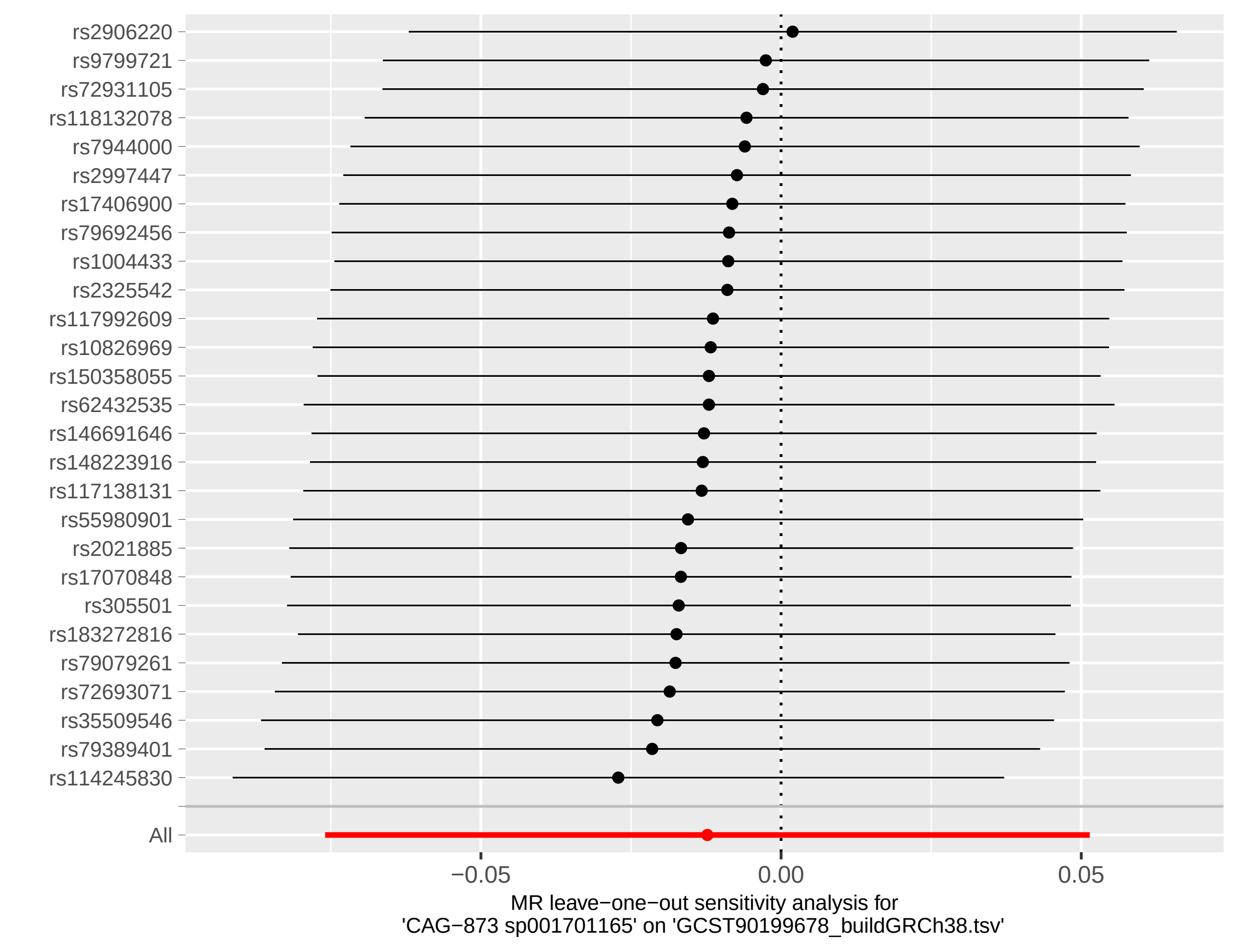
rs2906220
rs9799721
rs72931105
rs118132078
rs7944000
rs2997447
rs17406900
rs79692456
rs1004433
rs2325542
rs117992609
rs10826969
rs150358055
rs62432535
rs146691646
rs148223916
rs117138131
rs55980901
rs2021885
rs17070848
rs305501
rs183272816
rs79079261
rs72693071
rs35509546
rs79389401
rs114245830
All
−0.05
0.00
0.05
MR leave−one−out sensitivity analysis for
'CAG−873 sp001701165' on 'GCST90199678_buildGRCh38.tsv'

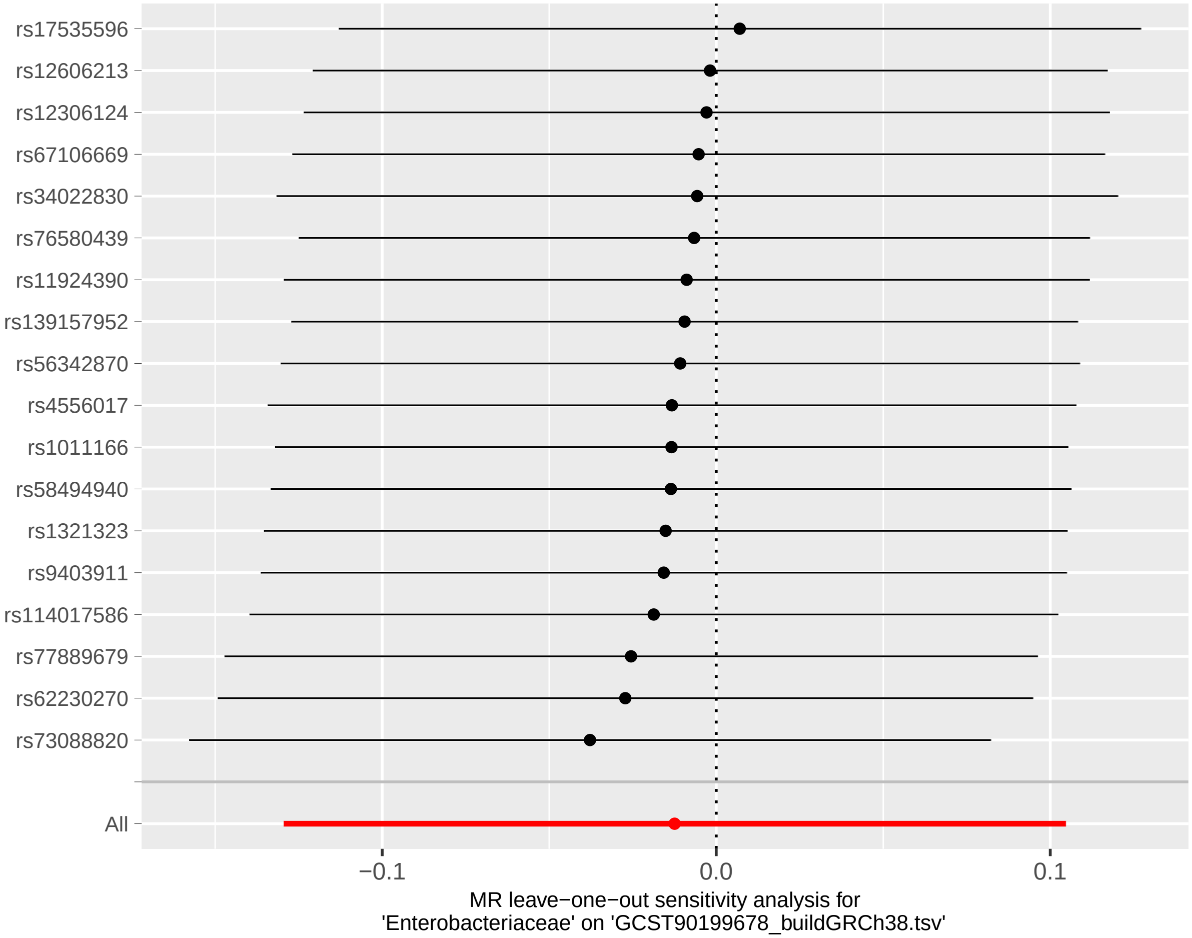
rs17535596
rs12606213
rs12306124
rs67106669
rs34022830
rs76580439
rs11924390
rs139157952
rs56342870
rs4556017
rs1011166
rs58494940
rs1321323
rs9403911
rs114017586
rs77889679
rs62230270
rs73088820
All
−0.1
0.0
0.1
MR leave−one−out sensitivity analysis for
'Enterobacteriaceae' on 'GCST90199678_buildGRCh38.tsv'

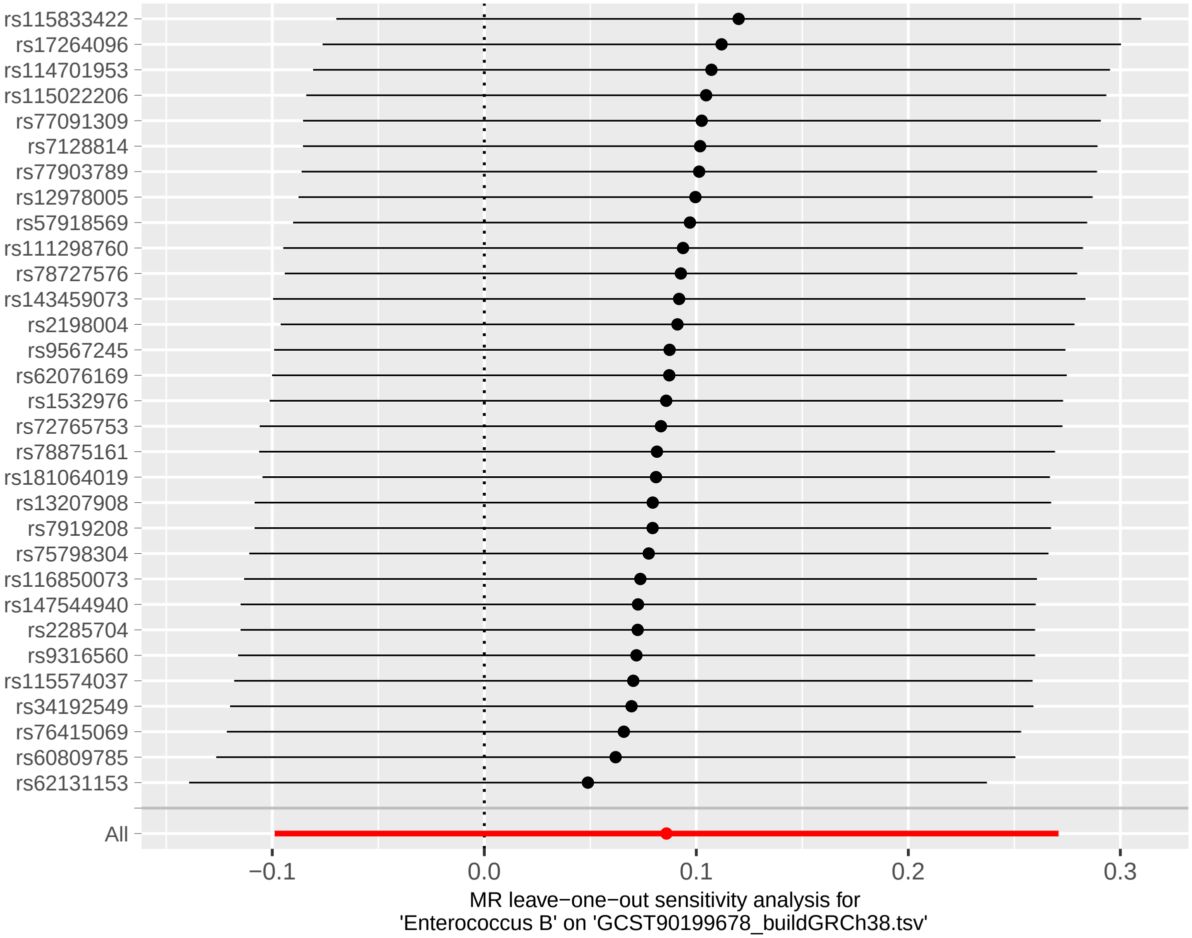

rs115833422
rs17264096
rs114701953
rs115022206
rs77091309
rs7128814
rs77903789
rs12978005
rs57918569
rs111298760
rs78727576
rs143459073
rs2198004
rs9567245
rs62076169
rs1532976
rs72765753
rs78875161
rs181064019
rs13207908
rs7919208
rs75798304
rs116850073
rs147544940
rs2285704
rs9316560
rs115574037
rs34192549
rs76415069
rs60809785
rs62131153
All
−0.1
0.0
0.1
0.2
0.3
MR leave−one−out sensitivity analysis for
'Enterococcus B' on 'GCST90199678_buildGRCh38.tsv'

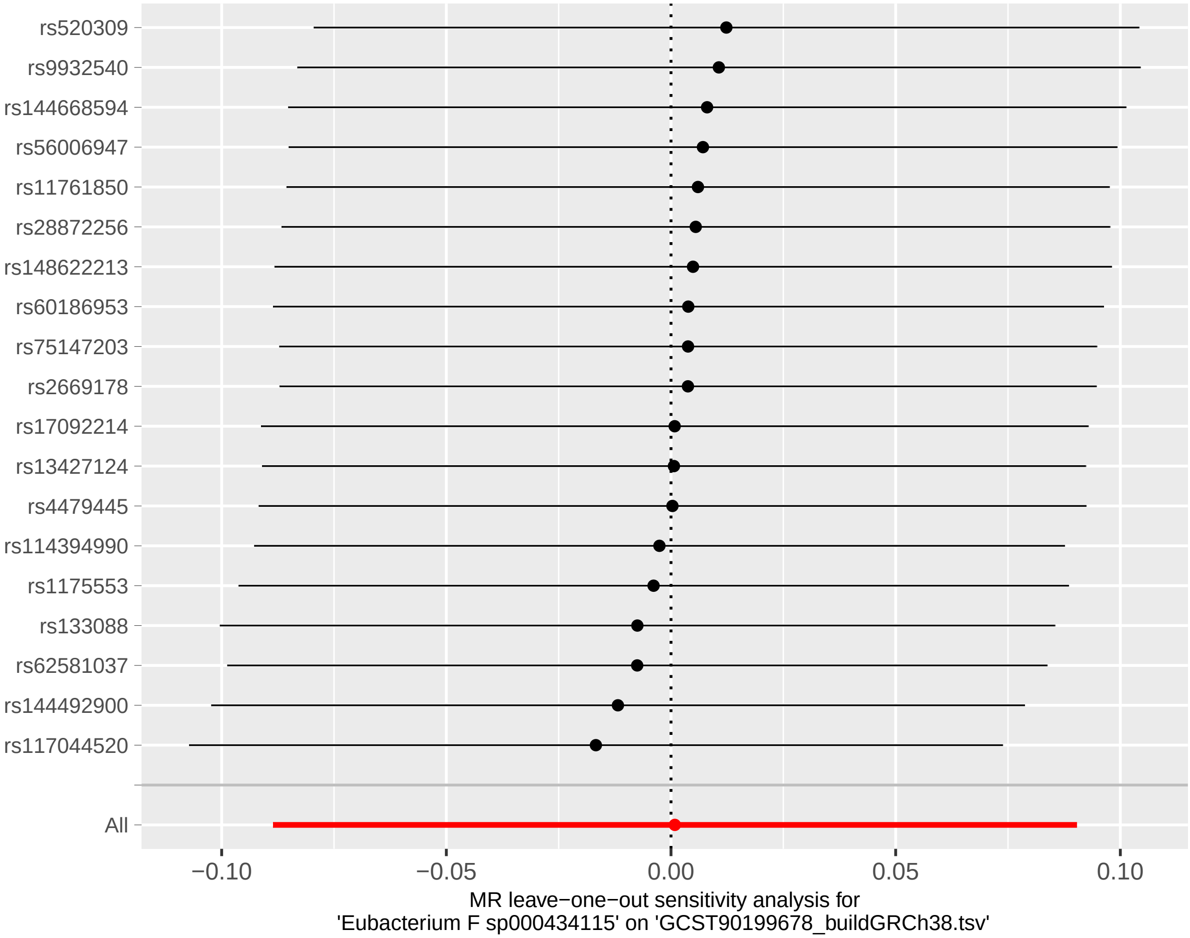

rs520309
rs9932540
rs144668594
rs56006947
rs11761850
rs28872256
rs148622213
rs60186953
rs75147203
rs2669178
rs17092214
rs13427124
rs4479445
rs114394990
rs1175553
rs133088
rs62581037
rs144492900
rs117044520
All
−0.10
−0.05
0.00
0.05
0.10
MR leave−one−out sensitivity analysis for
'Eubacterium F sp000434115' on 'GCST90199678_buildGRCh38.tsv'

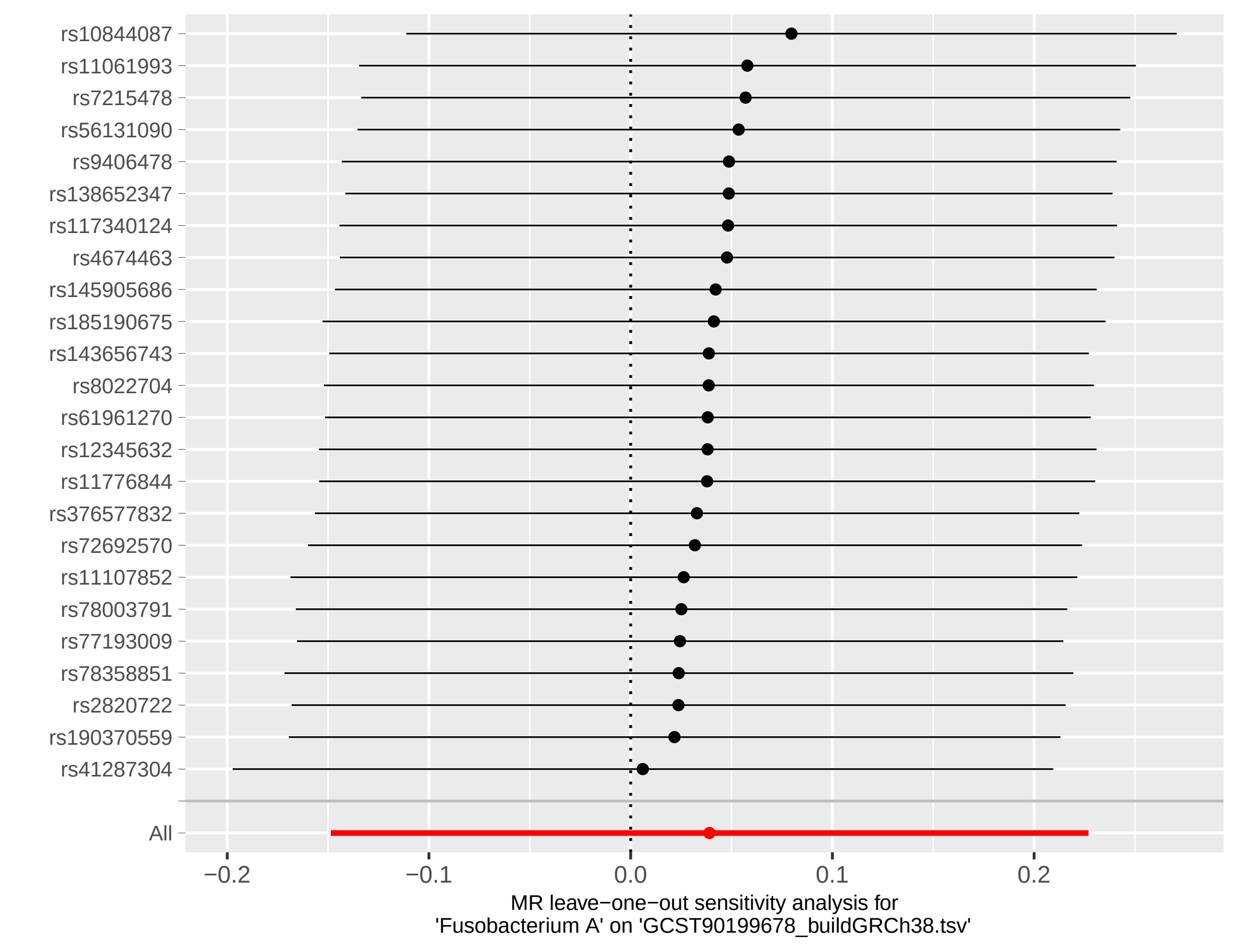

rs10844087
rs11061993
rs7215478
rs56131090
rs9406478
rs138652347
rs117340124
rs4674463
rs145905686
rs185190675
rs143656743
rs8022704
rs61961270
rs12345632
rs11776844
rs376577832
rs72692570
rs11107852
rs78003791
rs77193009
rs78358851
rs2820722
rs190370559
rs41287304
All
−0.2
−0.1
0.0
0.1
0.2
MR leave−one−out sensitivity analysis for
'Fusobacterium A' on 'GCST90199678_buildGRCh38.tsv'

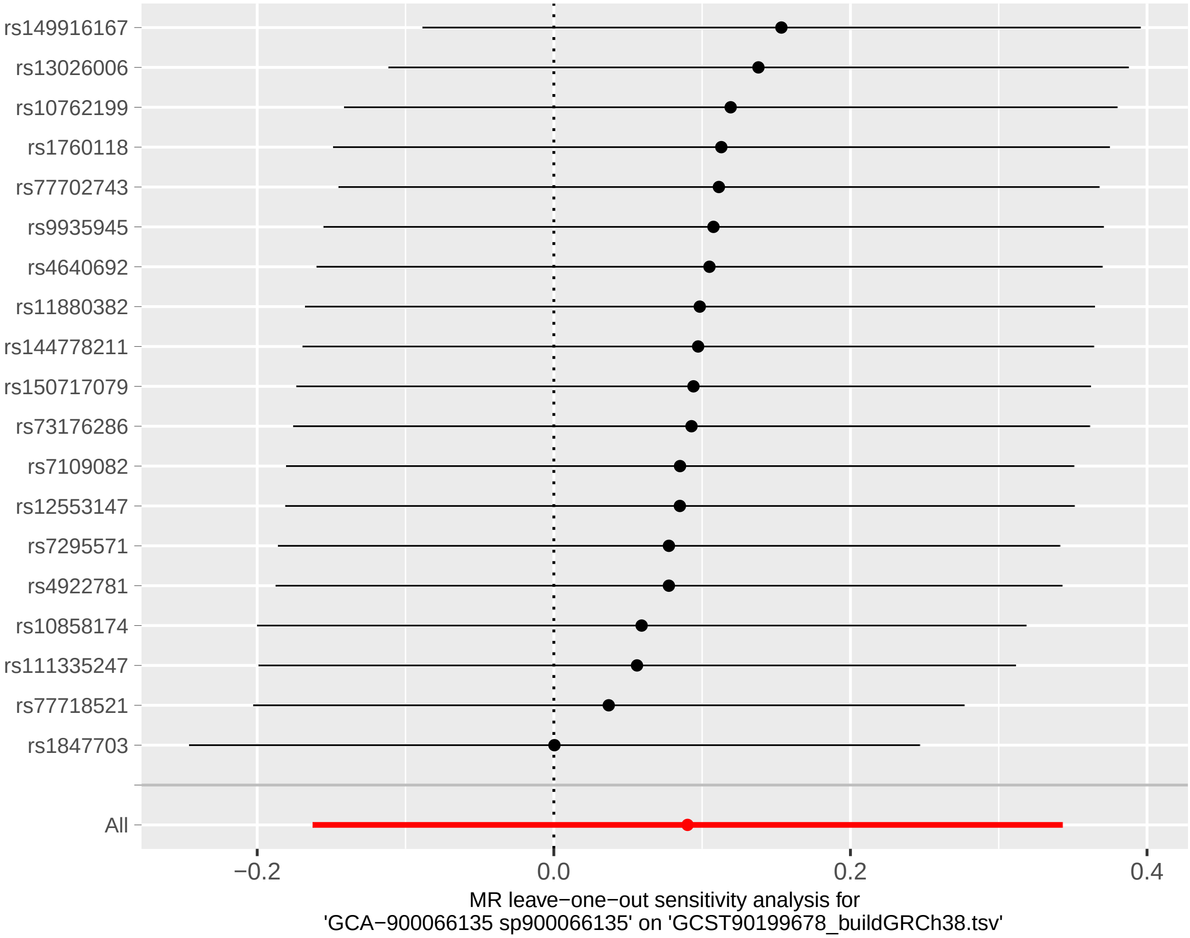

rs149916167
rs13026006
rs10762199
rs1760118
rs77702743
rs9935945
rs4640692
rs11880382
rs144778211
rs150717079
rs73176286
rs7109082
rs12553147
rs7295571
rs4922781
rs10858174
rs111335247
rs77718521
rs1847703
All
−0.2
0.0
0.2
0.4
MR leave−one−out sensitivity analysis for
'GCA−900066135 sp900066135' on 'GCST90199678_buildGRCh38.tsv'

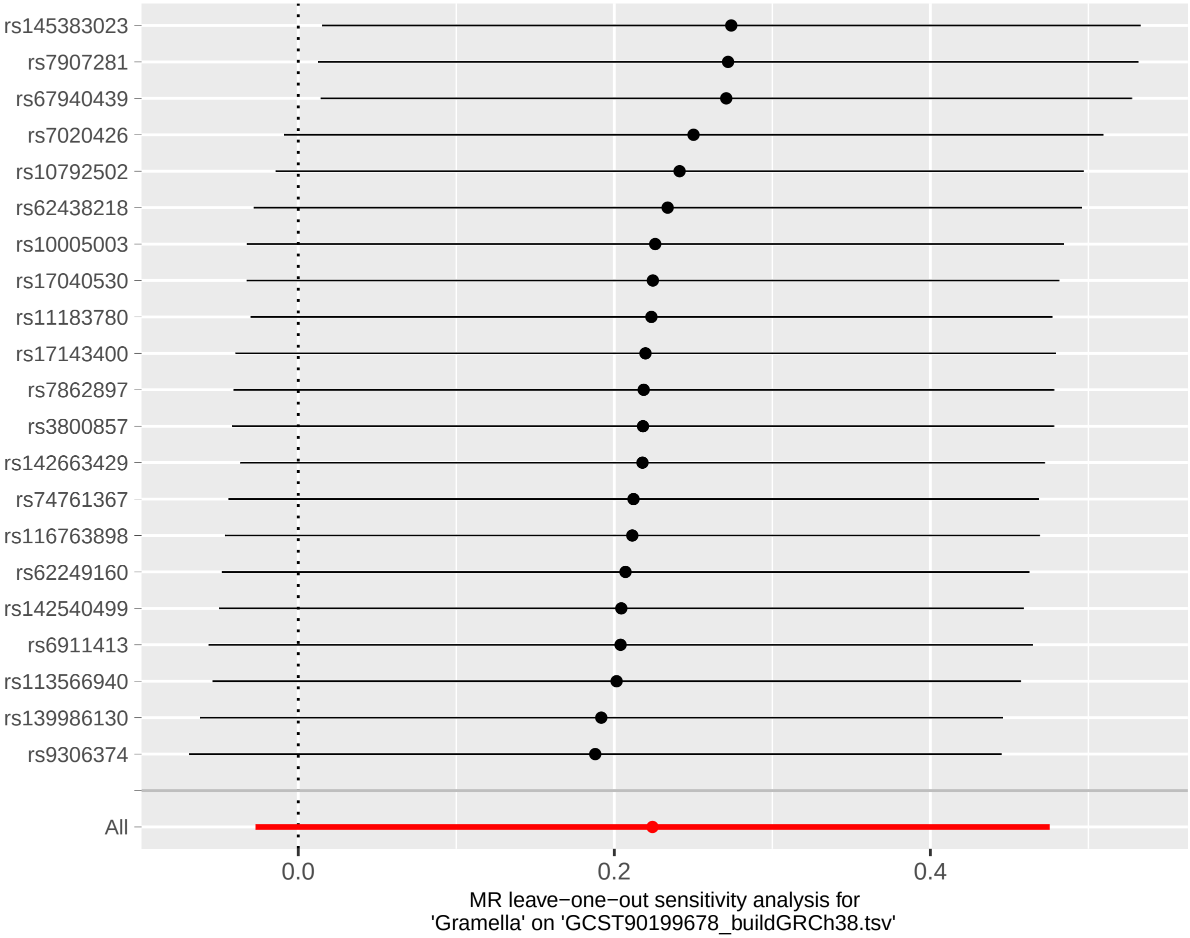
rs145383023
rs7907281
rs67940439
rs7020426
rs10792502
rs62438218
rs10005003
rs17040530
rs11183780
rs17143400
rs7862897
rs3800857
rs142663429
rs74761367
rs116763898
rs62249160
rs142540499
rs6911413
rs113566940
rs139986130
rs9306374
All
0.0
0.2
0.4
MR leave−one−out sensitivity analysis for
'Gramella' on 'GCST90199678_buildGRCh38.tsv'

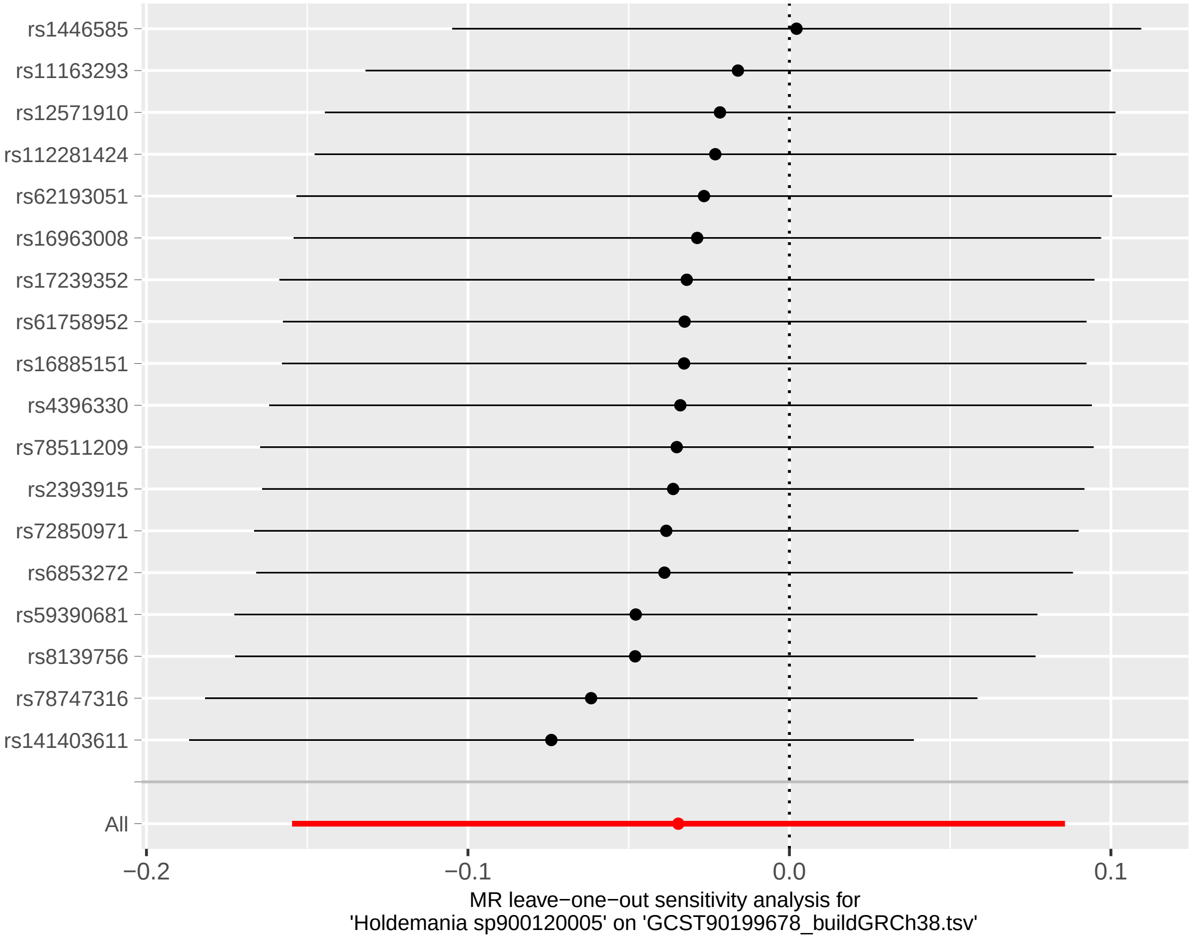
rs1446585
rs11163293
rs12571910
rs112281424
rs62193051
rs16963008
rs17239352
rs61758952
rs16885151
rs4396330
rs78511209
rs2393915
rs72850971
rs6853272
rs59390681
rs8139756
rs78747316
rs141403611
All
−0.2
−0.1
0.0
0.1
MR leave−one−out sensitivity analysis for
'Holdemania sp900120005' on 'GCST90199678_buildGRCh38.tsv'

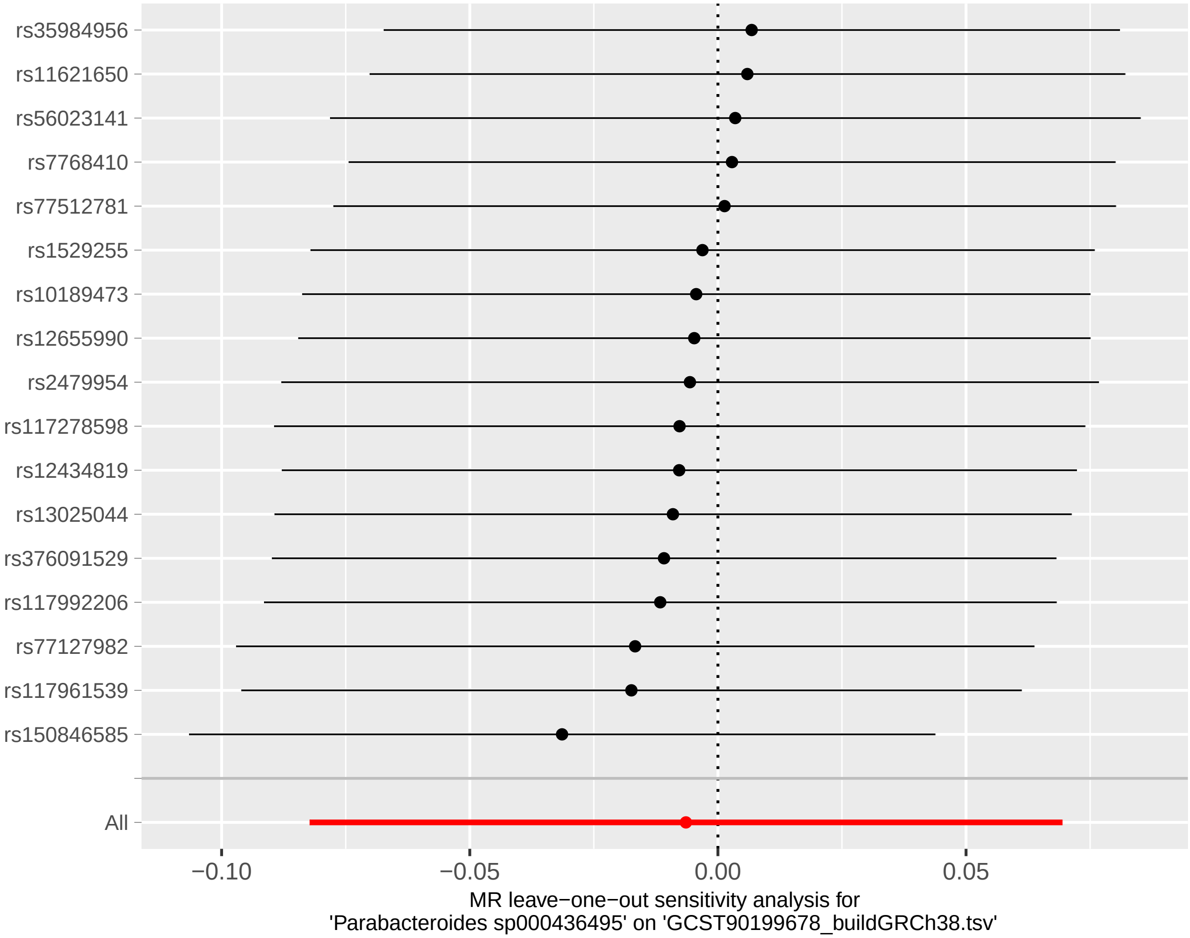

rs35984956
rs11621650
rs56023141
rs7768410
rs77512781
rs1529255
rs10189473
rs12655990
rs2479954
rs117278598
rs12434819
rs13025044
rs376091529
rs117992206
rs77127982
rs117961539
rs150846585
All
−0.10
−0.05
0.00
0.05
MR leave−one−out sensitivity analysis for
'Parabacteroides sp000436495' on 'GCST90199678_buildGRCh38.tsv'

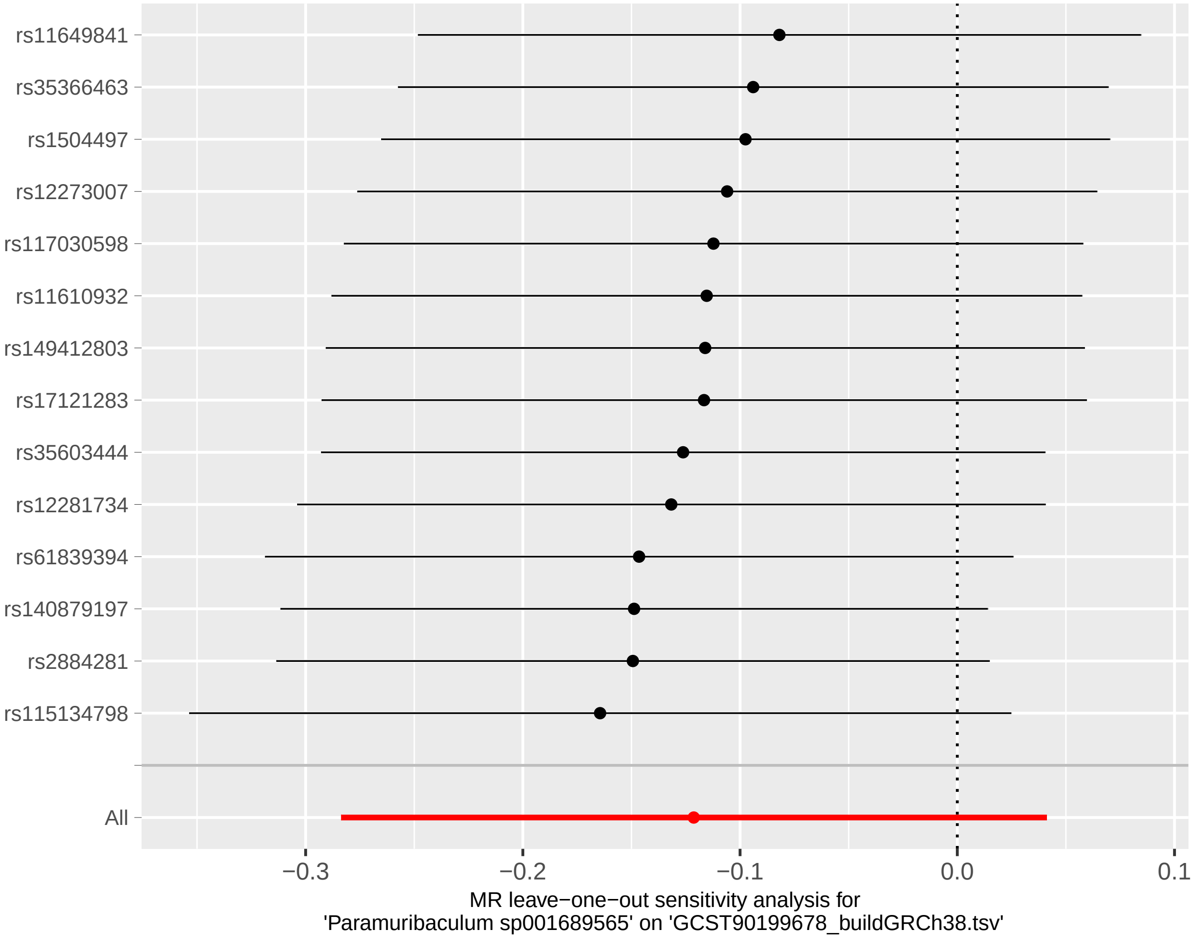
rs11649841
rs35366463
rs1504497
rs12273007
rs117030598
rs11610932
rs149412803
rs17121283
rs35603444
rs12281734
rs61839394
rs140879197
rs2884281
rs115134798
All
−0.3
−0.2
−0.1
0.0
0.1
MR leave−one−out sensitivity analysis for
'Paramuribaculum sp001689565' on 'GCST90199678_buildGRCh38.tsv'

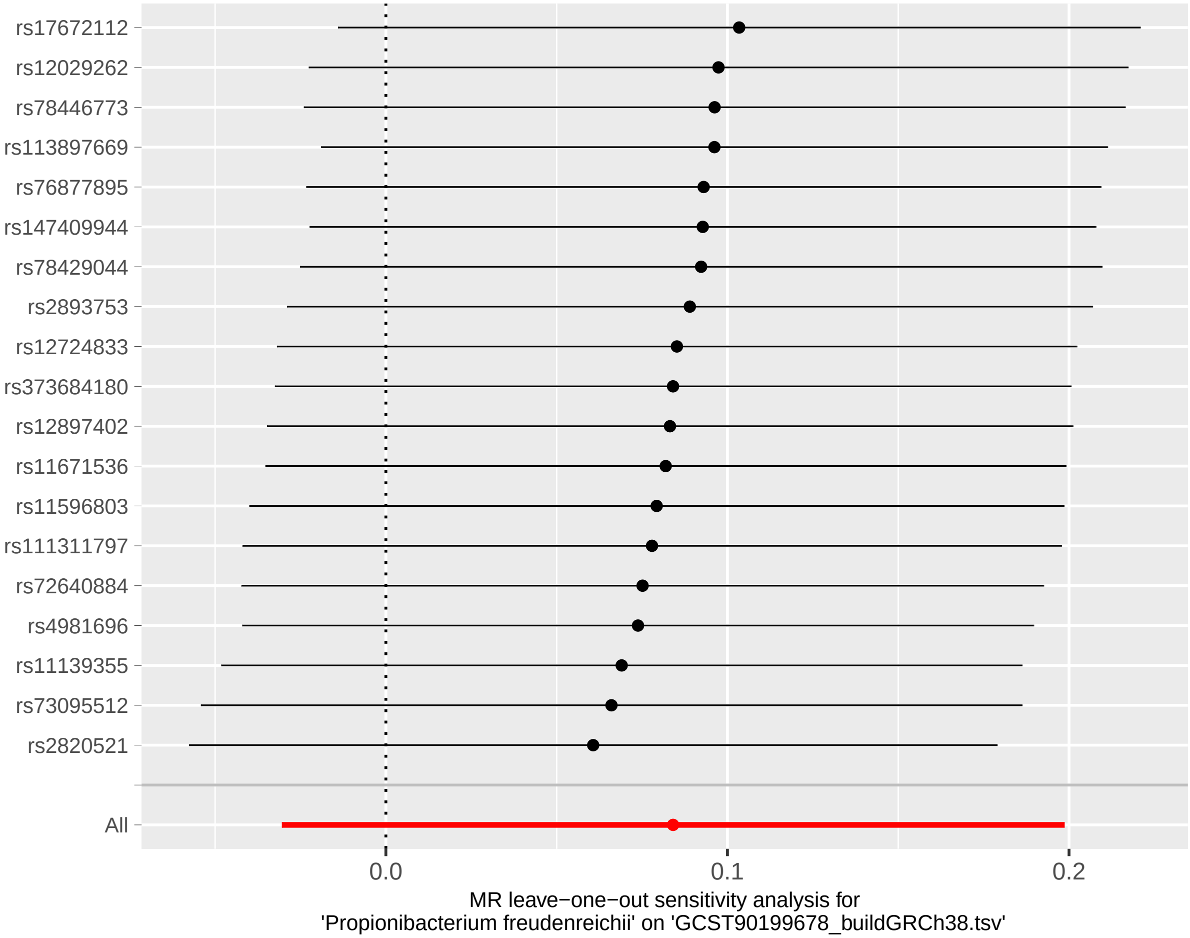
rs17672112
rs12029262
rs78446773
rs113897669
rs76877895
rs147409944
rs78429044
rs2893753
rs12724833
rs373684180
rs12897402
rs11671536
rs11596803
rs111311797
rs72640884
rs4981696
rs11139355
rs73095512
rs2820521
All
0.0
0.1
0.2
MR leave−one−out sensitivity analysis for
'Propionibacterium freudenreichii' on 'GCST90199678_buildGRCh38.tsv'

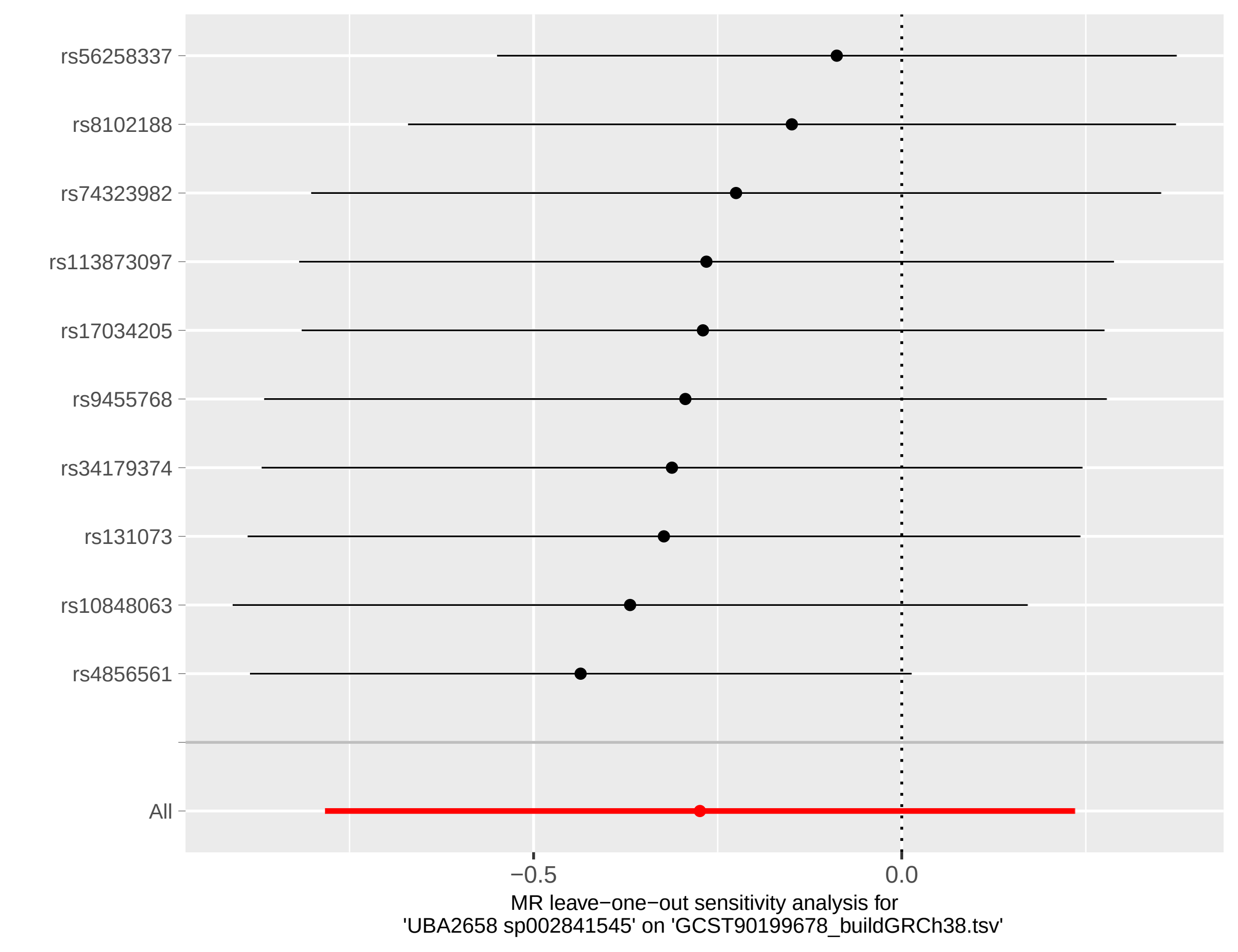
rs56258337
rs8102188
rs74323982
rs113873097
rs17034205
rs9455768
rs34179374
rs131073
rs10848063
rs4856561
All
−0.5
0.0
MR leave−one−out sensitivity analysis for
'UBA2658 sp002841545' on 'GCST90199678_buildGRCh38.tsv'

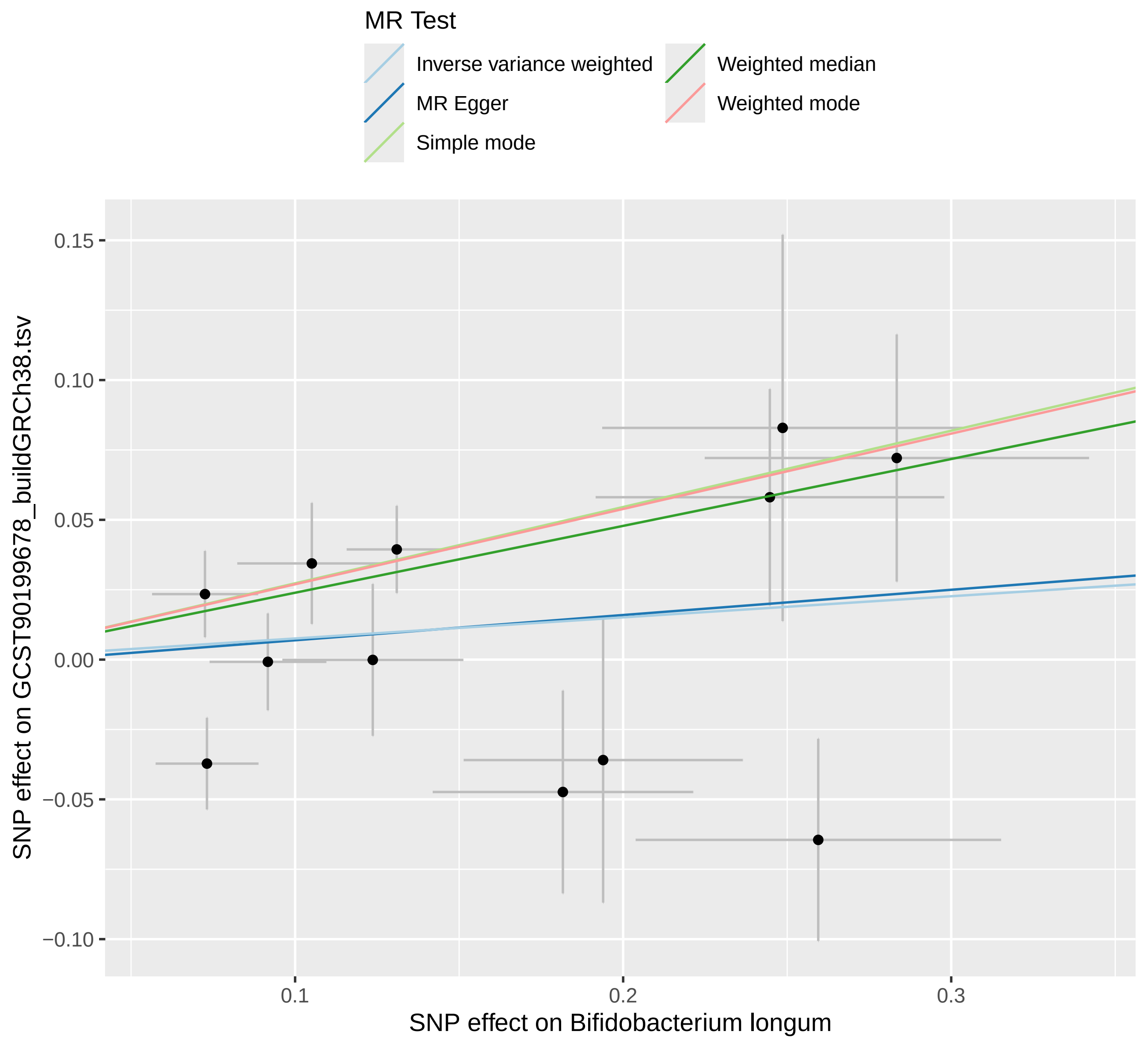

MR Test
Inverse variance weighted
MR Egger
Simple mode
Weighted median
Weighted mode
0.15
0.10
0.05
0.00
−0.05
−0.10
SNP effect on GCST90199678_buildGRCh38.tsv
0.1
0.2
0.3
SNP effect on Bifidobacterium longum

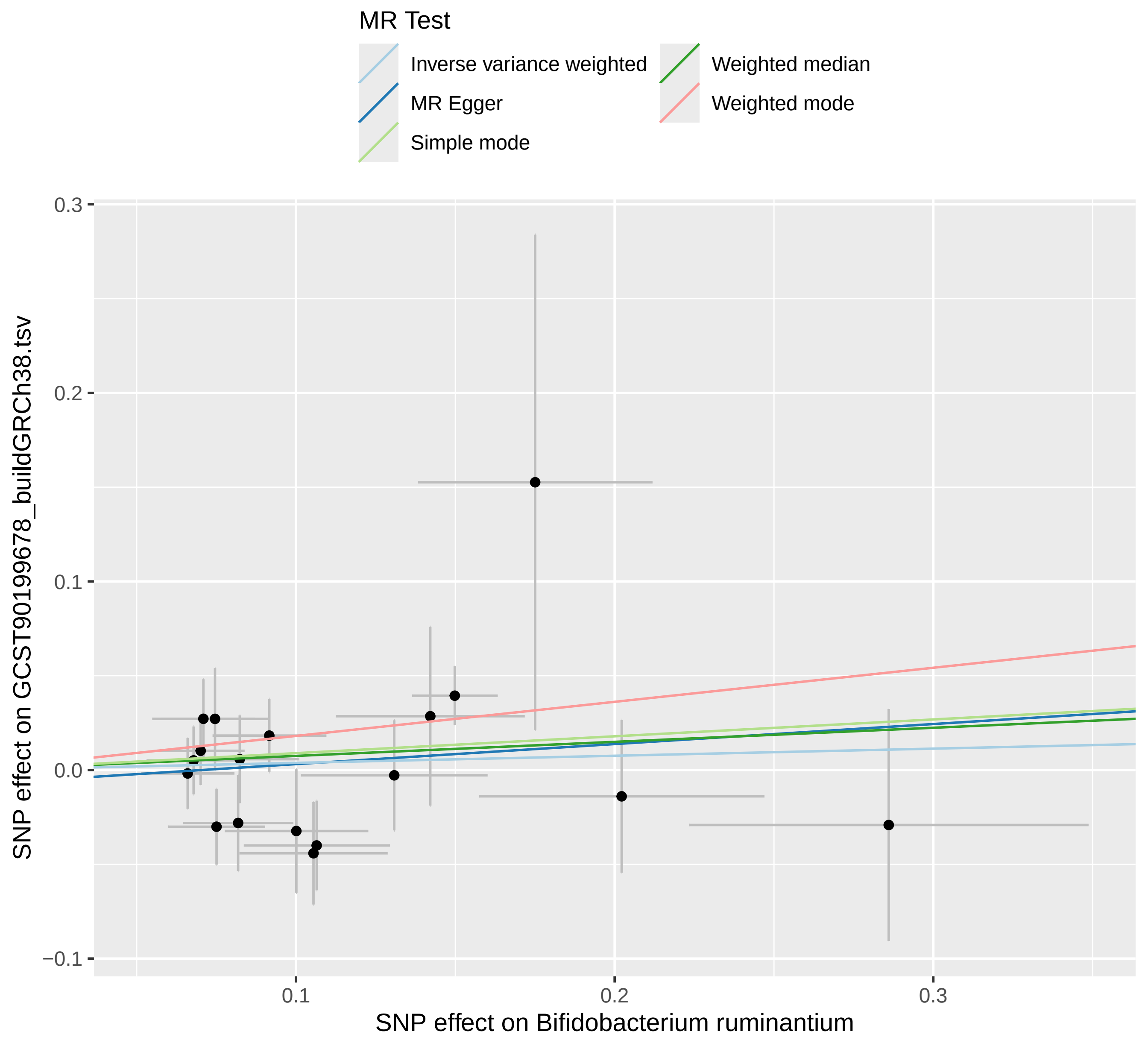

MR Test
Inverse variance weighted
MR Egger
Simple mode
Weighted median
Weighted mode
0.3
0.2
0.1
0.0
−0.1
0.1
0.2
0.3
SNP effect on GCST90199678_buildGRCh38.tsv
SNP effect on Bifidobacterium ruminantium

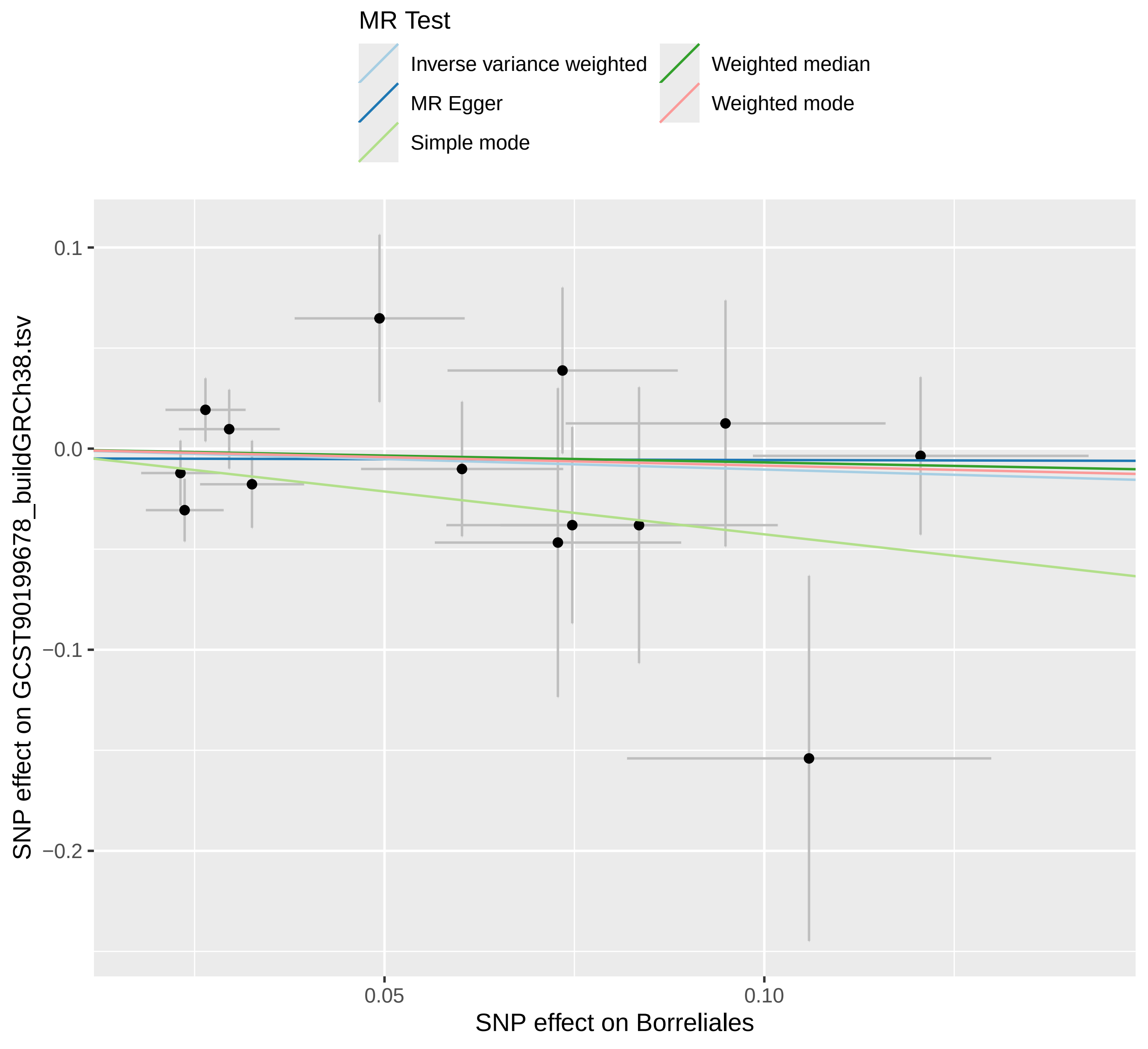

MR Test
Inverse variance weighted
MR Egger
Simple mode
Weighted median
Weighted mode
0.1
0.0
−0.1
−0.2
0.05
0.10
SNP effect on GCST90199678_buildGRCh38.tsv
SNP effect on Borreliales

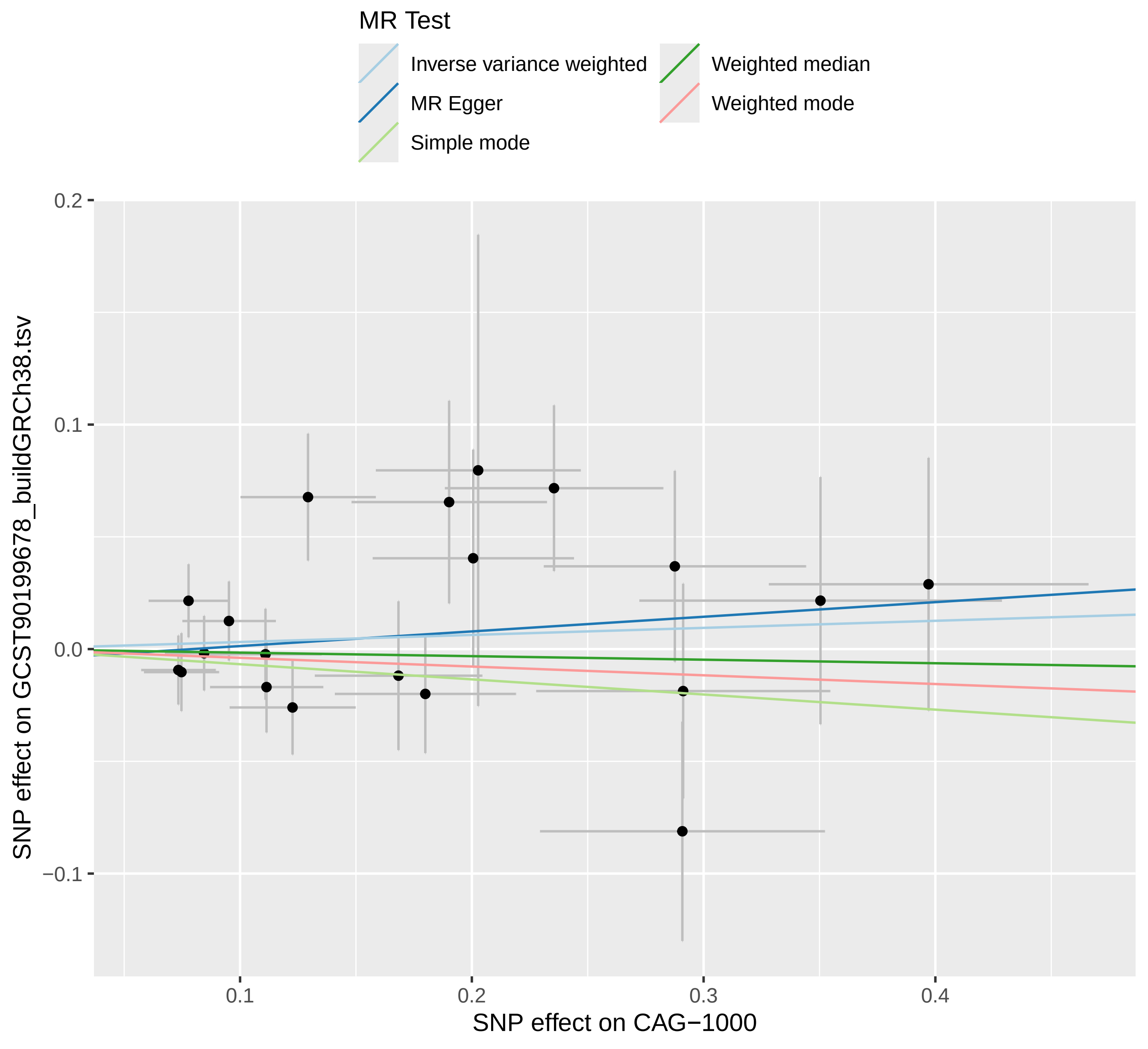
MR Test
Inverse variance weighted
MR Egger
Simple mode
Weighted median
Weighted mode
0.2
0.1
0.0
−0.1
0.1
0.2
0.3
0.4
SNP effect on GCST90199678_buildGRCh38.tsv
SNP effect on CAG−1000

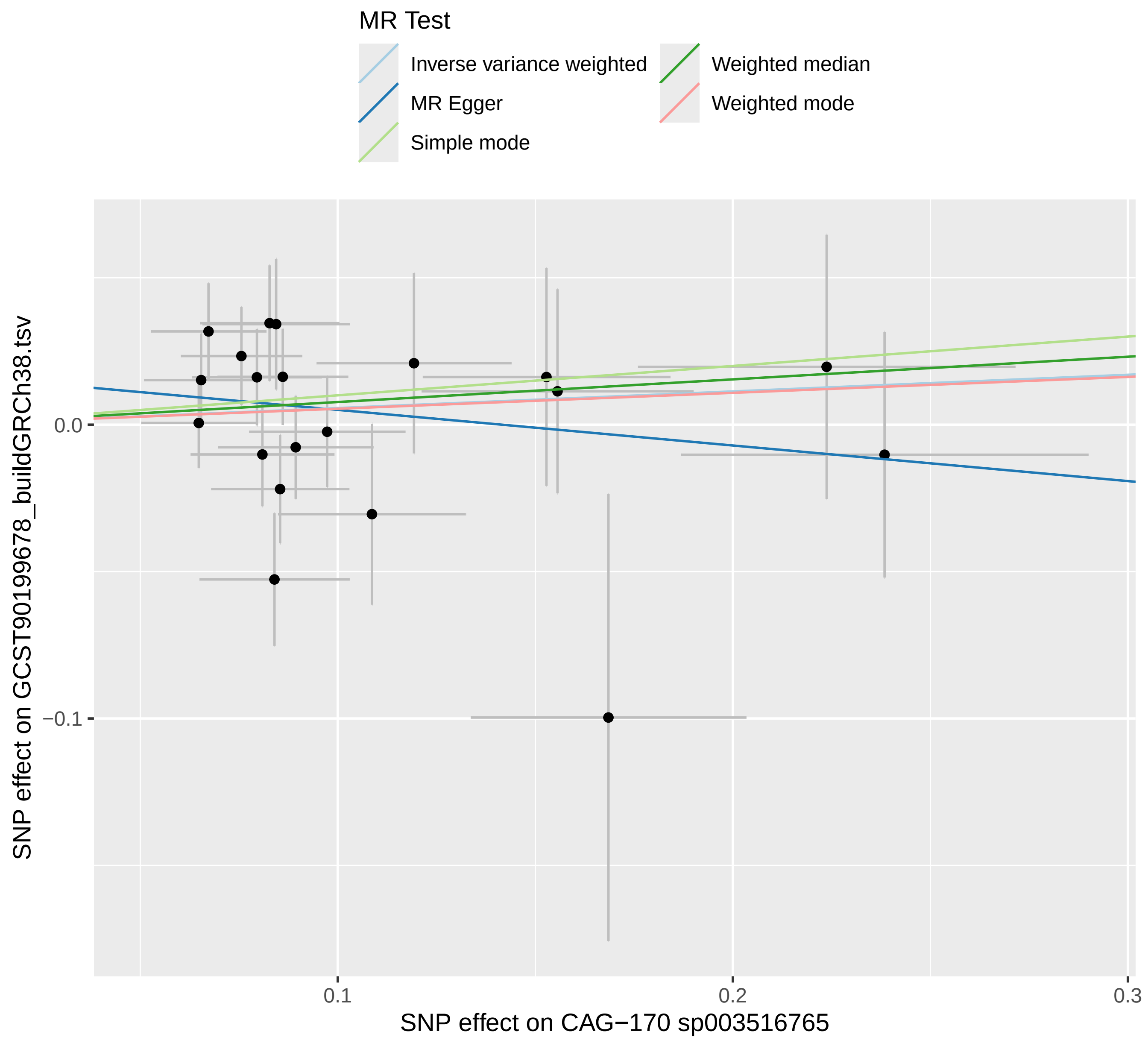
MR Test
Inverse variance weighted
MR Egger
Simple mode
Weighted median
Weighted mode
SNP effect on GCST90199678_buildGRCh38.tsv
0.0
−0.1
0.1
0.2
0.3
SNP effect on CAG−170 sp003516765

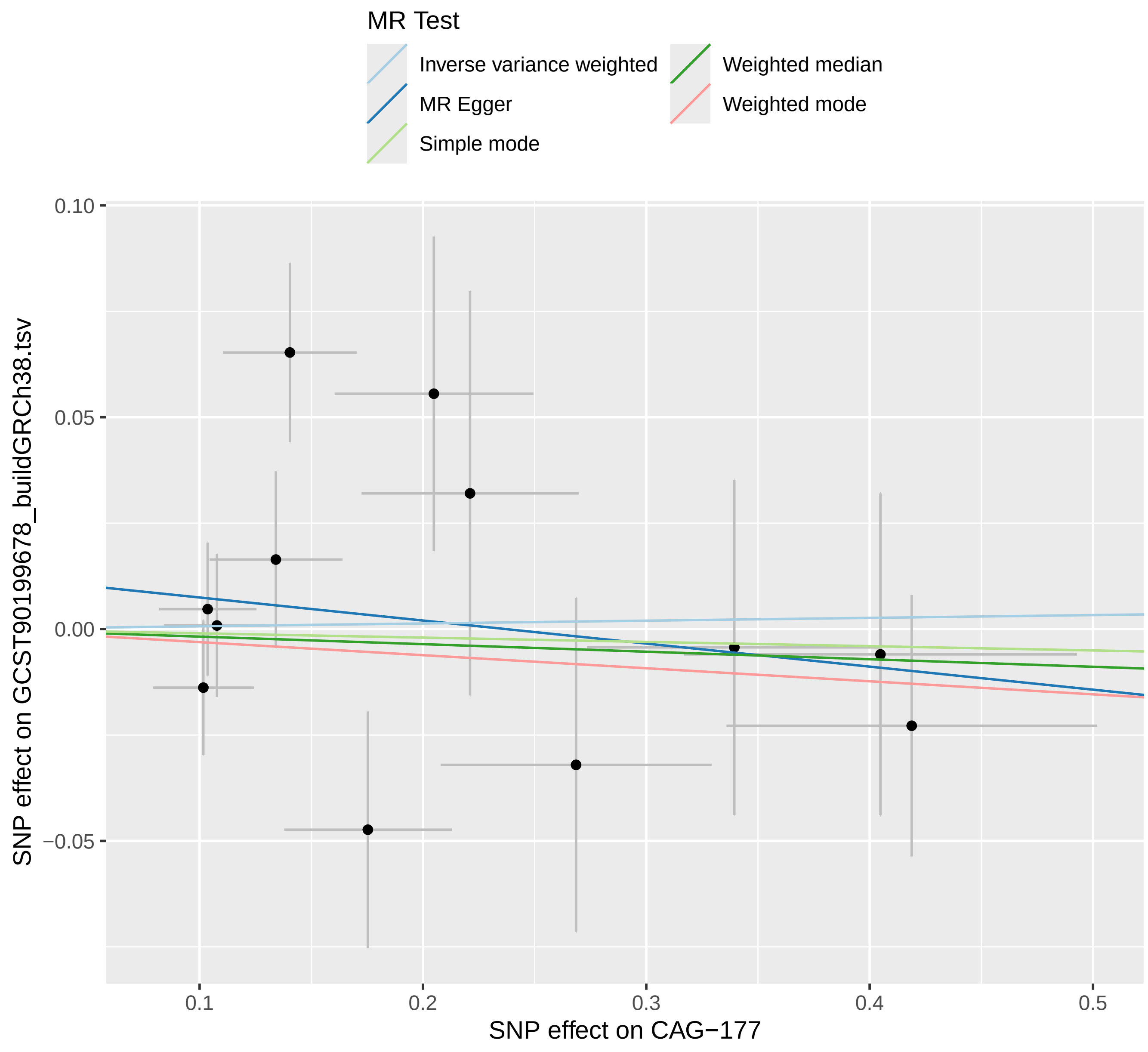
MR Test
Inverse variance weighted
MR Egger
Simple mode
Weighted median
Weighted mode
0.10
0.05
0.00
−0.05
0.1
0.2
0.3
0.4
0.5
SNP effect on CAG−177
SNP effect on GCST90199678_buildGRCh38.tsv

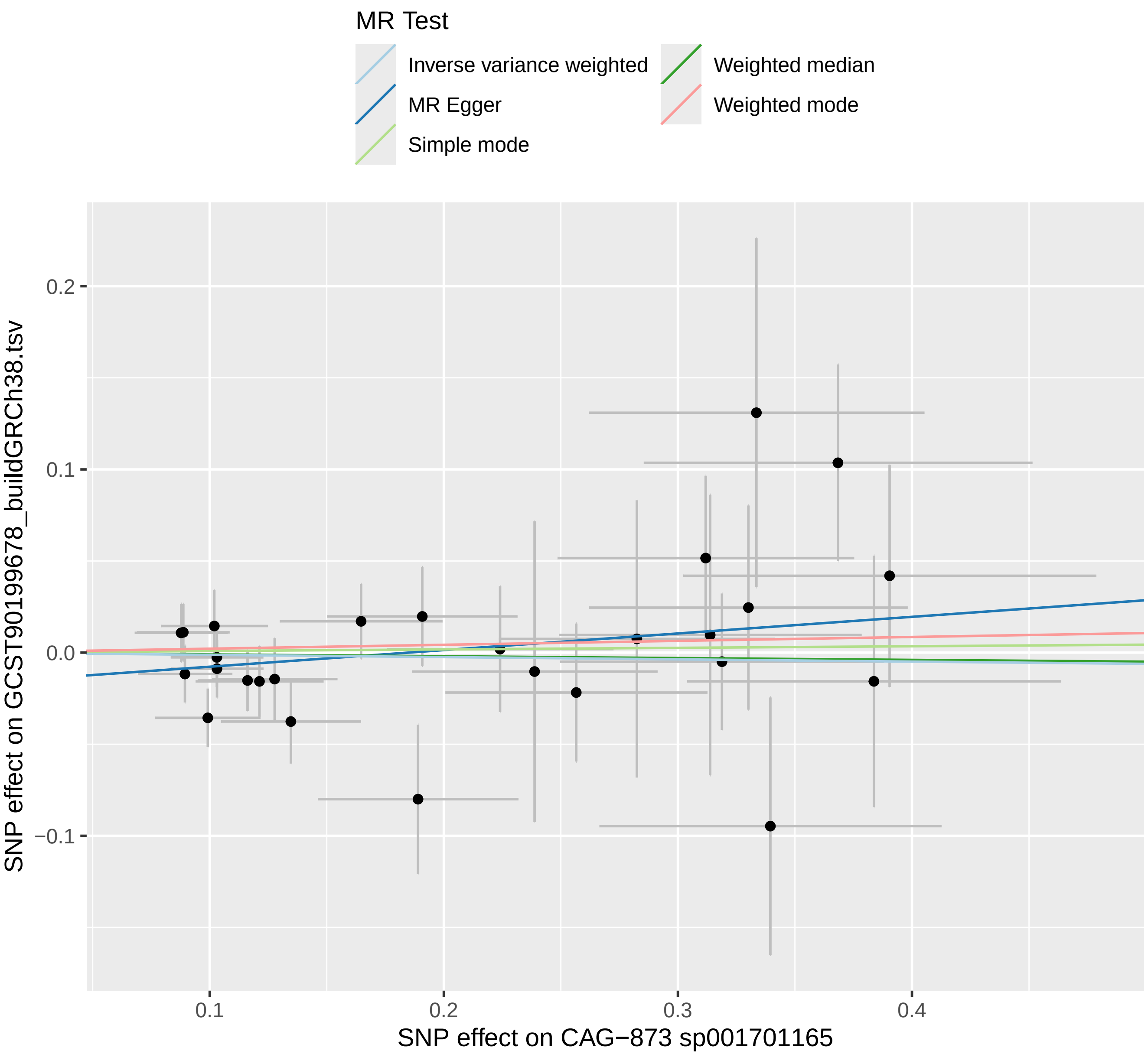

MR Test
Inverse variance weighted
MR Egger
Simple mode
Weighted median
Weighted mode
0.2
0.1
0.0
−0.1
SNP effect on GCST90199678_buildGRCh38.tsv
0.1
0.2
0.3
0.4
SNP effect on CAG−873 sp001701165

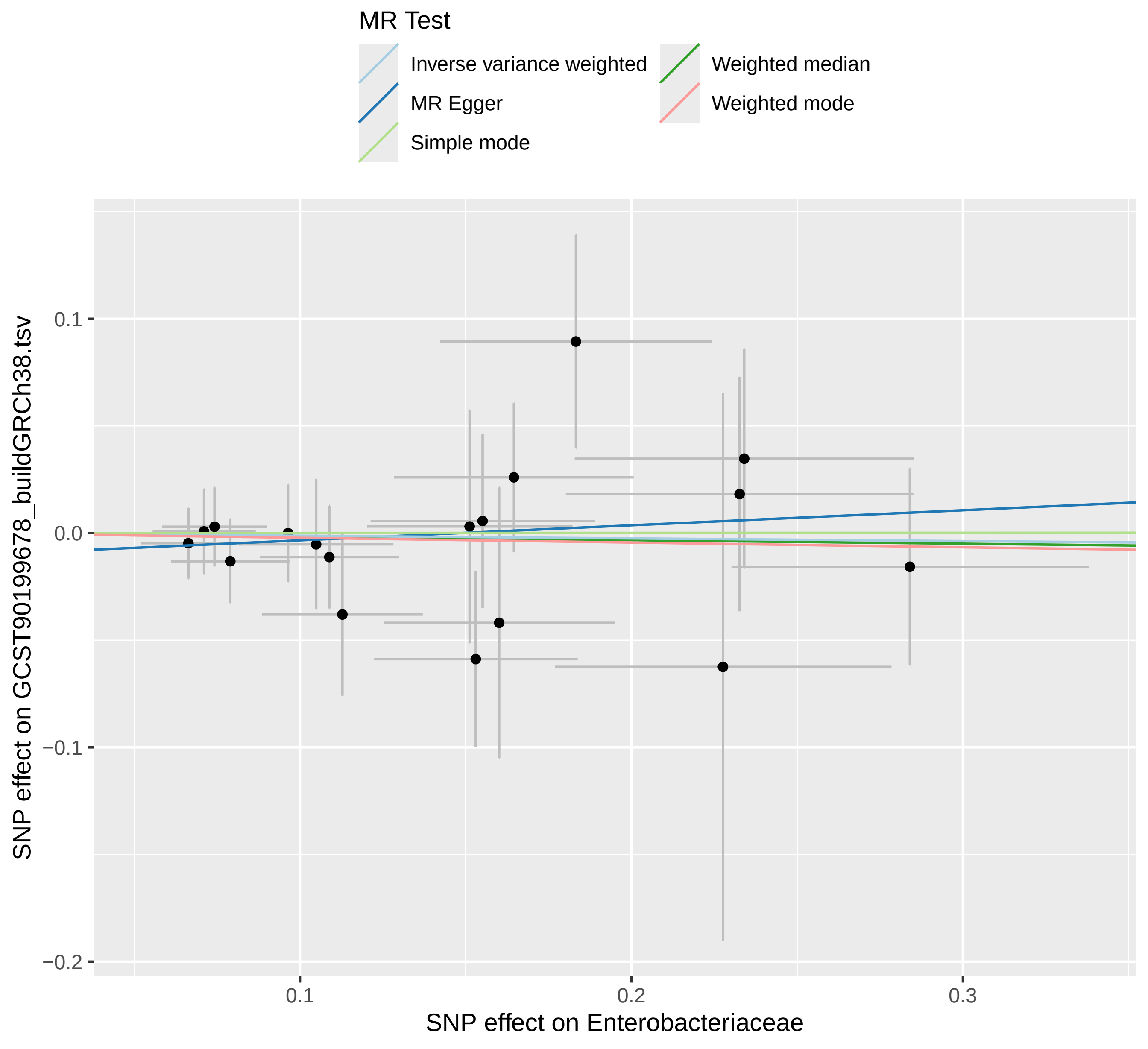
MR Test
Inverse variance weighted
MR Egger
Simple mode
Weighted median
Weighted mode
0.1
0.0
−0.1
−0.2
0.1
0.2
0.3
SNP effect on GCST90199678_buildGRCh38.tsv
SNP effect on Enterobacteriaceae

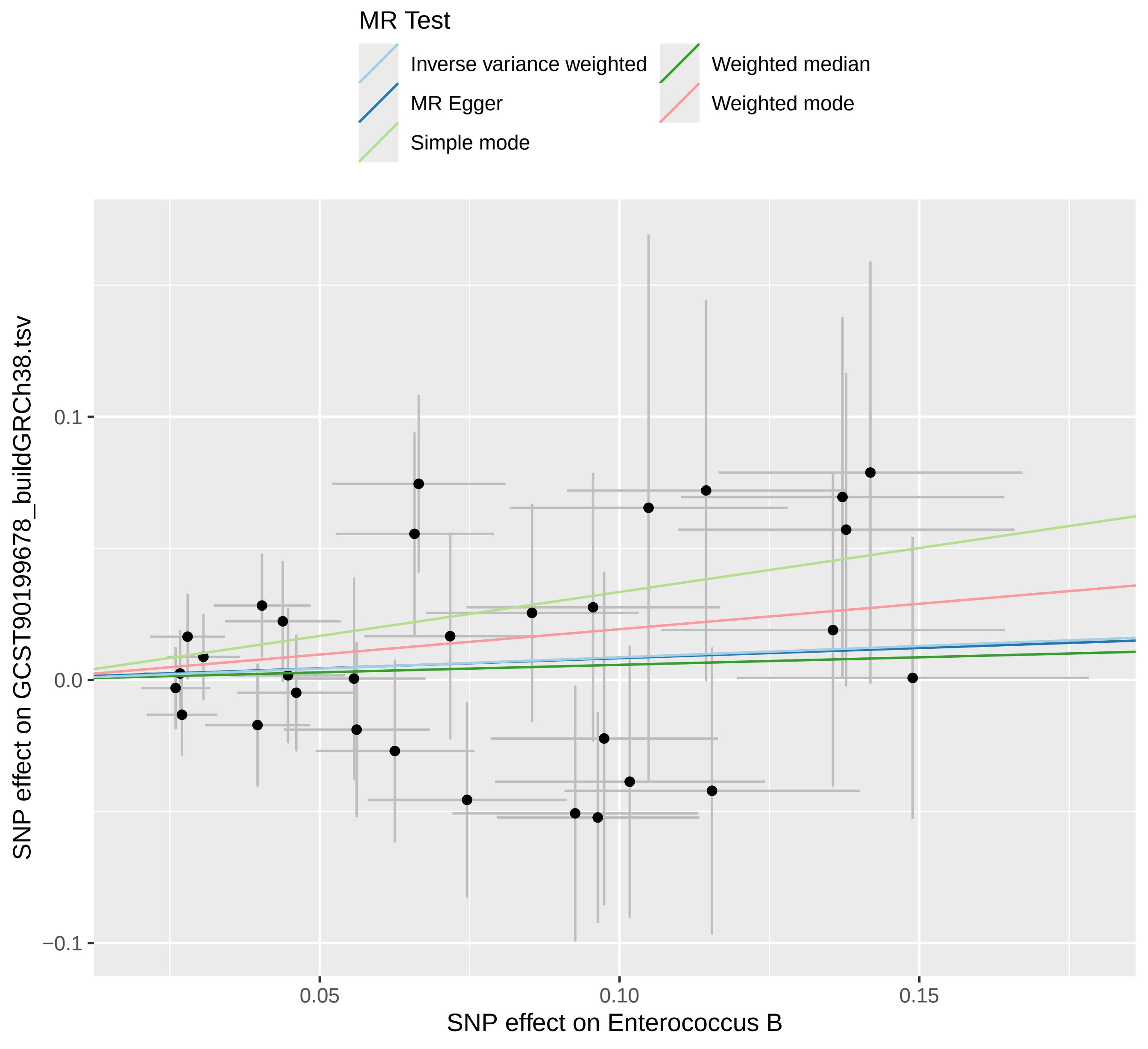

MR Test
Inverse variance weighted
MR Egger
Simple mode
Weighted median
Weighted mode
SNP effect on GCST90199678_buildGRCh38.tsv
0.1
0.0
−0.1
0.05
0.10
0.15
SNP effect on Enterococcus B

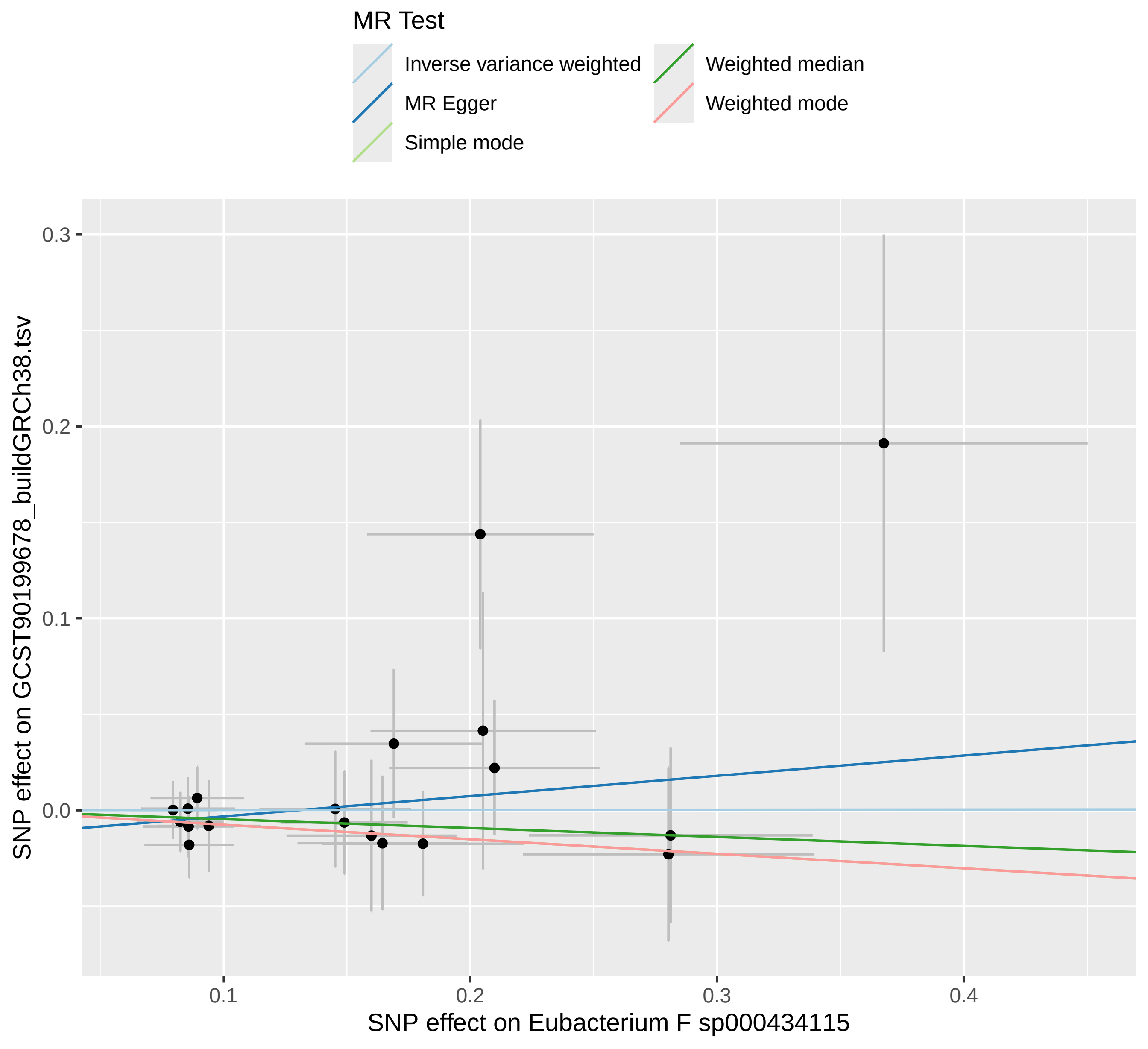
MR Test
Inverse variance weighted
MR Egger
Simple mode
Weighted median
Weighted mode
SNP effect on GCST90199678_buildGRCh38.tsv
0.3
0.2
0.1
0.0
0.1
0.2
0.3
0.4
SNP effect on Eubacterium F sp000434115

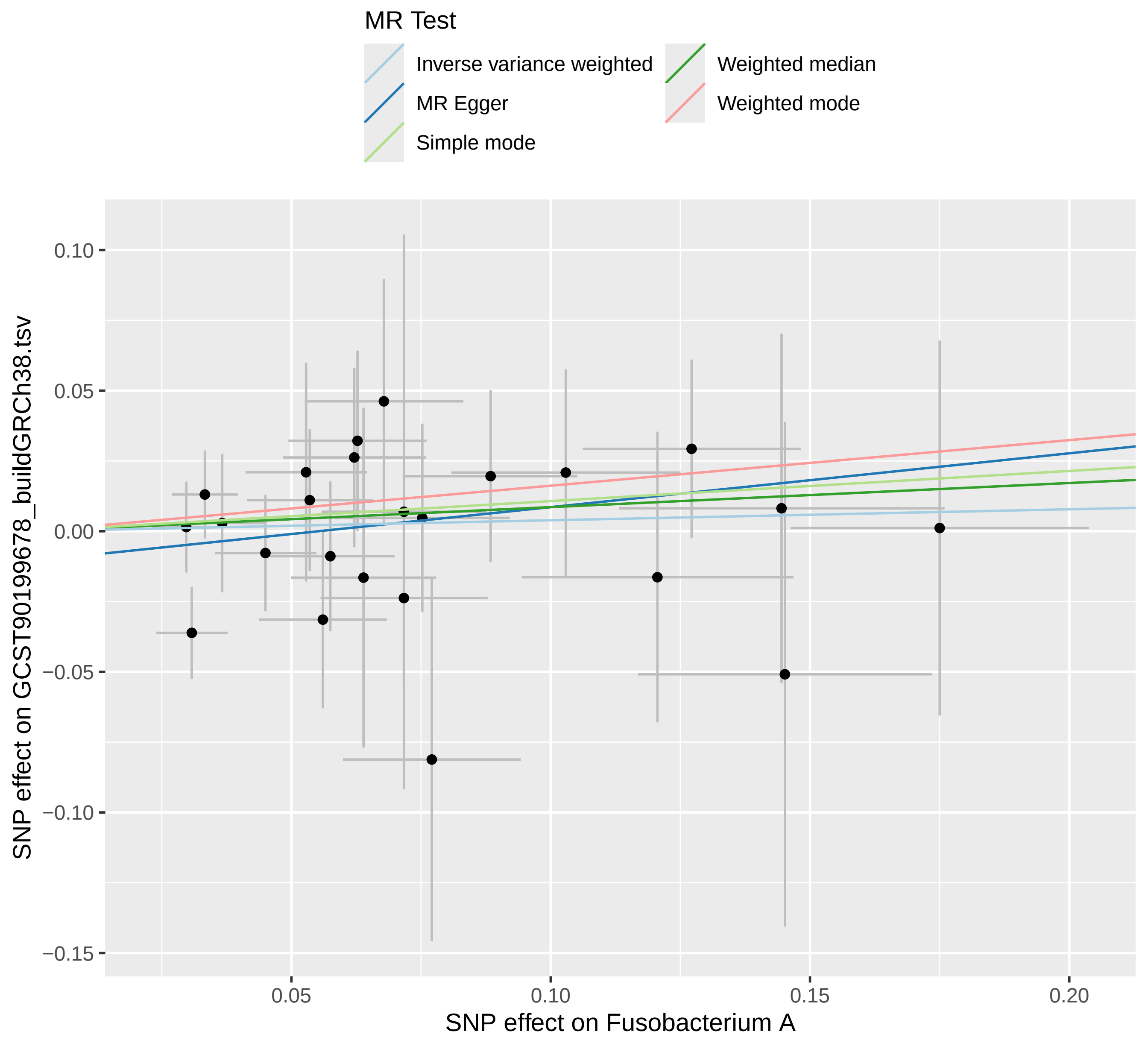
MR Test
Inverse variance weighted
MR Egger
Simple mode
Weighted median
Weighted mode
SNP effect on GCST90199678_buildGRCh38.tsv
0.10
0.05
0.00
−0.05
−0.10
−0.15
0.05
0.10
0.15
0.20
SNP effect on Fusobacterium A

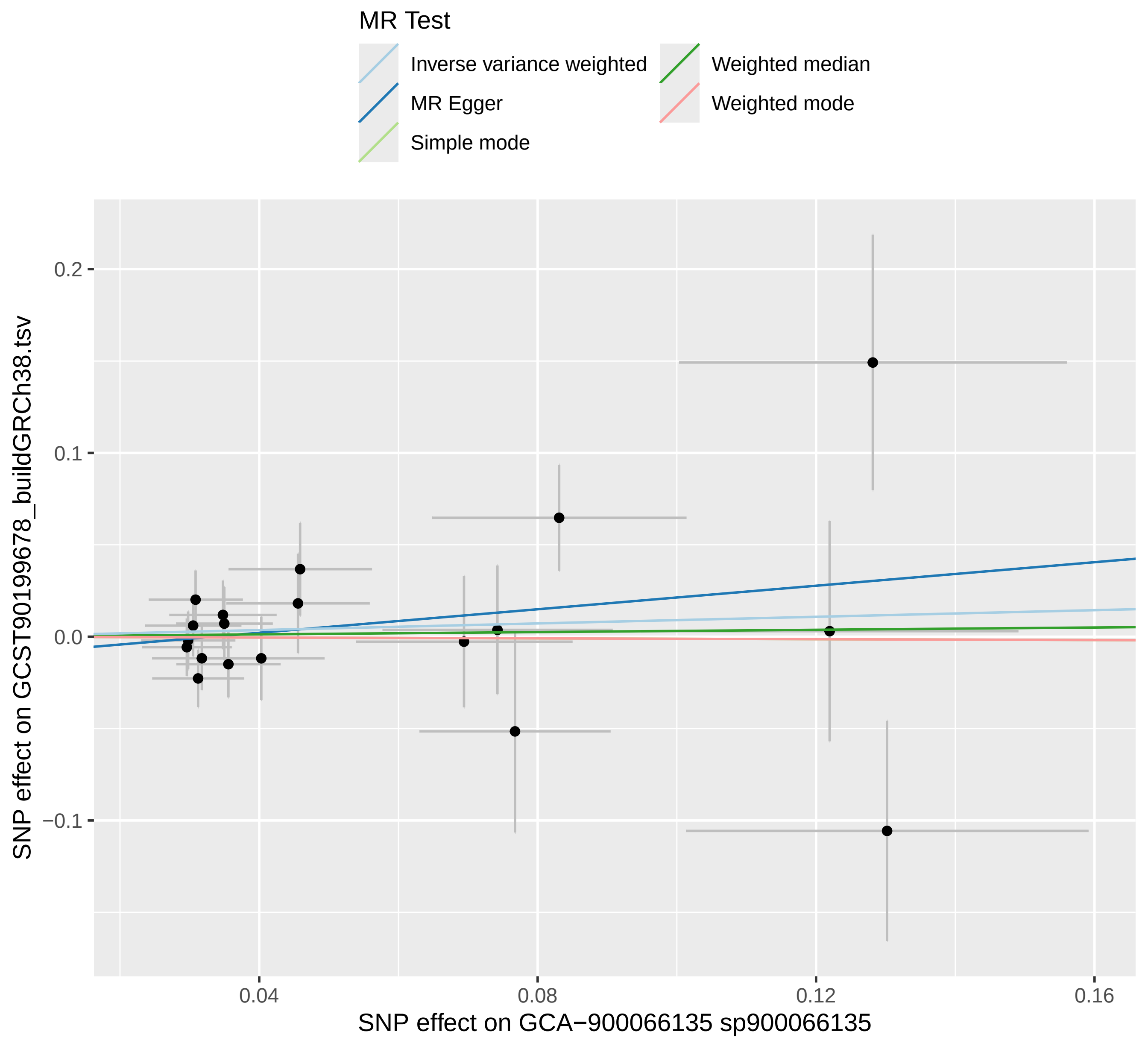

MR Test
Inverse variance weighted
MR Egger
Simple mode
Weighted median
Weighted mode
0.2
0.1
0.0
−0.1
0.04
0.08
0.12
0.16
SNP effect on GCST90199678_buildGRCh38.tsv
SNP effect on GCA−900066135 sp900066135

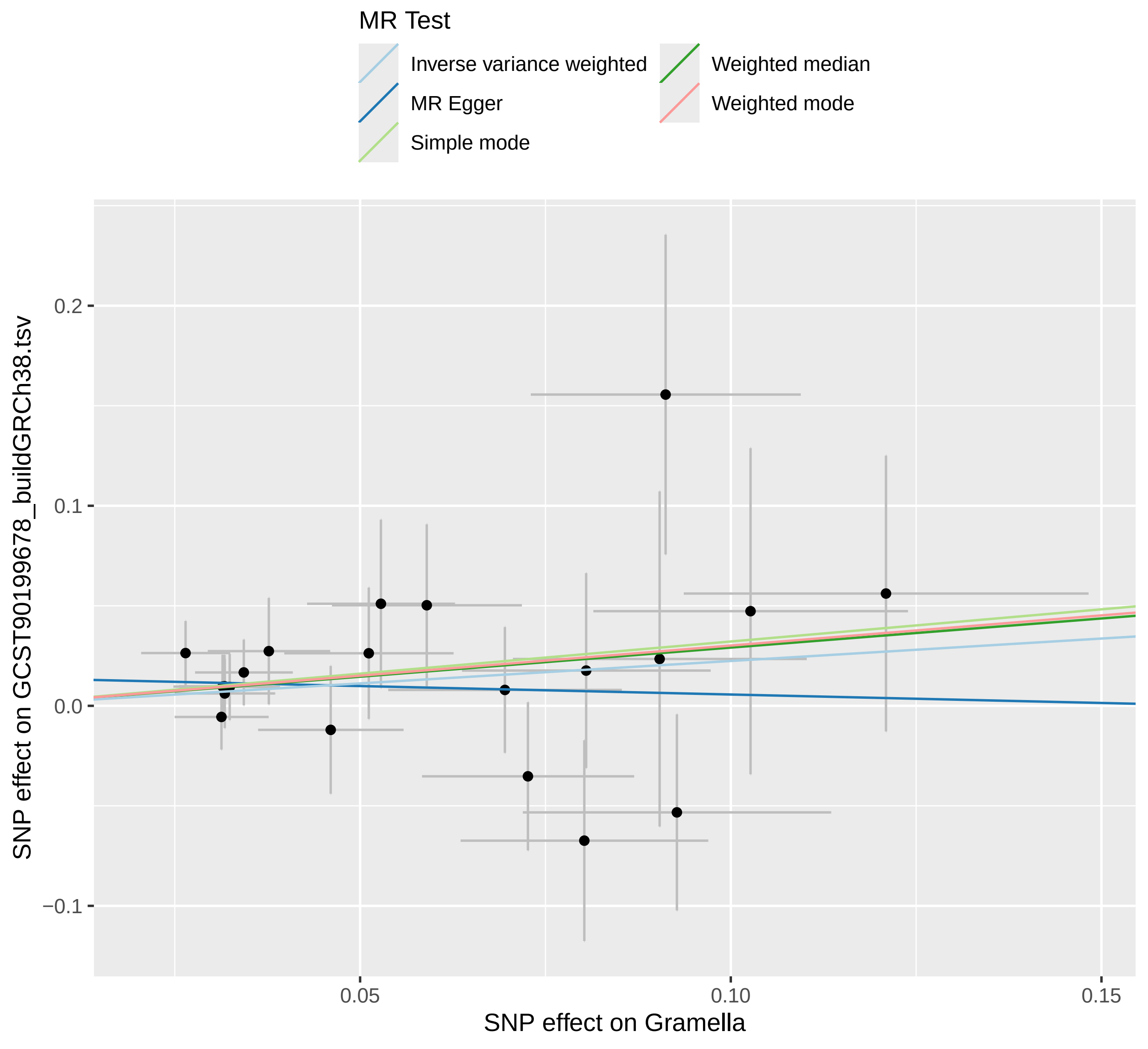
MR Test
Inverse variance weighted
MR Egger
Simple mode
Weighted median
Weighted mode
0.2
0.1
0.0
−0.1
0.05
0.10
0.15
SNP effect on GCST90199678_buildGRCh38.tsv
SNP effect on Gramella

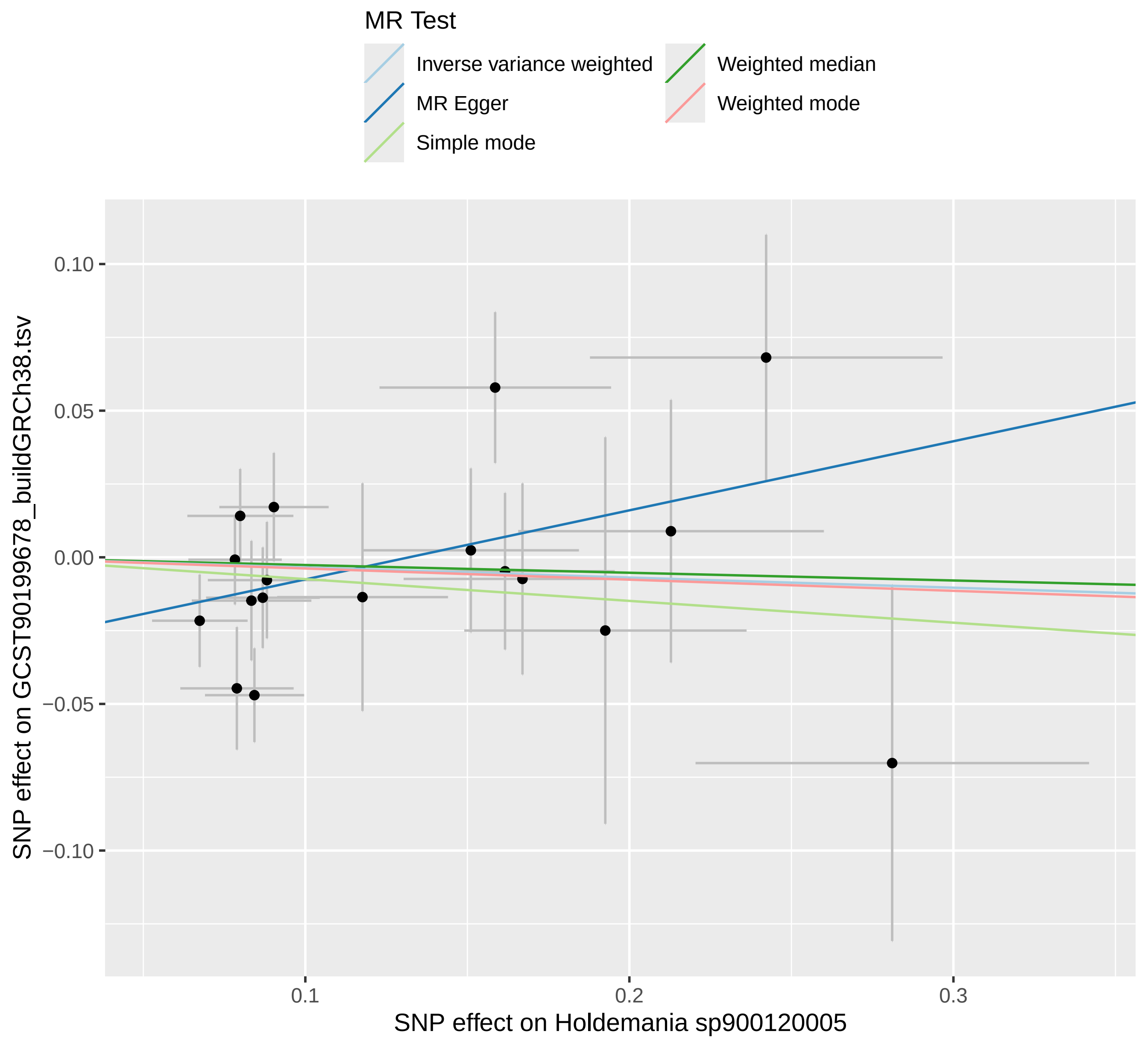

MR Test
Inverse variance weighted
MR Egger
Simple mode
Weighted median
Weighted mode
0.10
0.05
0.00
−0.05
−0.10
0.1
0.2
0.3
SNP effect on GCST90199678_buildGRCh38.tsv
SNP effect on Holdemania sp900120005

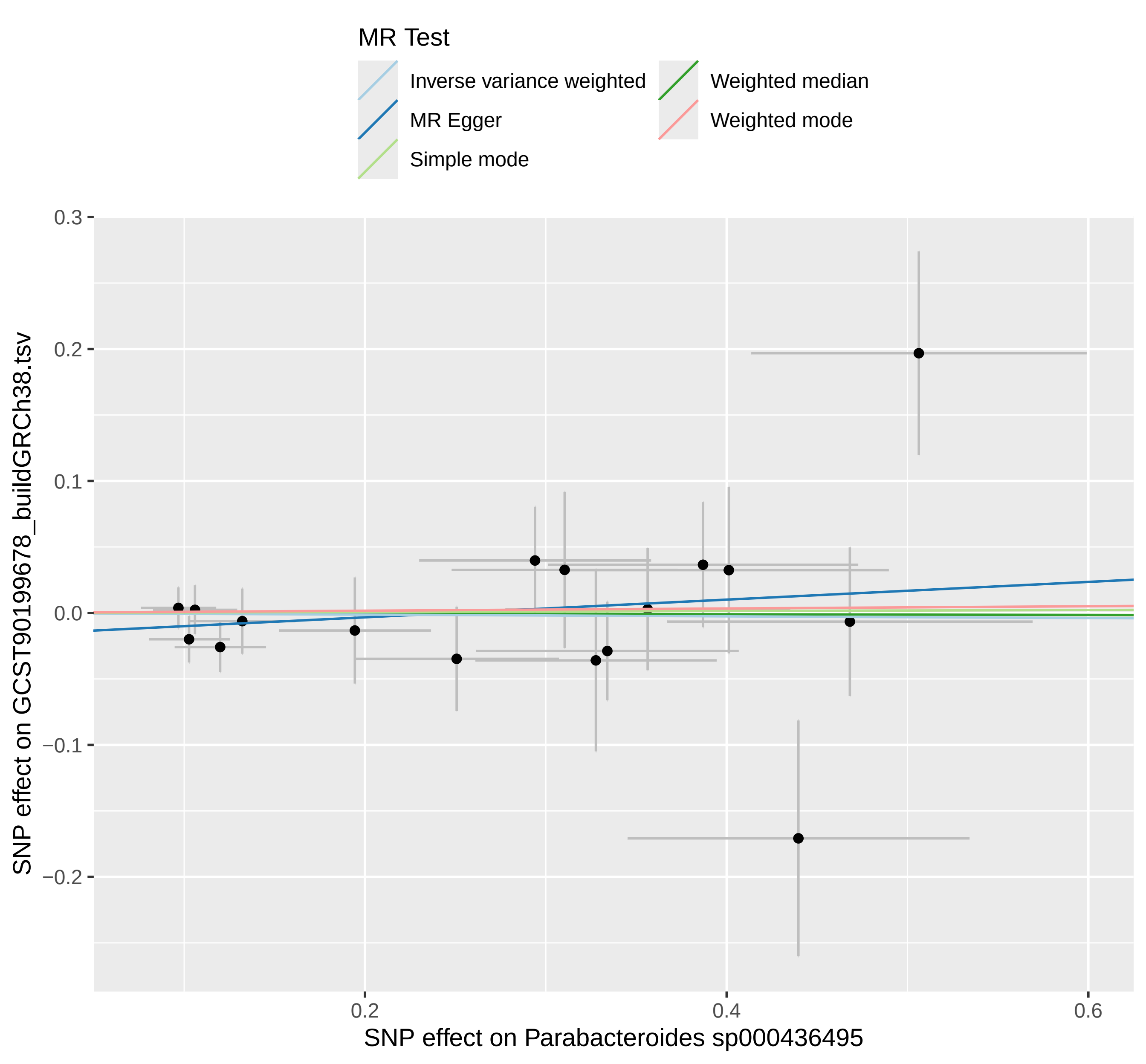

MR Test
Inverse variance weighted
MR Egger
Simple mode
Weighted median
Weighted mode
0.3
0.2
0.1
0.0
−0.1
−0.2
0.2
0.4
0.6
SNP effect on GCST90199678_buildGRCh38.tsv
SNP effect on Parabacteroides sp000436495

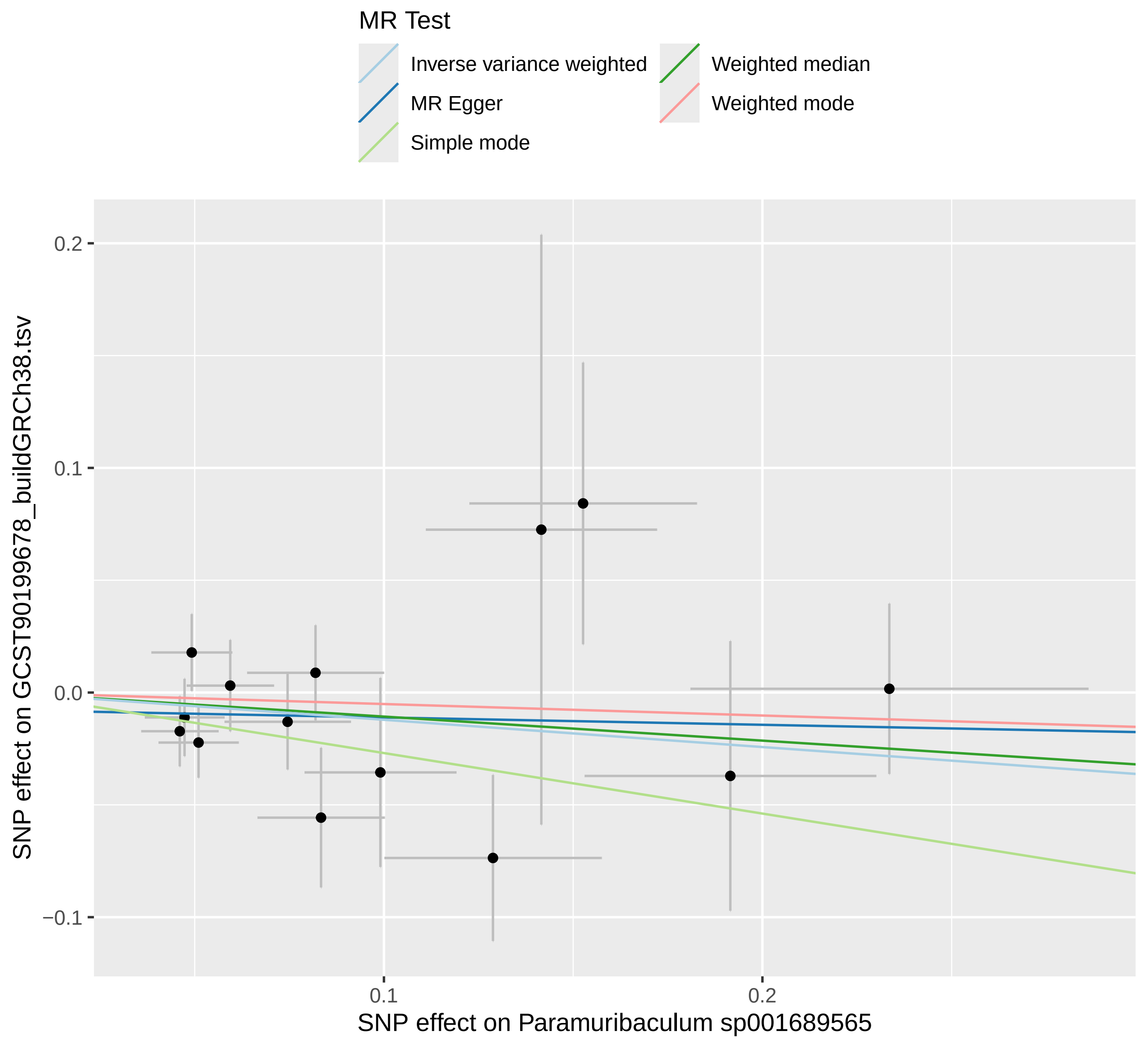
MR Test
Inverse variance weighted
MR Egger
Simple mode
Weighted median
Weighted mode
0.2
0.1
0.0
−0.1
0.1
0.2
SNP effect on GCST90199678_buildGRCh38.tsv
SNP effect on Paramuribaculum sp001689565

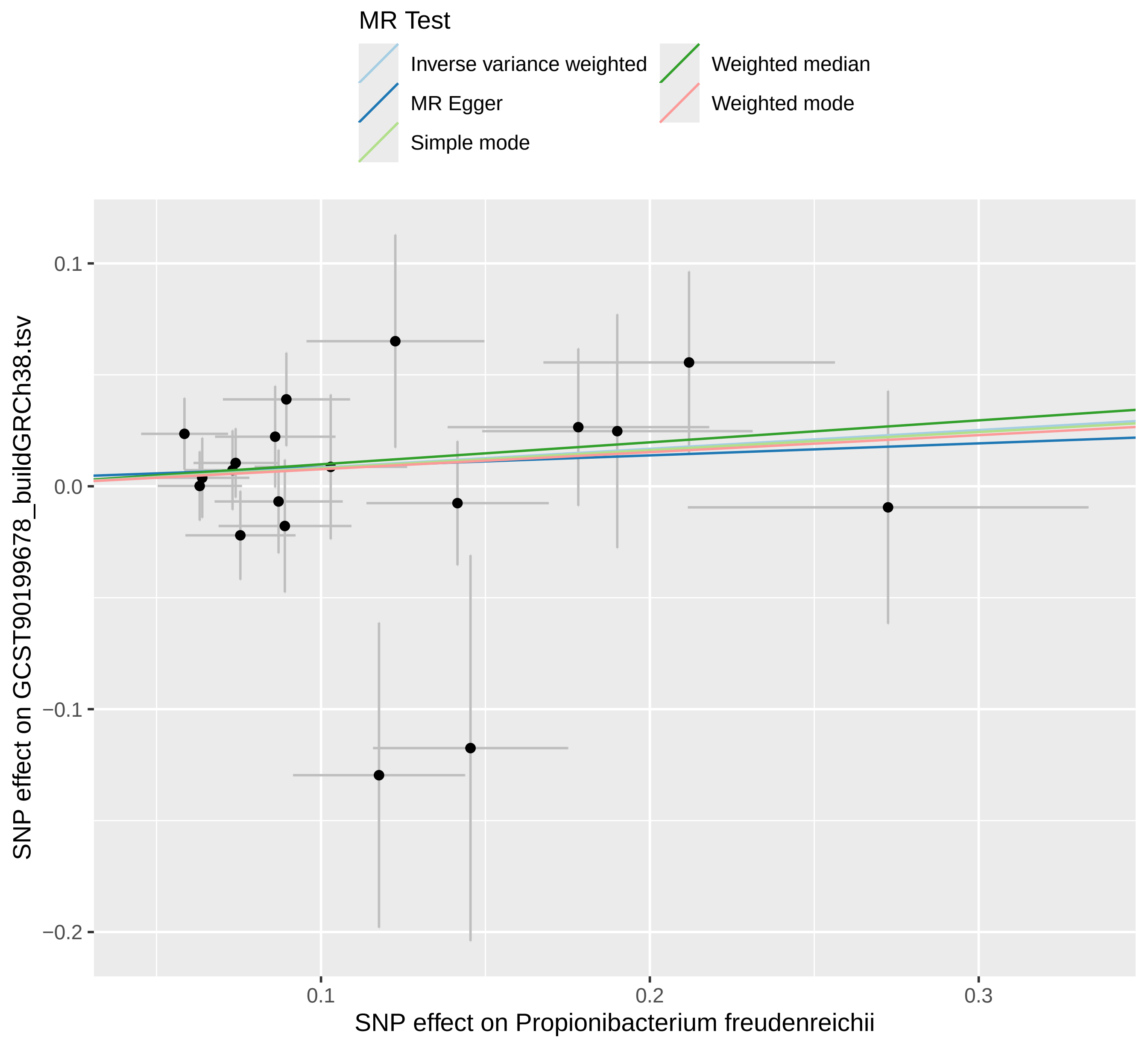

MR Test
Inverse variance weighted
MR Egger
Simple mode
Weighted median
Weighted mode
0.1
0.0
−0.1
−0.2
SNP effect on GCST90199678_buildGRCh38.tsv
0.1
0.2
0.3
SNP effect on Propionibacterium freudenreichii

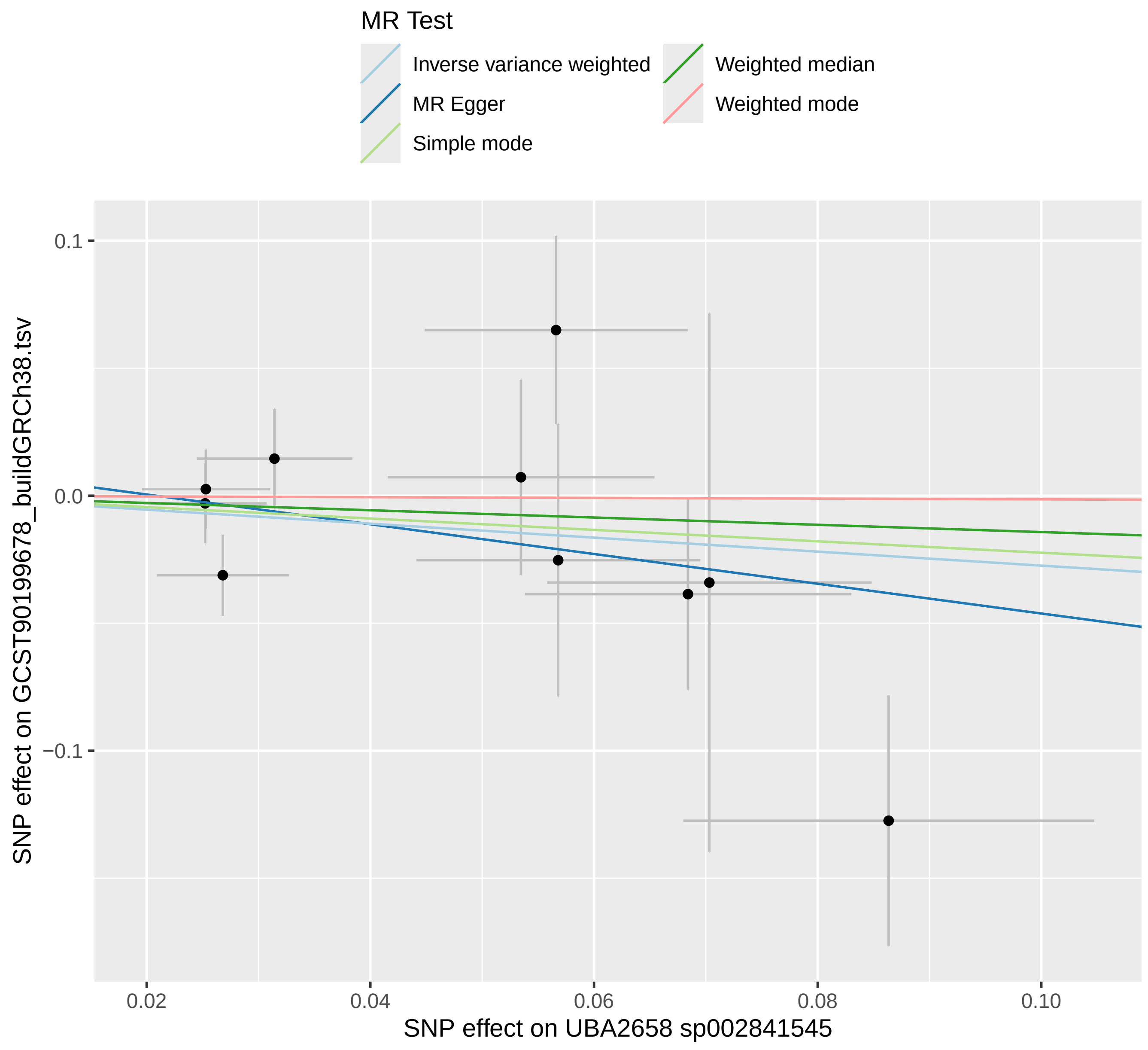
MR Test
Inverse variance weighted
MR Egger
Simple mode
Weighted median
Weighted mode
0.1
0.0
−0.1
0.02
0.04
0.06
0.08
0.10
SNP effect on GCST90199678_buildGRCh38.tsv
SNP effect on UBA2658 sp002841545

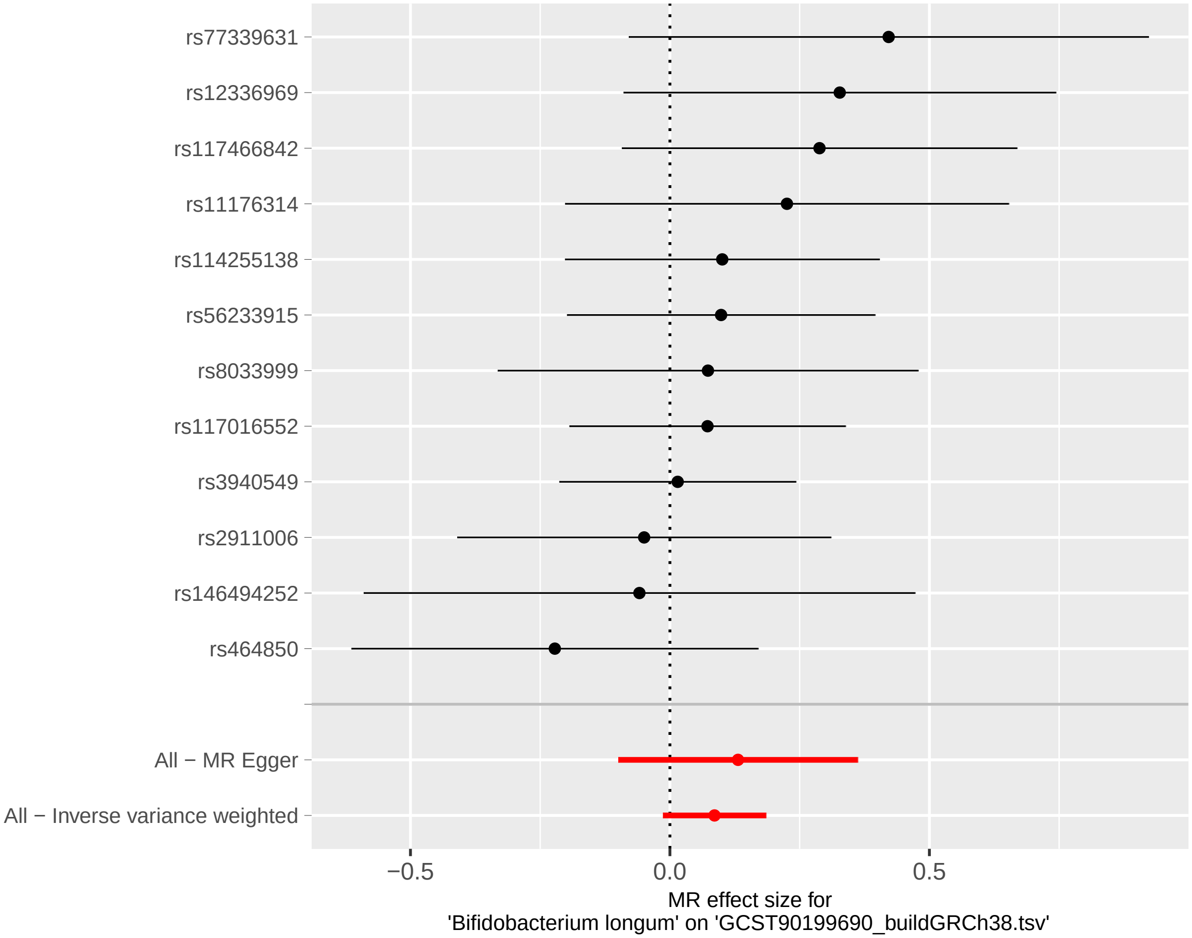
rs77339631
rs12336969
rs117466842
rs11176314
rs114255138
rs56233915
rs8033999
rs117016552
rs3940549
rs2911006
rs146494252
rs464850
All − MR Egger
All − Inverse variance weighted
−0.5
0.0
0.5
MR effect size for
'Bifidobacterium longum' on 'GCST90199690_buildGRCh38.tsv'

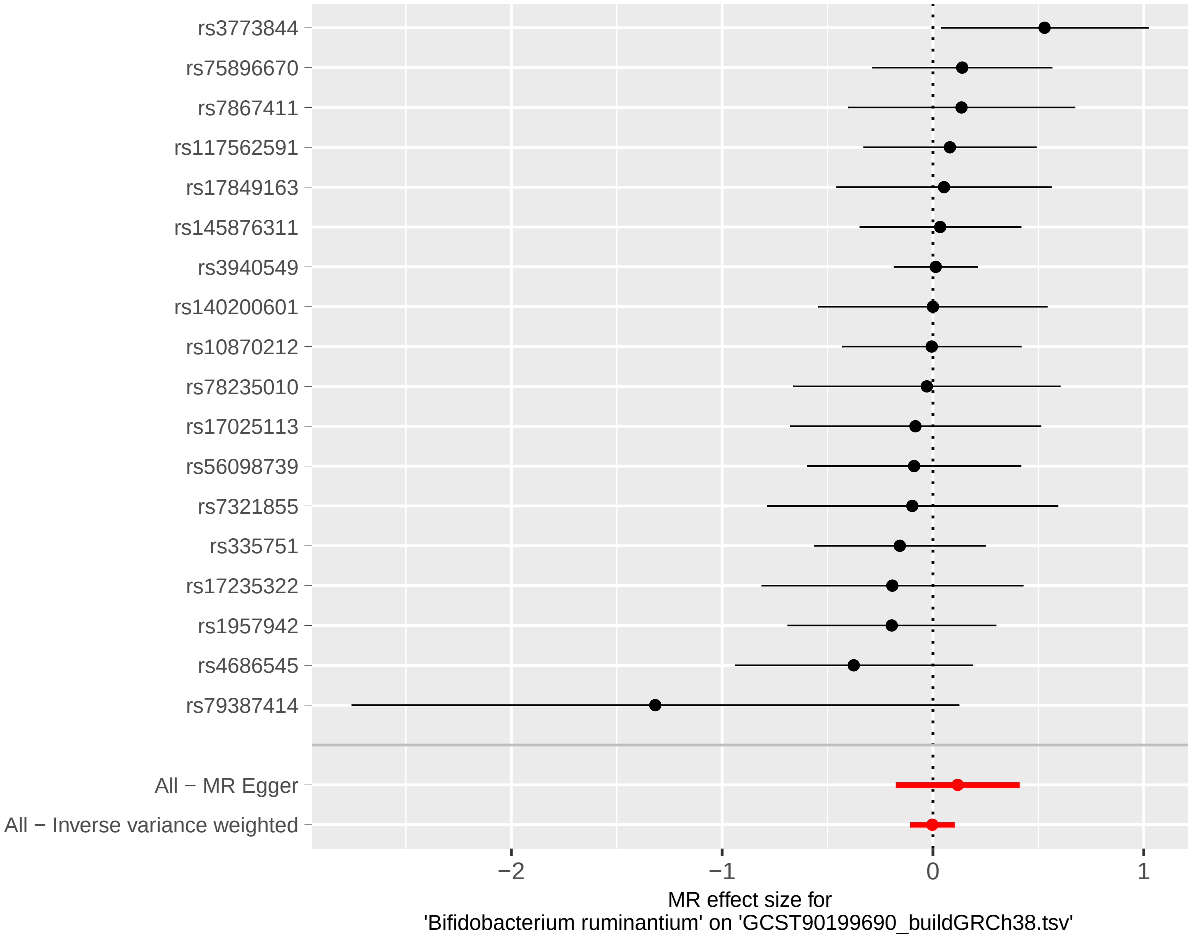

rs3773844
rs75896670
rs7867411
rs117562591
rs17849163
rs145876311
rs3940549
rs140200601
rs10870212
rs78235010
rs17025113
rs56098739
rs7321855
rs335751
rs17235322
rs1957942
rs4686545
rs79387414
All − MR Egger
All − Inverse variance weighted
−2
−1
0
1
MR effect size for
'Bifidobacterium ruminantium' on 'GCST90199690_buildGRCh38.tsv'

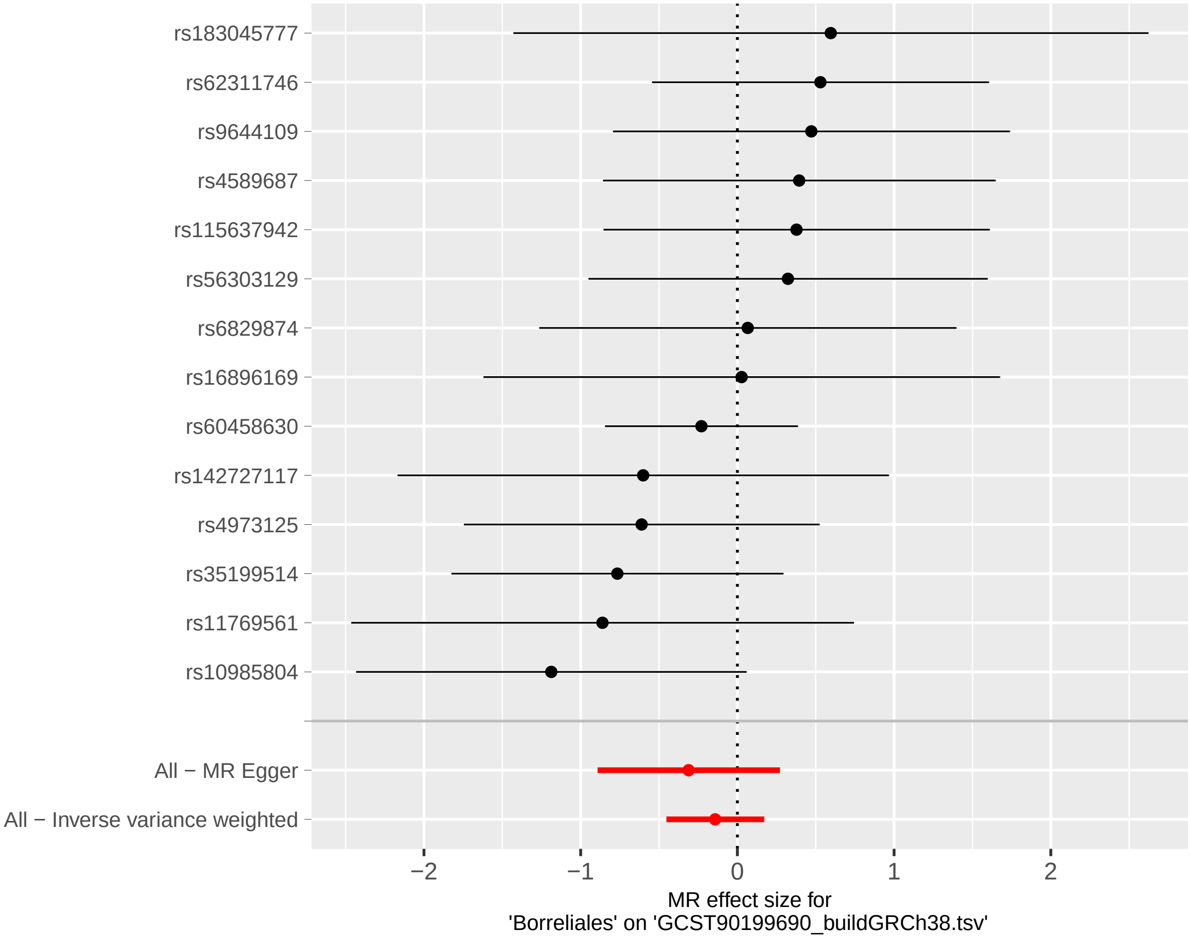

rs183045777
rs62311746
rs9644109
rs4589687
rs115637942
rs56303129
rs6829874
rs16896169
rs60458630
rs142727117
rs4973125
rs35199514
rs11769561
rs10985804
All − MR Egger
All − Inverse variance weighted
−2
−1
0
1
2
MR effect size for
'Borreliales' on 'GCST90199690_buildGRCh38.tsv'

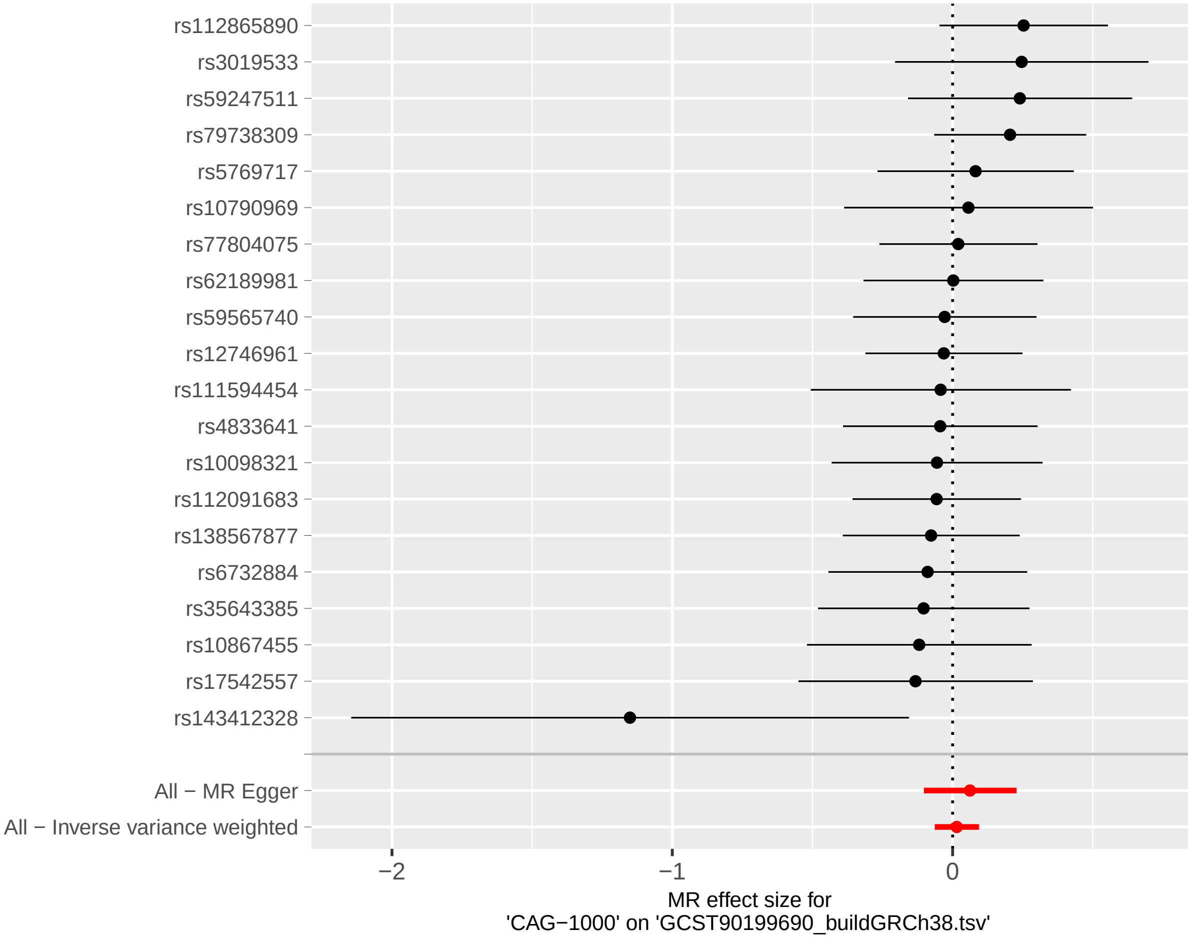
rs112865890
rs3019533
rs59247511
rs79738309
rs5769717
rs10790969
rs77804075
rs62189981
rs59565740
rs12746961
rs111594454
rs4833641
rs10098321
rs112091683
rs138567877
rs6732884
rs35643385
rs10867455
rs17542557
rs143412328
All − MR Egger
All − Inverse variance weighted
−2
−1
0
MR effect size for
'CAG−1000' on 'GCST90199690_buildGRCh38.tsv'

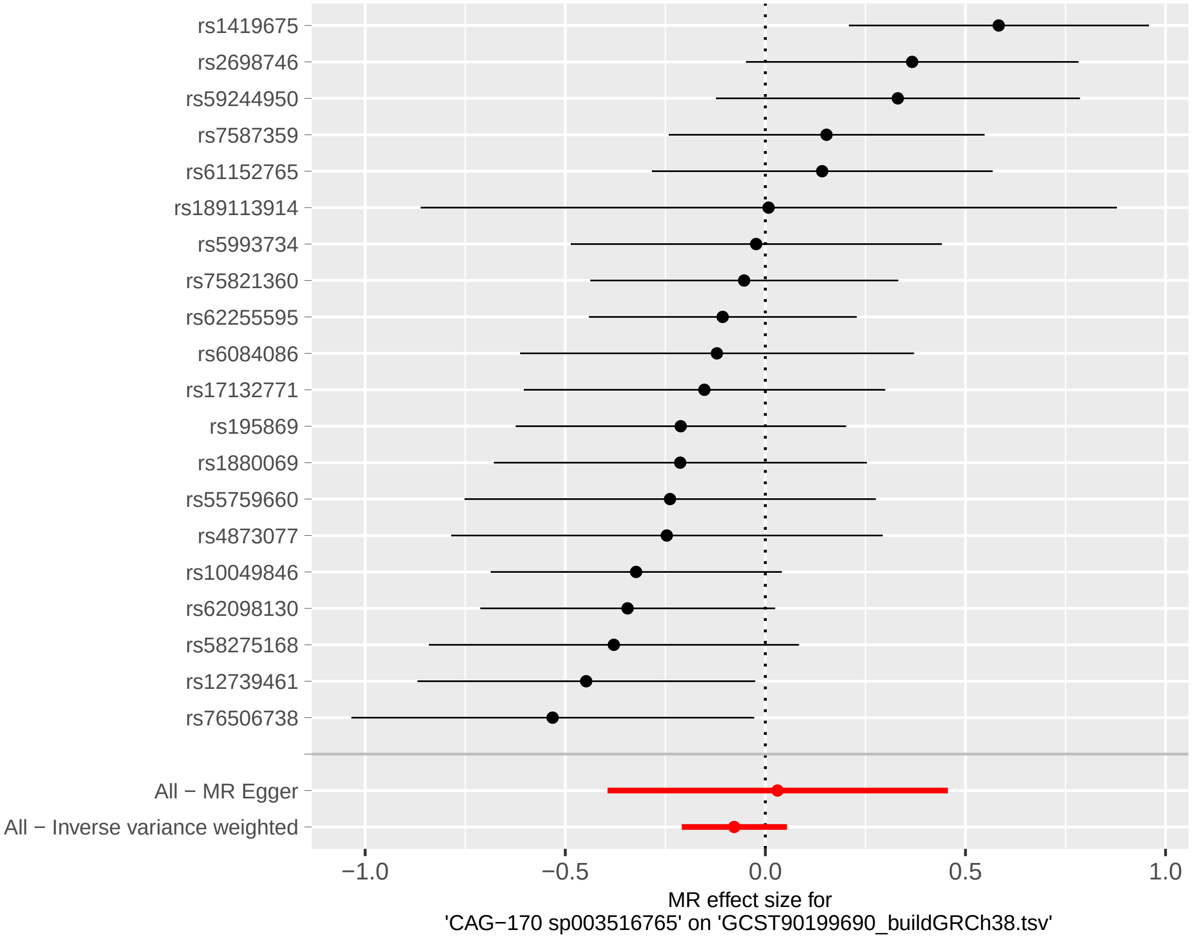
rs1419675
rs2698746
rs59244950
rs7587359
rs61152765
rs189113914
rs5993734
rs75821360
rs62255595
rs6084086
rs17132771
rs195869
rs1880069
rs55759660
rs4873077
rs10049846
rs62098130
rs58275168
rs12739461
rs76506738
All − MR Egger
All − Inverse variance weighted
−1.0
−0.5
0.0
0.5
1.0
MR effect size for
'CAG−170 sp003516765' on 'GCST90199690_buildGRCh38.tsv'

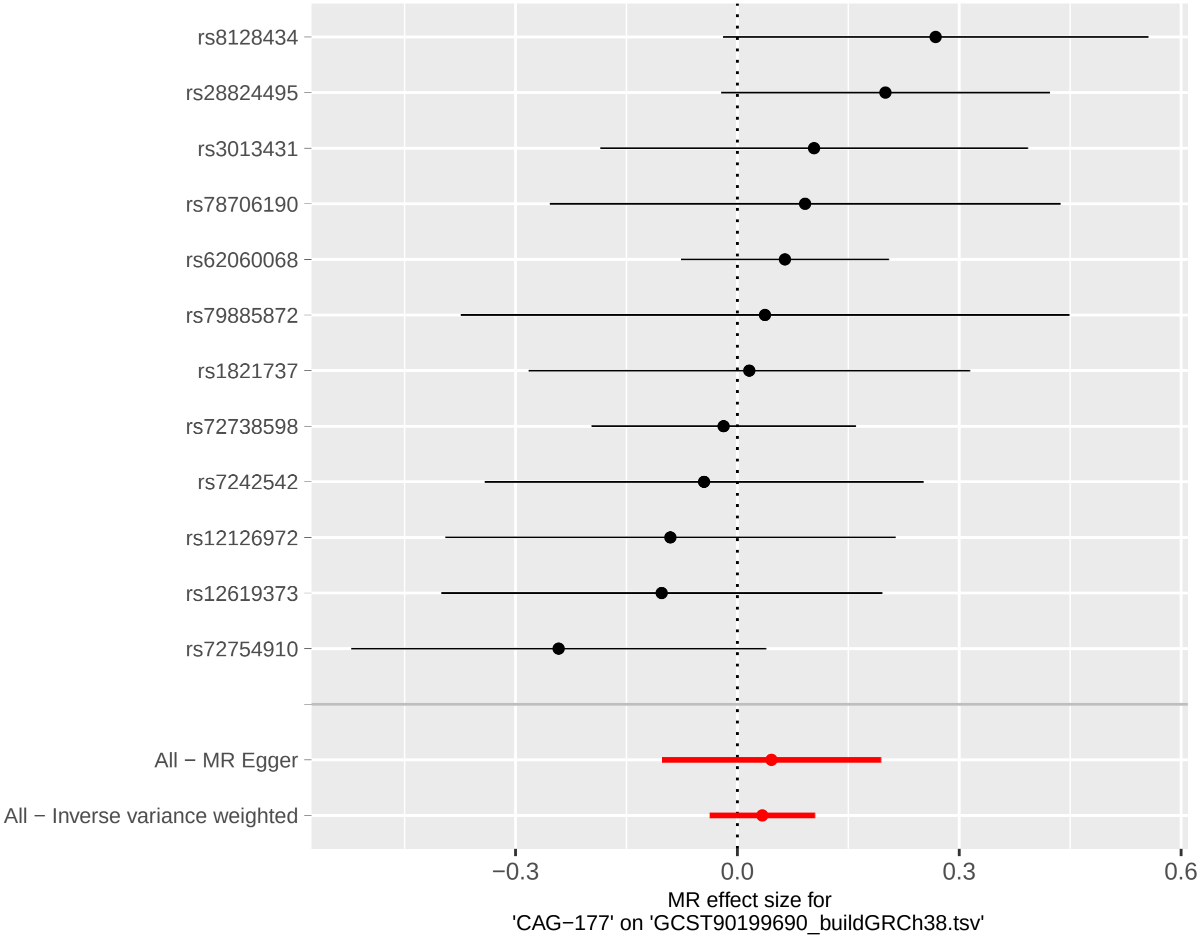

rs8128434
rs28824495
rs3013431
rs78706190
rs62060068
rs79885872
rs1821737
rs72738598
rs7242542
rs12126972
rs12619373
rs72754910
All − MR Egger
All − Inverse variance weighted
−0.3
0.0
0.3
0.6
MR effect size for
'CAG−177' on 'GCST90199690_buildGRCh38.tsv'

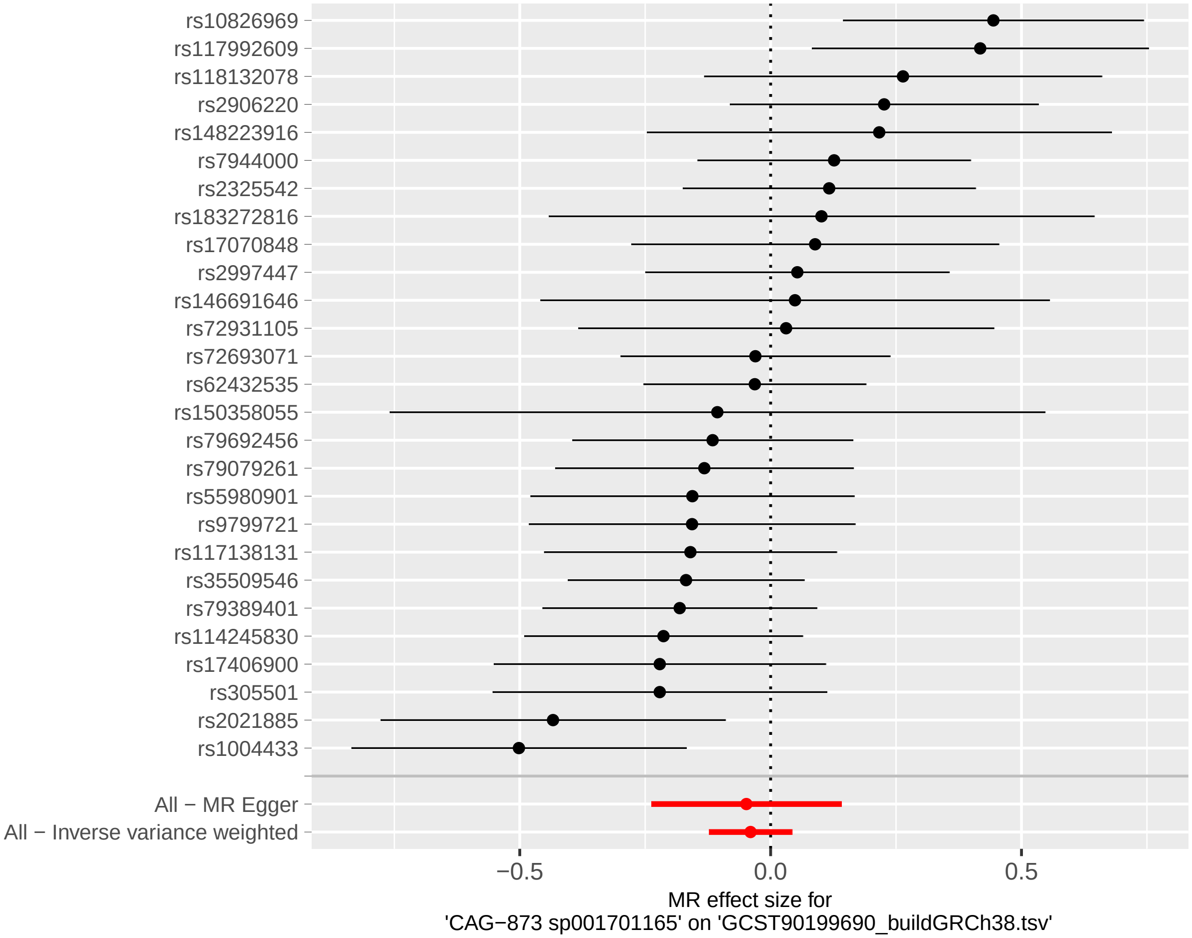
rs10826969
rs117992609
rs118132078
rs2906220
rs148223916
rs7944000
rs2325542
rs183272816
rs17070848
rs2997447
rs146691646
rs72931105
rs72693071
rs62432535
rs150358055
rs79692456
rs79079261
rs55980901
rs9799721
rs117138131
rs35509546
rs79389401
rs114245830
rs17406900
rs305501
rs2021885
rs1004433
All − MR Egger
All − Inverse variance weighted
−0.5
0.0
0.5
MR effect size for
'CAG−873 sp001701165' on 'GCST90199690_buildGRCh38.tsv'

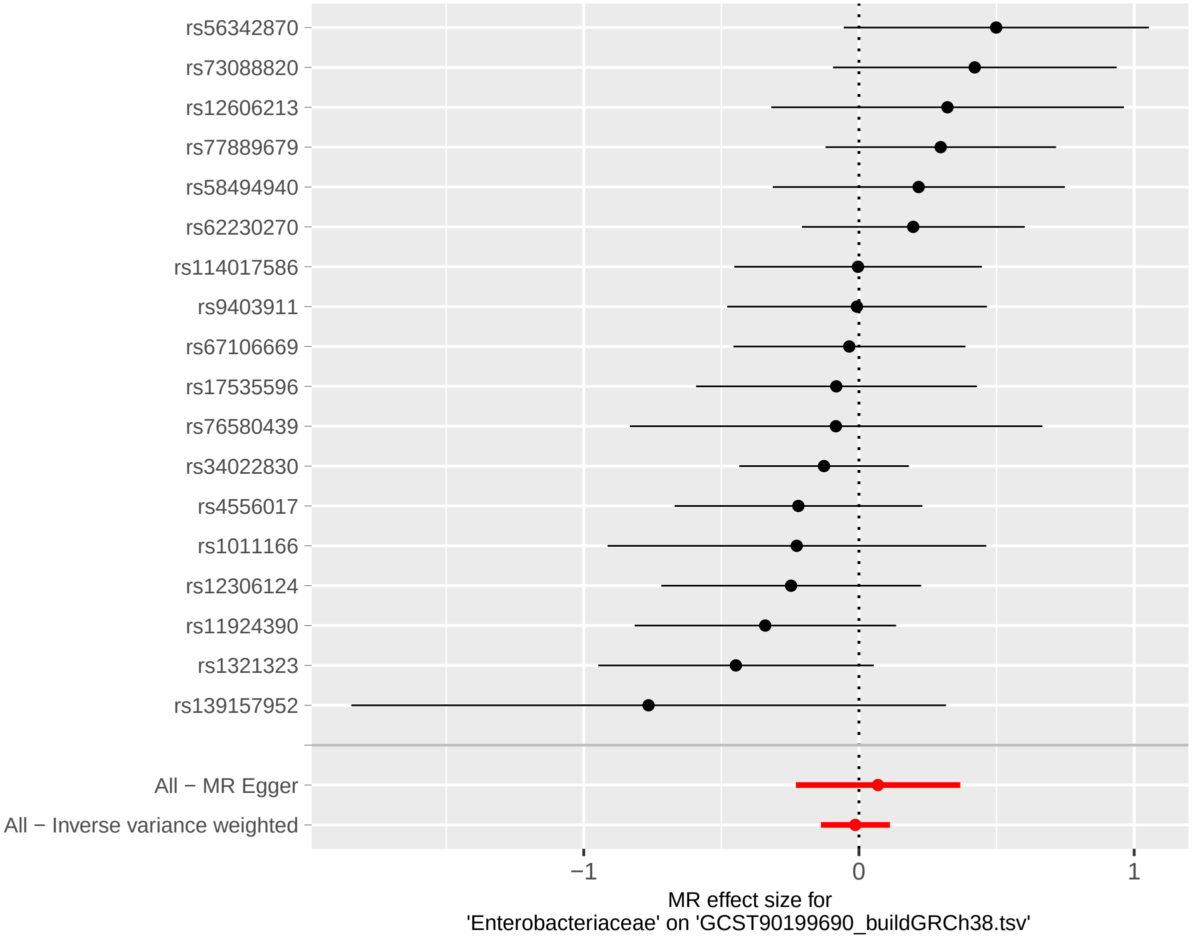
rs56342870
rs73088820
rs12606213
rs77889679
rs58494940
rs62230270
rs114017586
rs9403911
rs67106669
rs17535596
rs76580439
rs34022830
rs4556017
rs1011166
rs12306124
rs11924390
rs1321323
rs139157952
All − MR Egger
All − Inverse variance weighted
−1
0
1
MR effect size for
'Enterobacteriaceae' on 'GCST90199690_buildGRCh38.tsv'

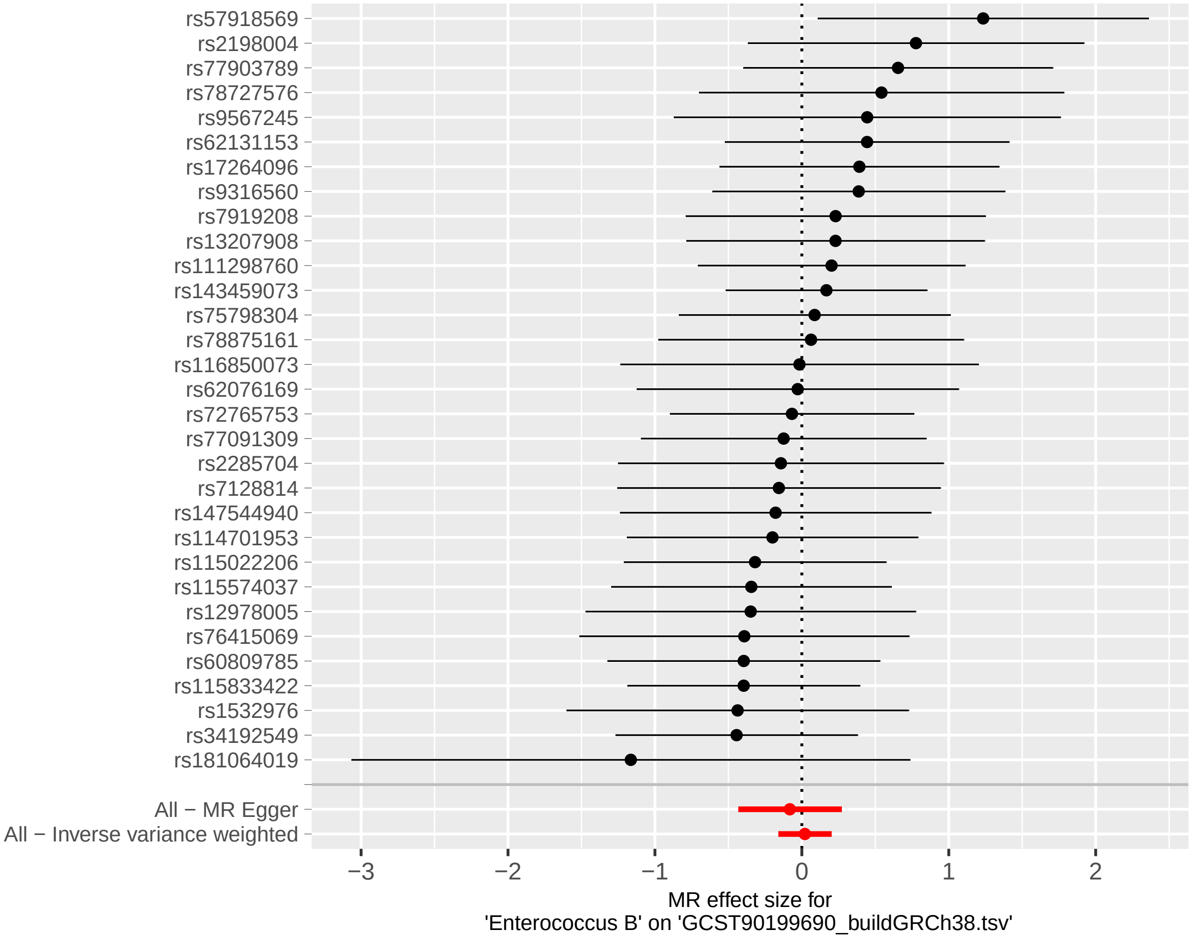

rs57918569
rs2198004
rs77903789
rs78727576
rs9567245
rs62131153
rs17264096
rs9316560
rs7919208
rs13207908
rs111298760
rs143459073
rs75798304
rs78875161
rs116850073
rs62076169
rs72765753
rs77091309
rs2285704
rs7128814
rs147544940
rs114701953
rs115022206
rs115574037
rs12978005
rs76415069
rs60809785
rs115833422
rs1532976
rs34192549
rs181064019
All − MR Egger
All − Inverse variance weighted
−3
−2
−1
0
1
2
MR effect size for
'Enterococcus B' on 'GCST90199690_buildGRCh38.tsv'

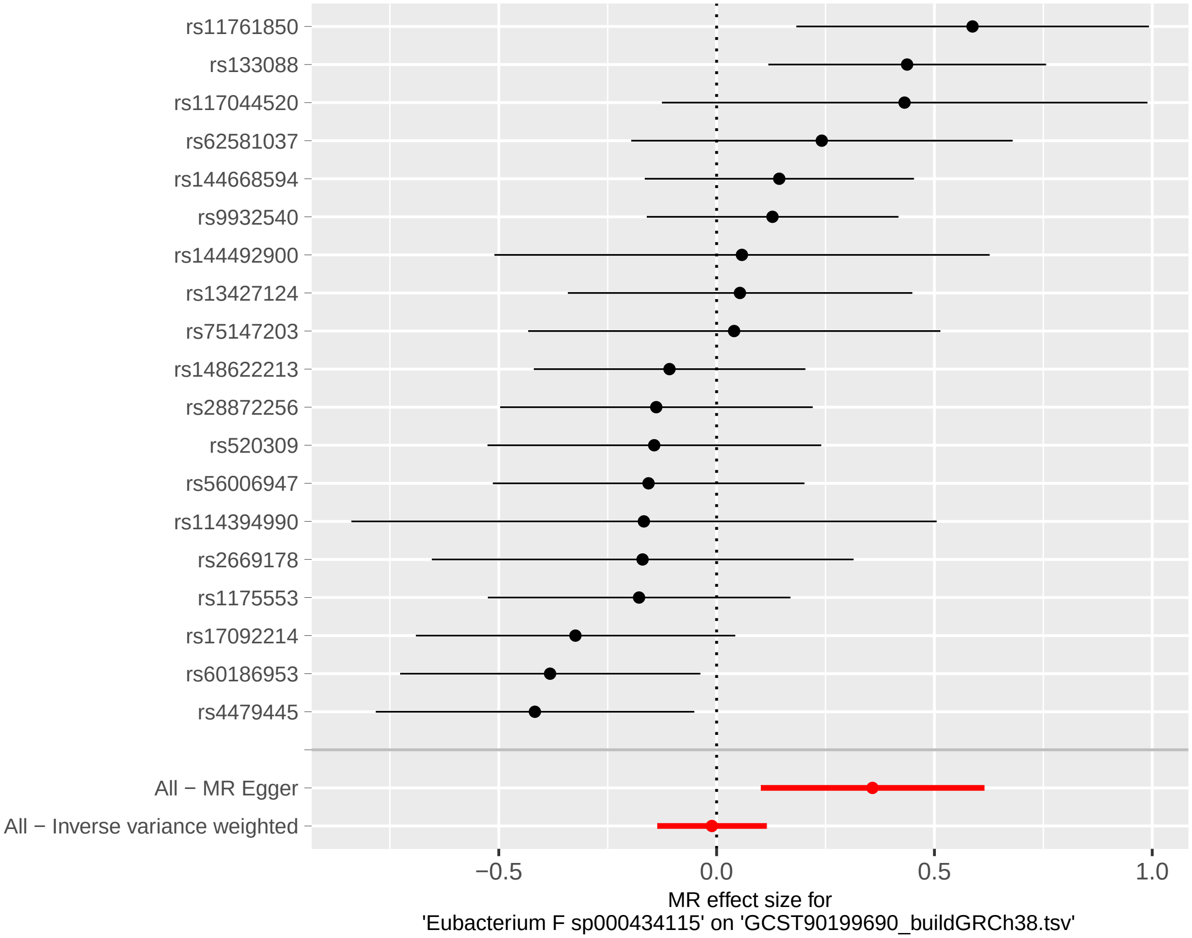

rs11761850
rs133088
rs117044520
rs62581037
rs144668594
rs9932540
rs144492900
rs13427124
rs75147203
rs148622213
rs28872256
rs520309
rs56006947
rs114394990
rs2669178
rs1175553
rs17092214
rs60186953
rs4479445
All − MR Egger
All − Inverse variance weighted
−0.5
0.0
0.5
1.0
MR effect size for
'Eubacterium F sp000434115' on 'GCST90199690_buildGRCh38.tsv'

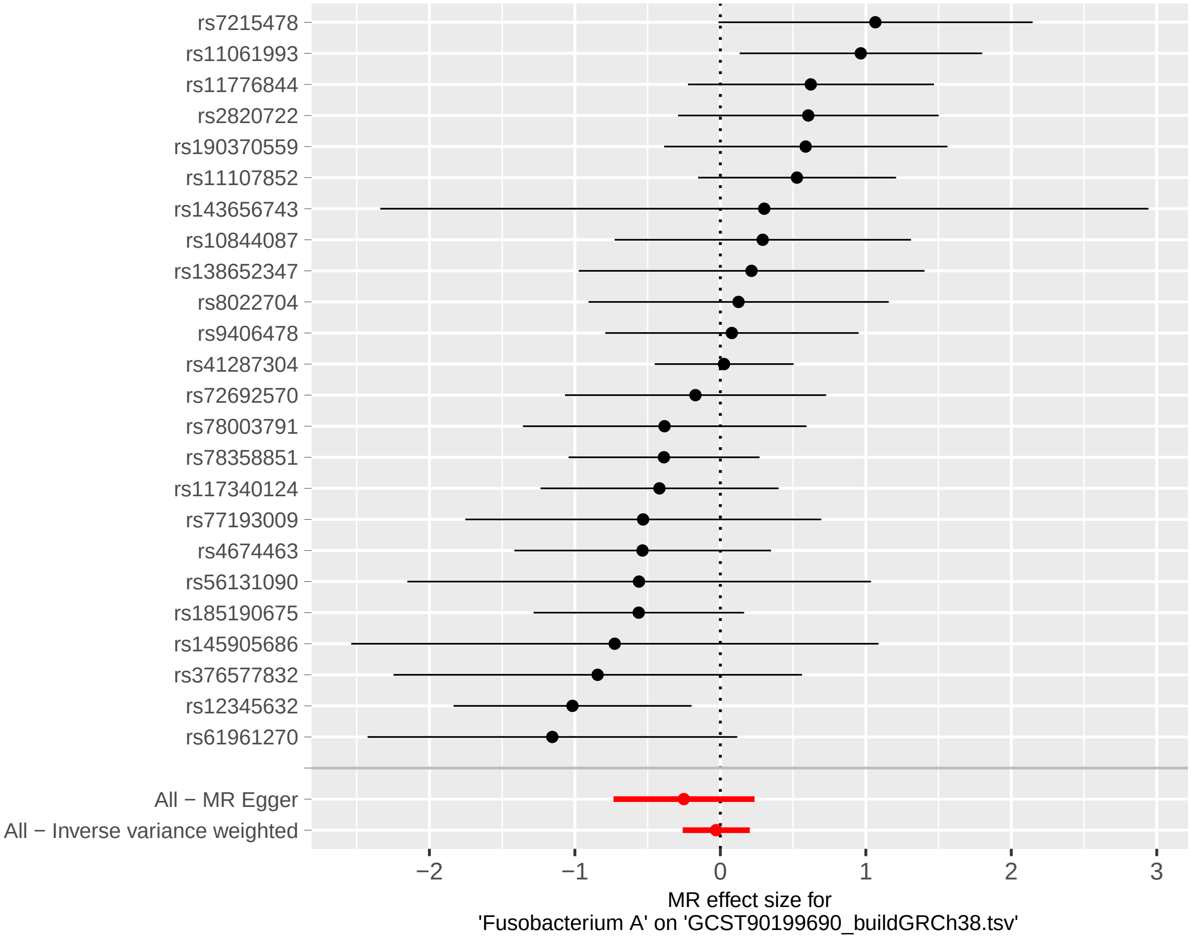

rs7215478
rs11061993
rs11776844
rs2820722
rs190370559
rs11107852
rs143656743
rs10844087
rs138652347
rs8022704
rs9406478
rs41287304
rs72692570
rs78003791
rs78358851
rs117340124
rs77193009
rs4674463
rs56131090
rs185190675
rs145905686
rs376577832
rs12345632
rs61961270
All − MR Egger
All − Inverse variance weighted
−2
−1
0
1
2
3
MR effect size for
'Fusobacterium A' on 'GCST90199690_buildGRCh38.tsv'

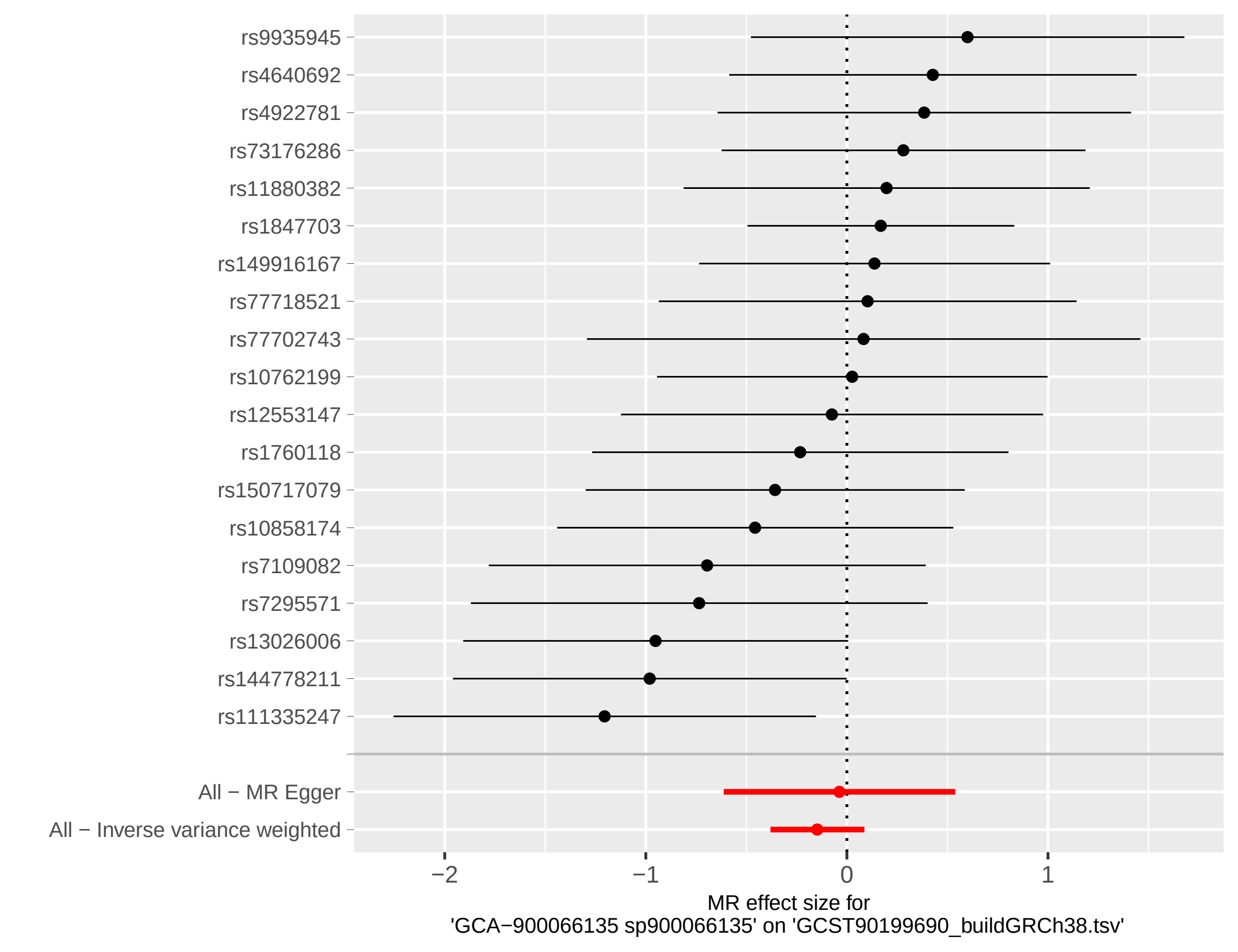
rs9935945
rs4640692
rs4922781
rs73176286
rs11880382
rs1847703
rs149916167
rs77718521
rs77702743
rs10762199
rs12553147
rs1760118
rs150717079
rs10858174
rs7109082
rs7295571
rs13026006
rs144778211
rs111335247
All − MR Egger
All − Inverse variance weighted
−2
−1
0
1
MR effect size for
'GCA−900066135 sp900066135' on 'GCST90199690_buildGRCh38.tsv'

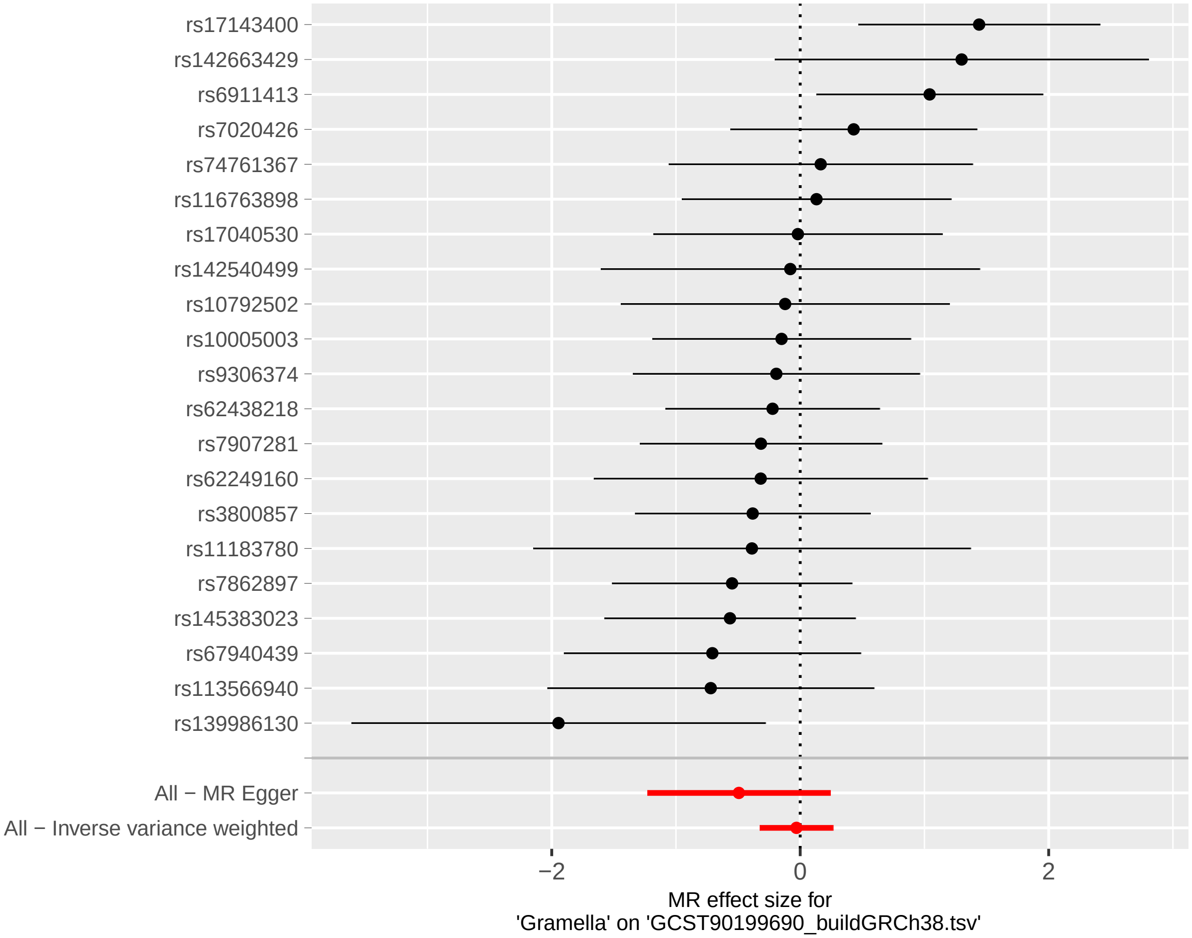
rs17143400
rs142663429
rs6911413
rs7020426
rs74761367
rs116763898
rs17040530
rs142540499
rs10792502
rs10005003
rs9306374
rs62438218
rs7907281
rs62249160
rs3800857
rs11183780
rs7862897
rs145383023
rs67940439
rs113566940
rs139986130
All − MR Egger
All − Inverse variance weighted
−2
0
2
MR effect size for
'Gramella' on 'GCST90199690_buildGRCh38.tsv'

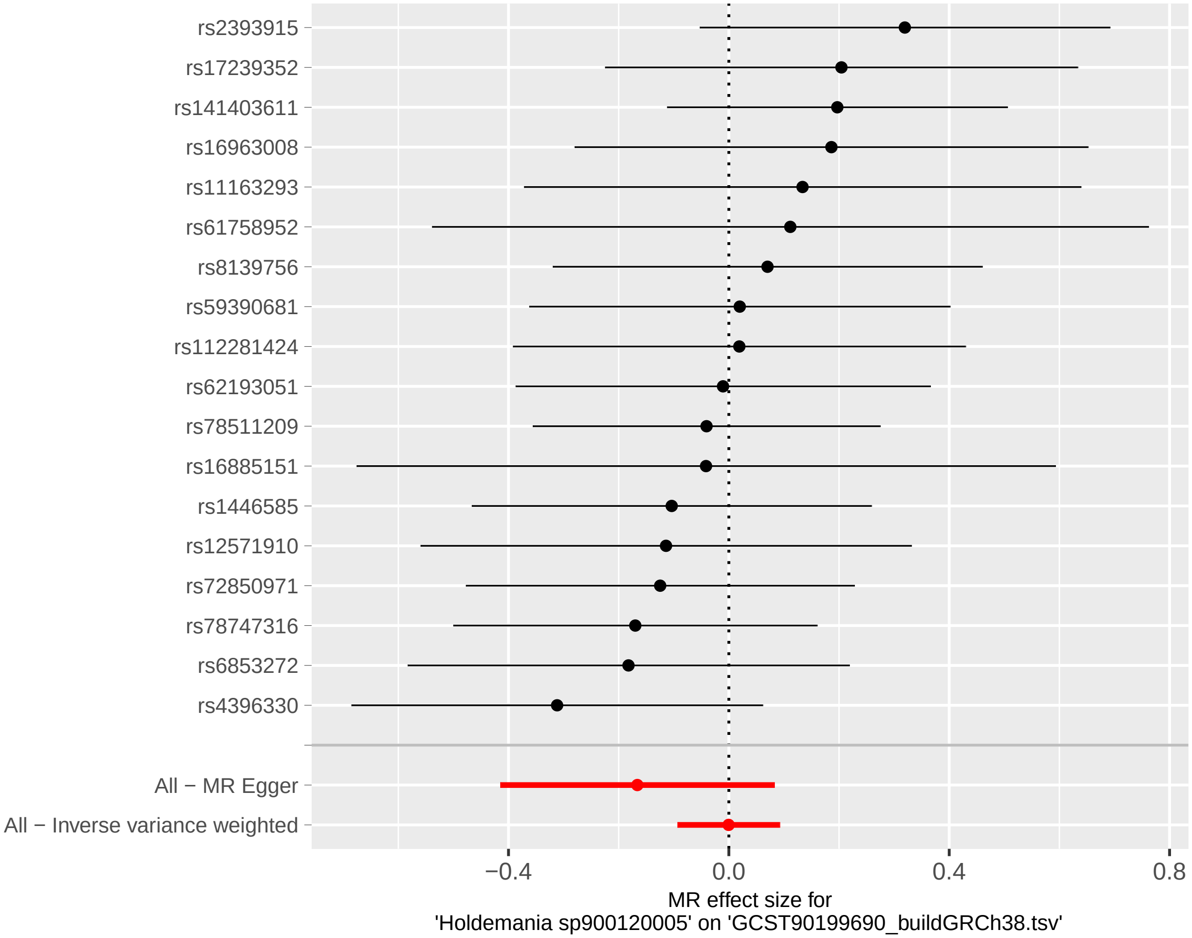
rs2393915
rs17239352
rs141403611
rs16963008
rs11163293
rs61758952
rs8139756
rs59390681
rs112281424
rs62193051
rs78511209
rs16885151
rs1446585
rs12571910
rs72850971
rs78747316
rs6853272
rs4396330
All − MR Egger
All − Inverse variance weighted
−0.4
0.0
0.4
0.8
MR effect size for
'Holdemania sp900120005' on 'GCST90199690_buildGRCh38.tsv'

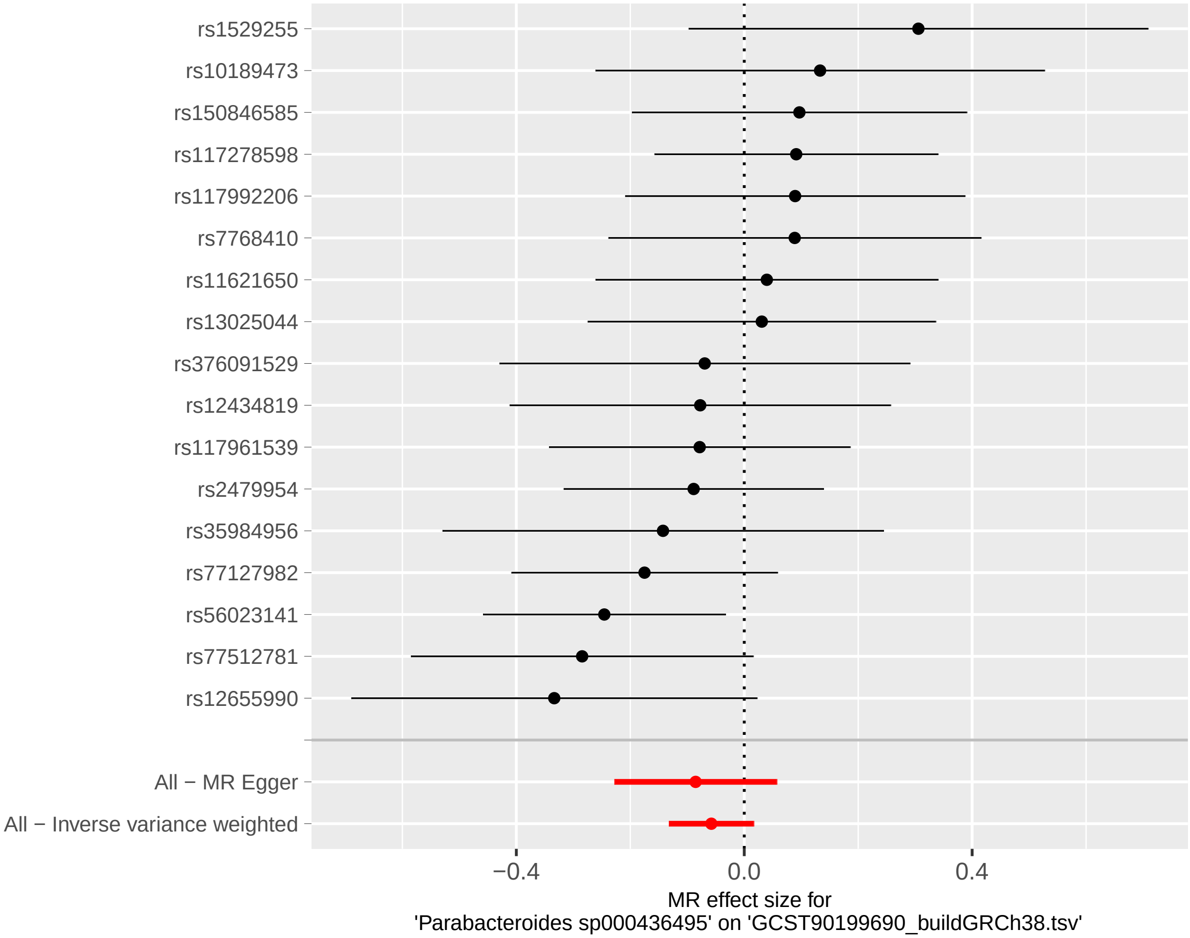

rs1529255
rs10189473
rs150846585
rs117278598
rs117992206
rs7768410
rs11621650
rs13025044
rs376091529
rs12434819
rs117961539
rs2479954
rs35984956
rs77127982
rs56023141
rs77512781
rs12655990
All − MR Egger
All − Inverse variance weighted
−0.4
0.0
0.4
MR effect size for
'Parabacteroides sp000436495' on 'GCST90199690_buildGRCh38.tsv'

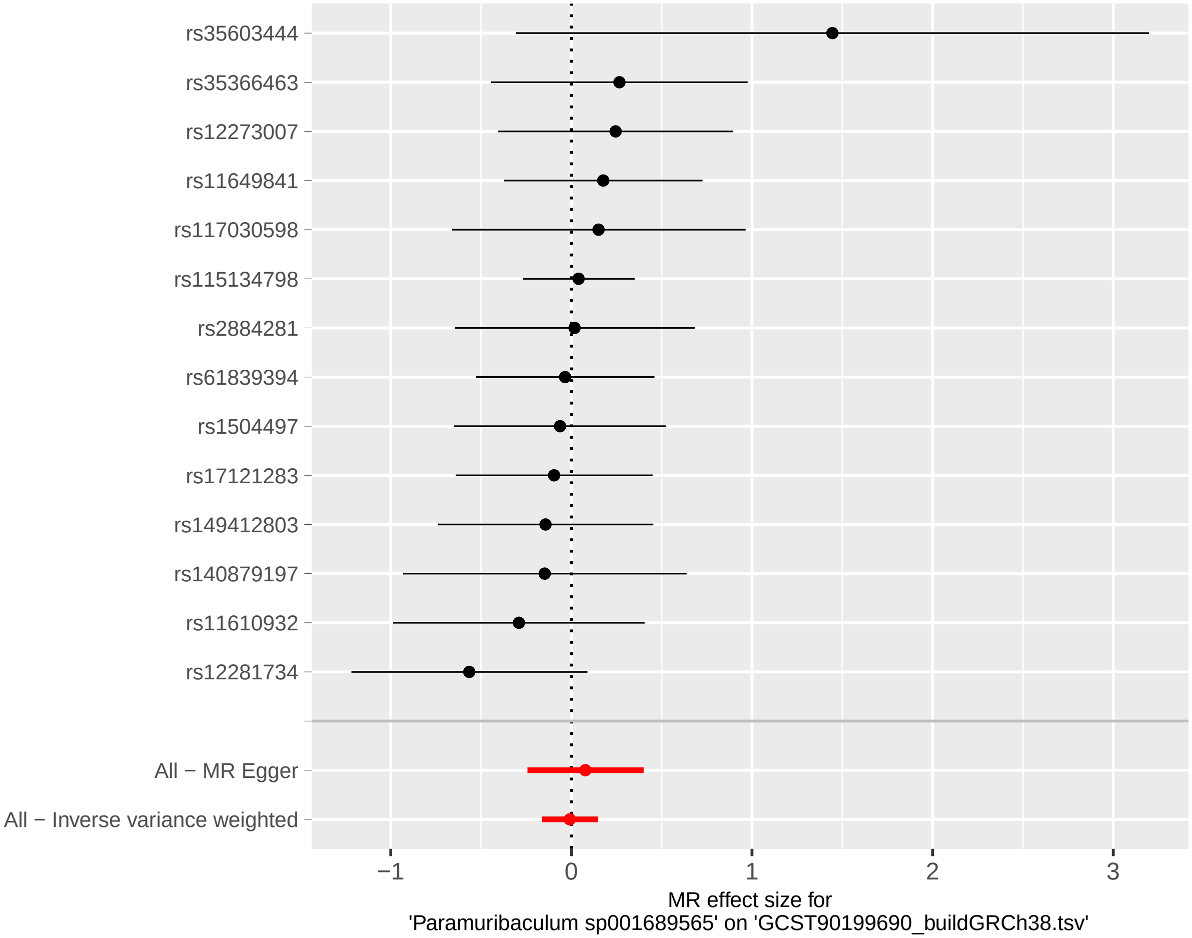
rs35603444
rs35366463
rs12273007
rs11649841
rs117030598
rs115134798
rs2884281
rs61839394
rs1504497
rs17121283
rs149412803
rs140879197
rs11610932
rs12281734
All − MR Egger
All − Inverse variance weighted
−1
0
1
2
3
MR effect size for
'Paramuribaculum sp001689565' on 'GCST90199690_buildGRCh38.tsv'

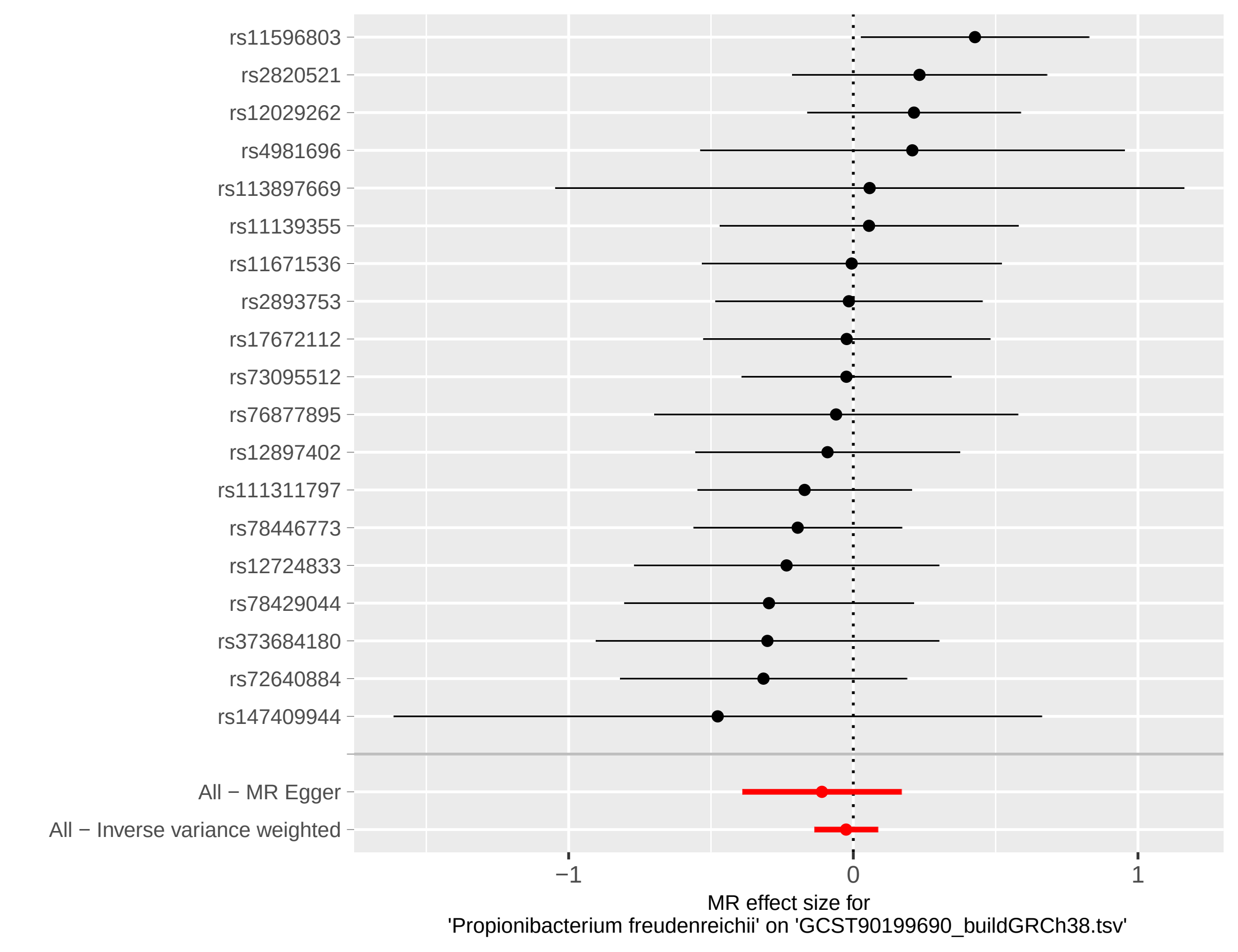
rs11596803
rs2820521
rs12029262
rs4981696
rs113897669
rs11139355
rs11671536
rs2893753
rs17672112
rs73095512
rs76877895
rs12897402
rs111311797
rs78446773
rs12724833
rs78429044
rs373684180
rs72640884
rs147409944
All − MR Egger
All − Inverse variance weighted
−1
0
1
MR effect size for
'Propionibacterium freudenreichii' on 'GCST90199690_buildGRCh38.tsv'

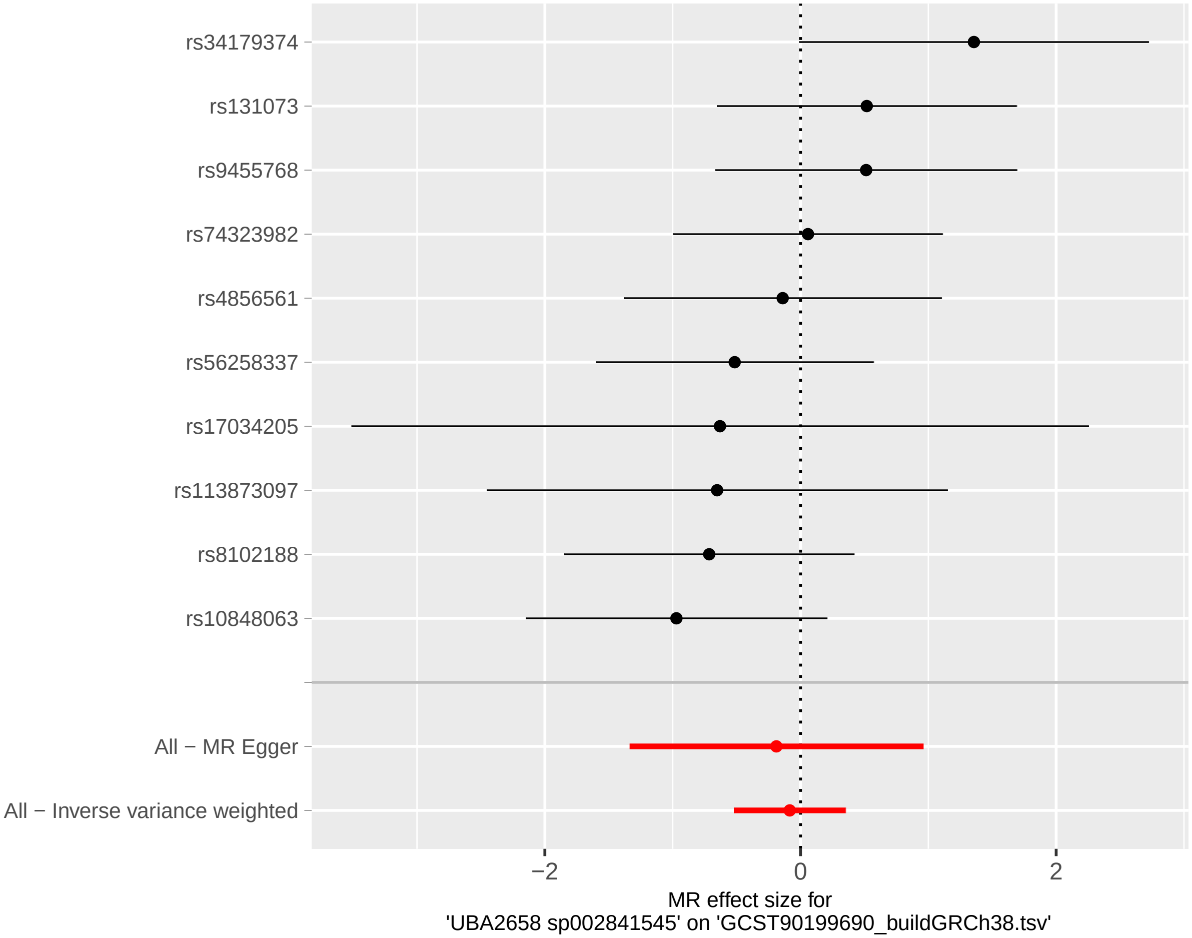

rs34179374
rs131073
rs9455768
rs74323982
rs4856561
rs56258337
rs17034205
rs113873097
rs8102188
rs10848063
All − MR Egger
All − Inverse variance weighted
−2
0
2
MR effect size for
'UBA2658 sp002841545' on 'GCST90199690_buildGRCh38.tsv'

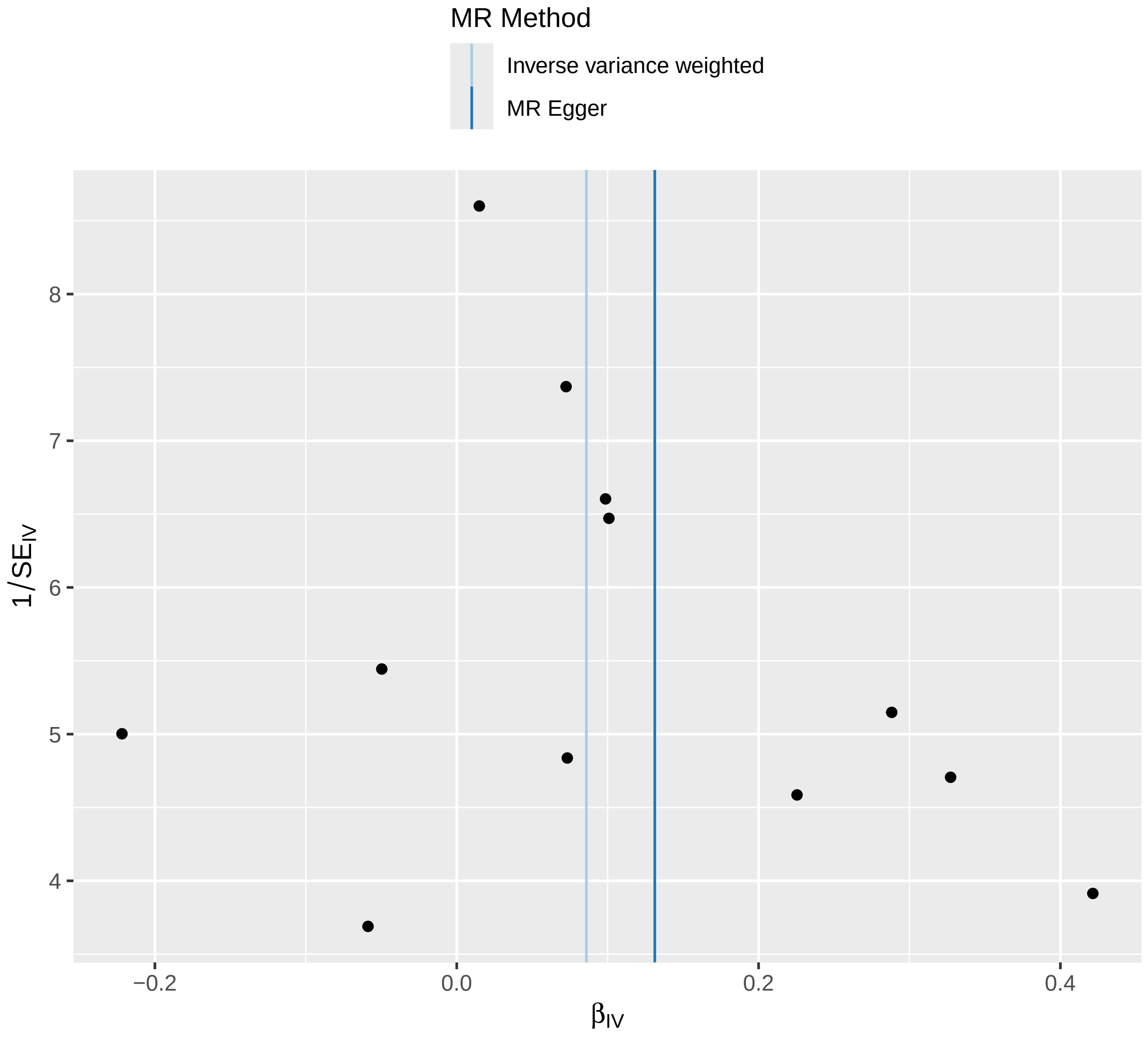
MR Method
Inverse variance weighted
MR Egger
8
7
6
5
4
1/SE$_{IV}$
−0.2
0.0
0.2
0.4
β$_{IV}$

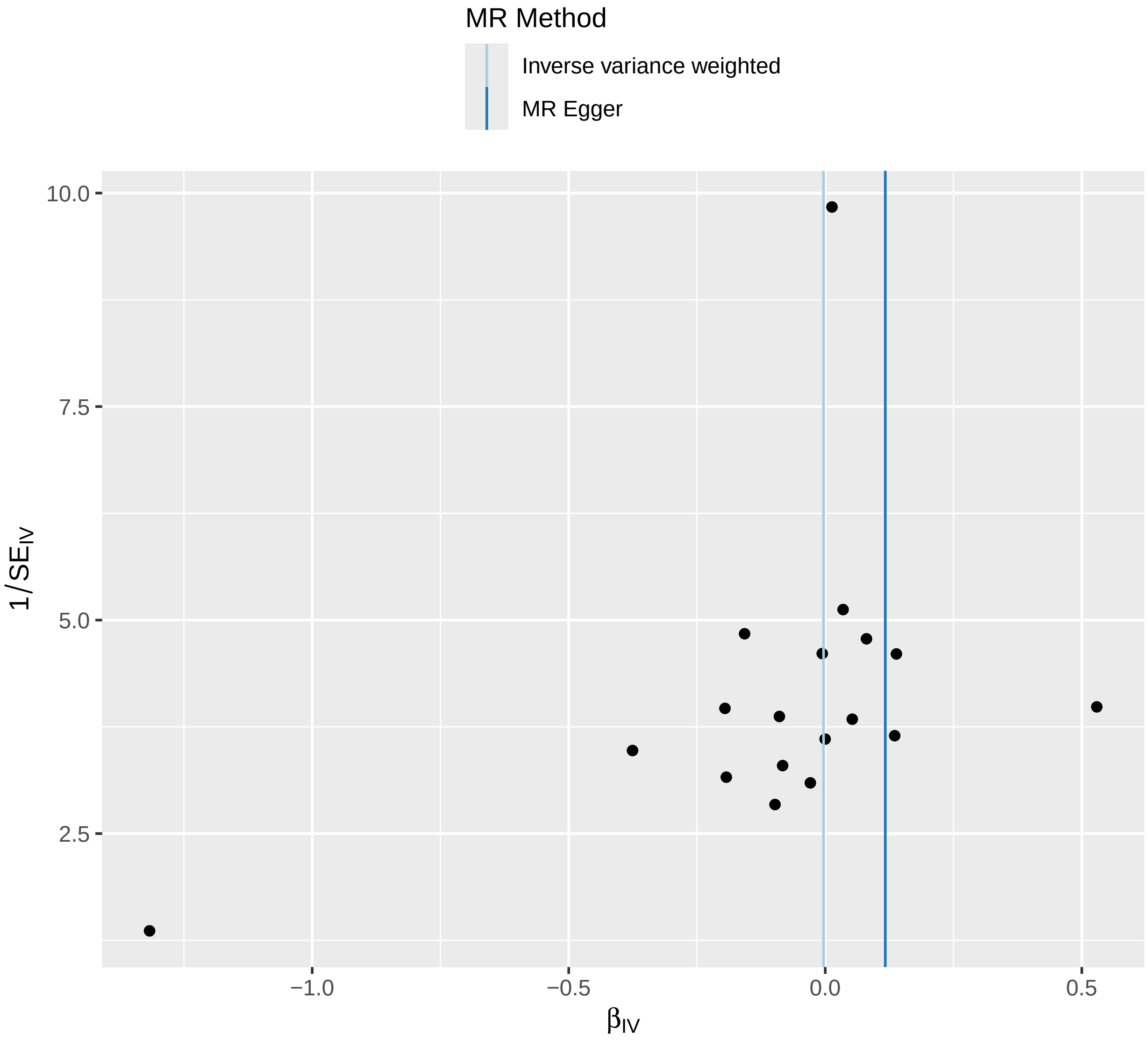

MR Method
Inverse variance weighted
MR Egger
10.0
7.5
5.0
2.5
1/SE$_{IV}$
−1.0
−0.5
0.0
0.5
β$_{IV}$

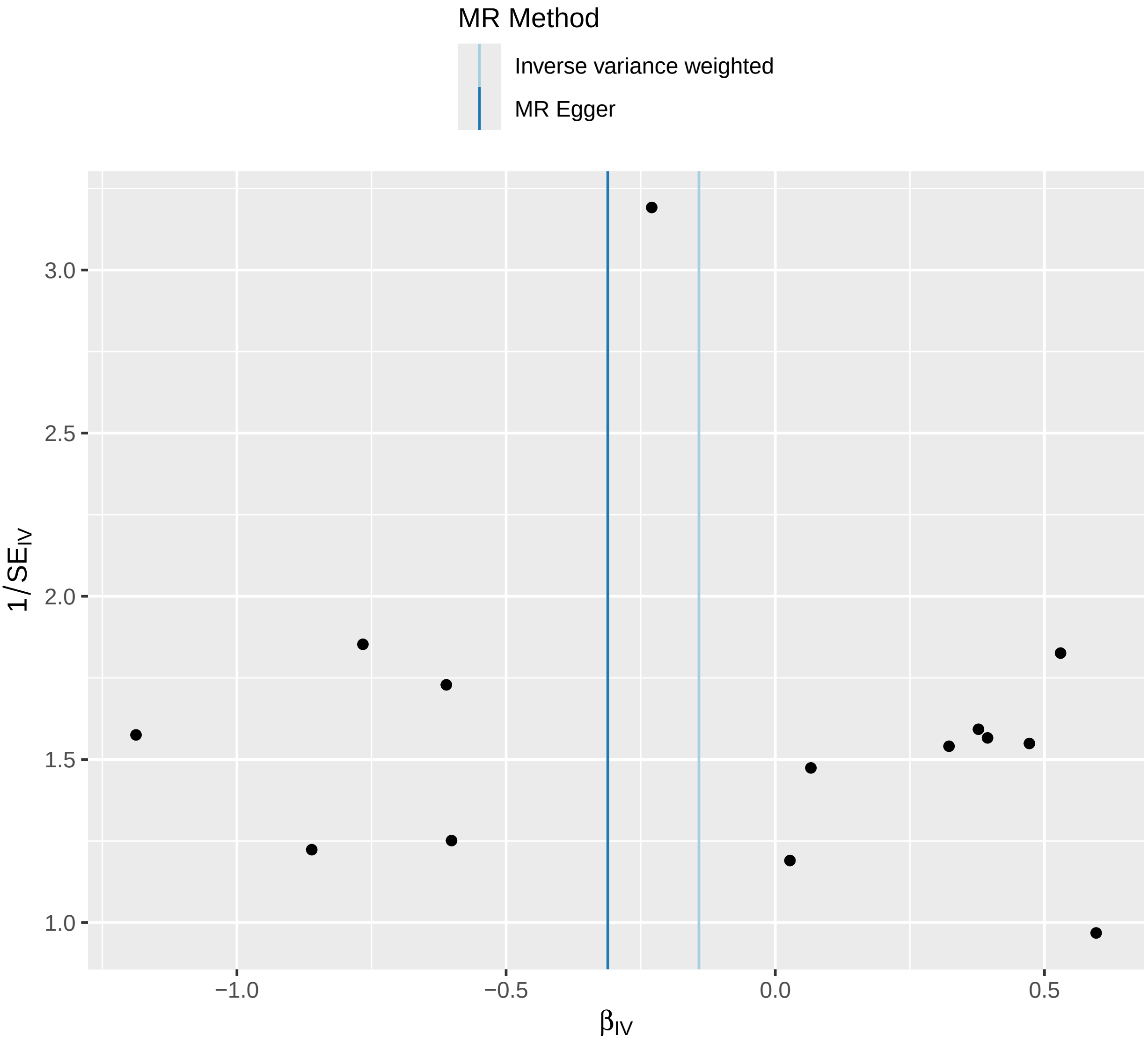

MR Method
Inverse variance weighted
MR Egger
3.0
2.5
2.0
1.5
1.0
$1/SE_{IV}$
−1.0
−0.5
0.0
0.5
β_{IV}

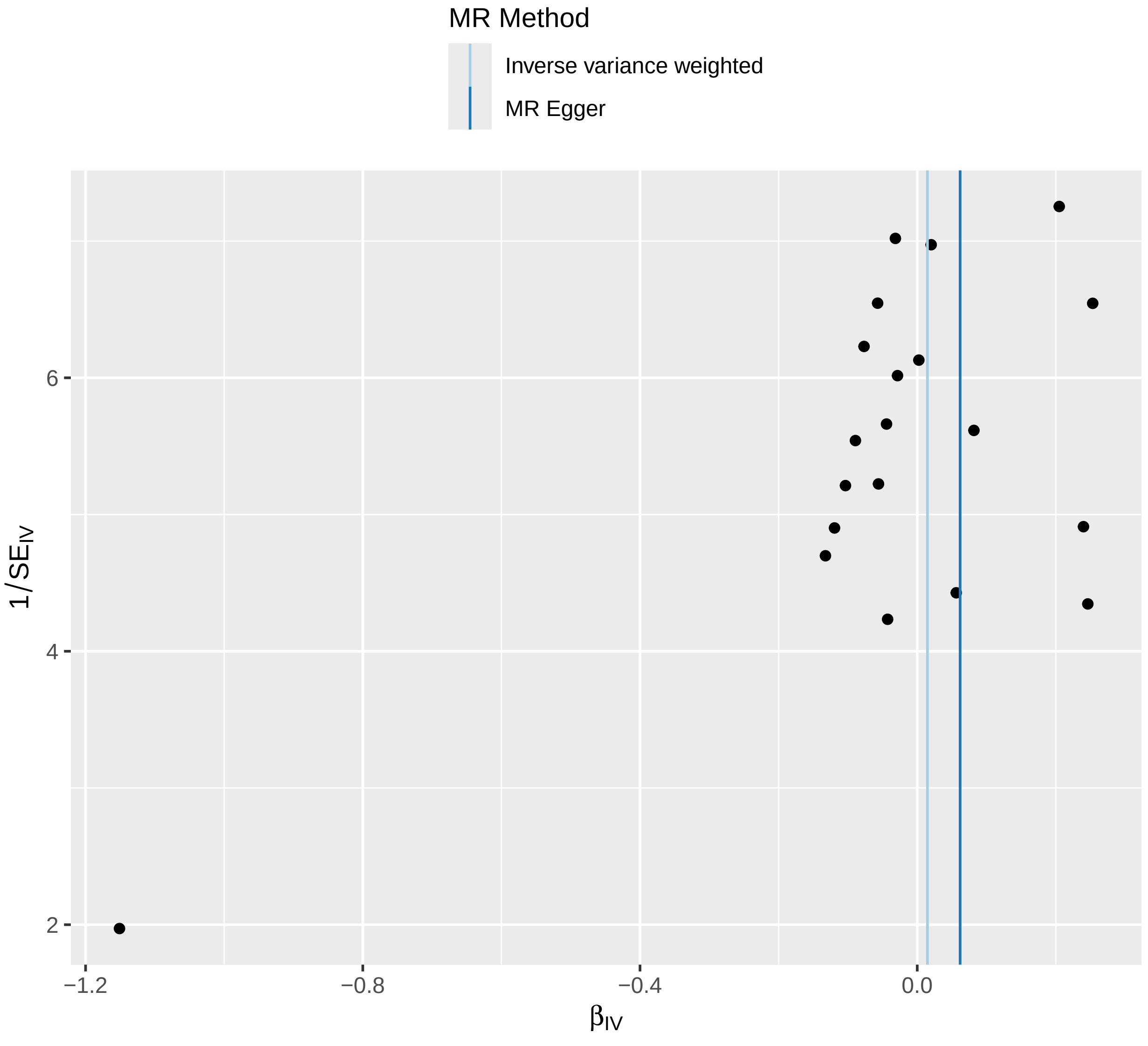

MR Method
Inverse variance weighted
MR Egger
6
4
2
$1/SE_{IV}$
−1.2
−0.8
−0.4
0.0
β_{IV}

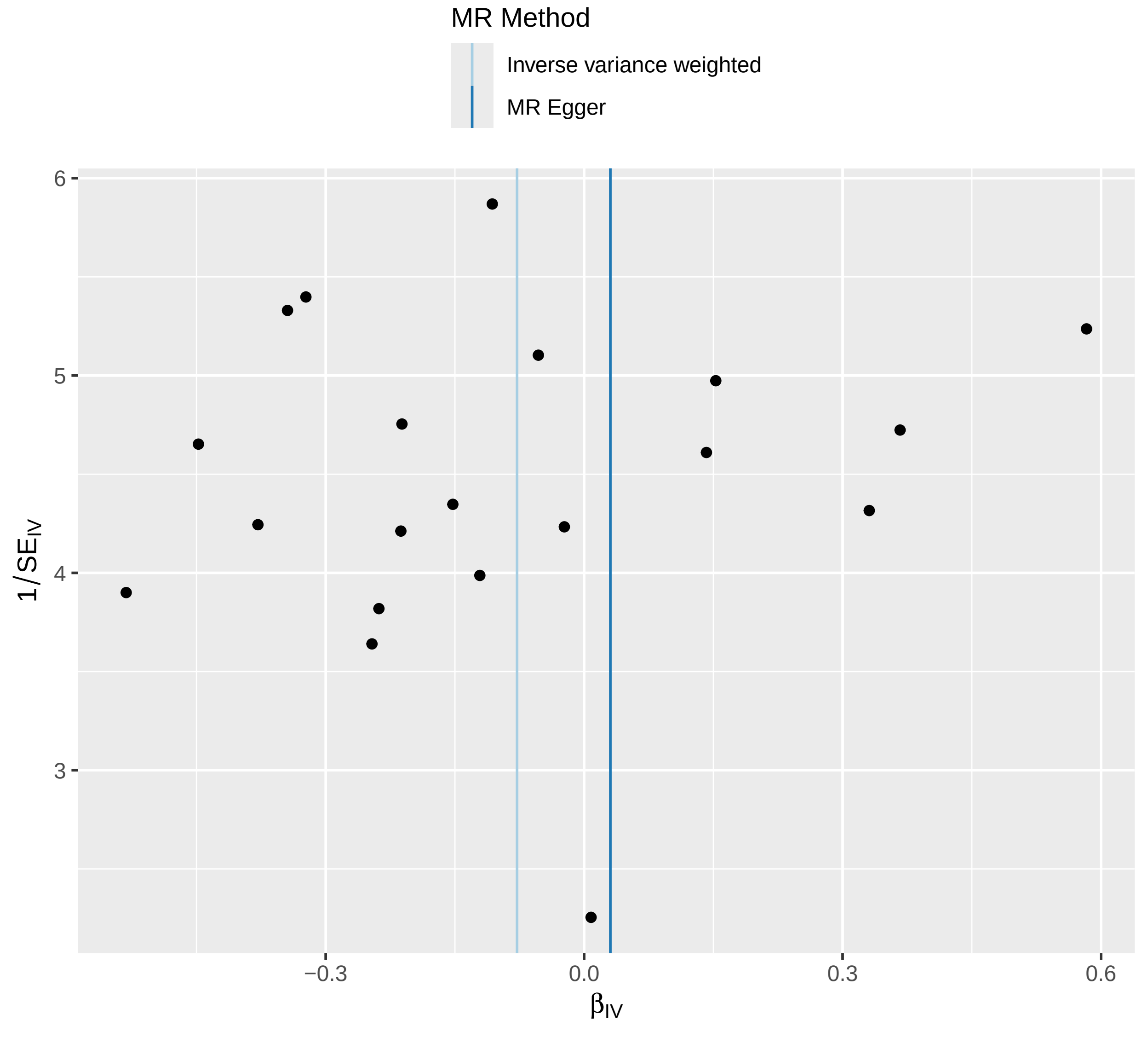
MR Method
Inverse variance weighted
MR Egger
6
5
4
3
1/SE_IV
−0.3
0.0
0.3
0.6
β_IV

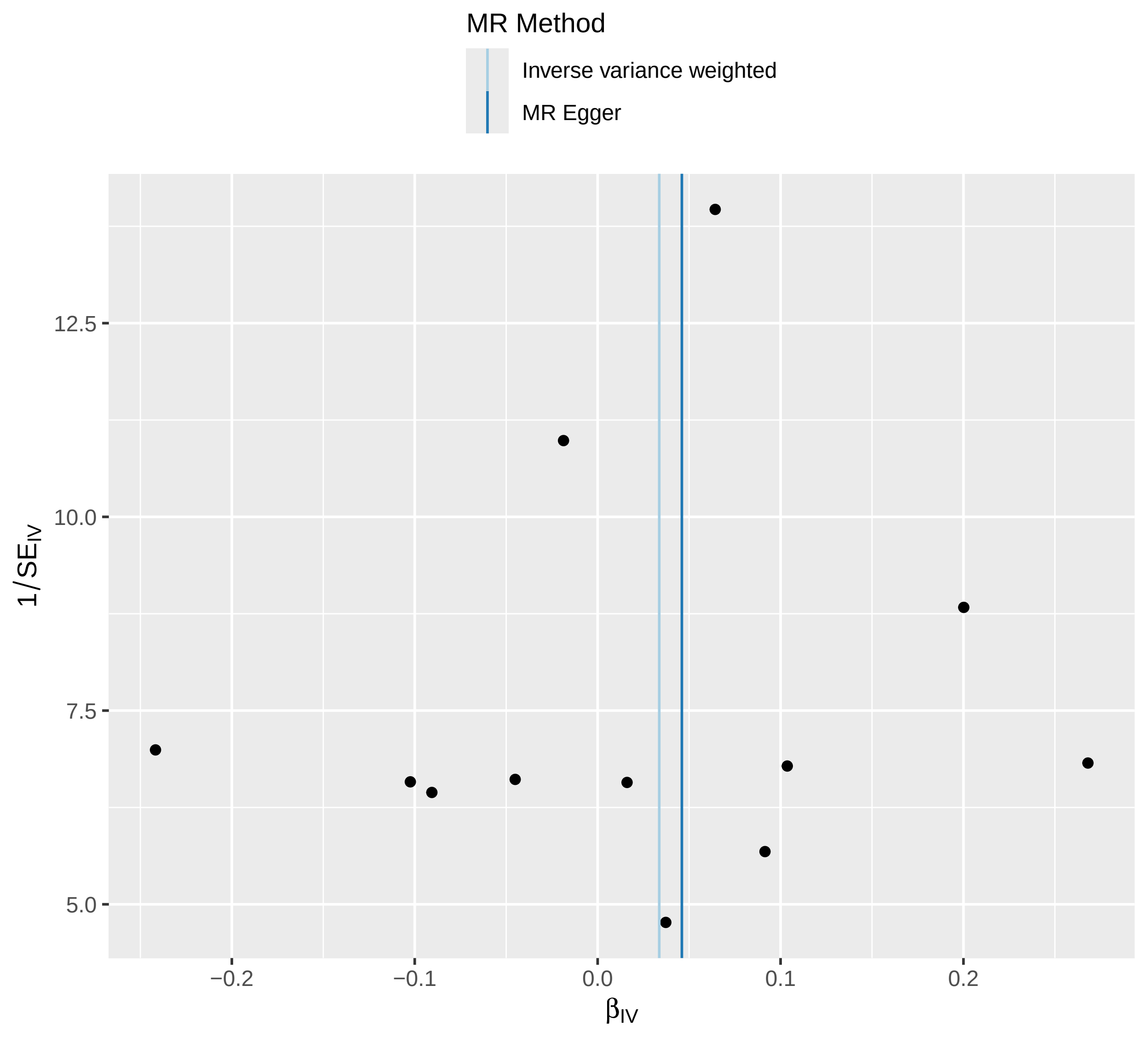

MR Method
Inverse variance weighted
MR Egger
12.5
10.0
7.5
5.0
−0.2
−0.1
0.0
0.1
0.2
1/SE_IV
β_IV

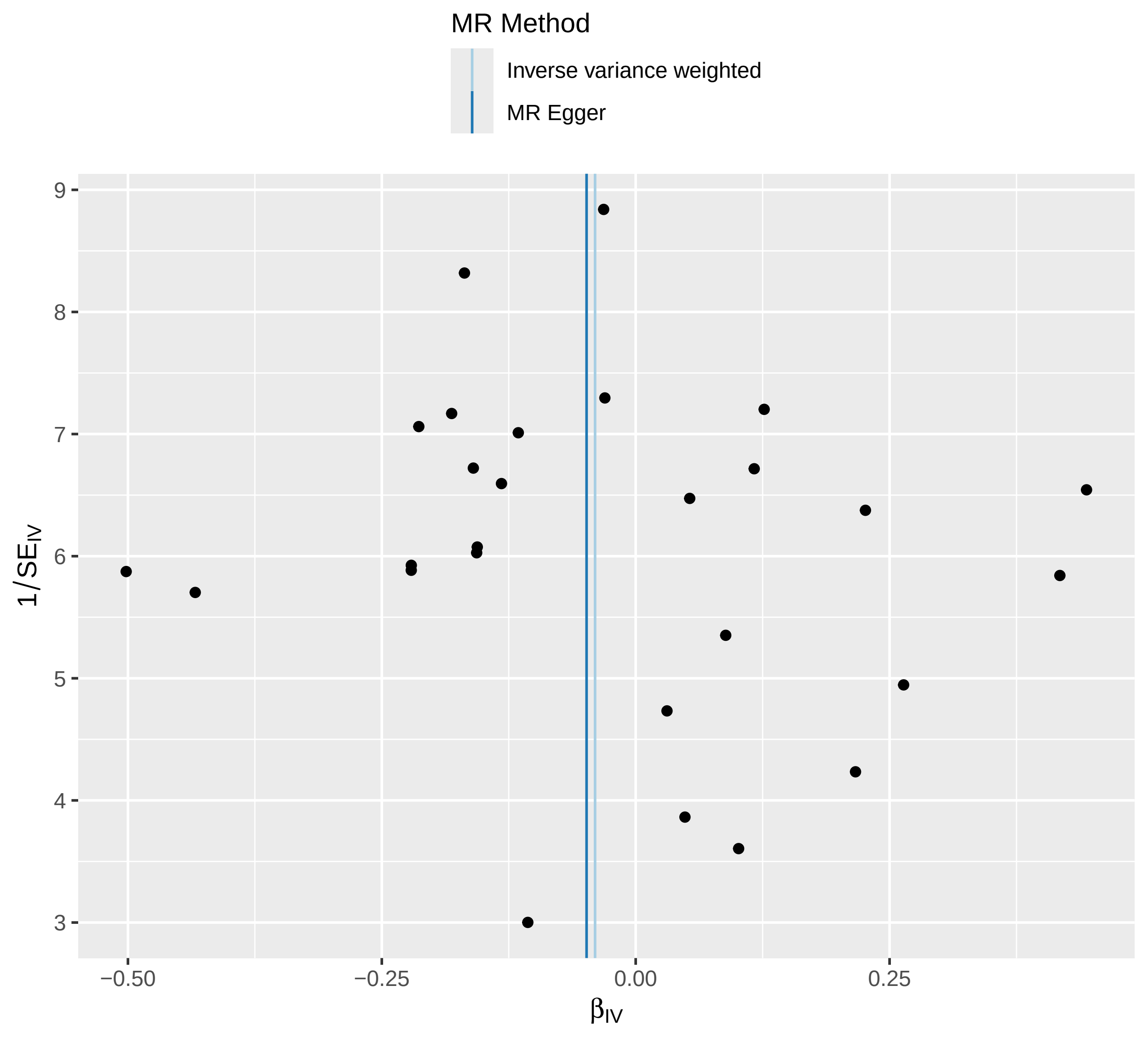

MR Method
Inverse variance weighted
MR Egger
9
8
7
6
5
4
3
1/SE_IV
−0.50
−0.25
0.00
0.25
β_IV

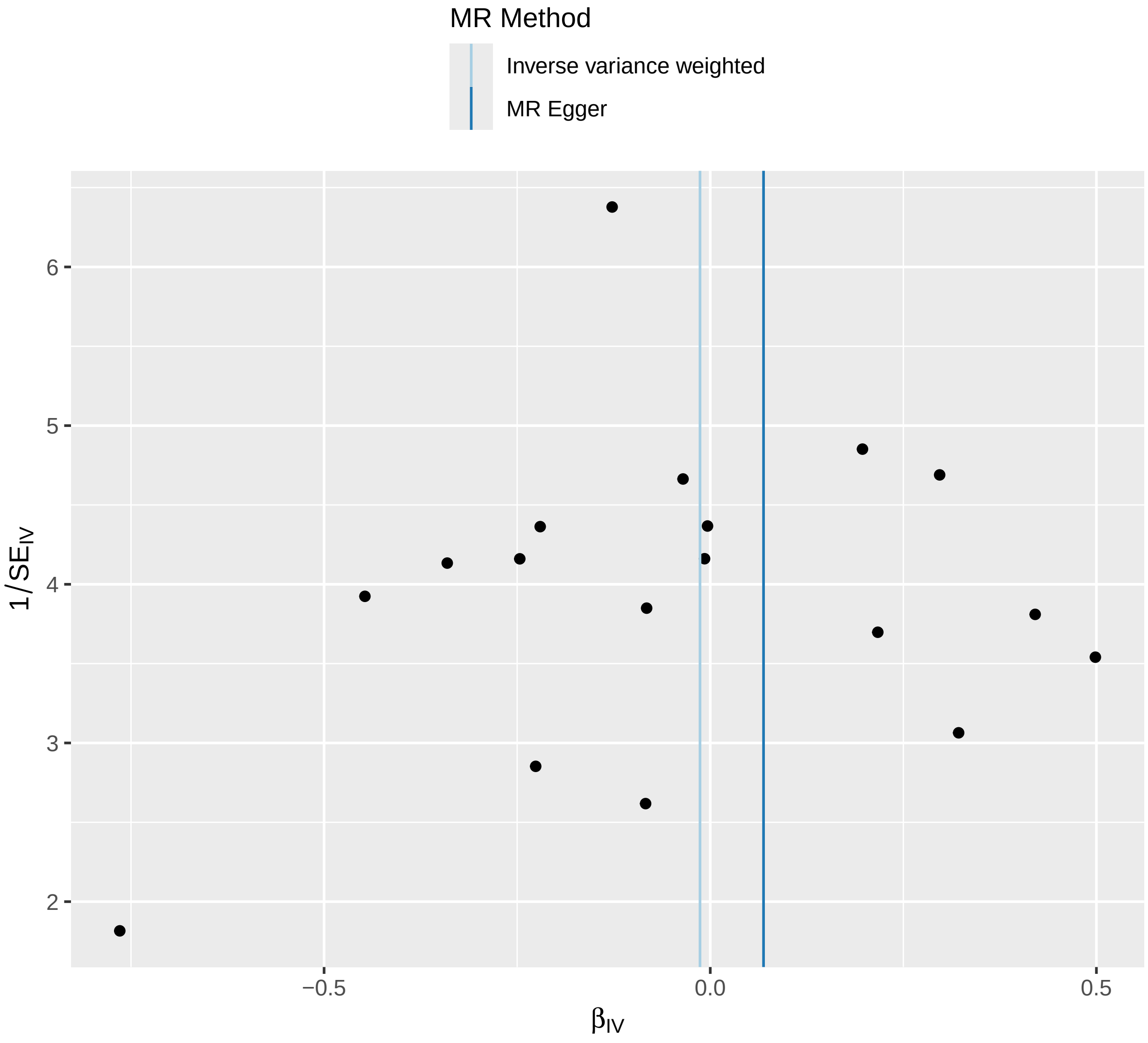
MR Method
Inverse variance weighted
MR Egger
6
5
4
3
2
1/SE_IV
−0.5
0.0
0.5
β_IV

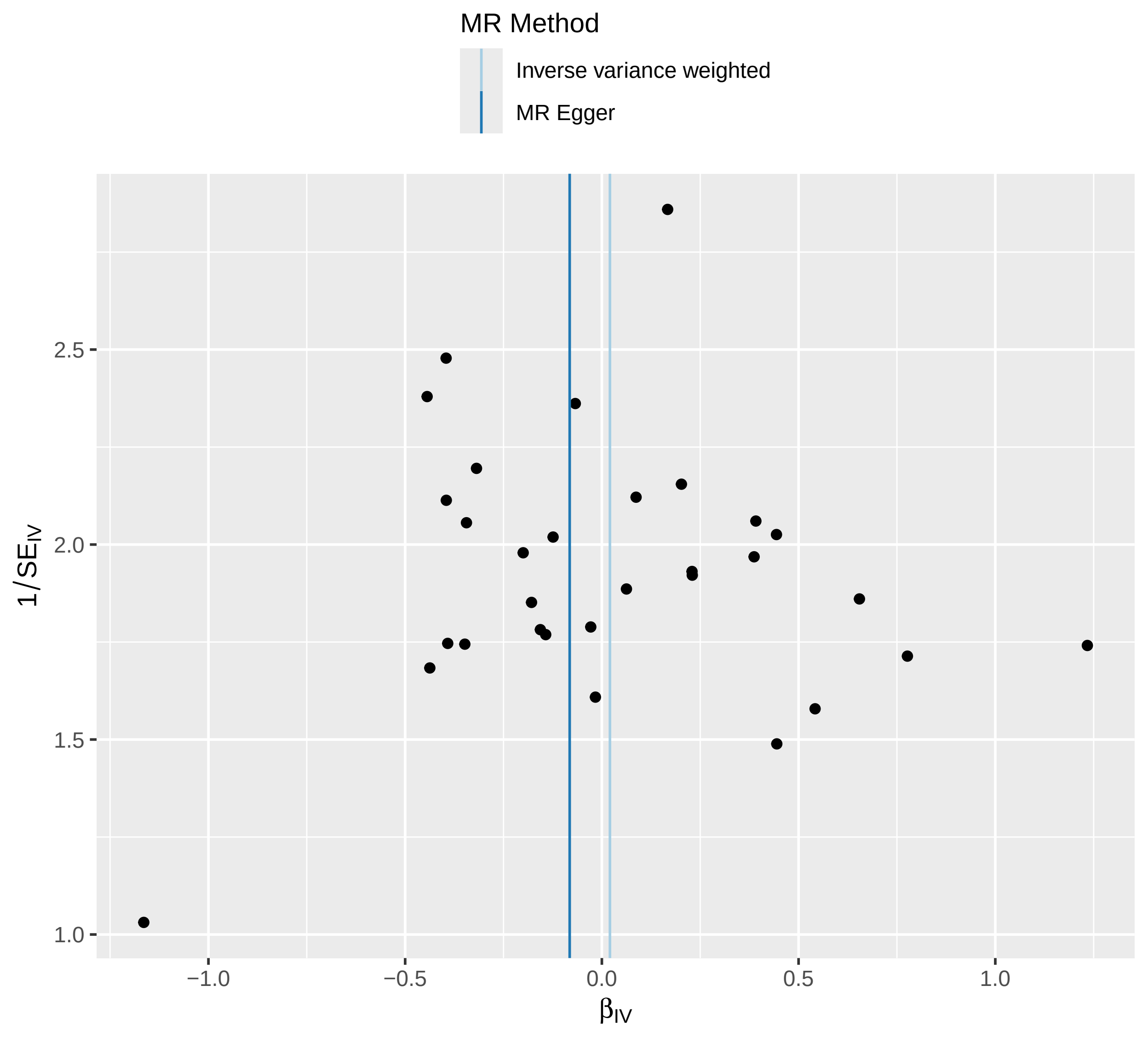
MR Method
Inverse variance weighted
MR Egger
2.5
2.0
1.5
1.0
1/SE$_{IV}$
−1.0
−0.5
0.0
0.5
1.0
β$_{IV}$

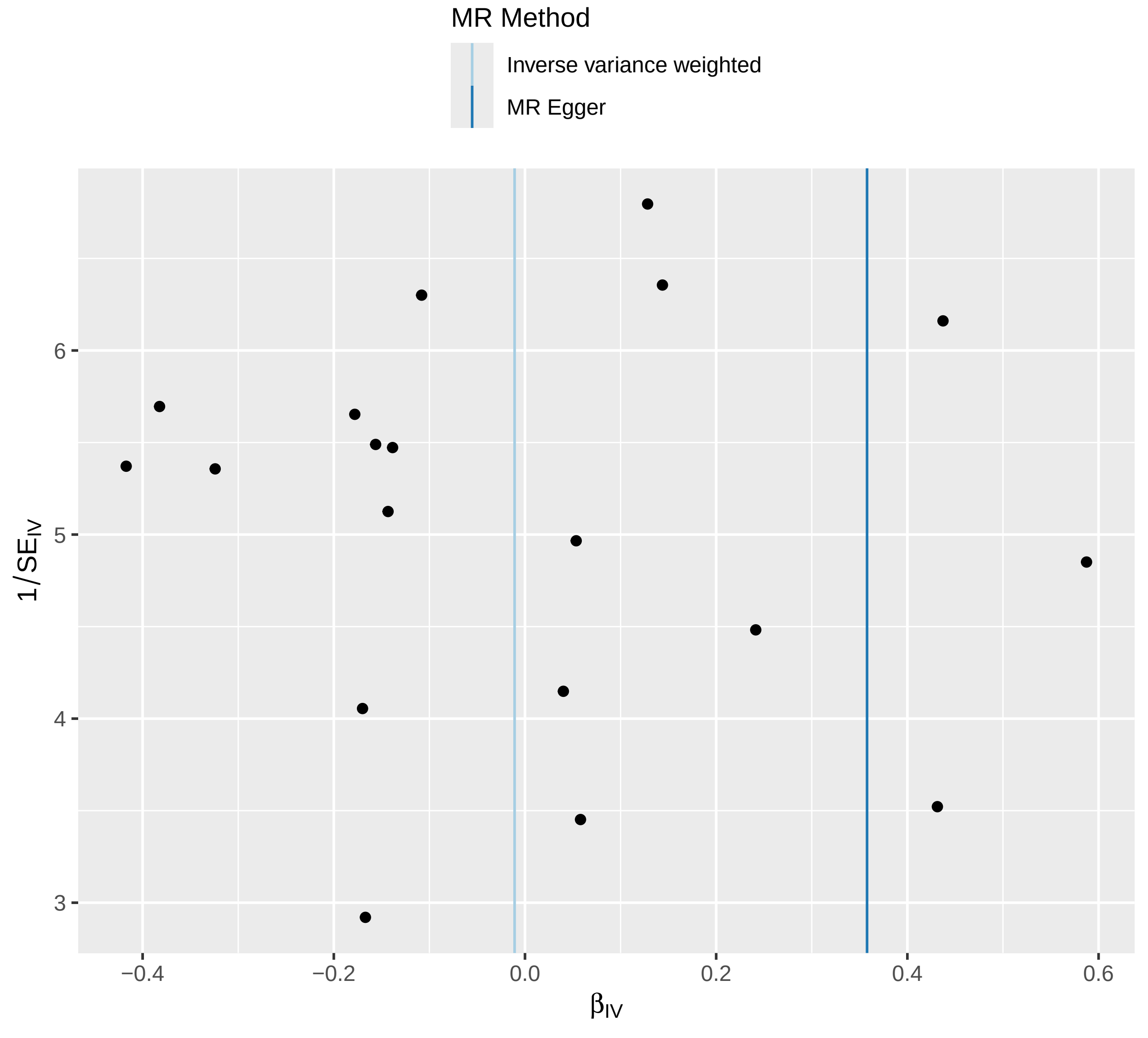

MR Method
Inverse variance weighted
MR Egger
6
5
4
3
1/SE_IV
−0.4
−0.2
0.0
0.2
0.4
0.6
β_IV

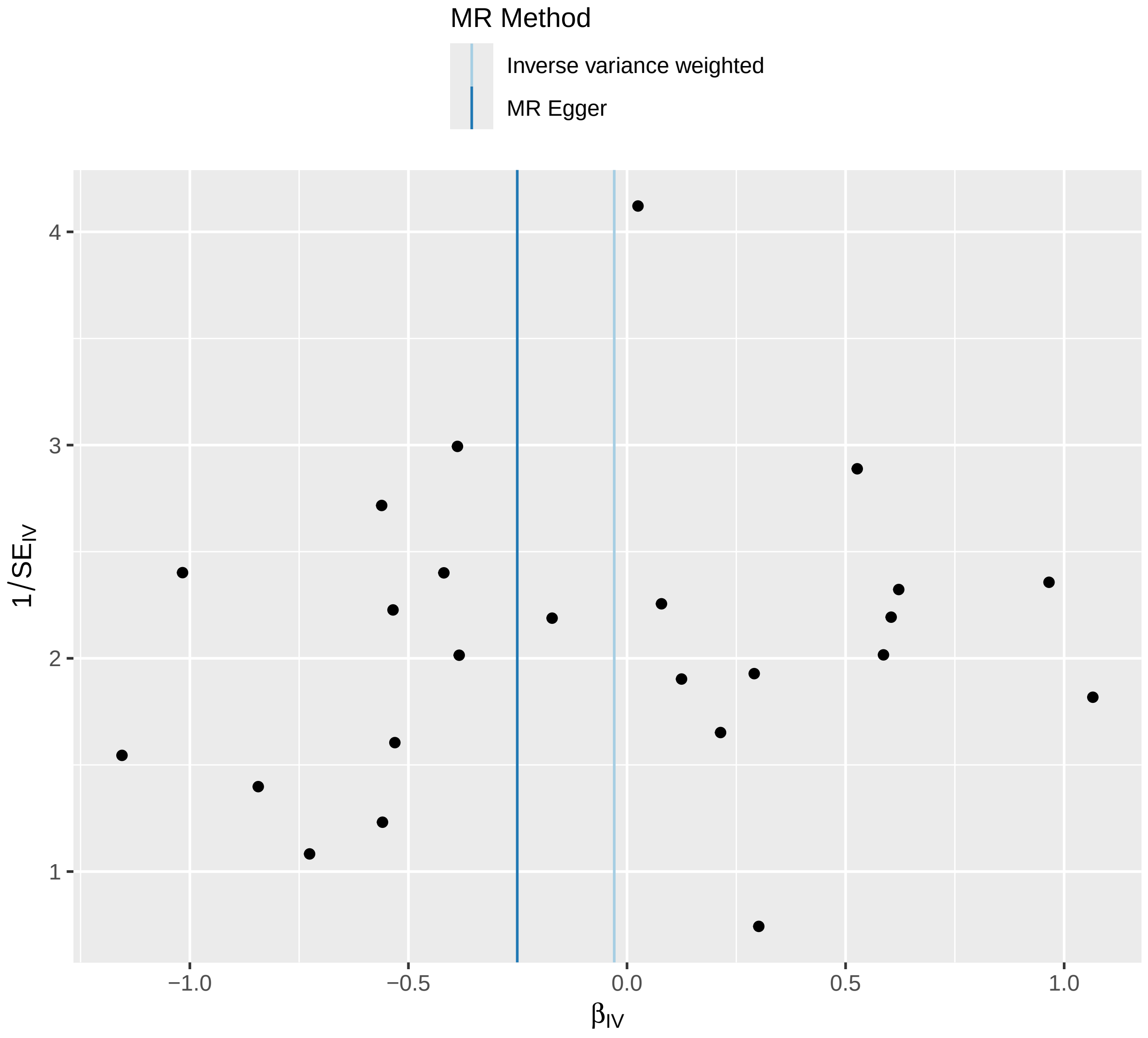
MR Method
Inverse variance weighted
MR Egger
4
3
2
1
$1/SE_{IV}$
−1.0
−0.5
0.0
0.5
1.0
β_{IV}

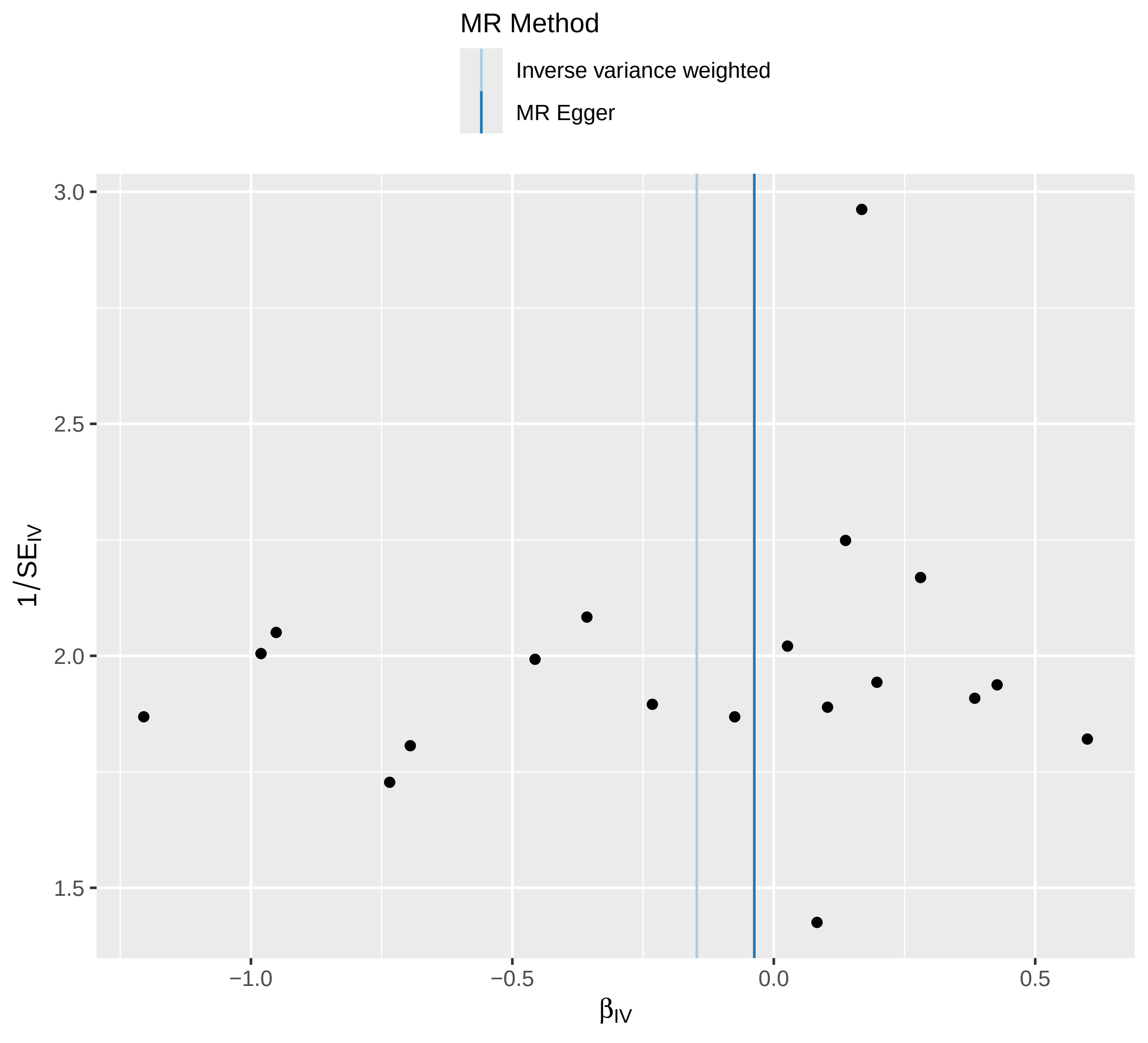
MR Method
Inverse variance weighted
MR Egger
3.0
2.5
2.0
1.5
$1/SE_{IV}$
−1.0
−0.5
0.0
0.5
β_{IV}

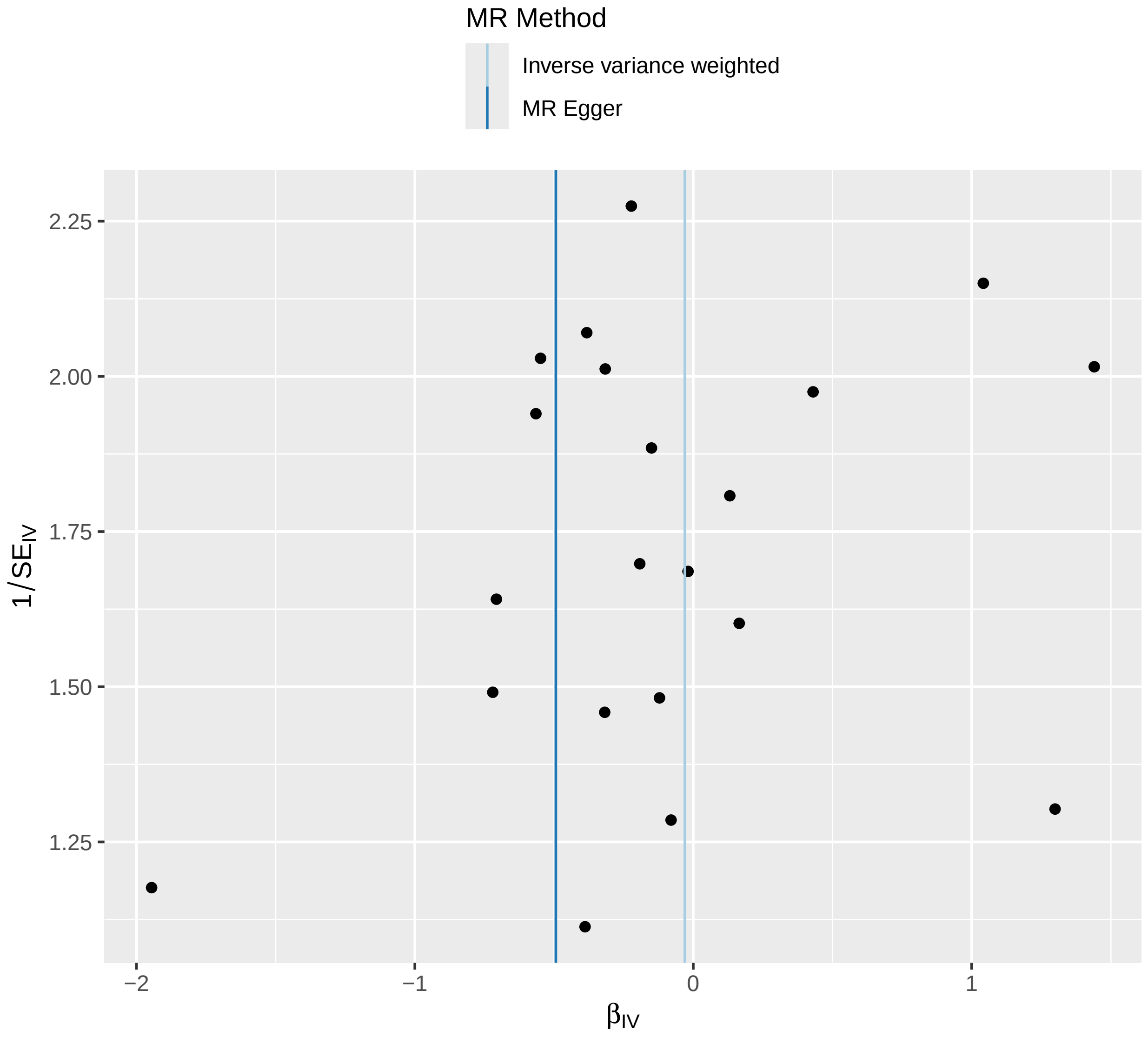

MR Method
Inverse variance weighted
MR Egger
2.25
2.00
1.75
1.50
1.25
$1/SE_{IV}$
−2
−1
0
1
β_{IV}

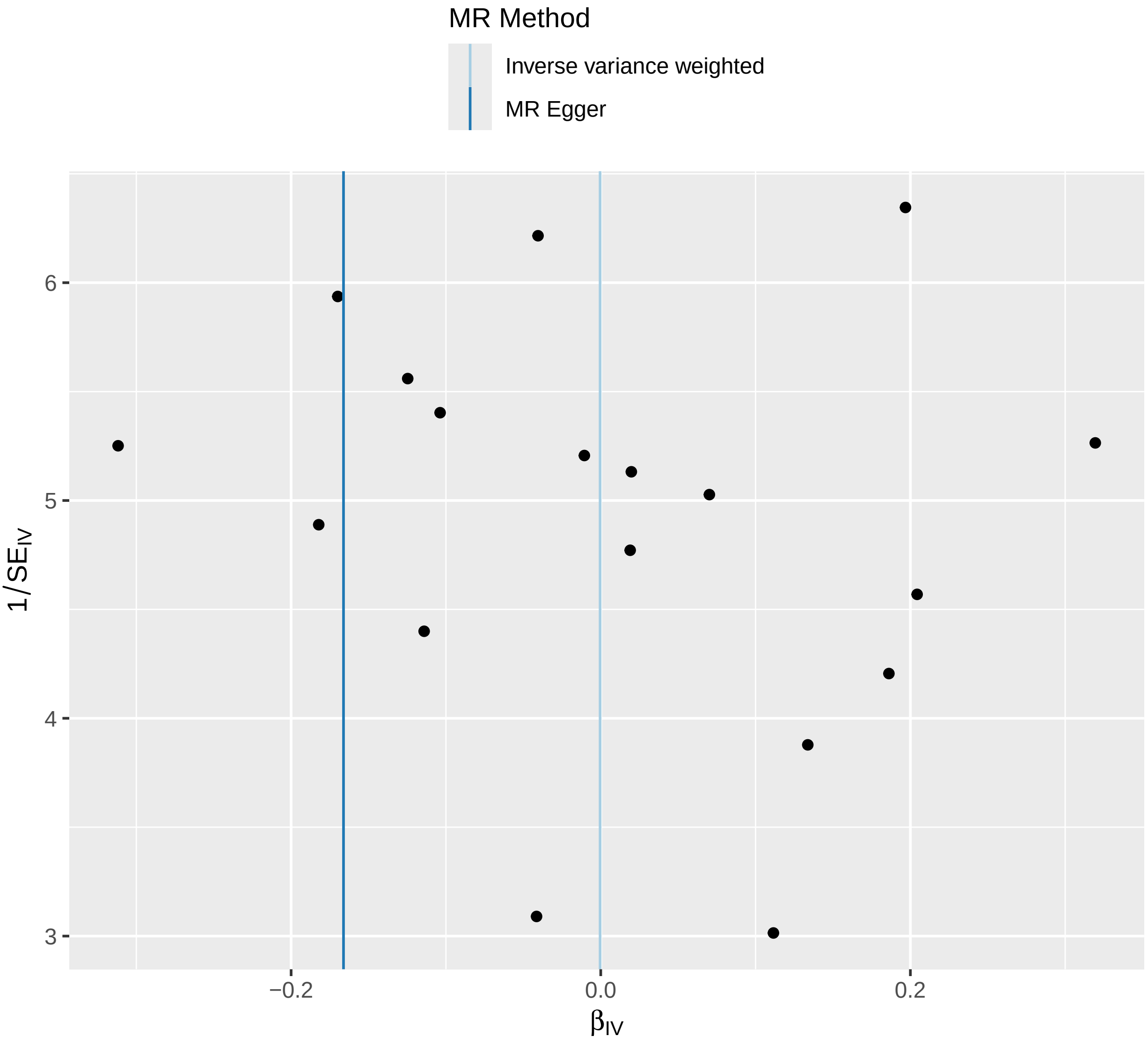

MR Method
Inverse variance weighted
MR Egger
6
5
4
3
1/SE_IV
−0.2
0.0
0.2
β_IV

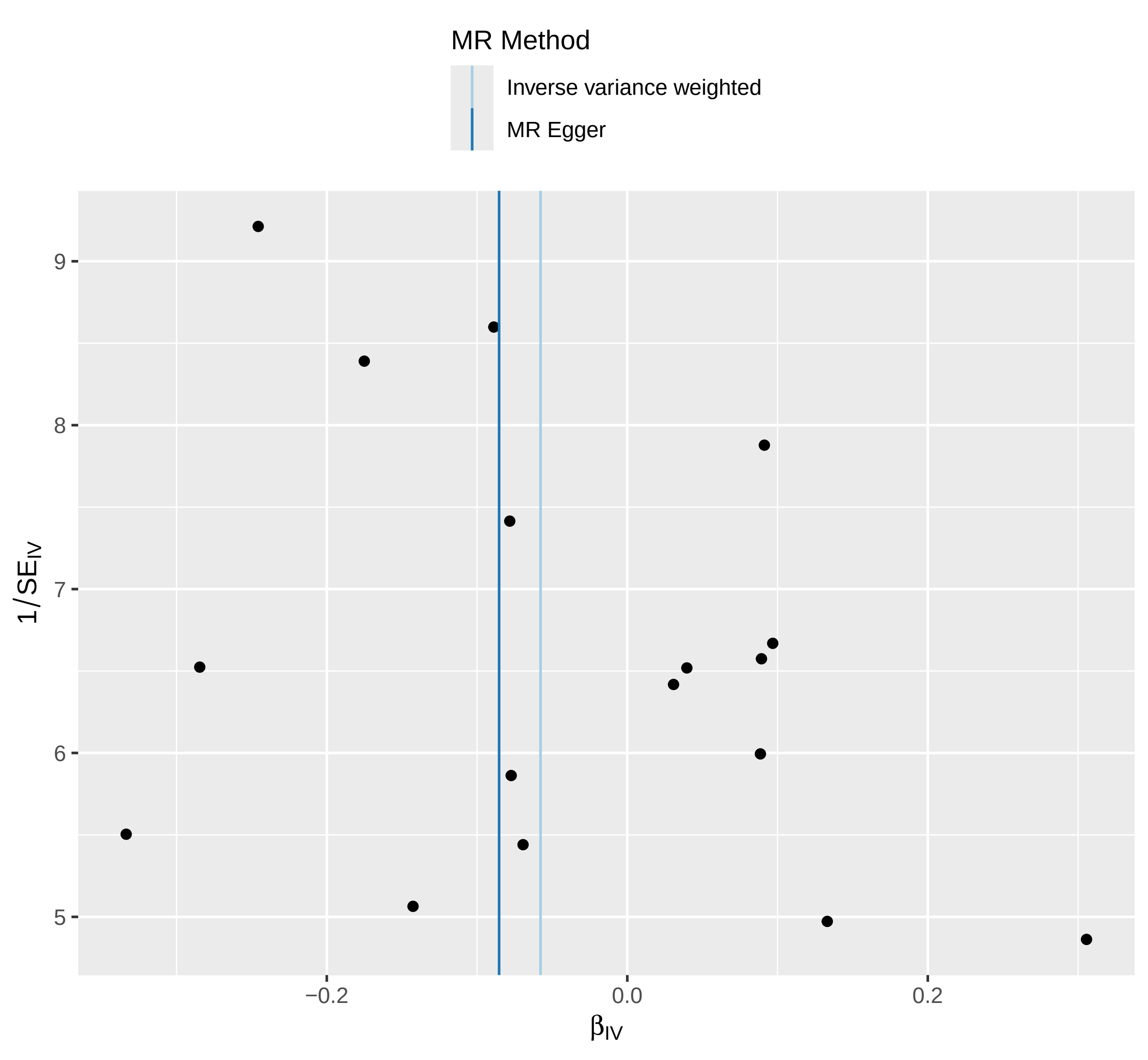
MR Method
Inverse variance weighted
MR Egger
1/SE_IV
β_IV
9
8
7
6
5
−0.2
0.0
0.2

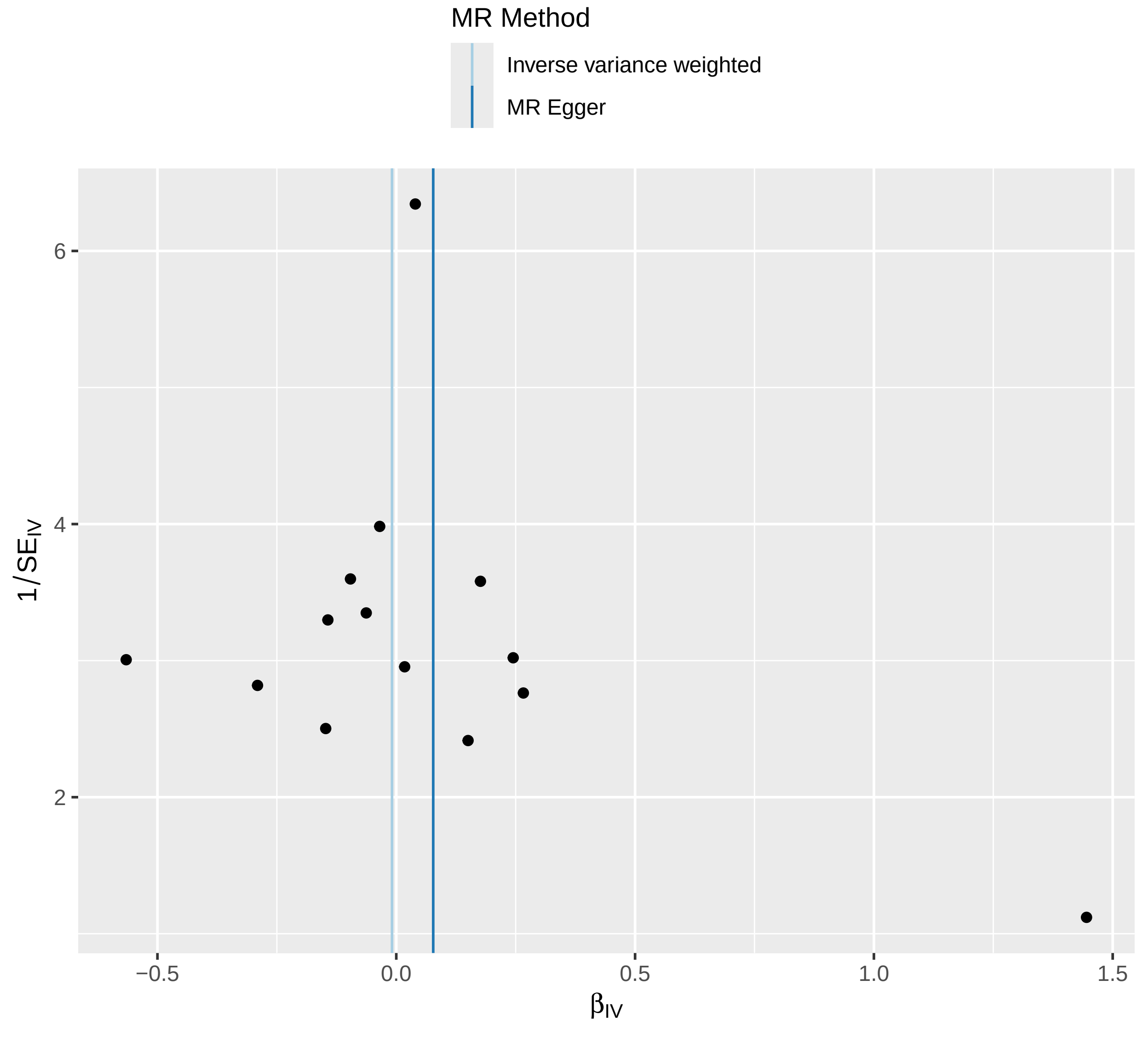

MR Method
Inverse variance weighted
MR Egger
6
4
2
1/SE_IV
−0.5
0.0
0.5
1.0
1.5
β_IV

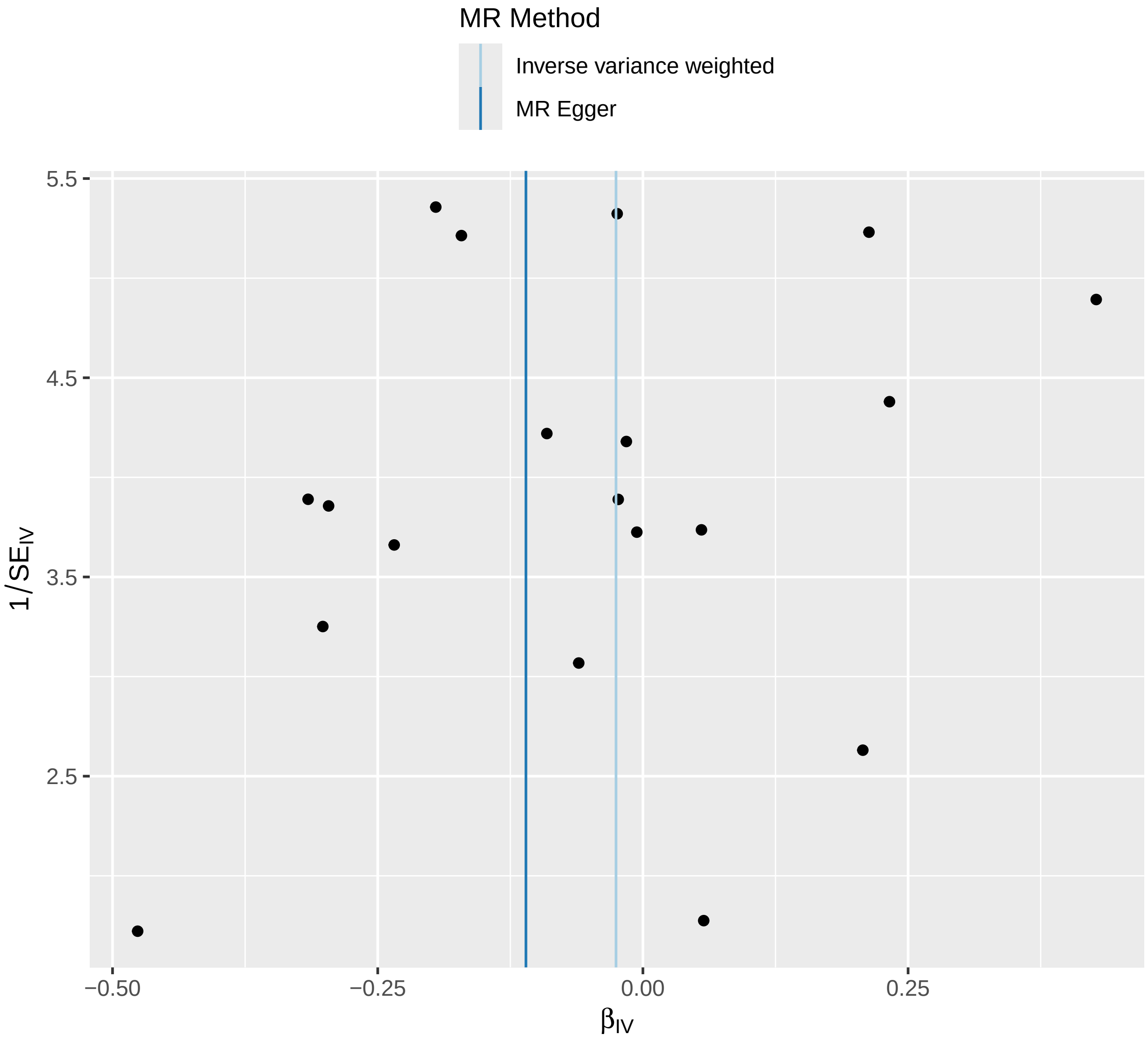

MR Method
Inverse variance weighted
MR Egger
5.5
4.5
3.5
2.5
1/SE_IV
−0.50
−0.25
0.00
0.25
β_IV

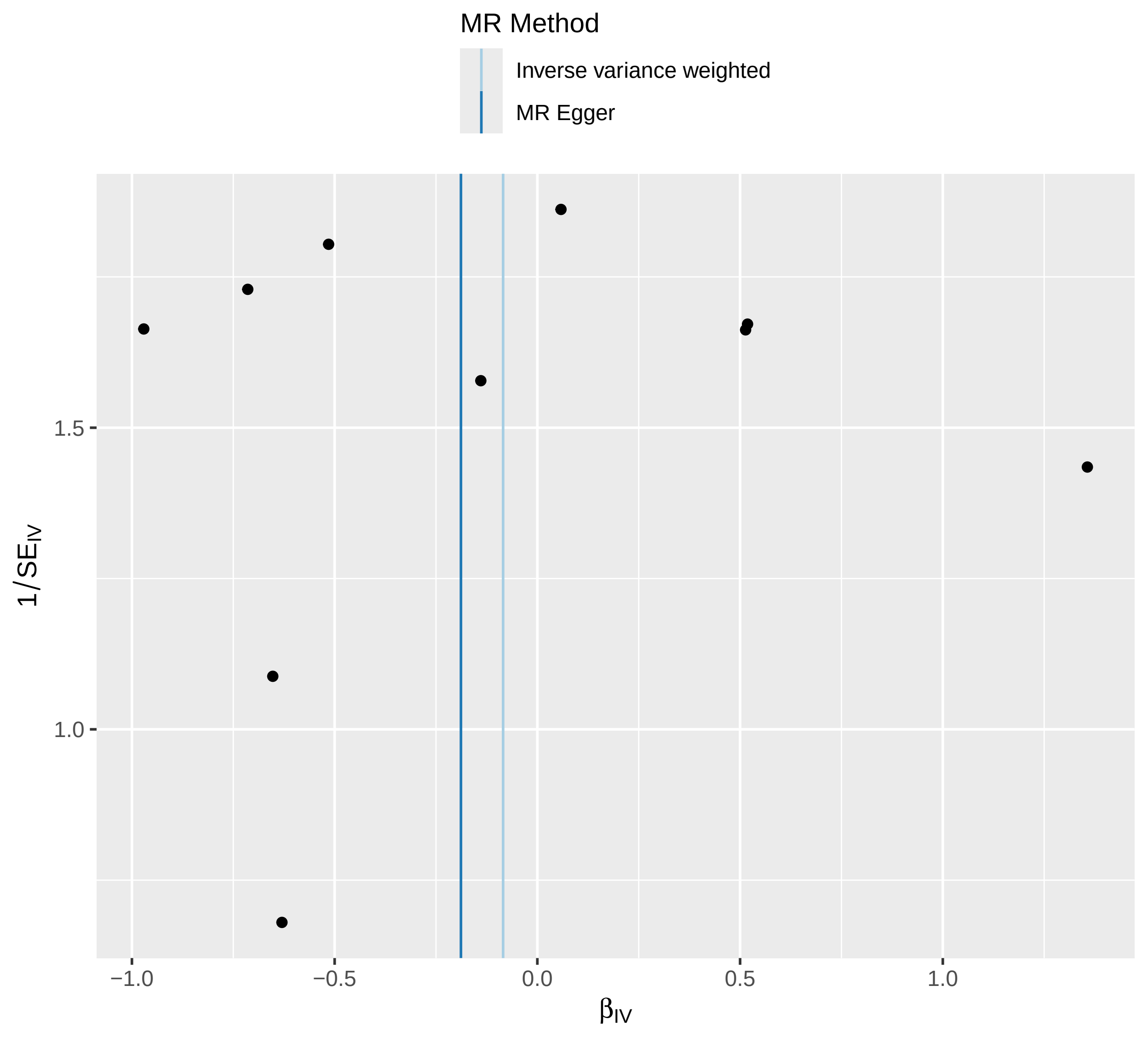

MR Method
Inverse variance weighted
MR Egger
1.5
1.0
1/SE$_{IV}$
−1.0
−0.5
0.0
0.5
1.0
β$_{IV}$

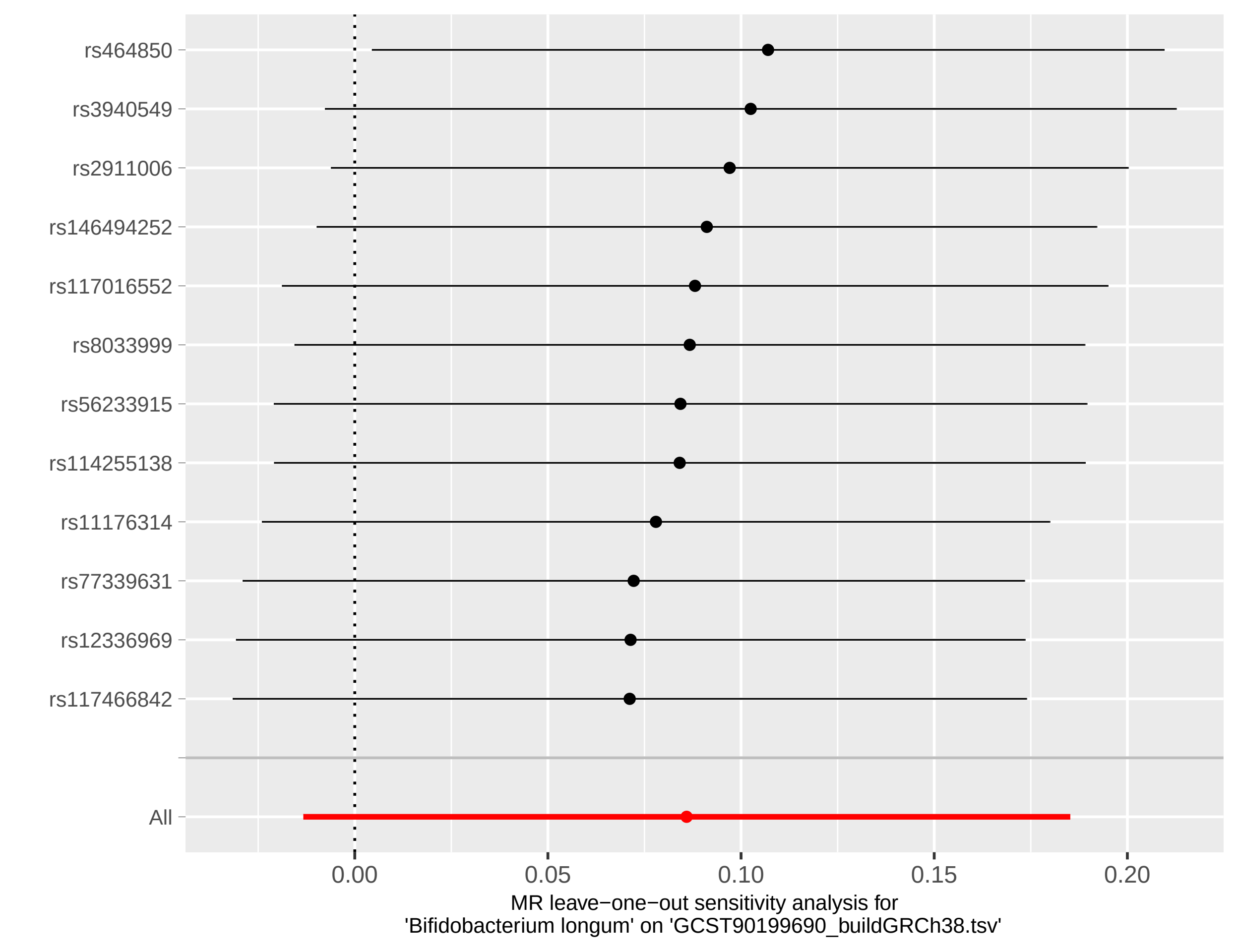
rs464850
rs3940549
rs2911006
rs146494252
rs117016552
rs8033999
rs56233915
rs114255138
rs11176314
rs77339631
rs12336969
rs117466842
All
0.00
0.05
0.10
0.15
0.20
MR leave−one−out sensitivity analysis for
'Bifidobacterium longum' on 'GCST90199690_buildGRCh38.tsv'

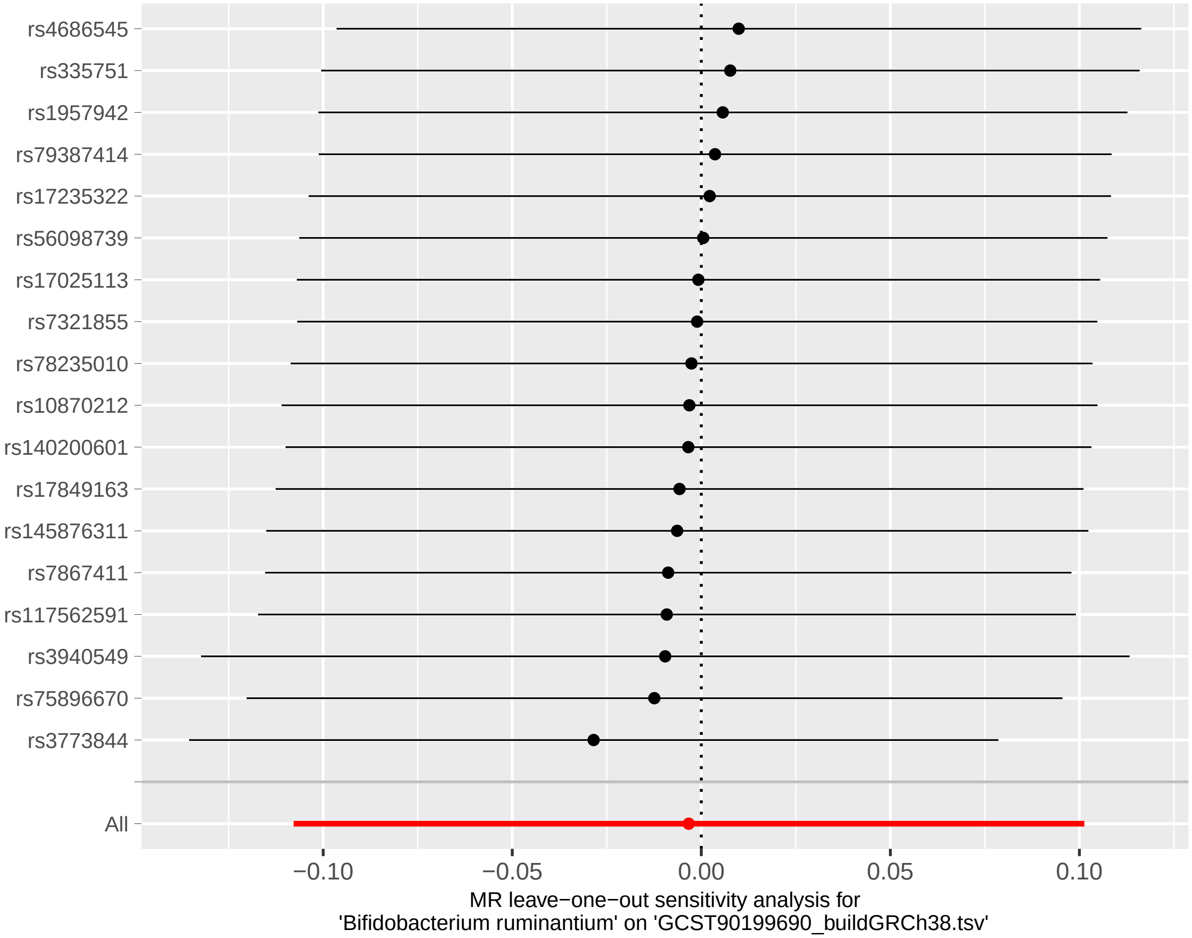
rs4686545
rs335751
rs1957942
rs79387414
rs17235322
rs56098739
rs17025113
rs7321855
rs78235010
rs10870212
rs140200601
rs17849163
rs145876311
rs7867411
rs117562591
rs3940549
rs75896670
rs3773844
All
−0.10
−0.05
0.00
0.05
0.10
MR leave−one−out sensitivity analysis for
'Bifidobacterium ruminantium' on 'GCST90199690_buildGRCh38.tsv'

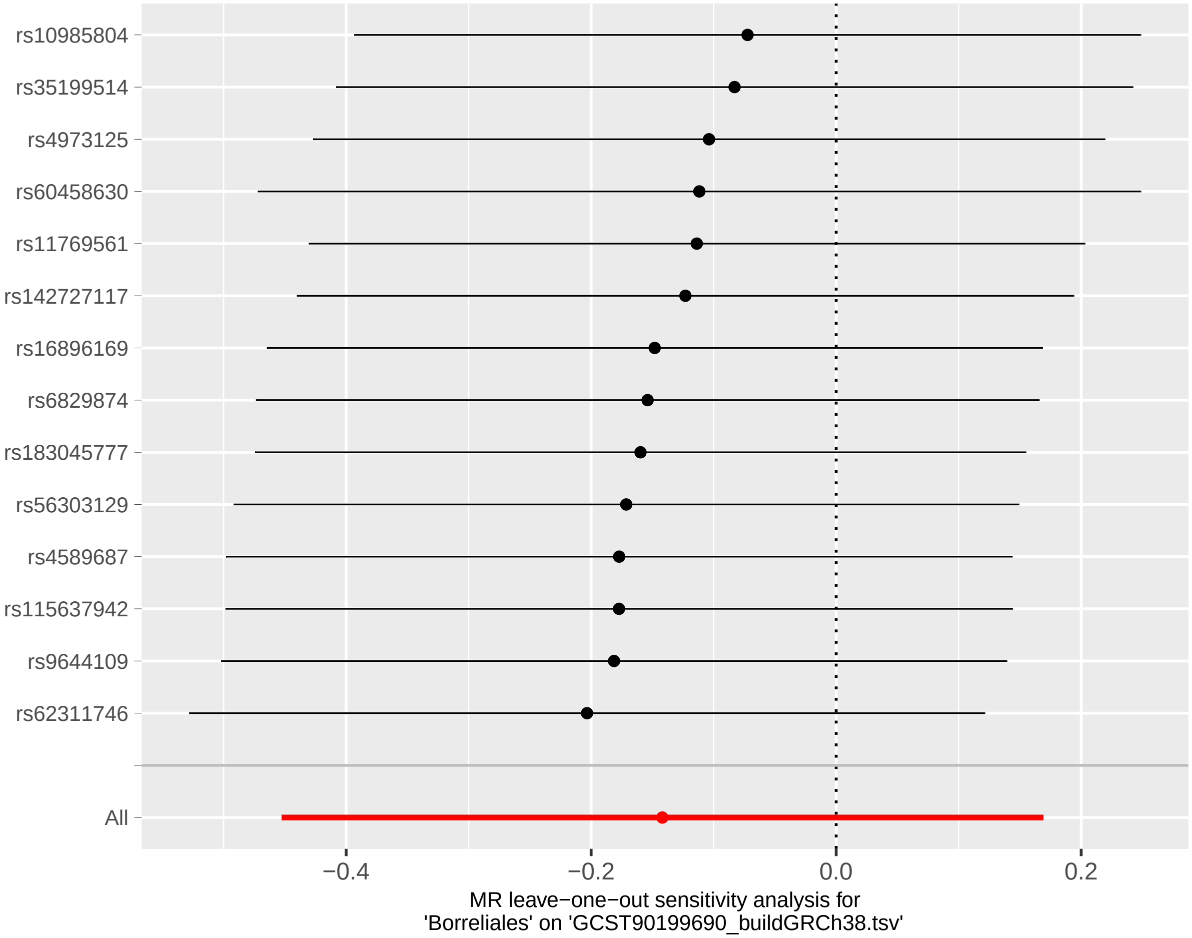
rs10985804
rs35199514
rs4973125
rs60458630
rs11769561
rs142727117
rs16896169
rs6829874
rs183045777
rs56303129
rs4589687
rs115637942
rs9644109
rs62311746
All
−0.4
−0.2
0.0
0.2
MR leave−one−out sensitivity analysis for
'Borreliales' on 'GCST90199690_buildGRCh38.tsv'

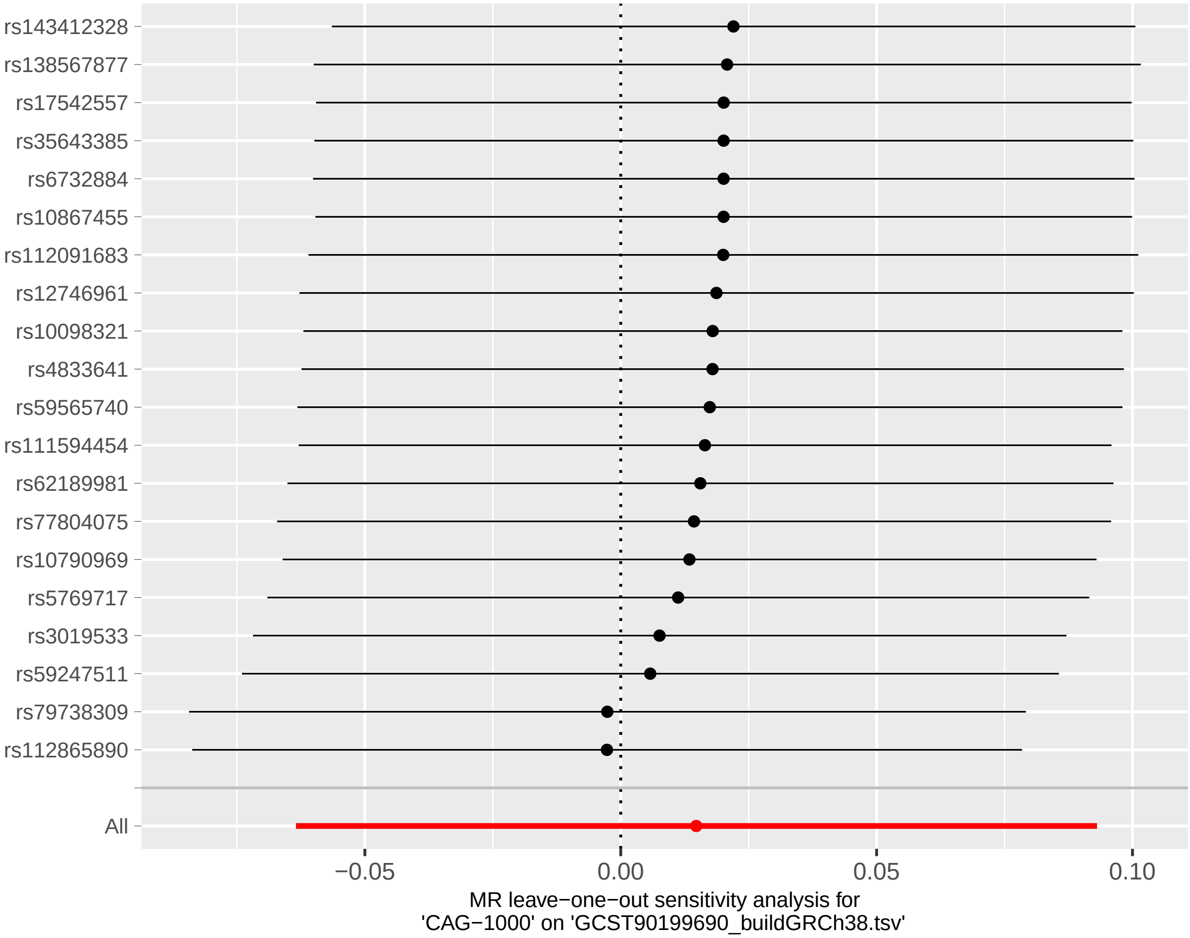
rs143412328
rs138567877
rs17542557
rs35643385
rs6732884
rs10867455
rs112091683
rs12746961
rs10098321
rs4833641
rs59565740
rs111594454
rs62189981
rs77804075
rs10790969
rs5769717
rs3019533
rs59247511
rs79738309
rs112865890
All
−0.05
0.00
0.05
0.10
MR leave−one−out sensitivity analysis for
'CAG−1000' on 'GCST90199690_buildGRCh38.tsv'

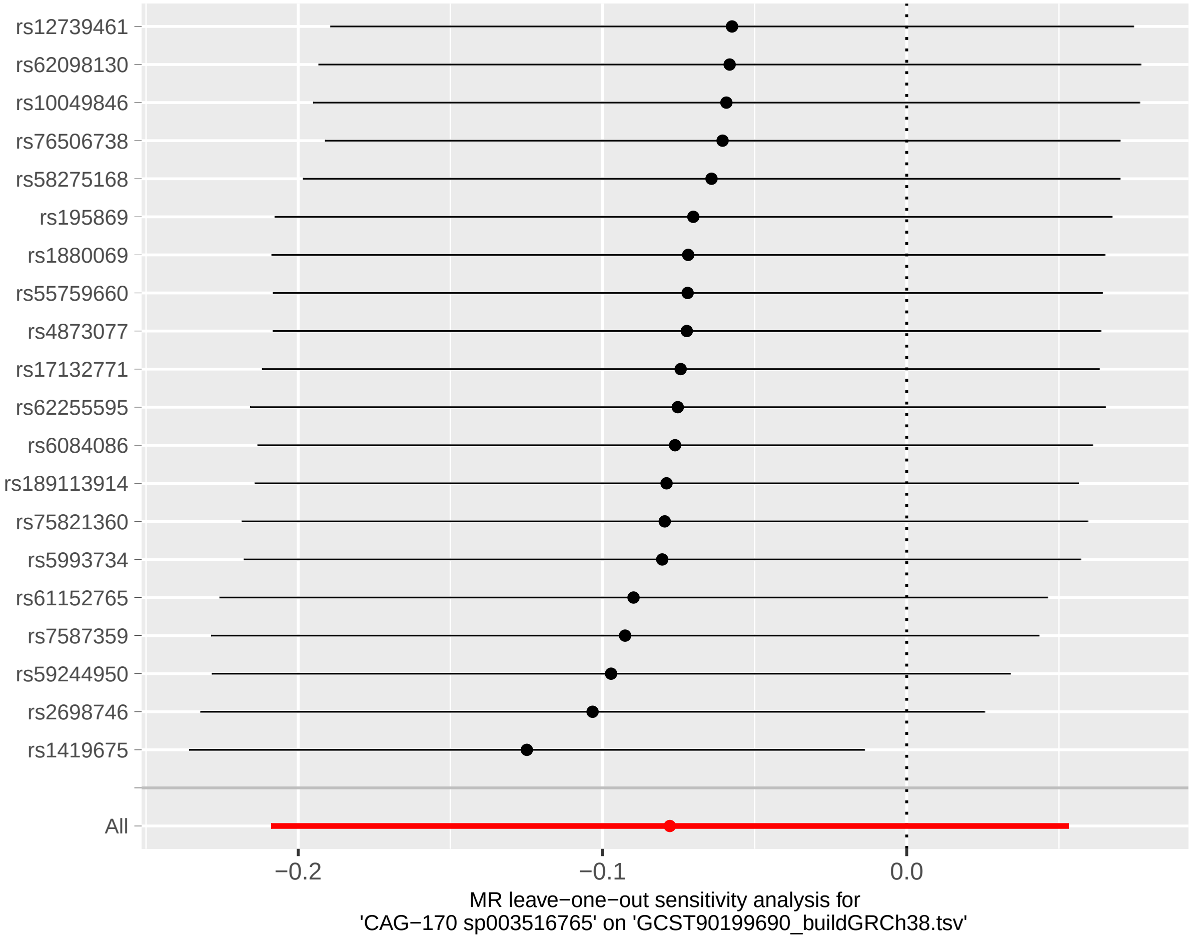

rs12739461
rs62098130
rs10049846
rs76506738
rs58275168
rs195869
rs1880069
rs55759660
rs4873077
rs17132771
rs62255595
rs6084086
rs189113914
rs75821360
rs5993734
rs61152765
rs7587359
rs59244950
rs2698746
rs1419675
All
−0.2
−0.1
0.0
MR leave−one−out sensitivity analysis for
'CAG−170 sp003516765' on 'GCST90199690_buildGRCh38.tsv'

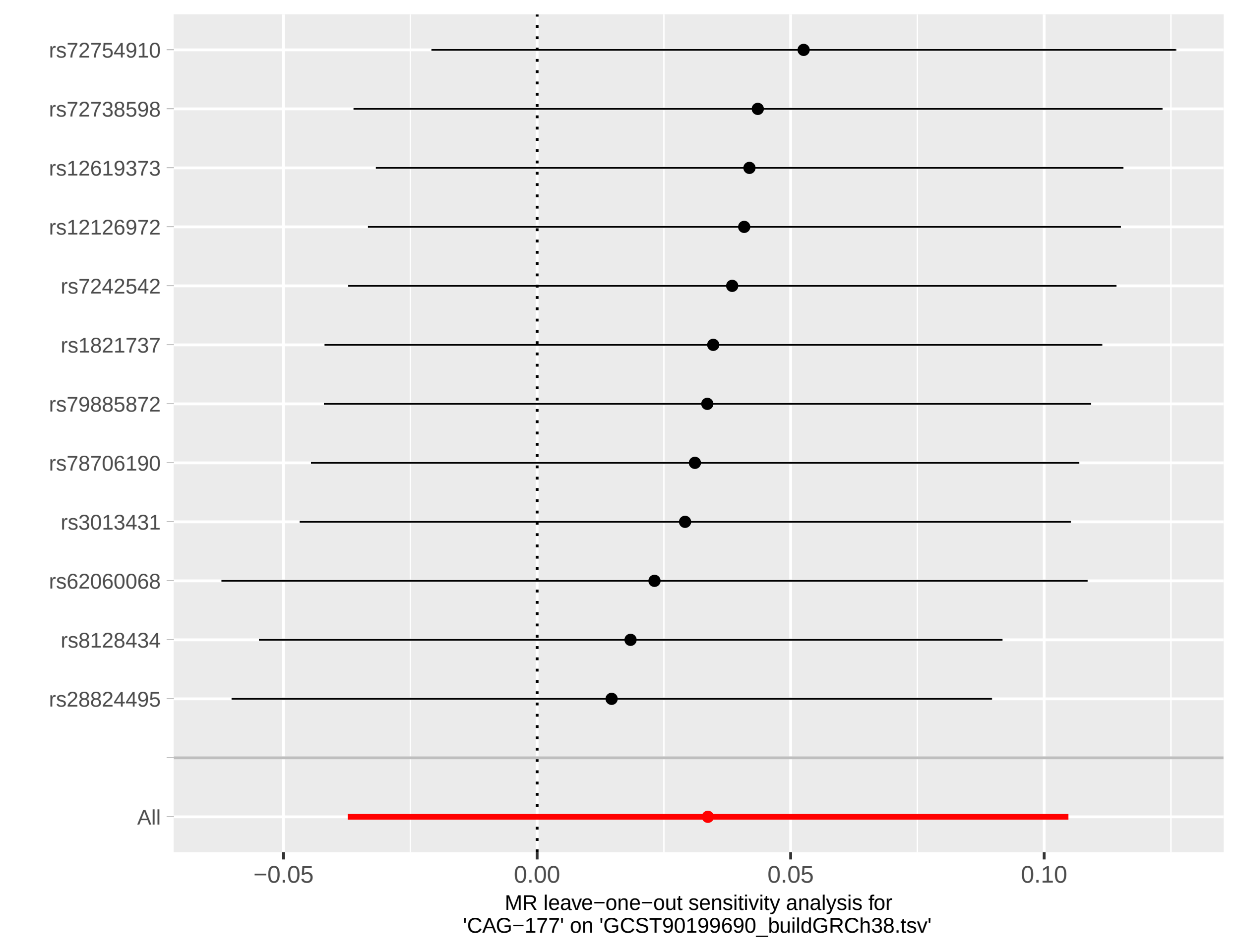
rs72754910
rs72738598
rs12619373
rs12126972
rs7242542
rs1821737
rs79885872
rs78706190
rs3013431
rs62060068
rs8128434
rs28824495
All
−0.05
0.00
0.05
0.10
MR leave−one−out sensitivity analysis for
'CAG−177' on 'GCST90199690_buildGRCh38.tsv'

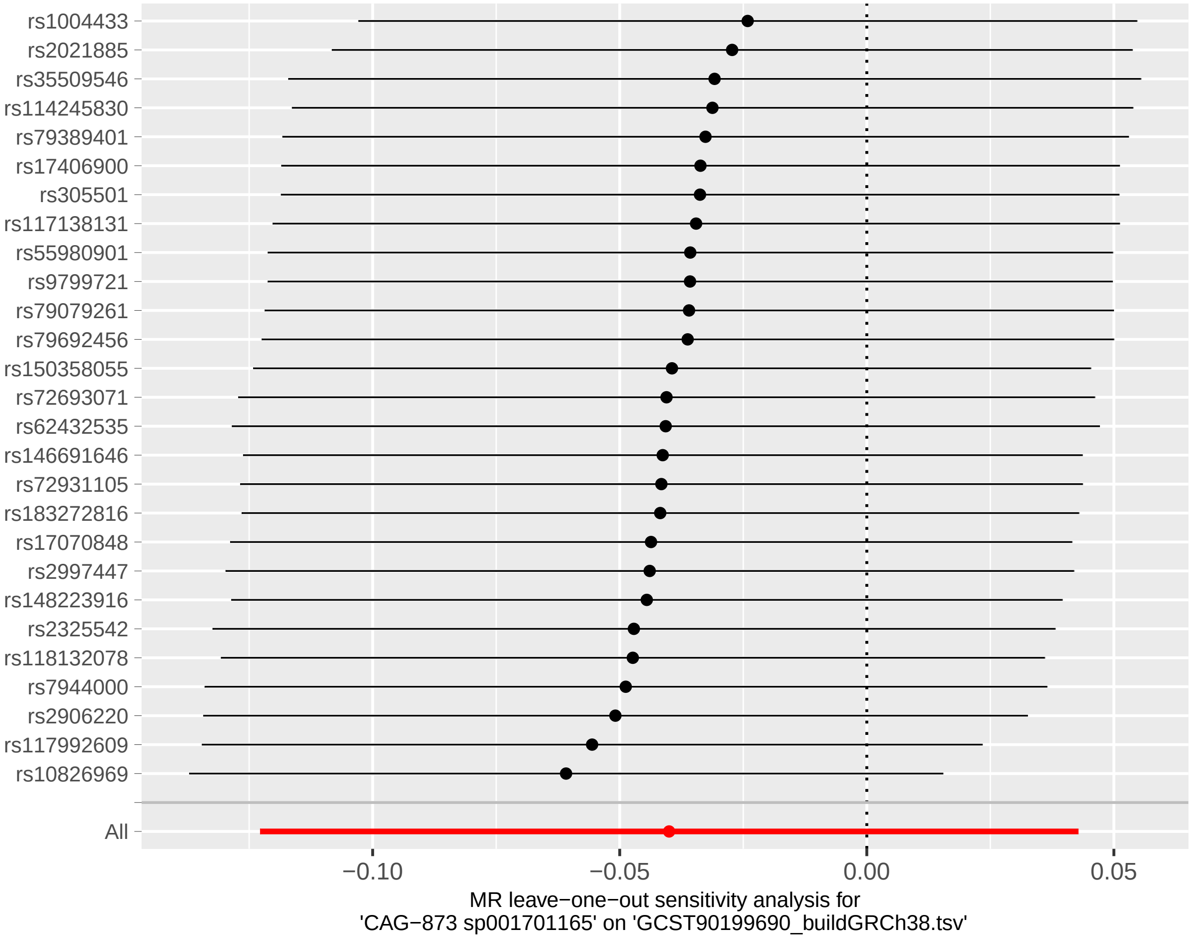
rs1004433
rs2021885
rs35509546
rs114245830
rs79389401
rs17406900
rs305501
rs117138131
rs55980901
rs9799721
rs79079261
rs79692456
rs150358055
rs72693071
rs62432535
rs146691646
rs72931105
rs183272816
rs17070848
rs2997447
rs148223916
rs2325542
rs118132078
rs7944000
rs2906220
rs117992609
rs10826969
All
−0.10
−0.05
0.00
0.05
MR leave−one−out sensitivity analysis for
'CAG−873 sp001701165' on 'GCST90199690_buildGRCh38.tsv'

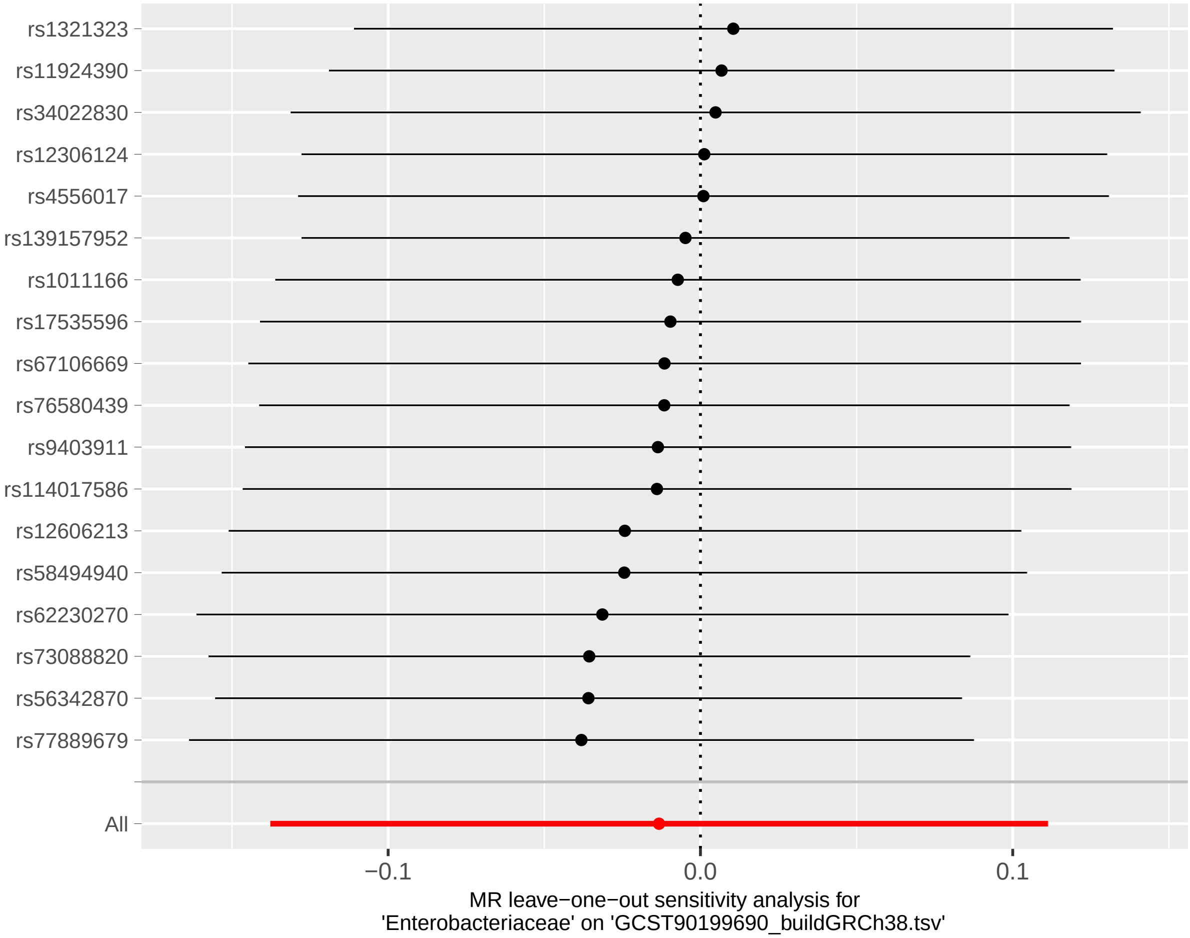
rs1321323
rs11924390
rs34022830
rs12306124
rs4556017
rs139157952
rs1011166
rs17535596
rs67106669
rs76580439
rs9403911
rs114017586
rs12606213
rs58494940
rs62230270
rs73088820
rs56342870
rs77889679
All
−0.1
0.0
0.1
MR leave−one−out sensitivity analysis for
'Enterobacteriaceae' on 'GCST90199690_buildGRCh38.tsv'

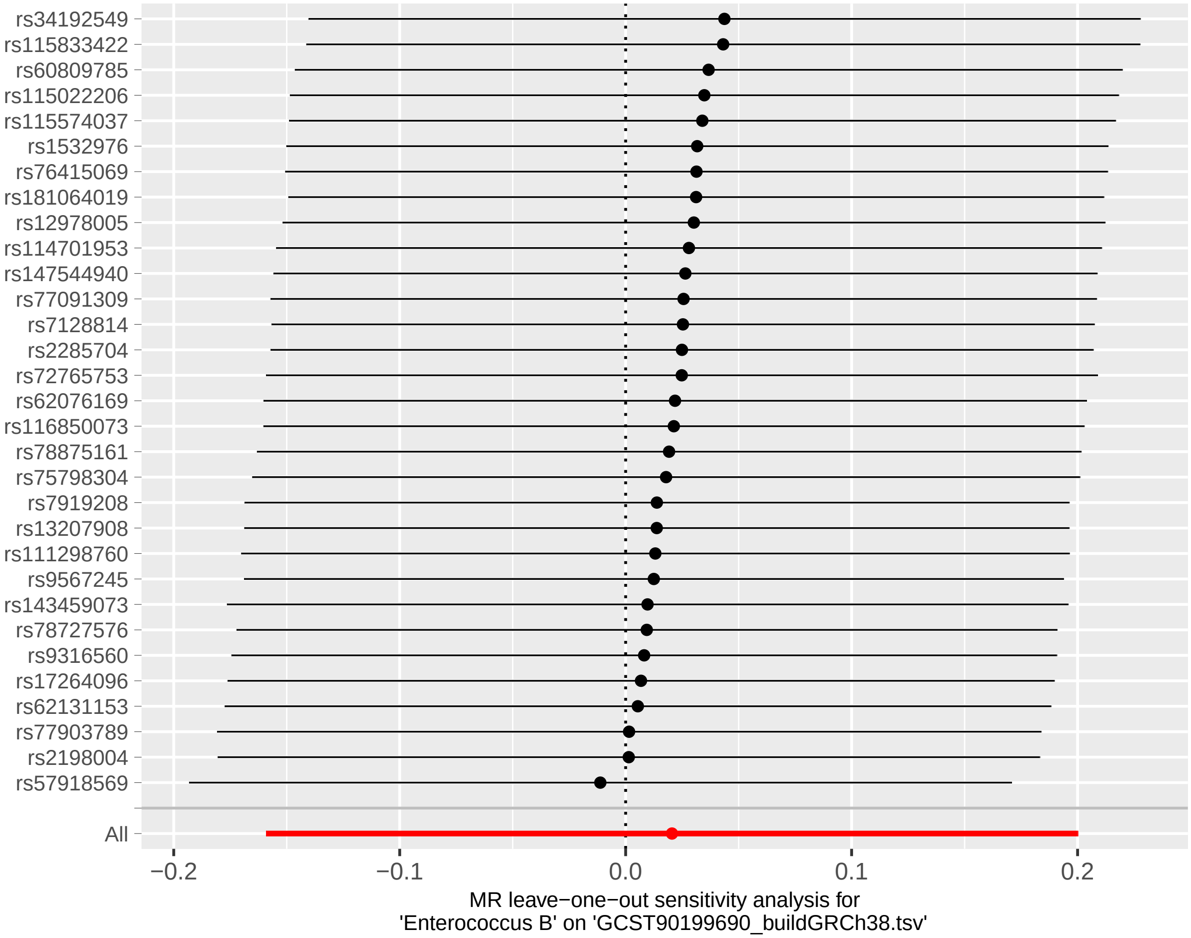
rs34192549
rs115833422
rs60809785
rs115022206
rs115574037
rs1532976
rs76415069
rs181064019
rs12978005
rs114701953
rs147544940
rs77091309
rs7128814
rs2285704
rs72765753
rs62076169
rs116850073
rs78875161
rs75798304
rs7919208
rs13207908
rs111298760
rs9567245
rs143459073
rs78727576
rs9316560
rs17264096
rs62131153
rs77903789
rs2198004
rs57918569
All
−0.2
−0.1
0.0
0.1
0.2
MR leave−one−out sensitivity analysis for
'Enterococcus B' on 'GCST90199690_buildGRCh38.tsv'

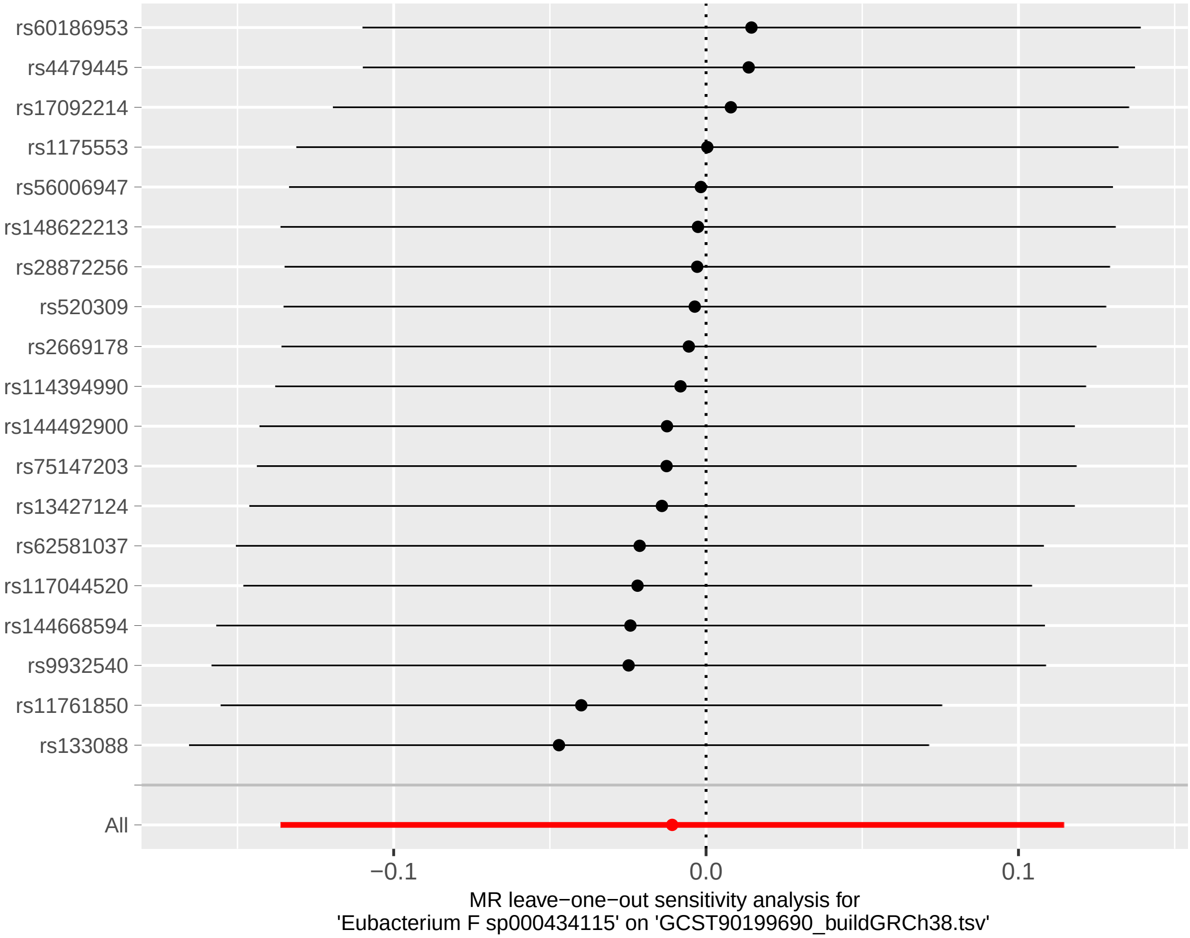

rs60186953
rs4479445
rs17092214
rs1175553
rs56006947
rs148622213
rs28872256
rs520309
rs2669178
rs114394990
rs144492900
rs75147203
rs13427124
rs62581037
rs117044520
rs144668594
rs9932540
rs11761850
rs133088
All
−0.1
0.0
0.1
MR leave−one−out sensitivity analysis for
'Eubacterium F sp000434115' on 'GCST90199690_buildGRCh38.tsv'

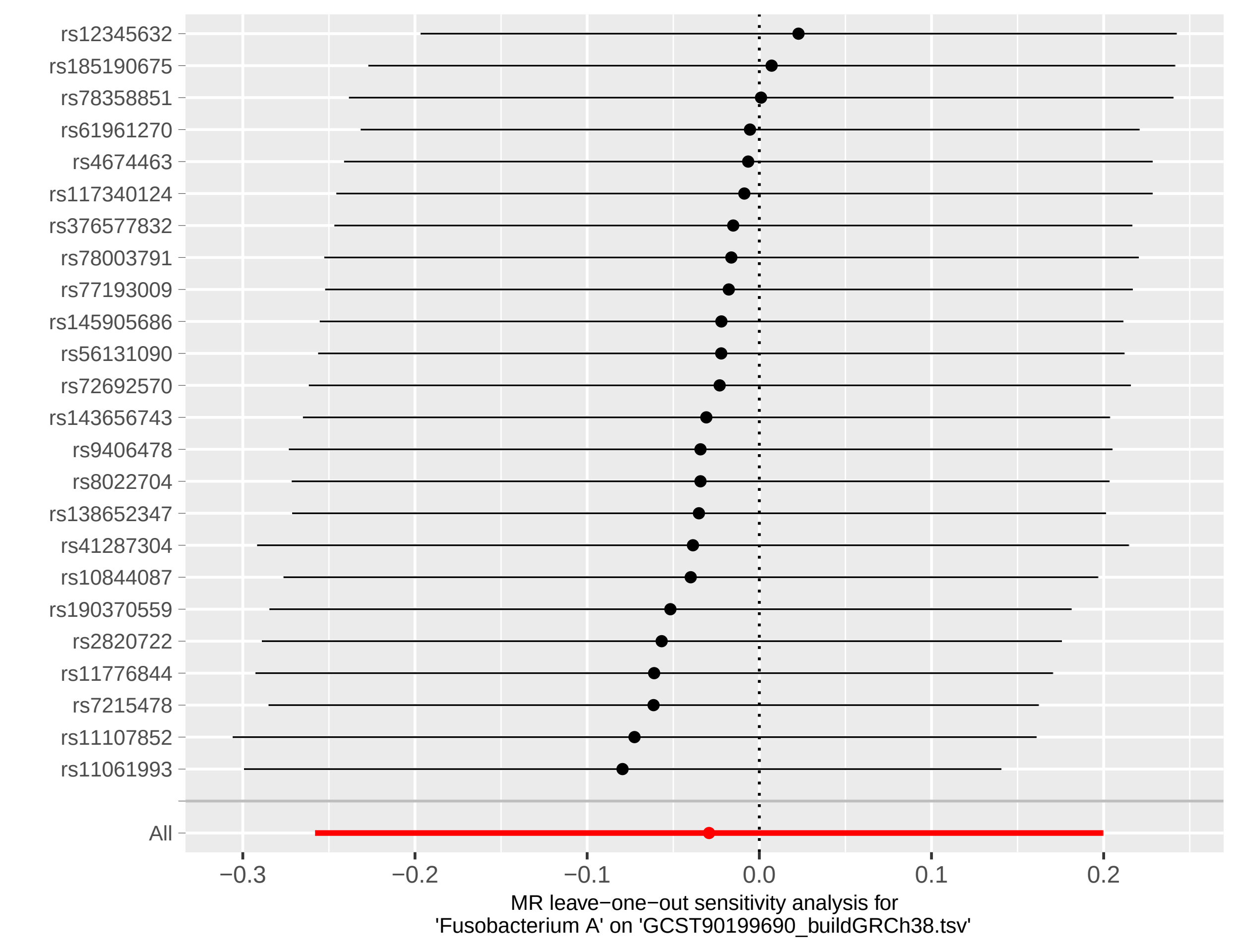
rs12345632
rs185190675
rs78358851
rs61961270
rs4674463
rs117340124
rs376577832
rs78003791
rs77193009
rs145905686
rs56131090
rs72692570
rs143656743
rs9406478
rs8022704
rs138652347
rs41287304
rs10844087
rs190370559
rs2820722
rs11776844
rs7215478
rs11107852
rs11061993
All
−0.3
−0.2
−0.1
0.0
0.1
0.2
MR leave−one−out sensitivity analysis for
'Fusobacterium A' on 'GCST90199690_buildGRCh38.tsv'

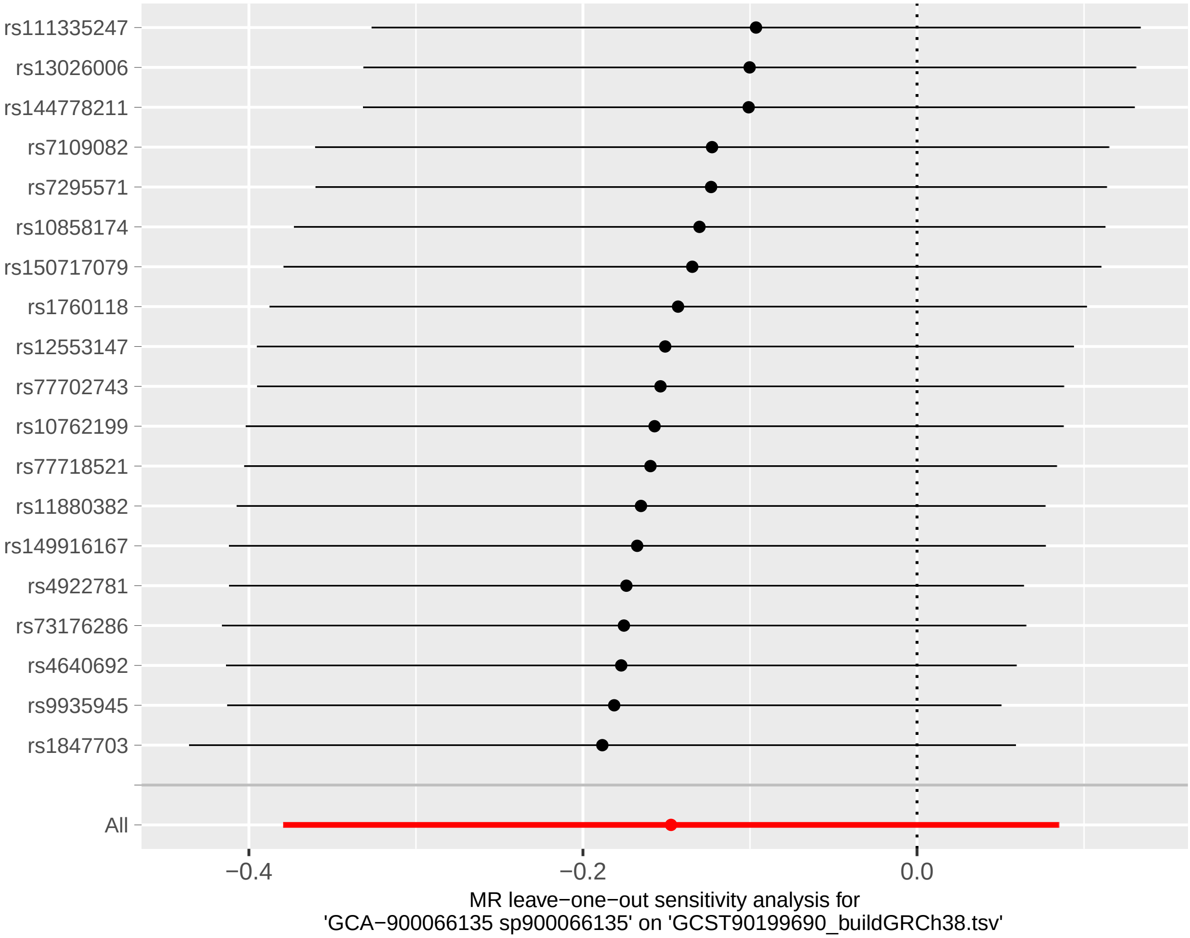

rs111335247
rs13026006
rs144778211
rs7109082
rs7295571
rs10858174
rs150717079
rs1760118
rs12553147
rs77702743
rs10762199
rs77718521
rs11880382
rs149916167
rs4922781
rs73176286
rs4640692
rs9935945
rs1847703
All
−0.4
−0.2
0.0
MR leave−one−out sensitivity analysis for
'GCA−900066135 sp900066135' on 'GCST90199690_buildGRCh38.tsv'

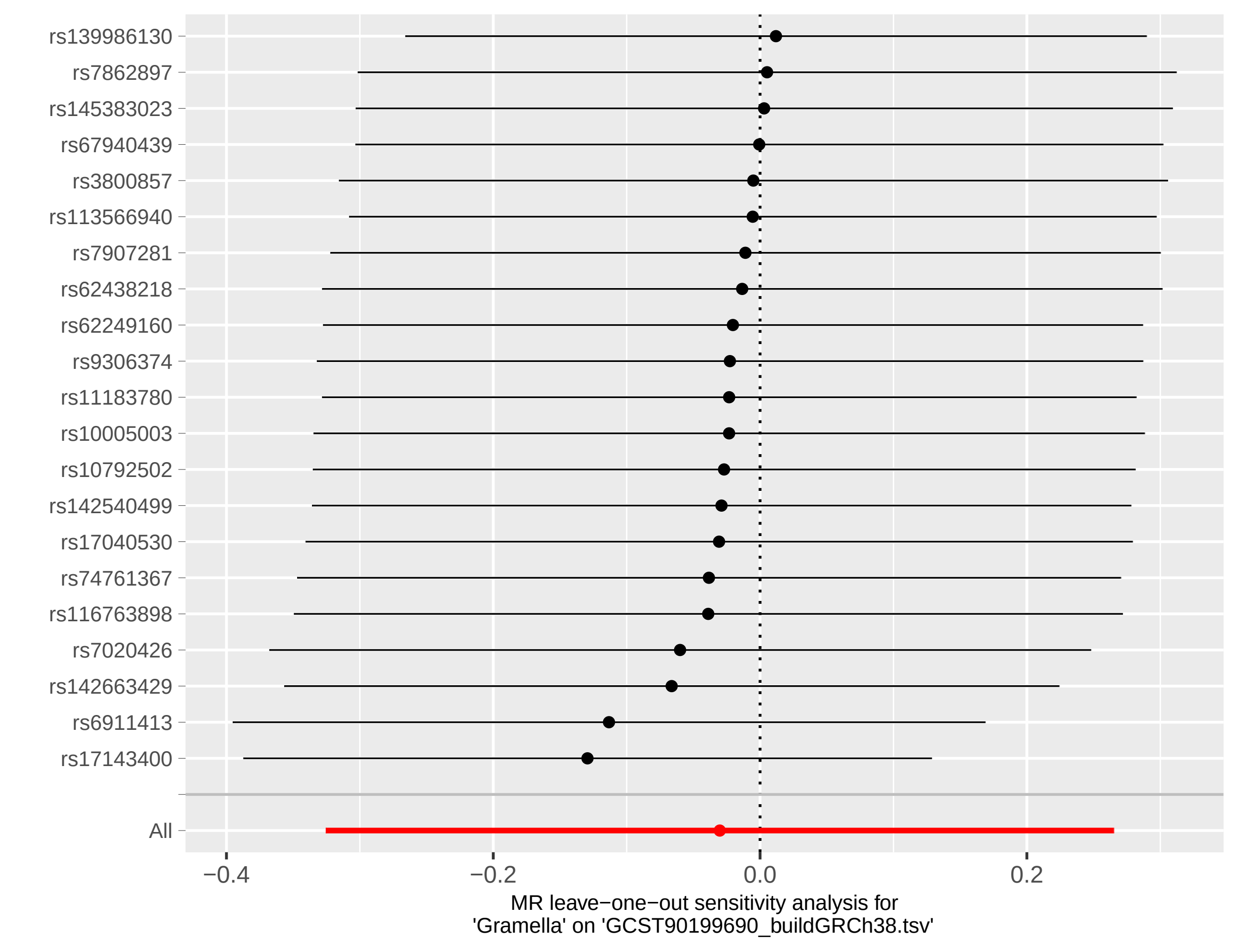

rs139986130
rs7862897
rs145383023
rs67940439
rs3800857
rs113566940
rs7907281
rs62438218
rs62249160
rs9306374
rs11183780
rs10005003
rs10792502
rs142540499
rs17040530
rs74761367
rs116763898
rs7020426
rs142663429
rs6911413
rs17143400
All
−0.4
−0.2
0.0
0.2
MR leave−one−out sensitivity analysis for
'Gramella' on 'GCST90199690_buildGRCh38.tsv'

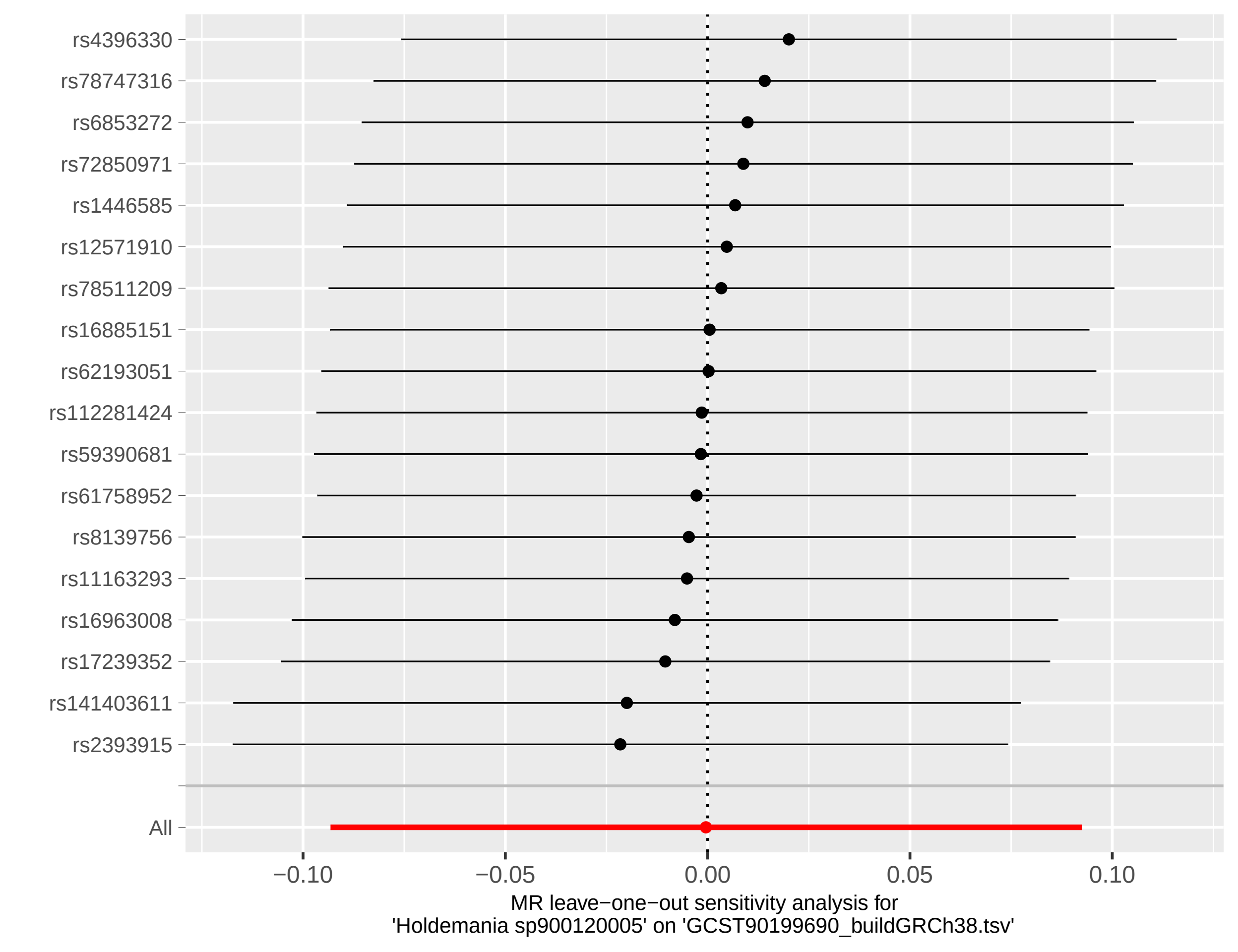

rs4396330
rs78747316
rs6853272
rs72850971
rs1446585
rs12571910
rs78511209
rs16885151
rs62193051
rs112281424
rs59390681
rs61758952
rs8139756
rs11163293
rs16963008
rs17239352
rs141403611
rs2393915
All
−0.10
−0.05
0.00
0.05
0.10
MR leave−one−out sensitivity analysis for
'Holdemania sp900120005' on 'GCST90199690_buildGRCh38.tsv'

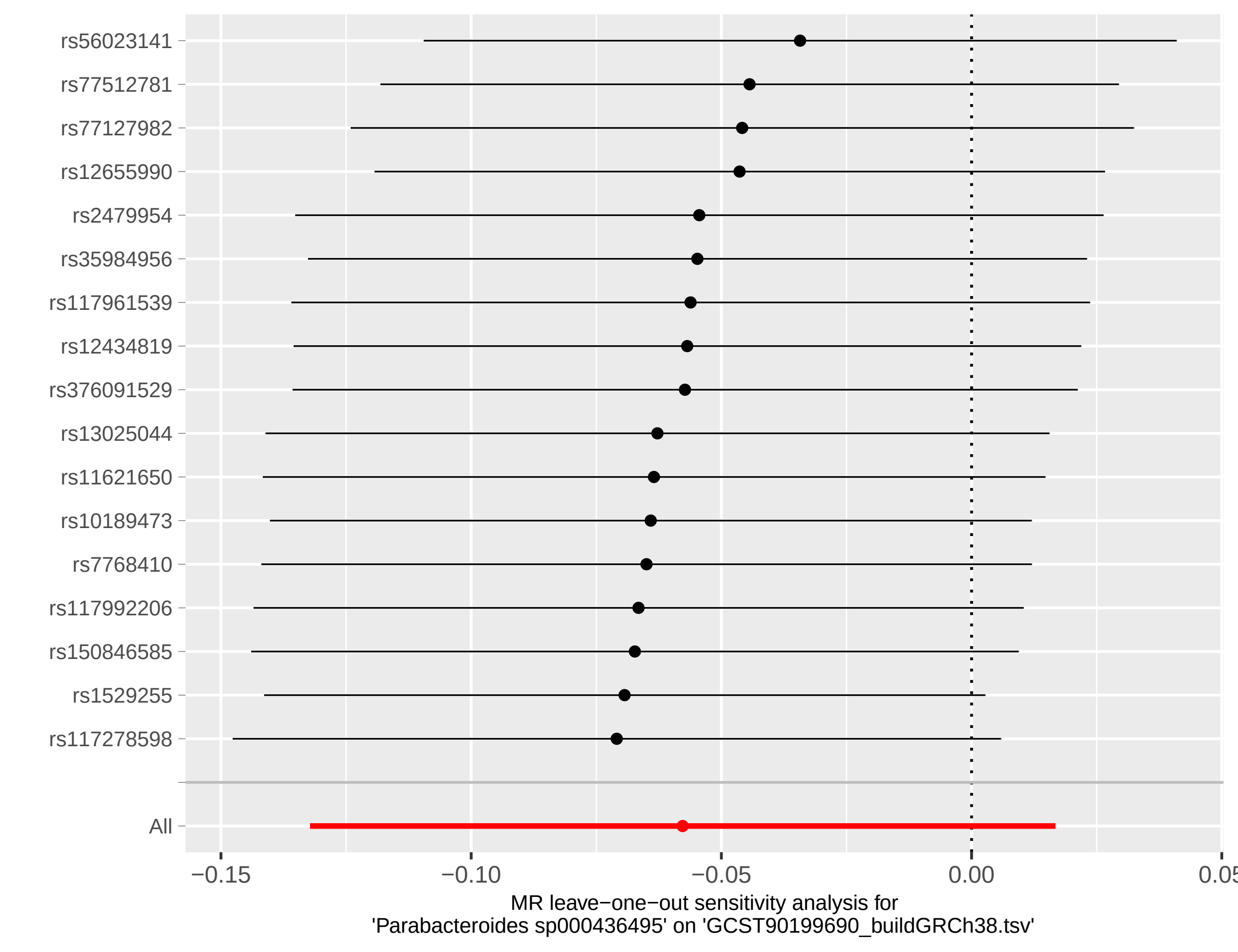
rs56023141
rs77512781
rs77127982
rs12655990
rs2479954
rs35984956
rs117961539
rs12434819
rs376091529
rs13025044
rs11621650
rs10189473
rs7768410
rs117992206
rs150846585
rs1529255
rs117278598
All
−0.15
−0.10
−0.05
0.00
0.05
MR leave−one−out sensitivity analysis for
'Parabacteroides sp000436495' on 'GCST90199690_buildGRCh38.tsv'

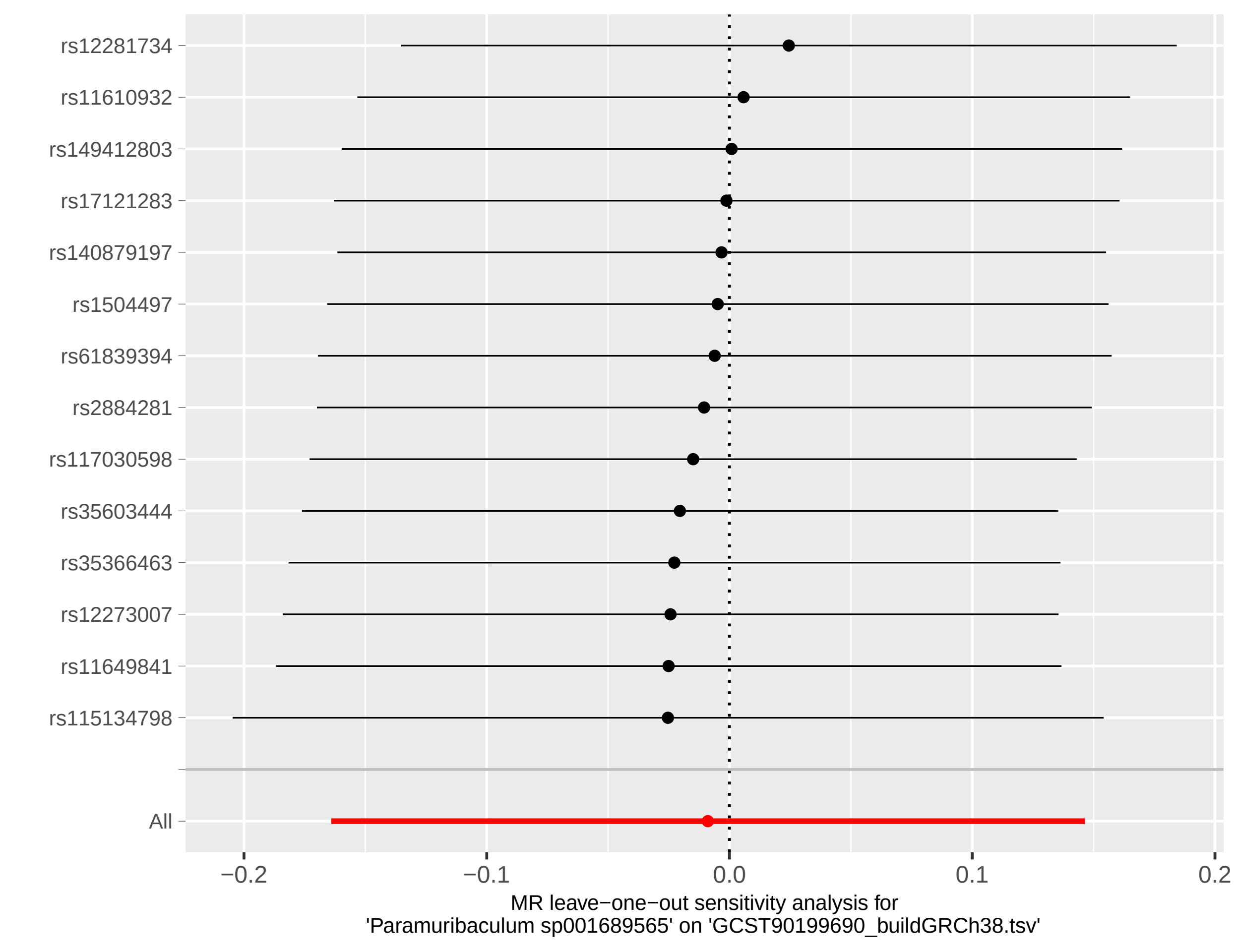
rs12281734
rs11610932
rs149412803
rs17121283
rs140879197
rs1504497
rs61839394
rs2884281
rs117030598
rs35603444
rs35366463
rs12273007
rs11649841
rs115134798
All
−0.2
−0.1
0.0
0.1
0.2
MR leave−one−out sensitivity analysis for
'Paramuribaculum sp001689565' on 'GCST90199690_buildGRCh38.tsv'

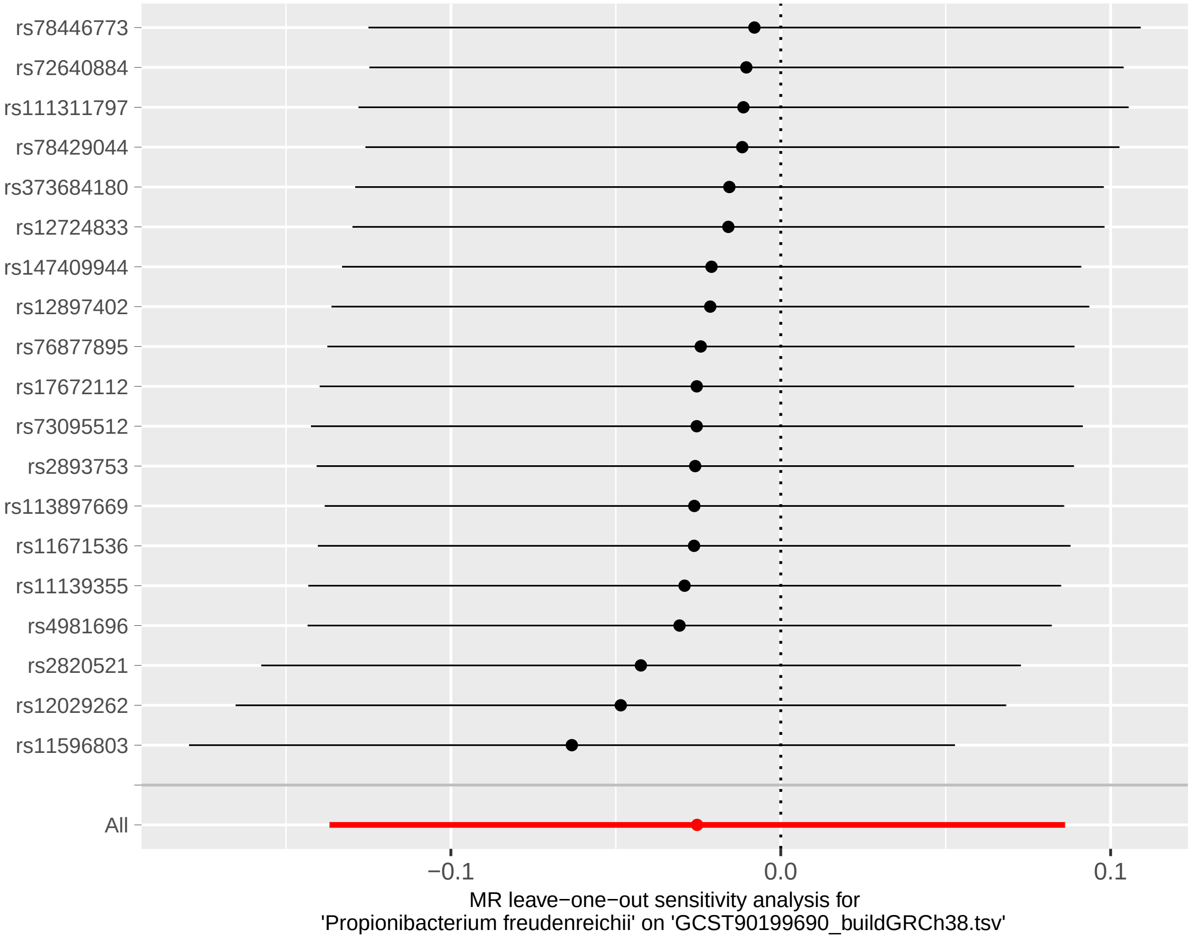

rs78446773
rs72640884
rs111311797
rs78429044
rs373684180
rs12724833
rs147409944
rs12897402
rs76877895
rs17672112
rs73095512
rs2893753
rs113897669
rs11671536
rs11139355
rs4981696
rs2820521
rs12029262
rs11596803
All
−0.1
0.0
0.1
MR leave−one−out sensitivity analysis for
'Propionibacterium freudenreichii' on 'GCST90199690_buildGRCh38.tsv'

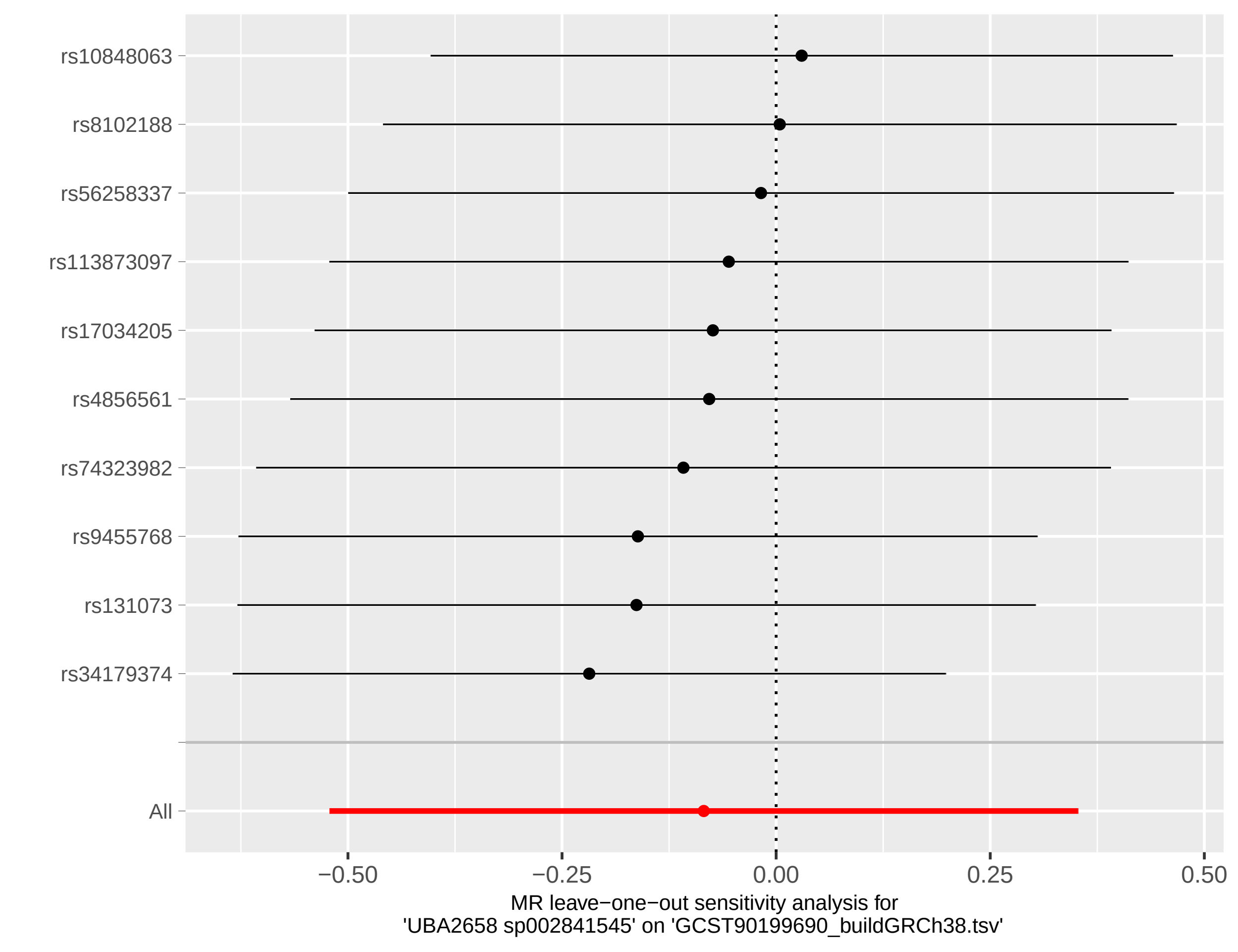
rs10848063
rs8102188
rs56258337
rs113873097
rs17034205
rs4856561
rs74323982
rs9455768
rs131073
rs34179374
All
−0.50
−0.25
0.00
0.25
0.50
MR leave−one−out sensitivity analysis for
'UBA2658 sp002841545' on 'GCST90199690_buildGRCh38.tsv'

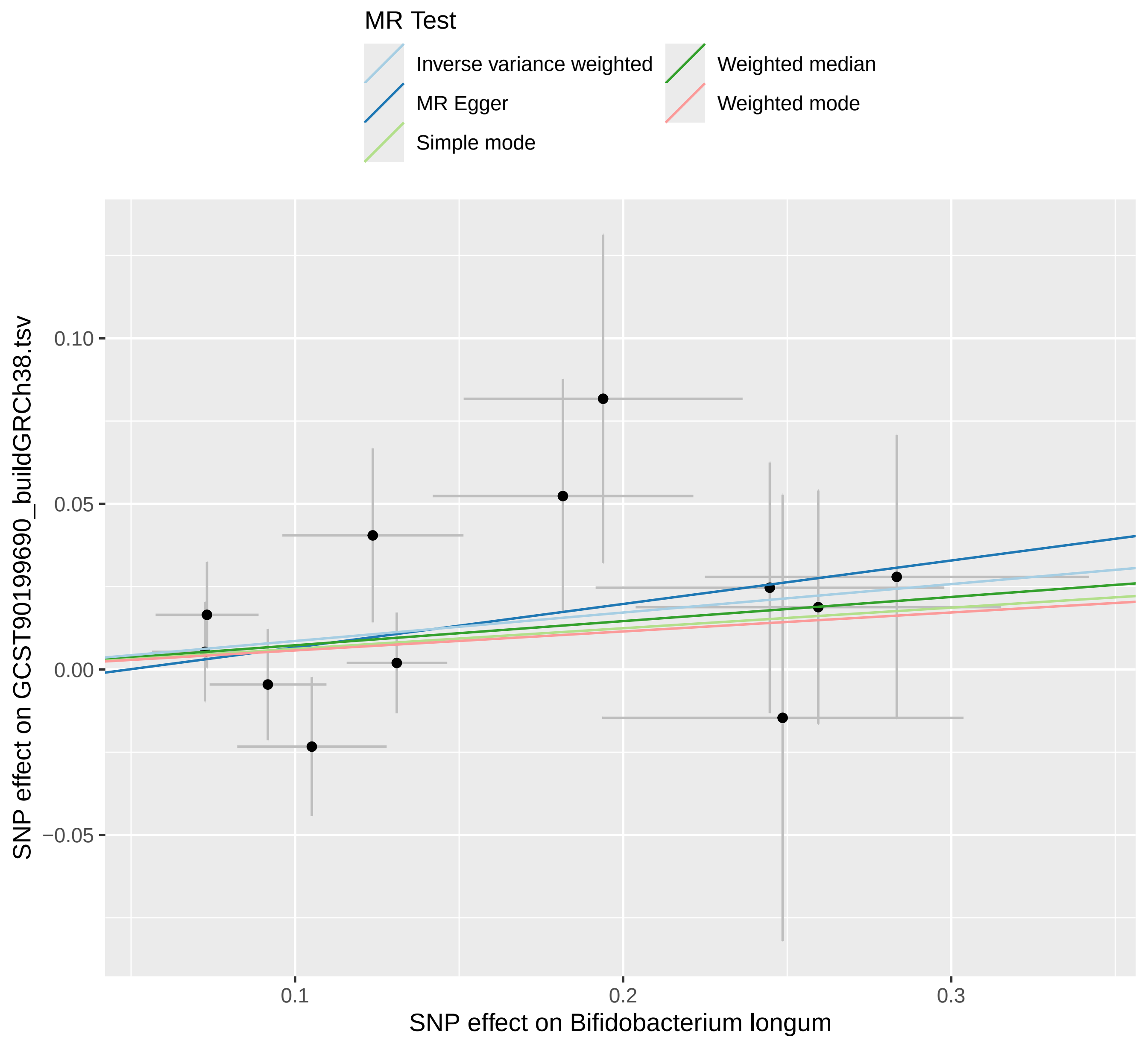

MR Test
Inverse variance weighted
MR Egger
Simple mode
Weighted median
Weighted mode
SNP effect on GCST90199690_buildGRCh38.tsv
0.10
0.05
0.00
−0.05
0.1
0.2
0.3
SNP effect on Bifidobacterium longum

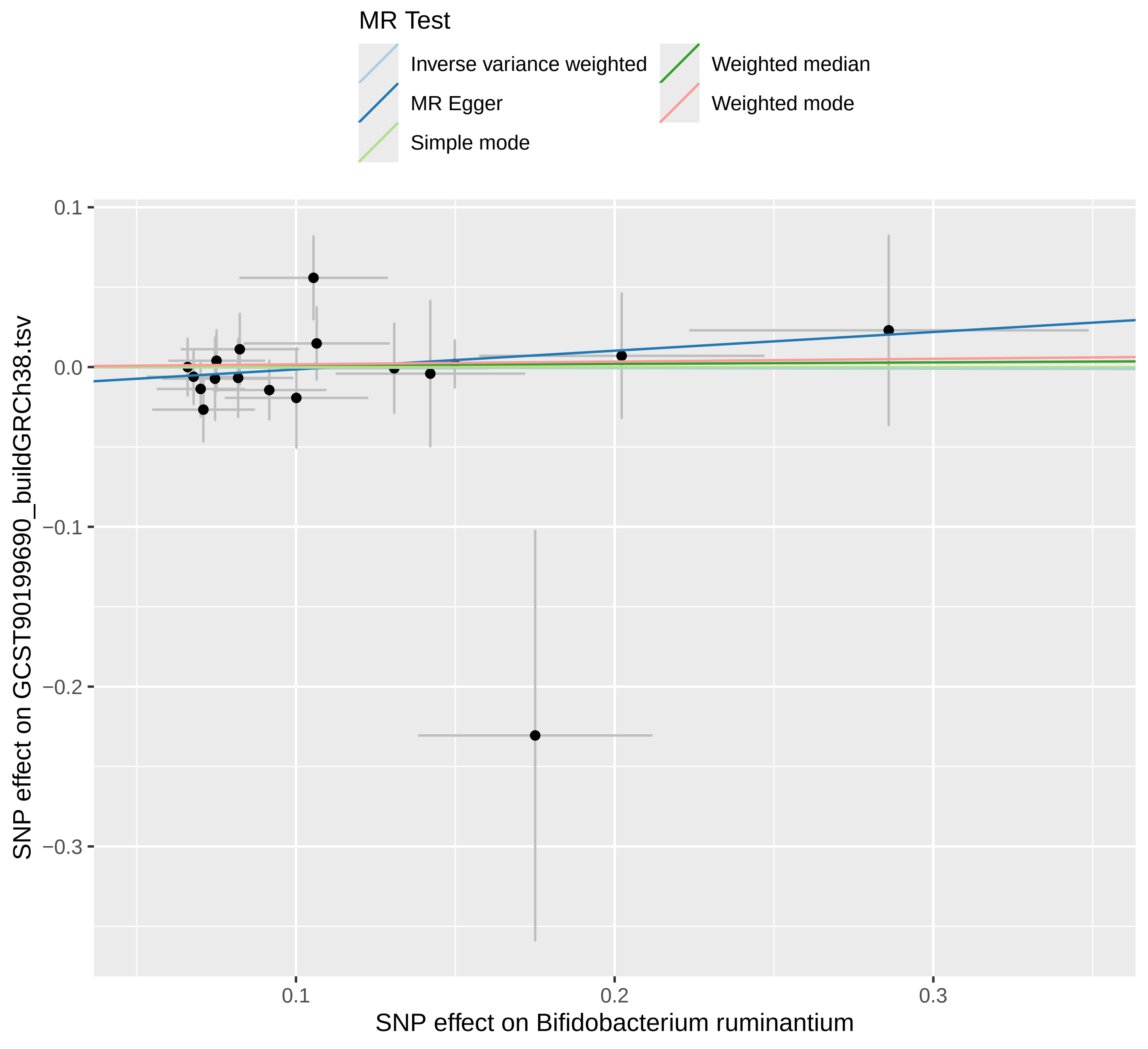
MR Test
Inverse variance weighted
MR Egger
Simple mode
Weighted median
Weighted mode
0.1
0.0
−0.1
−0.2
−0.3
0.1
0.2
0.3
SNP effect on GCST90199690_buildGRCh38.tsv
SNP effect on Bifidobacterium ruminantium

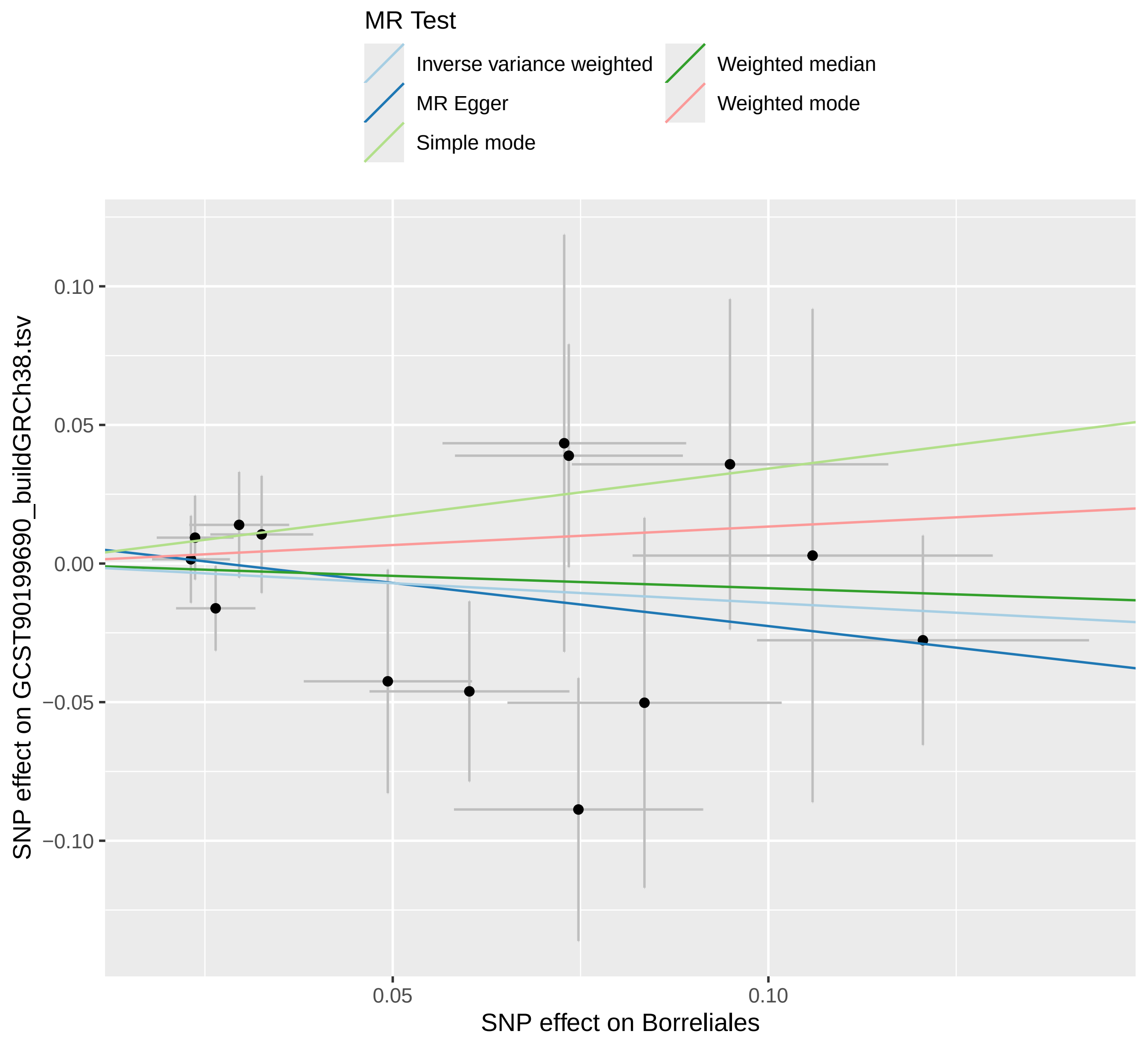
MR Test
Inverse variance weighted
MR Egger
Simple mode
Weighted median
Weighted mode
0.10
0.05
0.00
−0.05
−0.10
SNP effect on GCST90199690_buildGRCh38.tsv
0.05
0.10
SNP effect on Borreliales

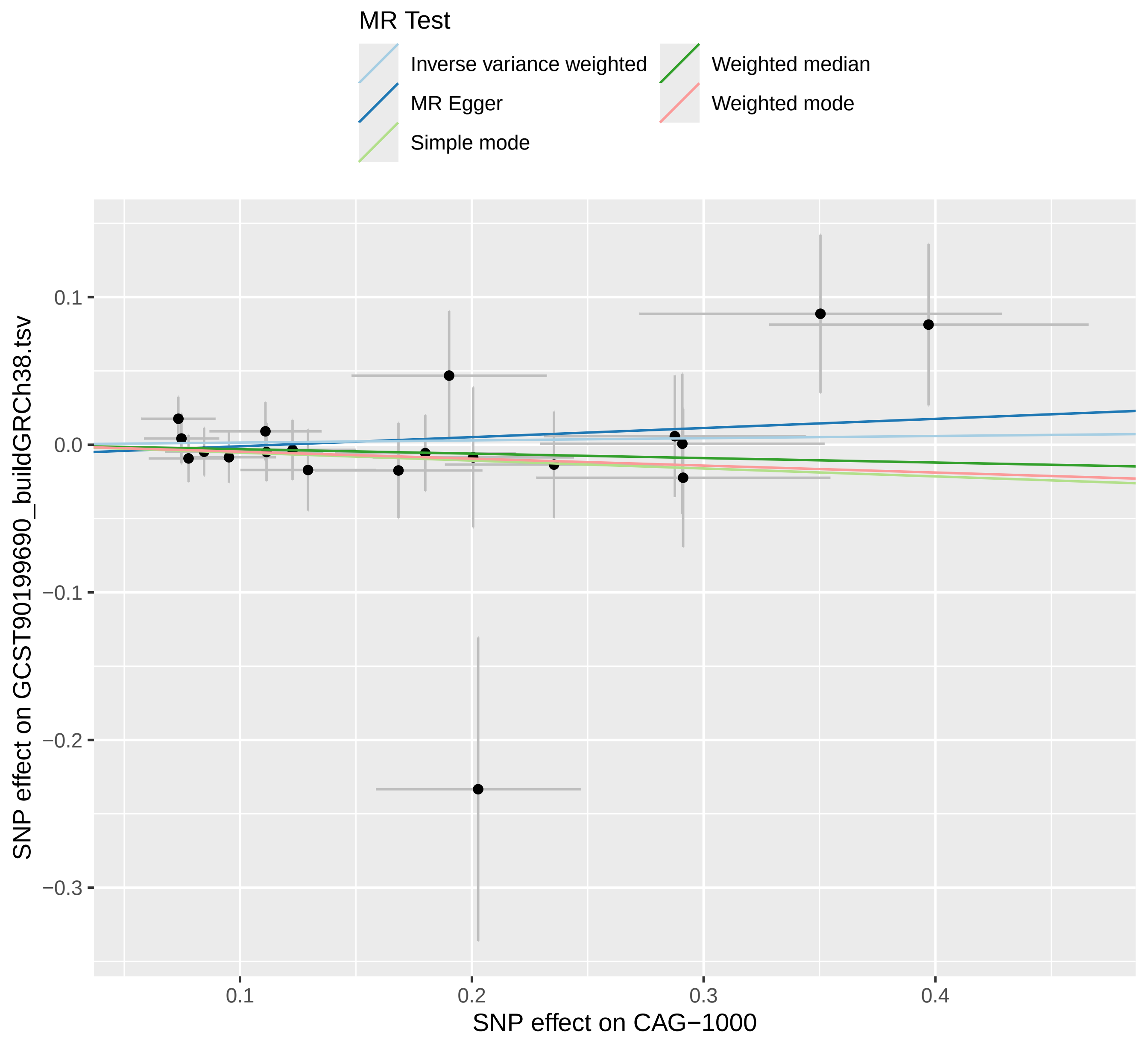

MR Test
Inverse variance weighted
MR Egger
Simple mode
Weighted median
Weighted mode
0.1
0.0
−0.1
−0.2
−0.3
0.1
0.2
0.3
0.4
SNP effect on GCST90199690_buildGRCh38.tsv
SNP effect on CAG−1000

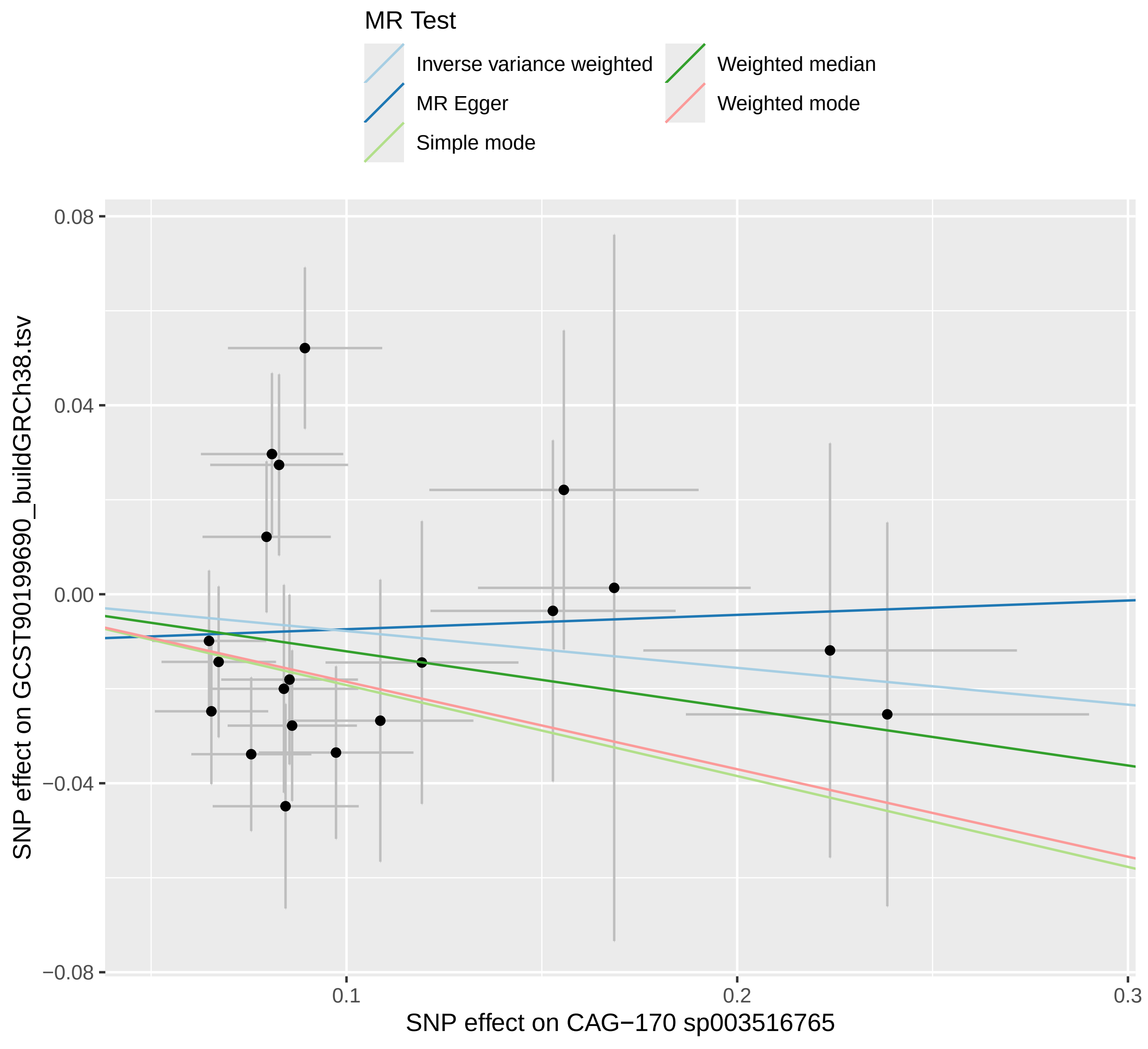
MR Test
Inverse variance weighted
MR Egger
Simple mode
Weighted median
Weighted mode
0.08
0.04
0.00
−0.04
−0.08
0.1
0.2
0.3
SNP effect on GCST90199690_buildGRCh38.tsv
SNP effect on CAG−170 sp003516765

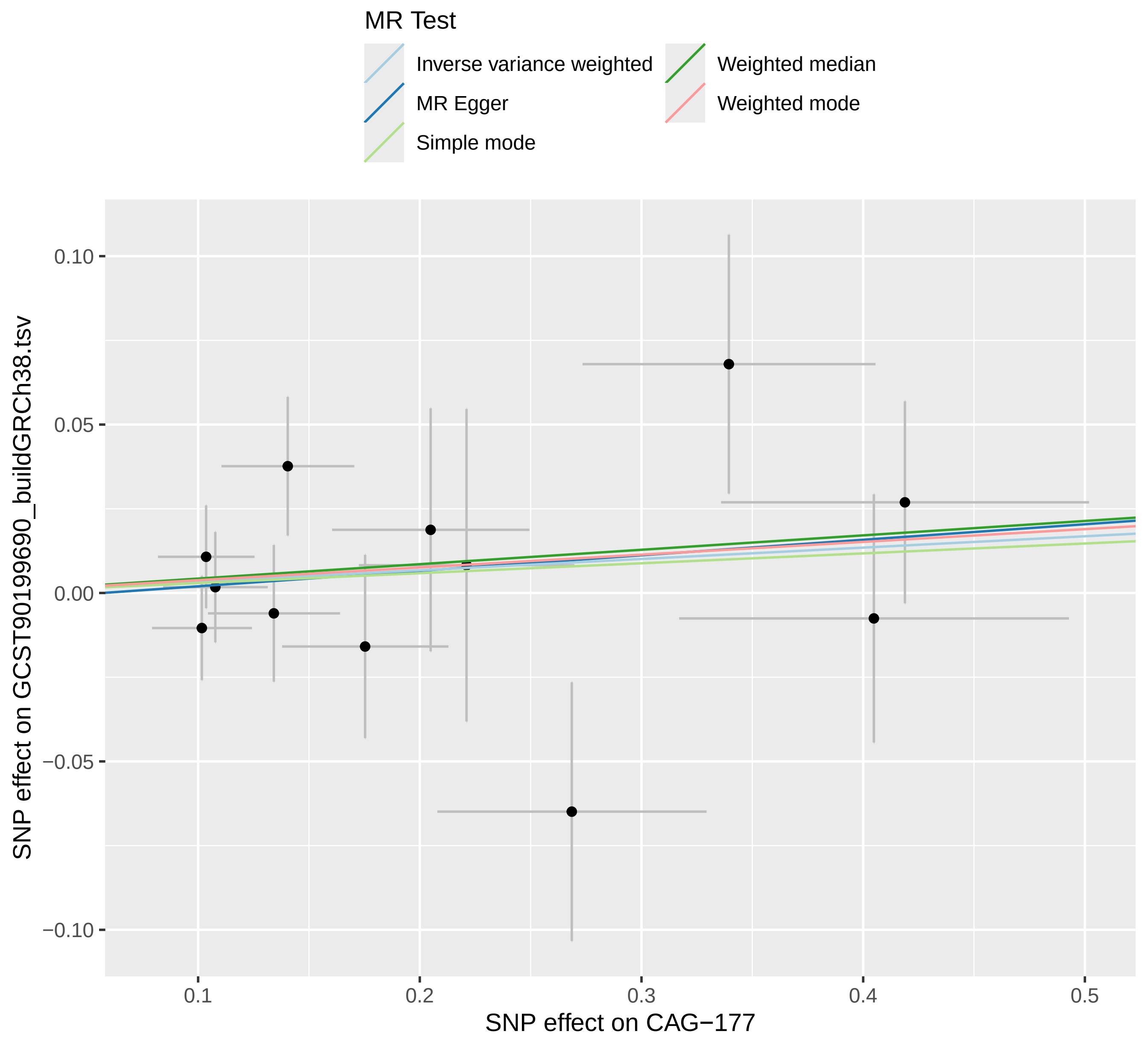

MR Test
Inverse variance weighted
MR Egger
Simple mode
Weighted median
Weighted mode
0.10
0.05
0.00
−0.05
−0.10
0.1
0.2
0.3
0.4
0.5
SNP effect on GCST90199690_buildGRCh38.tsv
SNP effect on CAG−177

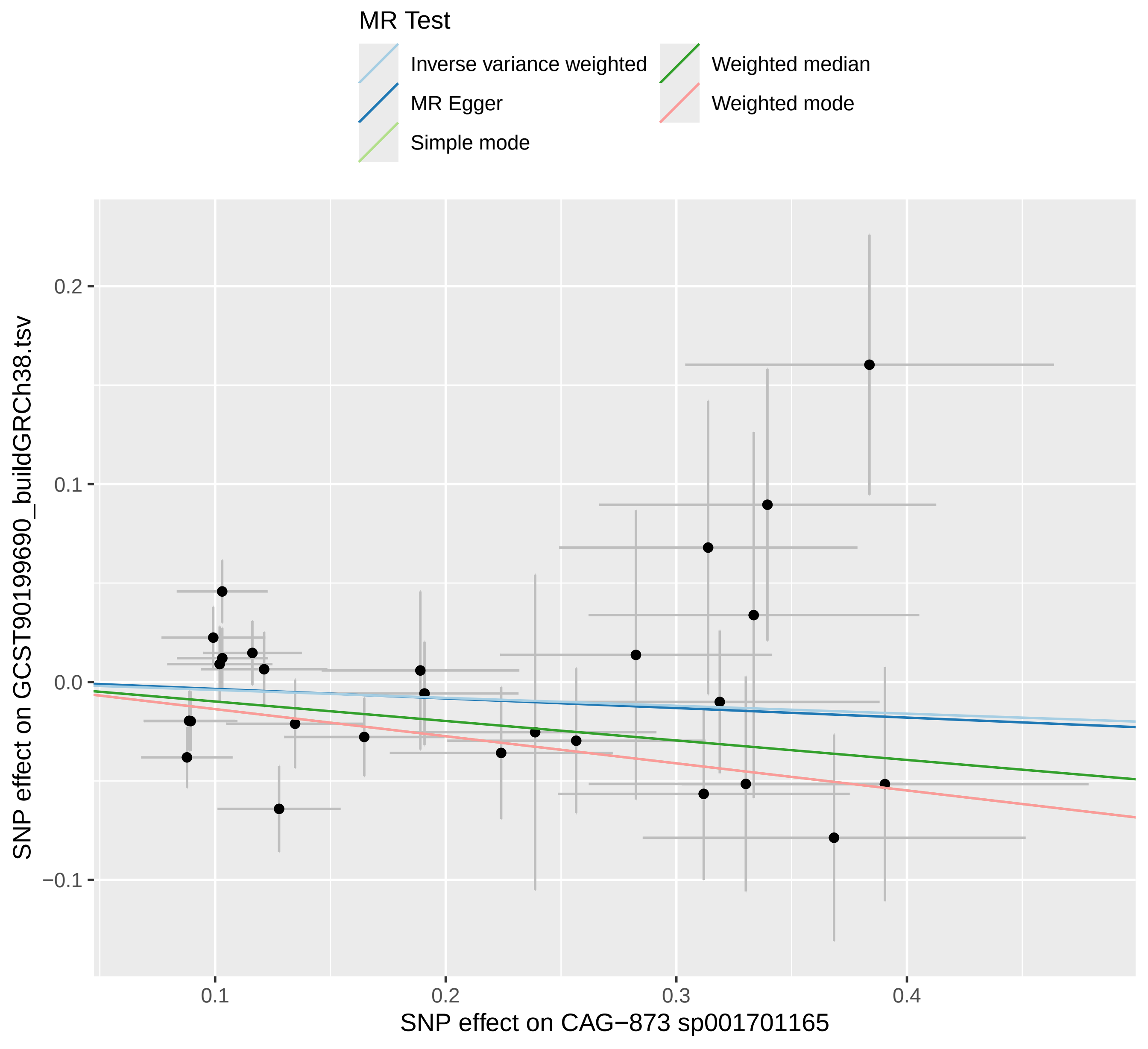
MR Test
Inverse variance weighted
MR Egger
Simple mode
Weighted median
Weighted mode
0.2
0.1
0.0
−0.1
0.1
0.2
0.3
0.4
SNP effect on GCST90199690_buildGRCh38.tsv
SNP effect on CAG−873 sp001701165

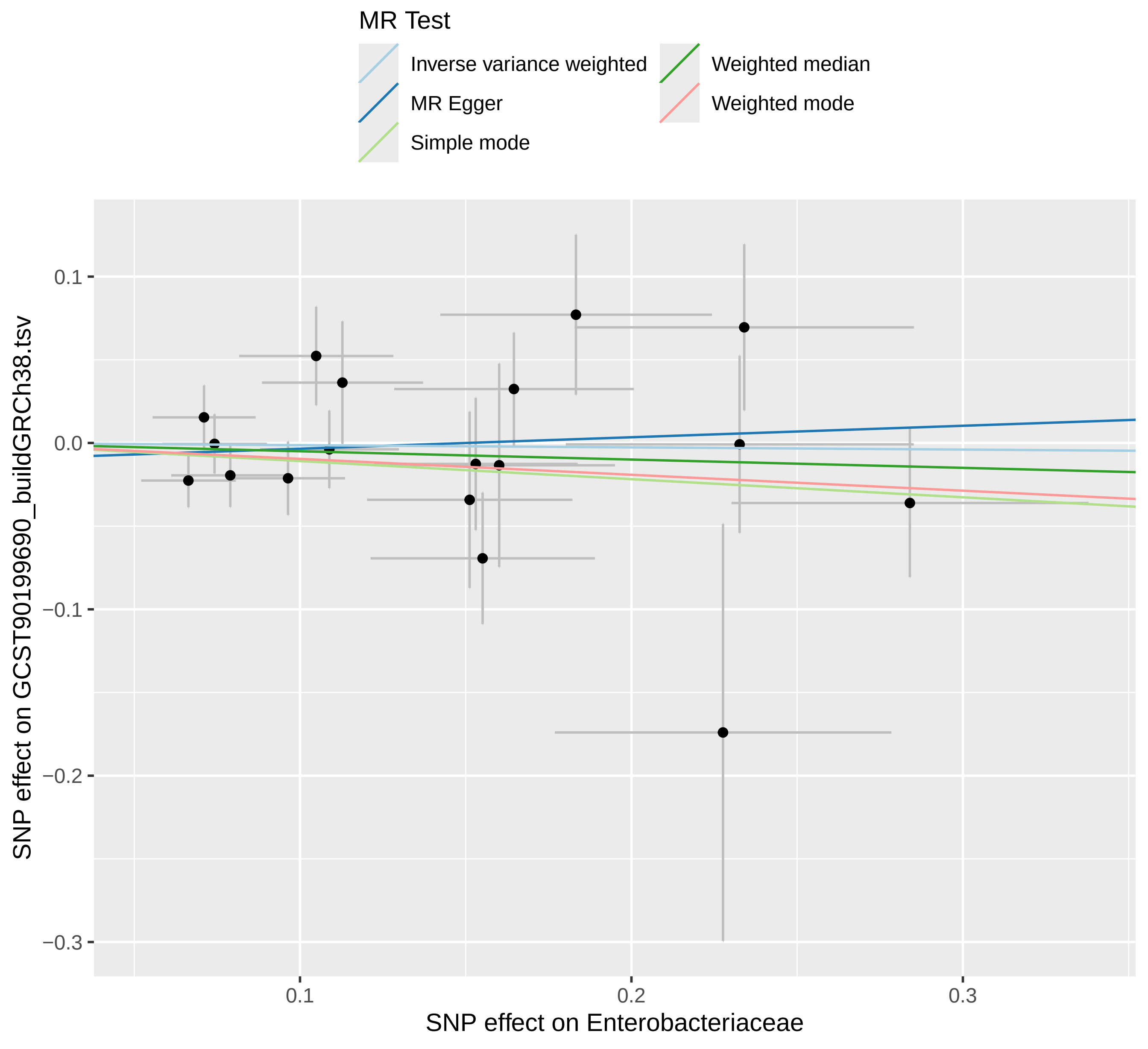
MR Test
Inverse variance weighted
MR Egger
Simple mode
Weighted median
Weighted mode
0.1
0.0
−0.1
−0.2
−0.3
SNP effect on GCST90199690_buildGRCh38.tsv
0.1
0.2
0.3
SNP effect on Enterobacteriaceae

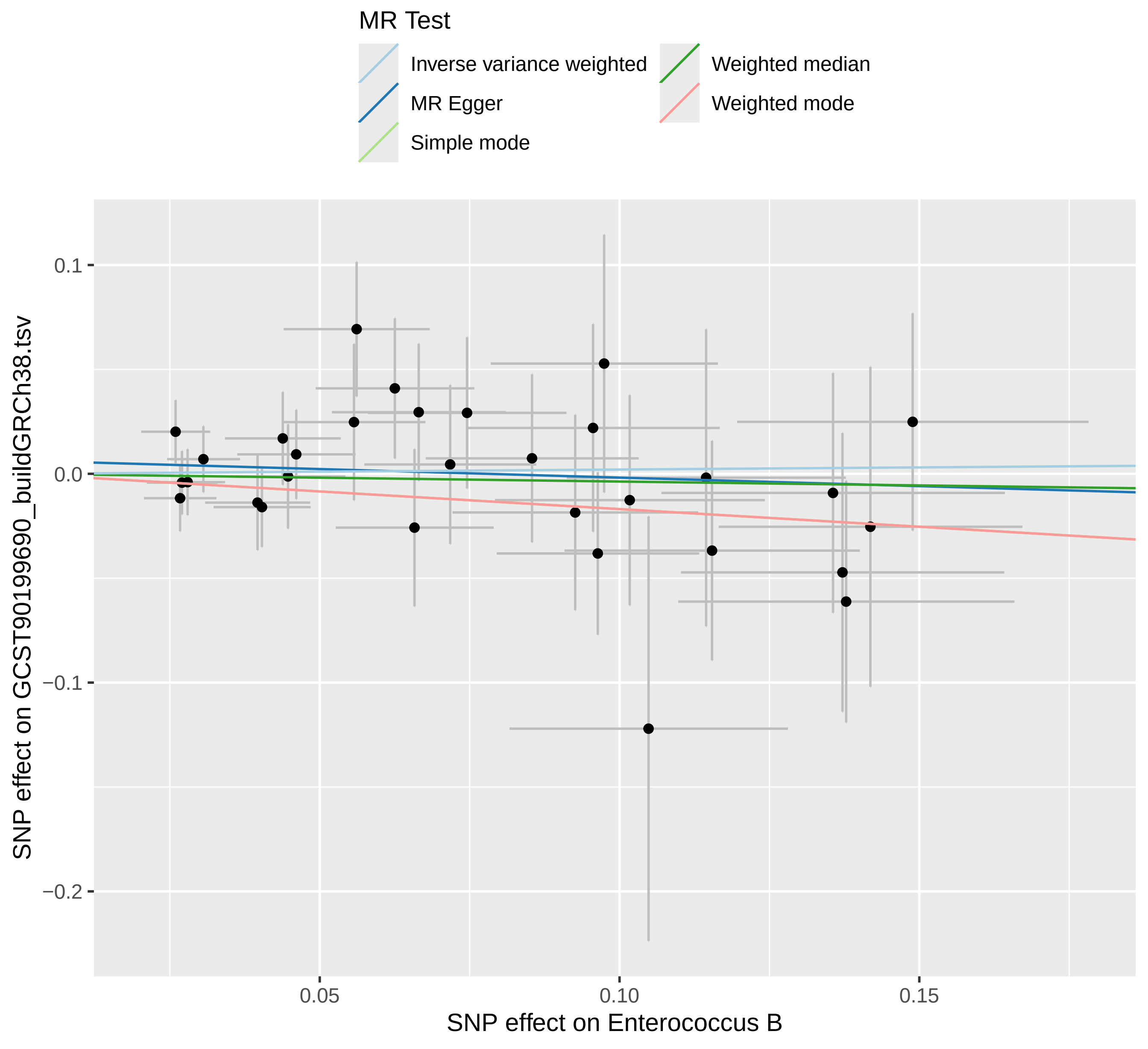
MR Test
Inverse variance weighted
MR Egger
Simple mode
Weighted median
Weighted mode
0.1
0.0
−0.1
−0.2
SNP effect on GCST90199690_buildGRCh38.tsv
0.05
0.10
0.15
SNP effect on Enterococcus B

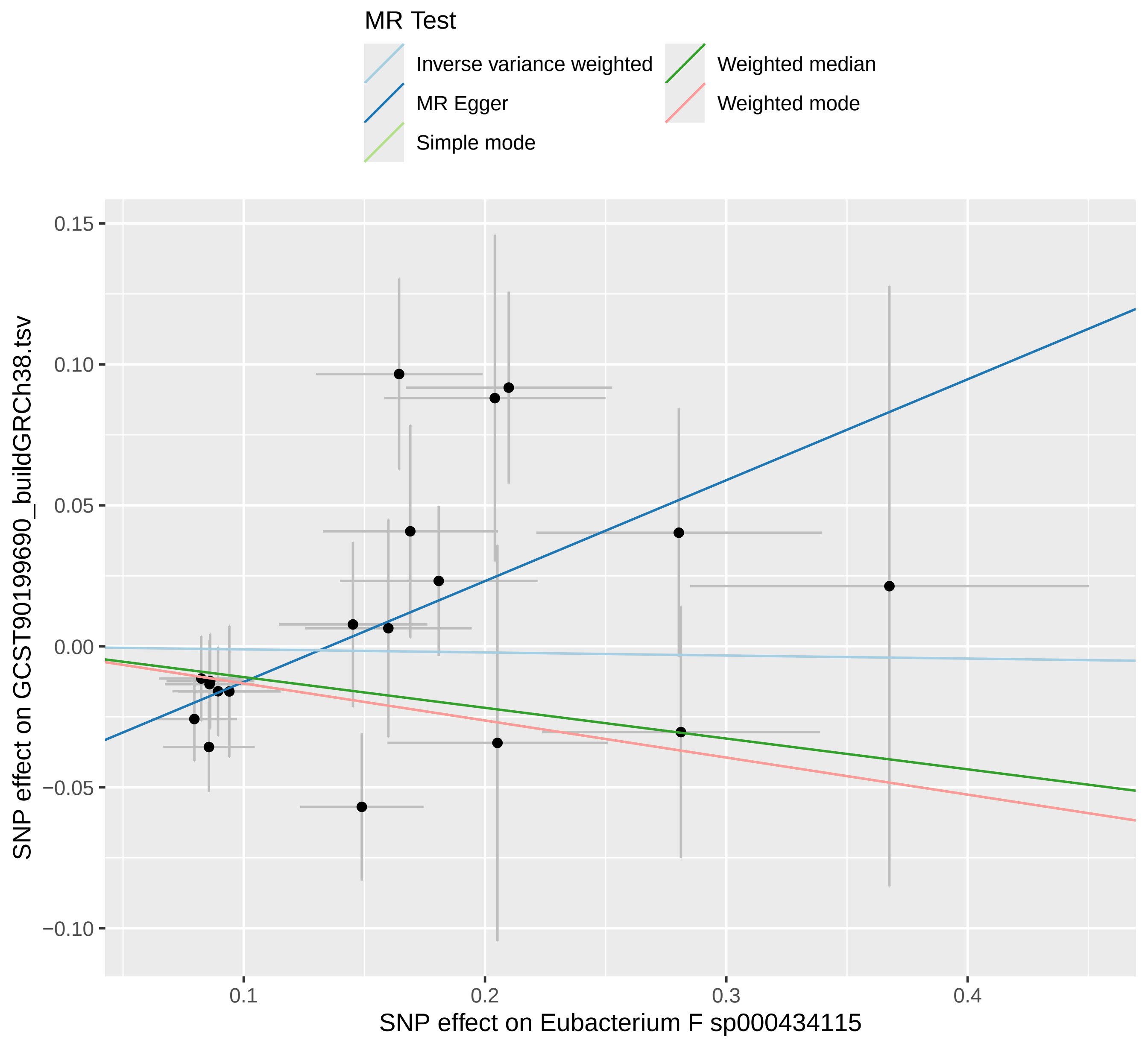
MR Test
Inverse variance weighted
MR Egger
Simple mode
Weighted median
Weighted mode
SNP effect on GCST90199690_buildGRCh38.tsv
0.15
0.10
0.05
0.00
−0.05
−0.10
0.1
0.2
0.3
0.4
SNP effect on Eubacterium F sp000434115

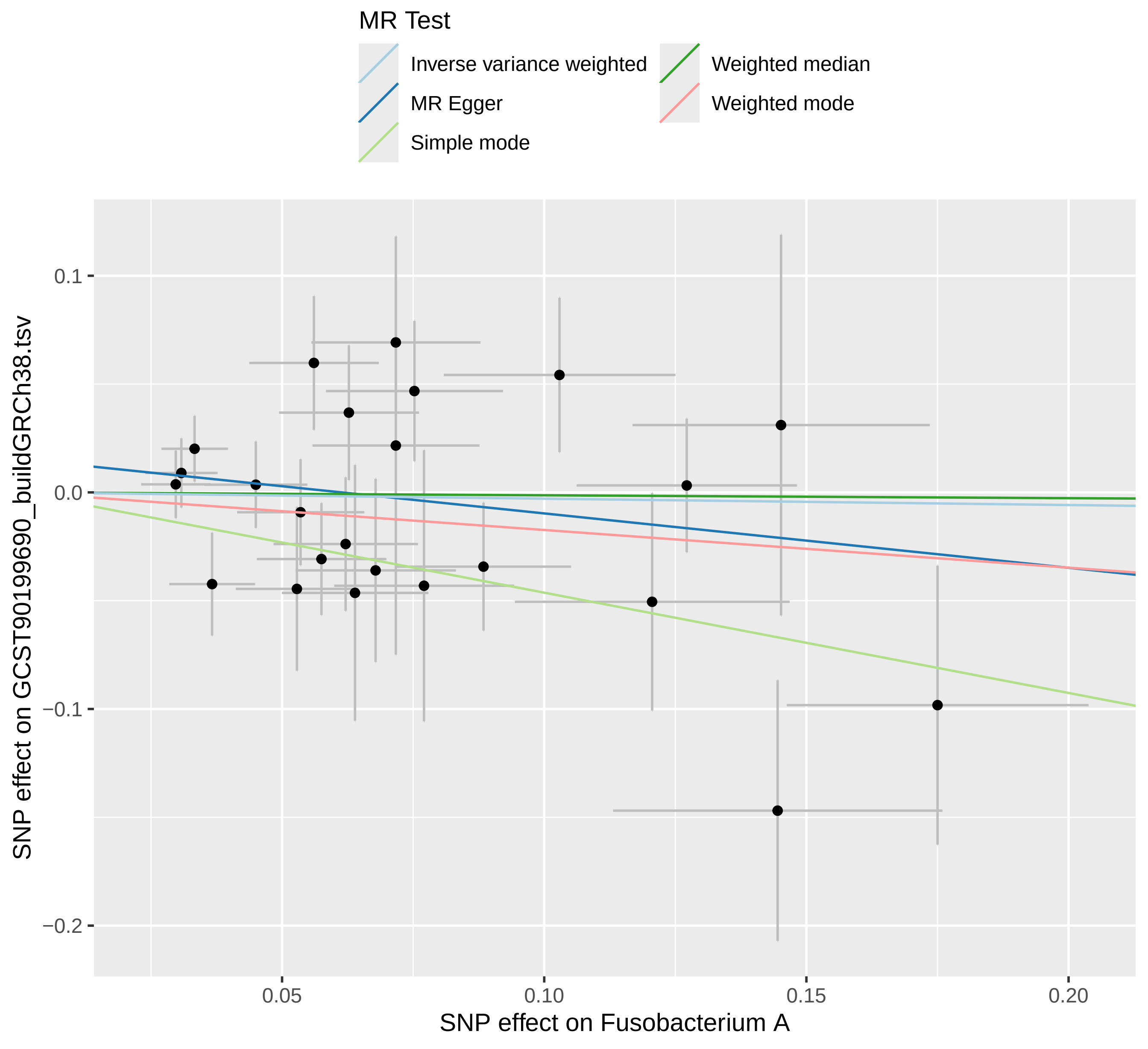
MR Test
Inverse variance weighted
MR Egger
Simple mode
Weighted median
Weighted mode
0.1
0.0
−0.1
−0.2
0.05
0.10
0.15
0.20
SNP effect on GCST90199690_buildGRCh38.tsv
SNP effect on Fusobacterium A

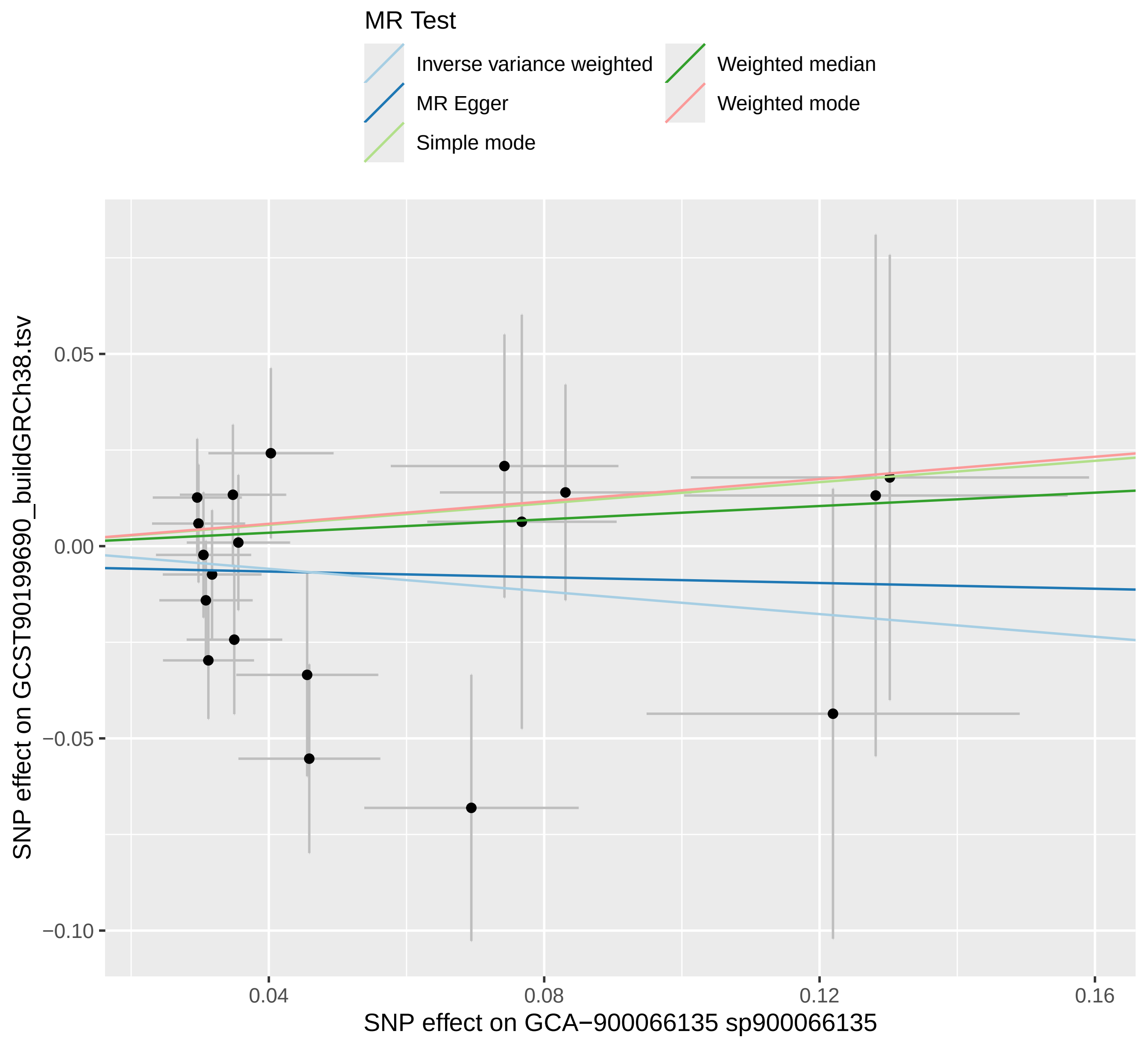
MR Test
Inverse variance weighted
MR Egger
Simple mode
Weighted median
Weighted mode
0.05
0.00
−0.05
−0.10
0.04
0.08
0.12
0.16
SNP effect on GCST90199690_buildGRCh38.tsv
SNP effect on GCA−900066135 sp900066135

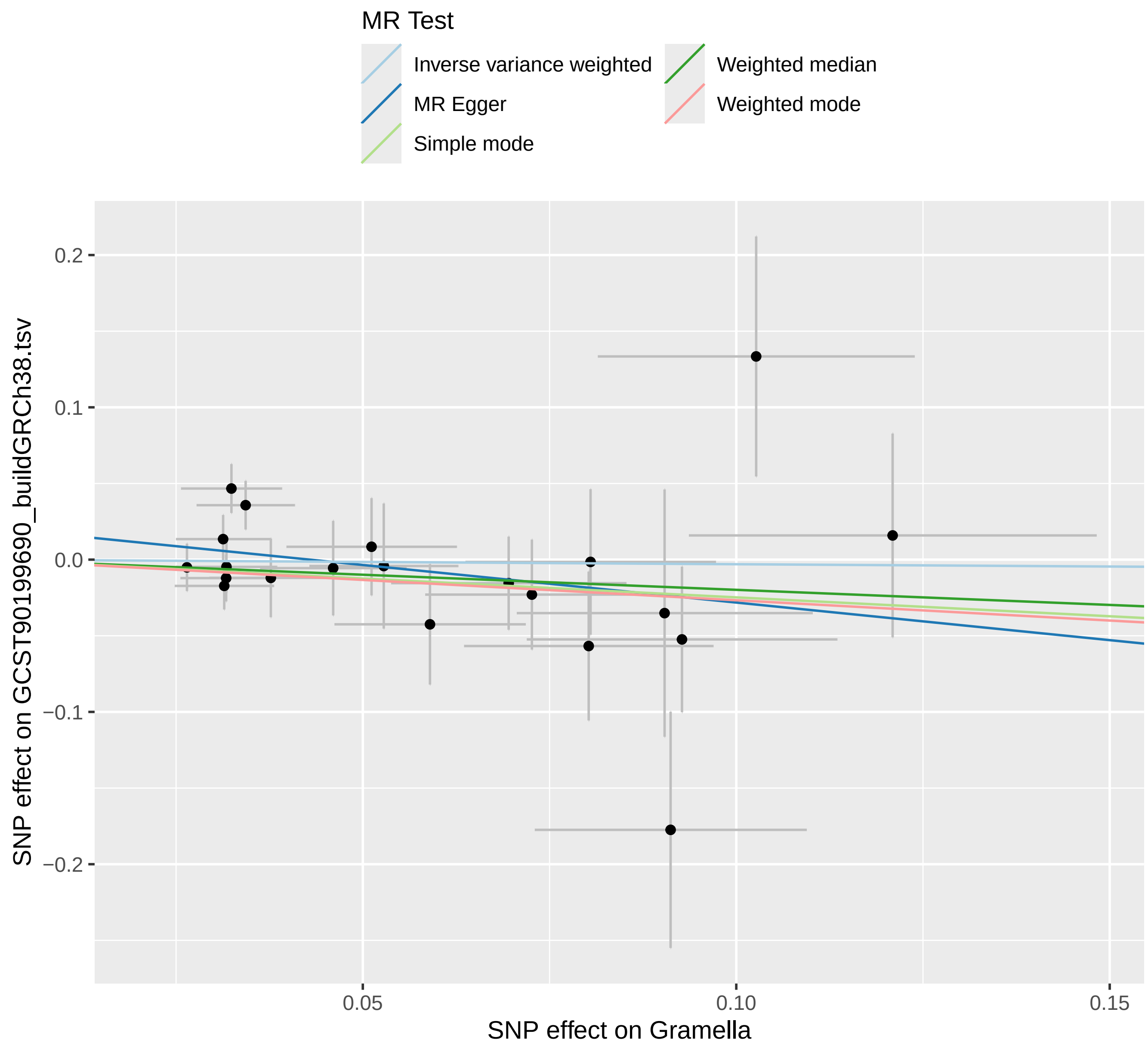

MR Test
Inverse variance weighted
MR Egger
Simple mode
Weighted median
Weighted mode
SNP effect on GCST90199690_buildGRCh38.tsv
0.2
0.1
0.0
−0.1
−0.2
0.05
0.10
0.15
SNP effect on Gramella

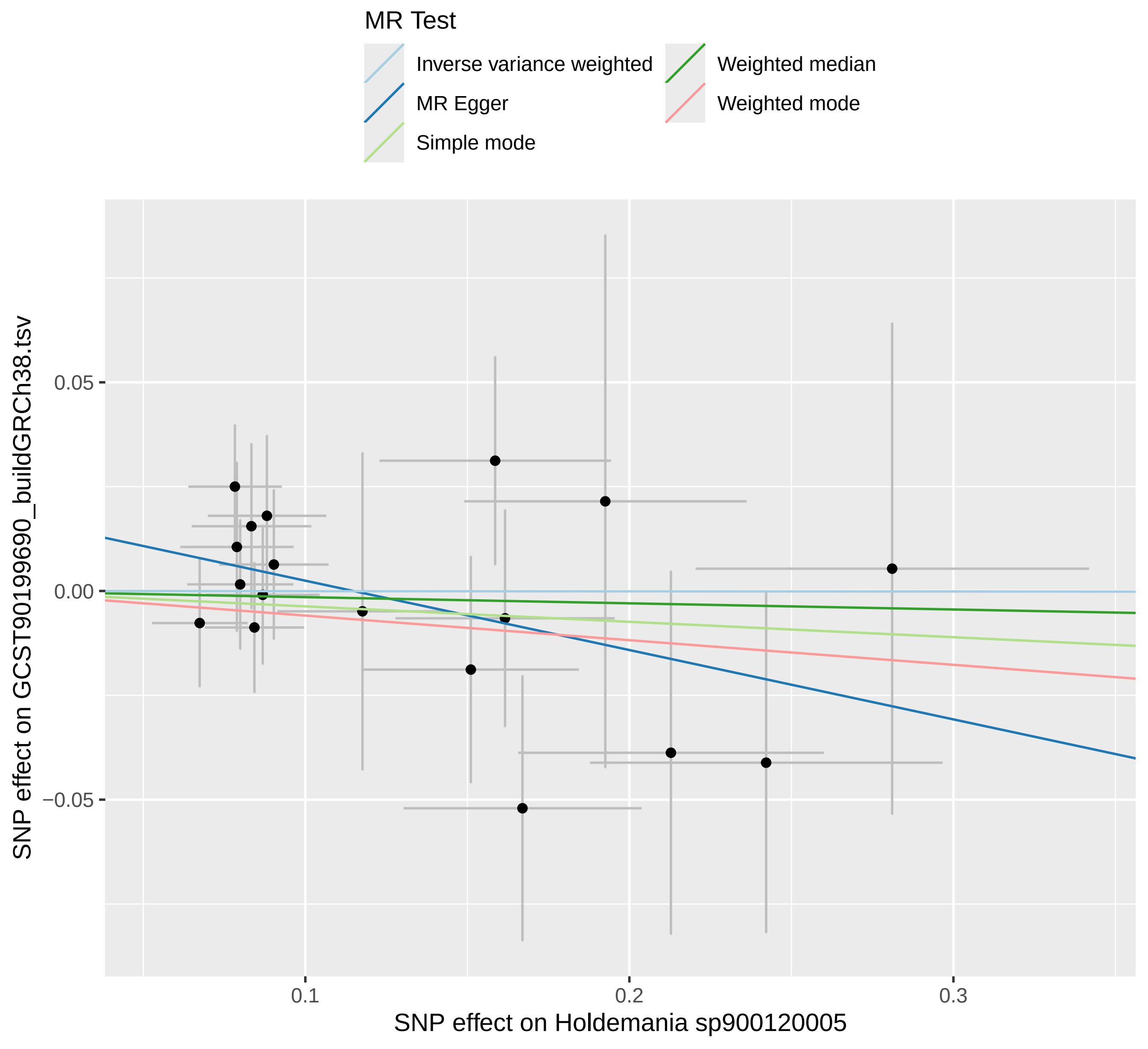

MR Test
Inverse variance weighted
MR Egger
Simple mode
Weighted median
Weighted mode
0.05
0.00
−0.05
0.1
0.2
0.3
SNP effect on GCST90199690_buildGRCh38.tsv
SNP effect on Holdemania sp900120005

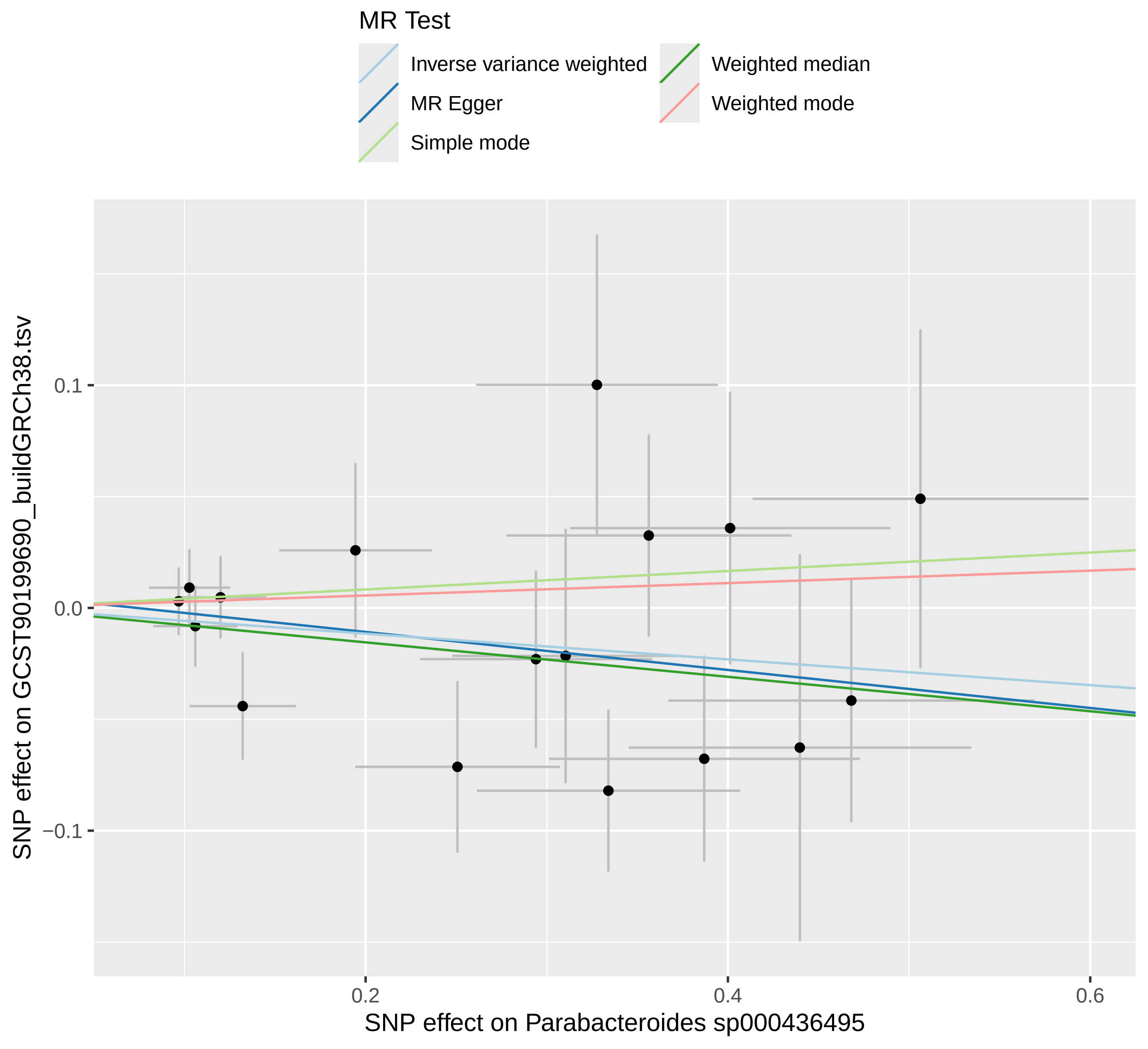

MR Test
Inverse variance weighted
MR Egger
Simple mode
Weighted median
Weighted mode
SNP effect on GCST90199690_buildGRCh38.tsv
0.1
0.0
−0.1
0.2
0.4
0.6
SNP effect on Parabacteroides sp000436495

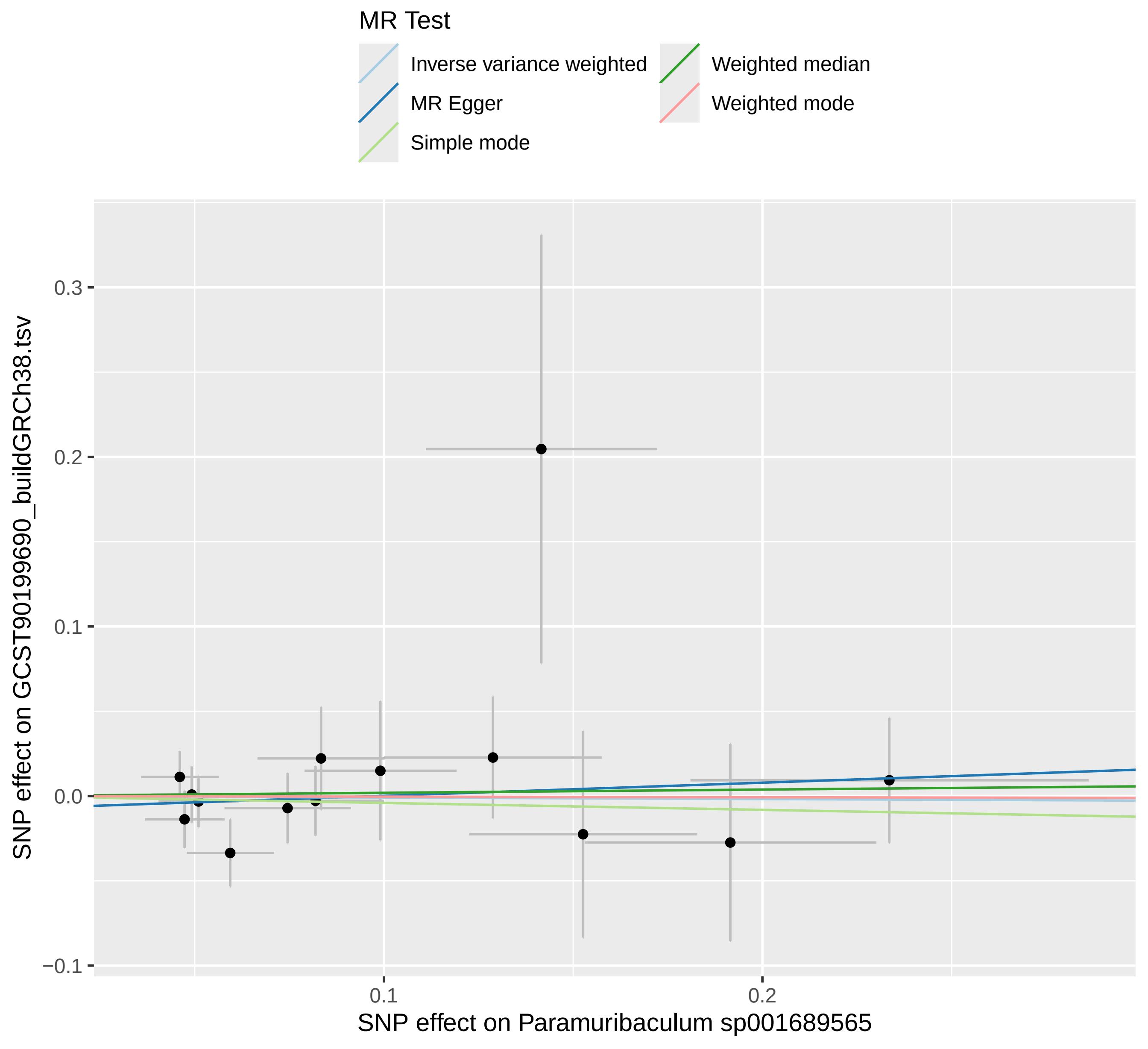
MR Test
Inverse variance weighted
MR Egger
Simple mode
Weighted median
Weighted mode
0.3
0.2
0.1
0.0
−0.1
0.1
0.2
SNP effect on GCST90199690_buildGRCh38.tsv
SNP effect on Paramuribaculum sp001689565

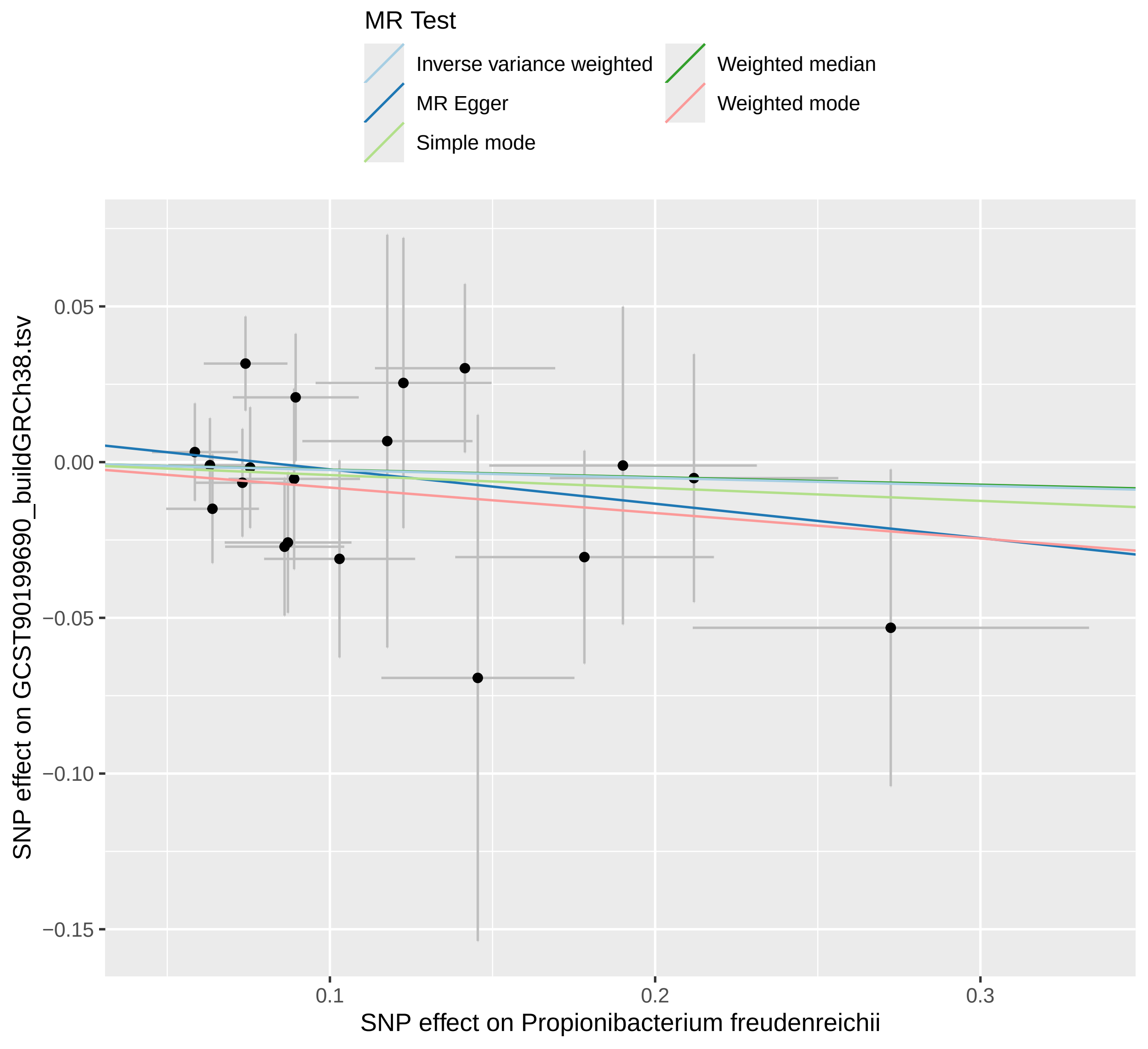
MR Test
Inverse variance weighted
MR Egger
Simple mode
Weighted median
Weighted mode
0.05
0.00
−0.05
−0.10
−0.15
0.1
0.2
0.3
SNP effect on GCST90199690_buildGRCh38.tsv
SNP effect on Propionibacterium freudenreichii

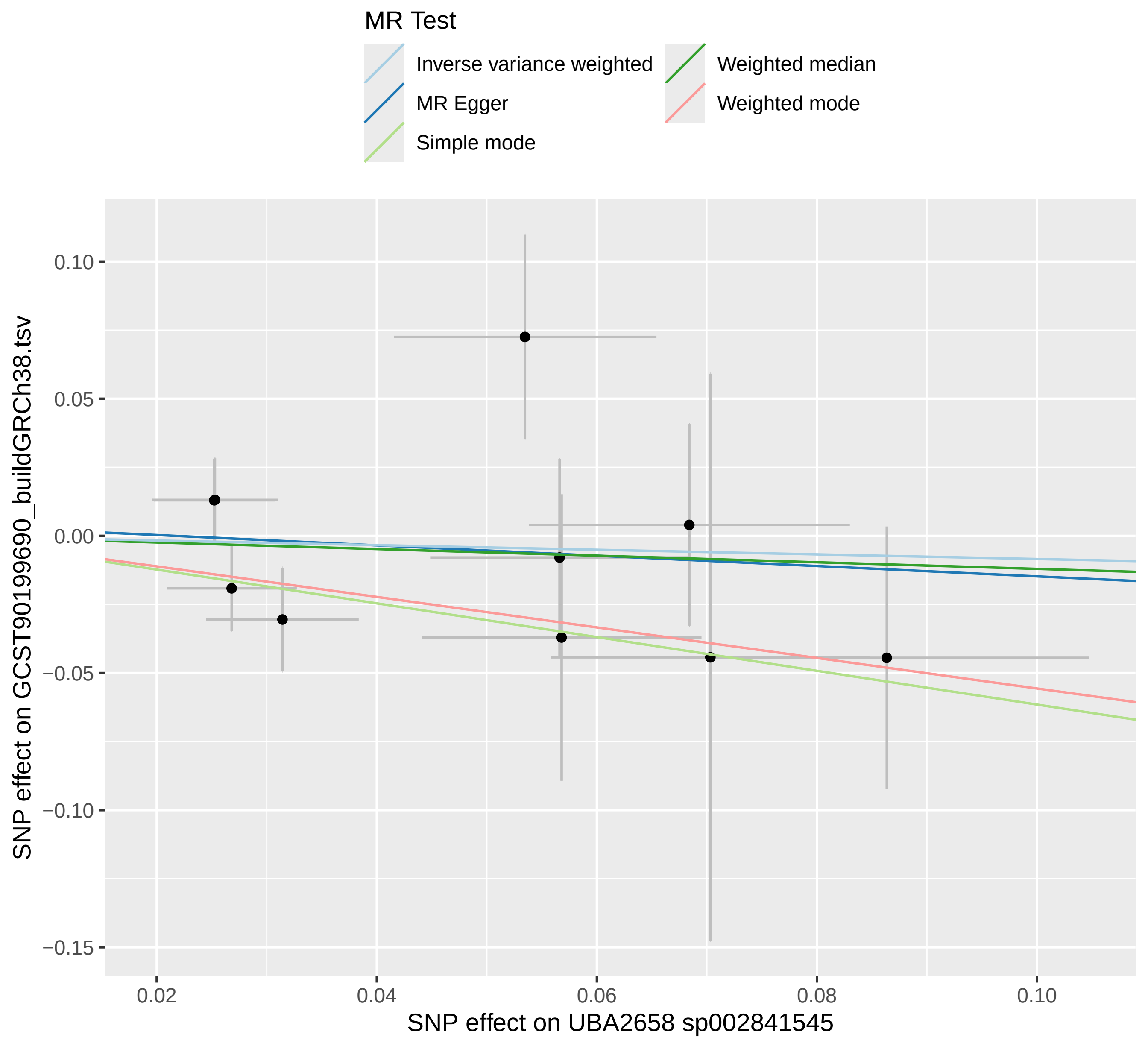

MR Test
Inverse variance weighted
MR Egger
Simple mode
Weighted median
Weighted mode
0.10
0.05
0.00
−0.05
−0.10
−0.15
SNP effect on GCST90199690_buildGRCh38.tsv
0.02
0.04
0.06
0.08
0.10
SNP effect on UBA2658 sp002841545

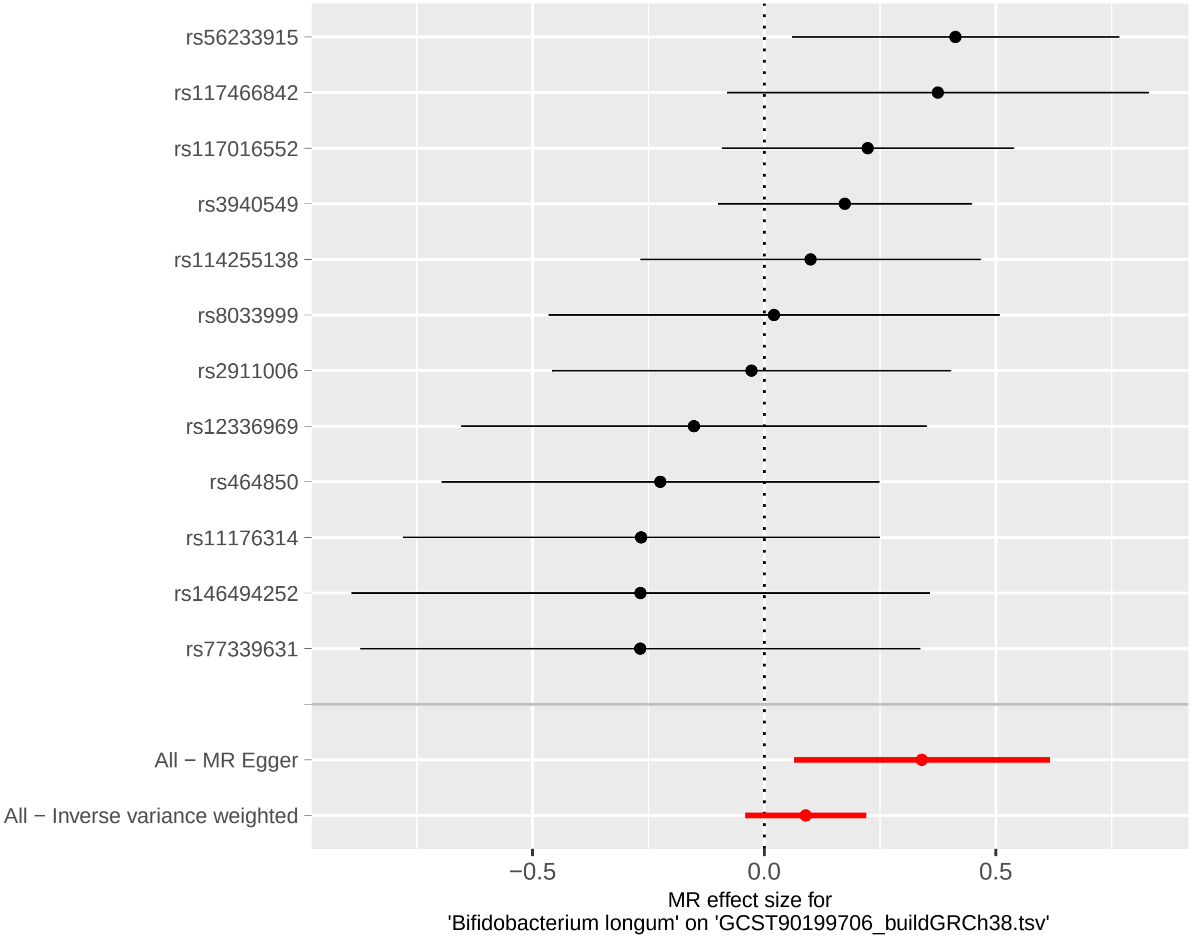
rs56233915
rs117466842
rs117016552
rs3940549
rs114255138
rs8033999
rs2911006
rs12336969
rs464850
rs11176314
rs146494252
rs77339631
All − MR Egger
All − Inverse variance weighted
−0.5
0.0
0.5
MR effect size for
'Bifidobacterium longum' on 'GCST90199706_buildGRCh38.tsv'

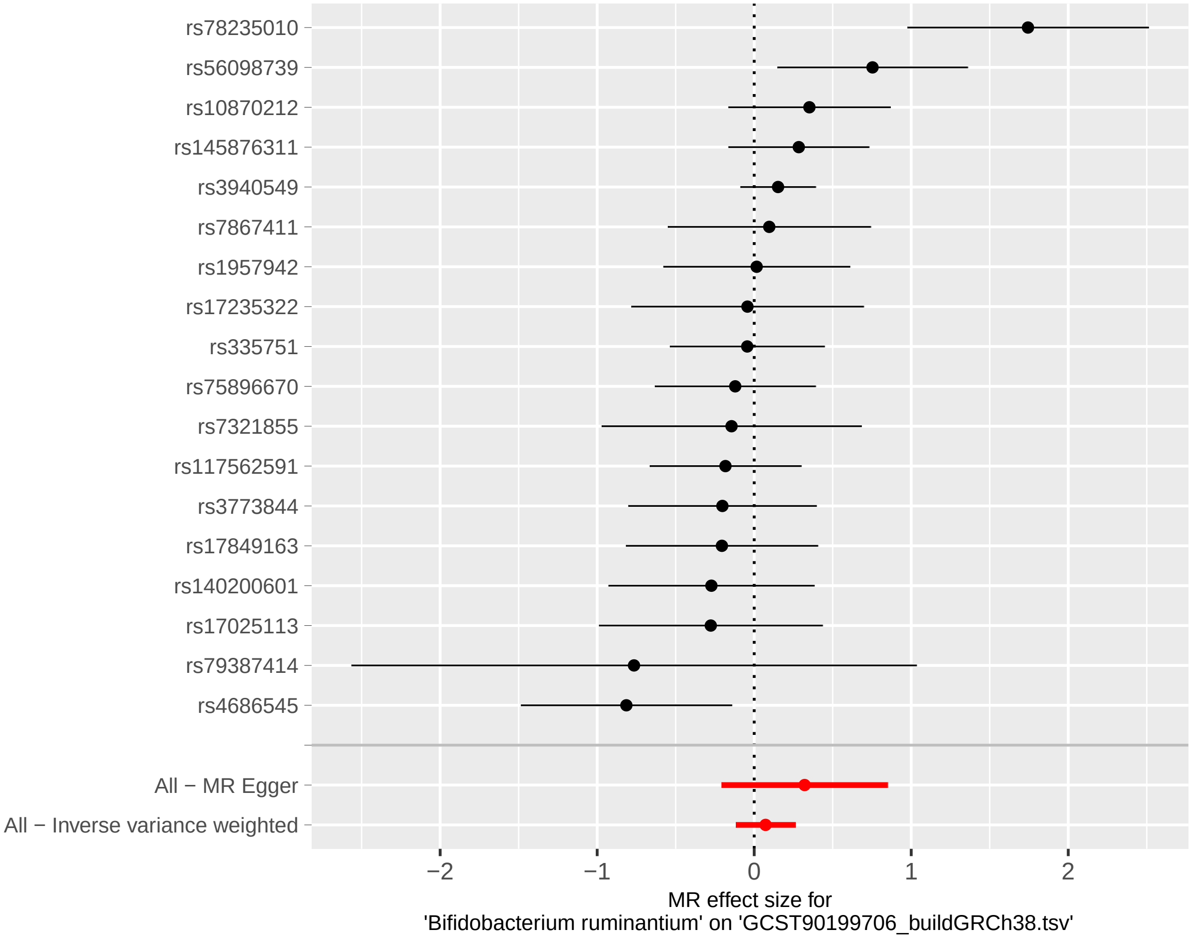

rs78235010
rs56098739
rs10870212
rs145876311
rs3940549
rs7867411
rs1957942
rs17235322
rs335751
rs75896670
rs7321855
rs117562591
rs3773844
rs17849163
rs140200601
rs17025113
rs79387414
rs4686545
All − MR Egger
All − Inverse variance weighted
−2
−1
0
1
2
MR effect size for
'Bifidobacterium ruminantium' on 'GCST90199706_buildGRCh38.tsv'

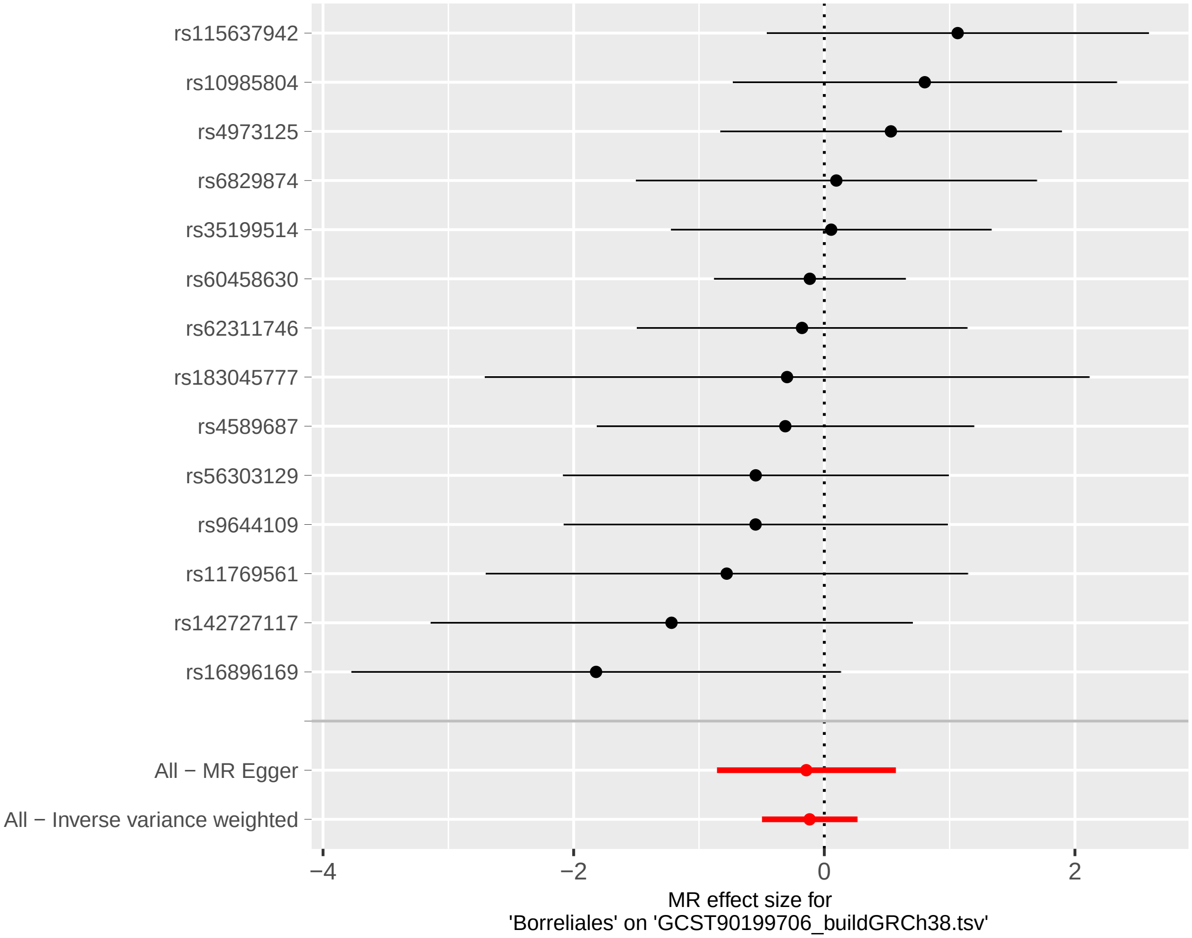
rs115637942
rs10985804
rs4973125
rs6829874
rs35199514
rs60458630
rs62311746
rs183045777
rs4589687
rs56303129
rs9644109
rs11769561
rs142727117
rs16896169
All − MR Egger
All − Inverse variance weighted
−4
−2
0
2
MR effect size for
'Borreliales' on 'GCST90199706_buildGRCh38.tsv'

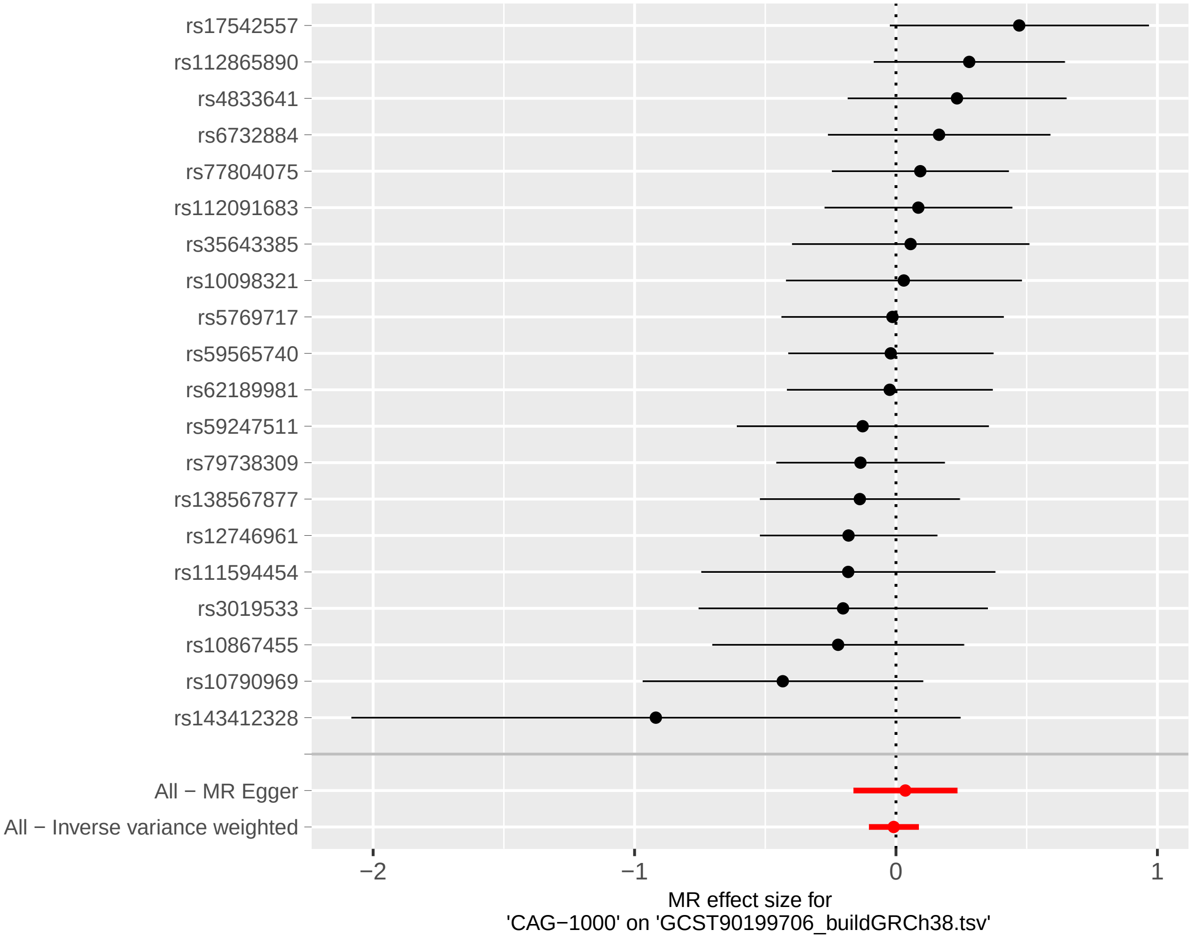

rs17542557
rs112865890
rs4833641
rs6732884
rs77804075
rs112091683
rs35643385
rs10098321
rs5769717
rs59565740
rs62189981
rs59247511
rs79738309
rs138567877
rs12746961
rs111594454
rs3019533
rs10867455
rs10790969
rs143412328
All − MR Egger
All − Inverse variance weighted
−2
−1
0
1
MR effect size for
'CAG−1000' on 'GCST90199706_buildGRCh38.tsv'

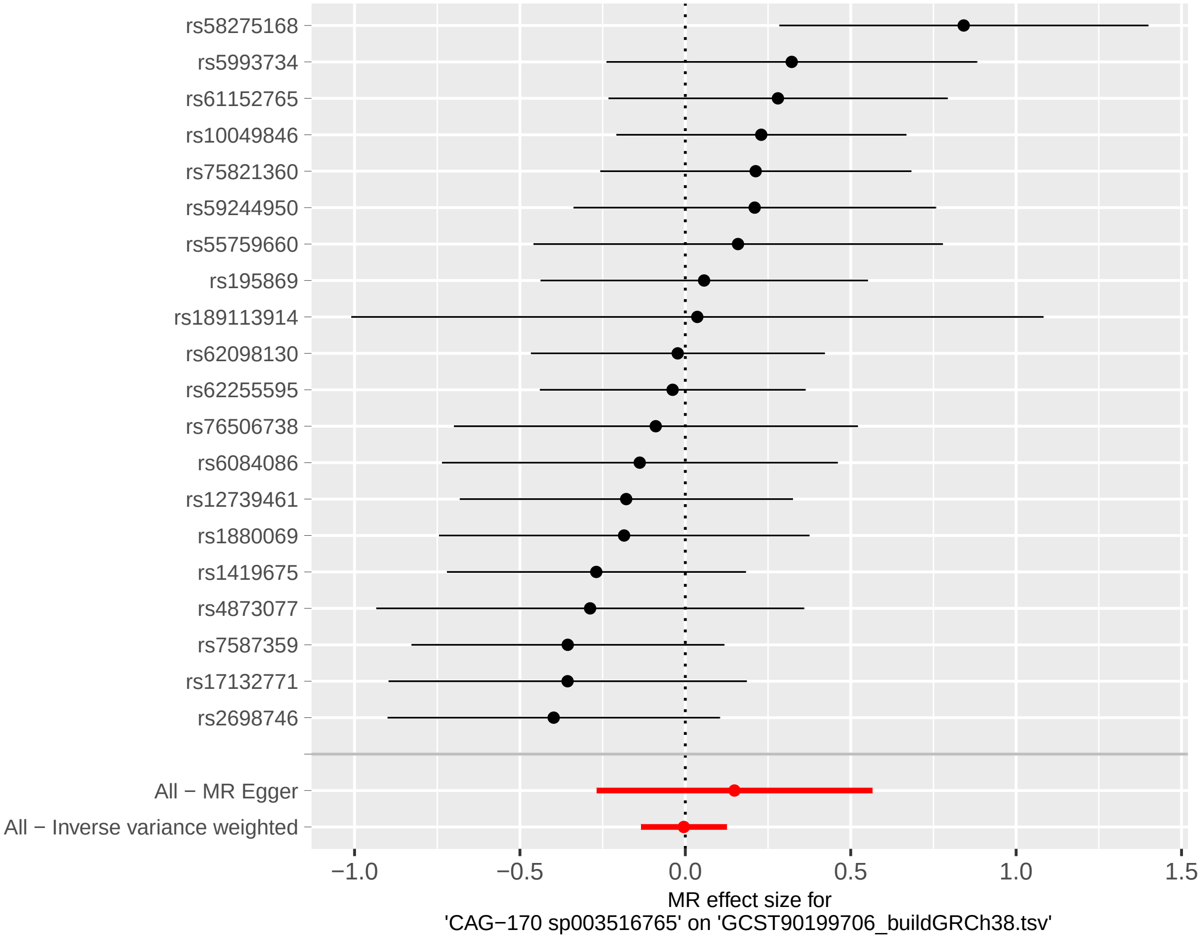
rs58275168
rs5993734
rs61152765
rs10049846
rs75821360
rs59244950
rs55759660
rs195869
rs189113914
rs62098130
rs62255595
rs76506738
rs6084086
rs12739461
rs1880069
rs1419675
rs4873077
rs7587359
rs17132771
rs2698746
All − MR Egger
All − Inverse variance weighted
−1.0
−0.5
0.0
0.5
1.0
1.5
MR effect size for
'CAG−170 sp003516765' on 'GCST90199706_buildGRCh38.tsv'

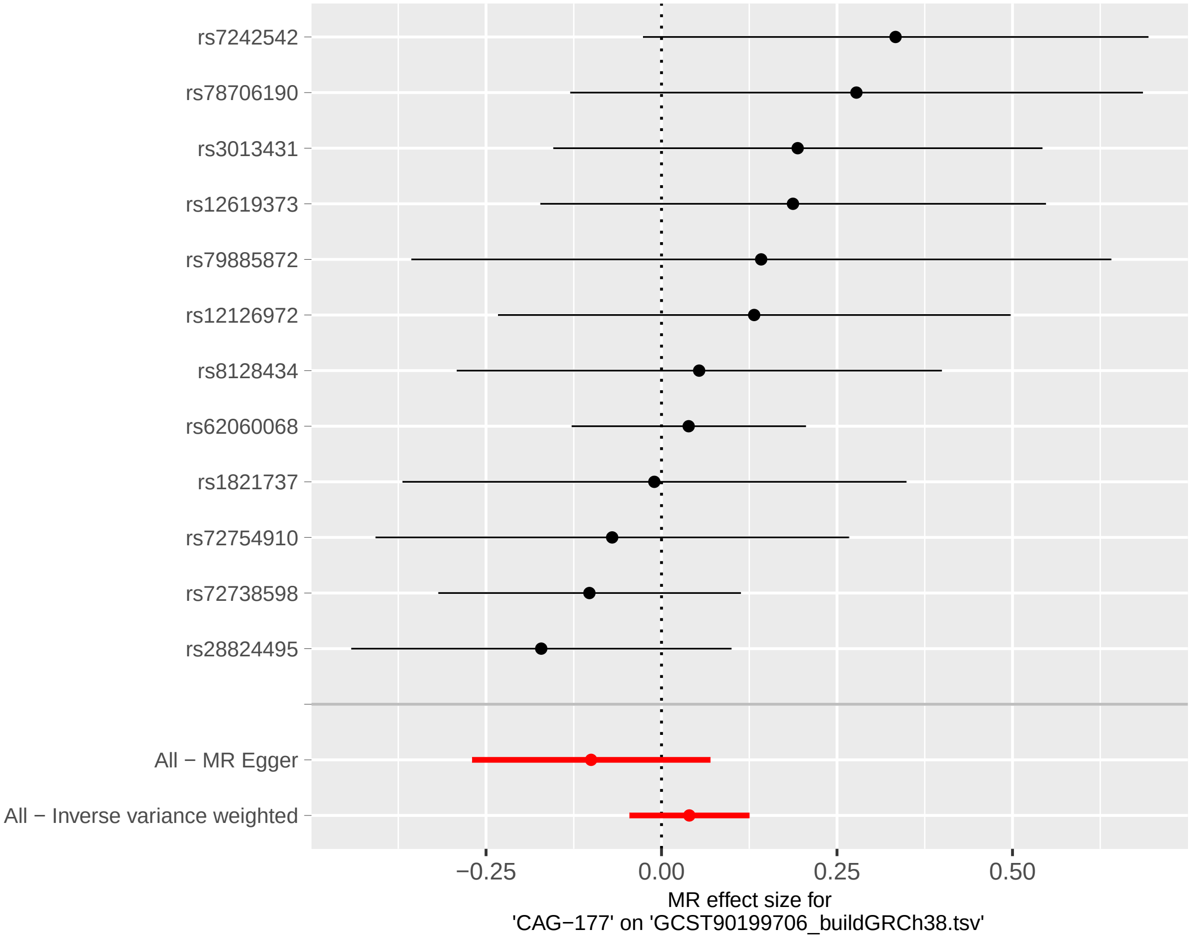
rs7242542
rs78706190
rs3013431
rs12619373
rs79885872
rs12126972
rs8128434
rs62060068
rs1821737
rs72754910
rs72738598
rs28824495
All − MR Egger
All − Inverse variance weighted
−0.25
0.00
0.25
0.50
MR effect size for
'CAG−177' on 'GCST90199706_buildGRCh38.tsv'

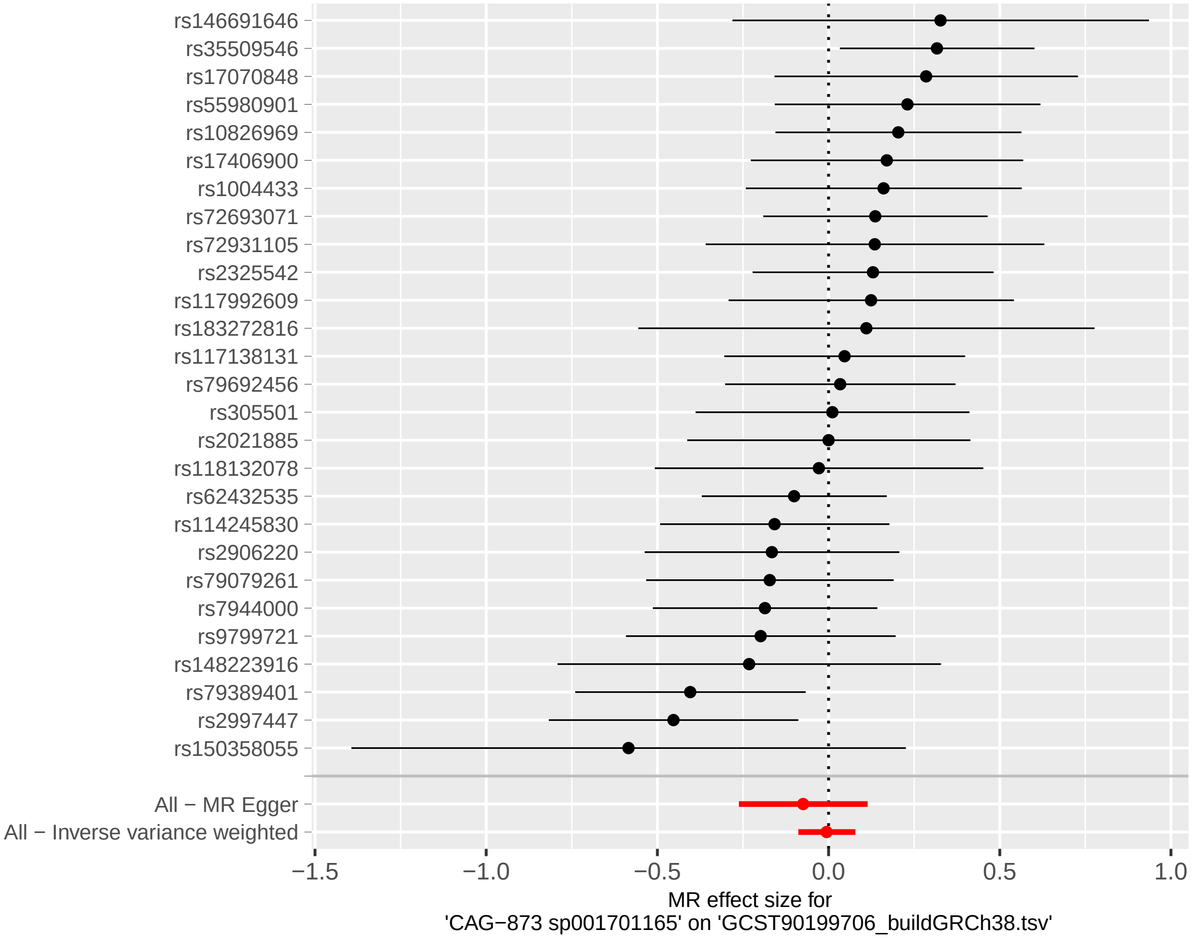
rs146691646
rs35509546
rs17070848
rs55980901
rs10826969
rs17406900
rs1004433
rs72693071
rs72931105
rs2325542
rs117992609
rs183272816
rs117138131
rs79692456
rs305501
rs2021885
rs118132078
rs62432535
rs114245830
rs2906220
rs79079261
rs7944000
rs9799721
rs148223916
rs79389401
rs2997447
rs150358055
All − MR Egger
All − Inverse variance weighted
−1.5
−1.0
−0.5
0.0
0.5
1.0
MR effect size for
'CAG−873 sp001701165' on 'GCST90199706_buildGRCh38.tsv'

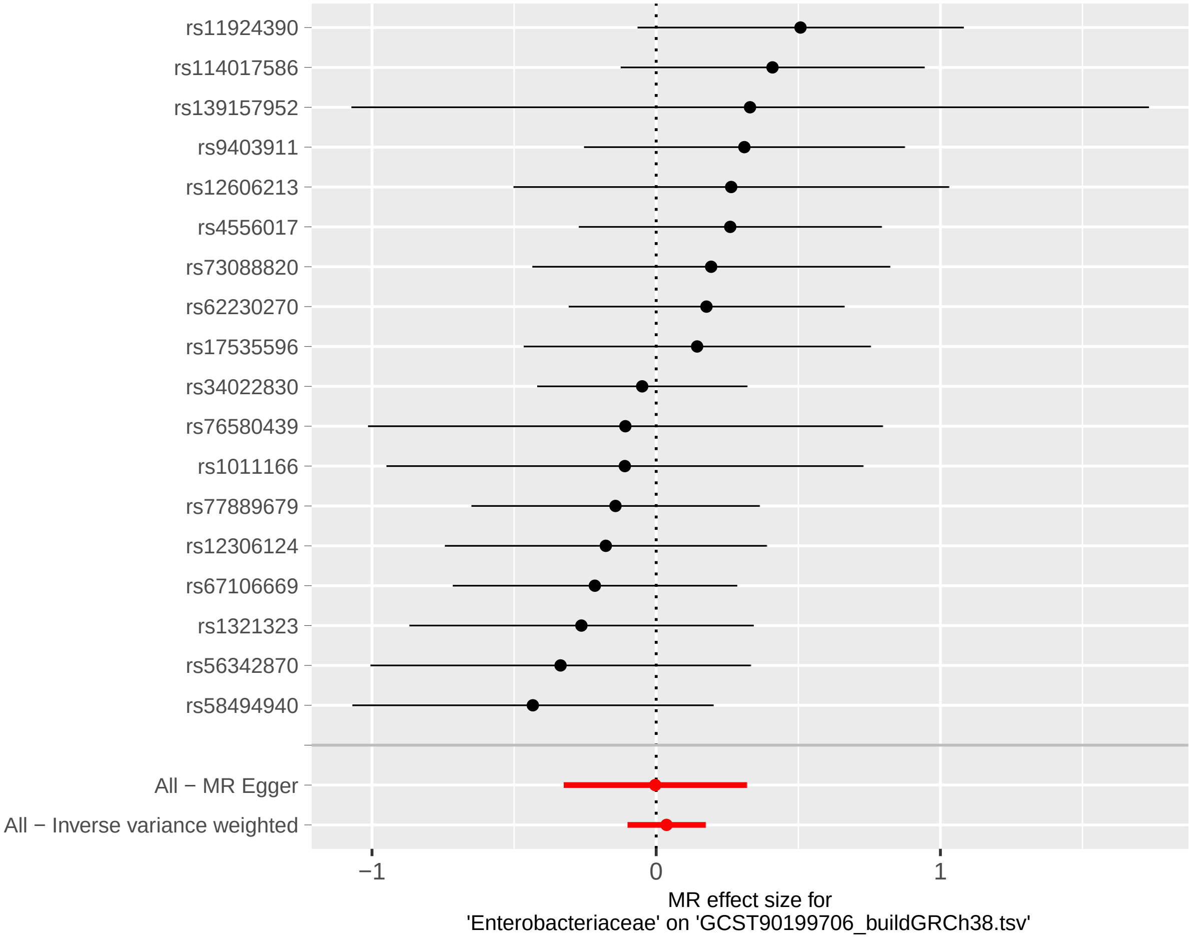

rs11924390
rs114017586
rs139157952
rs9403911
rs12606213
rs4556017
rs73088820
rs62230270
rs17535596
rs34022830
rs76580439
rs1011166
rs77889679
rs12306124
rs67106669
rs1321323
rs56342870
rs58494940
All − MR Egger
All − Inverse variance weighted
−1
0
1
MR effect size for
'Enterobacteriaceae' on 'GCST90199706_buildGRCh38.tsv'

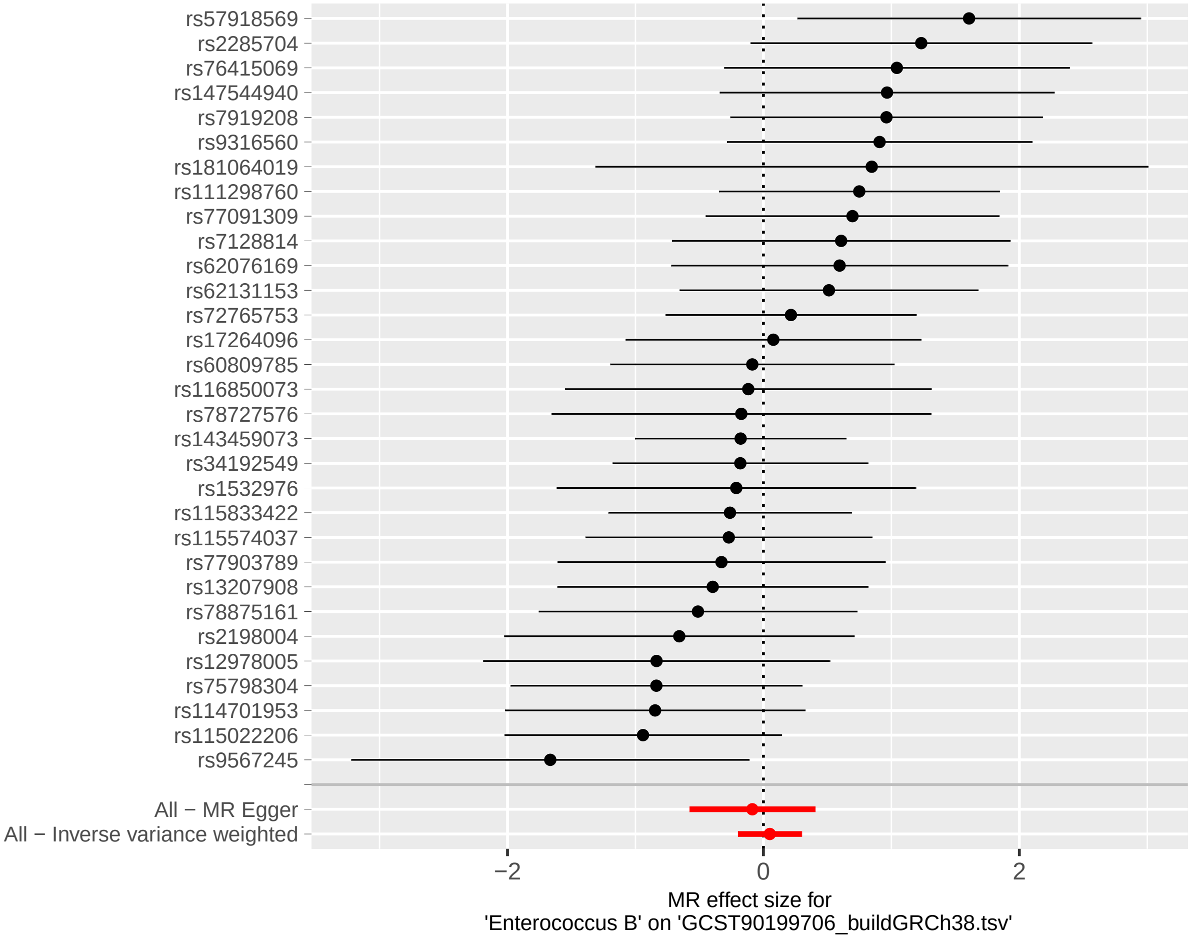
rs57918569
rs2285704
rs76415069
rs147544940
rs7919208
rs9316560
rs181064019
rs111298760
rs77091309
rs7128814
rs62076169
rs62131153
rs72765753
rs17264096
rs60809785
rs116850073
rs78727576
rs143459073
rs34192549
rs1532976
rs115833422
rs115574037
rs77903789
rs13207908
rs78875161
rs2198004
rs12978005
rs75798304
rs114701953
rs115022206
rs9567245
All − MR Egger
All − Inverse variance weighted
−2
0
2
MR effect size for
'Enterococcus B' on 'GCST90199706_buildGRCh38.tsv'

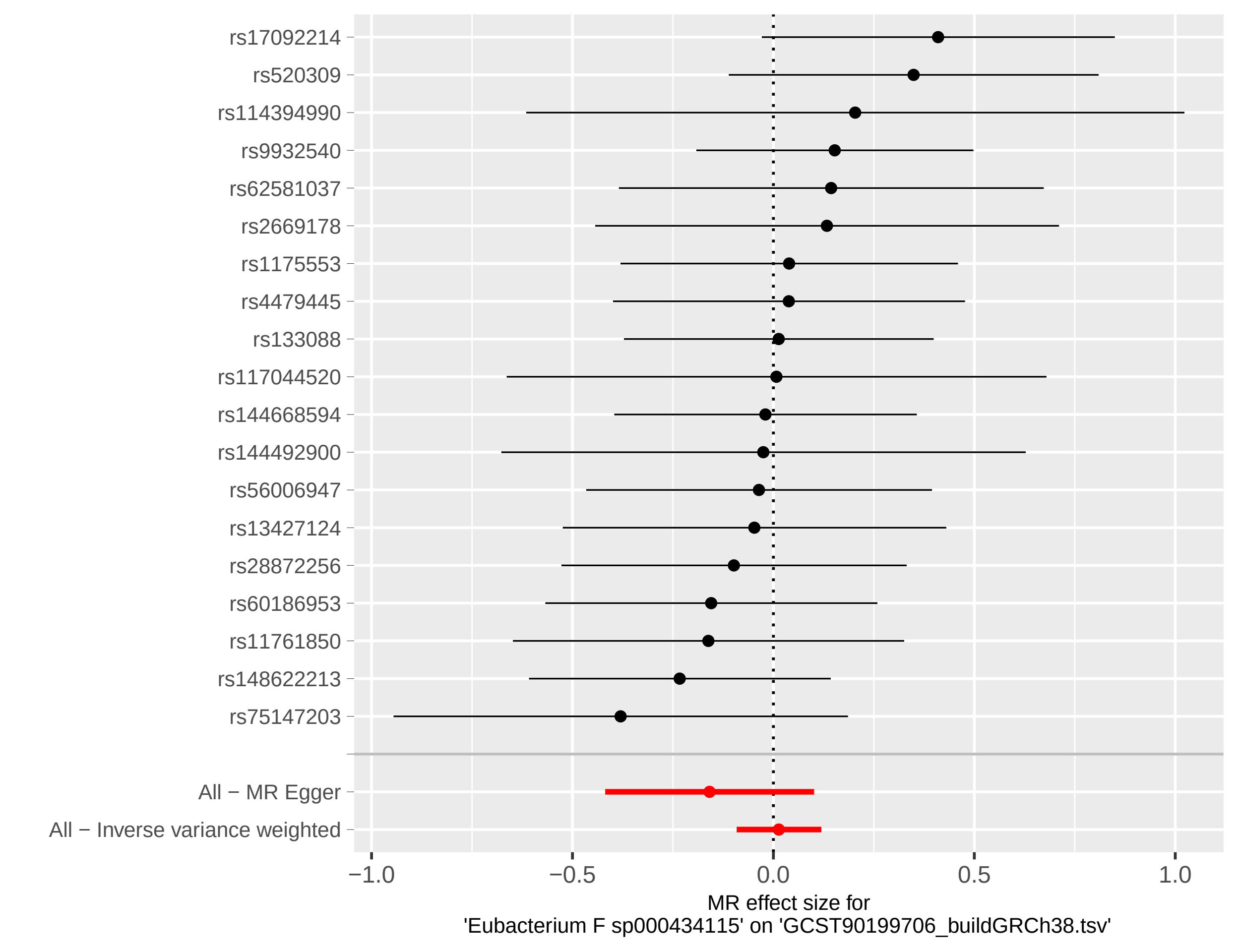

rs17092214
rs520309
rs114394990
rs9932540
rs62581037
rs2669178
rs1175553
rs4479445
rs133088
rs117044520
rs144668594
rs144492900
rs56006947
rs13427124
rs28872256
rs60186953
rs11761850
rs148622213
rs75147203
All − MR Egger
All − Inverse variance weighted
−1.0
−0.5
0.0
0.5
1.0
MR effect size for
'Eubacterium F sp000434115' on 'GCST90199706_buildGRCh38.tsv'

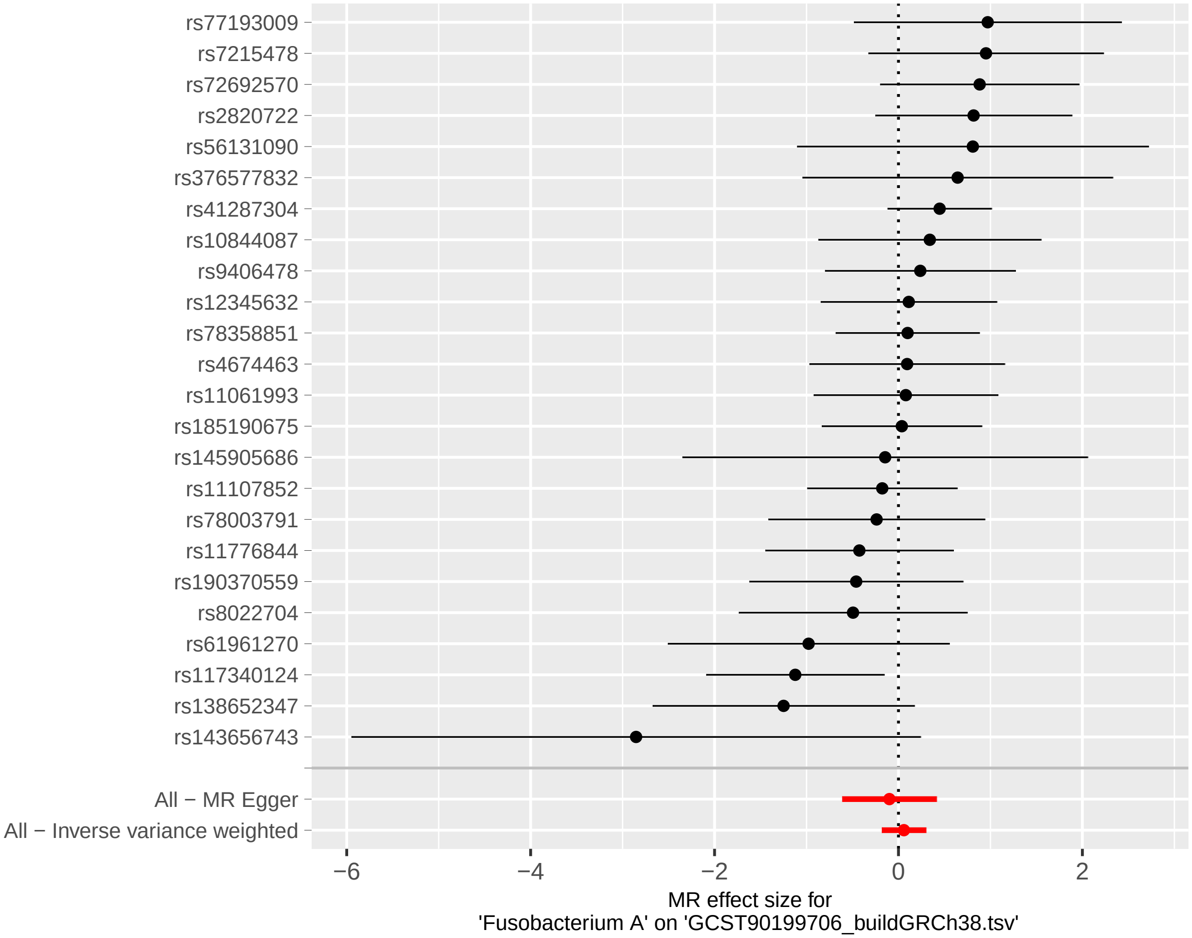

rs77193009
rs7215478
rs72692570
rs2820722
rs56131090
rs376577832
rs41287304
rs10844087
rs9406478
rs12345632
rs78358851
rs4674463
rs11061993
rs185190675
rs145905686
rs11107852
rs78003791
rs11776844
rs190370559
rs8022704
rs61961270
rs117340124
rs138652347
rs143656743
All − MR Egger
All − Inverse variance weighted
−6
−4
−2
0
2
MR effect size for
'Fusobacterium A' on 'GCST90199706_buildGRCh38.tsv'

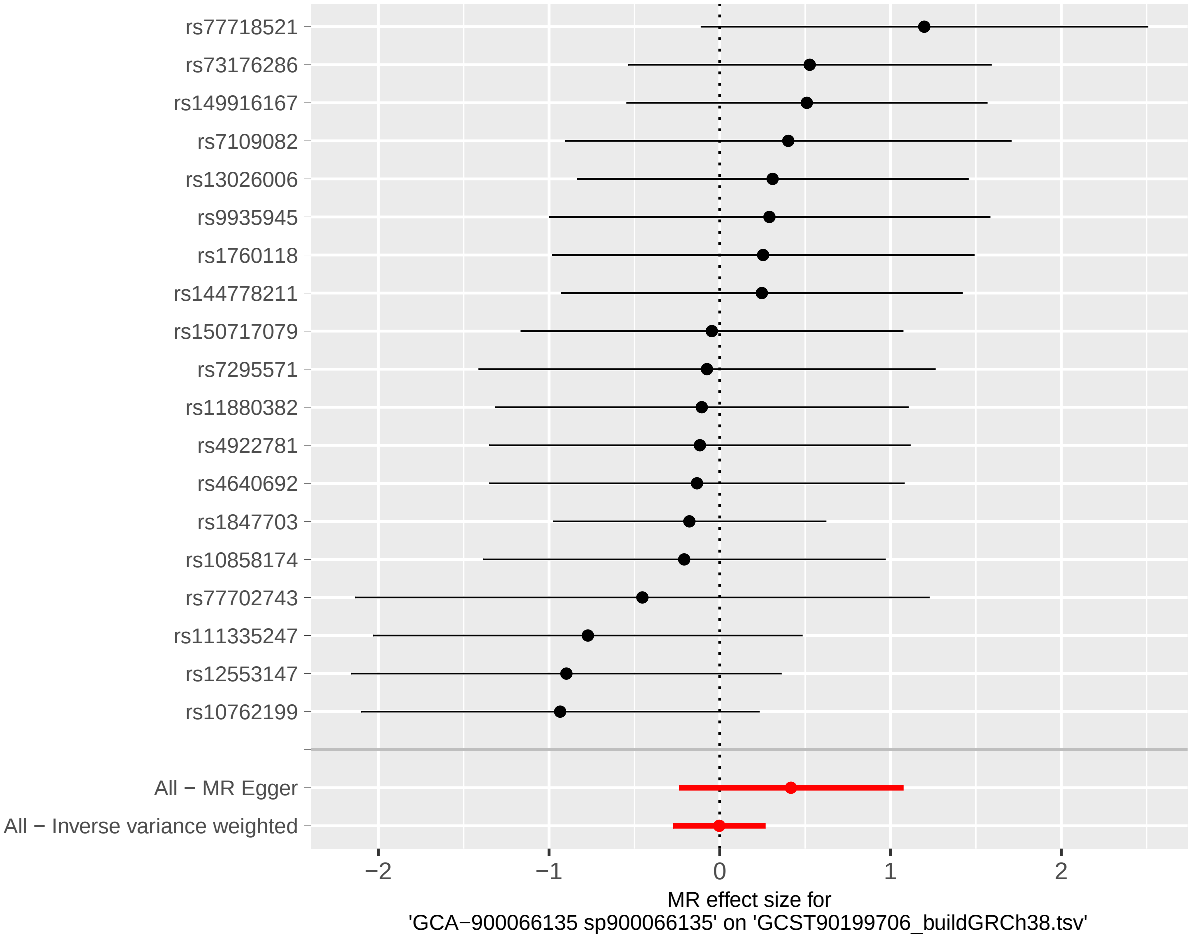
rs77718521
rs73176286
rs149916167
rs7109082
rs13026006
rs9935945
rs1760118
rs144778211
rs150717079
rs7295571
rs11880382
rs4922781
rs4640692
rs1847703
rs10858174
rs77702743
rs111335247
rs12553147
rs10762199
All − MR Egger
All − Inverse variance weighted
−2
−1
0
1
2
MR effect size for
'GCA−900066135 sp900066135' on 'GCST90199706_buildGRCh38.tsv'

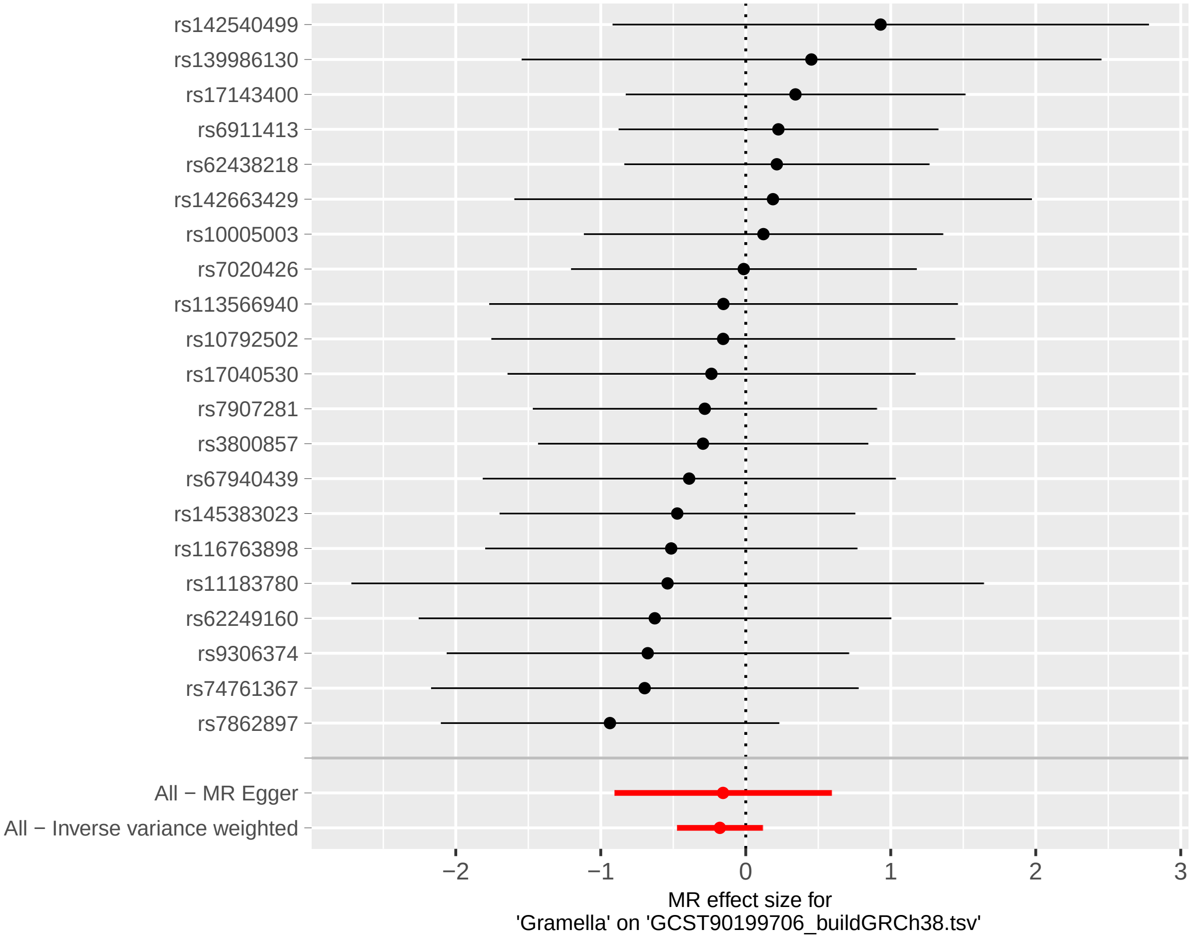
rs142540499
rs139986130
rs17143400
rs6911413
rs62438218
rs142663429
rs10005003
rs7020426
rs113566940
rs10792502
rs17040530
rs7907281
rs3800857
rs67940439
rs145383023
rs116763898
rs11183780
rs62249160
rs9306374
rs74761367
rs7862897
All − MR Egger
All − Inverse variance weighted
−2
−1
0
1
2
3
MR effect size for
'Gramella' on 'GCST90199706_buildGRCh38.tsv'

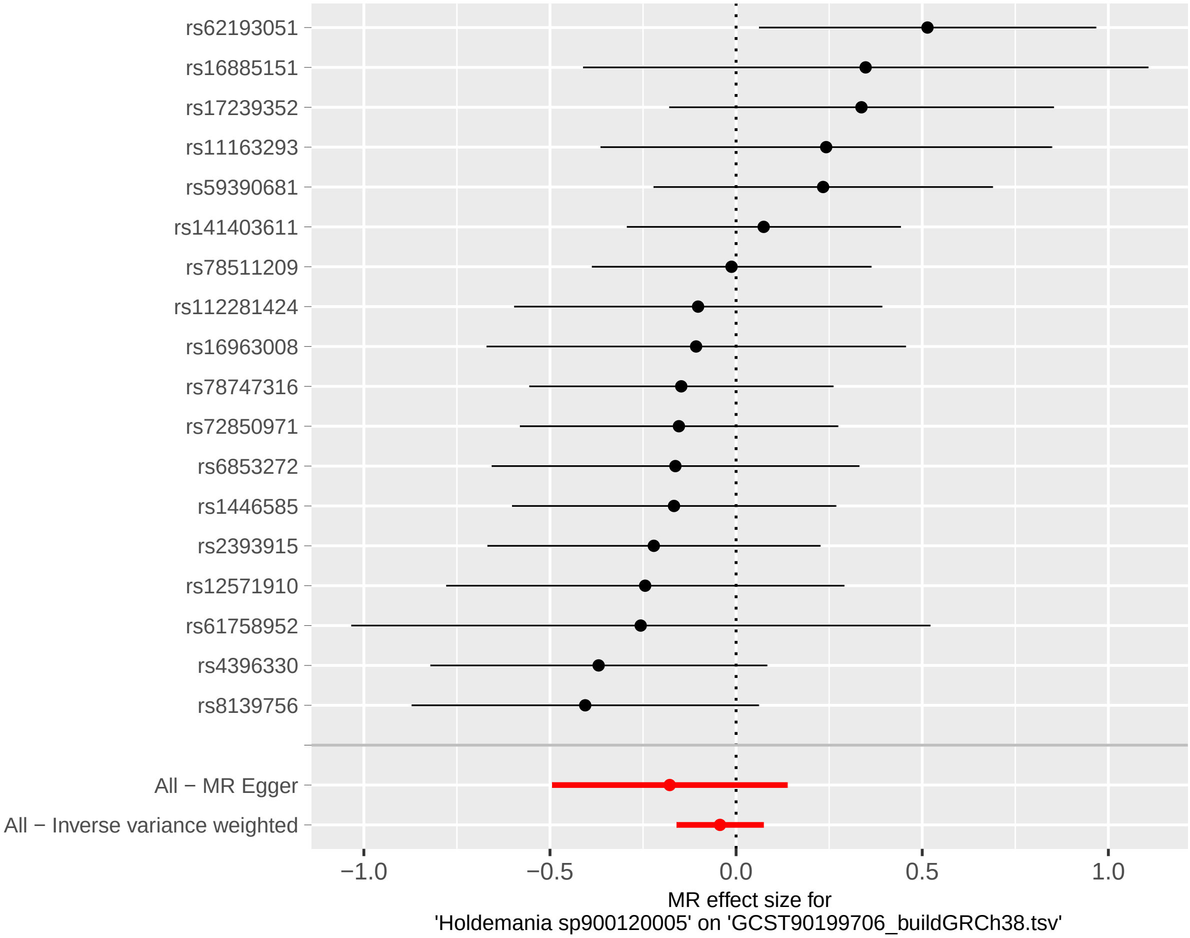
rs62193051
rs16885151
rs17239352
rs11163293
rs59390681
rs141403611
rs78511209
rs112281424
rs16963008
rs78747316
rs72850971
rs6853272
rs1446585
rs2393915
rs12571910
rs61758952
rs4396330
rs8139756
All − MR Egger
All − Inverse variance weighted
−1.0
−0.5
0.0
0.5
1.0
MR effect size for
'Holdemania sp900120005' on 'GCST90199706_buildGRCh38.tsv'

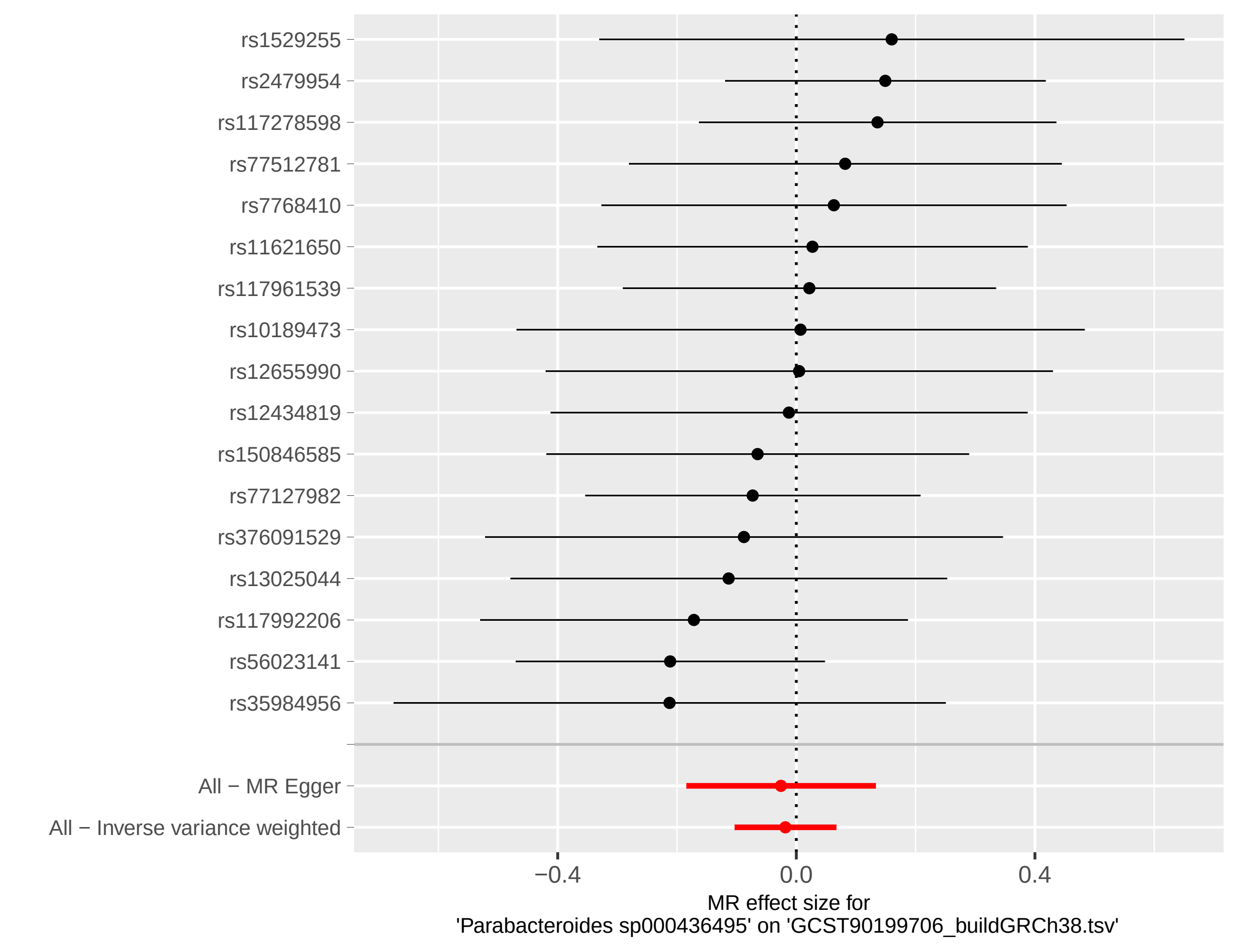

rs1529255
rs2479954
rs117278598
rs77512781
rs7768410
rs11621650
rs117961539
rs10189473
rs12655990
rs12434819
rs150846585
rs77127982
rs376091529
rs13025044
rs117992206
rs56023141
rs35984956
All − MR Egger
All − Inverse variance weighted
−0.4
0.0
0.4
MR effect size for
'Parabacteroides sp000436495' on 'GCST90199706_buildGRCh38.tsv'

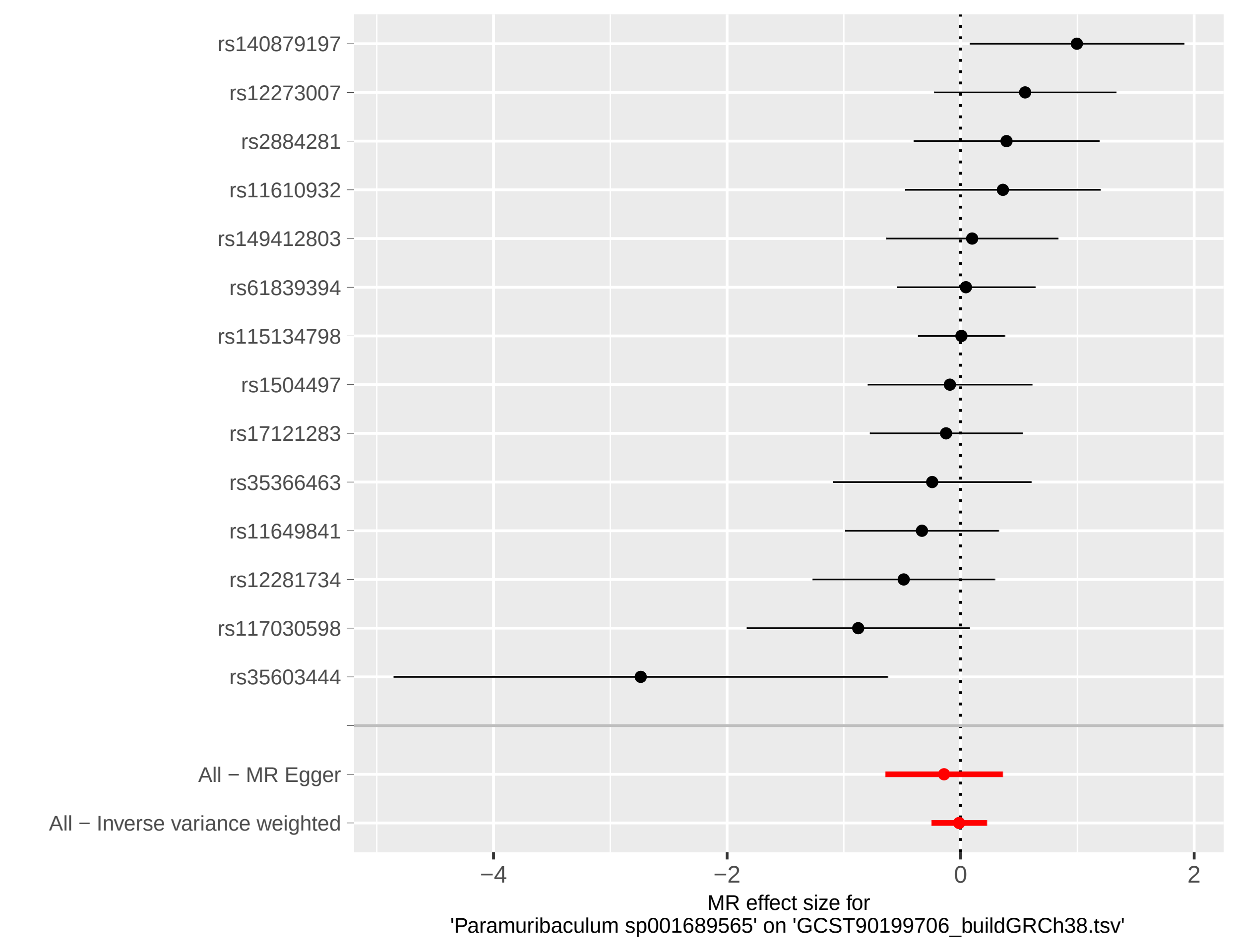

rs140879197
rs12273007
rs2884281
rs11610932
rs149412803
rs61839394
rs115134798
rs1504497
rs17121283
rs35366463
rs11649841
rs12281734
rs117030598
rs35603444
All − MR Egger
All − Inverse variance weighted
−4
−2
0
2
MR effect size for
'Paramuribaculum sp001689565' on 'GCST90199706_buildGRCh38.tsv'

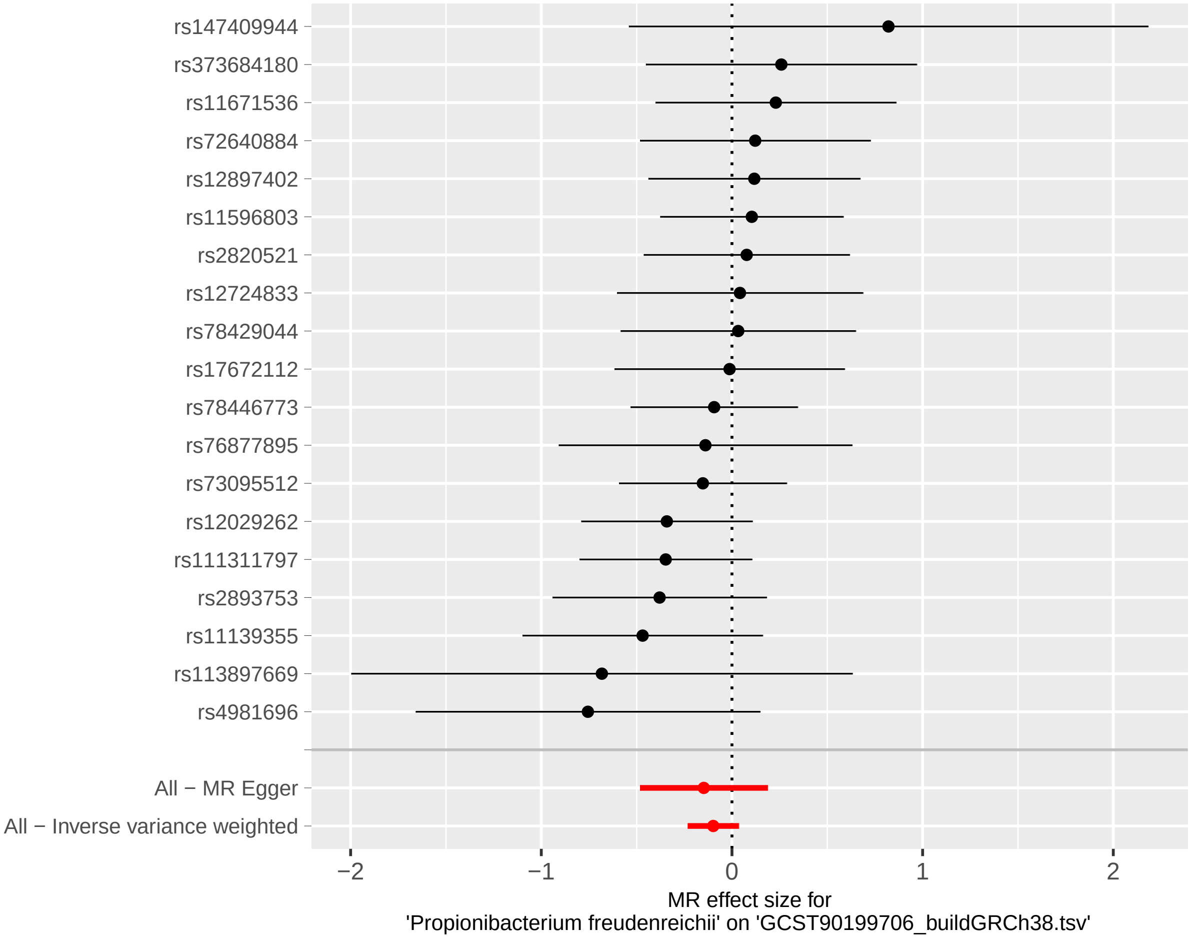
rs147409944
rs373684180
rs11671536
rs72640884
rs12897402
rs11596803
rs2820521
rs12724833
rs78429044
rs17672112
rs78446773
rs76877895
rs73095512
rs12029262
rs111311797
rs2893753
rs11139355
rs113897669
rs4981696
All − MR Egger
All − Inverse variance weighted
−2
−1
0
1
2
MR effect size for
'Propionibacterium freudenreichii' on 'GCST90199706_buildGRCh38.tsv'

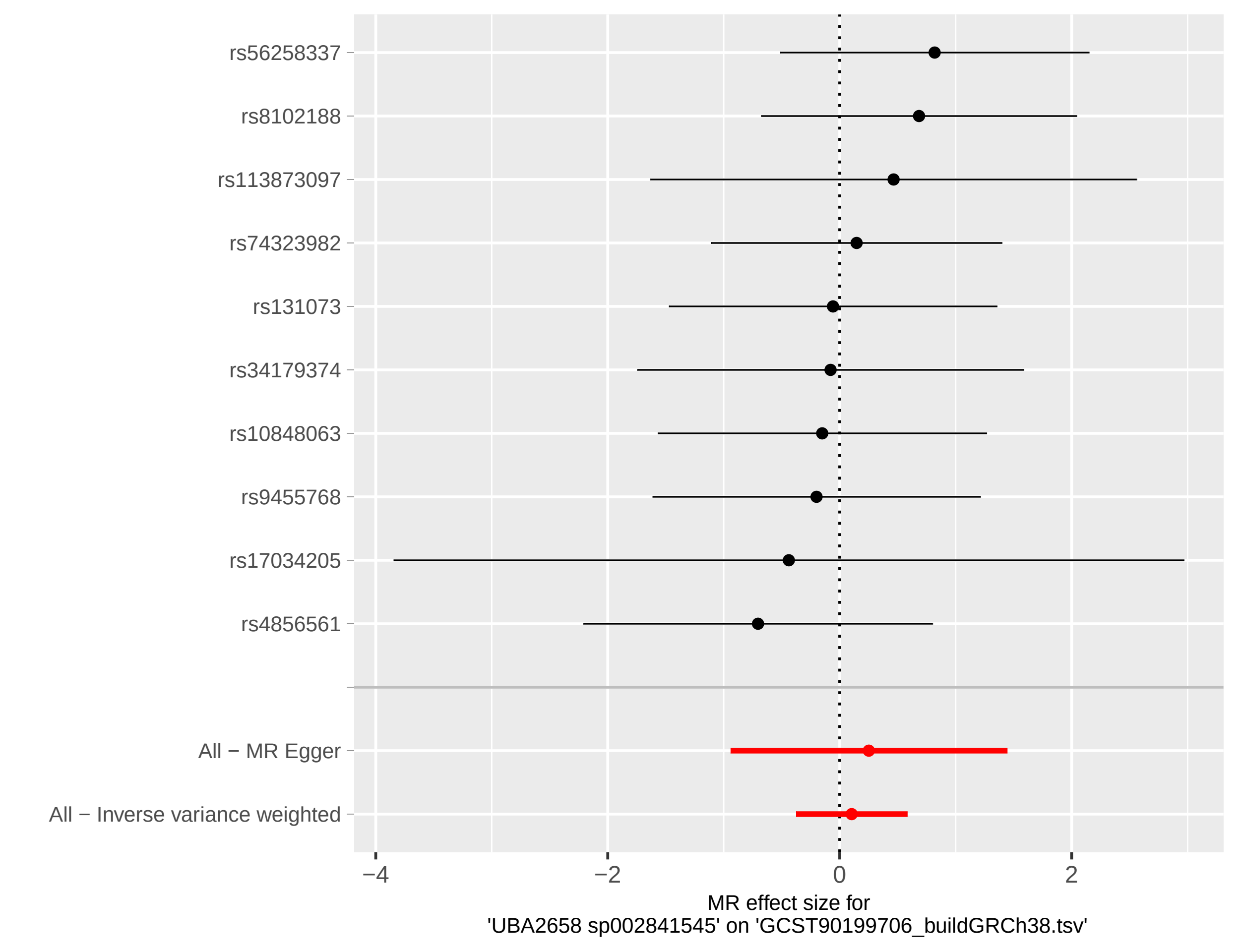

rs56258337
rs8102188
rs113873097
rs74323982
rs131073
rs34179374
rs10848063
rs9455768
rs17034205
rs4856561
All − MR Egger
All − Inverse variance weighted
−4
−2
0
2
MR effect size for
'UBA2658 sp002841545' on 'GCST90199706_buildGRCh38.tsv'

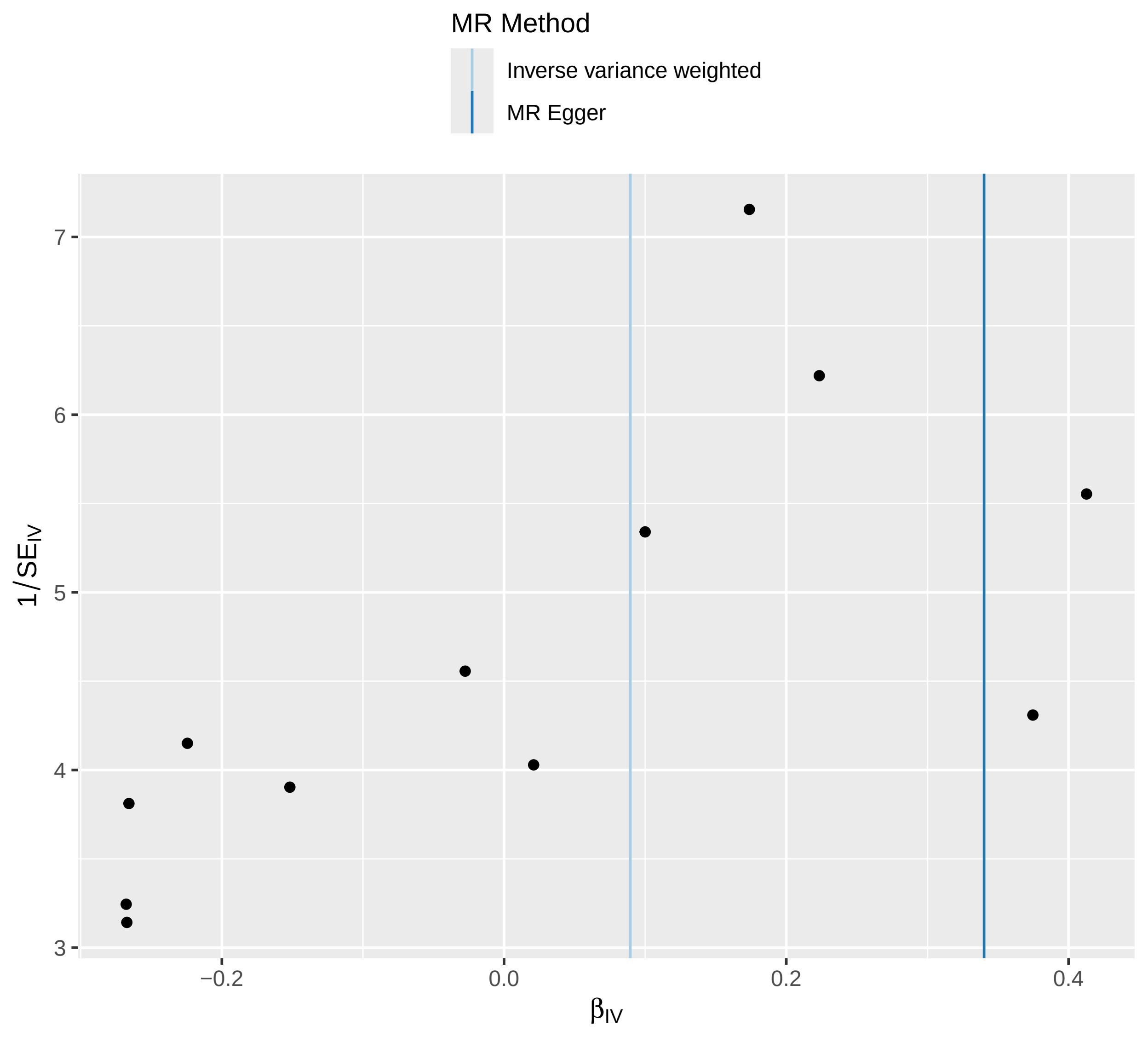
MR Method
Inverse variance weighted
MR Egger
1/SE_IV
β_IV
7
6
5
4
3
−0.2
0.0
0.2
0.4

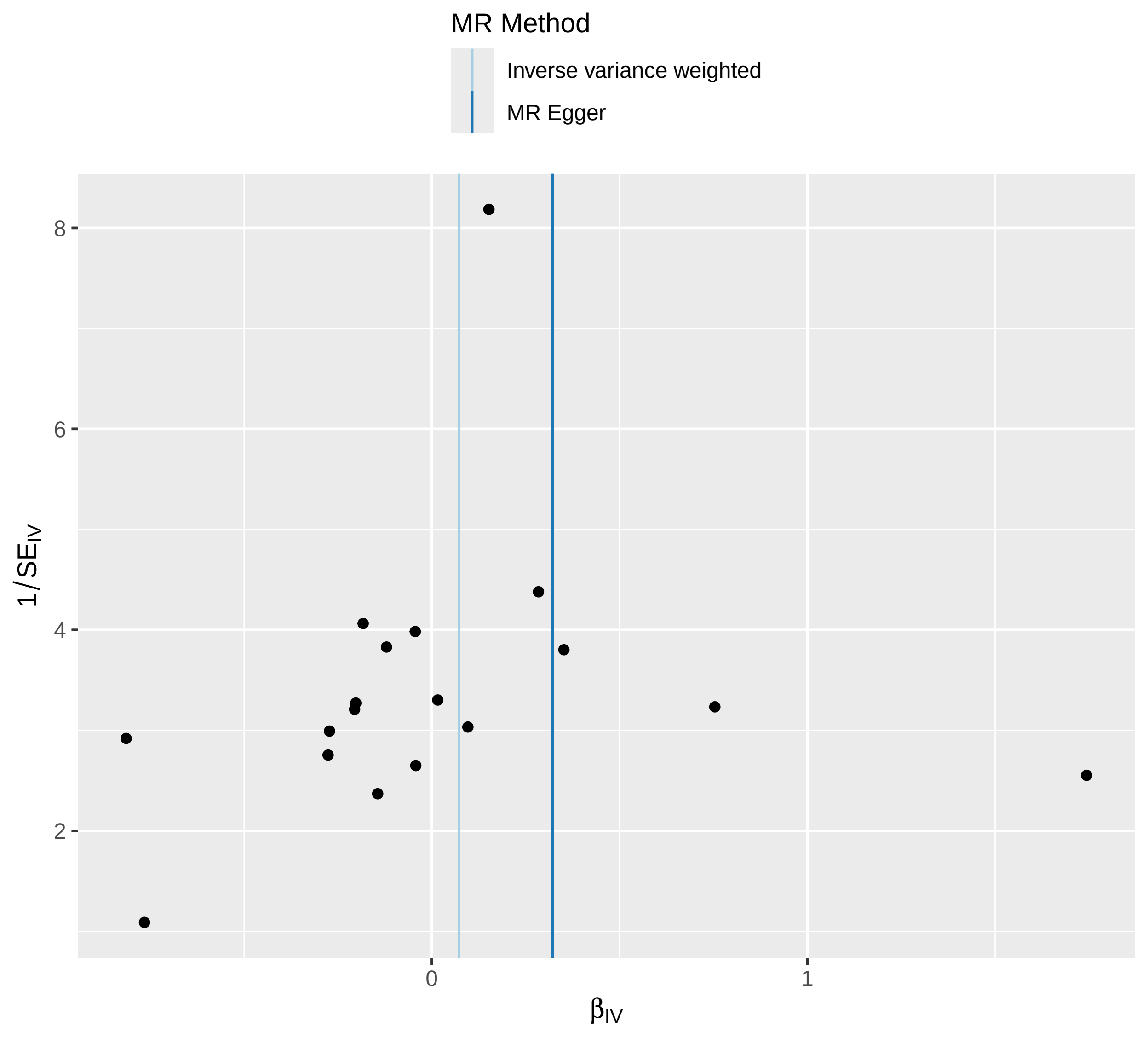
MR Method
Inverse variance weighted
MR Egger
8
6
4
2
0
1
$1/SE_{IV}$
β_{IV}

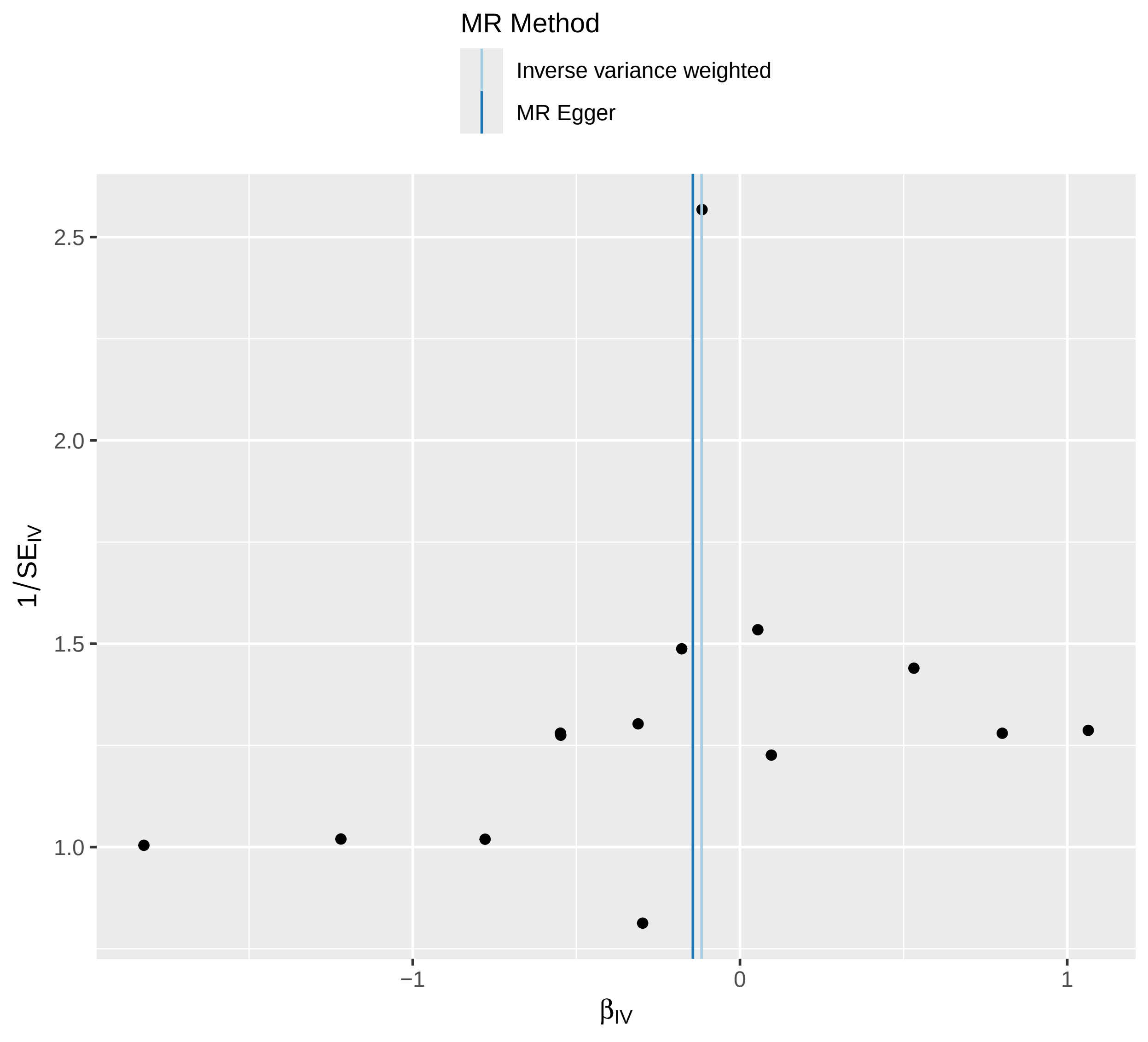

MR Method
Inverse variance weighted
MR Egger
2.5
2.0
1.5
1.0
$1/SE_{IV}$
−1
0
1
β_{IV}

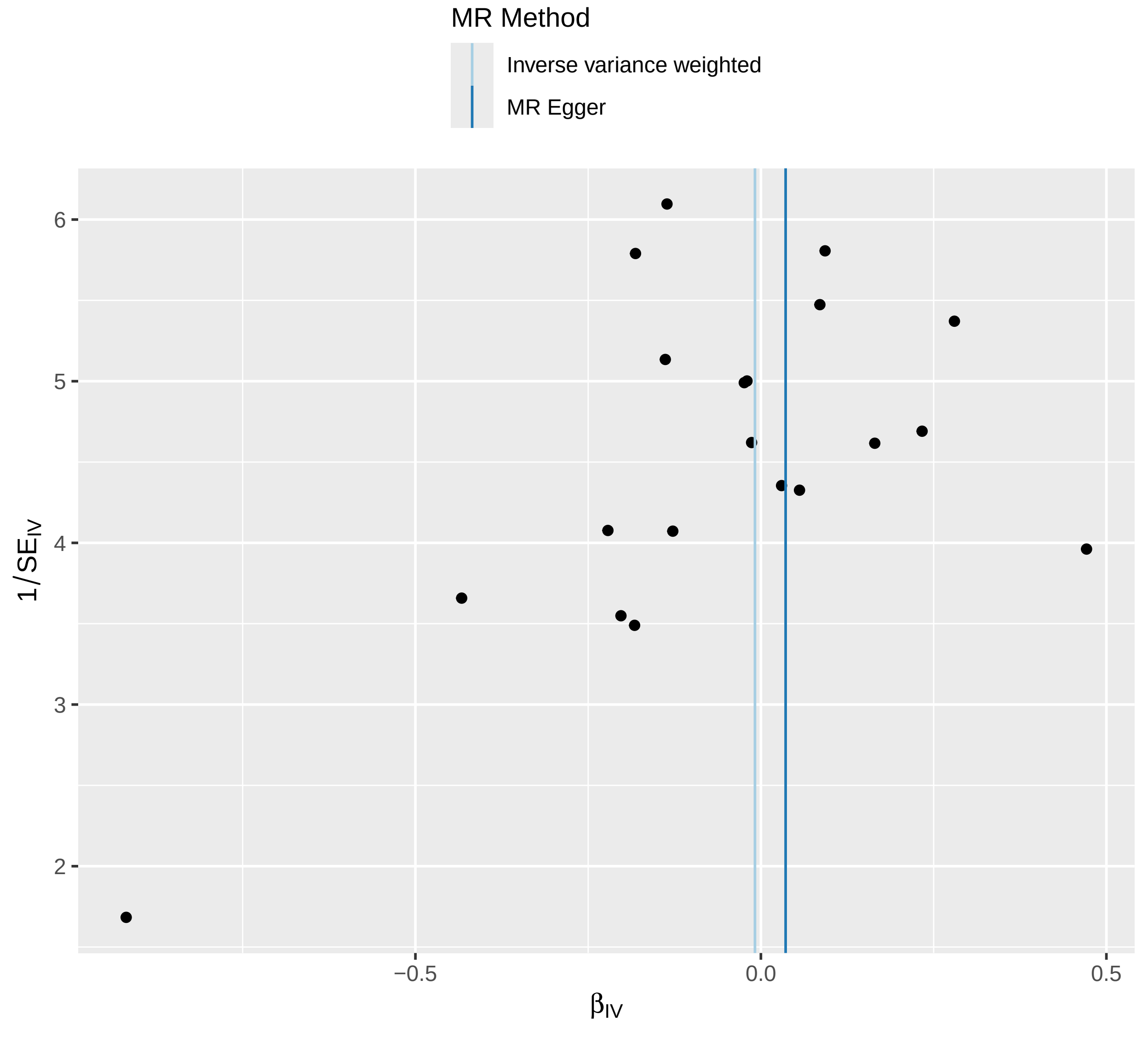

MR Method
Inverse variance weighted
MR Egger
6
5
4
3
2
1/SE_IV
−0.5
0.0
0.5
β_IV

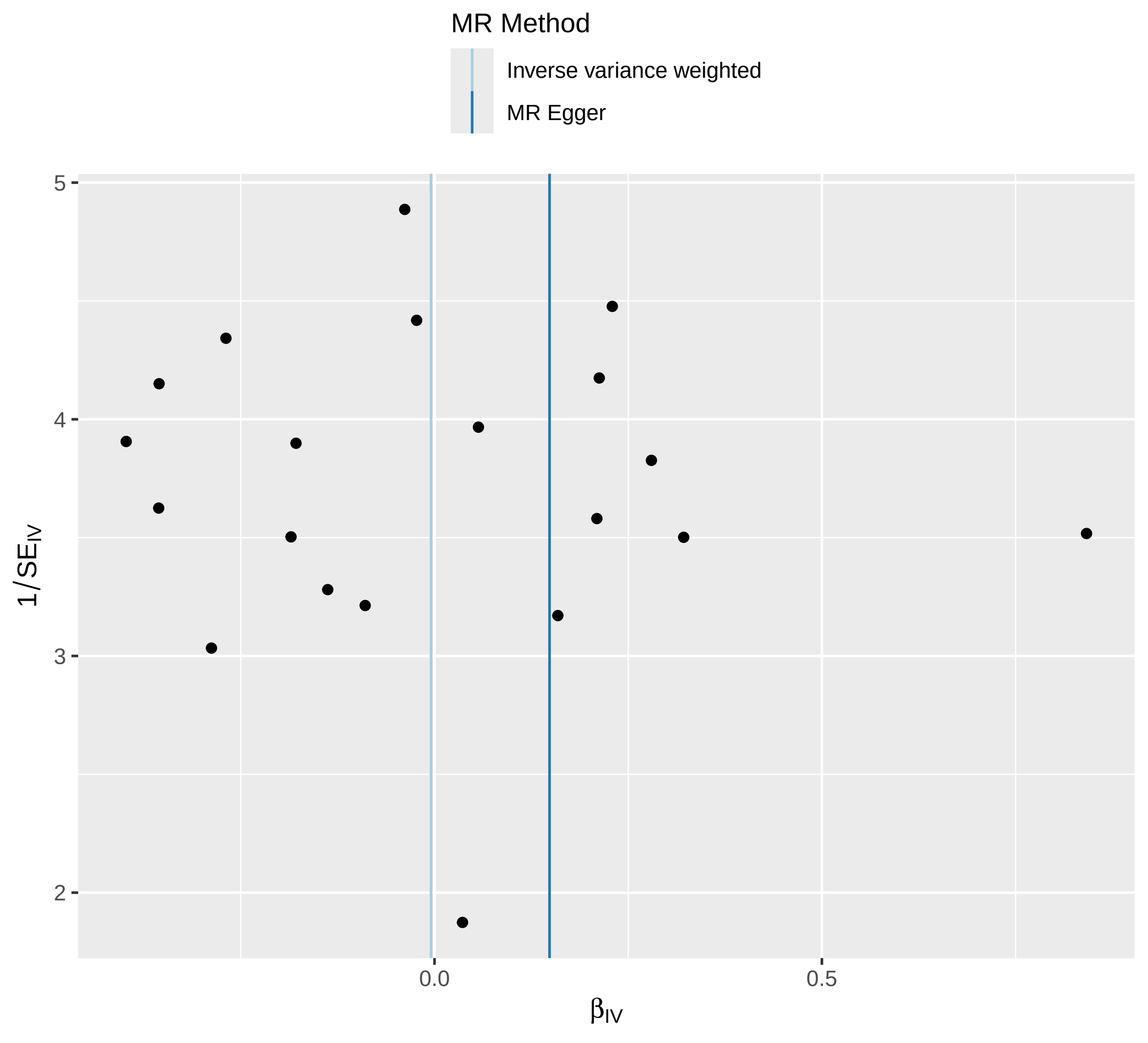
MR Method
Inverse variance weighted
MR Egger
5
4
3
2
1/SE_IV
0.0
0.5
β_IV

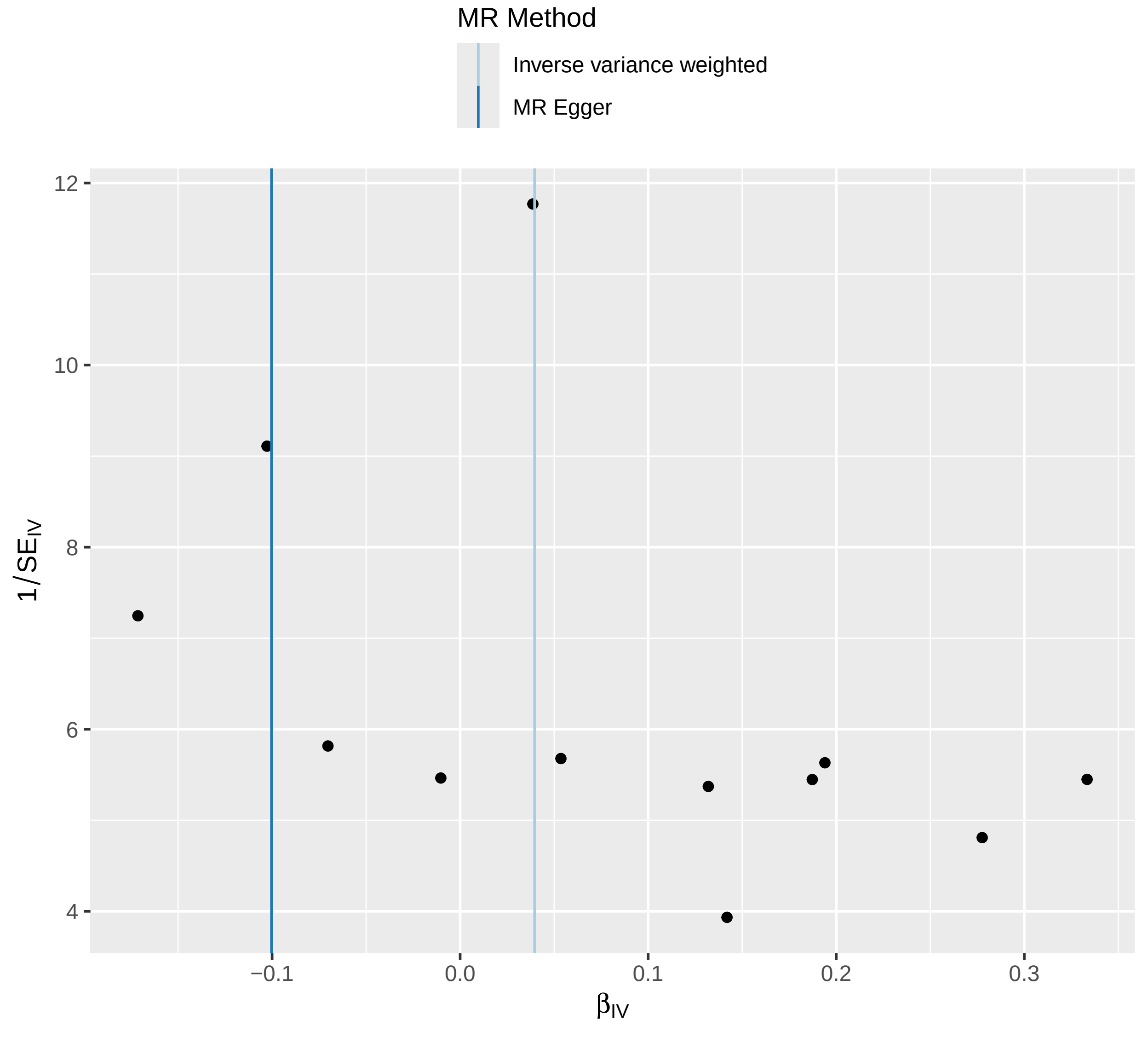
MR Method
Inverse variance weighted
MR Egger
12
10
8
6
4
1/SE_IV
−0.1
0.0
0.1
0.2
0.3
β_IV

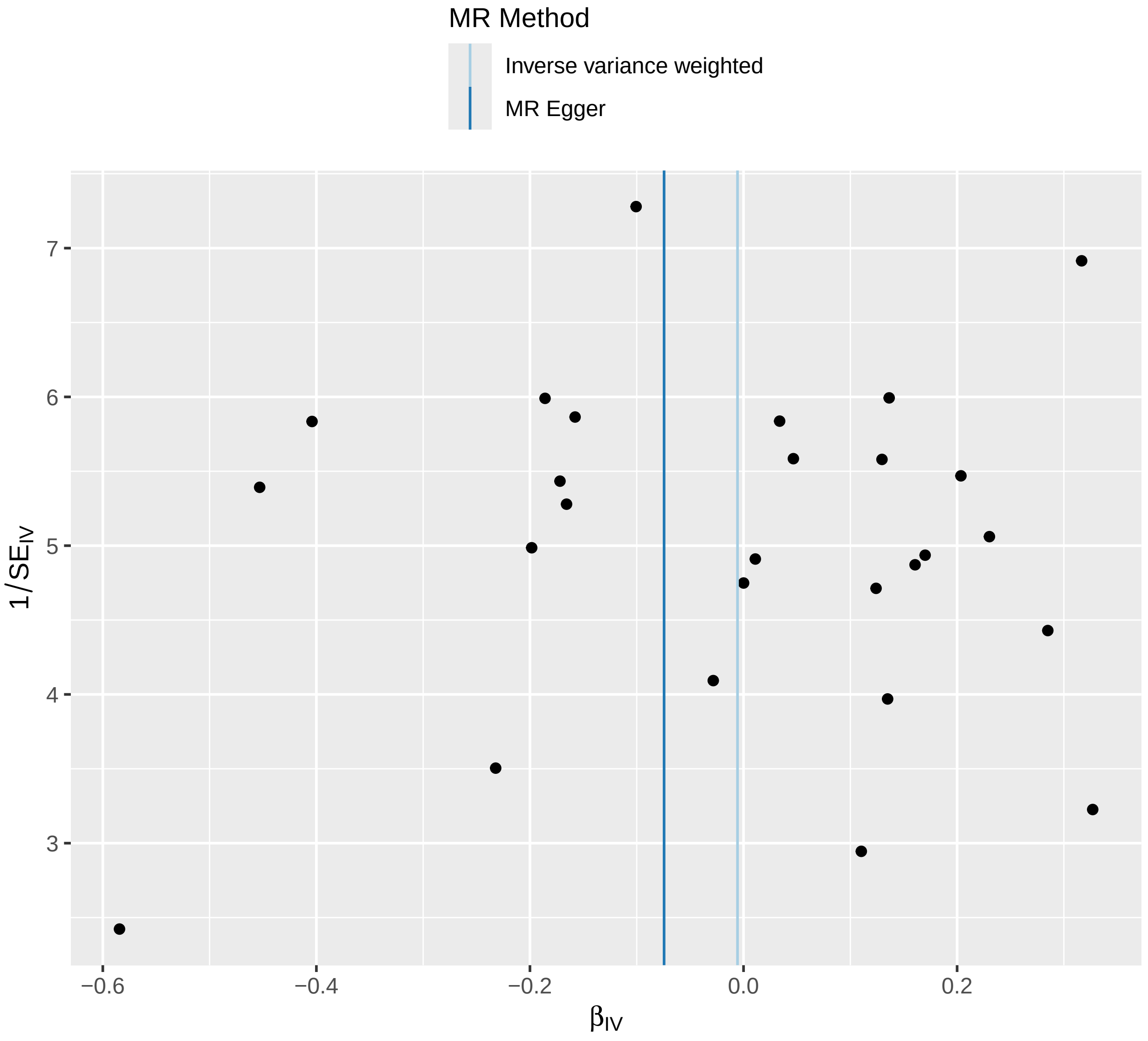

MR Method
Inverse variance weighted
MR Egger
7
6
5
4
3
1/SE$_{IV}$
−0.6
−0.4
−0.2
0.0
0.2
β$_{IV}$

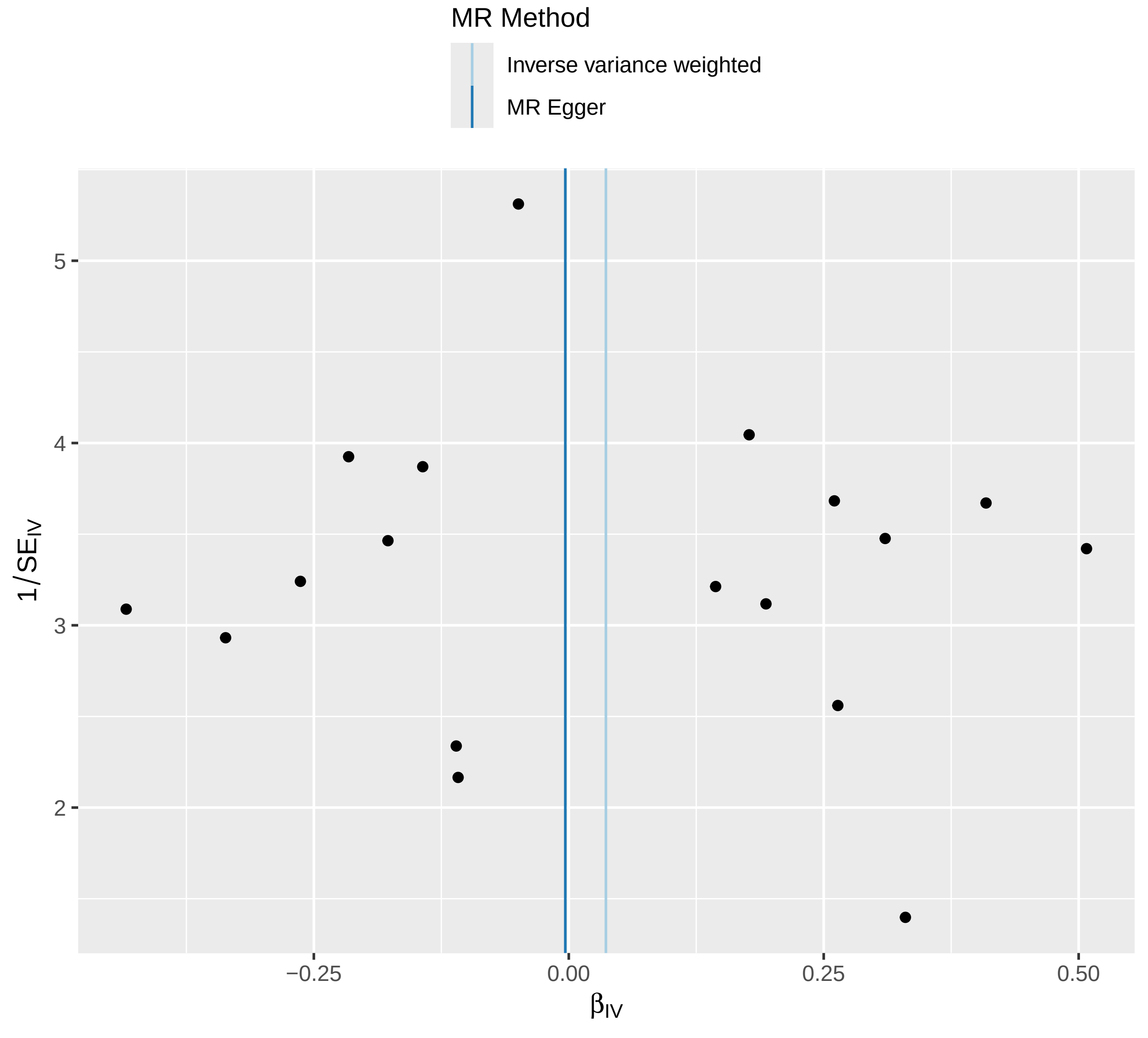

MR Method
Inverse variance weighted
MR Egger
5
4
3
2
1/SE_IV
−0.25
0.00
0.25
0.50
β_IV

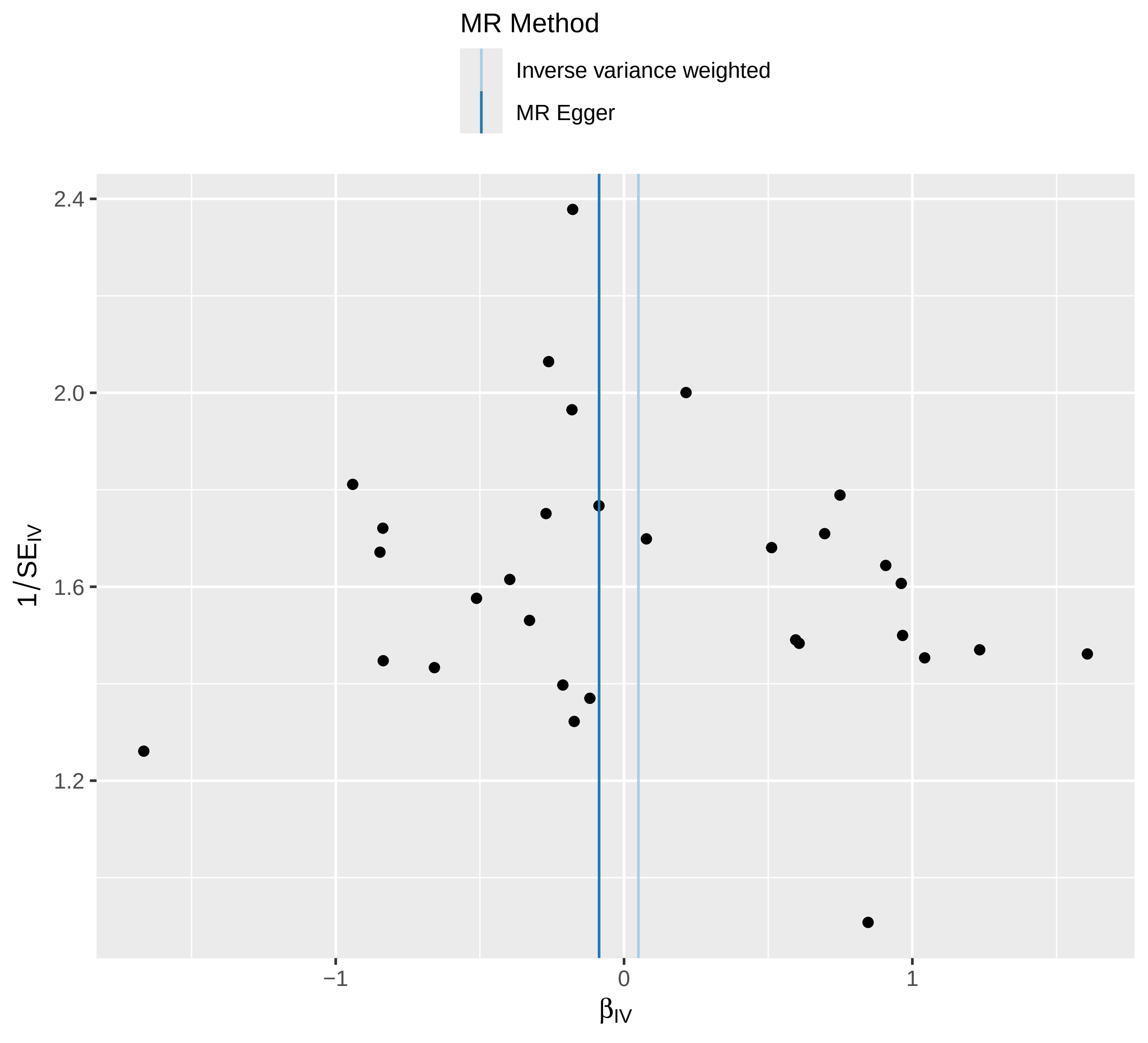
MR Method
Inverse variance weighted
MR Egger
2.4
2.0
1.6
1.2
$1/SE_{IV}$
−1
0
1
β_{IV}

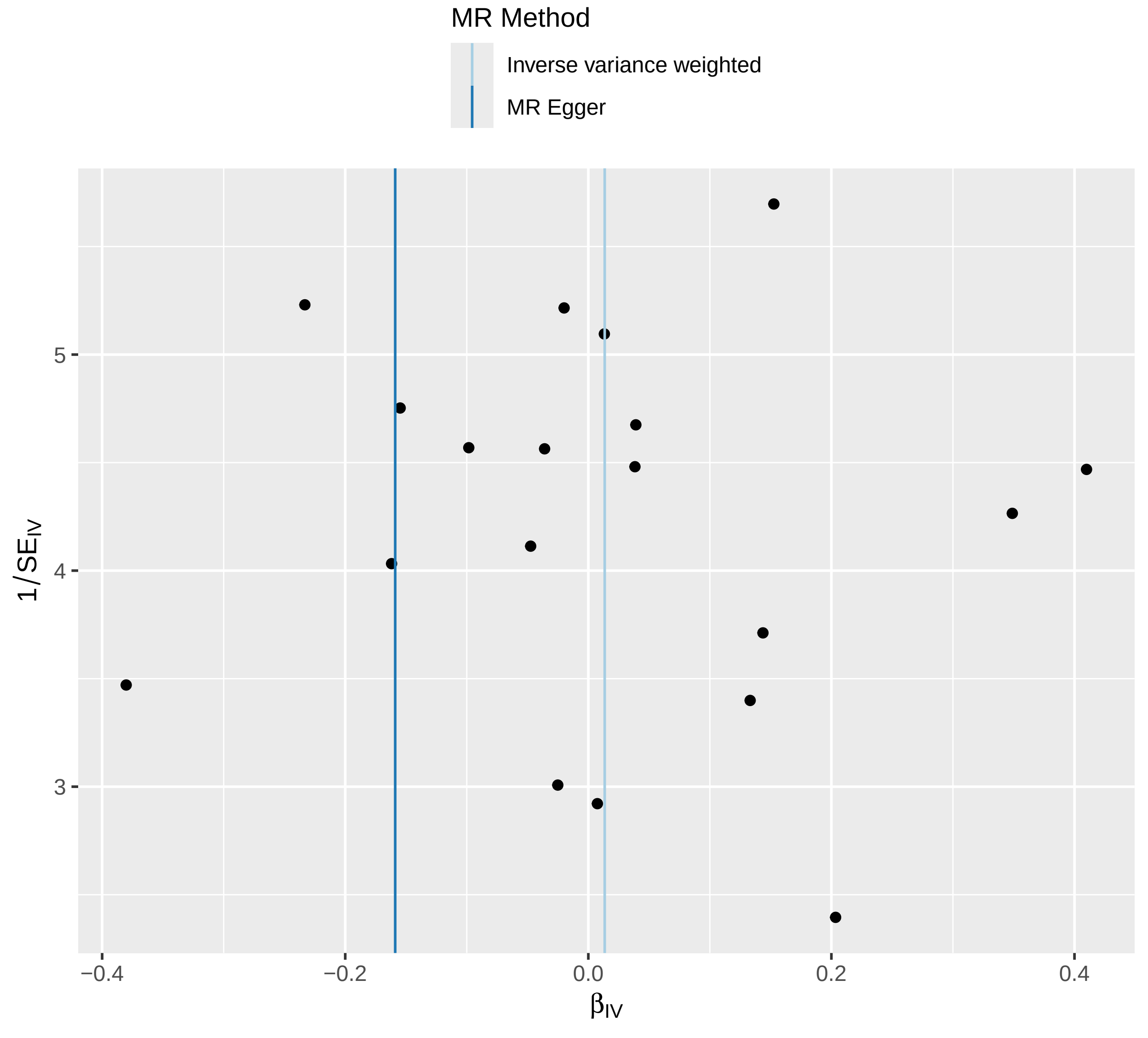

MR Method
Inverse variance weighted
MR Egger
5
4
3
1/SE_IV
−0.4
−0.2
0.0
0.2
0.4
β_IV

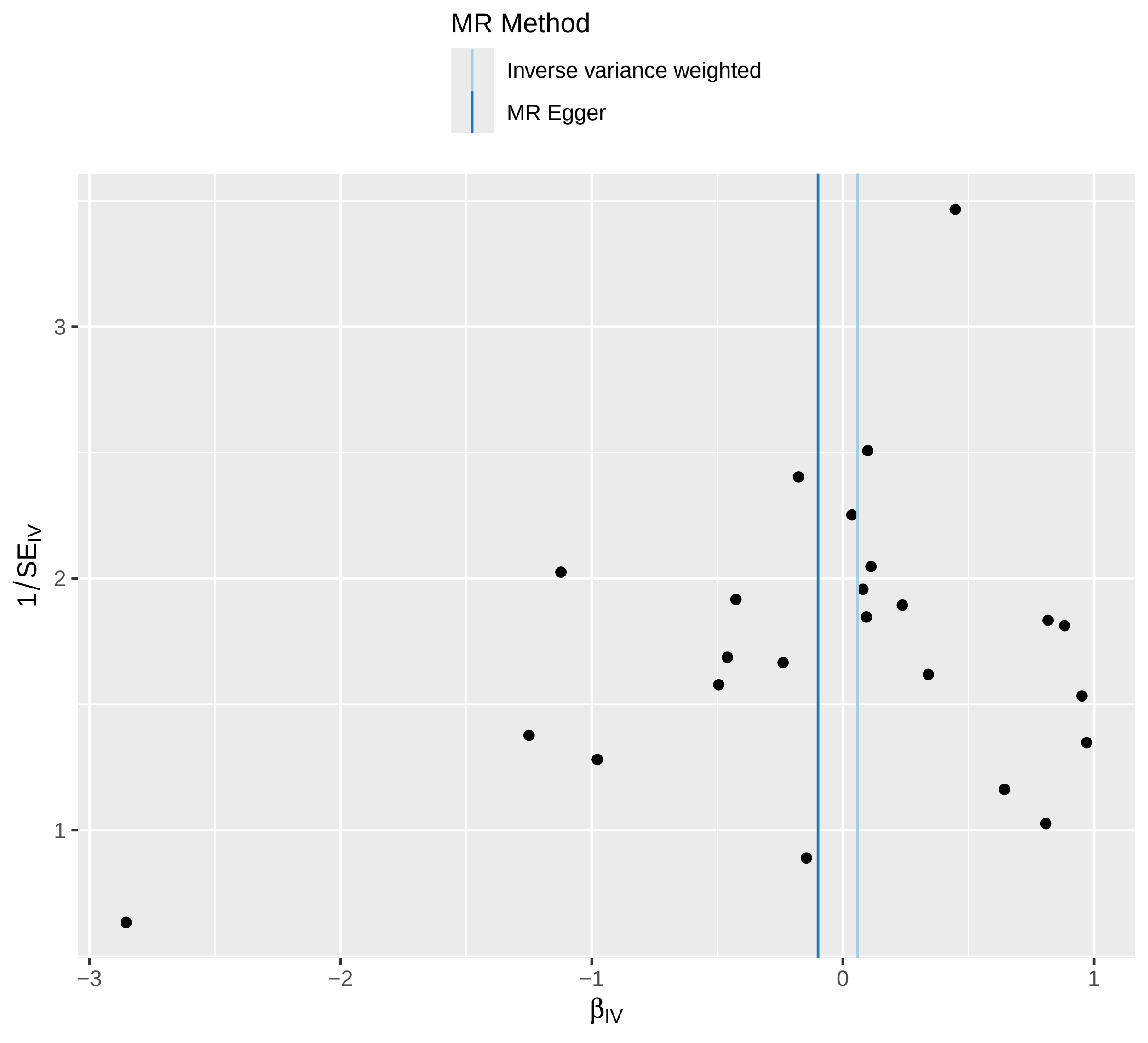
MR Method
Inverse variance weighted
MR Egger
$1/SE_{IV}$
3
2
1
−3
−2
−1
0
1
β_{IV}

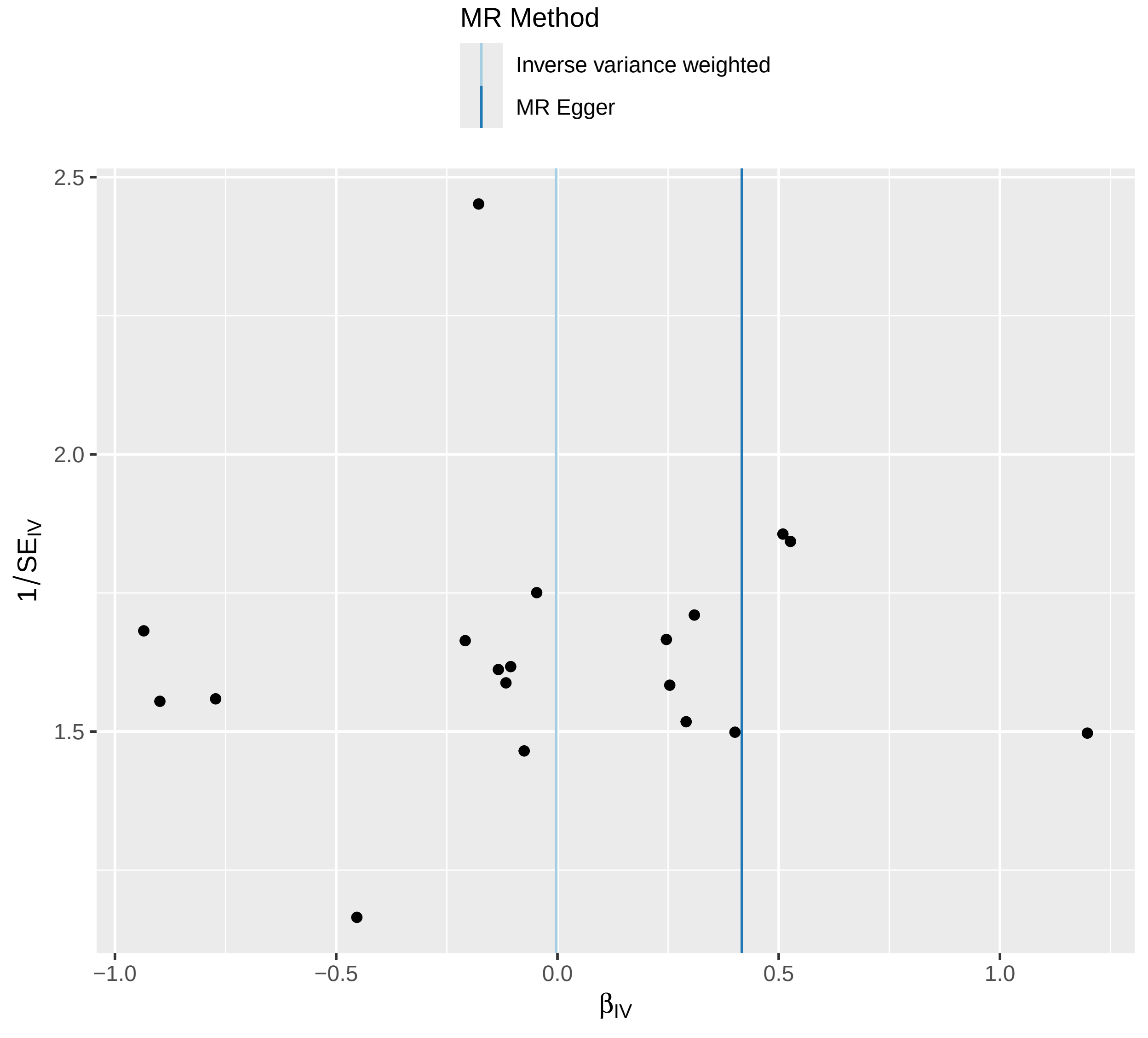

MR Method
Inverse variance weighted
MR Egger
2.5
2.0
1.5
1/SE_IV
−1.0
−0.5
0.0
0.5
1.0
β_IV

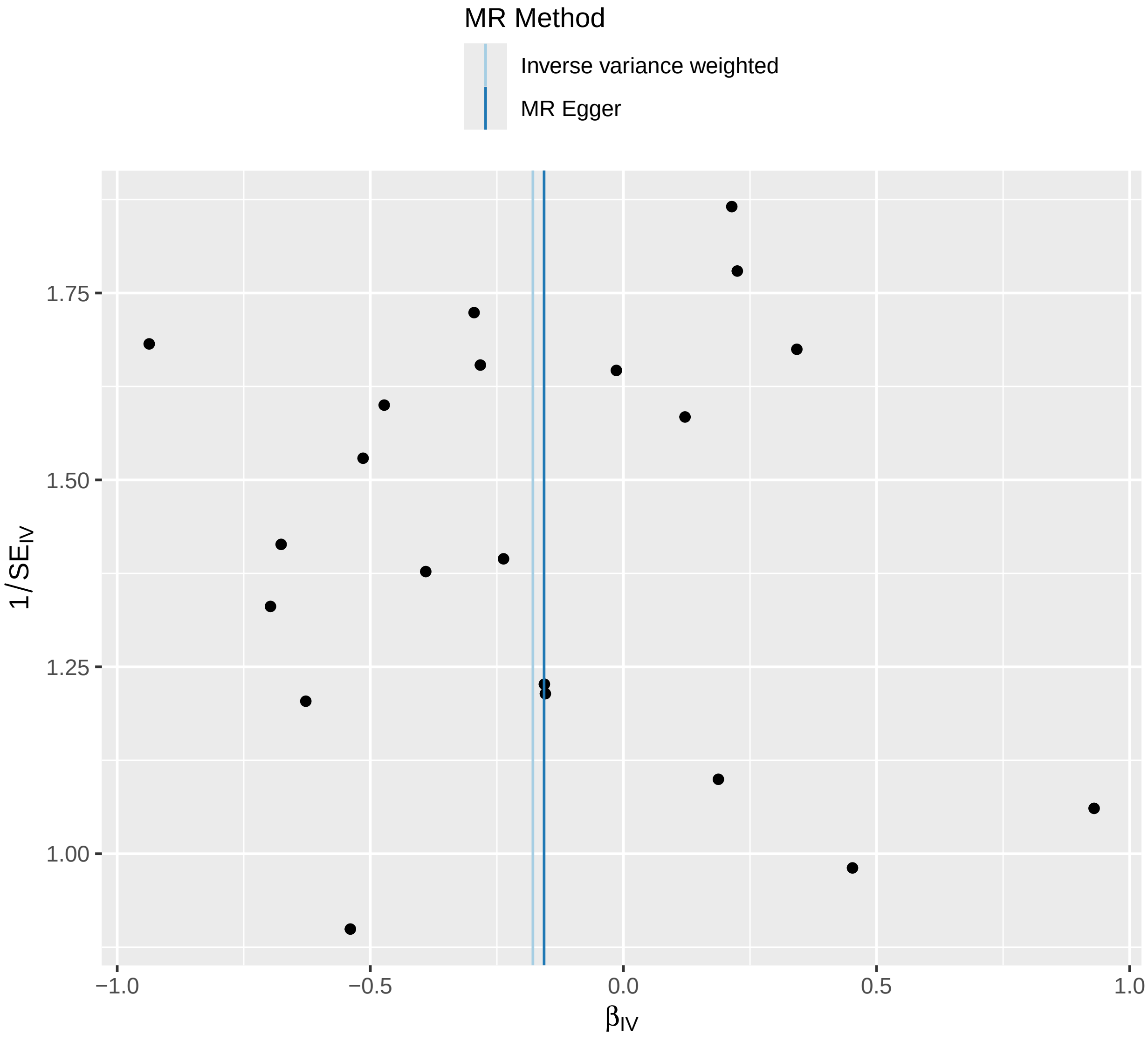

MR Method
Inverse variance weighted
MR Egger
1.75
1.50
1.25
1.00
1/SE_IV
−1.0
−0.5
0.0
0.5
1.0
β_IV

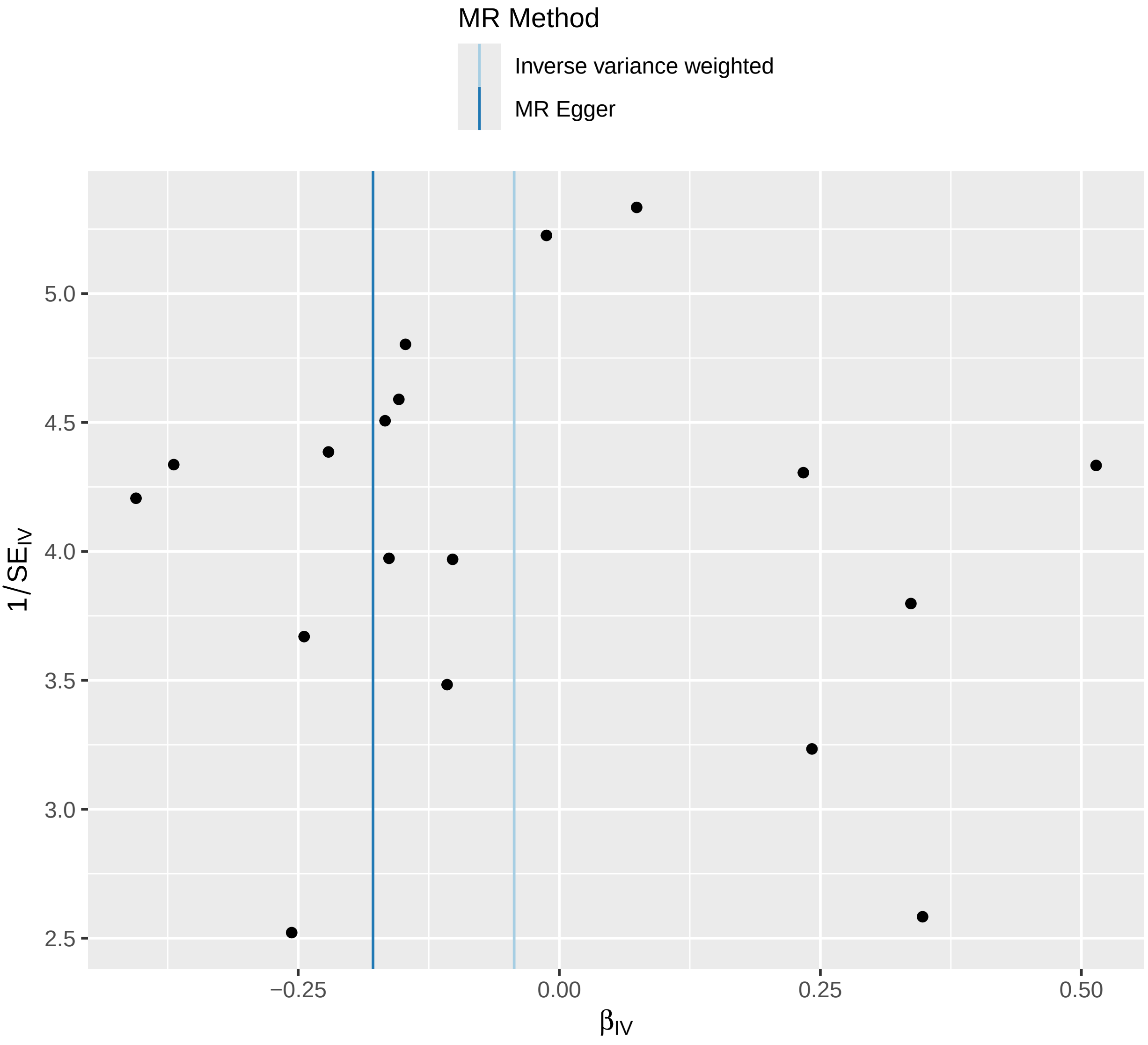
MR Method
Inverse variance weighted
MR Egger
5.0
4.5
4.0
3.5
3.0
2.5
1/SE_IV
−0.25
0.00
0.25
0.50
β_IV

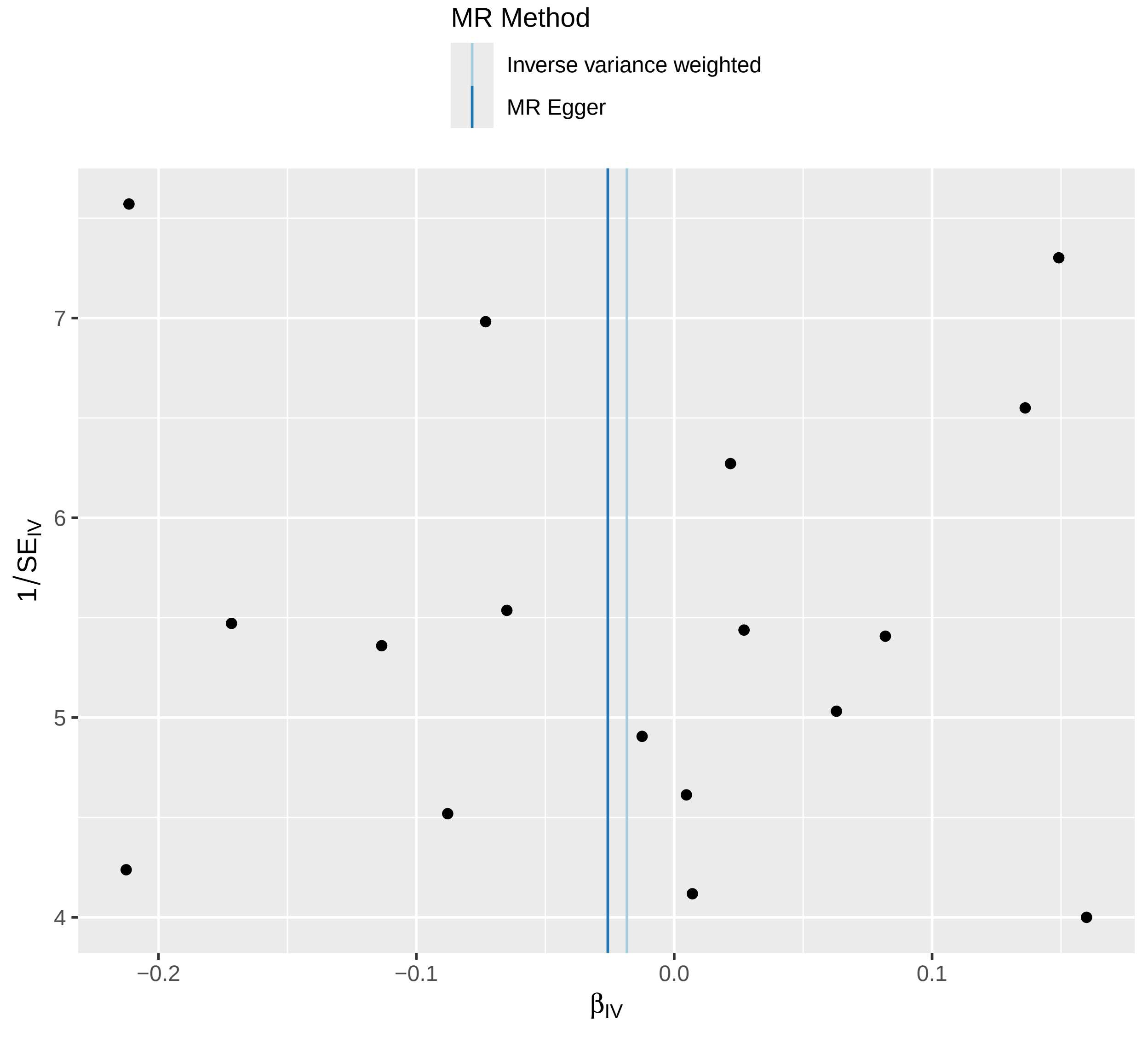

MR Method
Inverse variance weighted
MR Egger
1/SE_IV
7
6
5
4
−0.2
−0.1
0.0
0.1
β_IV

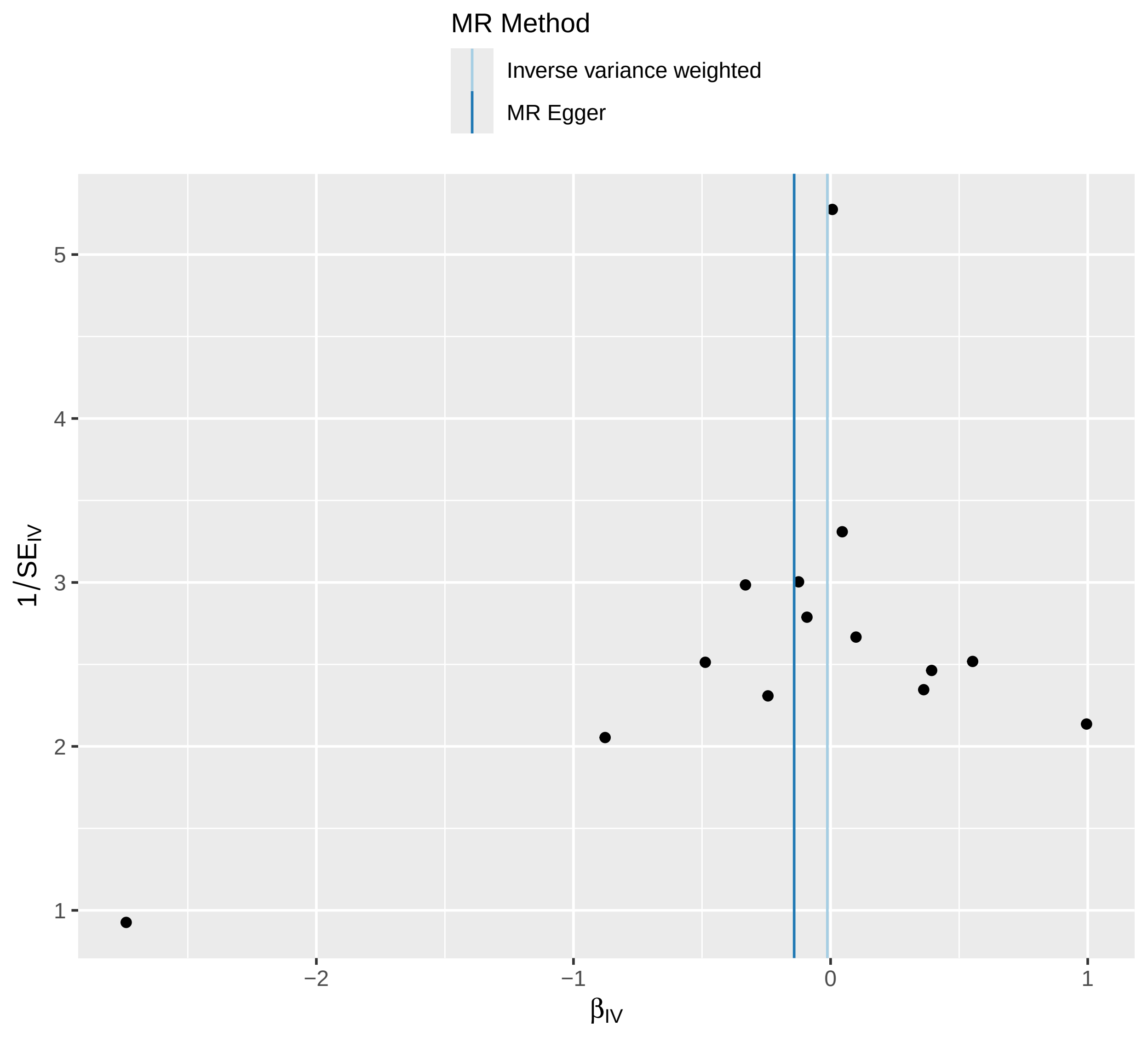

MR Method
Inverse variance weighted
MR Egger
5
4
3
2
1
1/SE$_{IV}$
−2
−1
0
1
β$_{IV}$

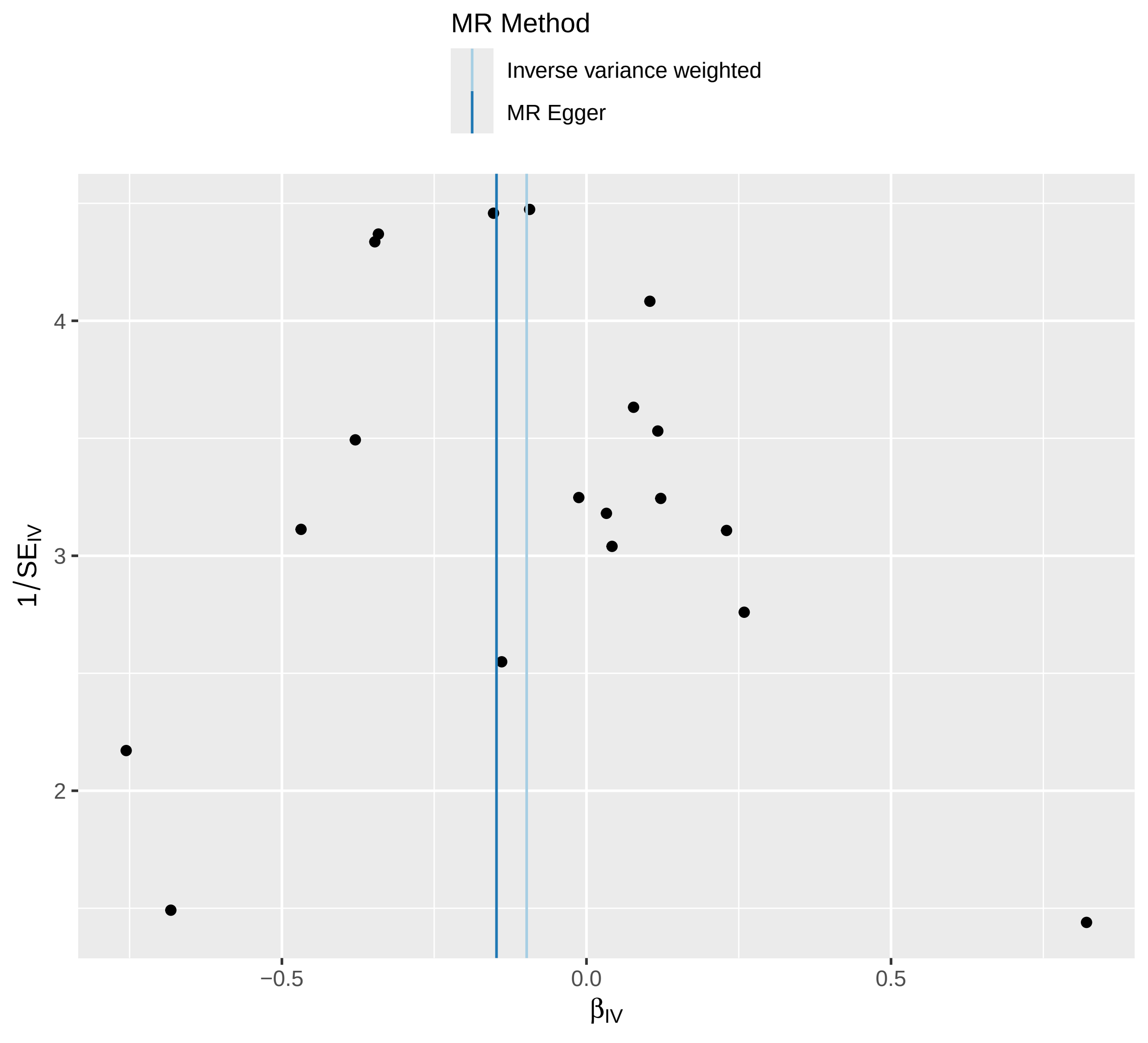
MR Method
Inverse variance weighted
MR Egger
4
3
2
1/SE_IV
−0.5
0.0
0.5
β_IV

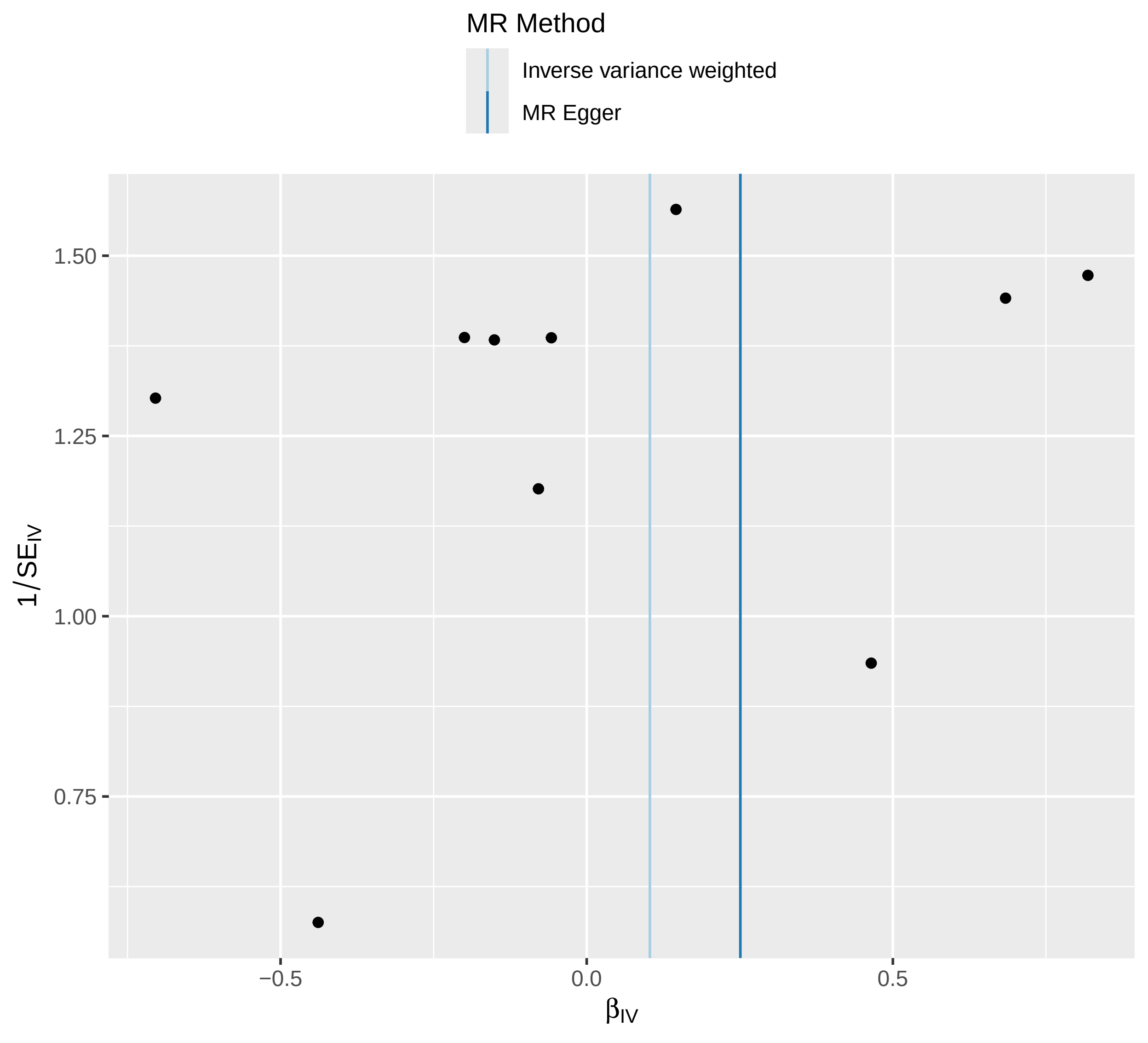
MR Method
Inverse variance weighted
MR Egger
1.50
1.25
1.00
0.75
1/SE_IV
−0.5
0.0
0.5
β_IV

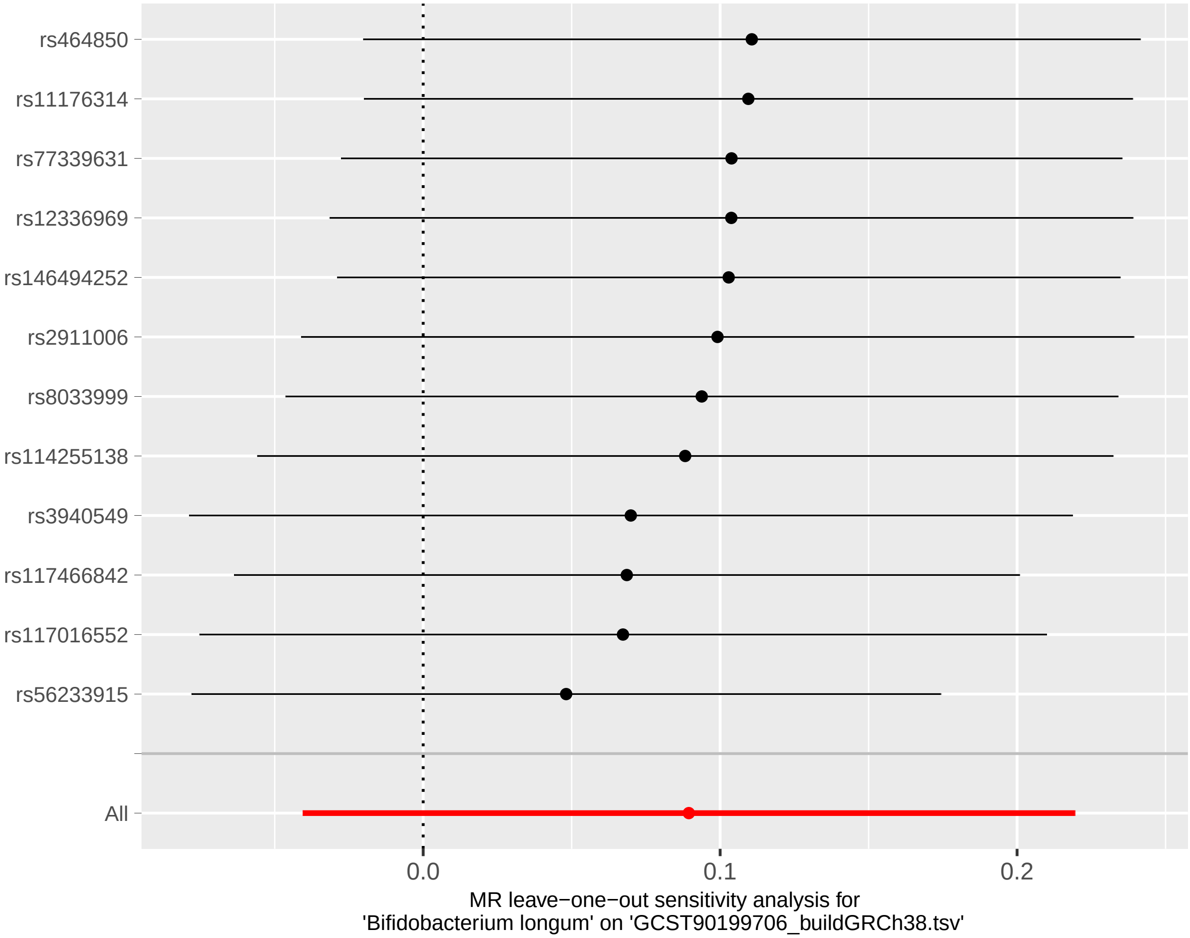

rs464850
rs11176314
rs77339631
rs12336969
rs146494252
rs2911006
rs8033999
rs114255138
rs3940549
rs117466842
rs117016552
rs56233915
All
0.0
0.1
0.2
MR leave−one−out sensitivity analysis for
'Bifidobacterium longum' on 'GCST90199706_buildGRCh38.tsv'

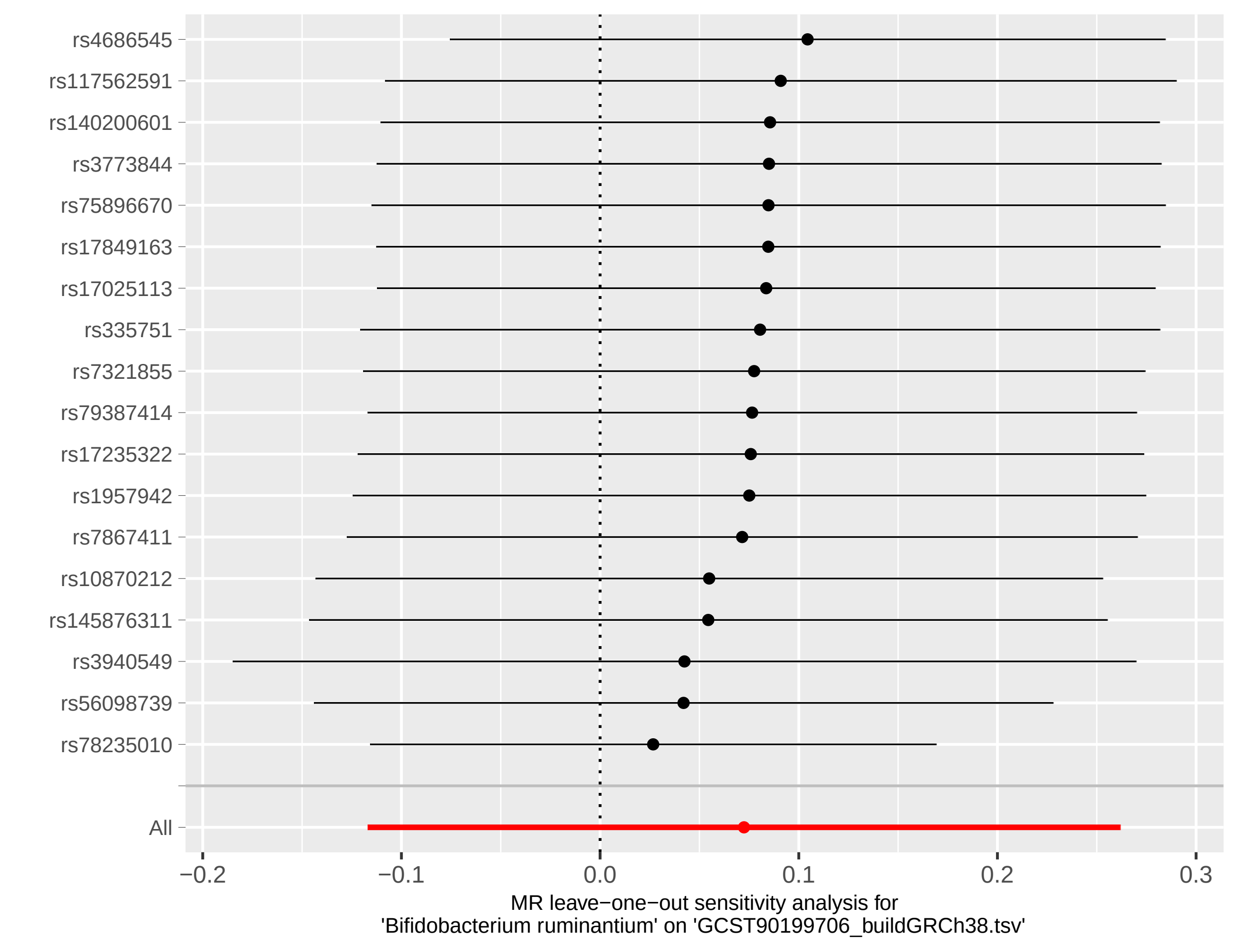

rs4686545
rs117562591
rs140200601
rs3773844
rs75896670
rs17849163
rs17025113
rs335751
rs7321855
rs79387414
rs17235322
rs1957942
rs7867411
rs10870212
rs145876311
rs3940549
rs56098739
rs78235010
All
−0.2
−0.1
0.0
0.1
0.2
0.3
MR leave−one−out sensitivity analysis for
'Bifidobacterium ruminantium' on 'GCST90199706_buildGRCh38.tsv'

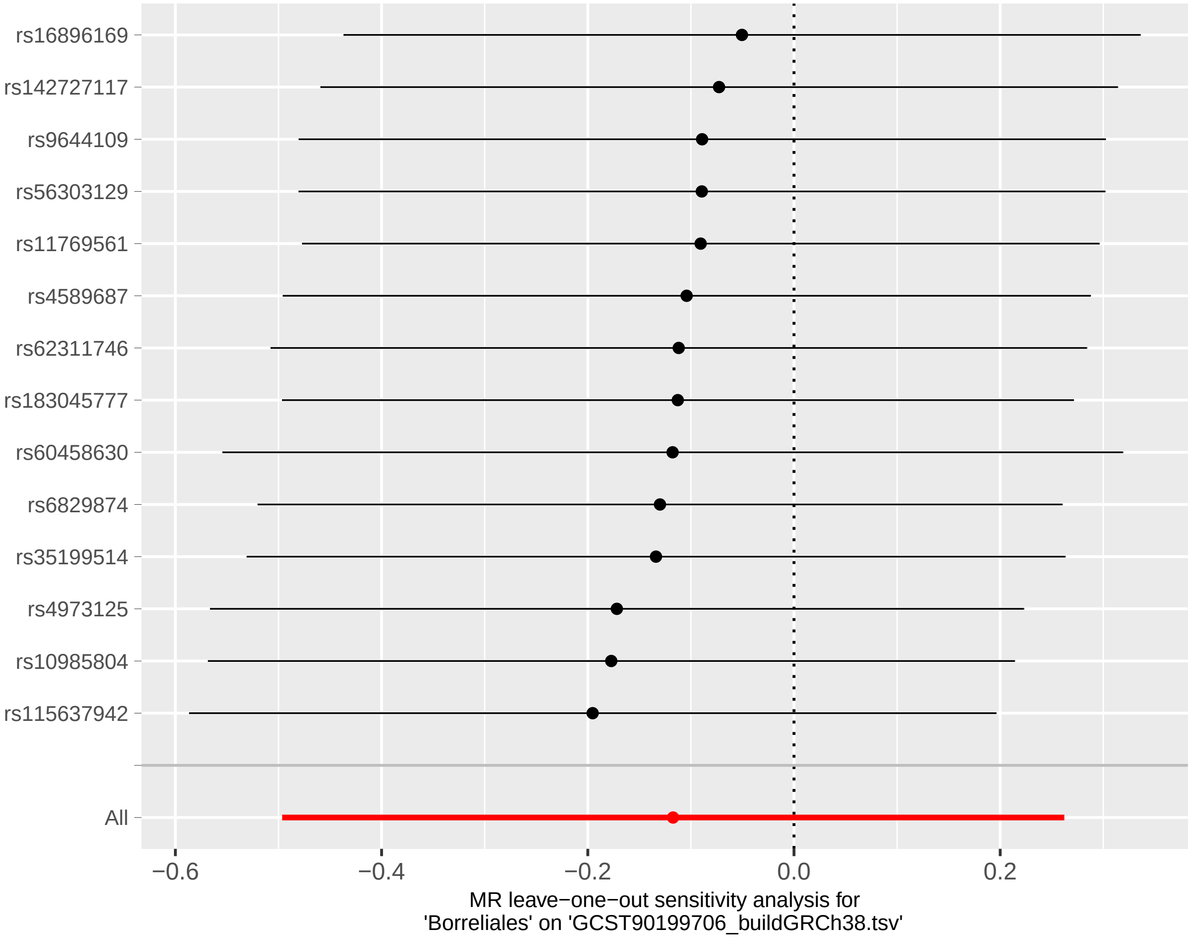

rs16896169
rs142727117
rs9644109
rs56303129
rs11769561
rs4589687
rs62311746
rs183045777
rs60458630
rs6829874
rs35199514
rs4973125
rs10985804
rs115637942
All
−0.6
−0.4
−0.2
0.0
0.2
MR leave−one−out sensitivity analysis for
'Borreliales' on 'GCST90199706_buildGRCh38.tsv'

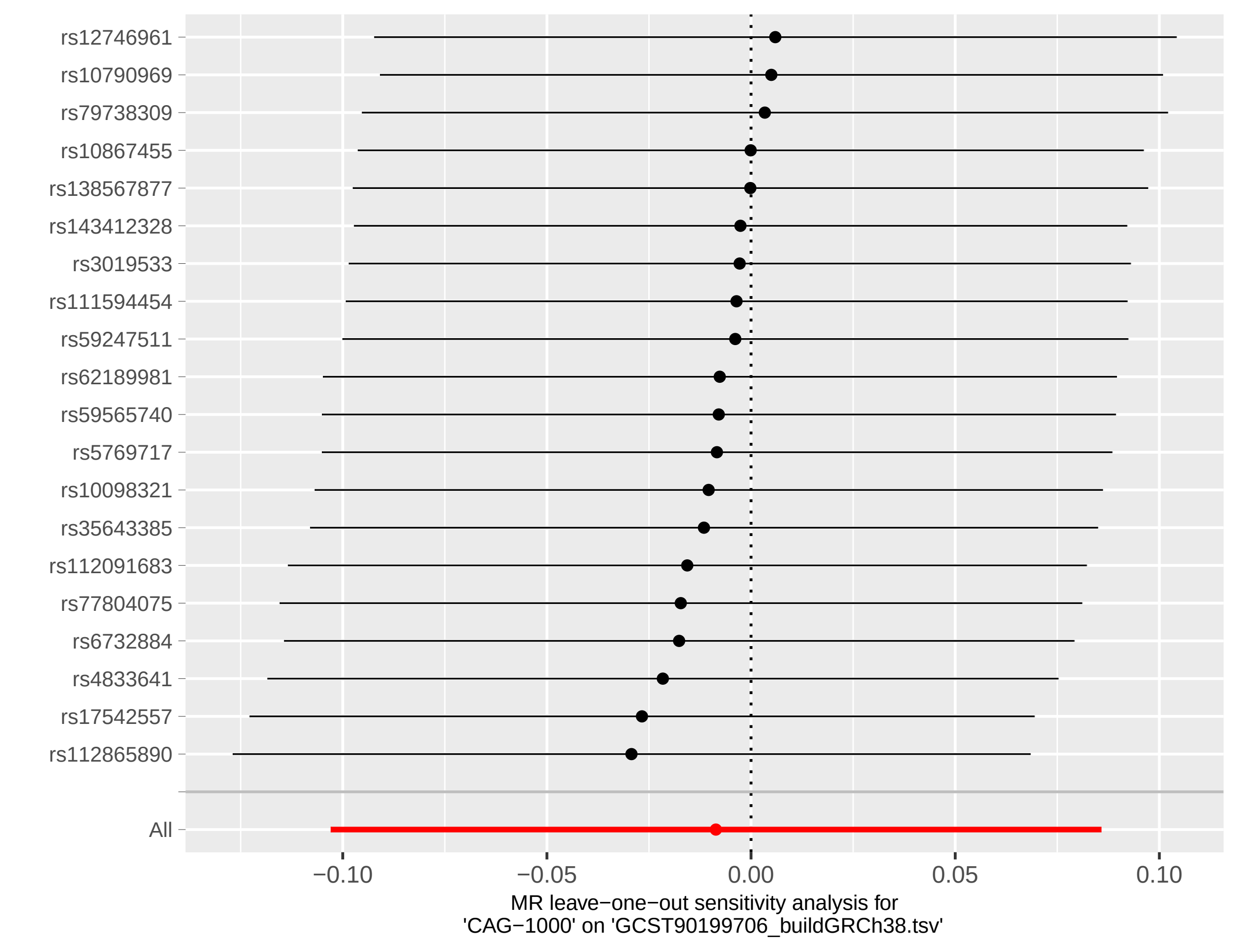
rs12746961
rs10790969
rs79738309
rs10867455
rs138567877
rs143412328
rs3019533
rs111594454
rs59247511
rs62189981
rs59565740
rs5769717
rs10098321
rs35643385
rs112091683
rs77804075
rs6732884
rs4833641
rs17542557
rs112865890
All
−0.10
−0.05
0.00
0.05
0.10
MR leave−one−out sensitivity analysis for
'CAG−1000' on 'GCST90199706_buildGRCh38.tsv'

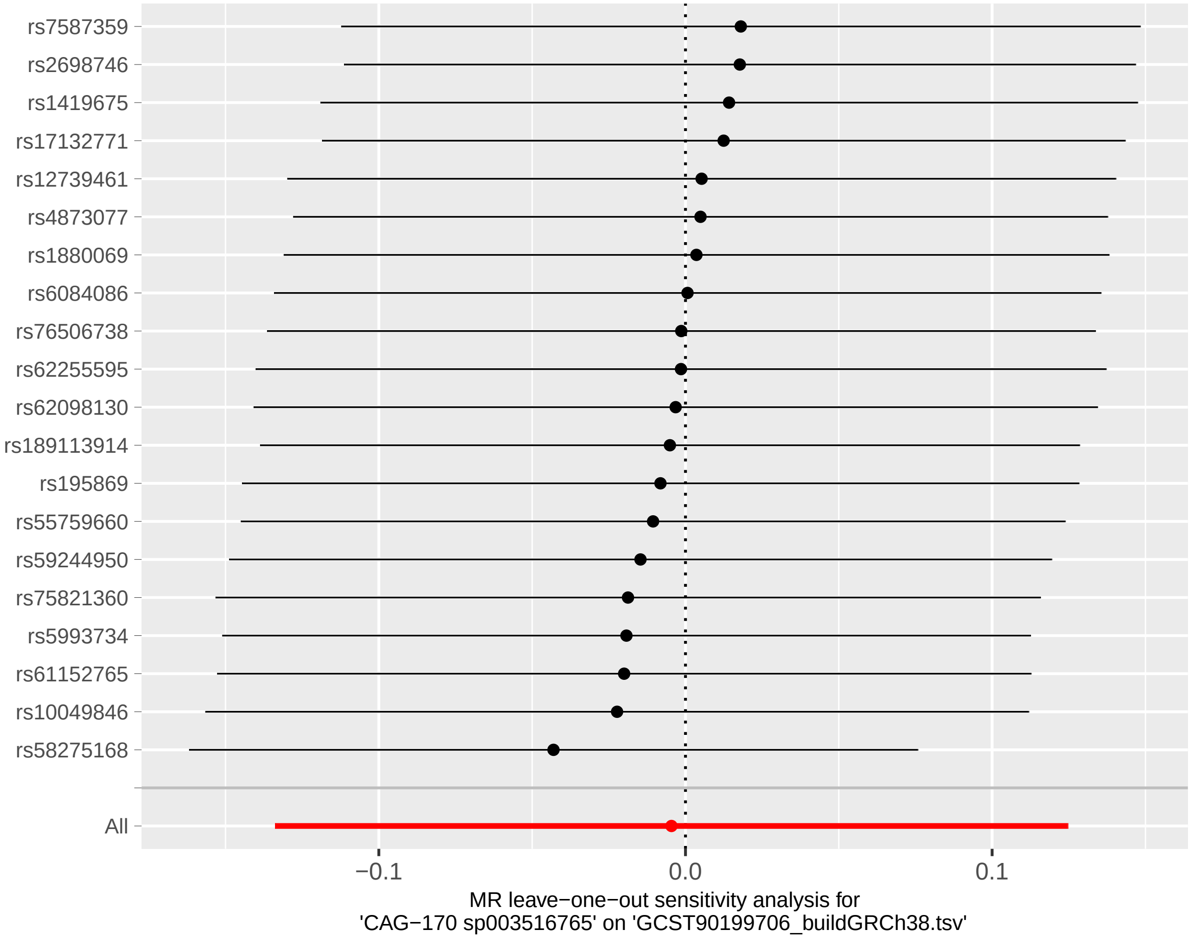
rs7587359
rs2698746
rs1419675
rs17132771
rs12739461
rs4873077
rs1880069
rs6084086
rs76506738
rs62255595
rs62098130
rs189113914
rs195869
rs55759660
rs59244950
rs75821360
rs5993734
rs61152765
rs10049846
rs58275168
All
−0.1
0.0
0.1
MR leave−one−out sensitivity analysis for
'CAG−170 sp003516765' on 'GCST90199706_buildGRCh38.tsv'

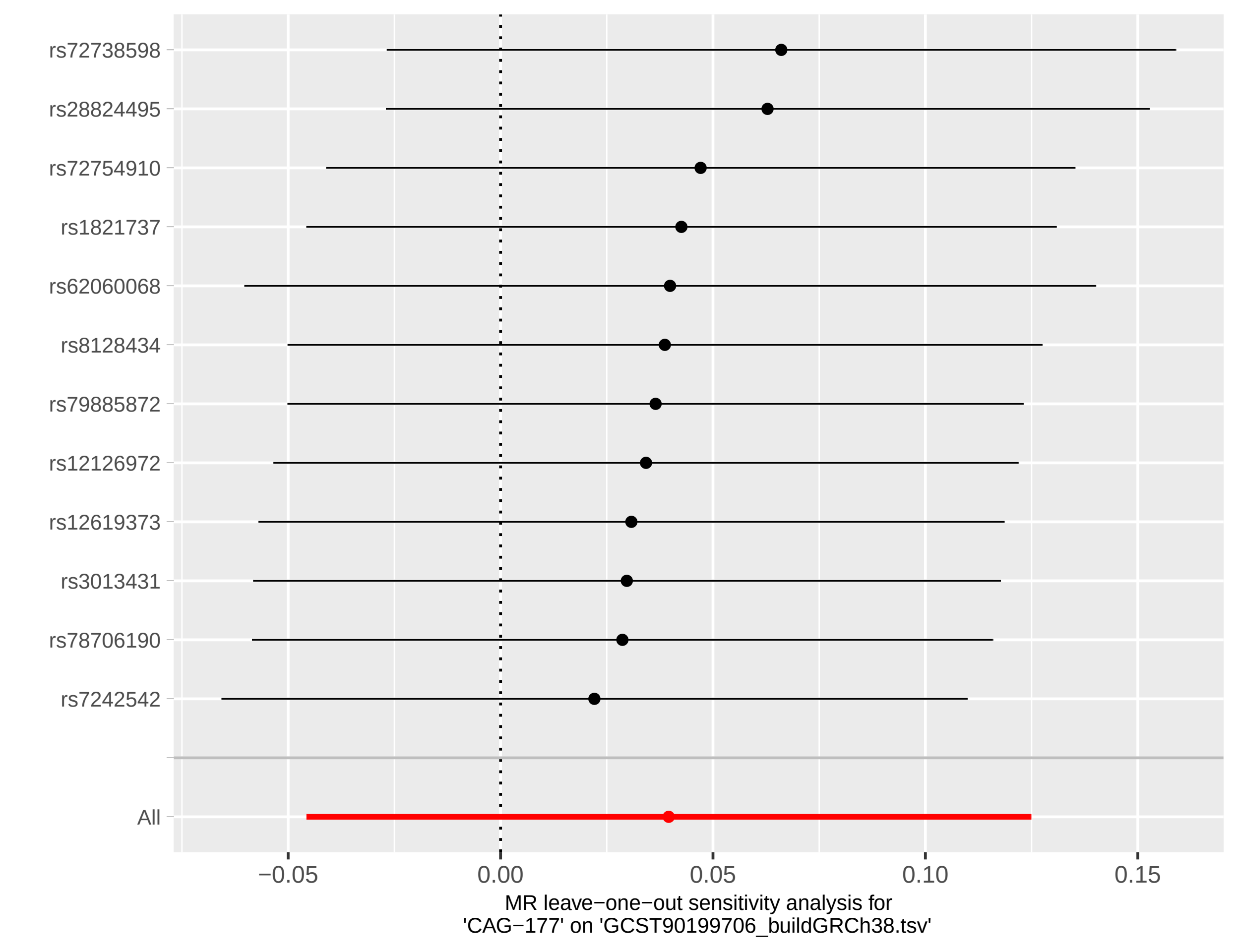
rs72738598
rs28824495
rs72754910
rs1821737
rs62060068
rs8128434
rs79885872
rs12126972
rs12619373
rs3013431
rs78706190
rs7242542
All
−0.05
0.00
0.05
0.10
0.15
MR leave−one−out sensitivity analysis for
'CAG−177' on 'GCST90199706_buildGRCh38.tsv'

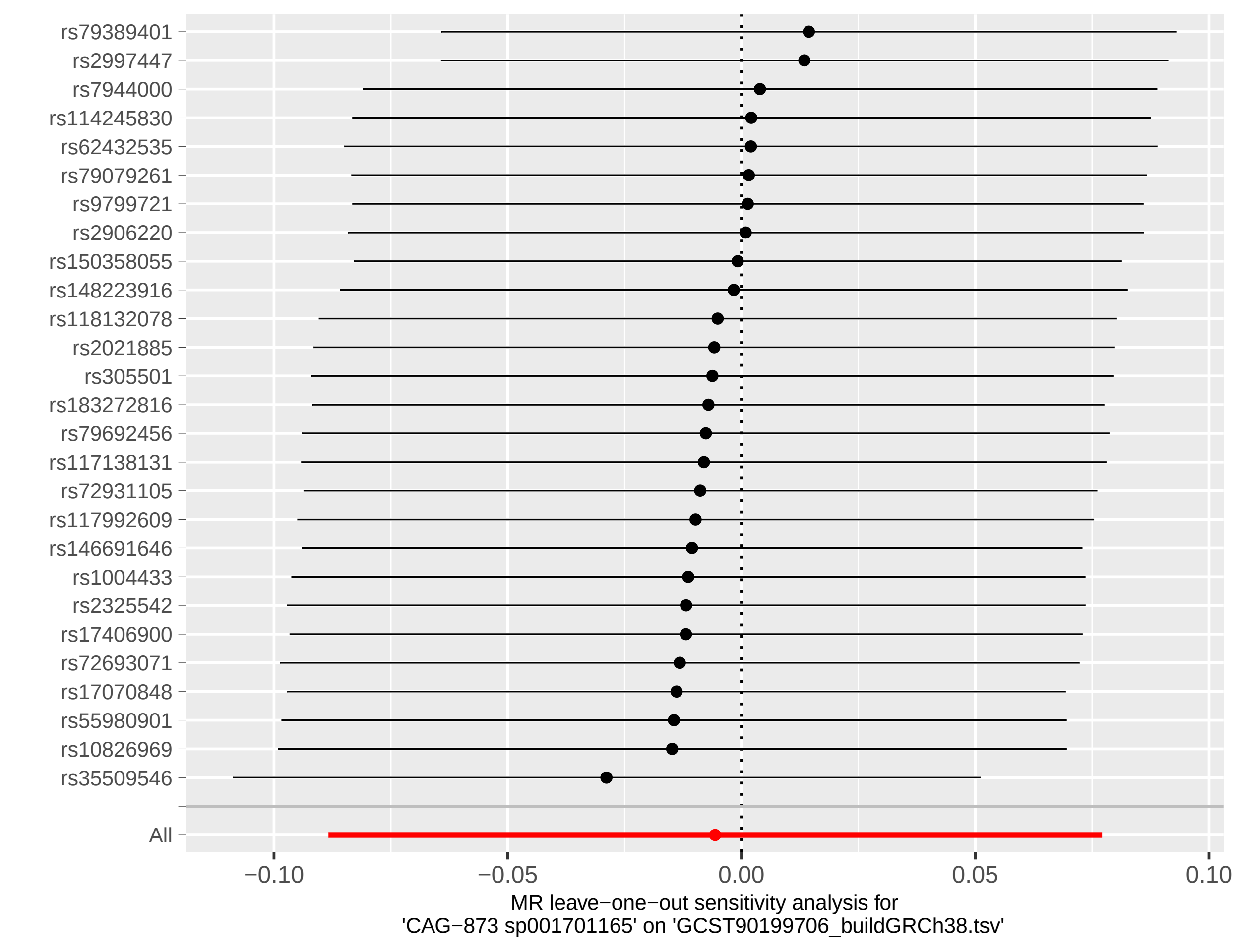
rs79389401
rs2997447
rs7944000
rs114245830
rs62432535
rs79079261
rs9799721
rs2906220
rs150358055
rs148223916
rs118132078
rs2021885
rs305501
rs183272816
rs79692456
rs117138131
rs72931105
rs117992609
rs146691646
rs1004433
rs2325542
rs17406900
rs72693071
rs17070848
rs55980901
rs10826969
rs35509546
All
−0.10
−0.05
0.00
0.05
0.10
MR leave−one−out sensitivity analysis for
'CAG−873 sp001701165' on 'GCST90199706_buildGRCh38.tsv'

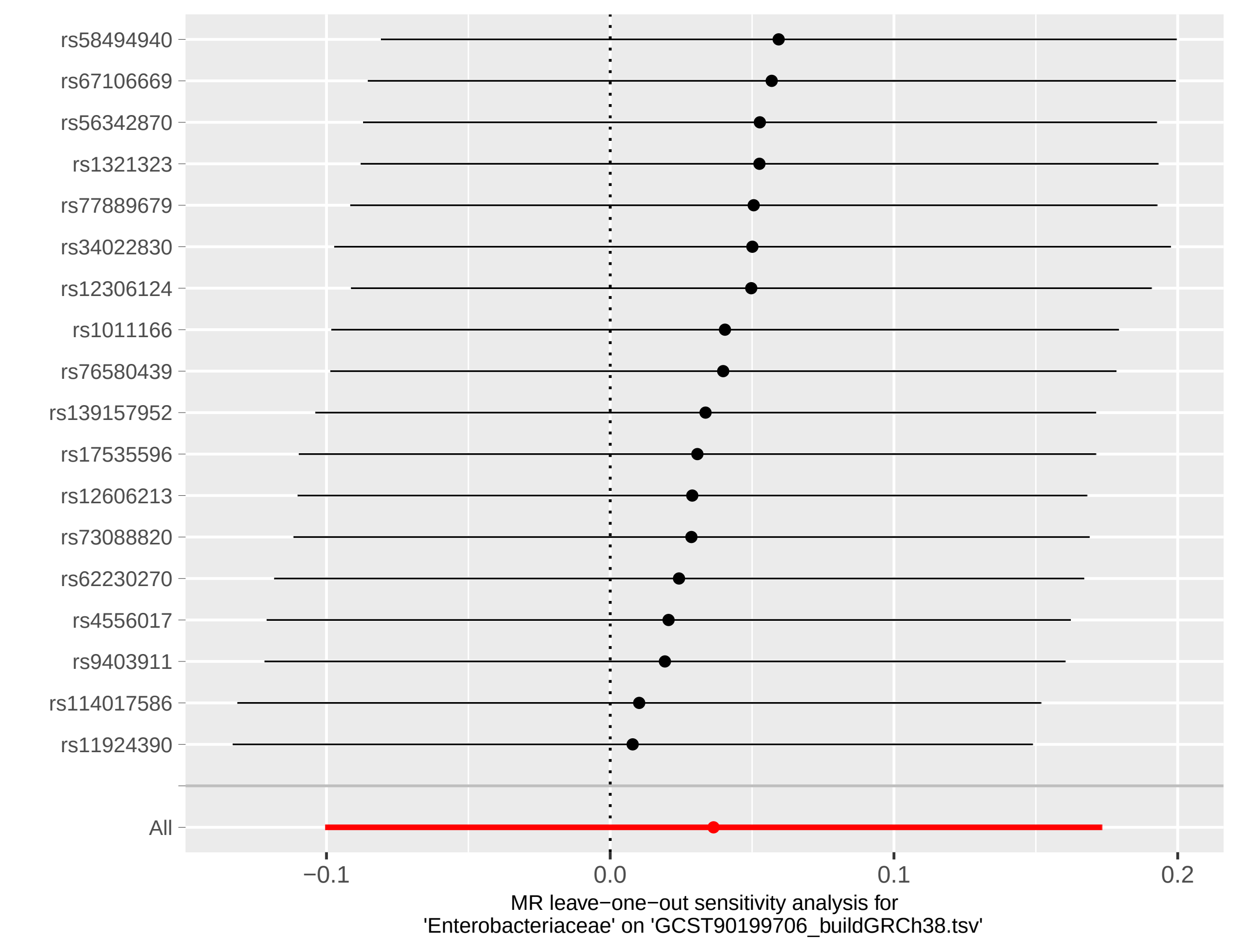
rs58494940
rs67106669
rs56342870
rs1321323
rs77889679
rs34022830
rs12306124
rs1011166
rs76580439
rs139157952
rs17535596
rs12606213
rs73088820
rs62230270
rs4556017
rs9403911
rs114017586
rs11924390
All
−0.1
0.0
0.1
0.2
MR leave−one−out sensitivity analysis for
'Enterobacteriaceae' on 'GCST90199706_buildGRCh38.tsv'

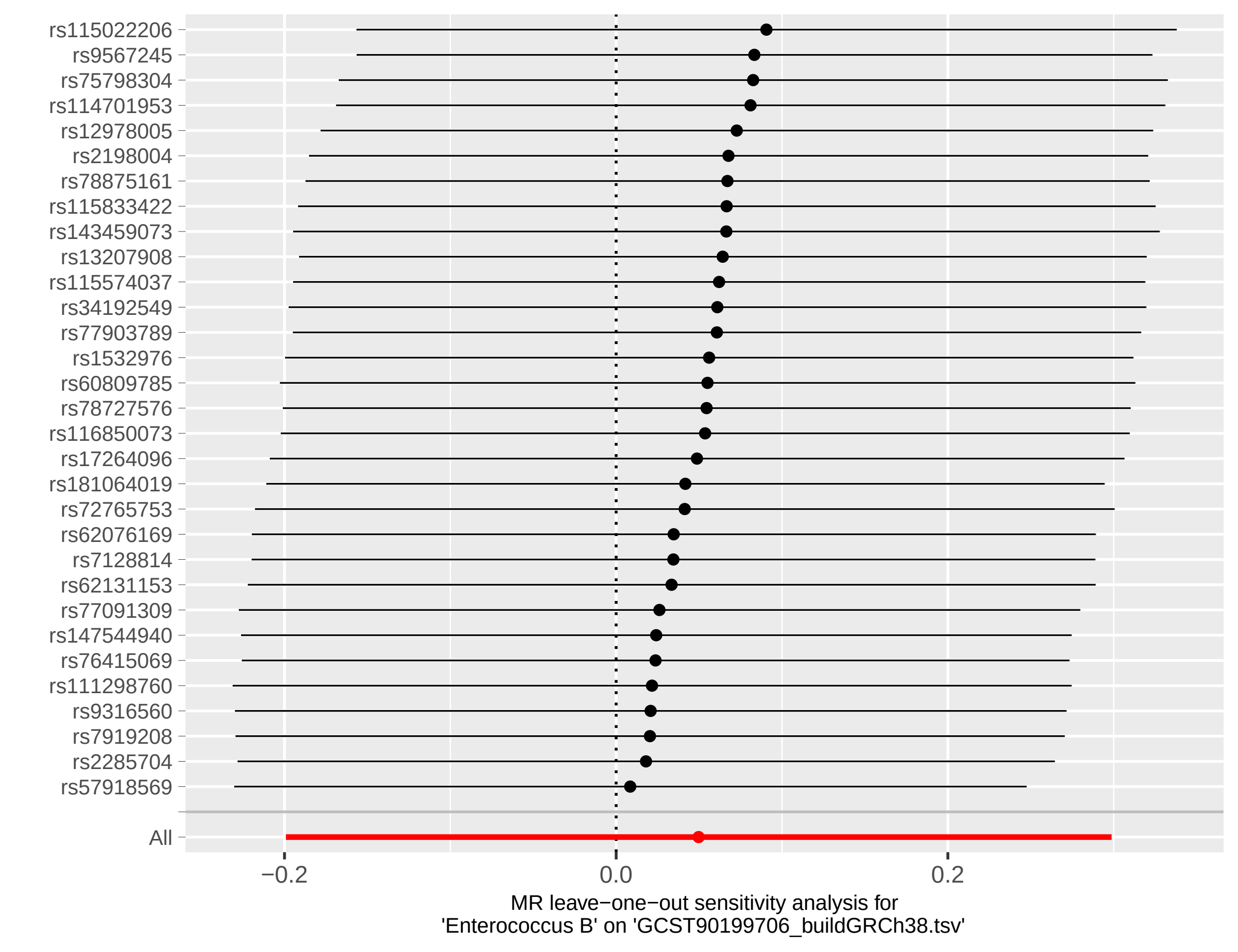
rs115022206
rs9567245
rs75798304
rs114701953
rs12978005
rs2198004
rs78875161
rs115833422
rs143459073
rs13207908
rs115574037
rs34192549
rs77903789
rs1532976
rs60809785
rs78727576
rs116850073
rs17264096
rs181064019
rs72765753
rs62076169
rs7128814
rs62131153
rs77091309
rs147544940
rs76415069
rs111298760
rs9316560
rs7919208
rs2285704
rs57918569
All
−0.2
0.0
0.2
MR leave−one−out sensitivity analysis for
'Enterococcus B' on 'GCST90199706_buildGRCh38.tsv'

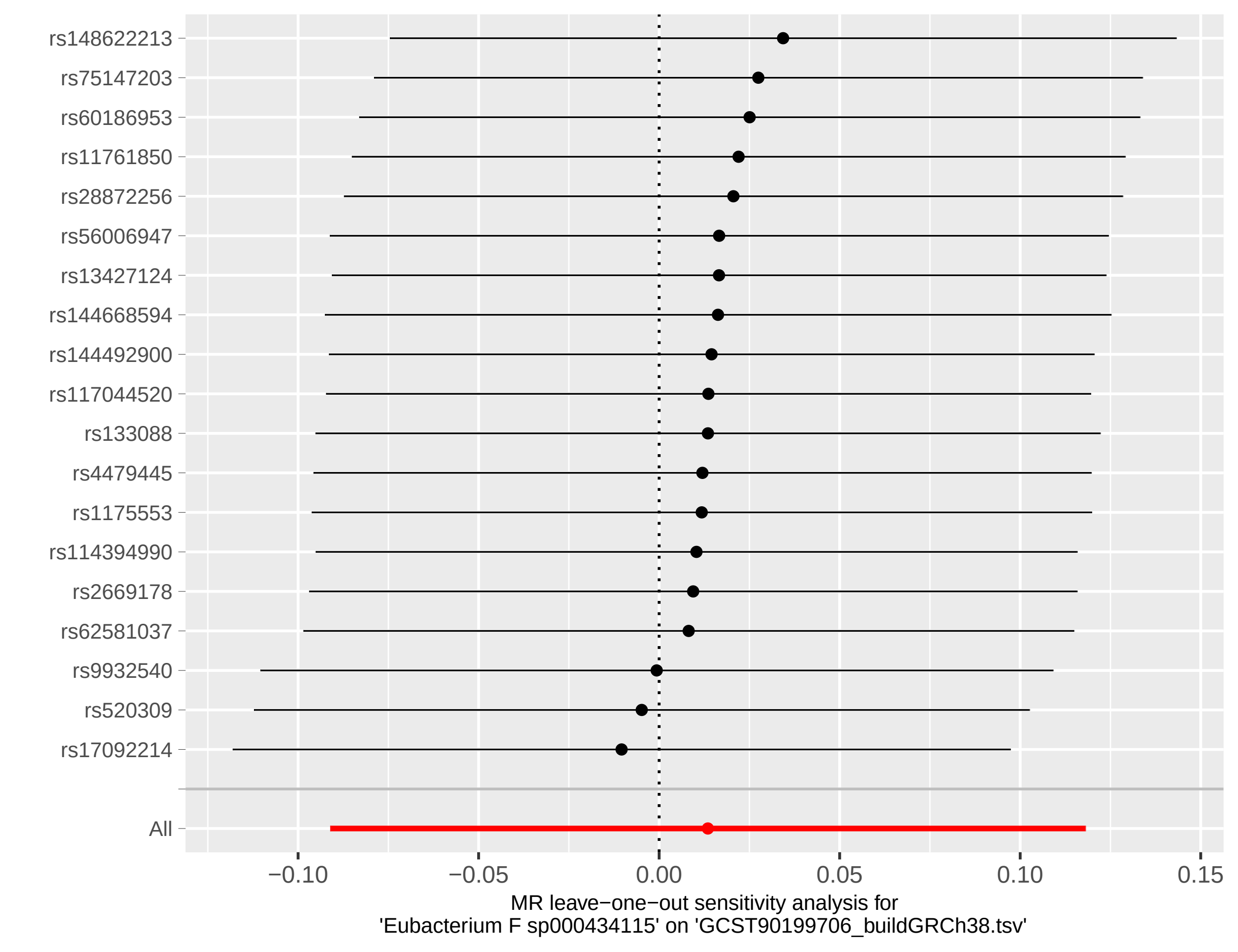
rs148622213
rs75147203
rs60186953
rs11761850
rs28872256
rs56006947
rs13427124
rs144668594
rs144492900
rs117044520
rs133088
rs4479445
rs1175553
rs114394990
rs2669178
rs62581037
rs9932540
rs520309
rs17092214
All
−0.10
−0.05
0.00
0.05
0.10
0.15
MR leave−one−out sensitivity analysis for
'Eubacterium F sp000434115' on 'GCST90199706_buildGRCh38.tsv'

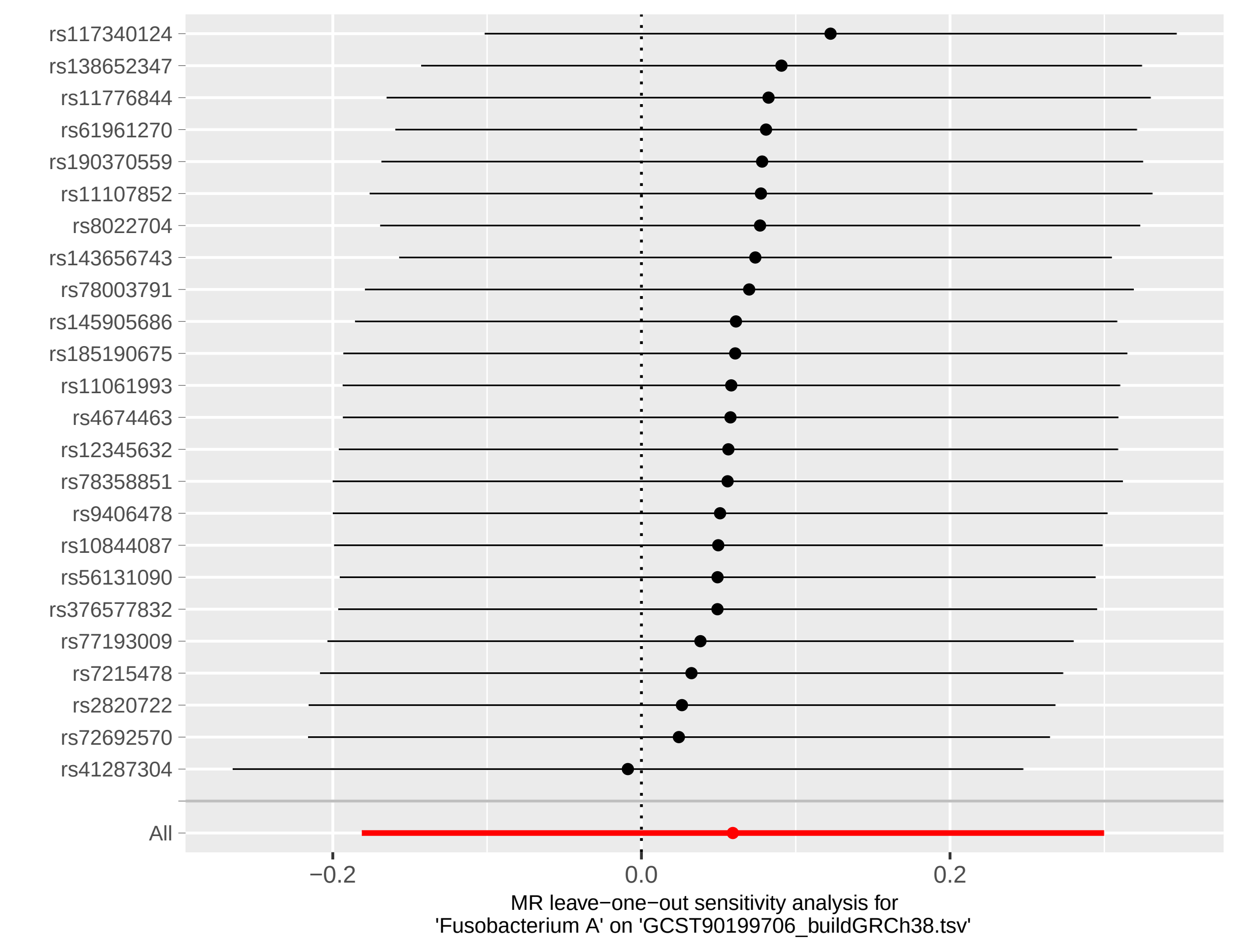
rs117340124
rs138652347
rs11776844
rs61961270
rs190370559
rs11107852
rs8022704
rs143656743
rs78003791
rs145905686
rs185190675
rs11061993
rs4674463
rs12345632
rs78358851
rs9406478
rs10844087
rs56131090
rs376577832
rs77193009
rs7215478
rs2820722
rs72692570
rs41287304
All
−0.2
0.0
0.2
MR leave−one−out sensitivity analysis for
'Fusobacterium A' on 'GCST90199706_buildGRCh38.tsv'

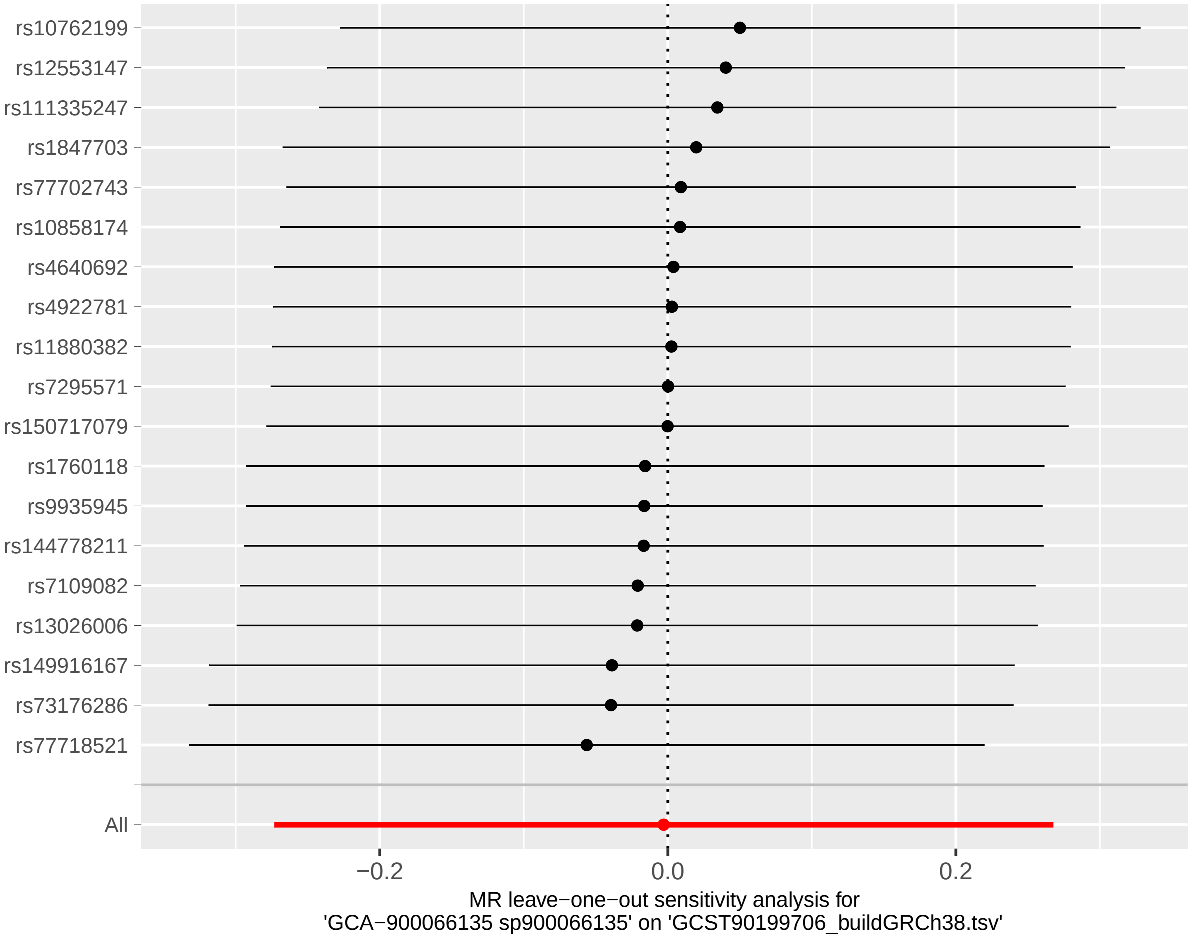
rs10762199
rs12553147
rs111335247
rs1847703
rs77702743
rs10858174
rs4640692
rs4922781
rs11880382
rs7295571
rs150717079
rs1760118
rs9935945
rs144778211
rs7109082
rs13026006
rs149916167
rs73176286
rs77718521
All
−0.2
0.0
0.2
MR leave−one−out sensitivity analysis for
'GCA−900066135 sp900066135' on 'GCST90199706_buildGRCh38.tsv'

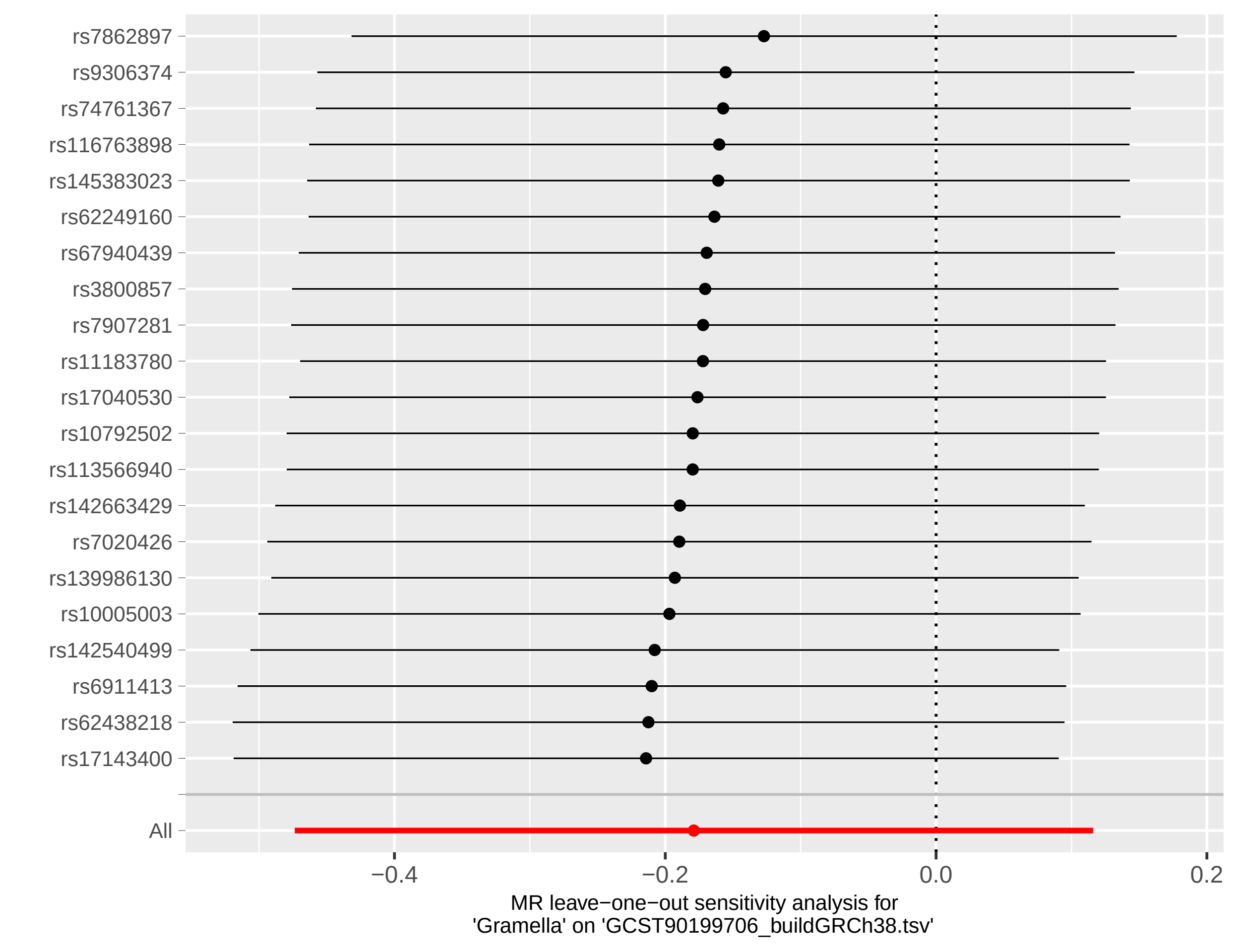
rs7862897
rs9306374
rs74761367
rs116763898
rs145383023
rs62249160
rs67940439
rs3800857
rs7907281
rs11183780
rs17040530
rs10792502
rs113566940
rs142663429
rs7020426
rs139986130
rs10005003
rs142540499
rs6911413
rs62438218
rs17143400
All
−0.4
−0.2
0.0
0.2
MR leave−one−out sensitivity analysis for
'Gramella' on 'GCST90199706_buildGRCh38.tsv'

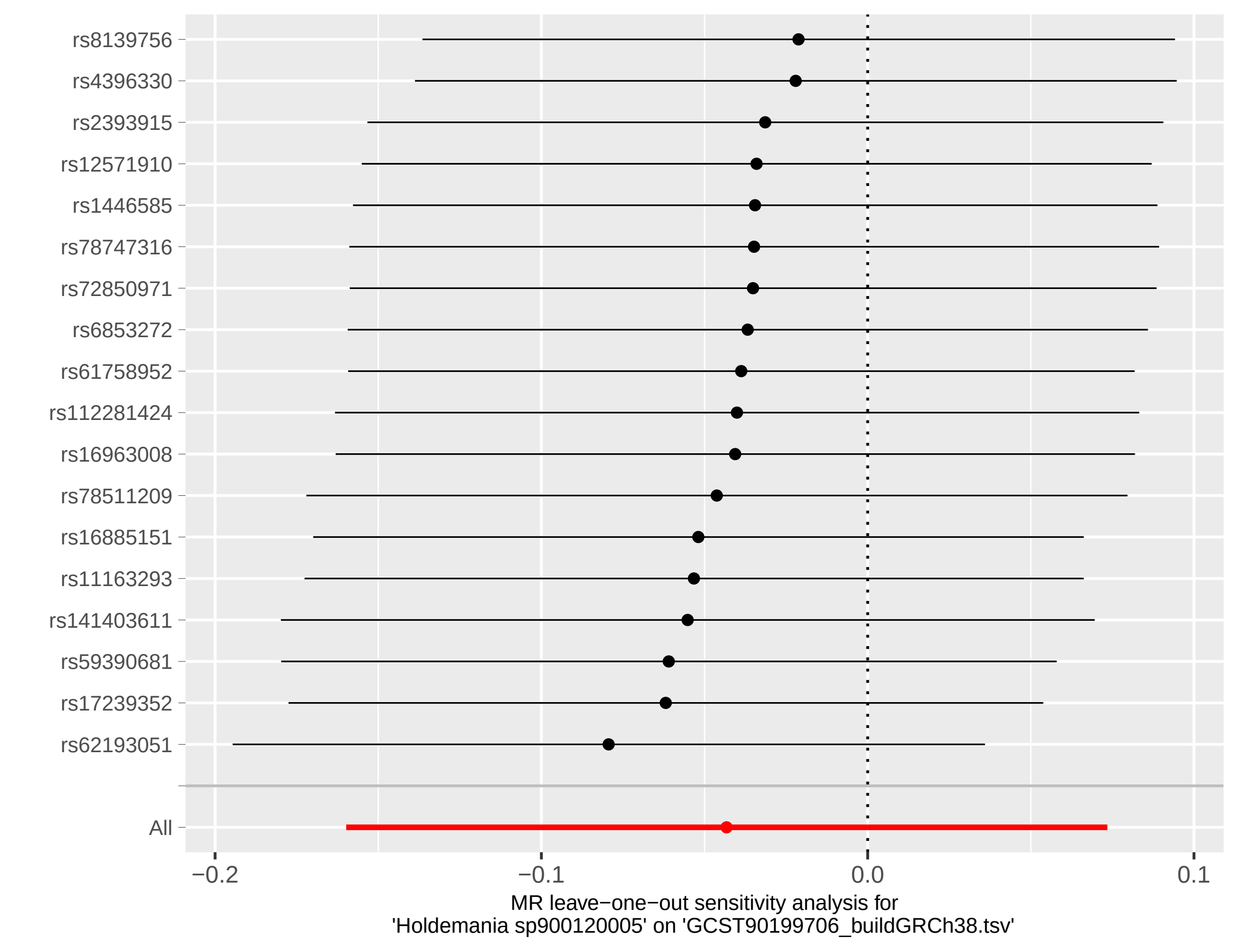
rs8139756
rs4396330
rs2393915
rs12571910
rs1446585
rs78747316
rs72850971
rs6853272
rs61758952
rs112281424
rs16963008
rs78511209
rs16885151
rs11163293
rs141403611
rs59390681
rs17239352
rs62193051
All
−0.2
−0.1
0.0
0.1
MR leave−one−out sensitivity analysis for
'Holdemania sp900120005' on 'GCST90199706_buildGRCh38.tsv'

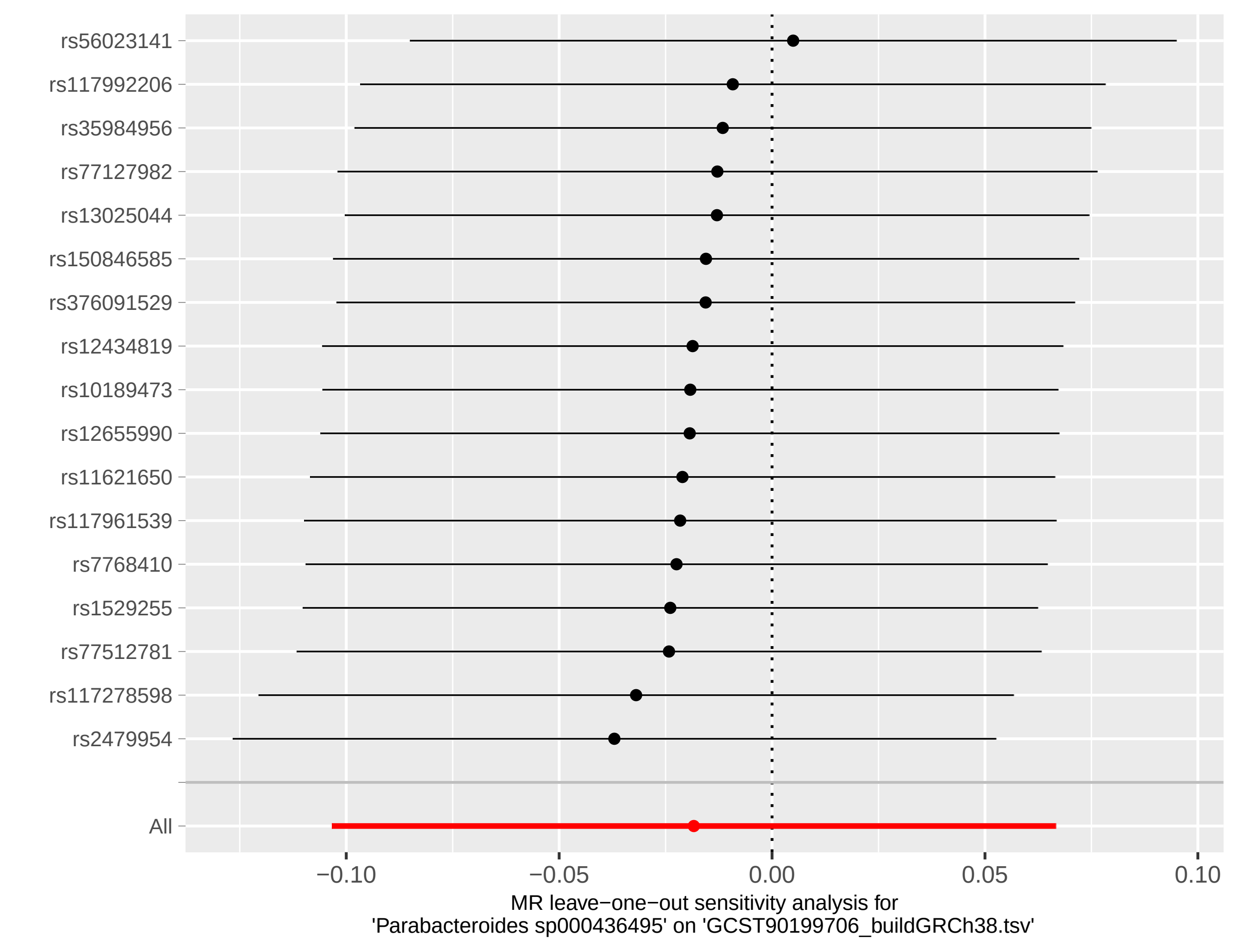
rs56023141
rs117992206
rs35984956
rs77127982
rs13025044
rs150846585
rs376091529
rs12434819
rs10189473
rs12655990
rs11621650
rs117961539
rs7768410
rs1529255
rs77512781
rs117278598
rs2479954
All
−0.10
−0.05
0.00
0.05
0.10
MR leave−one−out sensitivity analysis for
'Parabacteroides sp000436495' on 'GCST90199706_buildGRCh38.tsv'

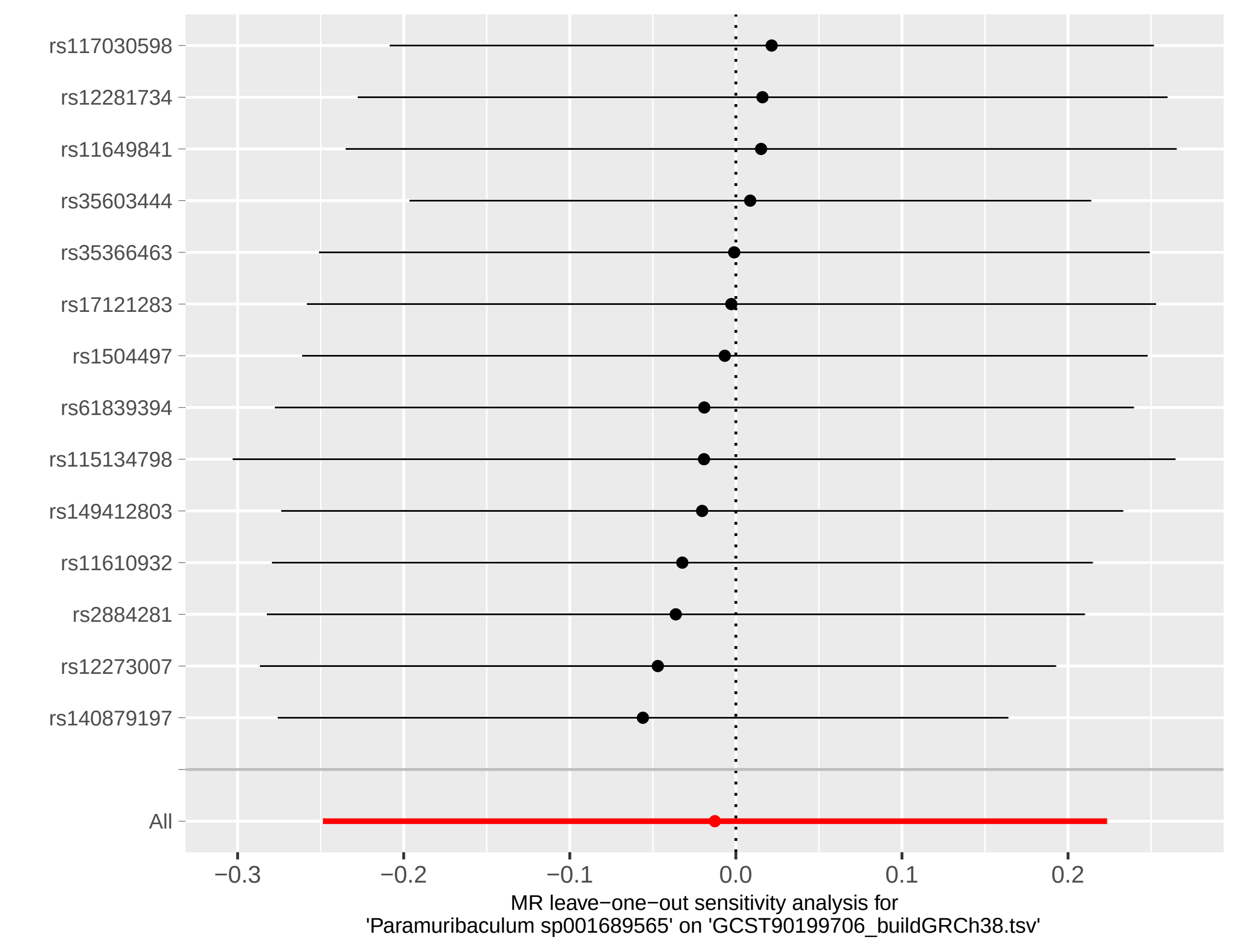
rs117030598
rs12281734
rs11649841
rs35603444
rs35366463
rs17121283
rs1504497
rs61839394
rs115134798
rs149412803
rs11610932
rs2884281
rs12273007
rs140879197
All
−0.3
−0.2
−0.1
0.0
0.1
0.2
MR leave−one−out sensitivity analysis for
'Paramuribaculum sp001689565' on 'GCST90199706_buildGRCh38.tsv'

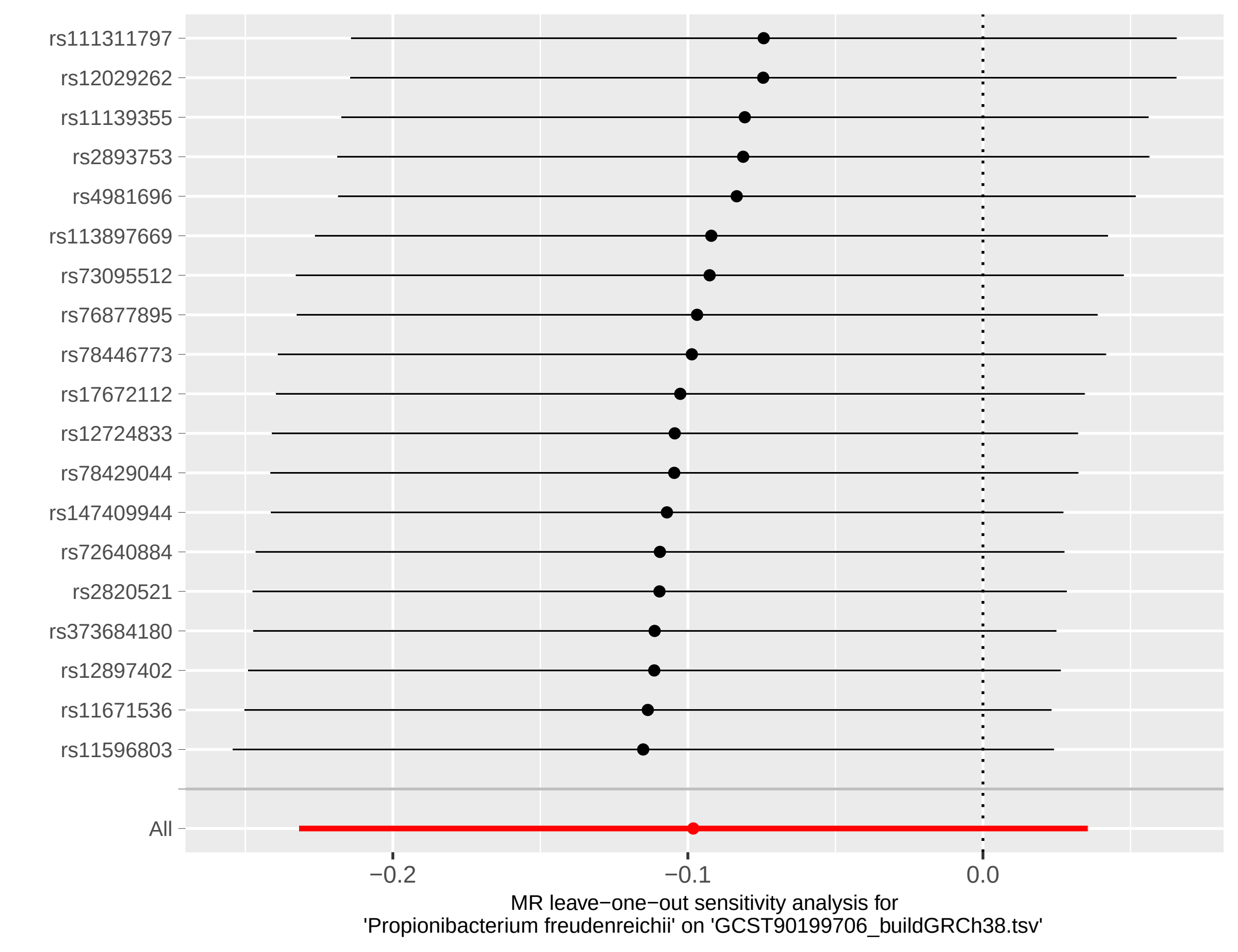
rs111311797
rs12029262
rs11139355
rs2893753
rs4981696
rs113897669
rs73095512
rs76877895
rs78446773
rs17672112
rs12724833
rs78429044
rs147409944
rs72640884
rs2820521
rs373684180
rs12897402
rs11671536
rs11596803
All
−0.2
−0.1
0.0
MR leave−one−out sensitivity analysis for
'Propionibacterium freudenreichii' on 'GCST90199706_buildGRCh38.tsv'

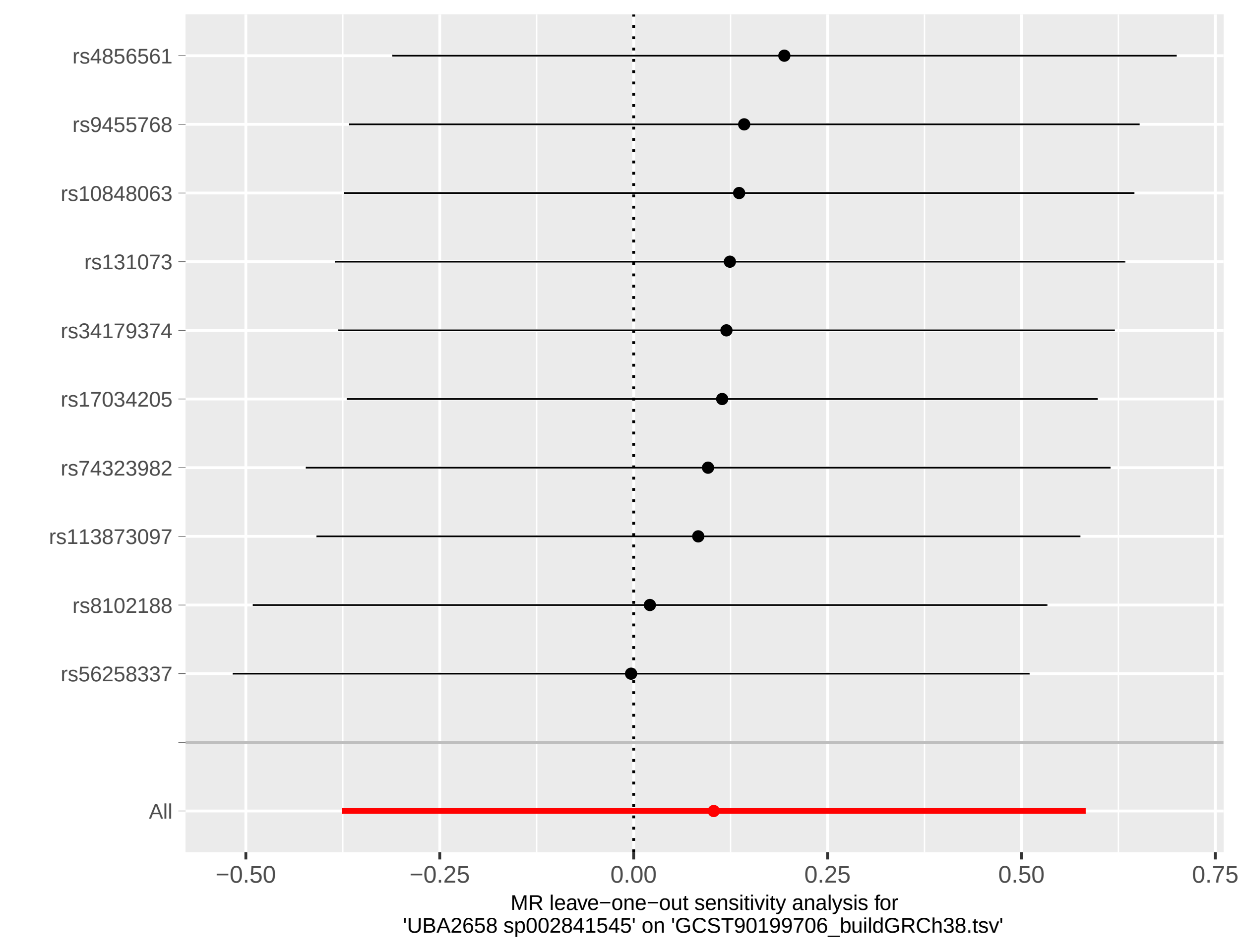

rs4856561
rs9455768
rs10848063
rs131073
rs34179374
rs17034205
rs74323982
rs113873097
rs8102188
rs56258337
All
−0.50
−0.25
0.00
0.25
0.50
0.75
MR leave−one−out sensitivity analysis for
'UBA2658 sp002841545' on 'GCST90199706_buildGRCh38.tsv'

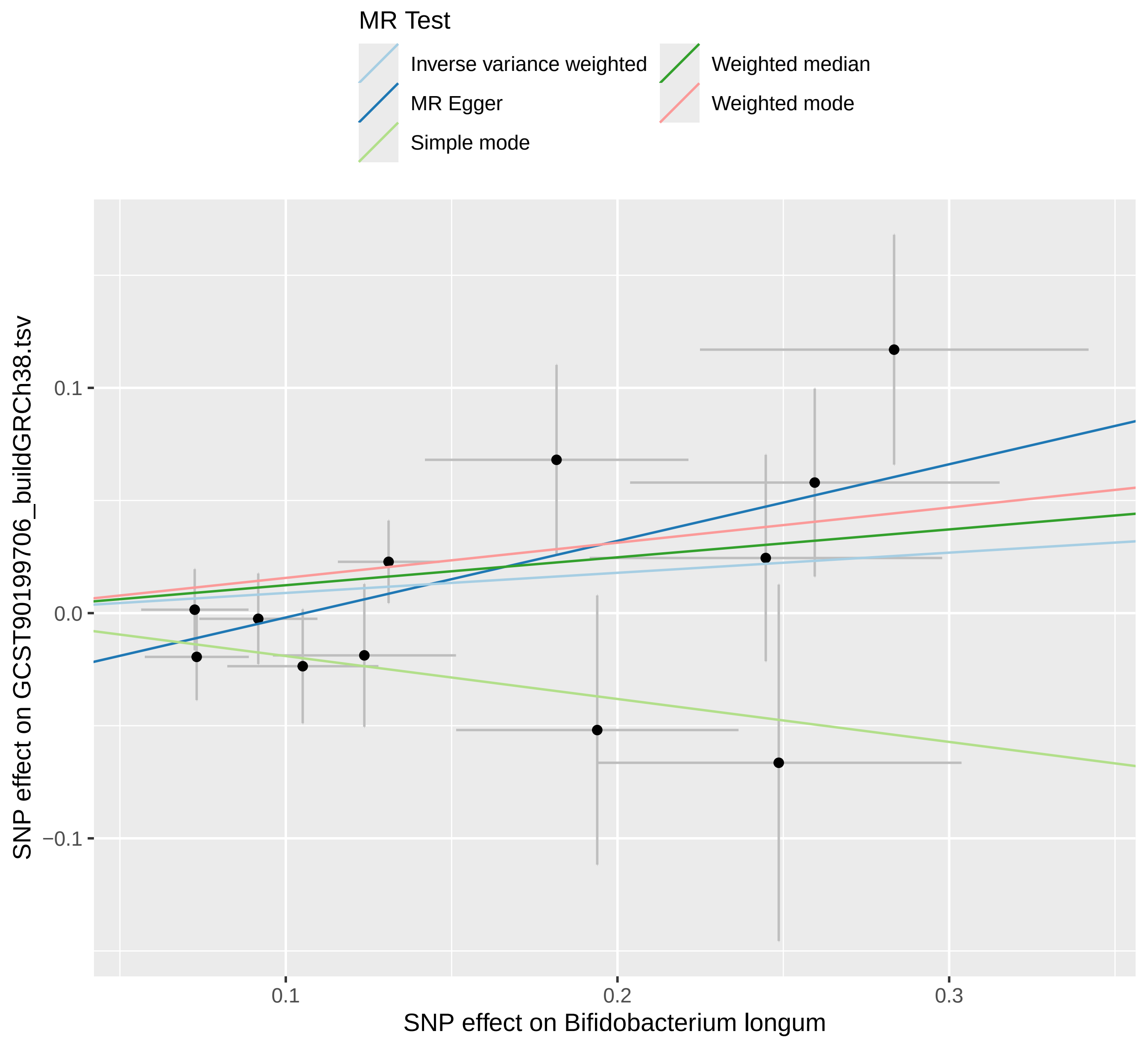
MR Test
Inverse variance weighted
MR Egger
Simple mode
Weighted median
Weighted mode
0.1
0.0
−0.1
0.1
0.2
0.3
SNP effect on GCST90199706_buildGRCh38.tsv
SNP effect on Bifidobacterium longum

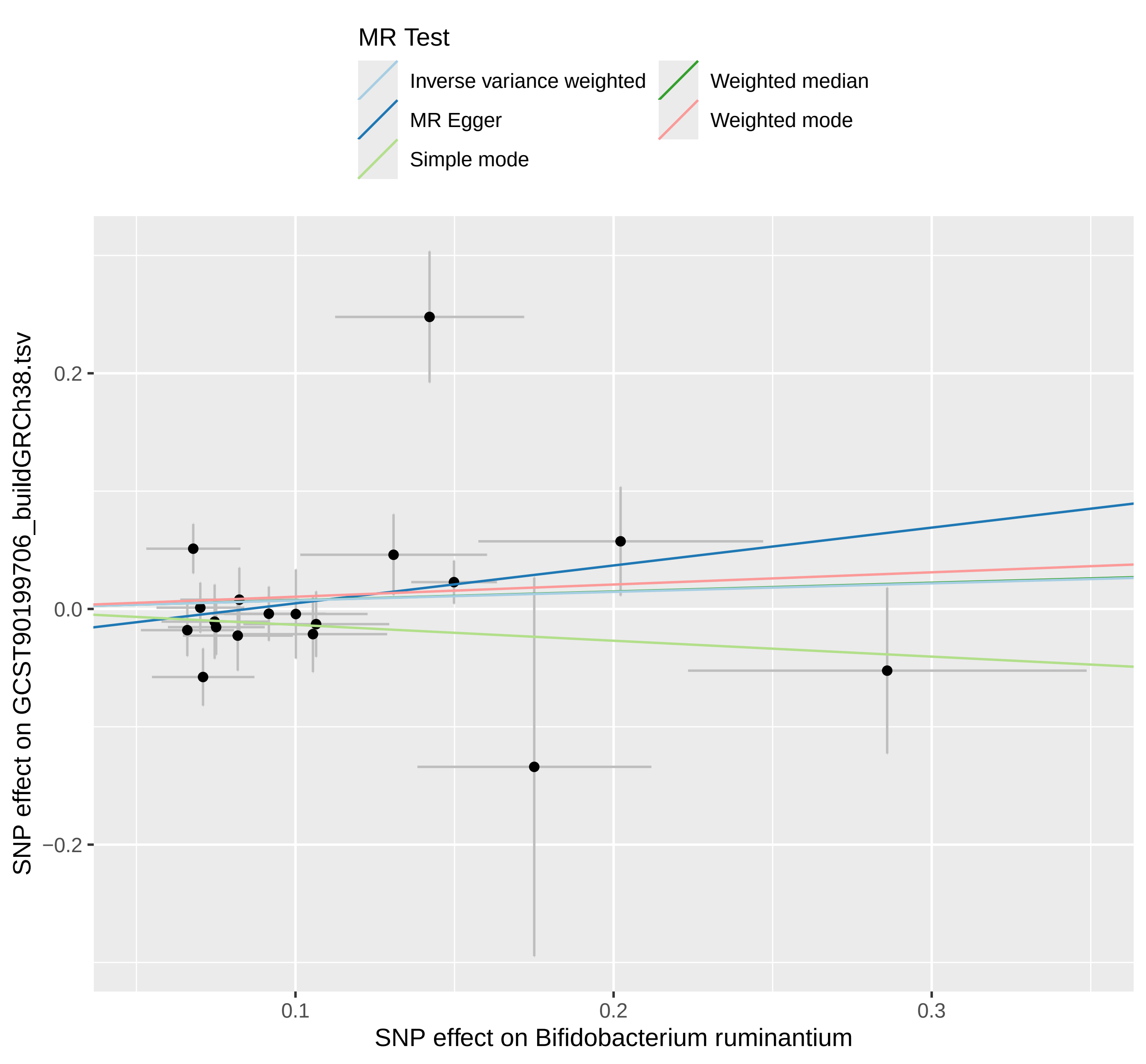
MR Test
Inverse variance weighted
MR Egger
Simple mode
Weighted median
Weighted mode
SNP effect on GCST90199706_buildGRCh38.tsv
0.2
0.0
−0.2
0.1
0.2
0.3
SNP effect on Bifidobacterium ruminantium

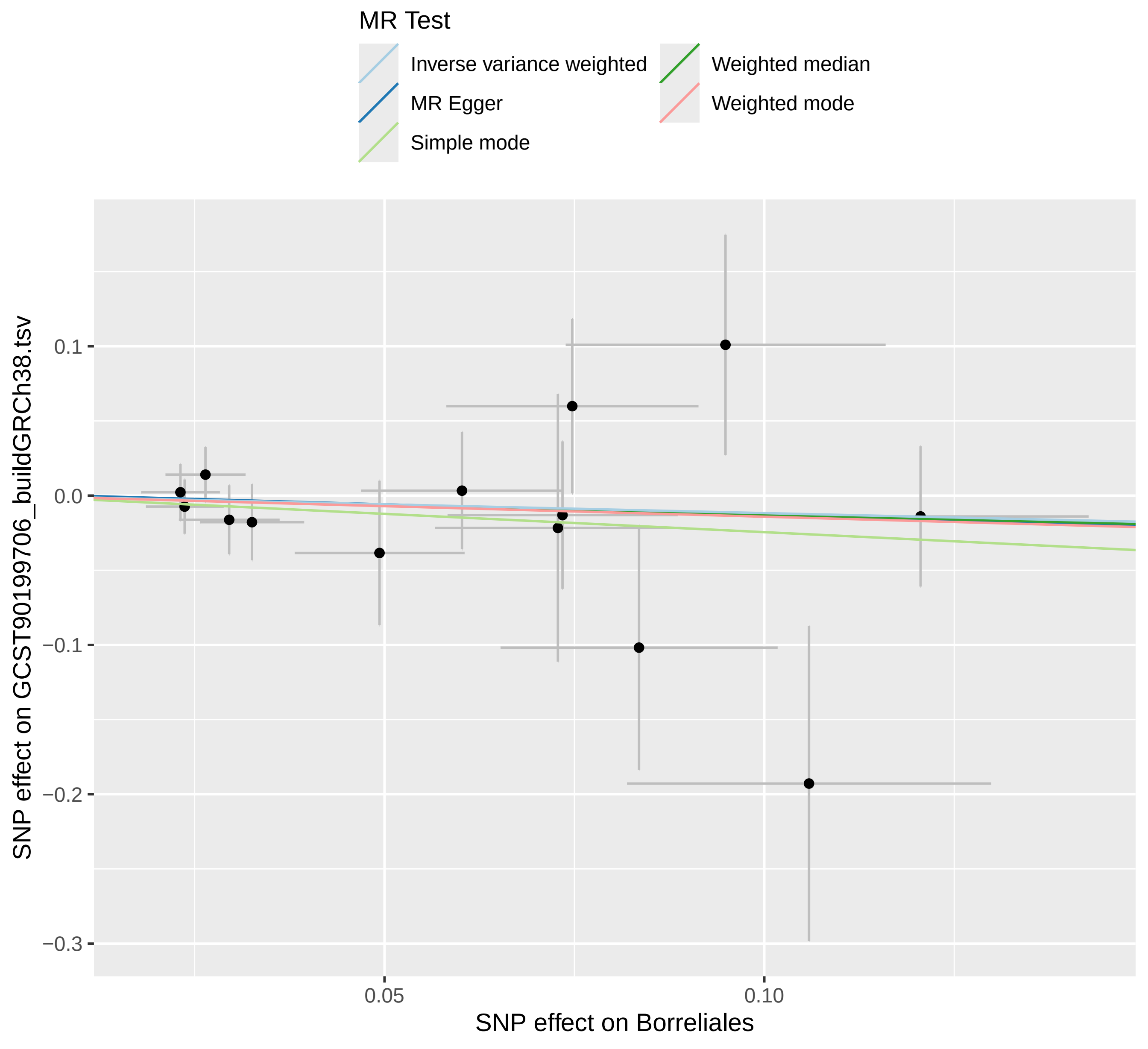
MR Test
Inverse variance weighted
MR Egger
Simple mode
Weighted median
Weighted mode
0.1
0.0
−0.1
−0.2
−0.3
SNP effect on GCST90199706_buildGRCh38.tsv
0.05
0.10
SNP effect on Borreliales

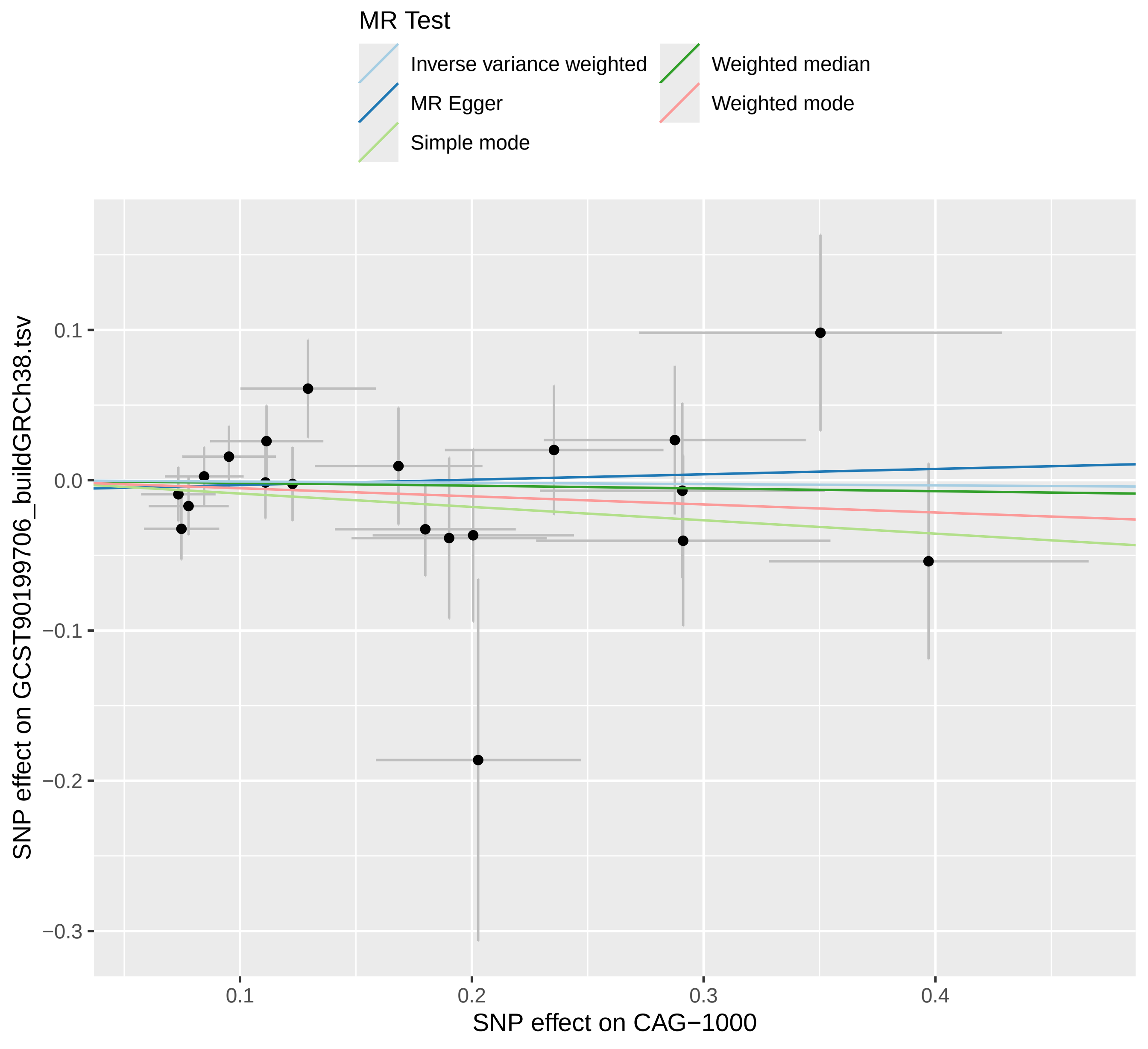

MR Test
Inverse variance weighted
MR Egger
Simple mode
Weighted median
Weighted mode
0.1
0.0
−0.1
−0.2
−0.3
0.1
0.2
0.3
0.4
SNP effect on GCST90199706_buildGRCh38.tsv
SNP effect on CAG−1000

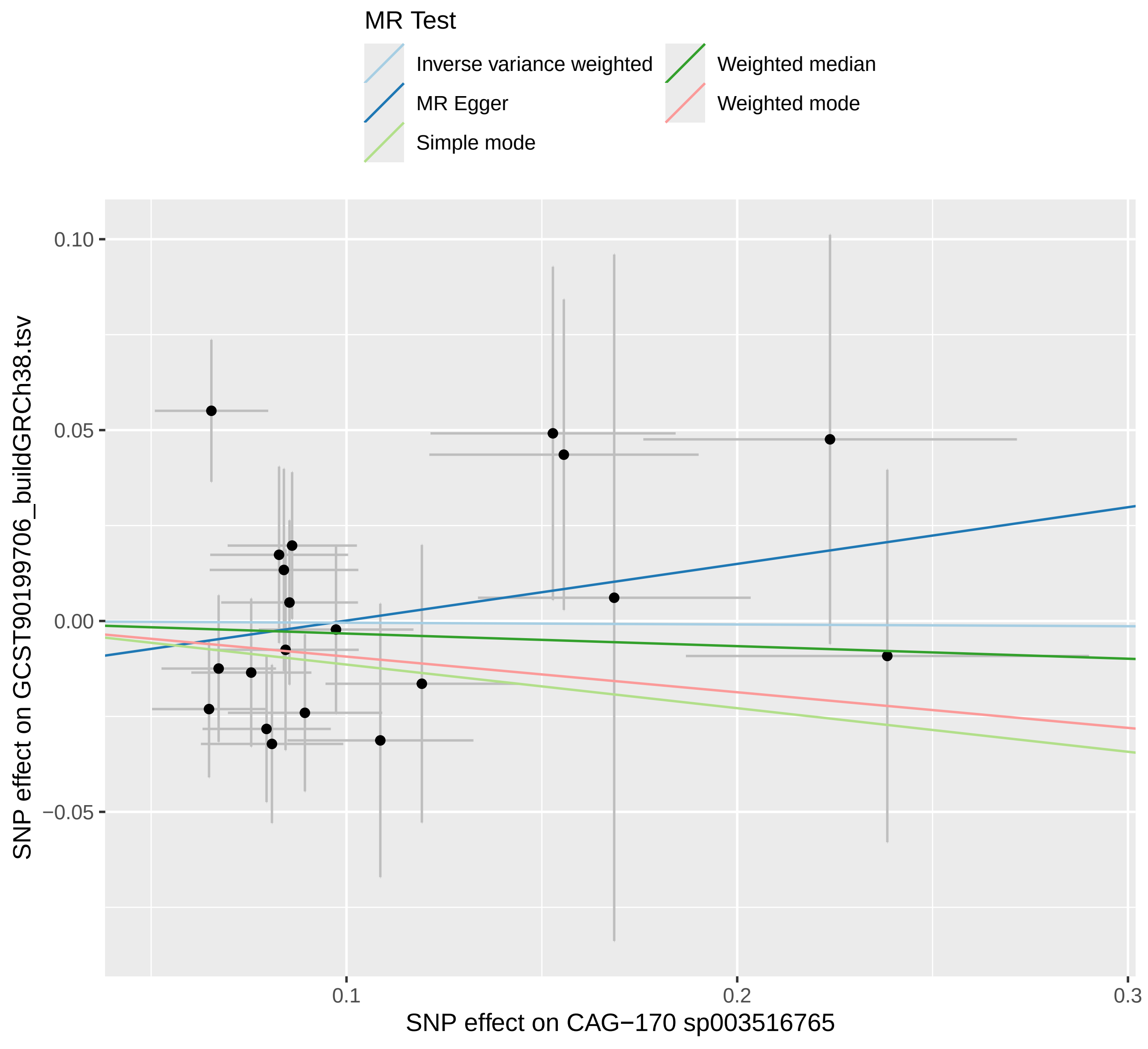
MR Test
Inverse variance weighted
MR Egger
Simple mode
Weighted median
Weighted mode
0.10
0.05
0.00
−0.05
0.1
0.2
0.3
SNP effect on GCST90199706_buildGRCh38.tsv
SNP effect on CAG−170 sp003516765

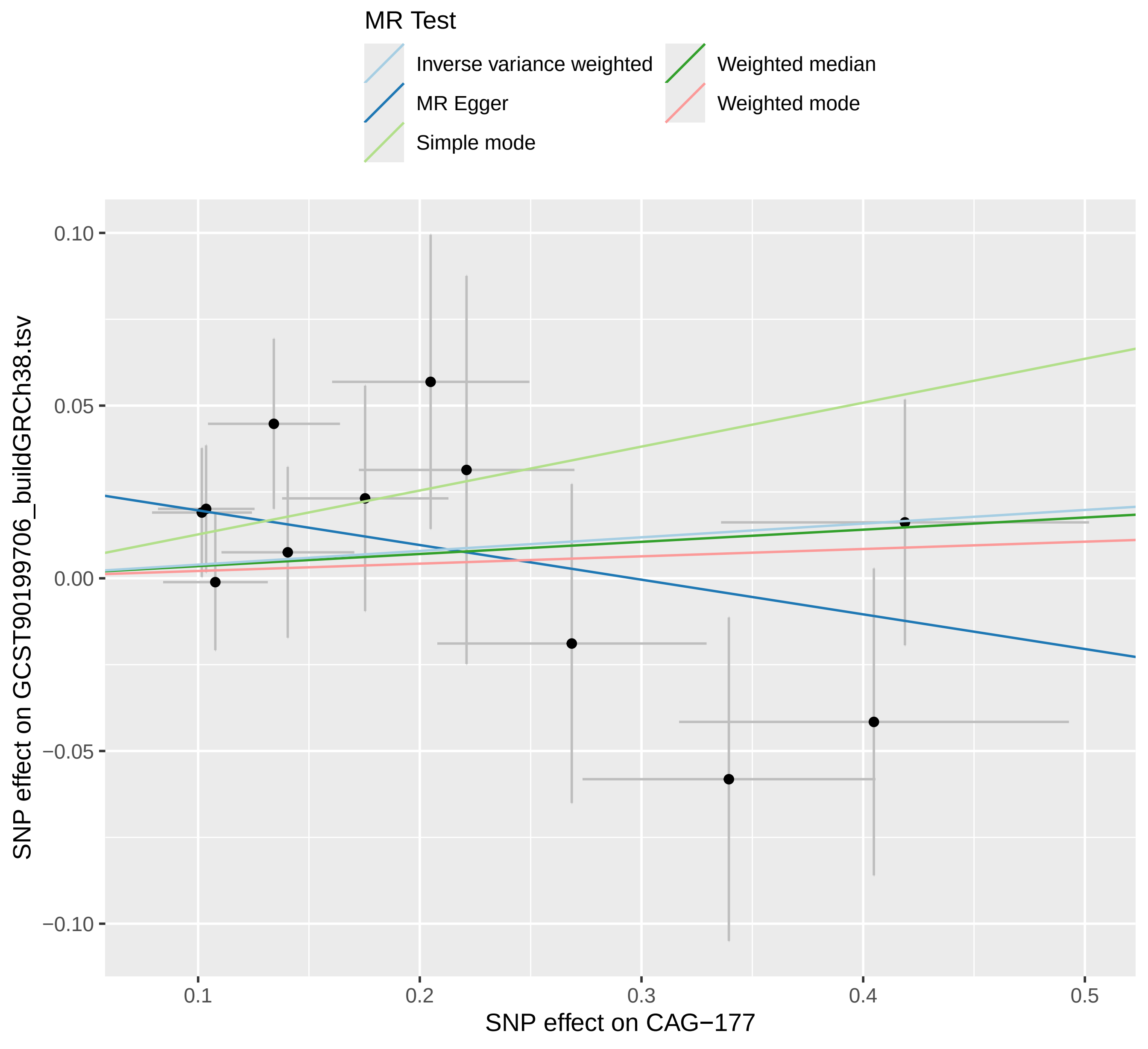

MR Test
Inverse variance weighted
MR Egger
Simple mode
Weighted median
Weighted mode
0.10
0.05
0.00
−0.05
−0.10
SNP effect on GCST90199706_buildGRCh38.tsv
0.1
0.2
0.3
0.4
0.5
SNP effect on CAG−177

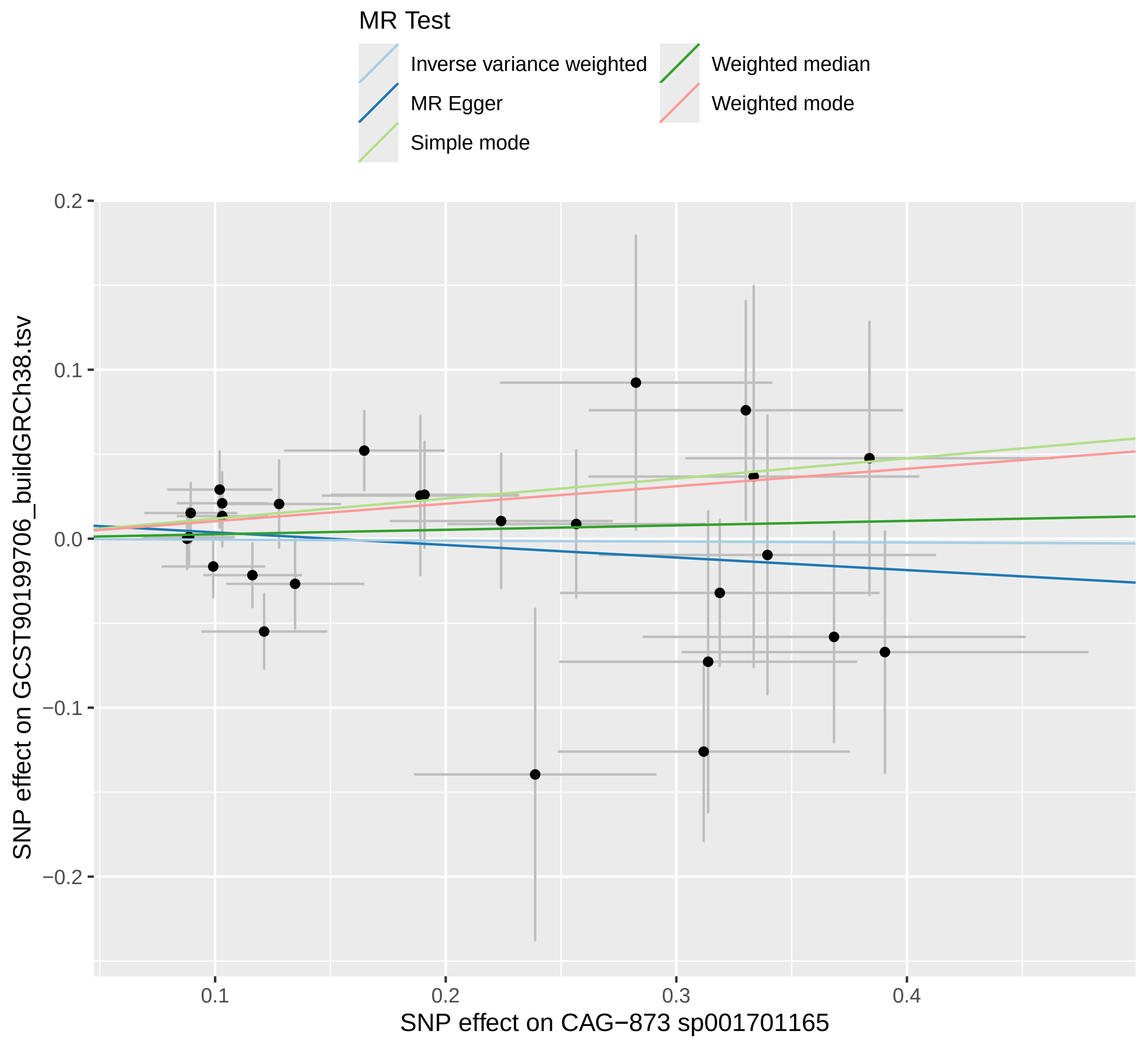
MR Test
Inverse variance weighted
MR Egger
Simple mode
Weighted median
Weighted mode
0.2
0.1
0.0
−0.1
−0.2
SNP effect on GCST90199706_buildGRCh38.tsv
0.1
0.2
0.3
0.4
SNP effect on CAG−873 sp001701165

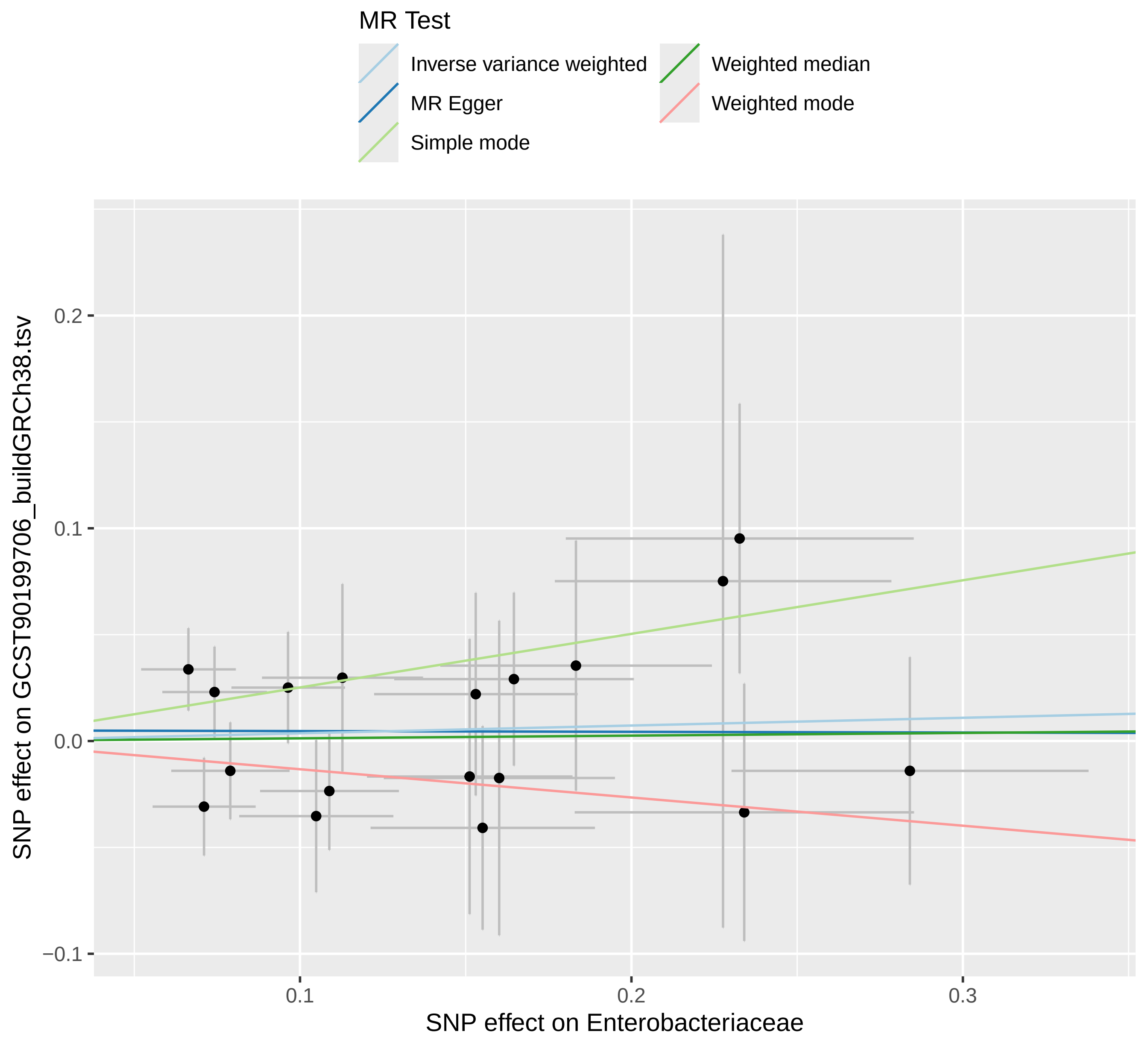
MR Test
Inverse variance weighted
MR Egger
Simple mode
Weighted median
Weighted mode
0.2
0.1
0.0
−0.1
0.1
0.2
0.3
SNP effect on GCST90199706_buildGRCh38.tsv
SNP effect on Enterobacteriaceae

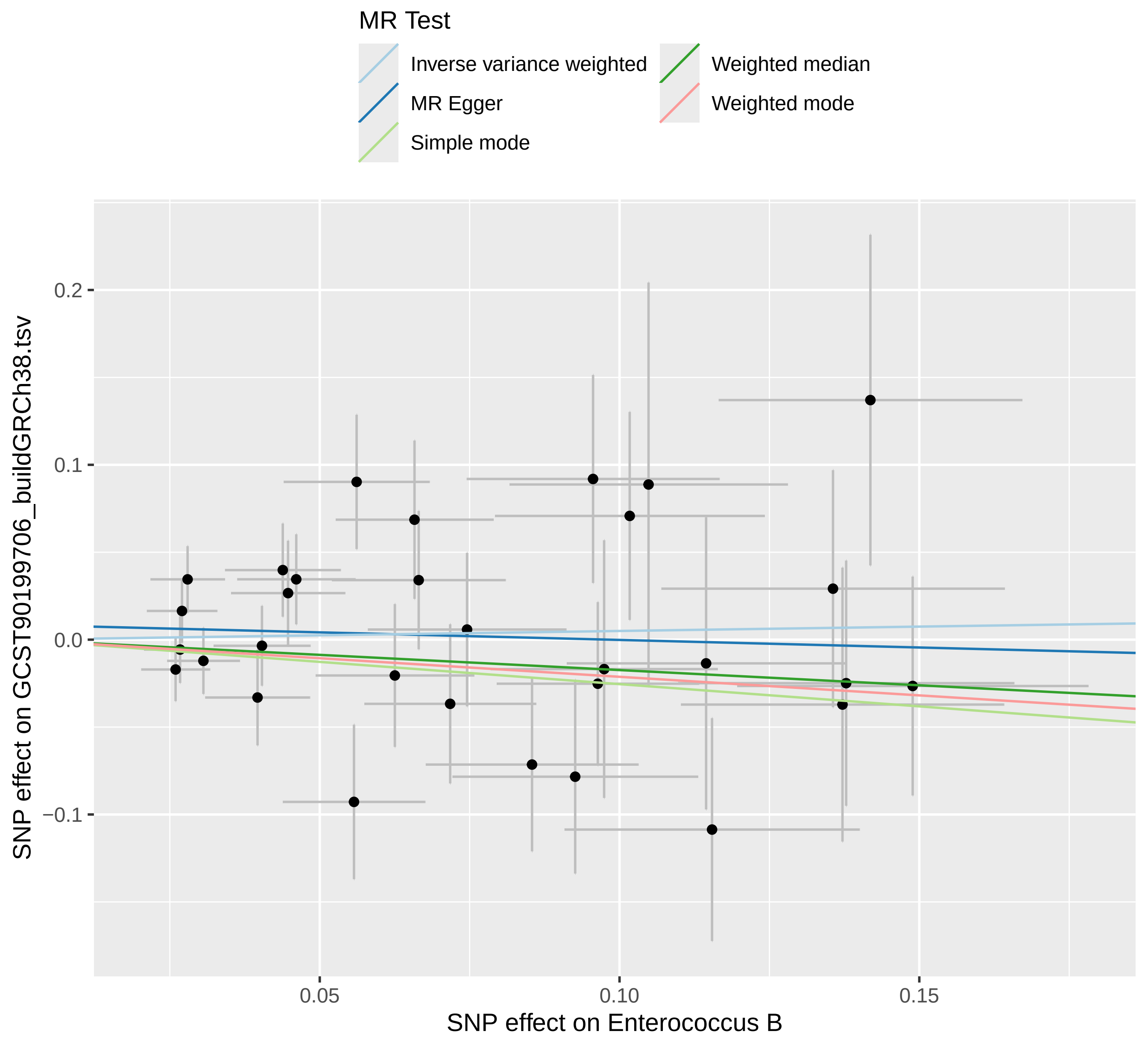
MR Test
Inverse variance weighted
MR Egger
Simple mode
Weighted median
Weighted mode
SNP effect on GCST90199706_buildGRCh38.tsv
0.2
0.1
0.0
−0.1
0.05
0.10
0.15
SNP effect on Enterococcus B

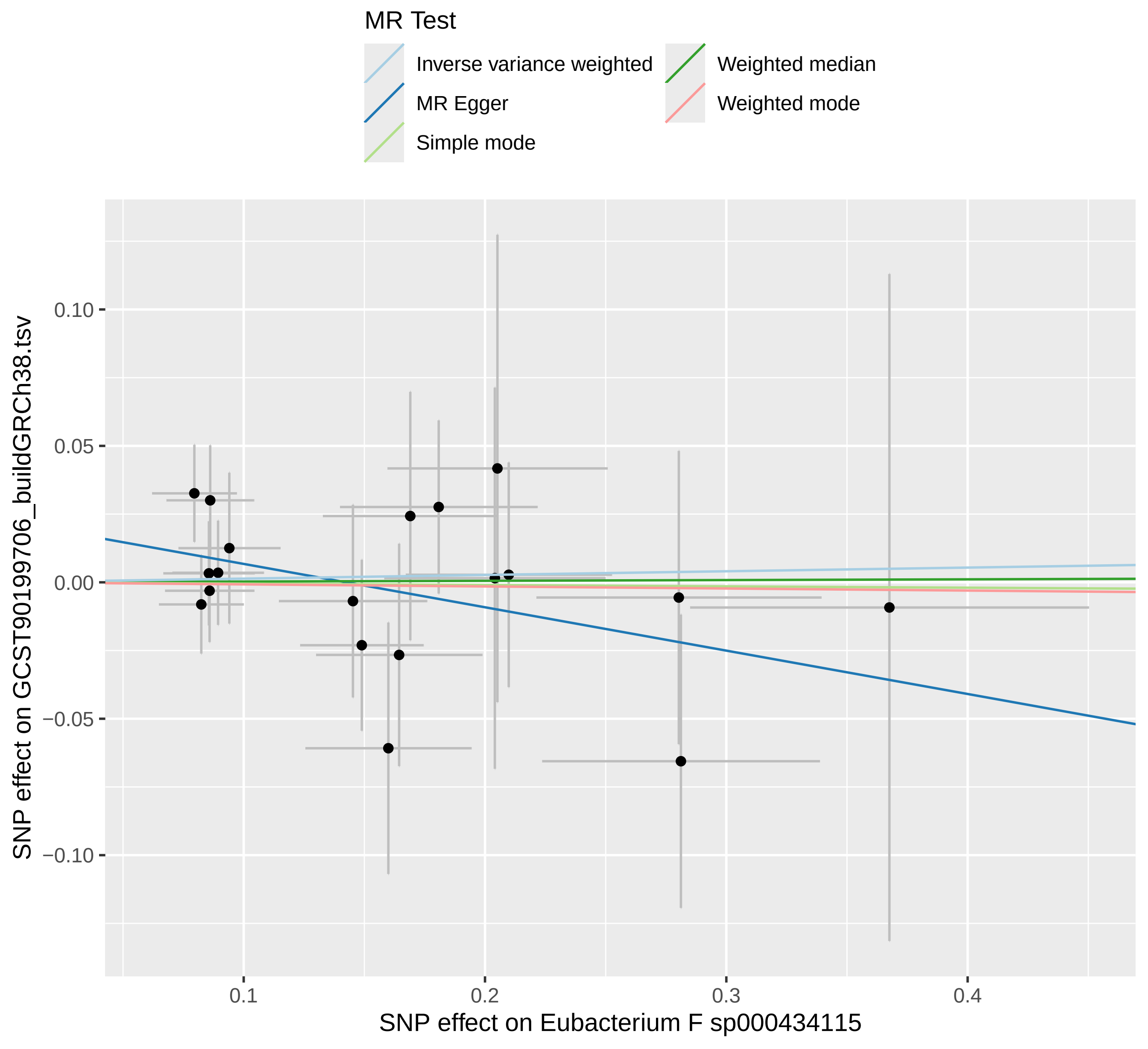
MR Test
Inverse variance weighted
MR Egger
Simple mode
Weighted median
Weighted mode
0.10
0.05
0.00
−0.05
−0.10
0.1
0.2
0.3
0.4
SNP effect on GCST90199706_buildGRCh38.tsv
SNP effect on Eubacterium F sp000434115

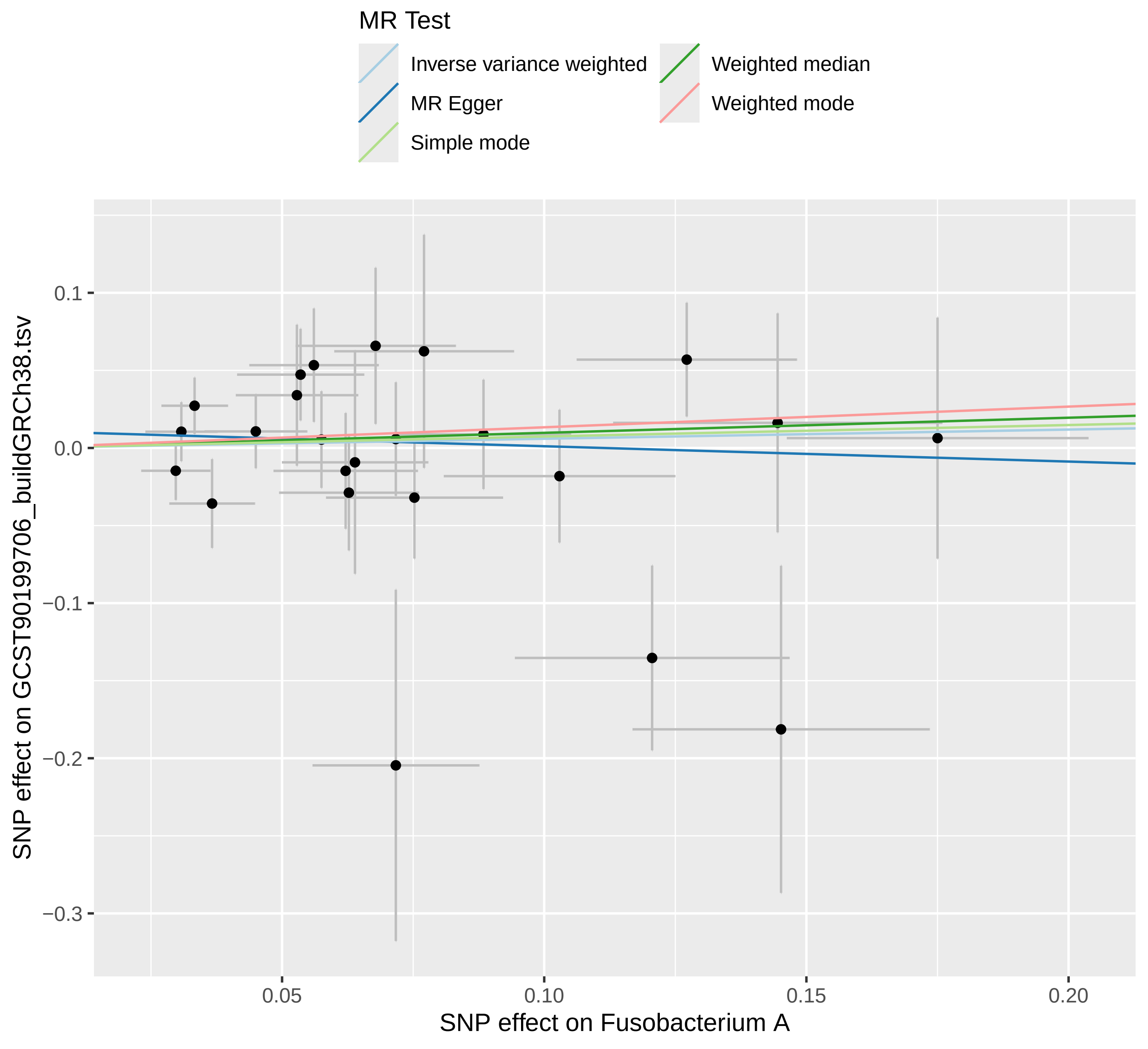
MR Test
Inverse variance weighted
MR Egger
Simple mode
Weighted median
Weighted mode
SNP effect on GCST90199706_buildGRCh38.tsv
0.1
0.0
−0.1
−0.2
−0.3
0.05
0.10
0.15
0.20
SNP effect on Fusobacterium A

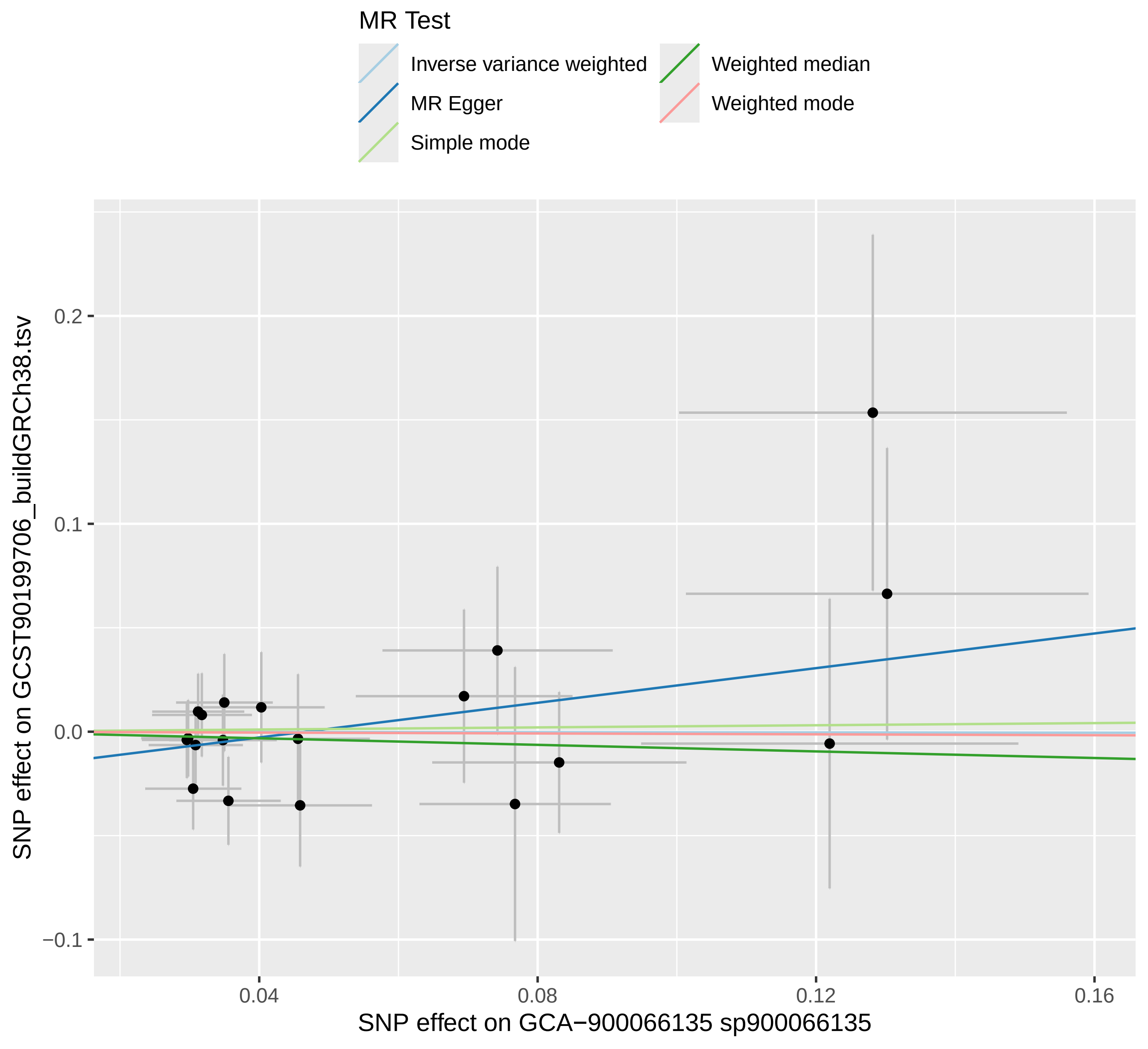

MR Test
Inverse variance weighted
MR Egger
Simple mode
Weighted median
Weighted mode
0.2
0.1
0.0
−0.1
0.04
0.08
0.12
0.16
SNP effect on GCST90199706_buildGRCh38.tsv
SNP effect on GCA−900066135 sp900066135

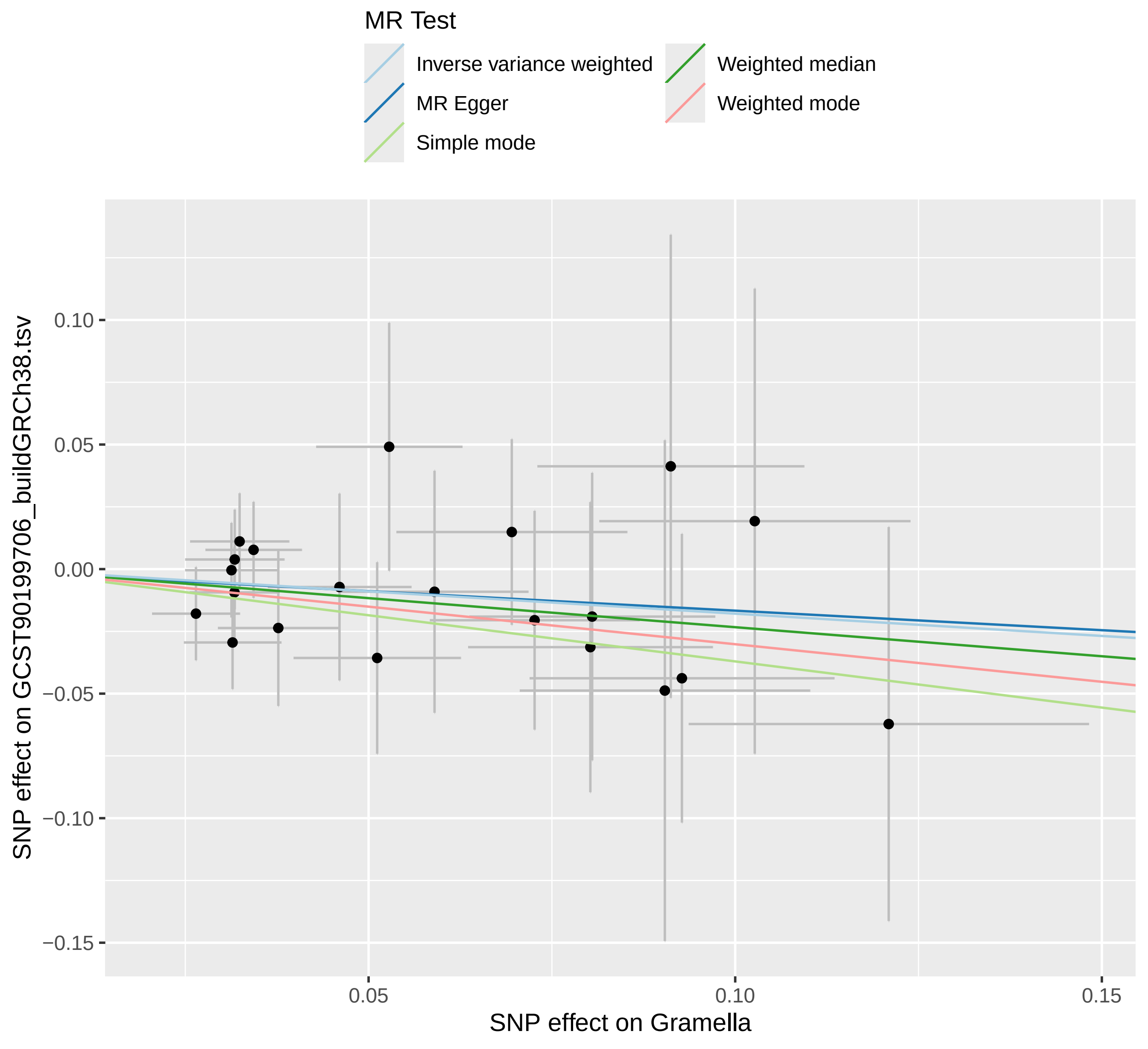

MR Test
Inverse variance weighted
MR Egger
Simple mode
Weighted median
Weighted mode
SNP effect on GCST90199706_buildGRCh38.tsv
0.10
0.05
0.00
−0.05
−0.10
−0.15
0.05
0.10
0.15
SNP effect on Gramella

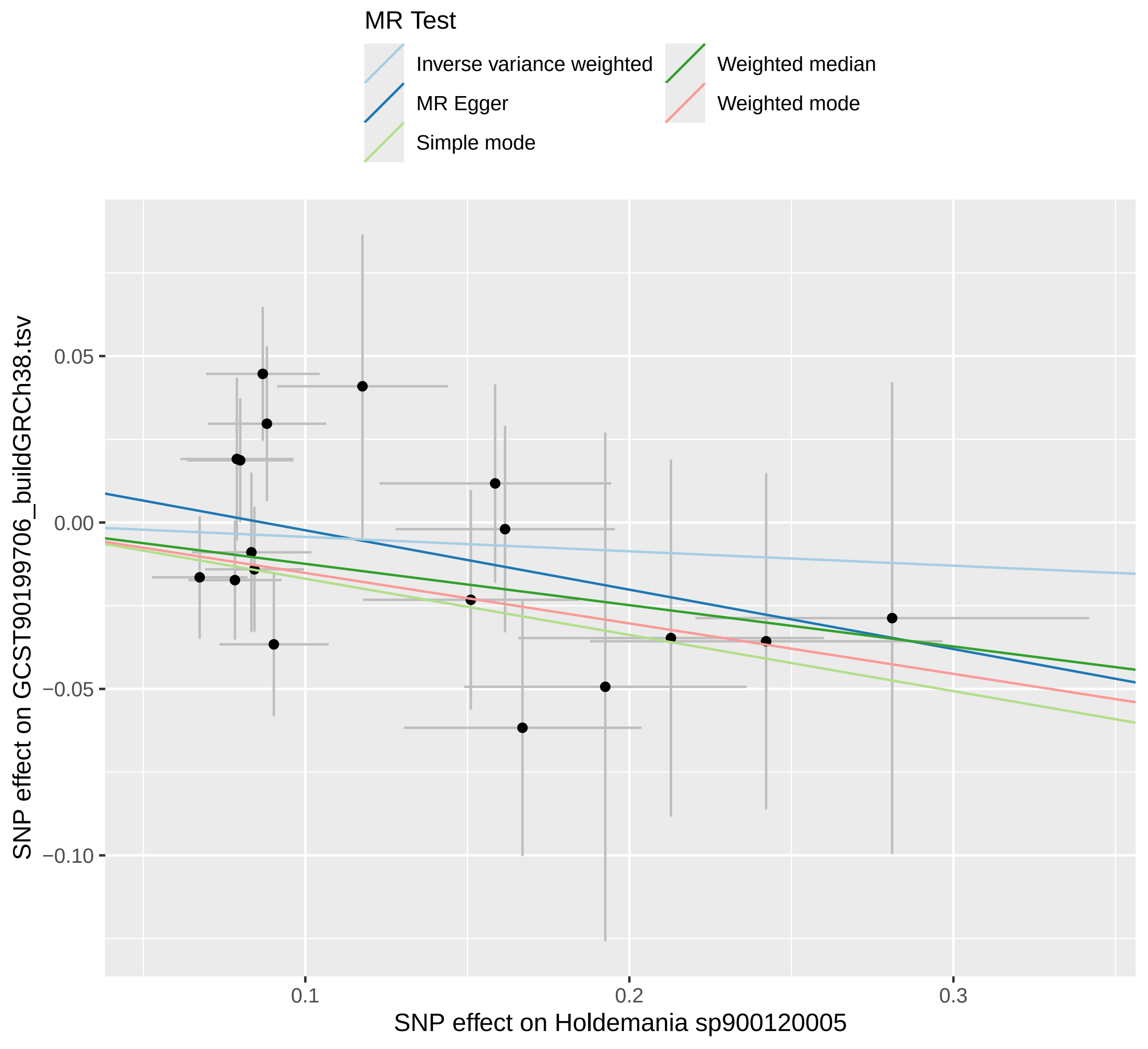

MR Test
Inverse variance weighted
MR Egger
Simple mode
Weighted median
Weighted mode
0.05
0.00
−0.05
−0.10
0.1
0.2
0.3
SNP effect on GCST90199706_buildGRCh38.tsv
SNP effect on Holdemania sp900120005

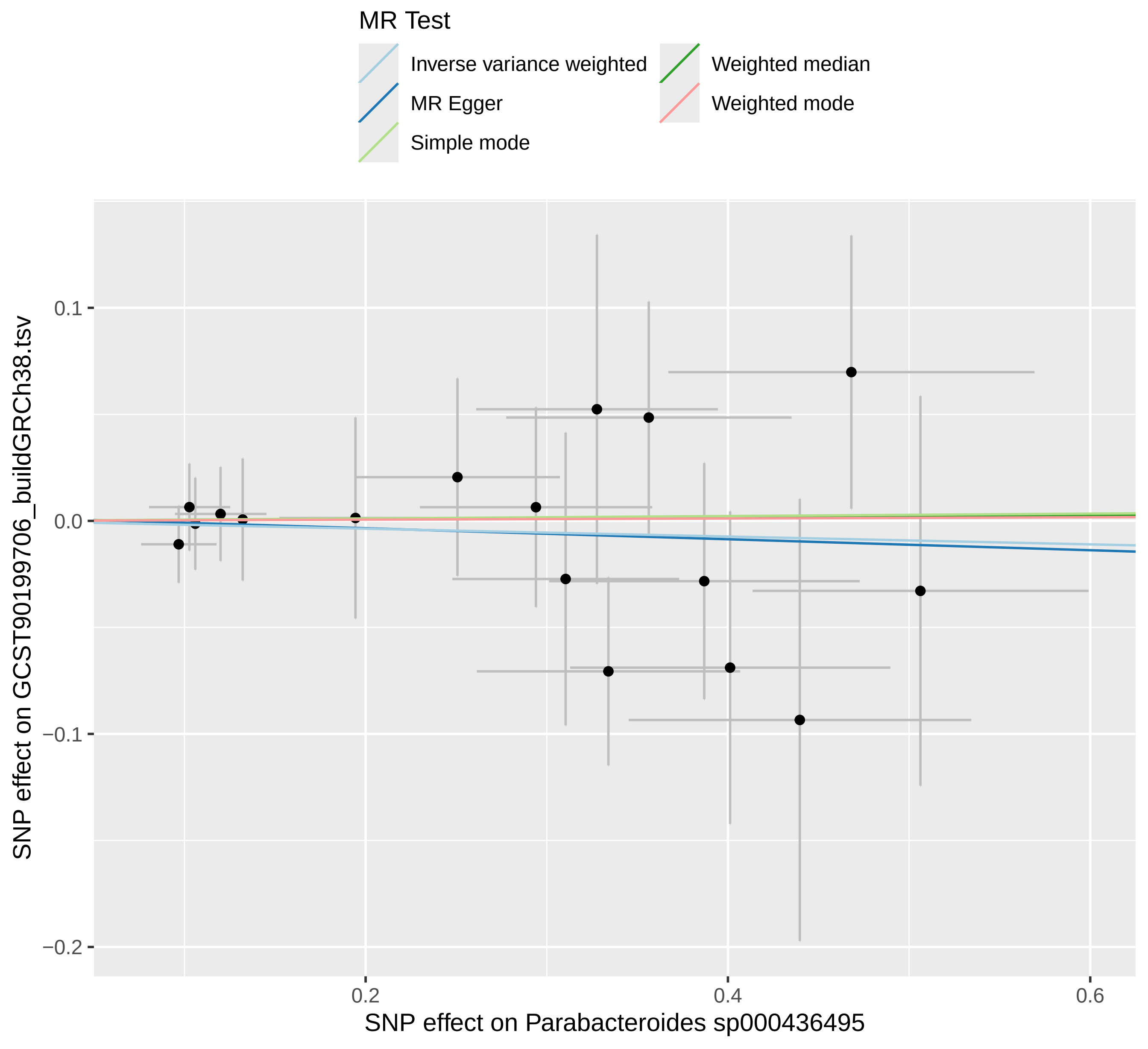

MR Test
Inverse variance weighted
MR Egger
Simple mode
Weighted median
Weighted mode
0.1
0.0
−0.1
−0.2
0.2
0.4
0.6
SNP effect on GCST90199706_buildGRCh38.tsv
SNP effect on Parabacteroides sp000436495

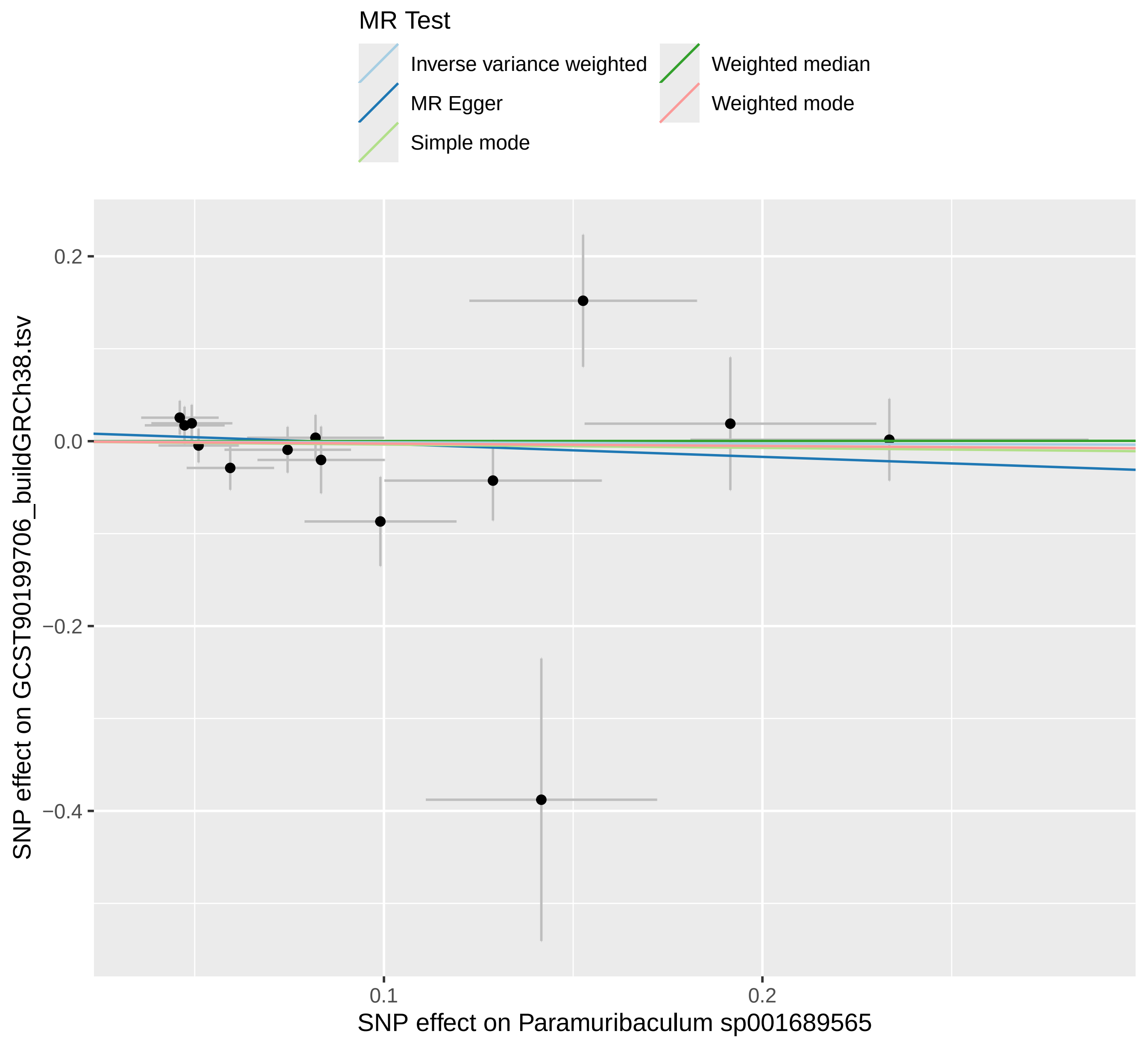
MR Test
Inverse variance weighted
MR Egger
Simple mode
Weighted median
Weighted mode
0.2
0.0
−0.2
−0.4
0.1
0.2
SNP effect on GCST90199706_buildGRCh38.tsv
SNP effect on Paramuribaculum sp001689565

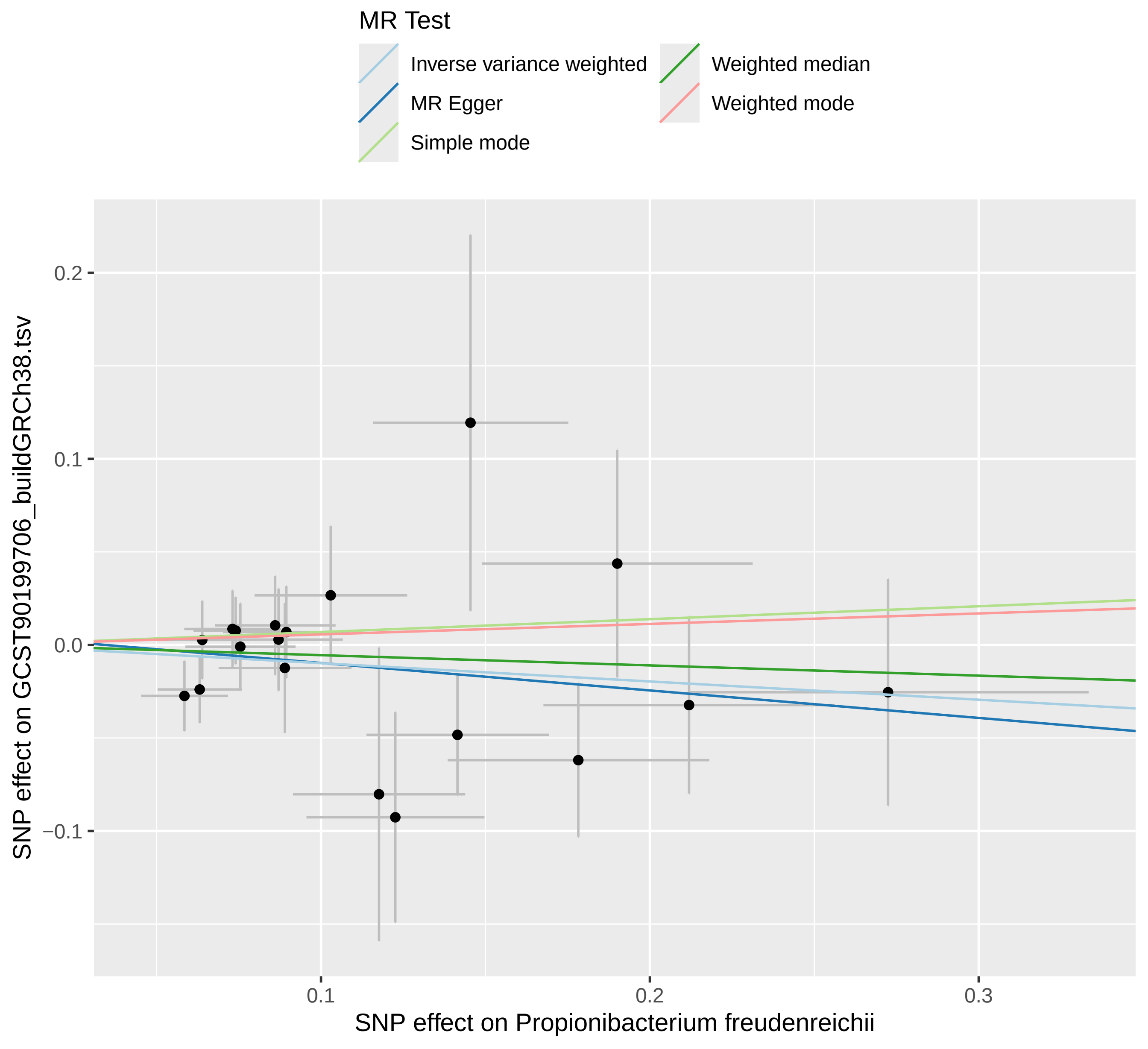
MR Test
Inverse variance weighted
MR Egger
Simple mode
Weighted median
Weighted mode
0.2
0.1
0.0
−0.1
0.1
0.2
0.3
SNP effect on GCST90199706_buildGRCh38.tsv
SNP effect on Propionibacterium freudenreichii

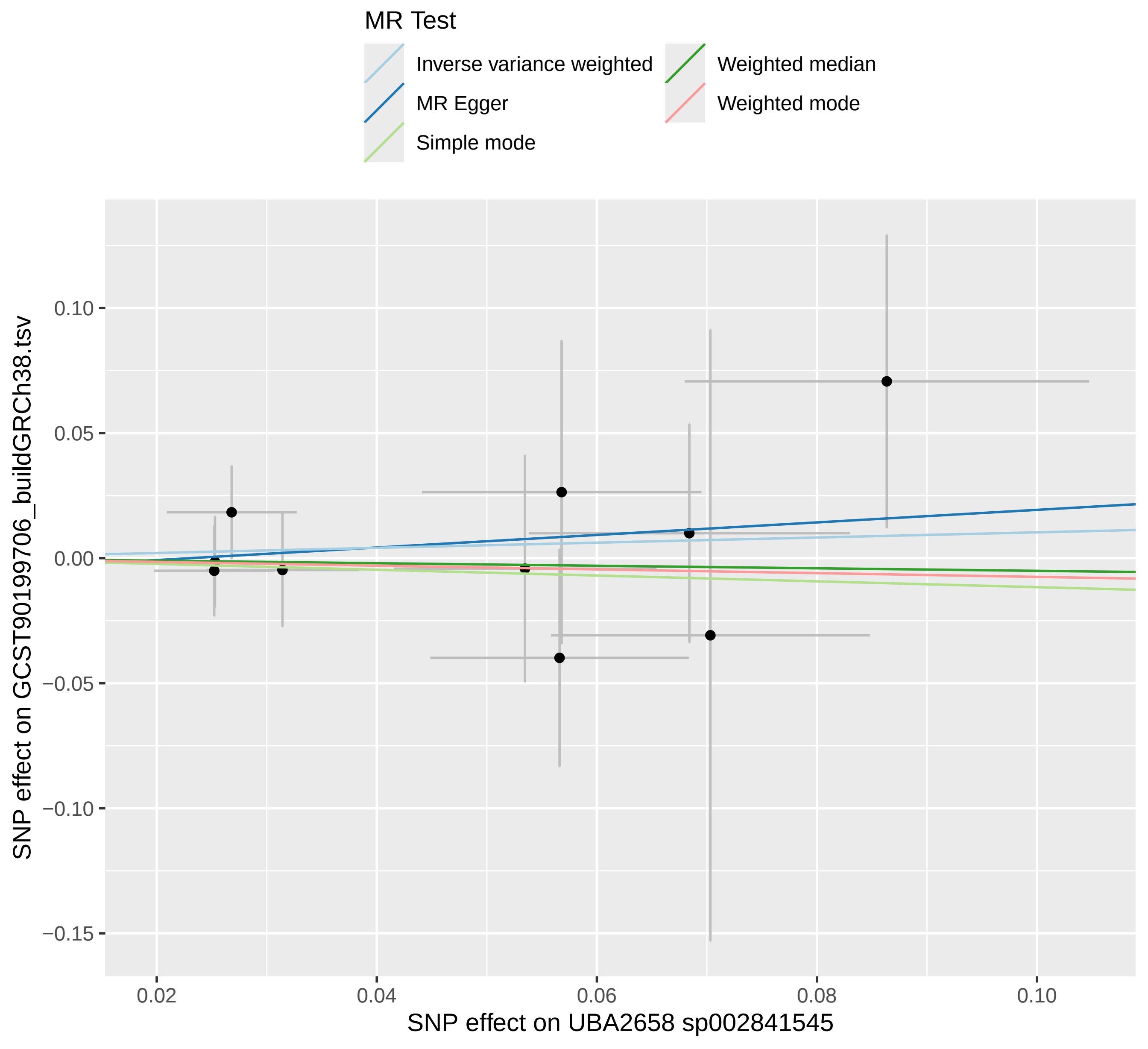
MR Test
Inverse variance weighted
MR Egger
Simple mode
Weighted median
Weighted mode
0.10
0.05
0.00
−0.05
−0.10
−0.15
0.02
0.04
0.06
0.08
0.10
SNP effect on GCST90199706_buildGRCh38.tsv
SNP effect on UBA2658 sp002841545

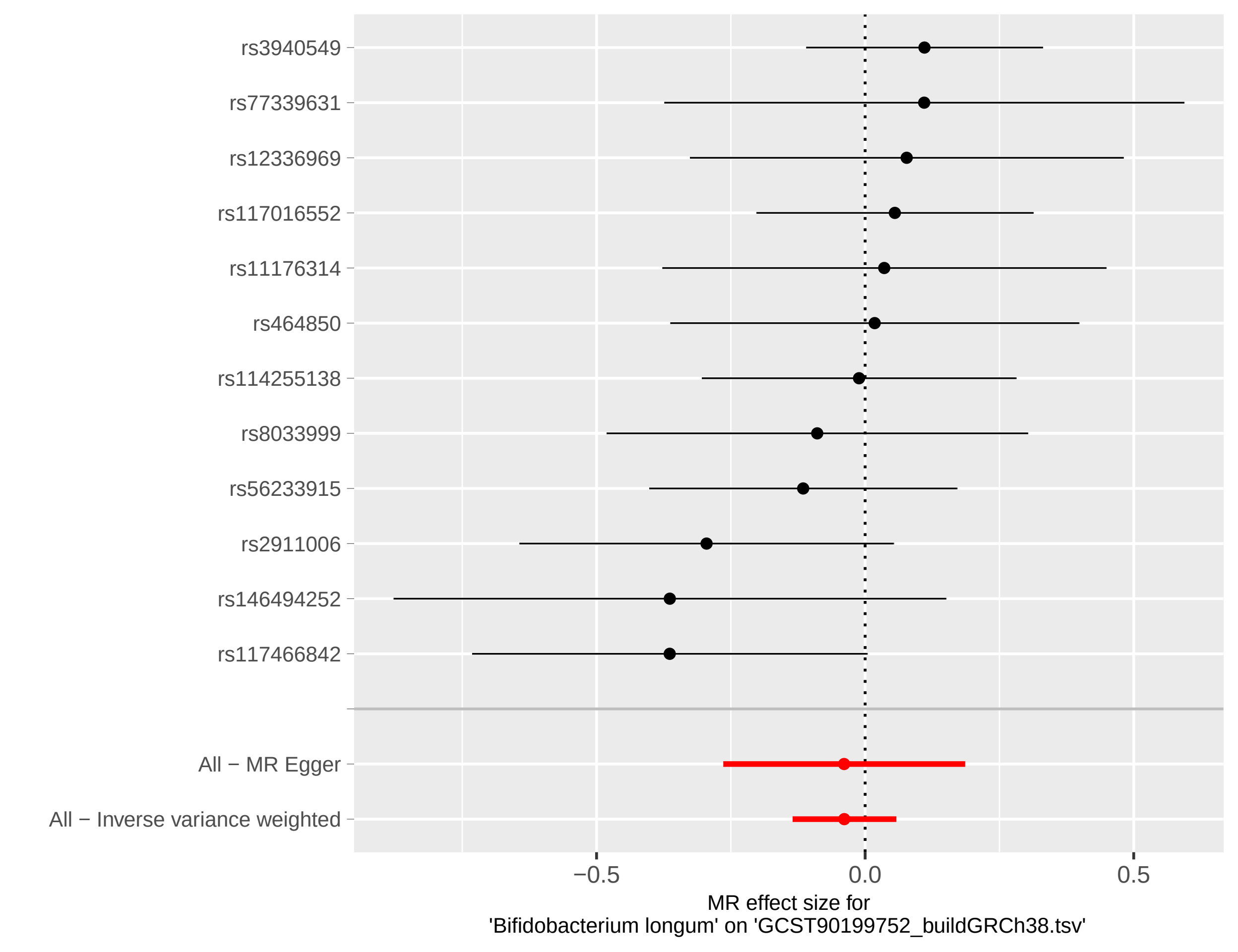
rs3940549
rs77339631
rs12336969
rs117016552
rs11176314
rs464850
rs114255138
rs8033999
rs56233915
rs2911006
rs146494252
rs117466842
All − MR Egger
All − Inverse variance weighted
−0.5
0.0
0.5
MR effect size for
'Bifidobacterium longum' on 'GCST90199752_buildGRCh38.tsv'

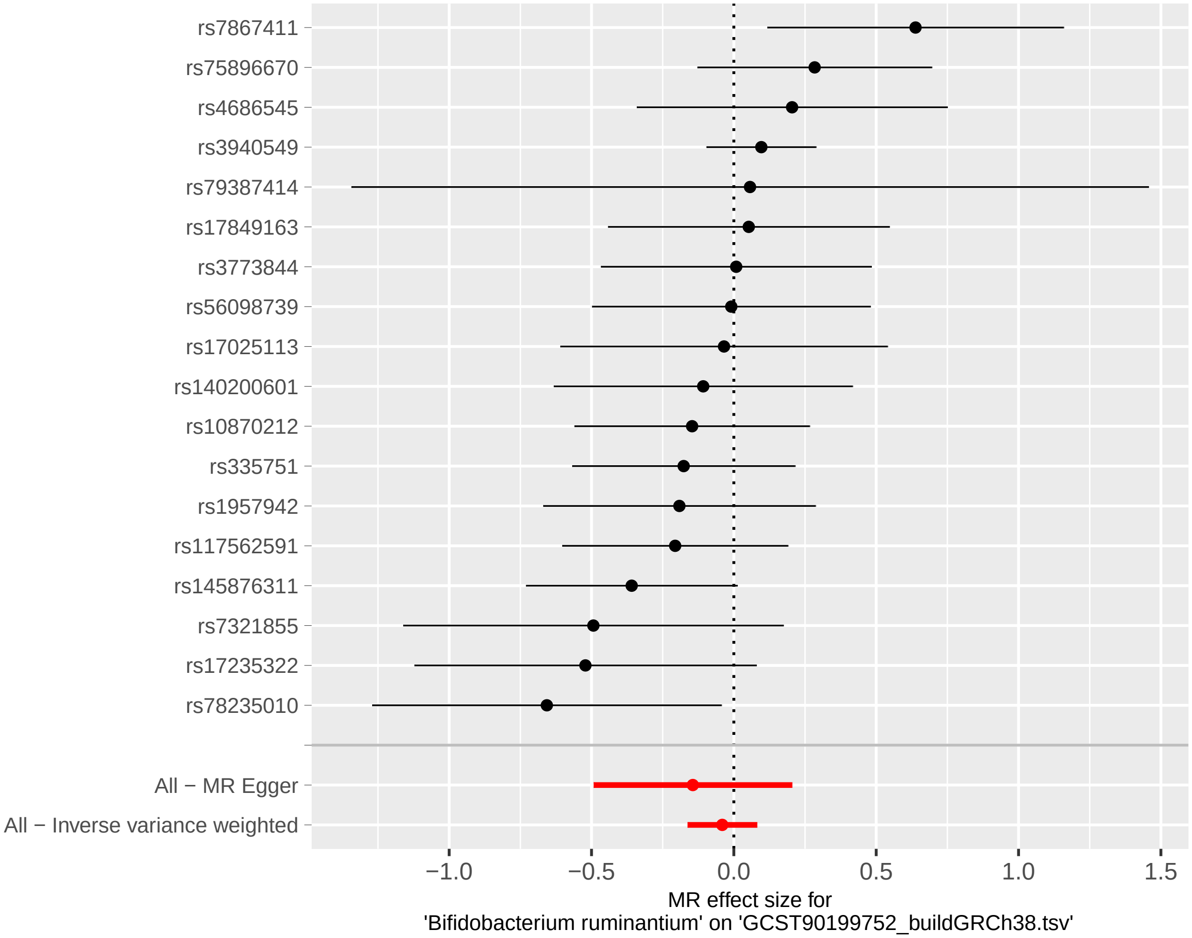
rs7867411
rs75896670
rs4686545
rs3940549
rs79387414
rs17849163
rs3773844
rs56098739
rs17025113
rs140200601
rs10870212
rs335751
rs1957942
rs117562591
rs145876311
rs7321855
rs17235322
rs78235010
All − MR Egger
All − Inverse variance weighted
−1.0
−0.5
0.0
0.5
1.0
1.5
MR effect size for
'Bifidobacterium ruminantium' on 'GCST90199752_buildGRCh38.tsv'

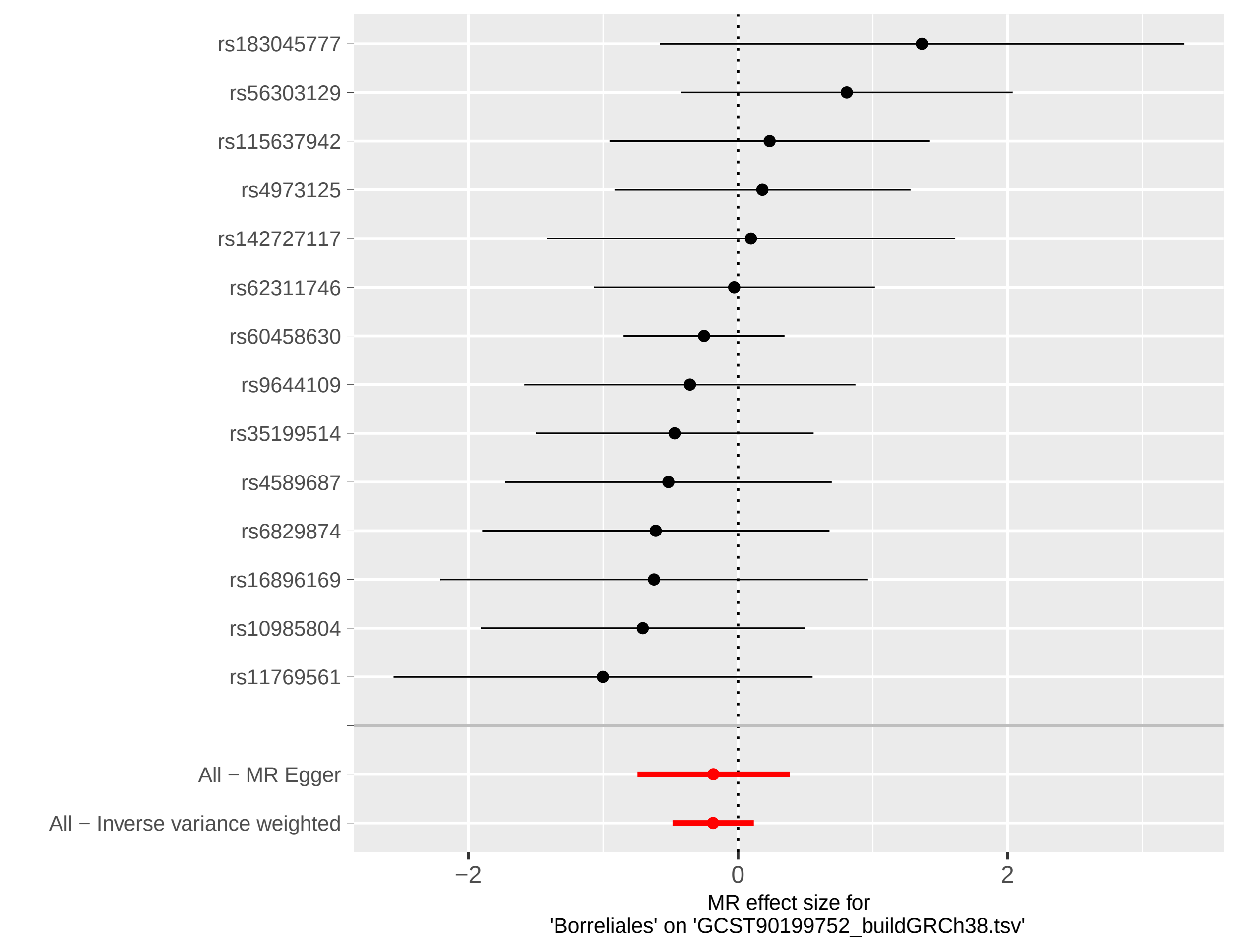
rs183045777
rs56303129
rs115637942
rs4973125
rs142727117
rs62311746
rs60458630
rs9644109
rs35199514
rs4589687
rs6829874
rs16896169
rs10985804
rs11769561
All − MR Egger
All − Inverse variance weighted
−2
0
2
MR effect size for
'Borreliales' on 'GCST90199752_buildGRCh38.tsv'

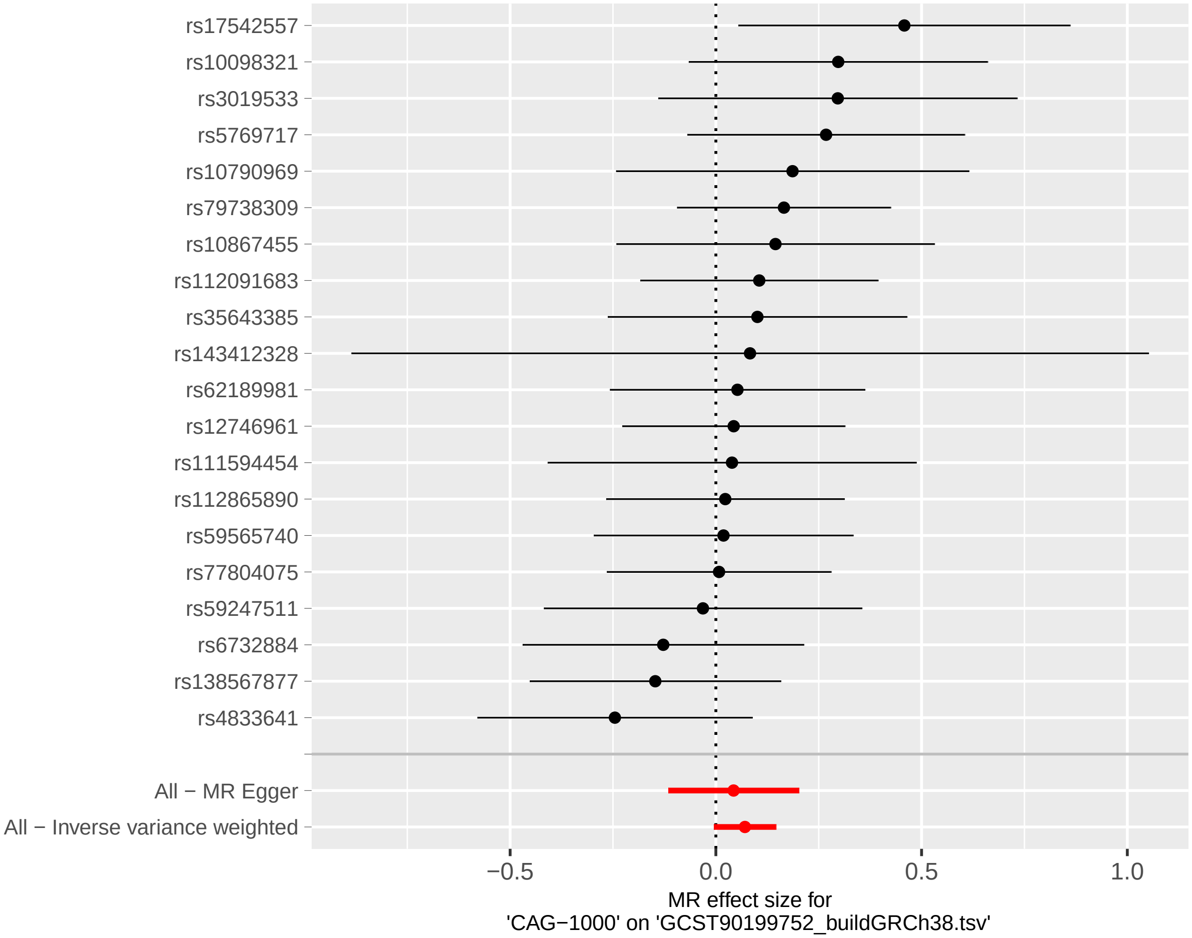
rs17542557
rs10098321
rs3019533
rs5769717
rs10790969
rs79738309
rs10867455
rs112091683
rs35643385
rs143412328
rs62189981
rs12746961
rs111594454
rs112865890
rs59565740
rs77804075
rs59247511
rs6732884
rs138567877
rs4833641
All − MR Egger
All − Inverse variance weighted
−0.5
0.0
0.5
1.0
MR effect size for
'CAG−1000' on 'GCST90199752_buildGRCh38.tsv'

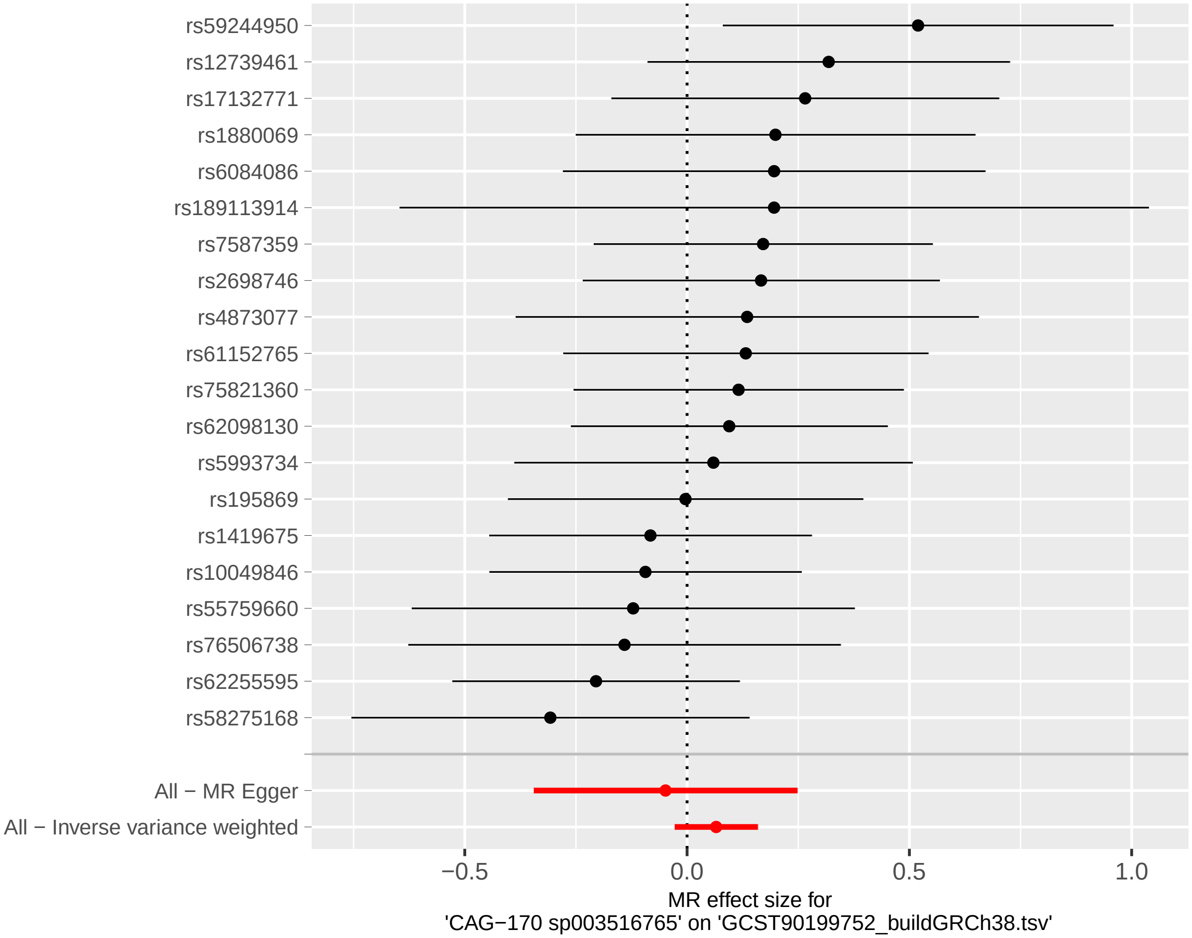
rs59244950
rs12739461
rs17132771
rs1880069
rs6084086
rs189113914
rs7587359
rs2698746
rs4873077
rs61152765
rs75821360
rs62098130
rs5993734
rs195869
rs1419675
rs10049846
rs55759660
rs76506738
rs62255595
rs58275168
All − MR Egger
All − Inverse variance weighted
−0.5
0.0
0.5
1.0
MR effect size for
'CAG−170 sp003516765' on 'GCST90199752_buildGRCh38.tsv'

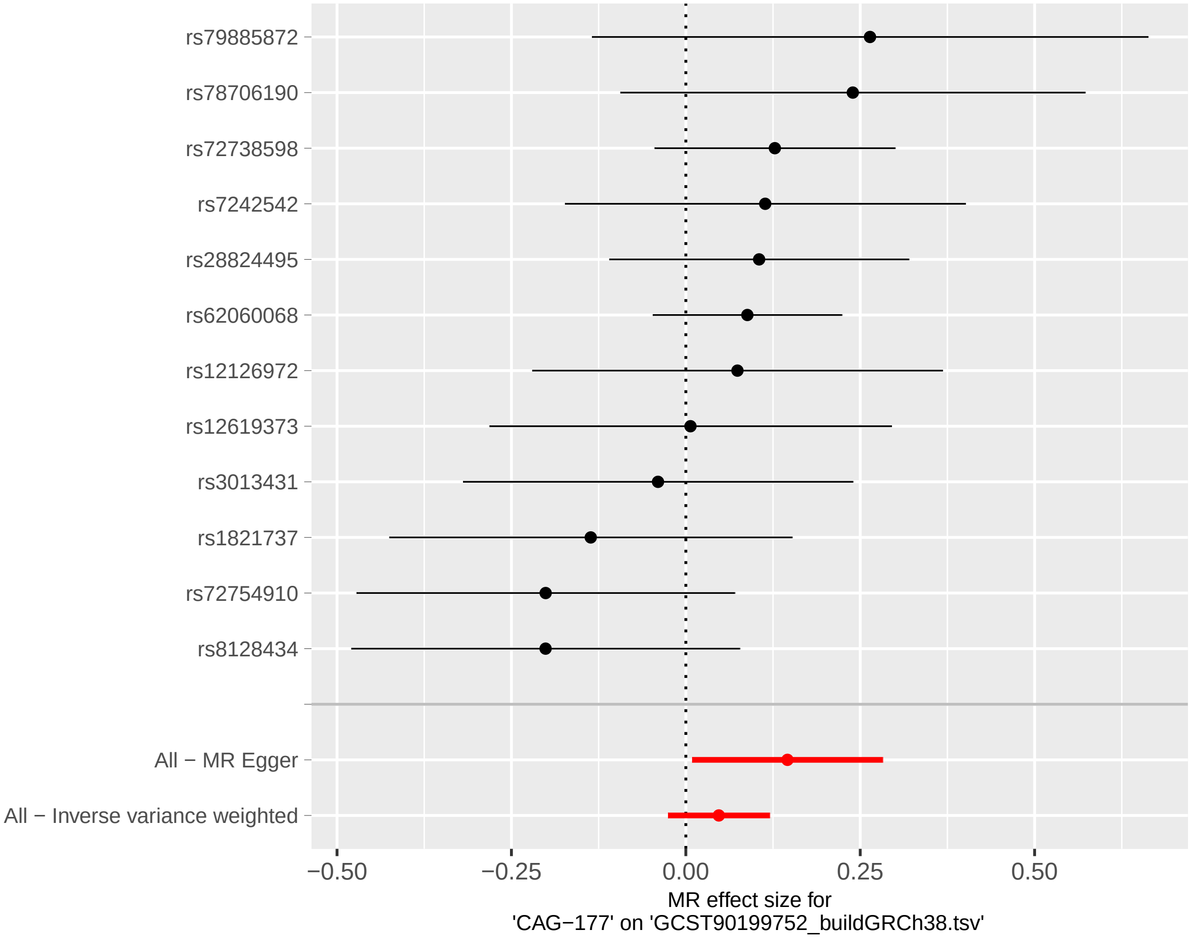
rs79885872
rs78706190
rs72738598
rs7242542
rs28824495
rs62060068
rs12126972
rs12619373
rs3013431
rs1821737
rs72754910
rs8128434
All − MR Egger
All − Inverse variance weighted
−0.50
−0.25
0.00
0.25
0.50
MR effect size for
'CAG−177' on 'GCST90199752_buildGRCh38.tsv'

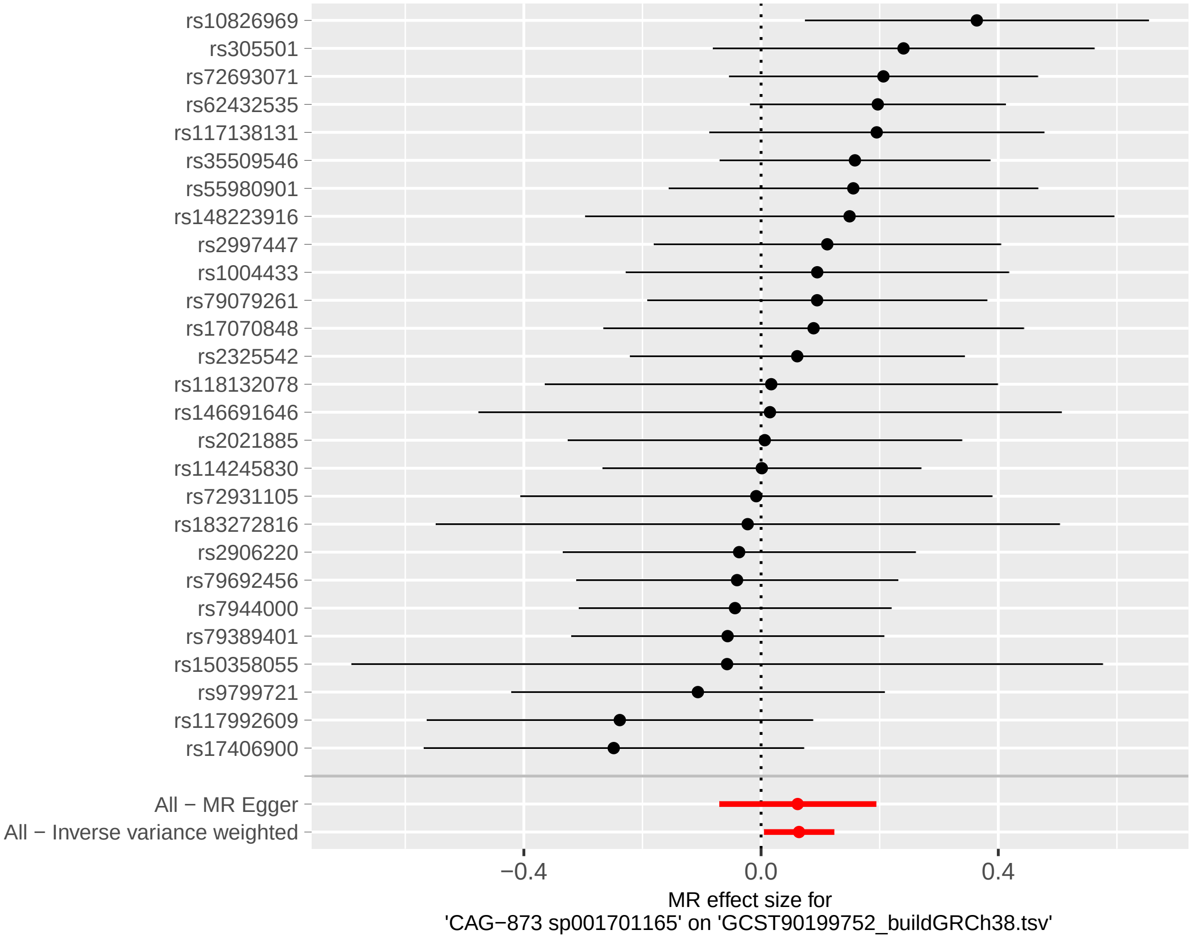
rs10826969
rs305501
rs72693071
rs62432535
rs117138131
rs35509546
rs55980901
rs148223916
rs2997447
rs1004433
rs79079261
rs17070848
rs2325542
rs118132078
rs146691646
rs2021885
rs114245830
rs72931105
rs183272816
rs2906220
rs79692456
rs7944000
rs79389401
rs150358055
rs9799721
rs117992609
rs17406900
All − MR Egger
All − Inverse variance weighted
−0.4
0.0
0.4
MR effect size for
'CAG−873 sp001701165' on 'GCST90199752_buildGRCh38.tsv'

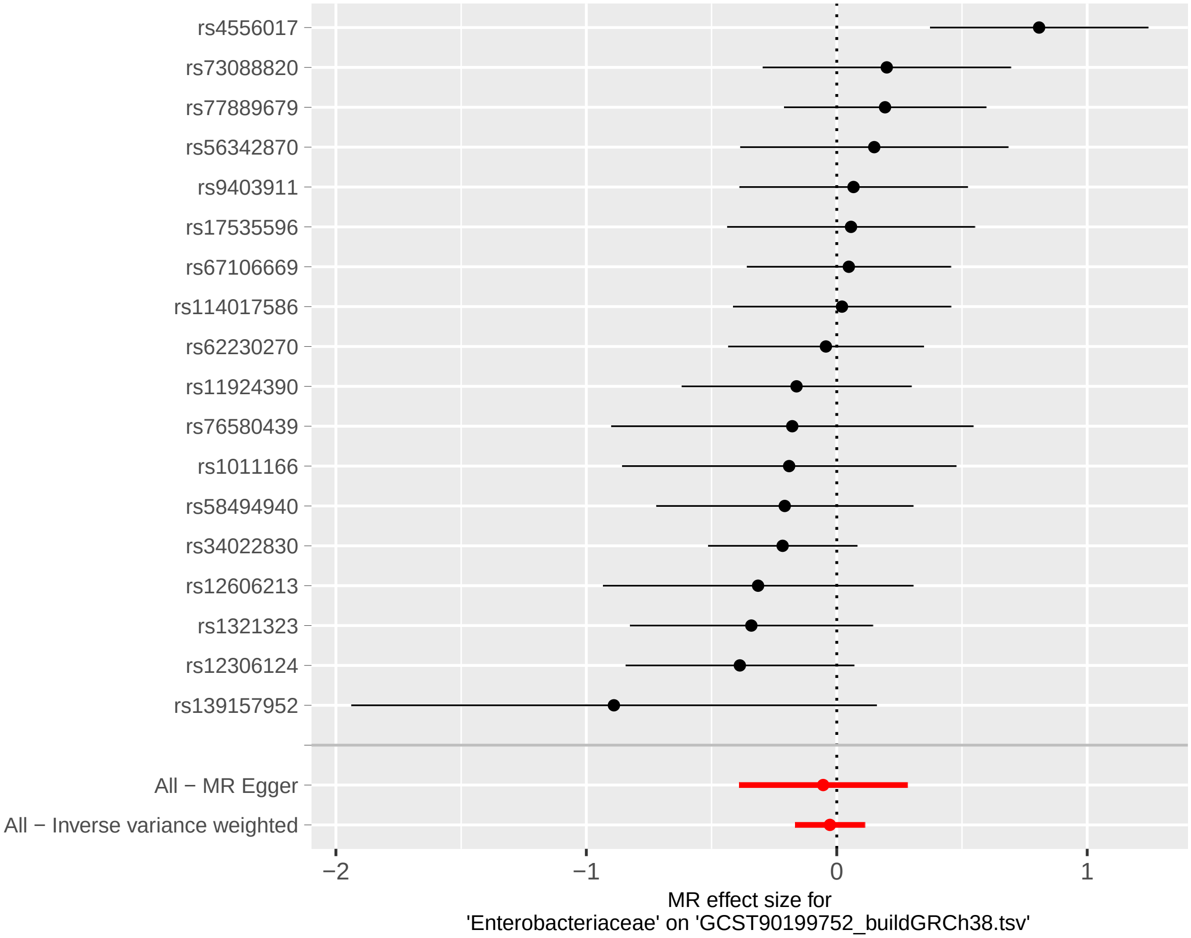
rs4556017
rs73088820
rs77889679
rs56342870
rs9403911
rs17535596
rs67106669
rs114017586
rs62230270
rs11924390
rs76580439
rs1011166
rs58494940
rs34022830
rs12606213
rs1321323
rs12306124
rs139157952
All − MR Egger
All − Inverse variance weighted
−2
−1
0
1
MR effect size for
'Enterobacteriaceae' on 'GCST90199752_buildGRCh38.tsv'

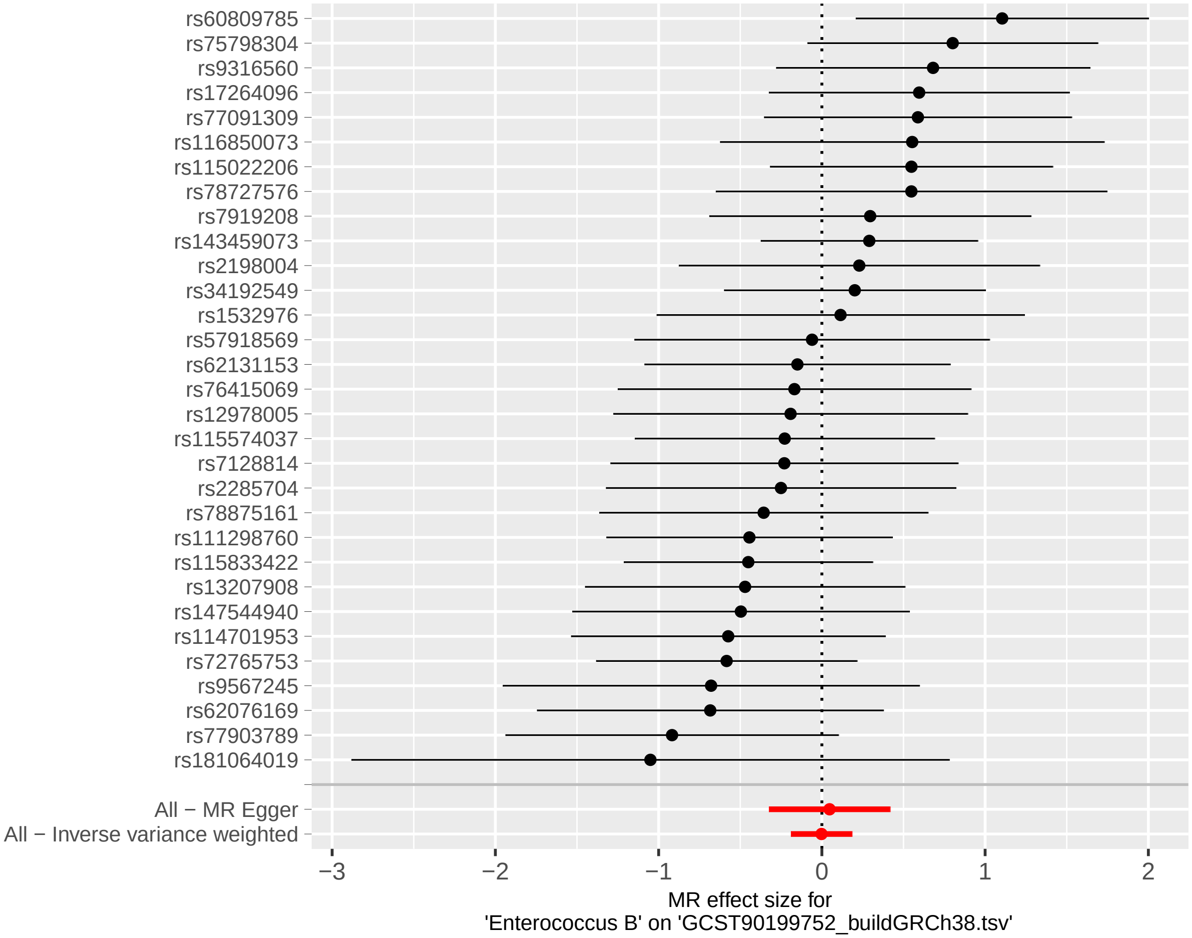

rs60809785
rs75798304
rs9316560
rs17264096
rs77091309
rs116850073
rs115022206
rs78727576
rs7919208
rs143459073
rs2198004
rs34192549
rs1532976
rs57918569
rs62131153
rs76415069
rs12978005
rs115574037
rs7128814
rs2285704
rs78875161
rs111298760
rs115833422
rs13207908
rs147544940
rs114701953
rs72765753
rs9567245
rs62076169
rs77903789
rs181064019
All − MR Egger
All − Inverse variance weighted
−3
−2
−1
0
1
2
MR effect size for
'Enterococcus B' on 'GCST90199752_buildGRCh38.tsv'

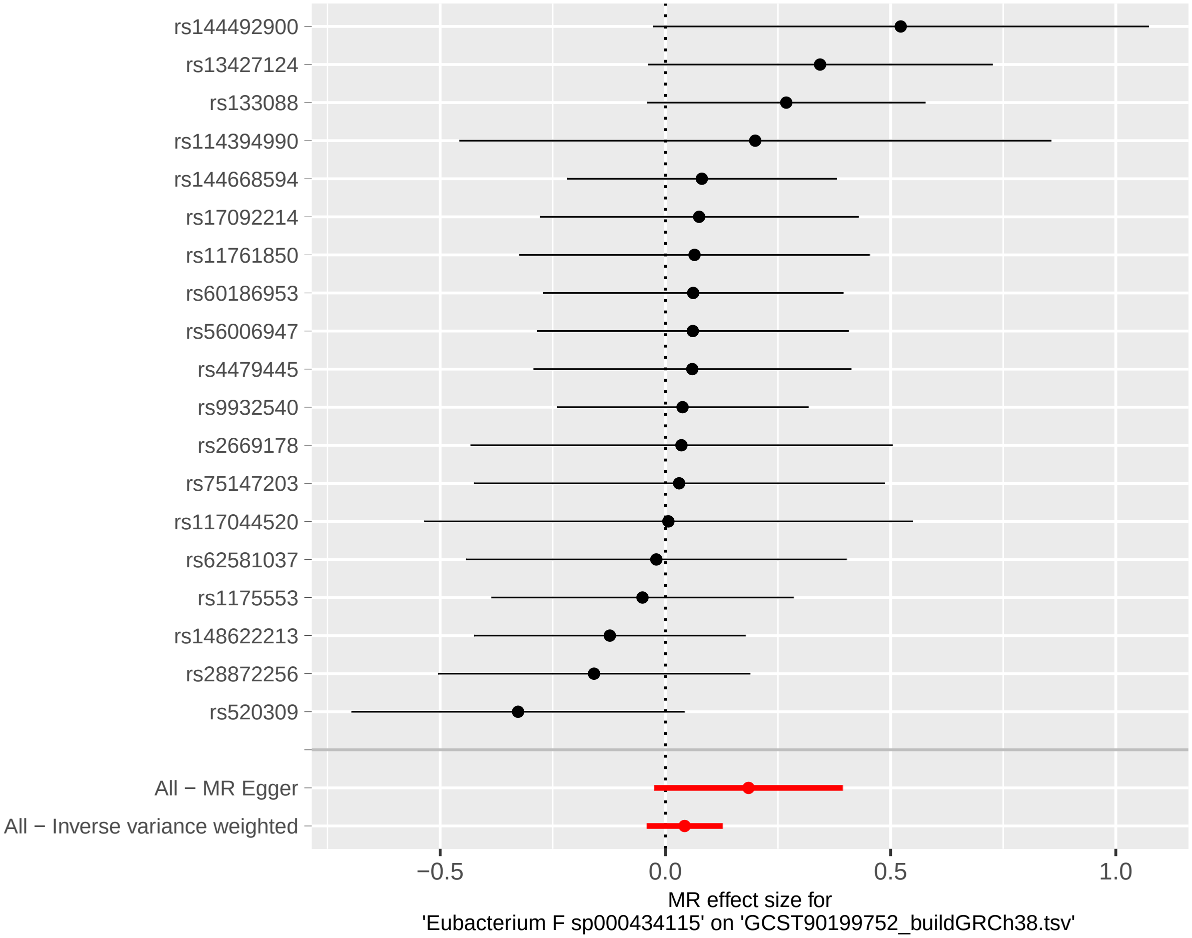
rs144492900
rs13427124
rs133088
rs114394990
rs144668594
rs17092214
rs11761850
rs60186953
rs56006947
rs4479445
rs9932540
rs2669178
rs75147203
rs117044520
rs62581037
rs1175553
rs148622213
rs28872256
rs520309
All − MR Egger
All − Inverse variance weighted
−0.5
0.0
0.5
1.0
MR effect size for
'Eubacterium F sp000434115' on 'GCST90199752_buildGRCh38.tsv'

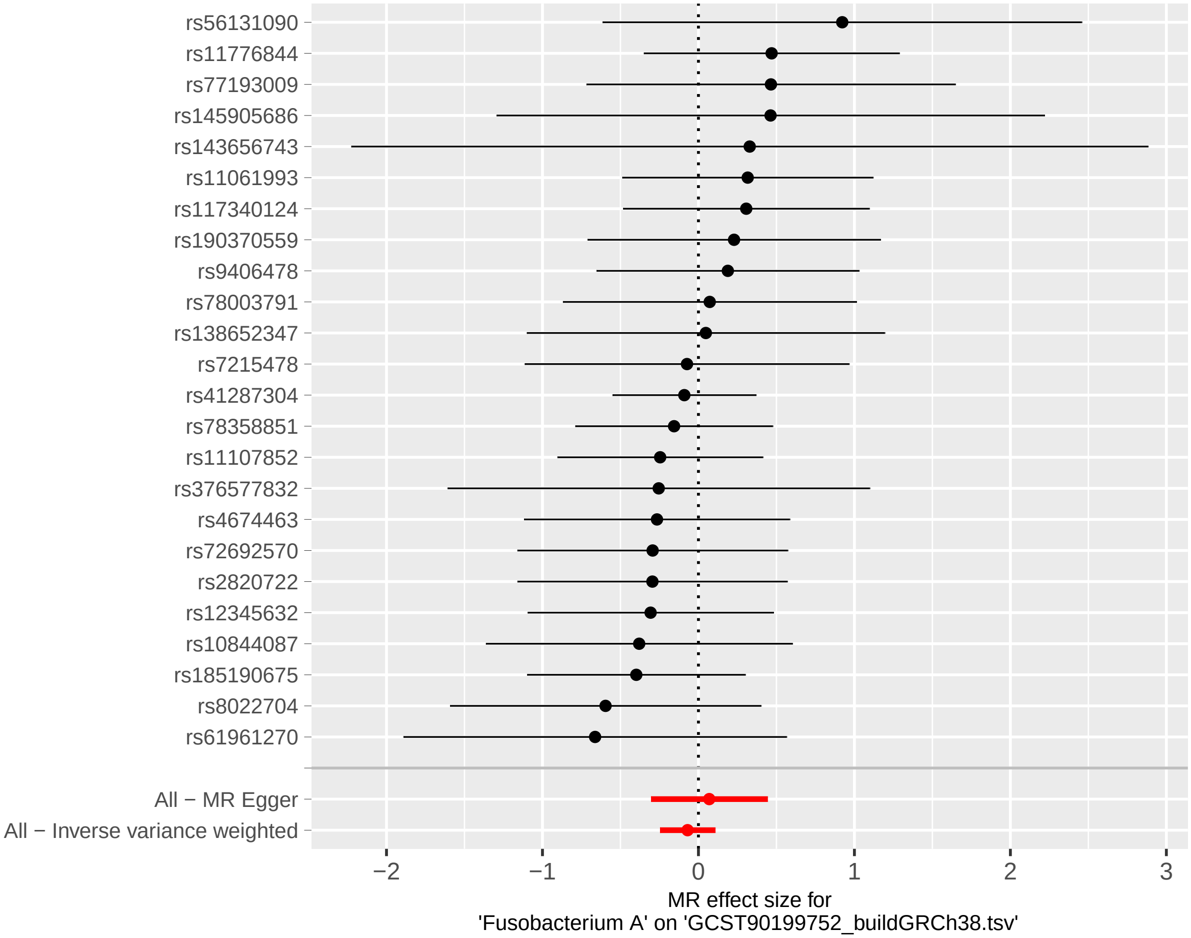

rs56131090
rs11776844
rs77193009
rs145905686
rs143656743
rs11061993
rs117340124
rs190370559
rs9406478
rs78003791
rs138652347
rs7215478
rs41287304
rs78358851
rs11107852
rs376577832
rs4674463
rs72692570
rs2820722
rs12345632
rs10844087
rs185190675
rs8022704
rs61961270
All − MR Egger
All − Inverse variance weighted
−2
−1
0
1
2
3
MR effect size for
'Fusobacterium A' on 'GCST90199752_buildGRCh38.tsv'

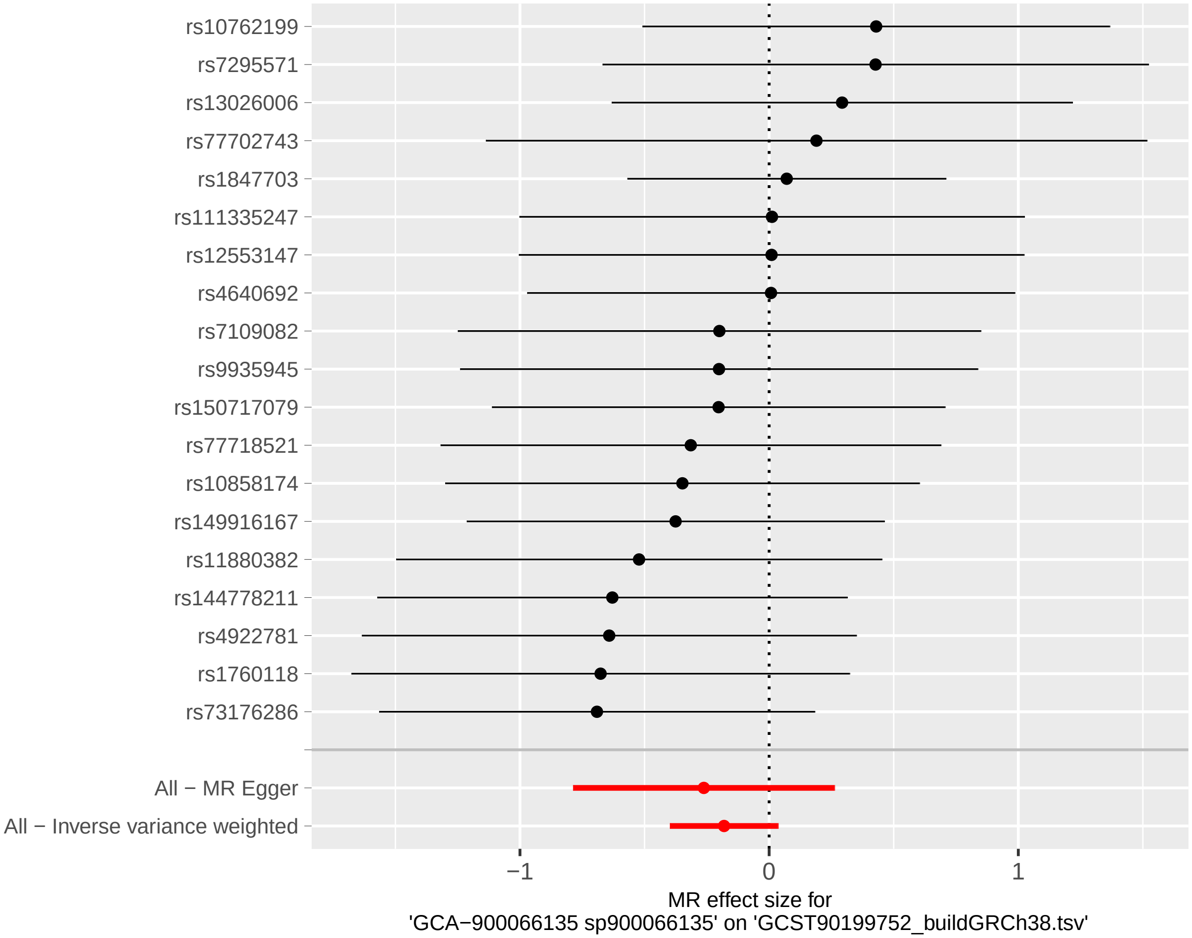
rs10762199
rs7295571
rs13026006
rs77702743
rs1847703
rs111335247
rs12553147
rs4640692
rs7109082
rs9935945
rs150717079
rs77718521
rs10858174
rs149916167
rs11880382
rs144778211
rs4922781
rs1760118
rs73176286
All − MR Egger
All − Inverse variance weighted
−1
0
1
MR effect size for
'GCA−900066135 sp900066135' on 'GCST90199752_buildGRCh38.tsv'

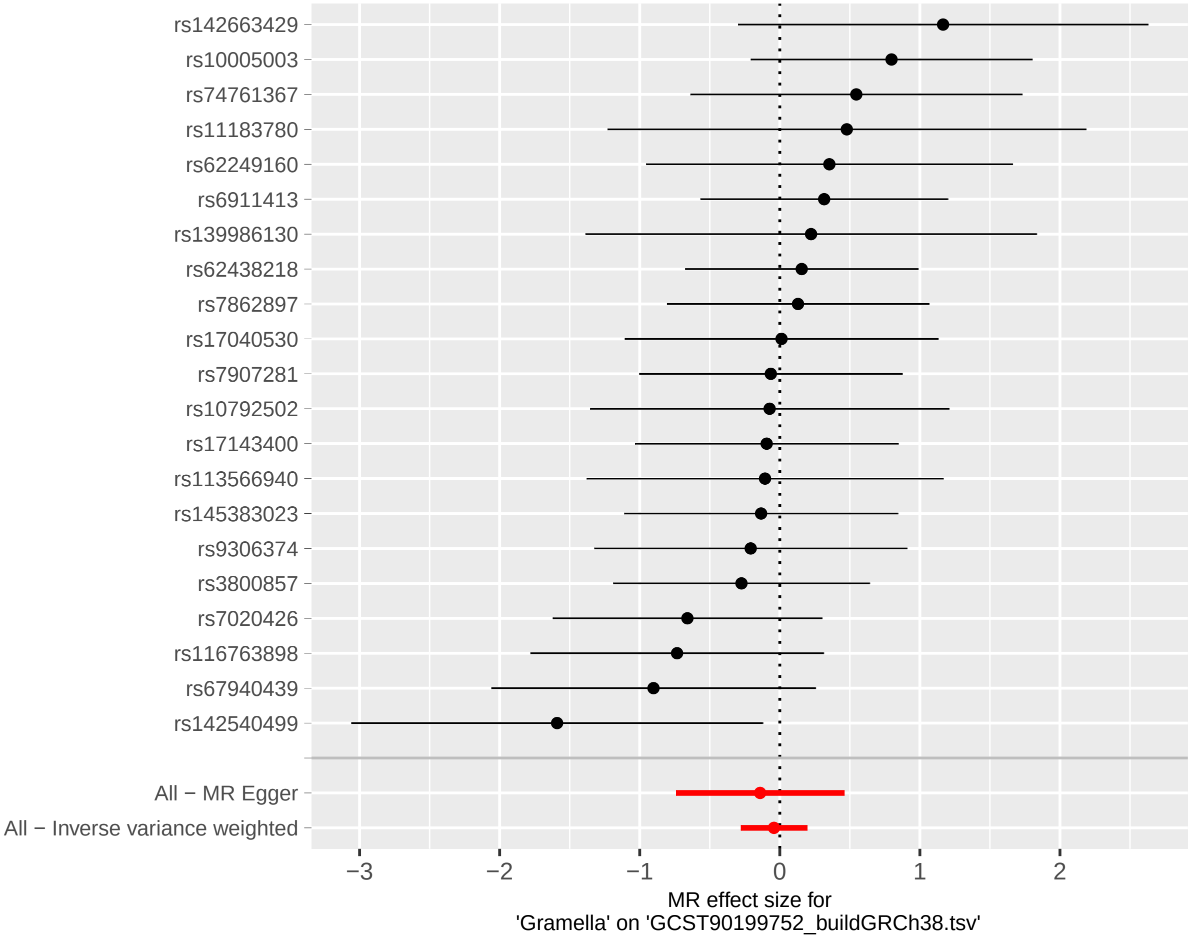
rs142663429
rs10005003
rs74761367
rs11183780
rs62249160
rs6911413
rs139986130
rs62438218
rs7862897
rs17040530
rs7907281
rs10792502
rs17143400
rs113566940
rs145383023
rs9306374
rs3800857
rs7020426
rs116763898
rs67940439
rs142540499
All − MR Egger
All − Inverse variance weighted
−3
−2
−1
0
1
2
MR effect size for
'Gramella' on 'GCST90199752_buildGRCh38.tsv'

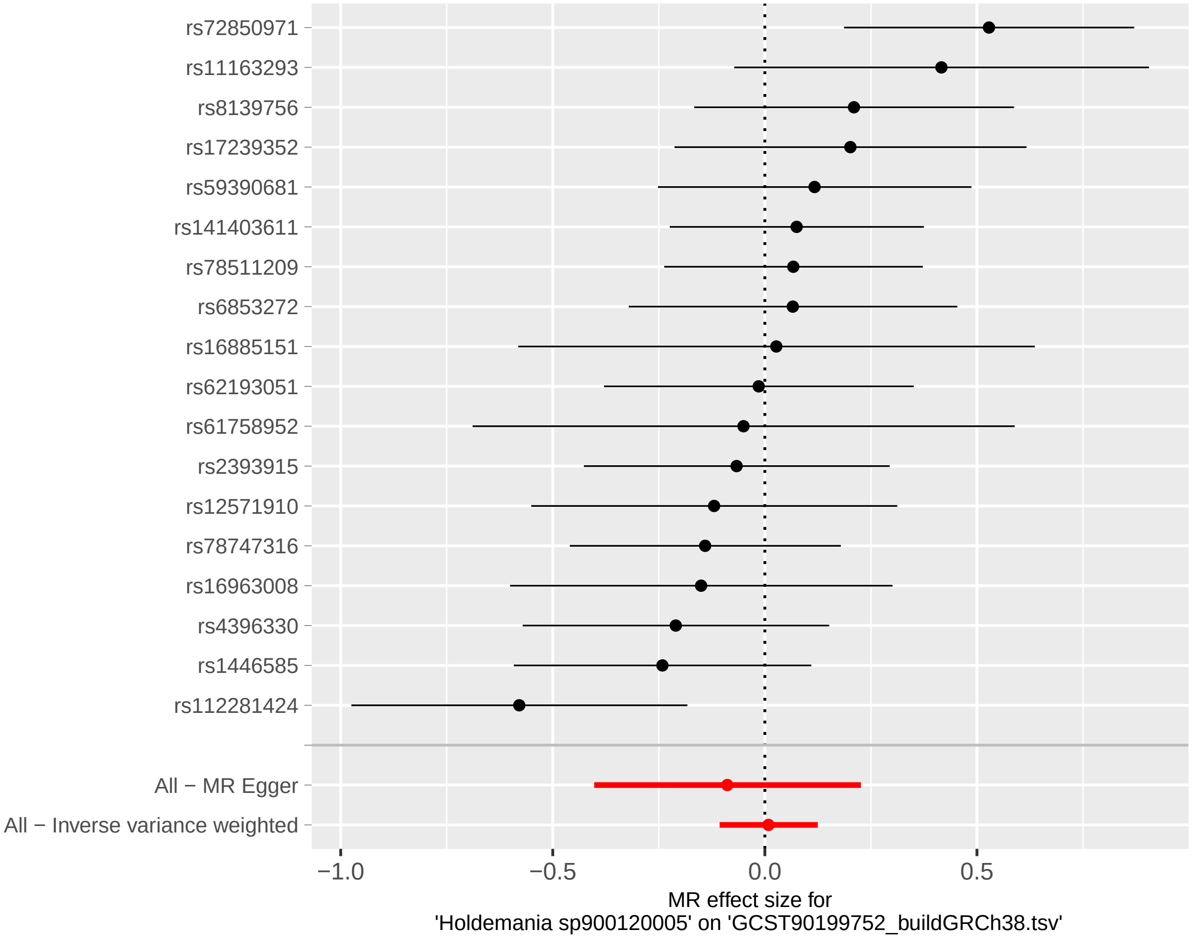
rs72850971
rs11163293
rs8139756
rs17239352
rs59390681
rs141403611
rs78511209
rs6853272
rs16885151
rs62193051
rs61758952
rs2393915
rs12571910
rs78747316
rs16963008
rs4396330
rs1446585
rs112281424
All − MR Egger
All − Inverse variance weighted
−1.0
−0.5
0.0
0.5
MR effect size for
'Holdemania sp900120005' on 'GCST90199752_buildGRCh38.tsv'

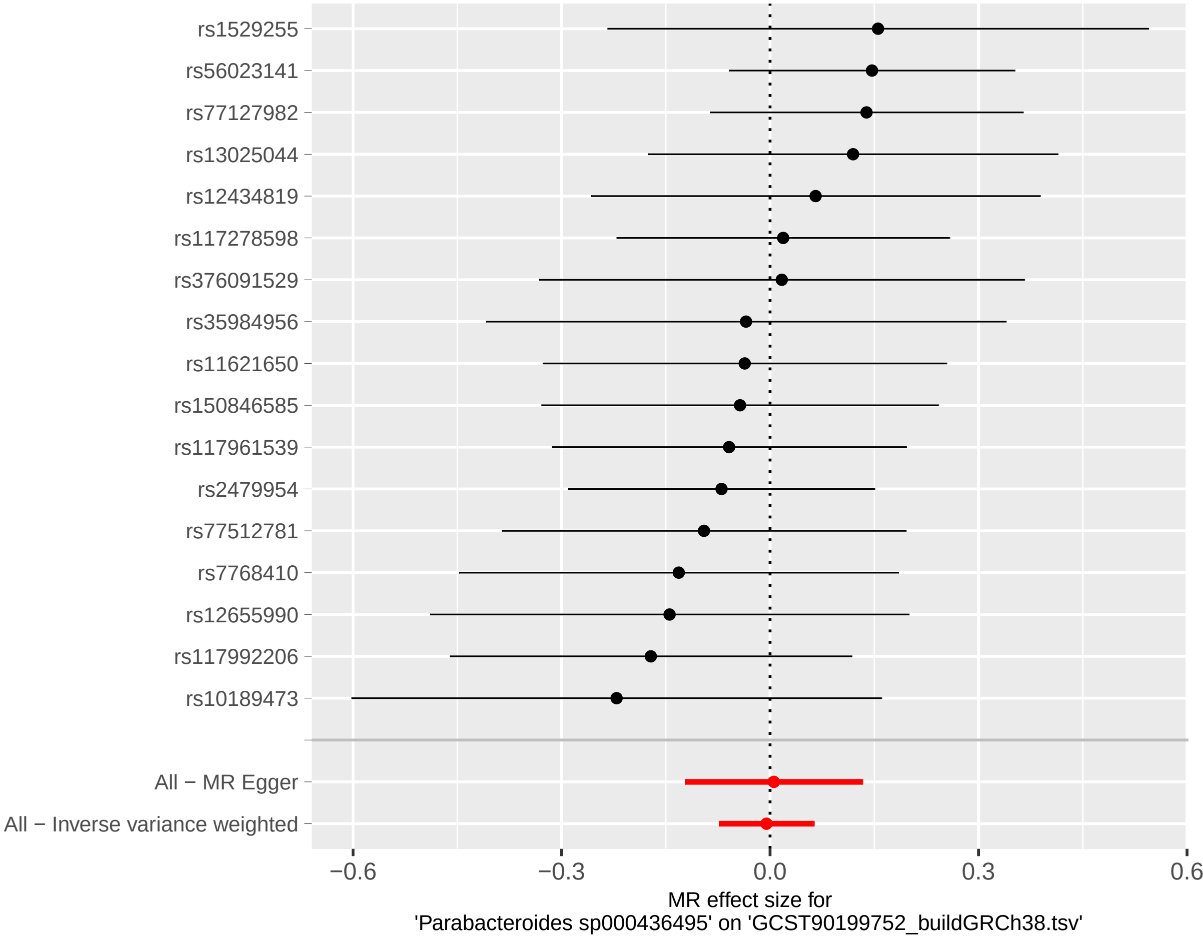
rs1529255
rs56023141
rs77127982
rs13025044
rs12434819
rs117278598
rs376091529
rs35984956
rs11621650
rs150846585
rs117961539
rs2479954
rs77512781
rs7768410
rs12655990
rs117992206
rs10189473
All − MR Egger
All − Inverse variance weighted
−0.6
−0.3
0.0
0.3
0.6
MR effect size for
'Parabacteroides sp000436495' on 'GCST90199752_buildGRCh38.tsv'

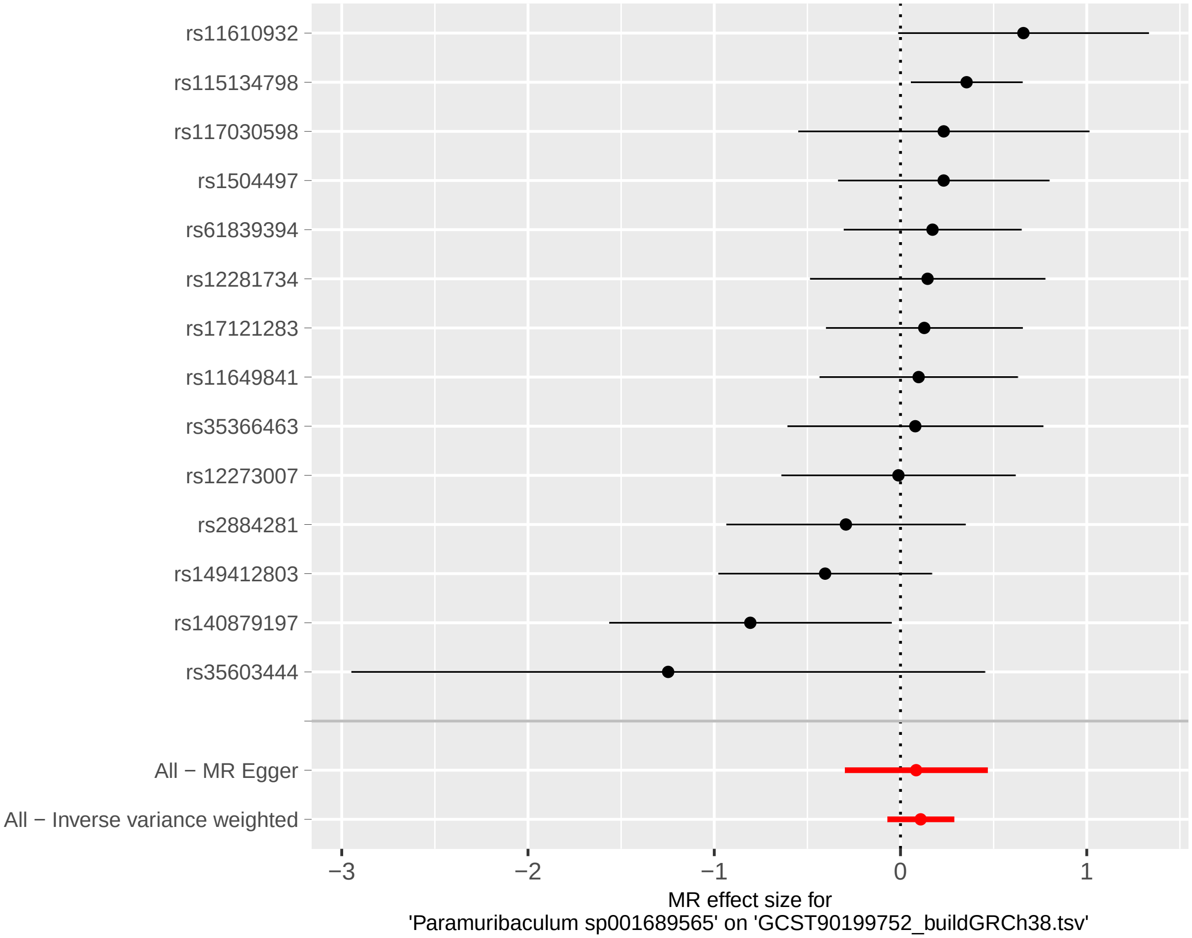
rs11610932
rs115134798
rs117030598
rs1504497
rs61839394
rs12281734
rs17121283
rs11649841
rs35366463
rs12273007
rs2884281
rs149412803
rs140879197
rs35603444
All − MR Egger
All − Inverse variance weighted
−3
−2
−1
0
1
MR effect size for
'Paramuribaculum sp001689565' on 'GCST90199752_buildGRCh38.tsv'

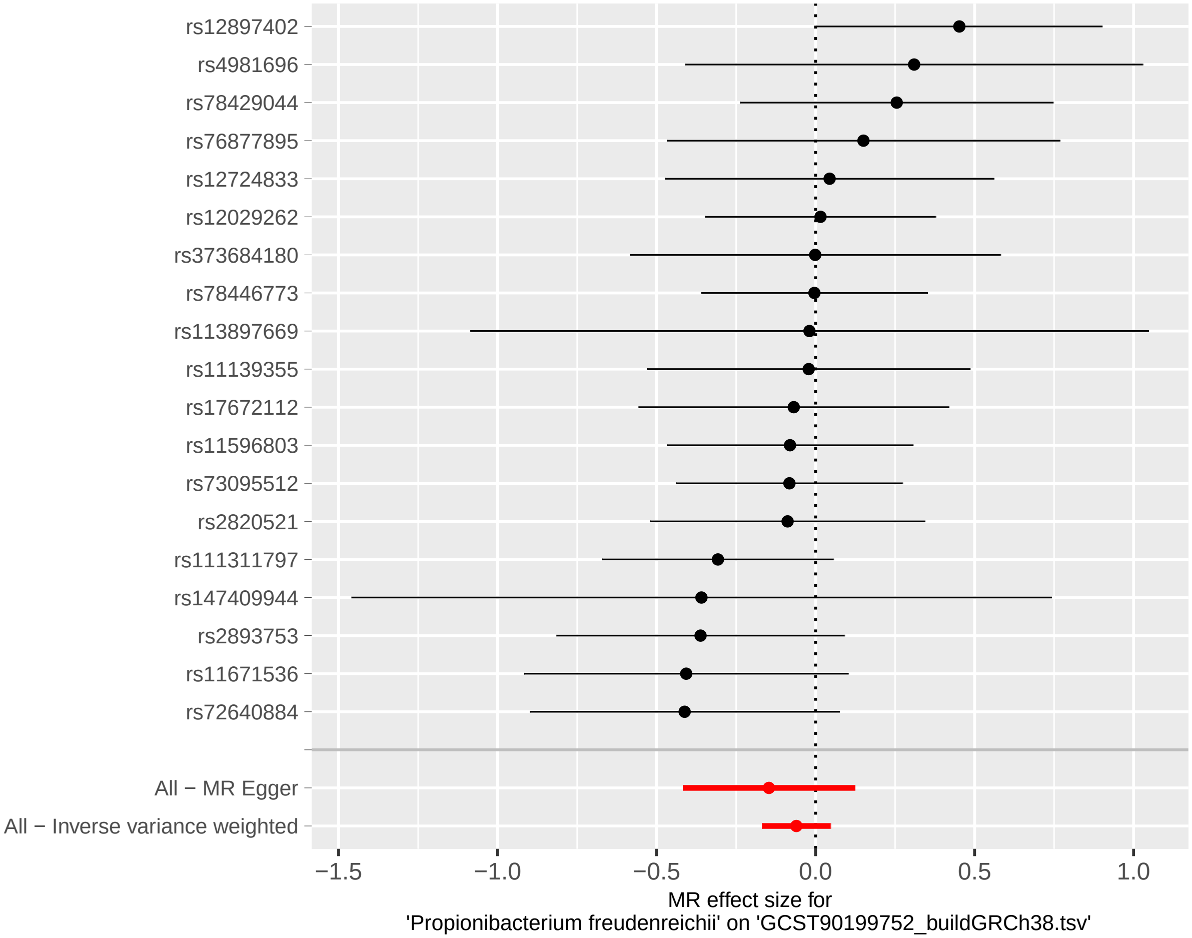

rs12897402
rs4981696
rs78429044
rs76877895
rs12724833
rs12029262
rs373684180
rs78446773
rs113897669
rs11139355
rs17672112
rs11596803
rs73095512
rs2820521
rs111311797
rs147409944
rs2893753
rs11671536
rs72640884
All − MR Egger
All − Inverse variance weighted
−1.5
−1.0
−0.5
0.0
0.5
1.0
MR effect size for
'Propionibacterium freudenreichii' on 'GCST90199752_buildGRCh38.tsv'

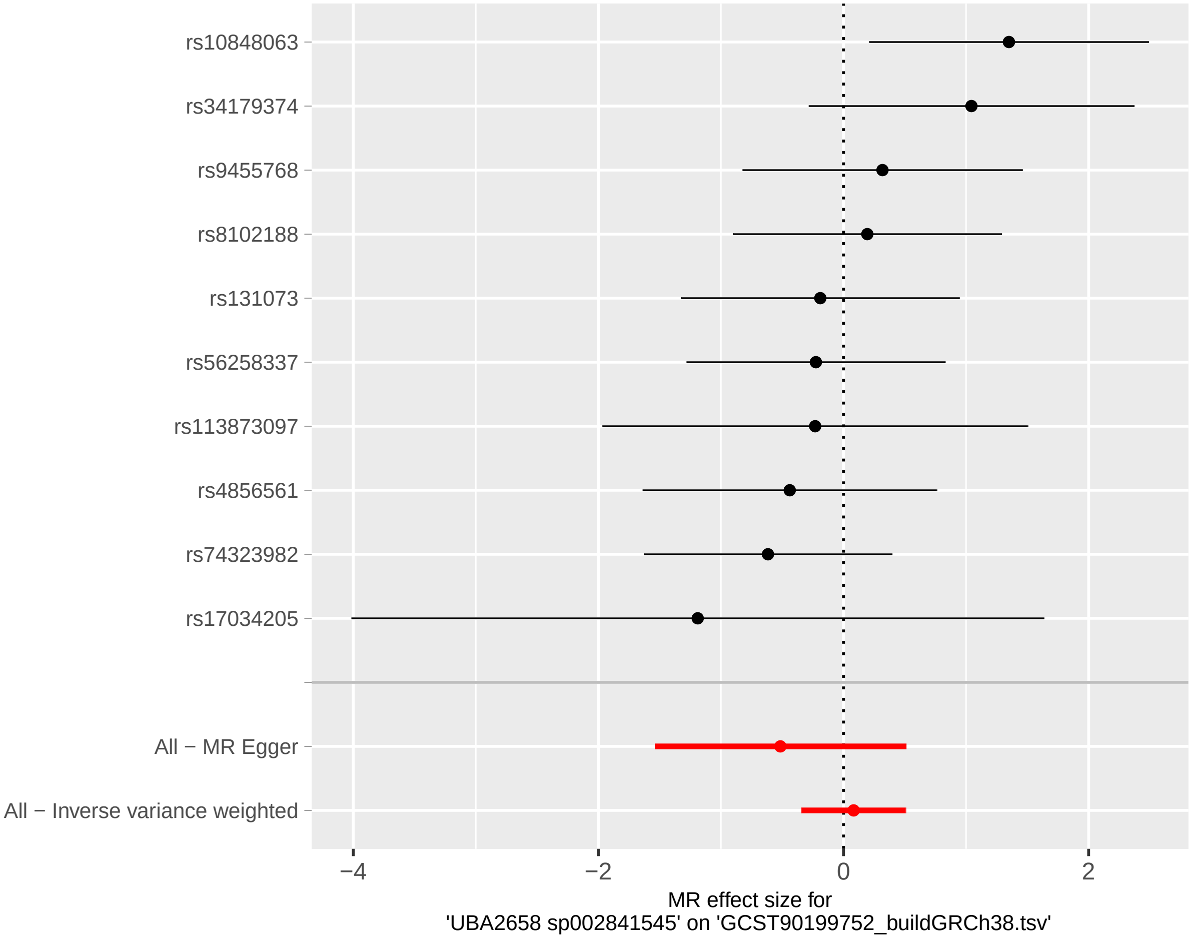
rs10848063
rs34179374
rs9455768
rs8102188
rs131073
rs56258337
rs113873097
rs4856561
rs74323982
rs17034205
All − MR Egger
All − Inverse variance weighted
−4
−2
0
2
MR effect size for
'UBA2658 sp002841545' on 'GCST90199752_buildGRCh38.tsv'

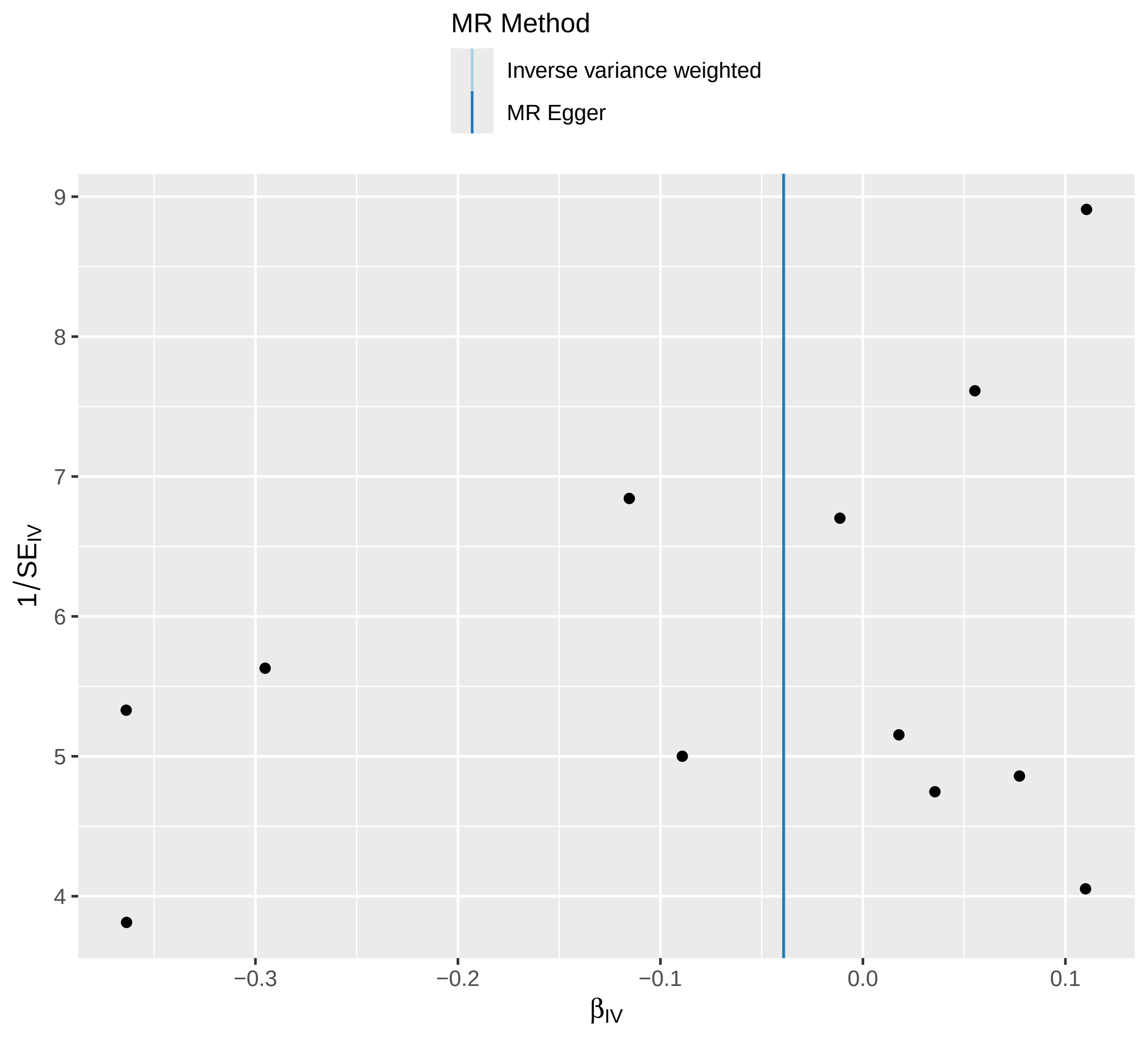
MR Method
Inverse variance weighted
MR Egger
9
8
7
6
5
4
$1/SE_{IV}$
−0.3
−0.2
−0.1
0.0
0.1
β_{IV}

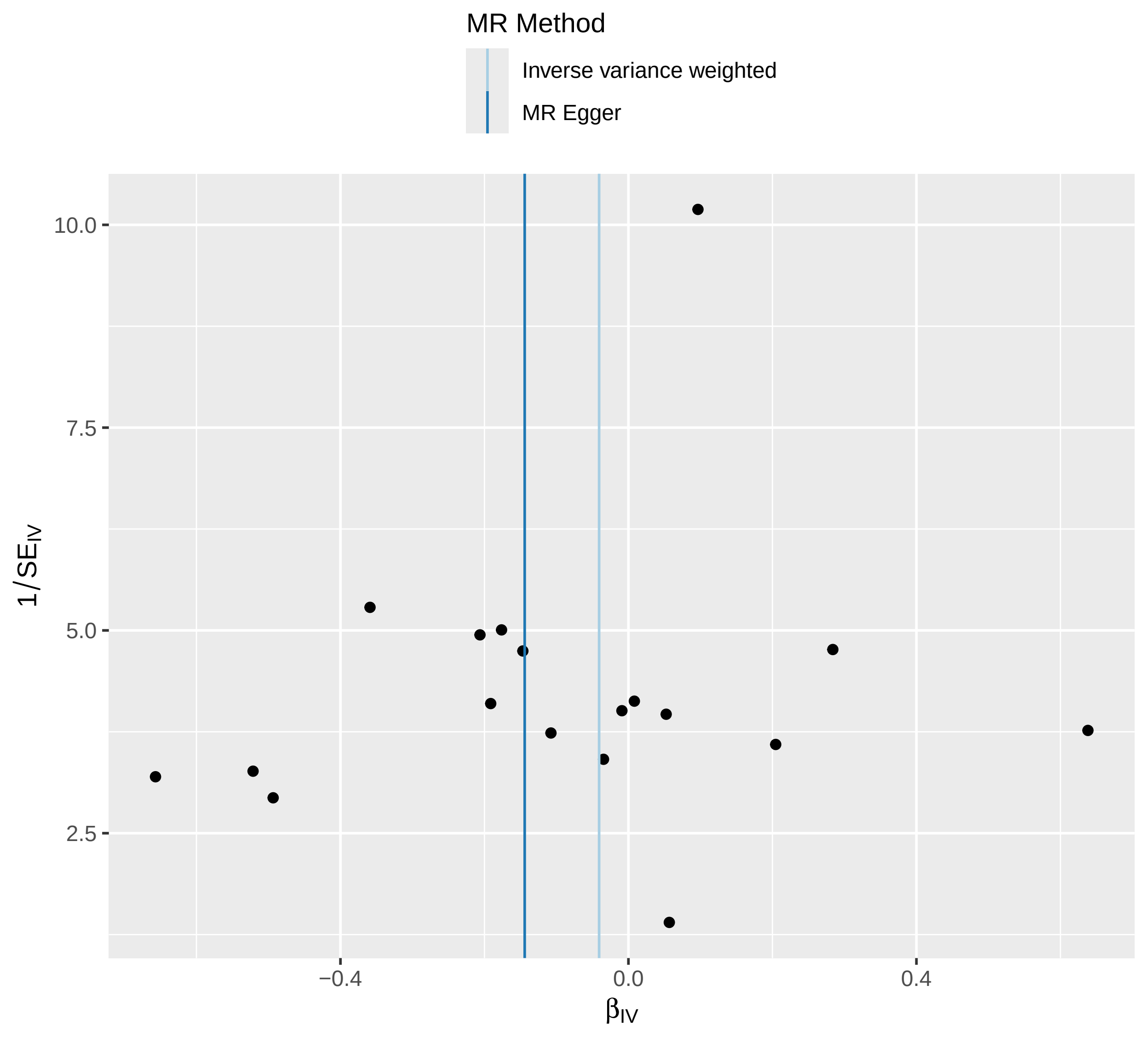
MR Method
Inverse variance weighted
MR Egger
10.0
7.5
5.0
2.5
1/SE_IV
−0.4
0.0
0.4
β_IV

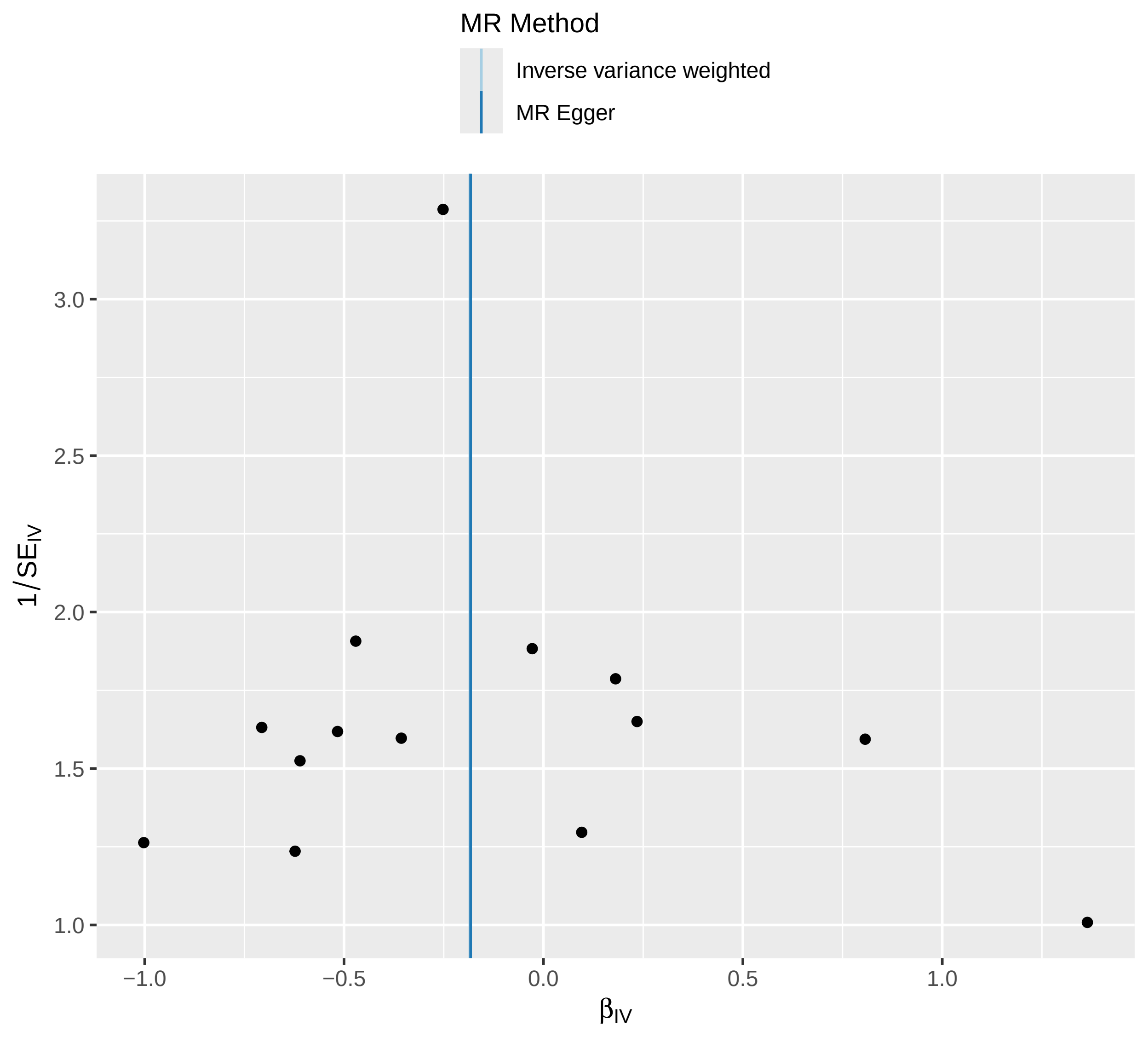
MR Method
Inverse variance weighted
MR Egger
3.0
2.5
2.0
1.5
1.0
1/SE_IV
−1.0
−0.5
0.0
0.5
1.0
β_IV

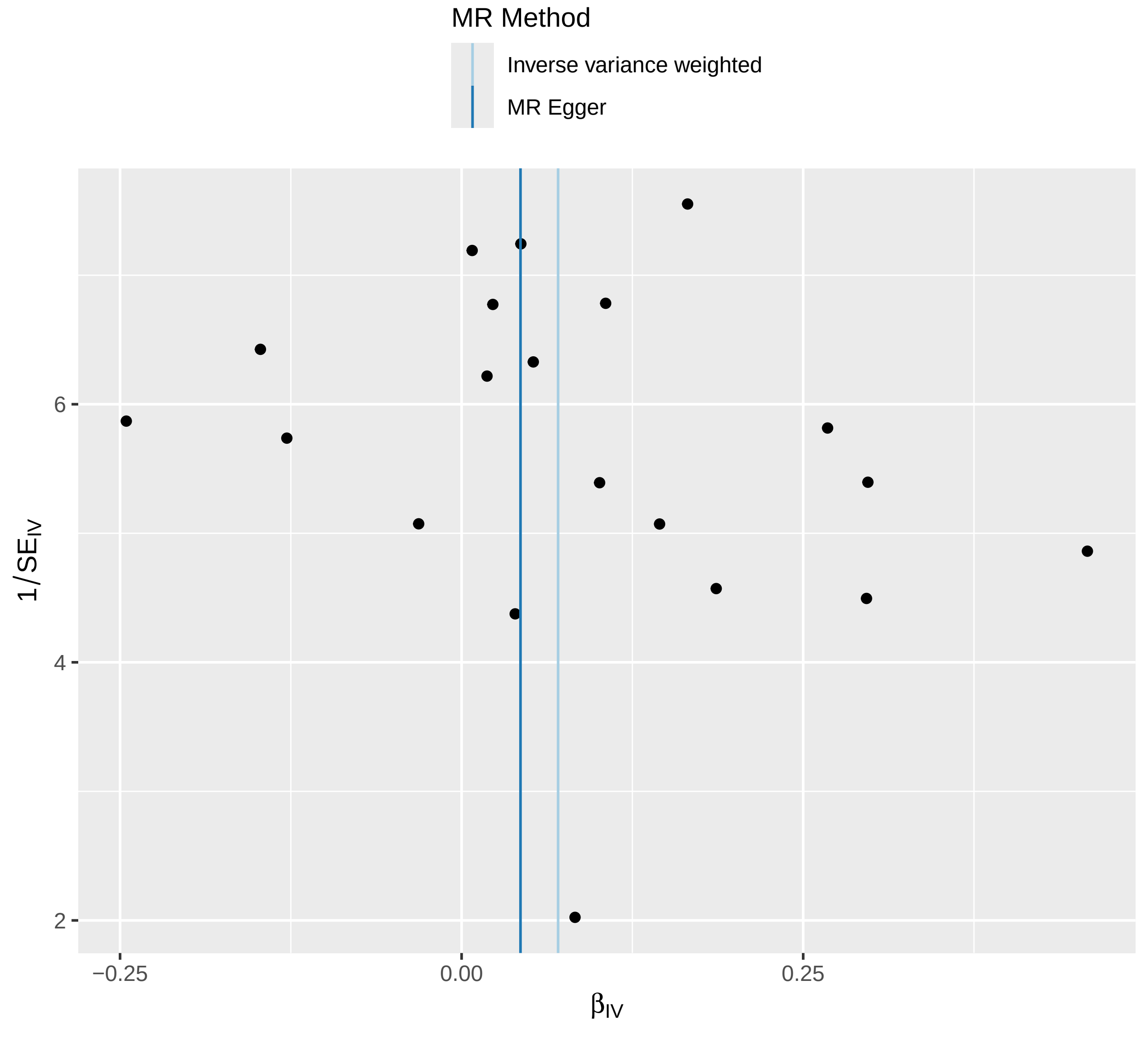

MR Method
Inverse variance weighted
MR Egger
6
4
2
1/SE$_{IV}$
−0.25
0.00
0.25
β_{IV}

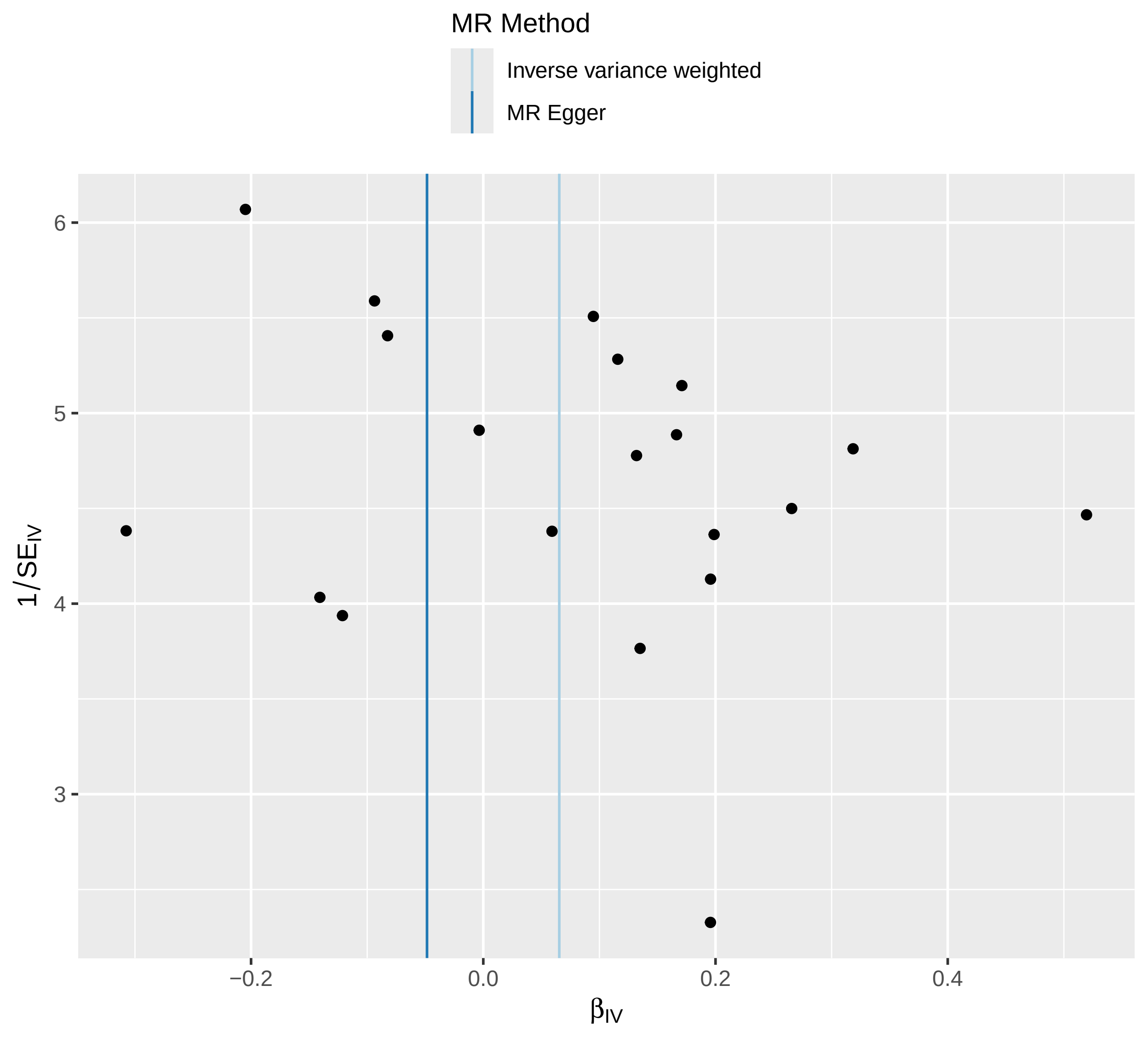
MR Method
Inverse variance weighted
MR Egger
6
5
4
3
1/SE_IV
−0.2
0.0
0.2
0.4
β_IV

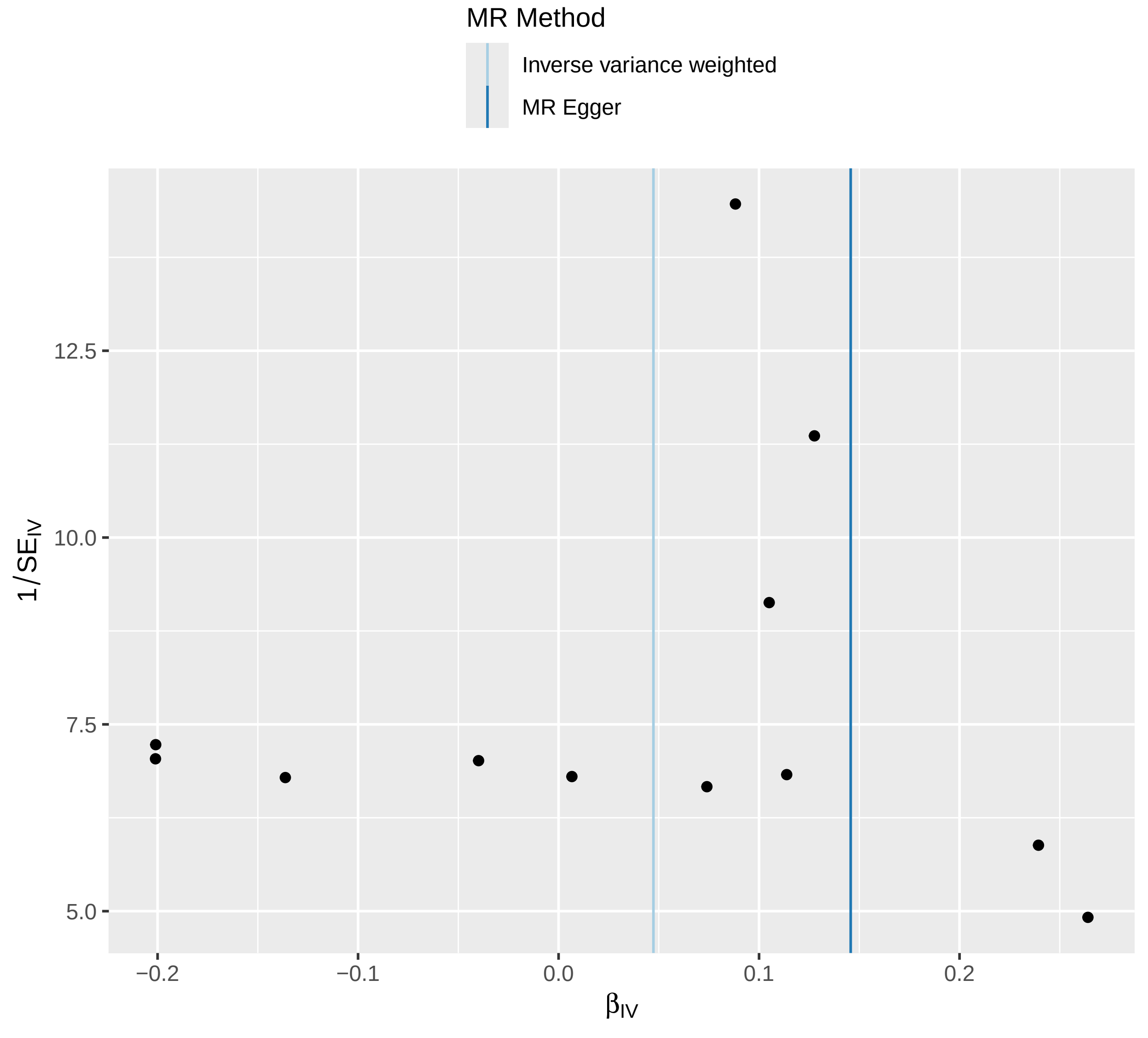

MR Method
Inverse variance weighted
MR Egger
12.5
10.0
7.5
5.0
1/SE_IV
−0.2
−0.1
0.0
0.1
0.2
β_IV

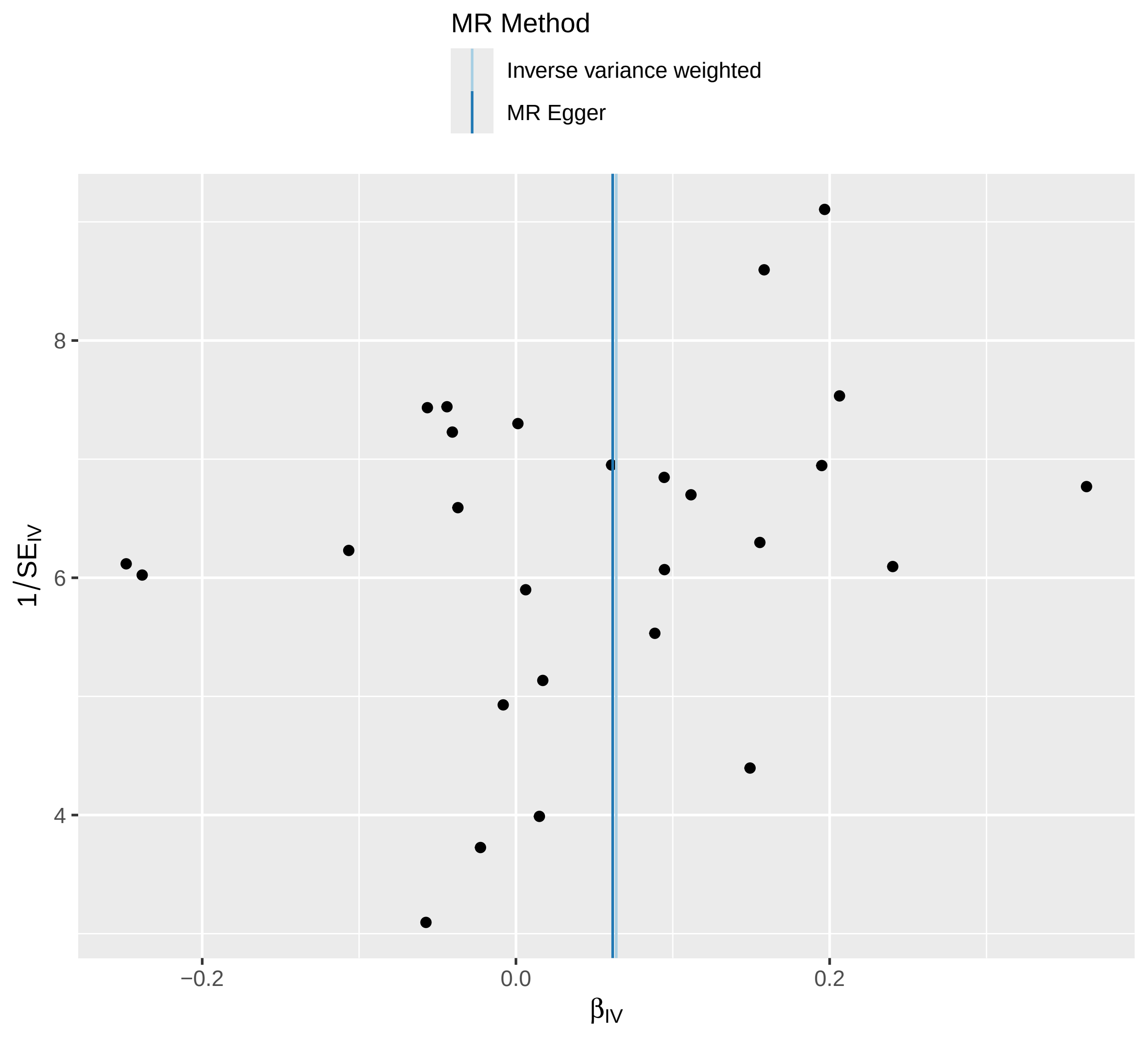
MR Method
Inverse variance weighted
MR Egger
8
6
4
$1/SE_{IV}$
−0.2
0.0
0.2
β_{IV}

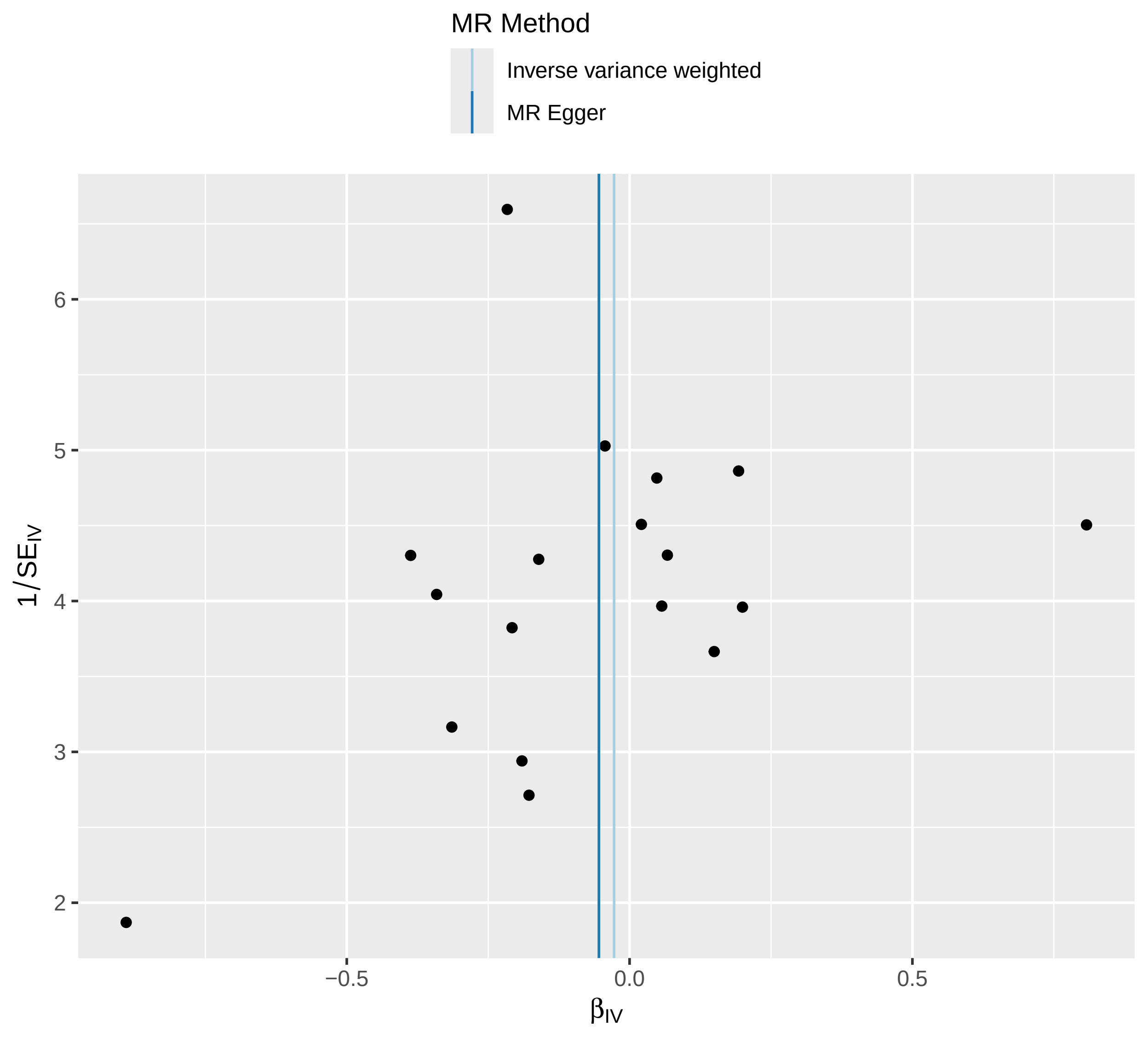

MR Method
Inverse variance weighted
MR Egger
6
5
4
3
2
1/SE$_{IV}$
−0.5
0.0
0.5
β$_{IV}$

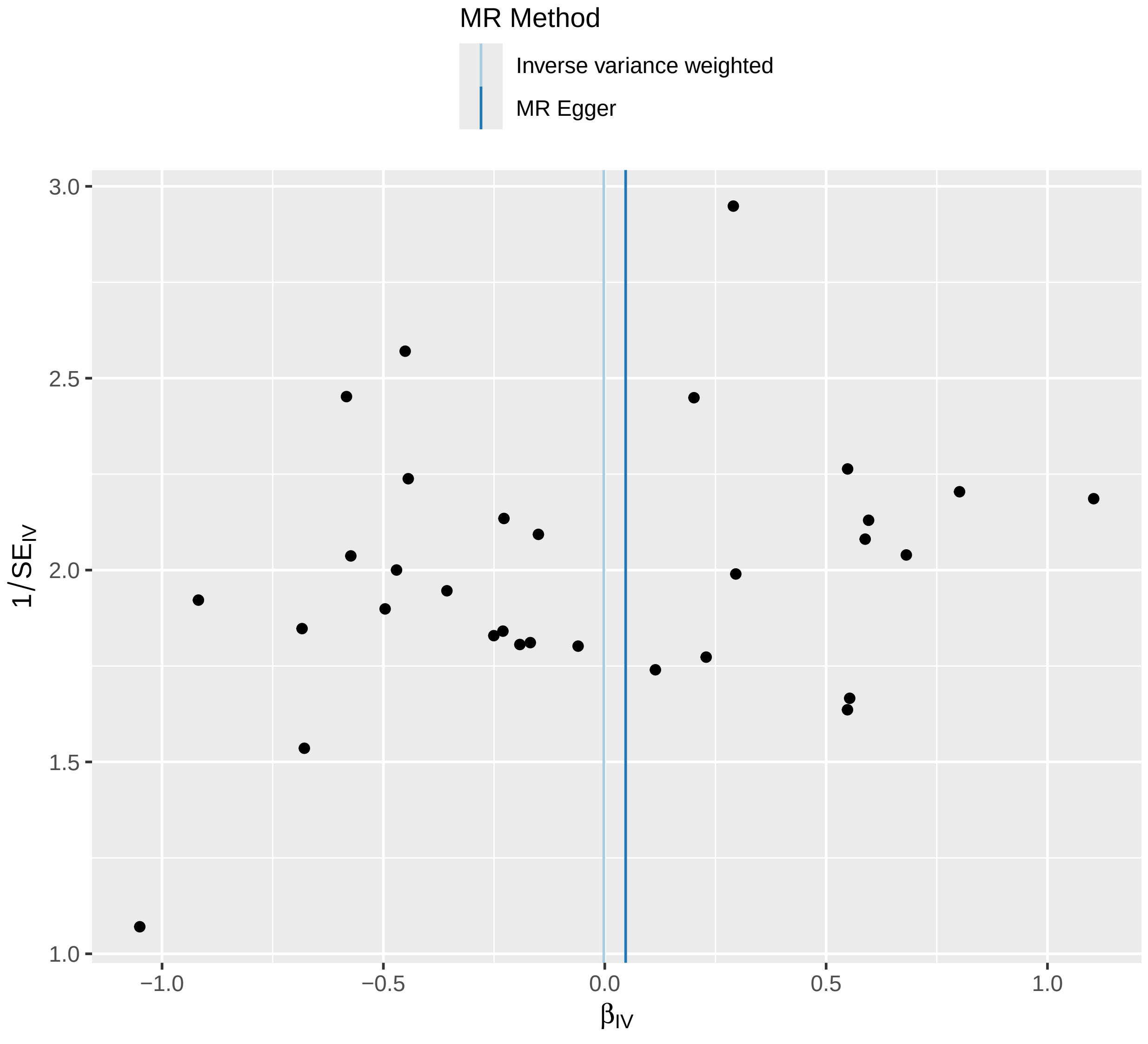

MR Method
Inverse variance weighted
MR Egger
3.0
2.5
2.0
1.5
1.0
1/SE_IV
−1.0
−0.5
0.0
0.5
1.0
β_IV

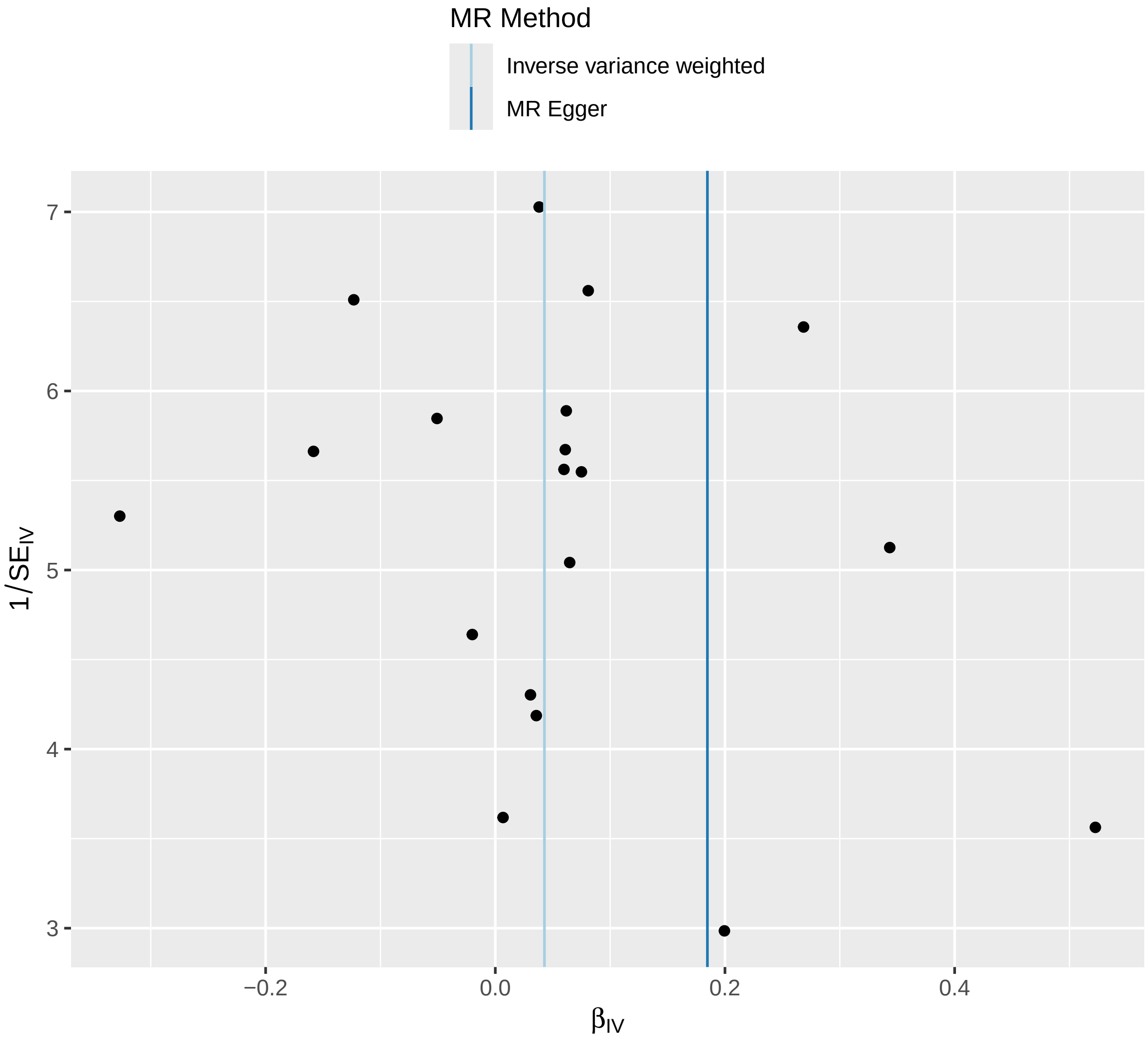

MR Method
Inverse variance weighted
MR Egger
7
6
5
4
3
1/SE$_{IV}$
−0.2
0.0
0.2
0.4
β$_{IV}$

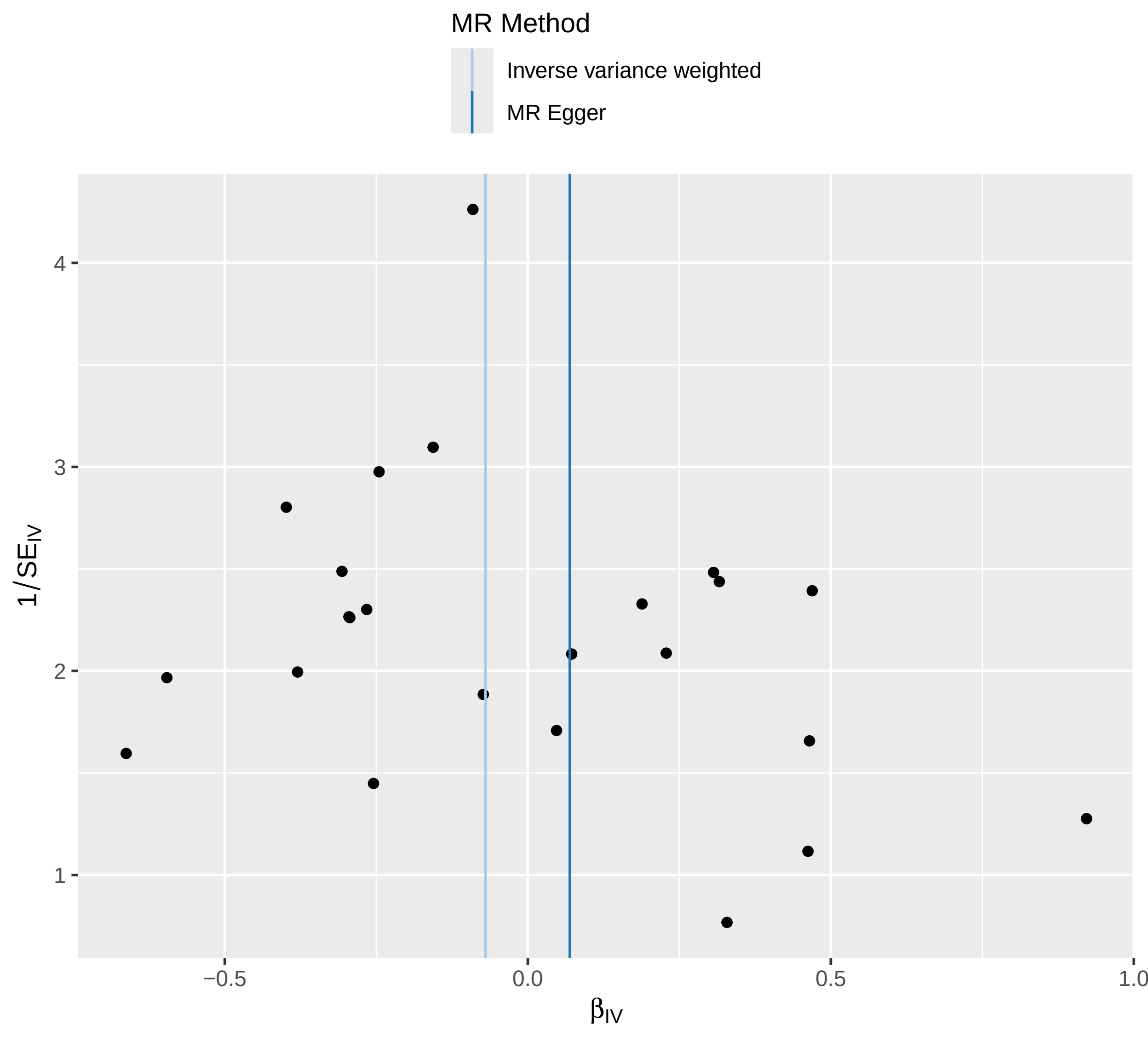
MR Method
Inverse variance weighted
MR Egger
4
3
2
1
1/SE$_{IV}$
−0.5
0.0
0.5
1.0
β$_{IV}$

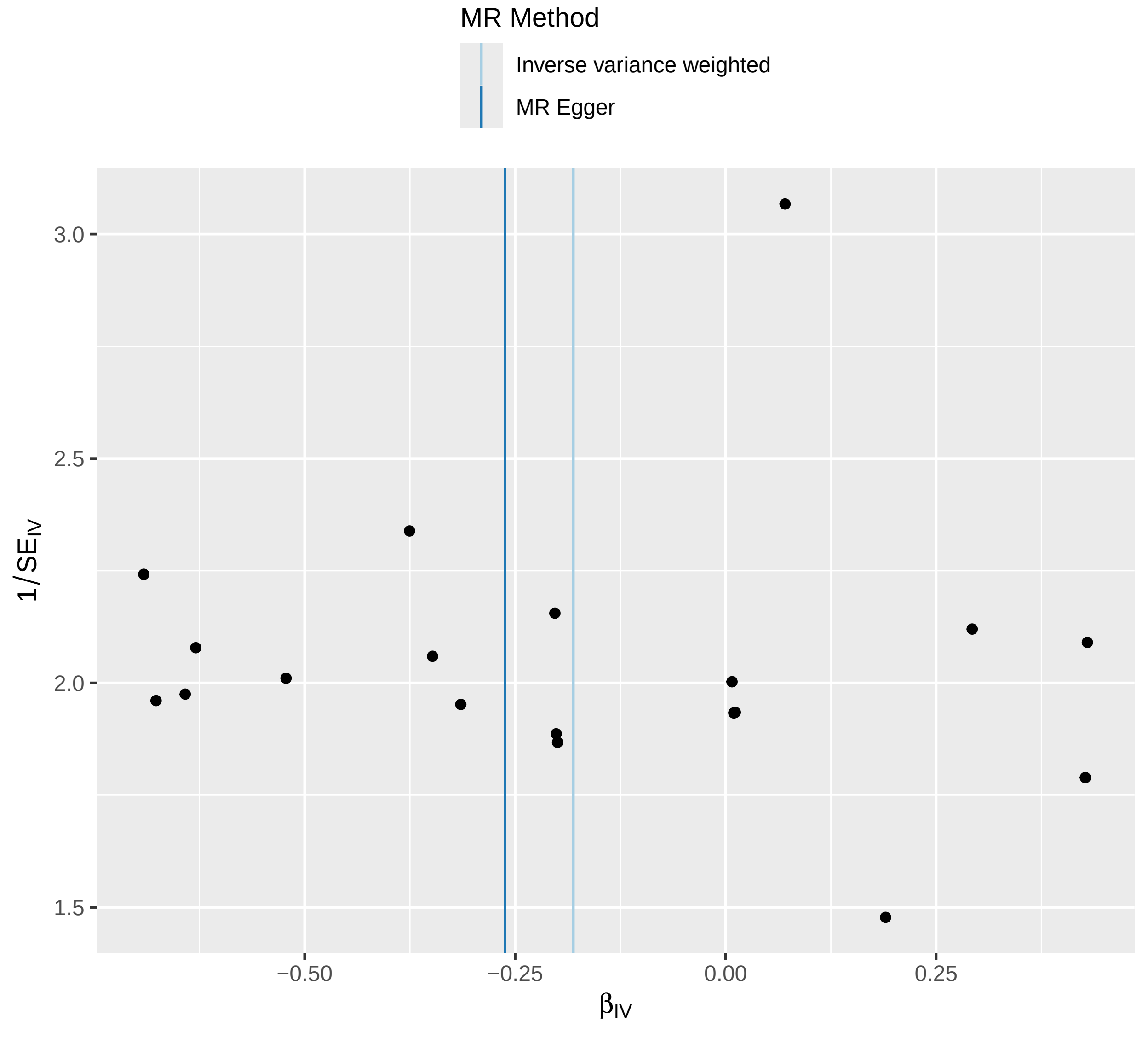
MR Method
Inverse variance weighted
MR Egger
3.0
2.5
2.0
1.5
−0.50
−0.25
0.00
0.25
$1/SE_{IV}$
β_{IV}

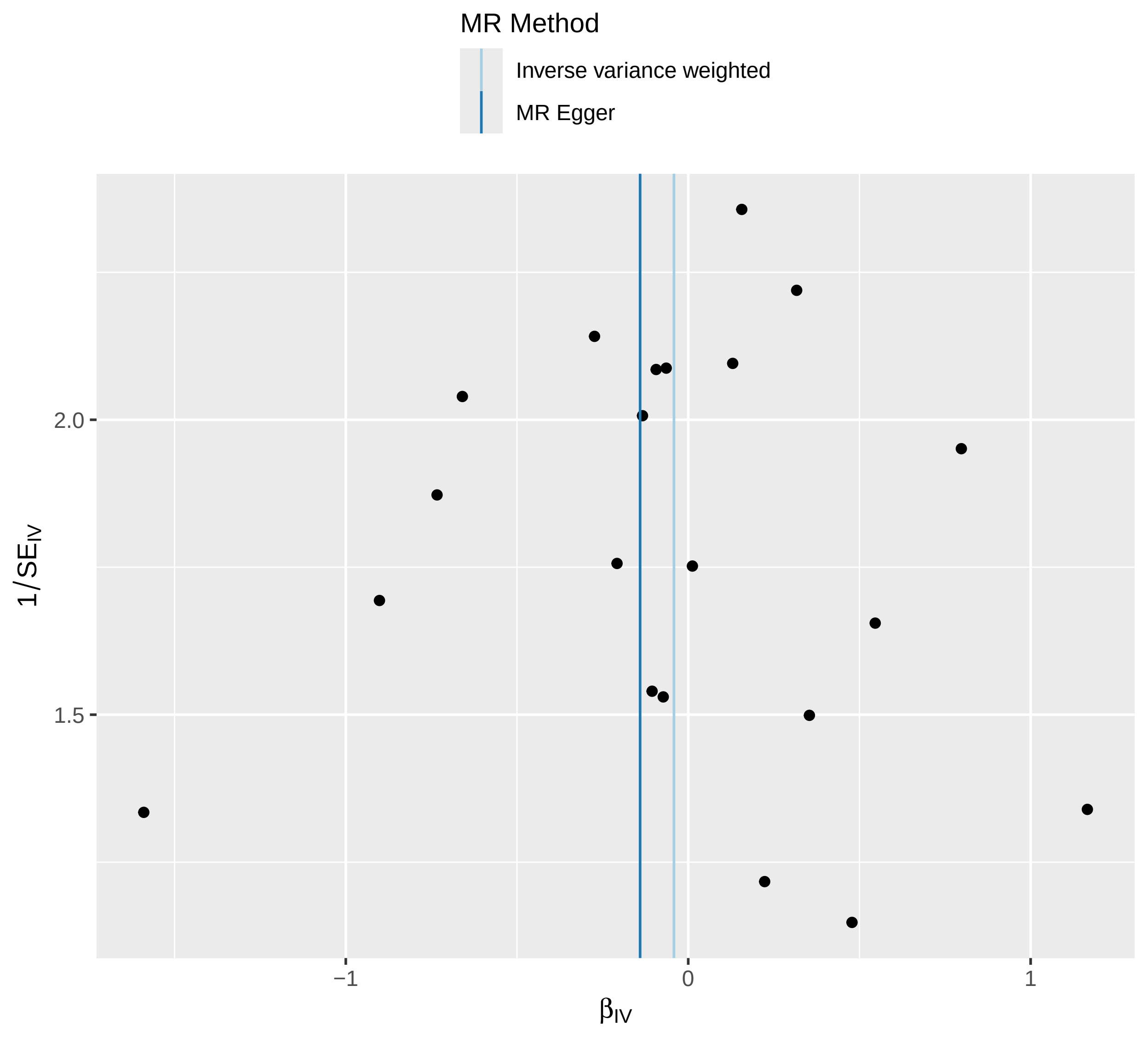

MR Method
Inverse variance weighted
MR Egger
2.0
1.5
1/SE_IV
−1
0
1
β_IV

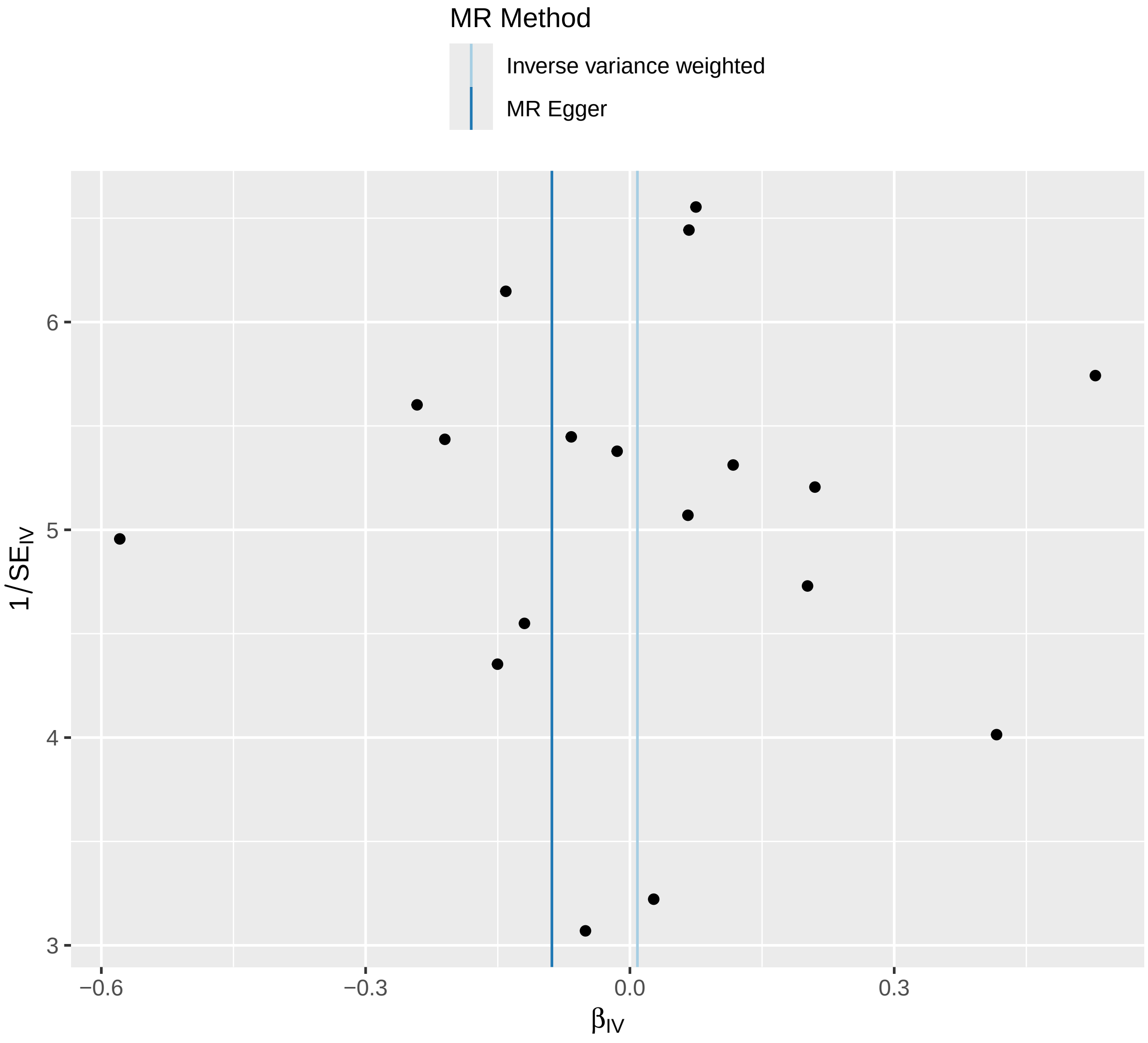

MR Method
Inverse variance weighted
MR Egger
1/SE_IV
6
5
4
3
−0.6
−0.3
0.0
0.3
β_IV

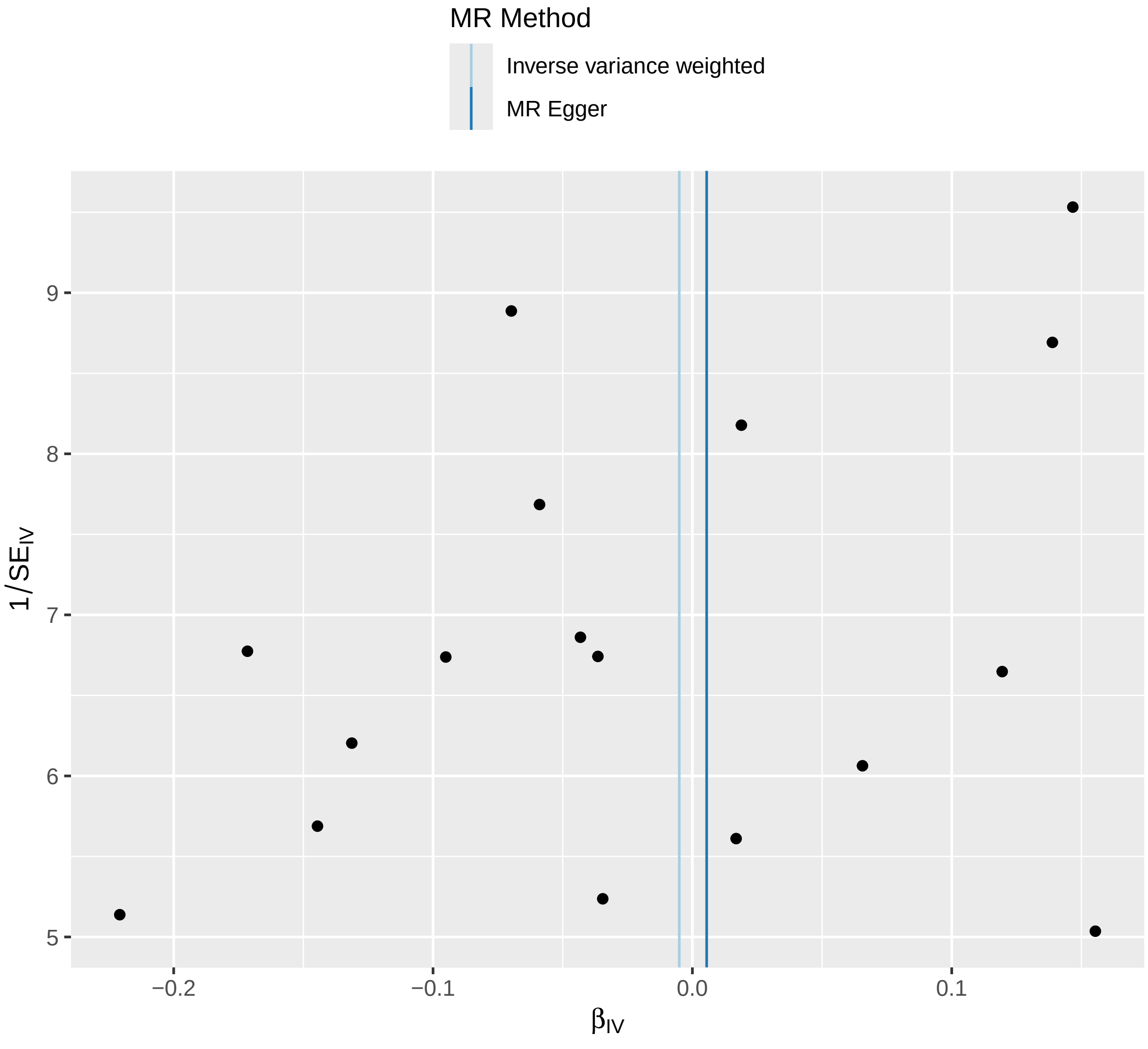
MR Method
Inverse variance weighted
MR Egger
1/SE_IV
9
8
7
6
5
−0.2
−0.1
0.0
0.1
β_IV

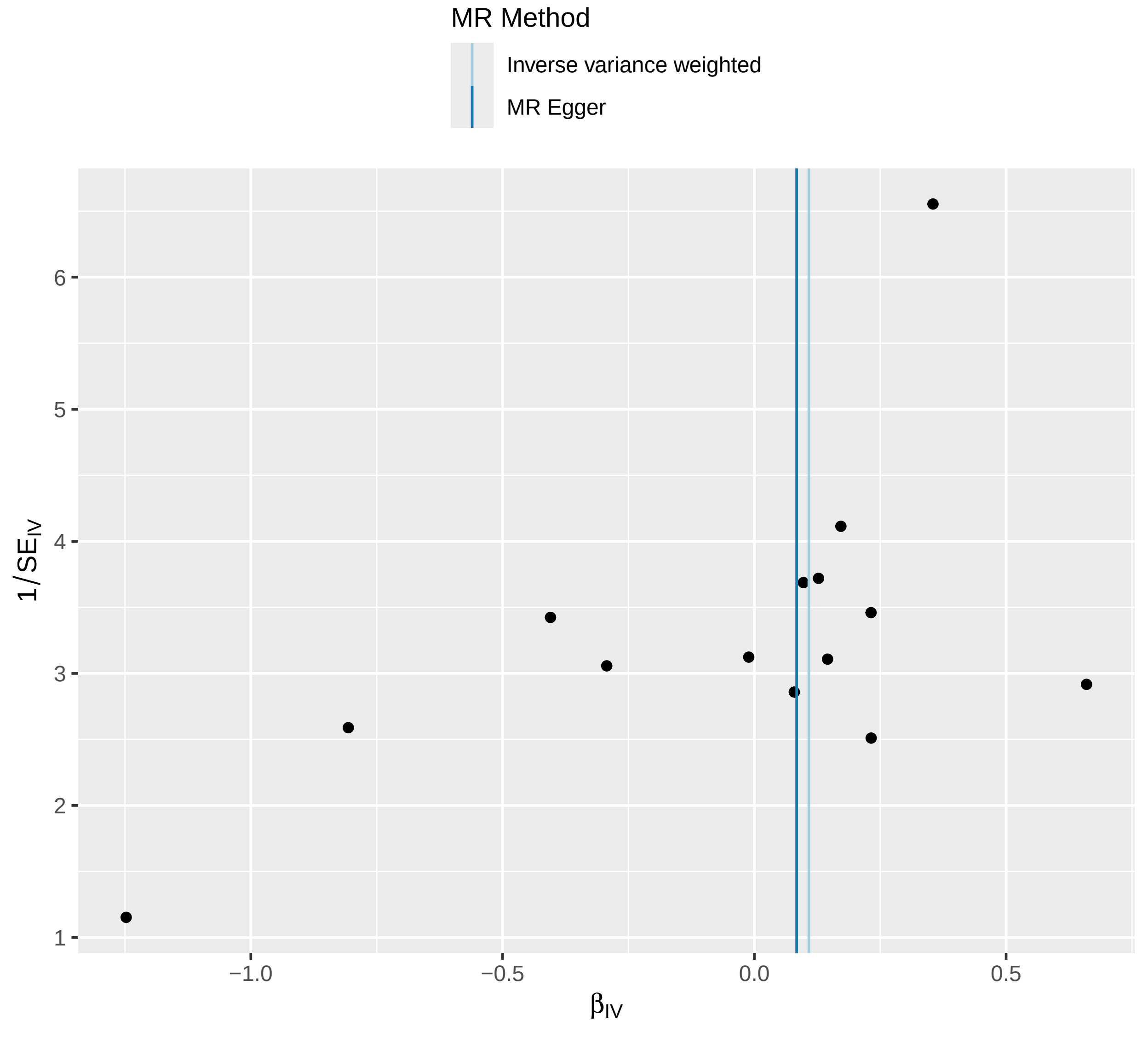

MR Method
Inverse variance weighted
MR Egger
1/SE$_{IV}$
β_{IV}
1
2
3
4
5
6
−1.0
−0.5
0.0
0.5

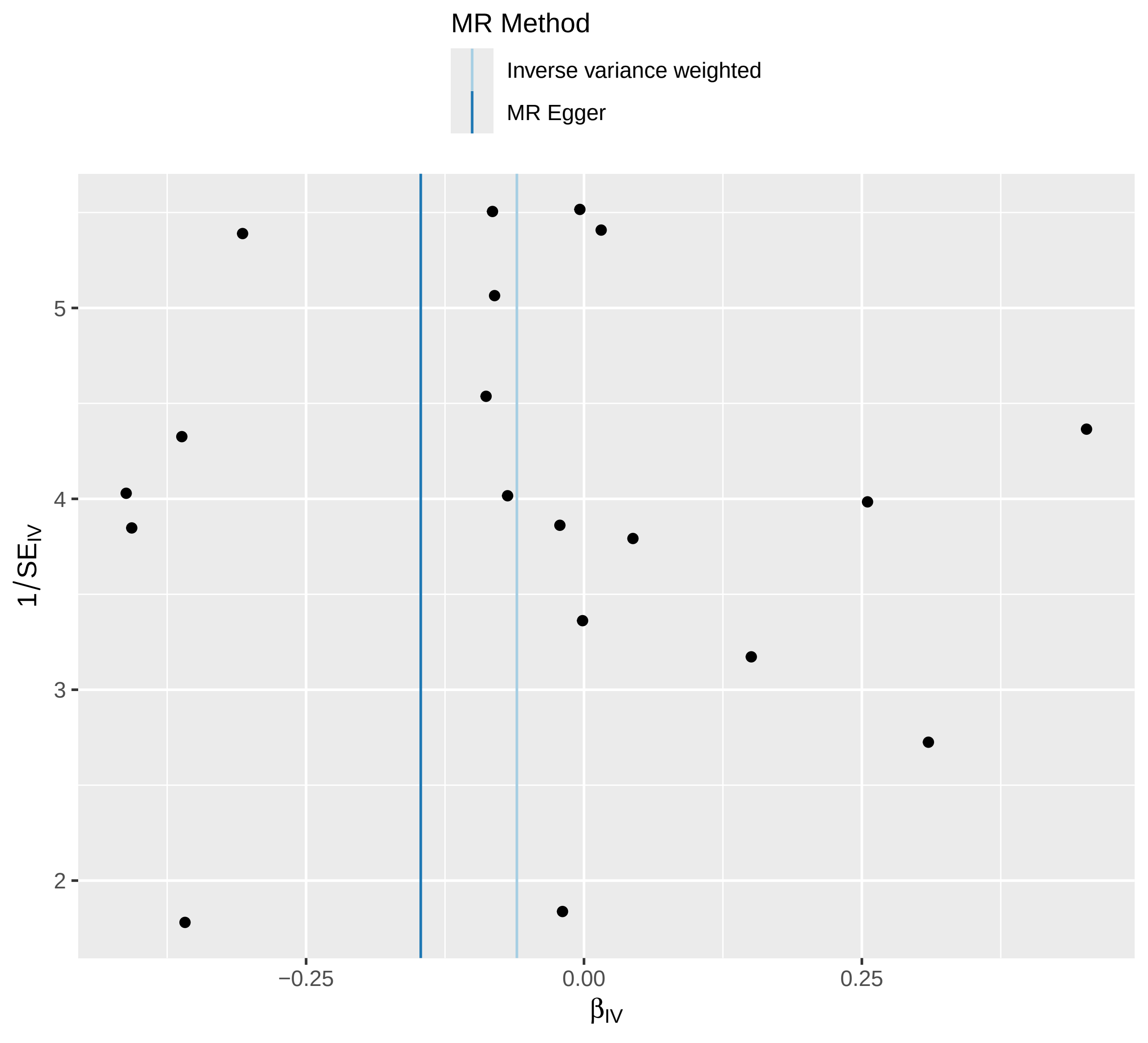

MR Method
Inverse variance weighted
MR Egger
5
4
3
2
1/SE_IV
−0.25
0.00
0.25
β_IV

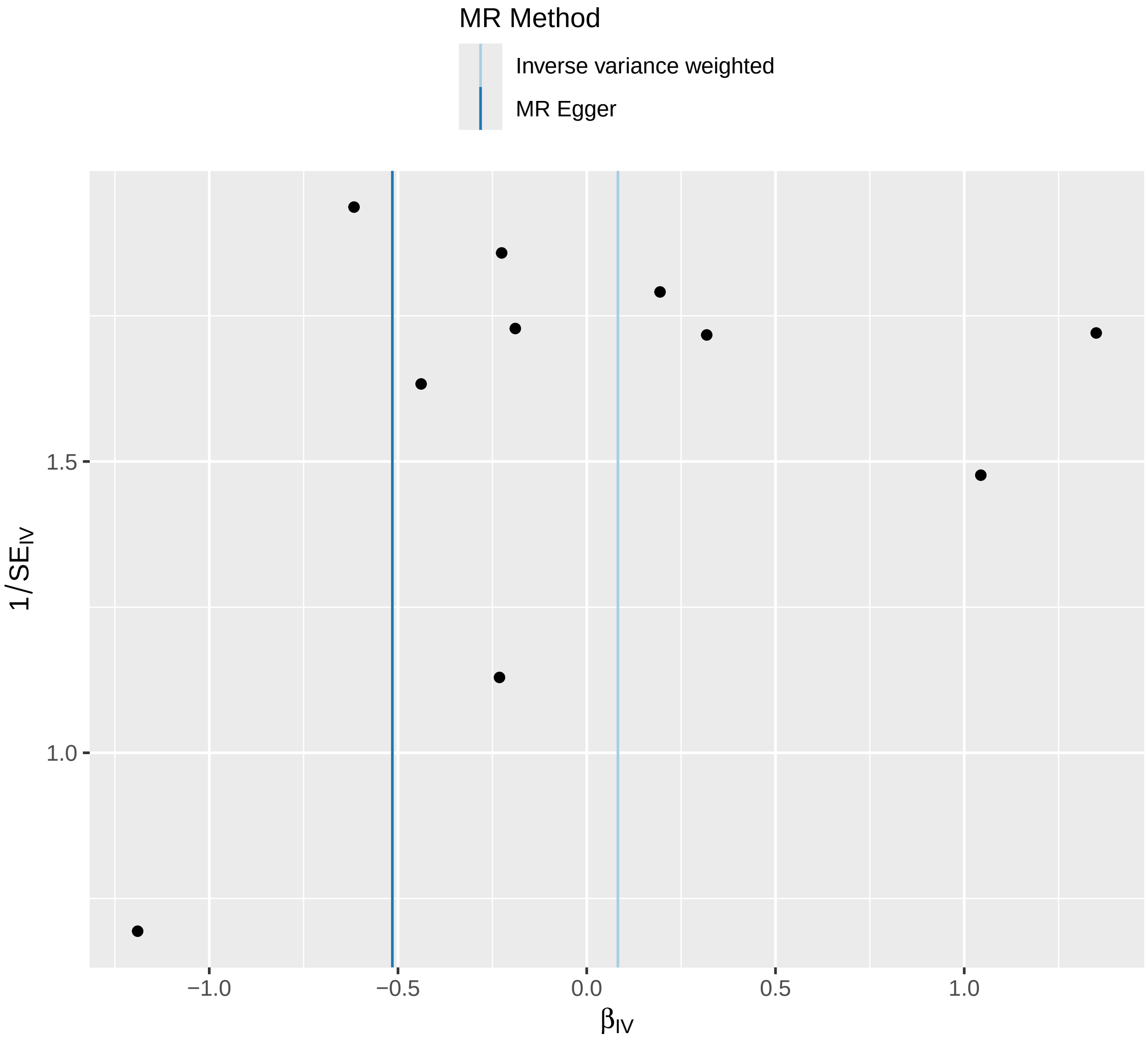

MR Method
Inverse variance weighted
MR Egger
1.5
1.0
−1.0
−0.5
0.0
0.5
1.0
$1/SE_{IV}$
β_{IV}

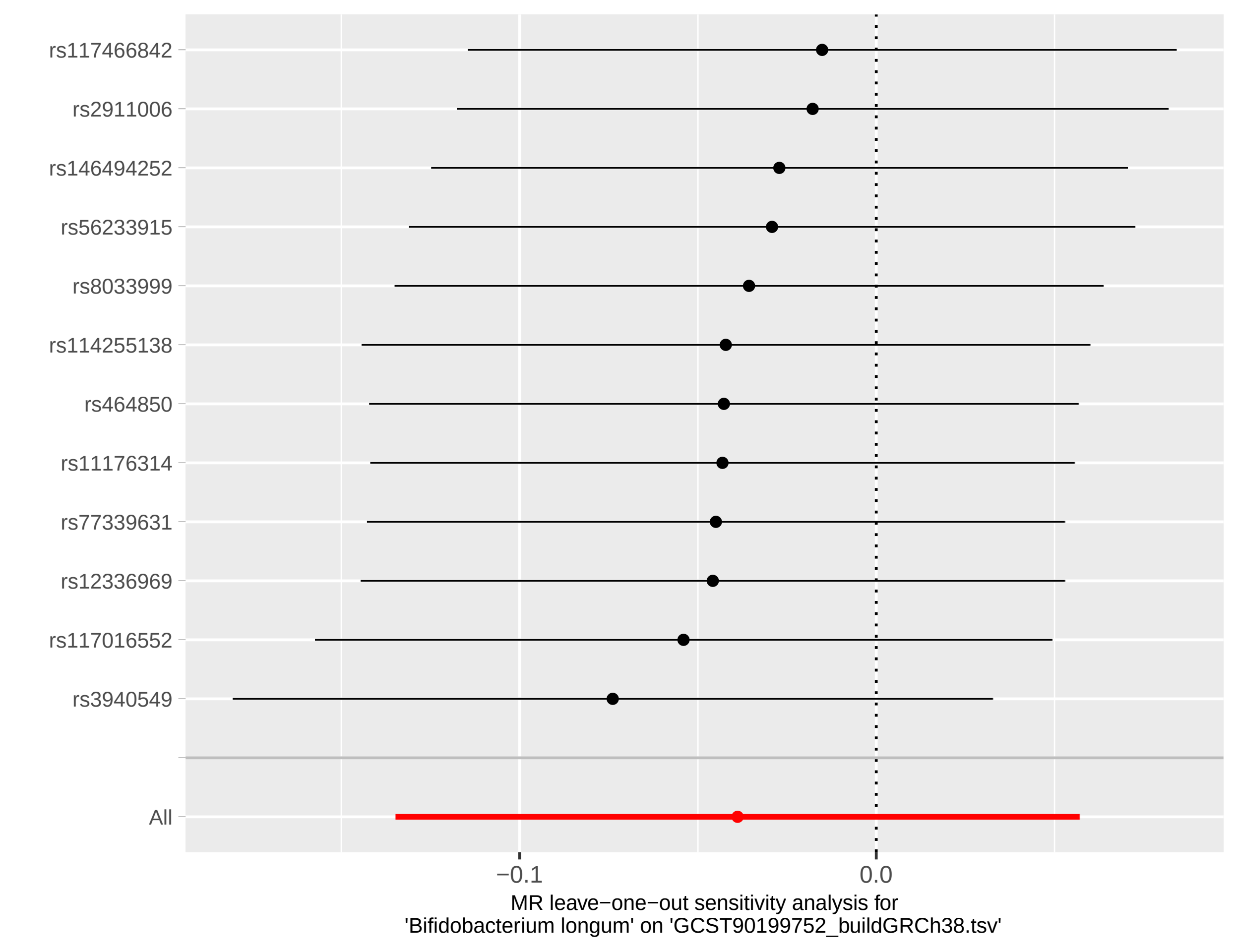

rs117466842
rs2911006
rs146494252
rs56233915
rs8033999
rs114255138
rs464850
rs11176314
rs77339631
rs12336969
rs117016552
rs3940549
All
−0.1
0.0
MR leave−one−out sensitivity analysis for
'Bifidobacterium longum' on 'GCST90199752_buildGRCh38.tsv'

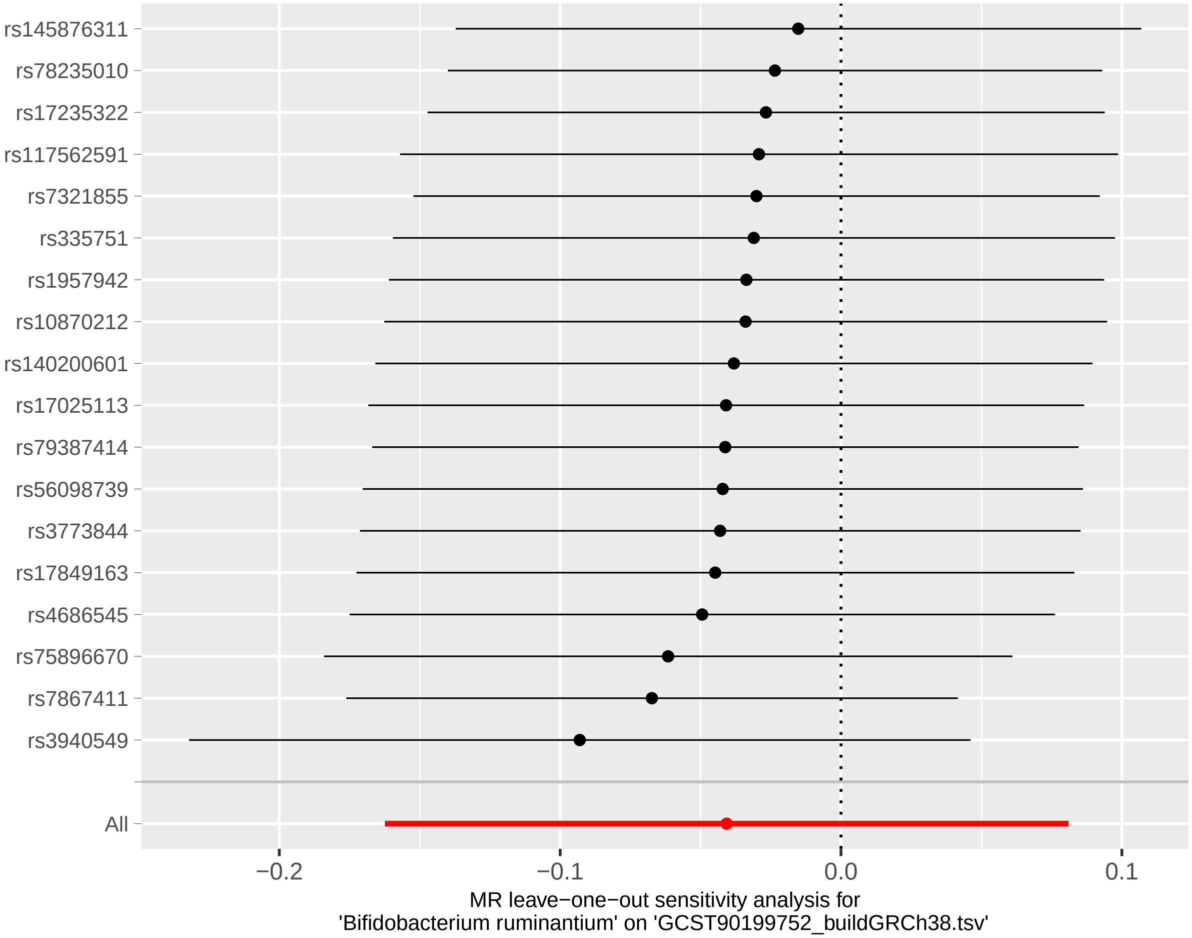
rs145876311
rs78235010
rs17235322
rs117562591
rs7321855
rs335751
rs1957942
rs10870212
rs140200601
rs17025113
rs79387414
rs56098739
rs3773844
rs17849163
rs4686545
rs75896670
rs7867411
rs3940549
All
−0.2
−0.1
0.0
0.1
MR leave−one−out sensitivity analysis for
'Bifidobacterium ruminantium' on 'GCST90199752_buildGRCh38.tsv'

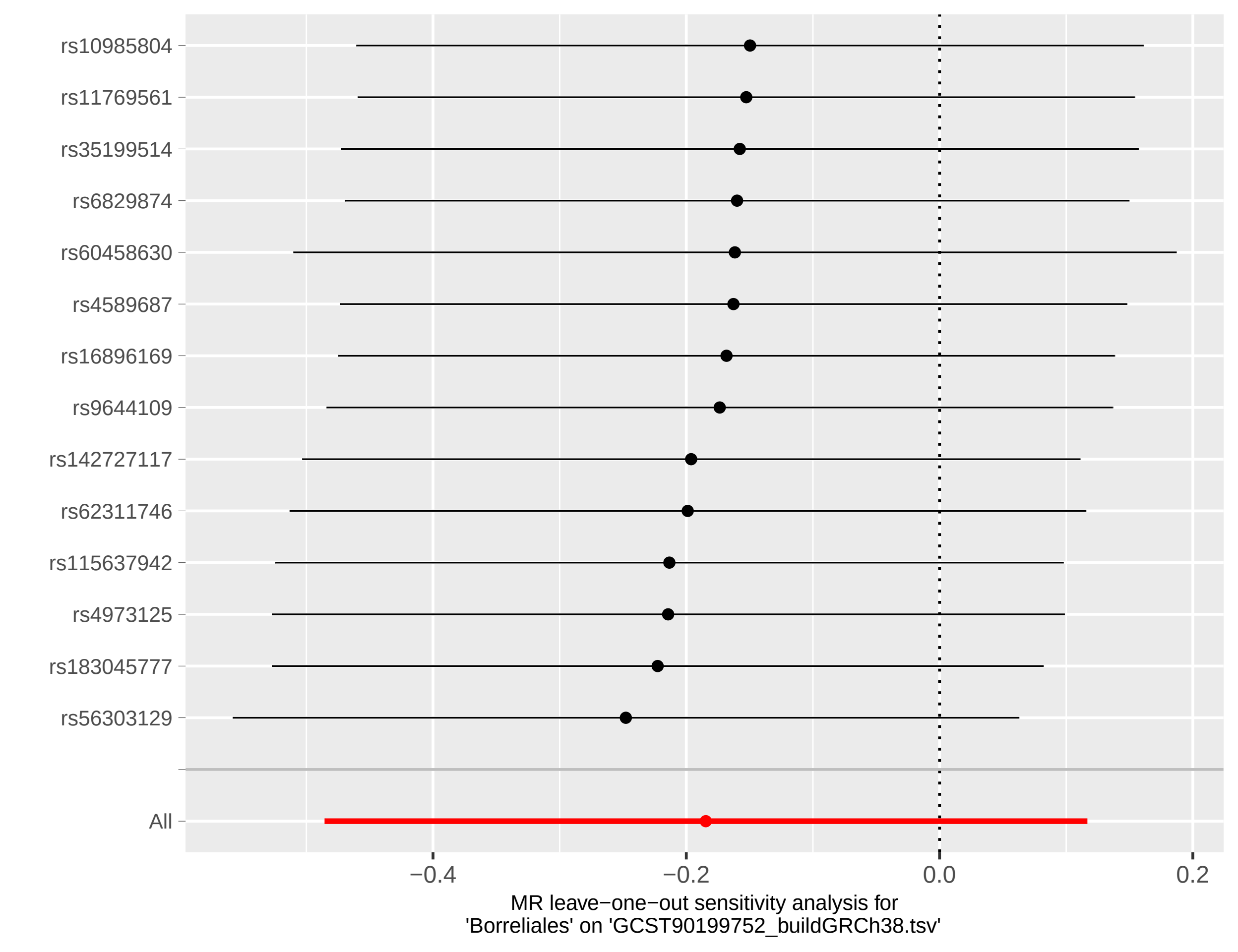

rs10985804
rs11769561
rs35199514
rs6829874
rs60458630
rs4589687
rs16896169
rs9644109
rs142727117
rs62311746
rs115637942
rs4973125
rs183045777
rs56303129
All
−0.4
−0.2
0.0
0.2
MR leave−one−out sensitivity analysis for
'Borreliales' on 'GCST90199752_buildGRCh38.tsv'

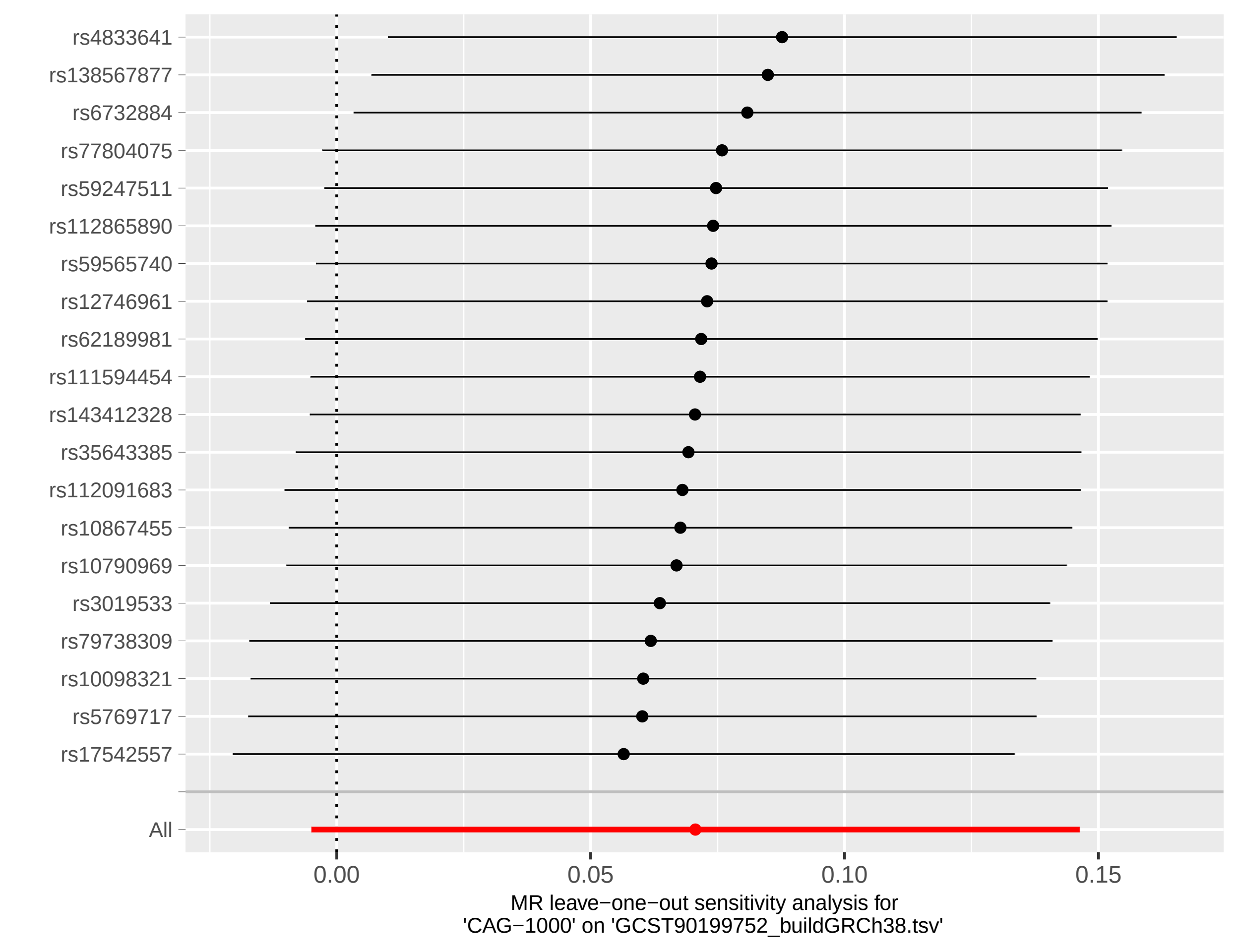

rs4833641
rs138567877
rs6732884
rs77804075
rs59247511
rs112865890
rs59565740
rs12746961
rs62189981
rs111594454
rs143412328
rs35643385
rs112091683
rs10867455
rs10790969
rs3019533
rs79738309
rs10098321
rs5769717
rs17542557
All
0.00
0.05
0.10
0.15
MR leave−one−out sensitivity analysis for
'CAG−1000' on 'GCST90199752_buildGRCh38.tsv'

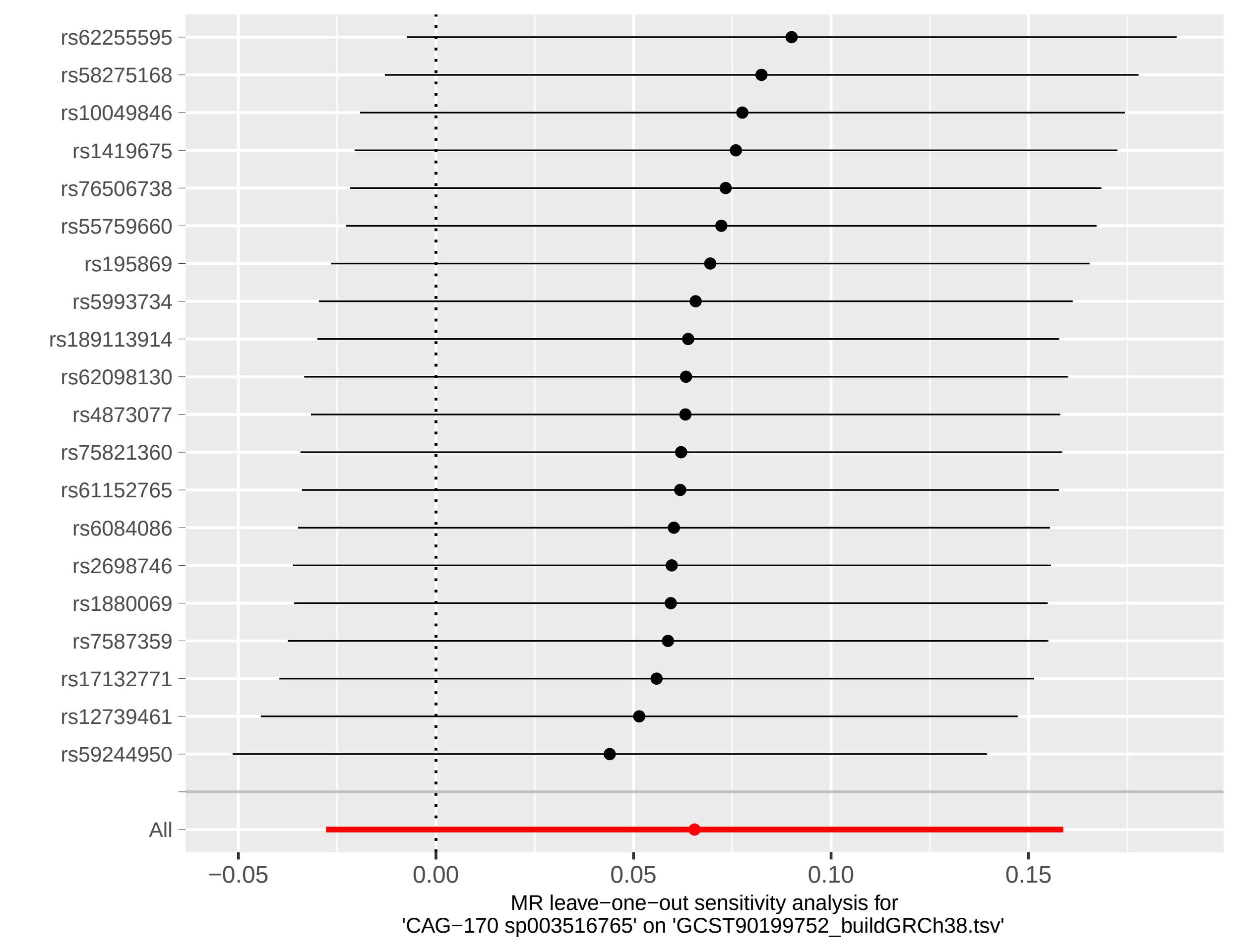

rs62255595
rs58275168
rs10049846
rs1419675
rs76506738
rs55759660
rs195869
rs5993734
rs189113914
rs62098130
rs4873077
rs75821360
rs61152765
rs6084086
rs2698746
rs1880069
rs7587359
rs17132771
rs12739461
rs59244950
All
−0.05
0.00
0.05
0.10
0.15
MR leave−one−out sensitivity analysis for
'CAG−170 sp003516765' on 'GCST90199752_buildGRCh38.tsv'

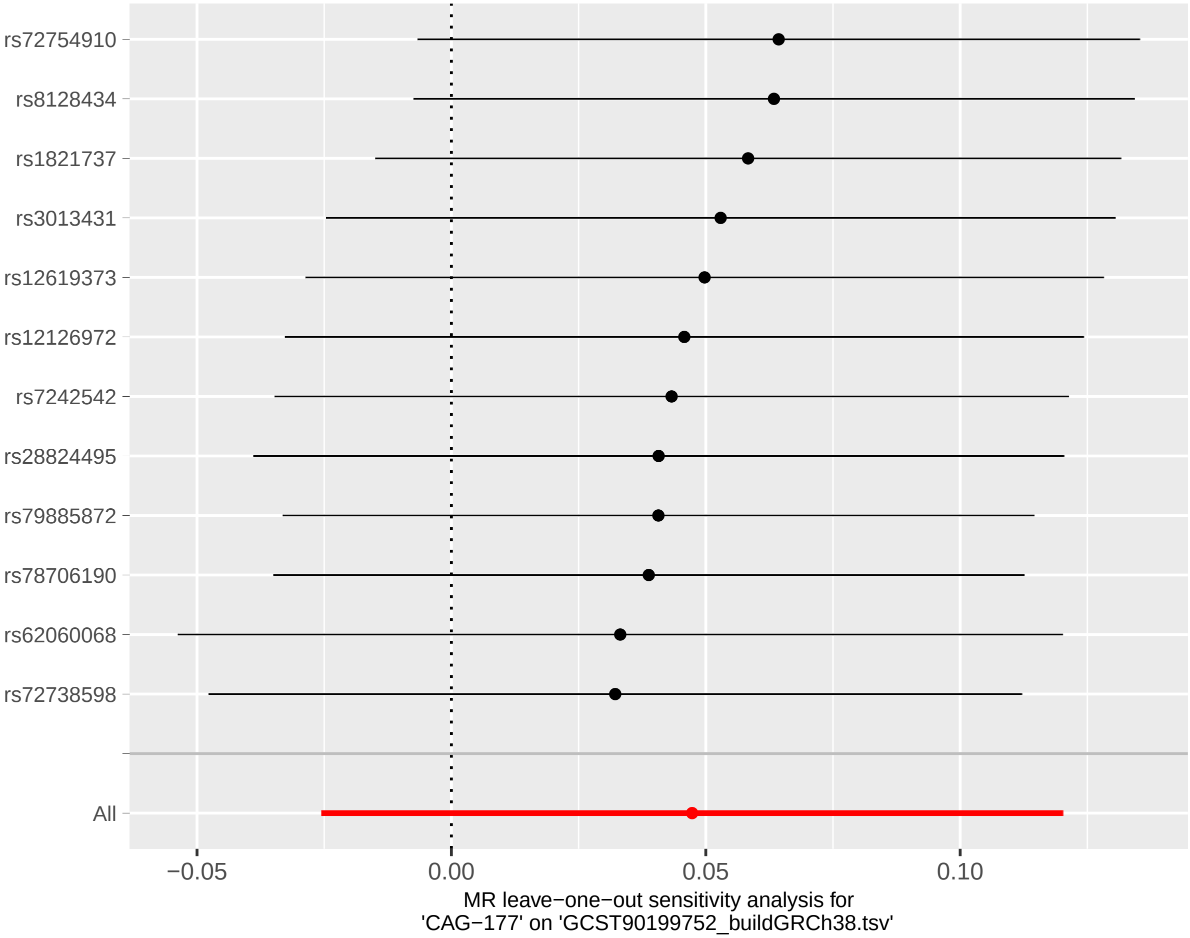
rs72754910
rs8128434
rs1821737
rs3013431
rs12619373
rs12126972
rs7242542
rs28824495
rs79885872
rs78706190
rs62060068
rs72738598
All
−0.05
0.00
0.05
0.10
MR leave−one−out sensitivity analysis for
'CAG−177' on 'GCST90199752_buildGRCh38.tsv'

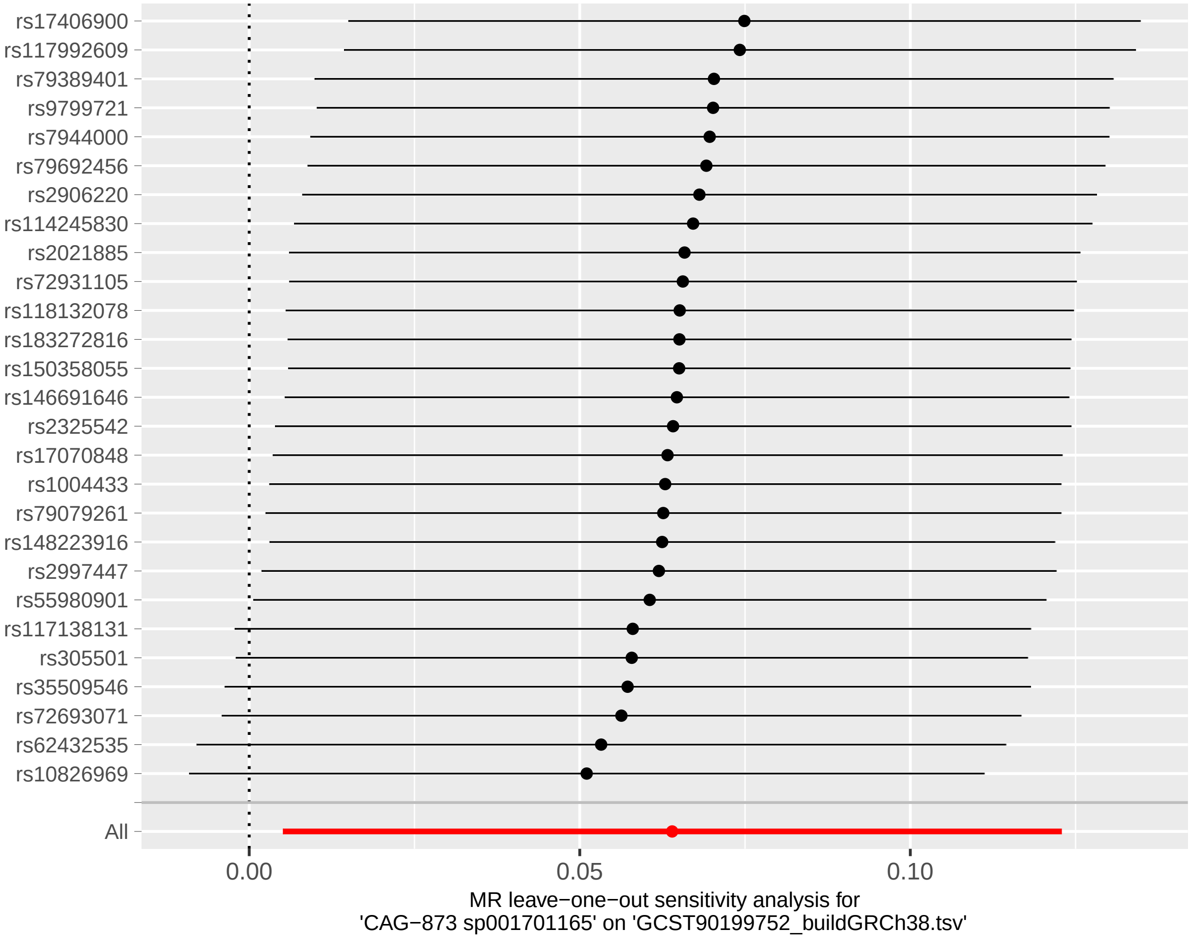
rs17406900
rs117992609
rs79389401
rs9799721
rs7944000
rs79692456
rs2906220
rs114245830
rs2021885
rs72931105
rs118132078
rs183272816
rs150358055
rs146691646
rs2325542
rs17070848
rs1004433
rs79079261
rs148223916
rs2997447
rs55980901
rs117138131
rs305501
rs35509546
rs72693071
rs62432535
rs10826969
All
0.00
0.05
0.10
MR leave−one−out sensitivity analysis for
'CAG−873 sp001701165' on 'GCST90199752_buildGRCh38.tsv'

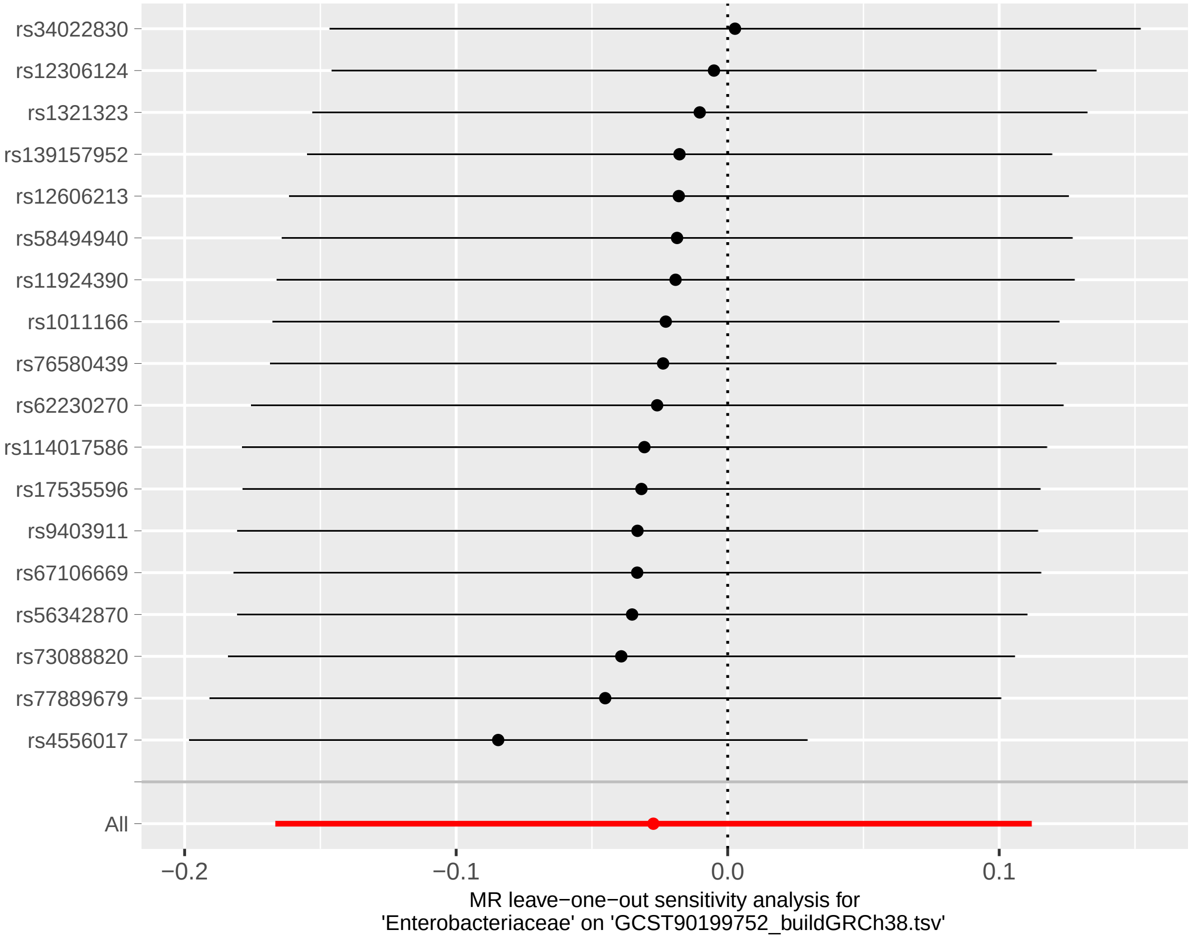

rs34022830
rs12306124
rs1321323
rs139157952
rs12606213
rs58494940
rs11924390
rs1011166
rs76580439
rs62230270
rs114017586
rs17535596
rs9403911
rs67106669
rs56342870
rs73088820
rs77889679
rs4556017
All
−0.2
−0.1
0.0
0.1
MR leave−one−out sensitivity analysis for
'Enterobacteriaceae' on 'GCST90199752_buildGRCh38.tsv'

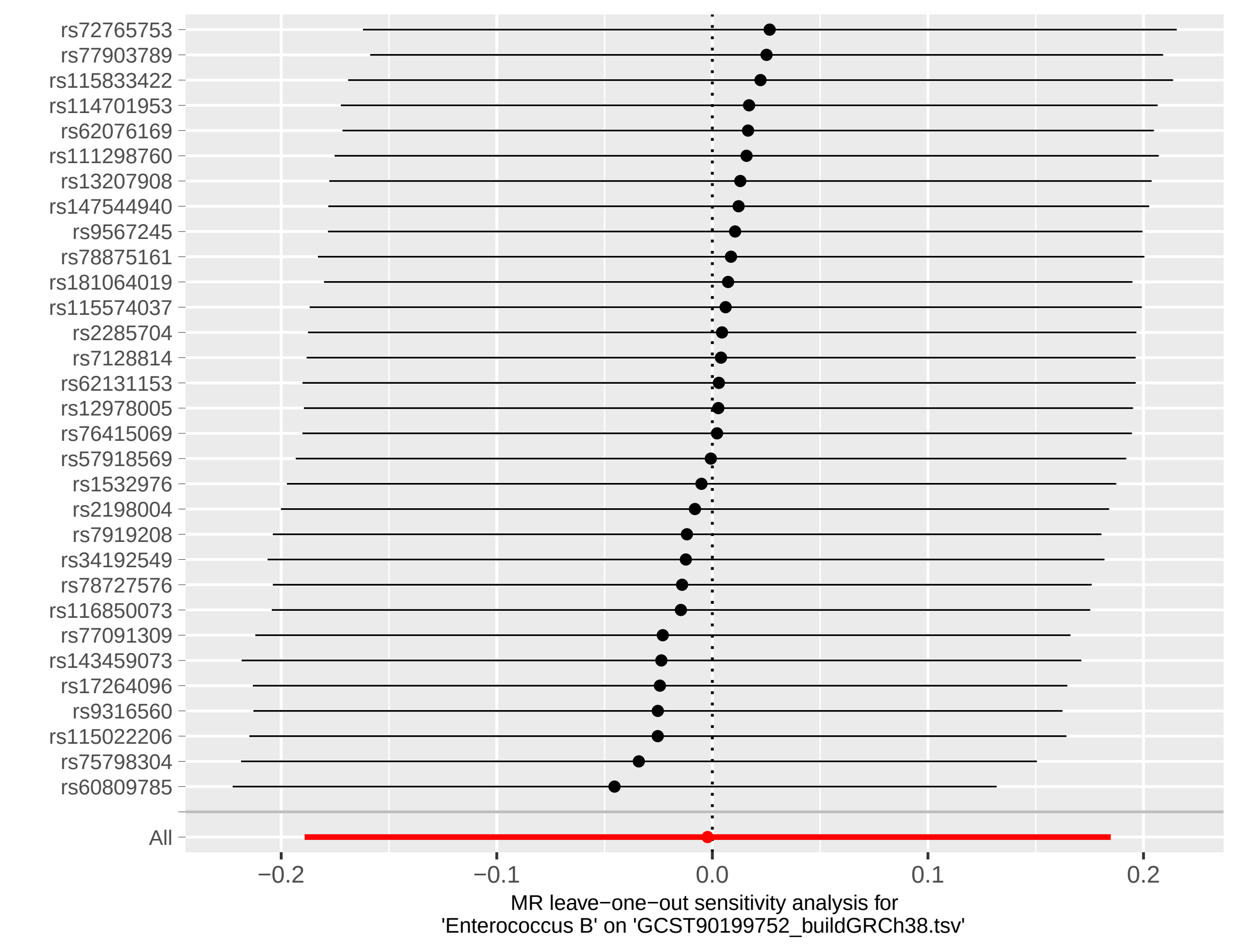

rs72765753
rs77903789
rs115833422
rs114701953
rs62076169
rs111298760
rs13207908
rs147544940
rs9567245
rs78875161
rs181064019
rs115574037
rs2285704
rs7128814
rs62131153
rs12978005
rs76415069
rs57918569
rs1532976
rs2198004
rs7919208
rs34192549
rs78727576
rs116850073
rs77091309
rs143459073
rs17264096
rs9316560
rs115022206
rs75798304
rs60809785
All
−0.2
−0.1
0.0
0.1
0.2
MR leave−one−out sensitivity analysis for
'Enterococcus B' on 'GCST90199752_buildGRCh38.tsv'

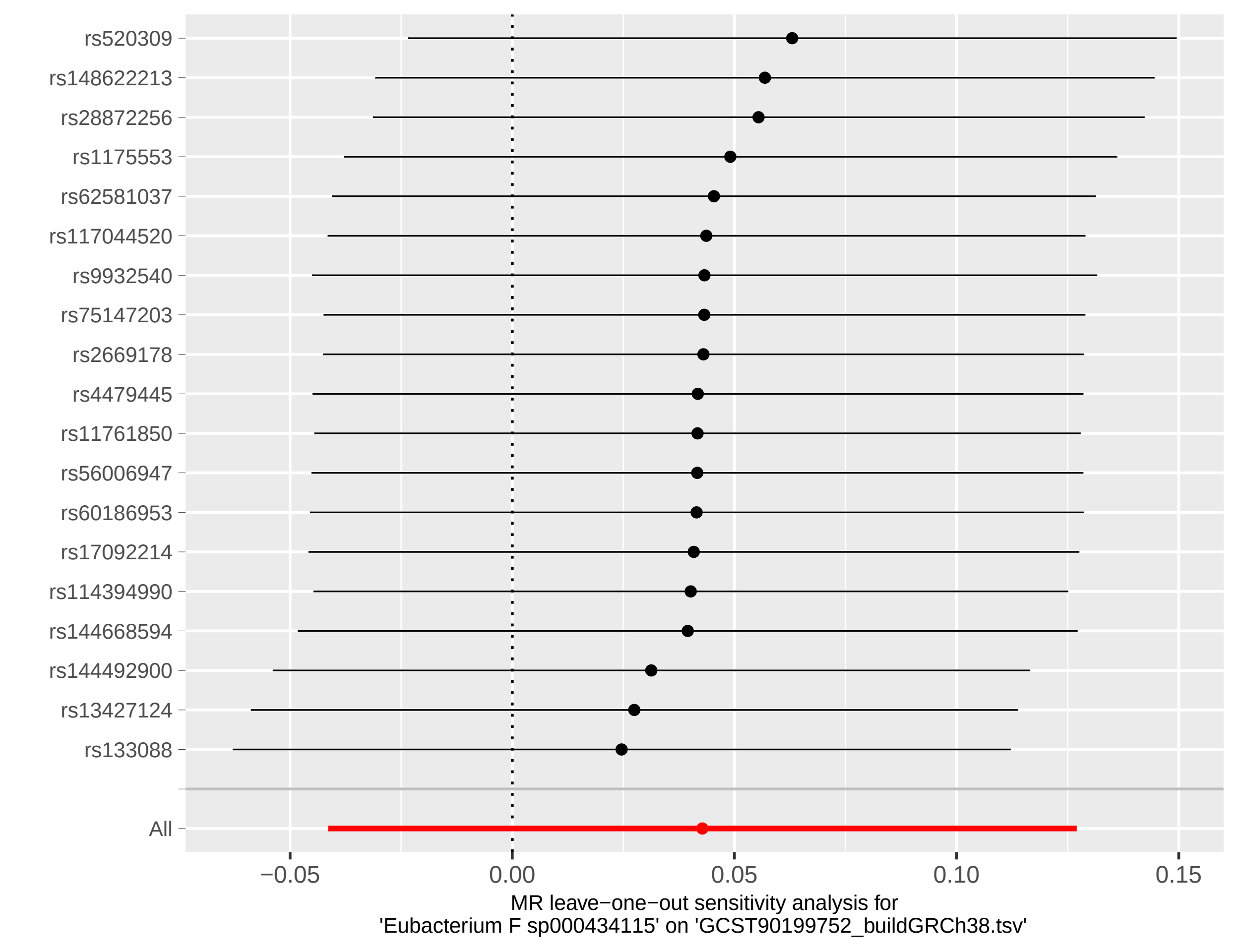
rs520309
rs148622213
rs28872256
rs1175553
rs62581037
rs117044520
rs9932540
rs75147203
rs2669178
rs4479445
rs11761850
rs56006947
rs60186953
rs17092214
rs114394990
rs144668594
rs144492900
rs13427124
rs133088
All
−0.05
0.00
0.05
0.10
0.15
MR leave−one−out sensitivity analysis for
'Eubacterium F sp000434115' on 'GCST90199752_buildGRCh38.tsv'

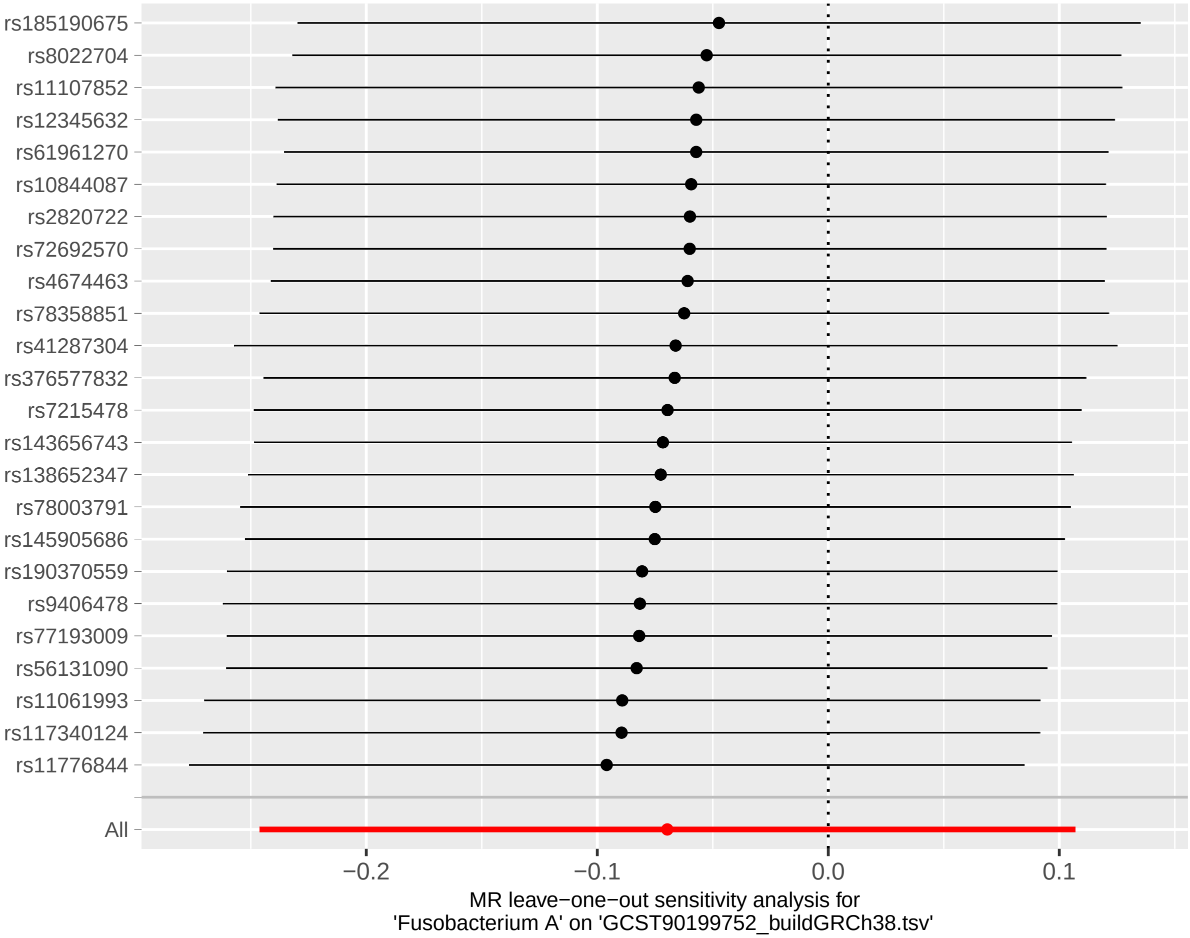

rs185190675
rs8022704
rs11107852
rs12345632
rs61961270
rs10844087
rs2820722
rs72692570
rs4674463
rs78358851
rs41287304
rs376577832
rs7215478
rs143656743
rs138652347
rs78003791
rs145905686
rs190370559
rs9406478
rs77193009
rs56131090
rs11061993
rs117340124
rs11776844
All
−0.2
−0.1
0.0
0.1
MR leave−one−out sensitivity analysis for
'Fusobacterium A' on 'GCST90199752_buildGRCh38.tsv'

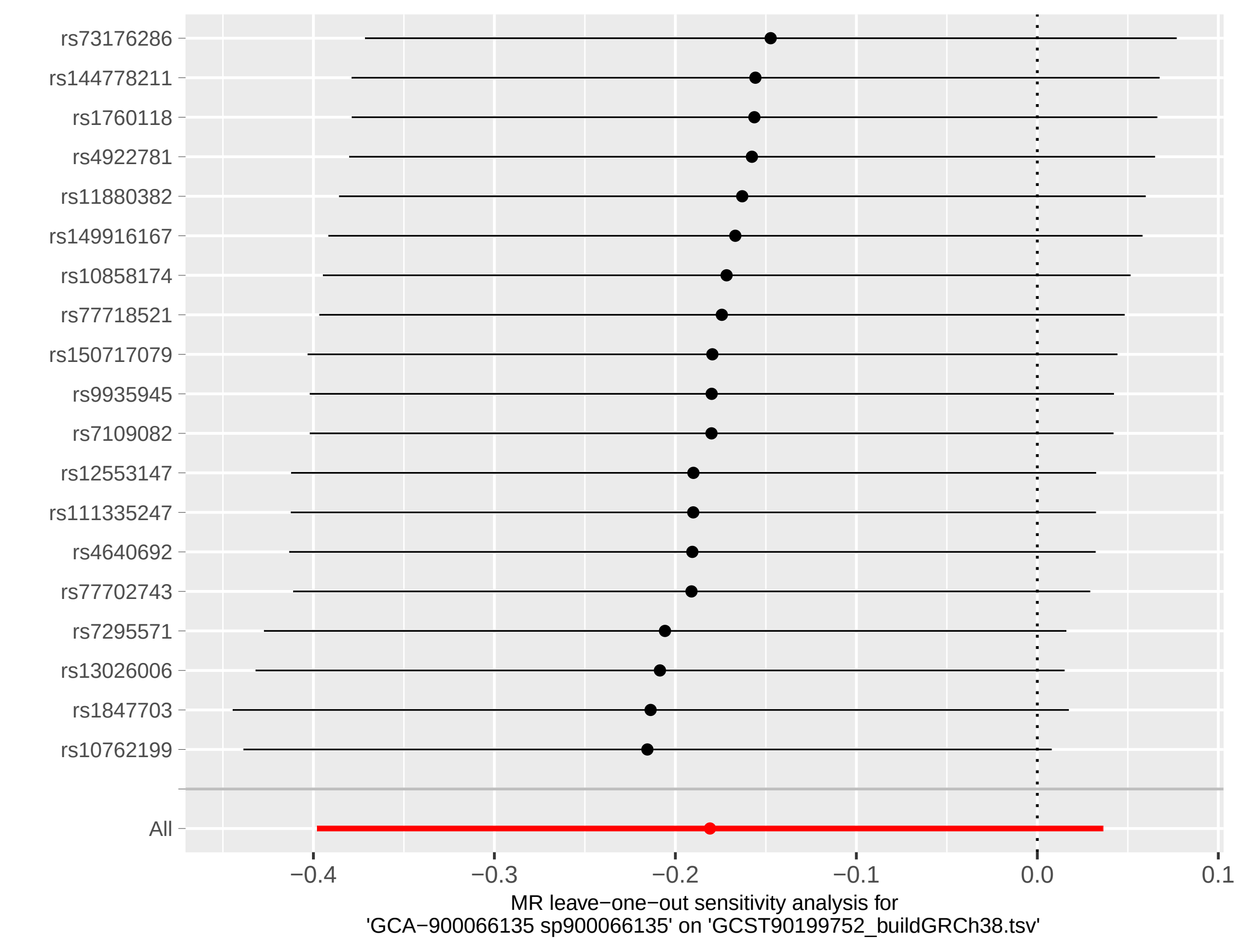
rs73176286
rs144778211
rs1760118
rs4922781
rs11880382
rs149916167
rs10858174
rs77718521
rs150717079
rs9935945
rs7109082
rs12553147
rs111335247
rs4640692
rs77702743
rs7295571
rs13026006
rs1847703
rs10762199
All
−0.4
−0.3
−0.2
−0.1
0.0
0.1
MR leave−one−out sensitivity analysis for
'GCA−900066135 sp900066135' on 'GCST90199752_buildGRCh38.tsv'

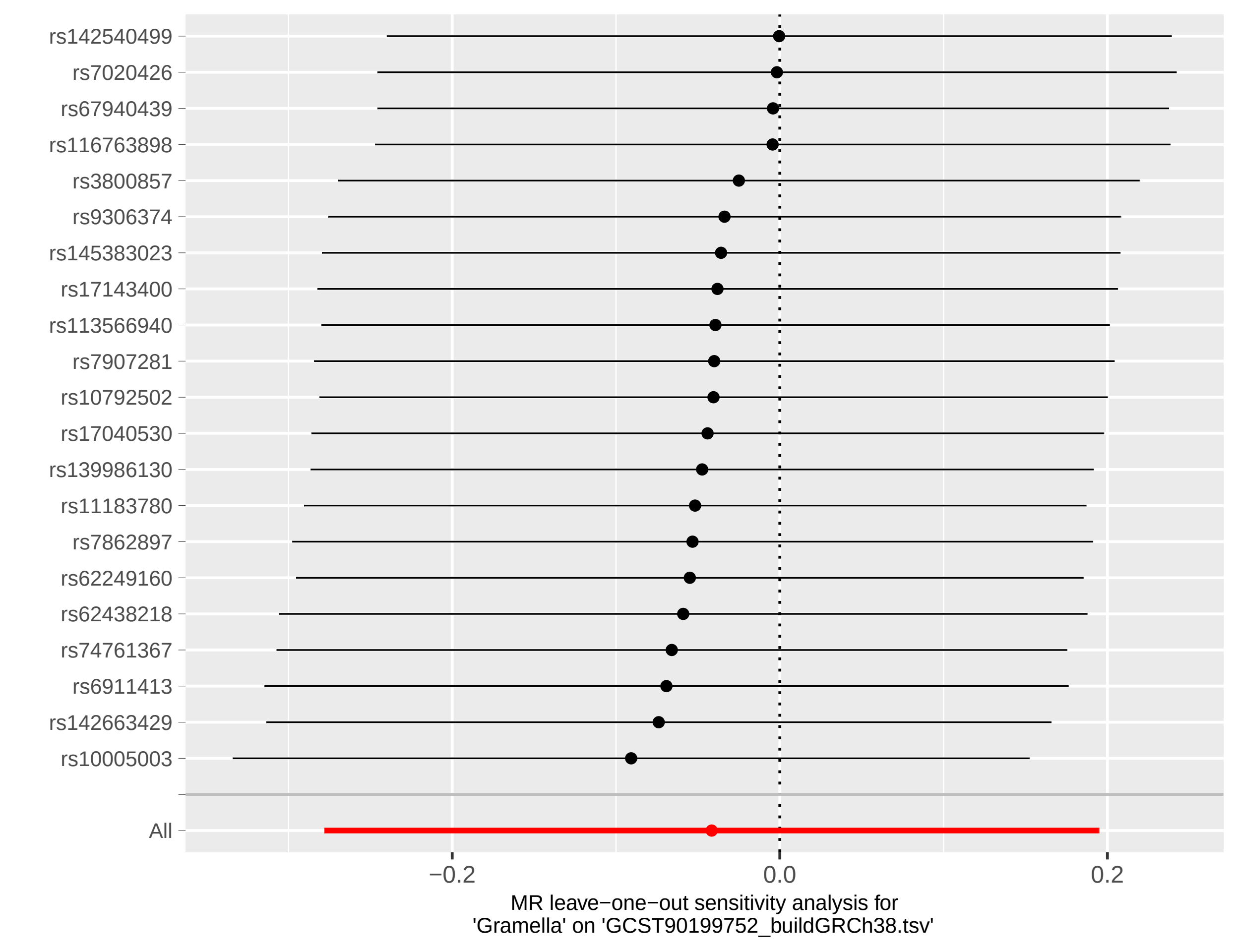
rs142540499
rs7020426
rs67940439
rs116763898
rs3800857
rs9306374
rs145383023
rs17143400
rs113566940
rs7907281
rs10792502
rs17040530
rs139986130
rs11183780
rs7862897
rs62249160
rs62438218
rs74761367
rs6911413
rs142663429
rs10005003
All
−0.2
0.0
0.2
MR leave−one−out sensitivity analysis for
'Gramella' on 'GCST90199752_buildGRCh38.tsv'

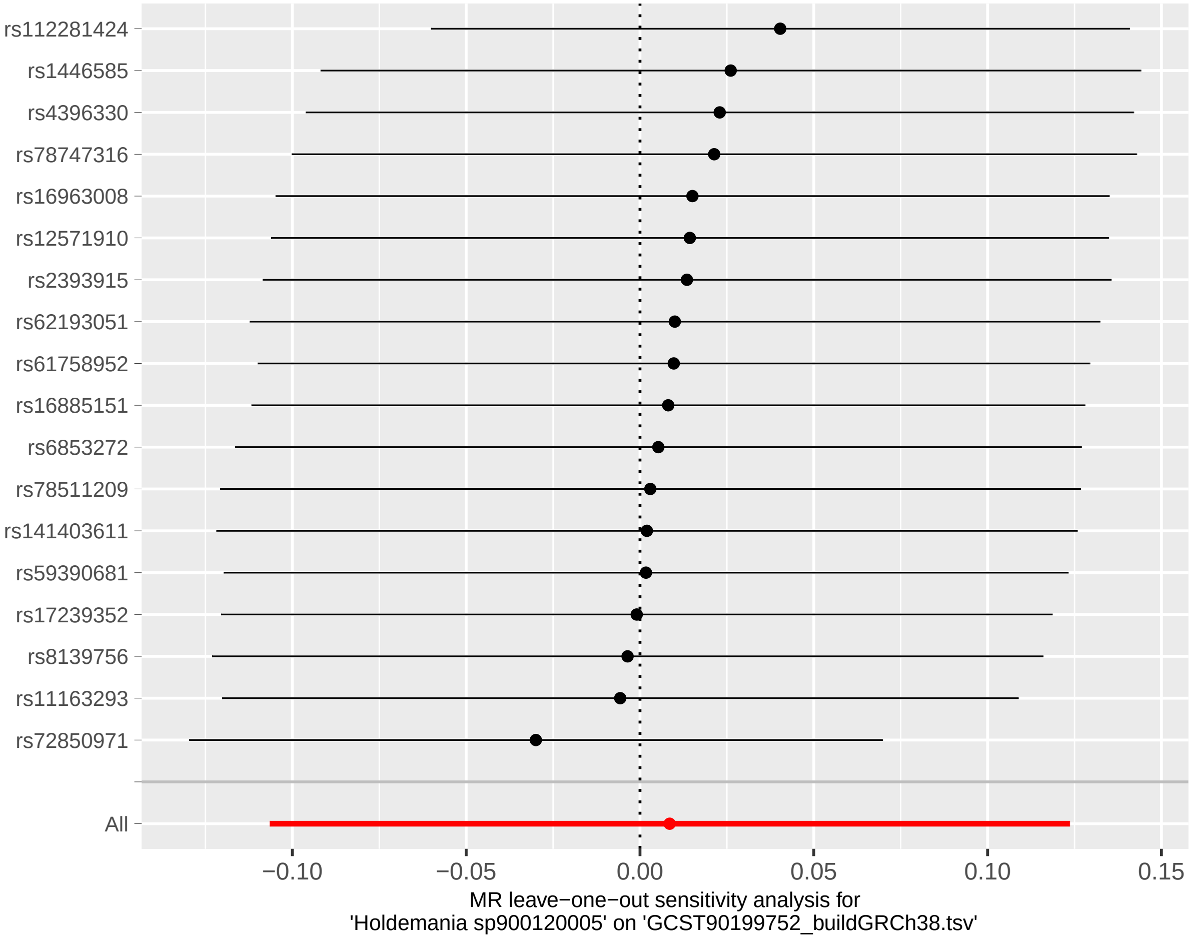

rs112281424
rs1446585
rs4396330
rs78747316
rs16963008
rs12571910
rs2393915
rs62193051
rs61758952
rs16885151
rs6853272
rs78511209
rs141403611
rs59390681
rs17239352
rs8139756
rs11163293
rs72850971
All
−0.10
−0.05
0.00
0.05
0.10
0.15
MR leave−one−out sensitivity analysis for
'Holdemania sp900120005' on 'GCST90199752_buildGRCh38.tsv'

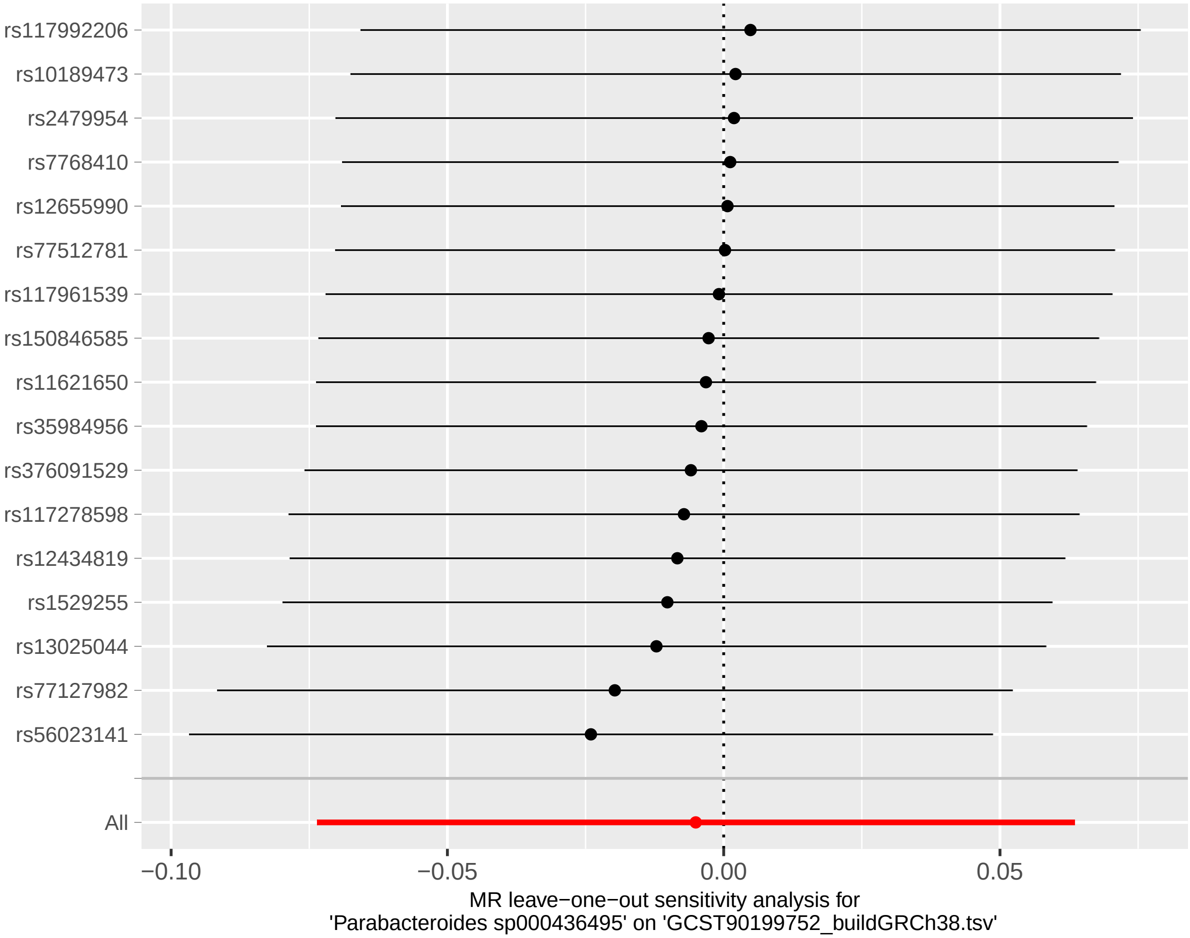
rs117992206
rs10189473
rs2479954
rs7768410
rs12655990
rs77512781
rs117961539
rs150846585
rs11621650
rs35984956
rs376091529
rs117278598
rs12434819
rs1529255
rs13025044
rs77127982
rs56023141
All
−0.10
−0.05
0.00
0.05
MR leave−one−out sensitivity analysis for
'Parabacteroides sp000436495' on 'GCST90199752_buildGRCh38.tsv'

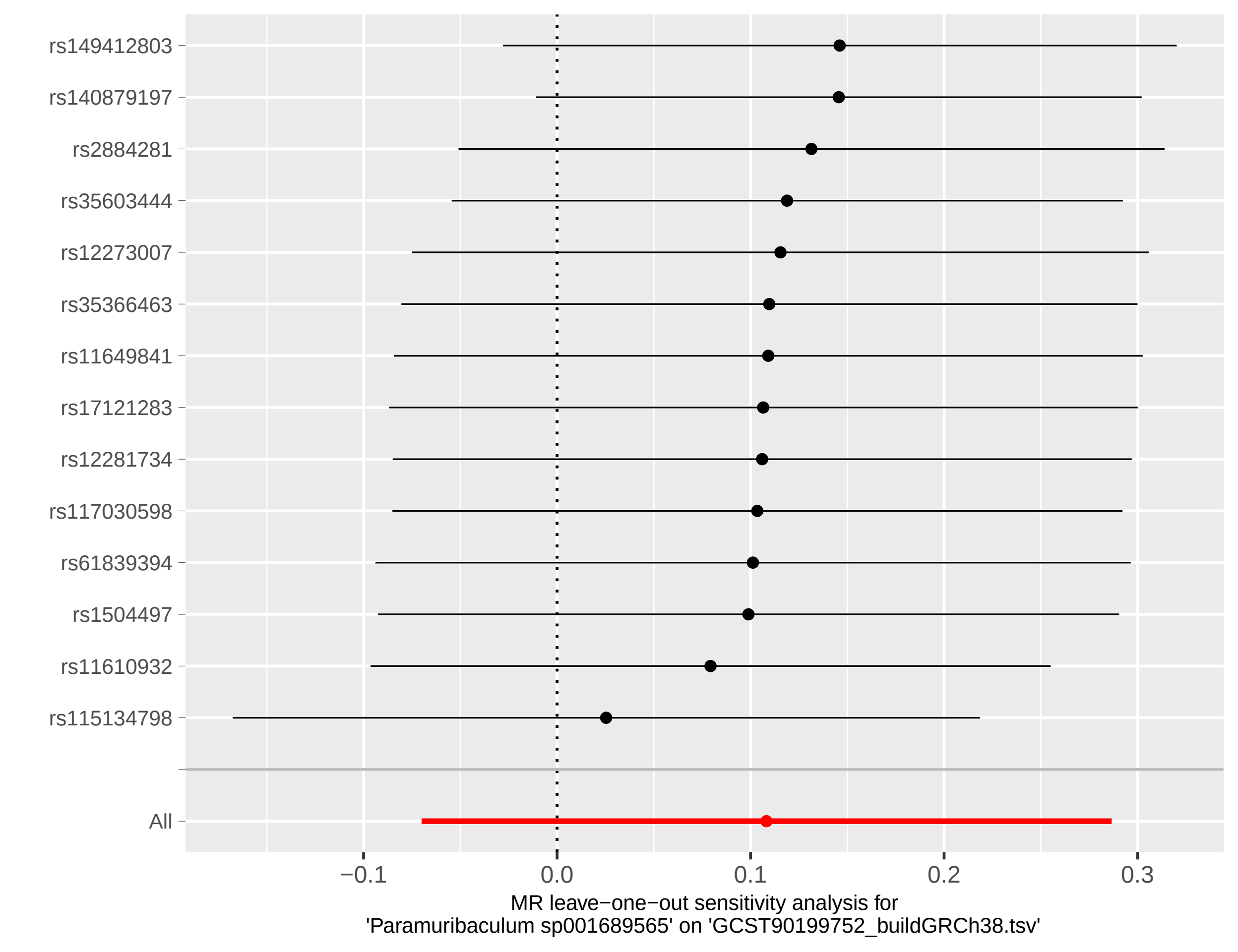
rs149412803
rs140879197
rs2884281
rs35603444
rs12273007
rs35366463
rs11649841
rs17121283
rs12281734
rs117030598
rs61839394
rs1504497
rs11610932
rs115134798
All
−0.1
0.0
0.1
0.2
0.3
MR leave−one−out sensitivity analysis for
'Paramuribaculum sp001689565' on 'GCST90199752_buildGRCh38.tsv'

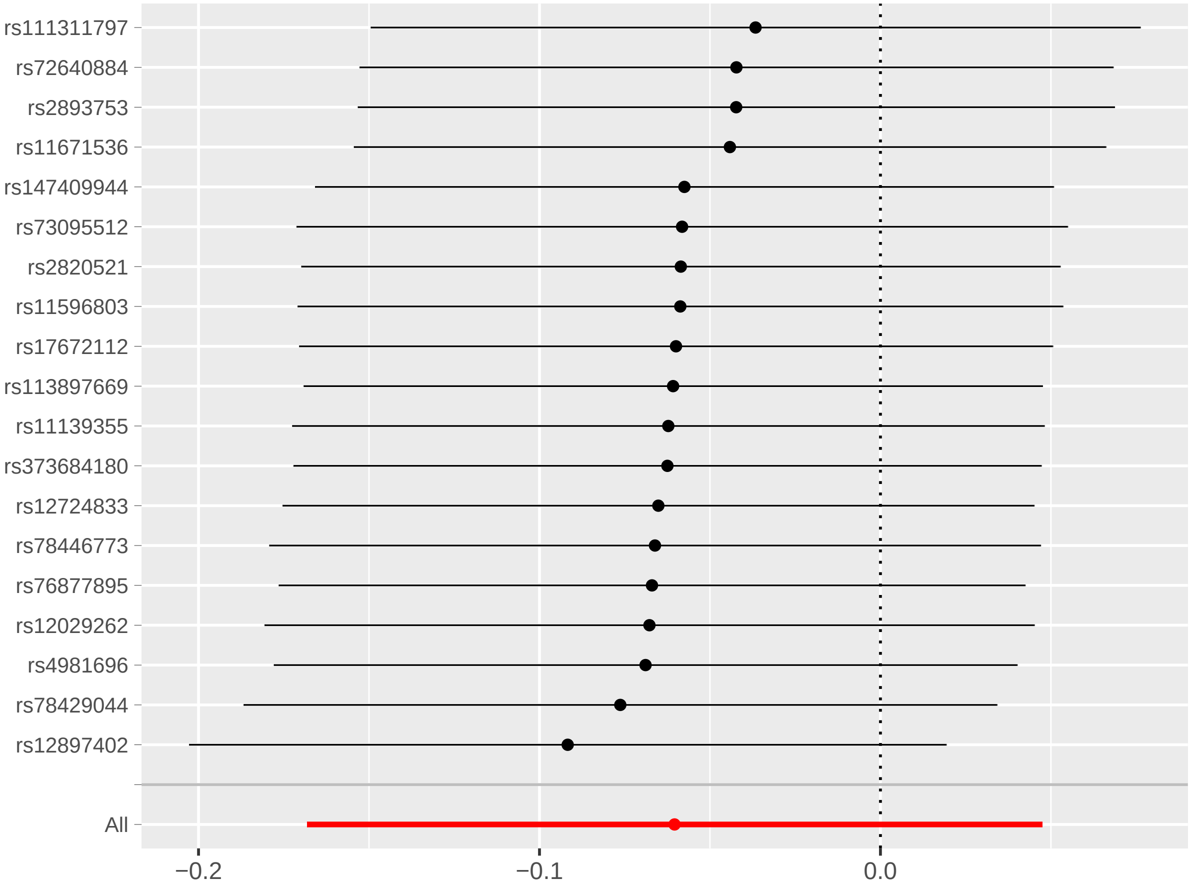

MR leave−one−out sensitivity analysis for
'Propionibacterium freudenreichii' on 'GCST90199752_buildGRCh38.tsv'

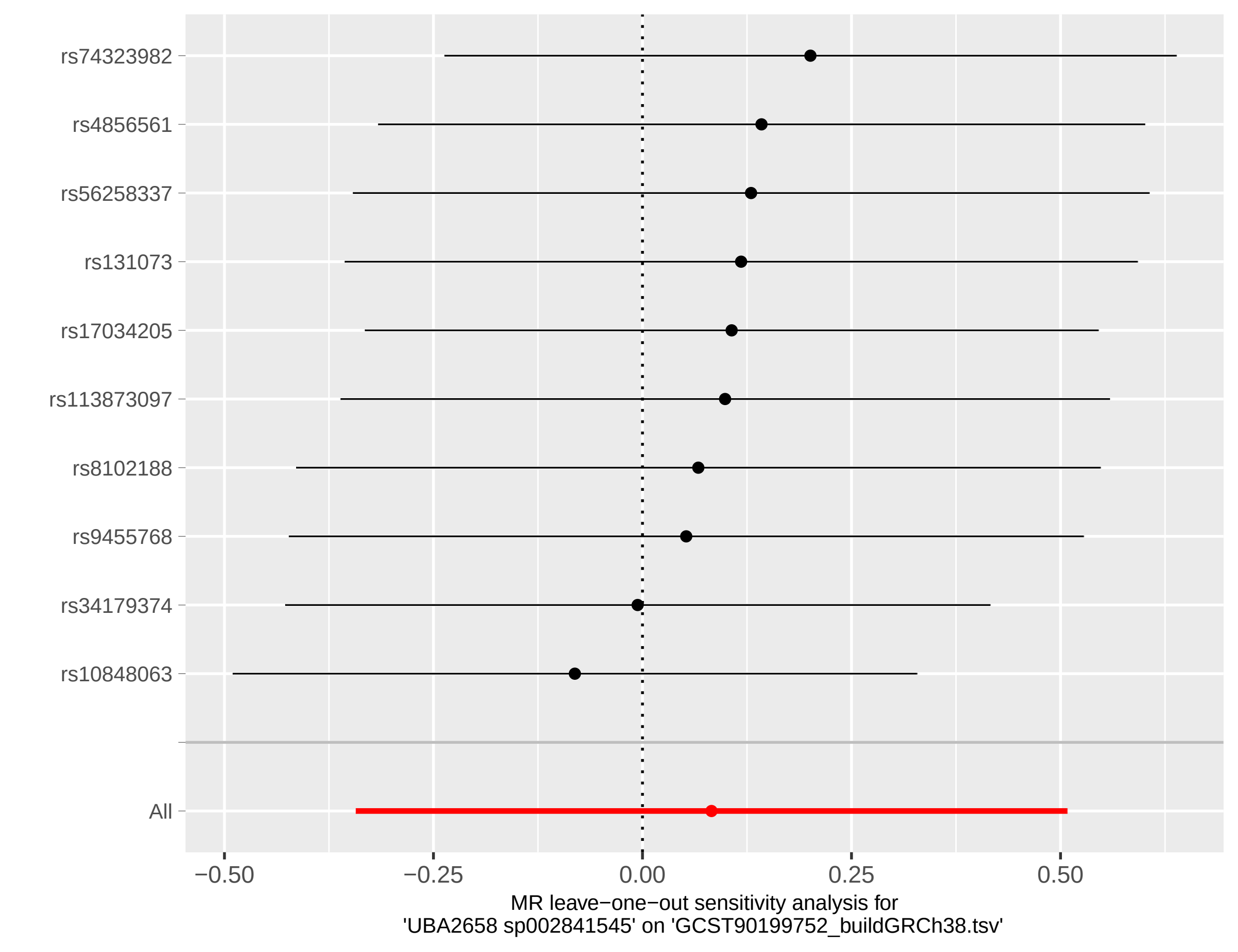
rs74323982
rs4856561
rs56258337
rs131073
rs17034205
rs113873097
rs8102188
rs9455768
rs34179374
rs10848063
All
−0.50
−0.25
0.00
0.25
0.50
MR leave−one−out sensitivity analysis for
'UBA2658 sp002841545' on 'GCST90199752_buildGRCh38.tsv'

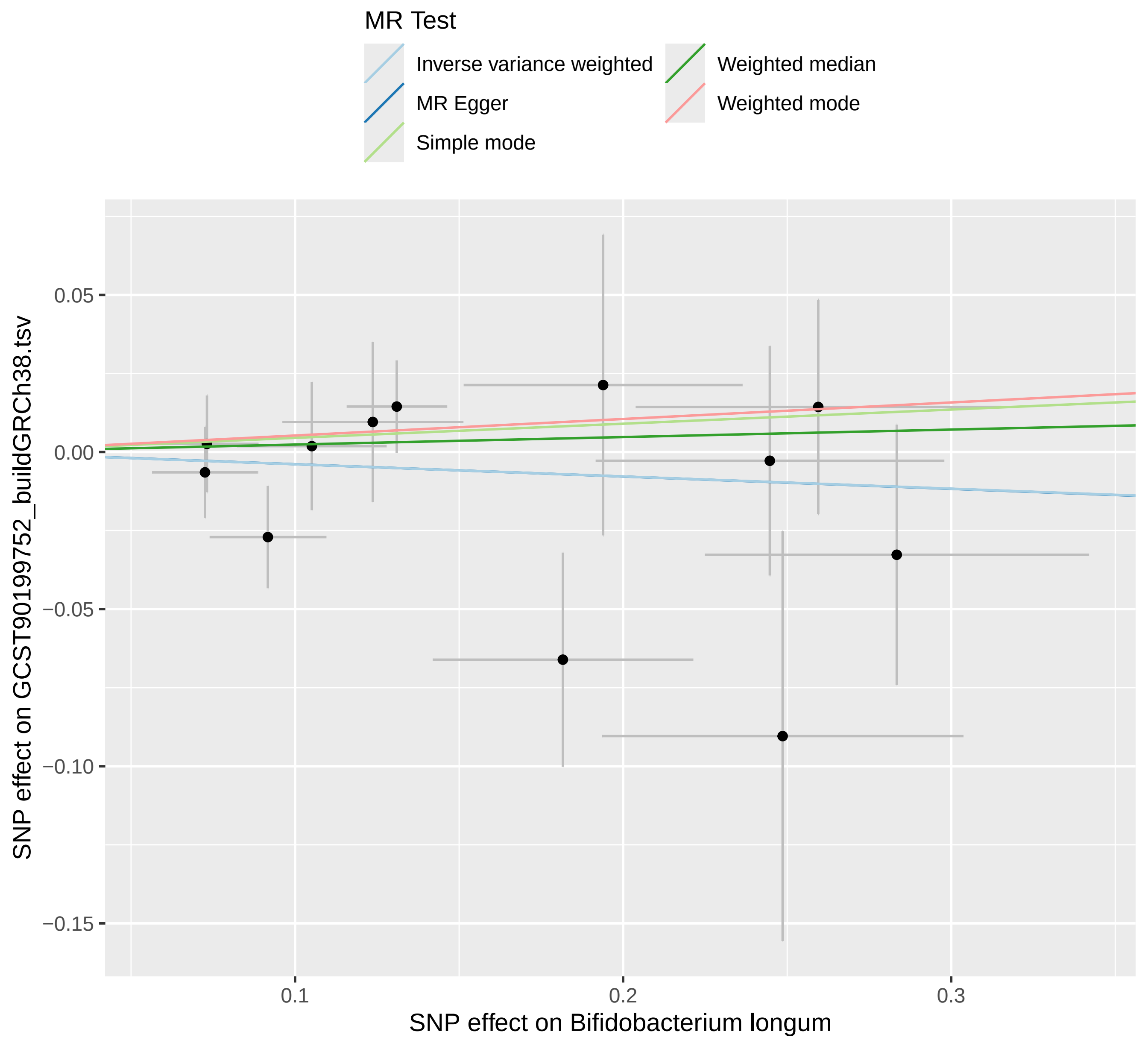
MR Test
Inverse variance weighted
MR Egger
Simple mode
Weighted median
Weighted mode
SNP effect on GCST90199752_buildGRCh38.tsv
0.05
0.00
−0.05
−0.10
−0.15
0.1
0.2
0.3
SNP effect on Bifidobacterium longum

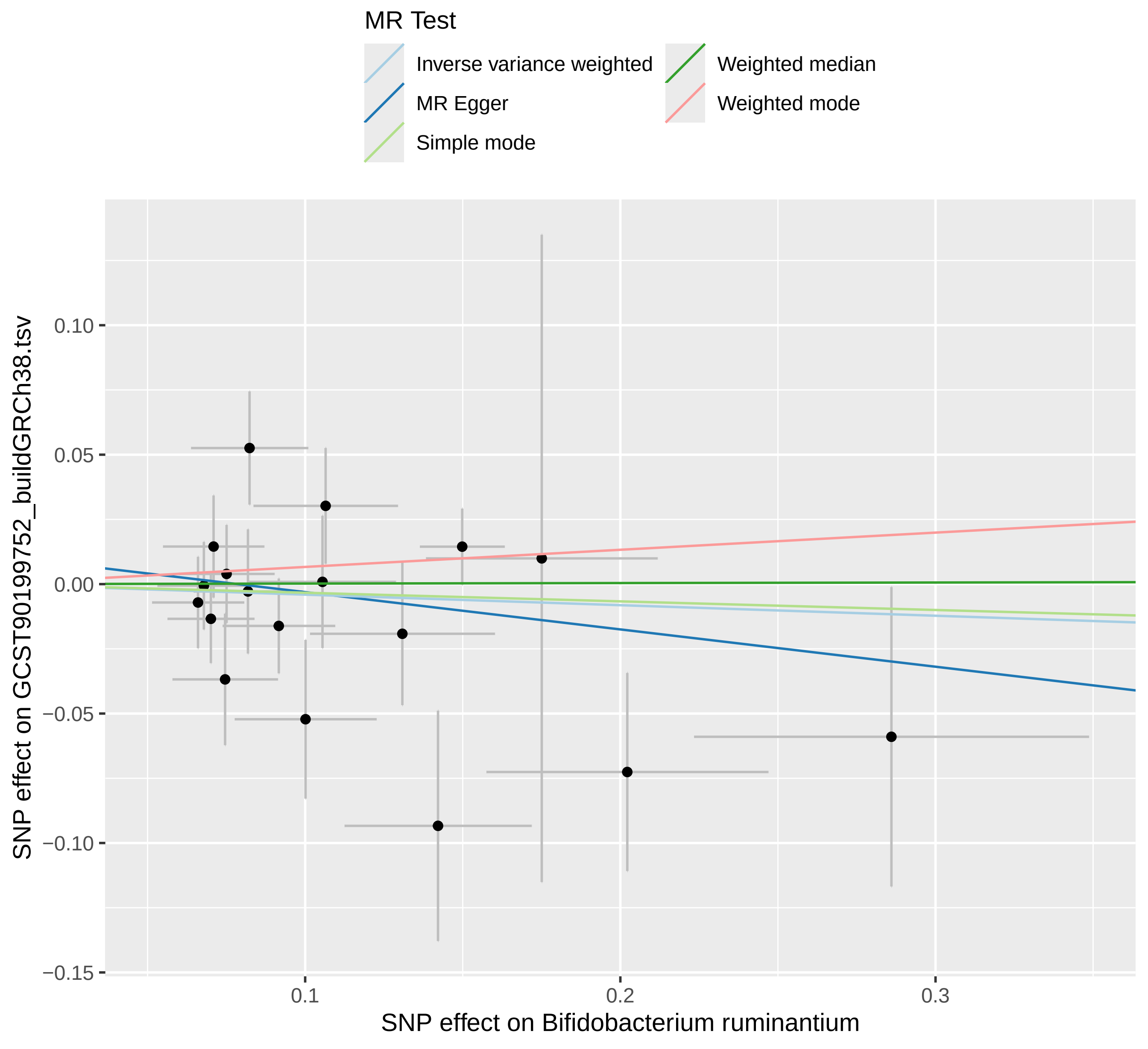
MR Test
Inverse variance weighted
MR Egger
Simple mode
Weighted median
Weighted mode
0.10
0.05
0.00
−0.05
−0.10
−0.15
0.1
0.2
0.3
SNP effect on GCST90199752_buildGRCh38.tsv
SNP effect on Bifidobacterium ruminantium

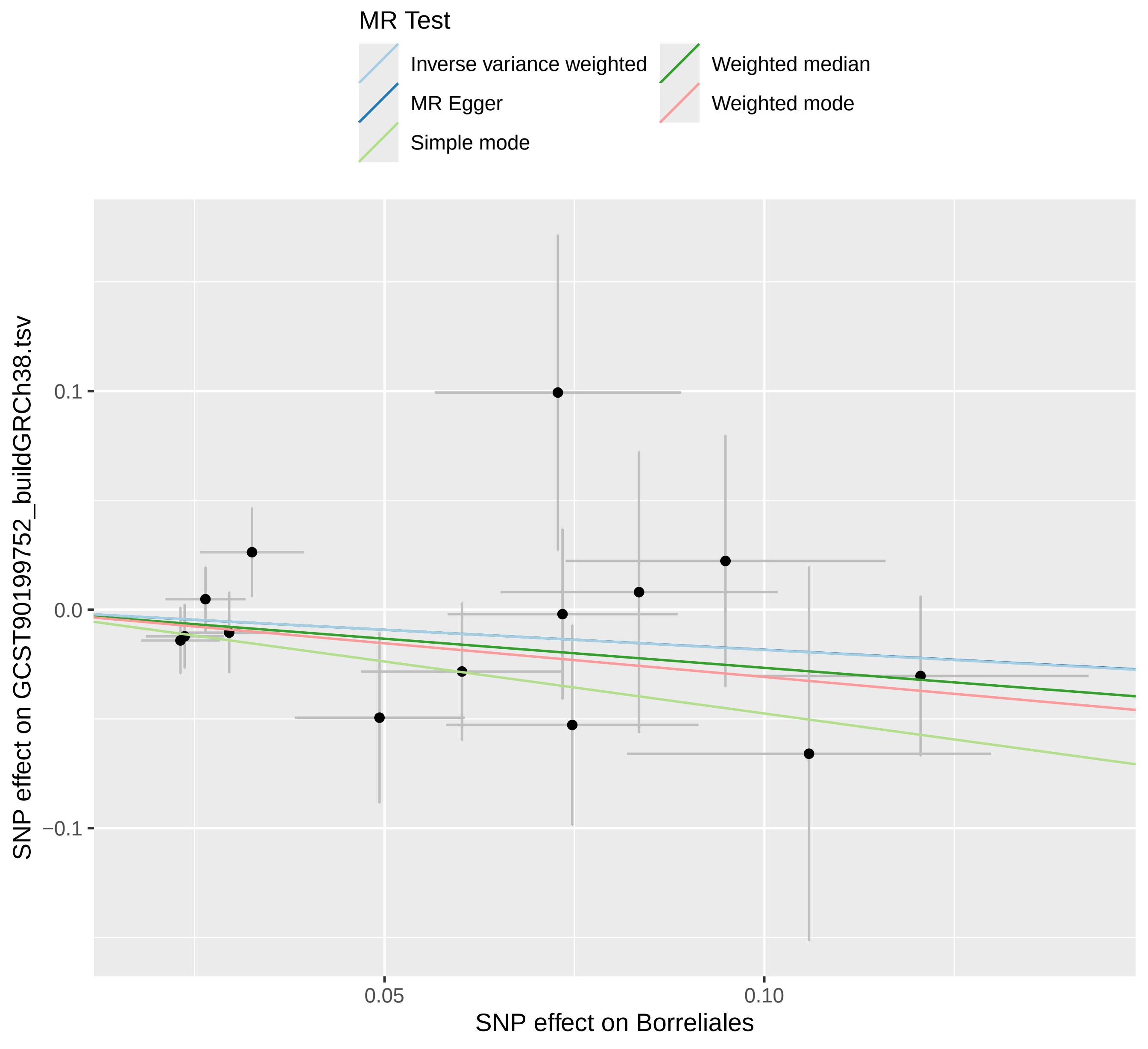
MR Test
Inverse variance weighted
MR Egger
Simple mode
Weighted median
Weighted mode
0.1
0.0
−0.1
0.05
0.10
SNP effect on GCST90199752_buildGRCh38.tsv
SNP effect on Borreliales

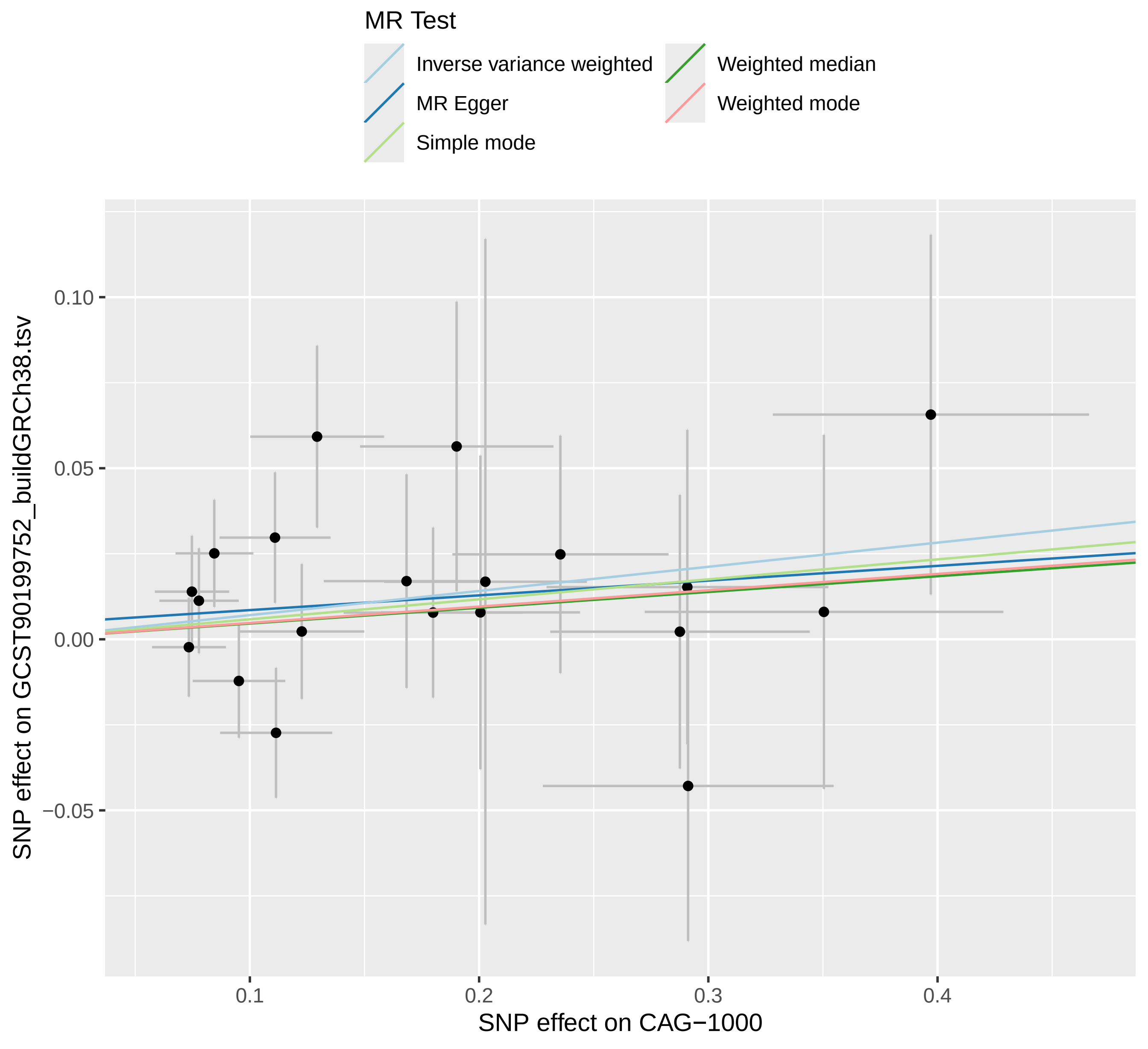
MR Test
Inverse variance weighted
MR Egger
Simple mode
Weighted median
Weighted mode
SNP effect on GCST90199752_buildGRCh38.tsv
0.10
0.05
0.00
−0.05
0.1
0.2
0.3
0.4
SNP effect on CAG−1000

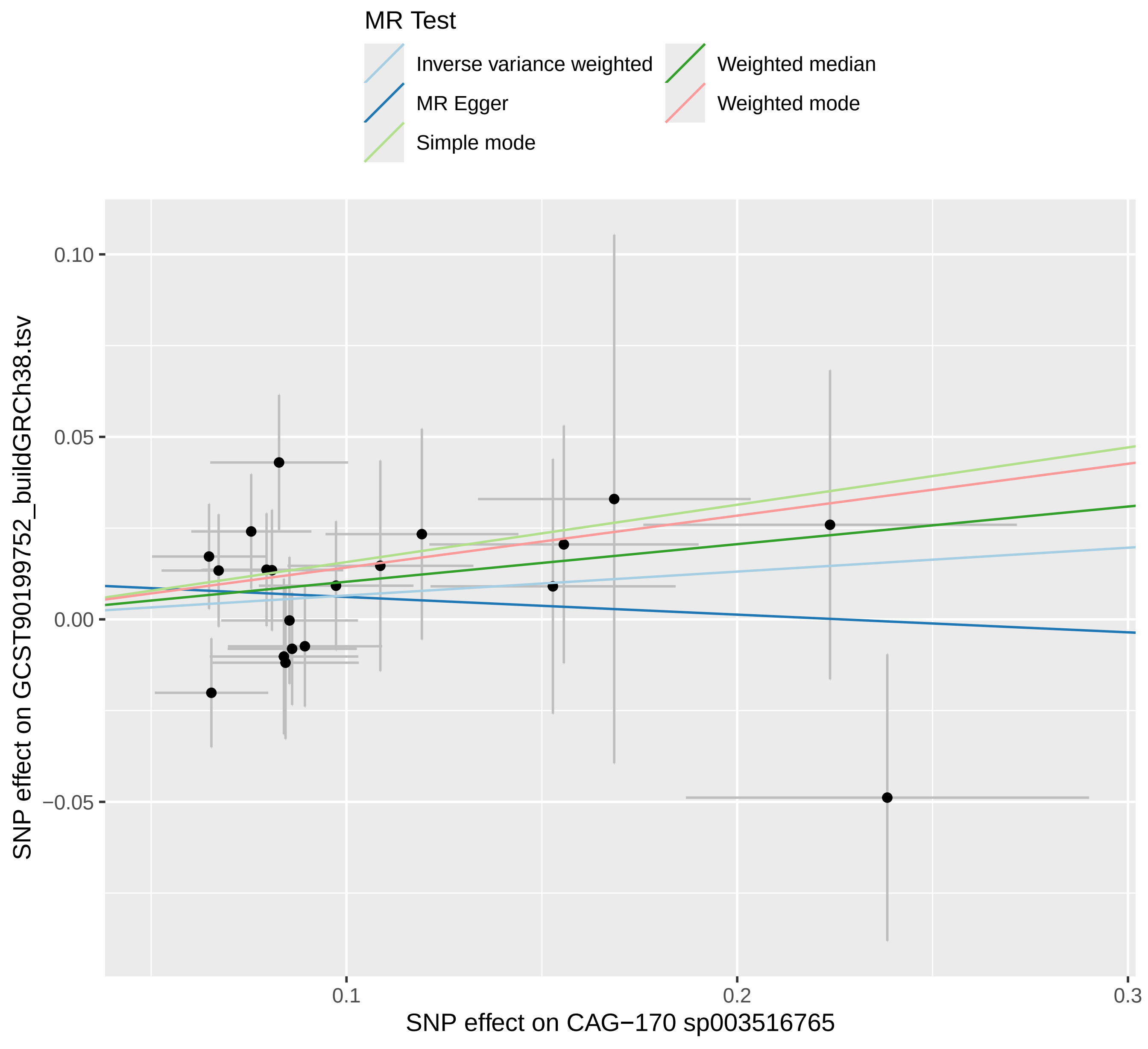

MR Test
Inverse variance weighted
MR Egger
Simple mode
Weighted median
Weighted mode
SNP effect on GCST90199752_buildGRCh38.tsv
0.10
0.05
0.00
−0.05
0.1
0.2
0.3
SNP effect on CAG−170 sp003516765

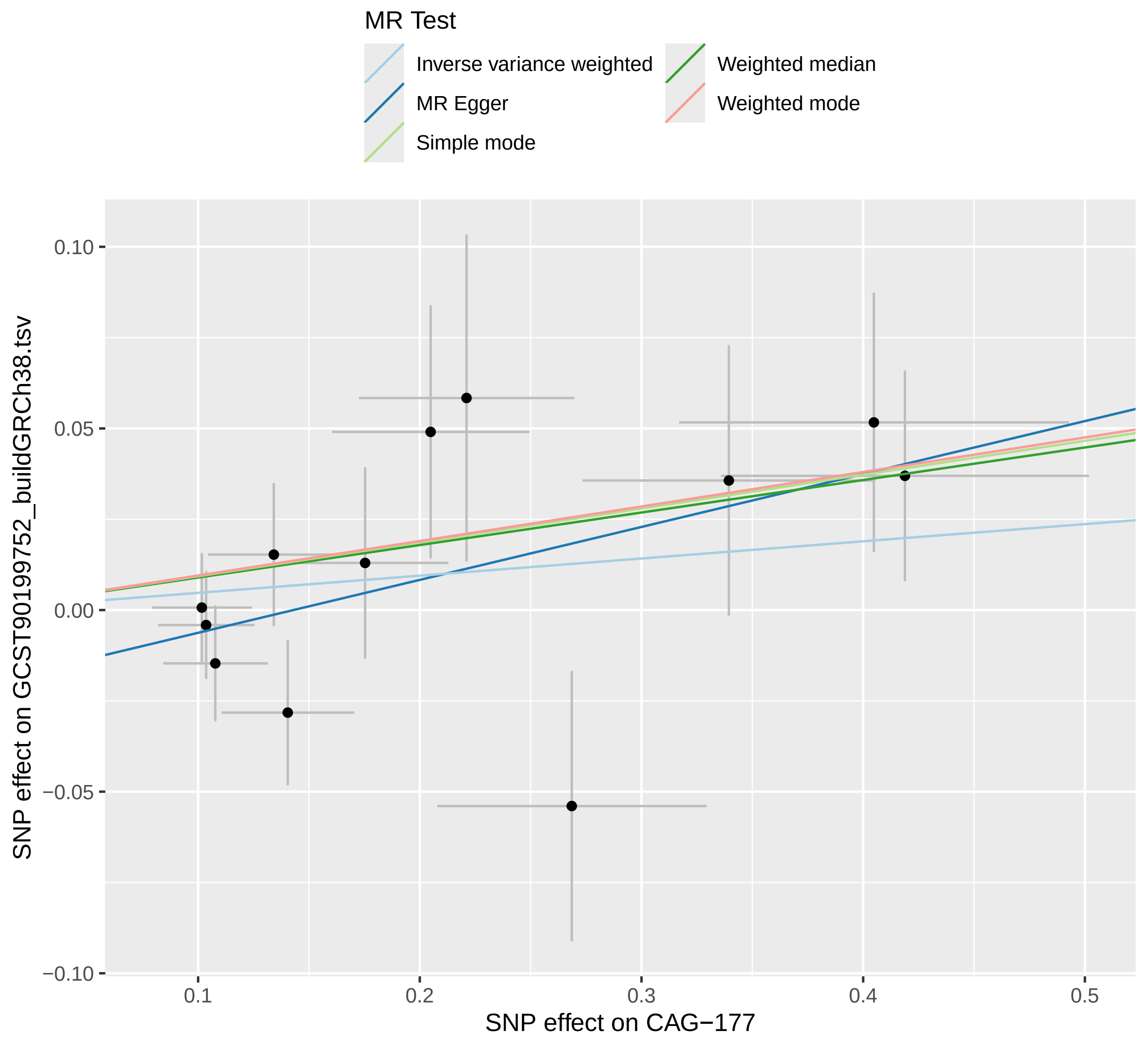
MR Test
Inverse variance weighted
MR Egger
Simple mode
Weighted median
Weighted mode
0.10
0.05
0.00
−0.05
−0.10
0.1
0.2
0.3
0.4
0.5
SNP effect on GCST90199752_buildGRCh38.tsv
SNP effect on CAG−177

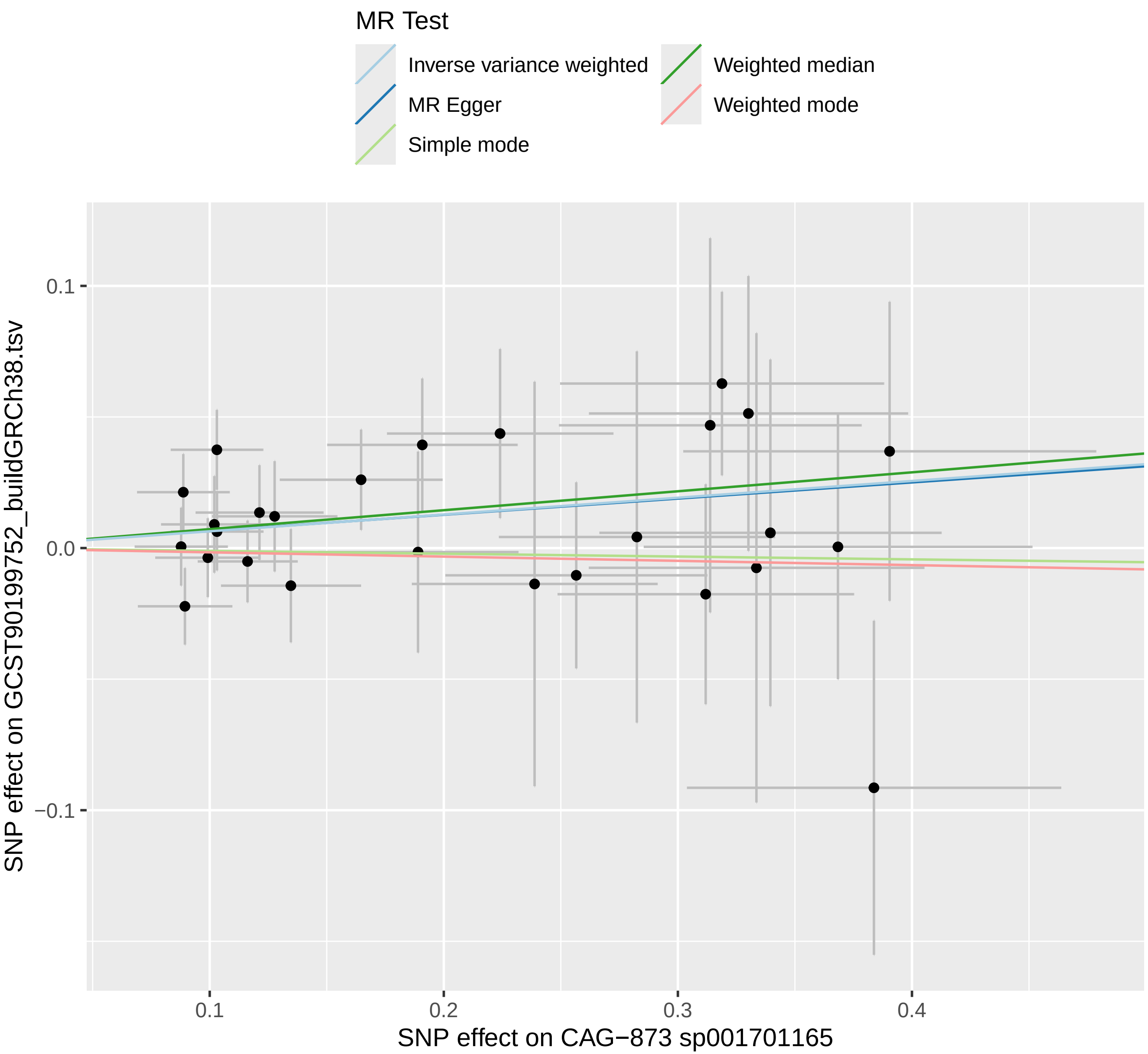
MR Test
Inverse variance weighted
MR Egger
Simple mode
Weighted median
Weighted mode
SNP effect on GCST90199752_buildGRCh38.tsv
SNP effect on CAG−873 sp001701165
0.1
0.0
−0.1
0.1
0.2
0.3
0.4

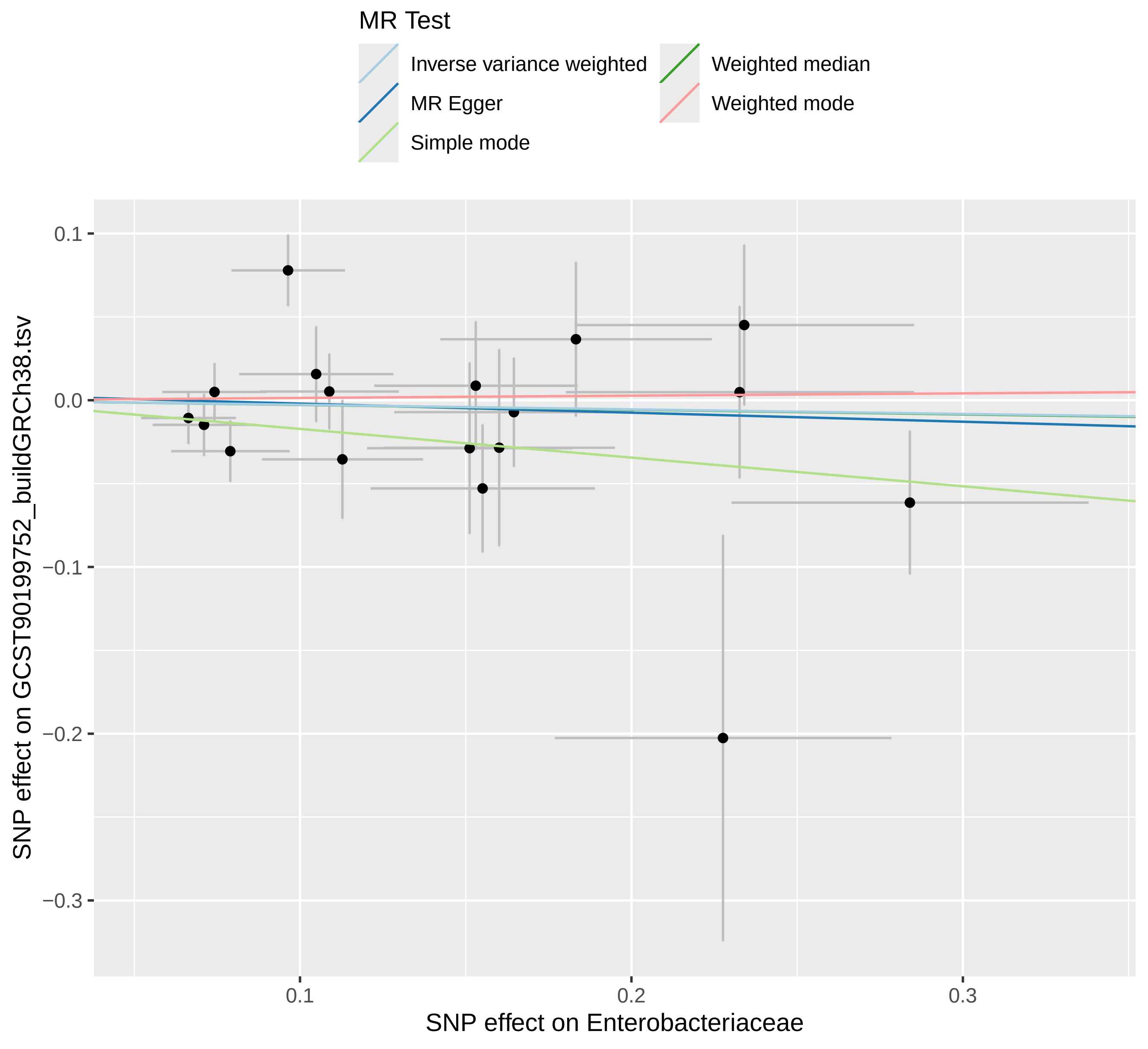

MR Test
Inverse variance weighted
MR Egger
Simple mode
Weighted median
Weighted mode
SNP effect on GCST90199752_buildGRCh38.tsv
0.1
0.0
−0.1
−0.2
−0.3
0.1
0.2
0.3
SNP effect on Enterobacteriaceae

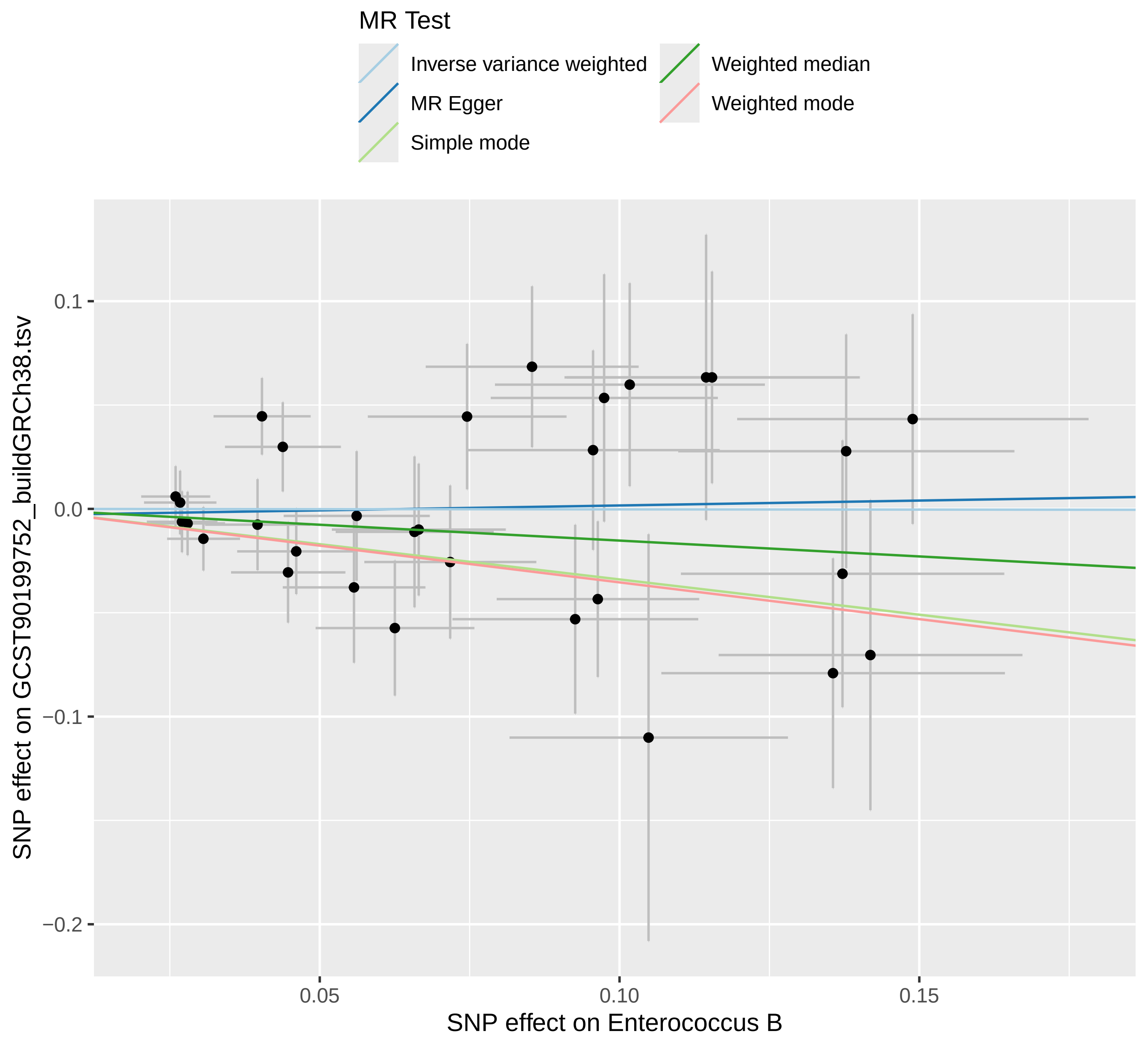

MR Test
Inverse variance weighted
MR Egger
Simple mode
Weighted median
Weighted mode
SNP effect on GCST90199752_buildGRCh38.tsv
0.1
0.0
−0.1
−0.2
0.05
0.10
0.15
SNP effect on Enterococcus B

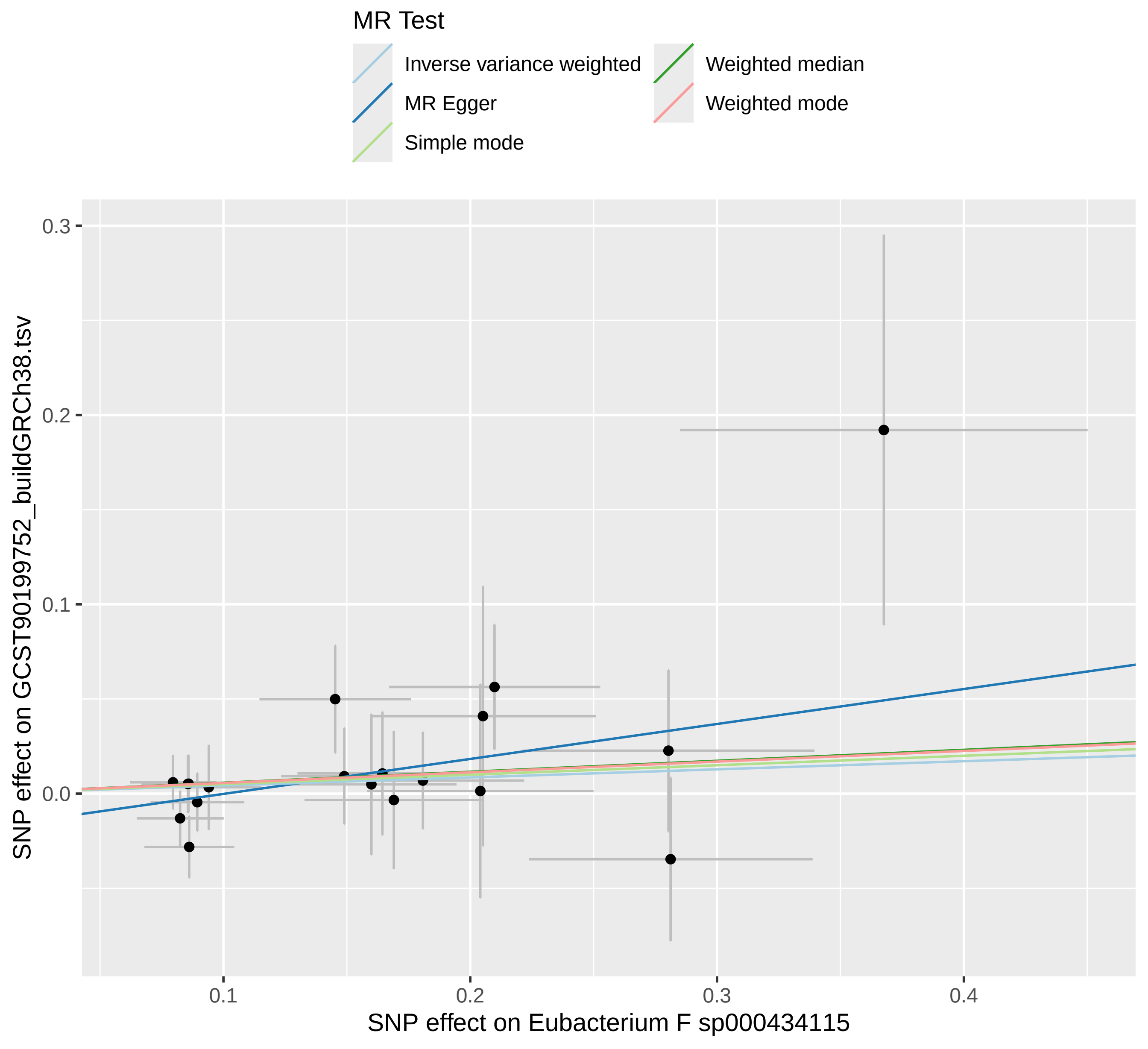
MR Test
Inverse variance weighted
MR Egger
Simple mode
Weighted median
Weighted mode
SNP effect on GCST90199752_buildGRCh38.tsv
0.3
0.2
0.1
0.0
0.1
0.2
0.3
0.4
SNP effect on Eubacterium F sp000434115

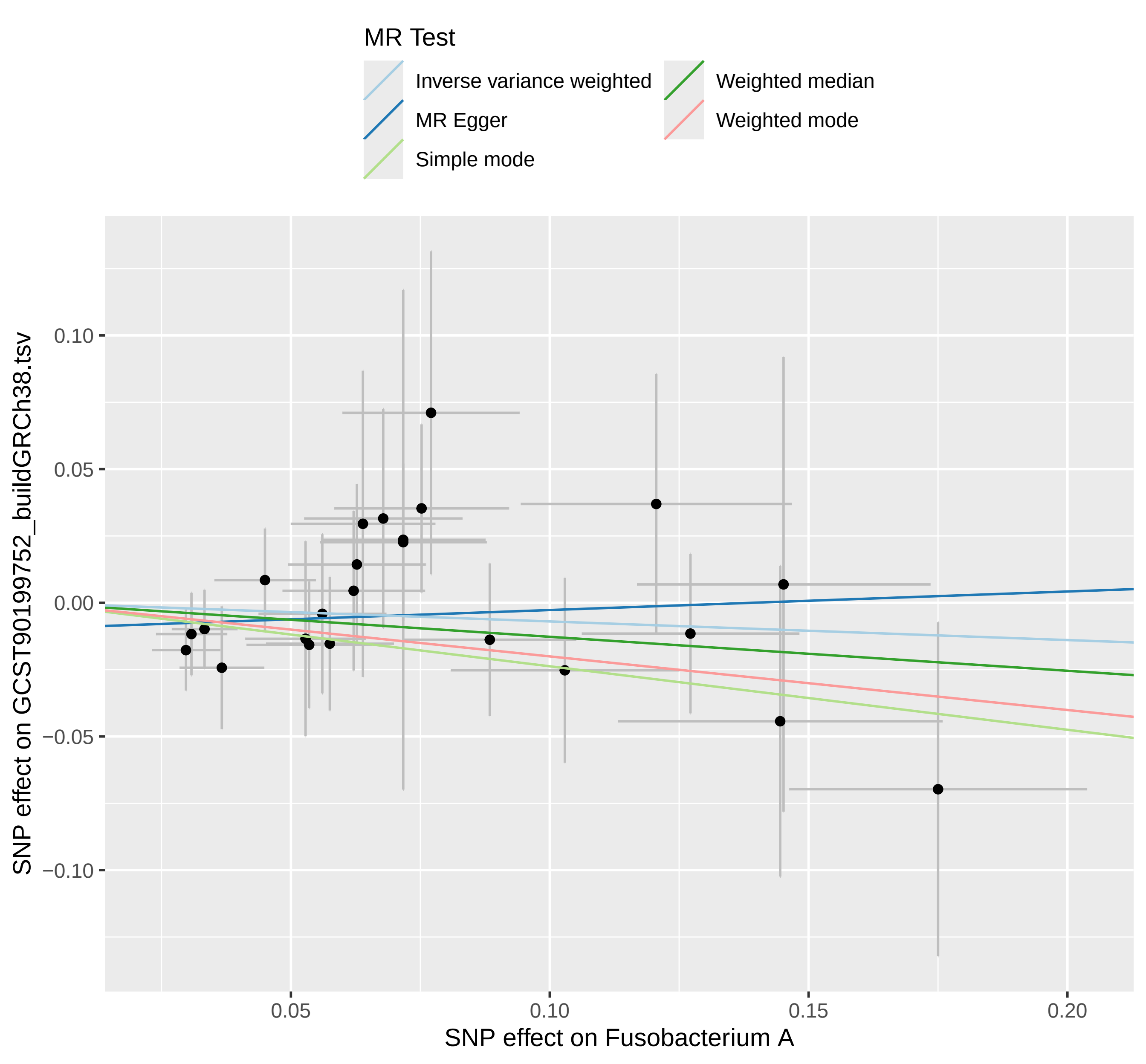

MR Test
Inverse variance weighted
MR Egger
Simple mode
Weighted median
Weighted mode
0.10
0.05
0.00
−0.05
−0.10
SNP effect on GCST90199752_buildGRCh38.tsv
0.05
0.10
0.15
0.20
SNP effect on Fusobacterium A

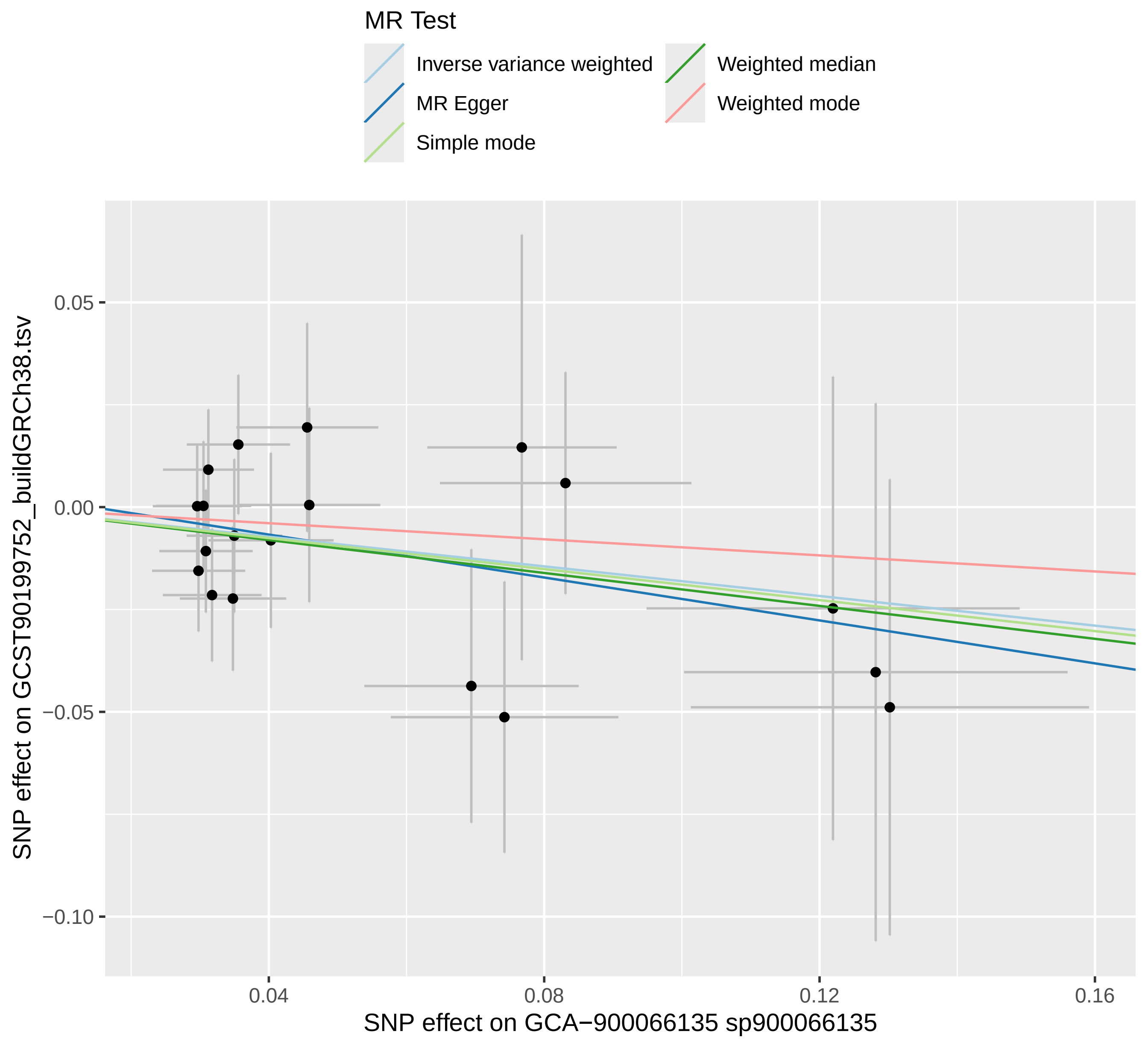
MR Test
Inverse variance weighted
MR Egger
Simple mode
Weighted median
Weighted mode
0.05
0.00
−0.05
−0.10
0.04
0.08
0.12
0.16
SNP effect on GCST90199752_buildGRCh38.tsv
SNP effect on GCA−900066135 sp900066135

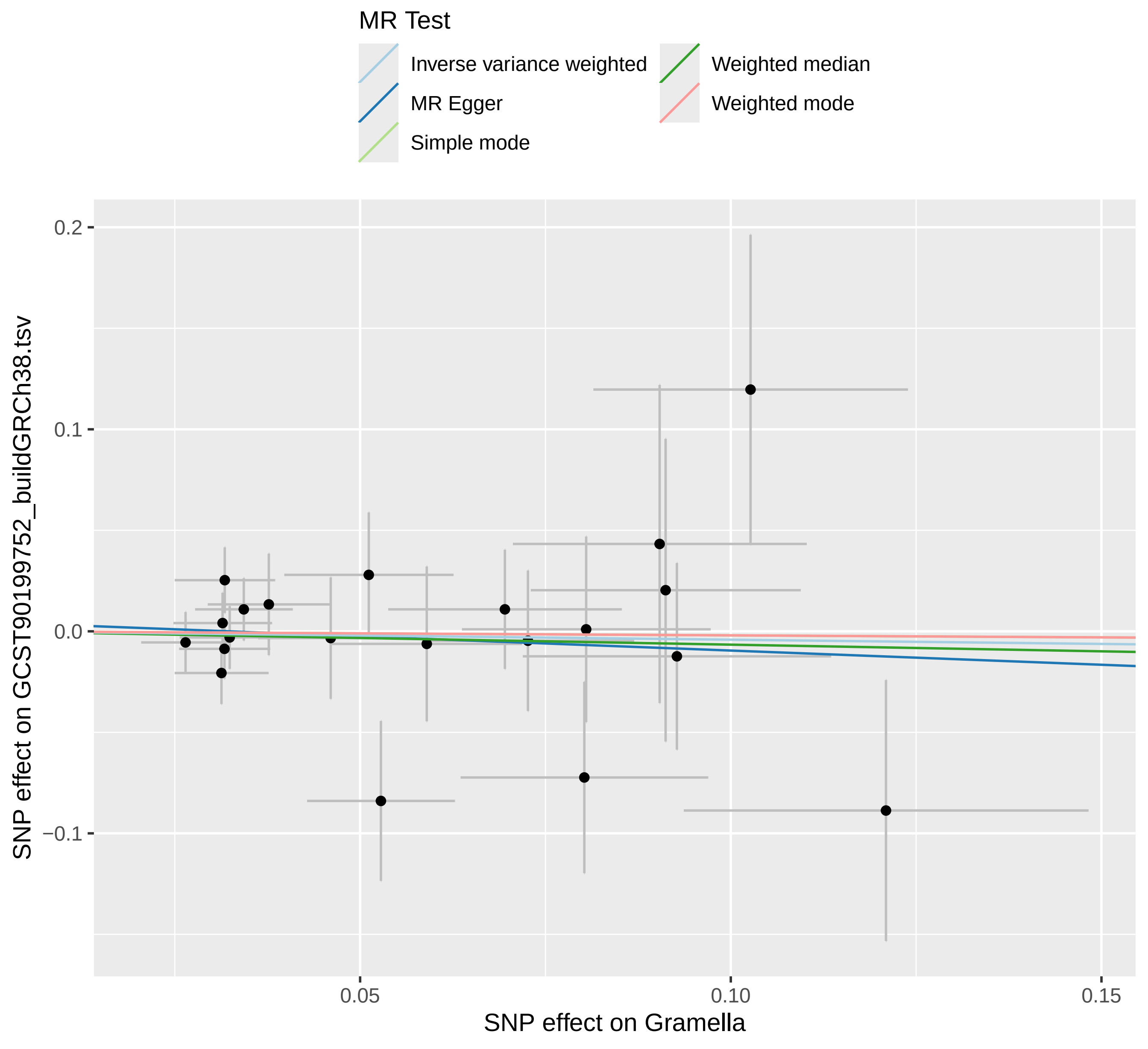

MR Test
Inverse variance weighted
MR Egger
Simple mode
Weighted median
Weighted mode
0.2
0.1
0.0
−0.1
0.05
0.10
0.15
SNP effect on GCST90199752_buildGRCh38.tsv
SNP effect on Gramella

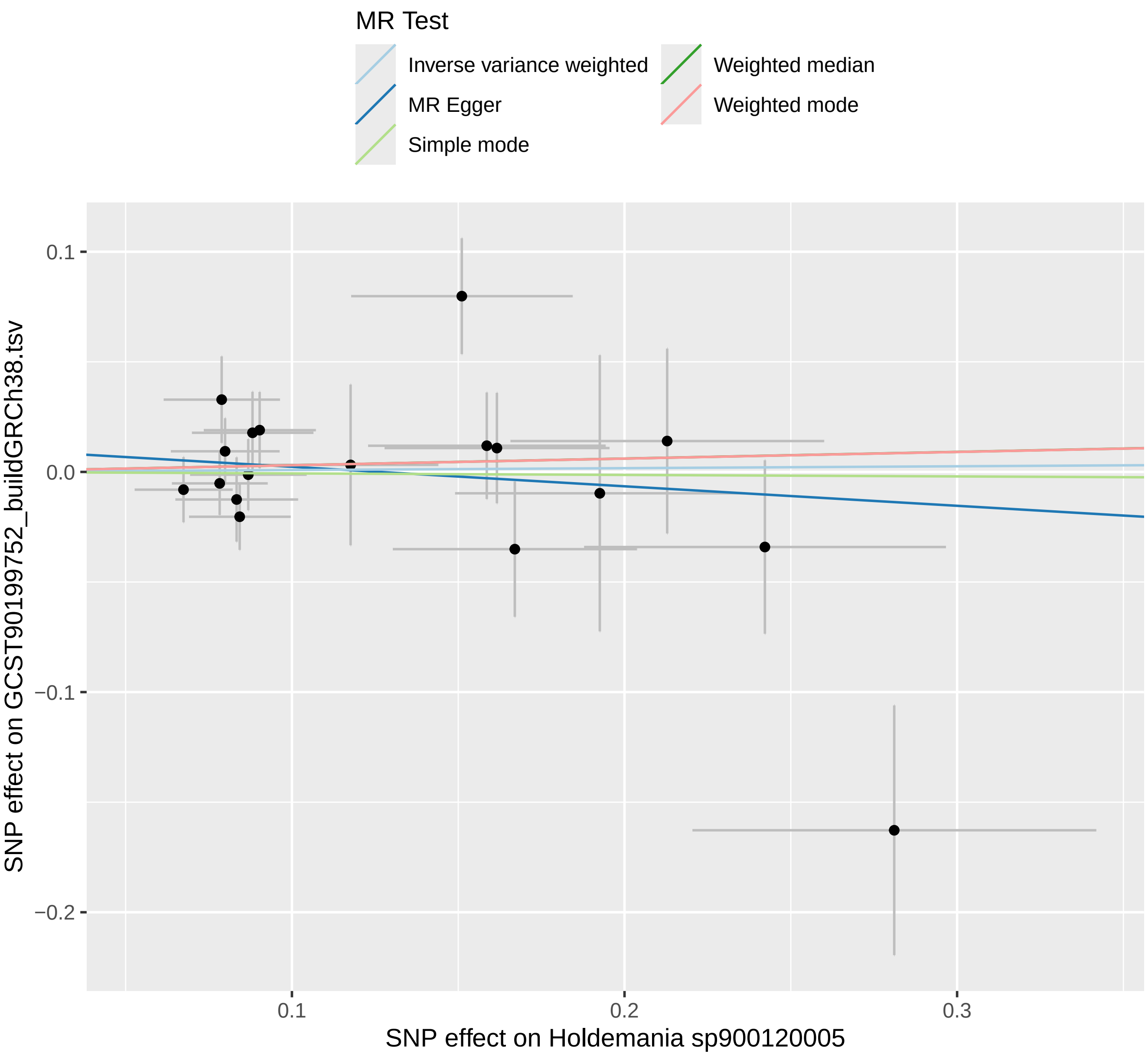

MR Test
Inverse variance weighted
MR Egger
Simple mode
Weighted median
Weighted mode
0.1
0.0
−0.1
−0.2
SNP effect on GCST90199752_buildGRCh38.tsv
0.1
0.2
0.3
SNP effect on Holdemania sp900120005

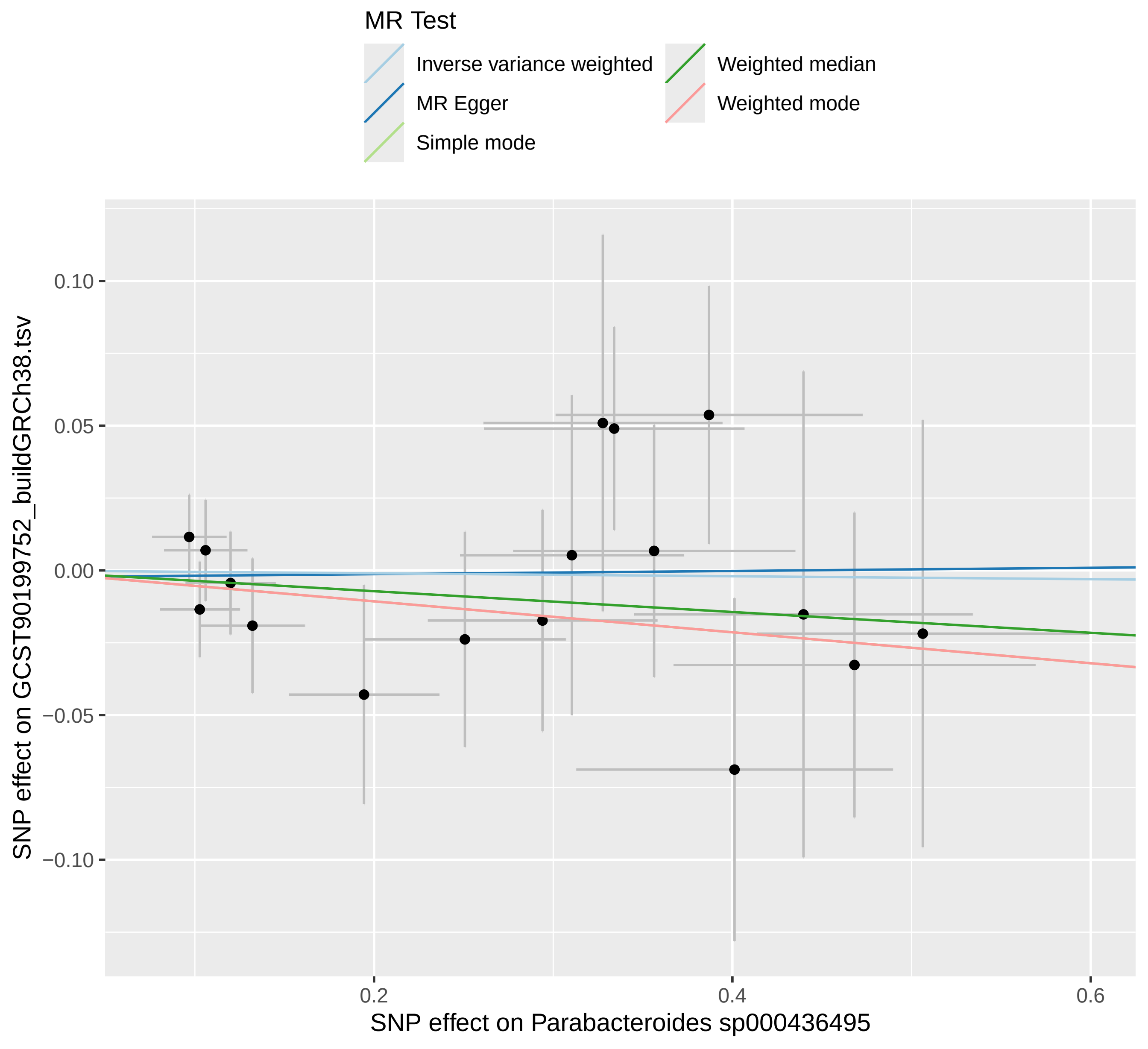

MR Test
Inverse variance weighted
MR Egger
Simple mode
Weighted median
Weighted mode
0.10
0.05
0.00
−0.05
−0.10
0.2
0.4
0.6
SNP effect on GCST90199752_buildGRCh38.tsv
SNP effect on Parabacteroides sp000436495

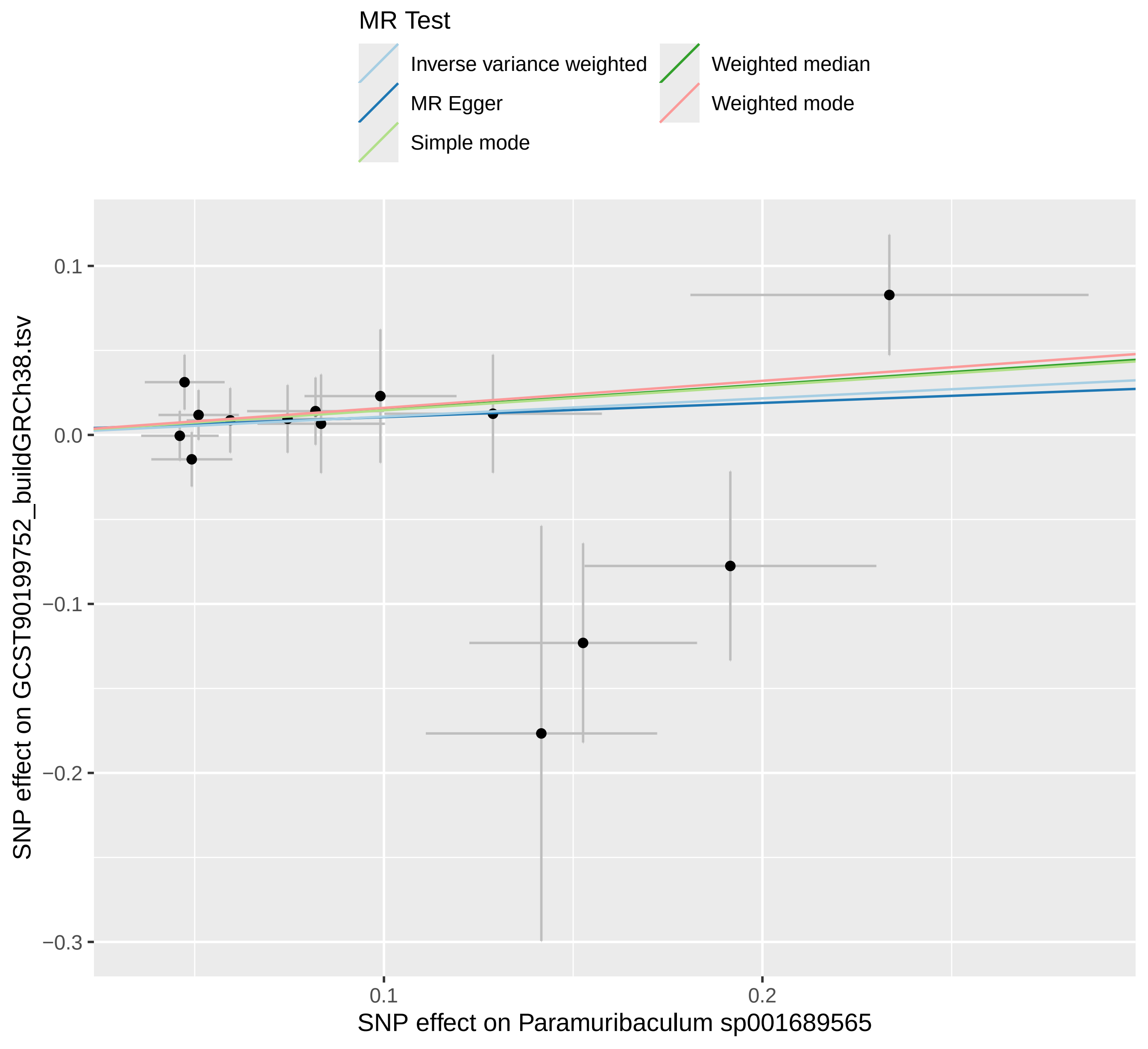

MR Test
Inverse variance weighted
MR Egger
Simple mode
Weighted median
Weighted mode
0.1
0.0
−0.1
−0.2
−0.3
0.1
0.2
SNP effect on GCST90199752_buildGRCh38.tsv
SNP effect on Paramuribaculum sp001689565

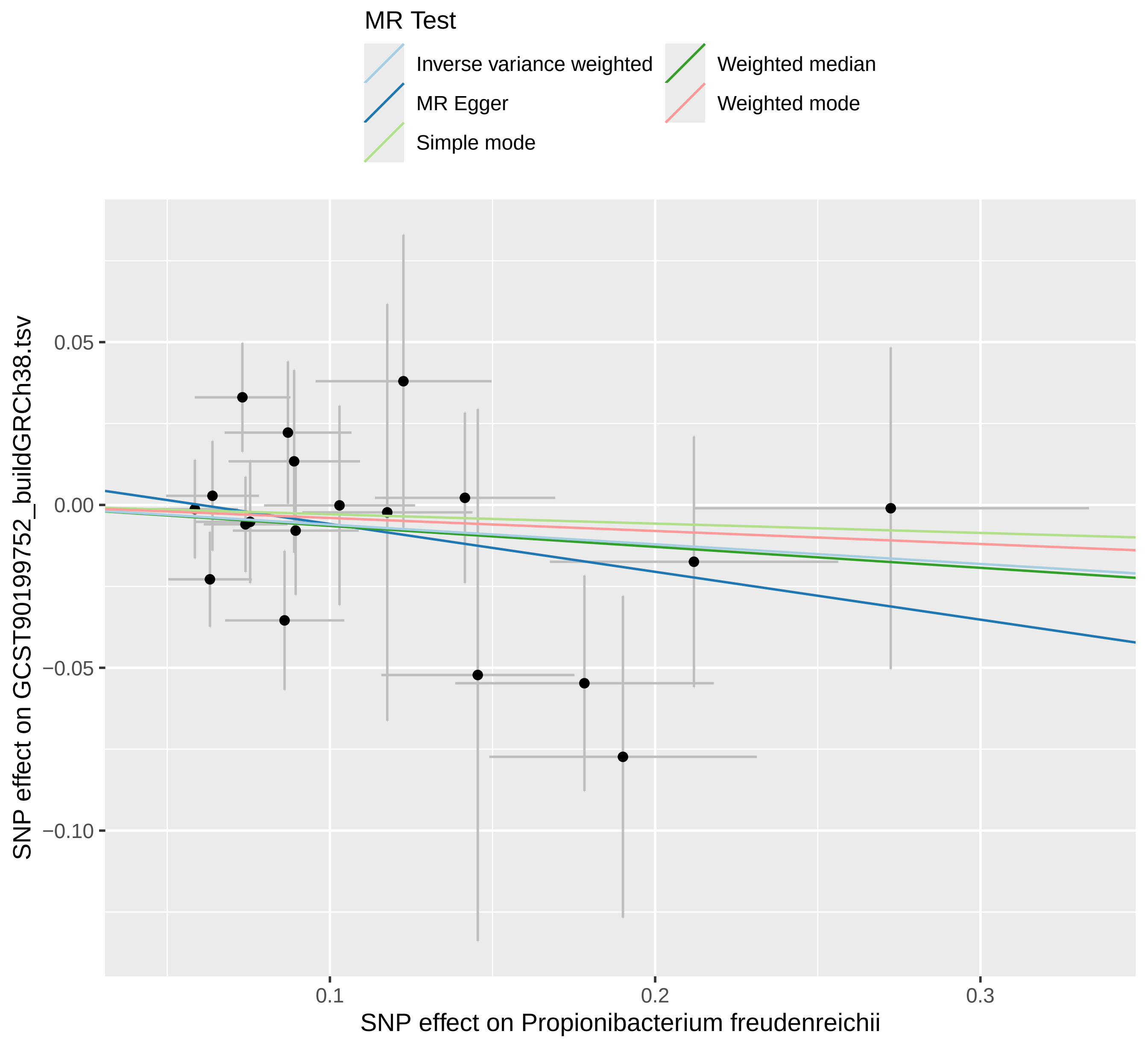
MR Test
Inverse variance weighted
MR Egger
Simple mode
Weighted median
Weighted mode
0.05
0.00
−0.05
−0.10
0.1
0.2
0.3
SNP effect on GCST90199752_buildGRCh38.tsv
SNP effect on Propionibacterium freudenreichii

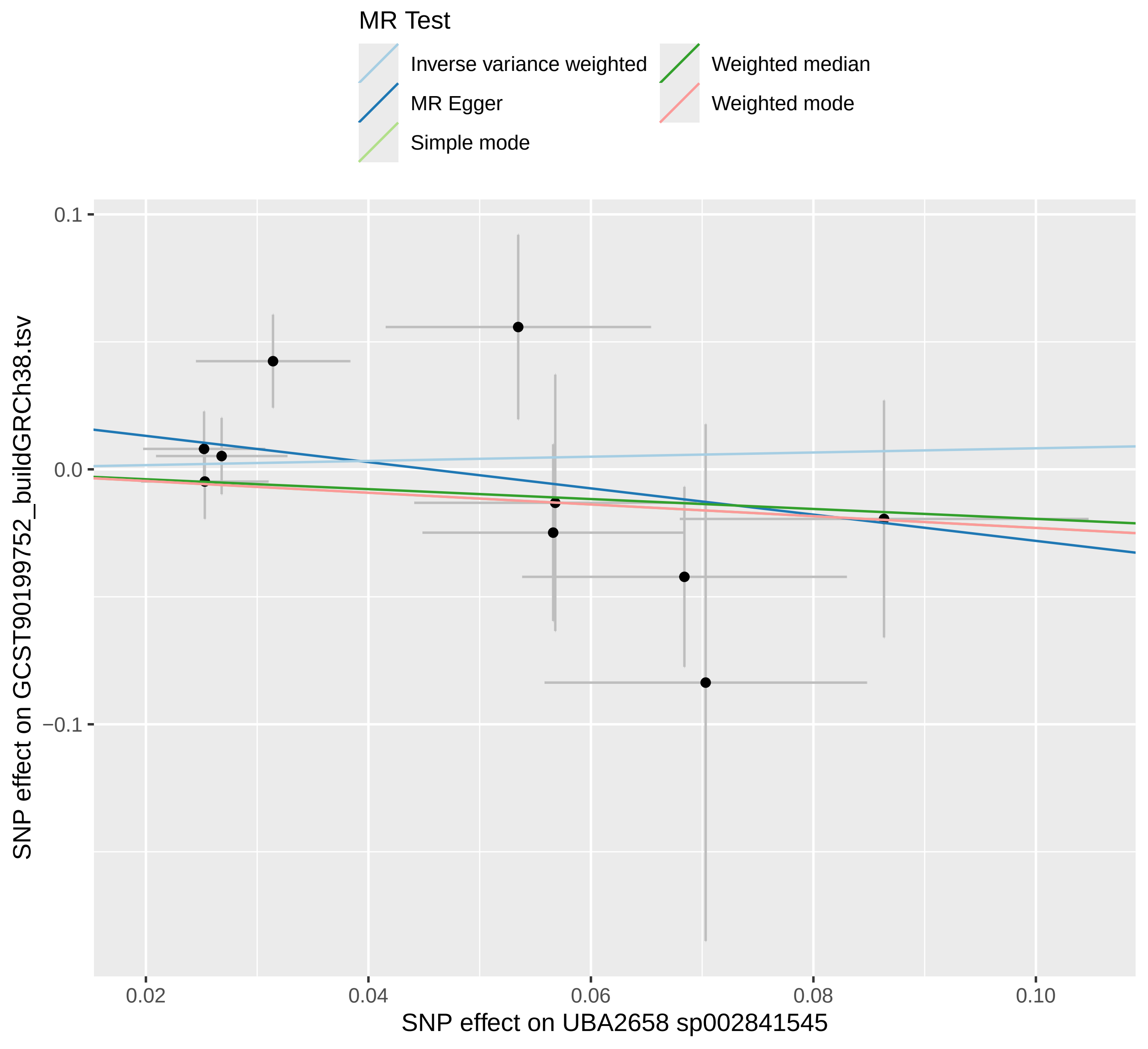
MR Test
Inverse variance weighted
MR Egger
Simple mode
Weighted median
Weighted mode
0.1
0.0
−0.1
0.02
0.04
0.06
0.08
0.10
SNP effect on GCST90199752_buildGRCh38.tsv
SNP effect on UBA2658 sp002841545

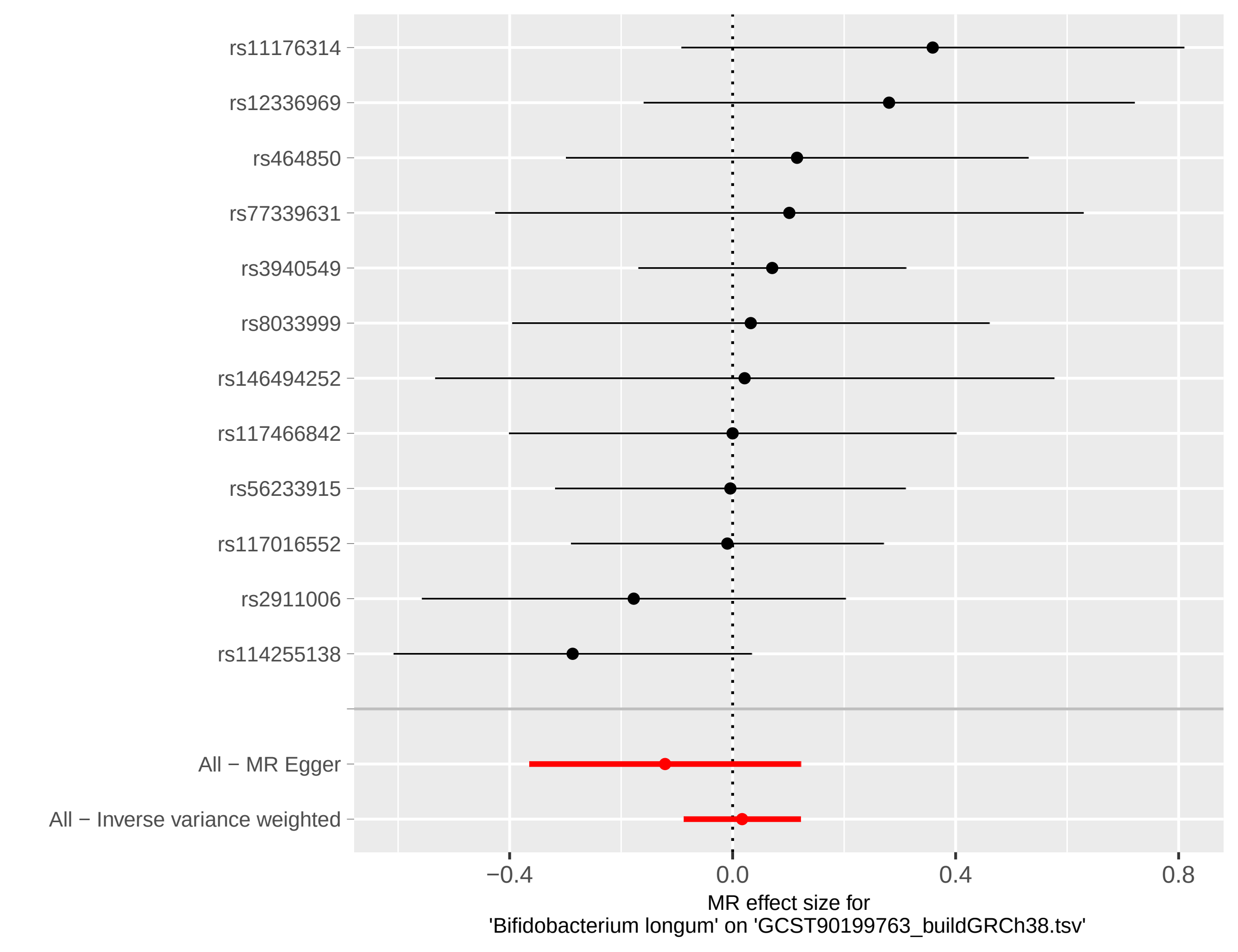
rs11176314
rs12336969
rs464850
rs77339631
rs3940549
rs8033999
rs146494252
rs117466842
rs56233915
rs117016552
rs2911006
rs114255138
All − MR Egger
All − Inverse variance weighted
−0.4
0.0
0.4
0.8
MR effect size for
'Bifidobacterium longum' on 'GCST90199763_buildGRCh38.tsv'

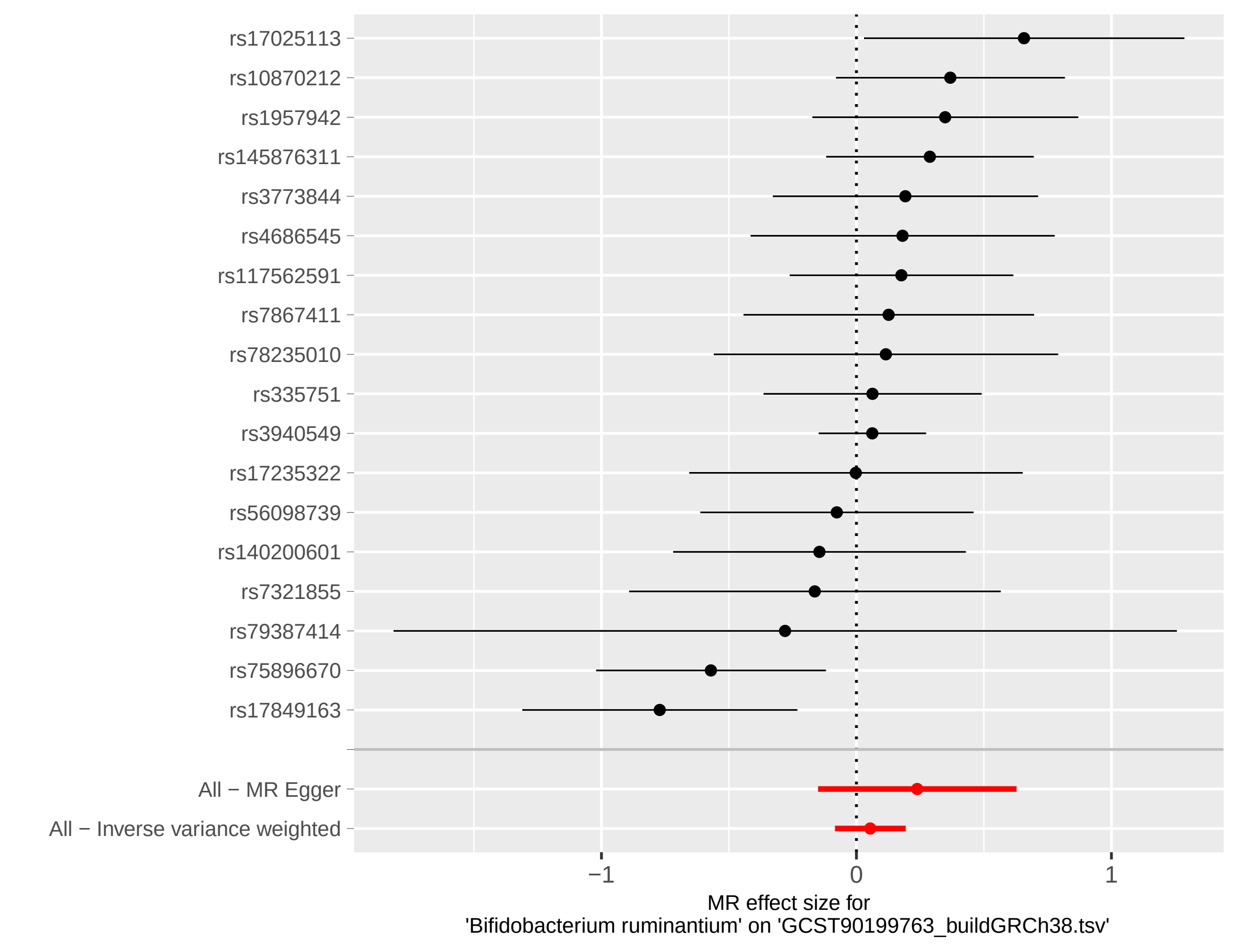
rs17025113
rs10870212
rs1957942
rs145876311
rs3773844
rs4686545
rs117562591
rs7867411
rs78235010
rs335751
rs3940549
rs17235322
rs56098739
rs140200601
rs7321855
rs79387414
rs75896670
rs17849163
All − MR Egger
All − Inverse variance weighted
−1
0
1
MR effect size for
'Bifidobacterium ruminantium' on 'GCST90199763_buildGRCh38.tsv'

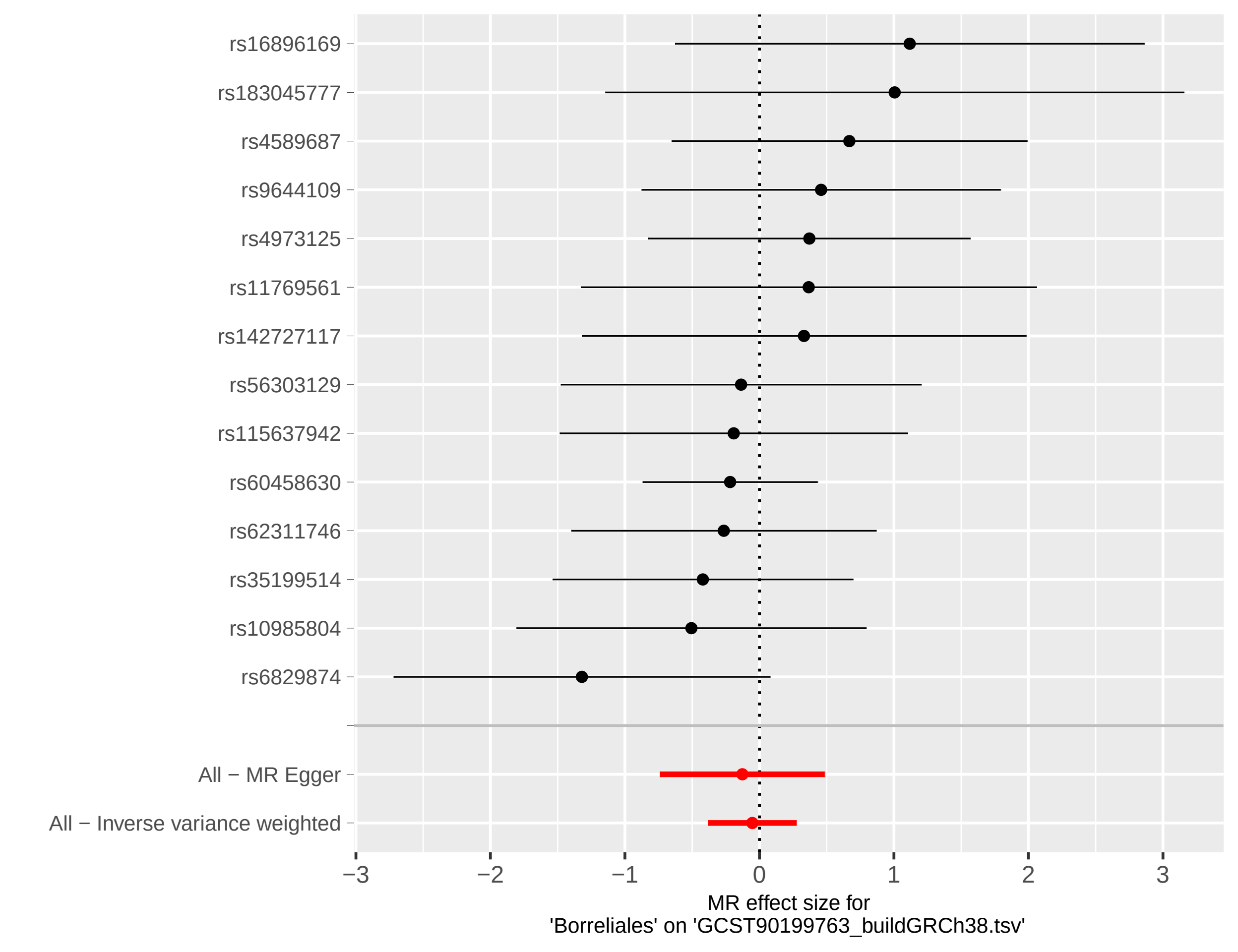
rs16896169
rs183045777
rs4589687
rs9644109
rs4973125
rs11769561
rs142727117
rs56303129
rs115637942
rs60458630
rs62311746
rs35199514
rs10985804
rs6829874
All − MR Egger
All − Inverse variance weighted
−3
−2
−1
0
1
2
3
MR effect size for
'Borreliales' on 'GCST90199763_buildGRCh38.tsv'

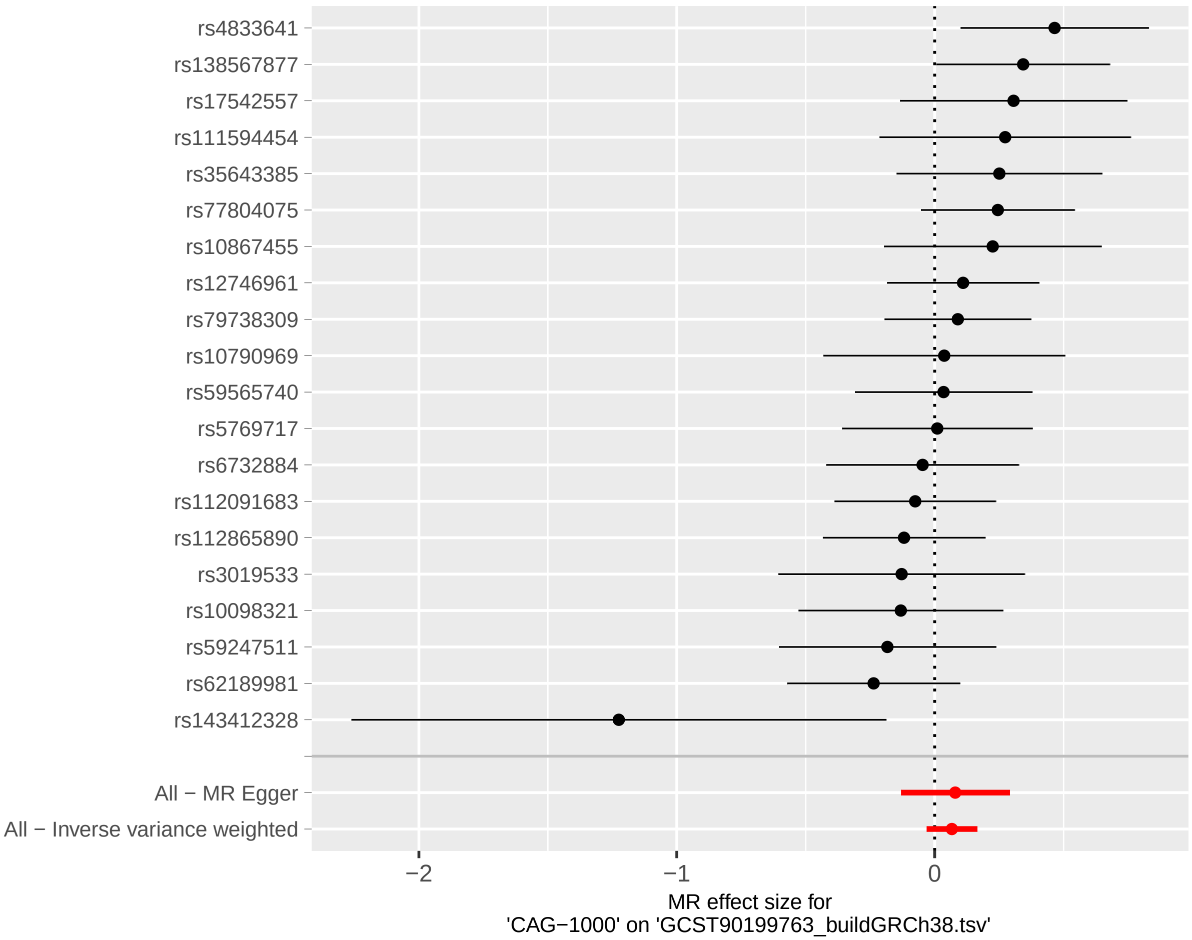

rs4833641
rs138567877
rs17542557
rs111594454
rs35643385
rs77804075
rs10867455
rs12746961
rs79738309
rs10790969
rs59565740
rs5769717
rs6732884
rs112091683
rs112865890
rs3019533
rs10098321
rs59247511
rs62189981
rs143412328
All − MR Egger
All − Inverse variance weighted
−2
−1
0
MR effect size for
'CAG−1000' on 'GCST90199763_buildGRCh38.tsv'

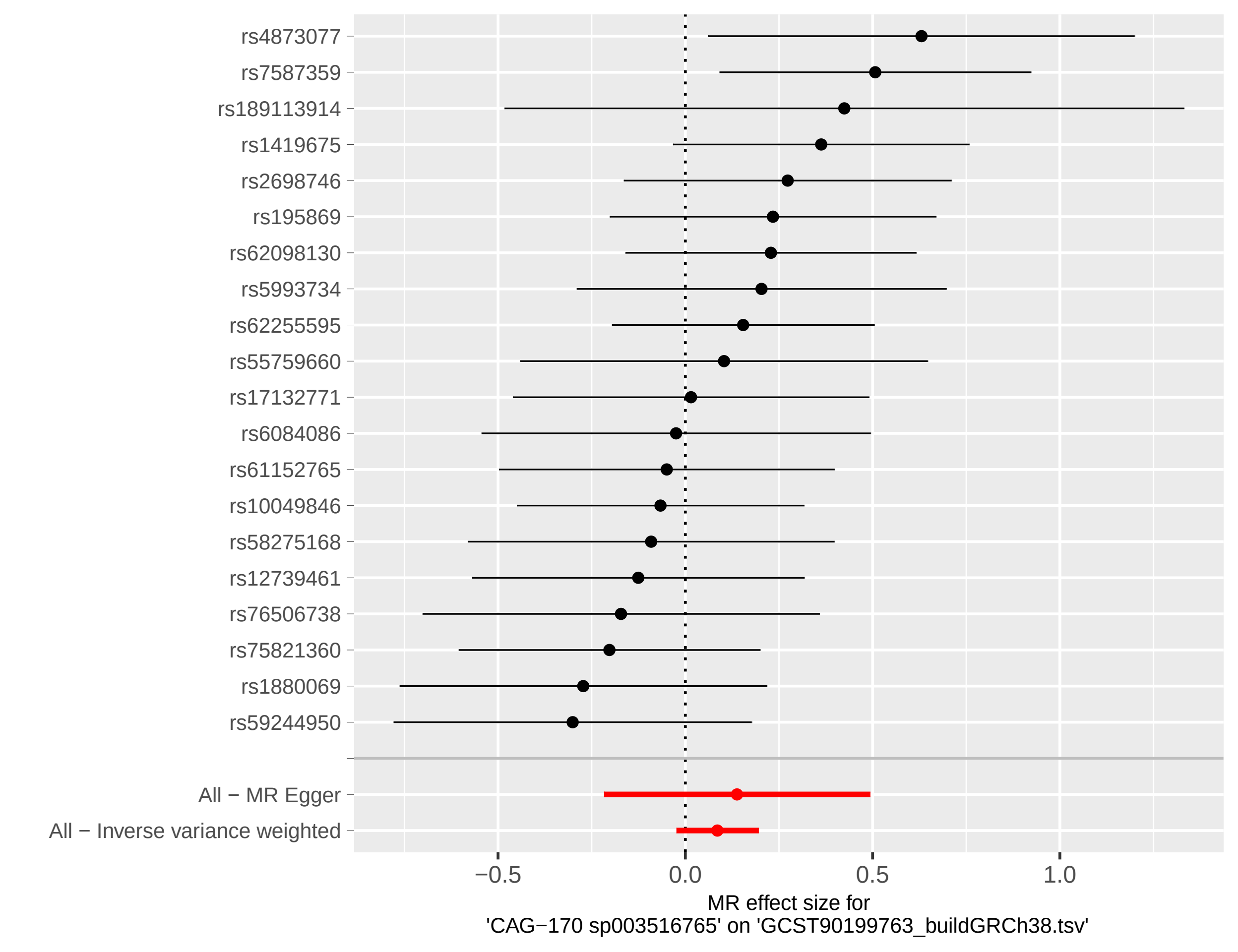
rs4873077
rs7587359
rs189113914
rs1419675
rs2698746
rs195869
rs62098130
rs5993734
rs62255595
rs55759660
rs17132771
rs6084086
rs61152765
rs10049846
rs58275168
rs12739461
rs76506738
rs75821360
rs1880069
rs59244950
All − MR Egger
All − Inverse variance weighted
−0.5
0.0
0.5
1.0
MR effect size for
'CAG−170 sp003516765' on 'GCST90199763_buildGRCh38.tsv'

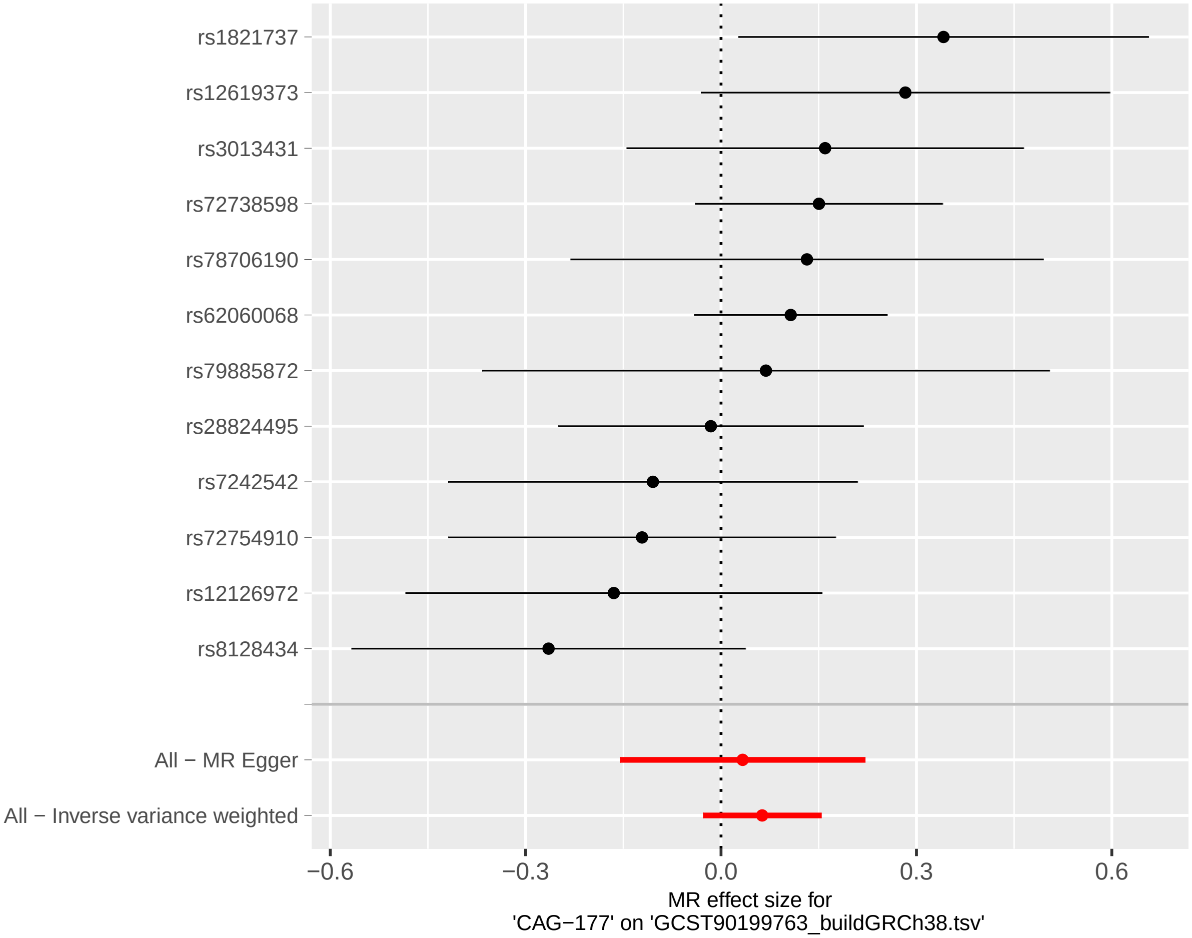
rs1821737
rs12619373
rs3013431
rs72738598
rs78706190
rs62060068
rs79885872
rs28824495
rs7242542
rs72754910
rs12126972
rs8128434
All − MR Egger
All − Inverse variance weighted
−0.6
−0.3
0.0
0.3
0.6
MR effect size for
'CAG−177' on 'GCST90199763_buildGRCh38.tsv'

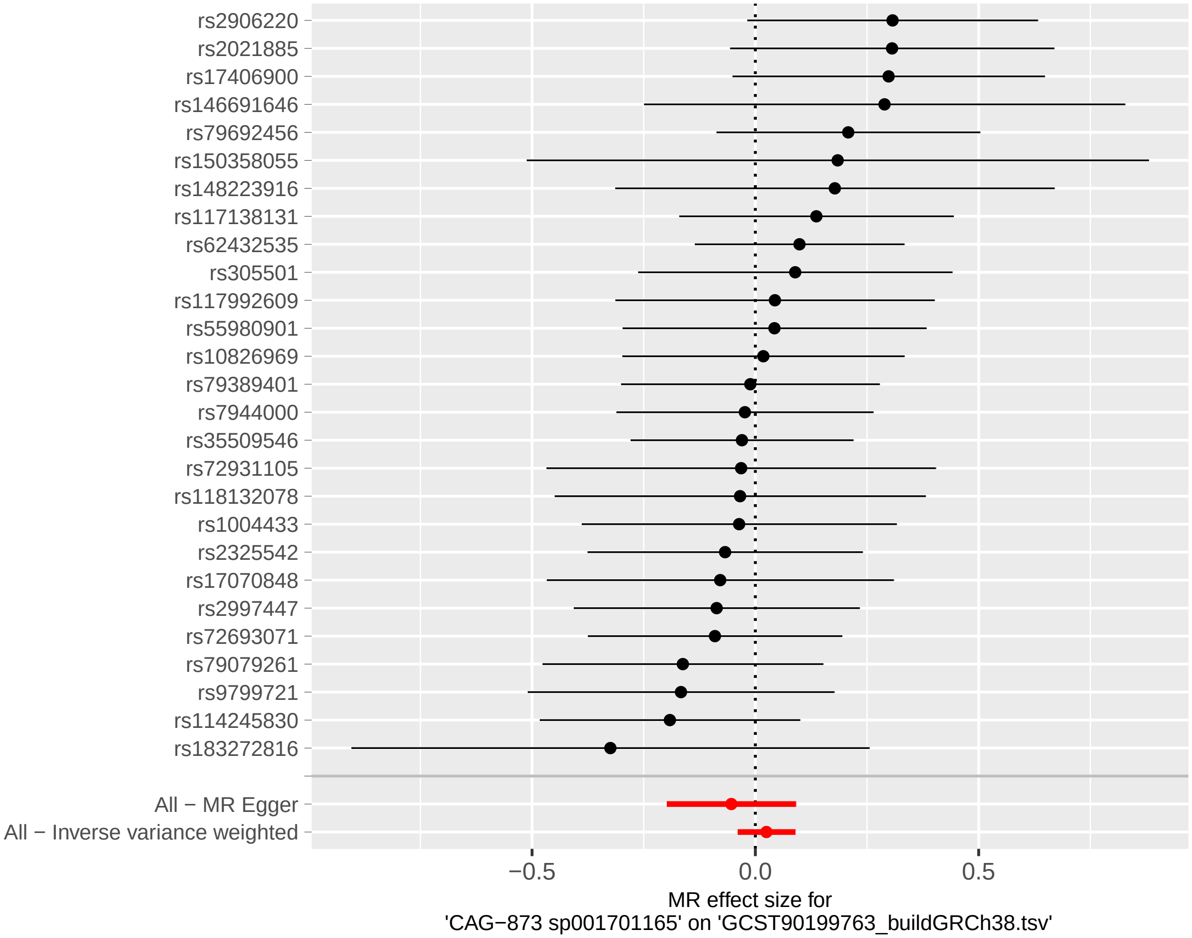
rs2906220
rs2021885
rs17406900
rs146691646
rs79692456
rs150358055
rs148223916
rs117138131
rs62432535
rs305501
rs117992609
rs55980901
rs10826969
rs79389401
rs7944000
rs35509546
rs72931105
rs118132078
rs1004433
rs2325542
rs17070848
rs2997447
rs72693071
rs79079261
rs9799721
rs114245830
rs183272816
All − MR Egger
All − Inverse variance weighted
−0.5
0.0
0.5
MR effect size for
'CAG−873 sp001701165' on 'GCST90199763_buildGRCh38.tsv'

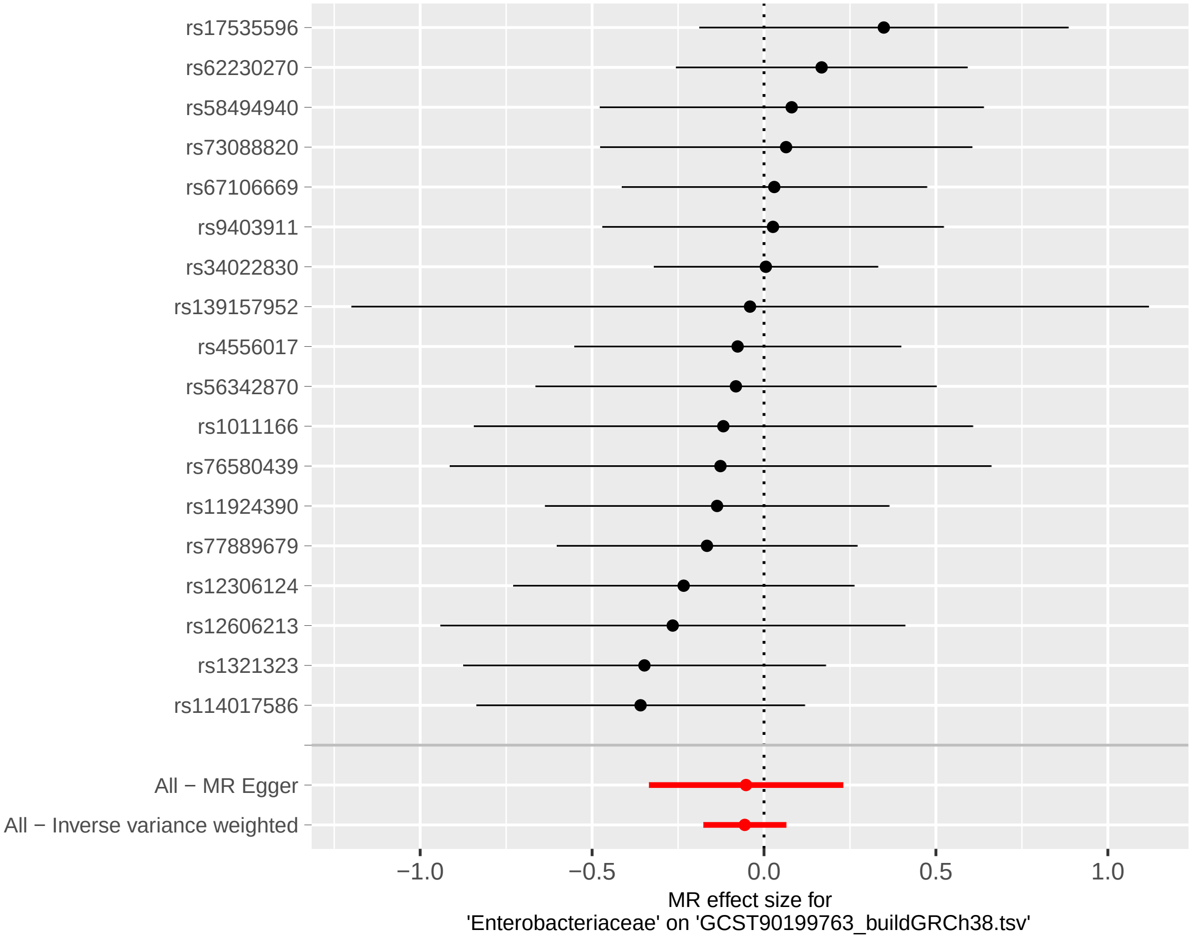
rs17535596
rs62230270
rs58494940
rs73088820
rs67106669
rs9403911
rs34022830
rs139157952
rs4556017
rs56342870
rs1011166
rs76580439
rs11924390
rs77889679
rs12306124
rs12606213
rs1321323
rs114017586
All − MR Egger
All − Inverse variance weighted
−1.0
−0.5
0.0
0.5
1.0
MR effect size for
'Enterobacteriaceae' on 'GCST90199763_buildGRCh38.tsv'

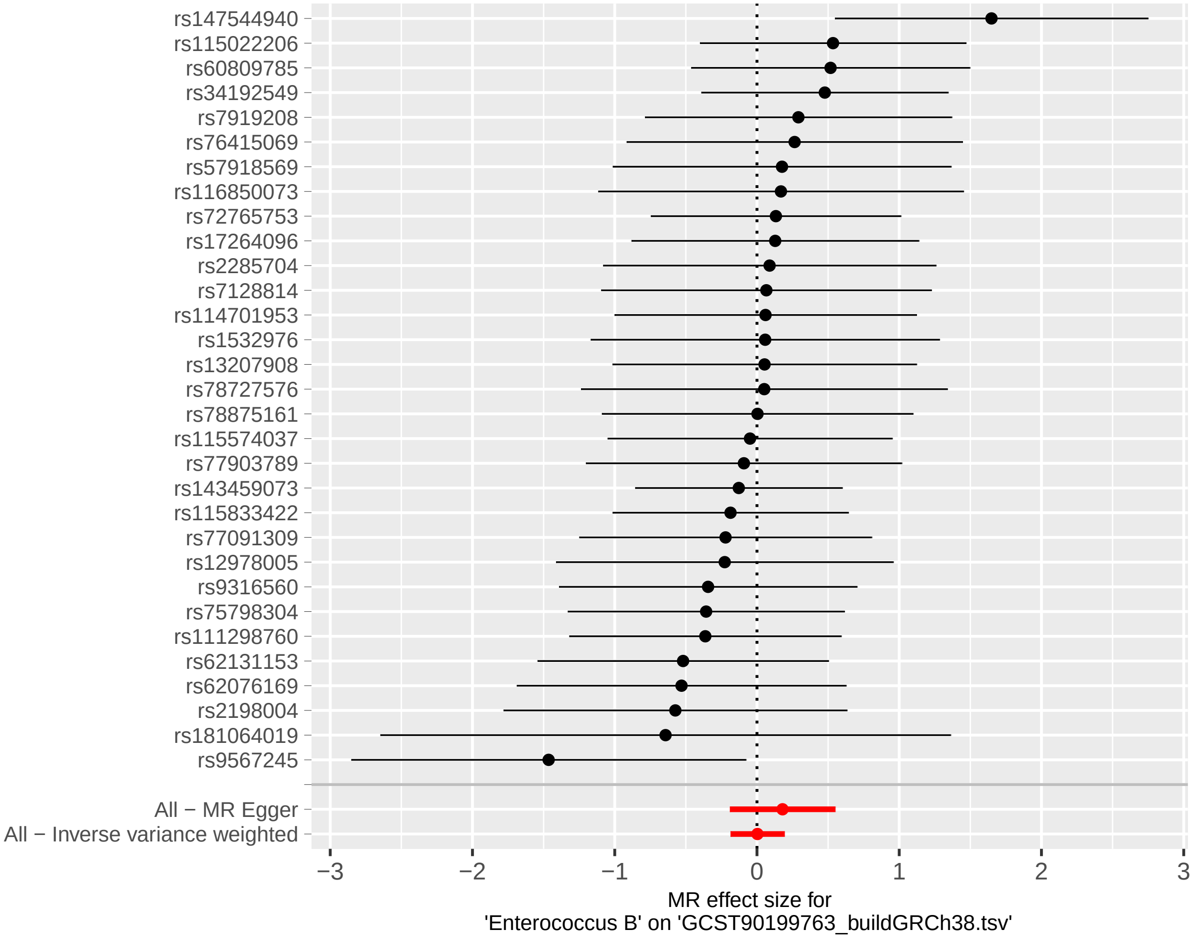
rs147544940
rs115022206
rs60809785
rs34192549
rs7919208
rs76415069
rs57918569
rs116850073
rs72765753
rs17264096
rs2285704
rs7128814
rs114701953
rs1532976
rs13207908
rs78727576
rs78875161
rs115574037
rs77903789
rs143459073
rs115833422
rs77091309
rs12978005
rs9316560
rs75798304
rs111298760
rs62131153
rs62076169
rs2198004
rs181064019
rs9567245
All − MR Egger
All − Inverse variance weighted
−3
−2
−1
0
1
2
3
MR effect size for
'Enterococcus B' on 'GCST90199763_buildGRCh38.tsv'

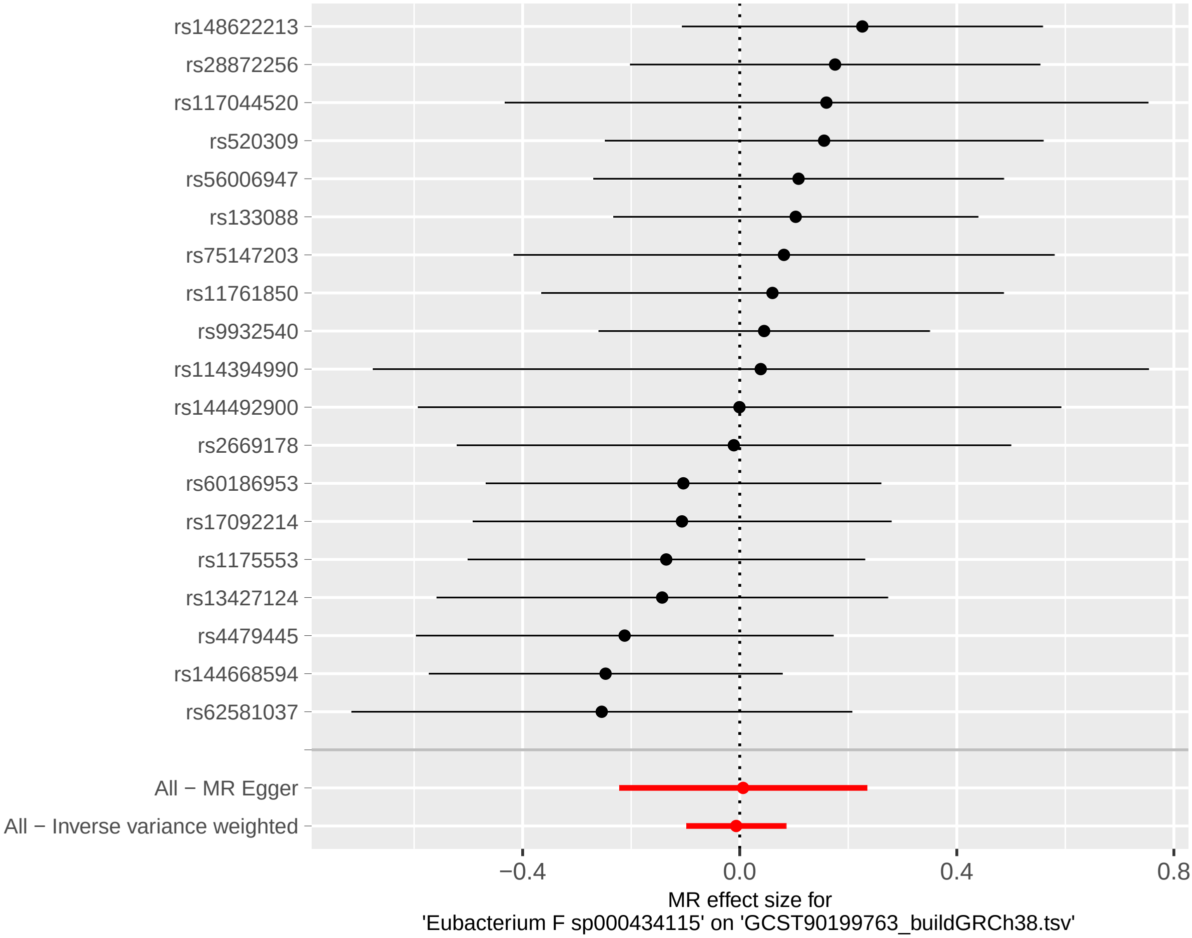
rs148622213
rs28872256
rs117044520
rs520309
rs56006947
rs133088
rs75147203
rs11761850
rs9932540
rs114394990
rs144492900
rs2669178
rs60186953
rs17092214
rs1175553
rs13427124
rs4479445
rs144668594
rs62581037
All − MR Egger
All − Inverse variance weighted
−0.4
0.0
0.4
0.8
MR effect size for
'Eubacterium F sp000434115' on 'GCST90199763_buildGRCh38.tsv'

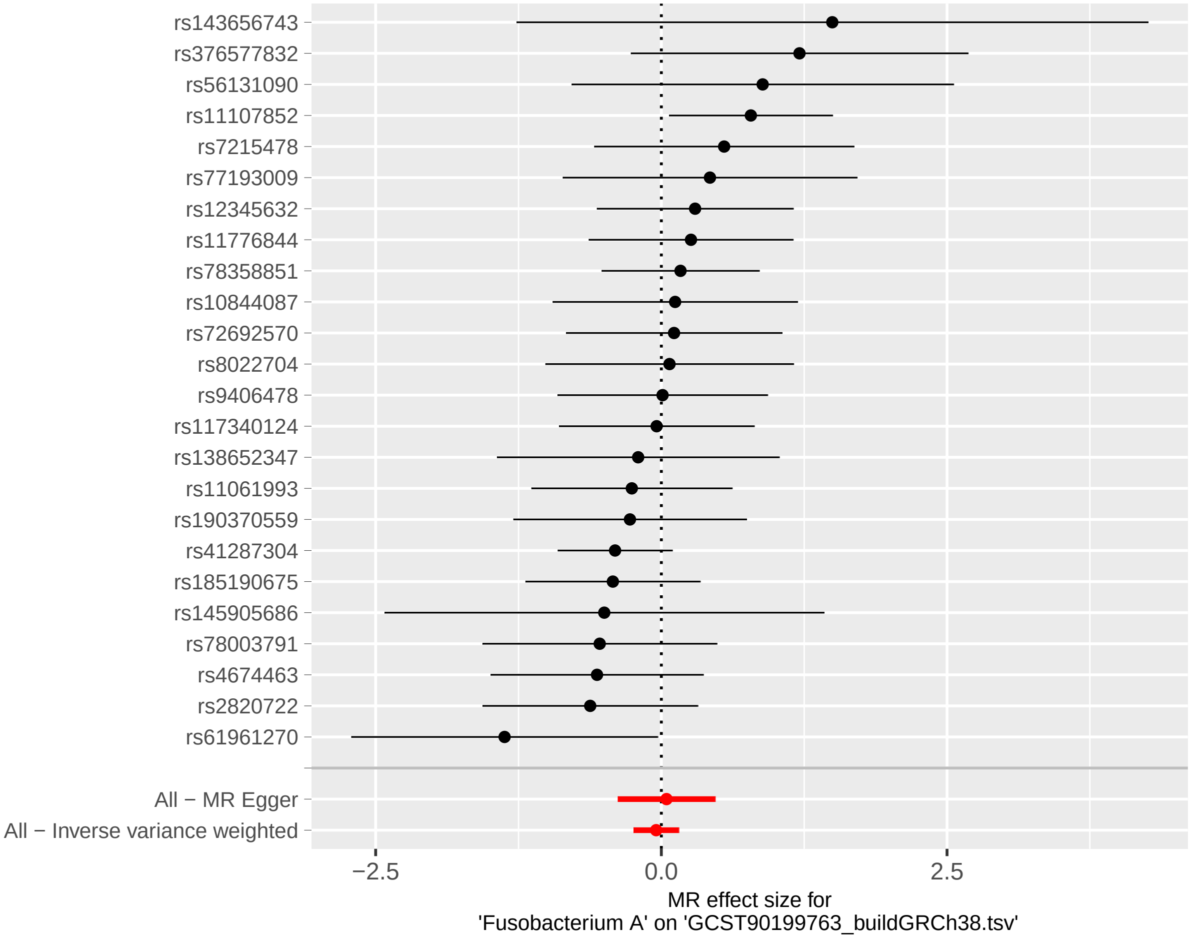
rs143656743
rs376577832
rs56131090
rs11107852
rs7215478
rs77193009
rs12345632
rs11776844
rs78358851
rs10844087
rs72692570
rs8022704
rs9406478
rs117340124
rs138652347
rs11061993
rs190370559
rs41287304
rs185190675
rs145905686
rs78003791
rs4674463
rs2820722
rs61961270
All − MR Egger
All − Inverse variance weighted
−2.5
0.0
2.5
MR effect size for
'Fusobacterium A' on 'GCST90199763_buildGRCh38.tsv'

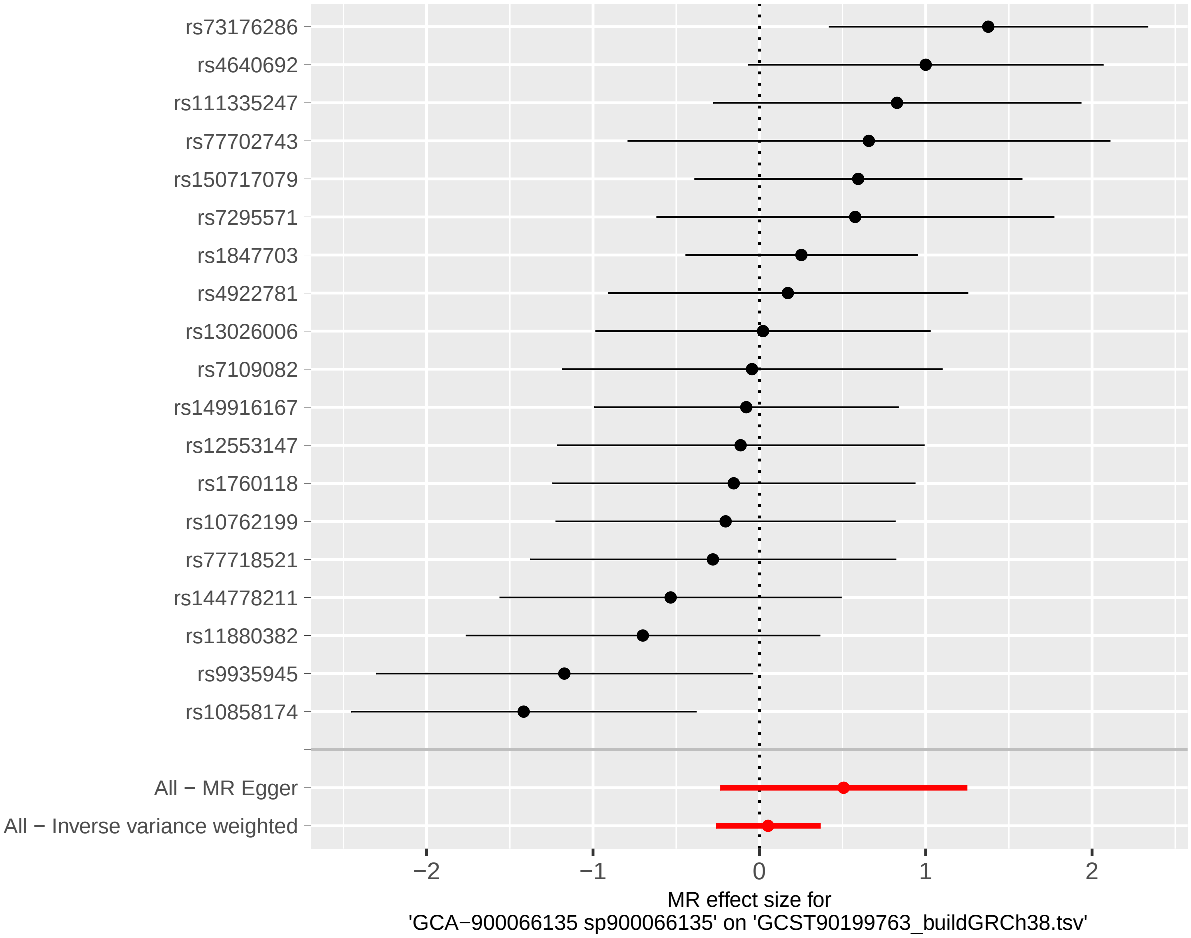
rs73176286
rs4640692
rs111335247
rs77702743
rs150717079
rs7295571
rs1847703
rs4922781
rs13026006
rs7109082
rs149916167
rs12553147
rs1760118
rs10762199
rs77718521
rs144778211
rs11880382
rs9935945
rs10858174
All − MR Egger
All − Inverse variance weighted
−2
−1
0
1
2
MR effect size for
'GCA−900066135 sp900066135' on 'GCST90199763_buildGRCh38.tsv'

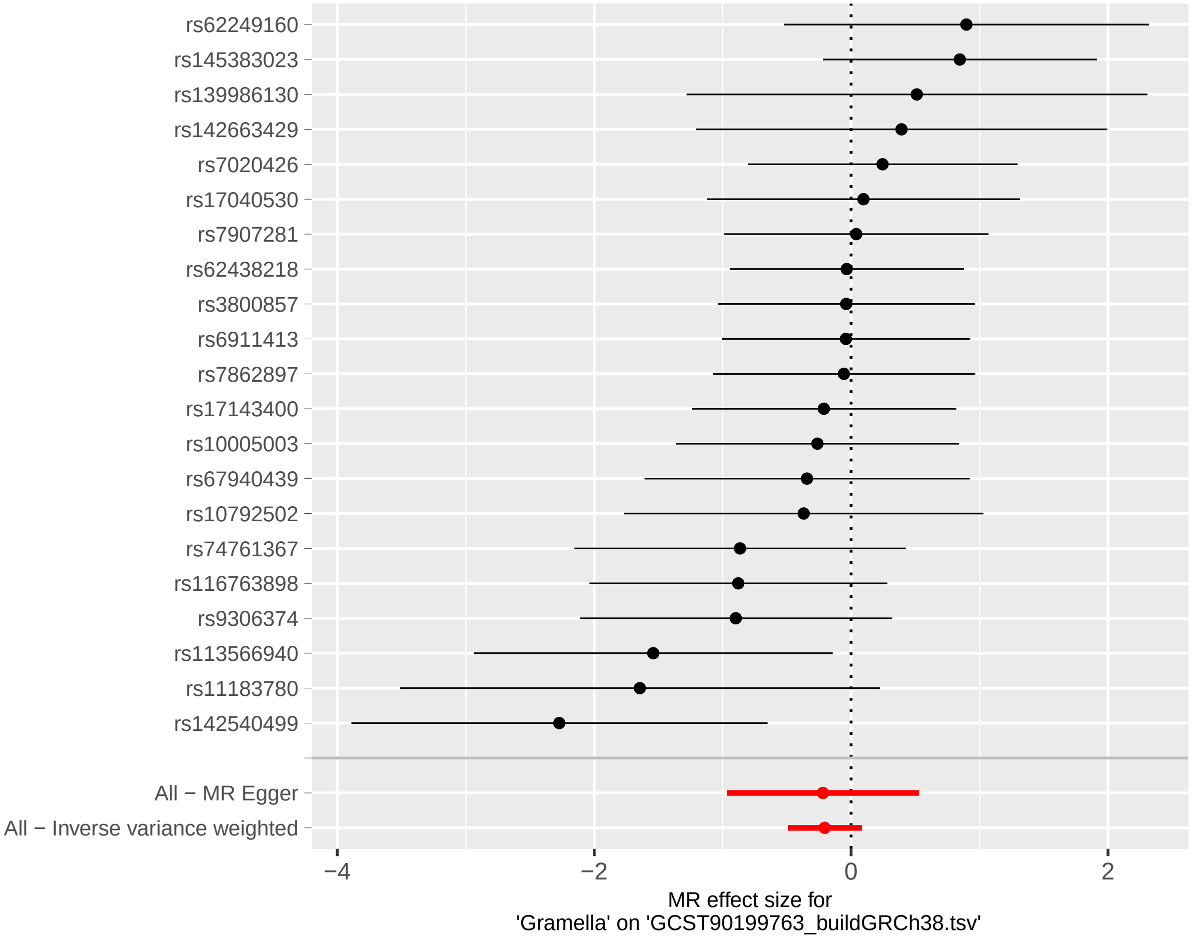
rs62249160
rs145383023
rs139986130
rs142663429
rs7020426
rs17040530
rs7907281
rs62438218
rs3800857
rs6911413
rs7862897
rs17143400
rs10005003
rs67940439
rs10792502
rs74761367
rs116763898
rs9306374
rs113566940
rs11183780
rs142540499
All − MR Egger
All − Inverse variance weighted
−4
−2
0
2
MR effect size for
'Gramella' on 'GCST90199763_buildGRCh38.tsv'

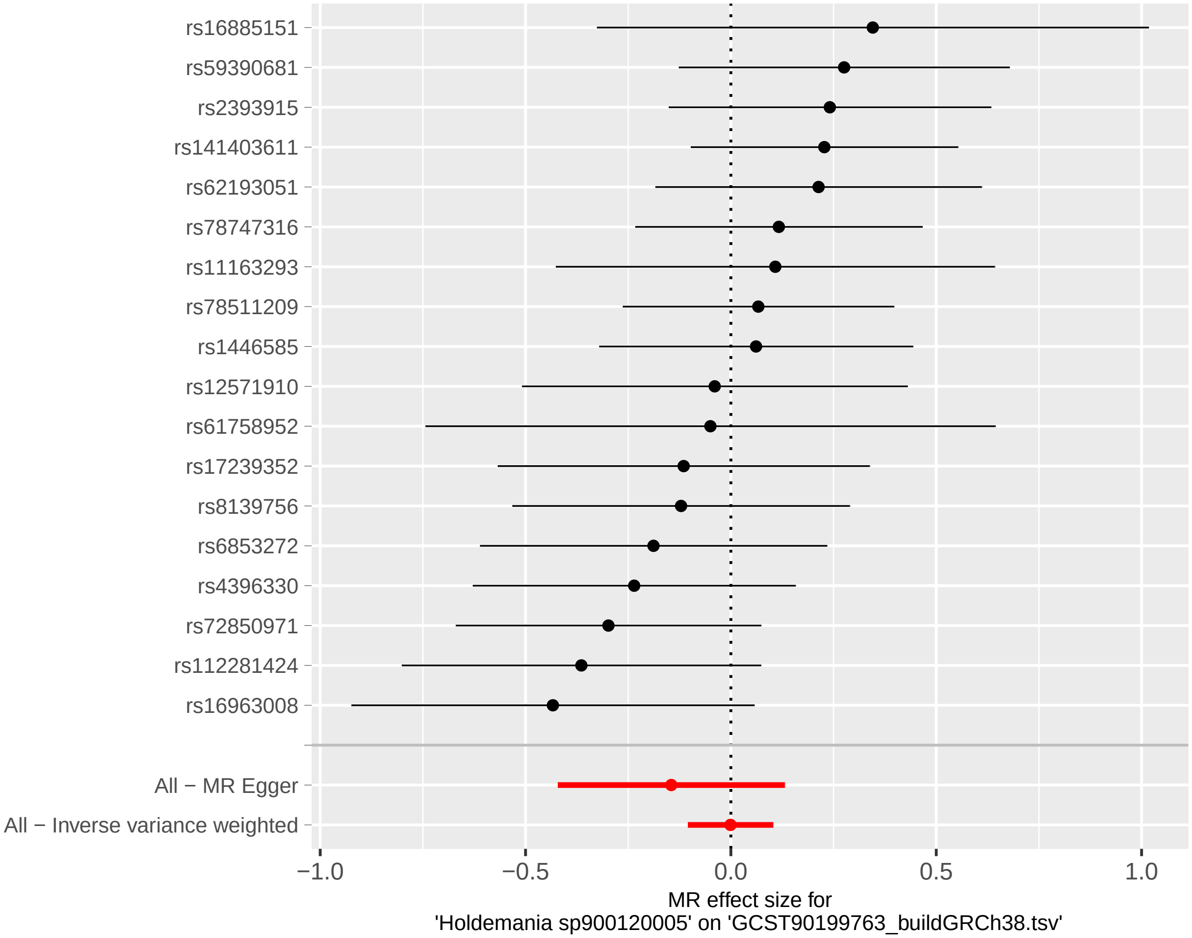

rs16885151
rs59390681
rs2393915
rs141403611
rs62193051
rs78747316
rs11163293
rs78511209
rs1446585
rs12571910
rs61758952
rs17239352
rs8139756
rs6853272
rs4396330
rs72850971
rs112281424
rs16963008
All − MR Egger
All − Inverse variance weighted
−1.0
−0.5
0.0
0.5
1.0
MR effect size for
'Holdemania sp900120005' on 'GCST90199763_buildGRCh38.tsv'

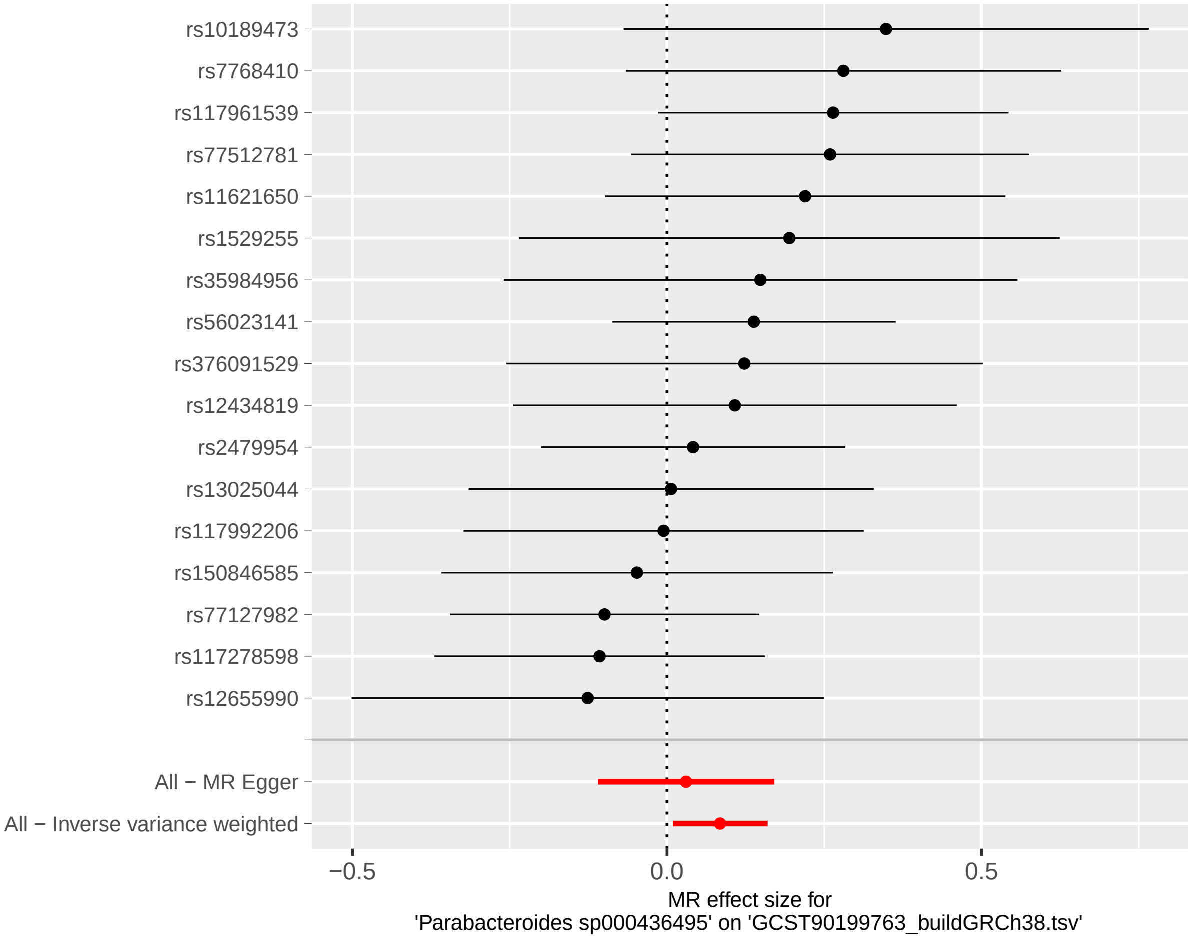

rs10189473
rs7768410
rs117961539
rs77512781
rs11621650
rs1529255
rs35984956
rs56023141
rs376091529
rs12434819
rs2479954
rs13025044
rs117992206
rs150846585
rs77127982
rs117278598
rs12655990
All − MR Egger
All − Inverse variance weighted
−0.5
0.0
0.5
MR effect size for
'Parabacteroides sp000436495' on 'GCST90199763_buildGRCh38.tsv'

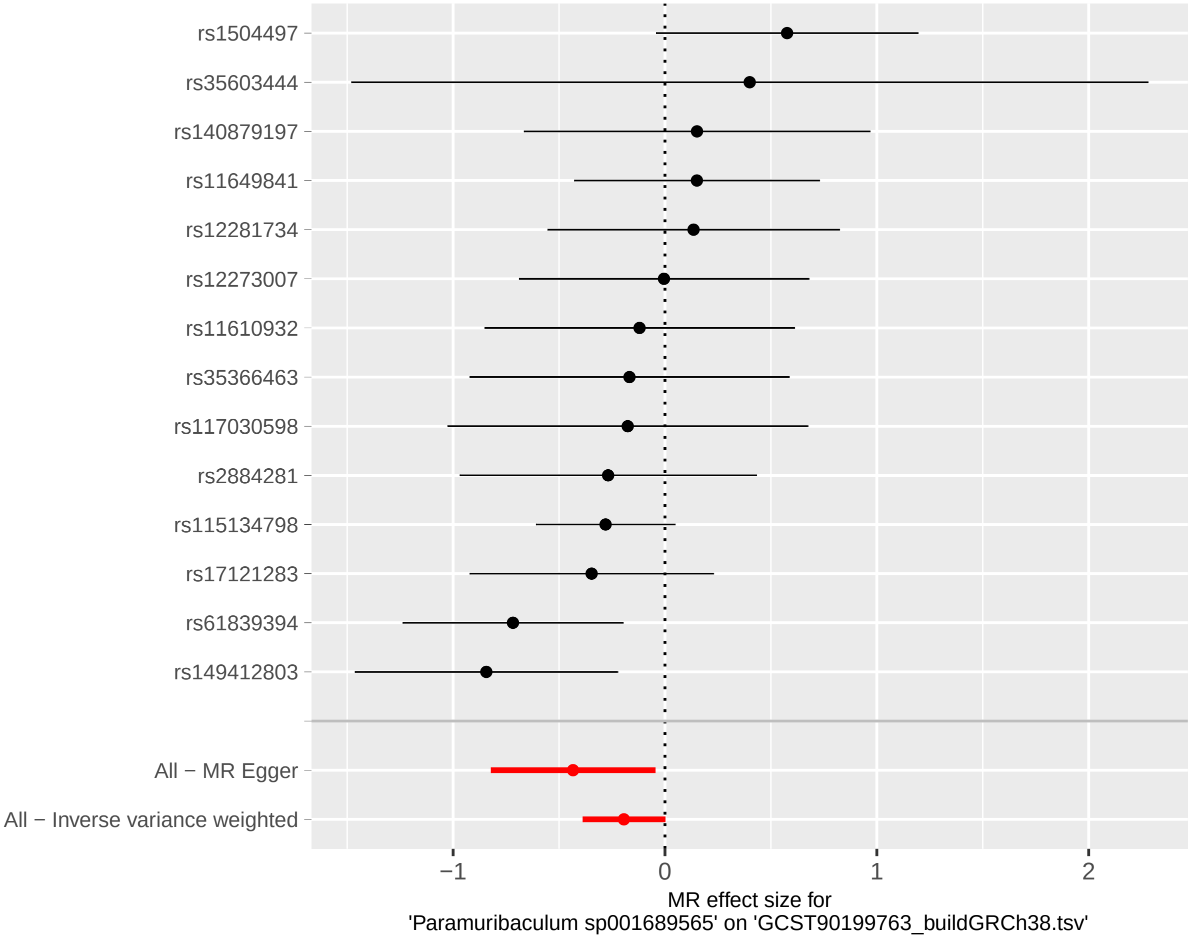
rs1504497
rs35603444
rs140879197
rs11649841
rs12281734
rs12273007
rs11610932
rs35366463
rs117030598
rs2884281
rs115134798
rs17121283
rs61839394
rs149412803
All − MR Egger
All − Inverse variance weighted
−1
0
1
2
MR effect size for
'Paramuribaculum sp001689565' on 'GCST90199763_buildGRCh38.tsv'

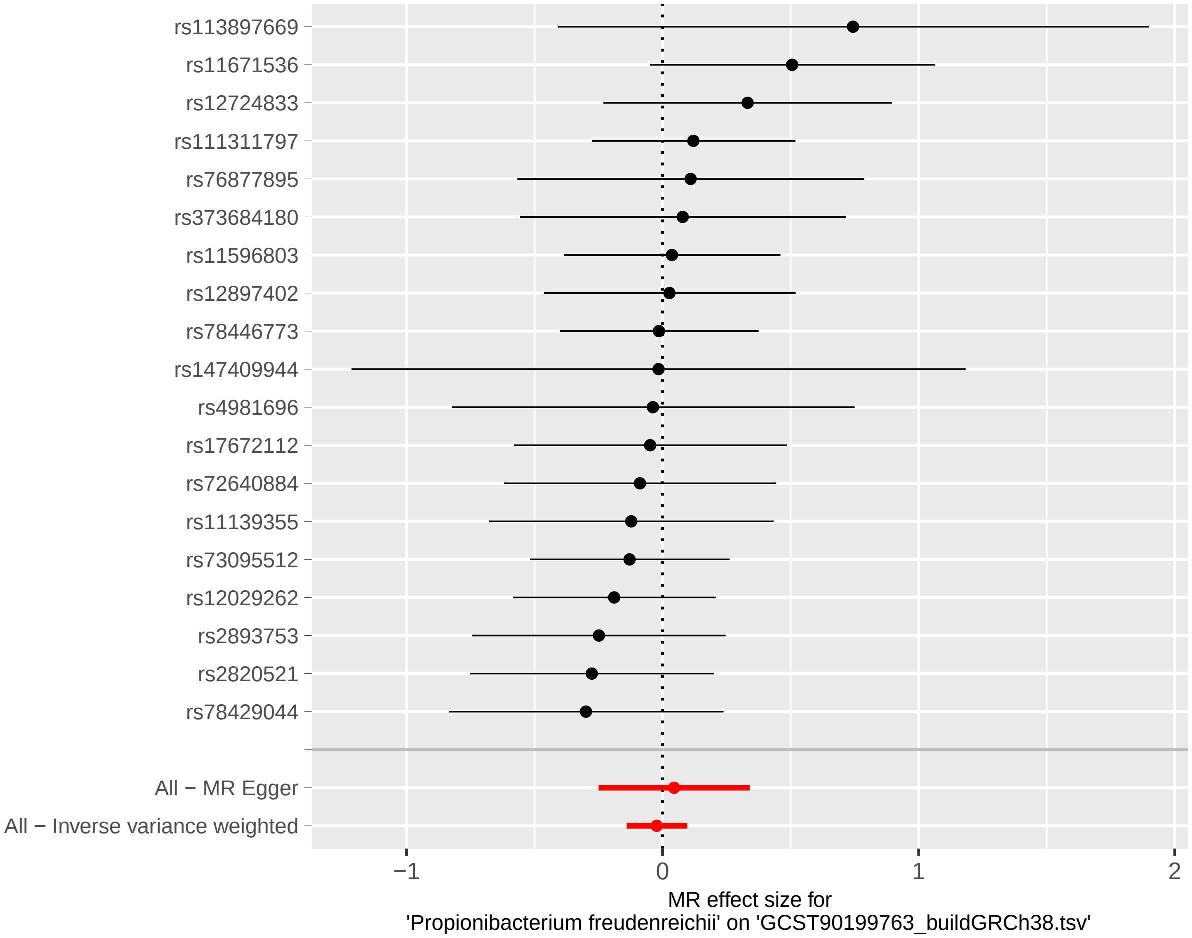

rs113897669
rs11671536
rs12724833
rs111311797
rs76877895
rs373684180
rs11596803
rs12897402
rs78446773
rs147409944
rs4981696
rs17672112
rs72640884
rs11139355
rs73095512
rs12029262
rs2893753
rs2820521
rs78429044
All − MR Egger
All − Inverse variance weighted
−1
0
1
2
MR effect size for
'Propionibacterium freudenreichii' on 'GCST90199763_buildGRCh38.tsv'

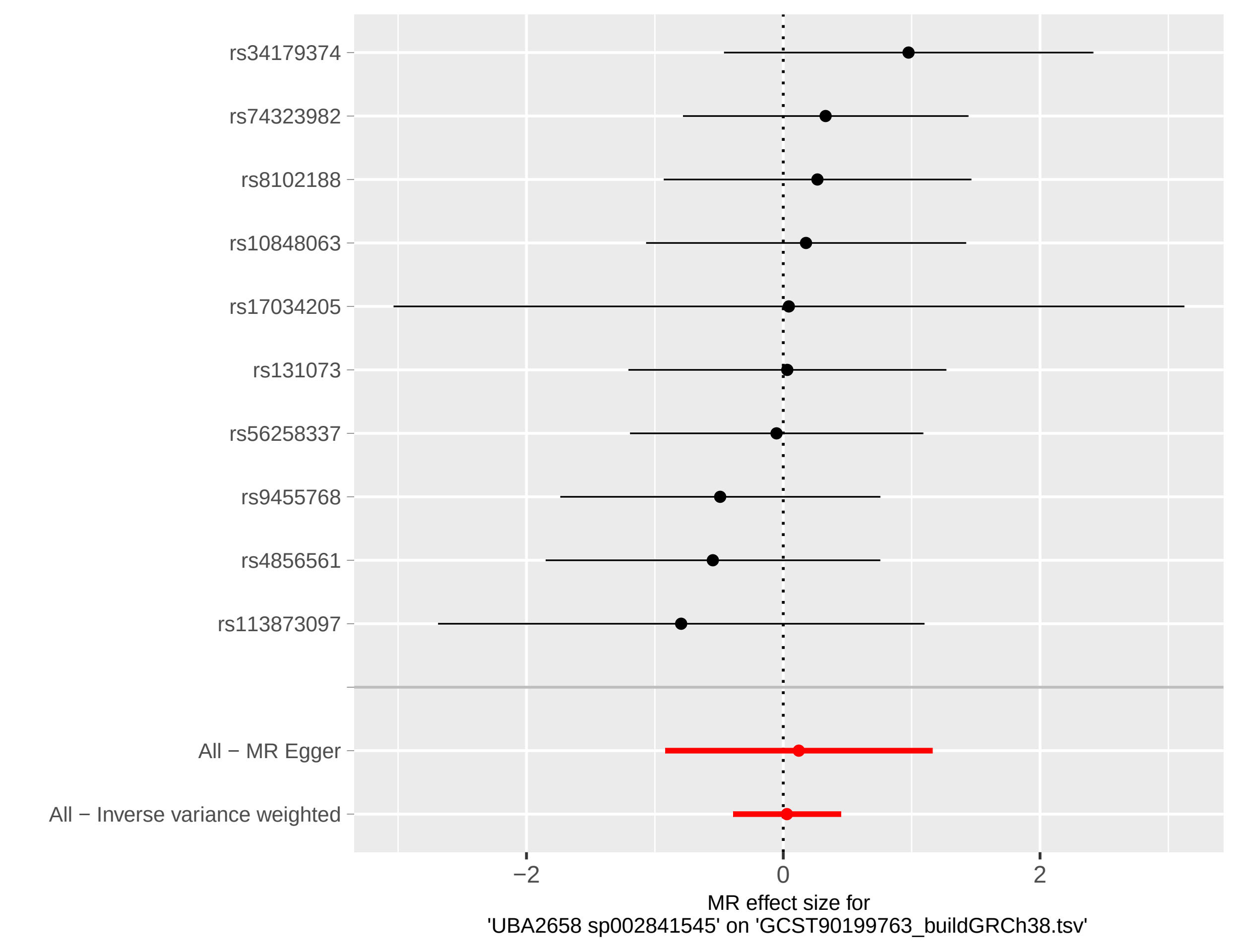

rs34179374
rs74323982
rs8102188
rs10848063
rs17034205
rs131073
rs56258337
rs9455768
rs4856561
rs113873097
All − MR Egger
All − Inverse variance weighted
−2
0
2
MR effect size for
'UBA2658 sp002841545' on 'GCST90199763_buildGRCh38.tsv'

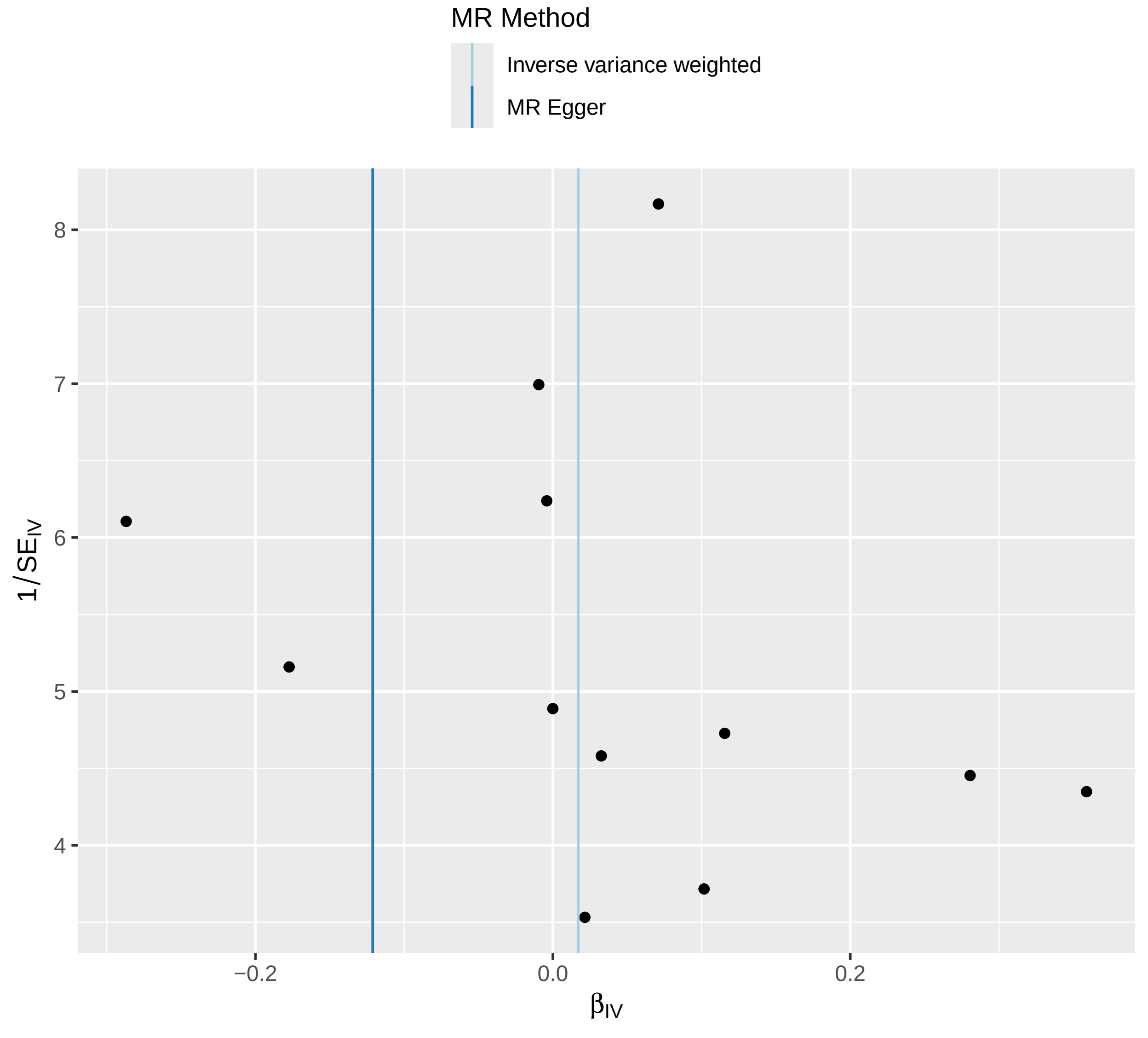
MR Method
Inverse variance weighted
MR Egger
8
7
6
5
4
1/SE_IV
−0.2
0.0
0.2
β_IV

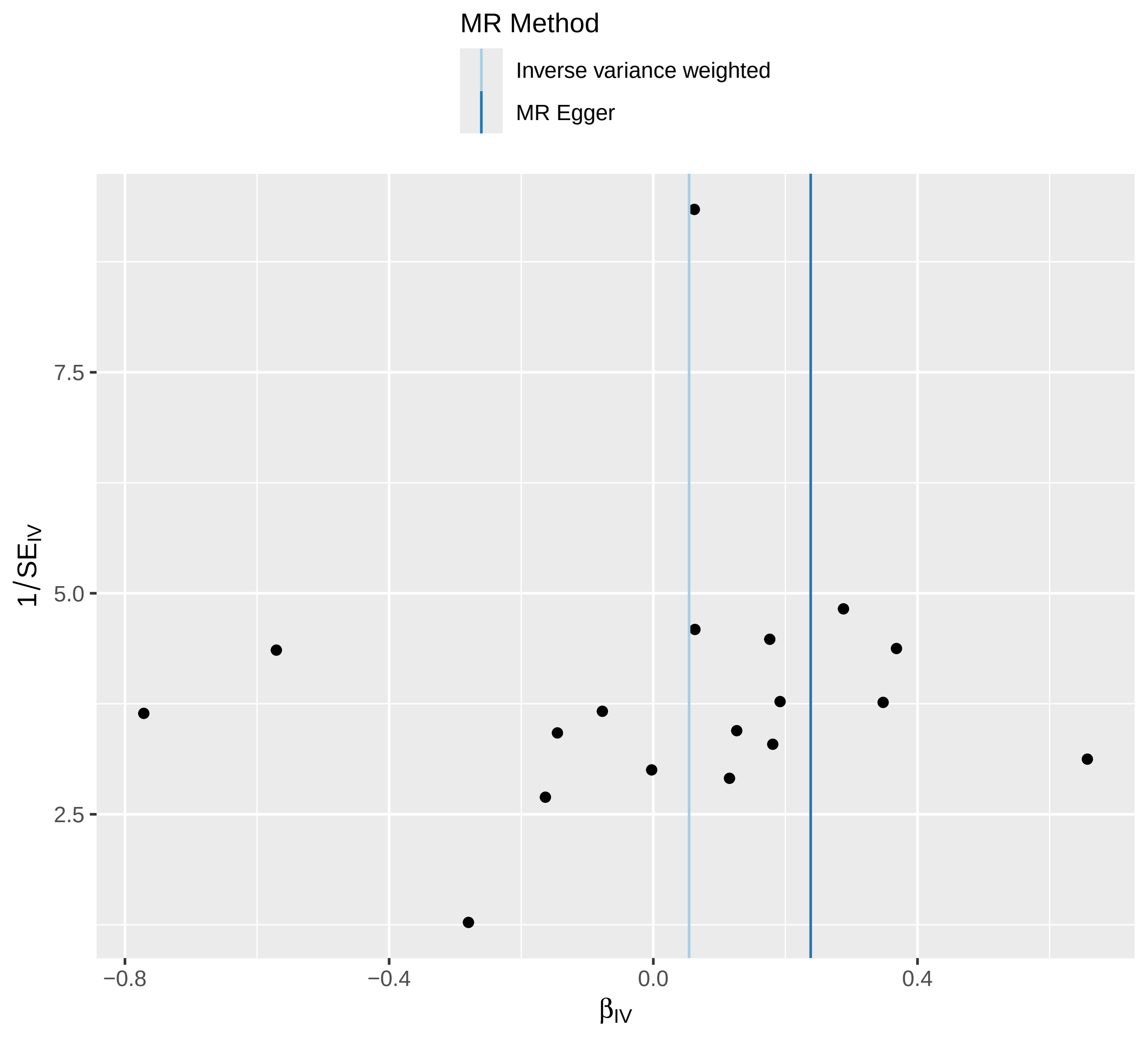
MR Method
Inverse variance weighted
MR Egger
7.5
5.0
2.5
$1/SE_{IV}$
−0.8
−0.4
0.0
0.4
β_{IV}

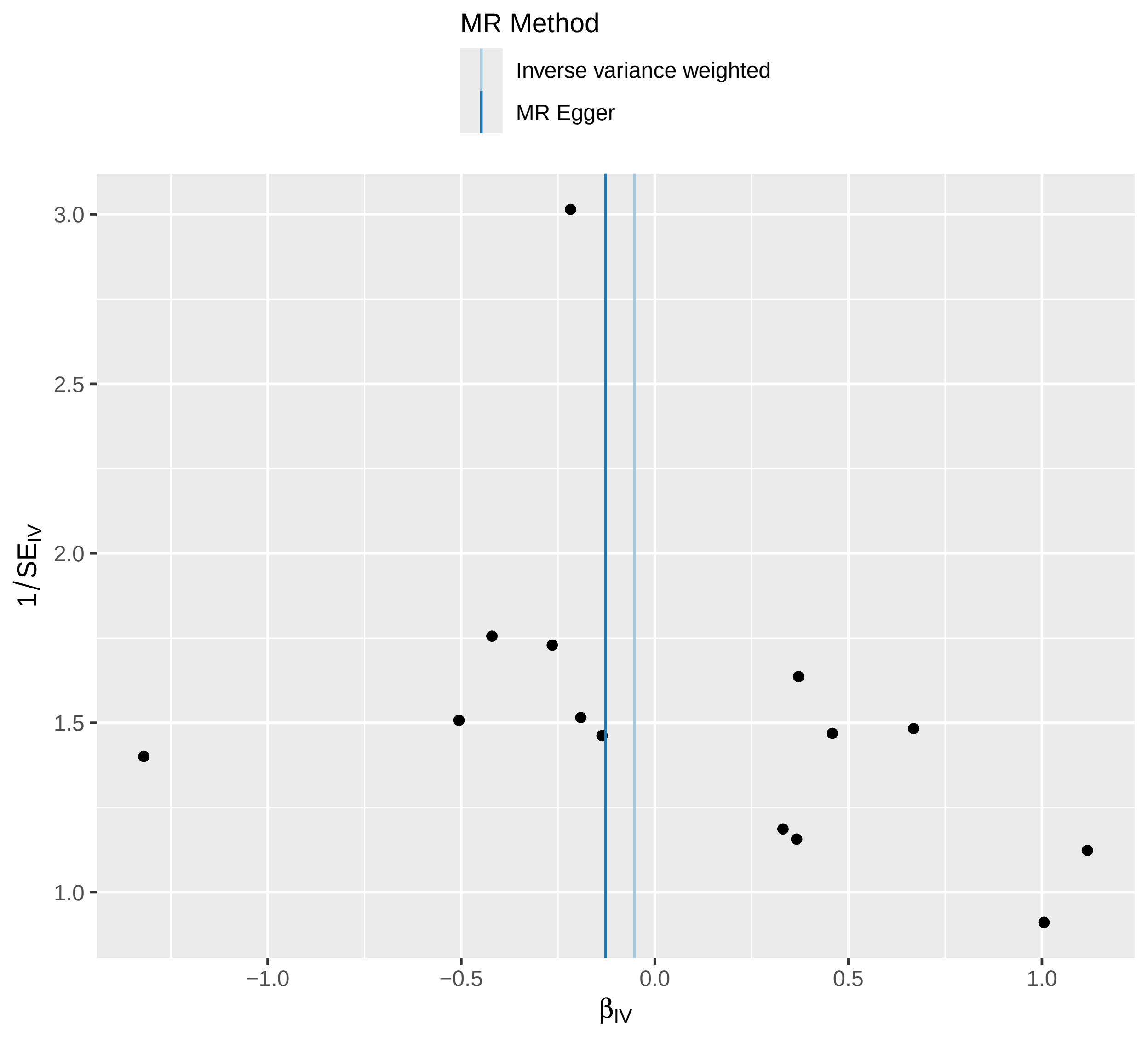
MR Method
Inverse variance weighted
MR Egger
3.0
2.5
2.0
1.5
1.0
1/SE_IV
−1.0
−0.5
0.0
0.5
1.0
β_IV

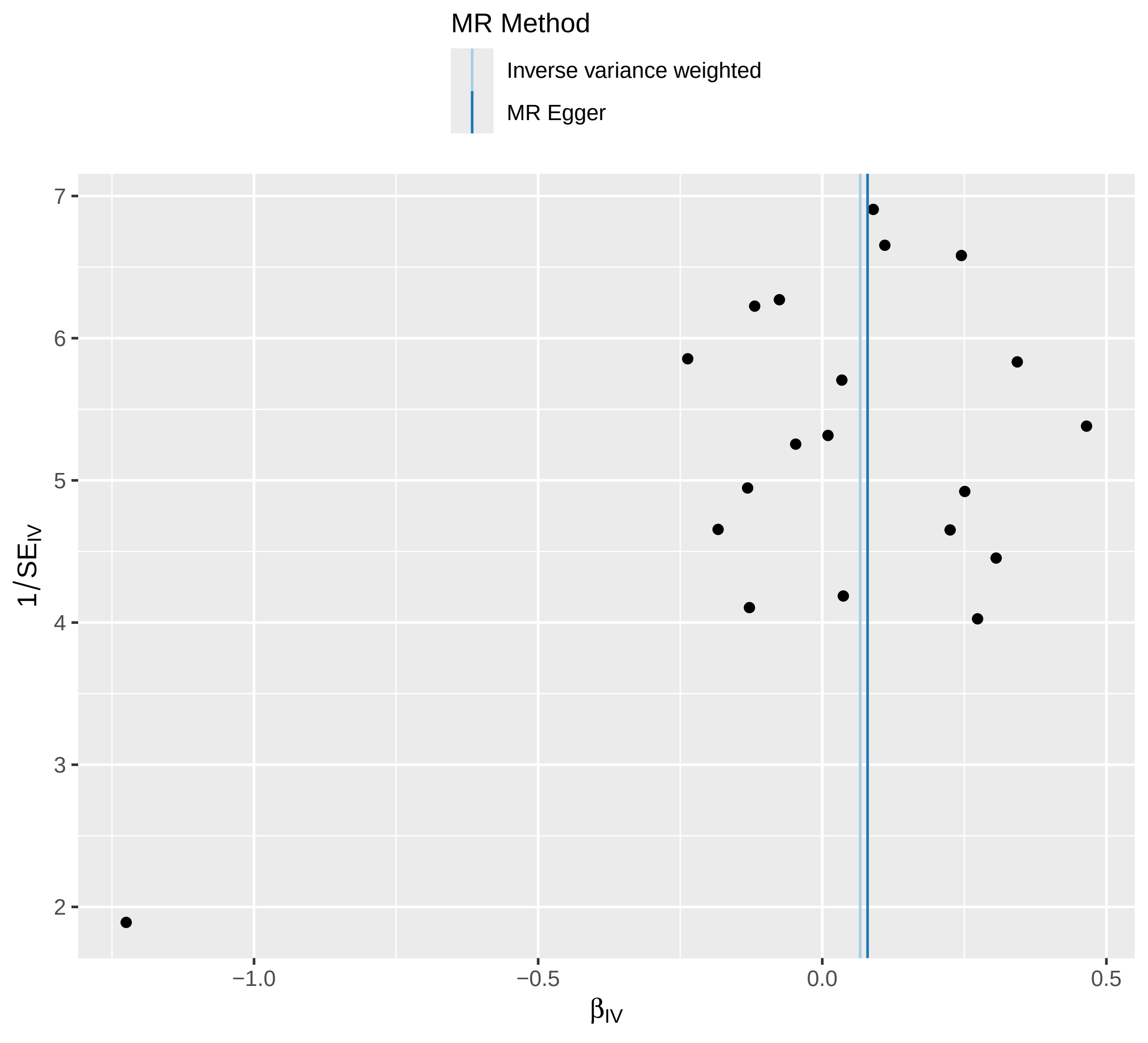
MR Method
Inverse variance weighted
MR Egger
7
6
5
4
3
2
−1.0
−0.5
0.0
0.5
1/SE_IV
β_IV

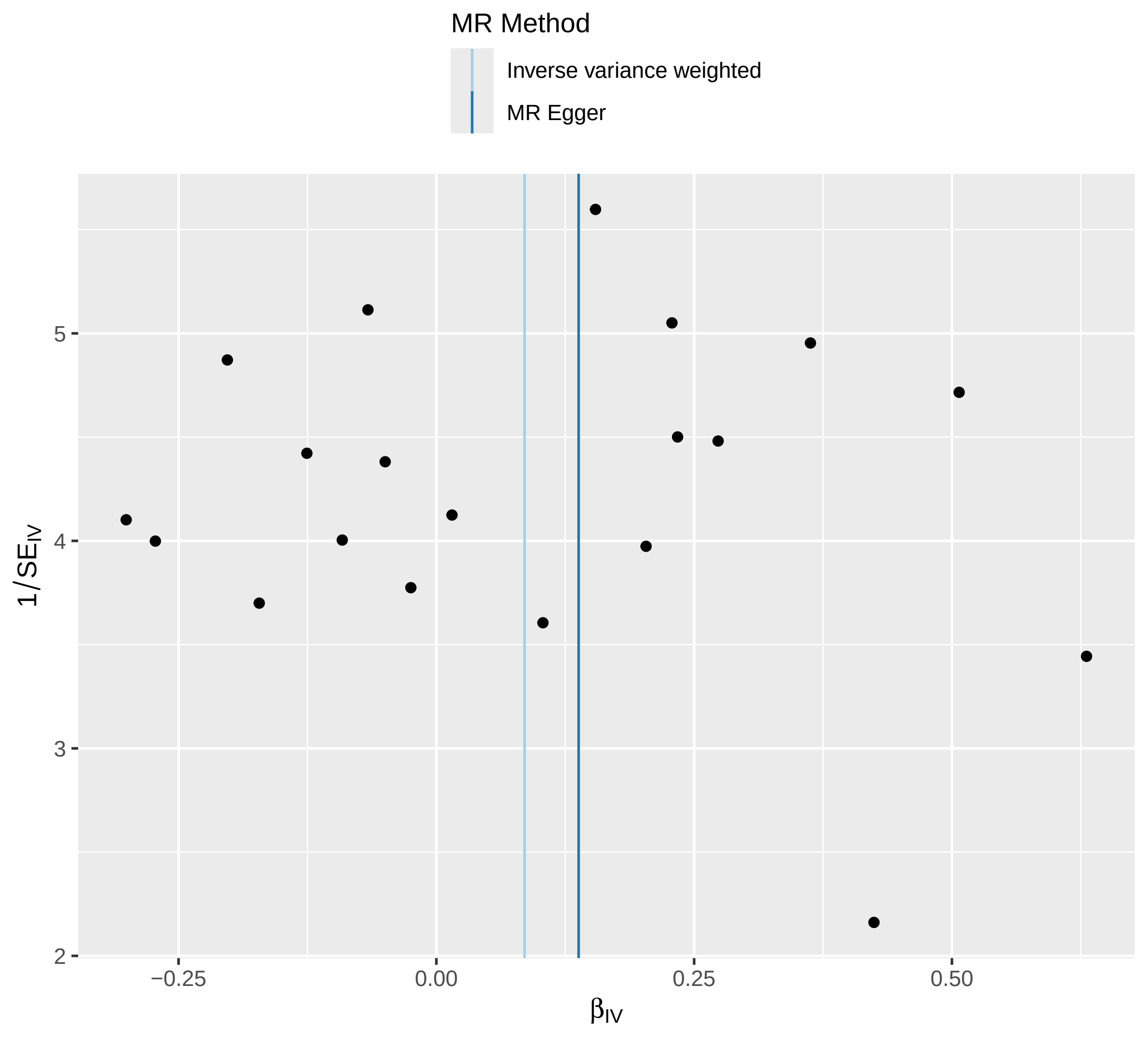
MR Method
Inverse variance weighted
MR Egger
5
4
3
2
1/SE_IV
−0.25
0.00
0.25
0.50
β_IV

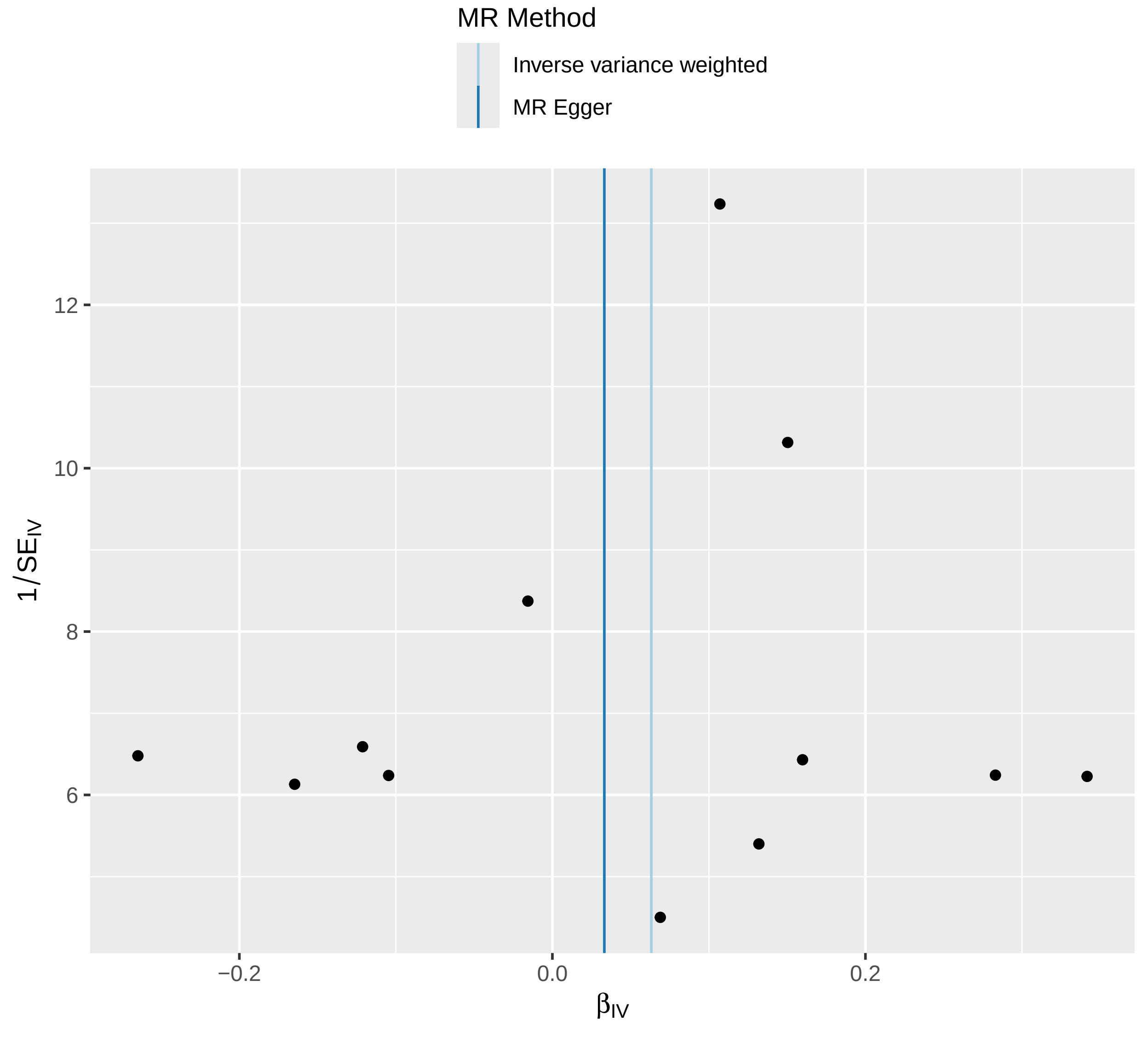

MR Method
Inverse variance weighted
MR Egger
12
10
8
6
1/SE$_{IV}$
−0.2
0.0
0.2
β$_{IV}$

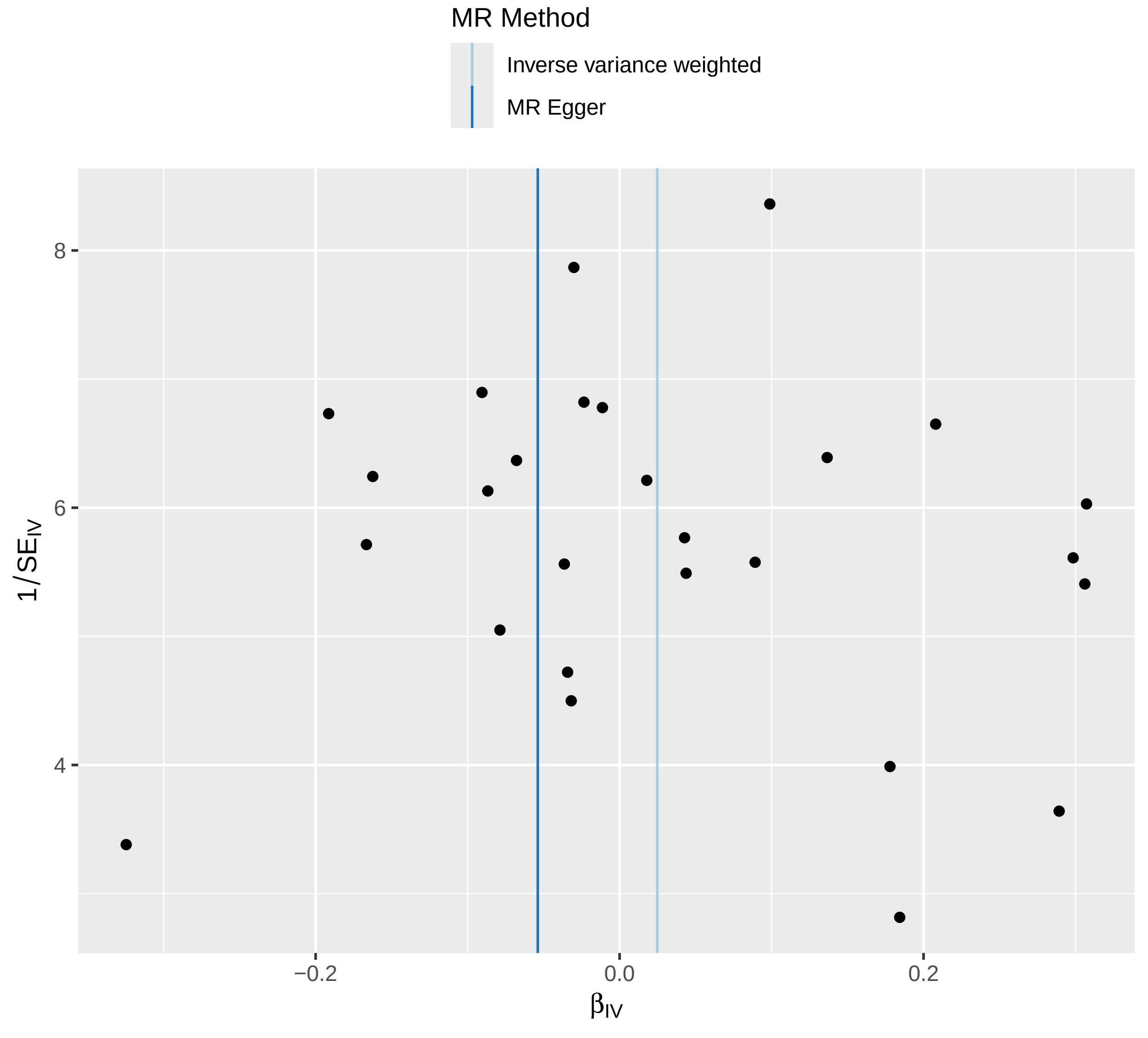
MR Method
Inverse variance weighted
MR Egger
8
6
4
1/SE_IV
−0.2
0.0
0.2
β_IV

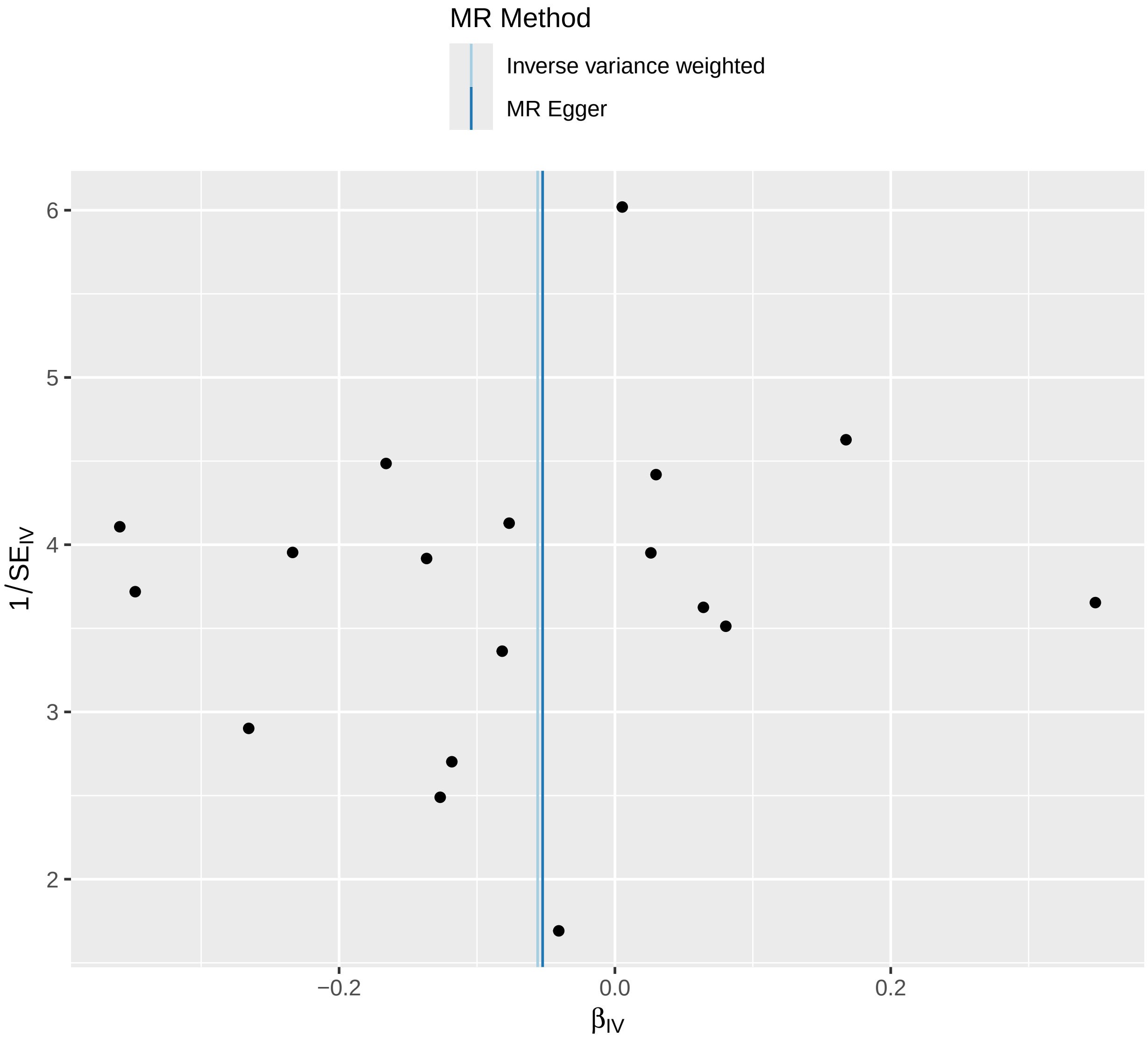
MR Method
Inverse variance weighted
MR Egger
6
5
4
3
2
1/SE_IV
−0.2
0.0
0.2
β_IV

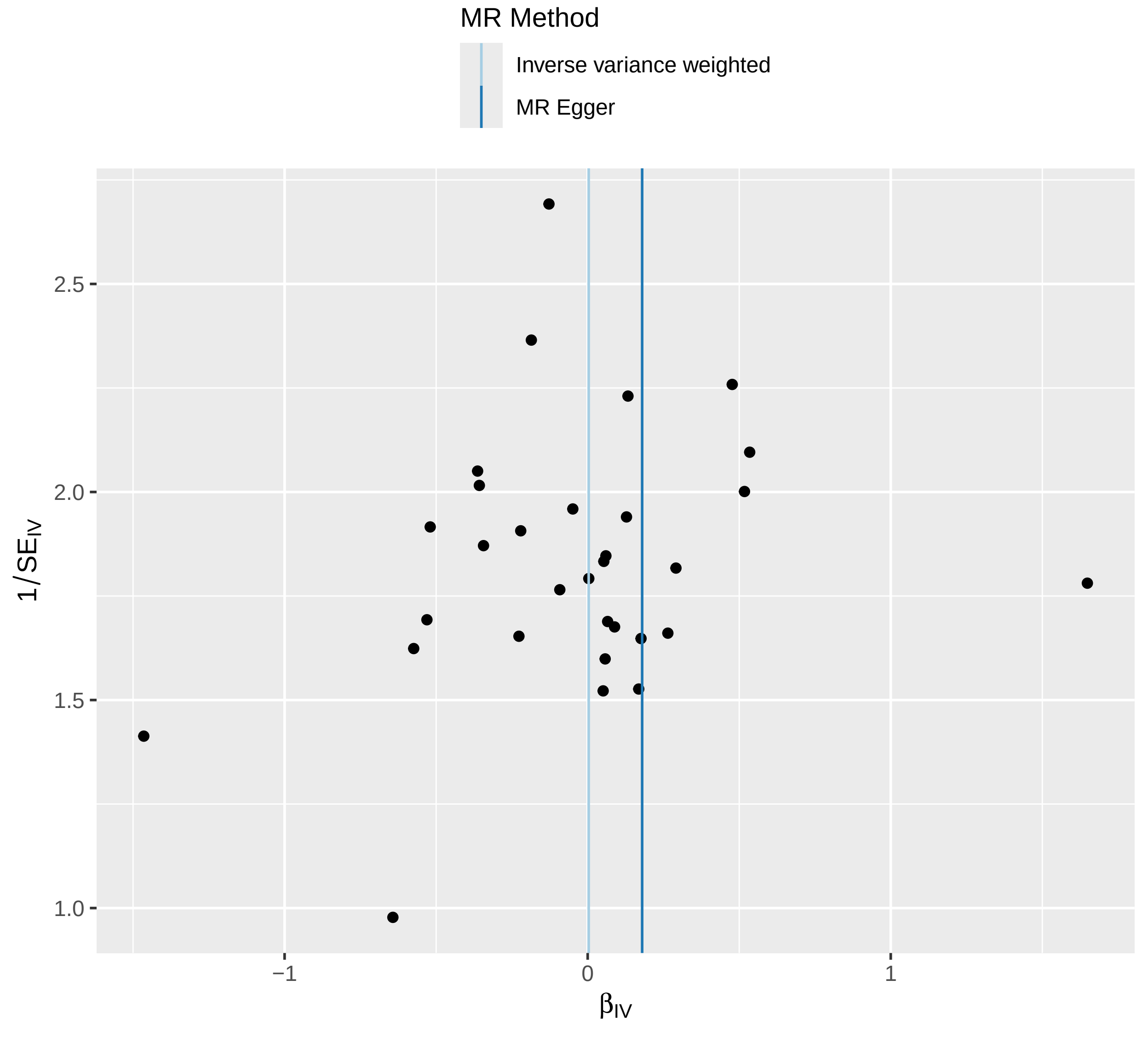
MR Method
Inverse variance weighted
MR Egger
2.5
2.0
1.5
1.0
$1/SE_{IV}$
−1
0
1
β_{IV}

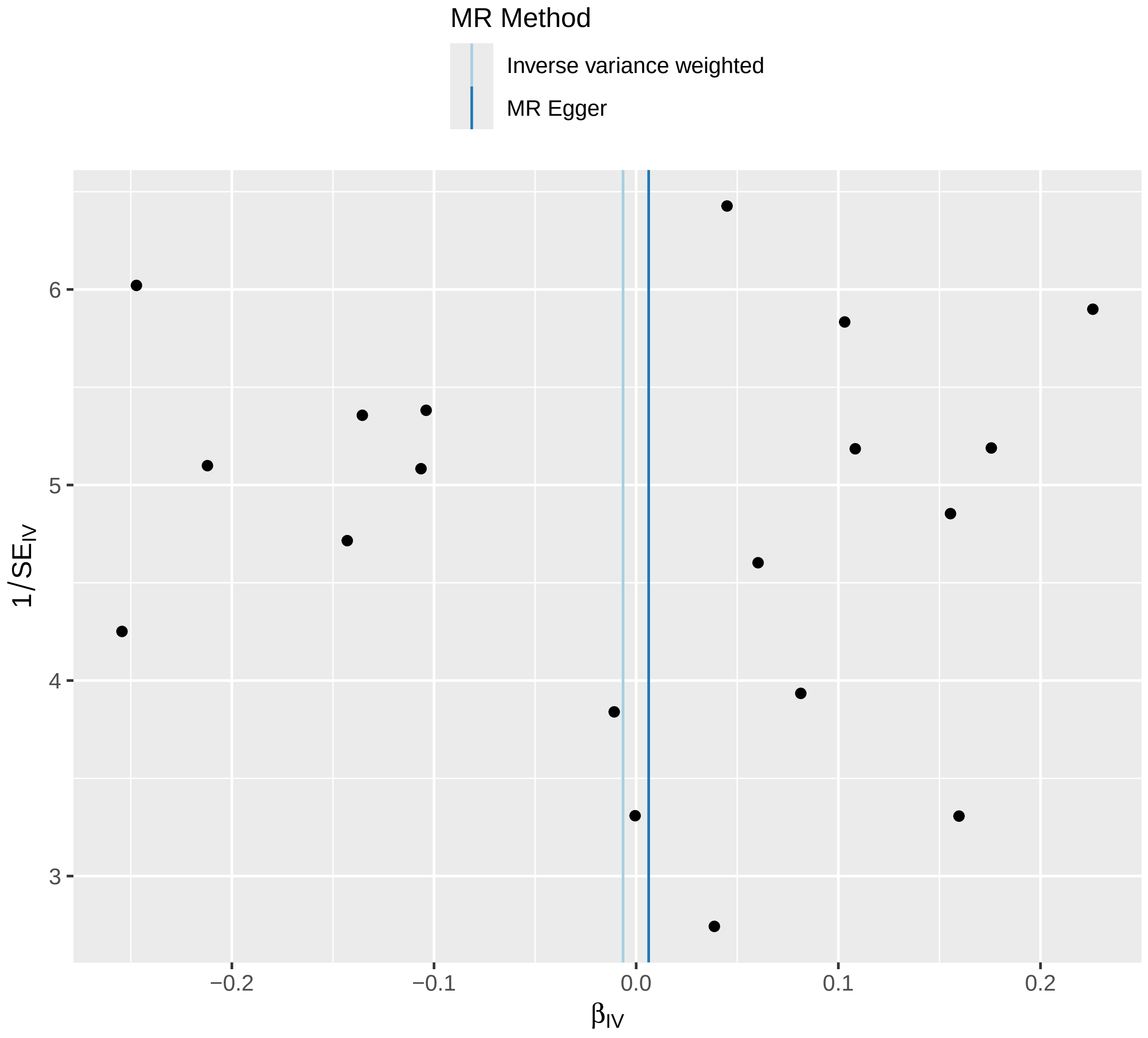
MR Method
Inverse variance weighted
MR Egger
6
5
4
3
1/SE_IV
−0.2
−0.1
0.0
0.1
0.2
β_IV

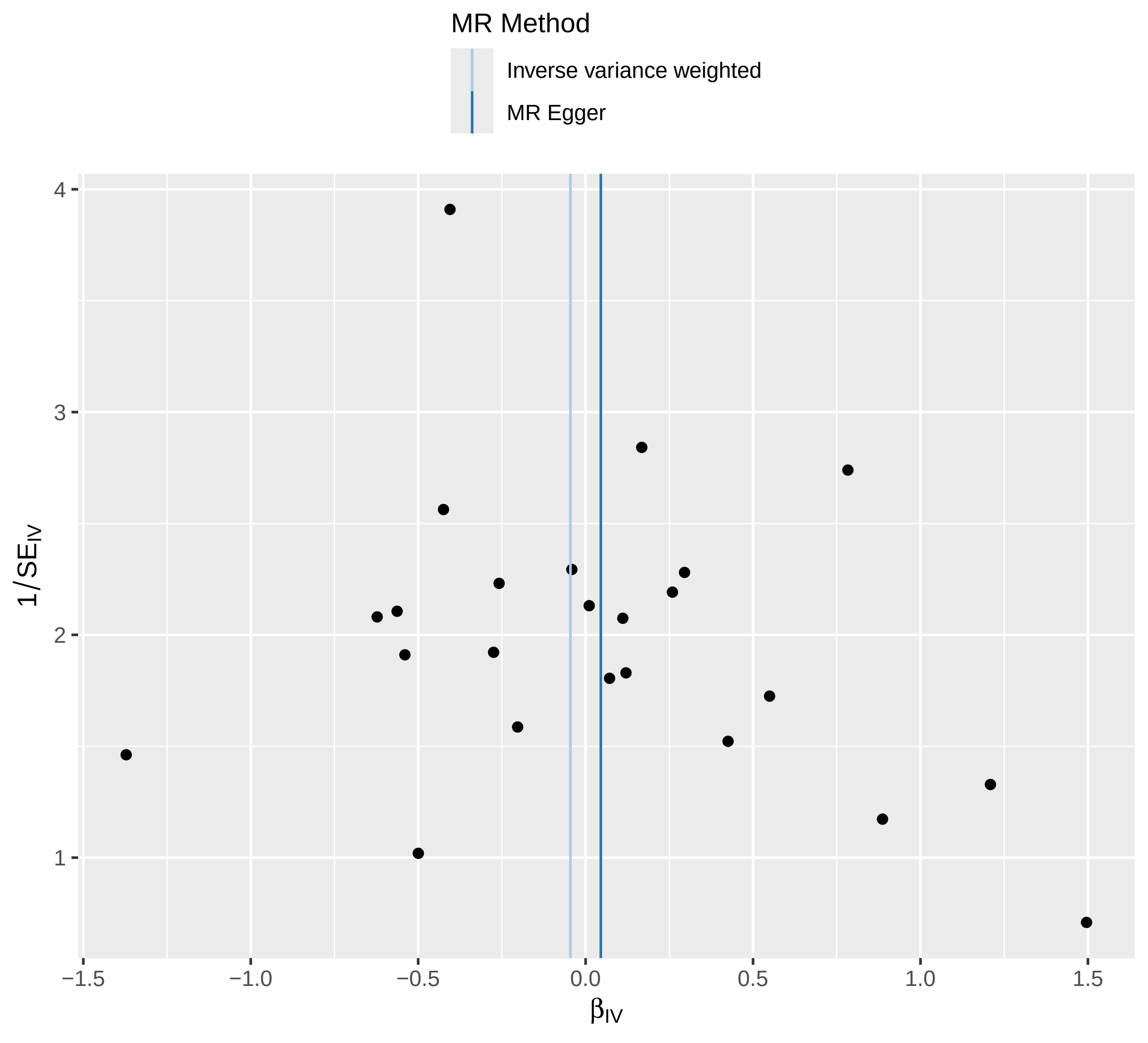

MR Method
Inverse variance weighted
MR Egger
4
3
2
1
1/SE_IV
−1.5
−1.0
−0.5
0.0
0.5
1.0
1.5
β_IV

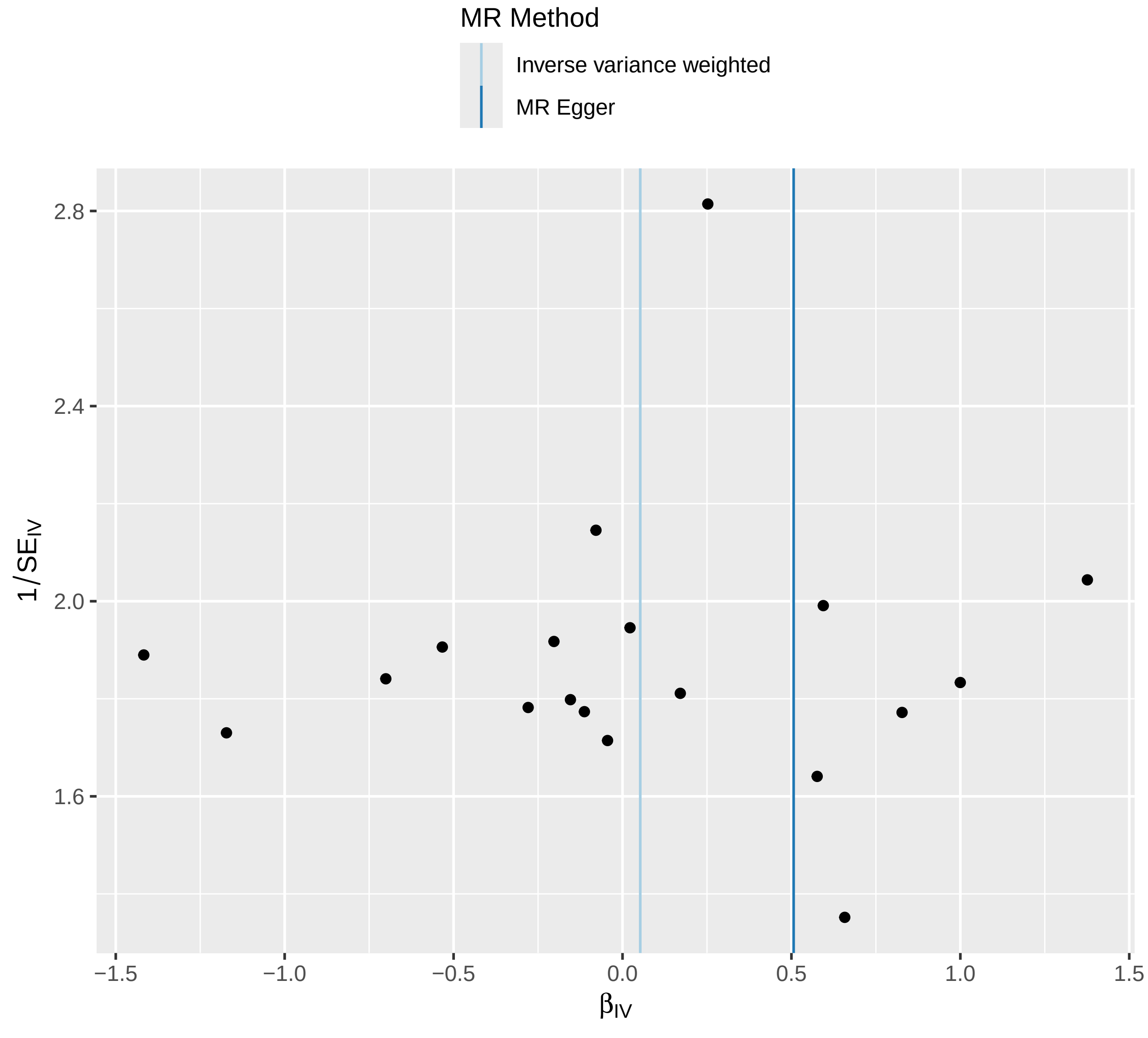

MR Method
Inverse variance weighted
MR Egger
2.8
2.4
2.0
1.6
1/SE_IV
−1.5
−1.0
−0.5
0.0
0.5
1.0
1.5
β_IV

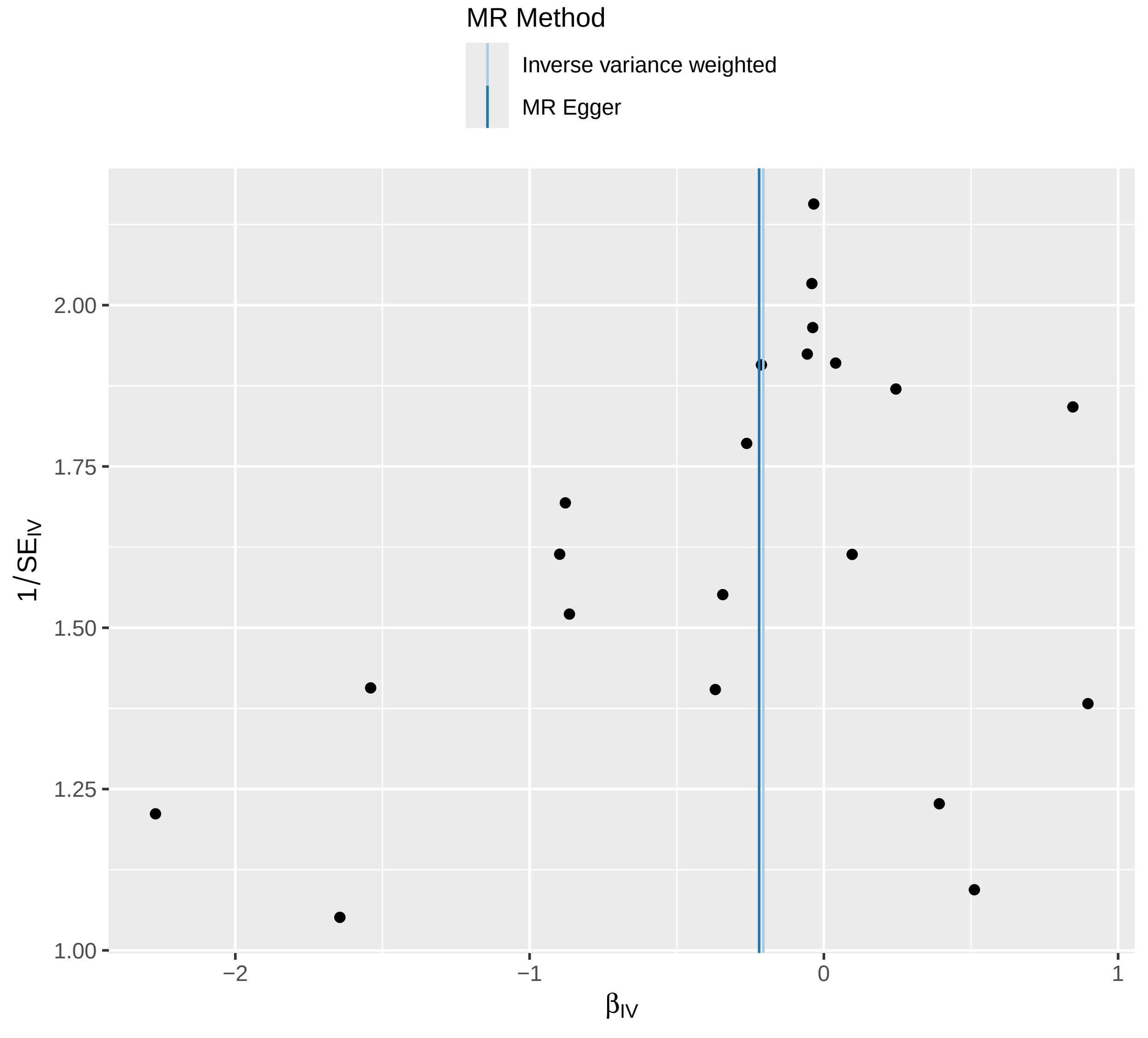
MR Method
Inverse variance weighted
MR Egger
2.00
1.75
1.50
1.25
1.00
1/SE_IV
−2
−1
0
1
β_IV

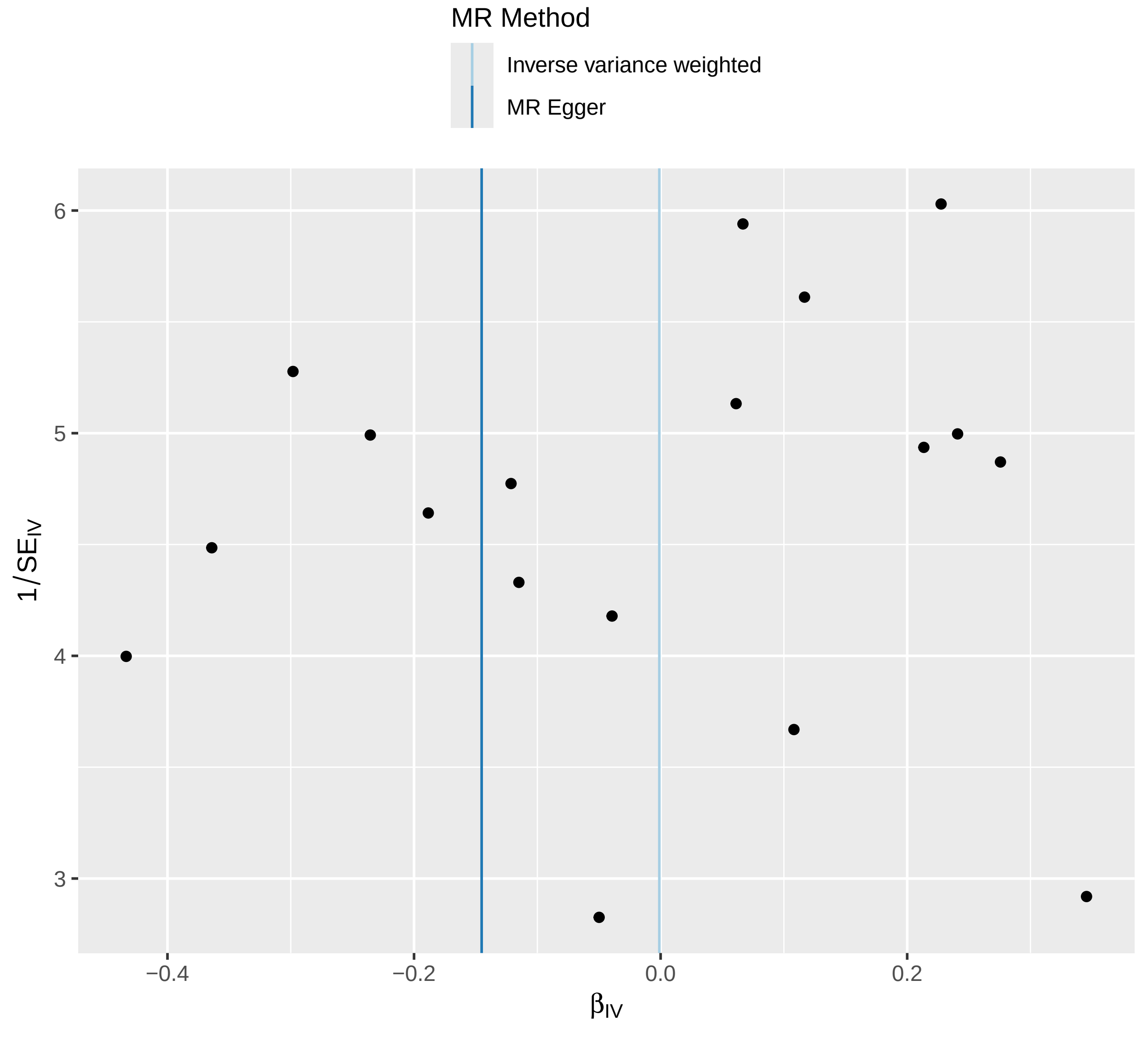

MR Method
Inverse variance weighted
MR Egger
6
5
4
3
1/SE$_{IV}$
−0.4
−0.2
0.0
0.2
β$_{IV}$

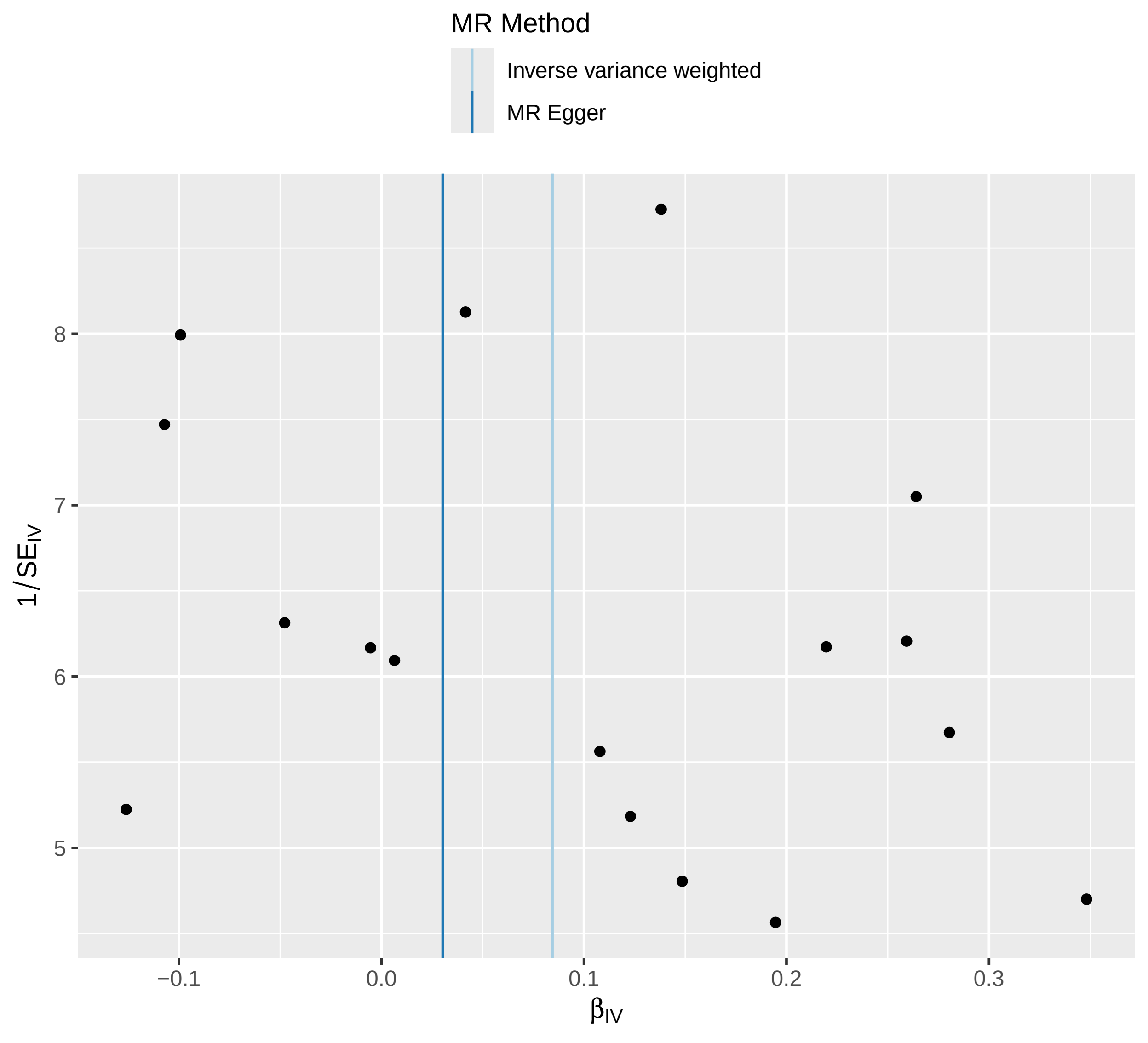
MR Method
Inverse variance weighted
MR Egger
8
7
6
5
$1/SE_{IV}$
−0.1
0.0
0.1
0.2
0.3
β_{IV}

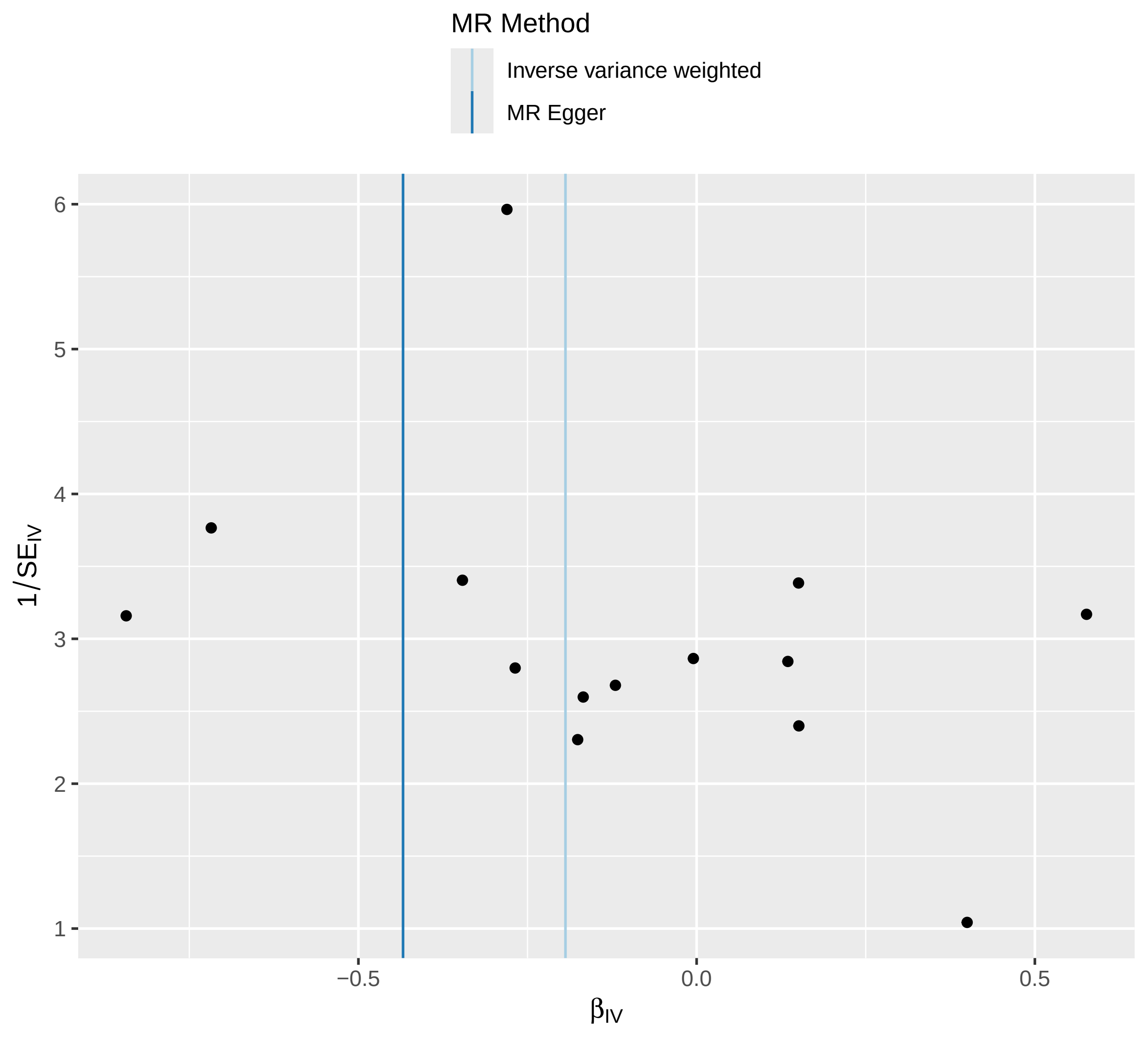
MR Method
Inverse variance weighted
MR Egger
6
5
4
3
2
1
$1/SE_{IV}$
−0.5
0.0
0.5
β_{IV}

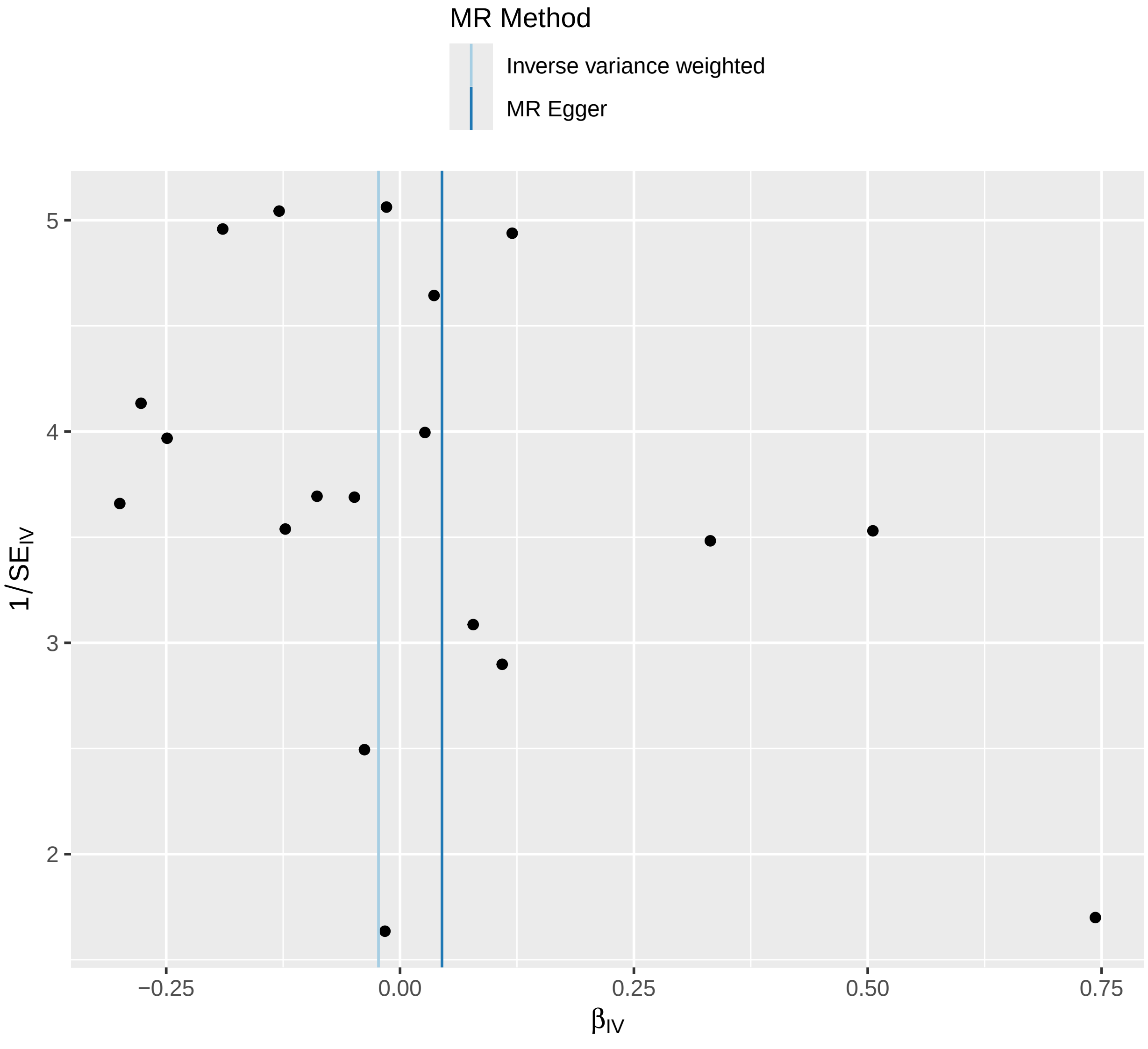

MR Method
Inverse variance weighted
MR Egger
5
4
3
2
1/SE_IV
−0.25
0.00
0.25
0.50
0.75
β_IV

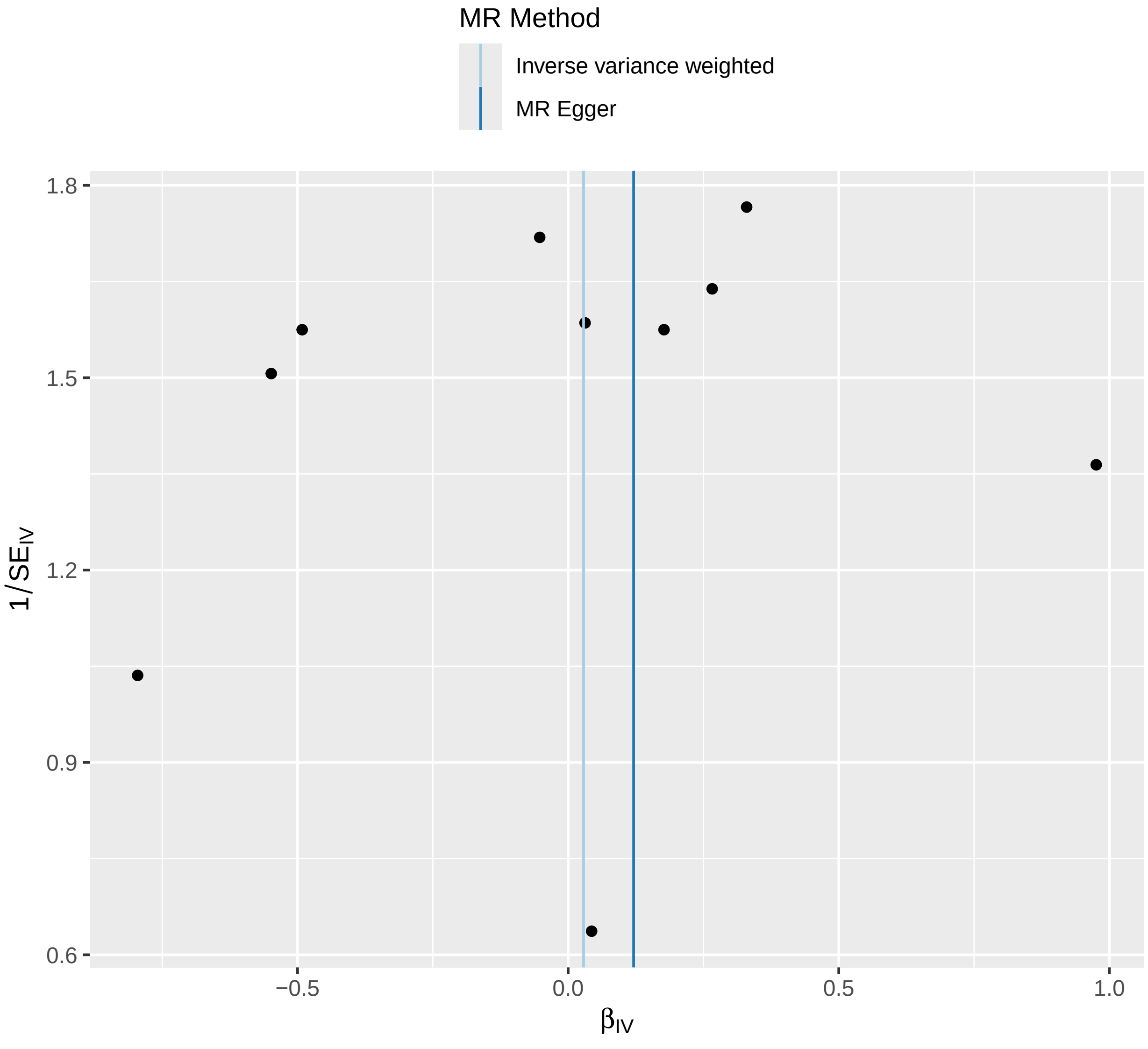
MR Method
Inverse variance weighted
MR Egger
1.8
1.5
1.2
0.9
0.6
1/SE_IV
−0.5
0.0
0.5
1.0
β_IV

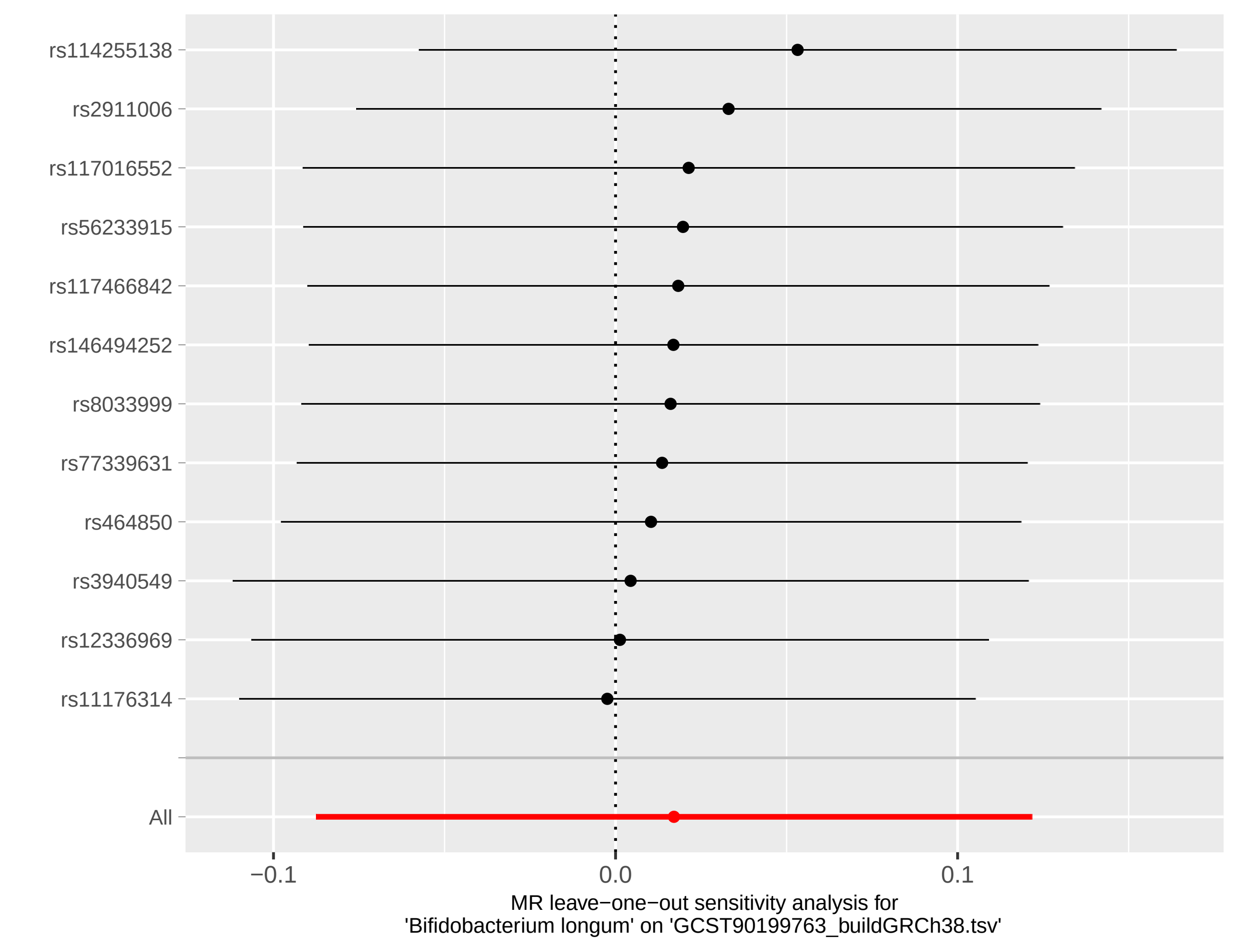
rs114255138
rs2911006
rs117016552
rs56233915
rs117466842
rs146494252
rs8033999
rs77339631
rs464850
rs3940549
rs12336969
rs11176314
All
−0.1
0.0
0.1
MR leave−one−out sensitivity analysis for
'Bifidobacterium longum' on 'GCST90199763_buildGRCh38.tsv'

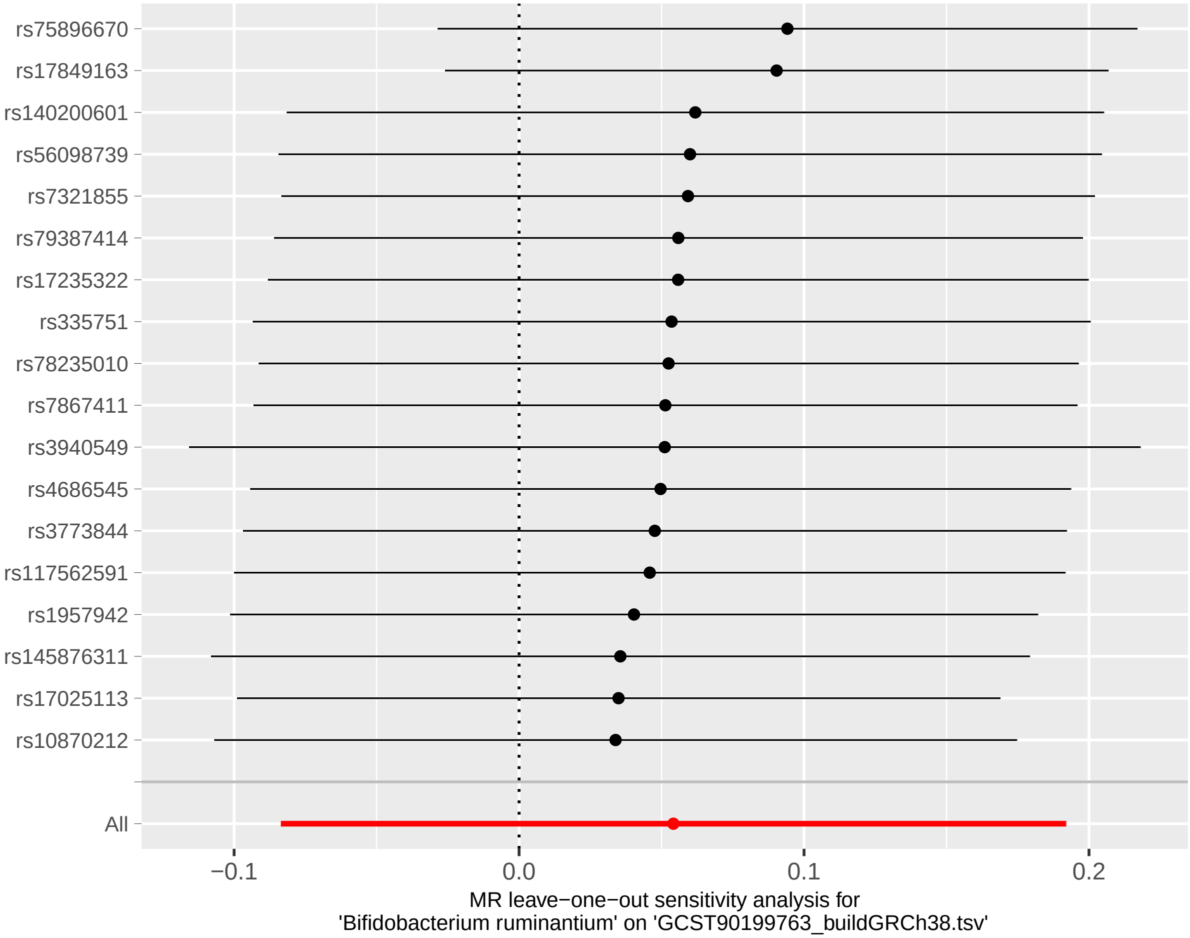
rs75896670
rs17849163
rs140200601
rs56098739
rs7321855
rs79387414
rs17235322
rs335751
rs78235010
rs7867411
rs3940549
rs4686545
rs3773844
rs117562591
rs1957942
rs145876311
rs17025113
rs10870212
All
−0.1
0.0
0.1
0.2
MR leave−one−out sensitivity analysis for
'Bifidobacterium ruminantium' on 'GCST90199763_buildGRCh38.tsv'

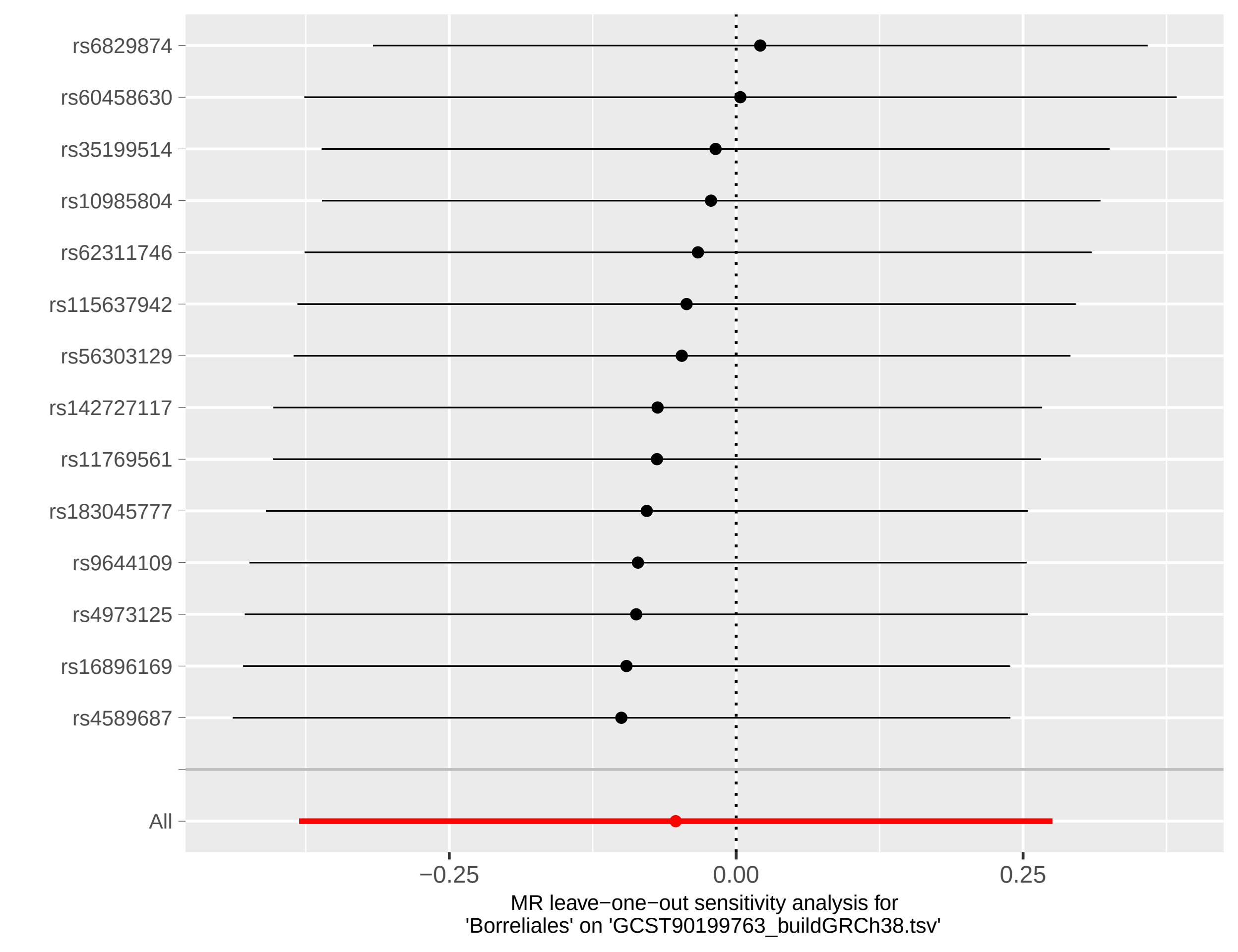

rs6829874
rs60458630
rs35199514
rs10985804
rs62311746
rs115637942
rs56303129
rs142727117
rs11769561
rs183045777
rs9644109
rs4973125
rs16896169
rs4589687
All
−0.25
0.00
0.25
MR leave−one−out sensitivity analysis for
'Borreliales' on 'GCST90199763_buildGRCh38.tsv'

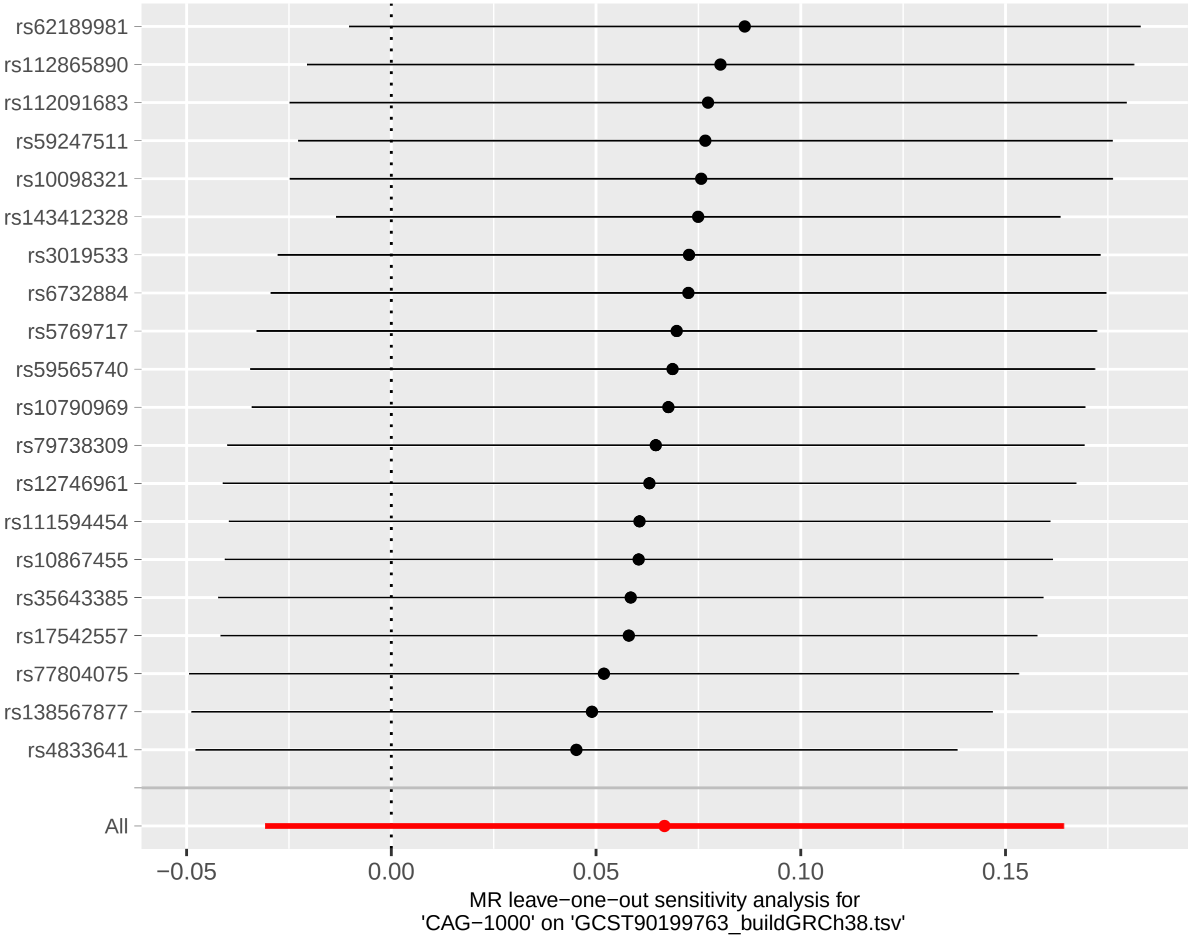
rs62189981
rs112865890
rs112091683
rs59247511
rs10098321
rs143412328
rs3019533
rs6732884
rs5769717
rs59565740
rs10790969
rs79738309
rs12746961
rs111594454
rs10867455
rs35643385
rs17542557
rs77804075
rs138567877
rs4833641
All
−0.05
0.00
0.05
0.10
0.15
MR leave−one−out sensitivity analysis for
'CAG−1000' on 'GCST90199763_buildGRCh38.tsv'

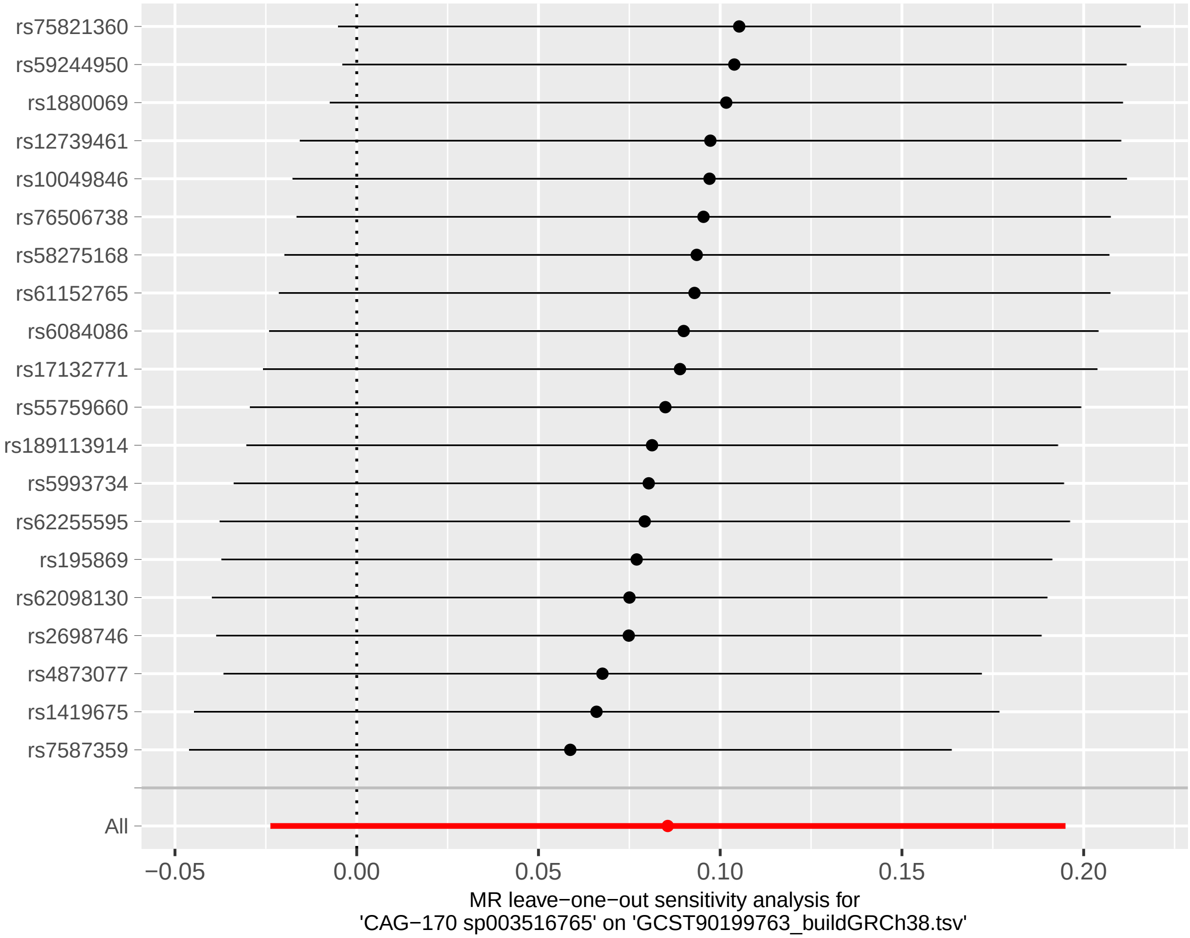
rs75821360
rs59244950
rs1880069
rs12739461
rs10049846
rs76506738
rs58275168
rs61152765
rs6084086
rs17132771
rs55759660
rs189113914
rs5993734
rs62255595
rs195869
rs62098130
rs2698746
rs4873077
rs1419675
rs7587359
All
−0.05
0.00
0.05
0.10
0.15
0.20
MR leave−one−out sensitivity analysis for
'CAG−170 sp003516765' on 'GCST90199763_buildGRCh38.tsv'

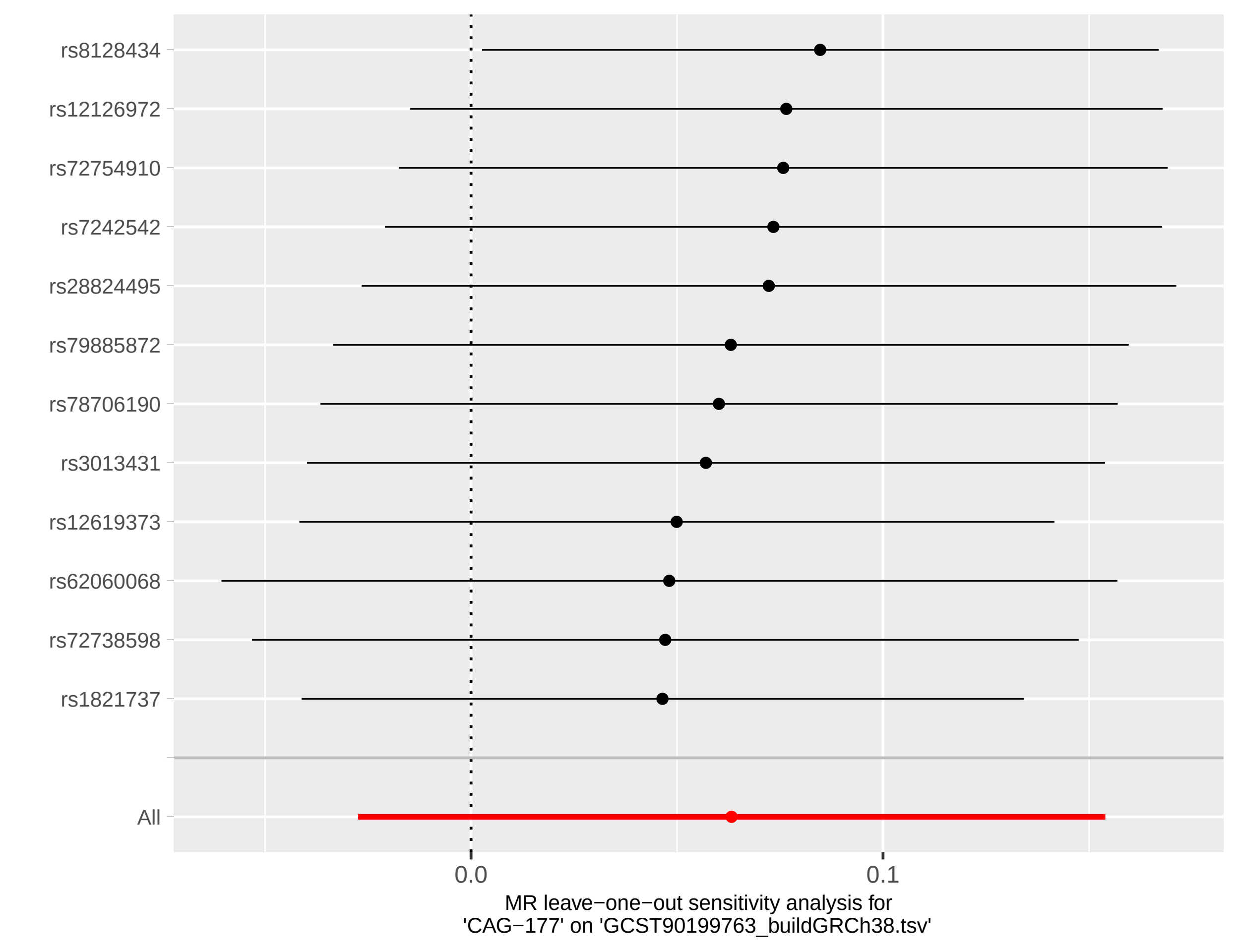
rs8128434
rs12126972
rs72754910
rs7242542
rs28824495
rs79885872
rs78706190
rs3013431
rs12619373
rs62060068
rs72738598
rs1821737
All
0.0
0.1
MR leave−one−out sensitivity analysis for
'CAG−177' on 'GCST90199763_buildGRCh38.tsv'

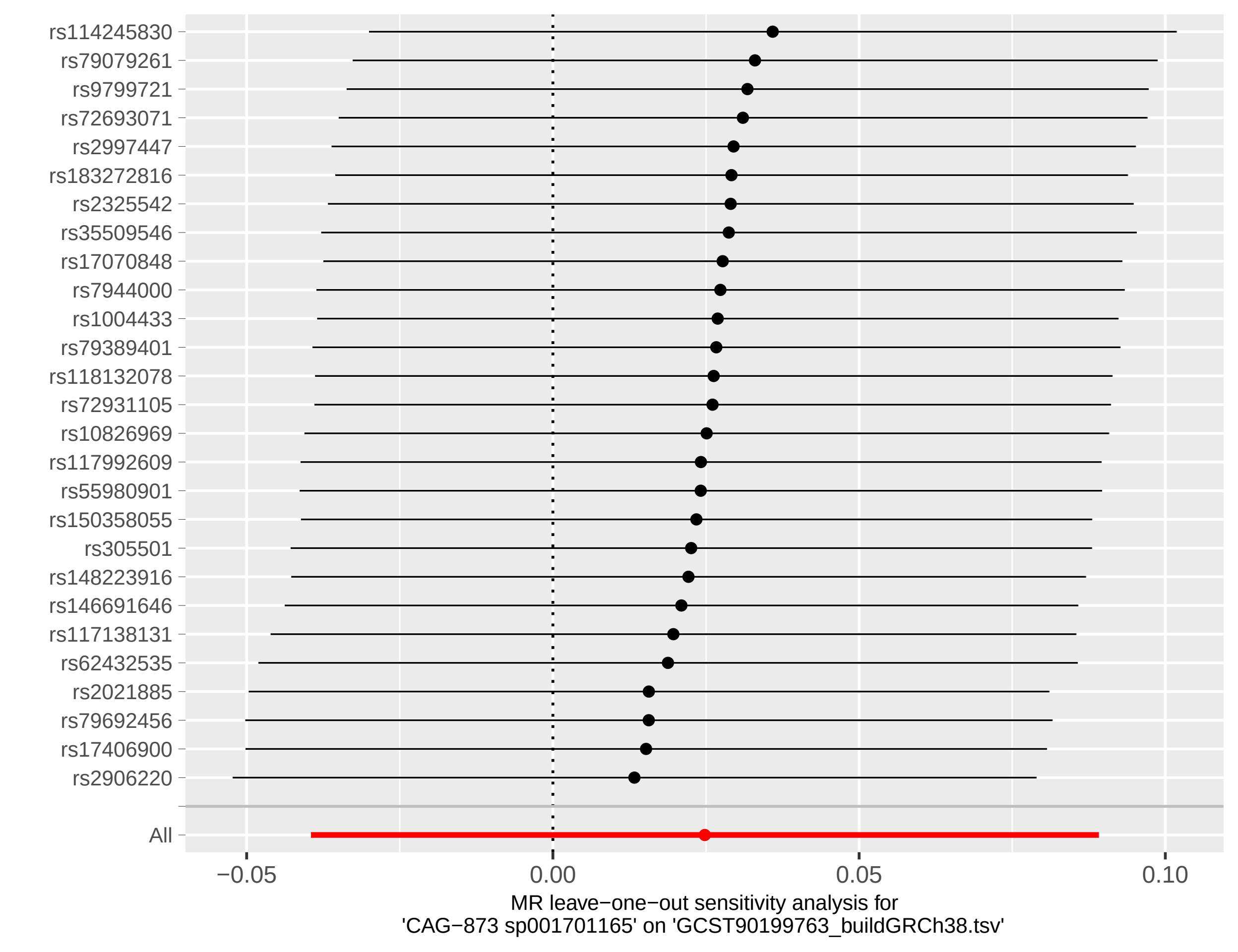

rs114245830
rs79079261
rs9799721
rs72693071
rs2997447
rs183272816
rs2325542
rs35509546
rs17070848
rs7944000
rs1004433
rs79389401
rs118132078
rs72931105
rs10826969
rs117992609
rs55980901
rs150358055
rs305501
rs148223916
rs146691646
rs117138131
rs62432535
rs2021885
rs79692456
rs17406900
rs2906220
All
−0.05
0.00
0.05
0.10
MR leave−one−out sensitivity analysis for
'CAG−873 sp001701165' on 'GCST90199763_buildGRCh38.tsv'

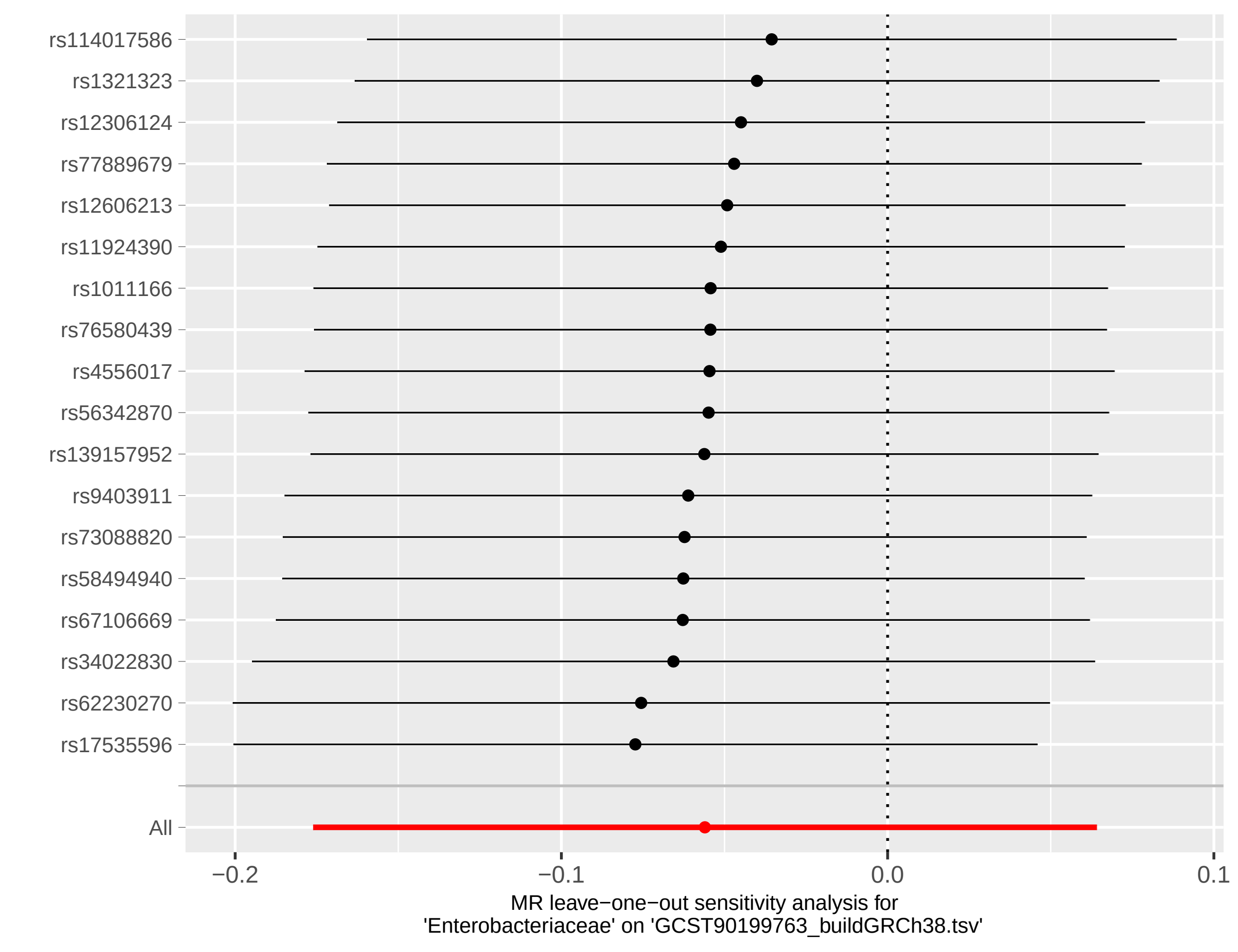
rs114017586
rs1321323
rs12306124
rs77889679
rs12606213
rs11924390
rs1011166
rs76580439
rs4556017
rs56342870
rs139157952
rs9403911
rs73088820
rs58494940
rs67106669
rs34022830
rs62230270
rs17535596
All
−0.2
−0.1
0.0
0.1
MR leave−one−out sensitivity analysis for
'Enterobacteriaceae' on 'GCST90199763_buildGRCh38.tsv'

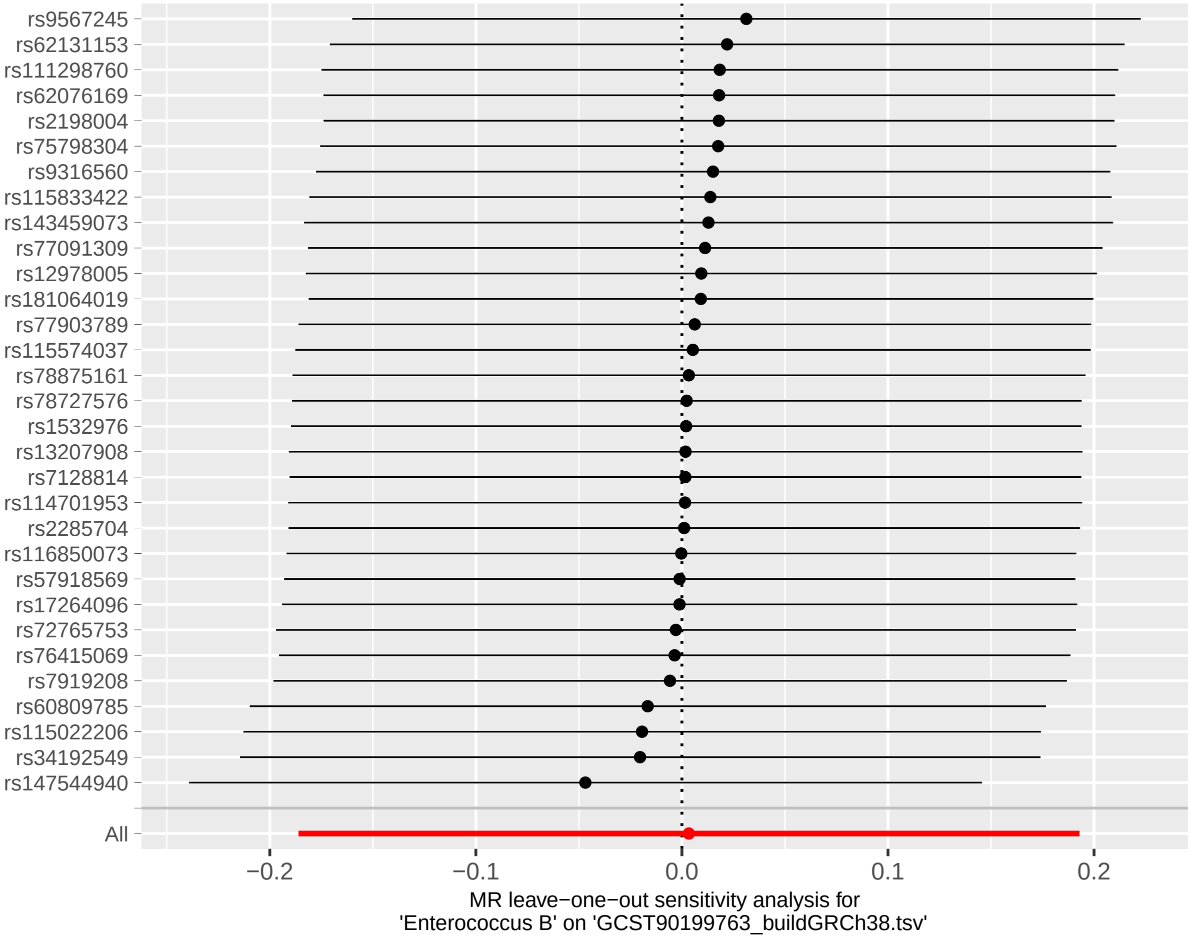
rs9567245
rs62131153
rs111298760
rs62076169
rs2198004
rs75798304
rs9316560
rs115833422
rs143459073
rs77091309
rs12978005
rs181064019
rs77903789
rs115574037
rs78875161
rs78727576
rs1532976
rs13207908
rs7128814
rs114701953
rs2285704
rs116850073
rs57918569
rs17264096
rs72765753
rs76415069
rs7919208
rs60809785
rs115022206
rs34192549
rs147544940
All
−0.2
−0.1
0.0
0.1
0.2
MR leave−one−out sensitivity analysis for
'Enterococcus B' on 'GCST90199763_buildGRCh38.tsv'

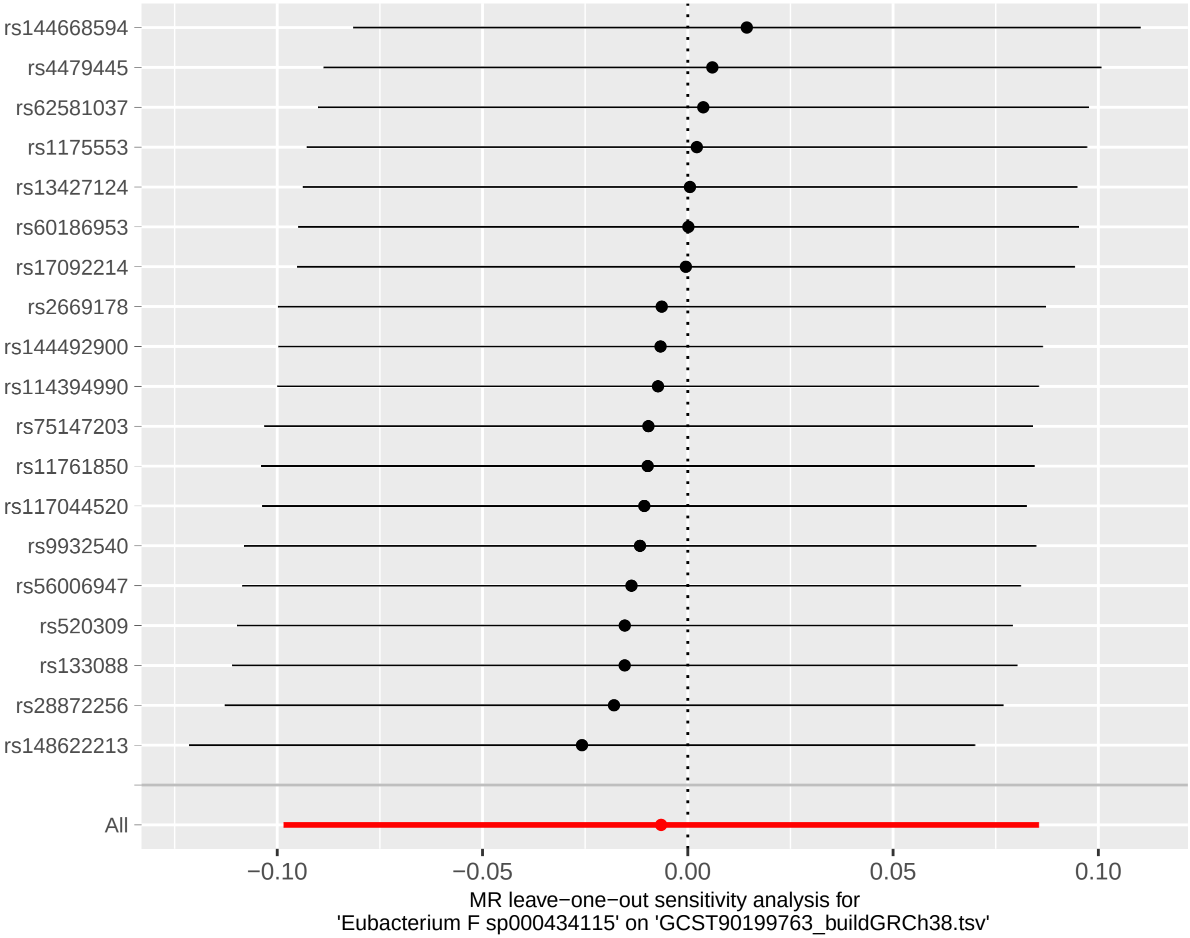
rs144668594
rs4479445
rs62581037
rs1175553
rs13427124
rs60186953
rs17092214
rs2669178
rs144492900
rs114394990
rs75147203
rs11761850
rs117044520
rs9932540
rs56006947
rs520309
rs133088
rs28872256
rs148622213
All
−0.10
−0.05
0.00
0.05
0.10
MR leave−one−out sensitivity analysis for
'Eubacterium F sp000434115' on 'GCST90199763_buildGRCh38.tsv'

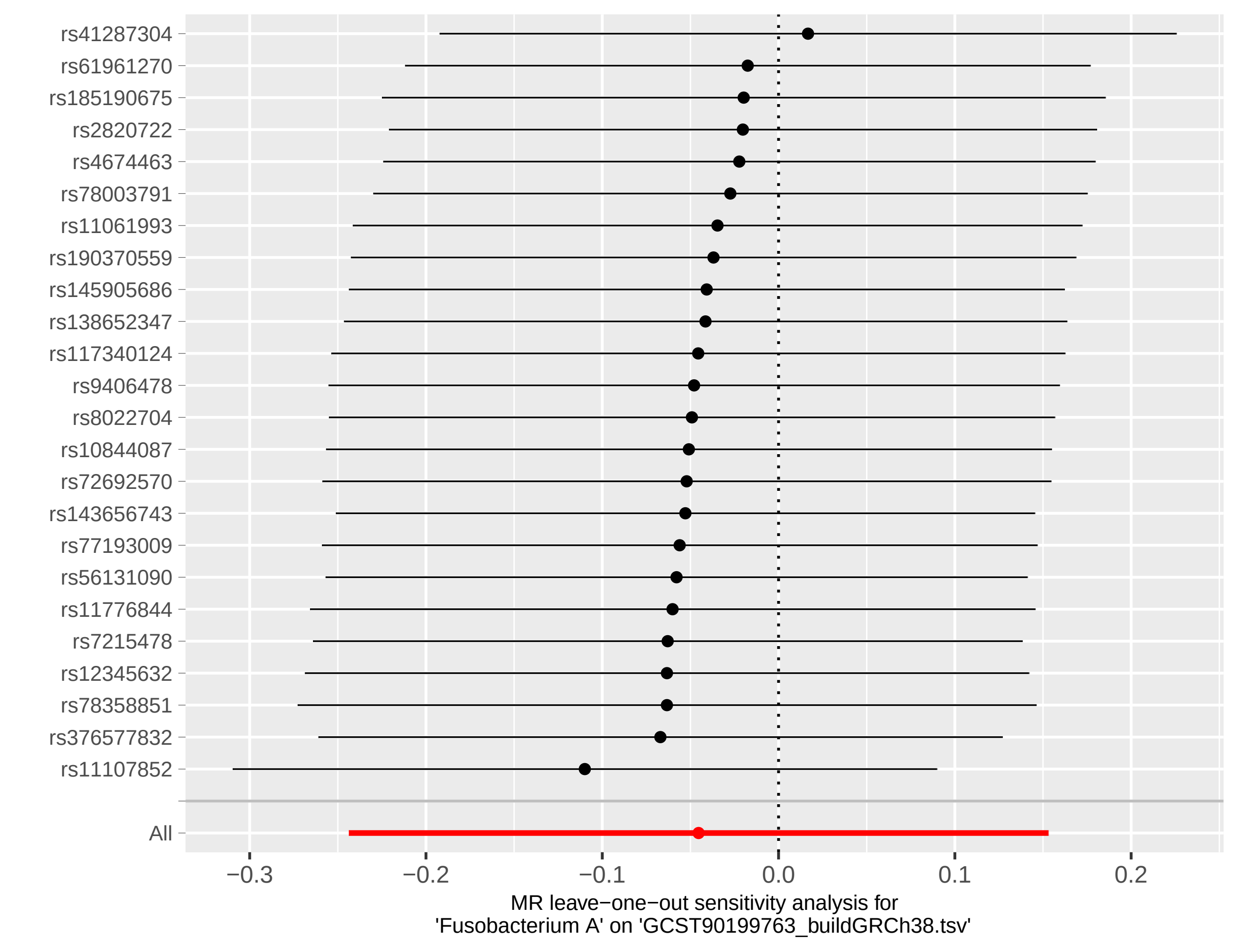

rs41287304
rs61961270
rs185190675
rs2820722
rs4674463
rs78003791
rs11061993
rs190370559
rs145905686
rs138652347
rs117340124
rs9406478
rs8022704
rs10844087
rs72692570
rs143656743
rs77193009
rs56131090
rs11776844
rs7215478
rs12345632
rs78358851
rs376577832
rs11107852
All
−0.3
−0.2
−0.1
0.0
0.1
0.2
MR leave−one−out sensitivity analysis for
'Fusobacterium A' on 'GCST90199763_buildGRCh38.tsv'

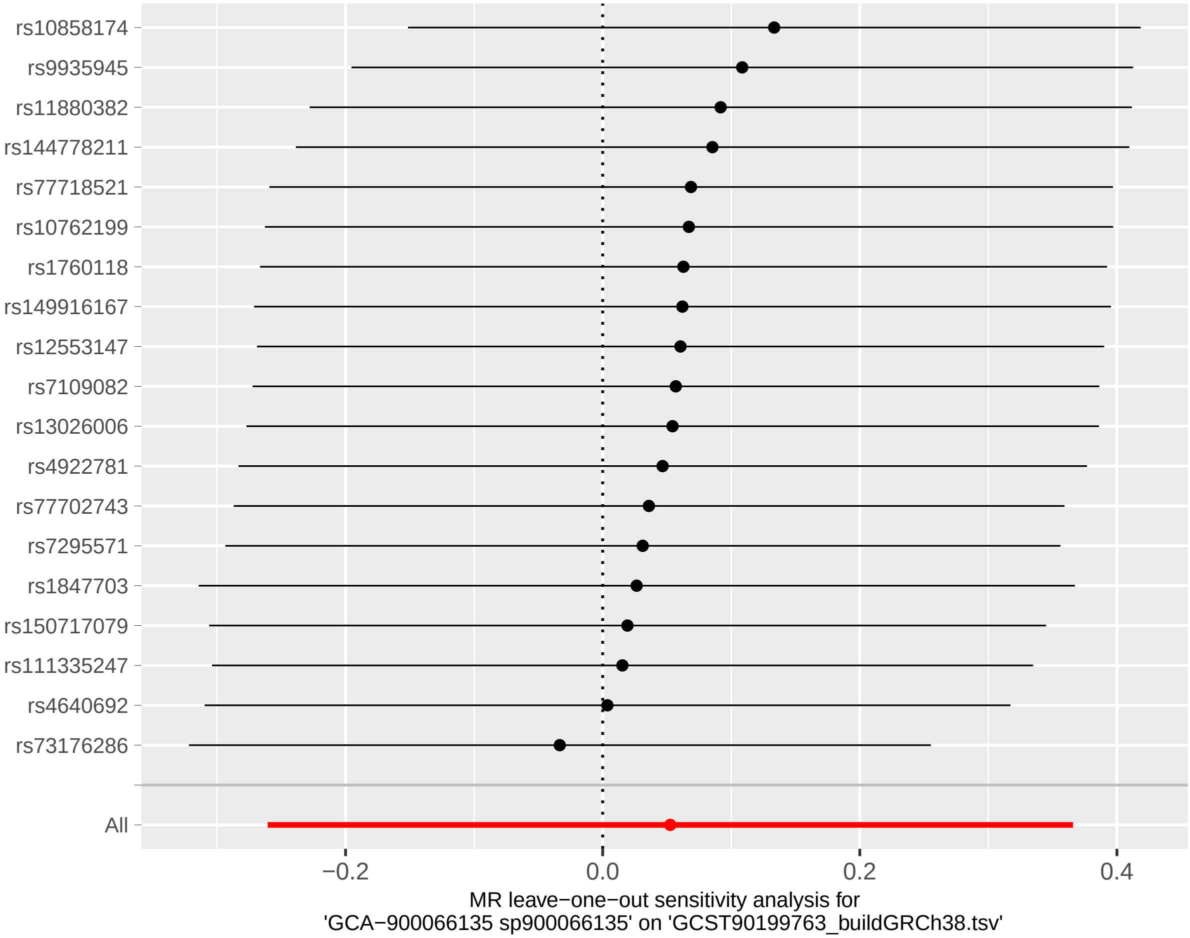
rs10858174
rs9935945
rs11880382
rs144778211
rs77718521
rs10762199
rs1760118
rs149916167
rs12553147
rs7109082
rs13026006
rs4922781
rs77702743
rs7295571
rs1847703
rs150717079
rs111335247
rs4640692
rs73176286
All
−0.2
0.0
0.2
0.4
MR leave−one−out sensitivity analysis for
'GCA−900066135 sp900066135' on 'GCST90199763_buildGRCh38.tsv'

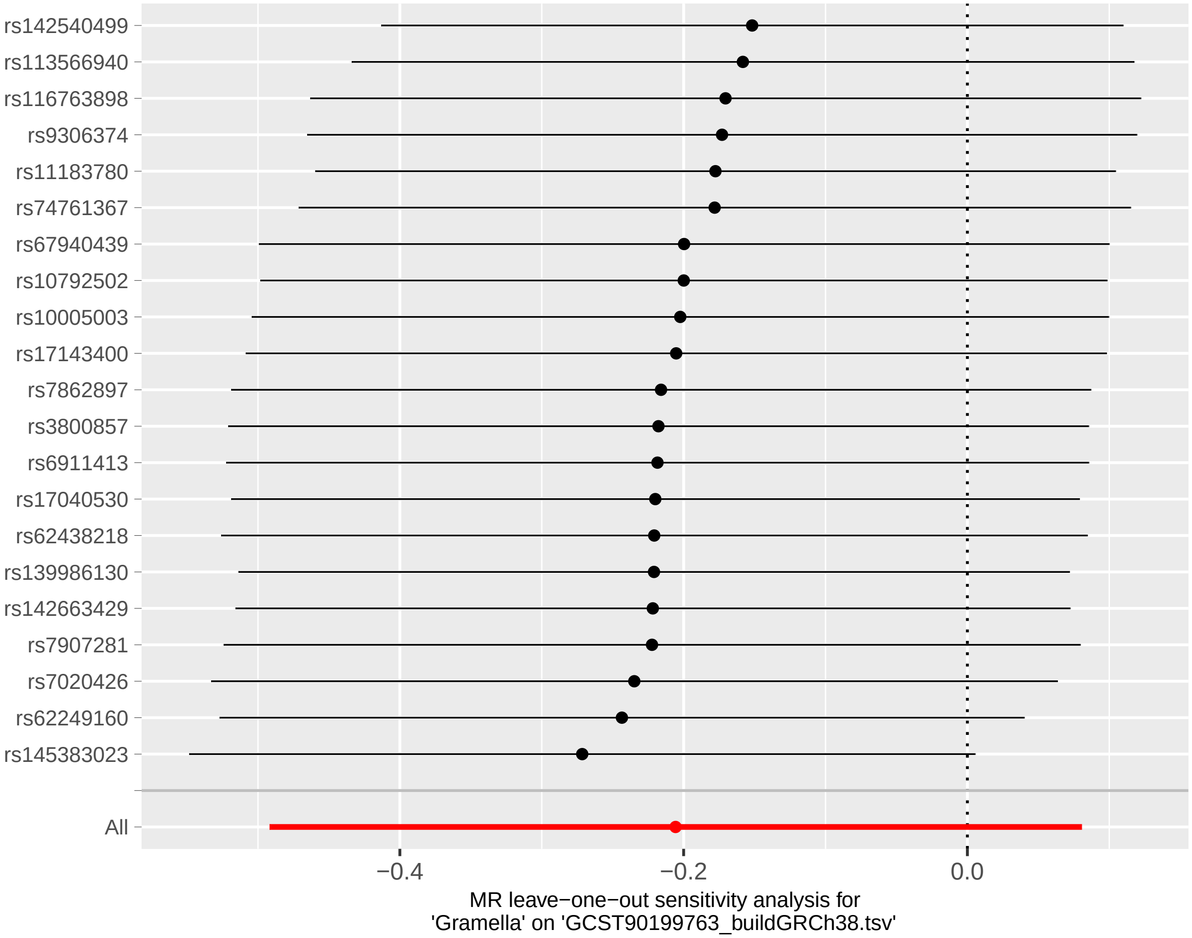

rs142540499
rs113566940
rs116763898
rs9306374
rs11183780
rs74761367
rs67940439
rs10792502
rs10005003
rs17143400
rs7862897
rs3800857
rs6911413
rs17040530
rs62438218
rs139986130
rs142663429
rs7907281
rs7020426
rs62249160
rs145383023
All
−0.4
−0.2
0.0
MR leave−one−out sensitivity analysis for
'Gramella' on 'GCST90199763_buildGRCh38.tsv'

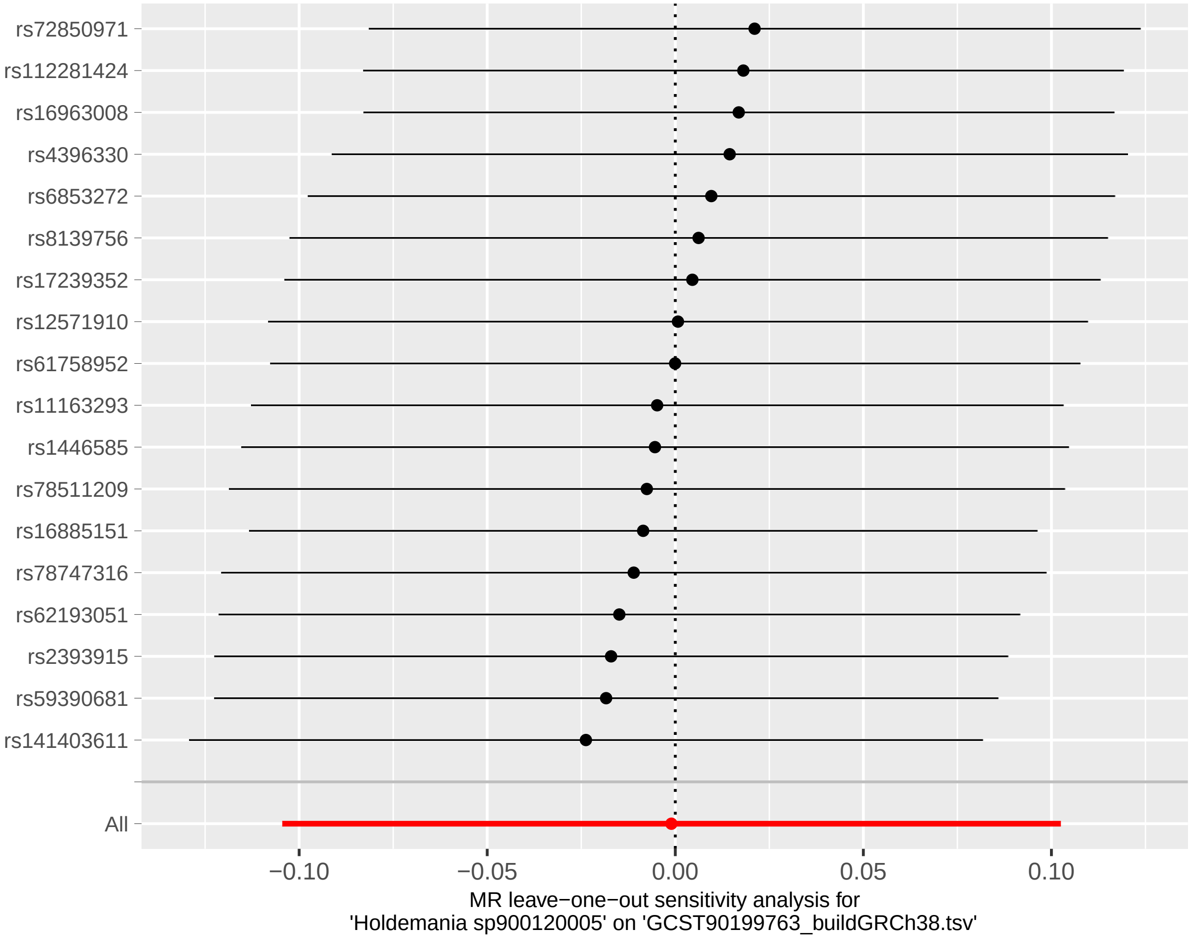
rs72850971
rs112281424
rs16963008
rs4396330
rs6853272
rs8139756
rs17239352
rs12571910
rs61758952
rs11163293
rs1446585
rs78511209
rs16885151
rs78747316
rs62193051
rs2393915
rs59390681
rs141403611
All
−0.10
−0.05
0.00
0.05
0.10
MR leave−one−out sensitivity analysis for
'Holdemania sp900120005' on 'GCST90199763_buildGRCh38.tsv'

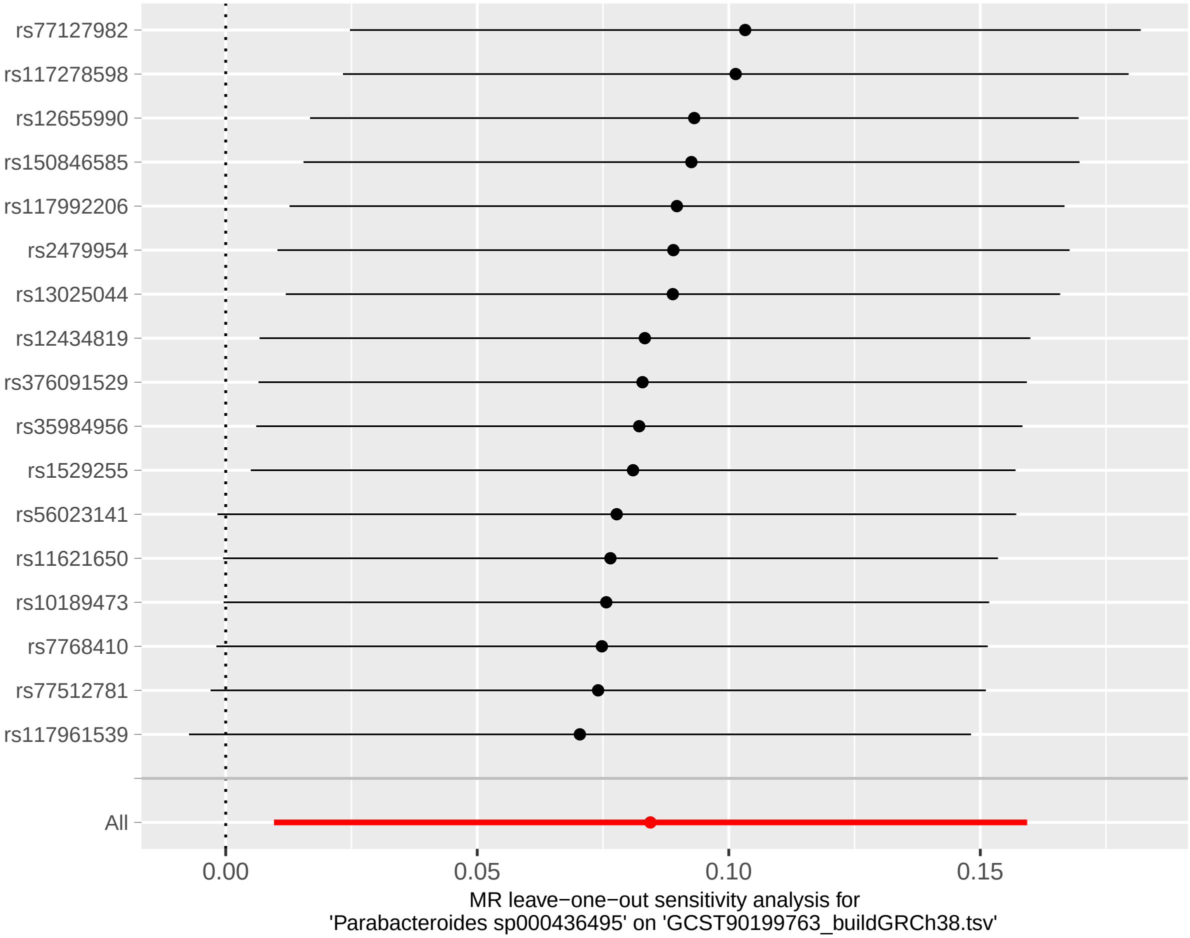
rs77127982
rs117278598
rs12655990
rs150846585
rs117992206
rs2479954
rs13025044
rs12434819
rs376091529
rs35984956
rs1529255
rs56023141
rs11621650
rs10189473
rs7768410
rs77512781
rs117961539
All
0.00
0.05
0.10
0.15
MR leave−one−out sensitivity analysis for
'Parabacteroides sp000436495' on 'GCST90199763_buildGRCh38.tsv'

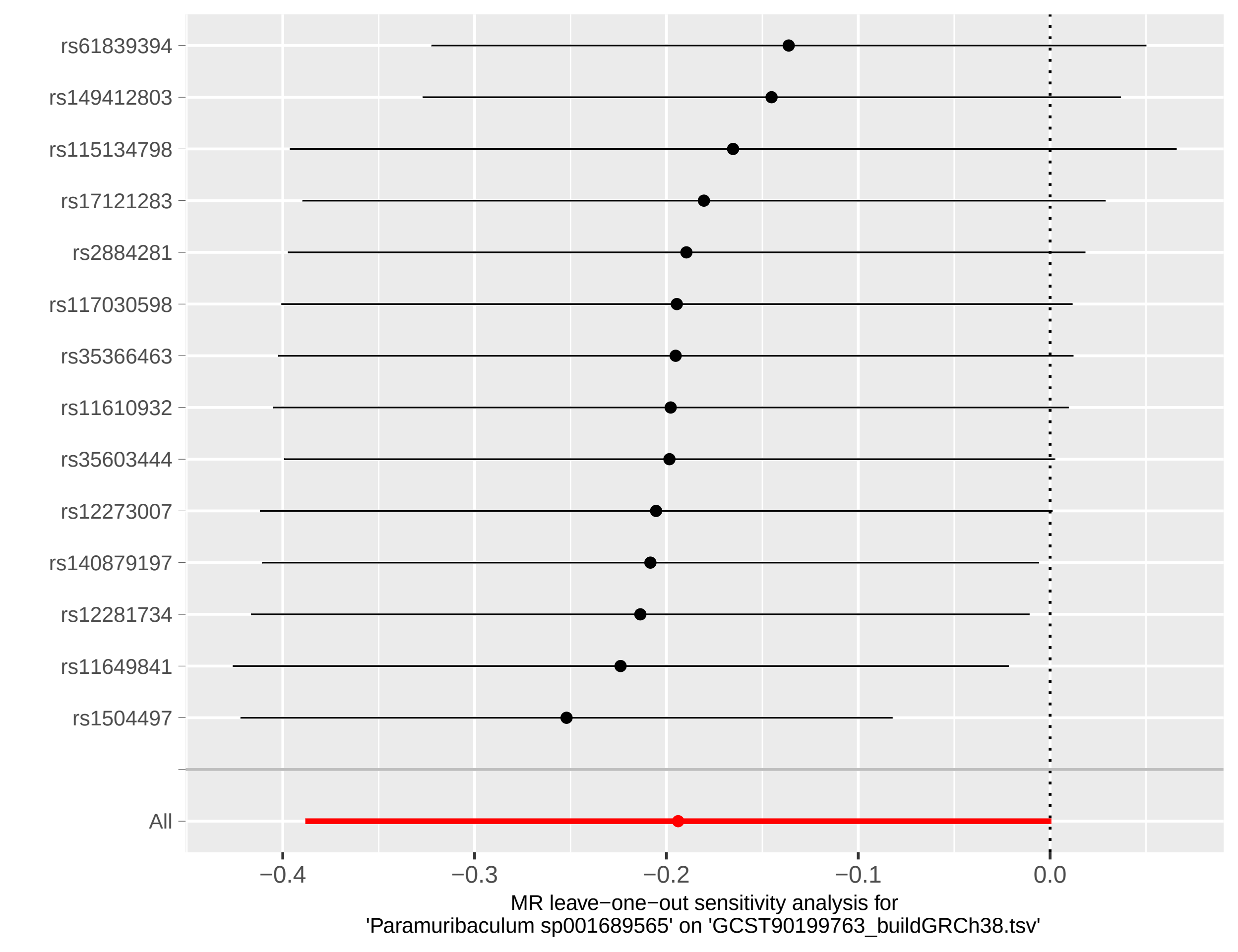
rs61839394
rs149412803
rs115134798
rs17121283
rs2884281
rs117030598
rs35366463
rs11610932
rs35603444
rs12273007
rs140879197
rs12281734
rs11649841
rs1504497
All
−0.4
−0.3
−0.2
−0.1
0.0
MR leave−one−out sensitivity analysis for
'Paramuribaculum sp001689565' on 'GCST90199763_buildGRCh38.tsv'

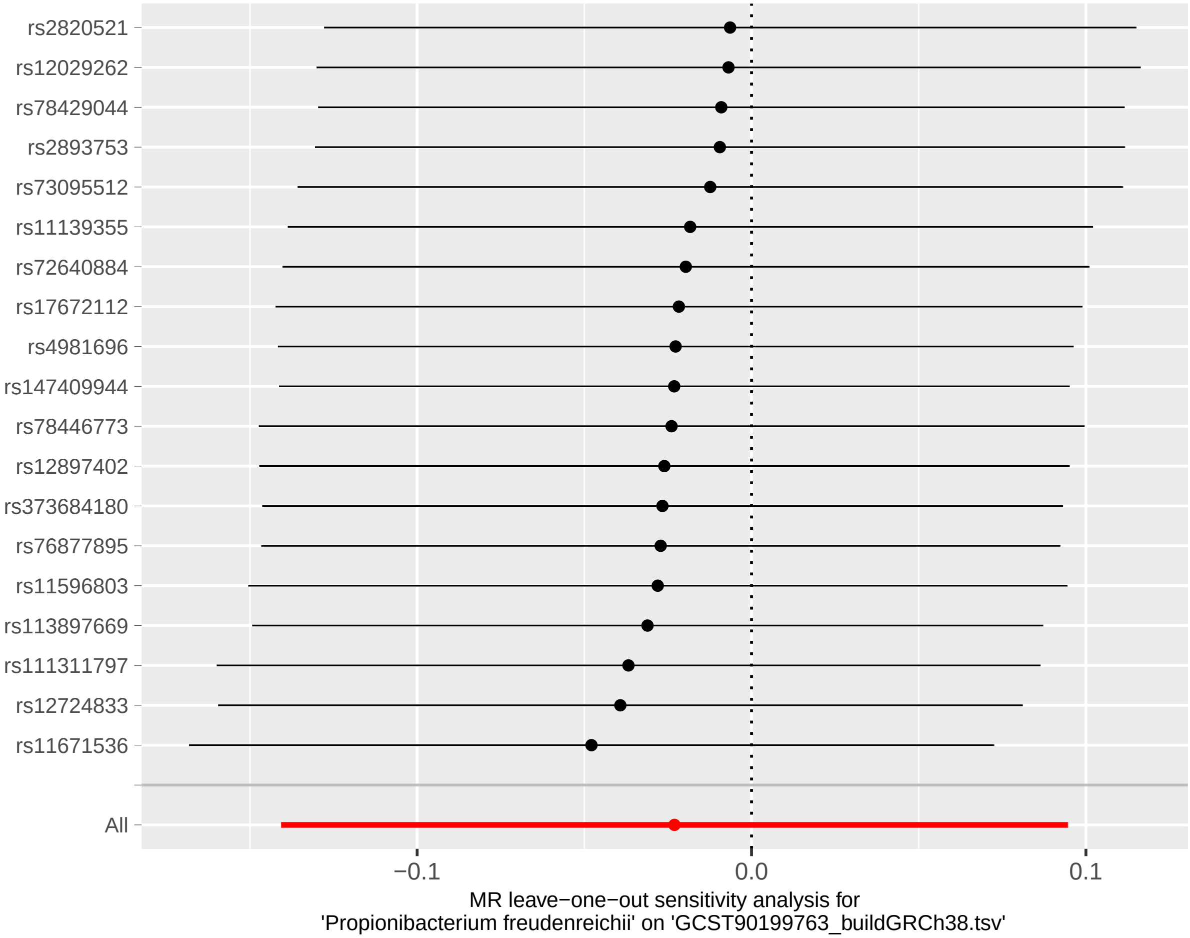

rs2820521
rs12029262
rs78429044
rs2893753
rs73095512
rs11139355
rs72640884
rs17672112
rs4981696
rs147409944
rs78446773
rs12897402
rs373684180
rs76877895
rs11596803
rs113897669
rs111311797
rs12724833
rs11671536
All
−0.1
0.0
0.1
MR leave−one−out sensitivity analysis for
'Propionibacterium freudenreichii' on 'GCST90199763_buildGRCh38.tsv'

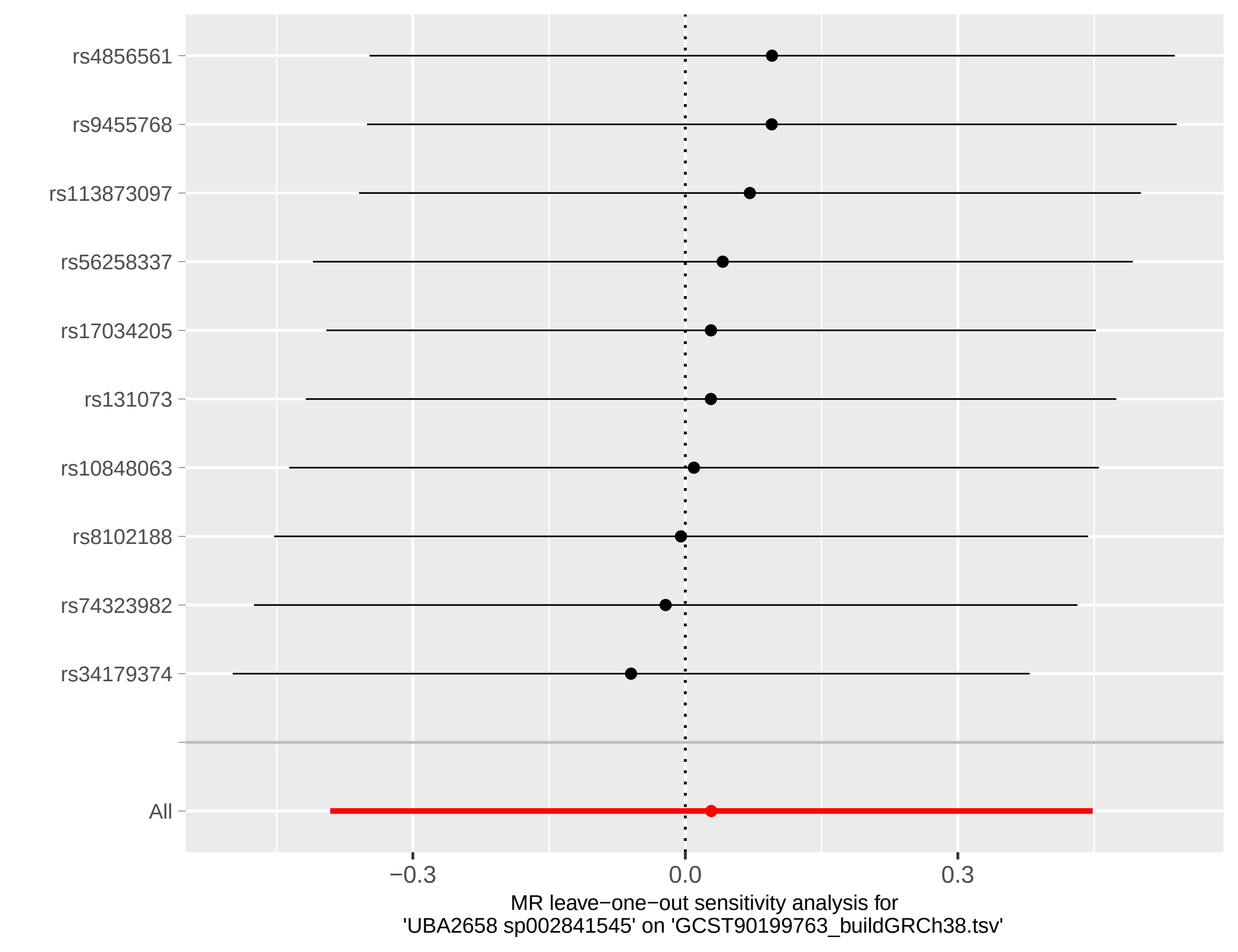
rs4856561
rs9455768
rs113873097
rs56258337
rs17034205
rs131073
rs10848063
rs8102188
rs74323982
rs34179374
All
−0.3
0.0
0.3
MR leave−one−out sensitivity analysis for
'UBA2658 sp002841545' on 'GCST90199763_buildGRCh38.tsv'

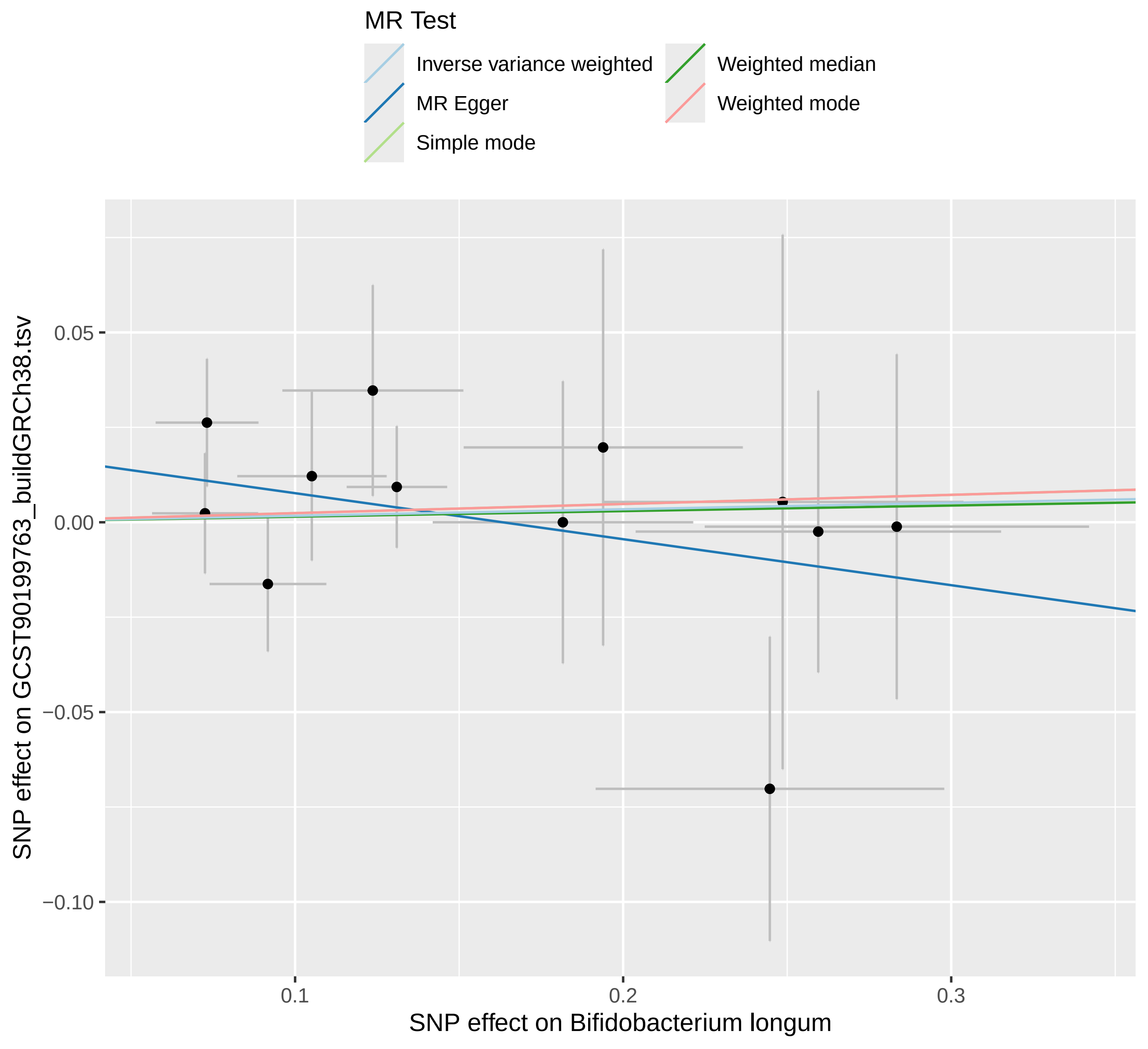

MR Test
Inverse variance weighted
MR Egger
Simple mode
Weighted median
Weighted mode
SNP effect on GCST90199763_buildGRCh38.tsv
0.05
0.00
−0.05
−0.10
0.1
0.2
0.3
SNP effect on Bifidobacterium longum

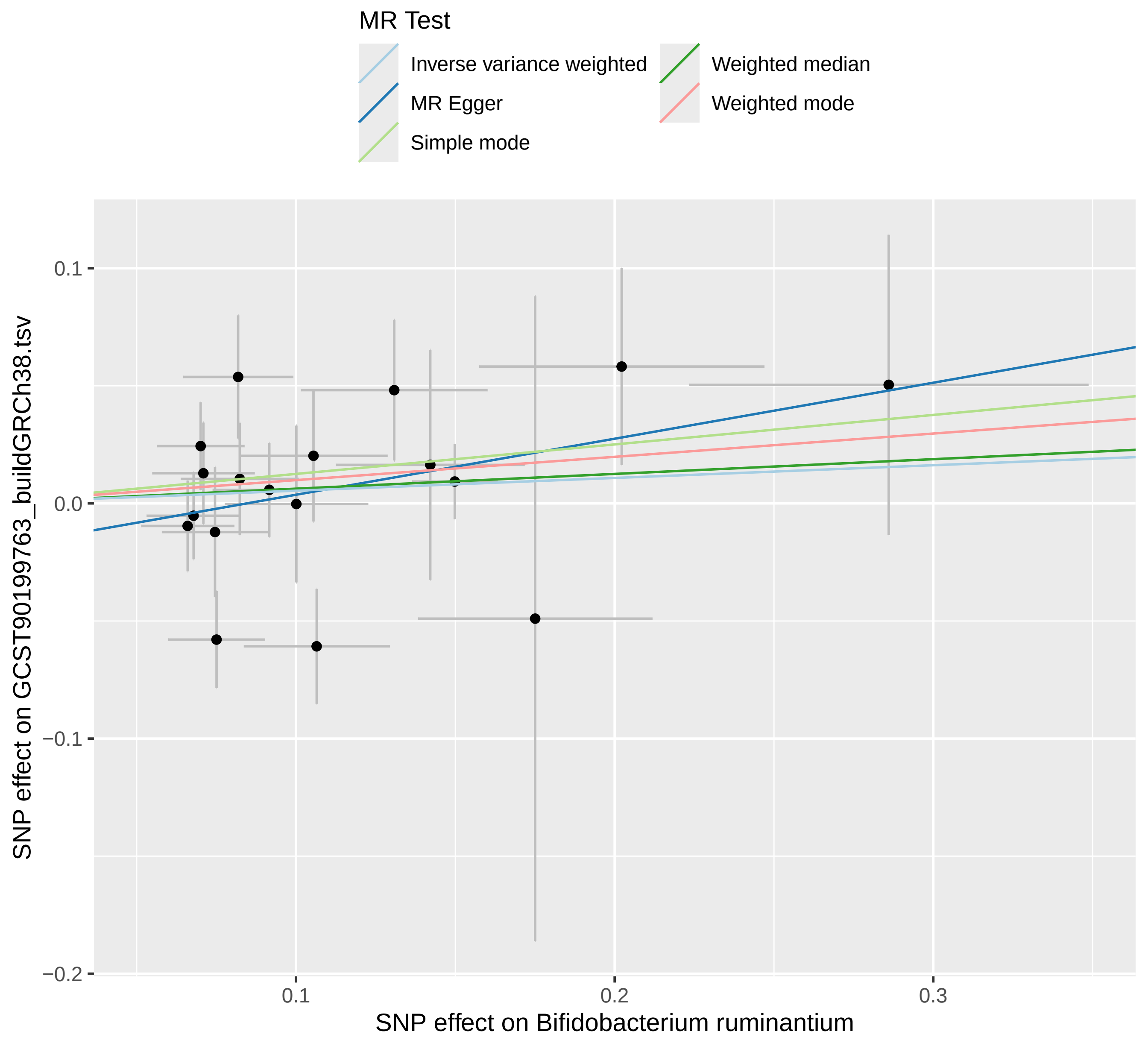
MR Test
Inverse variance weighted
MR Egger
Simple mode
Weighted median
Weighted mode
0.1
0.0
−0.1
−0.2
0.1
0.2
0.3
SNP effect on GCST90199763_buildGRCh38.tsv
SNP effect on Bifidobacterium ruminantium

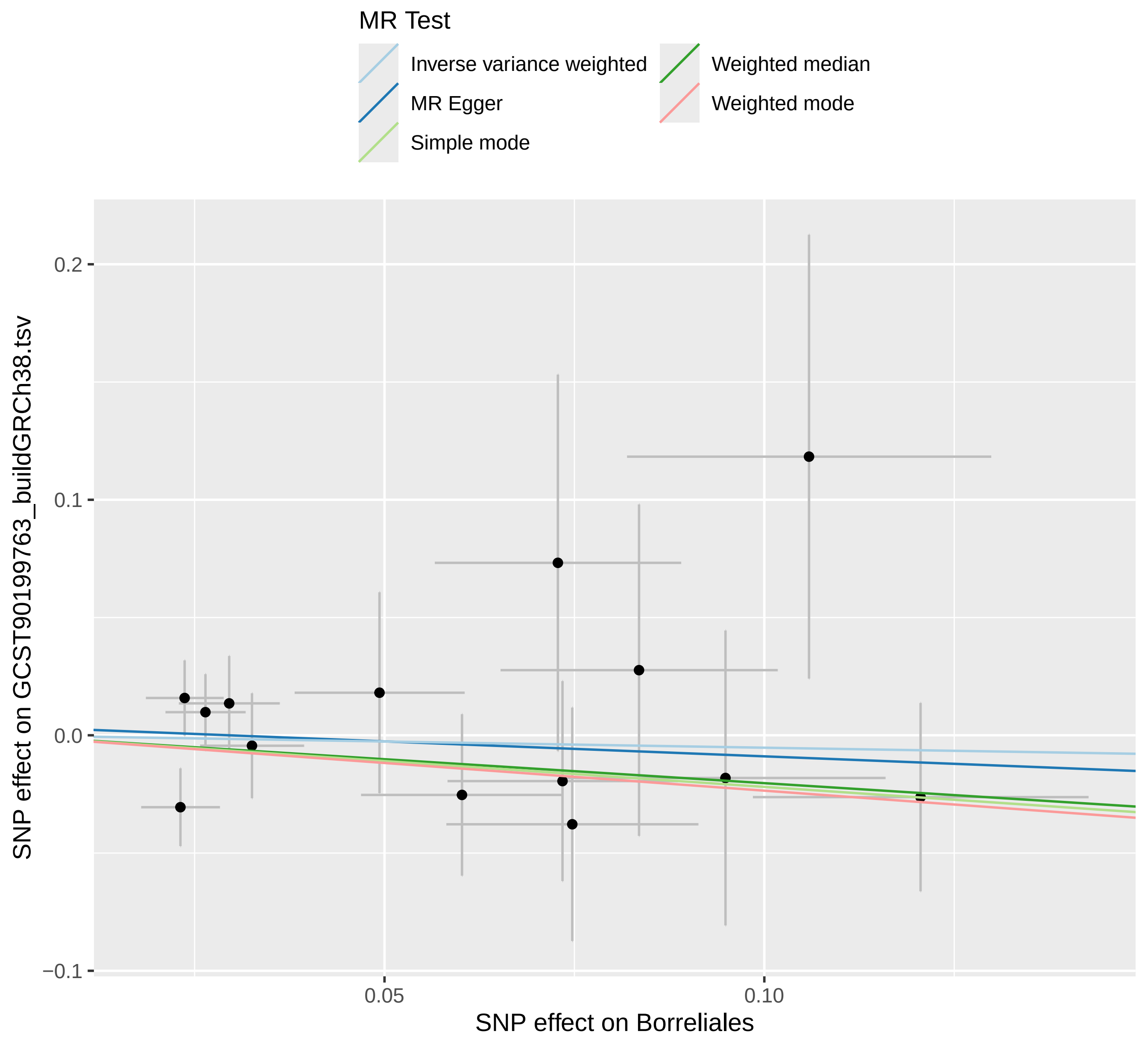
MR Test
Inverse variance weighted
MR Egger
Simple mode
Weighted median
Weighted mode
SNP effect on GCST90199763_buildGRCh38.tsv
0.2
0.1
0.0
−0.1
0.05
0.10
SNP effect on Borreliales

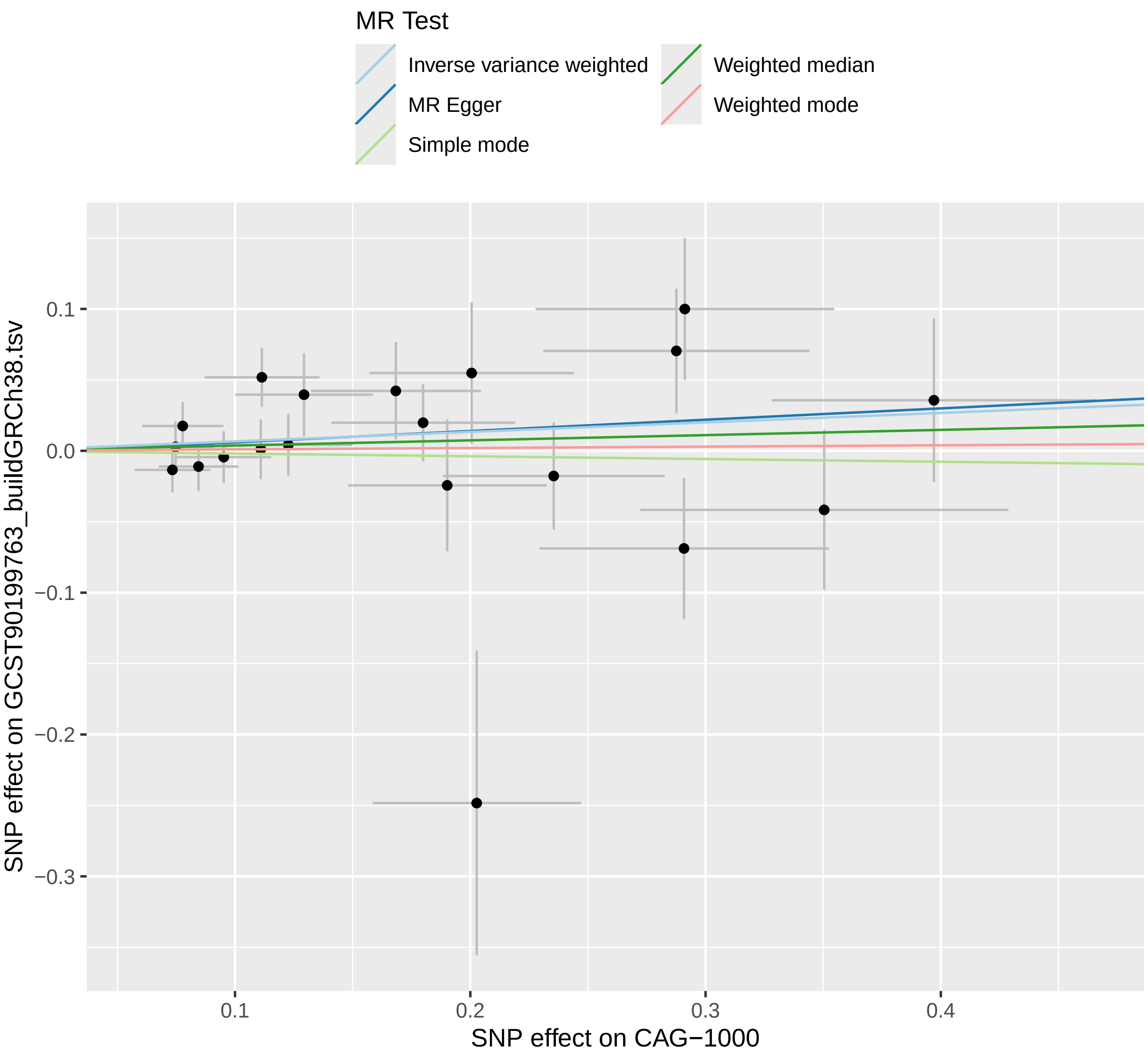
MR Test
Inverse variance weighted
MR Egger
Simple mode
Weighted median
Weighted mode
0.1
0.0
−0.1
−0.2
−0.3
SNP effect on GCST90199763_buildGRCh38.tsv
0.1
0.2
0.3
0.4
SNP effect on CAG−1000

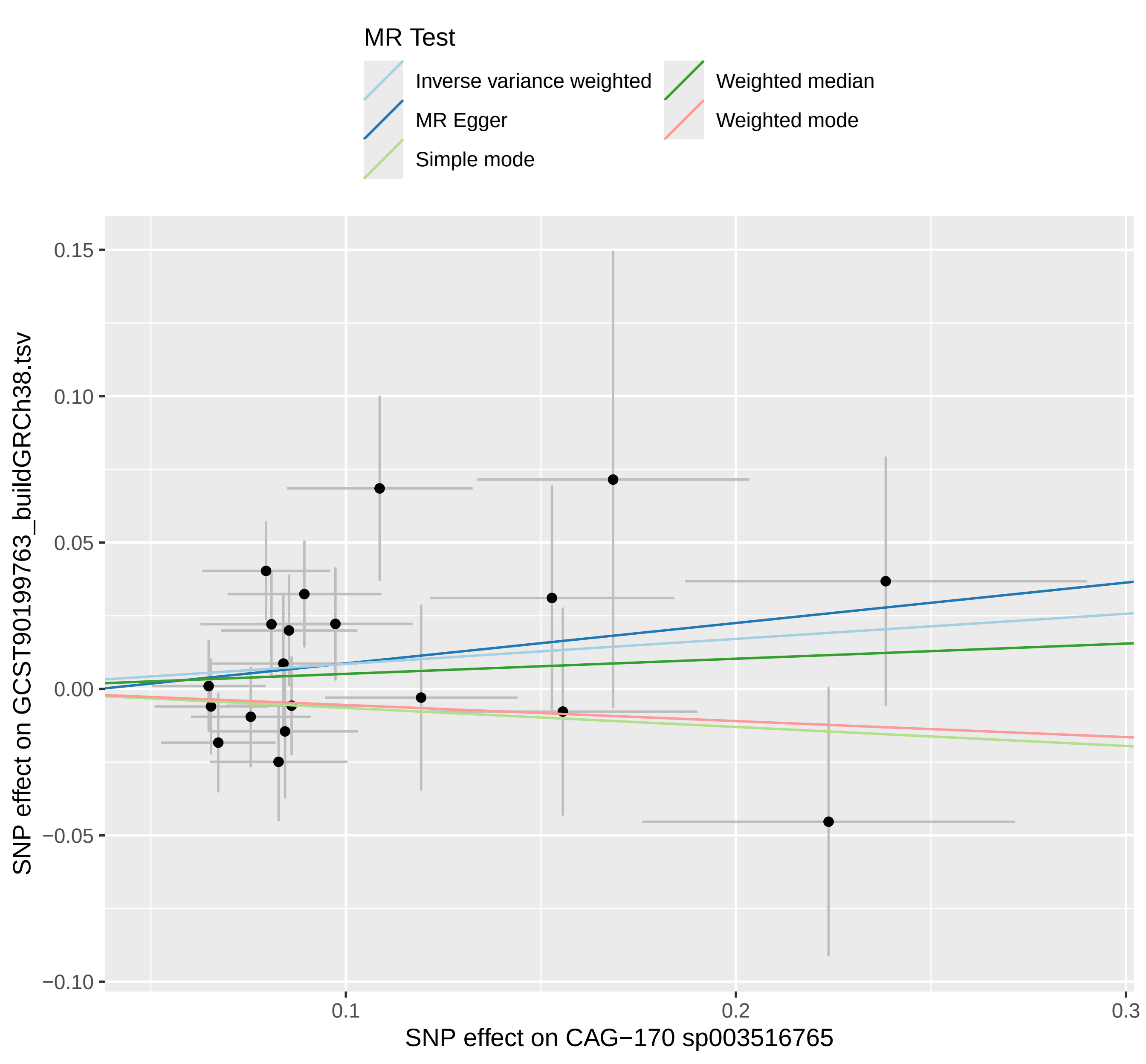

MR Test
Inverse variance weighted
MR Egger
Simple mode
Weighted median
Weighted mode
SNP effect on GCST90199763_buildGRCh38.tsv
0.15
0.10
0.05
0.00
−0.05
−0.10
0.1
0.2
0.3
SNP effect on CAG−170 sp003516765

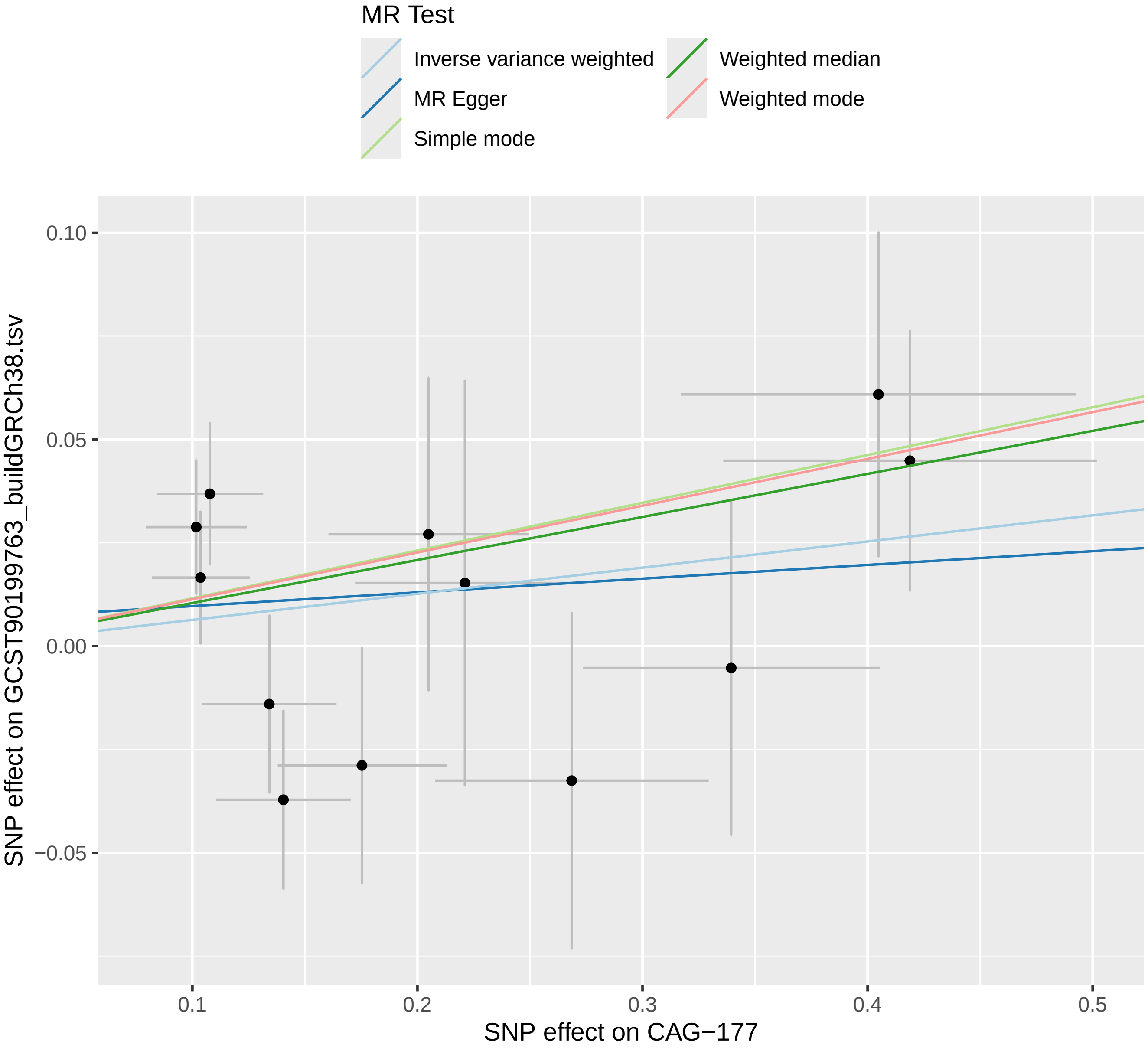

MR Test
Inverse variance weighted
MR Egger
Simple mode
Weighted median
Weighted mode
0.10
0.05
0.00
−0.05
0.1
0.2
0.3
0.4
0.5
SNP effect on GCST90199763_buildGRCh38.tsv
SNP effect on CAG−177

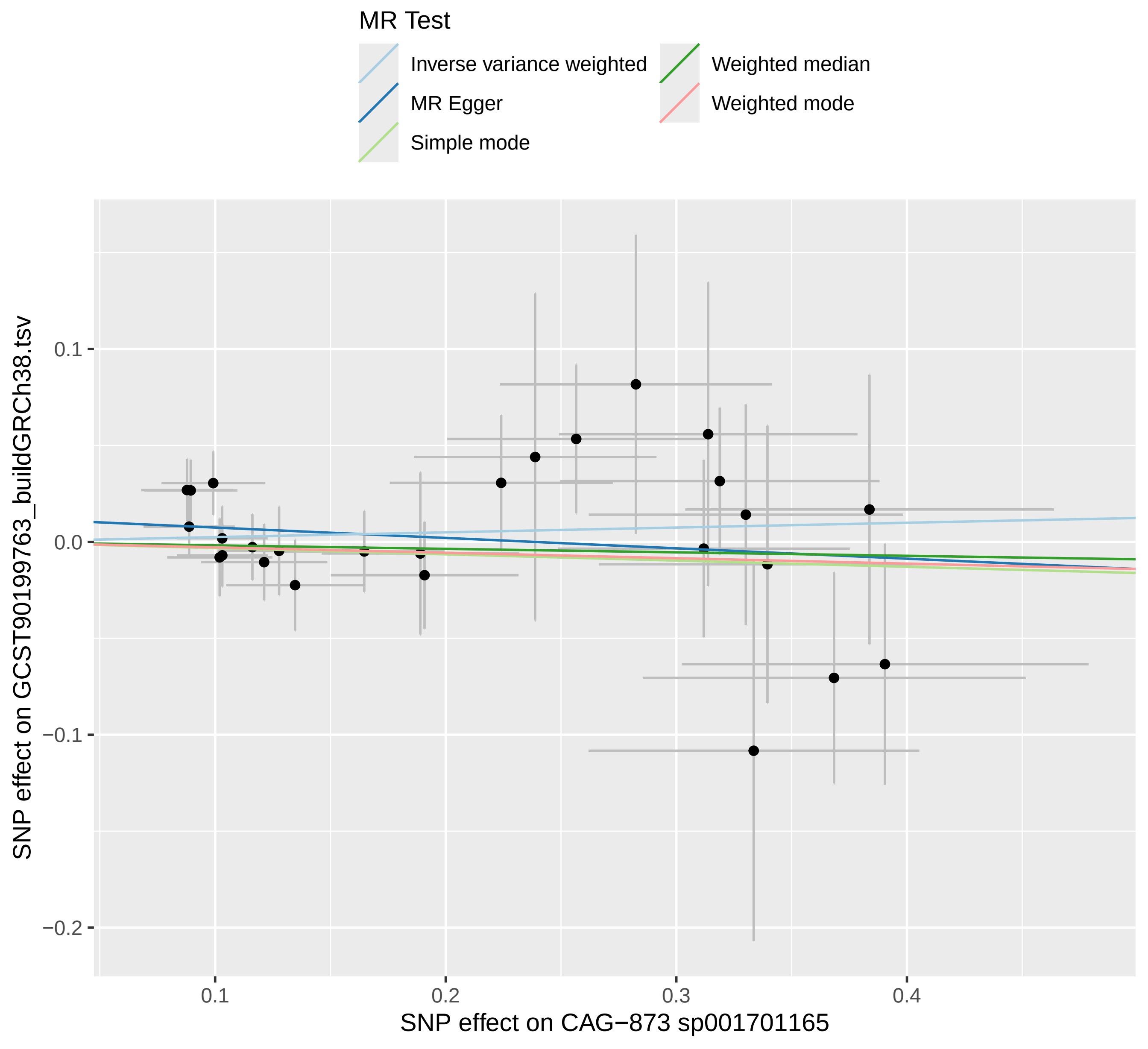

MR Test
Inverse variance weighted
MR Egger
Simple mode
Weighted median
Weighted mode
0.1
0.0
−0.1
−0.2
0.1
0.2
0.3
0.4
SNP effect on GCST90199763_buildGRCh38.tsv
SNP effect on CAG−873 sp001701165

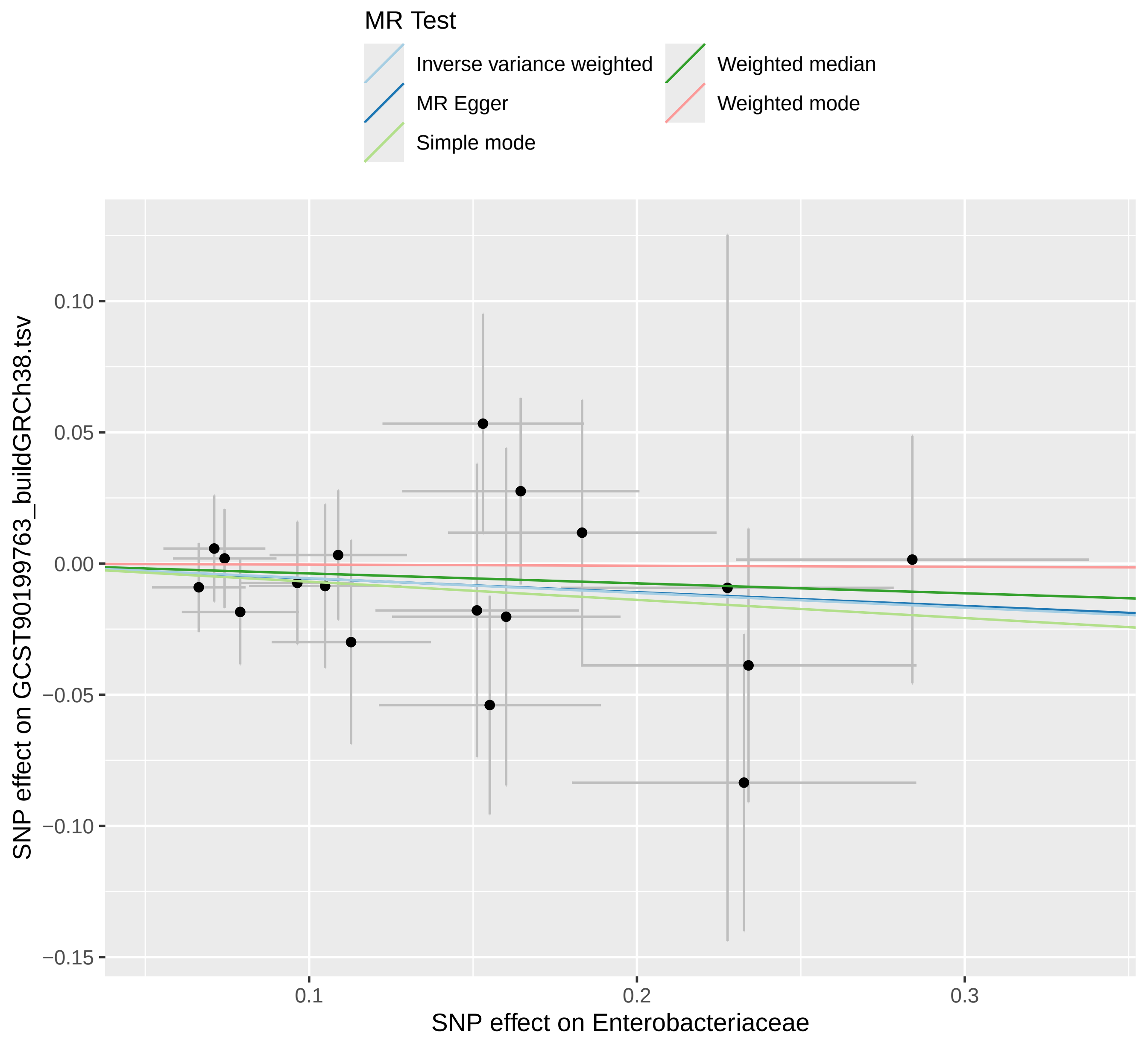
MR Test
Inverse variance weighted
MR Egger
Simple mode
Weighted median
Weighted mode
SNP effect on GCST90199763_buildGRCh38.tsv
0.10
0.05
0.00
−0.05
−0.10
−0.15
0.1
0.2
0.3
SNP effect on Enterobacteriaceae

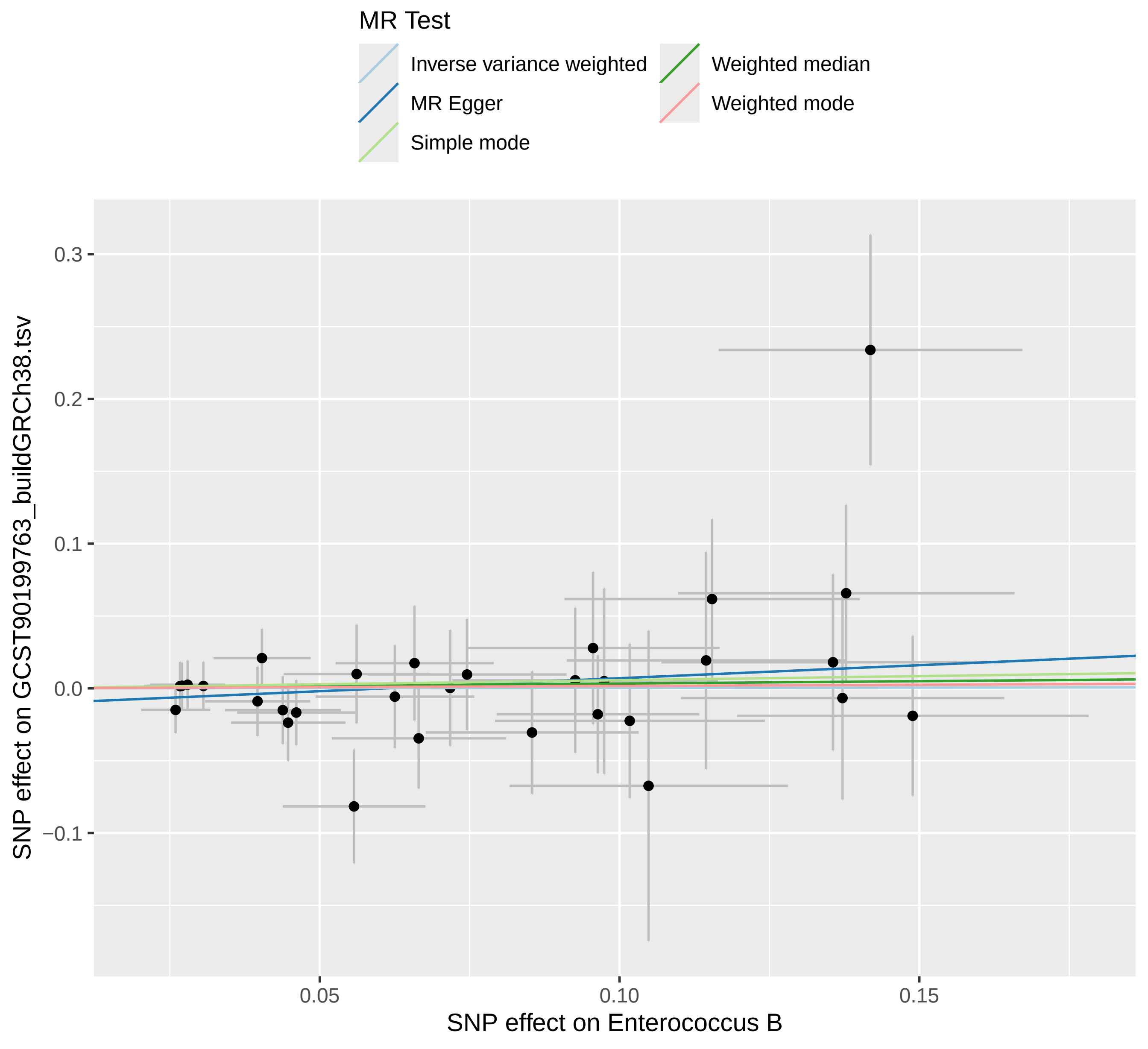
MR Test
Inverse variance weighted
MR Egger
Simple mode
Weighted median
Weighted mode
0.3
0.2
0.1
0.0
−0.1
0.05
0.10
0.15
SNP effect on GCST90199763_buildGRCh38.tsv
SNP effect on Enterococcus B

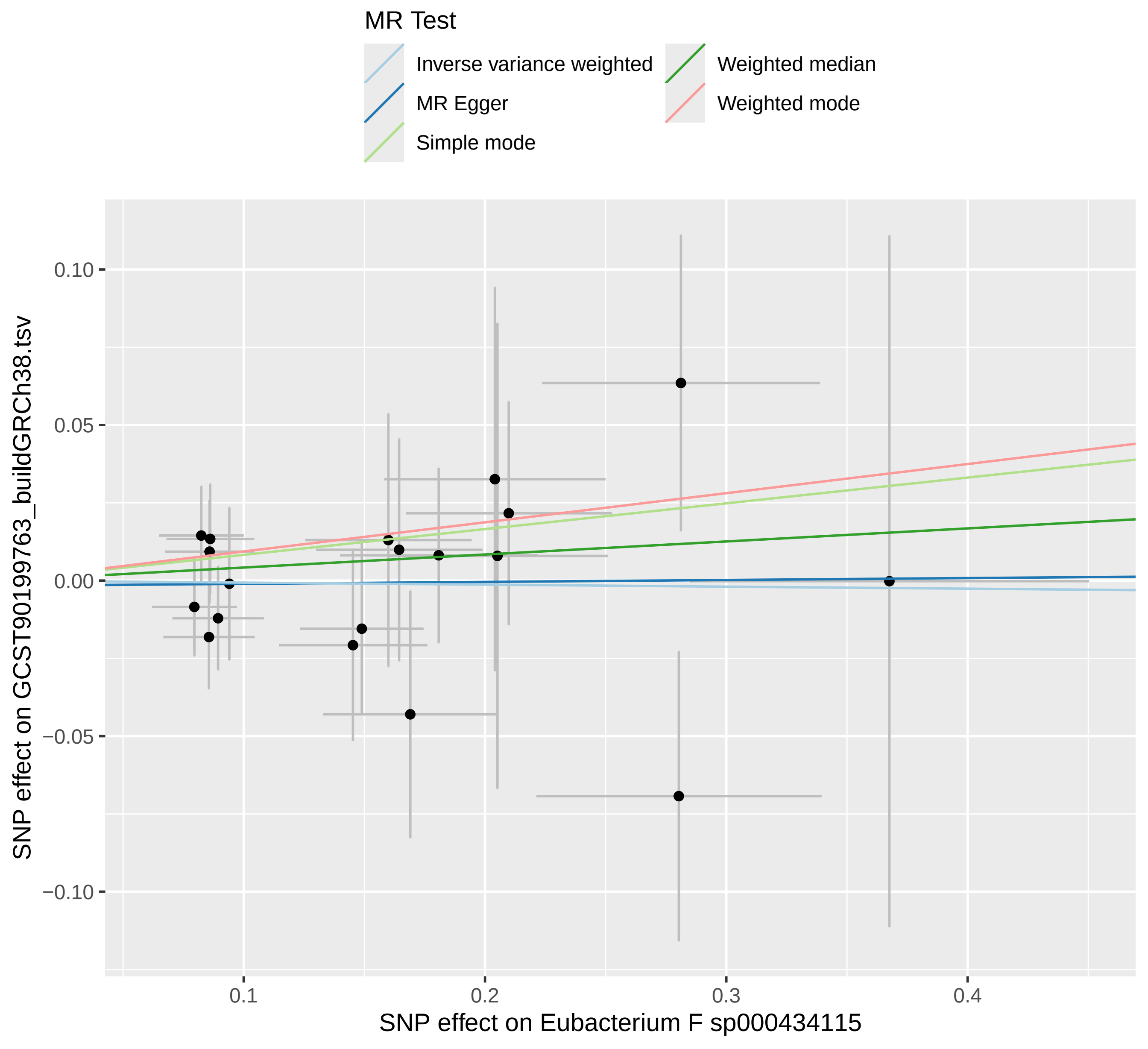

MR Test
Inverse variance weighted
MR Egger
Simple mode
Weighted median
Weighted mode
0.10
0.05
0.00
−0.05
−0.10
0.1
0.2
0.3
0.4
SNP effect on GCST90199763_buildGRCh38.tsv
SNP effect on Eubacterium F sp000434115

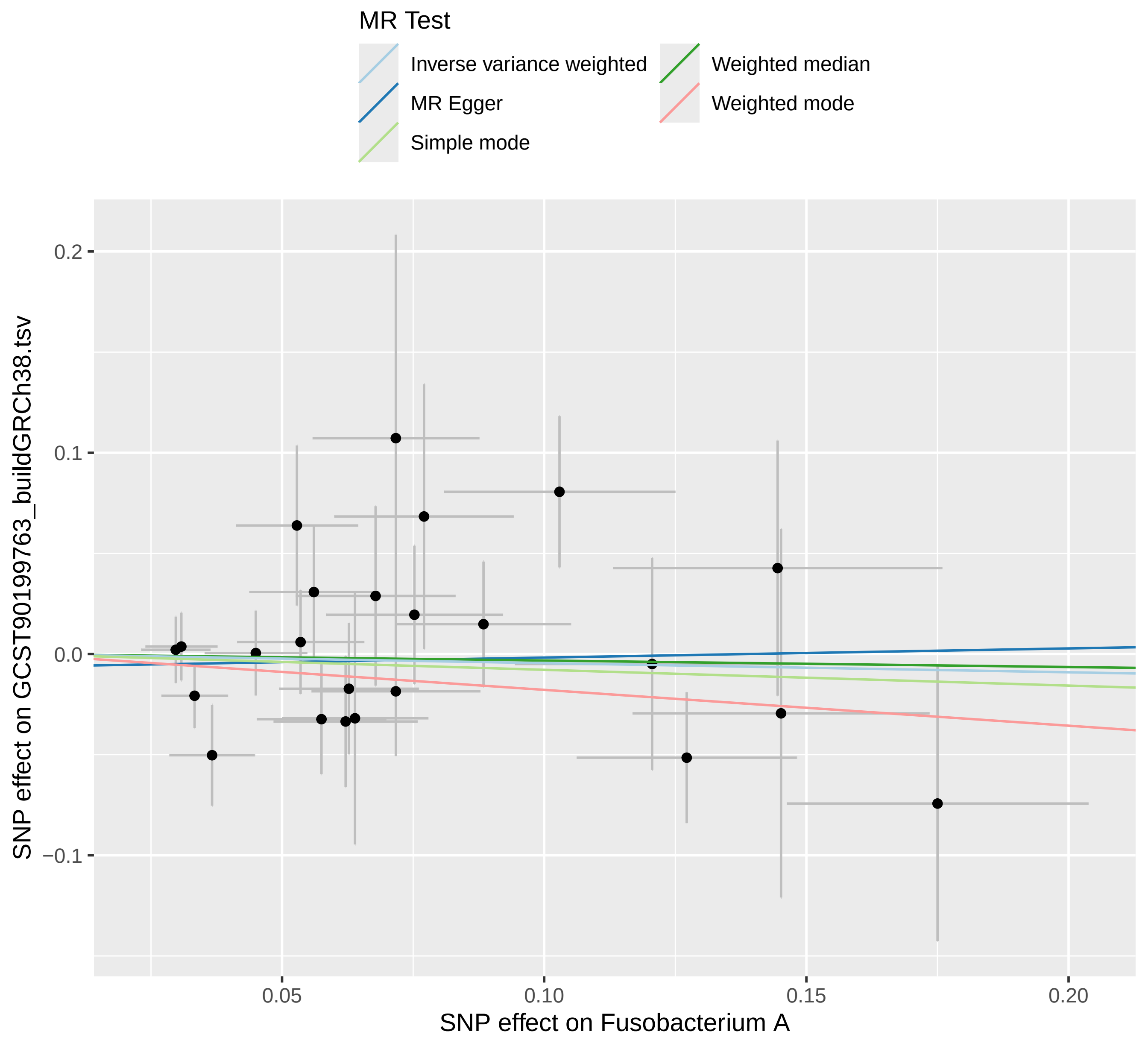

MR Test
Inverse variance weighted
MR Egger
Simple mode
Weighted median
Weighted mode
0.2
0.1
0.0
−0.1
0.05
0.10
0.15
0.20
SNP effect on GCST90199763_buildGRCh38.tsv
SNP effect on Fusobacterium A

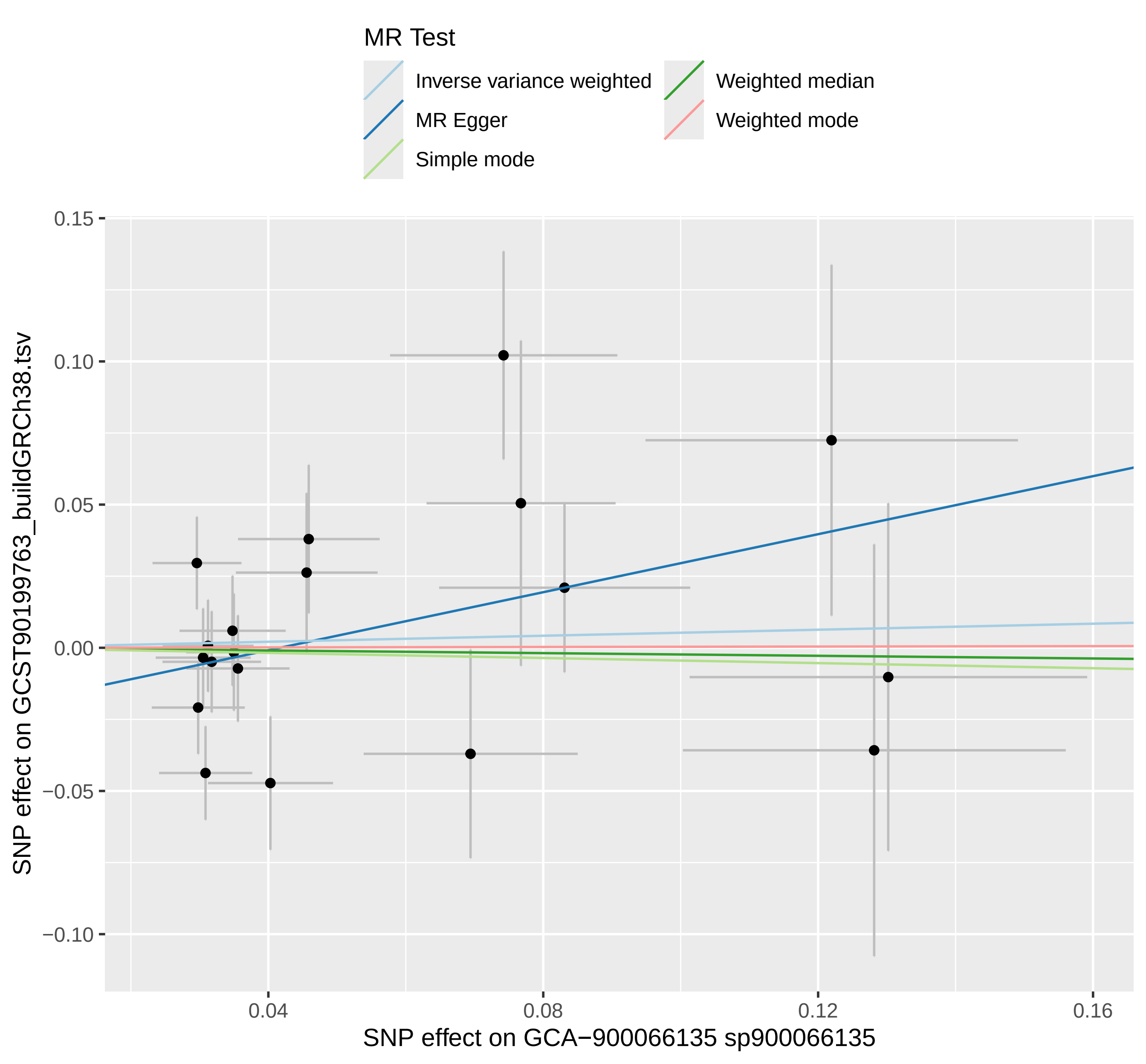

MR Test
Inverse variance weighted
MR Egger
Simple mode
Weighted median
Weighted mode
0.15
0.10
0.05
0.00
−0.05
−0.10
0.04
0.08
0.12
0.16
SNP effect on GCST90199763_buildGRCh38.tsv
SNP effect on GCA−900066135 sp900066135

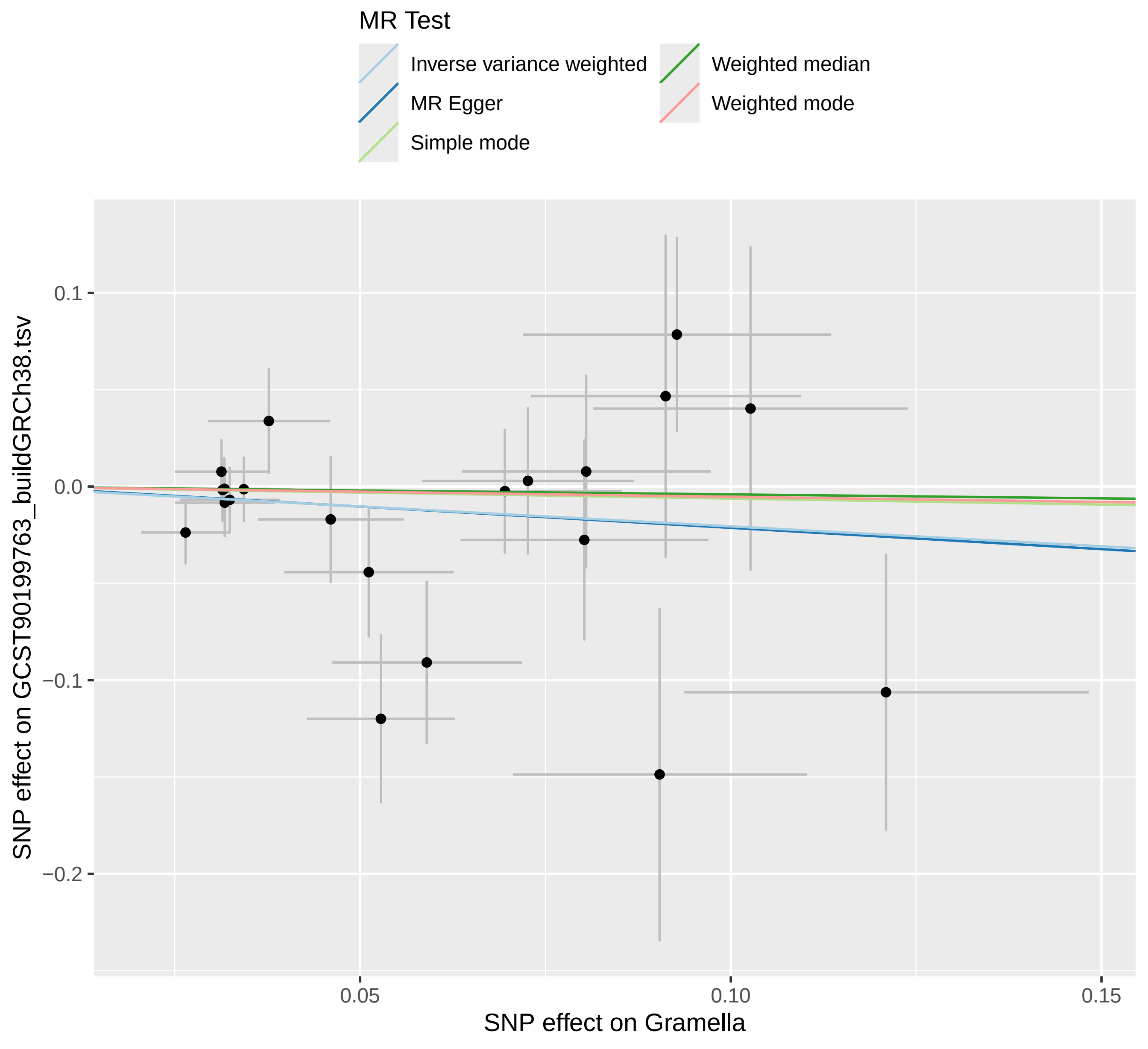
MR Test
Inverse variance weighted
MR Egger
Simple mode
Weighted median
Weighted mode
0.1
0.0
−0.1
−0.2
SNP effect on GCST90199763_buildGRCh38.tsv
0.05
0.10
0.15
SNP effect on Gramella

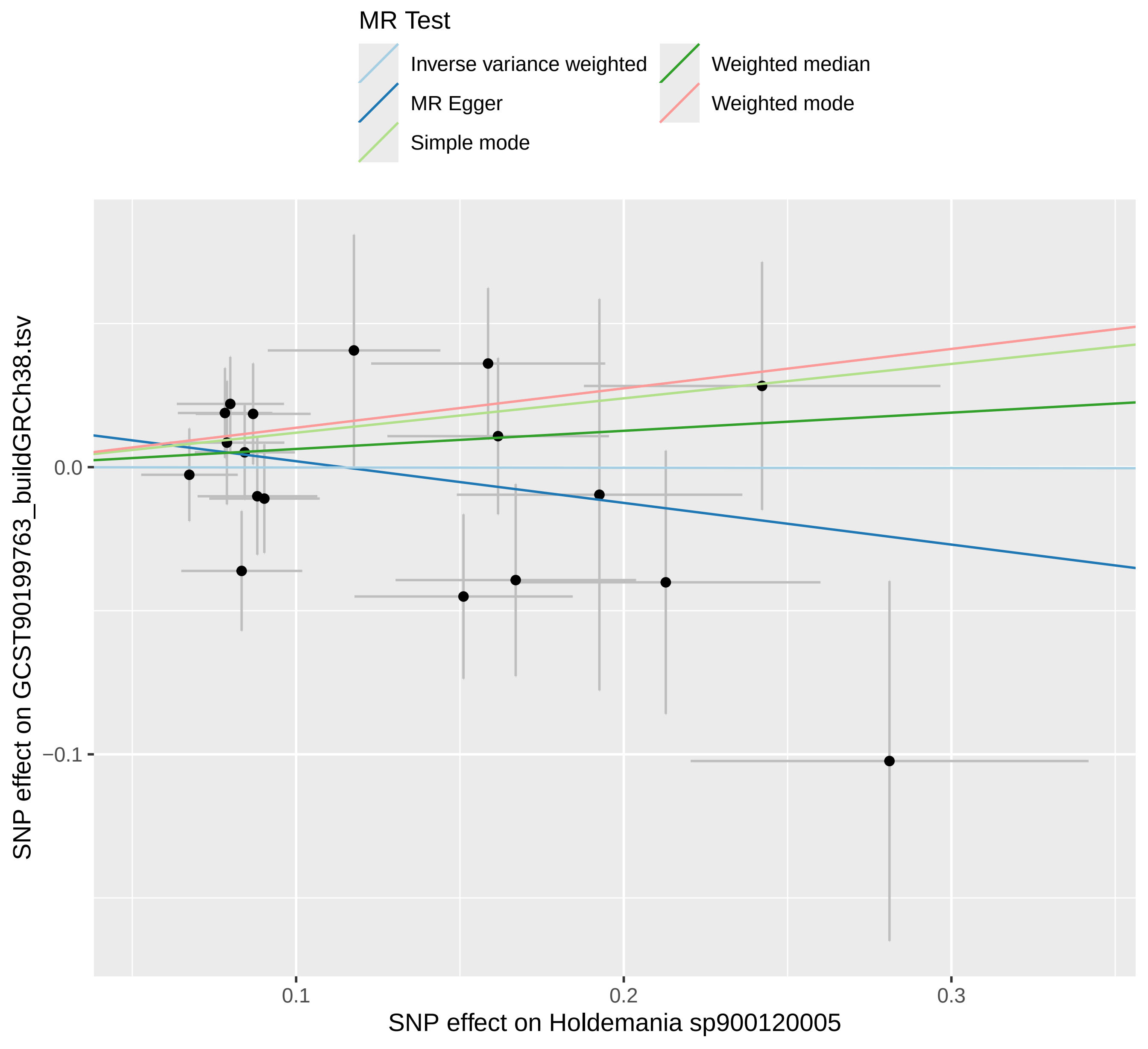
MR Test
Inverse variance weighted
MR Egger
Simple mode
Weighted median
Weighted mode
SNP effect on GCST90199763_buildGRCh38.tsv
0.0
−0.1
0.1
0.2
0.3
SNP effect on Holdemania sp900120005

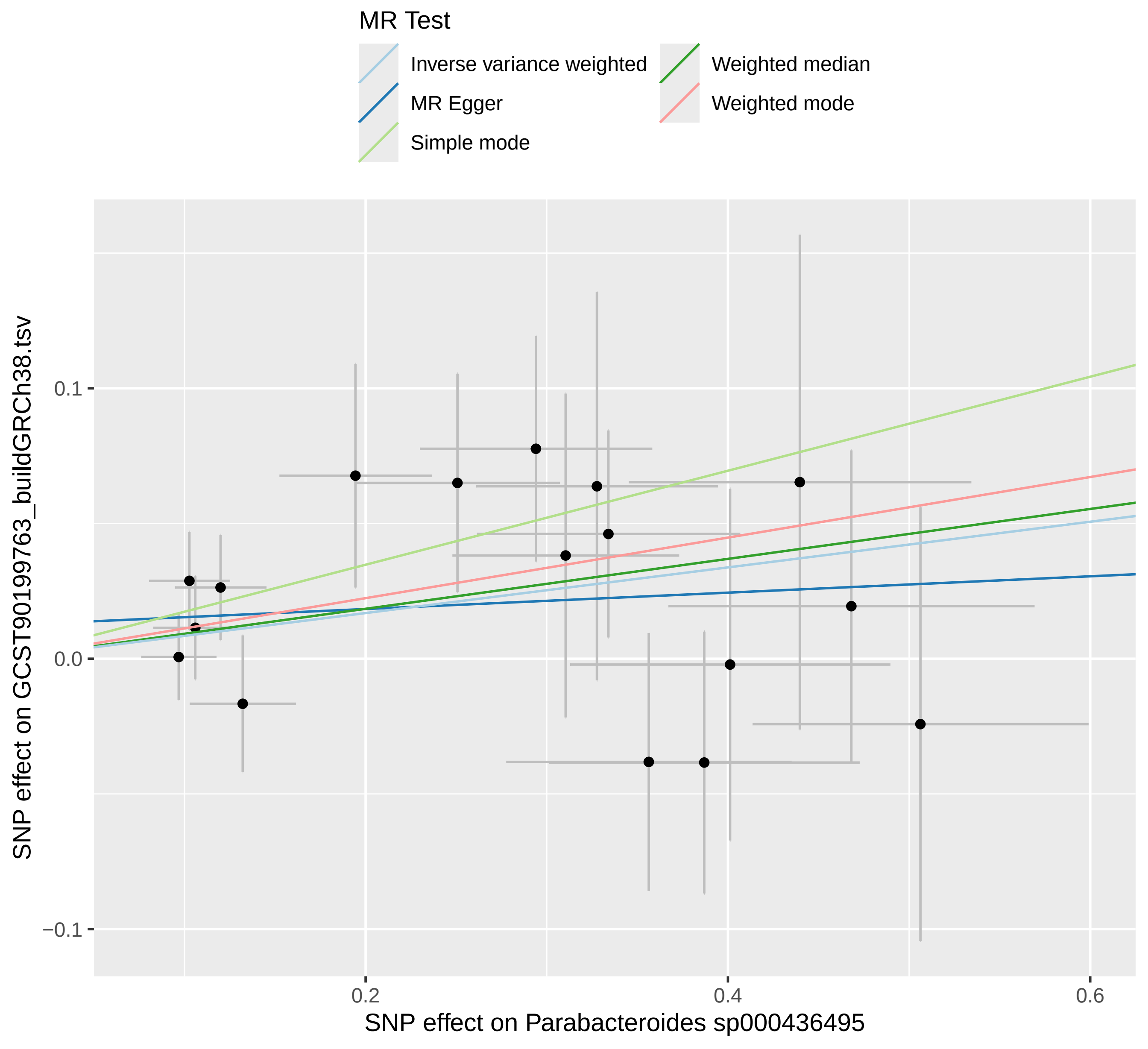

MR Test
Inverse variance weighted
MR Egger
Simple mode
Weighted median
Weighted mode
SNP effect on GCST90199763_buildGRCh38.tsv
0.1
0.0
−0.1
0.2
0.4
0.6
SNP effect on Parabacteroides sp000436495

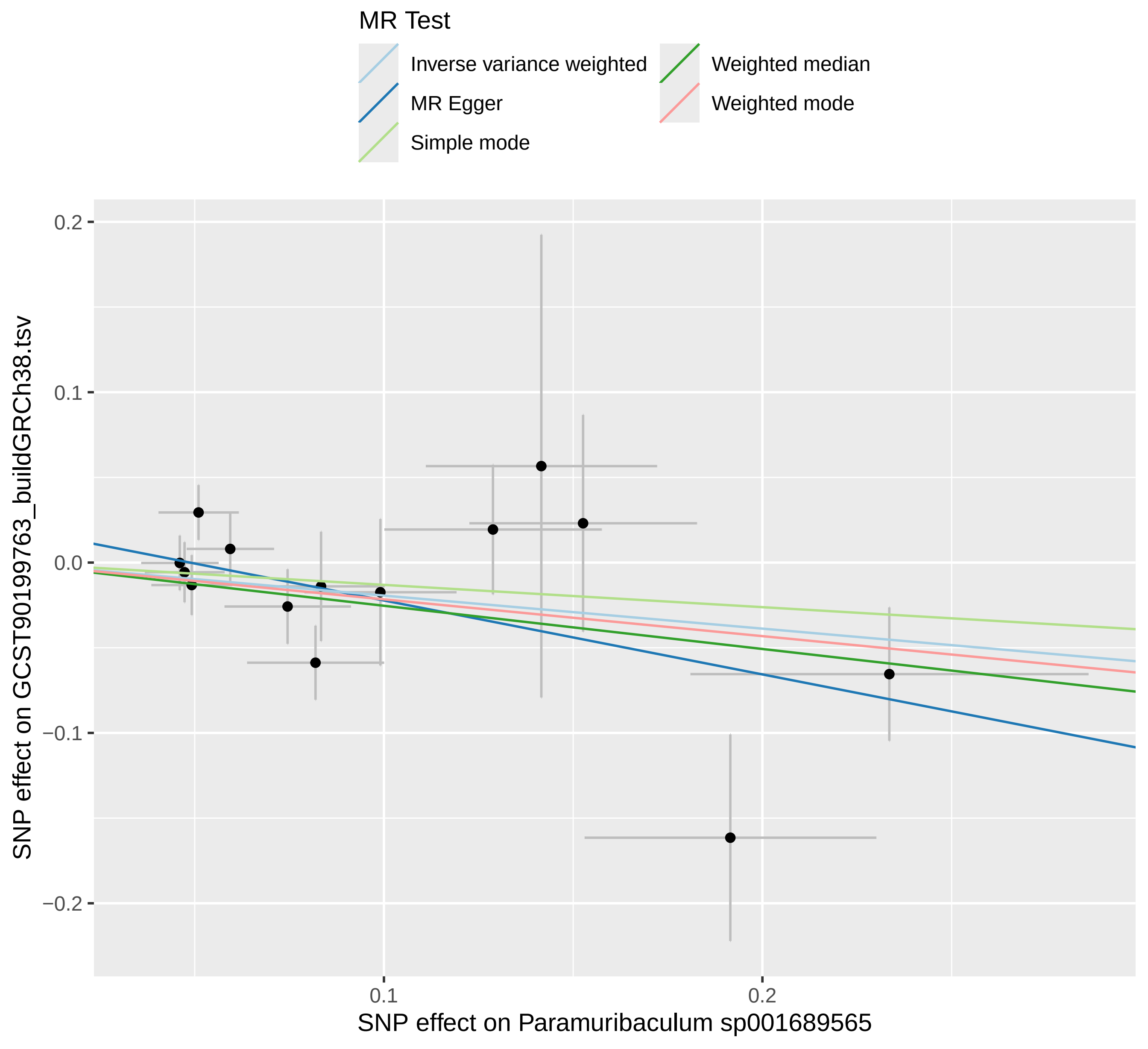
MR Test
Inverse variance weighted
MR Egger
Simple mode
Weighted median
Weighted mode
0.2
0.1
0.0
−0.1
−0.2
0.1
0.2
SNP effect on GCST90199763_buildGRCh38.tsv
SNP effect on Paramuribaculum sp001689565

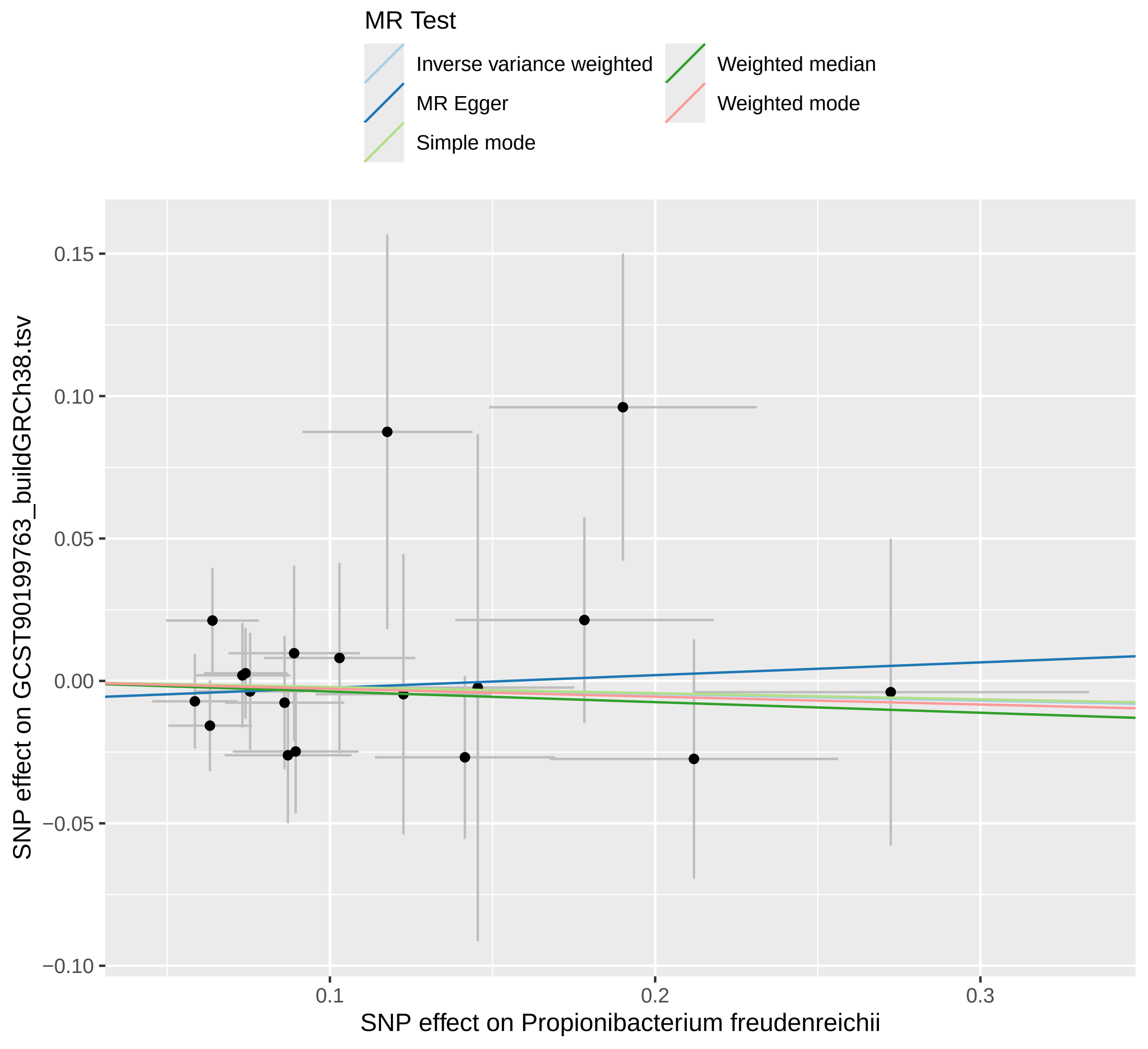
MR Test
Inverse variance weighted
MR Egger
Simple mode
Weighted median
Weighted mode
0.15
0.10
0.05
0.00
−0.05
−0.10
0.1
0.2
0.3
SNP effect on GCST90199763_buildGRCh38.tsv
SNP effect on Propionibacterium freudenreichii

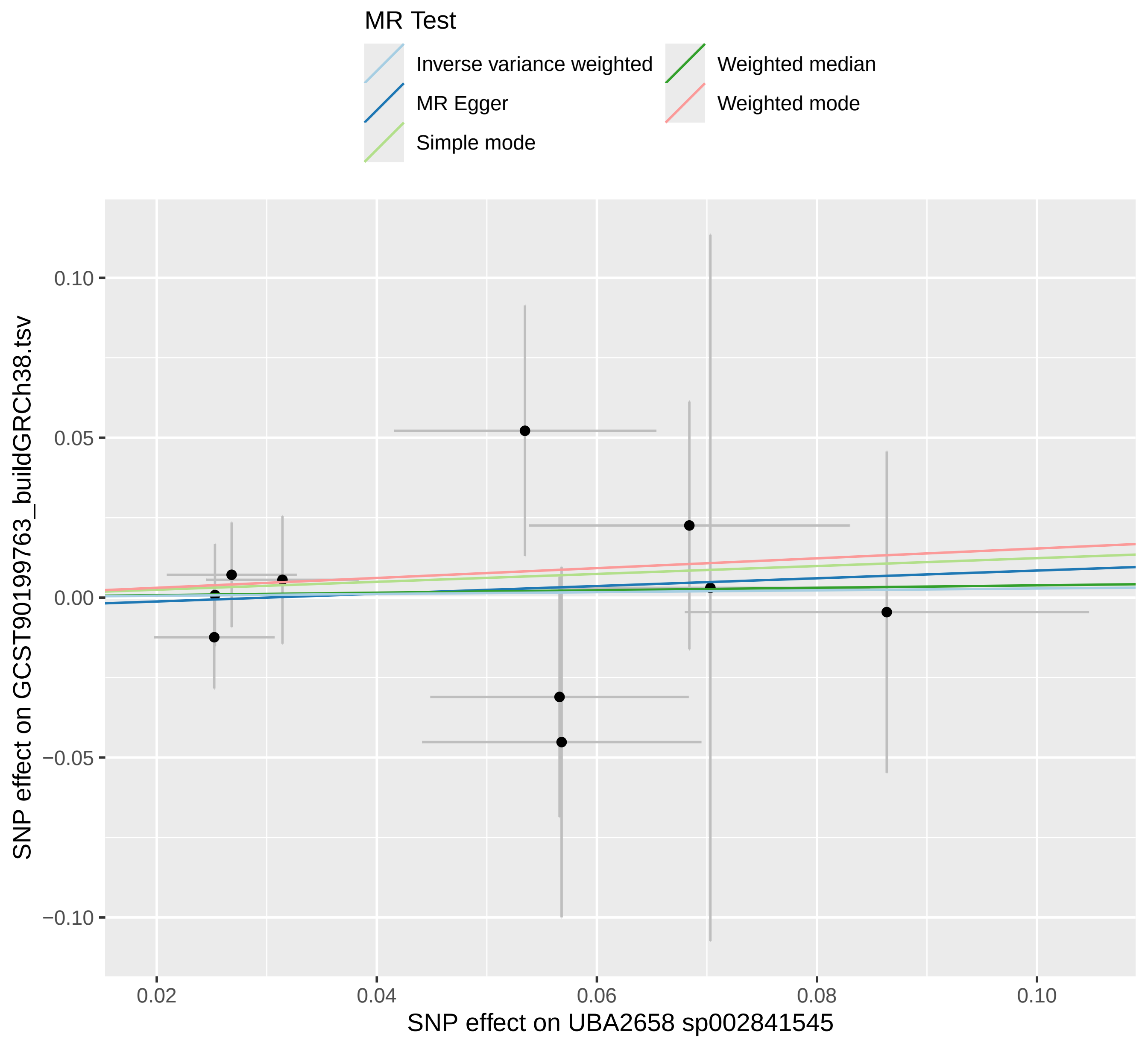

MR Test
Inverse variance weighted
MR Egger
Simple mode
Weighted median
Weighted mode
0.10
0.05
0.00
−0.05
−0.10
SNP effect on GCST90199763_buildGRCh38.tsv
0.02
0.04
0.06
0.08
0.10
SNP effect on UBA2658 sp002841545

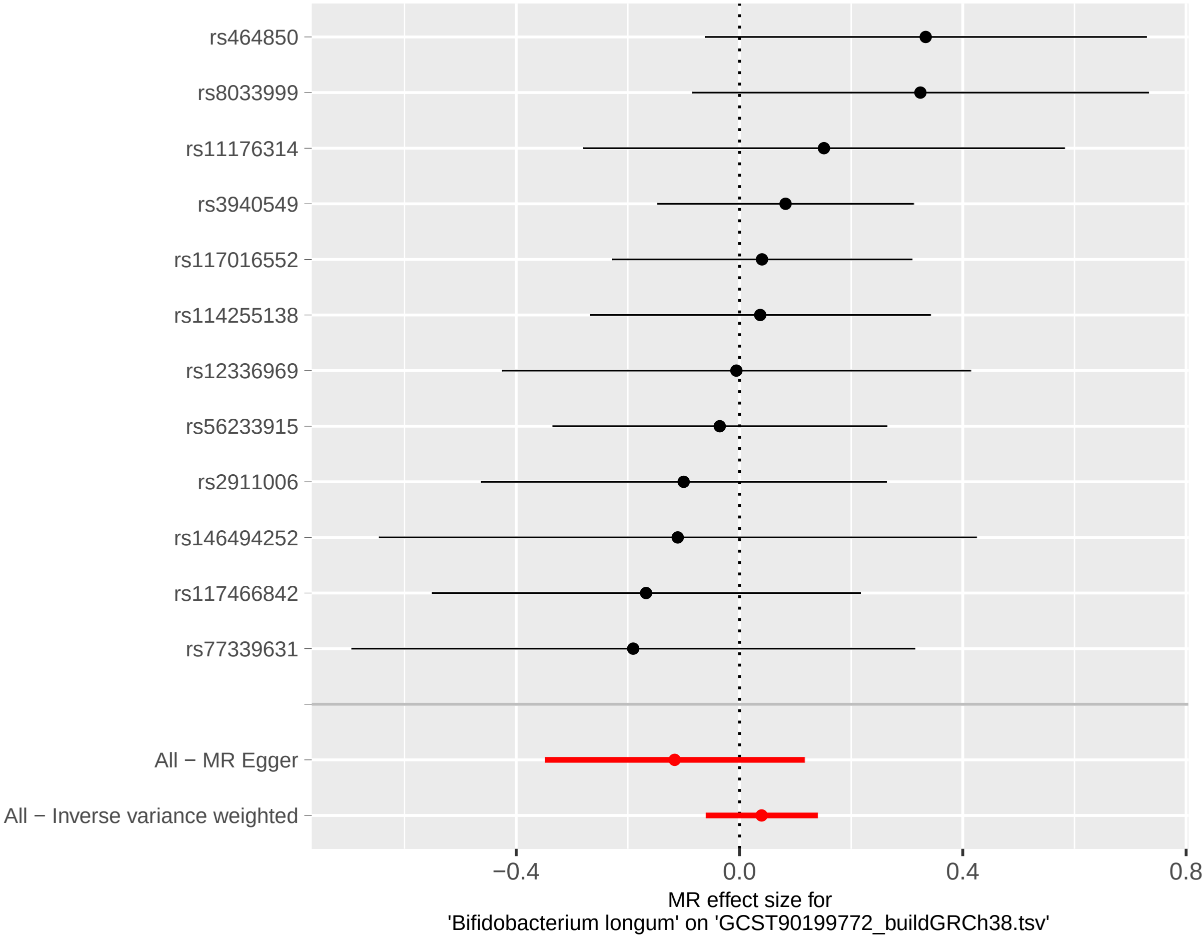
rs464850
rs8033999
rs11176314
rs3940549
rs117016552
rs114255138
rs12336969
rs56233915
rs2911006
rs146494252
rs117466842
rs77339631
All − MR Egger
All − Inverse variance weighted
−0.4
0.0
0.4
0.8
MR effect size for
'Bifidobacterium longum' on 'GCST90199772_buildGRCh38.tsv'

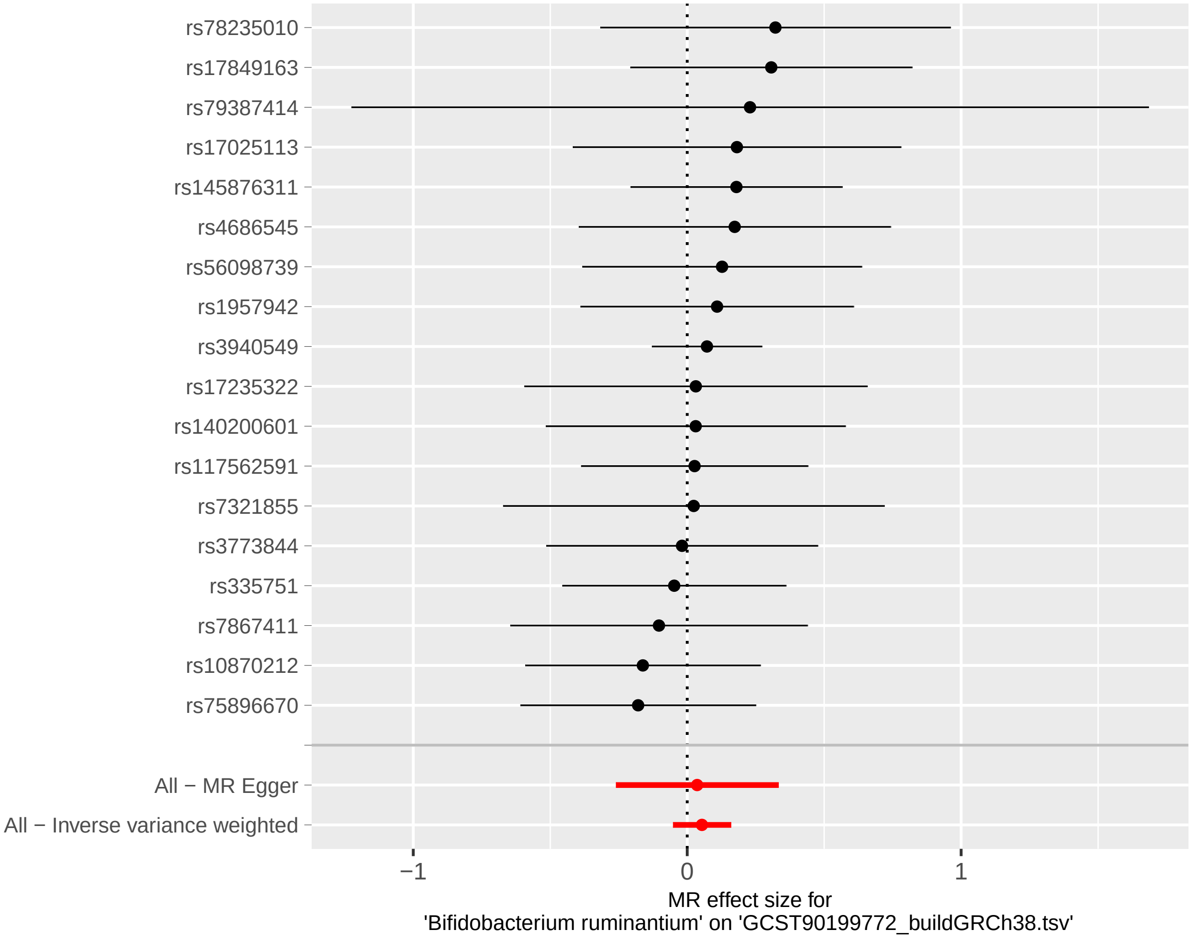
rs78235010
rs17849163
rs79387414
rs17025113
rs145876311
rs4686545
rs56098739
rs1957942
rs3940549
rs17235322
rs140200601
rs117562591
rs7321855
rs3773844
rs335751
rs7867411
rs10870212
rs75896670
All − MR Egger
All − Inverse variance weighted
−1
0
1
MR effect size for
'Bifidobacterium ruminantium' on 'GCST90199772_buildGRCh38.tsv'

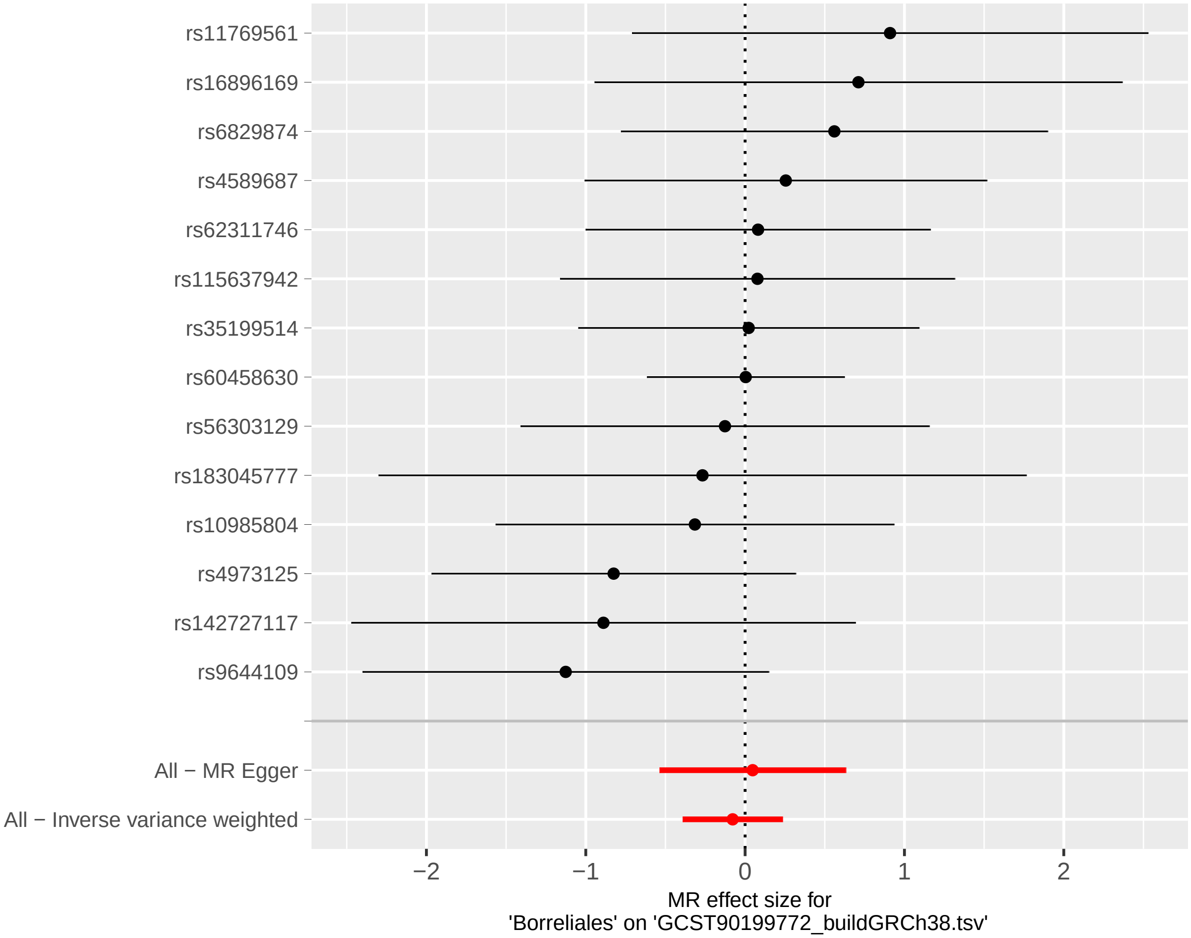

rs11769561
rs16896169
rs6829874
rs4589687
rs62311746
rs115637942
rs35199514
rs60458630
rs56303129
rs183045777
rs10985804
rs4973125
rs142727117
rs9644109
All − MR Egger
All − Inverse variance weighted
−2
−1
0
1
2
MR effect size for
'Borreliales' on 'GCST90199772_buildGRCh38.tsv'

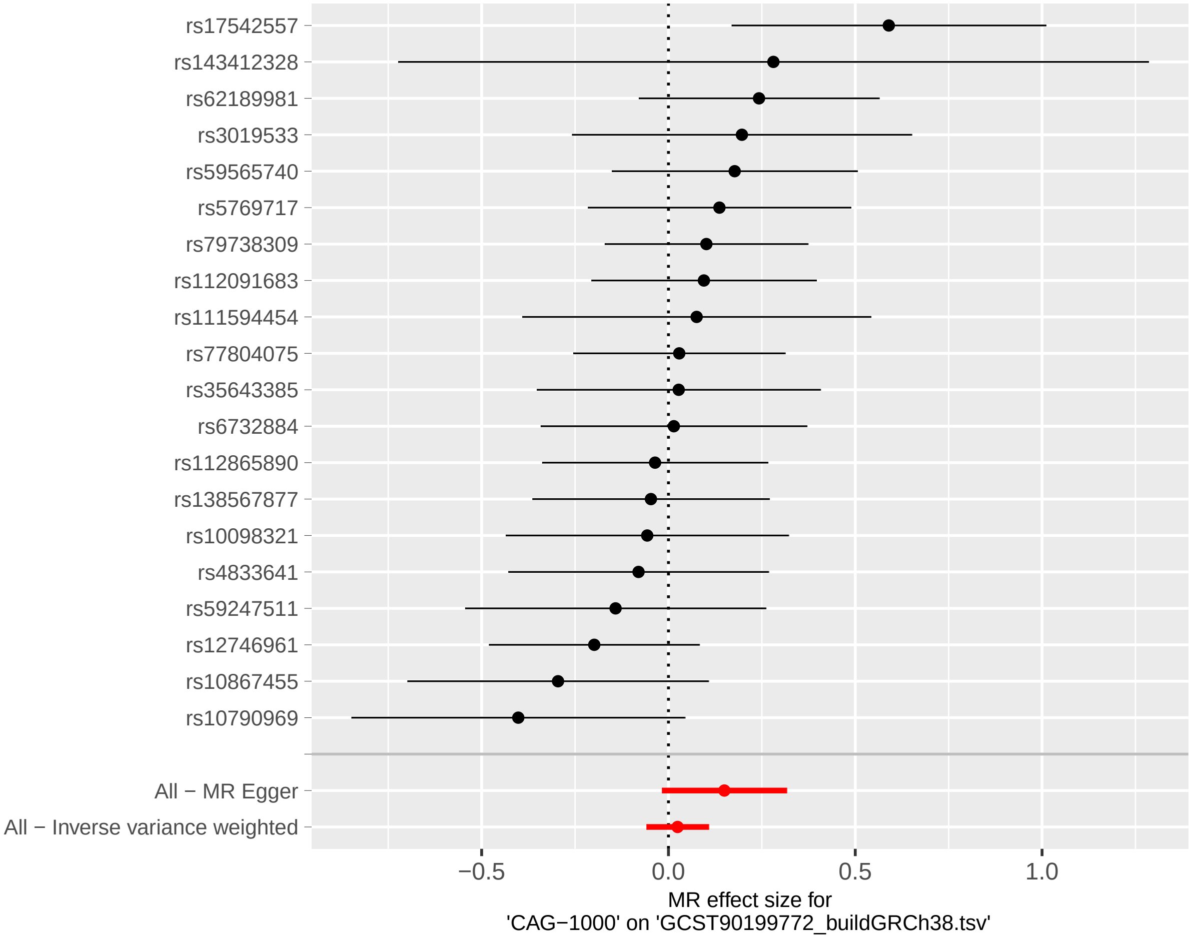

rs17542557
rs143412328
rs62189981
rs3019533
rs59565740
rs5769717
rs79738309
rs112091683
rs111594454
rs77804075
rs35643385
rs6732884
rs112865890
rs138567877
rs10098321
rs4833641
rs59247511
rs12746961
rs10867455
rs10790969
All − MR Egger
All − Inverse variance weighted
−0.5
0.0
0.5
1.0
MR effect size for
'CAG−1000' on 'GCST90199772_buildGRCh38.tsv'

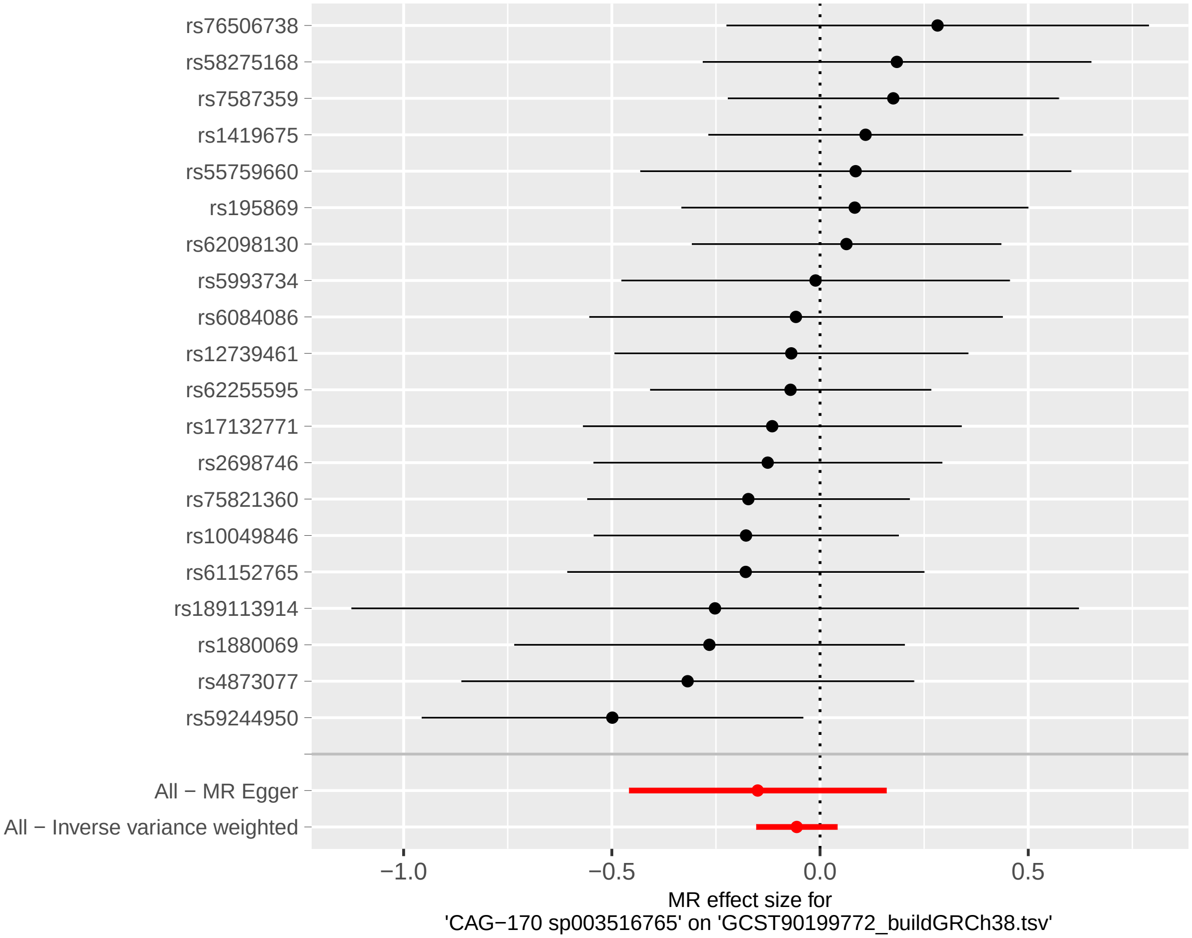
rs76506738
rs58275168
rs7587359
rs1419675
rs55759660
rs195869
rs62098130
rs5993734
rs6084086
rs12739461
rs62255595
rs17132771
rs2698746
rs75821360
rs10049846
rs61152765
rs189113914
rs1880069
rs4873077
rs59244950
All − MR Egger
All − Inverse variance weighted
−1.0
−0.5
0.0
0.5
MR effect size for
'CAG−170 sp003516765' on 'GCST90199772_buildGRCh38.tsv'

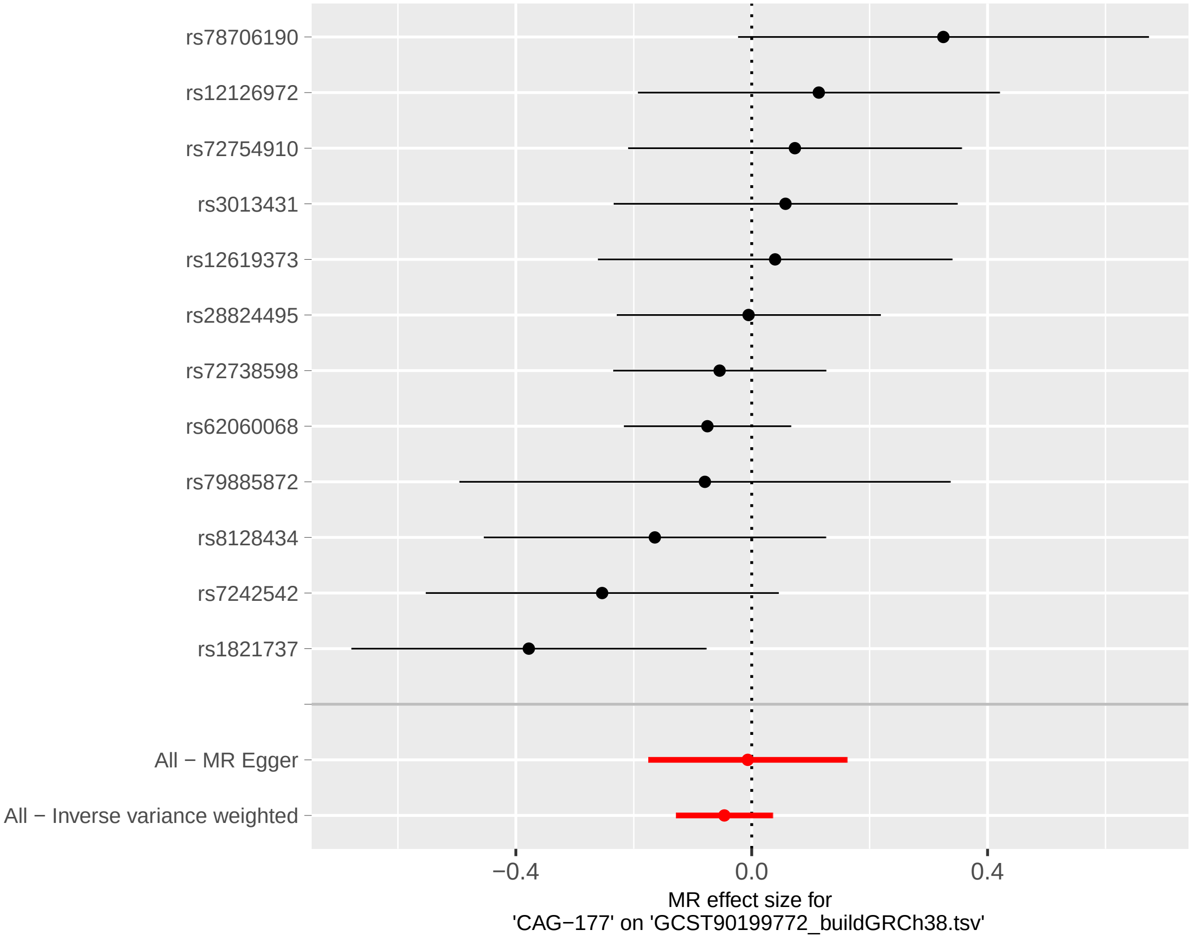
rs78706190
rs12126972
rs72754910
rs3013431
rs12619373
rs28824495
rs72738598
rs62060068
rs79885872
rs8128434
rs7242542
rs1821737
All − MR Egger
All − Inverse variance weighted
−0.4
0.0
0.4
MR effect size for
'CAG−177' on 'GCST90199772_buildGRCh38.tsv'

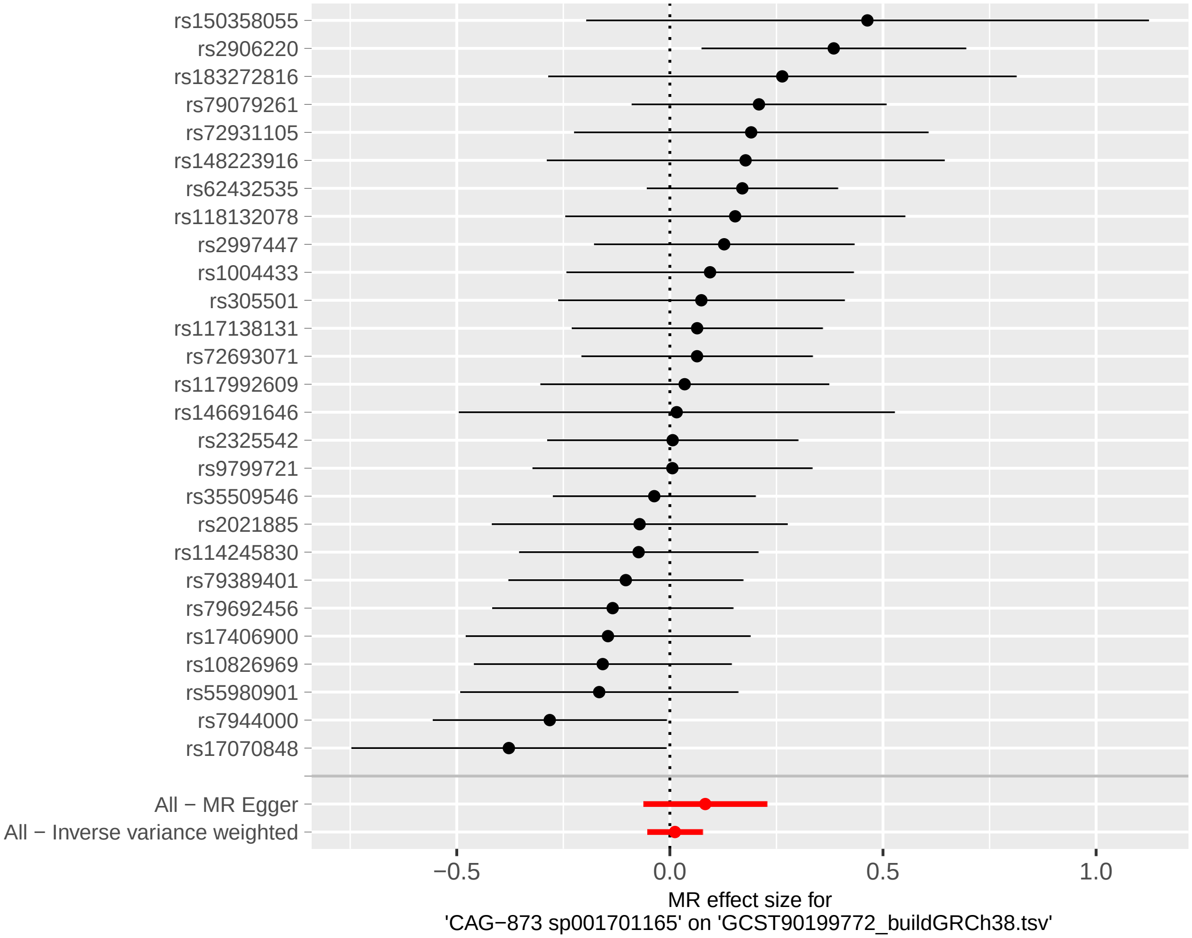

rs150358055
rs2906220
rs183272816
rs79079261
rs72931105
rs148223916
rs62432535
rs118132078
rs2997447
rs1004433
rs305501
rs117138131
rs72693071
rs117992609
rs146691646
rs2325542
rs9799721
rs35509546
rs2021885
rs114245830
rs79389401
rs79692456
rs17406900
rs10826969
rs55980901
rs7944000
rs17070848
All − MR Egger
All − Inverse variance weighted
−0.5
0.0
0.5
1.0
MR effect size for
'CAG−873 sp001701165' on 'GCST90199772_buildGRCh38.tsv'

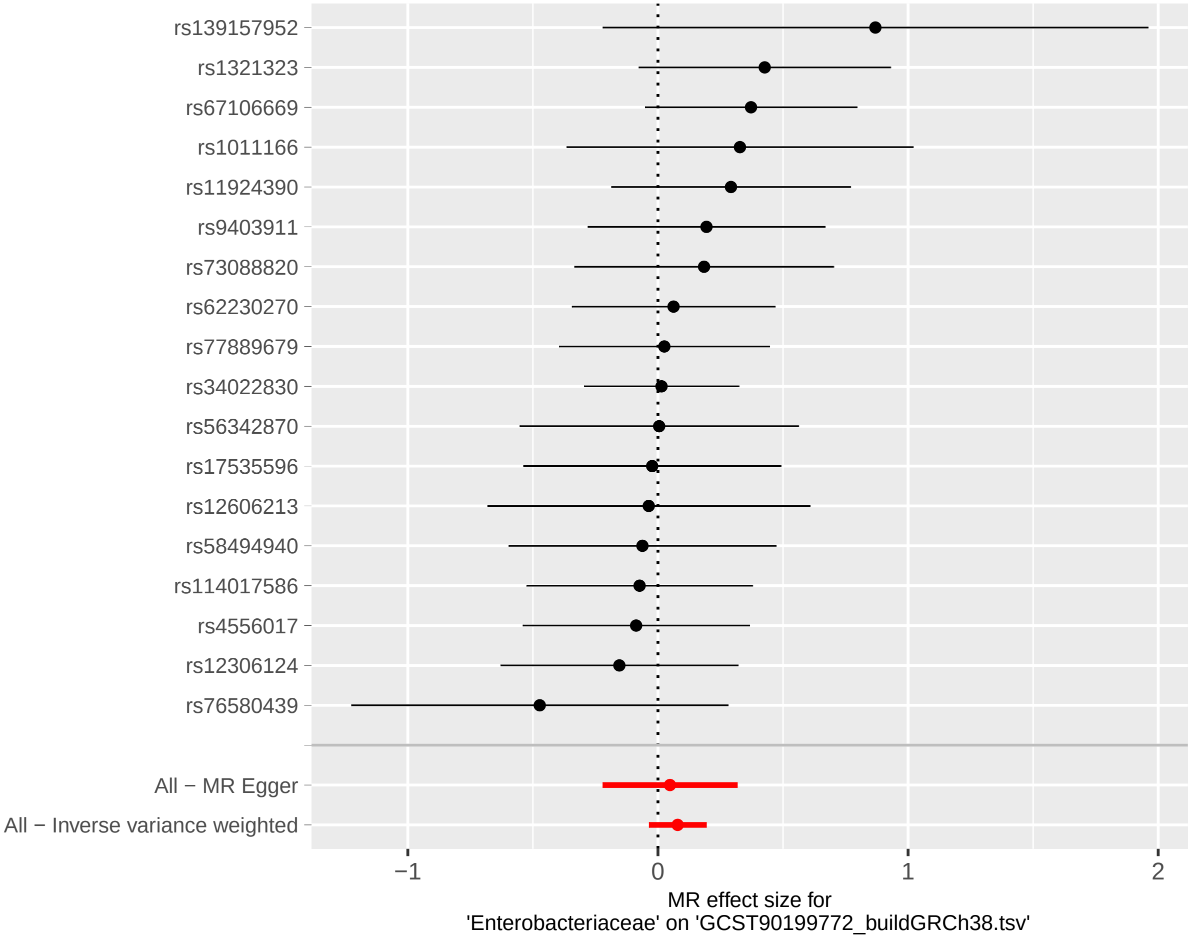

rs139157952
rs1321323
rs67106669
rs1011166
rs11924390
rs9403911
rs73088820
rs62230270
rs77889679
rs34022830
rs56342870
rs17535596
rs12606213
rs58494940
rs114017586
rs4556017
rs12306124
rs76580439
All − MR Egger
All − Inverse variance weighted
−1
0
1
2
MR effect size for
'Enterobacteriaceae' on 'GCST90199772_buildGRCh38.tsv'

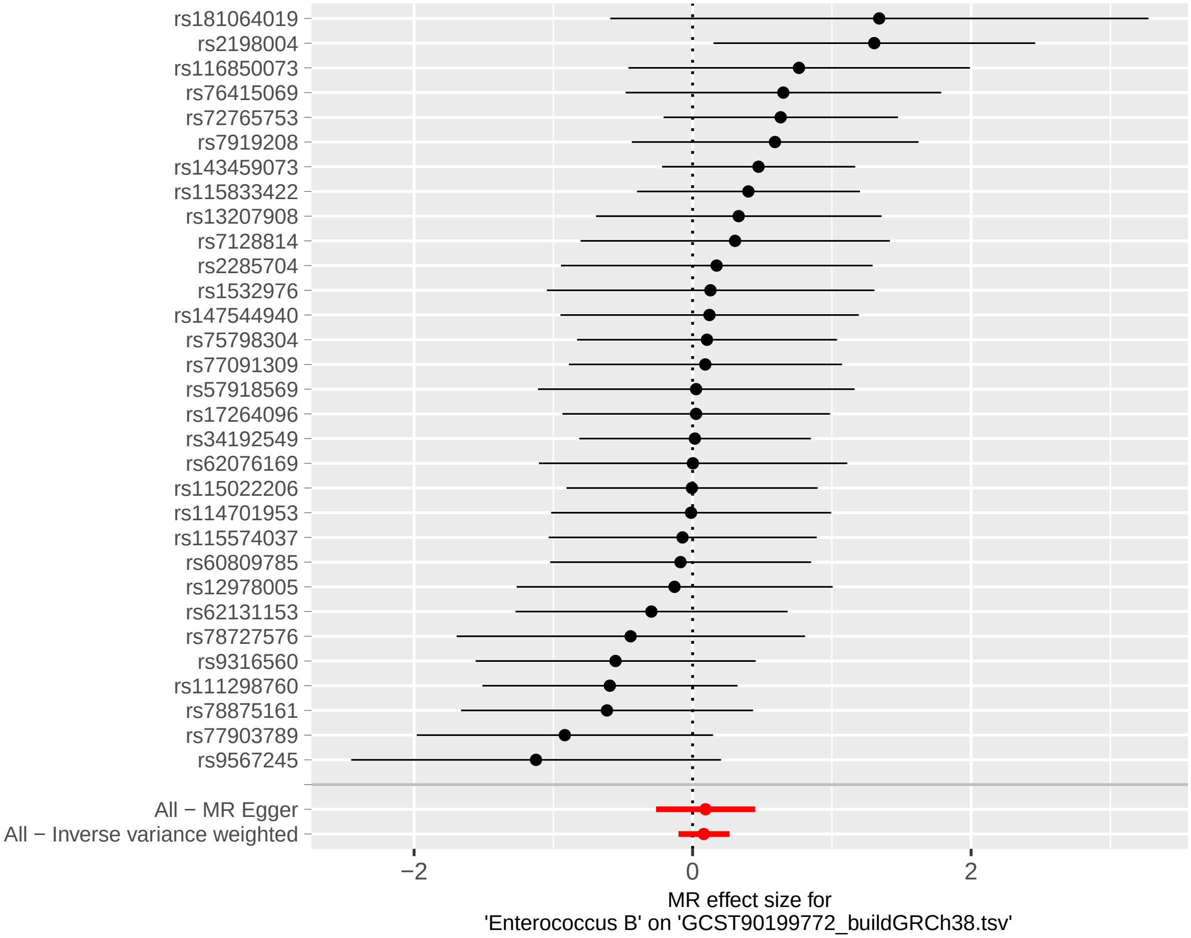

rs181064019
rs2198004
rs116850073
rs76415069
rs72765753
rs7919208
rs143459073
rs115833422
rs13207908
rs7128814
rs2285704
rs1532976
rs147544940
rs75798304
rs77091309
rs57918569
rs17264096
rs34192549
rs62076169
rs115022206
rs114701953
rs115574037
rs60809785
rs12978005
rs62131153
rs78727576
rs9316560
rs111298760
rs78875161
rs77903789
rs9567245
All − MR Egger
All − Inverse variance weighted
−2
0
2
MR effect size for
'Enterococcus B' on 'GCST90199772_buildGRCh38.tsv'

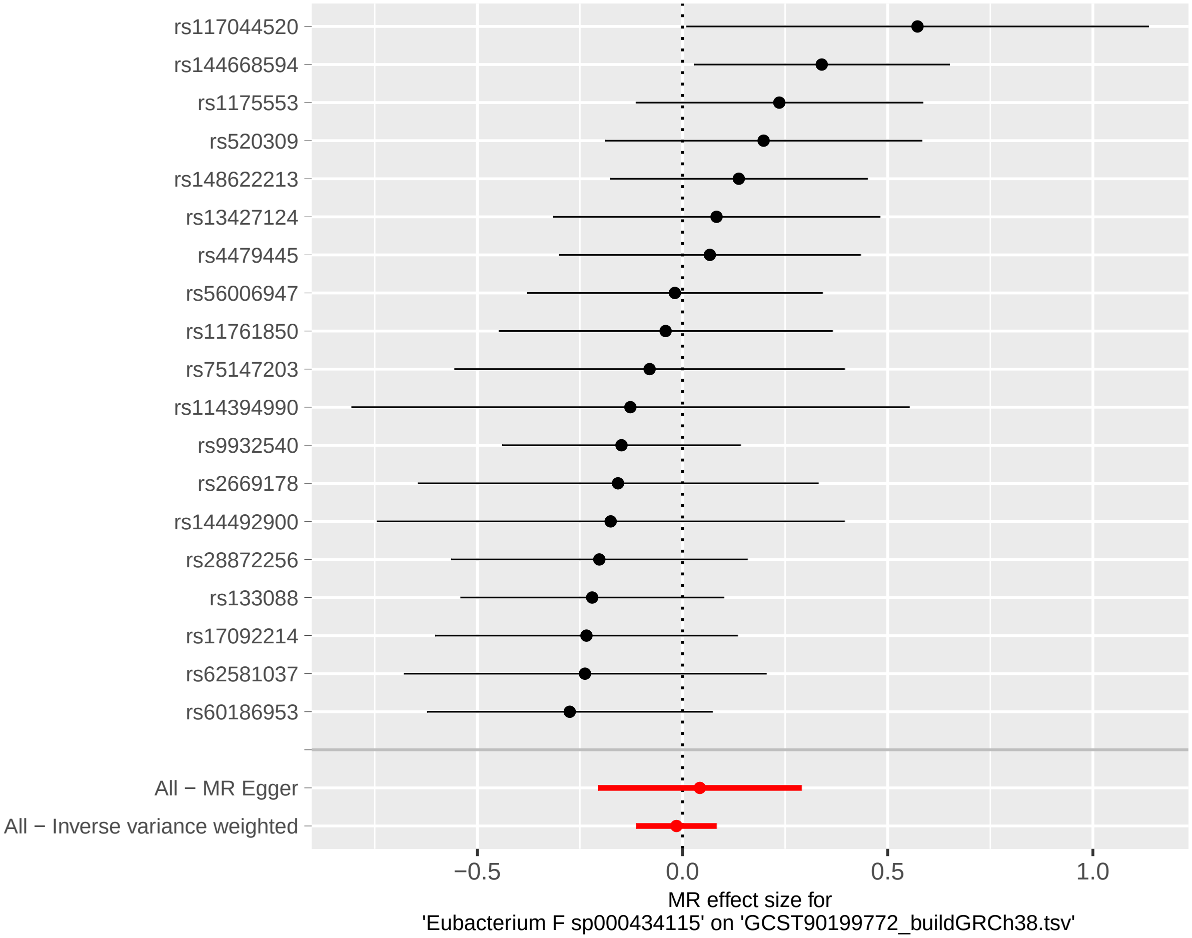

rs117044520
rs144668594
rs1175553
rs520309
rs148622213
rs13427124
rs4479445
rs56006947
rs11761850
rs75147203
rs114394990
rs9932540
rs2669178
rs144492900
rs28872256
rs133088
rs17092214
rs62581037
rs60186953
All − MR Egger
All − Inverse variance weighted
−0.5
0.0
0.5
1.0
MR effect size for
'Eubacterium F sp000434115' on 'GCST90199772_buildGRCh38.tsv'

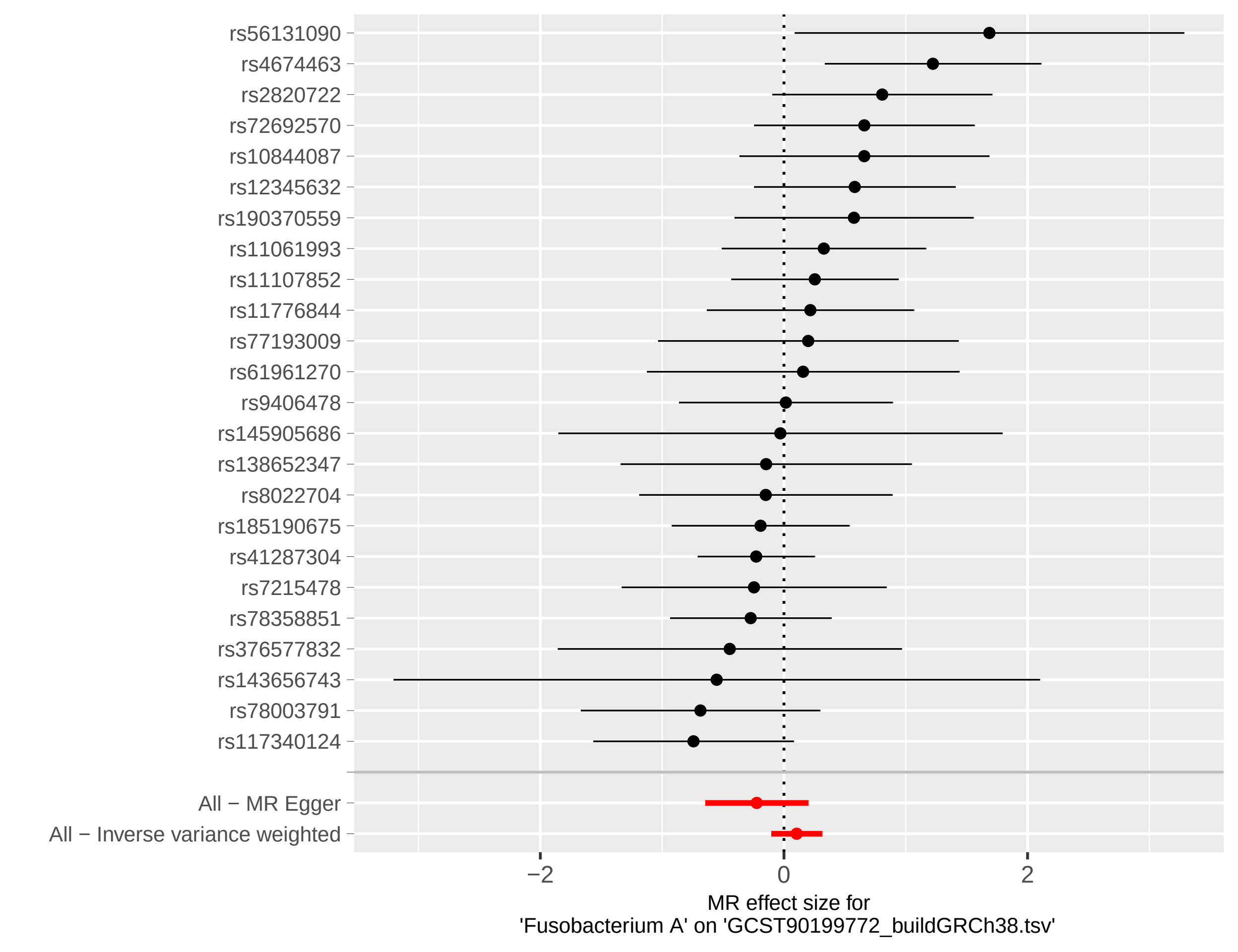
rs56131090
rs4674463
rs2820722
rs72692570
rs10844087
rs12345632
rs190370559
rs11061993
rs11107852
rs11776844
rs77193009
rs61961270
rs9406478
rs145905686
rs138652347
rs8022704
rs185190675
rs41287304
rs7215478
rs78358851
rs376577832
rs143656743
rs78003791
rs117340124
All − MR Egger
All − Inverse variance weighted
−2
0
2
MR effect size for
'Fusobacterium A' on 'GCST90199772_buildGRCh38.tsv'

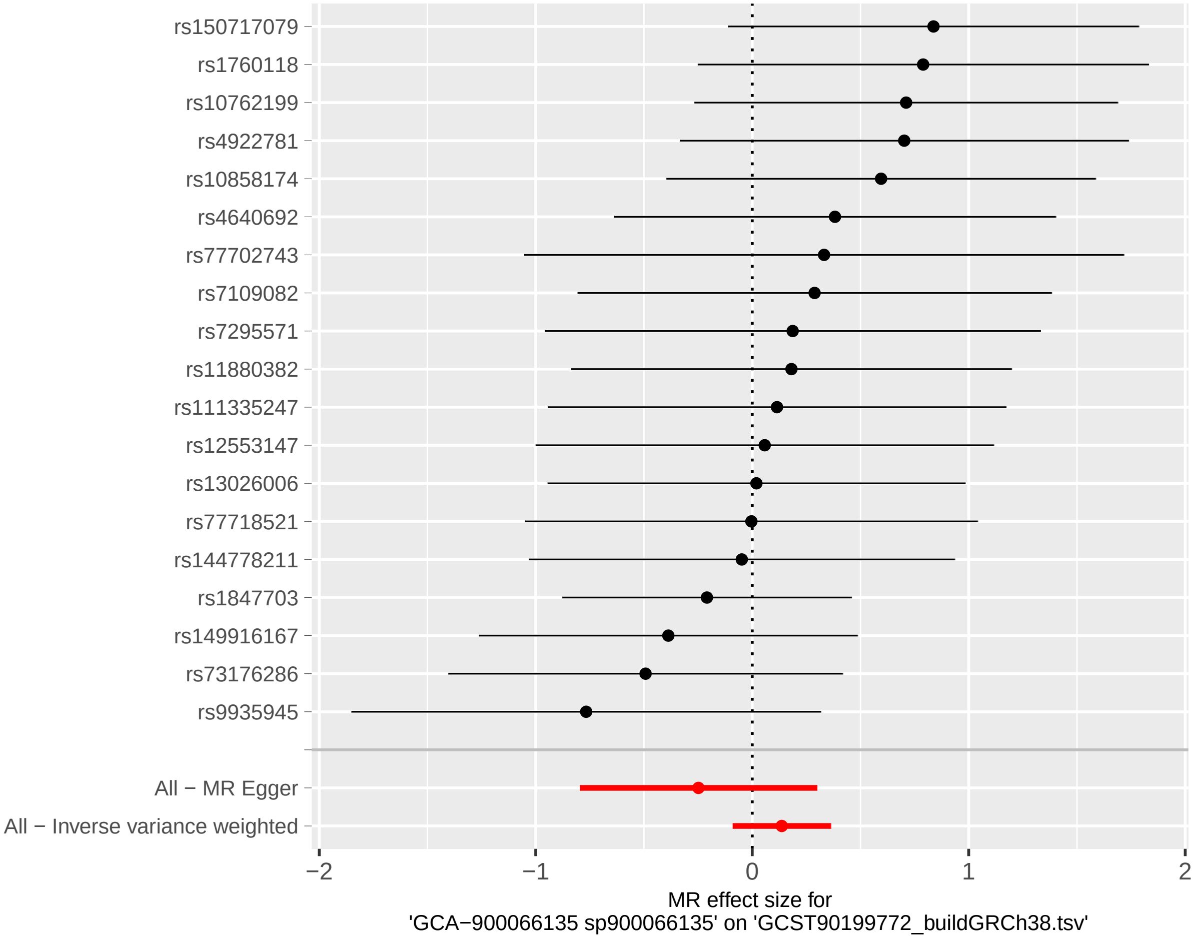

rs150717079
rs1760118
rs10762199
rs4922781
rs10858174
rs4640692
rs77702743
rs7109082
rs7295571
rs11880382
rs111335247
rs12553147
rs13026006
rs77718521
rs144778211
rs1847703
rs149916167
rs73176286
rs9935945
All − MR Egger
All − Inverse variance weighted
−2
−1
0
1
2
MR effect size for
'GCA−900066135 sp900066135' on 'GCST90199772_buildGRCh38.tsv'

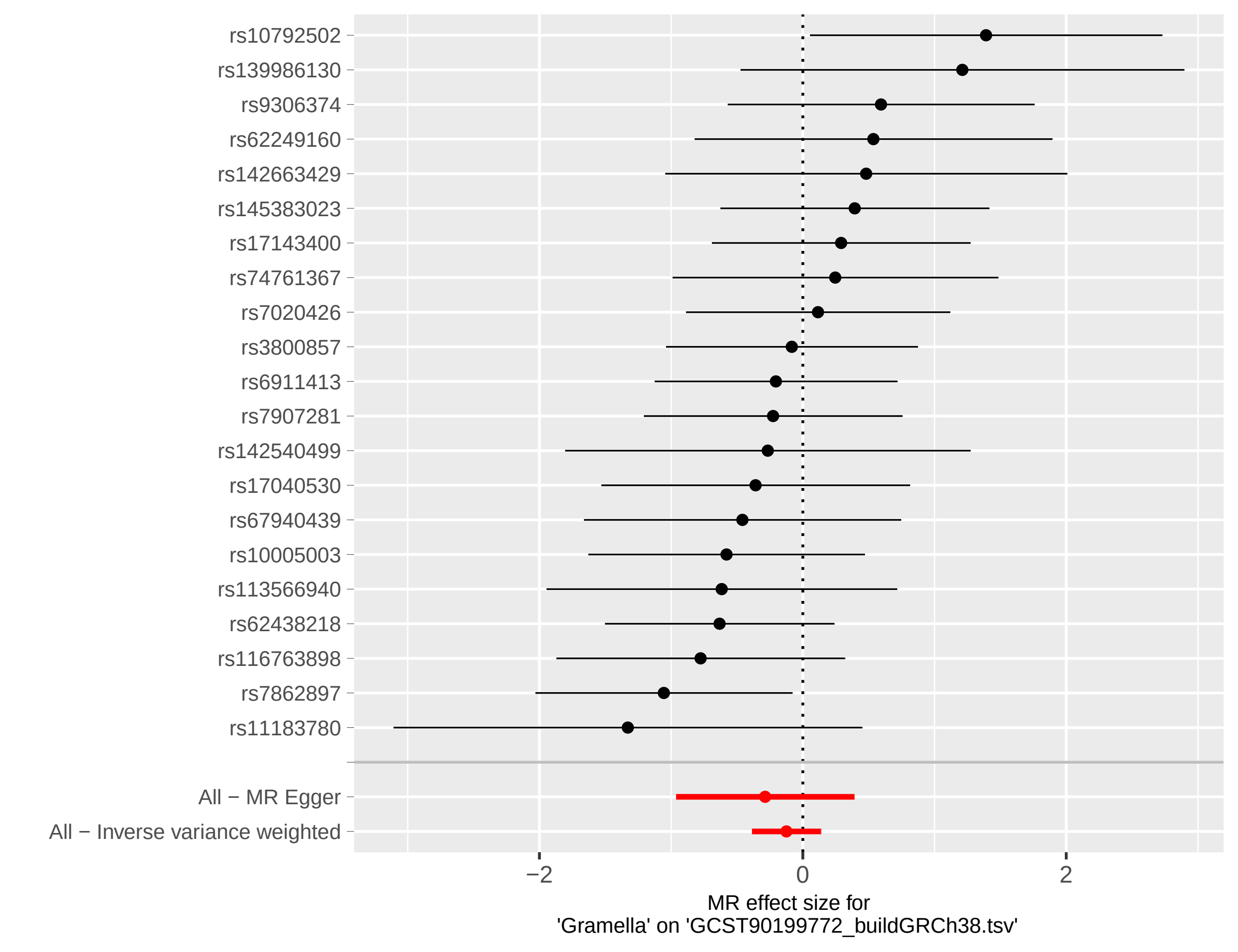

rs10792502
rs139986130
rs9306374
rs62249160
rs142663429
rs145383023
rs17143400
rs74761367
rs7020426
rs3800857
rs6911413
rs7907281
rs142540499
rs17040530
rs67940439
rs10005003
rs113566940
rs62438218
rs116763898
rs7862897
rs11183780
All − MR Egger
All − Inverse variance weighted
−2
0
2
MR effect size for
'Gramella' on 'GCST90199772_buildGRCh38.tsv'

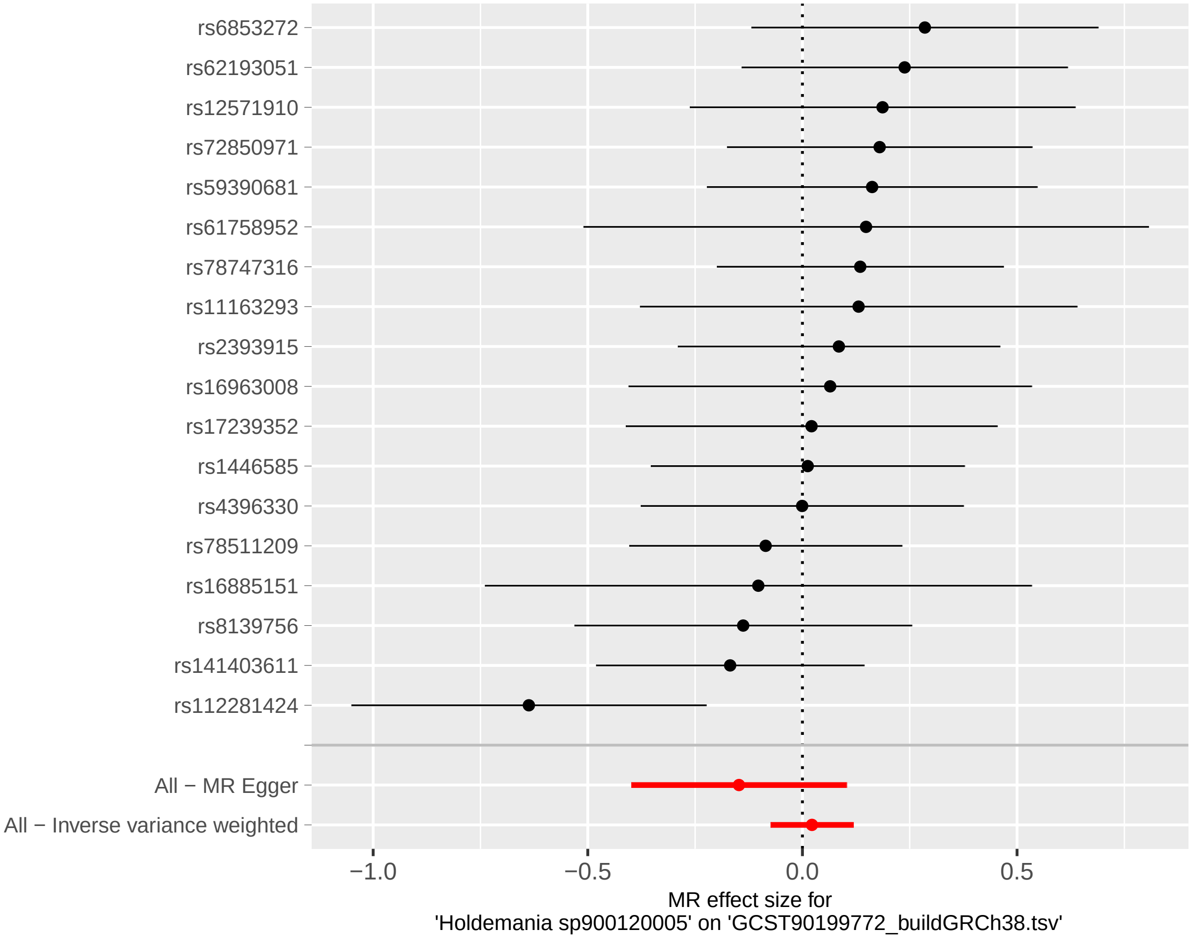
rs6853272
rs62193051
rs12571910
rs72850971
rs59390681
rs61758952
rs78747316
rs11163293
rs2393915
rs16963008
rs17239352
rs1446585
rs4396330
rs78511209
rs16885151
rs8139756
rs141403611
rs112281424
All − MR Egger
All − Inverse variance weighted
−1.0
−0.5
0.0
0.5
MR effect size for
'Holdemania sp900120005' on 'GCST90199772_buildGRCh38.tsv'

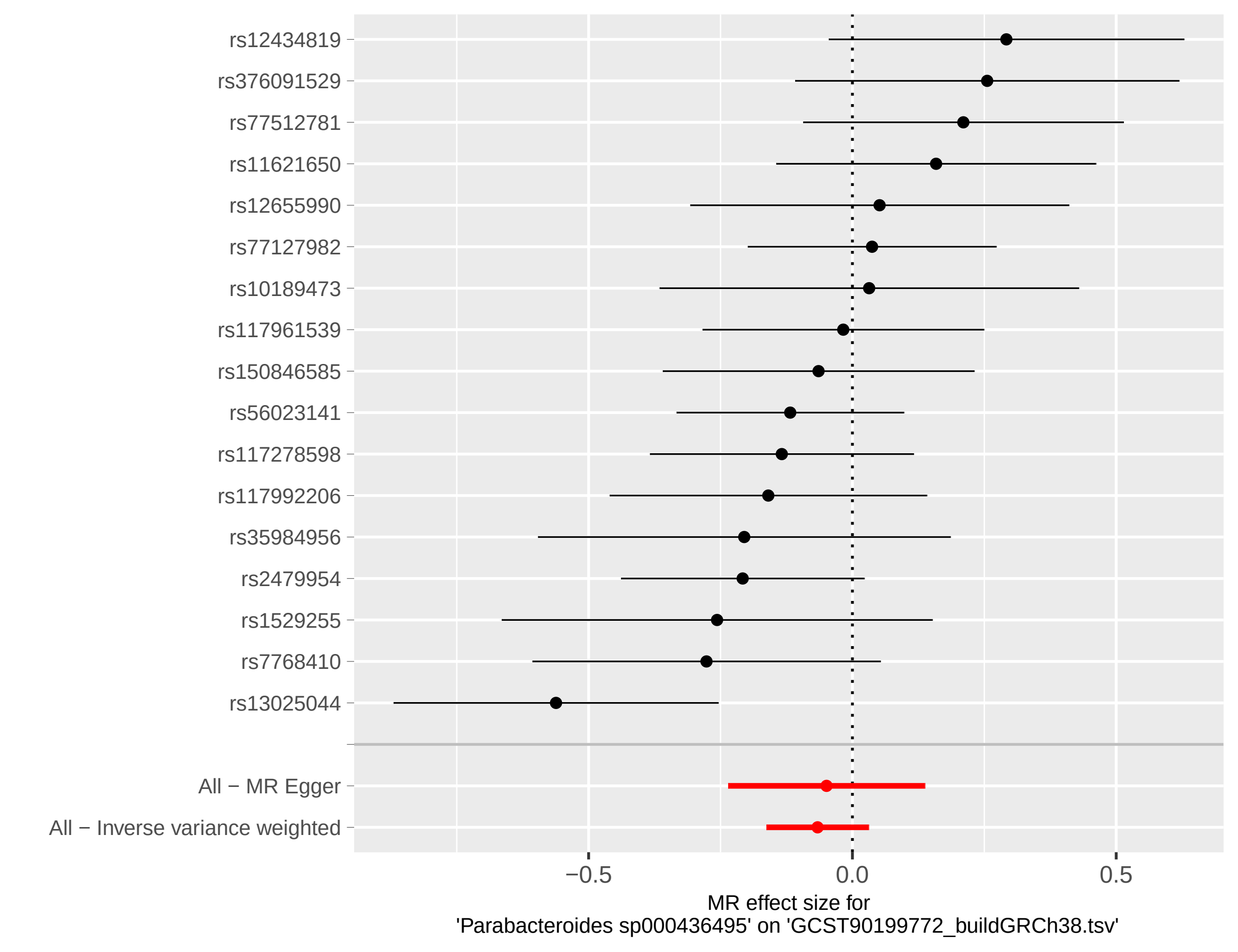
rs12434819
rs376091529
rs77512781
rs11621650
rs12655990
rs77127982
rs10189473
rs117961539
rs150846585
rs56023141
rs117278598
rs117992206
rs35984956
rs2479954
rs1529255
rs7768410
rs13025044
All − MR Egger
All − Inverse variance weighted
−0.5
0.0
0.5
MR effect size for
'Parabacteroides sp000436495' on 'GCST90199772_buildGRCh38.tsv'

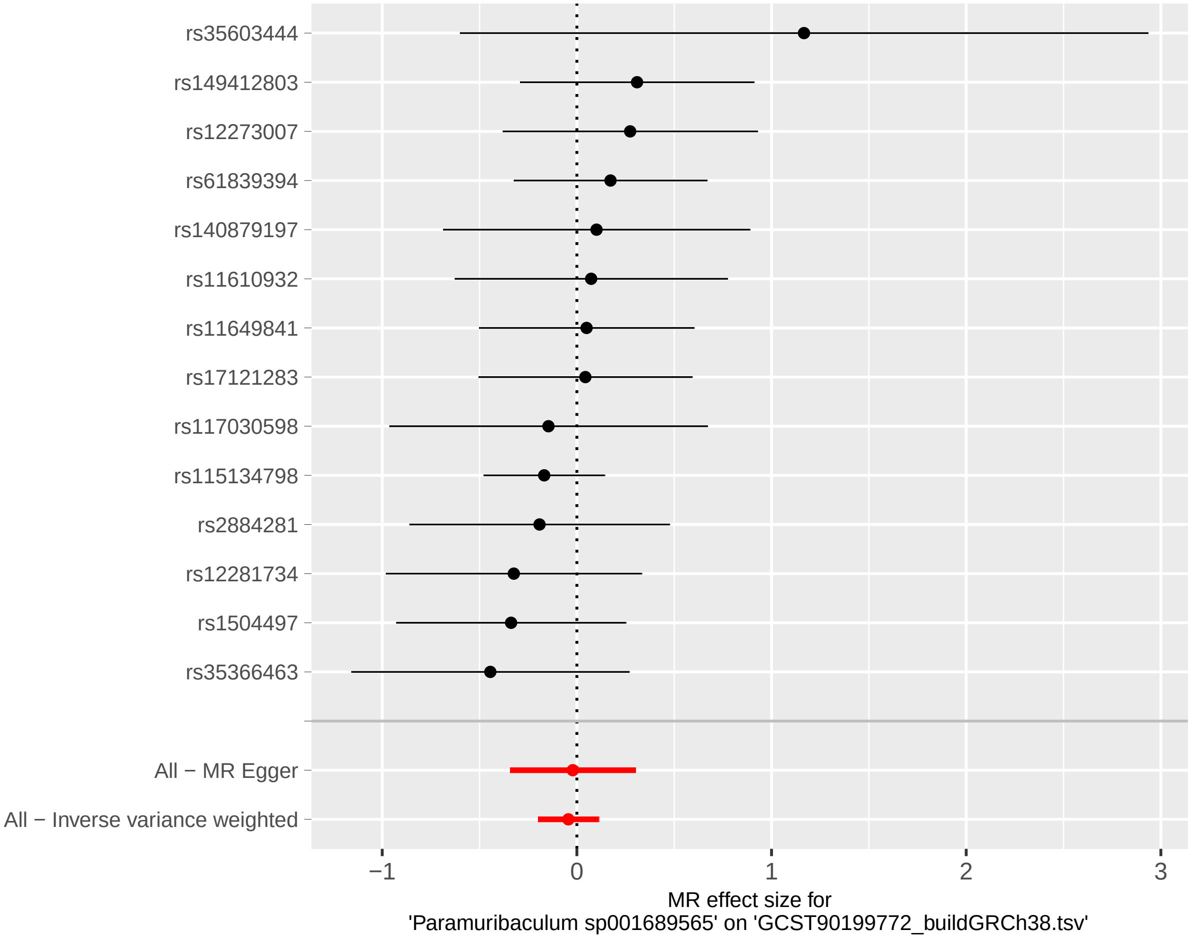
rs35603444
rs149412803
rs12273007
rs61839394
rs140879197
rs11610932
rs11649841
rs17121283
rs117030598
rs115134798
rs2884281
rs12281734
rs1504497
rs35366463
All − MR Egger
All − Inverse variance weighted
−1
0
1
2
3
MR effect size for
'Paramuribaculum sp001689565' on 'GCST90199772_buildGRCh38.tsv'

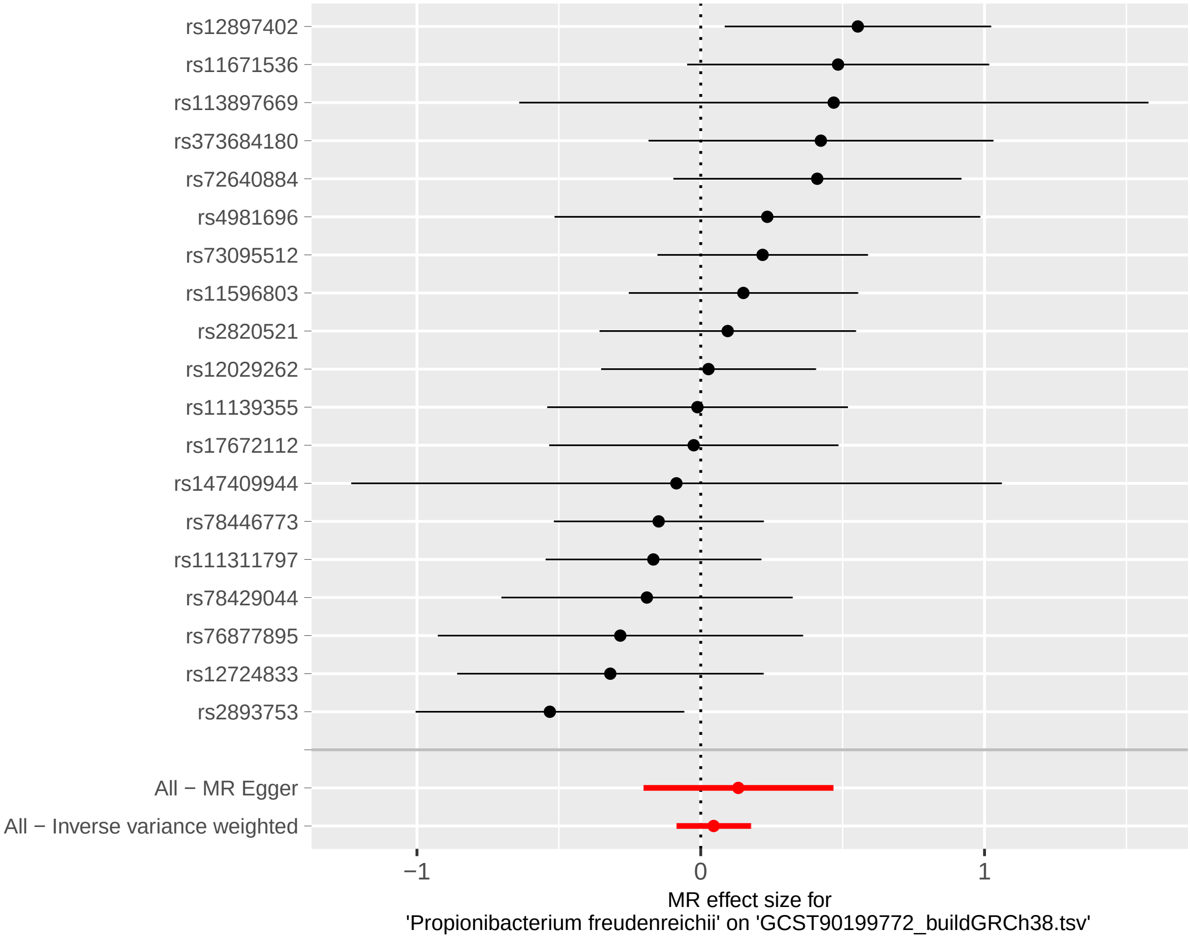
rs12897402
rs11671536
rs113897669
rs373684180
rs72640884
rs4981696
rs73095512
rs11596803
rs2820521
rs12029262
rs11139355
rs17672112
rs147409944
rs78446773
rs111311797
rs78429044
rs76877895
rs12724833
rs2893753
All − MR Egger
All − Inverse variance weighted
−1
0
1
MR effect size for
'Propionibacterium freudenreichii' on 'GCST90199772_buildGRCh38.tsv'

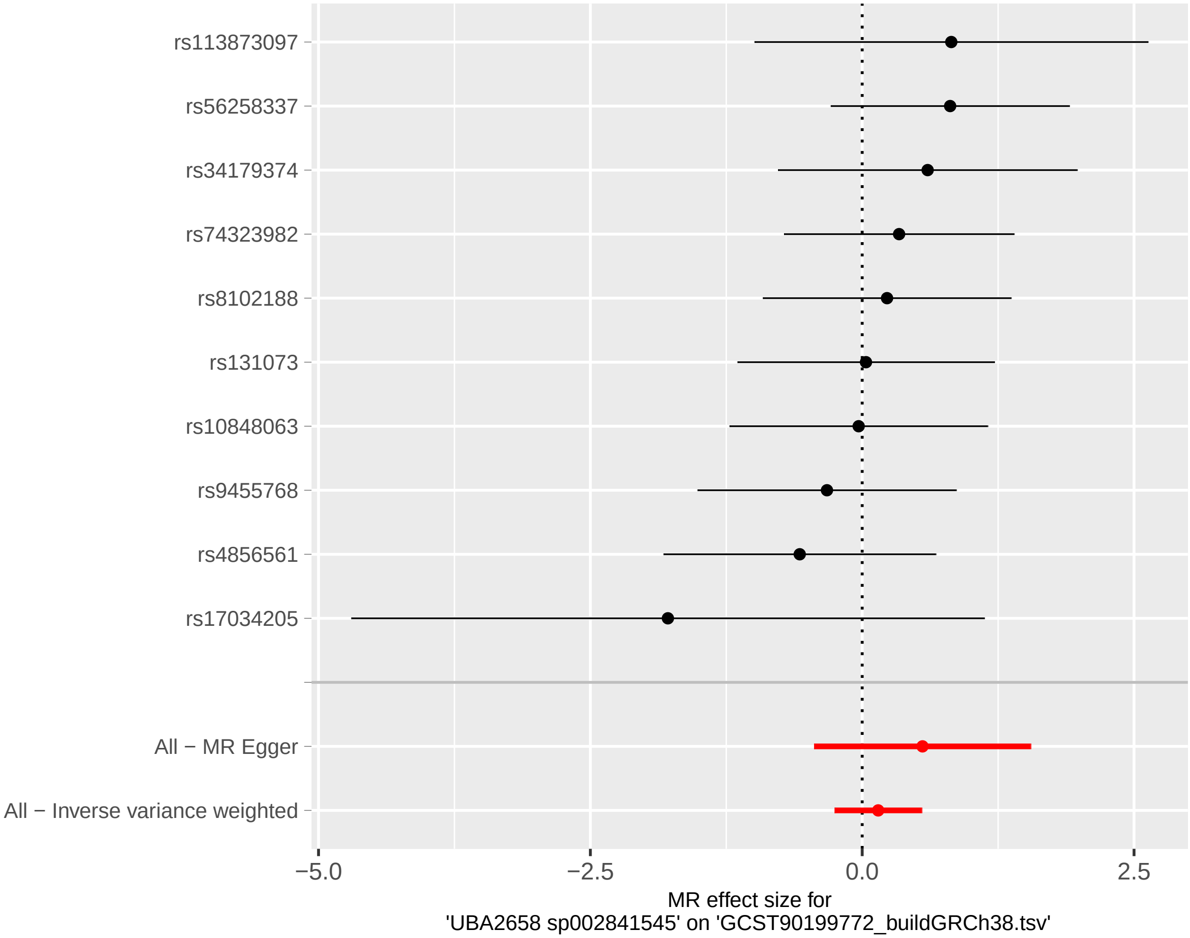
rs113873097
rs56258337
rs34179374
rs74323982
rs8102188
rs131073
rs10848063
rs9455768
rs4856561
rs17034205
All − MR Egger
All − Inverse variance weighted
−5.0
−2.5
0.0
2.5
MR effect size for
'UBA2658 sp002841545' on 'GCST90199772_buildGRCh38.tsv'

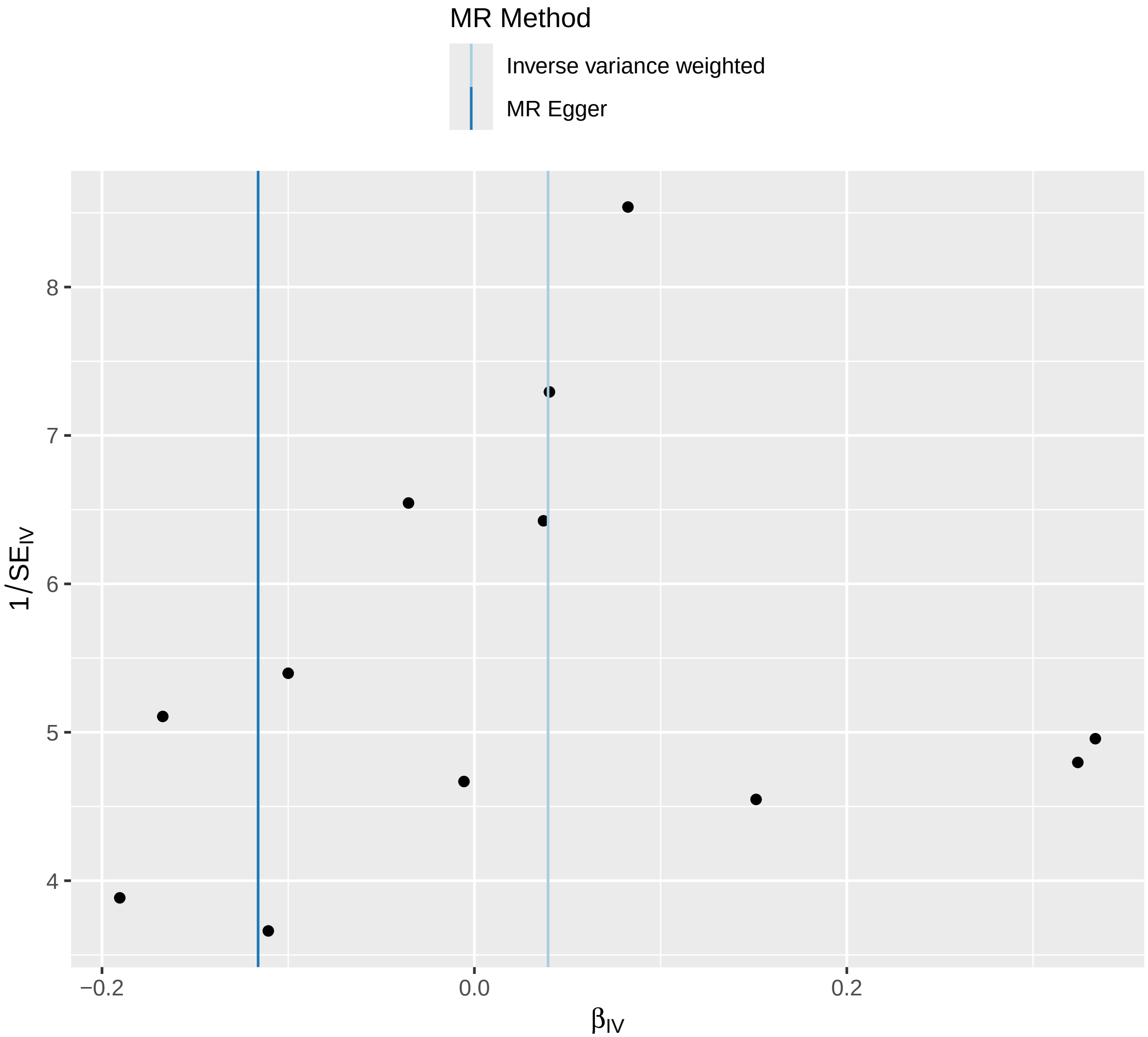

MR Method
Inverse variance weighted
MR Egger
1/SE$_{IV}$
8
7
6
5
4
−0.2
0.0
0.2
β_{IV}

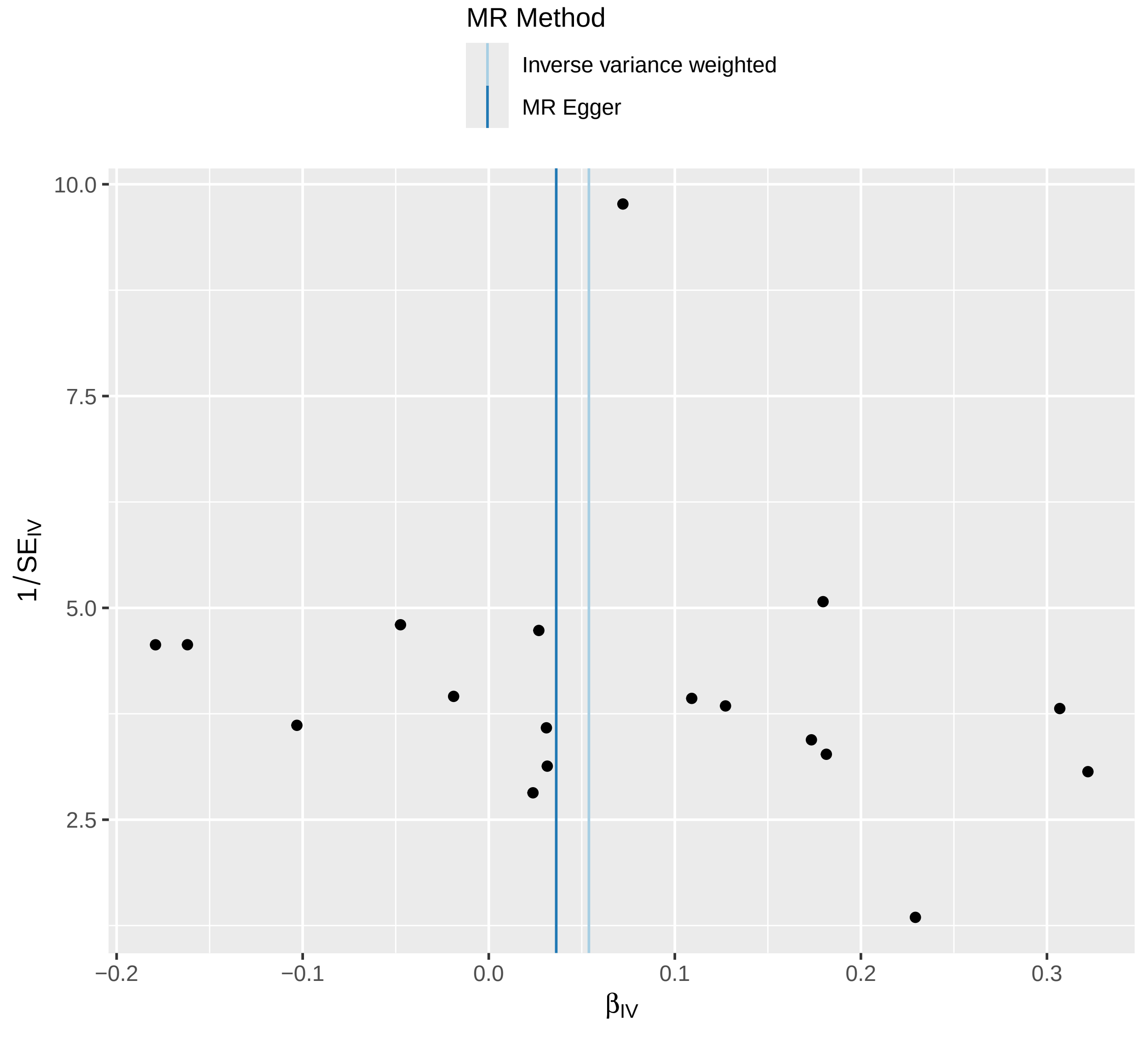

MR Method
Inverse variance weighted
MR Egger
10.0
7.5
5.0
2.5
$1/SE_{IV}$
−0.2
−0.1
0.0
0.1
0.2
0.3
β_{IV}

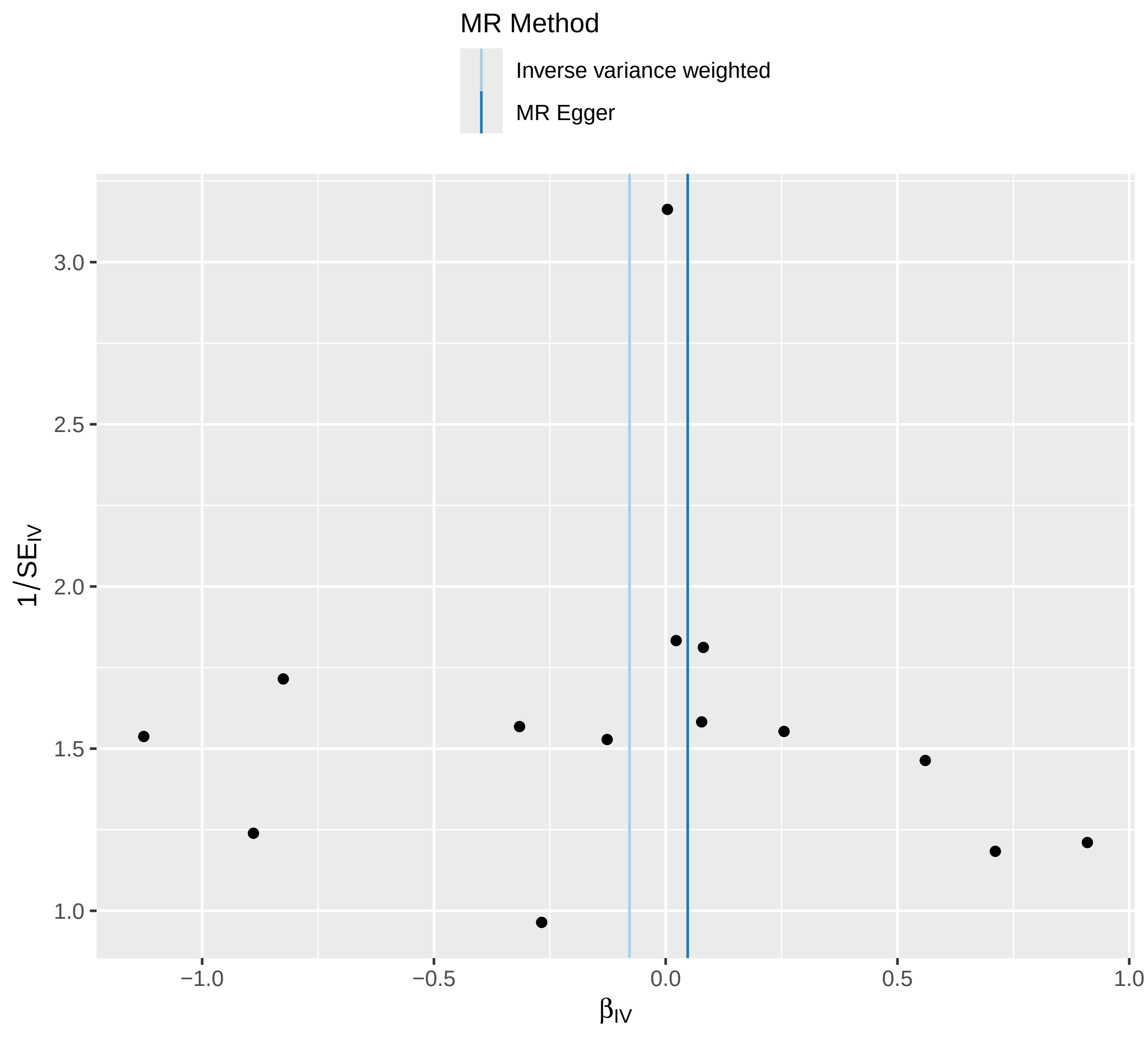

MR Method
Inverse variance weighted
MR Egger
1/SE$_{IV}$
3.0
2.5
2.0
1.5
1.0
−1.0
−0.5
0.0
0.5
1.0
β$_{IV}$

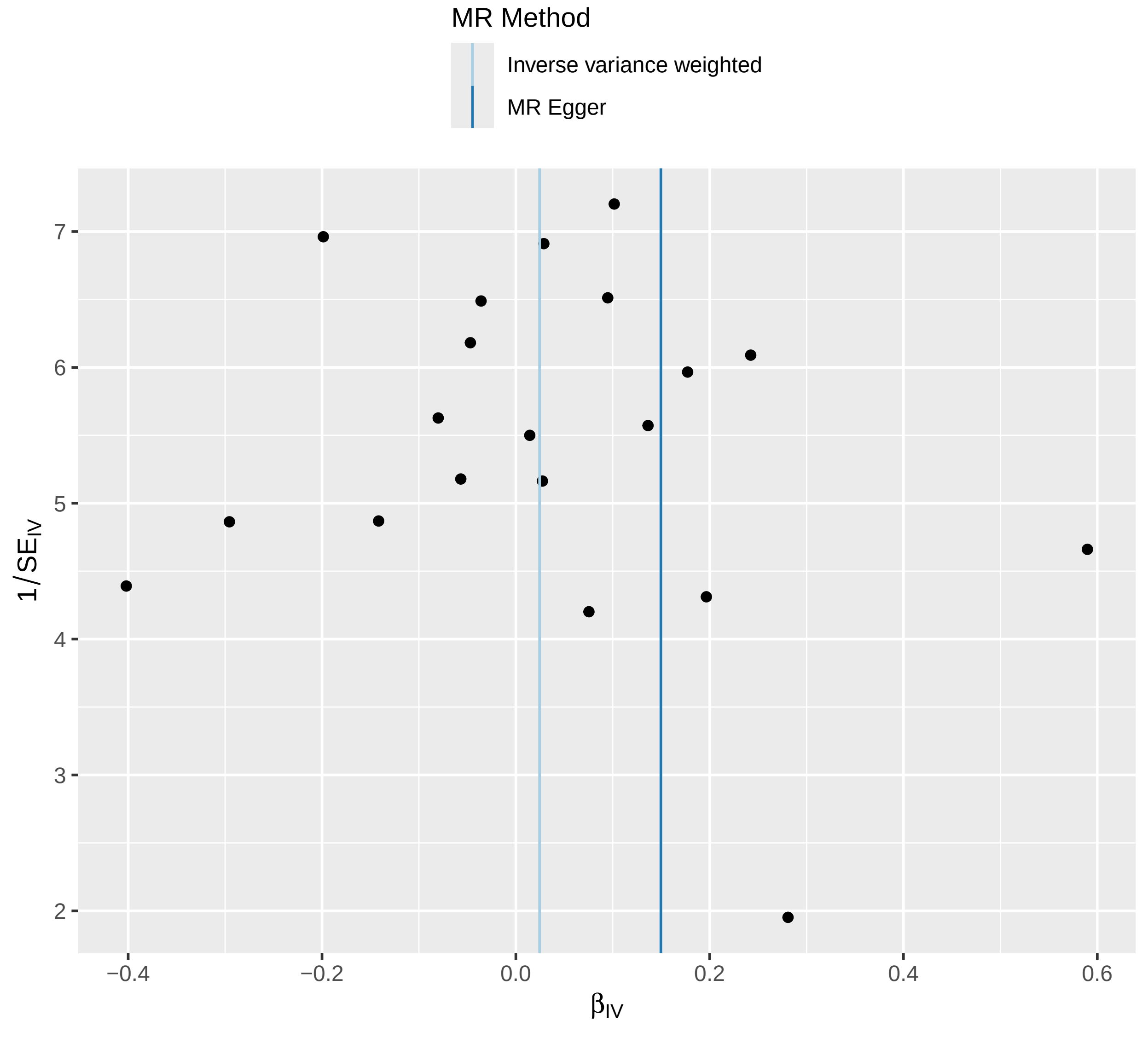

MR Method
Inverse variance weighted
MR Egger
7
6
5
4
3
2
$1/SE_{IV}$
−0.4
−0.2
0.0
0.2
0.4
0.6
β_{IV}

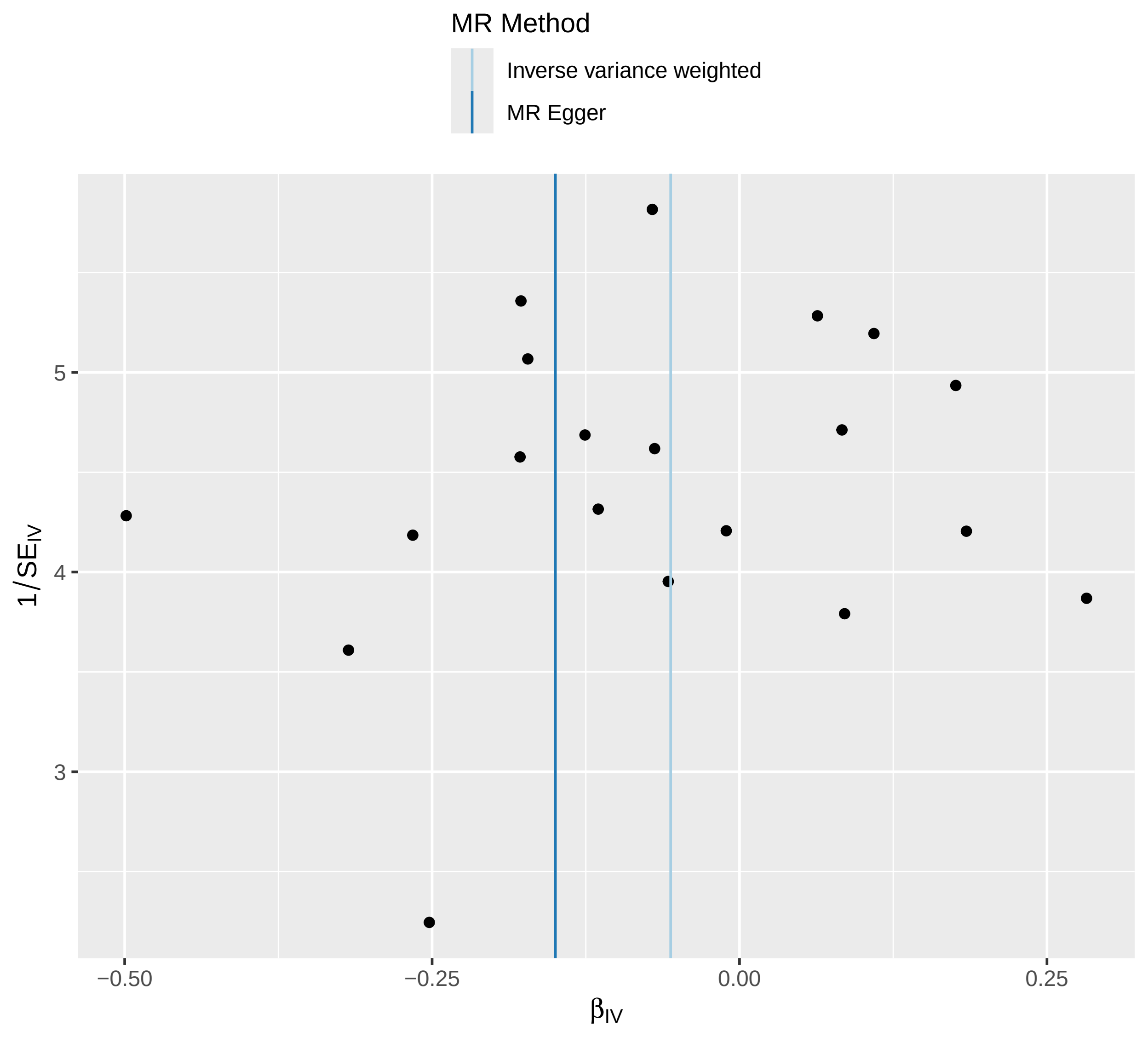

MR Method
Inverse variance weighted
MR Egger
5
4
3
$1/SE_{IV}$
−0.50
−0.25
0.00
0.25
β_{IV}

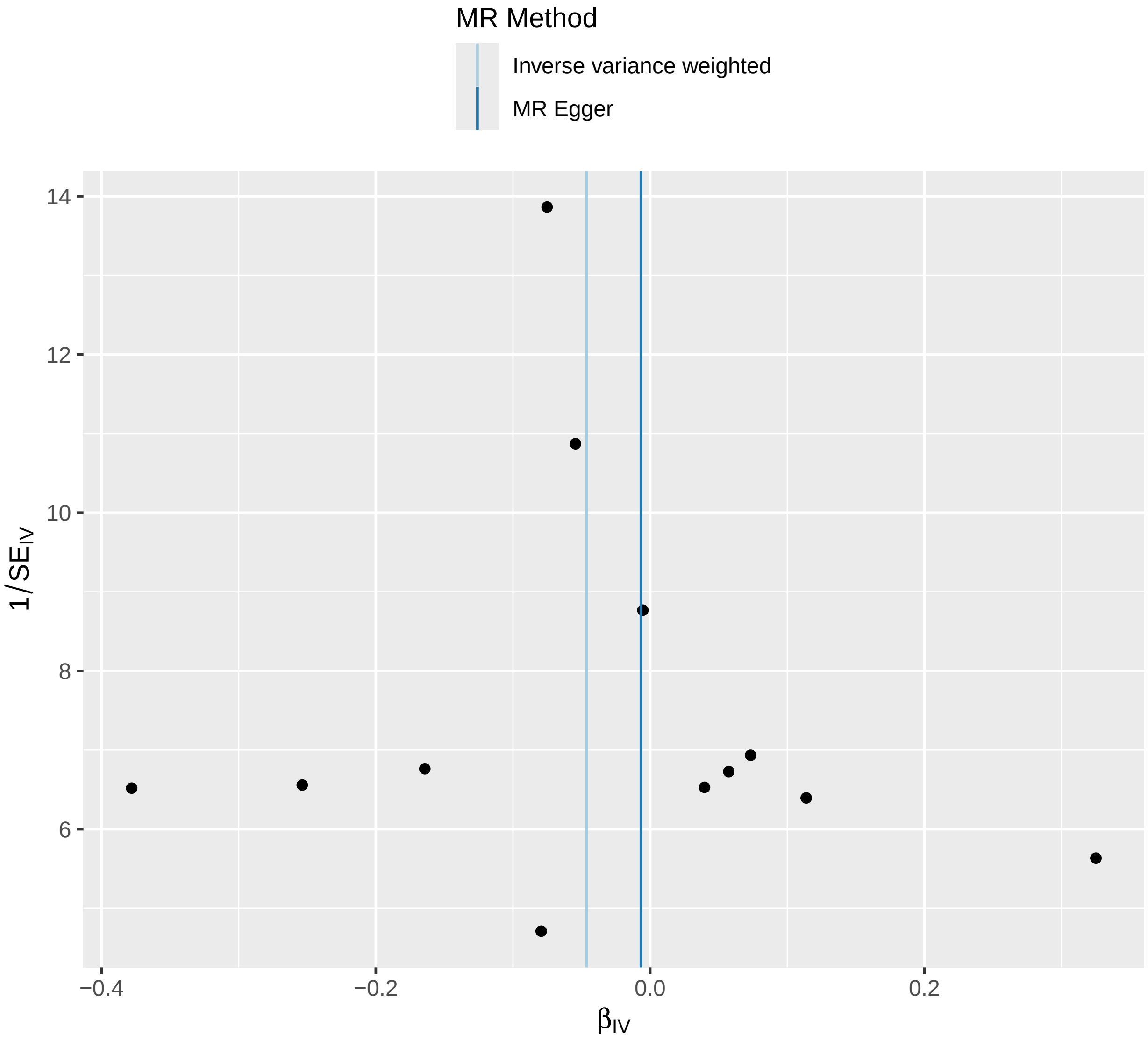

MR Method
Inverse variance weighted
MR Egger
14
12
10
8
6
1/SE$_{IV}$
−0.4
−0.2
0.0
0.2
β$_{IV}$

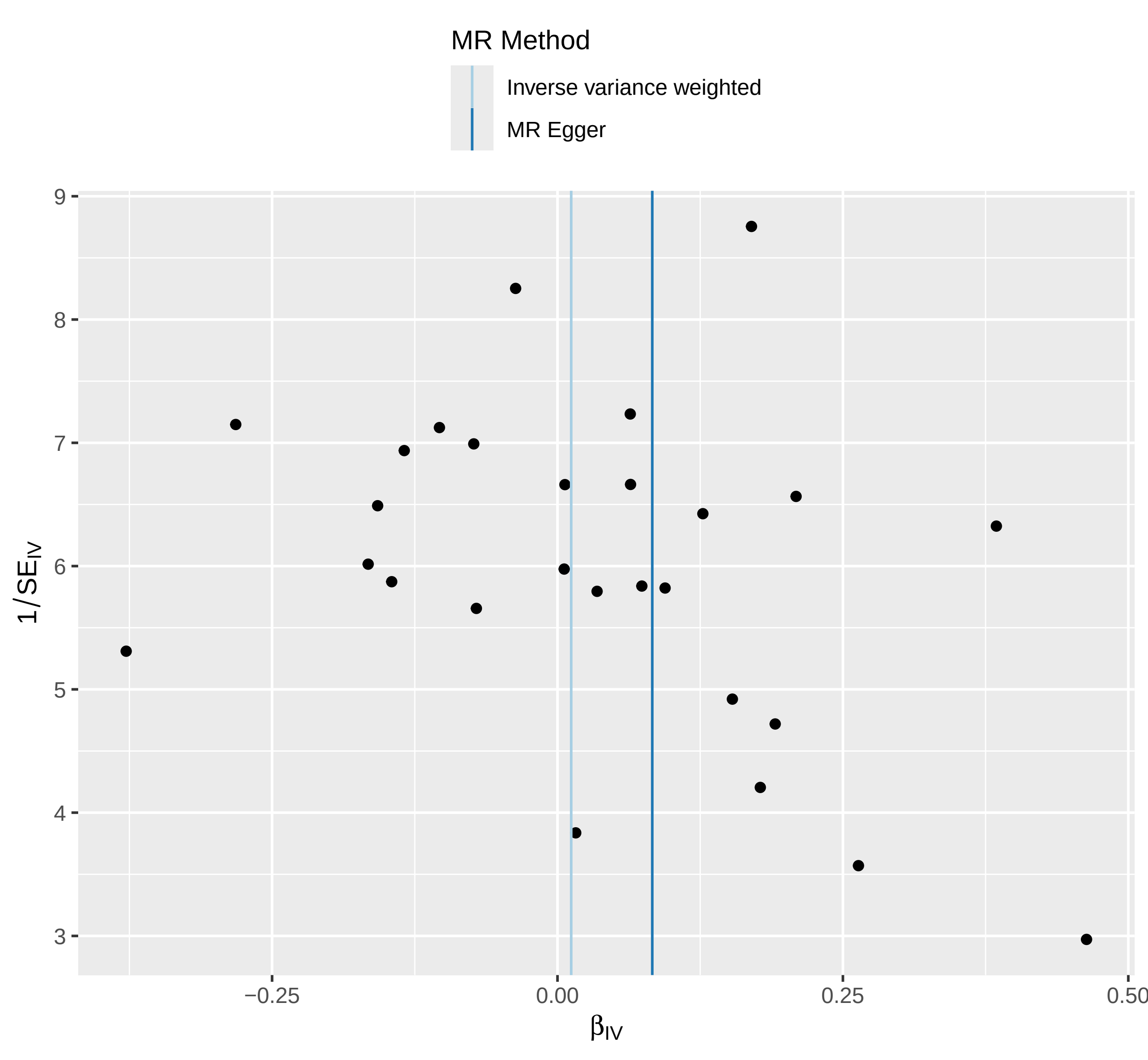
MR Method
Inverse variance weighted
MR Egger
9
8
7
6
5
4
3
1/SE_IV
−0.25
0.00
0.25
0.50
β_IV

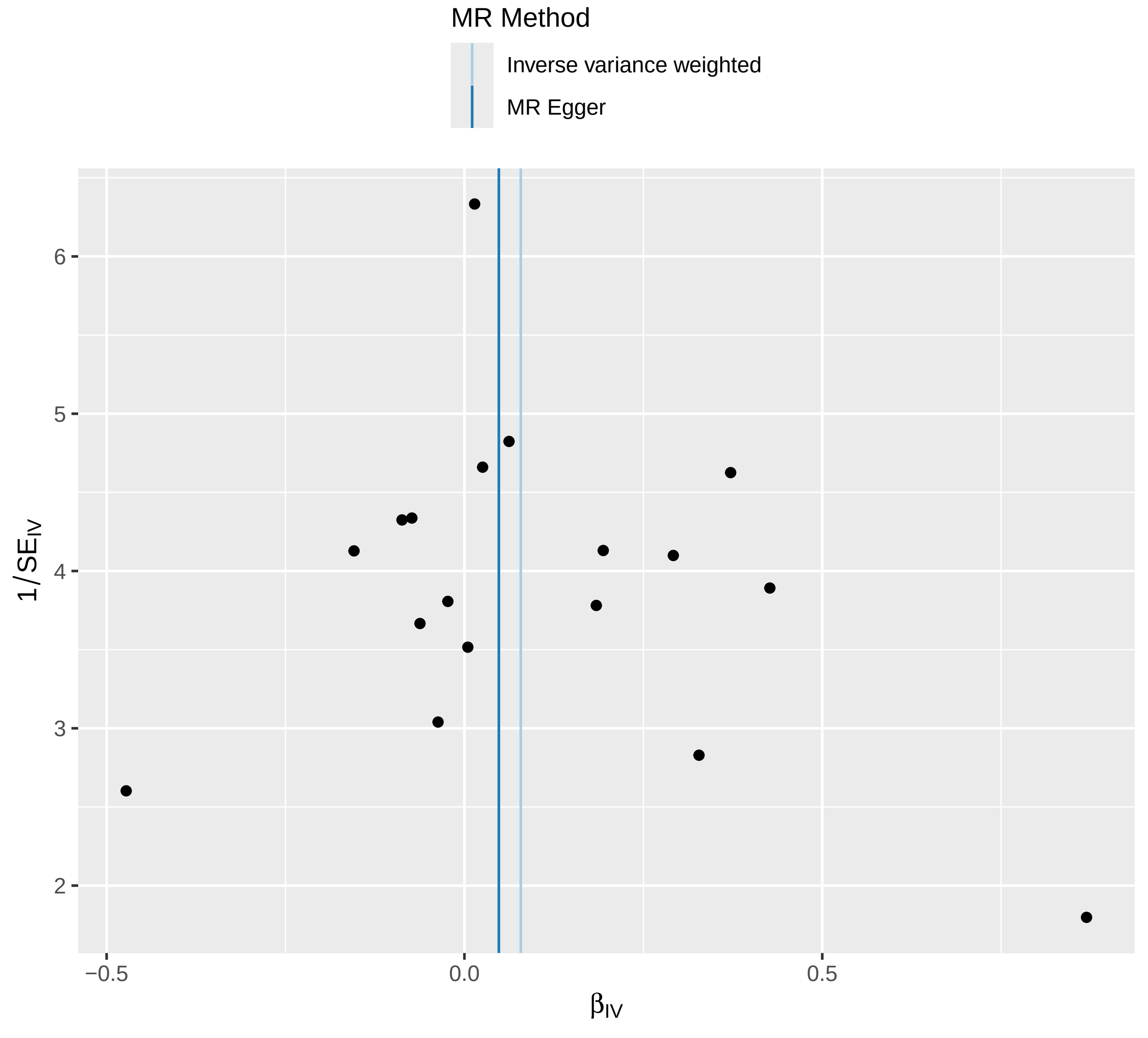

MR Method
Inverse variance weighted
MR Egger
6
5
4
3
2
1/SE$_{IV}$
−0.5
0.0
0.5
β$_{IV}$

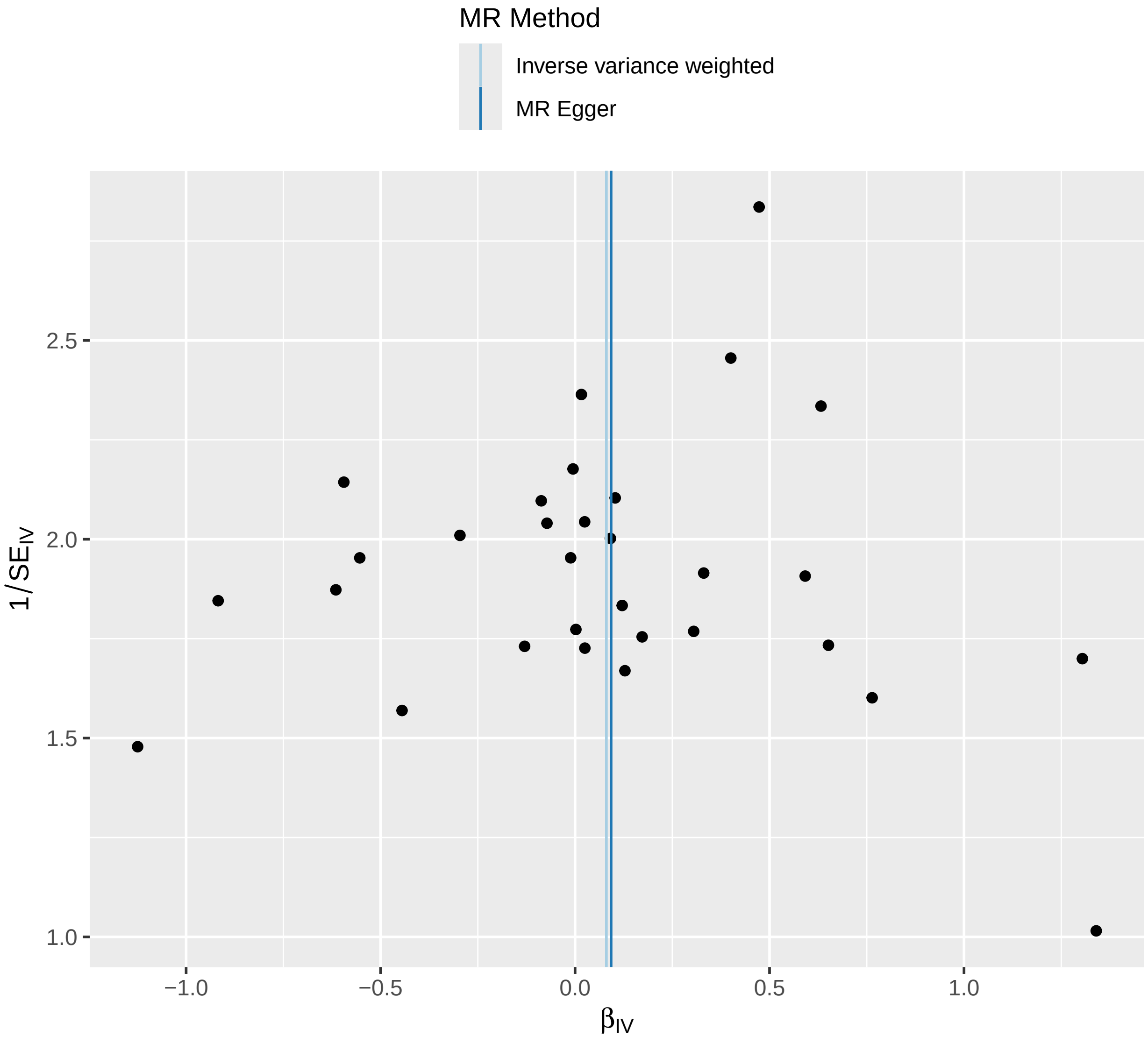
MR Method
Inverse variance weighted
MR Egger
1/SE$_{IV}$
2.5
2.0
1.5
1.0
−1.0
−0.5
0.0
0.5
1.0
β$_{IV}$

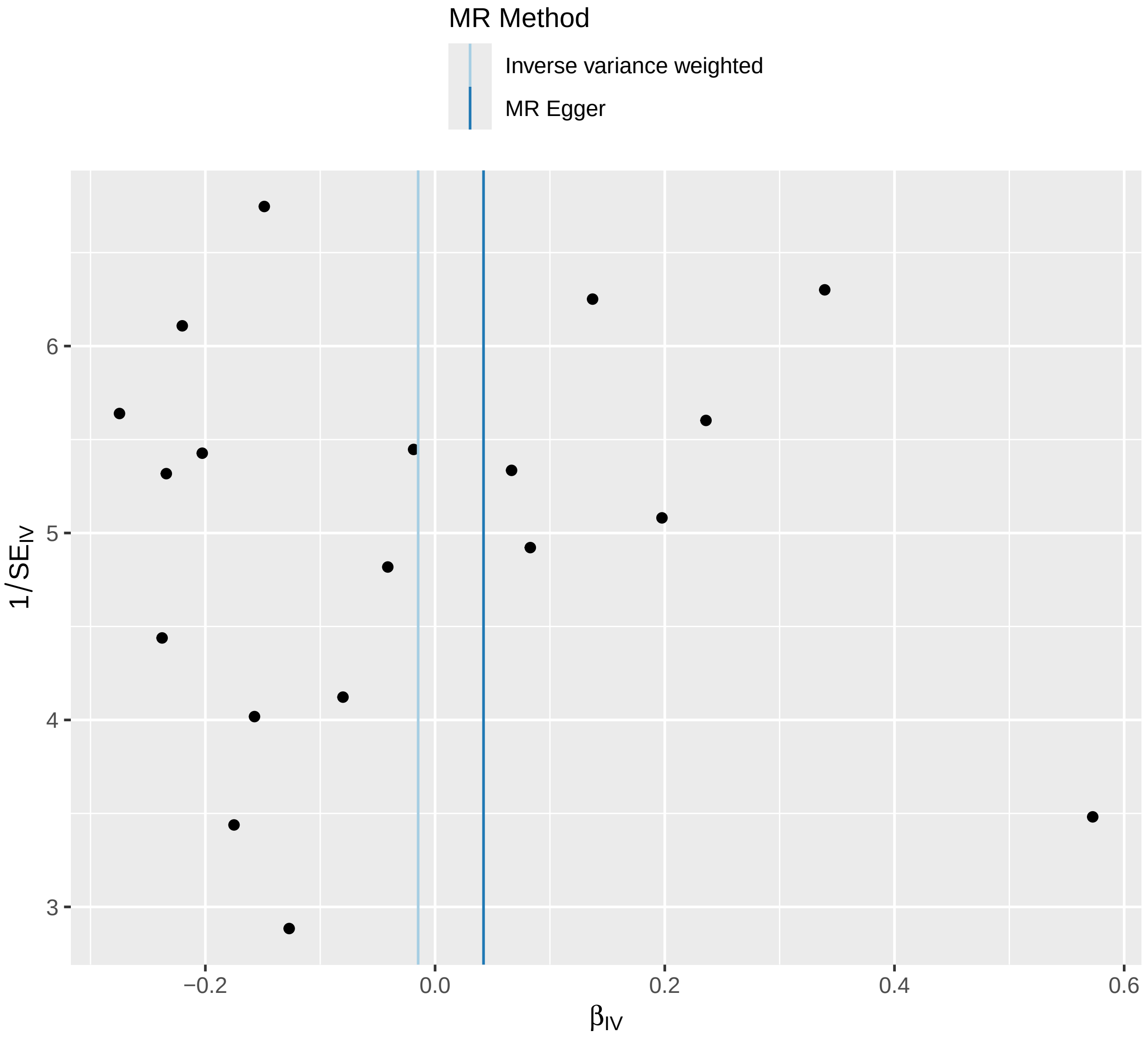
MR Method
Inverse variance weighted
MR Egger
6
5
4
3
1/SE_IV
−0.2
0.0
0.2
0.4
0.6
β_IV

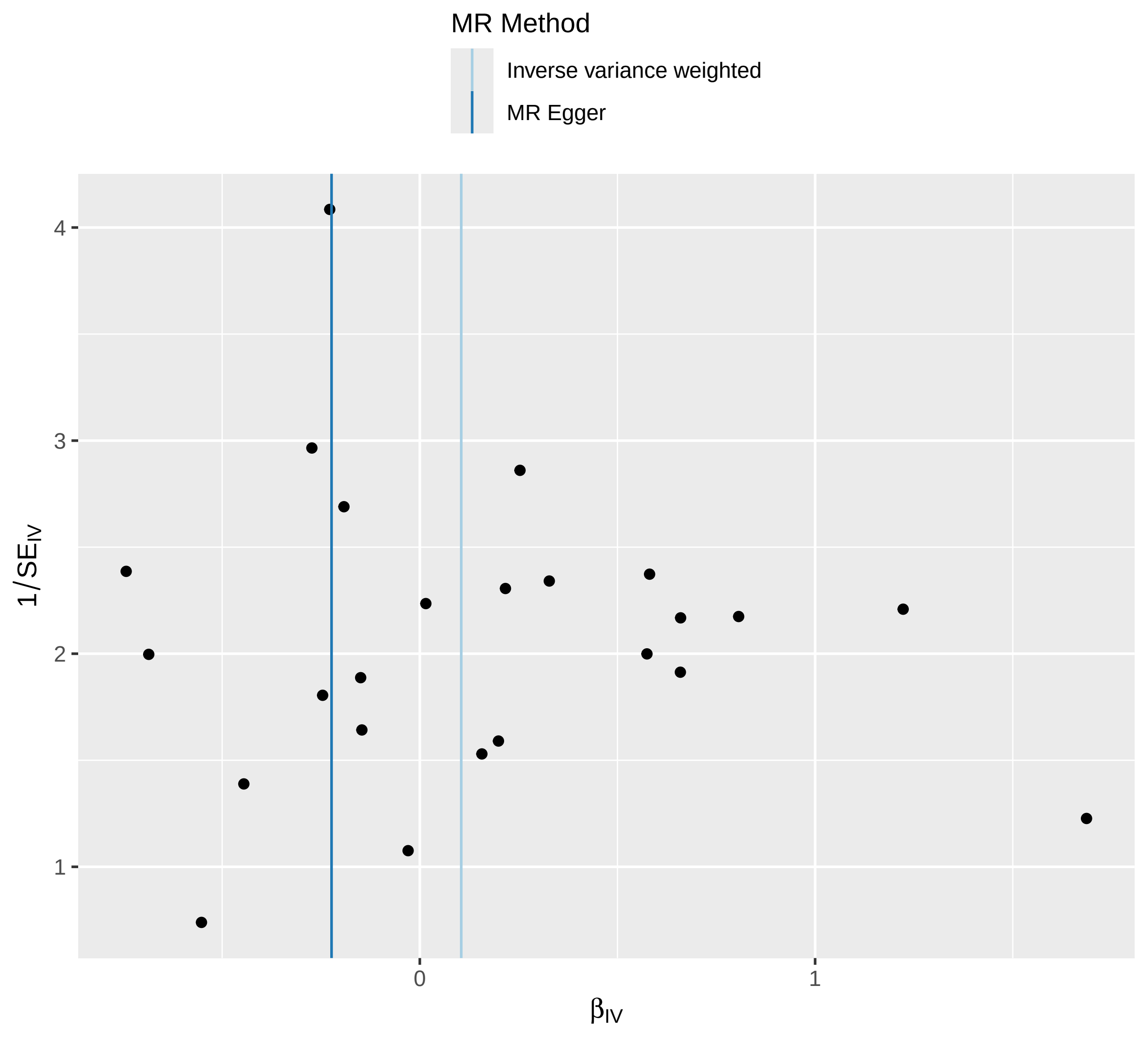
MR Method
Inverse variance weighted
MR Egger
1/SE$_{IV}$
4
3
2
1
0
1
β$_{IV}$

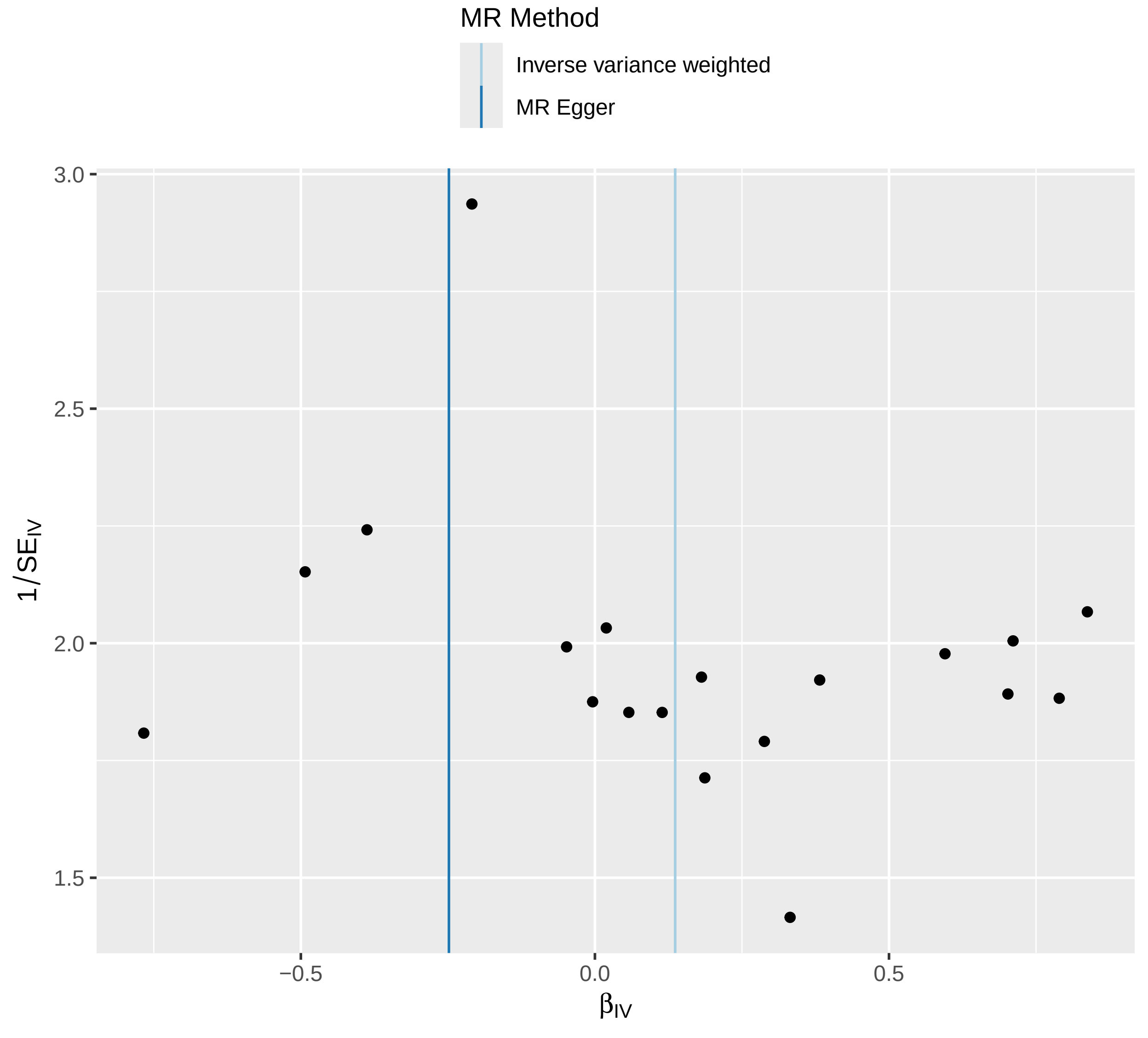
MR Method
Inverse variance weighted
MR Egger
3.0
2.5
2.0
1.5
1/SE$_{IV}$
−0.5
0.0
0.5
β$_{IV}$

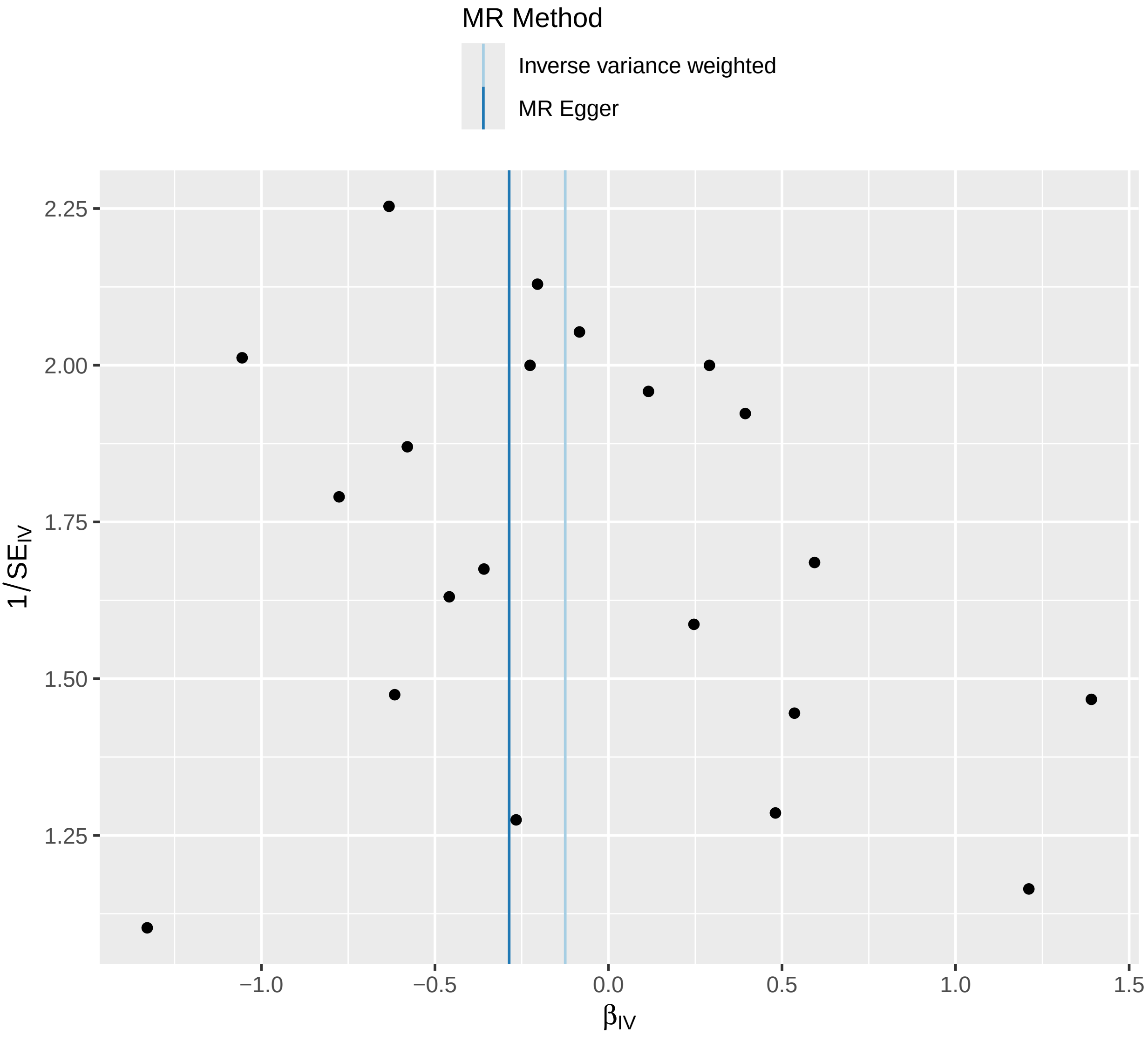
MR Method
Inverse variance weighted
MR Egger
2.25
2.00
1.75
1.50
1.25
$1/SE_{IV}$
−1.0
−0.5
0.0
0.5
1.0
1.5
β_{IV}

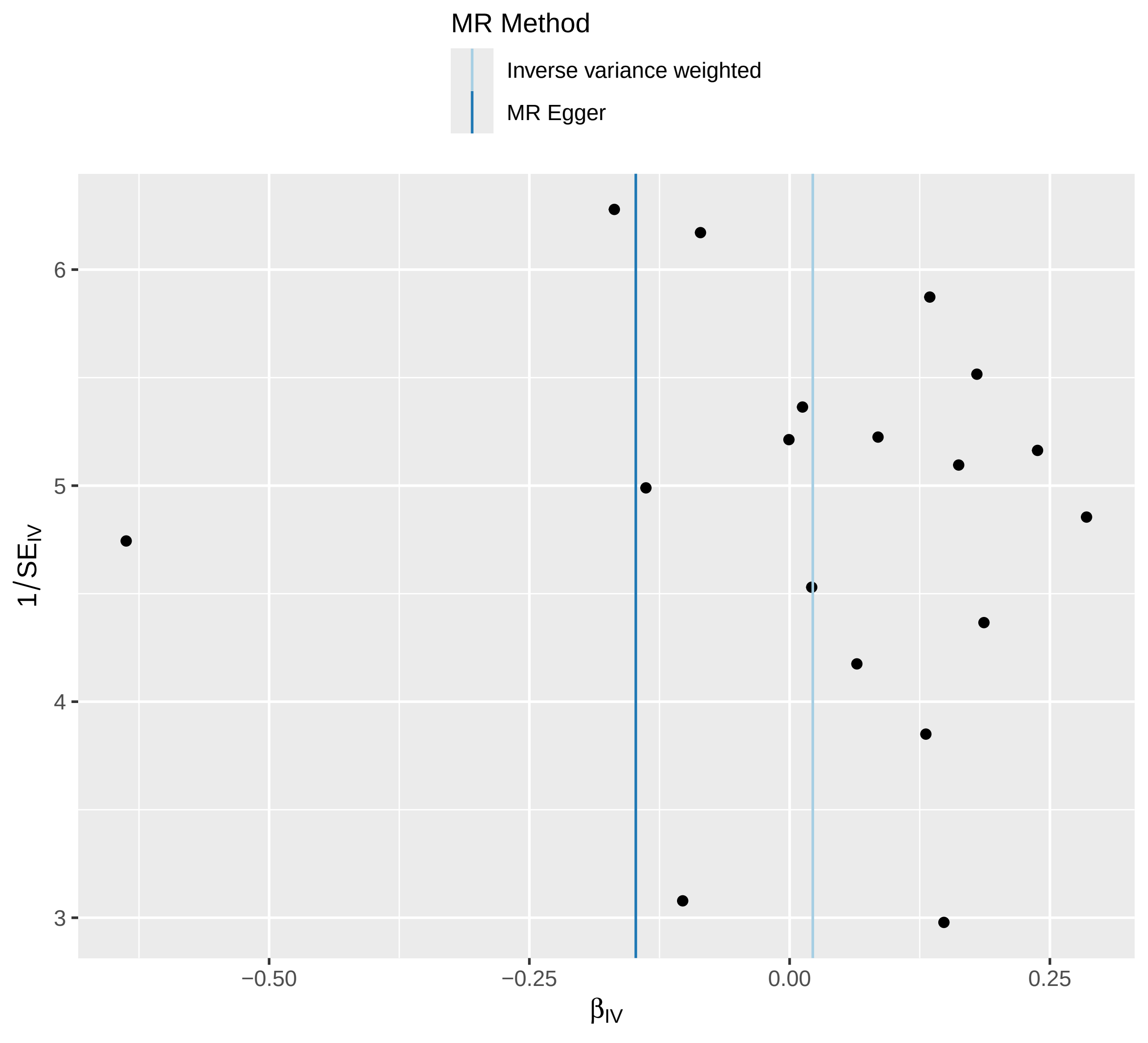
MR Method
Inverse variance weighted
MR Egger
1/SE_IV
6
5
4
3
β_IV
−0.50
−0.25
0.00
0.25

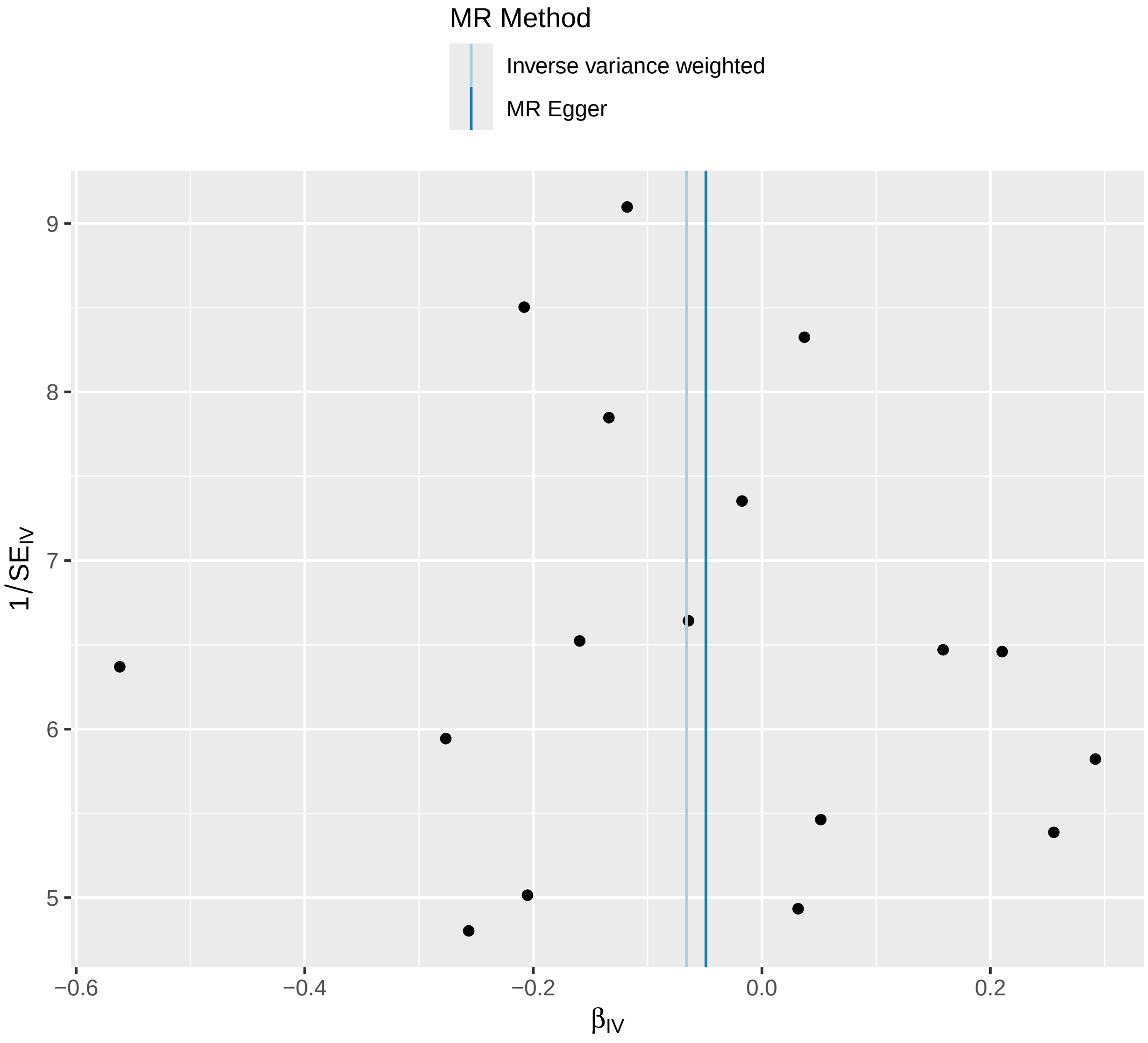
MR Method
Inverse variance weighted
MR Egger
9
8
7
6
5
$1/SE_{IV}$
−0.6
−0.4
−0.2
0.0
0.2
β_{IV}

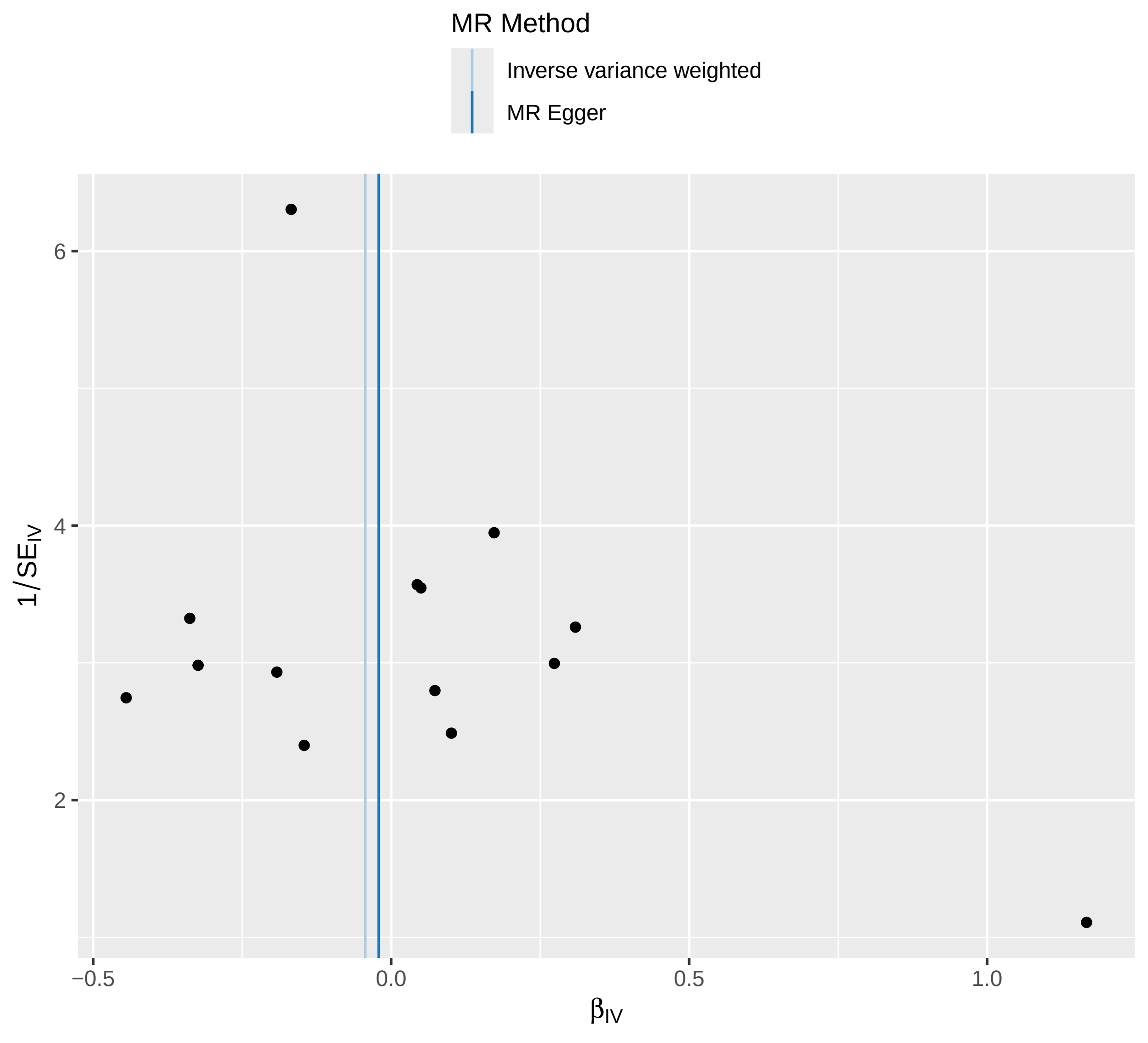

MR Method
Inverse variance weighted
MR Egger
6
4
2
$1/SE_{IV}$
−0.5
0.0
0.5
1.0
β_{IV}

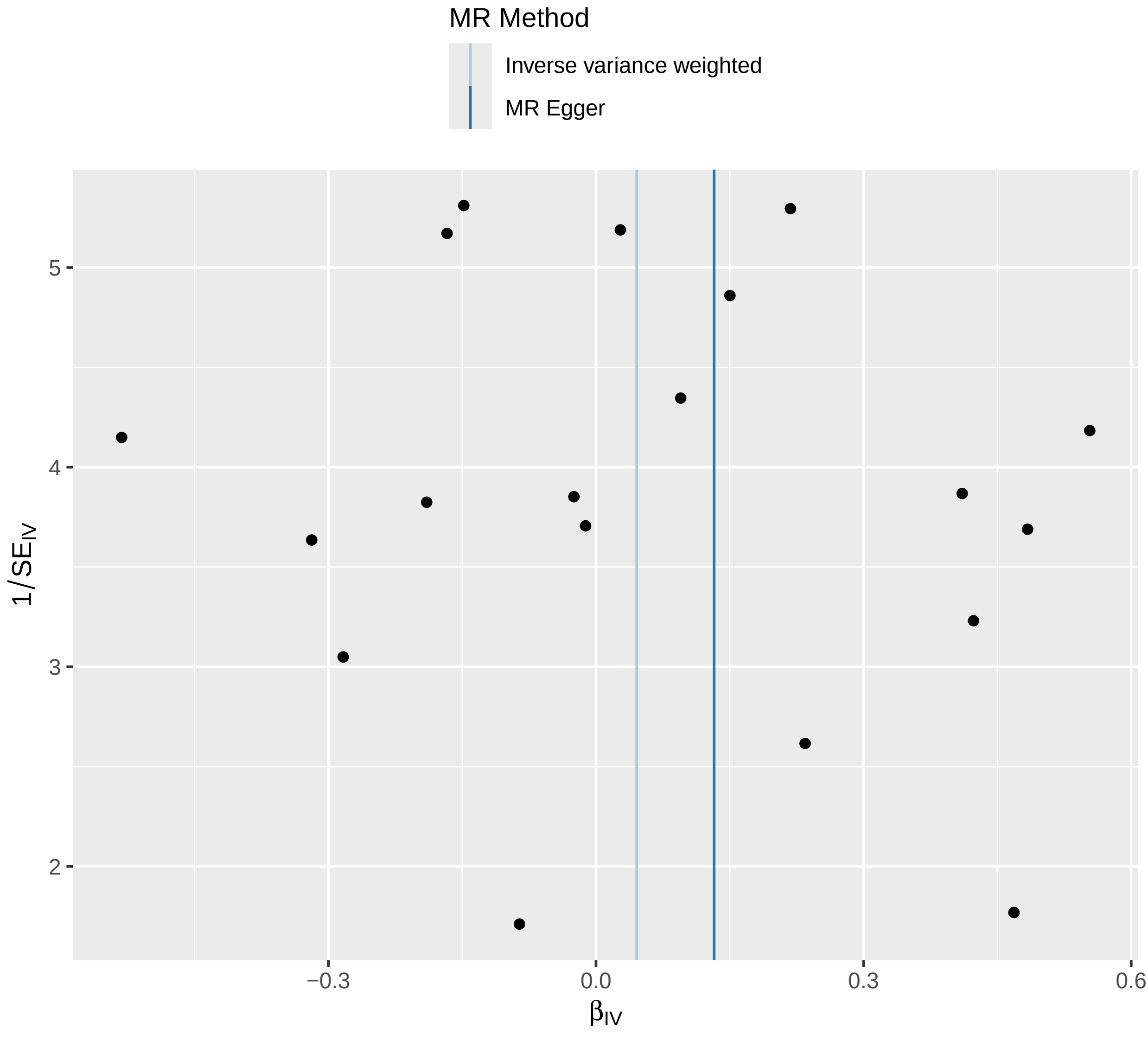
MR Method
Inverse variance weighted
MR Egger
5
4
3
2
$1/SE_{IV}$
−0.3
0.0
0.3
0.6
β_{IV}

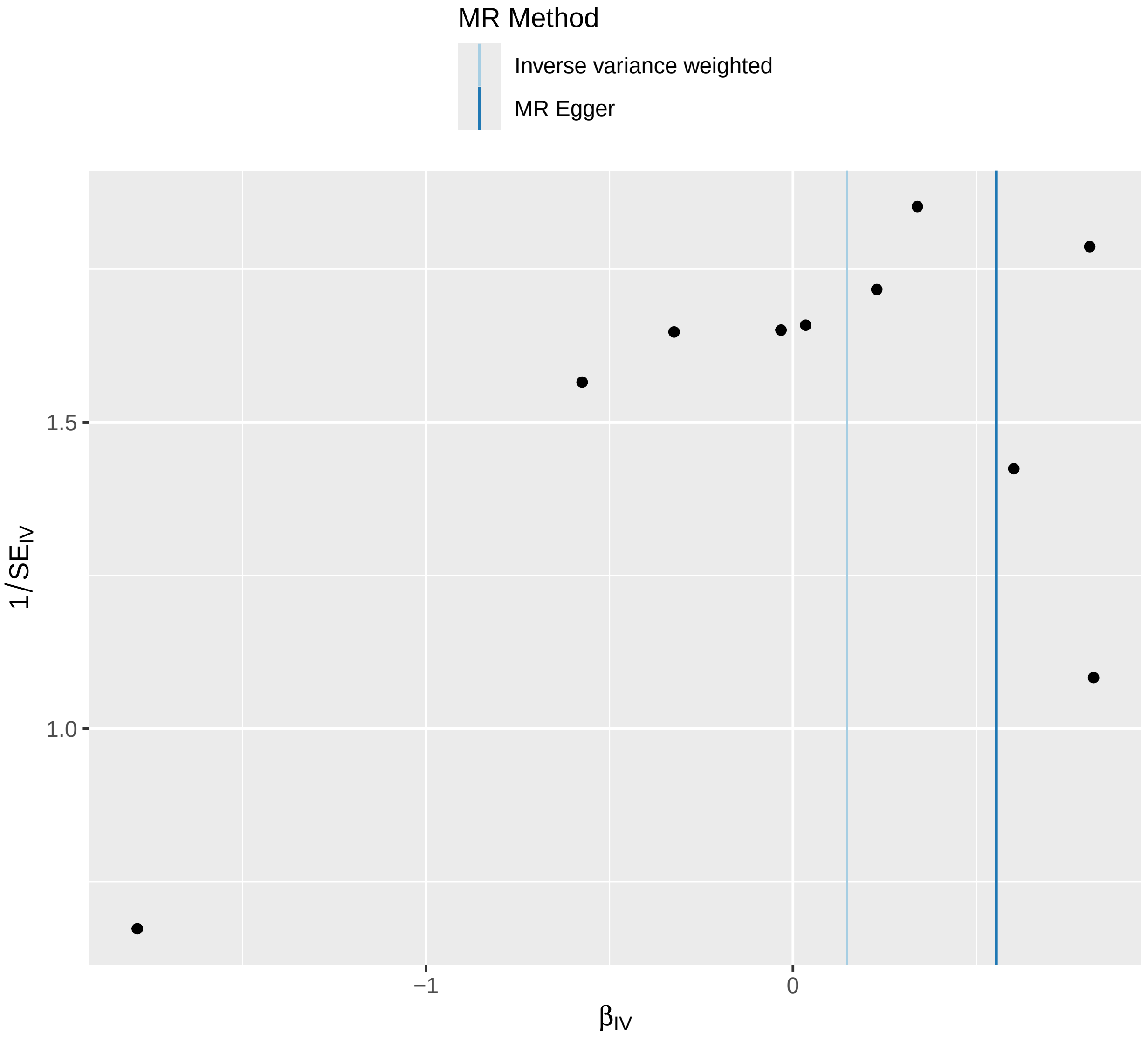

MR Method
Inverse variance weighted
MR Egger
1.5
1.0
1/SE$_{IV}$
−1
0
β_{IV}

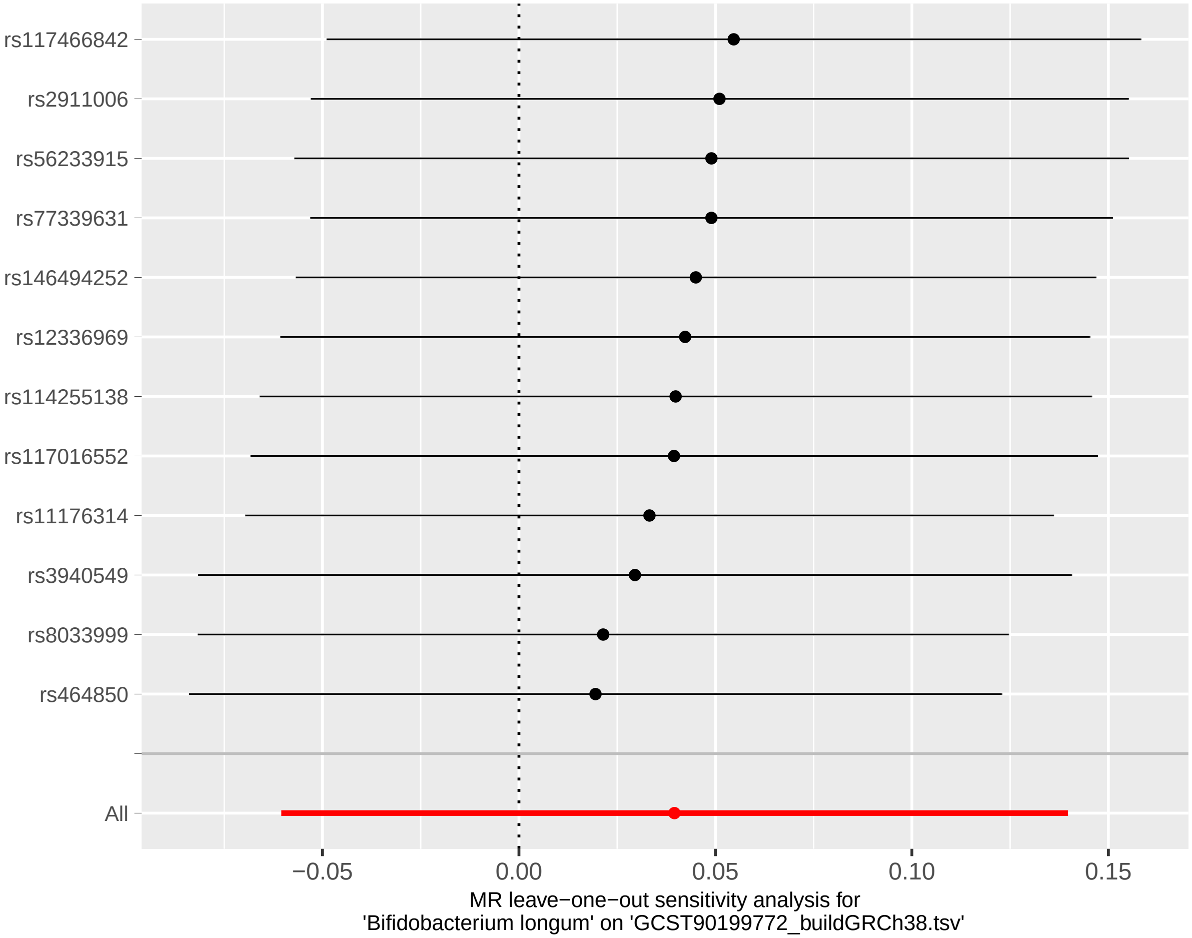
rs117466842
rs2911006
rs56233915
rs77339631
rs146494252
rs12336969
rs114255138
rs117016552
rs11176314
rs3940549
rs8033999
rs464850
All
−0.05
0.00
0.05
0.10
0.15
MR leave−one−out sensitivity analysis for
'Bifidobacterium longum' on 'GCST90199772_buildGRCh38.tsv'

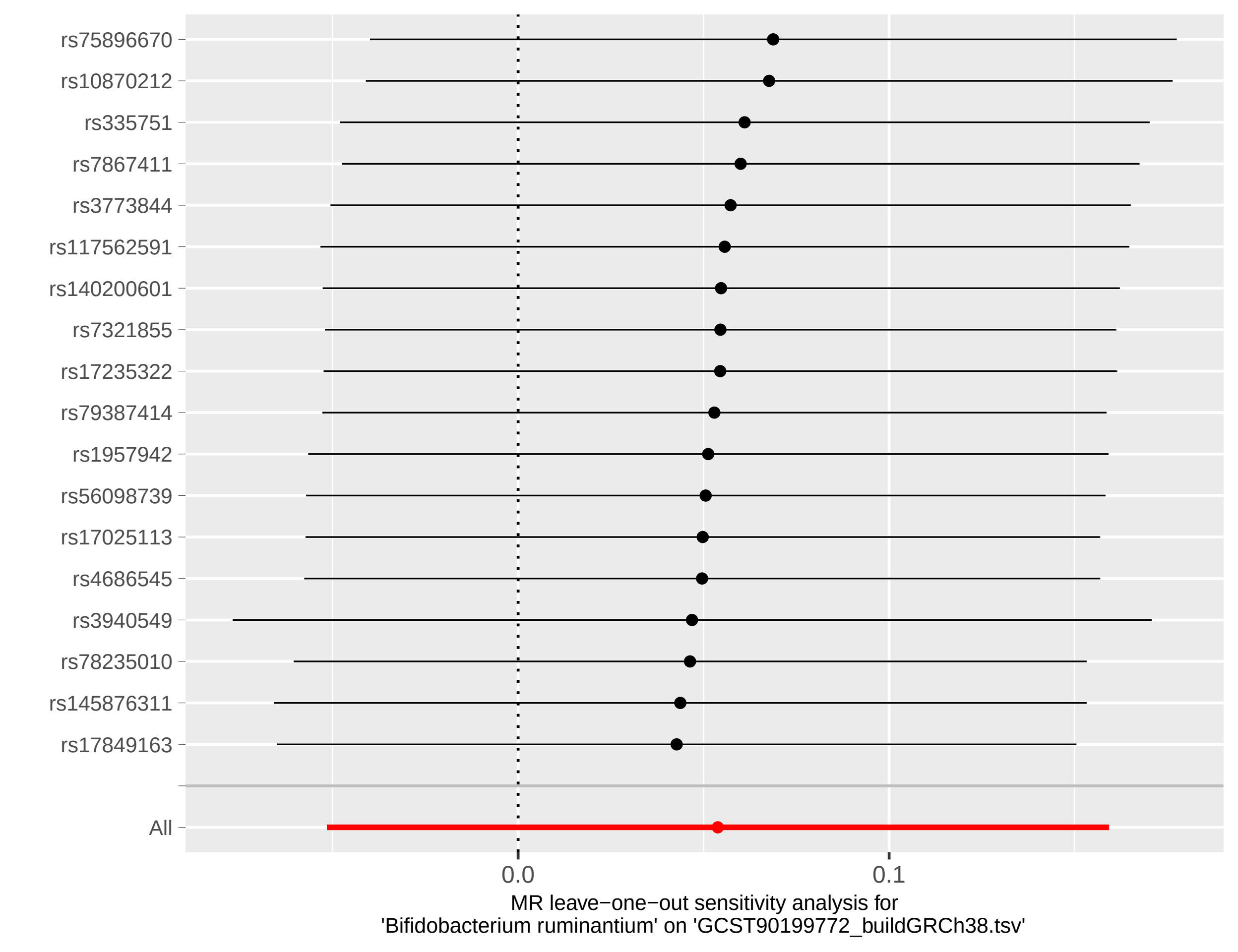
rs75896670
rs10870212
rs335751
rs7867411
rs3773844
rs117562591
rs140200601
rs7321855
rs17235322
rs79387414
rs1957942
rs56098739
rs17025113
rs4686545
rs3940549
rs78235010
rs145876311
rs17849163
All
0.0
0.1
MR leave−one−out sensitivity analysis for
'Bifidobacterium ruminantium' on 'GCST90199772_buildGRCh38.tsv'

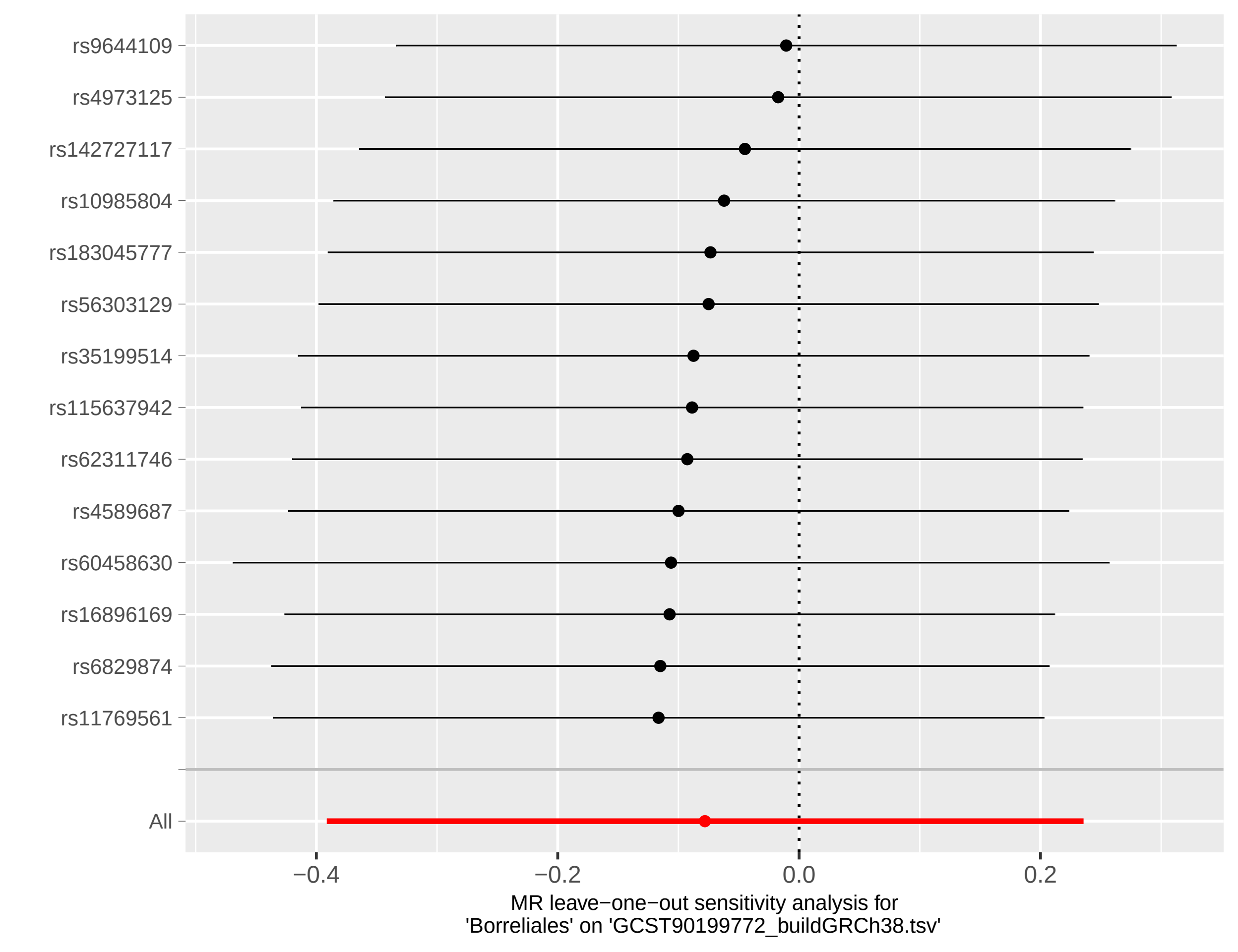

rs9644109
rs4973125
rs142727117
rs10985804
rs183045777
rs56303129
rs35199514
rs115637942
rs62311746
rs4589687
rs60458630
rs16896169
rs6829874
rs11769561
All
−0.4
−0.2
0.0
0.2
MR leave−one−out sensitivity analysis for
'Borreliales' on 'GCST90199772_buildGRCh38.tsv'

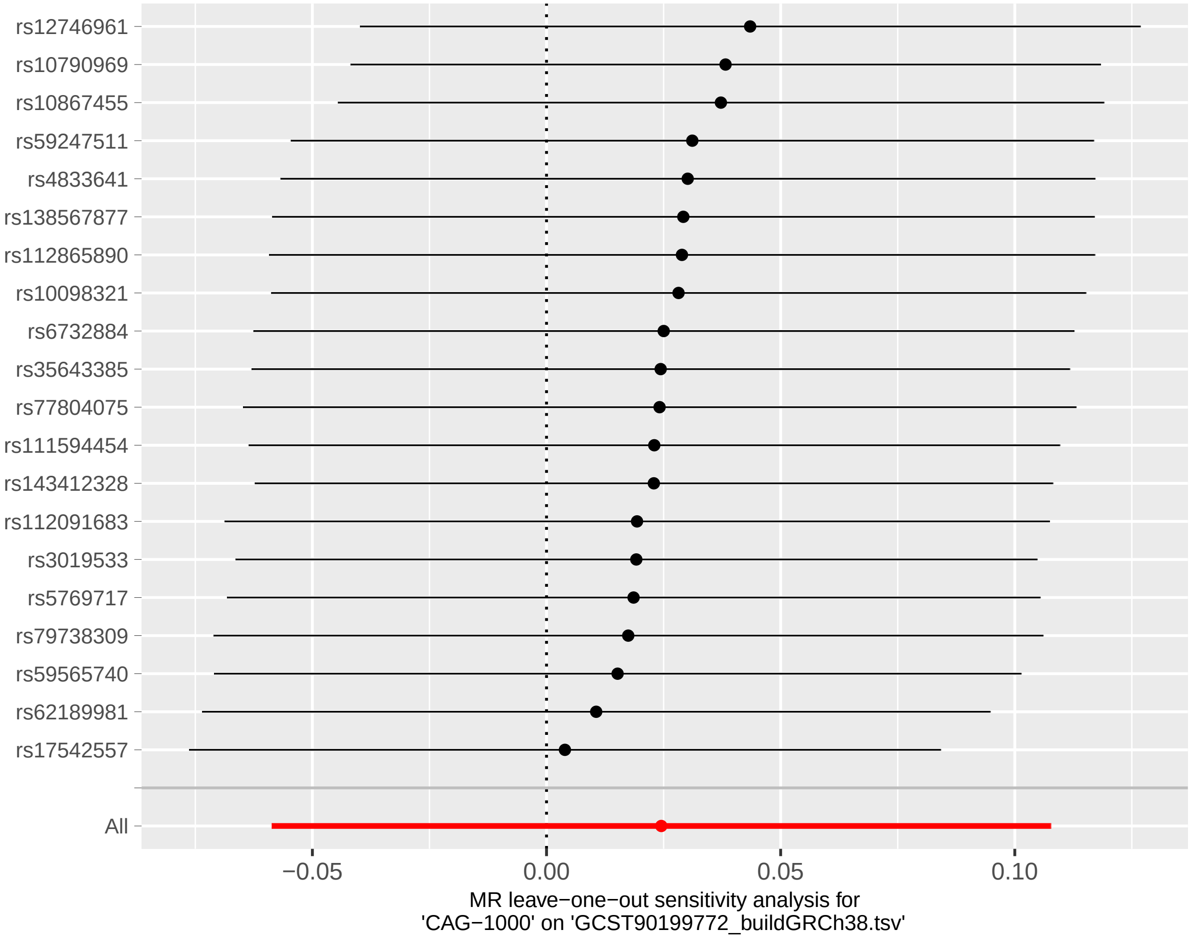
rs12746961
rs10790969
rs10867455
rs59247511
rs4833641
rs138567877
rs112865890
rs10098321
rs6732884
rs35643385
rs77804075
rs111594454
rs143412328
rs112091683
rs3019533
rs5769717
rs79738309
rs59565740
rs62189981
rs17542557
All
−0.05
0.00
0.05
0.10
MR leave−one−out sensitivity analysis for
'CAG−1000' on 'GCST90199772_buildGRCh38.tsv'

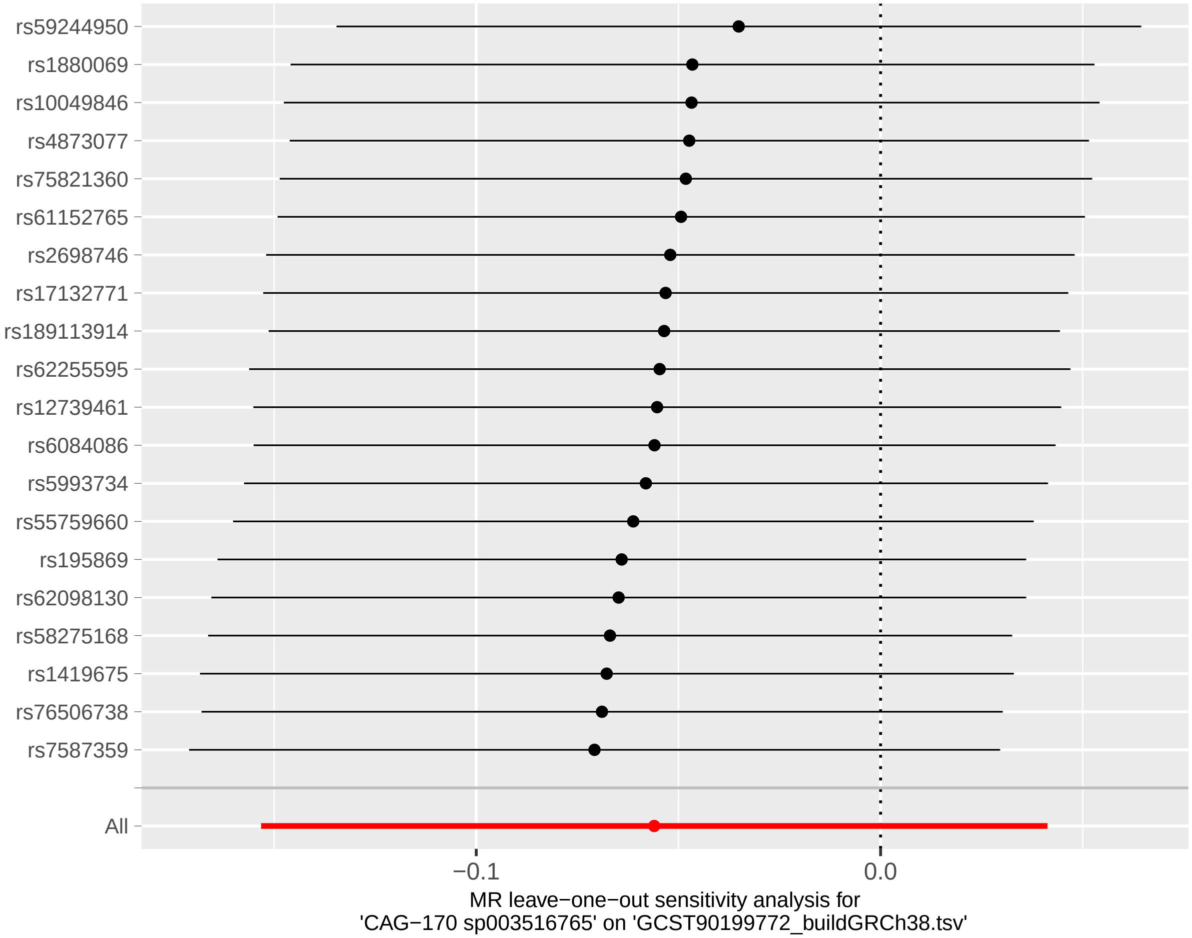
rs59244950
rs1880069
rs10049846
rs4873077
rs75821360
rs61152765
rs2698746
rs17132771
rs189113914
rs62255595
rs12739461
rs6084086
rs5993734
rs55759660
rs195869
rs62098130
rs58275168
rs1419675
rs76506738
rs7587359
All
−0.1
0.0
MR leave−one−out sensitivity analysis for
'CAG−170 sp003516765' on 'GCST90199772_buildGRCh38.tsv'

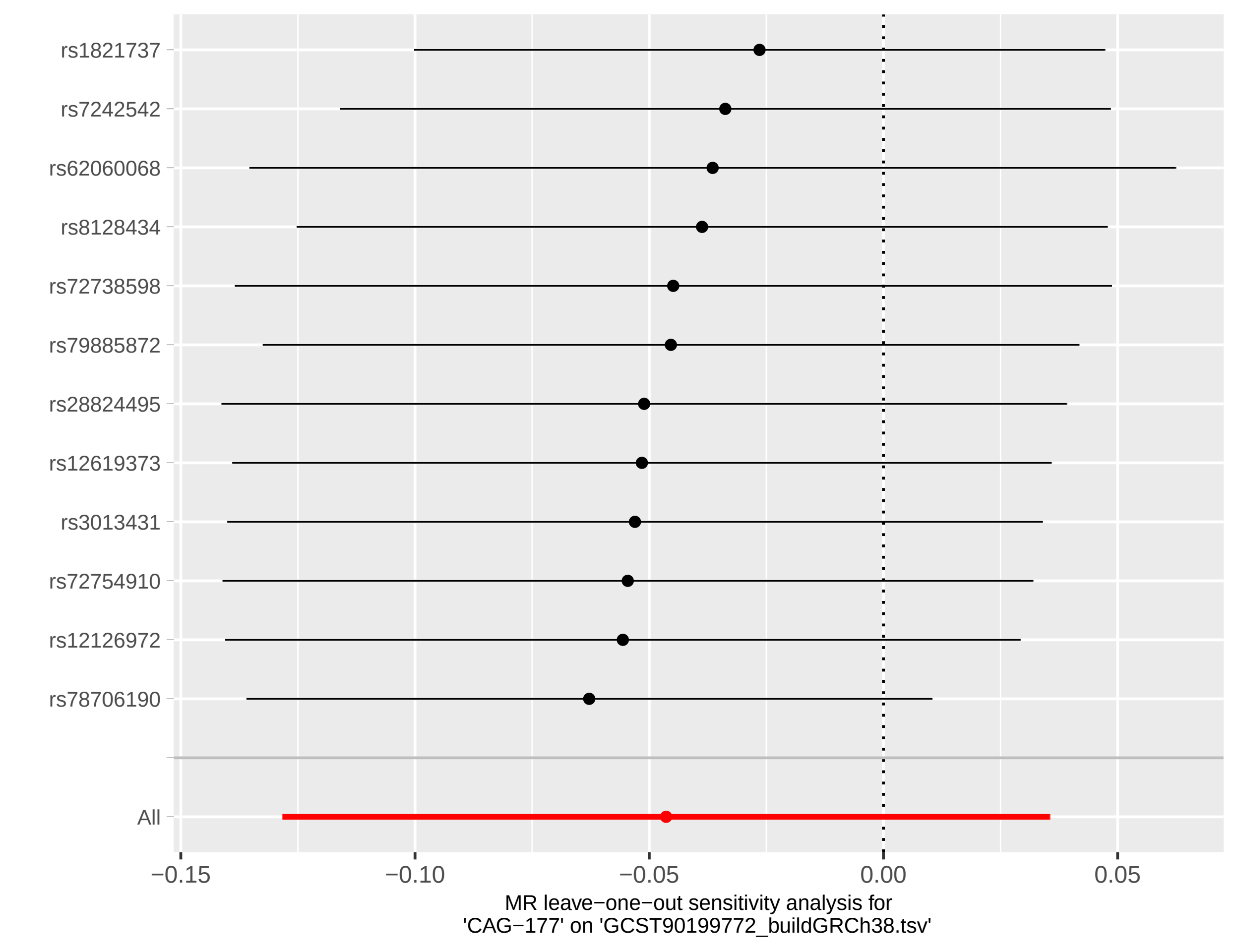

rs1821737
rs7242542
rs62060068
rs8128434
rs72738598
rs79885872
rs28824495
rs12619373
rs3013431
rs72754910
rs12126972
rs78706190
All
−0.15
−0.10
−0.05
0.00
0.05
MR leave−one−out sensitivity analysis for
'CAG−177' on 'GCST90199772_buildGRCh38.tsv'

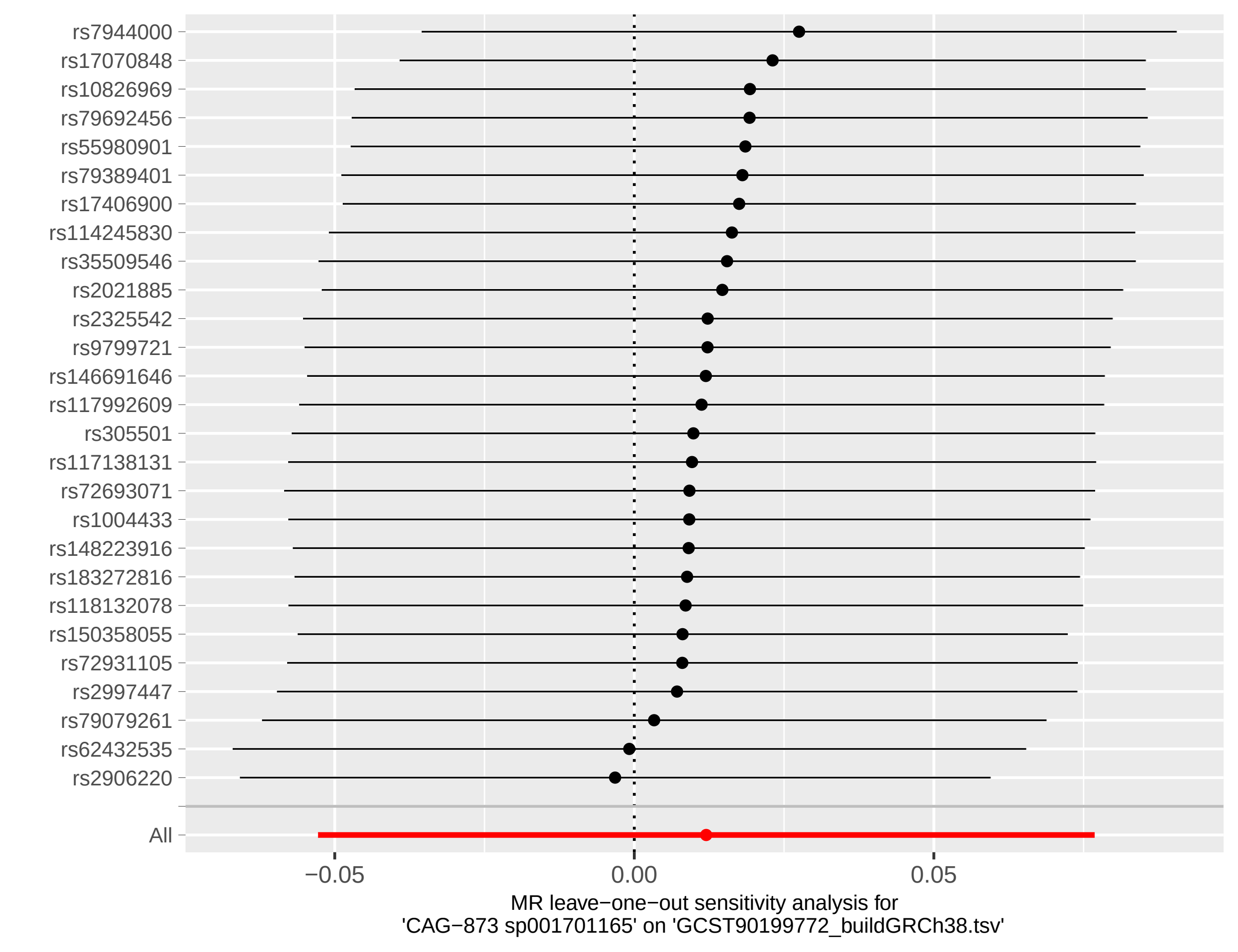

rs7944000
rs17070848
rs10826969
rs79692456
rs55980901
rs79389401
rs17406900
rs114245830
rs35509546
rs2021885
rs2325542
rs9799721
rs146691646
rs117992609
rs305501
rs117138131
rs72693071
rs1004433
rs148223916
rs183272816
rs118132078
rs150358055
rs72931105
rs2997447
rs79079261
rs62432535
rs2906220
All
−0.05
0.00
0.05
MR leave−one−out sensitivity analysis for
'CAG−873 sp001701165' on 'GCST90199772_buildGRCh38.tsv'

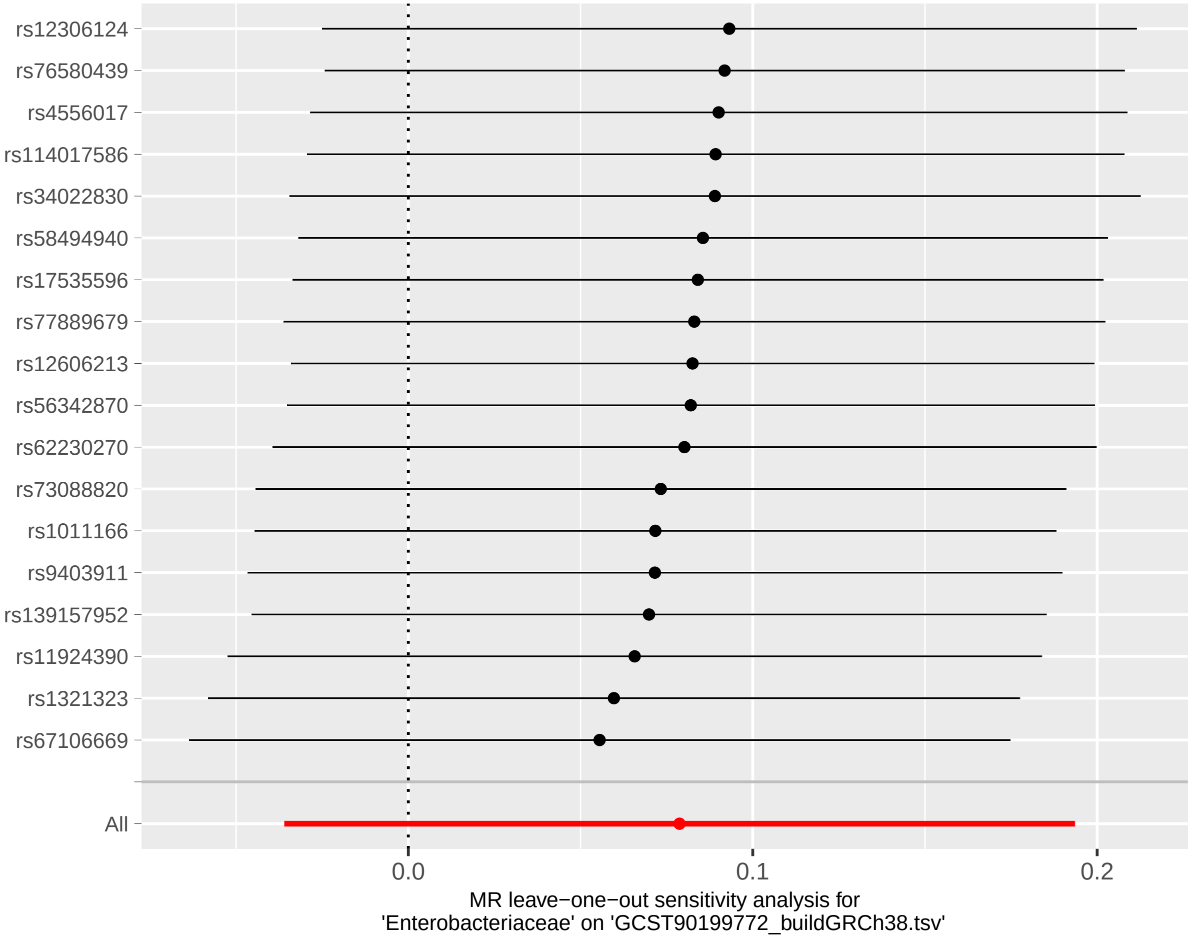

rs12306124
rs76580439
rs4556017
rs114017586
rs34022830
rs58494940
rs17535596
rs77889679
rs12606213
rs56342870
rs62230270
rs73088820
rs1011166
rs9403911
rs139157952
rs11924390
rs1321323
rs67106669
All
0.0
0.1
0.2
MR leave−one−out sensitivity analysis for
'Enterobacteriaceae' on 'GCST90199772_buildGRCh38.tsv'

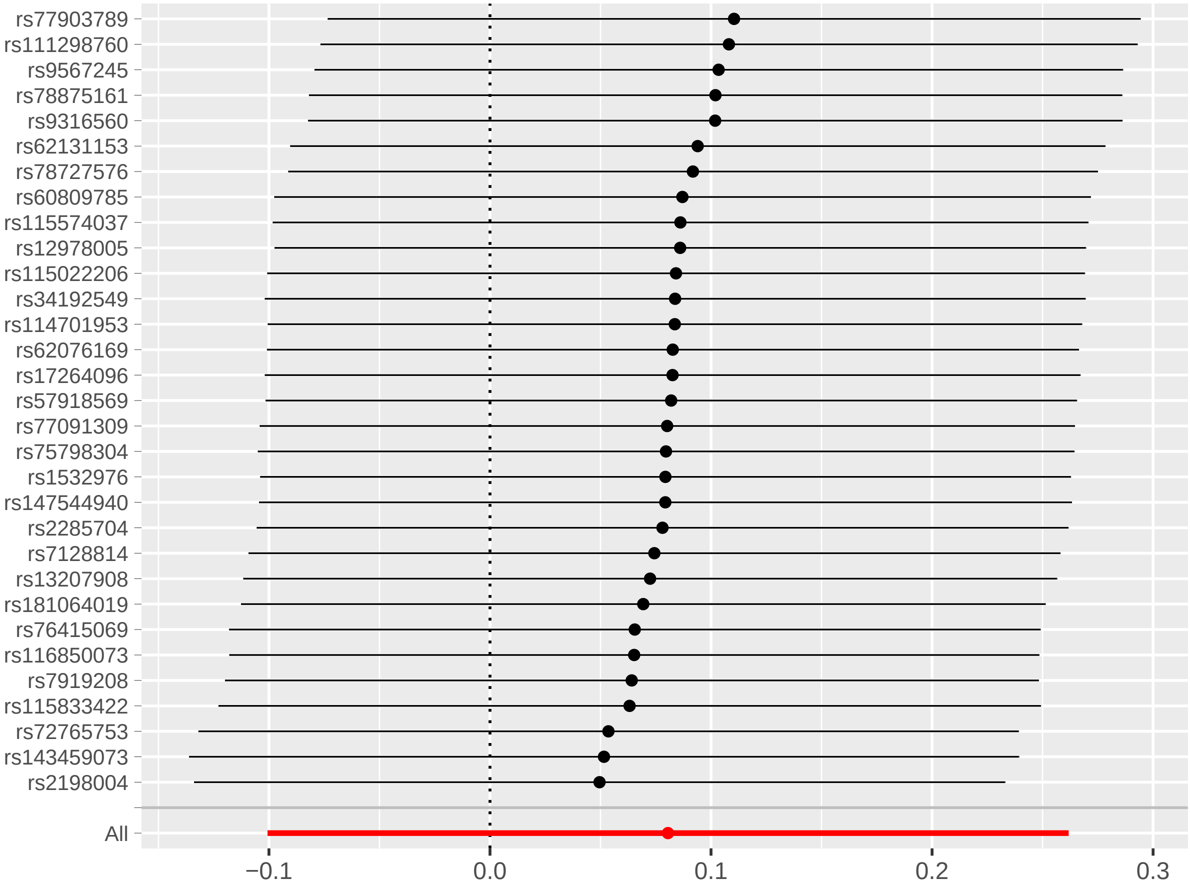

MR leave−one−out sensitivity analysis for
'Enterococcus B' on 'GCST90199772_buildGRCh38.tsv'

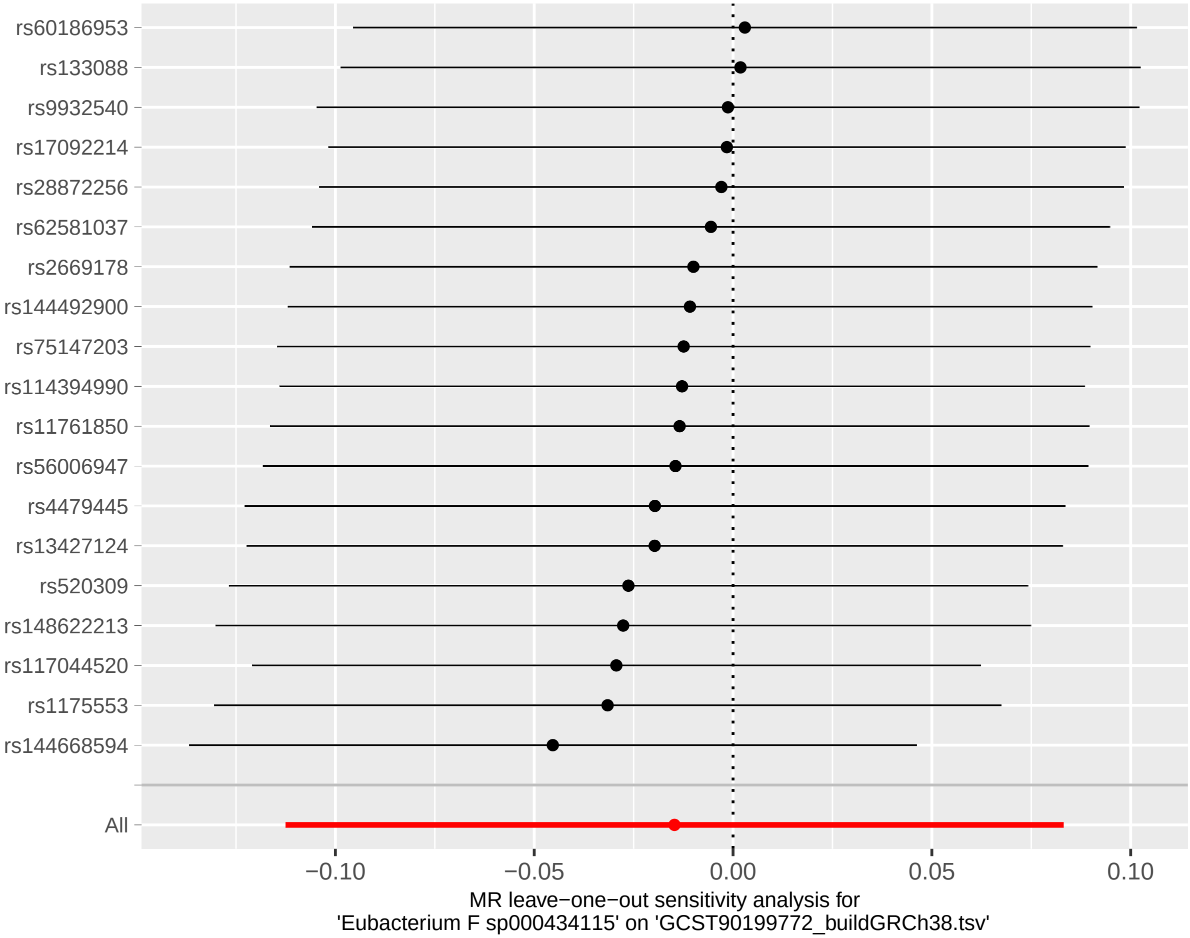
rs60186953
rs133088
rs9932540
rs17092214
rs28872256
rs62581037
rs2669178
rs144492900
rs75147203
rs114394990
rs11761850
rs56006947
rs4479445
rs13427124
rs520309
rs148622213
rs117044520
rs1175553
rs144668594
All
−0.10
−0.05
0.00
0.05
0.10
MR leave−one−out sensitivity analysis for
'Eubacterium F sp000434115' on 'GCST90199772_buildGRCh38.tsv'

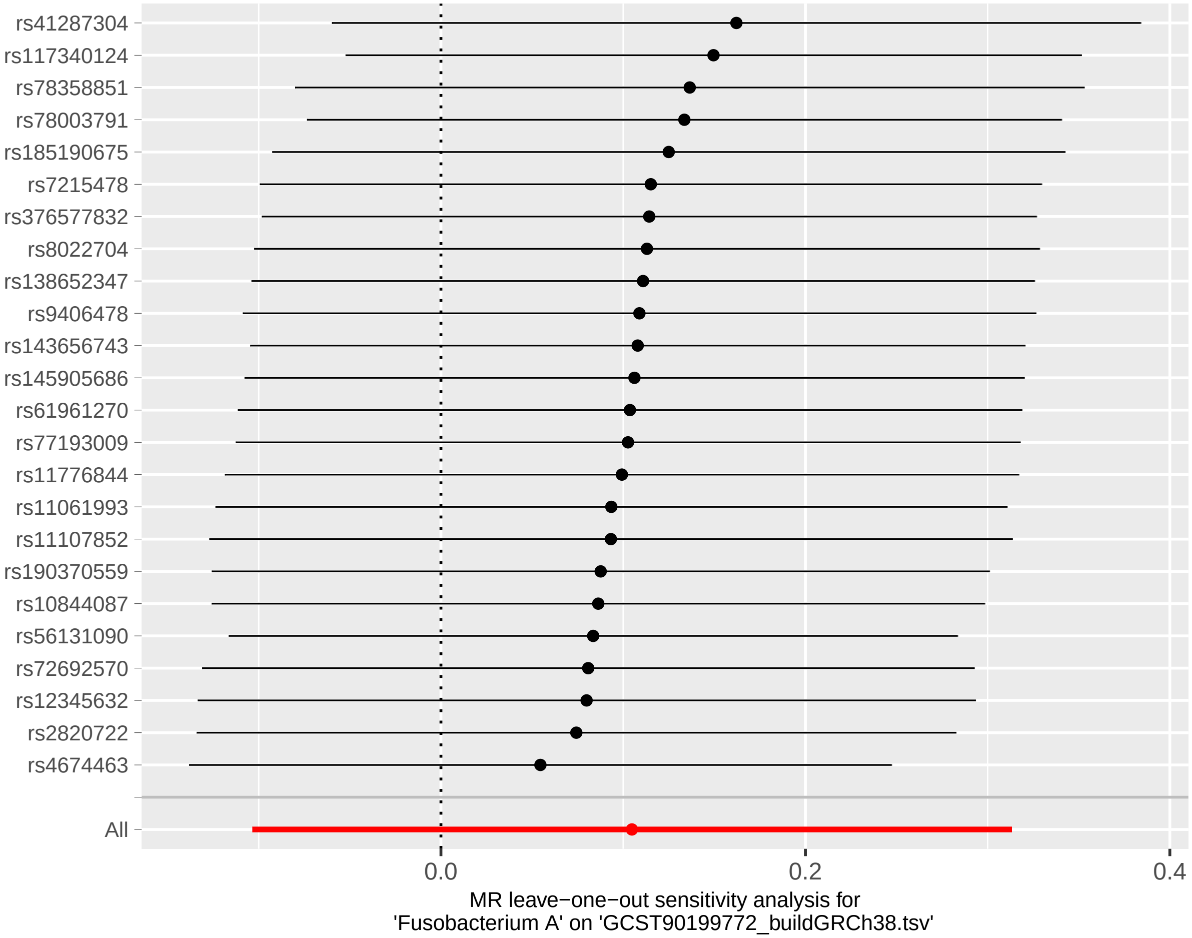
rs41287304
rs117340124
rs78358851
rs78003791
rs185190675
rs7215478
rs376577832
rs8022704
rs138652347
rs9406478
rs143656743
rs145905686
rs61961270
rs77193009
rs11776844
rs11061993
rs11107852
rs190370559
rs10844087
rs56131090
rs72692570
rs12345632
rs2820722
rs4674463
All
0.0
0.2
0.4
MR leave−one−out sensitivity analysis for
'Fusobacterium A' on 'GCST90199772_buildGRCh38.tsv'

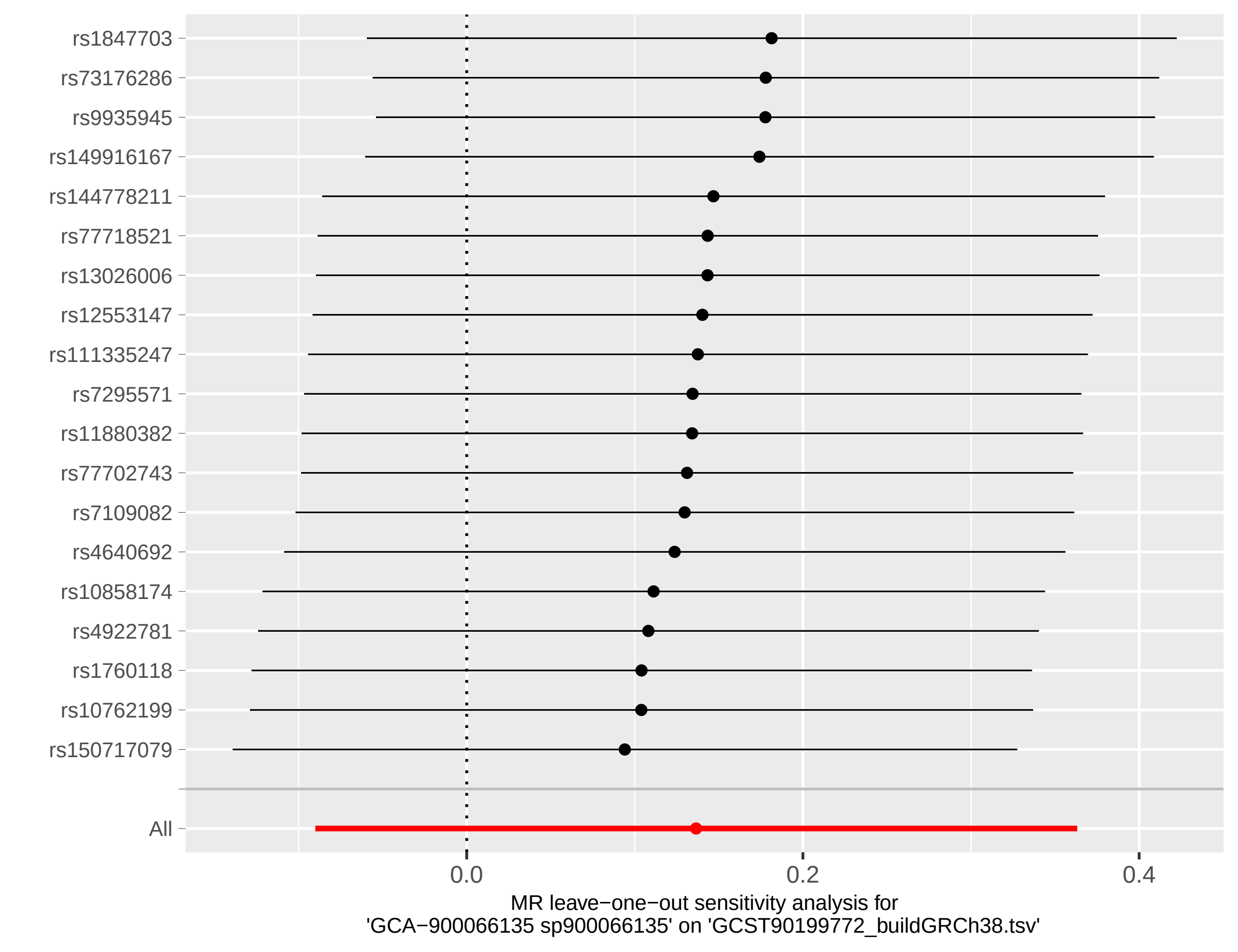
rs1847703
rs73176286
rs9935945
rs149916167
rs144778211
rs77718521
rs13026006
rs12553147
rs111335247
rs7295571
rs11880382
rs77702743
rs7109082
rs4640692
rs10858174
rs4922781
rs1760118
rs10762199
rs150717079
All
0.0
0.2
0.4
MR leave−one−out sensitivity analysis for
'GCA−900066135 sp900066135' on 'GCST90199772_buildGRCh38.tsv'

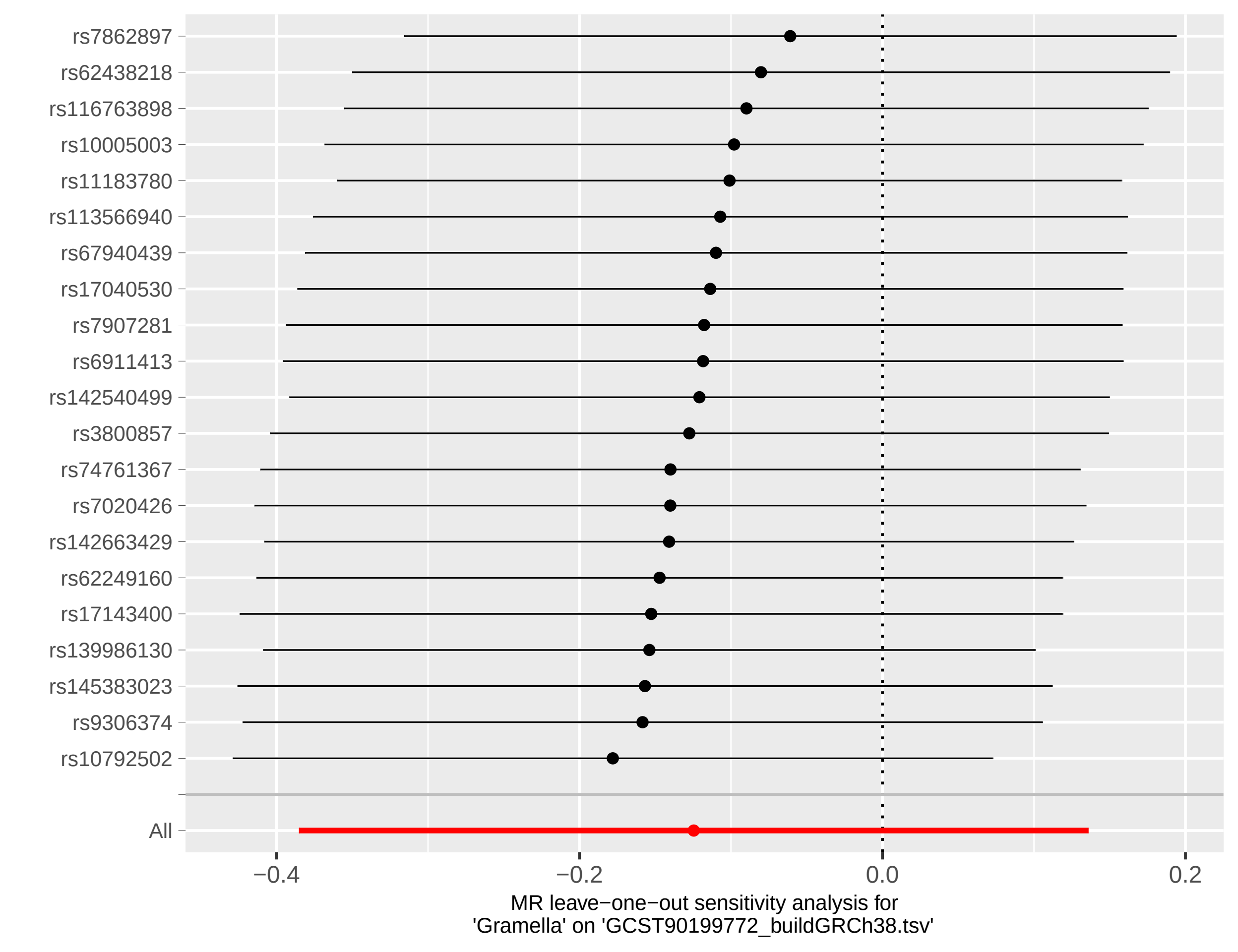
rs7862897
rs62438218
rs116763898
rs10005003
rs11183780
rs113566940
rs67940439
rs17040530
rs7907281
rs6911413
rs142540499
rs3800857
rs74761367
rs7020426
rs142663429
rs62249160
rs17143400
rs139986130
rs145383023
rs9306374
rs10792502
All
−0.4
−0.2
0.0
0.2
MR leave−one−out sensitivity analysis for
'Gramella' on 'GCST90199772_buildGRCh38.tsv'

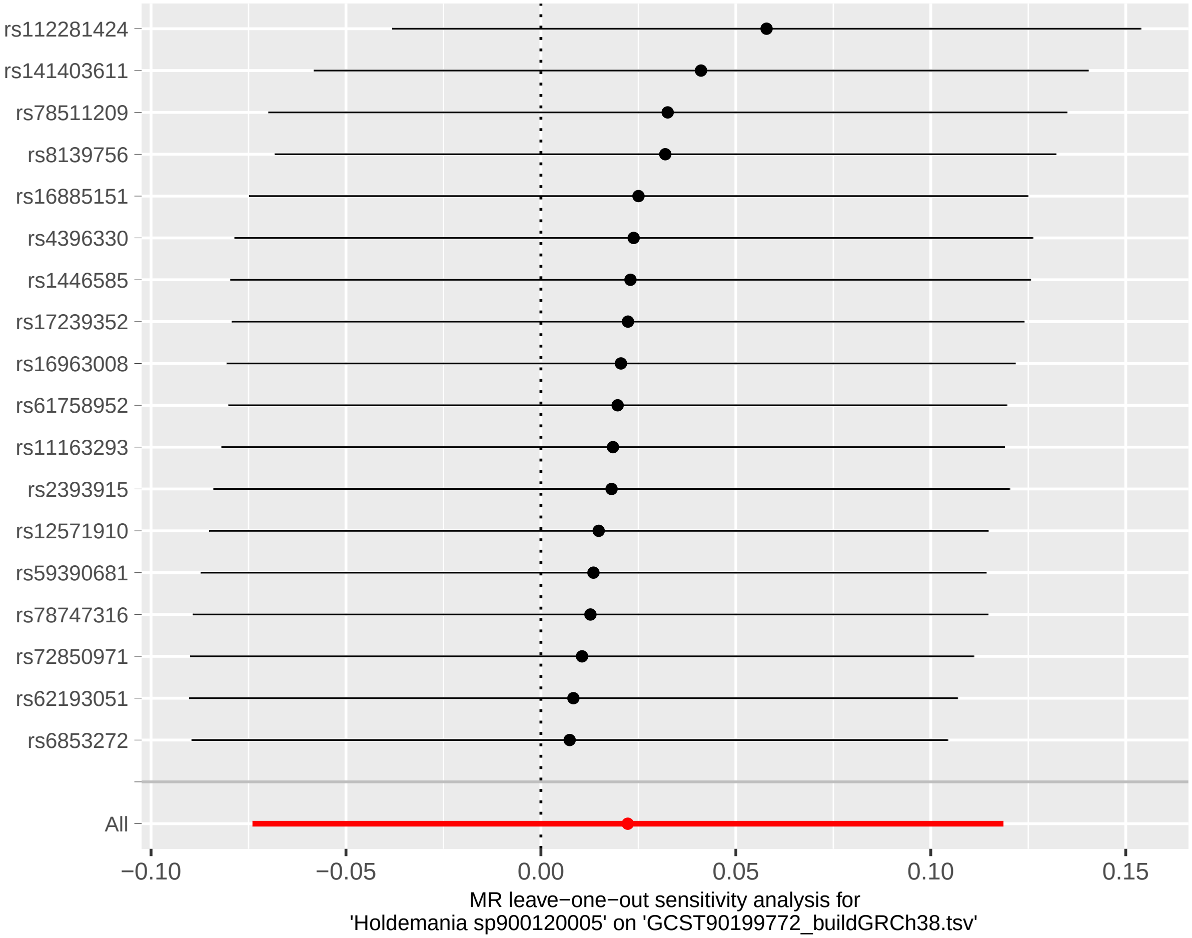

rs112281424
rs141403611
rs78511209
rs8139756
rs16885151
rs4396330
rs1446585
rs17239352
rs16963008
rs61758952
rs11163293
rs2393915
rs12571910
rs59390681
rs78747316
rs72850971
rs62193051
rs6853272
All
−0.10
−0.05
0.00
0.05
0.10
0.15
MR leave−one−out sensitivity analysis for
'Holdemania sp900120005' on 'GCST90199772_buildGRCh38.tsv'

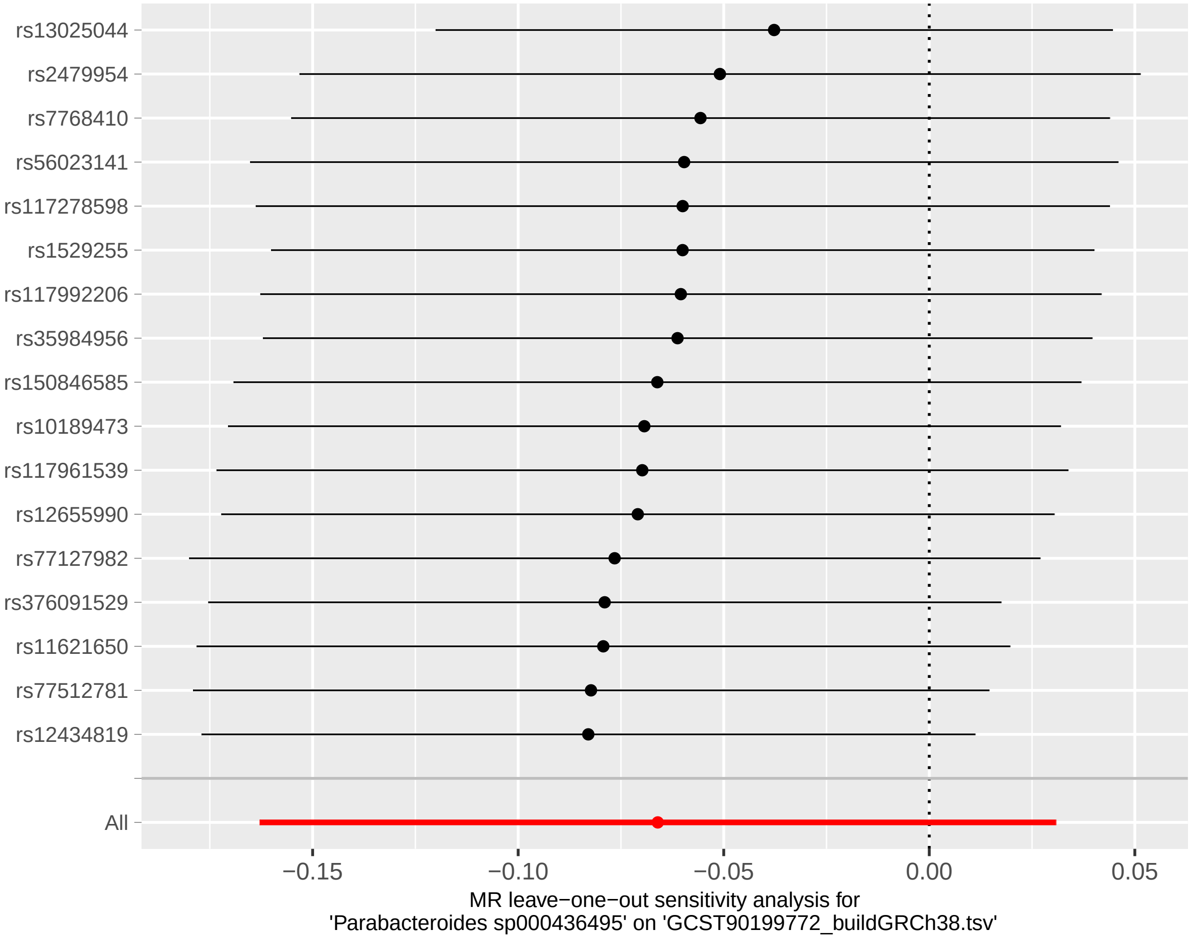
rs13025044
rs2479954
rs7768410
rs56023141
rs117278598
rs1529255
rs117992206
rs35984956
rs150846585
rs10189473
rs117961539
rs12655990
rs77127982
rs376091529
rs11621650
rs77512781
rs12434819
All
−0.15
−0.10
−0.05
0.00
0.05
MR leave−one−out sensitivity analysis for
'Parabacteroides sp000436495' on 'GCST90199772_buildGRCh38.tsv'

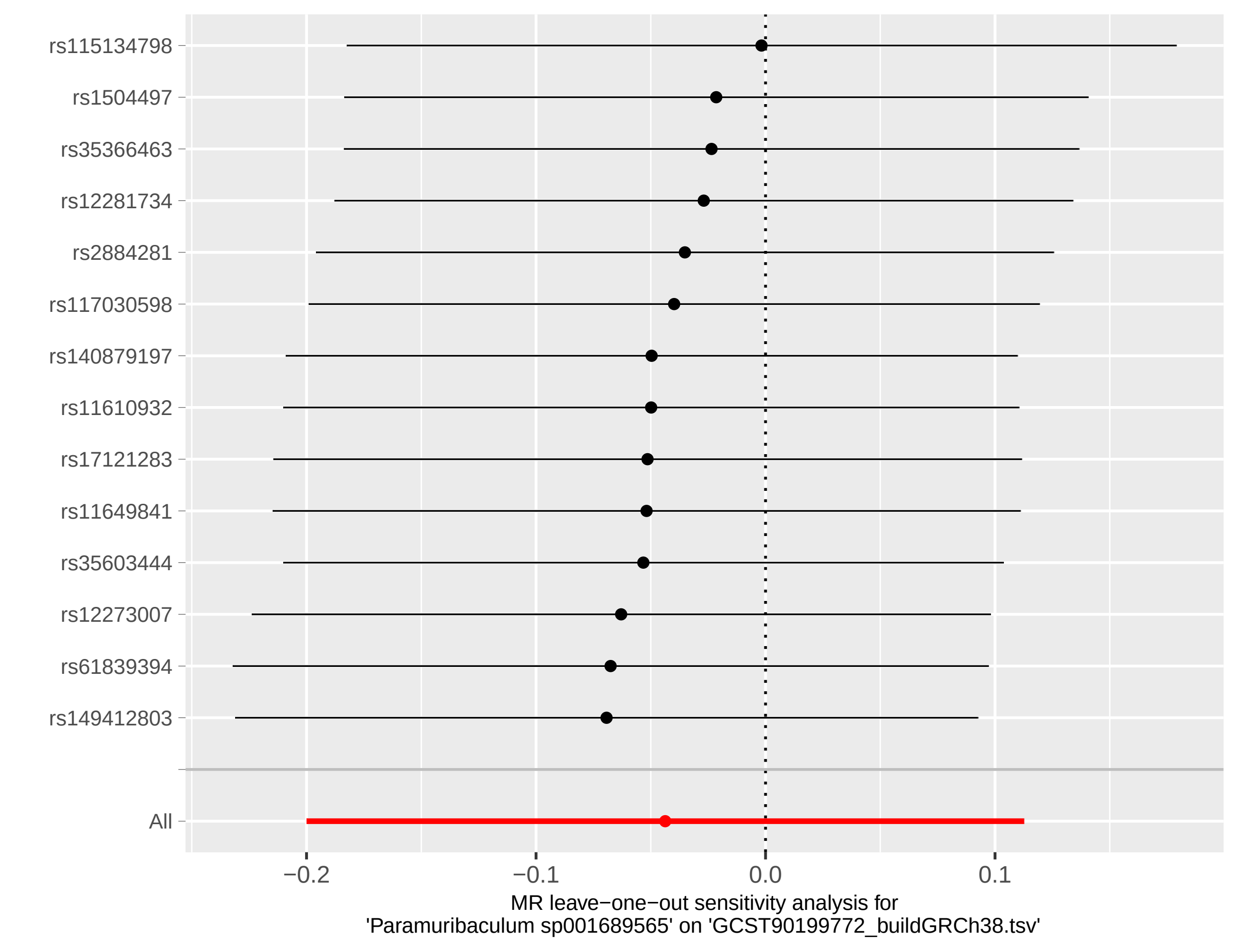

rs115134798
rs1504497
rs35366463
rs12281734
rs2884281
rs117030598
rs140879197
rs11610932
rs17121283
rs11649841
rs35603444
rs12273007
rs61839394
rs149412803
All
−0.2
−0.1
0.0
0.1
MR leave−one−out sensitivity analysis for
'Paramuribaculum sp001689565' on 'GCST90199772_buildGRCh38.tsv'

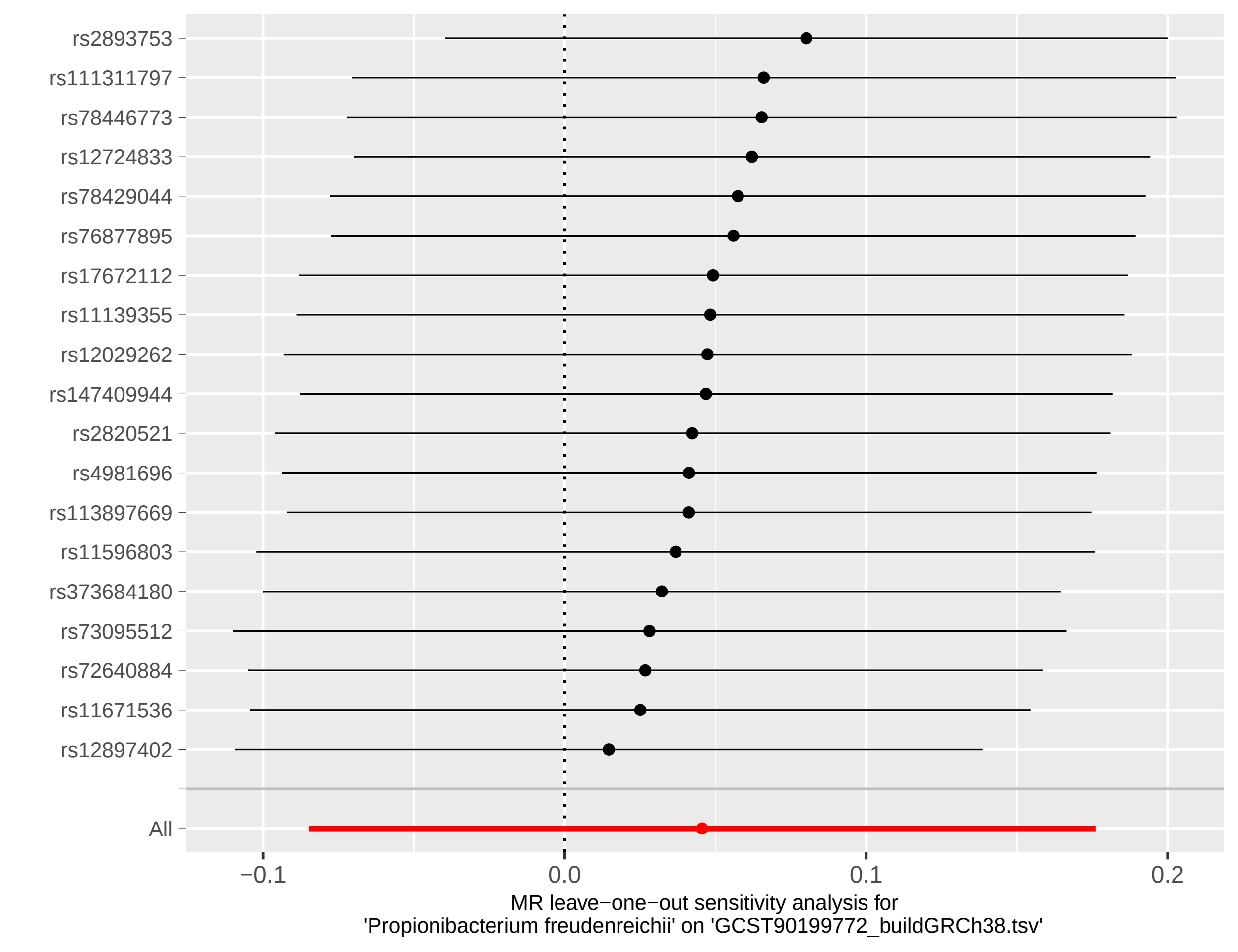

rs2893753
rs111311797
rs78446773
rs12724833
rs78429044
rs76877895
rs17672112
rs11139355
rs12029262
rs147409944
rs2820521
rs4981696
rs113897669
rs11596803
rs373684180
rs73095512
rs72640884
rs11671536
rs12897402
All
−0.1
0.0
0.1
0.2
MR leave−one−out sensitivity analysis for
'Propionibacterium freudenreichii' on 'GCST90199772_buildGRCh38.tsv'

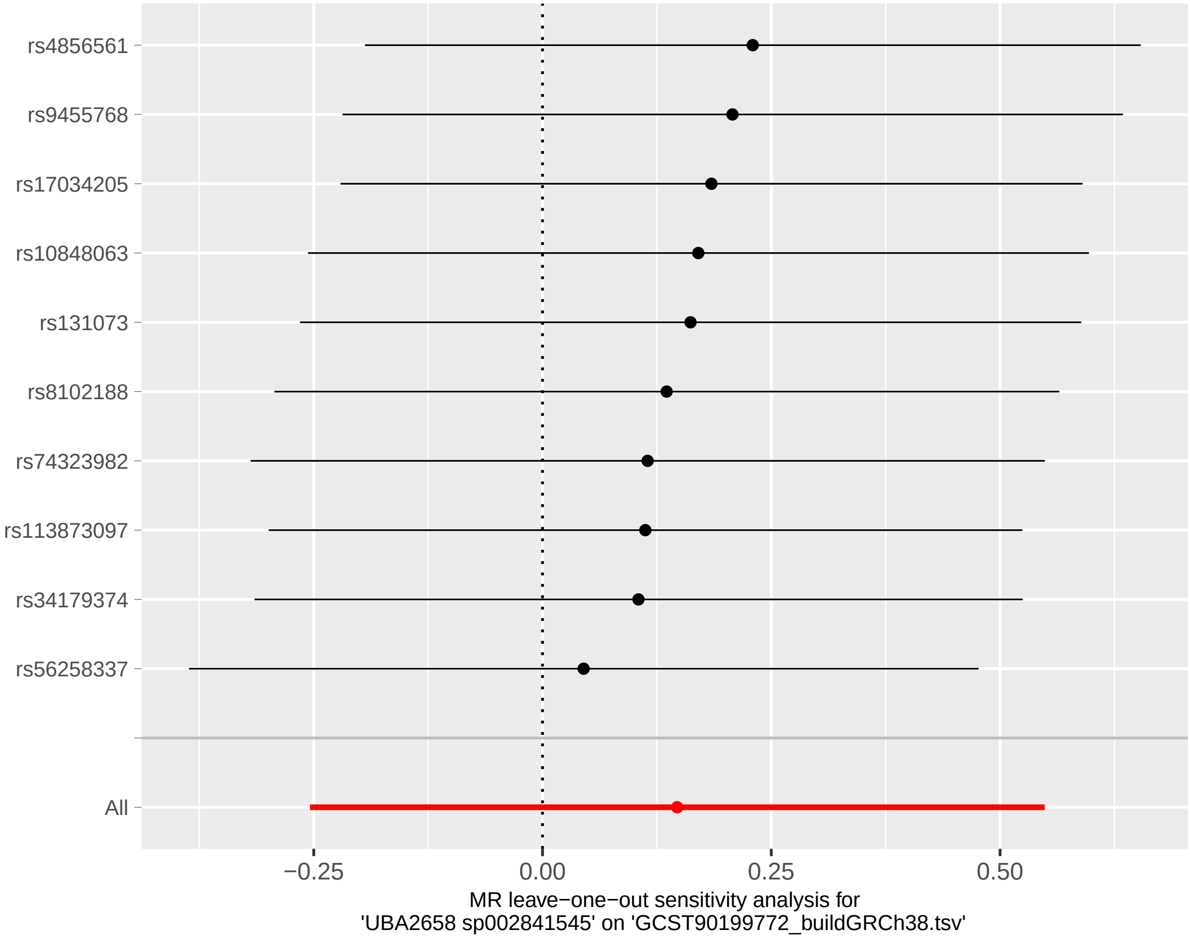
rs4856561
rs9455768
rs17034205
rs10848063
rs131073
rs8102188
rs74323982
rs113873097
rs34179374
rs56258337
All
−0.25
0.00
0.25
0.50
MR leave−one−out sensitivity analysis for
'UBA2658 sp002841545' on 'GCST90199772_buildGRCh38.tsv'

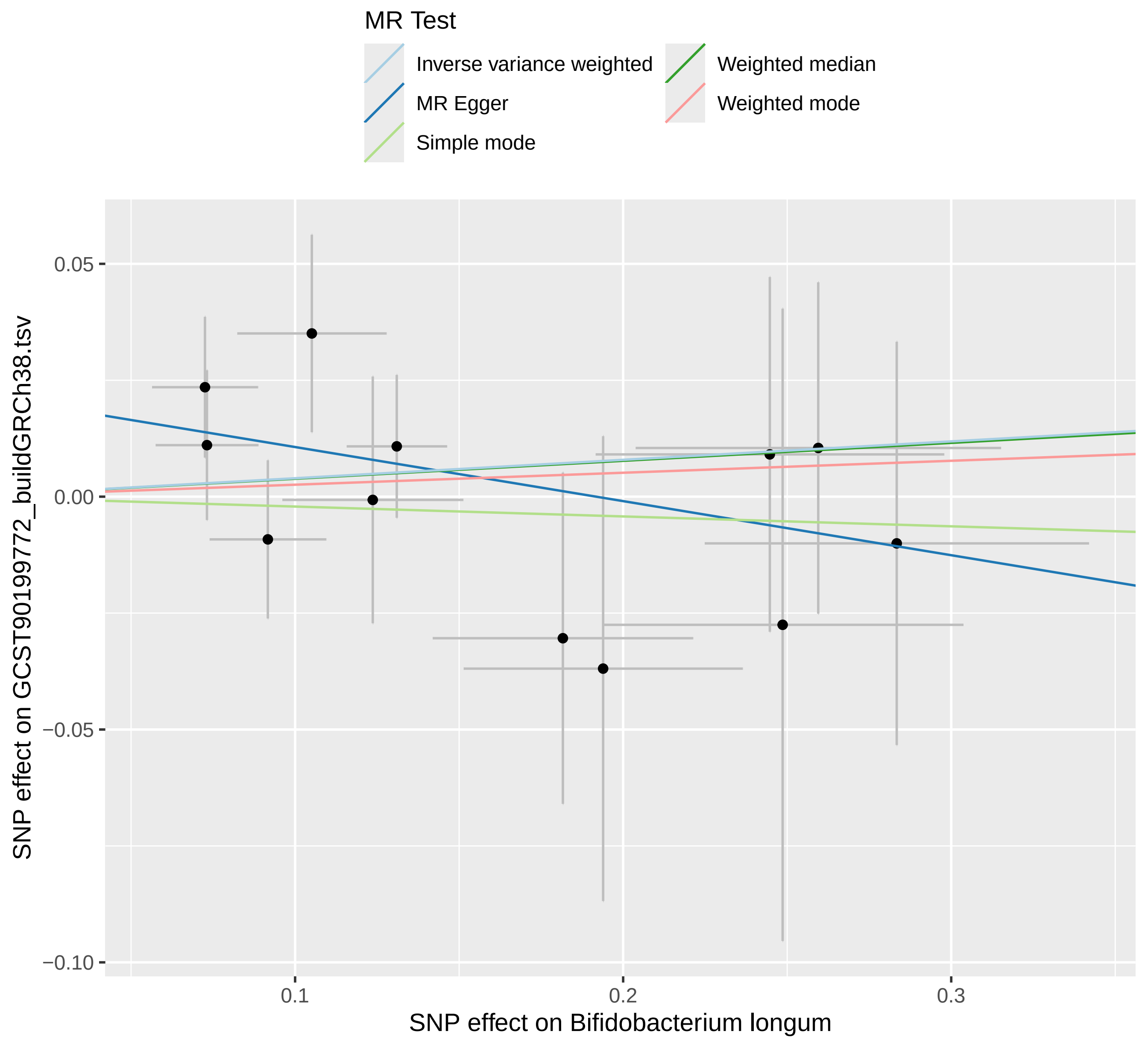
MR Test
Inverse variance weighted
MR Egger
Simple mode
Weighted median
Weighted mode
0.05
0.00
−0.05
−0.10
0.1
0.2
0.3
SNP effect on GCST90199772_buildGRCh38.tsv
SNP effect on Bifidobacterium longum

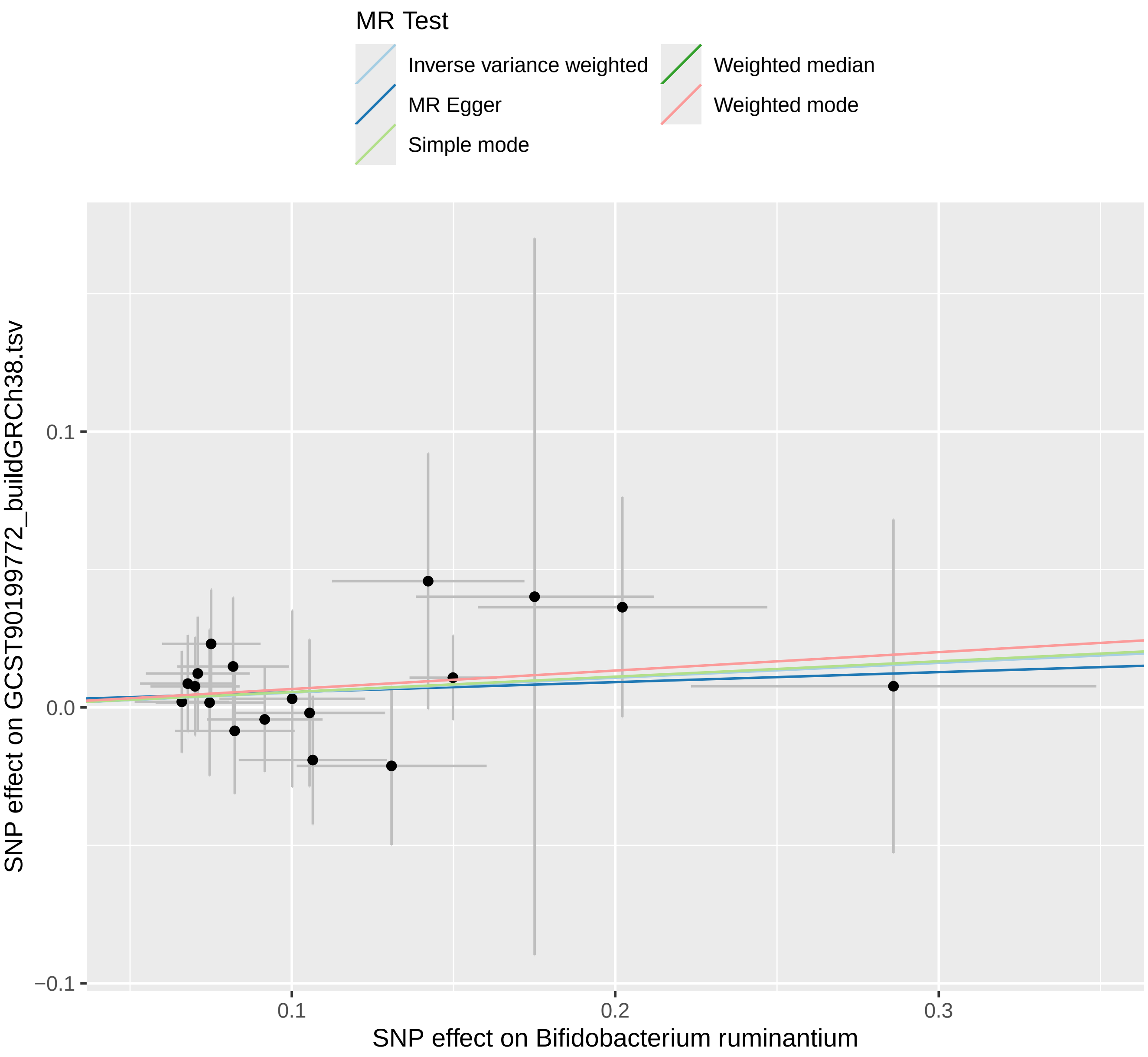
MR Test
Inverse variance weighted
MR Egger
Simple mode
Weighted median
Weighted mode
0.1
0.0
−0.1
0.1
0.2
0.3
SNP effect on GCST90199772_buildGRCh38.tsv
SNP effect on Bifidobacterium ruminantium

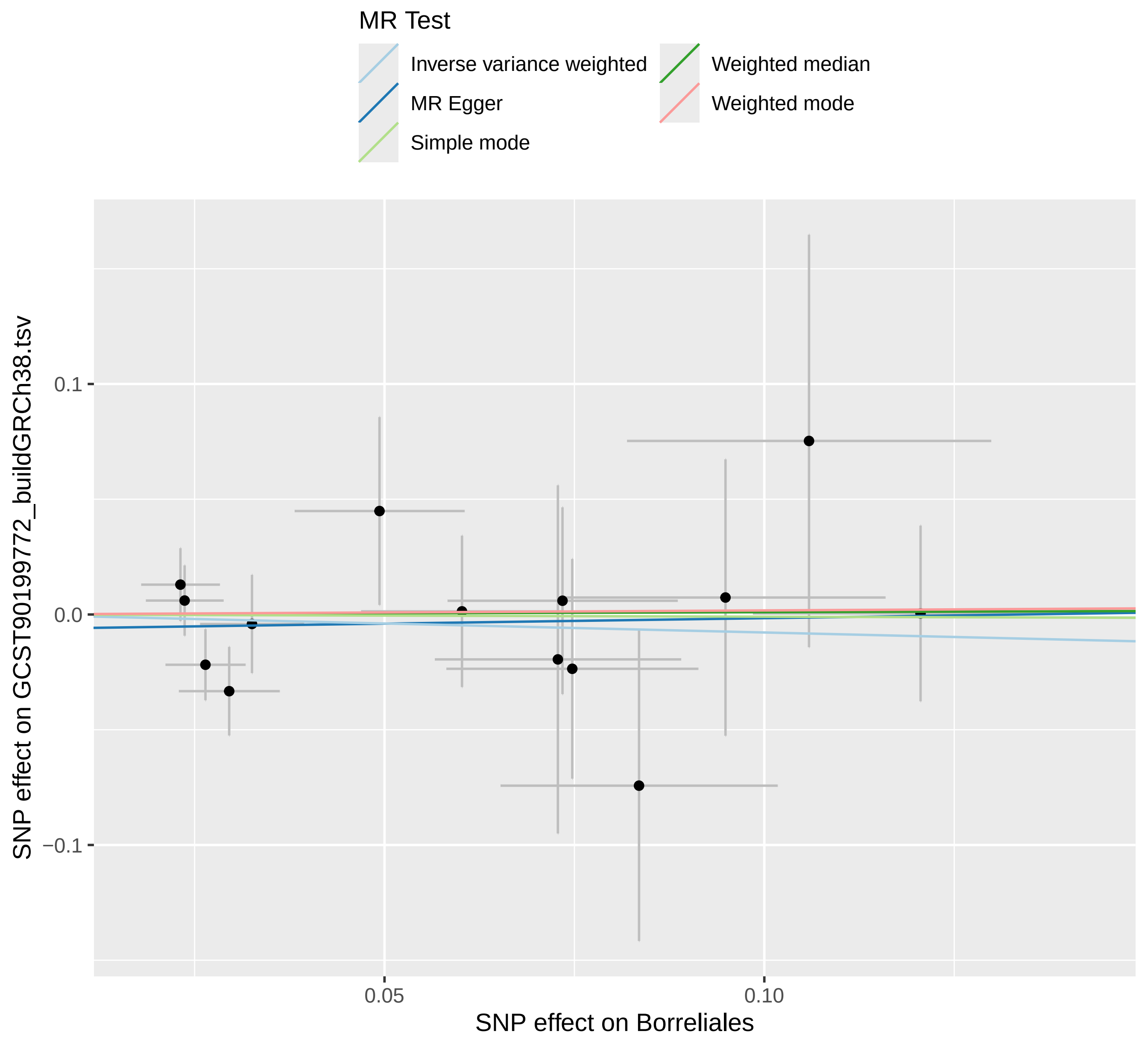

MR Test
Inverse variance weighted
MR Egger
Simple mode
Weighted median
Weighted mode
SNP effect on GCST90199772_buildGRCh38.tsv
0.1
0.0
−0.1
0.05
0.10
SNP effect on Borreliales

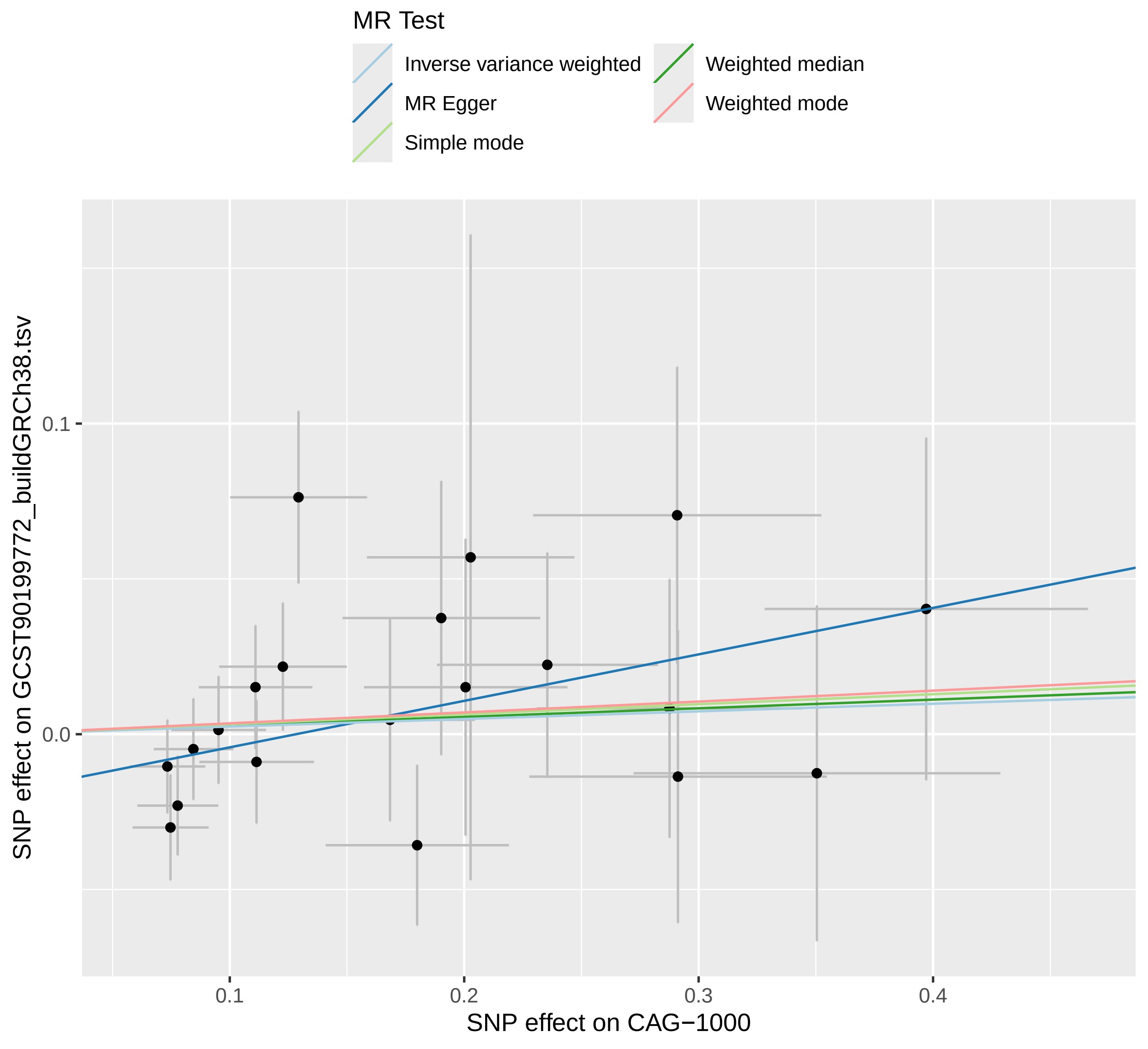

MR Test
Inverse variance weighted
MR Egger
Simple mode
Weighted median
Weighted mode
SNP effect on GCST90199772_buildGRCh38.tsv
0.1
0.0
0.1
0.2
0.3
0.4
SNP effect on CAG−1000

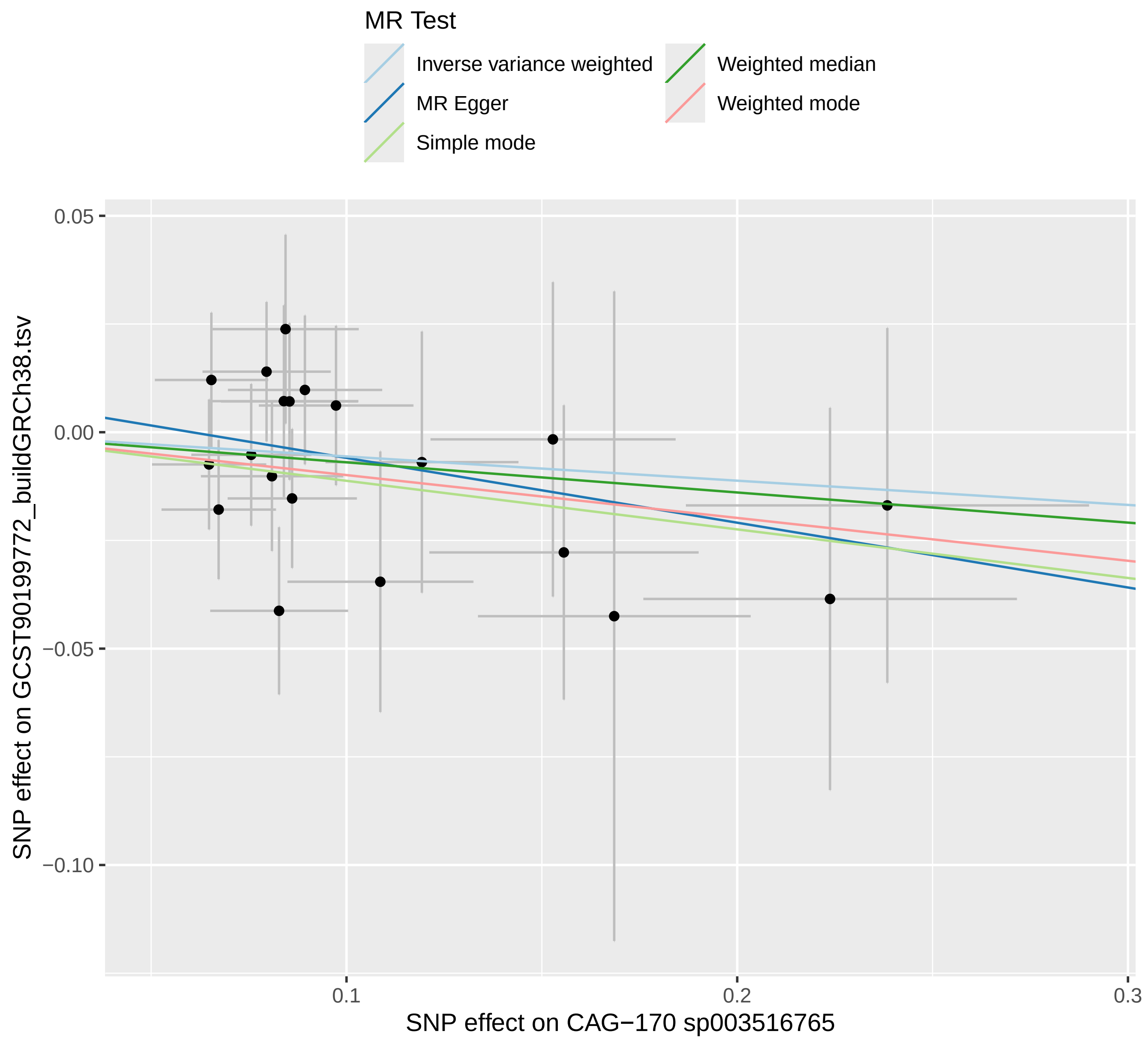

MR Test
Inverse variance weighted
MR Egger
Simple mode
Weighted median
Weighted mode
0.05
0.00
−0.05
−0.10
0.1
0.2
0.3
SNP effect on GCST90199772_buildGRCh38.tsv
SNP effect on CAG−170 sp003516765

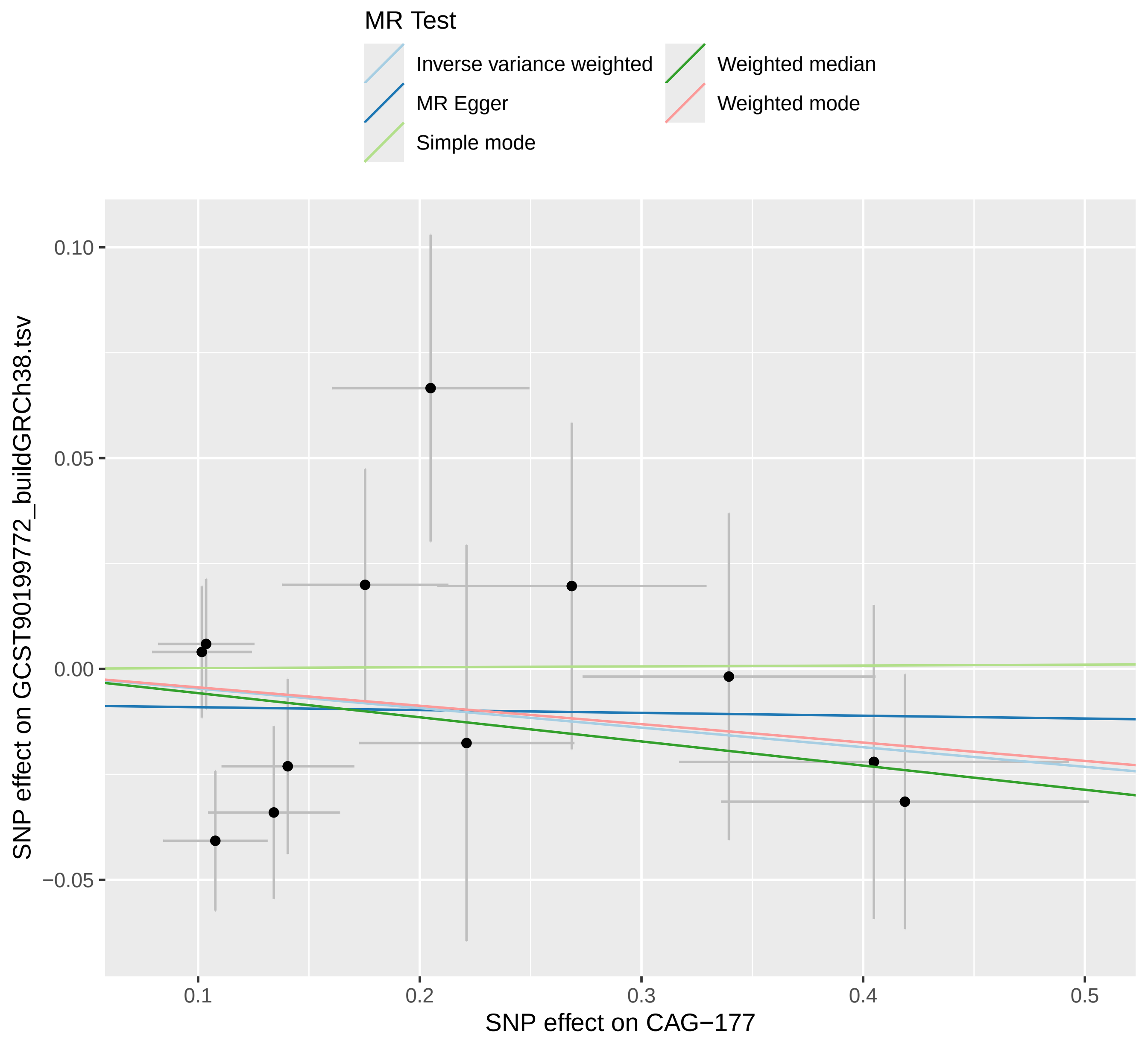
MR Test
Inverse variance weighted
MR Egger
Simple mode
Weighted median
Weighted mode
0.10
0.05
0.00
−0.05
SNP effect on GCST90199772_buildGRCh38.tsv
0.1
0.2
0.3
0.4
0.5
SNP effect on CAG−177

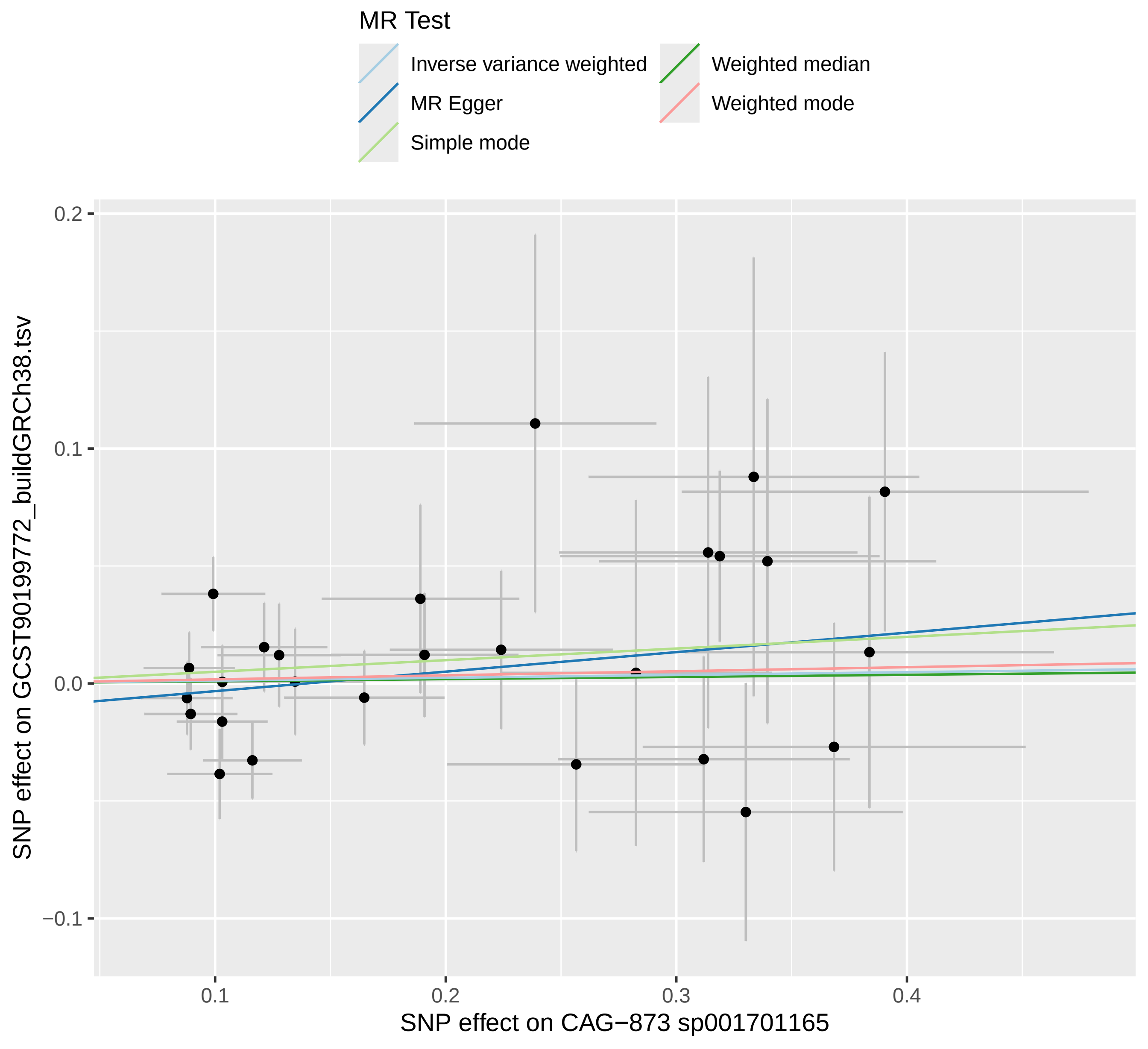
MR Test
Inverse variance weighted
MR Egger
Simple mode
Weighted median
Weighted mode
0.2
0.1
0.0
−0.1
0.1
0.2
0.3
0.4
SNP effect on GCST90199772_buildGRCh38.tsv
SNP effect on CAG−873 sp001701165

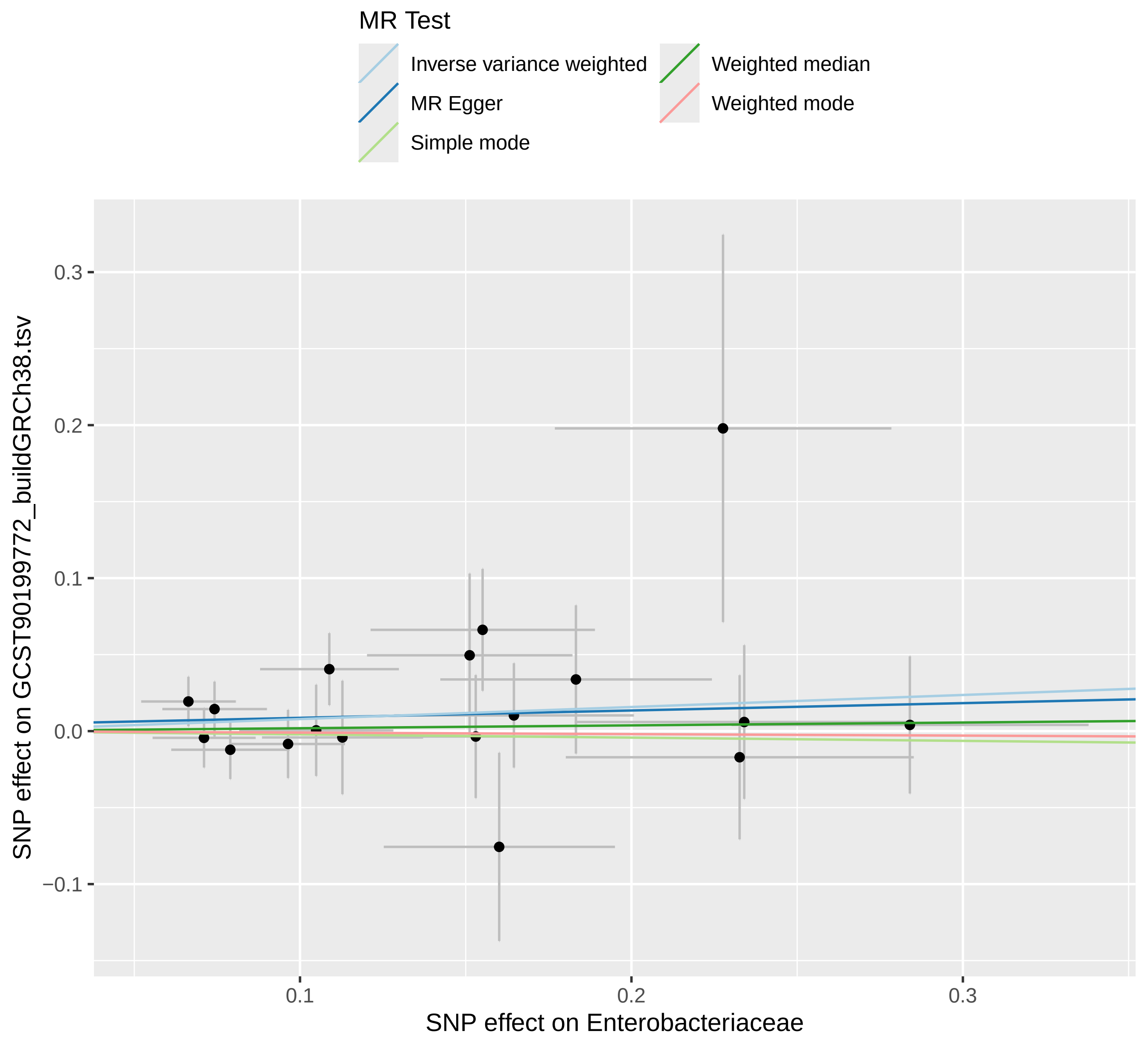

MR Test
Inverse variance weighted
MR Egger
Simple mode
Weighted median
Weighted mode
0.3
0.2
0.1
0.0
−0.1
0.1
0.2
0.3
SNP effect on GCST90199772_buildGRCh38.tsv
SNP effect on Enterobacteriaceae

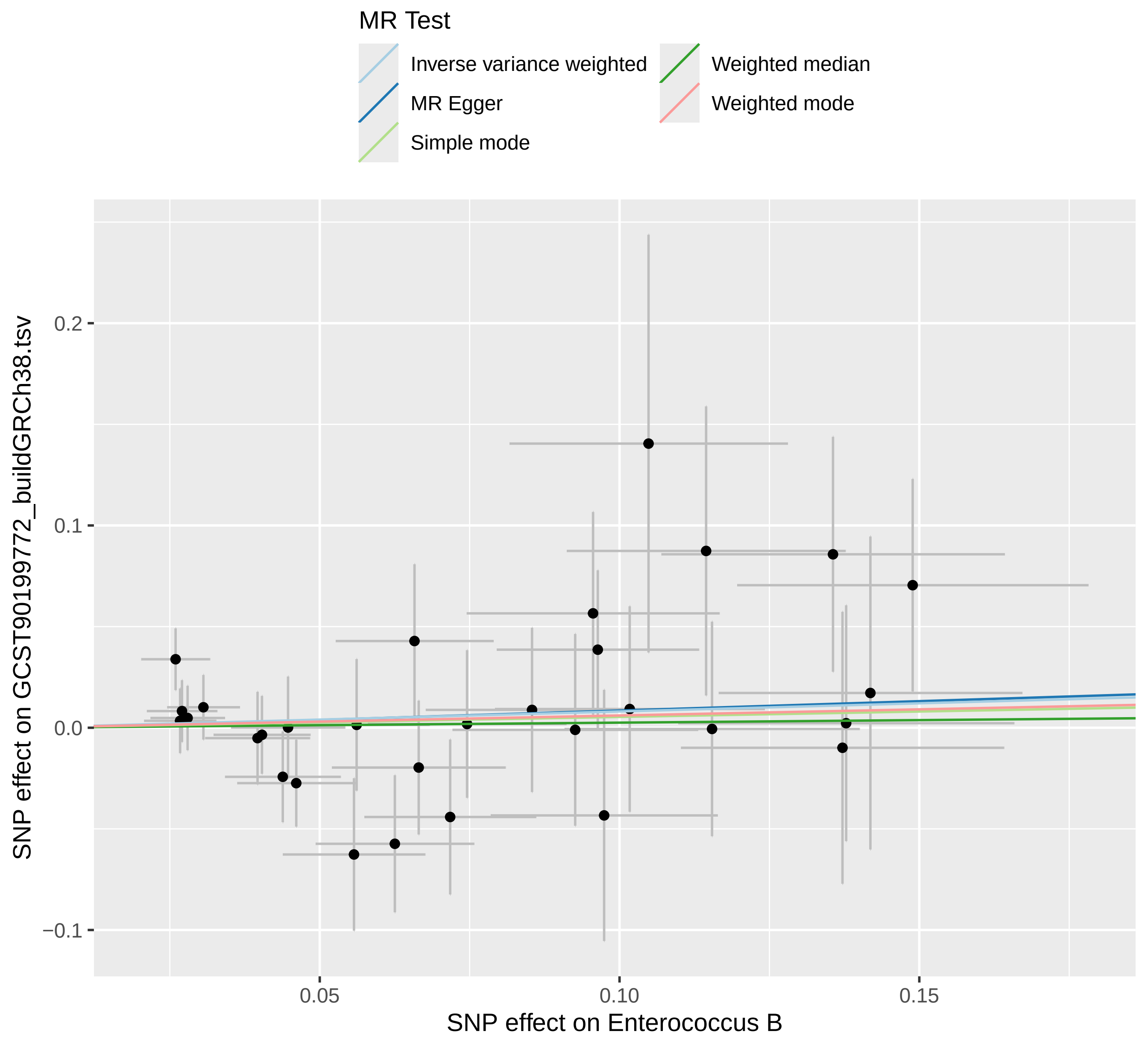
MR Test
Inverse variance weighted
MR Egger
Simple mode
Weighted median
Weighted mode
SNP effect on GCST90199772_buildGRCh38.tsv
0.2
0.1
0.0
−0.1
0.05
0.10
0.15
SNP effect on Enterococcus B

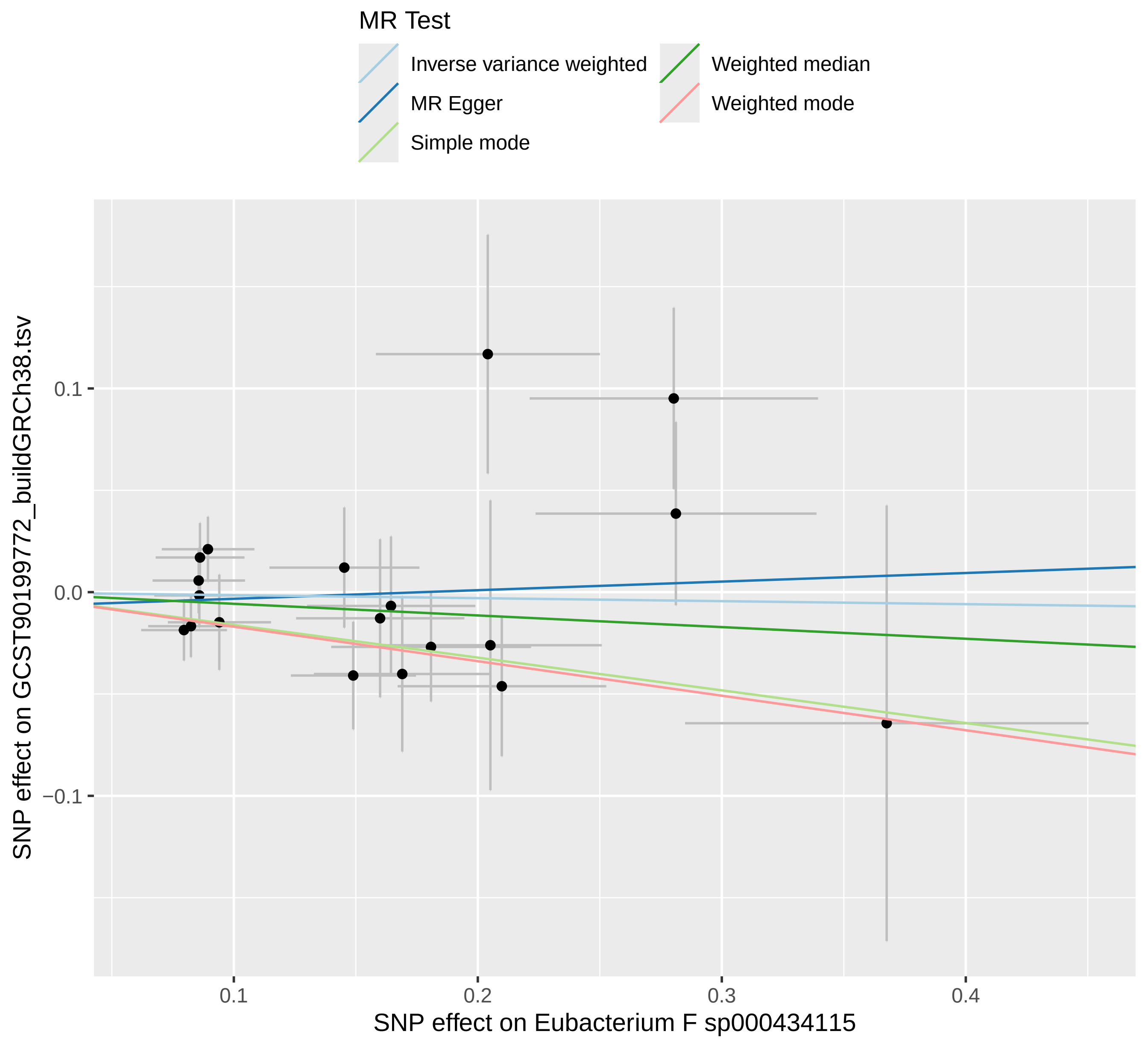

MR Test
Inverse variance weighted
MR Egger
Simple mode
Weighted median
Weighted mode
0.1
0.0
−0.1
0.1
0.2
0.3
0.4
SNP effect on GCST90199772_buildGRCh38.tsv
SNP effect on Eubacterium F sp000434115

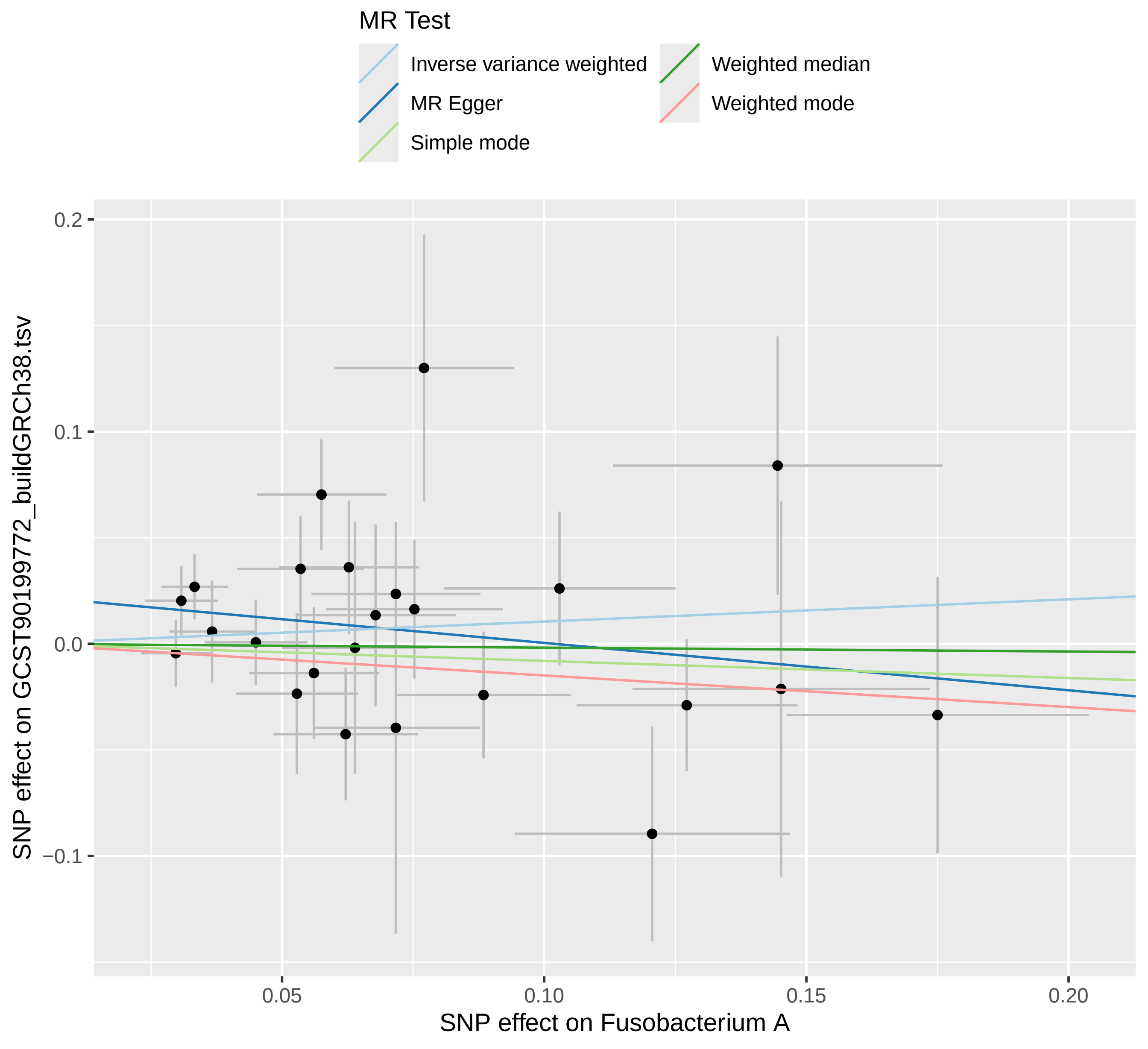

MR Test
Inverse variance weighted
MR Egger
Simple mode
Weighted median
Weighted mode
0.2
0.1
0.0
−0.1
SNP effect on GCST90199772_buildGRCh38.tsv
0.05
0.10
0.15
0.20
SNP effect on Fusobacterium A

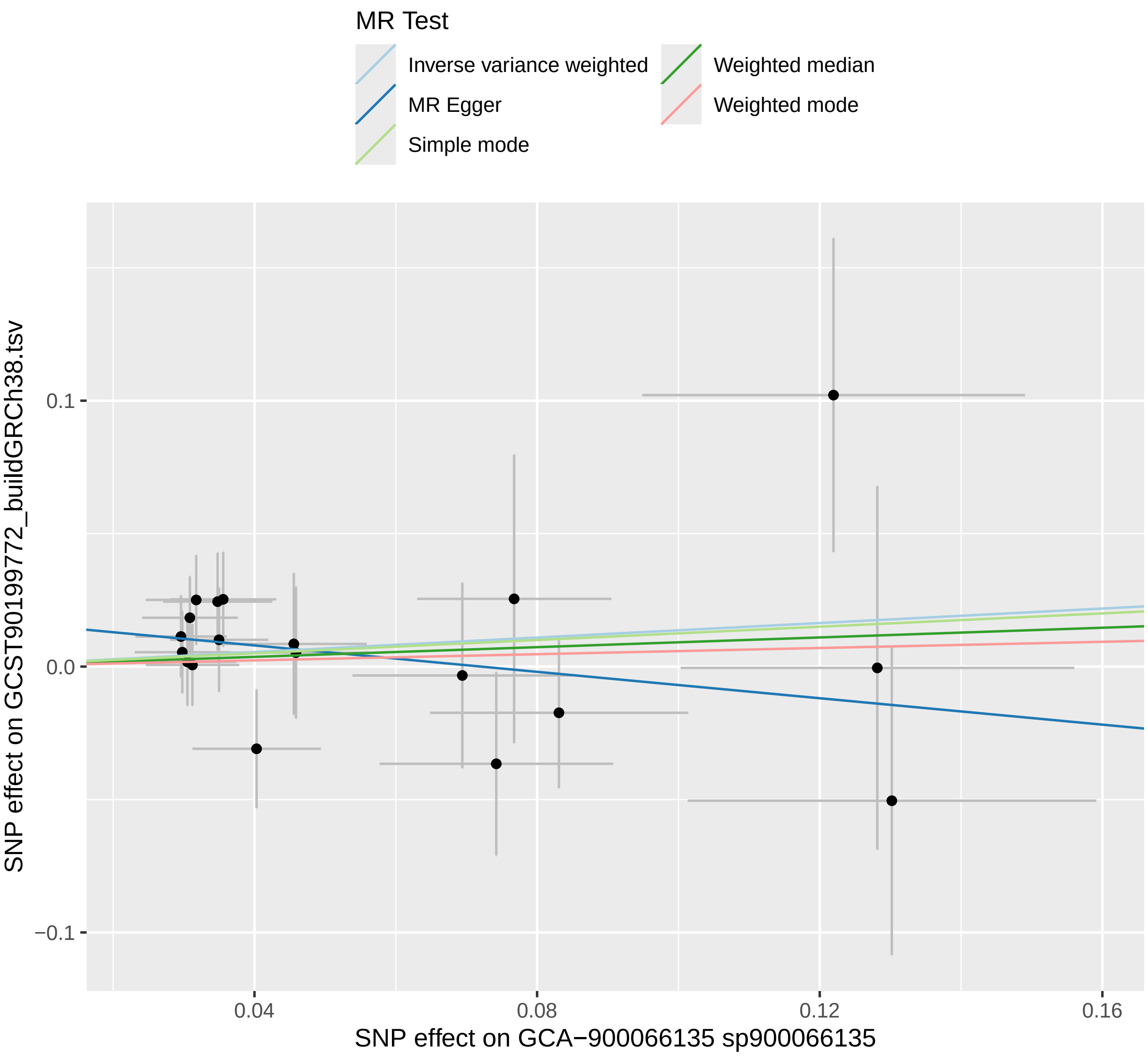
MR Test
Inverse variance weighted
MR Egger
Simple mode
Weighted median
Weighted mode
0.1
0.0
−0.1
0.04
0.08
0.12
0.16
SNP effect on GCST90199772_buildGRCh38.tsv
SNP effect on GCA−900066135 sp900066135

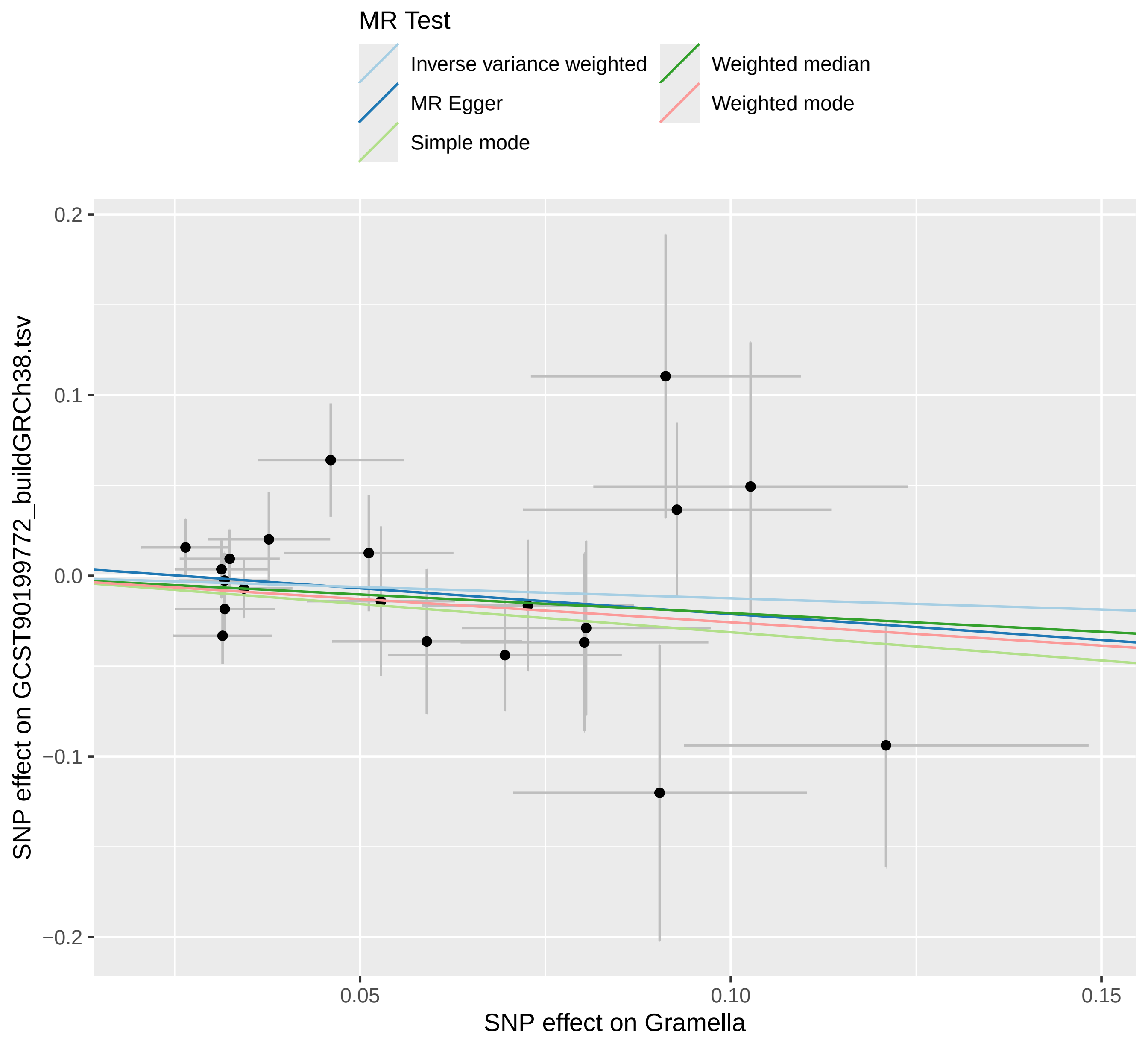
MR Test
Inverse variance weighted
MR Egger
Simple mode
Weighted median
Weighted mode
0.2
0.1
0.0
−0.1
−0.2
0.05
0.10
0.15
SNP effect on GCST90199772_buildGRCh38.tsv
SNP effect on Gramella

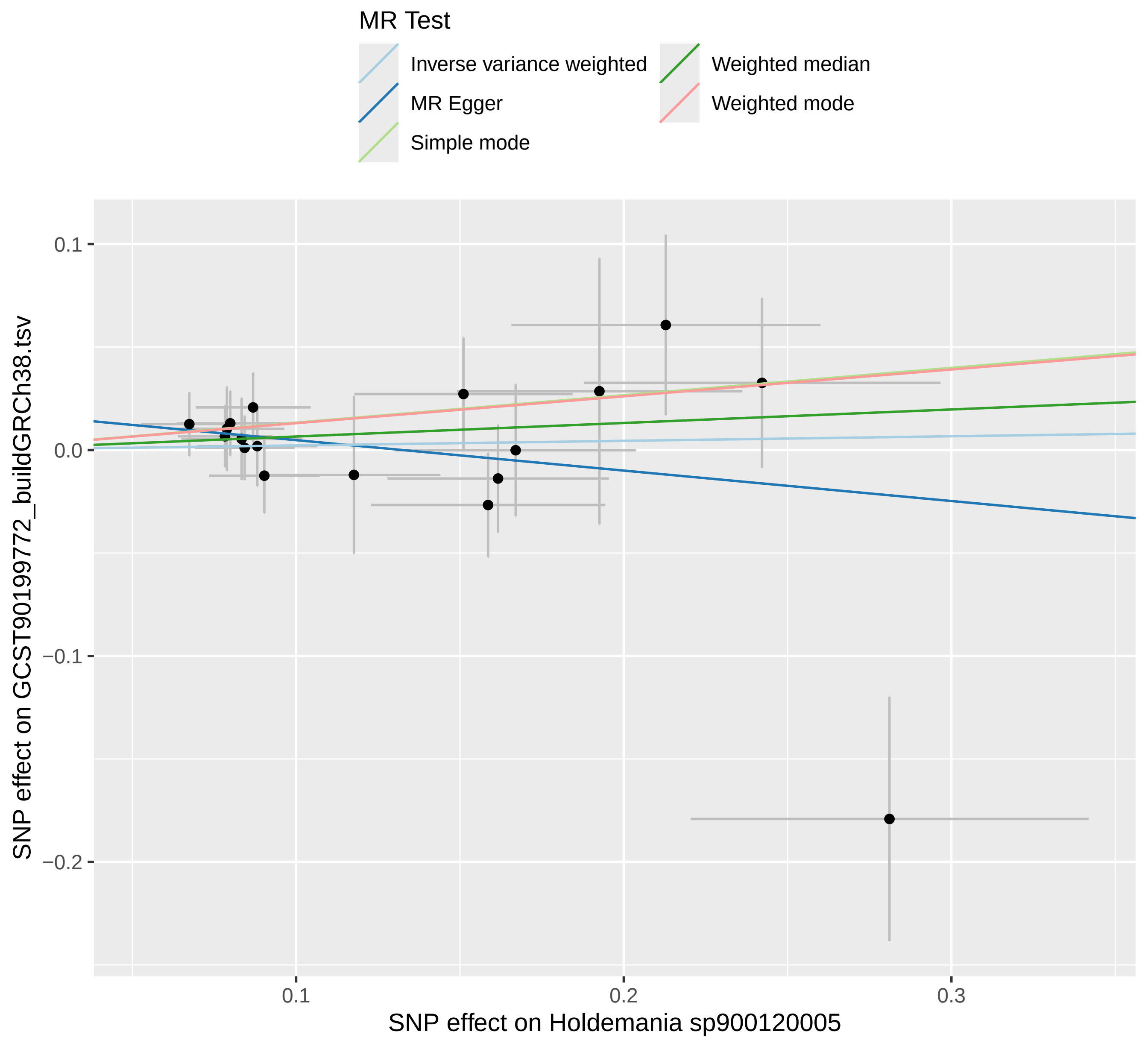

MR Test
Inverse variance weighted
MR Egger
Simple mode
Weighted median
Weighted mode
0.1
0.0
−0.1
−0.2
0.1
0.2
0.3
SNP effect on GCST90199772_buildGRCh38.tsv
SNP effect on Holdemania sp900120005

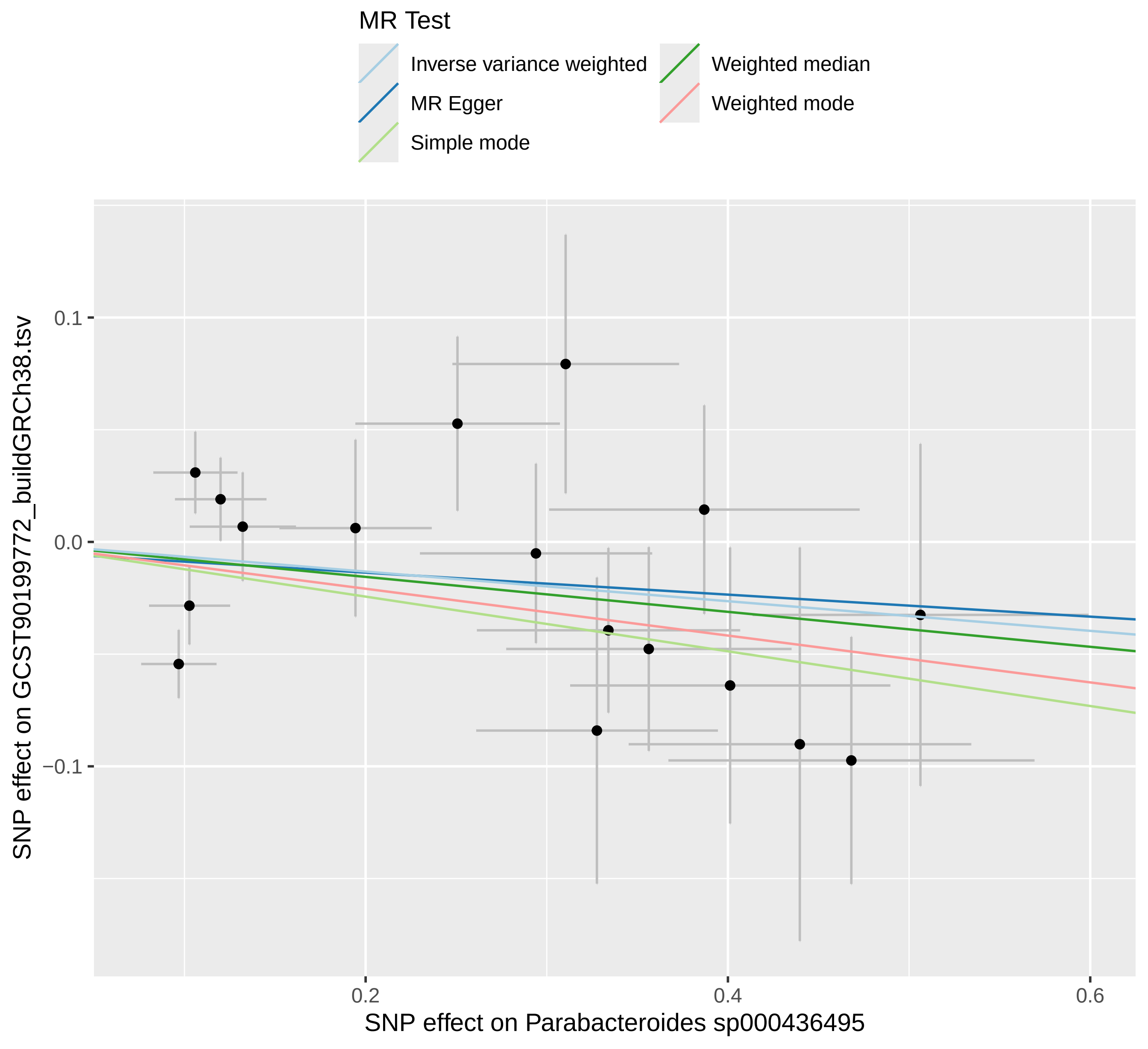

MR Test
Inverse variance weighted
MR Egger
Simple mode
Weighted median
Weighted mode
0.1
0.0
−0.1
0.2
0.4
0.6
SNP effect on GCST90199772_buildGRCh38.tsv
SNP effect on Parabacteroides sp000436495

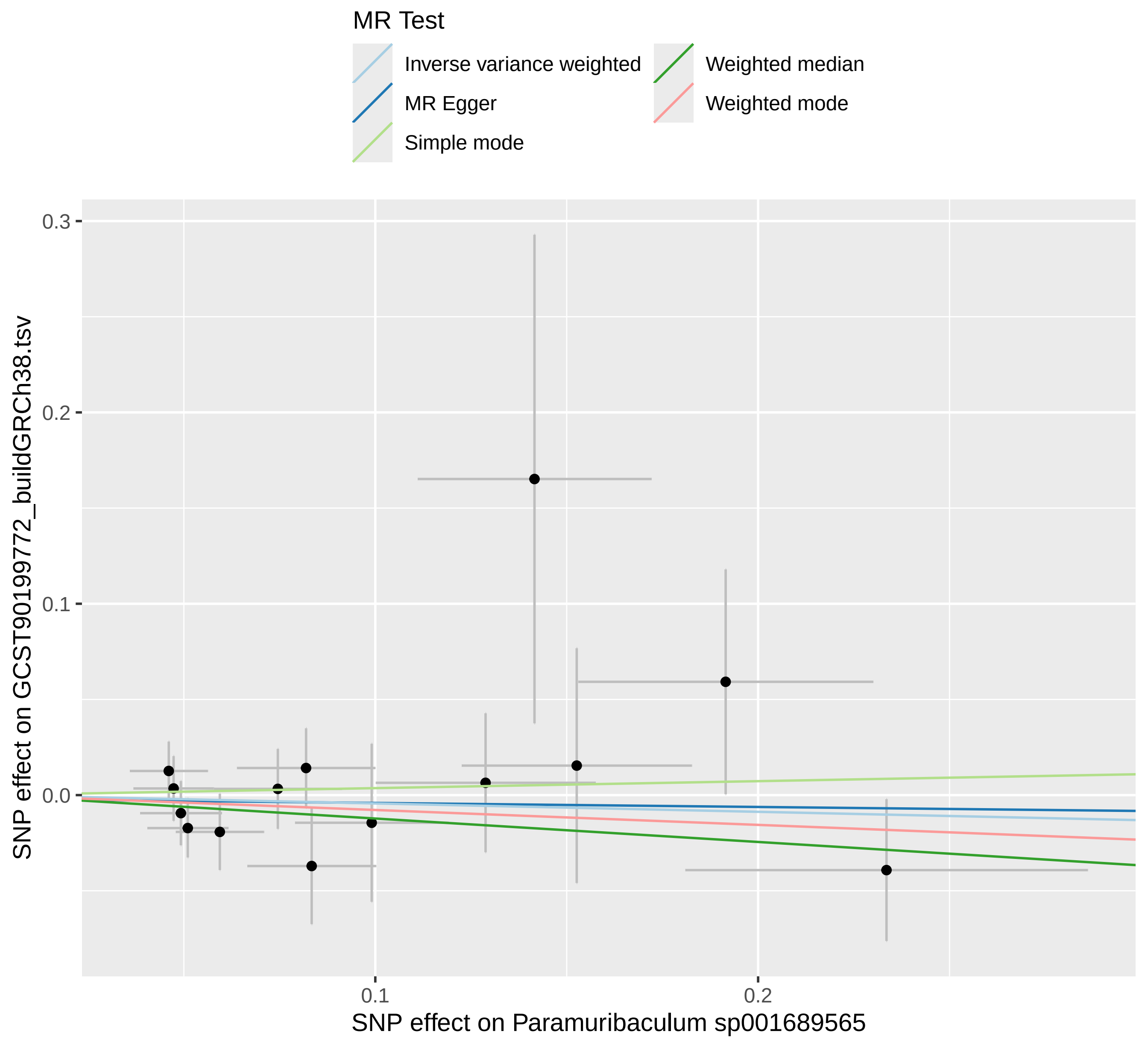
MR Test
Inverse variance weighted
MR Egger
Simple mode
Weighted median
Weighted mode
0.3
0.2
0.1
0.0
0.1
0.2
SNP effect on GCST90199772_buildGRCh38.tsv
SNP effect on Paramuribaculum sp001689565

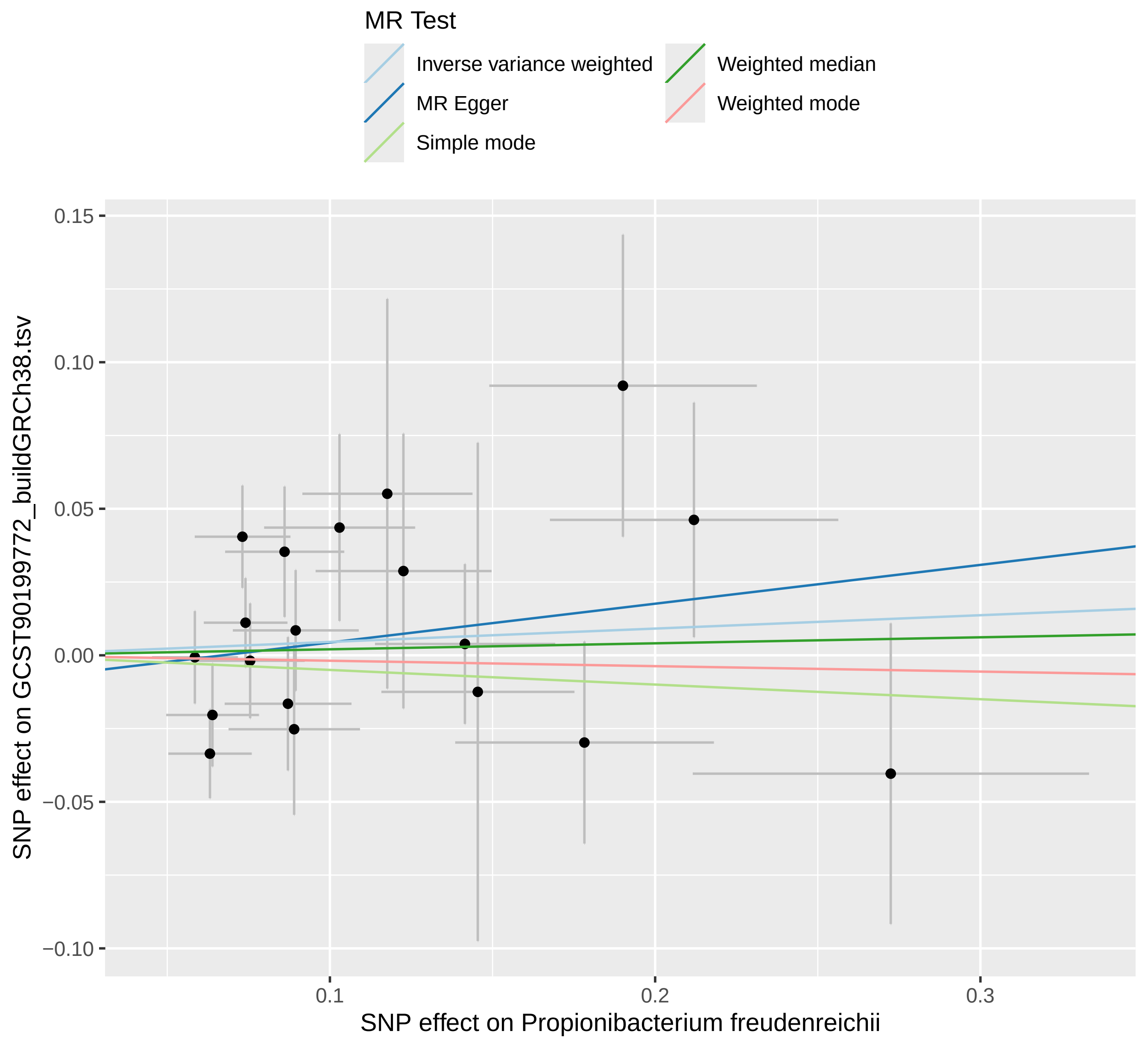

MR Test
Inverse variance weighted
MR Egger
Simple mode
Weighted median
Weighted mode
0.15
0.10
0.05
0.00
−0.05
−0.10
0.1
0.2
0.3
SNP effect on GCST90199772_buildGRCh38.tsv
SNP effect on Propionibacterium freudenreichii

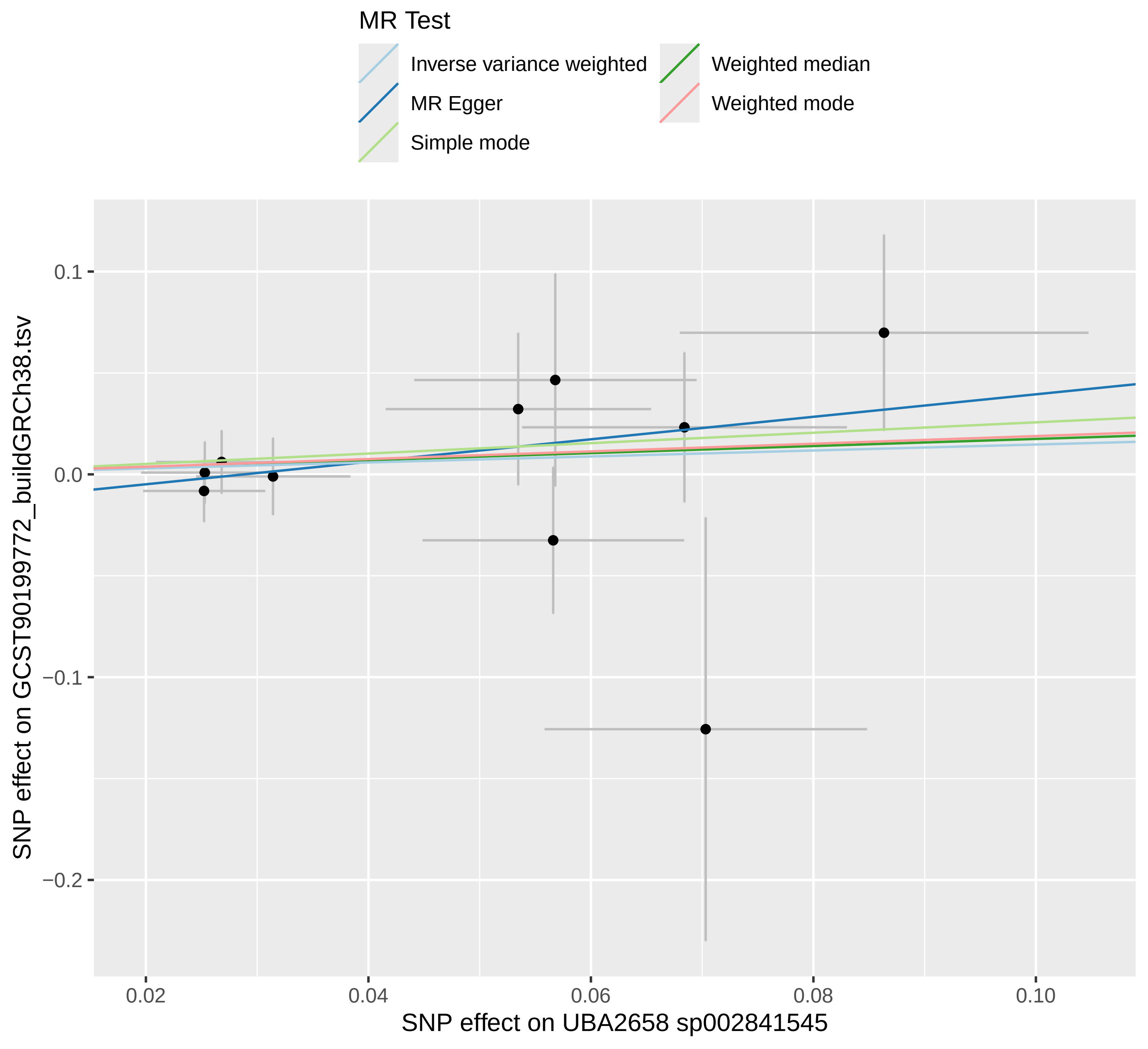

MR Test
Inverse variance weighted
MR Egger
Simple mode
Weighted median
Weighted mode
SNP effect on GCST90199772_buildGRCh38.tsv
0.1
0.0
−0.1
−0.2
0.02
0.04
0.06
0.08
0.10
SNP effect on UBA2658 sp002841545

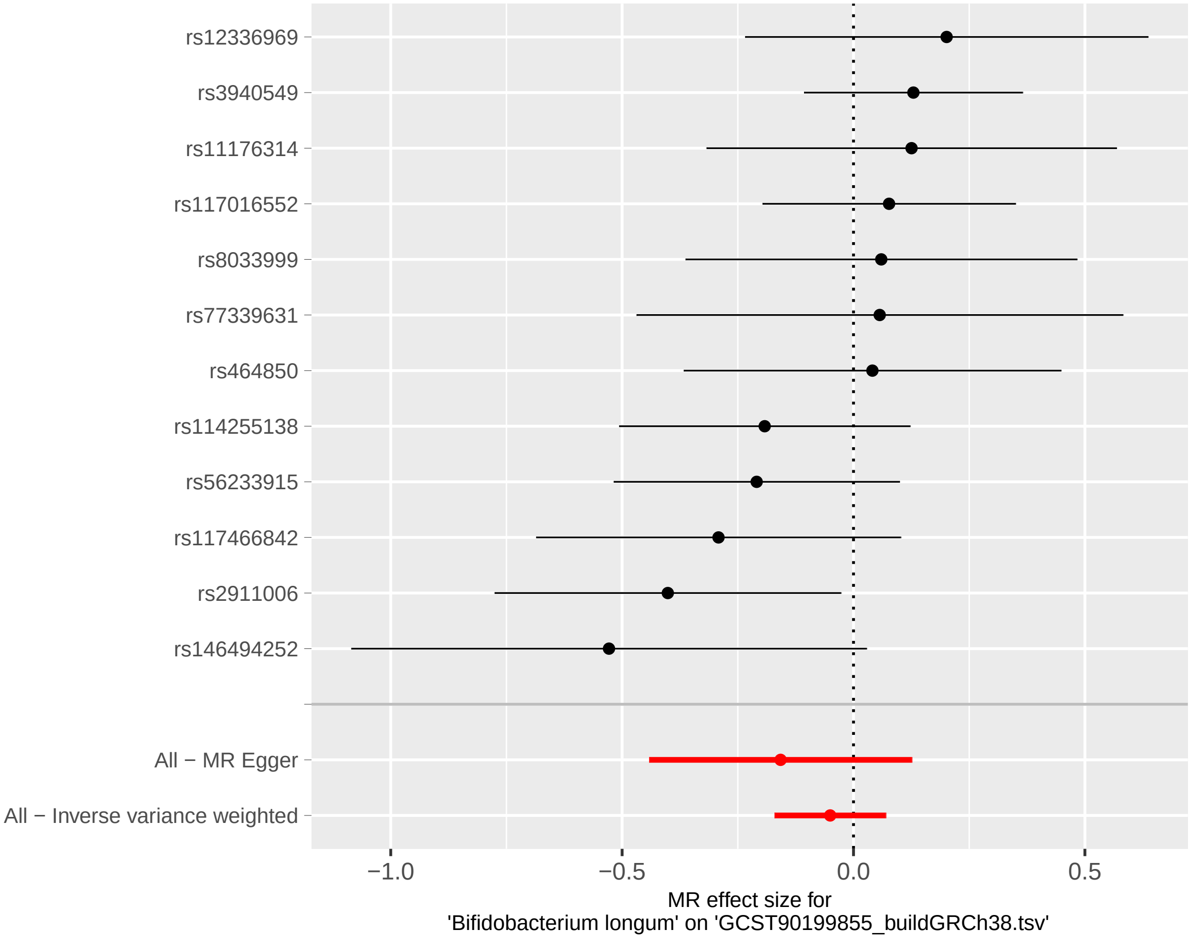
rs12336969
rs3940549
rs11176314
rs117016552
rs8033999
rs77339631
rs464850
rs114255138
rs56233915
rs117466842
rs2911006
rs146494252
All − MR Egger
All − Inverse variance weighted
−1.0
−0.5
0.0
0.5
MR effect size for
'Bifidobacterium longum' on 'GCST90199855_buildGRCh38.tsv'

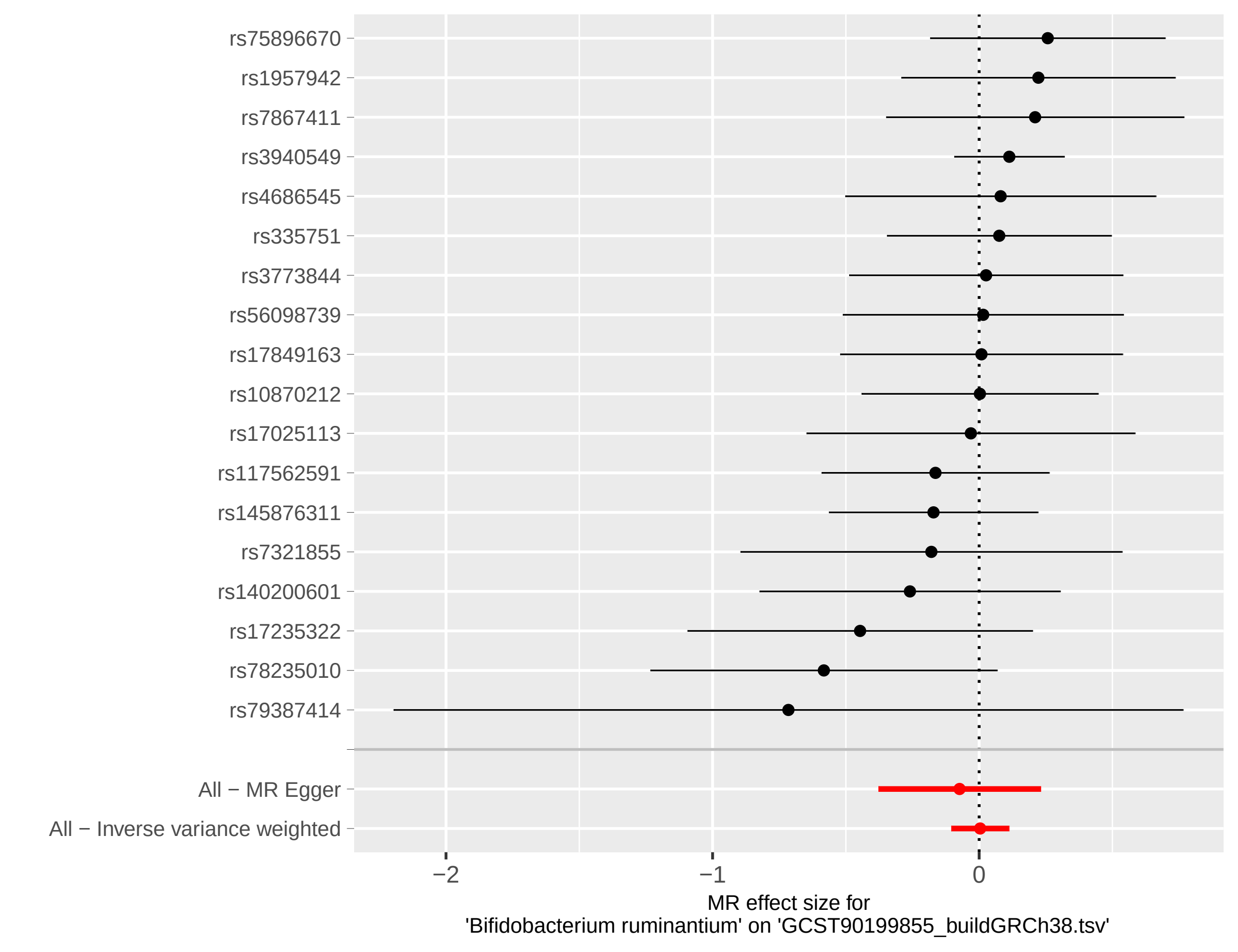
rs75896670
rs1957942
rs7867411
rs3940549
rs4686545
rs335751
rs3773844
rs56098739
rs17849163
rs10870212
rs17025113
rs117562591
rs145876311
rs7321855
rs140200601
rs17235322
rs78235010
rs79387414
All − MR Egger
All − Inverse variance weighted
−2
−1
0
MR effect size for
'Bifidobacterium ruminantium' on 'GCST90199855_buildGRCh38.tsv'

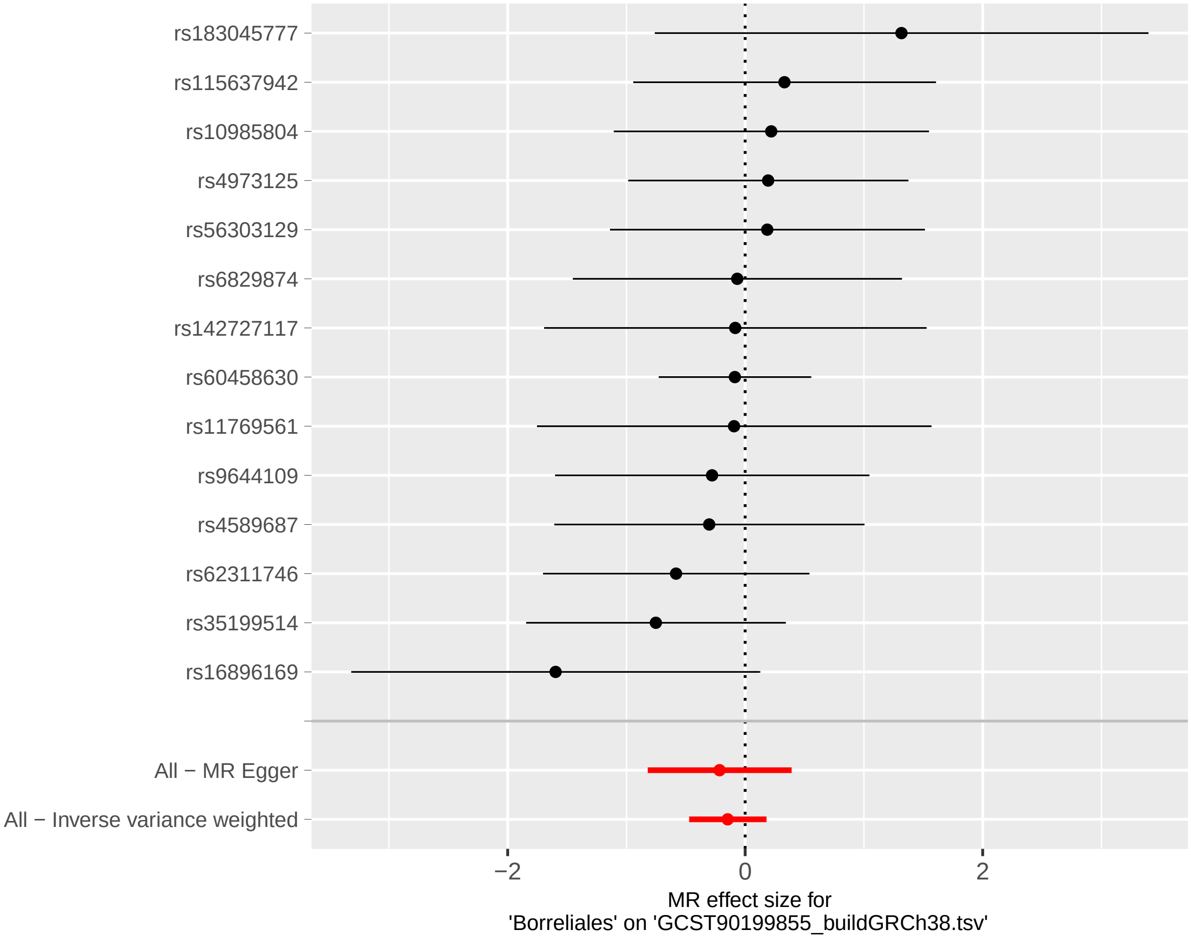

rs183045777
rs115637942
rs10985804
rs4973125
rs56303129
rs6829874
rs142727117
rs60458630
rs11769561
rs9644109
rs4589687
rs62311746
rs35199514
rs16896169
All − MR Egger
All − Inverse variance weighted
−2
0
2
MR effect size for
'Borreliales' on 'GCST90199855_buildGRCh38.tsv'

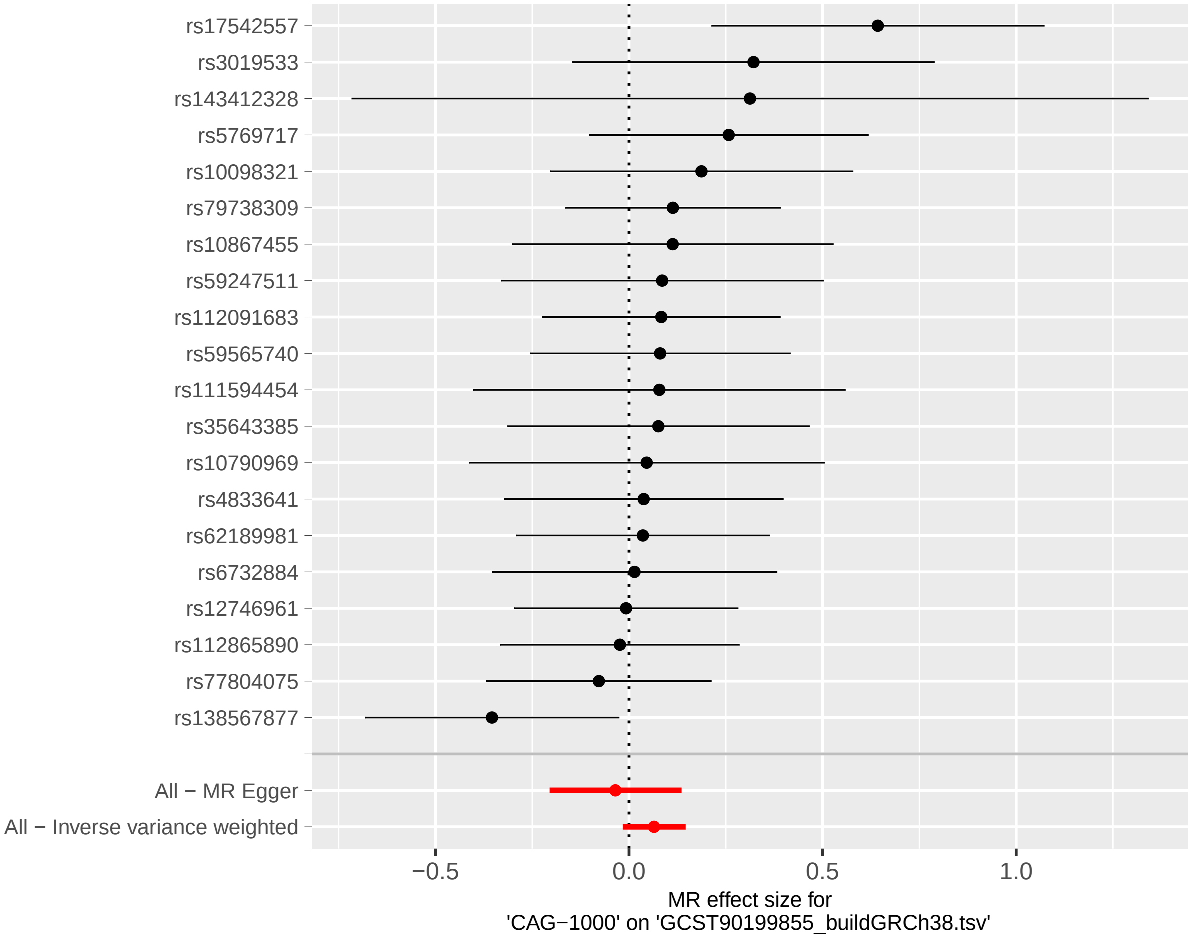
rs17542557
rs3019533
rs143412328
rs5769717
rs10098321
rs79738309
rs10867455
rs59247511
rs112091683
rs59565740
rs111594454
rs35643385
rs10790969
rs4833641
rs62189981
rs6732884
rs12746961
rs112865890
rs77804075
rs138567877
All − MR Egger
All − Inverse variance weighted
−0.5
0.0
0.5
1.0
MR effect size for
'CAG−1000' on 'GCST90199855_buildGRCh38.tsv'

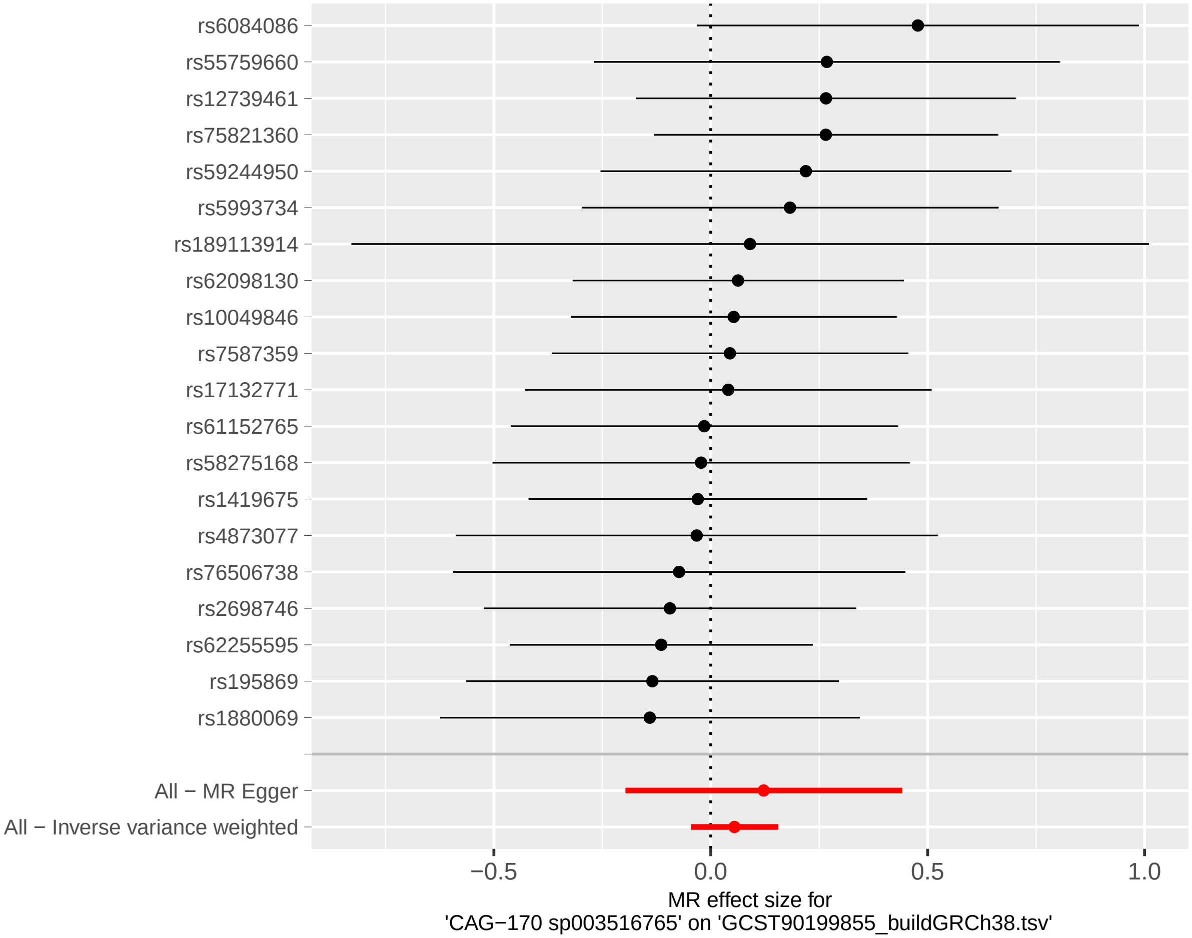
rs6084086
rs55759660
rs12739461
rs75821360
rs59244950
rs5993734
rs189113914
rs62098130
rs10049846
rs7587359
rs17132771
rs61152765
rs58275168
rs1419675
rs4873077
rs76506738
rs2698746
rs62255595
rs195869
rs1880069
All − MR Egger
All − Inverse variance weighted
−0.5
0.0
0.5
1.0
MR effect size for
'CAG−170 sp003516765' on 'GCST90199855_buildGRCh38.tsv'

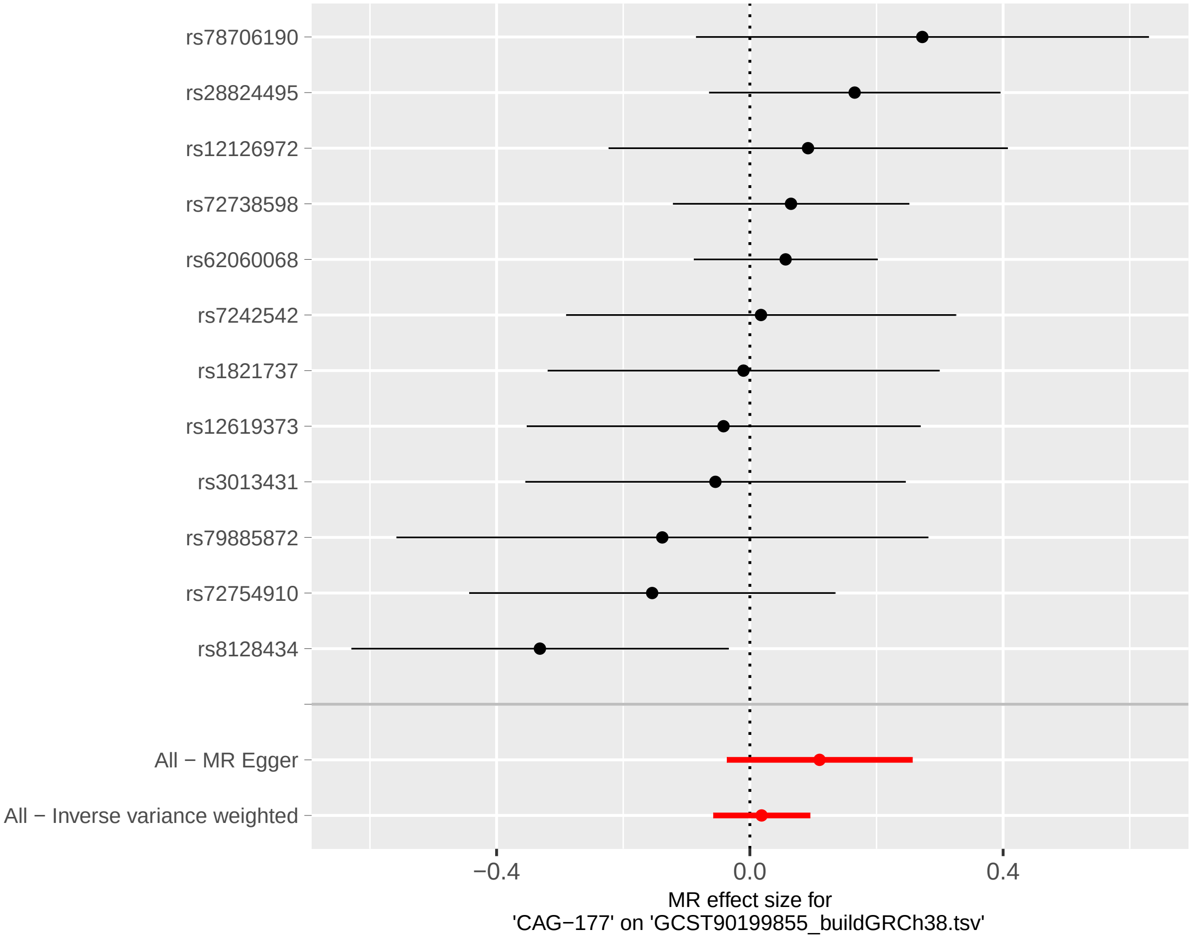

rs78706190
rs28824495
rs12126972
rs72738598
rs62060068
rs7242542
rs1821737
rs12619373
rs3013431
rs79885872
rs72754910
rs8128434
All − MR Egger
All − Inverse variance weighted
−0.4
0.0
0.4
MR effect size for
'CAG−177' on 'GCST90199855_buildGRCh38.tsv'

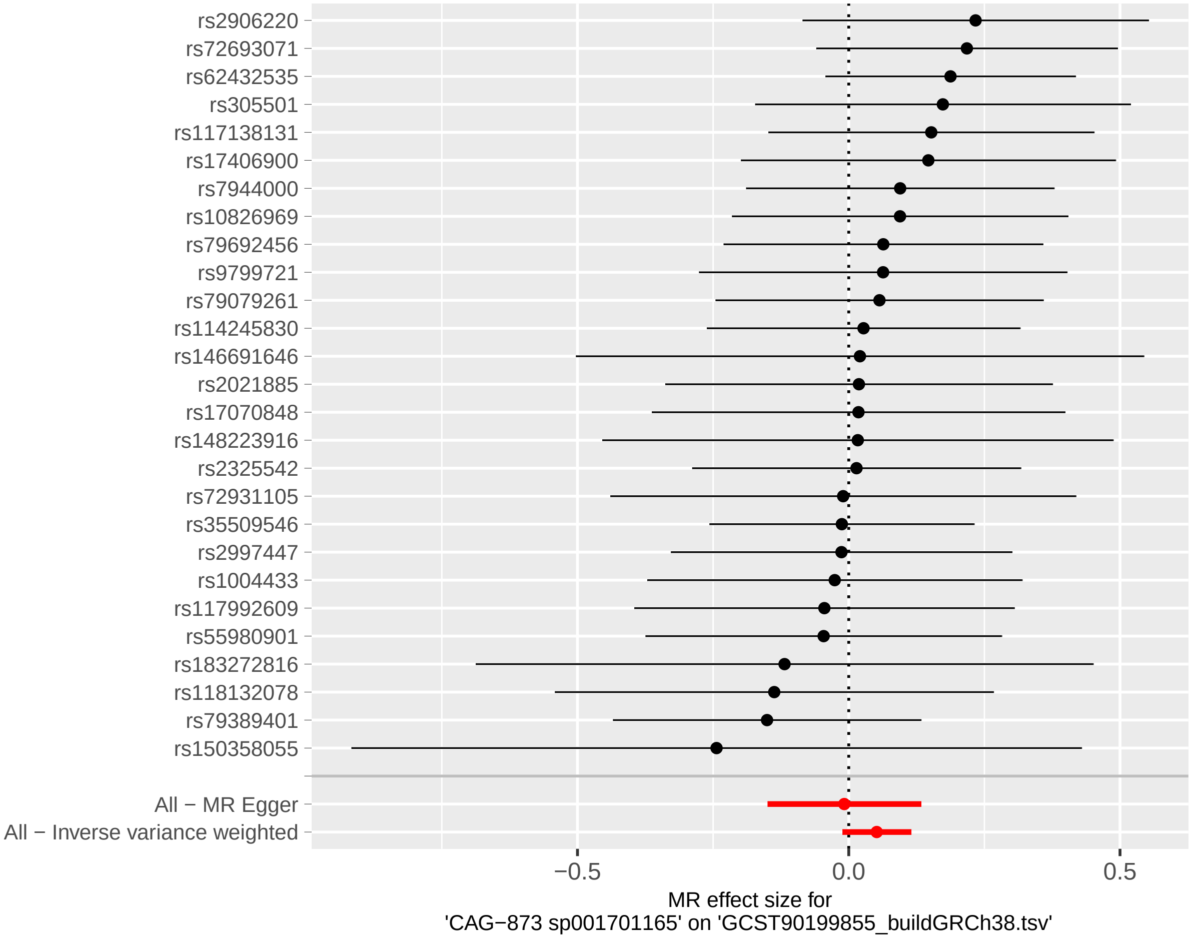
rs2906220
rs72693071
rs62432535
rs305501
rs117138131
rs17406900
rs7944000
rs10826969
rs79692456
rs9799721
rs79079261
rs114245830
rs146691646
rs2021885
rs17070848
rs148223916
rs2325542
rs72931105
rs35509546
rs2997447
rs1004433
rs117992609
rs55980901
rs183272816
rs118132078
rs79389401
rs150358055
All − MR Egger
All − Inverse variance weighted
−0.5
0.0
0.5
MR effect size for
'CAG−873 sp001701165' on 'GCST90199855_buildGRCh38.tsv'

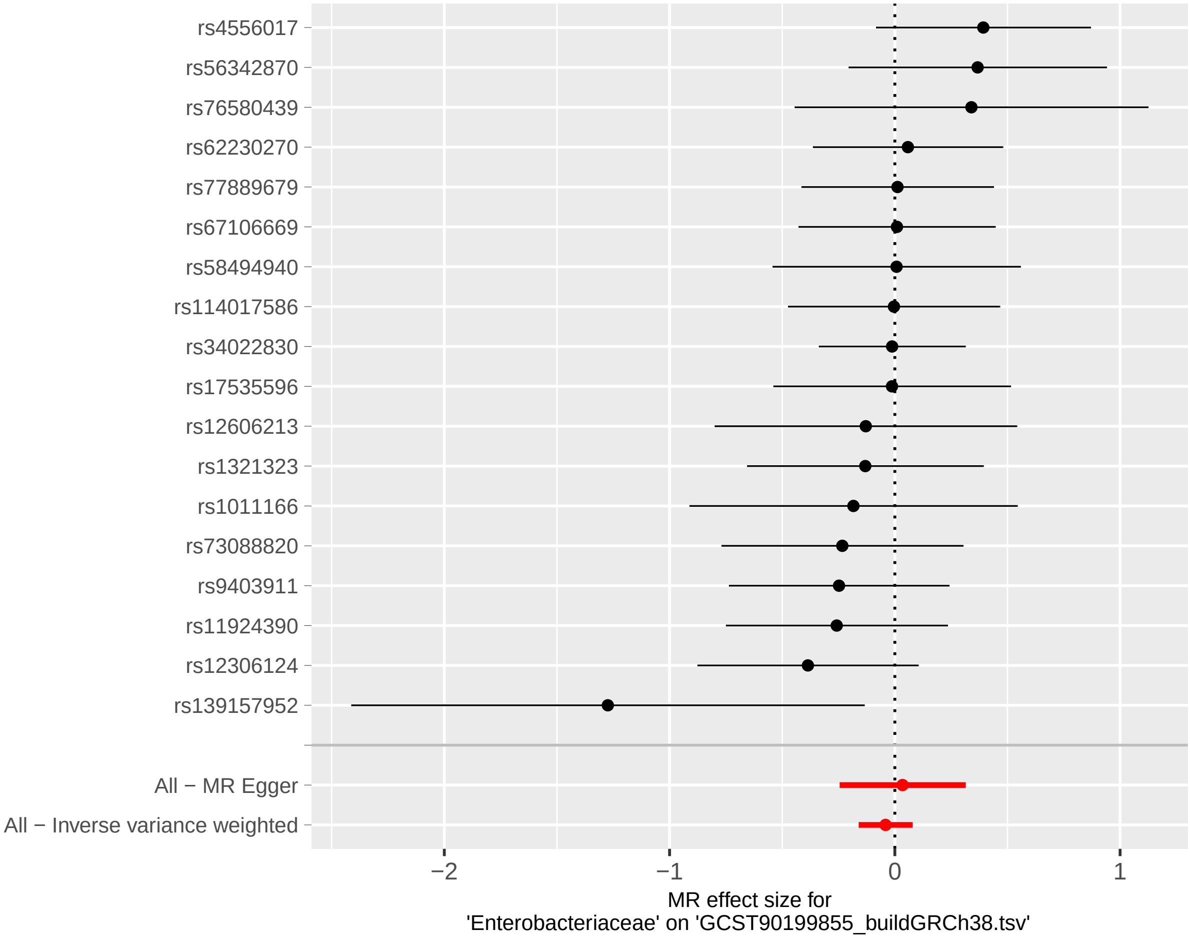
rs4556017
rs56342870
rs76580439
rs62230270
rs77889679
rs67106669
rs58494940
rs114017586
rs34022830
rs17535596
rs12606213
rs1321323
rs1011166
rs73088820
rs9403911
rs11924390
rs12306124
rs139157952
All − MR Egger
All − Inverse variance weighted
−2
−1
0
1
MR effect size for
'Enterobacteriaceae' on 'GCST90199855_buildGRCh38.tsv'

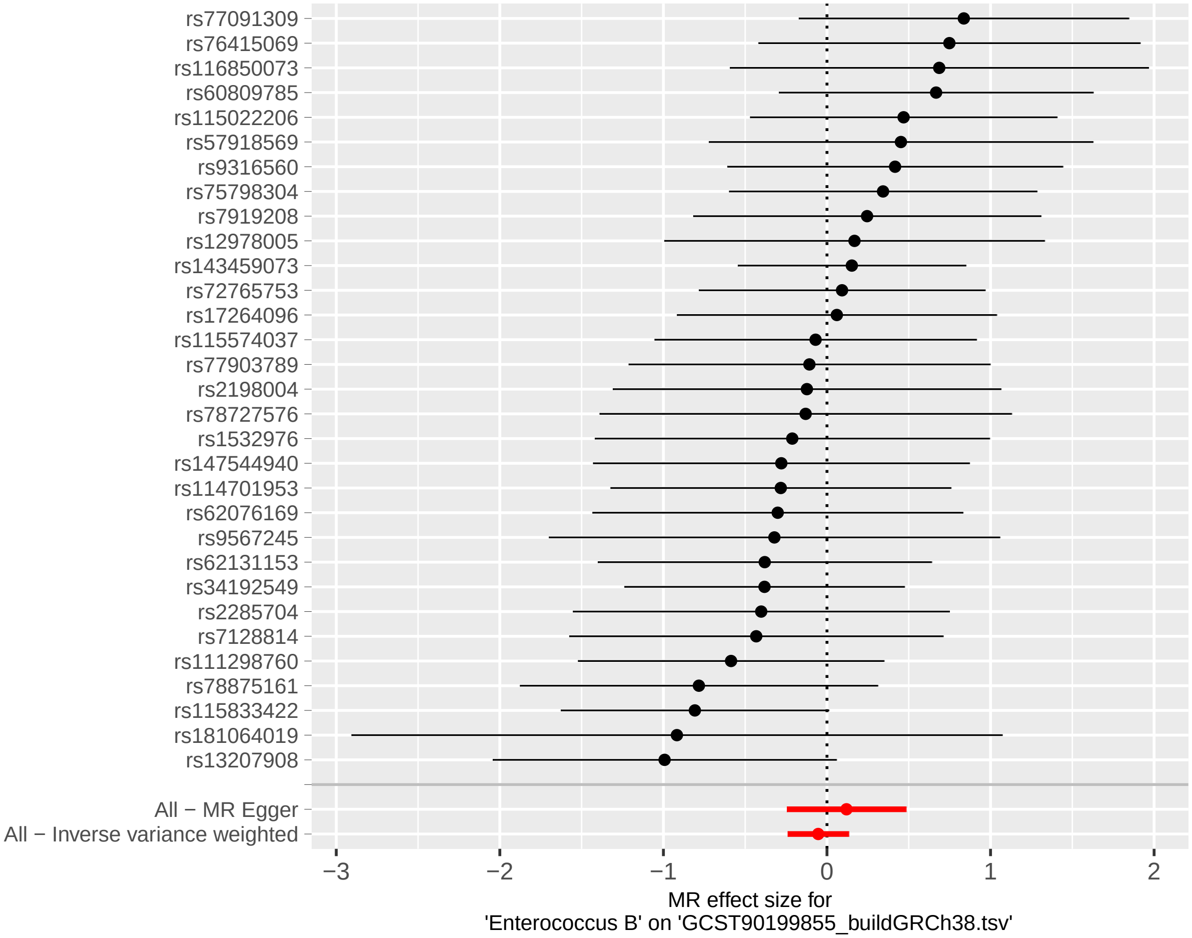
rs77091309
rs76415069
rs116850073
rs60809785
rs115022206
rs57918569
rs9316560
rs75798304
rs7919208
rs12978005
rs143459073
rs72765753
rs17264096
rs115574037
rs77903789
rs2198004
rs78727576
rs1532976
rs147544940
rs114701953
rs62076169
rs9567245
rs62131153
rs34192549
rs2285704
rs7128814
rs111298760
rs78875161
rs115833422
rs181064019
rs13207908
All − MR Egger
All − Inverse variance weighted
−3
−2
−1
0
1
2
MR effect size for
'Enterococcus B' on 'GCST90199855_buildGRCh38.tsv'

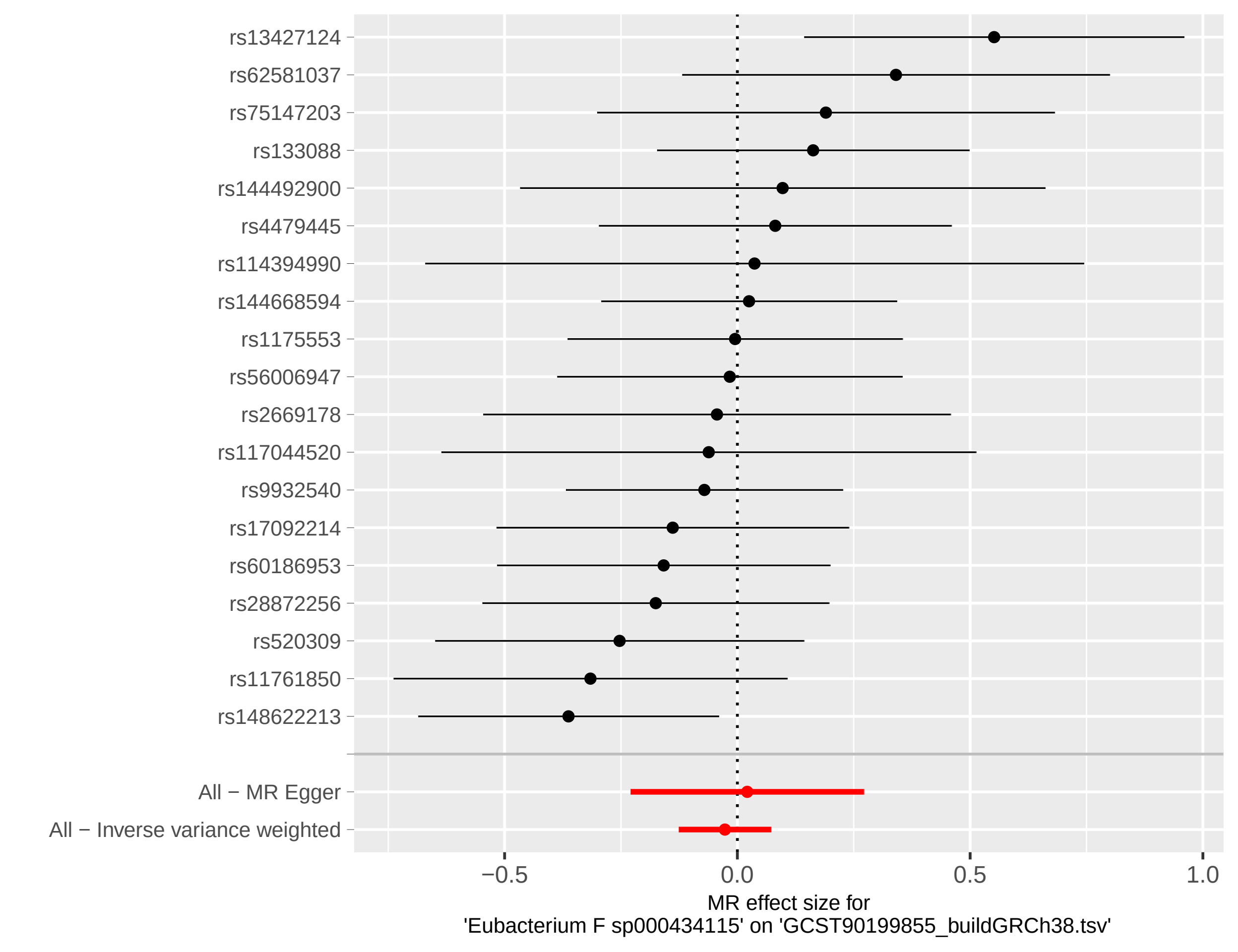
rs13427124
rs62581037
rs75147203
rs133088
rs144492900
rs4479445
rs114394990
rs144668594
rs1175553
rs56006947
rs2669178
rs117044520
rs9932540
rs17092214
rs60186953
rs28872256
rs520309
rs11761850
rs148622213
All − MR Egger
All − Inverse variance weighted
−0.5
0.0
0.5
1.0
MR effect size for
'Eubacterium F sp000434115' on 'GCST90199855_buildGRCh38.tsv'

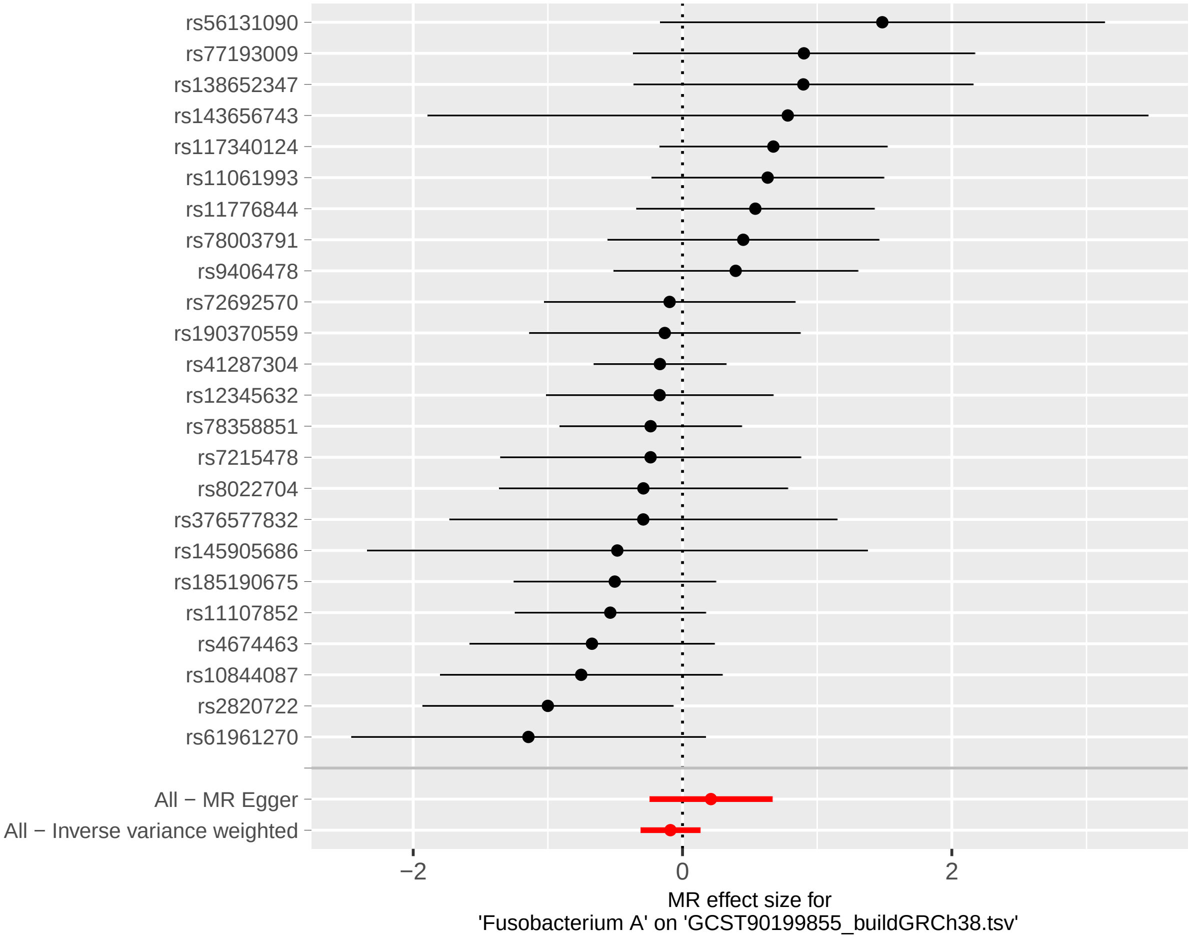
rs56131090
rs77193009
rs138652347
rs143656743
rs117340124
rs11061993
rs11776844
rs78003791
rs9406478
rs72692570
rs190370559
rs41287304
rs12345632
rs78358851
rs7215478
rs8022704
rs376577832
rs145905686
rs185190675
rs11107852
rs4674463
rs10844087
rs2820722
rs61961270
All − MR Egger
All − Inverse variance weighted
−2
0
2
MR effect size for
'Fusobacterium A' on 'GCST90199855_buildGRCh38.tsv'

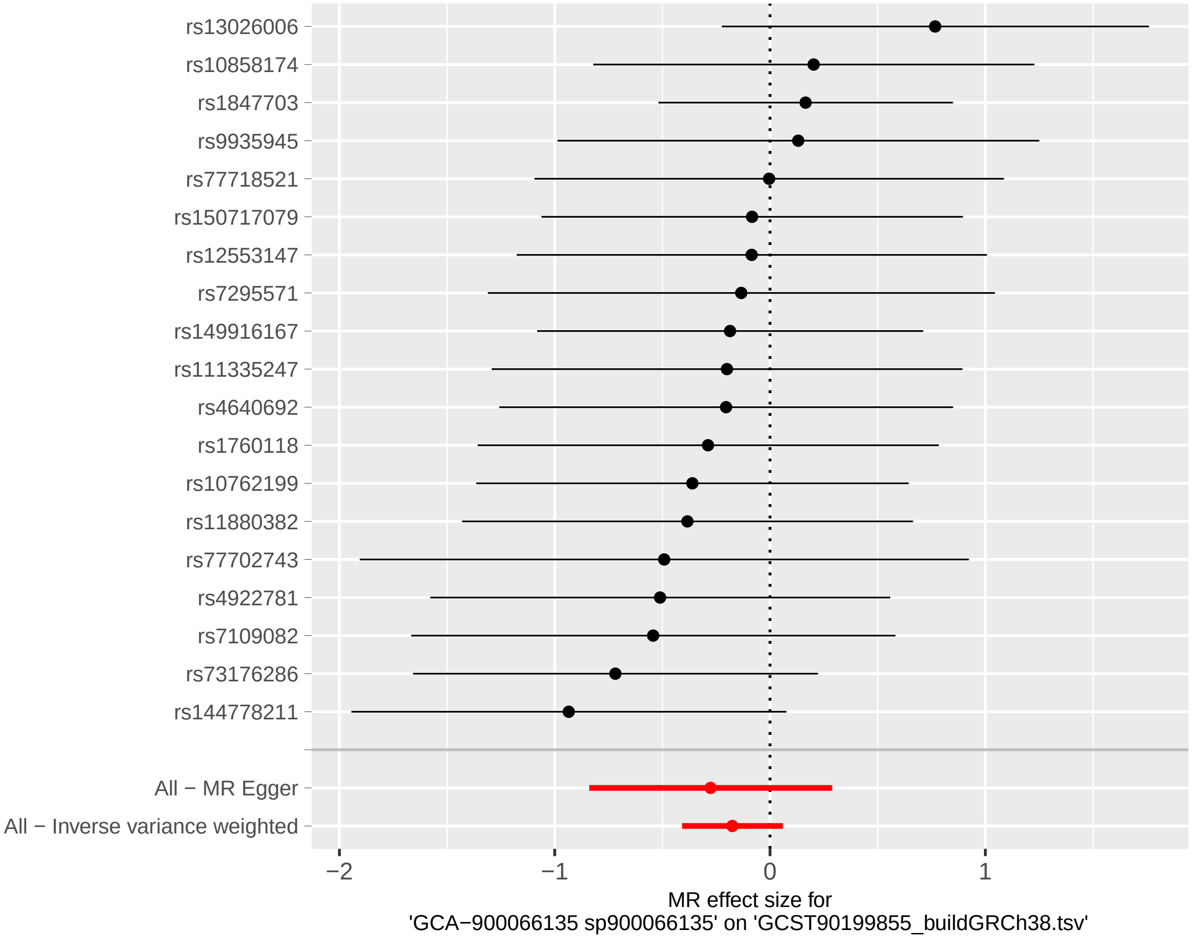
rs13026006
rs10858174
rs1847703
rs9935945
rs77718521
rs150717079
rs12553147
rs7295571
rs149916167
rs111335247
rs4640692
rs1760118
rs10762199
rs11880382
rs77702743
rs4922781
rs7109082
rs73176286
rs144778211
All − MR Egger
All − Inverse variance weighted
−2
−1
0
1
MR effect size for
'GCA−900066135 sp900066135' on 'GCST90199855_buildGRCh38.tsv'

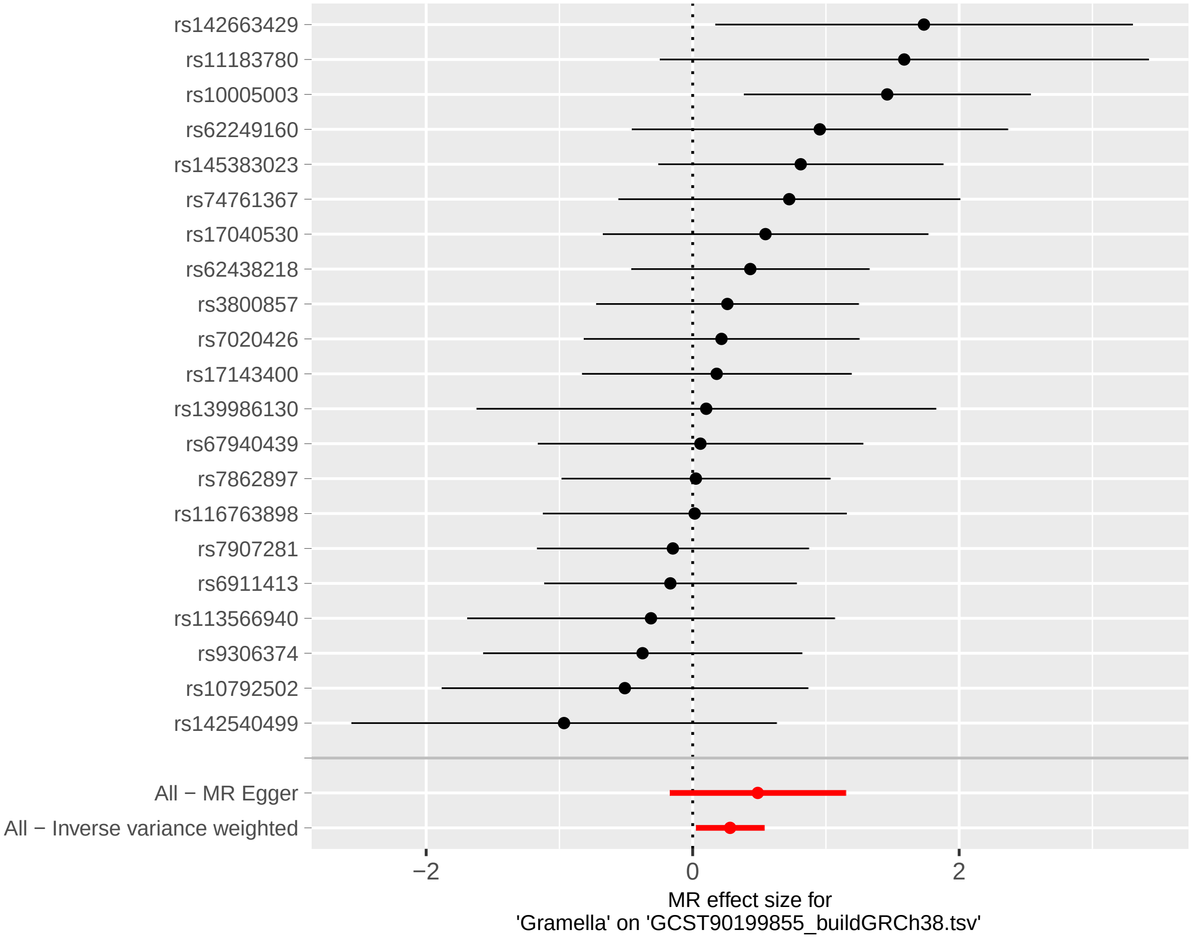
rs142663429
rs11183780
rs10005003
rs62249160
rs145383023
rs74761367
rs17040530
rs62438218
rs3800857
rs7020426
rs17143400
rs139986130
rs67940439
rs7862897
rs116763898
rs7907281
rs6911413
rs113566940
rs9306374
rs10792502
rs142540499
All − MR Egger
All − Inverse variance weighted
−2
0
2
MR effect size for
'Gramella' on 'GCST90199855_buildGRCh38.tsv'

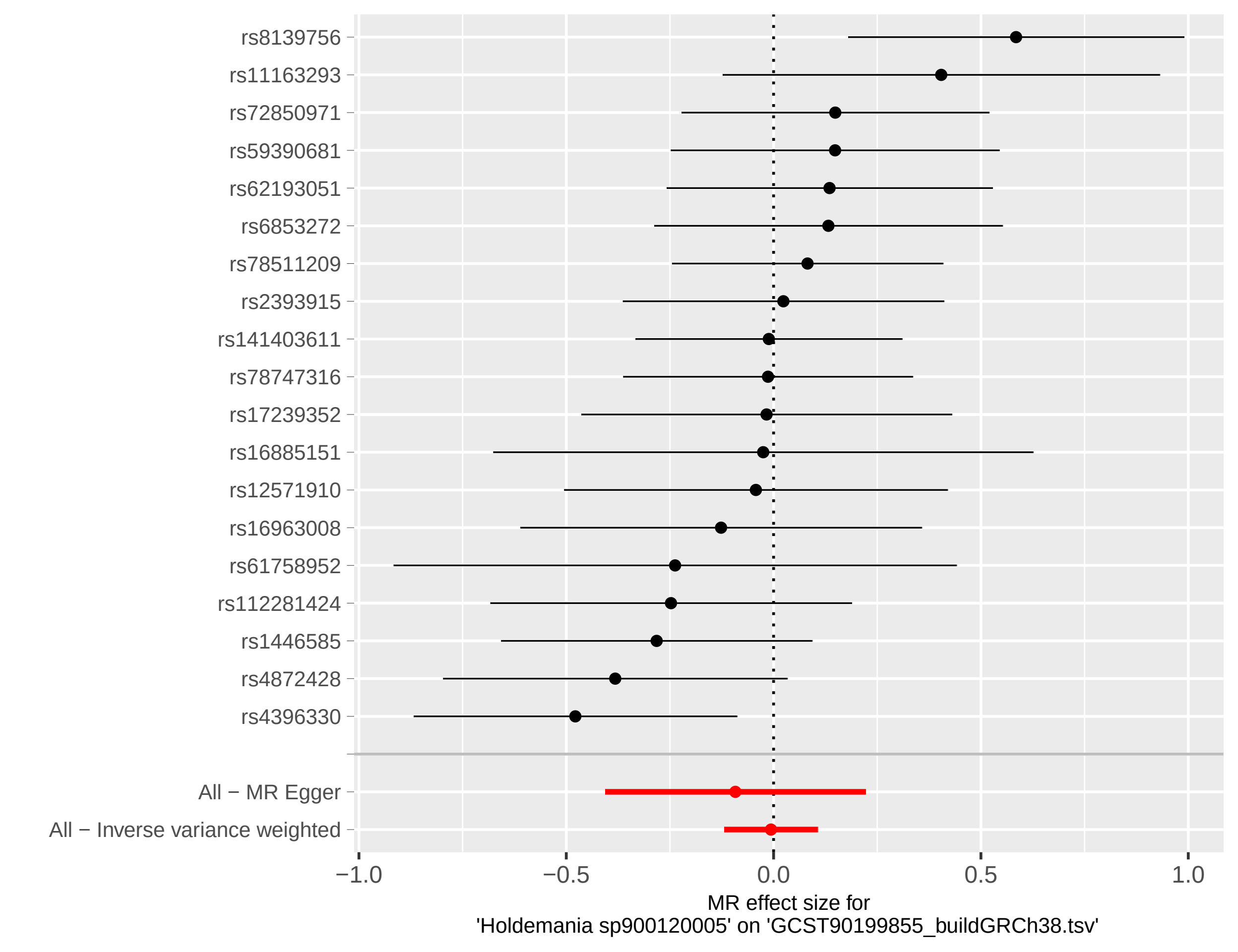

rs8139756
rs11163293
rs72850971
rs59390681
rs62193051
rs6853272
rs78511209
rs2393915
rs141403611
rs78747316
rs17239352
rs16885151
rs12571910
rs16963008
rs61758952
rs112281424
rs1446585
rs4872428
rs4396330
All − MR Egger
All − Inverse variance weighted
−1.0
−0.5
0.0
0.5
1.0
MR effect size for
'Holdemania sp900120005' on 'GCST90199855_buildGRCh38.tsv'

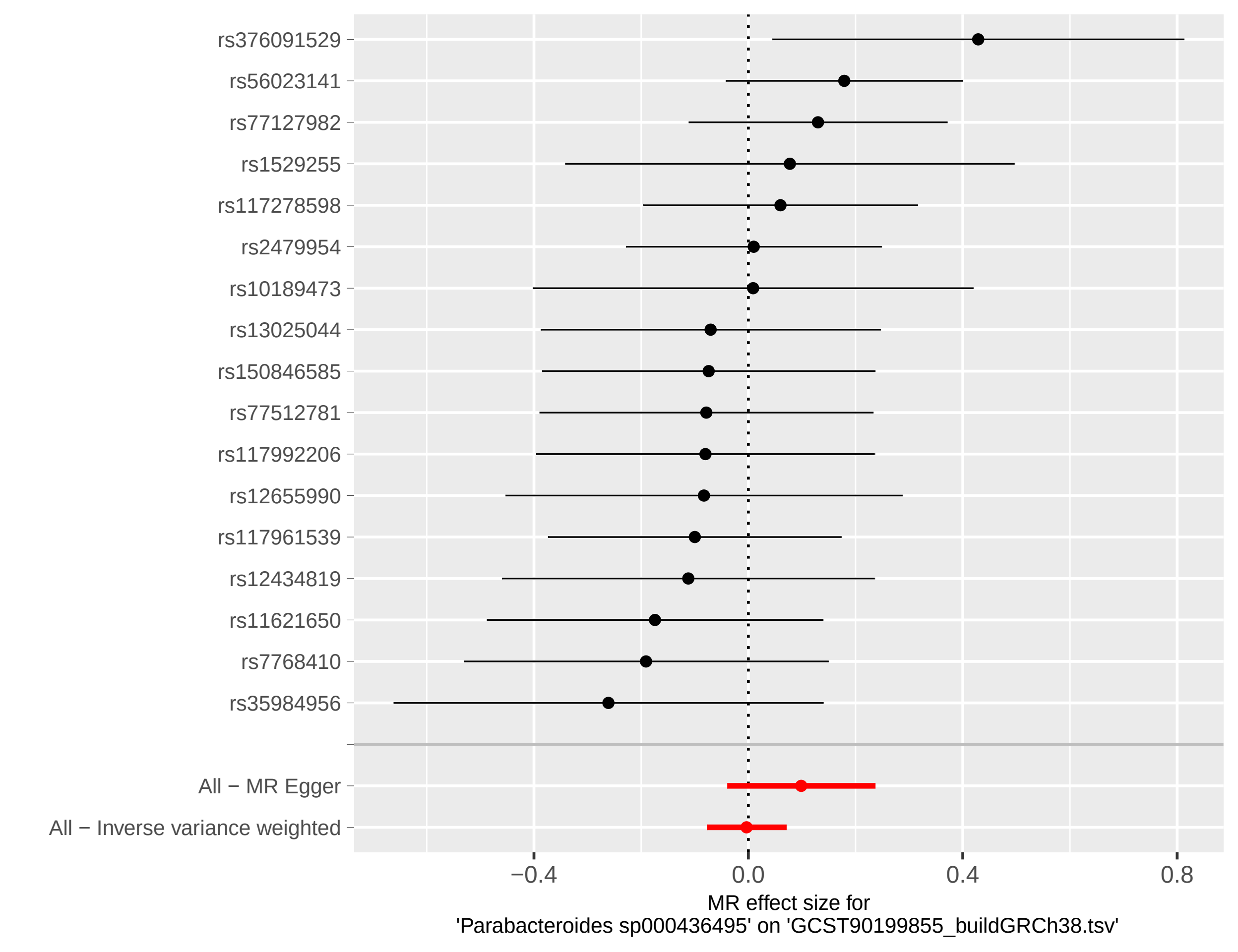
rs376091529
rs56023141
rs77127982
rs1529255
rs117278598
rs2479954
rs10189473
rs13025044
rs150846585
rs77512781
rs117992206
rs12655990
rs117961539
rs12434819
rs11621650
rs7768410
rs35984956
All − MR Egger
All − Inverse variance weighted
−0.4
0.0
0.4
0.8
MR effect size for
'Parabacteroides sp000436495' on 'GCST90199855_buildGRCh38.tsv'

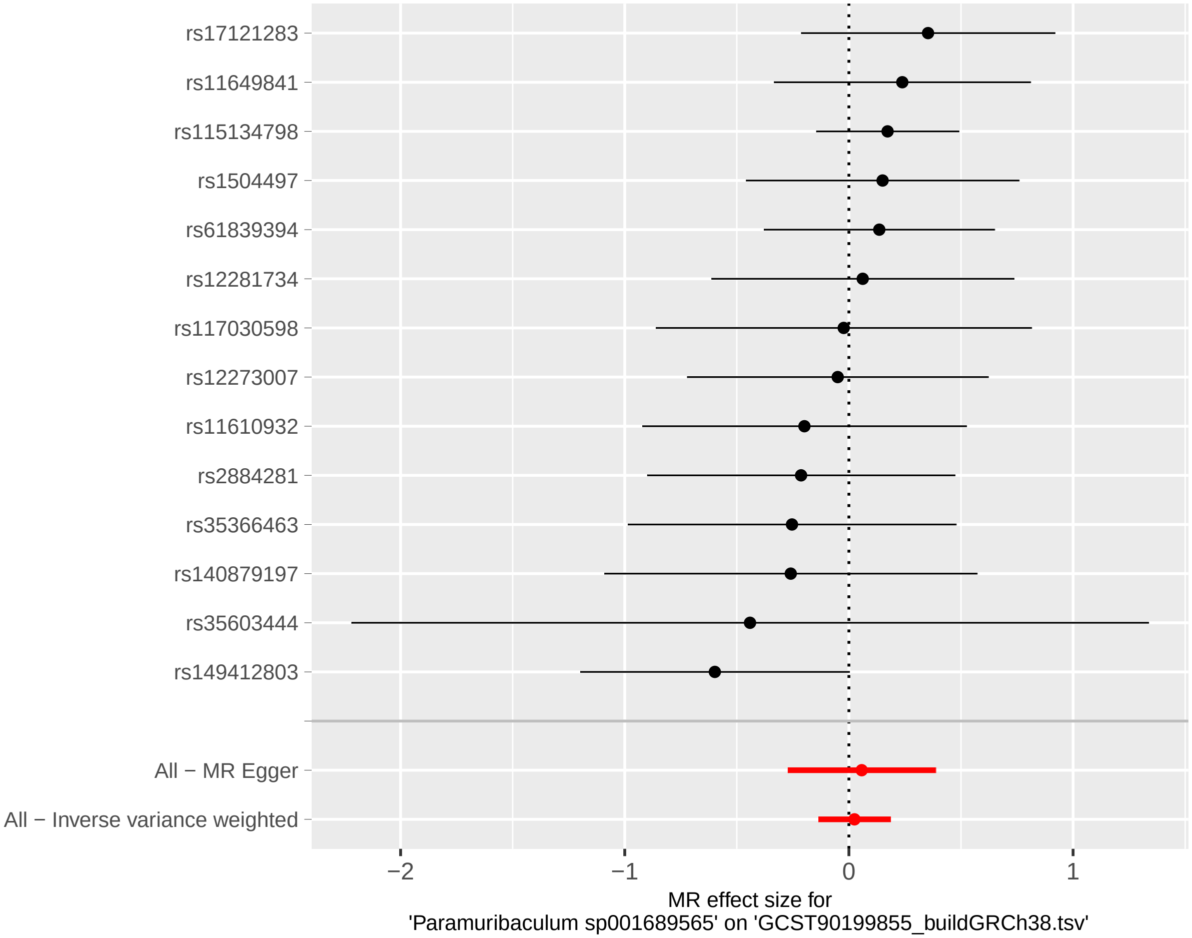

rs17121283
rs11649841
rs115134798
rs1504497
rs61839394
rs12281734
rs117030598
rs12273007
rs11610932
rs2884281
rs35366463
rs140879197
rs35603444
rs149412803
All − MR Egger
All − Inverse variance weighted
−2
−1
0
1
MR effect size for
'Paramuribaculum sp001689565' on 'GCST90199855_buildGRCh38.tsv'

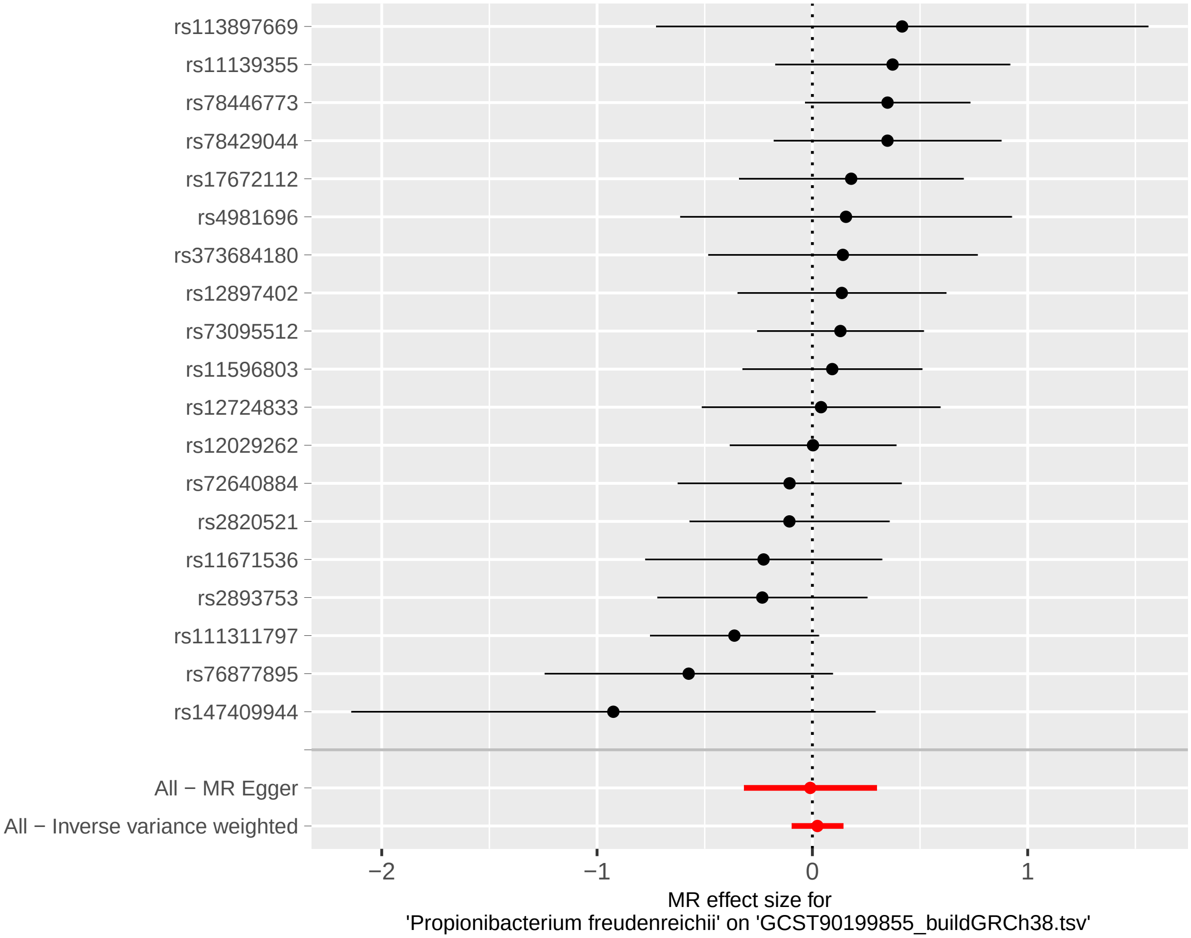
rs113897669
rs11139355
rs78446773
rs78429044
rs17672112
rs4981696
rs373684180
rs12897402
rs73095512
rs11596803
rs12724833
rs12029262
rs72640884
rs2820521
rs11671536
rs2893753
rs111311797
rs76877895
rs147409944
All − MR Egger
All − Inverse variance weighted
−2
−1
0
1
MR effect size for
'Propionibacterium freudenreichii' on 'GCST90199855_buildGRCh38.tsv'

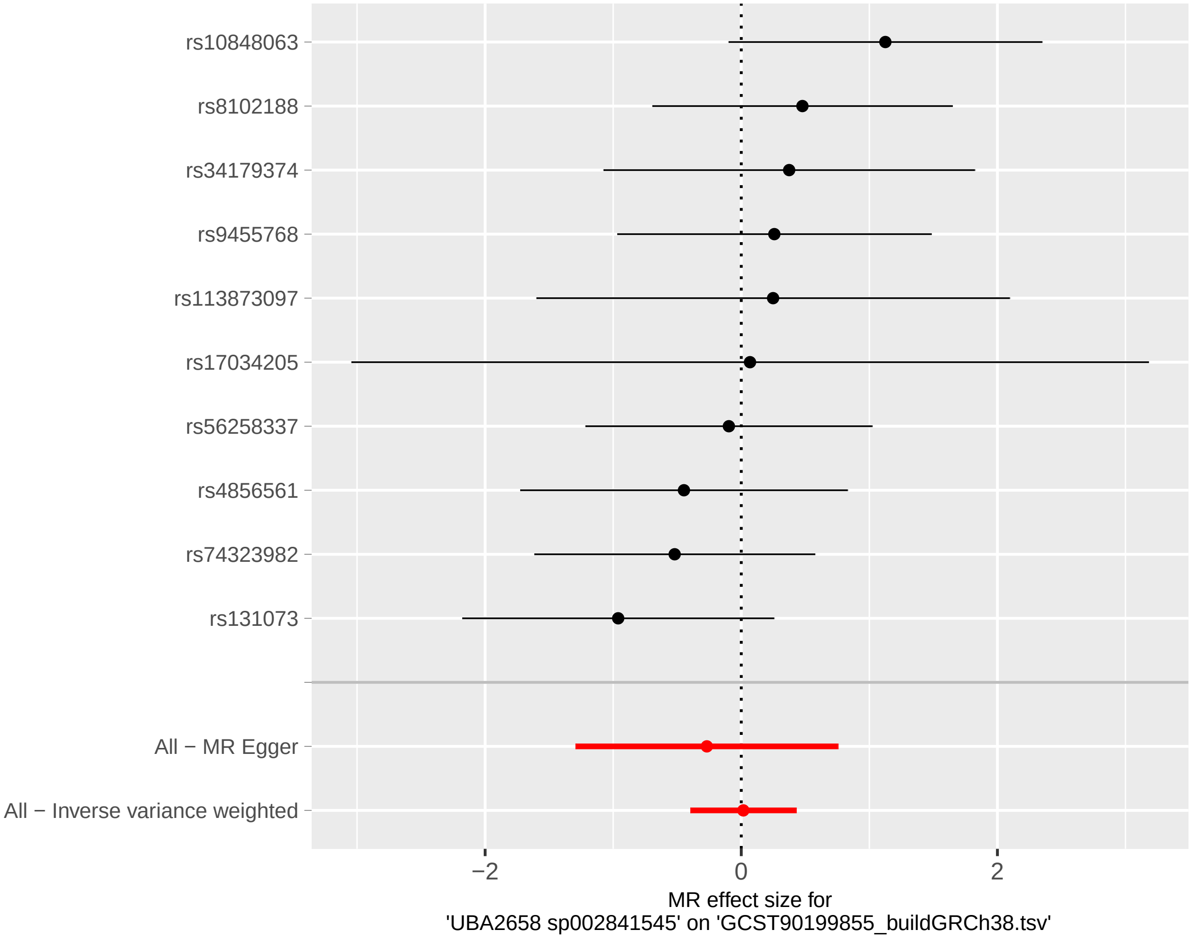
rs10848063
rs8102188
rs34179374
rs9455768
rs113873097
rs17034205
rs56258337
rs4856561
rs74323982
rs131073
All − MR Egger
All − Inverse variance weighted
−2
0
2
MR effect size for
'UBA2658 sp002841545' on 'GCST90199855_buildGRCh38.tsv'

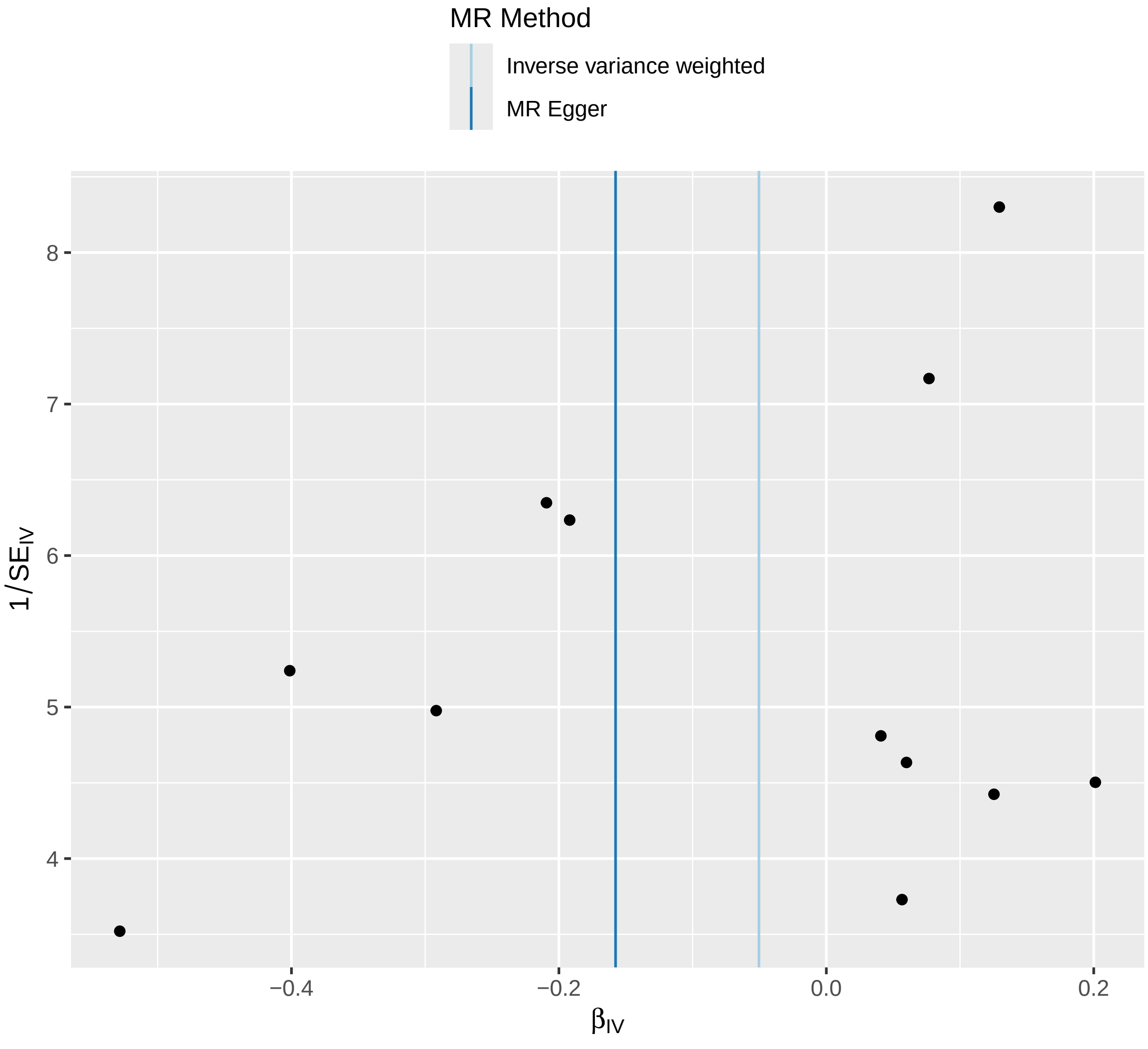
MR Method
Inverse variance weighted
MR Egger
8
7
6
5
4
1/SE_IV
−0.4
−0.2
0.0
0.2
β_IV

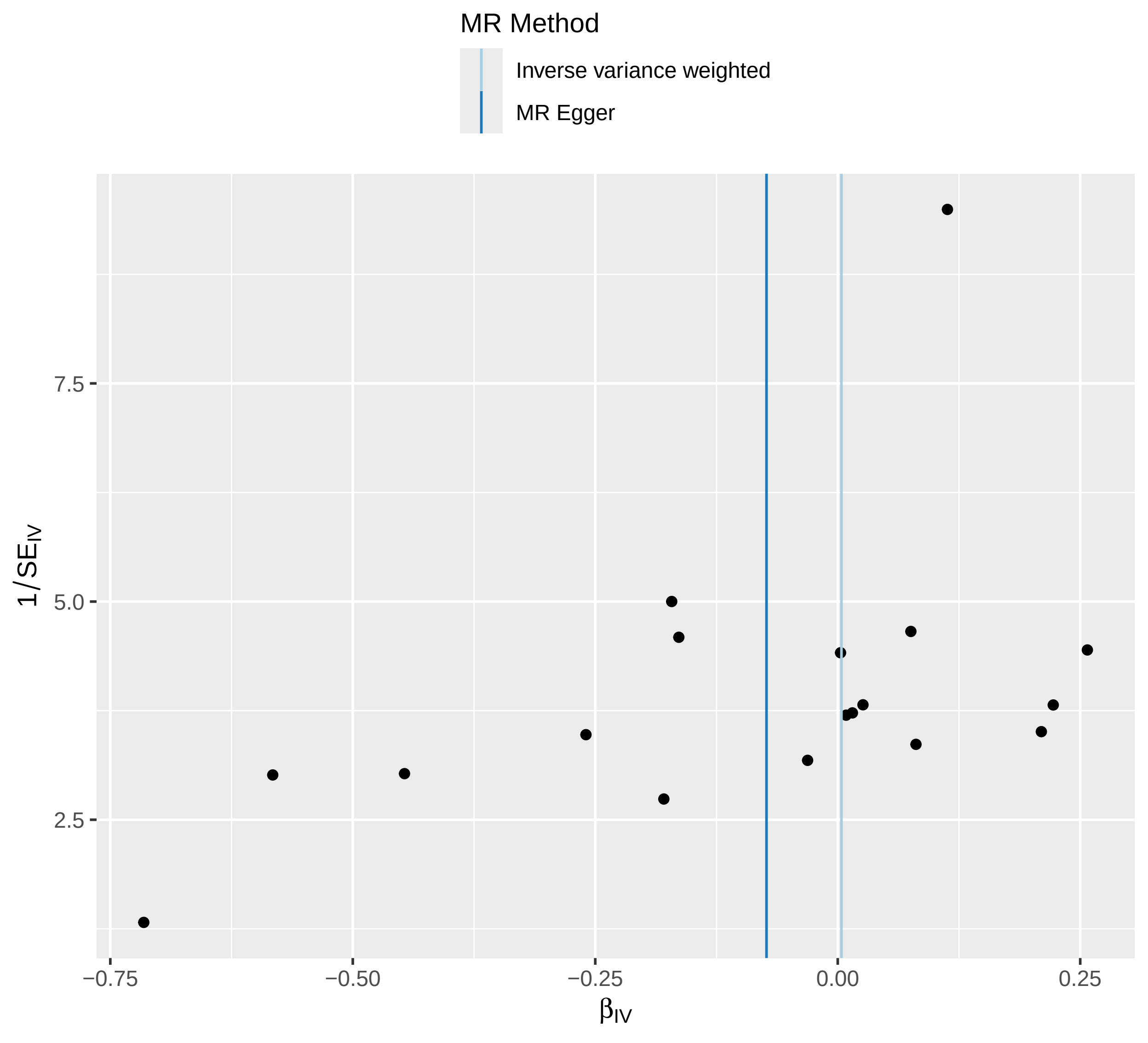

MR Method
Inverse variance weighted
MR Egger
7.5
5.0
2.5
1/SE_IV
−0.75
−0.50
−0.25
0.00
0.25
β_IV

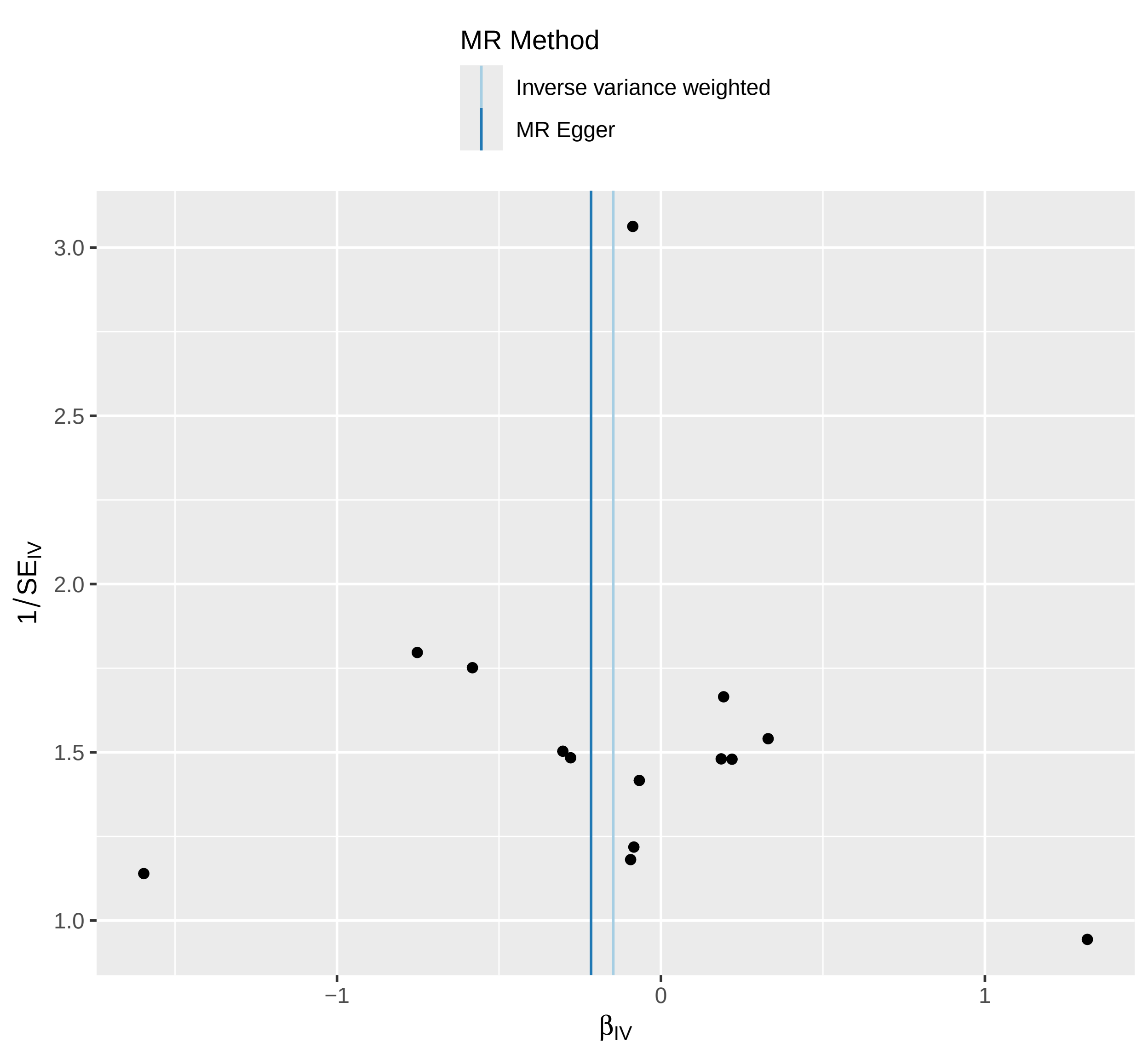

MR Method
Inverse variance weighted
MR Egger
3.0
2.5
2.0
1.5
1.0
1/SE_IV
−1
0
1
β_IV

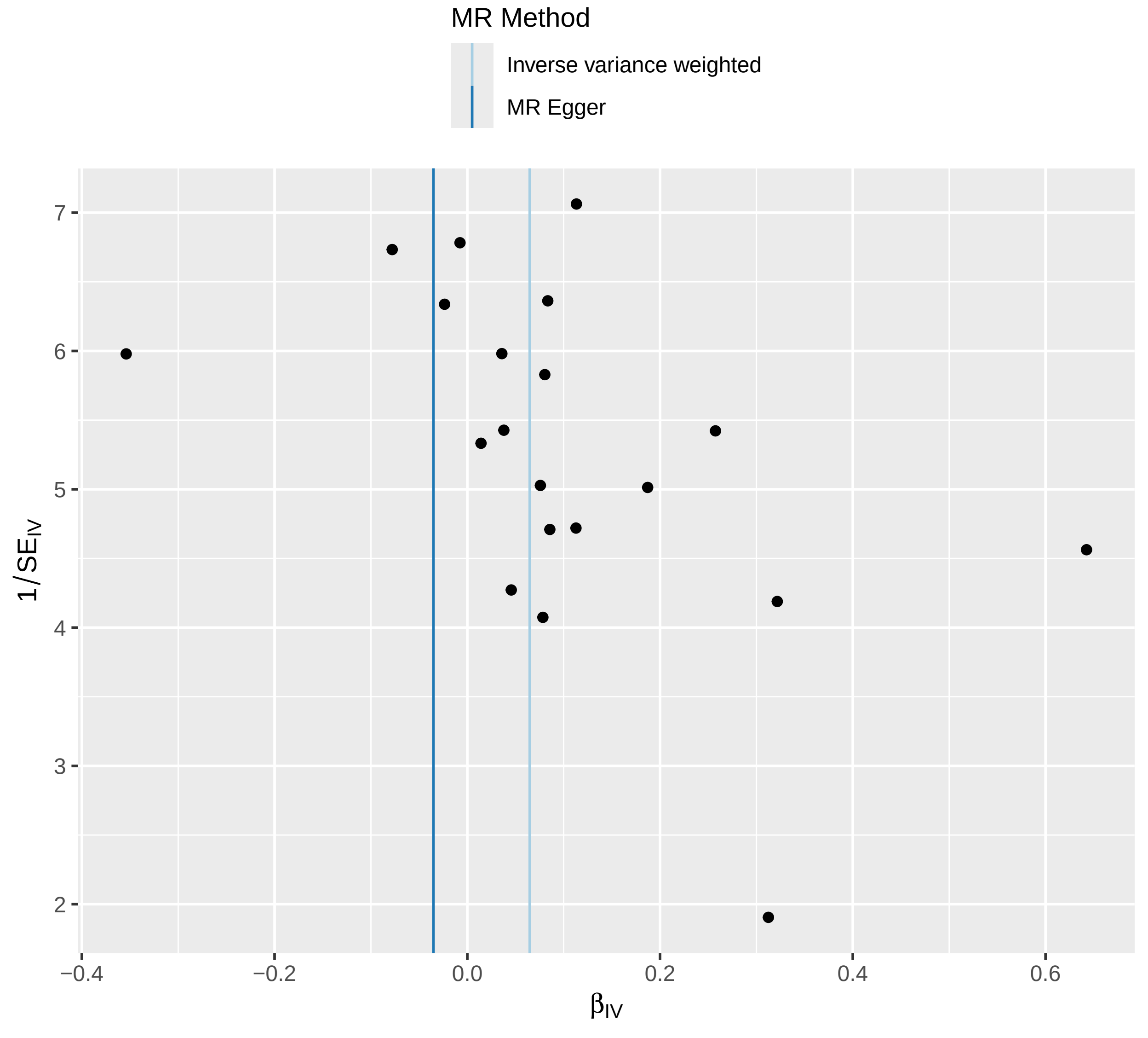

MR Method
Inverse variance weighted
MR Egger
7
6
5
4
3
2
1/SE_IV
−0.4
−0.2
0.0
0.2
0.4
0.6
β_IV

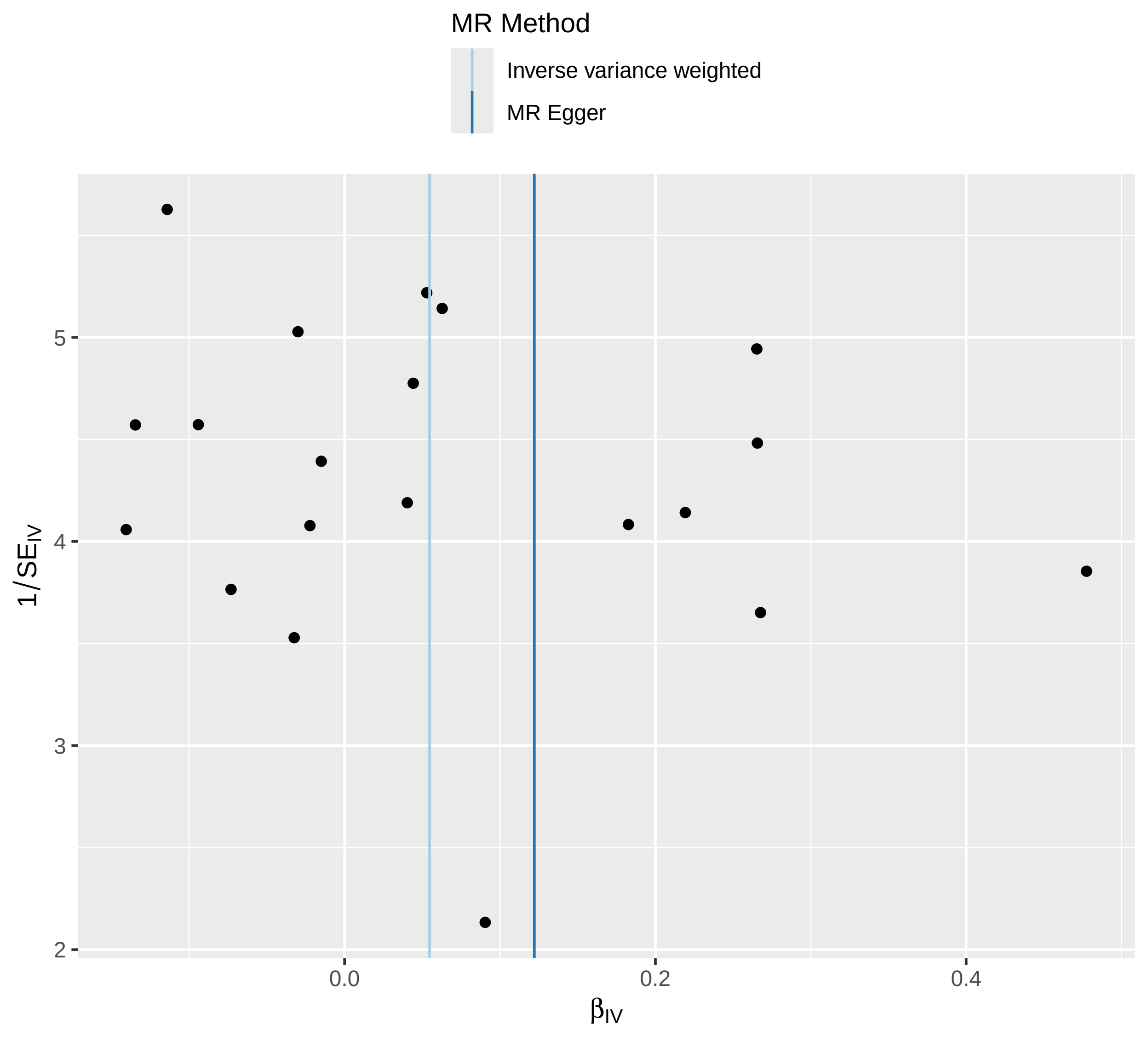

MR Method
Inverse variance weighted
MR Egger
5
4
3
2
1/SE_IV
0.0
0.2
0.4
β_IV

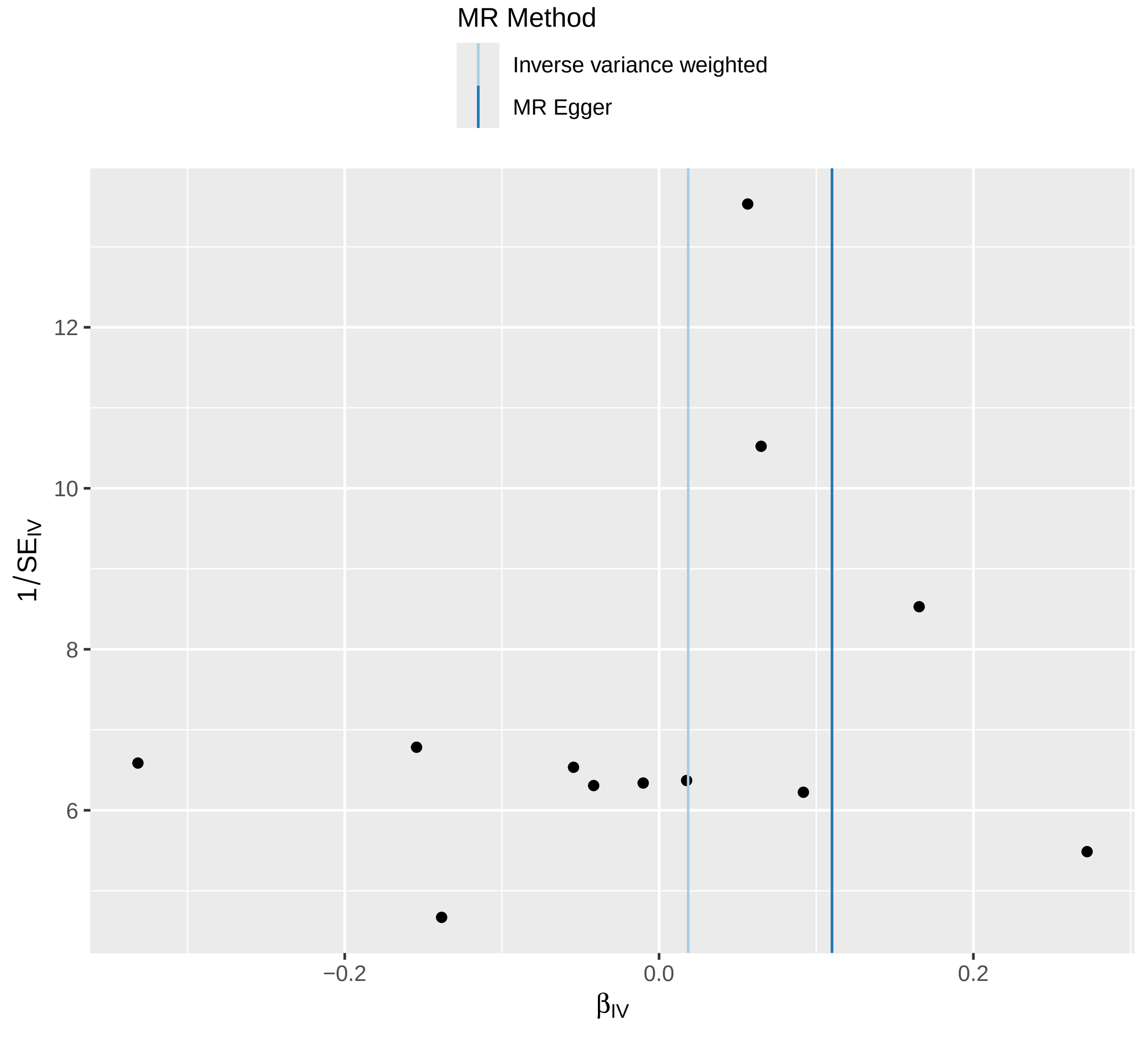
MR Method
Inverse variance weighted
MR Egger
12
10
8
6
1/SE_IV
−0.2
0.0
0.2
β_IV

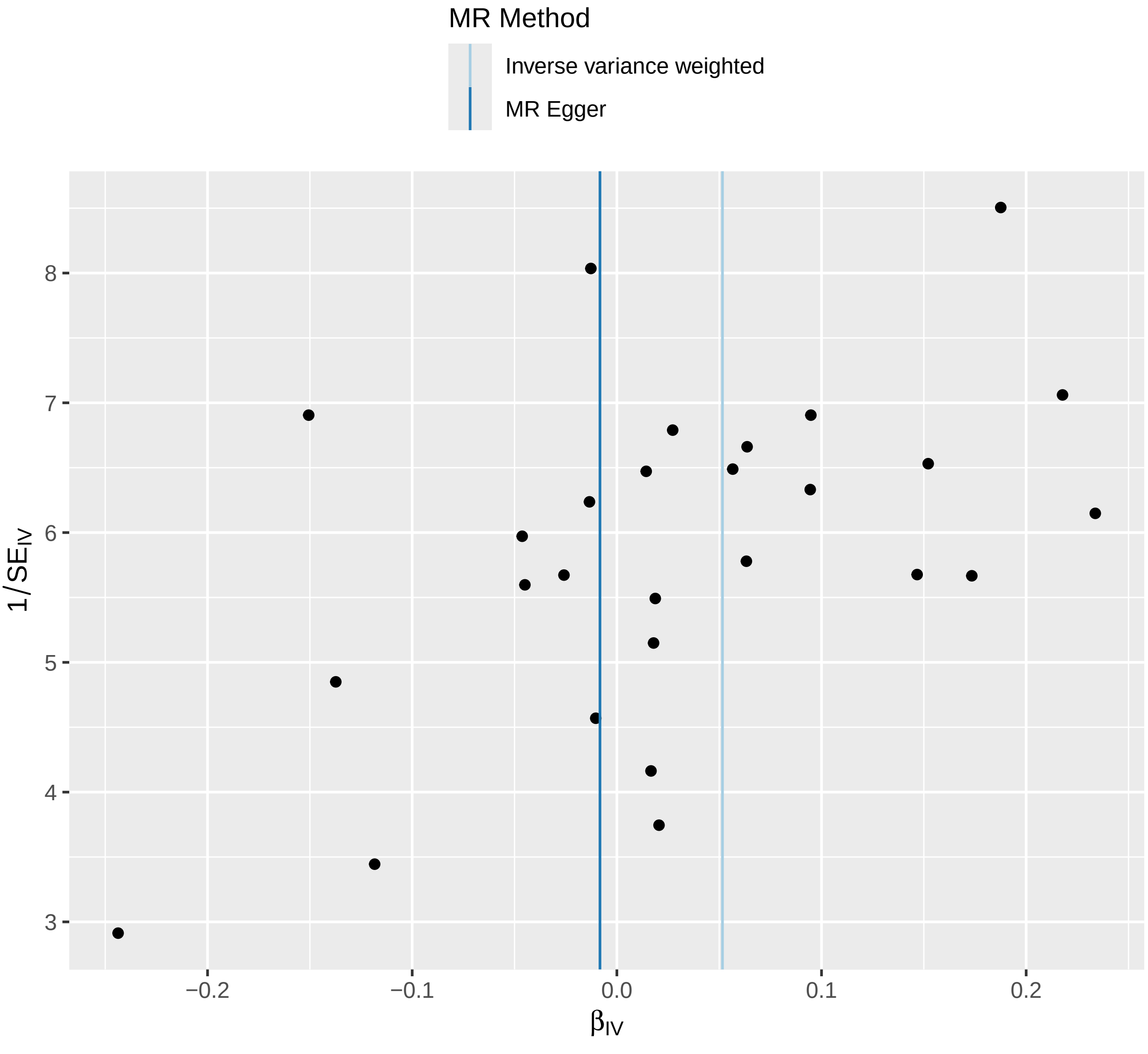
MR Method
Inverse variance weighted
MR Egger
8
7
6
5
4
3
1/SE_IV
−0.2
−0.1
0.0
0.1
0.2
β_IV

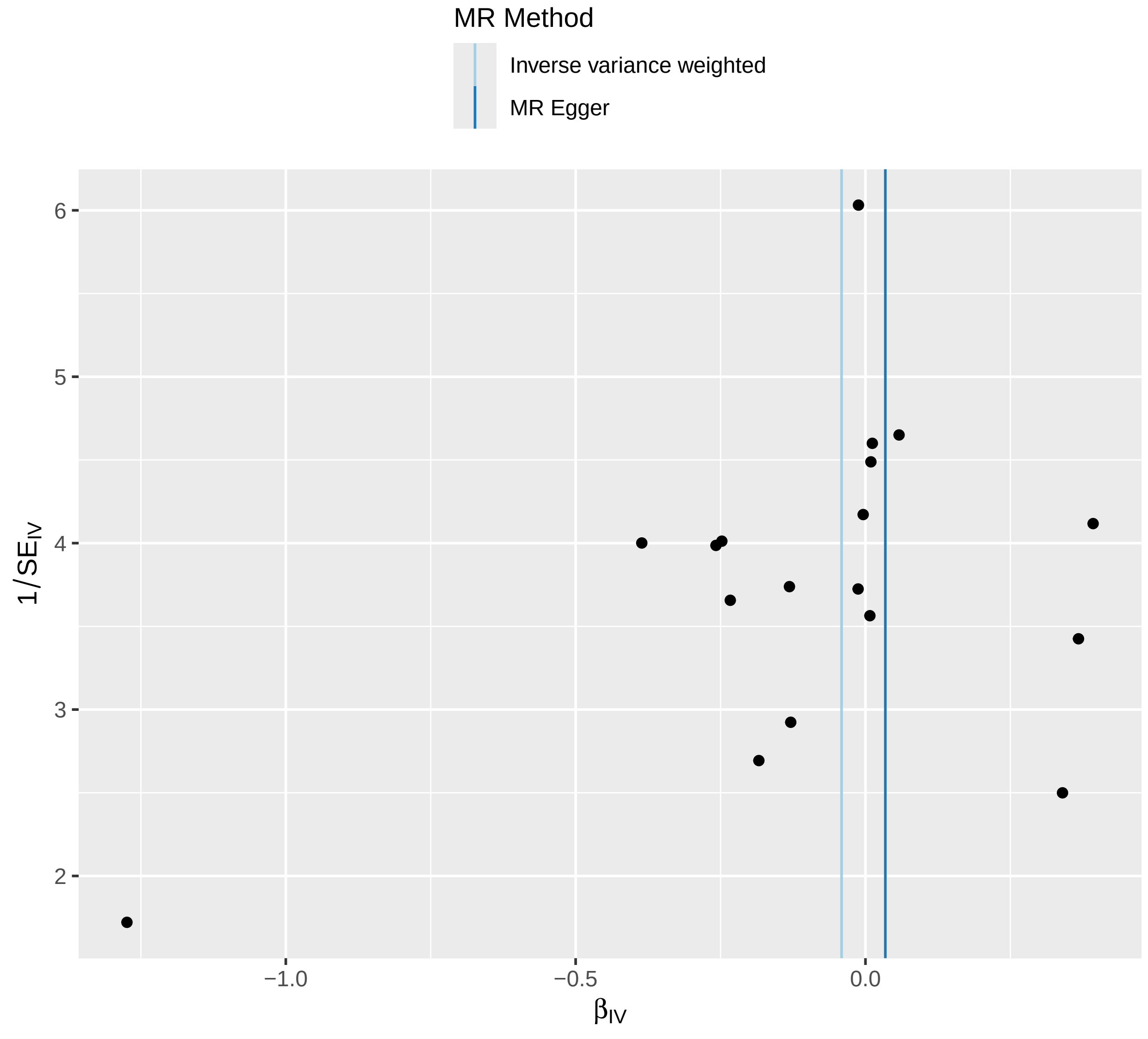
MR Method
Inverse variance weighted
MR Egger
1/SE_IV
6
5
4
3
2
−1.0
−0.5
0.0
β_IV

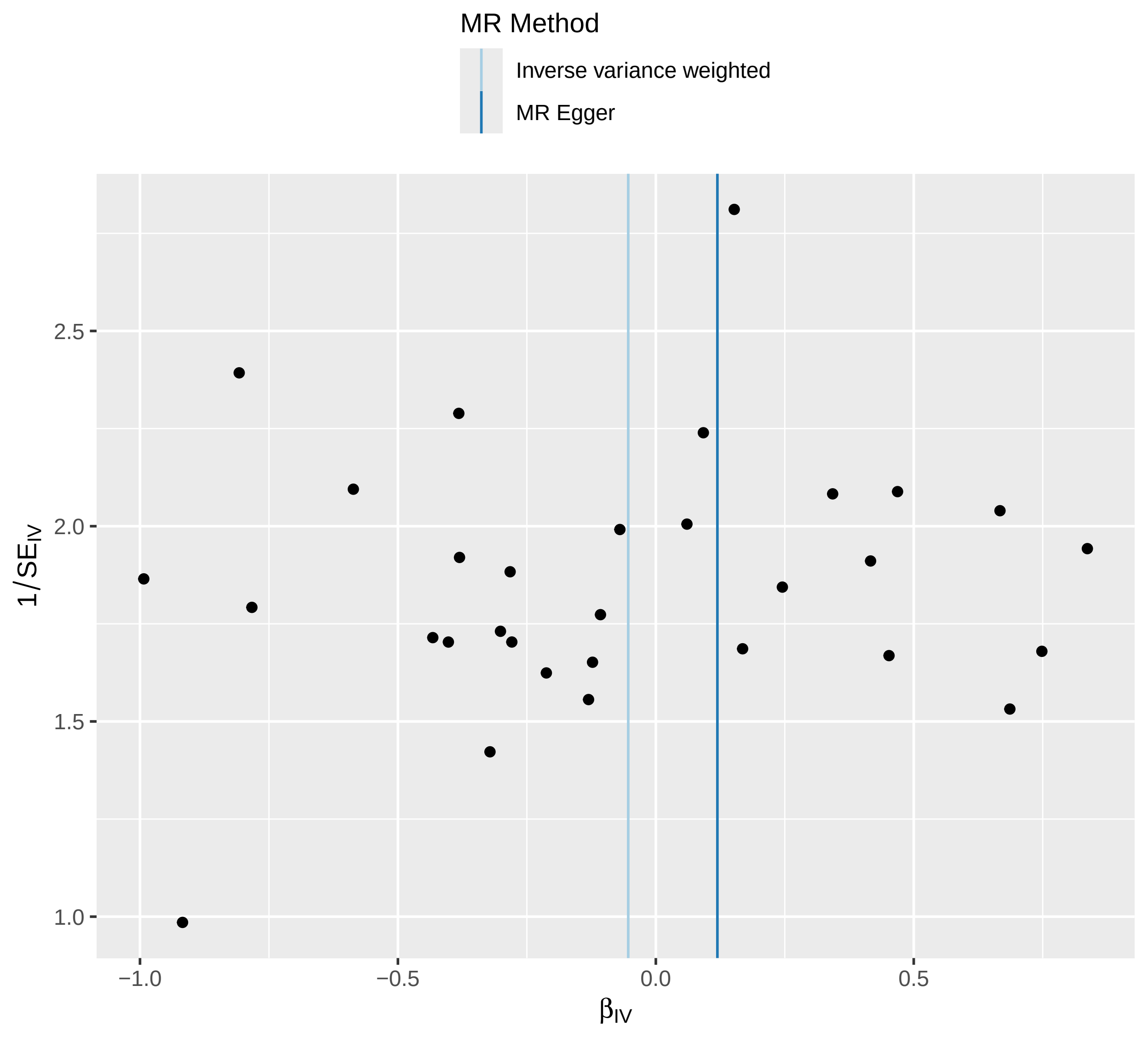
MR Method
Inverse variance weighted
MR Egger
2.5
2.0
1.5
1.0
−1.0
−0.5
0.0
0.5
$1/SE_{IV}$
β_{IV}

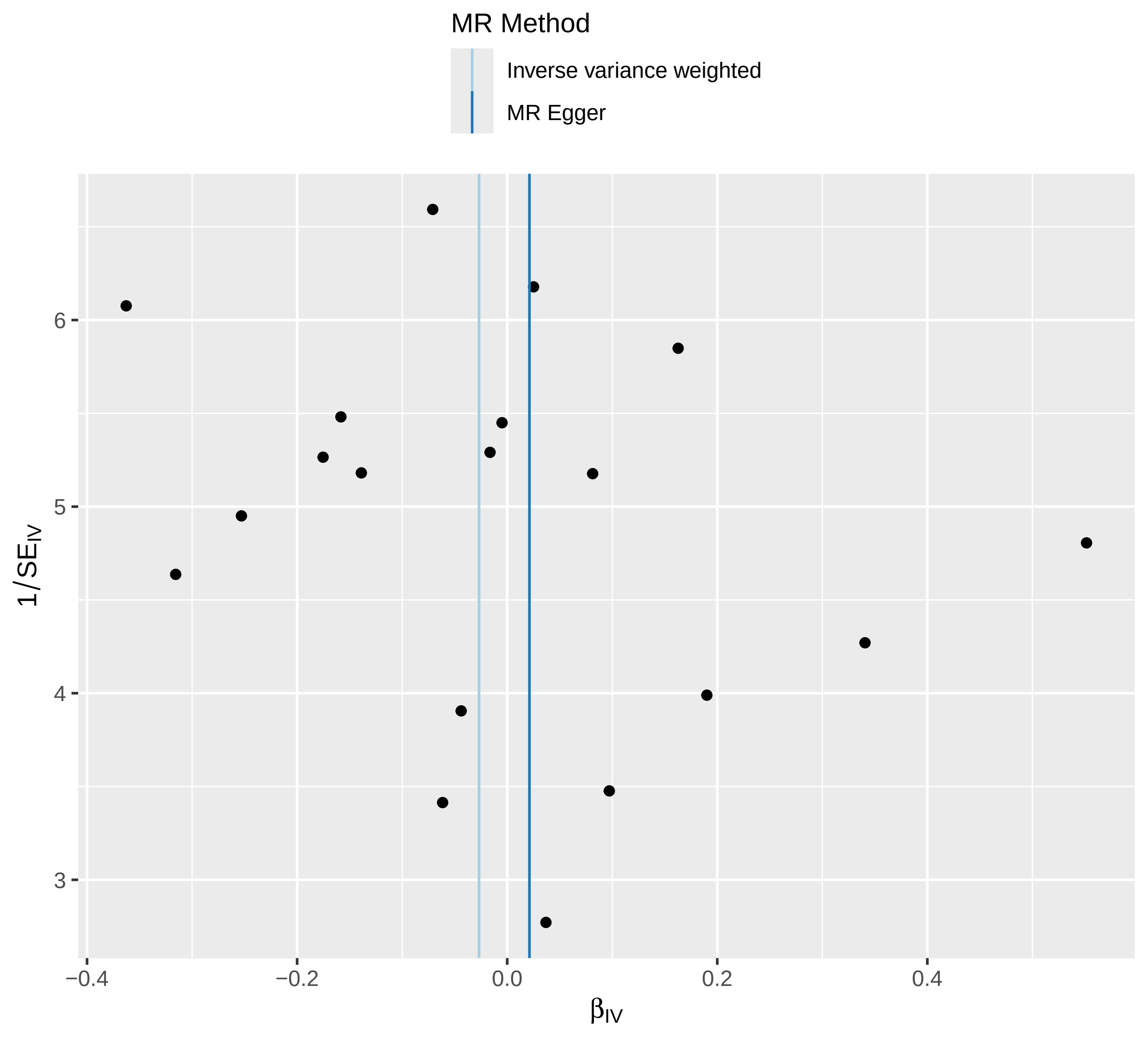

MR Method
Inverse variance weighted
MR Egger
6
5
4
3
1/SE$_{IV}$
−0.4
−0.2
0.0
0.2
0.4
β$_{IV}$

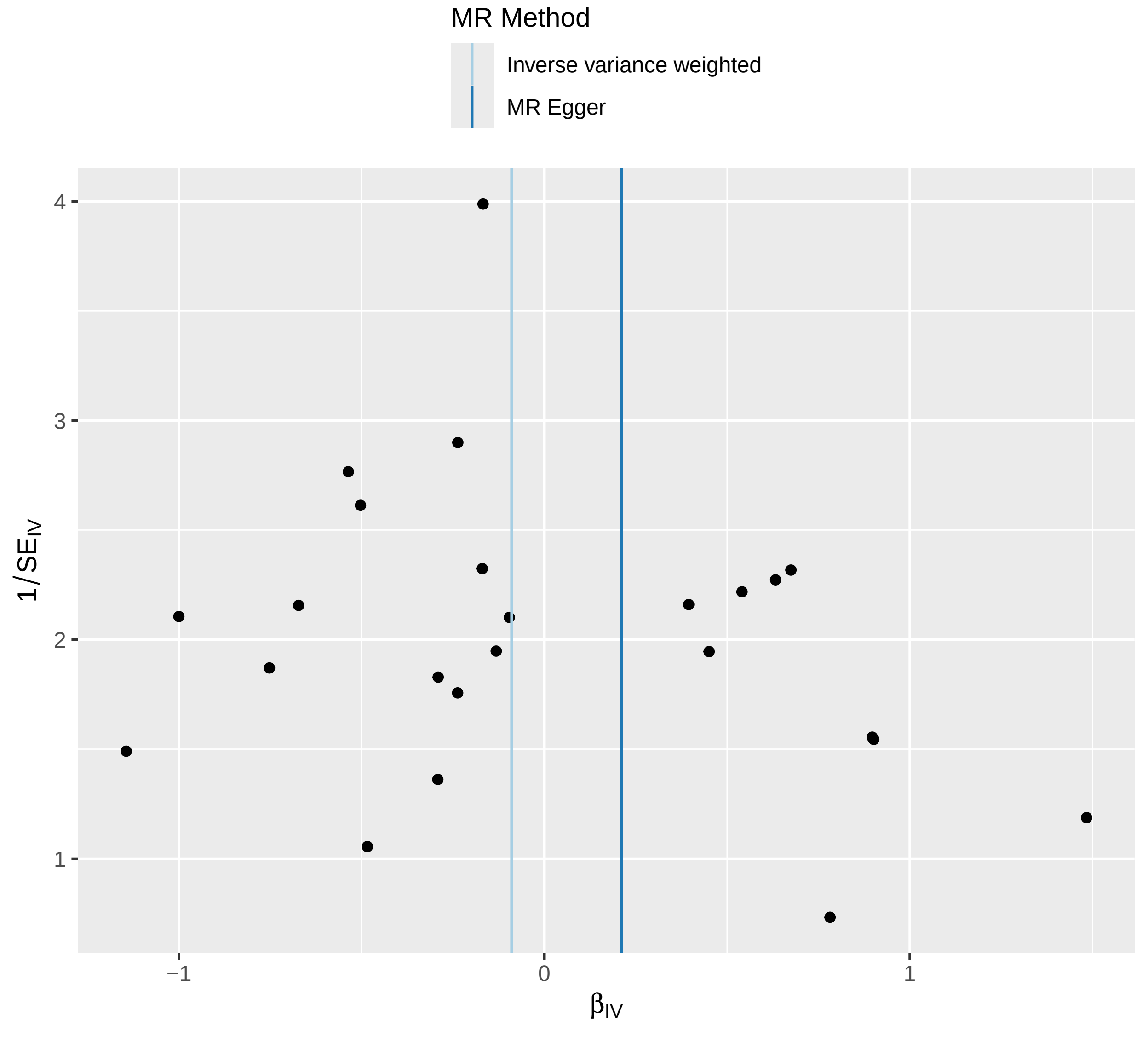

MR Method
Inverse variance weighted
MR Egger
4
3
2
1
1/SE_IV
−1
0
1
β_IV

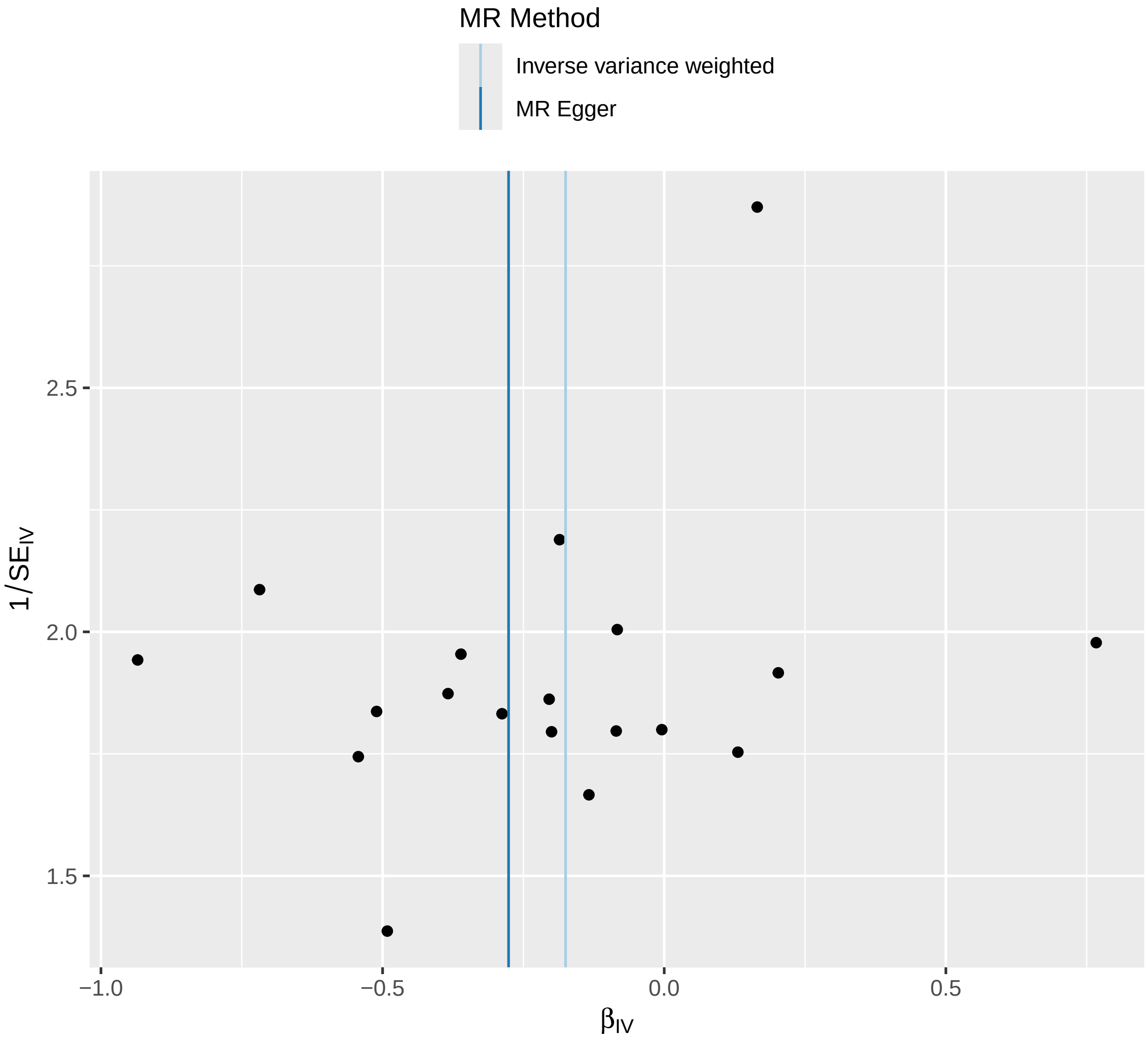

MR Method
Inverse variance weighted
MR Egger
2.5
2.0
1.5
$1/SE_{IV}$
−1.0
−0.5
0.0
0.5
β_{IV}

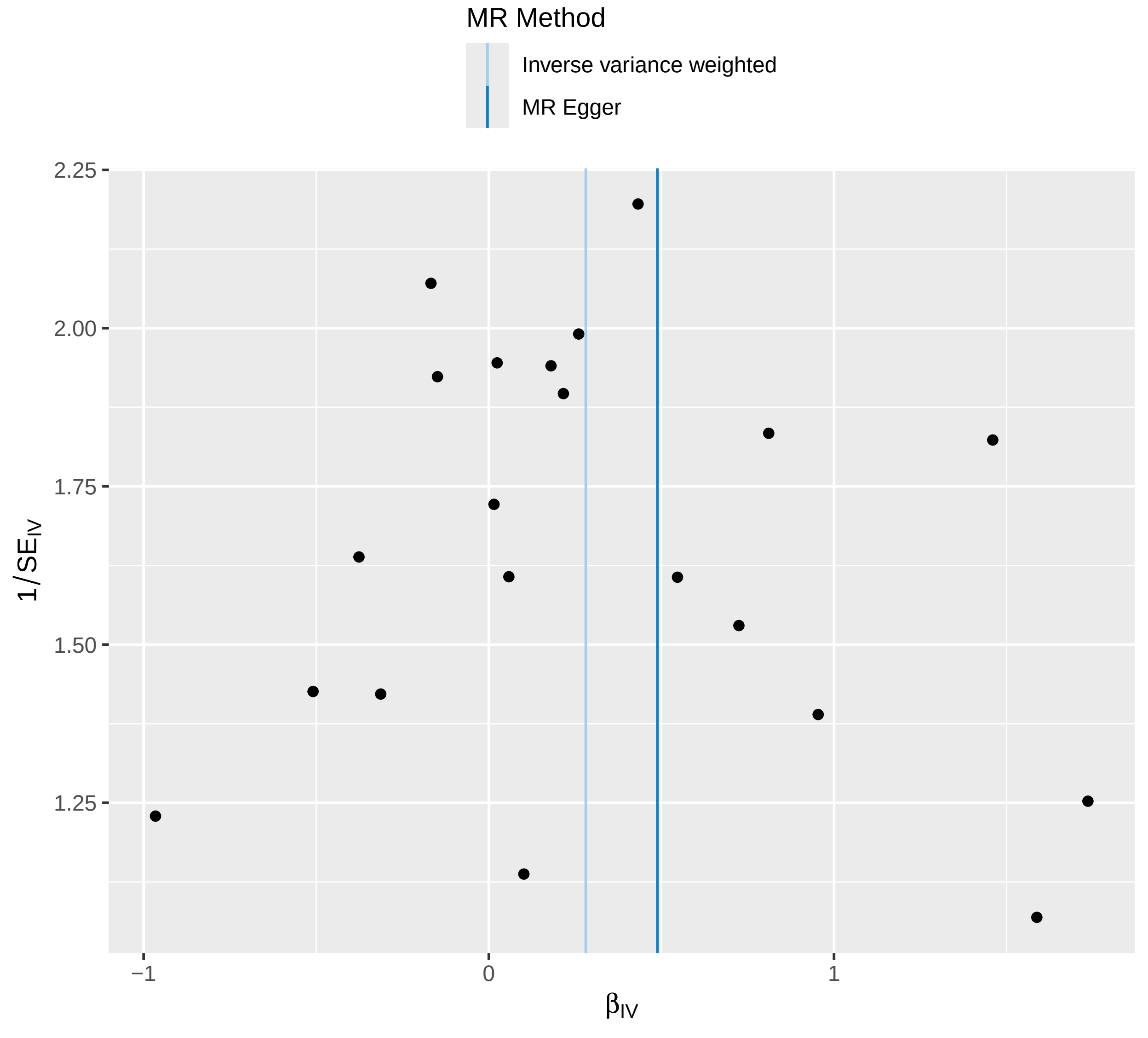
MR Method
Inverse variance weighted
MR Egger
2.25
2.00
1.75
1.50
1.25
−1
0
1
1/SE_IV
β_IV

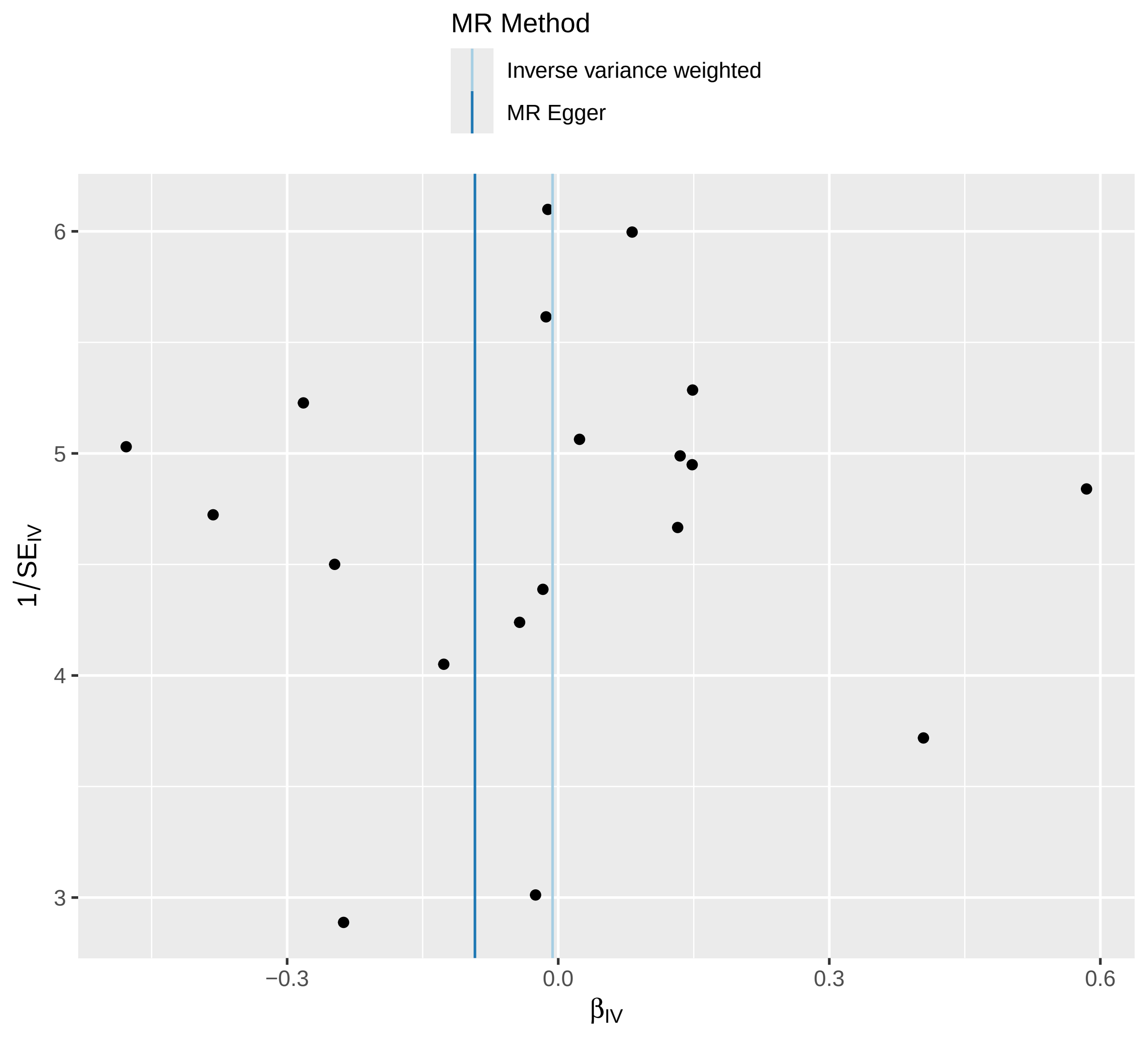
MR Method
Inverse variance weighted
MR Egger
6
5
4
3
1/SE$_{IV}$
−0.3
0.0
0.3
0.6
β_{IV}

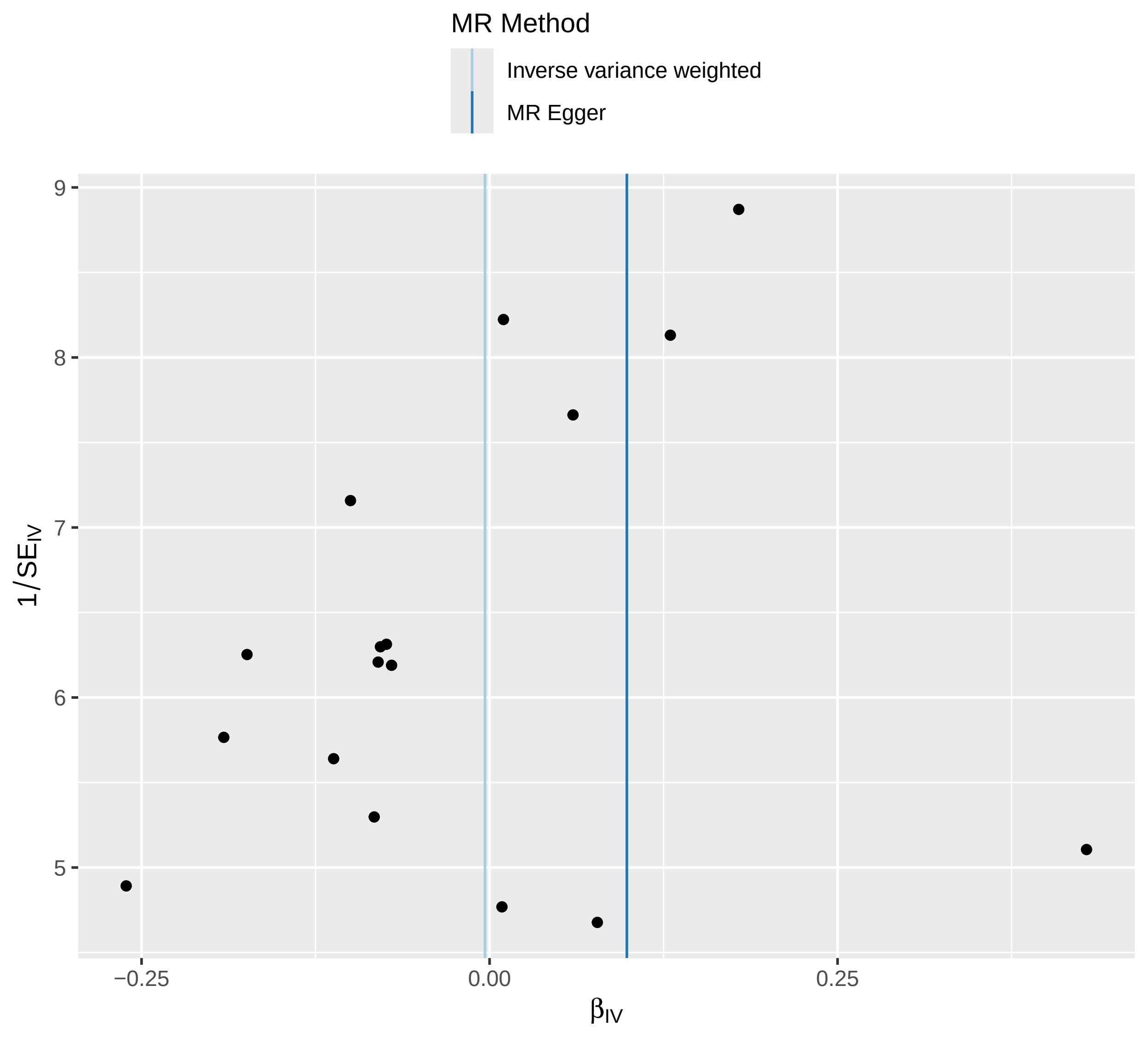
MR Method
Inverse variance weighted
MR Egger
1/SE_{IV}
9
8
7
6
5
−0.25
0.00
0.25
β_{IV}

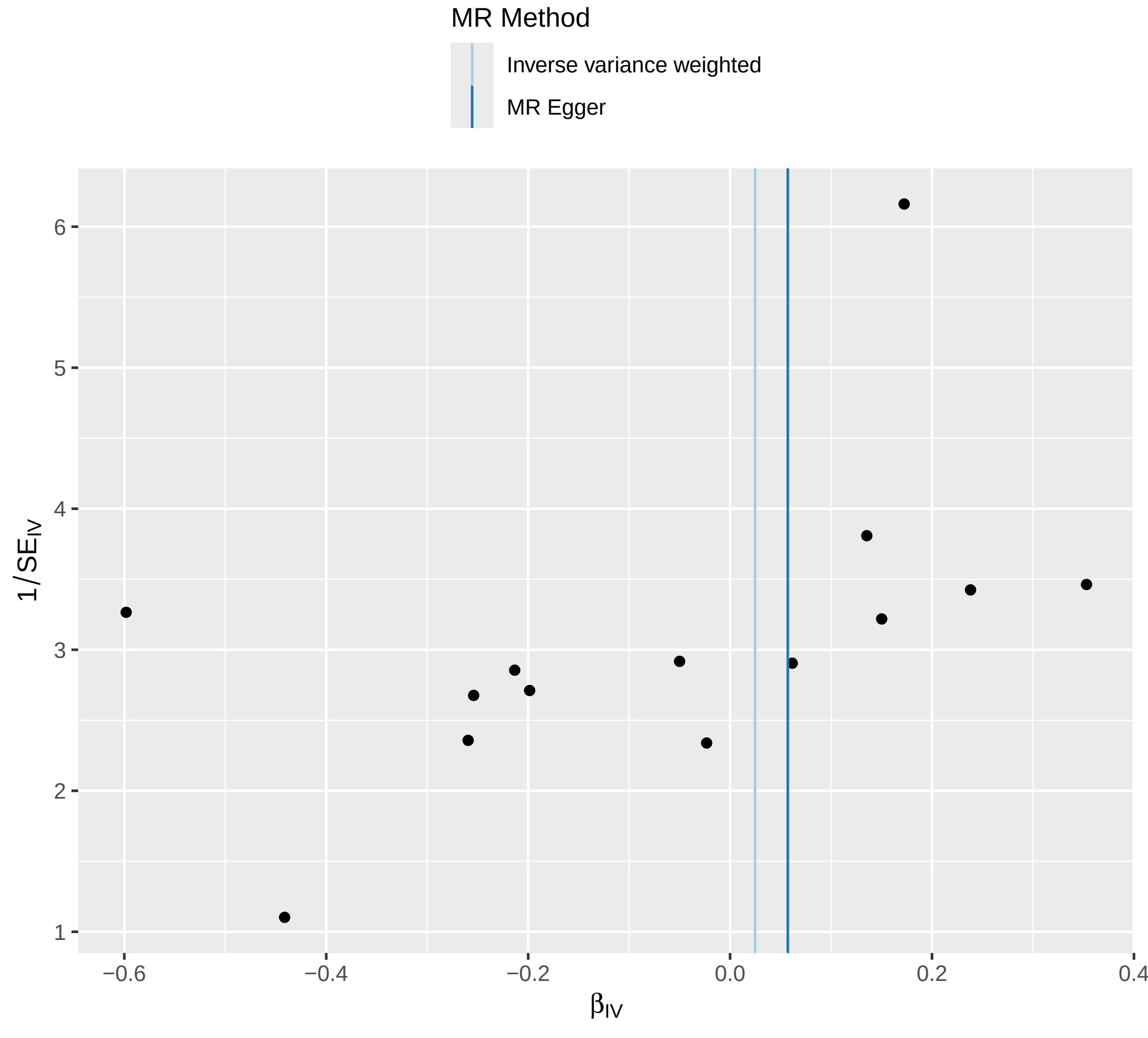
MR Method
Inverse variance weighted
MR Egger
1/SE_{IV}
β_{IV}
6
5
4
3
2
1
−0.6
−0.4
−0.2
0.0
0.2
0.4

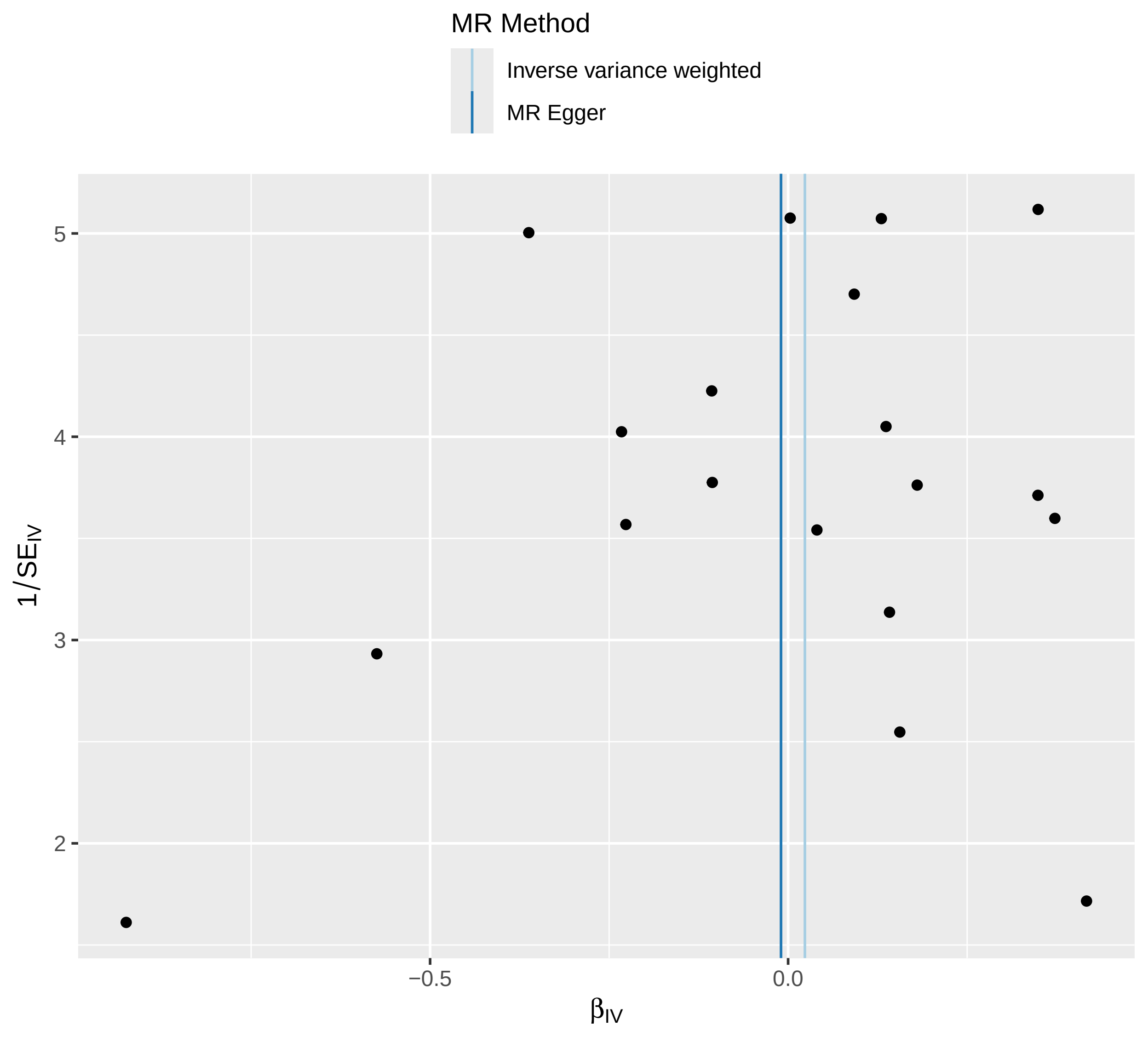
MR Method
Inverse variance weighted
MR Egger
5
4
3
2
1/SE$_{IV}$
−0.5
0.0
β$_{IV}$

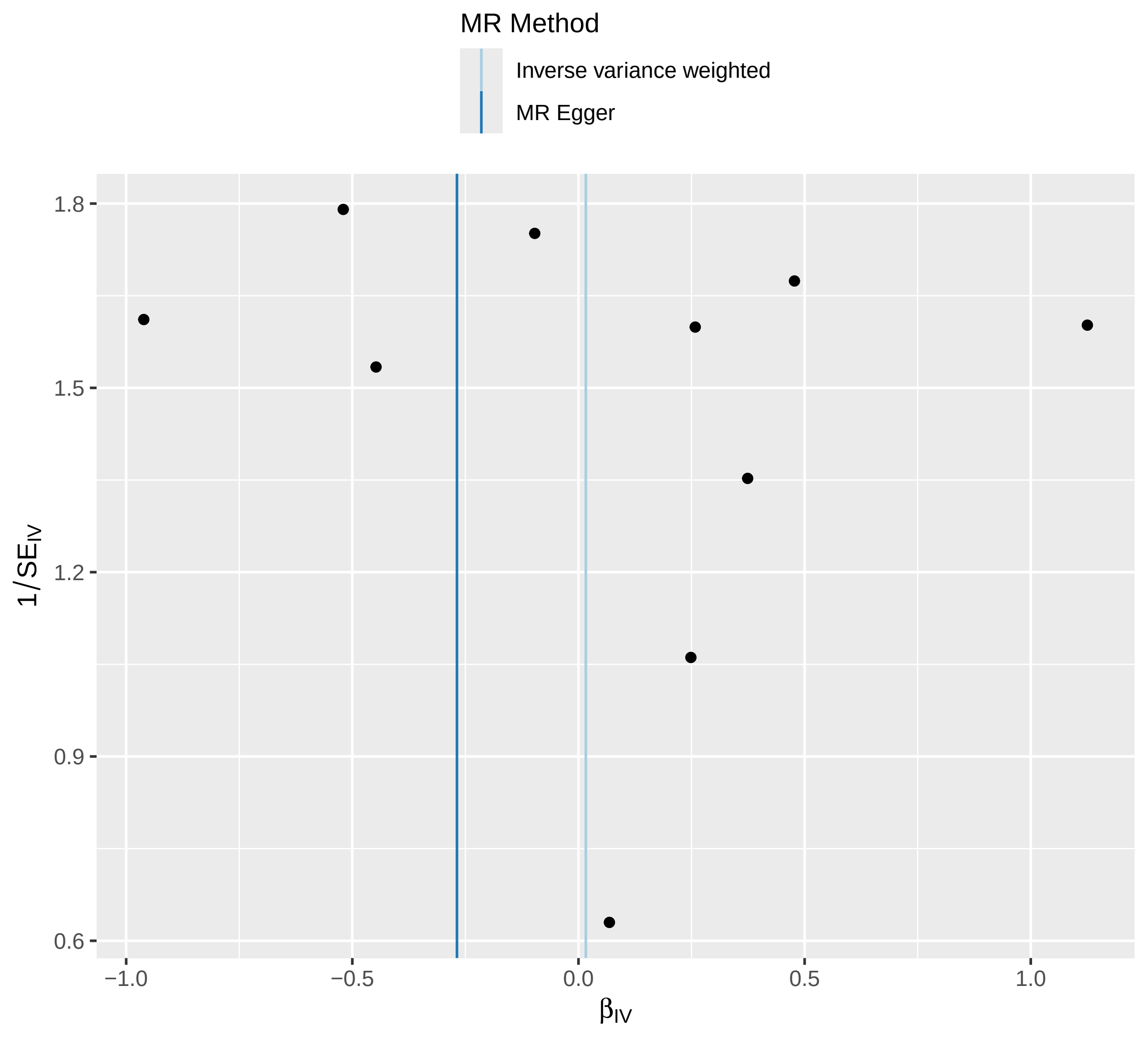
MR Method
Inverse variance weighted
MR Egger
1.8
1.5
1.2
0.9
0.6
$1/SE_{IV}$
−1.0
−0.5
0.0
0.5
1.0
β_{IV}

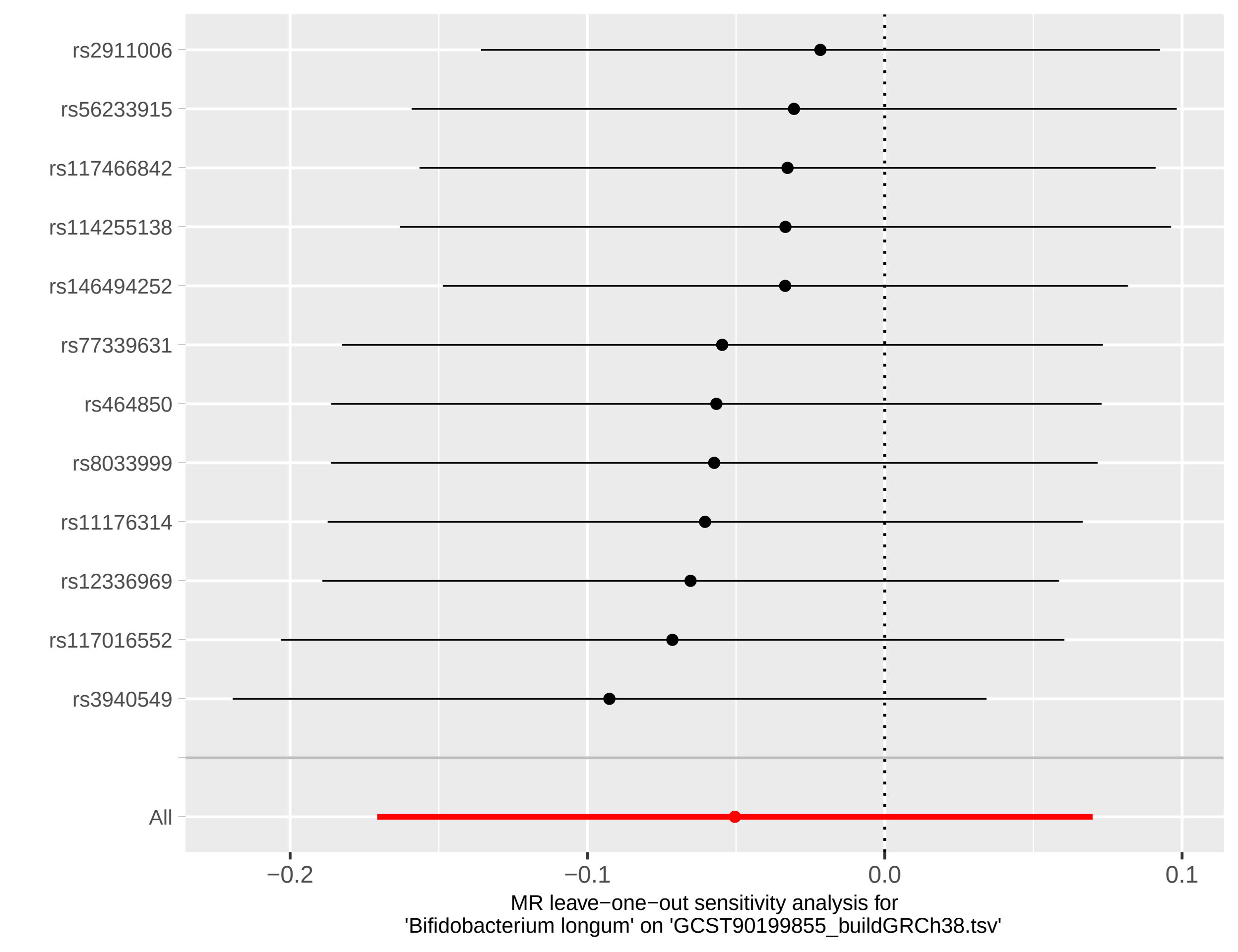

rs2911006
rs56233915
rs117466842
rs114255138
rs146494252
rs77339631
rs464850
rs8033999
rs11176314
rs12336969
rs117016552
rs3940549
All
−0.2
−0.1
0.0
0.1
MR leave−one−out sensitivity analysis for
'Bifidobacterium longum' on 'GCST90199855_buildGRCh38.tsv'

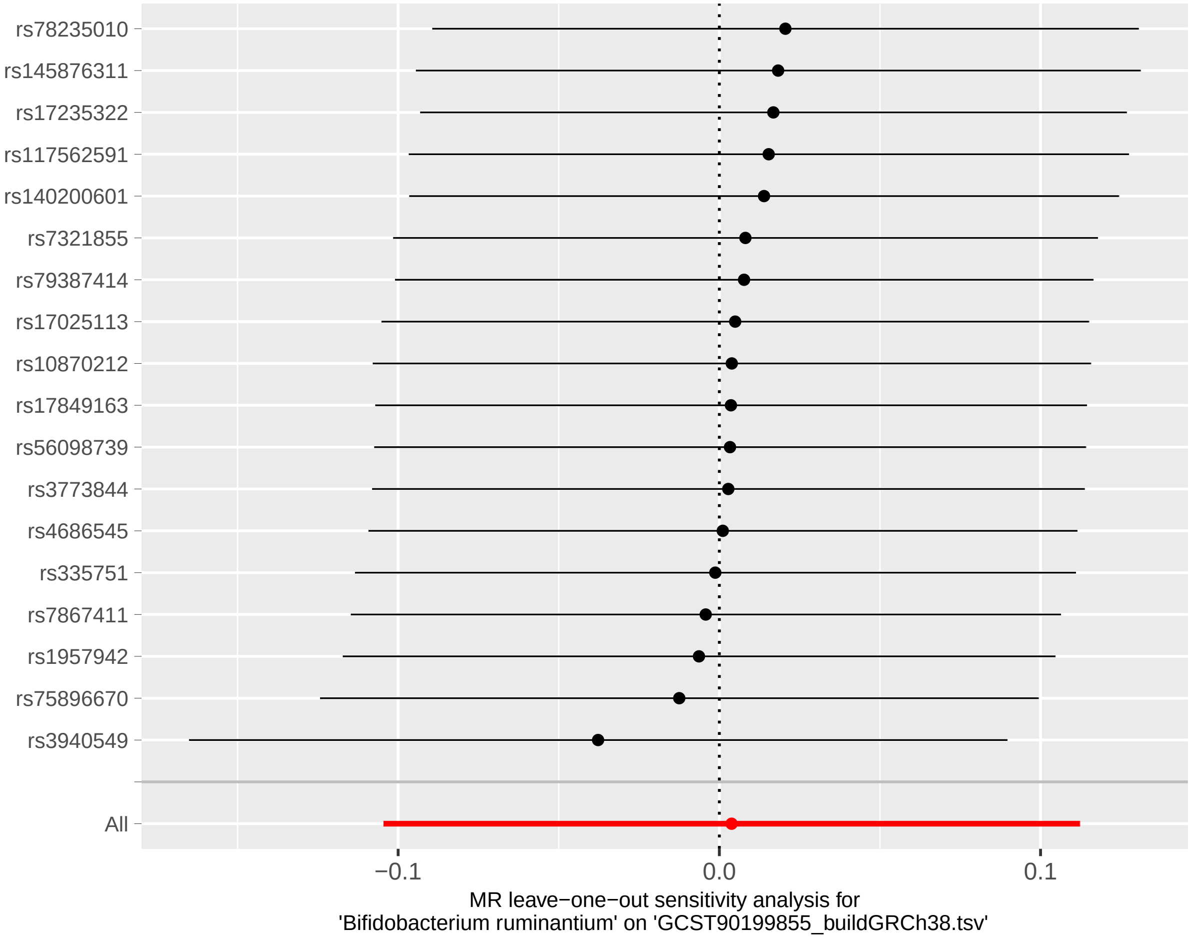
rs78235010
rs145876311
rs17235322
rs117562591
rs140200601
rs7321855
rs79387414
rs17025113
rs10870212
rs17849163
rs56098739
rs3773844
rs4686545
rs335751
rs7867411
rs1957942
rs75896670
rs3940549
All
−0.1
0.0
0.1
MR leave−one−out sensitivity analysis for
'Bifidobacterium ruminantium' on 'GCST90199855_buildGRCh38.tsv'

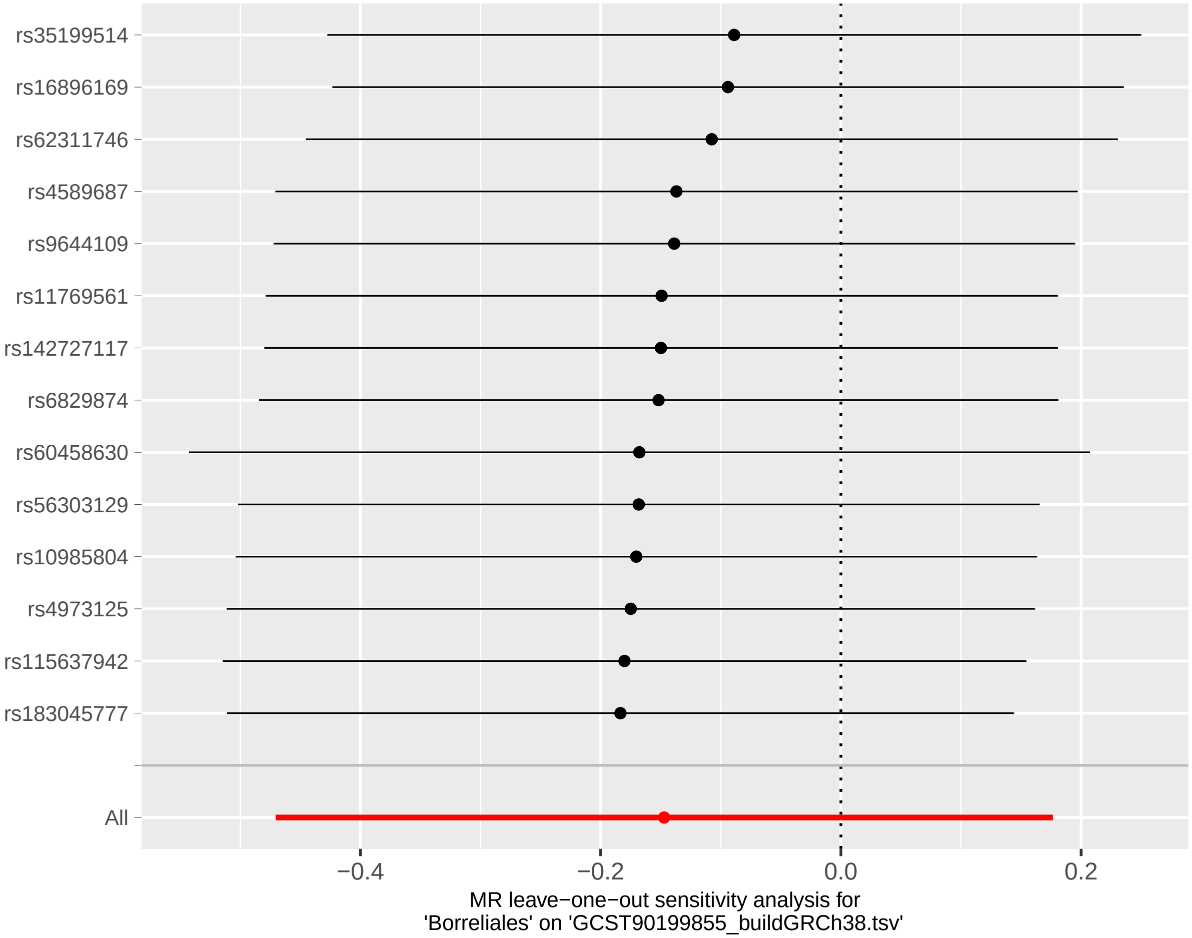
rs35199514
rs16896169
rs62311746
rs4589687
rs9644109
rs11769561
rs142727117
rs6829874
rs60458630
rs56303129
rs10985804
rs4973125
rs115637942
rs183045777
All
−0.4
−0.2
0.0
0.2
MR leave−one−out sensitivity analysis for
'Borreliales' on 'GCST90199855_buildGRCh38.tsv'

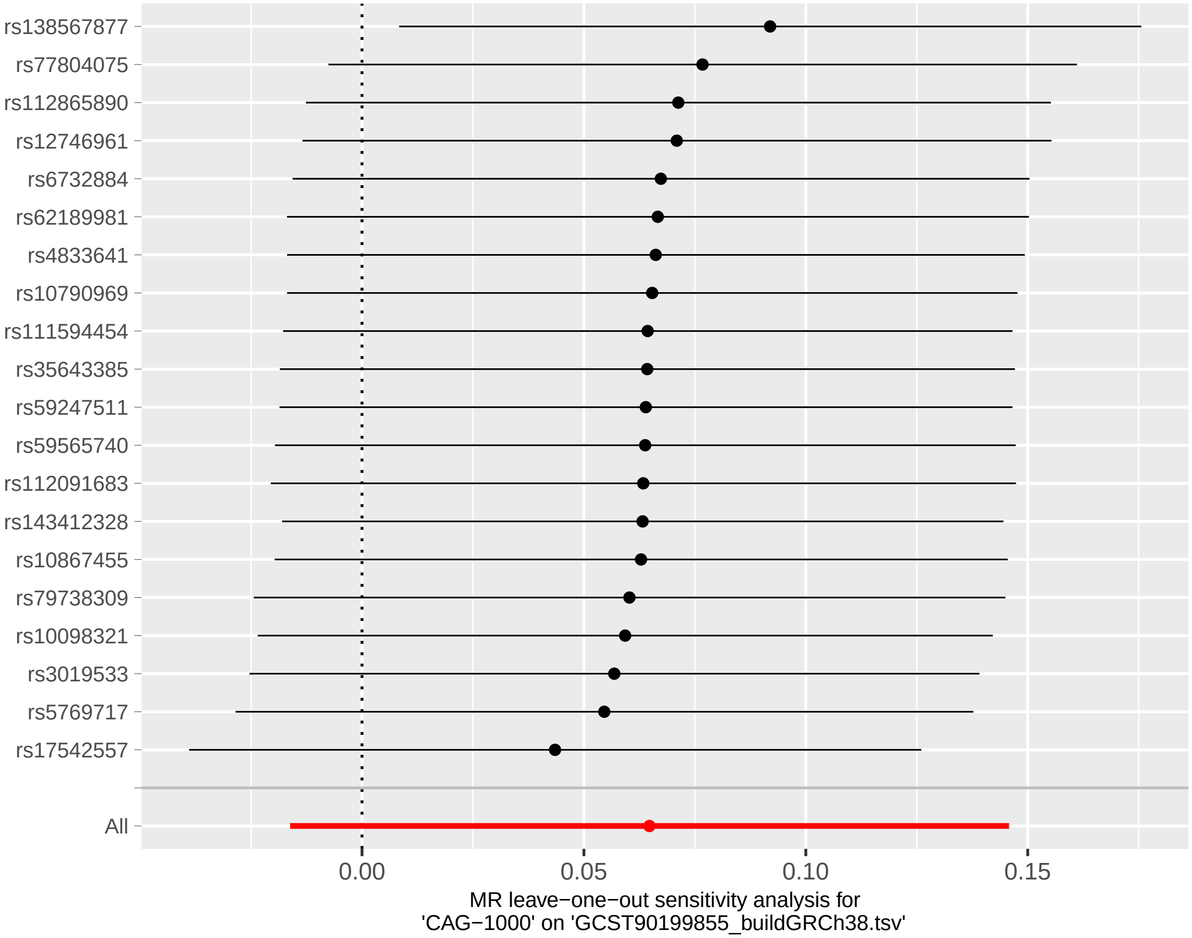
rs138567877
rs77804075
rs112865890
rs12746961
rs6732884
rs62189981
rs4833641
rs10790969
rs111594454
rs35643385
rs59247511
rs59565740
rs112091683
rs143412328
rs10867455
rs79738309
rs10098321
rs3019533
rs5769717
rs17542557
All
0.00
0.05
0.10
0.15
MR leave−one−out sensitivity analysis for
'CAG−1000' on 'GCST90199855_buildGRCh38.tsv'

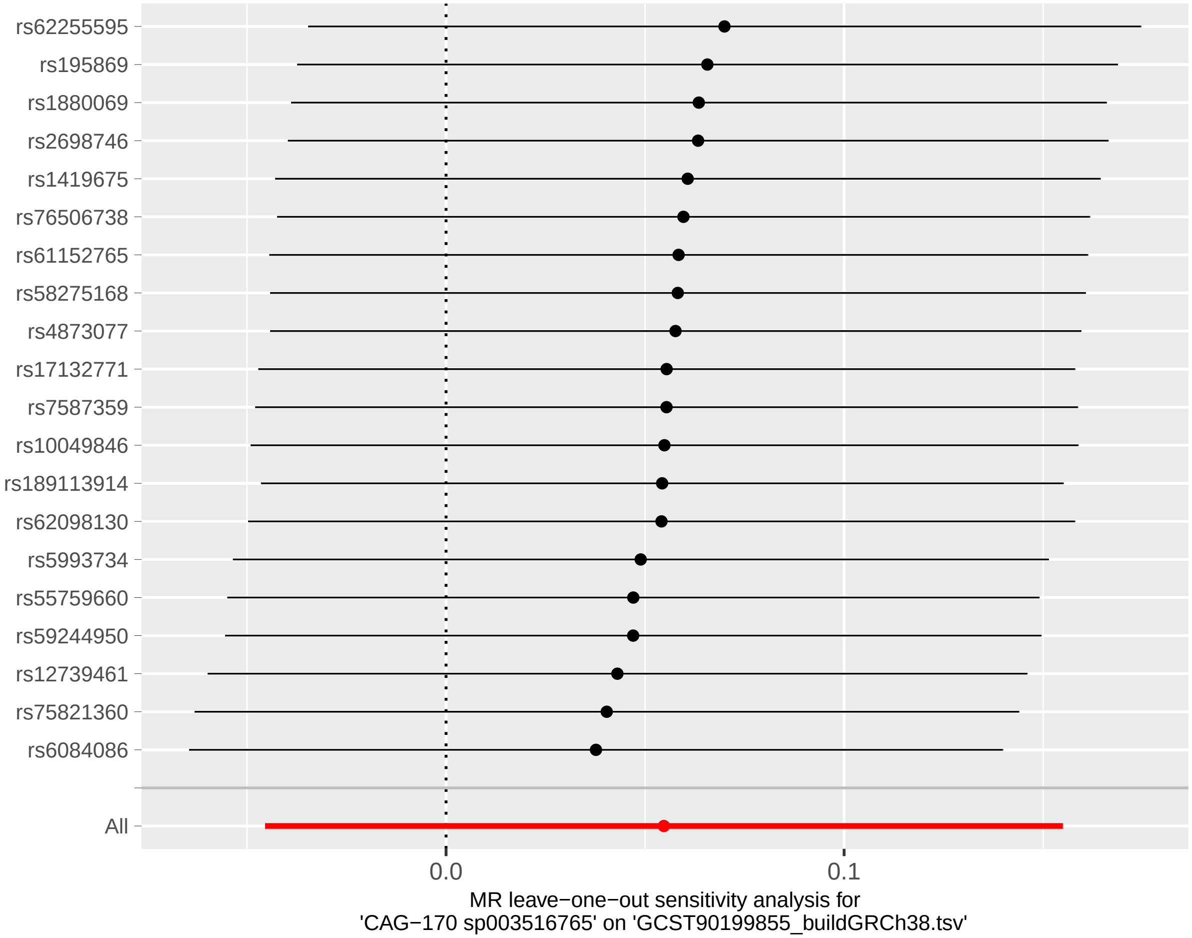

rs62255595
rs195869
rs1880069
rs2698746
rs1419675
rs76506738
rs61152765
rs58275168
rs4873077
rs17132771
rs7587359
rs10049846
rs189113914
rs62098130
rs5993734
rs55759660
rs59244950
rs12739461
rs75821360
rs6084086
All
0.0
0.1
MR leave−one−out sensitivity analysis for
'CAG−170 sp003516765' on 'GCST90199855_buildGRCh38.tsv'

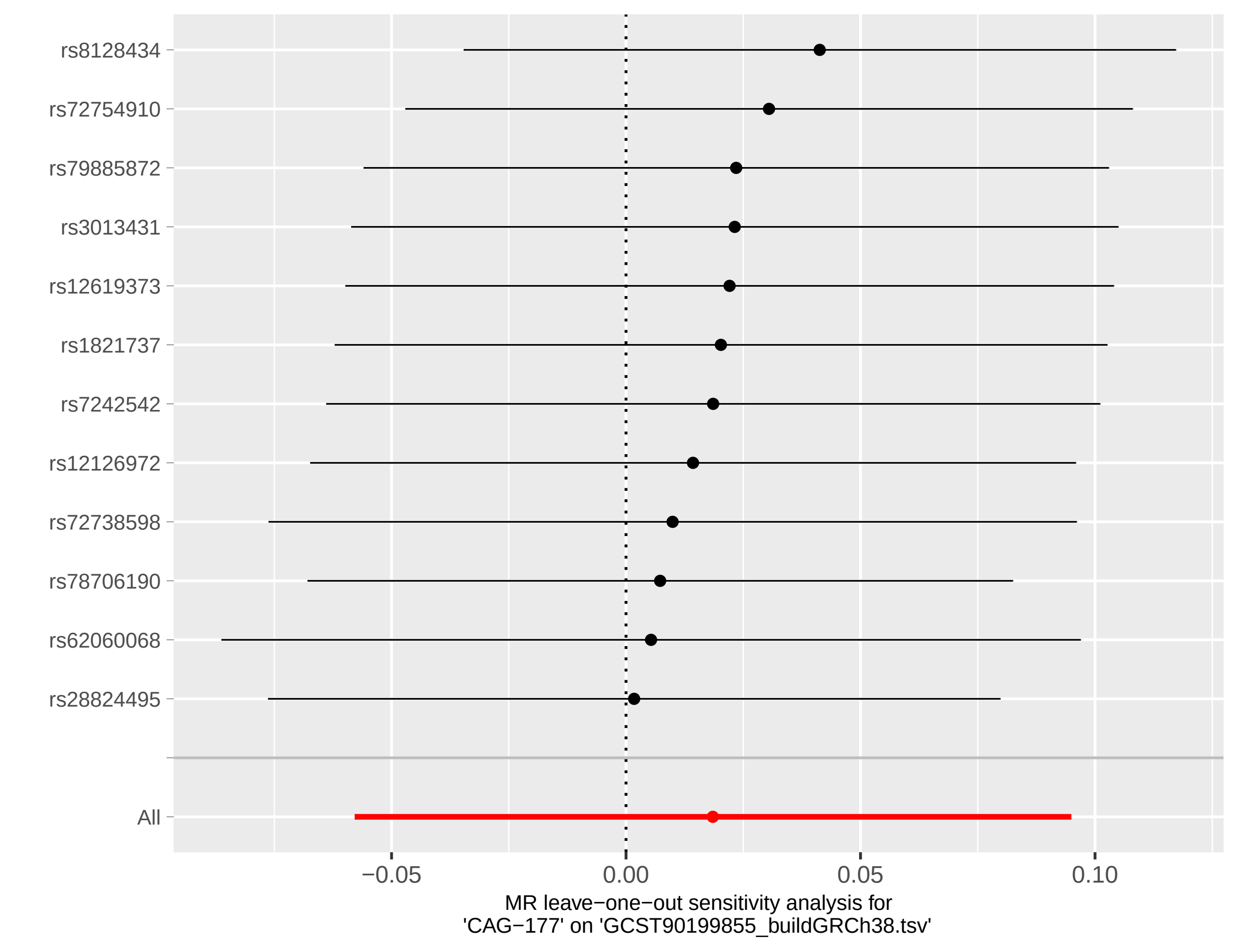
rs8128434
rs72754910
rs79885872
rs3013431
rs12619373
rs1821737
rs7242542
rs12126972
rs72738598
rs78706190
rs62060068
rs28824495
All
−0.05
0.00
0.05
0.10
MR leave−one−out sensitivity analysis for
'CAG−177' on 'GCST90199855_buildGRCh38.tsv'

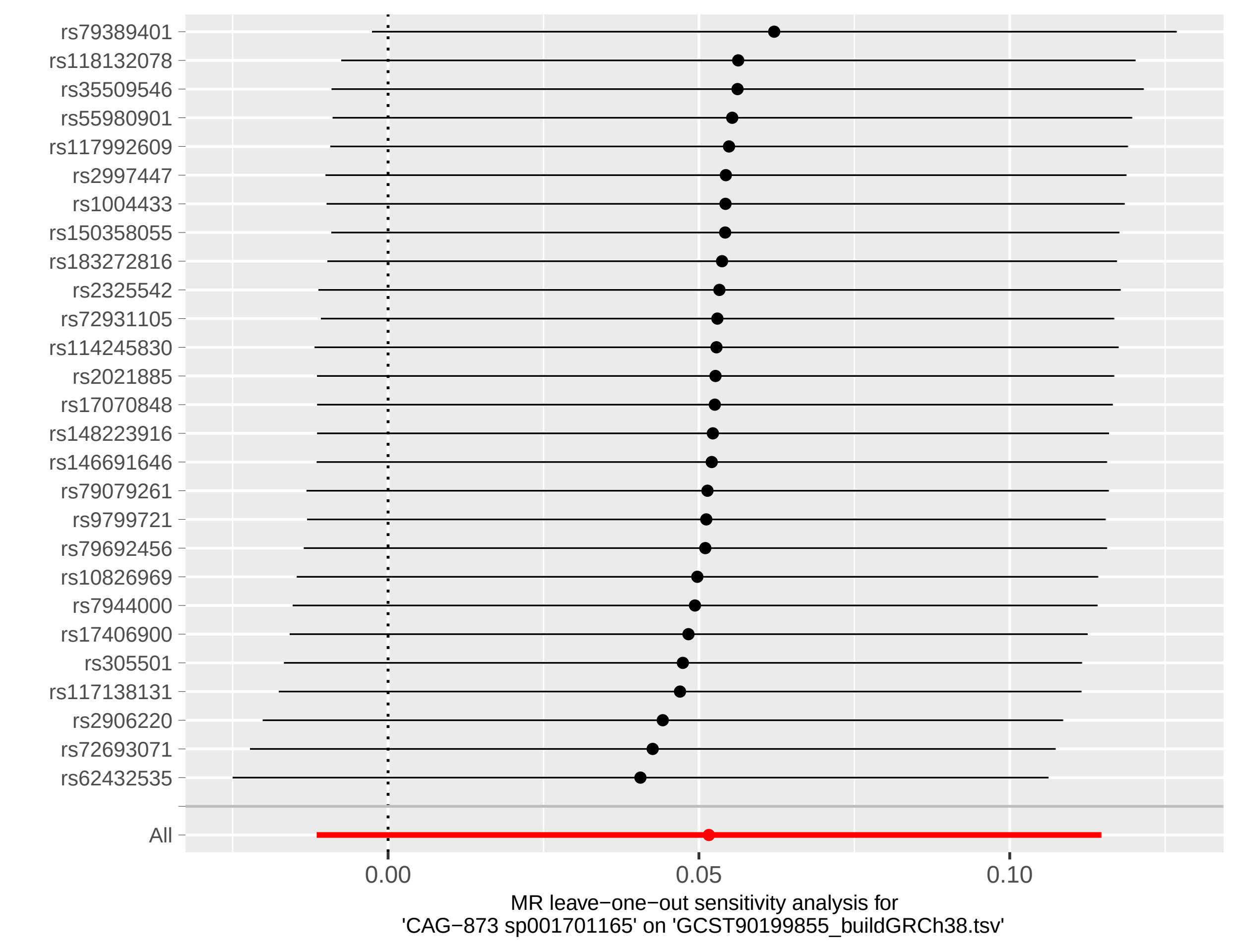
rs79389401
rs118132078
rs35509546
rs55980901
rs117992609
rs2997447
rs1004433
rs150358055
rs183272816
rs2325542
rs72931105
rs114245830
rs2021885
rs17070848
rs148223916
rs146691646
rs79079261
rs9799721
rs79692456
rs10826969
rs7944000
rs17406900
rs305501
rs117138131
rs2906220
rs72693071
rs62432535
All
0.00
0.05
0.10
MR leave−one−out sensitivity analysis for
'CAG−873 sp001701165' on 'GCST90199855_buildGRCh38.tsv'

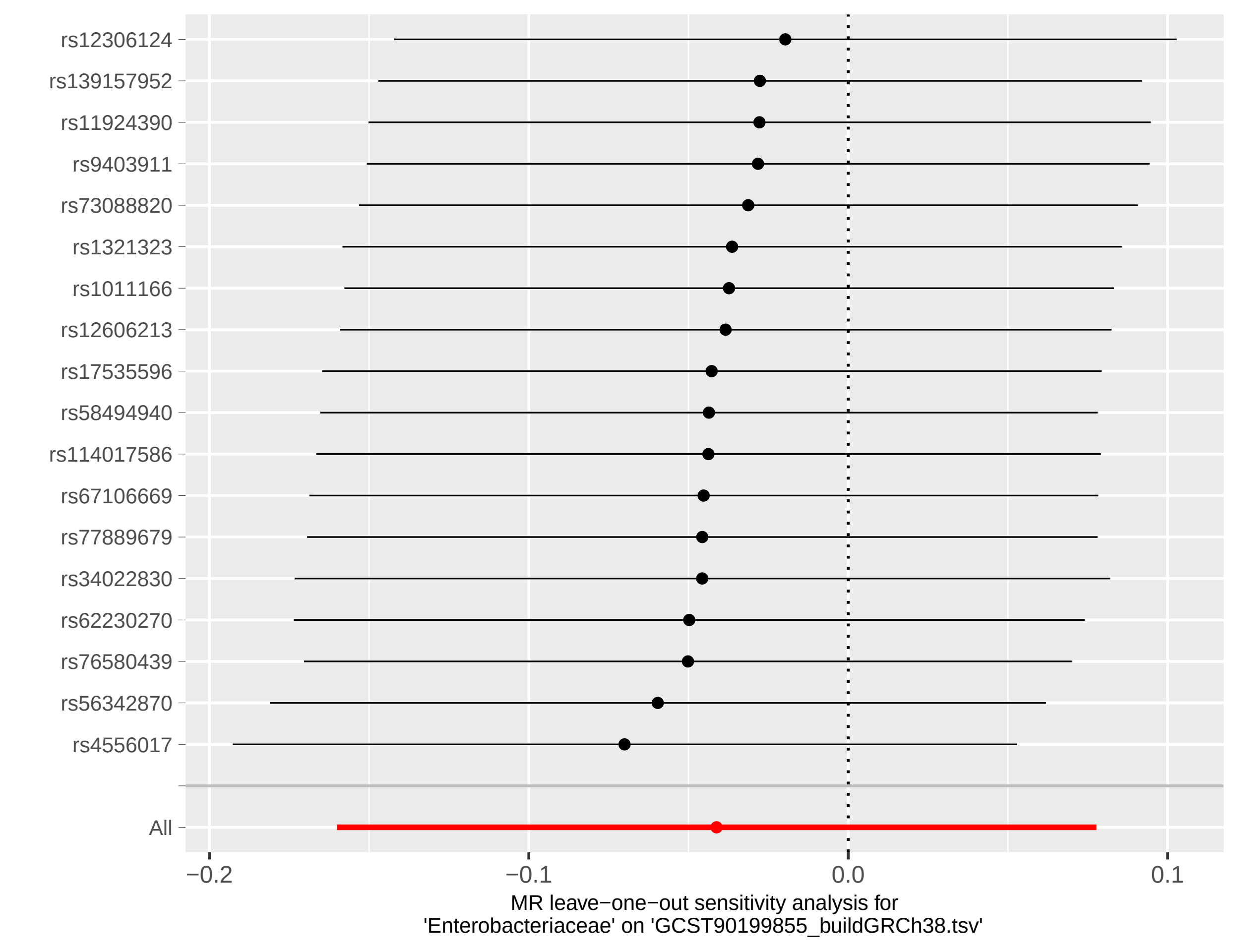

rs12306124
rs139157952
rs11924390
rs9403911
rs73088820
rs1321323
rs1011166
rs12606213
rs17535596
rs58494940
rs114017586
rs67106669
rs77889679
rs34022830
rs62230270
rs76580439
rs56342870
rs4556017
All
−0.2
−0.1
0.0
0.1
MR leave−one−out sensitivity analysis for
'Enterobacteriaceae' on 'GCST90199855_buildGRCh38.tsv'

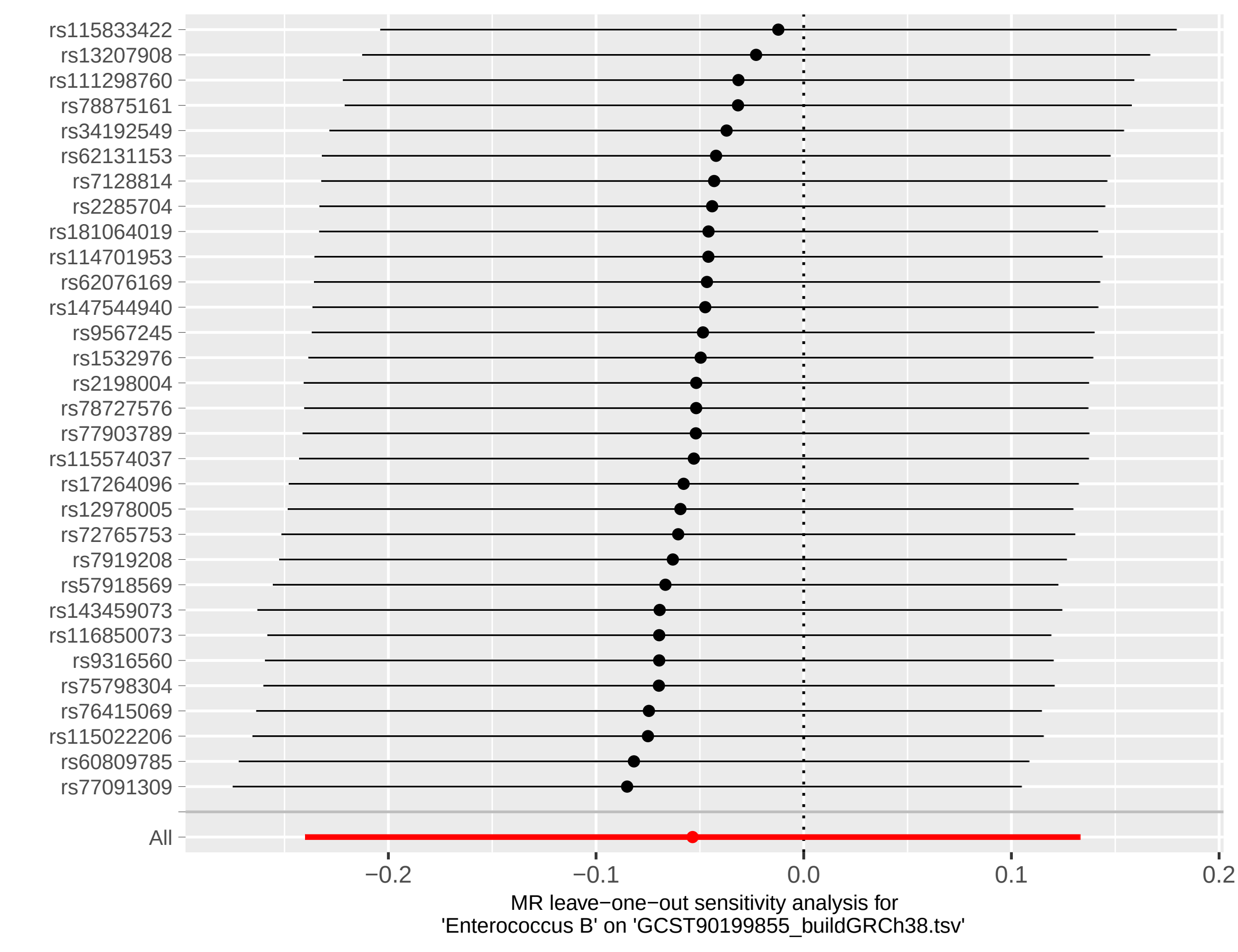
rs115833422
rs13207908
rs111298760
rs78875161
rs34192549
rs62131153
rs7128814
rs2285704
rs181064019
rs114701953
rs62076169
rs147544940
rs9567245
rs1532976
rs2198004
rs78727576
rs77903789
rs115574037
rs17264096
rs12978005
rs72765753
rs7919208
rs57918569
rs143459073
rs116850073
rs9316560
rs75798304
rs76415069
rs115022206
rs60809785
rs77091309
All
−0.2
−0.1
0.0
0.1
0.2
MR leave−one−out sensitivity analysis for
'Enterococcus B' on 'GCST90199855_buildGRCh38.tsv'

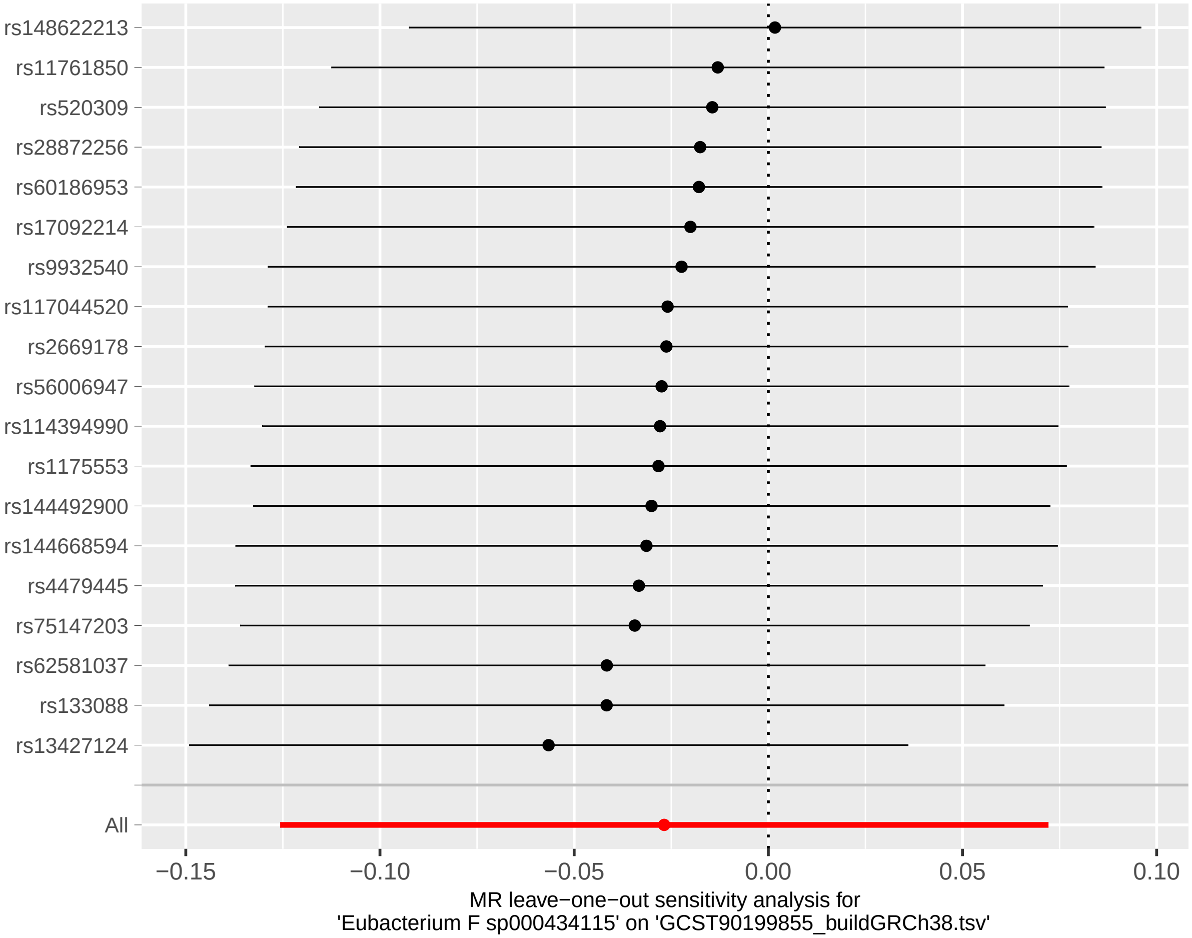
rs148622213
rs11761850
rs520309
rs28872256
rs60186953
rs17092214
rs9932540
rs117044520
rs2669178
rs56006947
rs114394990
rs1175553
rs144492900
rs144668594
rs4479445
rs75147203
rs62581037
rs133088
rs13427124
All
−0.15
−0.10
−0.05
0.00
0.05
0.10
MR leave−one−out sensitivity analysis for
'Eubacterium F sp000434115' on 'GCST90199855_buildGRCh38.tsv'

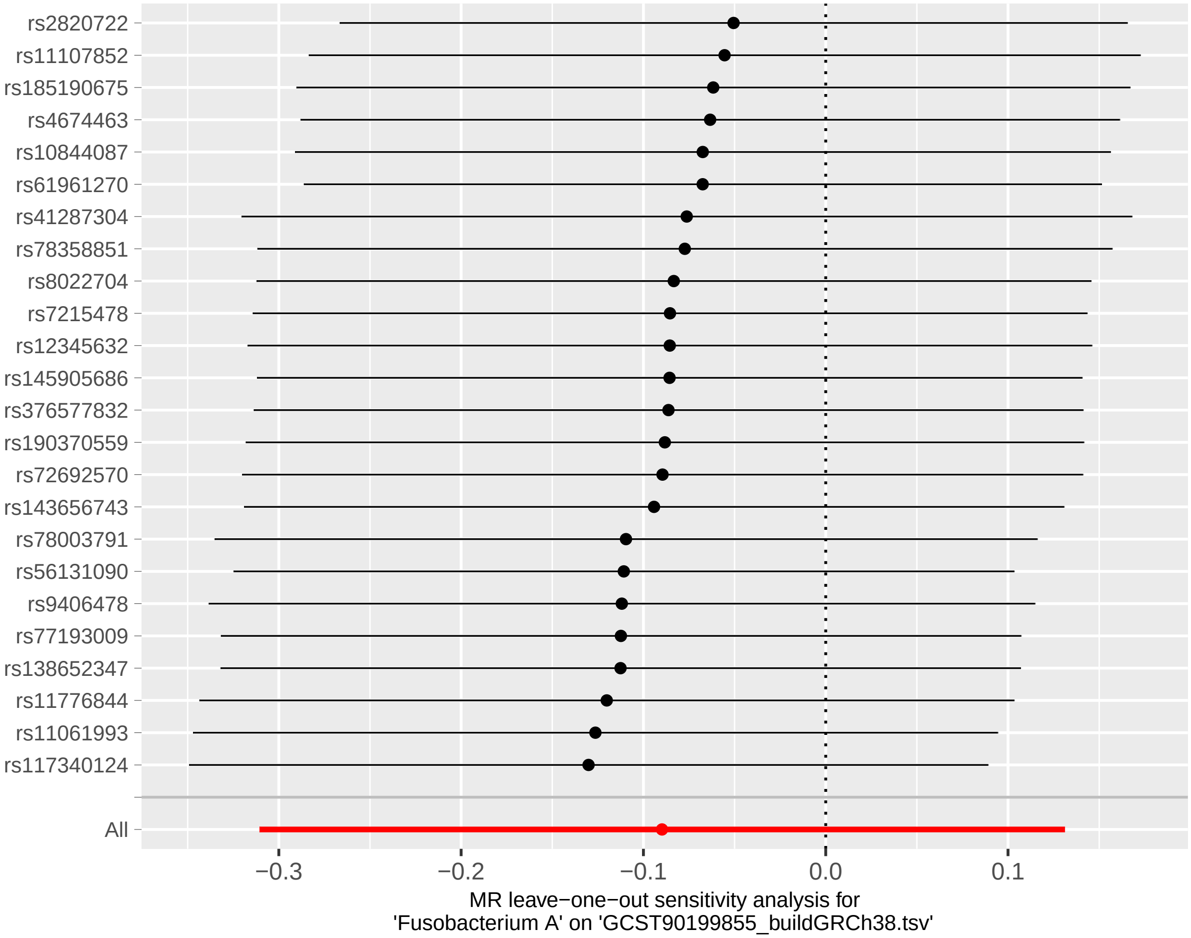
rs2820722
rs11107852
rs185190675
rs4674463
rs10844087
rs61961270
rs41287304
rs78358851
rs8022704
rs7215478
rs12345632
rs145905686
rs376577832
rs190370559
rs72692570
rs143656743
rs78003791
rs56131090
rs9406478
rs77193009
rs138652347
rs11776844
rs11061993
rs117340124
All
−0.3
−0.2
−0.1
0.0
0.1
MR leave−one−out sensitivity analysis for
'Fusobacterium A' on 'GCST90199855_buildGRCh38.tsv'

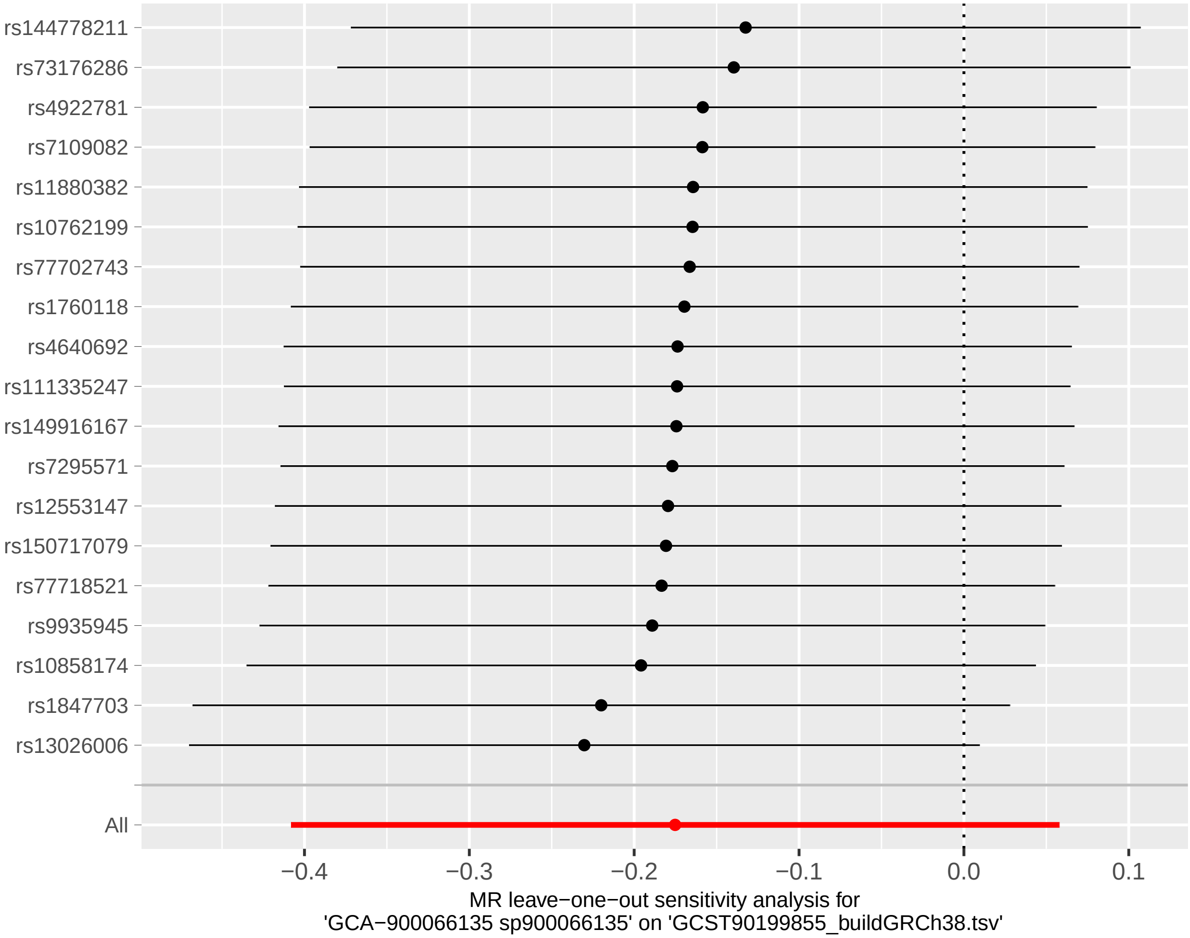
rs144778211
rs73176286
rs4922781
rs7109082
rs11880382
rs10762199
rs77702743
rs1760118
rs4640692
rs111335247
rs149916167
rs7295571
rs12553147
rs150717079
rs77718521
rs9935945
rs10858174
rs1847703
rs13026006
All
−0.4
−0.3
−0.2
−0.1
0.0
0.1
MR leave−one−out sensitivity analysis for
'GCA−900066135 sp900066135' on 'GCST90199855_buildGRCh38.tsv'

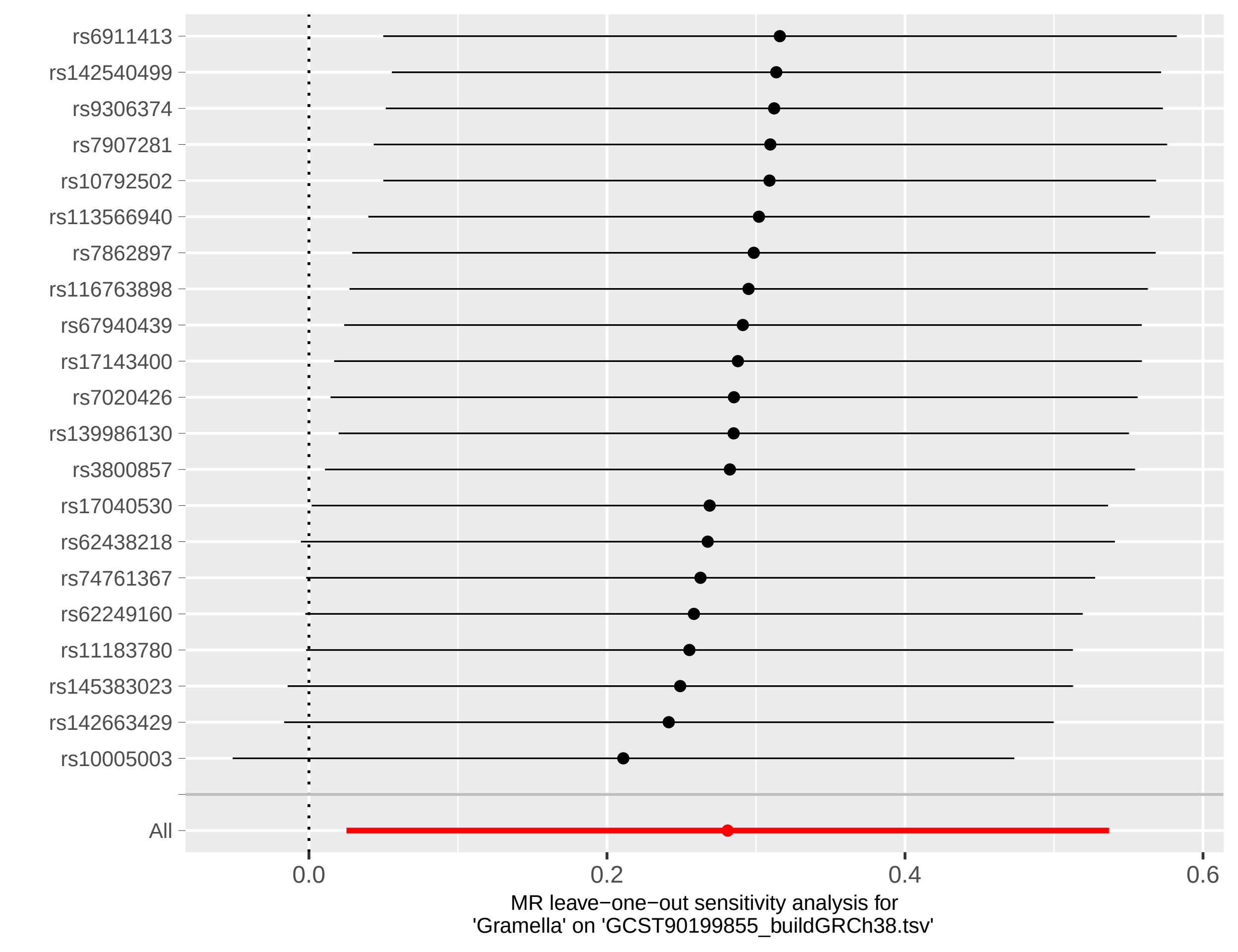

rs6911413
rs142540499
rs9306374
rs7907281
rs10792502
rs113566940
rs7862897
rs116763898
rs67940439
rs17143400
rs7020426
rs139986130
rs3800857
rs17040530
rs62438218
rs74761367
rs62249160
rs11183780
rs145383023
rs142663429
rs10005003
All
0.0
0.2
0.4
0.6
MR leave−one−out sensitivity analysis for
'Gramella' on 'GCST90199855_buildGRCh38.tsv'

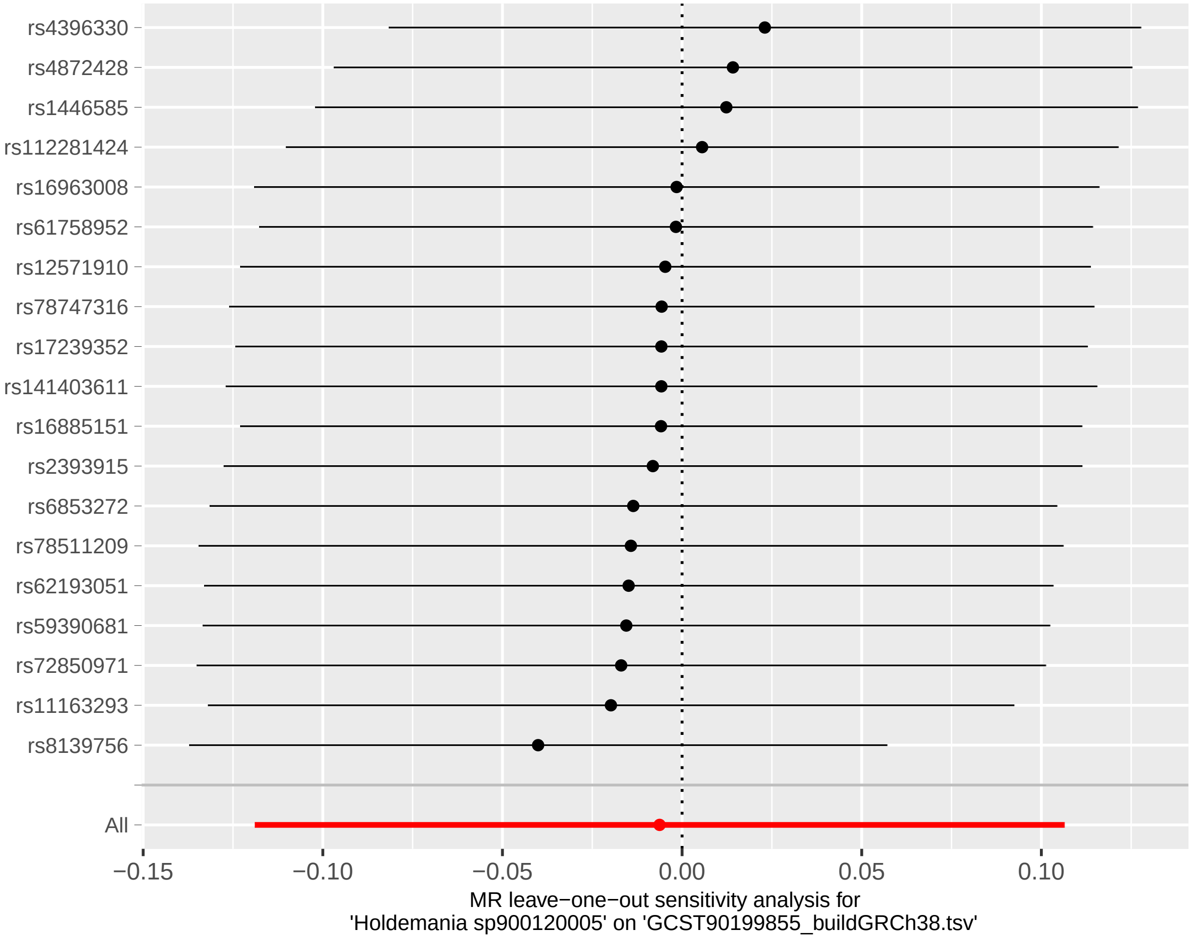

rs4396330
rs4872428
rs1446585
rs112281424
rs16963008
rs61758952
rs12571910
rs78747316
rs17239352
rs141403611
rs16885151
rs2393915
rs6853272
rs78511209
rs62193051
rs59390681
rs72850971
rs11163293
rs8139756
All
−0.15
−0.10
−0.05
0.00
0.05
0.10
MR leave−one−out sensitivity analysis for
'Holdemania sp900120005' on 'GCST90199855_buildGRCh38.tsv'

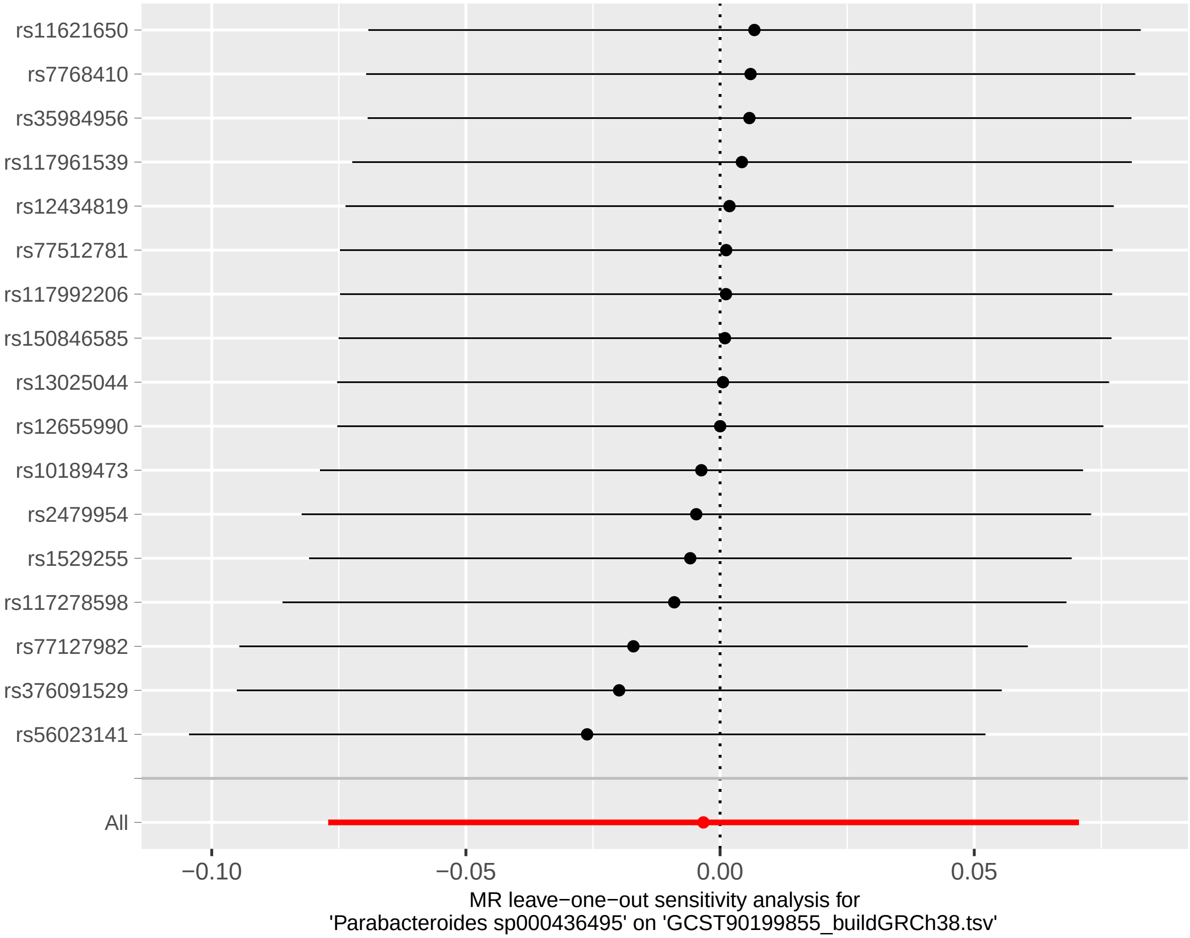
rs11621650
rs7768410
rs35984956
rs117961539
rs12434819
rs77512781
rs117992206
rs150846585
rs13025044
rs12655990
rs10189473
rs2479954
rs1529255
rs117278598
rs77127982
rs376091529
rs56023141
All
−0.10
−0.05
0.00
0.05
MR leave−one−out sensitivity analysis for
'Parabacteroides sp000436495' on 'GCST90199855_buildGRCh38.tsv'

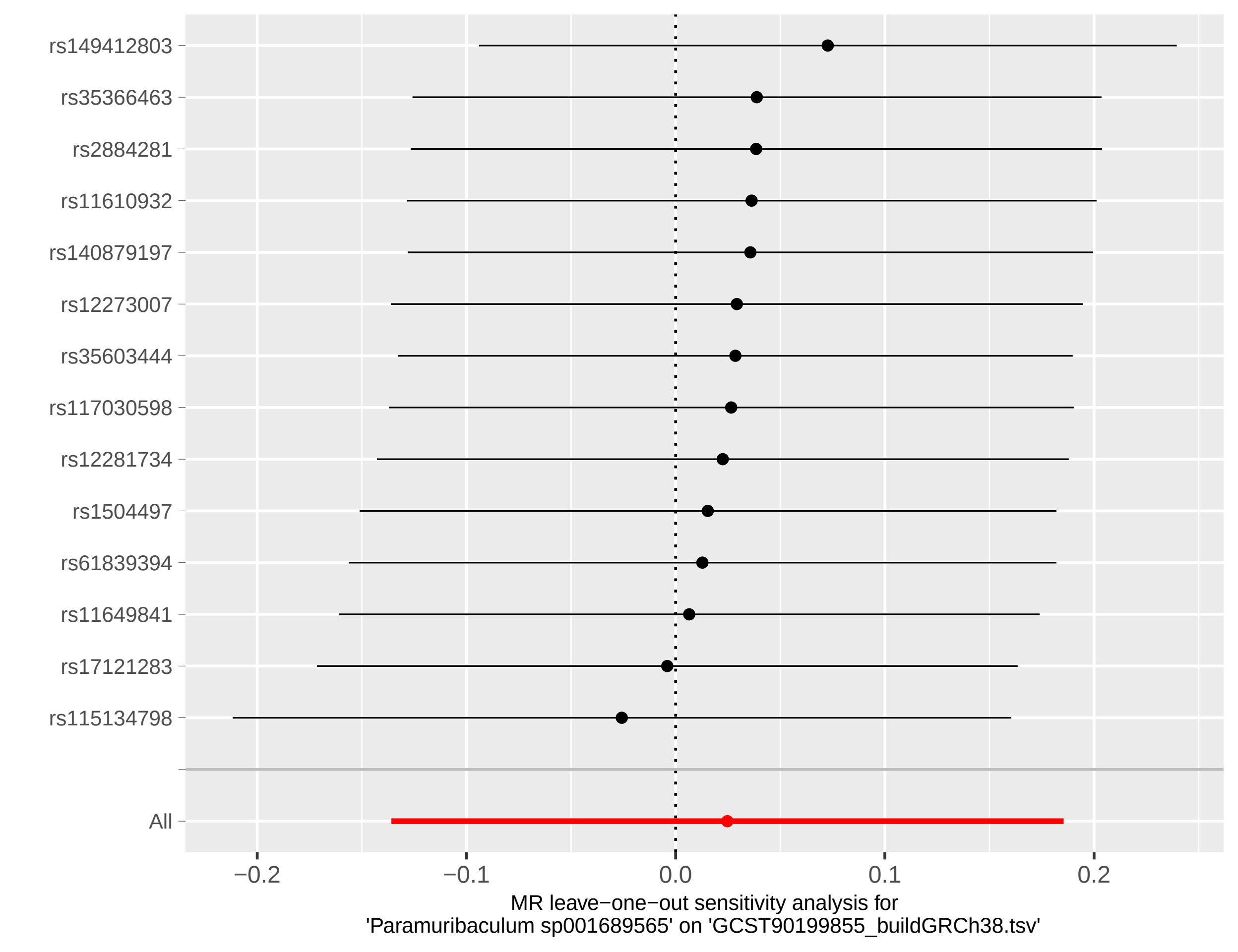

rs149412803
rs35366463
rs2884281
rs11610932
rs140879197
rs12273007
rs35603444
rs117030598
rs12281734
rs1504497
rs61839394
rs11649841
rs17121283
rs115134798
All
−0.2
−0.1
0.0
0.1
0.2
MR leave−one−out sensitivity analysis for
'Paramuribaculum sp001689565' on 'GCST90199855_buildGRCh38.tsv'

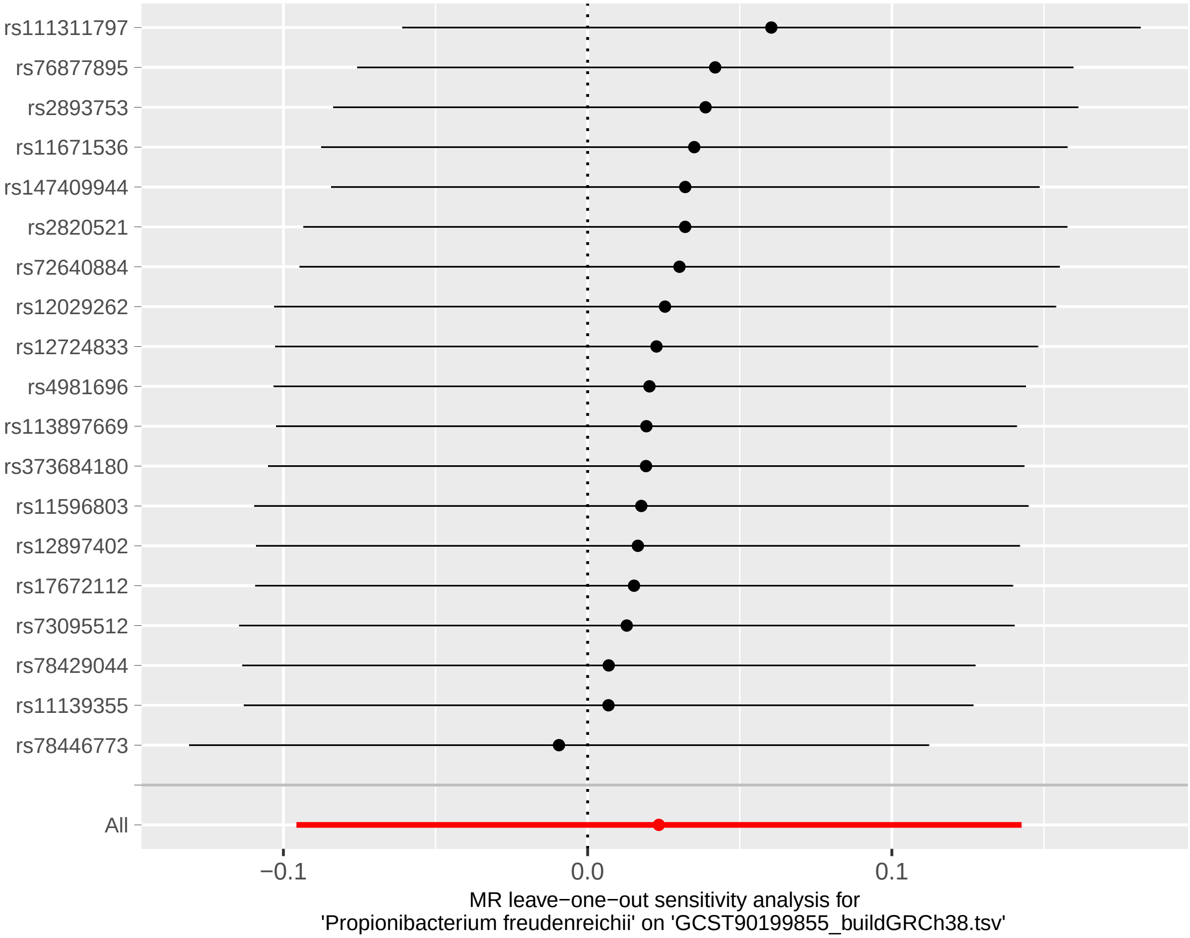
rs111311797
rs76877895
rs2893753
rs11671536
rs147409944
rs2820521
rs72640884
rs12029262
rs12724833
rs4981696
rs113897669
rs373684180
rs11596803
rs12897402
rs17672112
rs73095512
rs78429044
rs11139355
rs78446773
All
−0.1
0.0
0.1
MR leave−one−out sensitivity analysis for
'Propionibacterium freudenreichii' on 'GCST90199855_buildGRCh38.tsv'

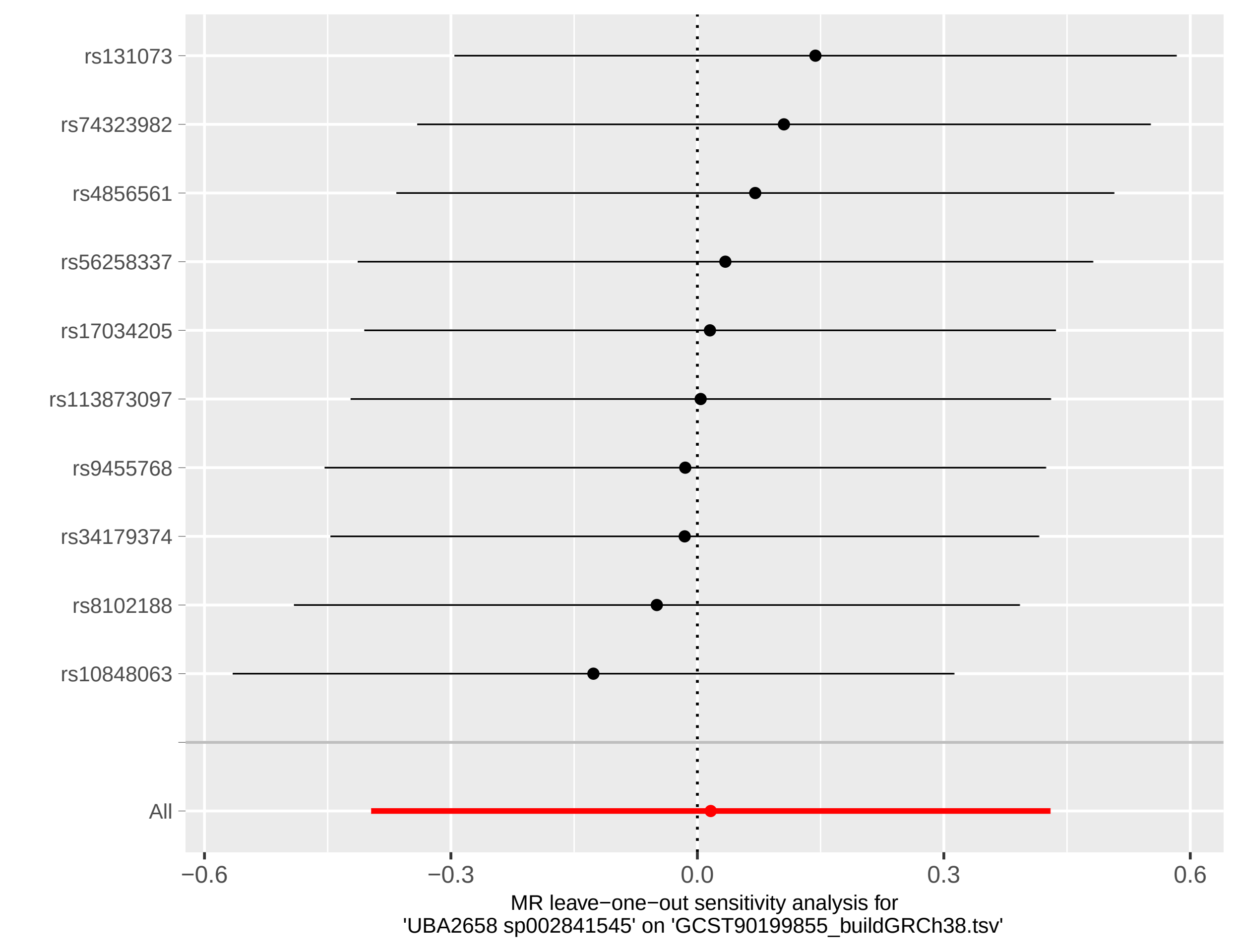

rs131073
rs74323982
rs4856561
rs56258337
rs17034205
rs113873097
rs9455768
rs34179374
rs8102188
rs10848063
All
−0.6
−0.3
0.0
0.3
0.6
MR leave−one−out sensitivity analysis for
'UBA2658 sp002841545' on 'GCST90199855_buildGRCh38.tsv'

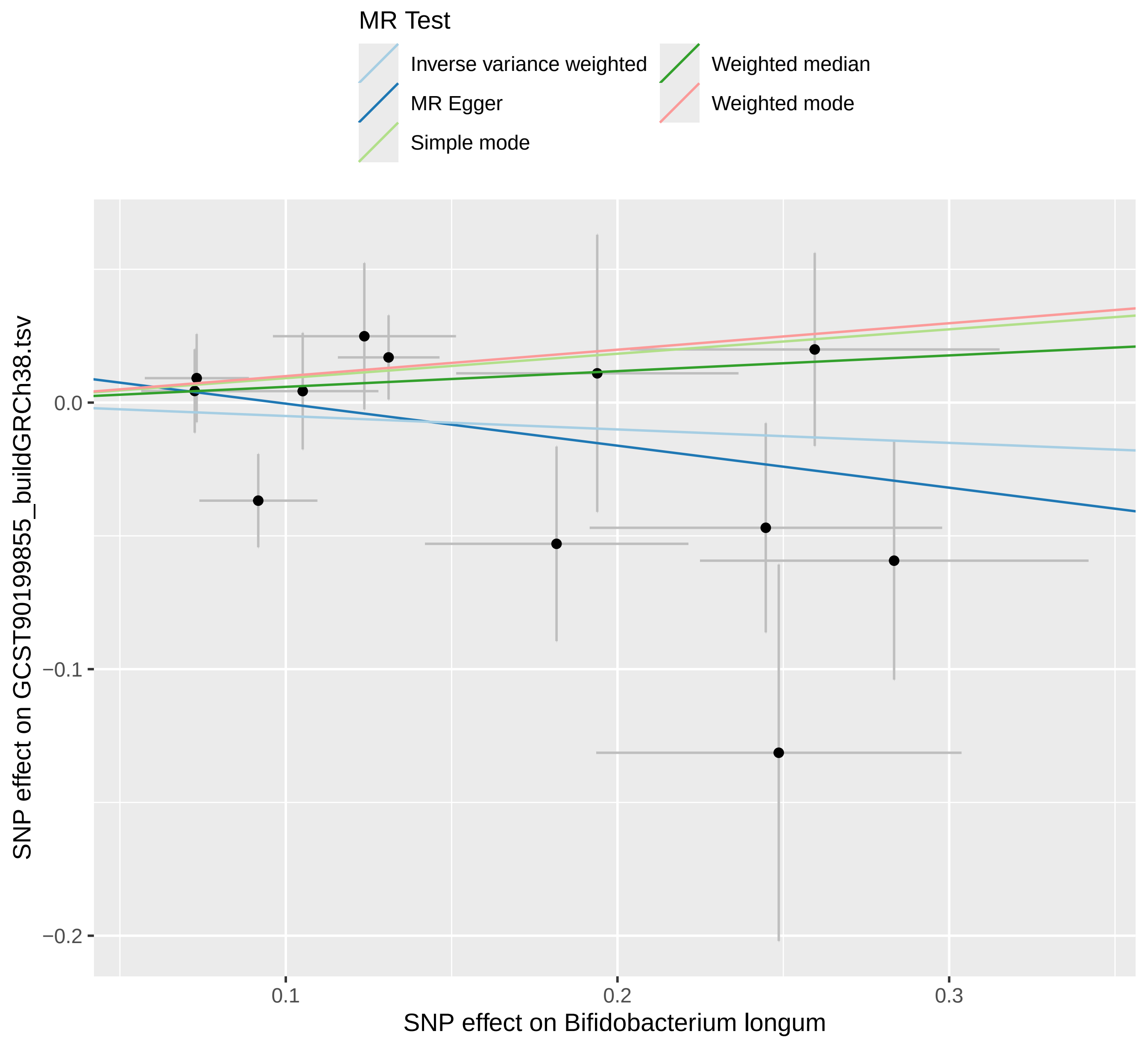
MR Test
Inverse variance weighted
MR Egger
Simple mode
Weighted median
Weighted mode
SNP effect on GCST90199855_buildGRCh38.tsv
0.0
−0.1
−0.2
0.1
0.2
0.3
SNP effect on Bifidobacterium longum

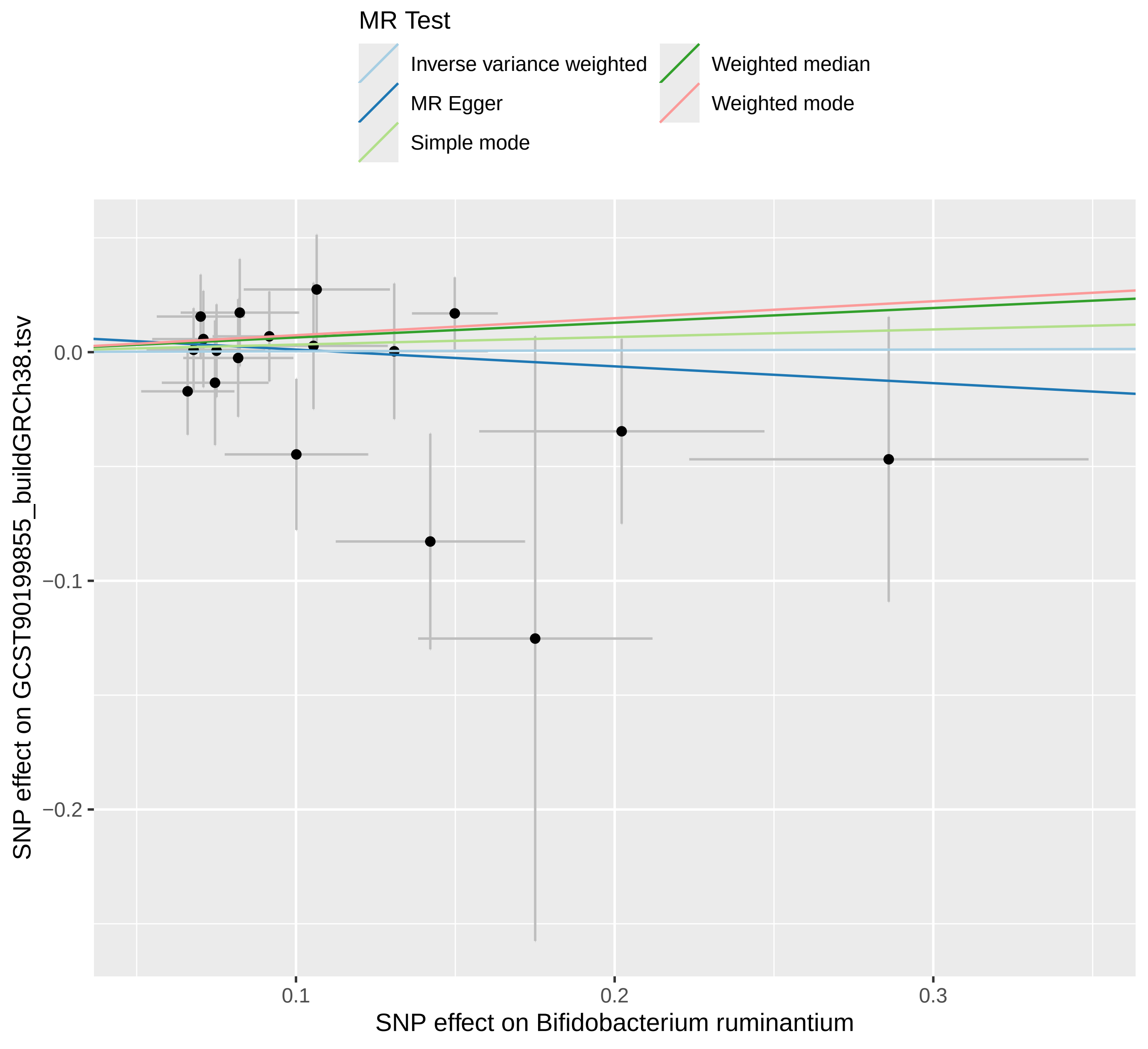

MR Test
Inverse variance weighted
MR Egger
Simple mode
Weighted median
Weighted mode
0.0
−0.1
−0.2
0.1
0.2
0.3
SNP effect on GCST90199855_buildGRCh38.tsv
SNP effect on Bifidobacterium ruminantium

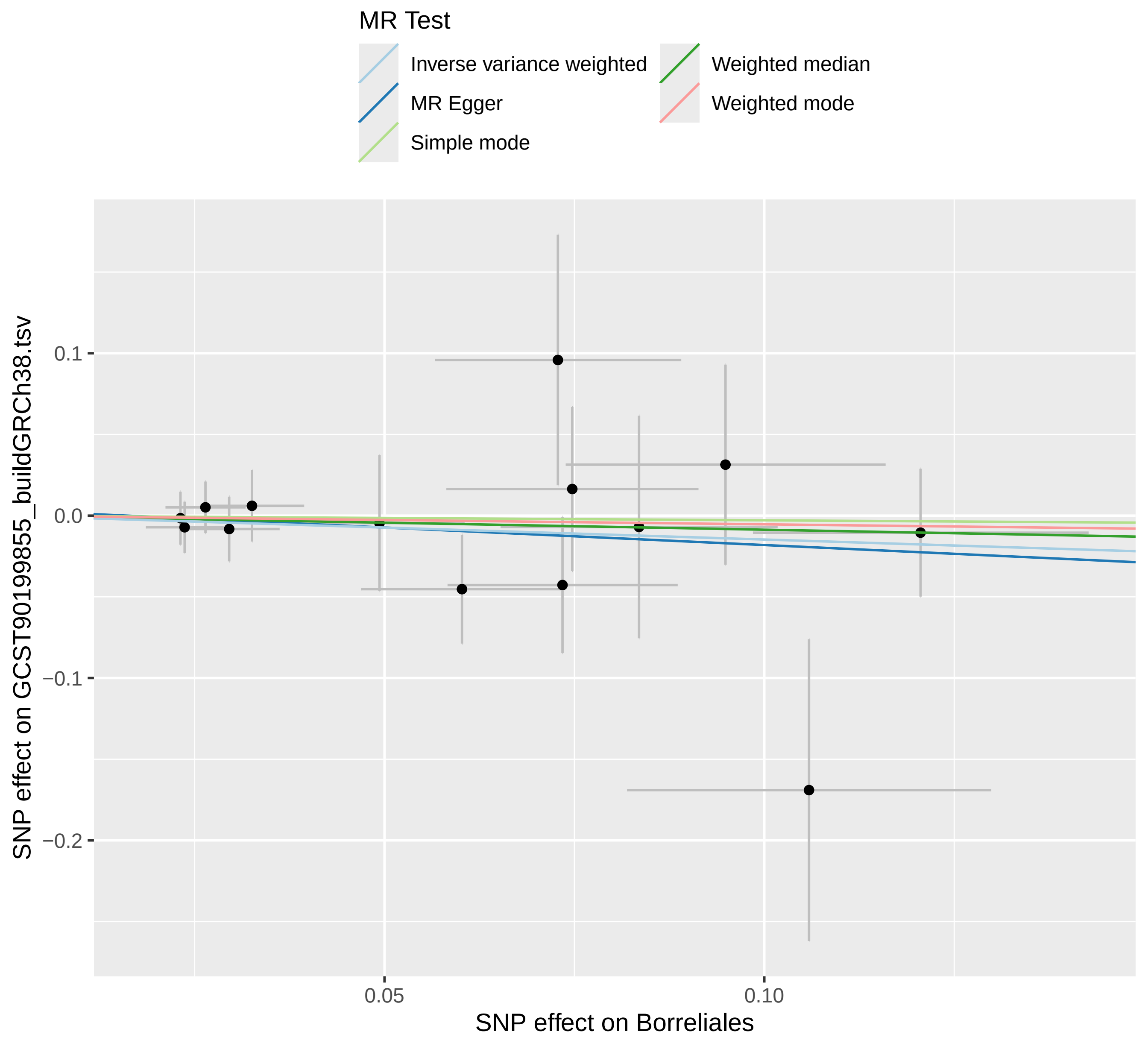
MR Test
Inverse variance weighted
MR Egger
Simple mode
Weighted median
Weighted mode
SNP effect on GCST90199855_buildGRCh38.tsv
0.1
0.0
−0.1
−0.2
0.05
0.10
SNP effect on Borreliales

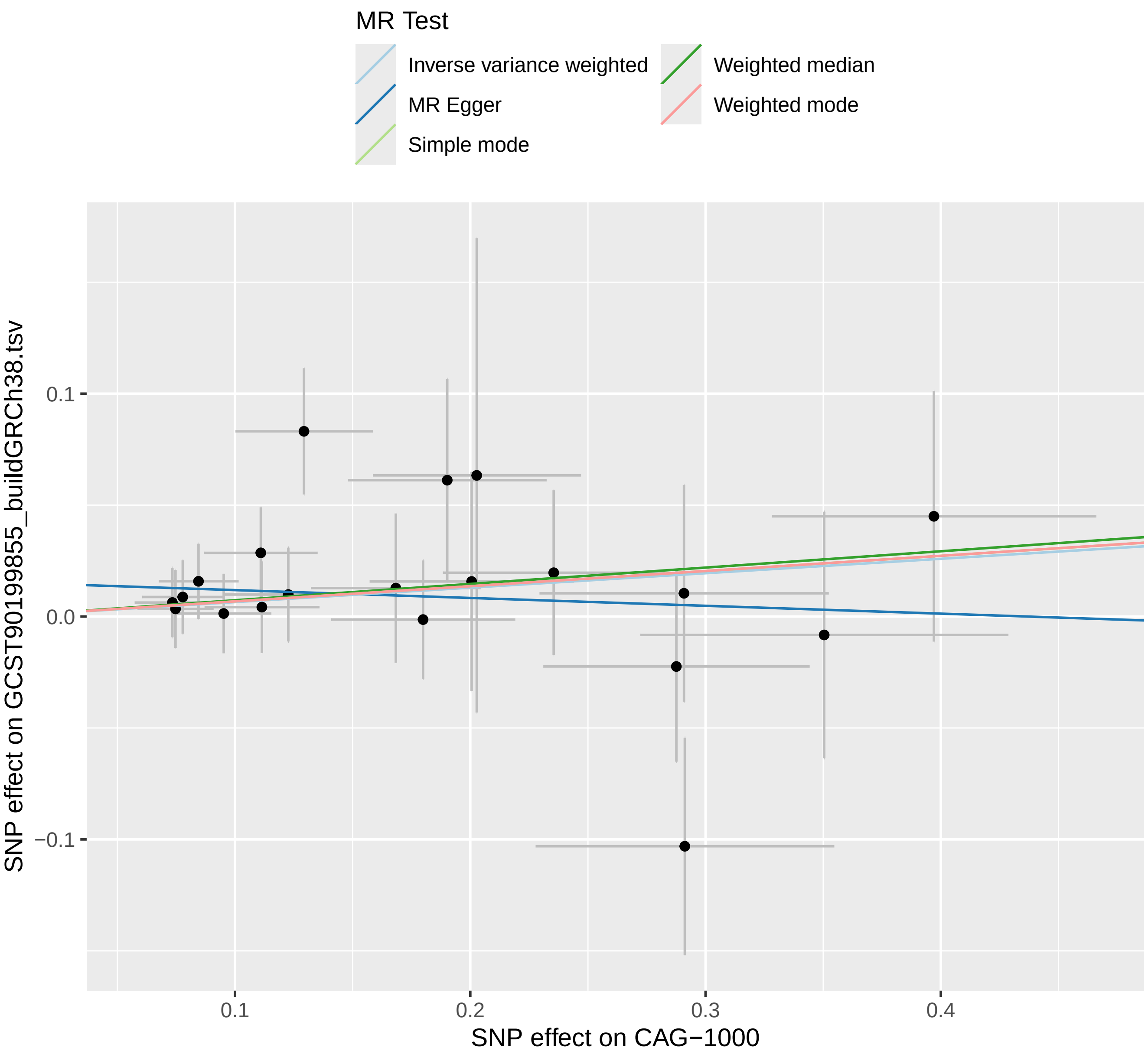
MR Test
Inverse variance weighted
MR Egger
Simple mode
Weighted median
Weighted mode
SNP effect on GCST90199855_buildGRCh38.tsv
0.1
0.0
−0.1
0.1
0.2
0.3
0.4
SNP effect on CAG−1000

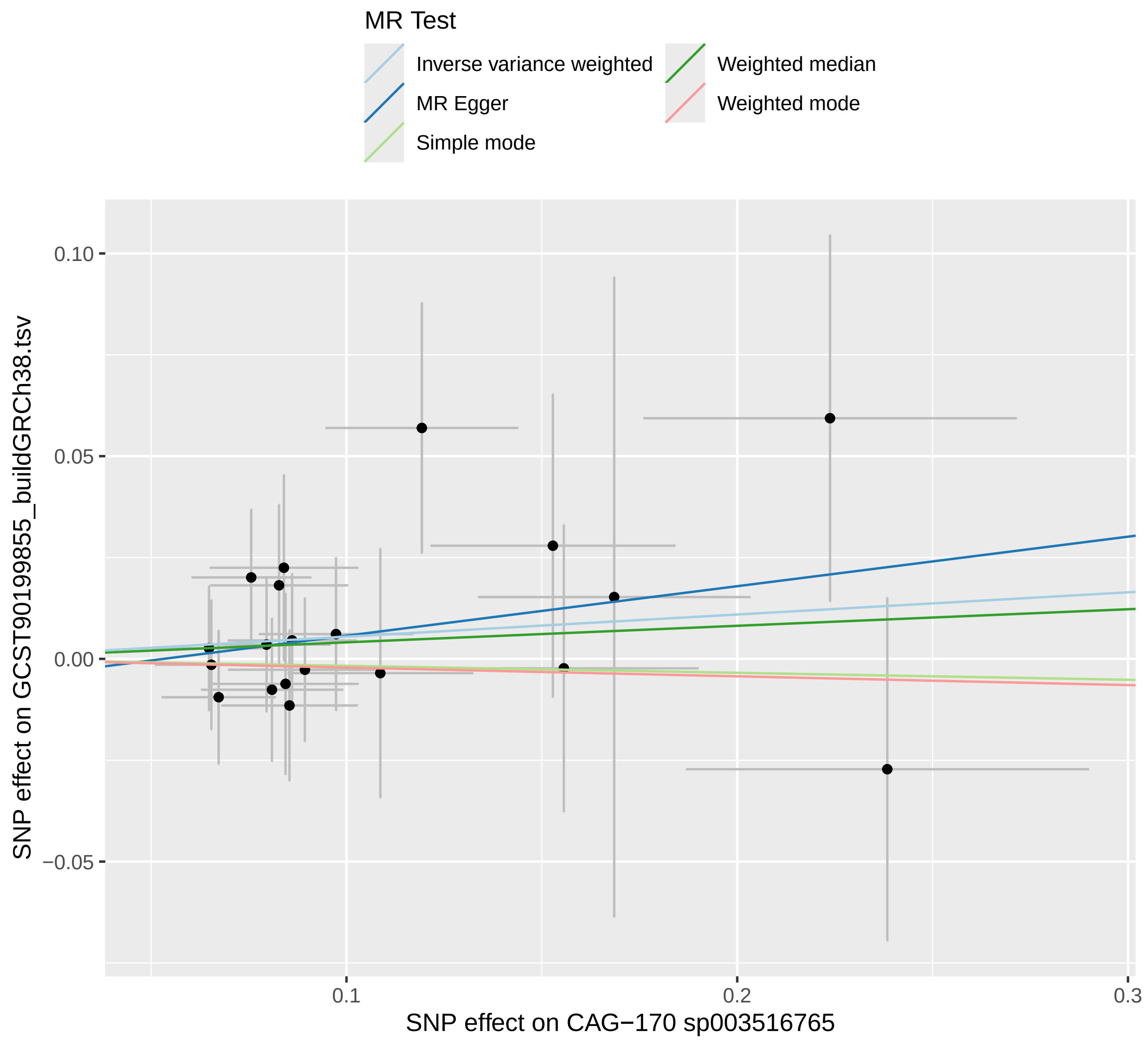
MR Test
Inverse variance weighted
MR Egger
Simple mode
Weighted median
Weighted mode
0.10
0.05
0.00
−0.05
0.1
0.2
0.3
SNP effect on GCST90199855_buildGRCh38.tsv
SNP effect on CAG−170 sp003516765

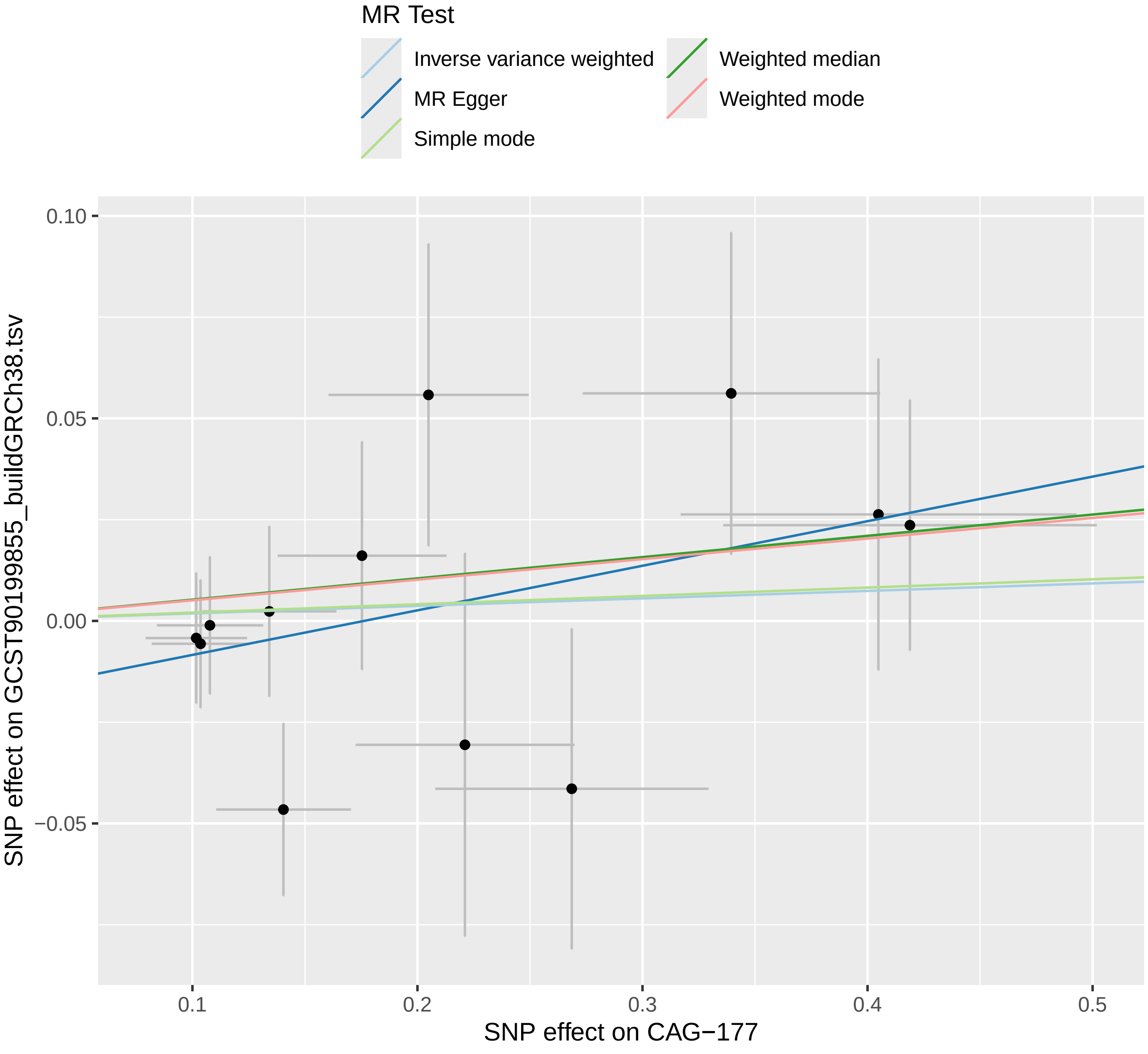
MR Test
Inverse variance weighted
MR Egger
Simple mode
Weighted median
Weighted mode
0.10
0.05
0.00
−0.05
SNP effect on GCST90199855_buildGRCh38.tsv
0.1
0.2
0.3
0.4
0.5
SNP effect on CAG−177

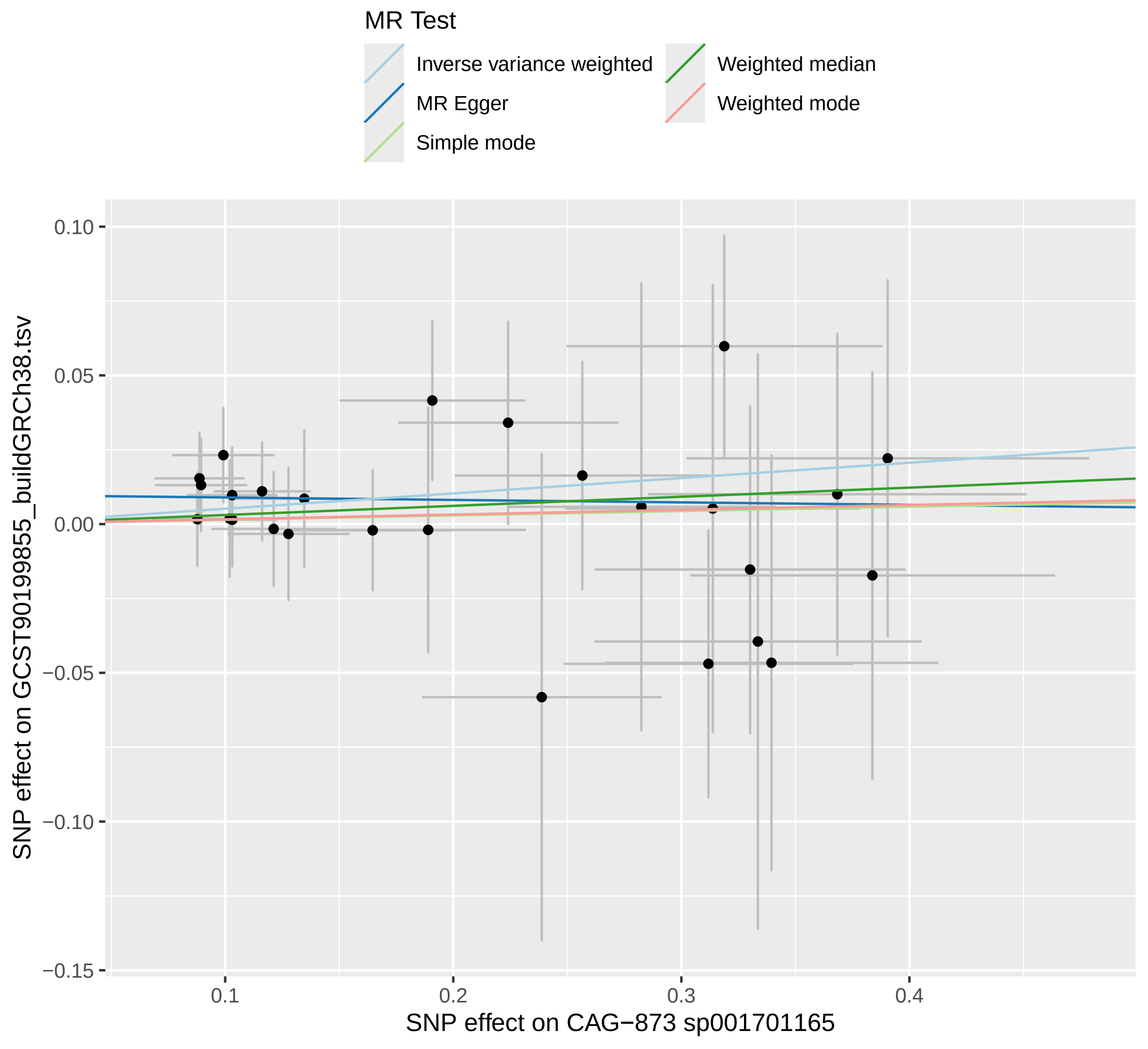
MR Test
Inverse variance weighted
MR Egger
Simple mode
Weighted median
Weighted mode
0.10
0.05
0.00
−0.05
−0.10
−0.15
0.1
0.2
0.3
0.4
SNP effect on GCST90199855_buildGRCh38.tsv
SNP effect on CAG−873 sp001701165

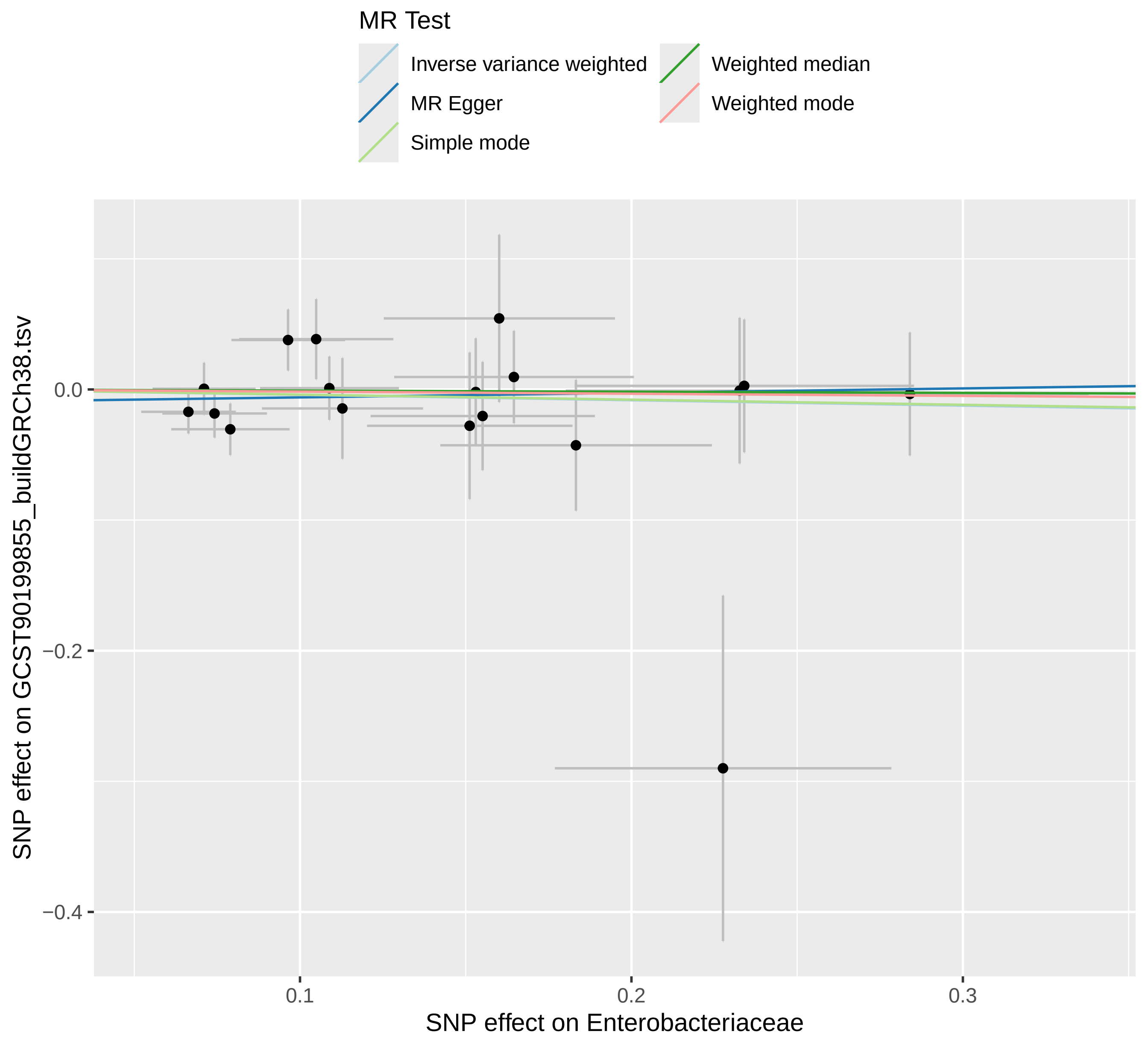

MR Test
Inverse variance weighted
MR Egger
Simple mode
Weighted median
Weighted mode
SNP effect on GCST90199855_buildGRCh38.tsv
0.0
−0.2
−0.4
0.1
0.2
0.3
SNP effect on Enterobacteriaceae

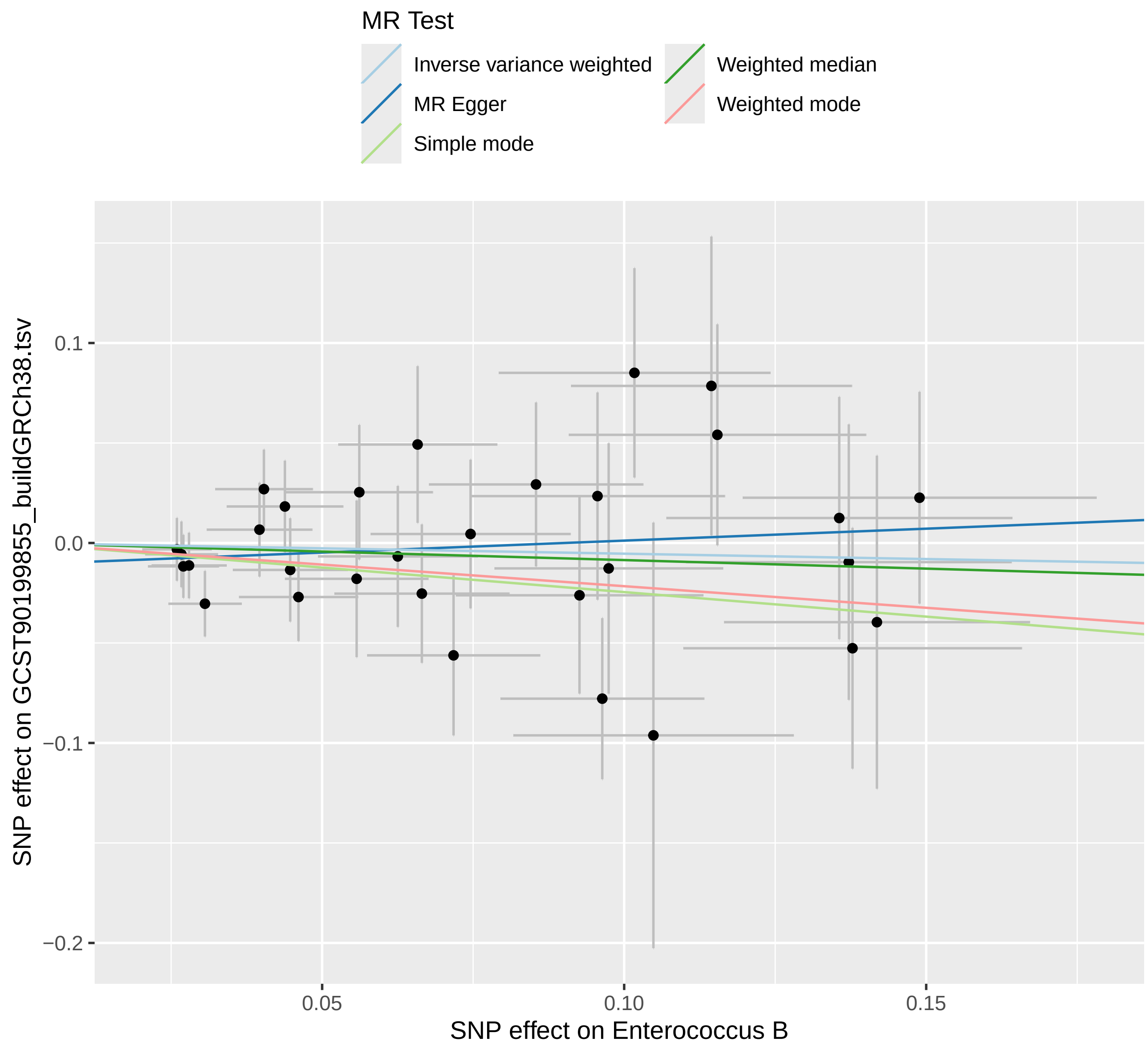

MR Test
Inverse variance weighted
MR Egger
Simple mode
Weighted median
Weighted mode
SNP effect on GCST90199855_buildGRCh38.tsv
0.1
0.0
−0.1
−0.2
0.05
0.10
0.15
SNP effect on Enterococcus B

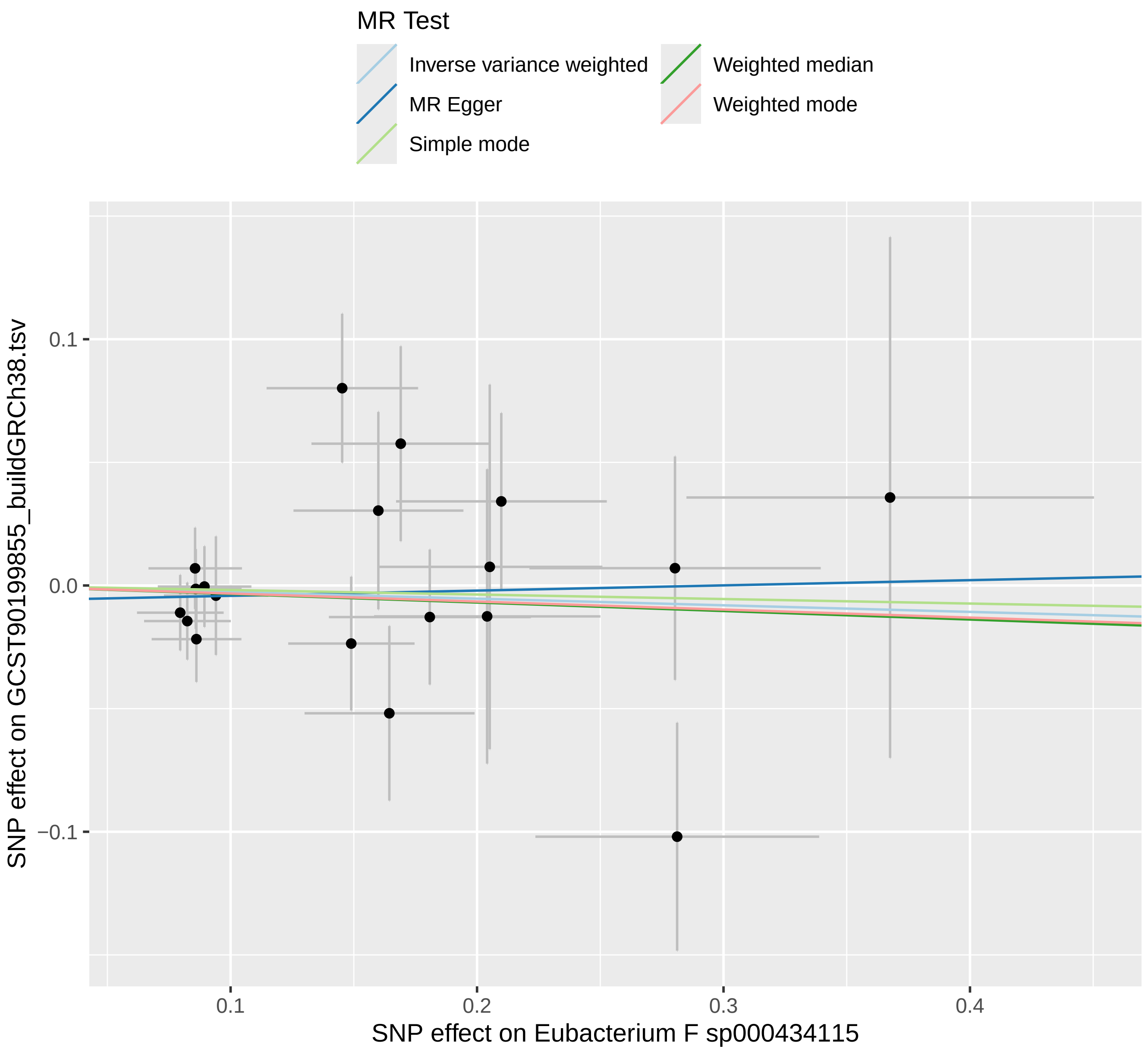
MR Test
Inverse variance weighted
MR Egger
Simple mode
Weighted median
Weighted mode
SNP effect on GCST90199855_buildGRCh38.tsv
0.1
0.0
−0.1
0.1
0.2
0.3
0.4
SNP effect on Eubacterium F sp000434115

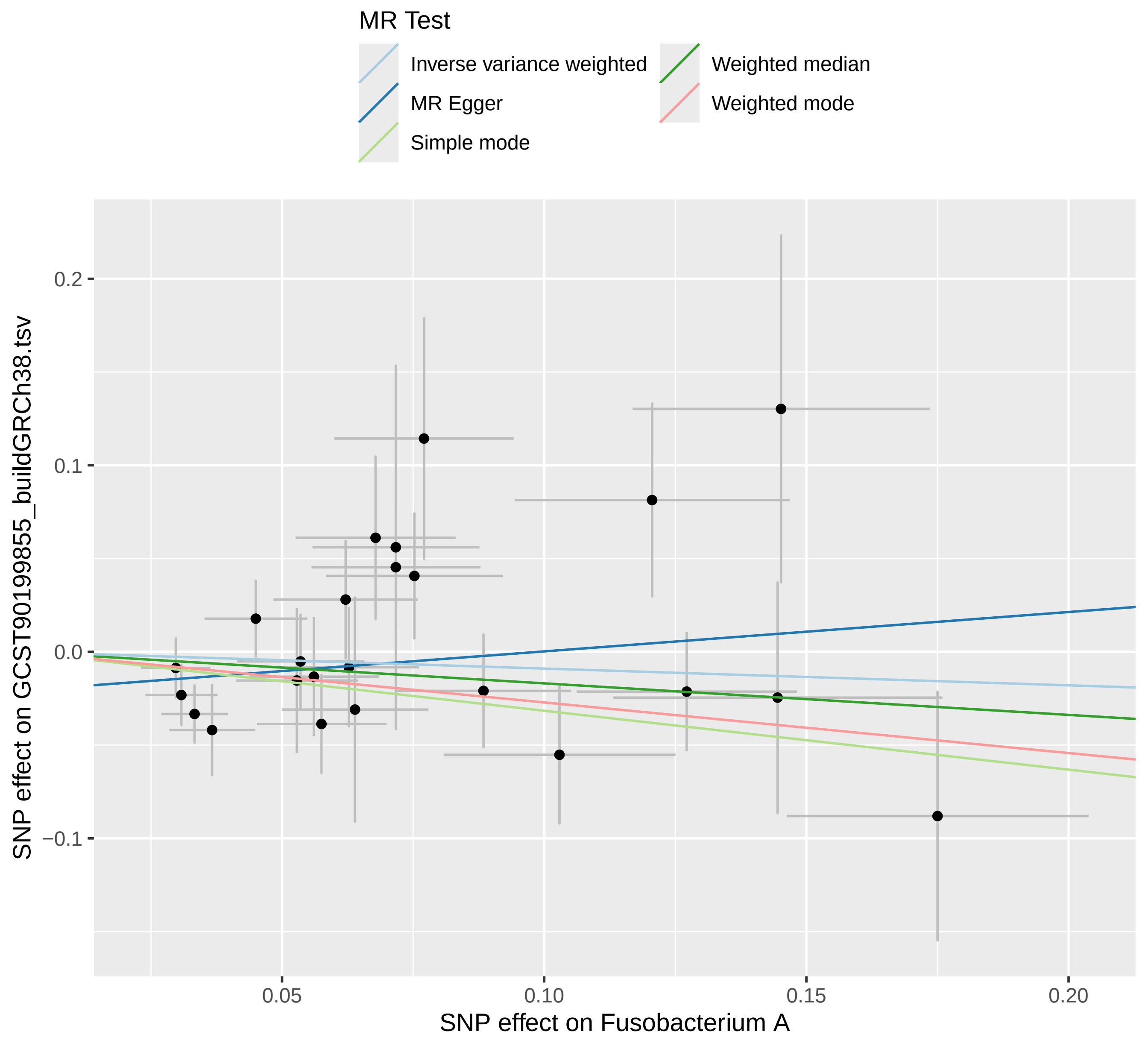
MR Test
Inverse variance weighted
MR Egger
Simple mode
Weighted median
Weighted mode
0.2
0.1
0.0
−0.1
0.05
0.10
0.15
0.20
SNP effect on GCST90199855_buildGRCh38.tsv
SNP effect on Fusobacterium A

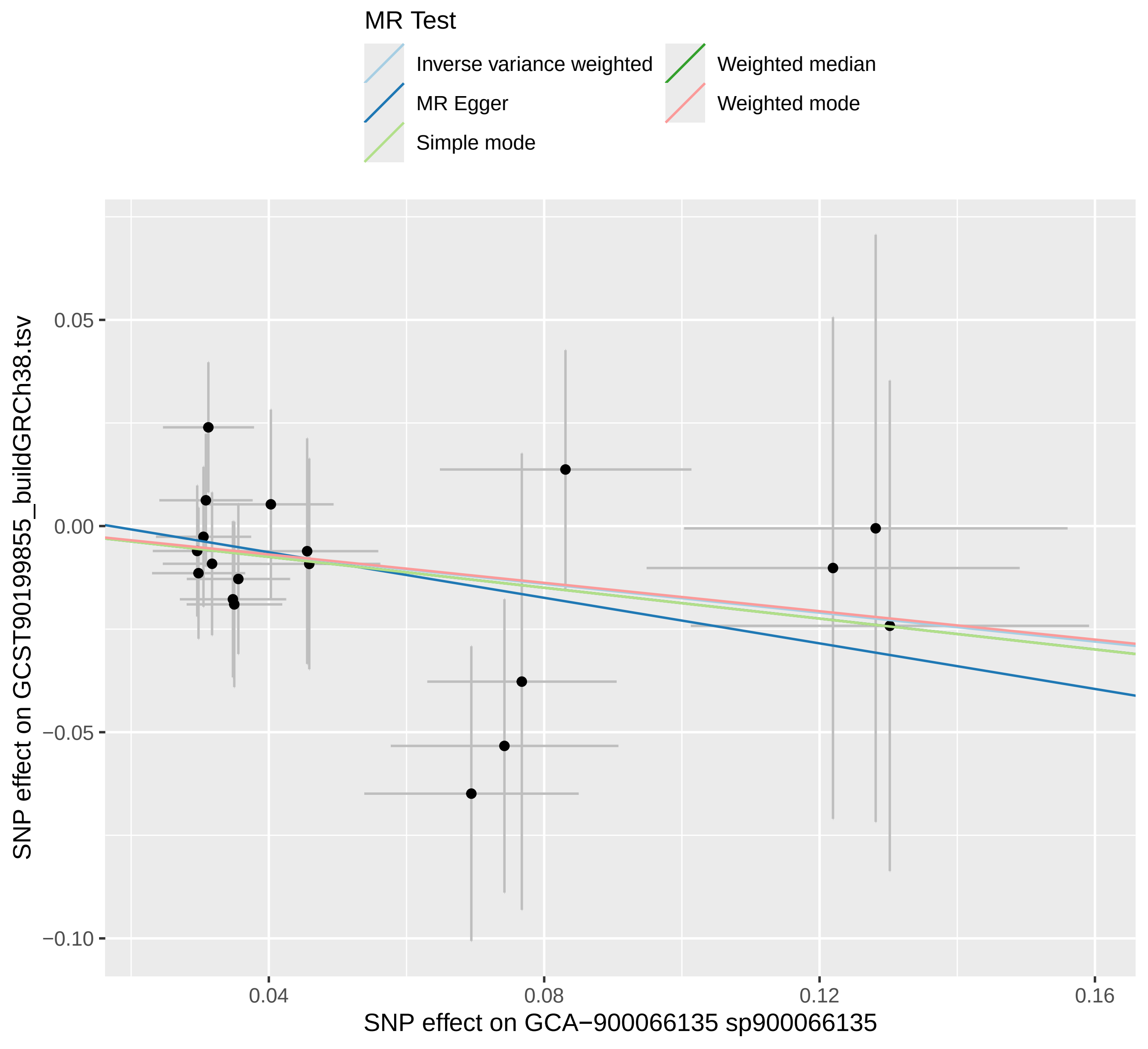
MR Test
Inverse variance weighted
MR Egger
Simple mode
Weighted median
Weighted mode
0.05
0.00
−0.05
−0.10
0.04
0.08
0.12
0.16
SNP effect on GCST90199855_buildGRCh38.tsv
SNP effect on GCA−900066135 sp900066135

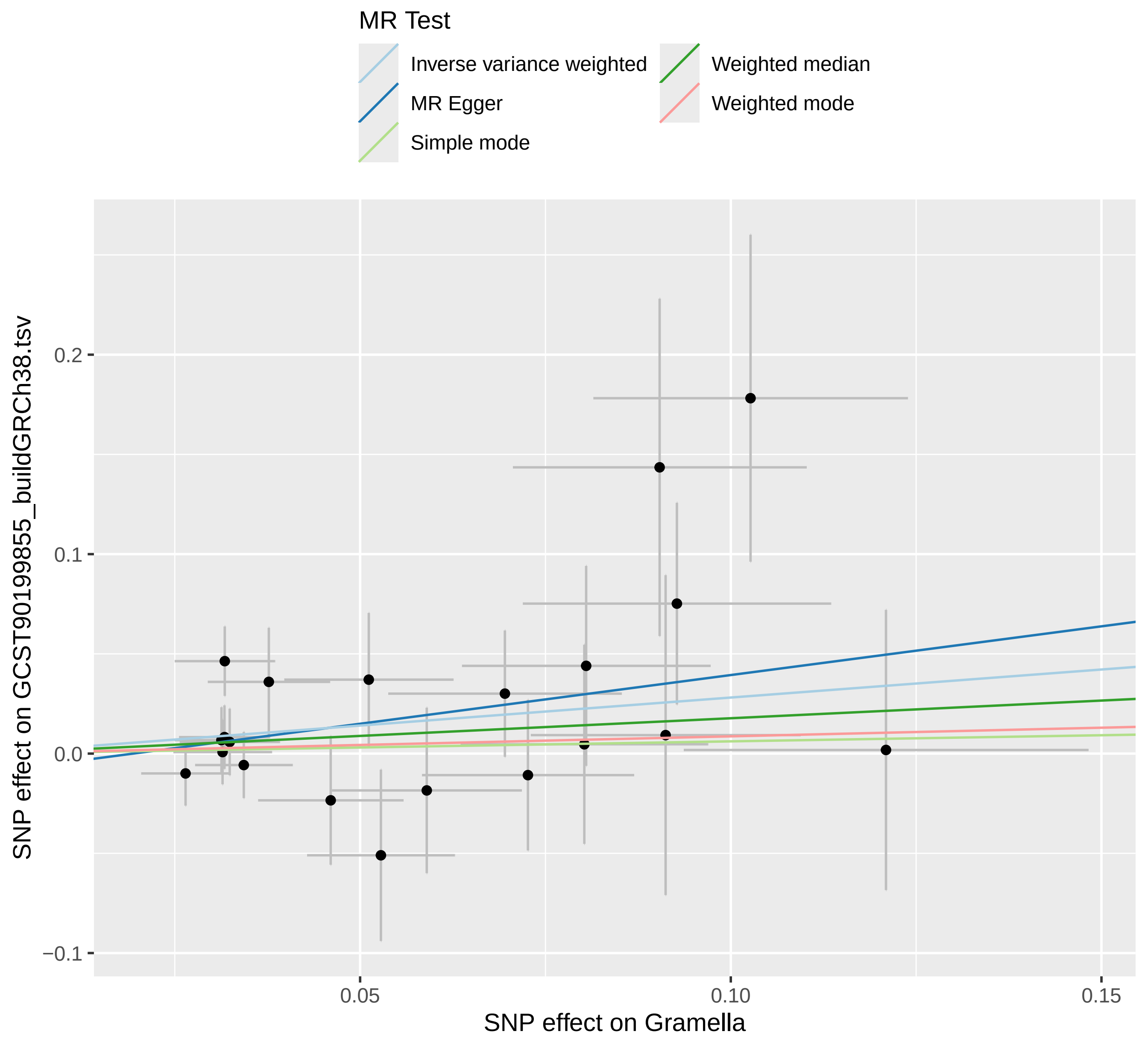

MR Test
Inverse variance weighted
MR Egger
Simple mode
Weighted median
Weighted mode
SNP effect on GCST90199855_buildGRCh38.tsv
0.2
0.1
0.0
−0.1
0.05
0.10
0.15
SNP effect on Gramella

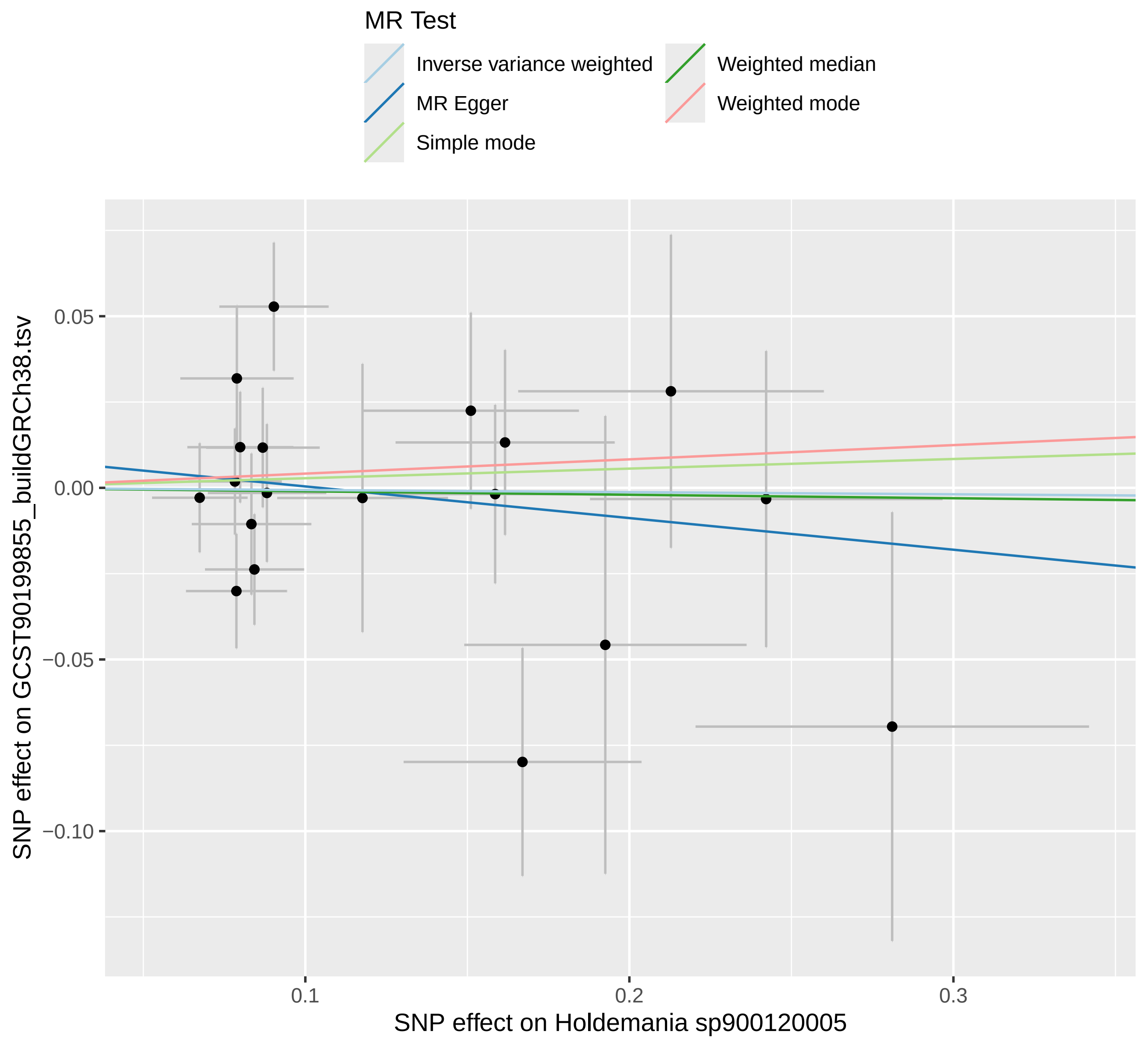

MR Test
Inverse variance weighted
MR Egger
Simple mode
Weighted median
Weighted mode
0.05
0.00
−0.05
−0.10
SNP effect on GCST90199855_buildGRCh38.tsv
0.1
0.2
0.3
SNP effect on Holdemania sp900120005

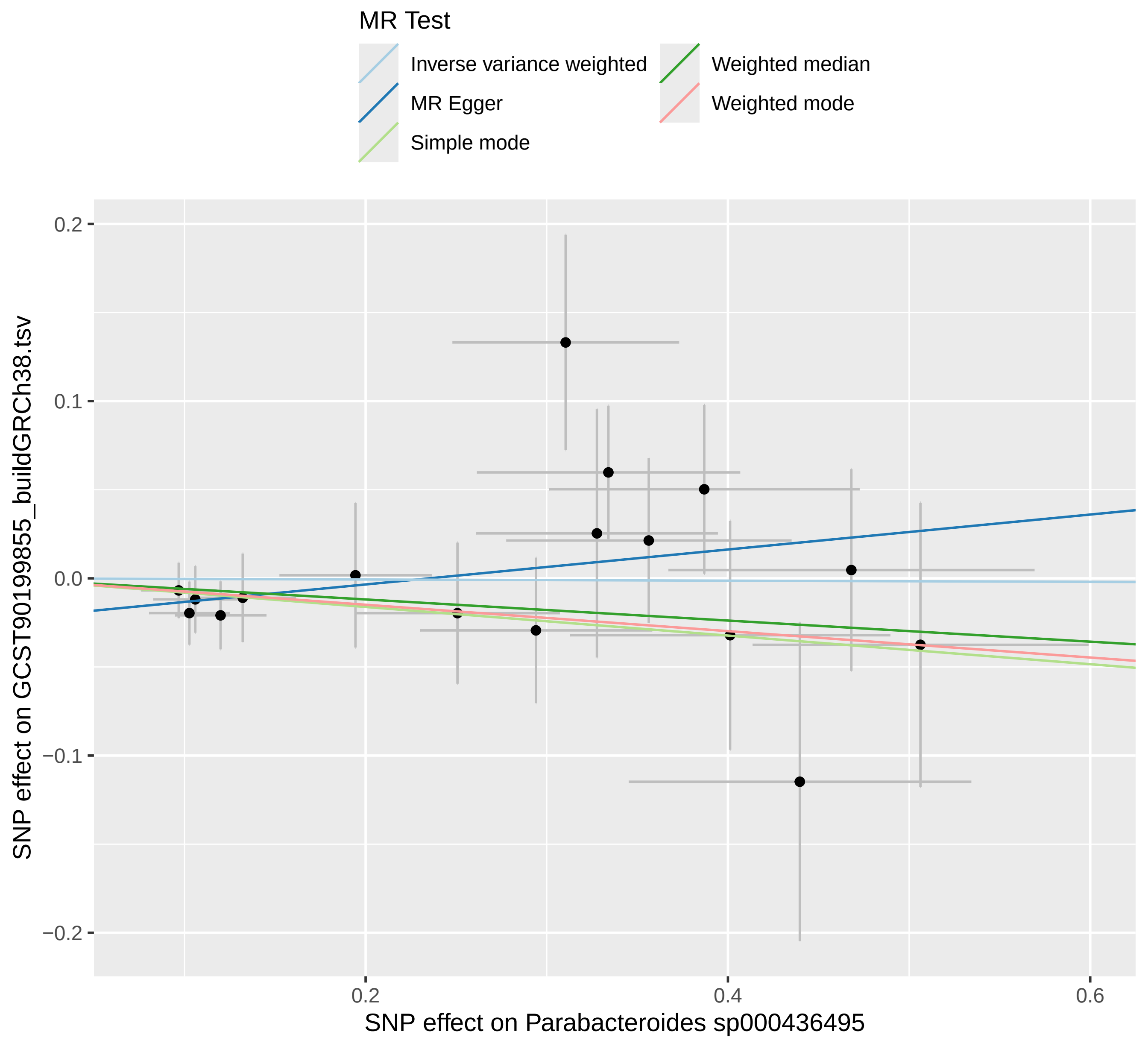
MR Test
Inverse variance weighted
MR Egger
Simple mode
Weighted median
Weighted mode
0.2
0.1
0.0
−0.1
−0.2
0.2
0.4
0.6
SNP effect on GCST90199855_buildGRCh38.tsv
SNP effect on Parabacteroides sp000436495

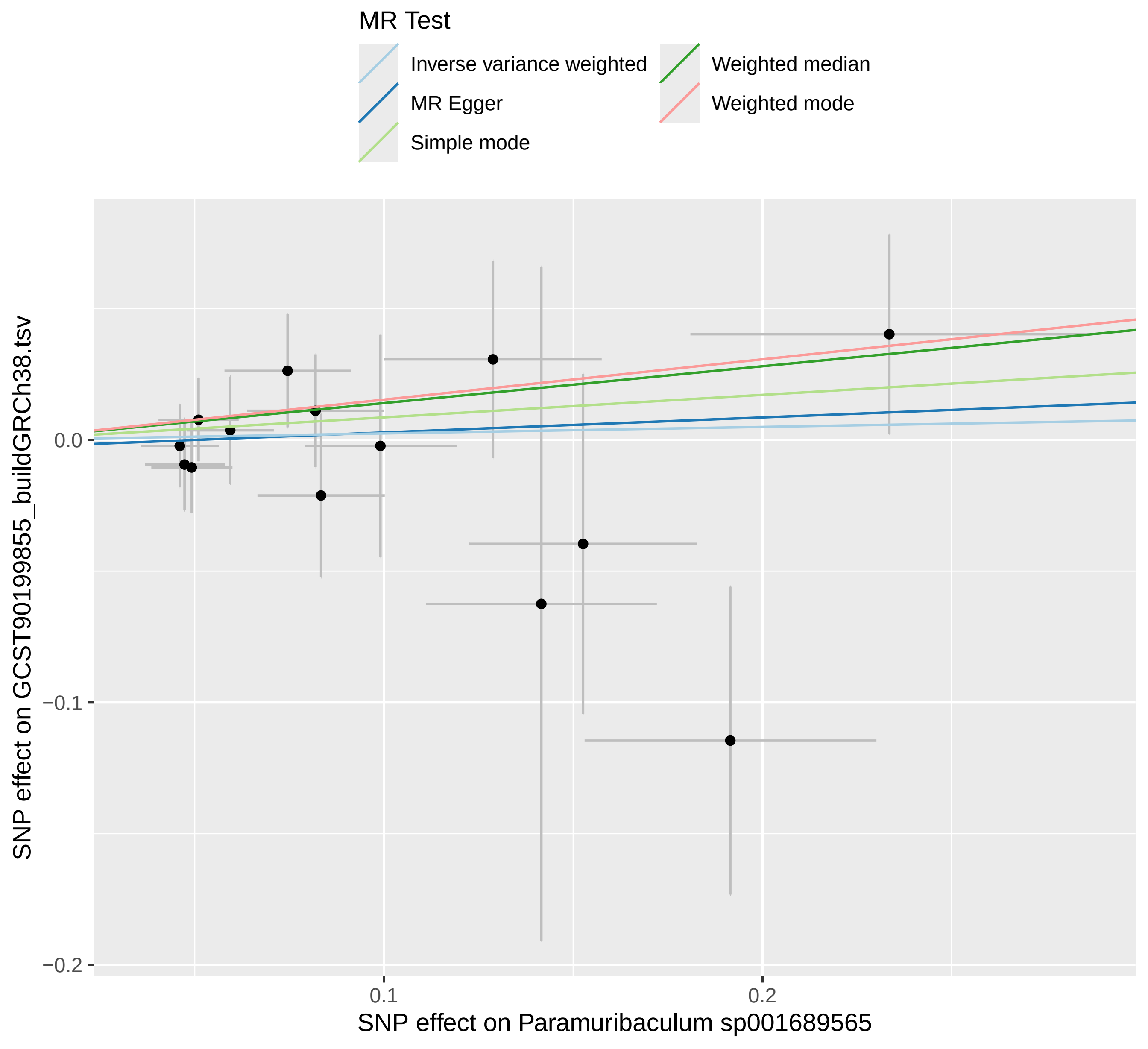
MR Test
Inverse variance weighted
MR Egger
Simple mode
Weighted median
Weighted mode
SNP effect on GCST90199855_buildGRCh38.tsv
0.0
−0.1
−0.2
0.1
0.2
SNP effect on Paramuribaculum sp001689565

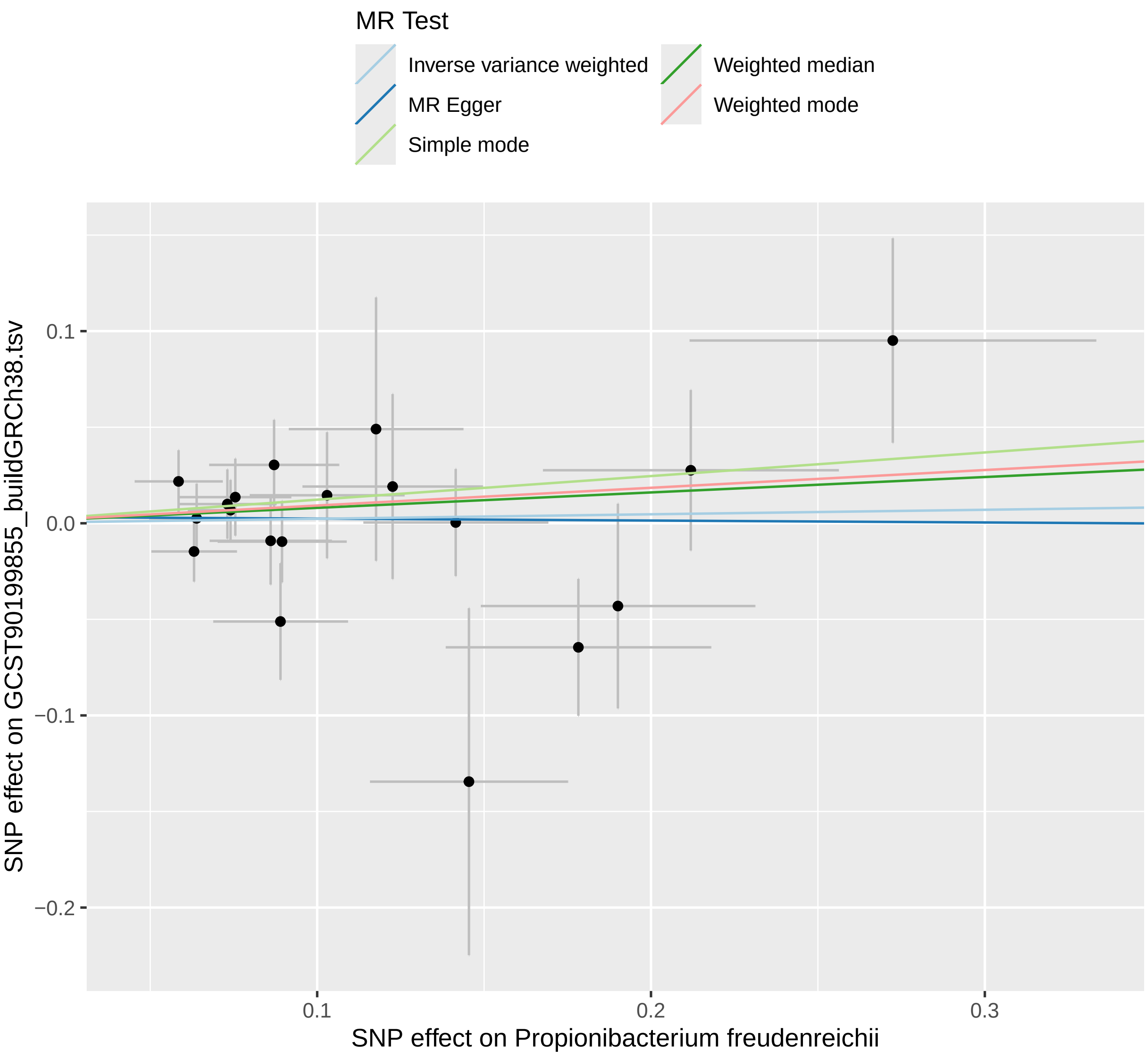
MR Test
Inverse variance weighted
MR Egger
Simple mode
Weighted median
Weighted mode
0.1
0.0
−0.1
−0.2
0.1
0.2
0.3
SNP effect on GCST90199855_buildGRCh38.tsv
SNP effect on Propionibacterium freudenreichii

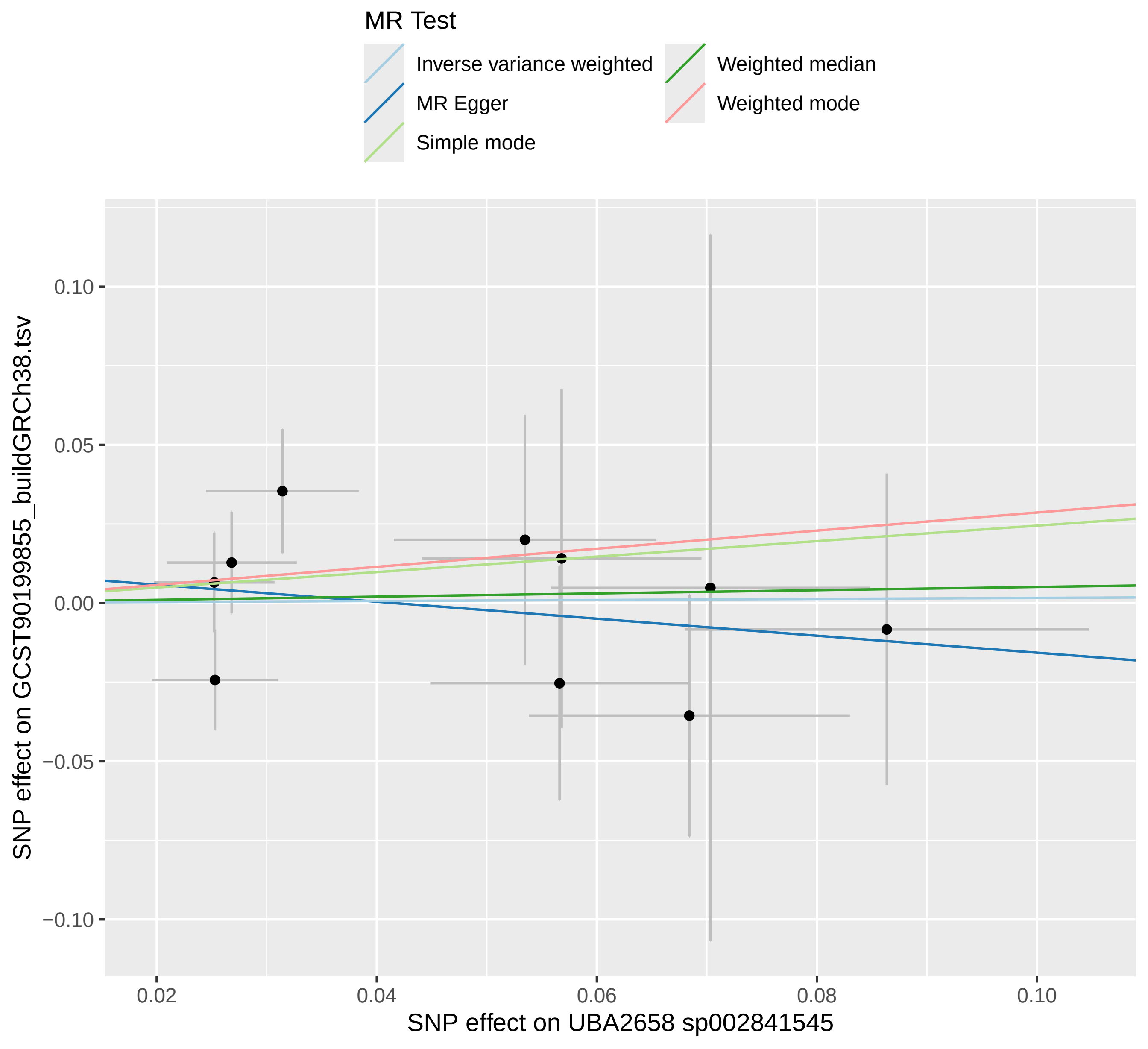
MR Test
Inverse variance weighted
MR Egger
Simple mode
Weighted median
Weighted mode
0.10
0.05
0.00
−0.05
−0.10
SNP effect on GCST90199855_buildGRCh38.tsv
0.02
0.04
0.06
0.08
0.10
SNP effect on UBA2658 sp002841545

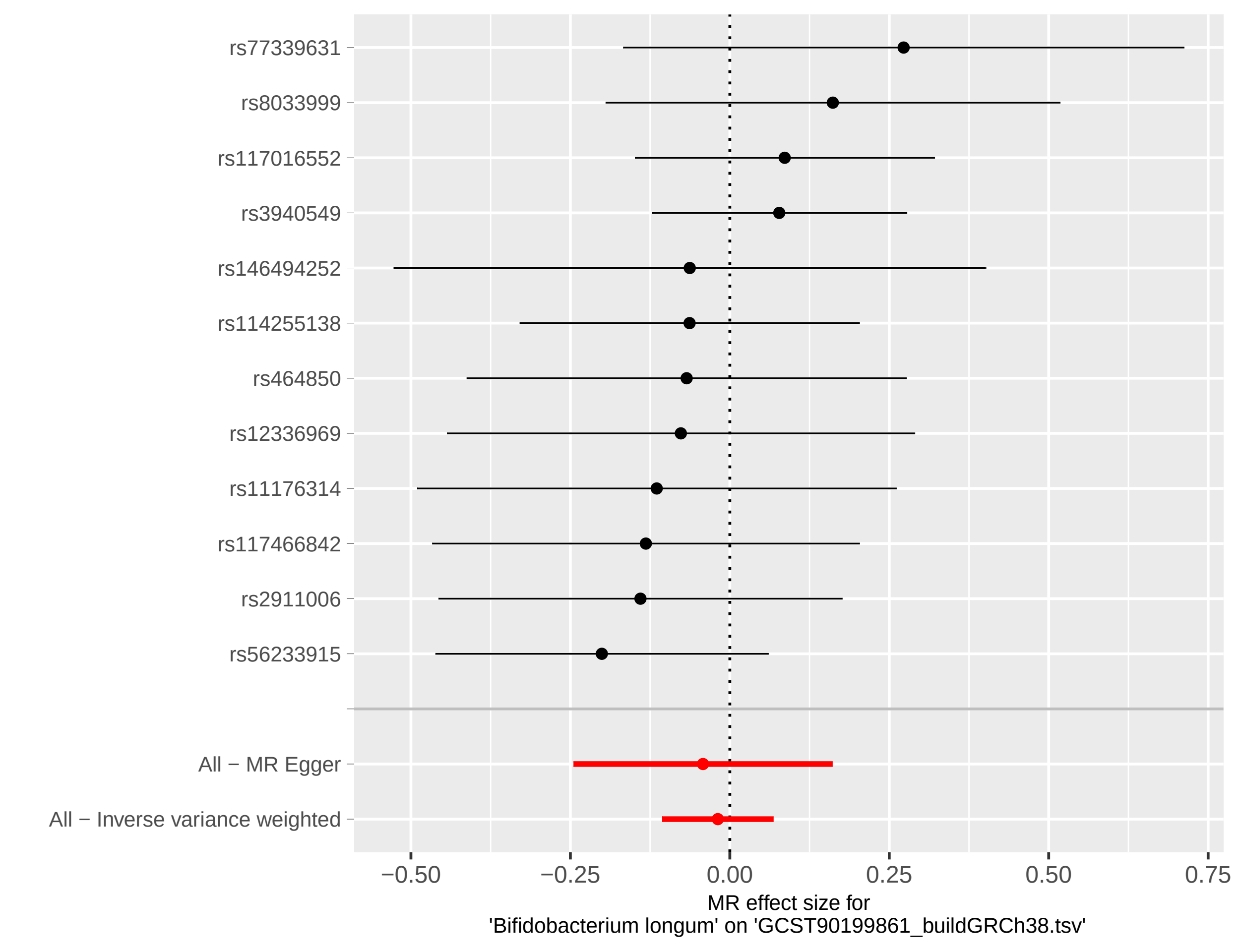
rs77339631
rs8033999
rs117016552
rs3940549
rs146494252
rs114255138
rs464850
rs12336969
rs11176314
rs117466842
rs2911006
rs56233915
All − MR Egger
All − Inverse variance weighted
−0.50
−0.25
0.00
0.25
0.50
0.75
MR effect size for
'Bifidobacterium longum' on 'GCST90199861_buildGRCh38.tsv'

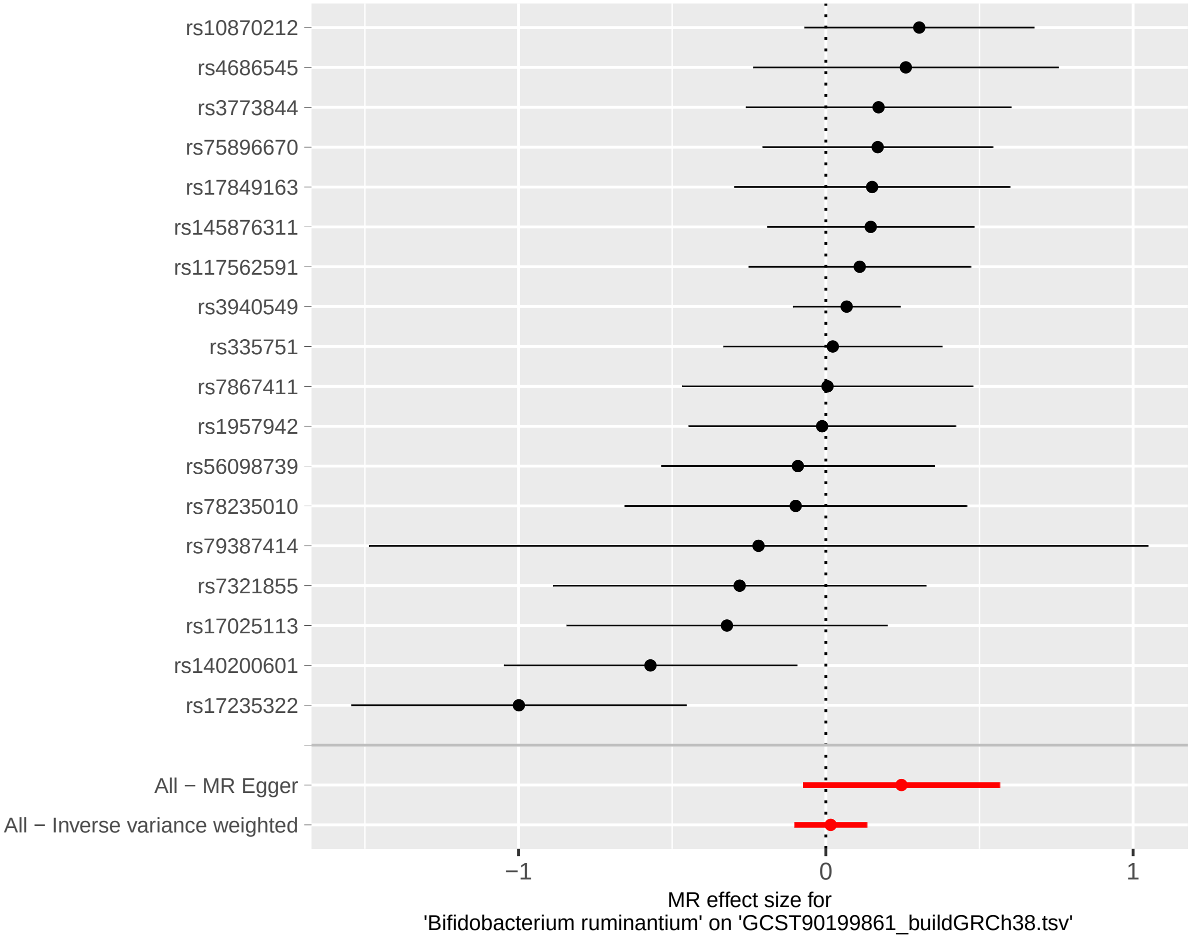

rs10870212
rs4686545
rs3773844
rs75896670
rs17849163
rs145876311
rs117562591
rs3940549
rs335751
rs7867411
rs1957942
rs56098739
rs78235010
rs79387414
rs7321855
rs17025113
rs140200601
rs17235322
All − MR Egger
All − Inverse variance weighted
−1
0
1
MR effect size for
'Bifidobacterium ruminantium' on 'GCST90199861_buildGRCh38.tsv'

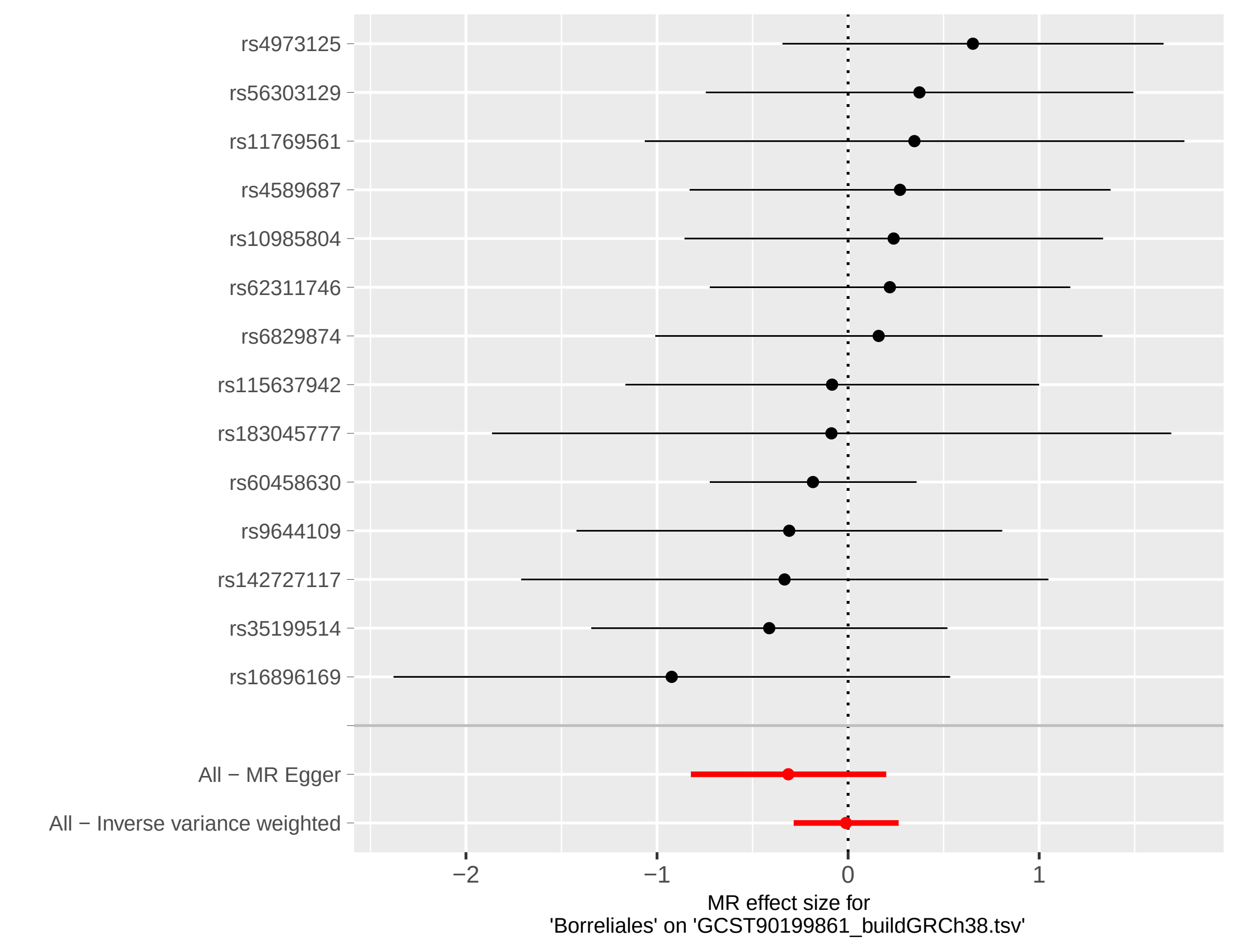
rs4973125
rs56303129
rs11769561
rs4589687
rs10985804
rs62311746
rs6829874
rs115637942
rs183045777
rs60458630
rs9644109
rs142727117
rs35199514
rs16896169
All − MR Egger
All − Inverse variance weighted
−2
−1
0
1
MR effect size for
'Borreliales' on 'GCST90199861_buildGRCh38.tsv'

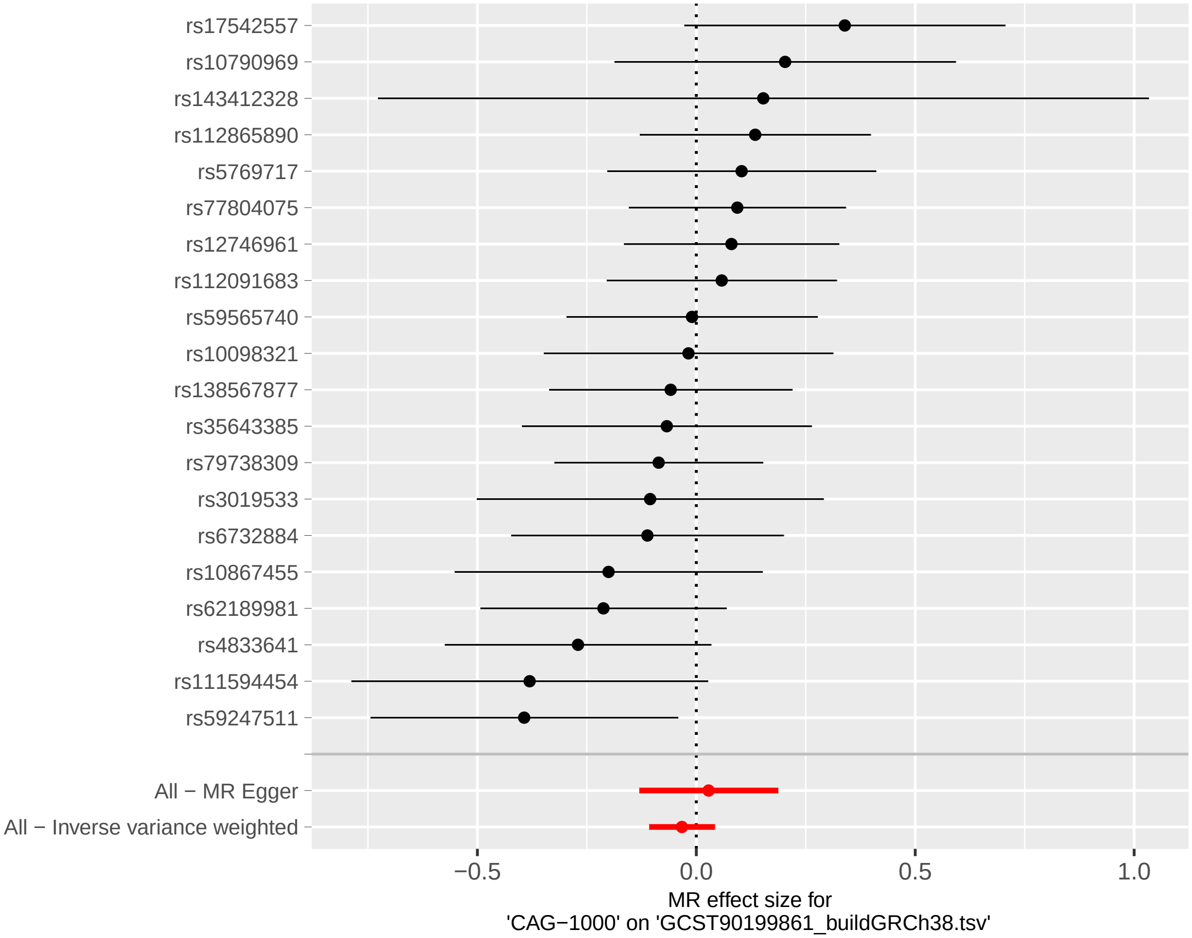

rs17542557
rs10790969
rs143412328
rs112865890
rs5769717
rs77804075
rs12746961
rs112091683
rs59565740
rs10098321
rs138567877
rs35643385
rs79738309
rs3019533
rs6732884
rs10867455
rs62189981
rs4833641
rs111594454
rs59247511
All − MR Egger
All − Inverse variance weighted
−0.5
0.0
0.5
1.0
MR effect size for
'CAG−1000' on 'GCST90199861_buildGRCh38.tsv'

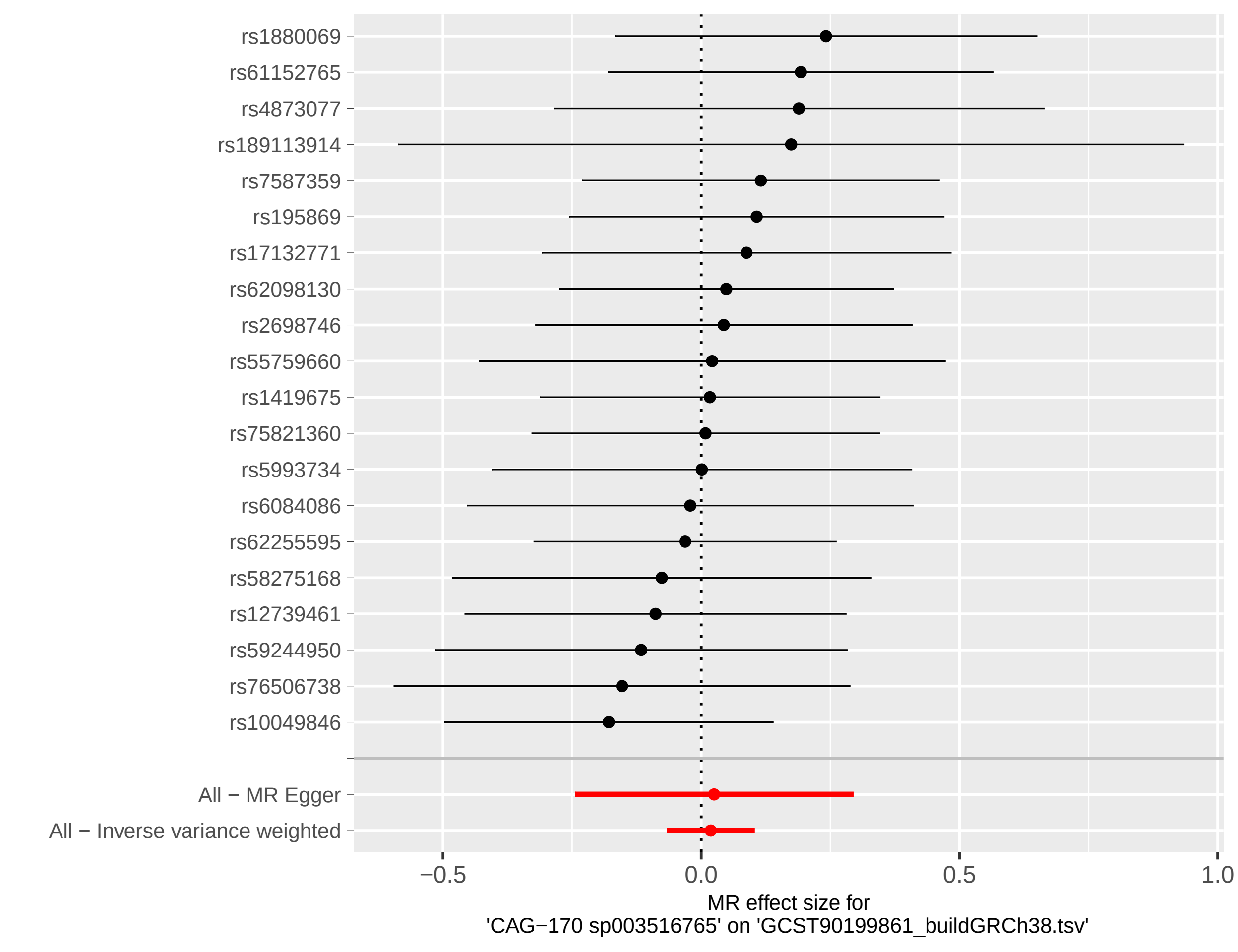
rs1880069
rs61152765
rs4873077
rs189113914
rs7587359
rs195869
rs17132771
rs62098130
rs2698746
rs55759660
rs1419675
rs75821360
rs5993734
rs6084086
rs62255595
rs58275168
rs12739461
rs59244950
rs76506738
rs10049846
All − MR Egger
All − Inverse variance weighted
−0.5
0.0
0.5
1.0
MR effect size for
'CAG−170 sp003516765' on 'GCST90199861_buildGRCh38.tsv'

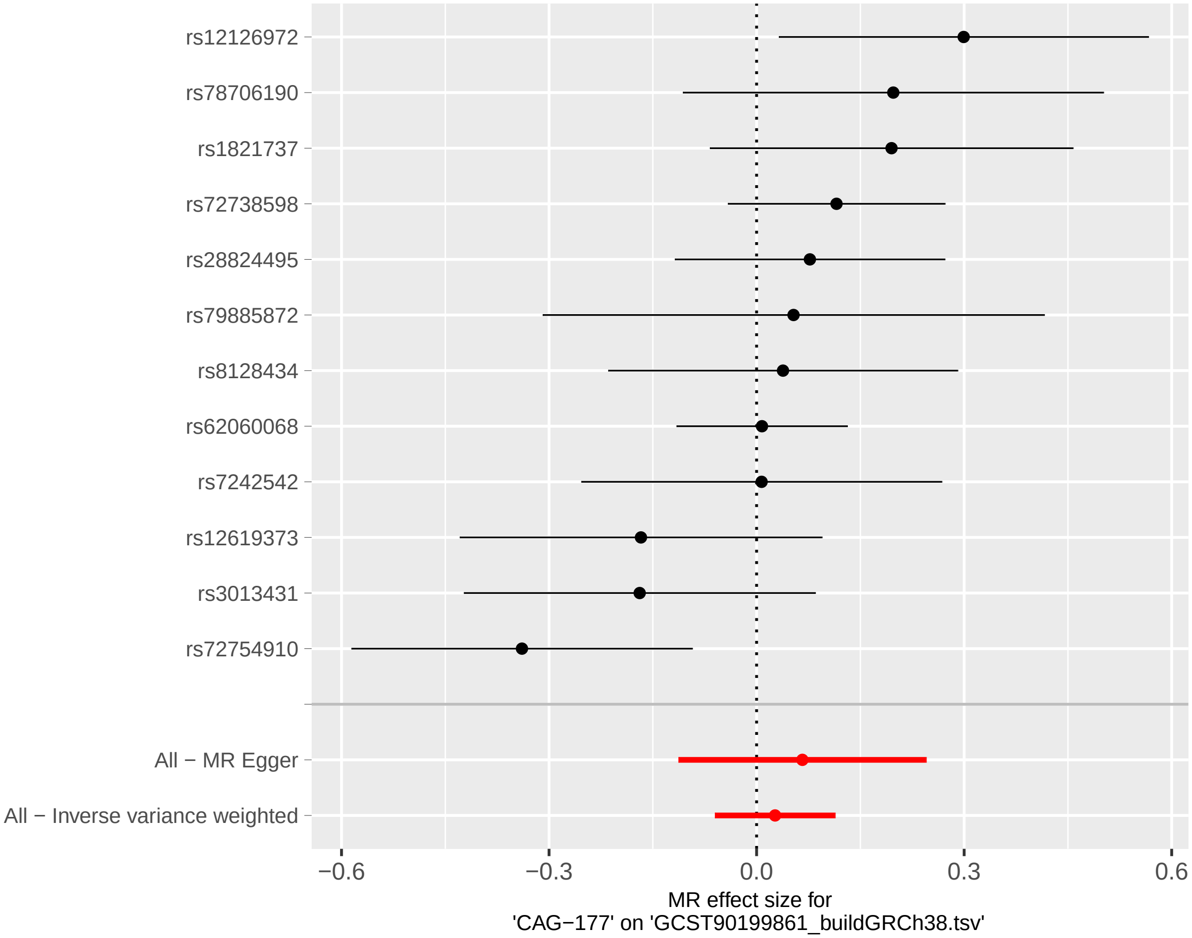
rs12126972
rs78706190
rs1821737
rs72738598
rs28824495
rs79885872
rs8128434
rs62060068
rs7242542
rs12619373
rs3013431
rs72754910
All − MR Egger
All − Inverse variance weighted
−0.6
−0.3
0.0
0.3
0.6
MR effect size for
'CAG−177' on 'GCST90199861_buildGRCh38.tsv'

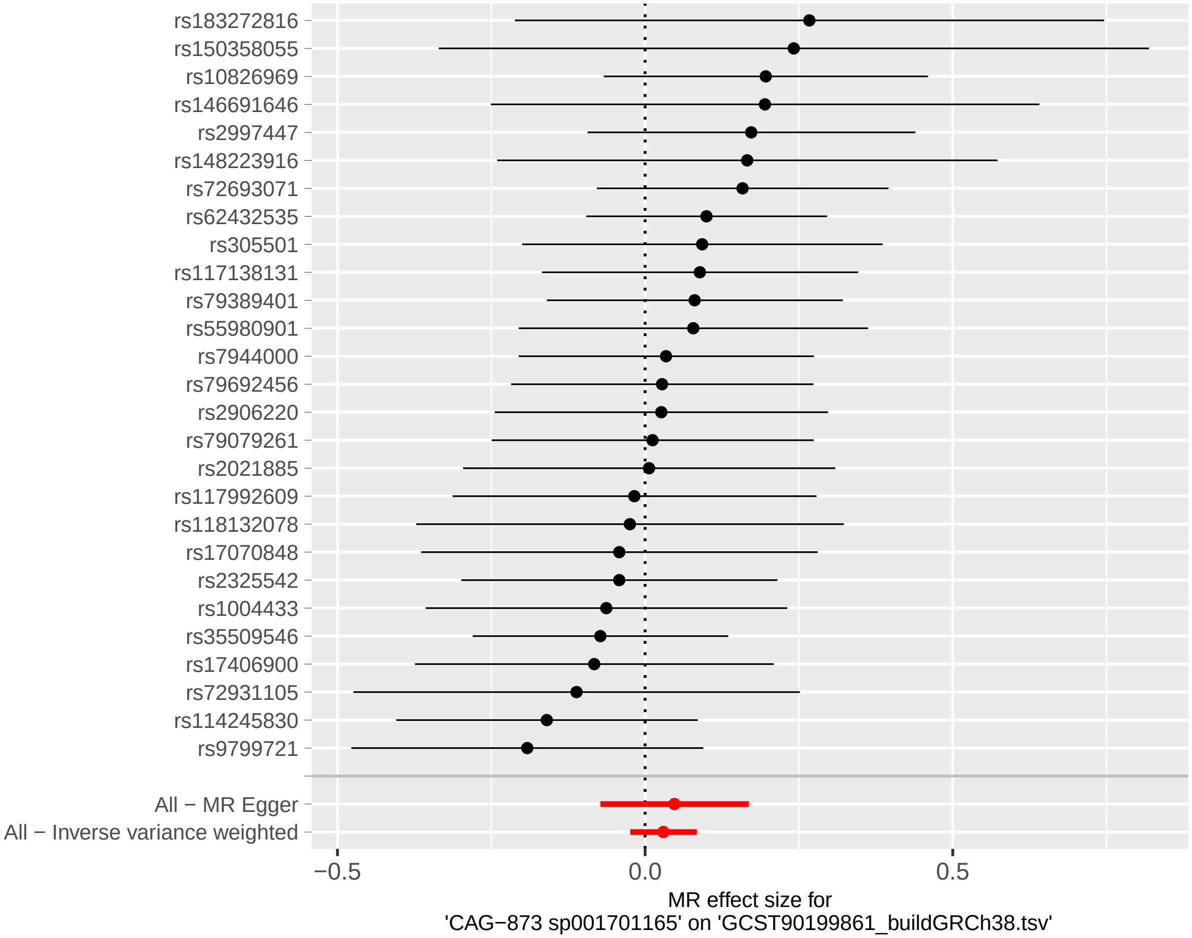
rs183272816
rs150358055
rs10826969
rs146691646
rs2997447
rs148223916
rs72693071
rs62432535
rs305501
rs117138131
rs79389401
rs55980901
rs7944000
rs79692456
rs2906220
rs79079261
rs2021885
rs117992609
rs118132078
rs17070848
rs2325542
rs1004433
rs35509546
rs17406900
rs72931105
rs114245830
rs9799721
All − MR Egger
All − Inverse variance weighted
−0.5
0.0
0.5
MR effect size for
'CAG−873 sp001701165' on 'GCST90199861_buildGRCh38.tsv'

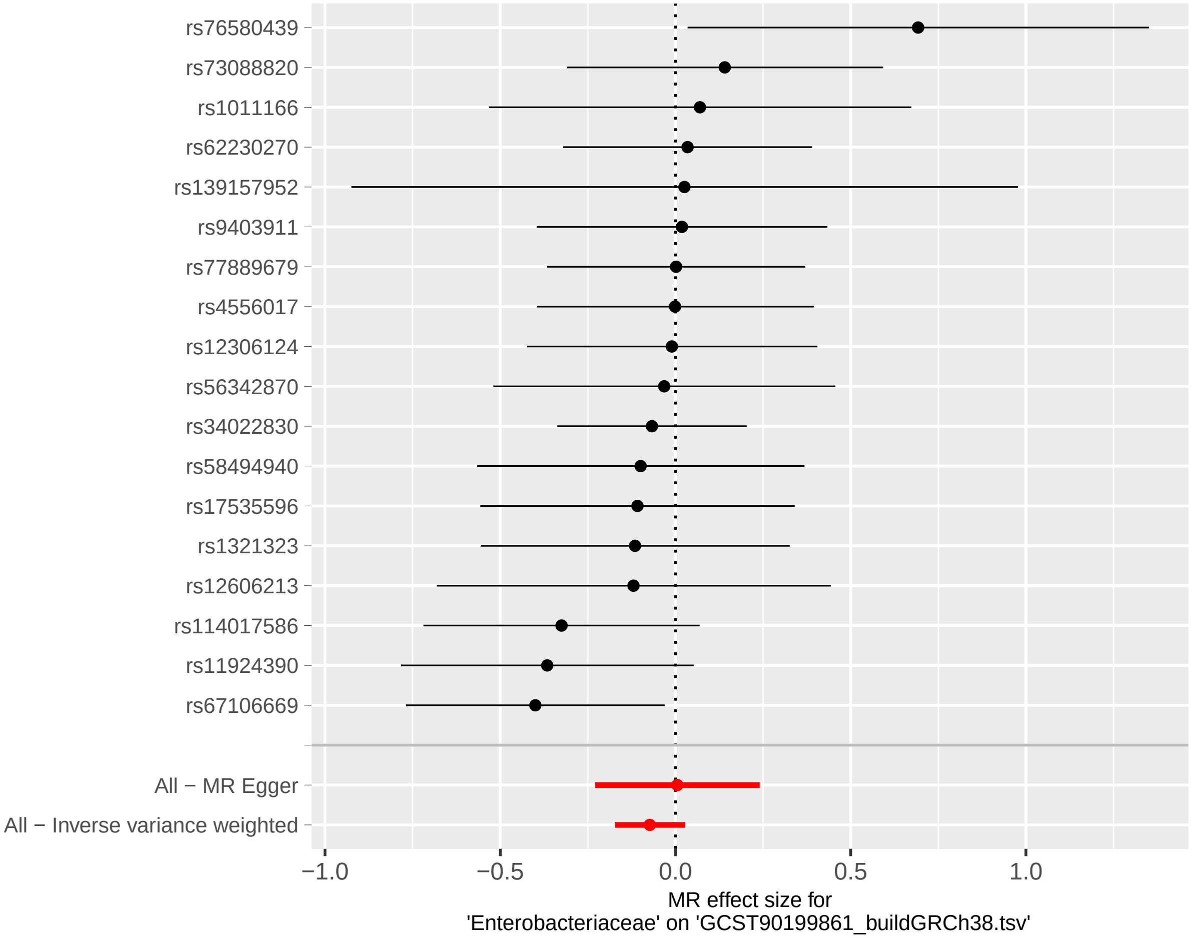
rs76580439
rs73088820
rs1011166
rs62230270
rs139157952
rs9403911
rs77889679
rs4556017
rs12306124
rs56342870
rs34022830
rs58494940
rs17535596
rs1321323
rs12606213
rs114017586
rs11924390
rs67106669
All − MR Egger
All − Inverse variance weighted
−1.0
−0.5
0.0
0.5
1.0
MR effect size for
'Enterobacteriaceae' on 'GCST90199861_buildGRCh38.tsv'

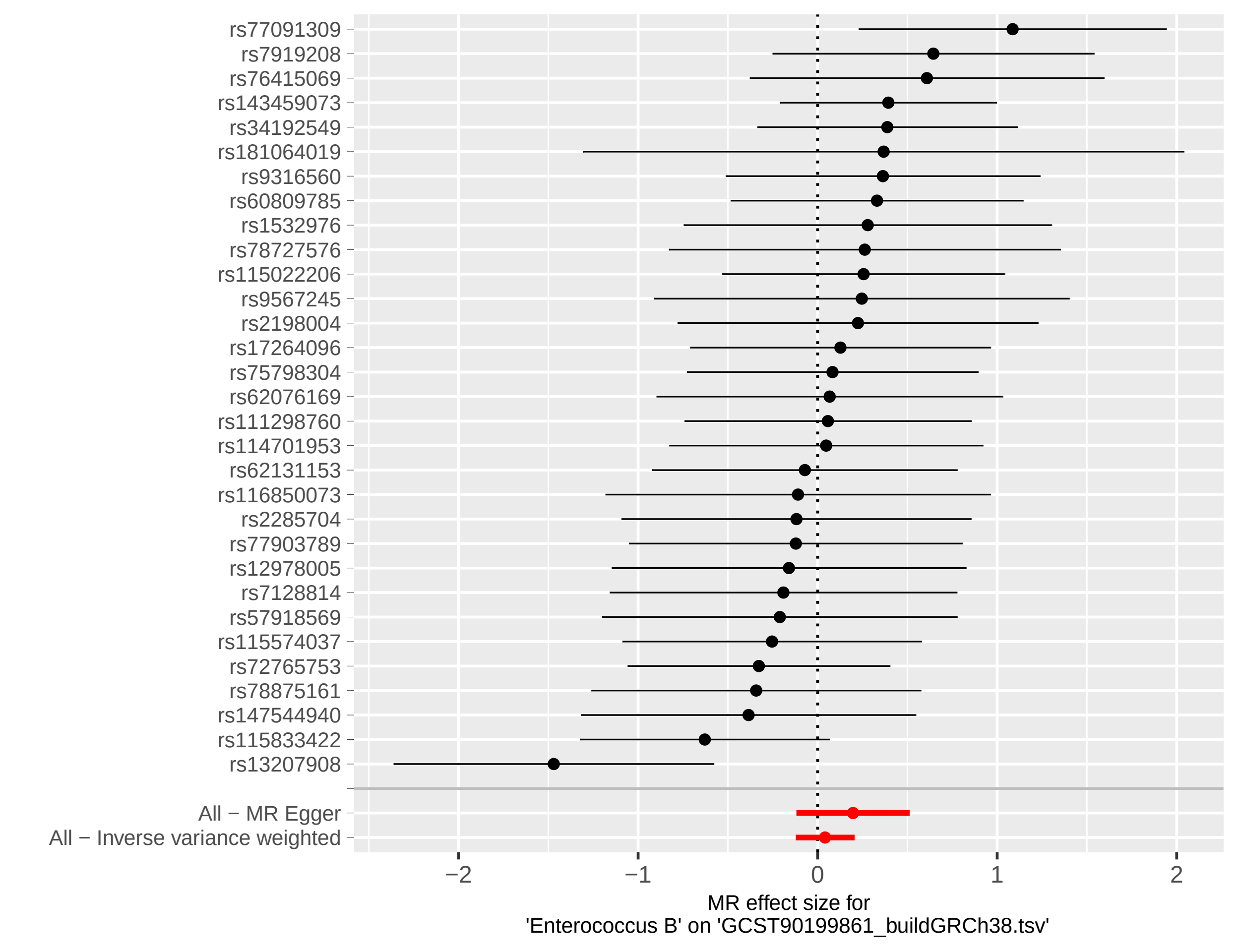

rs77091309
rs7919208
rs76415069
rs143459073
rs34192549
rs181064019
rs9316560
rs60809785
rs1532976
rs78727576
rs115022206
rs9567245
rs2198004
rs17264096
rs75798304
rs62076169
rs111298760
rs114701953
rs62131153
rs116850073
rs2285704
rs77903789
rs12978005
rs7128814
rs57918569
rs115574037
rs72765753
rs78875161
rs147544940
rs115833422
rs13207908
All − MR Egger
All − Inverse variance weighted
−2
−1
0
1
2
MR effect size for
'Enterococcus B' on 'GCST90199861_buildGRCh38.tsv'

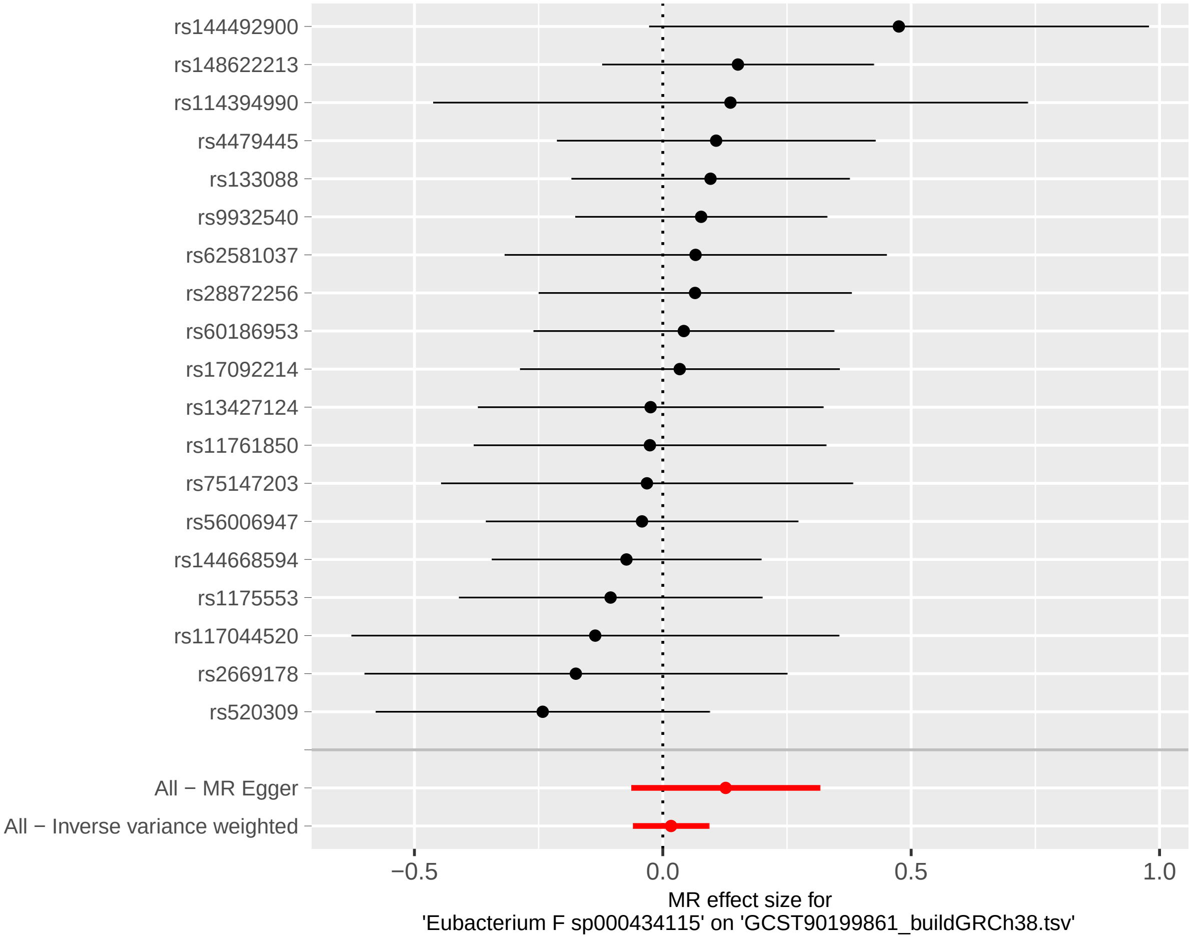

rs144492900
rs148622213
rs114394990
rs4479445
rs133088
rs9932540
rs62581037
rs28872256
rs60186953
rs17092214
rs13427124
rs11761850
rs75147203
rs56006947
rs144668594
rs1175553
rs117044520
rs2669178
rs520309
All − MR Egger
All − Inverse variance weighted
−0.5
0.0
0.5
1.0
MR effect size for
'Eubacterium F sp000434115' on 'GCST90199861_buildGRCh38.tsv'

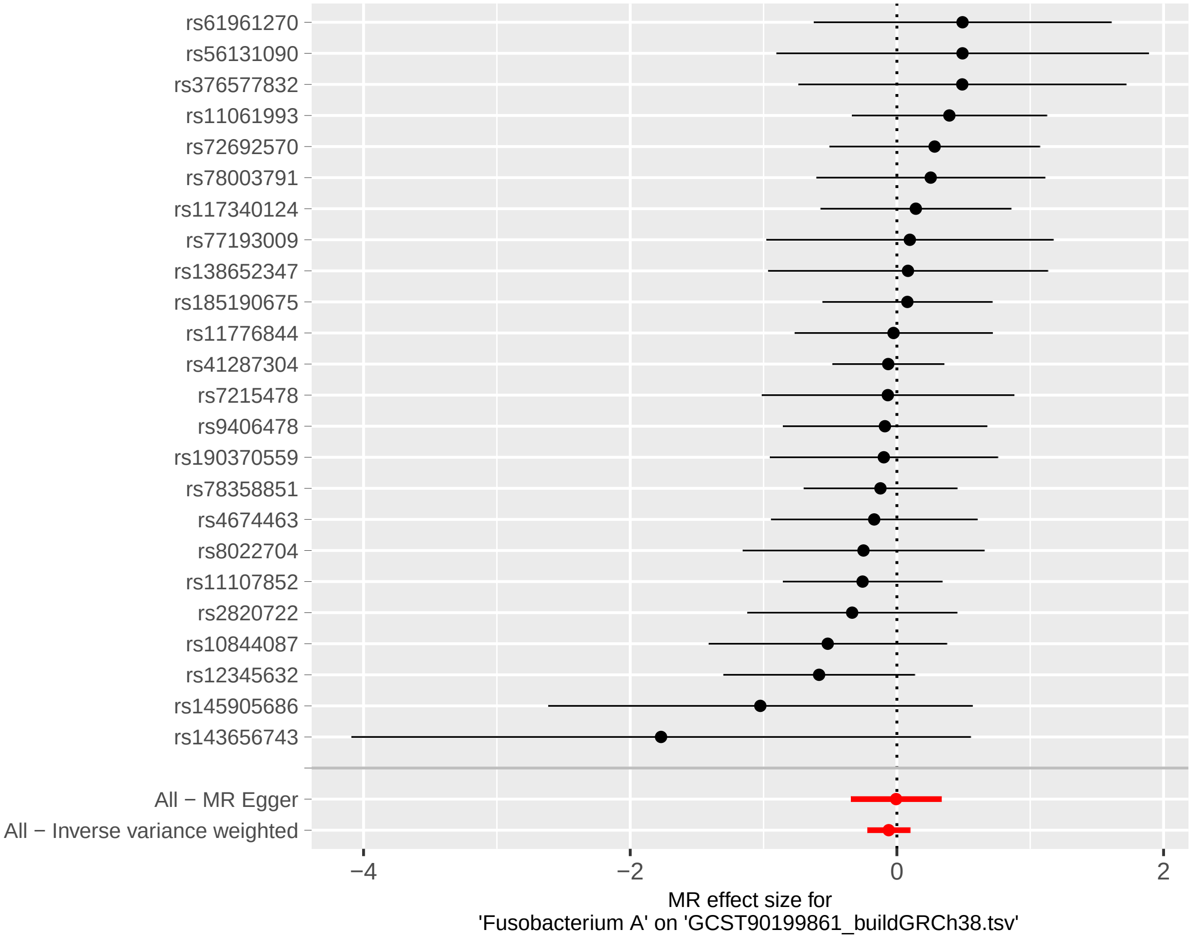

rs61961270
rs56131090
rs376577832
rs11061993
rs72692570
rs78003791
rs117340124
rs77193009
rs138652347
rs185190675
rs11776844
rs41287304
rs7215478
rs9406478
rs190370559
rs78358851
rs4674463
rs8022704
rs11107852
rs2820722
rs10844087
rs12345632
rs145905686
rs143656743
All − MR Egger
All − Inverse variance weighted
−4
−2
0
2
MR effect size for
'Fusobacterium A' on 'GCST90199861_buildGRCh38.tsv'

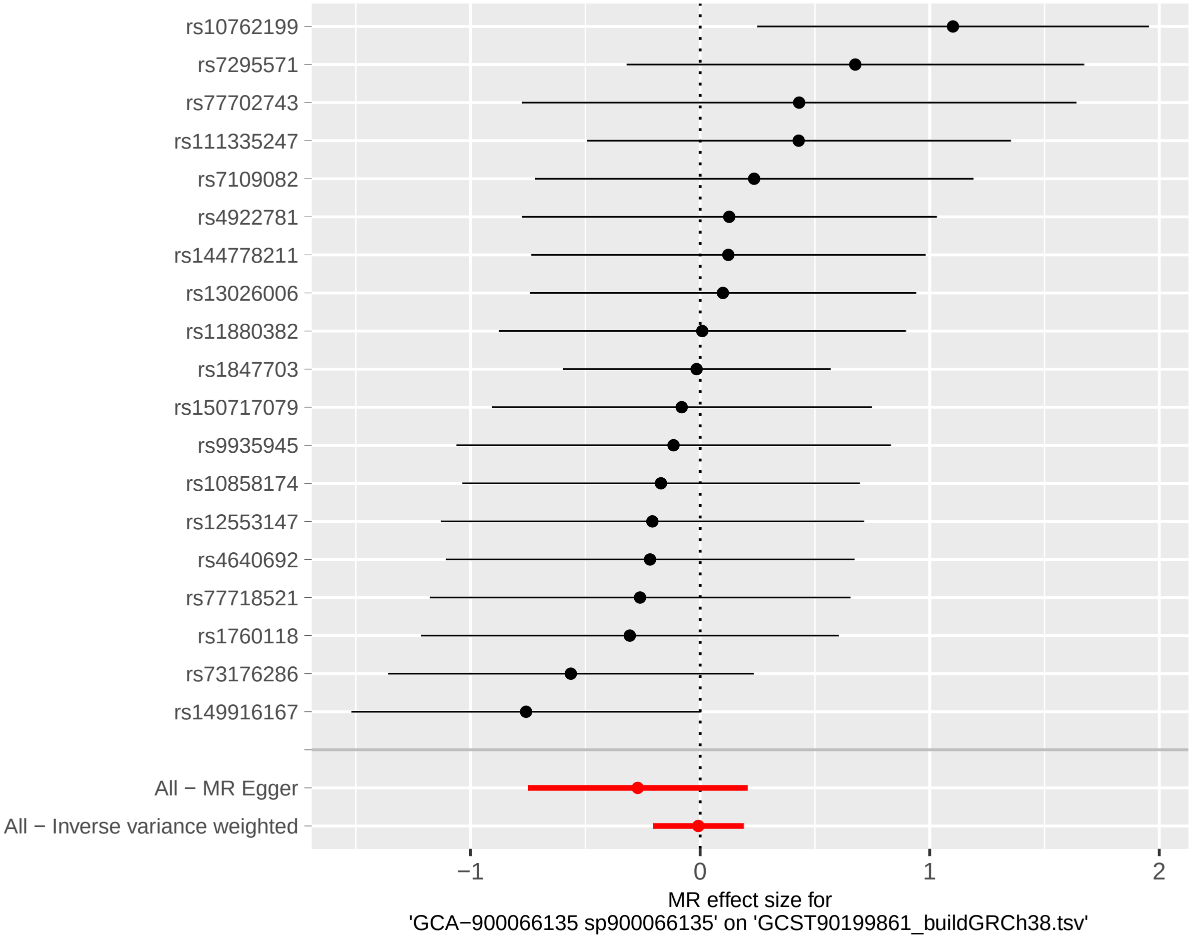
rs10762199
rs7295571
rs77702743
rs111335247
rs7109082
rs4922781
rs144778211
rs13026006
rs11880382
rs1847703
rs150717079
rs9935945
rs10858174
rs12553147
rs4640692
rs77718521
rs1760118
rs73176286
rs149916167
All − MR Egger
All − Inverse variance weighted
−1
0
1
2
MR effect size for
'GCA−900066135 sp900066135' on 'GCST90199861_buildGRCh38.tsv'

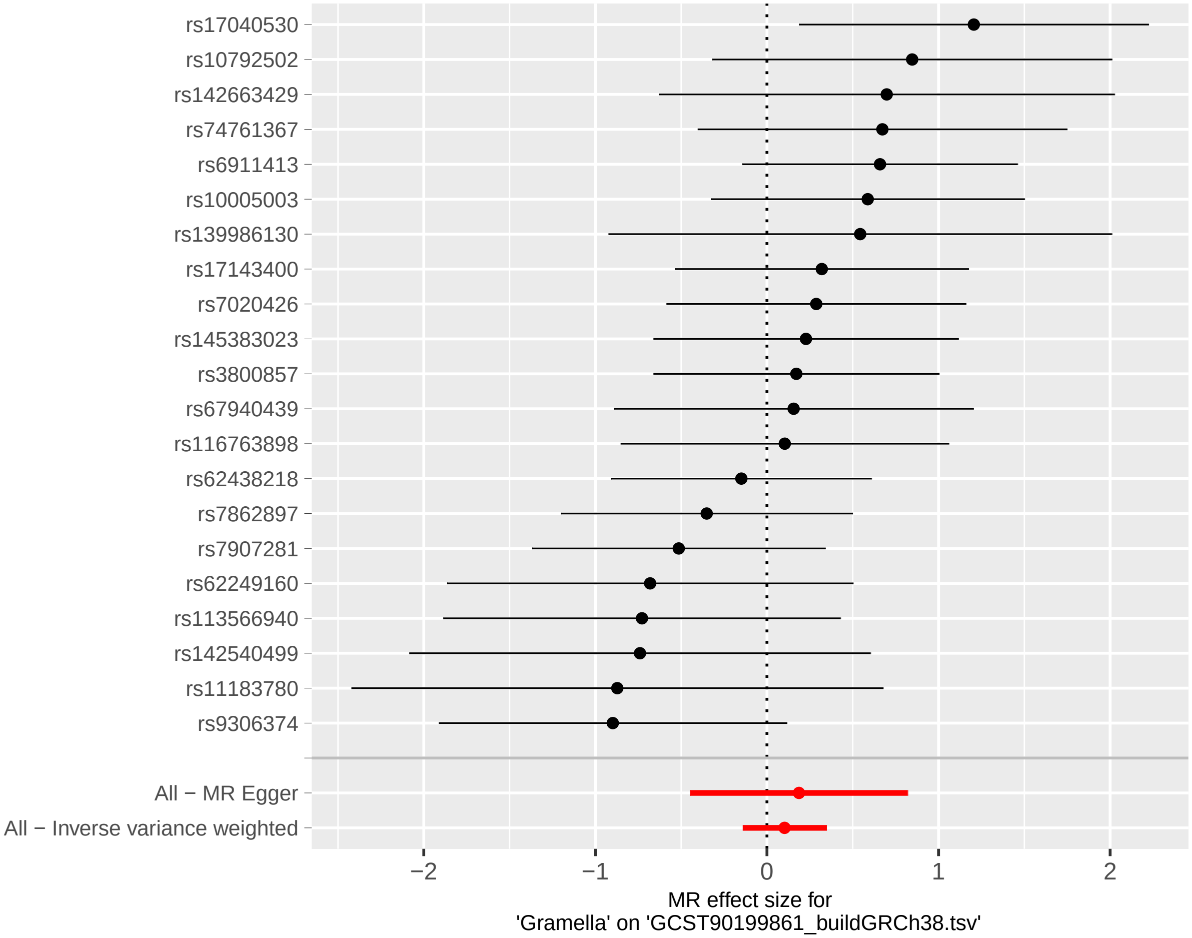
rs17040530
rs10792502
rs142663429
rs74761367
rs6911413
rs10005003
rs139986130
rs17143400
rs7020426
rs145383023
rs3800857
rs67940439
rs116763898
rs62438218
rs7862897
rs7907281
rs62249160
rs113566940
rs142540499
rs11183780
rs9306374
All − MR Egger
All − Inverse variance weighted
−2
−1
0
1
2
MR effect size for
'Gramella' on 'GCST90199861_buildGRCh38.tsv'

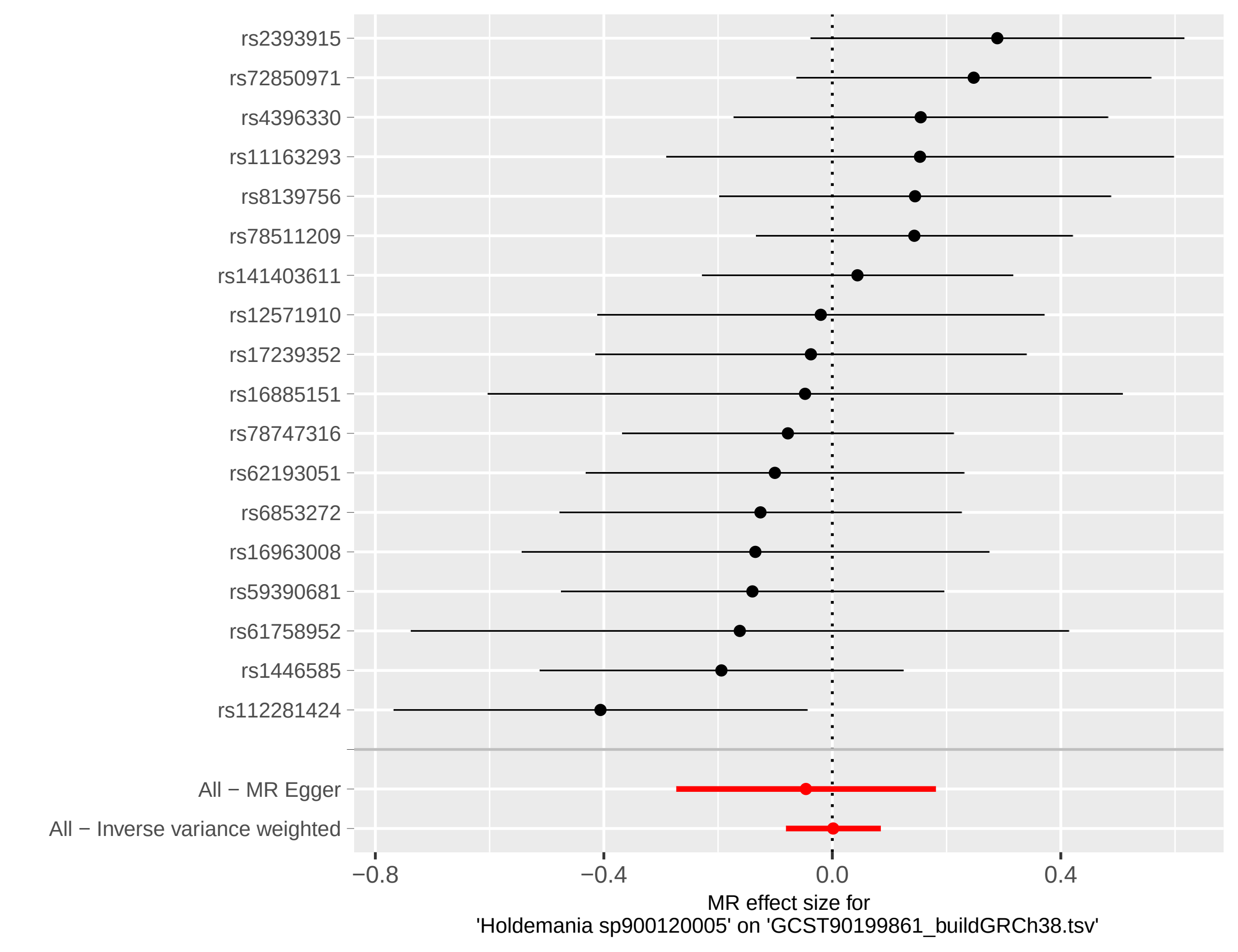
rs2393915
rs72850971
rs4396330
rs11163293
rs8139756
rs78511209
rs141403611
rs12571910
rs17239352
rs16885151
rs78747316
rs62193051
rs6853272
rs16963008
rs59390681
rs61758952
rs1446585
rs112281424
All − MR Egger
All − Inverse variance weighted
−0.8
−0.4
0.0
0.4
MR effect size for
'Holdemania sp900120005' on 'GCST90199861_buildGRCh38.tsv'

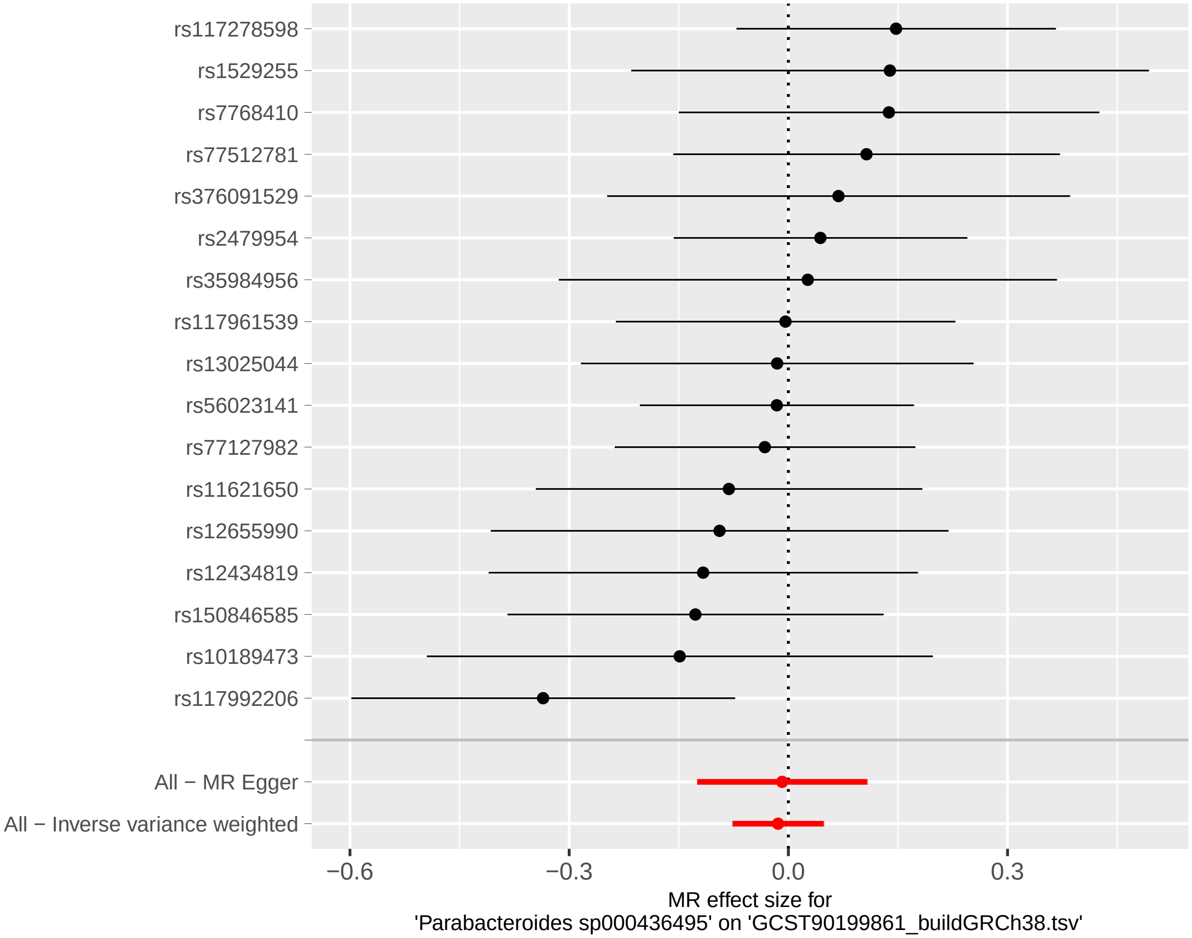

rs117278598
rs1529255
rs7768410
rs77512781
rs376091529
rs2479954
rs35984956
rs117961539
rs13025044
rs56023141
rs77127982
rs11621650
rs12655990
rs12434819
rs150846585
rs10189473
rs117992206
All − MR Egger
All − Inverse variance weighted
−0.6
−0.3
0.0
0.3
MR effect size for
'Parabacteroides sp000436495' on 'GCST90199861_buildGRCh38.tsv'

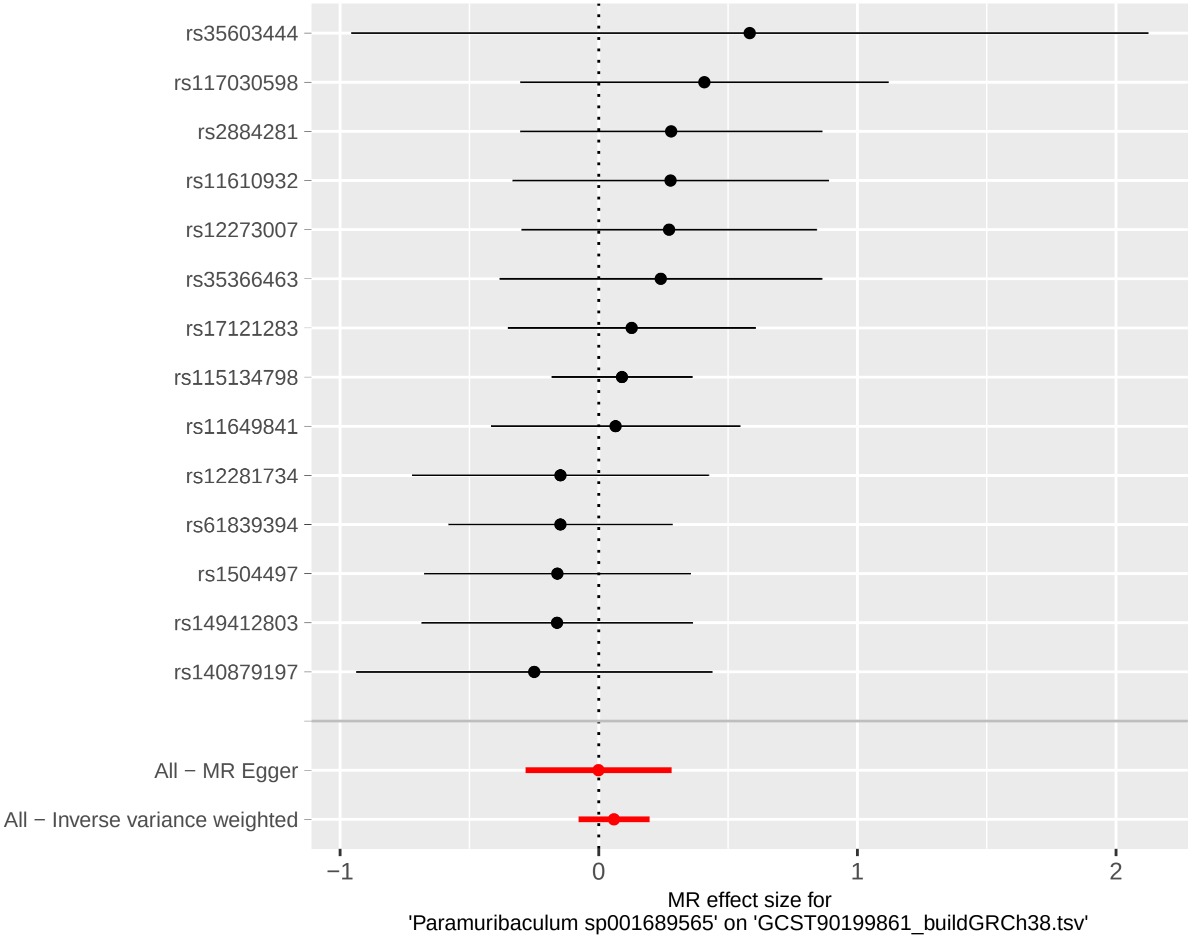
rs35603444
rs117030598
rs2884281
rs11610932
rs12273007
rs35366463
rs17121283
rs115134798
rs11649841
rs12281734
rs61839394
rs1504497
rs149412803
rs140879197
All − MR Egger
All − Inverse variance weighted
−1
0
1
2
MR effect size for
'Paramuribaculum sp001689565' on 'GCST90199861_buildGRCh38.tsv'

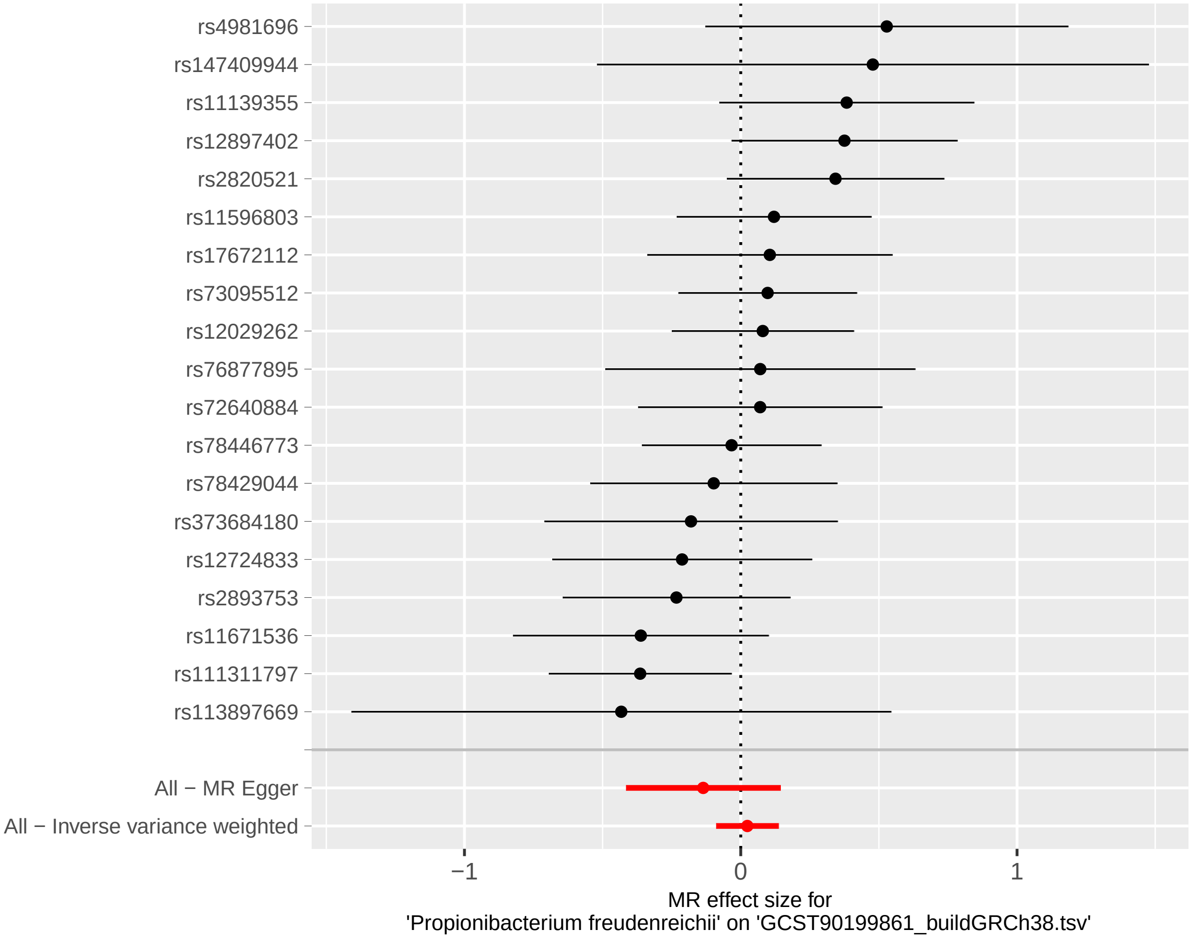
rs4981696
rs147409944
rs11139355
rs12897402
rs2820521
rs11596803
rs17672112
rs73095512
rs12029262
rs76877895
rs72640884
rs78446773
rs78429044
rs373684180
rs12724833
rs2893753
rs11671536
rs111311797
rs113897669
All − MR Egger
All − Inverse variance weighted
−1
0
1
MR effect size for
'Propionibacterium freudenreichii' on 'GCST90199861_buildGRCh38.tsv'

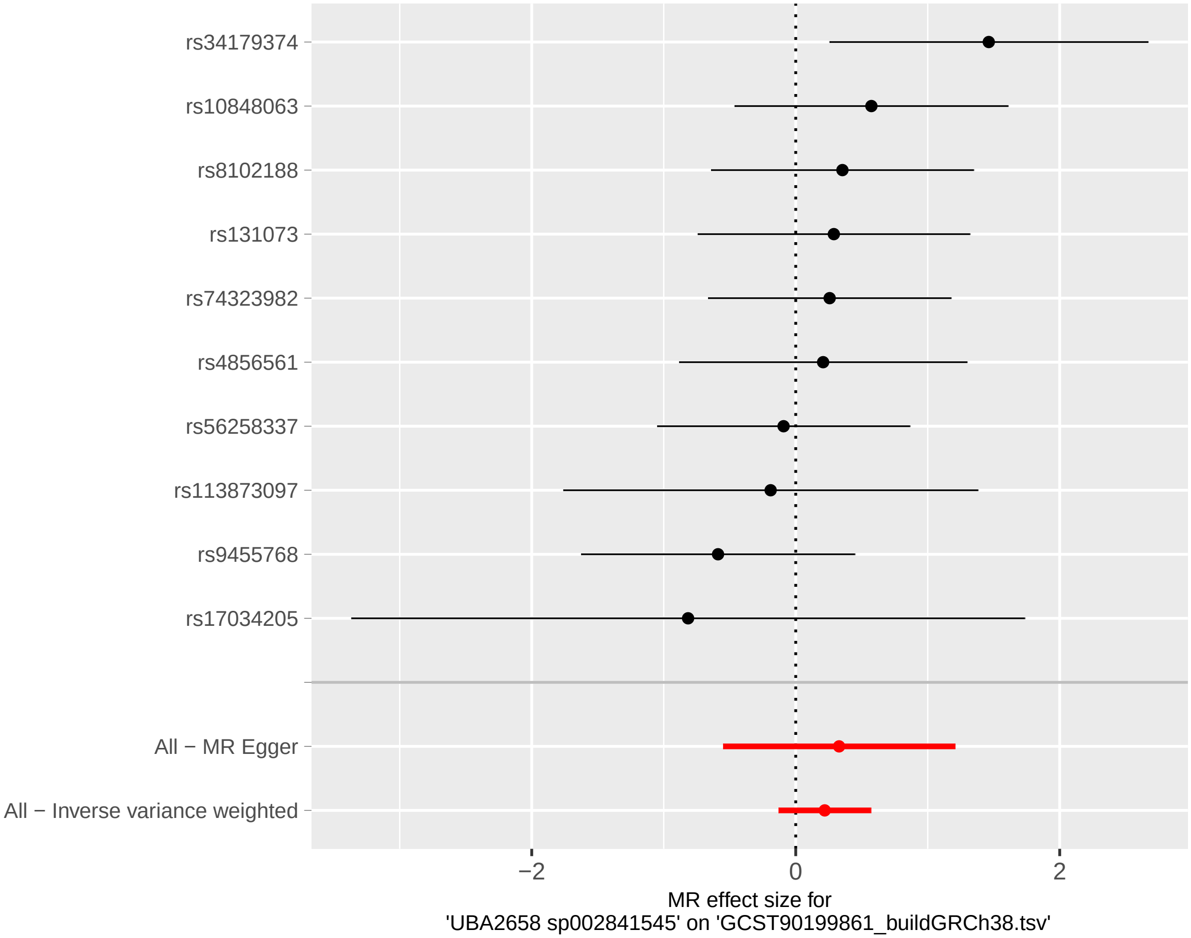
rs34179374
rs10848063
rs8102188
rs131073
rs74323982
rs4856561
rs56258337
rs113873097
rs9455768
rs17034205
All − MR Egger
All − Inverse variance weighted
−2
0
2
MR effect size for
'UBA2658 sp002841545' on 'GCST90199861_buildGRCh38.tsv'

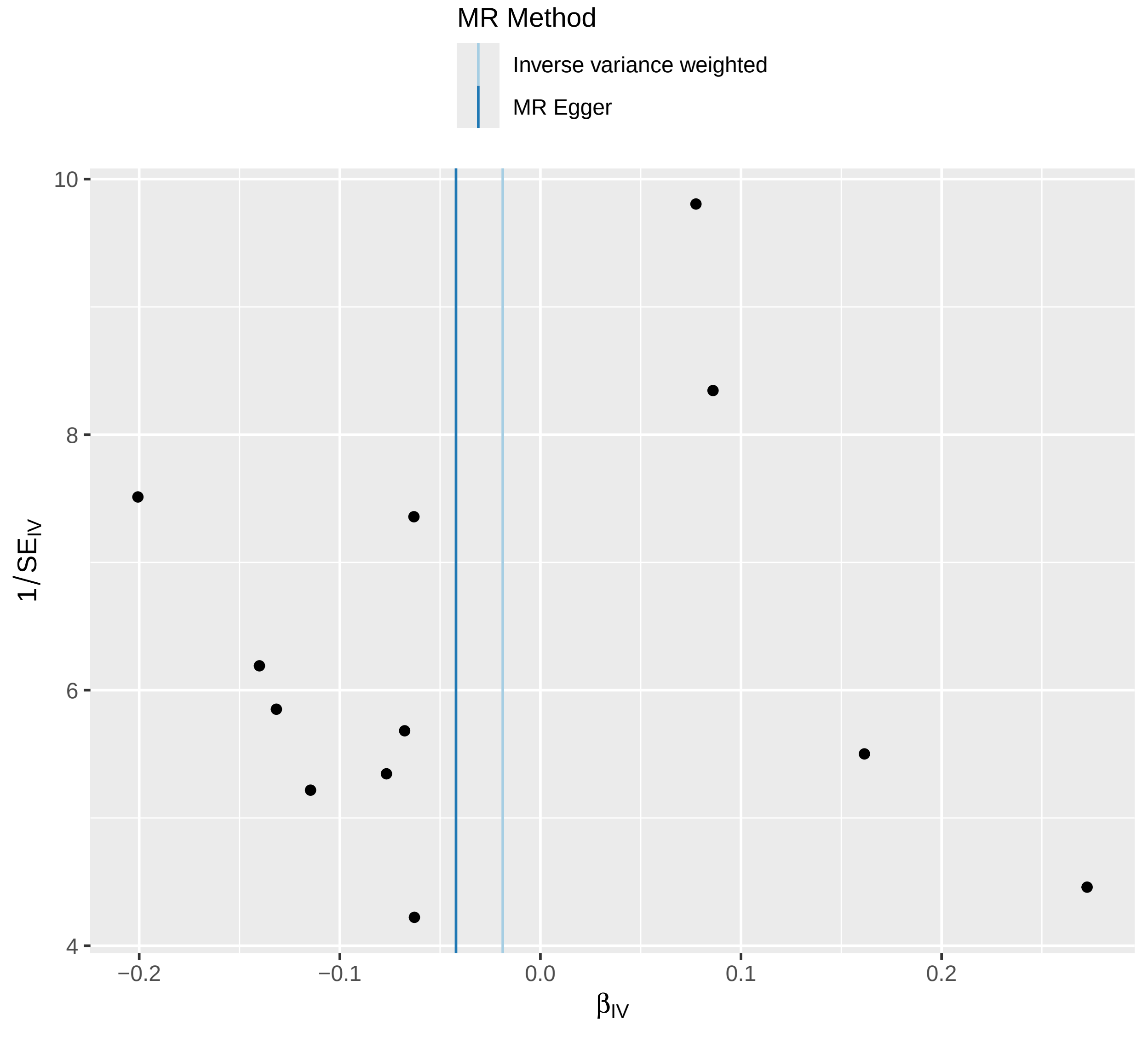

MR Method
Inverse variance weighted
MR Egger
10
8
6
4
1/SE_IV
−0.2
−0.1
0.0
0.1
0.2
β_IV

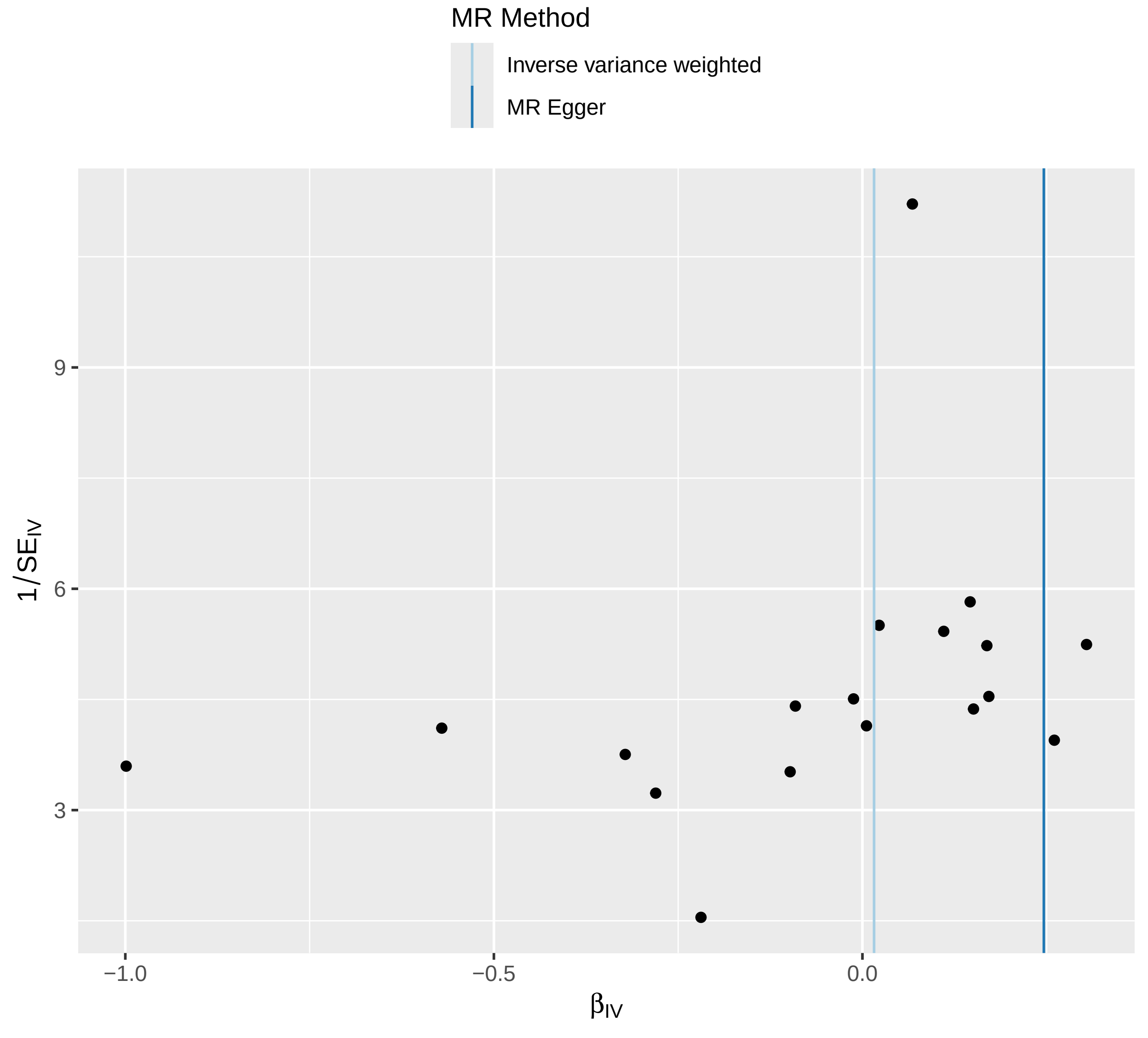

MR Method
Inverse variance weighted
MR Egger
9
6
3
1/SE$_{IV}$
−1.0
−0.5
0.0
β$_{IV}$

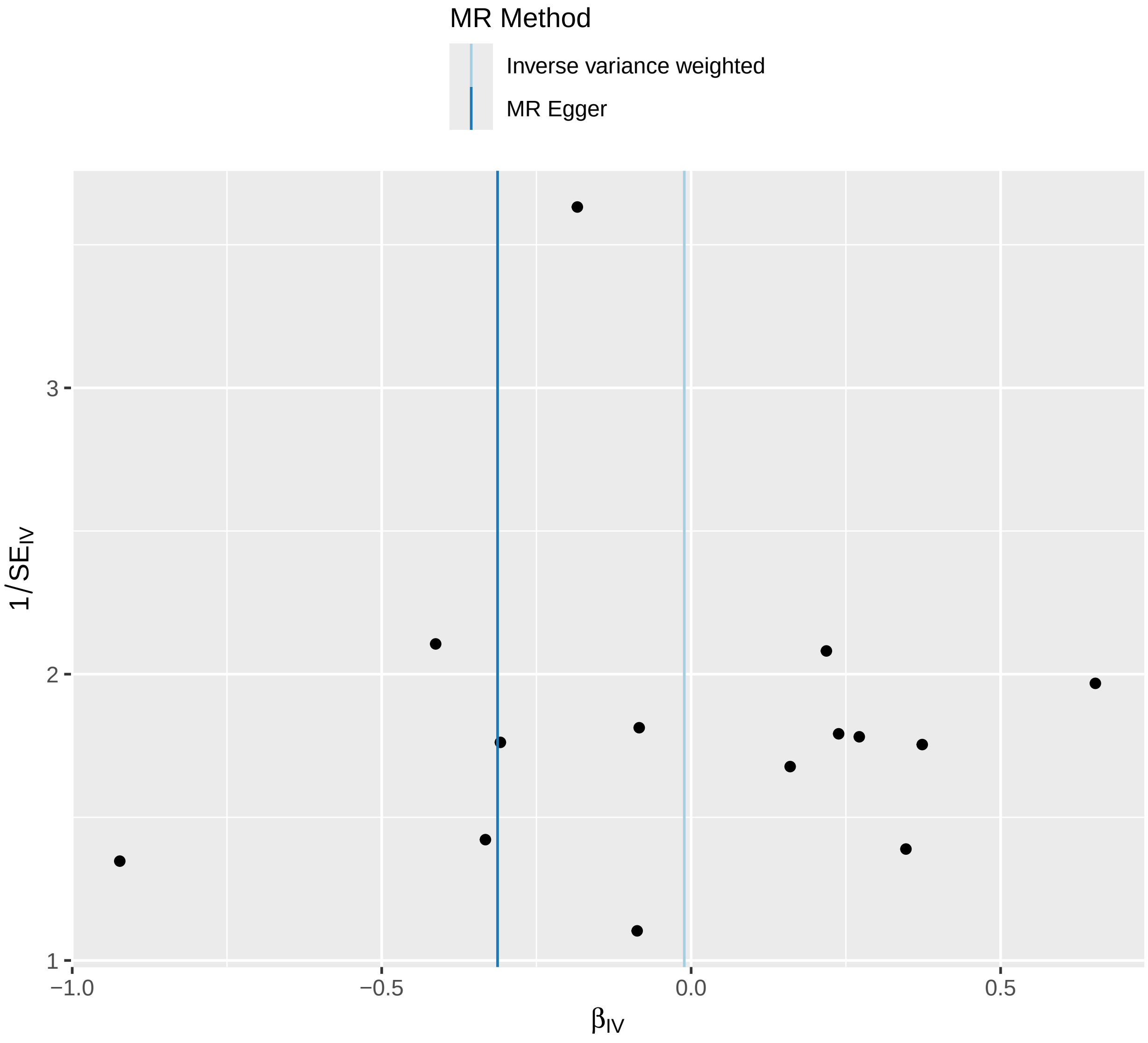
MR Method
Inverse variance weighted
MR Egger
3
2
1
−1.0
−0.5
0.0
0.5
1/SE_IV
β_IV

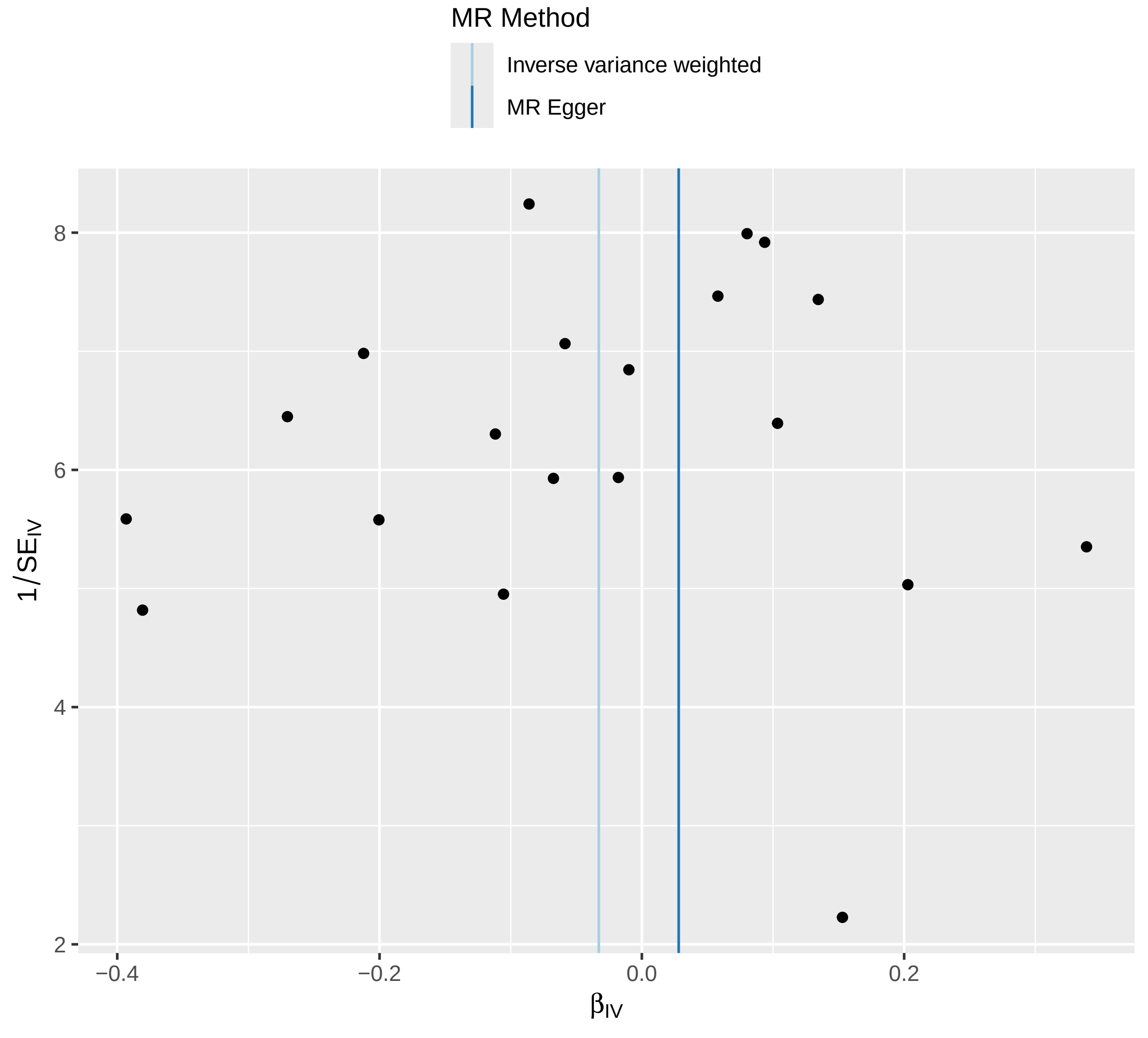
MR Method
Inverse variance weighted
MR Egger
8
6
4
2
1/SE_IV
−0.4
−0.2
0.0
0.2
β_IV

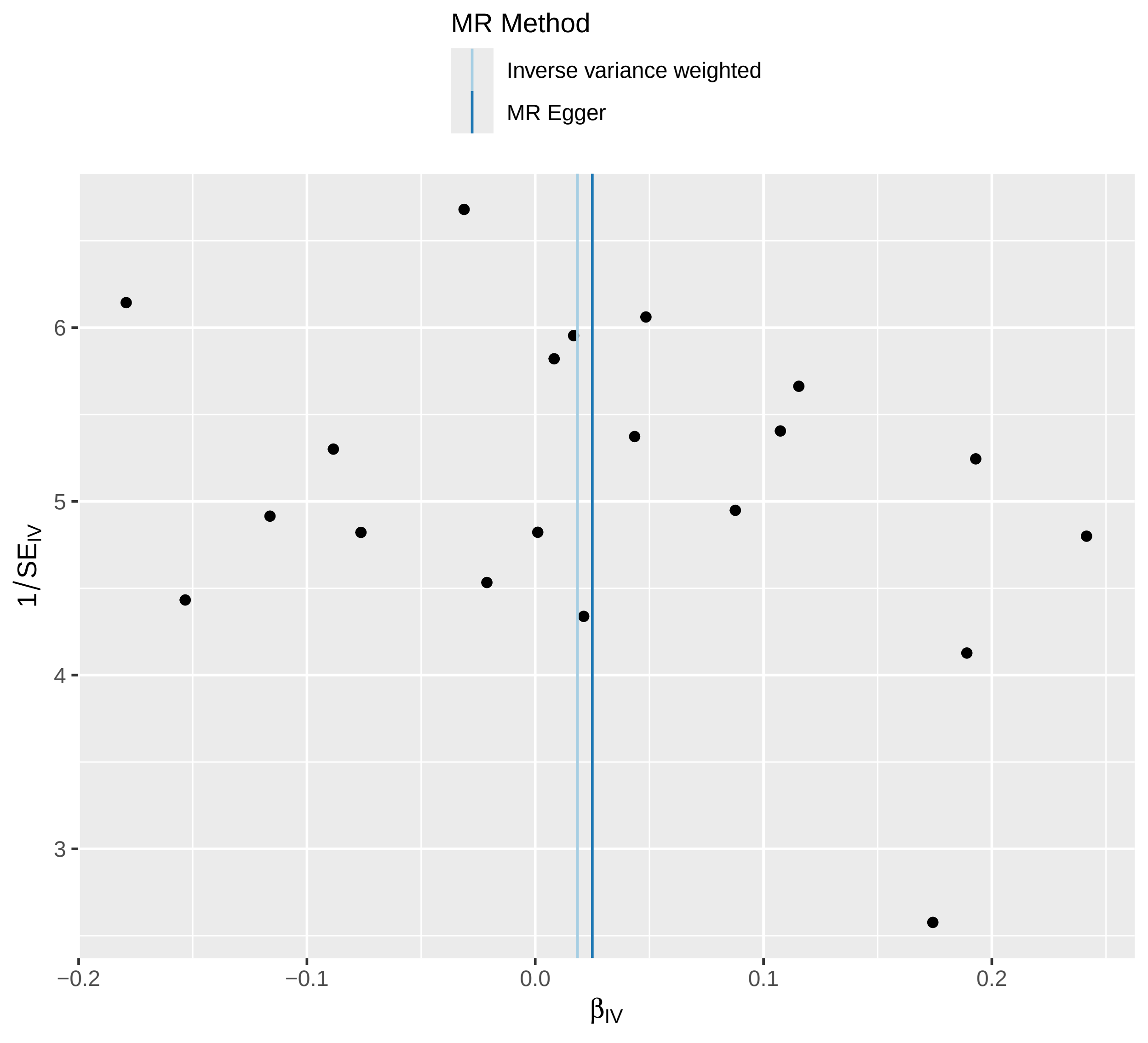

MR Method
Inverse variance weighted
MR Egger
1/SE$_{IV}$
6
5
4
3
−0.2
−0.1
0.0
0.1
0.2
β$_{IV}$

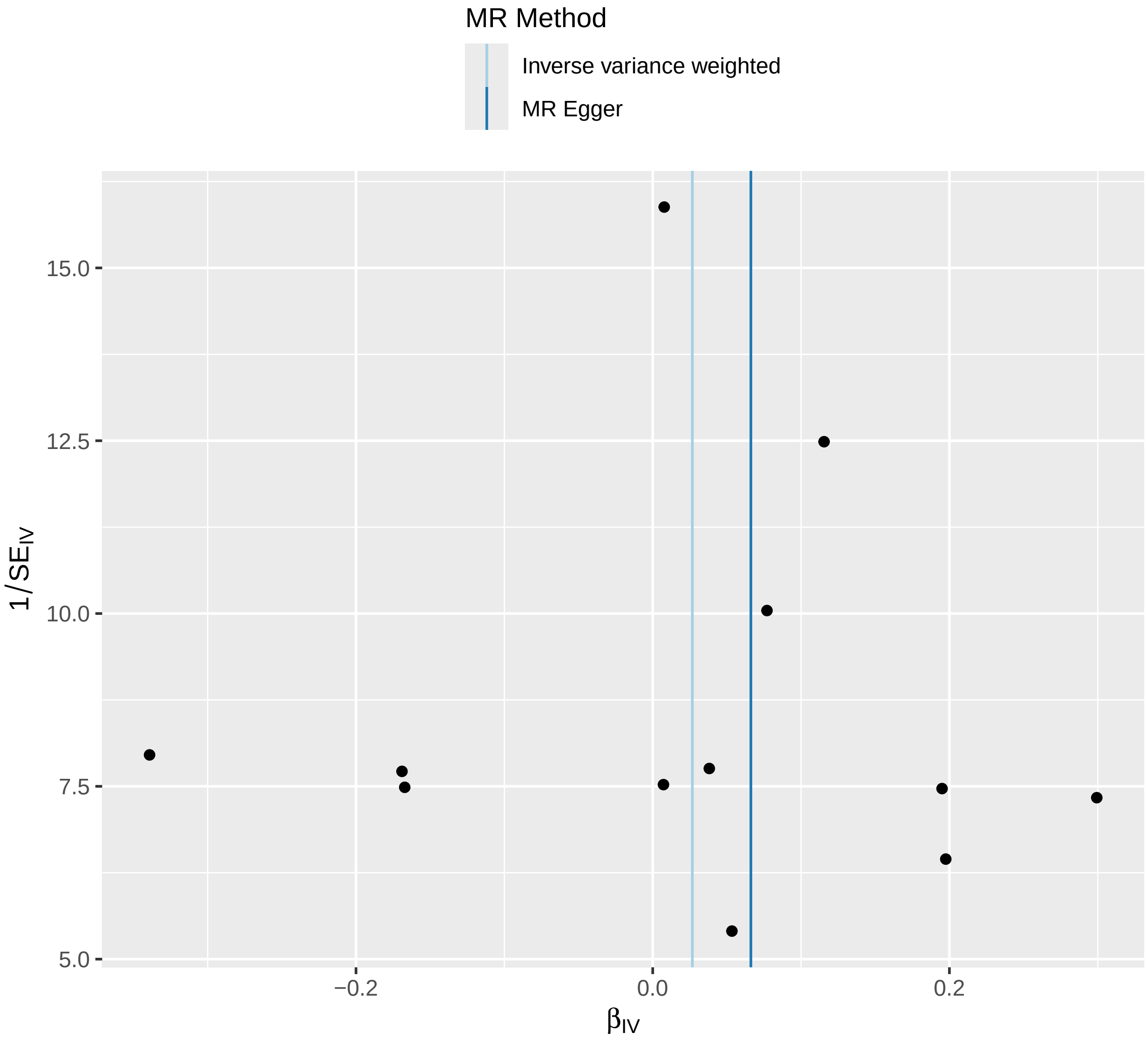

MR Method
Inverse variance weighted
MR Egger
15.0
12.5
10.0
7.5
5.0
$1/SE_{IV}$
−0.2
0.0
0.2
β_{IV}

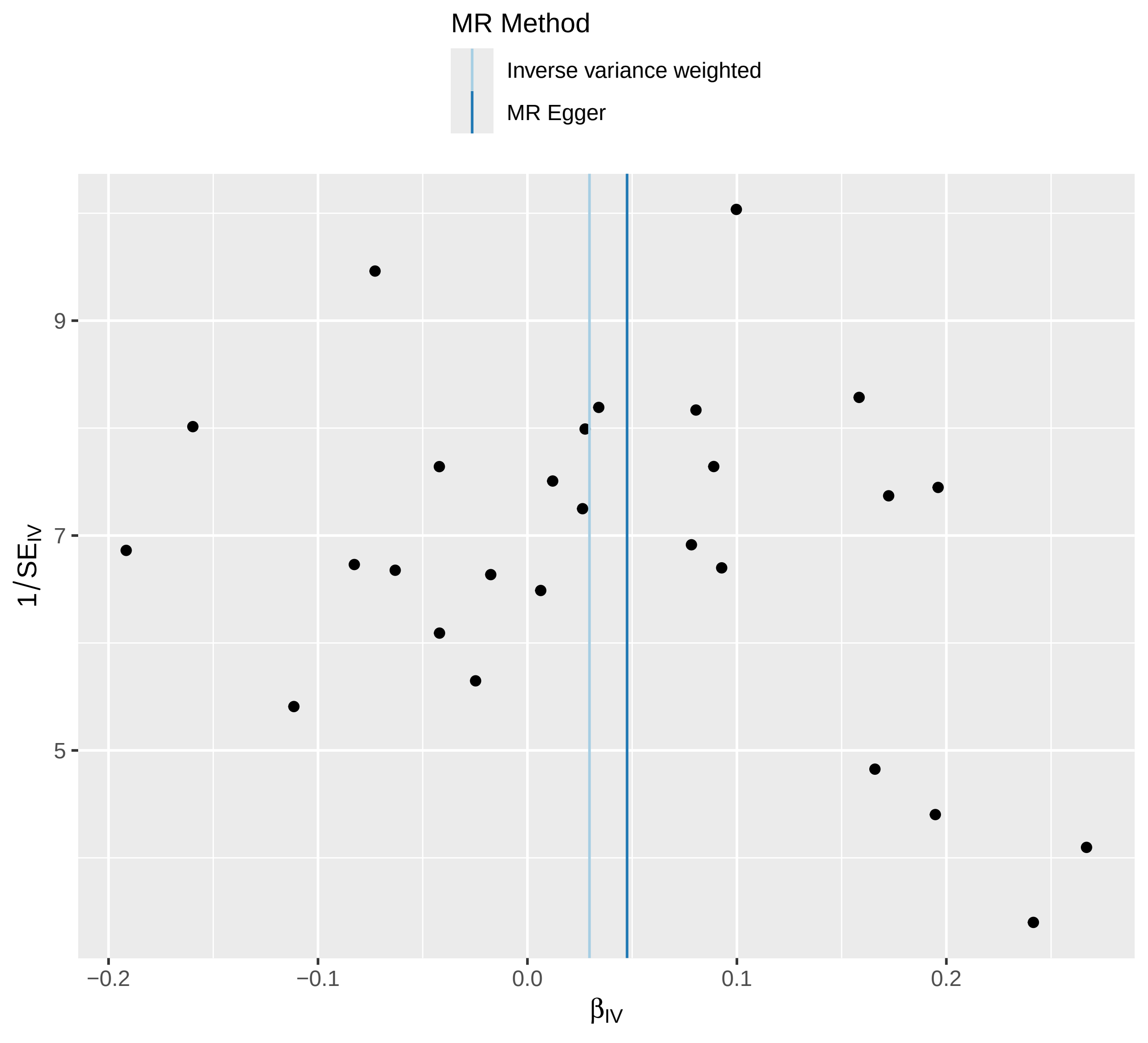
MR Method
Inverse variance weighted
MR Egger
9
7
5
1/SE_IV
−0.2
−0.1
0.0
0.1
0.2
β_IV

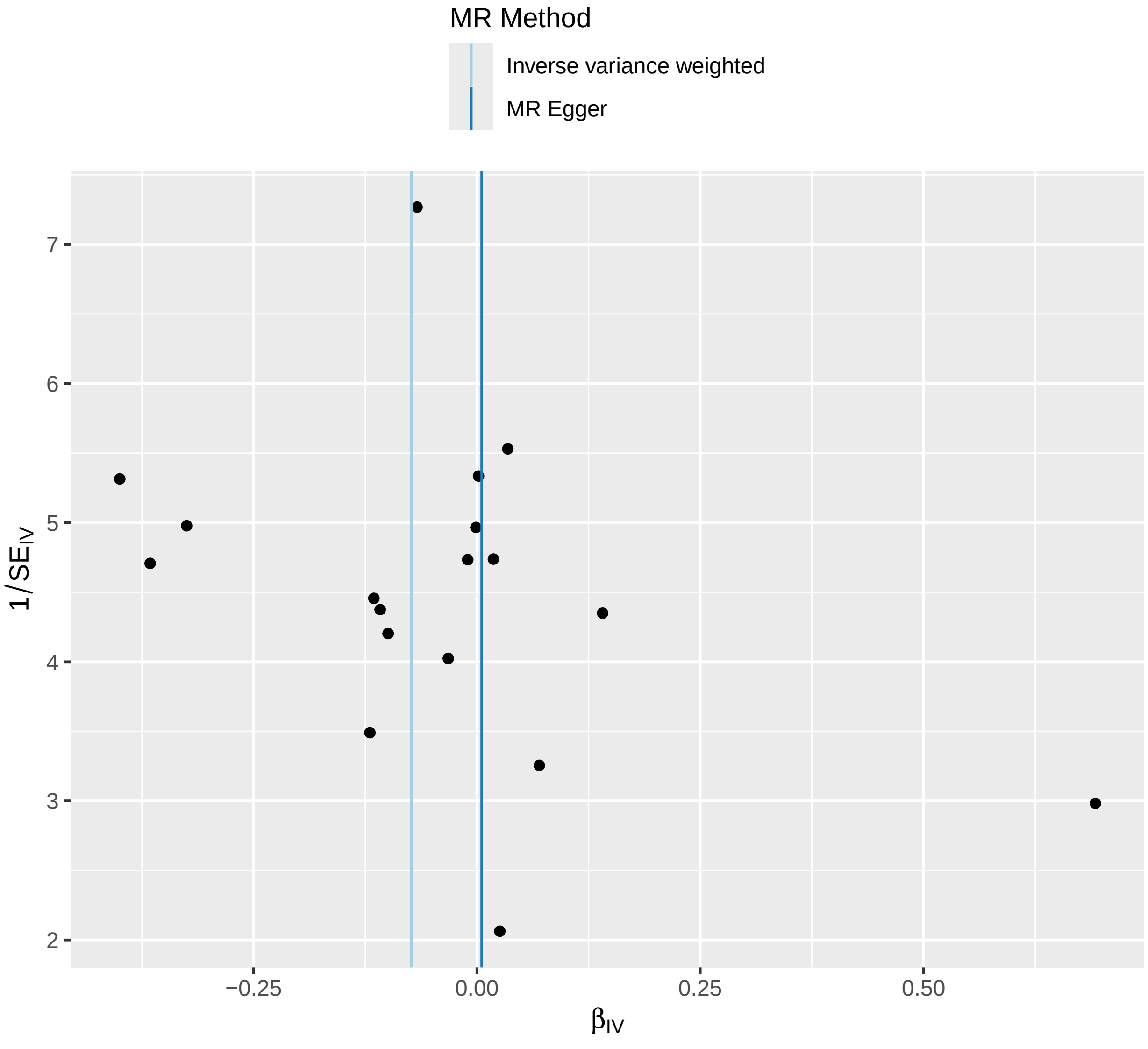

MR Method
Inverse variance weighted
MR Egger
1/SE_IV
7
6
5
4
3
2
−0.25
0.00
0.25
0.50
β_IV

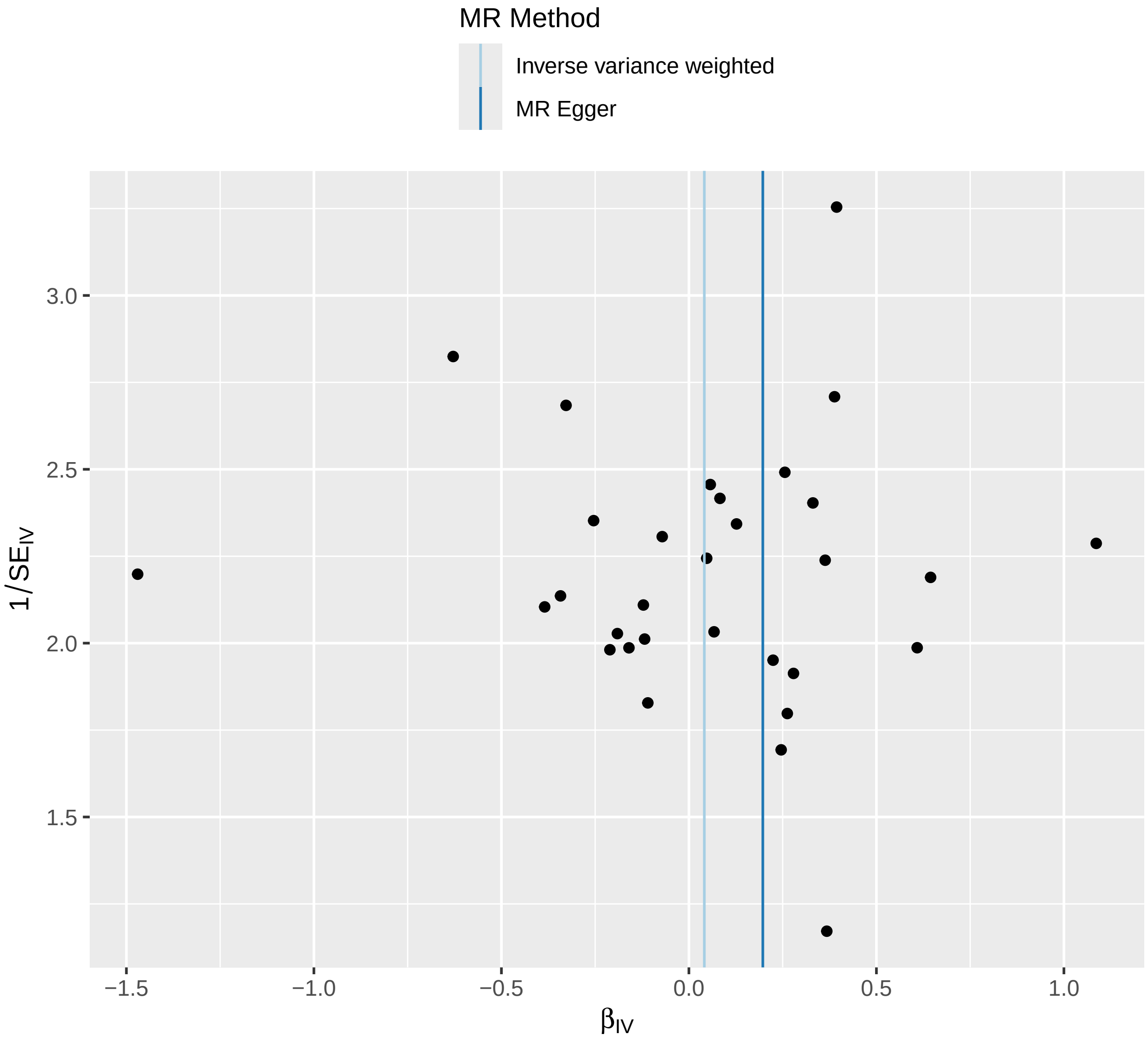

MR Method
Inverse variance weighted
MR Egger
3.0
2.5
2.0
1.5
1/SE$_{IV}$
−1.5
−1.0
−0.5
0.0
0.5
1.0
β$_{IV}$

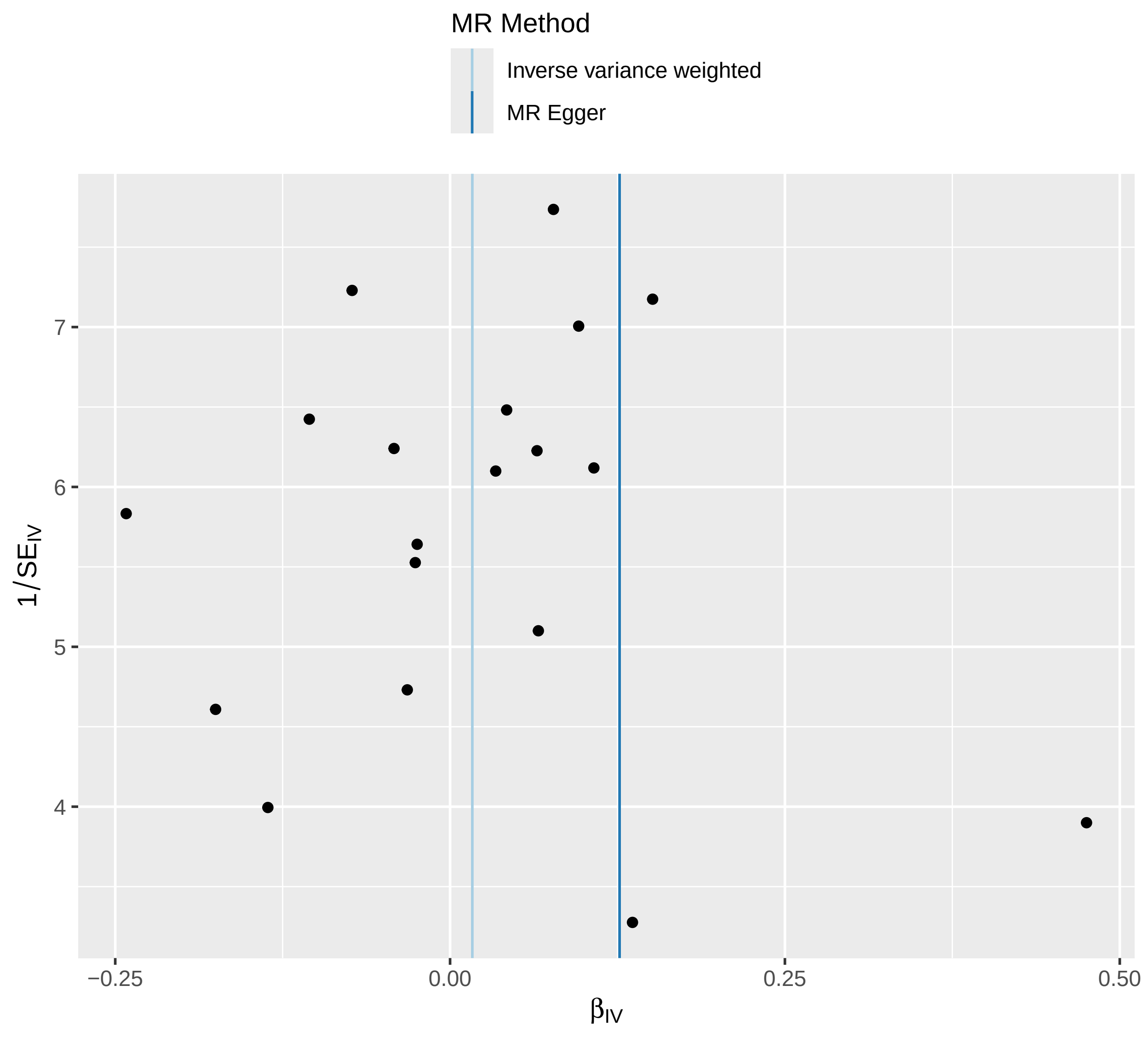
MR Method
Inverse variance weighted
MR Egger
7
6
5
4
1/SE$_{IV}$
−0.25
0.00
0.25
0.50
β$_{IV}$

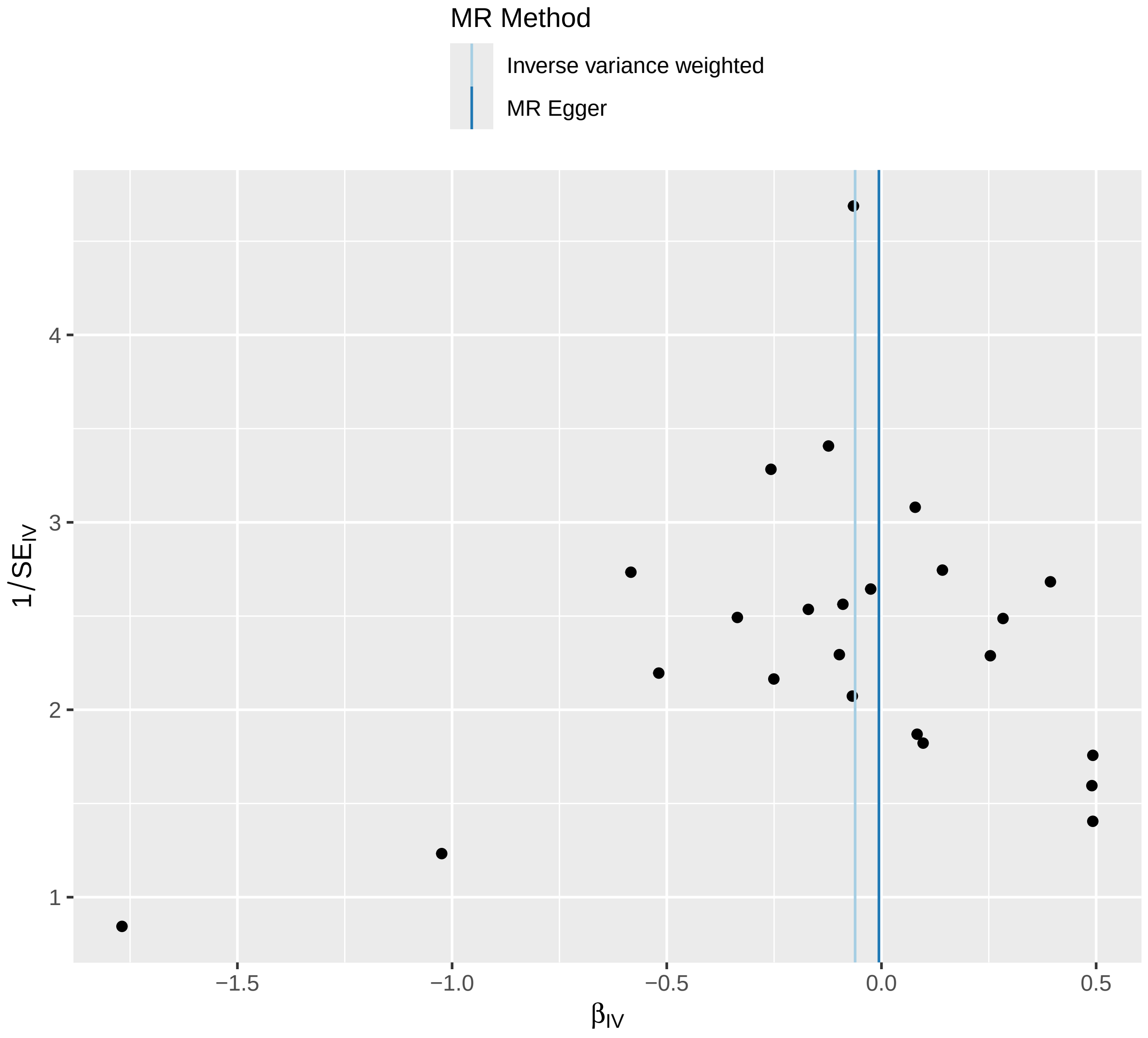
MR Method
Inverse variance weighted
MR Egger
1/SE_IV
β_IV
4
3
2
1
−1.5
−1.0
−0.5
0.0
0.5

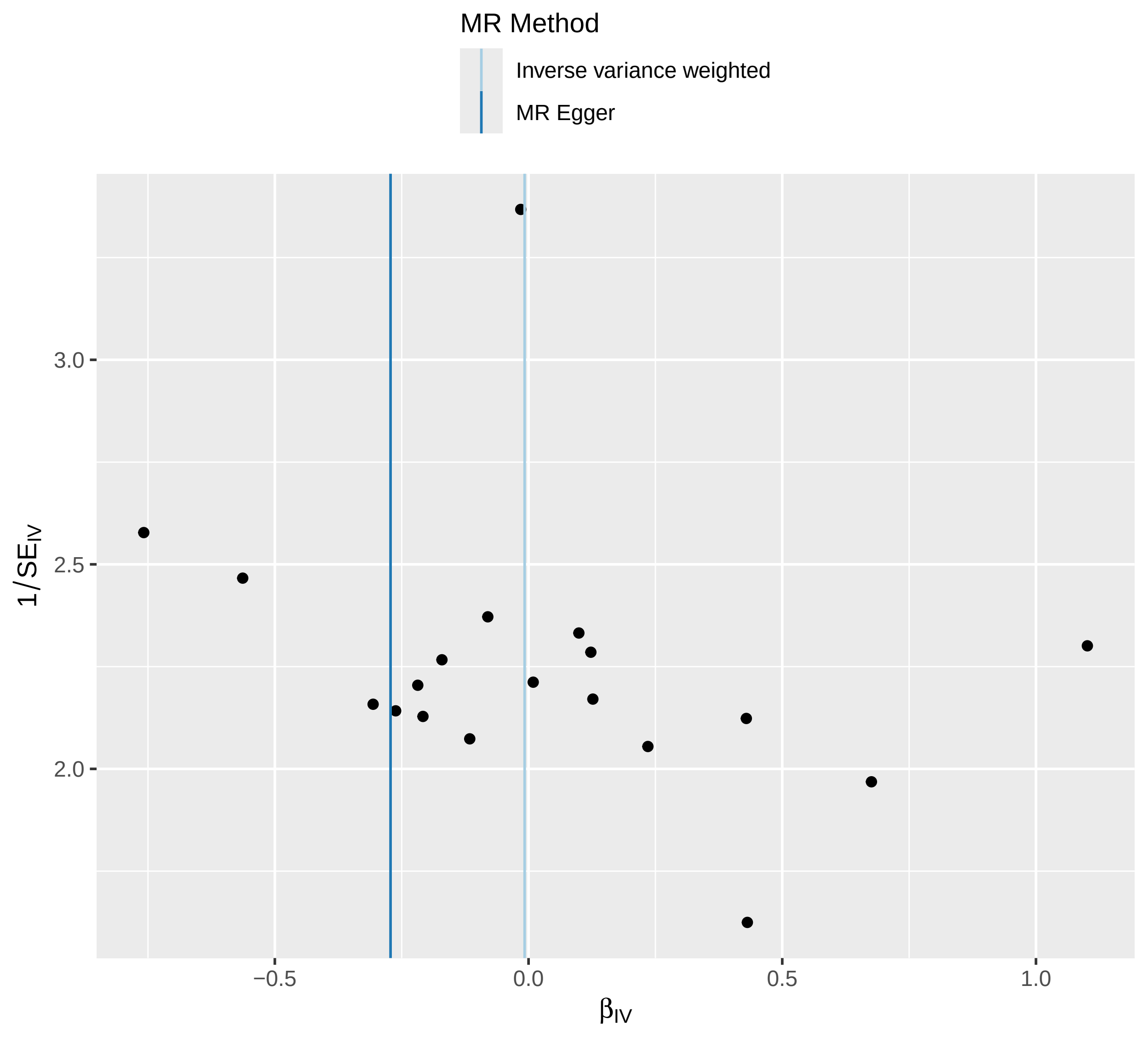
MR Method
Inverse variance weighted
MR Egger
3.0
2.5
2.0
$1/SE_{IV}$
−0.5
0.0
0.5
1.0
β_{IV}

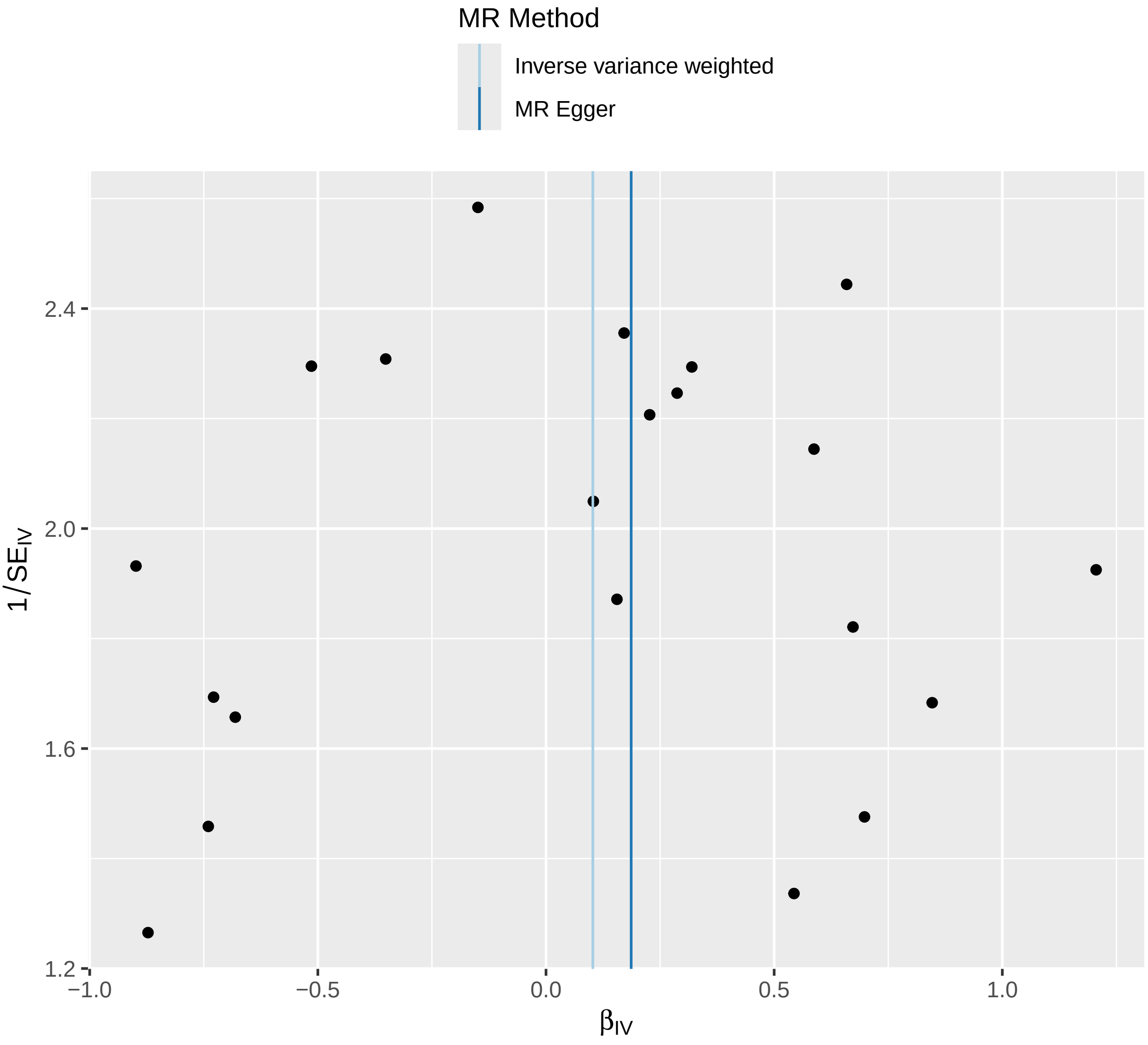
MR Method
Inverse variance weighted
MR Egger
2.4
2.0
1.6
1.2
$1/SE_{IV}$
−1.0
−0.5
0.0
0.5
1.0
β_{IV}

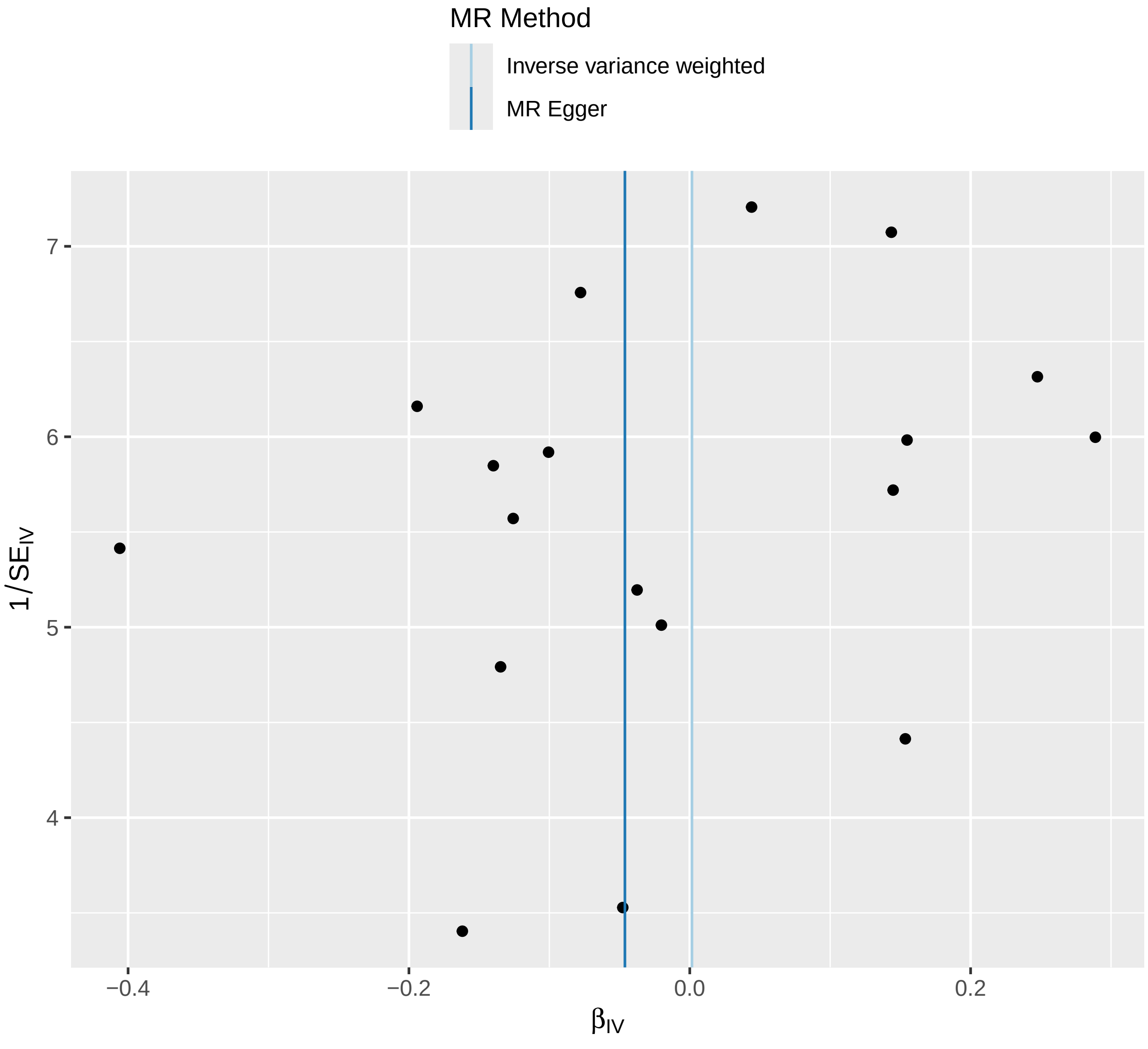

MR Method
Inverse variance weighted
MR Egger
7
6
5
4
1/SE_IV
−0.4
−0.2
0.0
0.2
β_IV

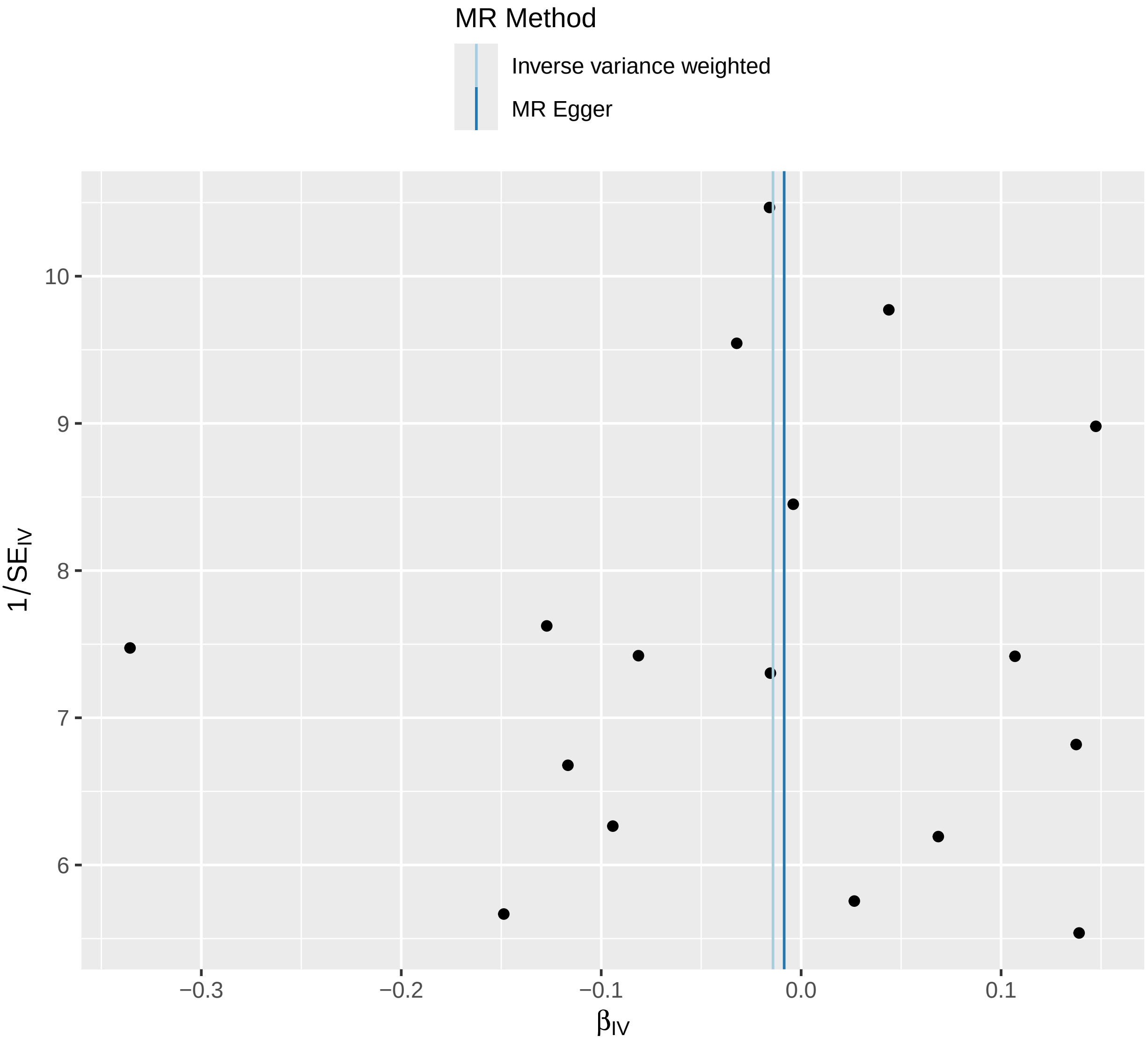
MR Method
Inverse variance weighted
MR Egger
10
9
8
7
6
1/SE$_{IV}$
−0.3
−0.2
−0.1
0.0
0.1
β$_{IV}$

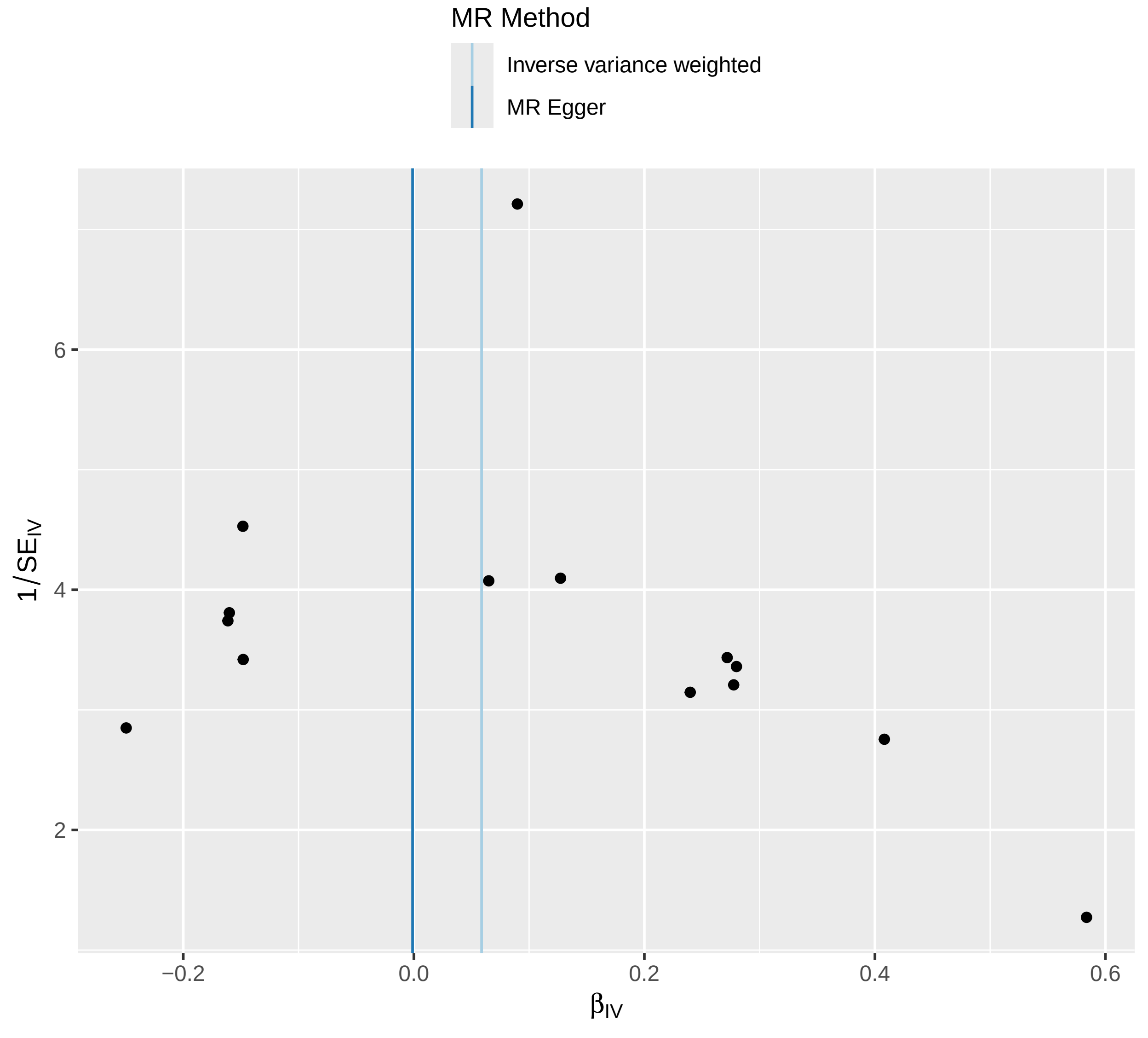

MR Method
Inverse variance weighted
MR Egger
6
4
2
1/SE_IV
−0.2
0.0
0.2
0.4
0.6
β_IV

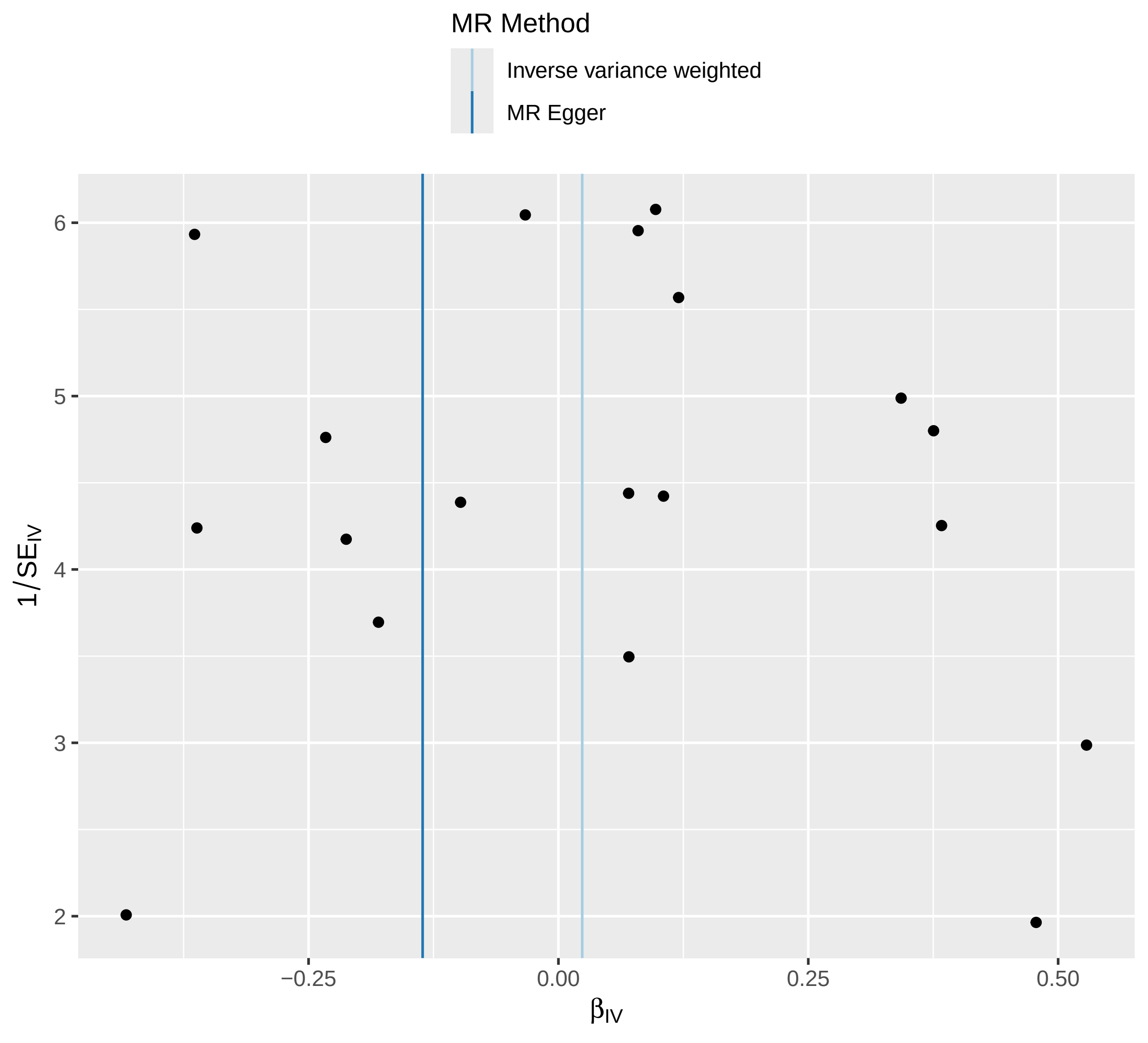
MR Method
Inverse variance weighted
MR Egger
6
5
4
3
2
1/SE_IV
−0.25
0.00
0.25
0.50
β_IV

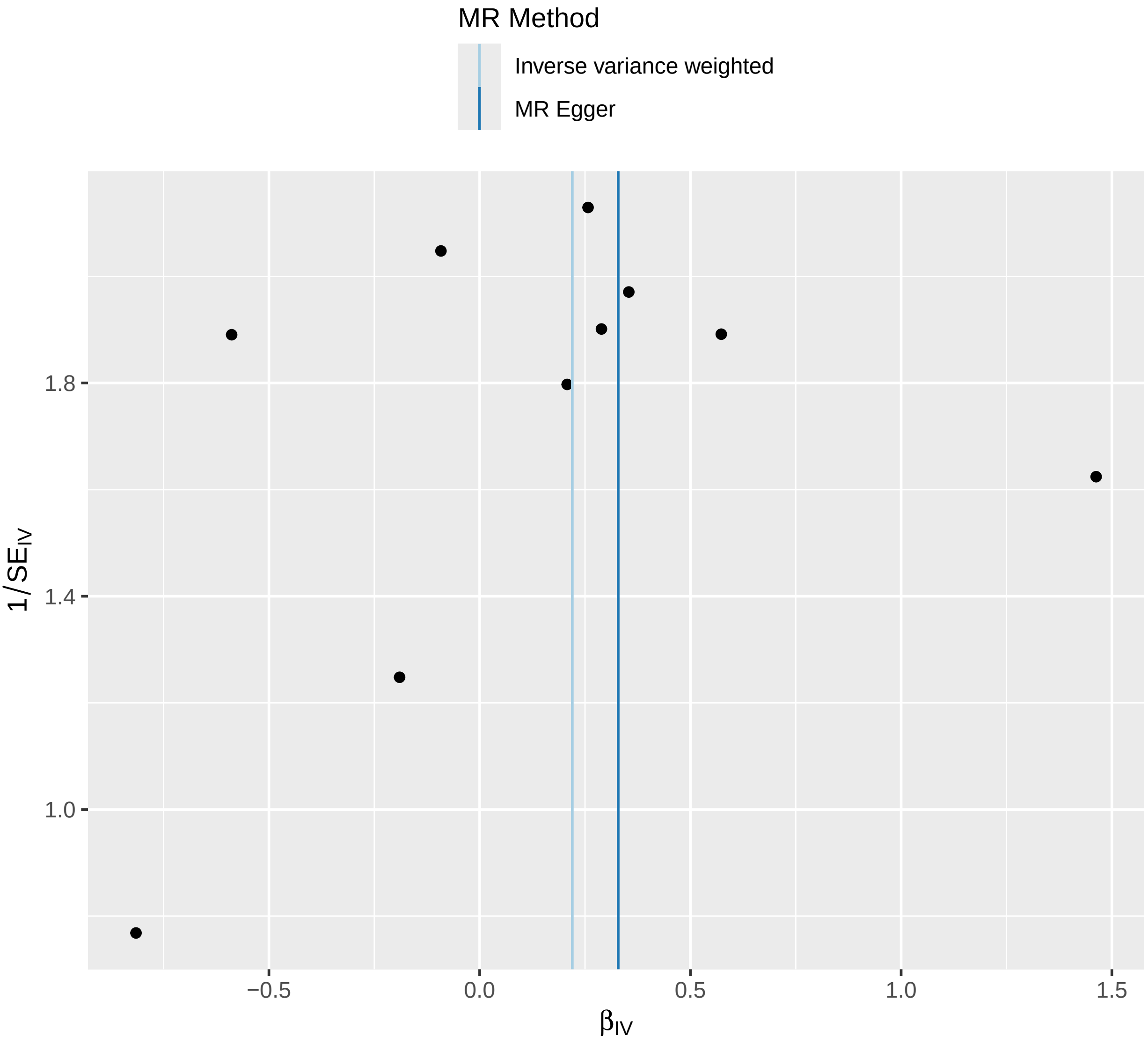
MR Method
Inverse variance weighted
MR Egger
1.8
1.4
1.0
1/SE_IV
−0.5
0.0
0.5
1.0
1.5
β_IV

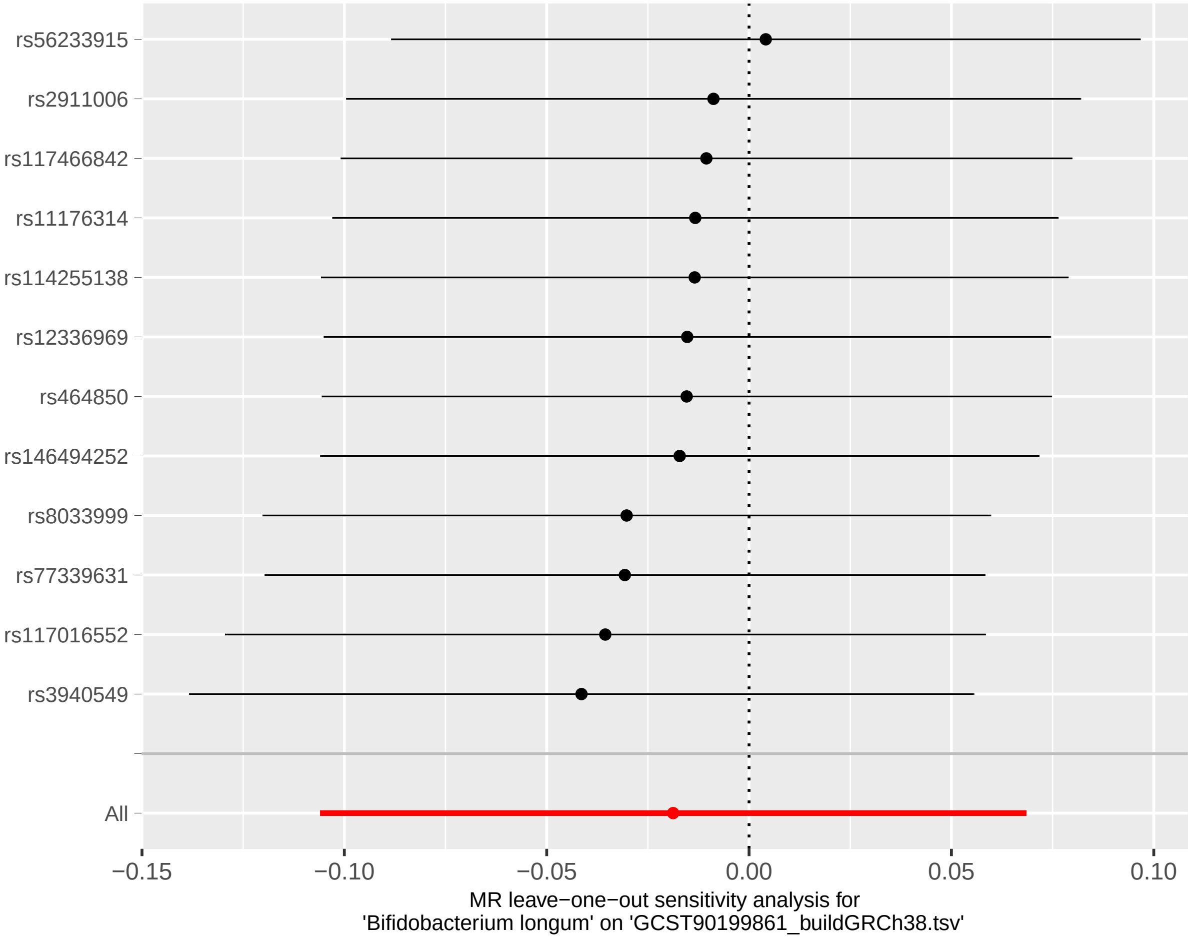
rs56233915
rs2911006
rs117466842
rs11176314
rs114255138
rs12336969
rs464850
rs146494252
rs8033999
rs77339631
rs117016552
rs3940549
All
−0.15
−0.10
−0.05
0.00
0.05
0.10
MR leave−one−out sensitivity analysis for
'Bifidobacterium longum' on 'GCST90199861_buildGRCh38.tsv'

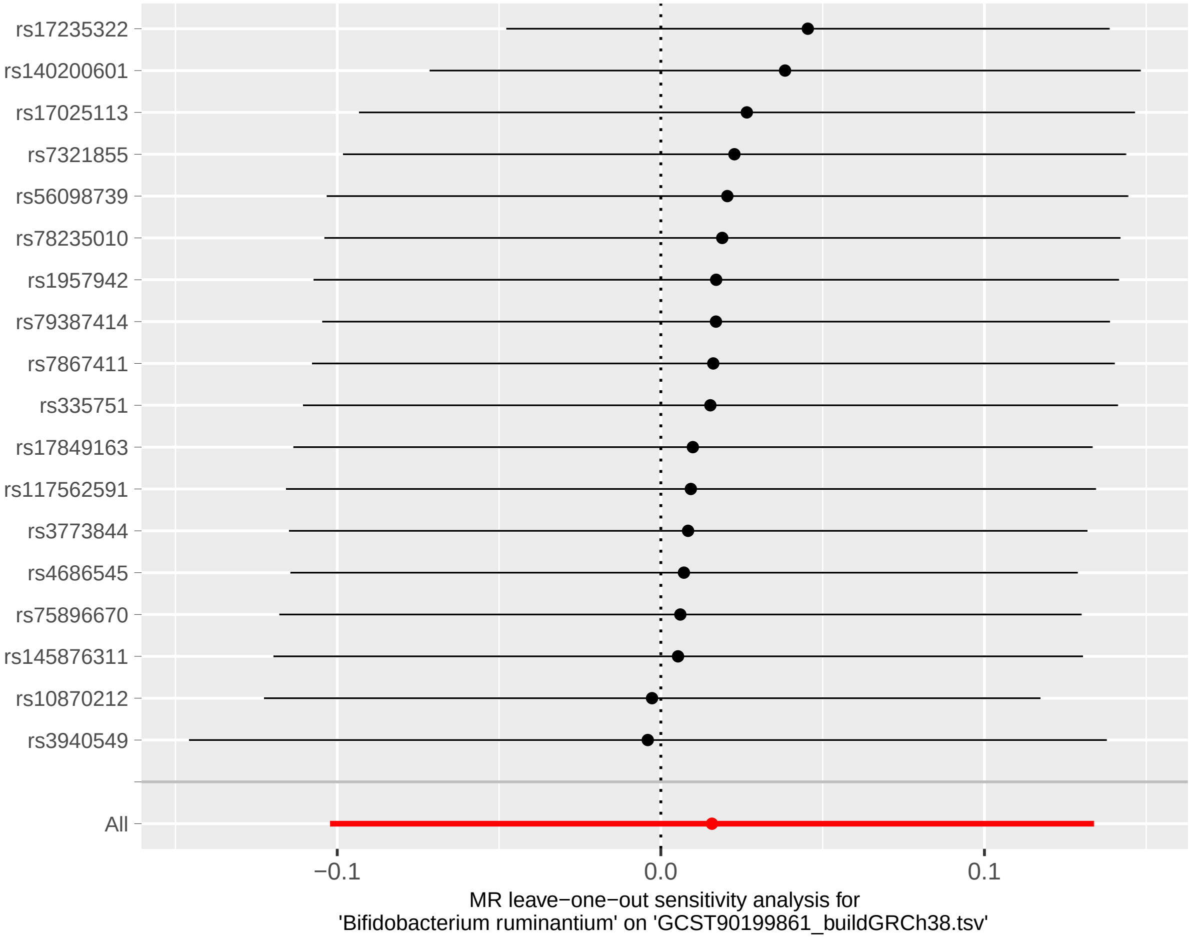
rs17235322
rs140200601
rs17025113
rs7321855
rs56098739
rs78235010
rs1957942
rs79387414
rs7867411
rs335751
rs17849163
rs117562591
rs3773844
rs4686545
rs75896670
rs145876311
rs10870212
rs3940549
All
−0.1
0.0
0.1
MR leave−one−out sensitivity analysis for
'Bifidobacterium ruminantium' on 'GCST90199861_buildGRCh38.tsv'

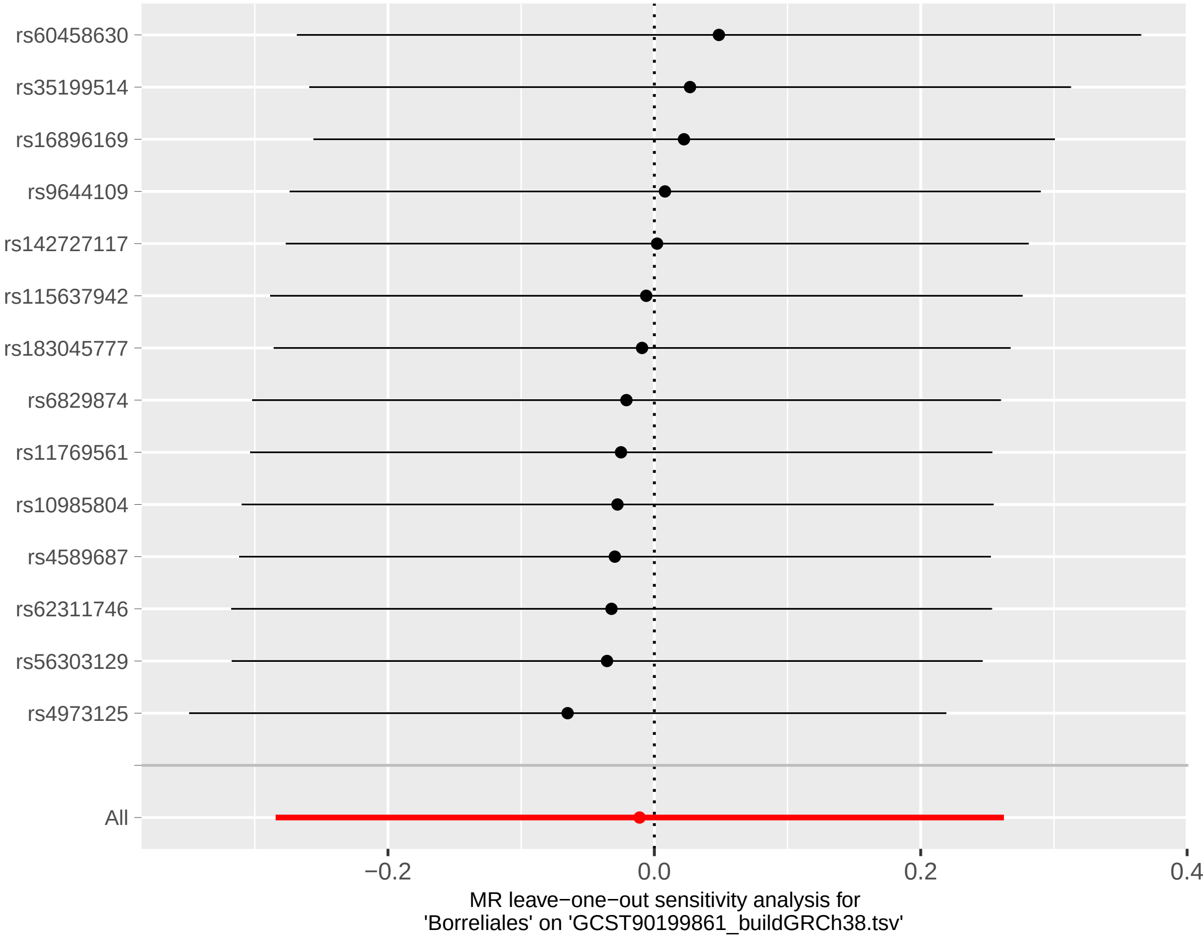
rs60458630
rs35199514
rs16896169
rs9644109
rs142727117
rs115637942
rs183045777
rs6829874
rs11769561
rs10985804
rs4589687
rs62311746
rs56303129
rs4973125
All
−0.2
0.0
0.2
0.4
MR leave−one−out sensitivity analysis for
'Borreliales' on 'GCST90199861_buildGRCh38.tsv'

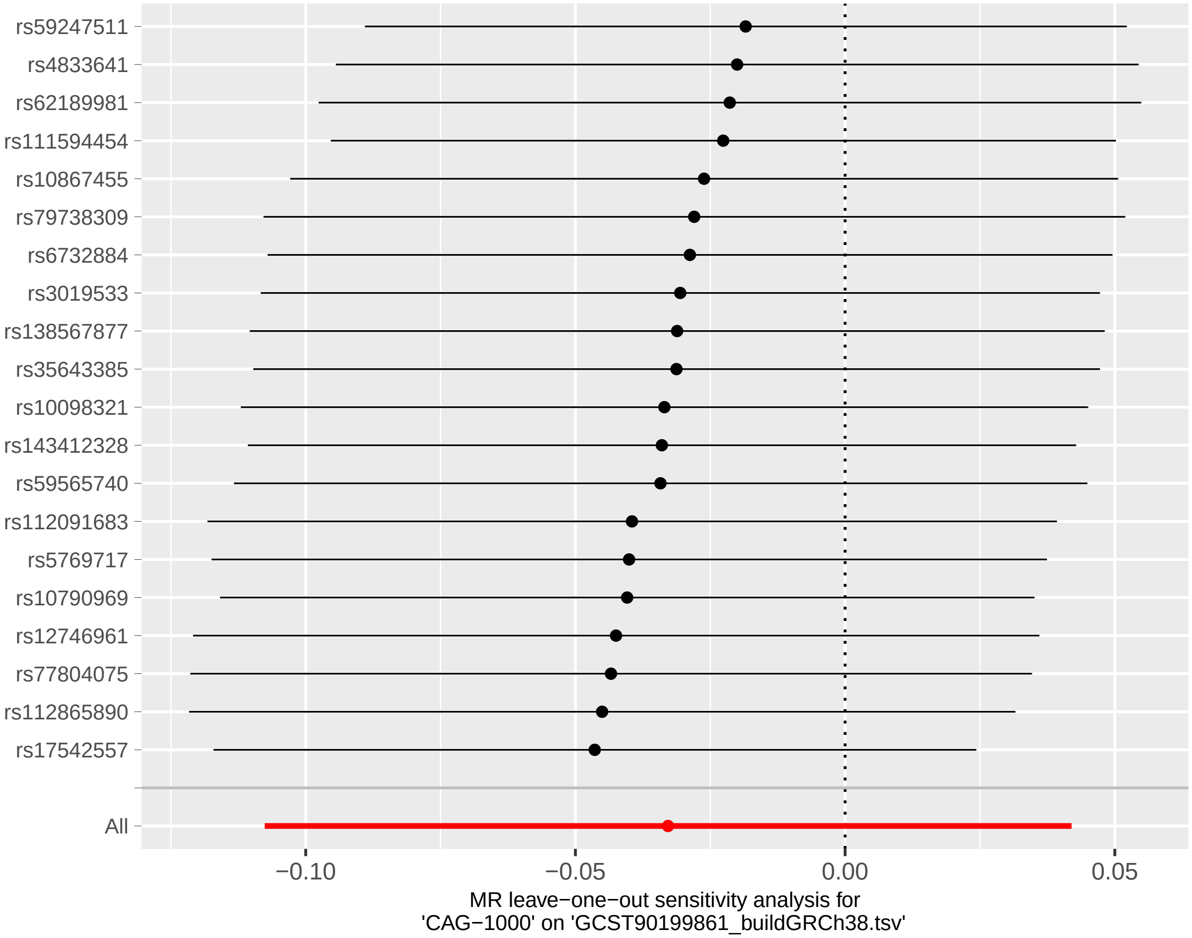

rs59247511
rs4833641
rs62189981
rs111594454
rs10867455
rs79738309
rs6732884
rs3019533
rs138567877
rs35643385
rs10098321
rs143412328
rs59565740
rs112091683
rs5769717
rs10790969
rs12746961
rs77804075
rs112865890
rs17542557
All
−0.10
−0.05
0.00
0.05
MR leave−one−out sensitivity analysis for
'CAG−1000' on 'GCST90199861_buildGRCh38.tsv'

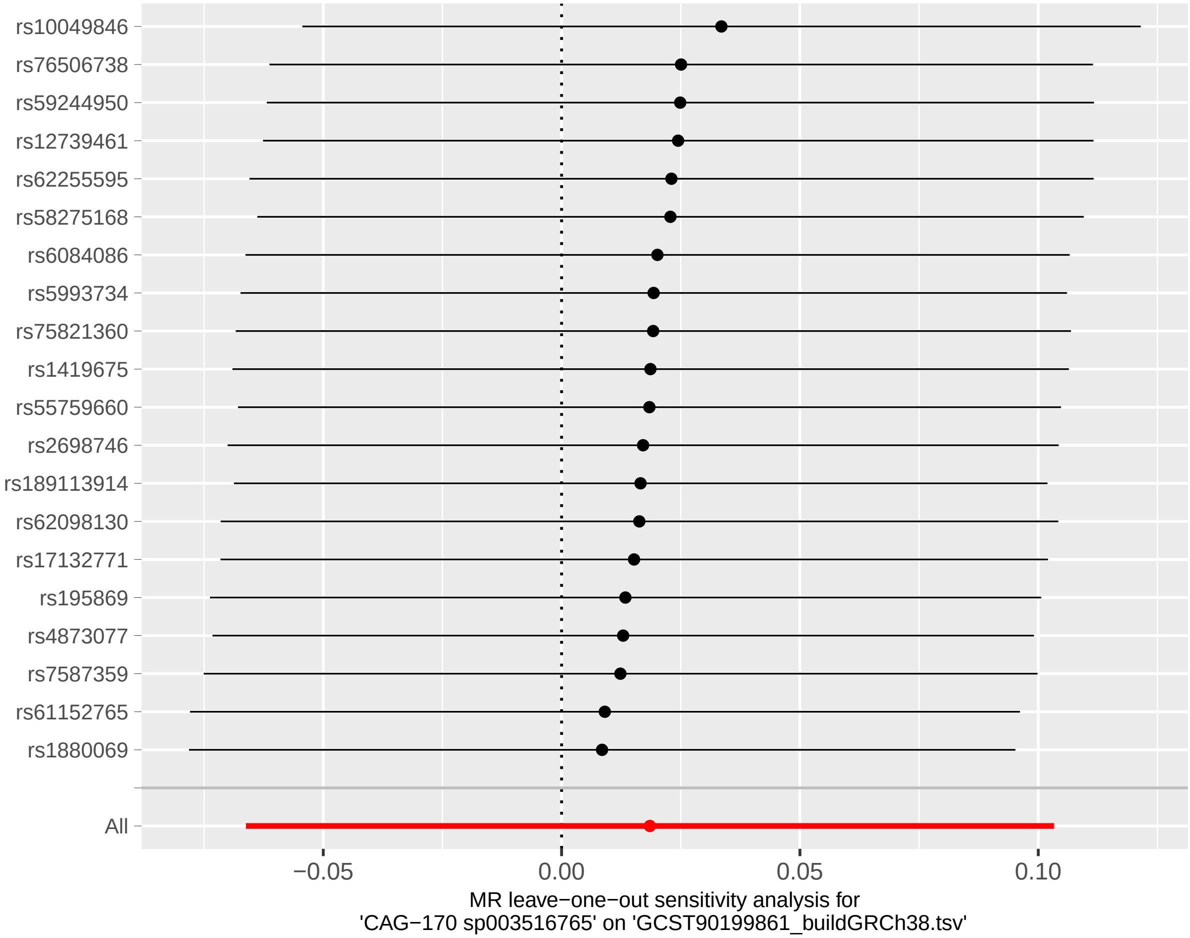
rs10049846
rs76506738
rs59244950
rs12739461
rs62255595
rs58275168
rs6084086
rs5993734
rs75821360
rs1419675
rs55759660
rs2698746
rs189113914
rs62098130
rs17132771
rs195869
rs4873077
rs7587359
rs61152765
rs1880069
All
−0.05
0.00
0.05
0.10
MR leave−one−out sensitivity analysis for
'CAG−170 sp003516765' on 'GCST90199861_buildGRCh38.tsv'

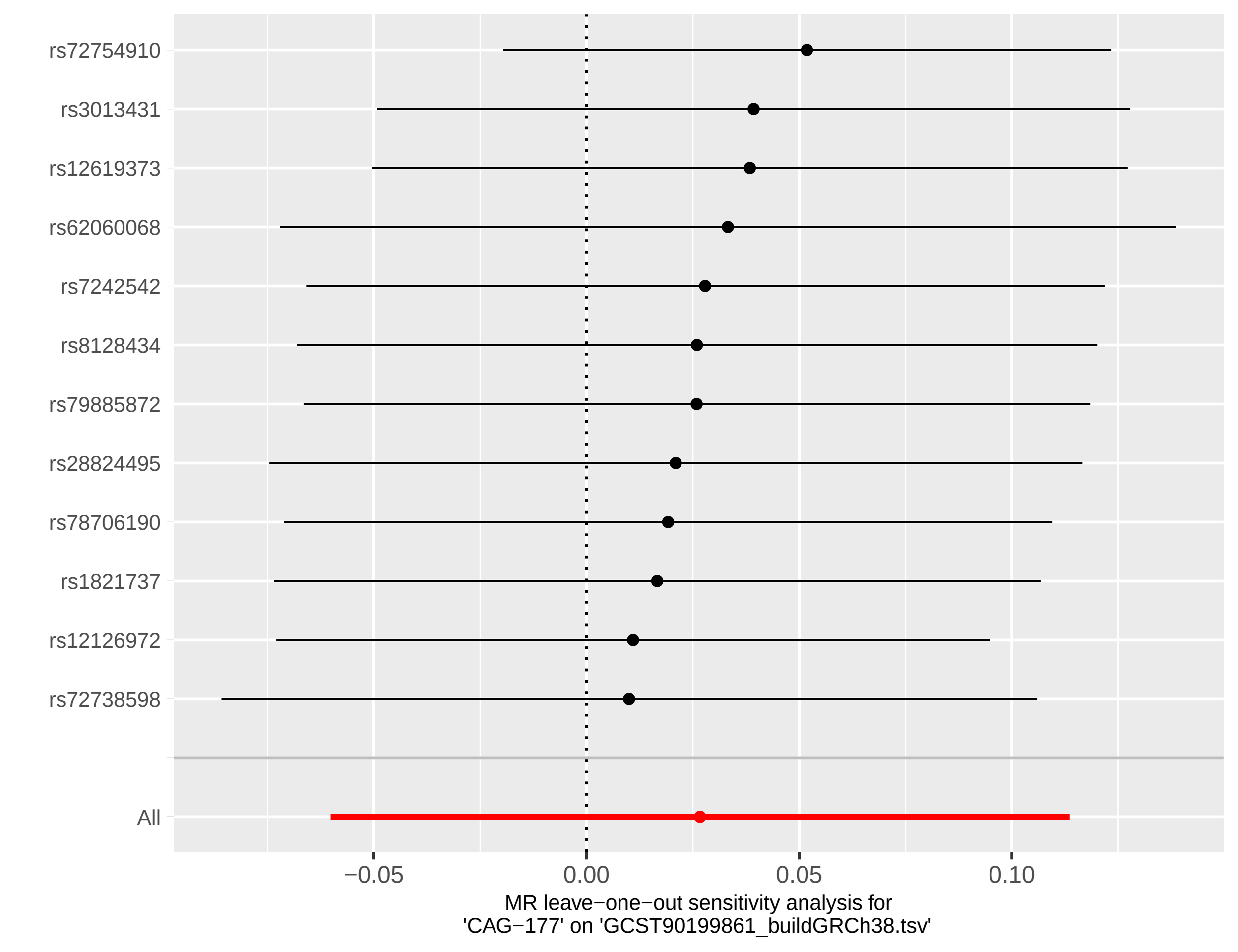
rs72754910
rs3013431
rs12619373
rs62060068
rs7242542
rs8128434
rs79885872
rs28824495
rs78706190
rs1821737
rs12126972
rs72738598
All
−0.05
0.00
0.05
0.10
MR leave−one−out sensitivity analysis for
'CAG−177' on 'GCST90199861_buildGRCh38.tsv'

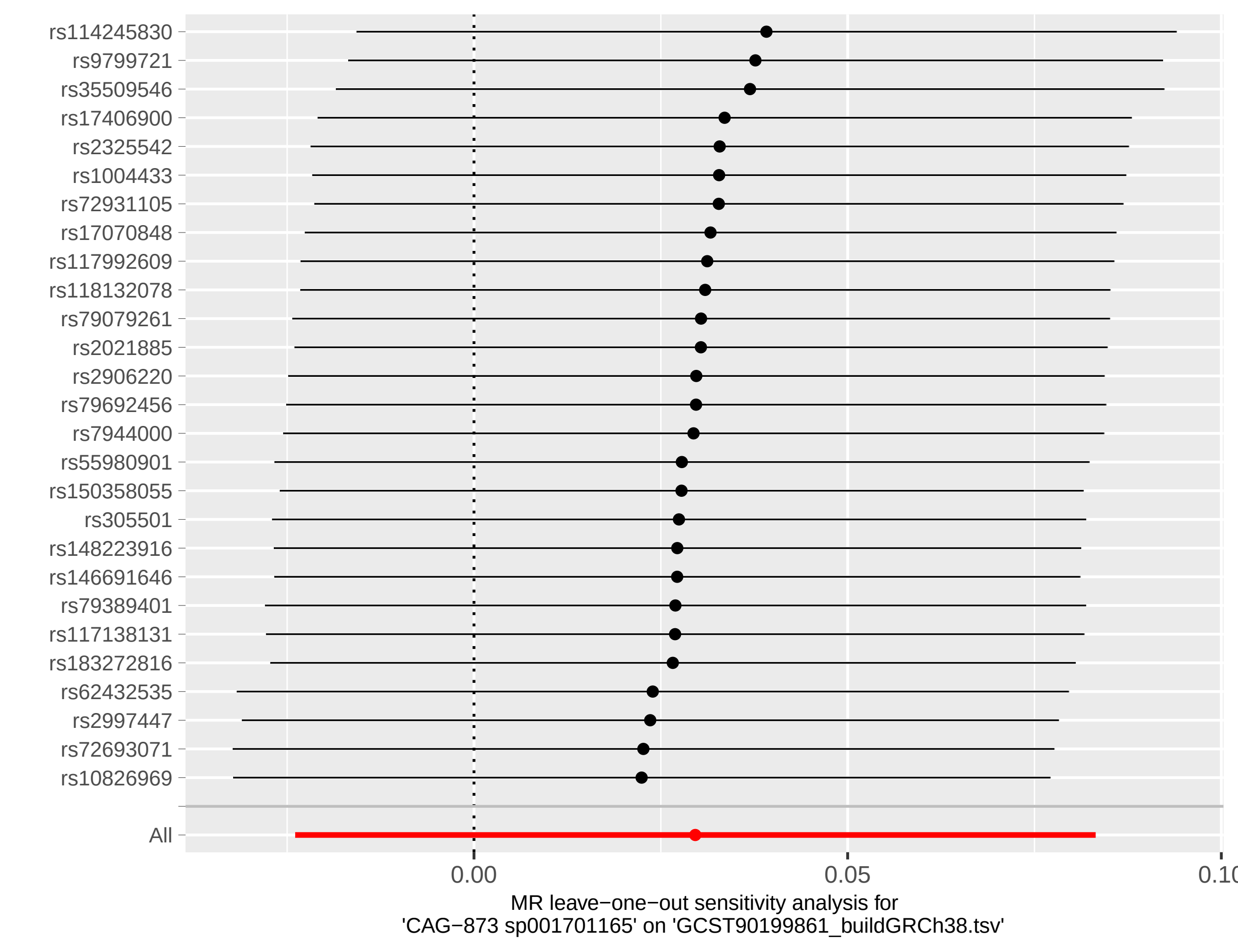
rs114245830
rs9799721
rs35509546
rs17406900
rs2325542
rs1004433
rs72931105
rs17070848
rs117992609
rs118132078
rs79079261
rs2021885
rs2906220
rs79692456
rs7944000
rs55980901
rs150358055
rs305501
rs148223916
rs146691646
rs79389401
rs117138131
rs183272816
rs62432535
rs2997447
rs72693071
rs10826969
All
0.00
0.05
0.10
MR leave−one−out sensitivity analysis for
'CAG−873 sp001701165' on 'GCST90199861_buildGRCh38.tsv'

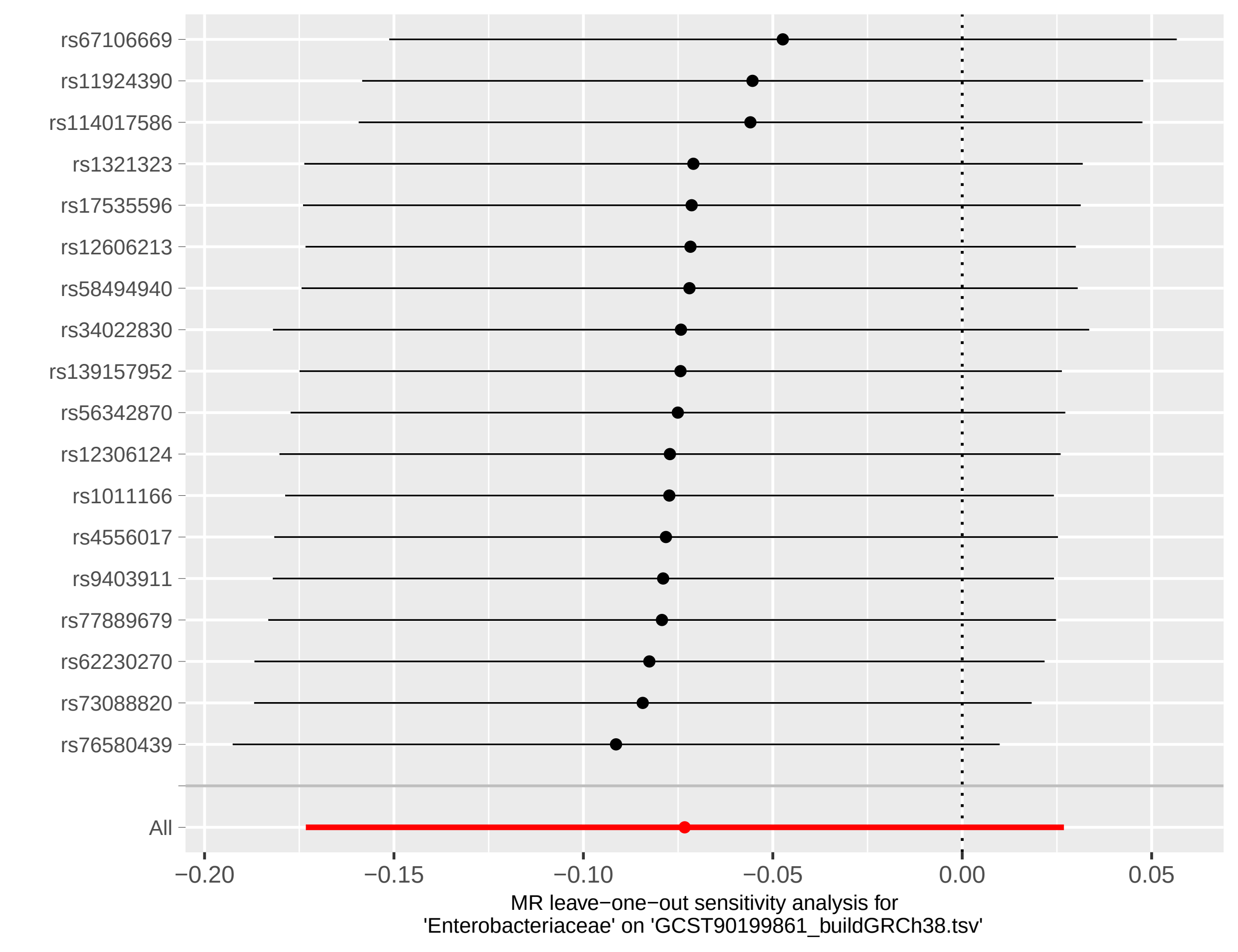

rs67106669
rs11924390
rs114017586
rs1321323
rs17535596
rs12606213
rs58494940
rs34022830
rs139157952
rs56342870
rs12306124
rs1011166
rs4556017
rs9403911
rs77889679
rs62230270
rs73088820
rs76580439
All
−0.20
−0.15
−0.10
−0.05
0.00
0.05
MR leave−one−out sensitivity analysis for
'Enterobacteriaceae' on 'GCST90199861_buildGRCh38.tsv'

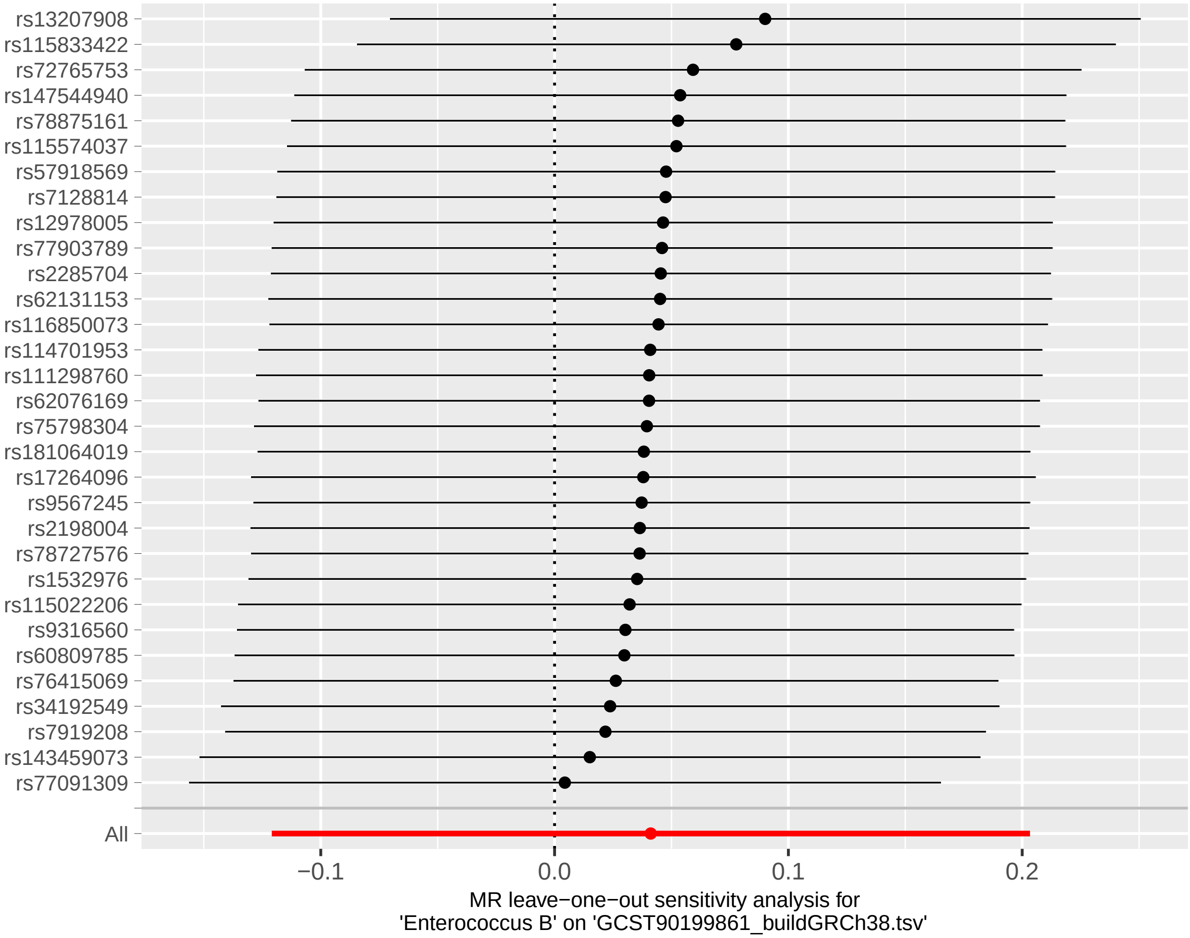

rs13207908
rs115833422
rs72765753
rs147544940
rs78875161
rs115574037
rs57918569
rs7128814
rs12978005
rs77903789
rs2285704
rs62131153
rs116850073
rs114701953
rs111298760
rs62076169
rs75798304
rs181064019
rs17264096
rs9567245
rs2198004
rs78727576
rs1532976
rs115022206
rs9316560
rs60809785
rs76415069
rs34192549
rs7919208
rs143459073
rs77091309
All
−0.1
0.0
0.1
0.2
MR leave−one−out sensitivity analysis for
'Enterococcus B' on 'GCST90199861_buildGRCh38.tsv'

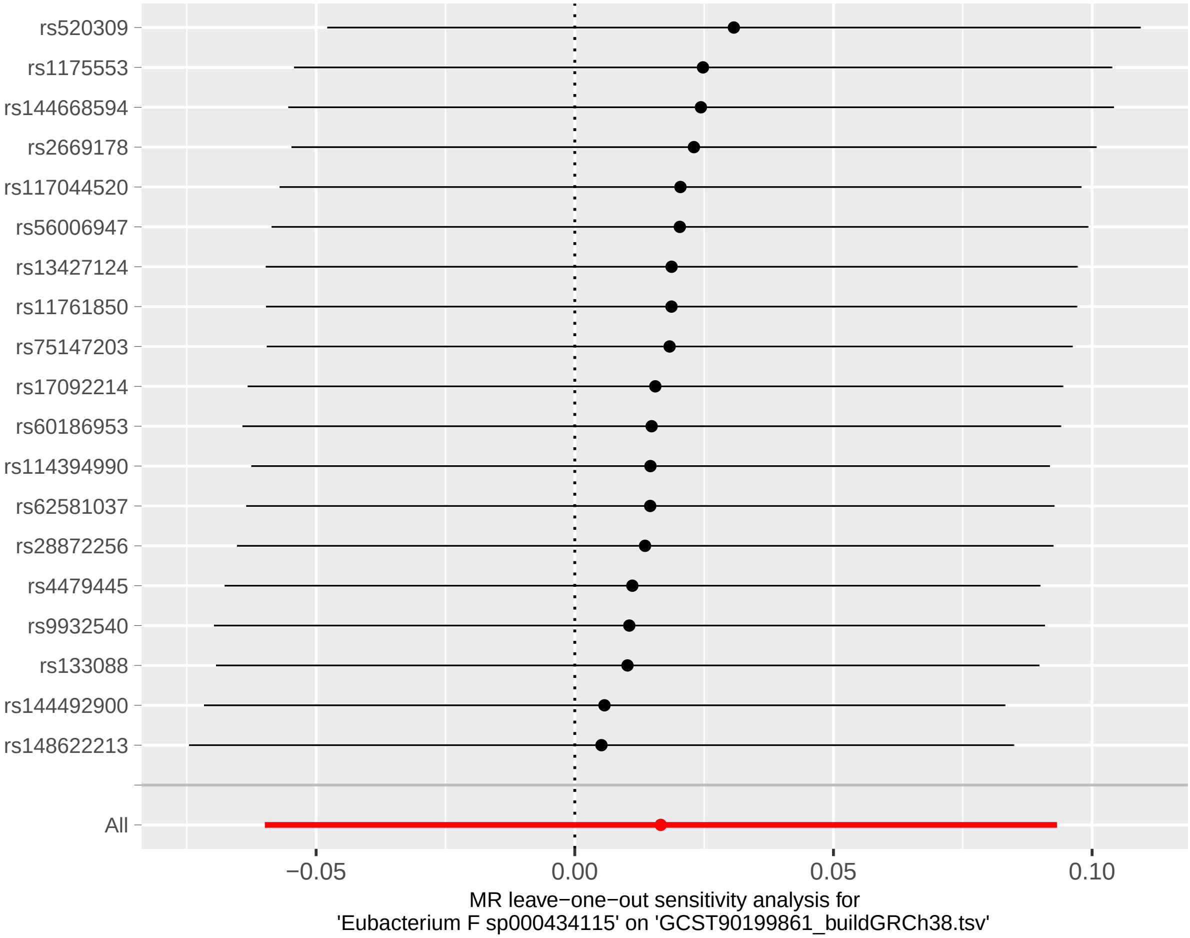

rs520309
rs1175553
rs144668594
rs2669178
rs117044520
rs56006947
rs13427124
rs11761850
rs75147203
rs17092214
rs60186953
rs114394990
rs62581037
rs28872256
rs4479445
rs9932540
rs133088
rs144492900
rs148622213
All
−0.05
0.00
0.05
0.10
MR leave−one−out sensitivity analysis for
'Eubacterium F sp000434115' on 'GCST90199861_buildGRCh38.tsv'

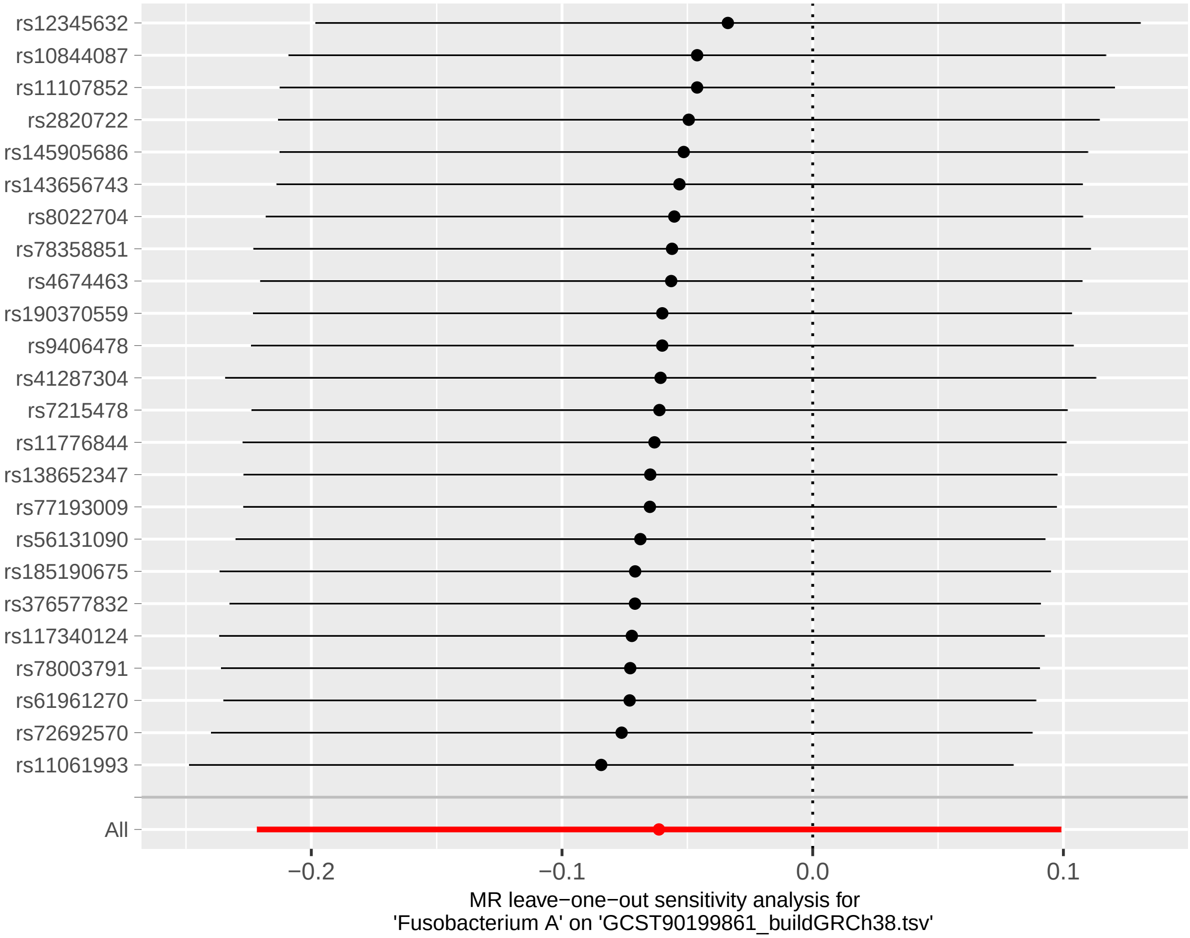

rs12345632
rs10844087
rs11107852
rs2820722
rs145905686
rs143656743
rs8022704
rs78358851
rs4674463
rs190370559
rs9406478
rs41287304
rs7215478
rs11776844
rs138652347
rs77193009
rs56131090
rs185190675
rs376577832
rs117340124
rs78003791
rs61961270
rs72692570
rs11061993
All
−0.2
−0.1
0.0
0.1
MR leave−one−out sensitivity analysis for
'Fusobacterium A' on 'GCST90199861_buildGRCh38.tsv'

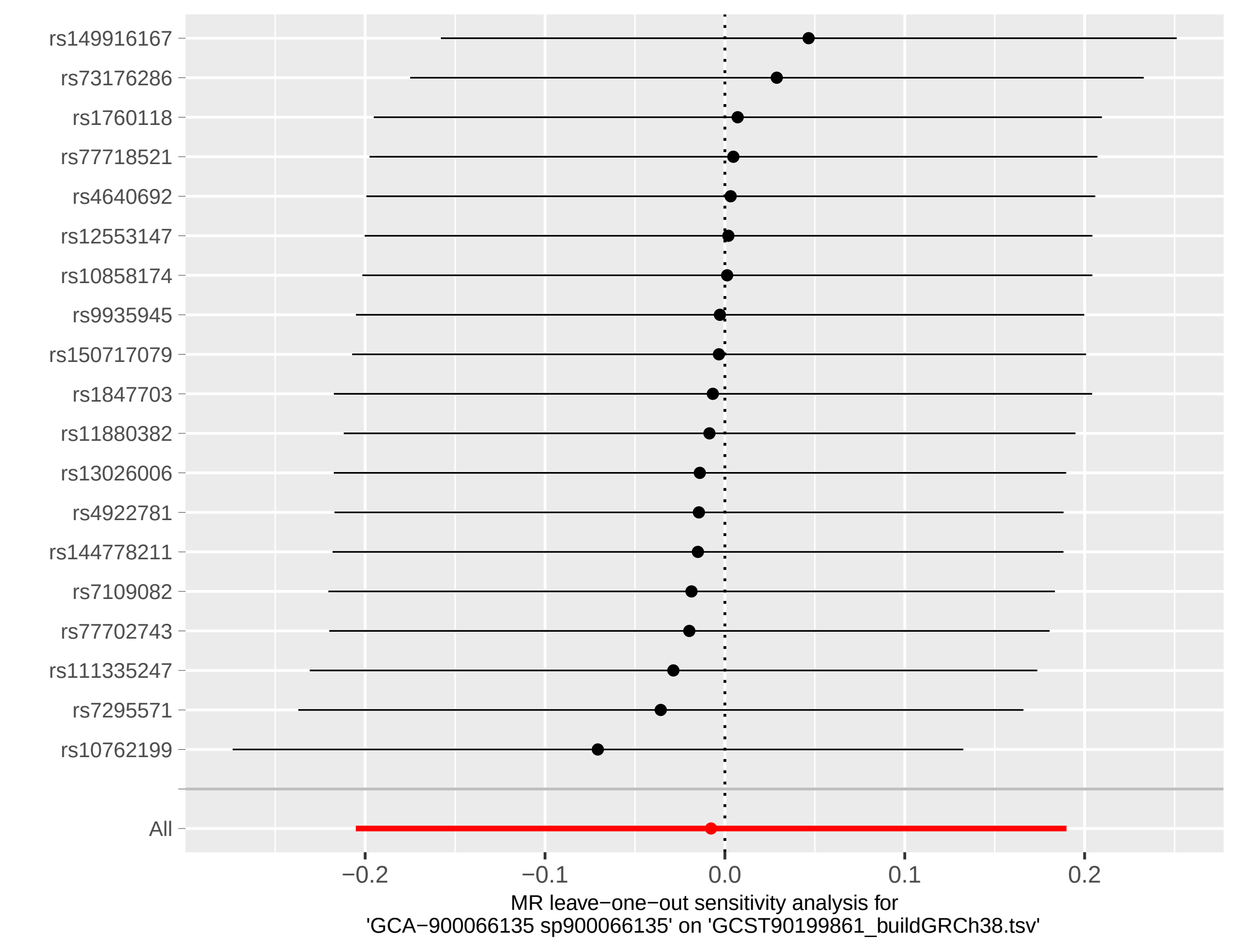

rs149916167
rs73176286
rs1760118
rs77718521
rs4640692
rs12553147
rs10858174
rs9935945
rs150717079
rs1847703
rs11880382
rs13026006
rs4922781
rs144778211
rs7109082
rs77702743
rs111335247
rs7295571
rs10762199
All
−0.2
−0.1
0.0
0.1
0.2
MR leave−one−out sensitivity analysis for
'GCA−900066135 sp900066135' on 'GCST90199861_buildGRCh38.tsv'

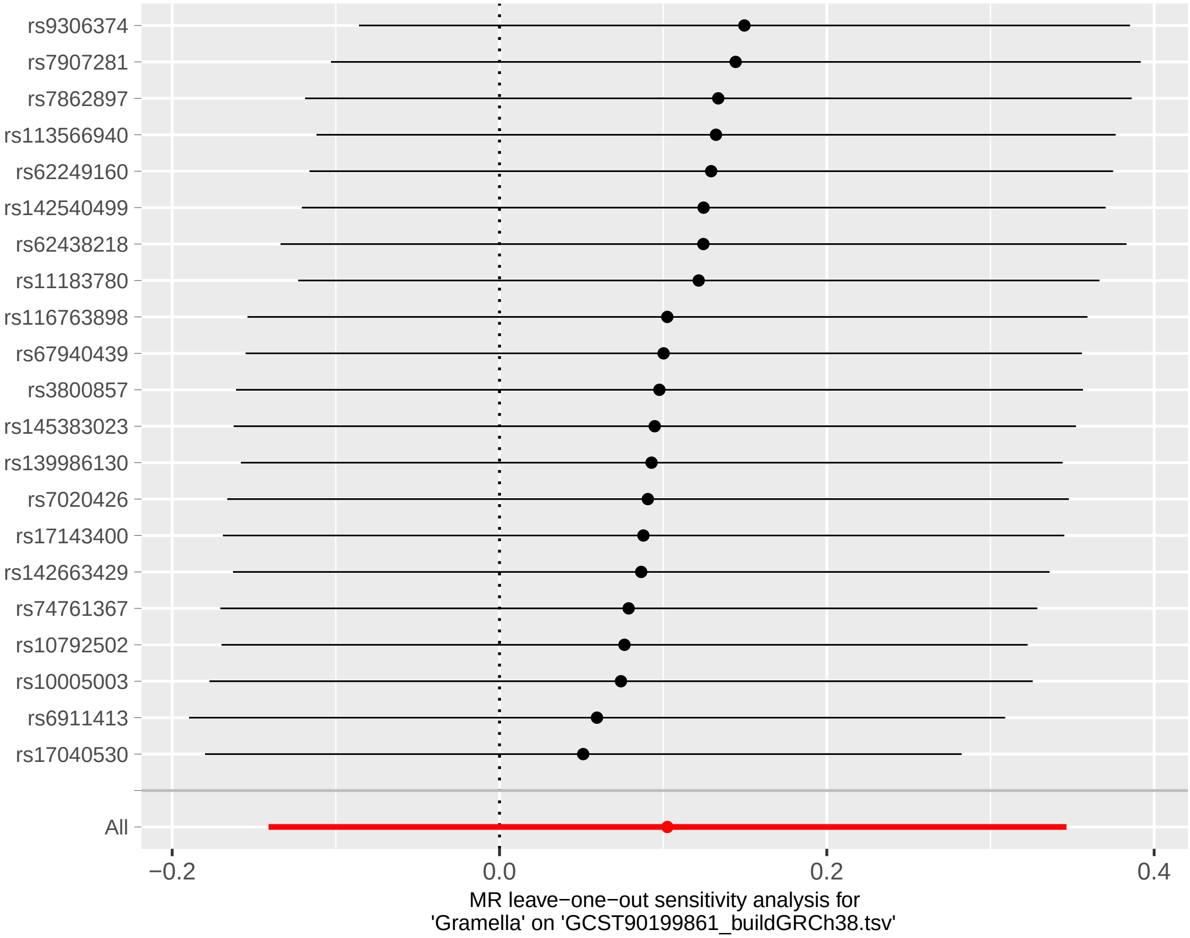
rs9306374
rs7907281
rs7862897
rs113566940
rs62249160
rs142540499
rs62438218
rs11183780
rs116763898
rs67940439
rs3800857
rs145383023
rs139986130
rs7020426
rs17143400
rs142663429
rs74761367
rs10792502
rs10005003
rs6911413
rs17040530
All
−0.2
0.0
0.2
0.4
MR leave−one−out sensitivity analysis for
'Gramella' on 'GCST90199861_buildGRCh38.tsv'

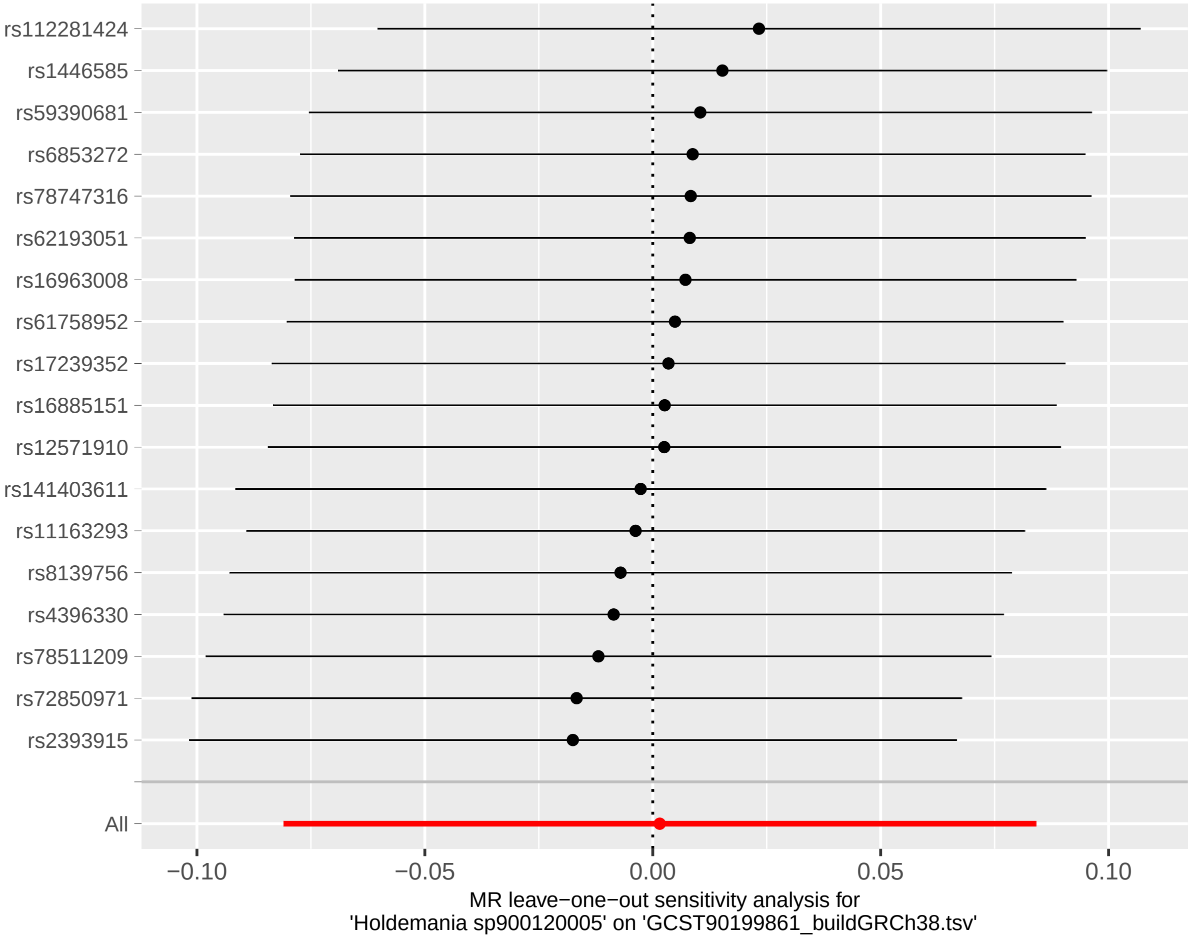

rs112281424
rs1446585
rs59390681
rs6853272
rs78747316
rs62193051
rs16963008
rs61758952
rs17239352
rs16885151
rs12571910
rs141403611
rs11163293
rs8139756
rs4396330
rs78511209
rs72850971
rs2393915
All
−0.10
−0.05
0.00
0.05
0.10
MR leave−one−out sensitivity analysis for
'Holdemania sp900120005' on 'GCST90199861_buildGRCh38.tsv'

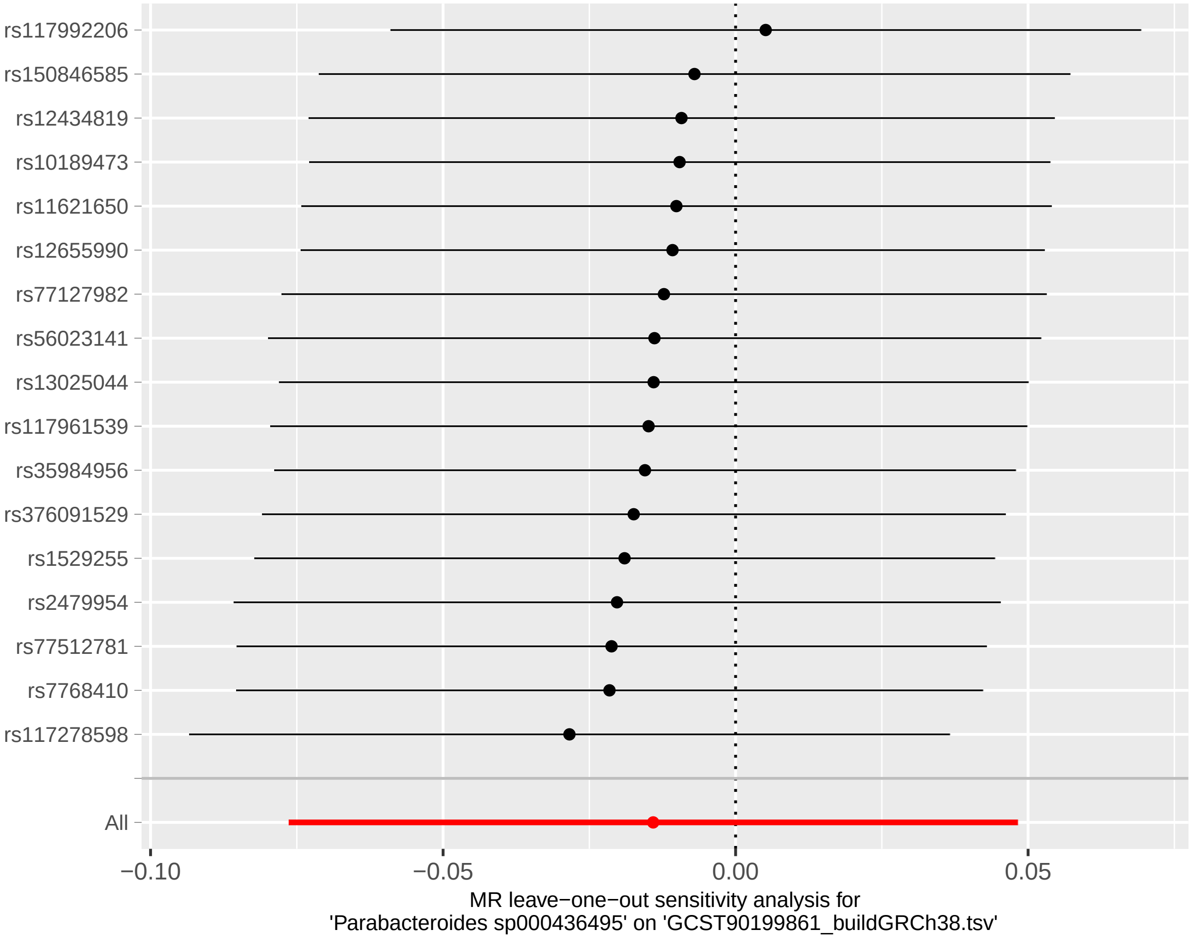
rs117992206
rs150846585
rs12434819
rs10189473
rs11621650
rs12655990
rs77127982
rs56023141
rs13025044
rs117961539
rs35984956
rs376091529
rs1529255
rs2479954
rs77512781
rs7768410
rs117278598
All
−0.10
−0.05
0.00
0.05
MR leave−one−out sensitivity analysis for
'Parabacteroides sp000436495' on 'GCST90199861_buildGRCh38.tsv'

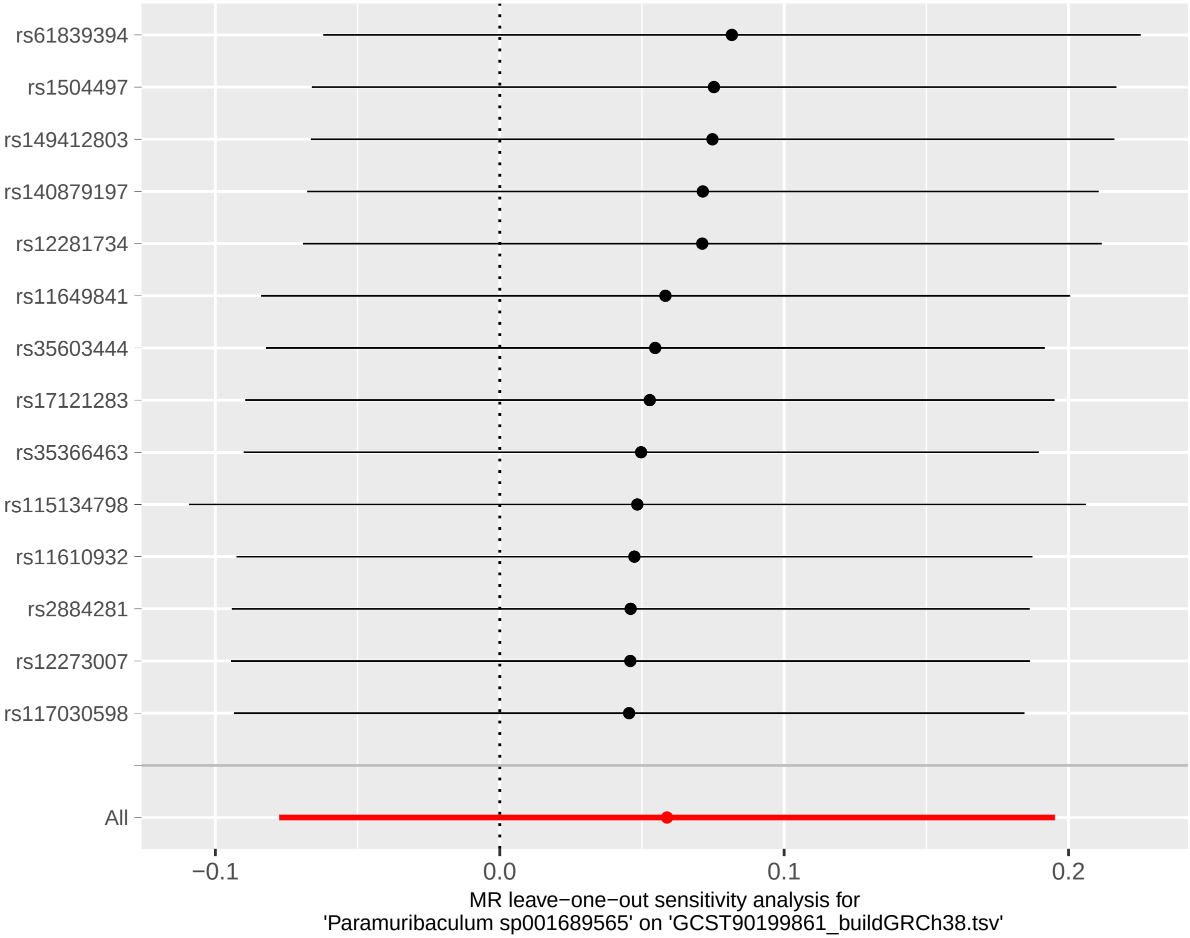

rs61839394
rs1504497
rs149412803
rs140879197
rs12281734
rs11649841
rs35603444
rs17121283
rs35366463
rs115134798
rs11610932
rs2884281
rs12273007
rs117030598
All
−0.1
0.0
0.1
0.2
MR leave−one−out sensitivity analysis for
'Paramuribaculum sp001689565' on 'GCST90199861_buildGRCh38.tsv'

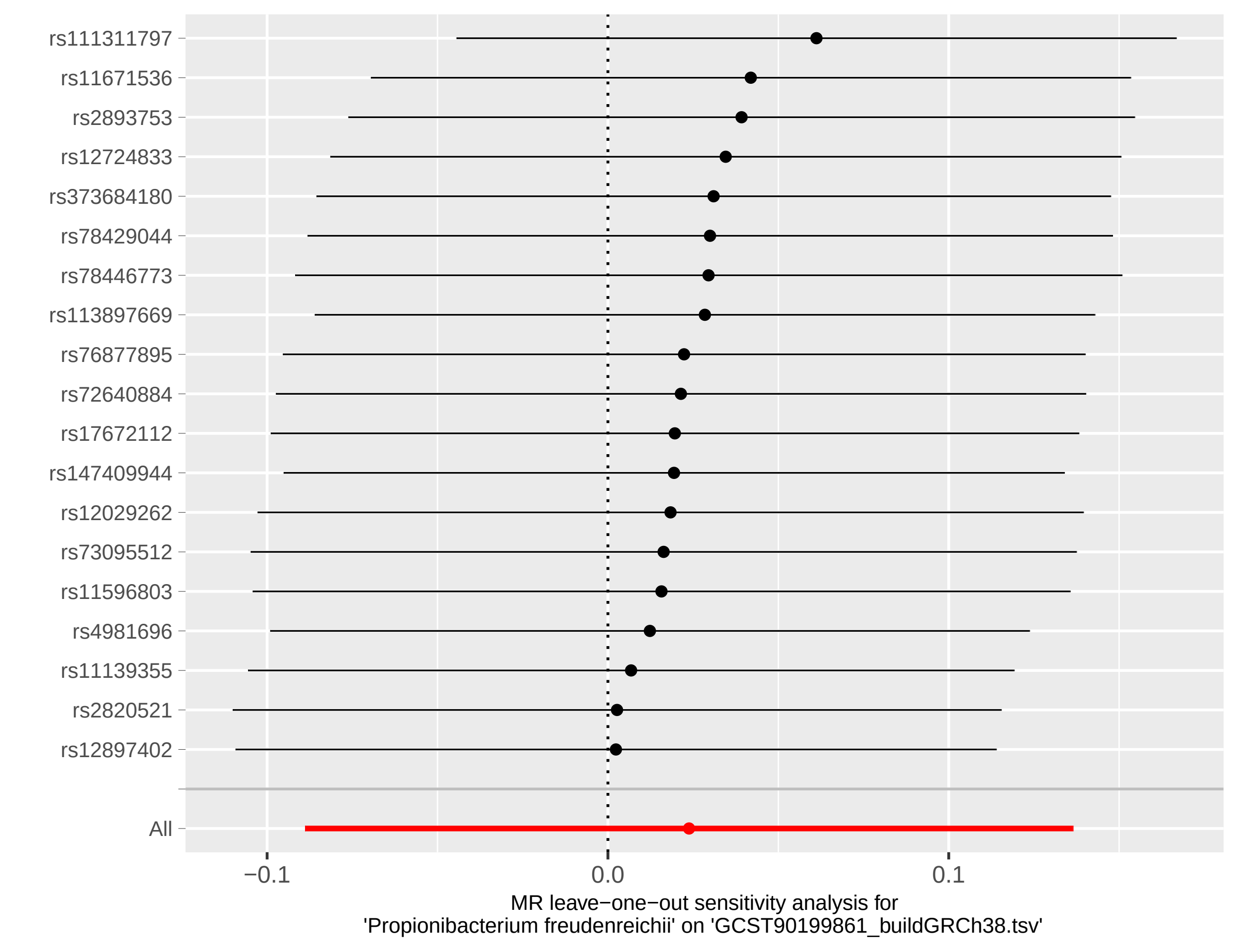

rs111311797
rs11671536
rs2893753
rs12724833
rs373684180
rs78429044
rs78446773
rs113897669
rs76877895
rs72640884
rs17672112
rs147409944
rs12029262
rs73095512
rs11596803
rs4981696
rs11139355
rs2820521
rs12897402
All
−0.1
0.0
0.1
MR leave−one−out sensitivity analysis for
'Propionibacterium freudenreichii' on 'GCST90199861_buildGRCh38.tsv'

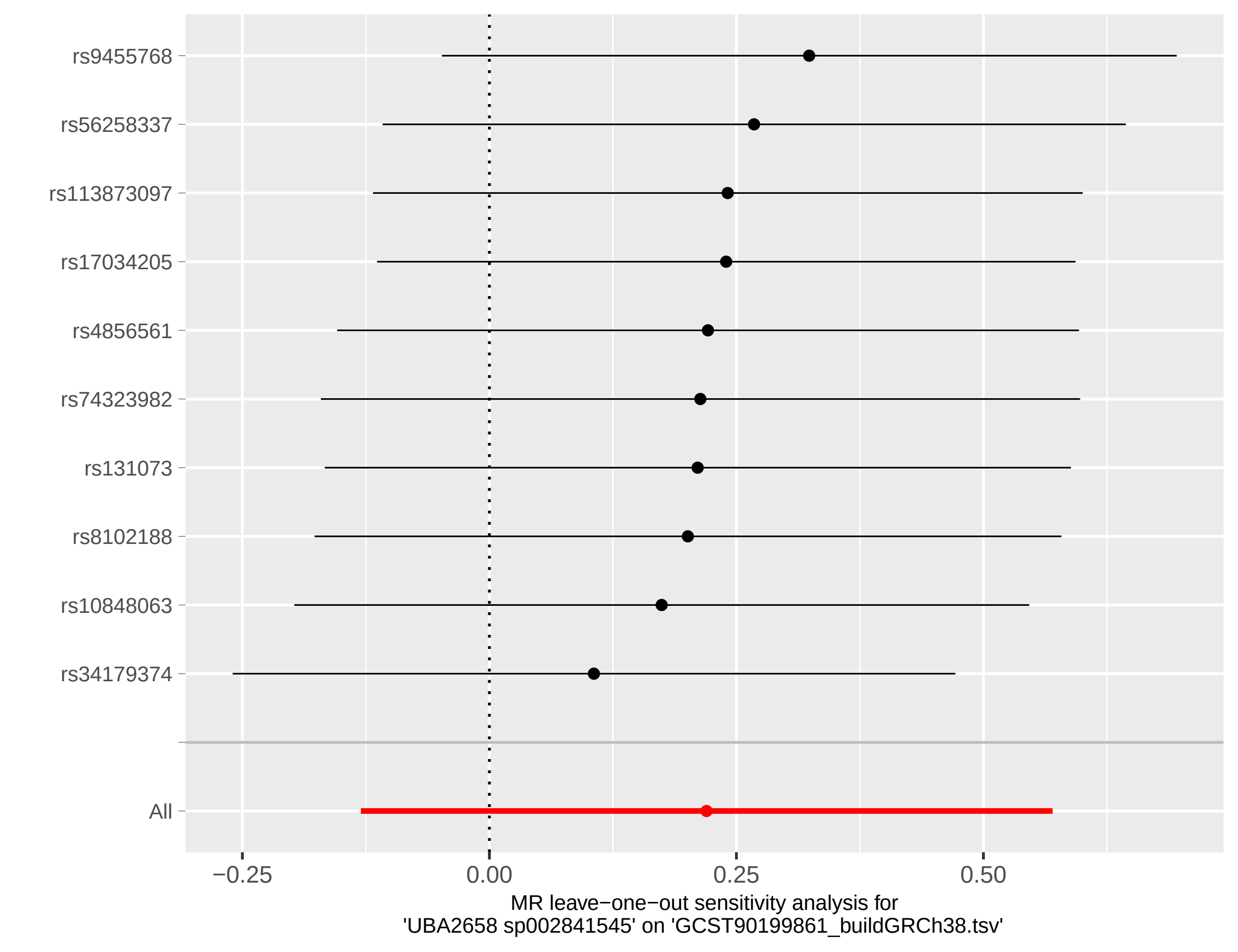

rs9455768
rs56258337
rs113873097
rs17034205
rs4856561
rs74323982
rs131073
rs8102188
rs10848063
rs34179374
All
−0.25
0.00
0.25
0.50
MR leave−one−out sensitivity analysis for
'UBA2658 sp002841545' on 'GCST90199861_buildGRCh38.tsv'

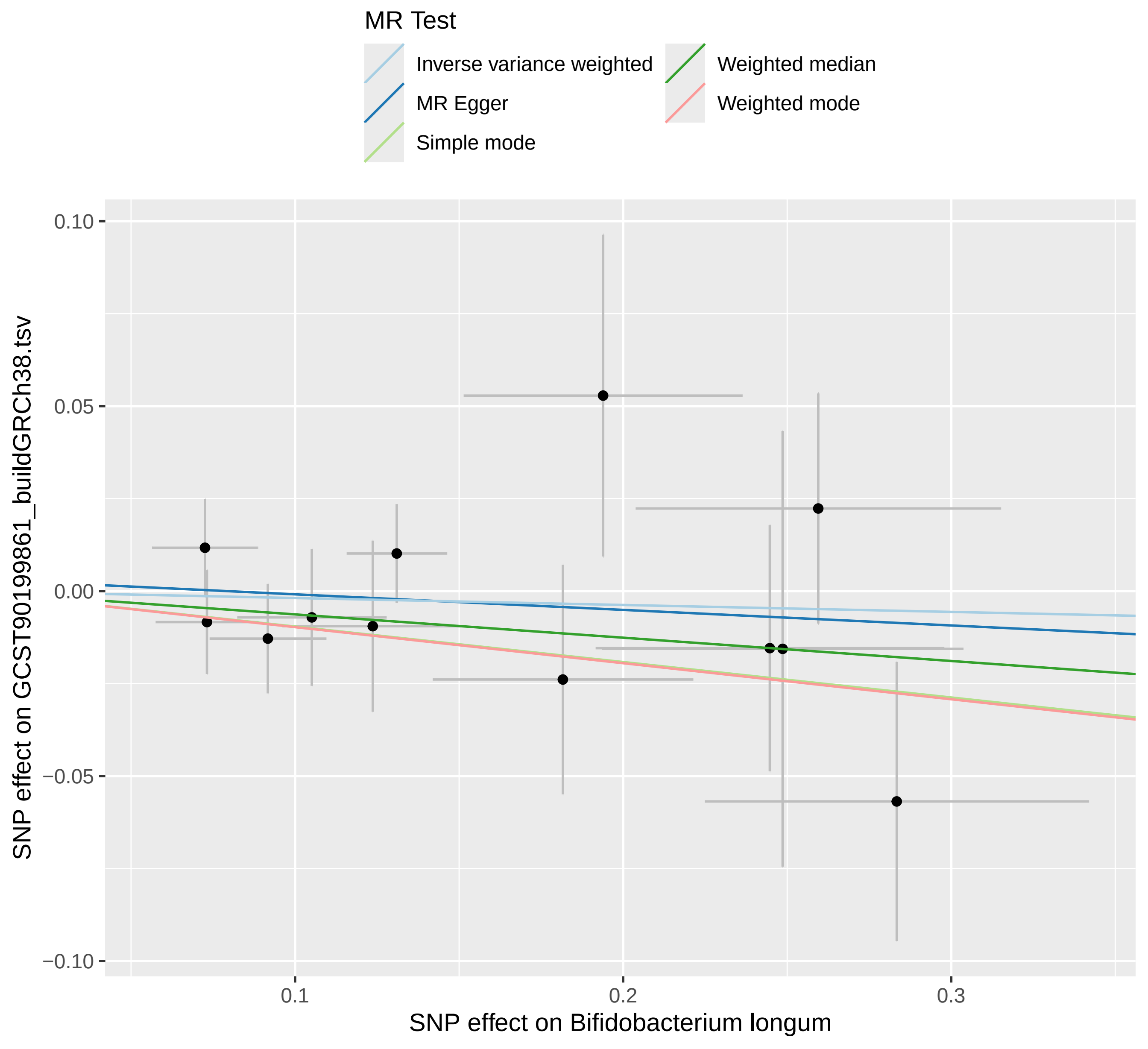
MR Test
Inverse variance weighted
MR Egger
Simple mode
Weighted median
Weighted mode
0.10
0.05
0.00
−0.05
−0.10
0.1
0.2
0.3
SNP effect on GCST90199861_buildGRCh38.tsv
SNP effect on Bifidobacterium longum

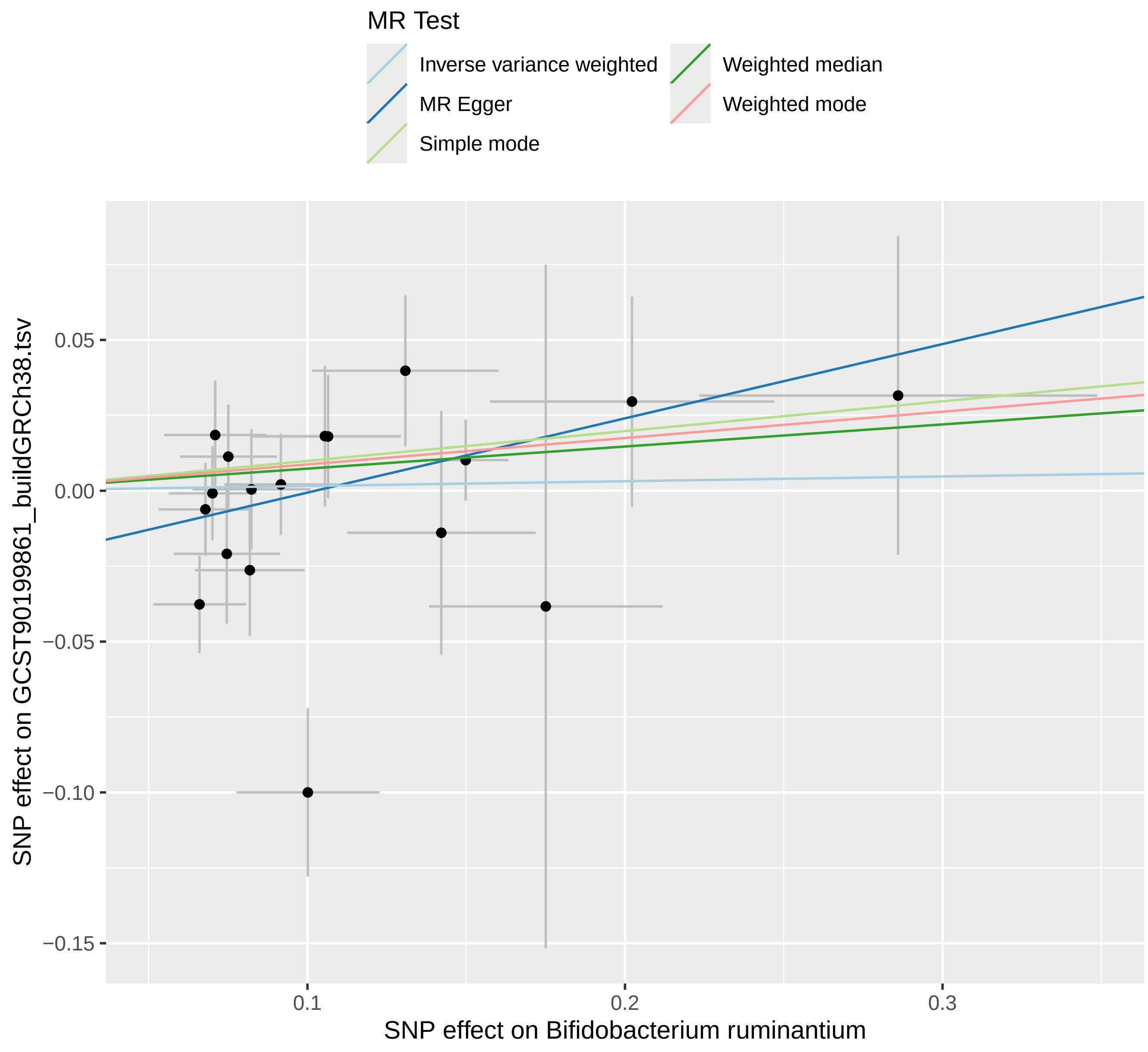

MR Test
Inverse variance weighted
MR Egger
Simple mode
Weighted median
Weighted mode
0.05
0.00
−0.05
−0.10
−0.15
0.1
0.2
0.3
SNP effect on GCST90199861_buildGRCh38.tsv
SNP effect on Bifidobacterium ruminantium

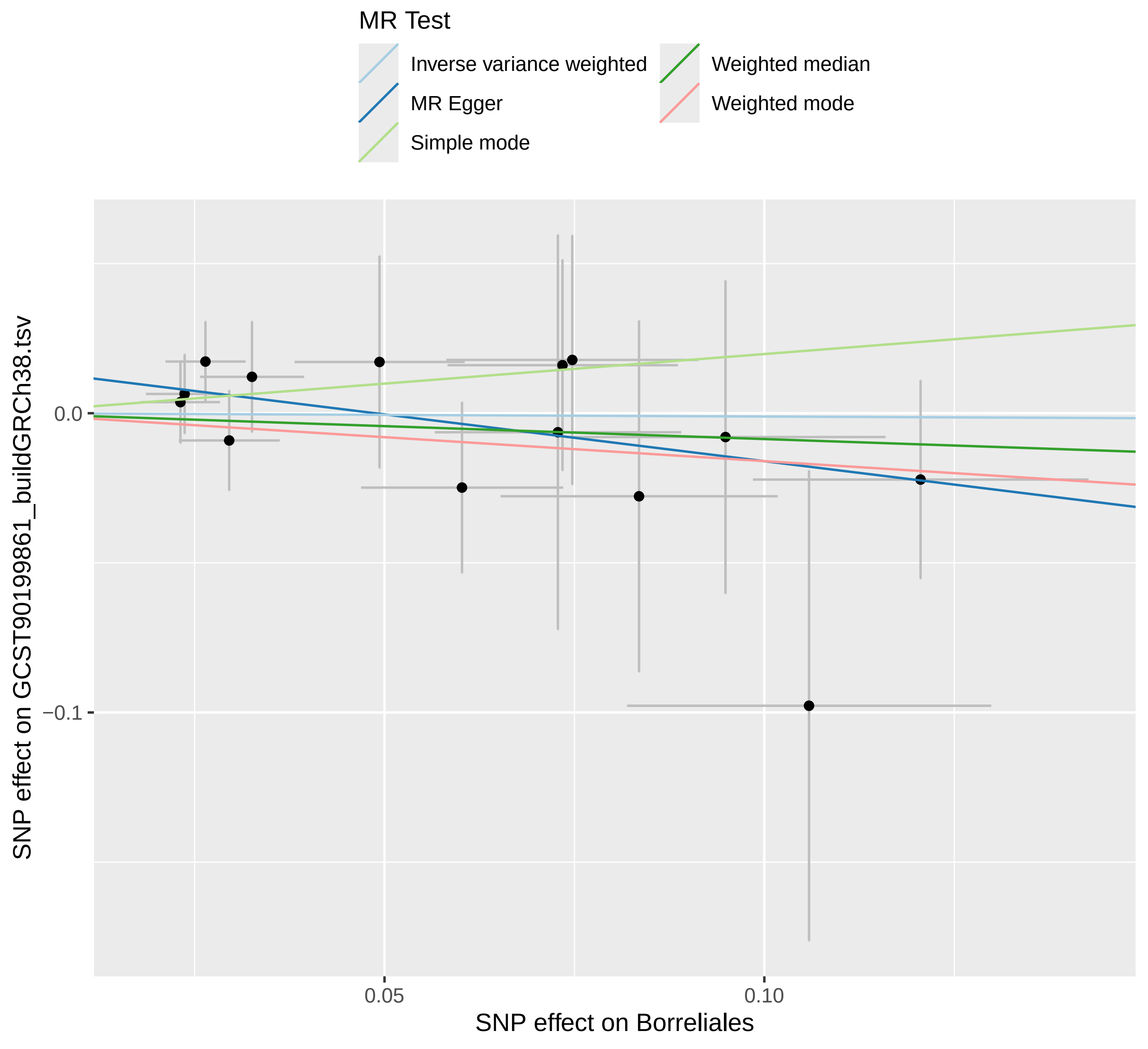

MR Test
Inverse variance weighted
MR Egger
Simple mode
Weighted median
Weighted mode
SNP effect on GCST90199861_buildGRCh38.tsv
0.0
−0.1
0.05
0.10
SNP effect on Borreliales

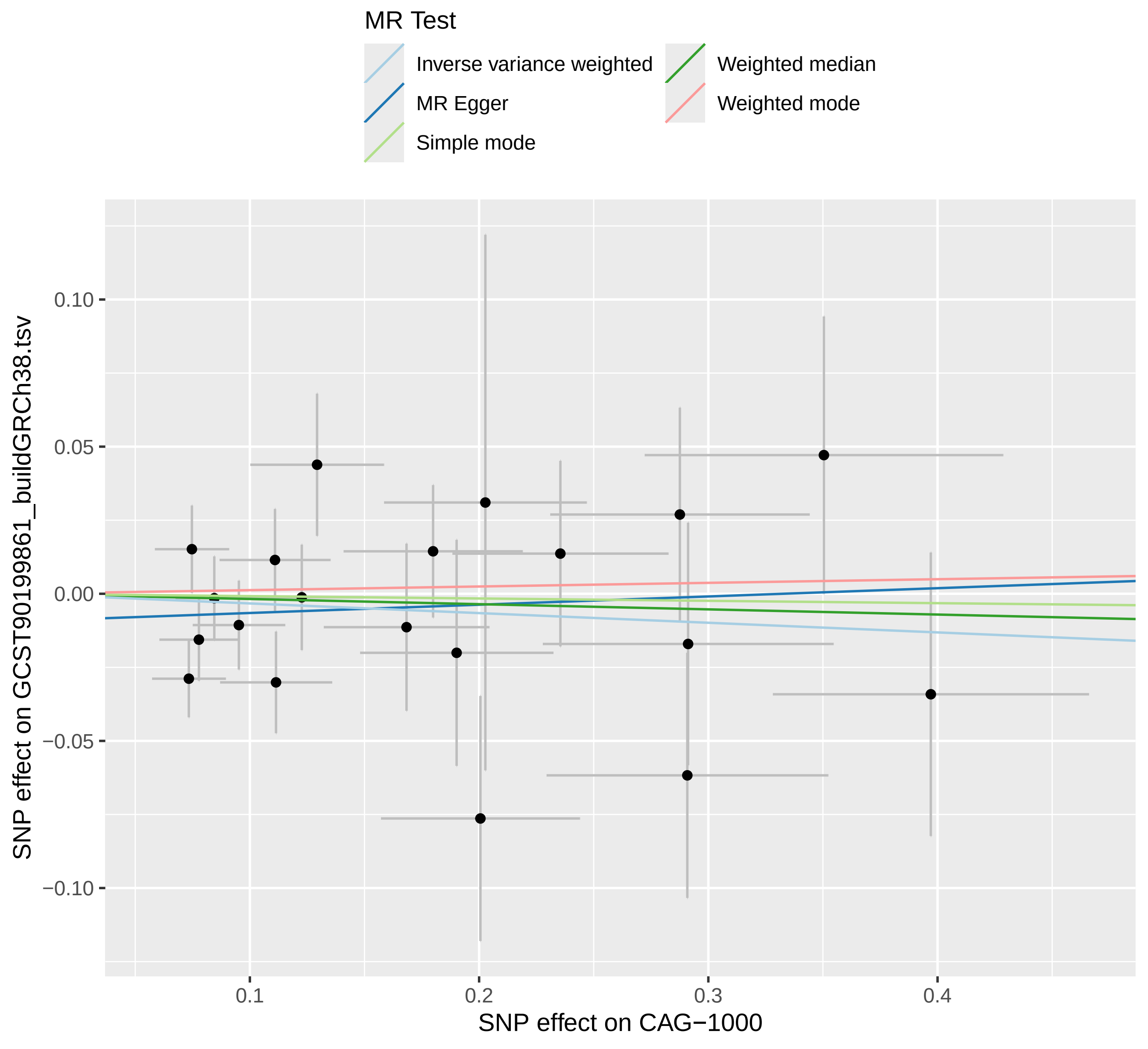
MR Test
Inverse variance weighted
MR Egger
Simple mode
Weighted median
Weighted mode
0.10
0.05
0.00
−0.05
−0.10
SNP effect on GCST90199861_buildGRCh38.tsv
0.1
0.2
0.3
0.4
SNP effect on CAG−1000

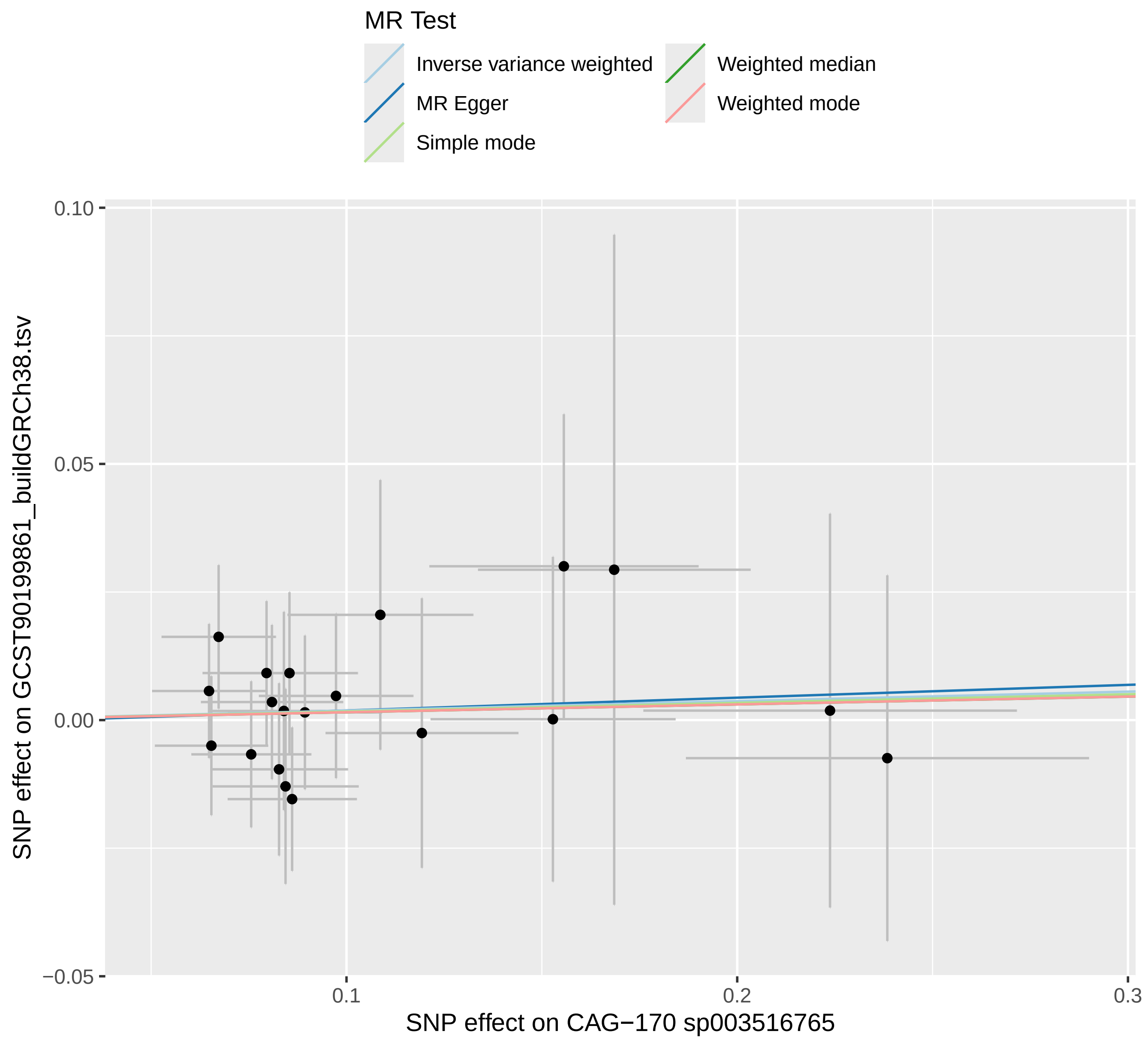
MR Test
Inverse variance weighted
MR Egger
Simple mode
Weighted median
Weighted mode
0.10
0.05
0.00
−0.05
0.1
0.2
0.3
SNP effect on GCST90199861_buildGRCh38.tsv
SNP effect on CAG−170 sp003516765

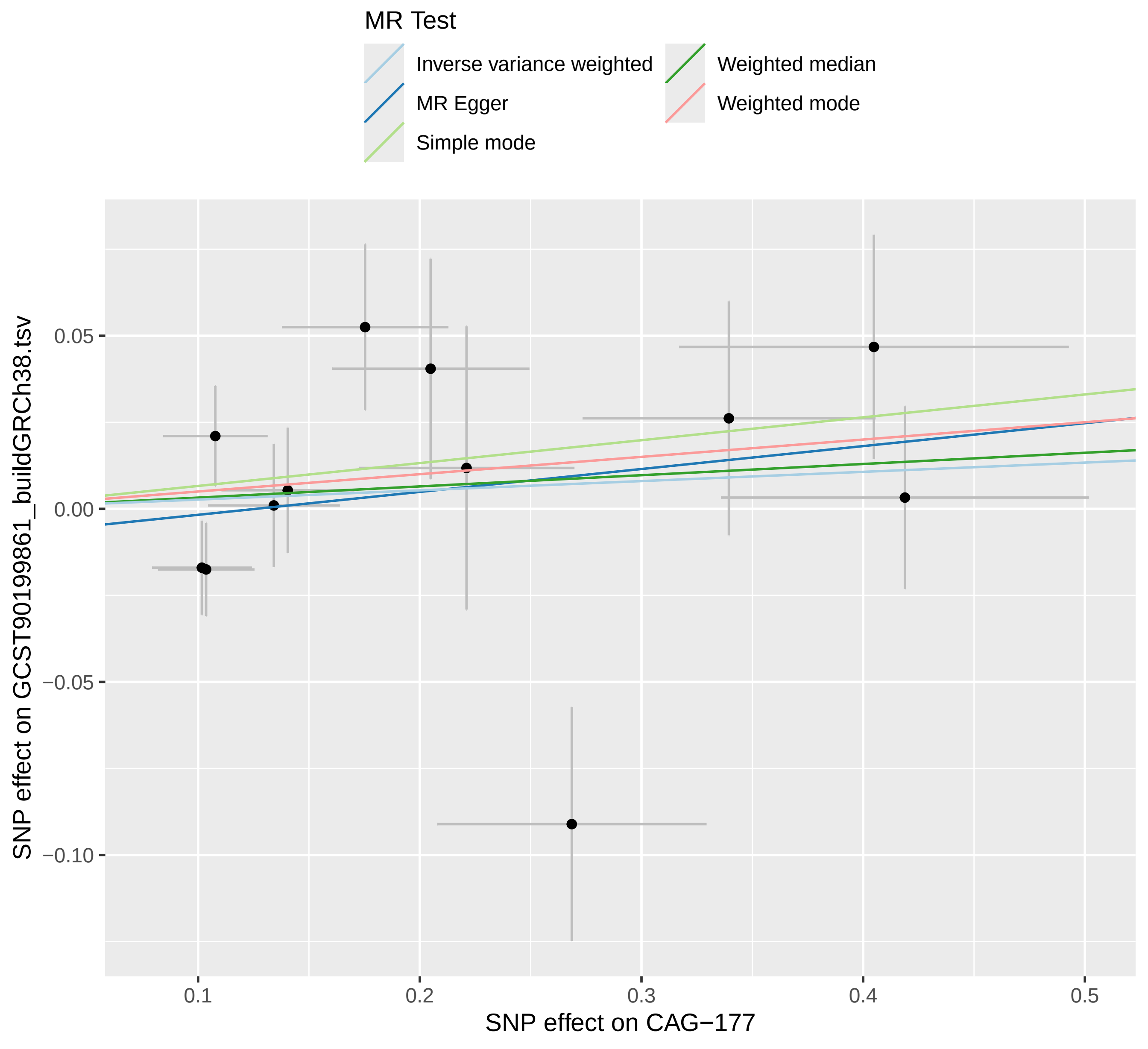

MR Test
Inverse variance weighted
MR Egger
Simple mode
Weighted median
Weighted mode
SNP effect on GCST90199861_buildGRCh38.tsv
0.05
0.00
−0.05
−0.10
0.1
0.2
0.3
0.4
0.5
SNP effect on CAG−177

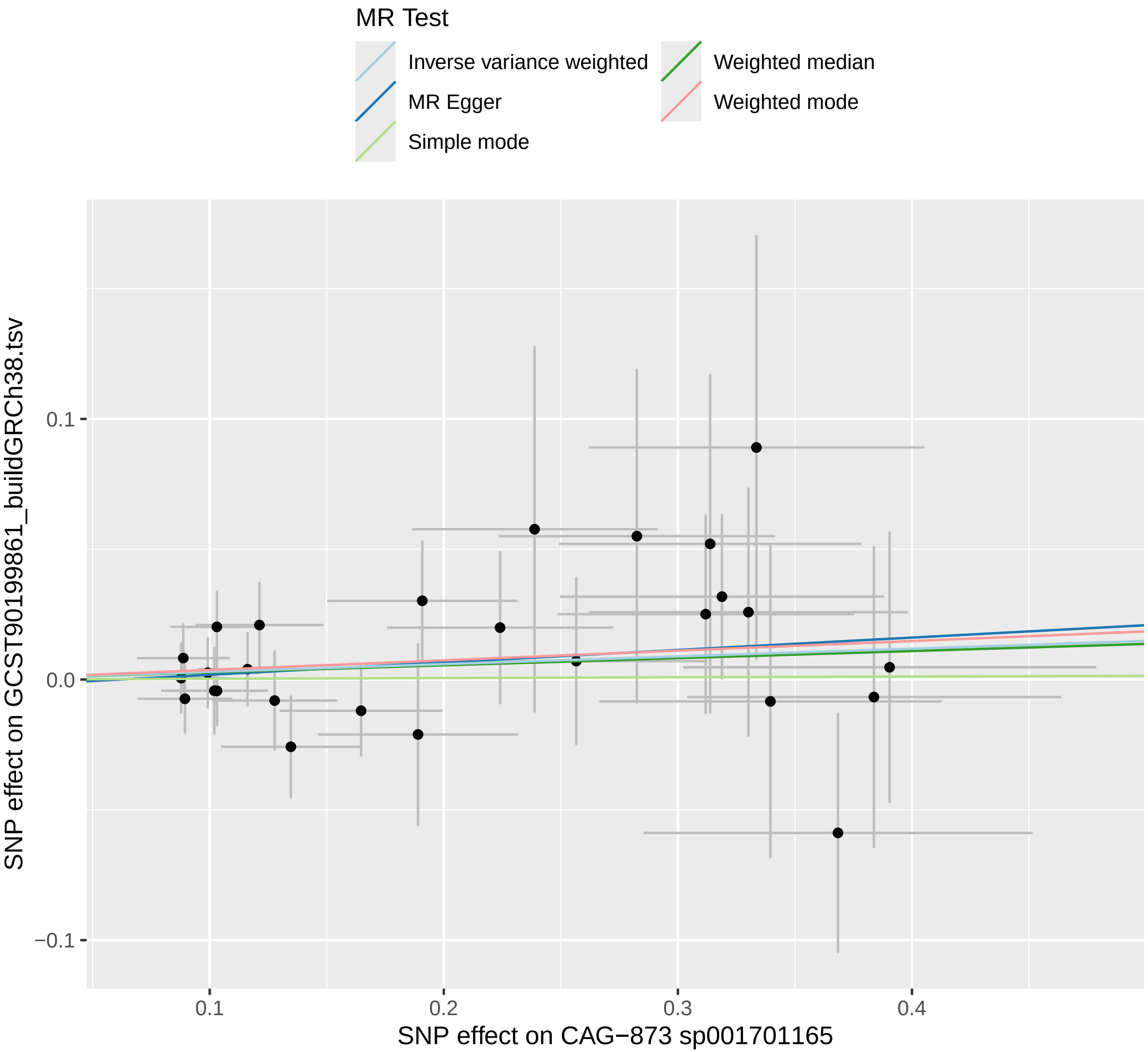
MR Test
Inverse variance weighted
MR Egger
Simple mode
Weighted median
Weighted mode
SNP effect on GCST90199861_buildGRCh38.tsv
0.1
0.0
−0.1
0.1
0.2
0.3
0.4
SNP effect on CAG−873 sp001701165

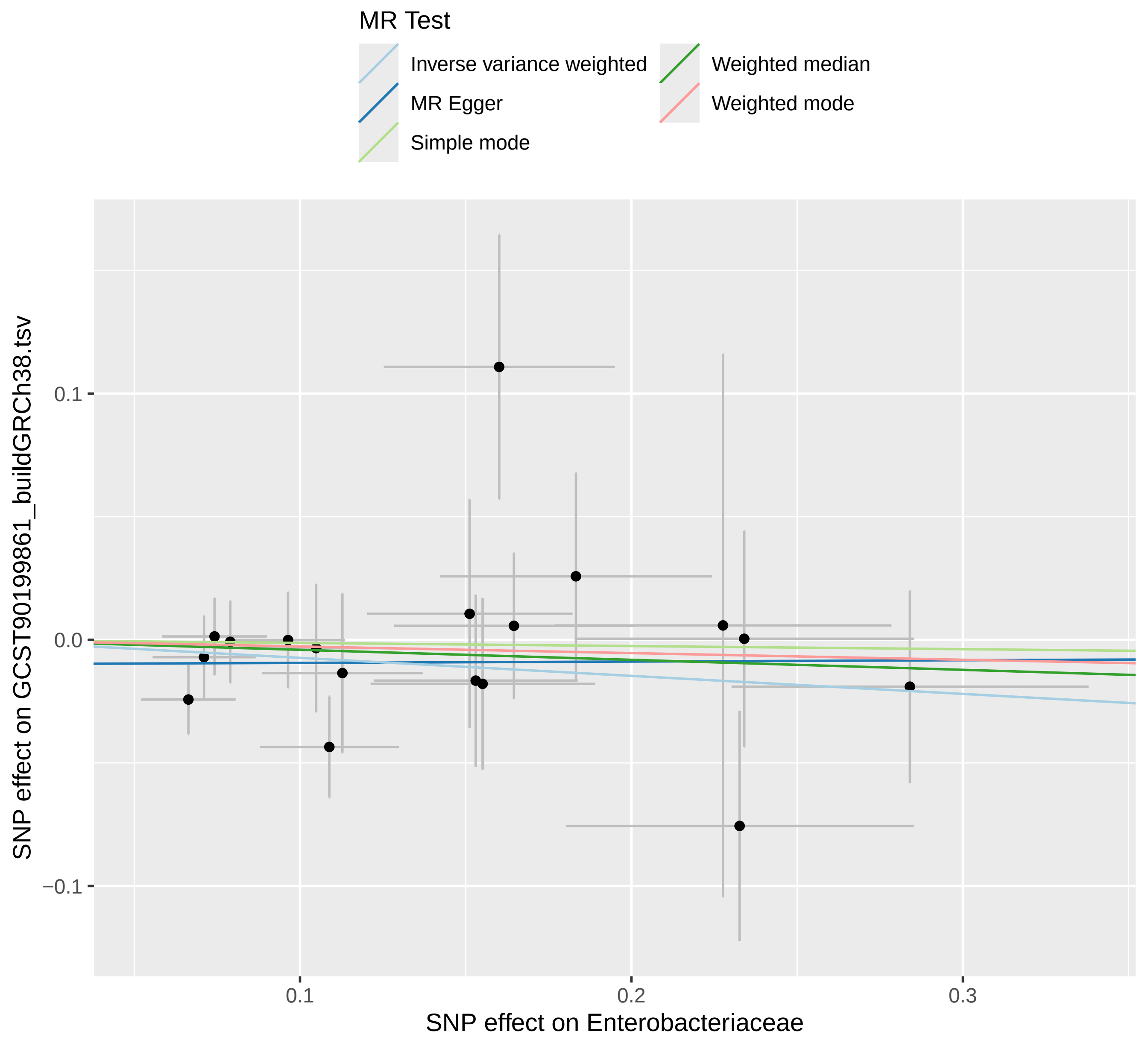

MR Test
Inverse variance weighted
MR Egger
Simple mode
Weighted median
Weighted mode
0.1
0.0
−0.1
0.1
0.2
0.3
SNP effect on GCST90199861_buildGRCh38.tsv
SNP effect on Enterobacteriaceae

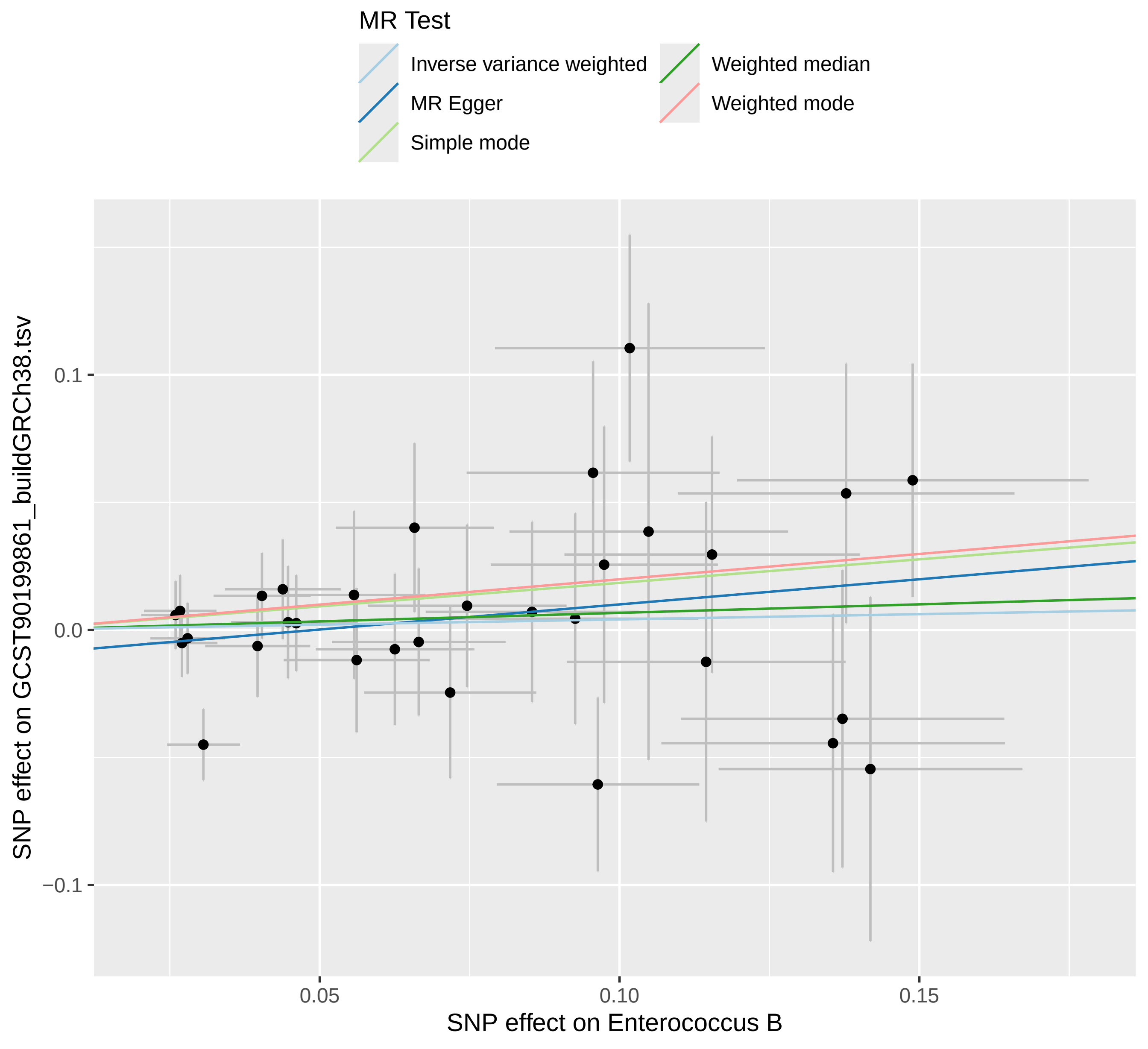

MR Test
Inverse variance weighted
MR Egger
Simple mode
Weighted median
Weighted mode
SNP effect on GCST90199861_buildGRCh38.tsv
0.1
0.0
−0.1
0.05
0.10
0.15
SNP effect on Enterococcus B

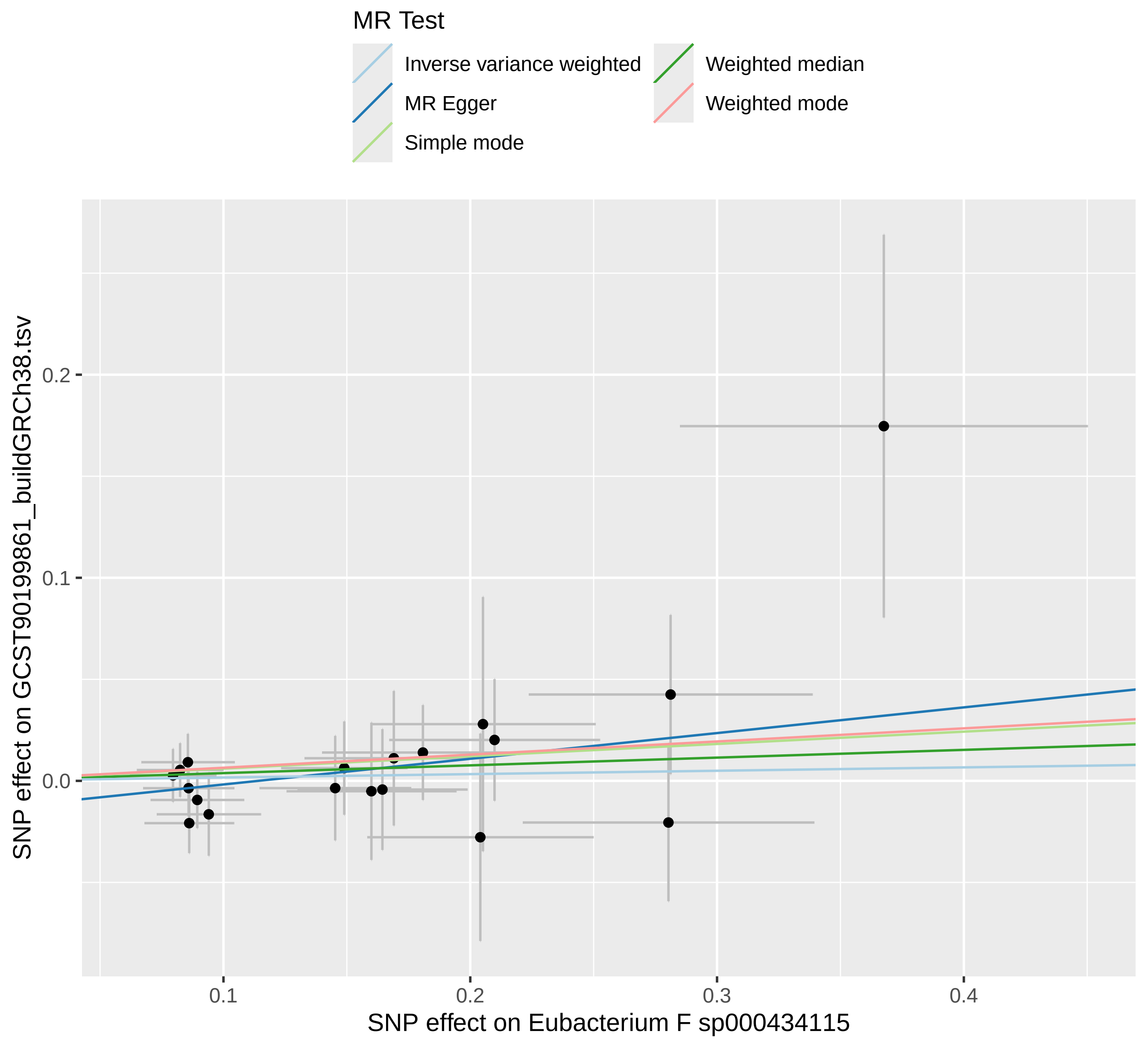

MR Test
Inverse variance weighted
MR Egger
Simple mode
Weighted median
Weighted mode
SNP effect on GCST90199861_buildGRCh38.tsv
0.2
0.1
0.0
0.1
0.2
0.3
0.4
SNP effect on Eubacterium F sp000434115

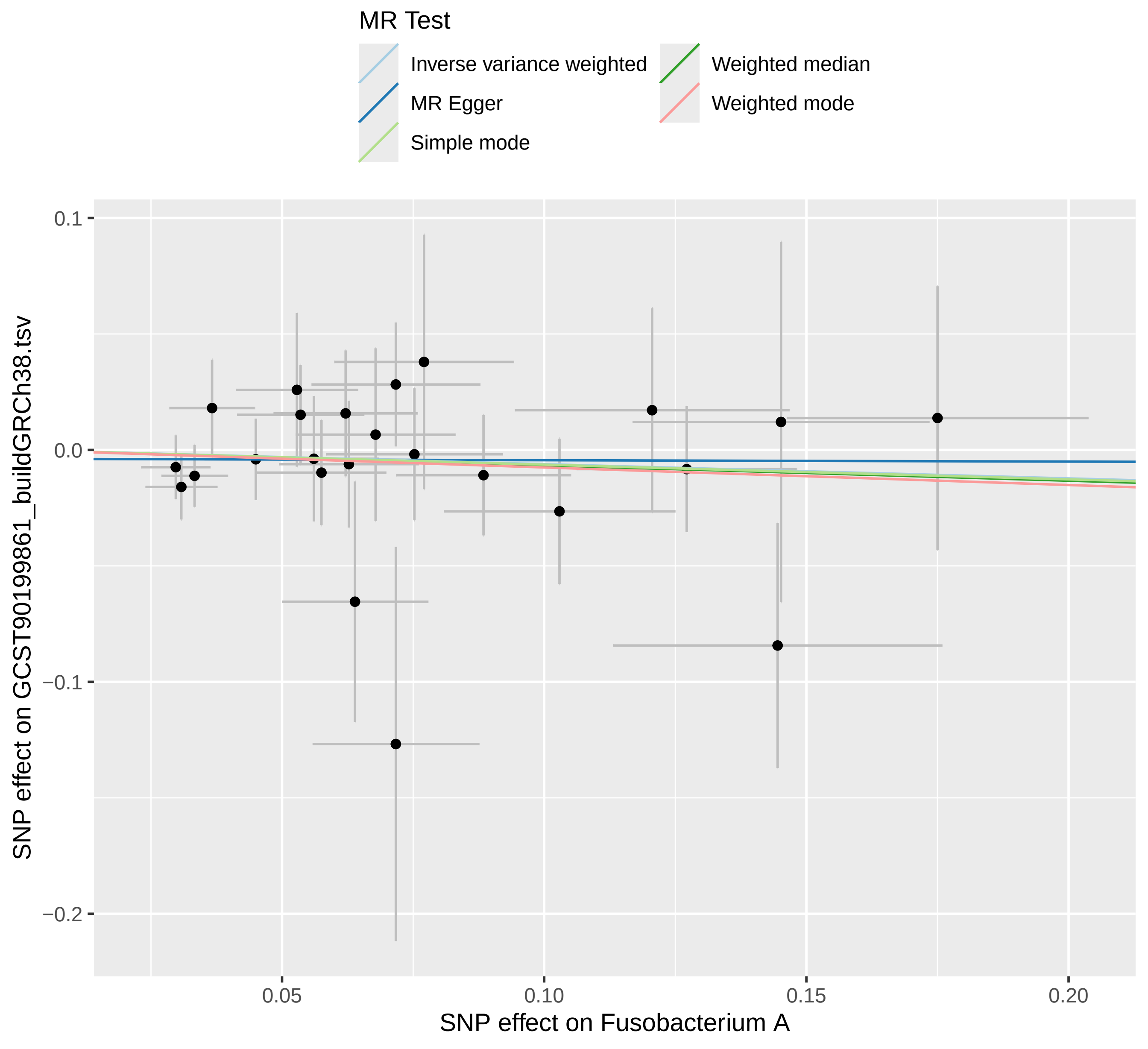
MR Test
Inverse variance weighted
MR Egger
Simple mode
Weighted median
Weighted mode
SNP effect on GCST90199861_buildGRCh38.tsv
0.1
0.0
−0.1
−0.2
0.05
0.10
0.15
0.20
SNP effect on Fusobacterium A

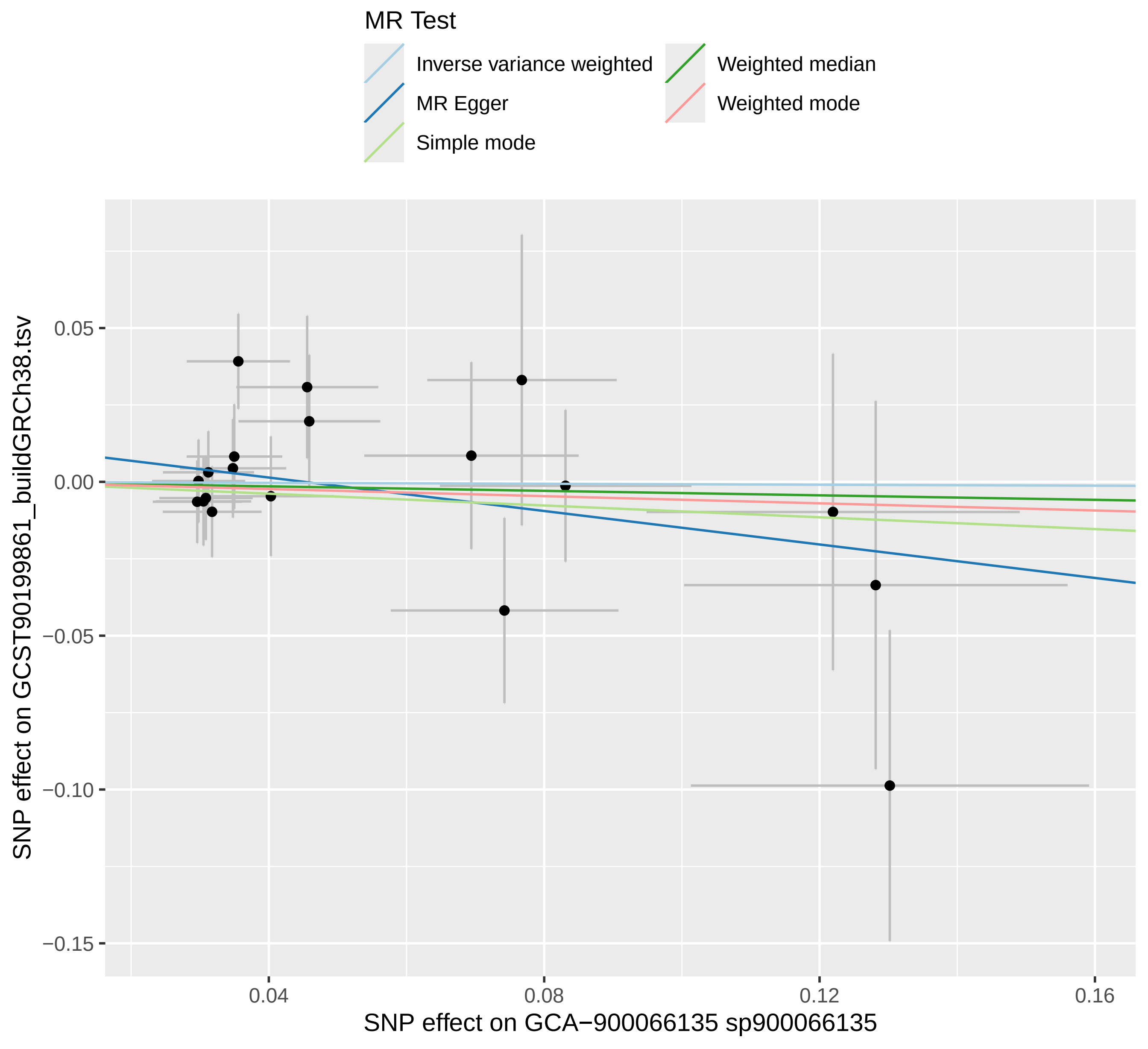

MR Test
Inverse variance weighted
MR Egger
Simple mode
Weighted median
Weighted mode
0.05
0.00
−0.05
−0.10
−0.15
0.04
0.08
0.12
0.16
SNP effect on GCST90199861_buildGRCh38.tsv
SNP effect on GCA−900066135 sp900066135

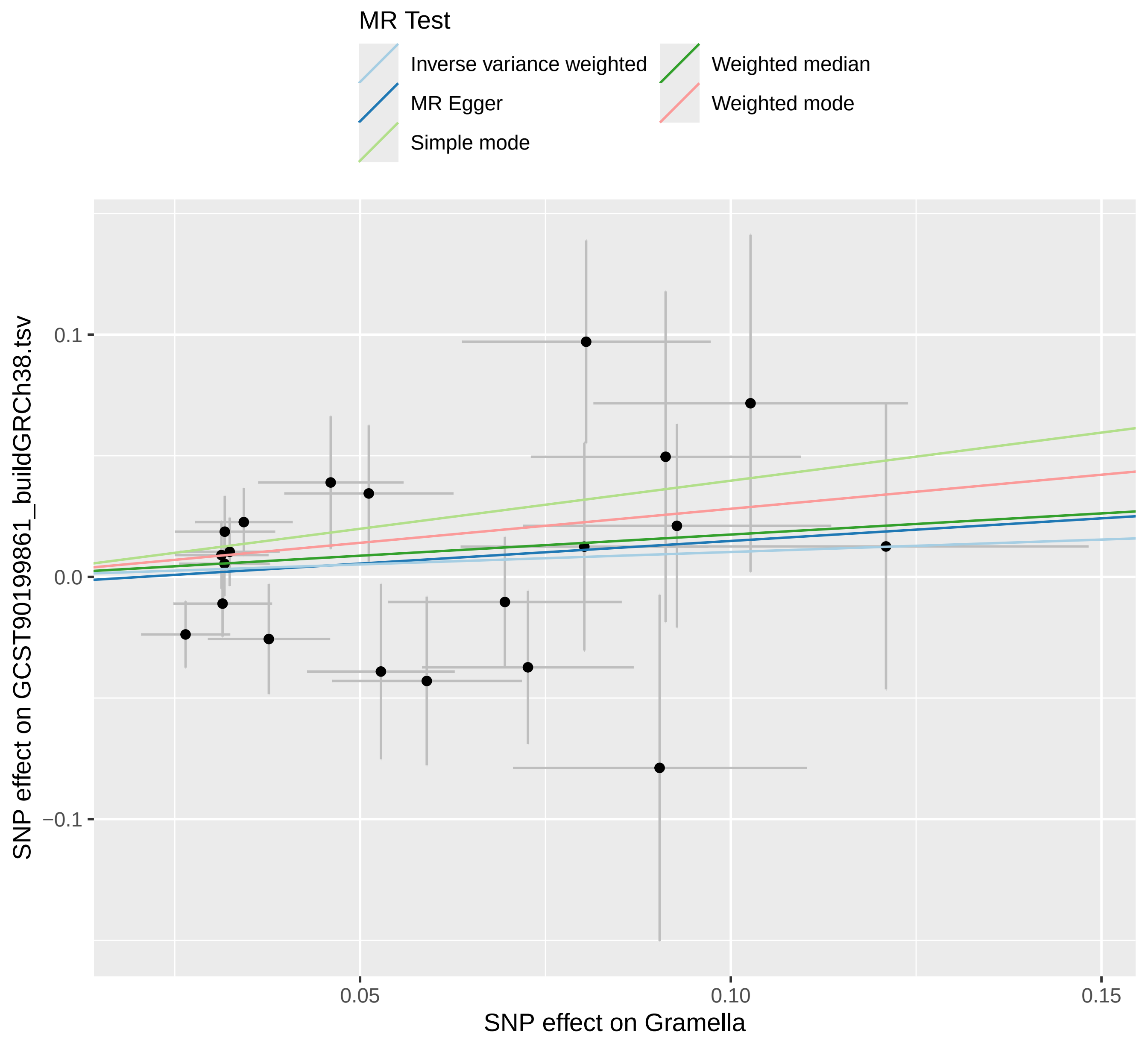

MR Test
Inverse variance weighted
MR Egger
Simple mode
Weighted median
Weighted mode
0.1
0.0
−0.1
0.05
0.10
0.15
SNP effect on GCST90199861_buildGRCh38.tsv
SNP effect on Gramella

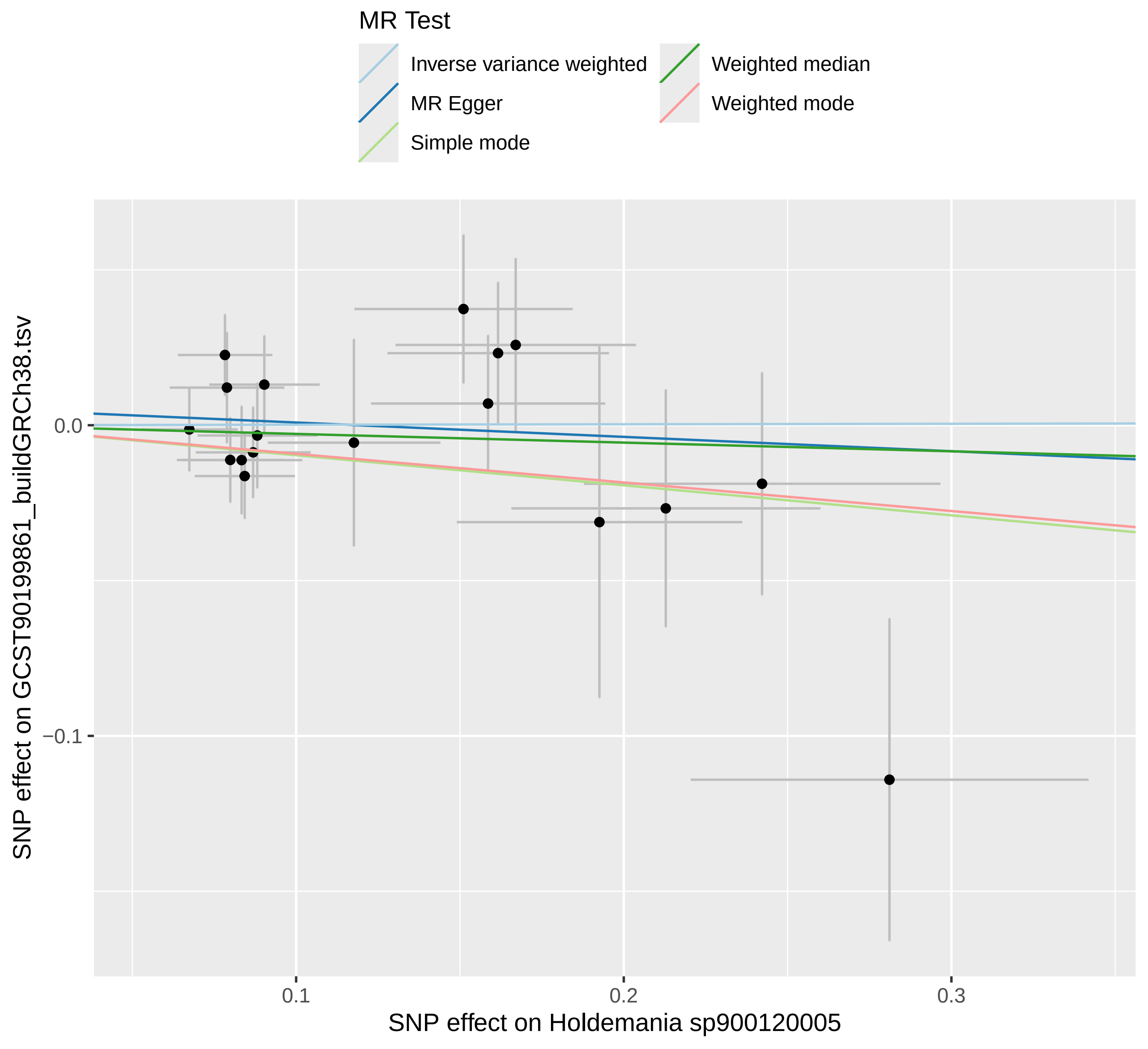
MR Test
Inverse variance weighted
MR Egger
Simple mode
Weighted median
Weighted mode
SNP effect on GCST90199861_buildGRCh38.tsv
0.0
−0.1
0.1
0.2
0.3
SNP effect on Holdemania sp900120005

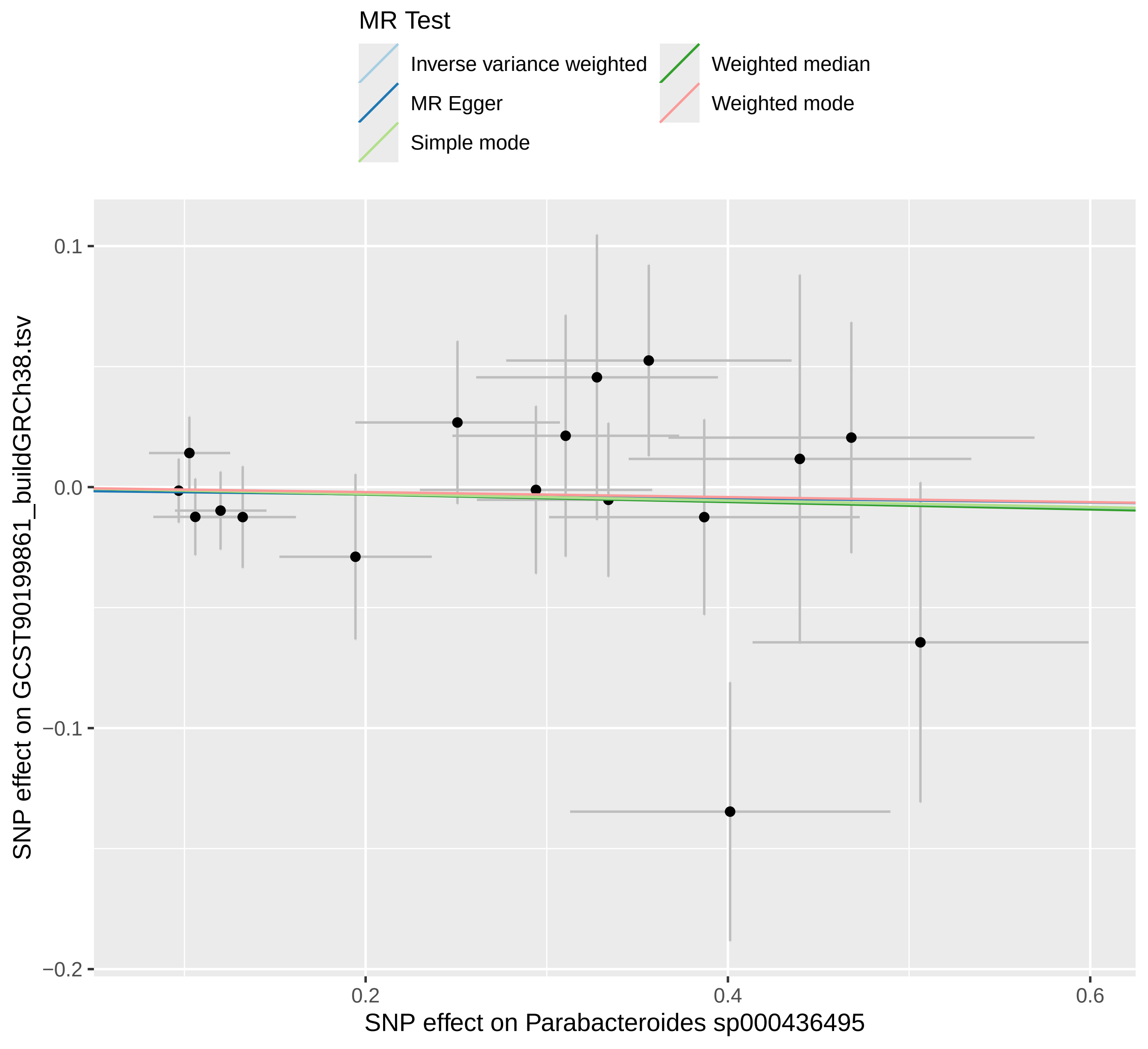
MR Test
Inverse variance weighted
MR Egger
Simple mode
Weighted median
Weighted mode
0.1
0.0
−0.1
−0.2
0.2
0.4
0.6
SNP effect on GCST90199861_buildGRCh38.tsv
SNP effect on Parabacteroides sp000436495

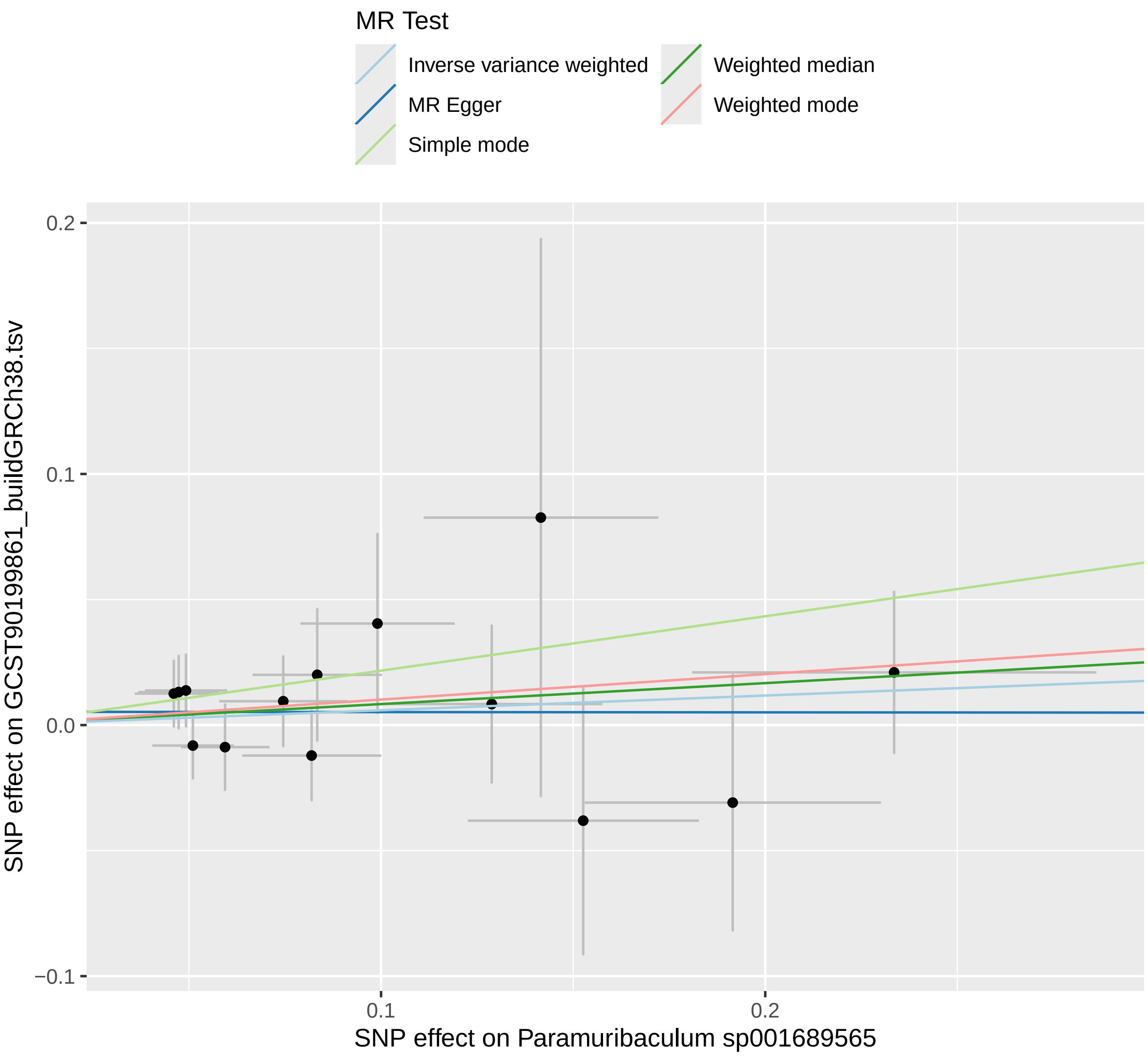
MR Test
Inverse variance weighted
MR Egger
Simple mode
Weighted median
Weighted mode
0.2
0.1
0.0
−0.1
0.1
0.2
SNP effect on GCST90199861_buildGRCh38.tsv
SNP effect on Paramuribaculum sp001689565

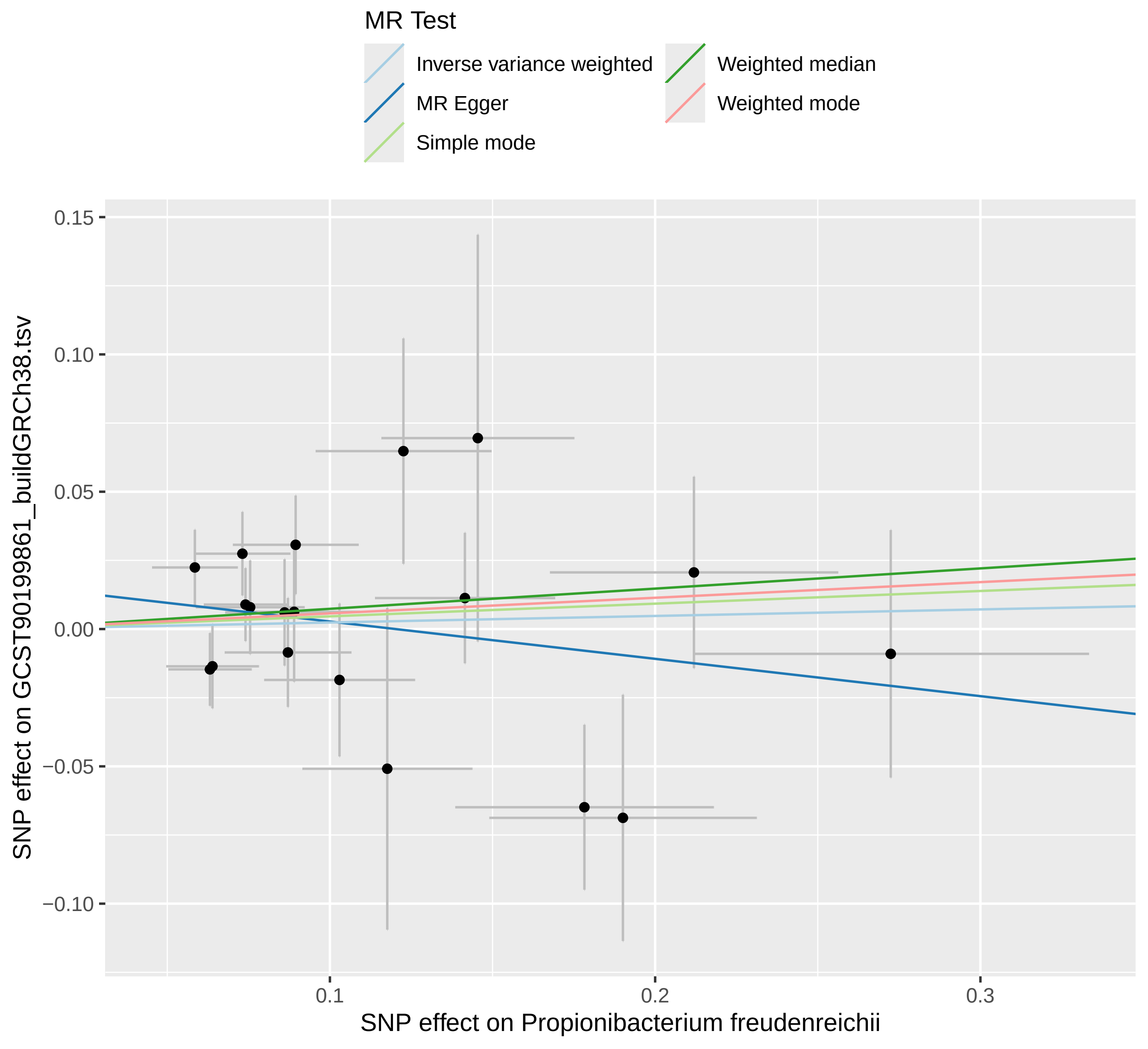

MR Test
Inverse variance weighted
MR Egger
Simple mode
Weighted median
Weighted mode
0.15
0.10
0.05
0.00
−0.05
−0.10
0.1
0.2
0.3
SNP effect on GCST90199861_buildGRCh38.tsv
SNP effect on Propionibacterium freudenreichii

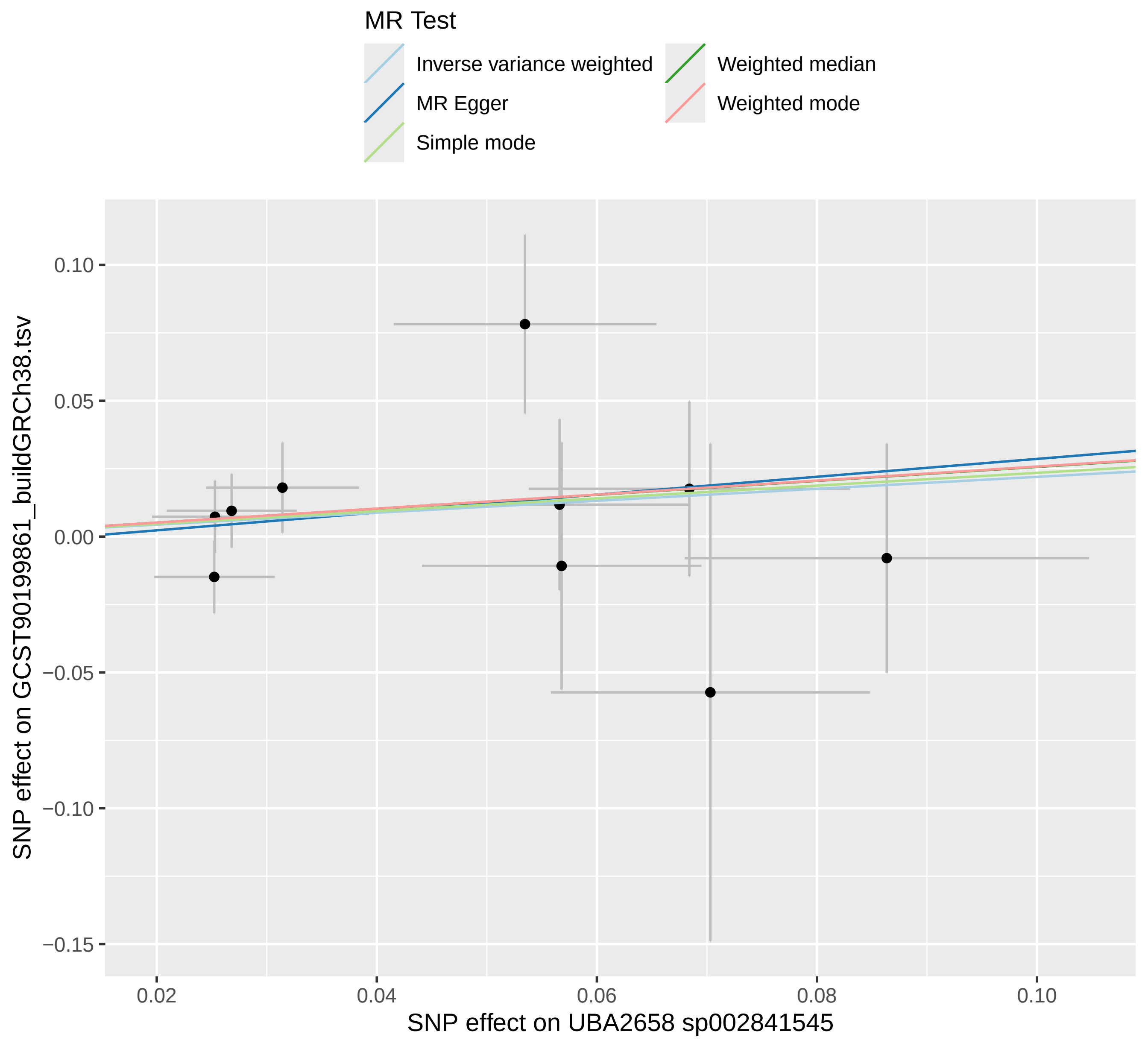
MR Test
Inverse variance weighted
MR Egger
Simple mode
Weighted median
Weighted mode
0.10
0.05
0.00
−0.05
−0.10
−0.15
0.02
0.04
0.06
0.08
0.10
SNP effect on GCST90199861_buildGRCh38.tsv
SNP effect on UBA2658 sp002841545

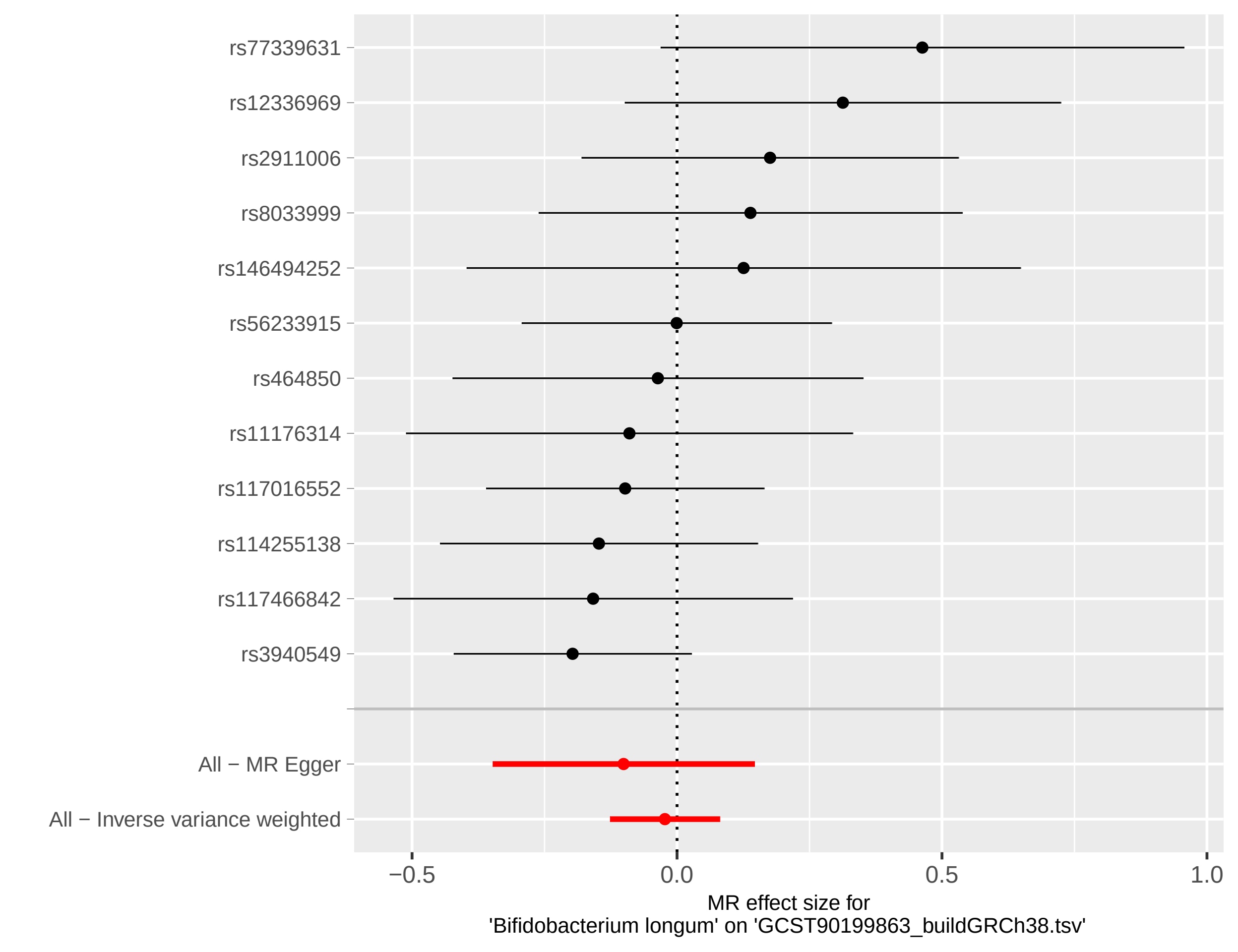

rs77339631
rs12336969
rs2911006
rs8033999
rs146494252
rs56233915
rs464850
rs11176314
rs117016552
rs114255138
rs117466842
rs3940549
All − MR Egger
All − Inverse variance weighted
−0.5
0.0
0.5
1.0
MR effect size for
'Bifidobacterium longum' on 'GCST90199863_buildGRCh38.tsv'

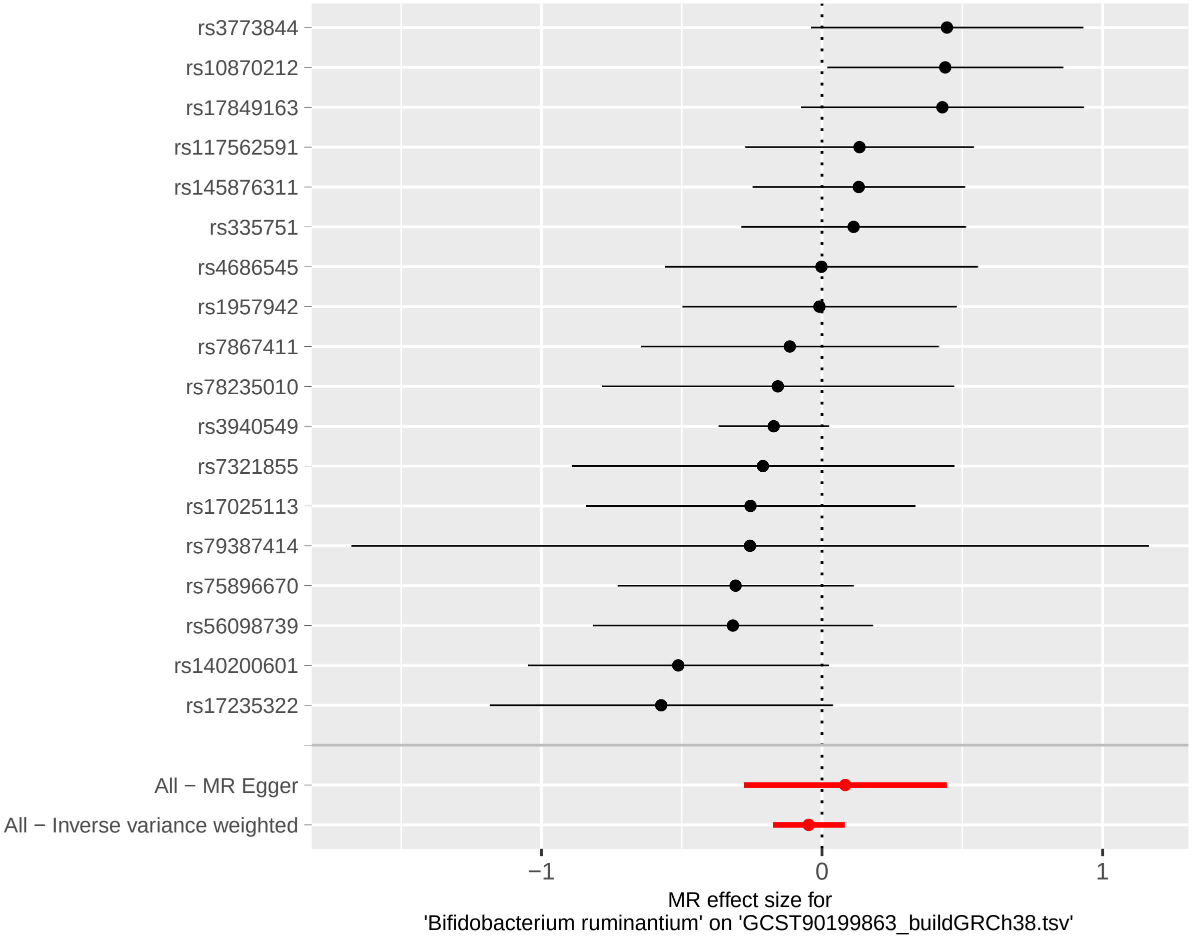

rs3773844
rs10870212
rs17849163
rs117562591
rs145876311
rs335751
rs4686545
rs1957942
rs7867411
rs78235010
rs3940549
rs7321855
rs17025113
rs79387414
rs75896670
rs56098739
rs140200601
rs17235322
All − MR Egger
All − Inverse variance weighted
−1
0
1
MR effect size for
'Bifidobacterium ruminantium' on 'GCST90199863_buildGRCh38.tsv'

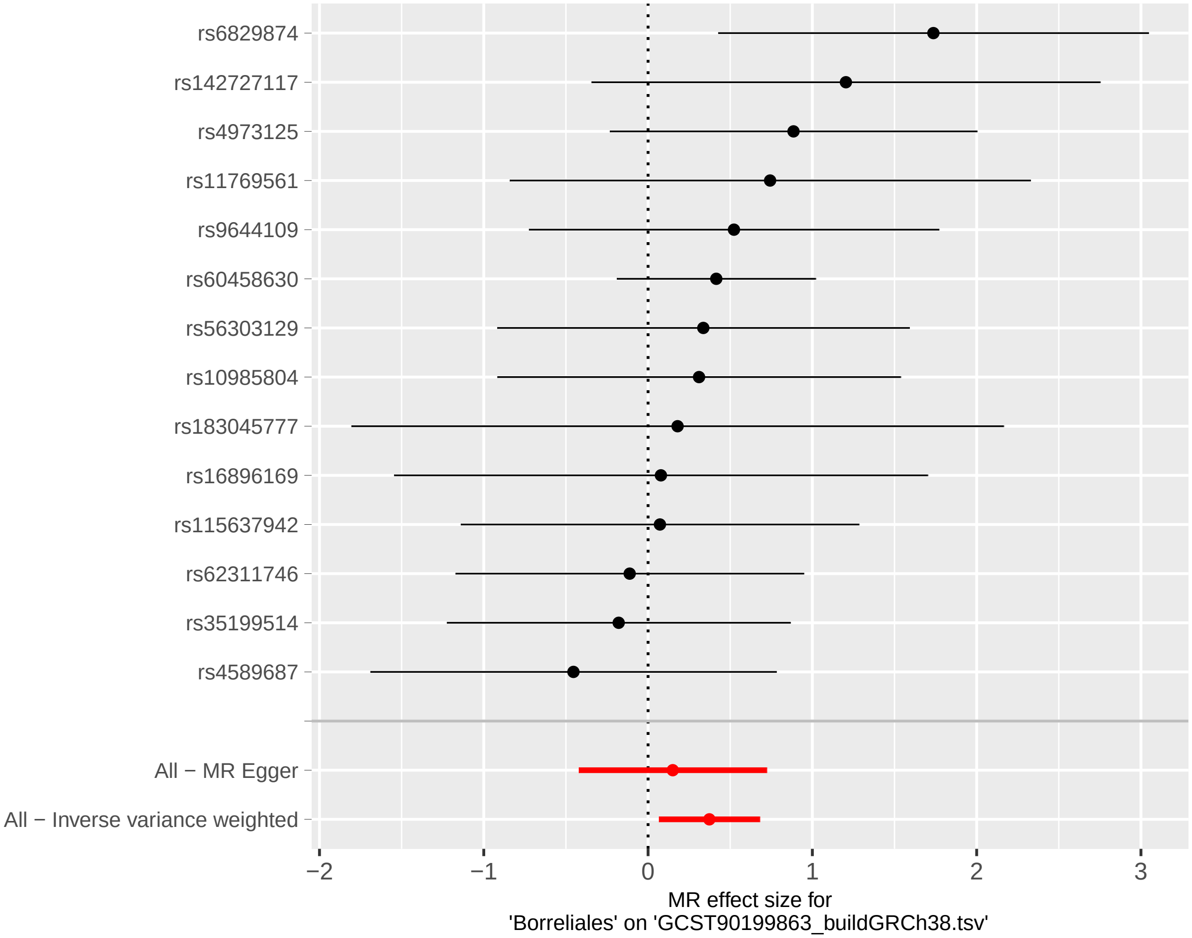
rs6829874
rs142727117
rs4973125
rs11769561
rs9644109
rs60458630
rs56303129
rs10985804
rs183045777
rs16896169
rs115637942
rs62311746
rs35199514
rs4589687
All − MR Egger
All − Inverse variance weighted
−2
−1
0
1
2
3
MR effect size for
'Borreliales' on 'GCST90199863_buildGRCh38.tsv'

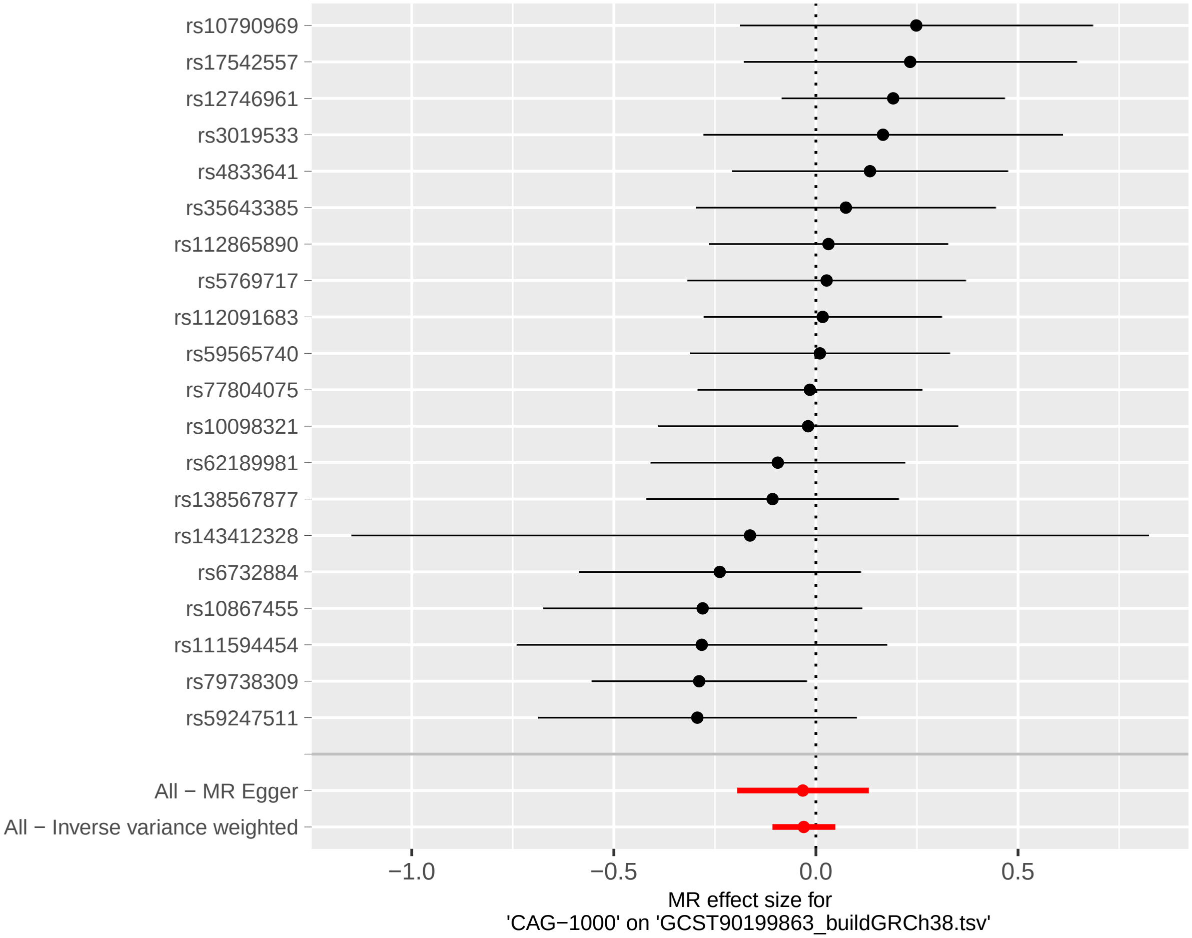

rs10790969
rs17542557
rs12746961
rs3019533
rs4833641
rs35643385
rs112865890
rs5769717
rs112091683
rs59565740
rs77804075
rs10098321
rs62189981
rs138567877
rs143412328
rs6732884
rs10867455
rs111594454
rs79738309
rs59247511
All − MR Egger
All − Inverse variance weighted
−1.0
−0.5
0.0
0.5
MR effect size for
'CAG−1000' on 'GCST90199863_buildGRCh38.tsv'

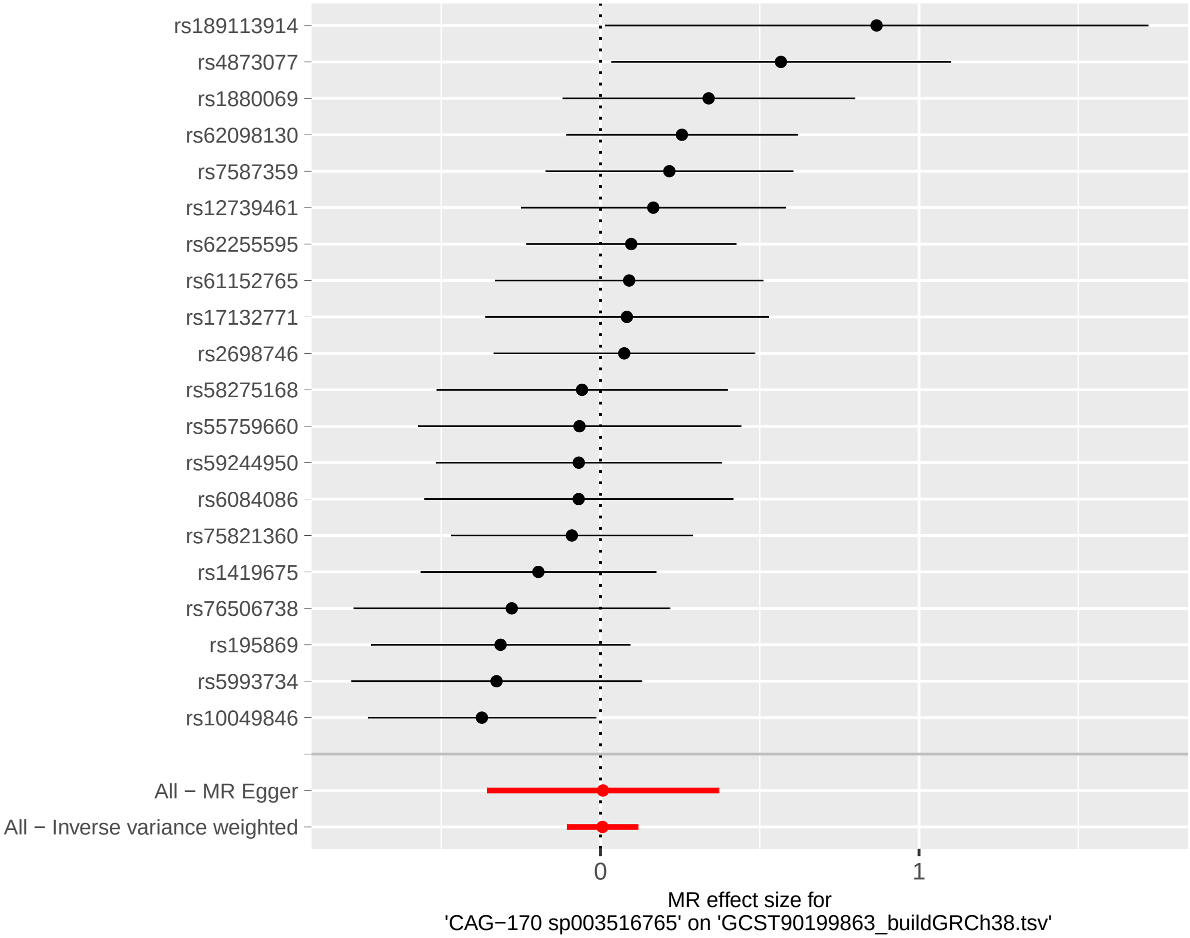
rs189113914
rs4873077
rs1880069
rs62098130
rs7587359
rs12739461
rs62255595
rs61152765
rs17132771
rs2698746
rs58275168
rs55759660
rs59244950
rs6084086
rs75821360
rs1419675
rs76506738
rs195869
rs5993734
rs10049846
All − MR Egger
All − Inverse variance weighted
0
1
MR effect size for
'CAG−170 sp003516765' on 'GCST90199863_buildGRCh38.tsv'

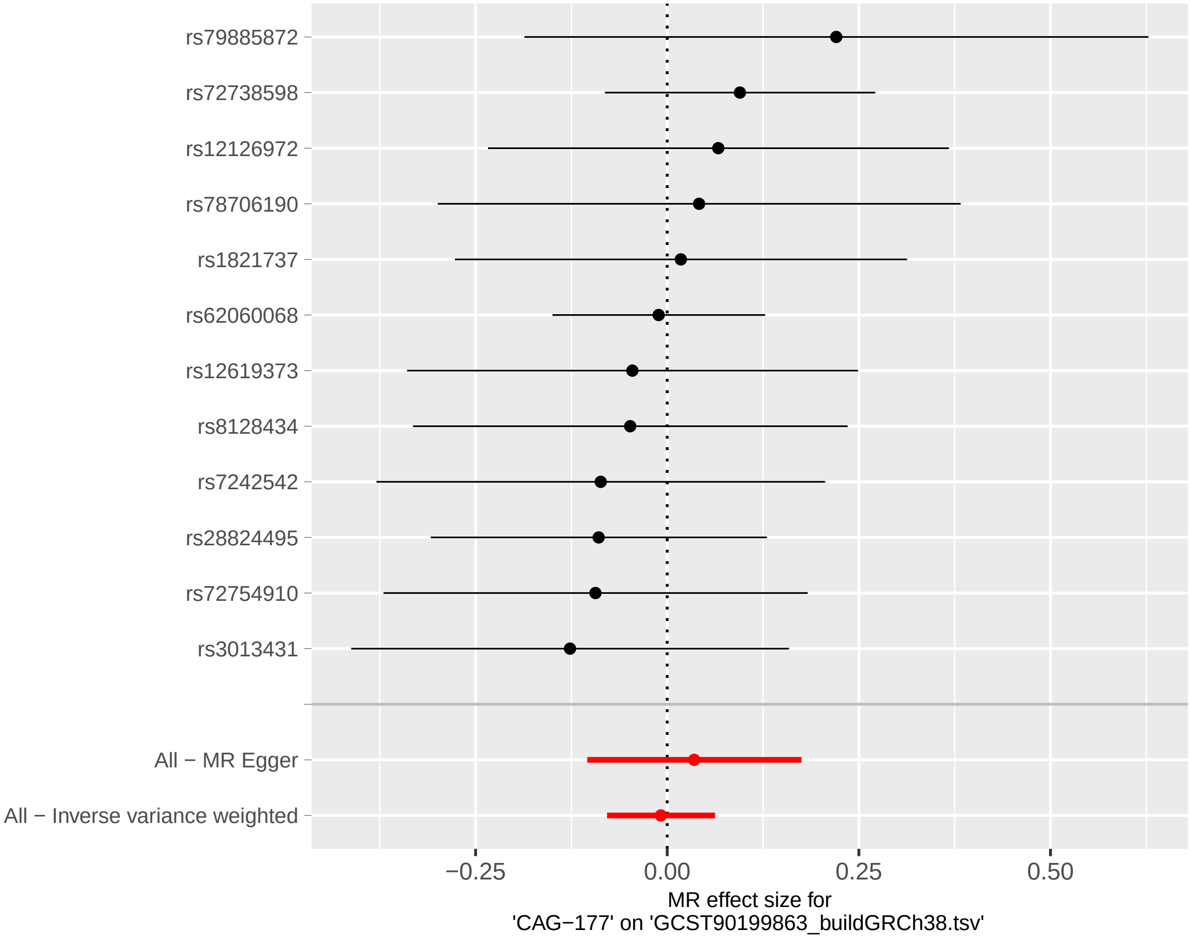

rs79885872
rs72738598
rs12126972
rs78706190
rs1821737
rs62060068
rs12619373
rs8128434
rs7242542
rs28824495
rs72754910
rs3013431
All − MR Egger
All − Inverse variance weighted
−0.25
0.00
0.25
0.50
MR effect size for
'CAG−177' on 'GCST90199863_buildGRCh38.tsv'

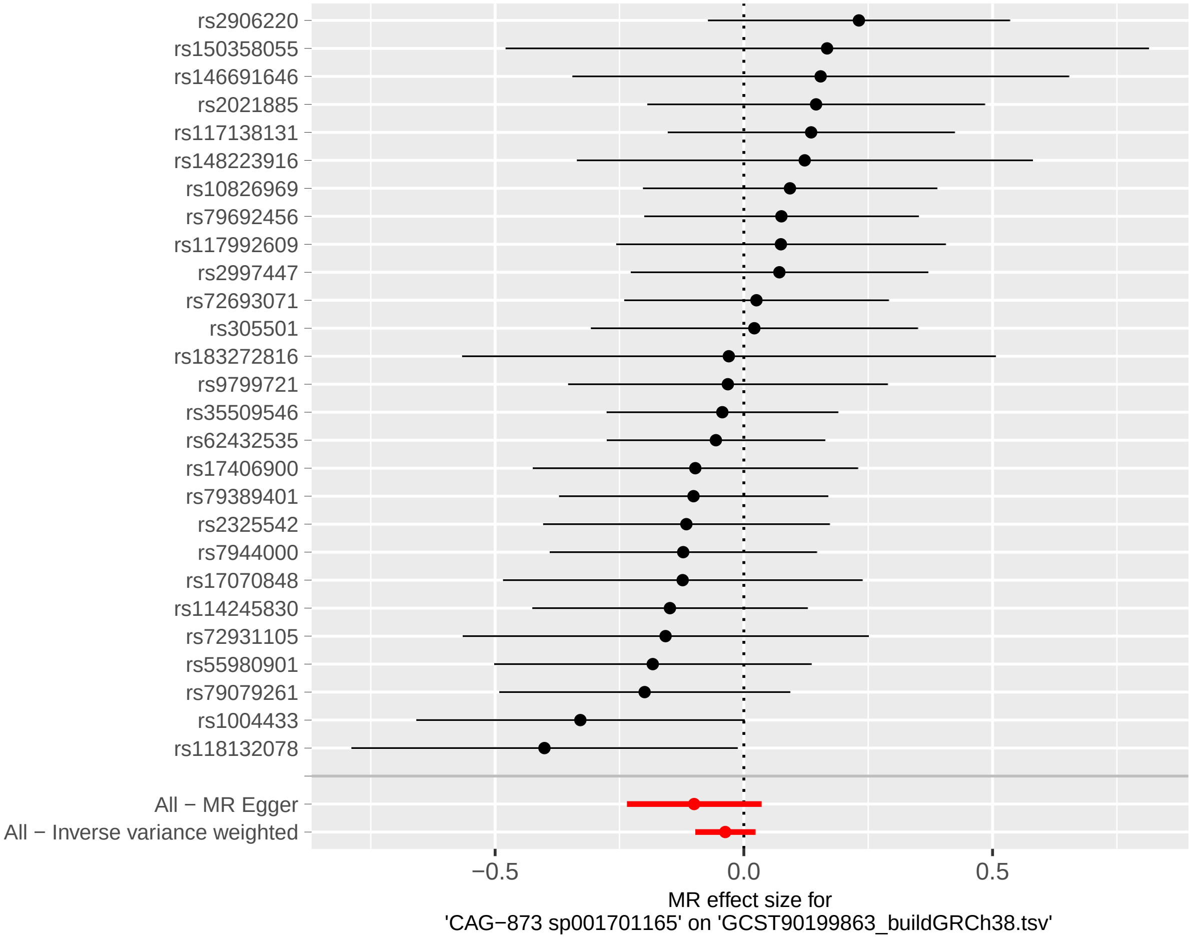
rs2906220
rs150358055
rs146691646
rs2021885
rs117138131
rs148223916
rs10826969
rs79692456
rs117992609
rs2997447
rs72693071
rs305501
rs183272816
rs9799721
rs35509546
rs62432535
rs17406900
rs79389401
rs2325542
rs7944000
rs17070848
rs114245830
rs72931105
rs55980901
rs79079261
rs1004433
rs118132078
All − MR Egger
All − Inverse variance weighted
−0.5
0.0
0.5
MR effect size for
'CAG−873 sp001701165' on 'GCST90199863_buildGRCh38.tsv'

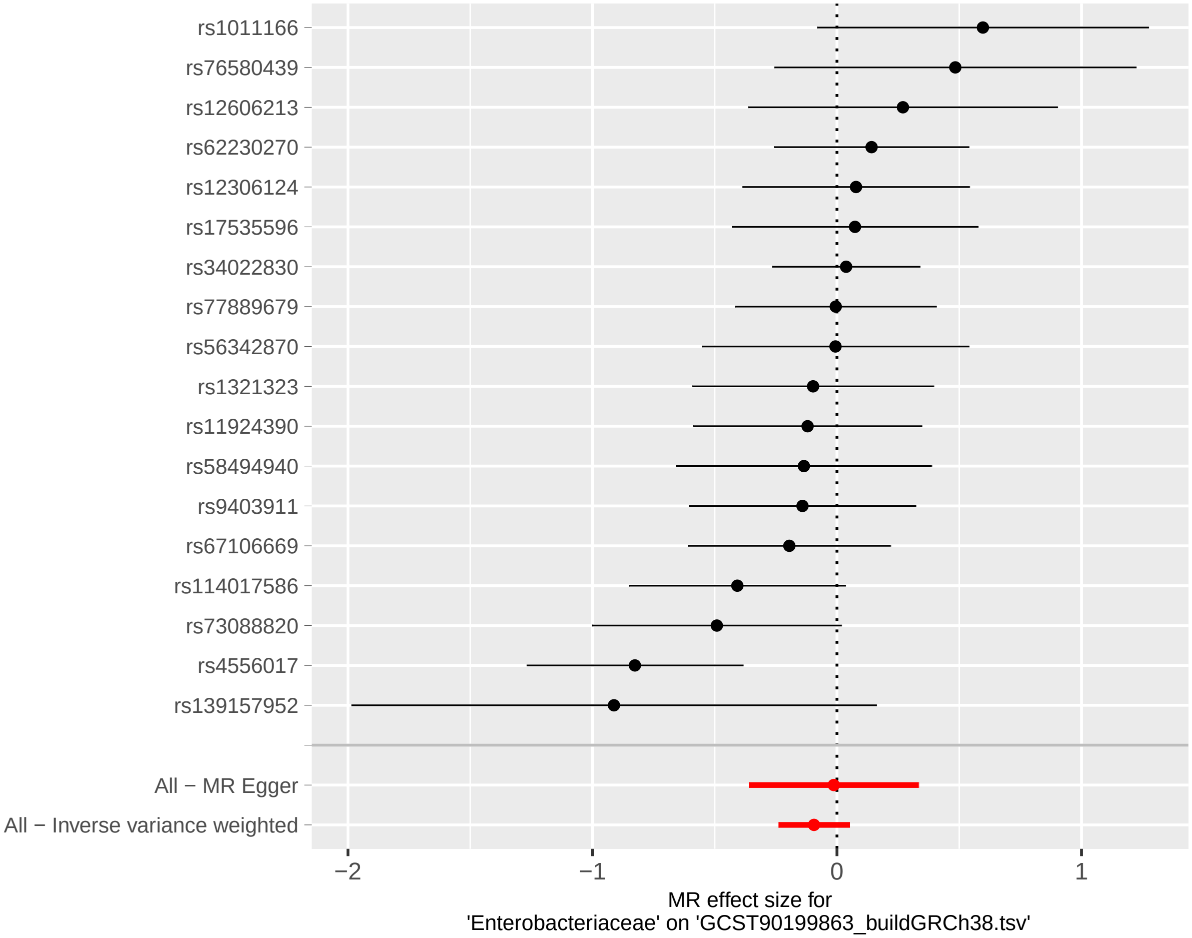
rs1011166
rs76580439
rs12606213
rs62230270
rs12306124
rs17535596
rs34022830
rs77889679
rs56342870
rs1321323
rs11924390
rs58494940
rs9403911
rs67106669
rs114017586
rs73088820
rs4556017
rs139157952
All − MR Egger
All − Inverse variance weighted
−2
−1
0
1
MR effect size for
'Enterobacteriaceae' on 'GCST90199863_buildGRCh38.tsv'

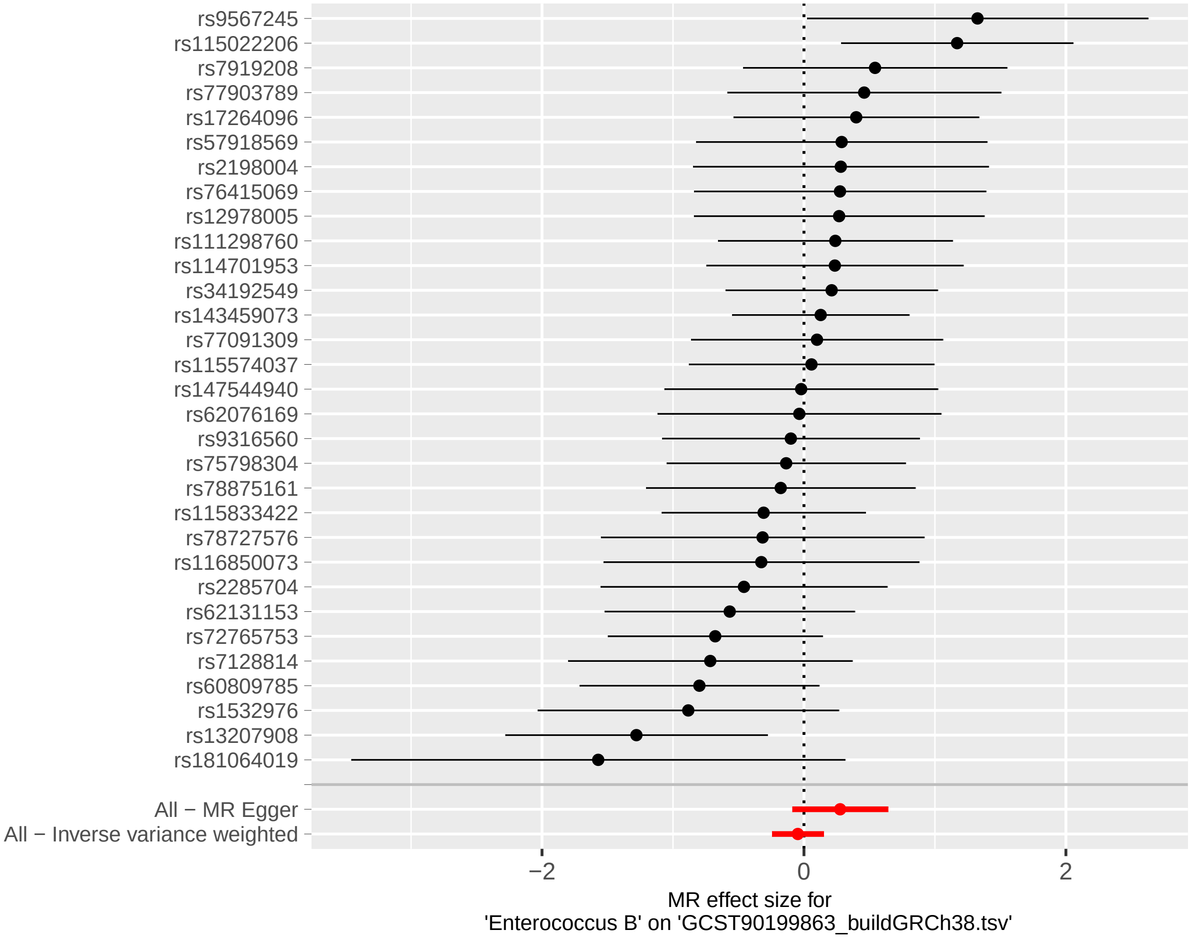

rs9567245
rs115022206
rs7919208
rs77903789
rs17264096
rs57918569
rs2198004
rs76415069
rs12978005
rs111298760
rs114701953
rs34192549
rs143459073
rs77091309
rs115574037
rs147544940
rs62076169
rs9316560
rs75798304
rs78875161
rs115833422
rs78727576
rs116850073
rs2285704
rs62131153
rs72765753
rs7128814
rs60809785
rs1532976
rs13207908
rs181064019
All − MR Egger
All − Inverse variance weighted
−2
0
2
MR effect size for
'Enterococcus B' on 'GCST90199863_buildGRCh38.tsv'

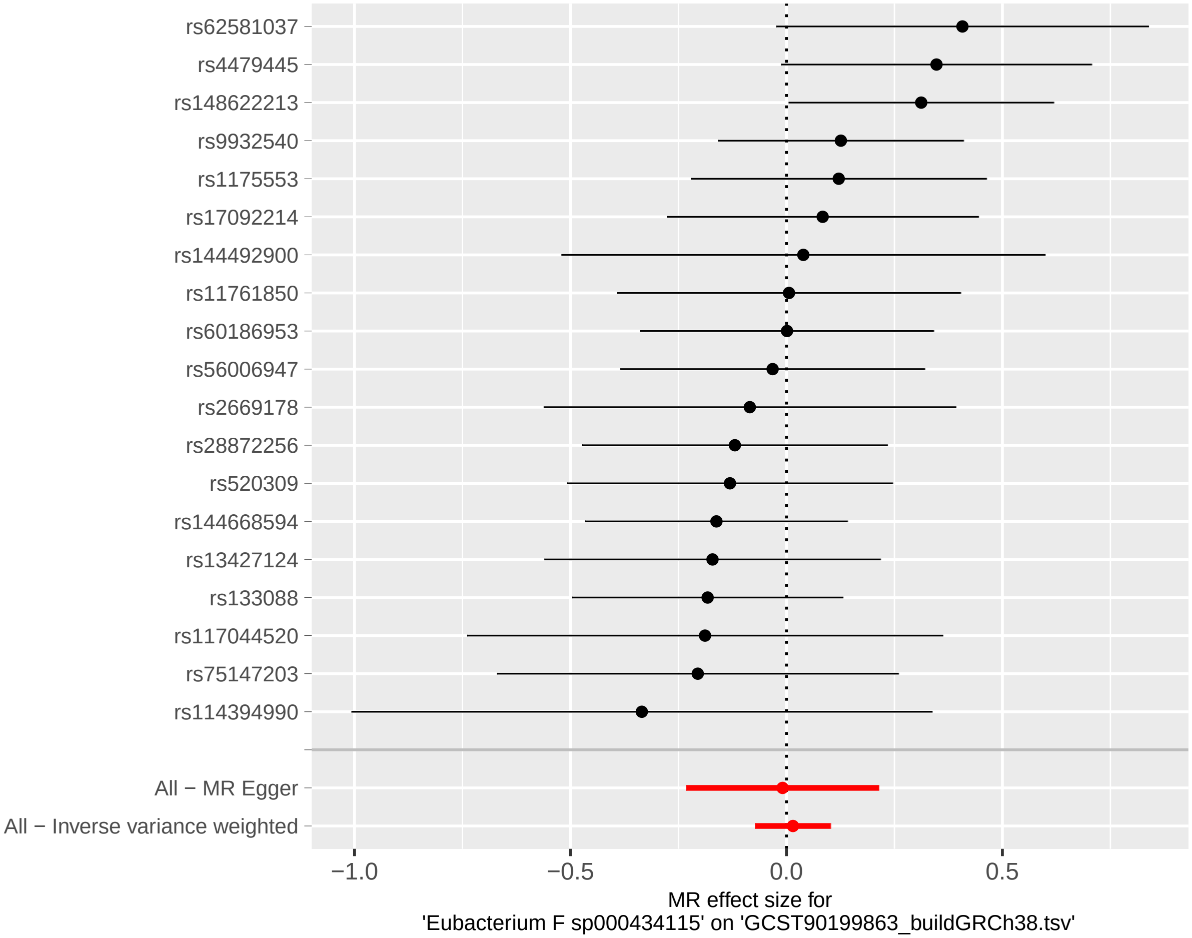
rs62581037
rs4479445
rs148622213
rs9932540
rs1175553
rs17092214
rs144492900
rs11761850
rs60186953
rs56006947
rs2669178
rs28872256
rs520309
rs144668594
rs13427124
rs133088
rs117044520
rs75147203
rs114394990
All − MR Egger
All − Inverse variance weighted
−1.0
−0.5
0.0
0.5
MR effect size for
'Eubacterium F sp000434115' on 'GCST90199863_buildGRCh38.tsv'

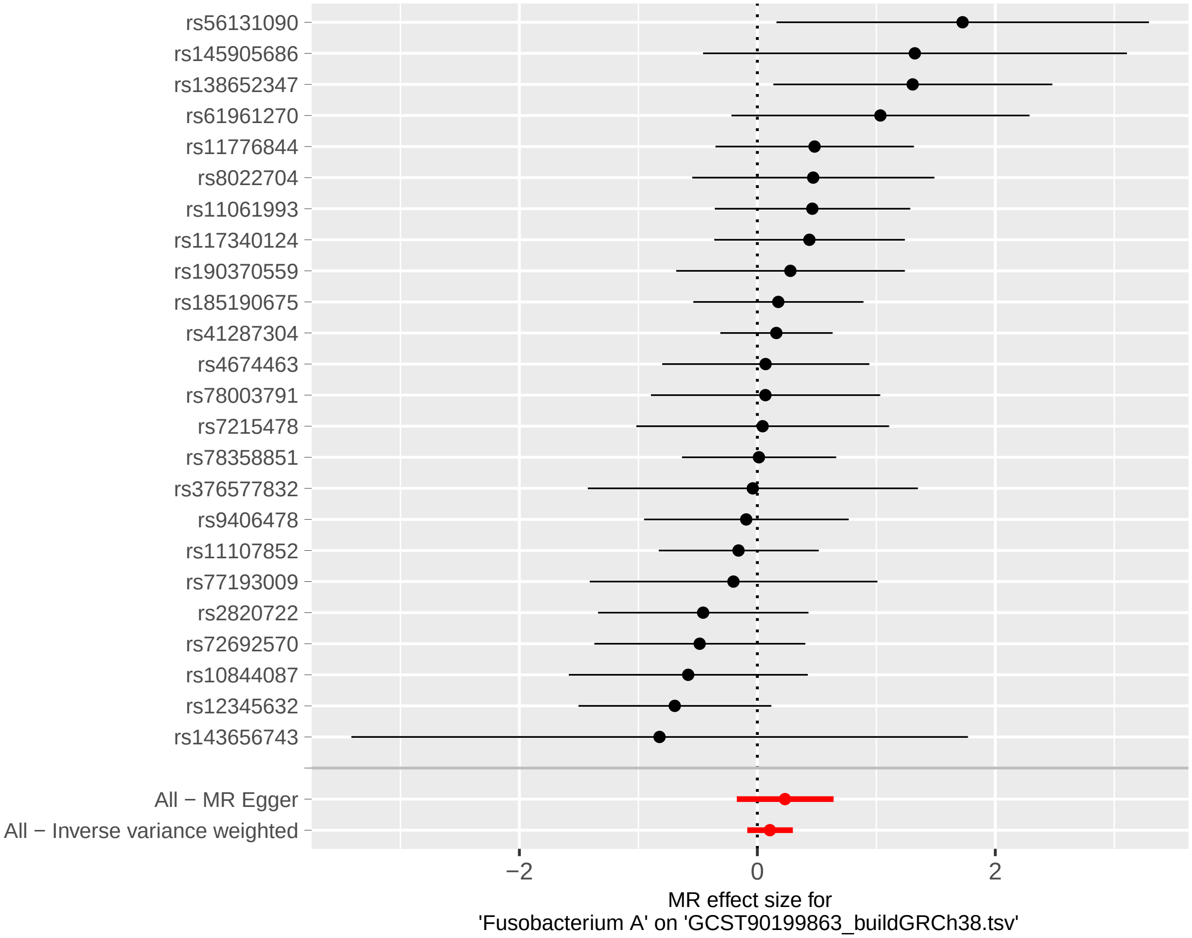
rs56131090
rs145905686
rs138652347
rs61961270
rs11776844
rs8022704
rs11061993
rs117340124
rs190370559
rs185190675
rs41287304
rs4674463
rs78003791
rs7215478
rs78358851
rs376577832
rs9406478
rs11107852
rs77193009
rs2820722
rs72692570
rs10844087
rs12345632
rs143656743
All − MR Egger
All − Inverse variance weighted
−2
0
2
MR effect size for
'Fusobacterium A' on 'GCST90199863_buildGRCh38.tsv'

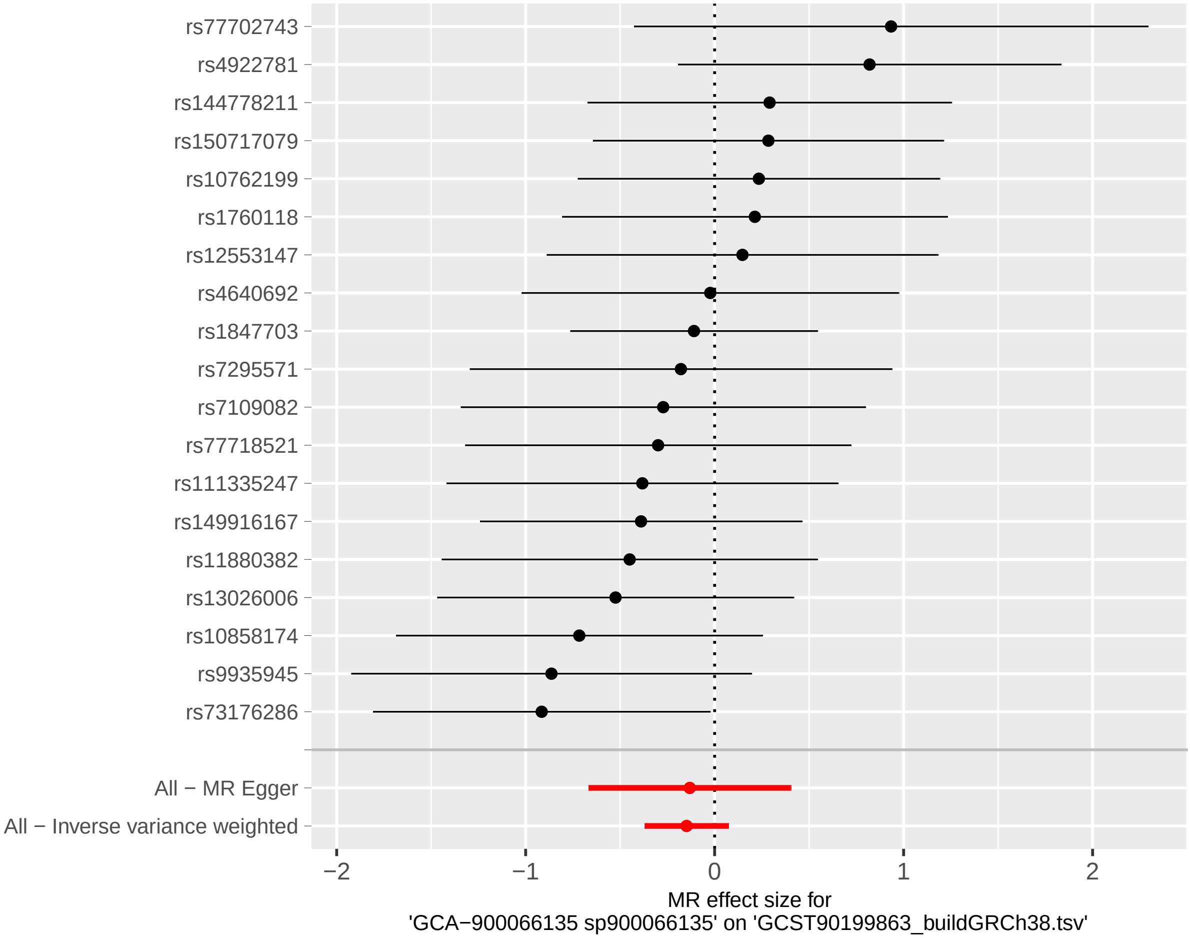

rs77702743
rs4922781
rs144778211
rs150717079
rs10762199
rs1760118
rs12553147
rs4640692
rs1847703
rs7295571
rs7109082
rs77718521
rs111335247
rs149916167
rs11880382
rs13026006
rs10858174
rs9935945
rs73176286
All − MR Egger
All − Inverse variance weighted
−2
−1
0
1
2
MR effect size for
'GCA−900066135 sp900066135' on 'GCST90199863_buildGRCh38.tsv'

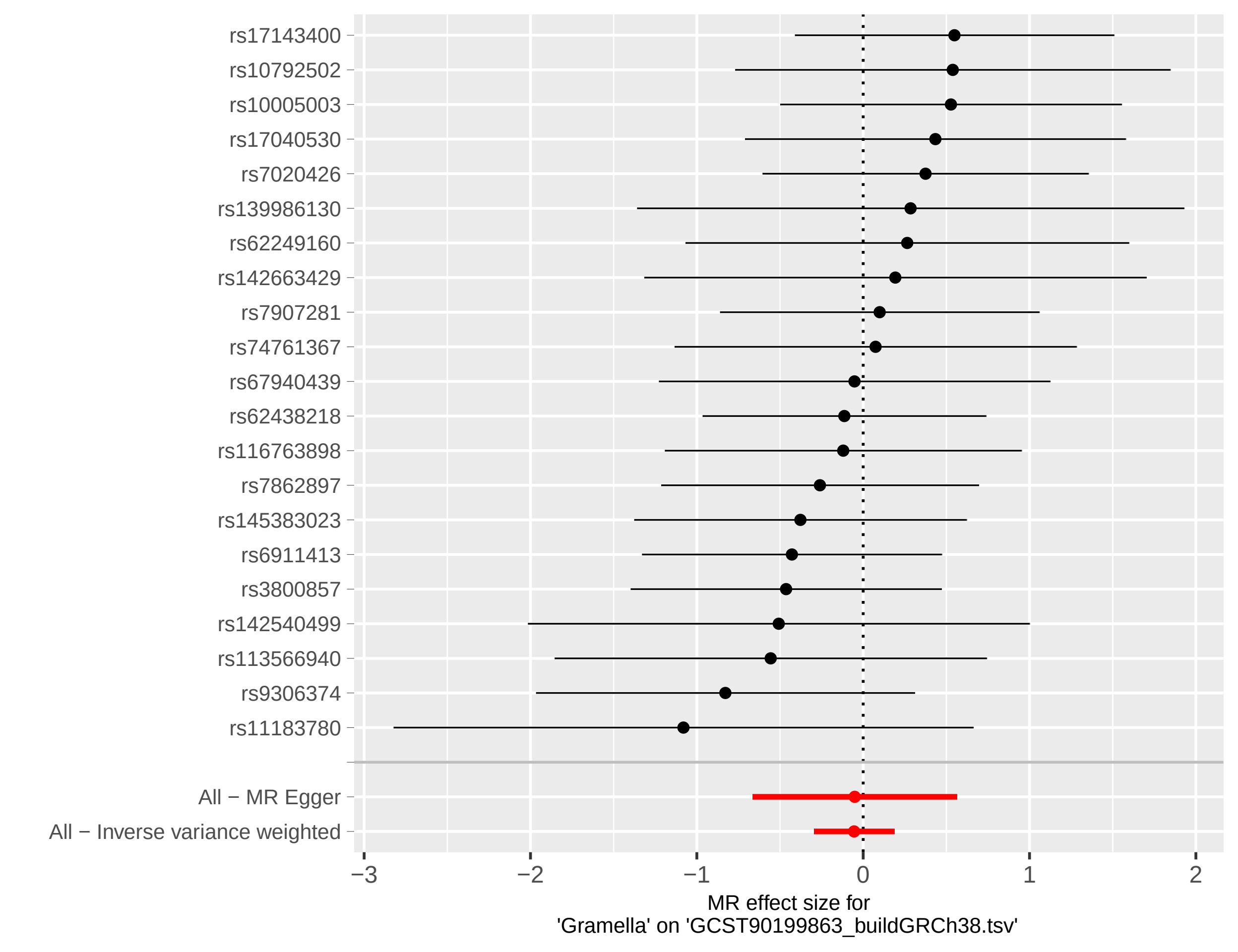
rs17143400
rs10792502
rs10005003
rs17040530
rs7020426
rs139986130
rs62249160
rs142663429
rs7907281
rs74761367
rs67940439
rs62438218
rs116763898
rs7862897
rs145383023
rs6911413
rs3800857
rs142540499
rs113566940
rs9306374
rs11183780
All − MR Egger
All − Inverse variance weighted
−3
−2
−1
0
1
2
MR effect size for
'Gramella' on 'GCST90199863_buildGRCh38.tsv'

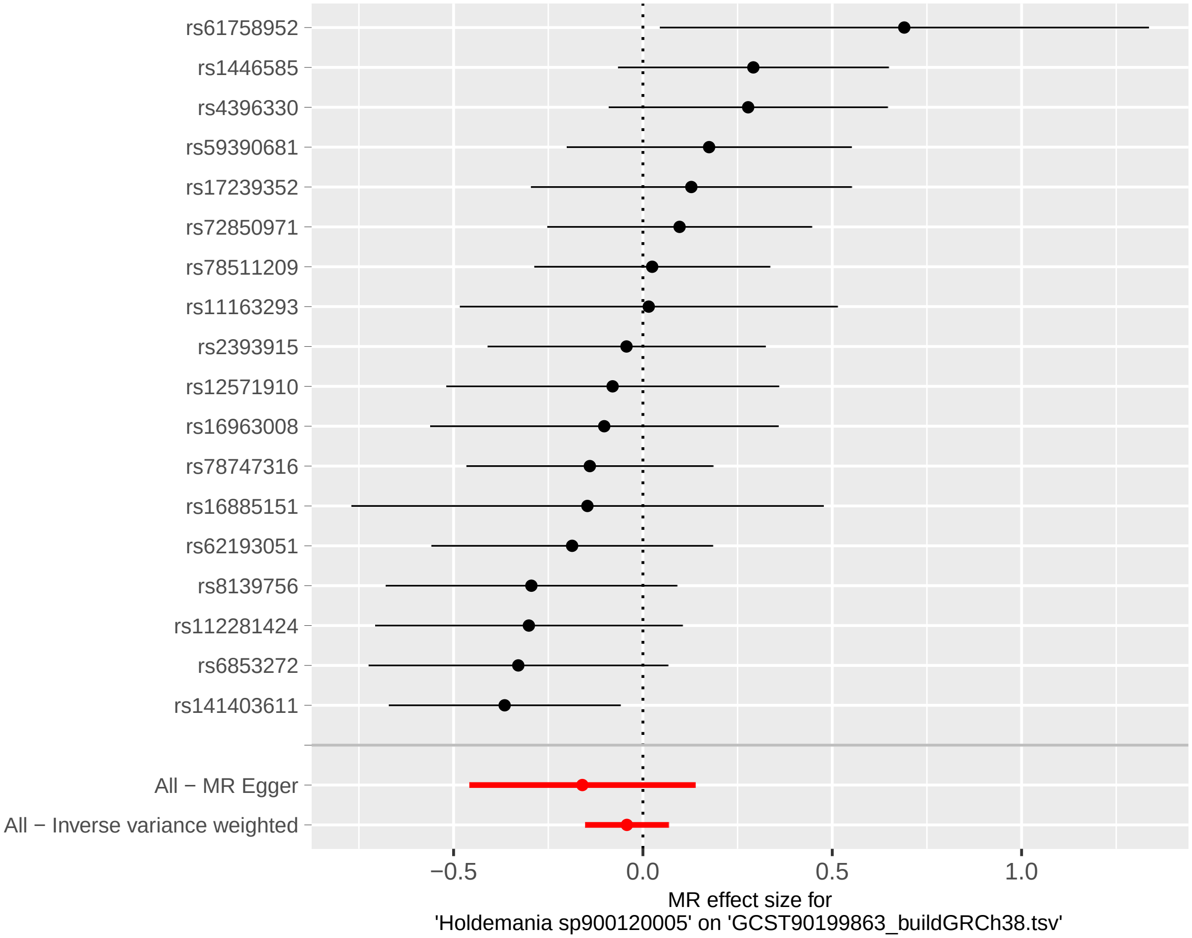
rs61758952
rs1446585
rs4396330
rs59390681
rs17239352
rs72850971
rs78511209
rs11163293
rs2393915
rs12571910
rs16963008
rs78747316
rs16885151
rs62193051
rs8139756
rs112281424
rs6853272
rs141403611
All − MR Egger
All − Inverse variance weighted
−0.5
0.0
0.5
1.0
MR effect size for
'Holdemania sp900120005' on 'GCST90199863_buildGRCh38.tsv'

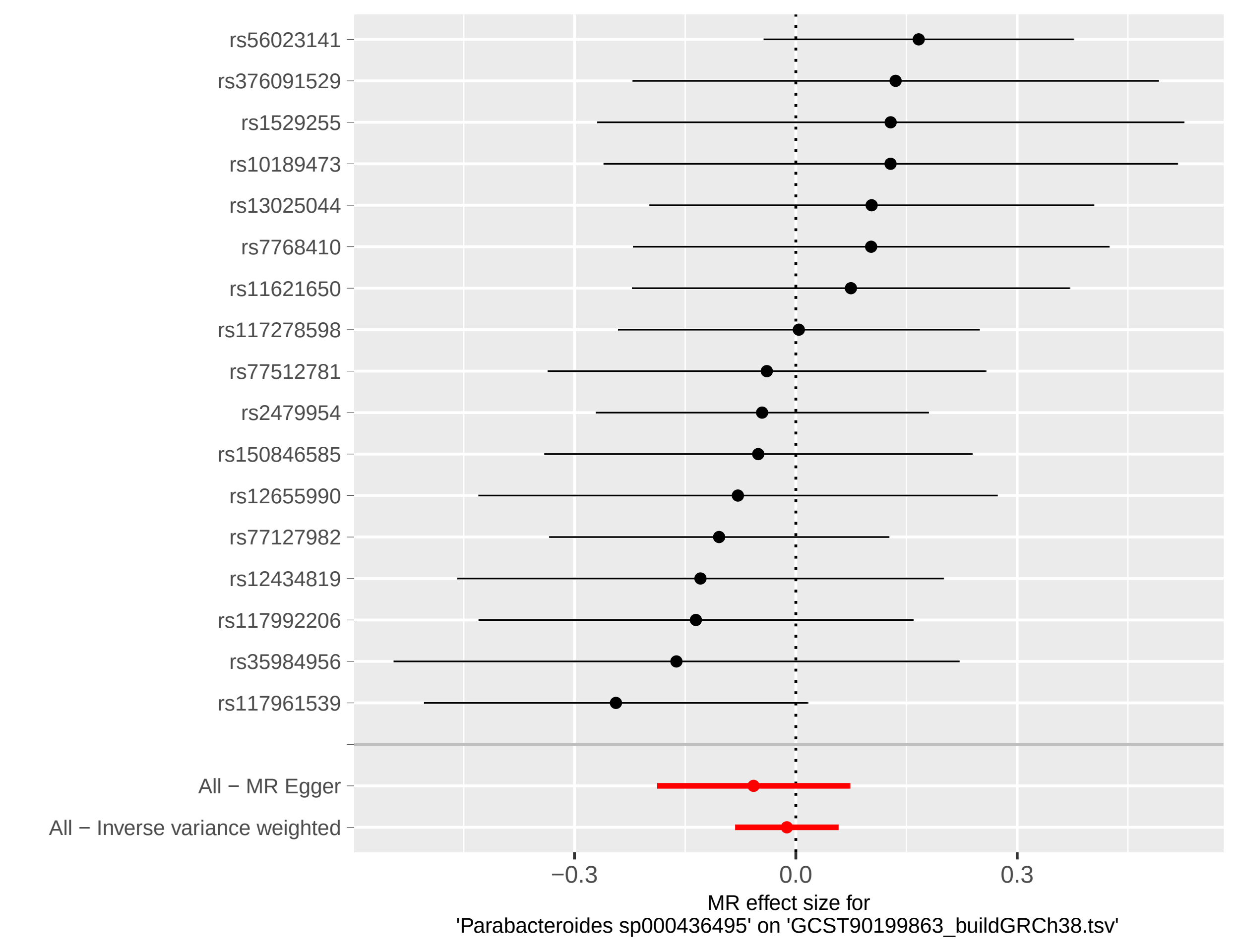
rs56023141
rs376091529
rs1529255
rs10189473
rs13025044
rs7768410
rs11621650
rs117278598
rs77512781
rs2479954
rs150846585
rs12655990
rs77127982
rs12434819
rs117992206
rs35984956
rs117961539
All − MR Egger
All − Inverse variance weighted
−0.3
0.0
0.3
MR effect size for
'Parabacteroides sp000436495' on 'GCST90199863_buildGRCh38.tsv'

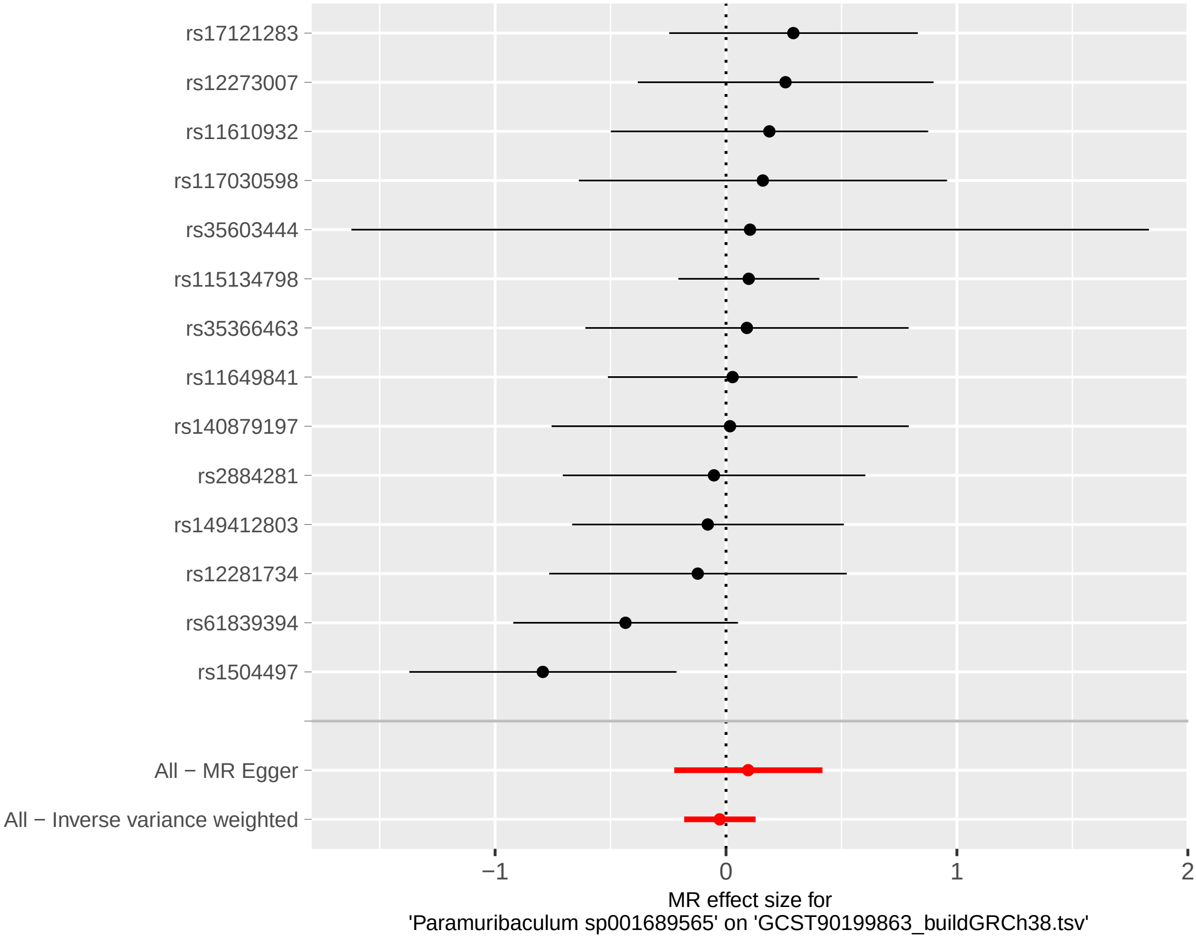
rs17121283
rs12273007
rs11610932
rs117030598
rs35603444
rs115134798
rs35366463
rs11649841
rs140879197
rs2884281
rs149412803
rs12281734
rs61839394
rs1504497
All − MR Egger
All − Inverse variance weighted
−1
0
1
2
MR effect size for
'Paramuribaculum sp001689565' on 'GCST90199863_buildGRCh38.tsv'

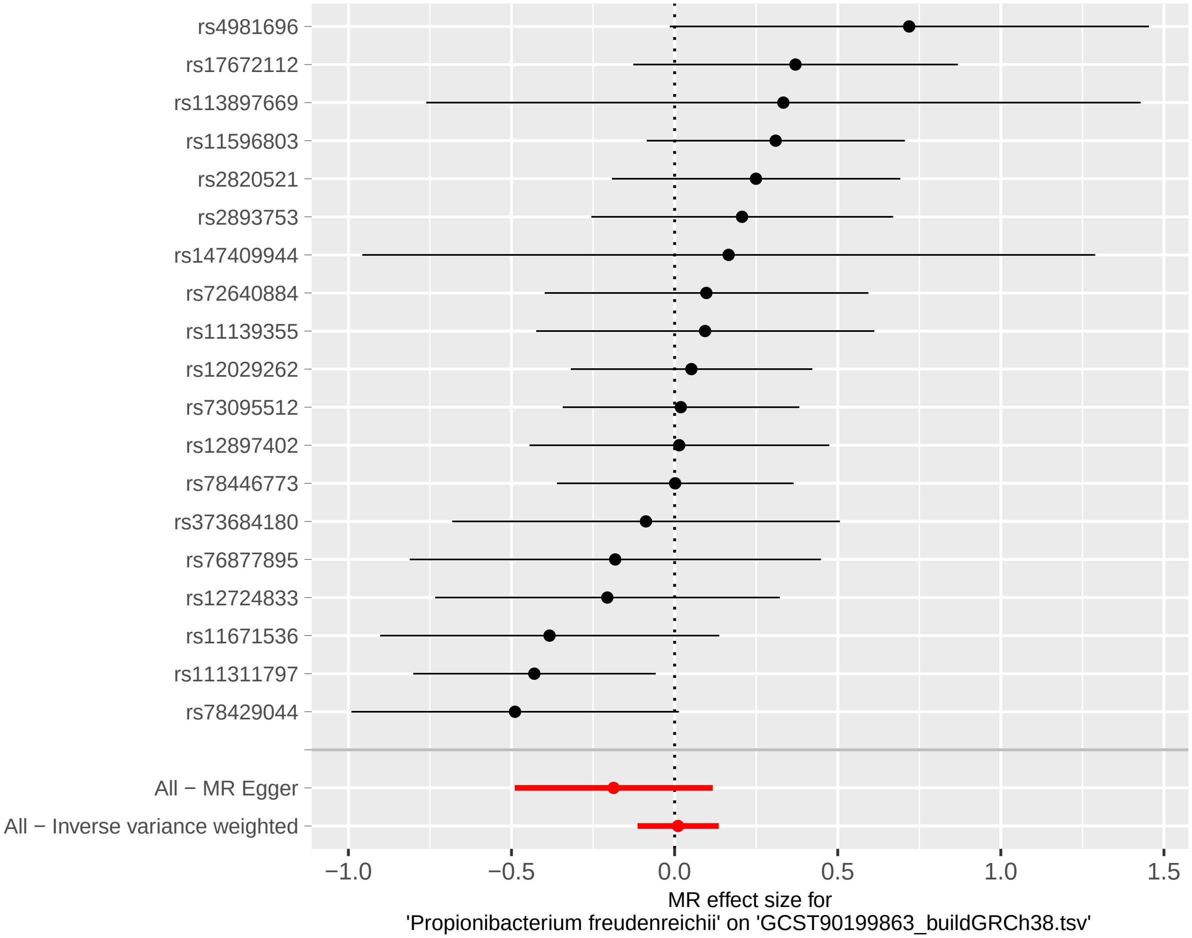
rs4981696
rs17672112
rs113897669
rs11596803
rs2820521
rs2893753
rs147409944
rs72640884
rs11139355
rs12029262
rs73095512
rs12897402
rs78446773
rs373684180
rs76877895
rs12724833
rs11671536
rs111311797
rs78429044
All − MR Egger
All − Inverse variance weighted
−1.0
−0.5
0.0
0.5
1.0
1.5
MR effect size for
'Propionibacterium freudenreichii' on 'GCST90199863_buildGRCh38.tsv'

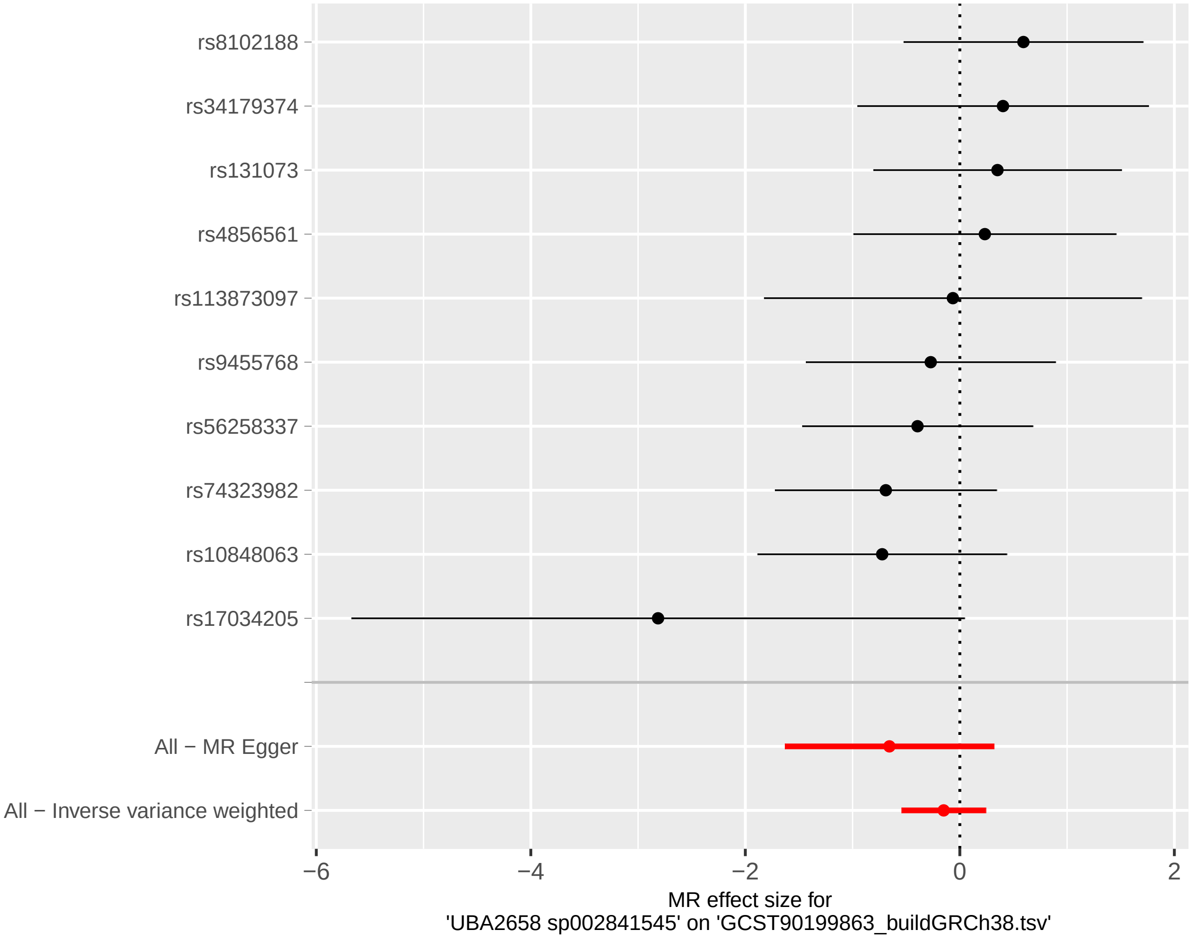

rs8102188
rs34179374
rs131073
rs4856561
rs113873097
rs9455768
rs56258337
rs74323982
rs10848063
rs17034205
All − MR Egger
All − Inverse variance weighted
−6
−4
−2
0
2
MR effect size for
'UBA2658 sp002841545' on 'GCST90199863_buildGRCh38.tsv'

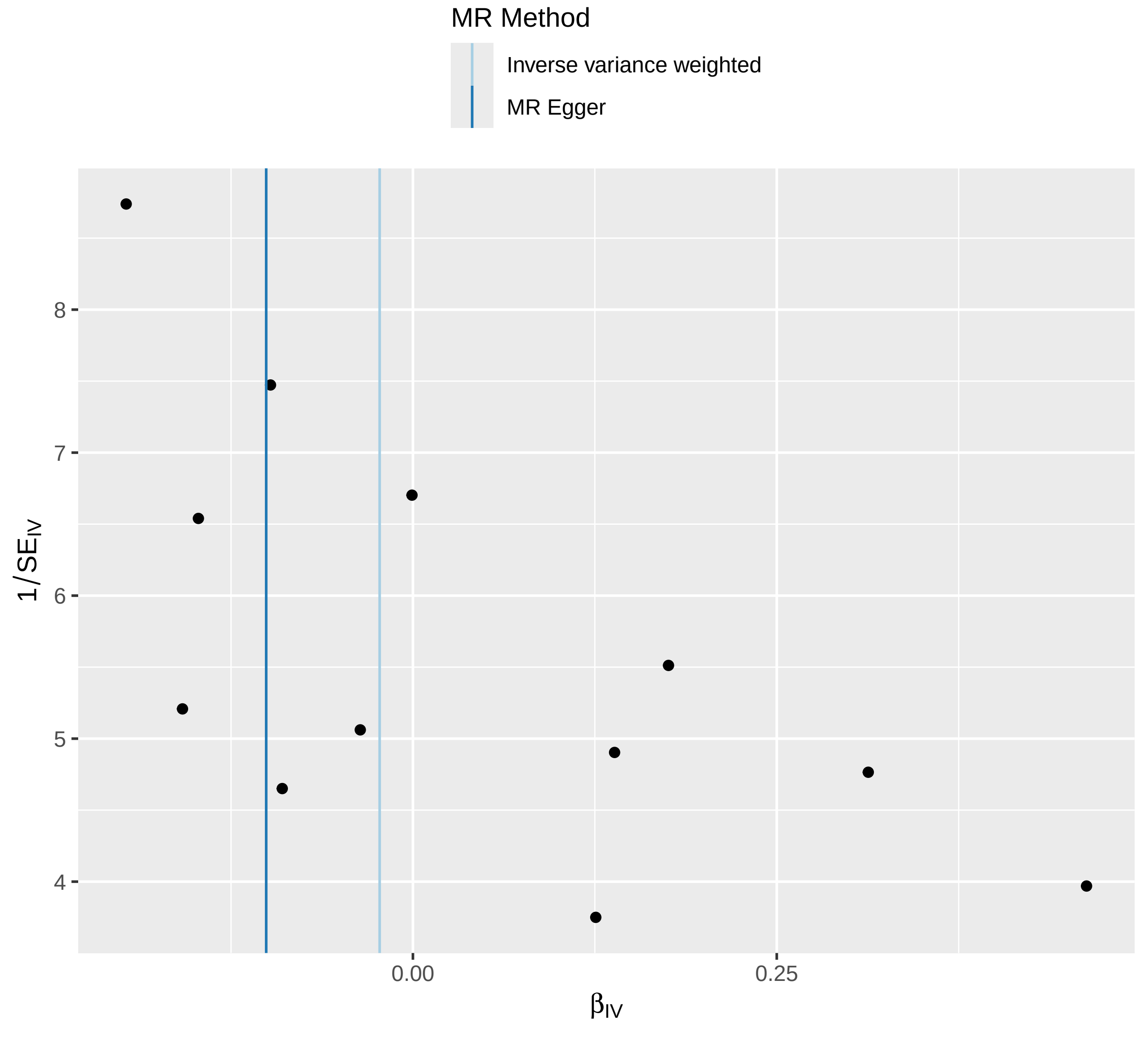
MR Method
Inverse variance weighted
MR Egger
8
7
6
5
4
1/SE_IV
0.00
0.25
β_IV

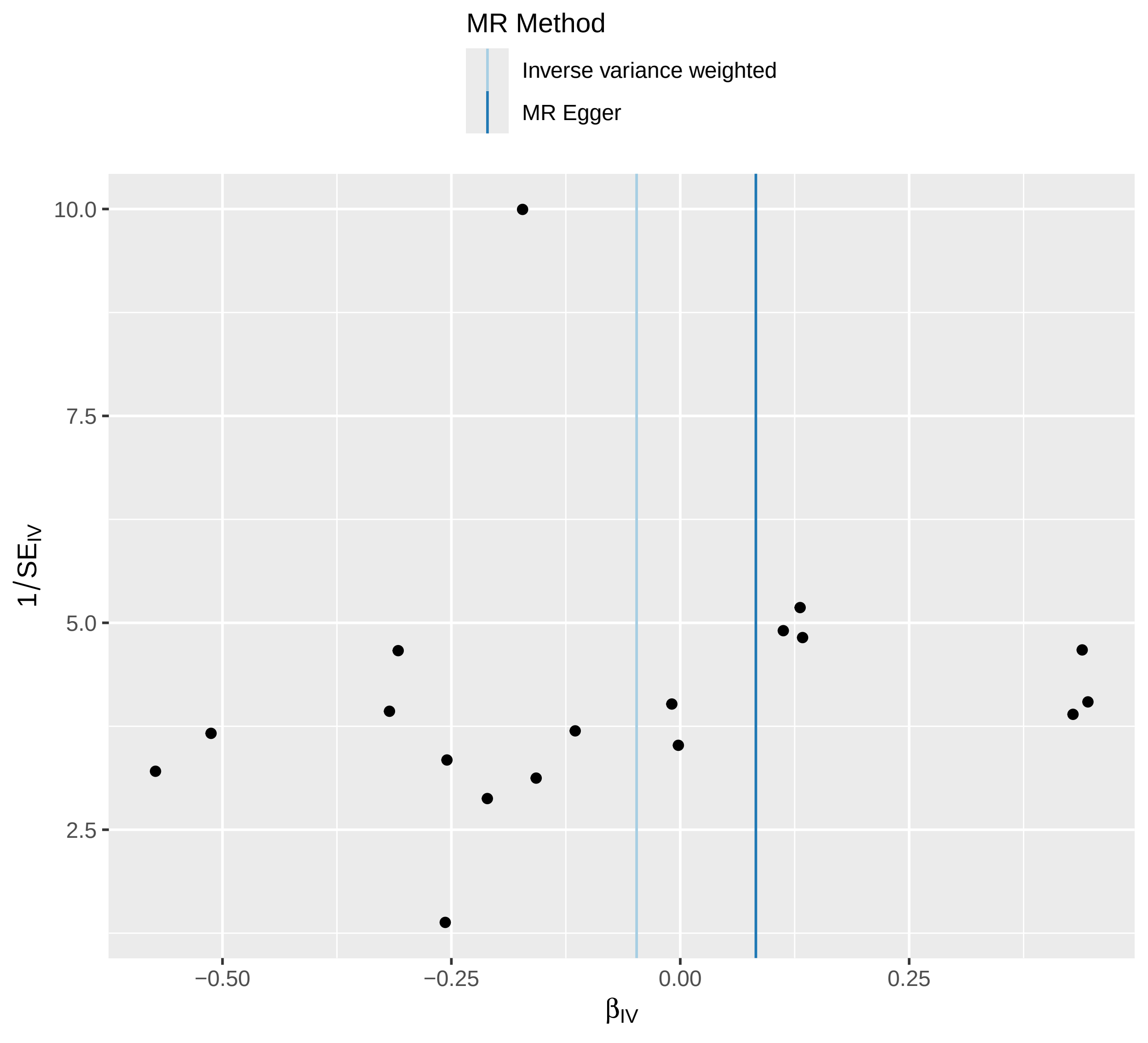
MR Method
Inverse variance weighted
MR Egger
10.0
7.5
5.0
2.5
−0.50
−0.25
0.00
0.25
$1/SE_{IV}$
β_{IV}

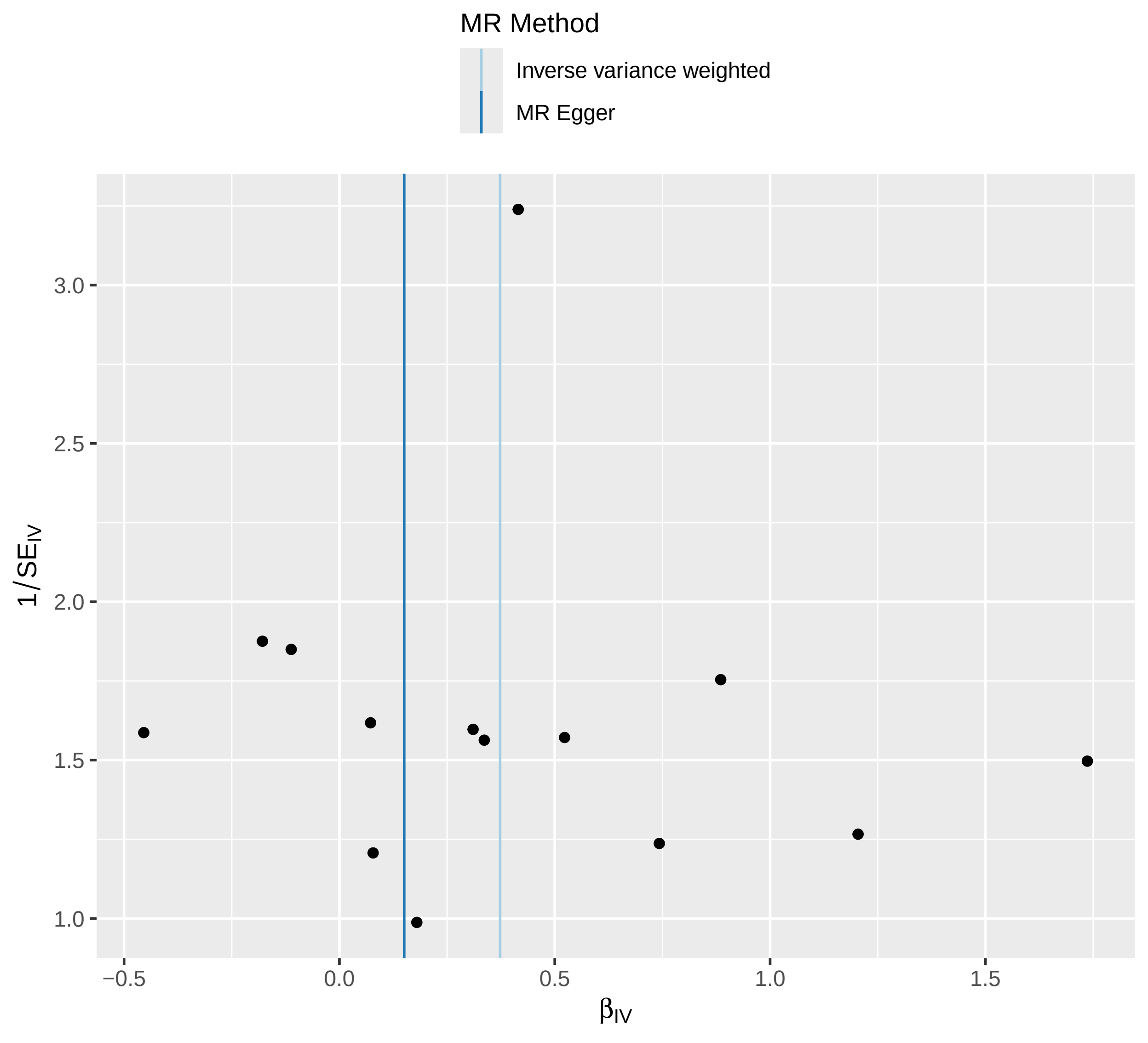

MR Method
Inverse variance weighted
MR Egger
3.0
2.5
2.0
1.5
1.0
1/SE$_{IV}$
−0.5
0.0
0.5
1.0
1.5
β$_{IV}$

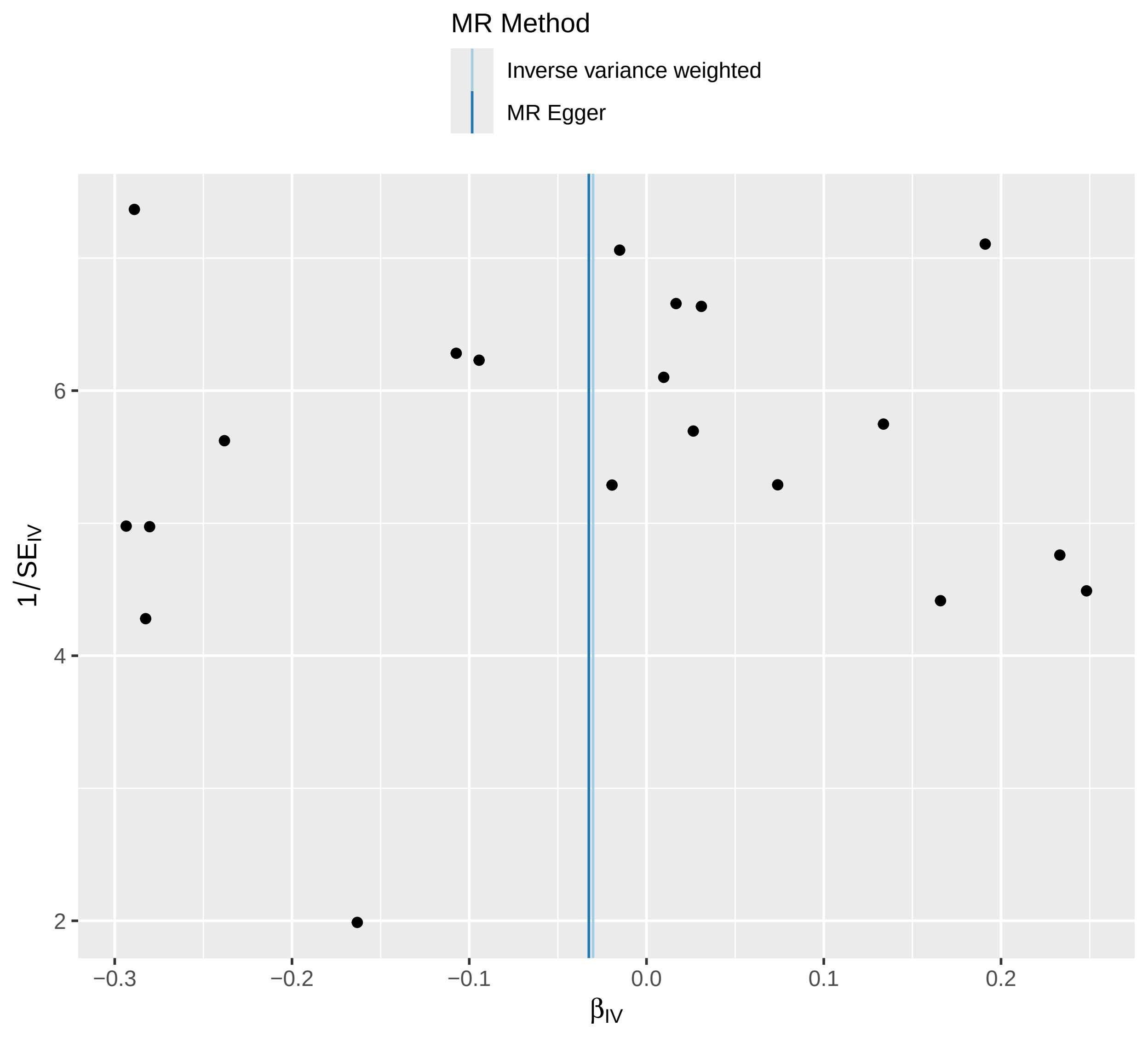

MR Method
Inverse variance weighted
MR Egger
6
4
2
1/SE_IV
−0.3
−0.2
−0.1
0.0
0.1
0.2
β_IV

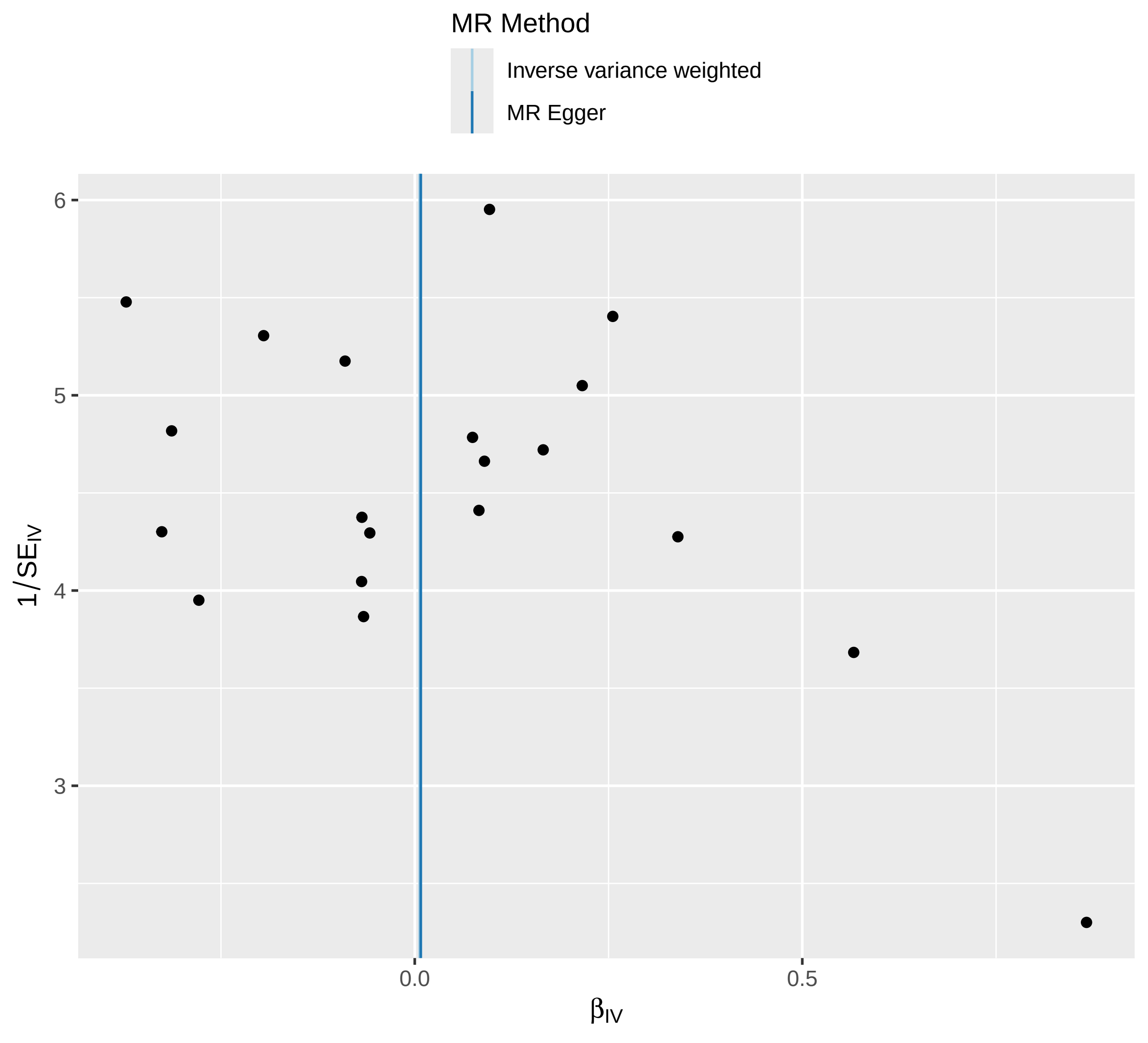

MR Method
Inverse variance weighted
MR Egger
6
5
4
3
$1/SE_{IV}$
0.0
0.5
β_{IV}

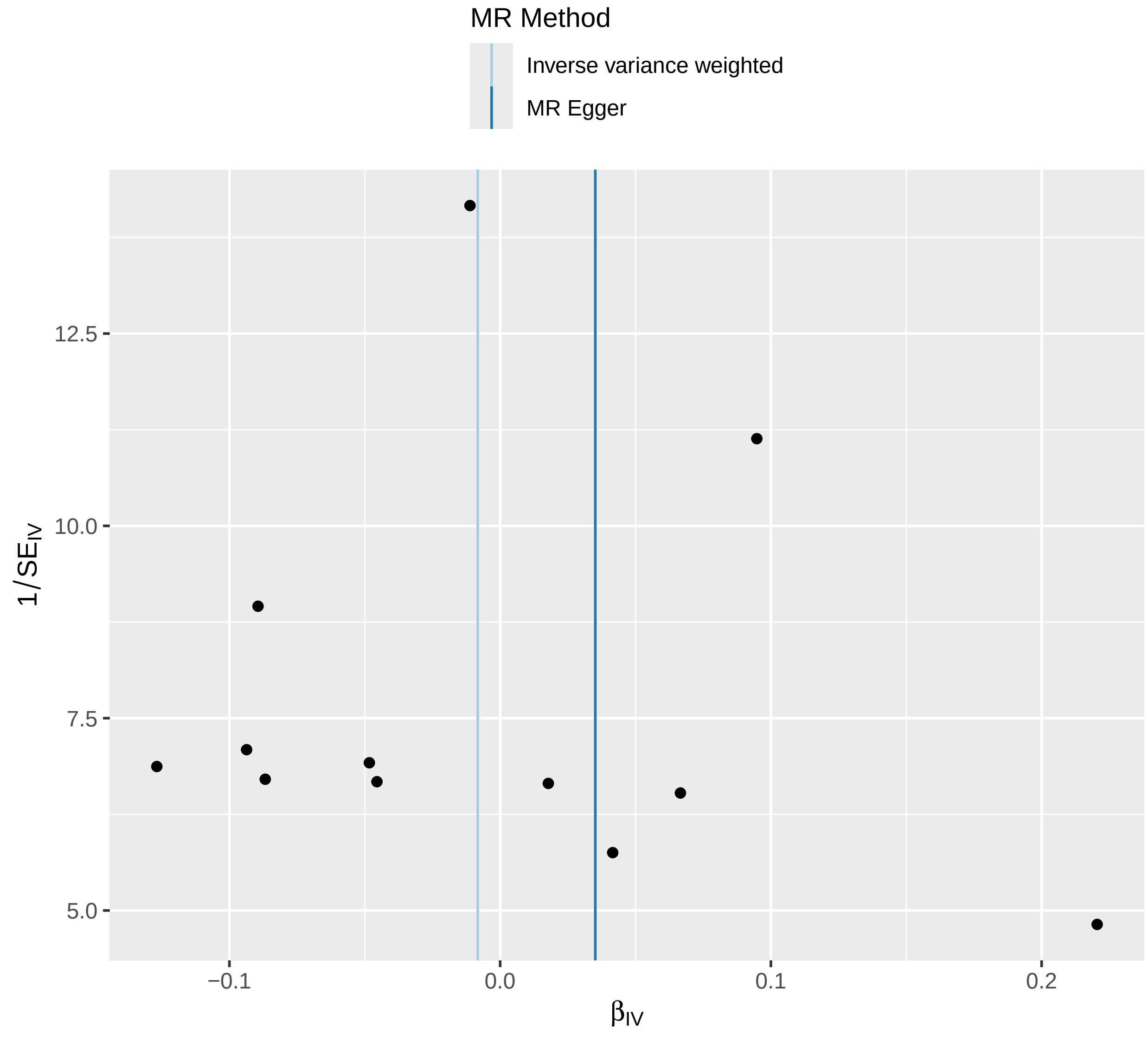

MR Method
Inverse variance weighted
MR Egger
12.5
10.0
7.5
5.0
1/SE$_{IV}$
−0.1
0.0
0.1
0.2
β$_{IV}$

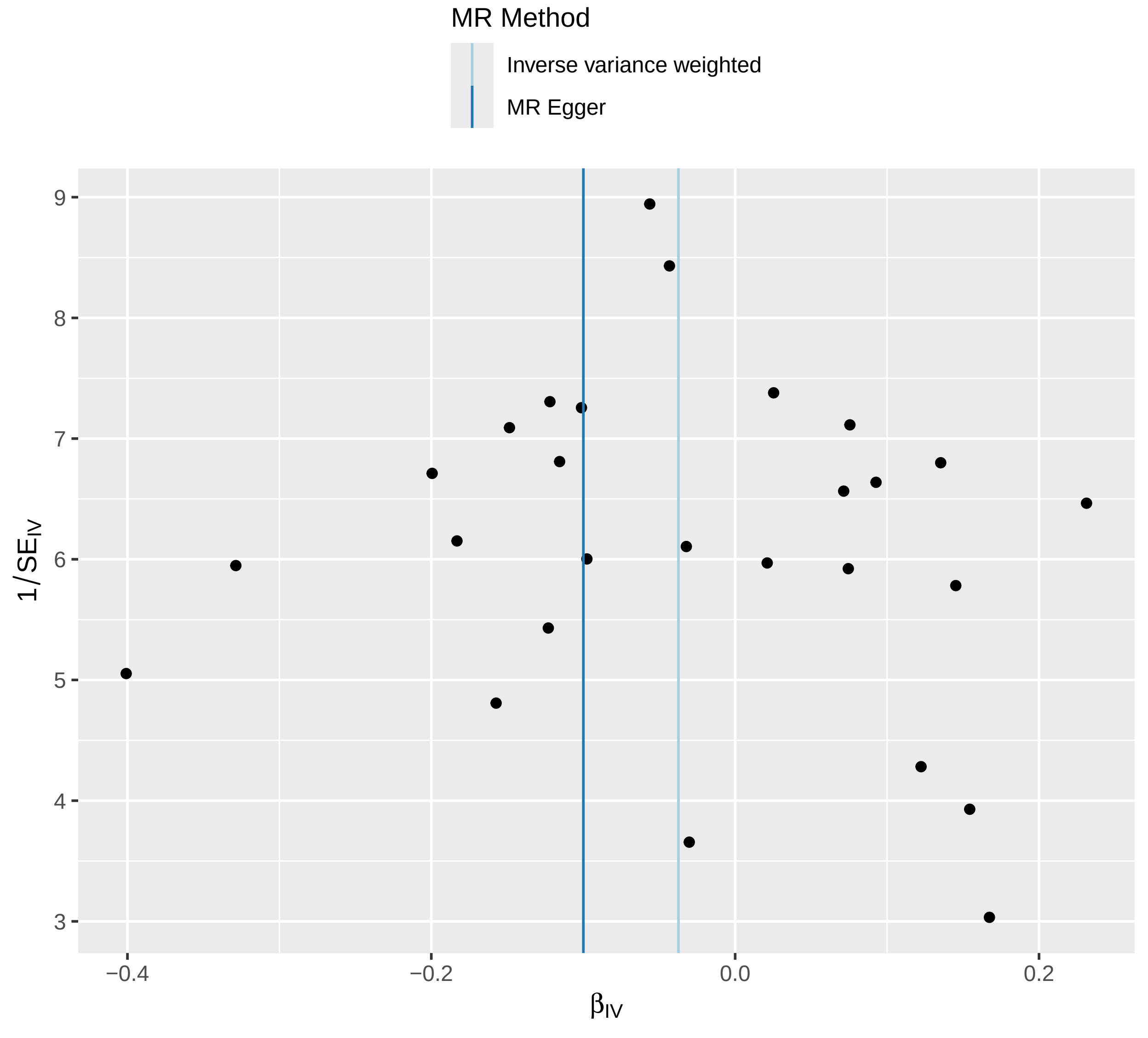
MR Method
Inverse variance weighted
MR Egger
1/SE_IV
β_IV
9
8
7
6
5
4
3
−0.4
−0.2
0.0
0.2

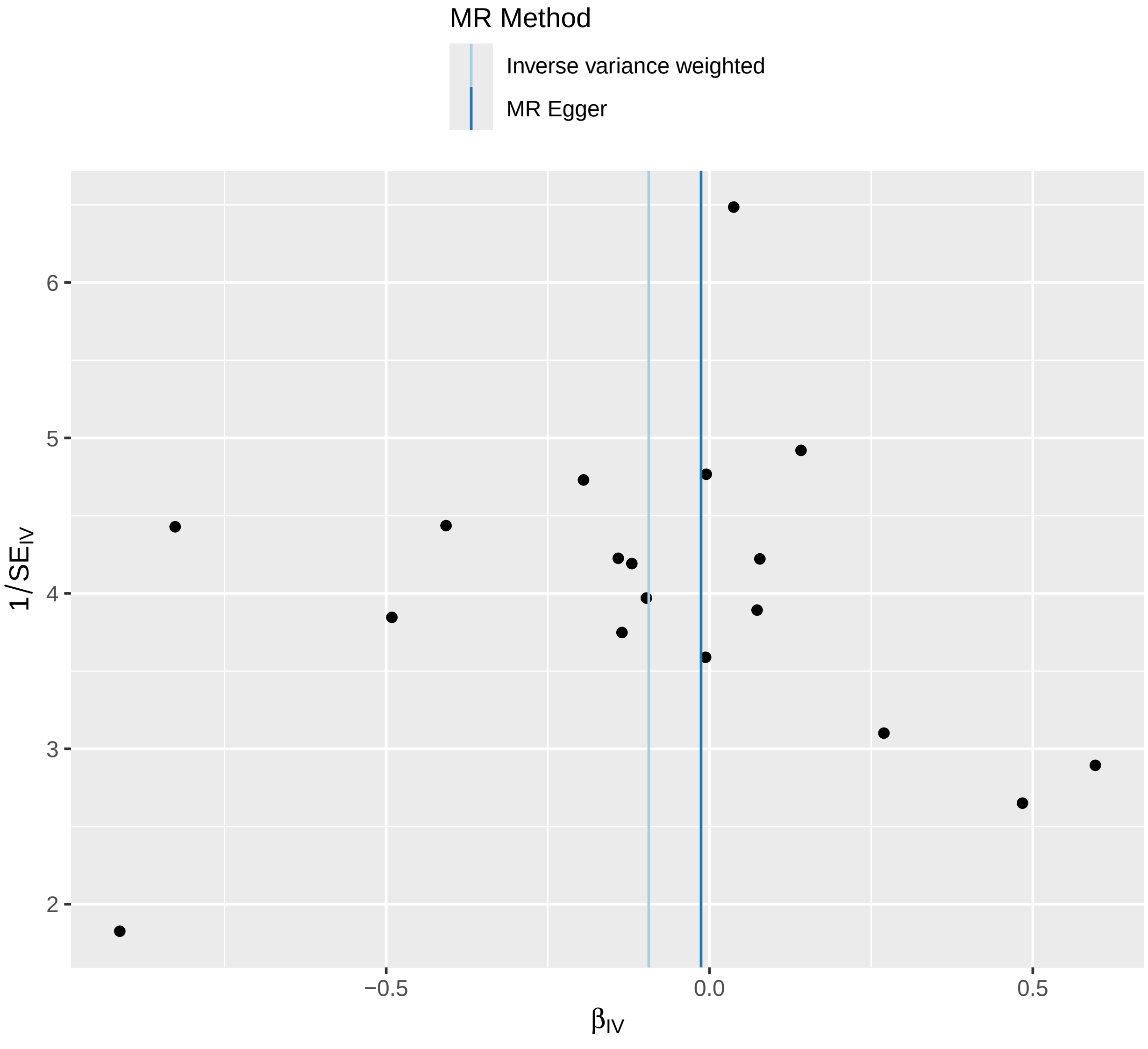
MR Method
Inverse variance weighted
MR Egger
1/SE_IV
β_IV
6
5
4
3
2
−0.5
0.0
0.5

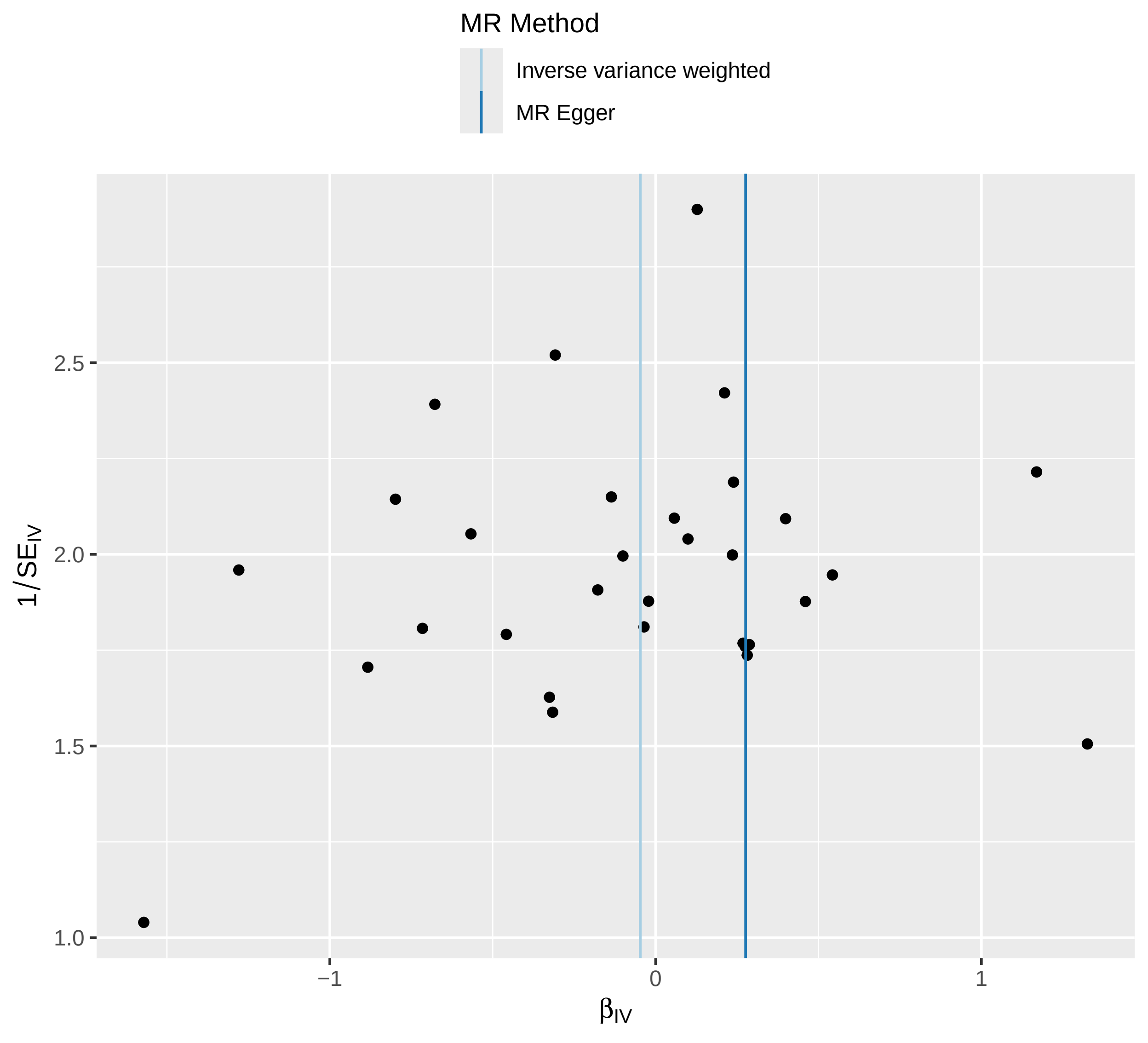

MR Method
Inverse variance weighted
MR Egger
2.5
2.0
1.5
1.0
−1
0
1
$1/SE_{IV}$
β_{IV}

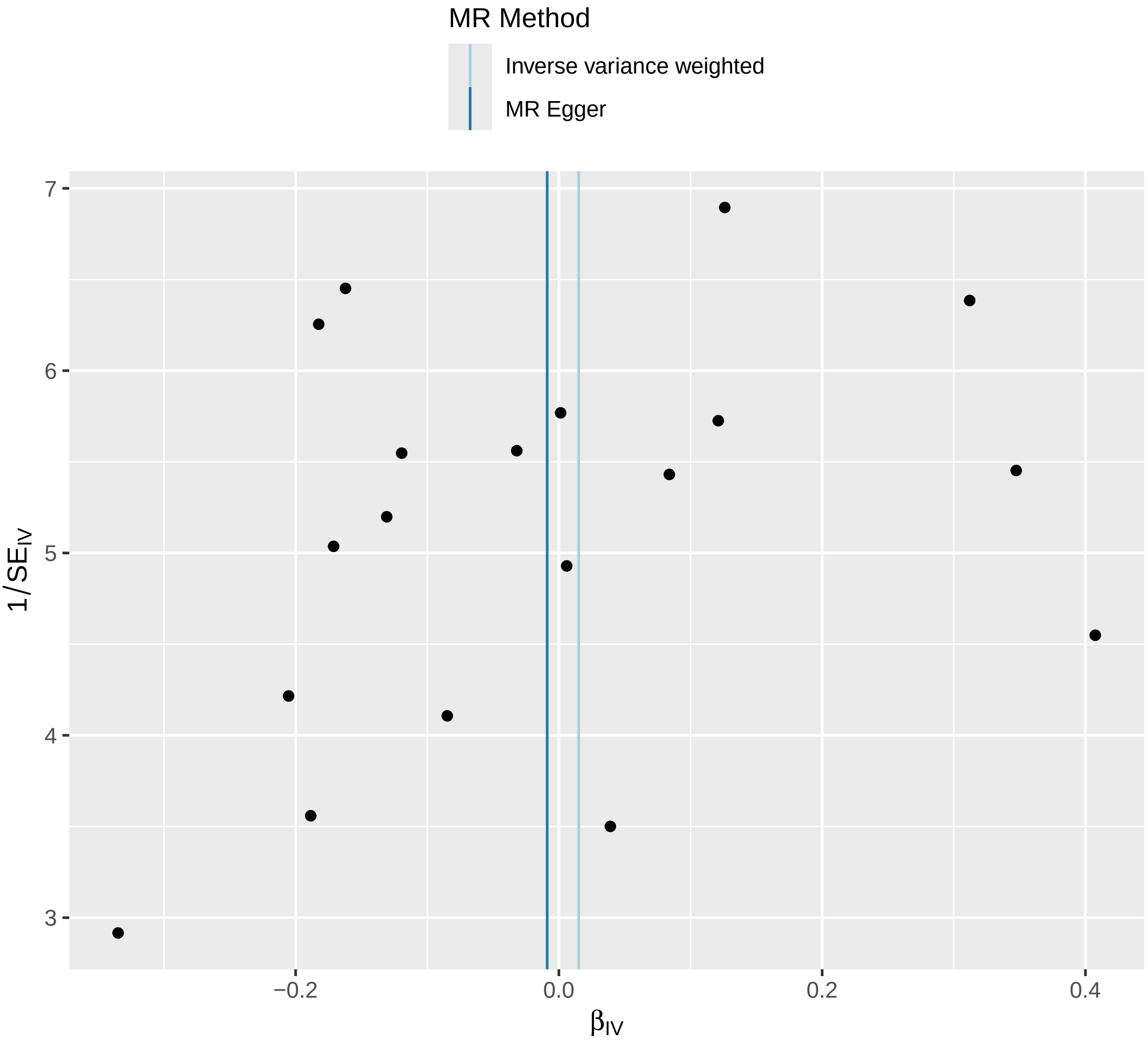

MR Method
Inverse variance weighted
MR Egger
7
6
5
4
3
1/SE_IV
−0.2
0.0
0.2
0.4
β_IV

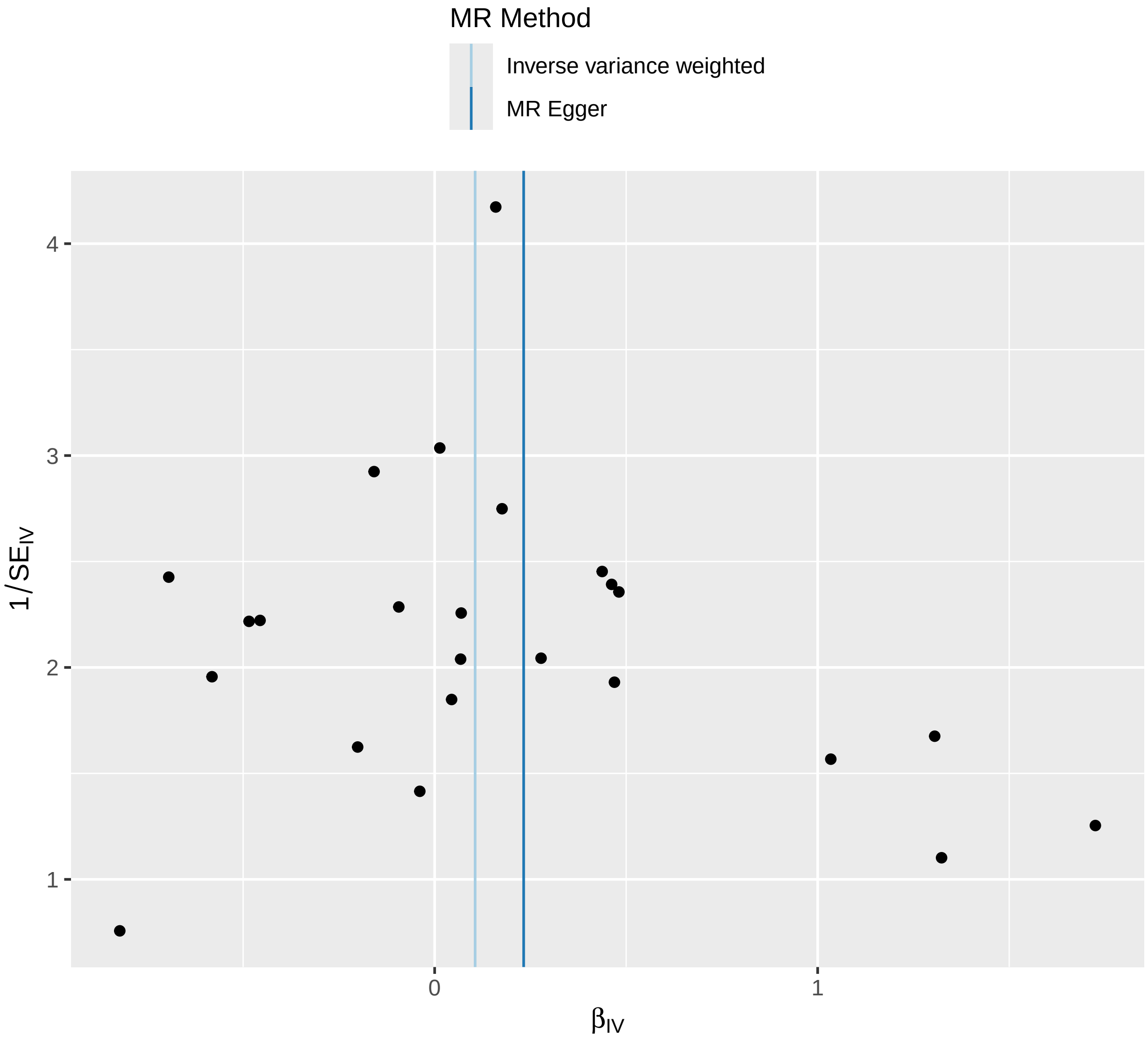
MR Method
Inverse variance weighted
MR Egger
4
3
2
1
1/SE_IV
0
1
β_IV

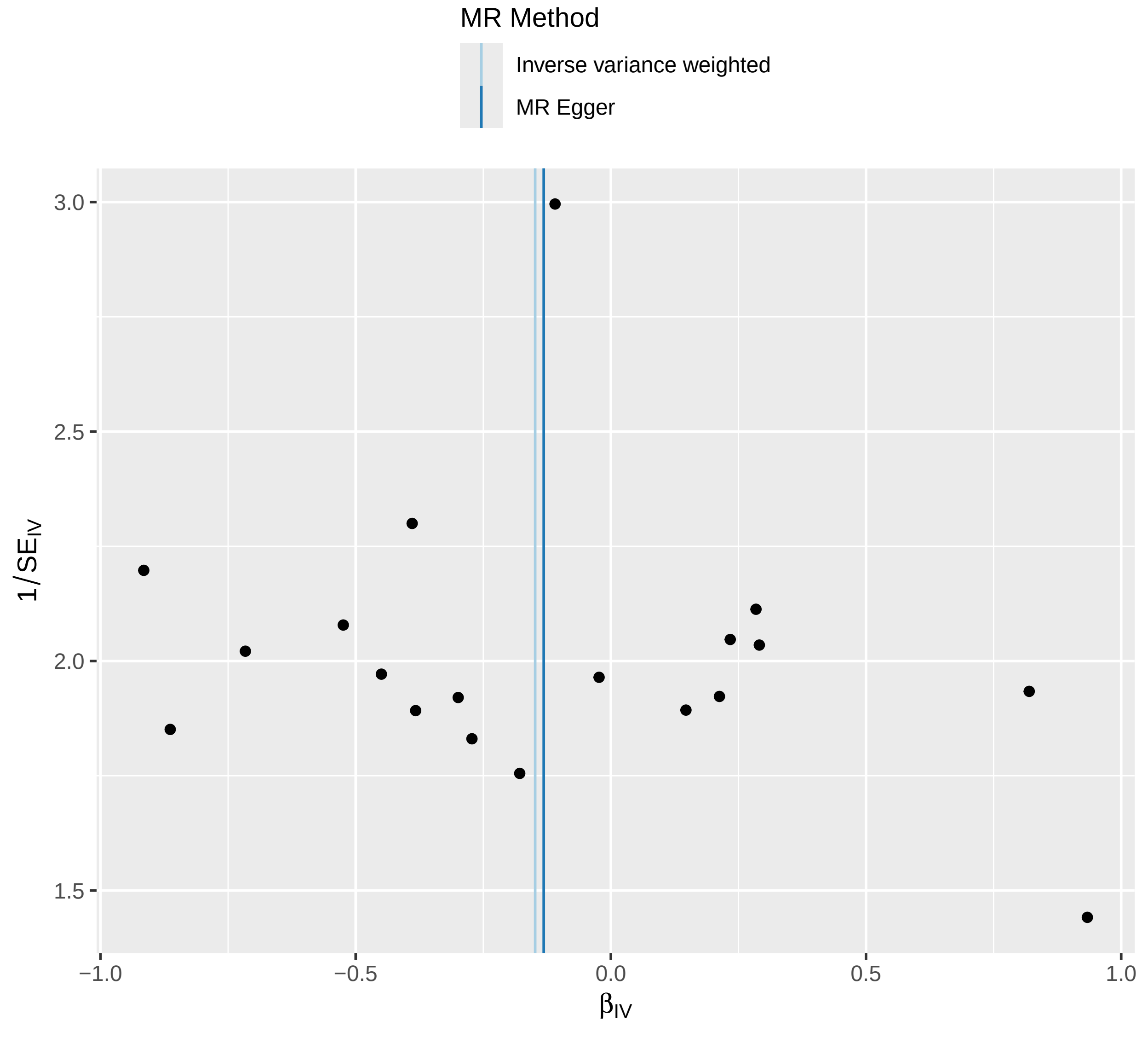

MR Method
Inverse variance weighted
MR Egger
3.0
2.5
2.0
1.5
$1/SE_{IV}$
−1.0
−0.5
0.0
0.5
1.0
β_{IV}

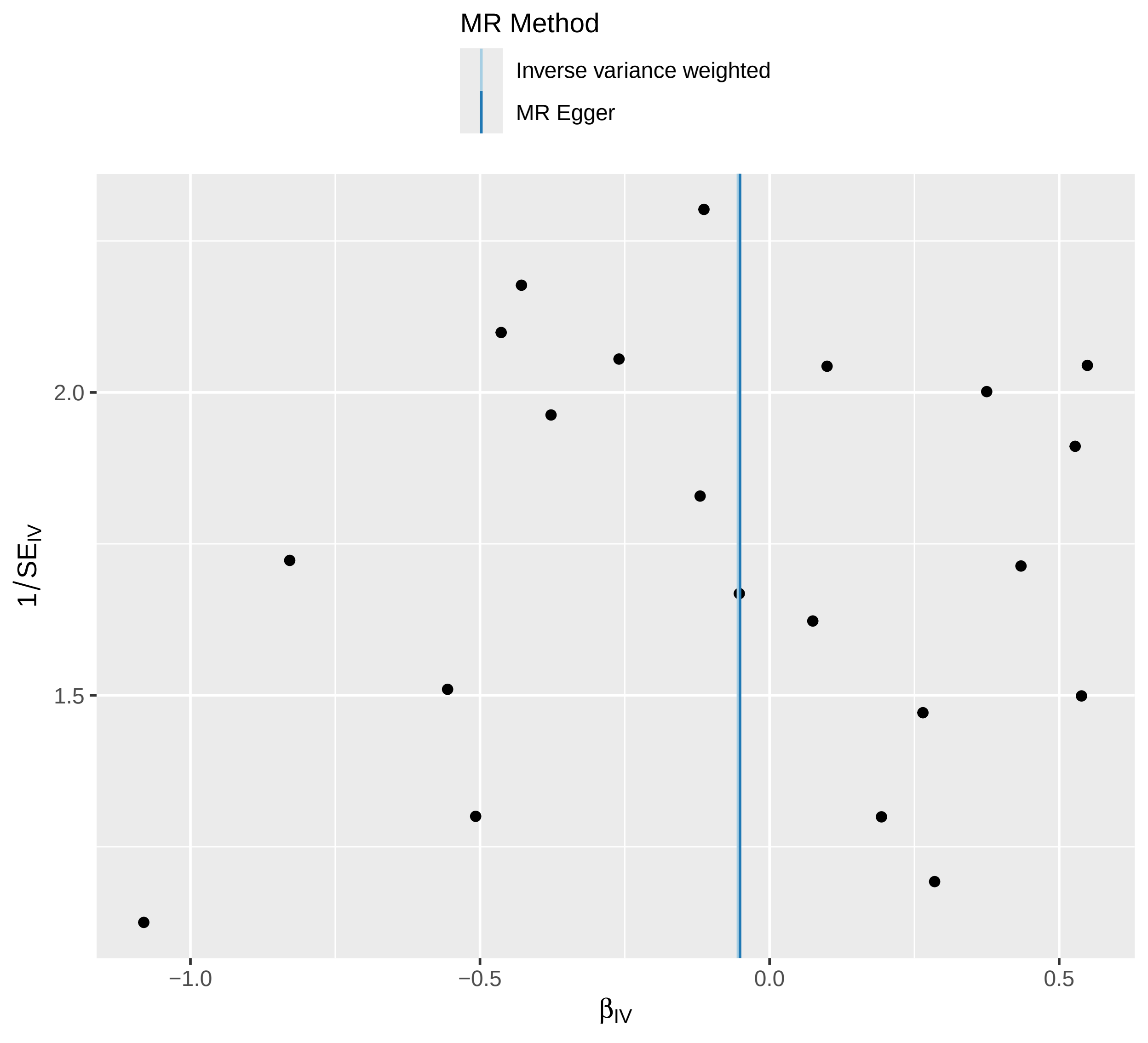

MR Method
Inverse variance weighted
MR Egger
2.0
1.5
−1.0
−0.5
0.0
0.5
$1/SE_{IV}$
β_{IV}

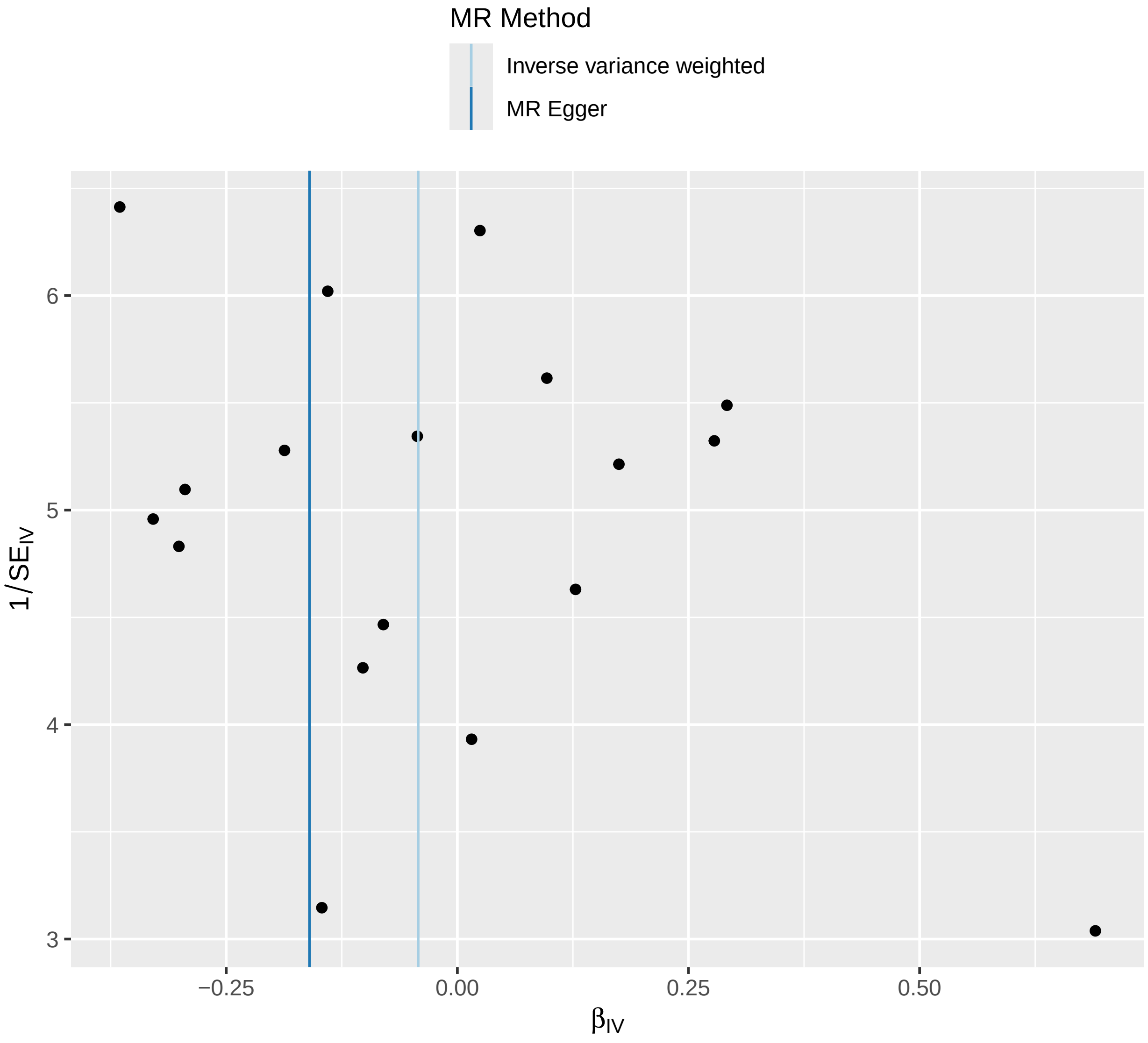

MR Method
Inverse variance weighted
MR Egger
6
5
4
3
$1/SE_{IV}$
−0.25
0.00
0.25
0.50
β_{IV}

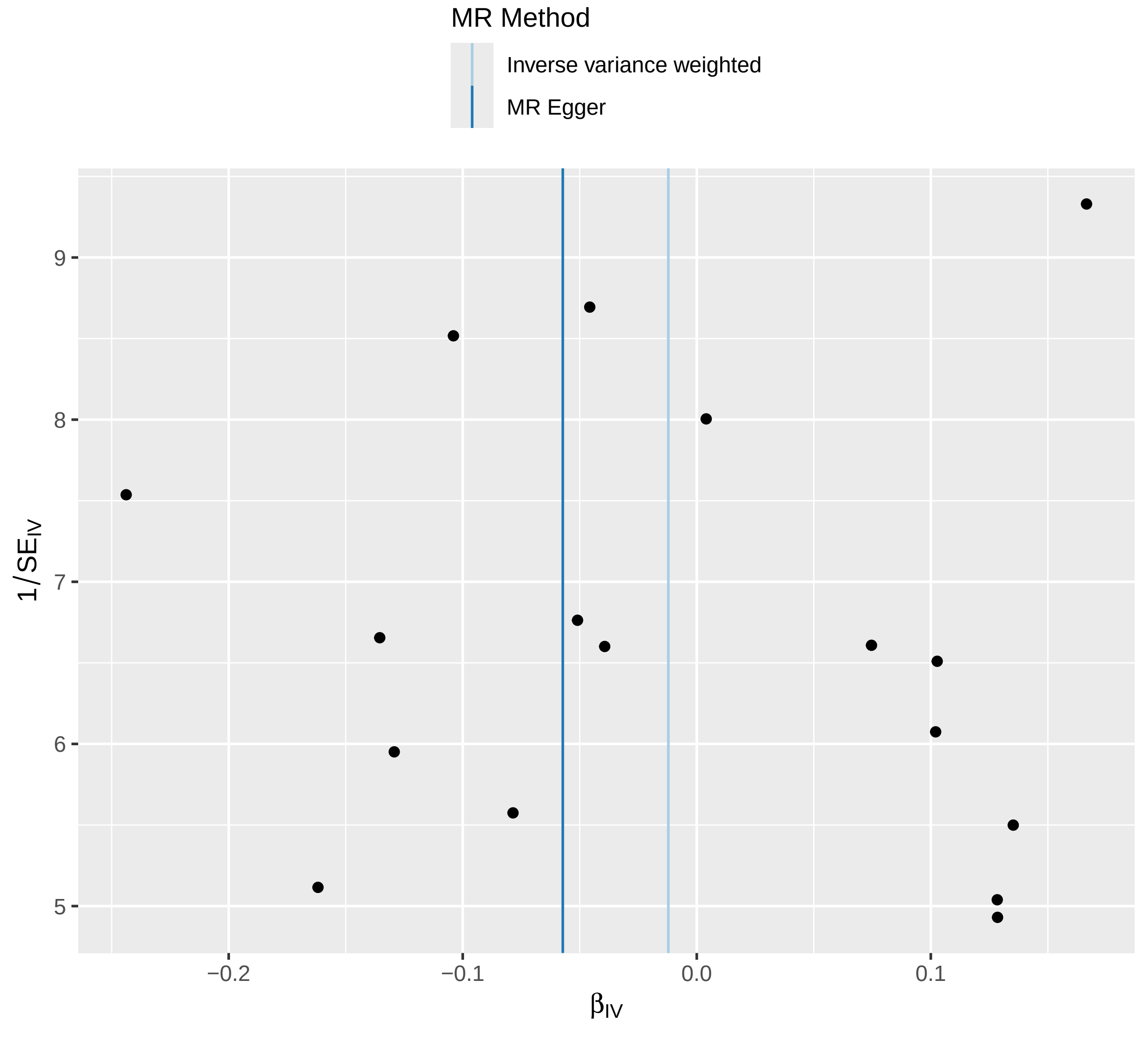

MR Method
Inverse variance weighted
MR Egger
9
8
7
6
5
1/SE_IV
−0.2
−0.1
0.0
0.1
β_IV

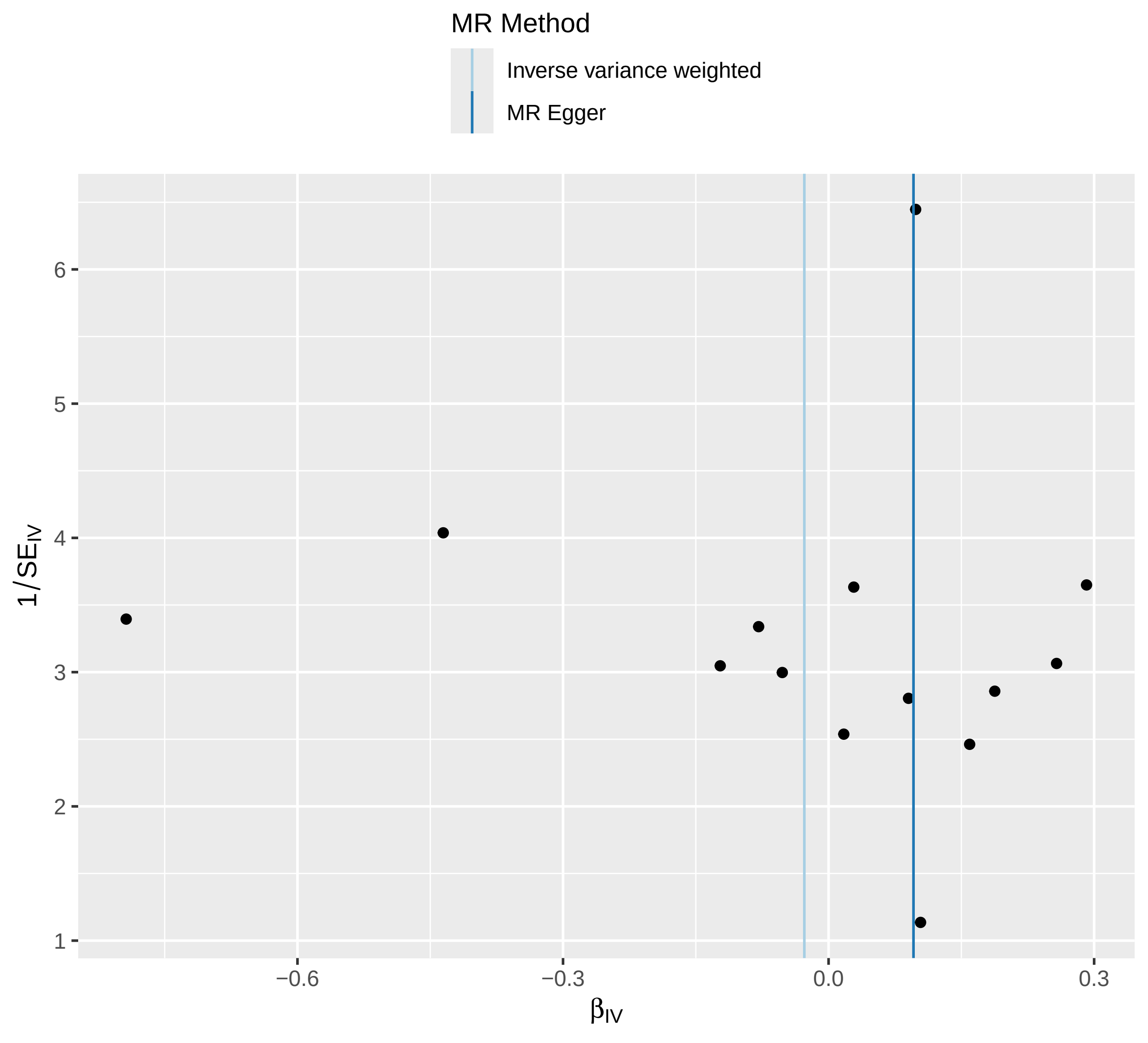

MR Method
Inverse variance weighted
MR Egger
6
5
4
3
2
1
1/SE_IV
−0.6
−0.3
0.0
0.3
β_IV

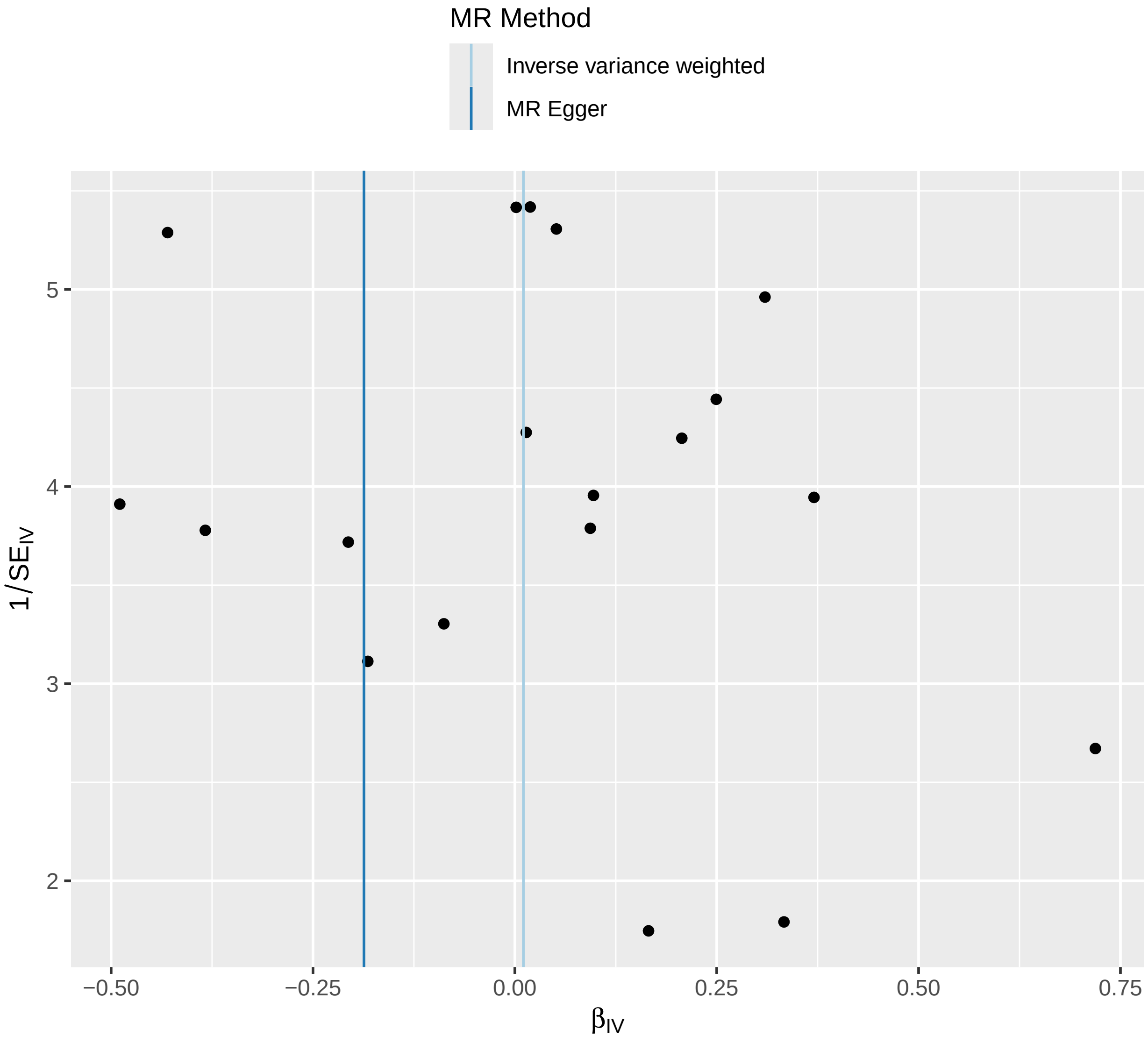
MR Method
Inverse variance weighted
MR Egger
1/SE$_{IV}$
β_{IV}
5
4
3
2
−0.50
−0.25
0.00
0.25
0.50
0.75

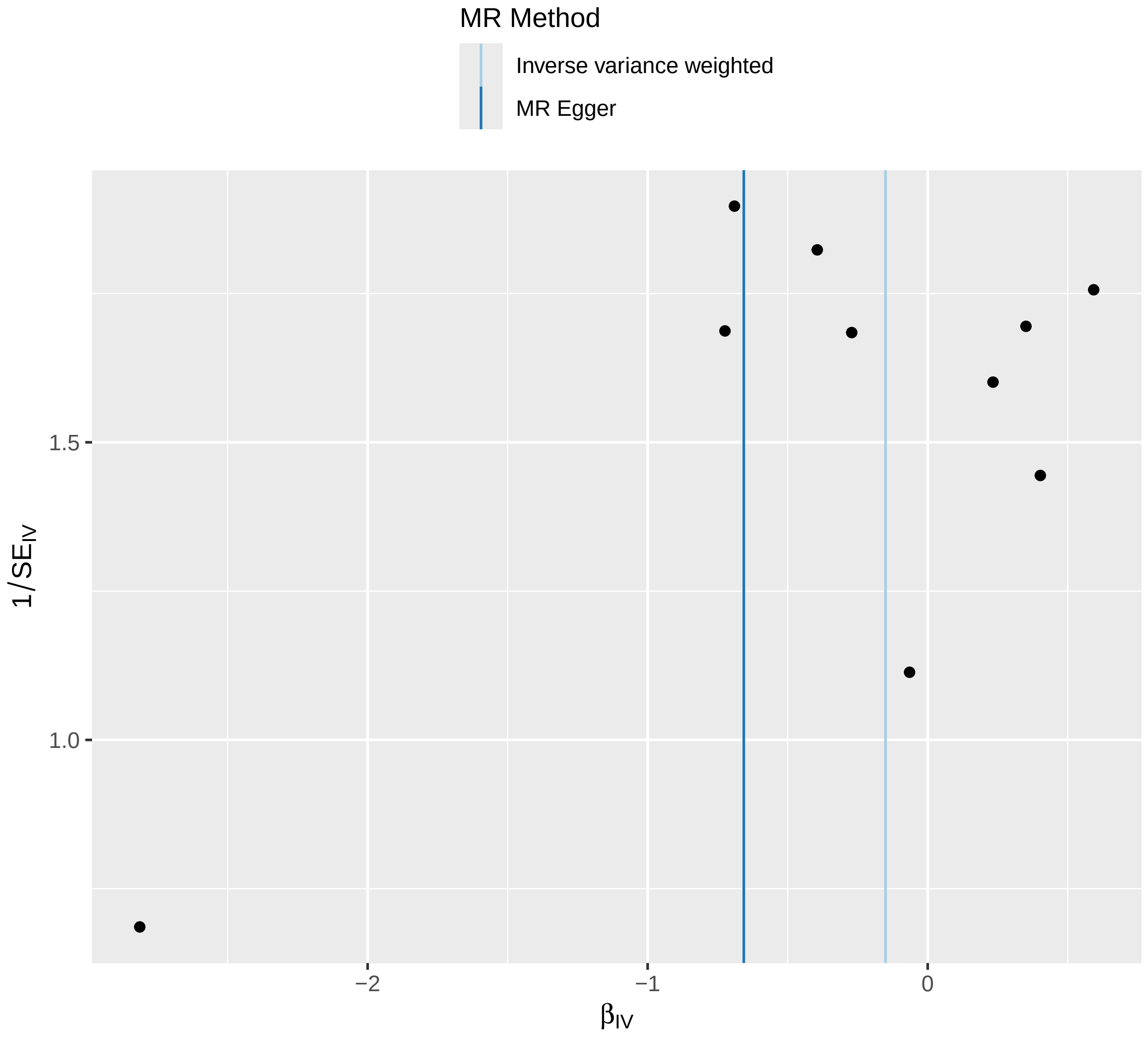

MR Method
Inverse variance weighted
MR Egger
1.5
1.0
1/SE_IV
−2
−1
0
β_IV

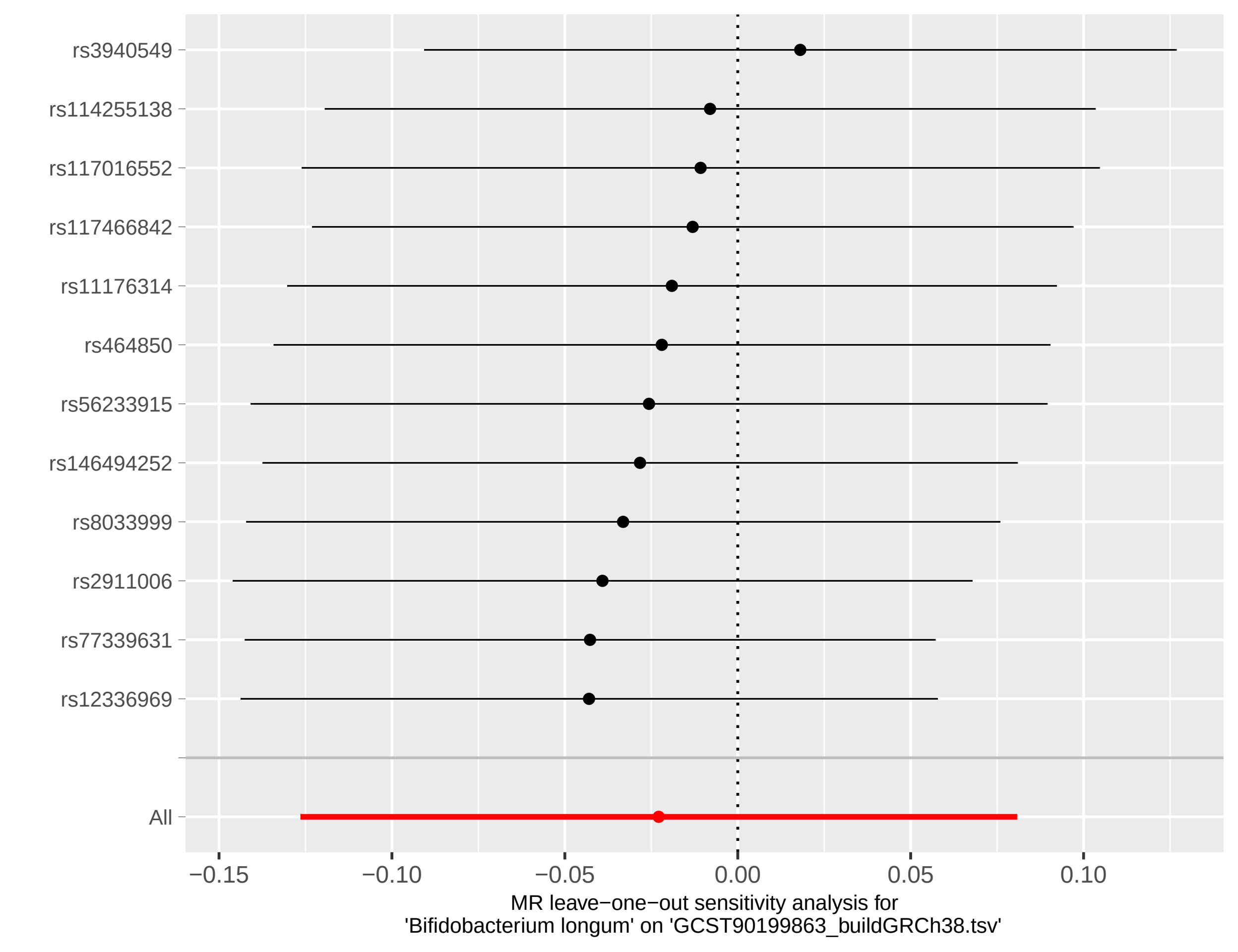

rs3940549
rs114255138
rs117016552
rs117466842
rs11176314
rs464850
rs56233915
rs146494252
rs8033999
rs2911006
rs77339631
rs12336969
All
−0.15
−0.10
−0.05
0.00
0.05
0.10
MR leave−one−out sensitivity analysis for
'Bifidobacterium longum' on 'GCST90199863_buildGRCh38.tsv'

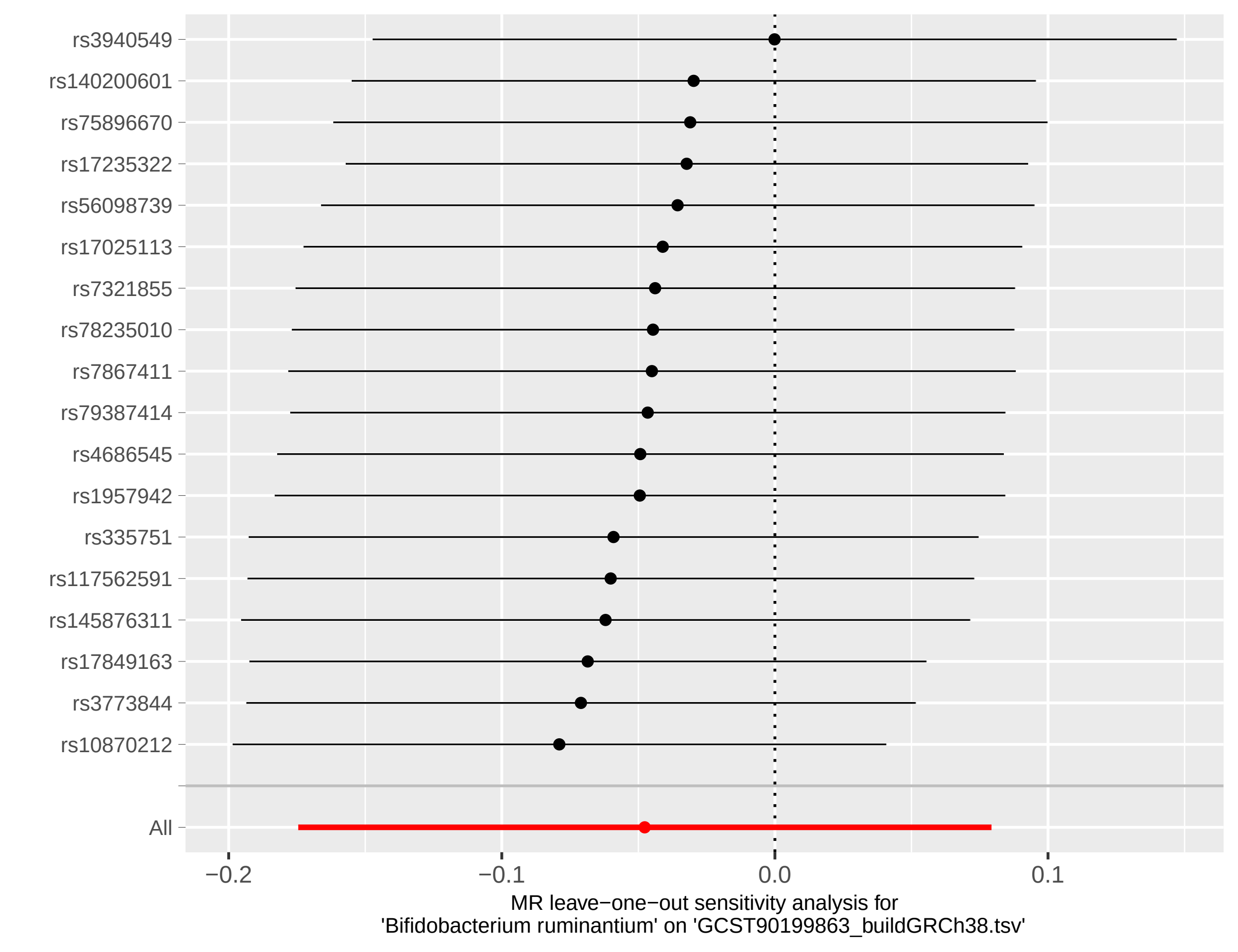

rs3940549
rs140200601
rs75896670
rs17235322
rs56098739
rs17025113
rs7321855
rs78235010
rs7867411
rs79387414
rs4686545
rs1957942
rs335751
rs117562591
rs145876311
rs17849163
rs3773844
rs10870212
All
−0.2
−0.1
0.0
0.1
MR leave−one−out sensitivity analysis for
'Bifidobacterium ruminantium' on 'GCST90199863_buildGRCh38.tsv'

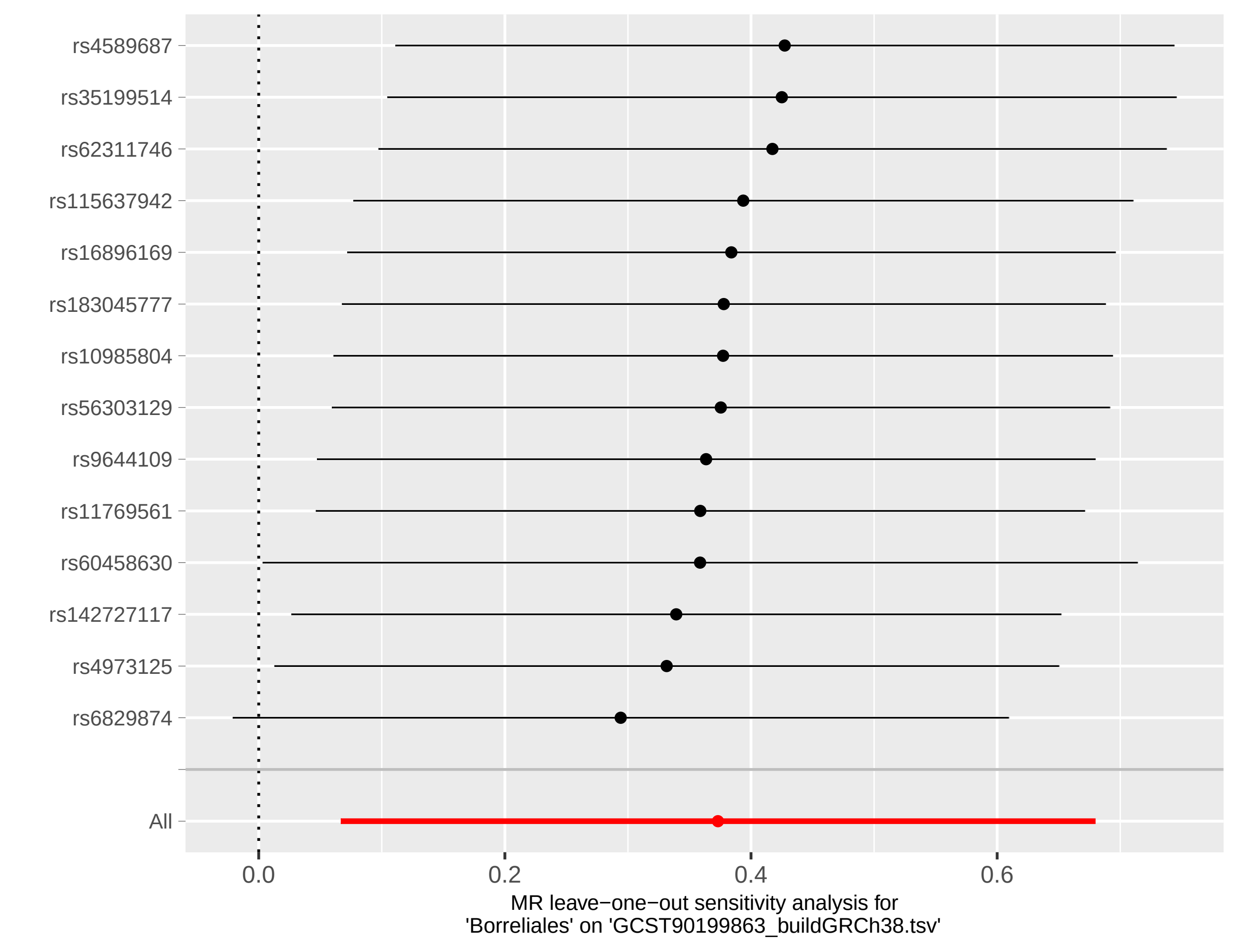
rs4589687
rs35199514
rs62311746
rs115637942
rs16896169
rs183045777
rs10985804
rs56303129
rs9644109
rs11769561
rs60458630
rs142727117
rs4973125
rs6829874
All
0.0
0.2
0.4
0.6
MR leave−one−out sensitivity analysis for
'Borreliales' on 'GCST90199863_buildGRCh38.tsv'

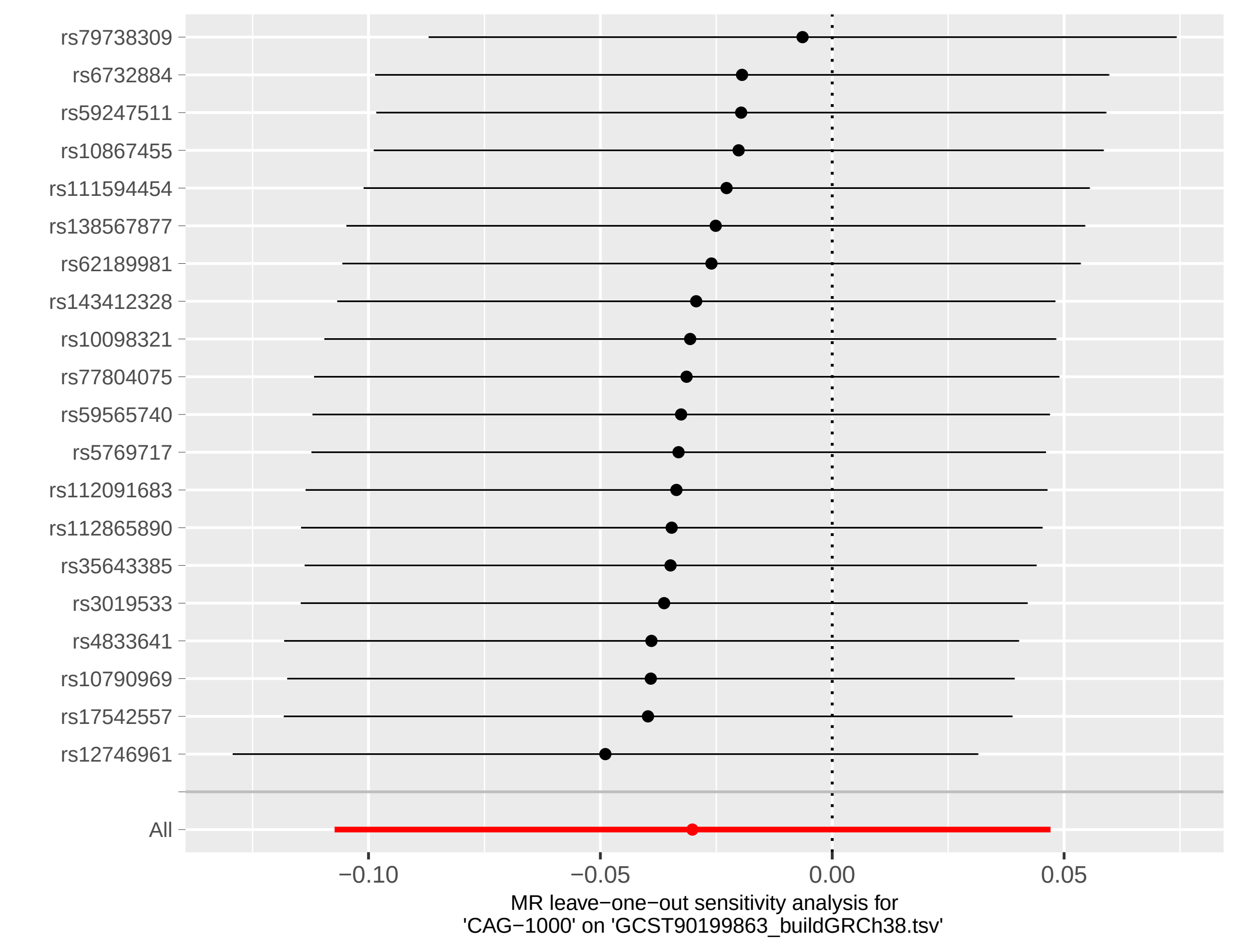
rs79738309
rs6732884
rs59247511
rs10867455
rs111594454
rs138567877
rs62189981
rs143412328
rs10098321
rs77804075
rs59565740
rs5769717
rs112091683
rs112865890
rs35643385
rs3019533
rs4833641
rs10790969
rs17542557
rs12746961
All
−0.10
−0.05
0.00
0.05
MR leave−one−out sensitivity analysis for
'CAG−1000' on 'GCST90199863_buildGRCh38.tsv'

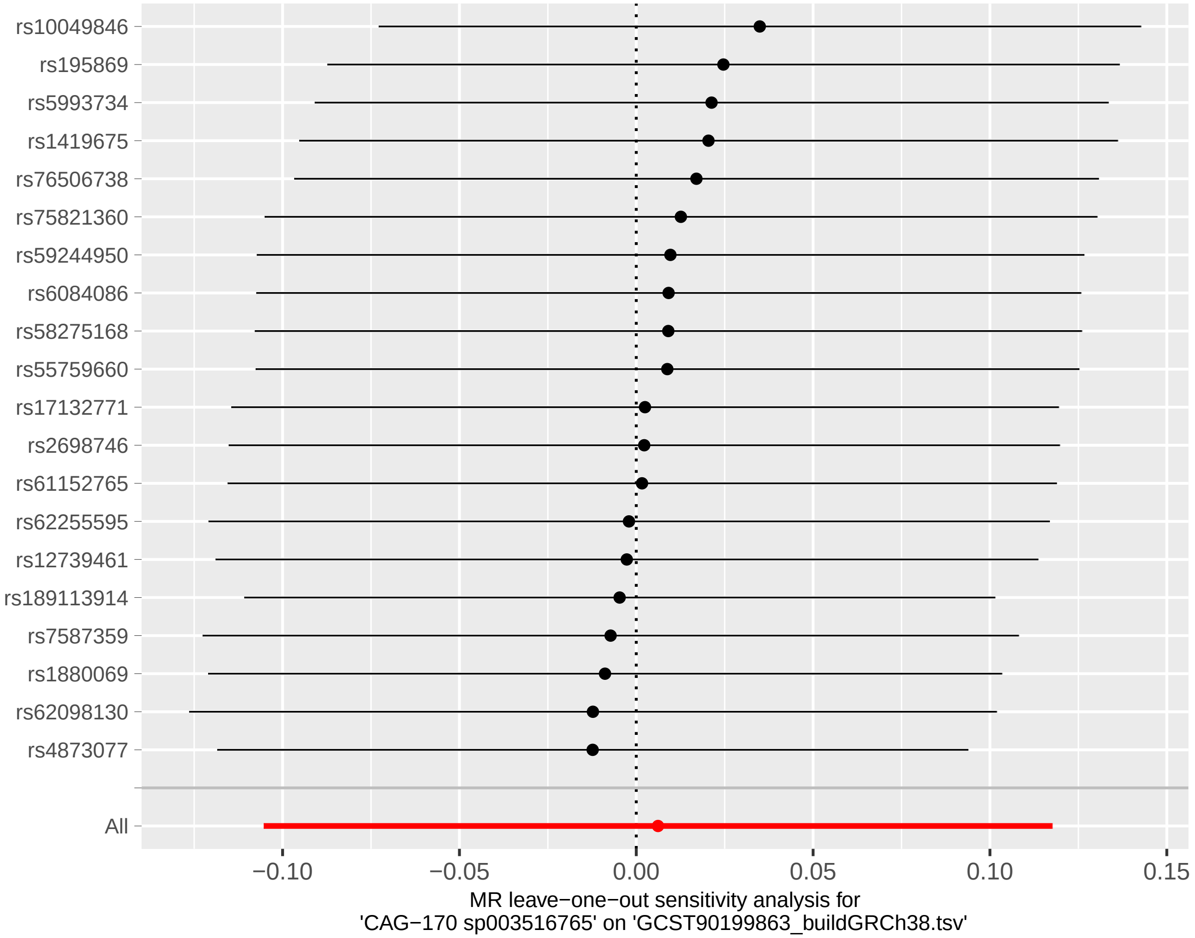

rs10049846
rs195869
rs5993734
rs1419675
rs76506738
rs75821360
rs59244950
rs6084086
rs58275168
rs55759660
rs17132771
rs2698746
rs61152765
rs62255595
rs12739461
rs189113914
rs7587359
rs1880069
rs62098130
rs4873077
All
−0.10
−0.05
0.00
0.05
0.10
0.15
MR leave−one−out sensitivity analysis for
'CAG−170 sp003516765' on 'GCST90199863_buildGRCh38.tsv'

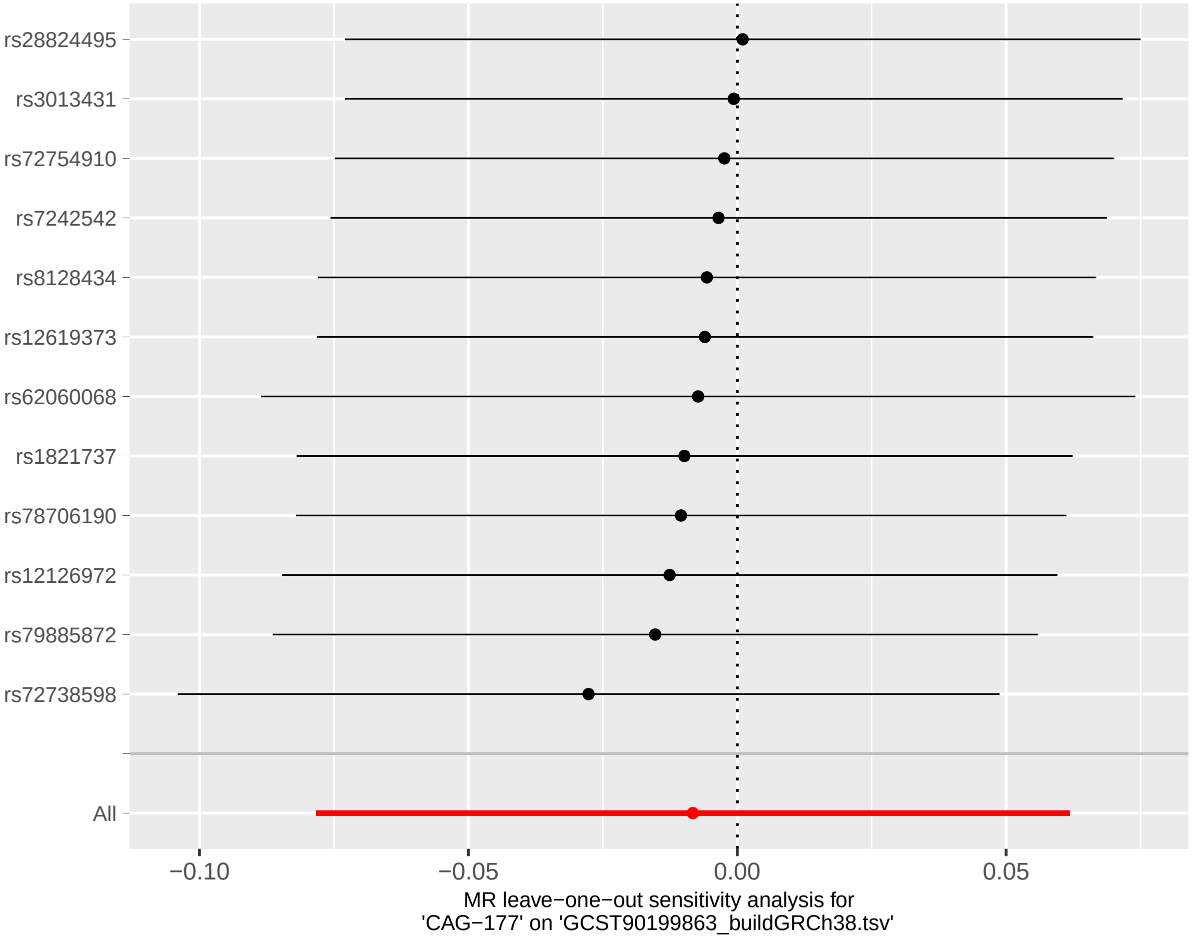
rs28824495
rs3013431
rs72754910
rs7242542
rs8128434
rs12619373
rs62060068
rs1821737
rs78706190
rs12126972
rs79885872
rs72738598
All
−0.10
−0.05
0.00
0.05
MR leave−one−out sensitivity analysis for
'CAG−177' on 'GCST90199863_buildGRCh38.tsv'

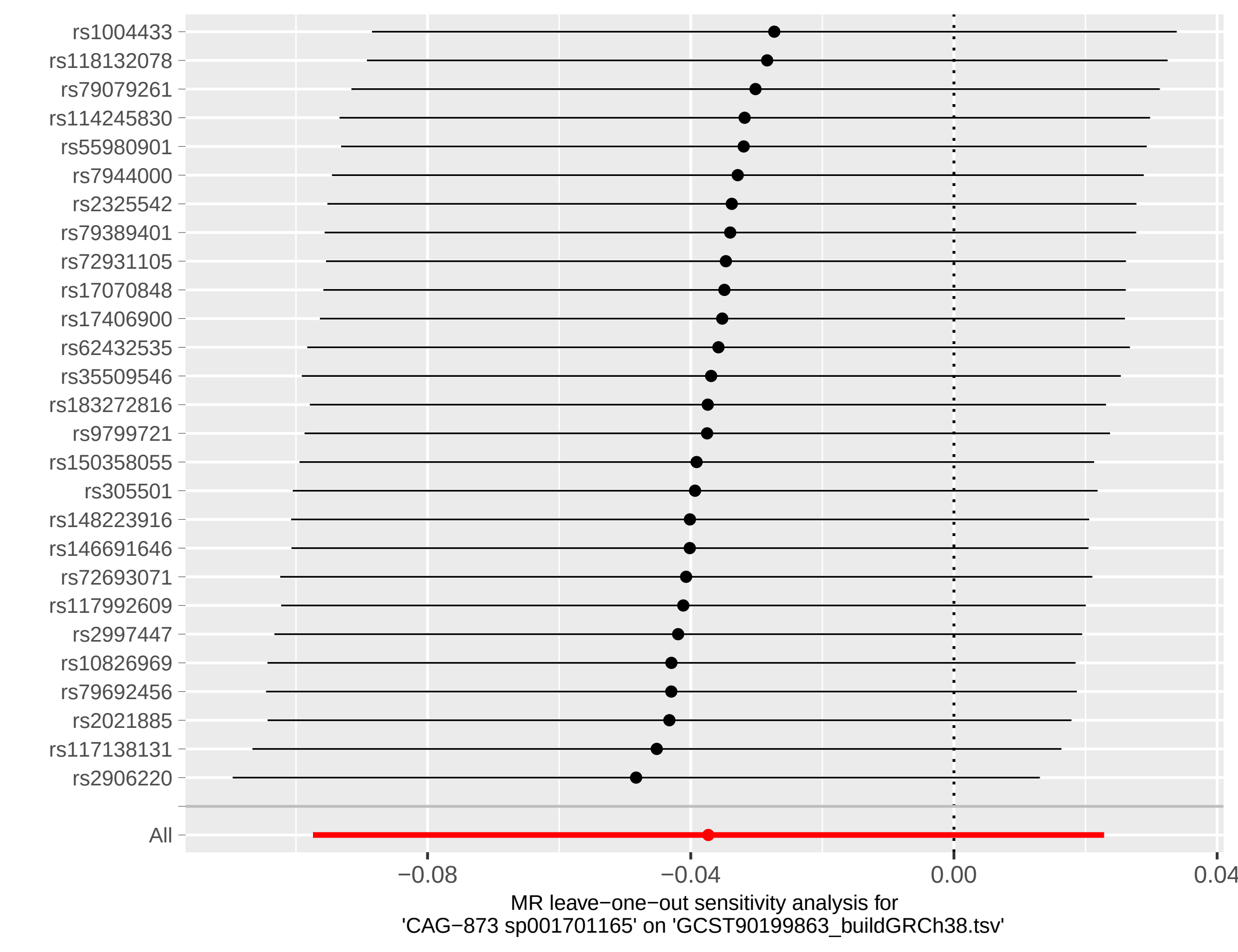
rs1004433
rs118132078
rs79079261
rs114245830
rs55980901
rs7944000
rs2325542
rs79389401
rs72931105
rs17070848
rs17406900
rs62432535
rs35509546
rs183272816
rs9799721
rs150358055
rs305501
rs148223916
rs146691646
rs72693071
rs117992609
rs2997447
rs10826969
rs79692456
rs2021885
rs117138131
rs2906220
All
−0.08
−0.04
0.00
0.04
MR leave−one−out sensitivity analysis for
'CAG−873 sp001701165' on 'GCST90199863_buildGRCh38.tsv'

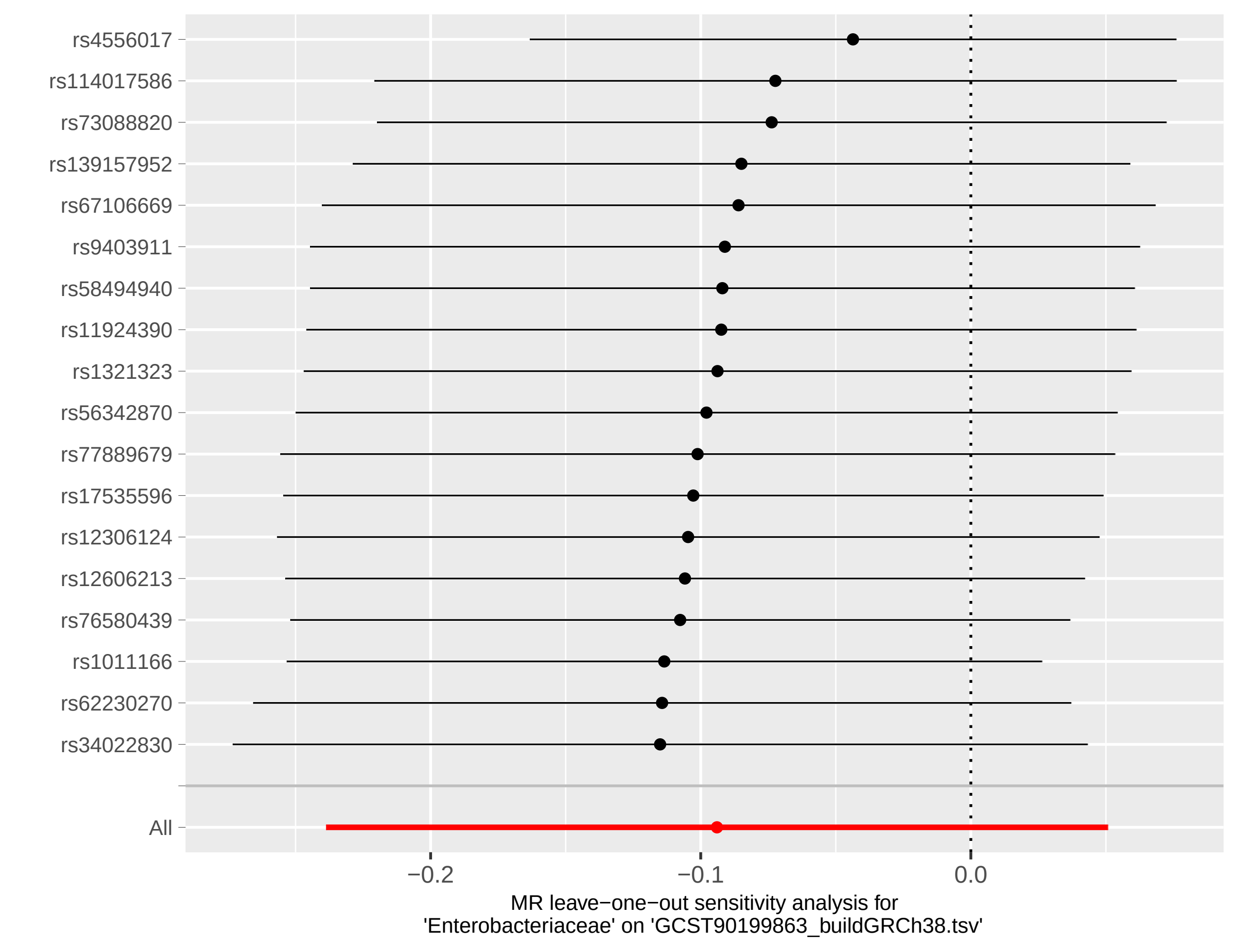

rs4556017
rs114017586
rs73088820
rs139157952
rs67106669
rs9403911
rs58494940
rs11924390
rs1321323
rs56342870
rs77889679
rs17535596
rs12306124
rs12606213
rs76580439
rs1011166
rs62230270
rs34022830
All
−0.2
−0.1
0.0
MR leave−one−out sensitivity analysis for
'Enterobacteriaceae' on 'GCST90199863_buildGRCh38.tsv'

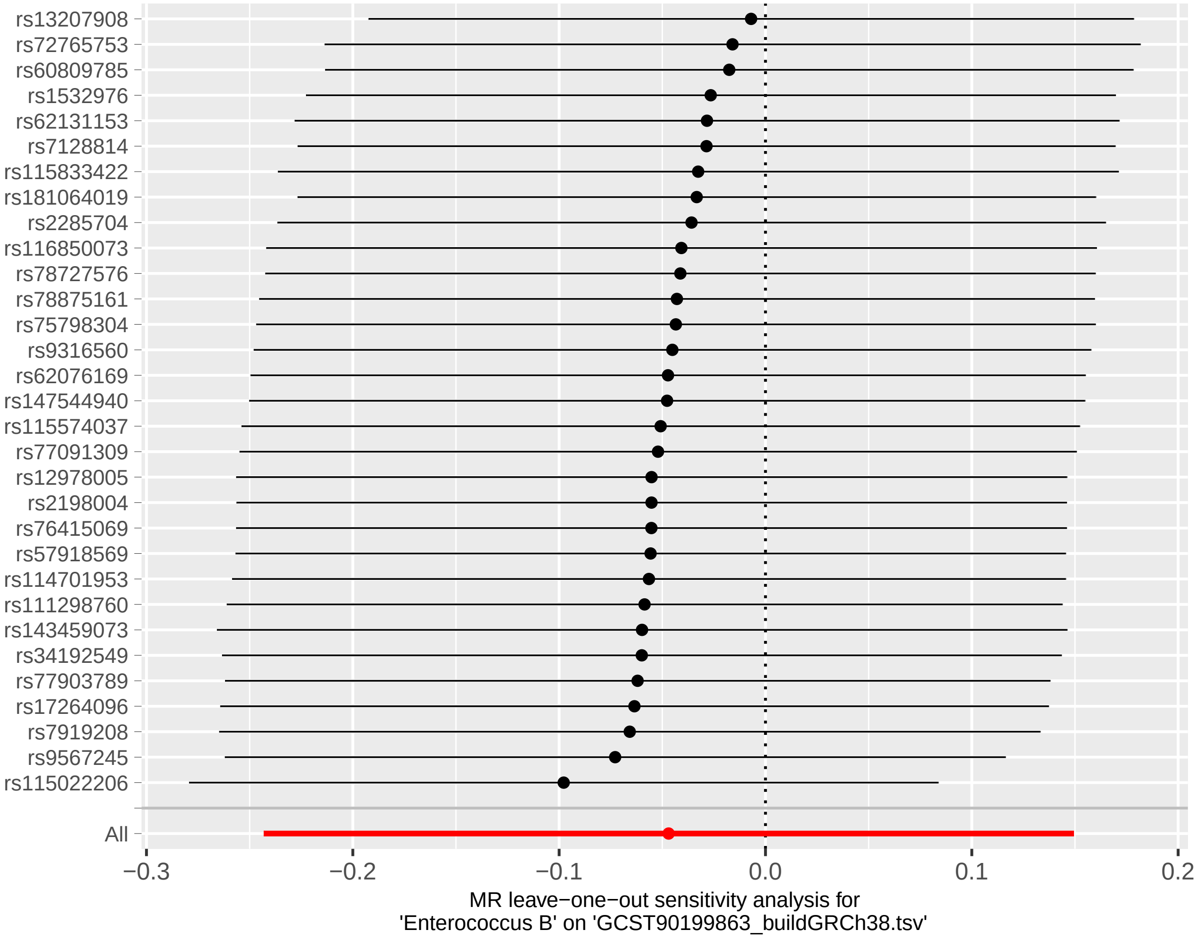
rs13207908
rs72765753
rs60809785
rs1532976
rs62131153
rs7128814
rs115833422
rs181064019
rs2285704
rs116850073
rs78727576
rs78875161
rs75798304
rs9316560
rs62076169
rs147544940
rs115574037
rs77091309
rs12978005
rs2198004
rs76415069
rs57918569
rs114701953
rs111298760
rs143459073
rs34192549
rs77903789
rs17264096
rs7919208
rs9567245
rs115022206
All
−0.3
−0.2
−0.1
0.0
0.1
0.2
MR leave−one−out sensitivity analysis for
'Enterococcus B' on 'GCST90199863_buildGRCh38.tsv'

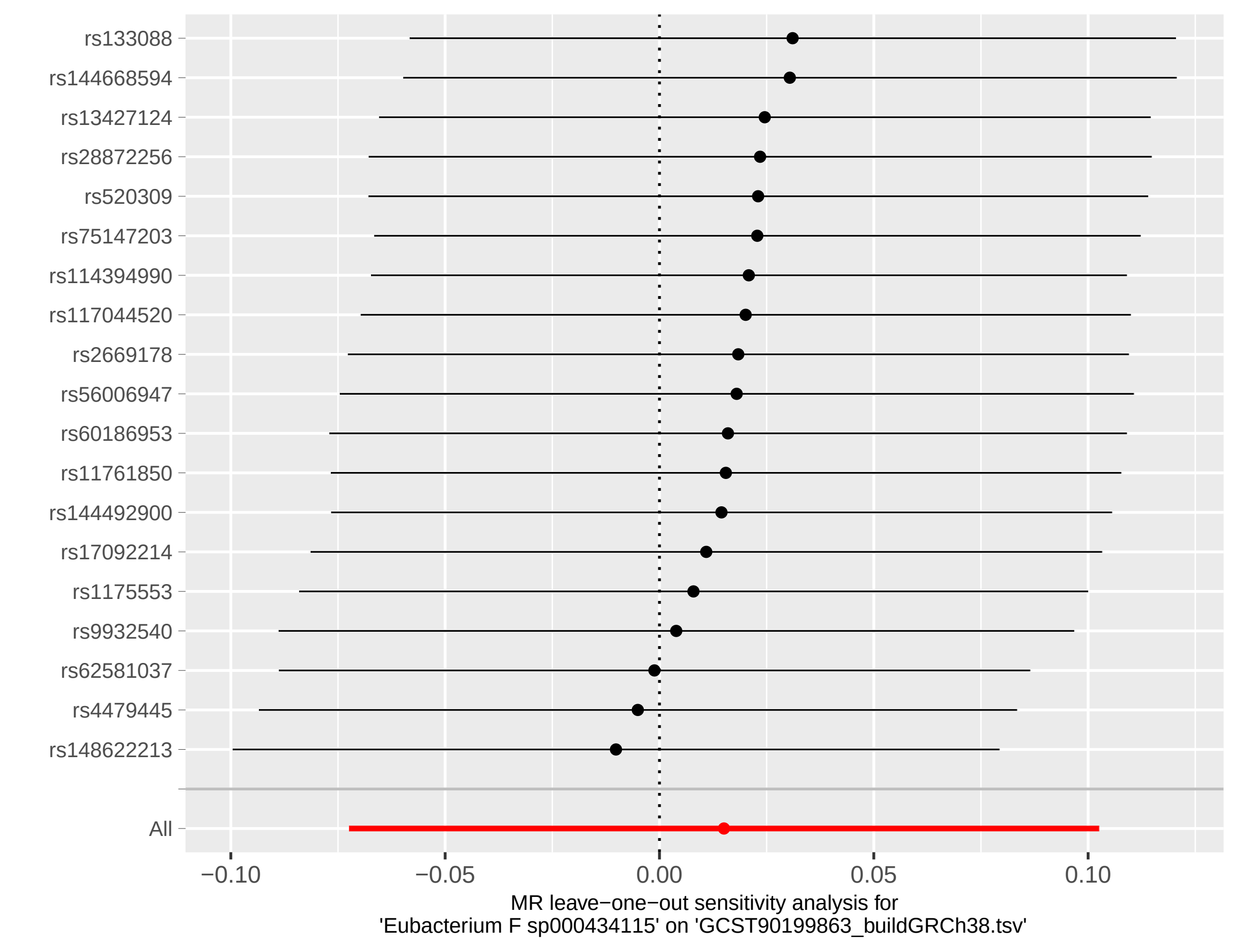

rs133088
rs144668594
rs13427124
rs28872256
rs520309
rs75147203
rs114394990
rs117044520
rs2669178
rs56006947
rs60186953
rs11761850
rs144492900
rs17092214
rs1175553
rs9932540
rs62581037
rs4479445
rs148622213
All
−0.10
−0.05
0.00
0.05
0.10
MR leave−one−out sensitivity analysis for
'Eubacterium F sp000434115' on 'GCST90199863_buildGRCh38.tsv'

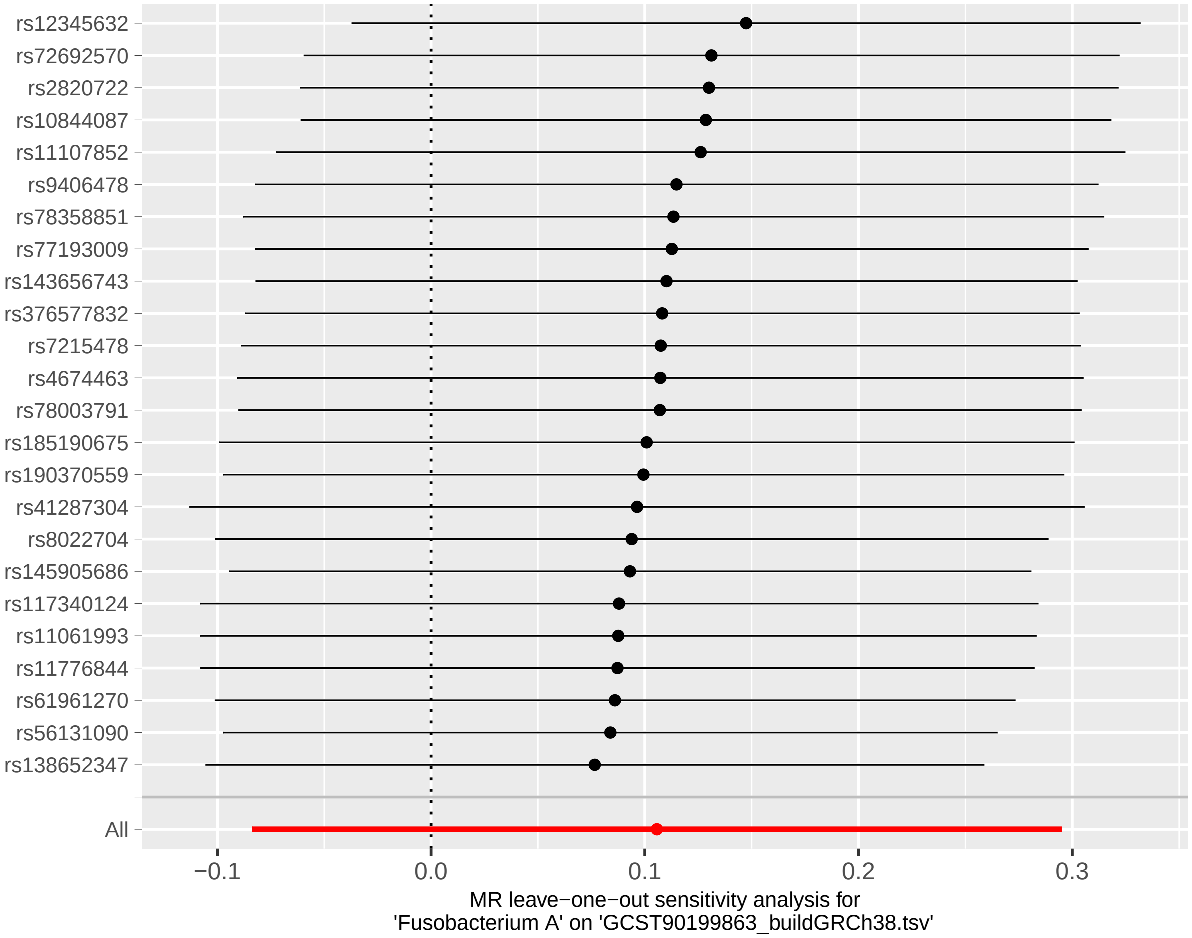
rs12345632
rs72692570
rs2820722
rs10844087
rs11107852
rs9406478
rs78358851
rs77193009
rs143656743
rs376577832
rs7215478
rs4674463
rs78003791
rs185190675
rs190370559
rs41287304
rs8022704
rs145905686
rs117340124
rs11061993
rs11776844
rs61961270
rs56131090
rs138652347
All
−0.1
0.0
0.1
0.2
0.3
MR leave−one−out sensitivity analysis for
'Fusobacterium A' on 'GCST90199863_buildGRCh38.tsv'

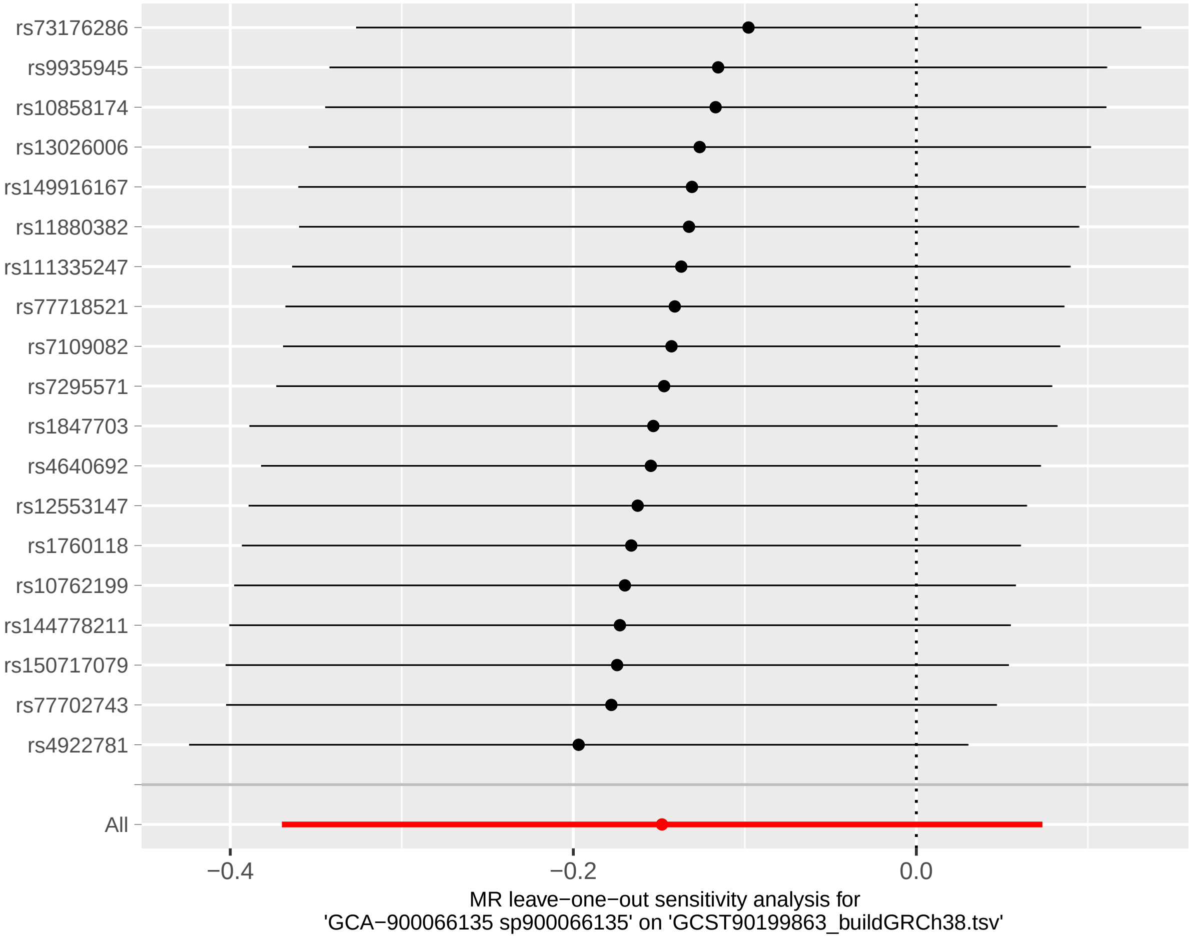
rs73176286
rs9935945
rs10858174
rs13026006
rs149916167
rs11880382
rs111335247
rs77718521
rs7109082
rs7295571
rs1847703
rs4640692
rs12553147
rs1760118
rs10762199
rs144778211
rs150717079
rs77702743
rs4922781
All
−0.4
−0.2
0.0
MR leave−one−out sensitivity analysis for
'GCA−900066135 sp900066135' on 'GCST90199863_buildGRCh38.tsv'

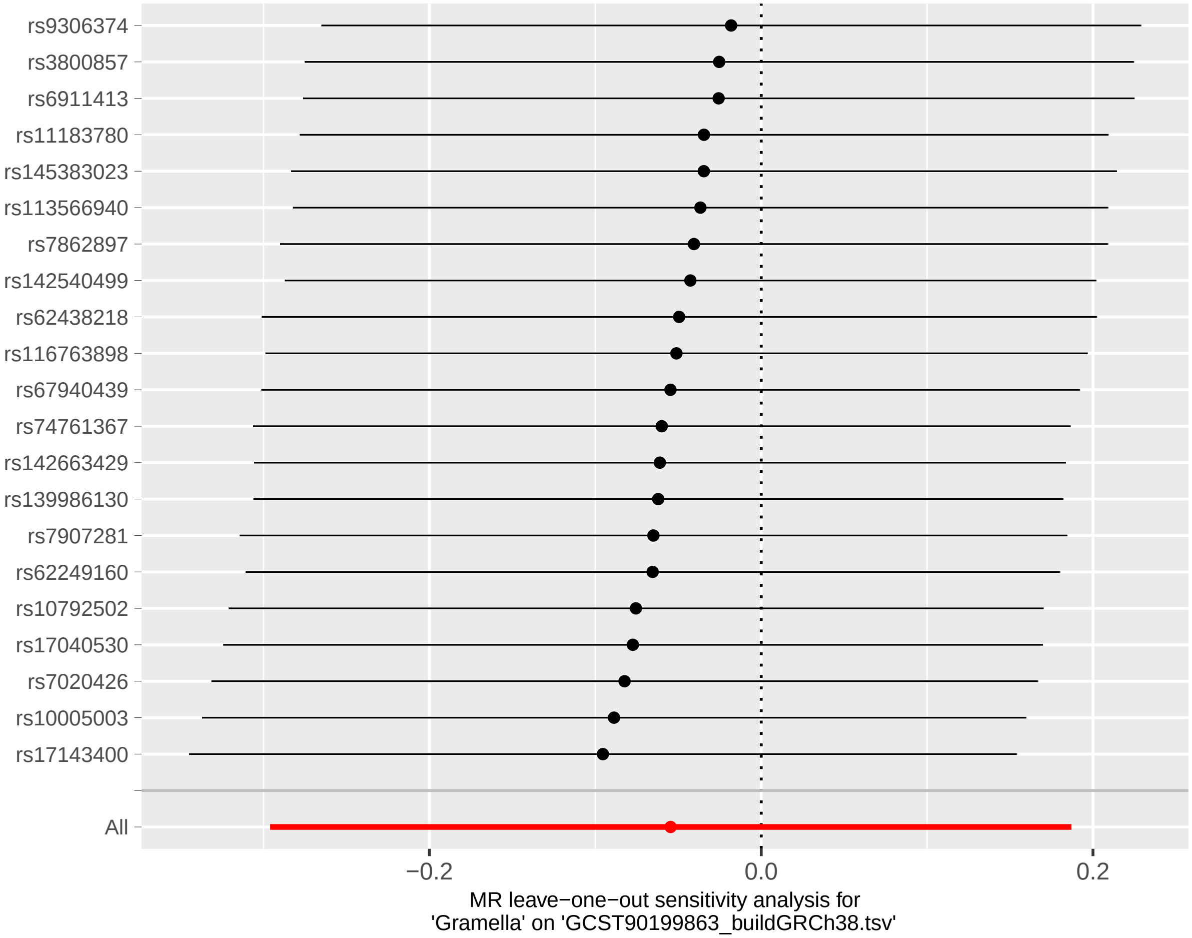

rs9306374
rs3800857
rs6911413
rs11183780
rs145383023
rs113566940
rs7862897
rs142540499
rs62438218
rs116763898
rs67940439
rs74761367
rs142663429
rs139986130
rs7907281
rs62249160
rs10792502
rs17040530
rs7020426
rs10005003
rs17143400
All
−0.2
0.0
0.2
MR leave−one−out sensitivity analysis for
'Gramella' on 'GCST90199863_buildGRCh38.tsv'

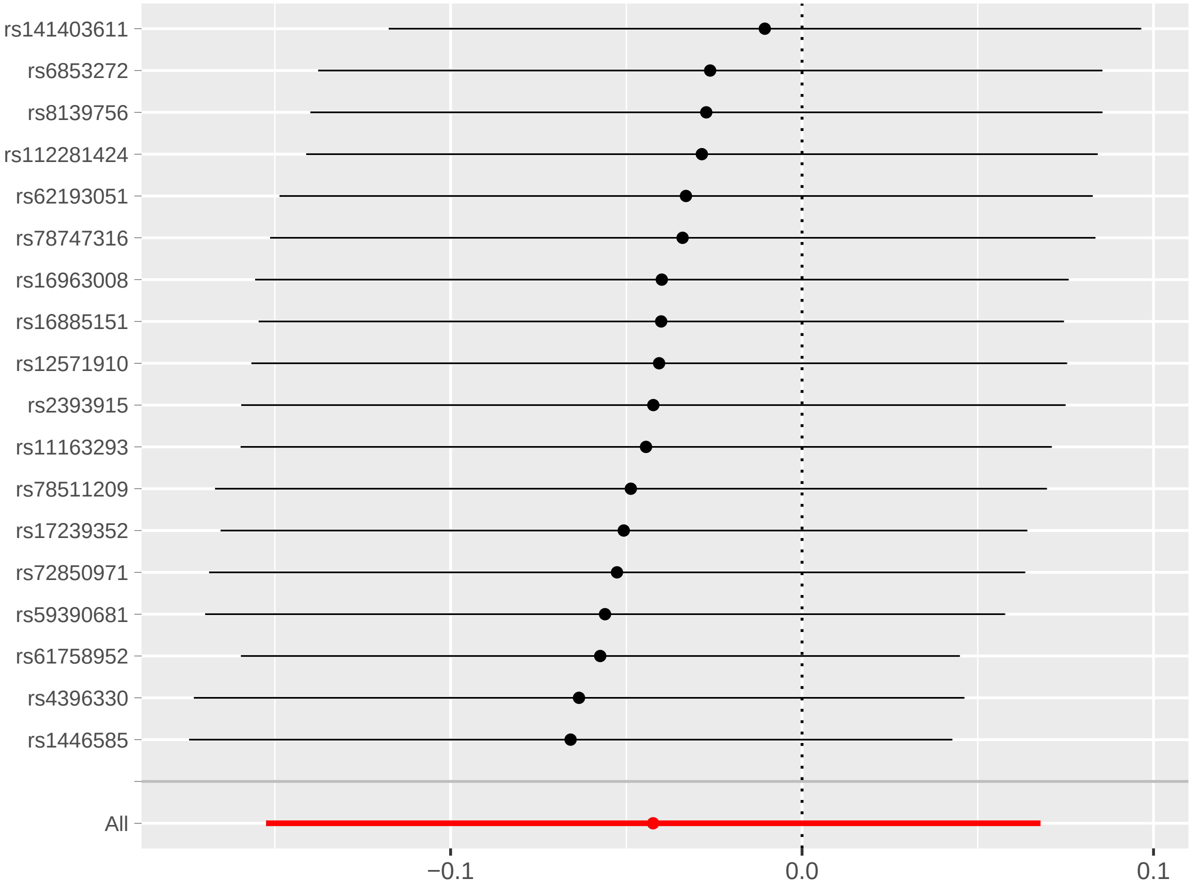

MR leave−one−out sensitivity analysis for
'Holdemania sp900120005' on 'GCST90199863_buildGRCh38.tsv'

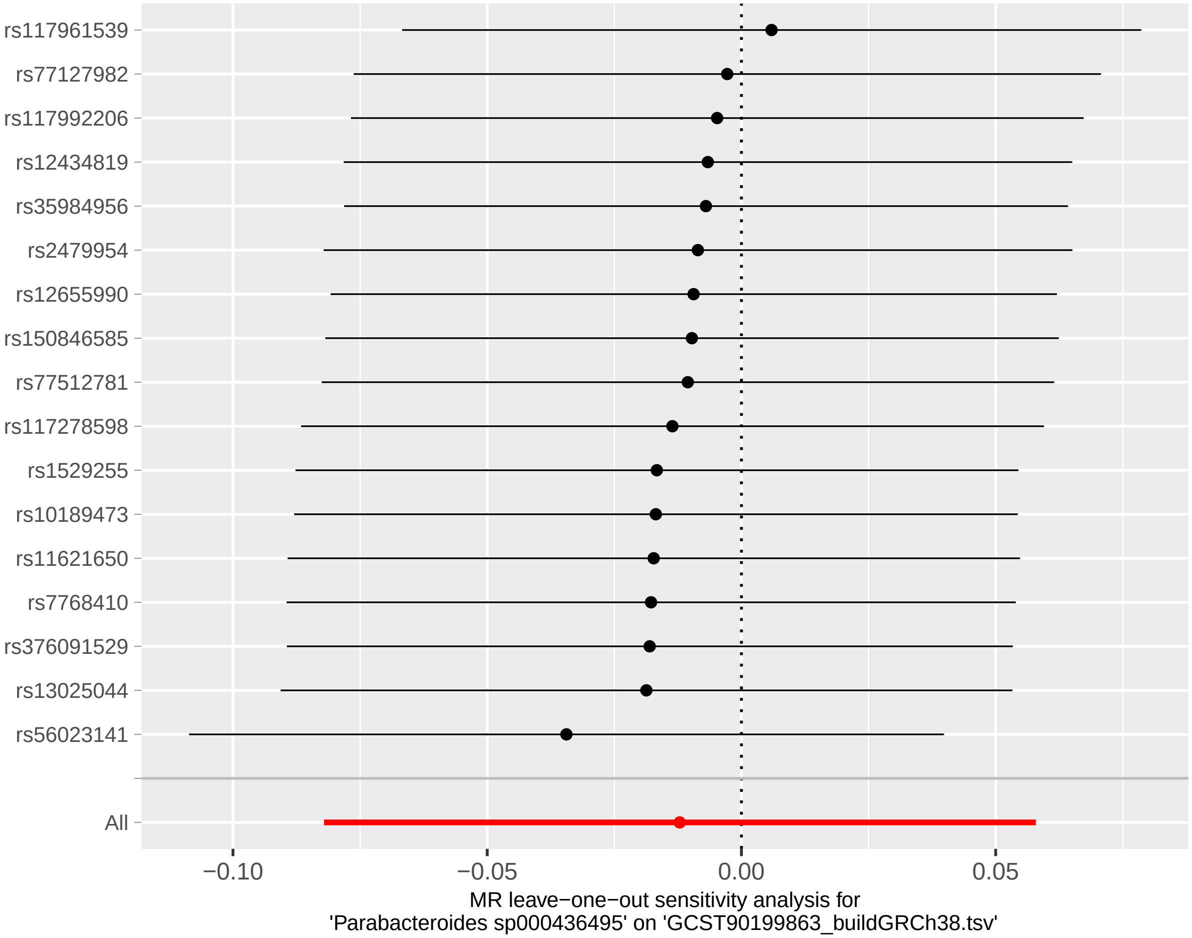
rs117961539
rs77127982
rs117992206
rs12434819
rs35984956
rs2479954
rs12655990
rs150846585
rs77512781
rs117278598
rs1529255
rs10189473
rs11621650
rs7768410
rs376091529
rs13025044
rs56023141
All
−0.10
−0.05
0.00
0.05
MR leave−one−out sensitivity analysis for
'Parabacteroides sp000436495' on 'GCST90199863_buildGRCh38.tsv'

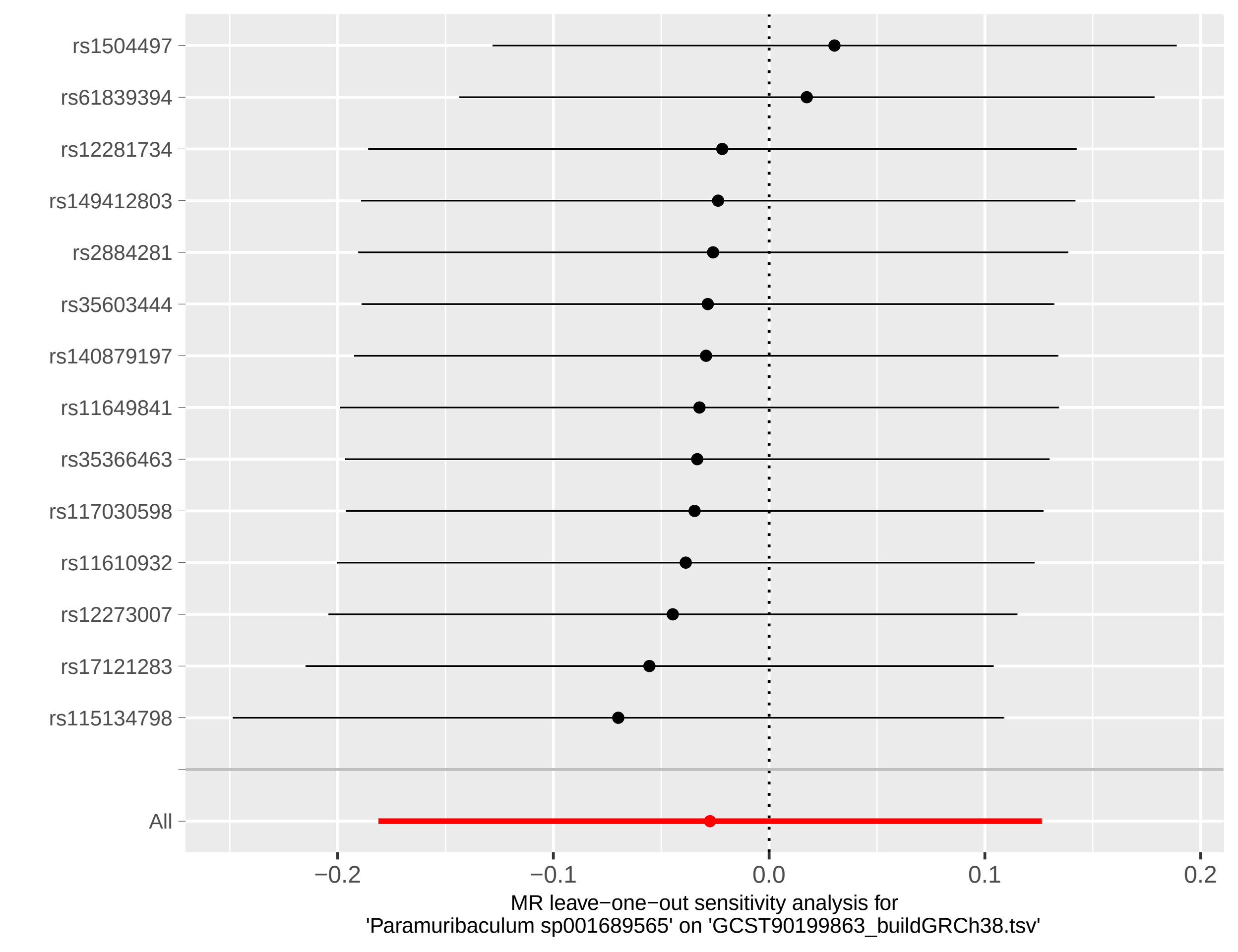
rs1504497
rs61839394
rs12281734
rs149412803
rs2884281
rs35603444
rs140879197
rs11649841
rs35366463
rs117030598
rs11610932
rs12273007
rs17121283
rs115134798
All
−0.2
−0.1
0.0
0.1
0.2
MR leave−one−out sensitivity analysis for
'Paramuribaculum sp001689565' on 'GCST90199863_buildGRCh38.tsv'

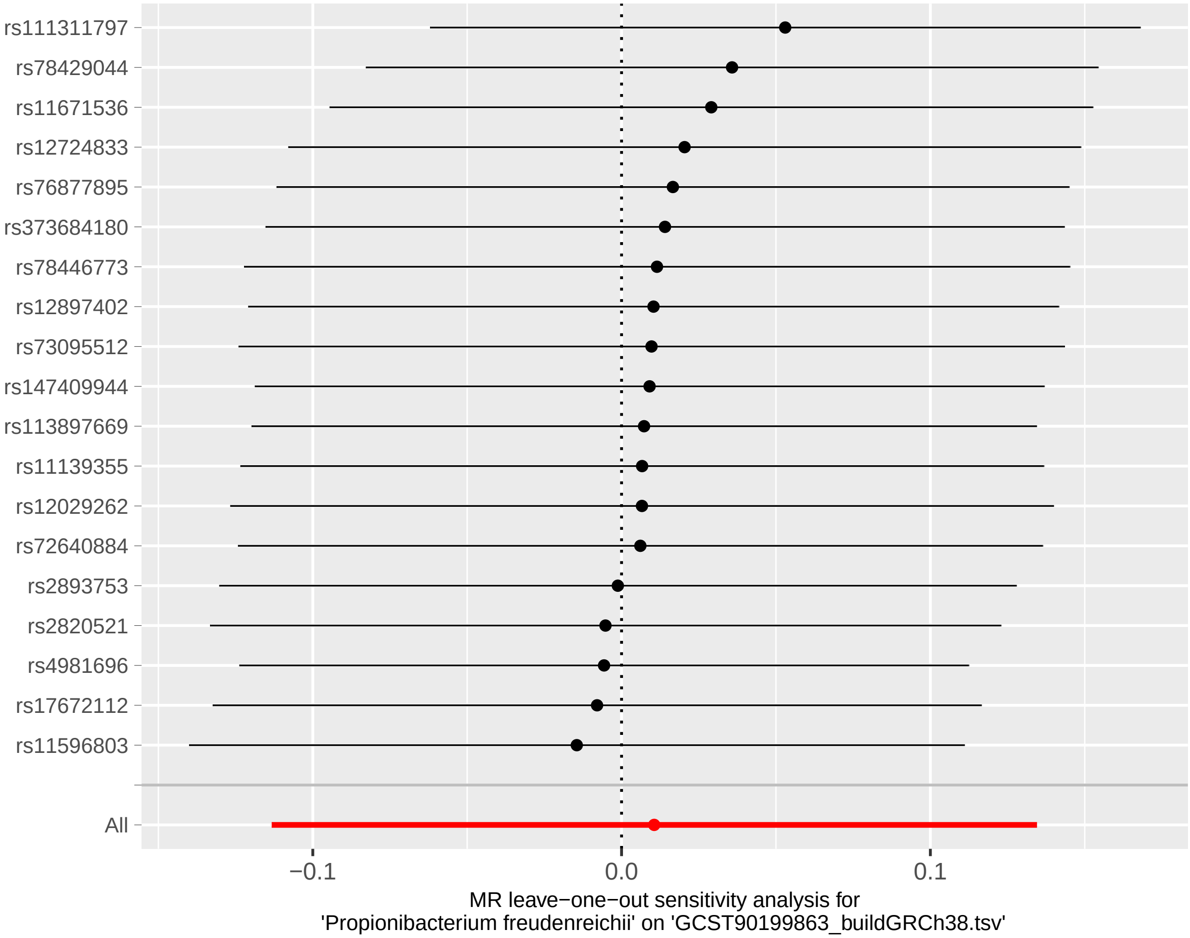

rs111311797
rs78429044
rs11671536
rs12724833
rs76877895
rs373684180
rs78446773
rs12897402
rs73095512
rs147409944
rs113897669
rs11139355
rs12029262
rs72640884
rs2893753
rs2820521
rs4981696
rs17672112
rs11596803
All
−0.1
0.0
0.1
MR leave−one−out sensitivity analysis for
'Propionibacterium freudenreichii' on 'GCST90199863_buildGRCh38.tsv'

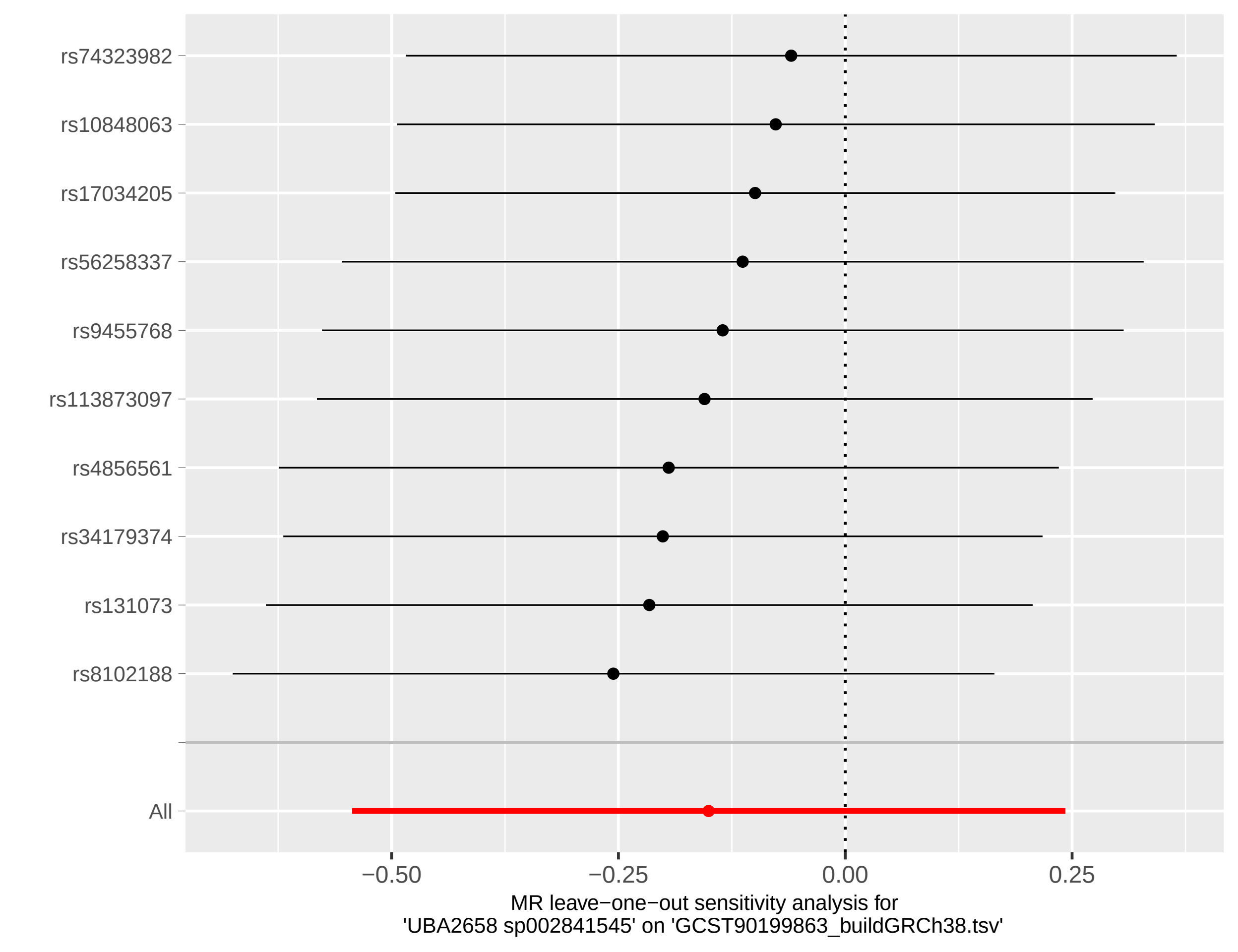
rs74323982
rs10848063
rs17034205
rs56258337
rs9455768
rs113873097
rs4856561
rs34179374
rs131073
rs8102188
All
−0.50
−0.25
0.00
0.25
MR leave−one−out sensitivity analysis for
'UBA2658 sp002841545' on 'GCST90199863_buildGRCh38.tsv'

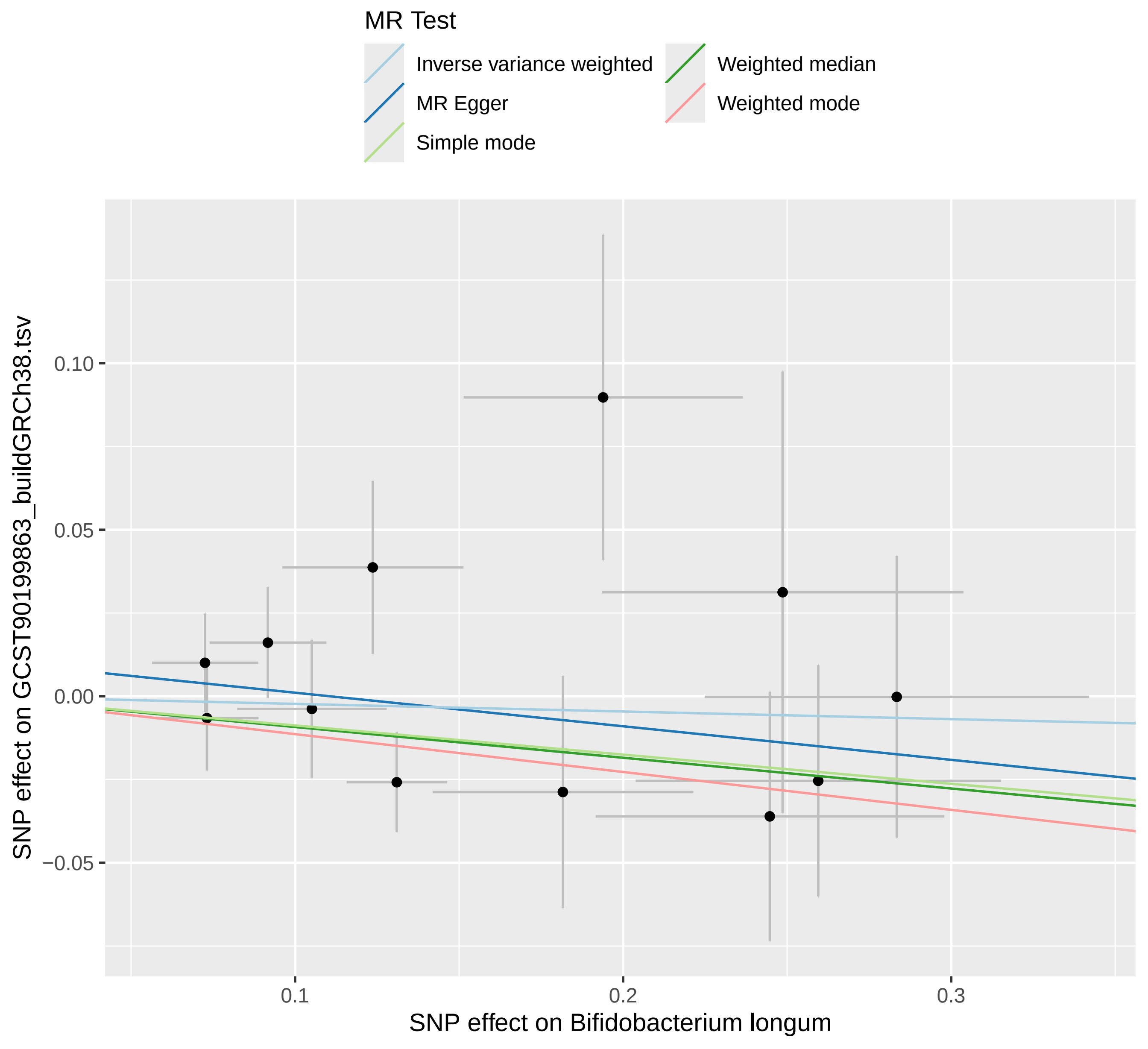
MR Test
Inverse variance weighted
MR Egger
Simple mode
Weighted median
Weighted mode
SNP effect on GCST90199863_buildGRCh38.tsv
0.10
0.05
0.00
−0.05
0.1
0.2
0.3
SNP effect on Bifidobacterium longum

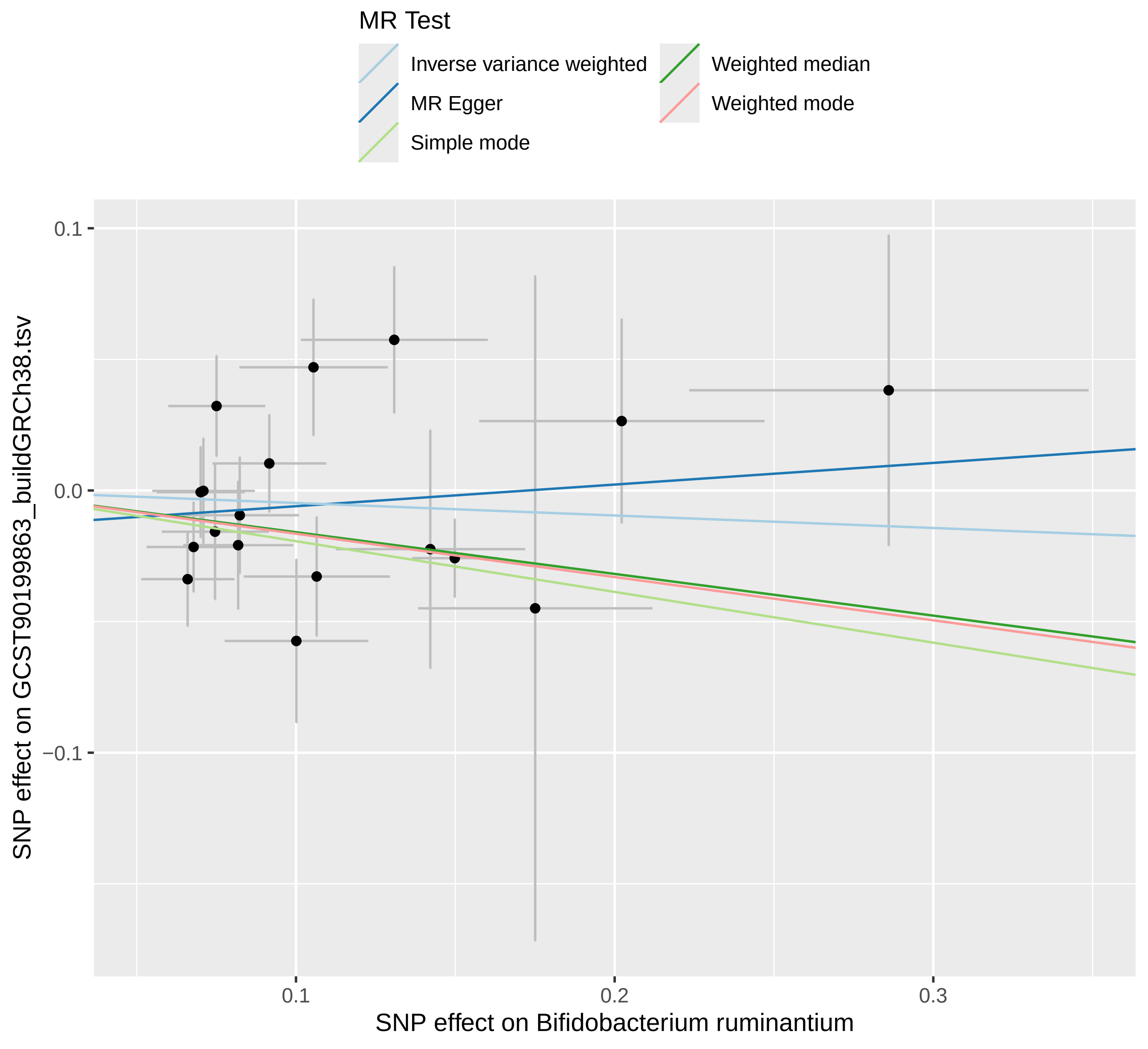
MR Test
Inverse variance weighted
MR Egger
Simple mode
Weighted median
Weighted mode
0.1
0.0
−0.1
0.1
0.2
0.3
SNP effect on GCST90199863_buildGRCh38.tsv
SNP effect on Bifidobacterium ruminantium

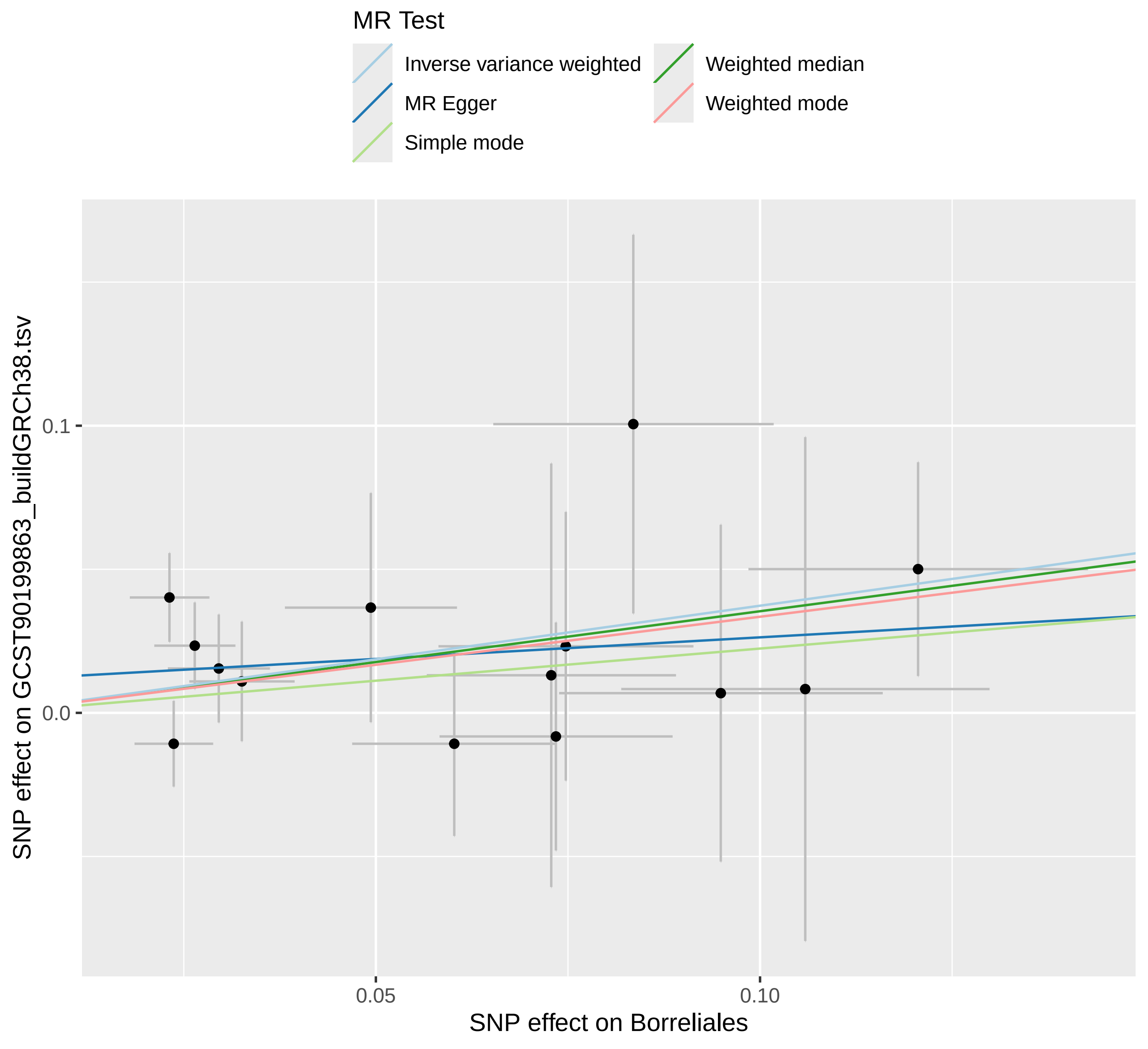
MR Test
Inverse variance weighted
MR Egger
Simple mode
Weighted median
Weighted mode
0.1
0.0
0.05
0.10
SNP effect on GCST90199863_buildGRCh38.tsv
SNP effect on Borreliales

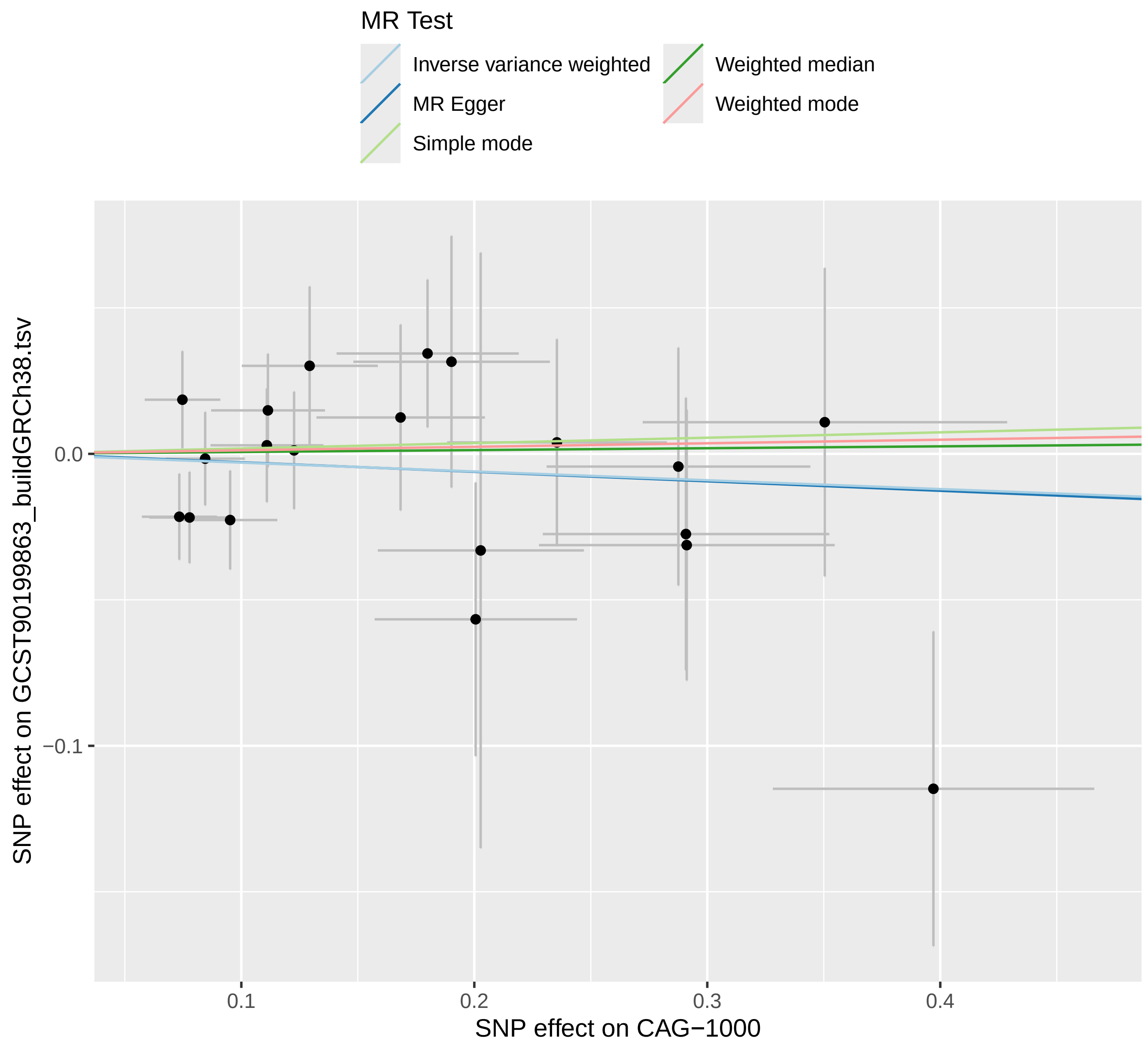

MR Test
Inverse variance weighted
MR Egger
Simple mode
Weighted median
Weighted mode
0.0
−0.1
0.1
0.2
0.3
0.4
SNP effect on GCST90199863_buildGRCh38.tsv
SNP effect on CAG−1000

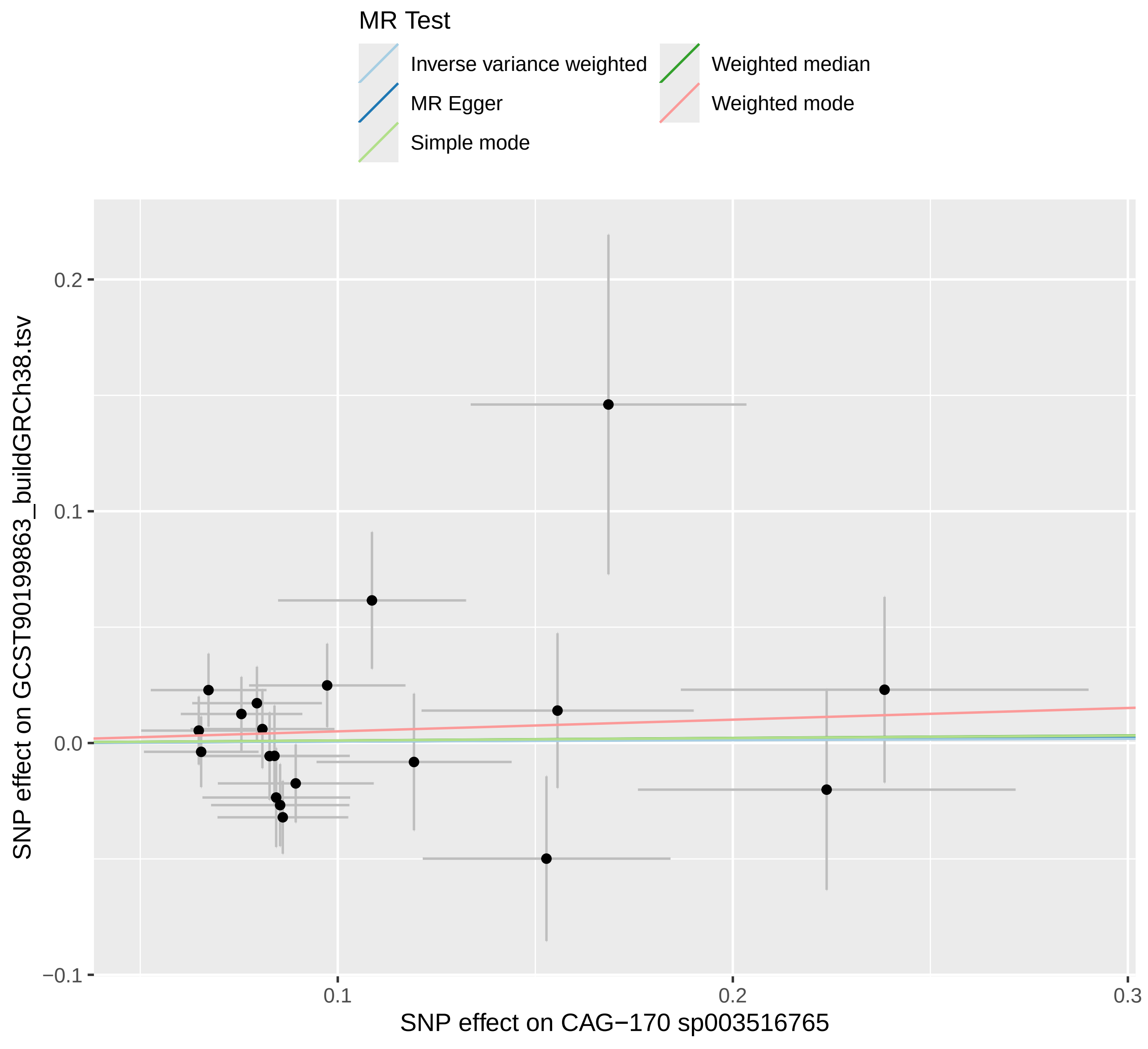
MR Test
Inverse variance weighted
MR Egger
Simple mode
Weighted median
Weighted mode
0.2
0.1
0.0
−0.1
0.1
0.2
0.3
SNP effect on GCST90199863_buildGRCh38.tsv
SNP effect on CAG−170 sp003516765

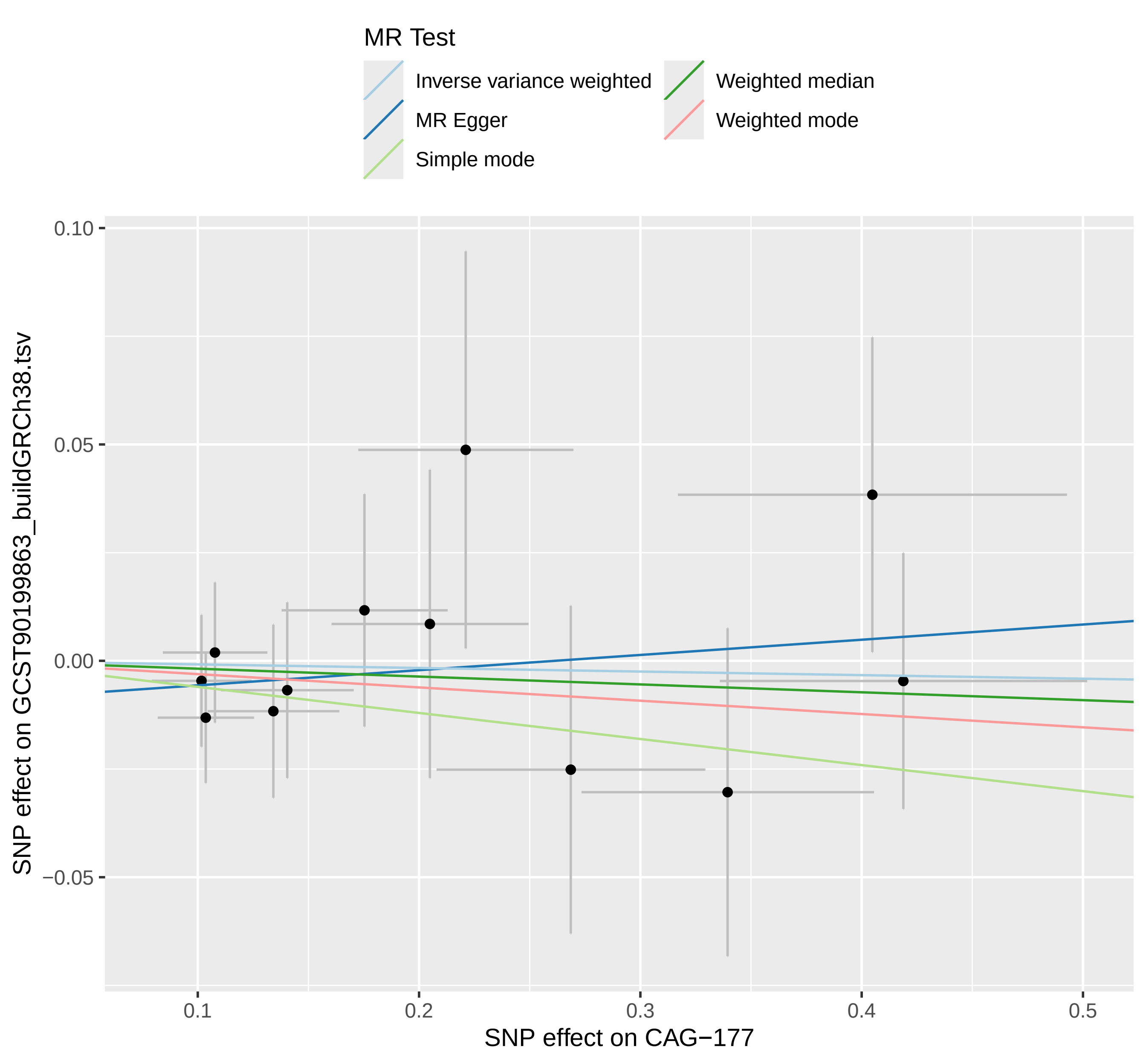
MR Test
Inverse variance weighted
MR Egger
Simple mode
Weighted median
Weighted mode
0.10
0.05
0.00
−0.05
0.1
0.2
0.3
0.4
0.5
SNP effect on GCST90199863_buildGRCh38.tsv
SNP effect on CAG−177

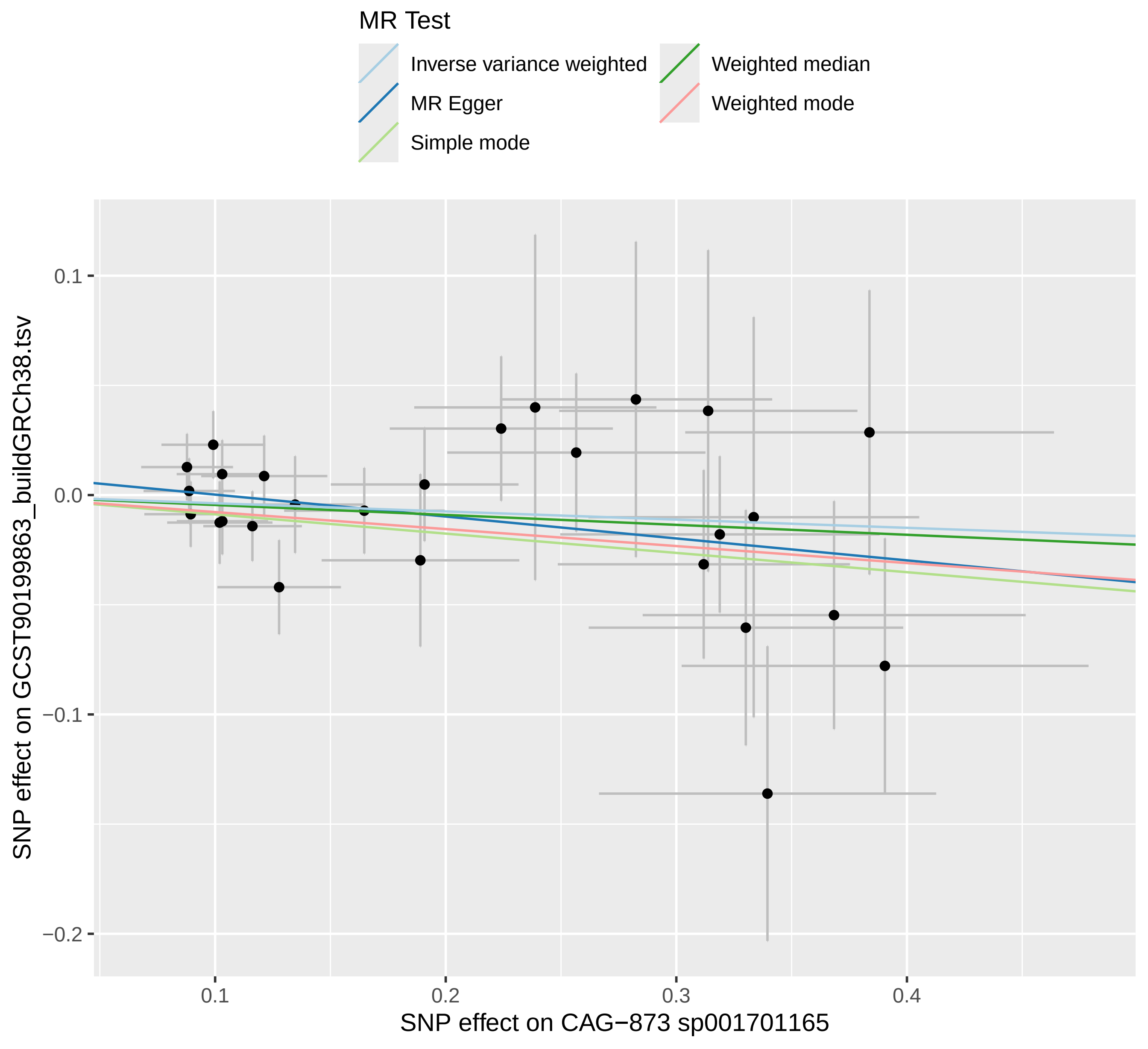

MR Test
Inverse variance weighted
MR Egger
Simple mode
Weighted median
Weighted mode
0.1
0.0
−0.1
−0.2
0.1
0.2
0.3
0.4
SNP effect on GCST90199863_buildGRCh38.tsv
SNP effect on CAG−873 sp001701165

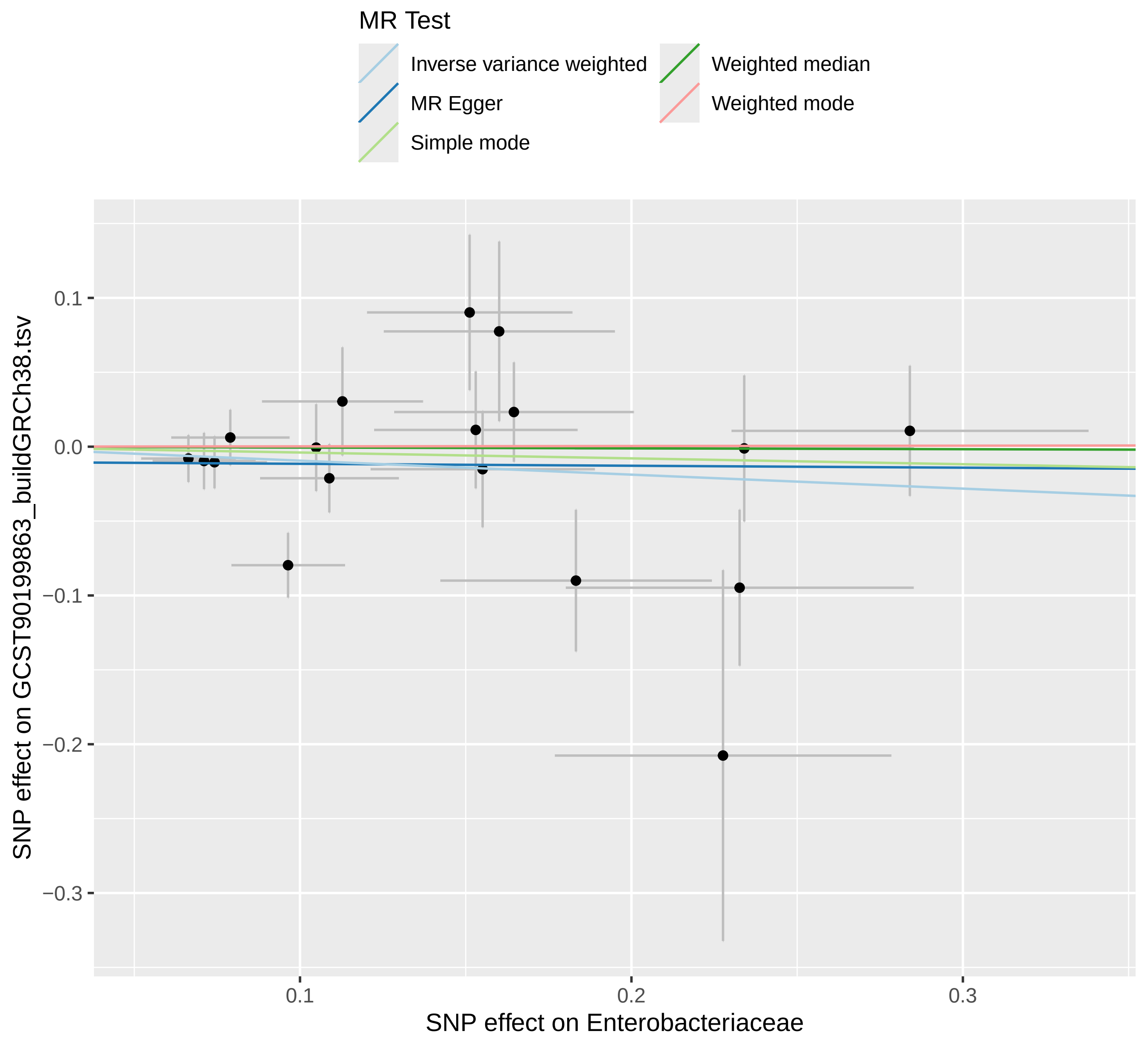
MR Test
Inverse variance weighted
MR Egger
Simple mode
Weighted median
Weighted mode
SNP effect on GCST90199863_buildGRCh38.tsv
0.1
0.0
−0.1
−0.2
−0.3
0.1
0.2
0.3
SNP effect on Enterobacteriaceae

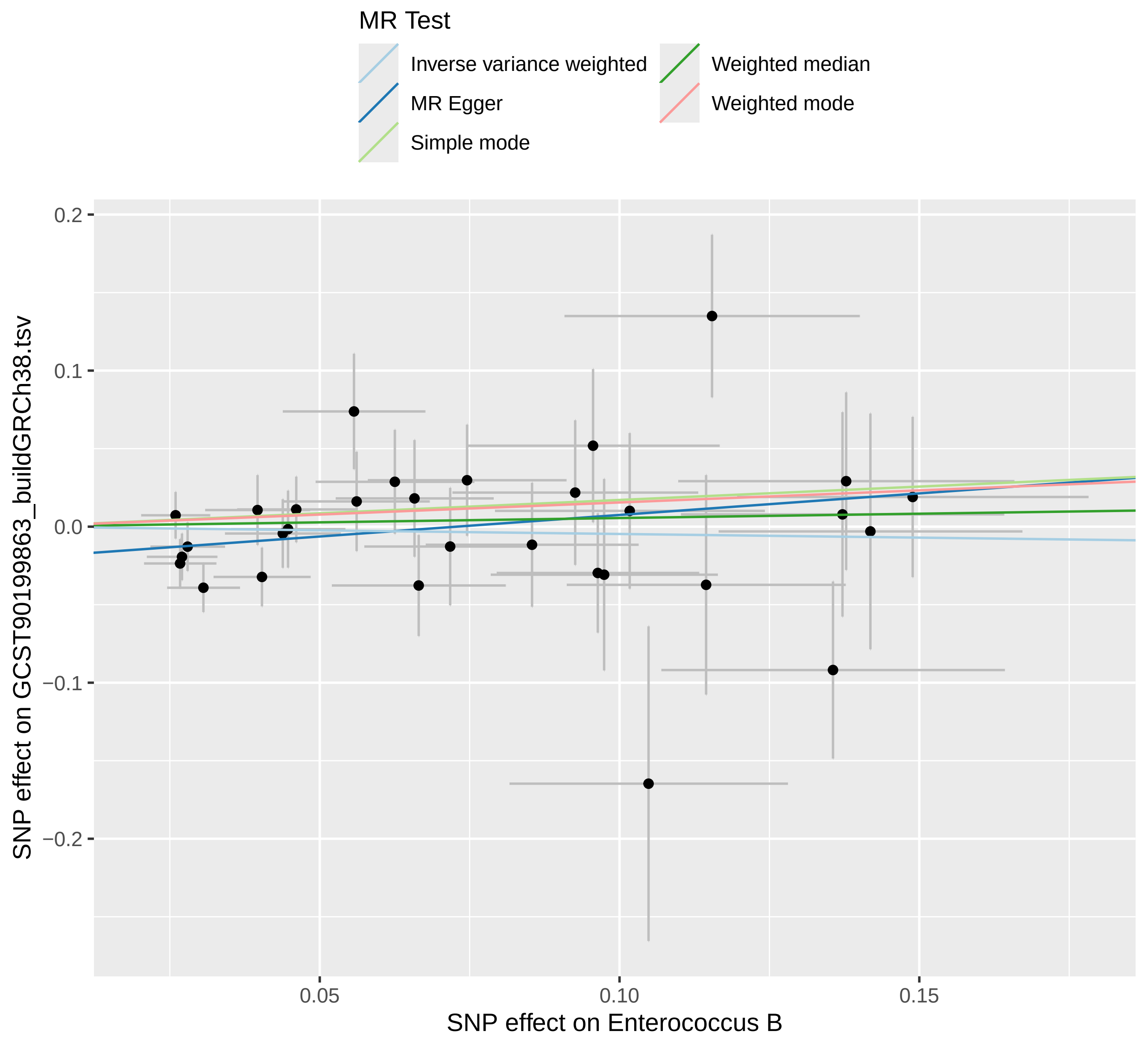
MR Test
Inverse variance weighted
MR Egger
Simple mode
Weighted median
Weighted mode
0.2
0.1
0.0
−0.1
−0.2
0.05
0.10
0.15
SNP effect on GCST90199863_buildGRCh38.tsv
SNP effect on Enterococcus B

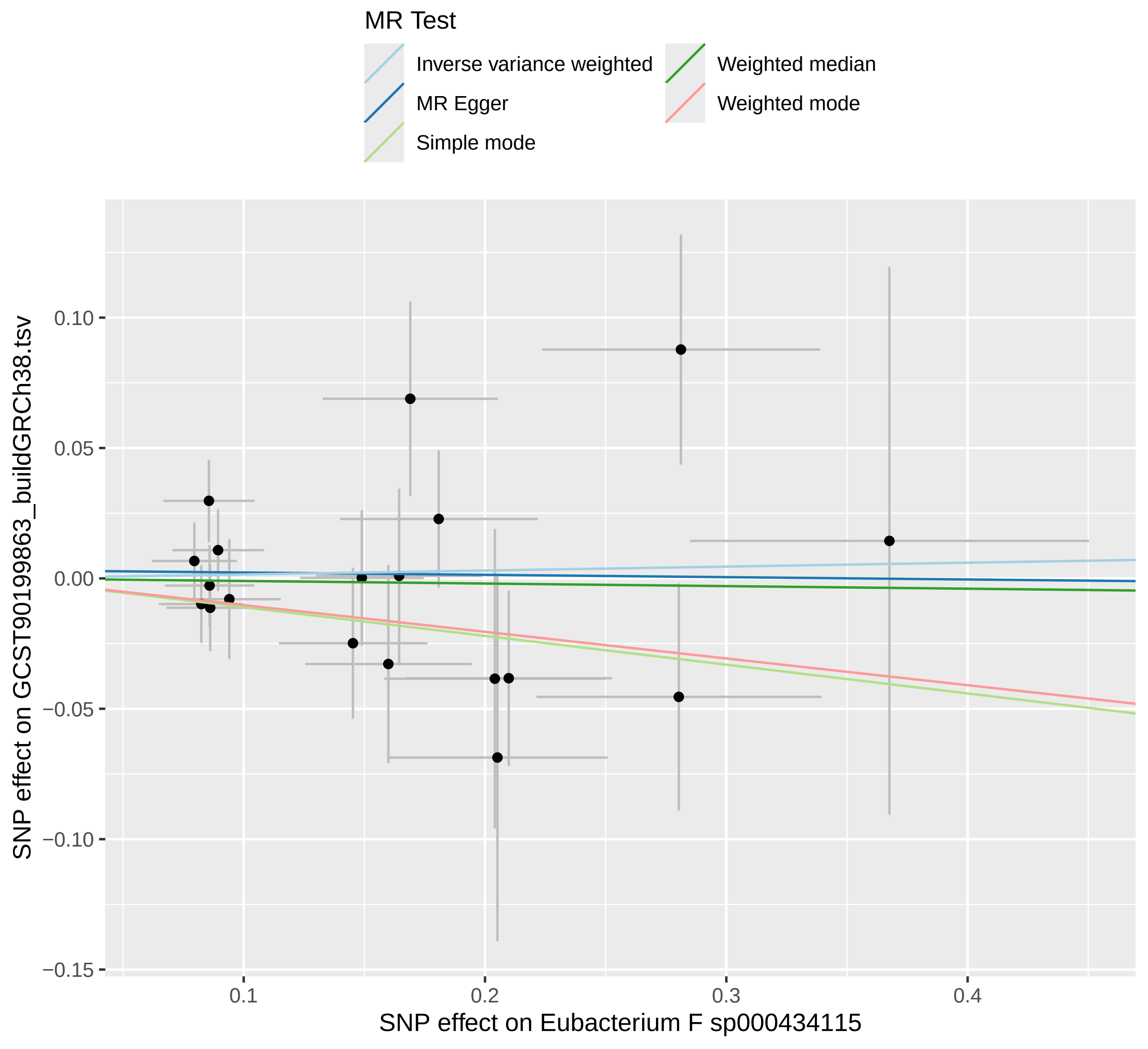

MR Test
Inverse variance weighted
MR Egger
Simple mode
Weighted median
Weighted mode
0.10
0.05
0.00
−0.05
−0.10
−0.15
0.1
0.2
0.3
0.4
SNP effect on GCST90199863_buildGRCh38.tsv
SNP effect on Eubacterium F sp000434115

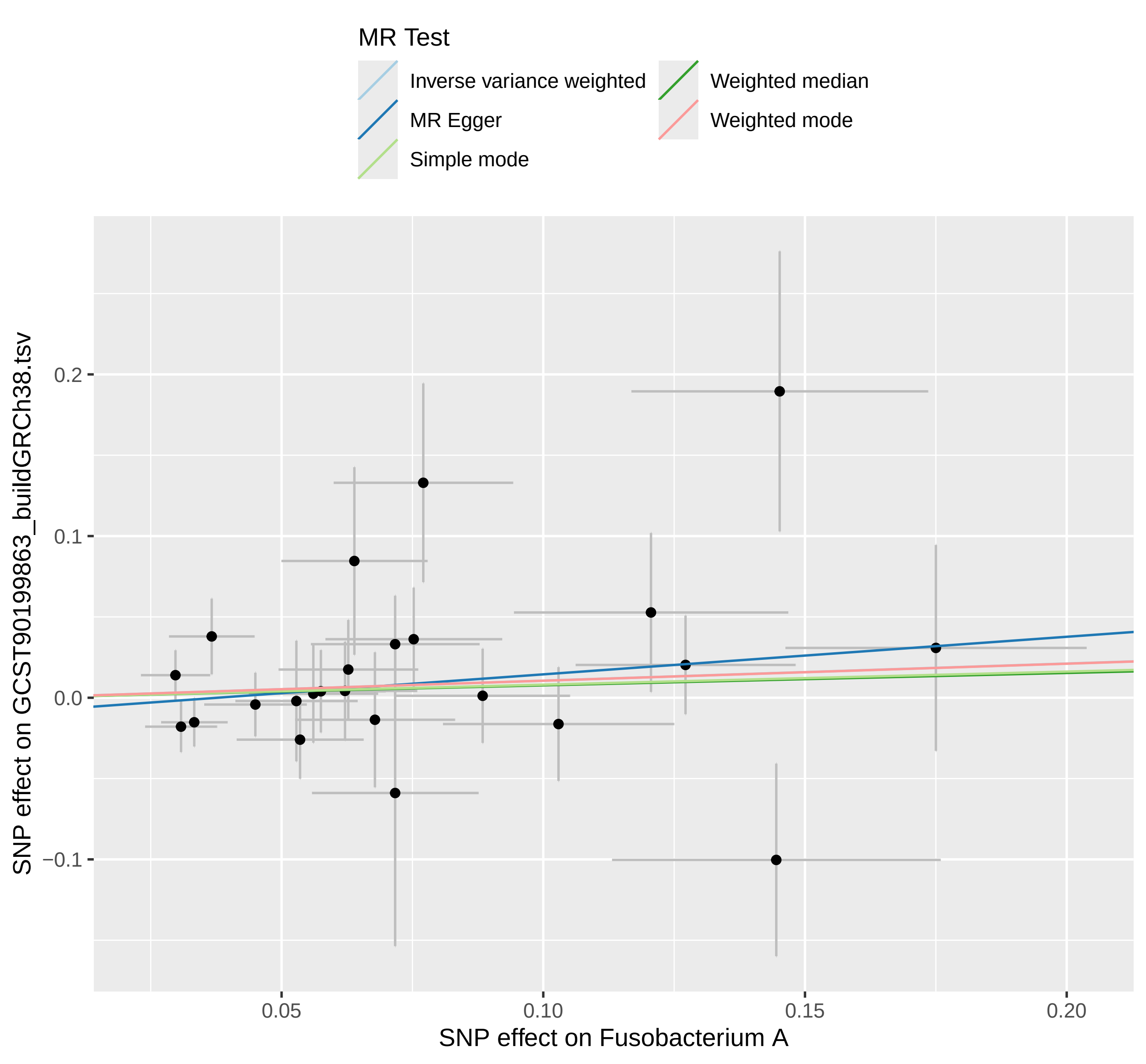
MR Test
Inverse variance weighted
MR Egger
Simple mode
Weighted median
Weighted mode
SNP effect on GCST90199863_buildGRCh38.tsv
0.2
0.1
0.0
−0.1
0.05
0.10
0.15
0.20
SNP effect on Fusobacterium A

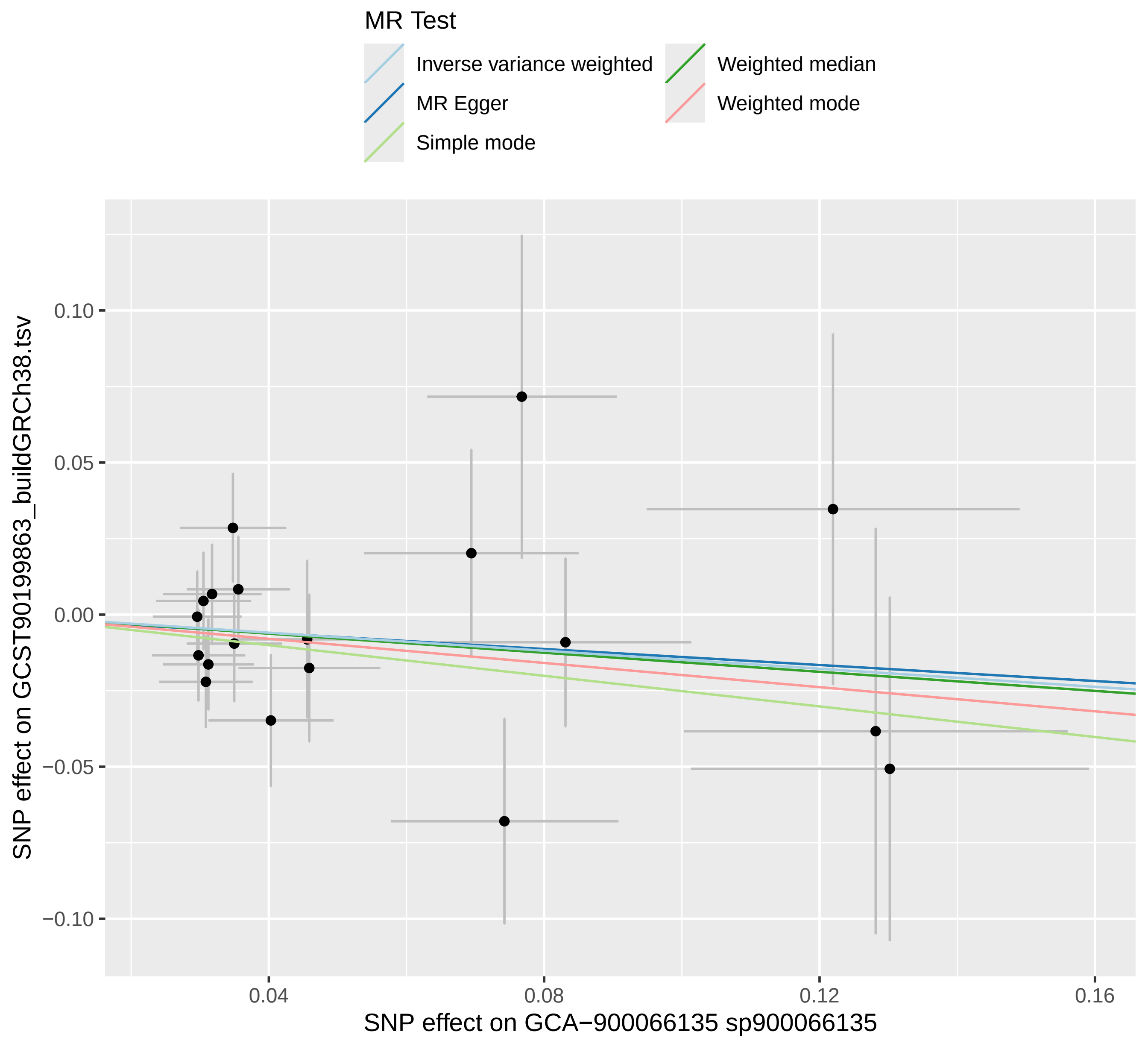
MR Test
Inverse variance weighted
MR Egger
Simple mode
Weighted median
Weighted mode
0.10
0.05
0.00
−0.05
−0.10
0.04
0.08
0.12
0.16
SNP effect on GCST90199863_buildGRCh38.tsv
SNP effect on GCA−900066135 sp900066135

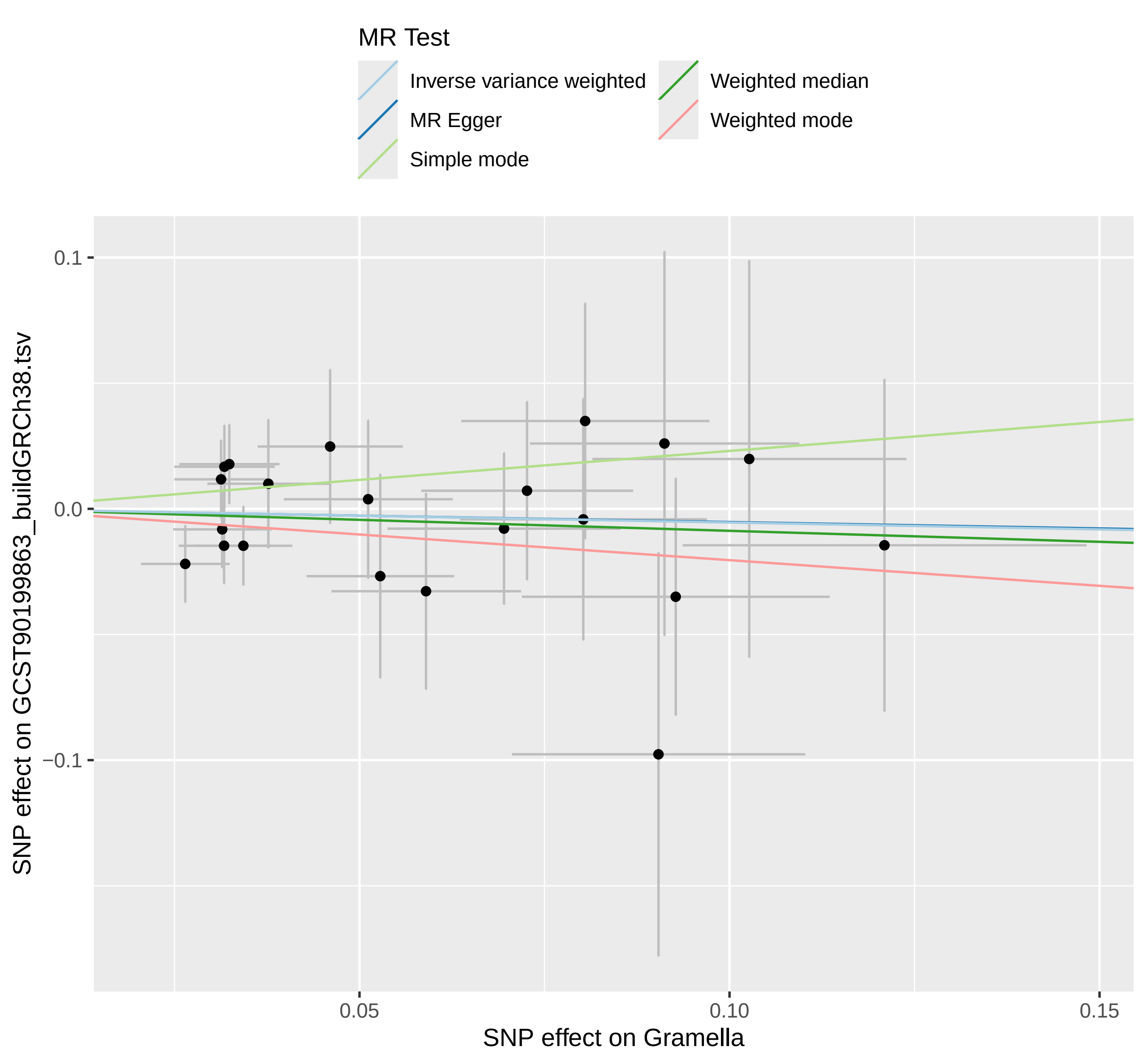
MR Test
Inverse variance weighted
MR Egger
Simple mode
Weighted median
Weighted mode
0.1
0.0
−0.1
0.05
0.10
0.15
SNP effect on GCST90199863_buildGRCh38.tsv
SNP effect on Gramella

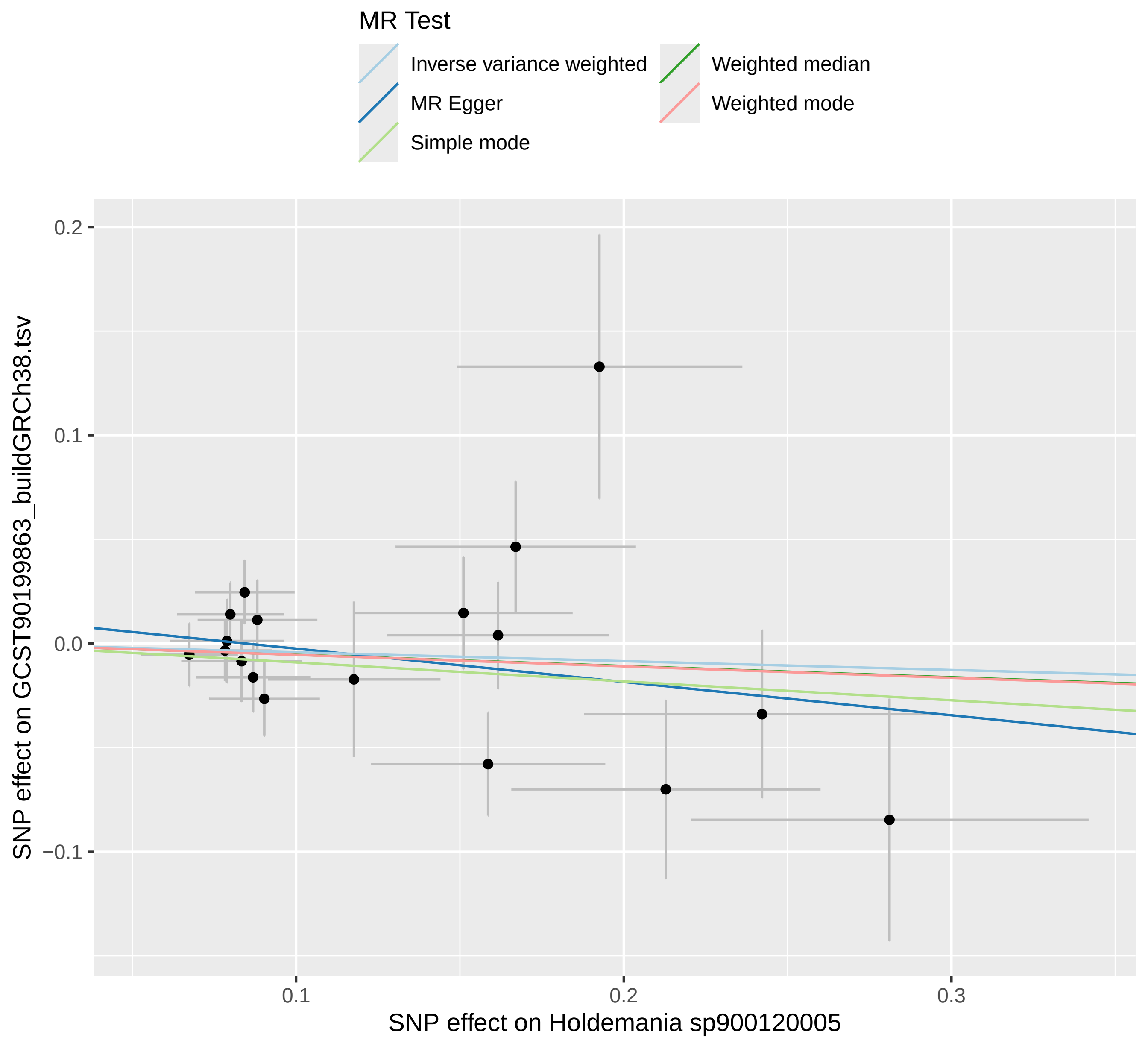
MR Test
Inverse variance weighted
MR Egger
Simple mode
Weighted median
Weighted mode
0.2
0.1
0.0
−0.1
0.1
0.2
0.3
SNP effect on GCST90199863_buildGRCh38.tsv
SNP effect on Holdemania sp900120005

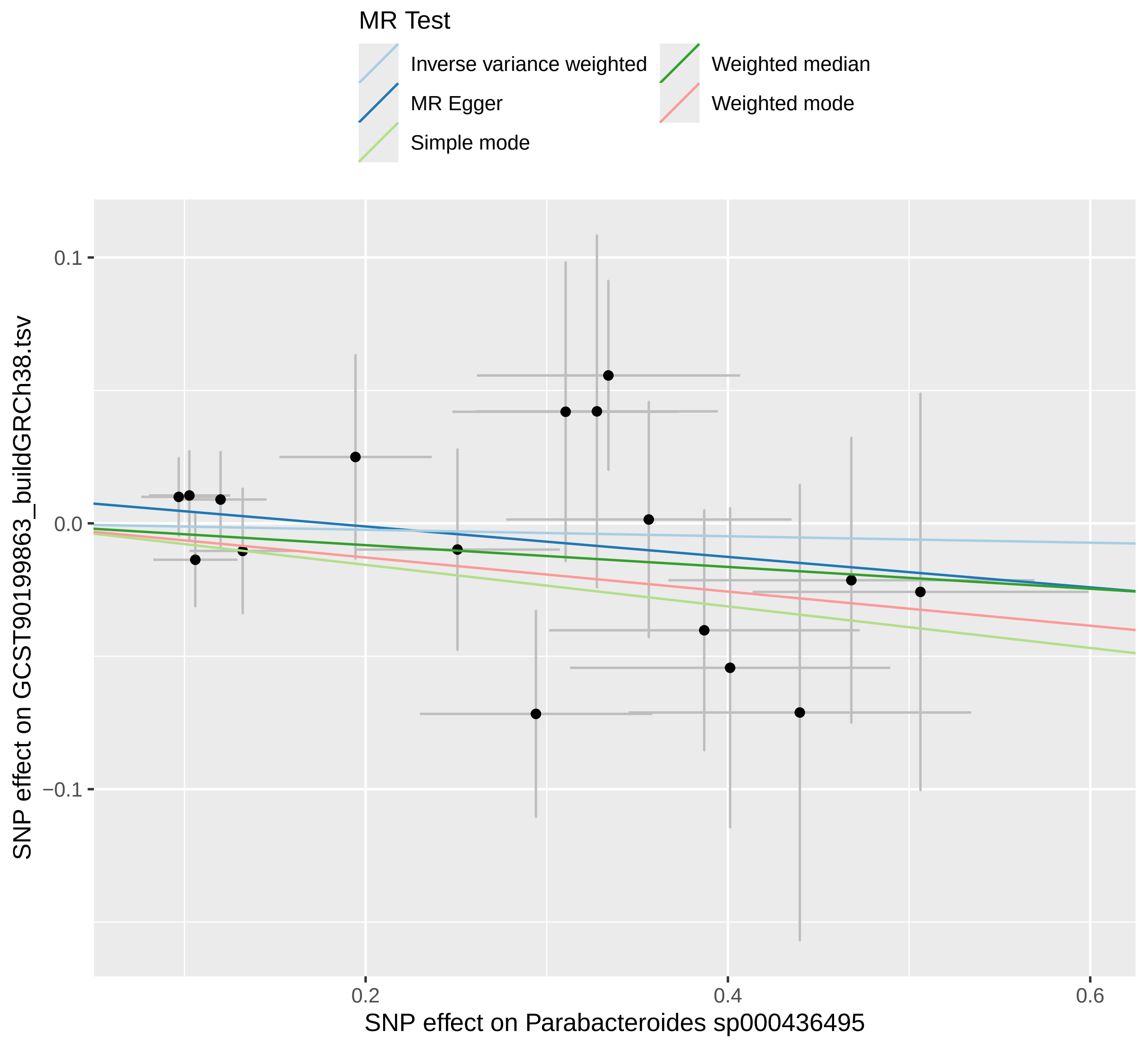

MR Test
Inverse variance weighted
MR Egger
Simple mode
Weighted median
Weighted mode
0.1
0.0
−0.1
0.2
0.4
0.6
SNP effect on GCST90199863_buildGRCh38.tsv
SNP effect on Parabacteroides sp000436495

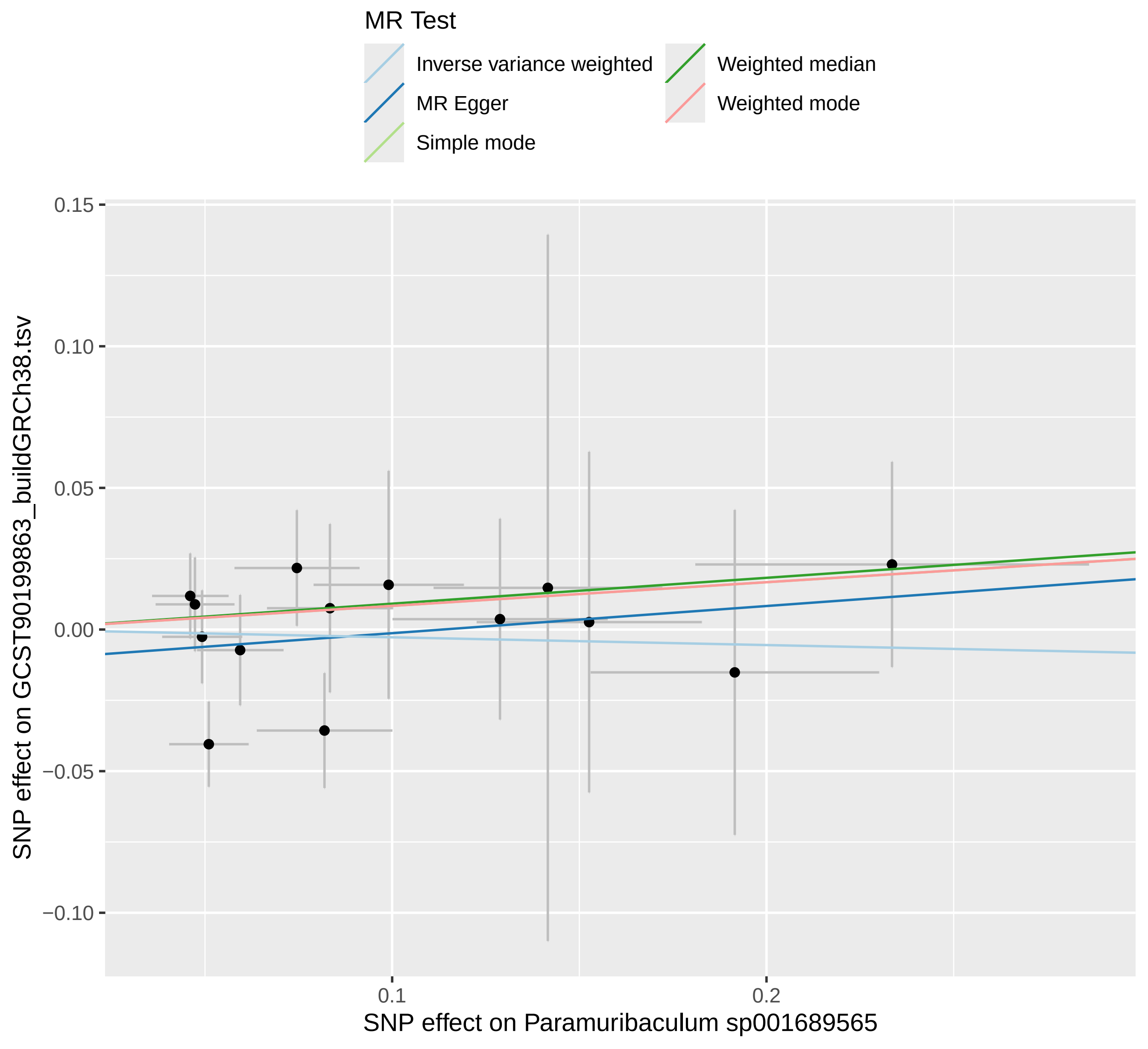

MR Test
Inverse variance weighted
MR Egger
Simple mode
Weighted median
Weighted mode
0.15
0.10
0.05
0.00
−0.05
−0.10
0.1
0.2
SNP effect on GCST90199863_buildGRCh38.tsv
SNP effect on Paramuribaculum sp001689565

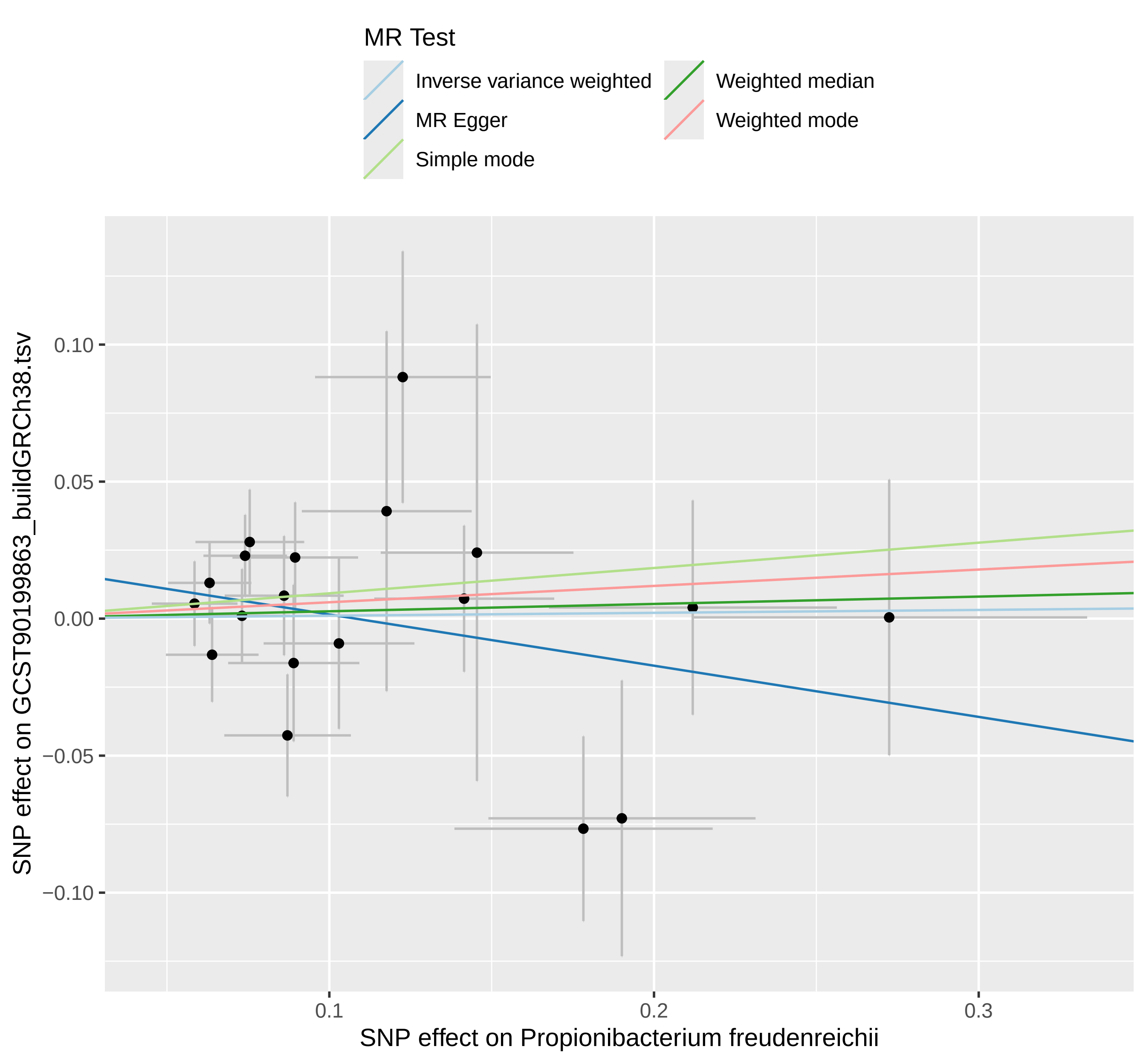
MR Test
Inverse variance weighted
MR Egger
Simple mode
Weighted median
Weighted mode
SNP effect on GCST90199863_buildGRCh38.tsv
0.10
0.05
0.00
−0.05
−0.10
0.1
0.2
0.3
SNP effect on Propionibacterium freudenreichii

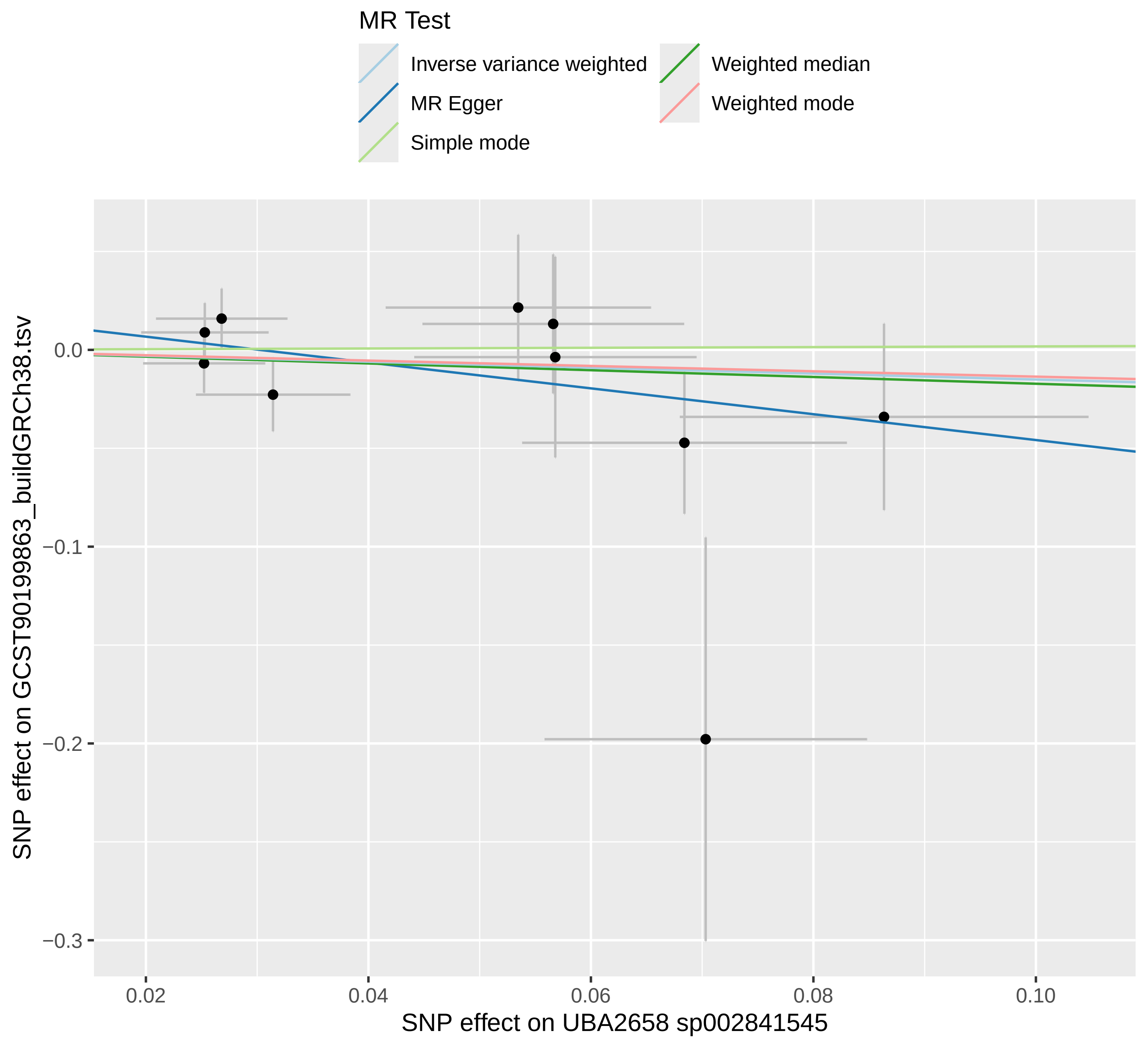

MR Test
Inverse variance weighted
MR Egger
Simple mode
Weighted median
Weighted mode
SNP effect on GCST90199863_buildGRCh38.tsv
0.0
−0.1
−0.2
−0.3
0.02
0.04
0.06
0.08
0.10
SNP effect on UBA2658 sp002841545

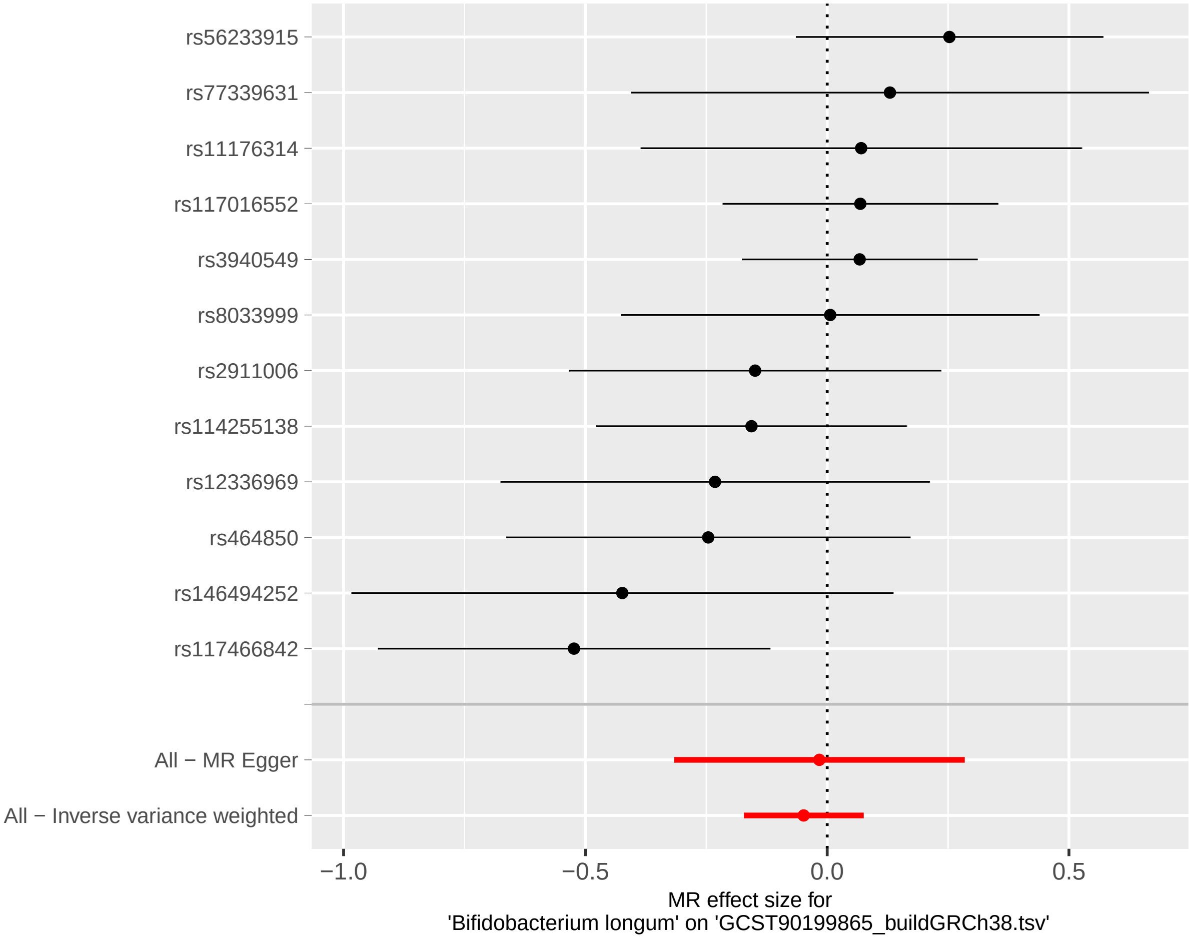
rs56233915
rs77339631
rs11176314
rs117016552
rs3940549
rs8033999
rs2911006
rs114255138
rs12336969
rs464850
rs146494252
rs117466842
All − MR Egger
All − Inverse variance weighted
−1.0
−0.5
0.0
0.5
MR effect size for
'Bifidobacterium longum' on 'GCST90199865_buildGRCh38.tsv'

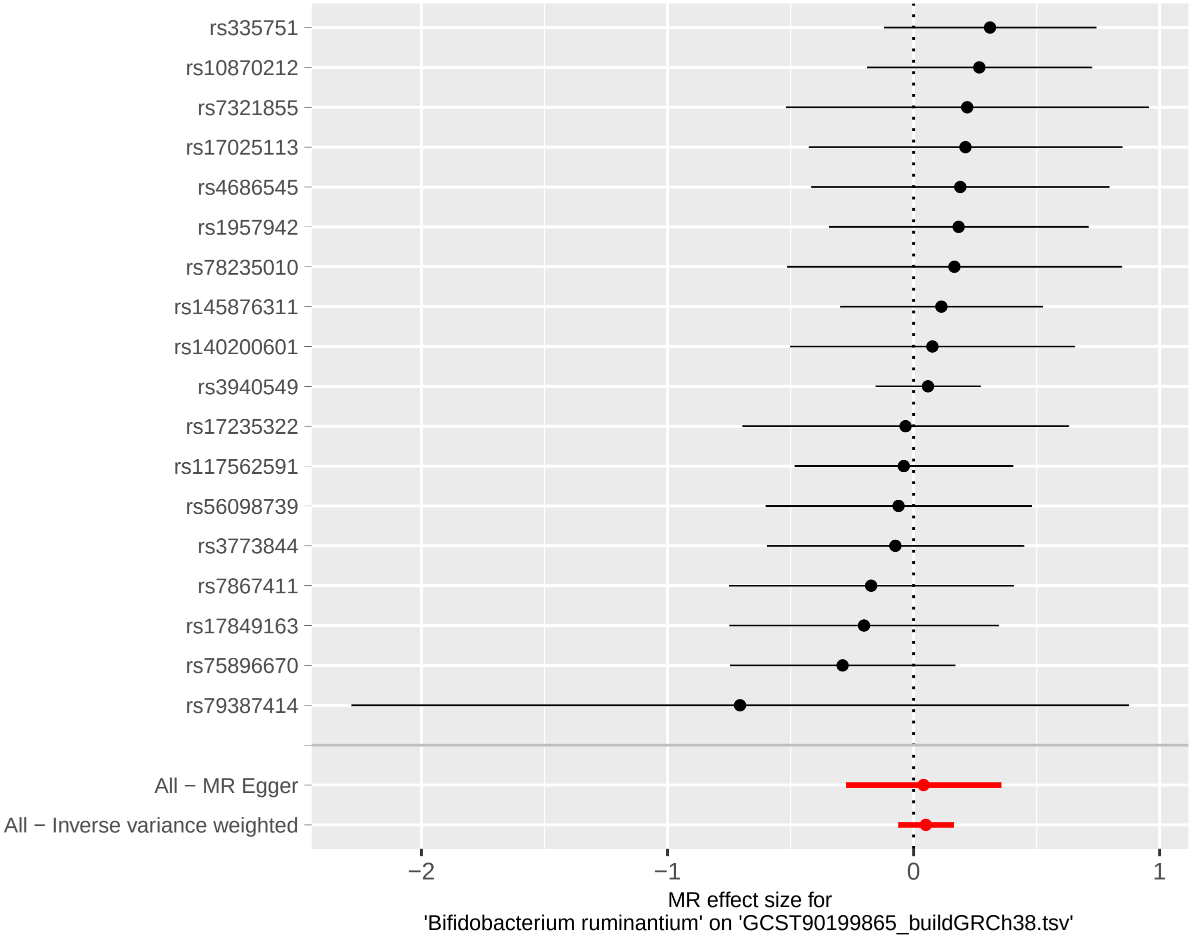
rs335751
rs10870212
rs7321855
rs17025113
rs4686545
rs1957942
rs78235010
rs145876311
rs140200601
rs3940549
rs17235322
rs117562591
rs56098739
rs3773844
rs7867411
rs17849163
rs75896670
rs79387414
All − MR Egger
All − Inverse variance weighted
−2
−1
0
1
MR effect size for
'Bifidobacterium ruminantium' on 'GCST90199865_buildGRCh38.tsv'

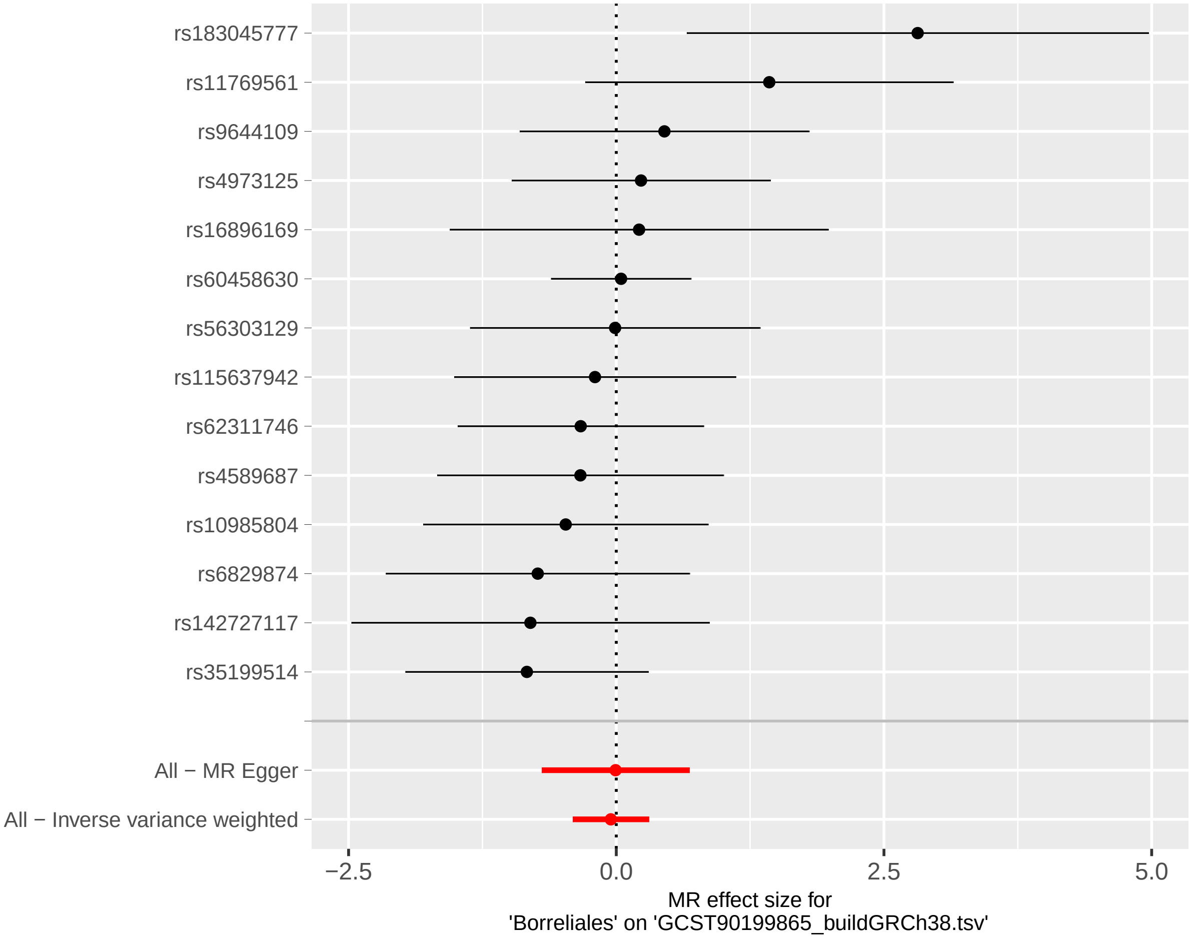
rs183045777
rs11769561
rs9644109
rs4973125
rs16896169
rs60458630
rs56303129
rs115637942
rs62311746
rs4589687
rs10985804
rs6829874
rs142727117
rs35199514
All − MR Egger
All − Inverse variance weighted
−2.5
0.0
2.5
5.0
MR effect size for
'Borreliales' on 'GCST90199865_buildGRCh38.tsv'

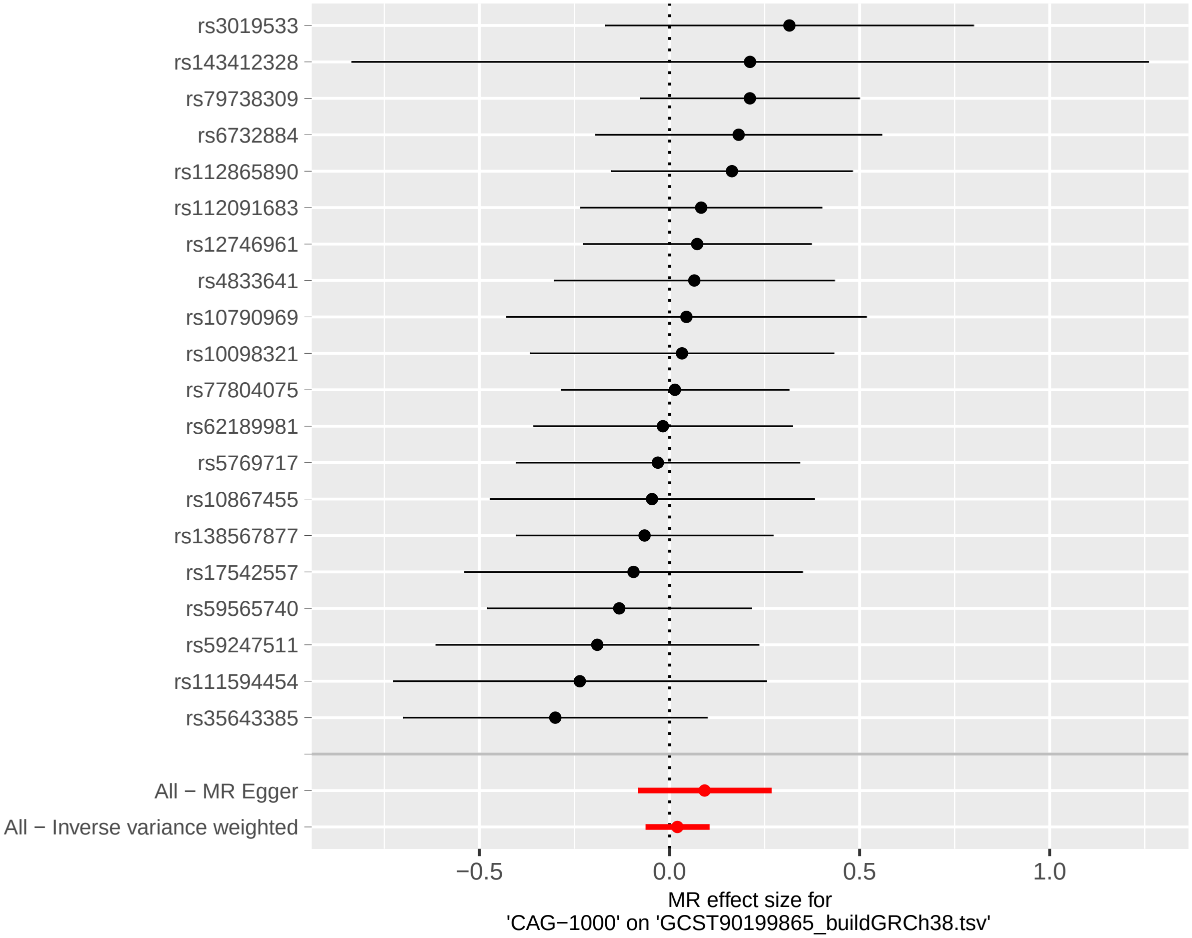
rs3019533
rs143412328
rs79738309
rs6732884
rs112865890
rs112091683
rs12746961
rs4833641
rs10790969
rs10098321
rs77804075
rs62189981
rs5769717
rs10867455
rs138567877
rs17542557
rs59565740
rs59247511
rs111594454
rs35643385
All − MR Egger
All − Inverse variance weighted
−0.5
0.0
0.5
1.0
MR effect size for
'CAG−1000' on 'GCST90199865_buildGRCh38.tsv'

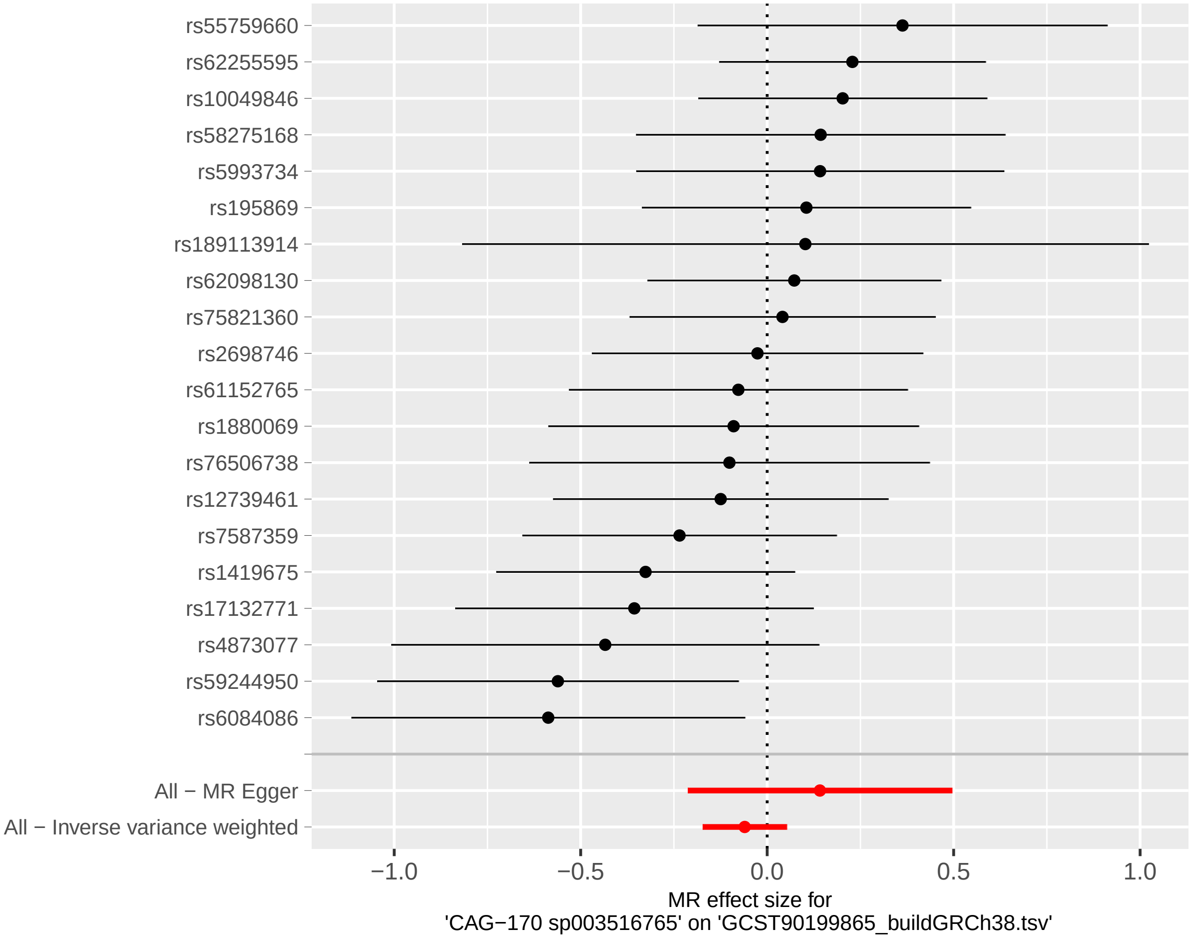
rs55759660
rs62255595
rs10049846
rs58275168
rs5993734
rs195869
rs189113914
rs62098130
rs75821360
rs2698746
rs61152765
rs1880069
rs76506738
rs12739461
rs7587359
rs1419675
rs17132771
rs4873077
rs59244950
rs6084086
All − MR Egger
All − Inverse variance weighted
−1.0
−0.5
0.0
0.5
1.0
MR effect size for
'CAG−170 sp003516765' on 'GCST90199865_buildGRCh38.tsv'

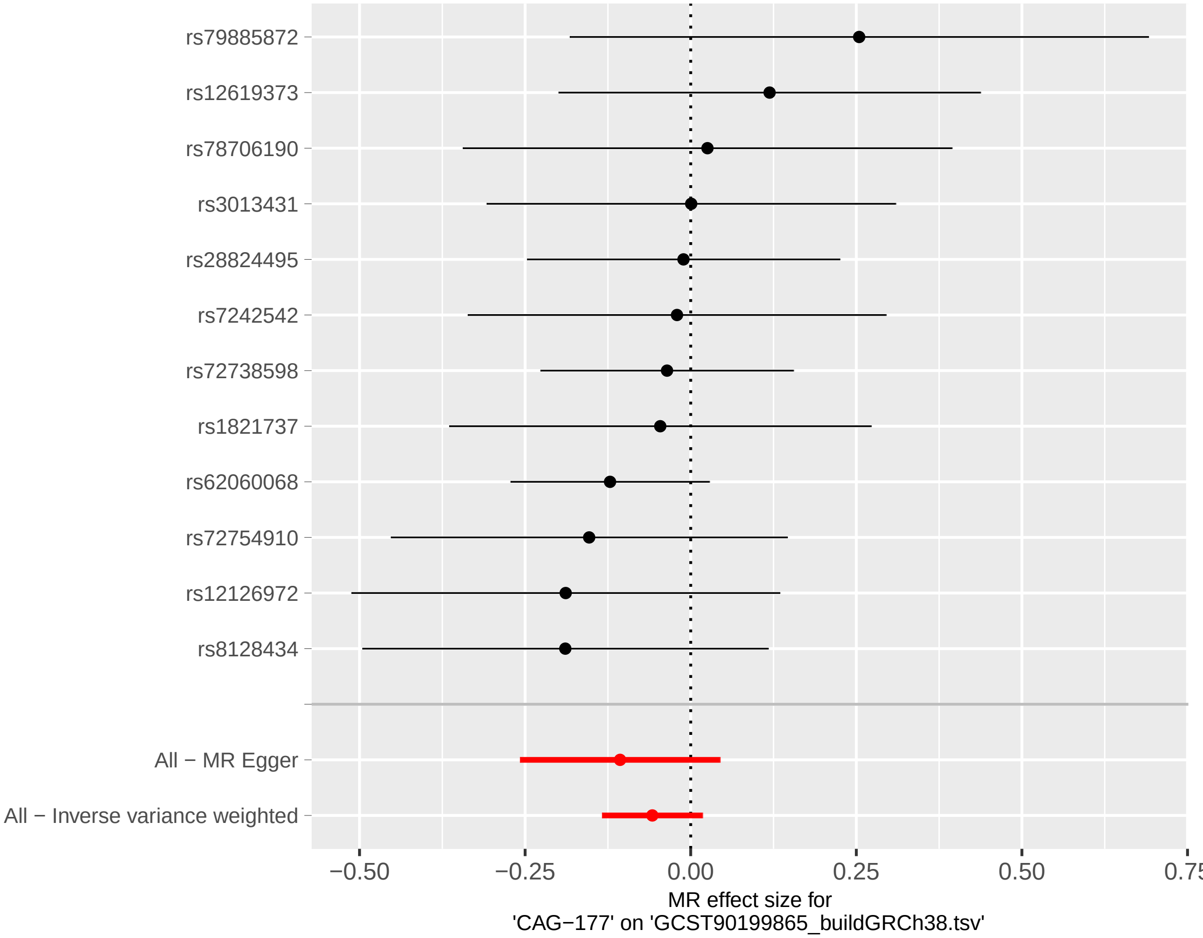
rs79885872
rs12619373
rs78706190
rs3013431
rs28824495
rs7242542
rs72738598
rs1821737
rs62060068
rs72754910
rs12126972
rs8128434
All − MR Egger
All − Inverse variance weighted
−0.50
−0.25
0.00
0.25
0.50
MR effect size for
'CAG−177' on 'GCST90199865_buildGRCh38.tsv'

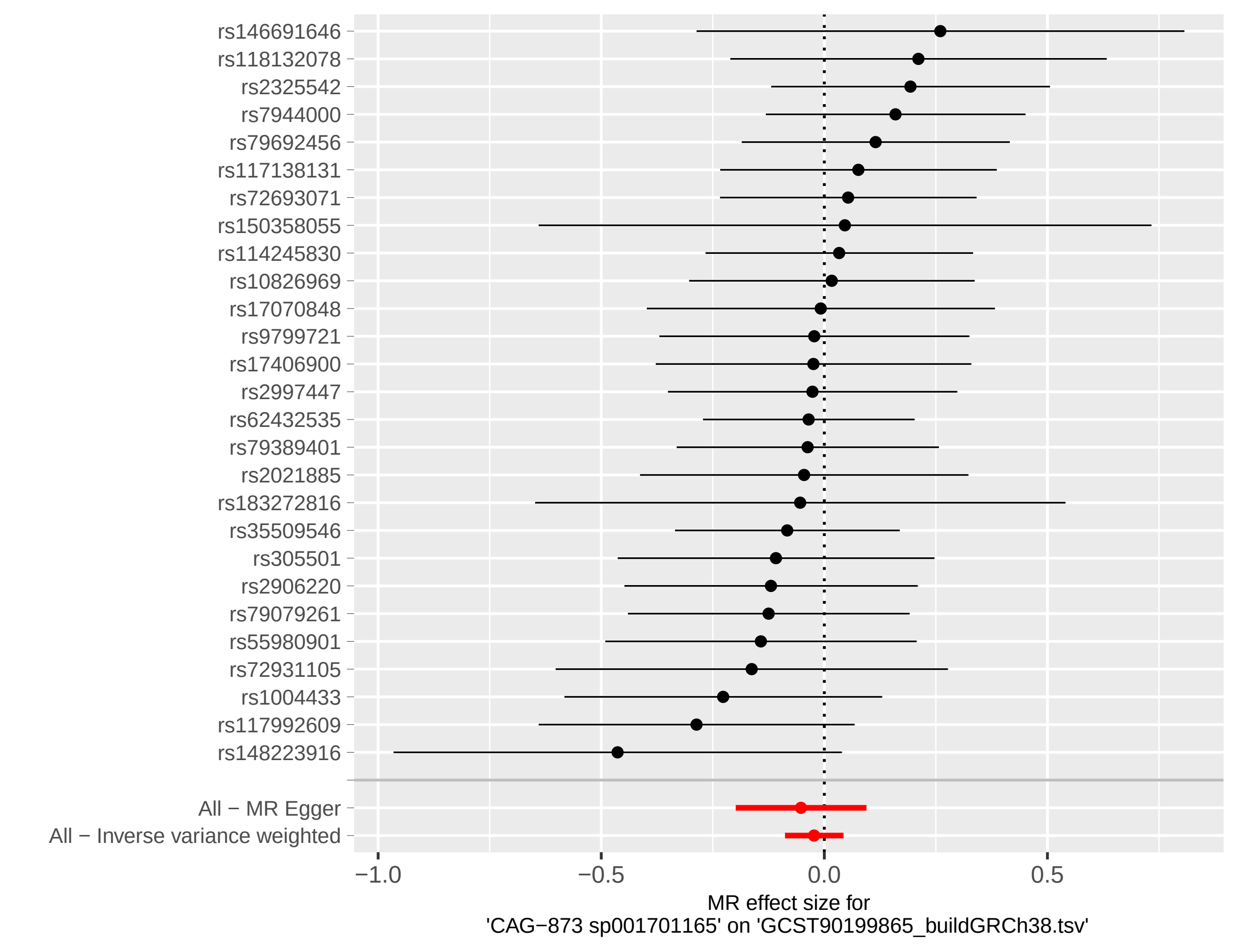

rs146691646
rs118132078
rs2325542
rs7944000
rs79692456
rs117138131
rs72693071
rs150358055
rs114245830
rs10826969
rs17070848
rs9799721
rs17406900
rs2997447
rs62432535
rs79389401
rs2021885
rs183272816
rs35509546
rs305501
rs2906220
rs79079261
rs55980901
rs72931105
rs1004433
rs117992609
rs148223916
All − MR Egger
All − Inverse variance weighted
−1.0
−0.5
0.0
0.5
MR effect size for
'CAG−873 sp001701165' on 'GCST90199865_buildGRCh38.tsv'

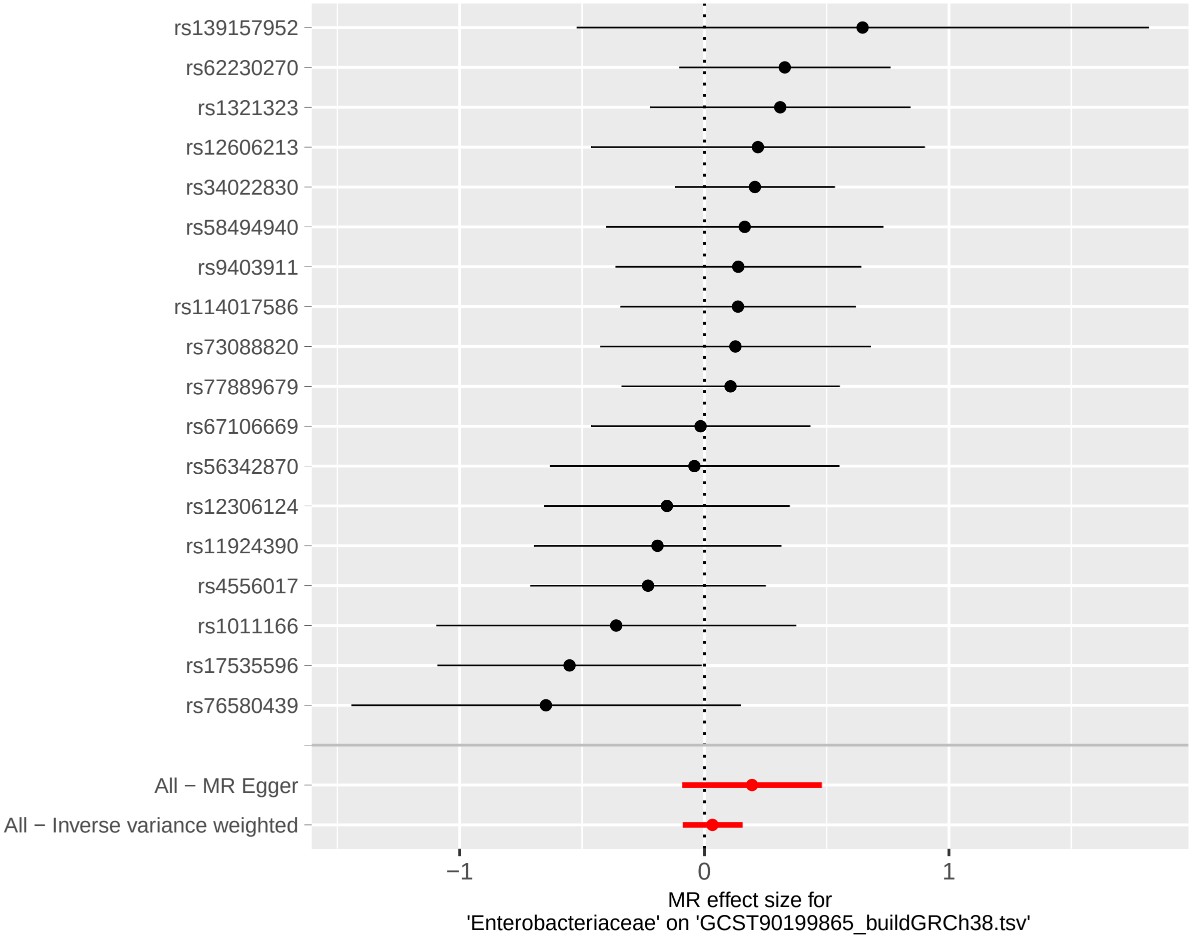
rs139157952
rs62230270
rs1321323
rs12606213
rs34022830
rs58494940
rs9403911
rs114017586
rs73088820
rs77889679
rs67106669
rs56342870
rs12306124
rs11924390
rs4556017
rs1011166
rs17535596
rs76580439
All − MR Egger
All − Inverse variance weighted
−1
0
1
MR effect size for
'Enterobacteriaceae' on 'GCST90199865_buildGRCh38.tsv'

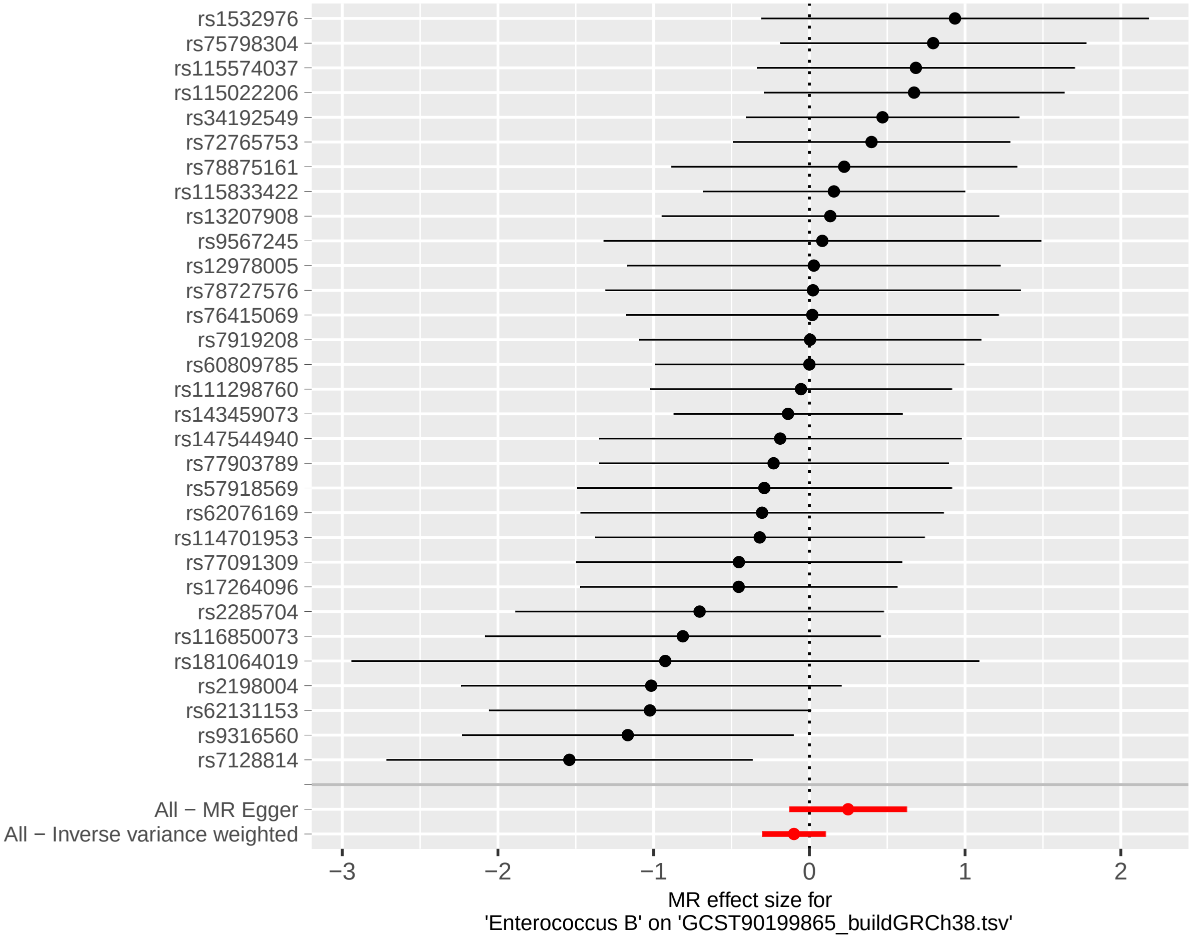
rs1532976
rs75798304
rs115574037
rs115022206
rs34192549
rs72765753
rs78875161
rs115833422
rs13207908
rs9567245
rs12978005
rs78727576
rs76415069
rs7919208
rs60809785
rs111298760
rs143459073
rs147544940
rs77903789
rs57918569
rs62076169
rs114701953
rs77091309
rs17264096
rs2285704
rs116850073
rs181064019
rs2198004
rs62131153
rs9316560
rs7128814
All − MR Egger
All − Inverse variance weighted
−3
−2
−1
0
1
2
MR effect size for
'Enterococcus B' on 'GCST90199865_buildGRCh38.tsv'

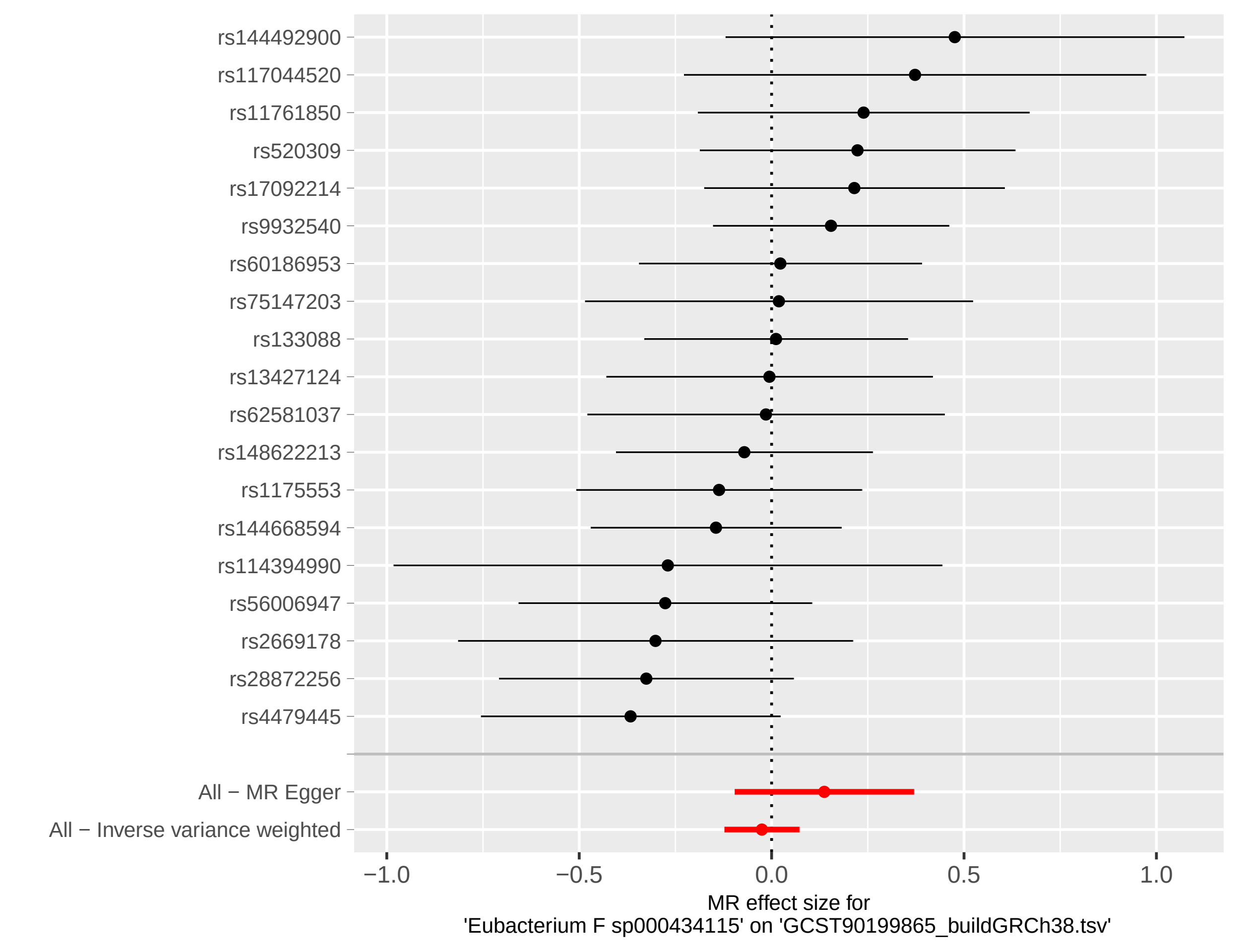

rs144492900
rs117044520
rs11761850
rs520309
rs17092214
rs9932540
rs60186953
rs75147203
rs133088
rs13427124
rs62581037
rs148622213
rs1175553
rs144668594
rs114394990
rs56006947
rs2669178
rs28872256
rs4479445
All − MR Egger
All − Inverse variance weighted
−1.0
−0.5
0.0
0.5
1.0
MR effect size for
'Eubacterium F sp000434115' on 'GCST90199865_buildGRCh38.tsv'

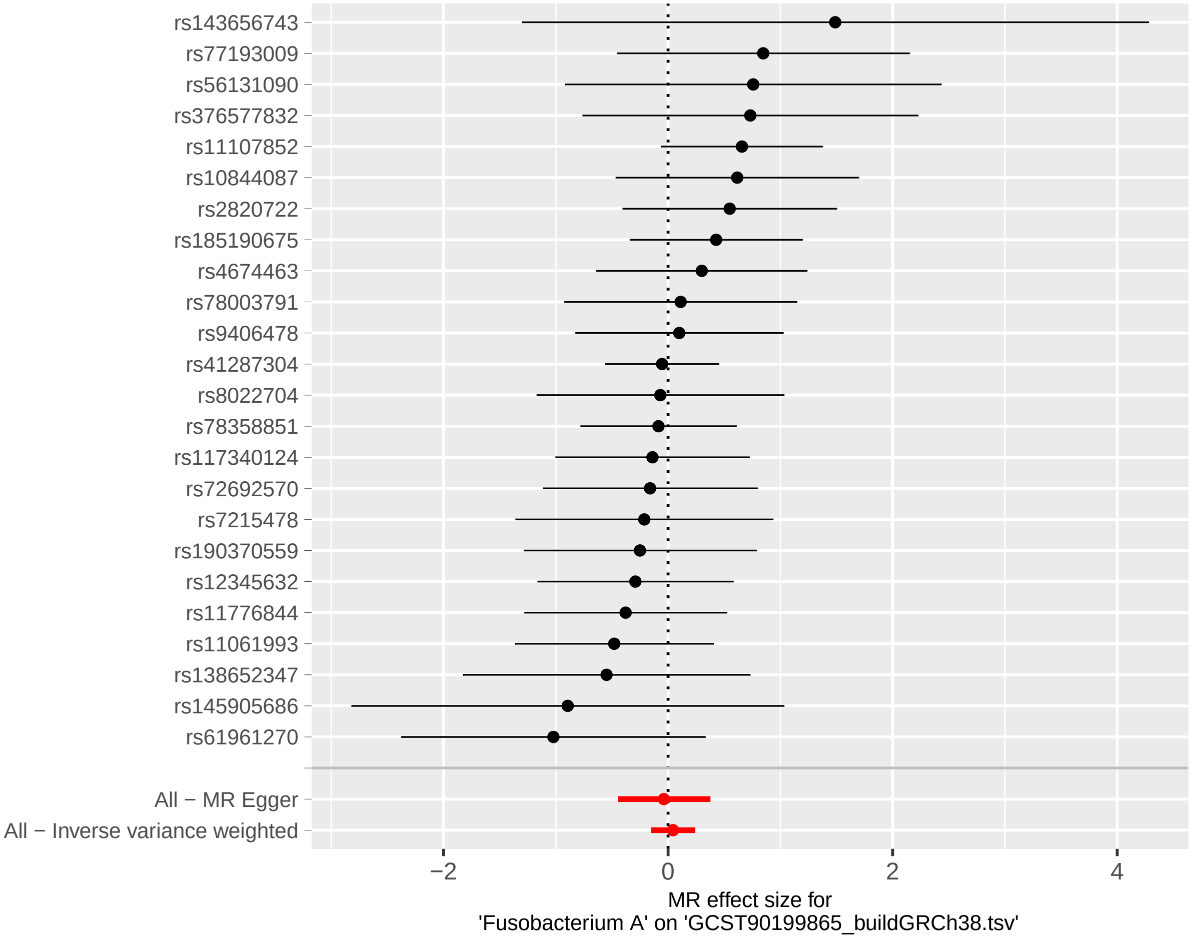

rs143656743
rs77193009
rs56131090
rs376577832
rs11107852
rs10844087
rs2820722
rs185190675
rs4674463
rs78003791
rs9406478
rs41287304
rs8022704
rs78358851
rs117340124
rs72692570
rs7215478
rs190370559
rs12345632
rs11776844
rs11061993
rs138652347
rs145905686
rs61961270
All − MR Egger
All − Inverse variance weighted
−2
0
2
4
MR effect size for
'Fusobacterium A' on 'GCST90199865_buildGRCh38.tsv'

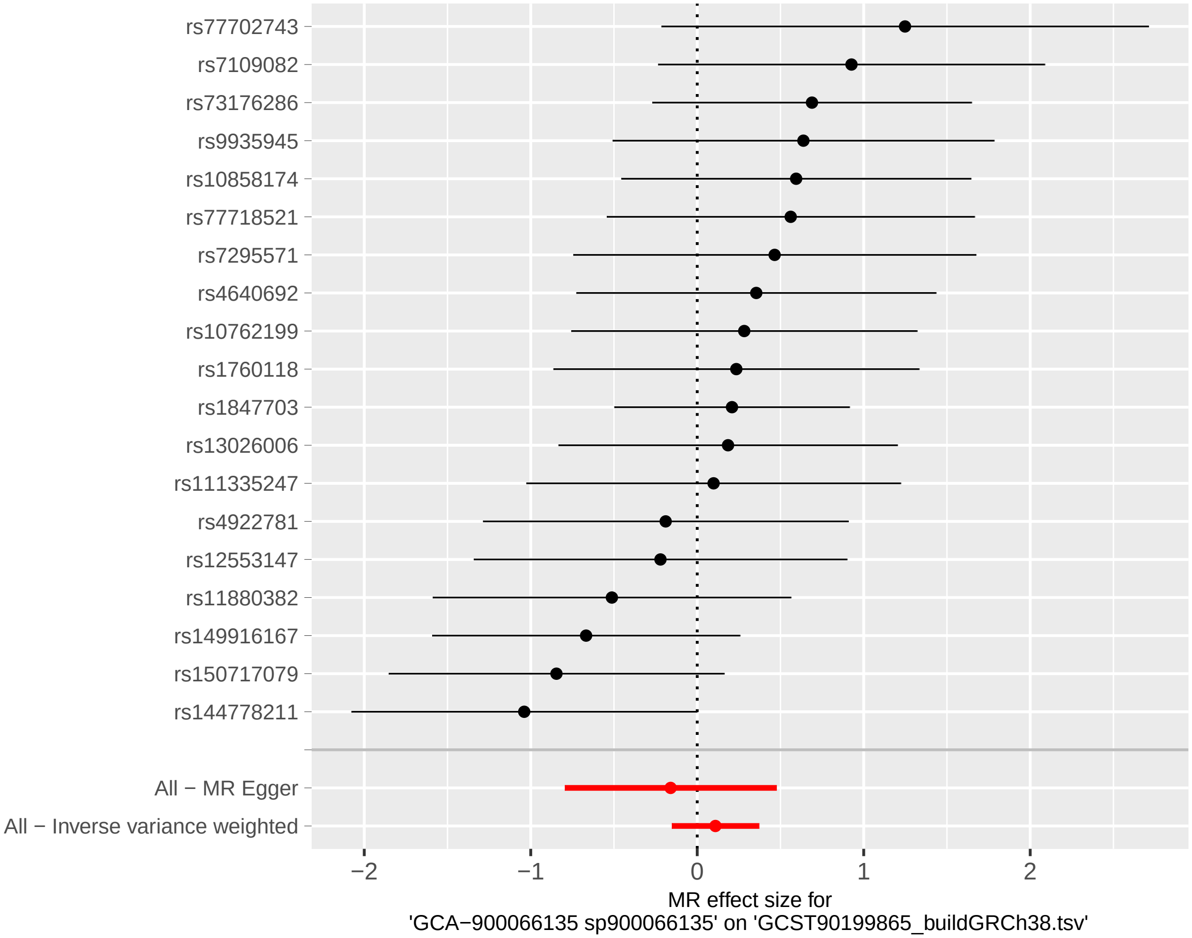
rs77702743
rs7109082
rs73176286
rs9935945
rs10858174
rs77718521
rs7295571
rs4640692
rs10762199
rs1760118
rs1847703
rs13026006
rs111335247
rs4922781
rs12553147
rs11880382
rs149916167
rs150717079
rs144778211
All − MR Egger
All − Inverse variance weighted
−2
−1
0
1
2
MR effect size for
'GCA−900066135 sp900066135' on 'GCST90199865_buildGRCh38.tsv'

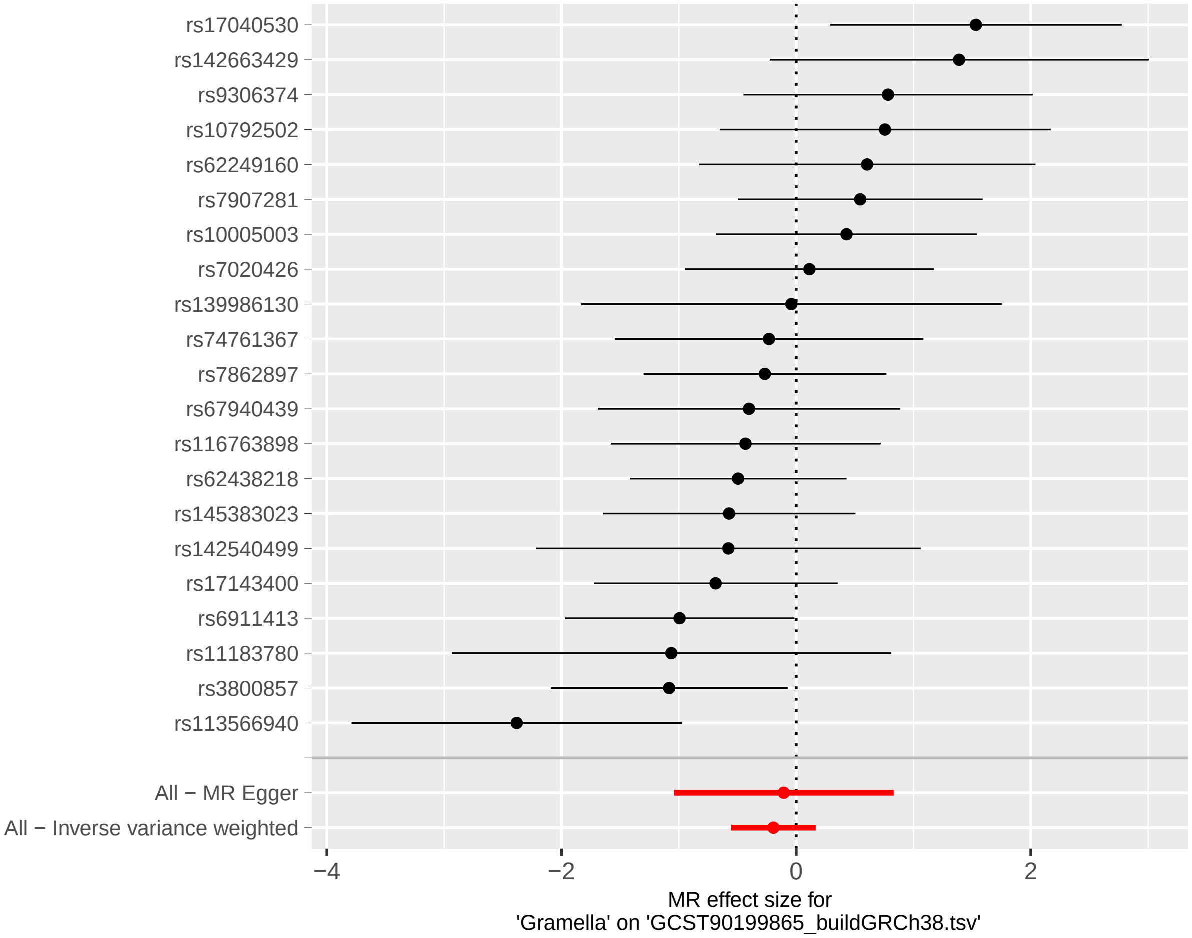

rs17040530
rs142663429
rs9306374
rs10792502
rs62249160
rs7907281
rs10005003
rs7020426
rs139986130
rs74761367
rs7862897
rs67940439
rs116763898
rs62438218
rs145383023
rs142540499
rs17143400
rs6911413
rs11183780
rs3800857
rs113566940
All − MR Egger
All − Inverse variance weighted
−4
−2
0
2
MR effect size for
'Gramella' on 'GCST90199865_buildGRCh38.tsv'

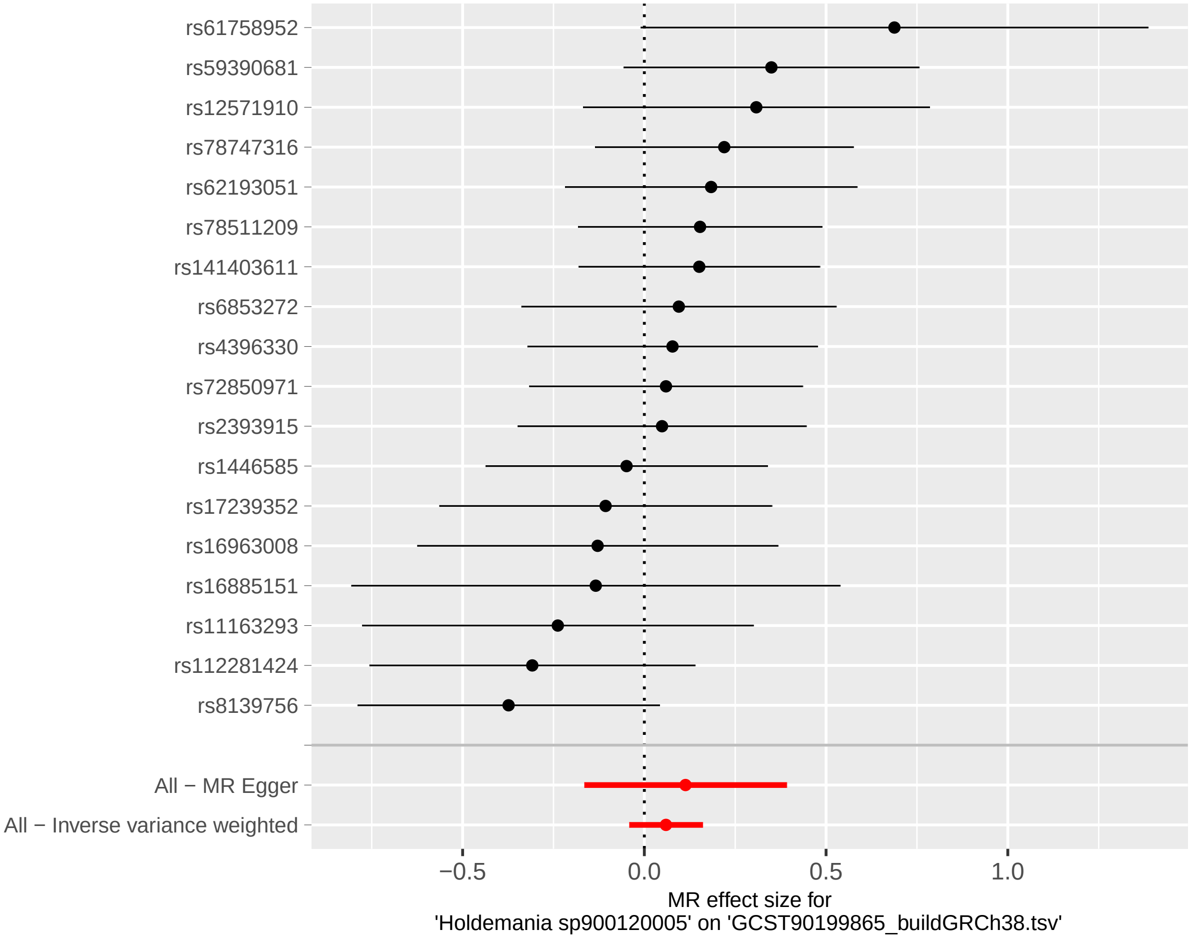
rs61758952
rs59390681
rs12571910
rs78747316
rs62193051
rs78511209
rs141403611
rs6853272
rs4396330
rs72850971
rs2393915
rs1446585
rs17239352
rs16963008
rs16885151
rs11163293
rs112281424
rs8139756
All − MR Egger
All − Inverse variance weighted
−0.5
0.0
0.5
1.0
MR effect size for
'Holdemania sp900120005' on 'GCST90199865_buildGRCh38.tsv'

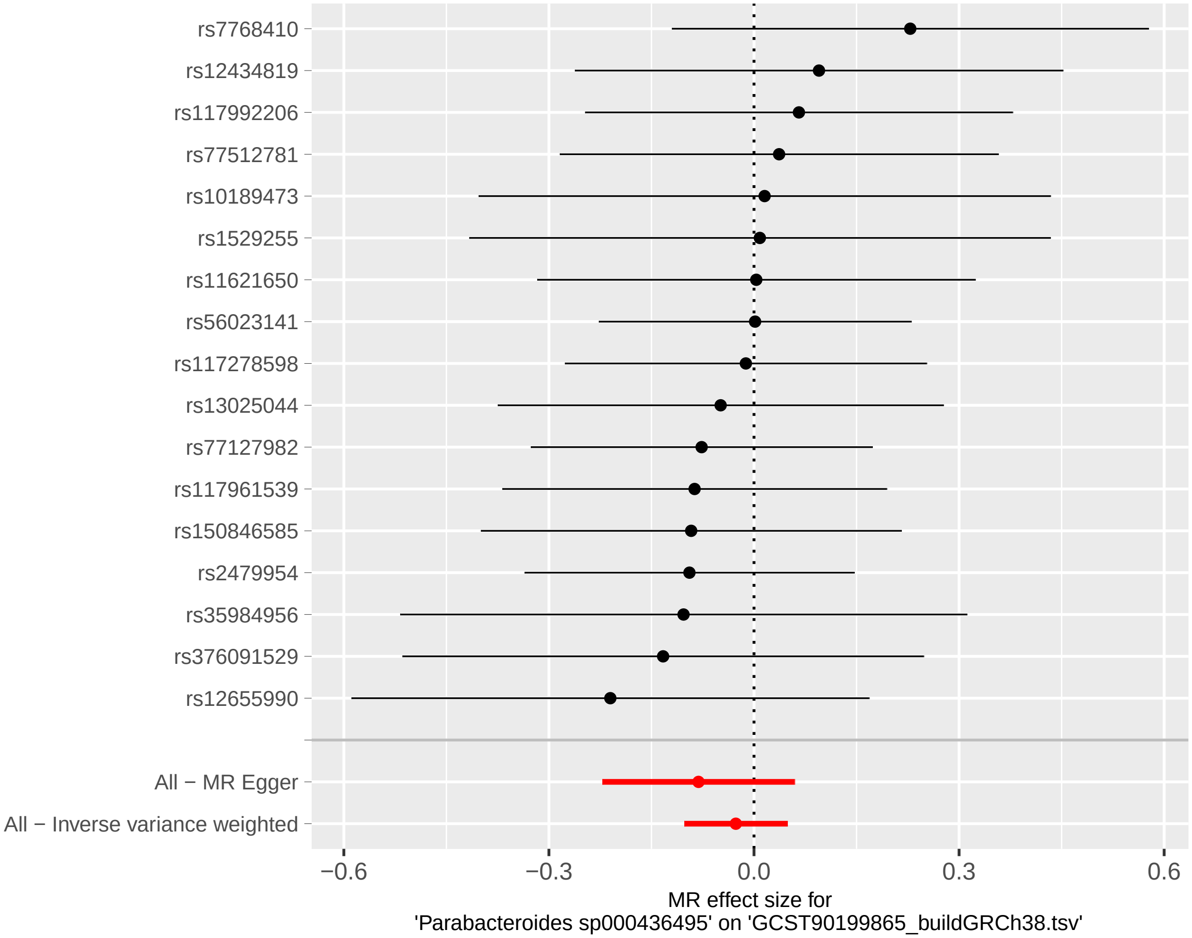
rs7768410
rs12434819
rs117992206
rs77512781
rs10189473
rs1529255
rs11621650
rs56023141
rs117278598
rs13025044
rs77127982
rs117961539
rs150846585
rs2479954
rs35984956
rs376091529
rs12655990
All − MR Egger
All − Inverse variance weighted
−0.6
−0.3
0.0
0.3
0.6
MR effect size for
'Parabacteroides sp000436495' on 'GCST90199865_buildGRCh38.tsv'

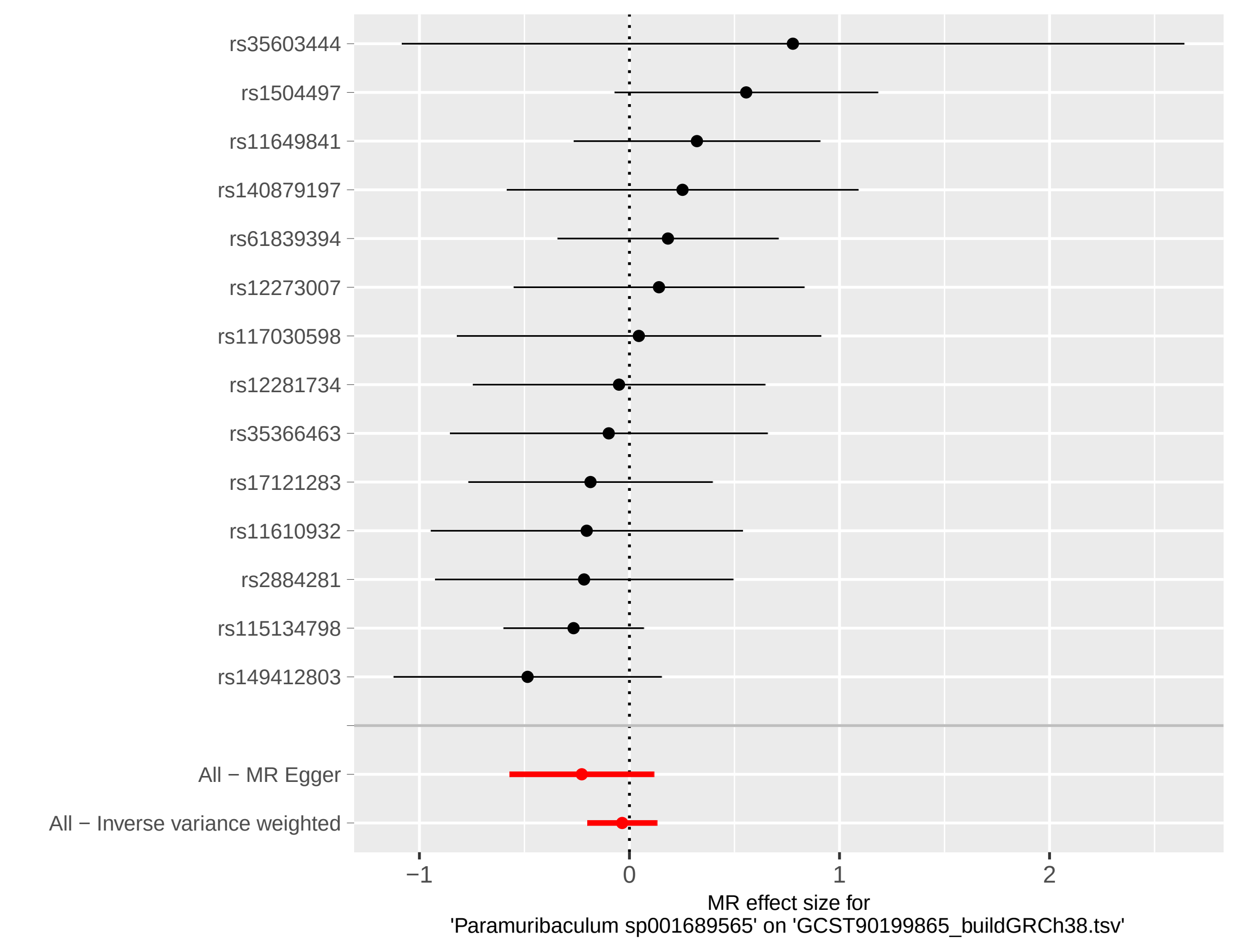
rs35603444
rs1504497
rs11649841
rs140879197
rs61839394
rs12273007
rs117030598
rs12281734
rs35366463
rs17121283
rs11610932
rs2884281
rs115134798
rs149412803
All − MR Egger
All − Inverse variance weighted
−1
0
1
2
MR effect size for
'Paramuribaculum sp001689565' on 'GCST90199865_buildGRCh38.tsv'

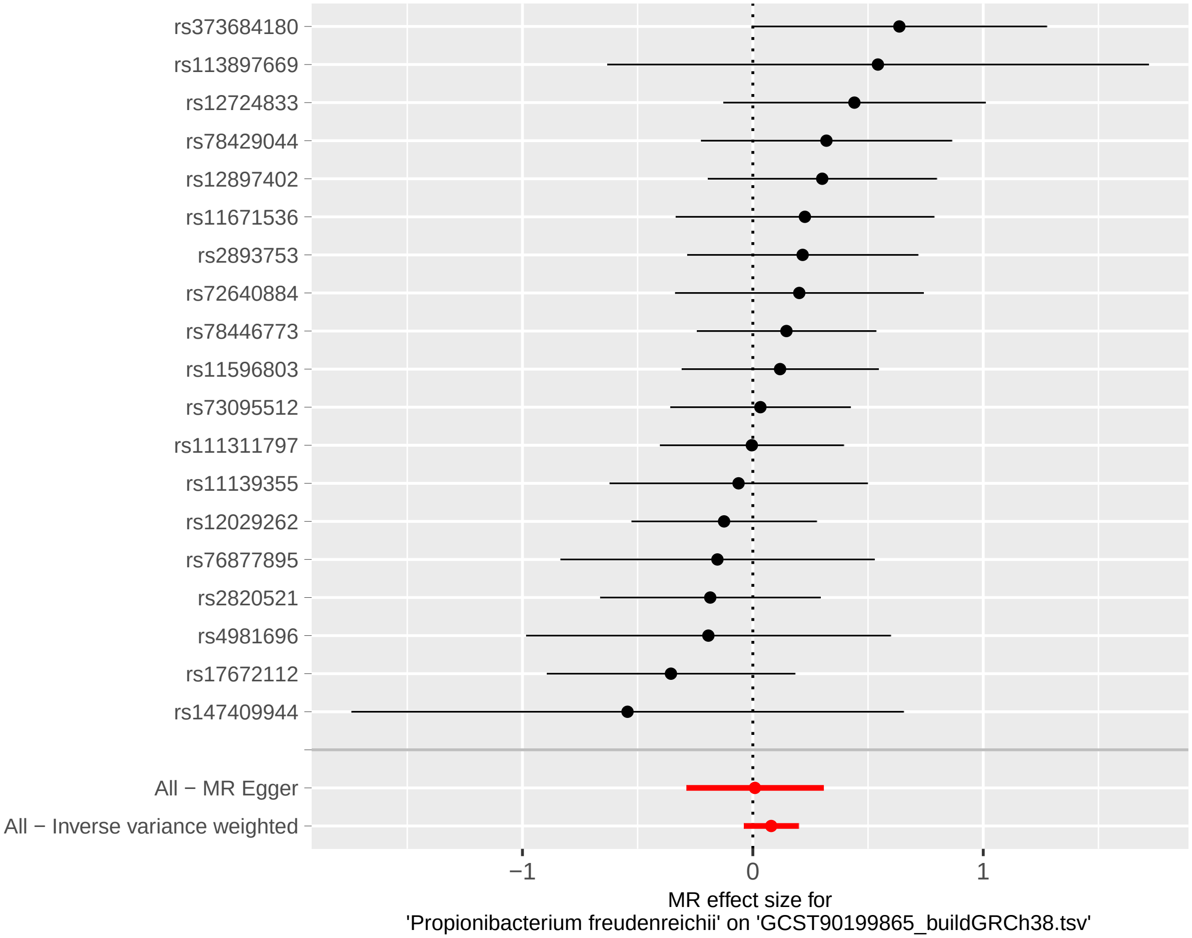
rs373684180
rs113897669
rs12724833
rs78429044
rs12897402
rs11671536
rs2893753
rs72640884
rs78446773
rs11596803
rs73095512
rs111311797
rs11139355
rs12029262
rs76877895
rs2820521
rs4981696
rs17672112
rs147409944
All − MR Egger
All − Inverse variance weighted
−1
0
1
MR effect size for
'Propionibacterium freudenreichii' on 'GCST90199865_buildGRCh38.tsv'

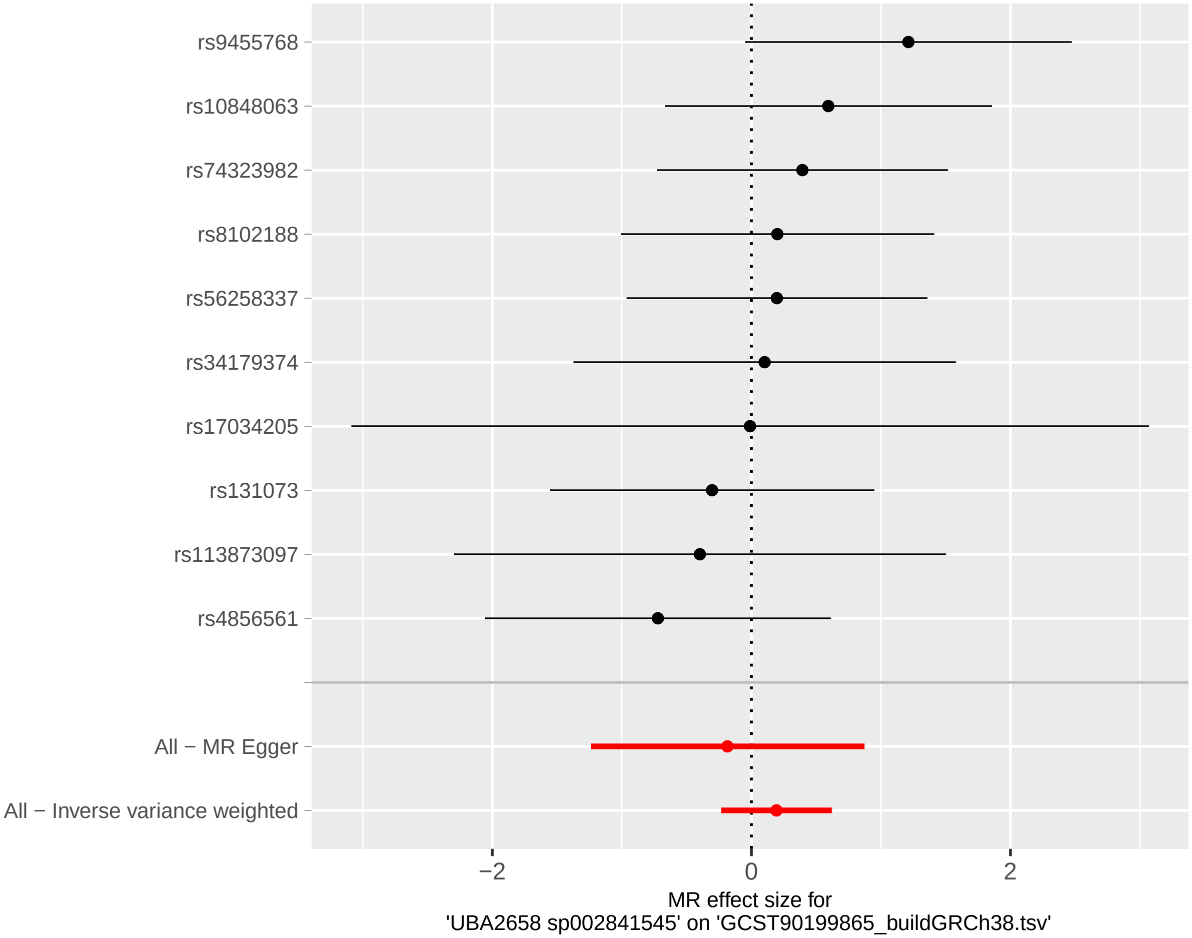
rs9455768
rs10848063
rs74323982
rs8102188
rs56258337
rs34179374
rs17034205
rs131073
rs113873097
rs4856561
All − MR Egger
All − Inverse variance weighted
−2
0
2
MR effect size for
'UBA2658 sp002841545' on 'GCST90199865_buildGRCh38.tsv'

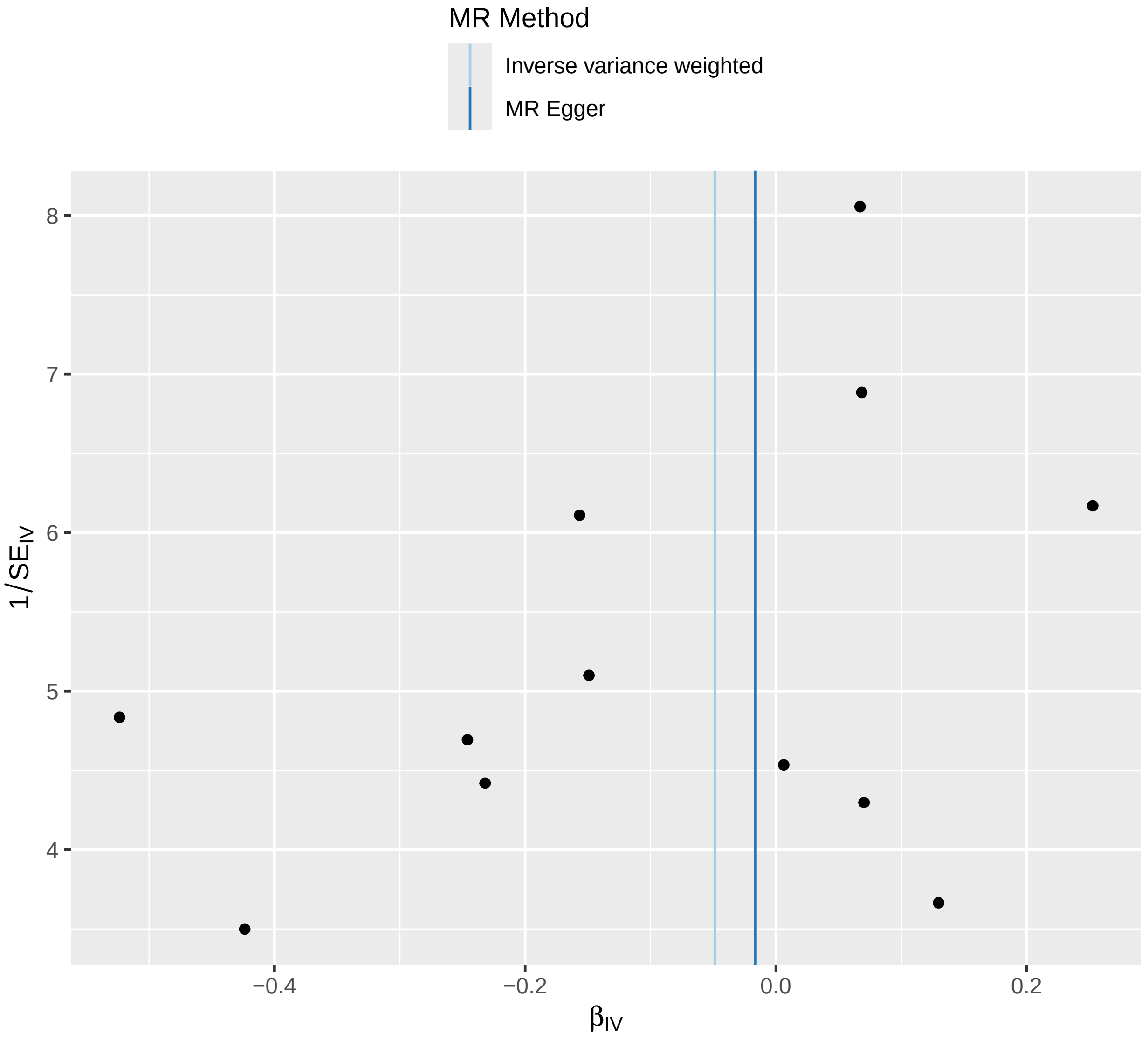
MR Method
Inverse variance weighted
MR Egger
8
7
6
5
4
1/SE$_{IV}$
−0.4
−0.2
0.0
0.2
β$_{IV}$

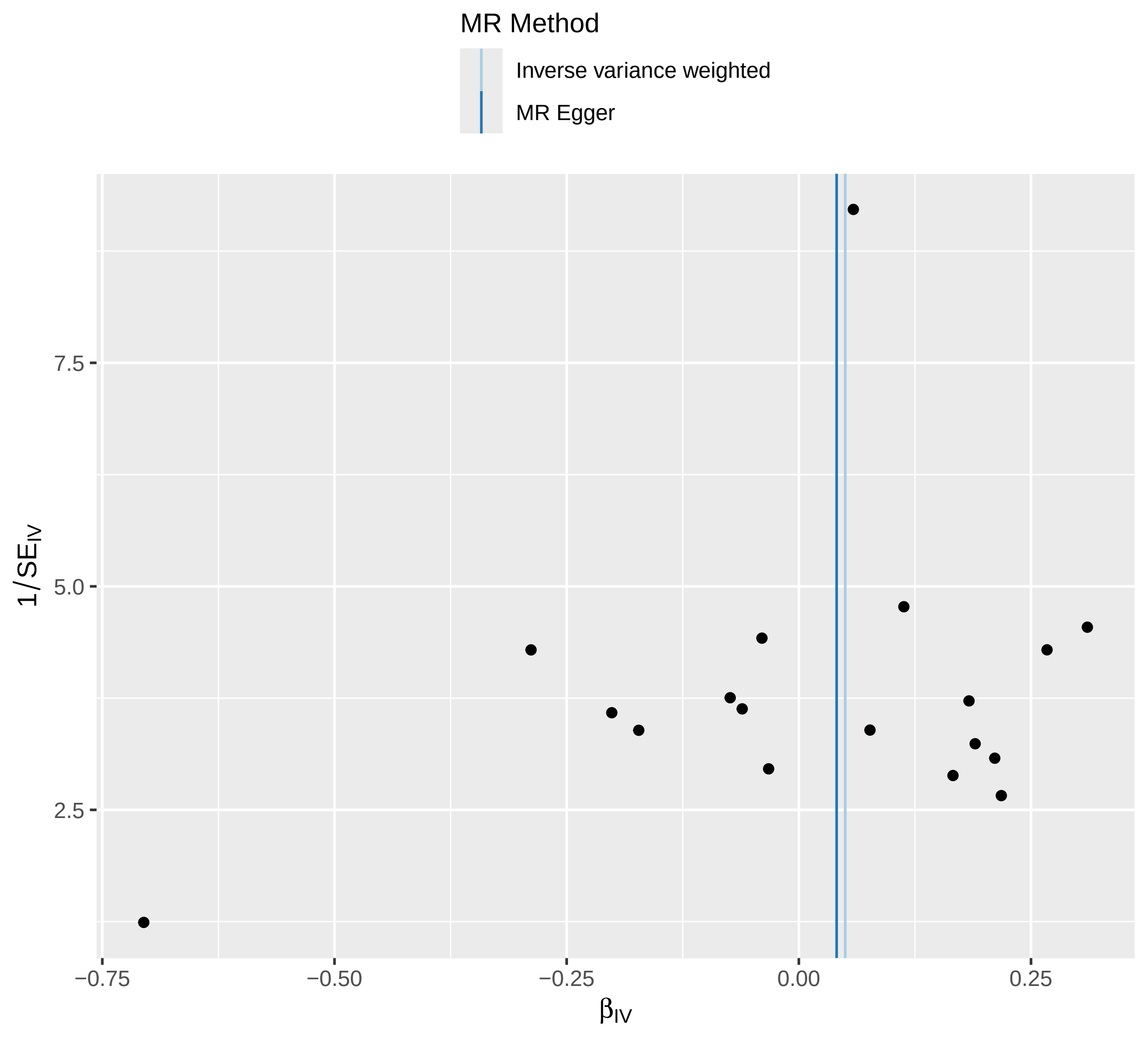
MR Method
Inverse variance weighted
MR Egger
7.5
5.0
2.5
1/SE$_{IV}$
−0.75
−0.50
−0.25
0.00
0.25
β$_{IV}$

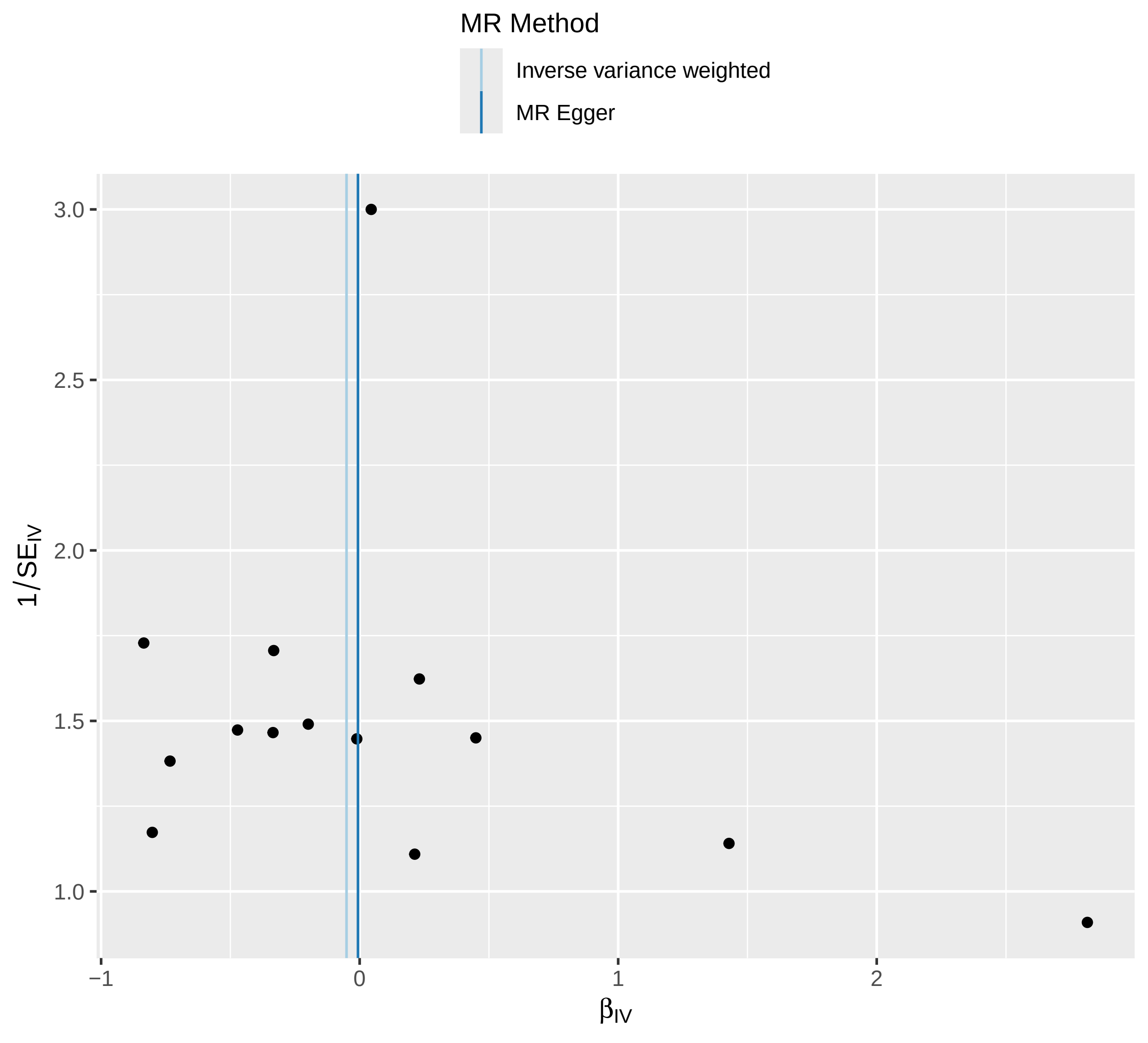
MR Method
Inverse variance weighted
MR Egger
3.0
2.5
2.0
1.5
1.0
$1/SE_{IV}$
−1
0
1
2
β_{IV}

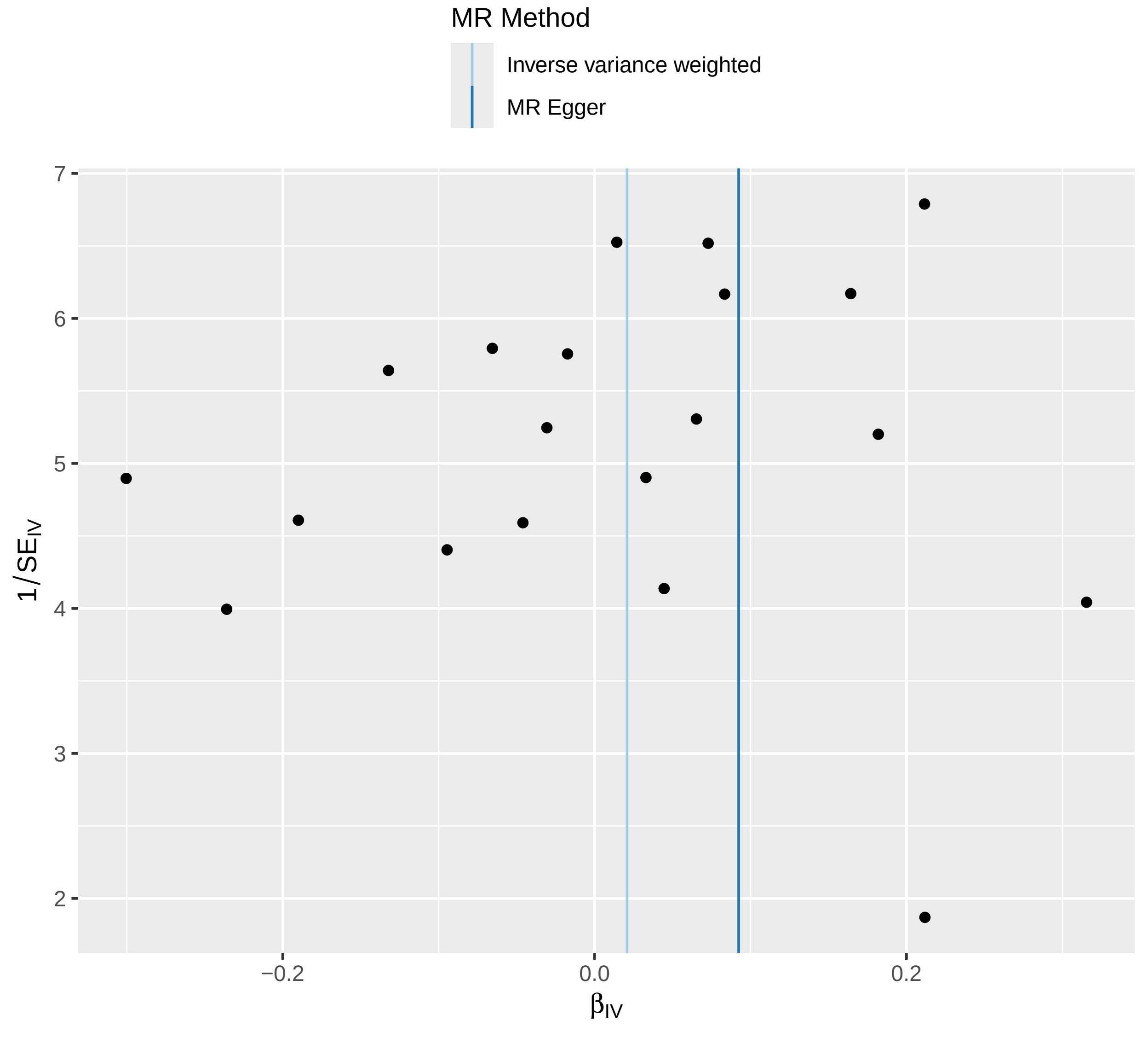

MR Method
Inverse variance weighted
MR Egger
7
6
5
4
3
2
1/SE_IV
−0.2
0.0
0.2
β_IV

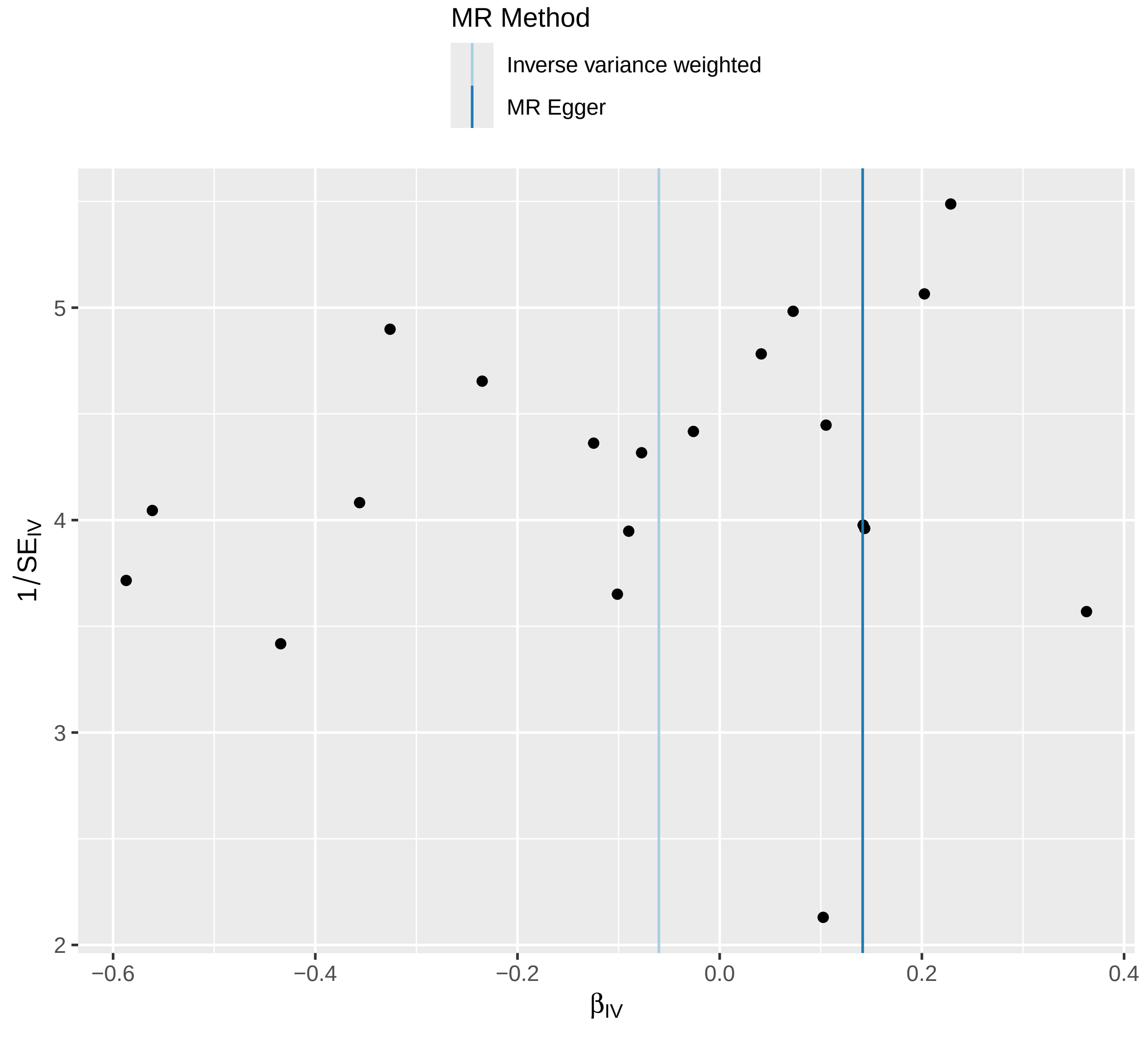
MR Method
Inverse variance weighted
MR Egger
5
4
3
2
1/SE$_{IV}$
−0.6
−0.4
−0.2
0.0
0.2
0.4
β$_{IV}$

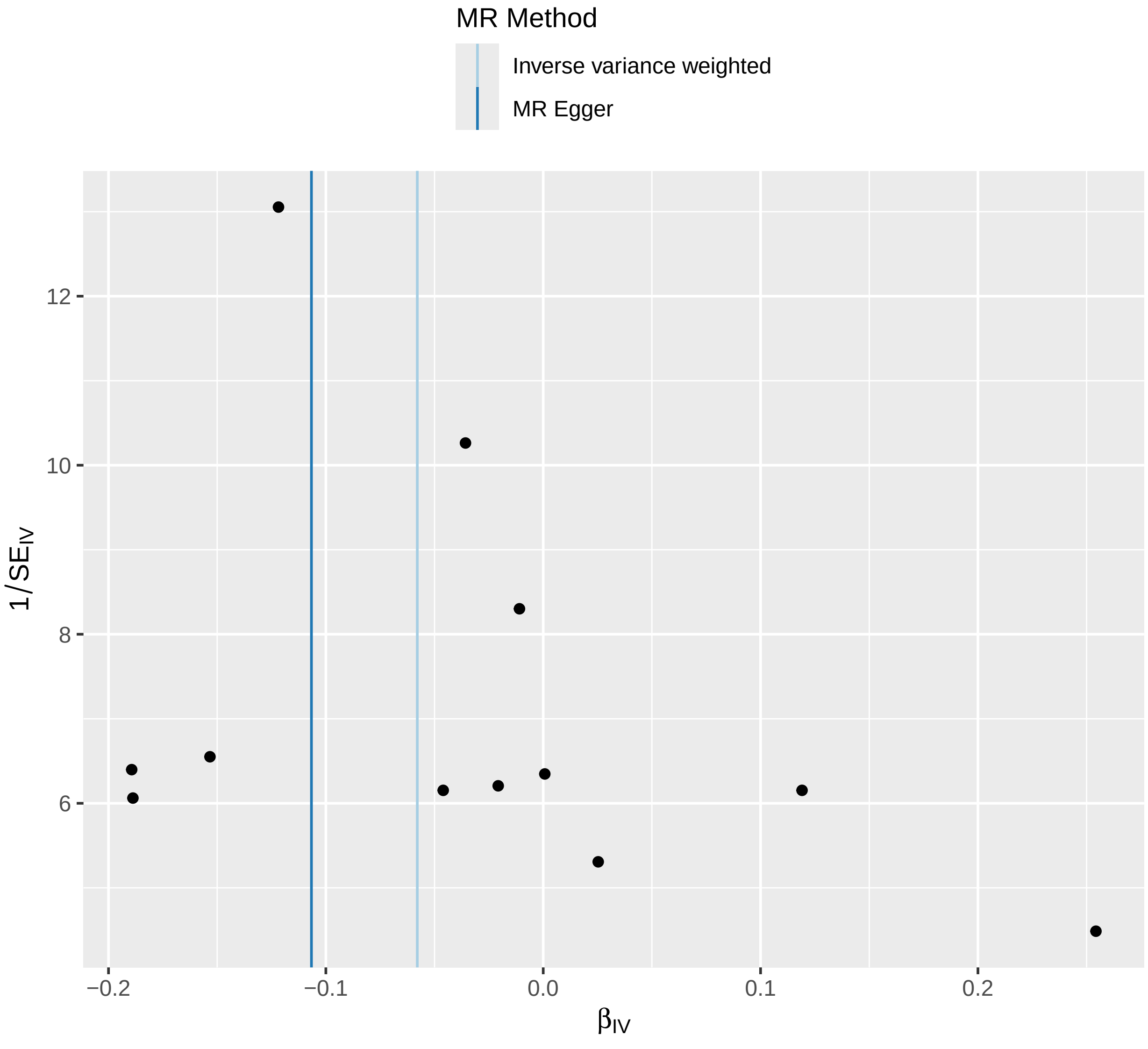
MR Method
Inverse variance weighted
MR Egger
12
10
8
6
1/SE$_{IV}$
−0.2
−0.1
0.0
0.1
0.2
β$_{IV}$

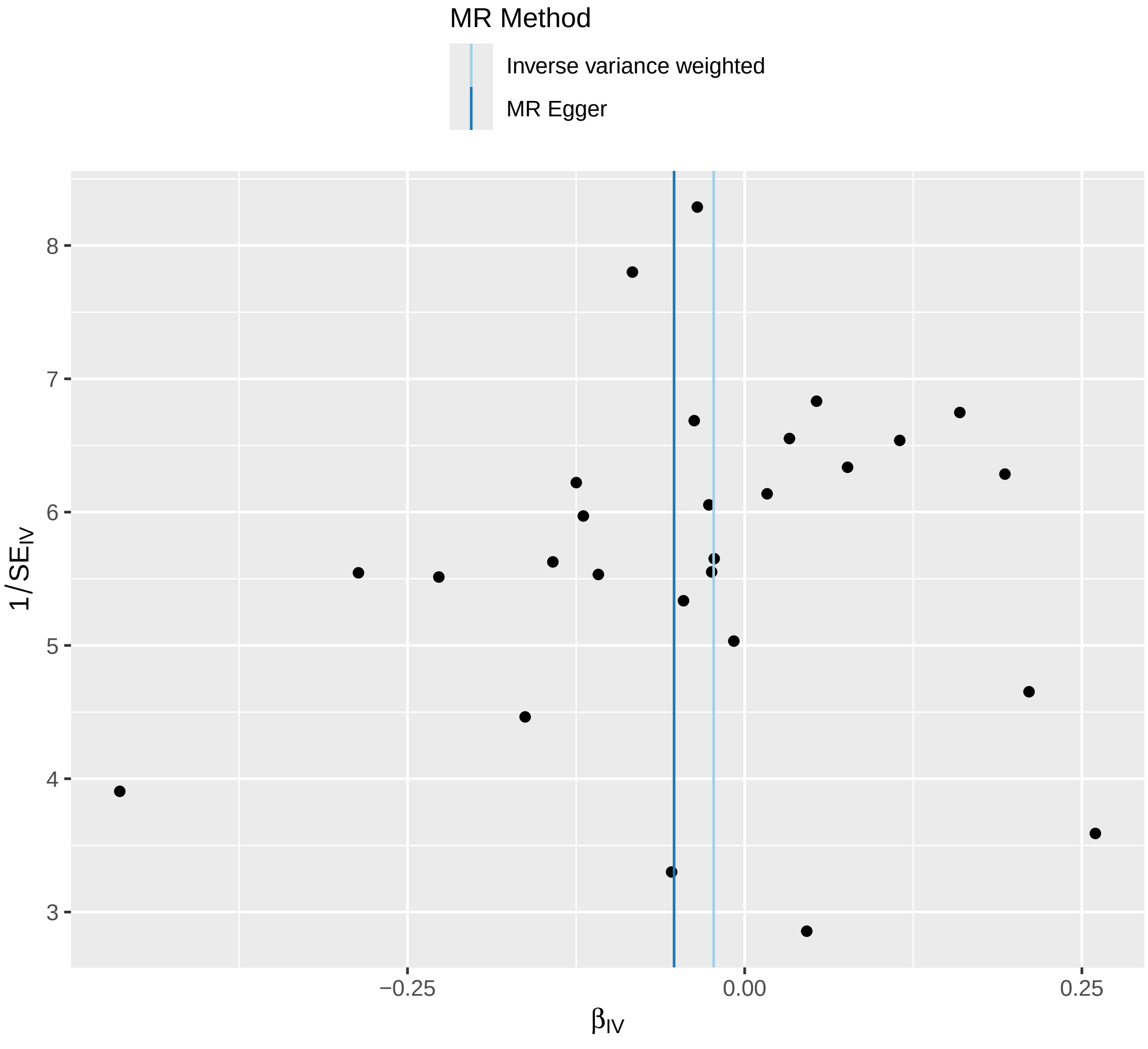

MR Method
Inverse variance weighted
MR Egger
8
7
6
5
4
3
1/SE_IV
−0.25
0.00
0.25
β_IV

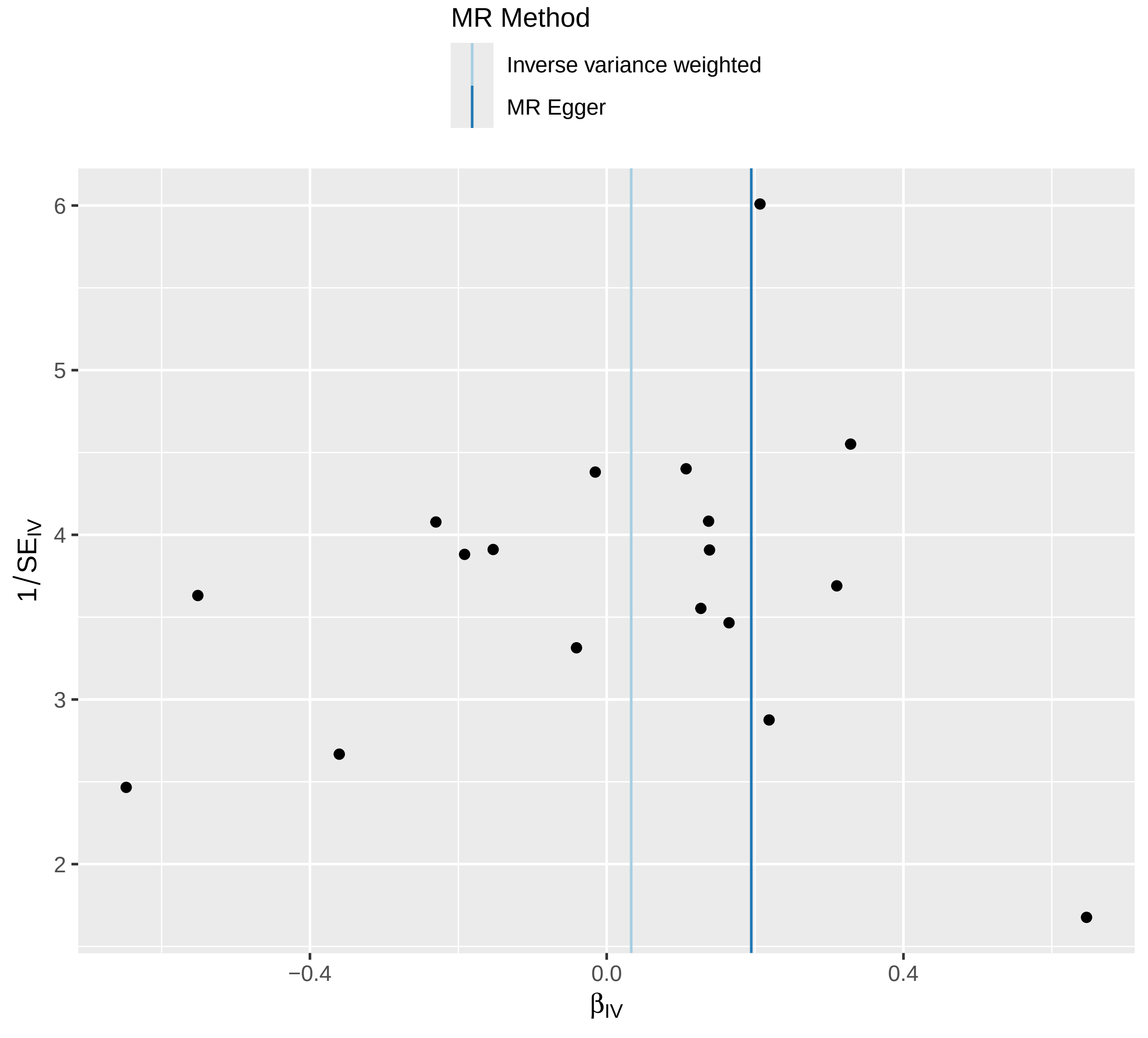
MR Method
Inverse variance weighted
MR Egger
6
5
4
3
2
1/SE$_{IV}$
−0.4
0.0
0.4
β$_{IV}$

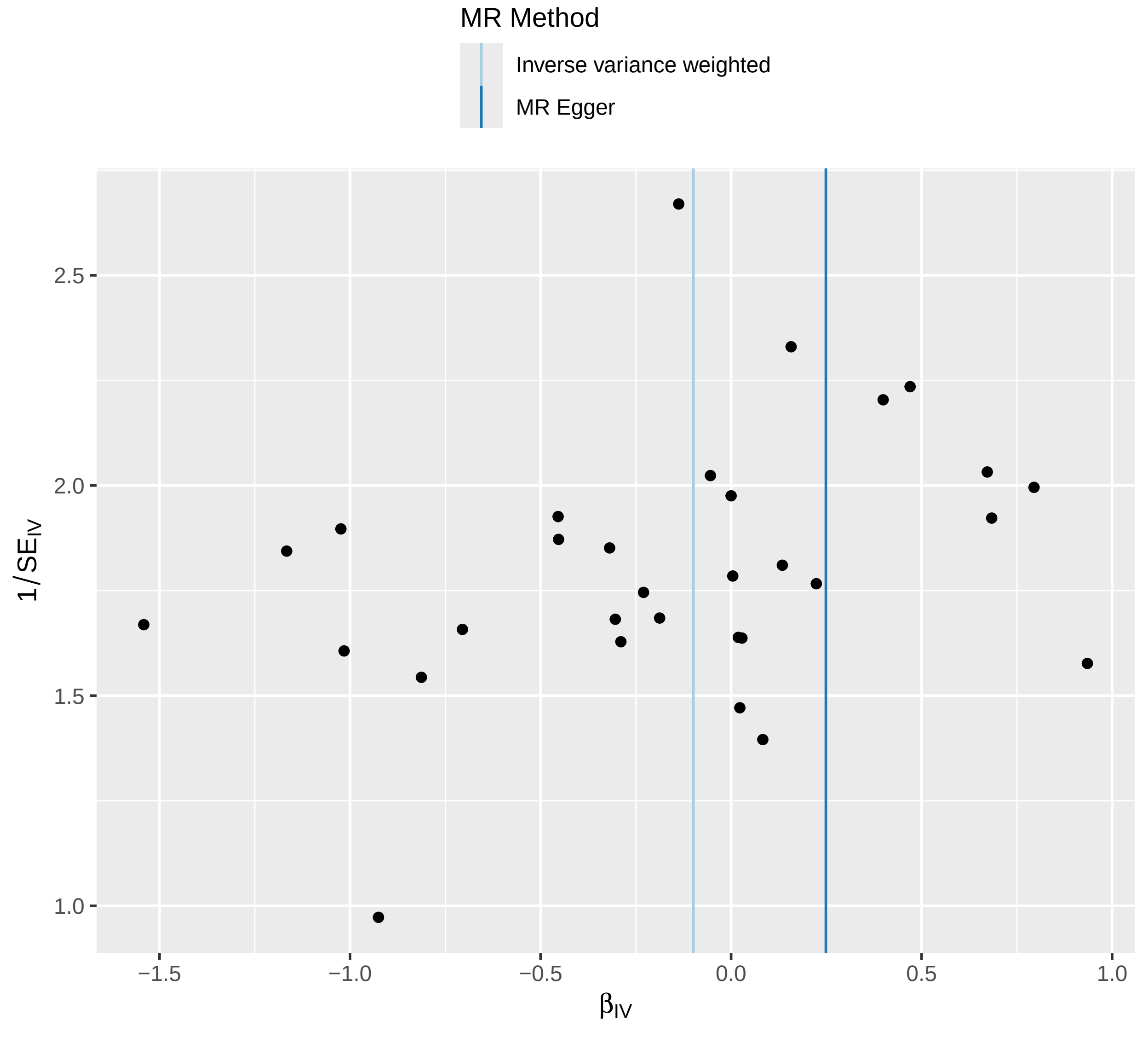

MR Method
Inverse variance weighted
MR Egger
2.5
2.0
1.5
1.0
1/SE_IV
−1.5
−1.0
−0.5
0.0
0.5
1.0
β_IV

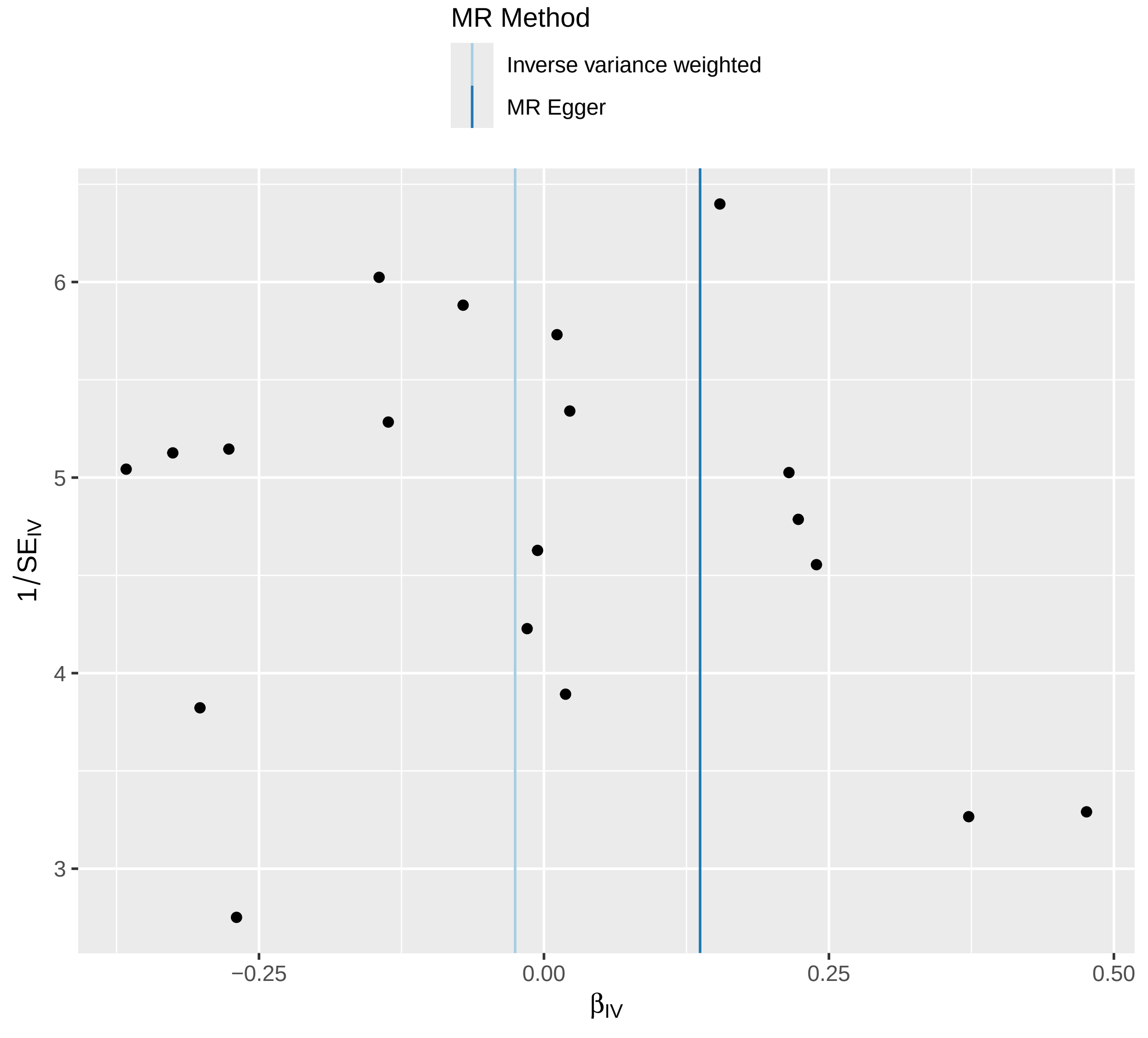

MR Method
Inverse variance weighted
MR Egger
6
5
4
3
1/SE$_{IV}$
−0.25
0.00
0.25
0.50
β$_{IV}$

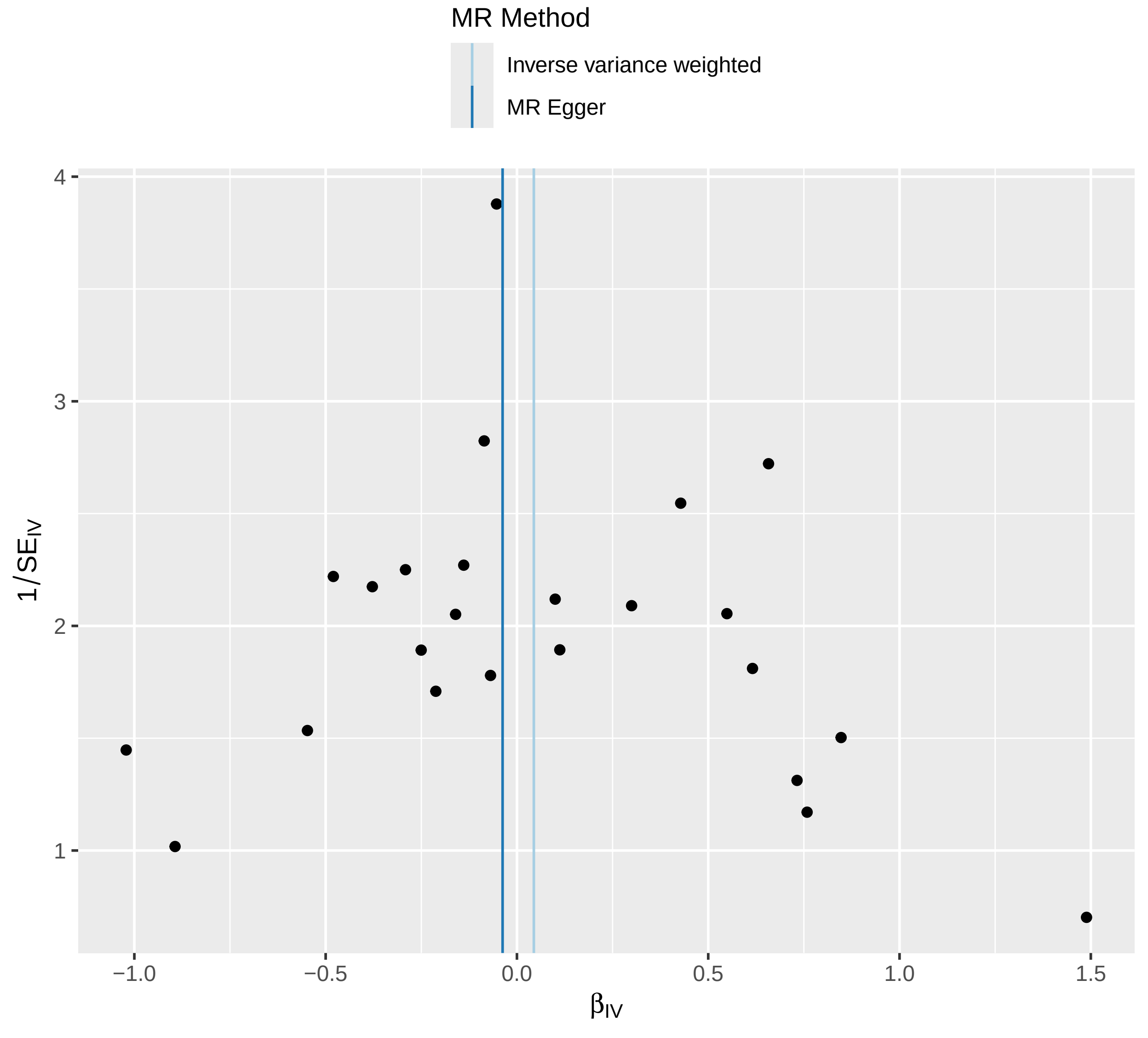
MR Method
Inverse variance weighted
MR Egger
4
3
2
1
1/SE_IV
−1.0
−0.5
0.0
0.5
1.0
1.5
β_IV

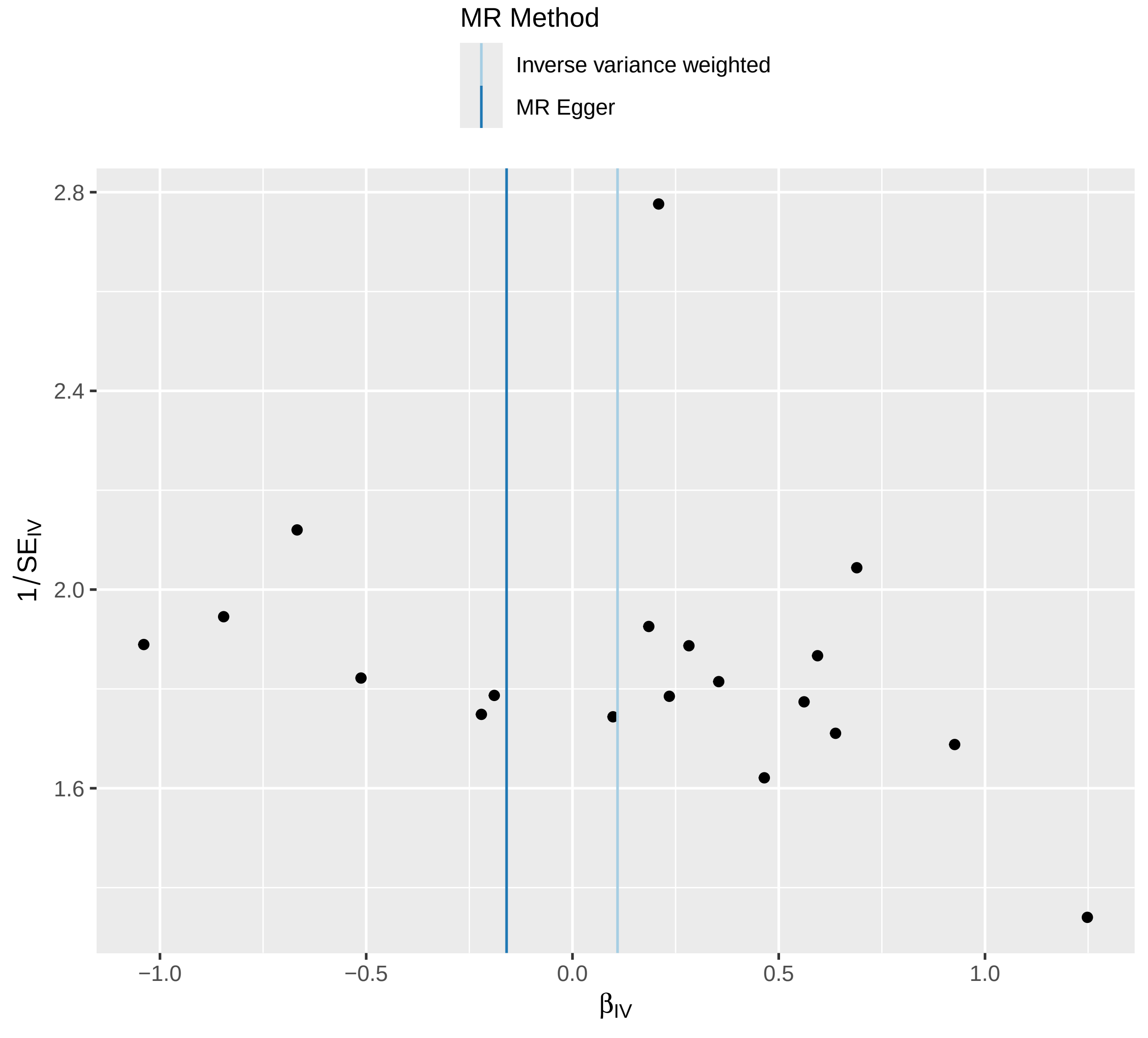
MR Method
Inverse variance weighted
MR Egger
2.8
2.4
2.0
1.6
$1/SE_{IV}$
−1.0
−0.5
0.0
0.5
1.0
β_{IV}

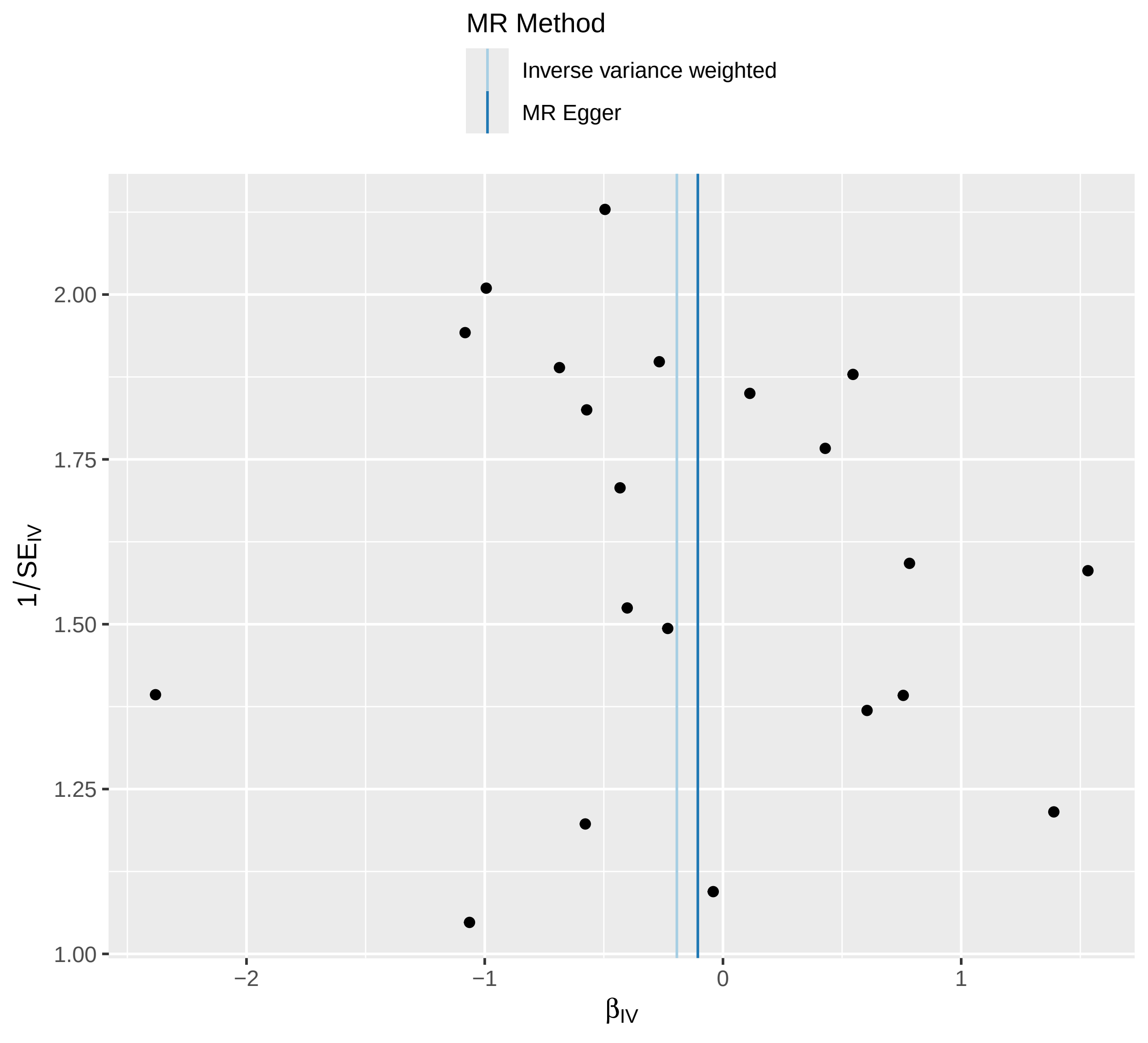

MR Method
Inverse variance weighted
MR Egger
2.00
1.75
1.50
1.25
1.00
1/SE_IV
−2
−1
0
1
β_IV

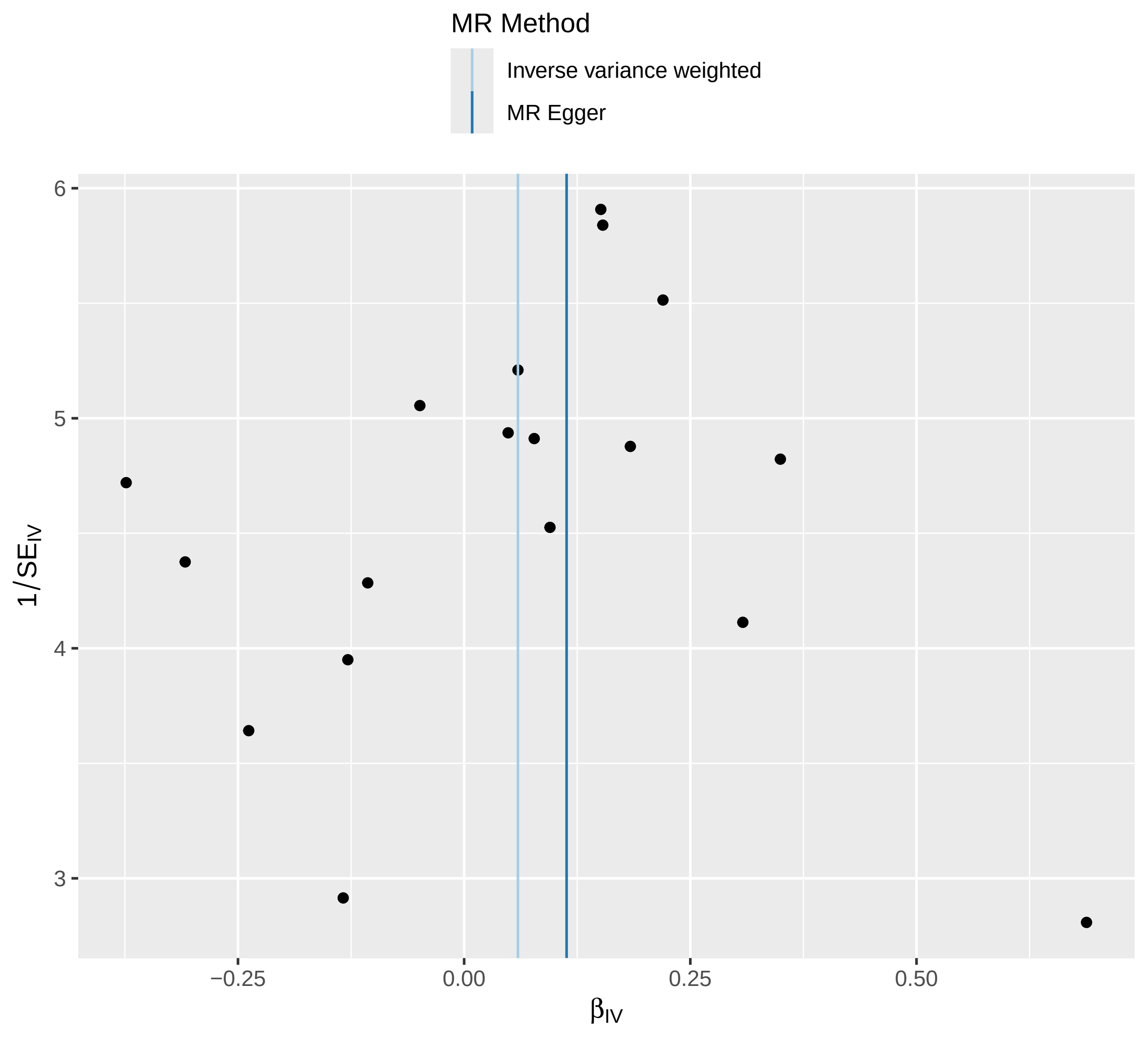

MR Method
Inverse variance weighted
MR Egger
6
5
4
3
1/SE_IV
−0.25
0.00
0.25
0.50
β_IV

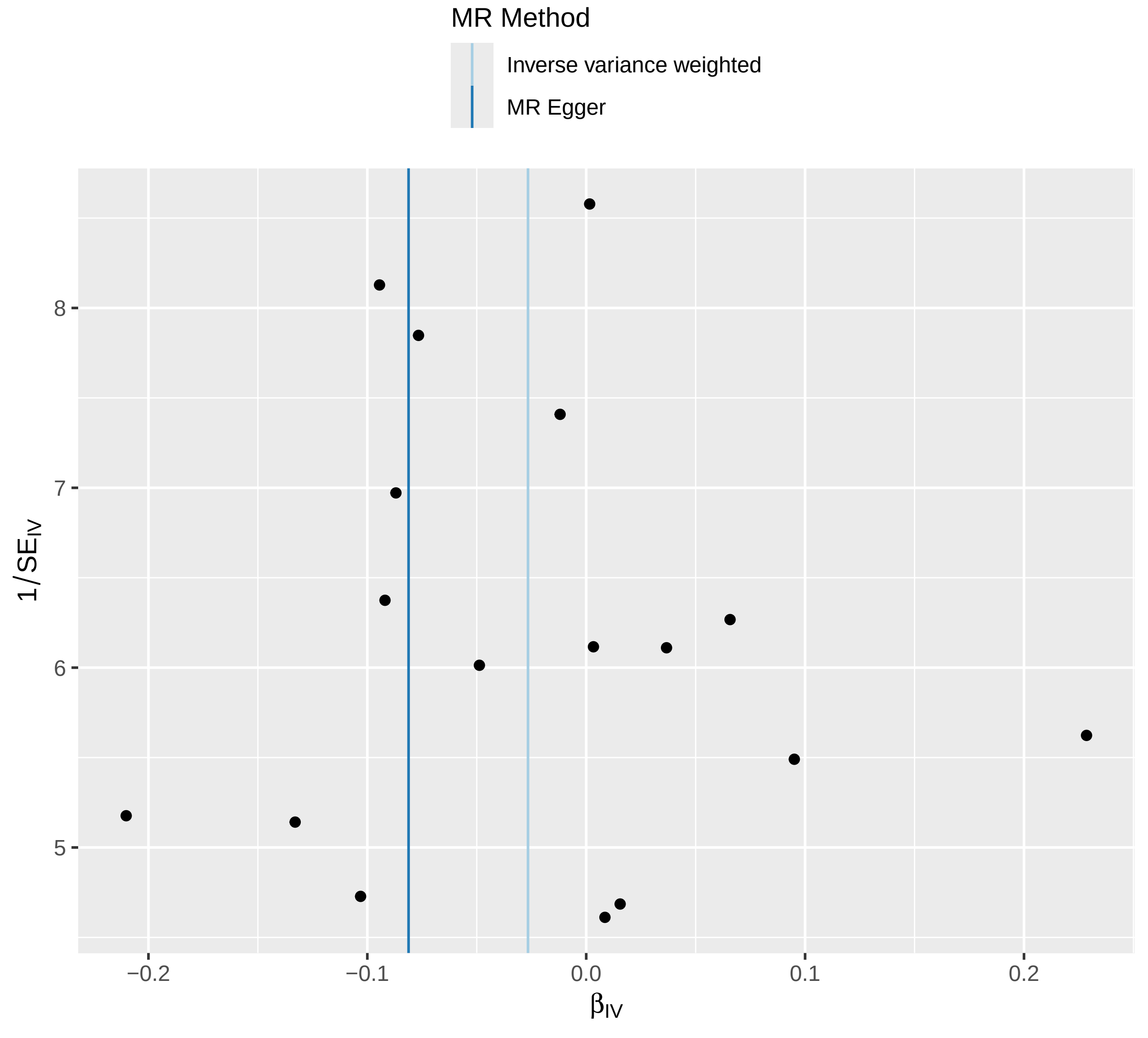
MR Method
Inverse variance weighted
MR Egger
8
7
6
5
1/SE_IV
−0.2
−0.1
0.0
0.1
0.2
β_IV

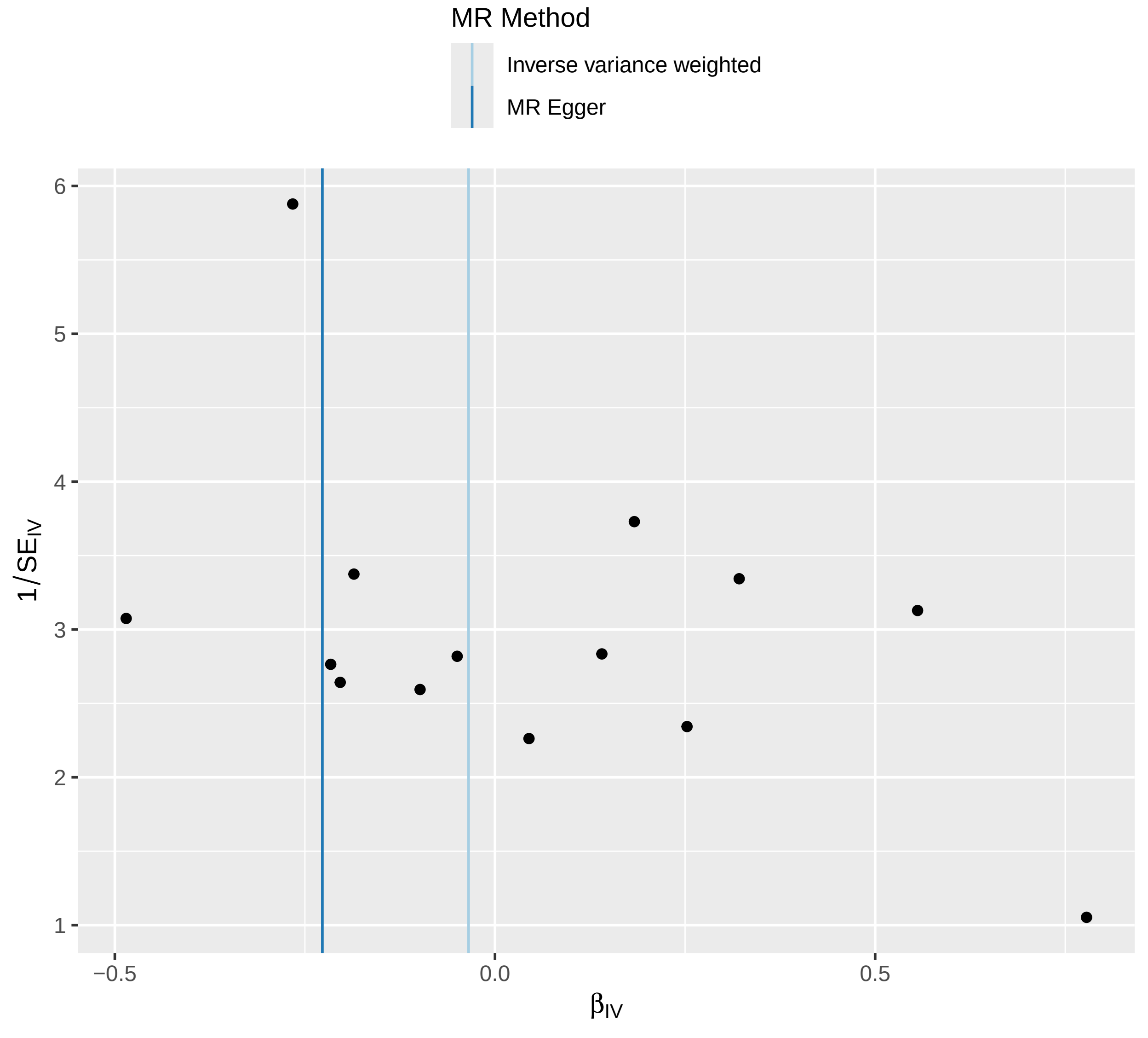
MR Method
Inverse variance weighted
MR Egger
1/SE$_{IV}$
6
5
4
3
2
1
−0.5
0.0
0.5
β$_{IV}$

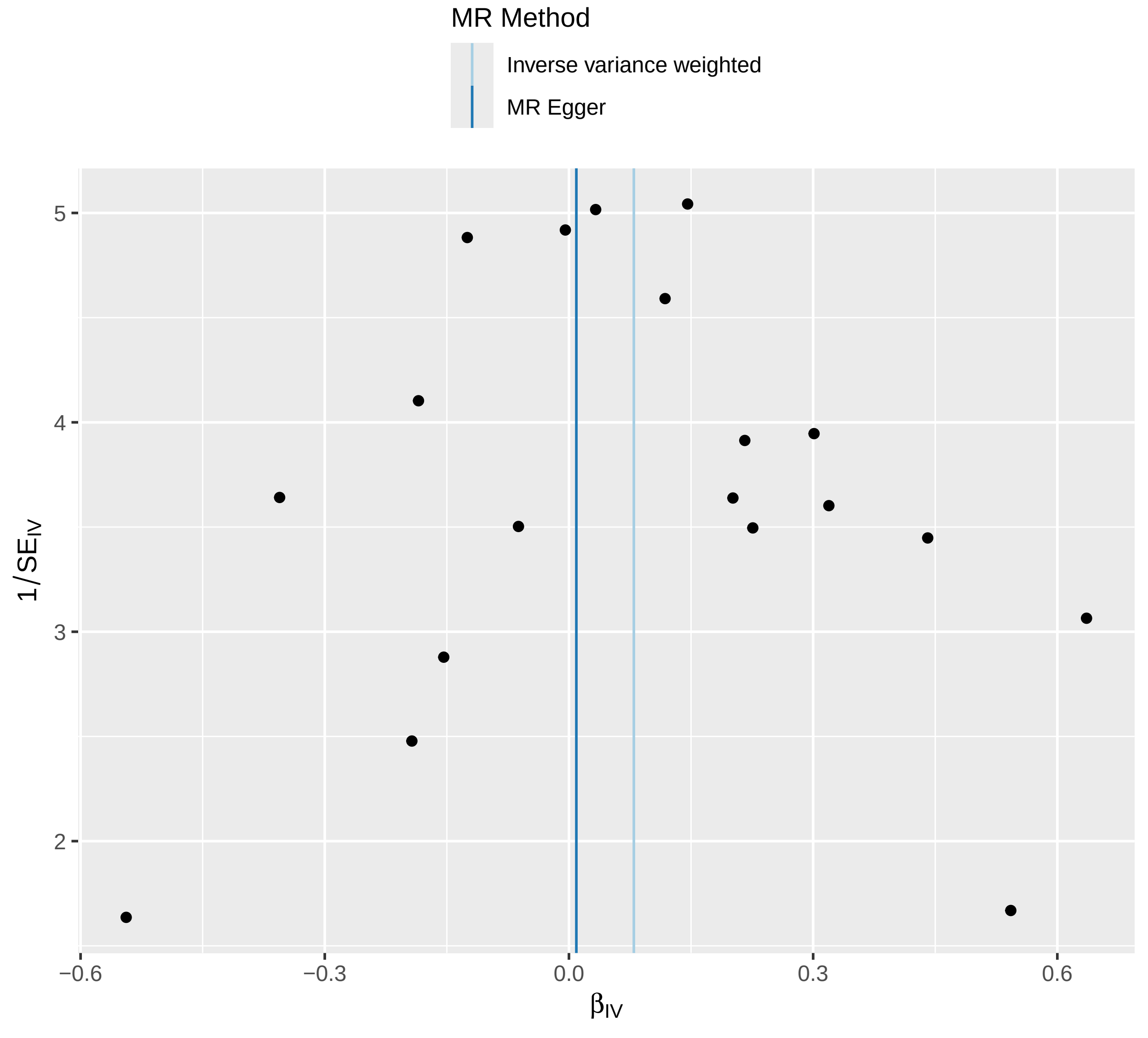

MR Method
Inverse variance weighted
MR Egger
5
4
3
2
1/SE$_{IV}$
−0.6
−0.3
0.0
0.3
0.6
β$_{IV}$

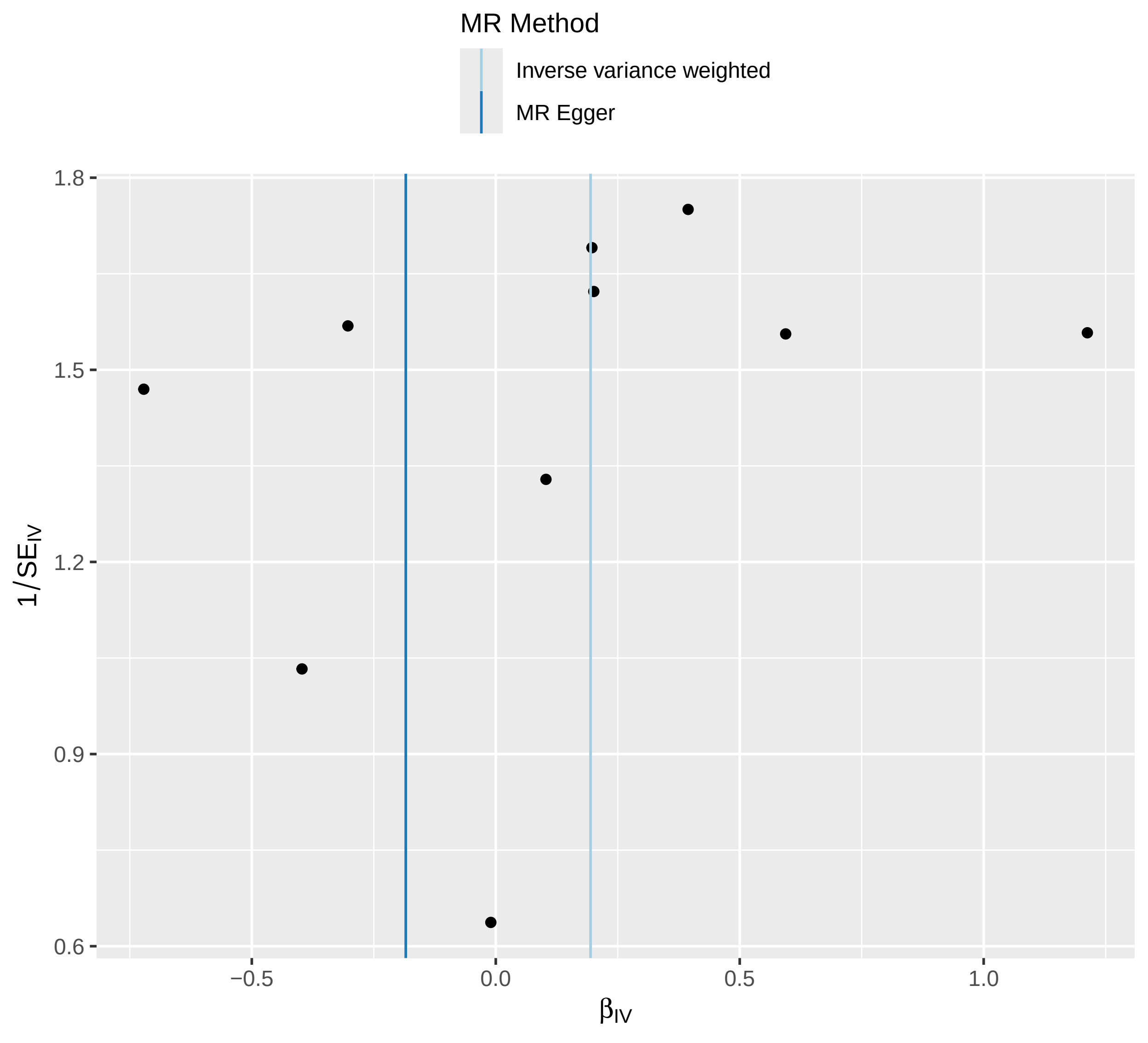

MR Method
Inverse variance weighted
MR Egger
1.8
1.5
1.2
0.9
0.6
1/SE$_{IV}$
−0.5
0.0
0.5
1.0
β$_{IV}$

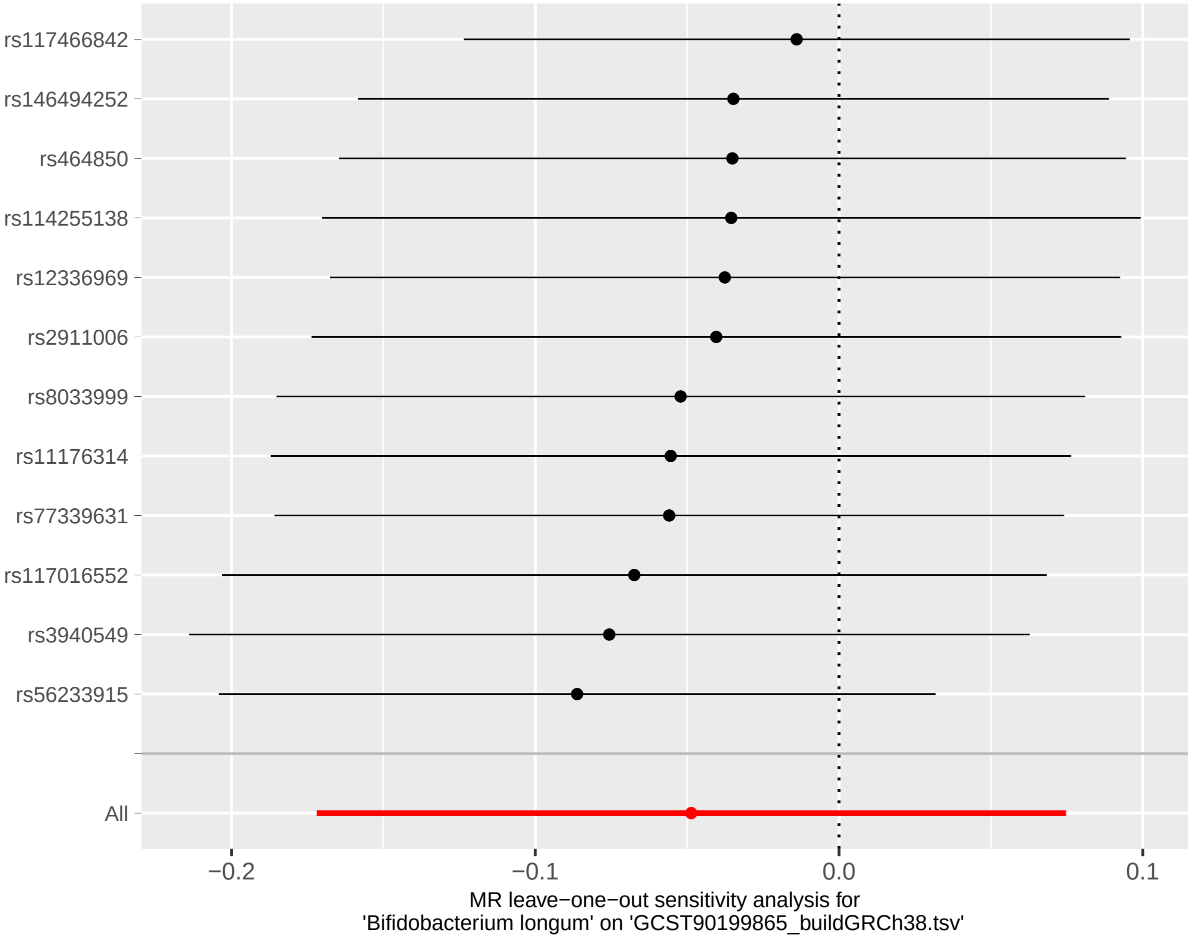

rs117466842
rs146494252
rs464850
rs114255138
rs12336969
rs2911006
rs8033999
rs11176314
rs77339631
rs117016552
rs3940549
rs56233915
All
−0.2
−0.1
0.0
0.1
MR leave−one−out sensitivity analysis for
'Bifidobacterium longum' on 'GCST90199865_buildGRCh38.tsv'

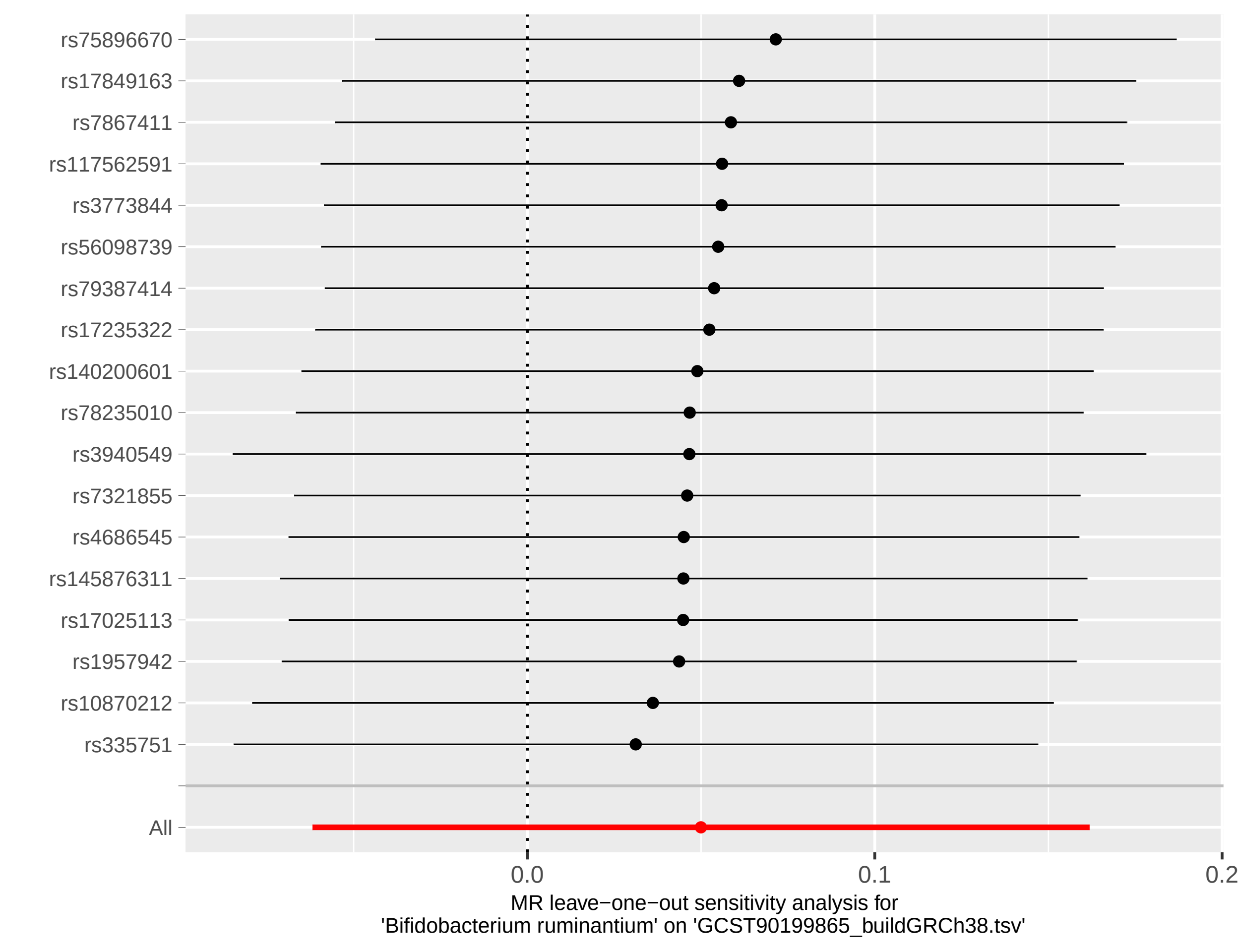
rs75896670
rs17849163
rs7867411
rs117562591
rs3773844
rs56098739
rs79387414
rs17235322
rs140200601
rs78235010
rs3940549
rs7321855
rs4686545
rs145876311
rs17025113
rs1957942
rs10870212
rs335751
All
0.0
0.1
0.2
MR leave−one−out sensitivity analysis for
'Bifidobacterium ruminantium' on 'GCST90199865_buildGRCh38.tsv'

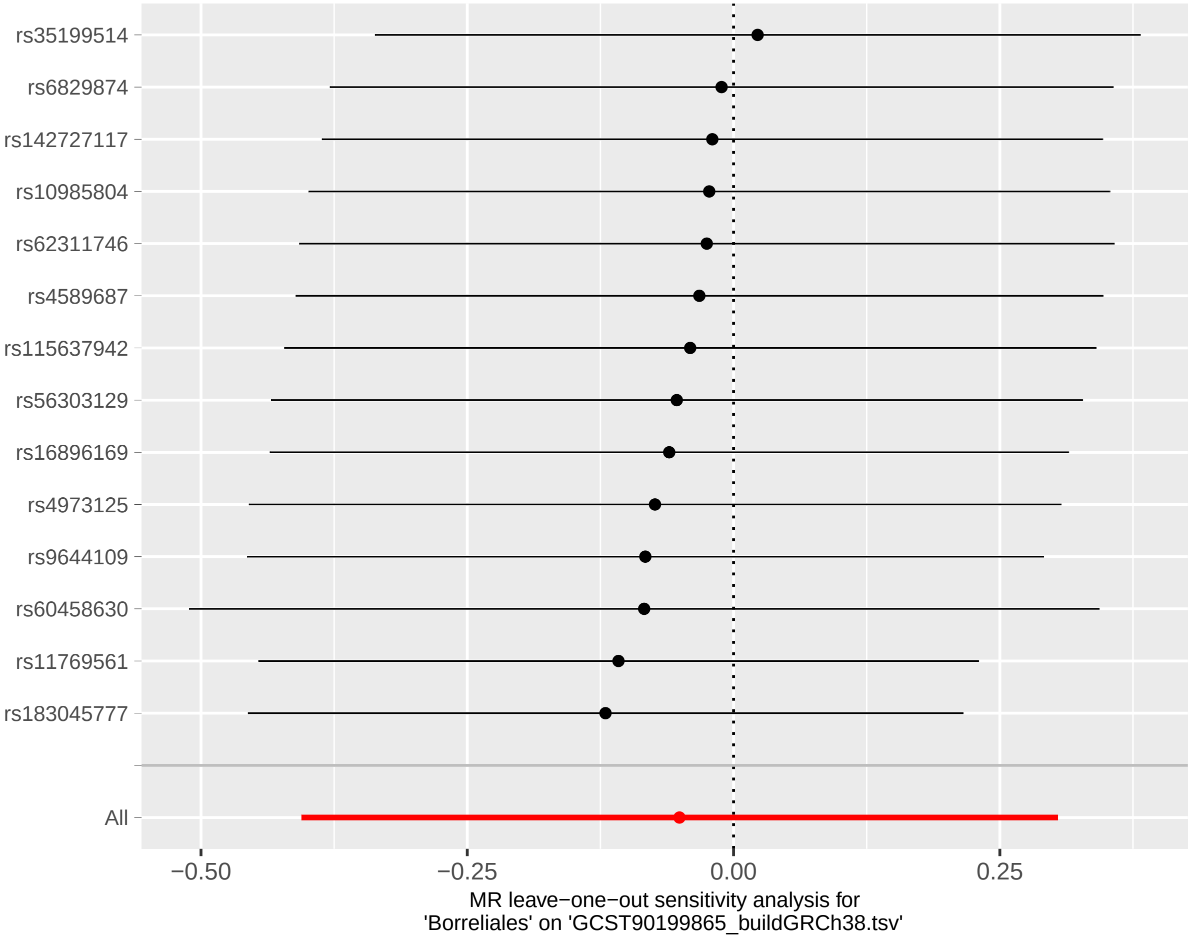

rs35199514
rs6829874
rs142727117
rs10985804
rs62311746
rs4589687
rs115637942
rs56303129
rs16896169
rs4973125
rs9644109
rs60458630
rs11769561
rs183045777
All
−0.50
−0.25
0.00
0.25
MR leave−one−out sensitivity analysis for
'Borreliales' on 'GCST90199865_buildGRCh38.tsv'

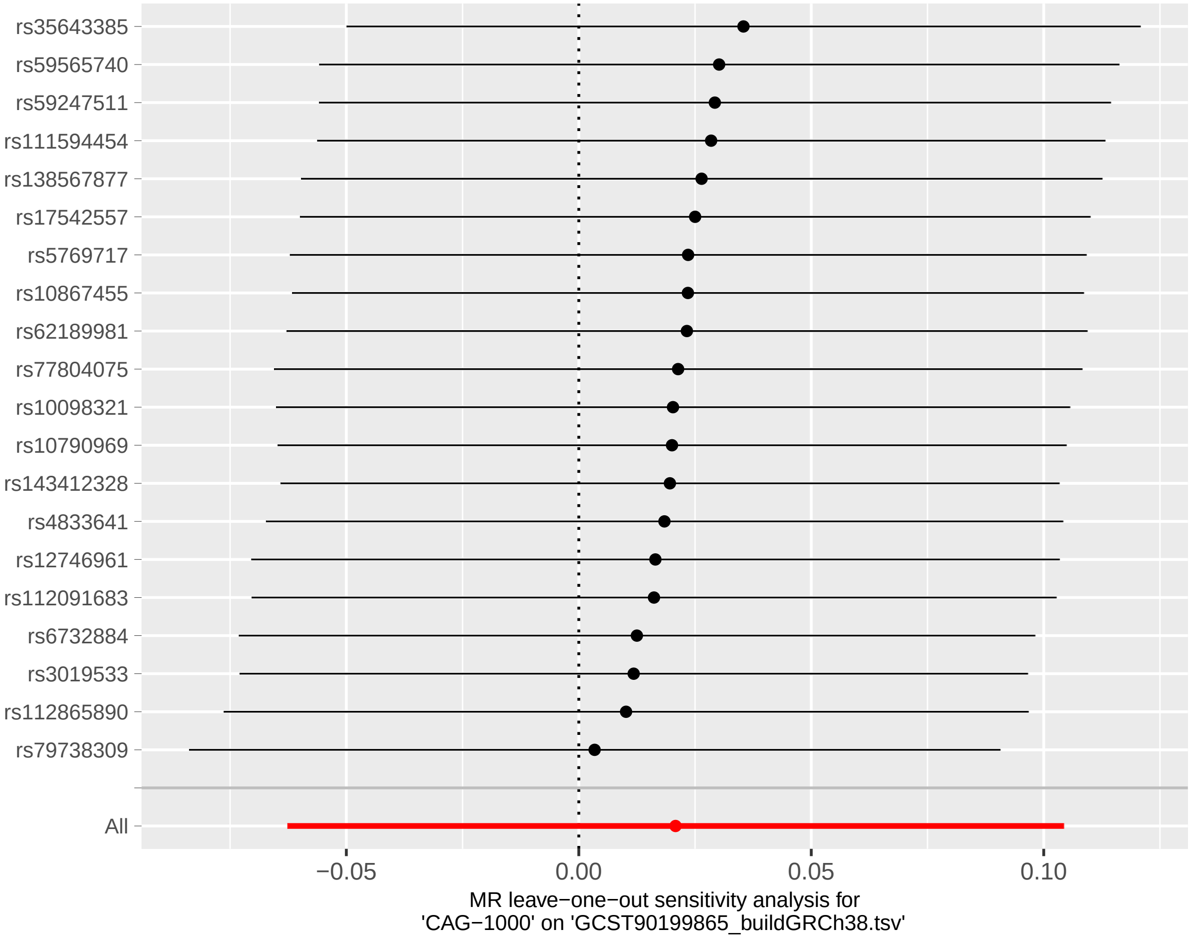

rs35643385
rs59565740
rs59247511
rs111594454
rs138567877
rs17542557
rs5769717
rs10867455
rs62189981
rs77804075
rs10098321
rs10790969
rs143412328
rs4833641
rs12746961
rs112091683
rs6732884
rs3019533
rs112865890
rs79738309
All
−0.05
0.00
0.05
0.10
MR leave−one−out sensitivity analysis for
'CAG−1000' on 'GCST90199865_buildGRCh38.tsv'

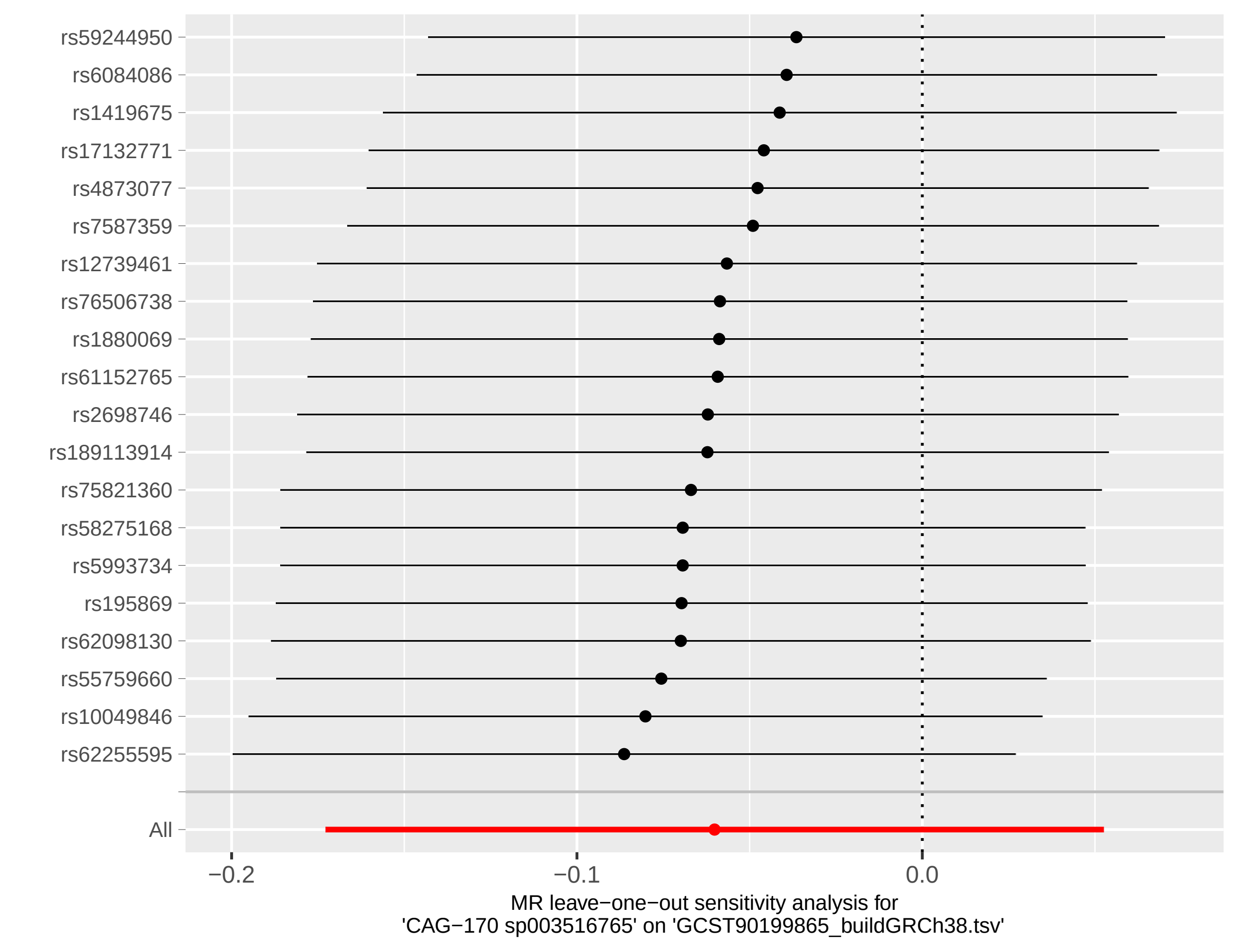
rs59244950
rs6084086
rs1419675
rs17132771
rs4873077
rs7587359
rs12739461
rs76506738
rs1880069
rs61152765
rs2698746
rs189113914
rs75821360
rs58275168
rs5993734
rs195869
rs62098130
rs55759660
rs10049846
rs62255595
All
−0.2
−0.1
0.0
MR leave−one−out sensitivity analysis for
'CAG−170 sp003516765' on 'GCST90199865_buildGRCh38.tsv'

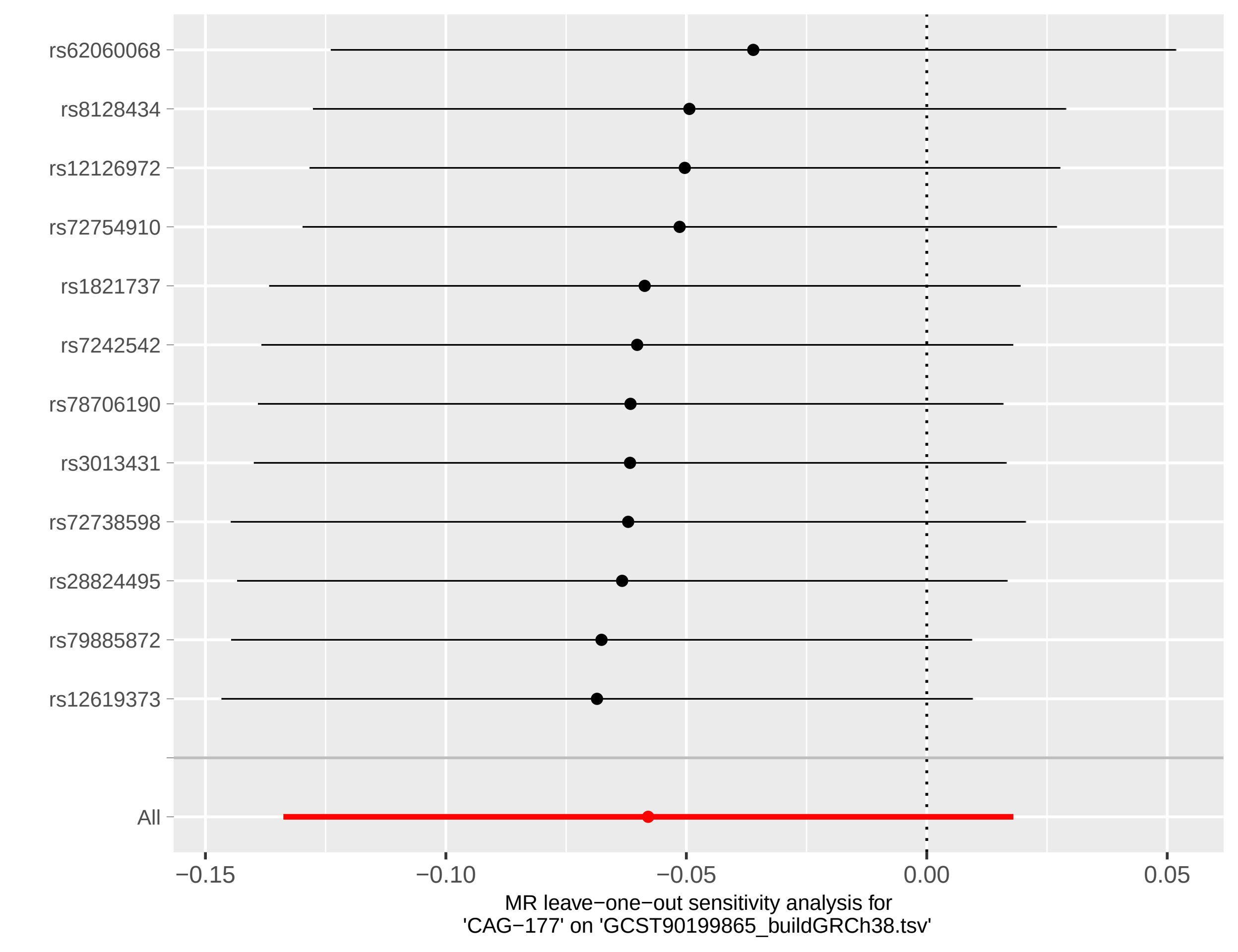

rs62060068
rs8128434
rs12126972
rs72754910
rs1821737
rs7242542
rs78706190
rs3013431
rs72738598
rs28824495
rs79885872
rs12619373
All
−0.15
−0.10
−0.05
0.00
0.05
MR leave−one−out sensitivity analysis for
'CAG−177' on 'GCST90199865_buildGRCh38.tsv'

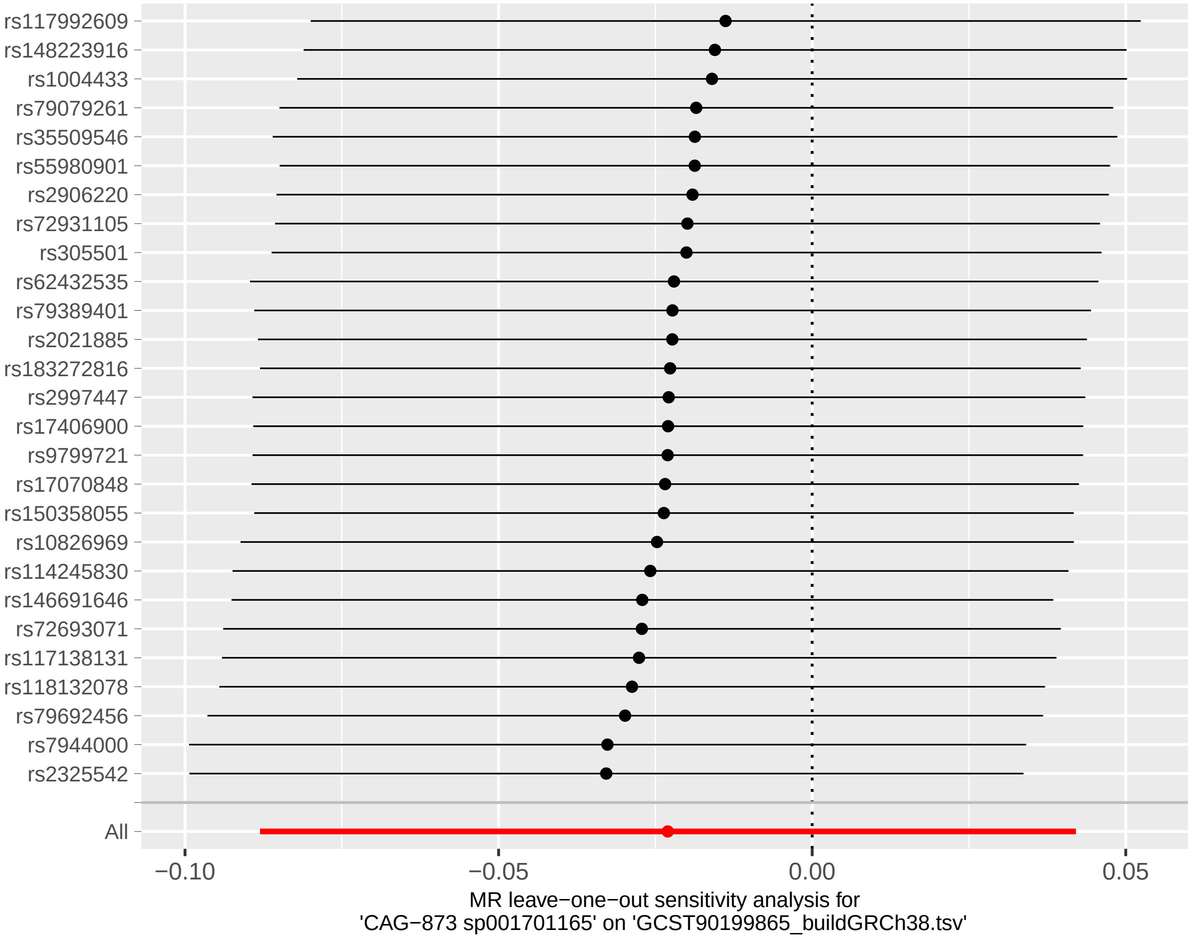
rs117992609
rs148223916
rs1004433
rs79079261
rs35509546
rs55980901
rs2906220
rs72931105
rs305501
rs62432535
rs79389401
rs2021885
rs183272816
rs2997447
rs17406900
rs9799721
rs17070848
rs150358055
rs10826969
rs114245830
rs146691646
rs72693071
rs117138131
rs118132078
rs79692456
rs7944000
rs2325542
All
−0.10
−0.05
0.00
0.05
MR leave−one−out sensitivity analysis for
'CAG−873 sp001701165' on 'GCST90199865_buildGRCh38.tsv'

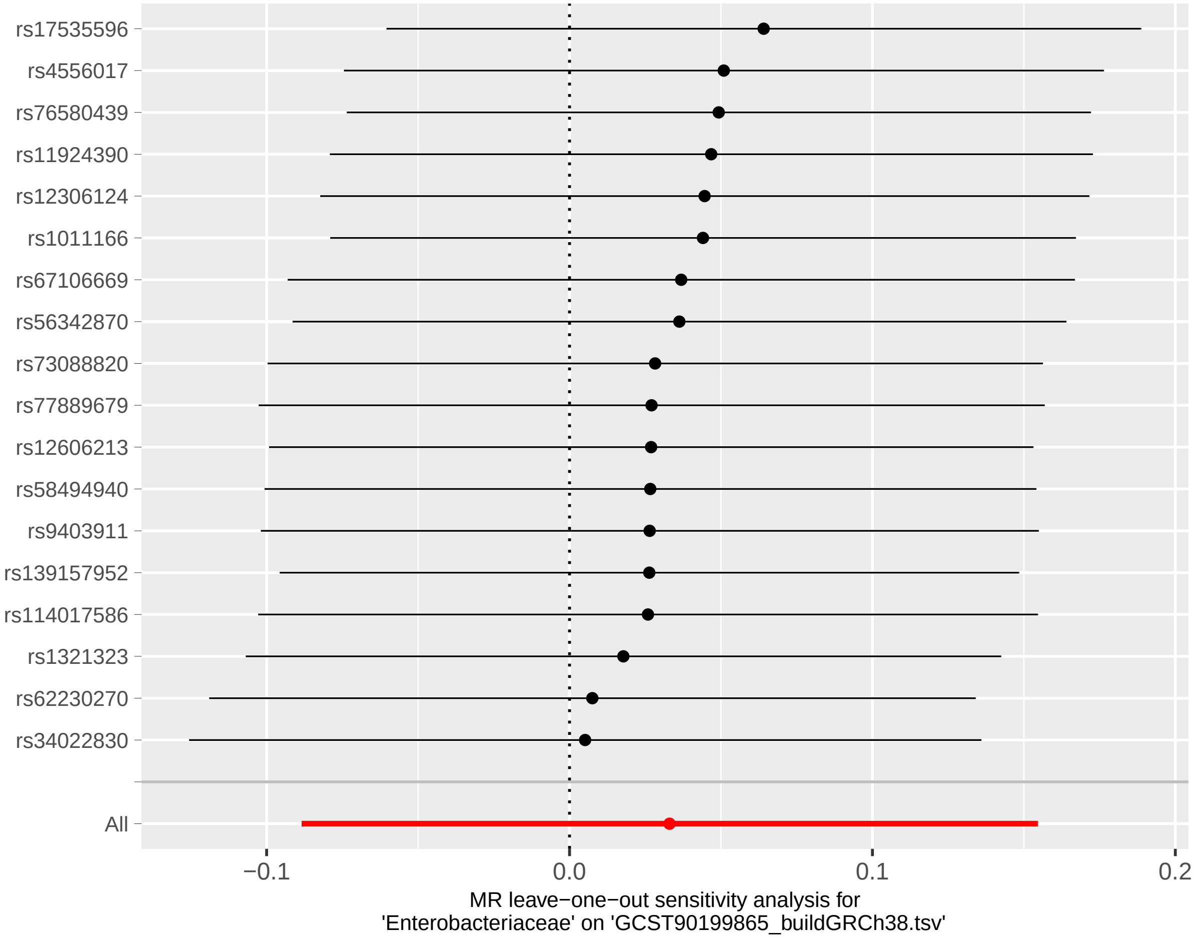
rs17535596
rs4556017
rs76580439
rs11924390
rs12306124
rs1011166
rs67106669
rs56342870
rs73088820
rs77889679
rs12606213
rs58494940
rs9403911
rs139157952
rs114017586
rs1321323
rs62230270
rs34022830
All
−0.1
0.0
0.1
0.2
MR leave−one−out sensitivity analysis for
'Enterobacteriaceae' on 'GCST90199865_buildGRCh38.tsv'

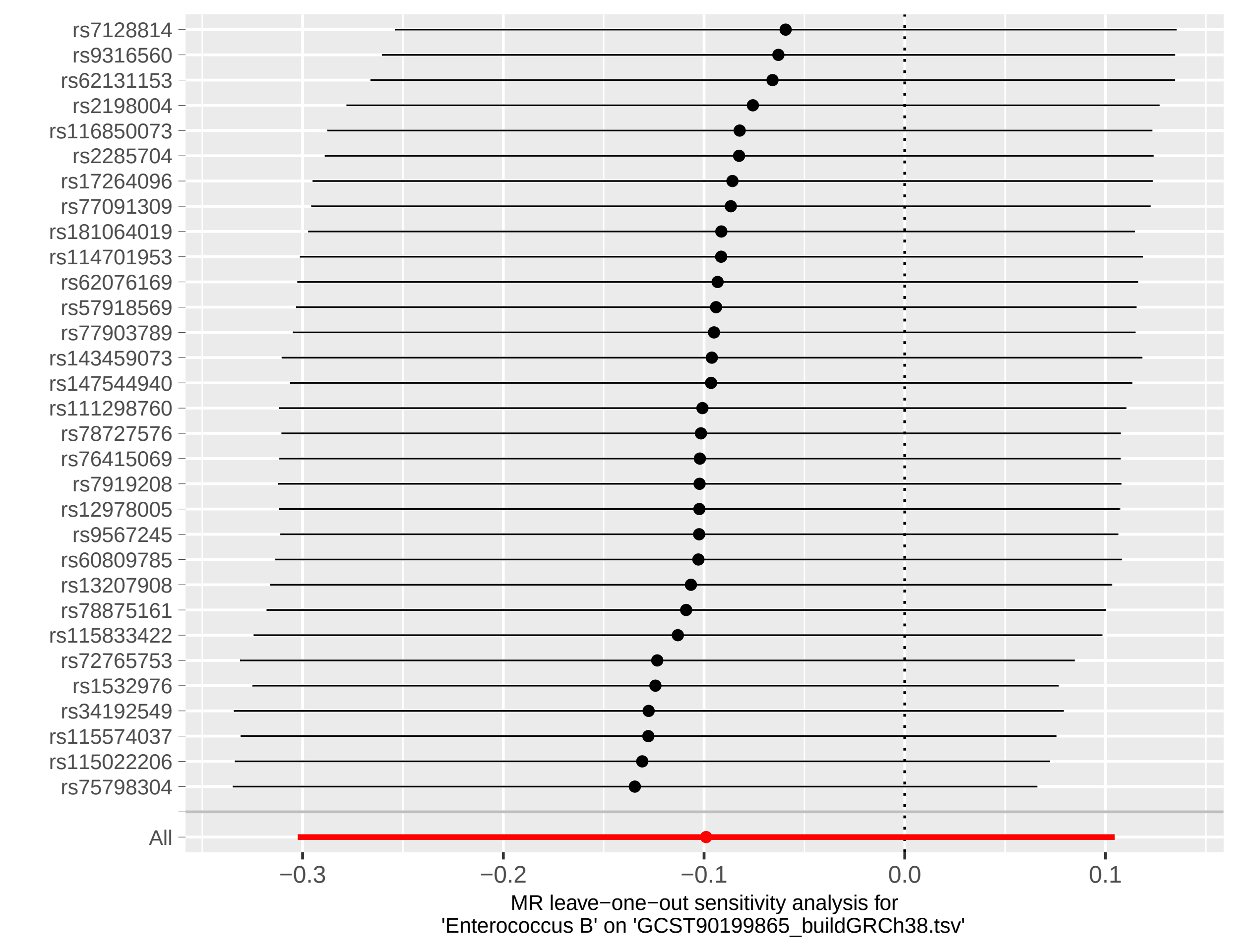
rs7128814
rs9316560
rs62131153
rs2198004
rs116850073
rs2285704
rs17264096
rs77091309
rs181064019
rs114701953
rs62076169
rs57918569
rs77903789
rs143459073
rs147544940
rs111298760
rs78727576
rs76415069
rs7919208
rs12978005
rs9567245
rs60809785
rs13207908
rs78875161
rs115833422
rs72765753
rs1532976
rs34192549
rs115574037
rs115022206
rs75798304
All
−0.3
−0.2
−0.1
0.0
0.1
MR leave−one−out sensitivity analysis for
'Enterococcus B' on 'GCST90199865_buildGRCh38.tsv'

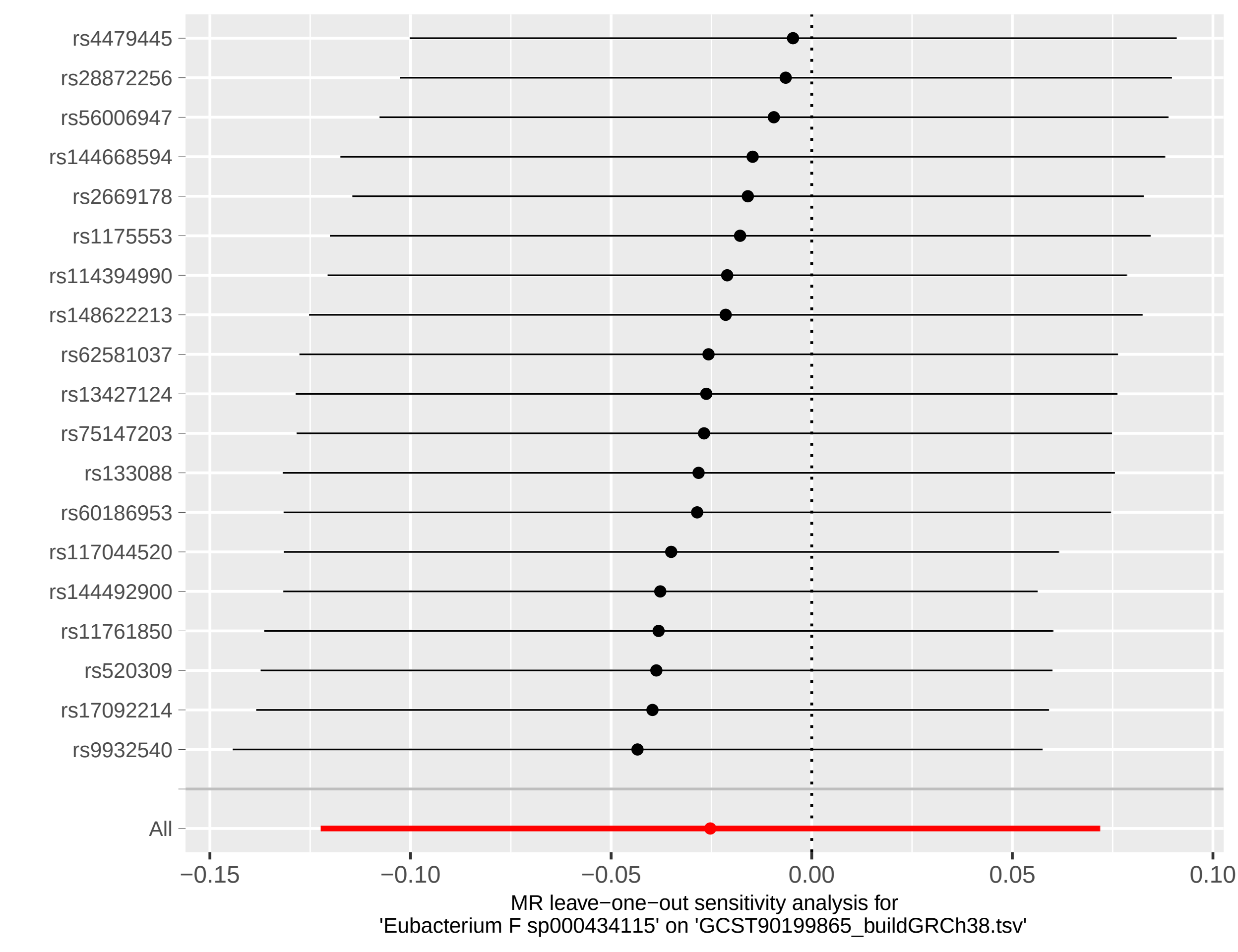
rs4479445
rs28872256
rs56006947
rs144668594
rs2669178
rs1175553
rs114394990
rs148622213
rs62581037
rs13427124
rs75147203
rs133088
rs60186953
rs117044520
rs144492900
rs11761850
rs520309
rs17092214
rs9932540
All
−0.15
−0.10
−0.05
0.00
0.05
0.10
MR leave−one−out sensitivity analysis for
'Eubacterium F sp000434115' on 'GCST90199865_buildGRCh38.tsv'

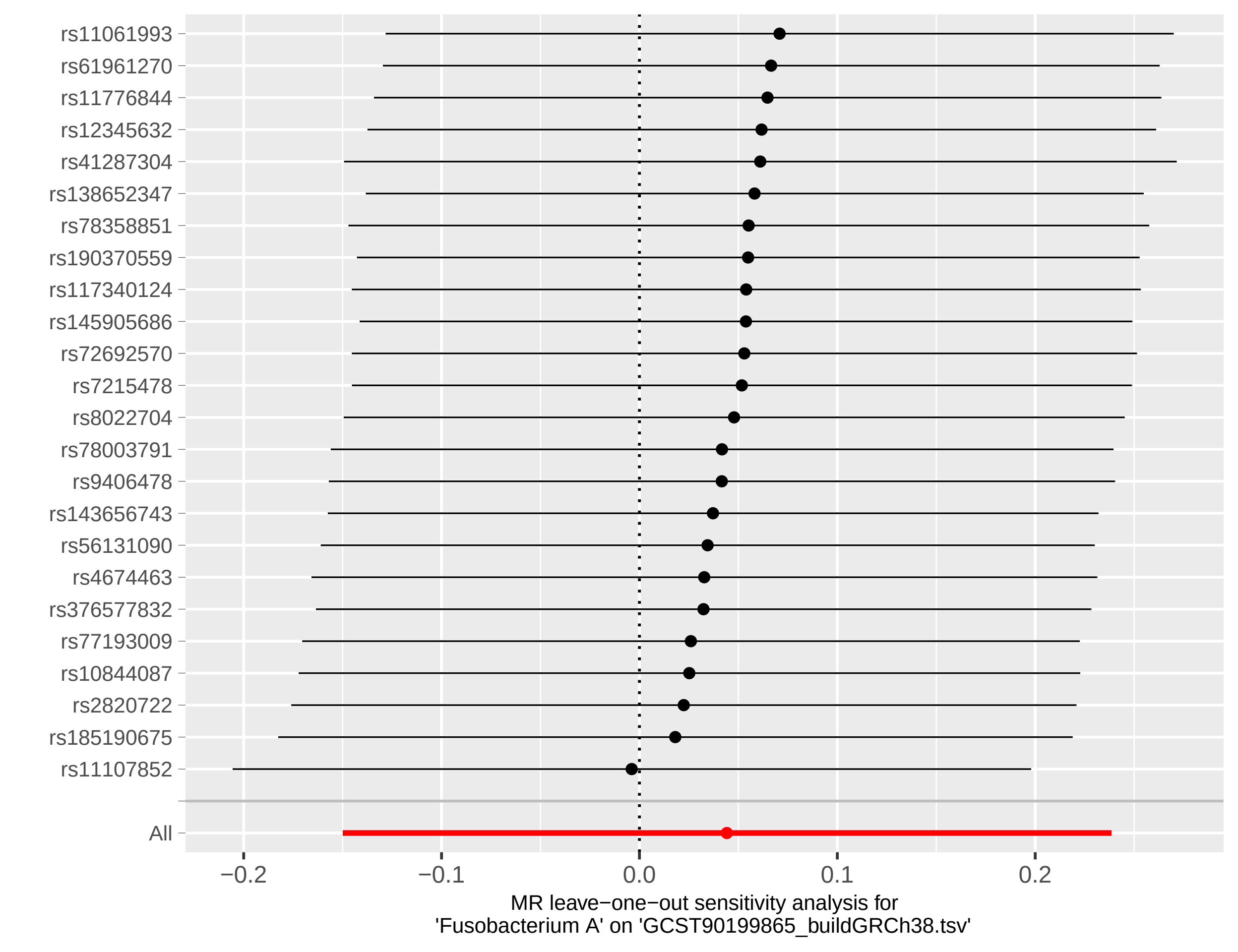
rs11061993
rs61961270
rs11776844
rs12345632
rs41287304
rs138652347
rs78358851
rs190370559
rs117340124
rs145905686
rs72692570
rs7215478
rs8022704
rs78003791
rs9406478
rs143656743
rs56131090
rs4674463
rs376577832
rs77193009
rs10844087
rs2820722
rs185190675
rs11107852
All
−0.2
−0.1
0.0
0.1
0.2
MR leave−one−out sensitivity analysis for
'Fusobacterium A' on 'GCST90199865_buildGRCh38.tsv'

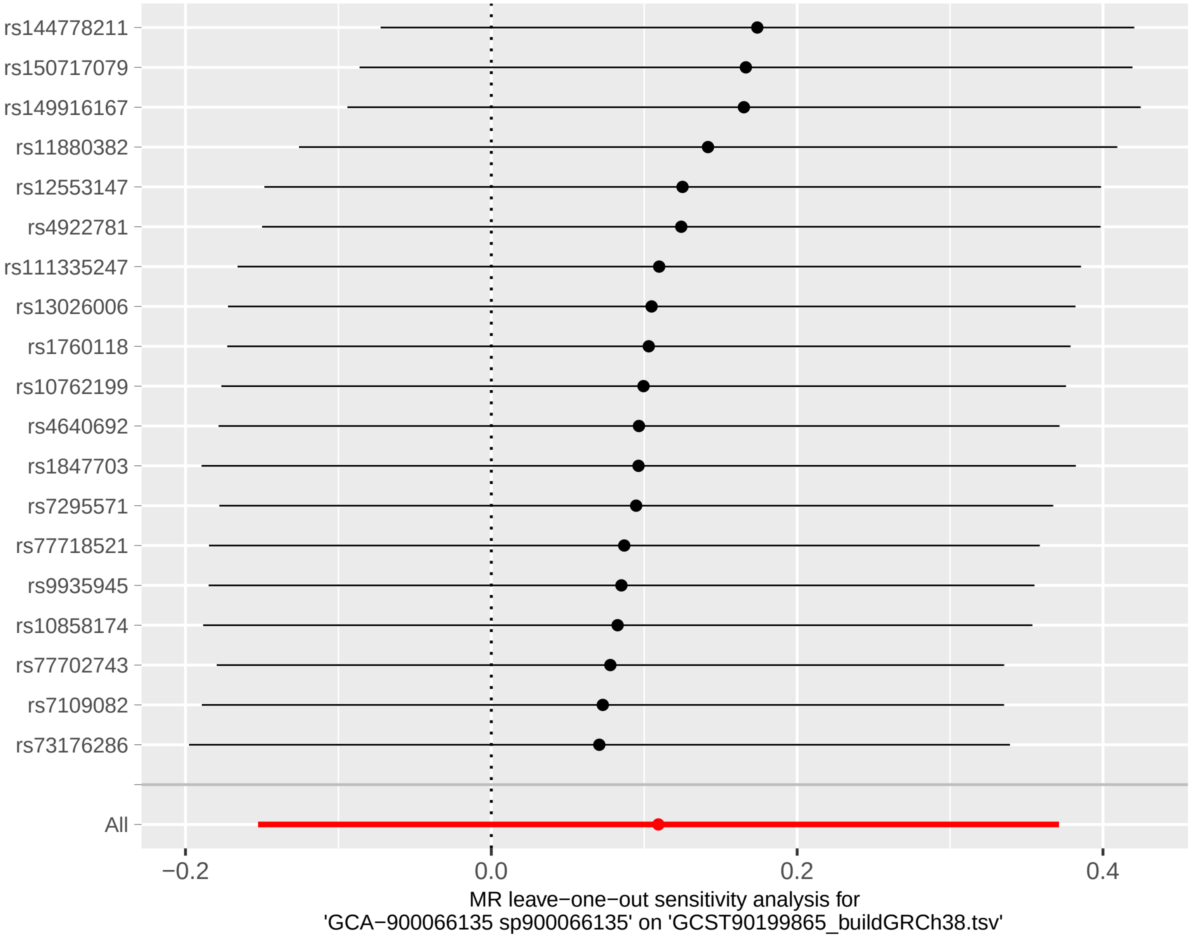
rs144778211
rs150717079
rs149916167
rs11880382
rs12553147
rs4922781
rs111335247
rs13026006
rs1760118
rs10762199
rs4640692
rs1847703
rs7295571
rs77718521
rs9935945
rs10858174
rs77702743
rs7109082
rs73176286
All
−0.2
0.0
0.2
0.4
MR leave−one−out sensitivity analysis for
'GCA−900066135 sp900066135' on 'GCST90199865_buildGRCh38.tsv'

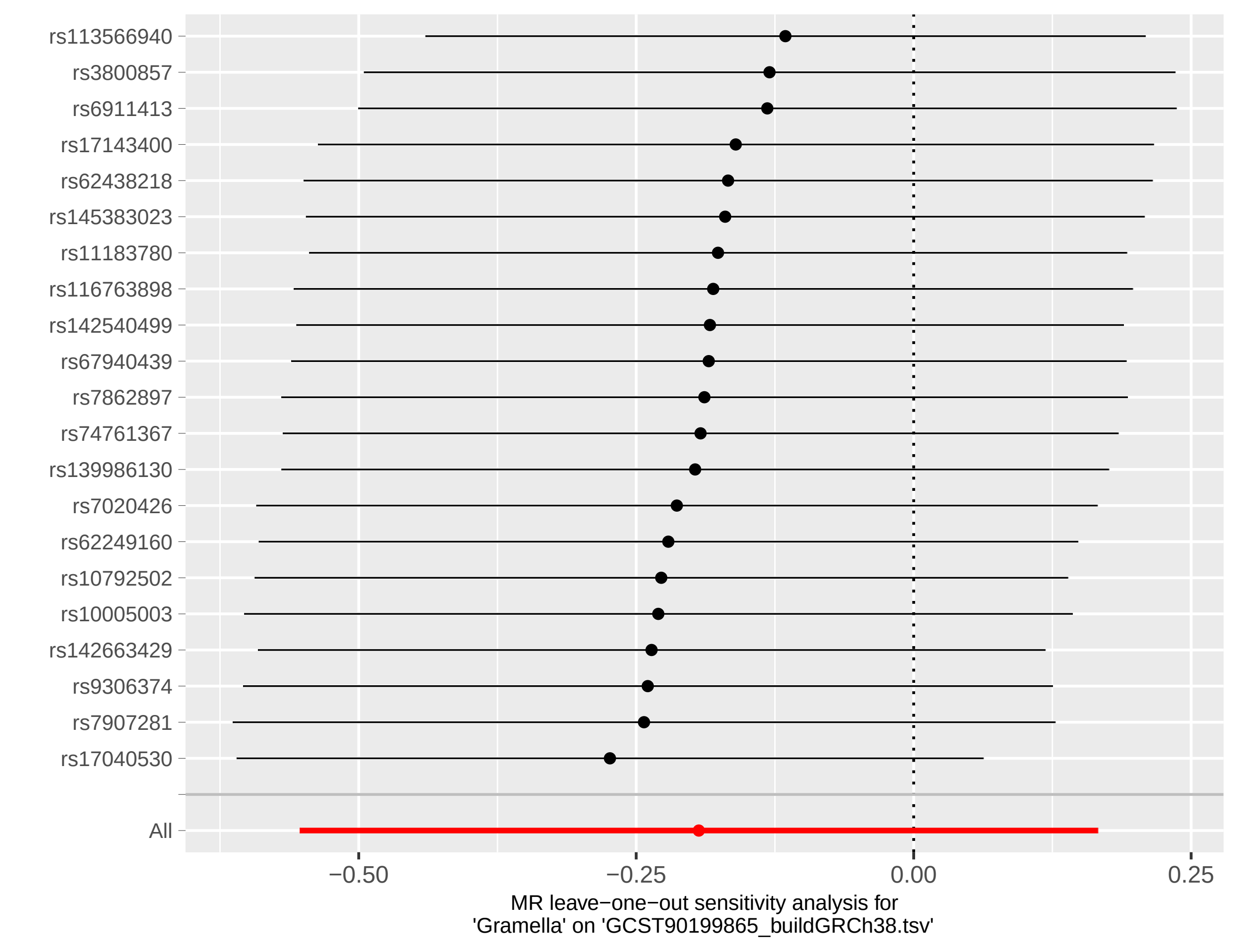
rs113566940
rs3800857
rs6911413
rs17143400
rs62438218
rs145383023
rs11183780
rs116763898
rs142540499
rs67940439
rs7862897
rs74761367
rs139986130
rs7020426
rs62249160
rs10792502
rs10005003
rs142663429
rs9306374
rs7907281
rs17040530
All
−0.50
−0.25
0.00
0.25
MR leave−one−out sensitivity analysis for
'Gramella' on 'GCST90199865_buildGRCh38.tsv'

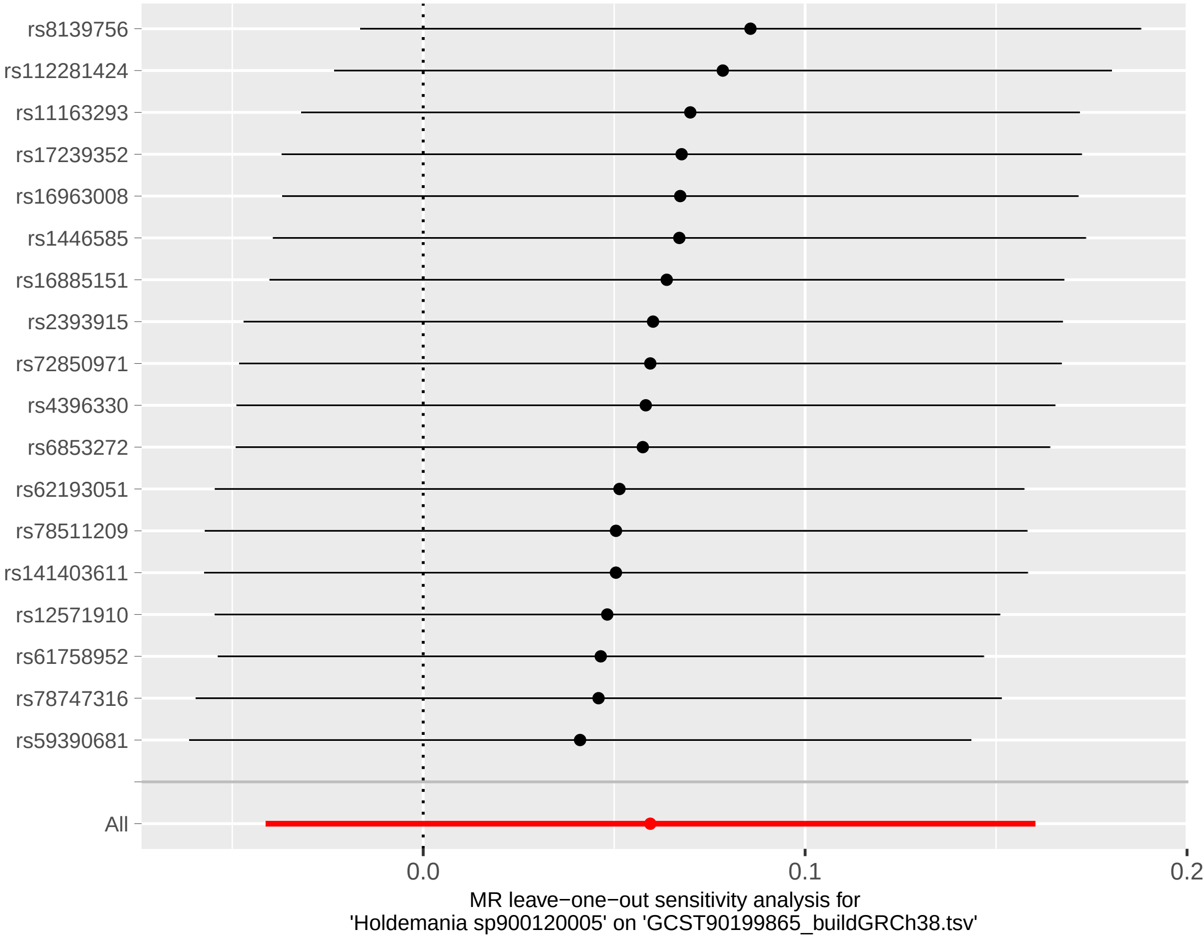
rs8139756
rs112281424
rs11163293
rs17239352
rs16963008
rs1446585
rs16885151
rs2393915
rs72850971
rs4396330
rs6853272
rs62193051
rs78511209
rs141403611
rs12571910
rs61758952
rs78747316
rs59390681
All
0.0
0.1
0.2
MR leave−one−out sensitivity analysis for
'Holdemania sp900120005' on 'GCST90199865_buildGRCh38.tsv'

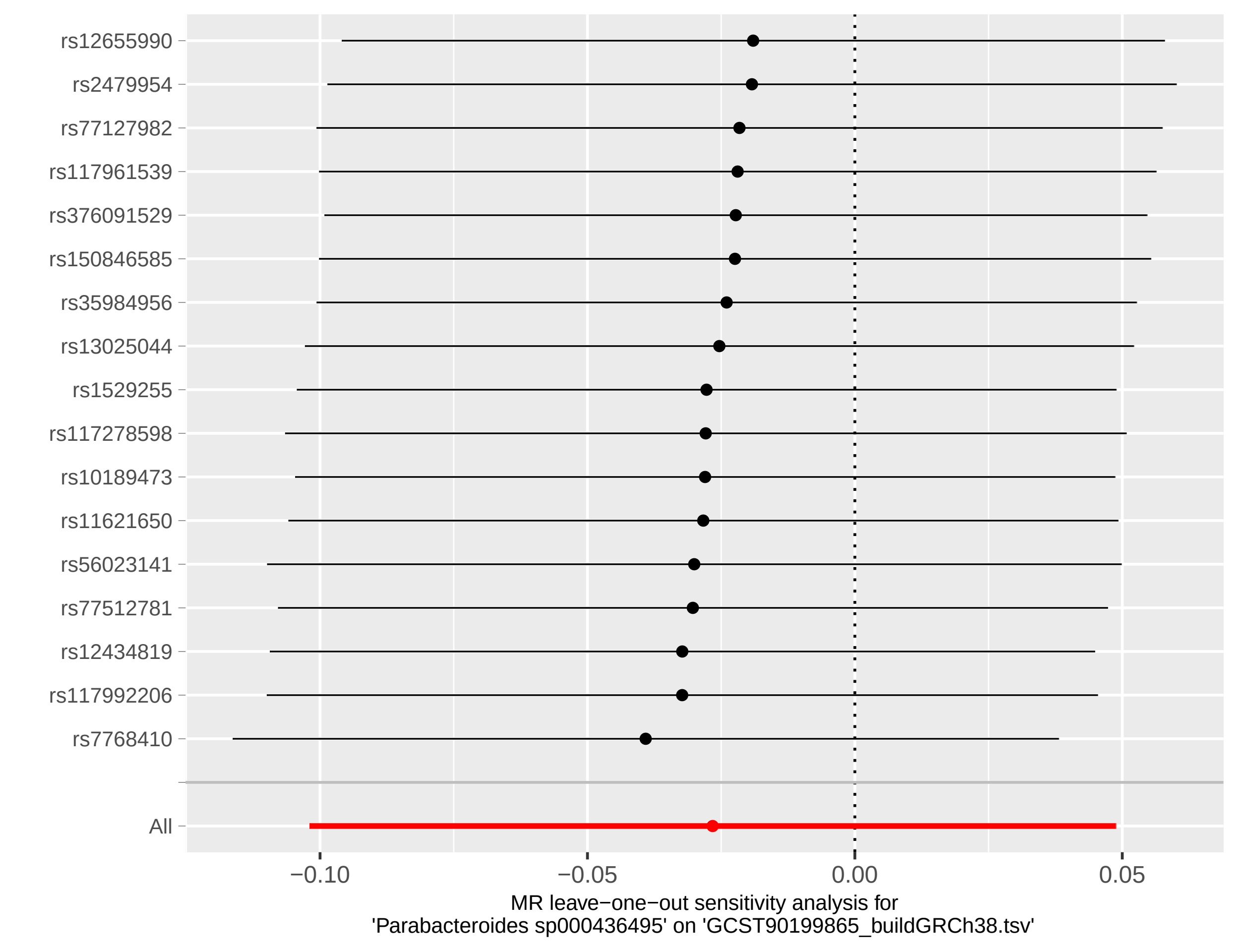
rs12655990
rs2479954
rs77127982
rs117961539
rs376091529
rs150846585
rs35984956
rs13025044
rs1529255
rs117278598
rs10189473
rs11621650
rs56023141
rs77512781
rs12434819
rs117992206
rs7768410
All
−0.10
−0.05
0.00
0.05
MR leave−one−out sensitivity analysis for
'Parabacteroides sp000436495' on 'GCST90199865_buildGRCh38.tsv'

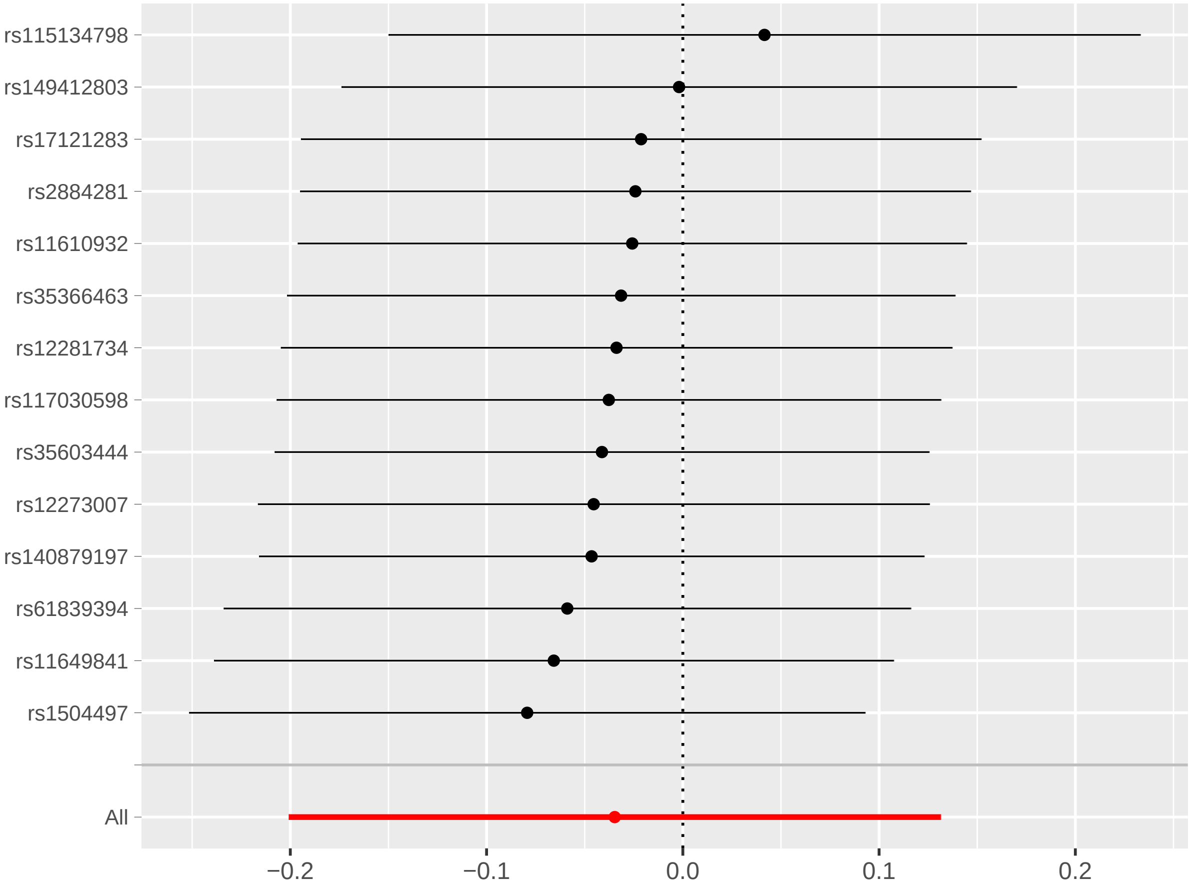

MR leave−one−out sensitivity analysis for
'Paramuribaculum sp001689565' on 'GCST90199865_buildGRCh38.tsv'

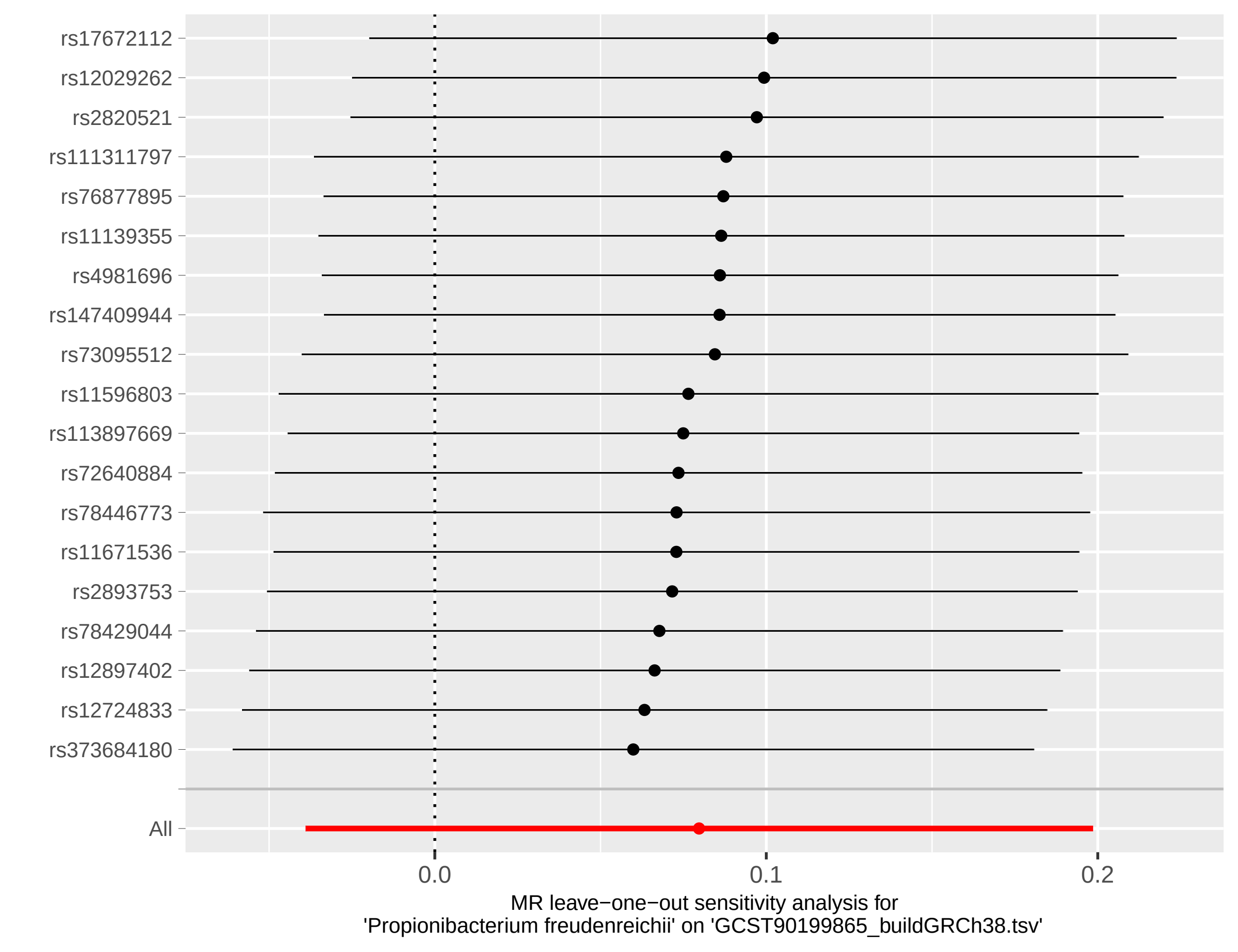
rs17672112
rs12029262
rs2820521
rs111311797
rs76877895
rs11139355
rs4981696
rs147409944
rs73095512
rs11596803
rs113897669
rs72640884
rs78446773
rs11671536
rs2893753
rs78429044
rs12897402
rs12724833
rs373684180
All
0.0
0.1
0.2
MR leave−one−out sensitivity analysis for
'Propionibacterium freudenreichii' on 'GCST90199865_buildGRCh38.tsv'

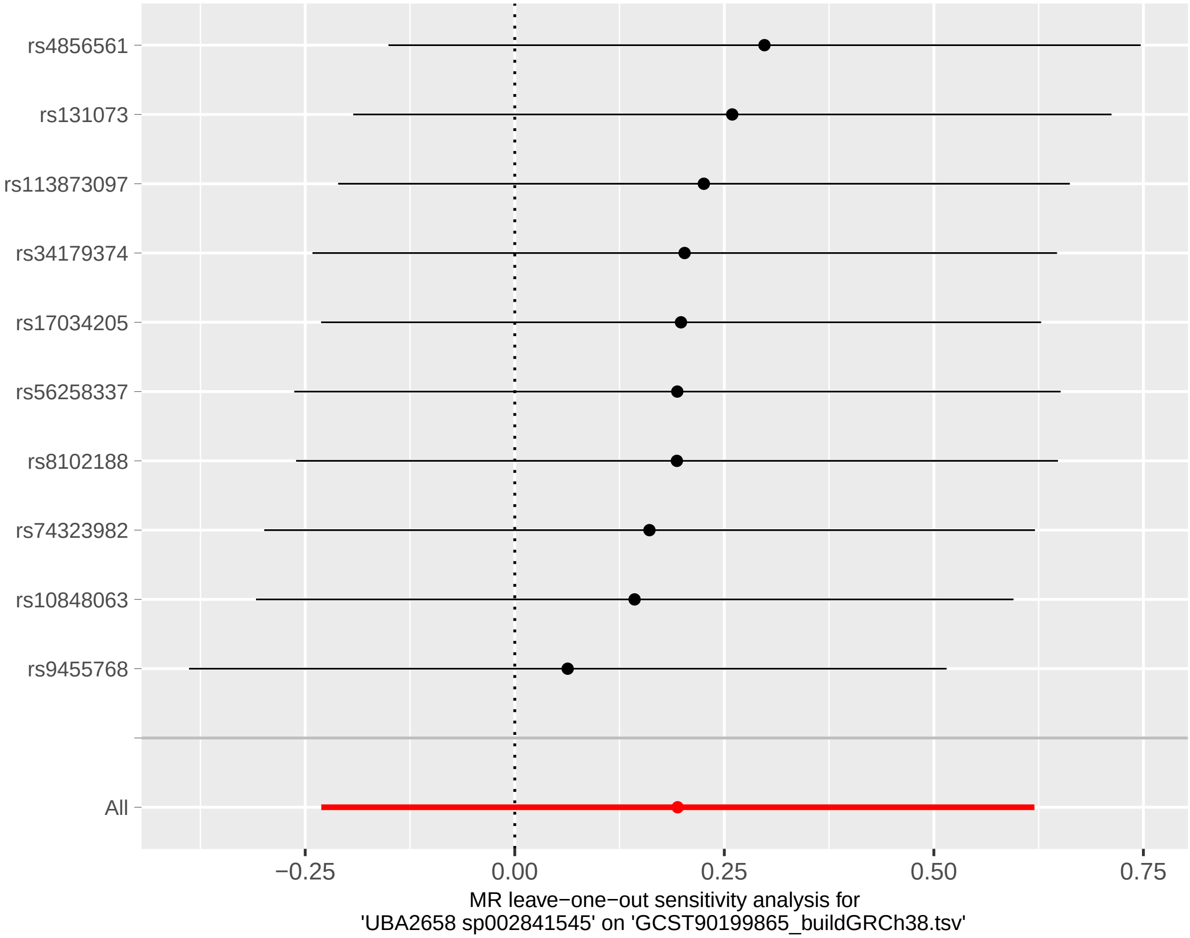
rs4856561
rs131073
rs113873097
rs34179374
rs17034205
rs56258337
rs8102188
rs74323982
rs10848063
rs9455768
All
−0.25
0.00
0.25
0.50
0.75
MR leave−one−out sensitivity analysis for
'UBA2658 sp002841545' on 'GCST90199865_buildGRCh38.tsv'

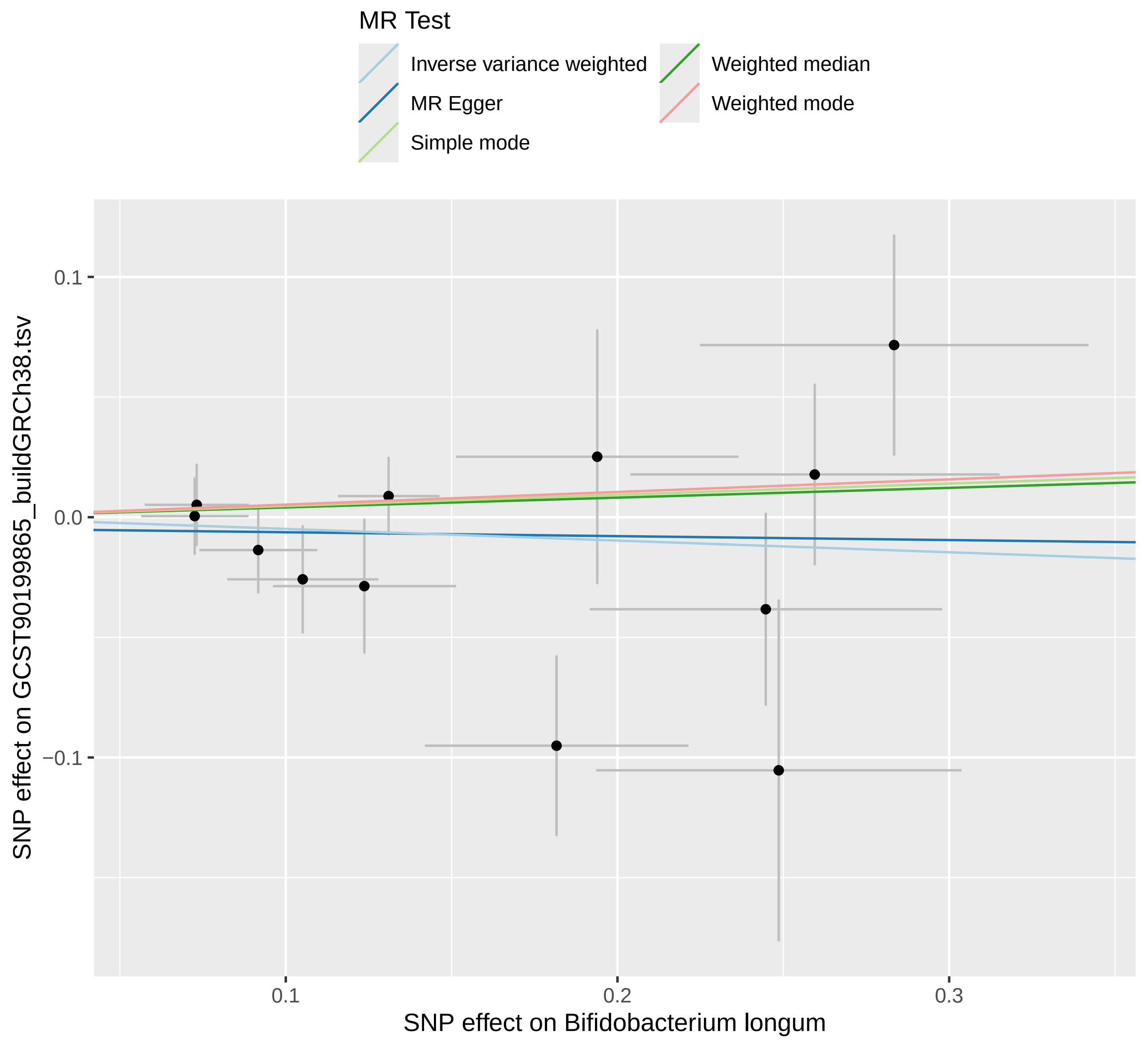
MR Test
Inverse variance weighted
MR Egger
Simple mode
Weighted median
Weighted mode
0.1
0.0
−0.1
0.1
0.2
0.3
SNP effect on GCST90199865_buildGRCh38.tsv
SNP effect on Bifidobacterium longum

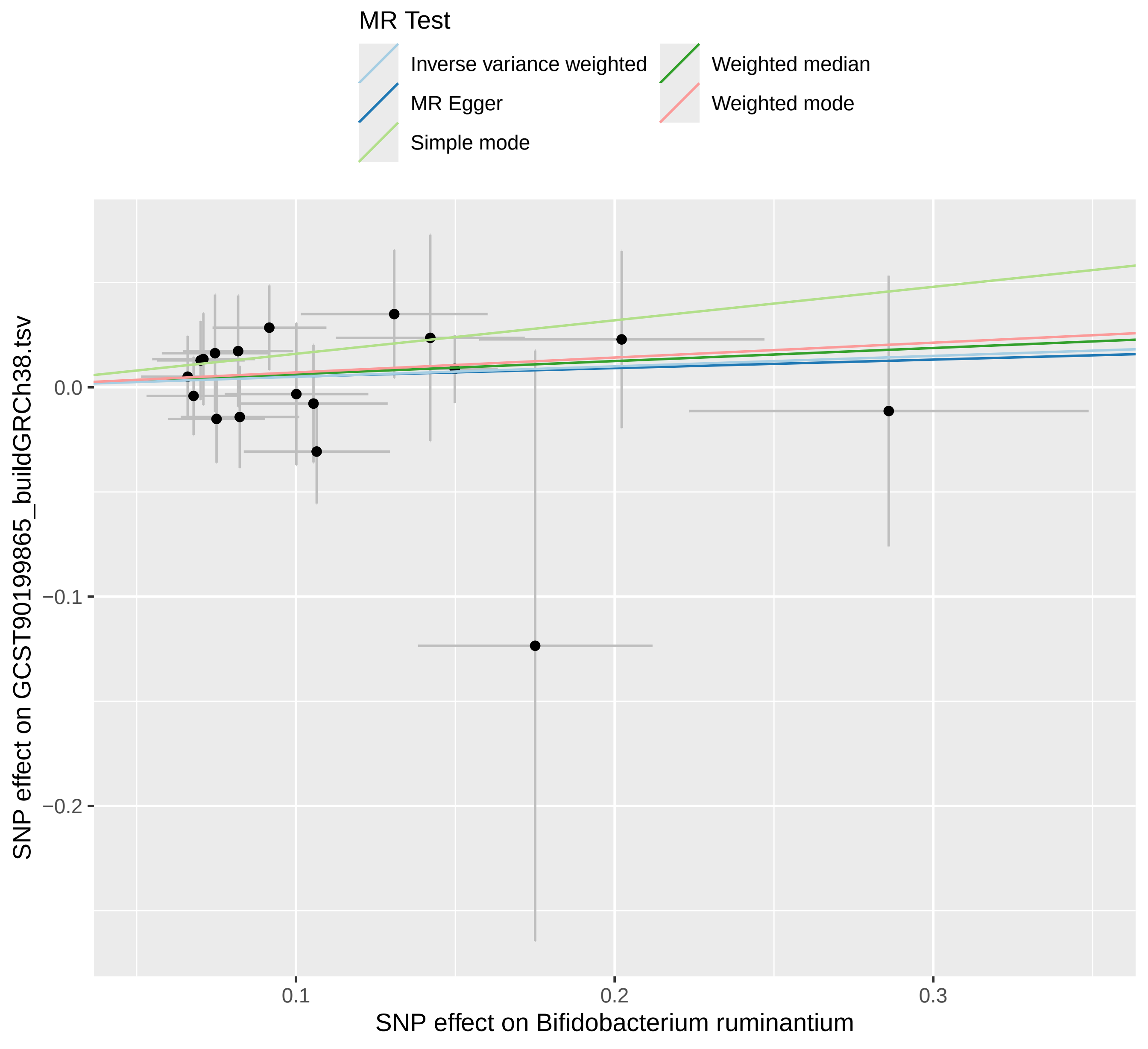
MR Test
Inverse variance weighted
MR Egger
Simple mode
Weighted median
Weighted mode
SNP effect on GCST90199865_buildGRCh38.tsv
0.0
−0.1
−0.2
0.1
0.2
0.3
SNP effect on Bifidobacterium ruminantium

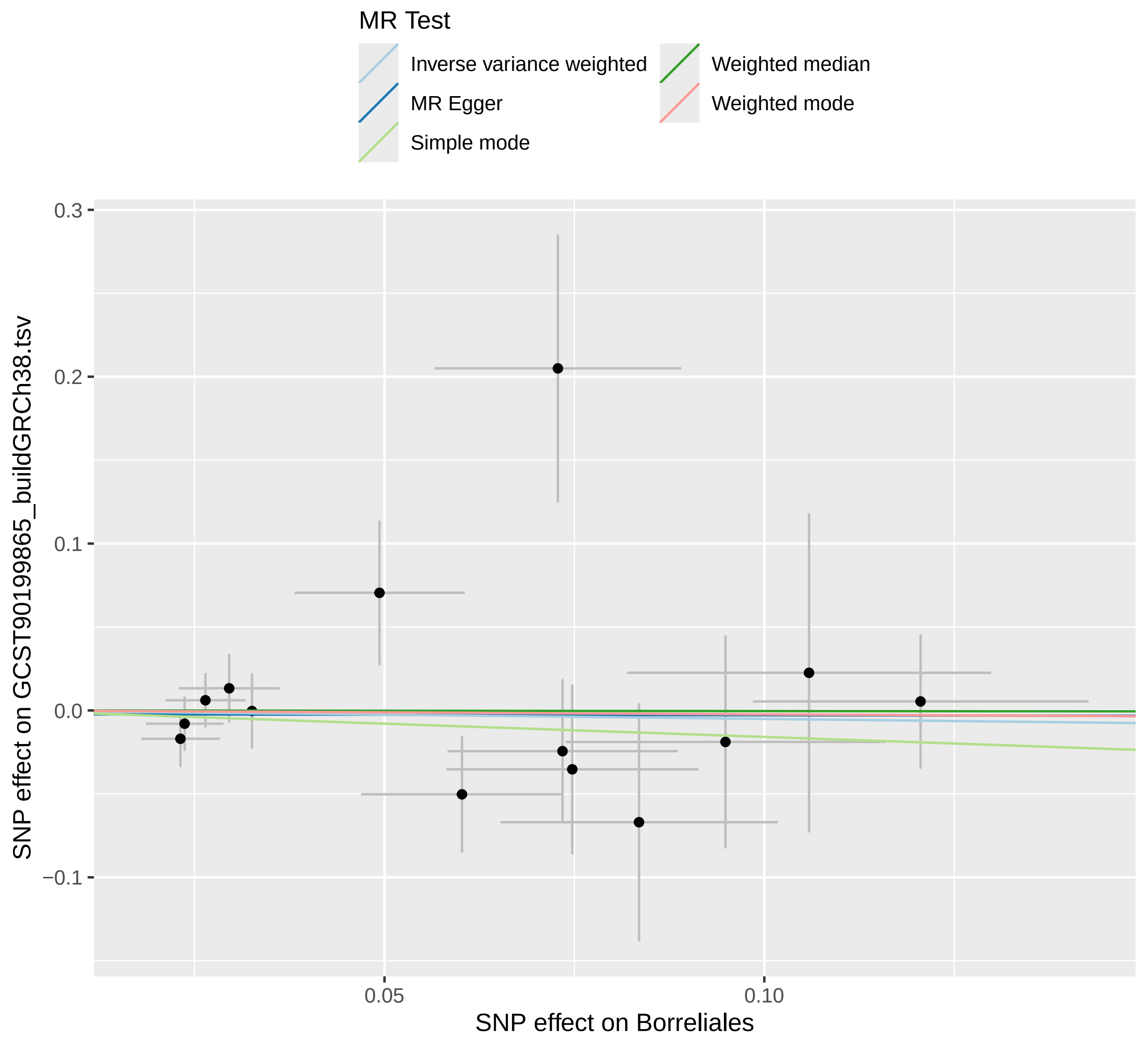
MR Test
Inverse variance weighted
MR Egger
Simple mode
Weighted median
Weighted mode
0.3
0.2
0.1
0.0
−0.1
0.05
0.10
SNP effect on GCST90199865_buildGRCh38.tsv
SNP effect on Borreliales

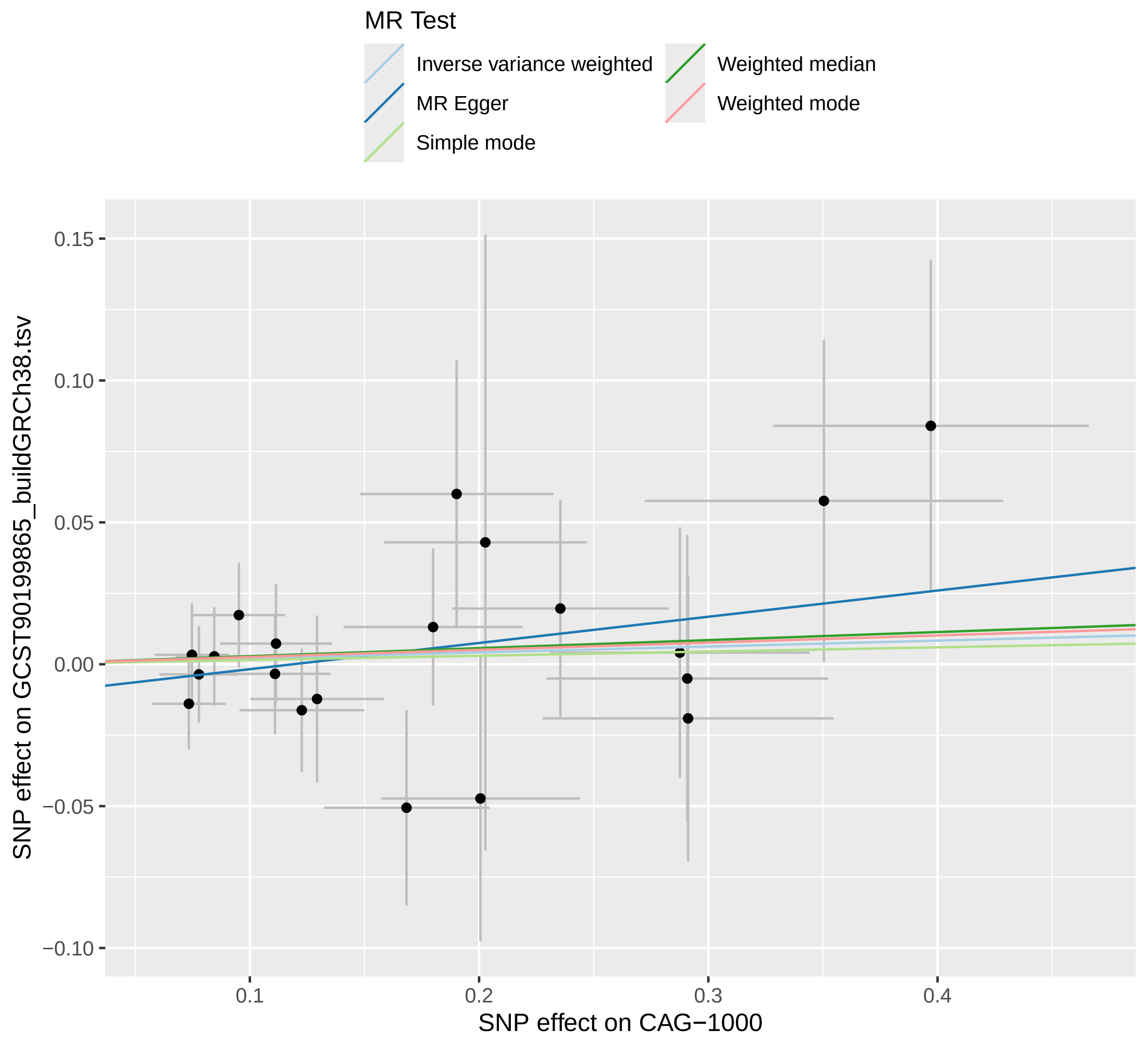
MR Test
Inverse variance weighted
MR Egger
Simple mode
Weighted median
Weighted mode
0.15
0.10
0.05
0.00
−0.05
−0.10
0.1
0.2
0.3
0.4
SNP effect on GCST90199865_buildGRCh38.tsv
SNP effect on CAG−1000

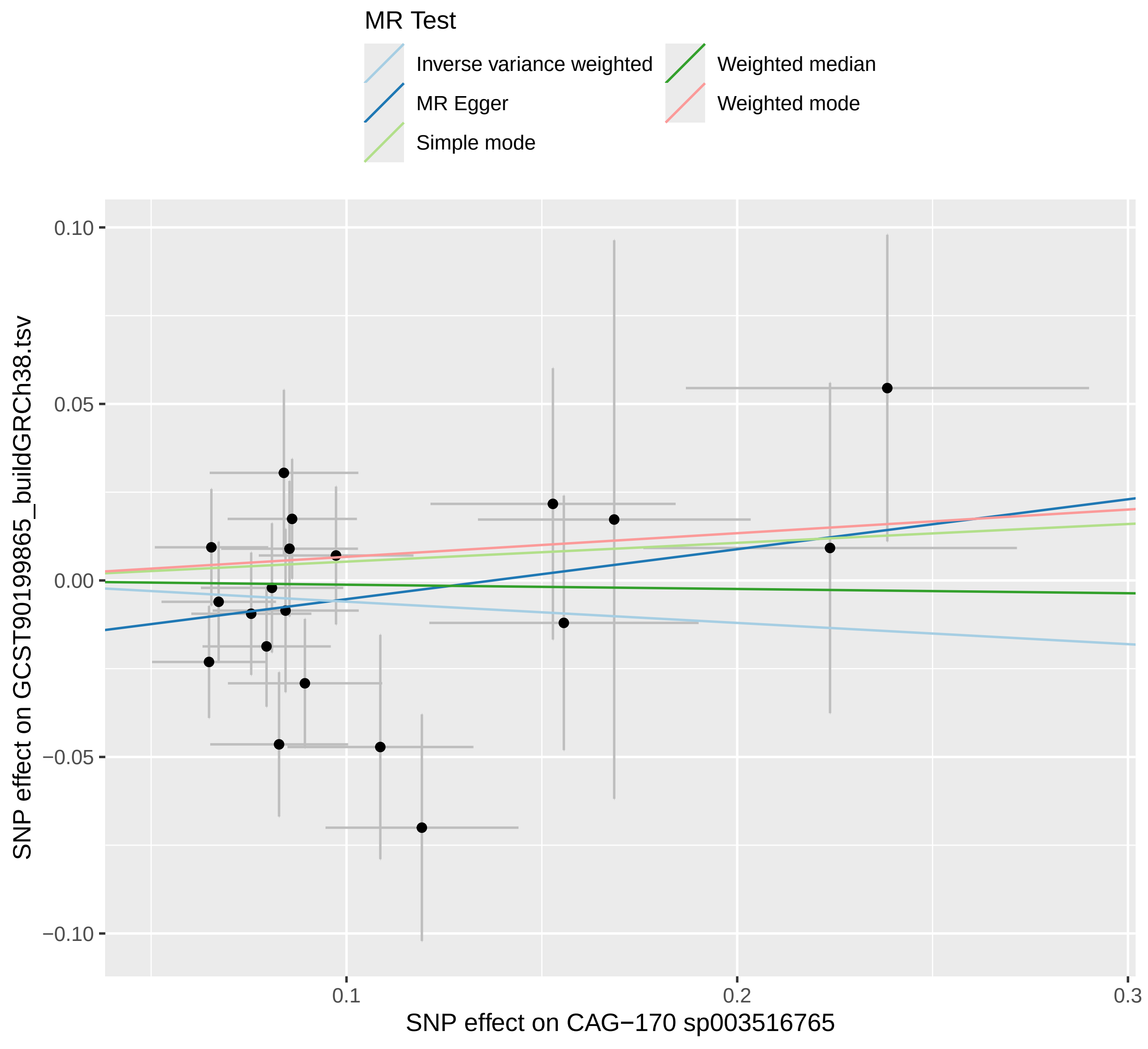
MR Test
Inverse variance weighted
MR Egger
Simple mode
Weighted median
Weighted mode
0.10
0.05
0.00
−0.05
−0.10
0.1
0.2
0.3
SNP effect on GCST90199865_buildGRCh38.tsv
SNP effect on CAG−170 sp003516765

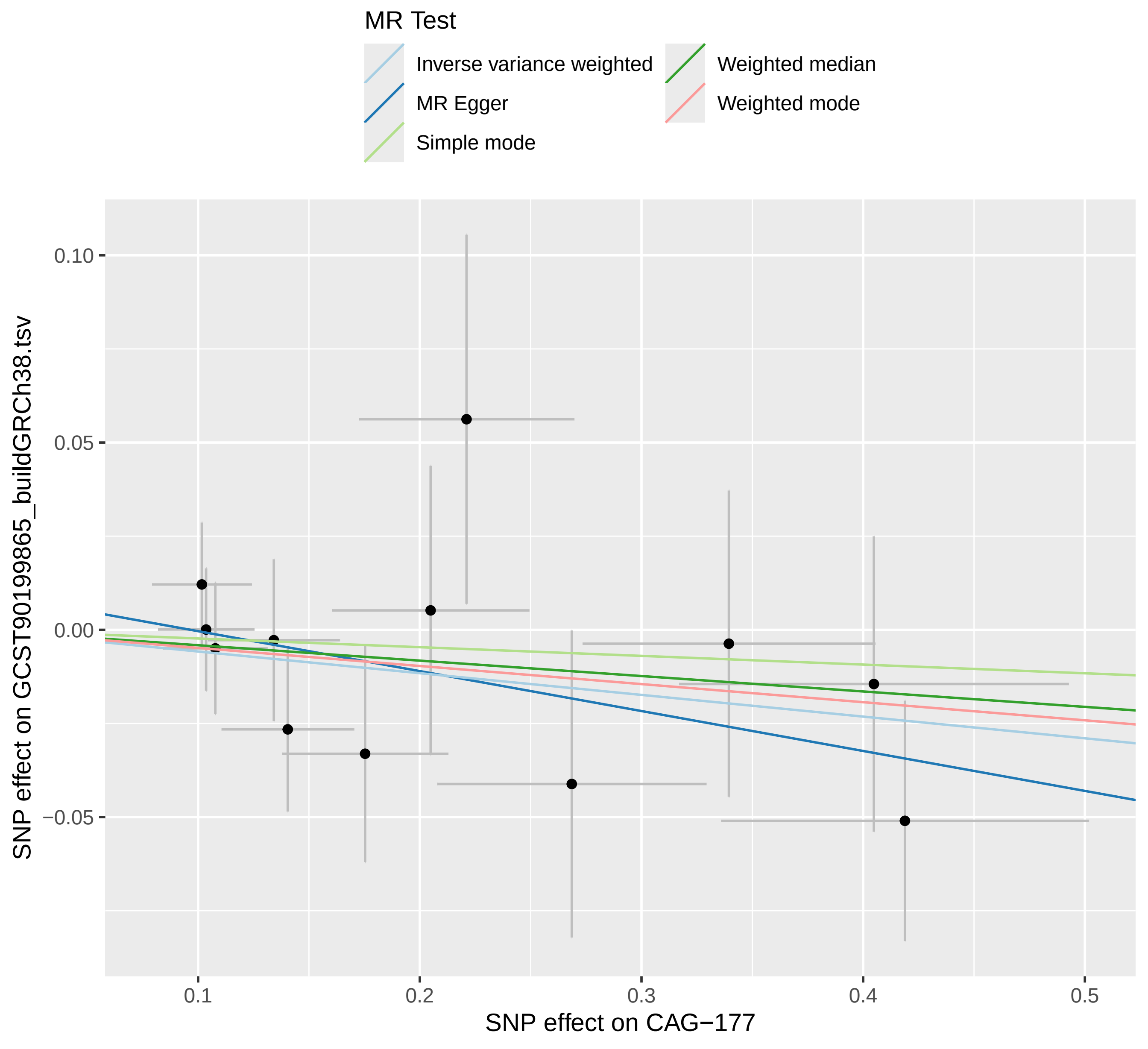

MR Test
Inverse variance weighted
MR Egger
Simple mode
Weighted median
Weighted mode
SNP effect on GCST90199865_buildGRCh38.tsv
0.10
0.05
0.00
−0.05
0.1
0.2
0.3
0.4
0.5
SNP effect on CAG−177

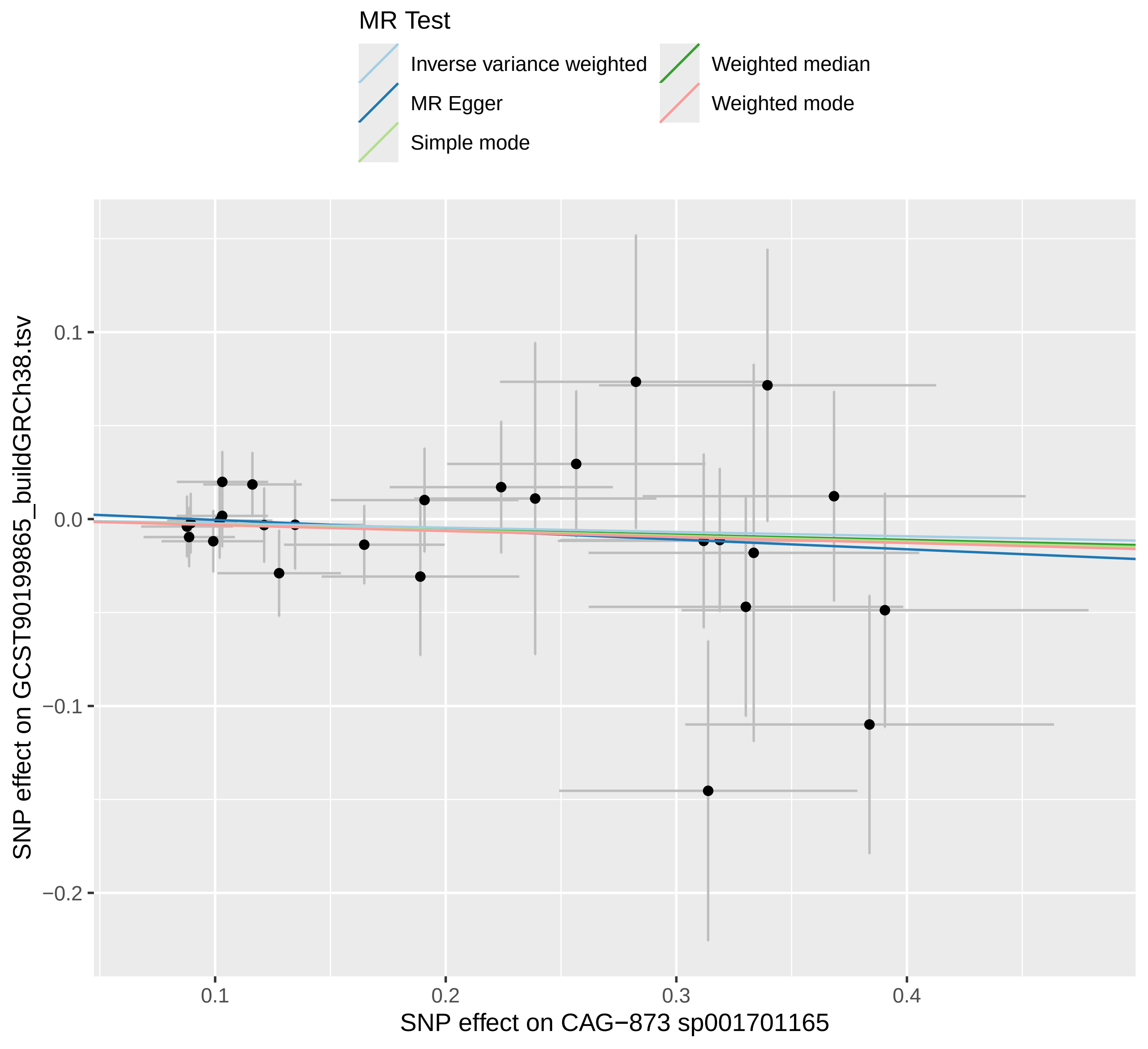

MR Test
Inverse variance weighted
MR Egger
Simple mode
Weighted median
Weighted mode
0.1
0.0
−0.1
−0.2
0.1
0.2
0.3
0.4
SNP effect on GCST90199865_buildGRCh38.tsv
SNP effect on CAG−873 sp001701165

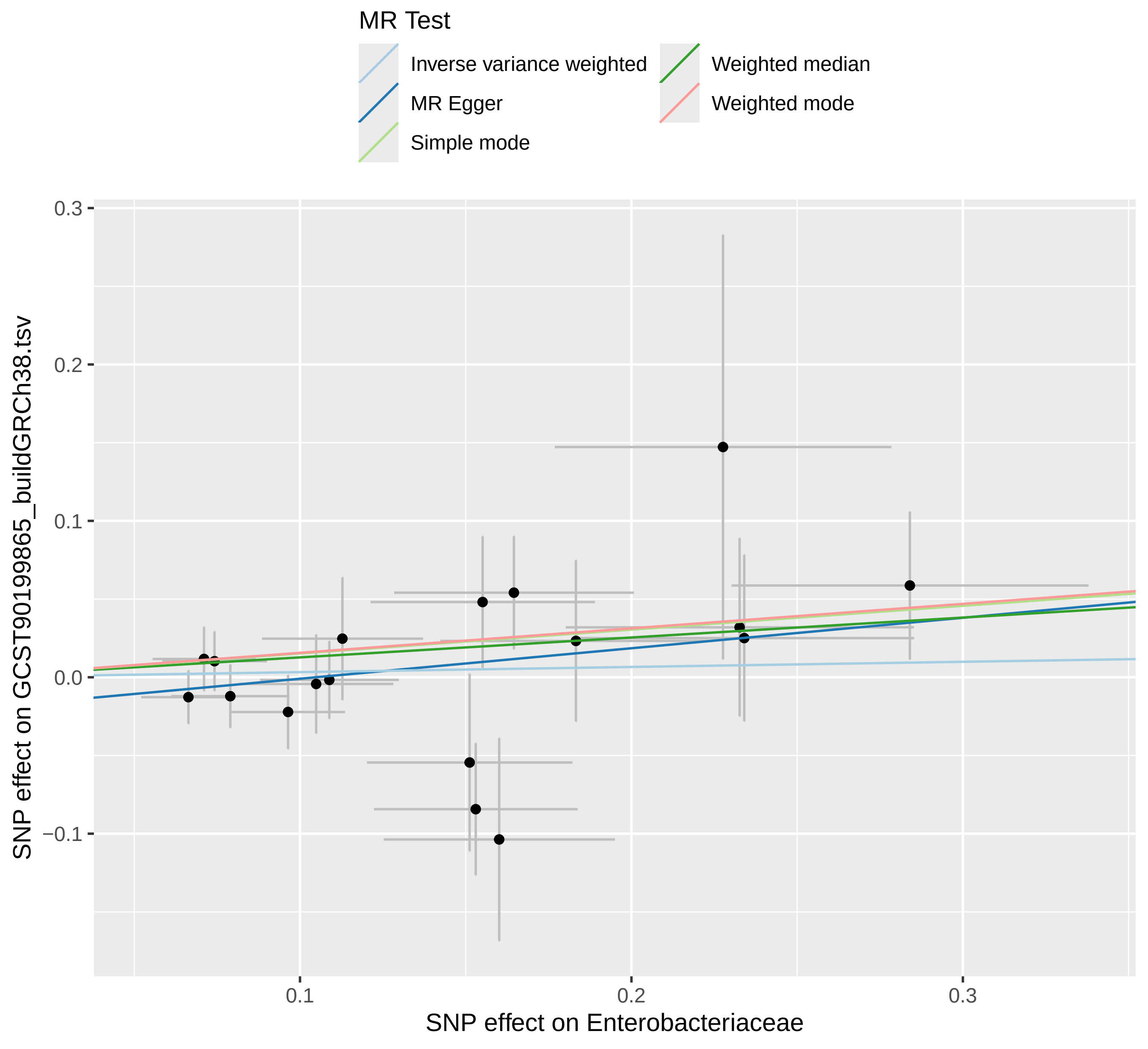
MR Test
Inverse variance weighted
MR Egger
Simple mode
Weighted median
Weighted mode
SNP effect on GCST90199865_buildGRCh38.tsv
0.3
0.2
0.1
0.0
−0.1
0.1
0.2
0.3
SNP effect on Enterobacteriaceae

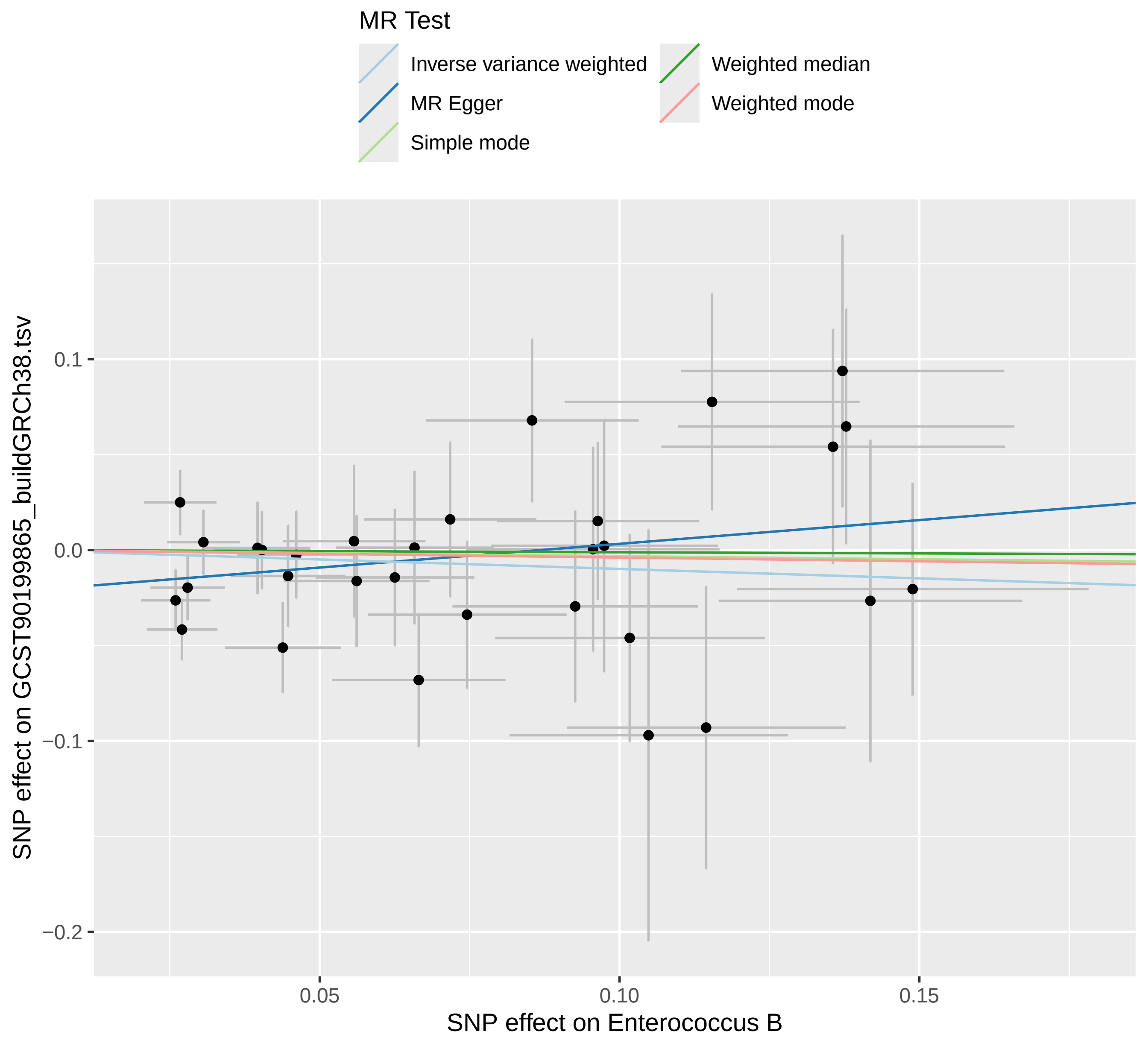
MR Test
Inverse variance weighted
MR Egger
Simple mode
Weighted median
Weighted mode
SNP effect on GCST90199865_buildGRCh38.tsv
0.1
0.0
−0.1
−0.2
0.05
0.10
0.15
SNP effect on Enterococcus B

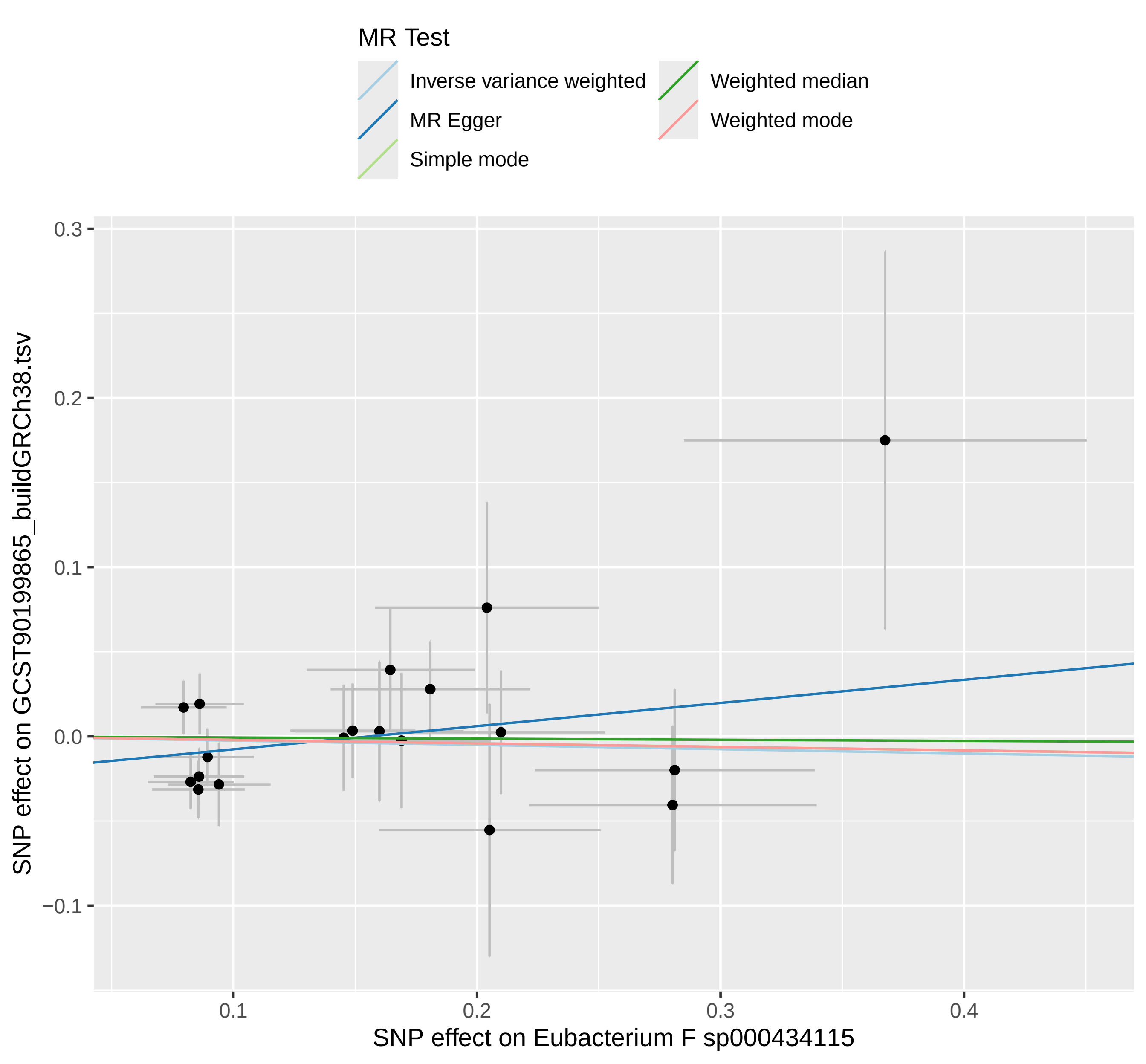

MR Test
Inverse variance weighted
MR Egger
Simple mode
Weighted median
Weighted mode
0.3
0.2
0.1
0.0
−0.1
0.1
0.2
0.3
0.4
SNP effect on GCST90199865_buildGRCh38.tsv
SNP effect on Eubacterium F sp000434115

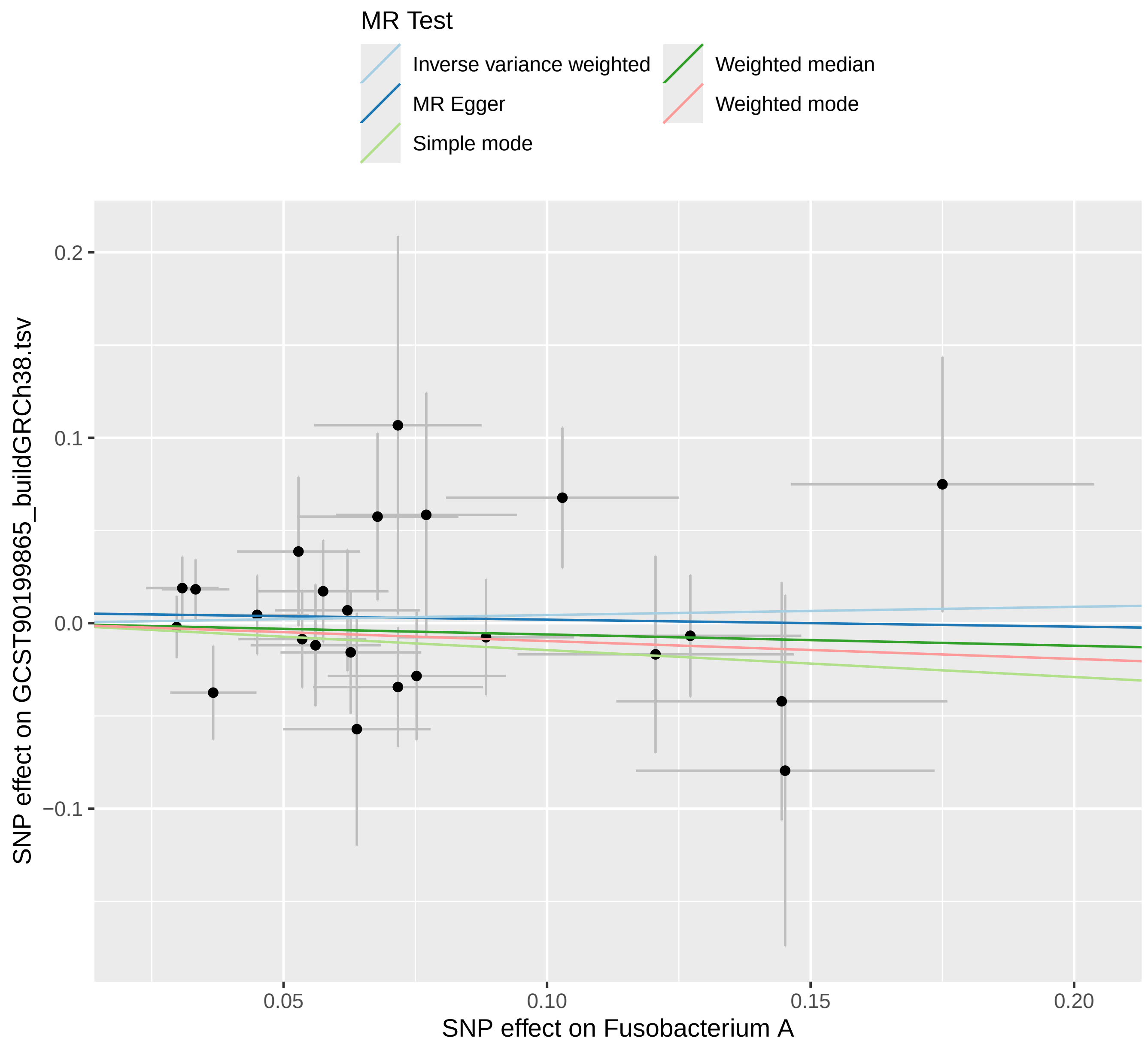
MR Test
Inverse variance weighted
MR Egger
Simple mode
Weighted median
Weighted mode
0.2
0.1
0.0
−0.1
SNP effect on GCST90199865_buildGRCh38.tsv
0.05
0.10
0.15
0.20
SNP effect on Fusobacterium A

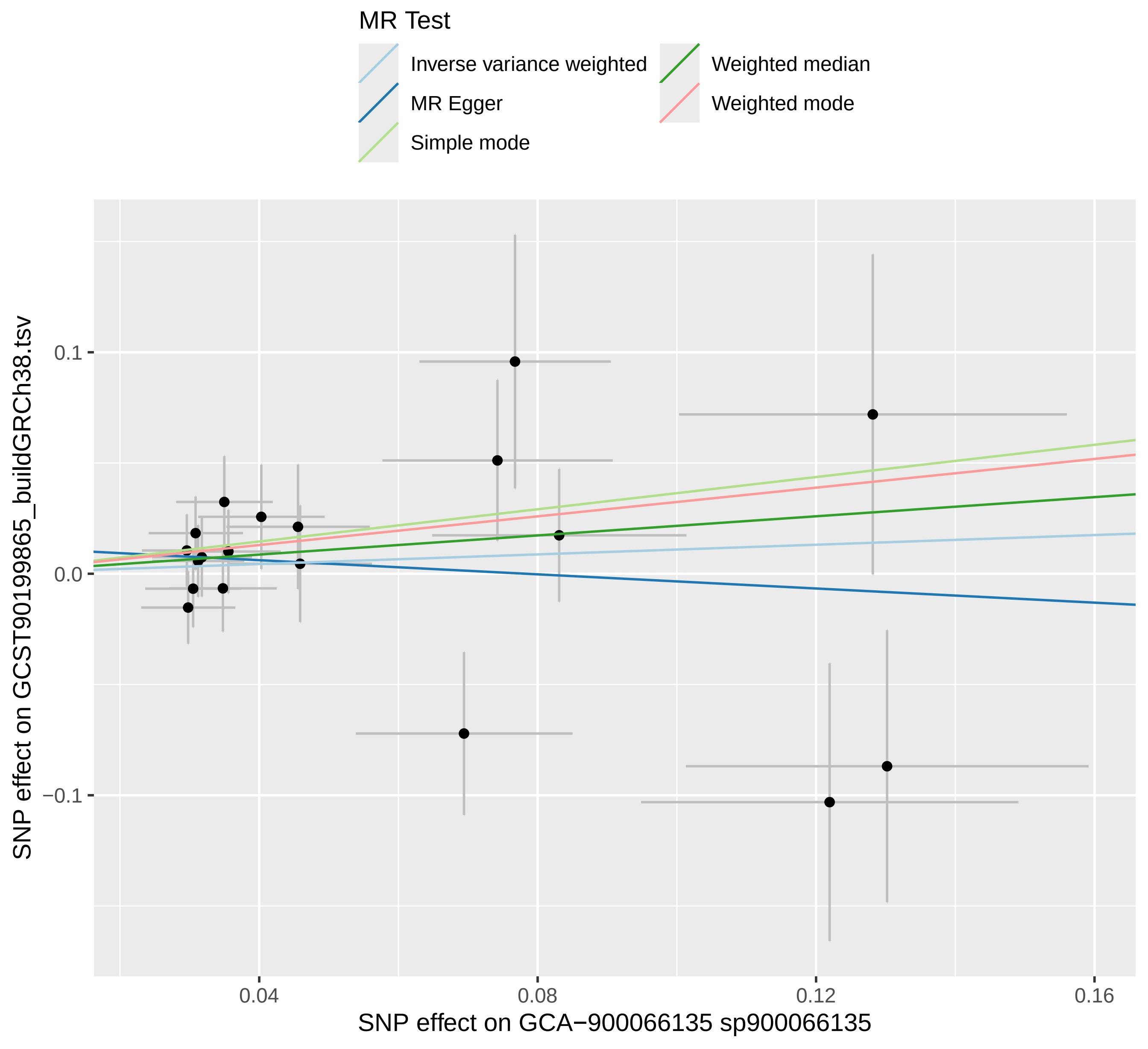

MR Test
Inverse variance weighted
MR Egger
Simple mode
Weighted median
Weighted mode
0.1
0.0
−0.1
0.04
0.08
0.12
0.16
SNP effect on GCST90199865_buildGRCh38.tsv
SNP effect on GCA−900066135 sp900066135

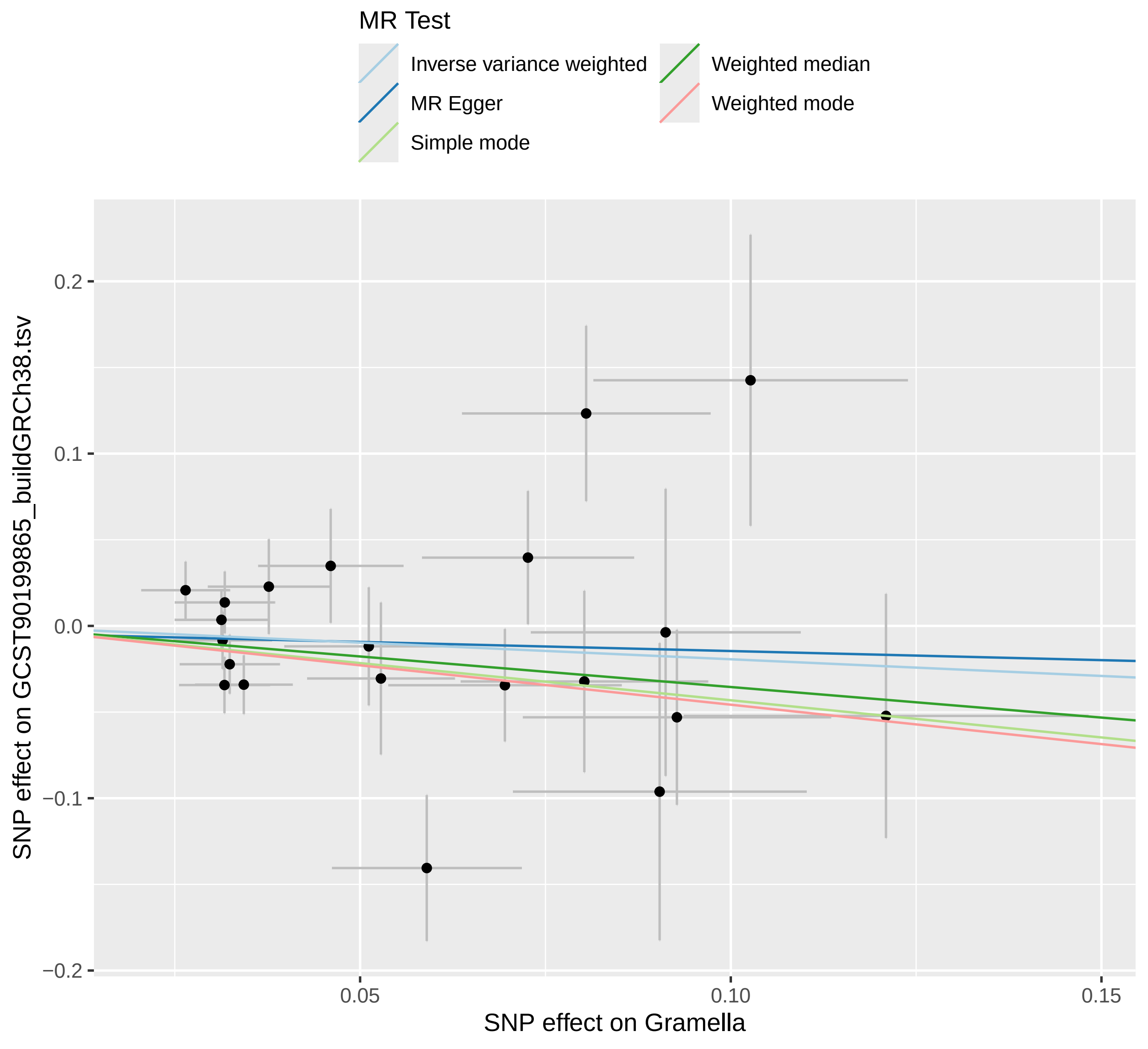

MR Test
Inverse variance weighted
MR Egger
Simple mode
Weighted median
Weighted mode
SNP effect on GCST90199865_buildGRCh38.tsv
0.2
0.1
0.0
−0.1
−0.2
0.05
0.10
0.15
SNP effect on Gramella

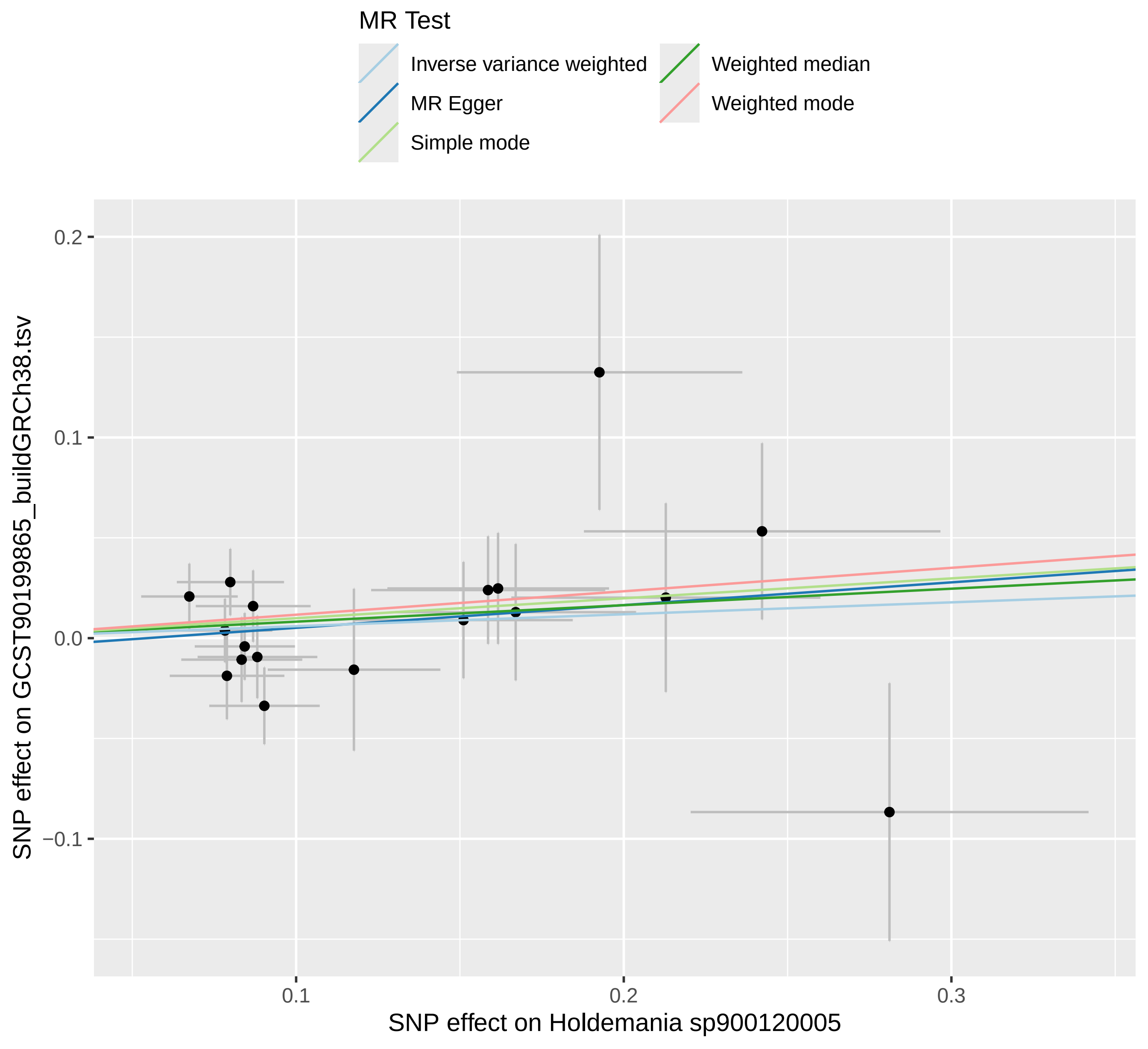
MR Test
Inverse variance weighted
MR Egger
Simple mode
Weighted median
Weighted mode
0.2
0.1
0.0
−0.1
0.1
0.2
0.3
SNP effect on GCST90199865_buildGRCh38.tsv
SNP effect on Holdemania sp900120005

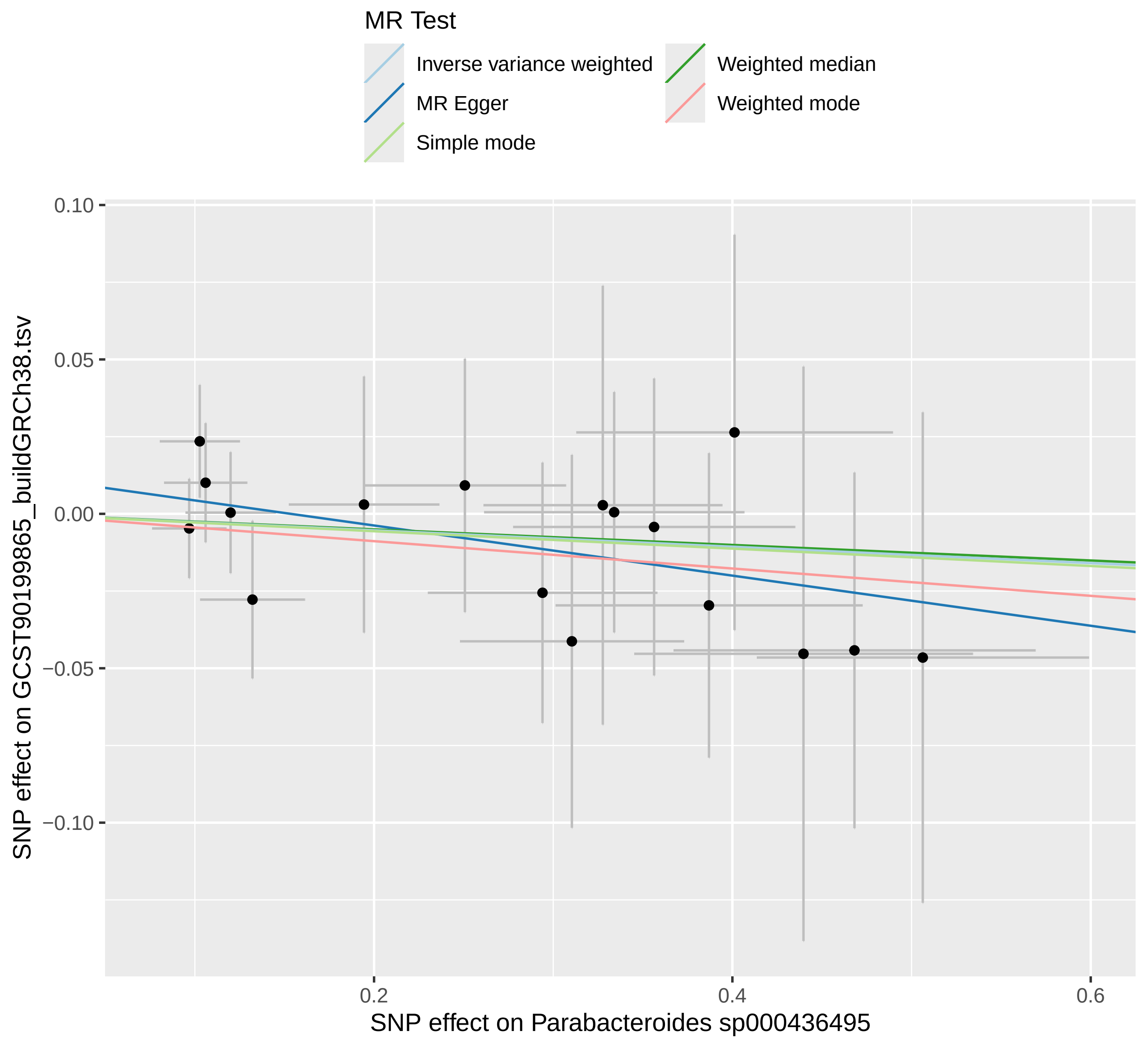

MR Test
Inverse variance weighted
MR Egger
Simple mode
Weighted median
Weighted mode
0.10
0.05
0.00
−0.05
−0.10
0.2
0.4
0.6
SNP effect on GCST90199865_buildGRCh38.tsv
SNP effect on Parabacteroides sp000436495

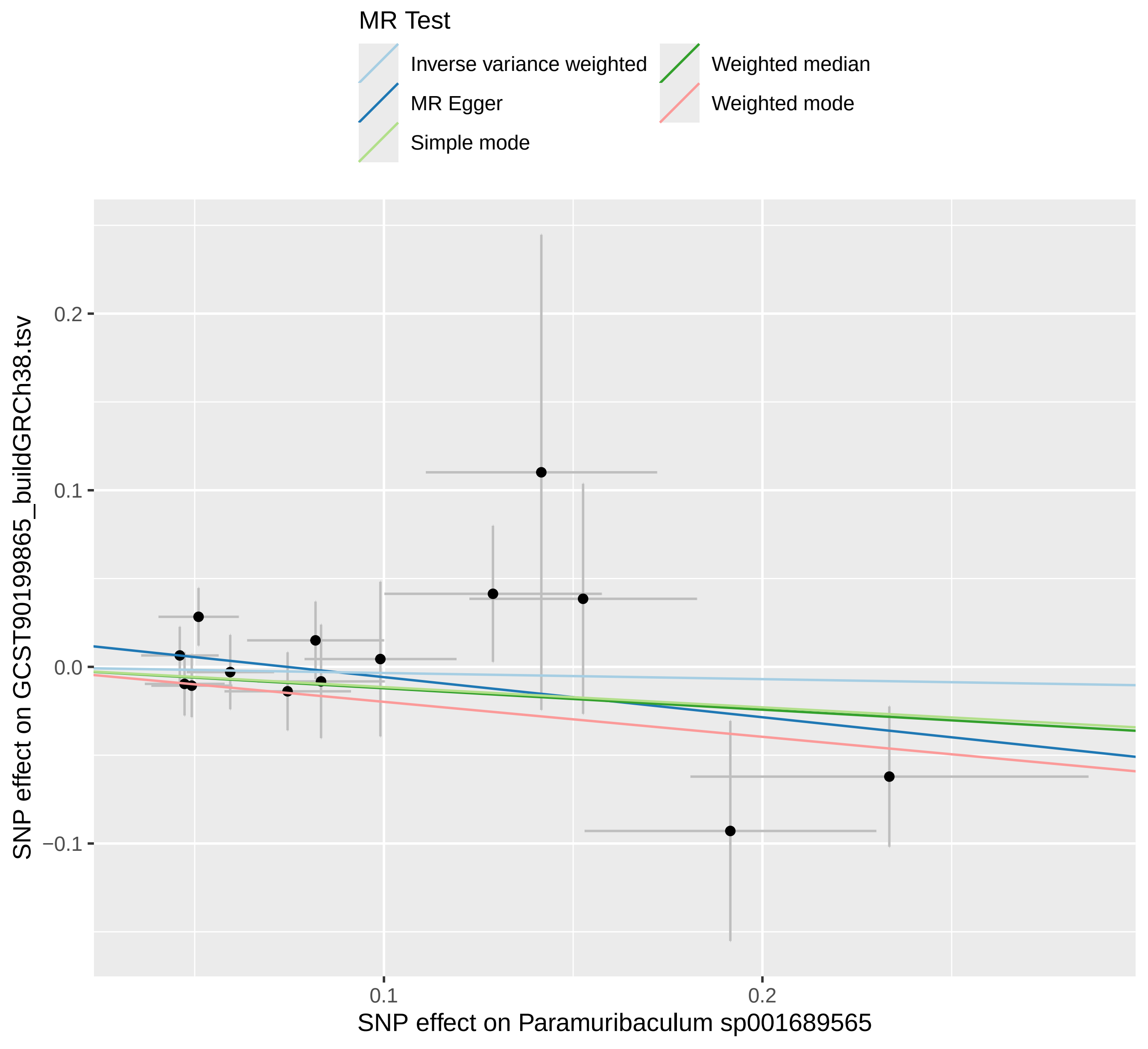
MR Test
Inverse variance weighted
MR Egger
Simple mode
Weighted median
Weighted mode
0.2
0.1
0.0
−0.1
0.1
0.2
SNP effect on GCST90199865_buildGRCh38.tsv
SNP effect on Paramuribaculum sp001689565

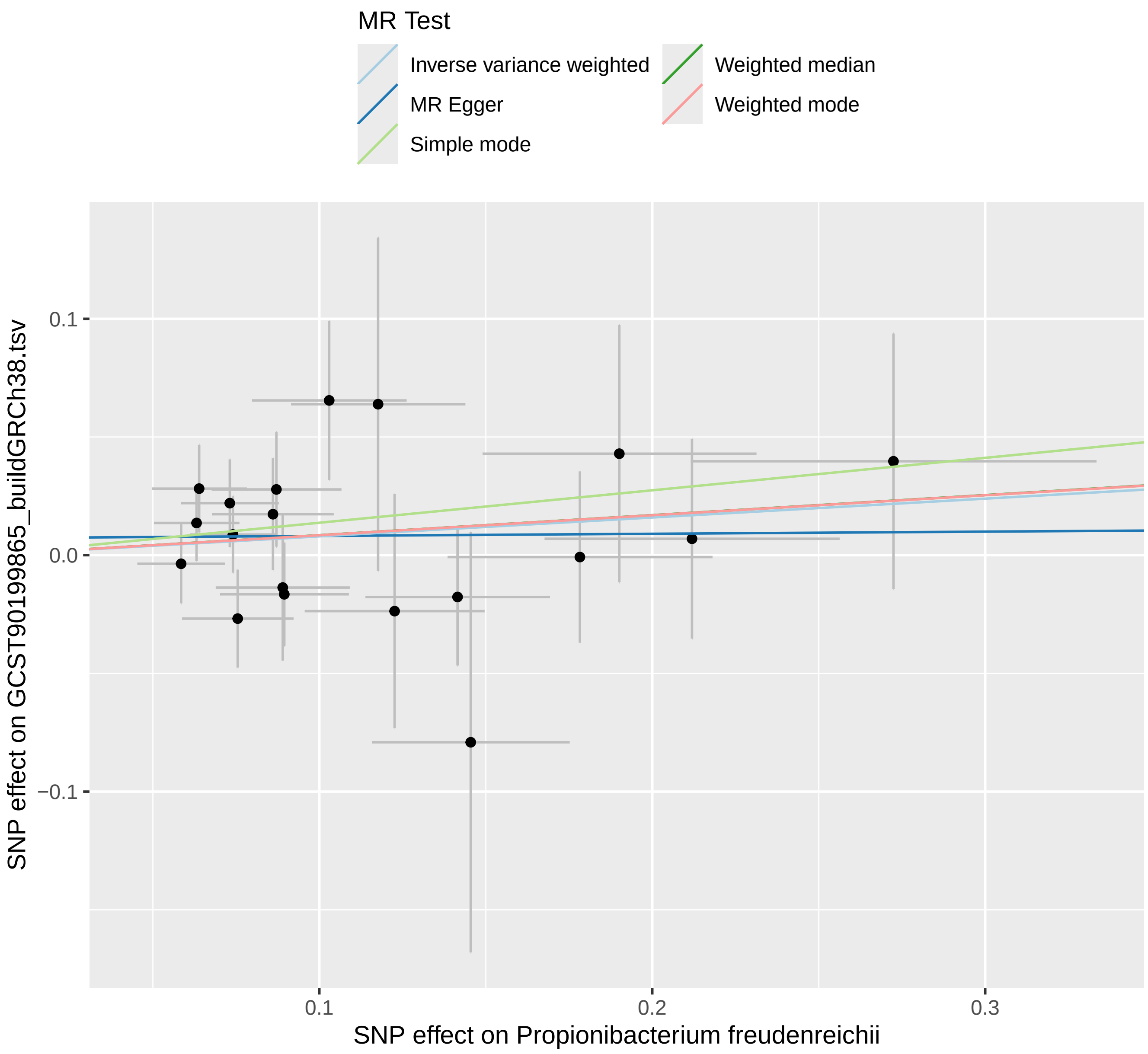

MR Test
Inverse variance weighted
MR Egger
Simple mode
Weighted median
Weighted mode
0.1
0.0
−0.1
0.1
0.2
0.3
SNP effect on GCST90199865_buildGRCh38.tsv
SNP effect on Propionibacterium freudenreichii

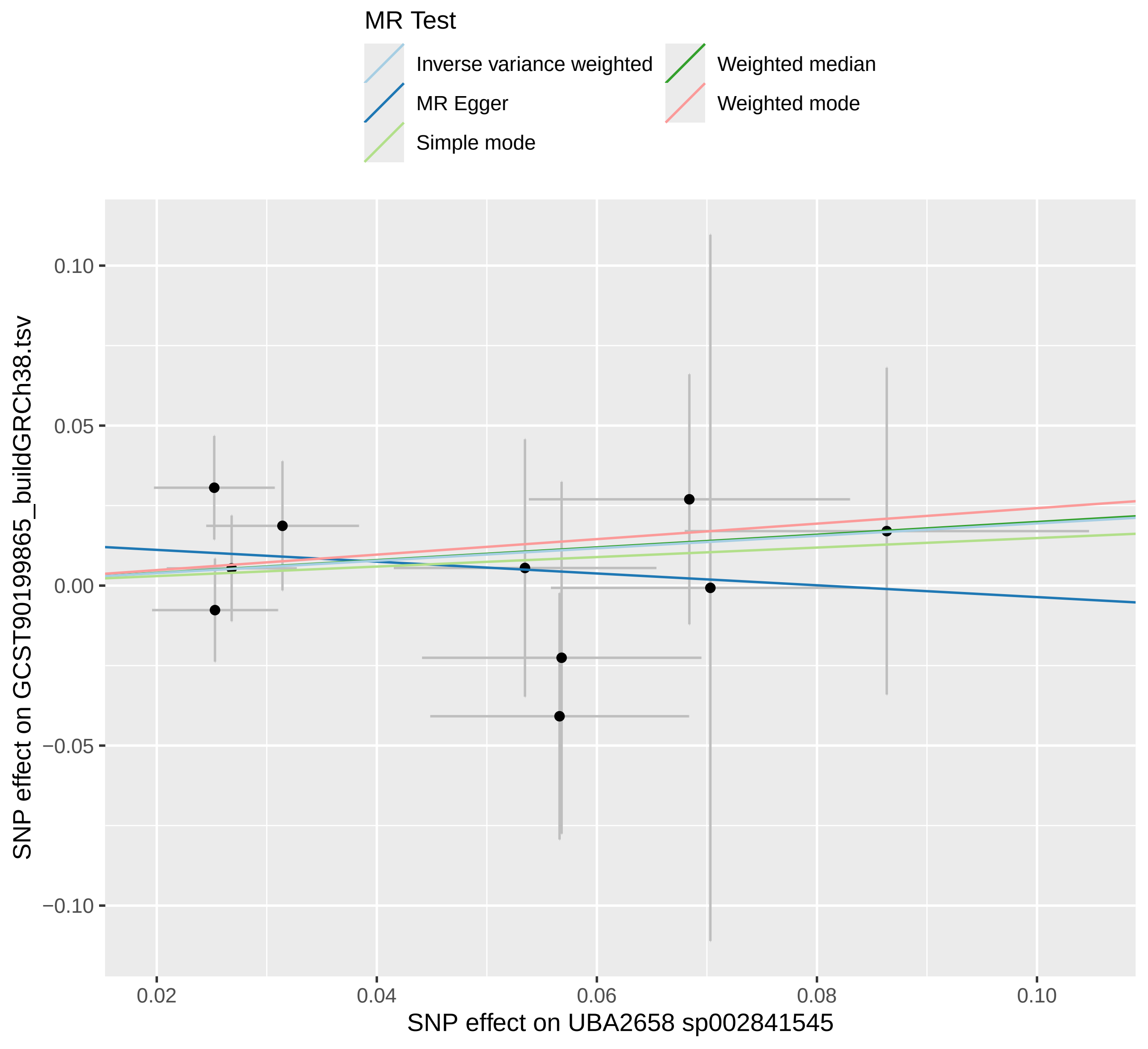
MR Test
Inverse variance weighted
MR Egger
Simple mode
Weighted median
Weighted mode
0.10
0.05
0.00
−0.05
−0.10
SNP effect on GCST90199865_buildGRCh38.tsv
0.02
0.04
0.06
0.08
0.10
SNP effect on UBA2658 sp002841545

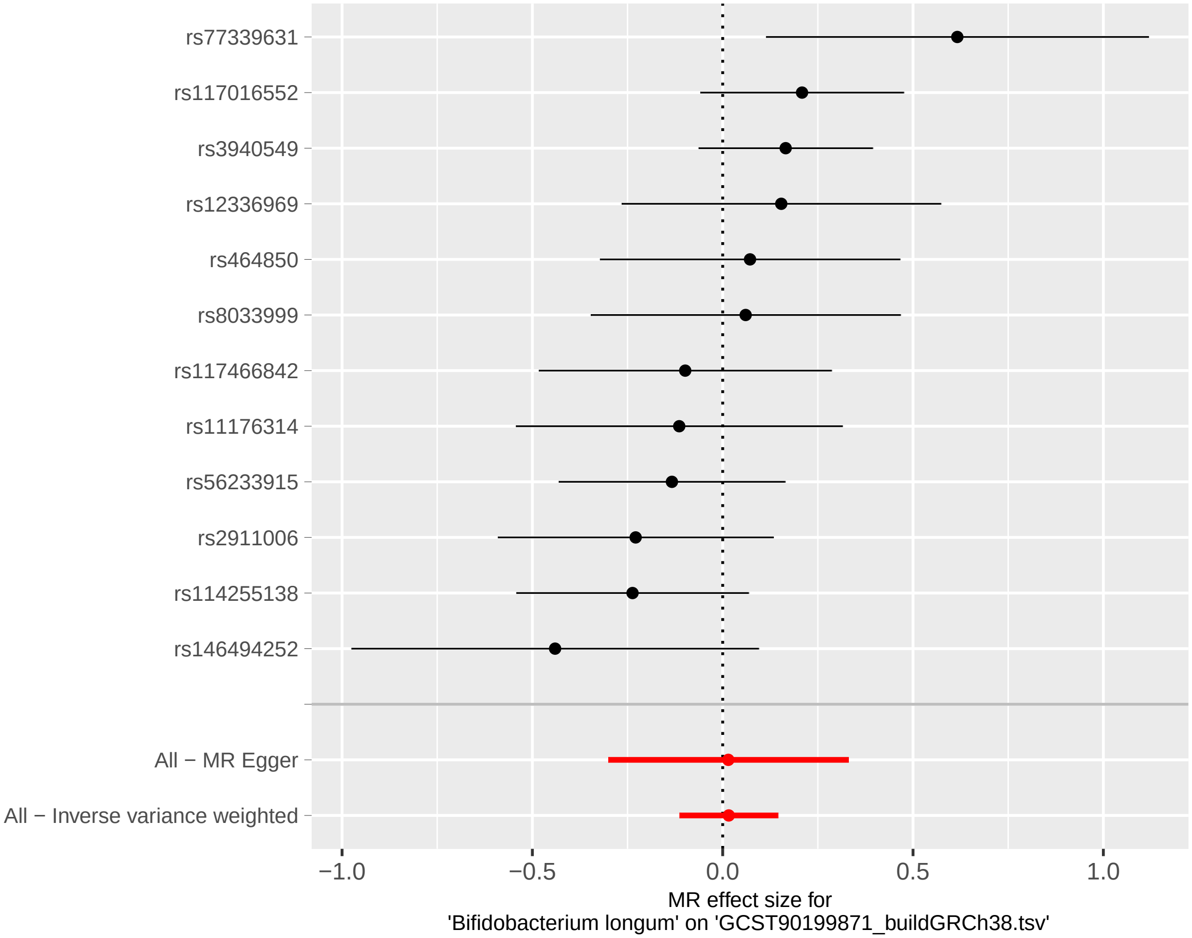
rs77339631
rs117016552
rs3940549
rs12336969
rs464850
rs8033999
rs117466842
rs11176314
rs56233915
rs2911006
rs114255138
rs146494252
All − MR Egger
All − Inverse variance weighted
−1.0
−0.5
0.0
0.5
1.0
MR effect size for
'Bifidobacterium longum' on 'GCST90199871_buildGRCh38.tsv'

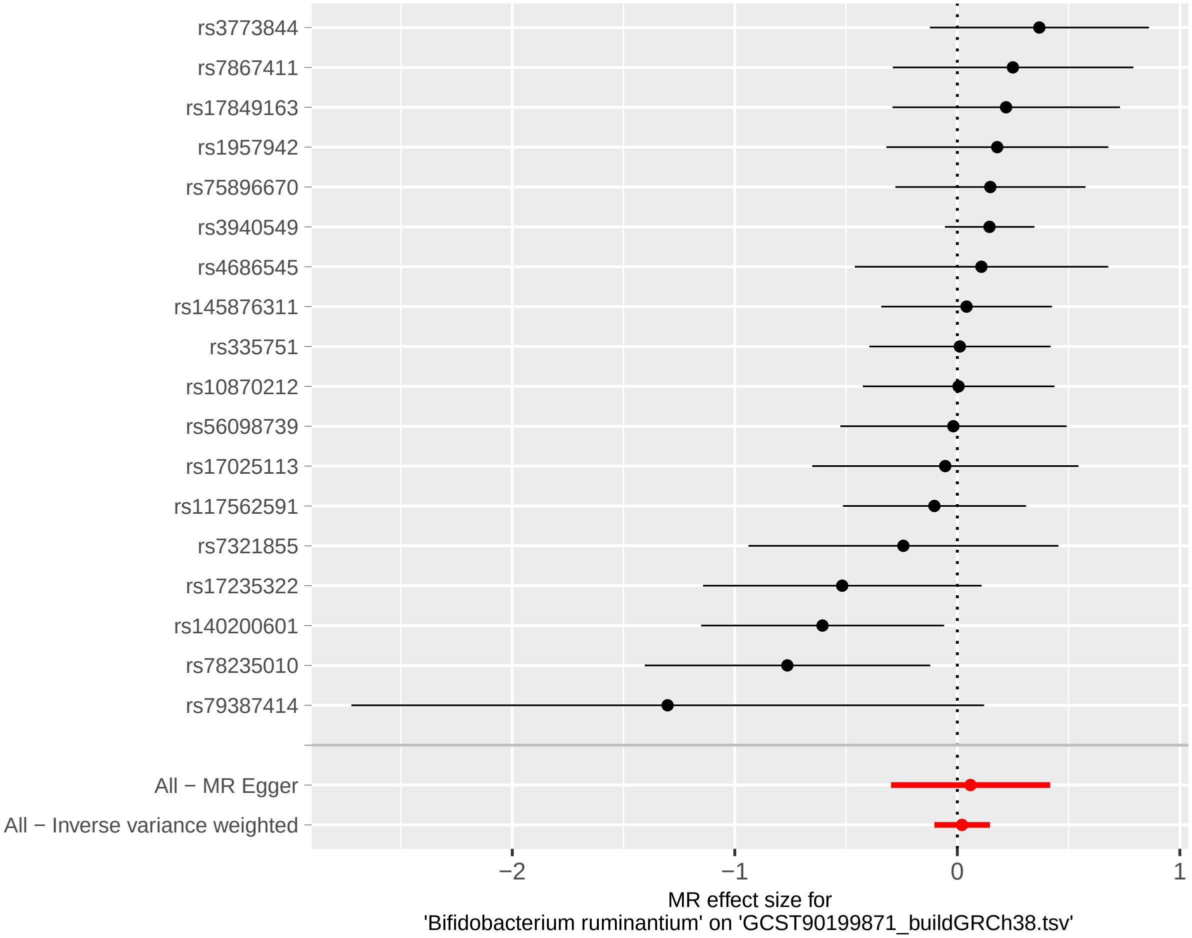
rs3773844
rs7867411
rs17849163
rs1957942
rs75896670
rs3940549
rs4686545
rs145876311
rs335751
rs10870212
rs56098739
rs17025113
rs117562591
rs7321855
rs17235322
rs140200601
rs78235010
rs79387414
All − MR Egger
All − Inverse variance weighted
−2
−1
0
1
MR effect size for
'Bifidobacterium ruminantium' on 'GCST90199871_buildGRCh38.tsv'

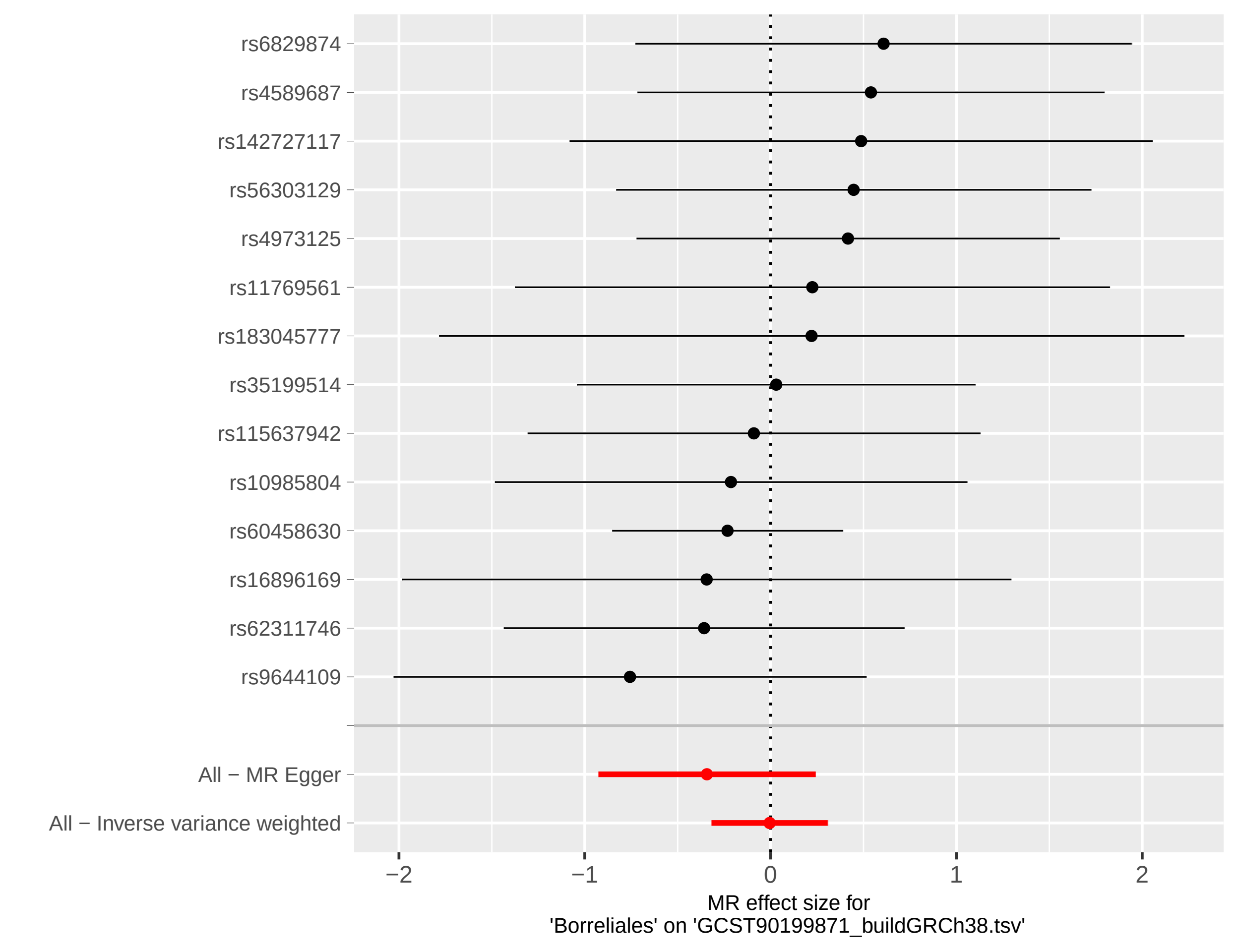
rs6829874
rs4589687
rs142727117
rs56303129
rs4973125
rs11769561
rs183045777
rs35199514
rs115637942
rs10985804
rs60458630
rs16896169
rs62311746
rs9644109
All − MR Egger
All − Inverse variance weighted
−2
−1
0
1
2
MR effect size for
'Borreliales' on 'GCST90199871_buildGRCh38.tsv'

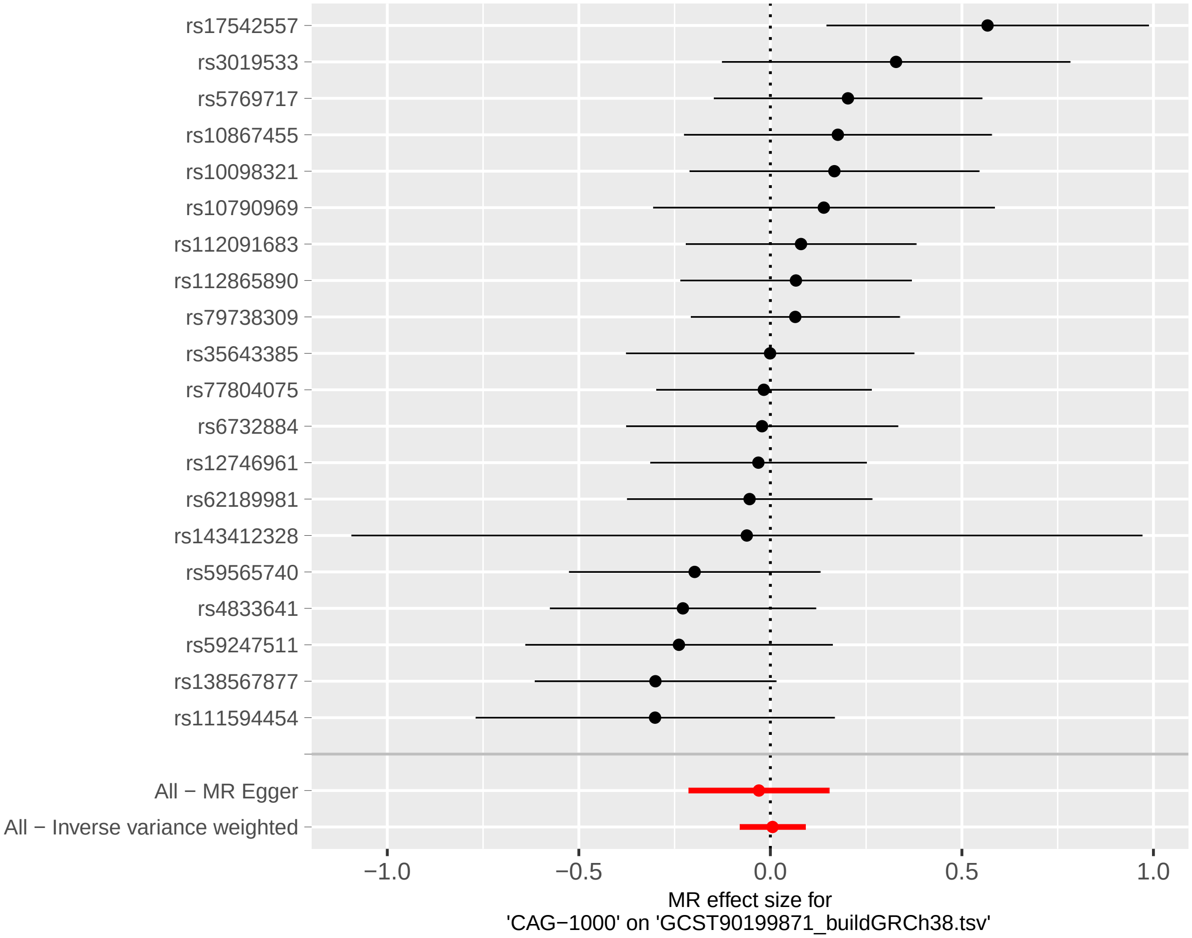

rs17542557
rs3019533
rs5769717
rs10867455
rs10098321
rs10790969
rs112091683
rs112865890
rs79738309
rs35643385
rs77804075
rs6732884
rs12746961
rs62189981
rs143412328
rs59565740
rs4833641
rs59247511
rs138567877
rs111594454
All − MR Egger
All − Inverse variance weighted
−1.0
−0.5
0.0
0.5
1.0
MR effect size for
'CAG−1000' on 'GCST90199871_buildGRCh38.tsv'

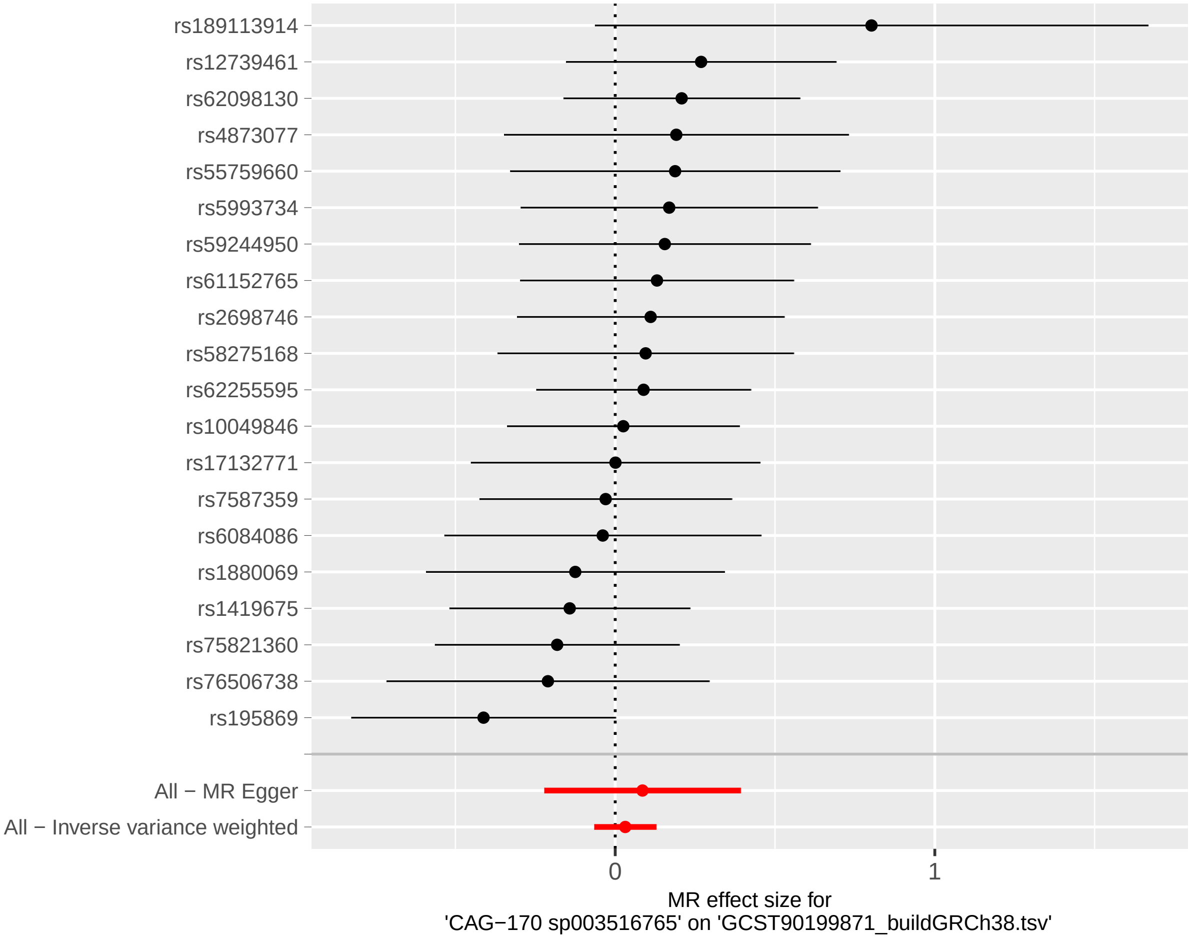

rs189113914
rs12739461
rs62098130
rs4873077
rs55759660
rs5993734
rs59244950
rs61152765
rs2698746
rs58275168
rs62255595
rs10049846
rs17132771
rs7587359
rs6084086
rs1880069
rs1419675
rs75821360
rs76506738
rs195869
All − MR Egger
All − Inverse variance weighted
0
1
MR effect size for
'CAG−170 sp003516765' on 'GCST90199871_buildGRCh38.tsv'

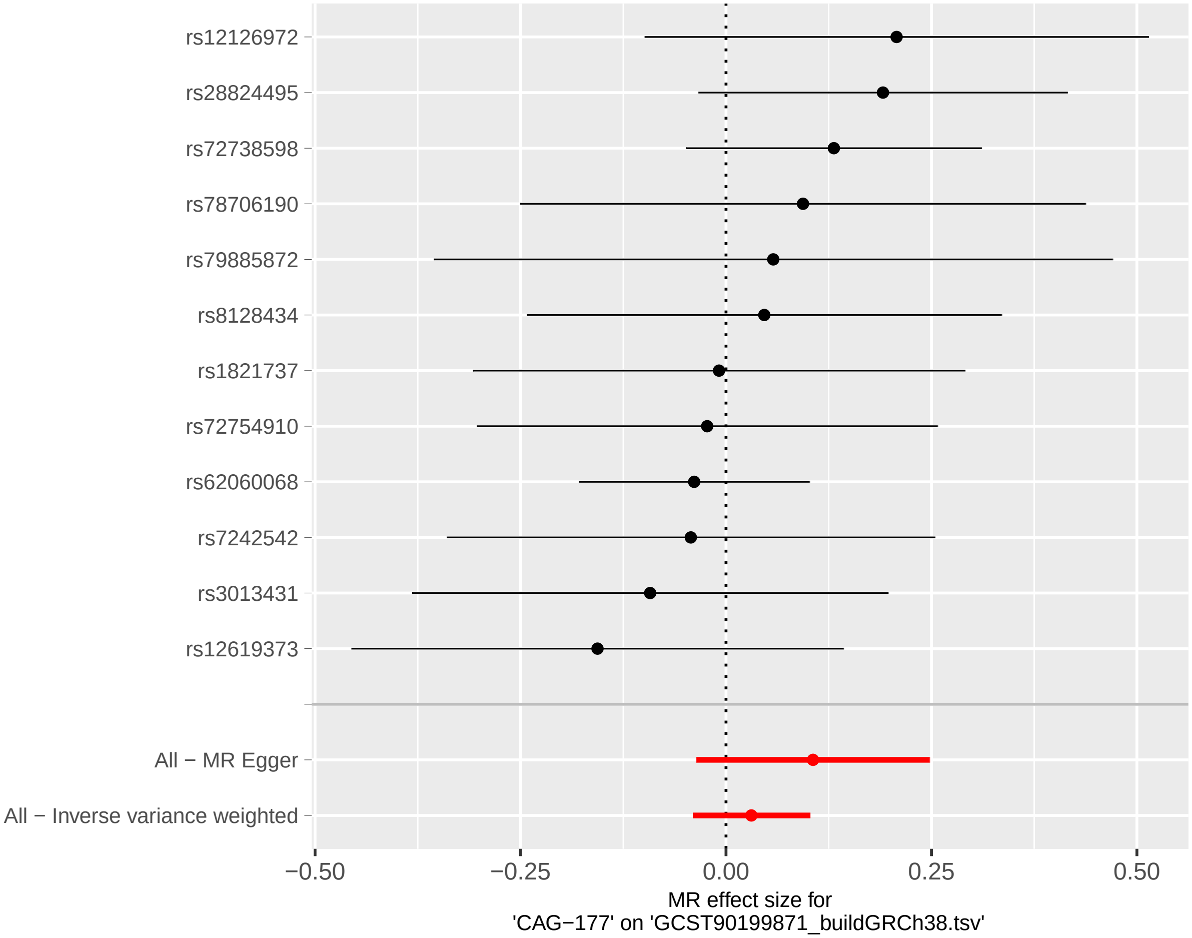
rs12126972
rs28824495
rs72738598
rs78706190
rs79885872
rs8128434
rs1821737
rs72754910
rs62060068
rs7242542
rs3013431
rs12619373
All − MR Egger
All − Inverse variance weighted
−0.50
−0.25
0.00
0.25
0.50
MR effect size for
'CAG−177' on 'GCST90199871_buildGRCh38.tsv'

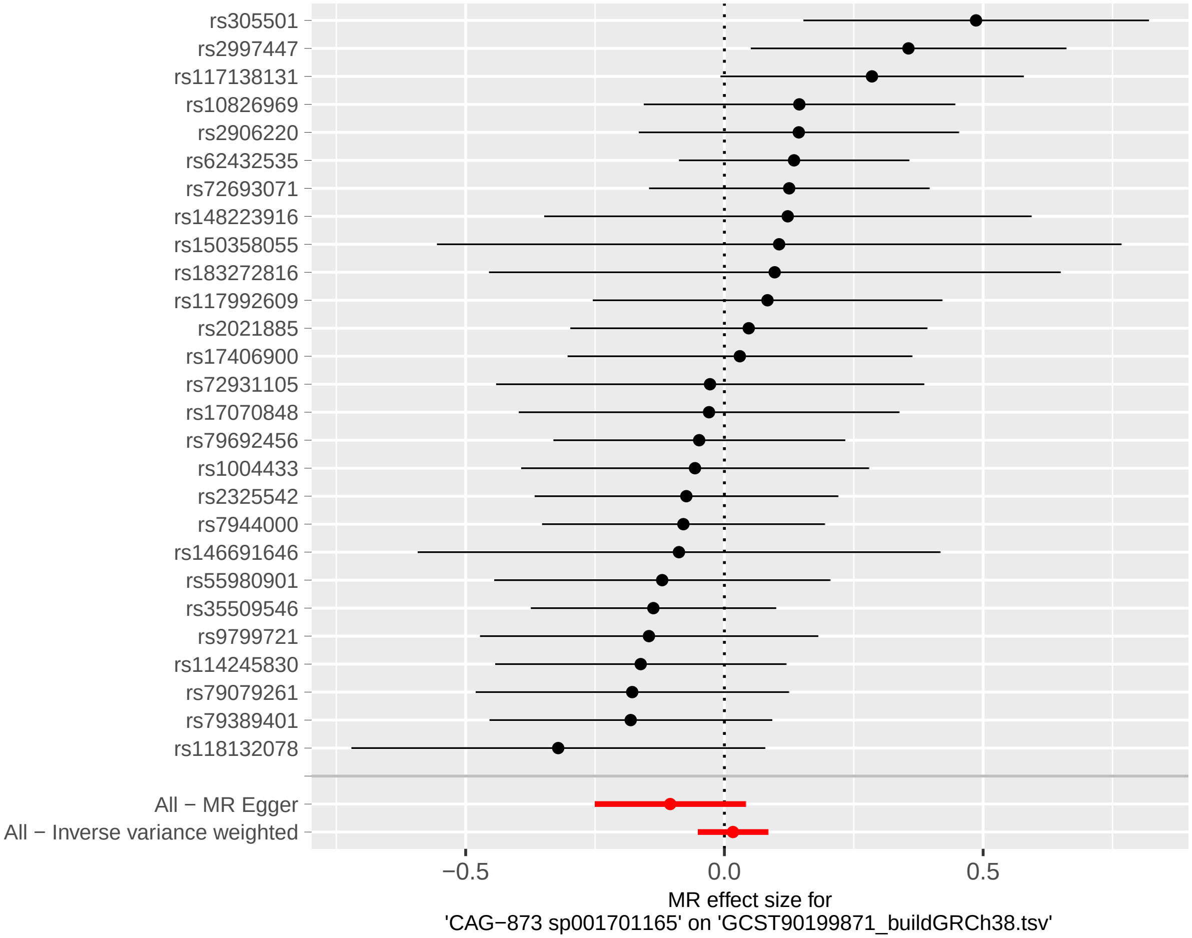
rs305501
rs2997447
rs117138131
rs10826969
rs2906220
rs62432535
rs72693071
rs148223916
rs150358055
rs183272816
rs117992609
rs2021885
rs17406900
rs72931105
rs17070848
rs79692456
rs1004433
rs2325542
rs7944000
rs146691646
rs55980901
rs35509546
rs9799721
rs114245830
rs79079261
rs79389401
rs118132078
All − MR Egger
All − Inverse variance weighted
−0.5
0.0
0.5
MR effect size for
'CAG−873 sp001701165' on 'GCST90199871_buildGRCh38.tsv'

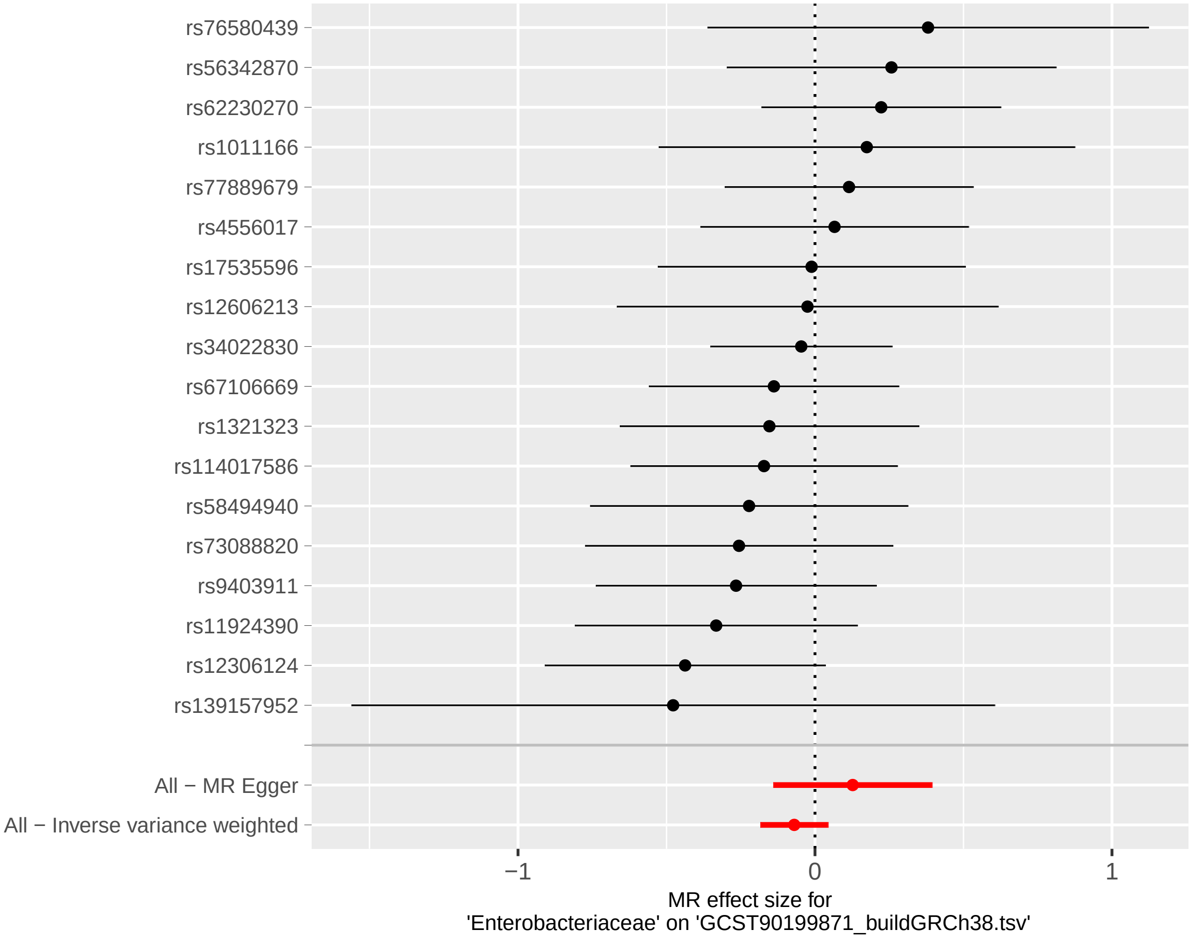
rs76580439
rs56342870
rs62230270
rs1011166
rs77889679
rs4556017
rs17535596
rs12606213
rs34022830
rs67106669
rs1321323
rs114017586
rs58494940
rs73088820
rs9403911
rs11924390
rs12306124
rs139157952
All − MR Egger
All − Inverse variance weighted
−1
0
1
MR effect size for
'Enterobacteriaceae' on 'GCST90199871_buildGRCh38.tsv'

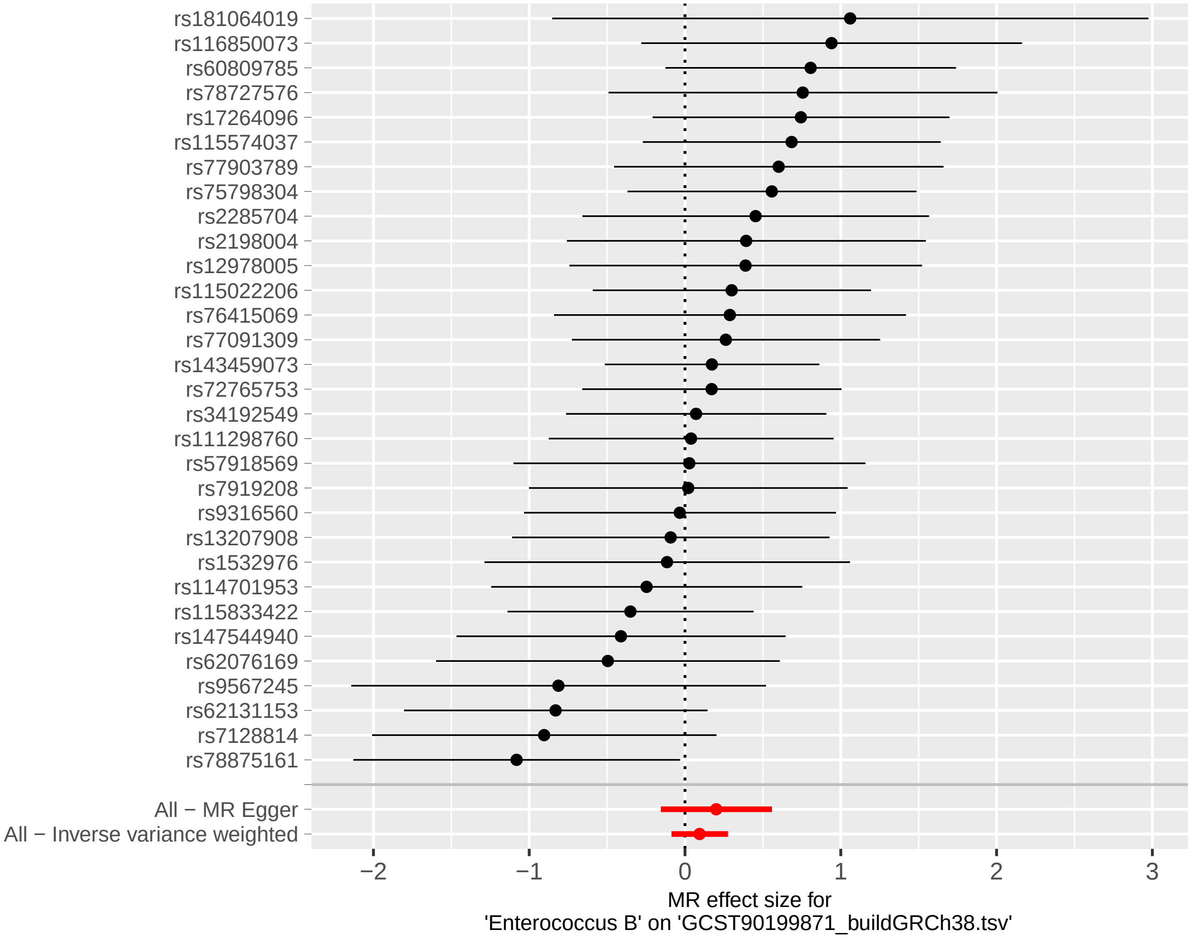
rs181064019
rs116850073
rs60809785
rs78727576
rs17264096
rs115574037
rs77903789
rs75798304
rs2285704
rs2198004
rs12978005
rs115022206
rs76415069
rs77091309
rs143459073
rs72765753
rs34192549
rs111298760
rs57918569
rs7919208
rs9316560
rs13207908
rs1532976
rs114701953
rs115833422
rs147544940
rs62076169
rs9567245
rs62131153
rs7128814
rs78875161
All − MR Egger
All − Inverse variance weighted
−2
−1
0
1
2
3
MR effect size for
'Enterococcus B' on 'GCST90199871_buildGRCh38.tsv'

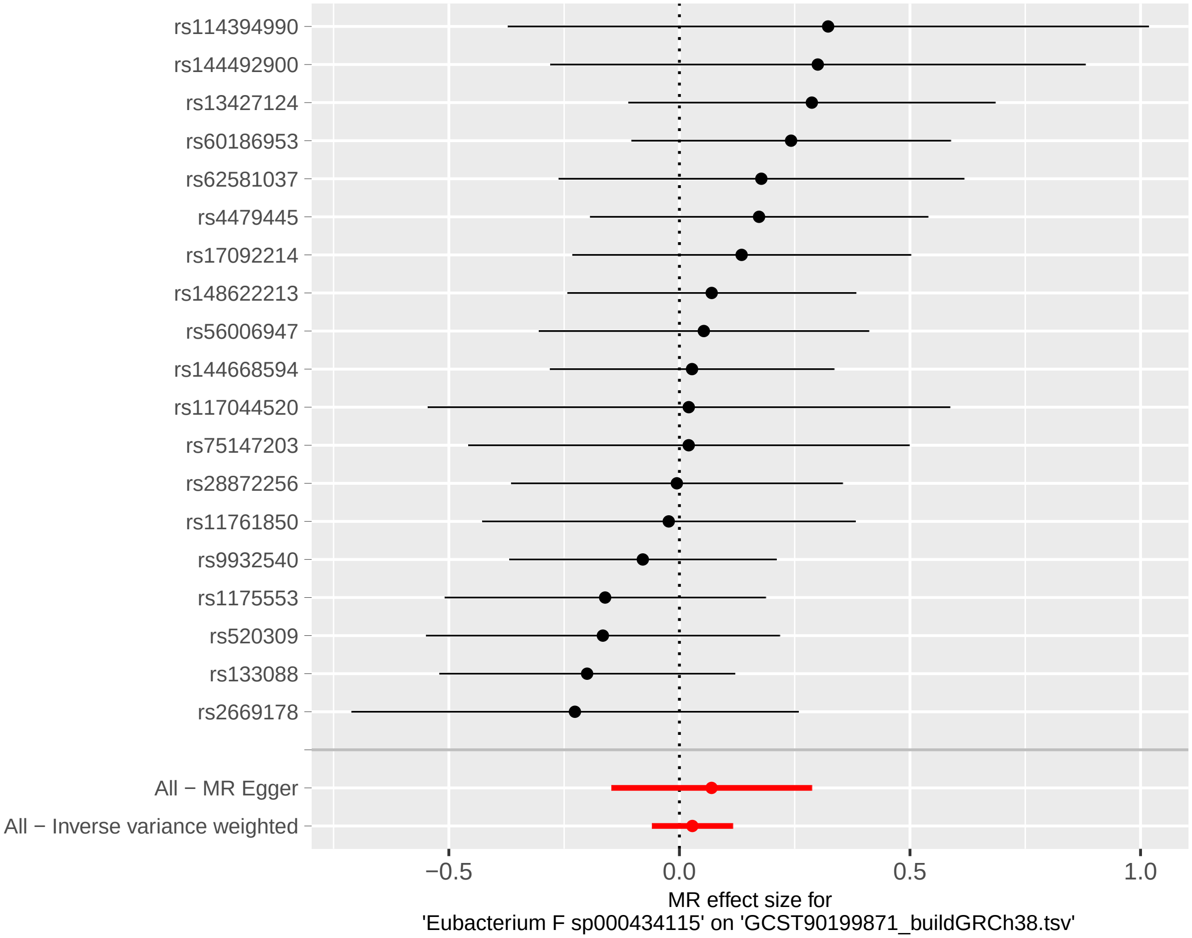
rs114394990
rs144492900
rs13427124
rs60186953
rs62581037
rs4479445
rs17092214
rs148622213
rs56006947
rs144668594
rs117044520
rs75147203
rs28872256
rs11761850
rs9932540
rs1175553
rs520309
rs133088
rs2669178
All − MR Egger
All − Inverse variance weighted
−0.5
0.0
0.5
1.0
MR effect size for
'Eubacterium F sp000434115' on 'GCST90199871_buildGRCh38.tsv'

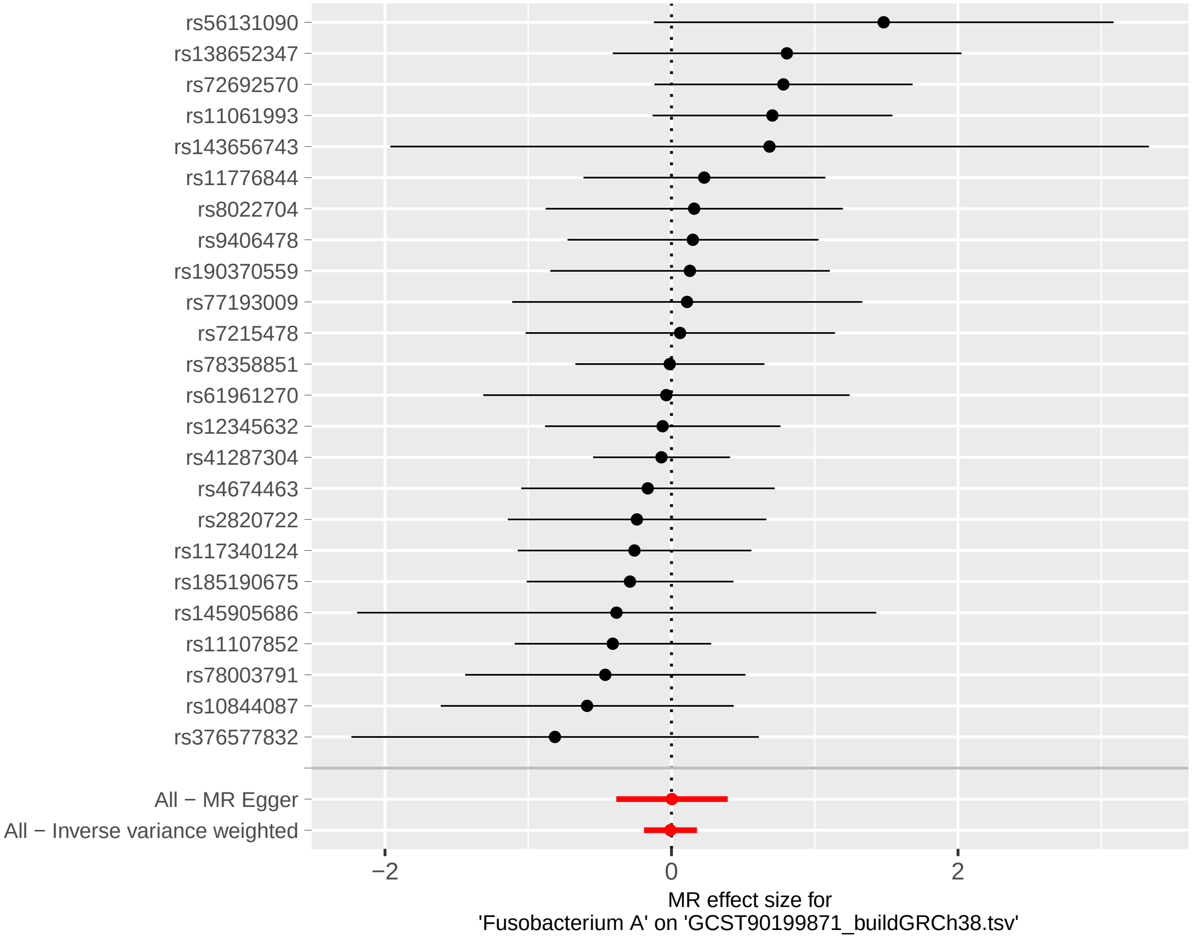
rs56131090
rs138652347
rs72692570
rs11061993
rs143656743
rs11776844
rs8022704
rs9406478
rs190370559
rs77193009
rs7215478
rs78358851
rs61961270
rs12345632
rs41287304
rs4674463
rs2820722
rs117340124
rs185190675
rs145905686
rs11107852
rs78003791
rs10844087
rs376577832
All − MR Egger
All − Inverse variance weighted
−2
0
2
MR effect size for
'Fusobacterium A' on 'GCST90199871_buildGRCh38.tsv'

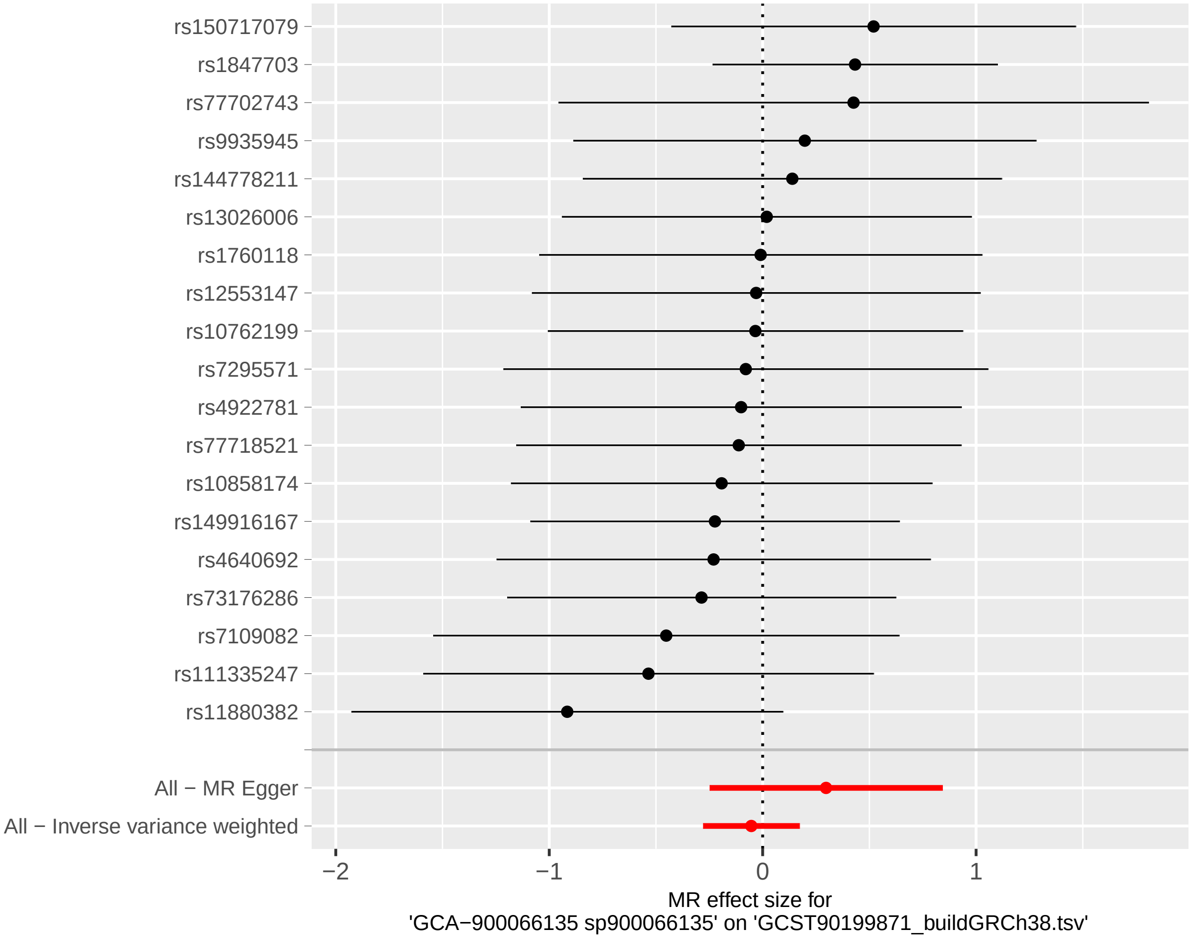

rs150717079
rs1847703
rs77702743
rs9935945
rs144778211
rs13026006
rs1760118
rs12553147
rs10762199
rs7295571
rs4922781
rs77718521
rs10858174
rs149916167
rs4640692
rs73176286
rs7109082
rs111335247
rs11880382
All − MR Egger
All − Inverse variance weighted
−2
−1
0
1
MR effect size for
'GCA−900066135 sp900066135' on 'GCST90199871_buildGRCh38.tsv'

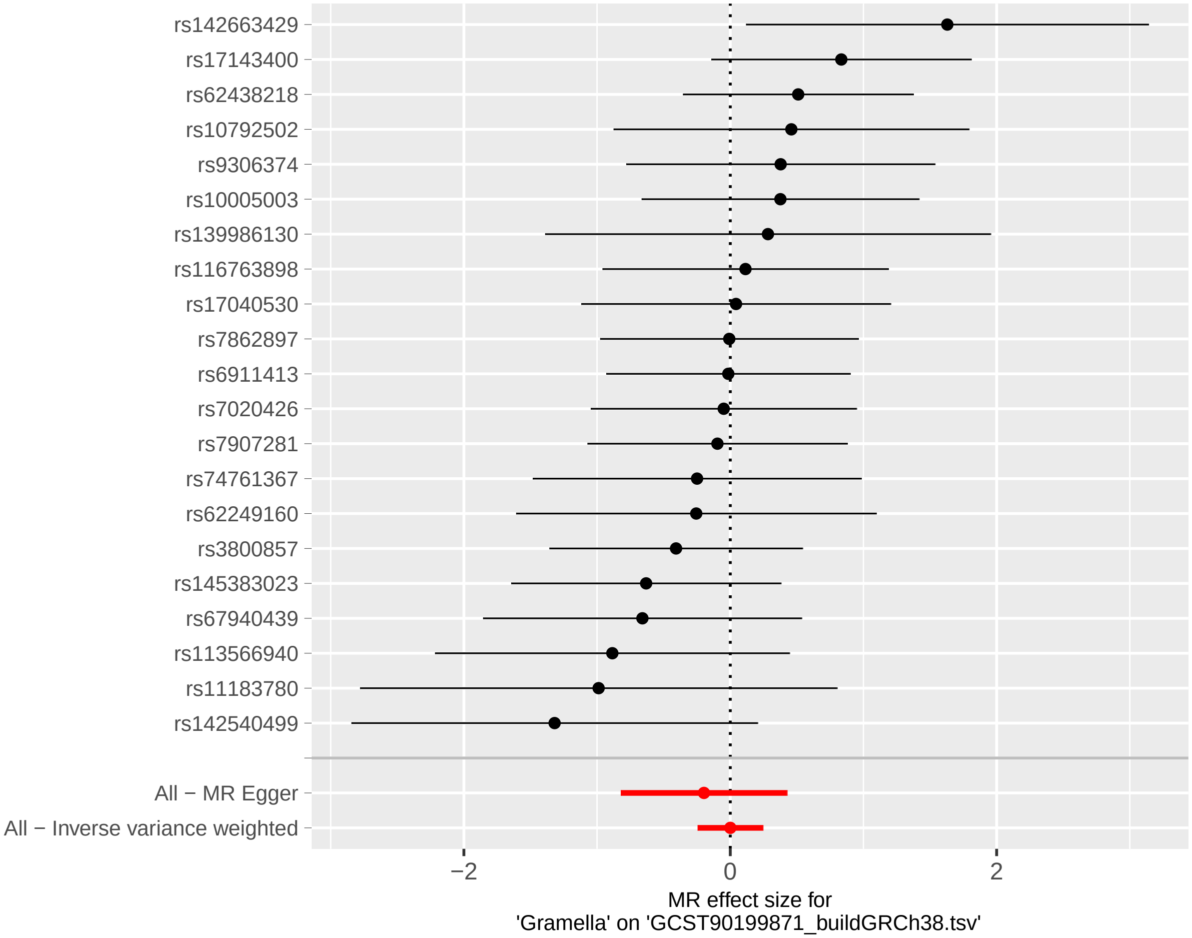
rs142663429
rs17143400
rs62438218
rs10792502
rs9306374
rs10005003
rs139986130
rs116763898
rs17040530
rs7862897
rs6911413
rs7020426
rs7907281
rs74761367
rs62249160
rs3800857
rs145383023
rs67940439
rs113566940
rs11183780
rs142540499
All − MR Egger
All − Inverse variance weighted
−2
0
2
MR effect size for
'Gramella' on 'GCST90199871_buildGRCh38.tsv'

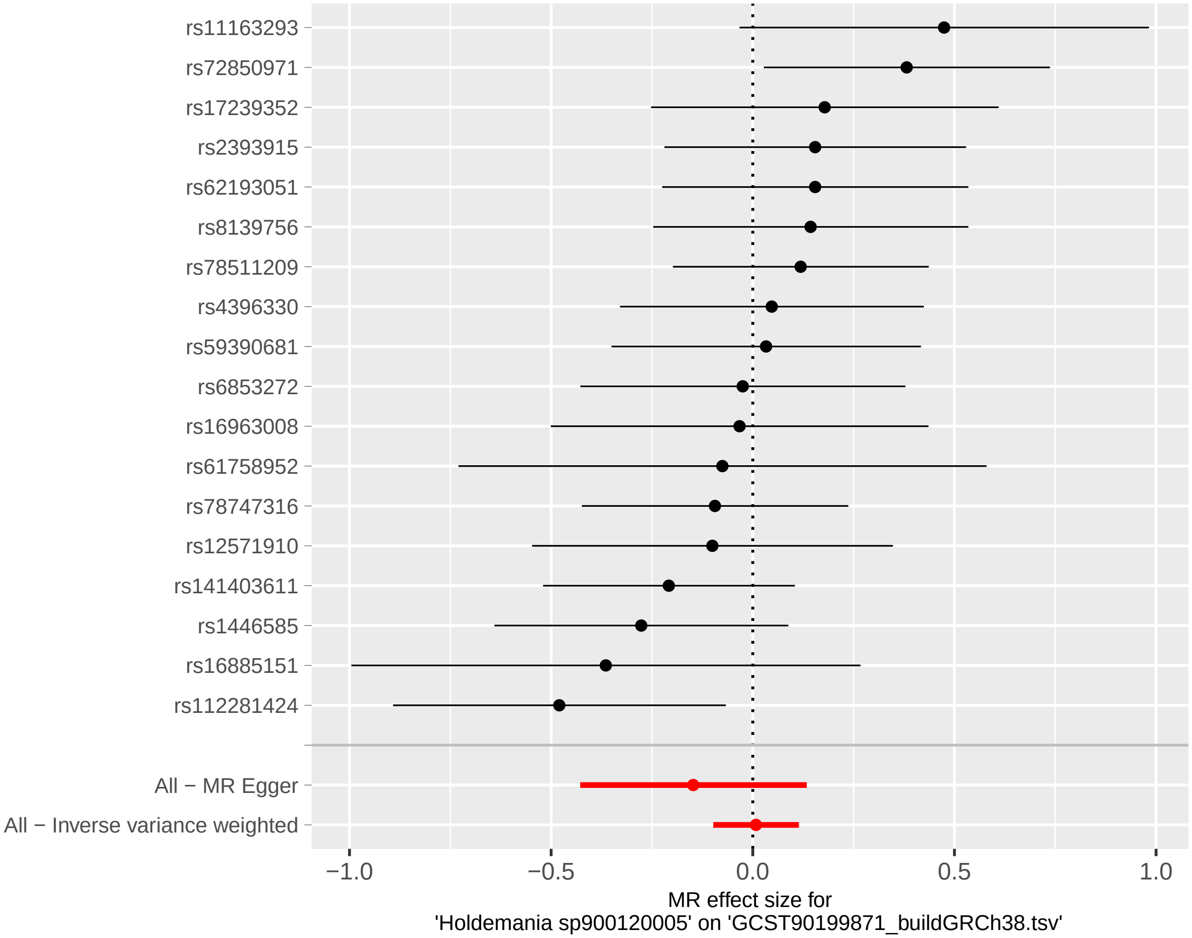
rs11163293
rs72850971
rs17239352
rs2393915
rs62193051
rs8139756
rs78511209
rs4396330
rs59390681
rs6853272
rs16963008
rs61758952
rs78747316
rs12571910
rs141403611
rs1446585
rs16885151
rs112281424
All − MR Egger
All − Inverse variance weighted
−1.0
−0.5
0.0
0.5
1.0
MR effect size for
'Holdemania sp900120005' on 'GCST90199871_buildGRCh38.tsv'

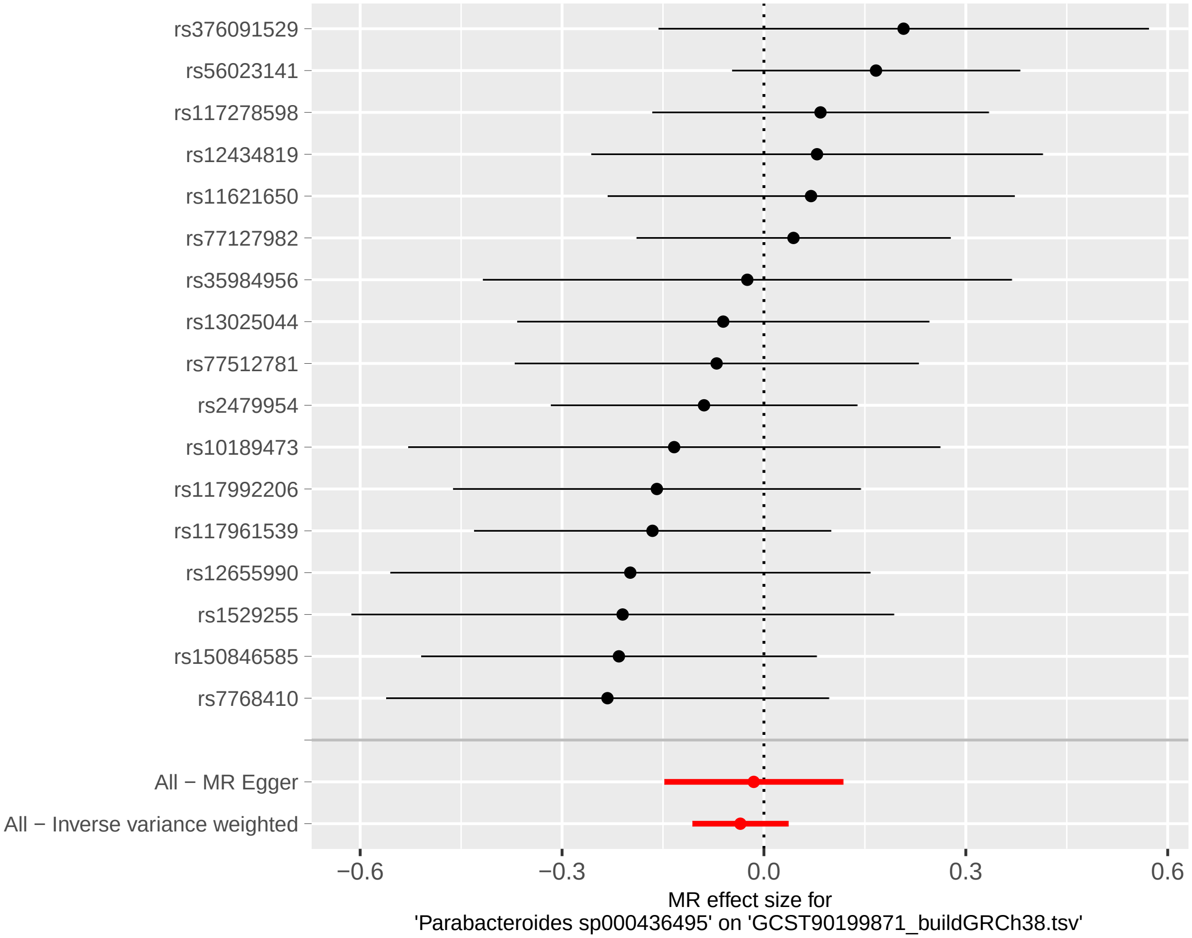
rs376091529
rs56023141
rs117278598
rs12434819
rs11621650
rs77127982
rs35984956
rs13025044
rs77512781
rs2479954
rs10189473
rs117992206
rs117961539
rs12655990
rs1529255
rs150846585
rs7768410
All − MR Egger
All − Inverse variance weighted
−0.6
−0.3
0.0
0.3
0.6
MR effect size for
'Parabacteroides sp000436495' on 'GCST90199871_buildGRCh38.tsv'

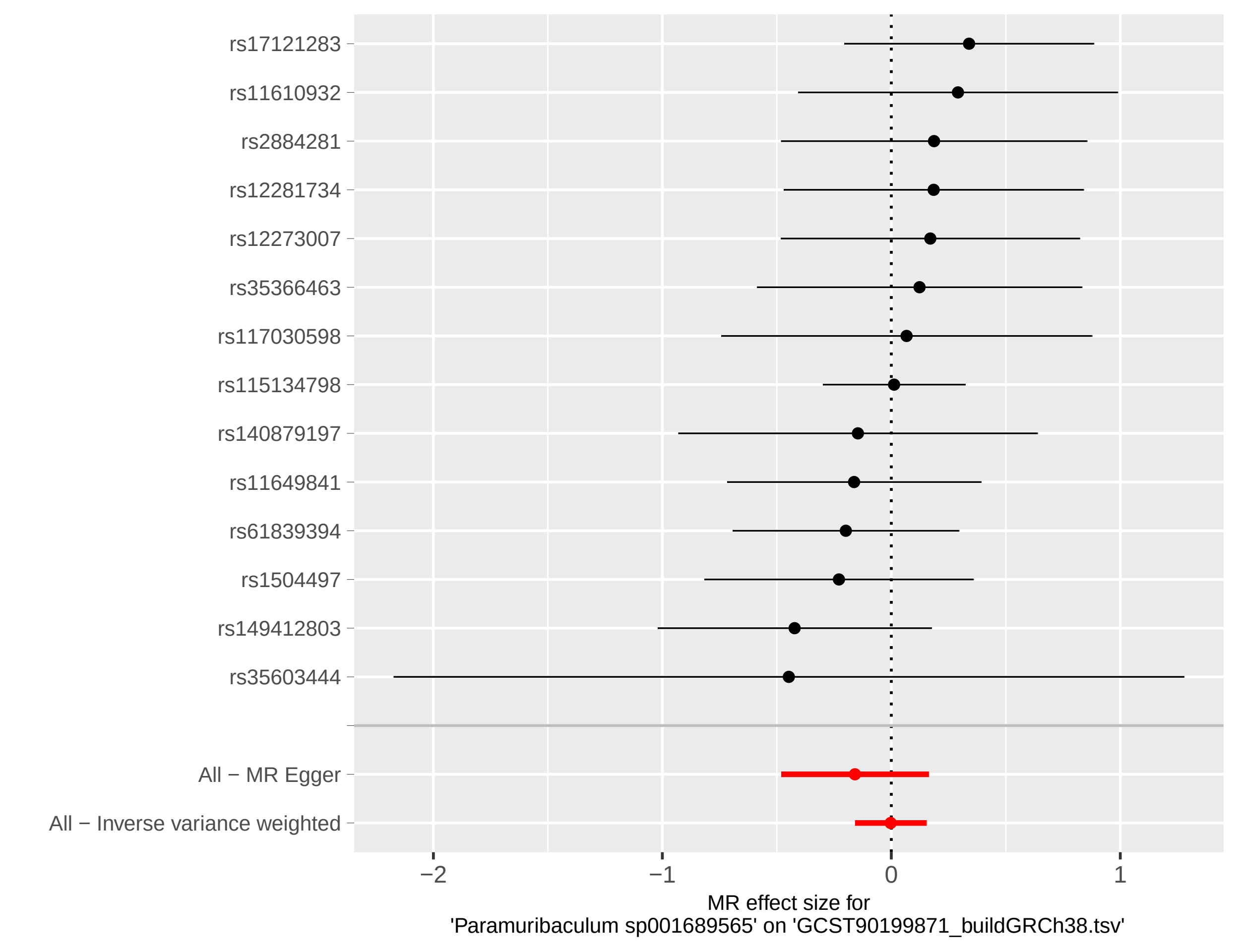
rs17121283
rs11610932
rs2884281
rs12281734
rs12273007
rs35366463
rs117030598
rs115134798
rs140879197
rs11649841
rs61839394
rs1504497
rs149412803
rs35603444
All − MR Egger
All − Inverse variance weighted
−2
−1
0
1
MR effect size for
'Paramuribaculum sp001689565' on 'GCST90199871_buildGRCh38.tsv'

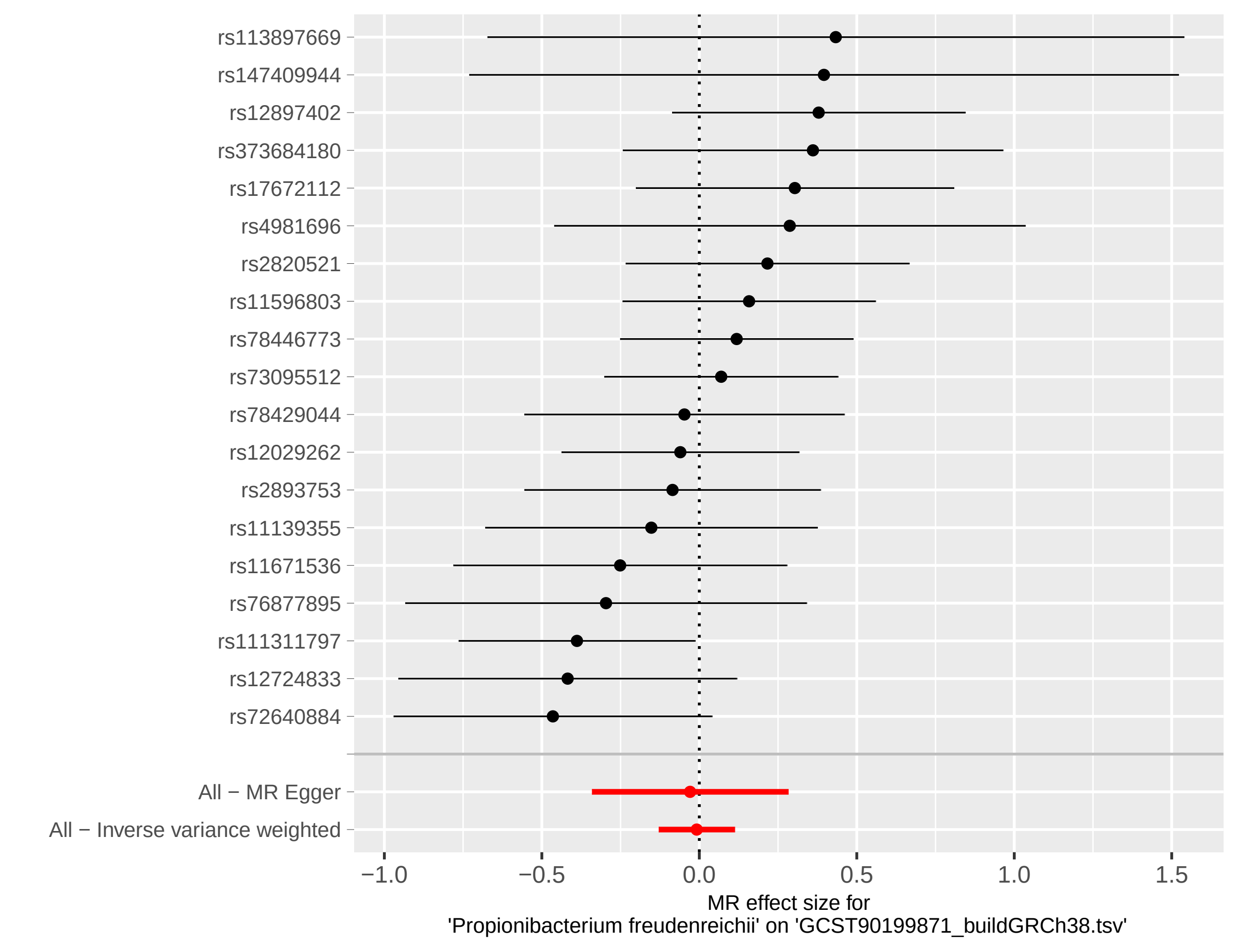
rs113897669
rs147409944
rs12897402
rs373684180
rs17672112
rs4981696
rs2820521
rs11596803
rs78446773
rs73095512
rs78429044
rs12029262
rs2893753
rs11139355
rs11671536
rs76877895
rs111311797
rs12724833
rs72640884
All − MR Egger
All − Inverse variance weighted
−1.0
−0.5
0.0
0.5
1.0
1.5
MR effect size for
'Propionibacterium freudenreichii' on 'GCST90199871_buildGRCh38.tsv'

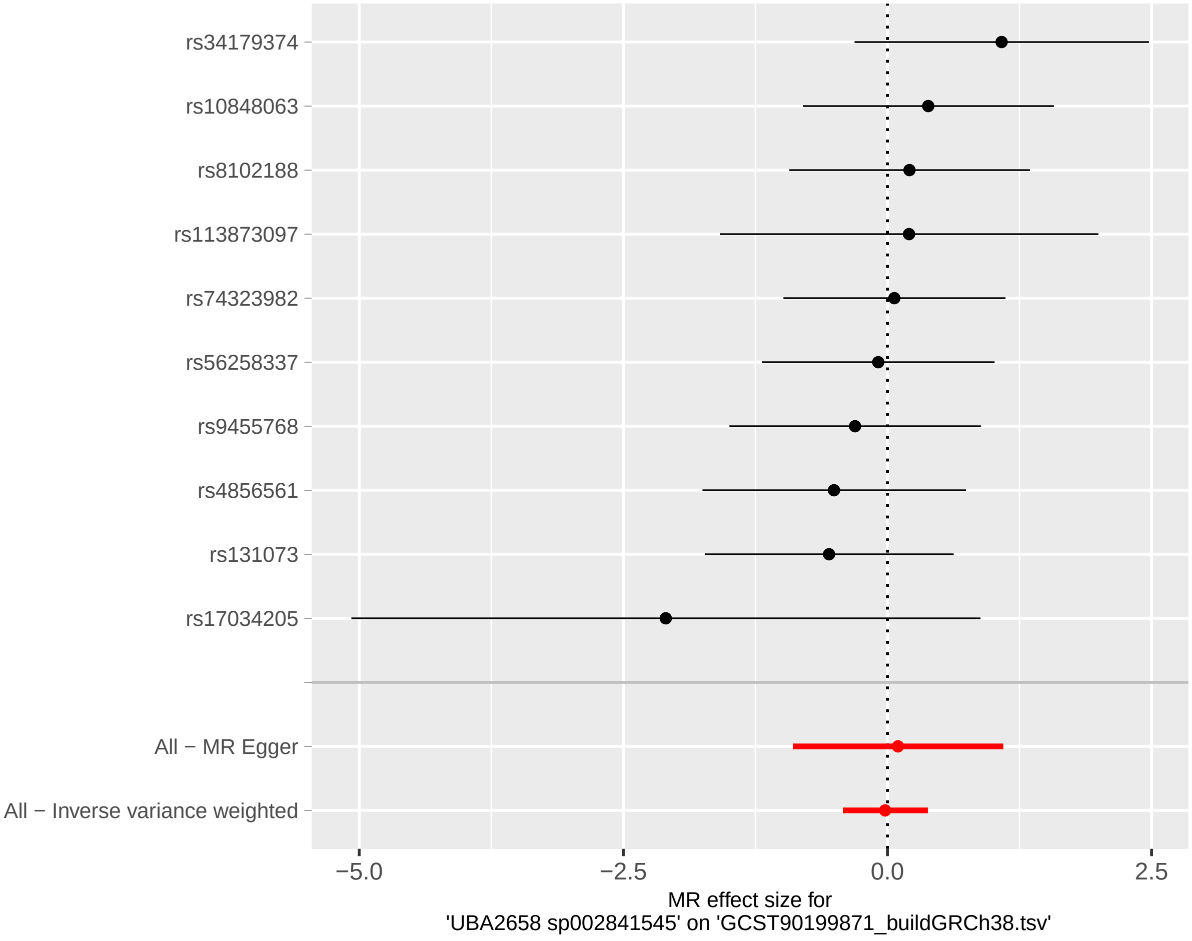

rs34179374
rs10848063
rs8102188
rs113873097
rs74323982
rs56258337
rs9455768
rs4856561
rs131073
rs17034205
All − MR Egger
All − Inverse variance weighted
−5.0
−2.5
0.0
2.5
MR effect size for
'UBA2658 sp002841545' on 'GCST90199871_buildGRCh38.tsv'

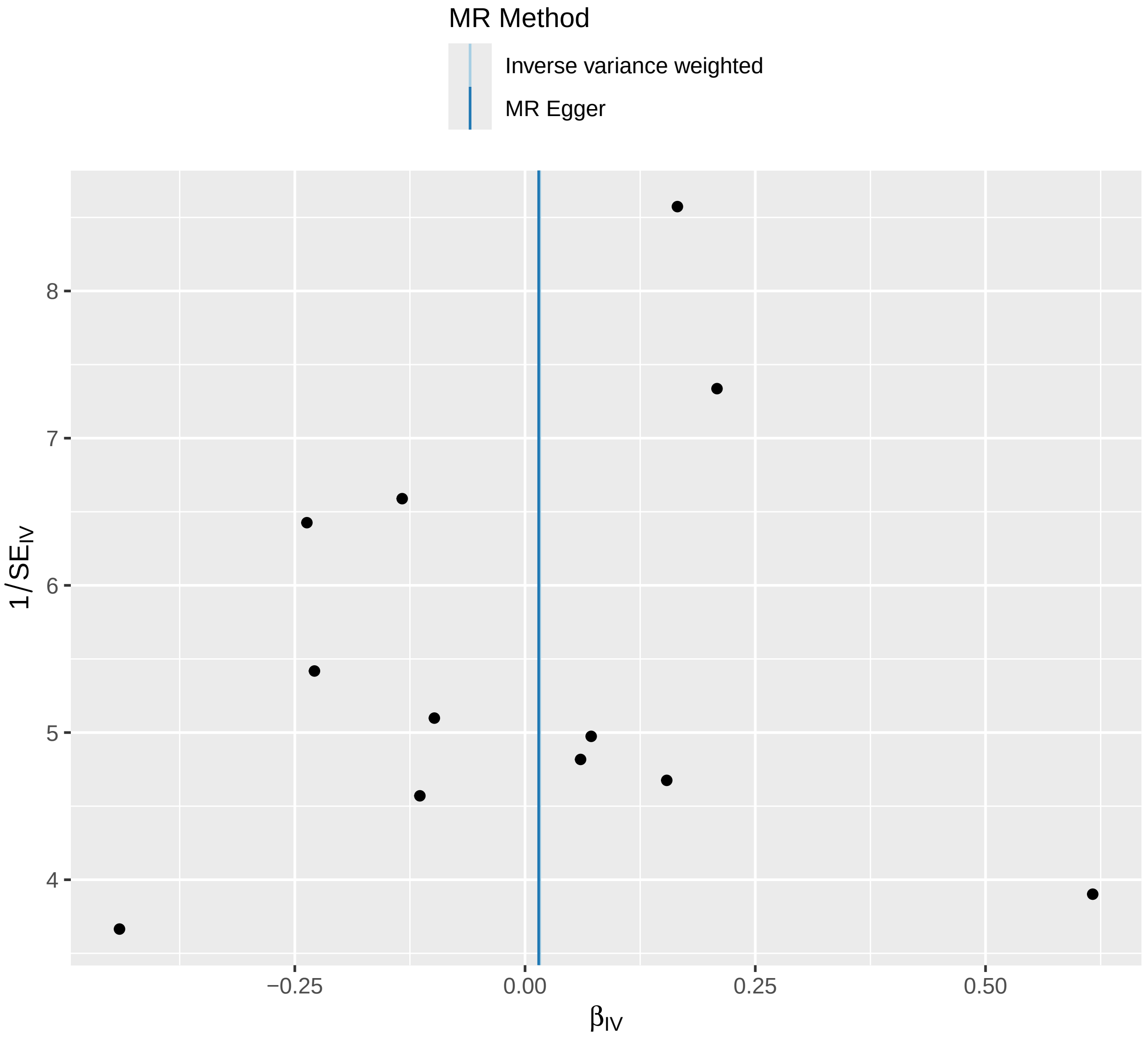
MR Method
Inverse variance weighted
MR Egger
8
7
6
5
4
1/SE_IV
−0.25
0.00
0.25
0.50
β_IV

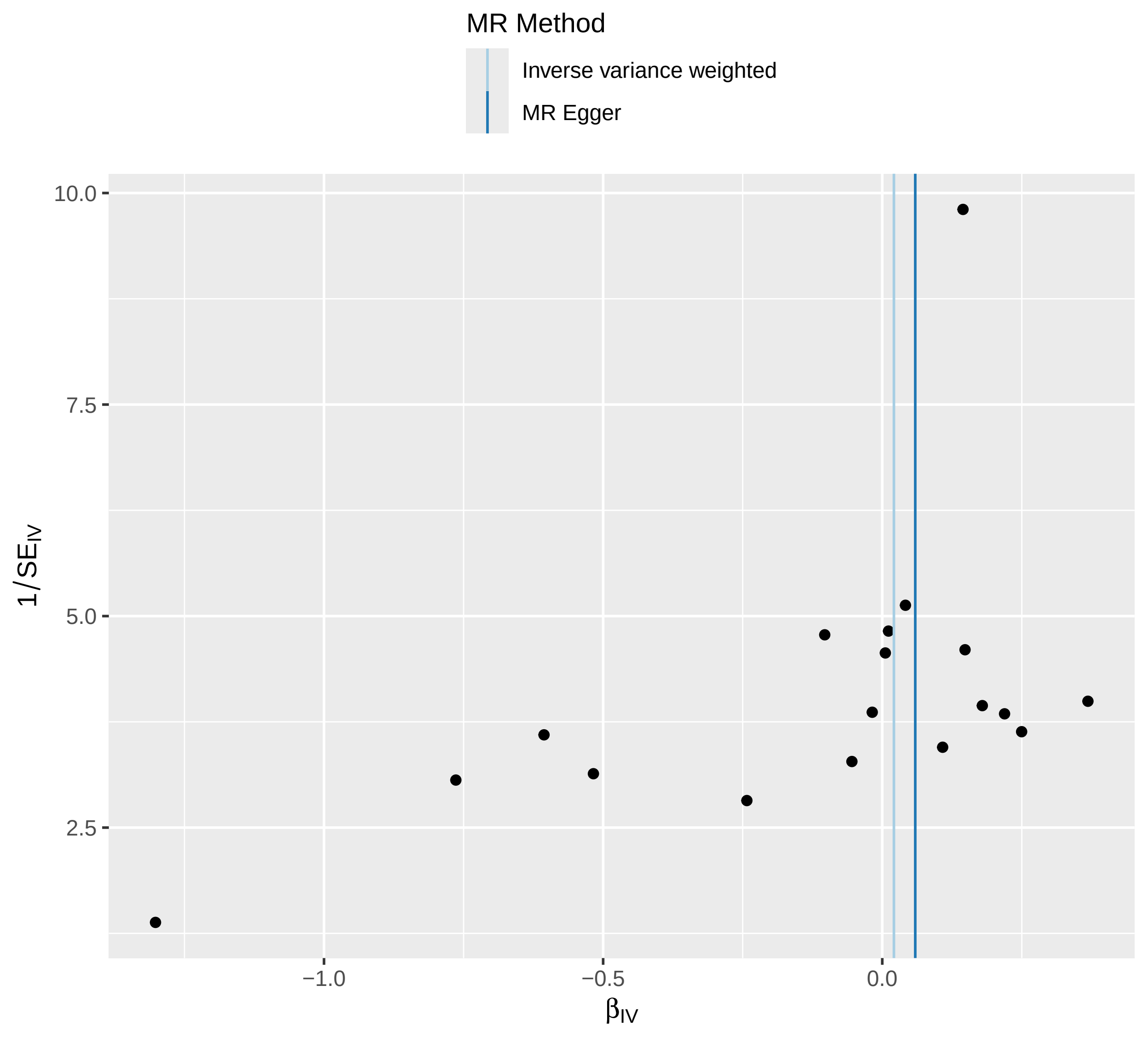

MR Method
Inverse variance weighted
MR Egger
10.0
7.5
5.0
2.5
$1/SE_{IV}$
−1.0
−0.5
0.0
β_{IV}

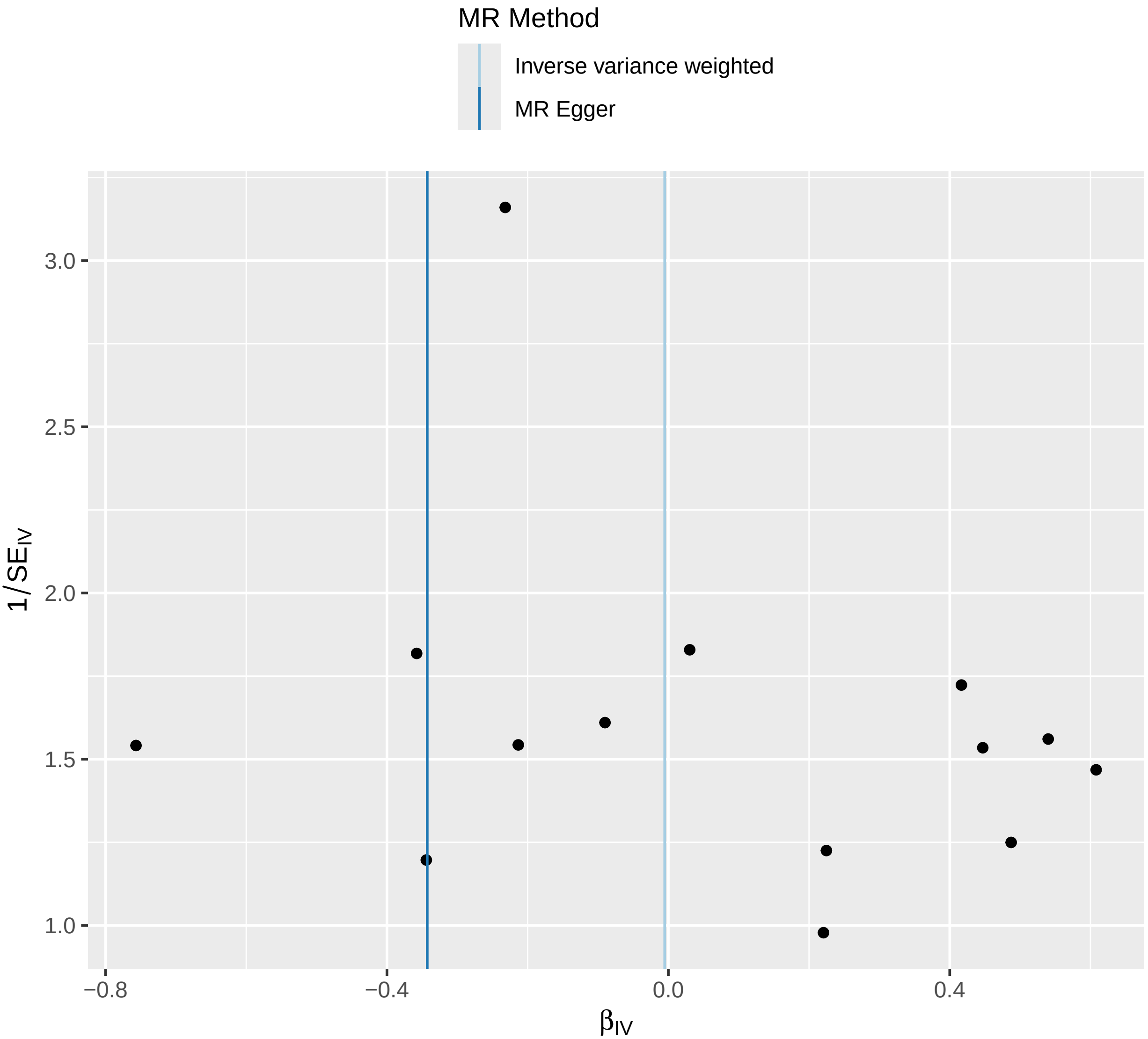
MR Method
Inverse variance weighted
MR Egger
3.0
2.5
2.0
1.5
1.0
$1/SE_{IV}$
−0.8
−0.4
0.0
0.4
β_{IV}

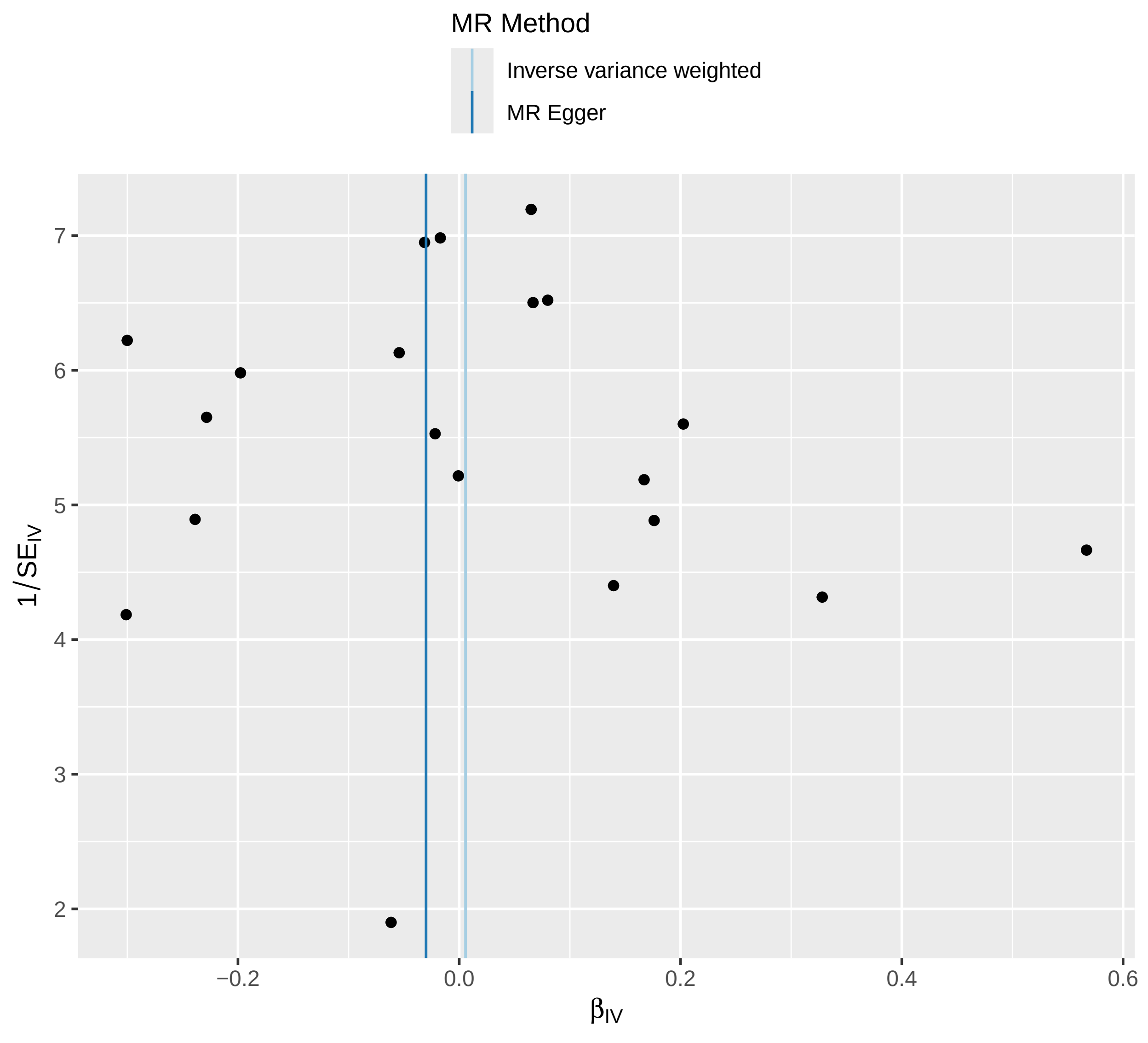
MR Method
Inverse variance weighted
MR Egger
7
6
5
4
3
2
1/SE_IV
−0.2
0.0
0.2
0.4
0.6
β_IV

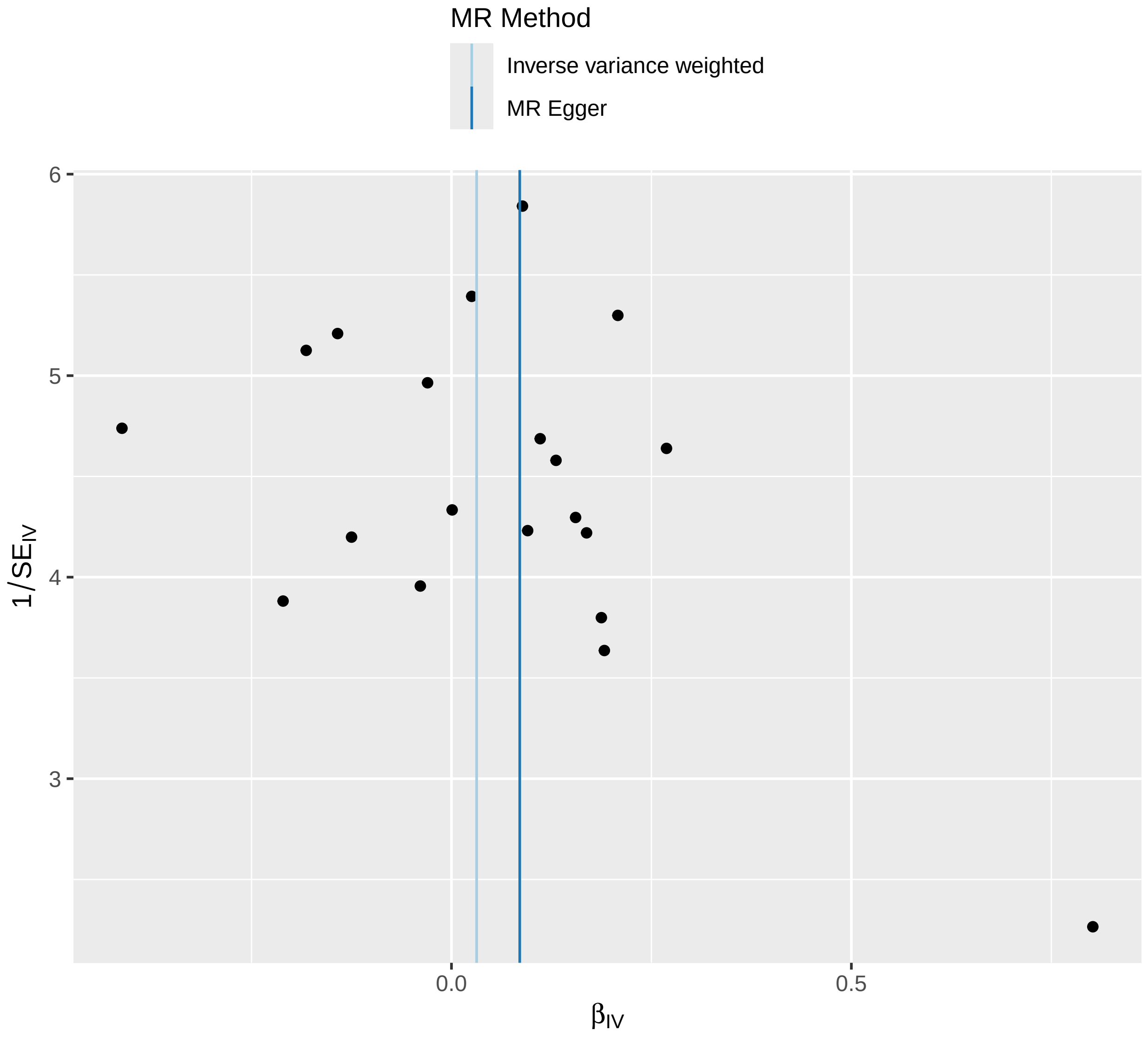
MR Method
Inverse variance weighted
MR Egger
6
5
4
3
1/SE$_{IV}$
0.0
0.5
β_{IV}

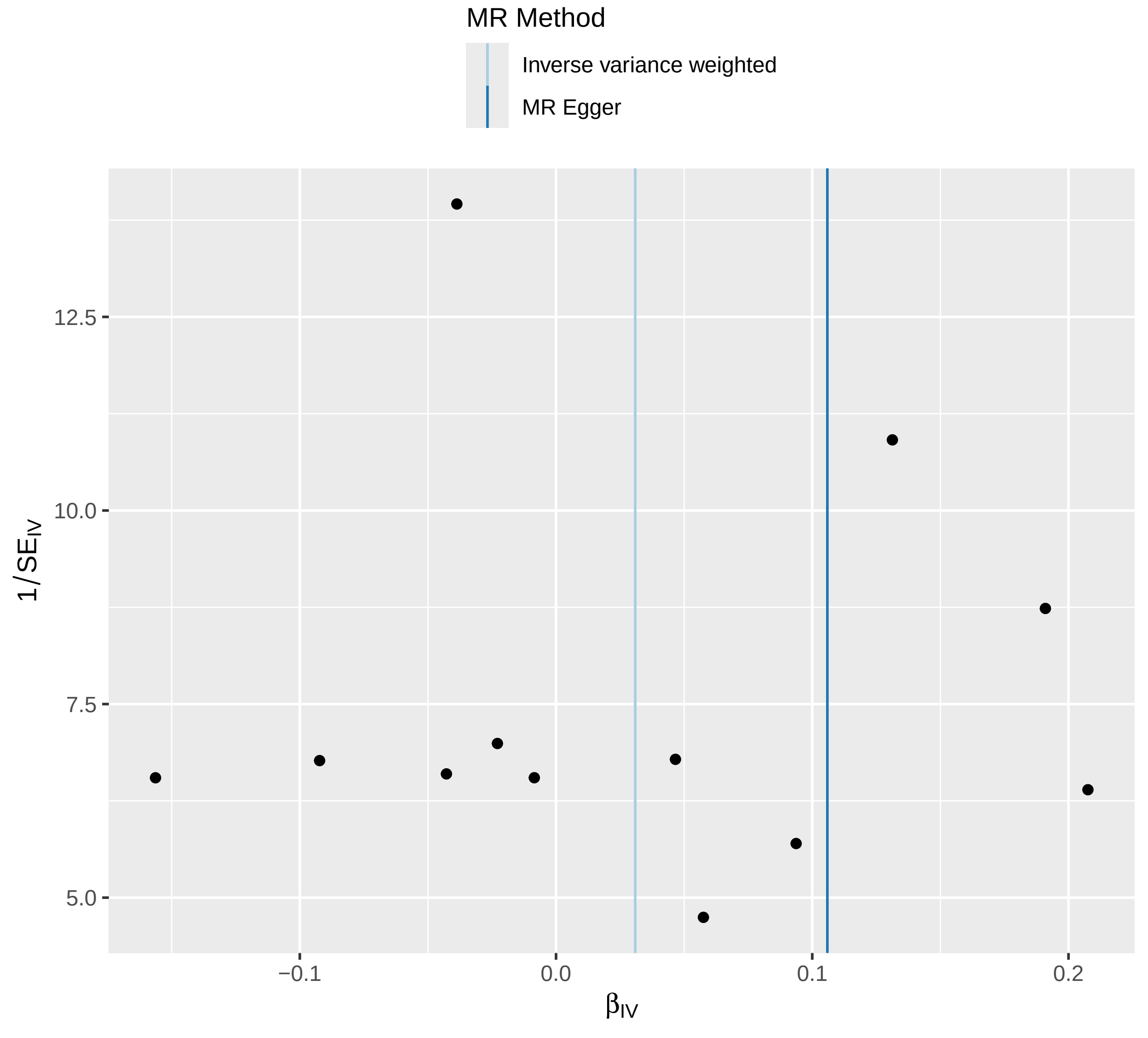
MR Method
Inverse variance weighted
MR Egger
12.5
10.0
7.5
5.0
1/SE_IV
−0.1
0.0
0.1
0.2
β_IV

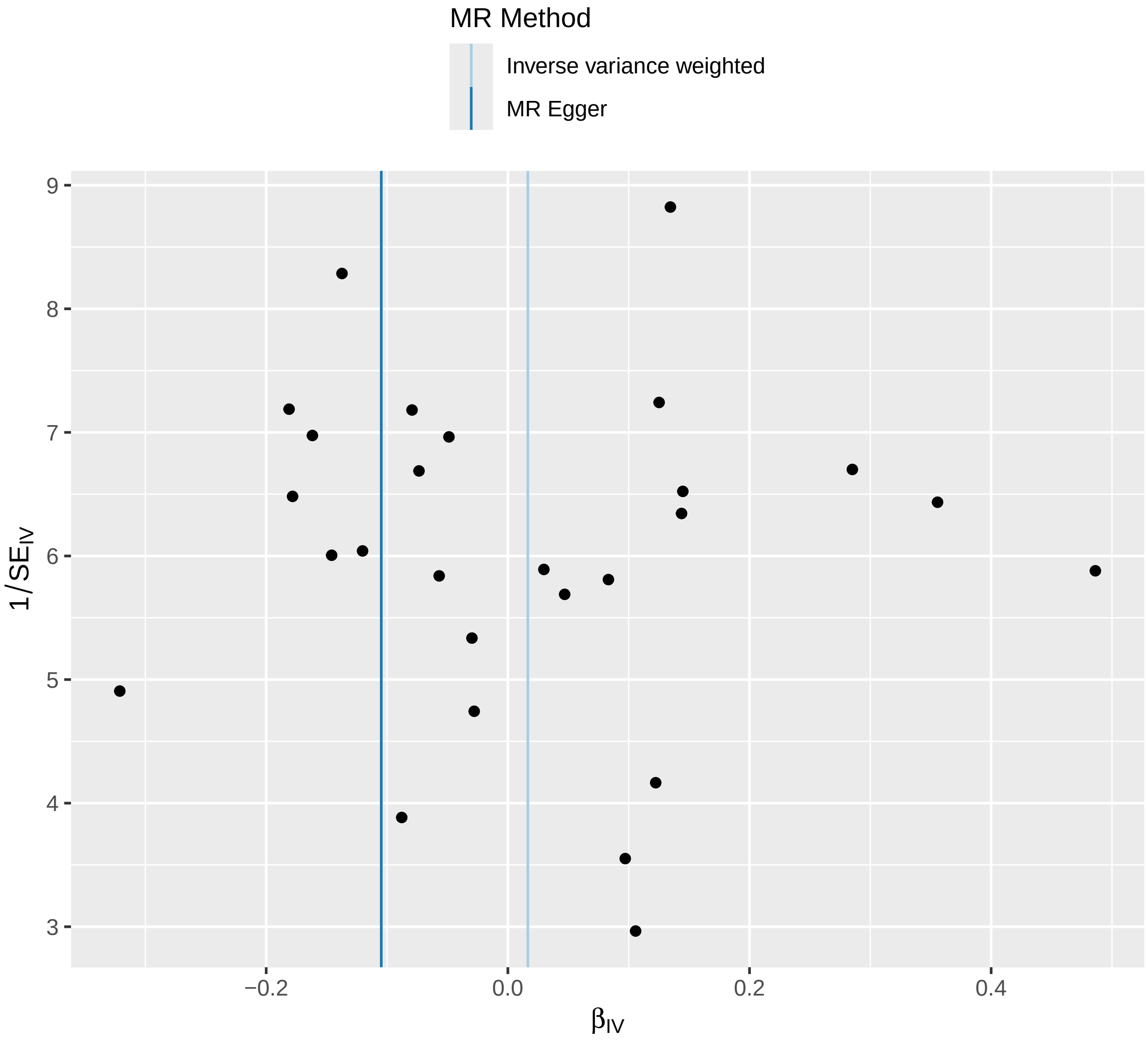
MR Method
Inverse variance weighted
MR Egger
9
8
7
6
5
4
3
1/SE$_{IV}$
−0.2
0.0
0.2
0.4
β$_{IV}$

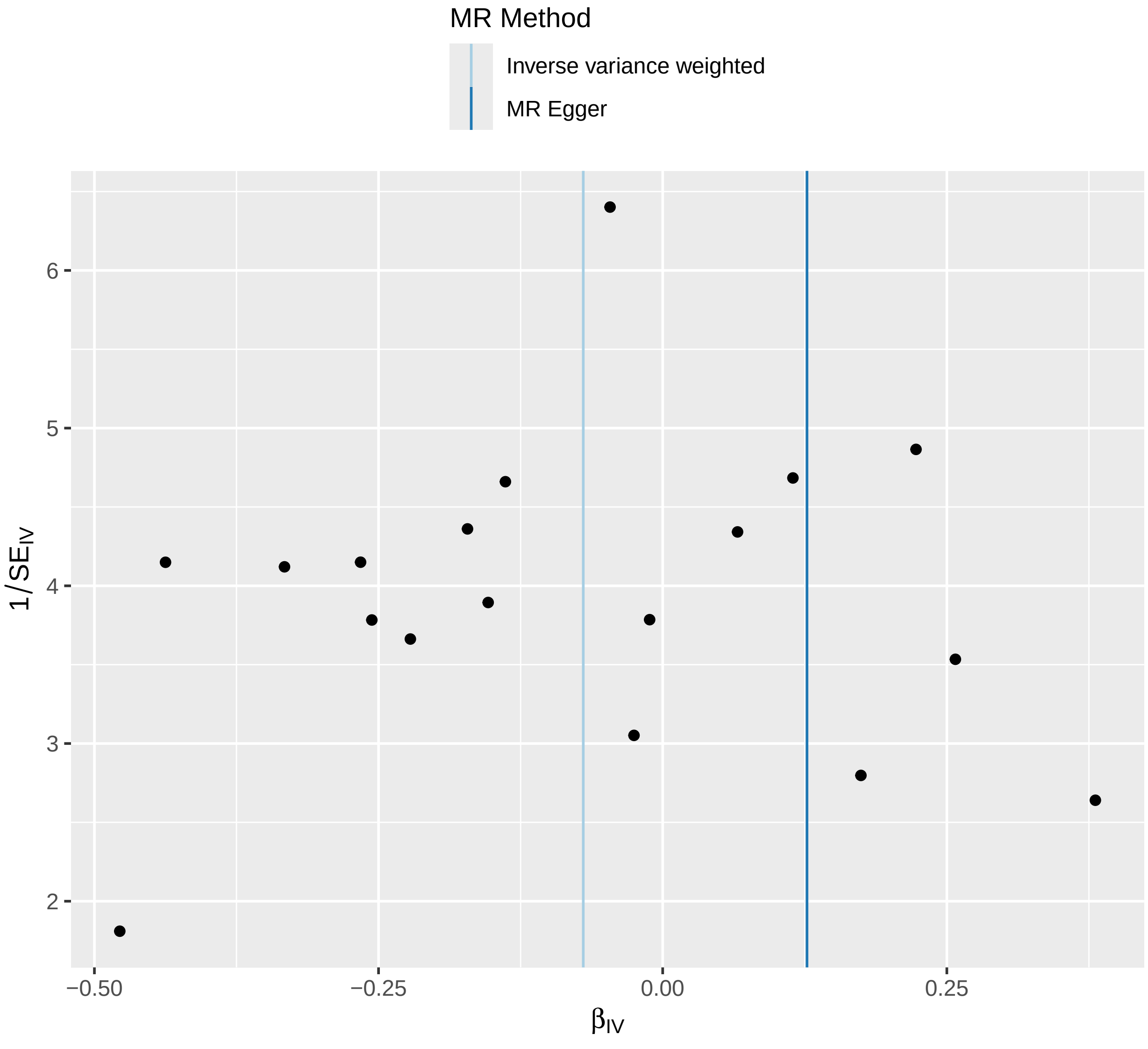

MR Method
Inverse variance weighted
MR Egger
6
5
4
3
2
$1/SE_{IV}$
−0.50
−0.25
0.00
0.25
β_{IV}

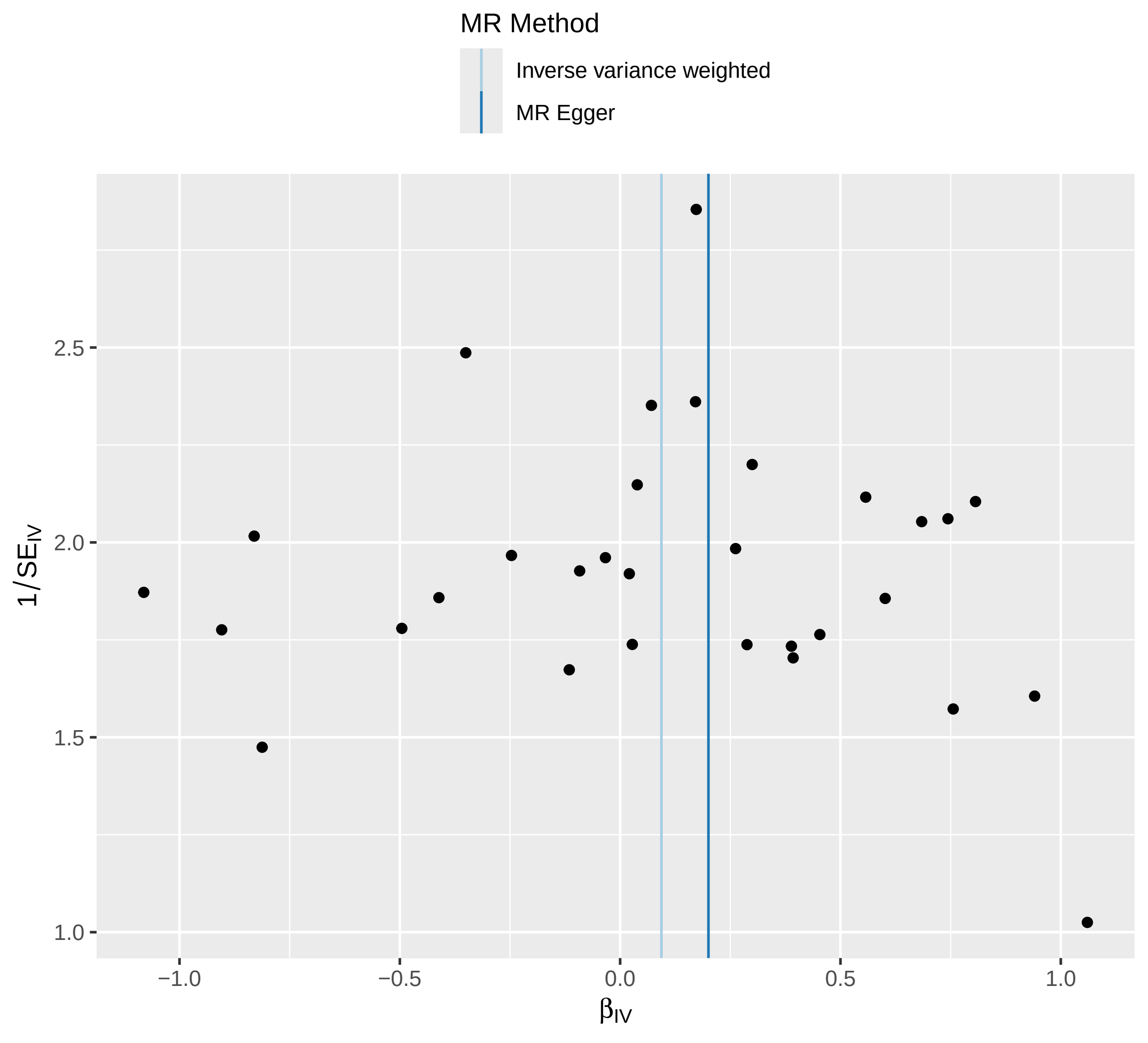
MR Method
Inverse variance weighted
MR Egger
2.5
2.0
1.5
1.0
1/SE$_{IV}$
−1.0
−0.5
0.0
0.5
1.0
β_{IV}

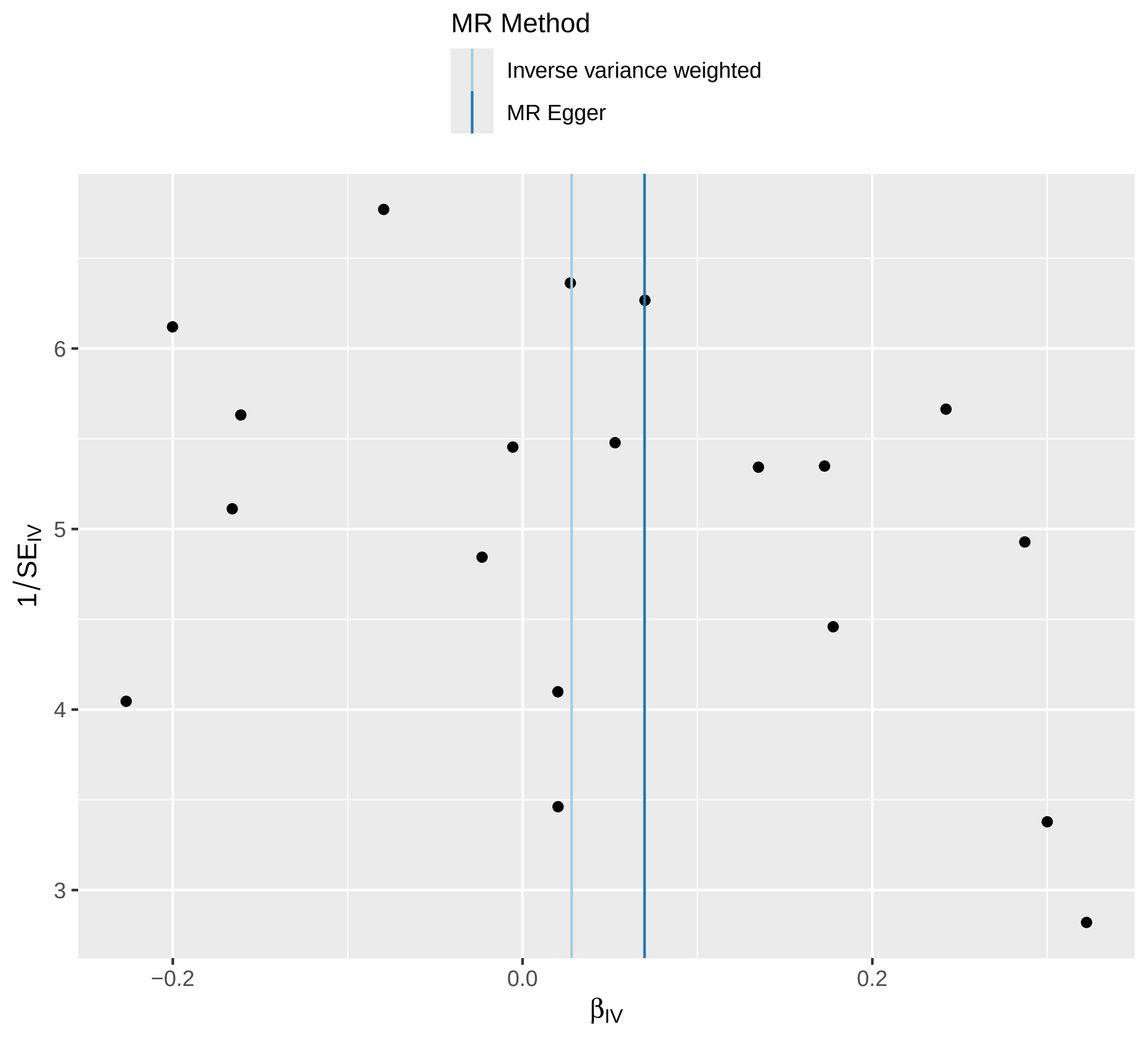
MR Method
Inverse variance weighted
MR Egger
6
5
4
3
1/SE_IV
−0.2
0.0
0.2
β_IV

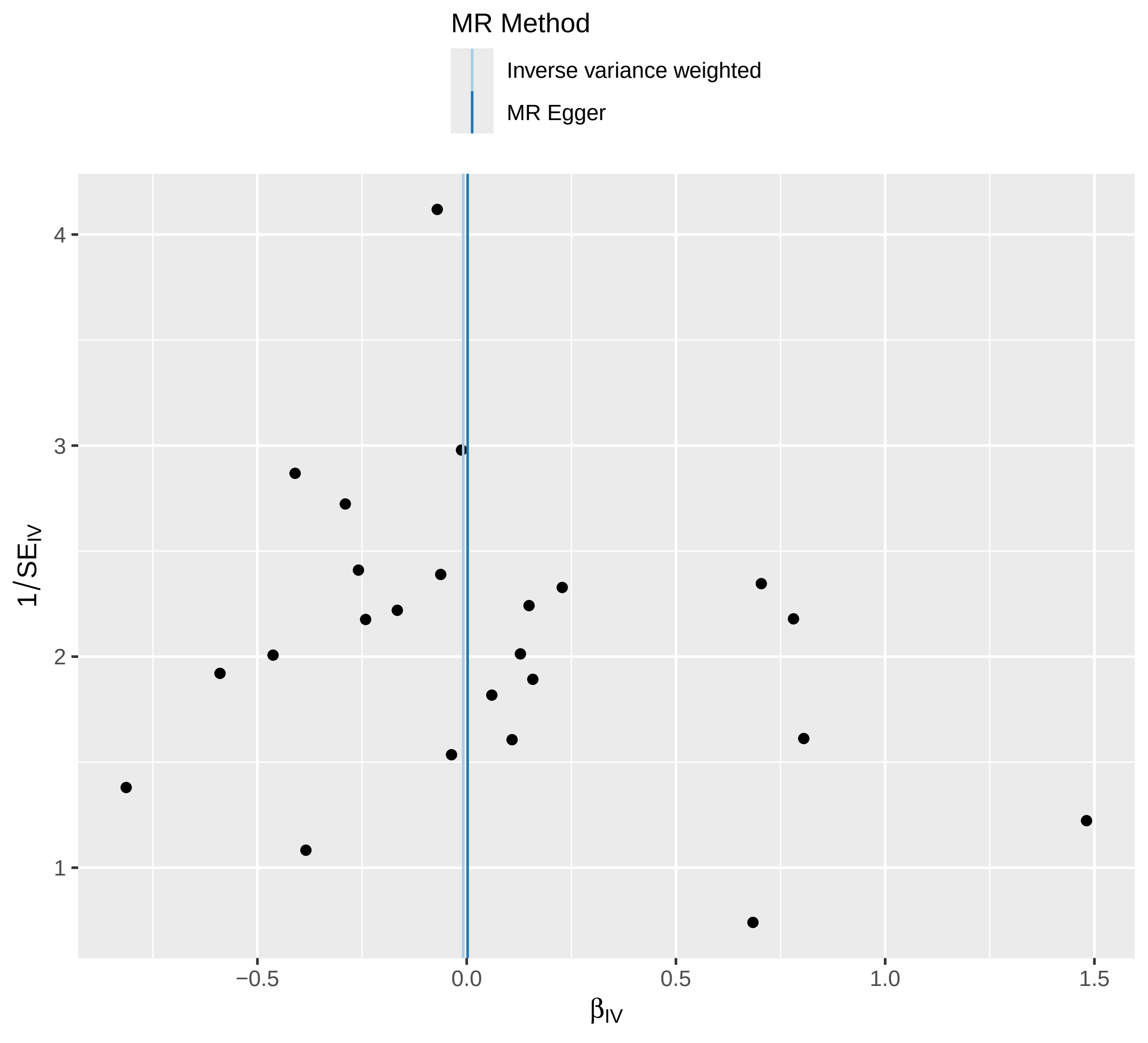

MR Method
Inverse variance weighted
MR Egger
4
3
2
1
1/SE$_{IV}$
−0.5
0.0
0.5
1.0
1.5
β$_{IV}$

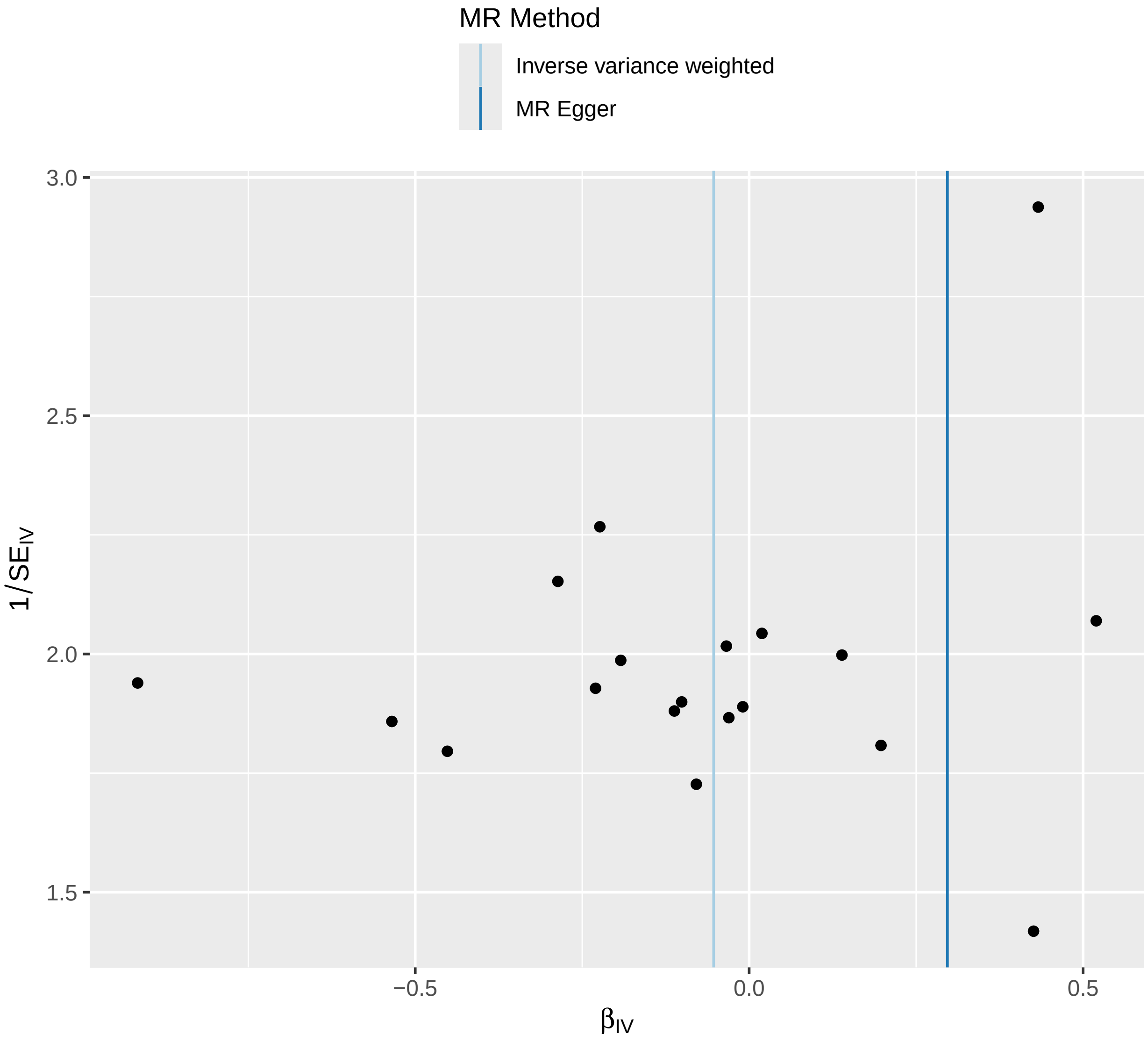

MR Method
Inverse variance weighted
MR Egger
3.0
2.5
2.0
1.5
1/SE_IV
−0.5
0.0
0.5
β_IV

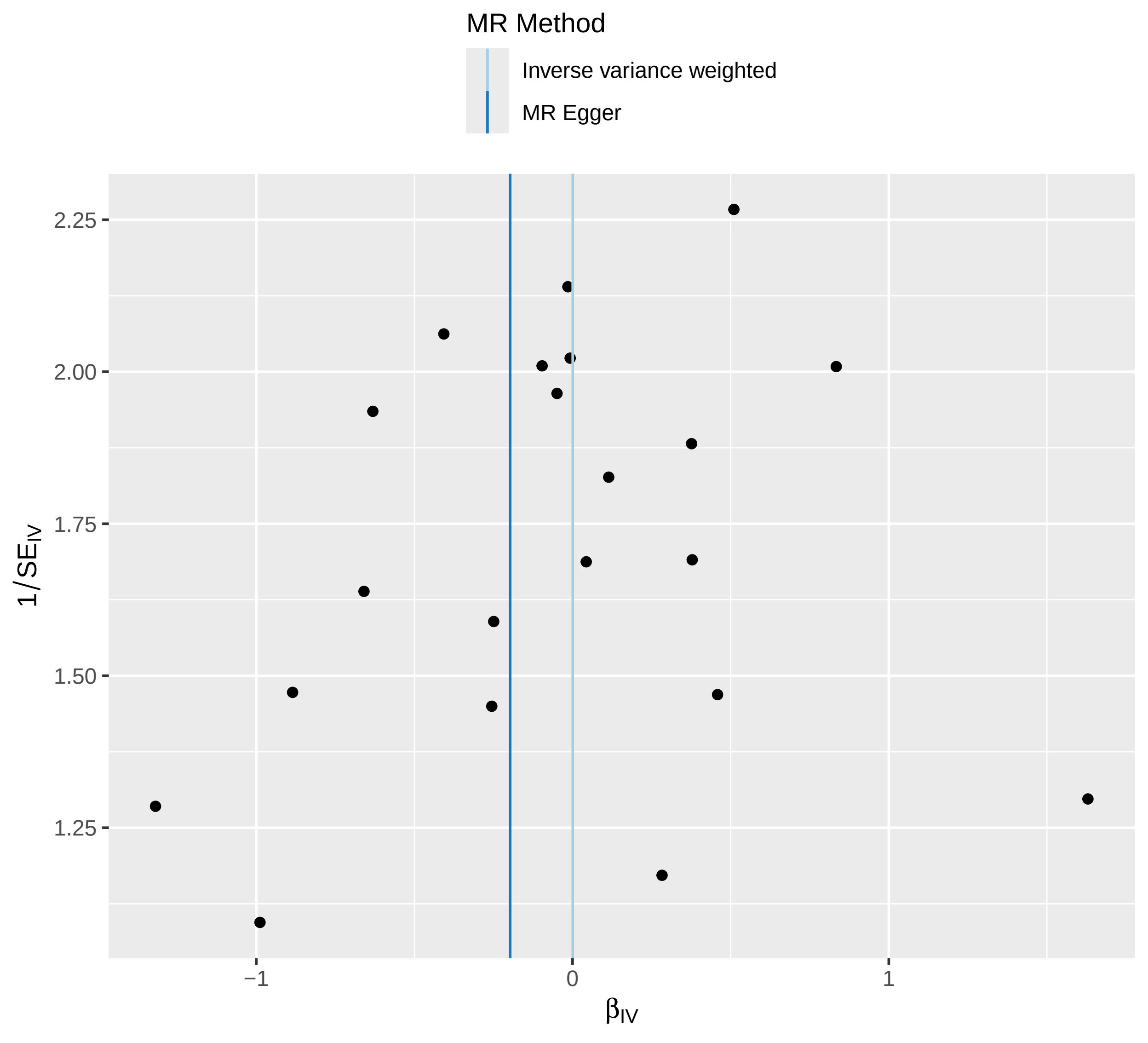
MR Method
Inverse variance weighted
MR Egger
2.25
2.00
1.75
1.50
1.25
1/SE_IV
−1
0
1
β_IV

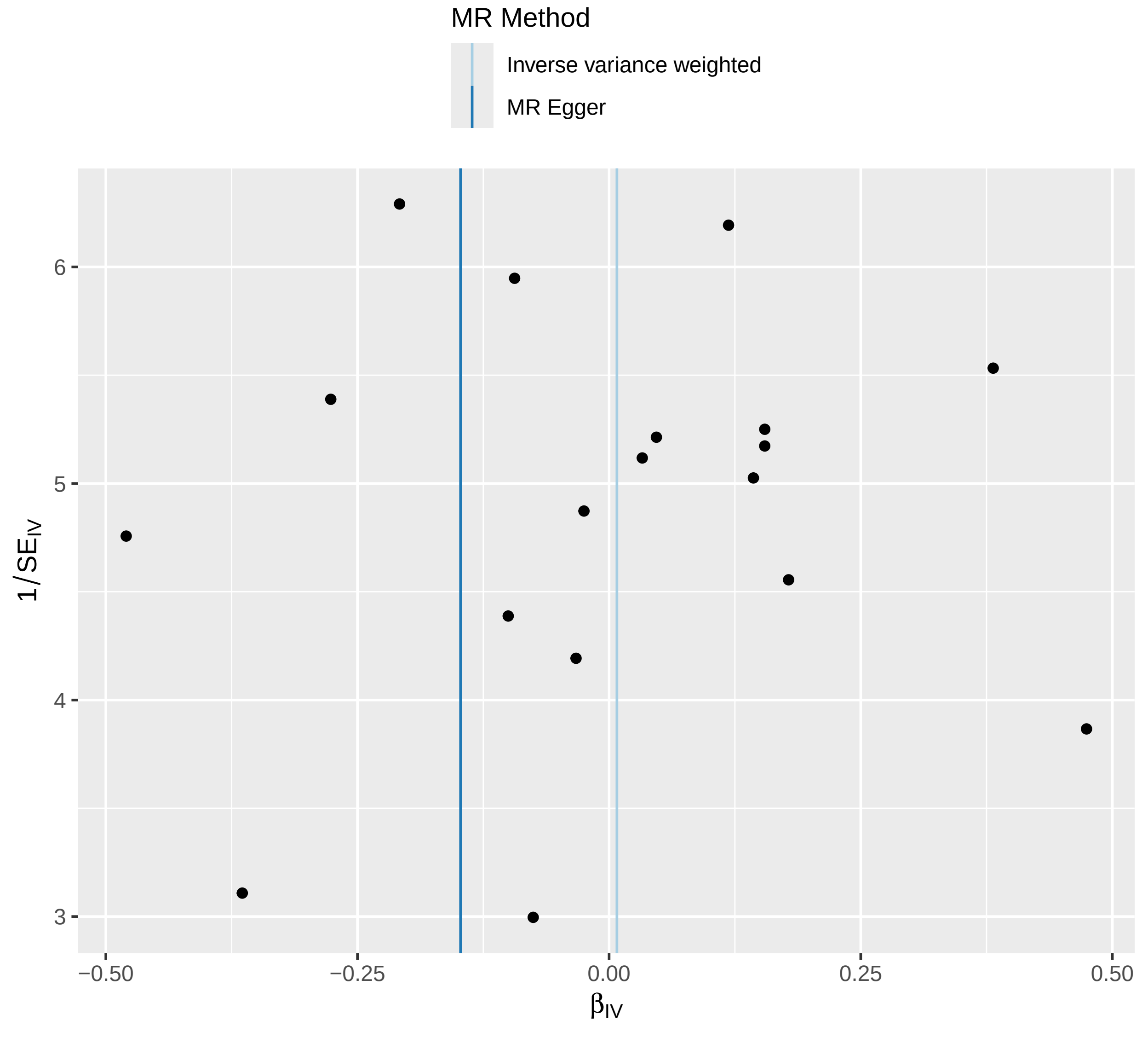

MR Method
Inverse variance weighted
MR Egger
6
5
4
3
1/SE_IV
−0.50
−0.25
0.00
0.25
0.50
β_IV

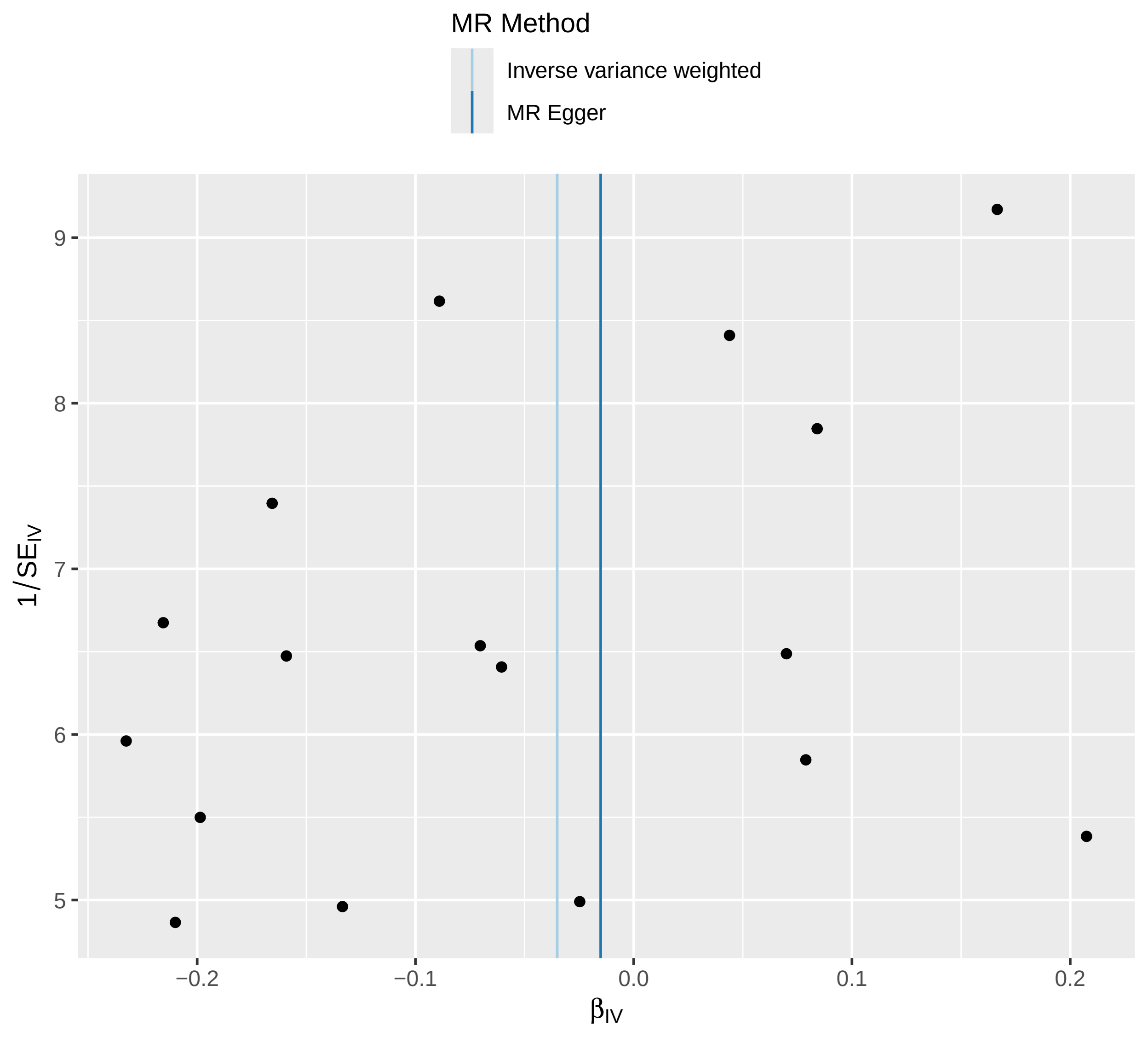
MR Method
Inverse variance weighted
MR Egger
9
8
7
6
5
1/SE_IV
−0.2
−0.1
0.0
0.1
0.2
β_IV

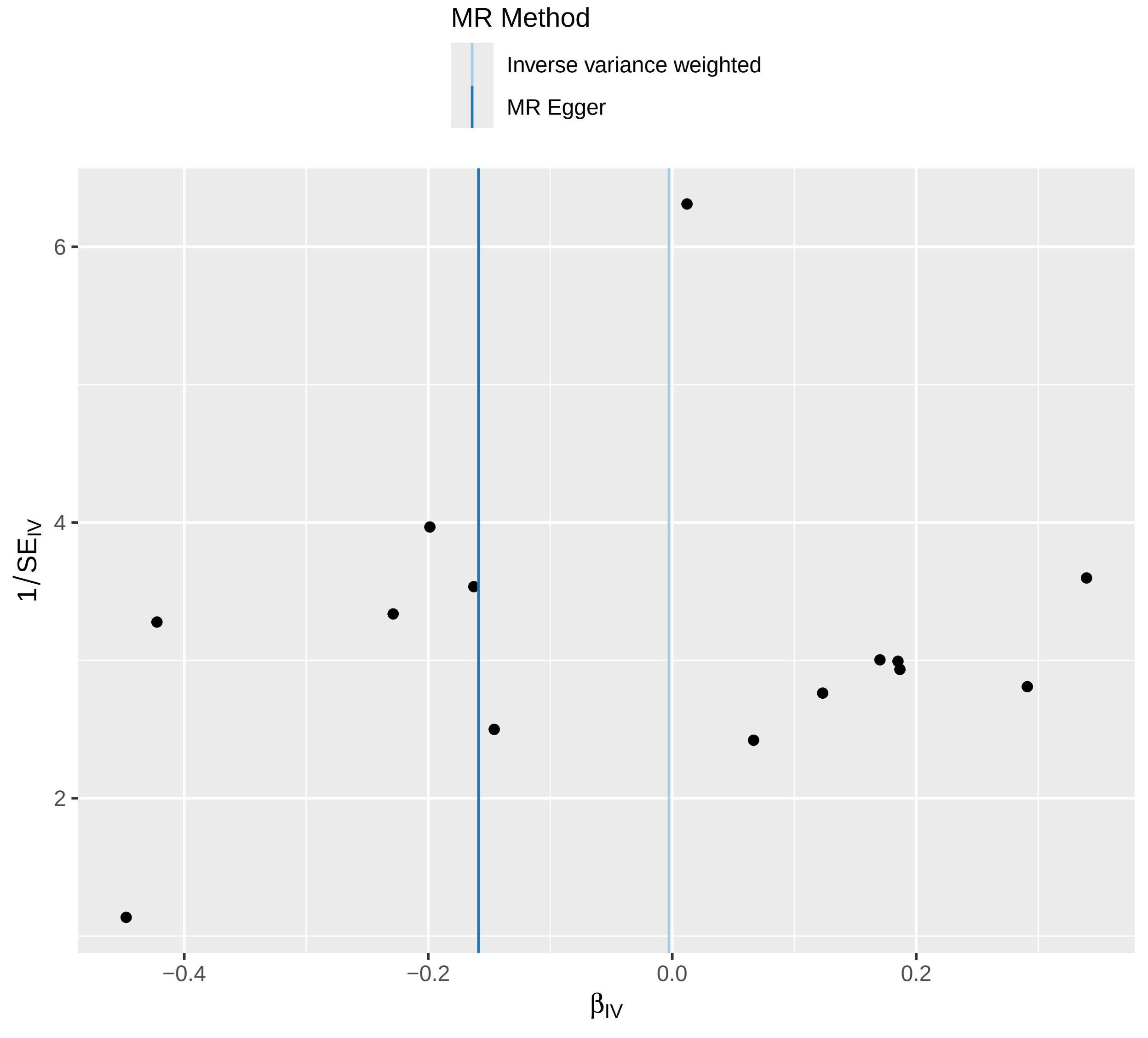
MR Method
Inverse variance weighted
MR Egger
6
4
2
1/SE$_{IV}$
−0.4
−0.2
0.0
0.2
β$_{IV}$

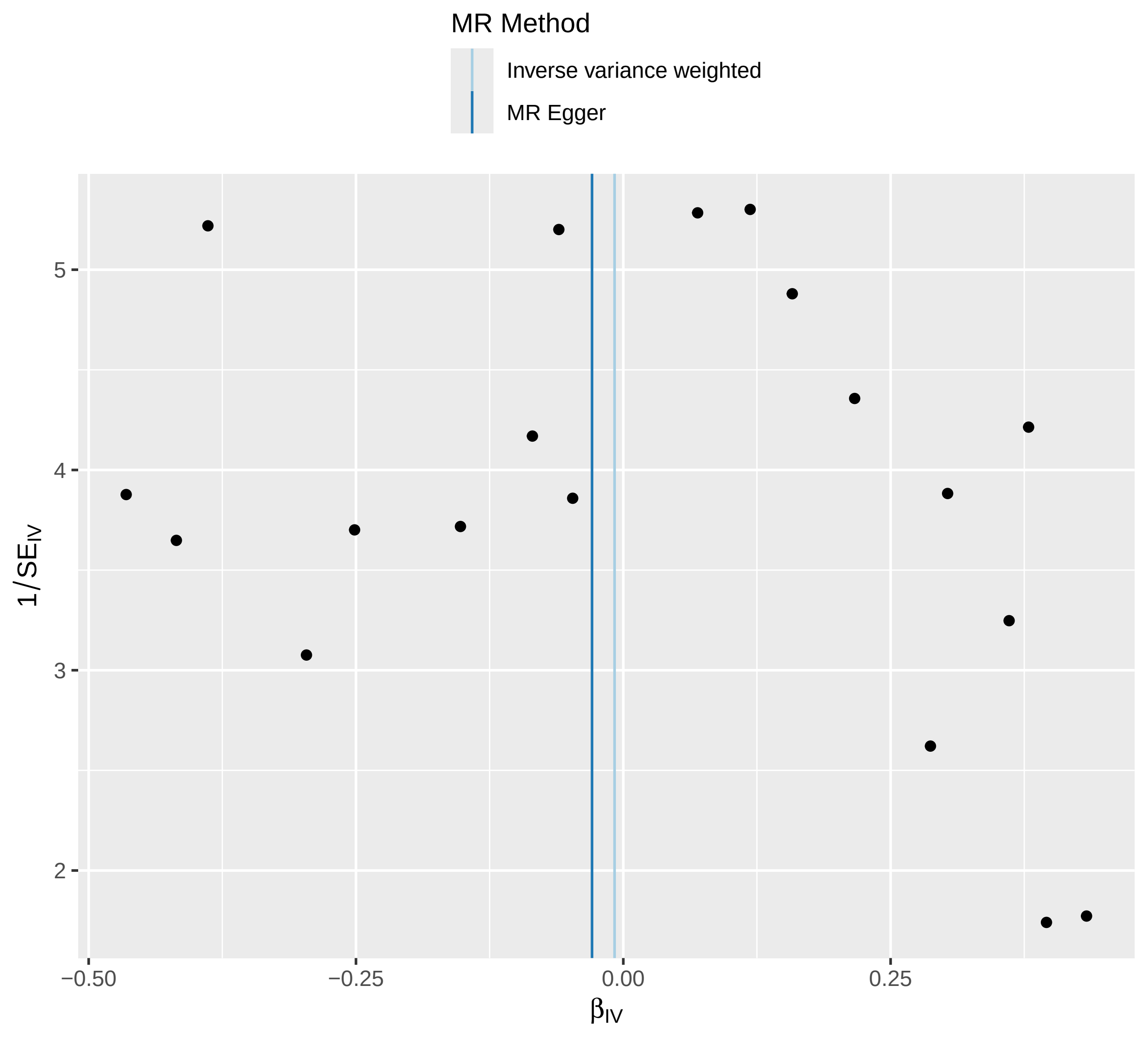

MR Method
Inverse variance weighted
MR Egger
5
4
3
2
$1/SE_{IV}$
−0.50
−0.25
0.00
0.25
β_{IV}

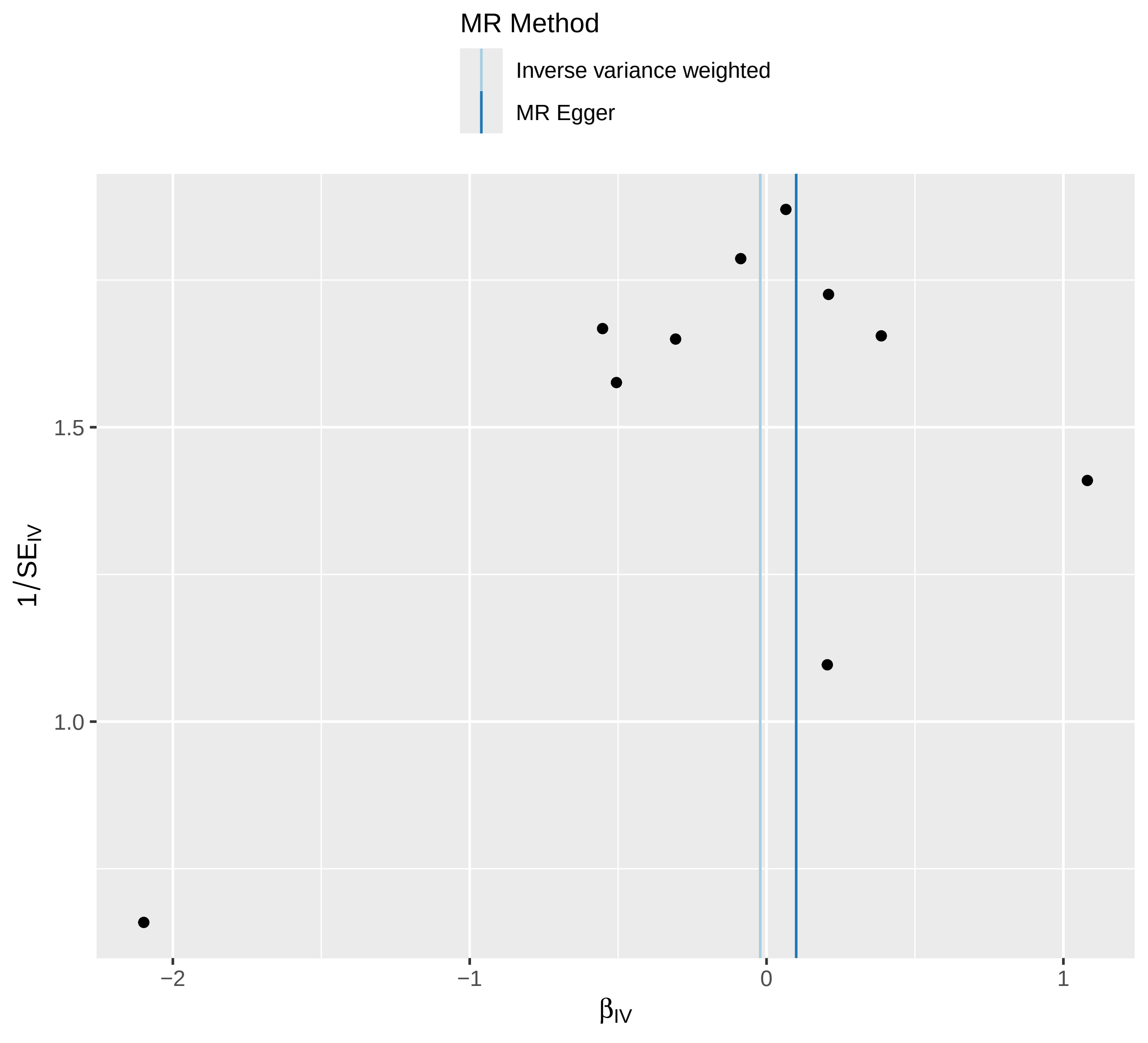

MR Method
Inverse variance weighted
MR Egger
1.5
1.0
1/SE_IV
−2
−1
0
1
β_IV

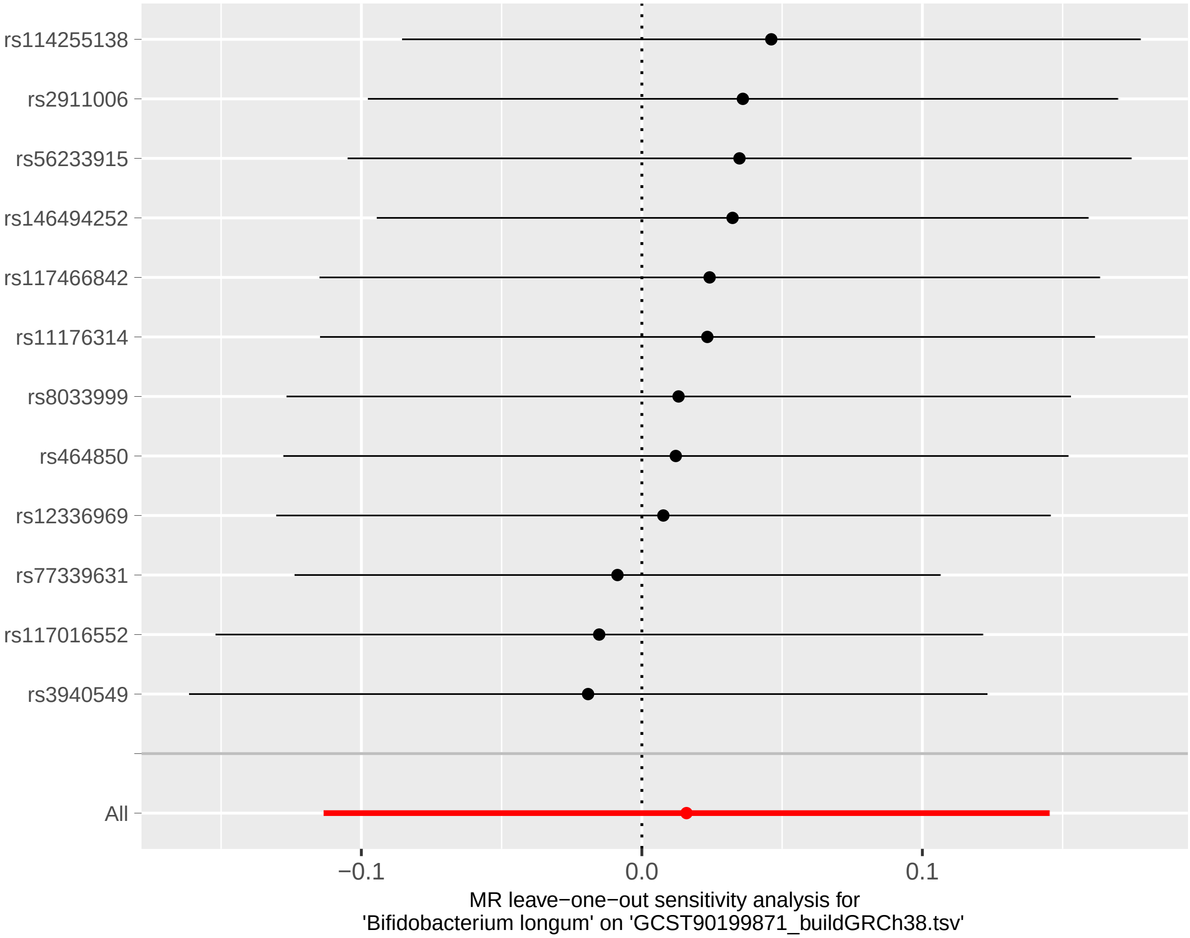
rs114255138
rs2911006
rs56233915
rs146494252
rs117466842
rs11176314
rs8033999
rs464850
rs12336969
rs77339631
rs117016552
rs3940549
All
−0.1
0.0
0.1
MR leave−one−out sensitivity analysis for
'Bifidobacterium longum' on 'GCST90199871_buildGRCh38.tsv'

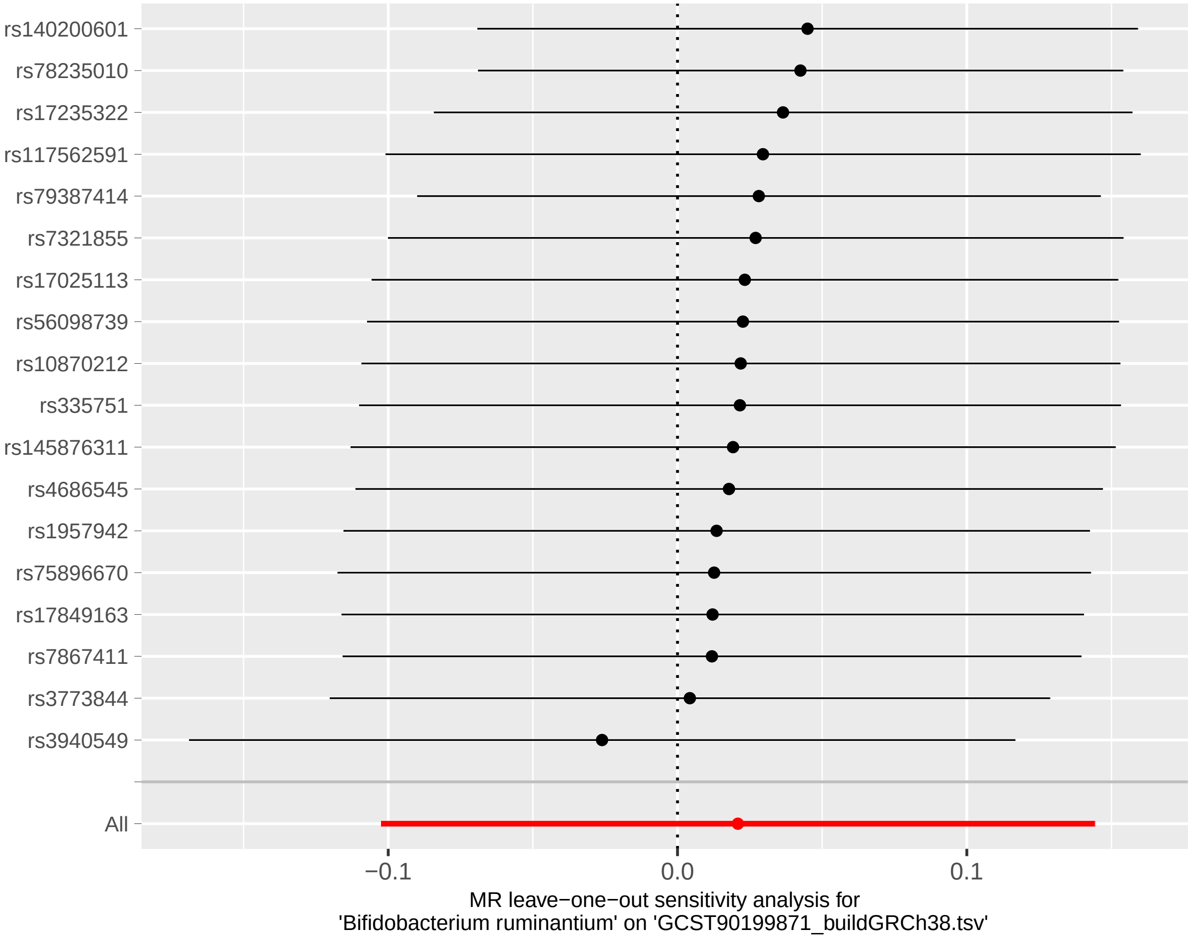
rs140200601
rs78235010
rs17235322
rs117562591
rs79387414
rs7321855
rs17025113
rs56098739
rs10870212
rs335751
rs145876311
rs4686545
rs1957942
rs75896670
rs17849163
rs7867411
rs3773844
rs3940549
All
−0.1
0.0
0.1
MR leave−one−out sensitivity analysis for
'Bifidobacterium ruminantium' on 'GCST90199871_buildGRCh38.tsv'

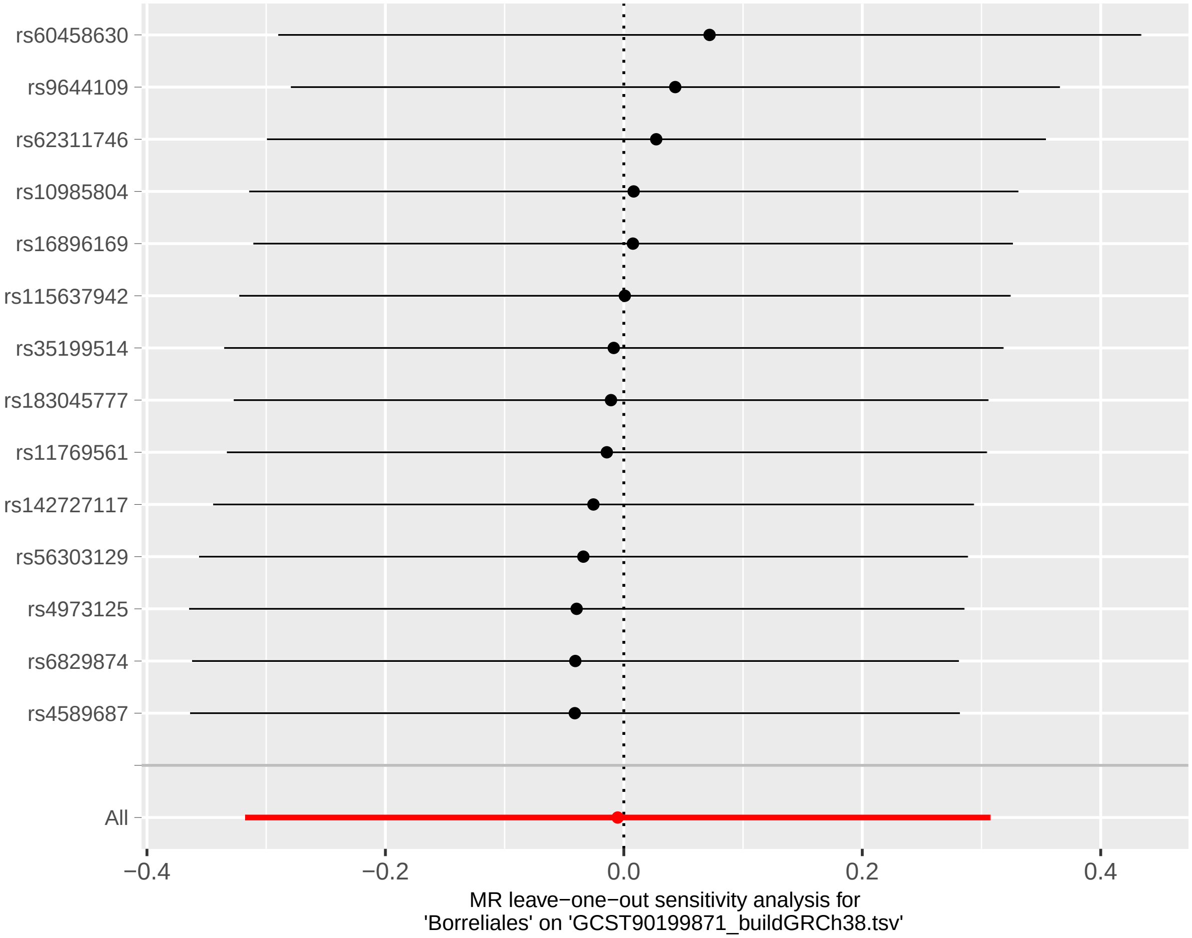

rs60458630
rs9644109
rs62311746
rs10985804
rs16896169
rs115637942
rs35199514
rs183045777
rs11769561
rs142727117
rs56303129
rs4973125
rs6829874
rs4589687
All
−0.4
−0.2
0.0
0.2
0.4
MR leave−one−out sensitivity analysis for
'Borreliales' on 'GCST90199871_buildGRCh38.tsv'

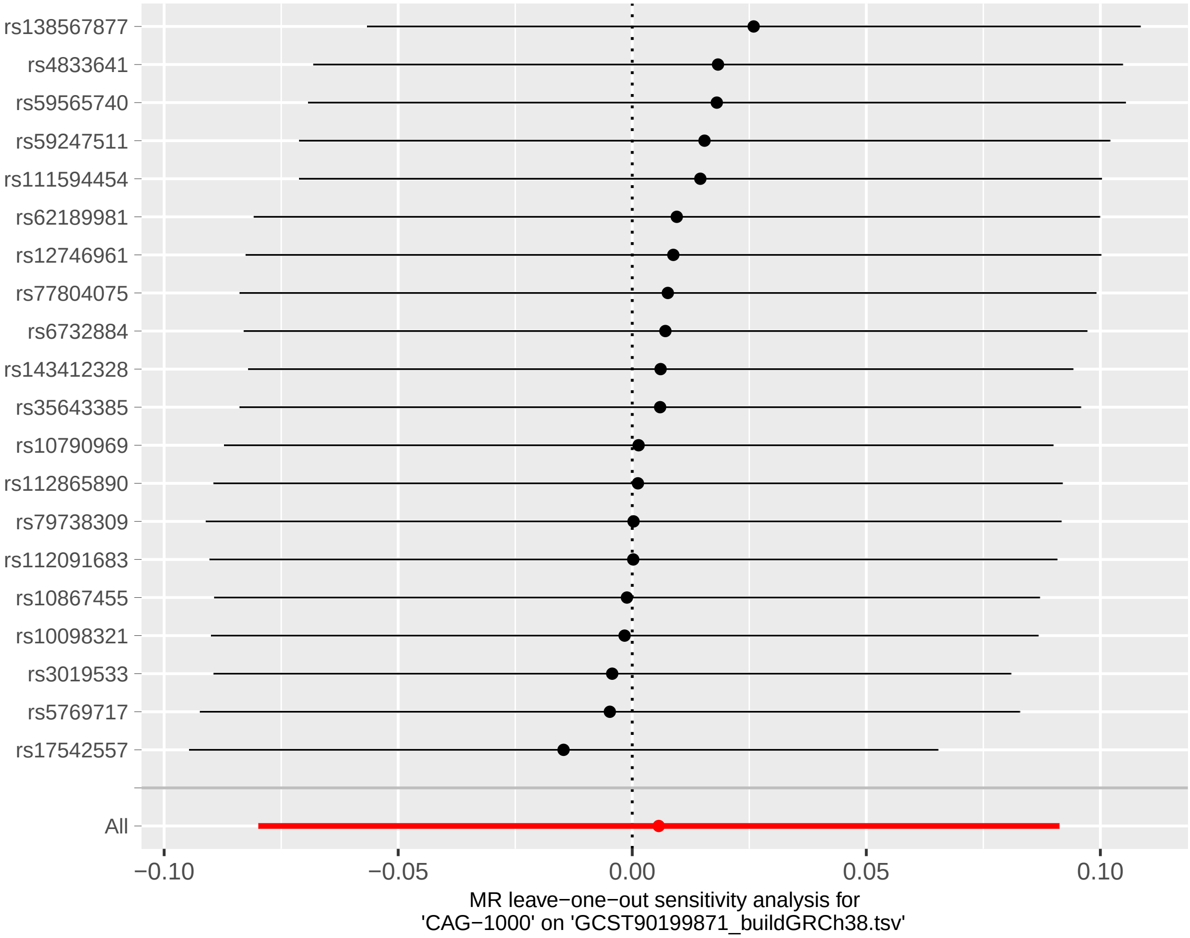
rs138567877
rs4833641
rs59565740
rs59247511
rs111594454
rs62189981
rs12746961
rs77804075
rs6732884
rs143412328
rs35643385
rs10790969
rs112865890
rs79738309
rs112091683
rs10867455
rs10098321
rs3019533
rs5769717
rs17542557
All
−0.10
−0.05
0.00
0.05
0.10
MR leave−one−out sensitivity analysis for
'CAG−1000' on 'GCST90199871_buildGRCh38.tsv'

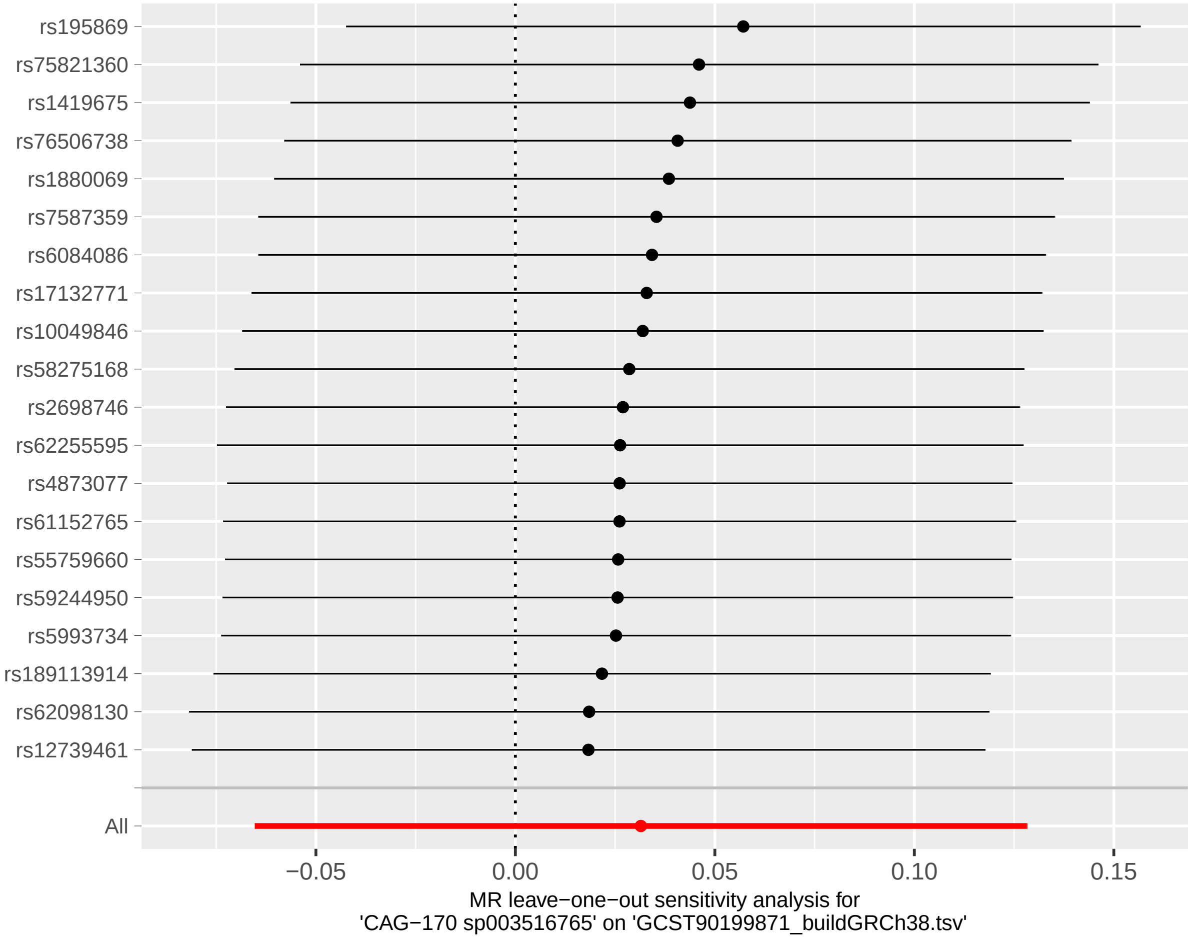
rs195869
rs75821360
rs1419675
rs76506738
rs1880069
rs7587359
rs6084086
rs17132771
rs10049846
rs58275168
rs2698746
rs62255595
rs4873077
rs61152765
rs55759660
rs59244950
rs5993734
rs189113914
rs62098130
rs12739461
All
−0.05
0.00
0.05
0.10
0.15
MR leave−one−out sensitivity analysis for
'CAG−170 sp003516765' on 'GCST90199871_buildGRCh38.tsv'

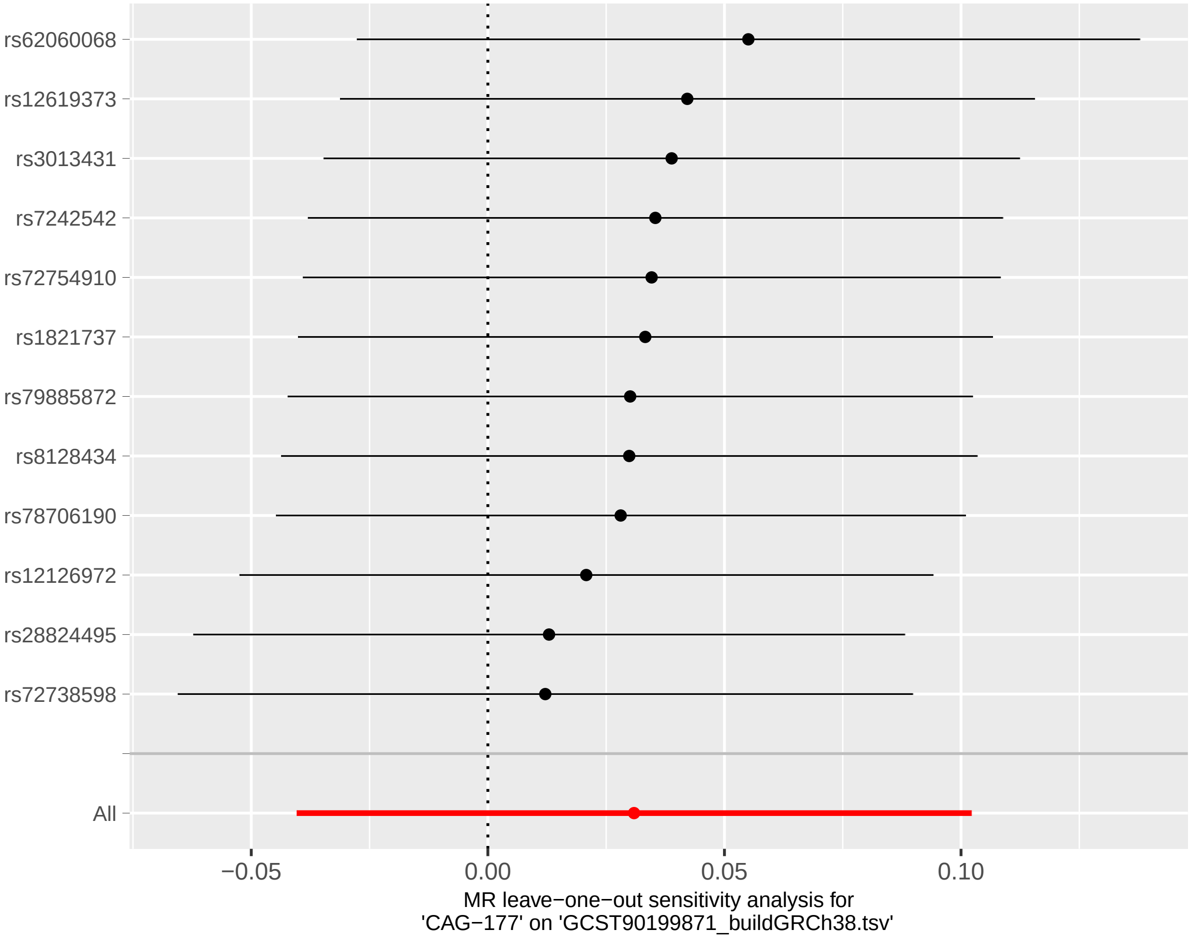

rs62060068
rs12619373
rs3013431
rs7242542
rs72754910
rs1821737
rs79885872
rs8128434
rs78706190
rs12126972
rs28824495
rs72738598
All
−0.05
0.00
0.05
0.10
MR leave−one−out sensitivity analysis for
'CAG−177' on 'GCST90199871_buildGRCh38.tsv'

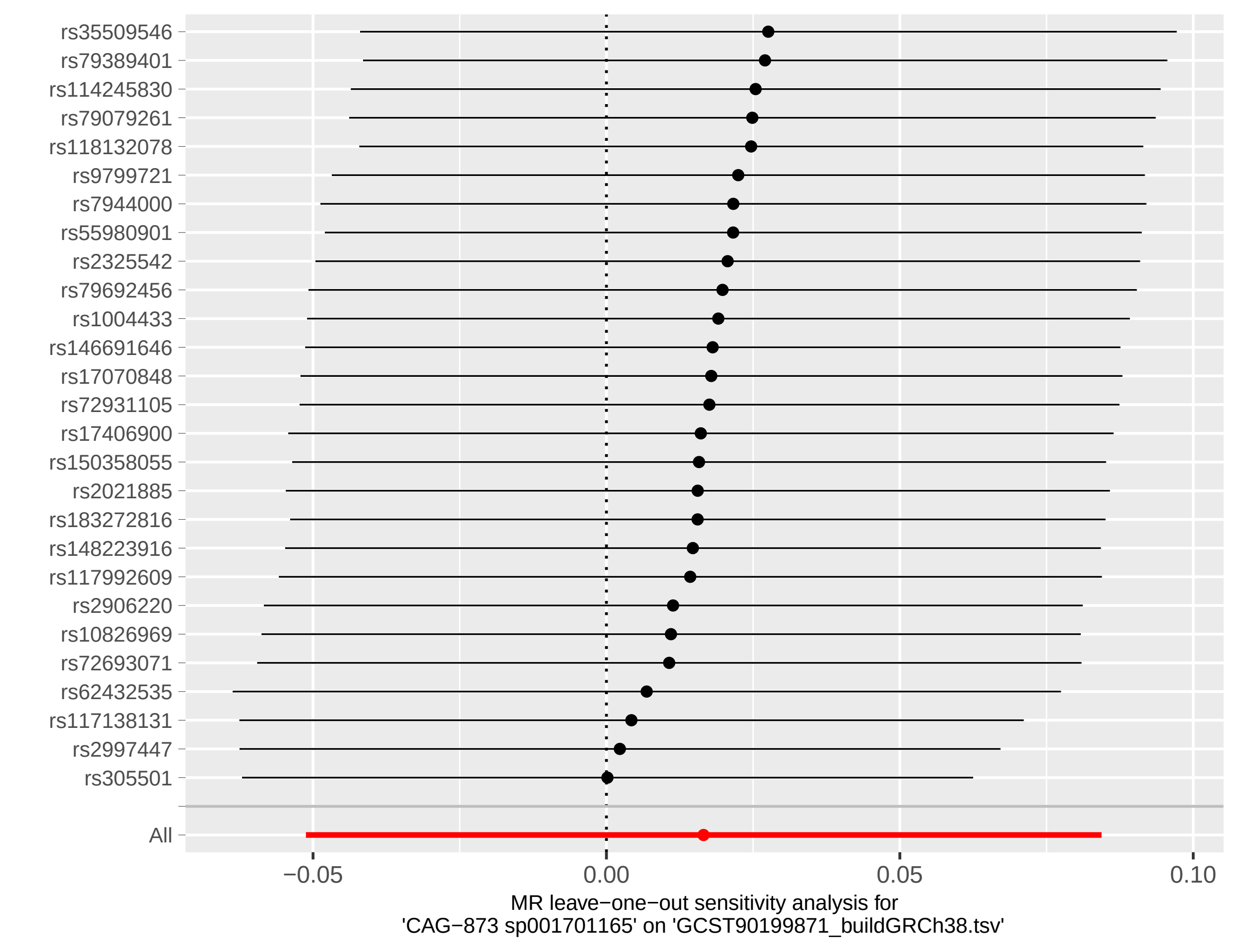
rs35509546
rs79389401
rs114245830
rs79079261
rs118132078
rs9799721
rs7944000
rs55980901
rs2325542
rs79692456
rs1004433
rs146691646
rs17070848
rs72931105
rs17406900
rs150358055
rs2021885
rs183272816
rs148223916
rs117992609
rs2906220
rs10826969
rs72693071
rs62432535
rs117138131
rs2997447
rs305501
All
−0.05
0.00
0.05
0.10
MR leave−one−out sensitivity analysis for
'CAG−873 sp001701165' on 'GCST90199871_buildGRCh38.tsv'

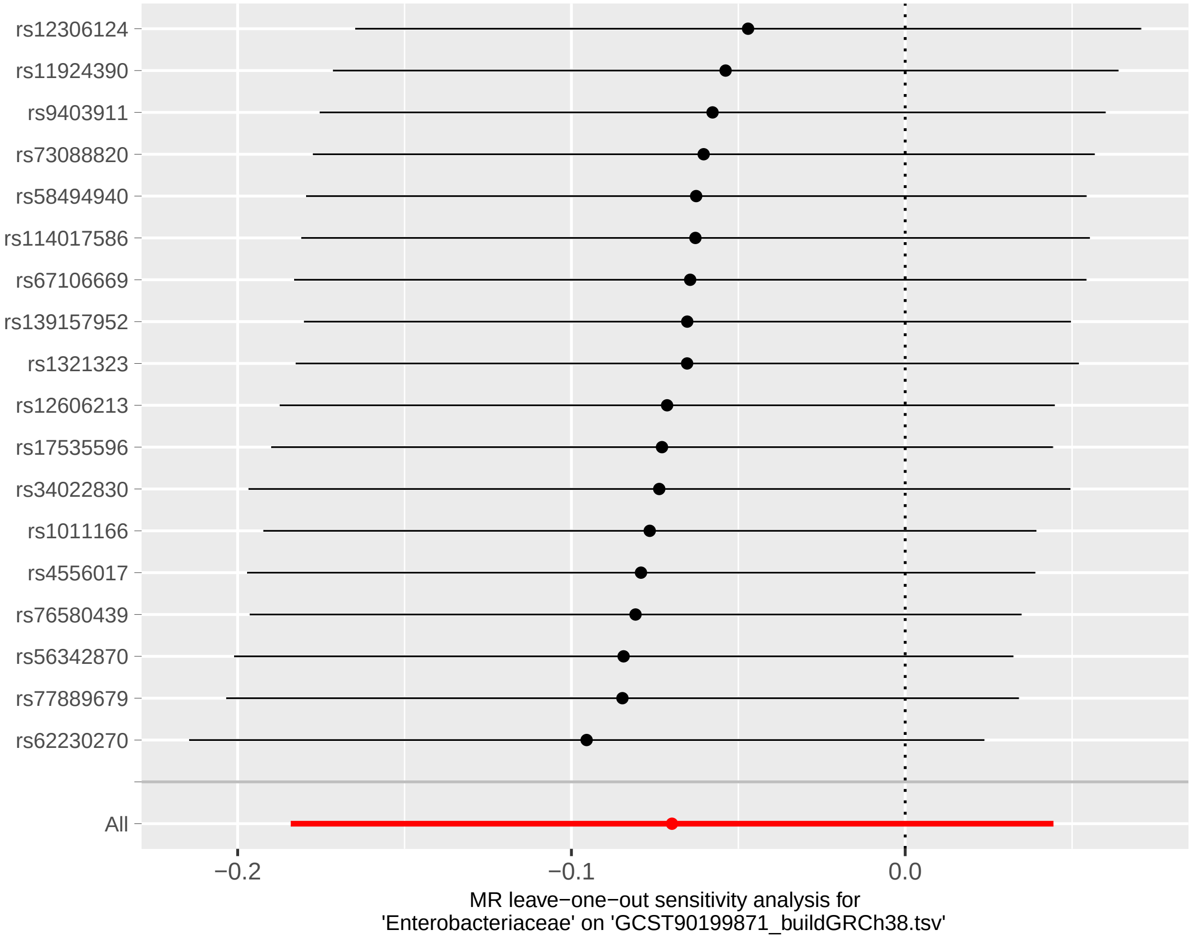

rs12306124
rs11924390
rs9403911
rs73088820
rs58494940
rs114017586
rs67106669
rs139157952
rs1321323
rs12606213
rs17535596
rs34022830
rs1011166
rs4556017
rs76580439
rs56342870
rs77889679
rs62230270
All
−0.2
−0.1
0.0
MR leave−one−out sensitivity analysis for
'Enterobacteriaceae' on 'GCST90199871_buildGRCh38.tsv'

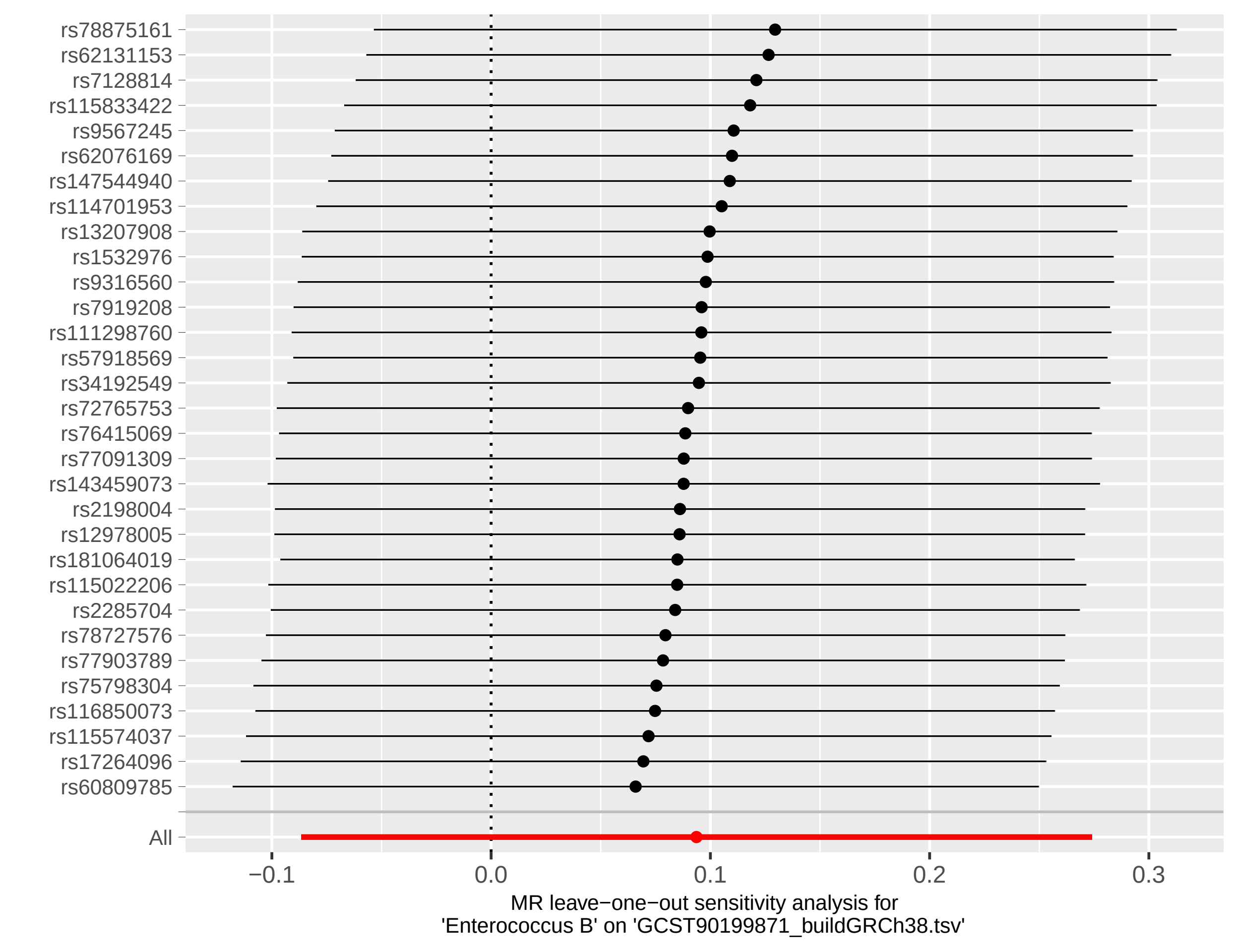
rs78875161
rs62131153
rs7128814
rs115833422
rs9567245
rs62076169
rs147544940
rs114701953
rs13207908
rs1532976
rs9316560
rs7919208
rs111298760
rs57918569
rs34192549
rs72765753
rs76415069
rs77091309
rs143459073
rs2198004
rs12978005
rs181064019
rs115022206
rs2285704
rs78727576
rs77903789
rs75798304
rs116850073
rs115574037
rs17264096
rs60809785
All
−0.1
0.0
0.1
0.2
0.3
MR leave−one−out sensitivity analysis for
'Enterococcus B' on 'GCST90199871_buildGRCh38.tsv'

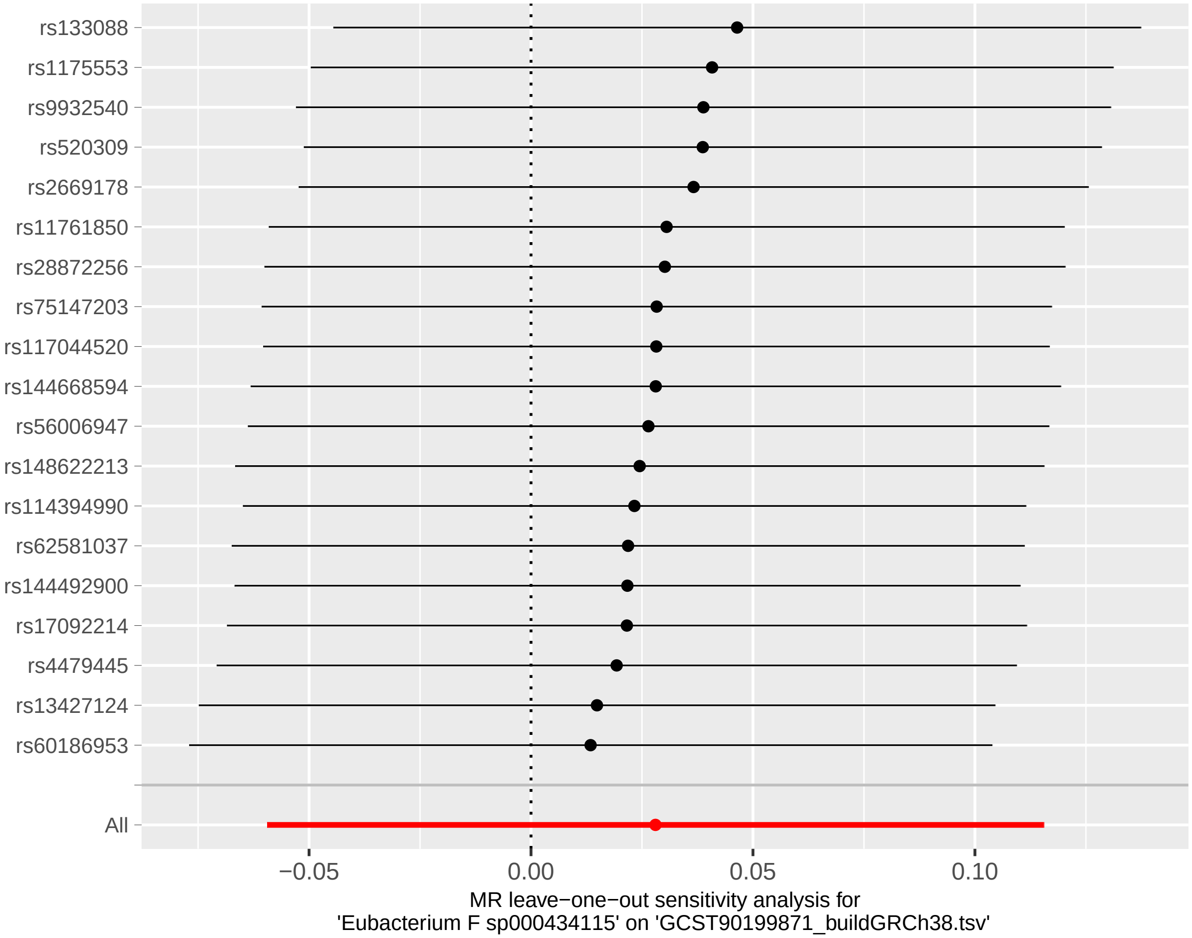

rs133088
rs1175553
rs9932540
rs520309
rs2669178
rs11761850
rs28872256
rs75147203
rs117044520
rs144668594
rs56006947
rs148622213
rs114394990
rs62581037
rs144492900
rs17092214
rs4479445
rs13427124
rs60186953
All
−0.05
0.00
0.05
0.10
MR leave−one−out sensitivity analysis for
'Eubacterium F sp000434115' on 'GCST90199871_buildGRCh38.tsv'

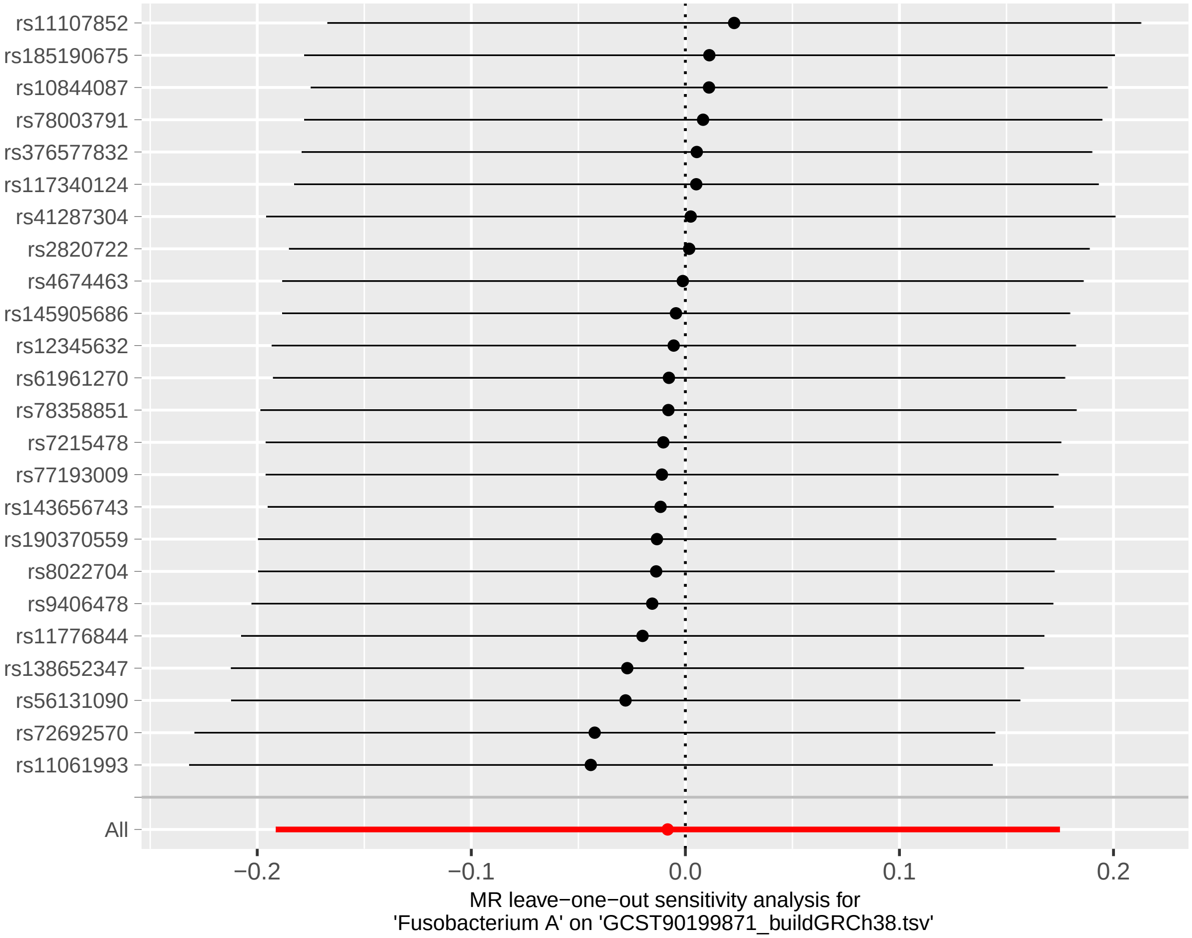
rs11107852
rs185190675
rs10844087
rs78003791
rs376577832
rs117340124
rs41287304
rs2820722
rs4674463
rs145905686
rs12345632
rs61961270
rs78358851
rs7215478
rs77193009
rs143656743
rs190370559
rs8022704
rs9406478
rs11776844
rs138652347
rs56131090
rs72692570
rs11061993
All
−0.2
−0.1
0.0
0.1
0.2
MR leave−one−out sensitivity analysis for
'Fusobacterium A' on 'GCST90199871_buildGRCh38.tsv'

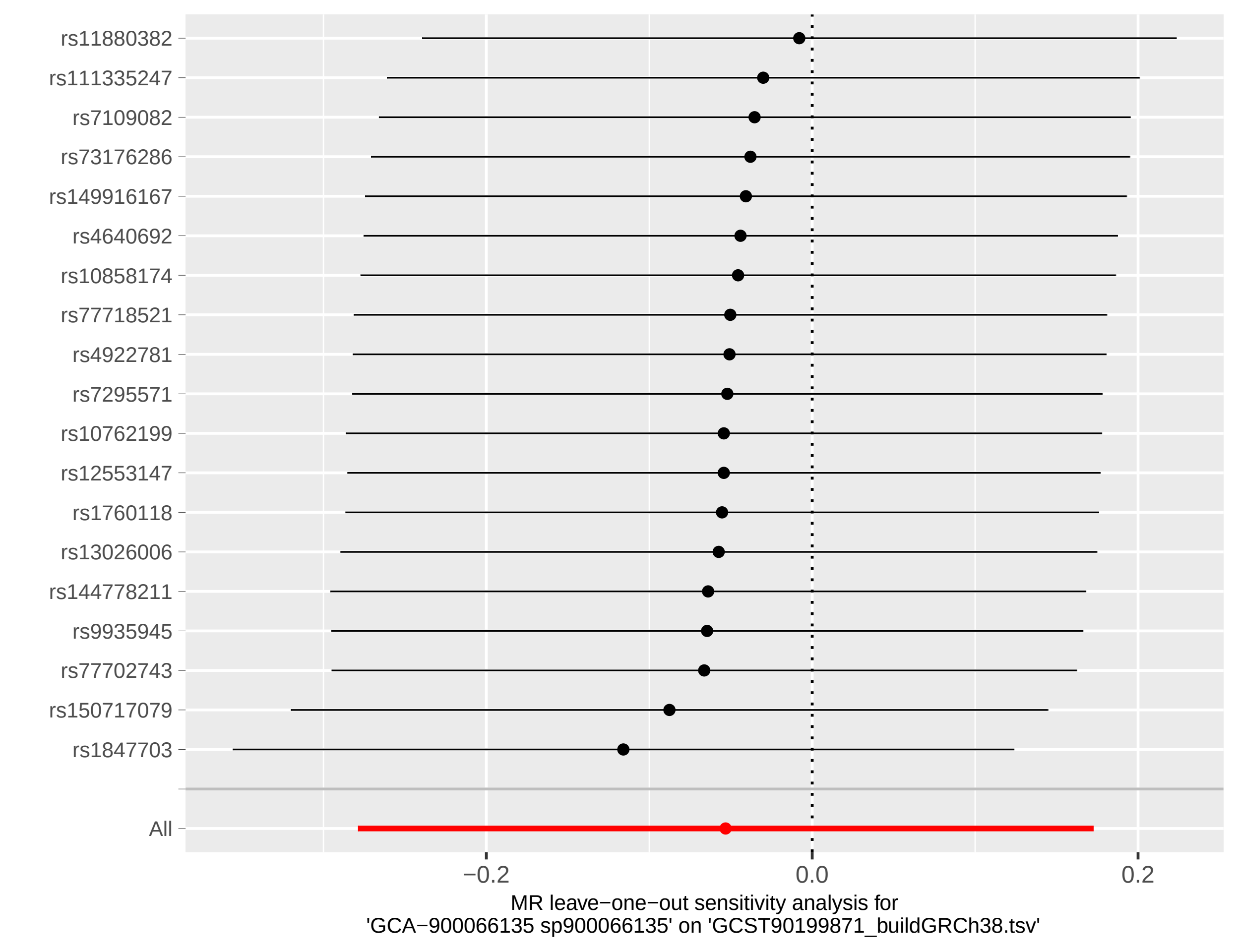
rs11880382
rs111335247
rs7109082
rs73176286
rs149916167
rs4640692
rs10858174
rs77718521
rs4922781
rs7295571
rs10762199
rs12553147
rs1760118
rs13026006
rs144778211
rs9935945
rs77702743
rs150717079
rs1847703
All
−0.2
0.0
0.2
MR leave−one−out sensitivity analysis for
'GCA−900066135 sp900066135' on 'GCST90199871_buildGRCh38.tsv'

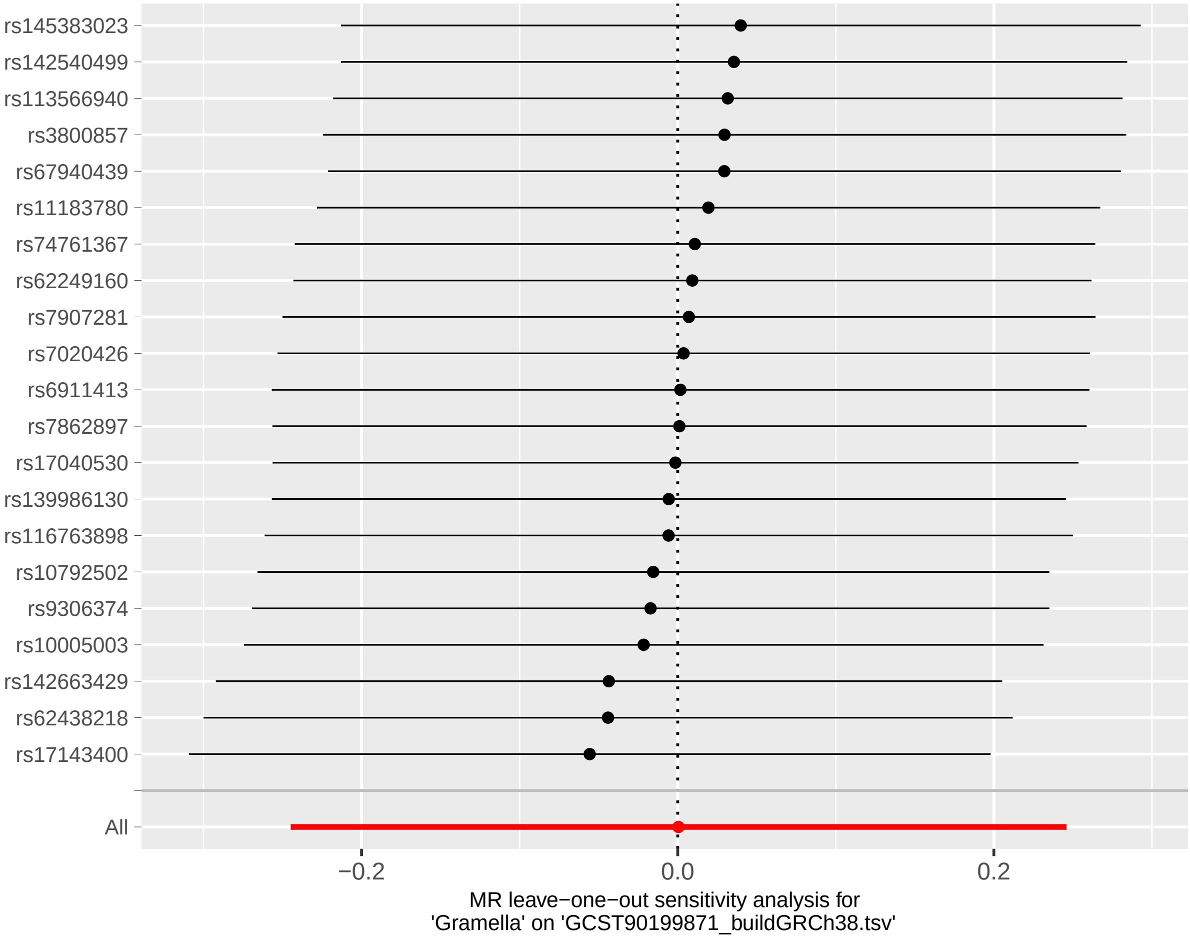

rs145383023
rs142540499
rs113566940
rs3800857
rs67940439
rs11183780
rs74761367
rs62249160
rs7907281
rs7020426
rs6911413
rs7862897
rs17040530
rs139986130
rs116763898
rs10792502
rs9306374
rs10005003
rs142663429
rs62438218
rs17143400
All
−0.2
0.0
0.2
MR leave−one−out sensitivity analysis for
'Gramella' on 'GCST90199871_buildGRCh38.tsv'

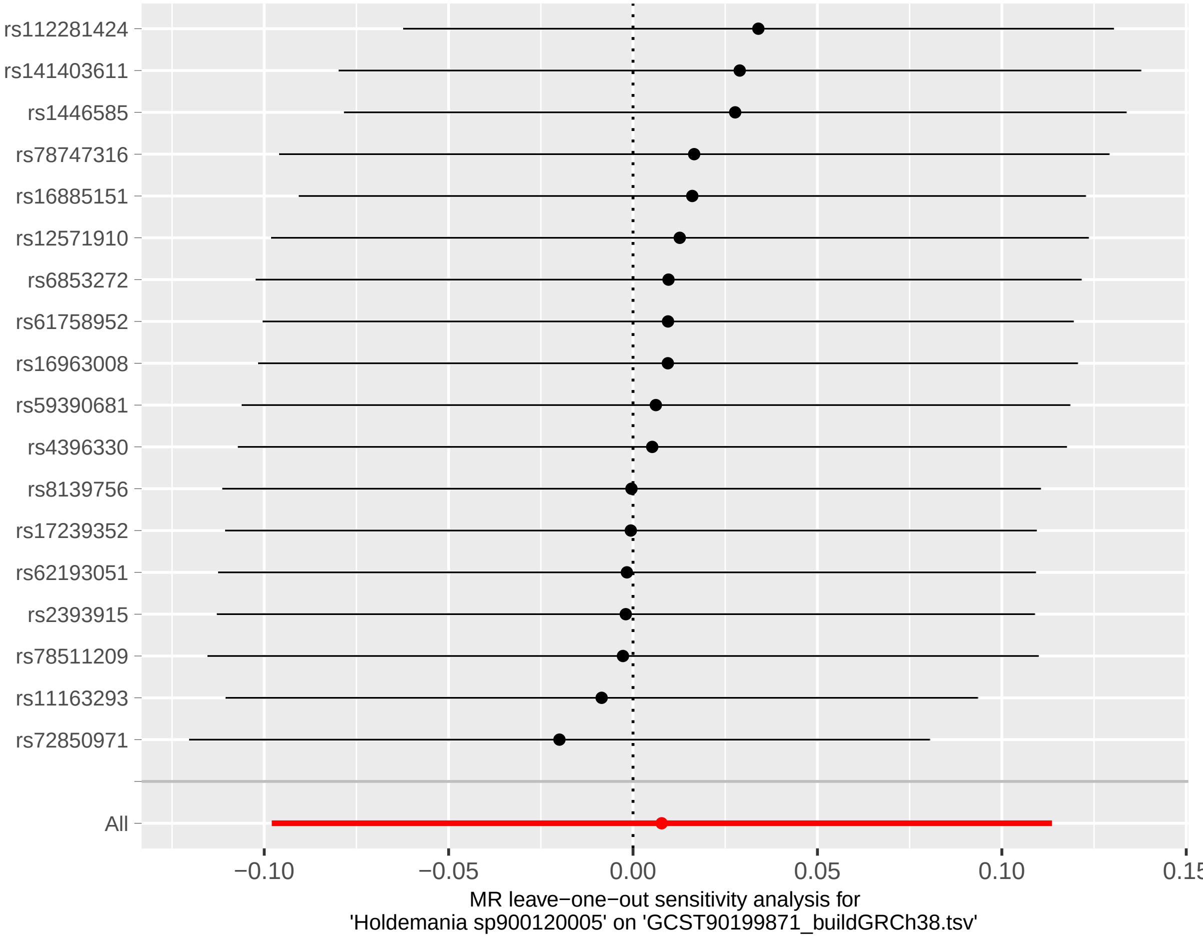
rs112281424
rs141403611
rs1446585
rs78747316
rs16885151
rs12571910
rs6853272
rs61758952
rs16963008
rs59390681
rs4396330
rs8139756
rs17239352
rs62193051
rs2393915
rs78511209
rs11163293
rs72850971
All
−0.10
−0.05
0.00
0.05
0.10
MR leave−one−out sensitivity analysis for
'Holdemania sp900120005' on 'GCST90199871_buildGRCh38.tsv'

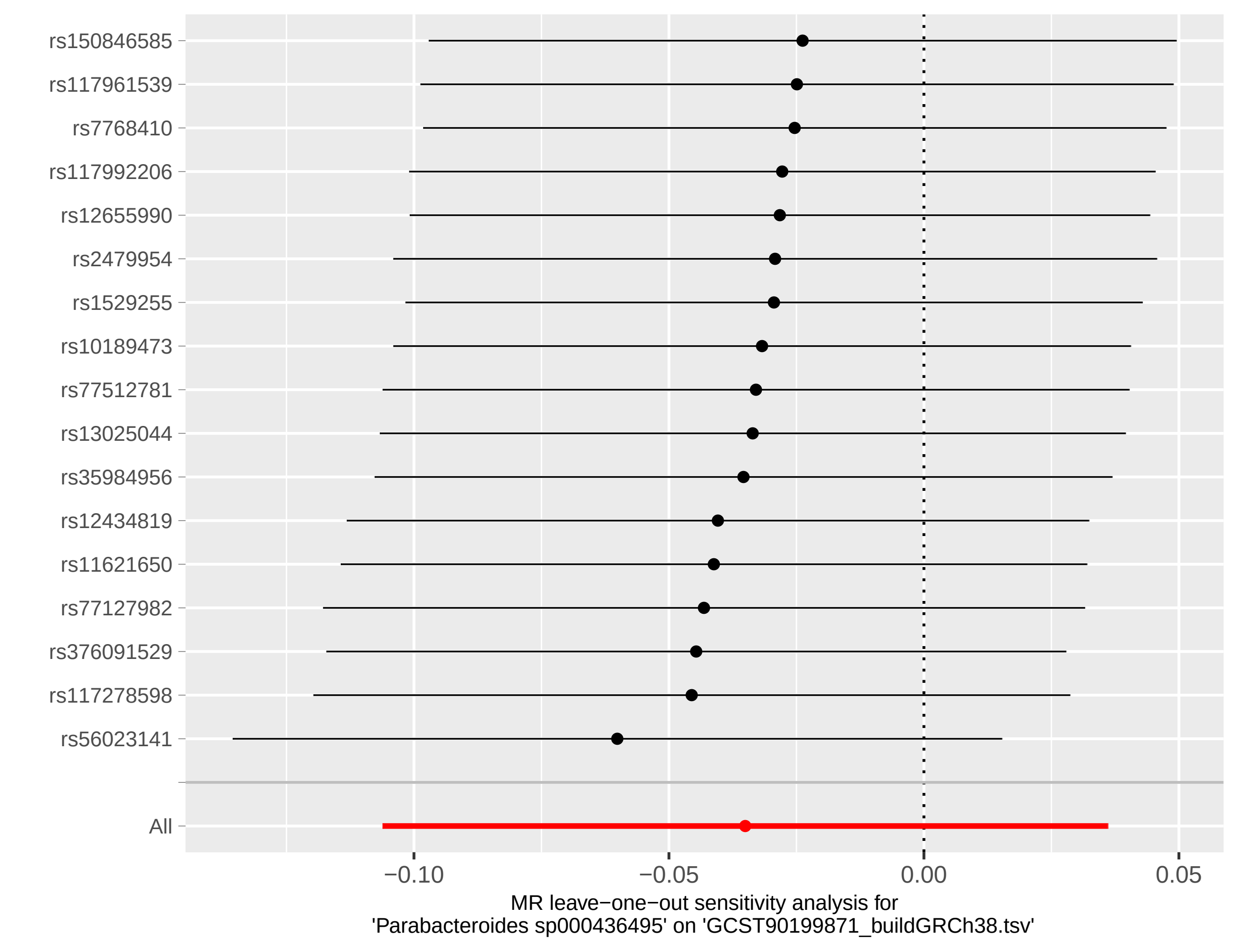
rs150846585
rs117961539
rs7768410
rs117992206
rs12655990
rs2479954
rs1529255
rs10189473
rs77512781
rs13025044
rs35984956
rs12434819
rs11621650
rs77127982
rs376091529
rs117278598
rs56023141
All
−0.10
−0.05
0.00
0.05
MR leave−one−out sensitivity analysis for
'Parabacteroides sp000436495' on 'GCST90199871_buildGRCh38.tsv'

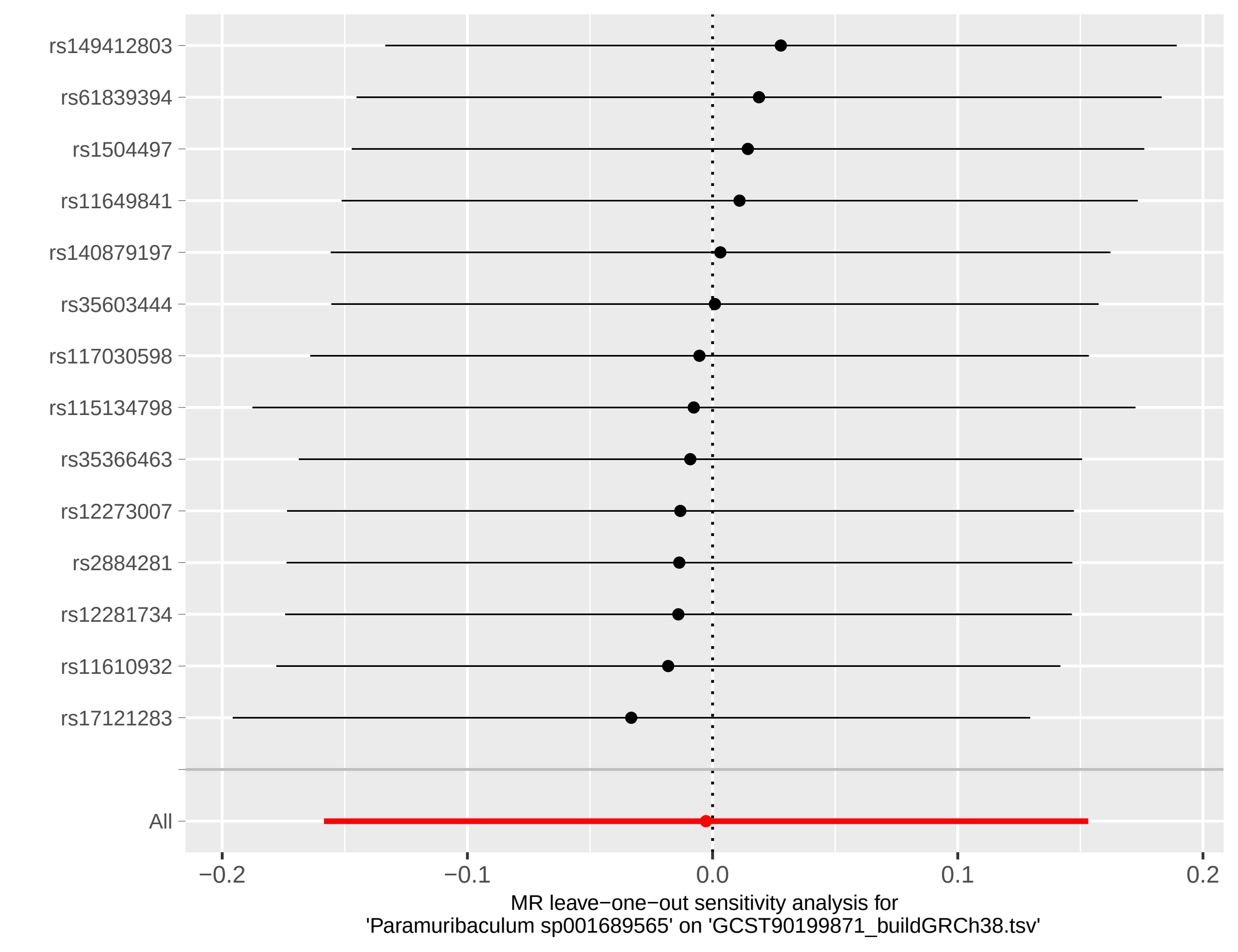

rs149412803
rs61839394
rs1504497
rs11649841
rs140879197
rs35603444
rs117030598
rs115134798
rs35366463
rs12273007
rs2884281
rs12281734
rs11610932
rs17121283
All
−0.2
−0.1
0.0
0.1
0.2
MR leave−one−out sensitivity analysis for
'Paramuribaculum sp001689565' on 'GCST90199871_buildGRCh38.tsv'

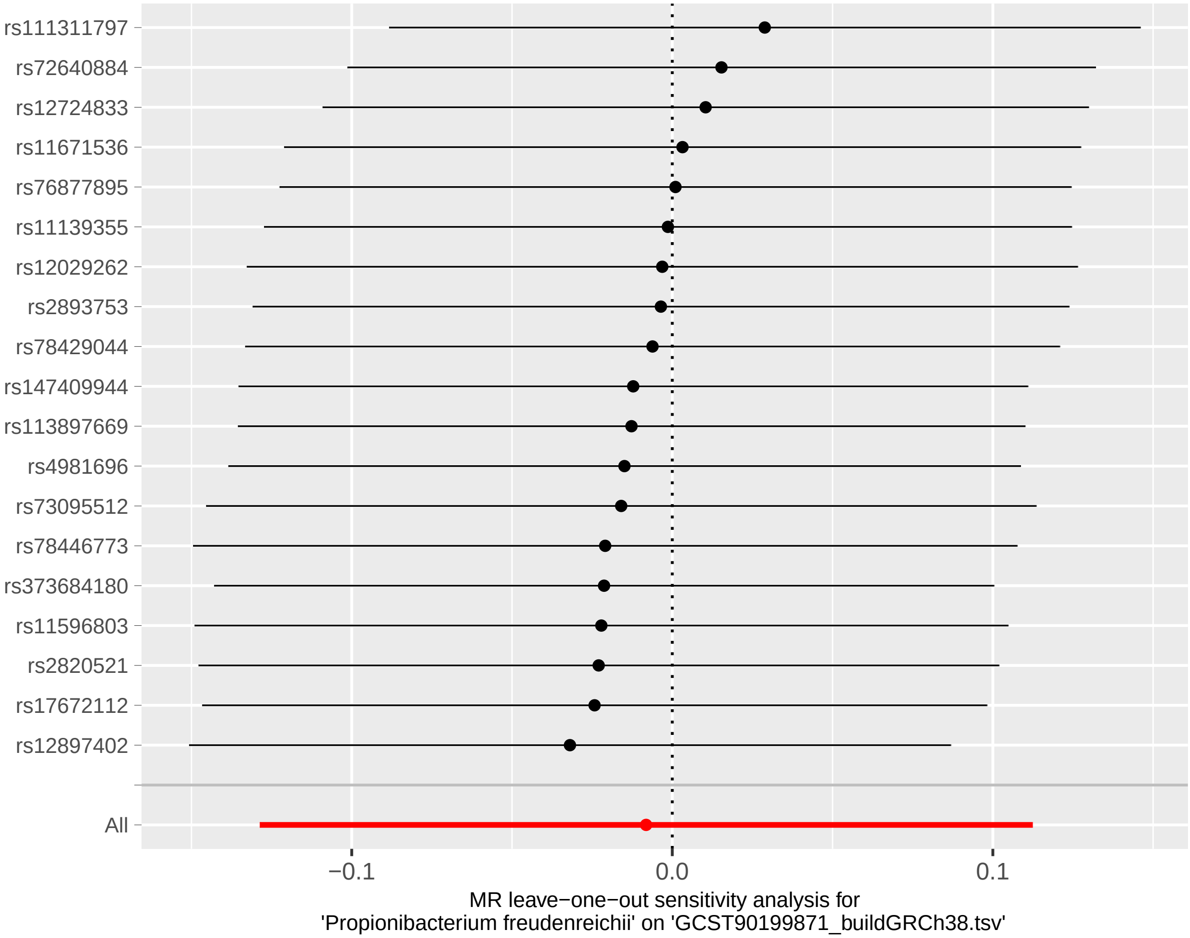

rs111311797
rs72640884
rs12724833
rs11671536
rs76877895
rs11139355
rs12029262
rs2893753
rs78429044
rs147409944
rs113897669
rs4981696
rs73095512
rs78446773
rs373684180
rs11596803
rs2820521
rs17672112
rs12897402
All
−0.1
0.0
0.1
MR leave−one−out sensitivity analysis for
'Propionibacterium freudenreichii' on 'GCST90199871_buildGRCh38.tsv'

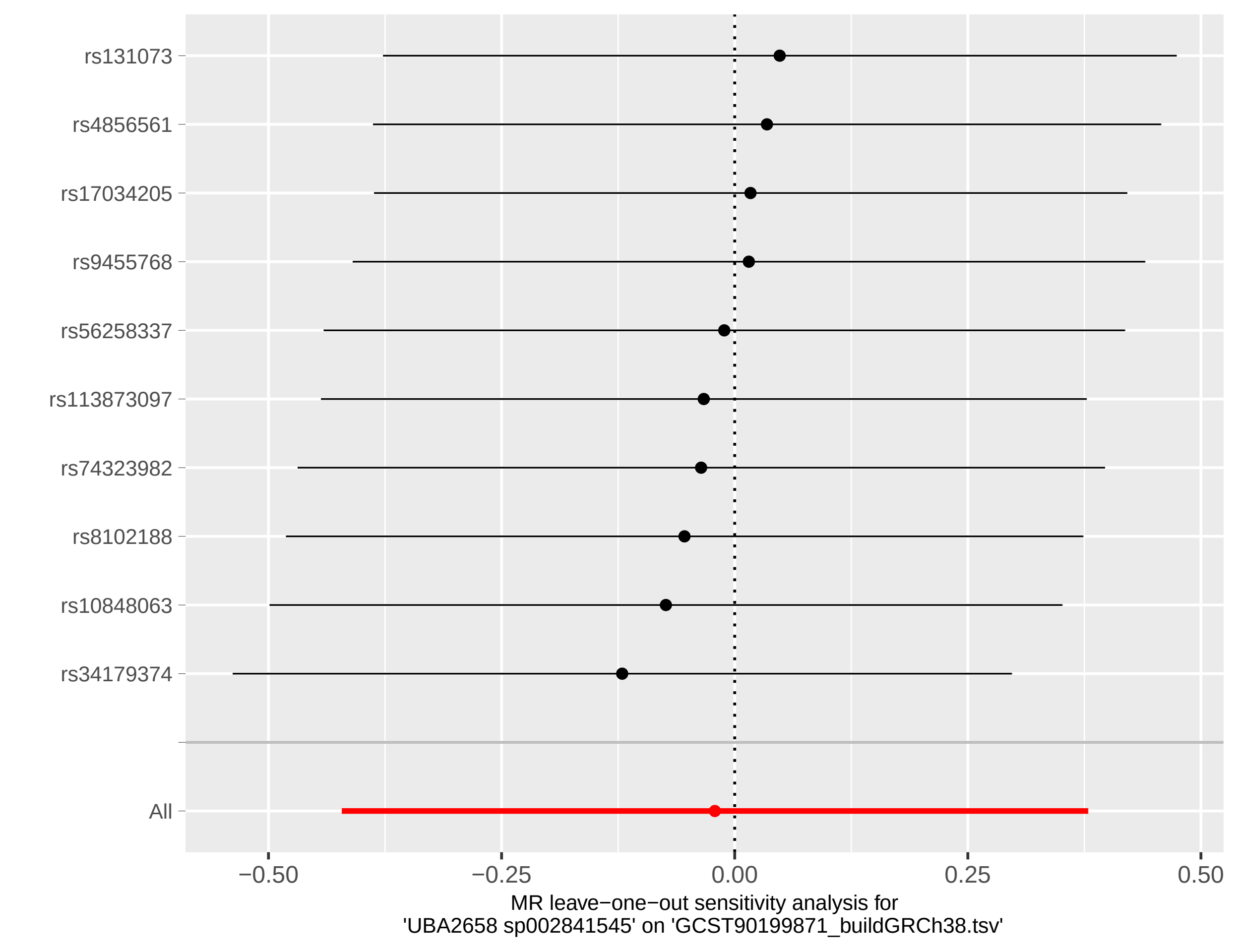

rs131073
rs4856561
rs17034205
rs9455768
rs56258337
rs113873097
rs74323982
rs8102188
rs10848063
rs34179374
All
−0.50
−0.25
0.00
0.25
0.50
MR leave−one−out sensitivity analysis for
'UBA2658 sp002841545' on 'GCST90199871_buildGRCh38.tsv'

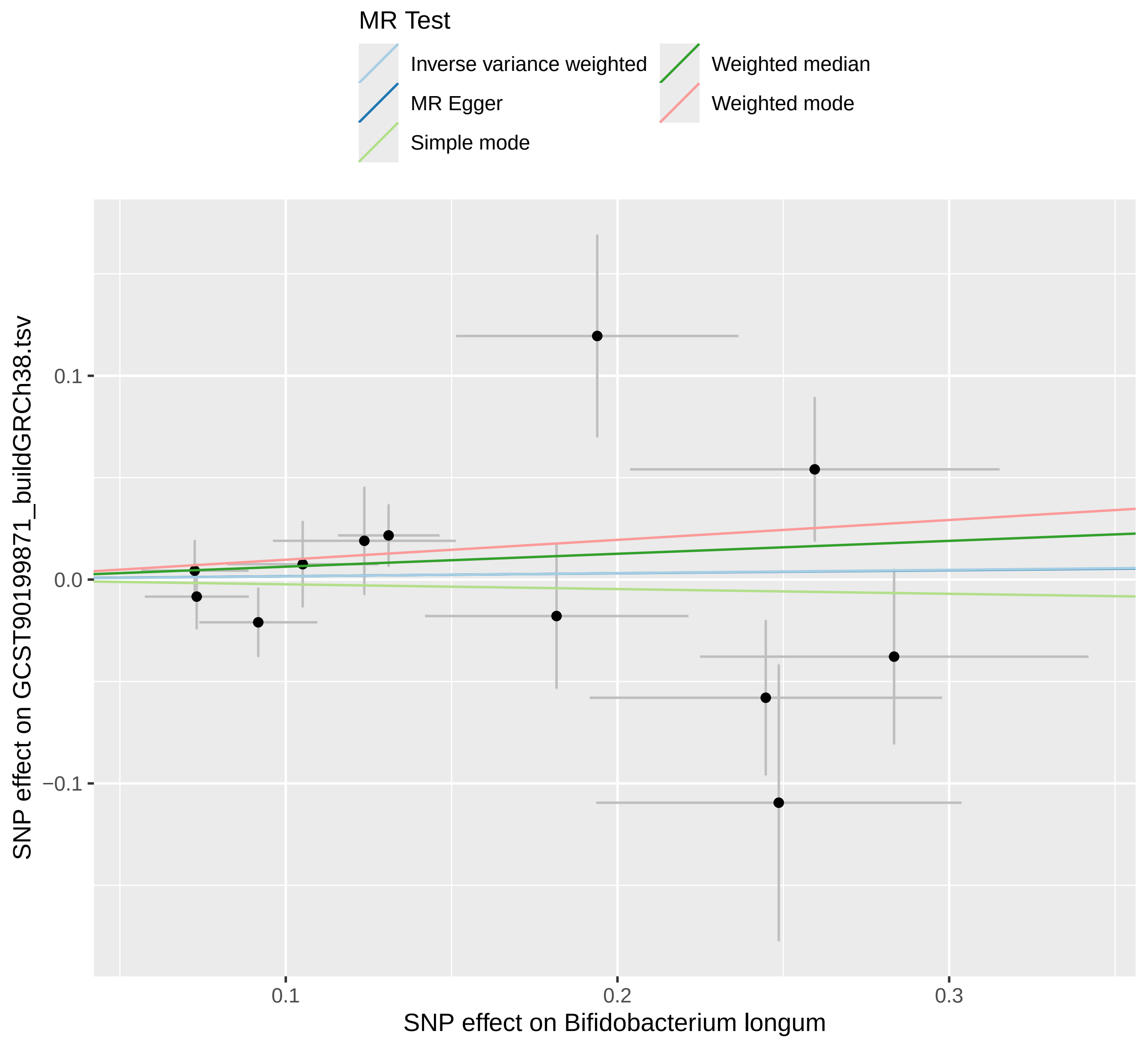
MR Test
Inverse variance weighted
MR Egger
Simple mode
Weighted median
Weighted mode
0.1
0.0
−0.1
SNP effect on GCST90199871_buildGRCh38.tsv
0.1
0.2
0.3
SNP effect on Bifidobacterium longum

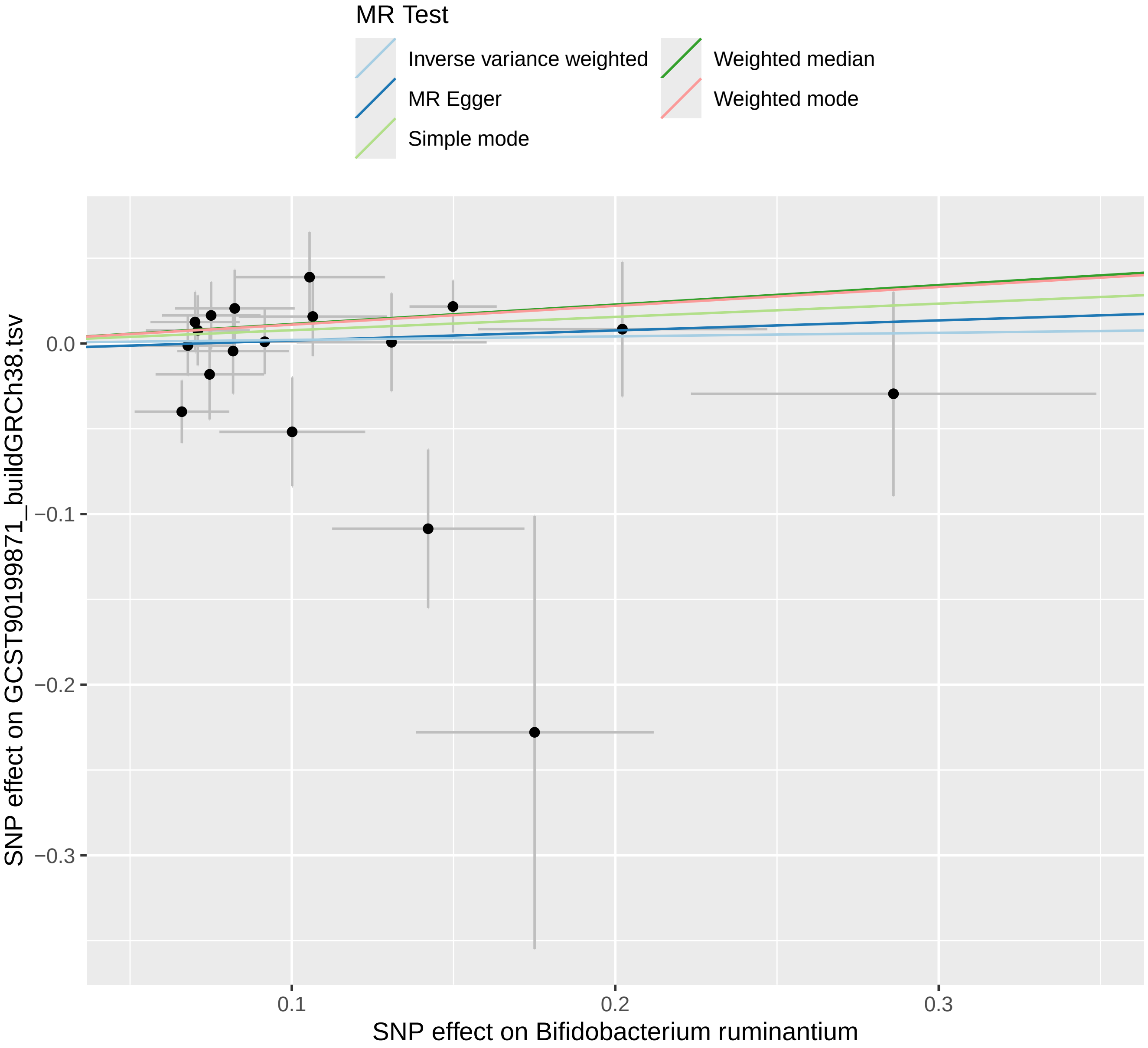
MR Test
Inverse variance weighted
MR Egger
Simple mode
Weighted median
Weighted mode
SNP effect on GCST90199871_buildGRCh38.tsv
0.0
−0.1
−0.2
−0.3
0.1
0.2
0.3
SNP effect on Bifidobacterium ruminantium

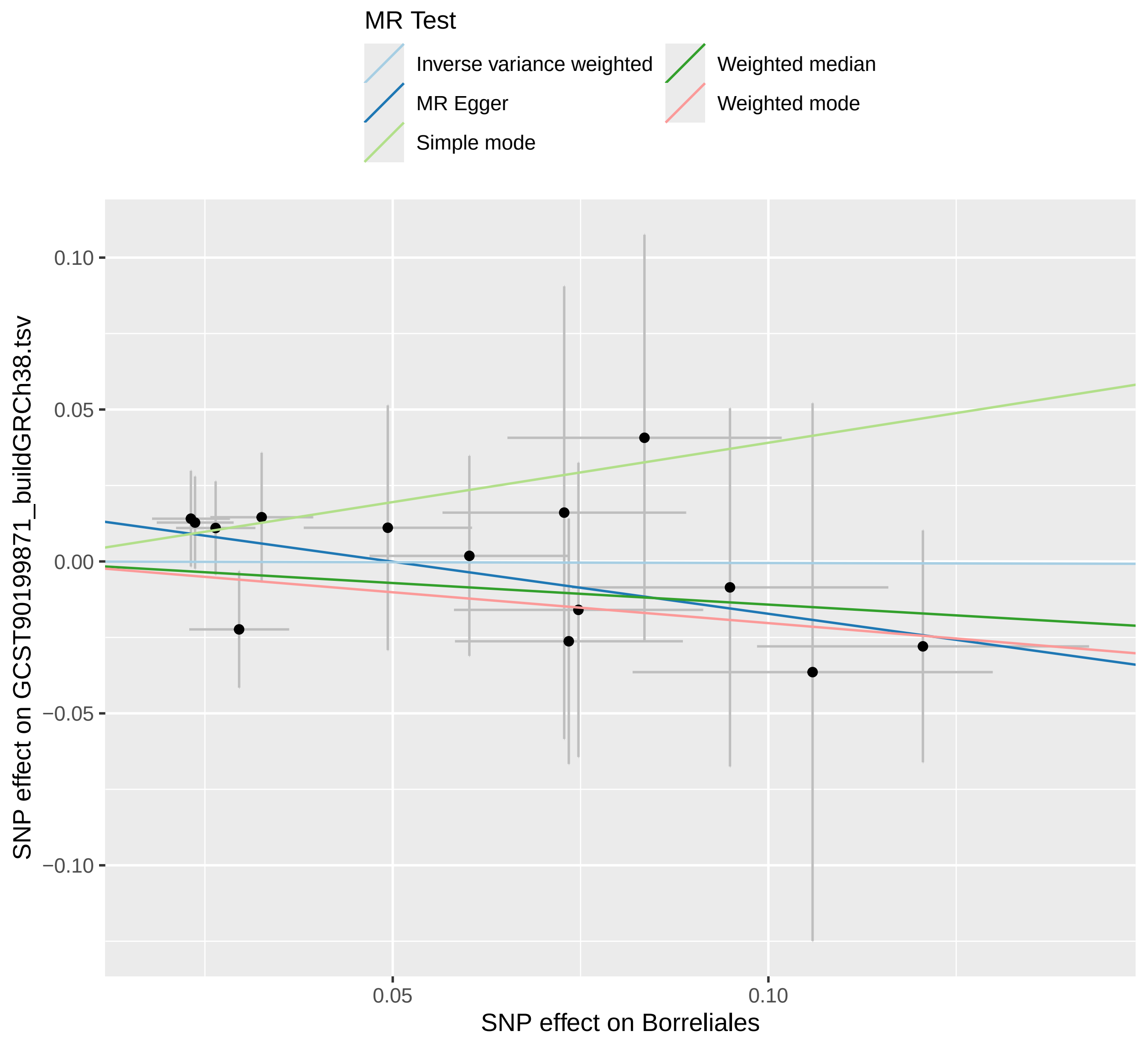
MR Test
Inverse variance weighted
MR Egger
Simple mode
Weighted median
Weighted mode
0.10
0.05
0.00
−0.05
−0.10
SNP effect on GCST90199871_buildGRCh38.tsv
0.05
0.10
SNP effect on Borreliales

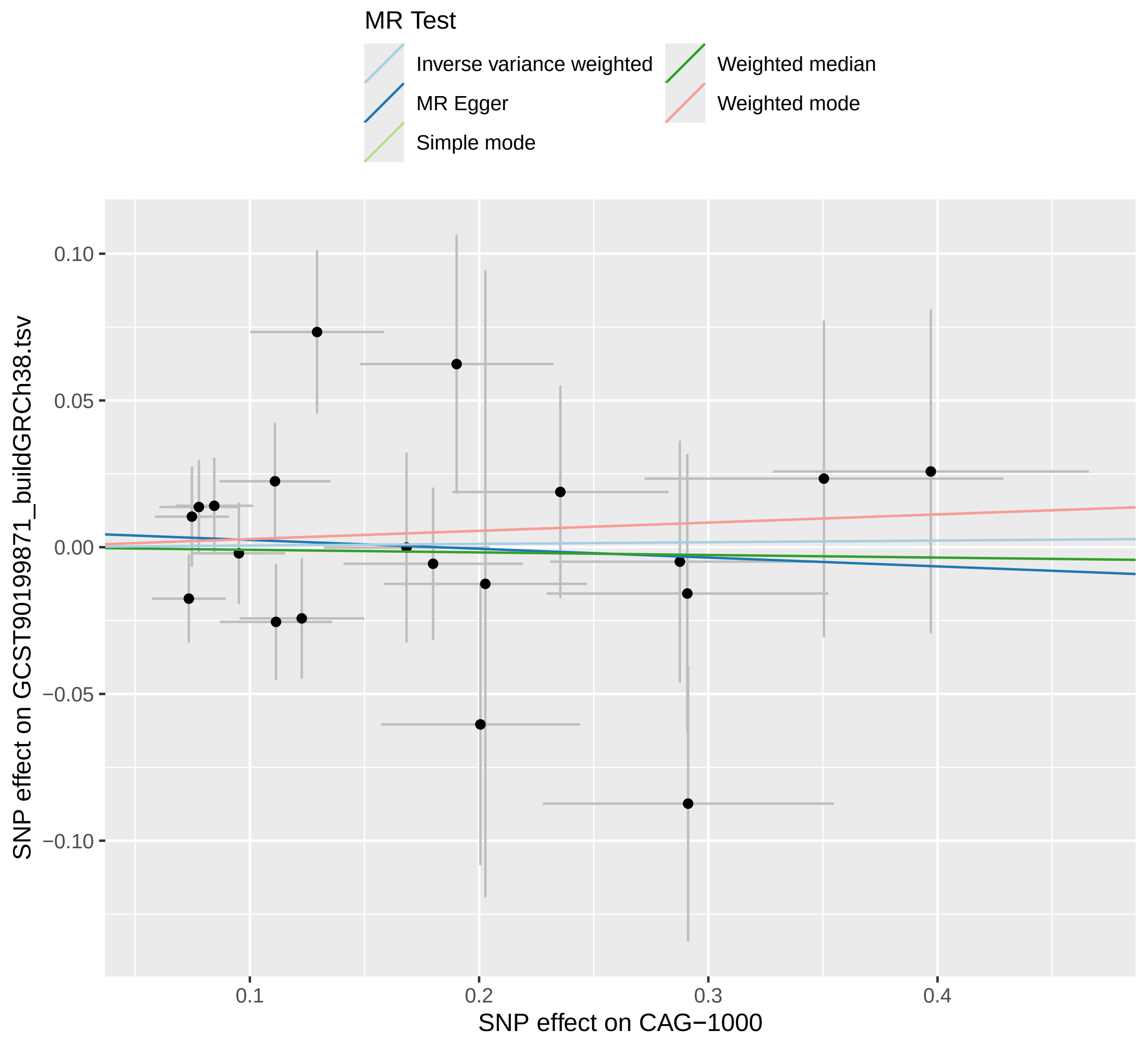

MR Test
Inverse variance weighted
MR Egger
Simple mode
Weighted median
Weighted mode
0.10
0.05
0.00
−0.05
−0.10
0.1
0.2
0.3
0.4
SNP effect on GCST90199871_buildGRCh38.tsv
SNP effect on CAG−1000

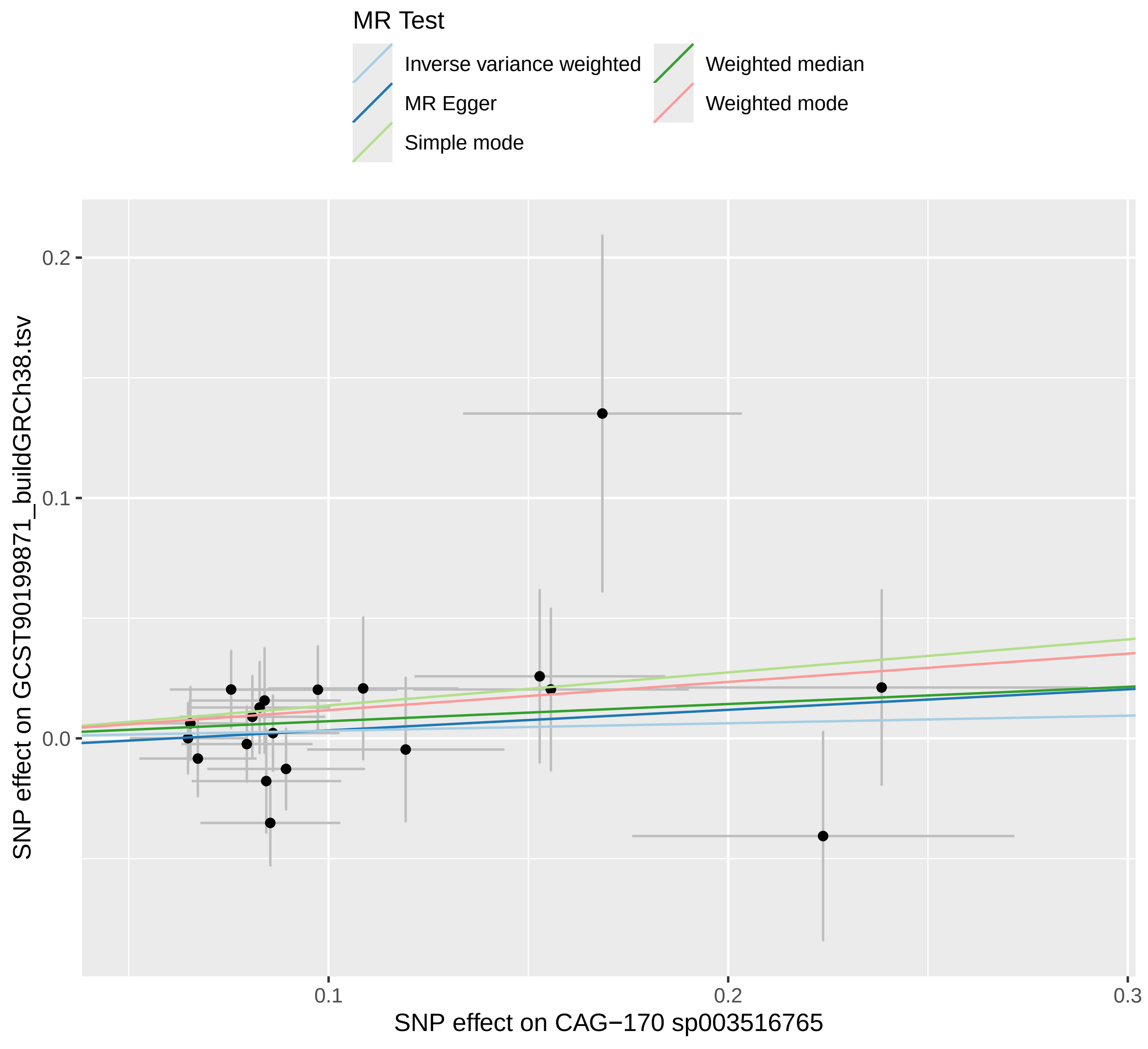

MR Test
Inverse variance weighted
MR Egger
Simple mode
Weighted median
Weighted mode
0.2
0.1
0.0
0.1
0.2
0.3
SNP effect on GCST90199871_buildGRCh38.tsv
SNP effect on CAG−170 sp003516765

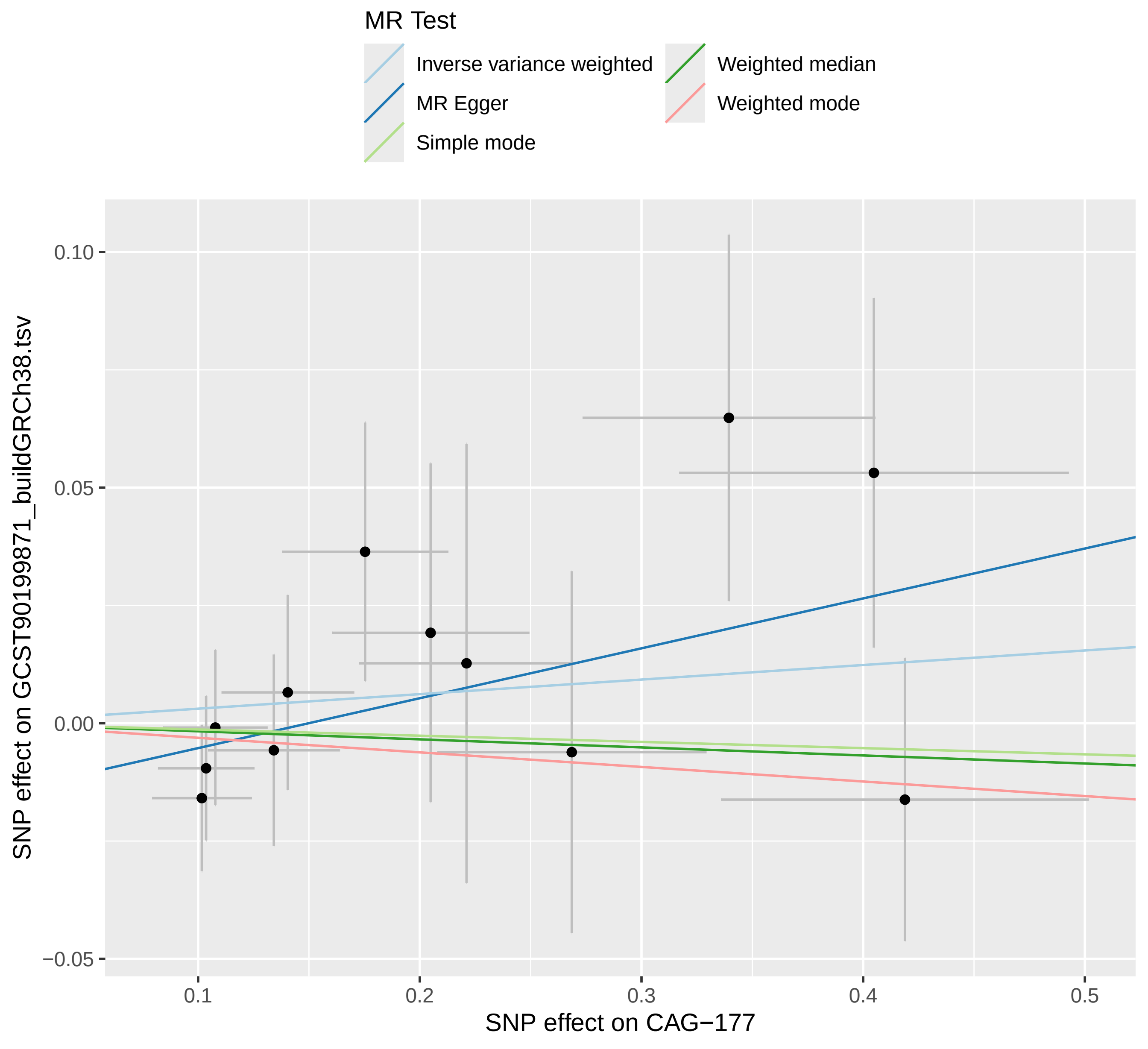

MR Test
Inverse variance weighted
MR Egger
Simple mode
Weighted median
Weighted mode
0.10
0.05
0.00
−0.05
0.1
0.2
0.3
0.4
0.5
SNP effect on CAG−177
SNP effect on GCST90199871_buildGRCh38.tsv

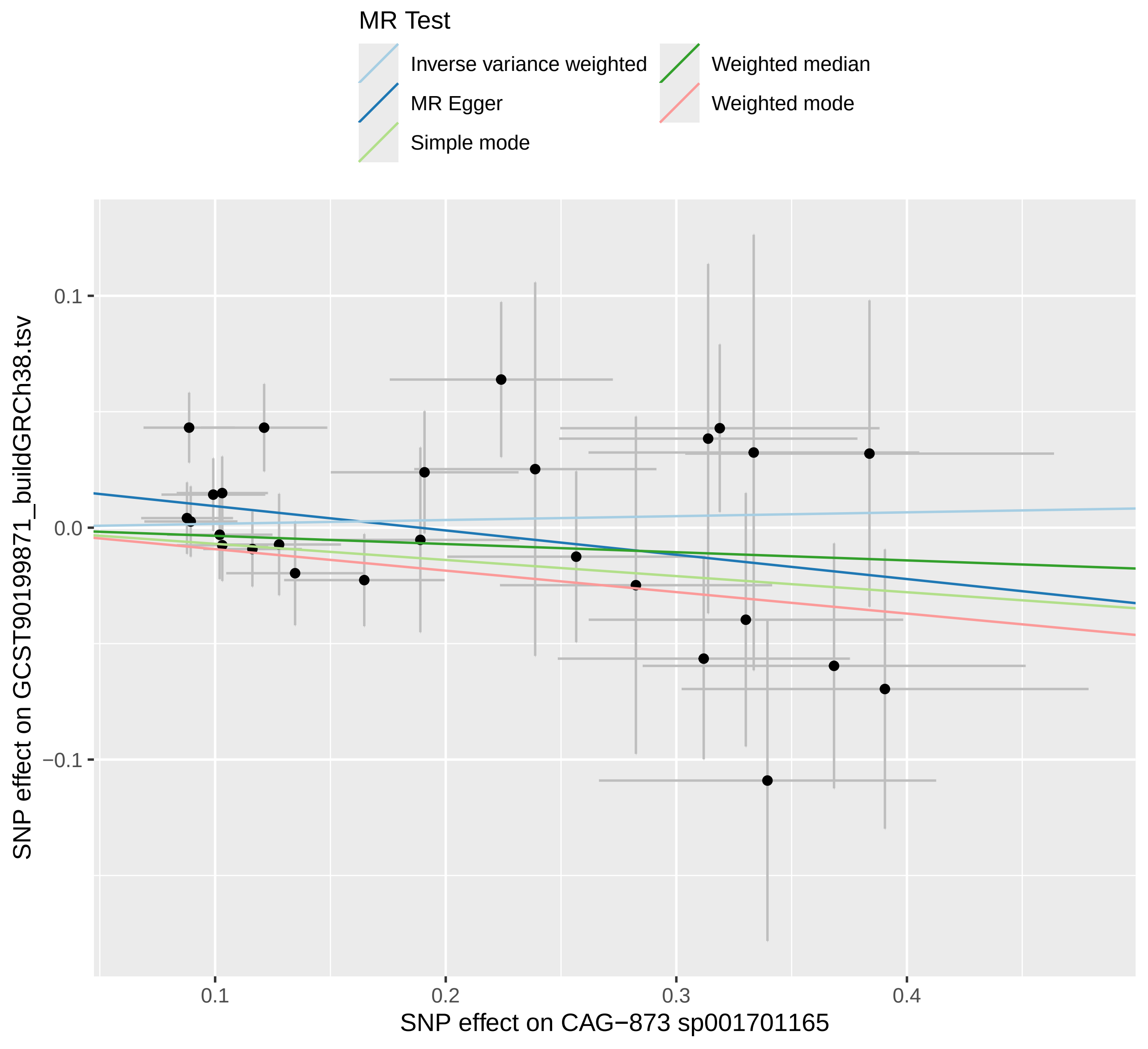

MR Test
Inverse variance weighted
MR Egger
Simple mode
Weighted median
Weighted mode
0.1
0.0
−0.1
0.1
0.2
0.3
0.4
SNP effect on GCST90199871_buildGRCh38.tsv
SNP effect on CAG−873 sp001701165

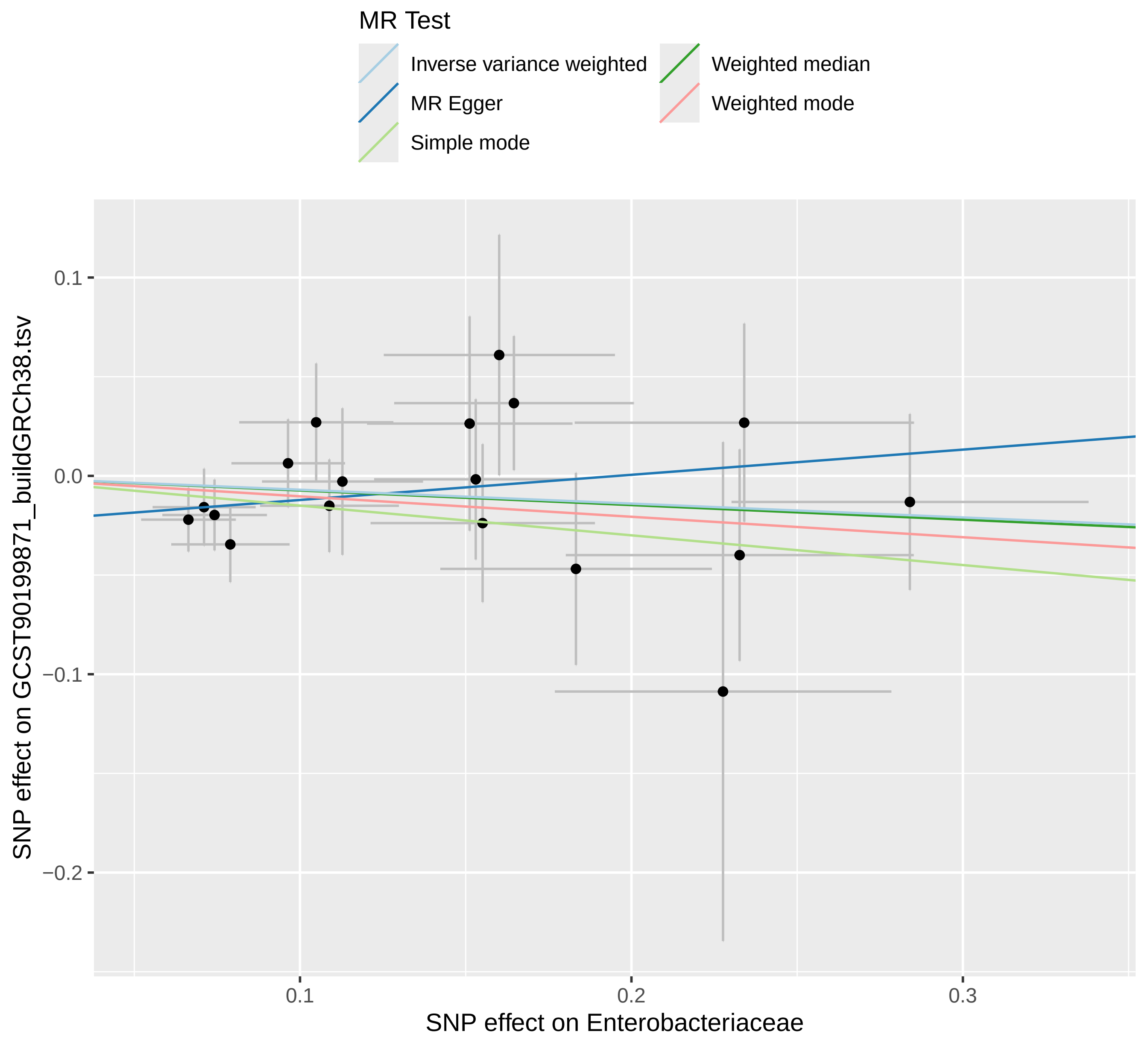
MR Test
Inverse variance weighted
MR Egger
Simple mode
Weighted median
Weighted mode
SNP effect on GCST90199871_buildGRCh38.tsv
0.1
0.0
−0.1
−0.2
0.1
0.2
0.3
SNP effect on Enterobacteriaceae

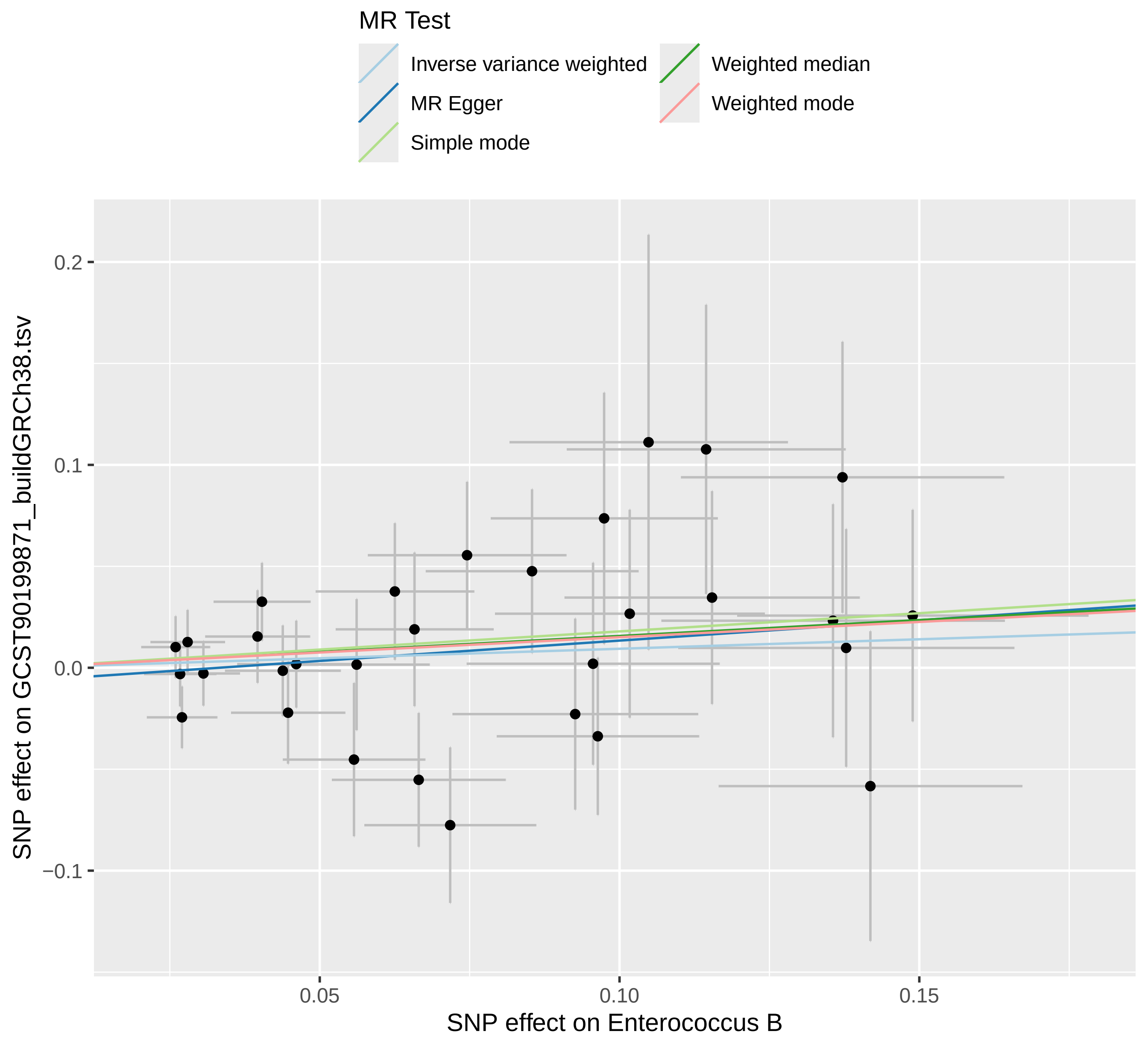

MR Test
Inverse variance weighted
MR Egger
Simple mode
Weighted median
Weighted mode
0.2
0.1
0.0
−0.1
SNP effect on GCST90199871_buildGRCh38.tsv
0.05
0.10
0.15
SNP effect on Enterococcus B

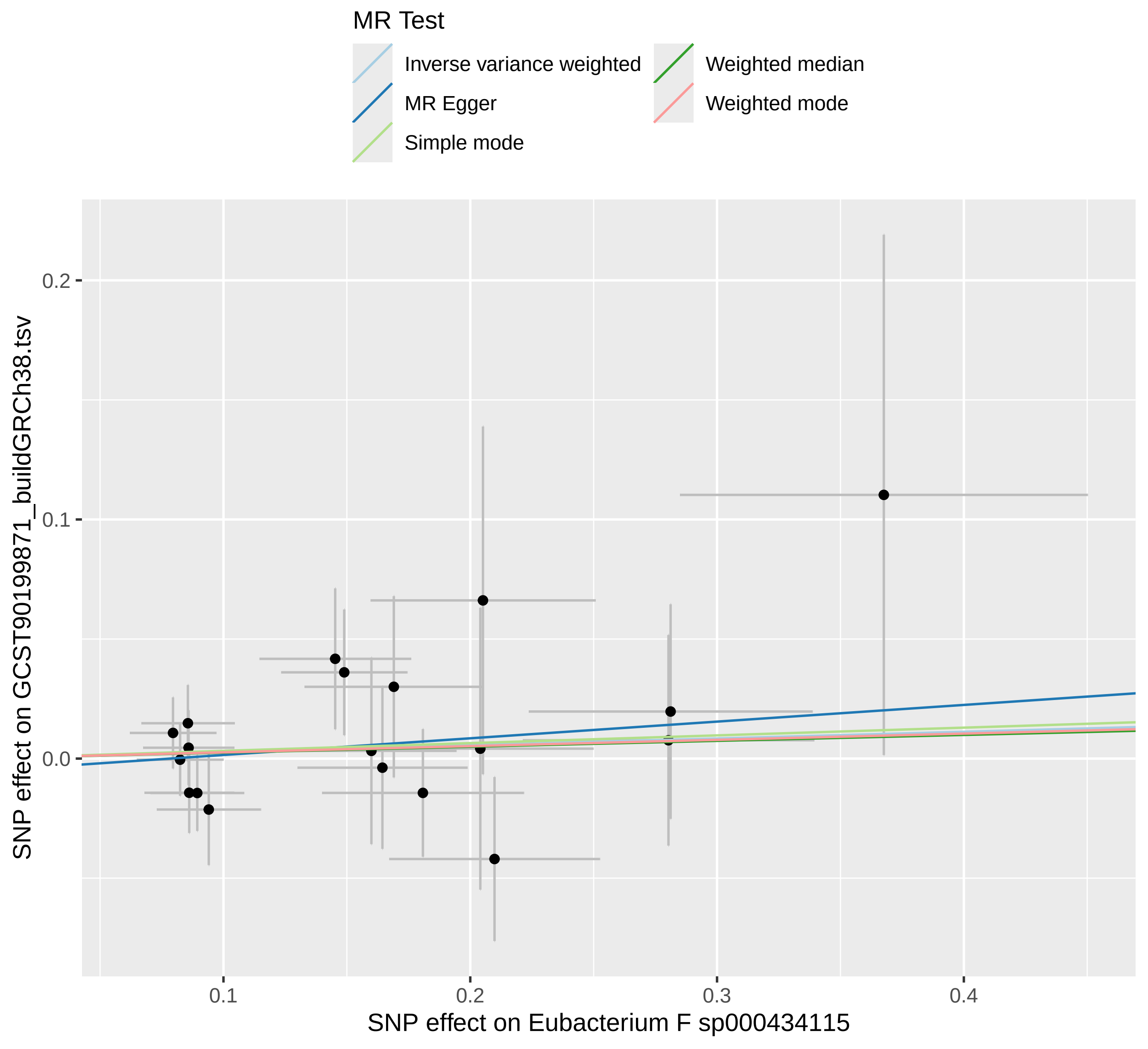

MR Test
Inverse variance weighted
MR Egger
Simple mode
Weighted median
Weighted mode
0.2
0.1
0.0
0.1
0.2
0.3
0.4
SNP effect on GCST90199871_buildGRCh38.tsv
SNP effect on Eubacterium F sp000434115

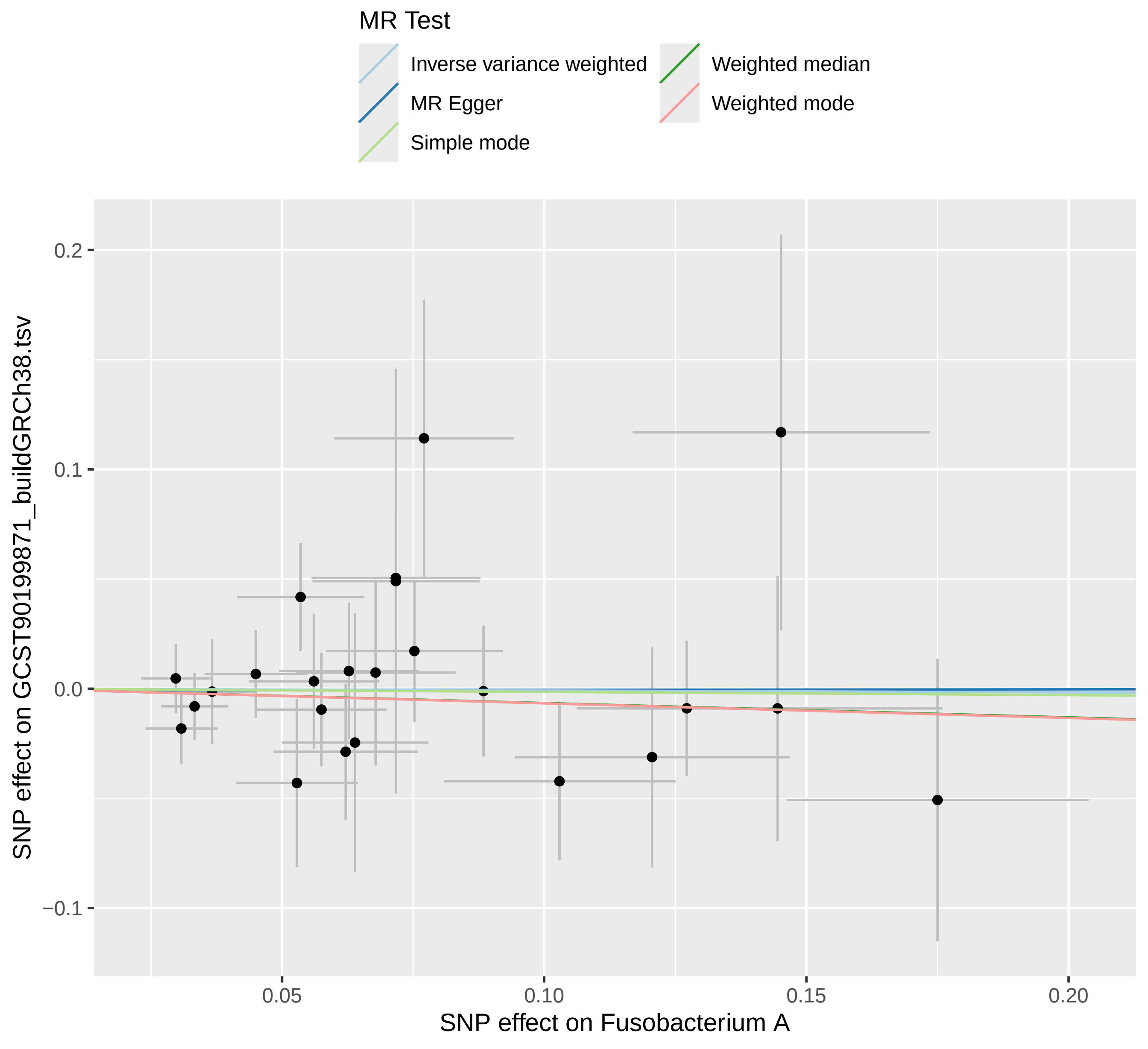

MR Test
Inverse variance weighted
MR Egger
Simple mode
Weighted median
Weighted mode
SNP effect on GCST90199871_buildGRCh38.tsv
0.2
0.1
0.0
−0.1
0.05
0.10
0.15
0.20
SNP effect on Fusobacterium A

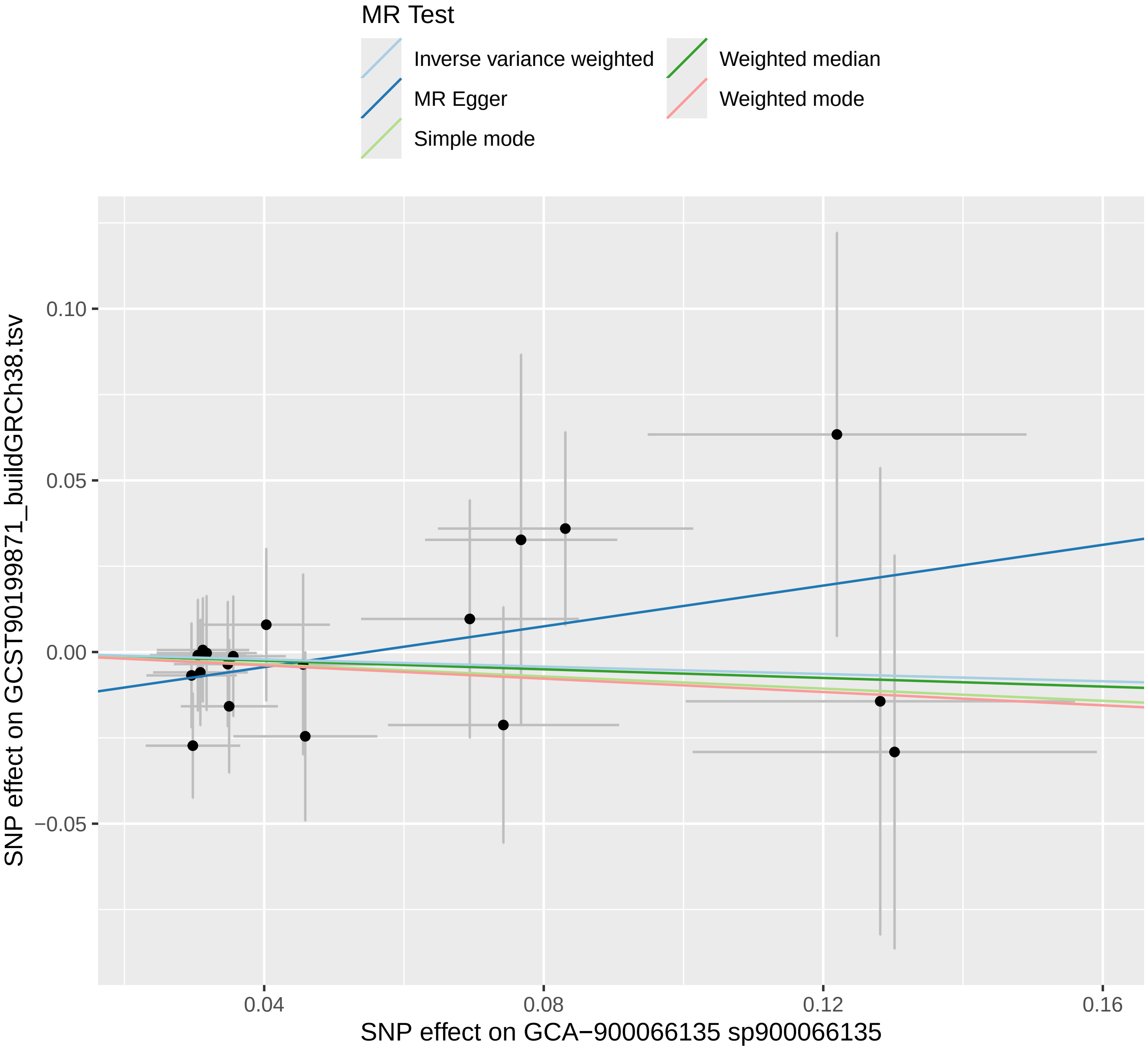

MR Test
Inverse variance weighted
MR Egger
Simple mode
Weighted median
Weighted mode
0.10
0.05
0.00
−0.05
0.04
0.08
0.12
0.16
SNP effect on GCST90199871_buildGRCh38.tsv
SNP effect on GCA−900066135 sp900066135

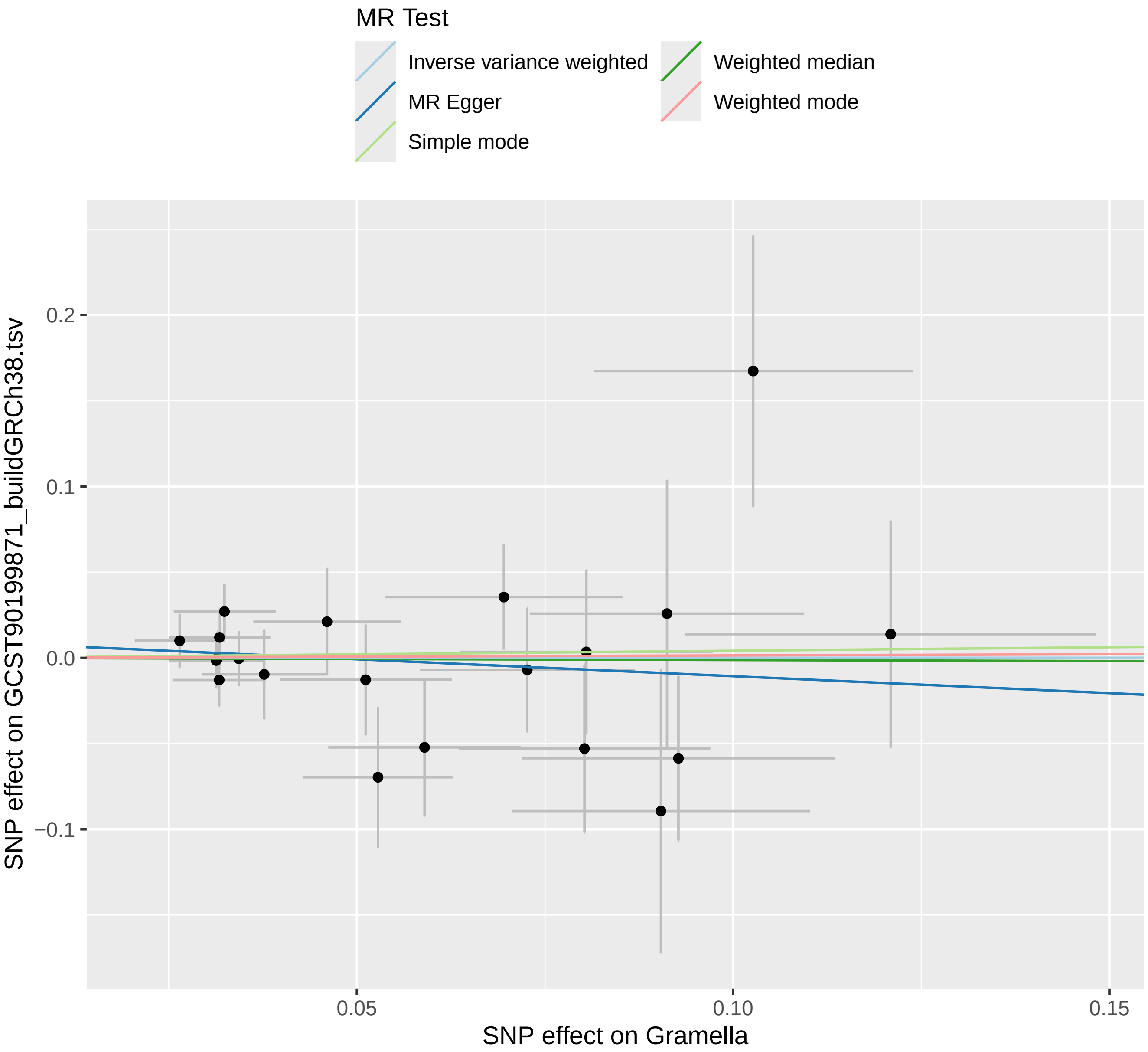

MR Test
Inverse variance weighted
MR Egger
Simple mode
Weighted median
Weighted mode
0.2
0.1
0.0
−0.1
0.05
0.10
0.15
SNP effect on GCST90199871_buildGRCh38.tsv
SNP effect on Gramella

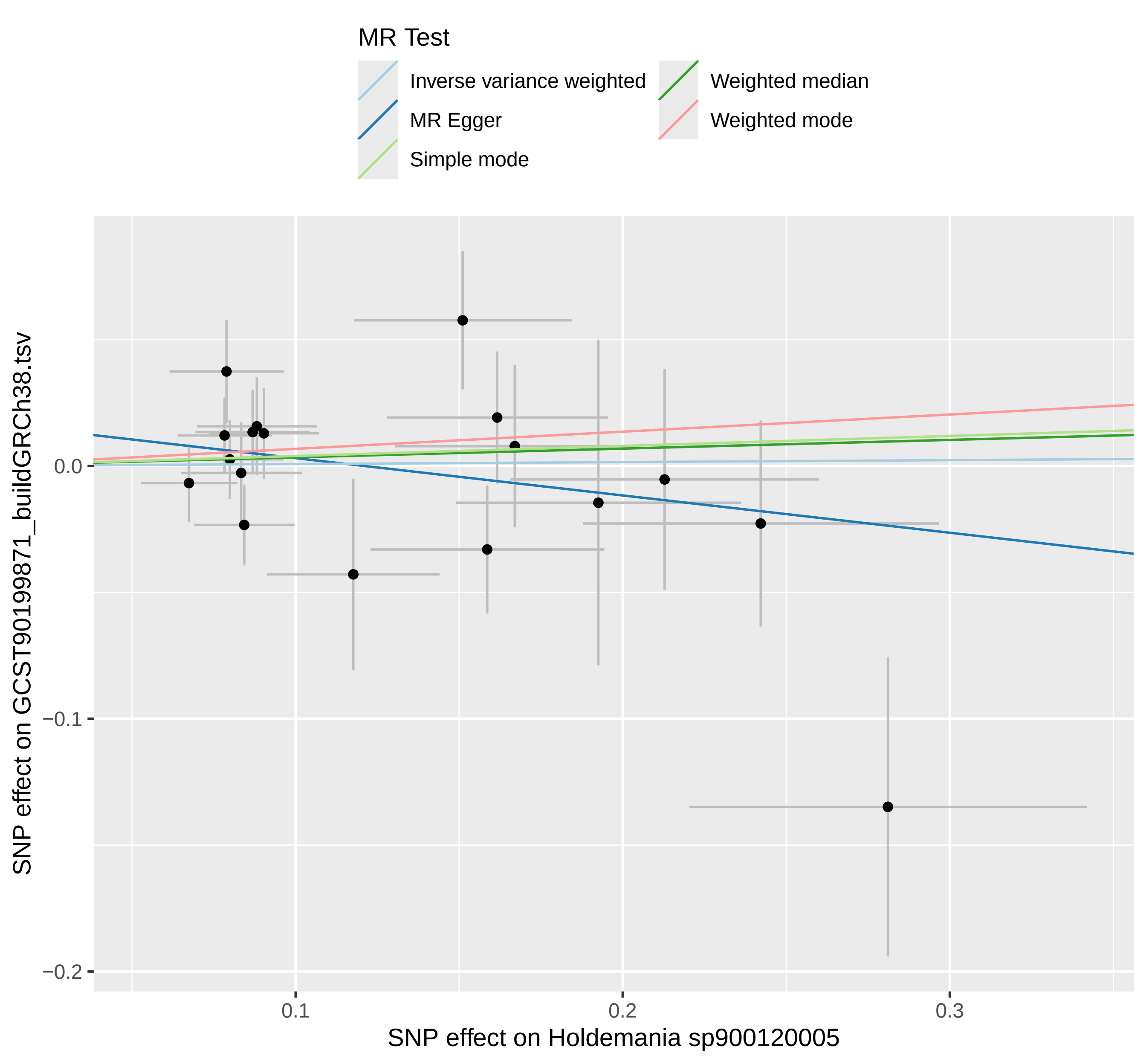

MR Test
Inverse variance weighted
MR Egger
Simple mode
Weighted median
Weighted mode
SNP effect on GCST90199871_buildGRCh38.tsv
0.0
−0.1
−0.2
0.1
0.2
0.3
SNP effect on Holdemania sp900120005

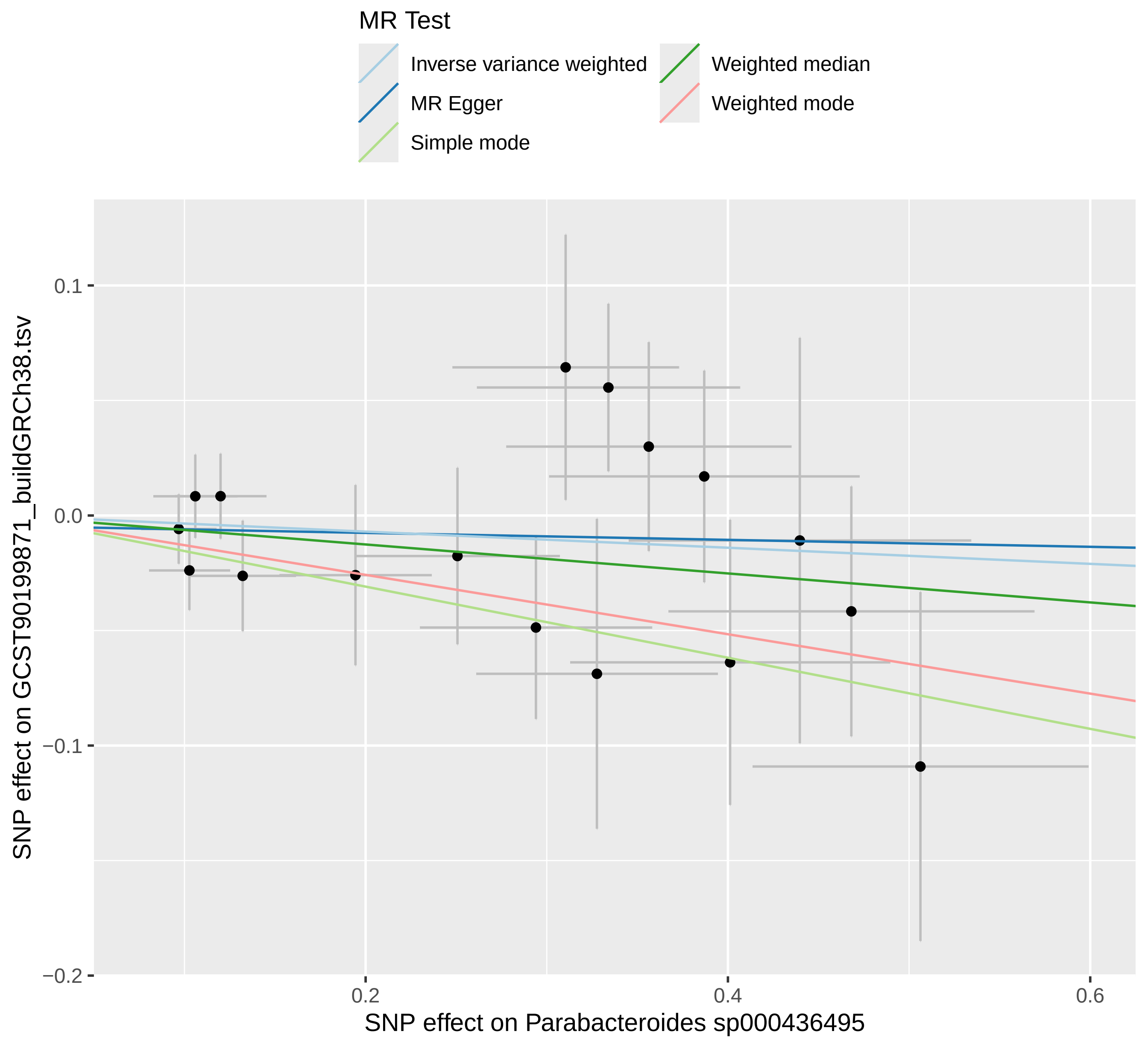

MR Test
Inverse variance weighted
MR Egger
Simple mode
Weighted median
Weighted mode
0.1
0.0
−0.1
−0.2
0.2
0.4
0.6
SNP effect on GCST90199871_buildGRCh38.tsv
SNP effect on Parabacteroides sp000436495

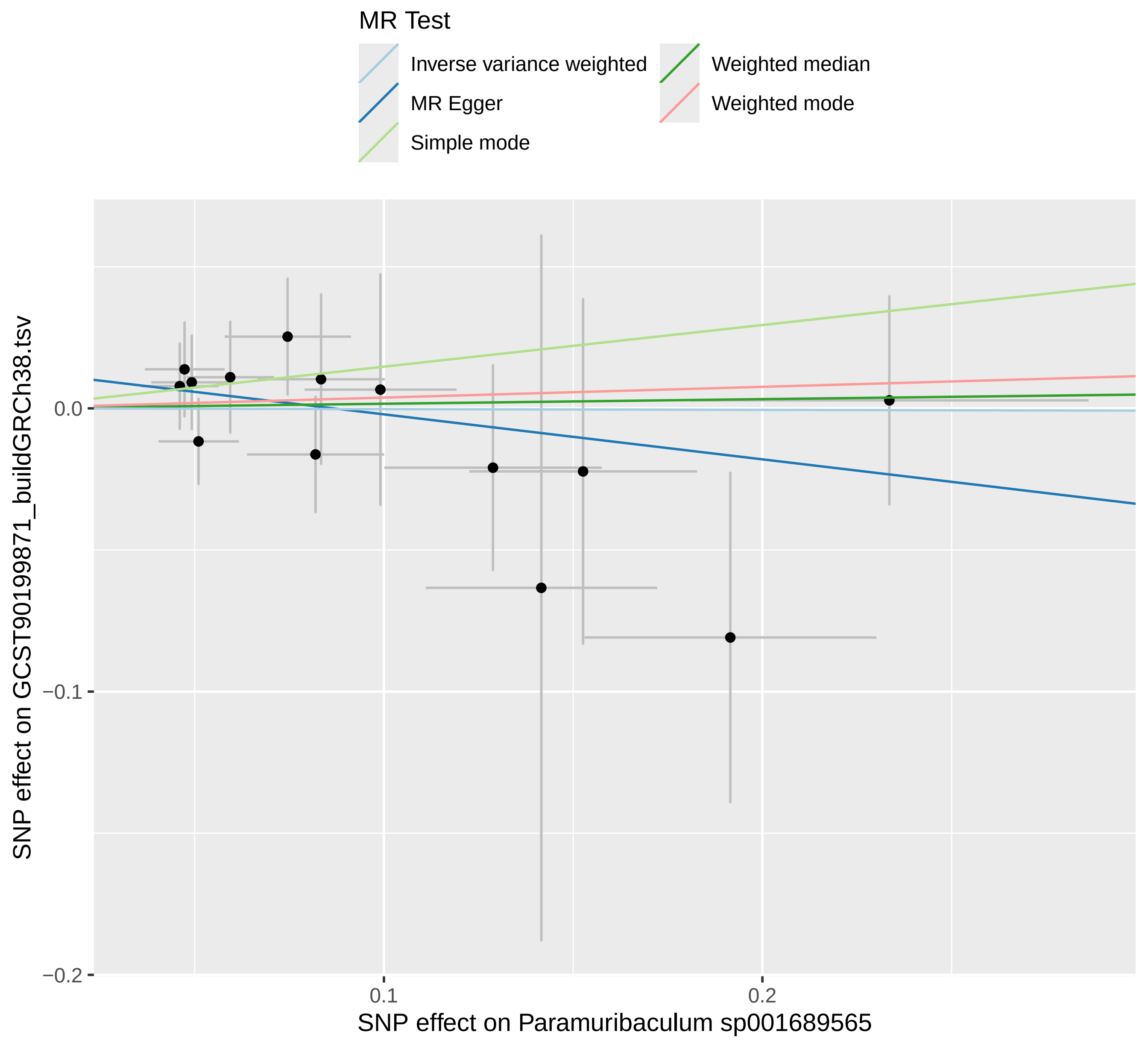
MR Test
Inverse variance weighted
MR Egger
Simple mode
Weighted median
Weighted mode
SNP effect on GCST90199871_buildGRCh38.tsv
0.0
−0.1
−0.2
0.1
0.2
SNP effect on Paramuribaculum sp001689565

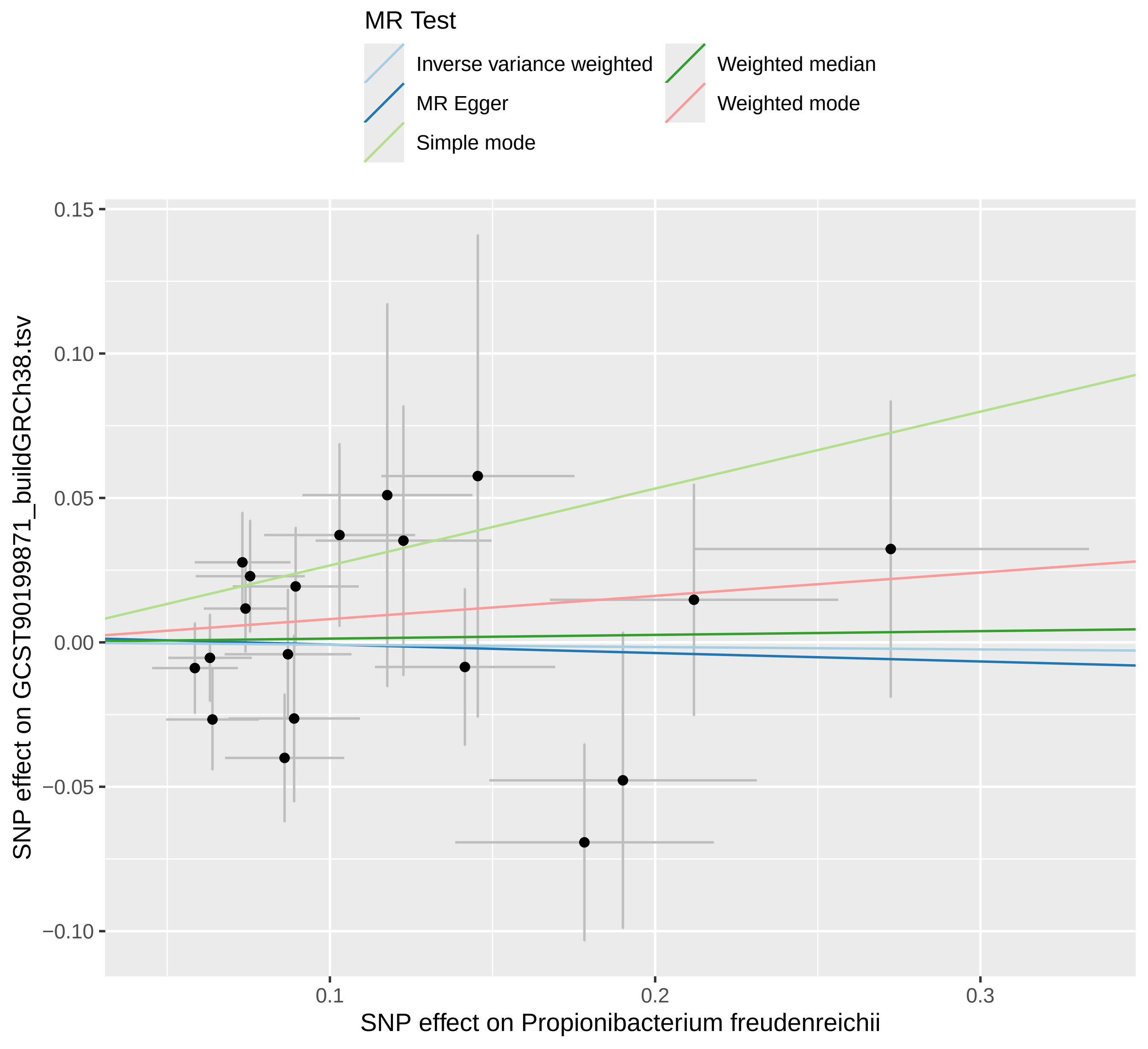

MR Test
Inverse variance weighted
MR Egger
Simple mode
Weighted median
Weighted mode
0.15
0.10
0.05
0.00
−0.05
−0.10
0.1
0.2
0.3
SNP effect on GCST90199871_buildGRCh38.tsv
SNP effect on Propionibacterium freudenreichii

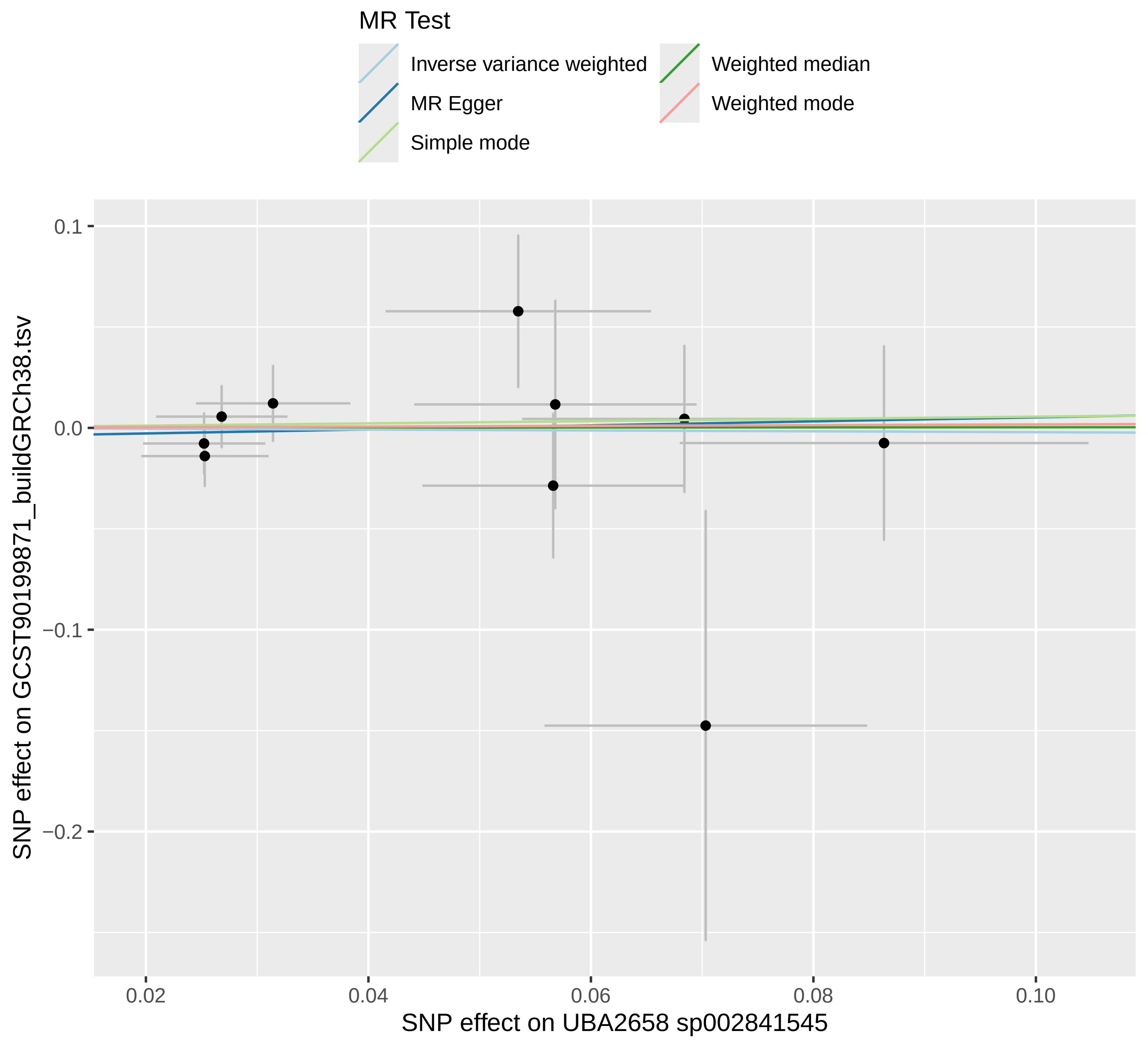
MR Test
Inverse variance weighted
MR Egger
Simple mode
Weighted median
Weighted mode
0.1
0.0
−0.1
−0.2
0.02
0.04
0.06
0.08
0.10
SNP effect on UBA2658 sp002841545
SNP effect on GCST90199871_buildGRCh38.tsv

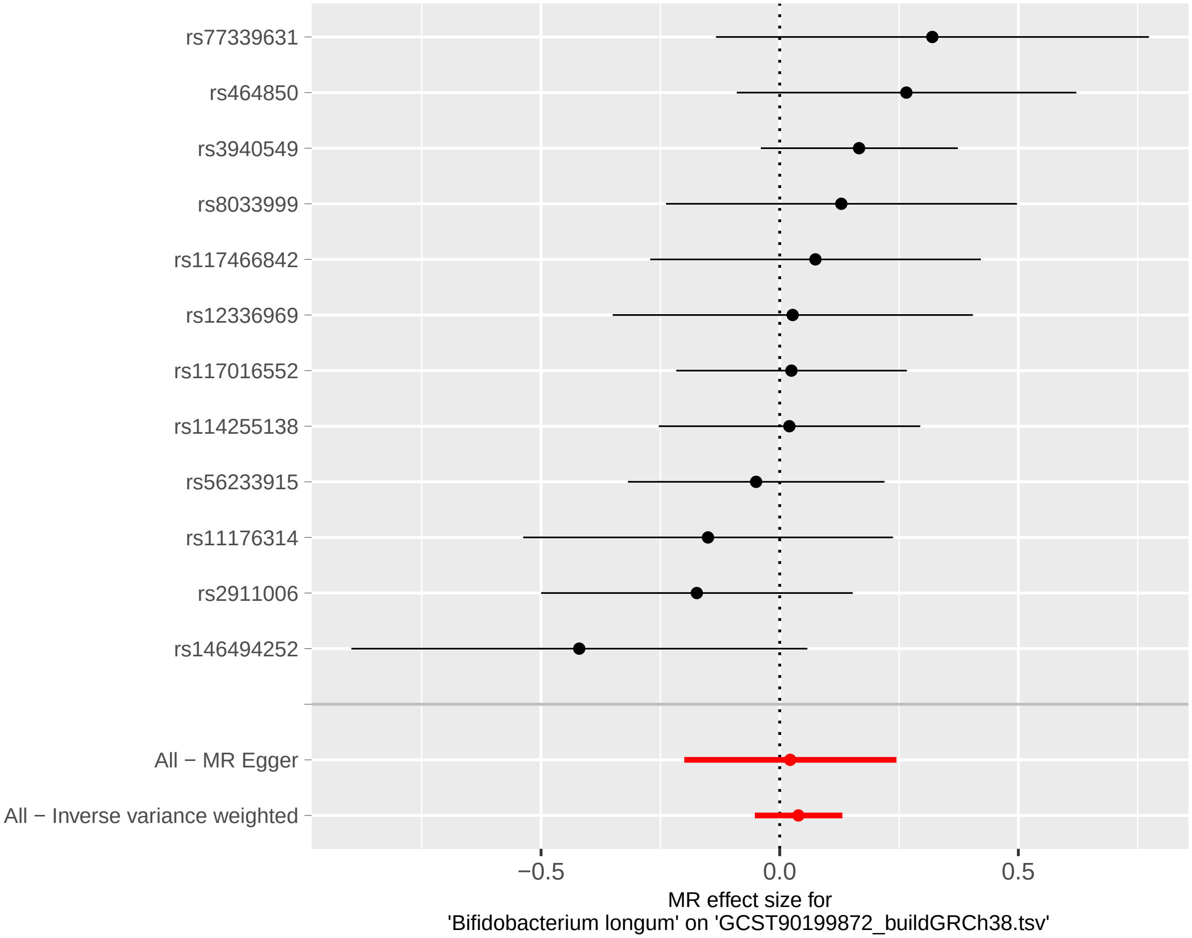
rs77339631
rs464850
rs3940549
rs8033999
rs117466842
rs12336969
rs117016552
rs114255138
rs56233915
rs11176314
rs2911006
rs146494252
All − MR Egger
All − Inverse variance weighted
−0.5
0.0
0.5
MR effect size for
'Bifidobacterium longum' on 'GCST90199872_buildGRCh38.tsv'

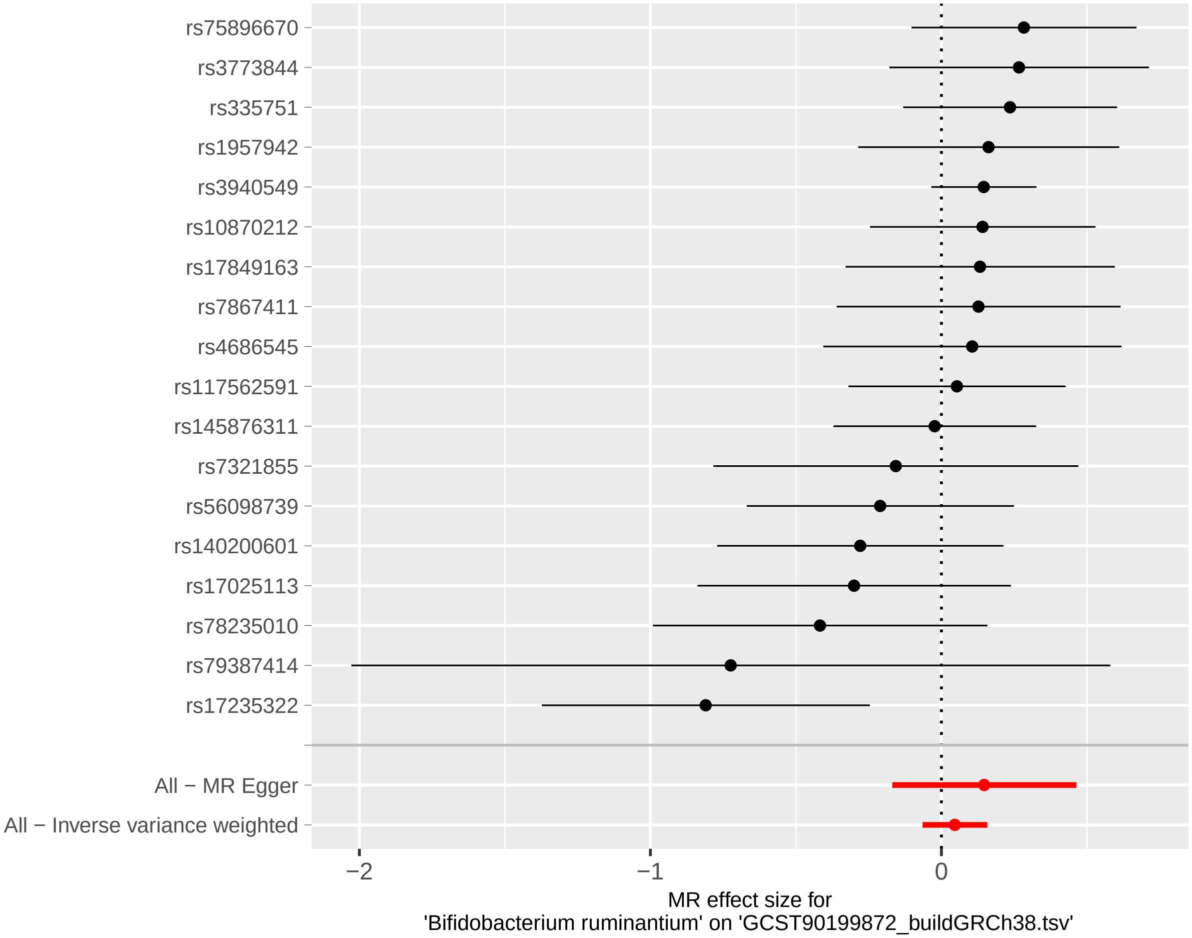
rs75896670
rs3773844
rs335751
rs1957942
rs3940549
rs10870212
rs17849163
rs7867411
rs4686545
rs117562591
rs145876311
rs7321855
rs56098739
rs140200601
rs17025113
rs78235010
rs79387414
rs17235322
All − MR Egger
All − Inverse variance weighted
−2
−1
0
MR effect size for
'Bifidobacterium ruminantium' on 'GCST90199872_buildGRCh38.tsv'

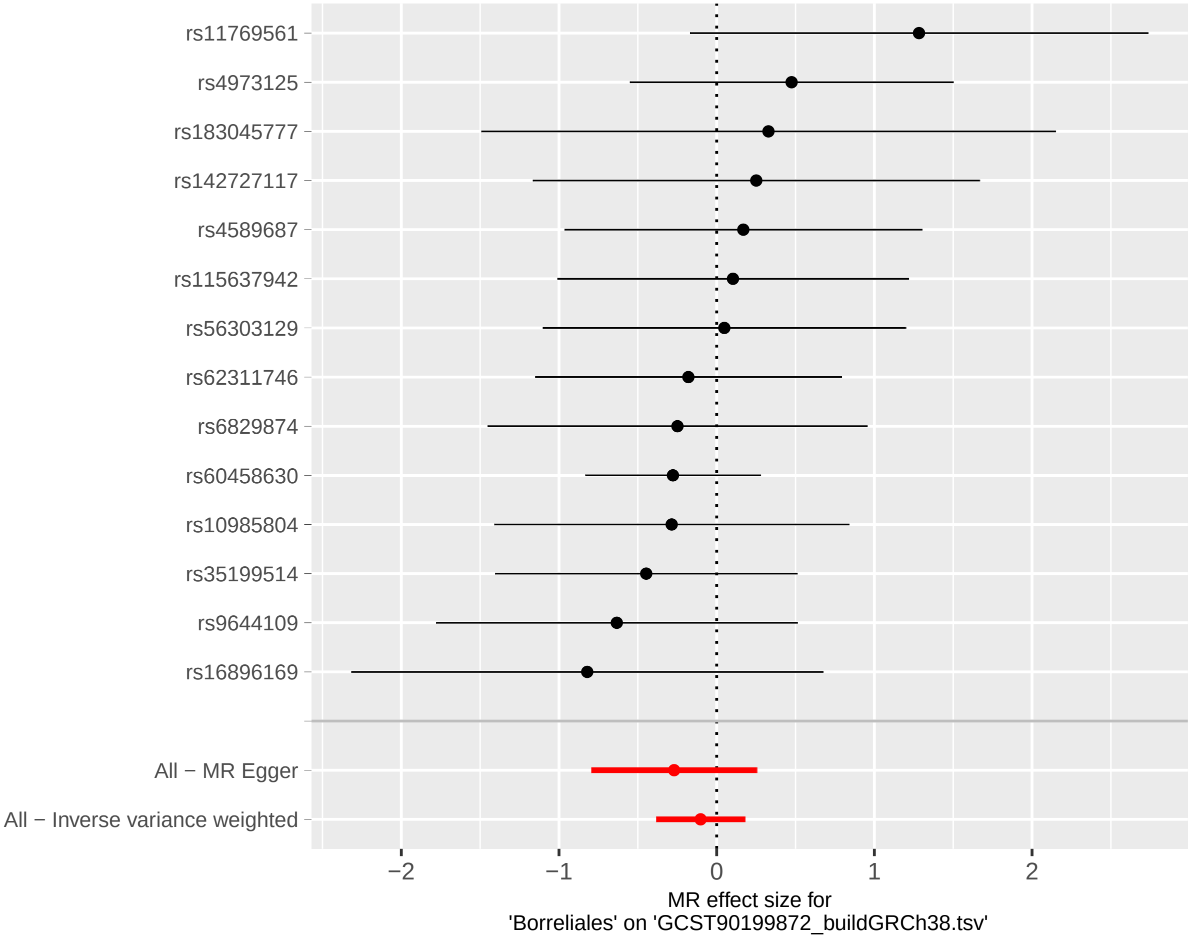

rs11769561
rs4973125
rs183045777
rs142727117
rs4589687
rs115637942
rs56303129
rs62311746
rs6829874
rs60458630
rs10985804
rs35199514
rs9644109
rs16896169
All − MR Egger
All − Inverse variance weighted
−2
−1
0
1
2
MR effect size for
'Borreliales' on 'GCST90199872_buildGRCh38.tsv'

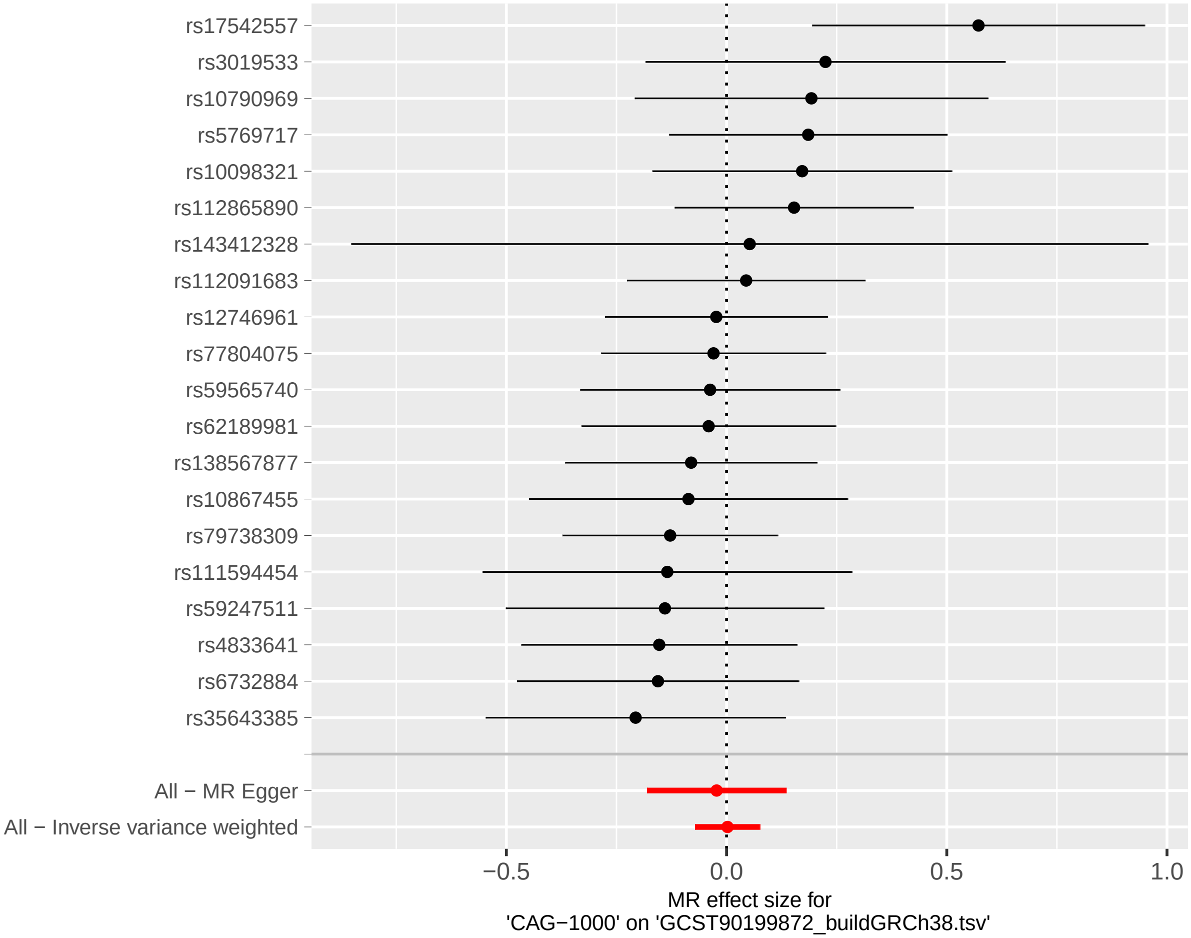

rs17542557
rs3019533
rs10790969
rs5769717
rs10098321
rs112865890
rs143412328
rs112091683
rs12746961
rs77804075
rs59565740
rs62189981
rs138567877
rs10867455
rs79738309
rs111594454
rs59247511
rs4833641
rs6732884
rs35643385
All − MR Egger
All − Inverse variance weighted
−0.5
0.0
0.5
1.0
MR effect size for
'CAG−1000' on 'GCST90199872_buildGRCh38.tsv'

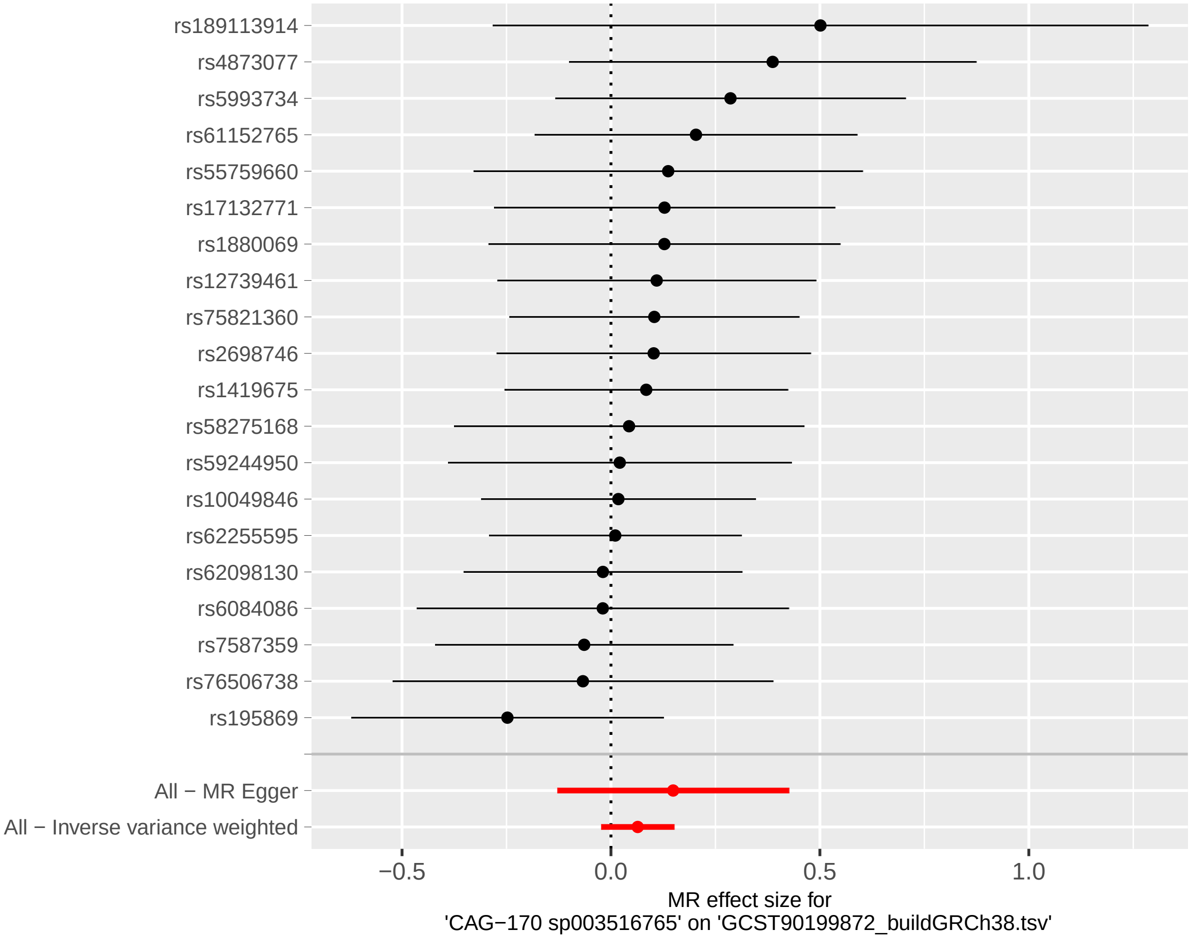
rs189113914
rs4873077
rs5993734
rs61152765
rs55759660
rs17132771
rs1880069
rs12739461
rs75821360
rs2698746
rs1419675
rs58275168
rs59244950
rs10049846
rs62255595
rs62098130
rs6084086
rs7587359
rs76506738
rs195869
All − MR Egger
All − Inverse variance weighted
−0.5
0.0
0.5
1.0
MR effect size for
'CAG−170 sp003516765' on 'GCST90199872_buildGRCh38.tsv'

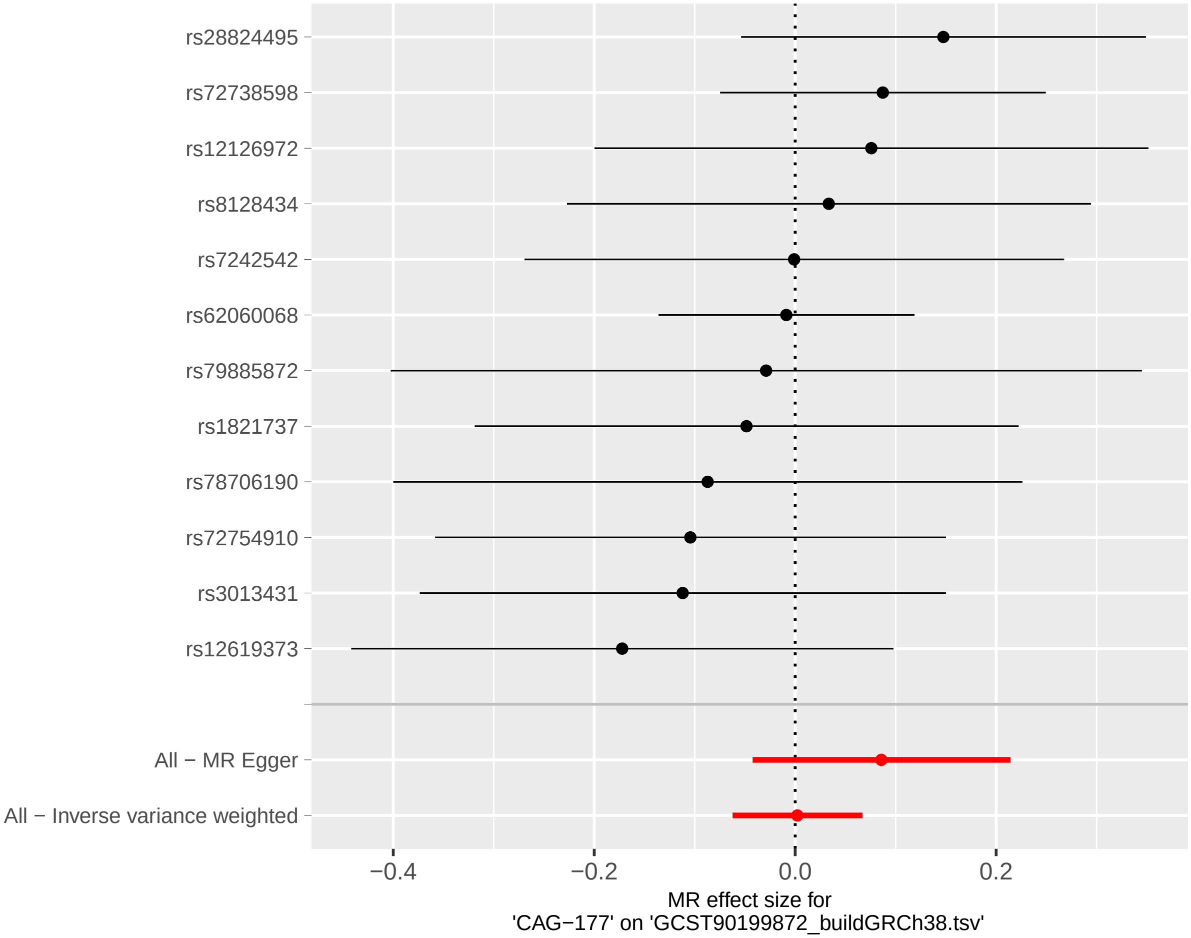
rs28824495
rs72738598
rs12126972
rs8128434
rs7242542
rs62060068
rs79885872
rs1821737
rs78706190
rs72754910
rs3013431
rs12619373
All − MR Egger
All − Inverse variance weighted
−0.4
−0.2
0.0
0.2
MR effect size for
'CAG−177' on 'GCST90199872_buildGRCh38.tsv'

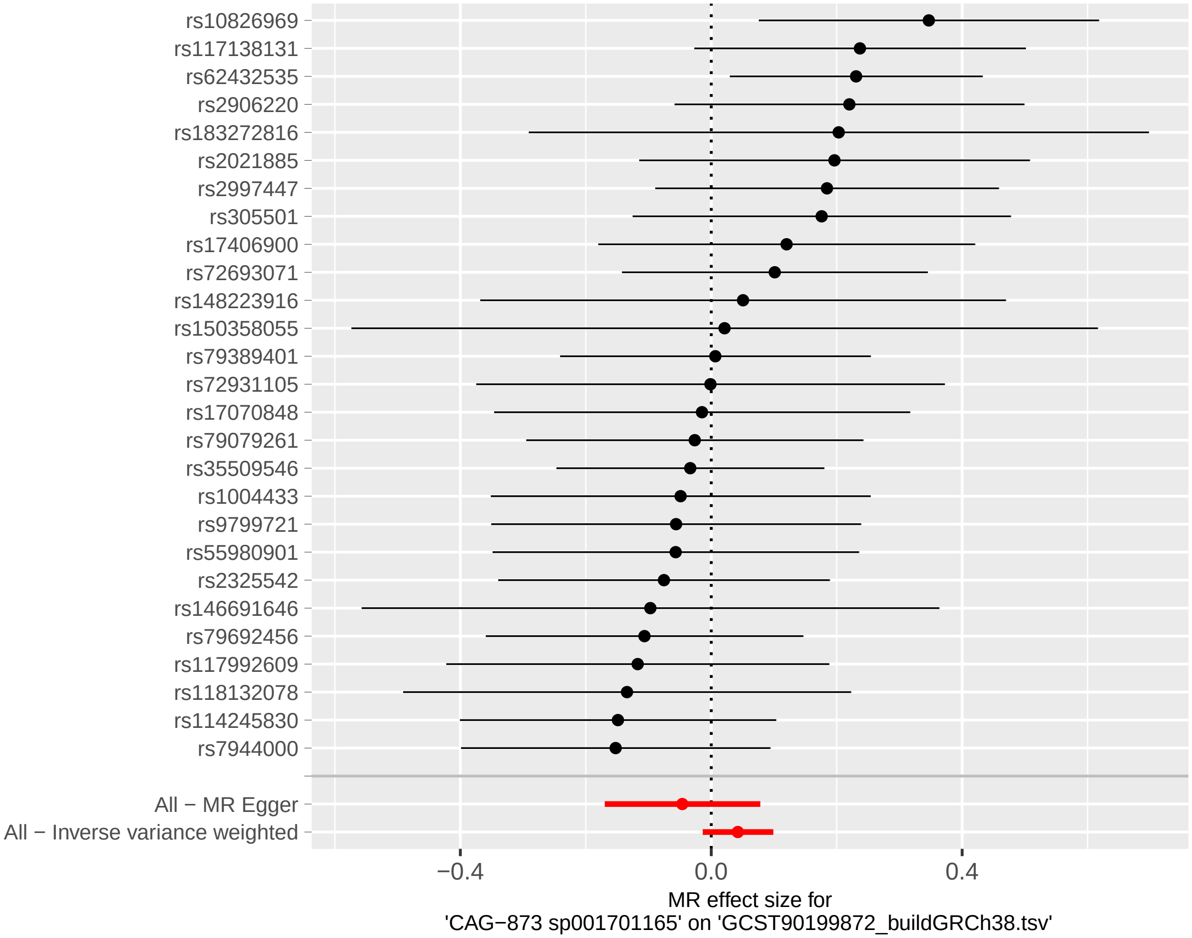
rs10826969
rs117138131
rs62432535
rs2906220
rs183272816
rs2021885
rs2997447
rs305501
rs17406900
rs72693071
rs148223916
rs150358055
rs79389401
rs72931105
rs17070848
rs79079261
rs35509546
rs1004433
rs9799721
rs55980901
rs2325542
rs146691646
rs79692456
rs117992609
rs118132078
rs114245830
rs7944000
All − MR Egger
All − Inverse variance weighted
−0.4
0.0
0.4
MR effect size for
'CAG−873 sp001701165' on 'GCST90199872_buildGRCh38.tsv'

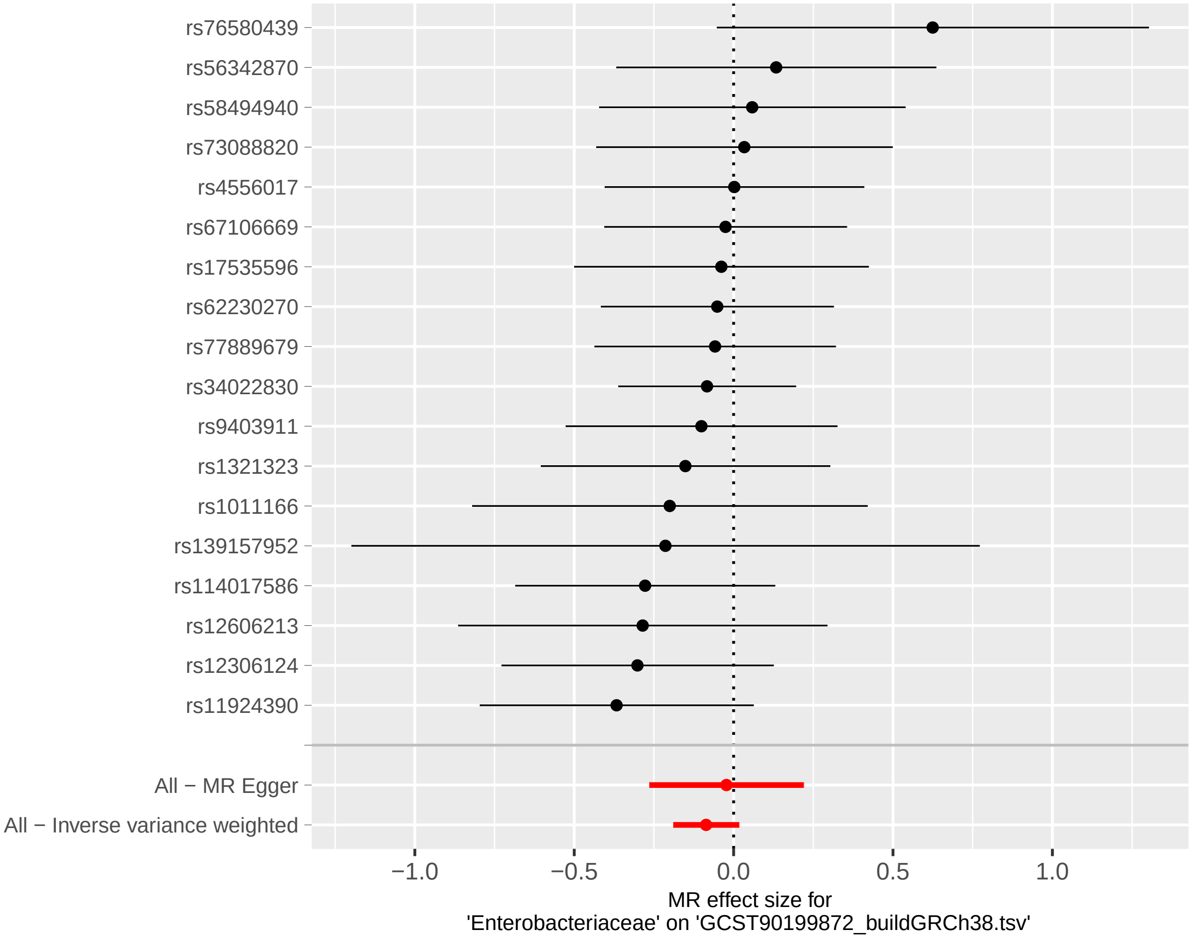
rs76580439
rs56342870
rs58494940
rs73088820
rs4556017
rs67106669
rs17535596
rs62230270
rs77889679
rs34022830
rs9403911
rs1321323
rs1011166
rs139157952
rs114017586
rs12606213
rs12306124
rs11924390
All − MR Egger
All − Inverse variance weighted
−1.0
−0.5
0.0
0.5
1.0
MR effect size for
'Enterobacteriaceae' on 'GCST90199872_buildGRCh38.tsv'

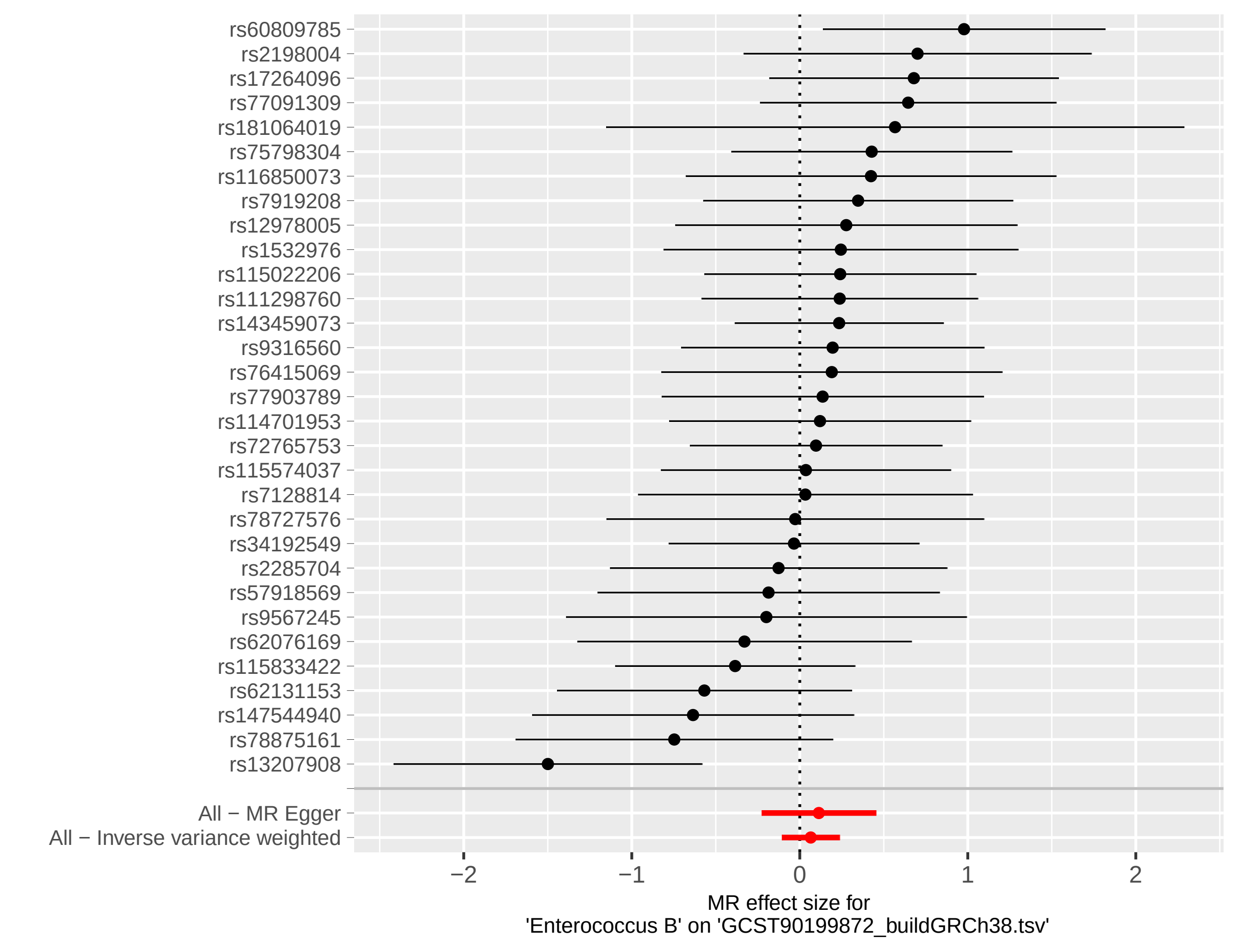

rs60809785
rs2198004
rs17264096
rs77091309
rs181064019
rs75798304
rs116850073
rs7919208
rs12978005
rs1532976
rs115022206
rs111298760
rs143459073
rs9316560
rs76415069
rs77903789
rs114701953
rs72765753
rs115574037
rs7128814
rs78727576
rs34192549
rs2285704
rs57918569
rs9567245
rs62076169
rs115833422
rs62131153
rs147544940
rs78875161
rs13207908
All − MR Egger
All − Inverse variance weighted
−2
−1
0
1
2
MR effect size for
'Enterococcus B' on 'GCST90199872_buildGRCh38.tsv'

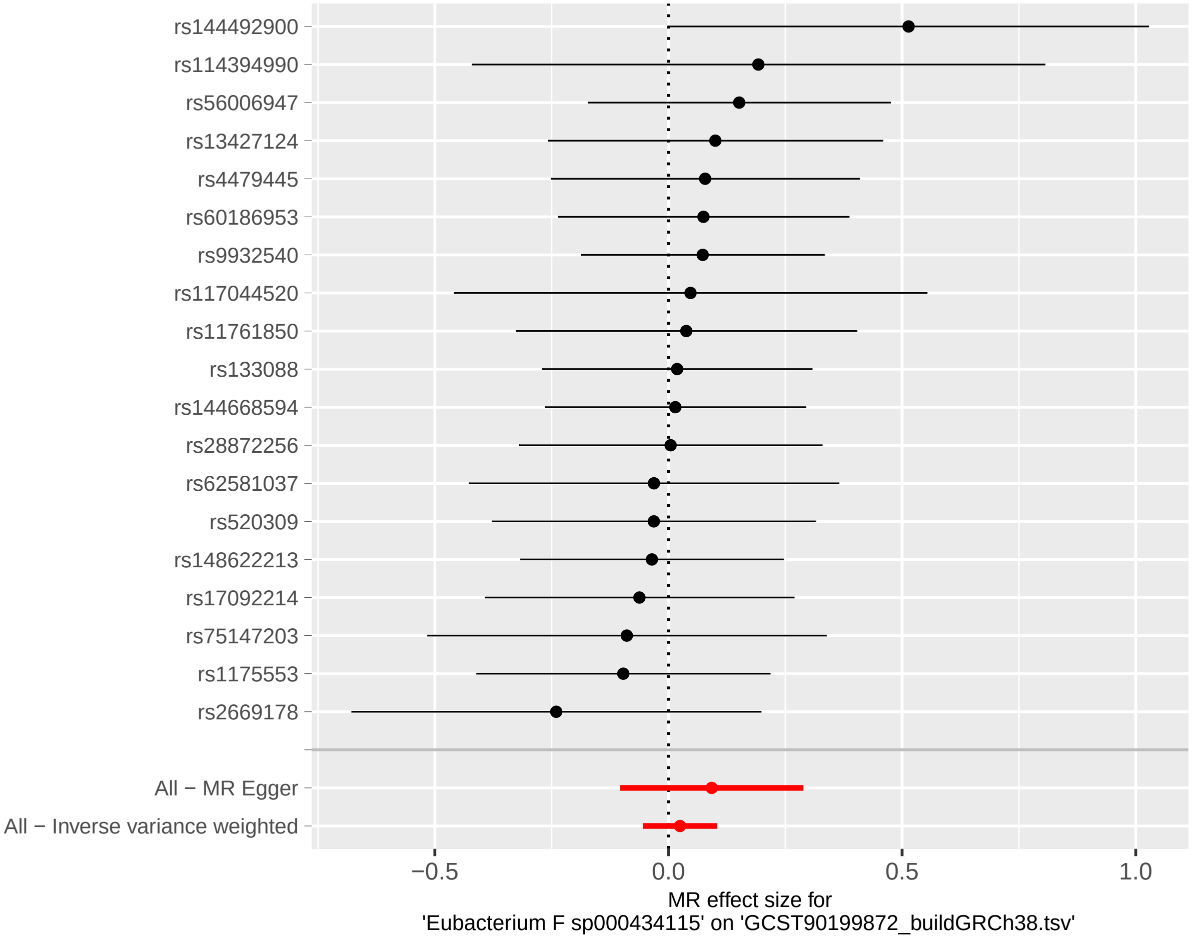
rs144492900
rs114394990
rs56006947
rs13427124
rs4479445
rs60186953
rs9932540
rs117044520
rs11761850
rs133088
rs144668594
rs28872256
rs62581037
rs520309
rs148622213
rs17092214
rs75147203
rs1175553
rs2669178
All − MR Egger
All − Inverse variance weighted
−0.5
0.0
0.5
1.0
MR effect size for
'Eubacterium F sp000434115' on 'GCST90199872_buildGRCh38.tsv'

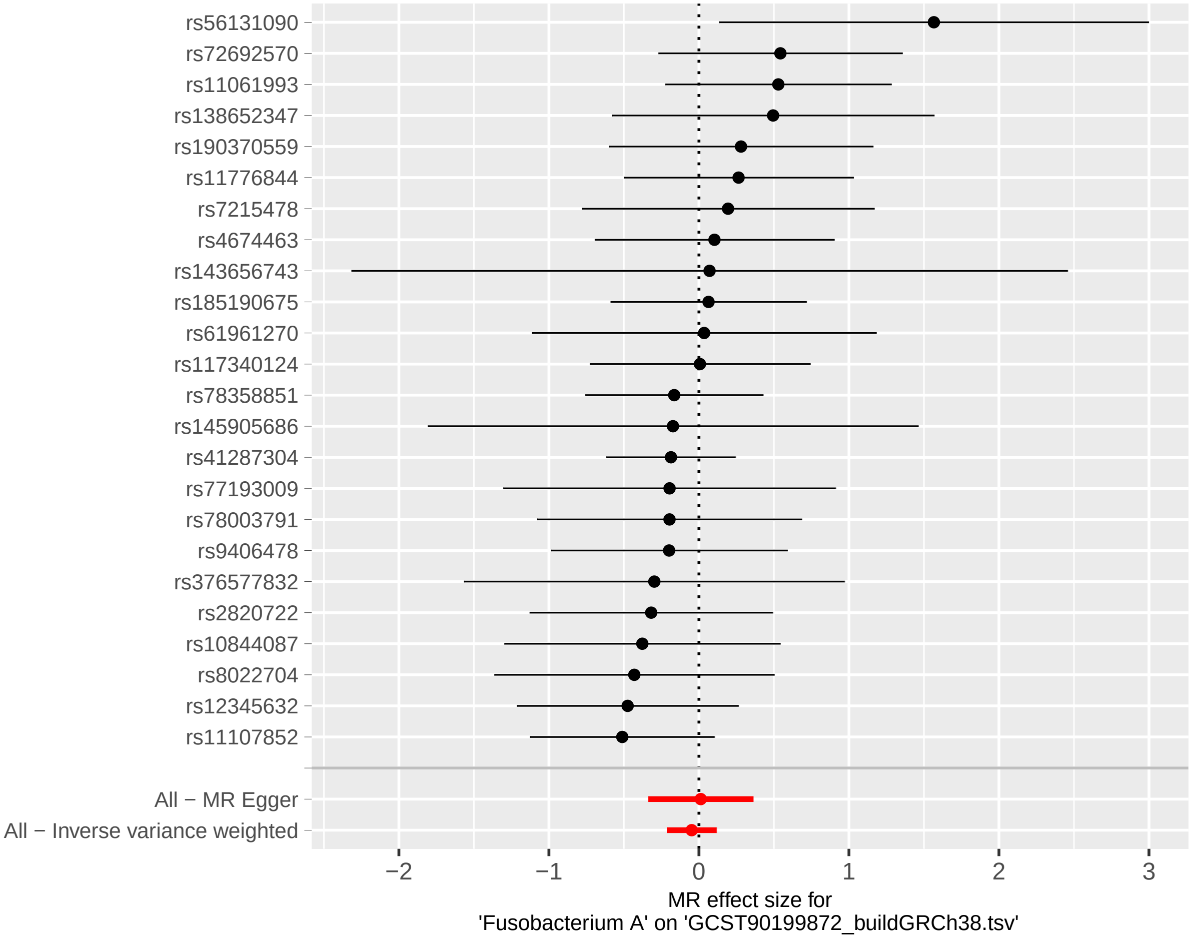
rs56131090
rs72692570
rs11061993
rs138652347
rs190370559
rs11776844
rs7215478
rs4674463
rs143656743
rs185190675
rs61961270
rs117340124
rs78358851
rs145905686
rs41287304
rs77193009
rs78003791
rs9406478
rs376577832
rs2820722
rs10844087
rs8022704
rs12345632
rs11107852
All − MR Egger
All − Inverse variance weighted
−2
−1
0
1
2
3
MR effect size for
'Fusobacterium A' on 'GCST90199872_buildGRCh38.tsv'

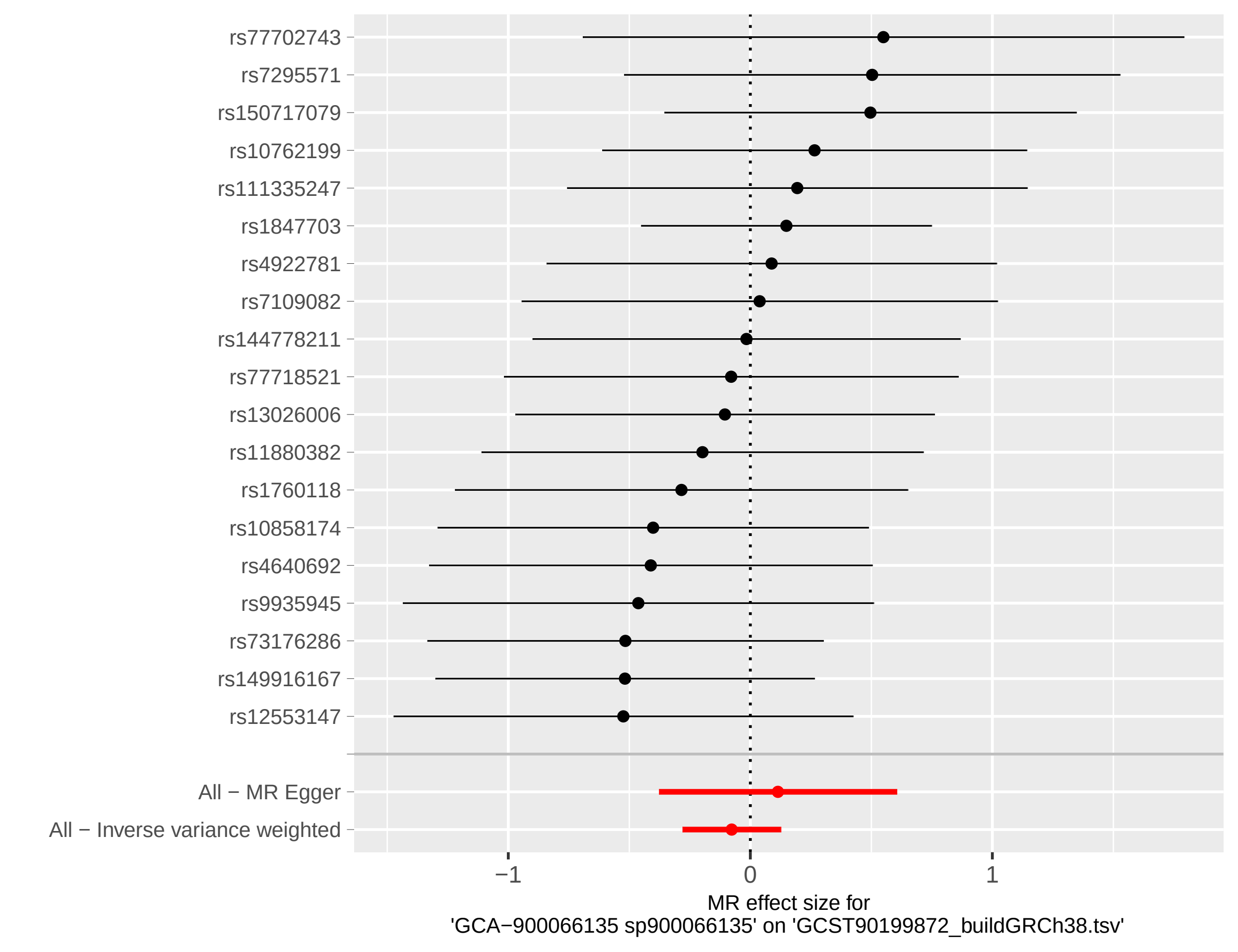

rs77702743
rs7295571
rs150717079
rs10762199
rs111335247
rs1847703
rs4922781
rs7109082
rs144778211
rs77718521
rs13026006
rs11880382
rs1760118
rs10858174
rs4640692
rs9935945
rs73176286
rs149916167
rs12553147
All − MR Egger
All − Inverse variance weighted
−1
0
1
MR effect size for
'GCA−900066135 sp900066135' on 'GCST90199872_buildGRCh38.tsv'

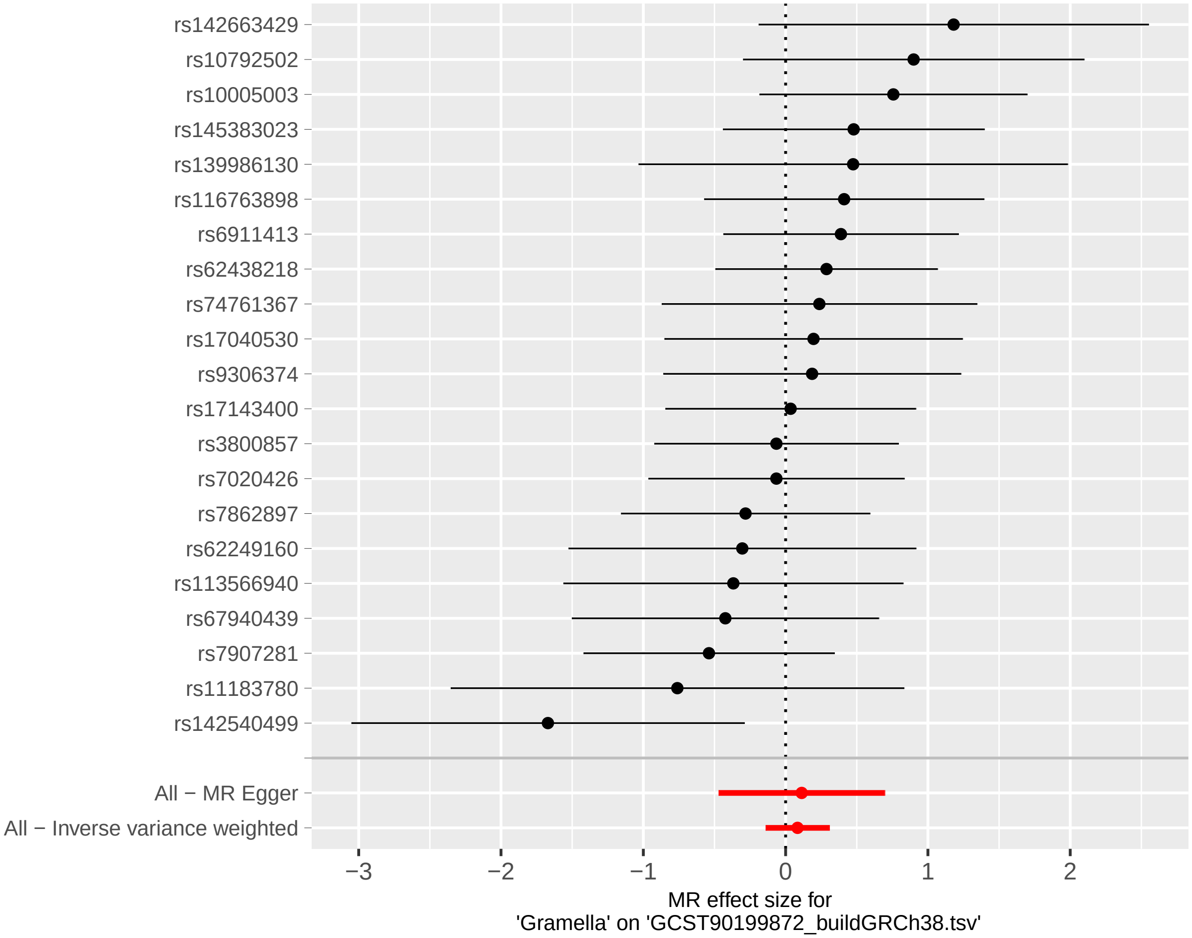
rs142663429
rs10792502
rs10005003
rs145383023
rs139986130
rs116763898
rs6911413
rs62438218
rs74761367
rs17040530
rs9306374
rs17143400
rs3800857
rs7020426
rs7862897
rs62249160
rs113566940
rs67940439
rs7907281
rs11183780
rs142540499
All − MR Egger
All − Inverse variance weighted
−3
−2
−1
0
1
2
MR effect size for
'Gramella' on 'GCST90199872_buildGRCh38.tsv'

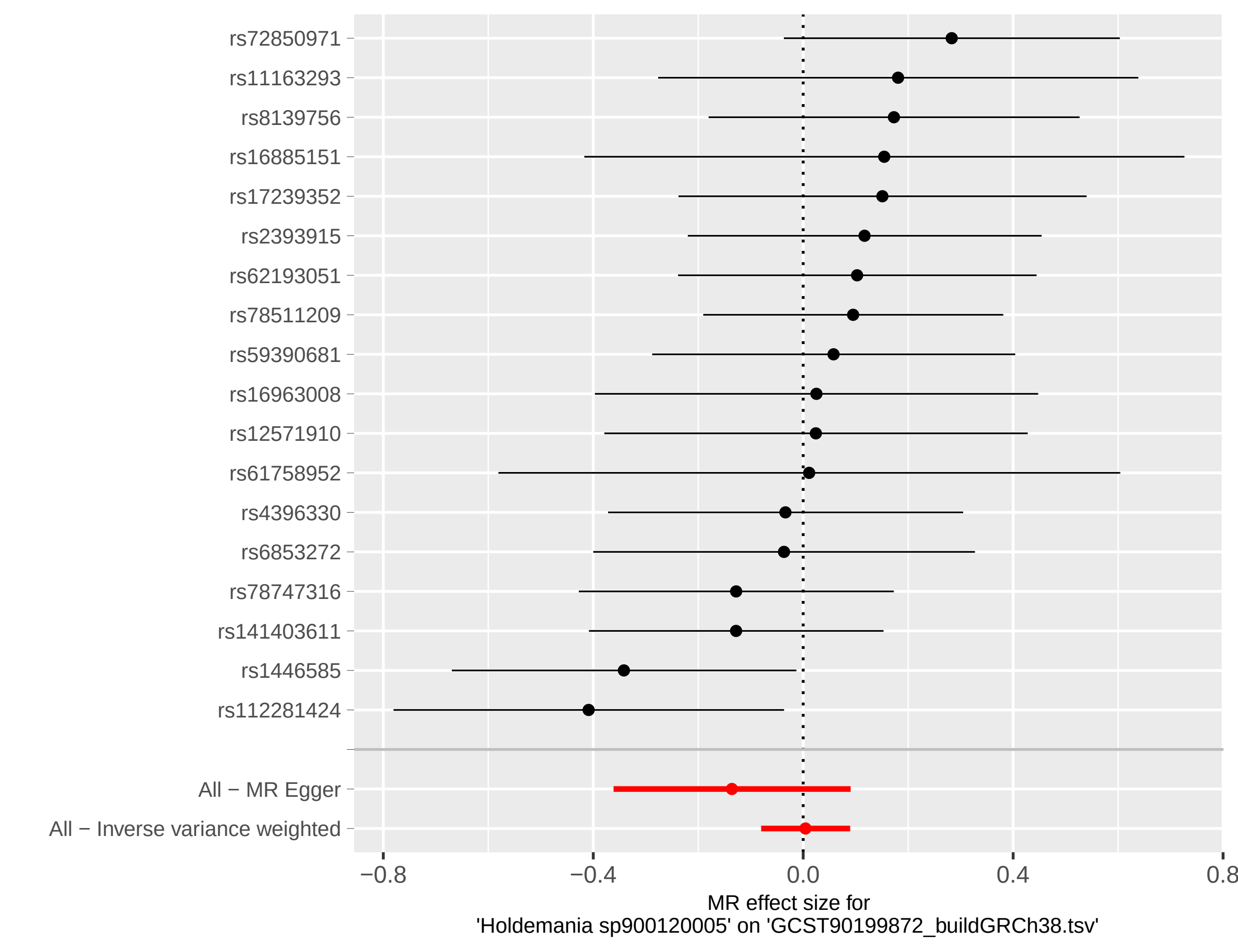
rs72850971
rs11163293
rs8139756
rs16885151
rs17239352
rs2393915
rs62193051
rs78511209
rs59390681
rs16963008
rs12571910
rs61758952
rs4396330
rs6853272
rs78747316
rs141403611
rs1446585
rs112281424
All − MR Egger
All − Inverse variance weighted
−0.8
−0.4
0.0
0.4
0.8
MR effect size for
'Holdemania sp900120005' on 'GCST90199872_buildGRCh38.tsv'

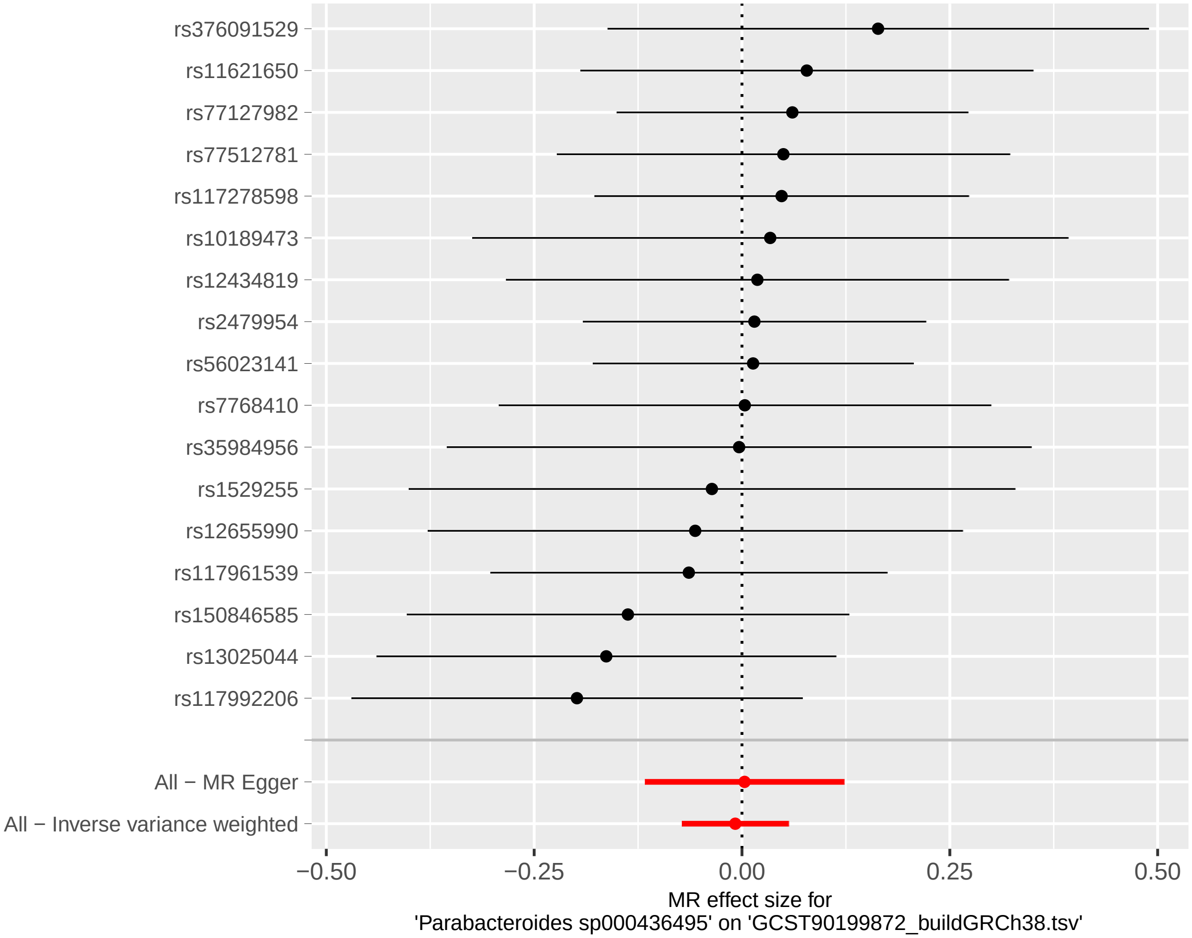

rs376091529
rs11621650
rs77127982
rs77512781
rs117278598
rs10189473
rs12434819
rs2479954
rs56023141
rs7768410
rs35984956
rs1529255
rs12655990
rs117961539
rs150846585
rs13025044
rs117992206
All − MR Egger
All − Inverse variance weighted
−0.50
−0.25
0.00
0.25
0.50
MR effect size for
'Parabacteroides sp000436495' on 'GCST90199872_buildGRCh38.tsv'

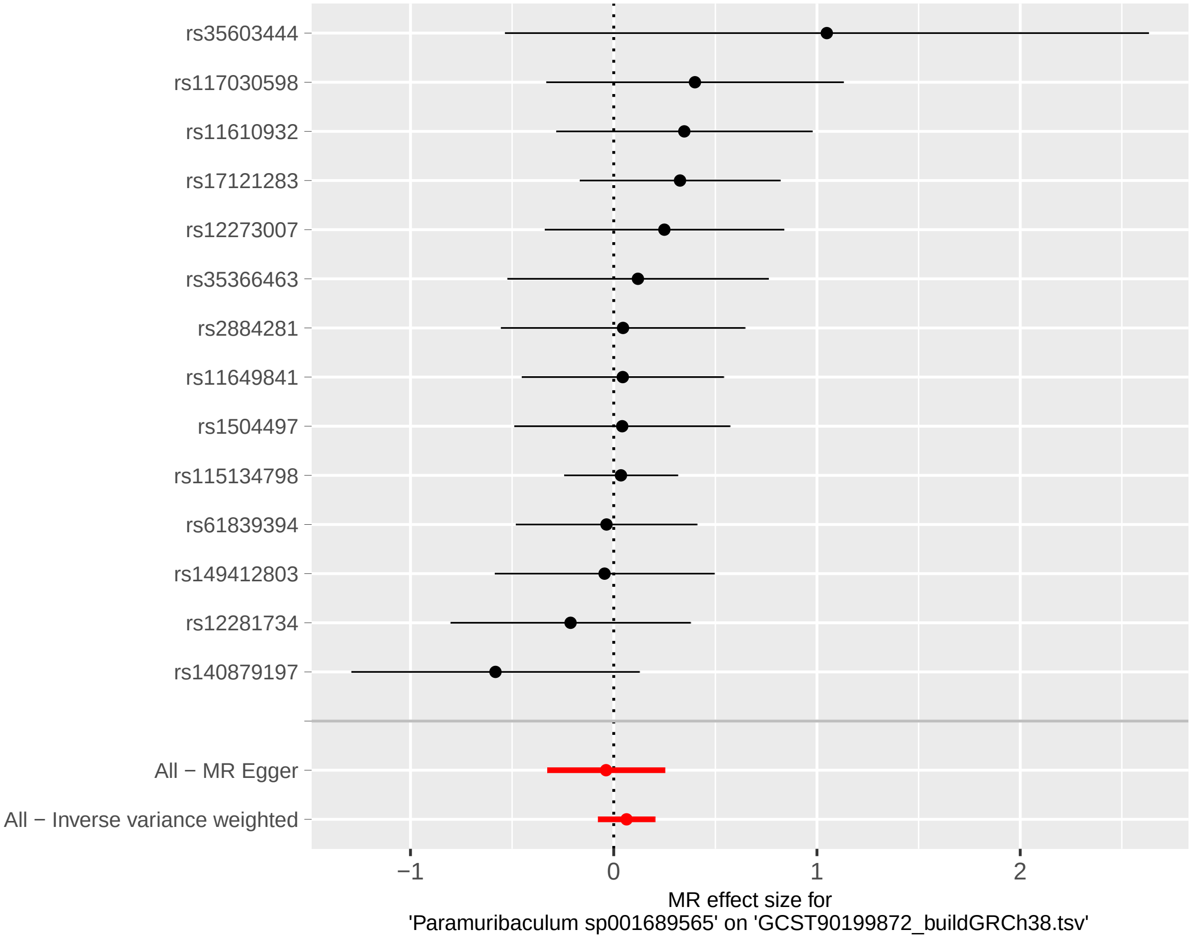
rs35603444
rs117030598
rs11610932
rs17121283
rs12273007
rs35366463
rs2884281
rs11649841
rs1504497
rs115134798
rs61839394
rs149412803
rs12281734
rs140879197
All − MR Egger
All − Inverse variance weighted
−1
0
1
2
MR effect size for
'Paramuribaculum sp001689565' on 'GCST90199872_buildGRCh38.tsv'

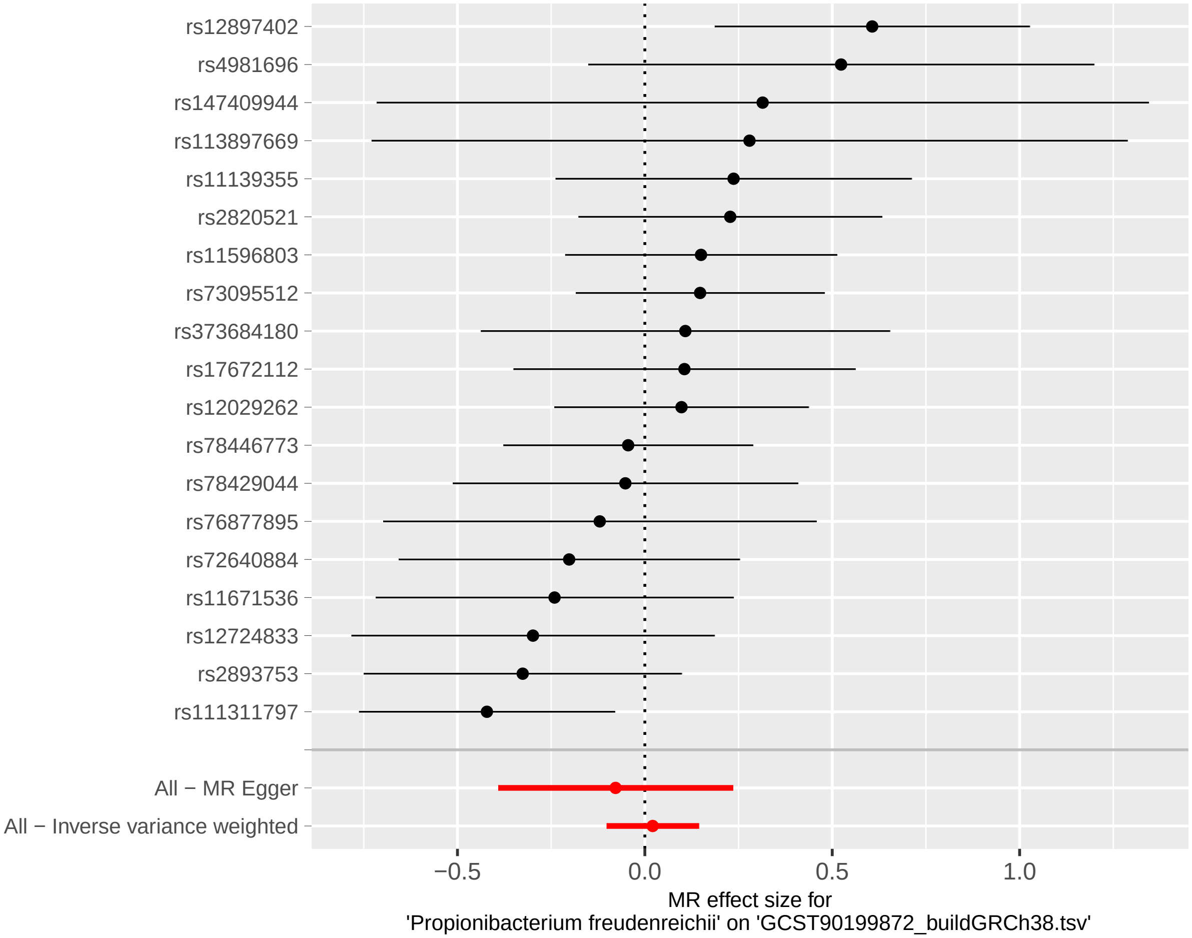
rs12897402
rs4981696
rs147409944
rs113897669
rs11139355
rs2820521
rs11596803
rs73095512
rs373684180
rs17672112
rs12029262
rs78446773
rs78429044
rs76877895
rs72640884
rs11671536
rs12724833
rs2893753
rs111311797
All − MR Egger
All − Inverse variance weighted
−0.5
0.0
0.5
1.0
MR effect size for
'Propionibacterium freudenreichii' on 'GCST90199872_buildGRCh38.tsv'

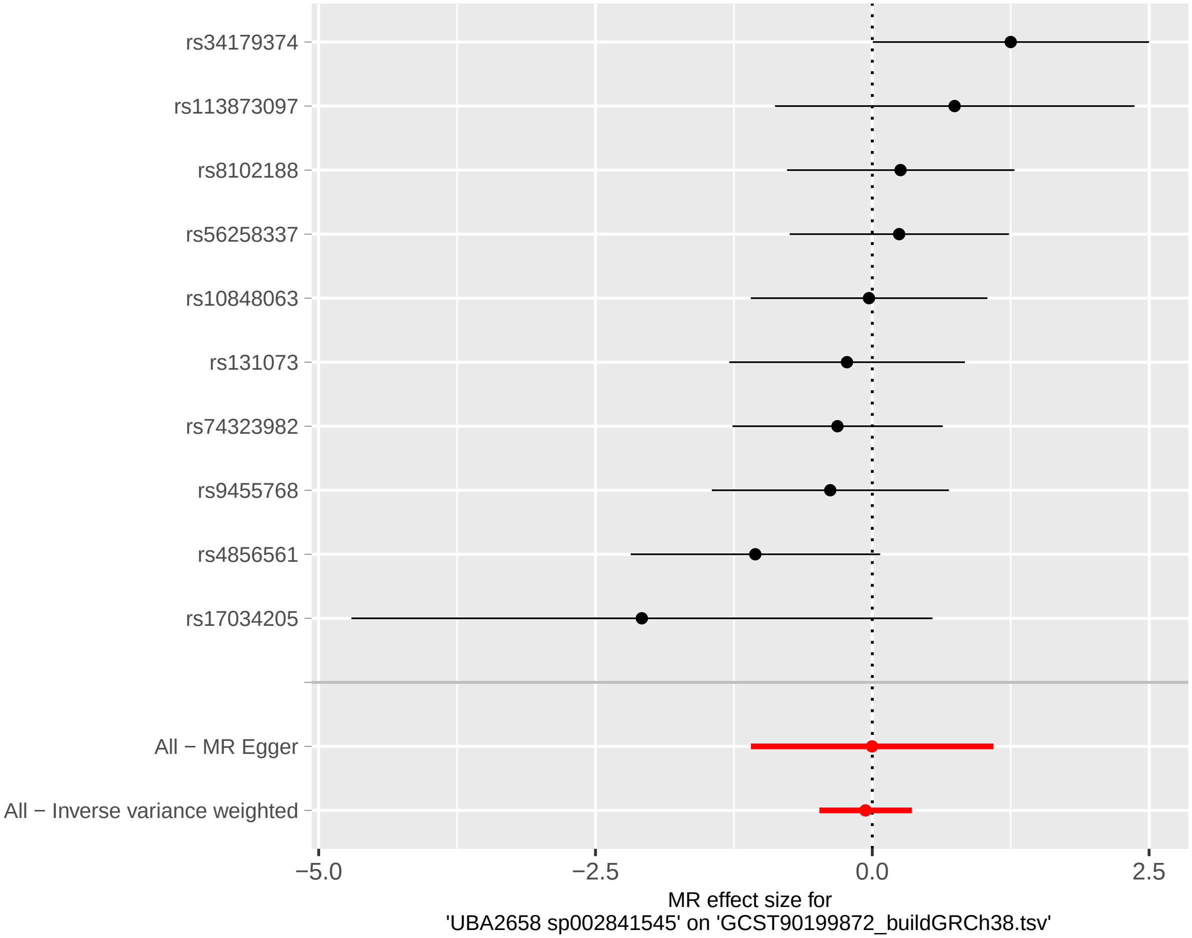
rs34179374
rs113873097
rs8102188
rs56258337
rs10848063
rs131073
rs74323982
rs9455768
rs4856561
rs17034205
All − MR Egger
All − Inverse variance weighted
−5.0
−2.5
0.0
2.5
MR effect size for
'UBA2658 sp002841545' on 'GCST90199872_buildGRCh38.tsv'

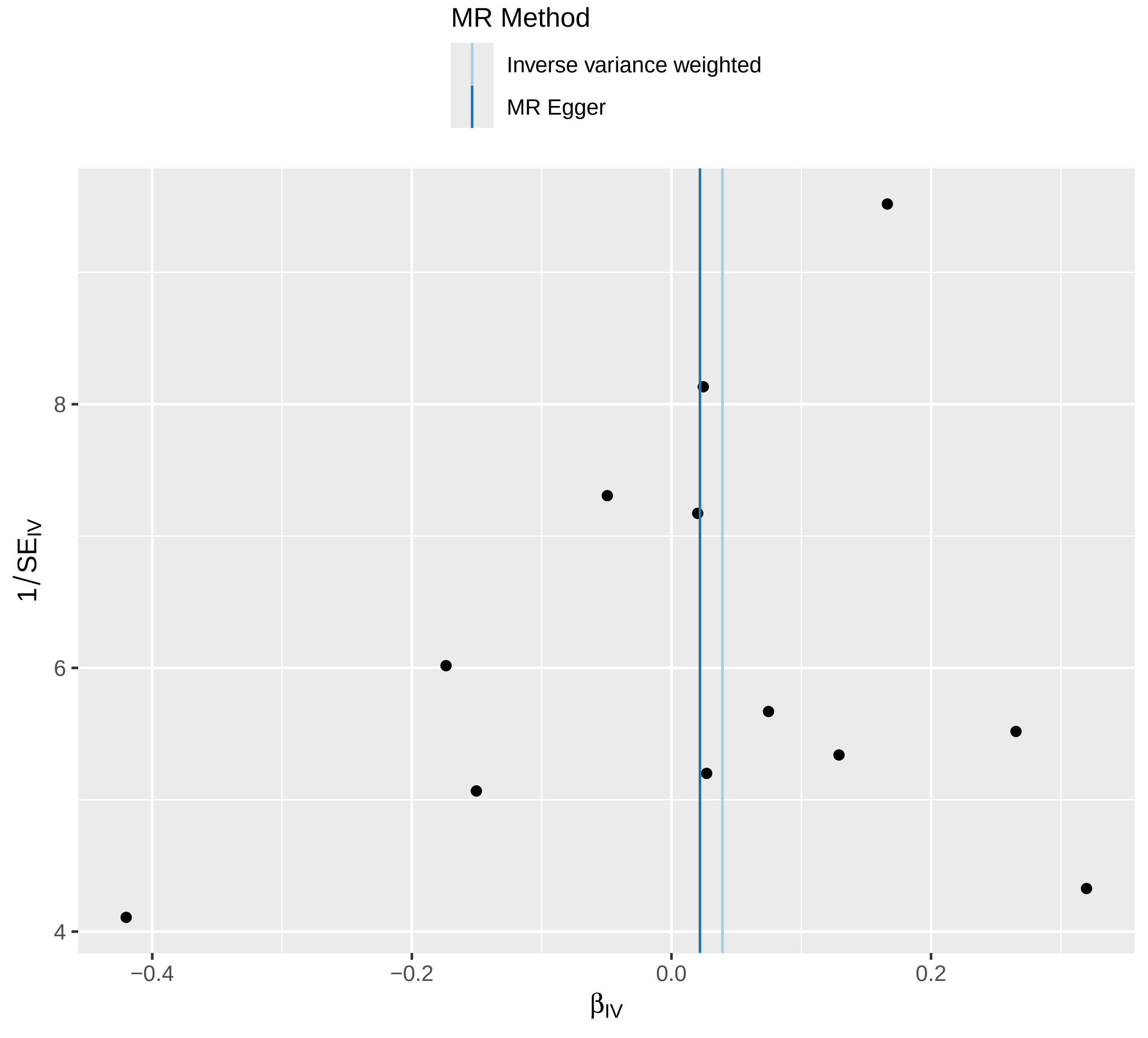

MR Method
Inverse variance weighted
MR Egger
8
6
4
$1/SE_{IV}$
−0.4
−0.2
0.0
0.2
β_{IV}

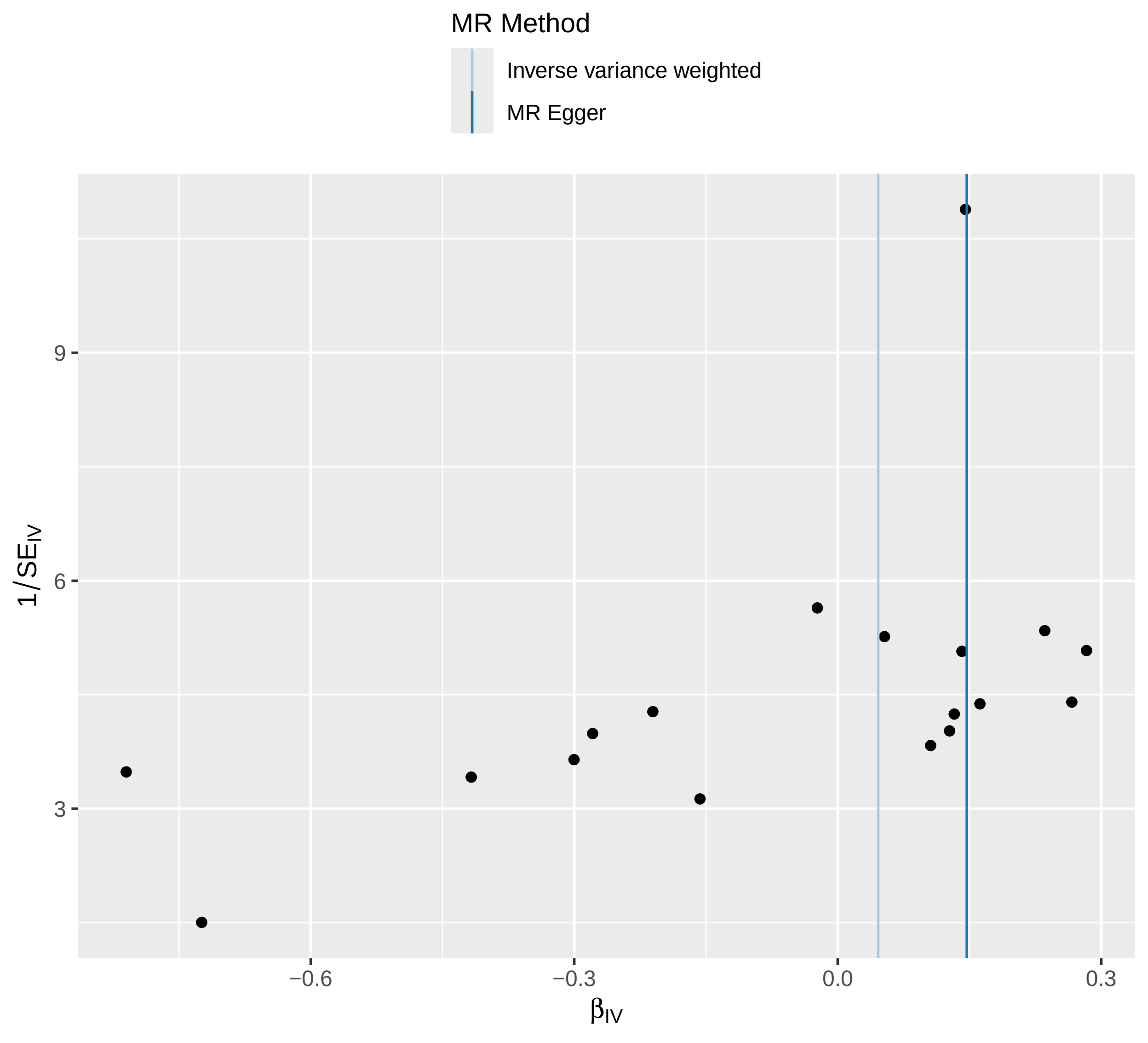

MR Method
Inverse variance weighted
MR Egger
9
6
3
1/SE$_{IV}$
−0.6
−0.3
0.0
0.3
β$_{IV}$

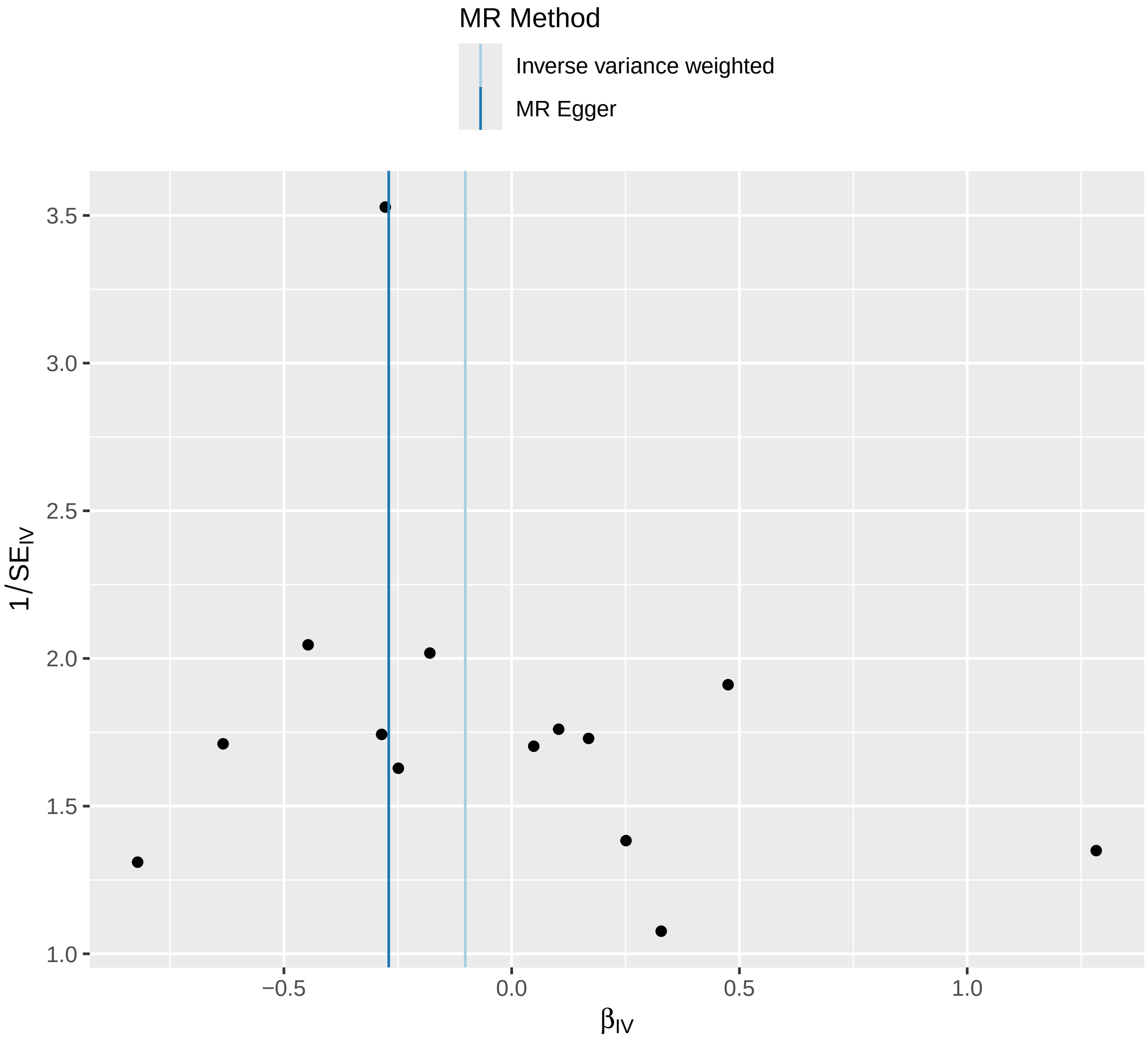
MR Method
Inverse variance weighted
MR Egger
3.5
3.0
2.5
2.0
1.5
1.0
1/SE_IV
−0.5
0.0
0.5
1.0
β_IV

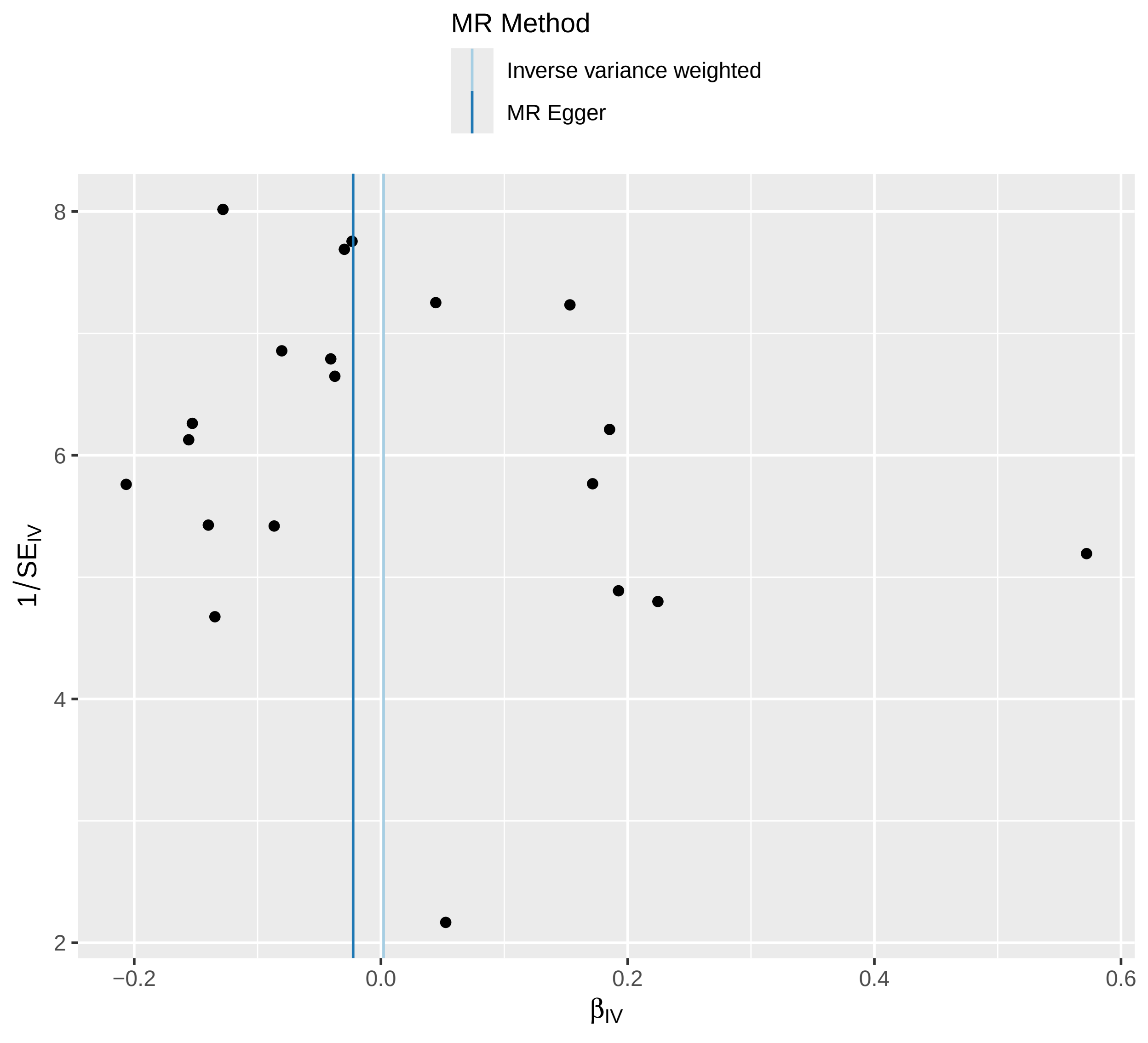

MR Method
Inverse variance weighted
MR Egger
8
6
4
2
1/SE$_{IV}$
−0.2
0.0
0.2
0.4
0.6
β$_{IV}$

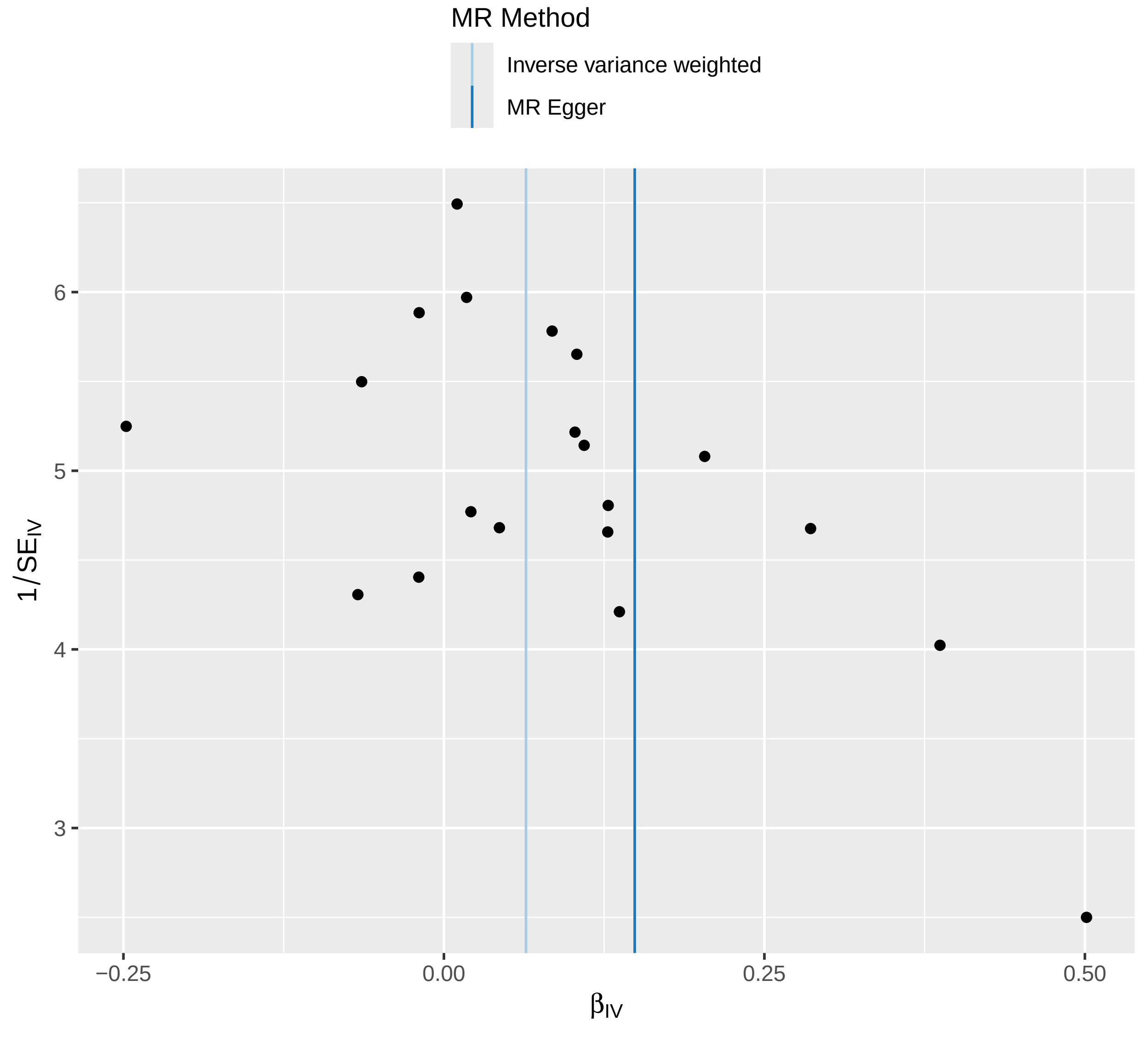

MR Method
Inverse variance weighted
MR Egger
6
5
4
3
1/SE_IV
−0.25
0.00
0.25
0.50
β_IV

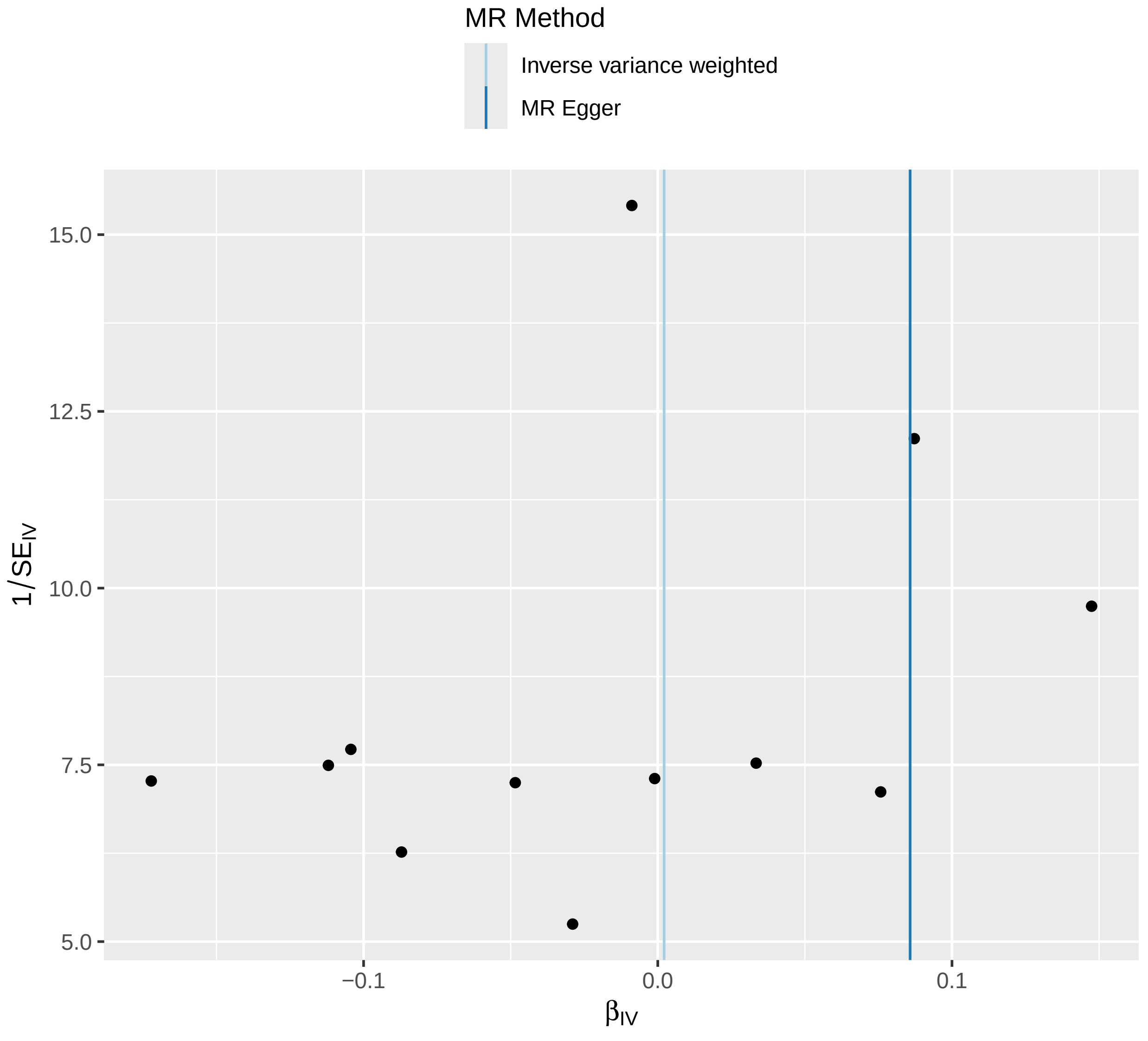
MR Method
Inverse variance weighted
MR Egger
15.0
12.5
10.0
7.5
5.0
1/SE_IV
−0.1
0.0
0.1
β_IV

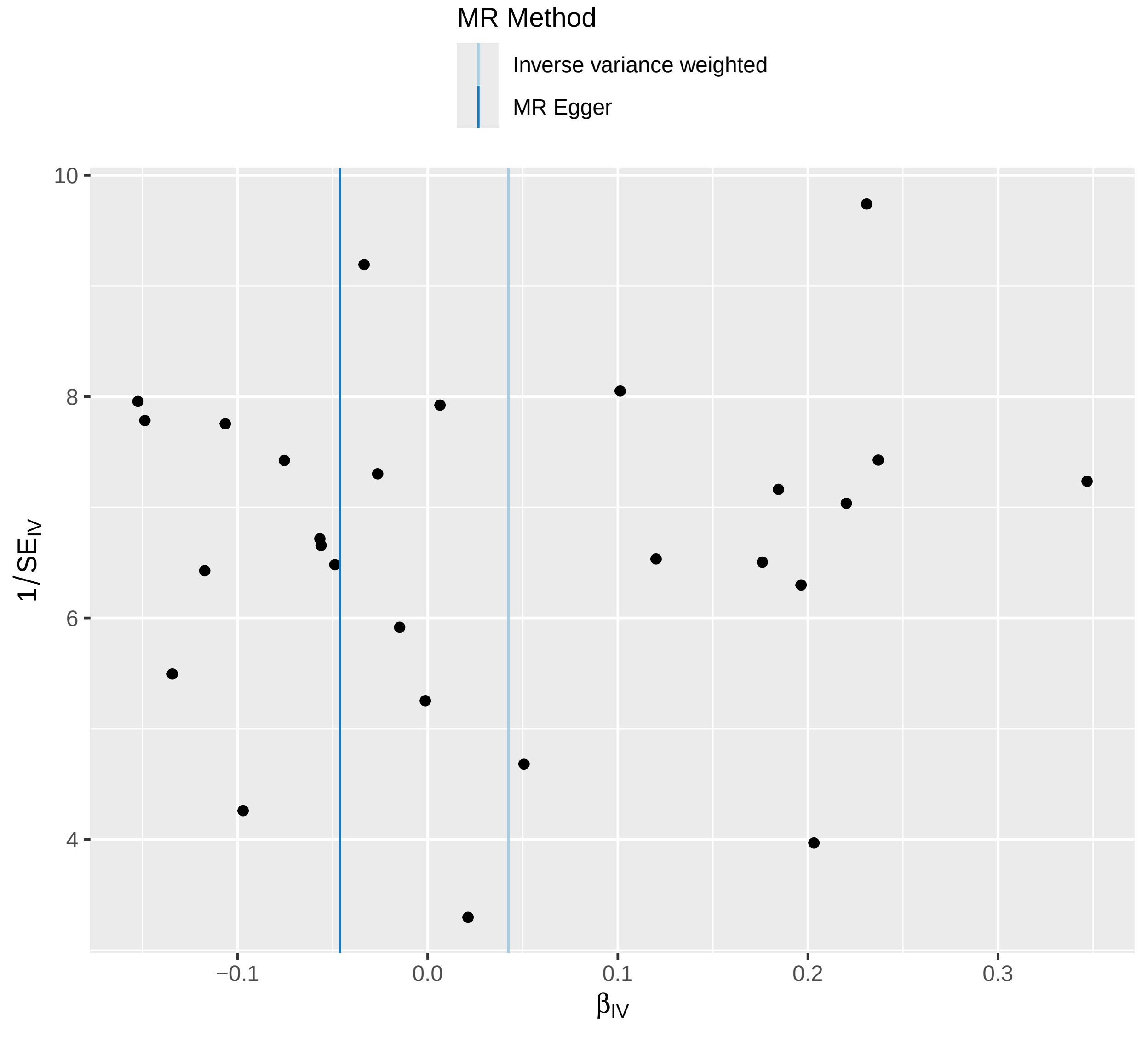
MR Method
Inverse variance weighted
MR Egger
10
8
6
4
1/SE$_{IV}$
−0.1
0.0
0.1
0.2
0.3
β$_{IV}$

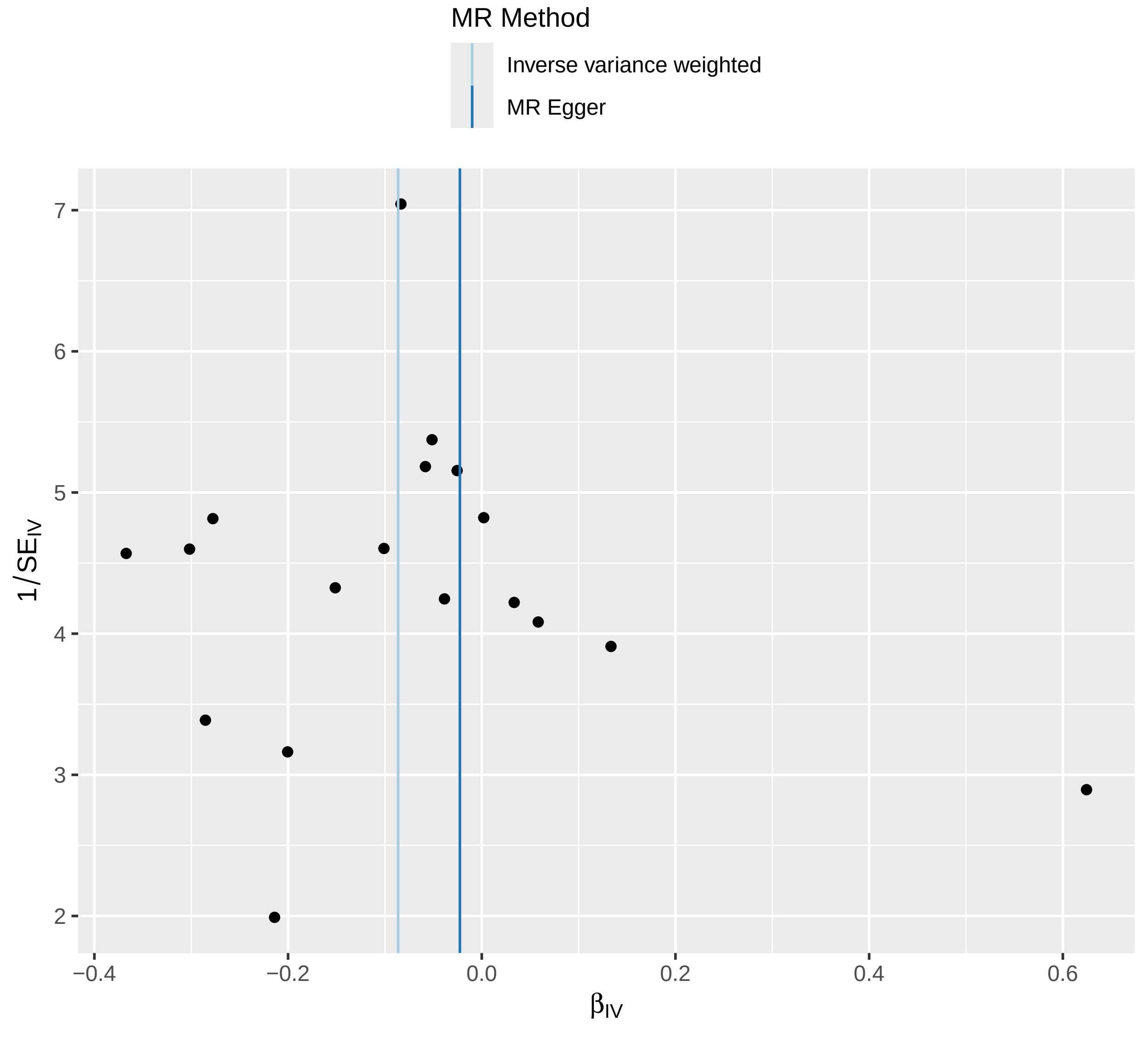
MR Method
Inverse variance weighted
MR Egger
7
6
5
4
3
2
1/SE_IV
−0.4
−0.2
0.0
0.2
0.4
0.6
β_IV

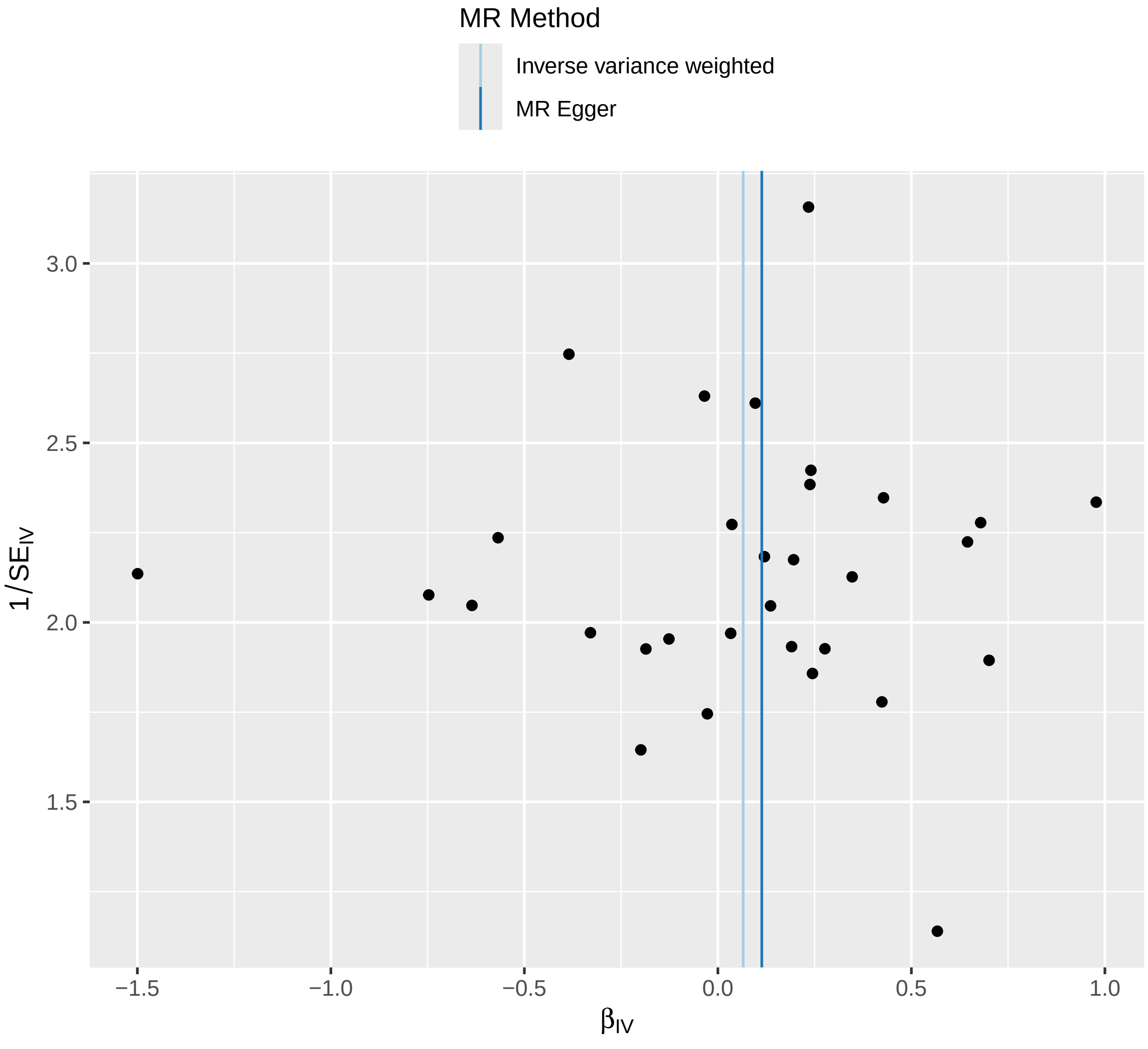
MR Method
Inverse variance weighted
MR Egger
3.0
2.5
2.0
1.5
1/SE$_{IV}$
−1.5
−1.0
−0.5
0.0
0.5
1.0
β$_{IV}$

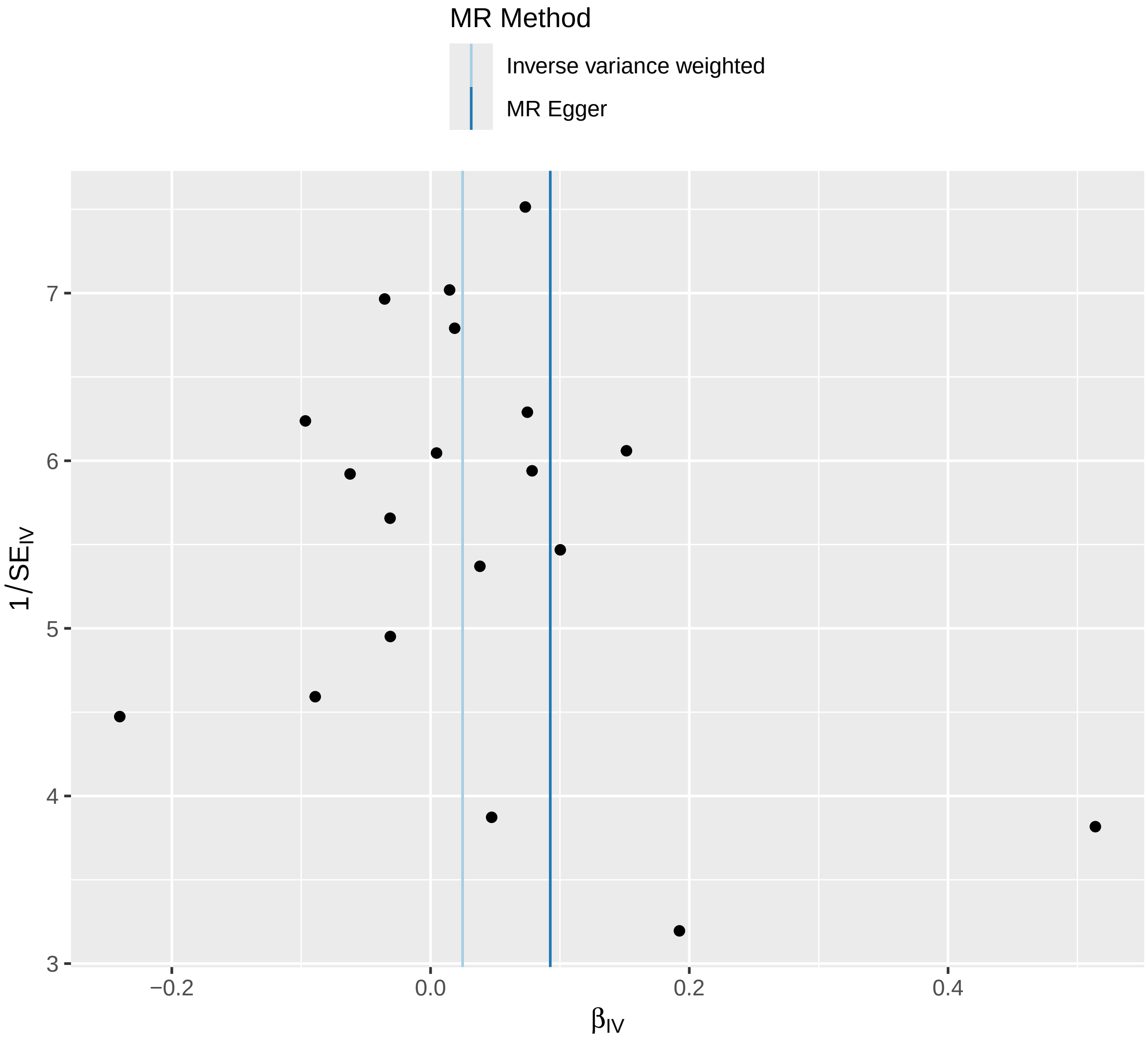

MR Method
Inverse variance weighted
MR Egger
7
6
5
4
3
$1/SE_{IV}$
−0.2
0.0
0.2
0.4
β_{IV}

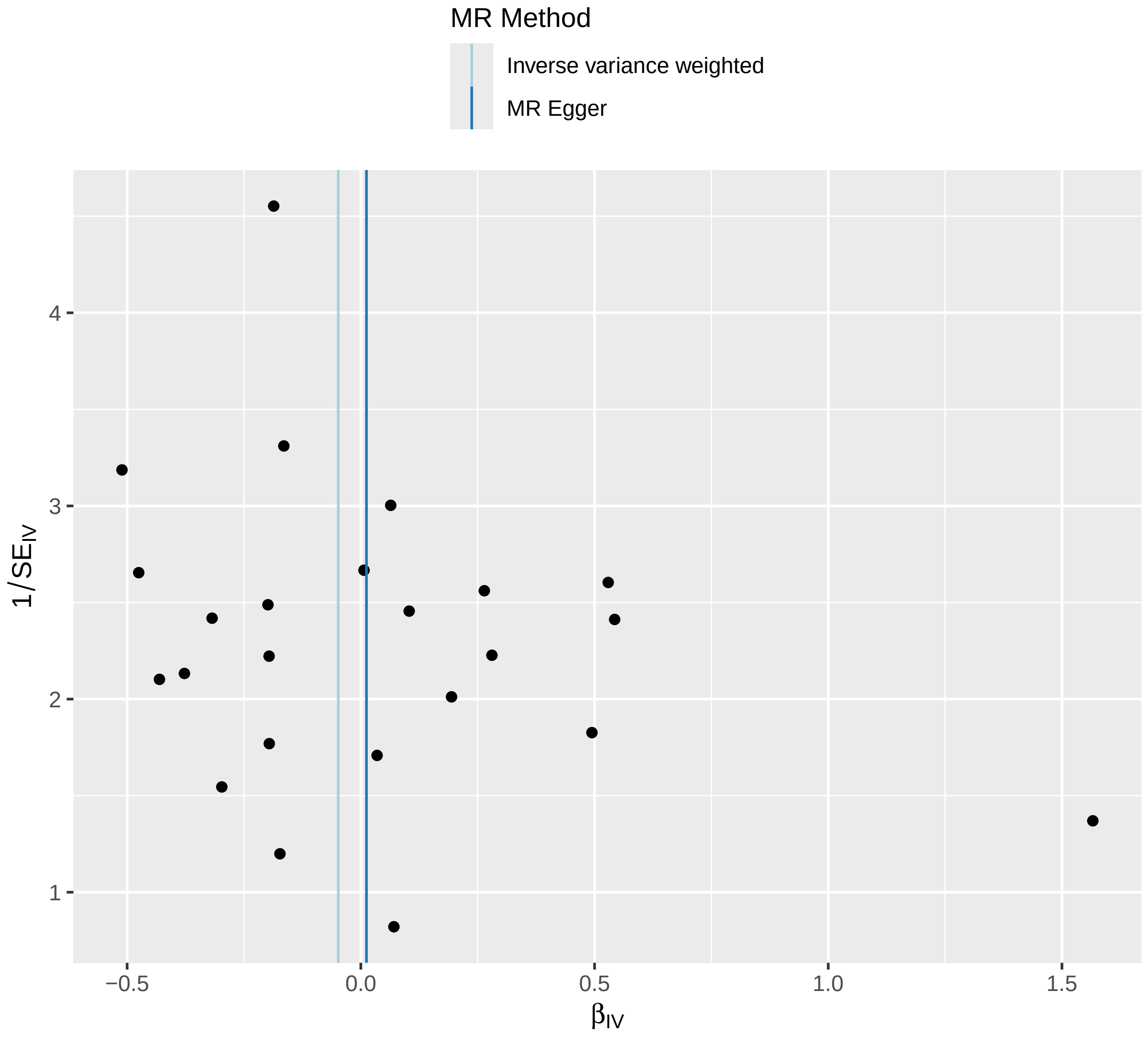
MR Method
Inverse variance weighted
MR Egger
$1/SE_{IV}$
4
3
2
1
−0.5
0.0
0.5
1.0
1.5
β_{IV}

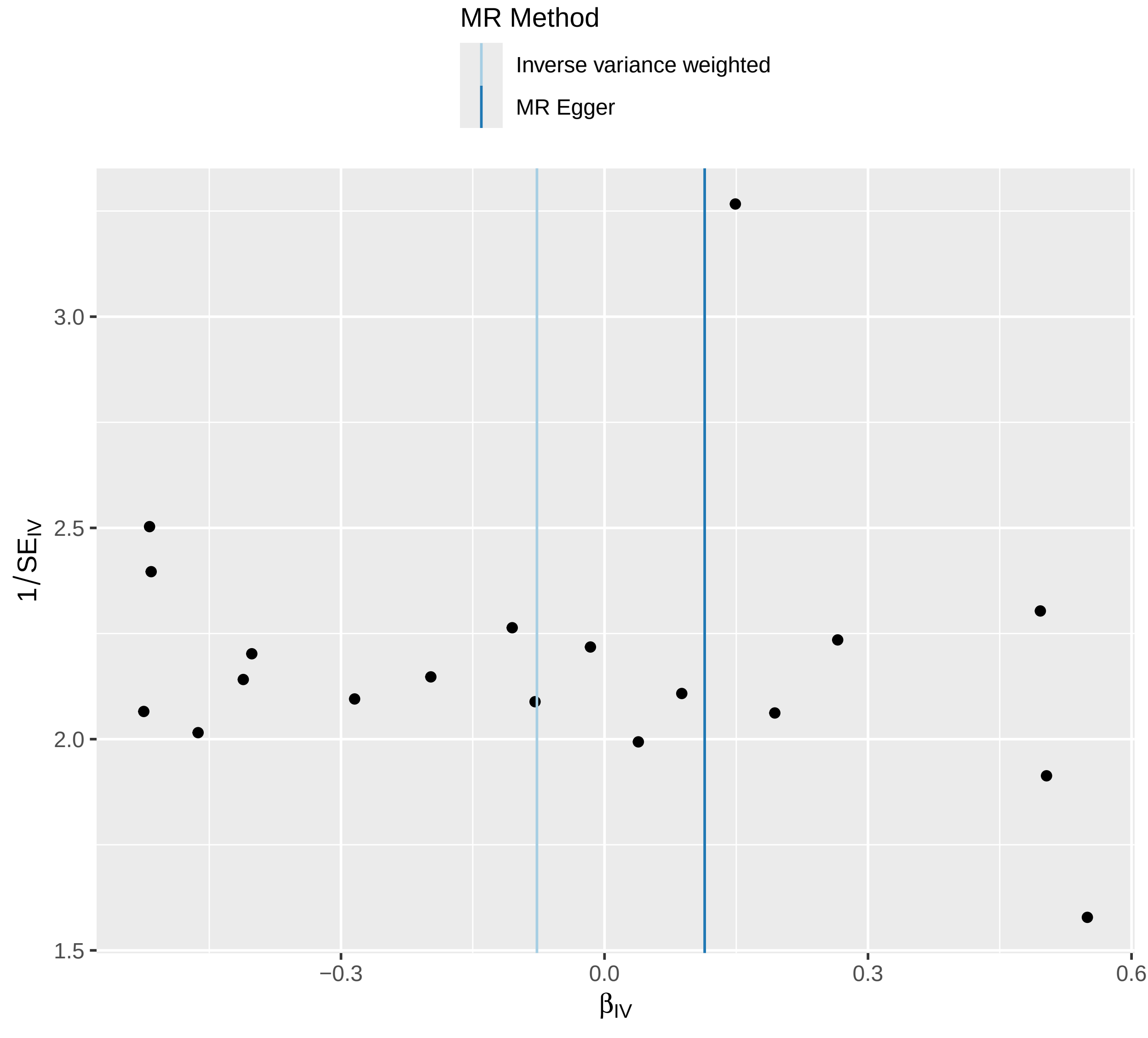

MR Method
Inverse variance weighted
MR Egger
3.0
2.5
2.0
1.5
1/SE$_{IV}$
−0.3
0.0
0.3
0.6
β_{IV}

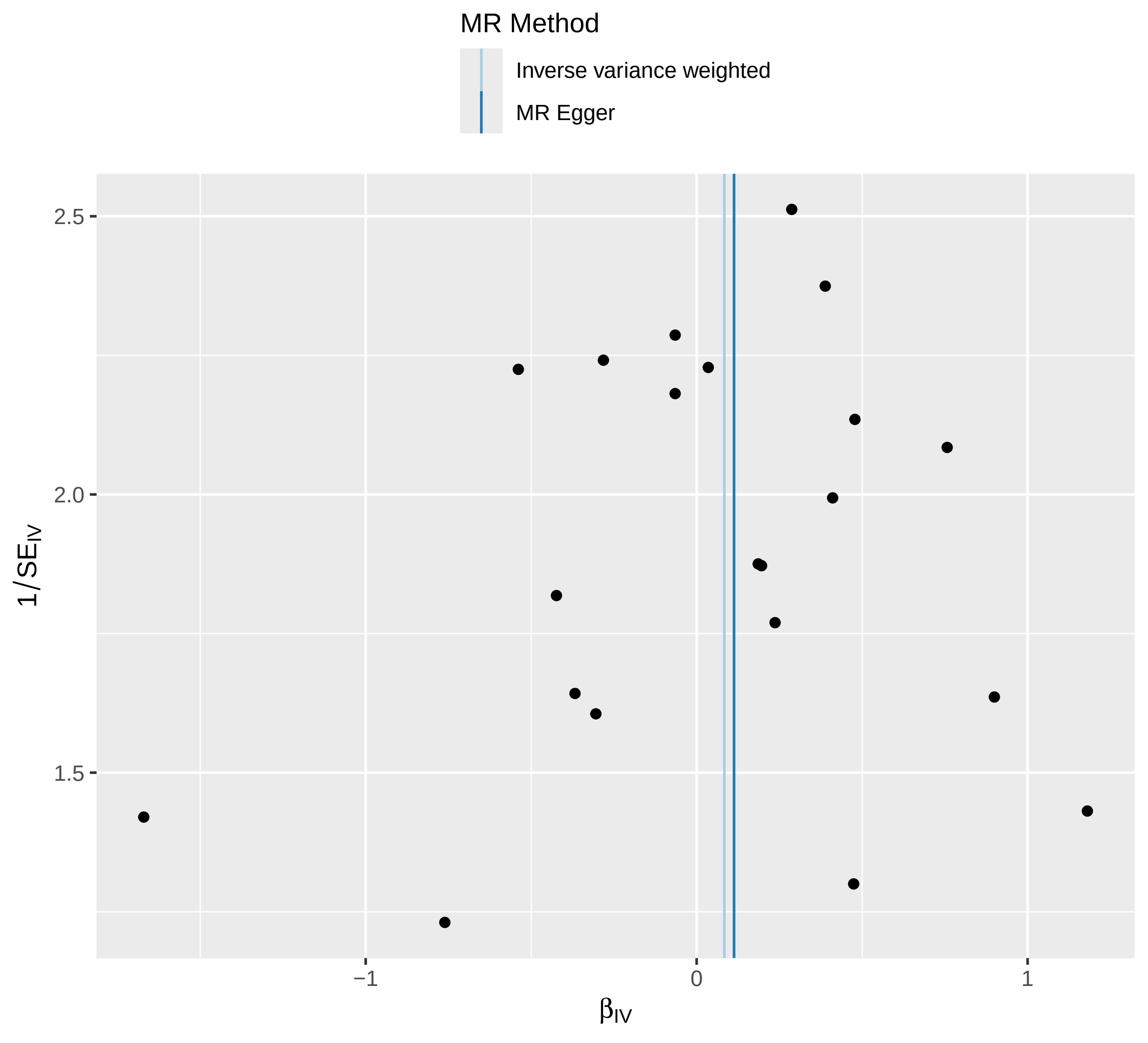

MR Method
Inverse variance weighted
MR Egger
2.5
2.0
1.5
1/SE_IV
−1
0
1
β_IV

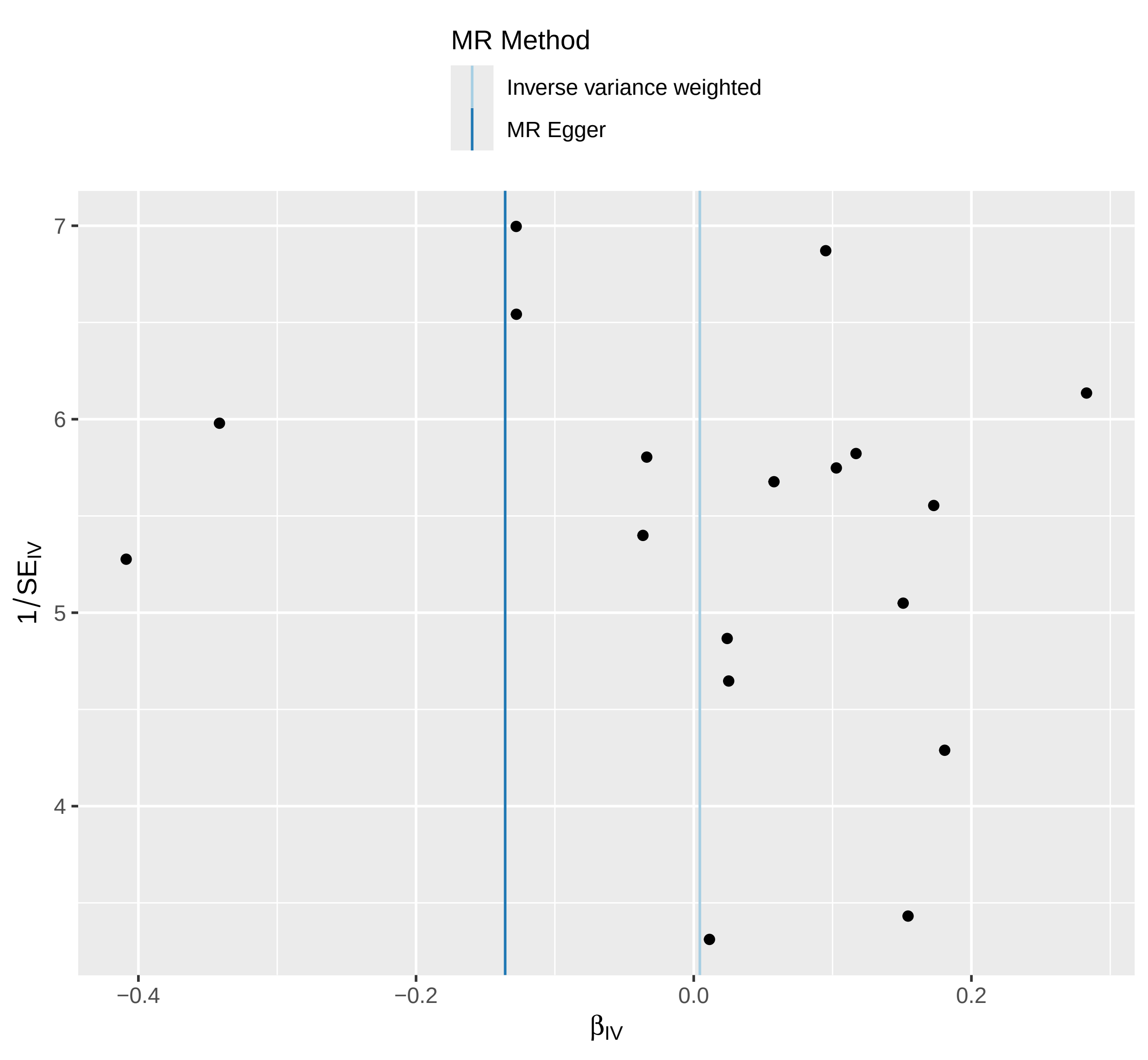
MR Method
Inverse variance weighted
MR Egger
7
6
5
4
1/SE_IV
−0.4
−0.2
0.0
0.2
β_IV

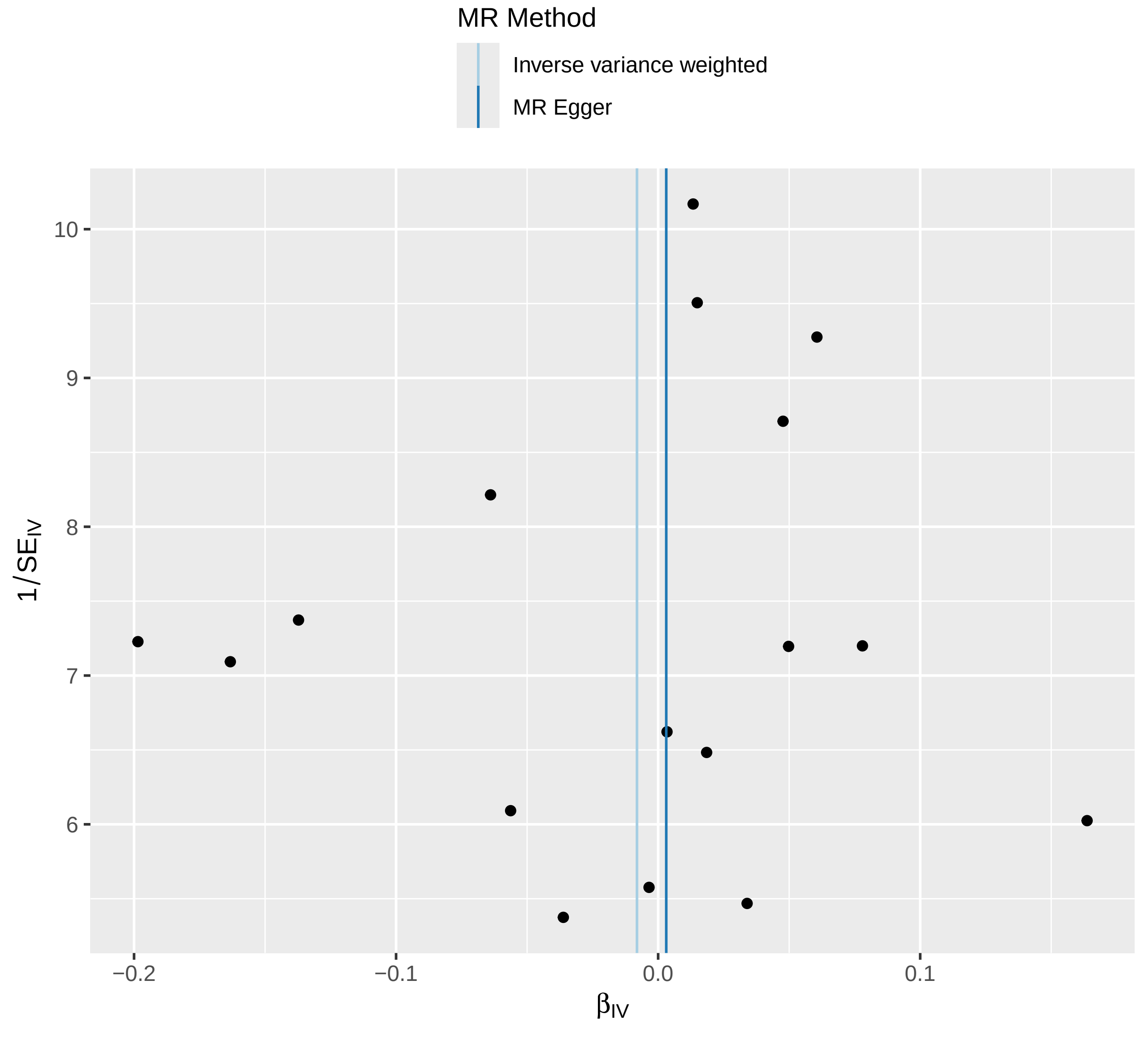
MR Method
Inverse variance weighted
MR Egger
10
9
8
7
6
1/SE_IV
−0.2
−0.1
0.0
0.1
β_IV

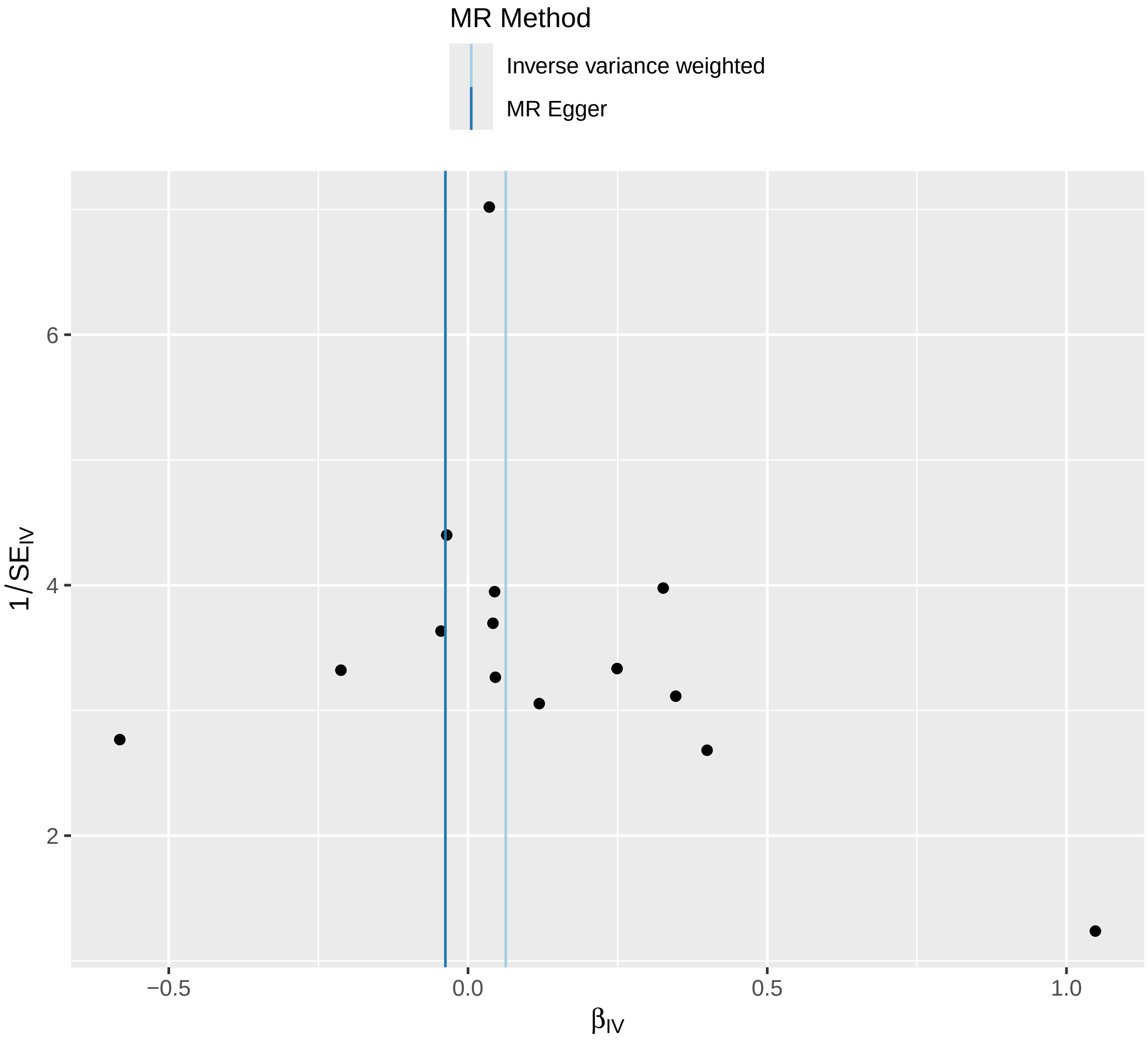
MR Method
Inverse variance weighted
MR Egger
6
4
2
$1/SE_{IV}$
−0.5
0.0
0.5
1.0
β_{IV}

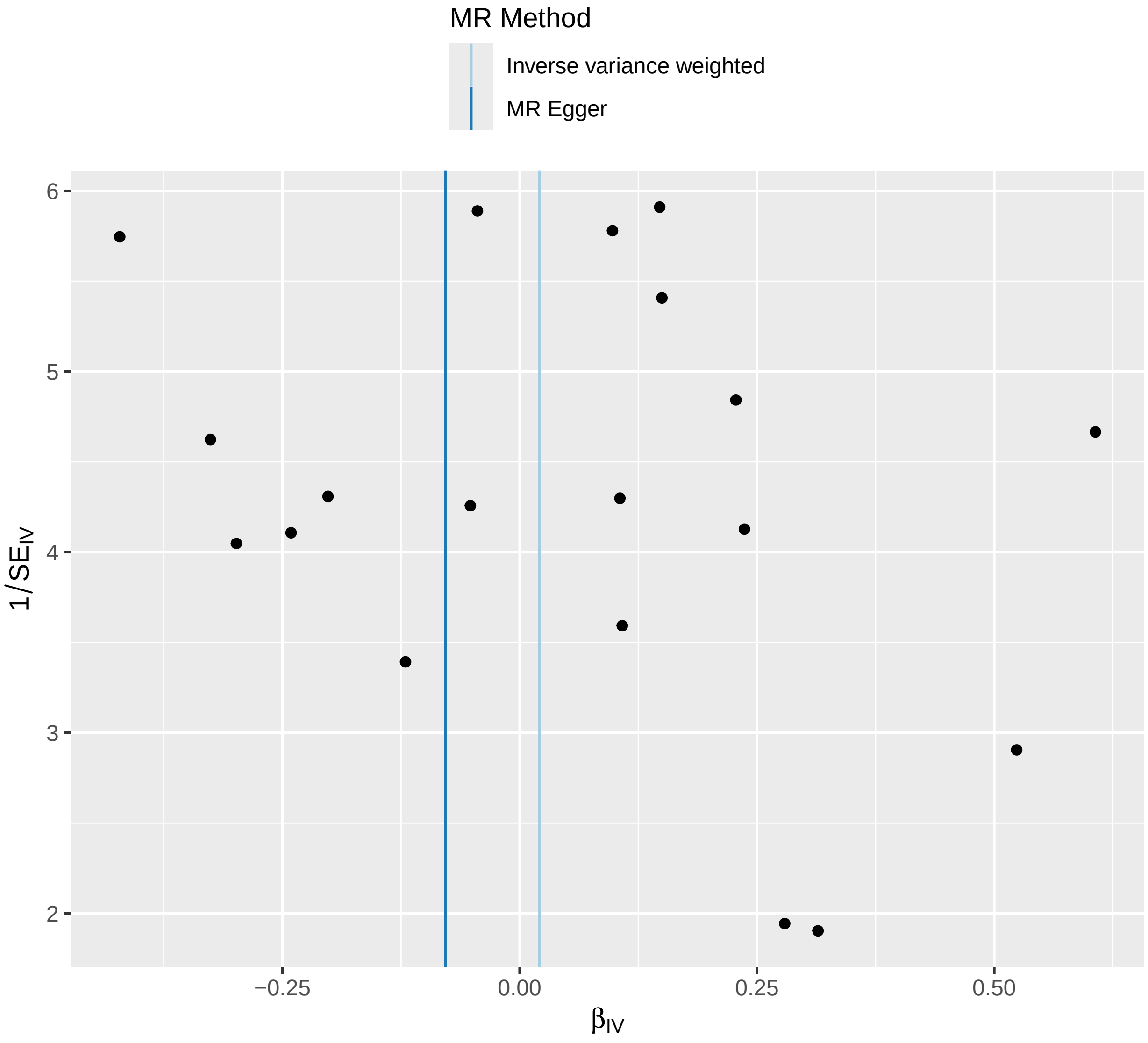
MR Method
Inverse variance weighted
MR Egger
6
5
4
3
2
1/SE$_{IV}$
−0.25
0.00
0.25
0.50
β$_{IV}$

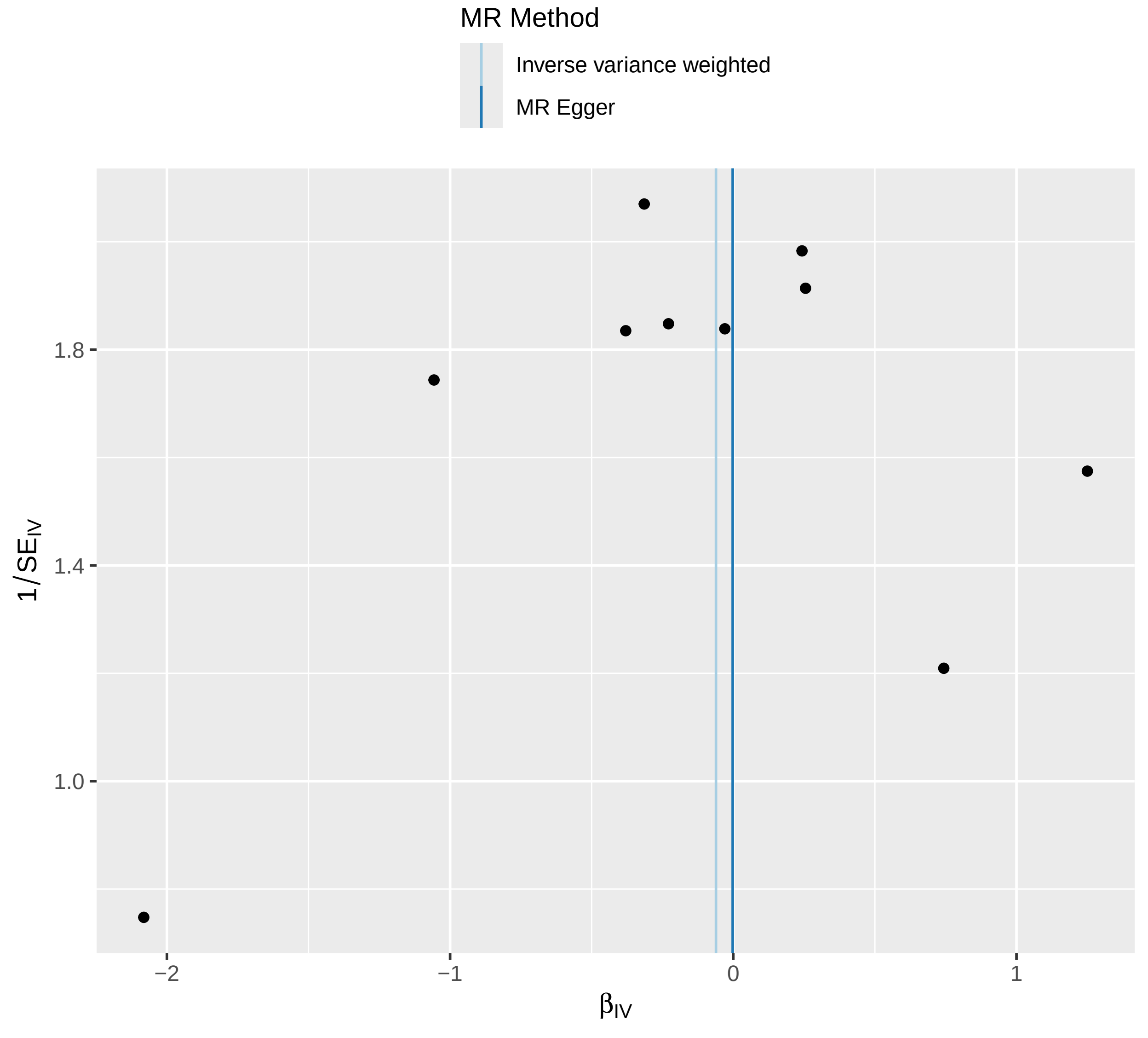

MR Method
Inverse variance weighted
MR Egger
1.8
1.4
1.0
$1/SE_{IV}$
−2
−1
0
1
β_{IV}

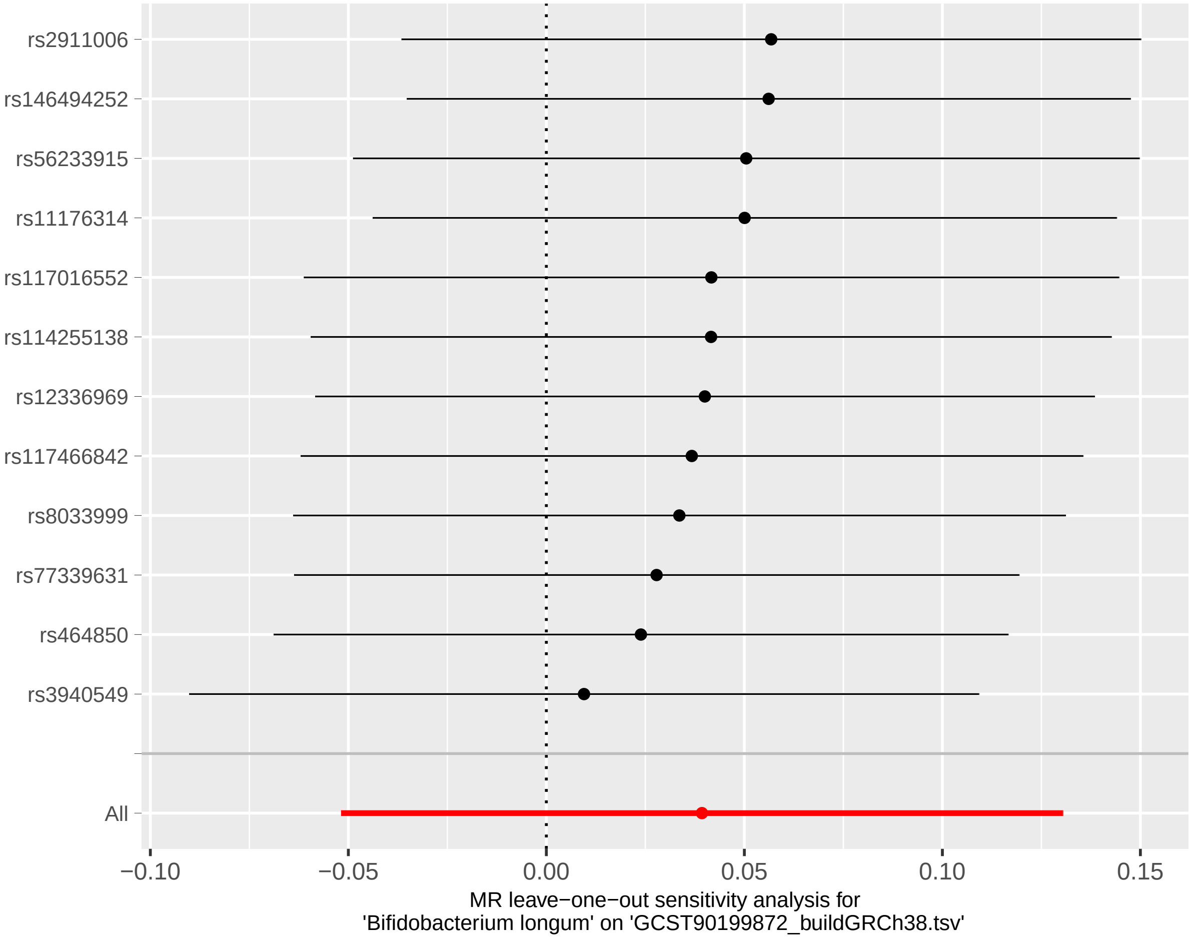
rs2911006
rs146494252
rs56233915
rs11176314
rs117016552
rs114255138
rs12336969
rs117466842
rs8033999
rs77339631
rs464850
rs3940549
All
−0.10
−0.05
0.00
0.05
0.10
0.15
MR leave−one−out sensitivity analysis for
'Bifidobacterium longum' on 'GCST90199872_buildGRCh38.tsv'

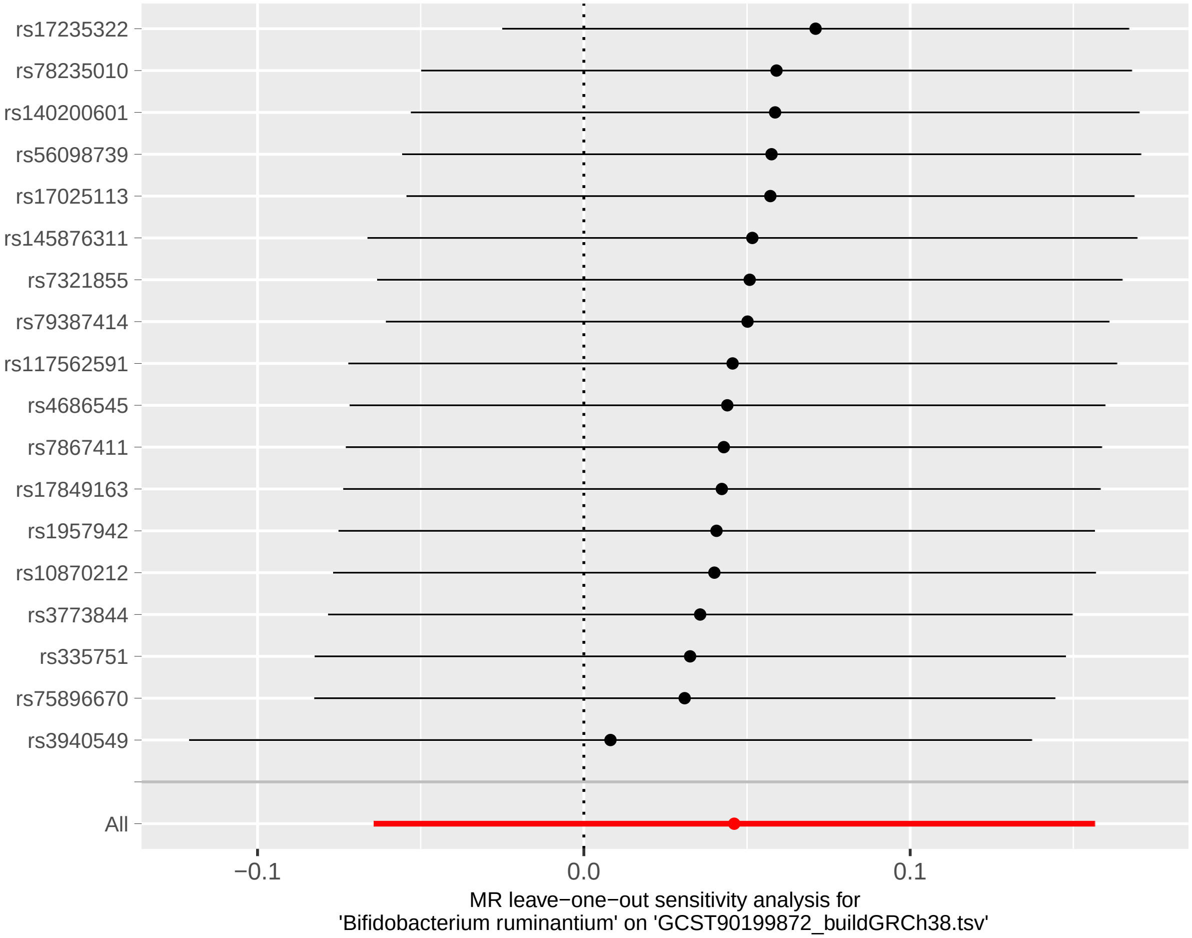
rs17235322
rs78235010
rs140200601
rs56098739
rs17025113
rs145876311
rs7321855
rs79387414
rs117562591
rs4686545
rs7867411
rs17849163
rs1957942
rs10870212
rs3773844
rs335751
rs75896670
rs3940549
All
−0.1
0.0
0.1
MR leave−one−out sensitivity analysis for
'Bifidobacterium ruminantium' on 'GCST90199872_buildGRCh38.tsv'

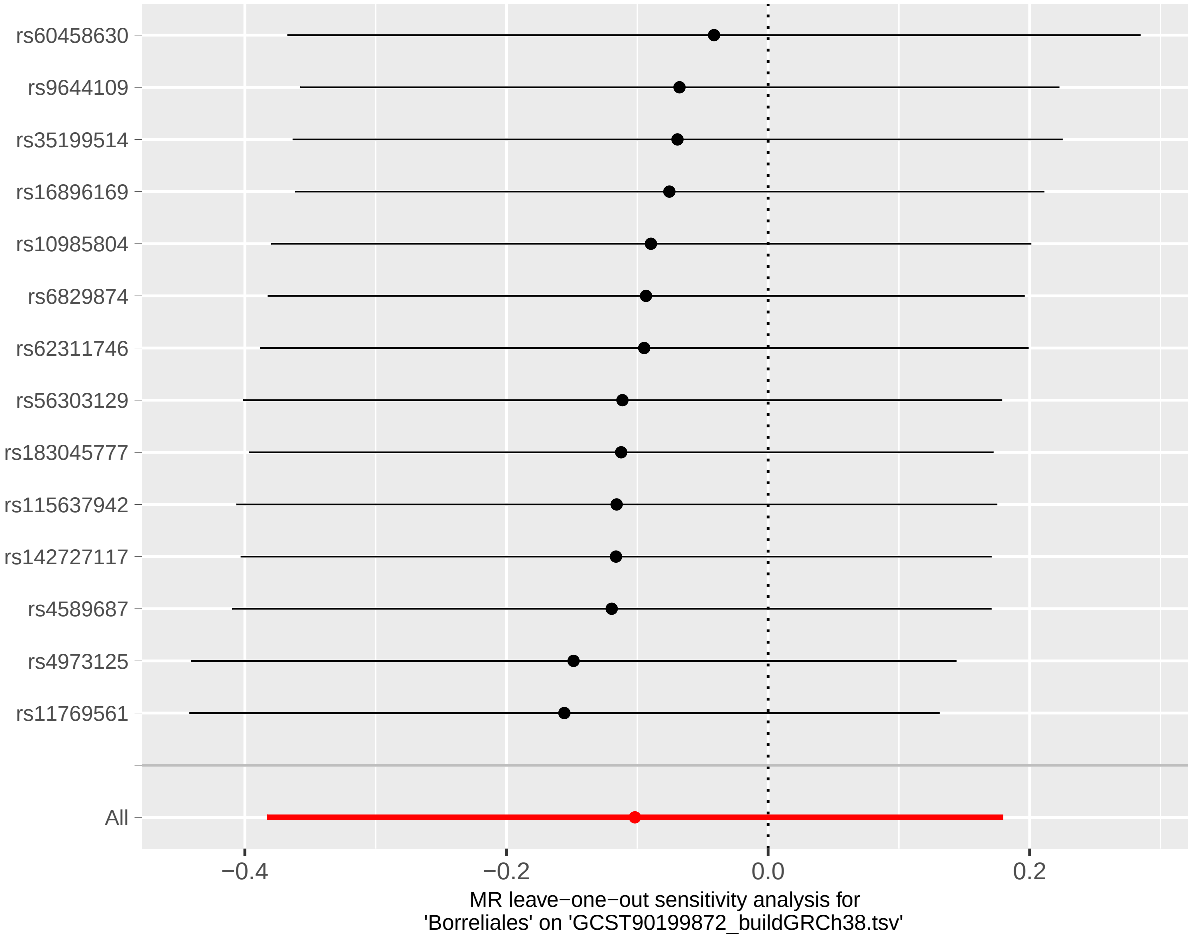

rs60458630
rs9644109
rs35199514
rs16896169
rs10985804
rs6829874
rs62311746
rs56303129
rs183045777
rs115637942
rs142727117
rs4589687
rs4973125
rs11769561
All
−0.4
−0.2
0.0
0.2
MR leave−one−out sensitivity analysis for
'Borreliales' on 'GCST90199872_buildGRCh38.tsv'

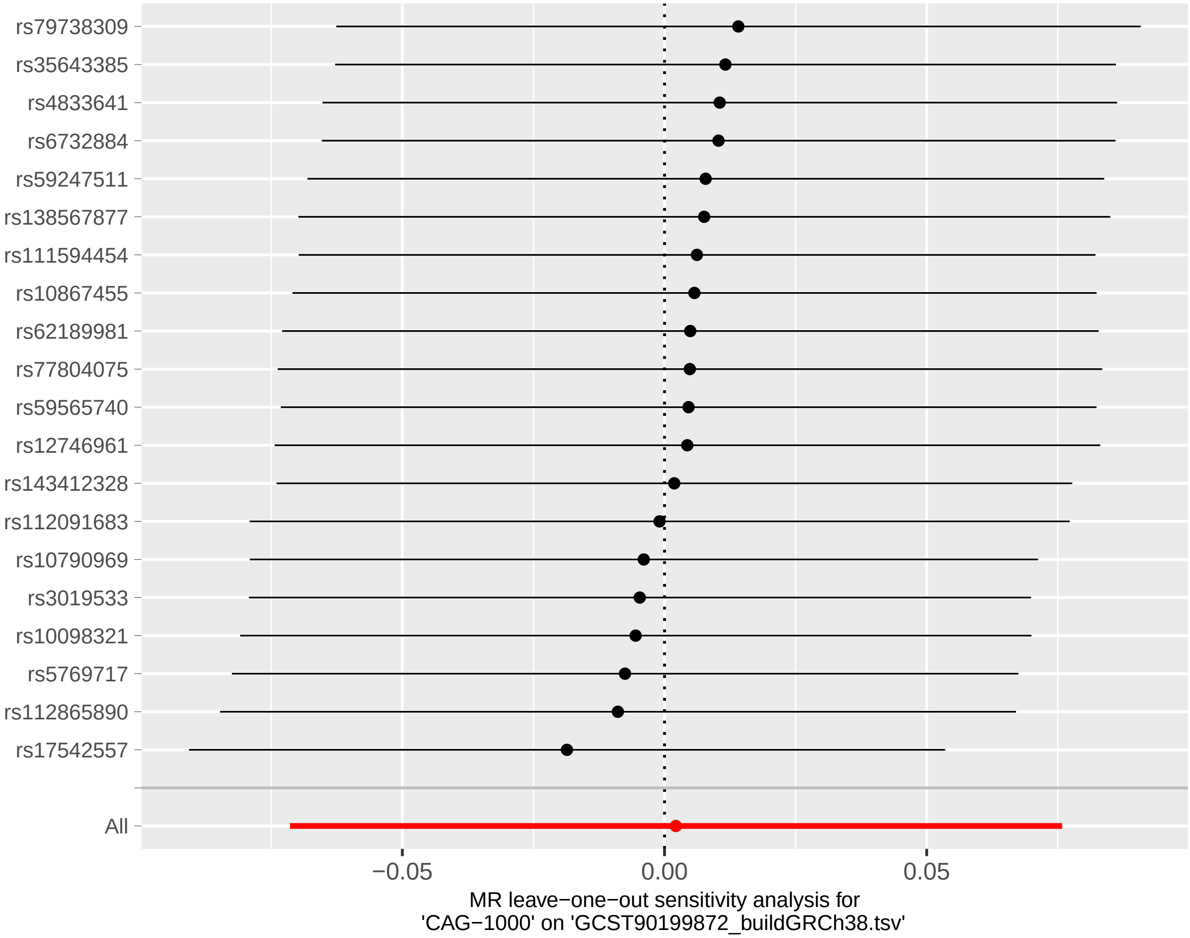

rs79738309
rs35643385
rs4833641
rs6732884
rs59247511
rs138567877
rs111594454
rs10867455
rs62189981
rs77804075
rs59565740
rs12746961
rs143412328
rs112091683
rs10790969
rs3019533
rs10098321
rs5769717
rs112865890
rs17542557
All
−0.05
0.00
0.05
MR leave−one−out sensitivity analysis for
'CAG−1000' on 'GCST90199872_buildGRCh38.tsv'

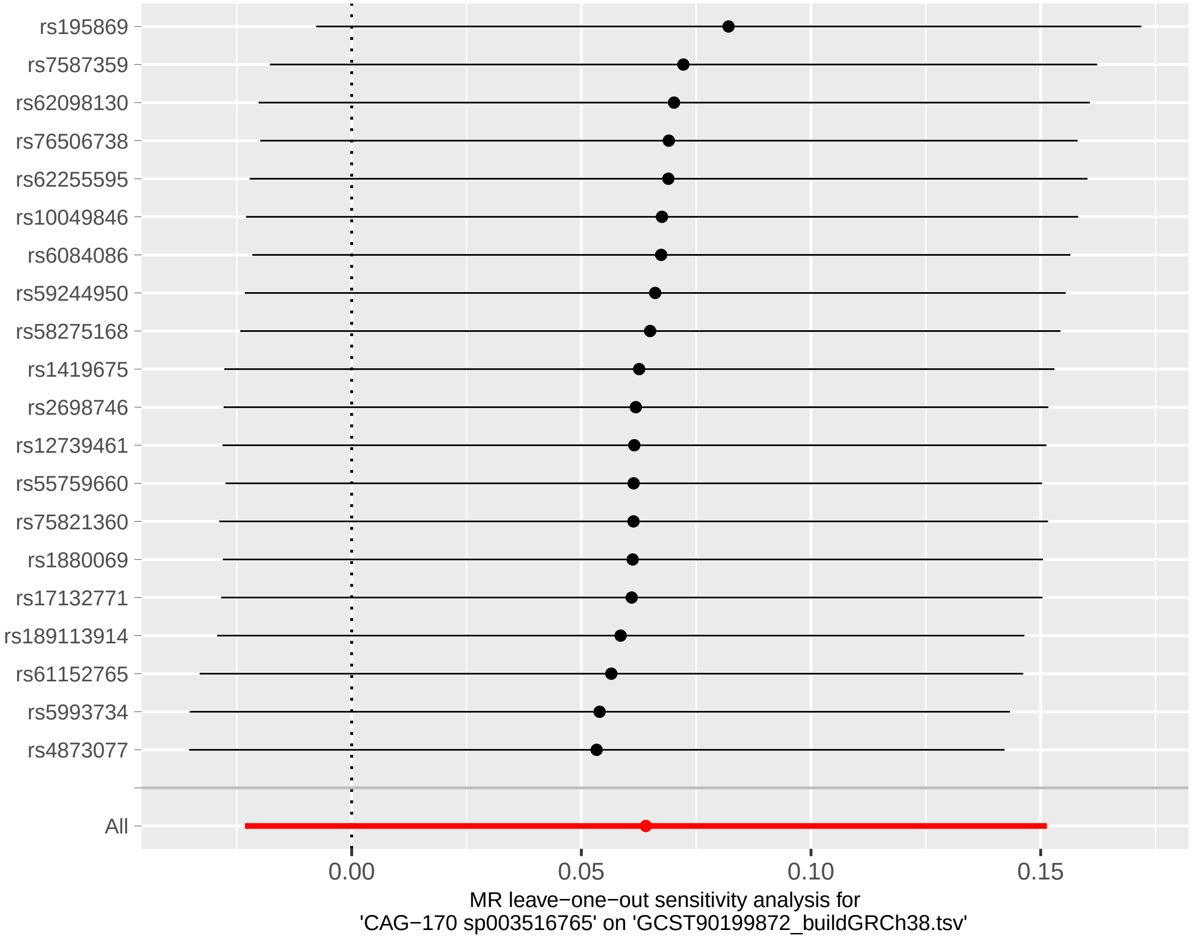
rs195869
rs7587359
rs62098130
rs76506738
rs62255595
rs10049846
rs6084086
rs59244950
rs58275168
rs1419675
rs2698746
rs12739461
rs55759660
rs75821360
rs1880069
rs17132771
rs189113914
rs61152765
rs5993734
rs4873077
All
0.00
0.05
0.10
0.15
MR leave−one−out sensitivity analysis for
'CAG−170 sp003516765' on 'GCST90199872_buildGRCh38.tsv'

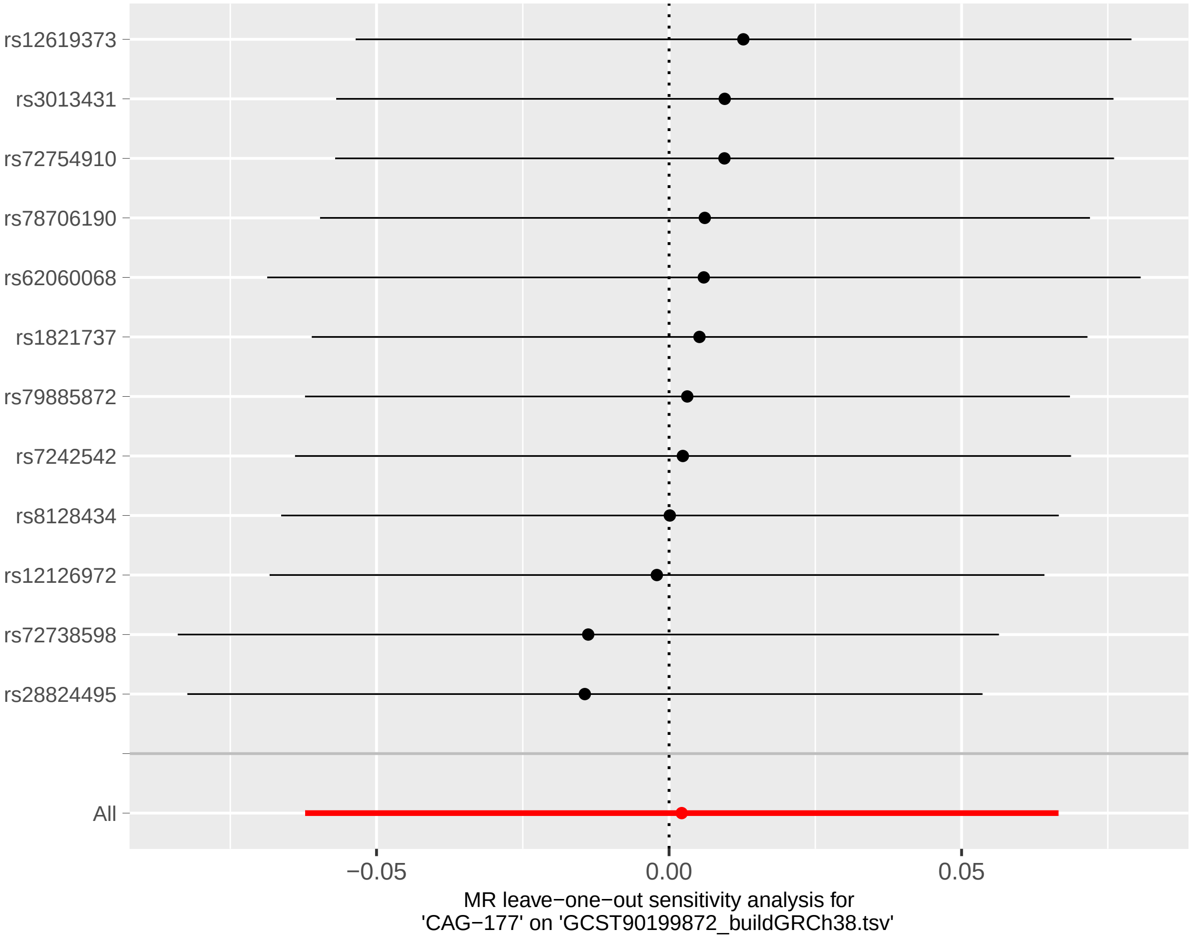

rs12619373
rs3013431
rs72754910
rs78706190
rs62060068
rs1821737
rs79885872
rs7242542
rs8128434
rs12126972
rs72738598
rs28824495
All
−0.05
0.00
0.05
MR leave−one−out sensitivity analysis for
'CAG−177' on 'GCST90199872_buildGRCh38.tsv'

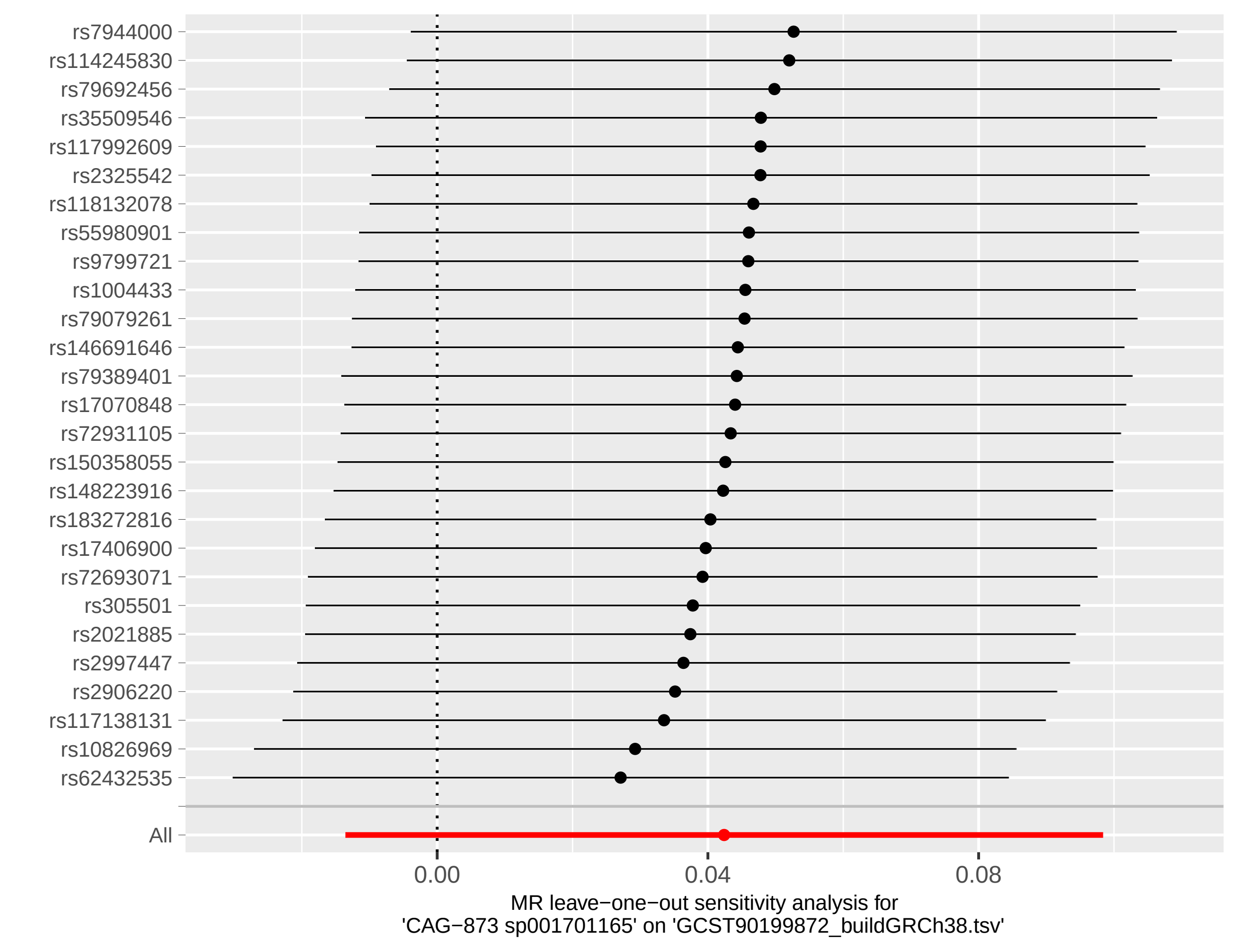
rs7944000
rs114245830
rs79692456
rs35509546
rs117992609
rs2325542
rs118132078
rs55980901
rs9799721
rs1004433
rs79079261
rs146691646
rs79389401
rs17070848
rs72931105
rs150358055
rs148223916
rs183272816
rs17406900
rs72693071
rs305501
rs2021885
rs2997447
rs2906220
rs117138131
rs10826969
rs62432535
All
0.00
0.04
0.08
MR leave−one−out sensitivity analysis for
'CAG−873 sp001701165' on 'GCST90199872_buildGRCh38.tsv'

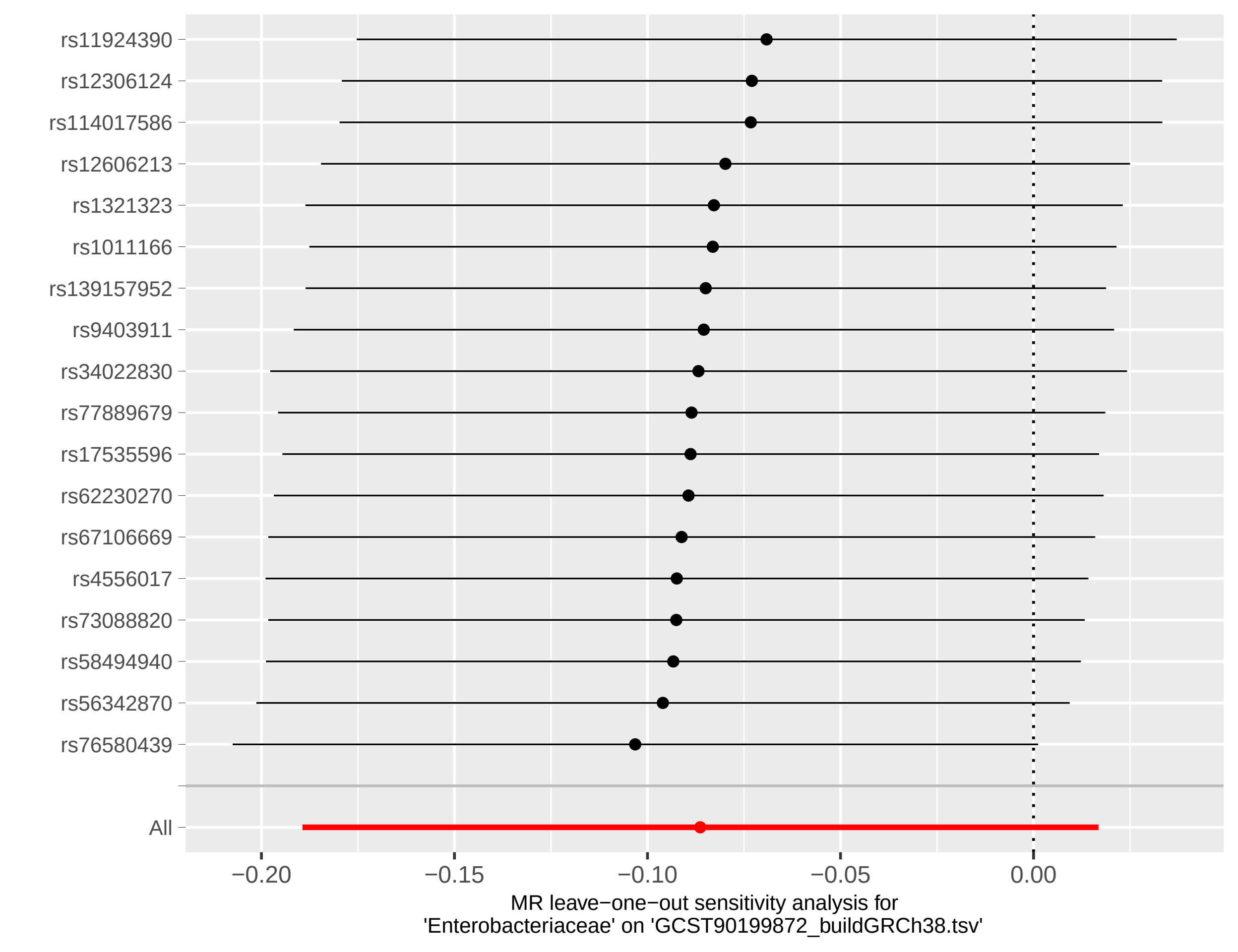
rs11924390
rs12306124
rs114017586
rs12606213
rs1321323
rs1011166
rs139157952
rs9403911
rs34022830
rs77889679
rs17535596
rs62230270
rs67106669
rs4556017
rs73088820
rs58494940
rs56342870
rs76580439
All
−0.20
−0.15
−0.10
−0.05
0.00
MR leave−one−out sensitivity analysis for
'Enterobacteriaceae' on 'GCST90199872_buildGRCh38.tsv'

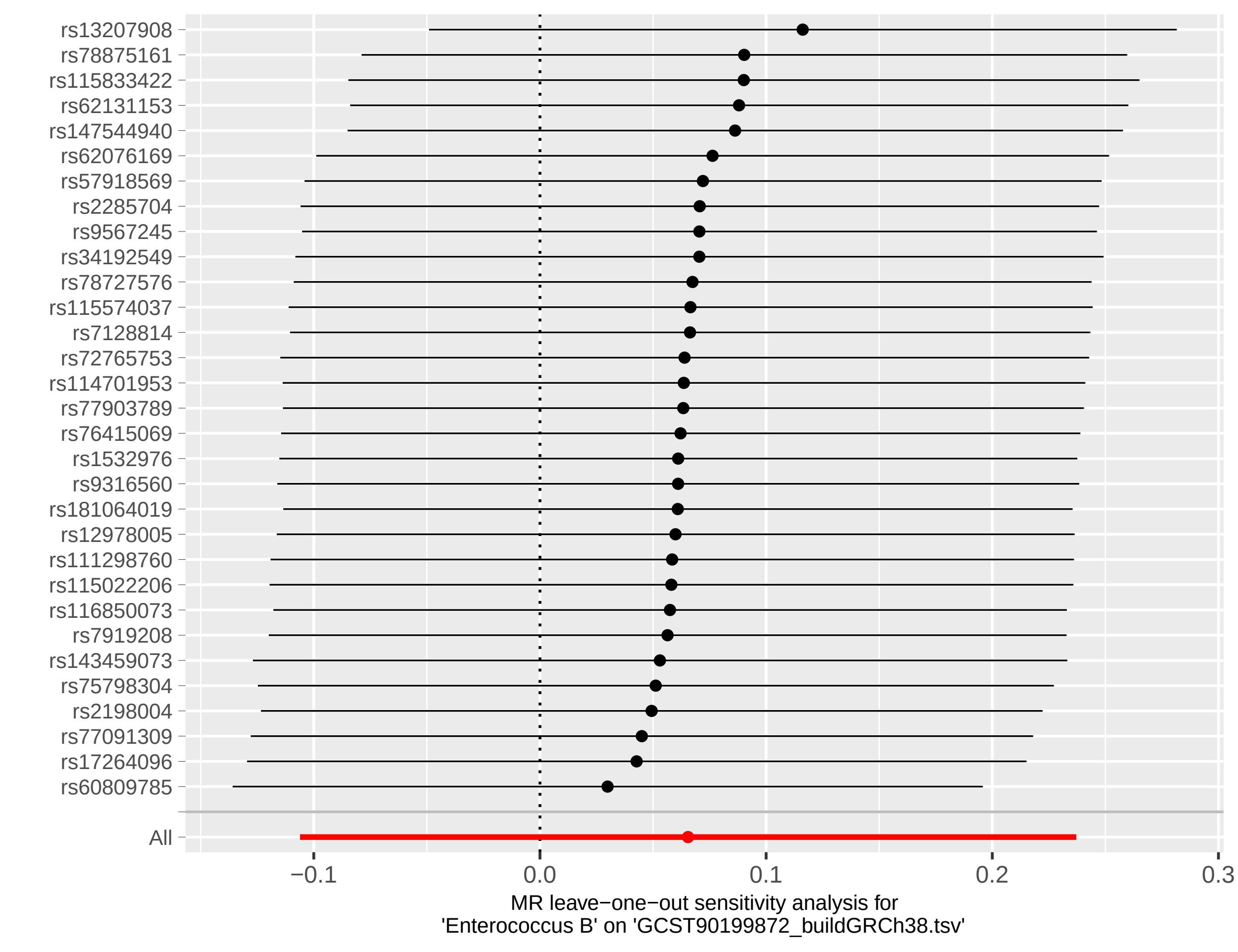
rs13207908
rs78875161
rs115833422
rs62131153
rs147544940
rs62076169
rs57918569
rs2285704
rs9567245
rs34192549
rs78727576
rs115574037
rs7128814
rs72765753
rs114701953
rs77903789
rs76415069
rs1532976
rs9316560
rs181064019
rs12978005
rs111298760
rs115022206
rs116850073
rs7919208
rs143459073
rs75798304
rs2198004
rs77091309
rs17264096
rs60809785
All
−0.1
0.0
0.1
0.2
0.3
MR leave−one−out sensitivity analysis for
'Enterococcus B' on 'GCST90199872_buildGRCh38.tsv'

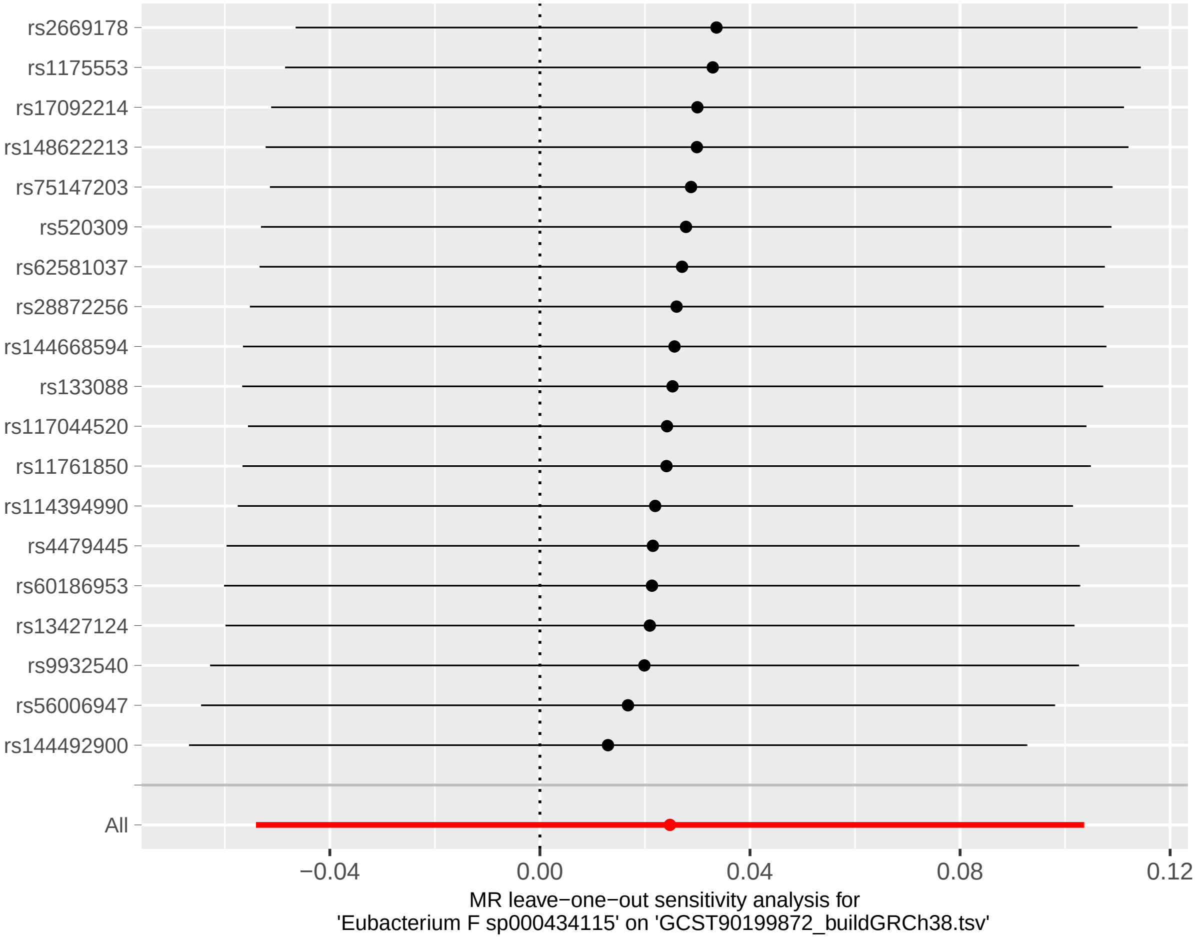
rs2669178
rs1175553
rs17092214
rs148622213
rs75147203
rs520309
rs62581037
rs28872256
rs144668594
rs133088
rs117044520
rs11761850
rs114394990
rs4479445
rs60186953
rs13427124
rs9932540
rs56006947
rs144492900
All
−0.04
0.00
0.04
0.08
0.12
MR leave−one−out sensitivity analysis for
'Eubacterium F sp000434115' on 'GCST90199872_buildGRCh38.tsv'

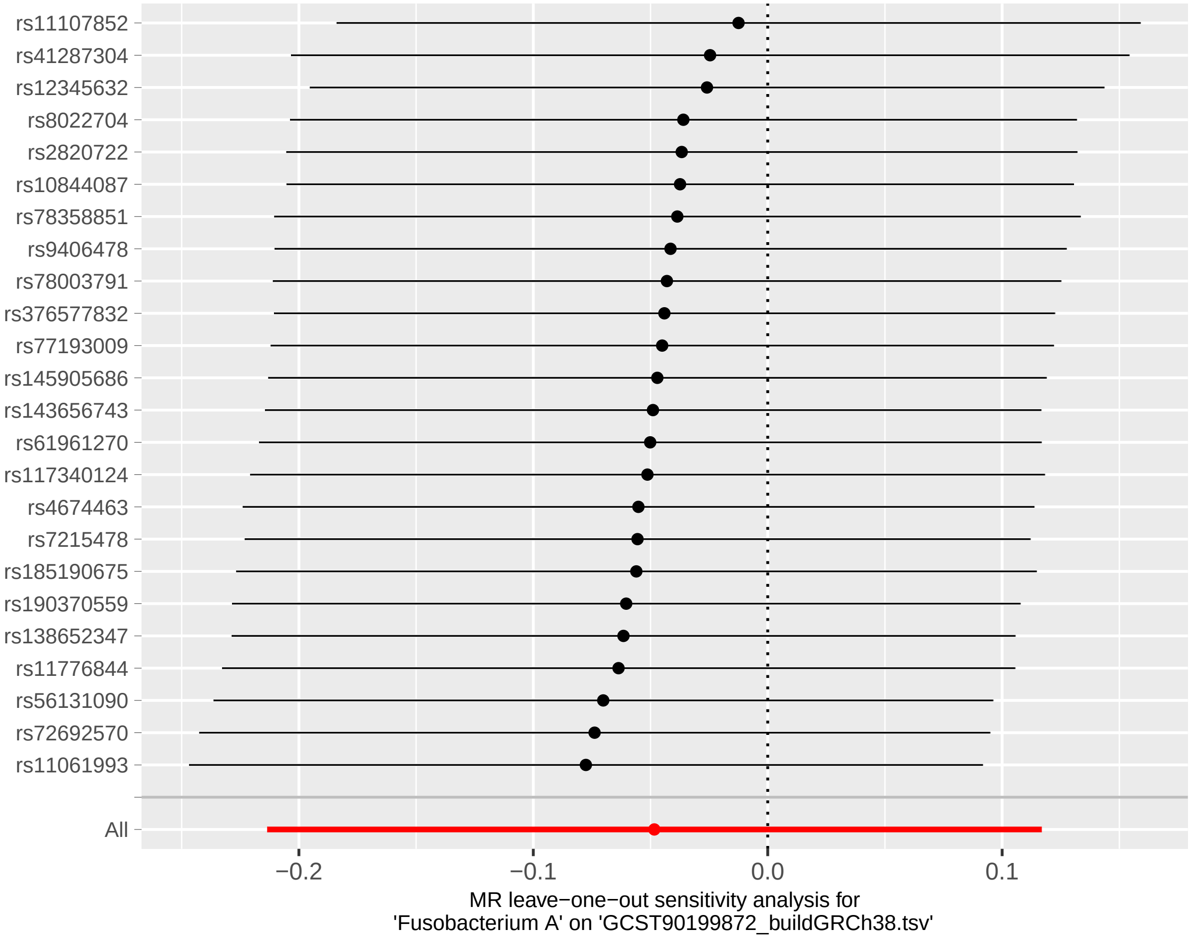
rs11107852
rs41287304
rs12345632
rs8022704
rs2820722
rs10844087
rs78358851
rs9406478
rs78003791
rs376577832
rs77193009
rs145905686
rs143656743
rs61961270
rs117340124
rs4674463
rs7215478
rs185190675
rs190370559
rs138652347
rs11776844
rs56131090
rs72692570
rs11061993
All
−0.2
−0.1
0.0
0.1
MR leave−one−out sensitivity analysis for
'Fusobacterium A' on 'GCST90199872_buildGRCh38.tsv'

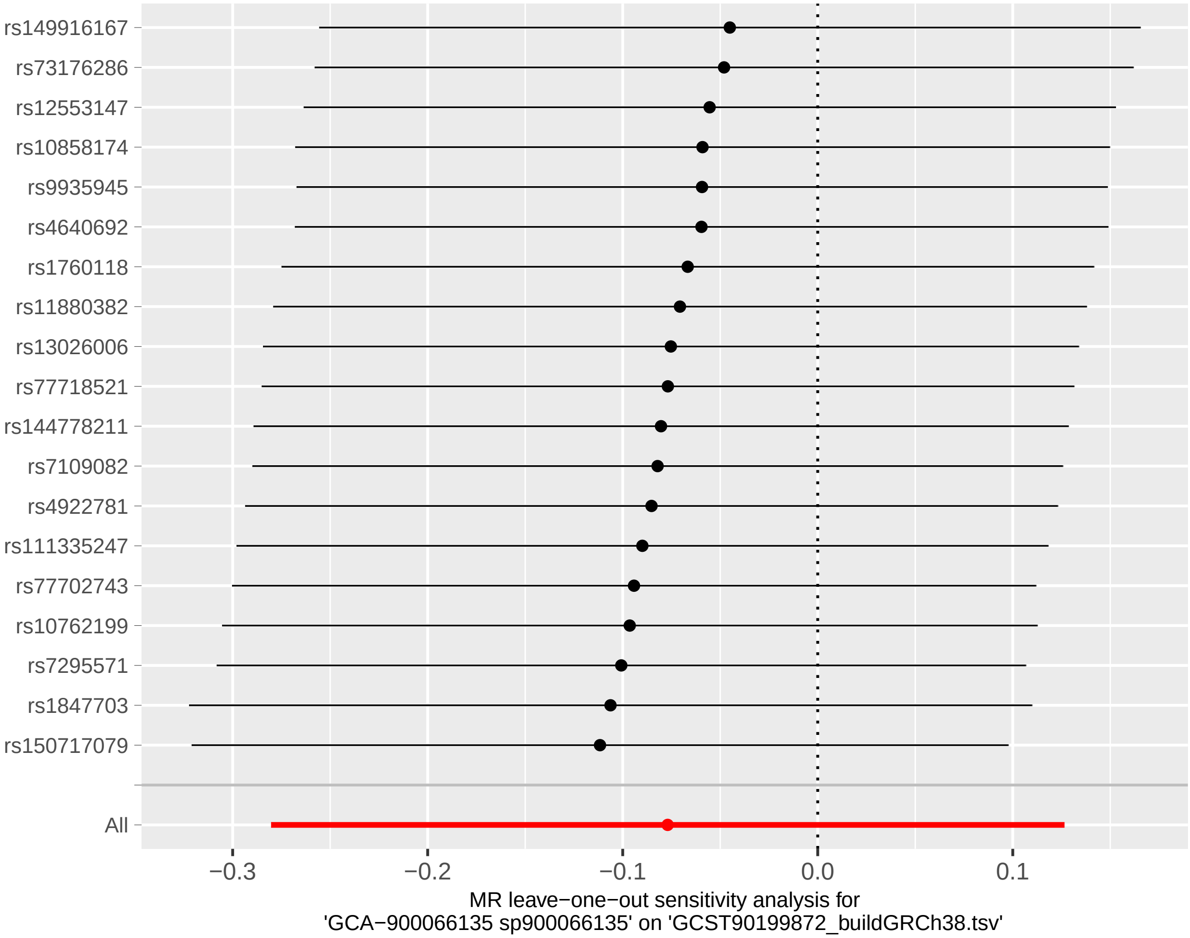

rs149916167
rs73176286
rs12553147
rs10858174
rs9935945
rs4640692
rs1760118
rs11880382
rs13026006
rs77718521
rs144778211
rs7109082
rs4922781
rs111335247
rs77702743
rs10762199
rs7295571
rs1847703
rs150717079
All
−0.3
−0.2
−0.1
0.0
0.1
MR leave−one−out sensitivity analysis for
'GCA−900066135 sp900066135' on 'GCST90199872_buildGRCh38.tsv'

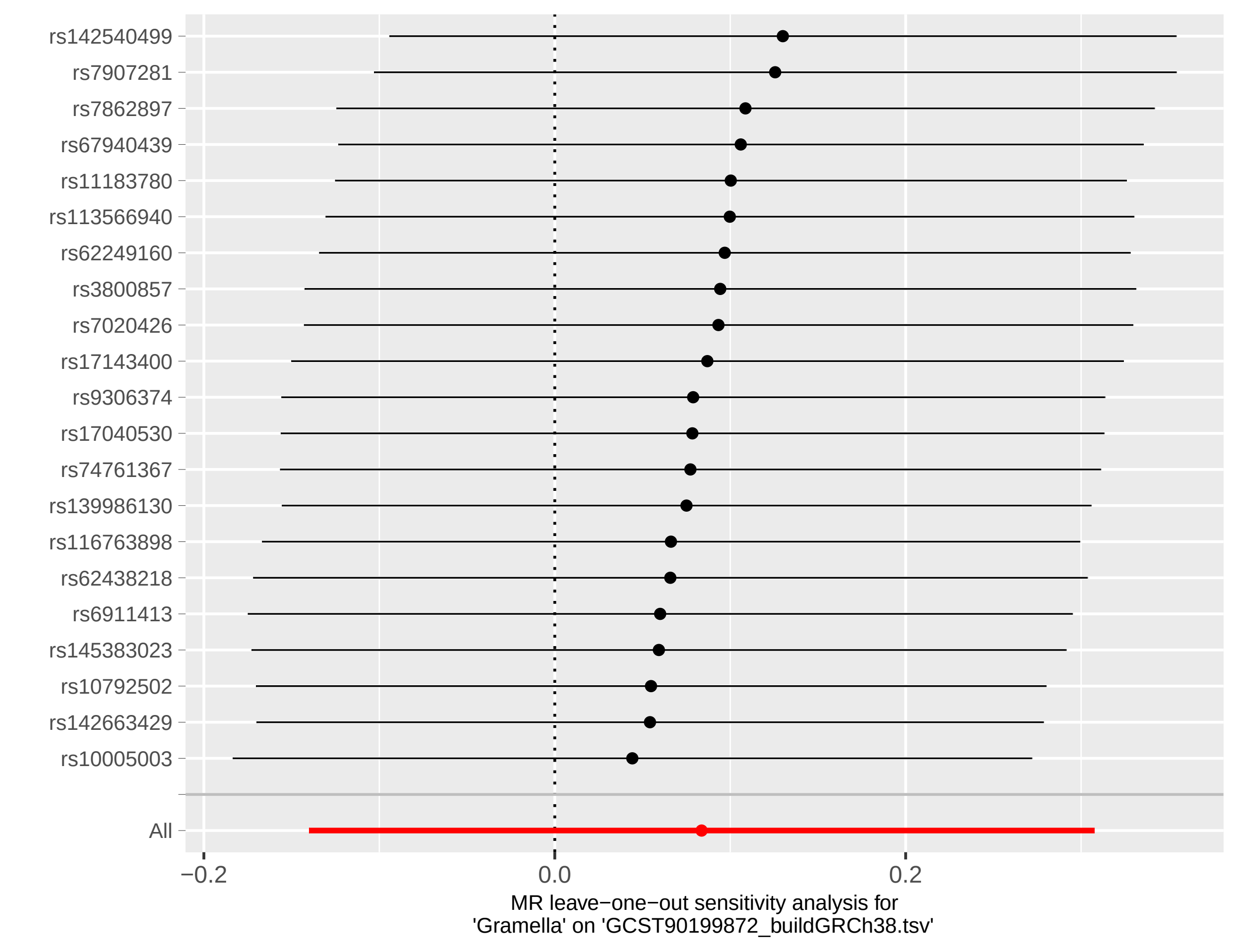
rs142540499
rs7907281
rs7862897
rs67940439
rs11183780
rs113566940
rs62249160
rs3800857
rs7020426
rs17143400
rs9306374
rs17040530
rs74761367
rs139986130
rs116763898
rs62438218
rs6911413
rs145383023
rs10792502
rs142663429
rs10005003
All
−0.2
0.0
0.2
MR leave−one−out sensitivity analysis for
'Gramella' on 'GCST90199872_buildGRCh38.tsv'

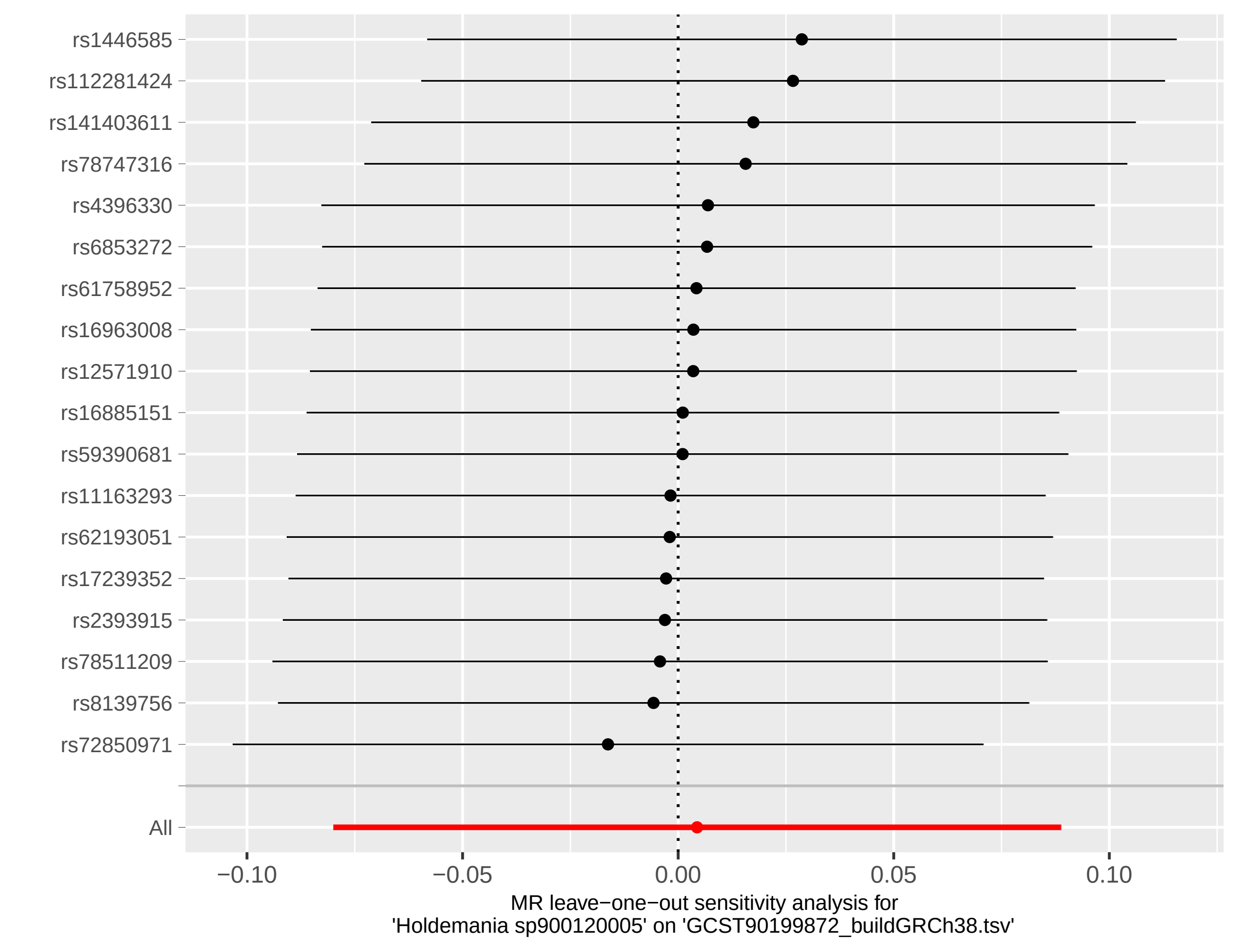
rs1446585
rs112281424
rs141403611
rs78747316
rs4396330
rs6853272
rs61758952
rs16963008
rs12571910
rs16885151
rs59390681
rs11163293
rs62193051
rs17239352
rs2393915
rs78511209
rs8139756
rs72850971
All
−0.10
−0.05
0.00
0.05
0.10
MR leave−one−out sensitivity analysis for
'Holdemania sp900120005' on 'GCST90199872_buildGRCh38.tsv'

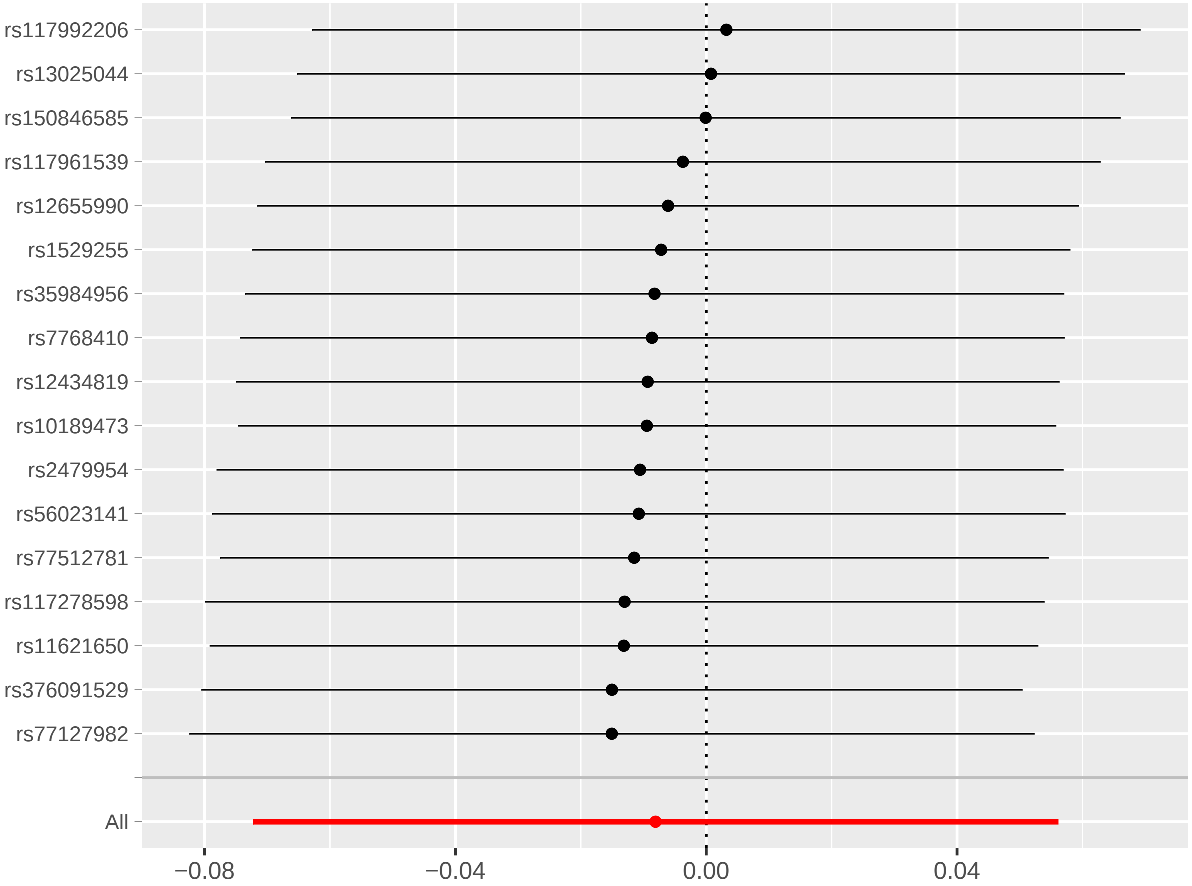

MR leave−one−out sensitivity analysis for
'Parabacteroides sp000436495' on 'GCST90199872_buildGRCh38.tsv'

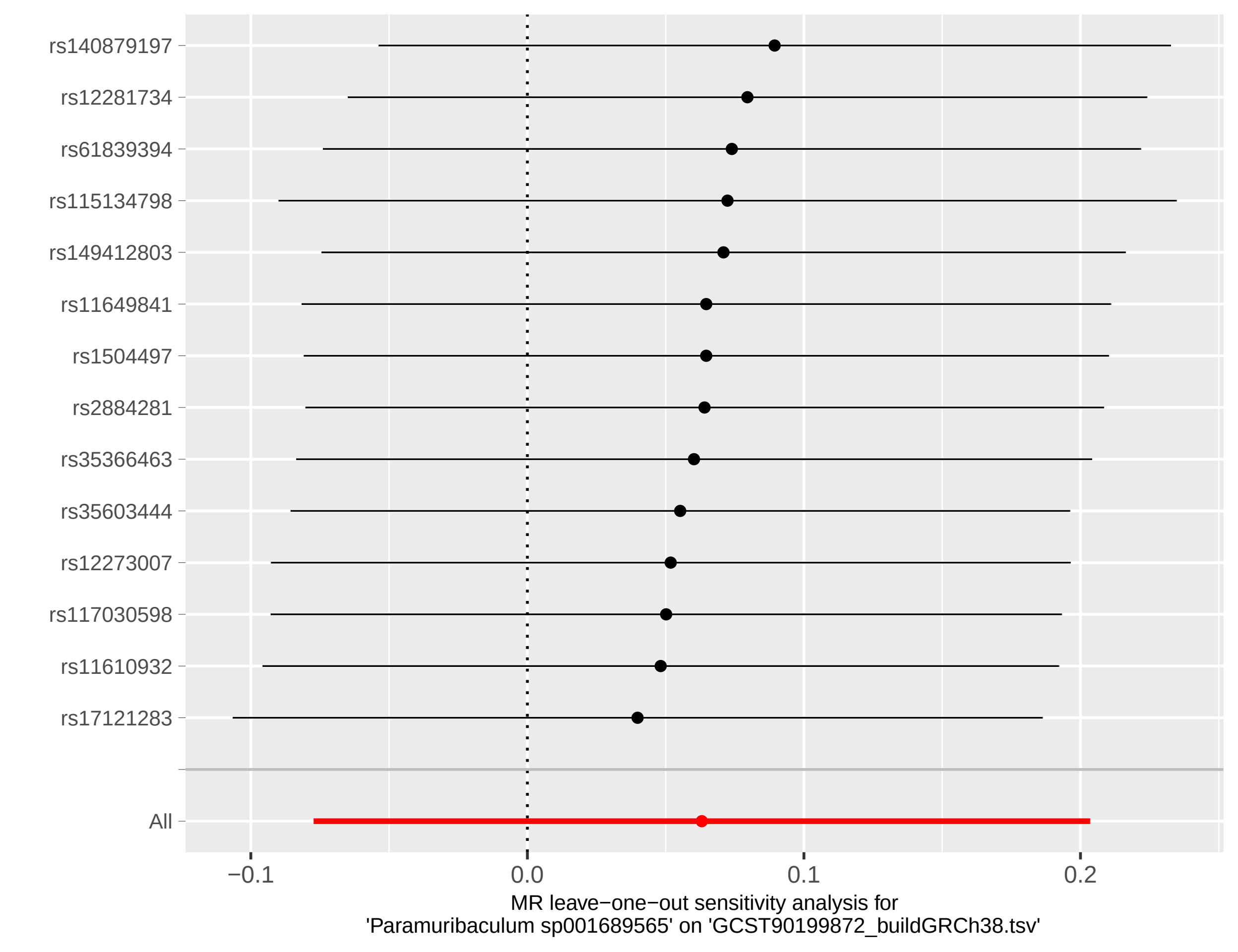

rs140879197
rs12281734
rs61839394
rs115134798
rs149412803
rs11649841
rs1504497
rs2884281
rs35366463
rs35603444
rs12273007
rs117030598
rs11610932
rs17121283
All
−0.1
0.0
0.1
0.2
MR leave−one−out sensitivity analysis for
'Paramuribaculum sp001689565' on 'GCST90199872_buildGRCh38.tsv'

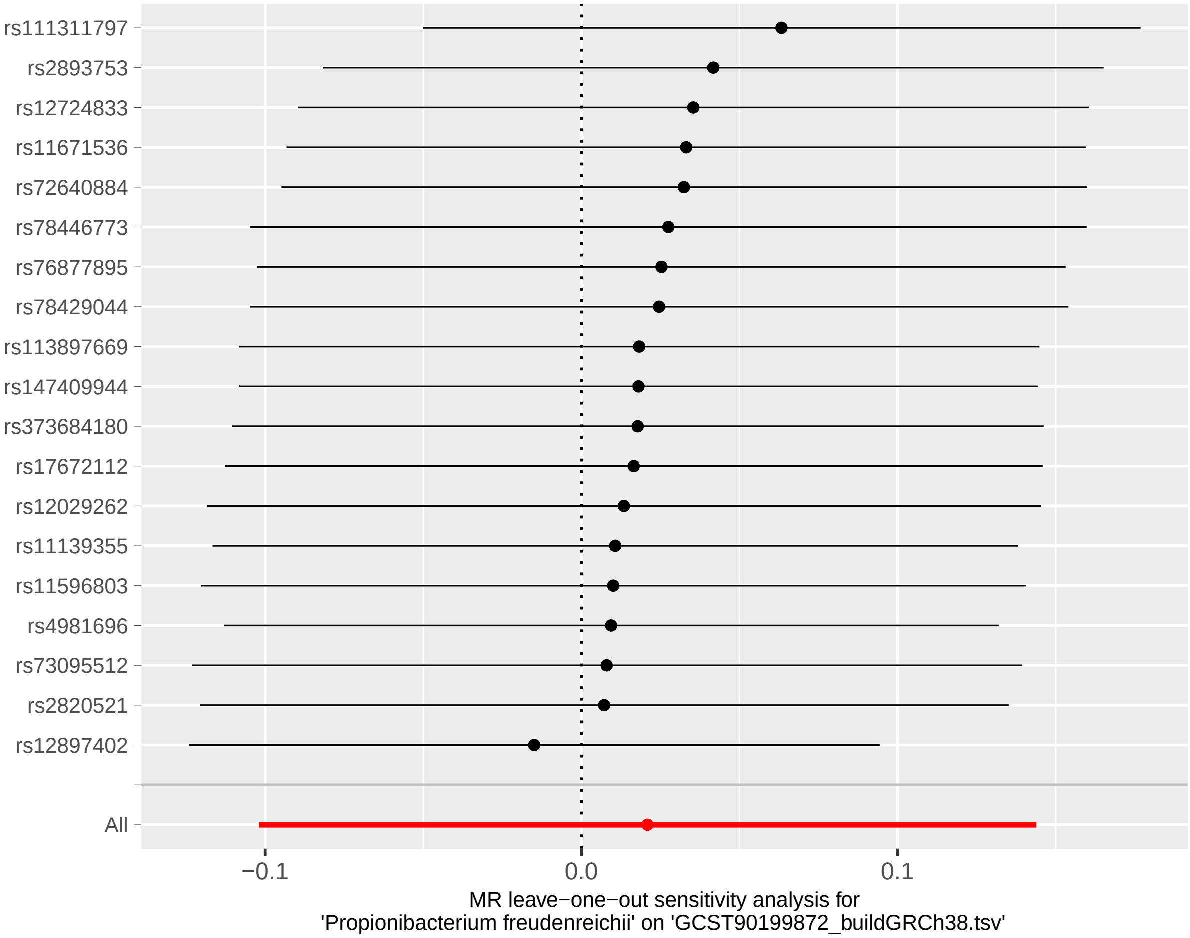
rs111311797
rs2893753
rs12724833
rs11671536
rs72640884
rs78446773
rs76877895
rs78429044
rs113897669
rs147409944
rs373684180
rs17672112
rs12029262
rs11139355
rs11596803
rs4981696
rs73095512
rs2820521
rs12897402
All
−0.1
0.0
0.1
MR leave−one−out sensitivity analysis for
'Propionibacterium freudenreichii' on 'GCST90199872_buildGRCh38.tsv'

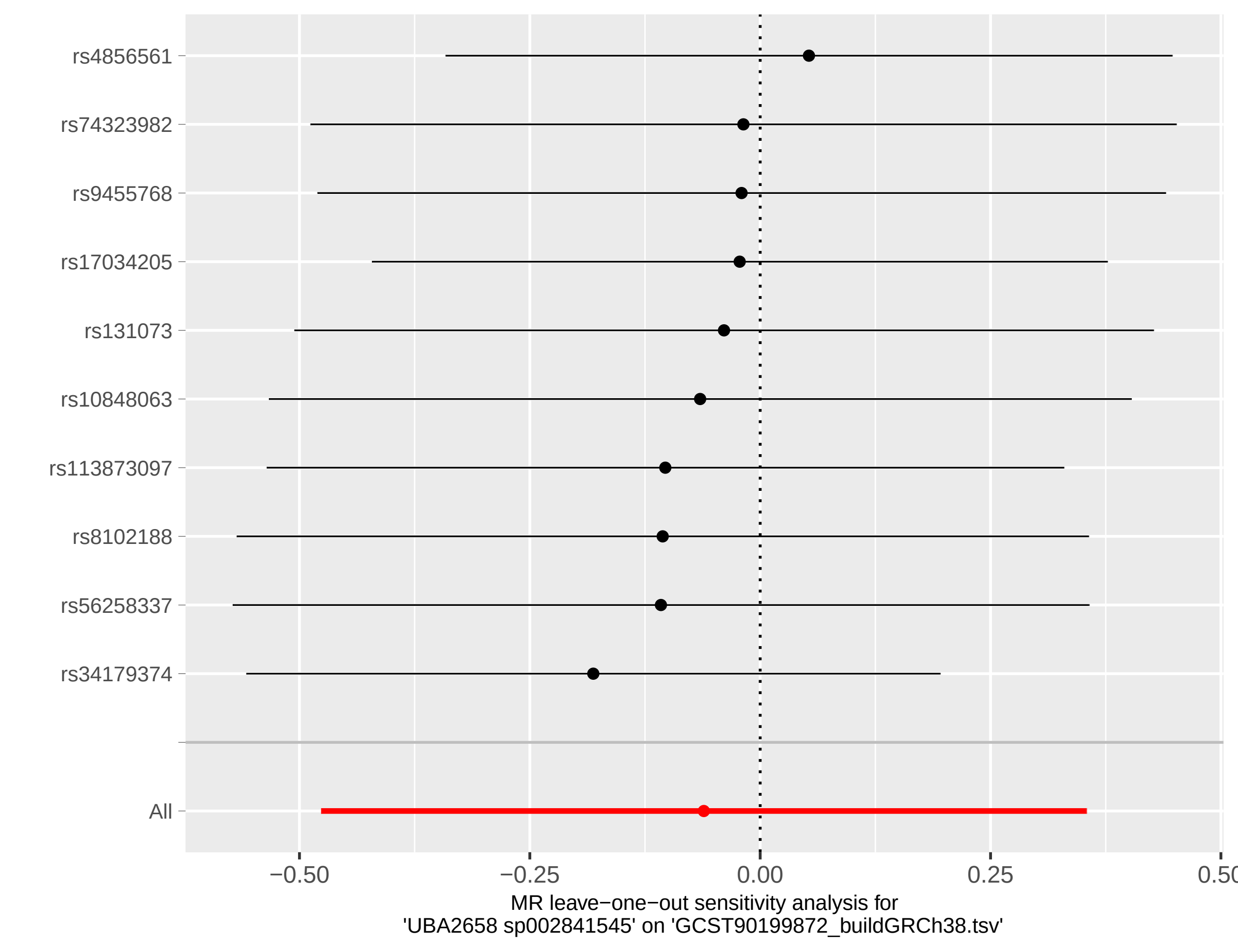
rs4856561
rs74323982
rs9455768
rs17034205
rs131073
rs10848063
rs113873097
rs8102188
rs56258337
rs34179374
All
−0.50
−0.25
0.00
0.25
0.50
MR leave−one−out sensitivity analysis for
'UBA2658 sp002841545' on 'GCST90199872_buildGRCh38.tsv'

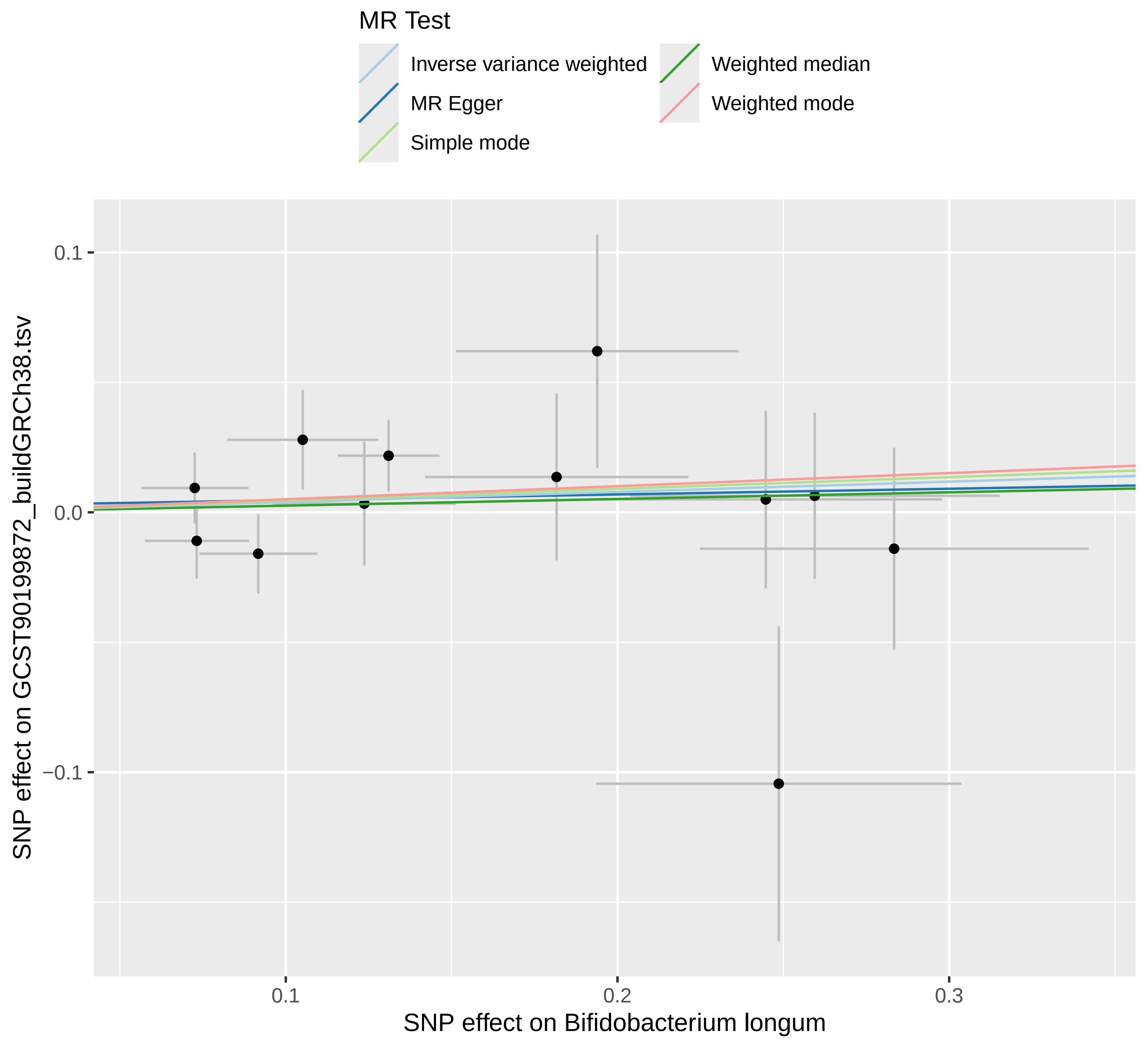
MR Test
Inverse variance weighted
MR Egger
Simple mode
Weighted median
Weighted mode
0.1
0.0
−0.1
0.1
0.2
0.3
SNP effect on GCST90199872_buildGRCh38.tsv
SNP effect on Bifidobacterium longum

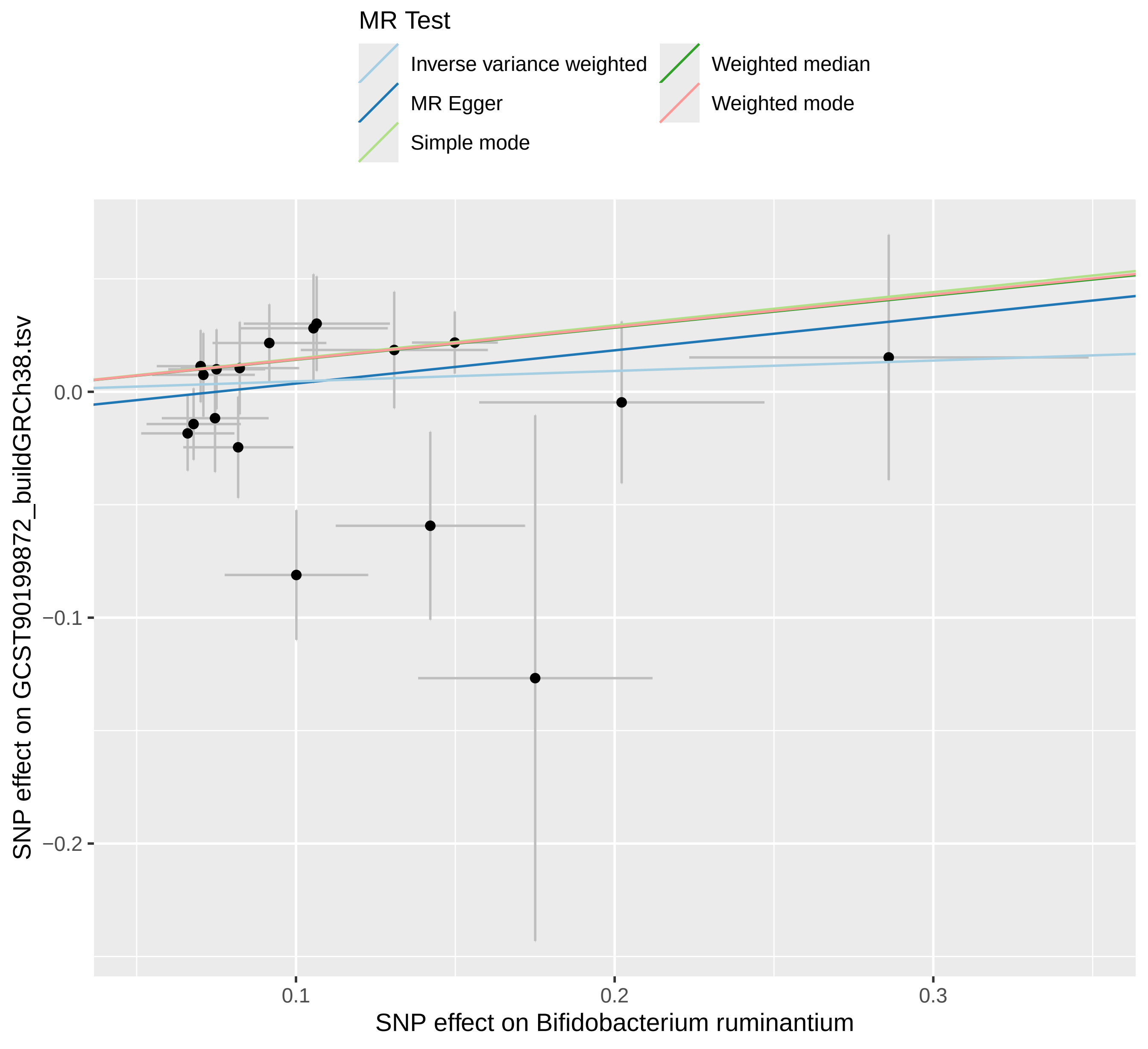

MR Test
Inverse variance weighted
MR Egger
Simple mode
Weighted median
Weighted mode
0.0
−0.1
−0.2
0.1
0.2
0.3
SNP effect on GCST90199872_buildGRCh38.tsv
SNP effect on Bifidobacterium ruminantium

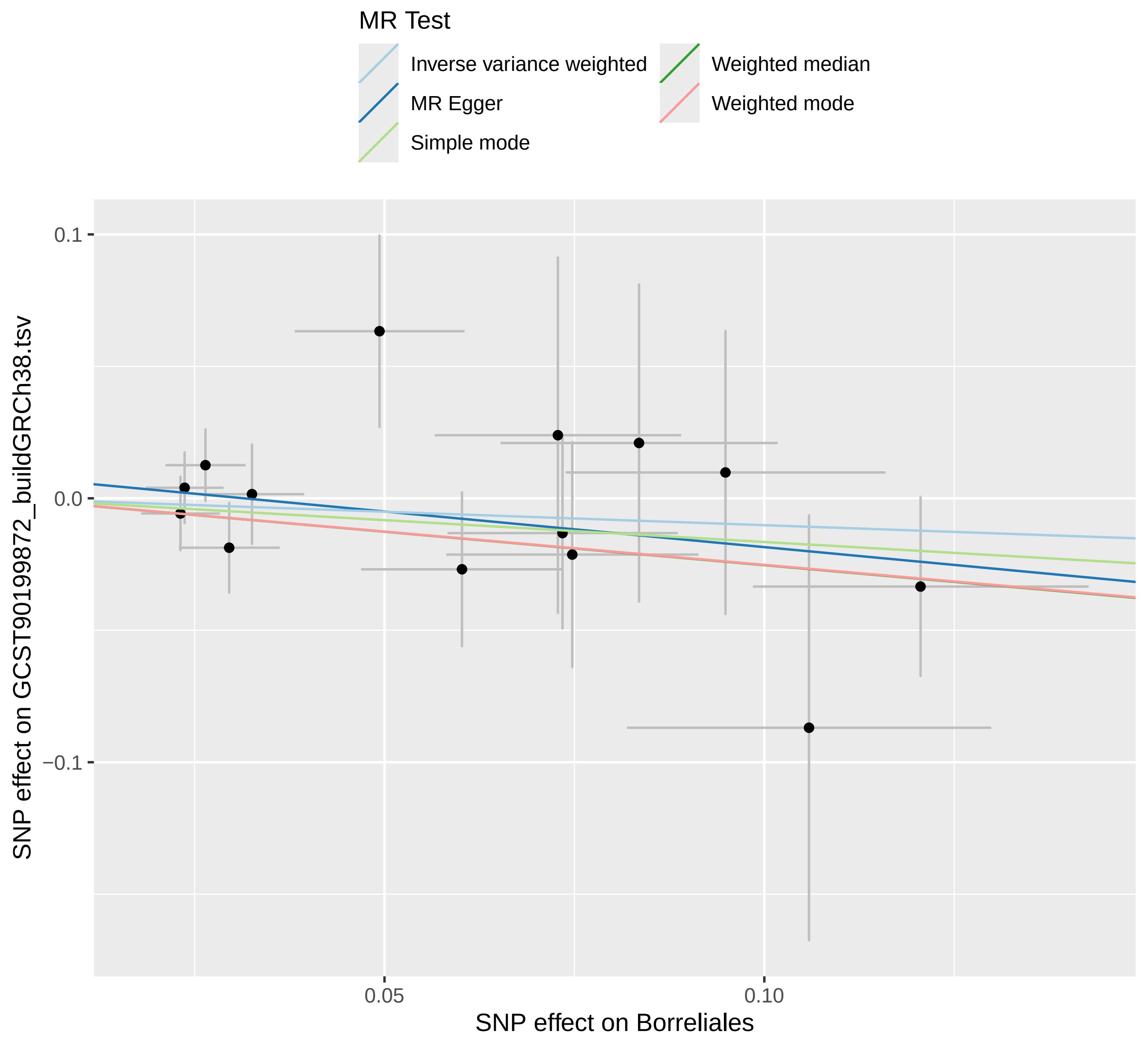
MR Test
Inverse variance weighted
MR Egger
Simple mode
Weighted median
Weighted mode
0.1
0.0
−0.1
0.05
0.10
SNP effect on Borreliales
SNP effect on GCST90199872_buildGRCh38.tsv

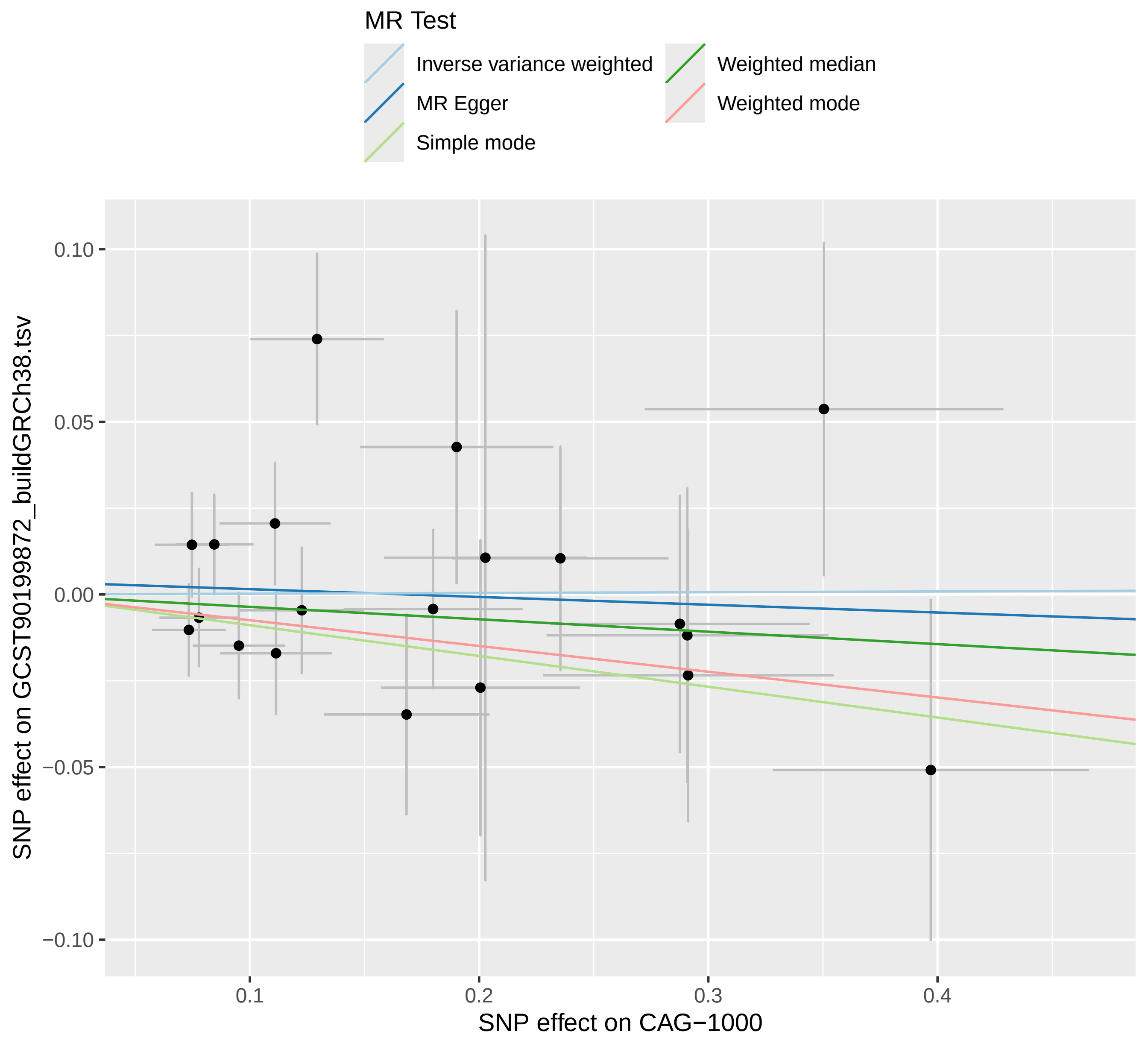

MR Test
Inverse variance weighted
MR Egger
Simple mode
Weighted median
Weighted mode
0.10
0.05
0.00
−0.05
−0.10
SNP effect on GCST90199872_buildGRCh38.tsv
0.1
0.2
0.3
0.4
SNP effect on CAG−1000

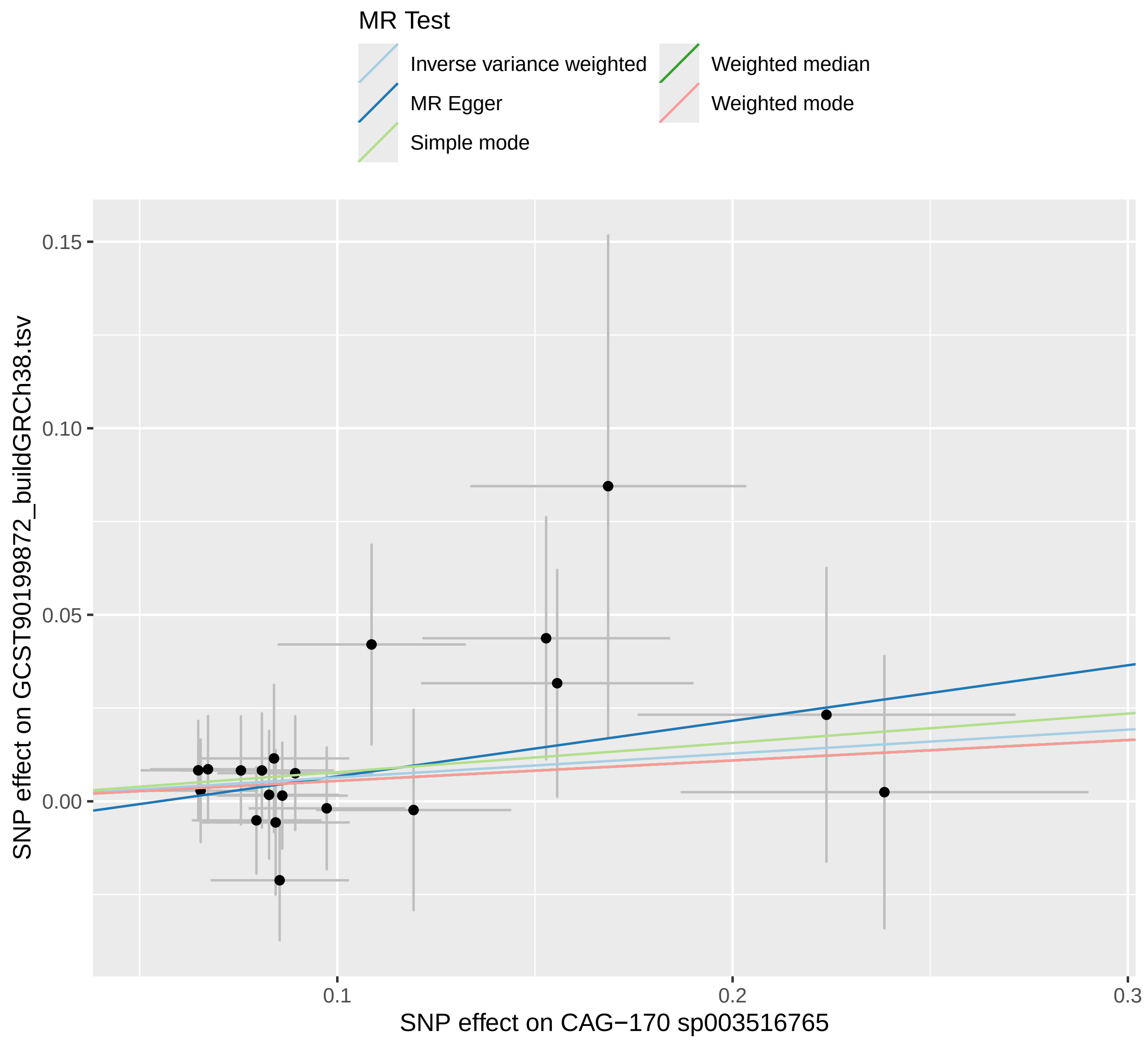
MR Test
Inverse variance weighted
MR Egger
Simple mode
Weighted median
Weighted mode
0.15
0.10
0.05
0.00
0.1
0.2
0.3
SNP effect on GCST90199872_buildGRCh38.tsv
SNP effect on CAG−170 sp003516765

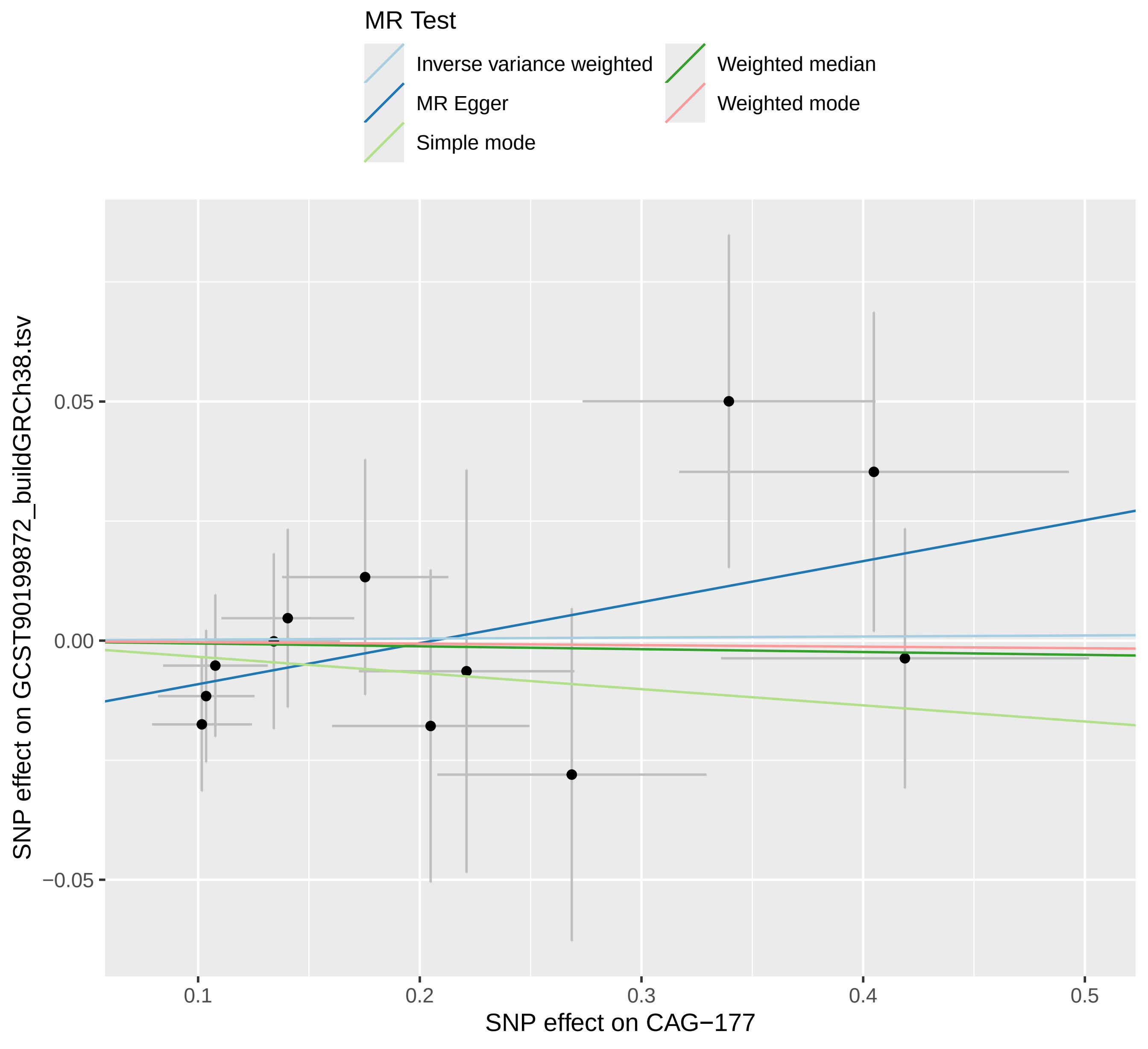
MR Test
Inverse variance weighted
MR Egger
Simple mode
Weighted median
Weighted mode
SNP effect on GCST90199872_buildGRCh38.tsv
0.05
0.00
−0.05
0.1
0.2
0.3
0.4
0.5
SNP effect on CAG−177

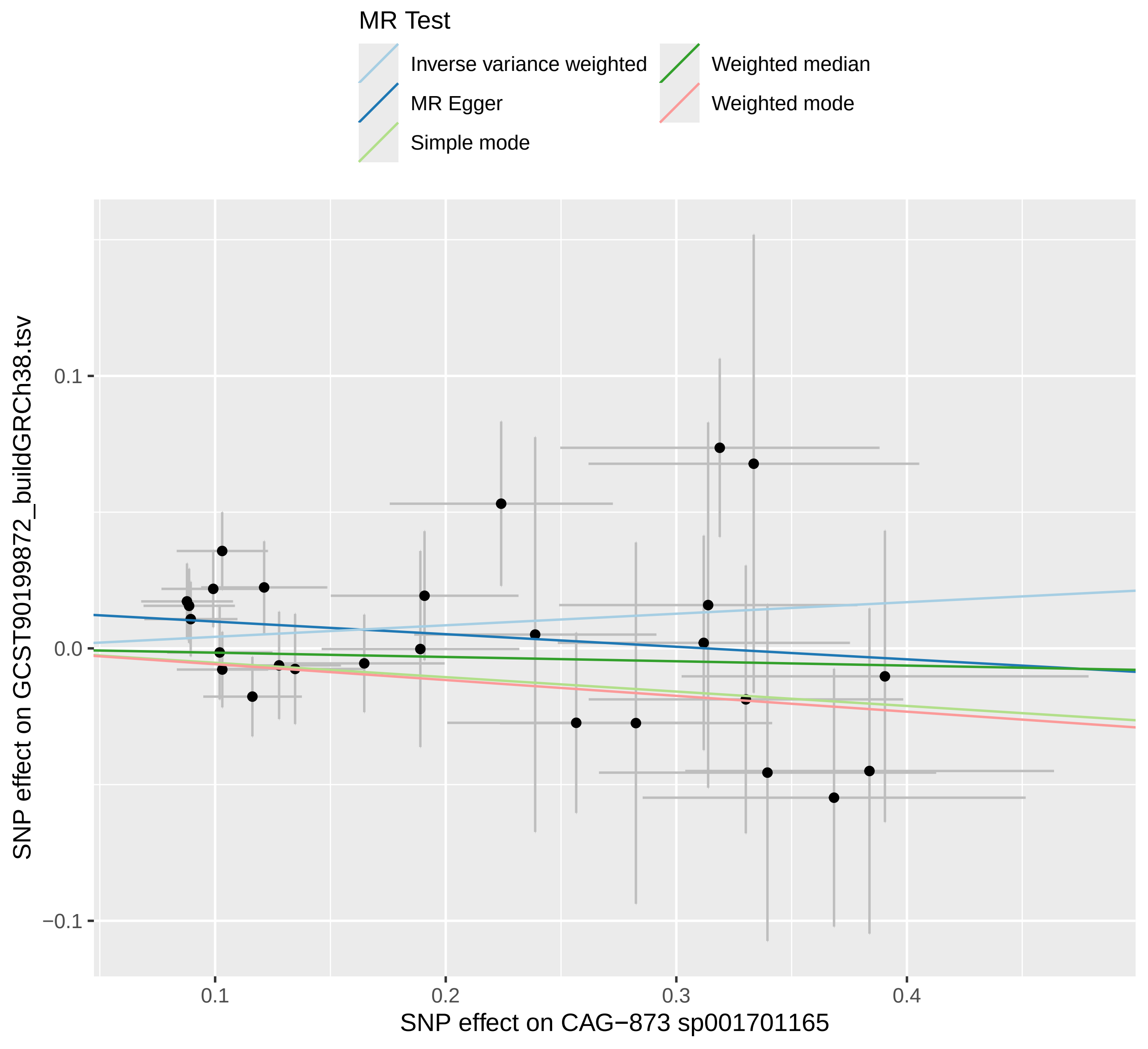

MR Test
Inverse variance weighted
MR Egger
Simple mode
Weighted median
Weighted mode
0.1
0.0
−0.1
0.1
0.2
0.3
0.4
SNP effect on GCST90199872_buildGRCh38.tsv
SNP effect on CAG−873 sp001701165

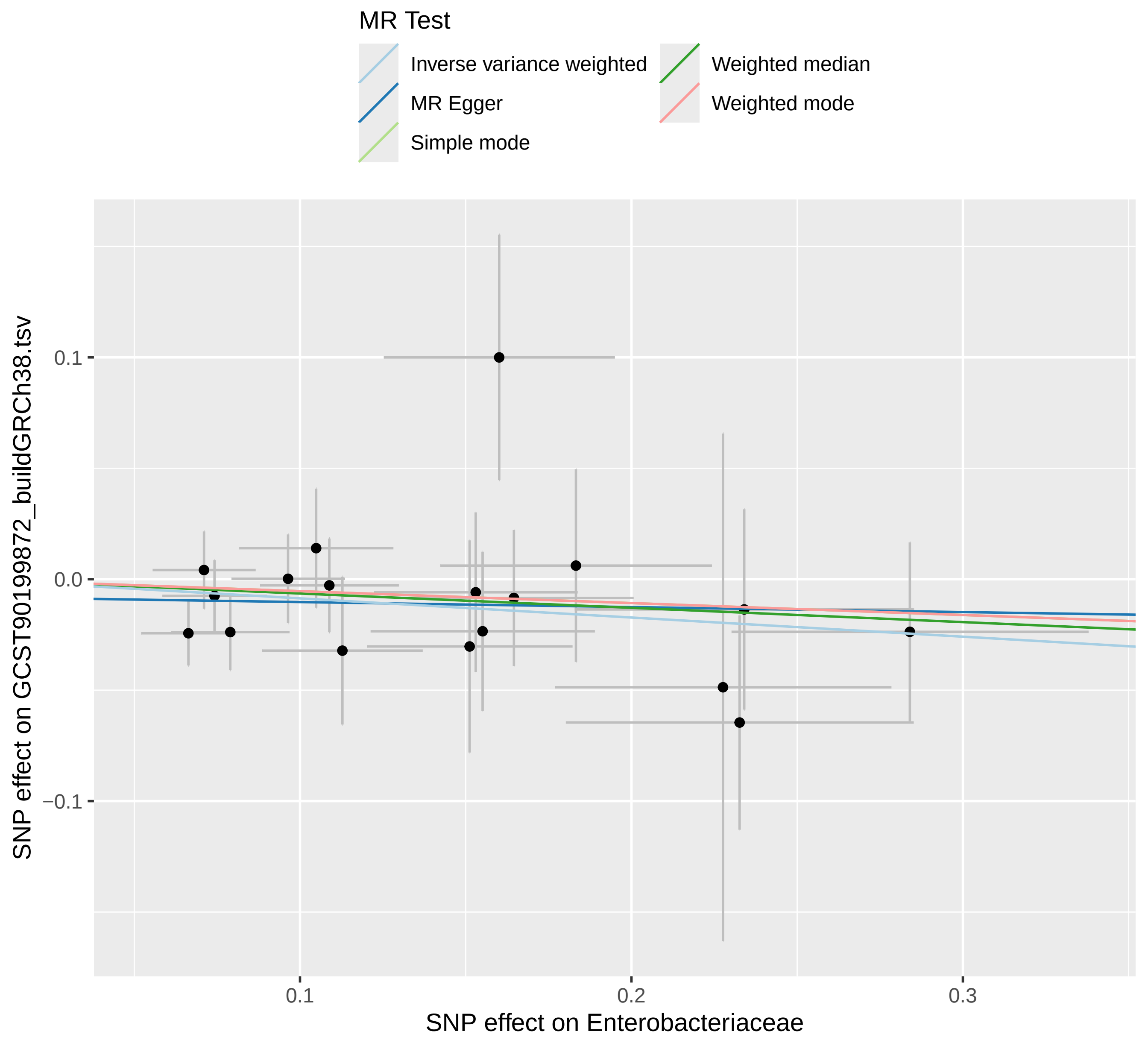

MR Test
Inverse variance weighted
MR Egger
Simple mode
Weighted median
Weighted mode
SNP effect on GCST90199872_buildGRCh38.tsv
0.1
0.0
−0.1
0.1
0.2
0.3
SNP effect on Enterobacteriaceae

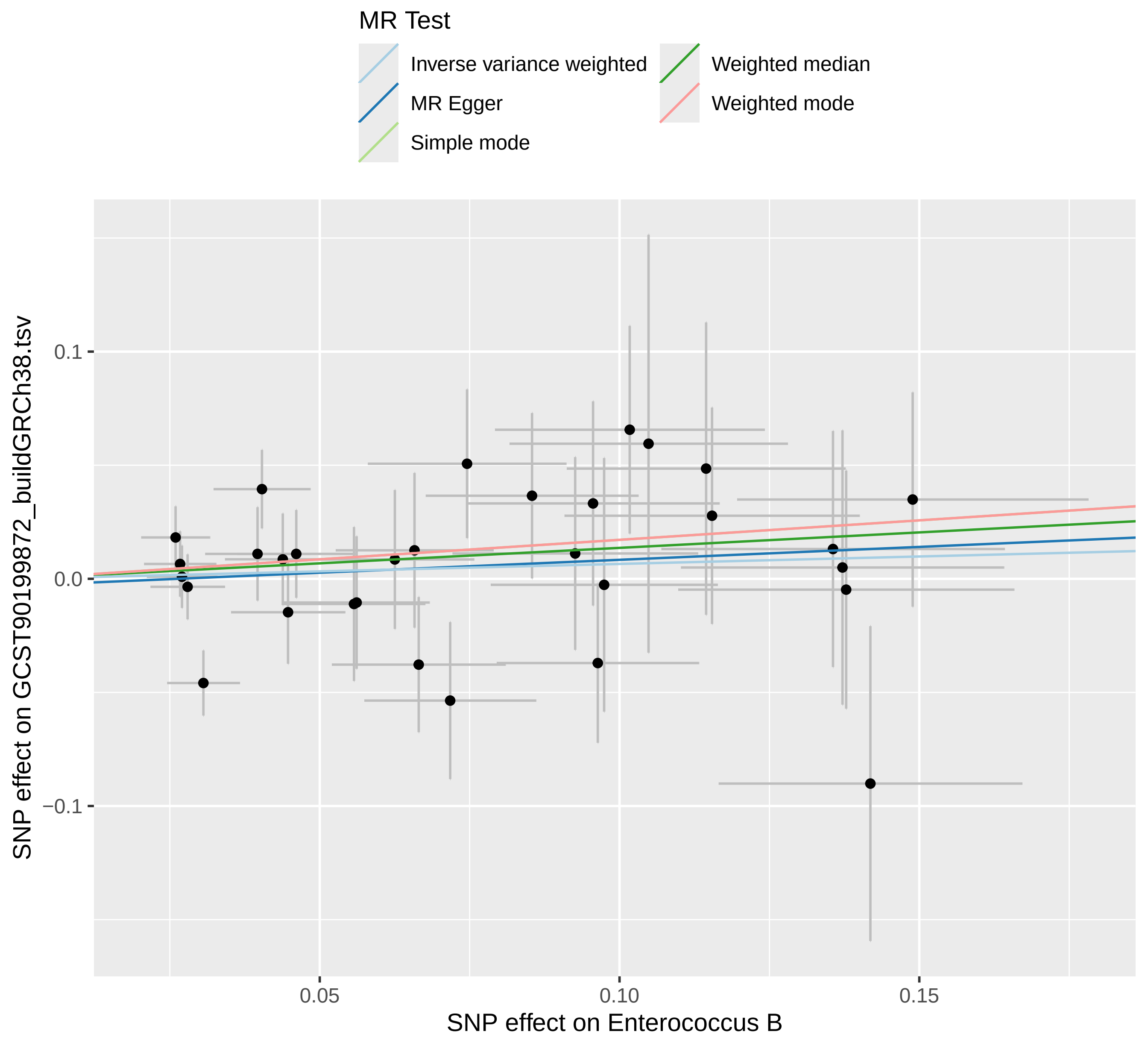

MR Test
Inverse variance weighted
MR Egger
Simple mode
Weighted median
Weighted mode
SNP effect on GCST90199872_buildGRCh38.tsv
0.1
0.0
−0.1
0.05
0.10
0.15
SNP effect on Enterococcus B

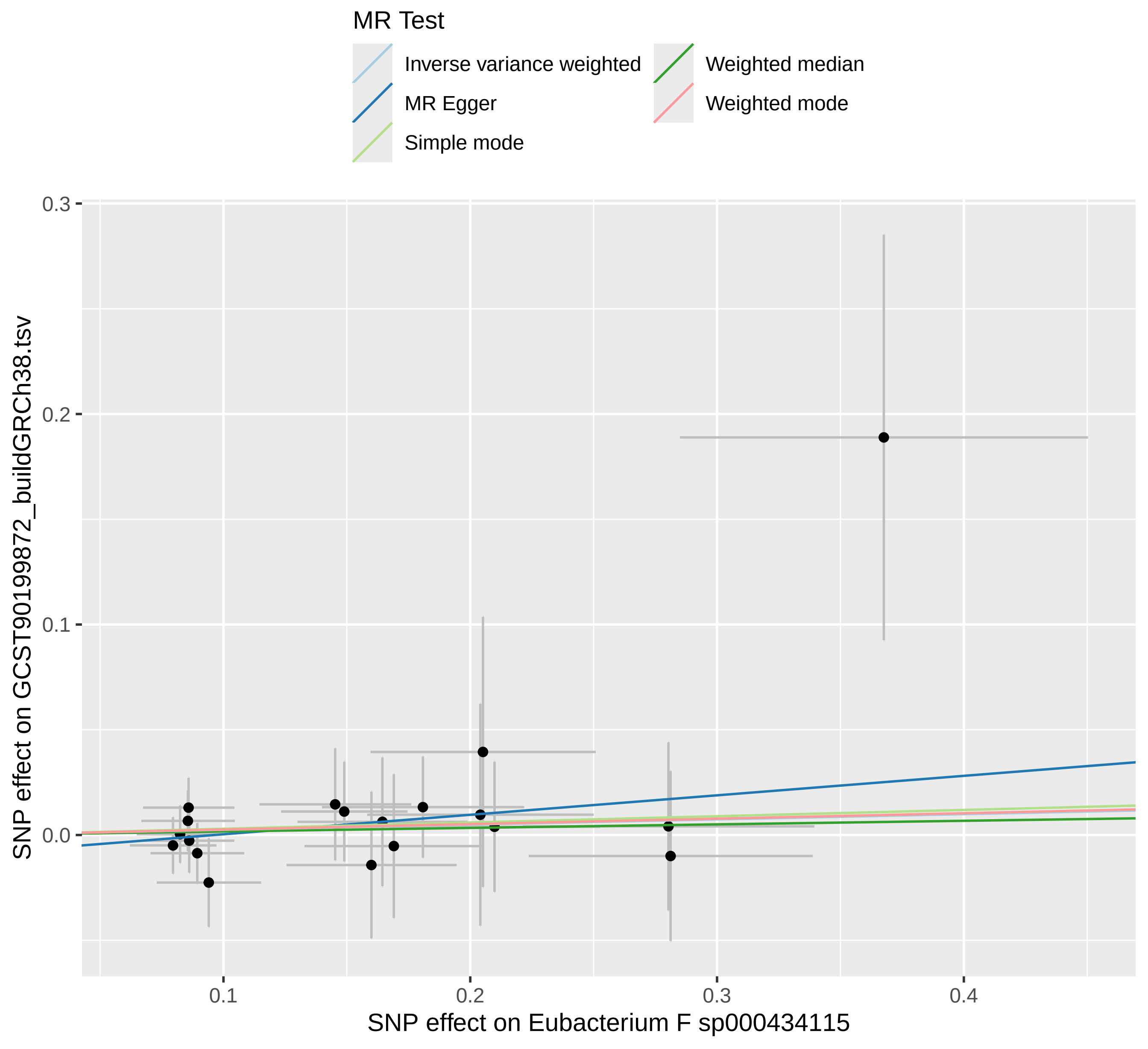

MR Test
Inverse variance weighted
MR Egger
Simple mode
Weighted median
Weighted mode
0.3
0.2
0.1
0.0
0.1
0.2
0.3
0.4
SNP effect on GCST90199872_buildGRCh38.tsv
SNP effect on Eubacterium F sp000434115

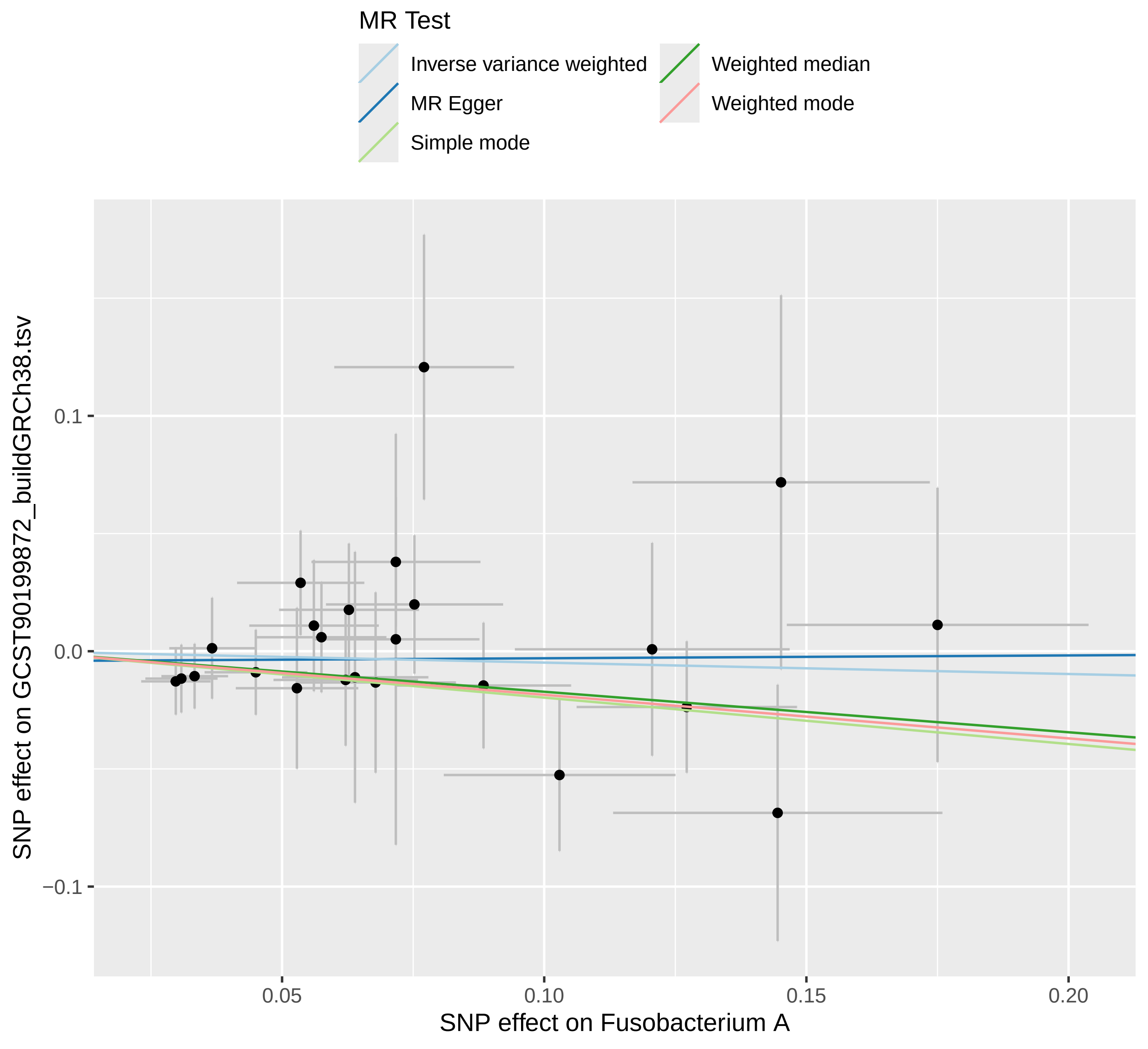
MR Test
Inverse variance weighted
MR Egger
Simple mode
Weighted median
Weighted mode
SNP effect on GCST90199872_buildGRCh38.tsv
0.1
0.0
−0.1
0.05
0.10
0.15
0.20
SNP effect on Fusobacterium A

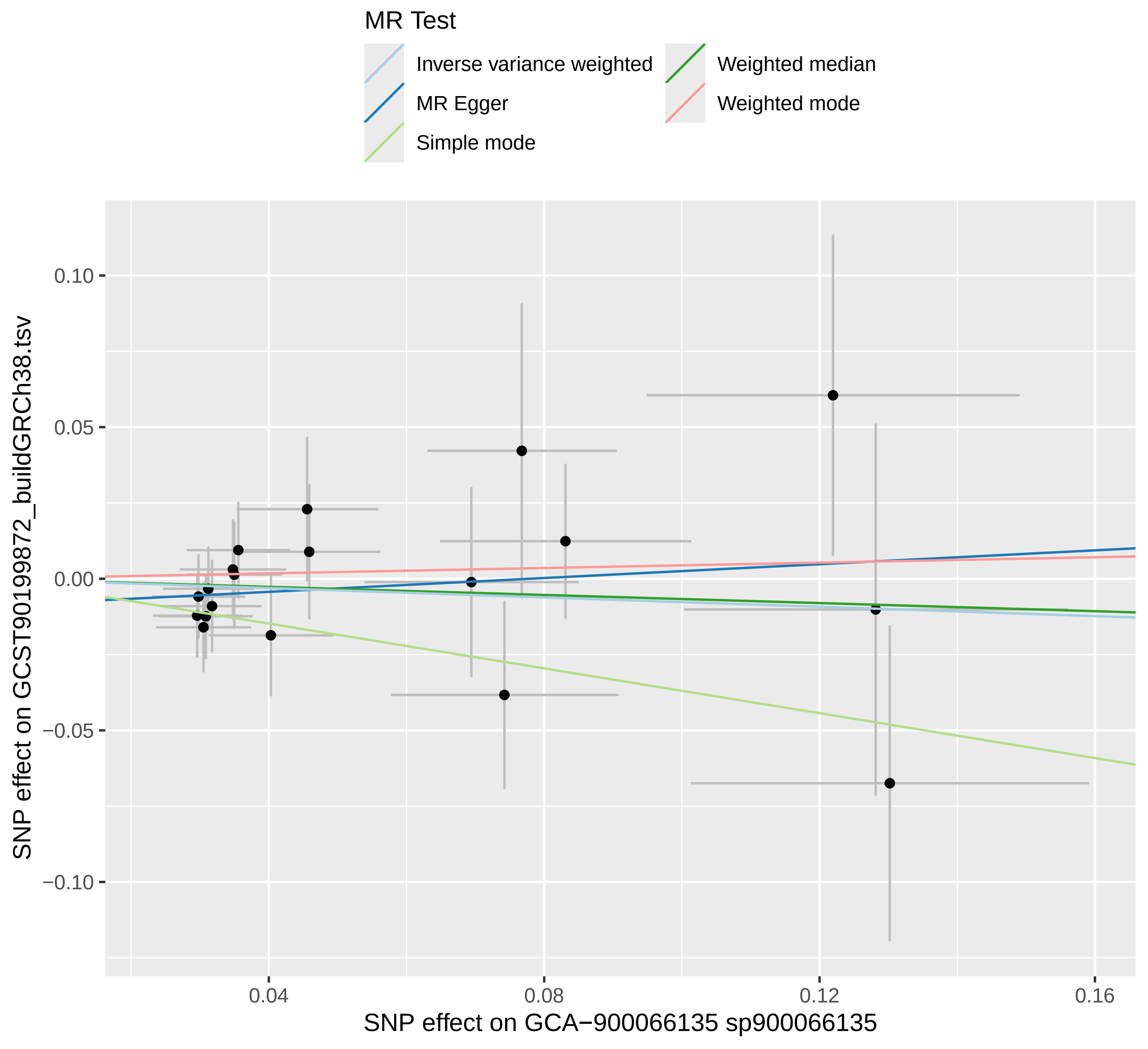

MR Test
Inverse variance weighted
MR Egger
Simple mode
Weighted median
Weighted mode
0.10
0.05
0.00
−0.05
−0.10
0.04
0.08
0.12
0.16
SNP effect on GCST90199872_buildGRCh38.tsv
SNP effect on GCA−900066135 sp900066135

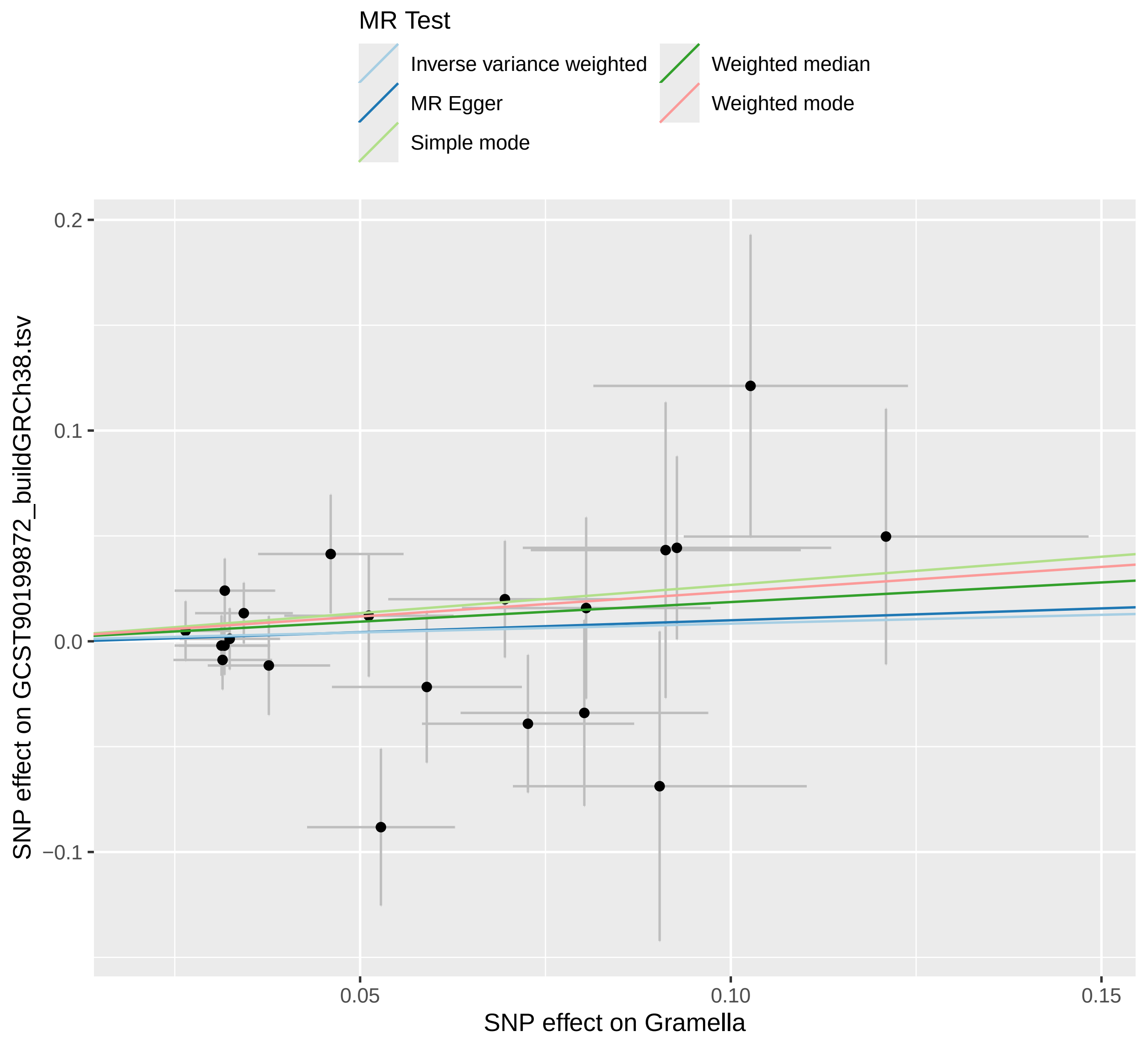

MR Test
Inverse variance weighted
MR Egger
Simple mode
Weighted median
Weighted mode
0.2
0.1
0.0
−0.1
0.05
0.10
0.15
SNP effect on GCST90199872_buildGRCh38.tsv
SNP effect on Gramella

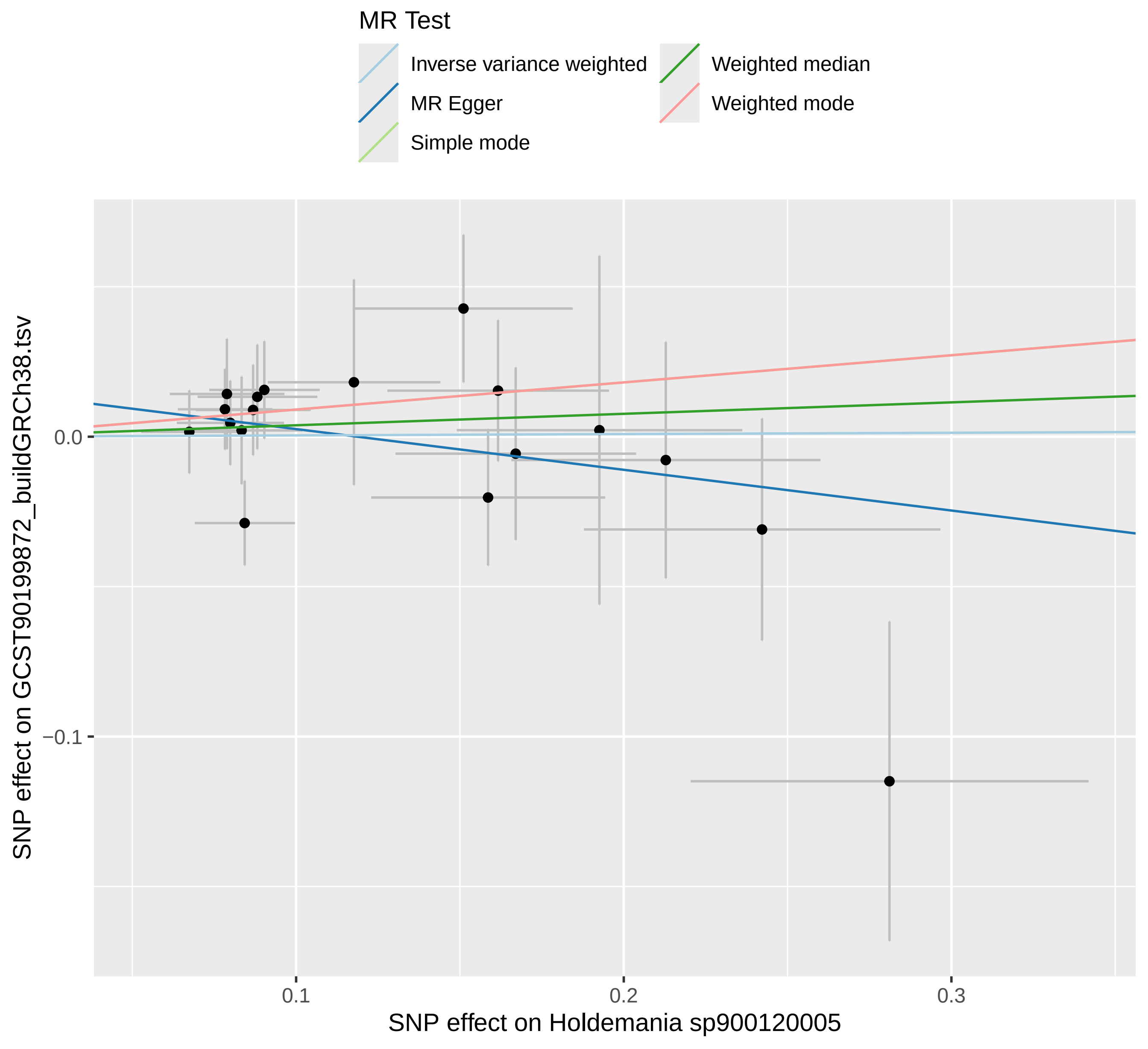
MR Test
Inverse variance weighted
MR Egger
Simple mode
Weighted median
Weighted mode
SNP effect on GCST90199872_buildGRCh38.tsv
0.0
−0.1
0.1
0.2
0.3
SNP effect on Holdemania sp900120005

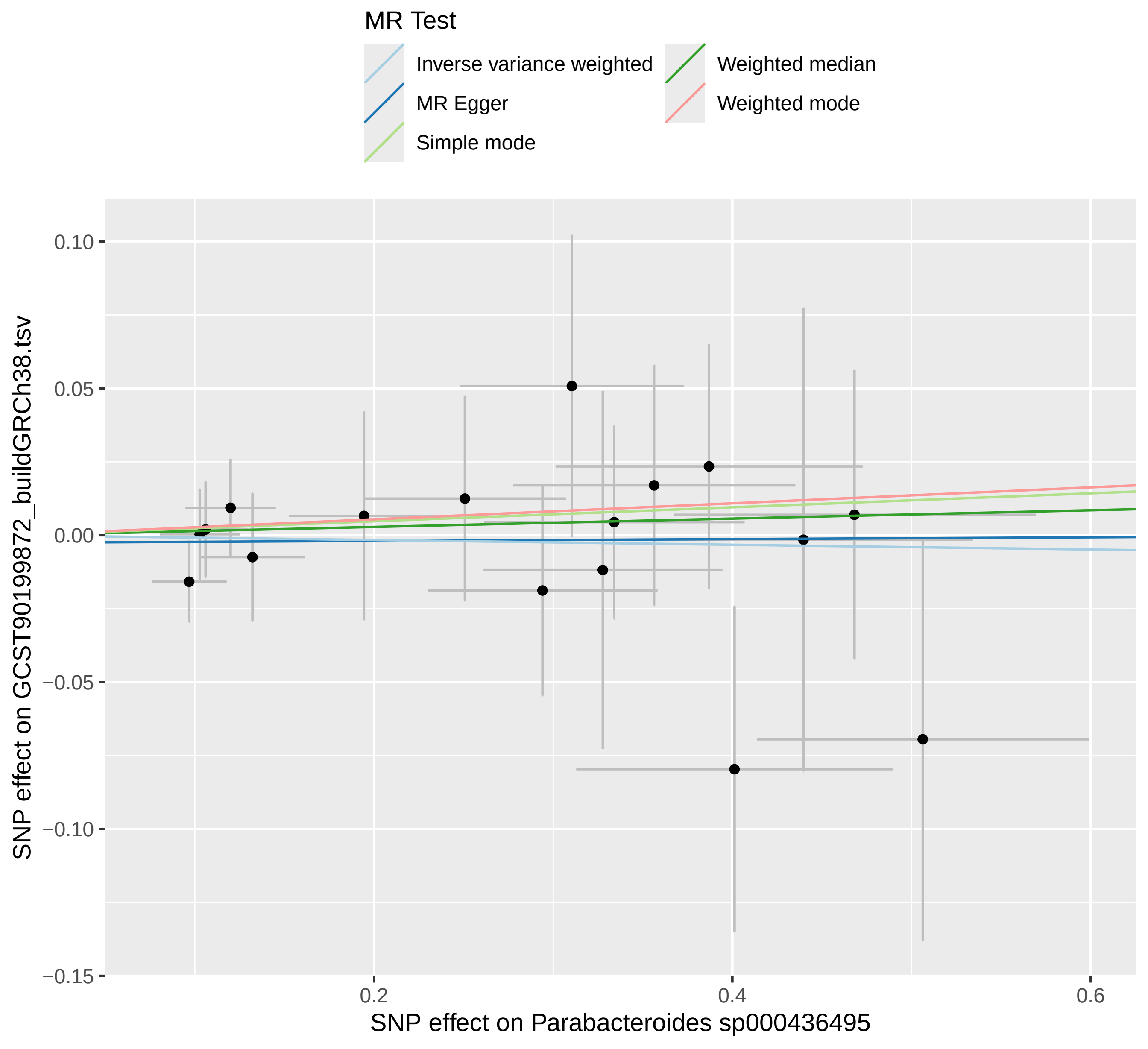
MR Test
Inverse variance weighted
MR Egger
Simple mode
Weighted median
Weighted mode
0.10
0.05
0.00
−0.05
−0.10
−0.15
0.2
0.4
0.6
SNP effect on GCST90199872_buildGRCh38.tsv
SNP effect on Parabacteroides sp000436495

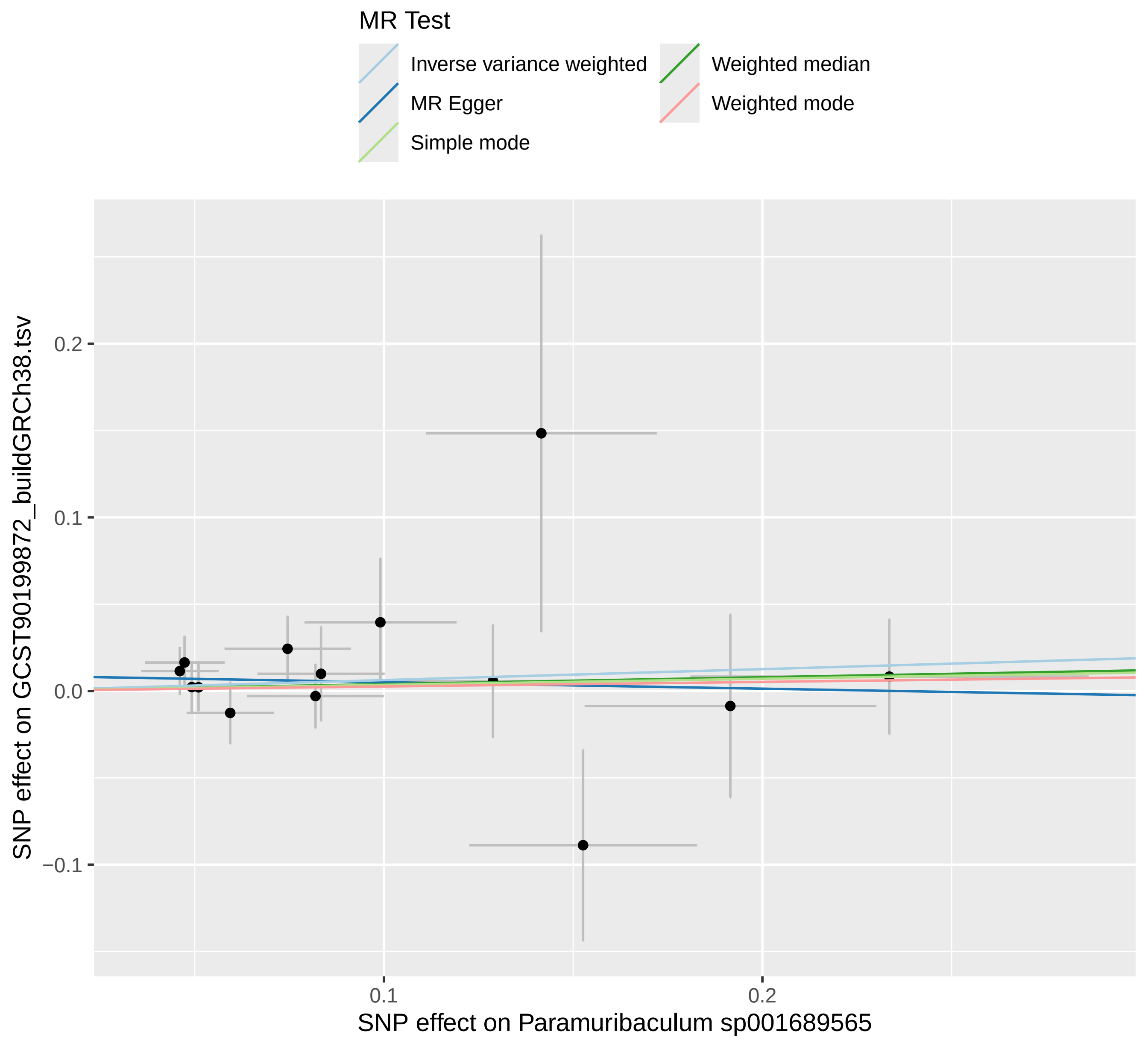

MR Test
Inverse variance weighted
MR Egger
Simple mode
Weighted median
Weighted mode
0.2
0.1
0.0
−0.1
0.1
0.2
SNP effect on GCST90199872_buildGRCh38.tsv
SNP effect on Paramuribaculum sp001689565

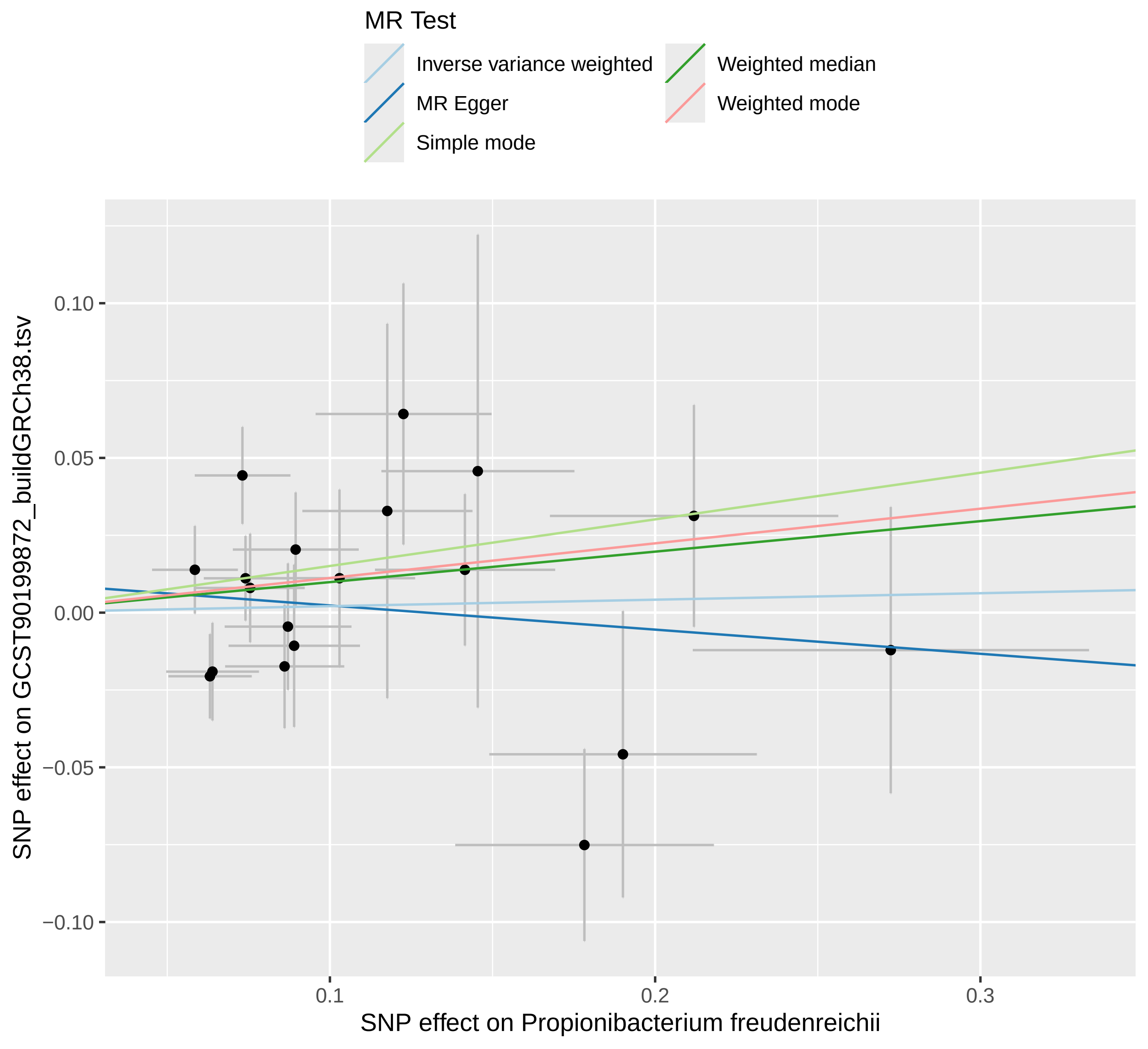

MR Test
Inverse variance weighted
MR Egger
Simple mode
Weighted median
Weighted mode
0.10
0.05
0.00
−0.05
−0.10
0.1
0.2
0.3
SNP effect on GCST90199872_buildGRCh38.tsv
SNP effect on Propionibacterium freudenreichii

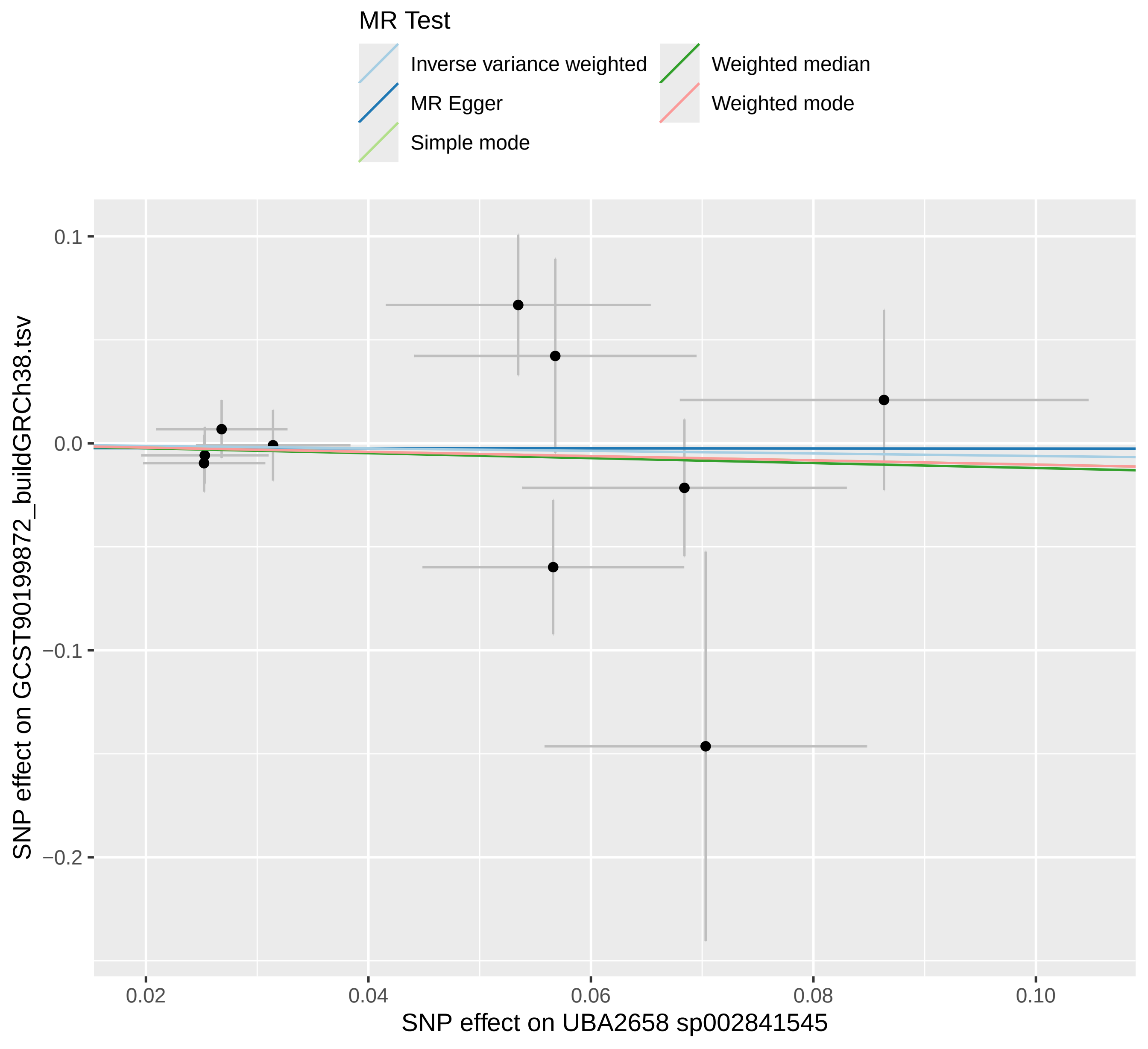
MR Test
Inverse variance weighted
MR Egger
Simple mode
Weighted median
Weighted mode
0.1
0.0
−0.1
−0.2
SNP effect on GCST90199872_buildGRCh38.tsv
0.02
0.04
0.06
0.08
0.10
SNP effect on UBA2658 sp002841545

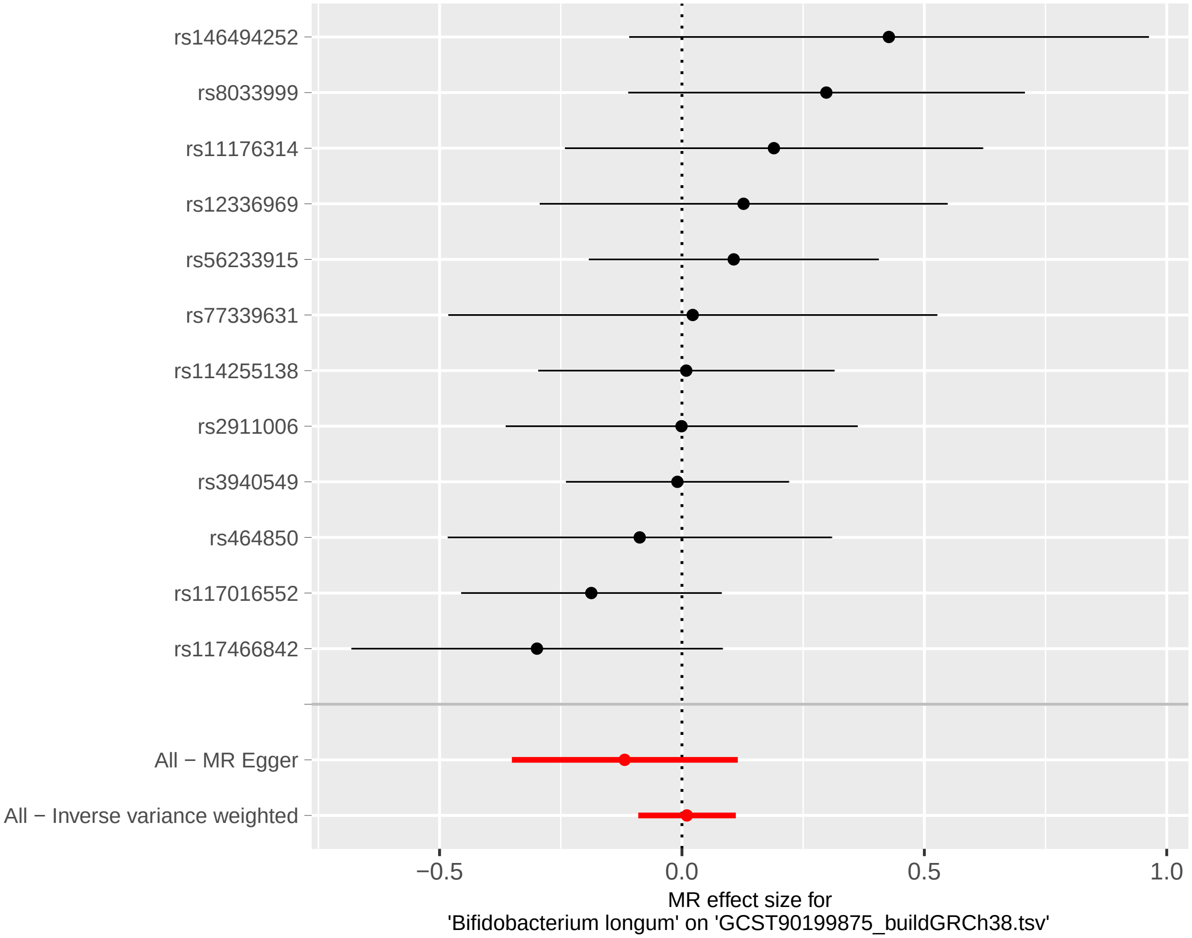
rs146494252
rs8033999
rs11176314
rs12336969
rs56233915
rs77339631
rs114255138
rs2911006
rs3940549
rs464850
rs117016552
rs117466842
All − MR Egger
All − Inverse variance weighted
−0.5
0.0
0.5
1.0
MR effect size for
'Bifidobacterium longum' on 'GCST90199875_buildGRCh38.tsv'

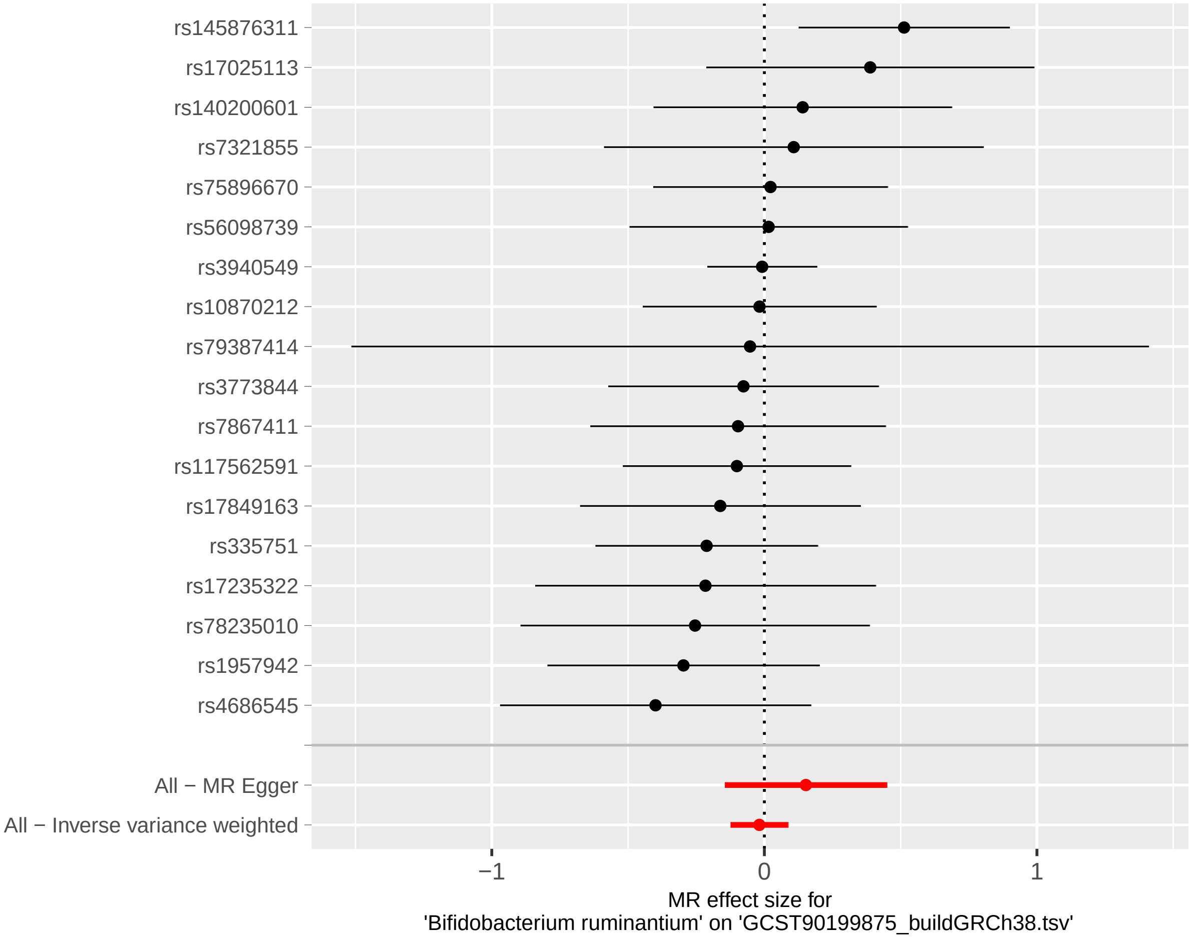
rs145876311
rs17025113
rs140200601
rs7321855
rs75896670
rs56098739
rs3940549
rs10870212
rs79387414
rs3773844
rs7867411
rs117562591
rs17849163
rs335751
rs17235322
rs78235010
rs1957942
rs4686545
All − MR Egger
All − Inverse variance weighted
−1
0
1
MR effect size for
'Bifidobacterium ruminantium' on 'GCST90199875_buildGRCh38.tsv'

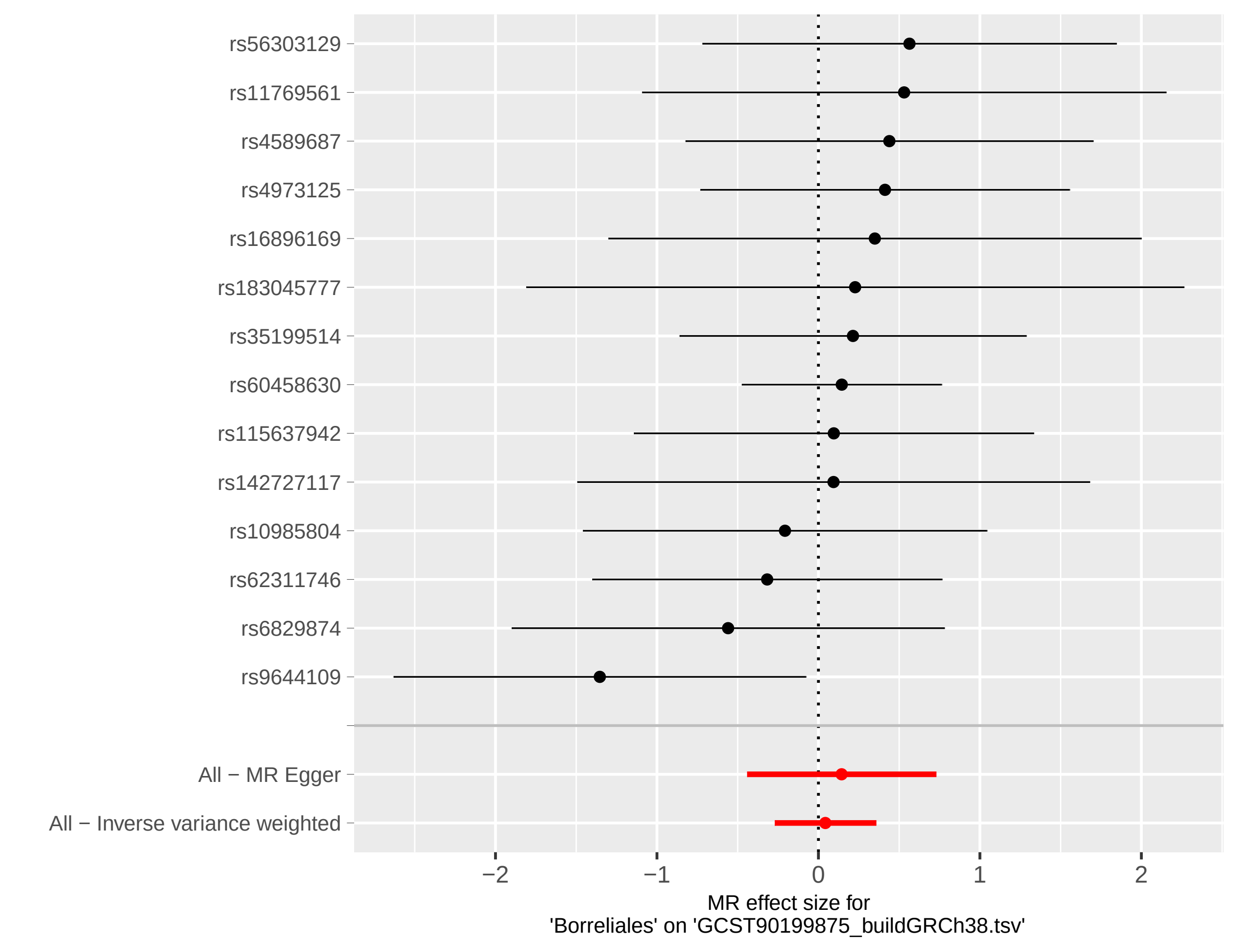
rs56303129
rs11769561
rs4589687
rs4973125
rs16896169
rs183045777
rs35199514
rs60458630
rs115637942
rs142727117
rs10985804
rs62311746
rs6829874
rs9644109
All − MR Egger
All − Inverse variance weighted
−2
−1
0
1
2
MR effect size for
'Borreliales' on 'GCST90199875_buildGRCh38.tsv'

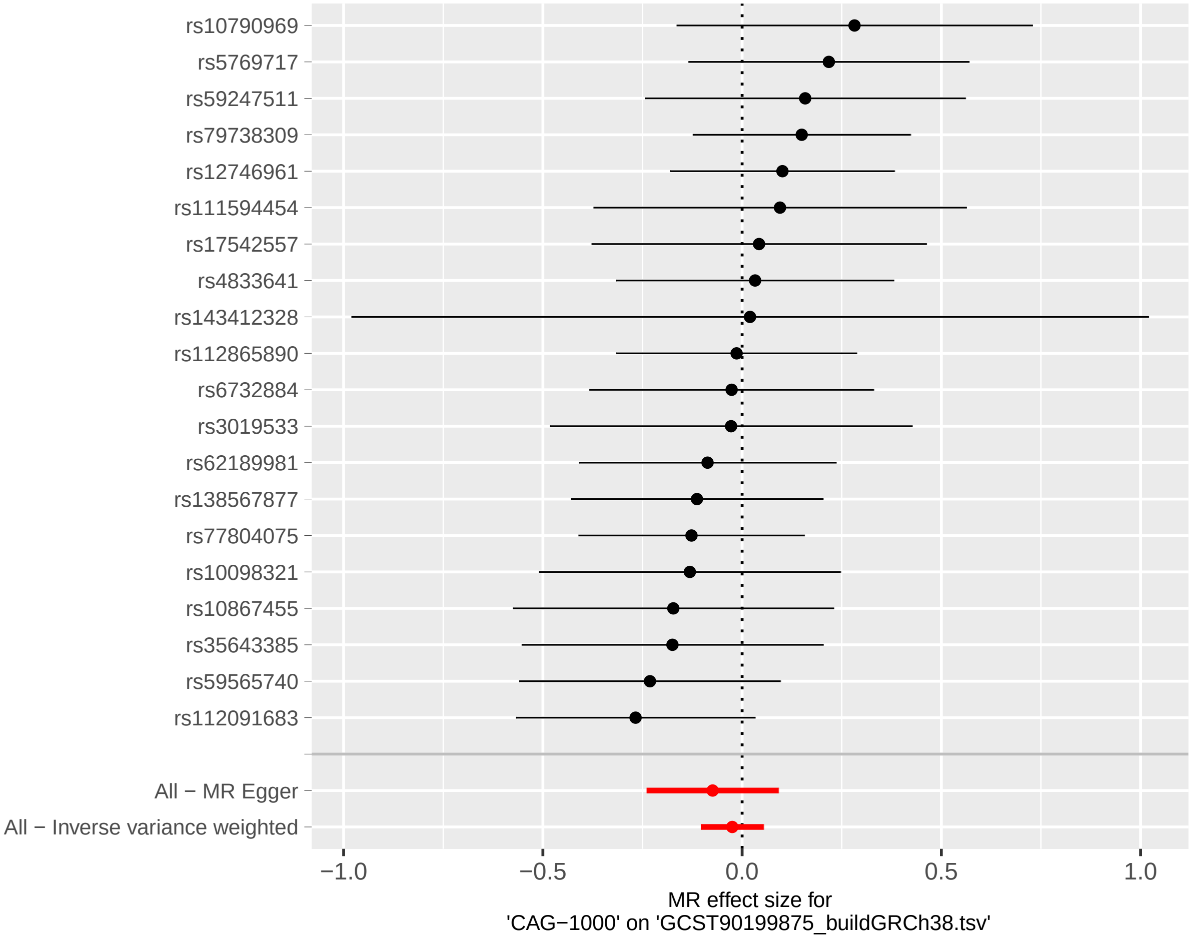
rs10790969
rs5769717
rs59247511
rs79738309
rs12746961
rs111594454
rs17542557
rs4833641
rs143412328
rs112865890
rs6732884
rs3019533
rs62189981
rs138567877
rs77804075
rs10098321
rs10867455
rs35643385
rs59565740
rs112091683
All − MR Egger
All − Inverse variance weighted
−1.0
−0.5
0.0
0.5
1.0
MR effect size for
'CAG−1000' on 'GCST90199875_buildGRCh38.tsv'

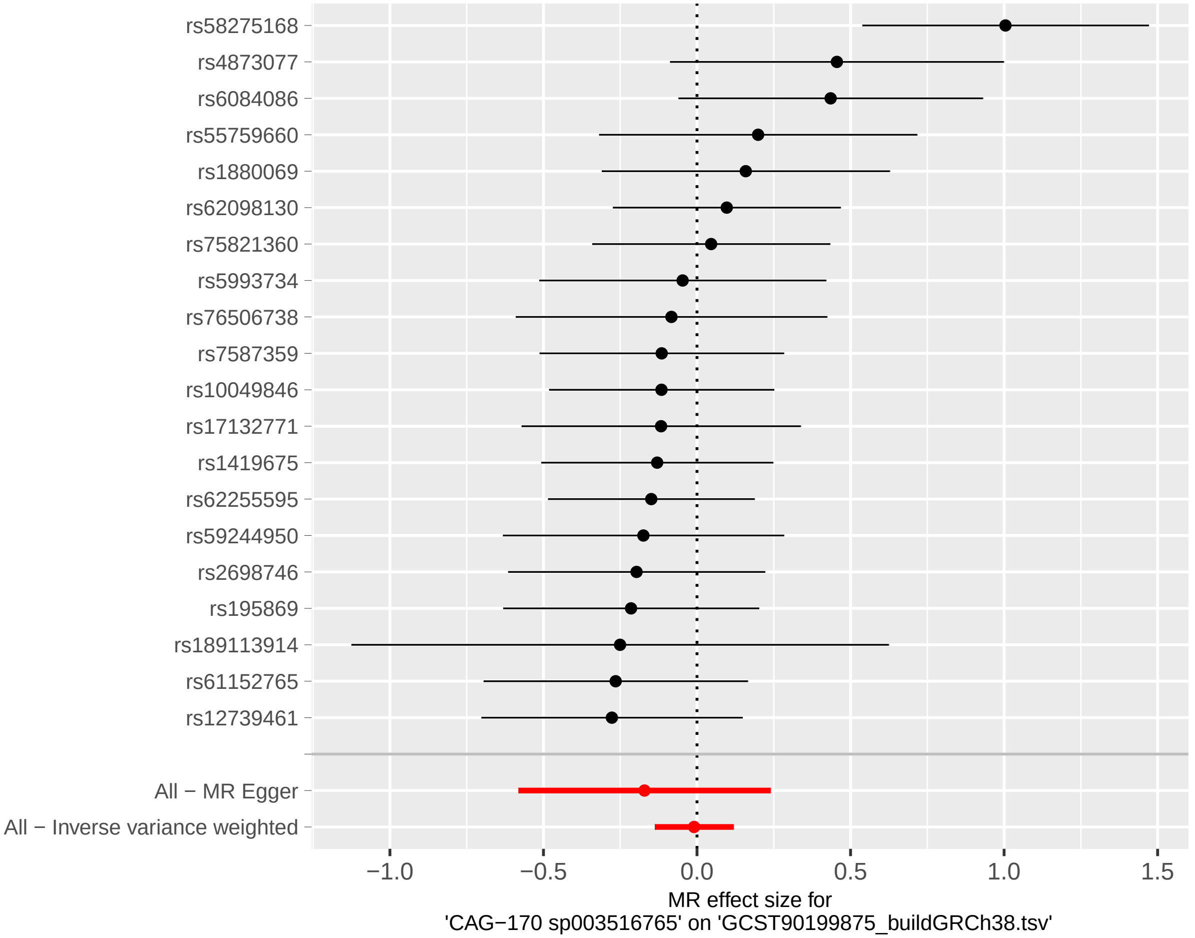
rs58275168
rs4873077
rs6084086
rs55759660
rs1880069
rs62098130
rs75821360
rs5993734
rs76506738
rs7587359
rs10049846
rs17132771
rs1419675
rs62255595
rs59244950
rs2698746
rs195869
rs189113914
rs61152765
rs12739461
All − MR Egger
All − Inverse variance weighted
−1.0
−0.5
0.0
0.5
1.0
1.5
MR effect size for
'CAG−170 sp003516765' on 'GCST90199875_buildGRCh38.tsv'

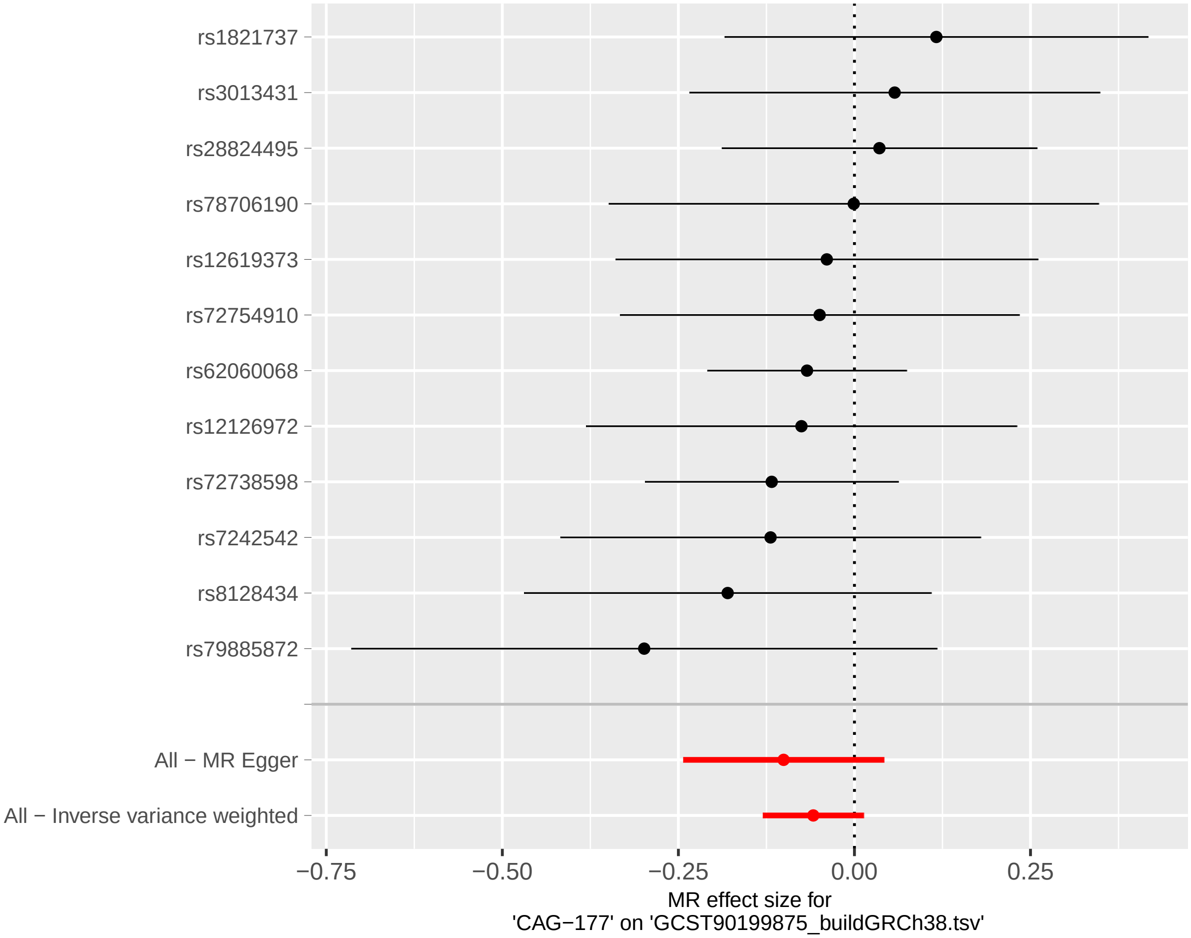

rs1821737
rs3013431
rs28824495
rs78706190
rs12619373
rs72754910
rs62060068
rs12126972
rs72738598
rs7242542
rs8128434
rs79885872
All − MR Egger
All − Inverse variance weighted
−0.75
−0.50
−0.25
0.00
0.25
MR effect size for
'CAG−177' on 'GCST90199875_buildGRCh38.tsv'

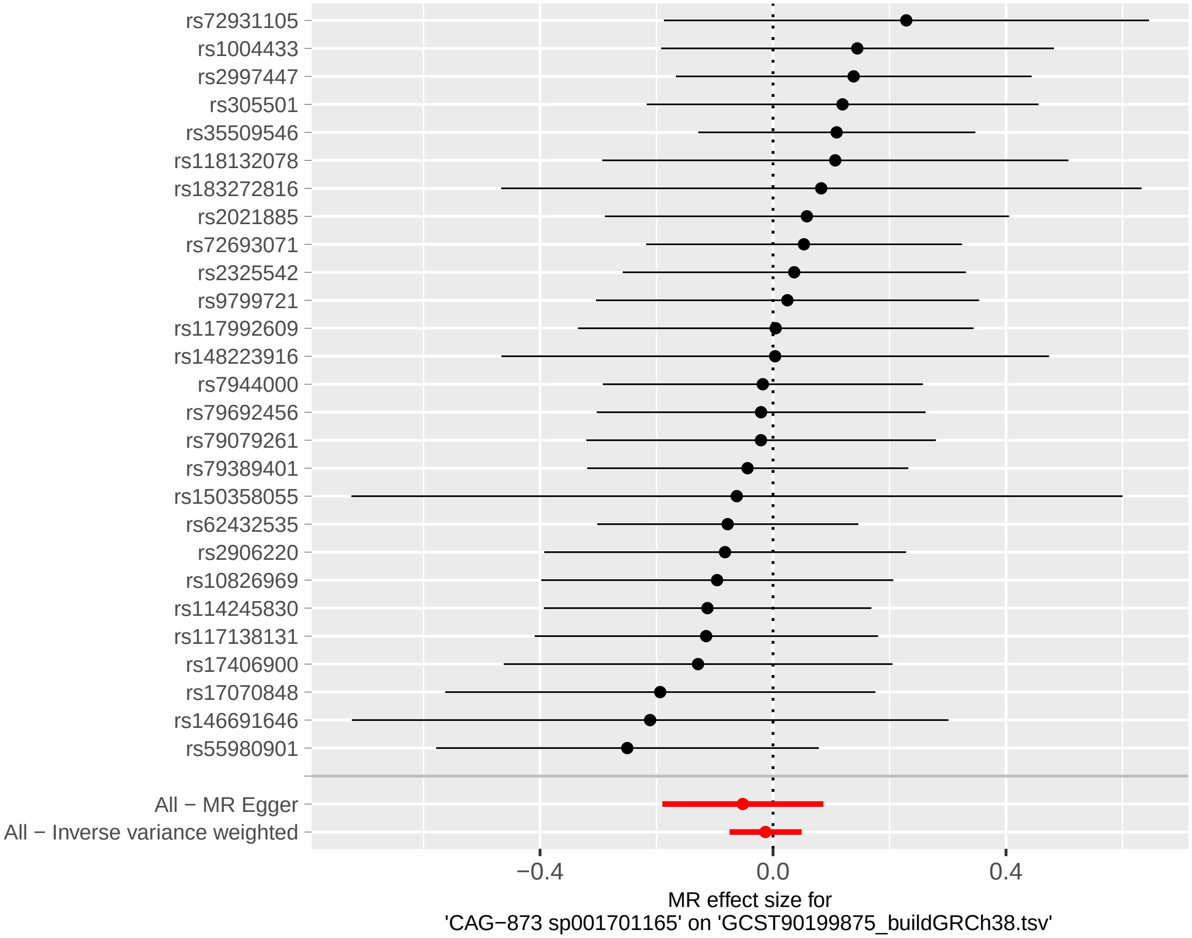

rs72931105
rs1004433
rs2997447
rs305501
rs35509546
rs118132078
rs183272816
rs2021885
rs72693071
rs2325542
rs9799721
rs117992609
rs148223916
rs7944000
rs79692456
rs79079261
rs79389401
rs150358055
rs62432535
rs2906220
rs10826969
rs114245830
rs117138131
rs17406900
rs17070848
rs146691646
rs55980901
All − MR Egger
All − Inverse variance weighted
−0.4
0.0
0.4
MR effect size for
'CAG−873 sp001701165' on 'GCST90199875_buildGRCh38.tsv'

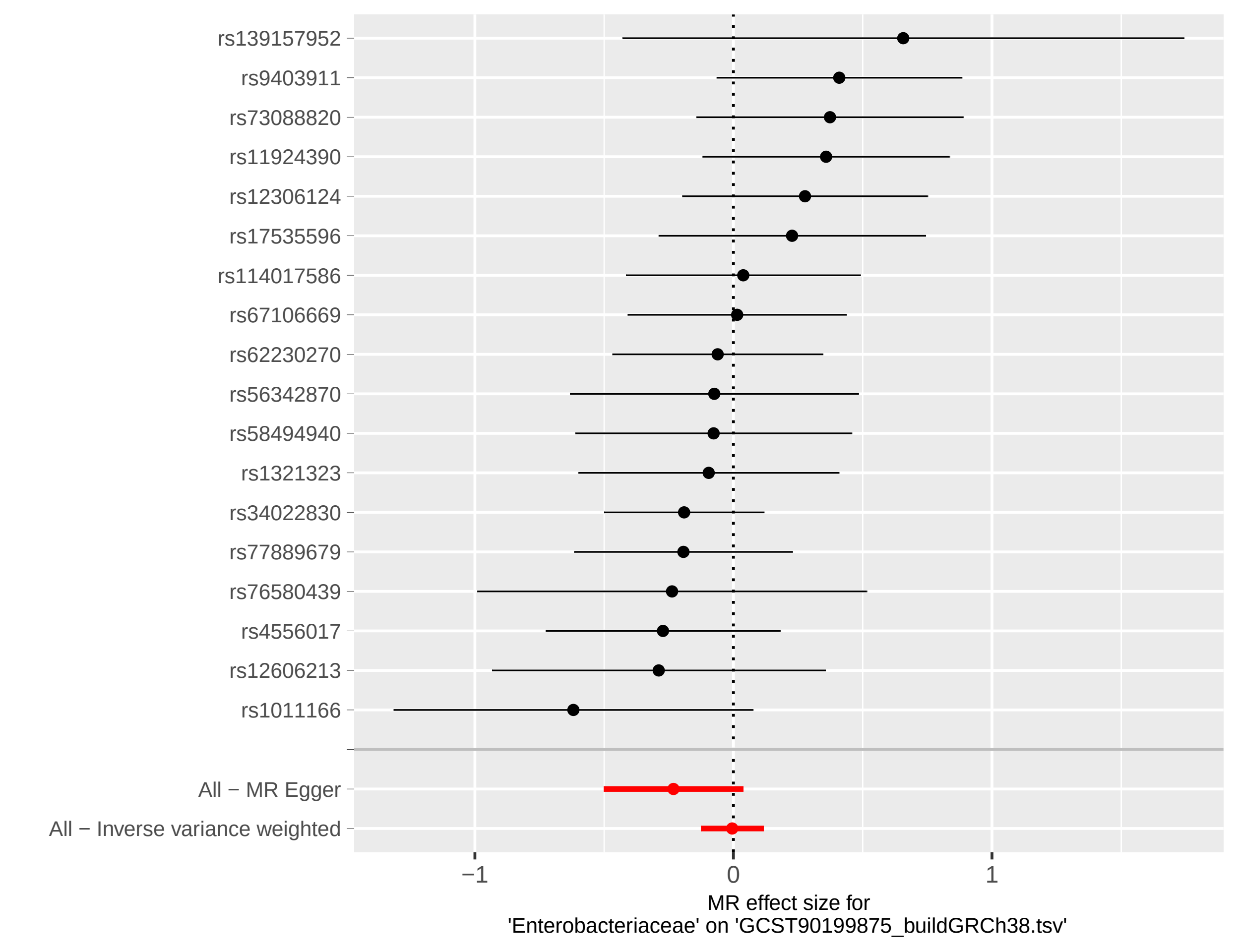
rs139157952
rs9403911
rs73088820
rs11924390
rs12306124
rs17535596
rs114017586
rs67106669
rs62230270
rs56342870
rs58494940
rs1321323
rs34022830
rs77889679
rs76580439
rs4556017
rs12606213
rs1011166
All − MR Egger
All − Inverse variance weighted
−1
0
1
MR effect size for
'Enterobacteriaceae' on 'GCST90199875_buildGRCh38.tsv'

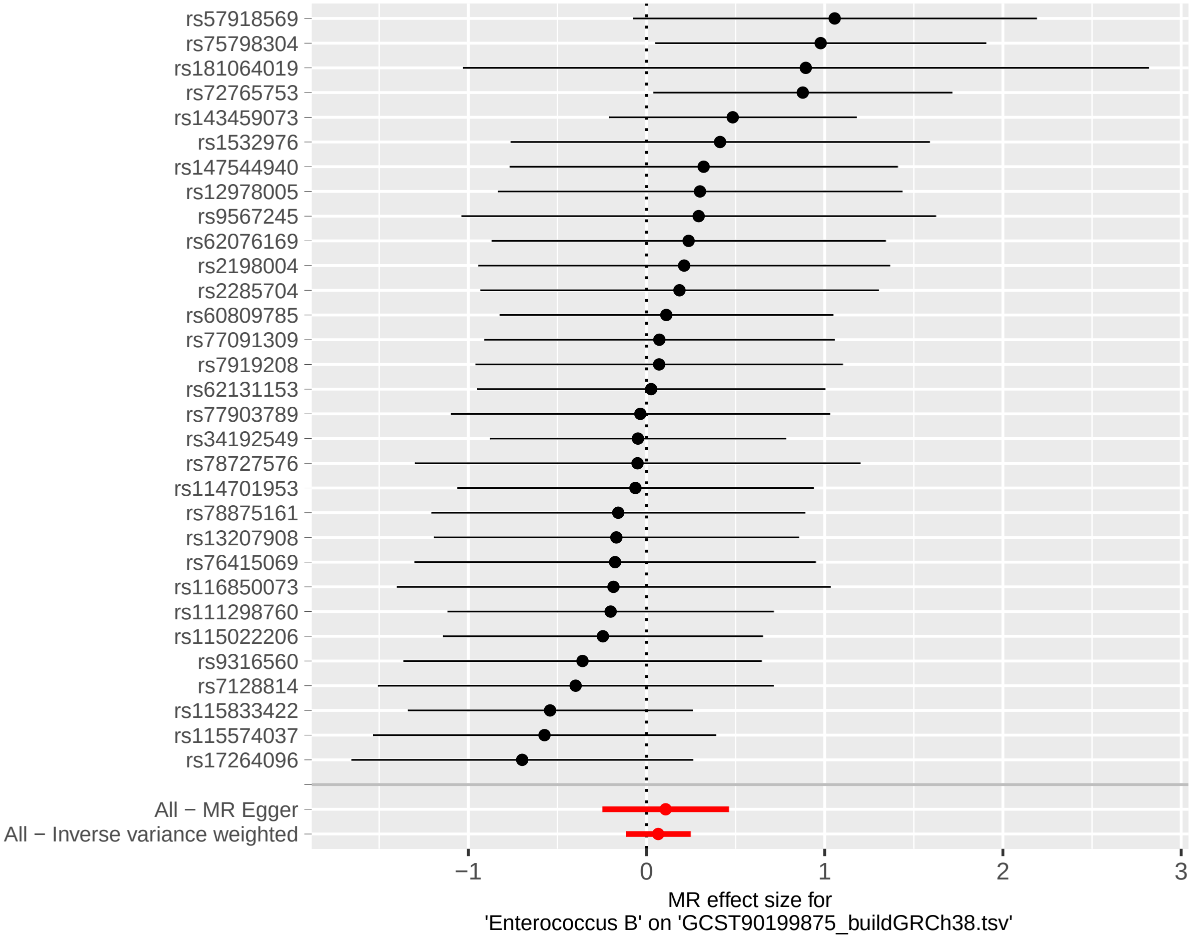

rs57918569
rs75798304
rs181064019
rs72765753
rs143459073
rs1532976
rs147544940
rs12978005
rs9567245
rs62076169
rs2198004
rs2285704
rs60809785
rs77091309
rs7919208
rs62131153
rs77903789
rs34192549
rs78727576
rs114701953
rs78875161
rs13207908
rs76415069
rs116850073
rs111298760
rs115022206
rs9316560
rs7128814
rs115833422
rs115574037
rs17264096
All − MR Egger
All − Inverse variance weighted
−1
0
1
2
3
MR effect size for
'Enterococcus B' on 'GCST90199875_buildGRCh38.tsv'

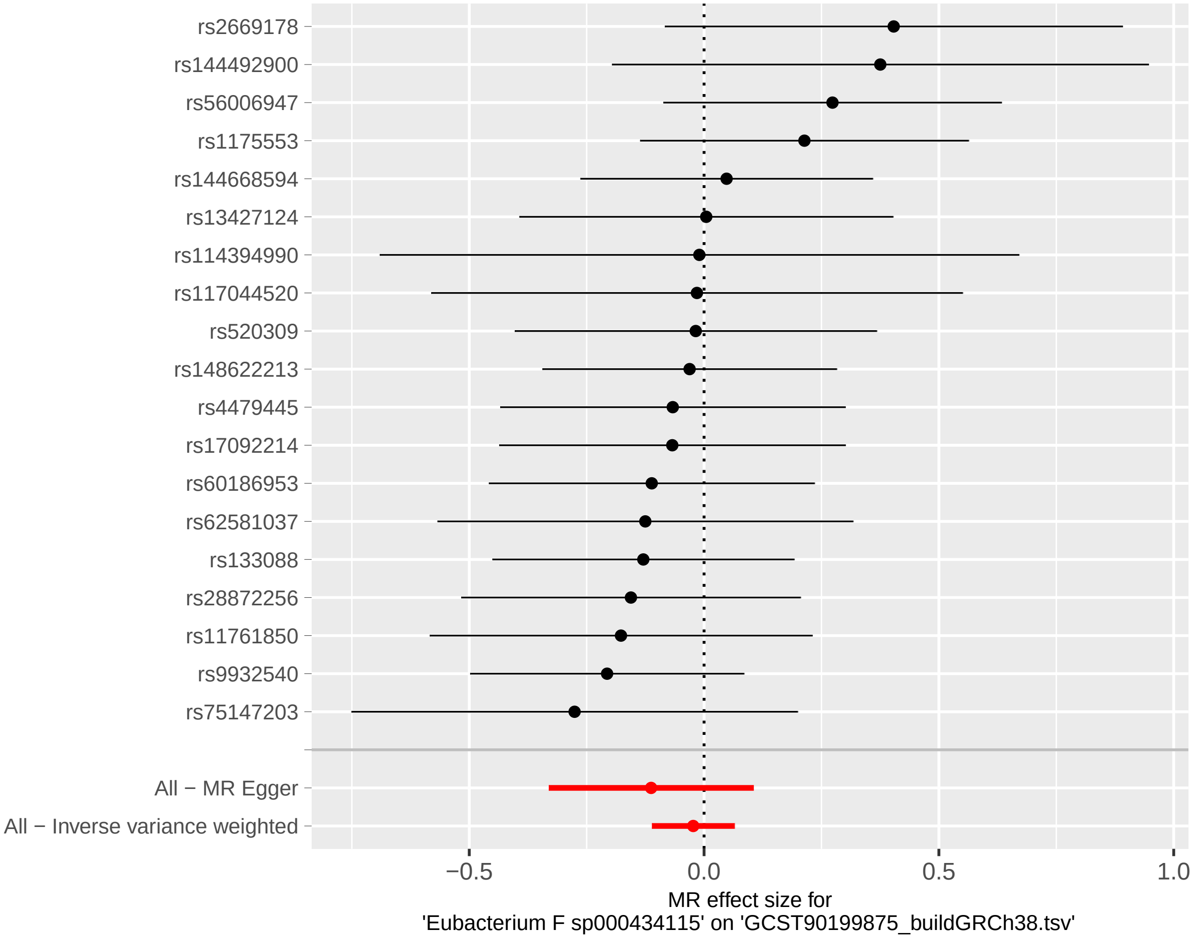

rs2669178
rs144492900
rs56006947
rs1175553
rs144668594
rs13427124
rs114394990
rs117044520
rs520309
rs148622213
rs4479445
rs17092214
rs60186953
rs62581037
rs133088
rs28872256
rs11761850
rs9932540
rs75147203
All − MR Egger
All − Inverse variance weighted
−0.5
0.0
0.5
1.0
MR effect size for
'Eubacterium F sp000434115' on 'GCST90199875_buildGRCh38.tsv'

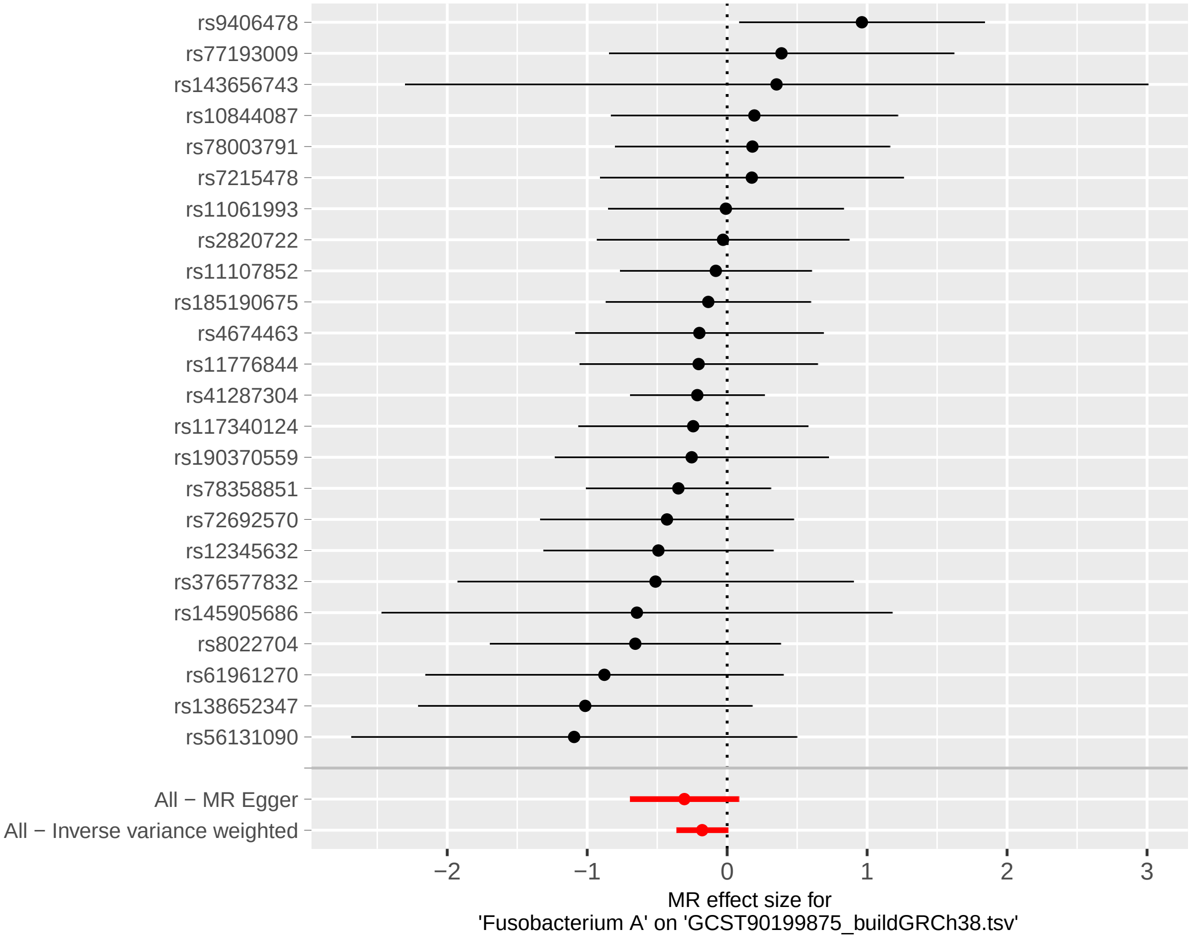
rs9406478
rs77193009
rs143656743
rs10844087
rs78003791
rs7215478
rs11061993
rs2820722
rs11107852
rs185190675
rs4674463
rs11776844
rs41287304
rs117340124
rs190370559
rs78358851
rs72692570
rs12345632
rs376577832
rs145905686
rs8022704
rs61961270
rs138652347
rs56131090
All − MR Egger
All − Inverse variance weighted
−2
−1
0
1
2
3
MR effect size for
'Fusobacterium A' on 'GCST90199875_buildGRCh38.tsv'

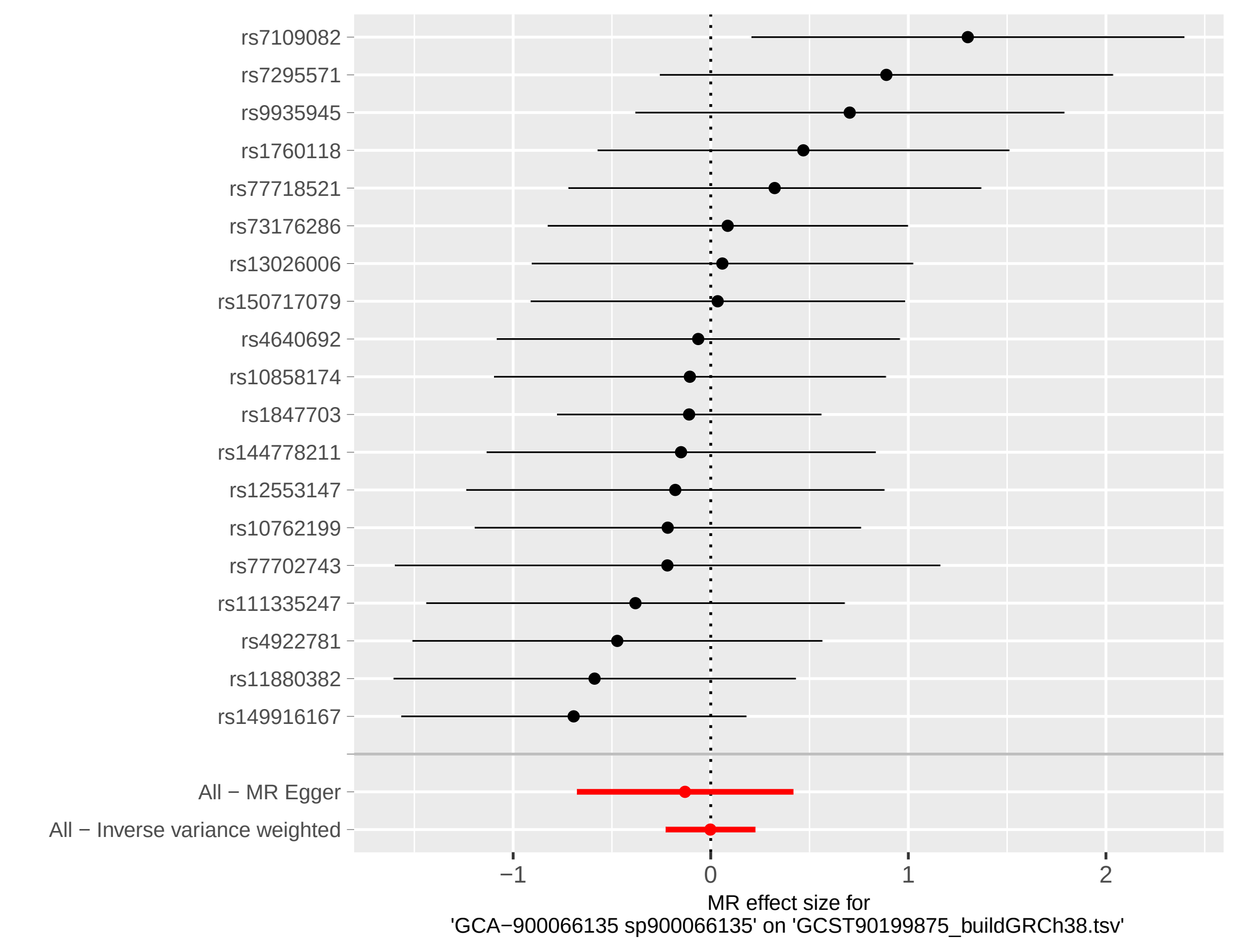

rs7109082
rs7295571
rs9935945
rs1760118
rs77718521
rs73176286
rs13026006
rs150717079
rs4640692
rs10858174
rs1847703
rs144778211
rs12553147
rs10762199
rs77702743
rs111335247
rs4922781
rs11880382
rs149916167
All − MR Egger
All − Inverse variance weighted
−1
0
1
2
MR effect size for
'GCA−900066135 sp900066135' on 'GCST90199875_buildGRCh38.tsv'

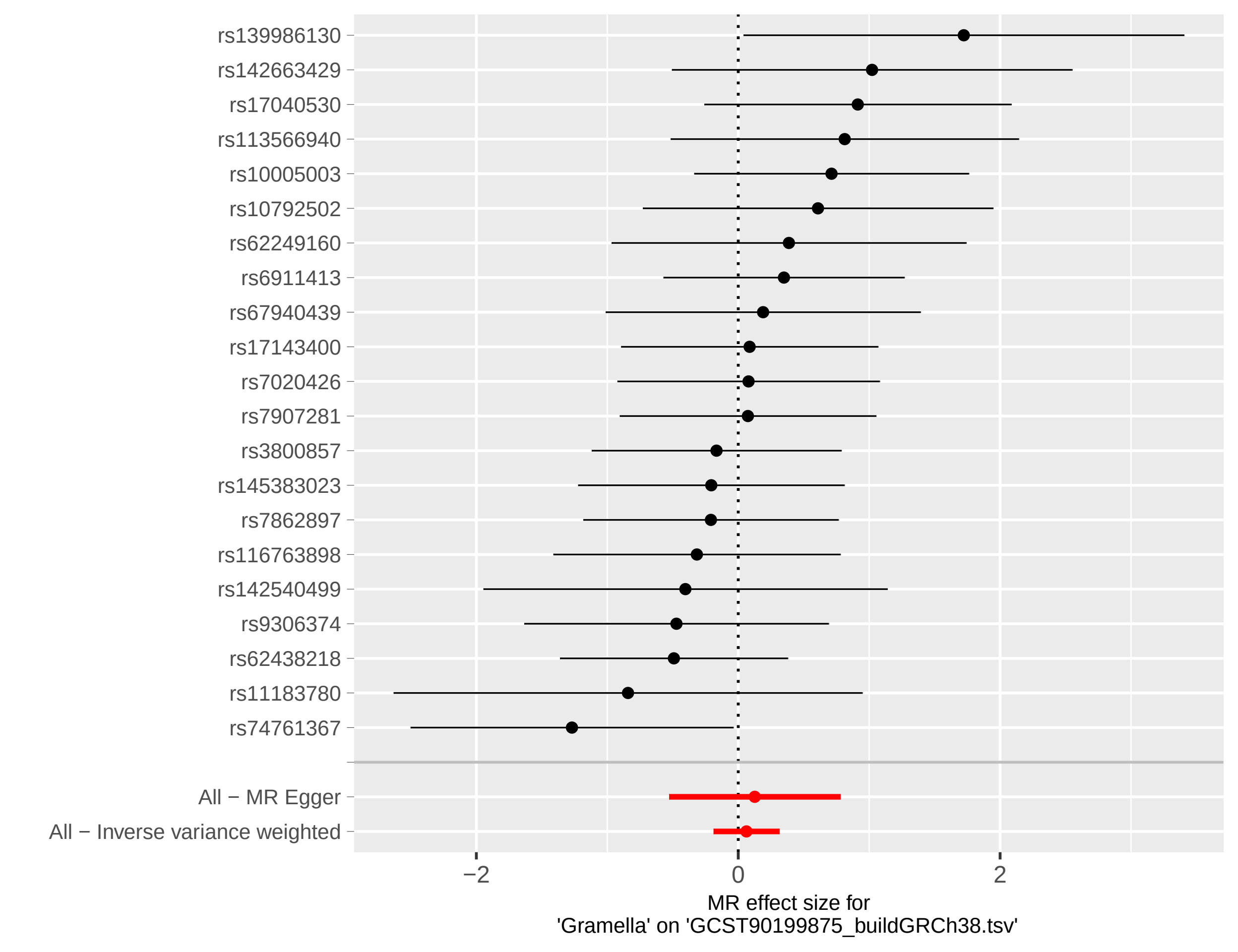
rs139986130
rs142663429
rs17040530
rs113566940
rs10005003
rs10792502
rs62249160
rs6911413
rs67940439
rs17143400
rs7020426
rs7907281
rs3800857
rs145383023
rs7862897
rs116763898
rs142540499
rs9306374
rs62438218
rs11183780
rs74761367
All − MR Egger
All − Inverse variance weighted
−2
0
2
MR effect size for
'Gramella' on 'GCST90199875_buildGRCh38.tsv'

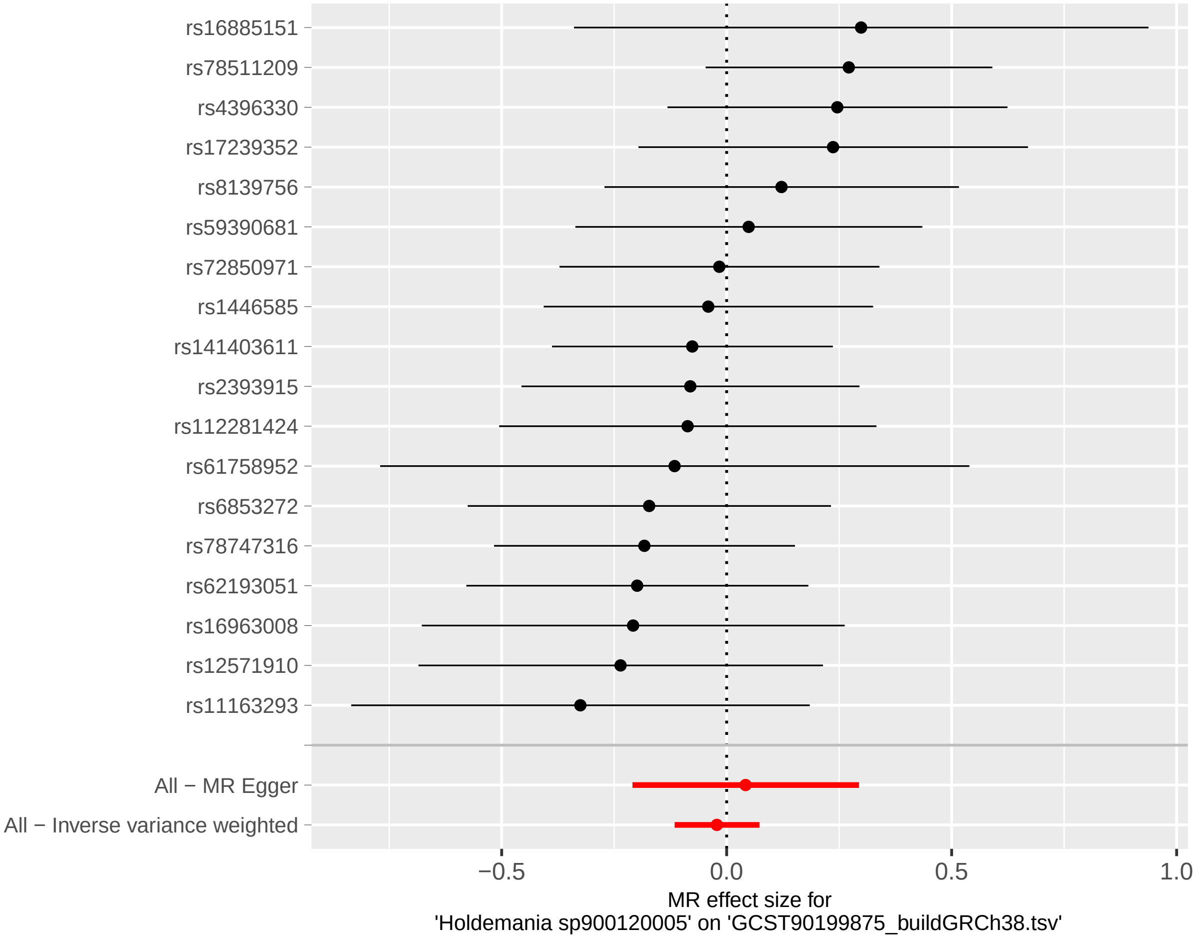

rs16885151
rs78511209
rs4396330
rs17239352
rs8139756
rs59390681
rs72850971
rs1446585
rs141403611
rs2393915
rs112281424
rs61758952
rs6853272
rs78747316
rs62193051
rs16963008
rs12571910
rs11163293
All − MR Egger
All − Inverse variance weighted
−0.5
0.0
0.5
1.0
MR effect size for
'Holdemania sp900120005' on 'GCST90199875_buildGRCh38.tsv'

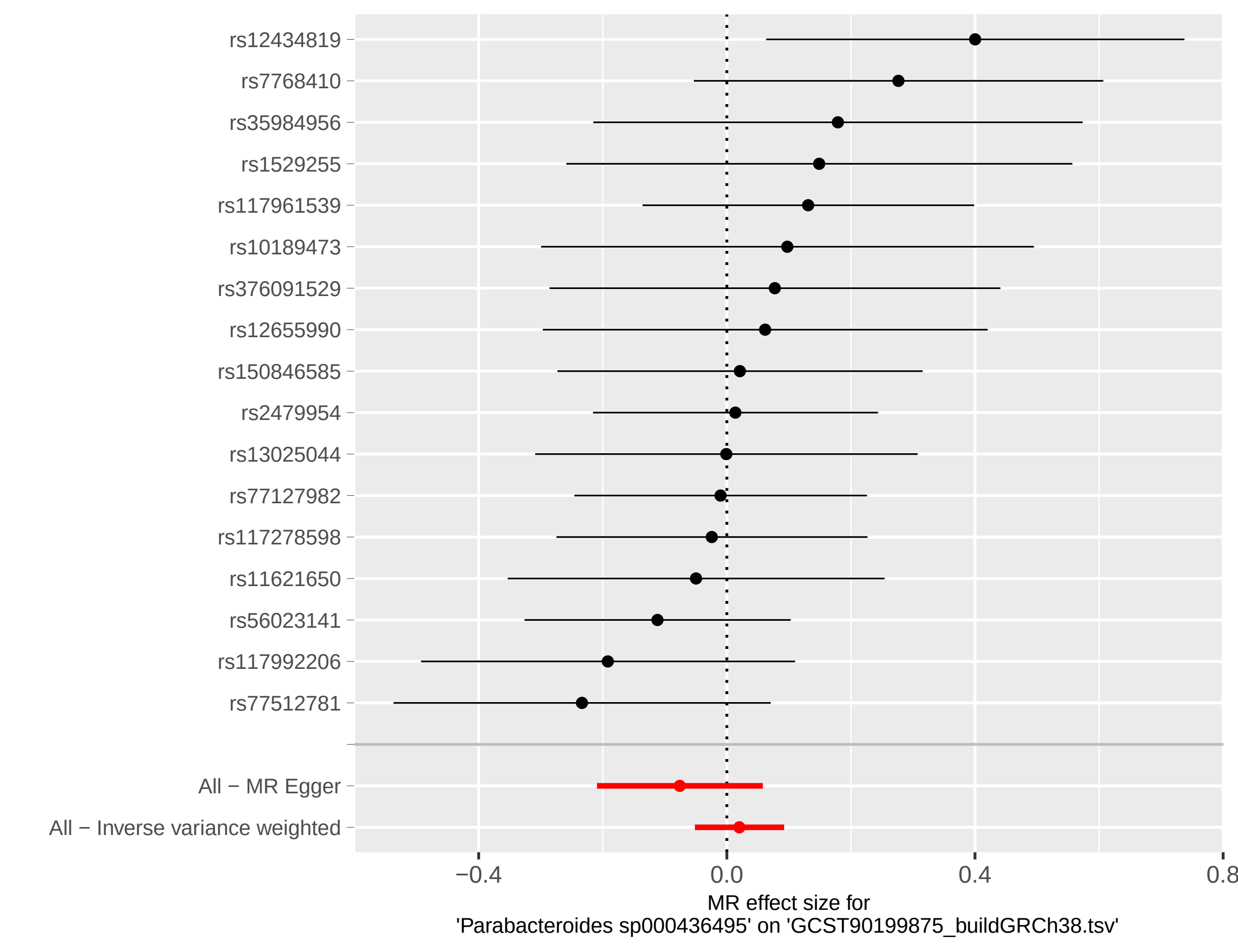
rs12434819
rs7768410
rs35984956
rs1529255
rs117961539
rs10189473
rs376091529
rs12655990
rs150846585
rs2479954
rs13025044
rs77127982
rs117278598
rs11621650
rs56023141
rs117992206
rs77512781
All − MR Egger
All − Inverse variance weighted
−0.4
0.0
0.4
0.8
MR effect size for
'Parabacteroides sp000436495' on 'GCST90199875_buildGRCh38.tsv'

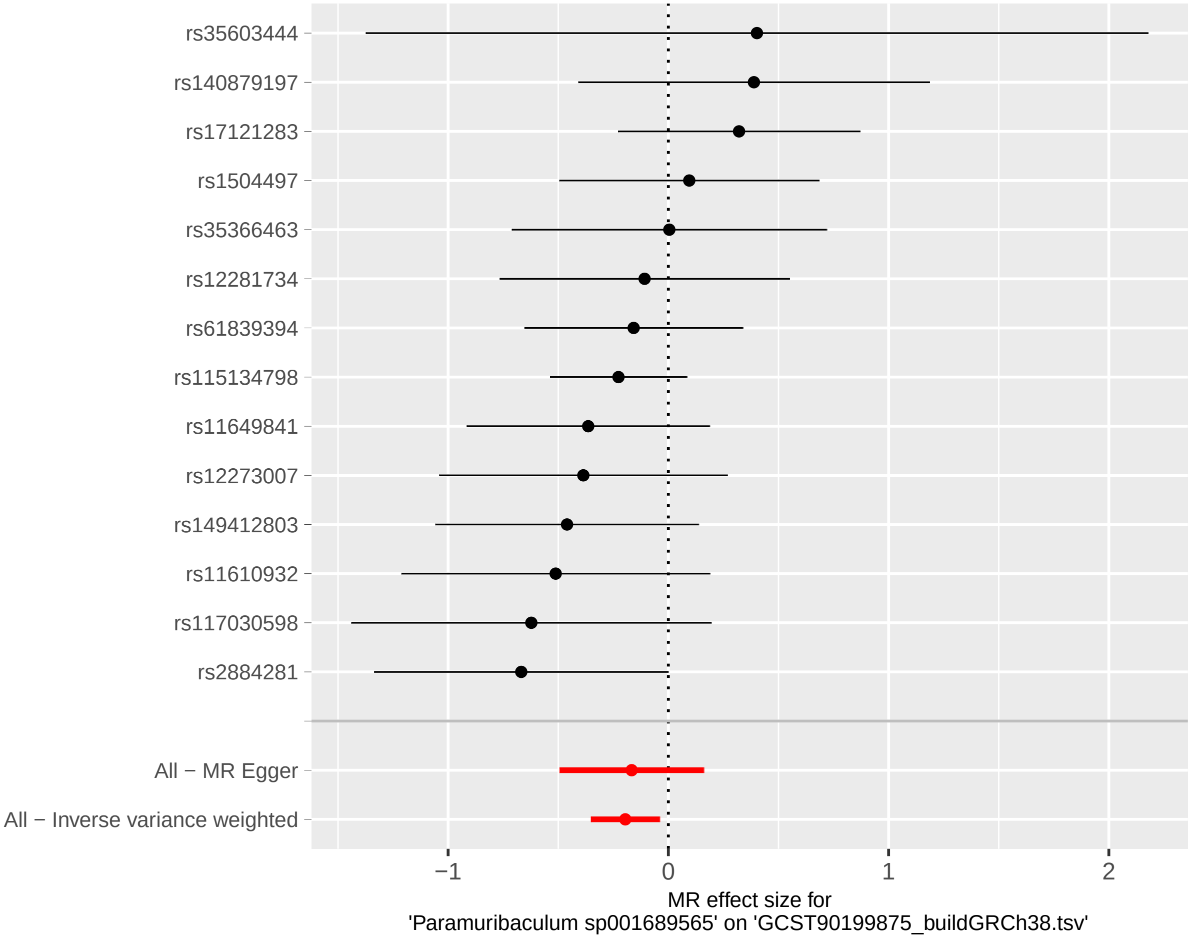

rs35603444
rs140879197
rs17121283
rs1504497
rs35366463
rs12281734
rs61839394
rs115134798
rs11649841
rs12273007
rs149412803
rs11610932
rs117030598
rs2884281
All − MR Egger
All − Inverse variance weighted
−1
0
1
2
MR effect size for
'Paramuribaculum sp001689565' on 'GCST90199875_buildGRCh38.tsv'

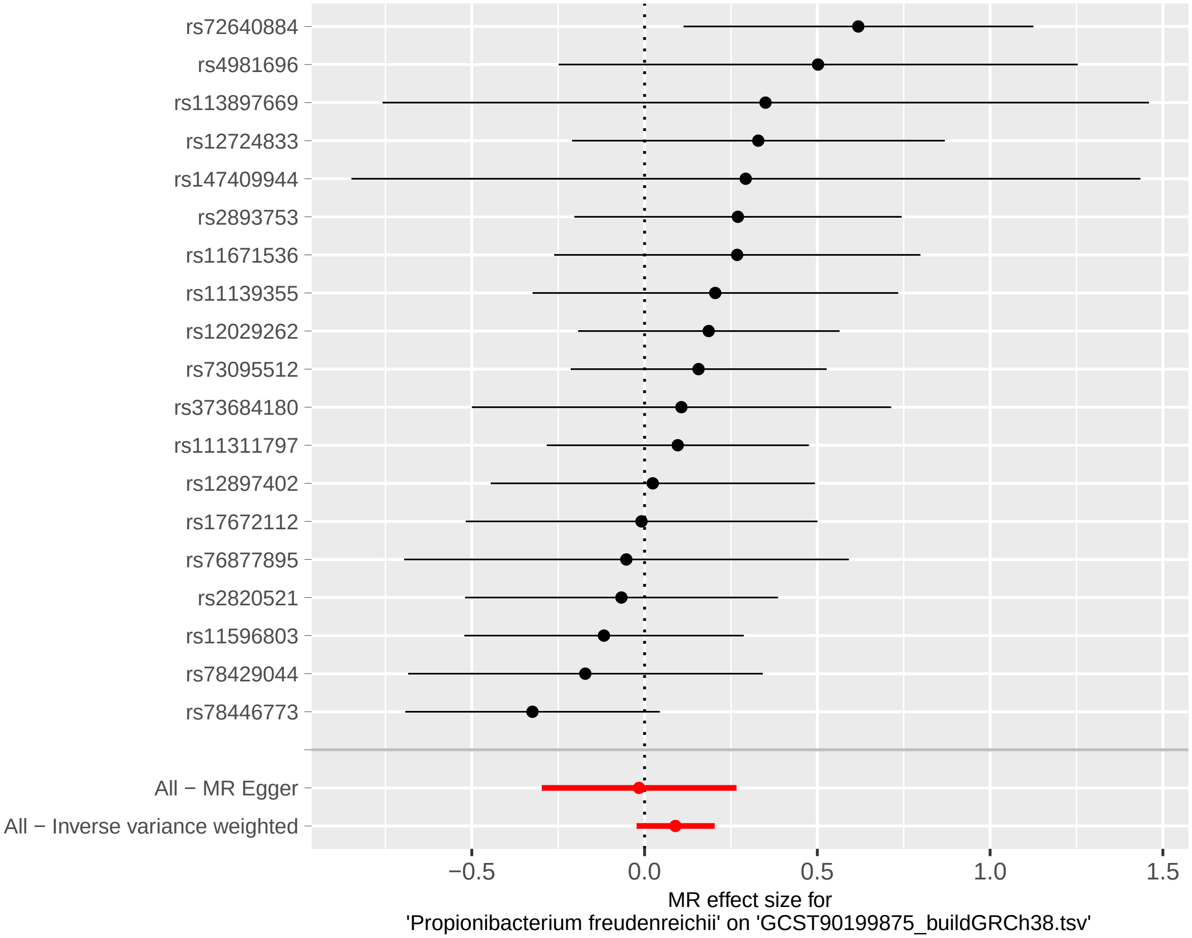
rs72640884
rs4981696
rs113897669
rs12724833
rs147409944
rs2893753
rs11671536
rs11139355
rs12029262
rs73095512
rs373684180
rs111311797
rs12897402
rs17672112
rs76877895
rs2820521
rs11596803
rs78429044
rs78446773
All − MR Egger
All − Inverse variance weighted
−0.5
0.0
0.5
1.0
1.5
MR effect size for
'Propionibacterium freudenreichii' on 'GCST90199875_buildGRCh38.tsv'

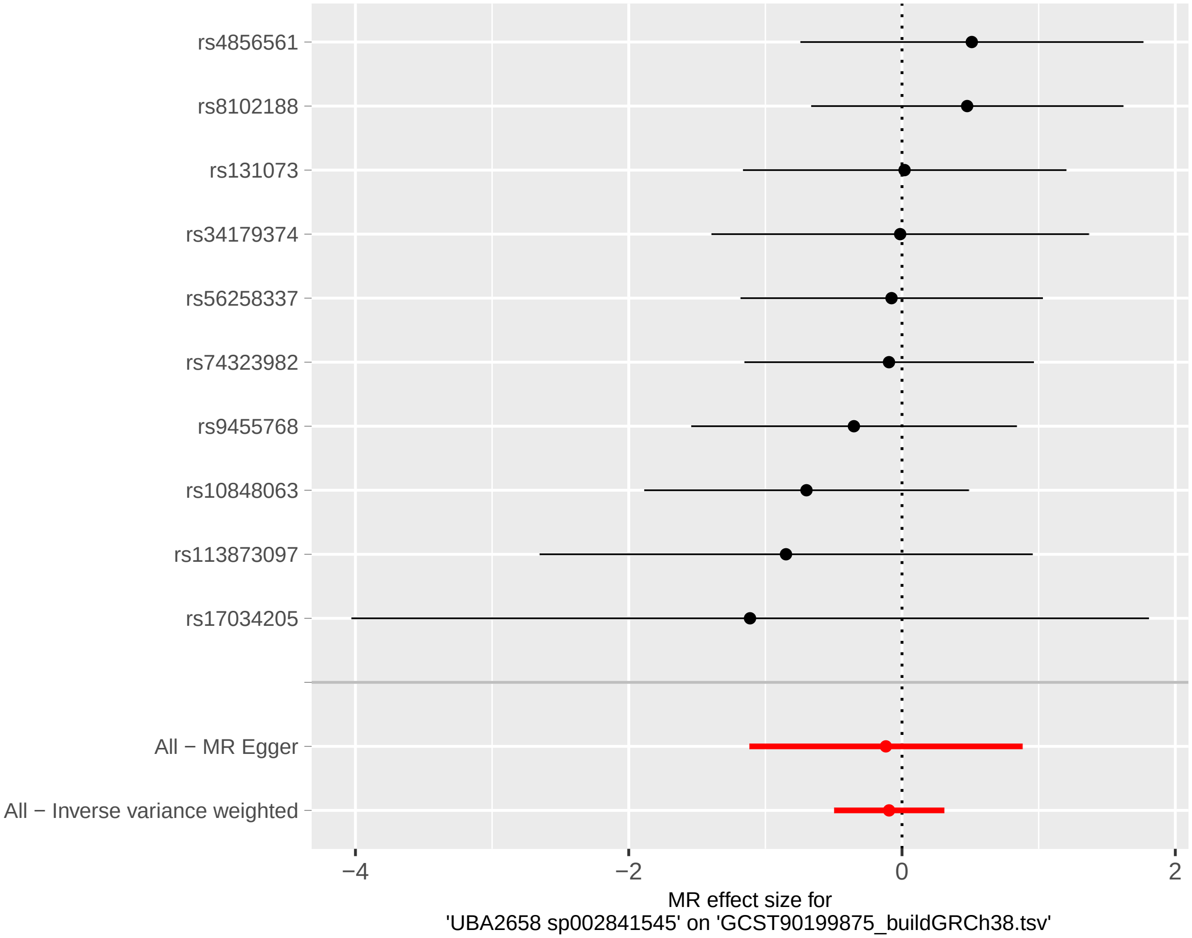

rs4856561
rs8102188
rs131073
rs34179374
rs56258337
rs74323982
rs9455768
rs10848063
rs113873097
rs17034205
All − MR Egger
All − Inverse variance weighted
−4
−2
0
2
MR effect size for
'UBA2658 sp002841545' on 'GCST90199875_buildGRCh38.tsv'

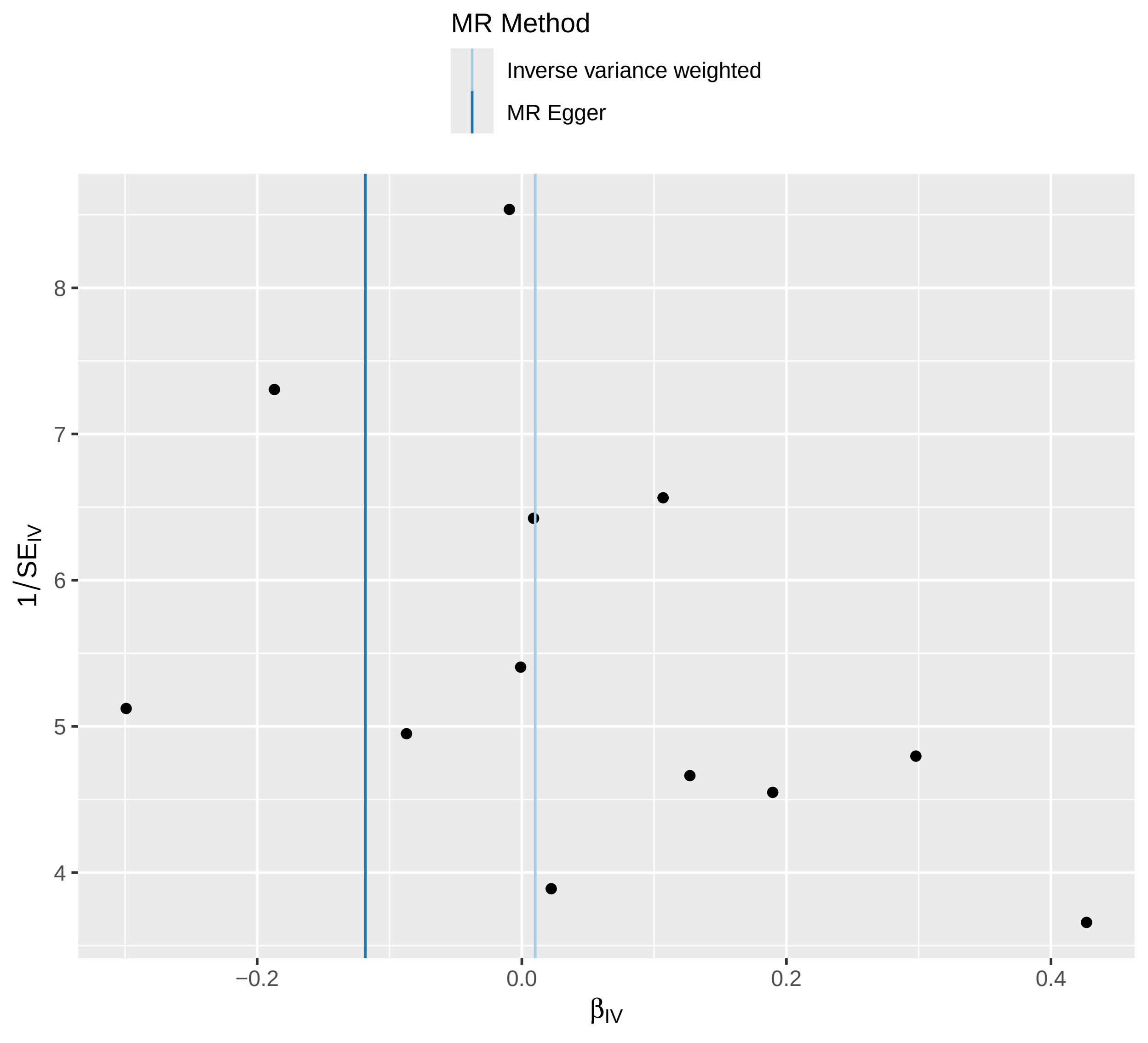
MR Method
Inverse variance weighted
MR Egger
8
7
6
5
4
1/SE_IV
−0.2
0.0
0.2
0.4
β_IV

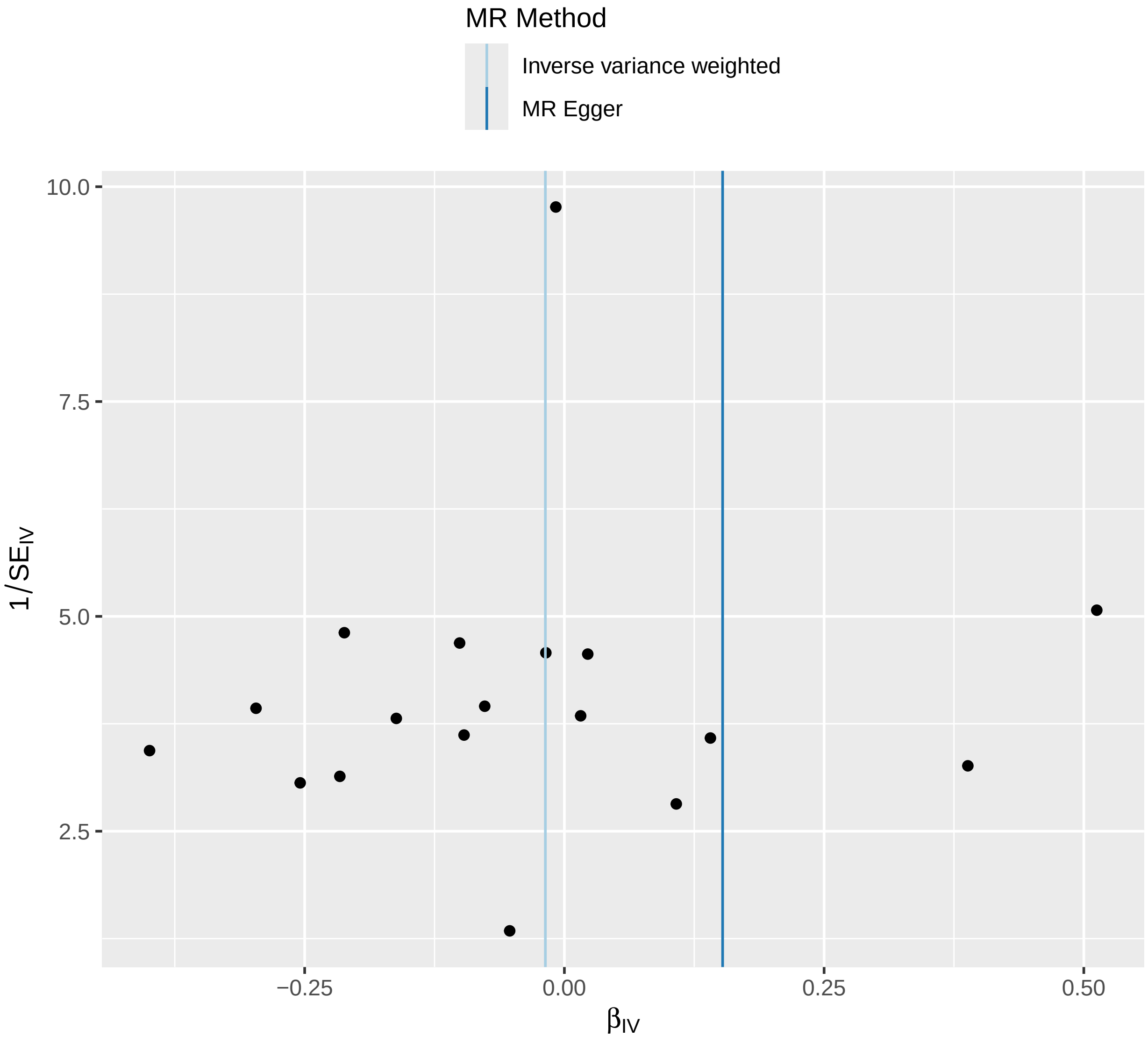
MR Method
Inverse variance weighted
MR Egger
10.0
7.5
5.0
2.5
1/SE_IV
−0.25
0.00
0.25
0.50
β_IV

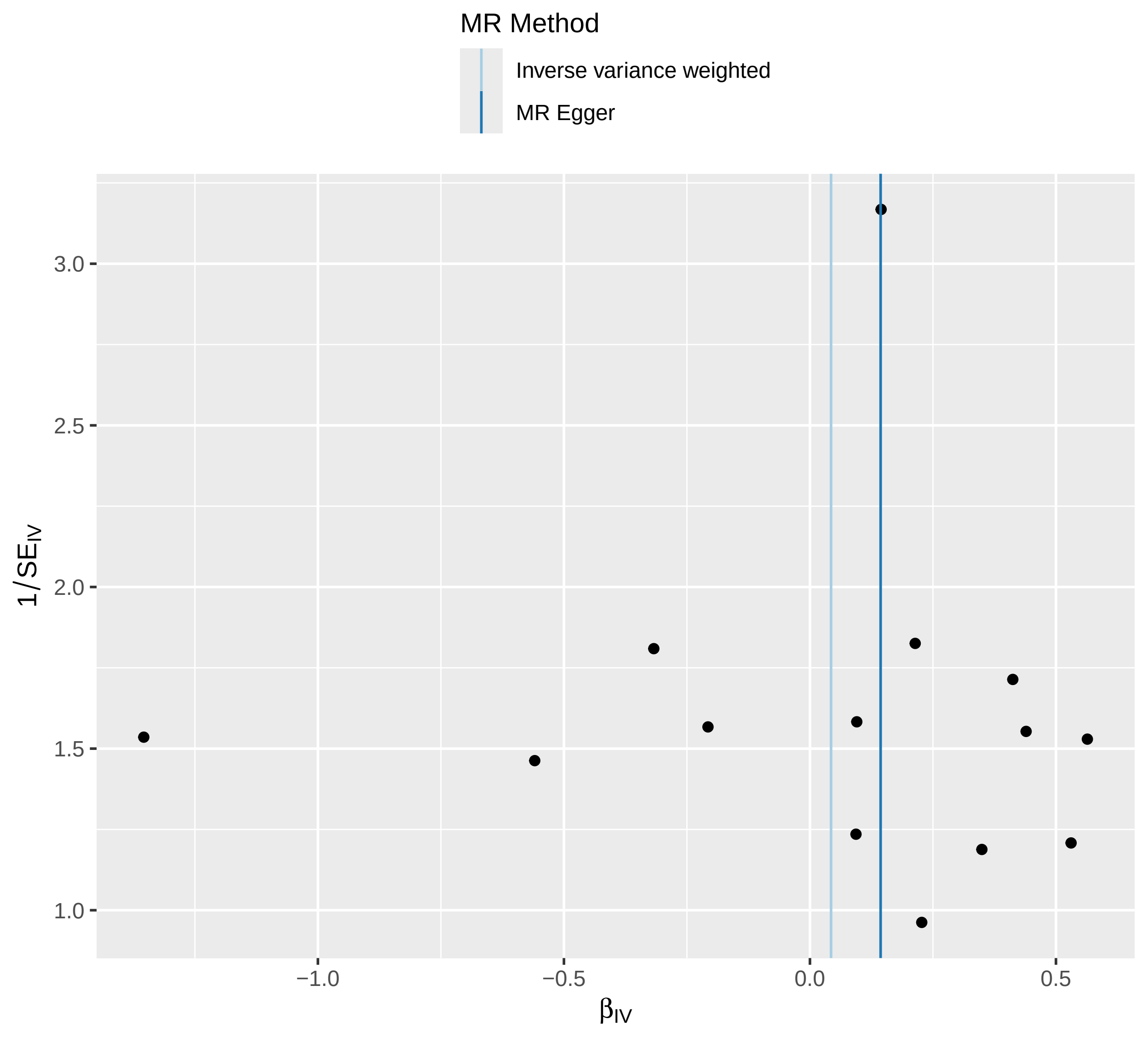
MR Method
Inverse variance weighted
MR Egger
1/SE_IV
3.0
2.5
2.0
1.5
1.0
−1.0
−0.5
0.0
0.5
β_IV

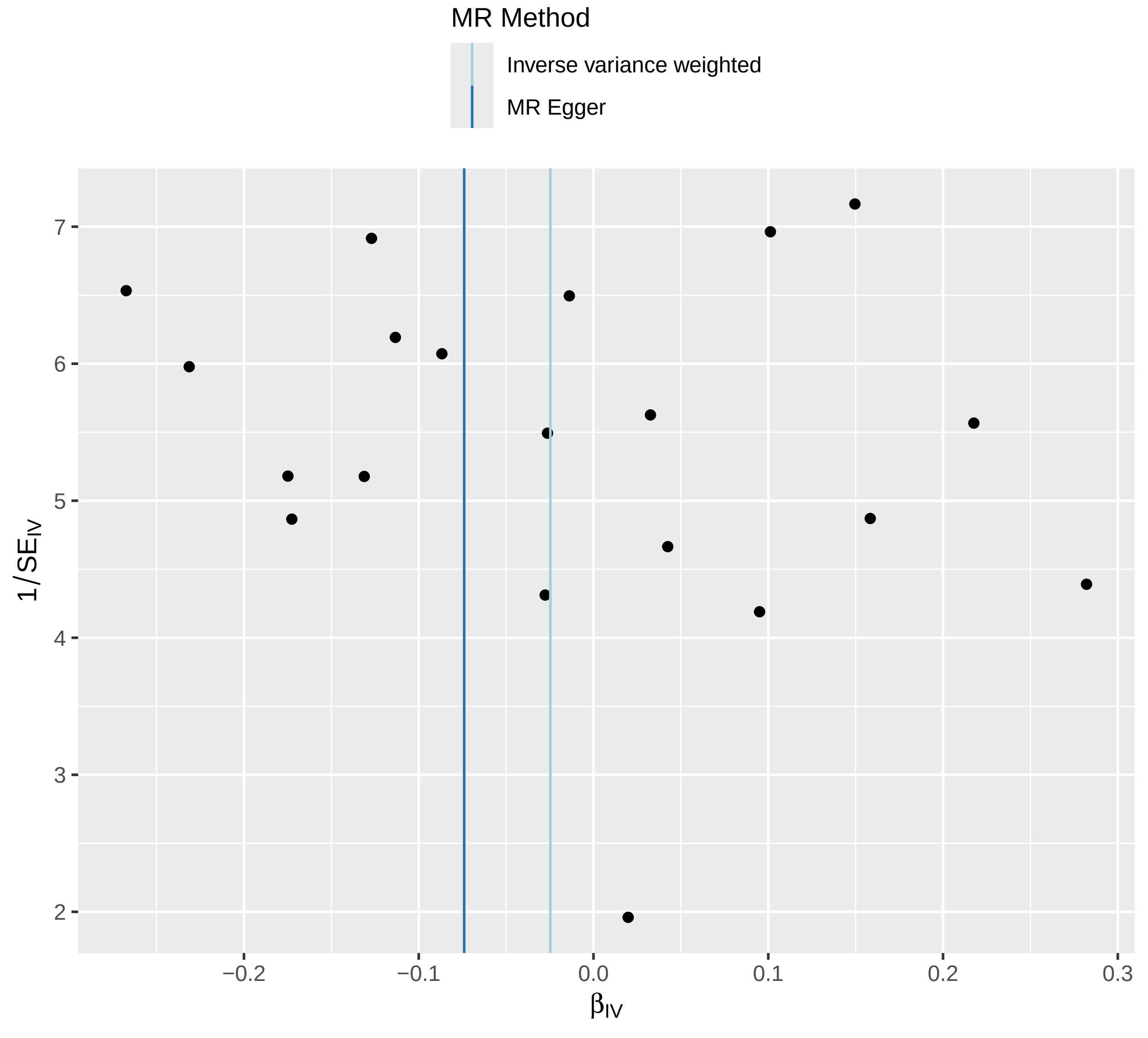

MR Method
Inverse variance weighted
MR Egger
1/SE_IV
β_IV
7
6
5
4
3
2
−0.2
−0.1
0.0
0.1
0.2
0.3

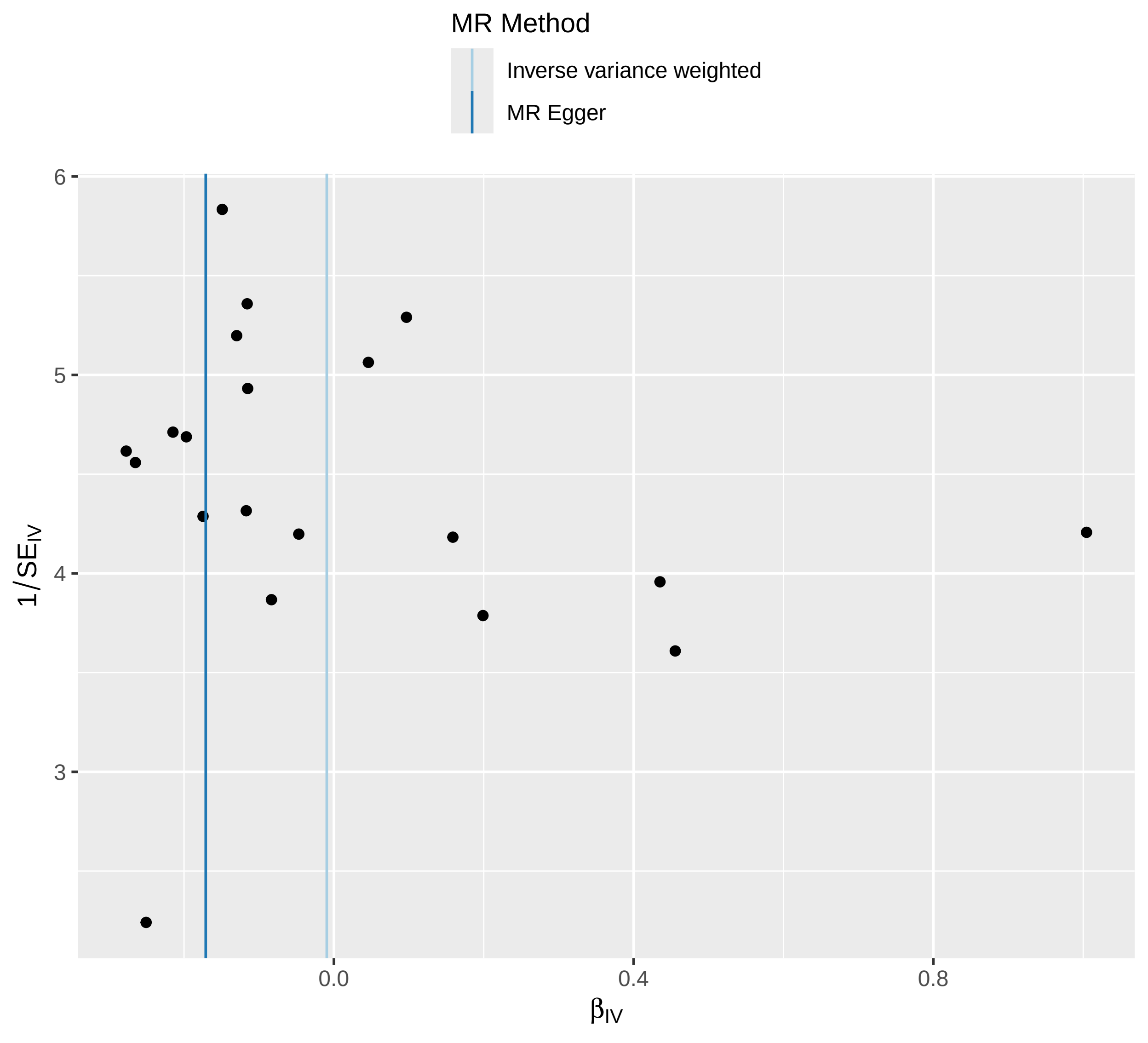

MR Method
Inverse variance weighted
MR Egger
6
5
4
3
1/SE_IV
0.0
0.4
0.8
β_IV

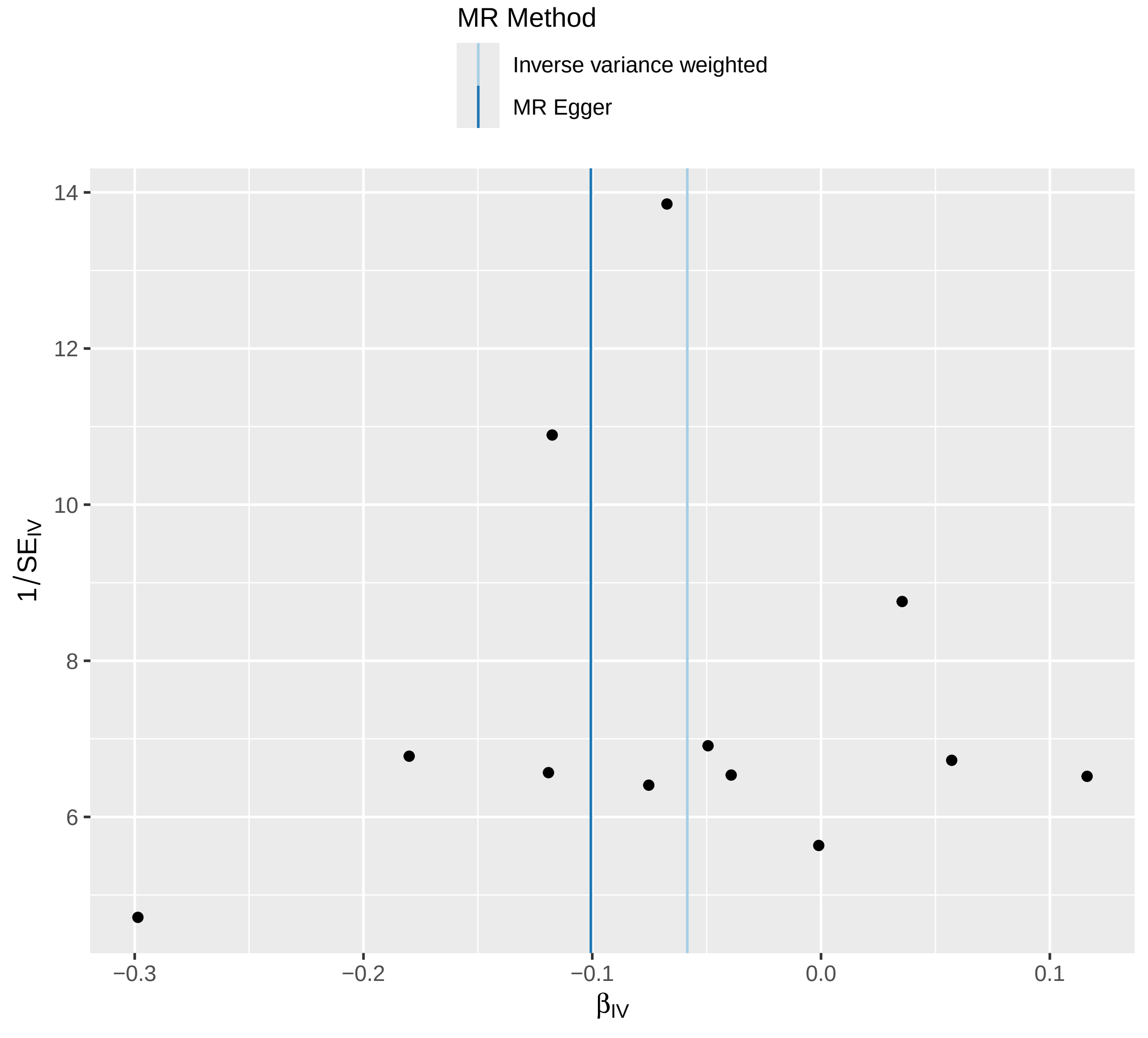
MR Method
Inverse variance weighted
MR Egger
14
12
10
8
6
1/SE_IV
−0.3
−0.2
−0.1
0.0
0.1
β_IV

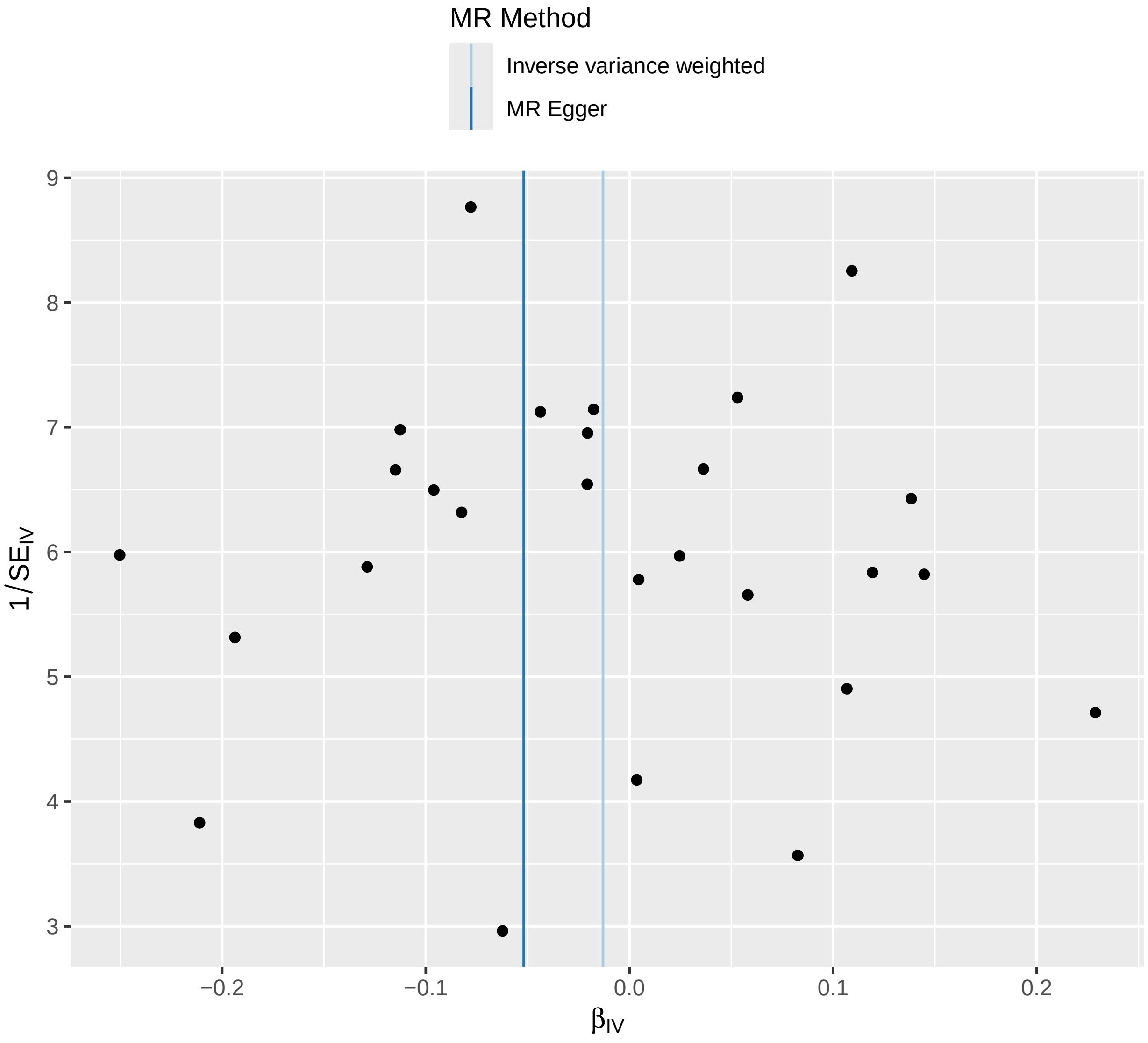
MR Method
Inverse variance weighted
MR Egger
9
8
7
6
5
4
3
1/SE$_{IV}$
−0.2
−0.1
0.0
0.1
0.2
β$_{IV}$

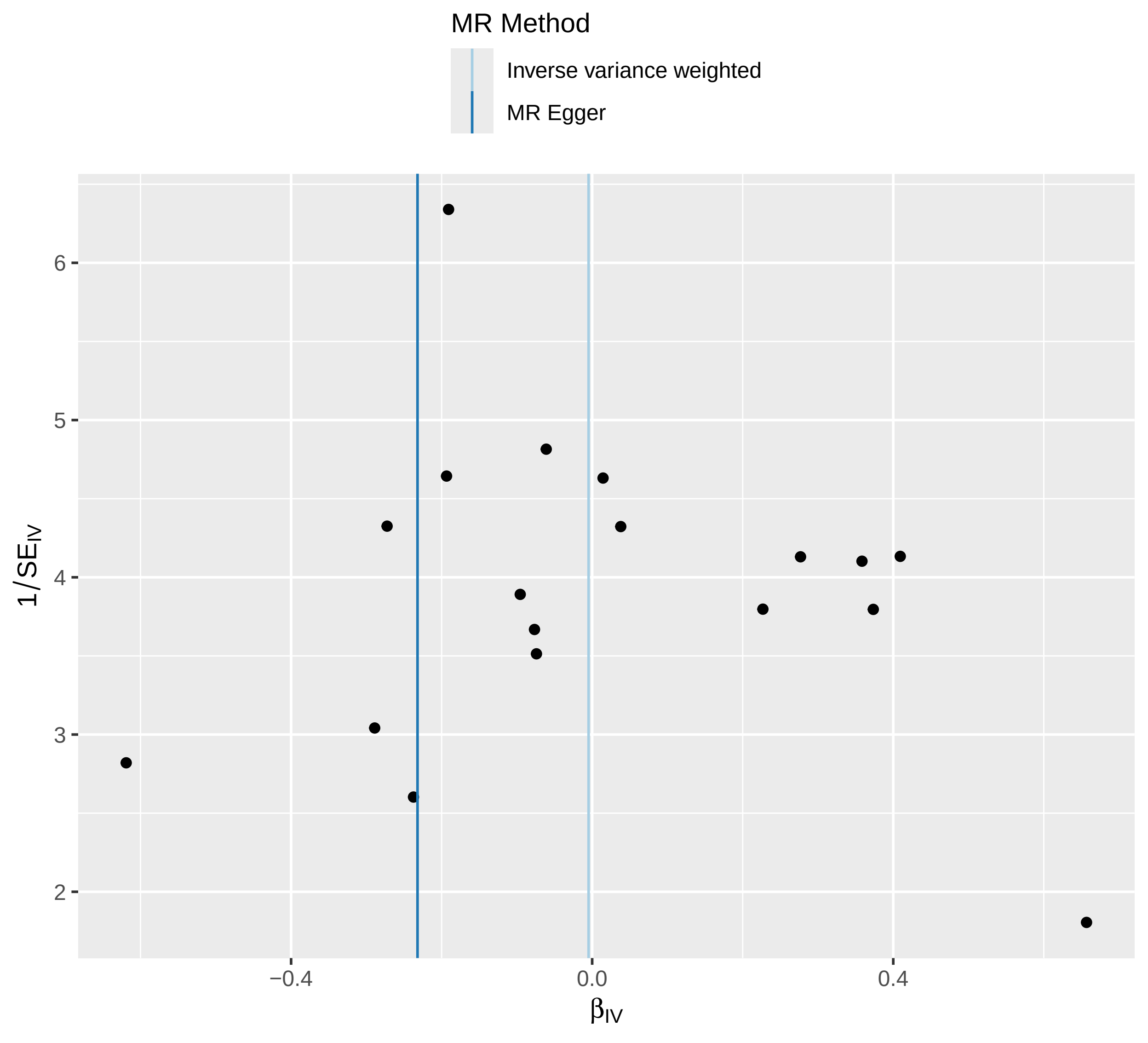
MR Method
Inverse variance weighted
MR Egger
6
5
4
3
2
1/SE_IV
−0.4
0.0
0.4
β_IV

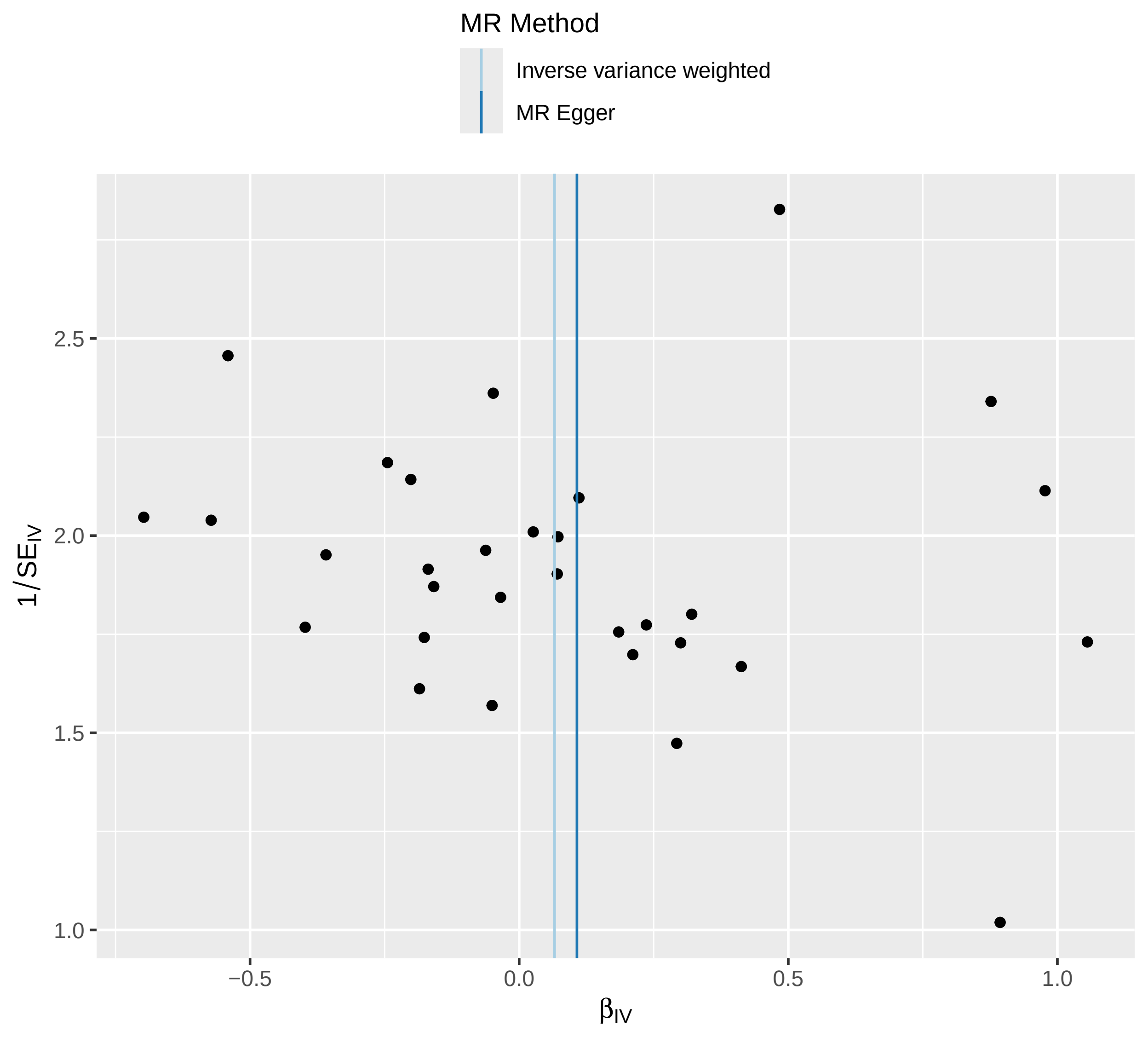
MR Method
Inverse variance weighted
MR Egger
2.5
2.0
1.5
1.0
1/SE_IV
−0.5
0.0
0.5
1.0
β_IV

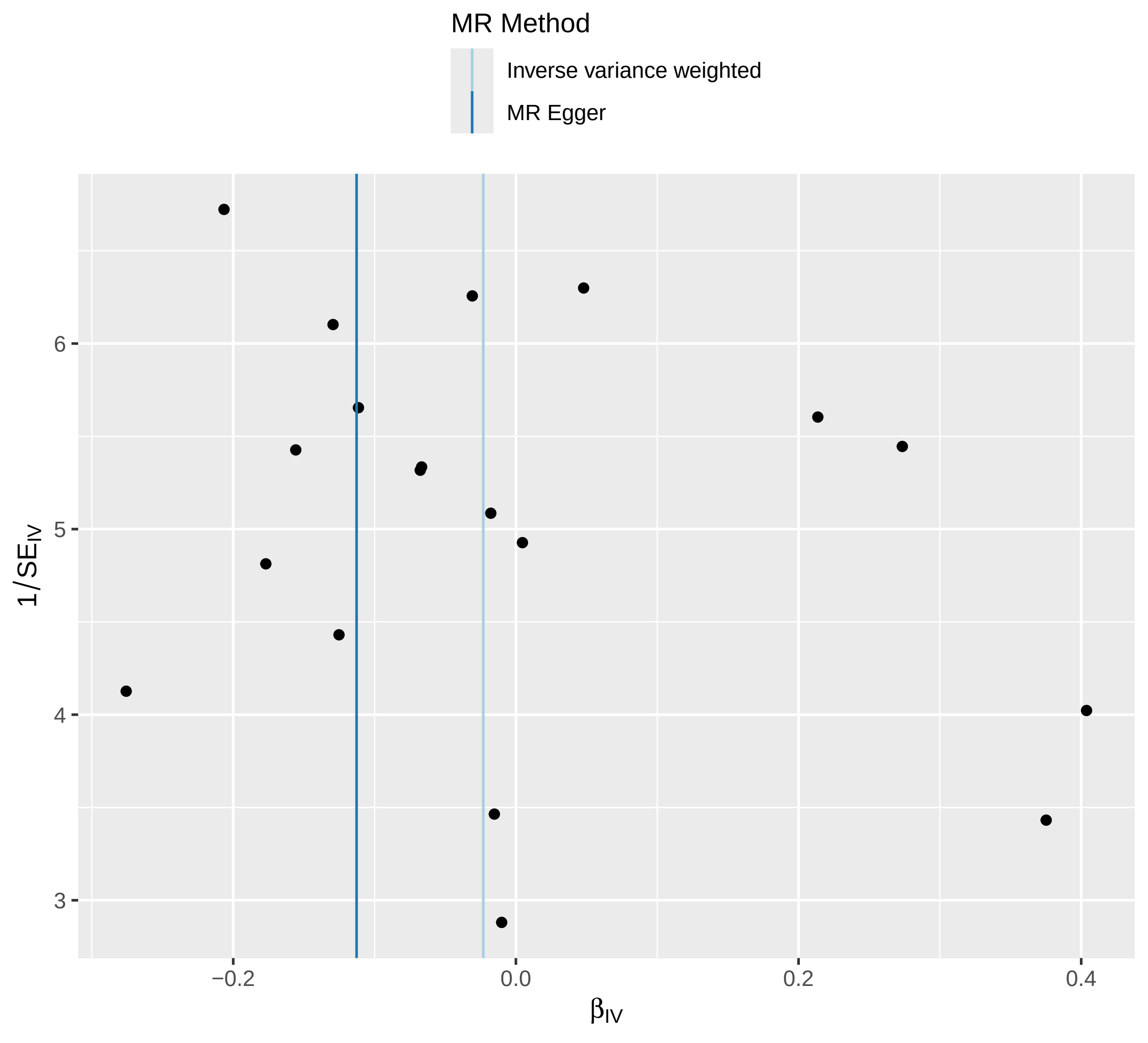

MR Method
Inverse variance weighted
MR Egger
1/SE_IV
6
5
4
3
−0.2
0.0
0.2
0.4
β_IV

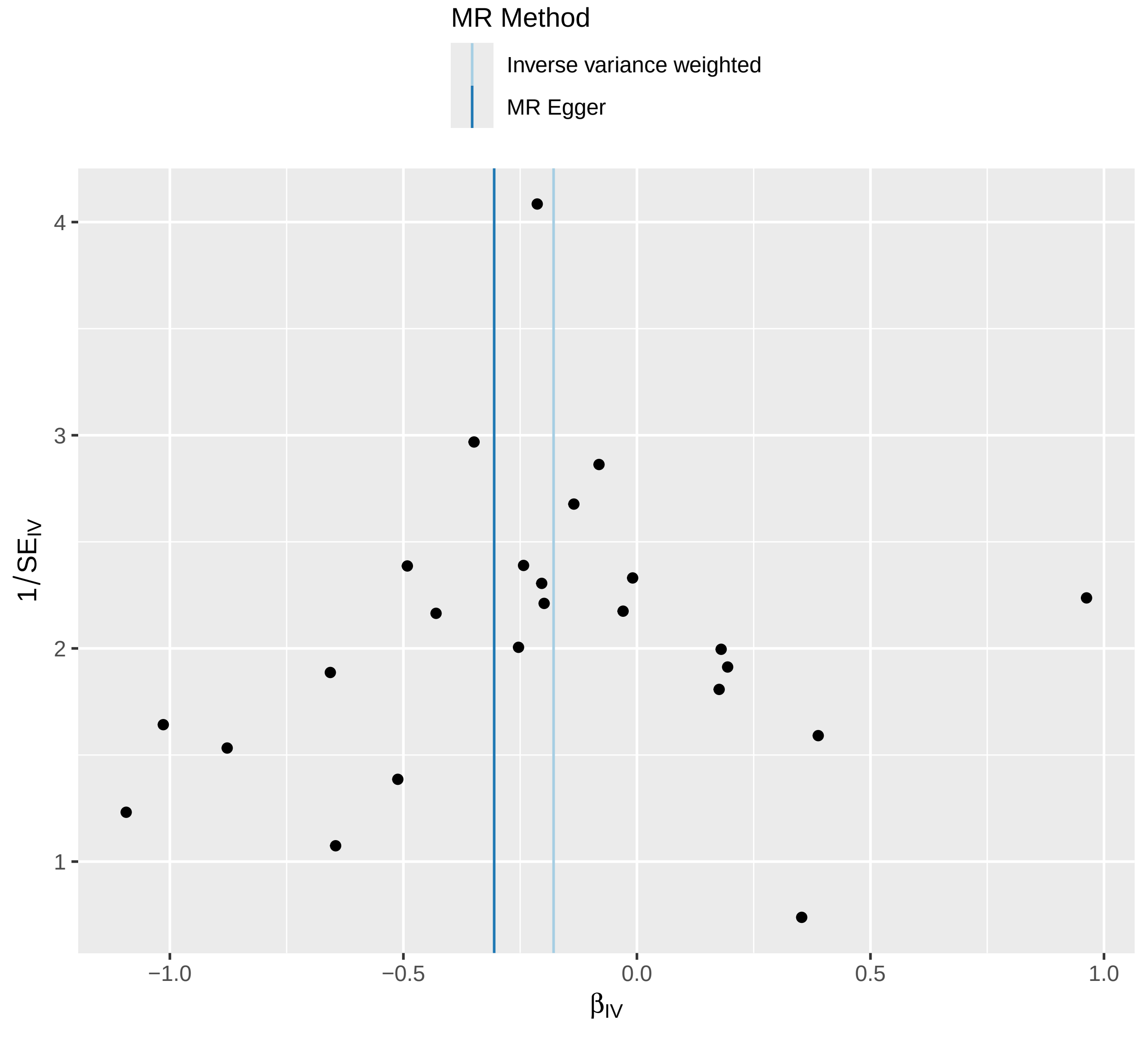

MR Method
Inverse variance weighted
MR Egger
4
3
2
1
1/SE_IV
−1.0
−0.5
0.0
0.5
1.0
β_IV

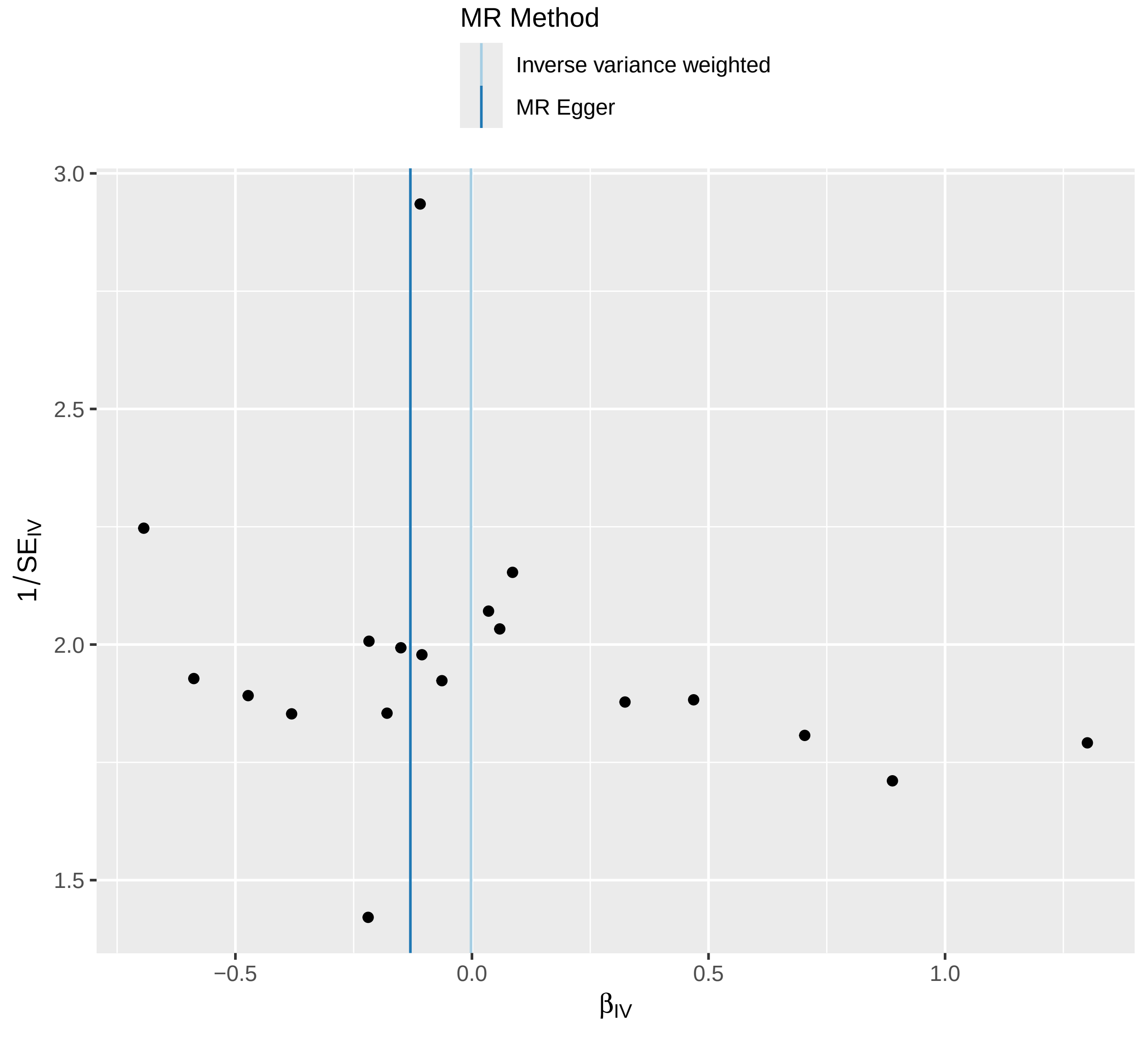
MR Method
Inverse variance weighted
MR Egger
3.0
2.5
2.0
1.5
−0.5
0.0
0.5
1.0
1/SE_IV
β_IV

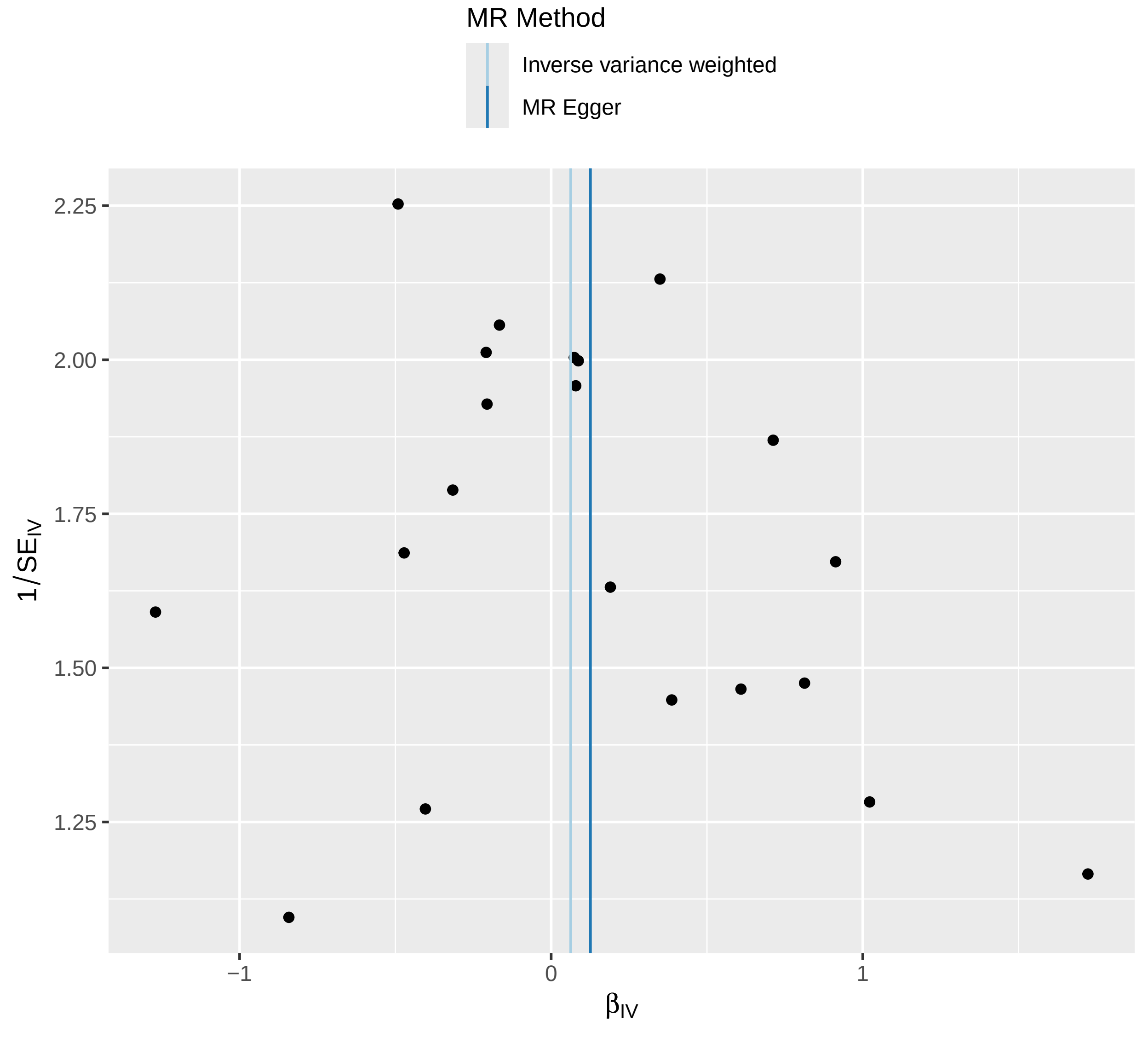

MR Method
Inverse variance weighted
MR Egger
2.25
2.00
1.75
1.50
1.25
1/SE$_{IV}$
−1
0
1
β$_{IV}$

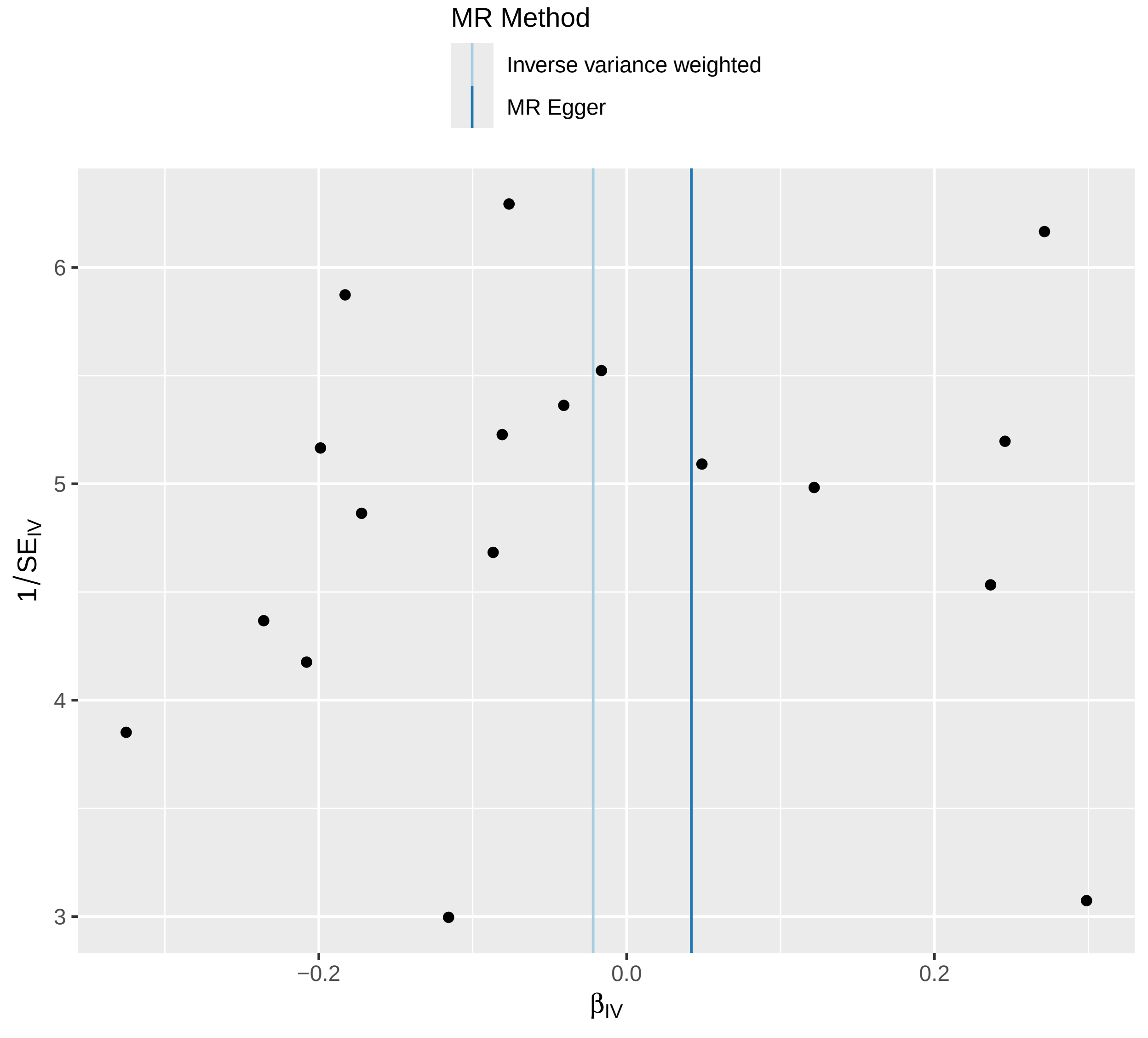
MR Method
Inverse variance weighted
MR Egger
6
5
4
3
1/SE$_{IV}$
−0.2
0.0
0.2
β$_{IV}$

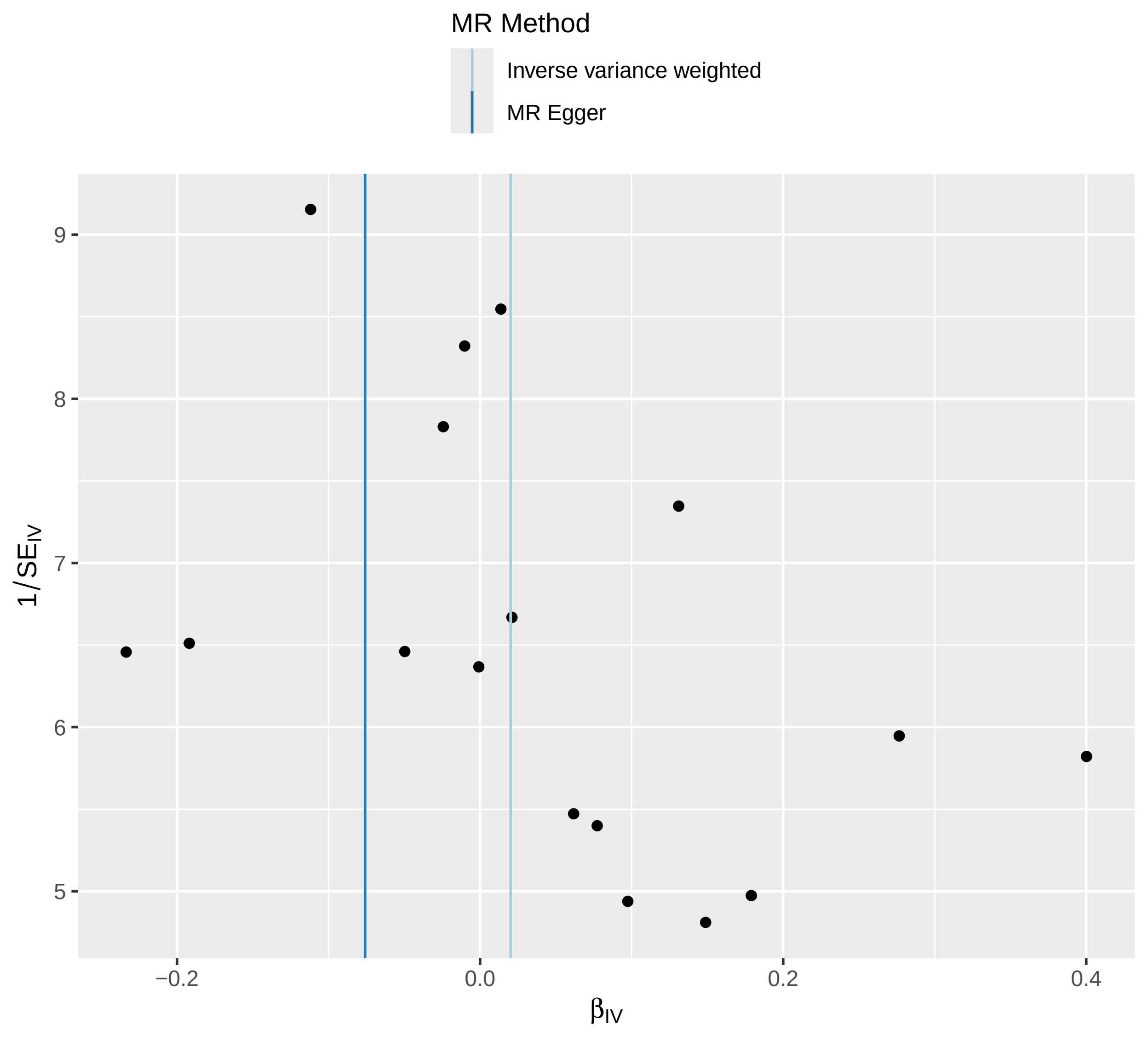

MR Method
Inverse variance weighted
MR Egger
9
8
7
6
5
1/SE_IV
−0.2
0.0
0.2
0.4
β_IV

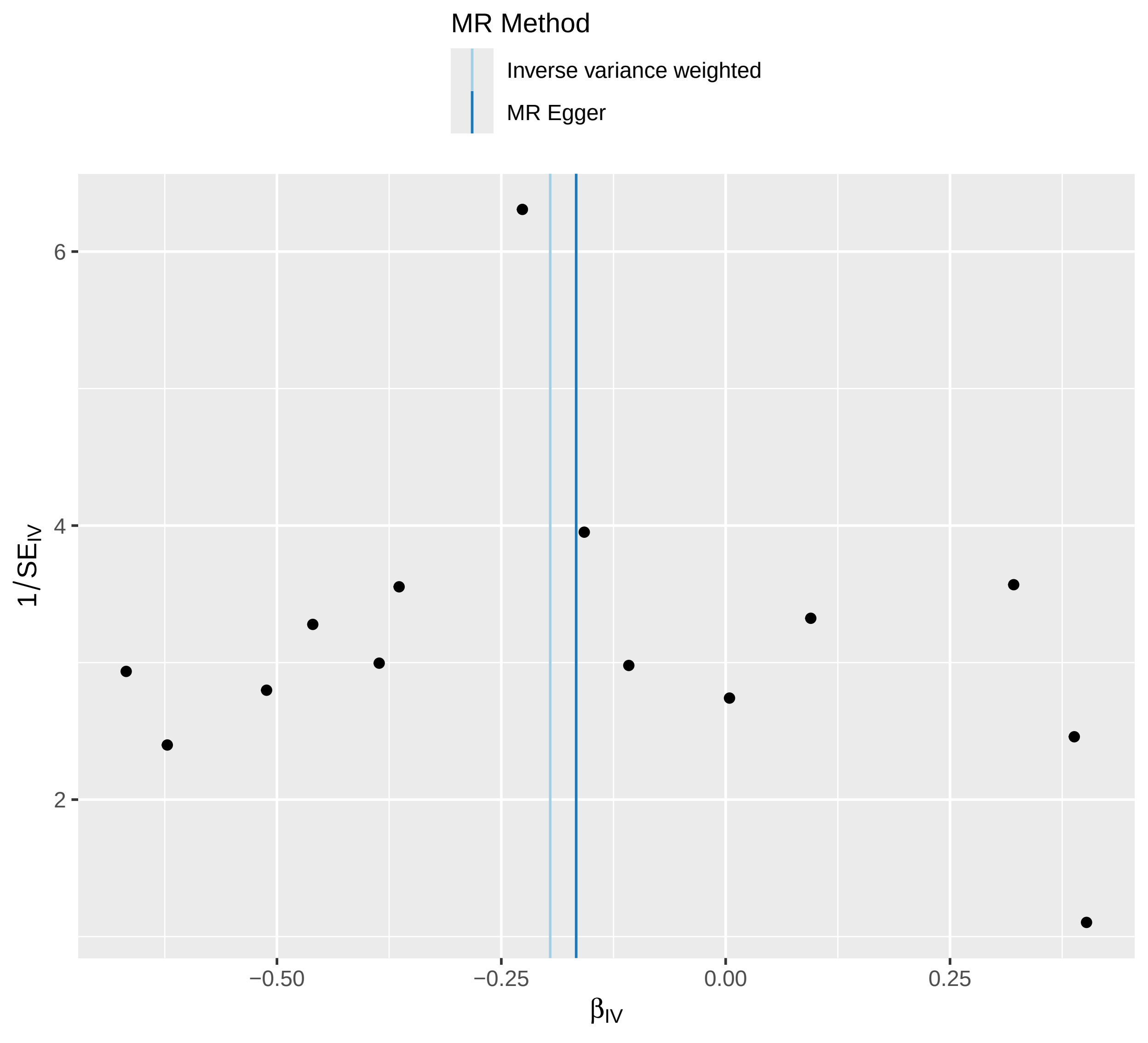

MR Method
Inverse variance weighted
MR Egger
6
4
2
1/SE$_{IV}$
−0.50
−0.25
0.00
0.25
β$_{IV}$

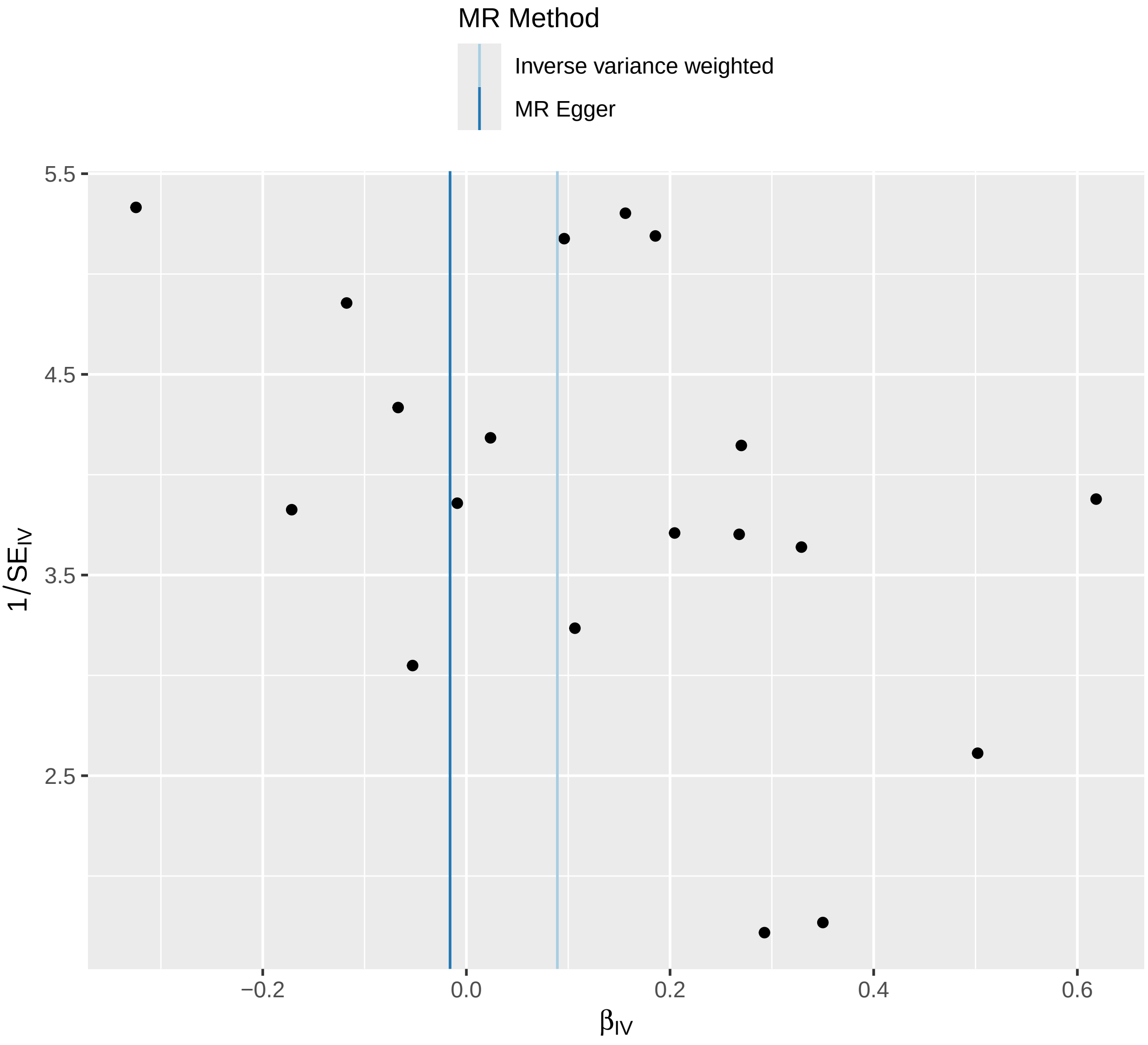
MR Method
Inverse variance weighted
MR Egger
5.5
4.5
3.5
2.5
1/SE_IV
−0.2
0.0
0.2
0.4
0.6
β_IV

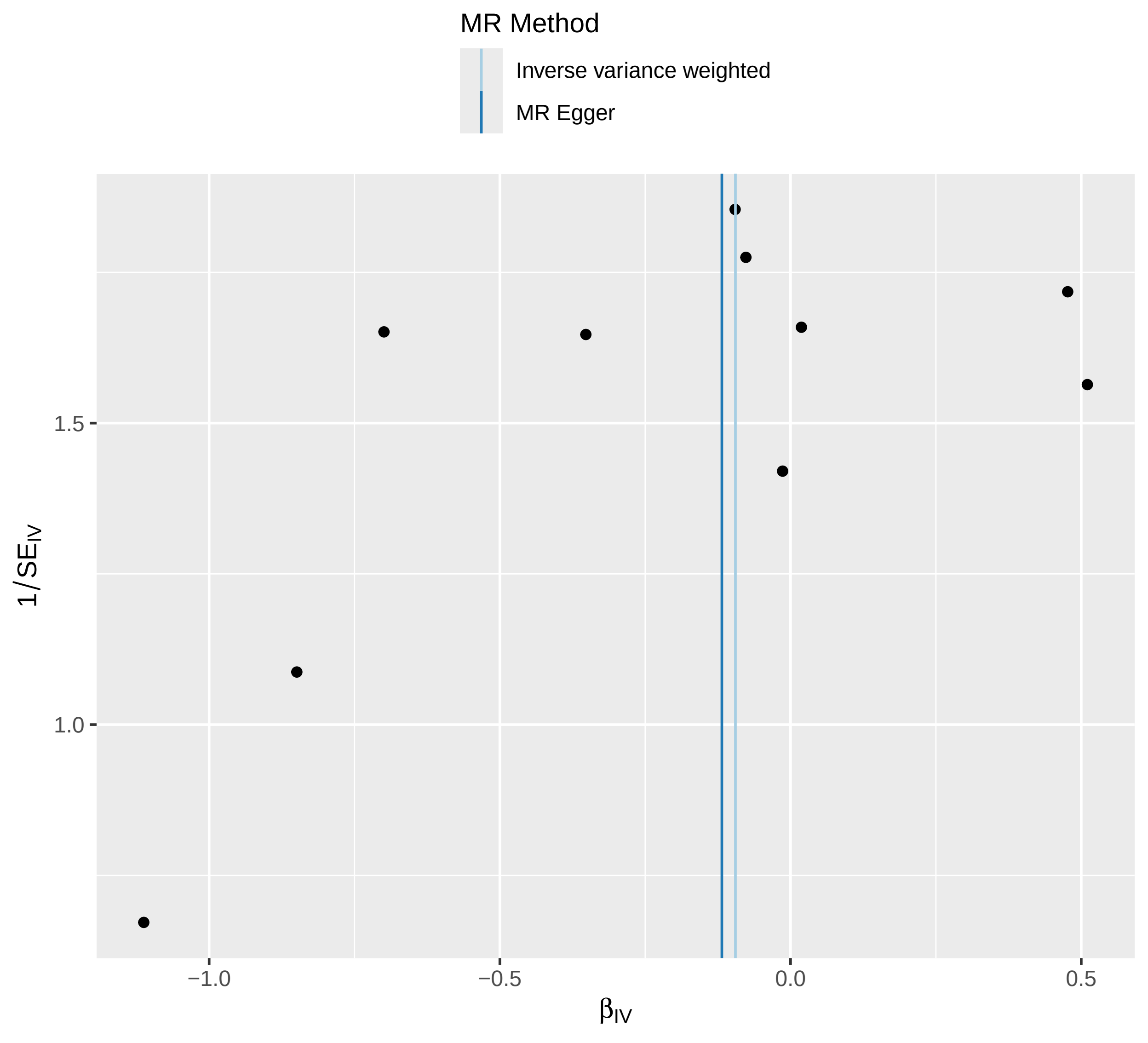

MR Method
Inverse variance weighted
MR Egger
1.5
1.0
$1/SE_{IV}$
−1.0
−0.5
0.0
0.5
β_{IV}

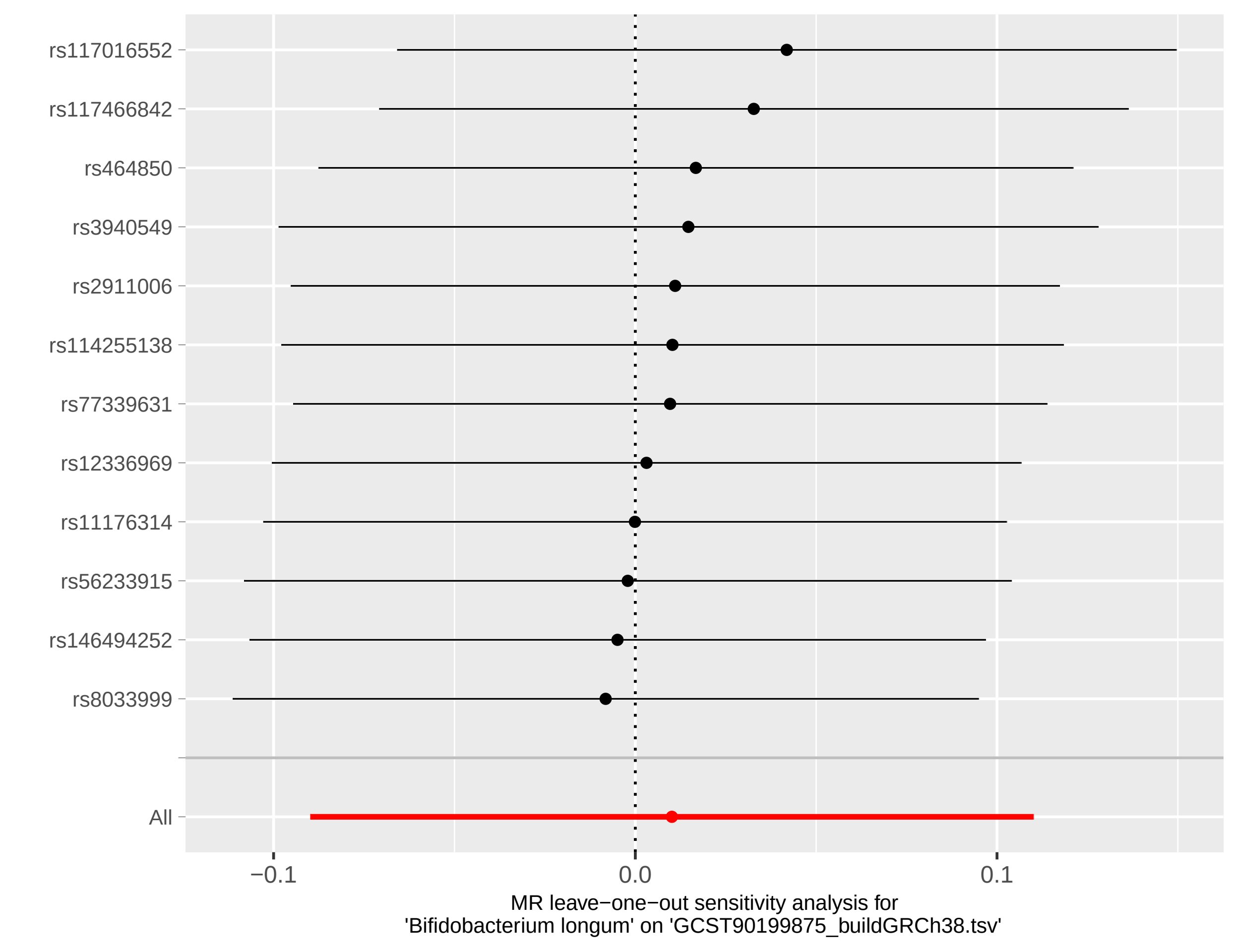

rs117016552
rs117466842
rs464850
rs3940549
rs2911006
rs114255138
rs77339631
rs12336969
rs11176314
rs56233915
rs146494252
rs8033999
All
−0.1
0.0
0.1
MR leave−one−out sensitivity analysis for
'Bifidobacterium longum' on 'GCST90199875_buildGRCh38.tsv'

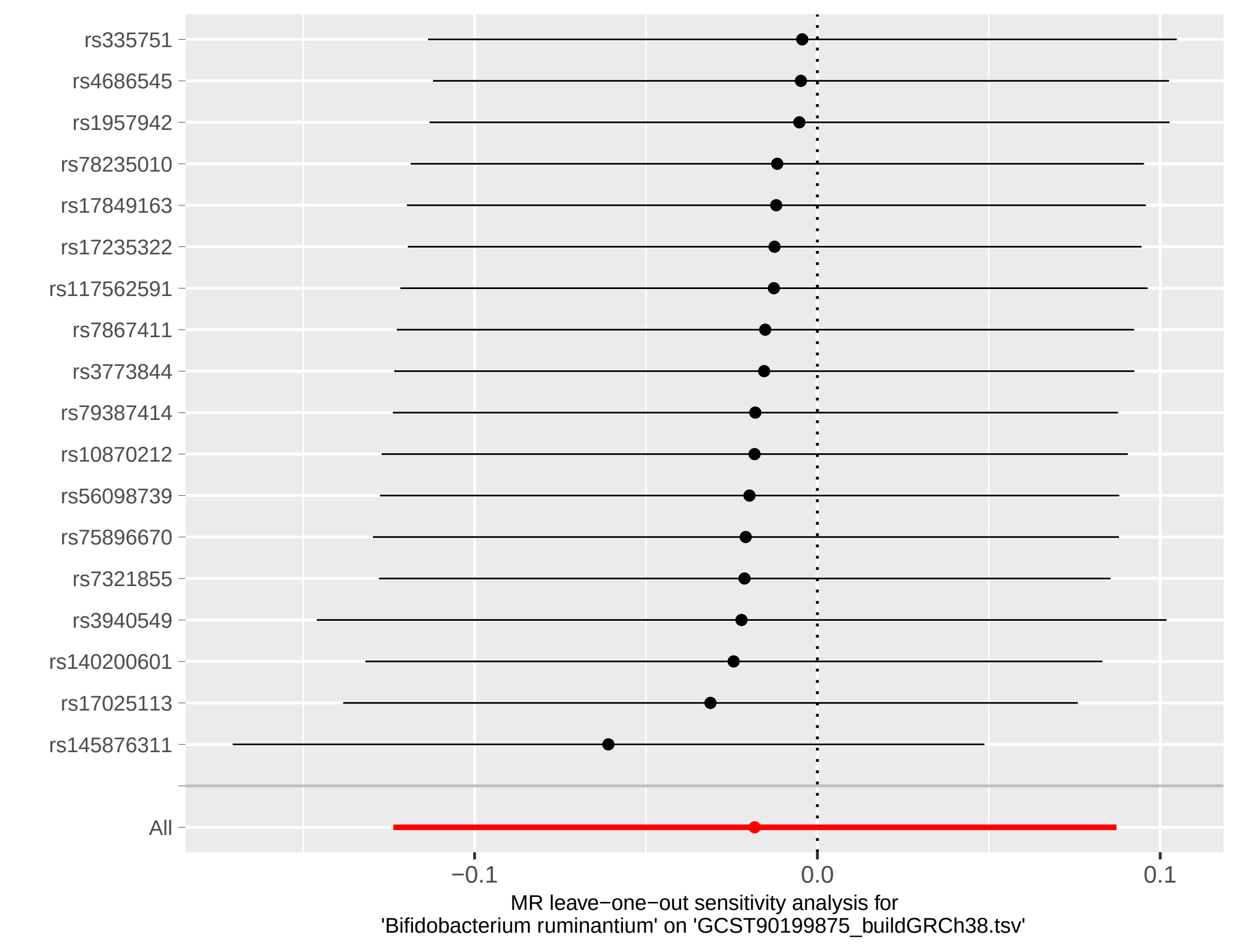

rs335751
rs4686545
rs1957942
rs78235010
rs17849163
rs17235322
rs117562591
rs7867411
rs3773844
rs79387414
rs10870212
rs56098739
rs75896670
rs7321855
rs3940549
rs140200601
rs17025113
rs145876311
All
−0.1
0.0
0.1
MR leave−one−out sensitivity analysis for
'Bifidobacterium ruminantium' on 'GCST90199875_buildGRCh38.tsv'

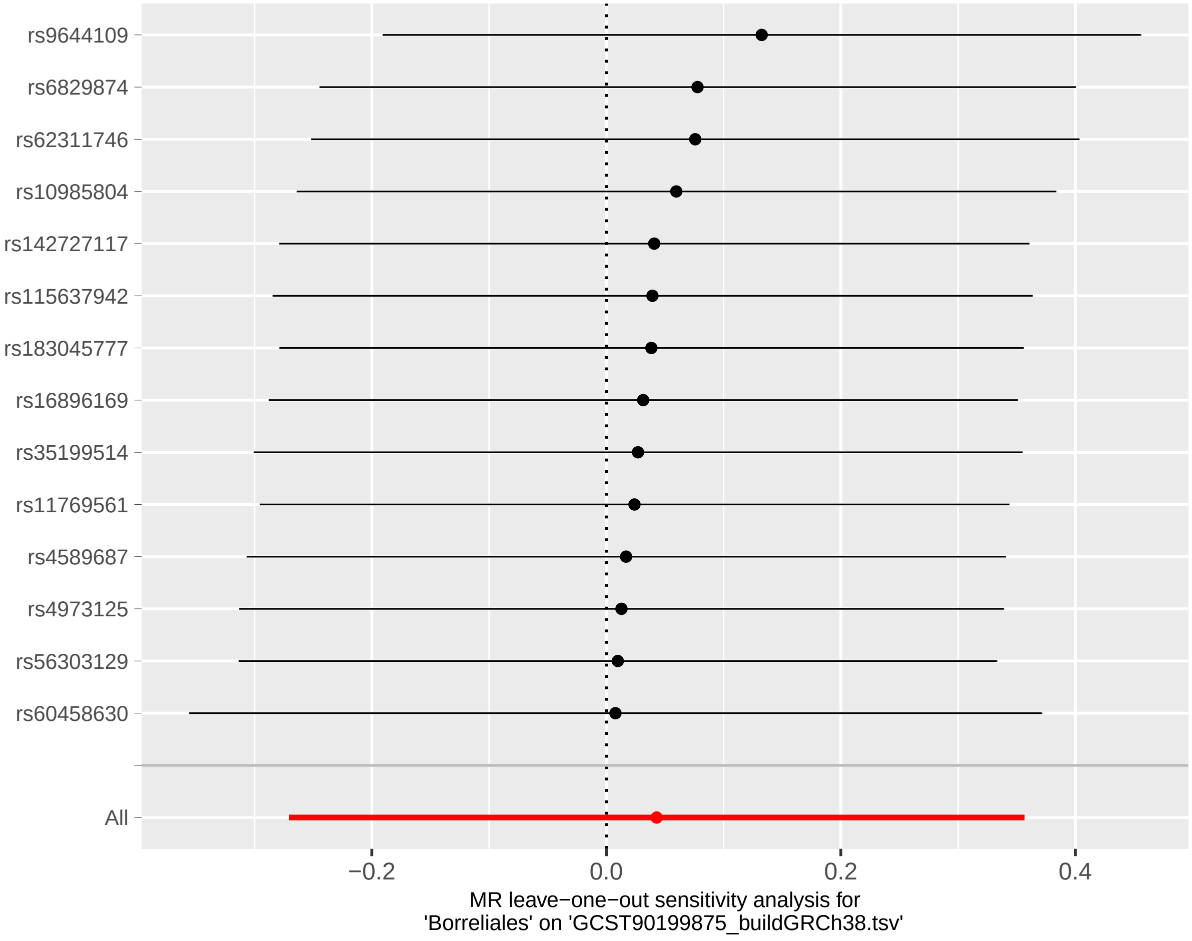
rs9644109
rs6829874
rs62311746
rs10985804
rs142727117
rs115637942
rs183045777
rs16896169
rs35199514
rs11769561
rs4589687
rs4973125
rs56303129
rs60458630
All
−0.2
0.0
0.2
0.4
MR leave−one−out sensitivity analysis for
'Borreliales' on 'GCST90199875_buildGRCh38.tsv'

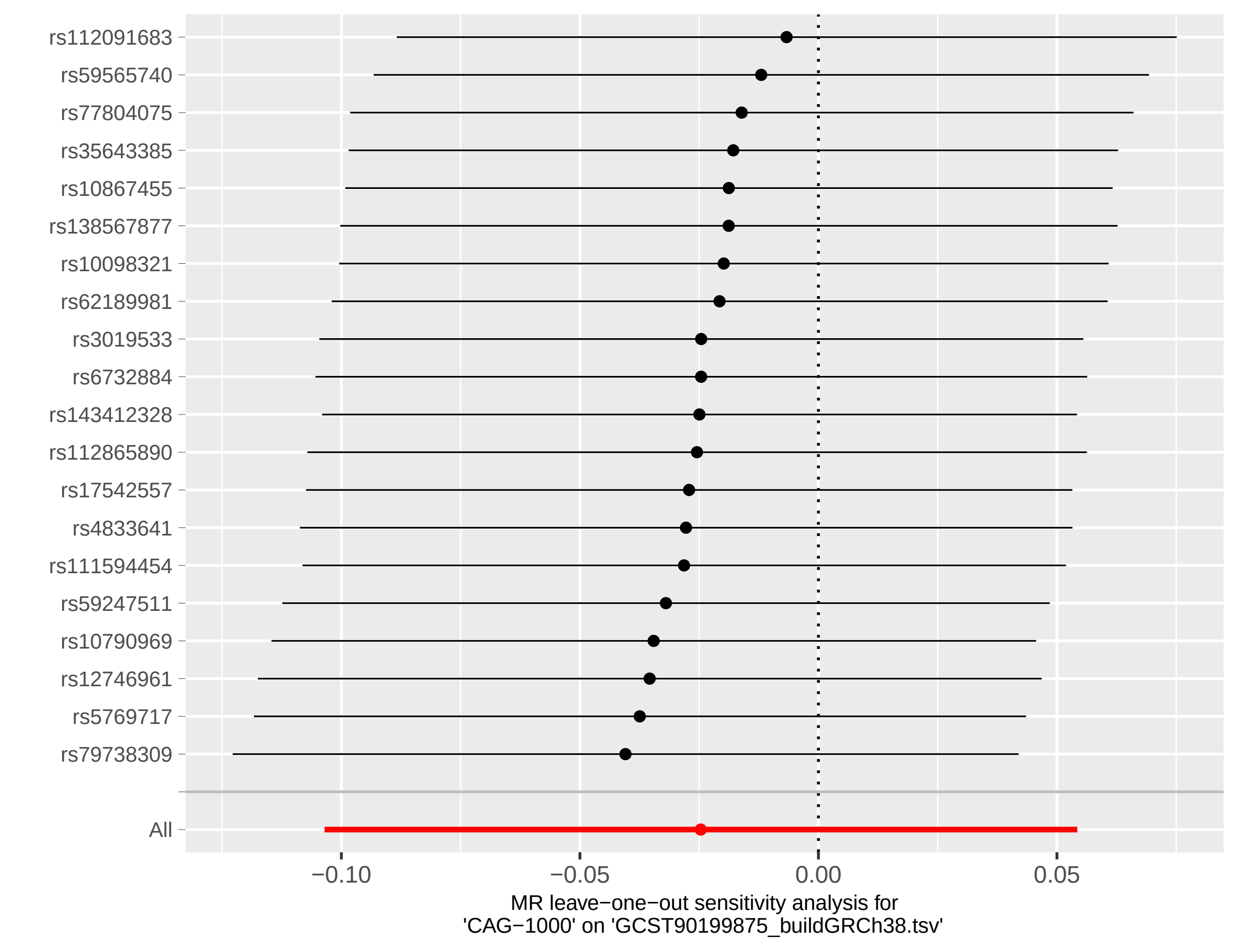

rs112091683
rs59565740
rs77804075
rs35643385
rs10867455
rs138567877
rs10098321
rs62189981
rs3019533
rs6732884
rs143412328
rs112865890
rs17542557
rs4833641
rs111594454
rs59247511
rs10790969
rs12746961
rs5769717
rs79738309
All
−0.10
−0.05
0.00
0.05
MR leave−one−out sensitivity analysis for
'CAG−1000' on 'GCST90199875_buildGRCh38.tsv'

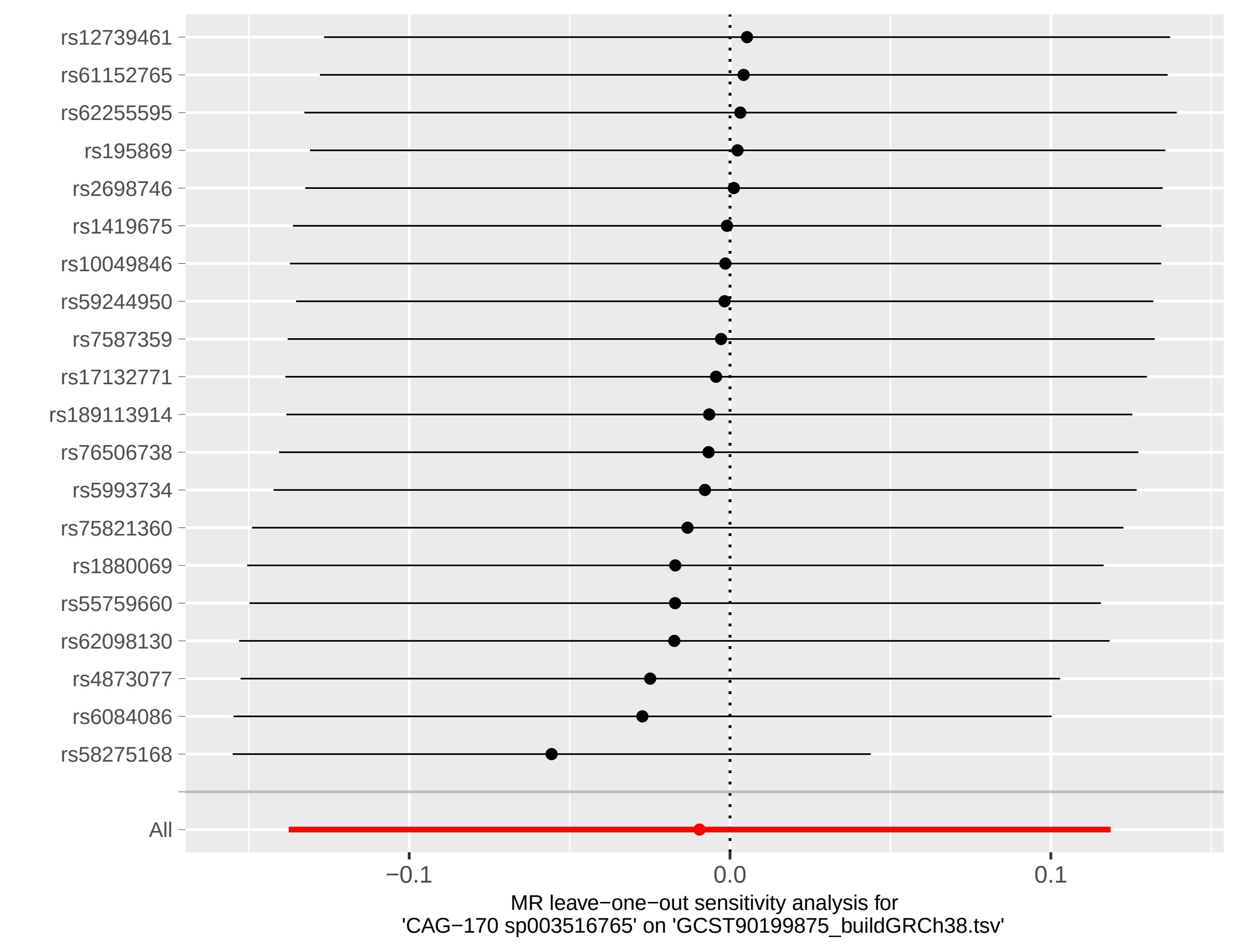
rs12739461
rs61152765
rs62255595
rs195869
rs2698746
rs1419675
rs10049846
rs59244950
rs7587359
rs17132771
rs189113914
rs76506738
rs5993734
rs75821360
rs1880069
rs55759660
rs62098130
rs4873077
rs6084086
rs58275168
All
−0.1
0.0
0.1
MR leave−one−out sensitivity analysis for
'CAG−170 sp003516765' on 'GCST90199875_buildGRCh38.tsv'

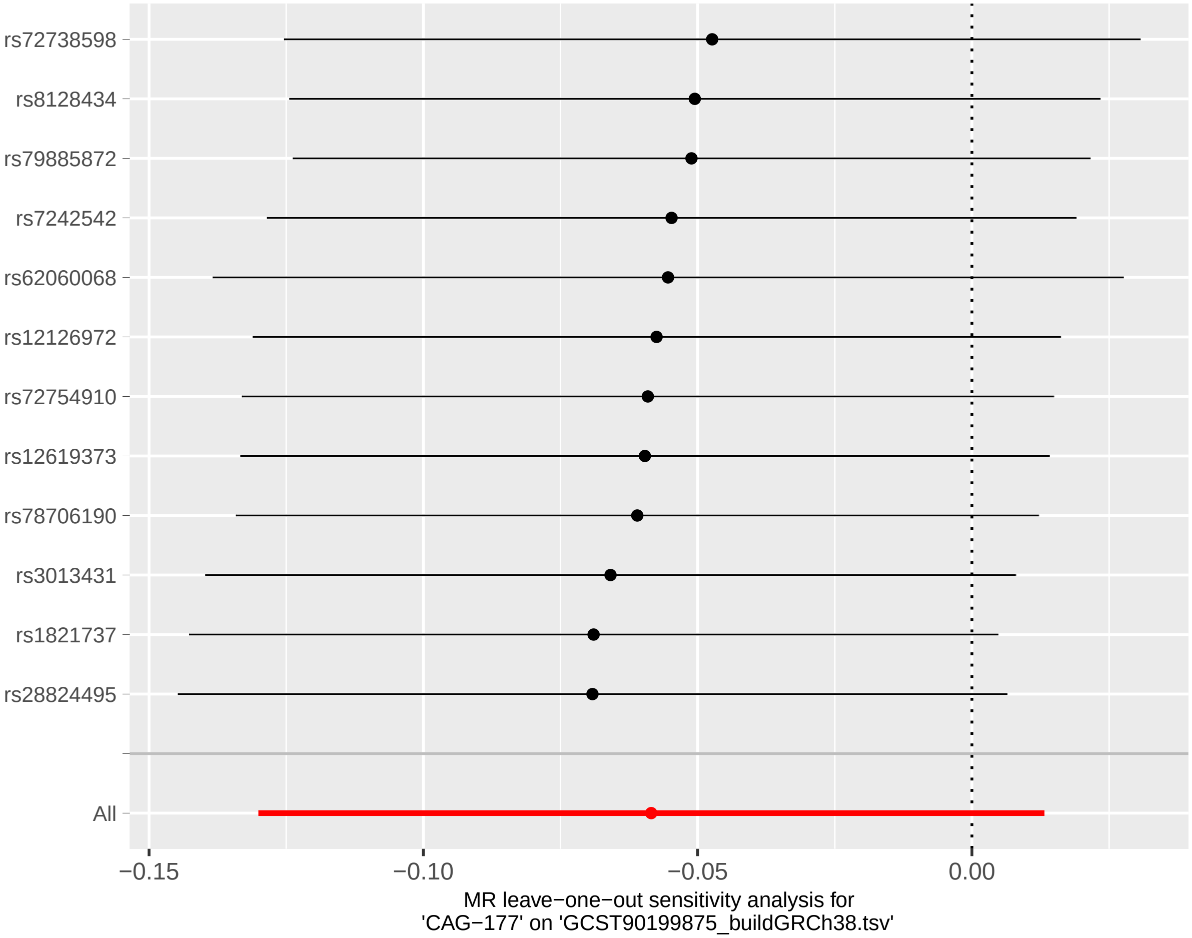
rs72738598
rs8128434
rs79885872
rs7242542
rs62060068
rs12126972
rs72754910
rs12619373
rs78706190
rs3013431
rs1821737
rs28824495
All
−0.15
−0.10
−0.05
0.00
MR leave−one−out sensitivity analysis for
'CAG−177' on 'GCST90199875_buildGRCh38.tsv'

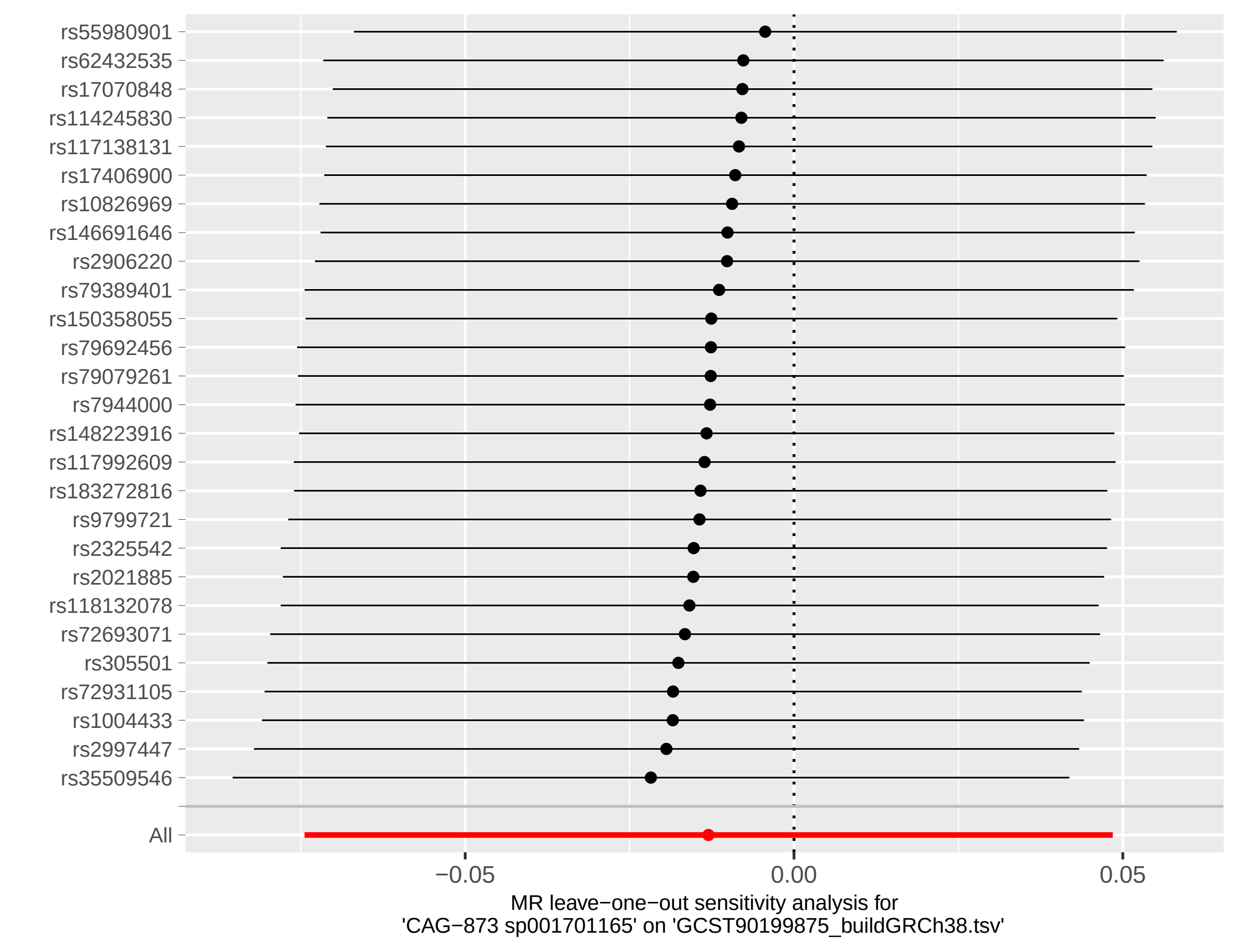
rs55980901
rs62432535
rs17070848
rs114245830
rs117138131
rs17406900
rs10826969
rs146691646
rs2906220
rs79389401
rs150358055
rs79692456
rs79079261
rs7944000
rs148223916
rs117992609
rs183272816
rs9799721
rs2325542
rs2021885
rs118132078
rs72693071
rs305501
rs72931105
rs1004433
rs2997447
rs35509546
All
−0.05
0.00
0.05
MR leave−one−out sensitivity analysis for
'CAG−873 sp001701165' on 'GCST90199875_buildGRCh38.tsv'

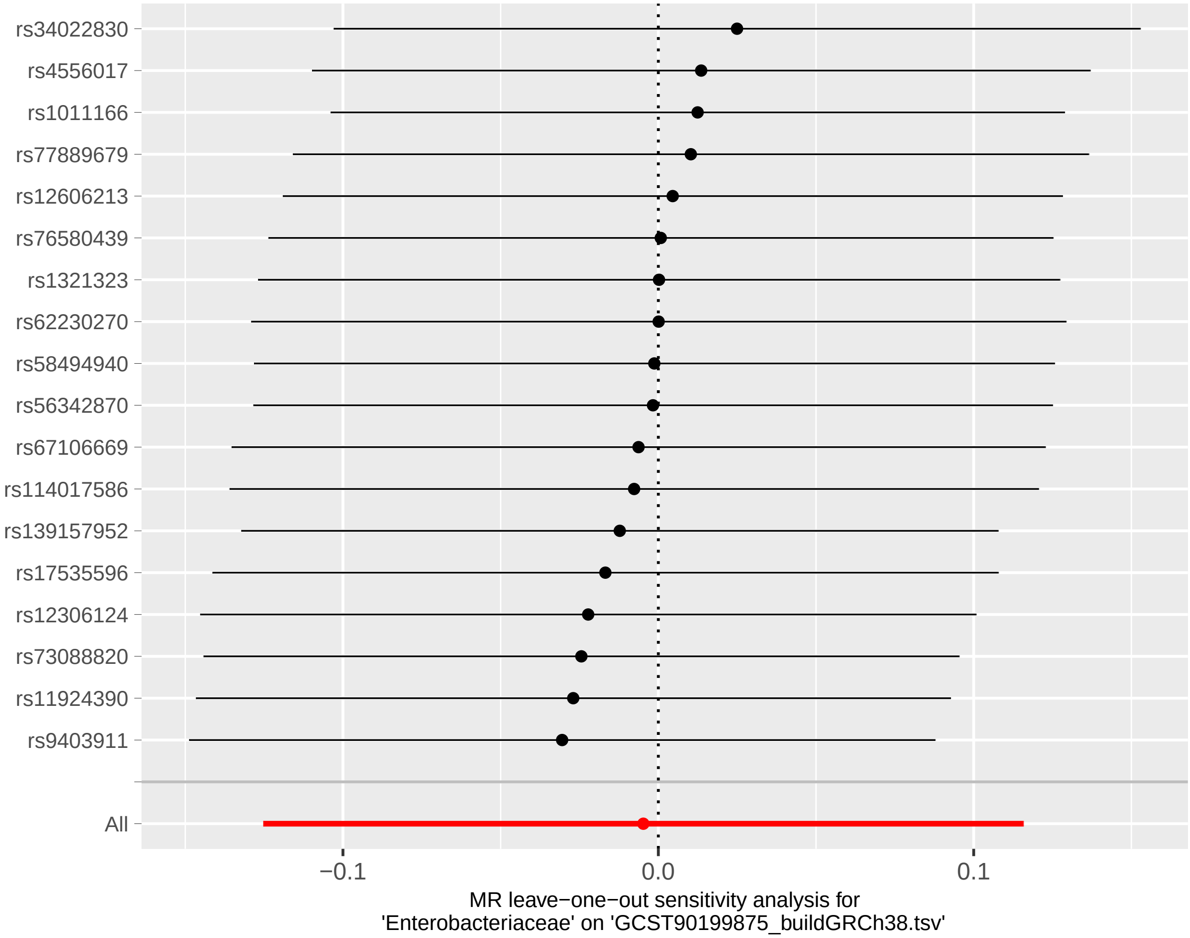

rs34022830
rs4556017
rs1011166
rs77889679
rs12606213
rs76580439
rs1321323
rs62230270
rs58494940
rs56342870
rs67106669
rs114017586
rs139157952
rs17535596
rs12306124
rs73088820
rs11924390
rs9403911
All
−0.1
0.0
0.1
MR leave−one−out sensitivity analysis for
'Enterobacteriaceae' on 'GCST90199875_buildGRCh38.tsv'

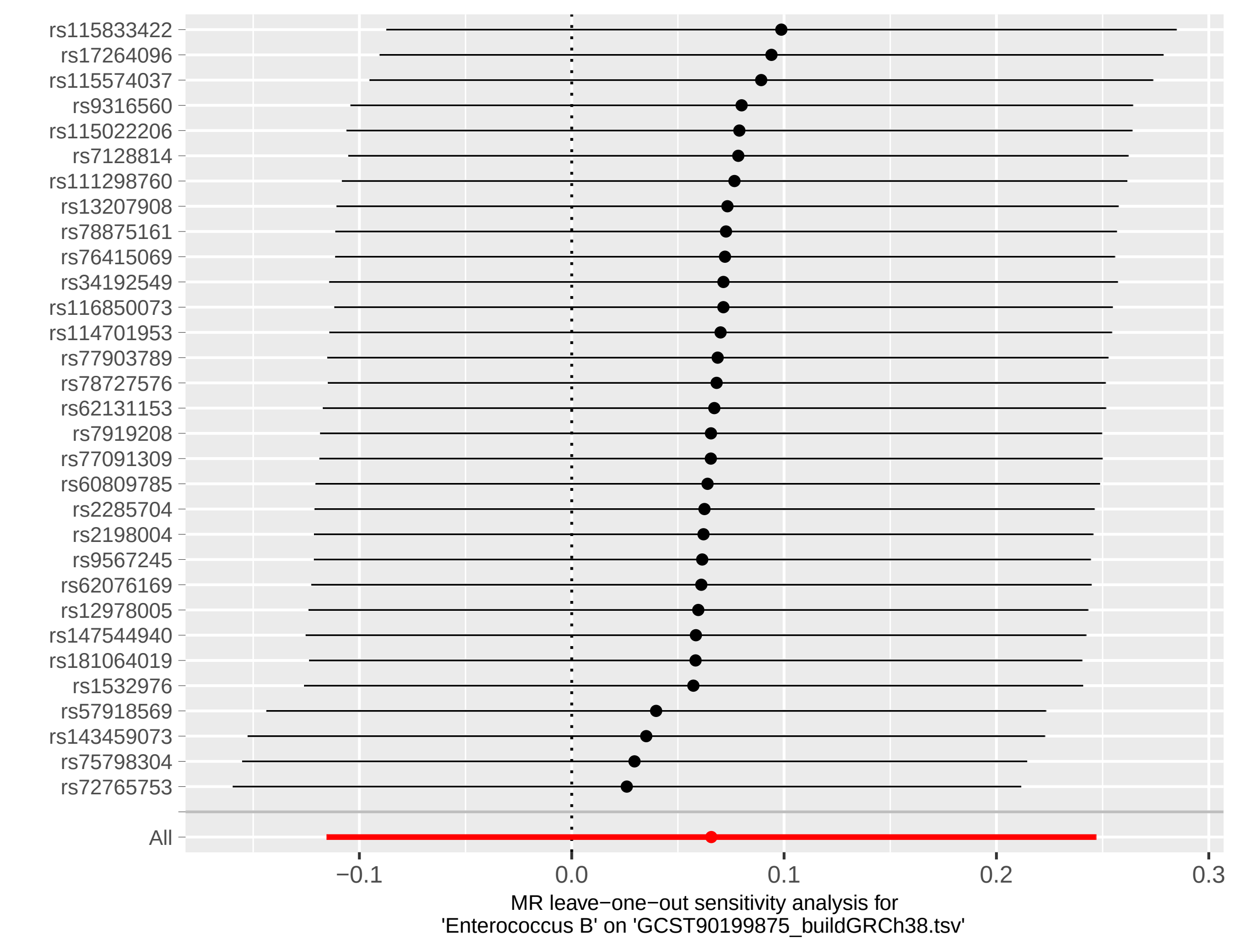
rs115833422
rs17264096
rs115574037
rs9316560
rs115022206
rs7128814
rs111298760
rs13207908
rs78875161
rs76415069
rs34192549
rs116850073
rs114701953
rs77903789
rs78727576
rs62131153
rs7919208
rs77091309
rs60809785
rs2285704
rs2198004
rs9567245
rs62076169
rs12978005
rs147544940
rs181064019
rs1532976
rs57918569
rs143459073
rs75798304
rs72765753
All
−0.1
0.0
0.1
0.2
0.3
MR leave−one−out sensitivity analysis for
'Enterococcus B' on 'GCST90199875_buildGRCh38.tsv'

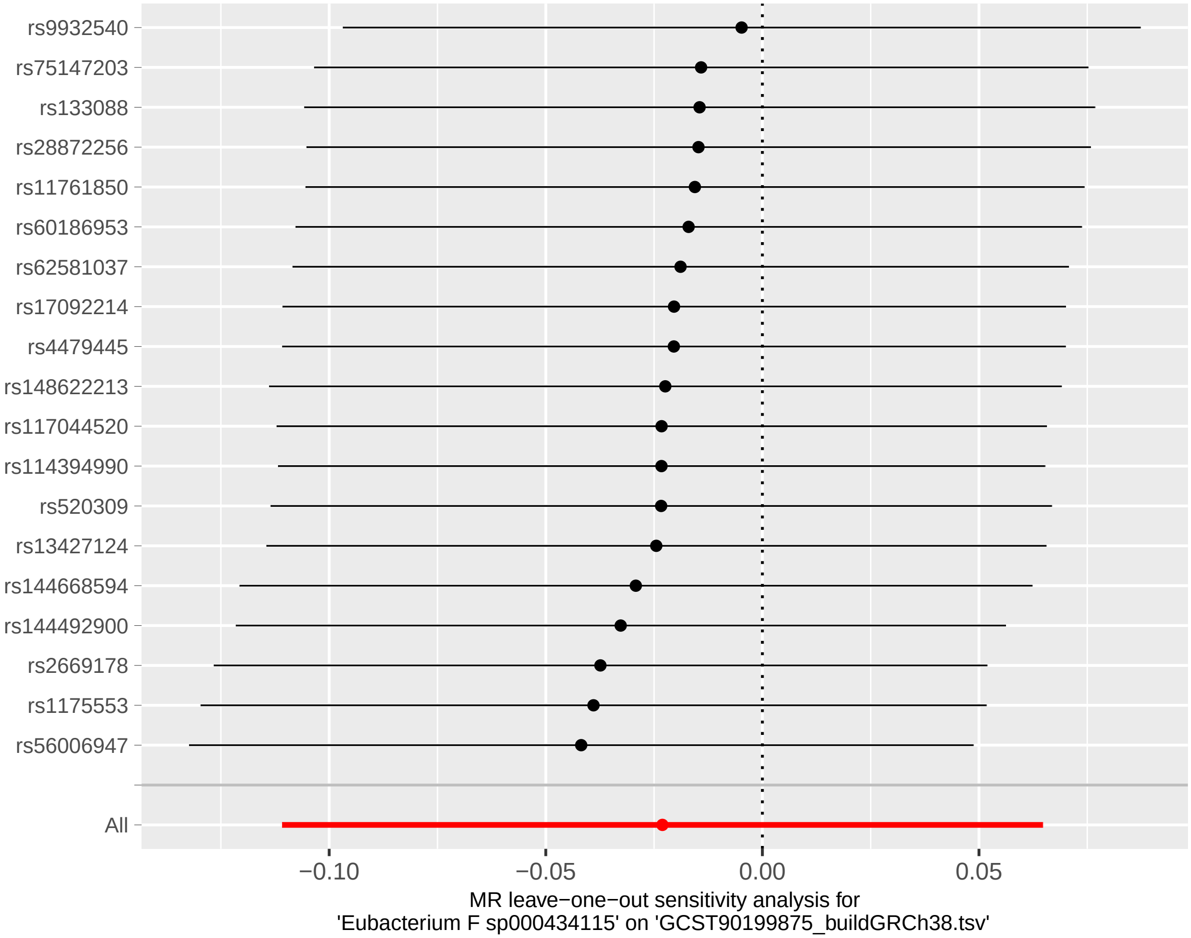
rs9932540
rs75147203
rs133088
rs28872256
rs11761850
rs60186953
rs62581037
rs17092214
rs4479445
rs148622213
rs117044520
rs114394990
rs520309
rs13427124
rs144668594
rs144492900
rs2669178
rs1175553
rs56006947
All
−0.10
−0.05
0.00
0.05
MR leave−one−out sensitivity analysis for
'Eubacterium F sp000434115' on 'GCST90199875_buildGRCh38.tsv'

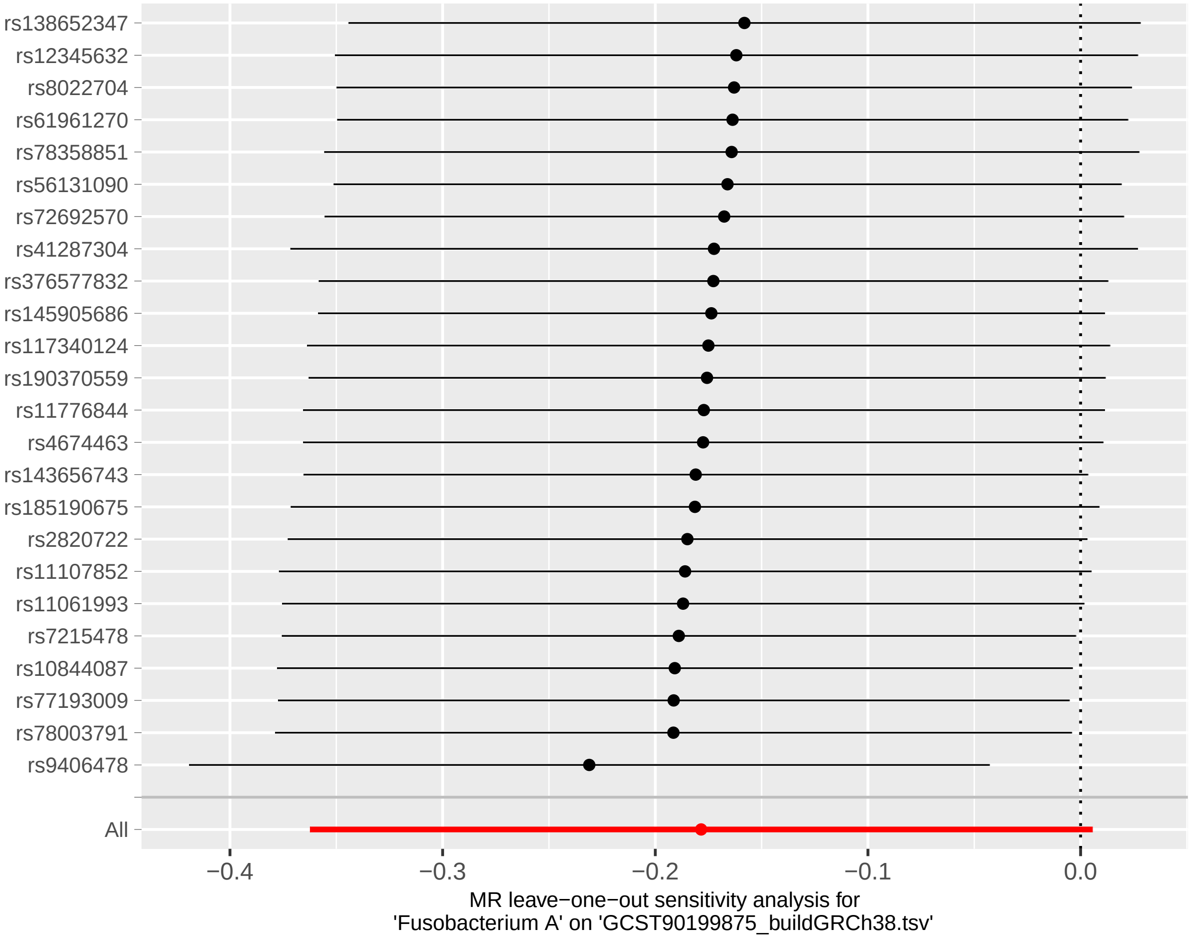
rs138652347
rs12345632
rs8022704
rs61961270
rs78358851
rs56131090
rs72692570
rs41287304
rs376577832
rs145905686
rs117340124
rs190370559
rs11776844
rs4674463
rs143656743
rs185190675
rs2820722
rs11107852
rs11061993
rs7215478
rs10844087
rs77193009
rs78003791
rs9406478
All
−0.4
−0.3
−0.2
−0.1
0.0
MR leave−one−out sensitivity analysis for
'Fusobacterium A' on 'GCST90199875_buildGRCh38.tsv'

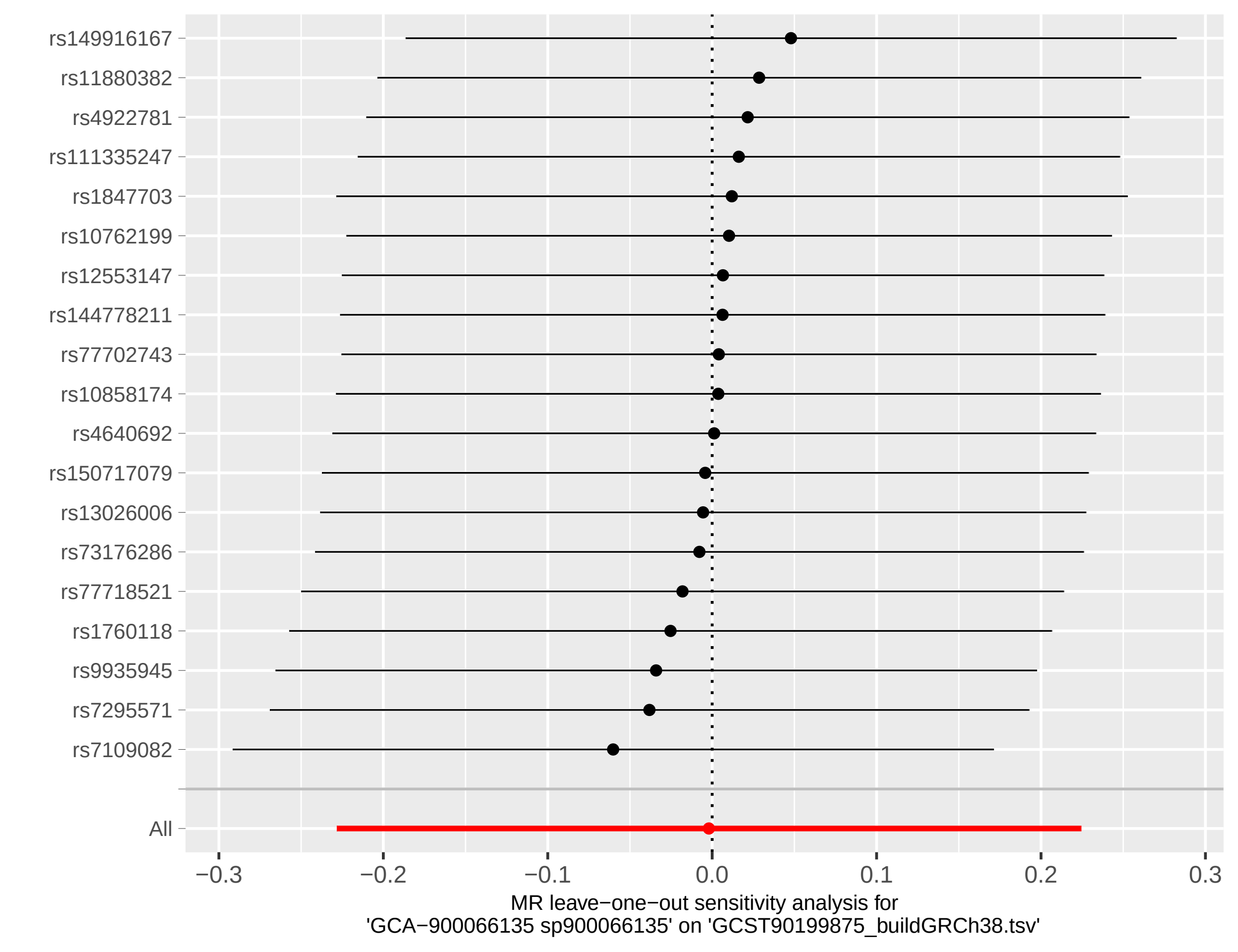

rs149916167
rs11880382
rs4922781
rs111335247
rs1847703
rs10762199
rs12553147
rs144778211
rs77702743
rs10858174
rs4640692
rs150717079
rs13026006
rs73176286
rs77718521
rs1760118
rs9935945
rs7295571
rs7109082
All
−0.3
−0.2
−0.1
0.0
0.1
0.2
0.3
MR leave−one−out sensitivity analysis for
'GCA−900066135 sp900066135' on 'GCST90199875_buildGRCh38.tsv'

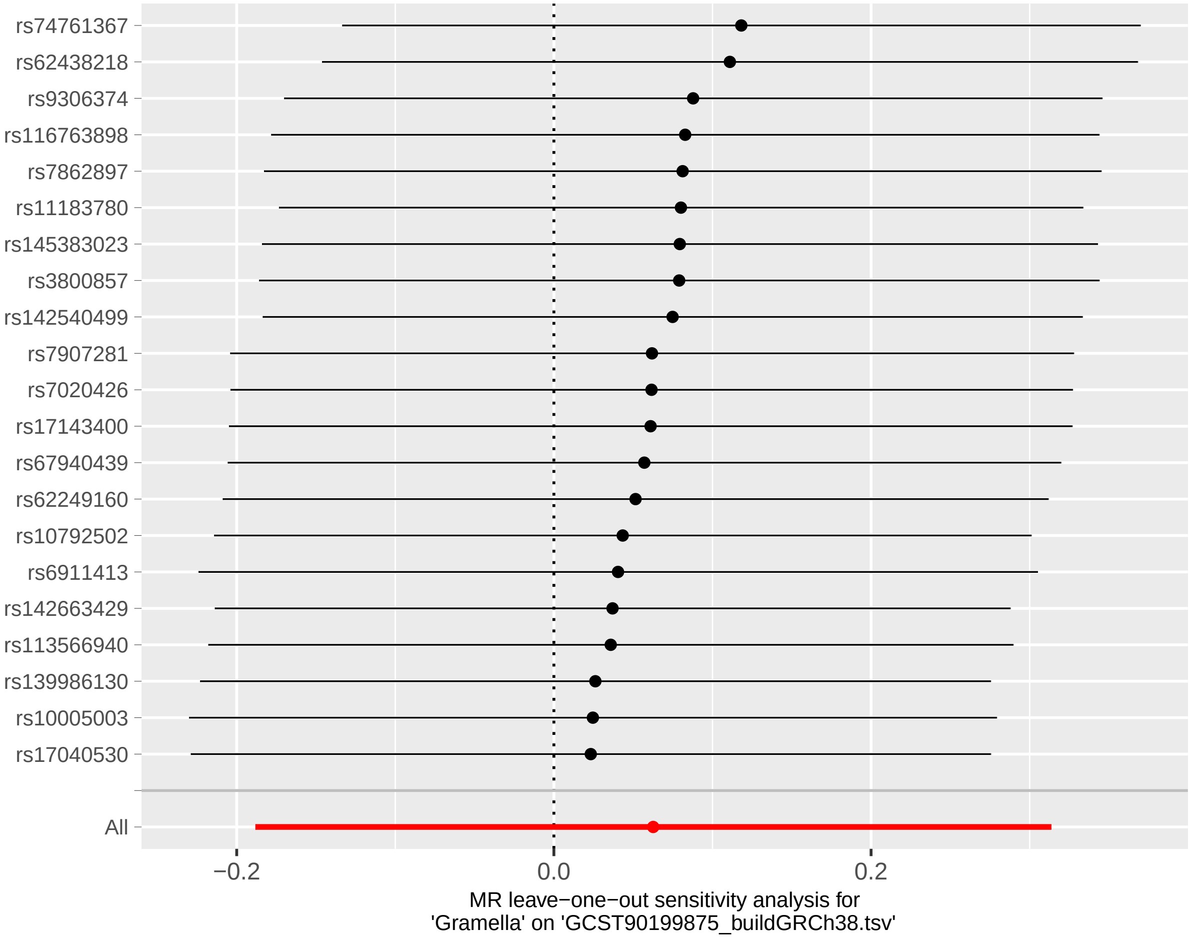
rs74761367
rs62438218
rs9306374
rs116763898
rs7862897
rs11183780
rs145383023
rs3800857
rs142540499
rs7907281
rs7020426
rs17143400
rs67940439
rs62249160
rs10792502
rs6911413
rs142663429
rs113566940
rs139986130
rs10005003
rs17040530
All
−0.2
0.0
0.2
MR leave−one−out sensitivity analysis for
'Gramella' on 'GCST90199875_buildGRCh38.tsv'

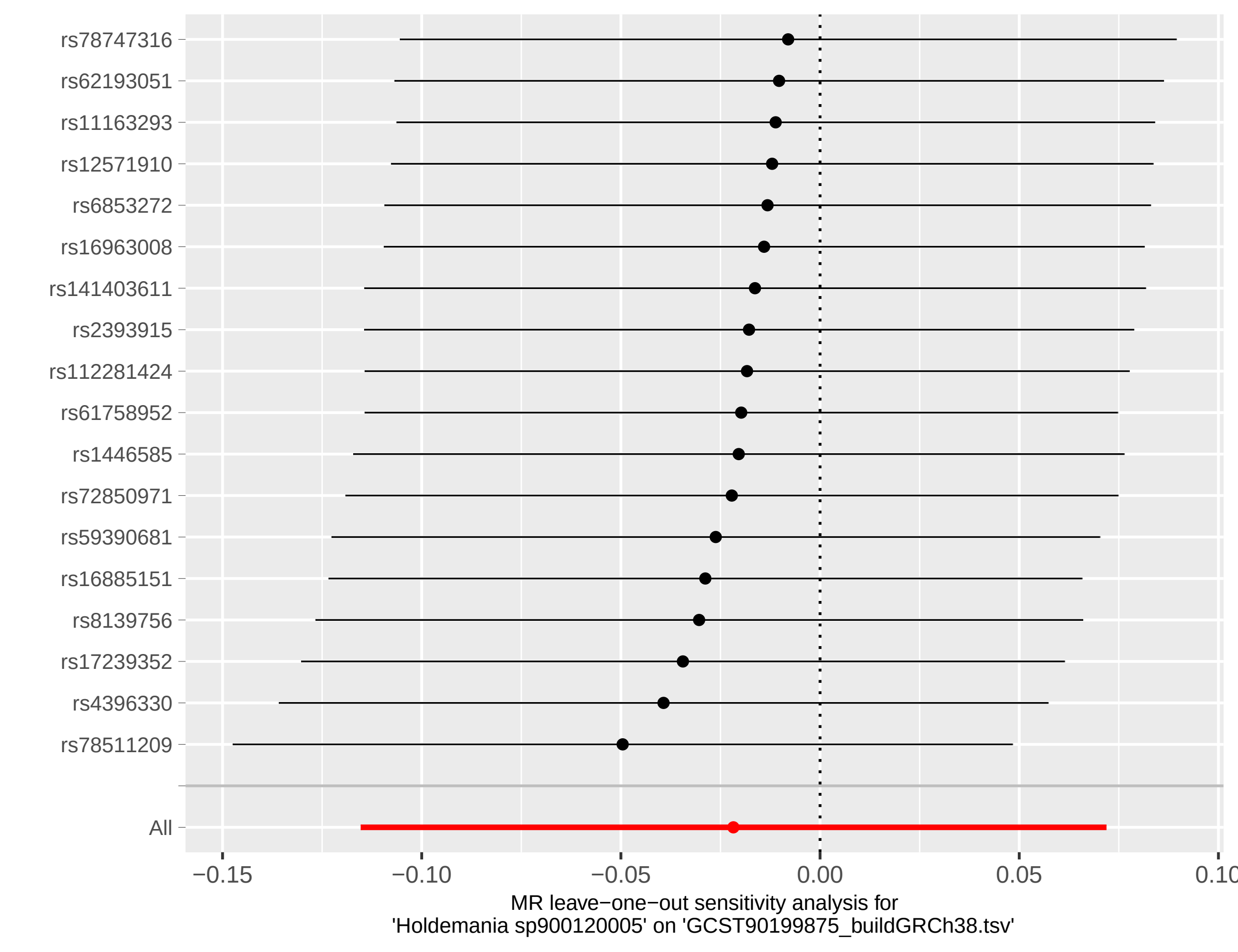
rs78747316
rs62193051
rs11163293
rs12571910
rs6853272
rs16963008
rs141403611
rs2393915
rs112281424
rs61758952
rs1446585
rs72850971
rs59390681
rs16885151
rs8139756
rs17239352
rs4396330
rs78511209
All
−0.15
−0.10
−0.05
0.00
0.05
0.10
MR leave−one−out sensitivity analysis for
'Holdemania sp900120005' on 'GCST90199875_buildGRCh38.tsv'

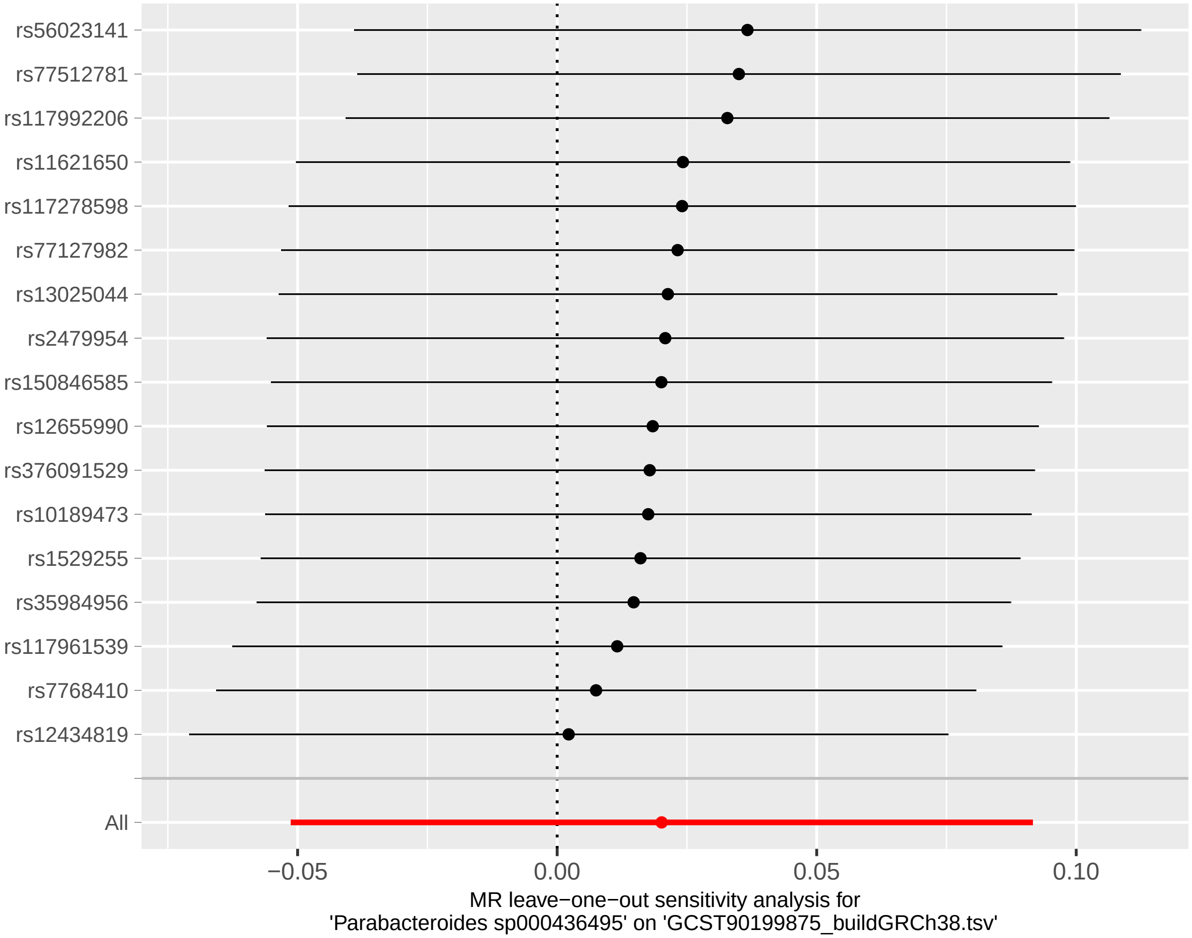
rs56023141
rs77512781
rs117992206
rs11621650
rs117278598
rs77127982
rs13025044
rs2479954
rs150846585
rs12655990
rs376091529
rs10189473
rs1529255
rs35984956
rs117961539
rs7768410
rs12434819
All
−0.05
0.00
0.05
0.10
MR leave−one−out sensitivity analysis for
'Parabacteroides sp000436495' on 'GCST90199875_buildGRCh38.tsv'

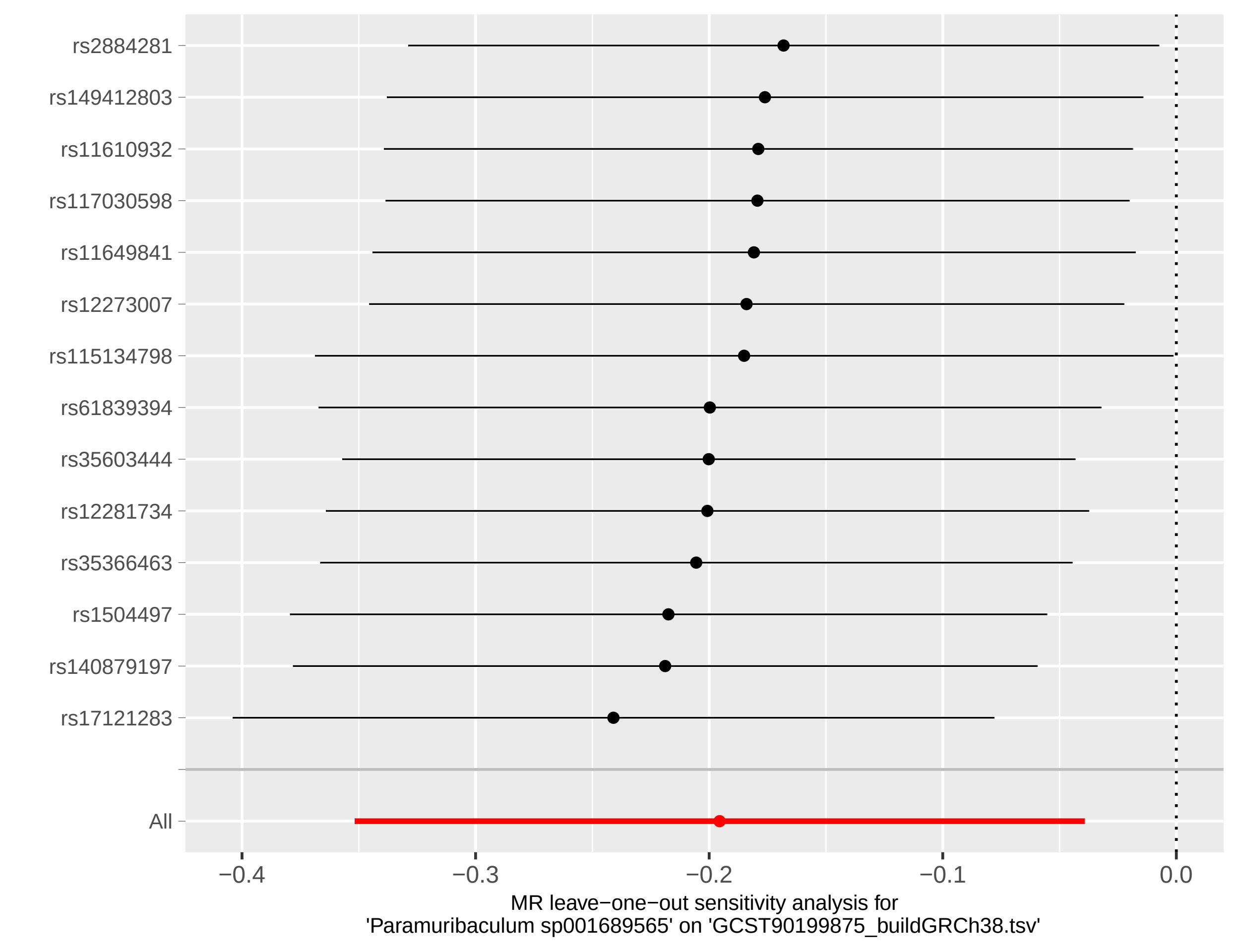

rs2884281
rs149412803
rs11610932
rs117030598
rs11649841
rs12273007
rs115134798
rs61839394
rs35603444
rs12281734
rs35366463
rs1504497
rs140879197
rs17121283
All
−0.4
−0.3
−0.2
−0.1
0.0
MR leave−one−out sensitivity analysis for
'Paramuribaculum sp001689565' on 'GCST90199875_buildGRCh38.tsv'

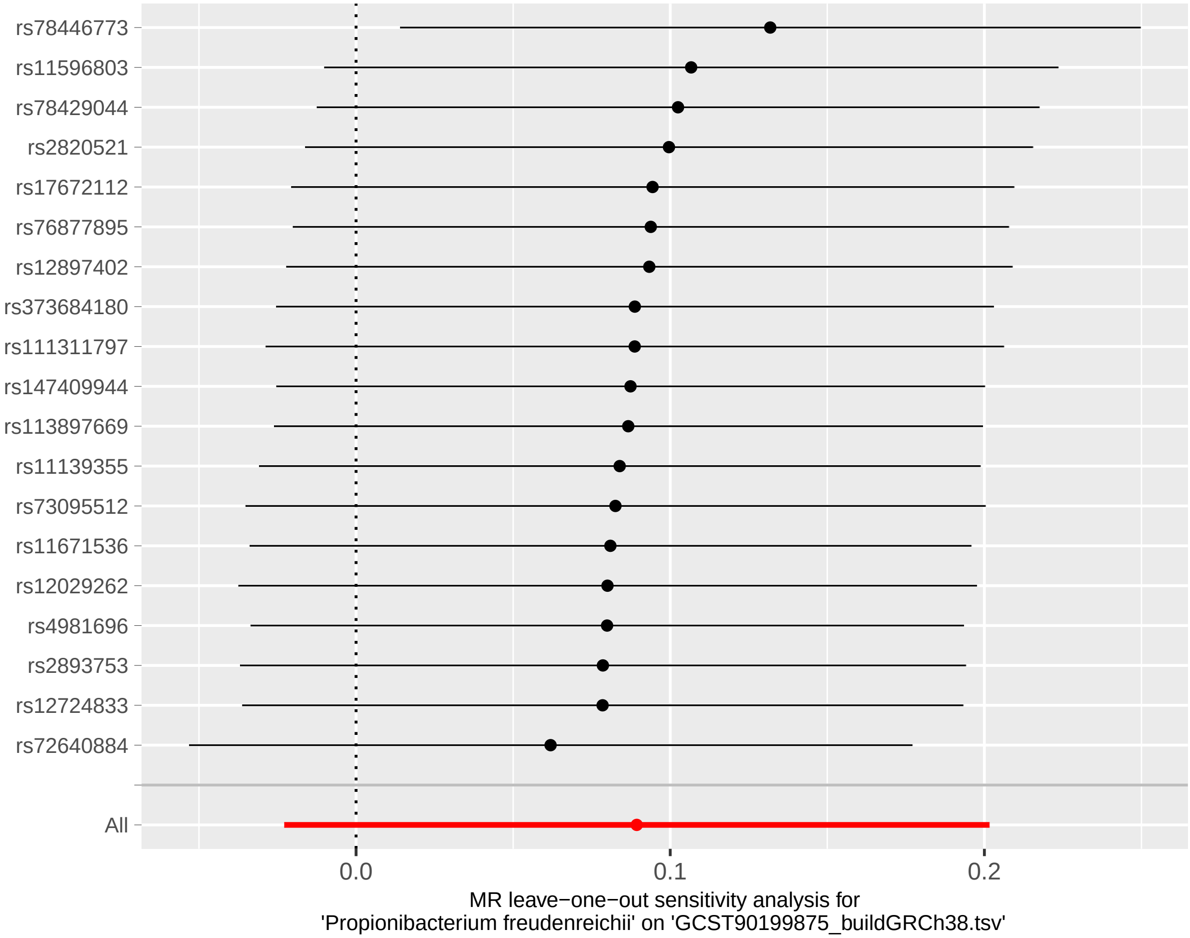
rs78446773
rs11596803
rs78429044
rs2820521
rs17672112
rs76877895
rs12897402
rs373684180
rs111311797
rs147409944
rs113897669
rs11139355
rs73095512
rs11671536
rs12029262
rs4981696
rs2893753
rs12724833
rs72640884
All
0.0
0.1
0.2
MR leave−one−out sensitivity analysis for
'Propionibacterium freudenreichii' on 'GCST90199875_buildGRCh38.tsv'

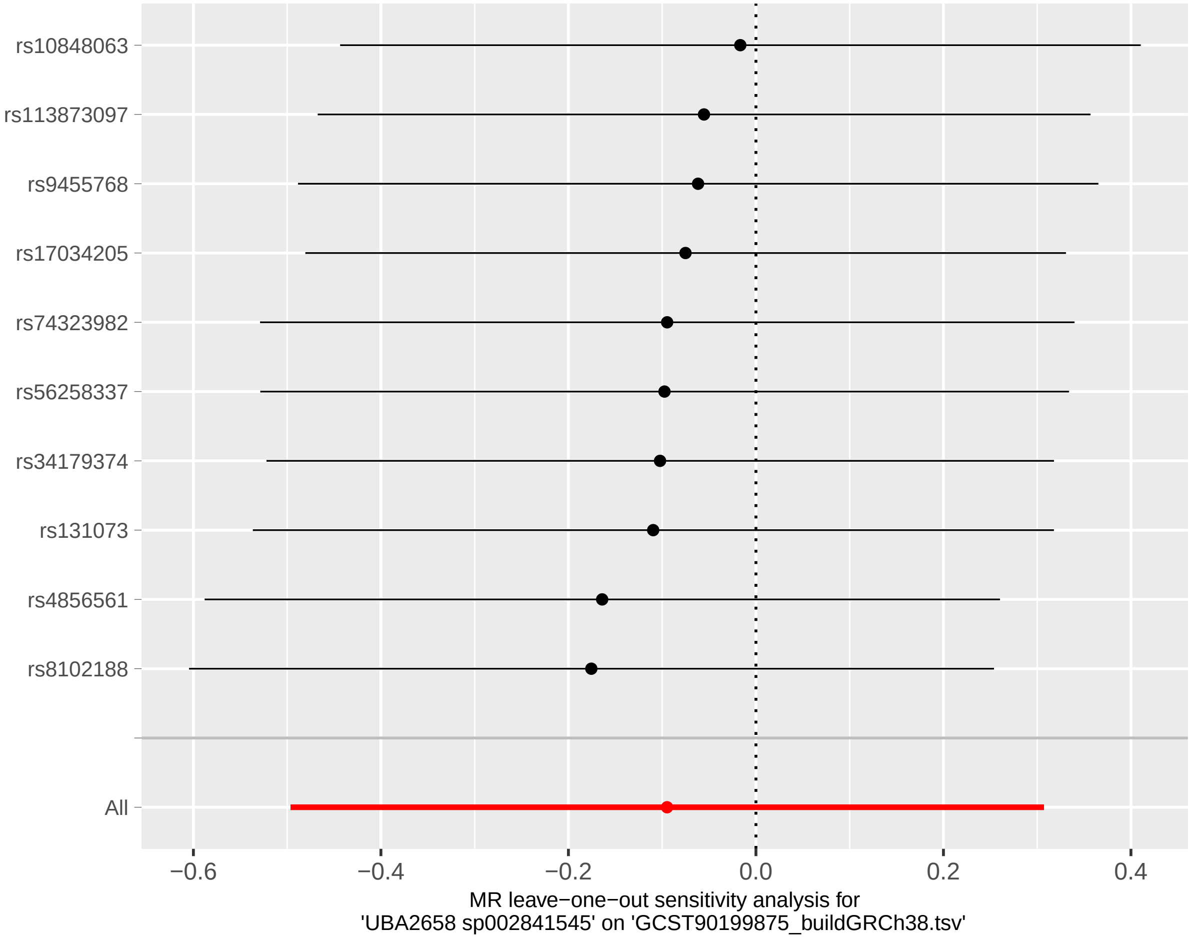

rs10848063
rs113873097
rs9455768
rs17034205
rs74323982
rs56258337
rs34179374
rs131073
rs4856561
rs8102188
All
−0.6
−0.4
−0.2
0.0
0.2
0.4
MR leave−one−out sensitivity analysis for
'UBA2658 sp002841545' on 'GCST90199875_buildGRCh38.tsv'

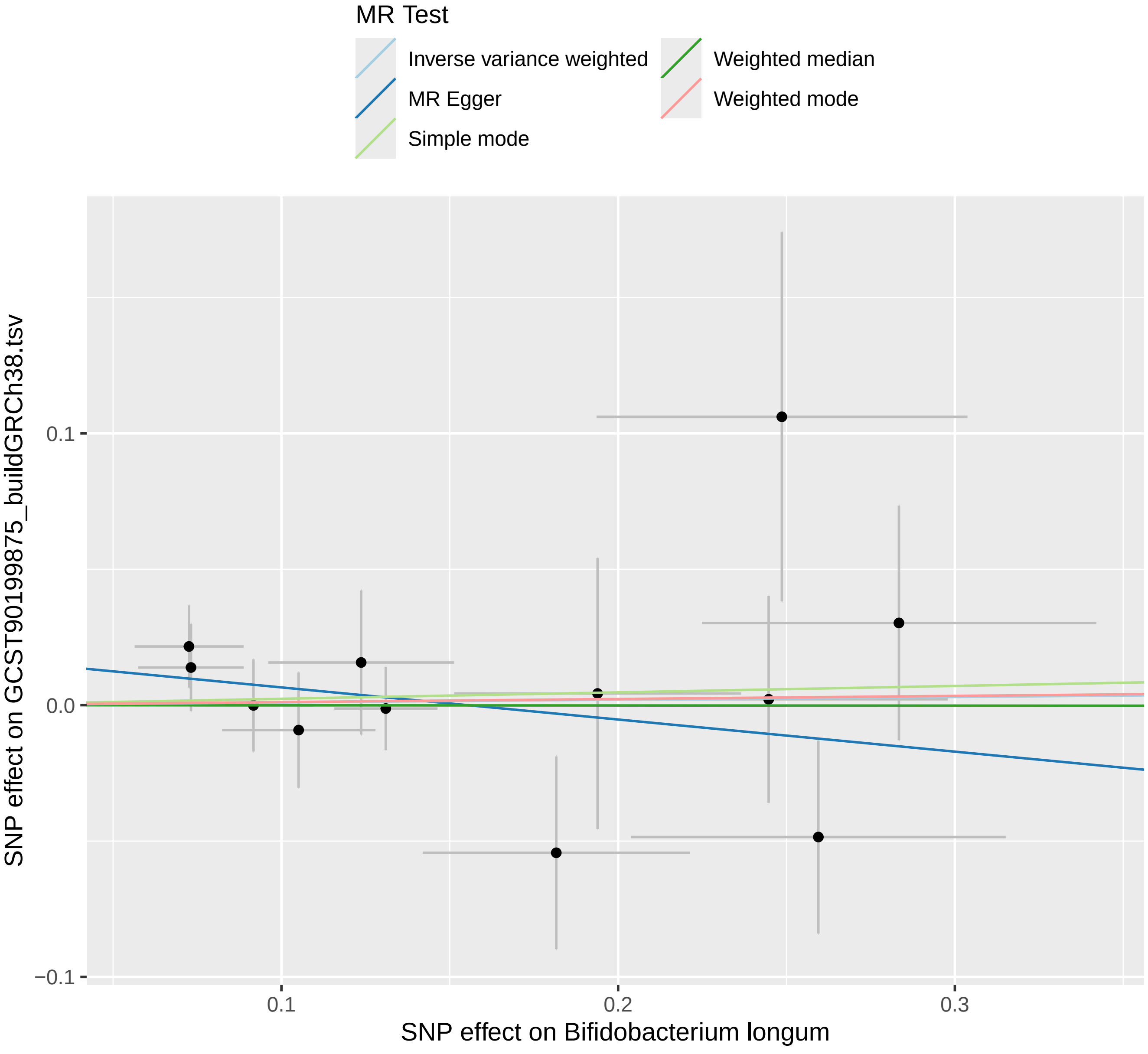
MR Test
Inverse variance weighted
MR Egger
Simple mode
Weighted median
Weighted mode
SNP effect on GCST90199875_buildGRCh38.tsv
0.1
0.0
−0.1
0.1
0.2
0.3
SNP effect on Bifidobacterium longum

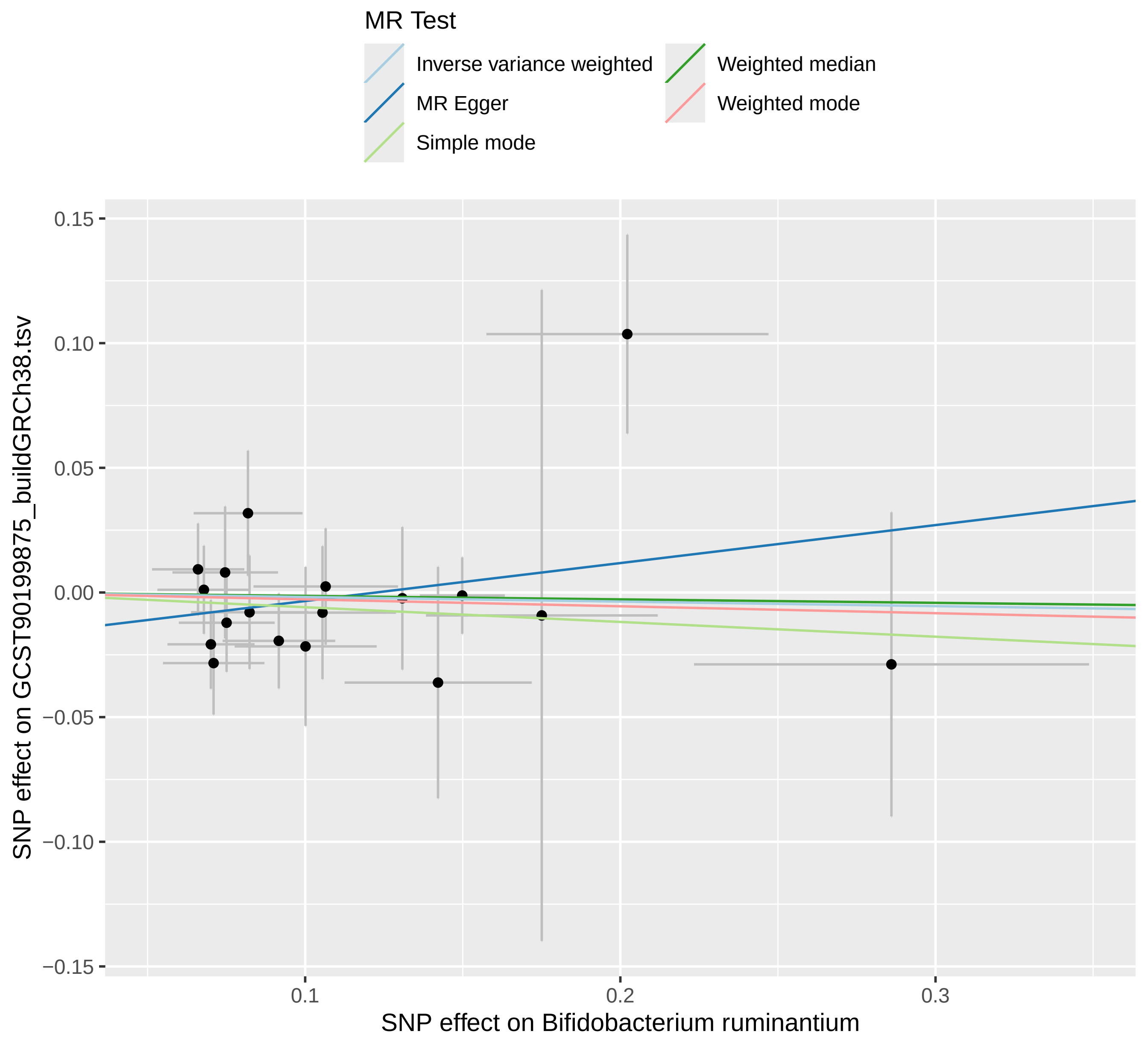
MR Test
Inverse variance weighted
MR Egger
Simple mode
Weighted median
Weighted mode
0.15
0.10
0.05
0.00
−0.05
−0.10
−0.15
SNP effect on GCST90199875_buildGRCh38.tsv
0.1
0.2
0.3
SNP effect on Bifidobacterium ruminantium

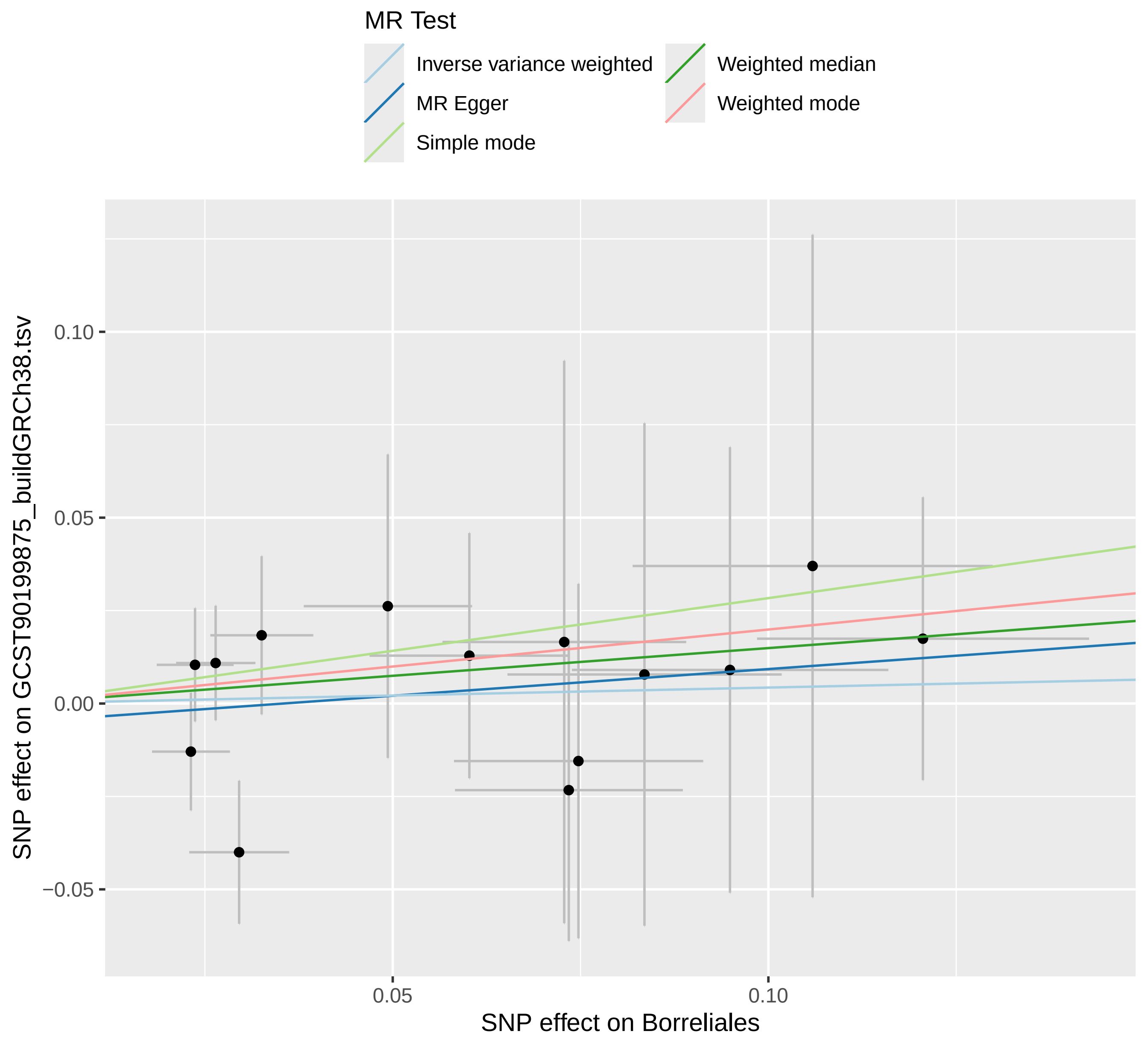

MR Test
Inverse variance weighted
MR Egger
Simple mode
Weighted median
Weighted mode
0.10
0.05
0.00
−0.05
SNP effect on GCST90199875_buildGRCh38.tsv
0.05
0.10
SNP effect on Borreliales

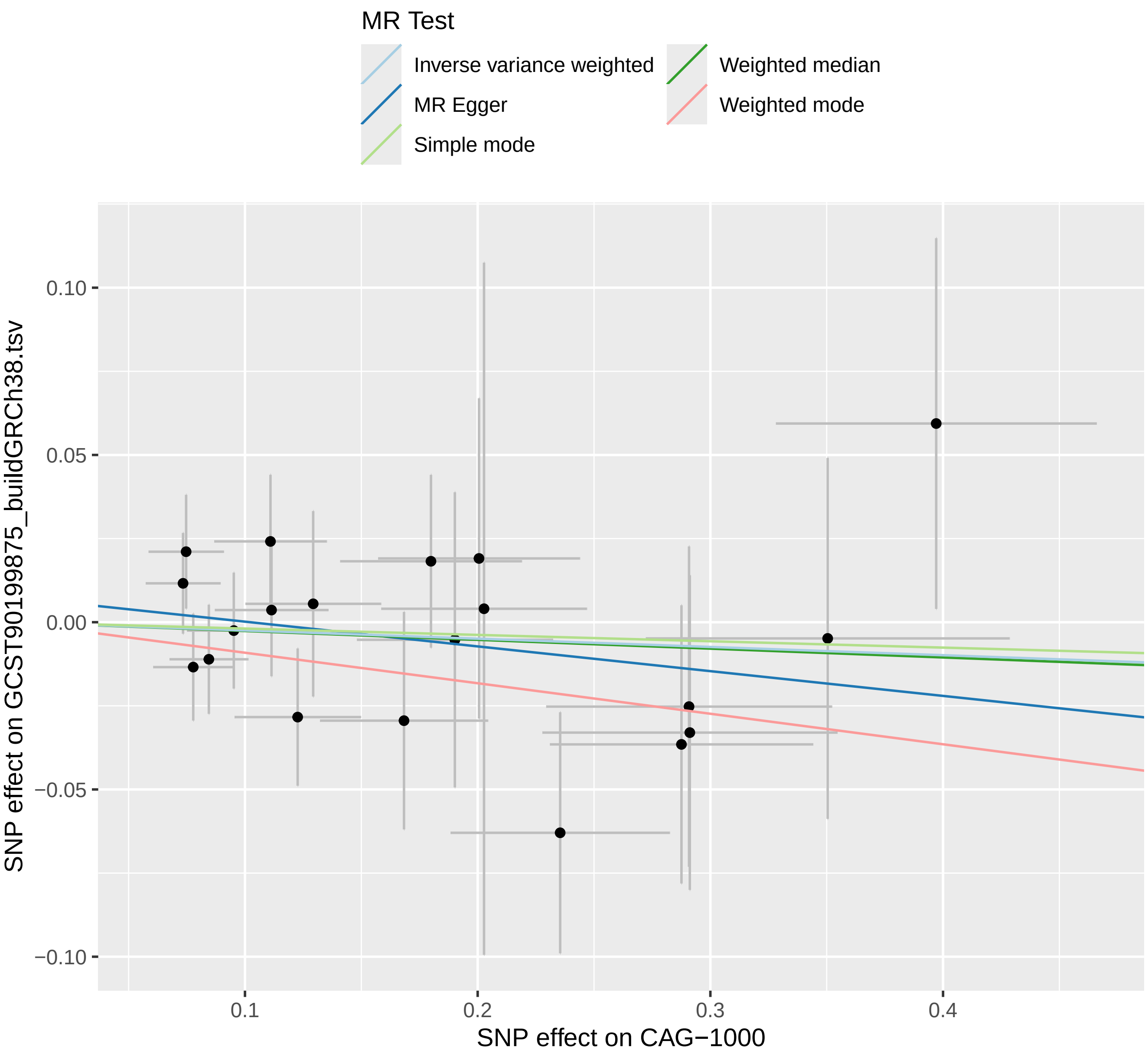
MR Test
Inverse variance weighted
MR Egger
Simple mode
Weighted median
Weighted mode
0.10
0.05
0.00
−0.05
−0.10
SNP effect on GCST90199875_buildGRCh38.tsv
0.1
0.2
0.3
0.4
SNP effect on CAG−1000

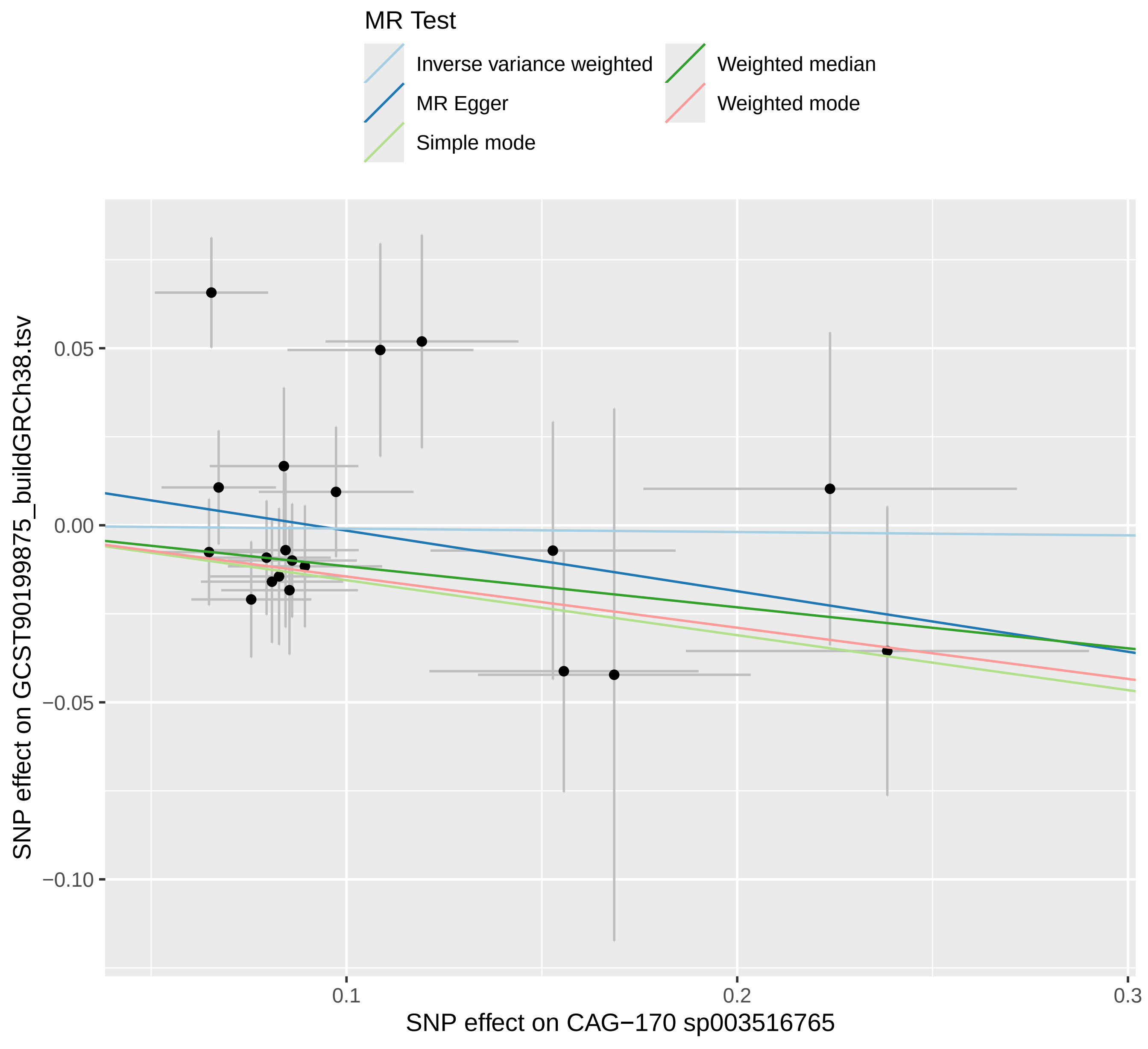
MR Test
Inverse variance weighted
MR Egger
Simple mode
Weighted median
Weighted mode
0.05
0.00
−0.05
−0.10
SNP effect on GCST90199875_buildGRCh38.tsv
0.1
0.2
0.3
SNP effect on CAG−170 sp003516765

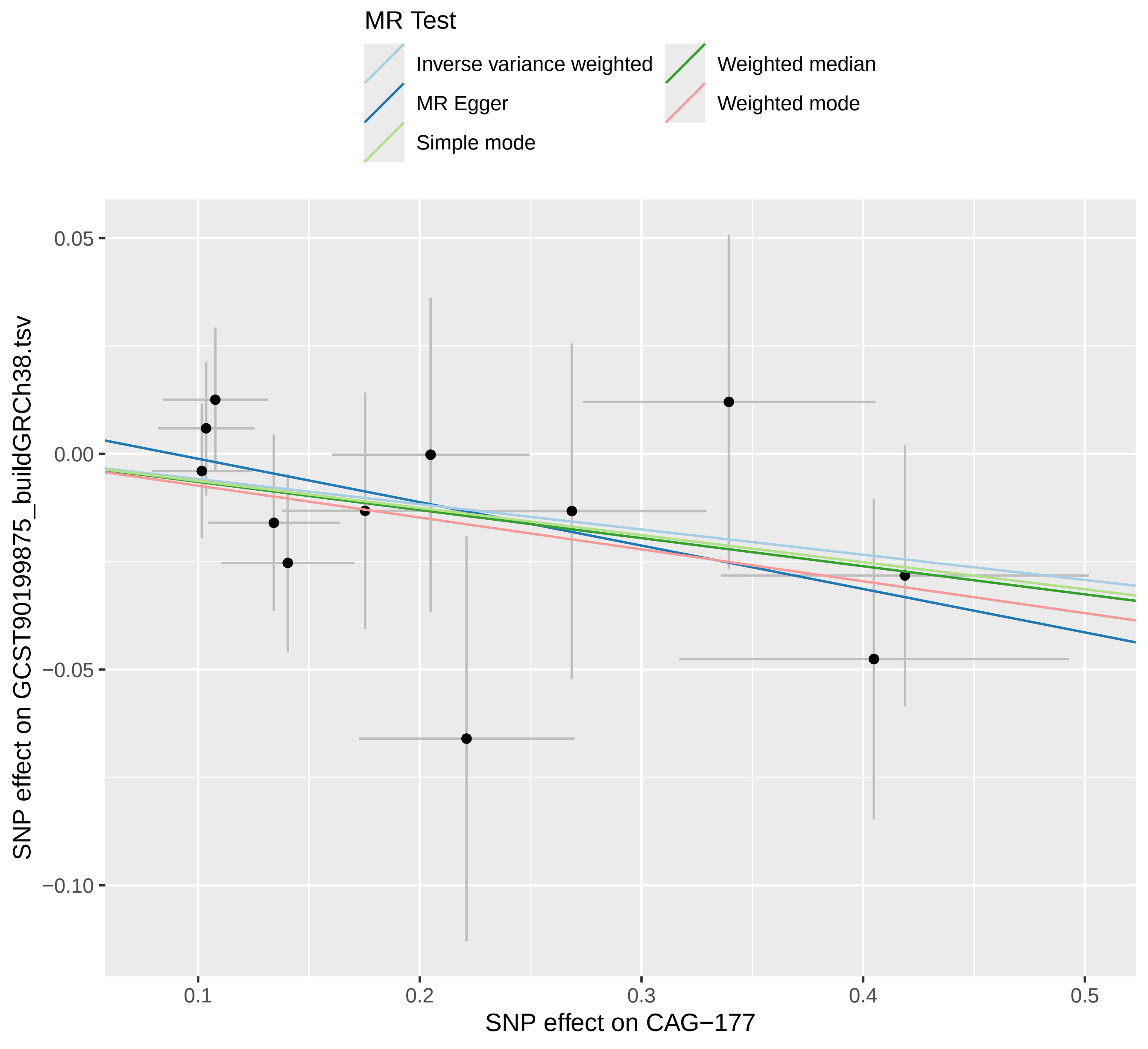

MR Test
Inverse variance weighted
MR Egger
Simple mode
Weighted median
Weighted mode
0.05
0.00
−0.05
−0.10
0.1
0.2
0.3
0.4
0.5
SNP effect on GCST90199875_buildGRCh38.tsv
SNP effect on CAG−177

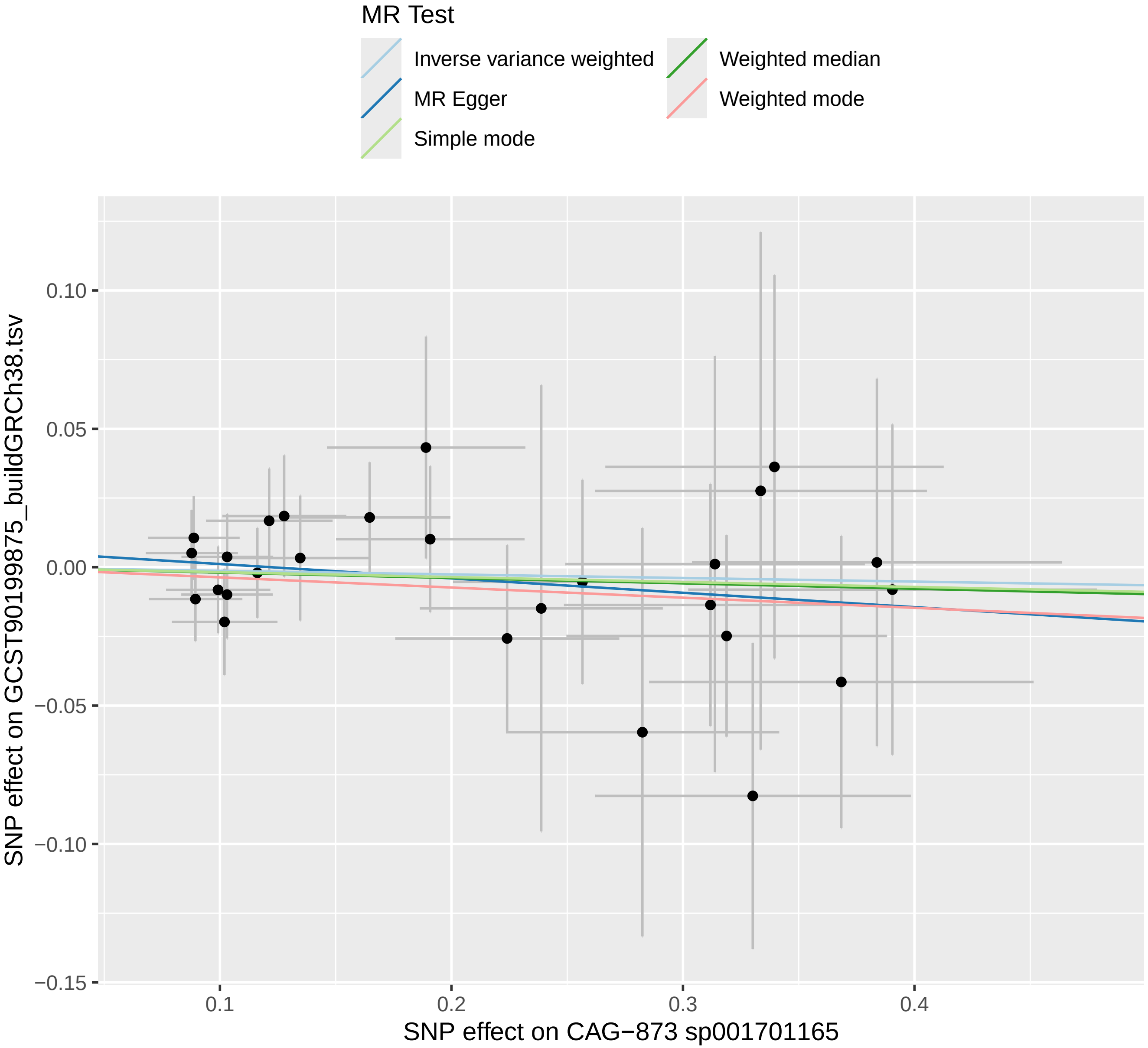

MR Test
Inverse variance weighted
MR Egger
Simple mode
Weighted median
Weighted mode
0.10
0.05
0.00
−0.05
−0.10
−0.15
SNP effect on GCST90199875_buildGRCh38.tsv
0.1
0.2
0.3
0.4
SNP effect on CAG−873 sp001701165

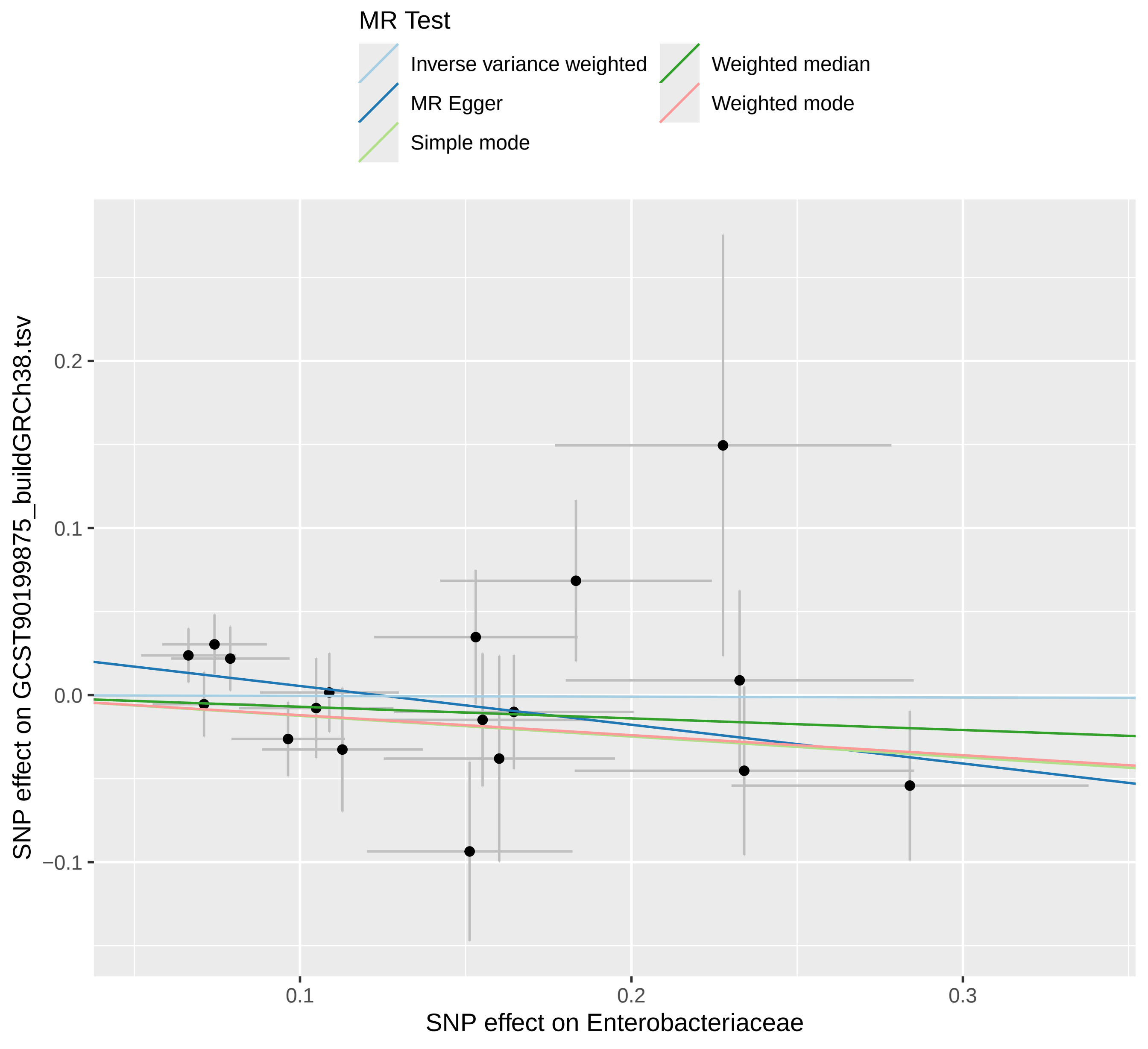
MR Test
Inverse variance weighted
MR Egger
Simple mode
Weighted median
Weighted mode
0.2
0.1
0.0
−0.1
0.1
0.2
0.3
SNP effect on GCST90199875_buildGRCh38.tsv
SNP effect on Enterobacteriaceae

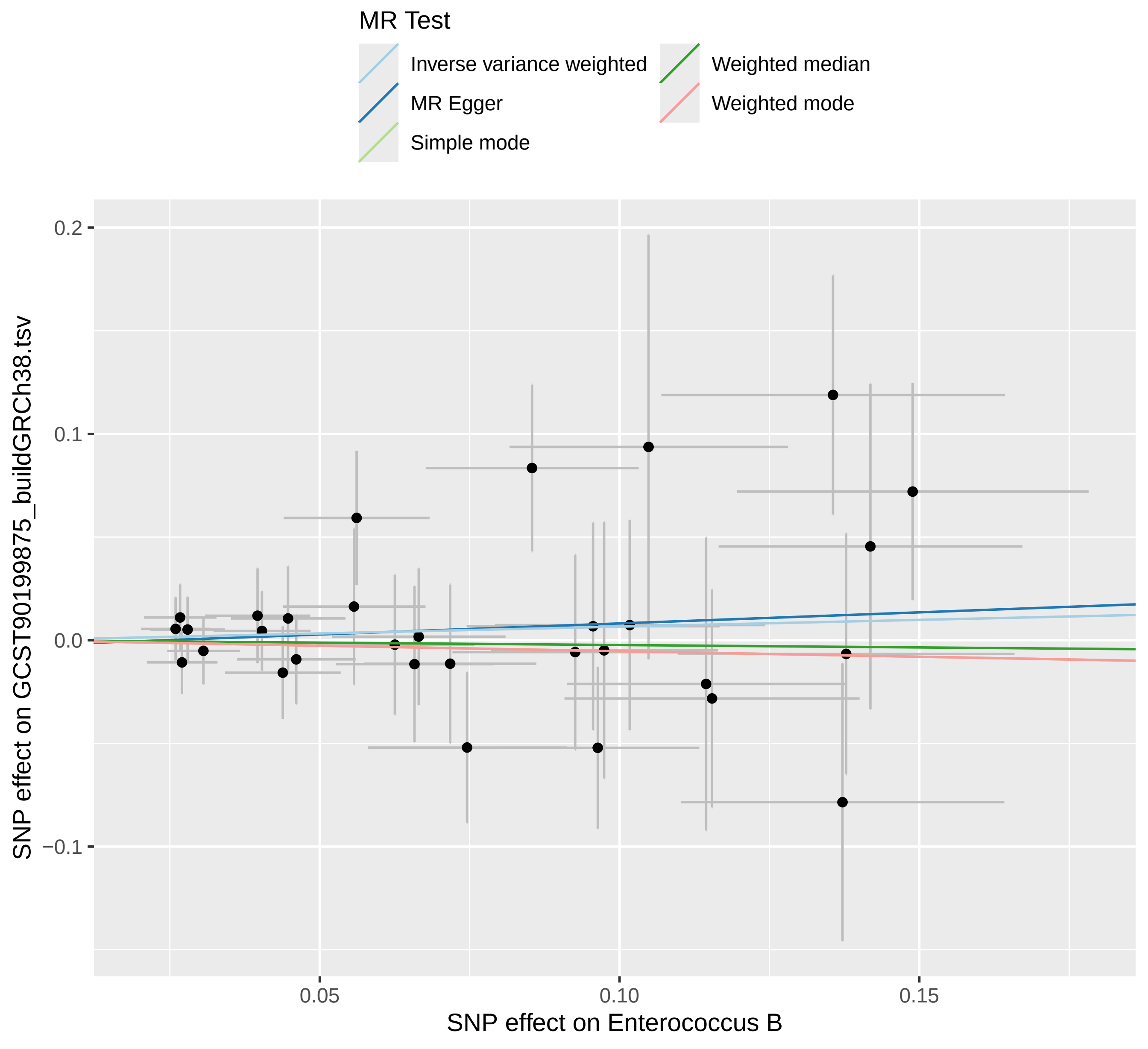
MR Test
Inverse variance weighted
MR Egger
Simple mode
Weighted median
Weighted mode
SNP effect on GCST90199875_buildGRCh38.tsv
0.2
0.1
0.0
−0.1
0.05
0.10
0.15
SNP effect on Enterococcus B

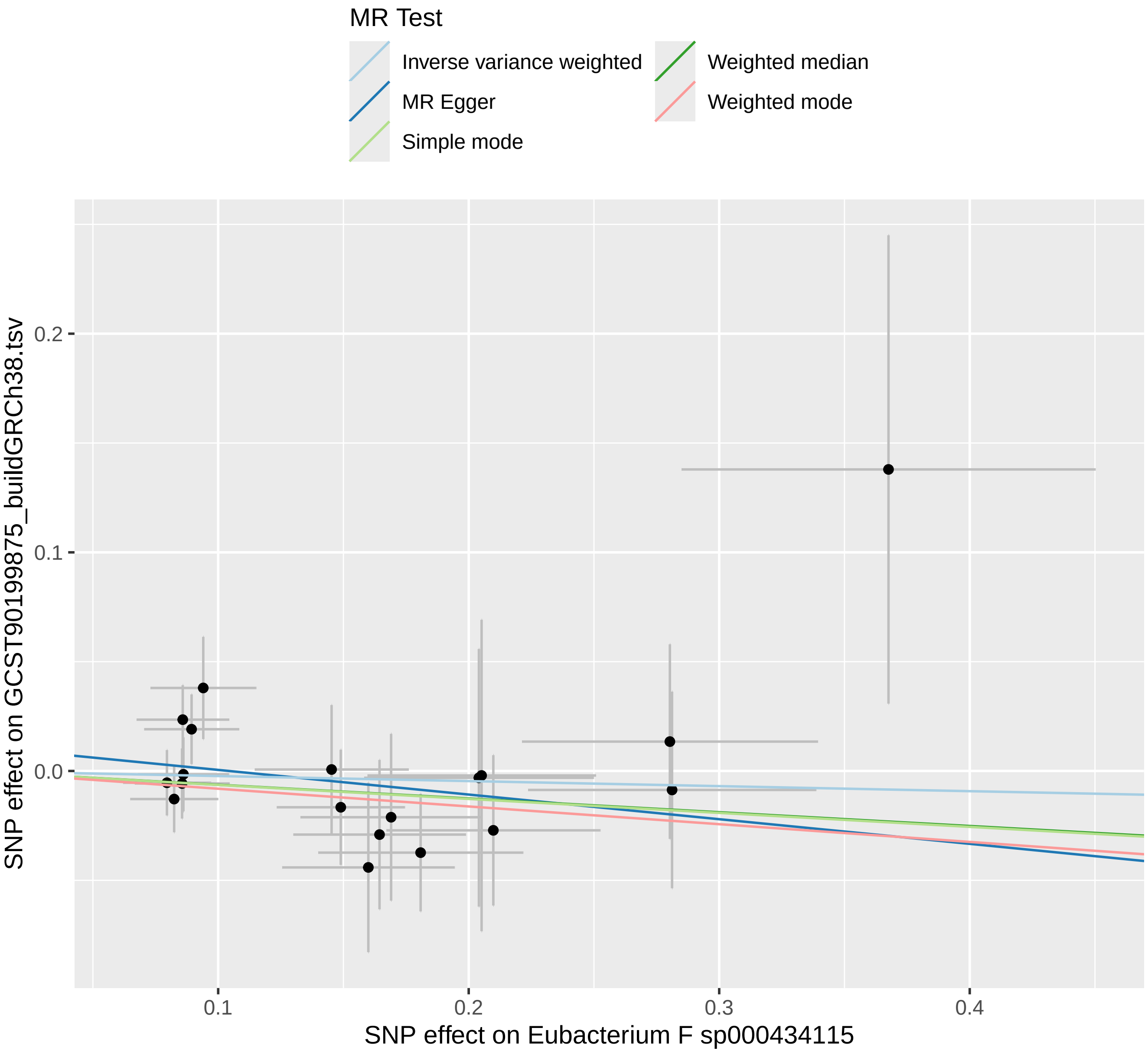

MR Test
Inverse variance weighted
MR Egger
Simple mode
Weighted median
Weighted mode
SNP effect on GCST90199875_buildGRCh38.tsv
0.2
0.1
0.0
0.1
0.2
0.3
0.4
SNP effect on Eubacterium F sp000434115

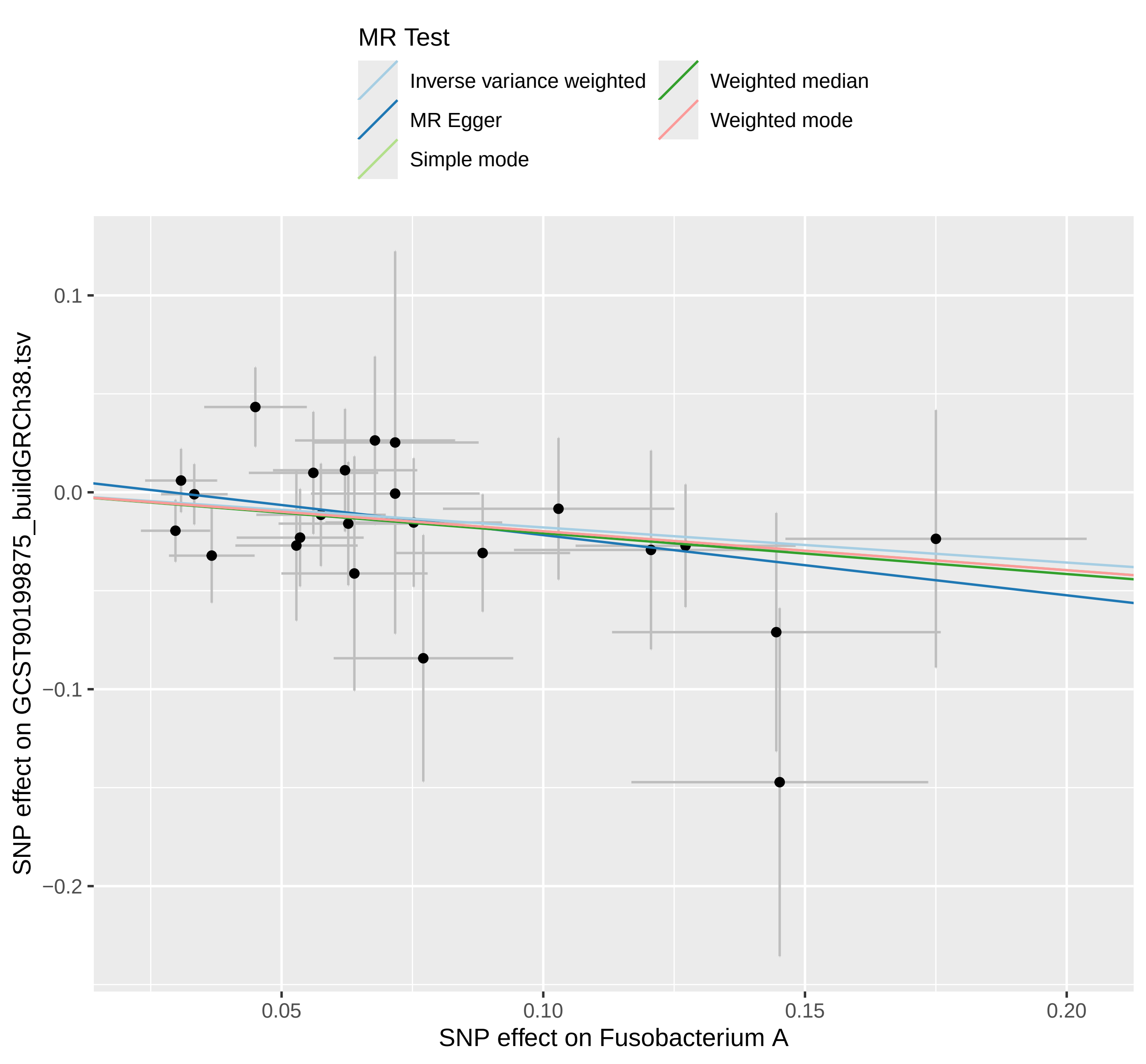
MR Test
Inverse variance weighted
MR Egger
Simple mode
Weighted median
Weighted mode
0.1
0.0
−0.1
−0.2
SNP effect on GCST90199875_buildGRCh38.tsv
0.05
0.10
0.15
0.20
SNP effect on Fusobacterium A

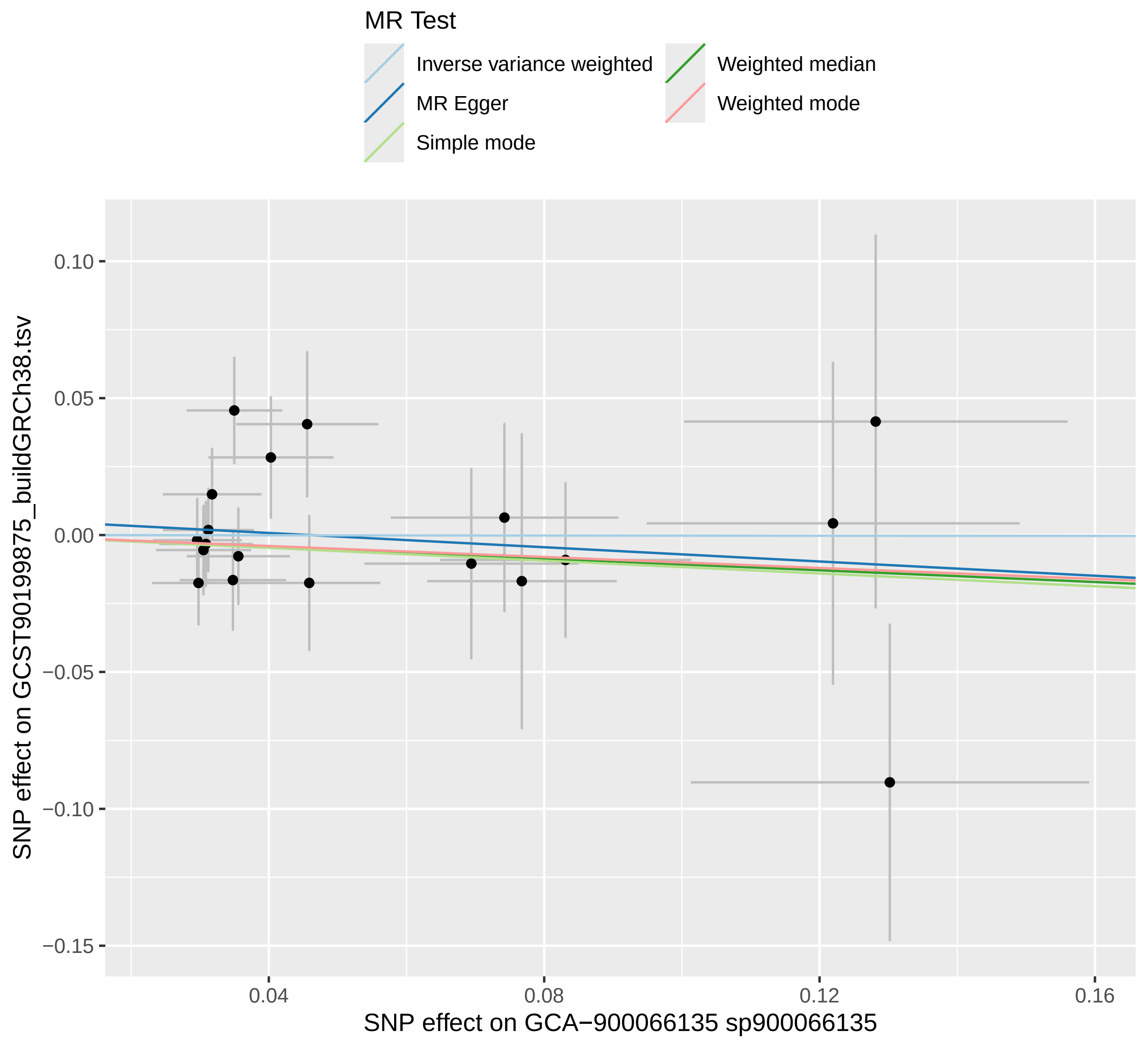
MR Test
Inverse variance weighted
MR Egger
Simple mode
Weighted median
Weighted mode
SNP effect on GCST90199875_buildGRCh38.tsv
0.10
0.05
0.00
−0.05
−0.10
−0.15
0.04
0.08
0.12
0.16
SNP effect on GCA−900066135 sp900066135

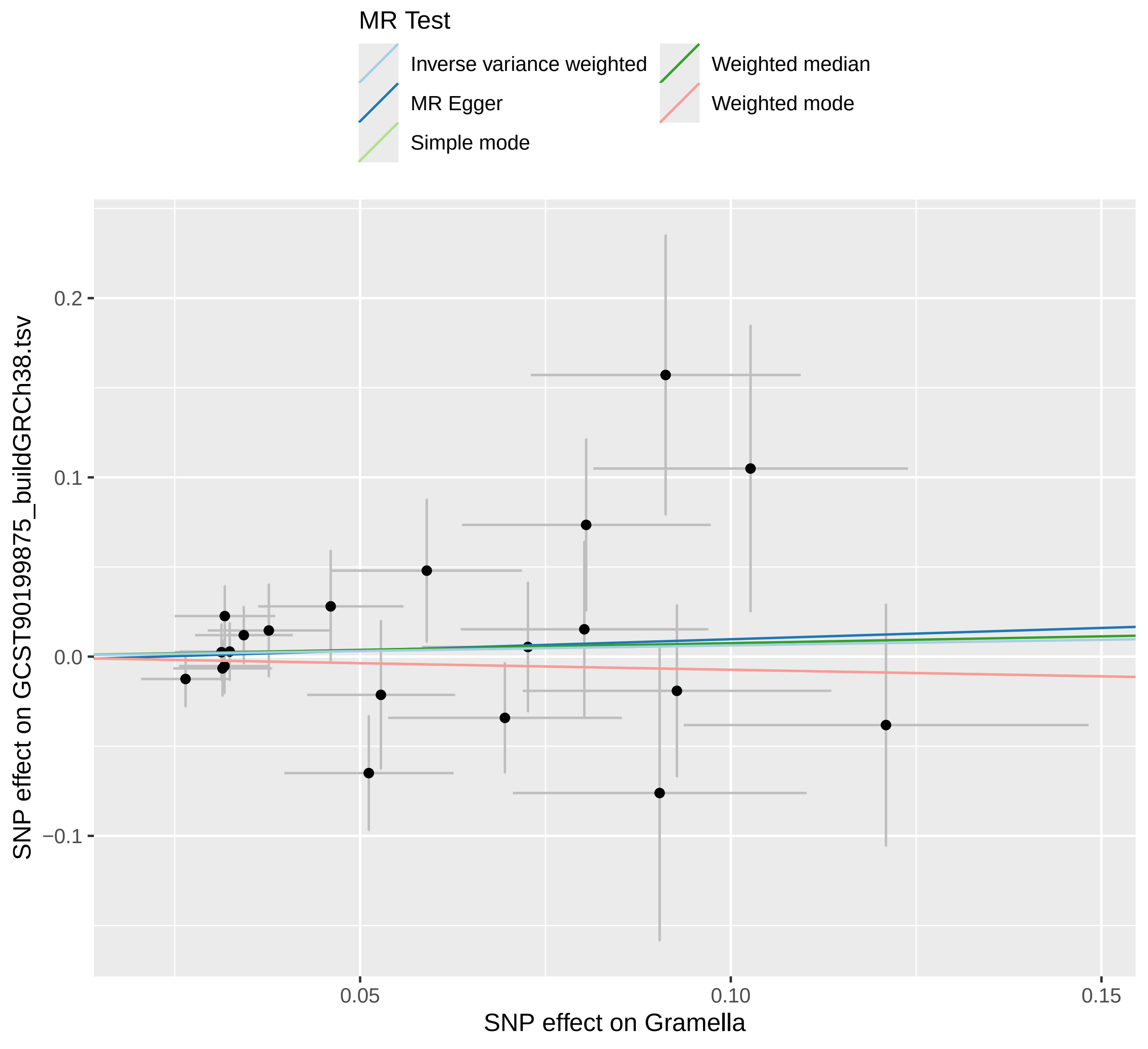

MR Test
Inverse variance weighted
MR Egger
Simple mode
Weighted median
Weighted mode
0.2
0.1
0.0
−0.1
0.05
0.10
0.15
SNP effect on GCST90199875_buildGRCh38.tsv
SNP effect on Gramella

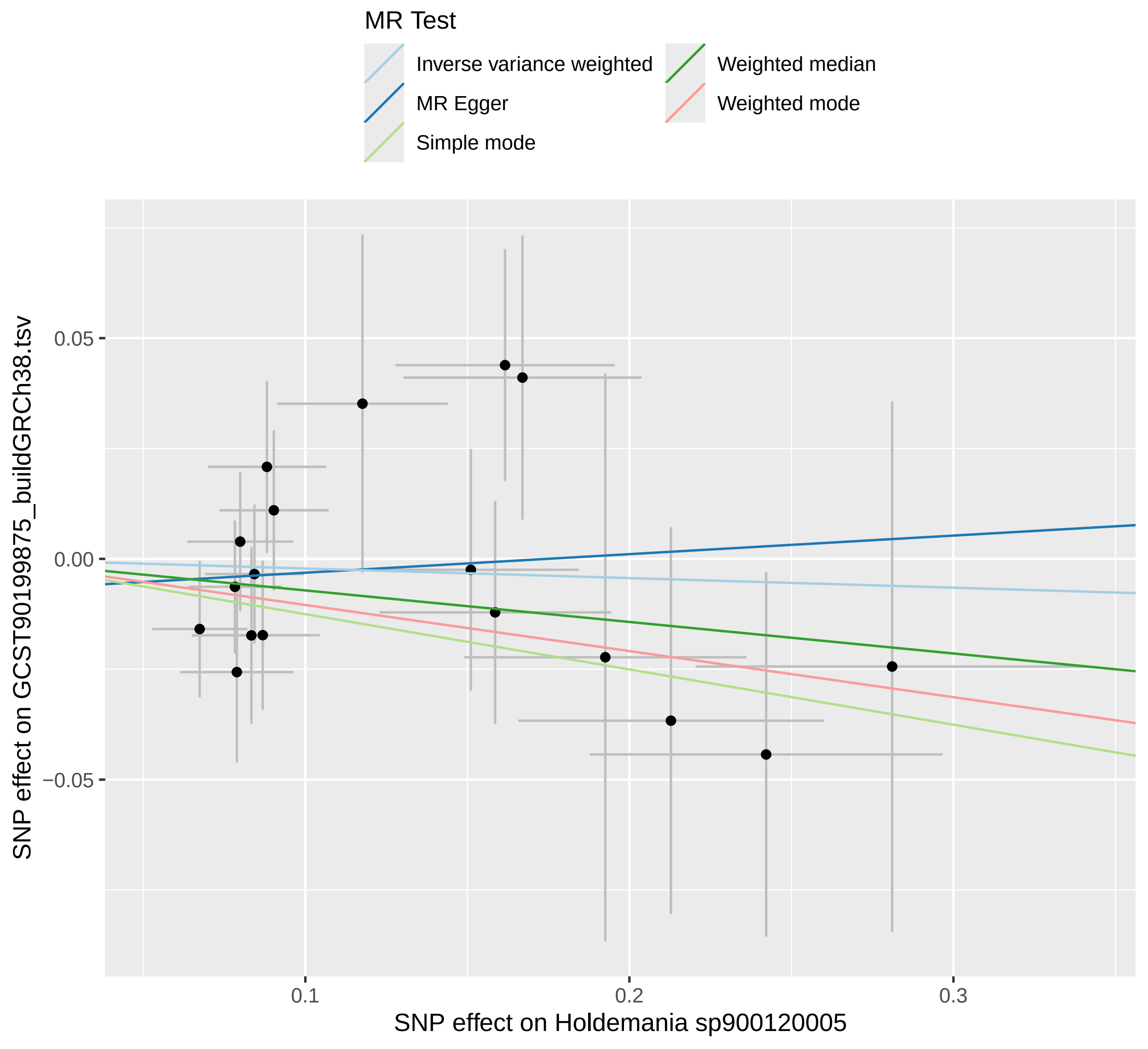
MR Test
Inverse variance weighted
MR Egger
Simple mode
Weighted median
Weighted mode
0.05
0.00
−0.05
0.1
0.2
0.3
SNP effect on GCST90199875_buildGRCh38.tsv
SNP effect on Holdemania sp900120005

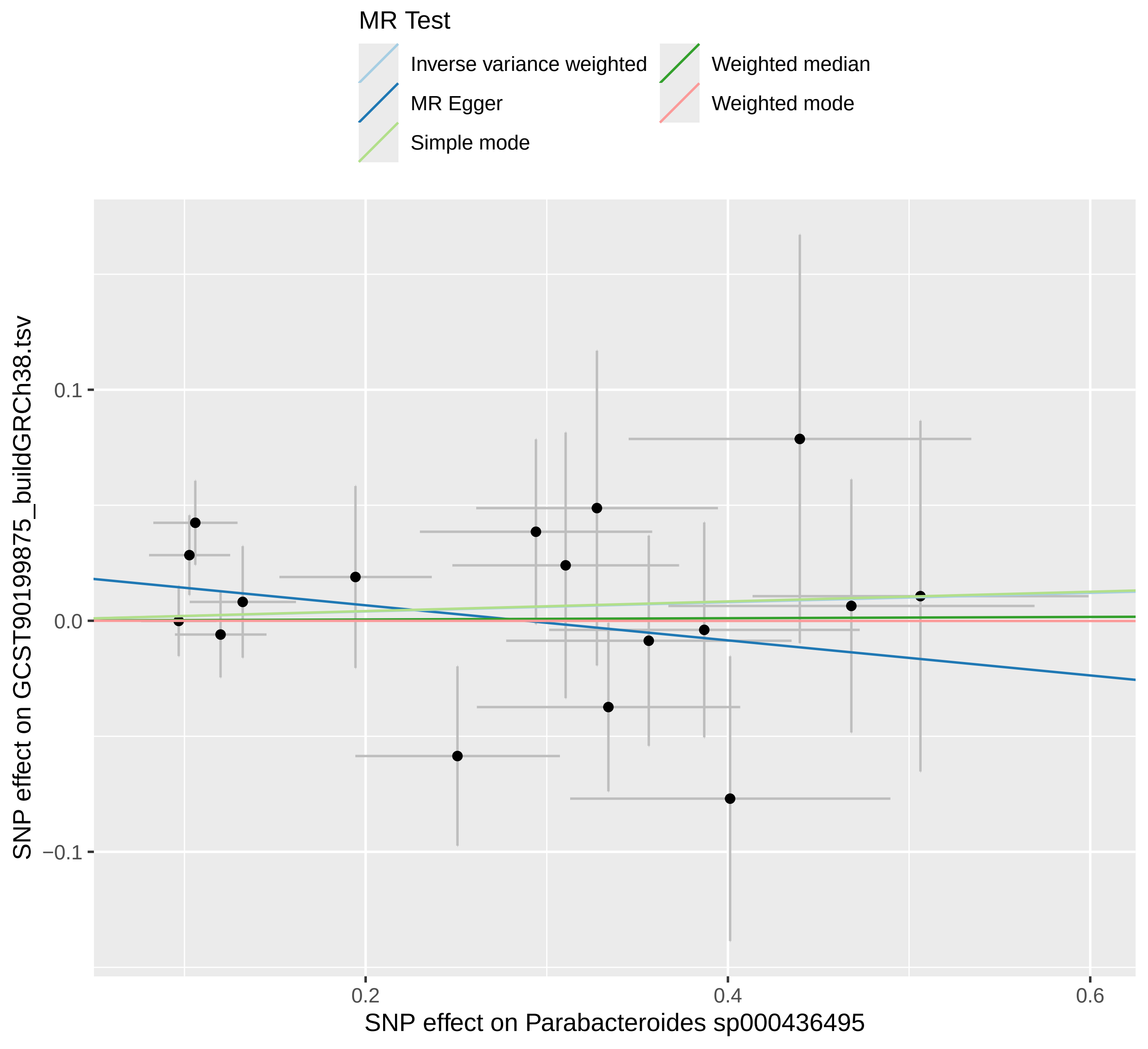

MR Test
Inverse variance weighted
MR Egger
Simple mode
Weighted median
Weighted mode
SNP effect on GCST90199875_buildGRCh38.tsv
0.1
0.0
−0.1
0.2
0.4
0.6
SNP effect on Parabacteroides sp000436495

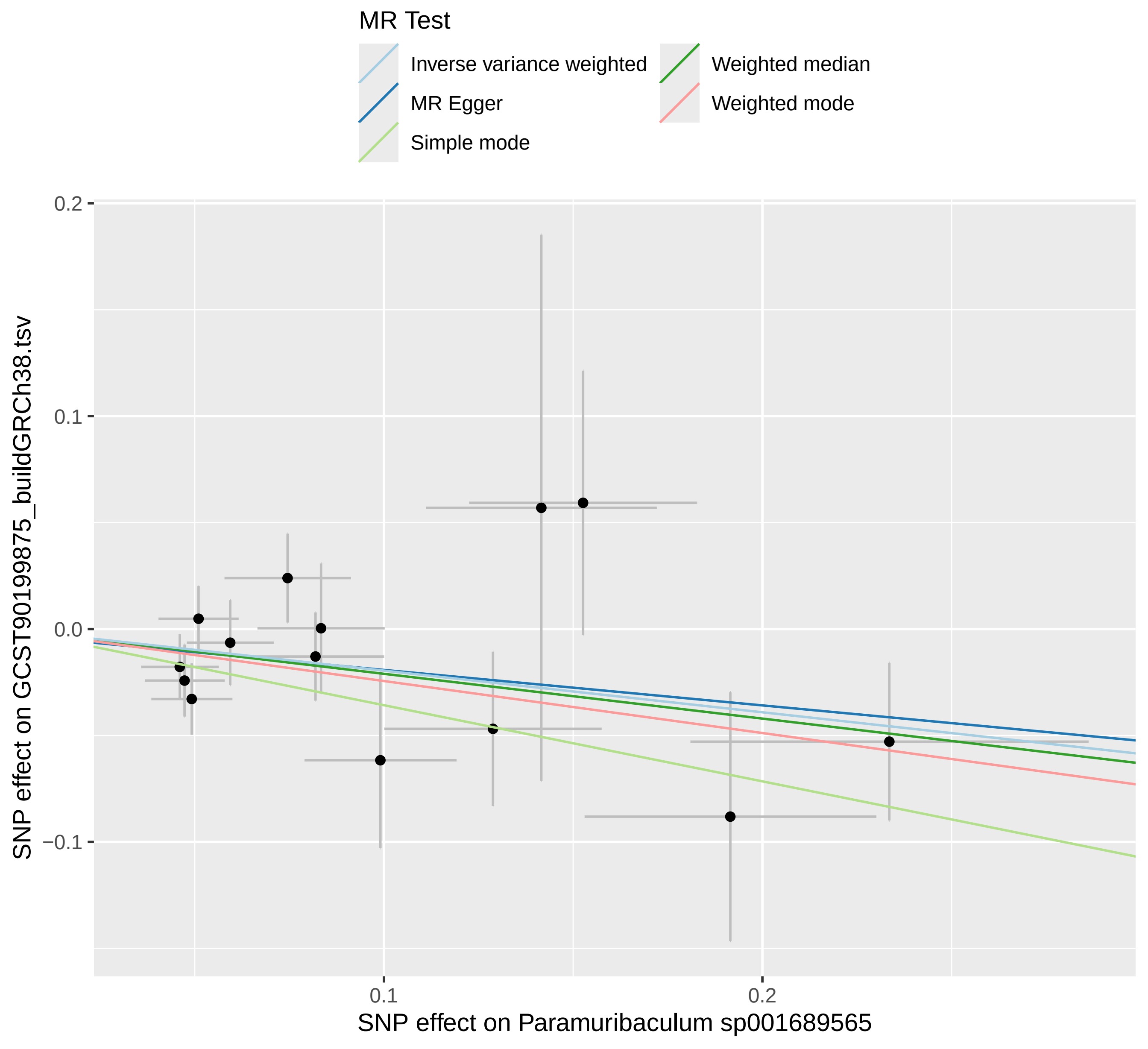

MR Test
Inverse variance weighted
MR Egger
Simple mode
Weighted median
Weighted mode
0.2
0.1
0.0
−0.1
0.1
0.2
SNP effect on GCST90199875_buildGRCh38.tsv
SNP effect on Paramuribaculum sp001689565

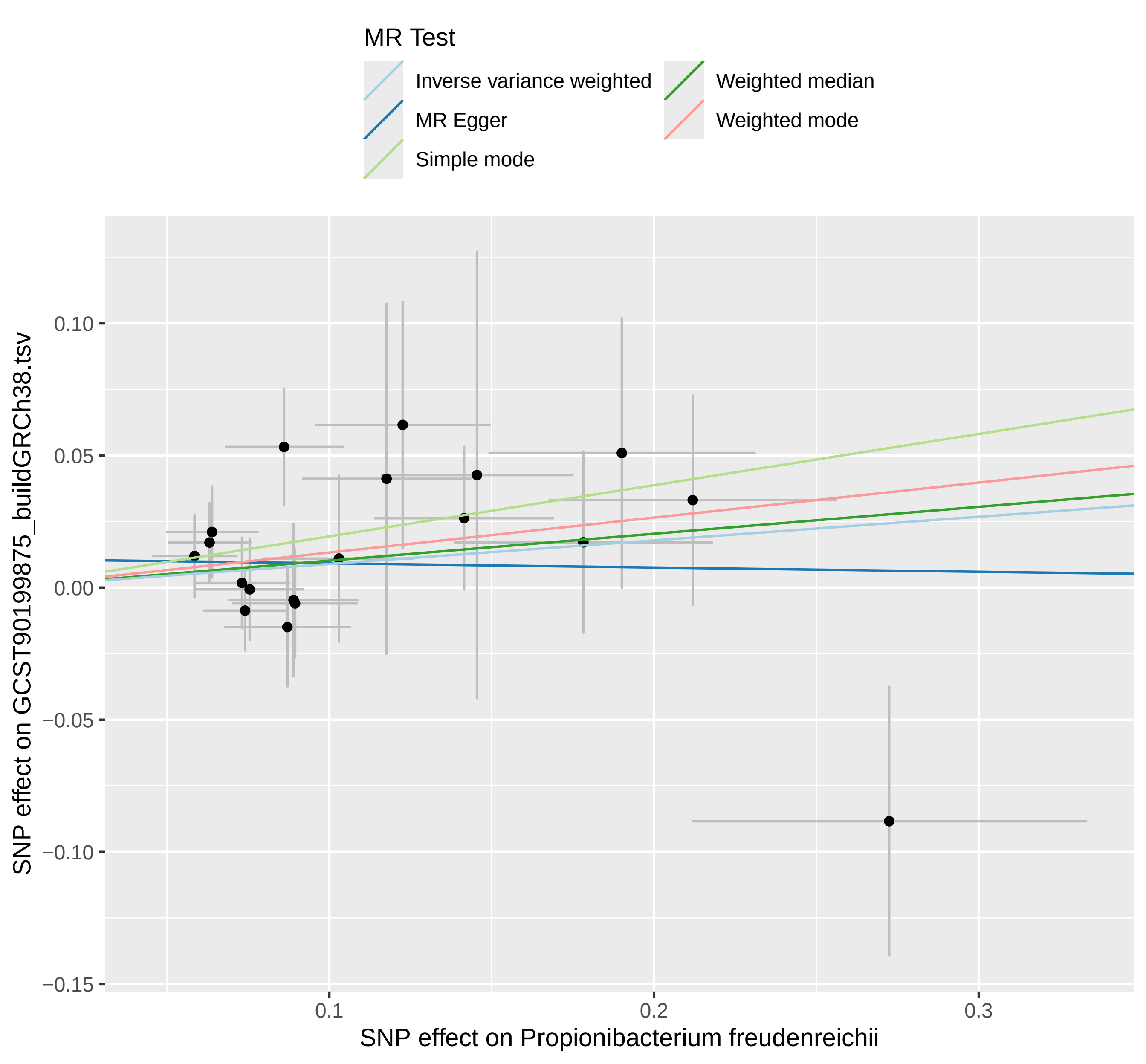

MR Test
Inverse variance weighted
MR Egger
Simple mode
Weighted median
Weighted mode
0.10
0.05
0.00
−0.05
−0.10
−0.15
0.1
0.2
0.3
SNP effect on GCST90199875_buildGRCh38.tsv
SNP effect on Propionibacterium freudenreichii

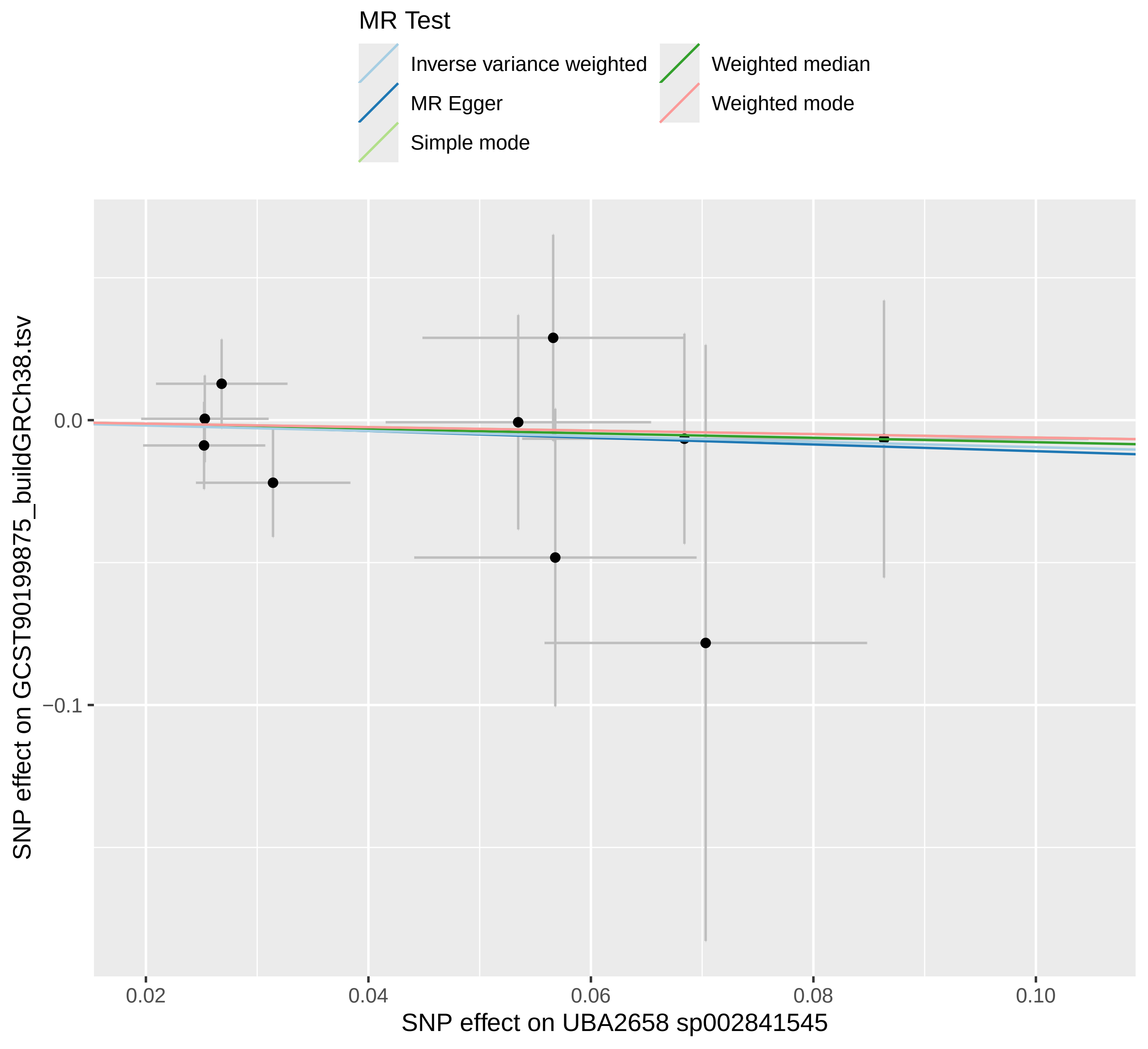

MR Test
Inverse variance weighted
MR Egger
Simple mode
Weighted median
Weighted mode
0.0
−0.1
0.02
0.04
0.06
0.08
0.10
SNP effect on UBA2658 sp002841545
SNP effect on GCST90199875_buildGRCh38.tsv

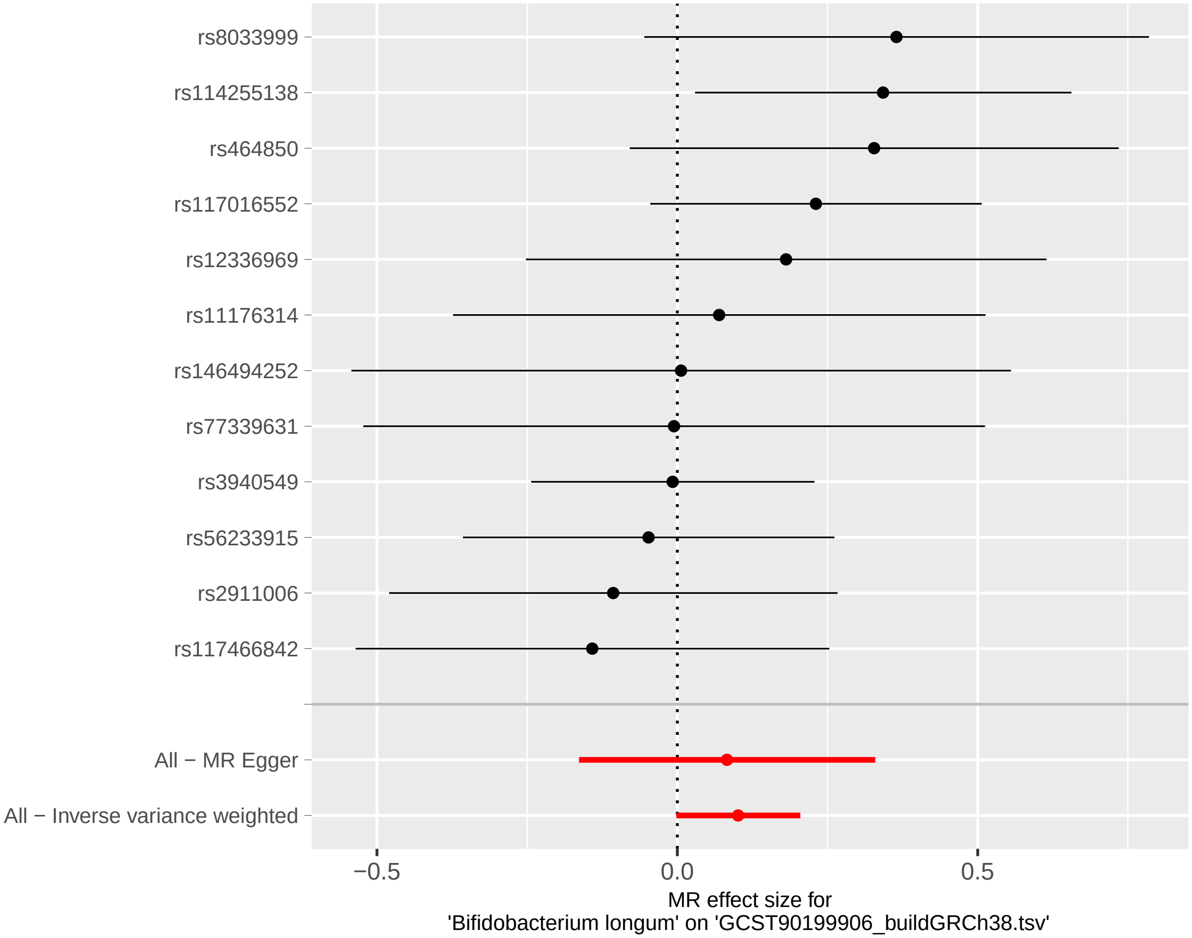
rs8033999
rs114255138
rs464850
rs117016552
rs12336969
rs11176314
rs146494252
rs77339631
rs3940549
rs56233915
rs2911006
rs117466842
All − MR Egger
All − Inverse variance weighted
−0.5
0.0
0.5
MR effect size for
'Bifidobacterium longum' on 'GCST90199906_buildGRCh38.tsv'

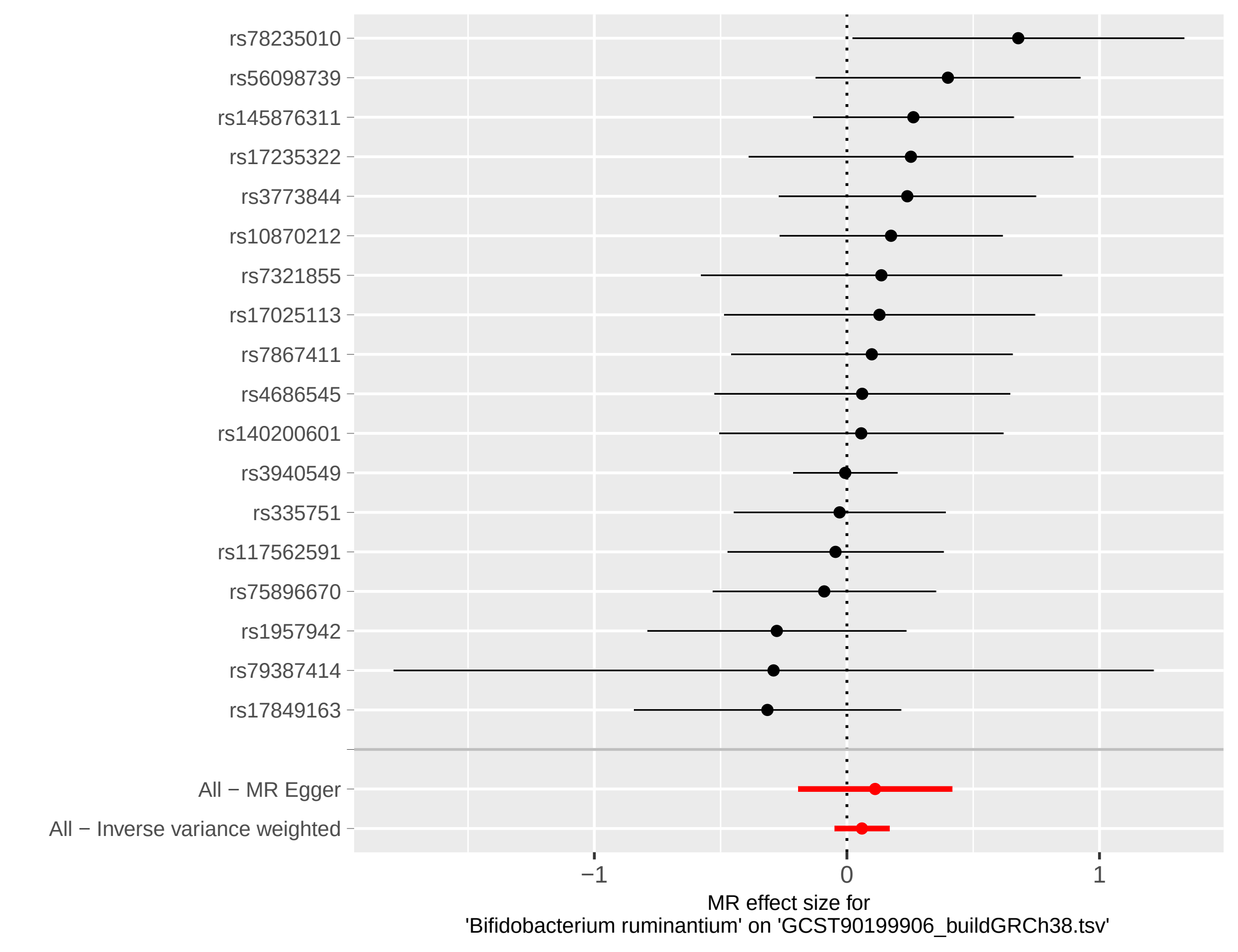

rs78235010
rs56098739
rs145876311
rs17235322
rs3773844
rs10870212
rs7321855
rs17025113
rs7867411
rs4686545
rs140200601
rs3940549
rs335751
rs117562591
rs75896670
rs1957942
rs79387414
rs17849163
All − MR Egger
All − Inverse variance weighted
−1
0
1
MR effect size for
'Bifidobacterium ruminantium' on 'GCST90199906_buildGRCh38.tsv'

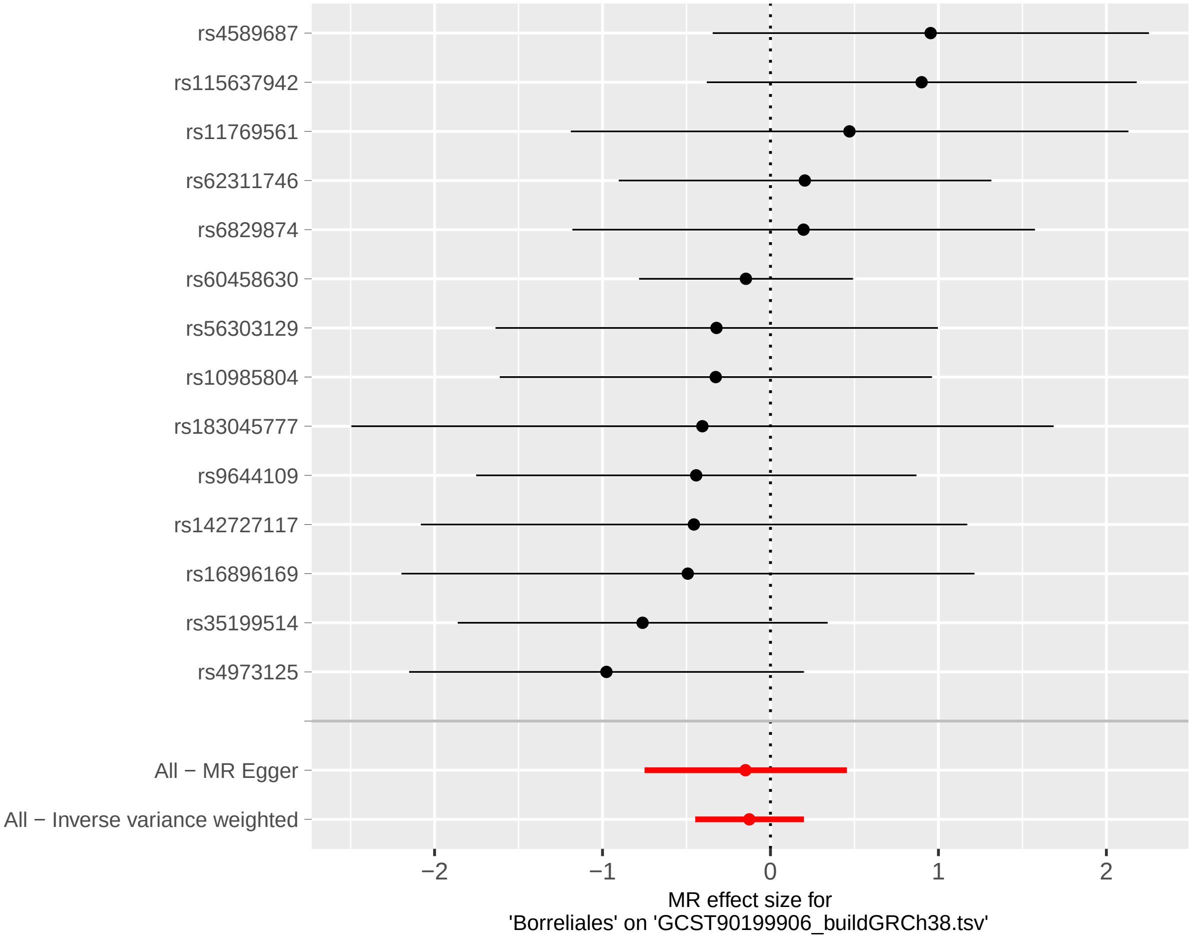
rs4589687
rs115637942
rs11769561
rs62311746
rs6829874
rs60458630
rs56303129
rs10985804
rs183045777
rs9644109
rs142727117
rs16896169
rs35199514
rs4973125
All − MR Egger
All − Inverse variance weighted
−2
−1
0
1
2
MR effect size for
'Borreliales' on 'GCST90199906_buildGRCh38.tsv'

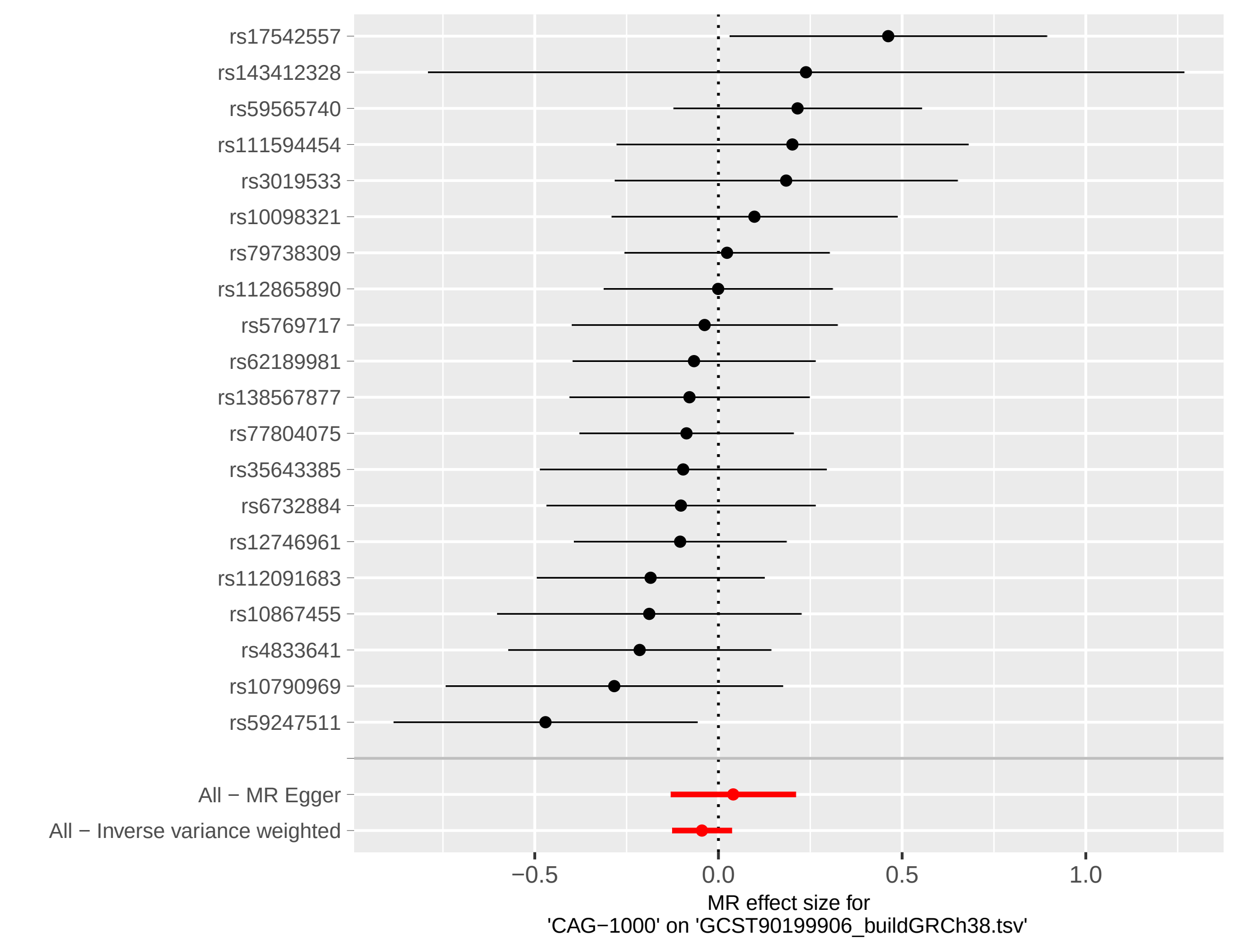

rs17542557
rs143412328
rs59565740
rs111594454
rs3019533
rs10098321
rs79738309
rs112865890
rs5769717
rs62189981
rs138567877
rs77804075
rs35643385
rs6732884
rs12746961
rs112091683
rs10867455
rs4833641
rs10790969
rs59247511
All − MR Egger
All − Inverse variance weighted
−0.5
0.0
0.5
1.0
MR effect size for
'CAG−1000' on 'GCST90199906_buildGRCh38.tsv'

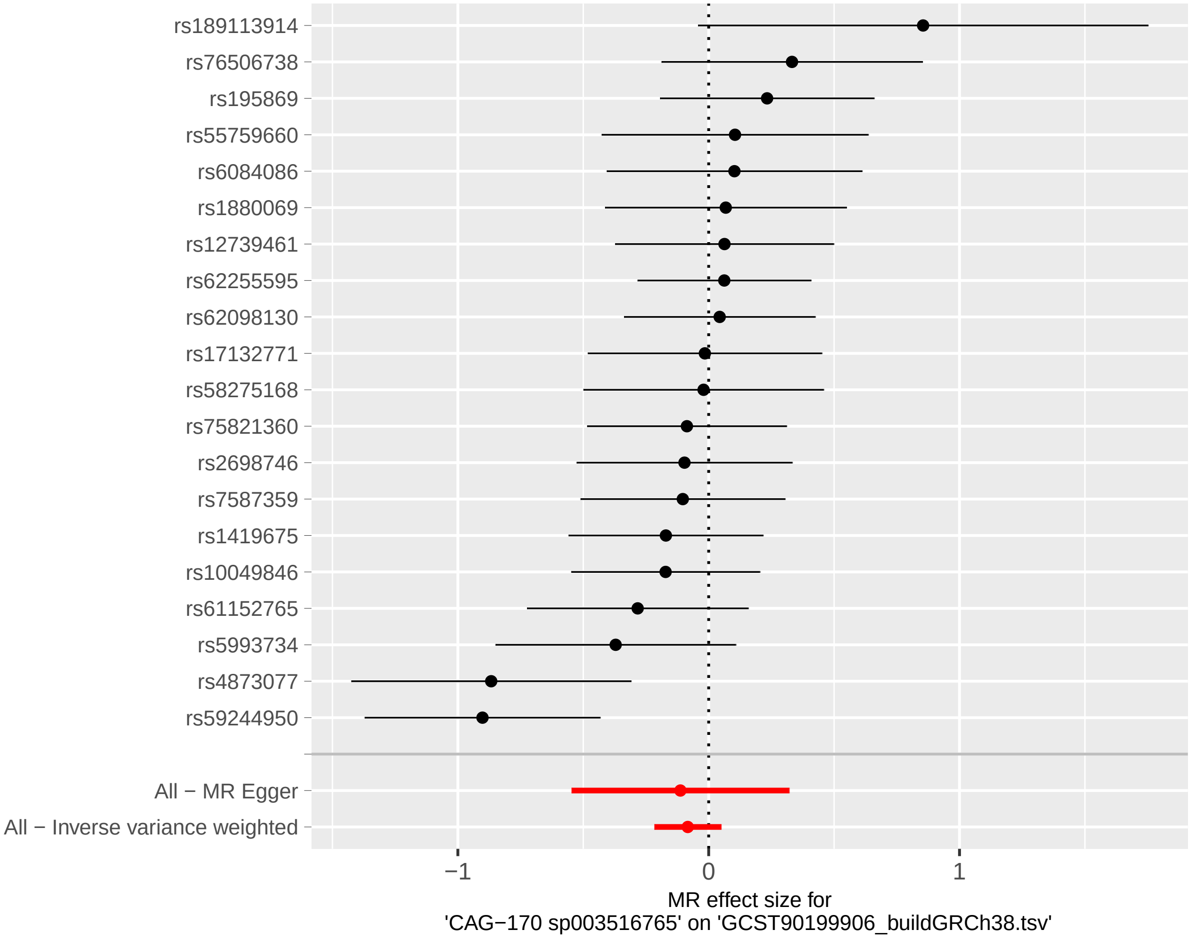

rs189113914
rs76506738
rs195869
rs55759660
rs6084086
rs1880069
rs12739461
rs62255595
rs62098130
rs17132771
rs58275168
rs75821360
rs2698746
rs7587359
rs1419675
rs10049846
rs61152765
rs5993734
rs4873077
rs59244950
All − MR Egger
All − Inverse variance weighted
−1
0
1
MR effect size for
'CAG−170 sp003516765' on 'GCST90199906_buildGRCh38.tsv'

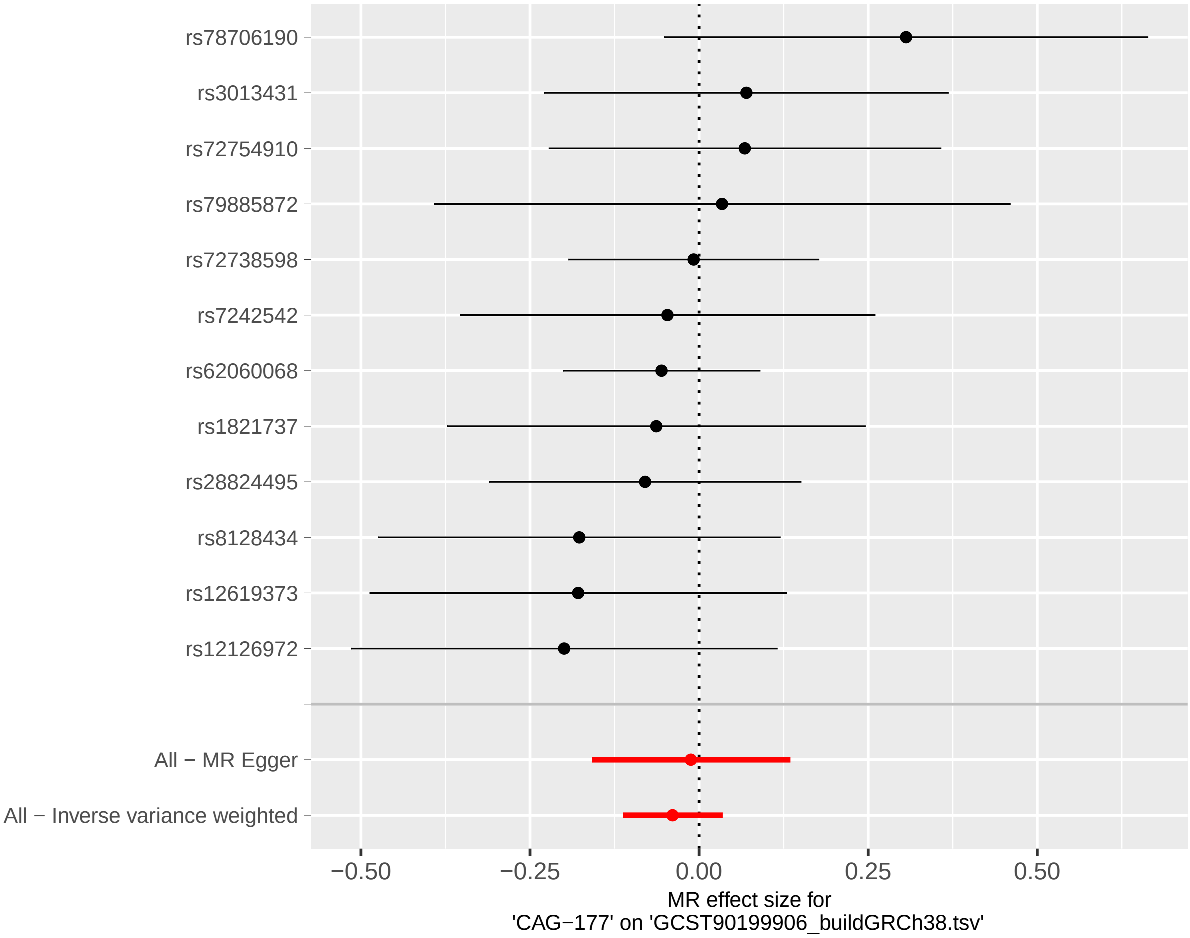

rs78706190
rs3013431
rs72754910
rs79885872
rs72738598
rs7242542
rs62060068
rs1821737
rs28824495
rs8128434
rs12619373
rs12126972
All − MR Egger
All − Inverse variance weighted
−0.50
−0.25
0.00
0.25
0.50
MR effect size for
'CAG−177' on 'GCST90199906_buildGRCh38.tsv'

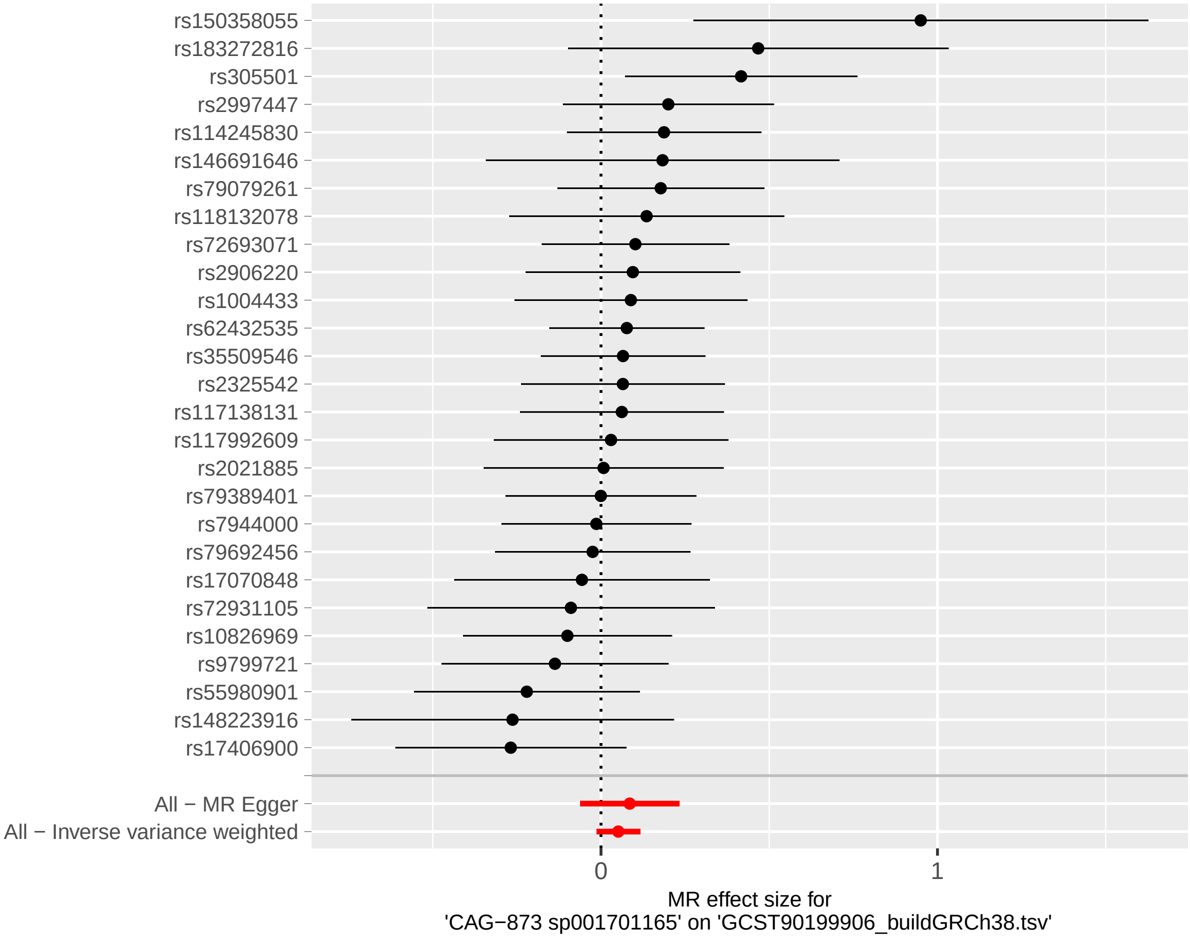

rs150358055
rs183272816
rs305501
rs2997447
rs114245830
rs146691646
rs79079261
rs118132078
rs72693071
rs2906220
rs1004433
rs62432535
rs35509546
rs2325542
rs117138131
rs117992609
rs2021885
rs79389401
rs7944000
rs79692456
rs17070848
rs72931105
rs10826969
rs9799721
rs55980901
rs148223916
rs17406900
All − MR Egger
All − Inverse variance weighted
0
1
MR effect size for
'CAG−873 sp001701165' on 'GCST90199906_buildGRCh38.tsv'

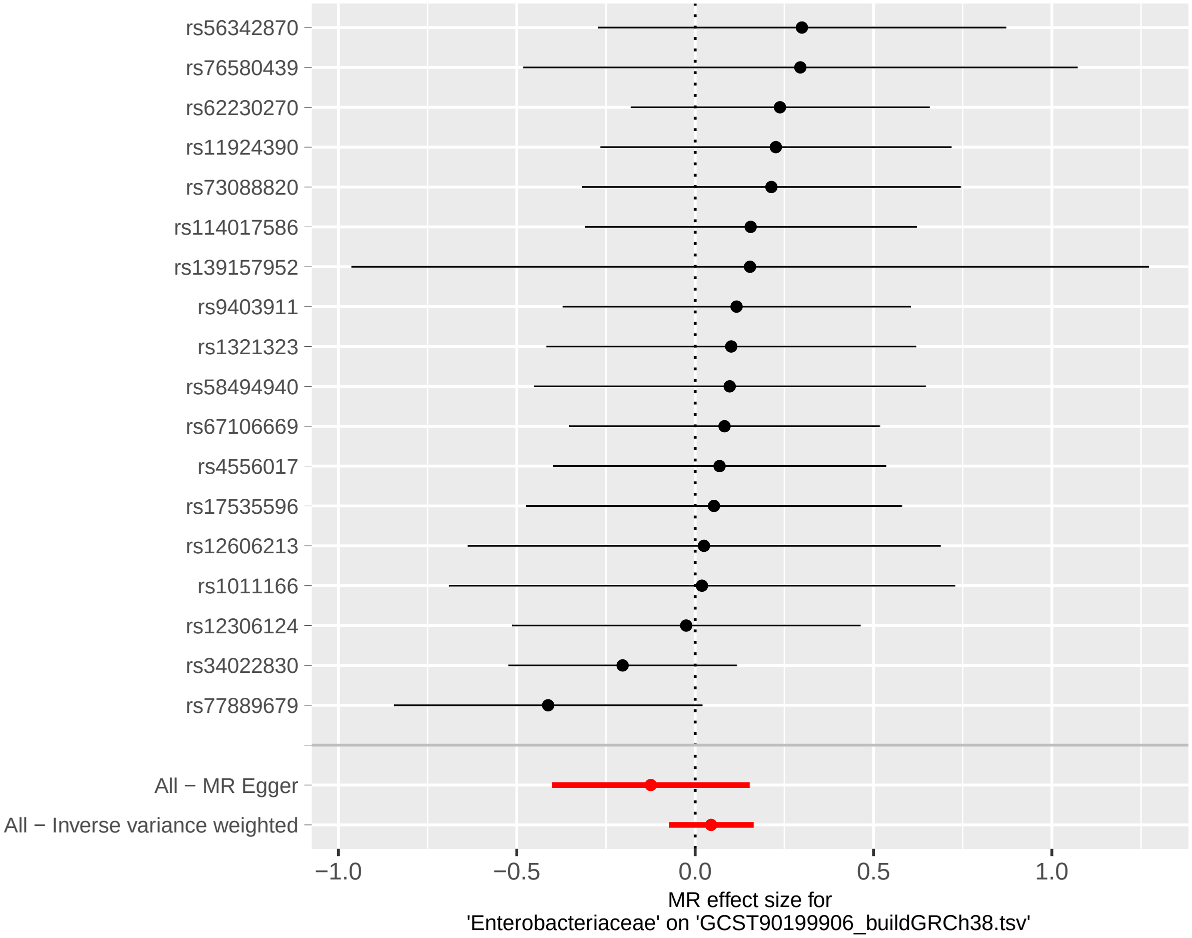
rs56342870
rs76580439
rs62230270
rs11924390
rs73088820
rs114017586
rs139157952
rs9403911
rs1321323
rs58494940
rs67106669
rs4556017
rs17535596
rs12606213
rs1011166
rs12306124
rs34022830
rs77889679
All − MR Egger
All − Inverse variance weighted
−1.0
−0.5
0.0
0.5
1.0
MR effect size for
'Enterobacteriaceae' on 'GCST90199906_buildGRCh38.tsv'

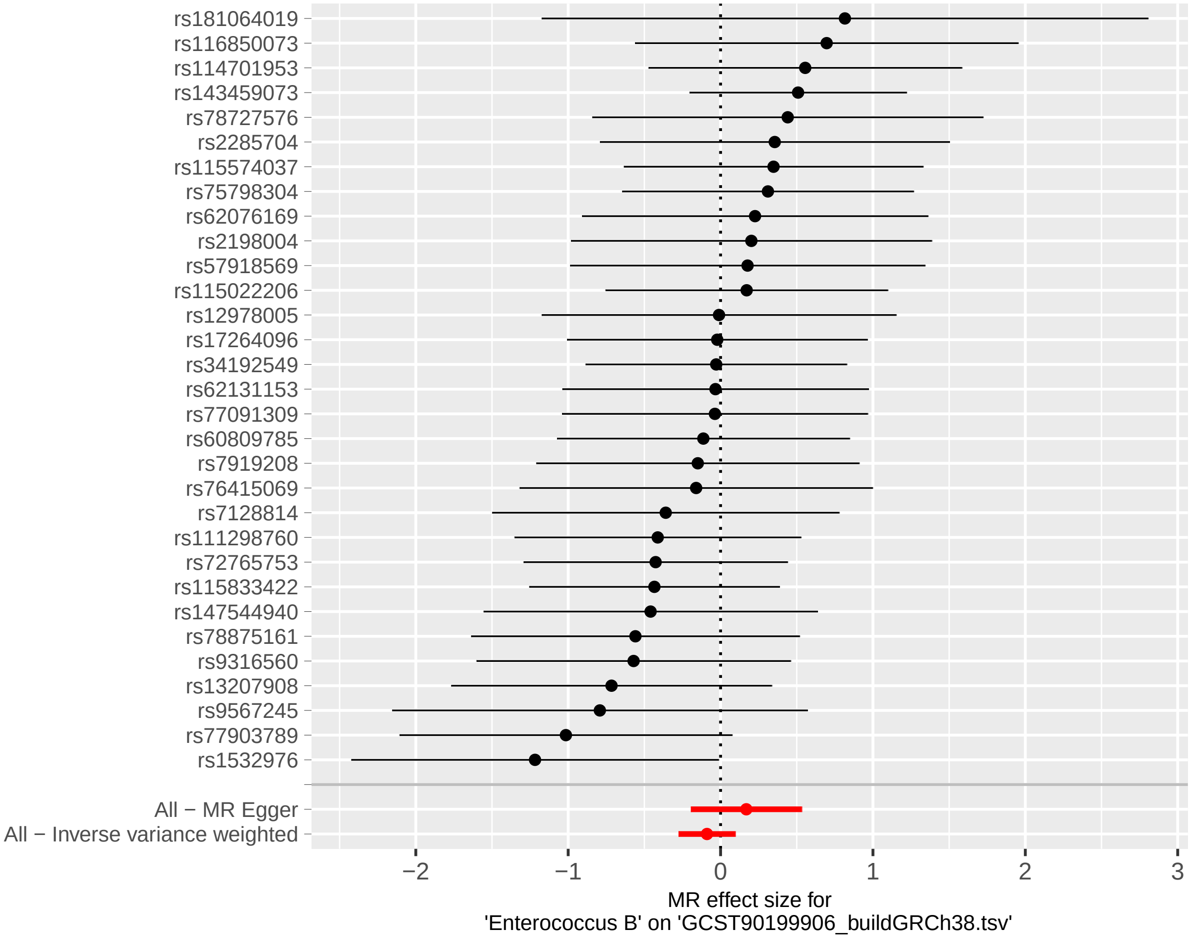
rs181064019
rs116850073
rs114701953
rs143459073
rs78727576
rs2285704
rs115574037
rs75798304
rs62076169
rs2198004
rs57918569
rs115022206
rs12978005
rs17264096
rs34192549
rs62131153
rs77091309
rs60809785
rs7919208
rs76415069
rs7128814
rs111298760
rs72765753
rs115833422
rs147544940
rs78875161
rs9316560
rs13207908
rs9567245
rs77903789
rs1532976
All − MR Egger
All − Inverse variance weighted
−2
−1
0
1
2
3
MR effect size for
'Enterococcus B' on 'GCST90199906_buildGRCh38.tsv'

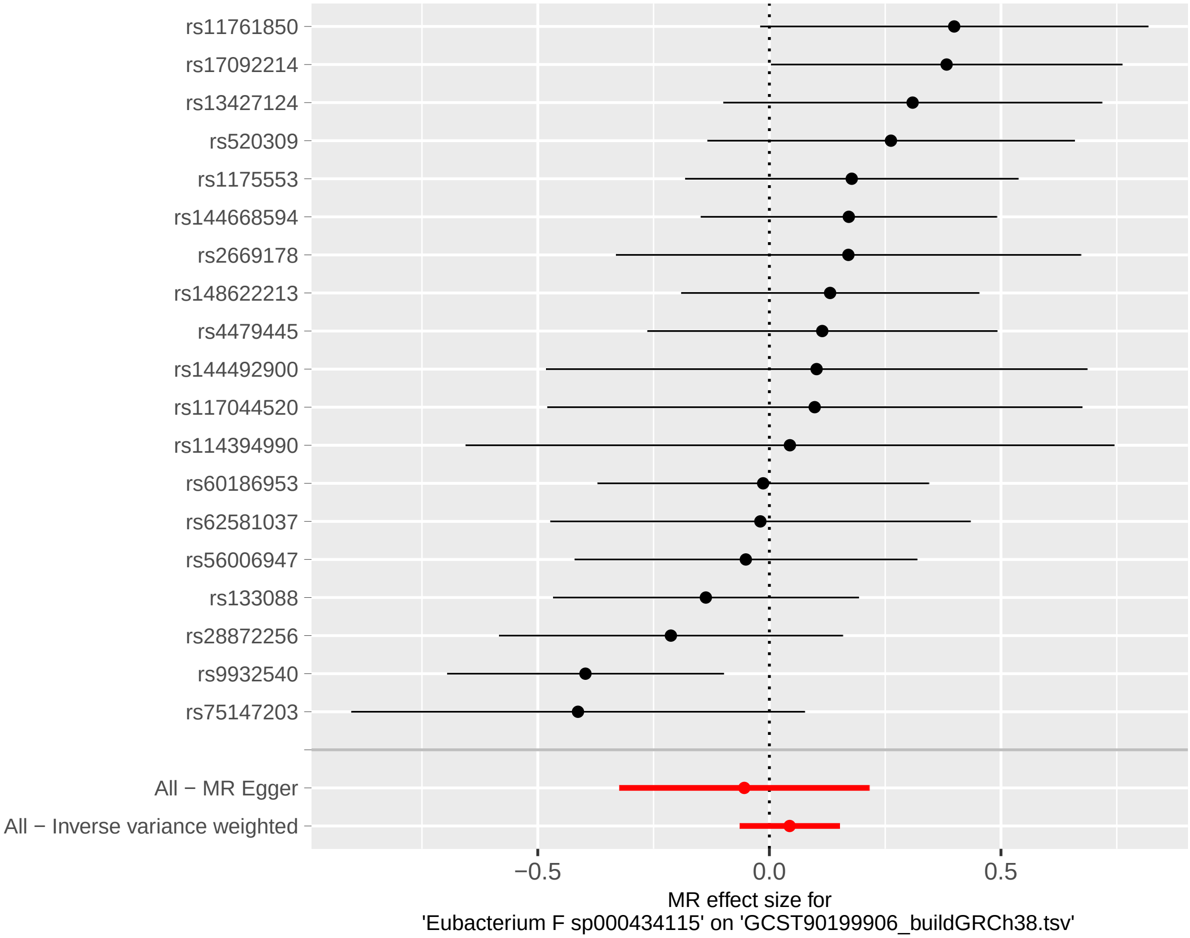

rs11761850
rs17092214
rs13427124
rs520309
rs1175553
rs144668594
rs2669178
rs148622213
rs4479445
rs144492900
rs117044520
rs114394990
rs60186953
rs62581037
rs56006947
rs133088
rs28872256
rs9932540
rs75147203
All − MR Egger
All − Inverse variance weighted
−0.5
0.0
0.5
MR effect size for
'Eubacterium F sp000434115' on 'GCST90199906_buildGRCh38.tsv'

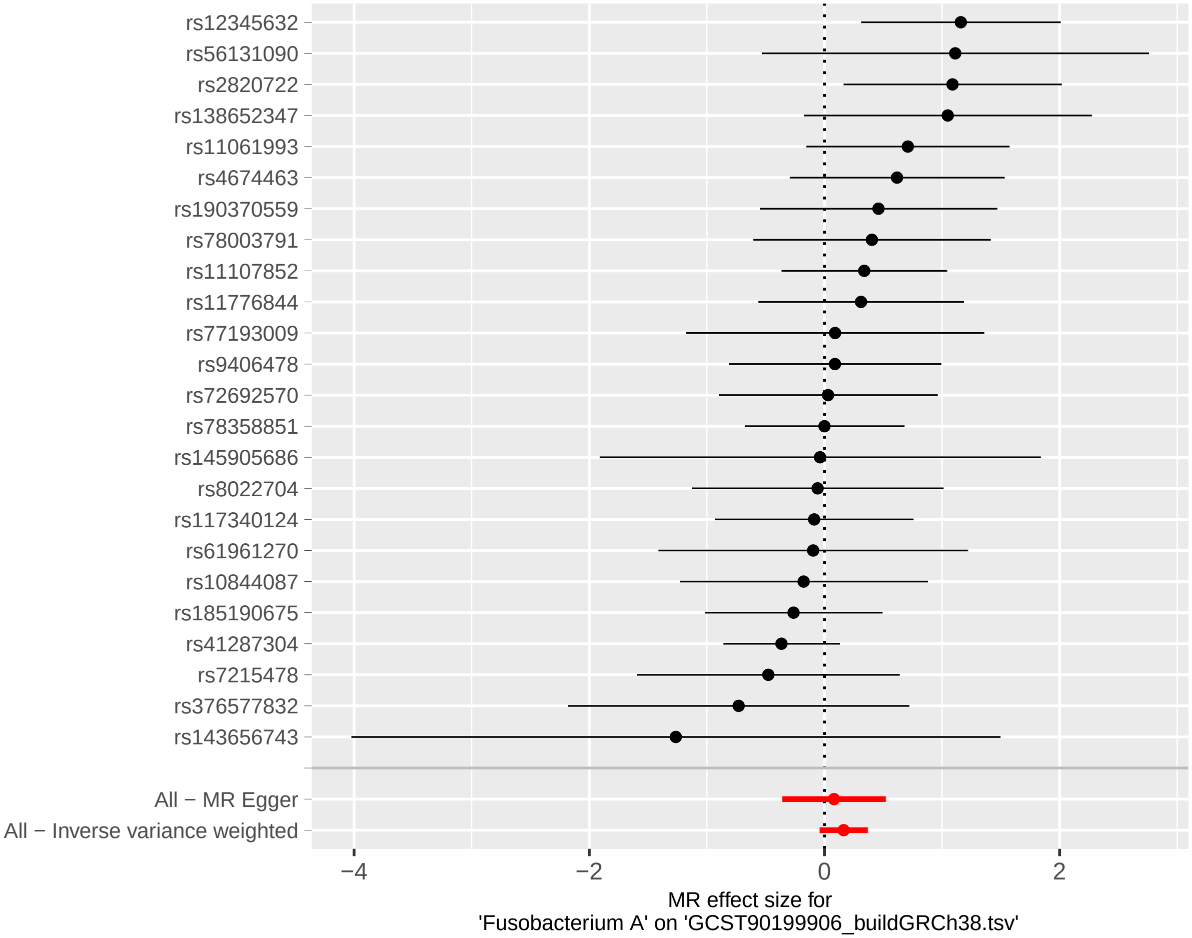
rs12345632
rs56131090
rs2820722
rs138652347
rs11061993
rs4674463
rs190370559
rs78003791
rs11107852
rs11776844
rs77193009
rs9406478
rs72692570
rs78358851
rs145905686
rs8022704
rs117340124
rs61961270
rs10844087
rs185190675
rs41287304
rs7215478
rs376577832
rs143656743
All − MR Egger
All − Inverse variance weighted
−4
−2
0
2
MR effect size for
'Fusobacterium A' on 'GCST90199906_buildGRCh38.tsv'

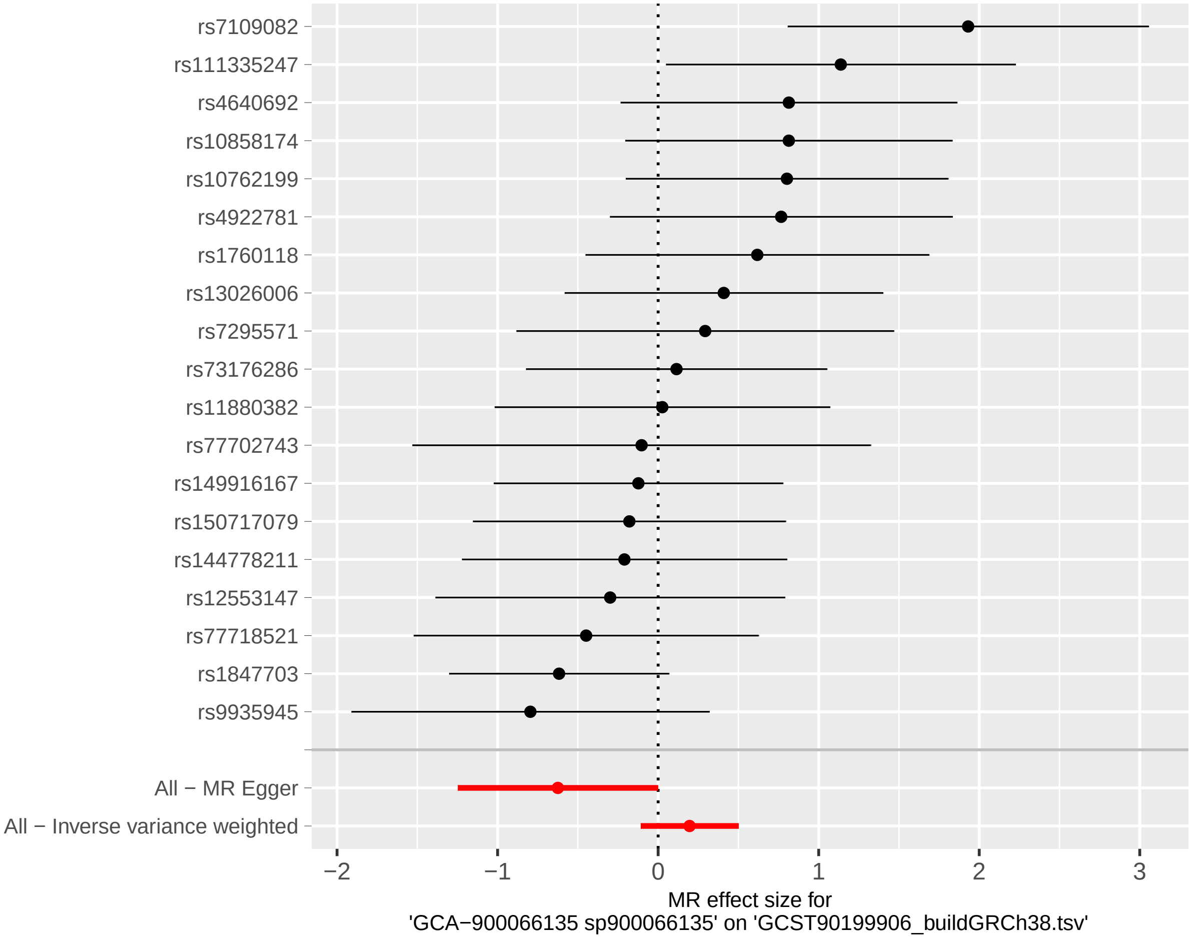

rs7109082
rs111335247
rs4640692
rs10858174
rs10762199
rs4922781
rs1760118
rs13026006
rs7295571
rs73176286
rs11880382
rs77702743
rs149916167
rs150717079
rs144778211
rs12553147
rs77718521
rs1847703
rs9935945
All − MR Egger
All − Inverse variance weighted
−2
−1
0
1
2
3
MR effect size for
'GCA−900066135 sp900066135' on 'GCST90199906_buildGRCh38.tsv'

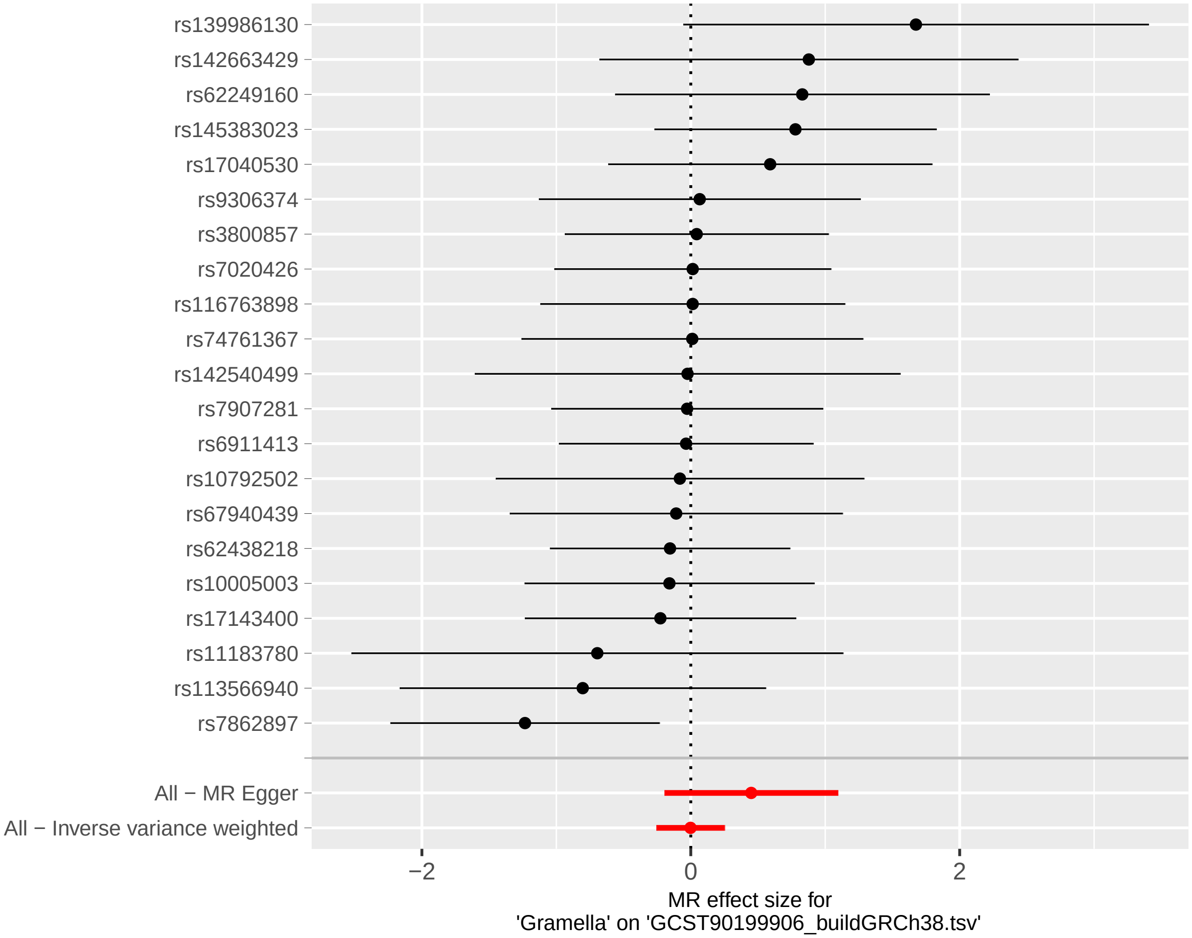
rs139986130
rs142663429
rs62249160
rs145383023
rs17040530
rs9306374
rs3800857
rs7020426
rs116763898
rs74761367
rs142540499
rs7907281
rs6911413
rs10792502
rs67940439
rs62438218
rs10005003
rs17143400
rs11183780
rs113566940
rs7862897
All − MR Egger
All − Inverse variance weighted
−2
0
2
MR effect size for
'Gramella' on 'GCST90199906_buildGRCh38.tsv'

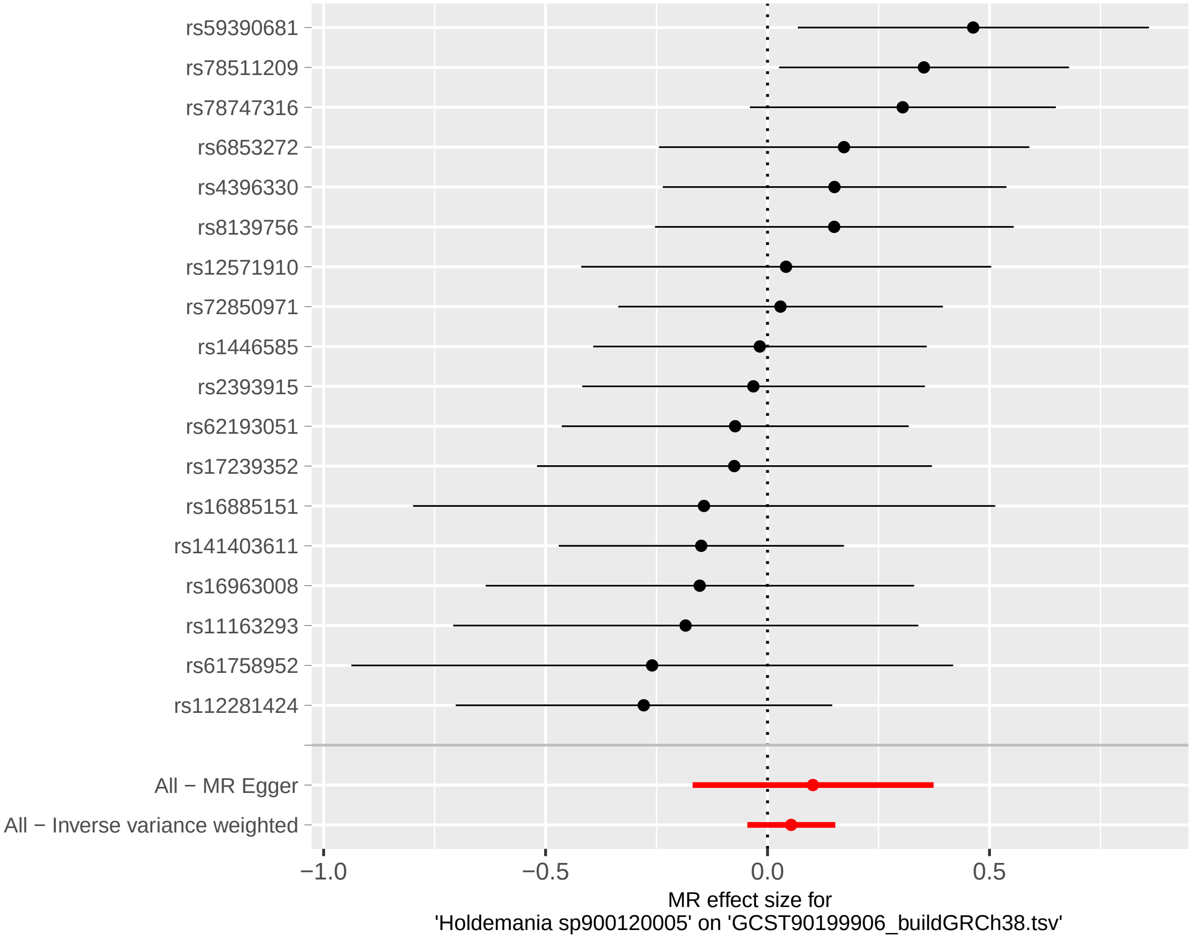
rs59390681
rs78511209
rs78747316
rs6853272
rs4396330
rs8139756
rs12571910
rs72850971
rs1446585
rs2393915
rs62193051
rs17239352
rs16885151
rs141403611
rs16963008
rs11163293
rs61758952
rs112281424
All − MR Egger
All − Inverse variance weighted
−1.0
−0.5
0.0
0.5
MR effect size for
'Holdemania sp900120005' on 'GCST90199906_buildGRCh38.tsv'

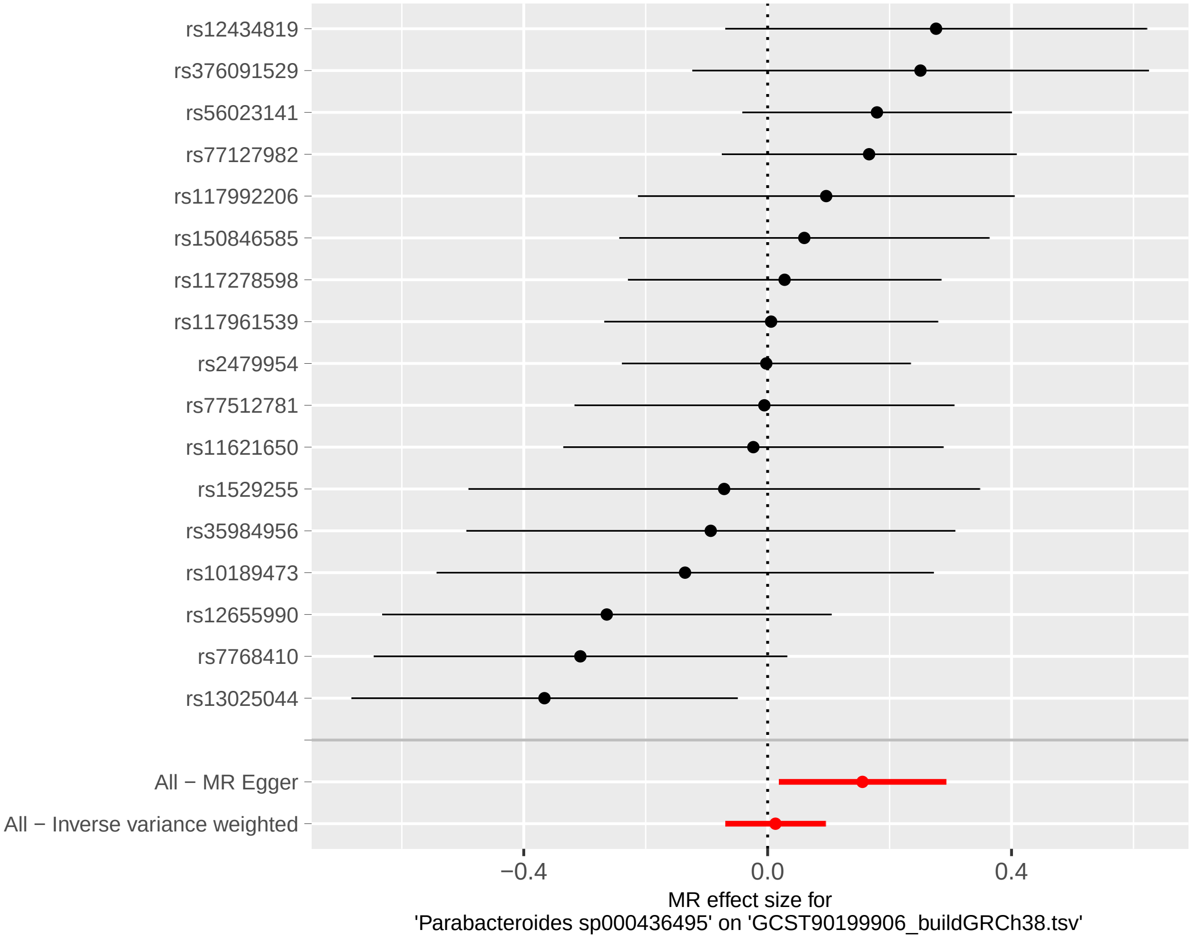
rs12434819
rs376091529
rs56023141
rs77127982
rs117992206
rs150846585
rs117278598
rs117961539
rs2479954
rs77512781
rs11621650
rs1529255
rs35984956
rs10189473
rs12655990
rs7768410
rs13025044
All − MR Egger
All − Inverse variance weighted
−0.4
0.0
0.4
MR effect size for
'Parabacteroides sp000436495' on 'GCST90199906_buildGRCh38.tsv'

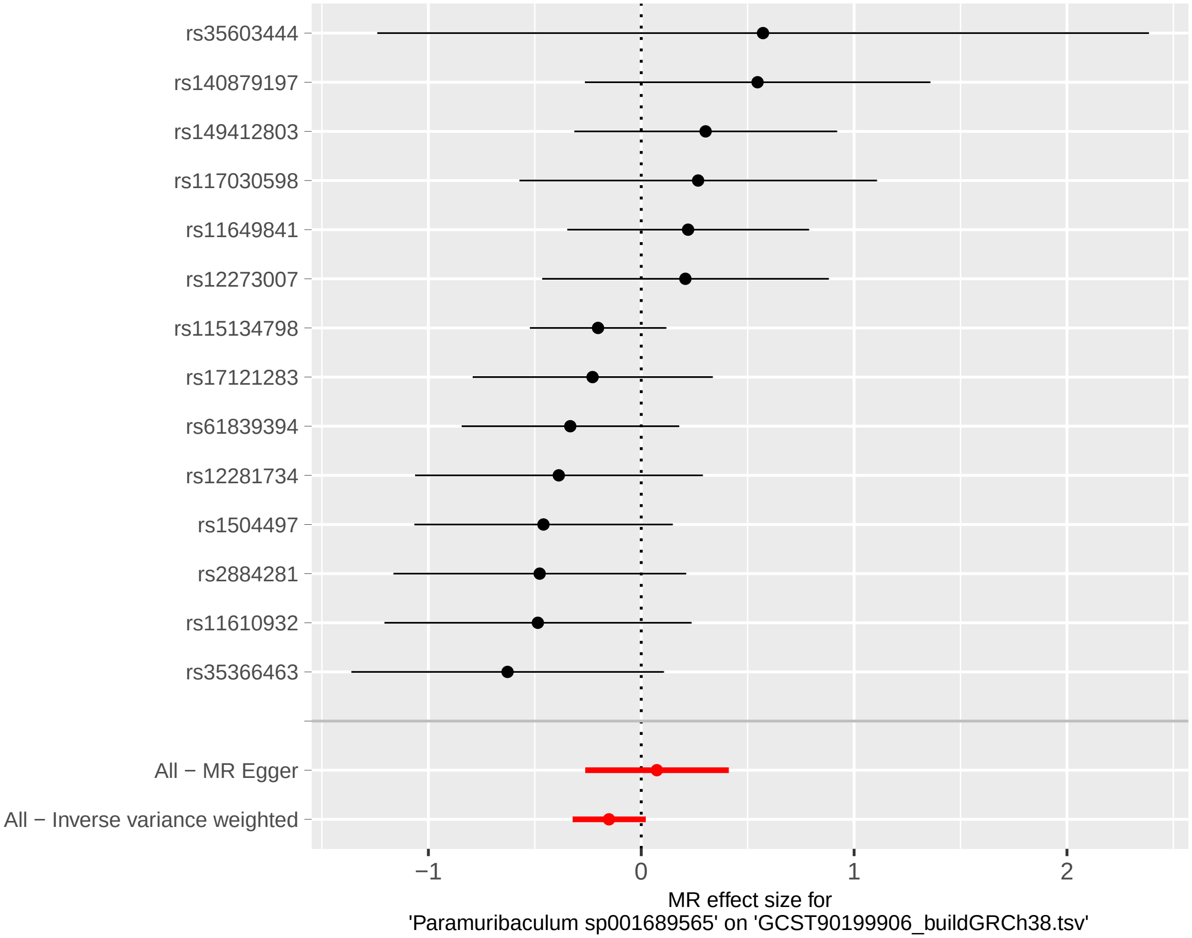
rs35603444
rs140879197
rs149412803
rs117030598
rs11649841
rs12273007
rs115134798
rs17121283
rs61839394
rs12281734
rs1504497
rs2884281
rs11610932
rs35366463
All − MR Egger
All − Inverse variance weighted
−1
0
1
2
MR effect size for
'Paramuribaculum sp001689565' on 'GCST90199906_buildGRCh38.tsv'

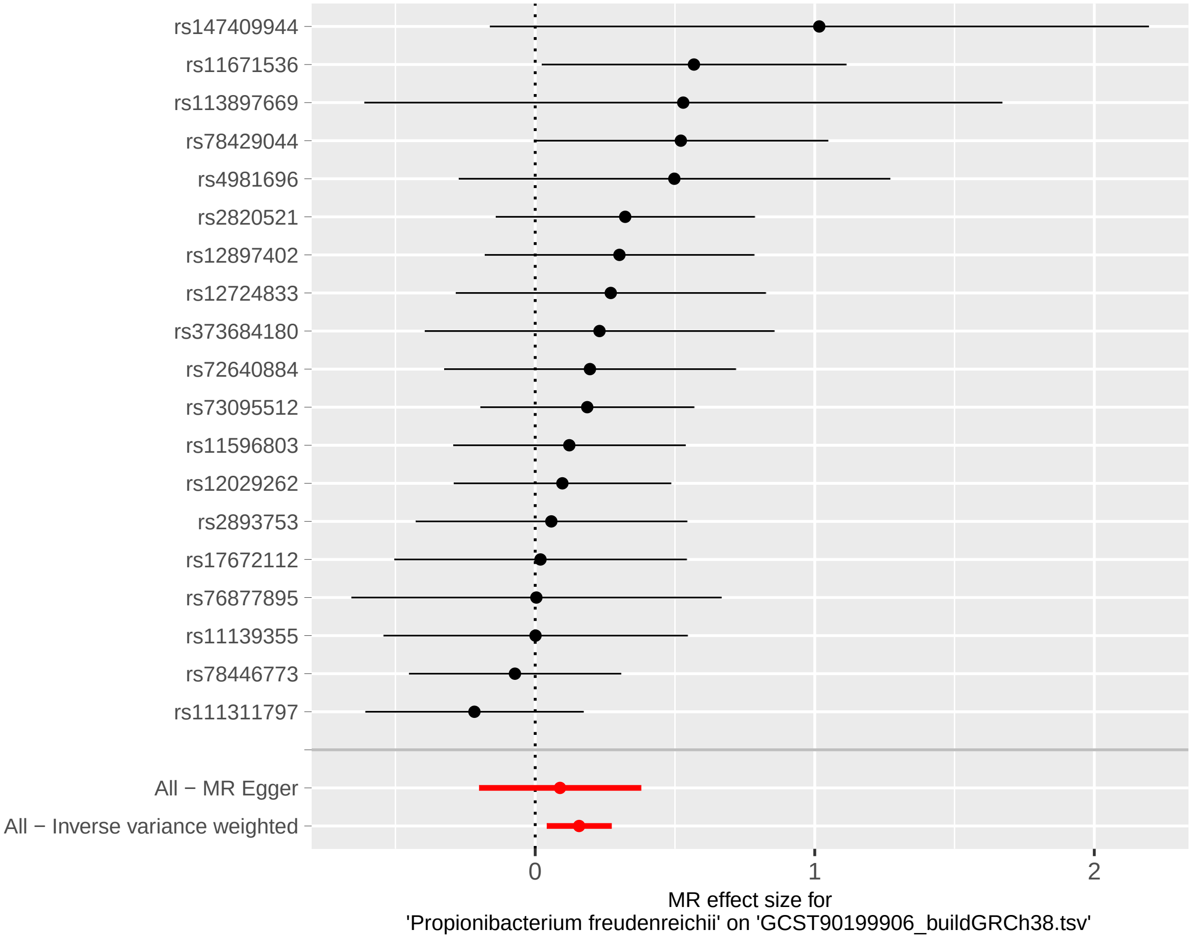

rs147409944
rs11671536
rs113897669
rs78429044
rs4981696
rs2820521
rs12897402
rs12724833
rs373684180
rs72640884
rs73095512
rs11596803
rs12029262
rs2893753
rs17672112
rs76877895
rs11139355
rs78446773
rs111311797
All − MR Egger
All − Inverse variance weighted
0
1
2
MR effect size for
'Propionibacterium freudenreichii' on 'GCST90199906_buildGRCh38.tsv'

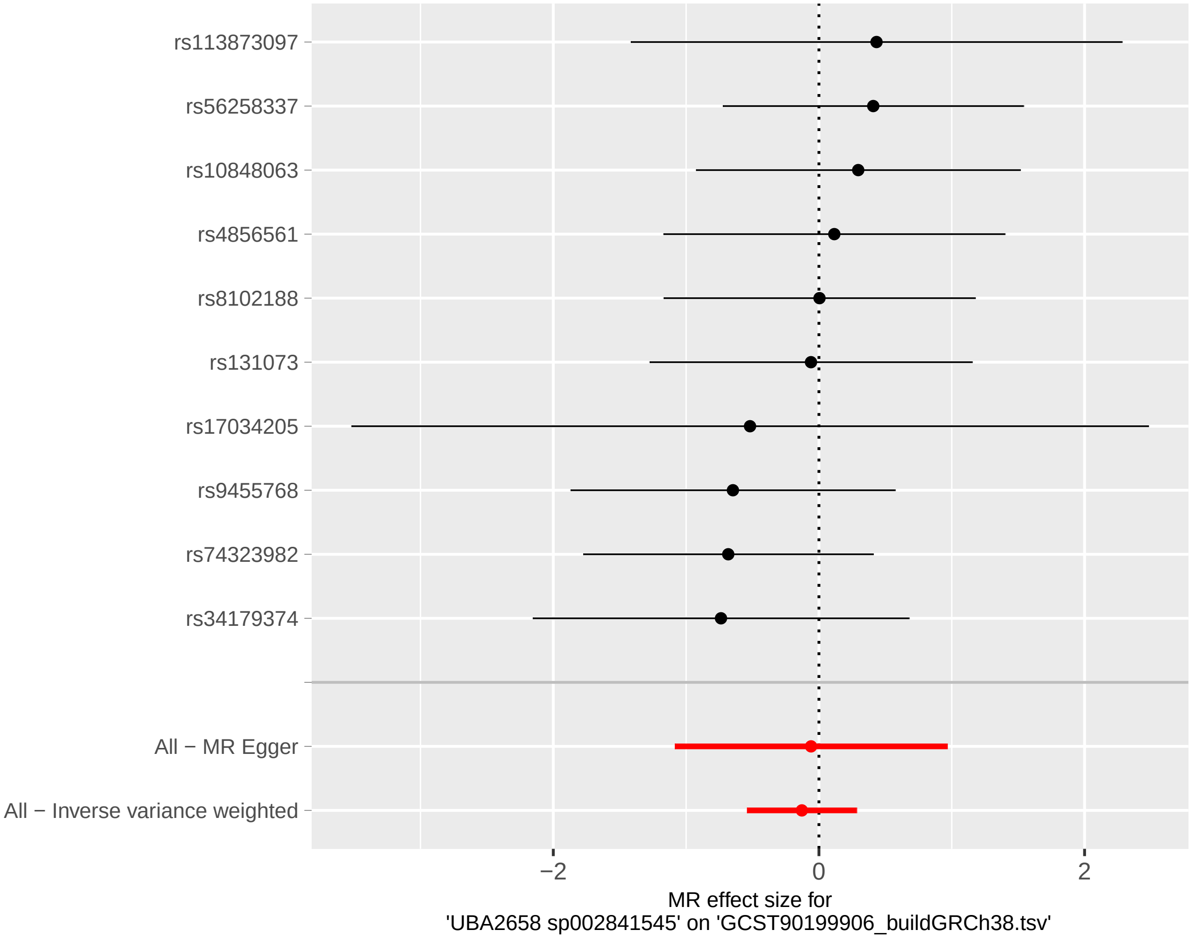

rs113873097
rs56258337
rs10848063
rs4856561
rs8102188
rs131073
rs17034205
rs9455768
rs74323982
rs34179374
All − MR Egger
All − Inverse variance weighted
−2
0
2
MR effect size for
'UBA2658 sp002841545' on 'GCST90199906_buildGRCh38.tsv'

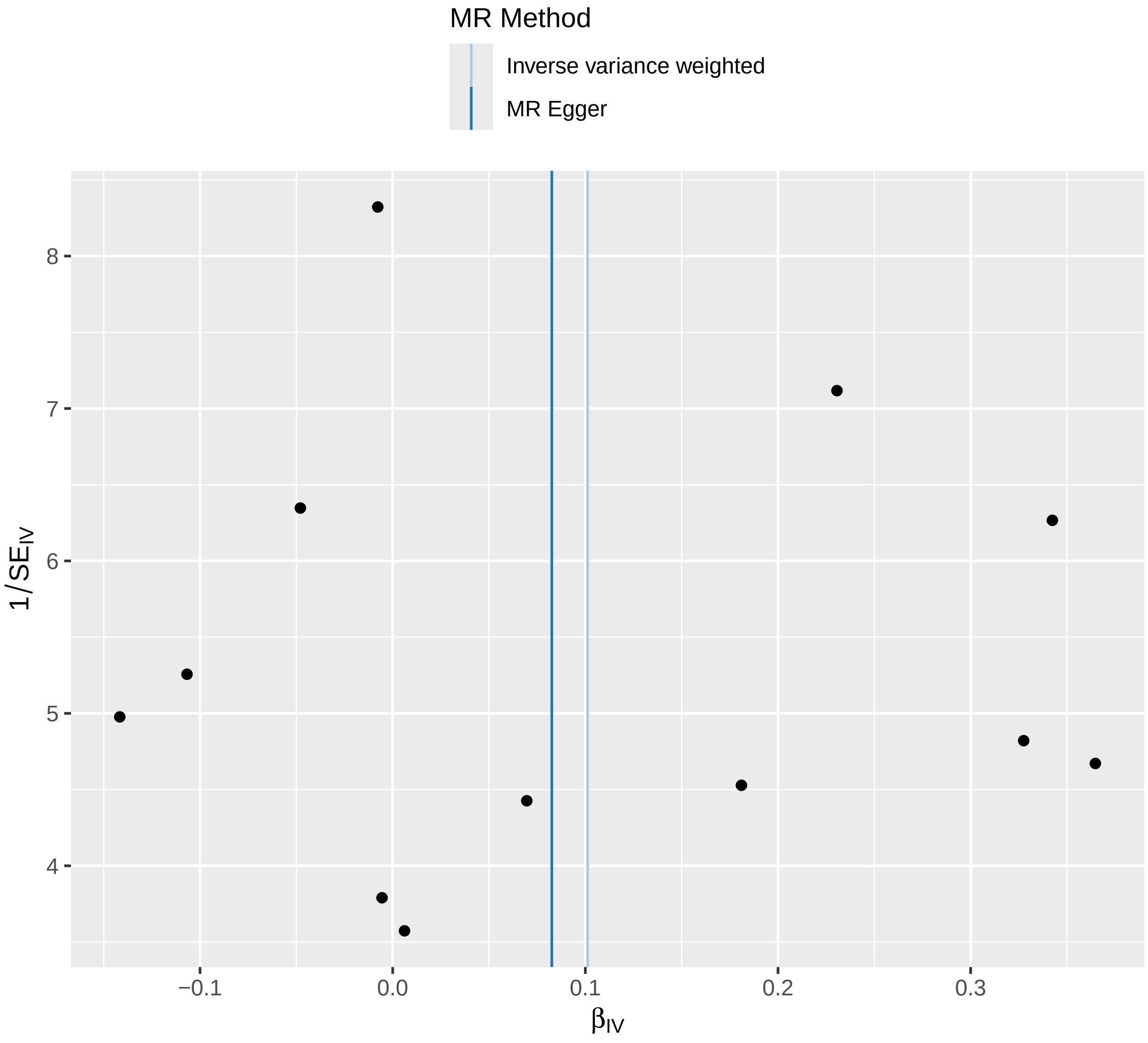
MR Method
Inverse variance weighted
MR Egger
8
7
6
5
4
$1/SE_{IV}$
−0.1
0.0
0.1
0.2
0.3
β_{IV}

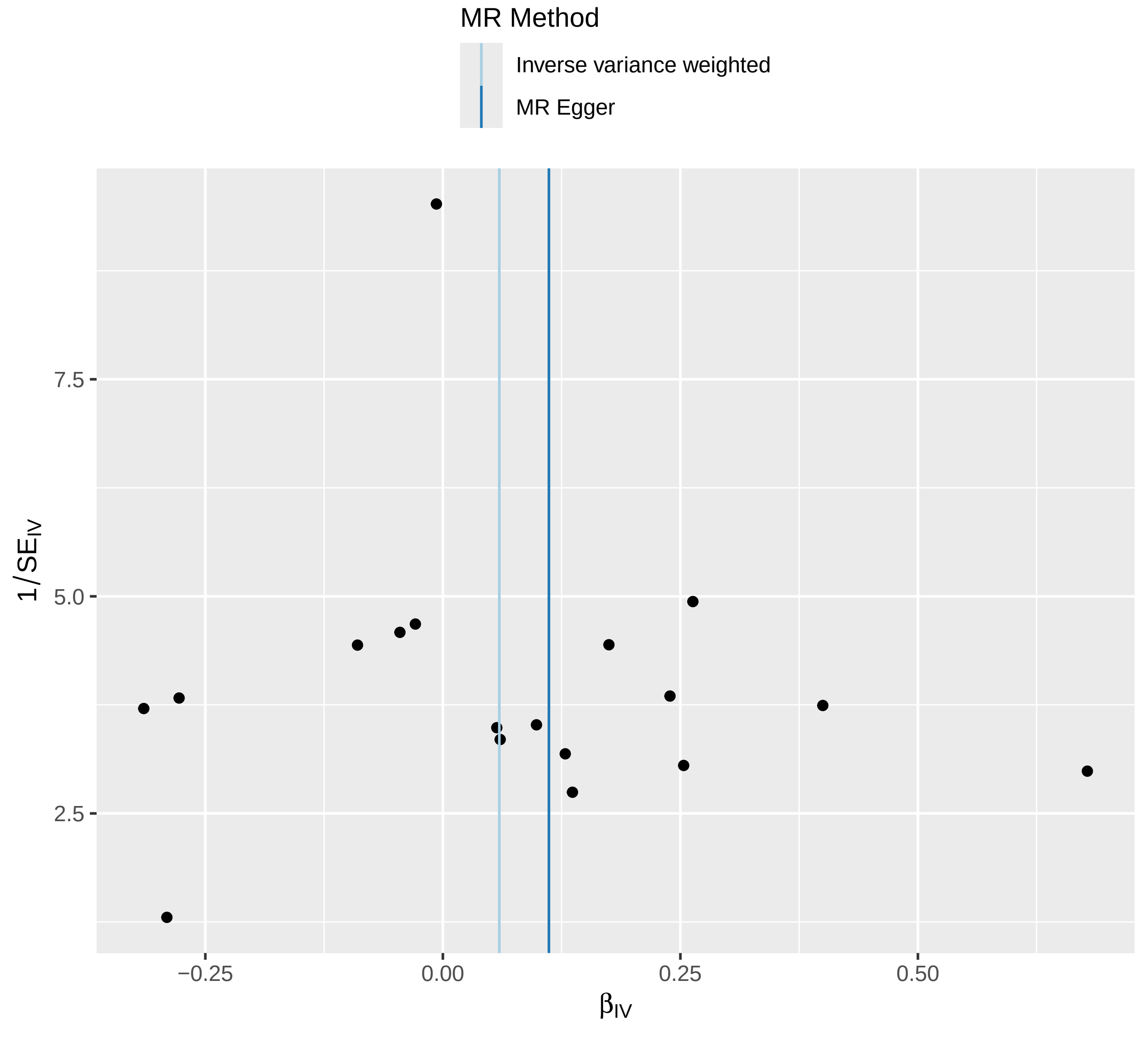

MR Method
Inverse variance weighted
MR Egger
7.5
5.0
2.5
$1/SE_{IV}$
−0.25
0.00
0.25
0.50
β_{IV}

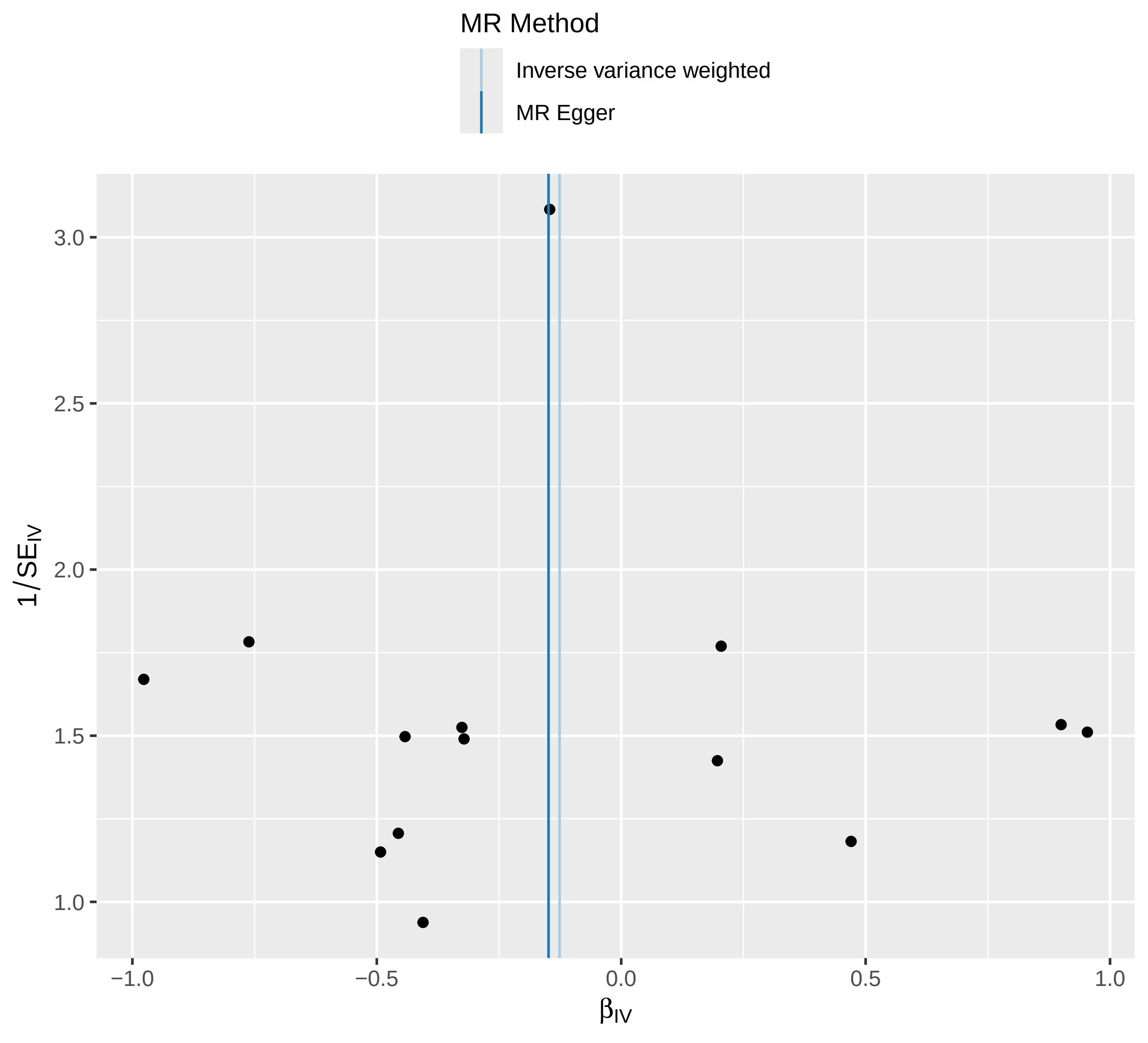

MR Method
Inverse variance weighted
MR Egger
1/SE_IV
3.0
2.5
2.0
1.5
1.0
−1.0
−0.5
0.0
0.5
1.0
β_IV

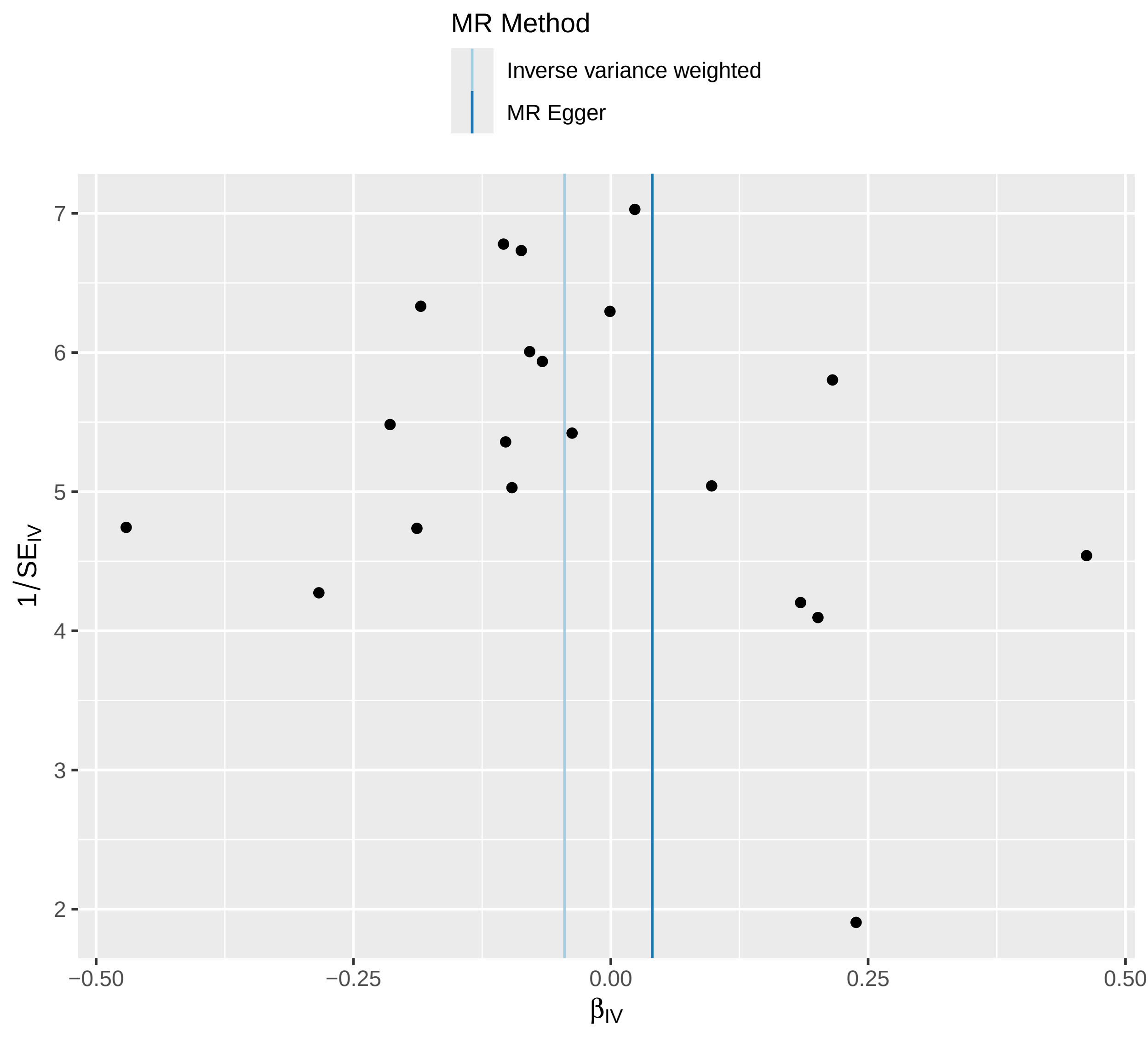

MR Method
Inverse variance weighted
MR Egger
1/SE_IV
β_IV
7
6
5
4
3
2
−0.50
−0.25
0.00
0.25
0.50

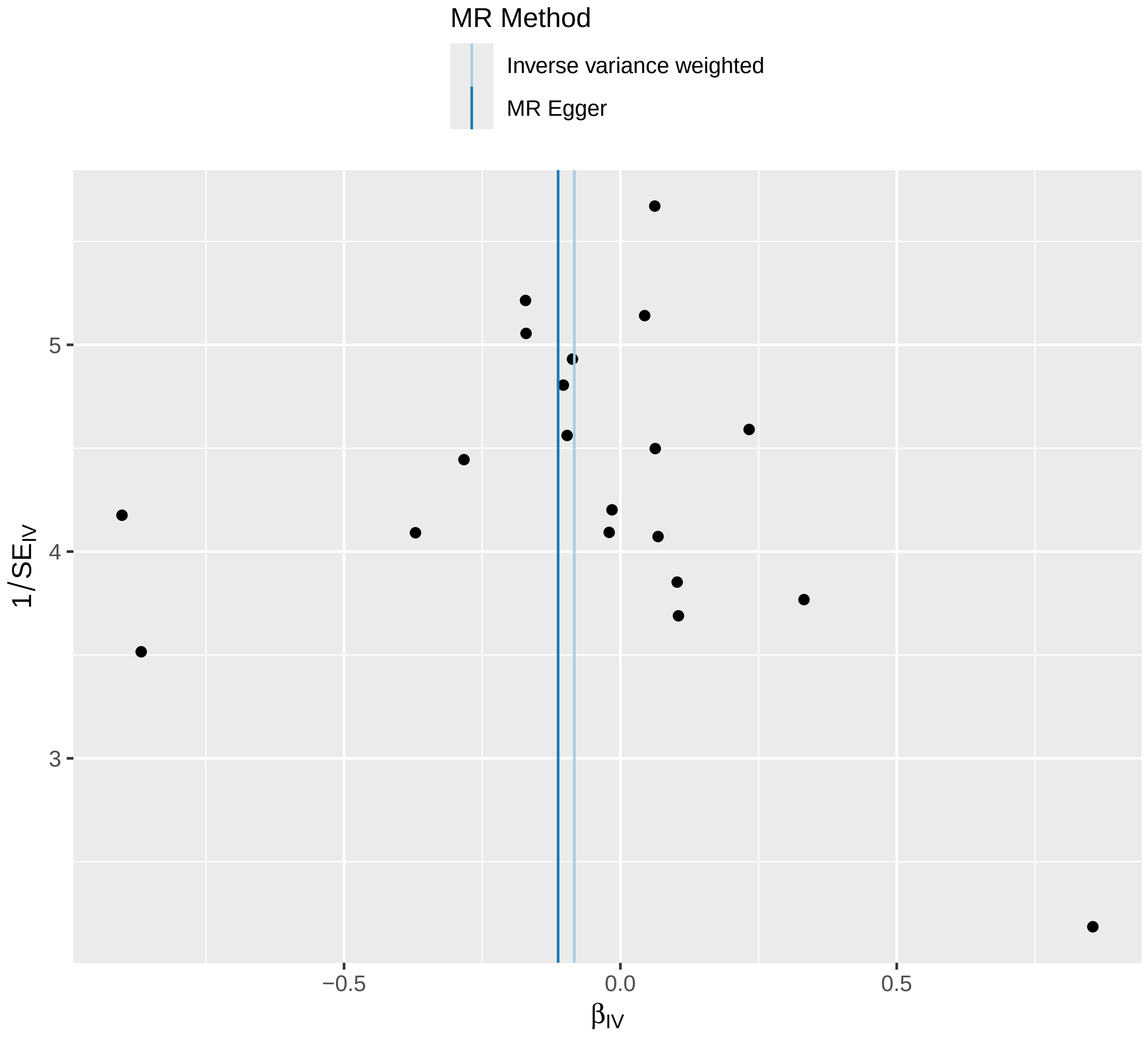

MR Method
Inverse variance weighted
MR Egger
5
4
3
1/SE_IV
−0.5
0.0
0.5
β_IV

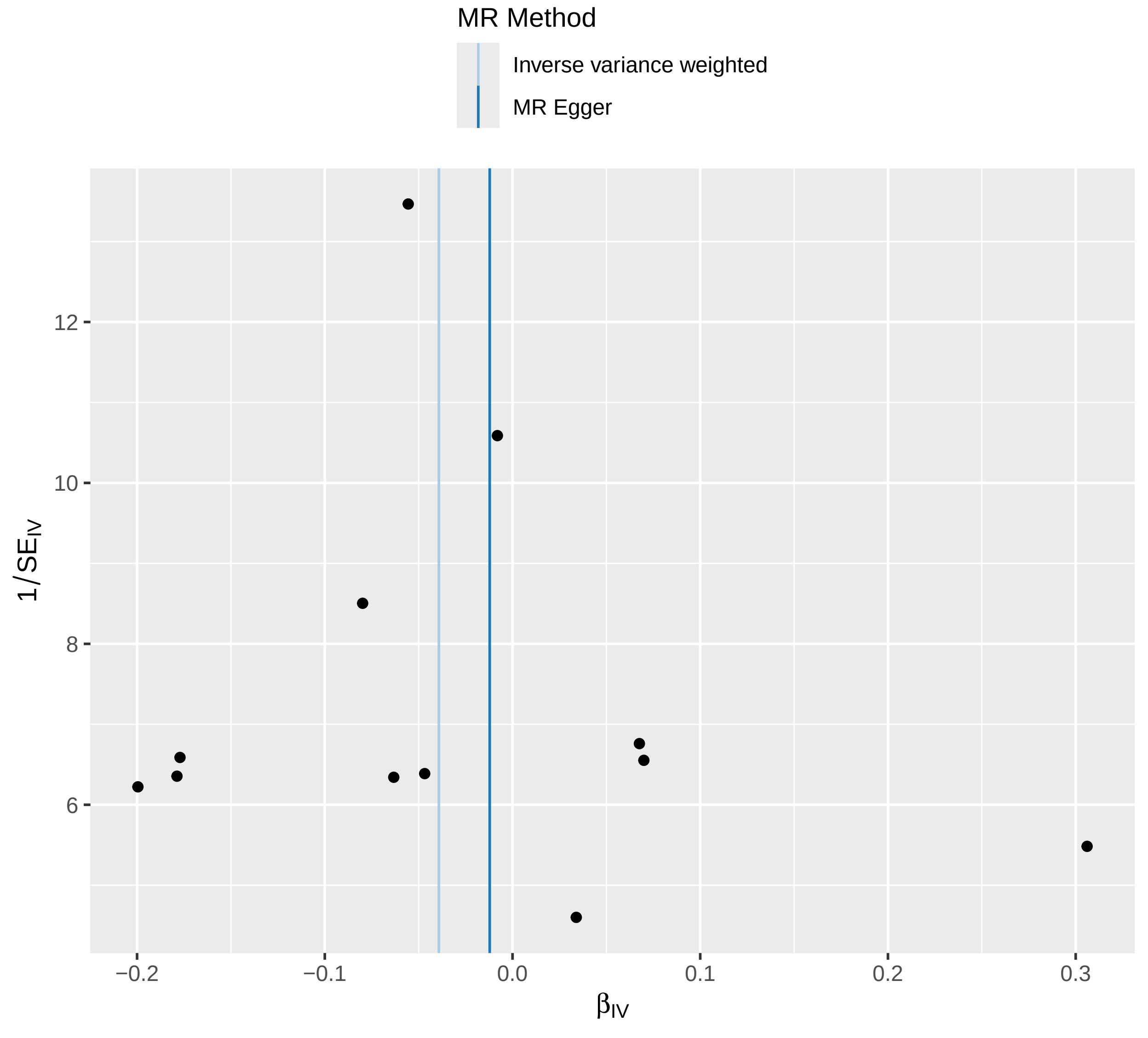
MR Method
Inverse variance weighted
MR Egger
12
10
8
6
1/SE_IV
−0.2
−0.1
0.0
0.1
0.2
0.3
β_IV

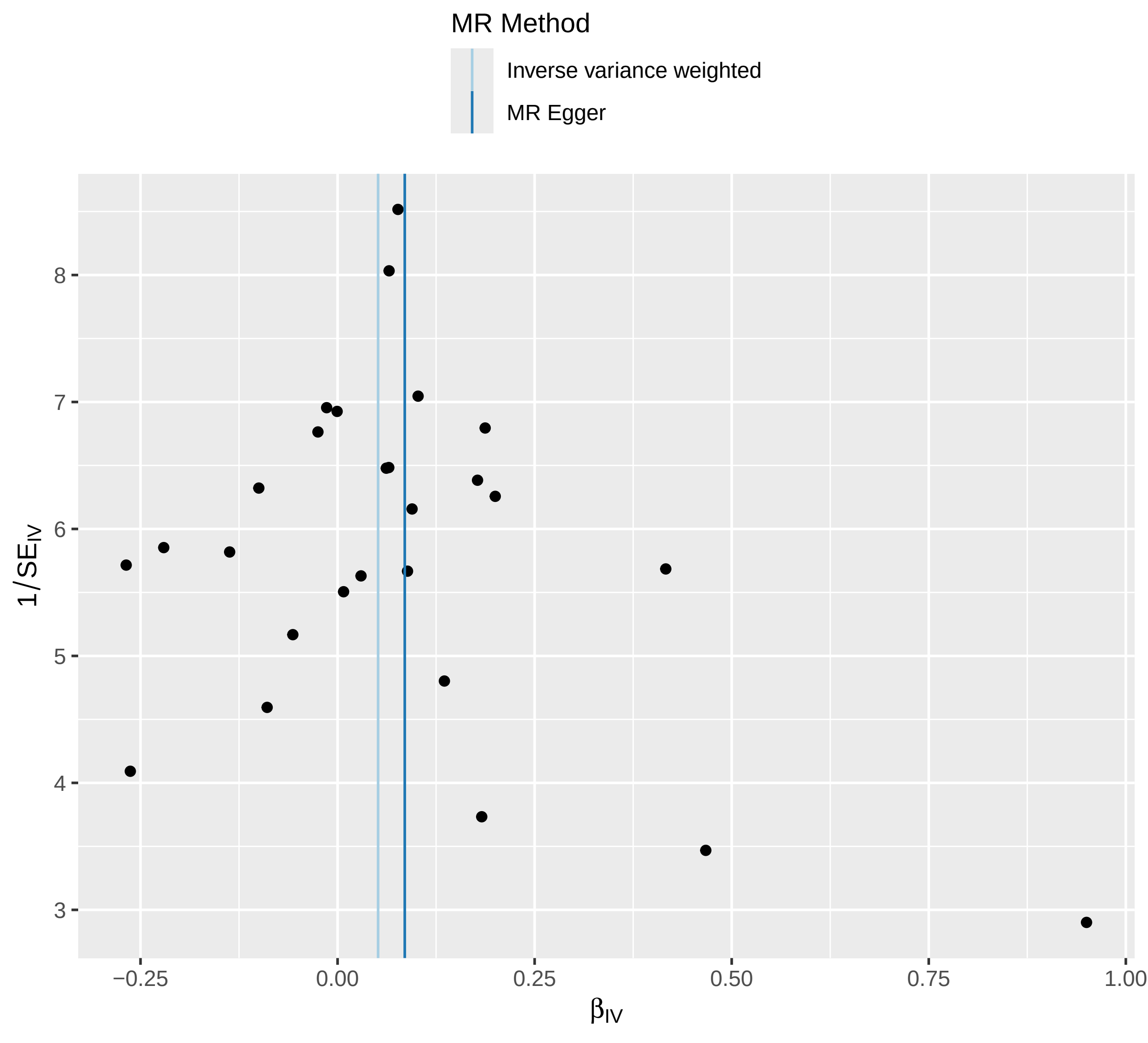

MR Method
Inverse variance weighted
MR Egger
8
7
6
5
4
3
1/SE$_{IV}$
−0.25
0.00
0.25
0.50
0.75
1.00
β$_{IV}$

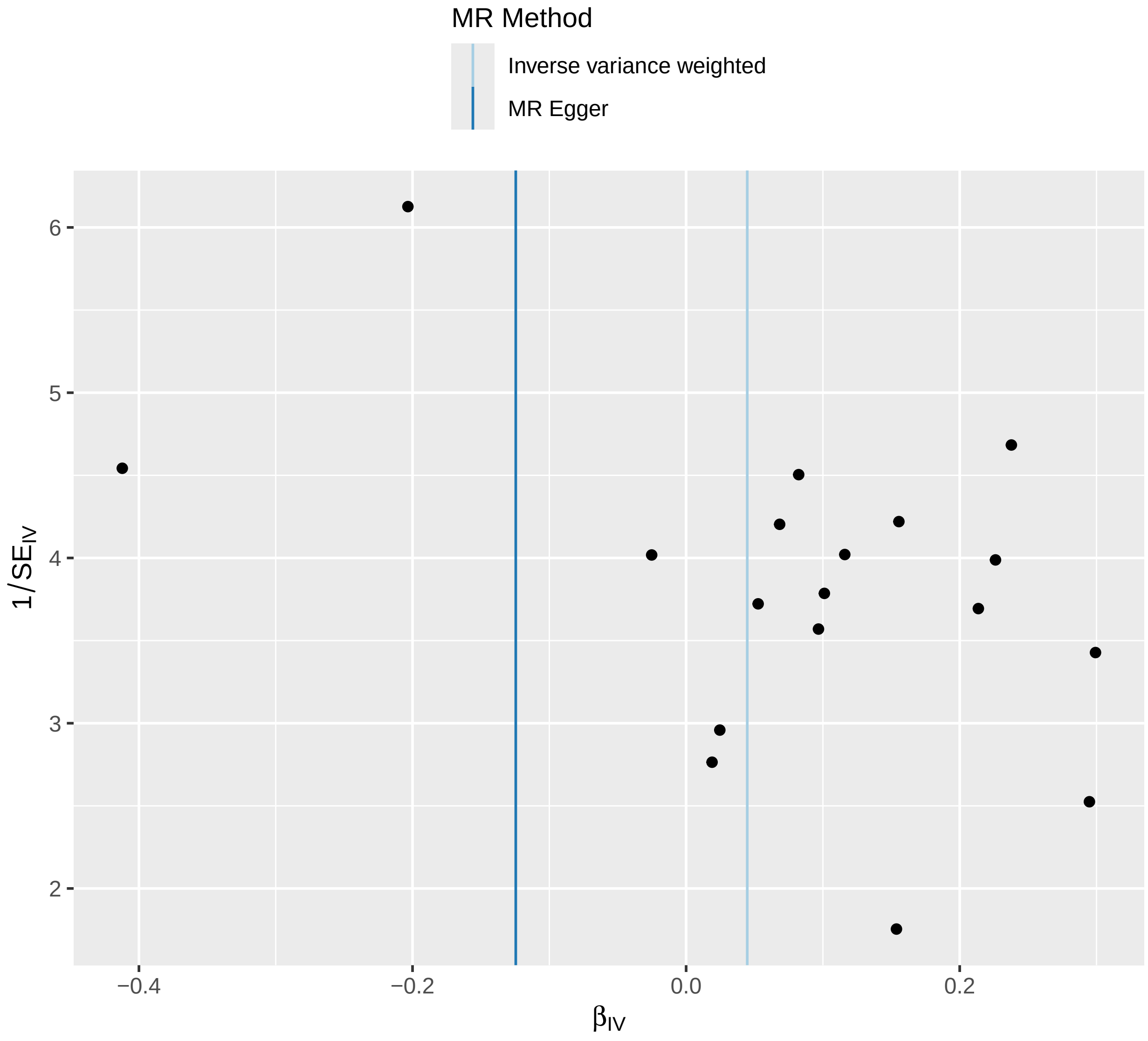
MR Method
Inverse variance weighted
MR Egger
6
5
4
3
2
1/SE_IV
−0.4
−0.2
0.0
0.2
β_IV

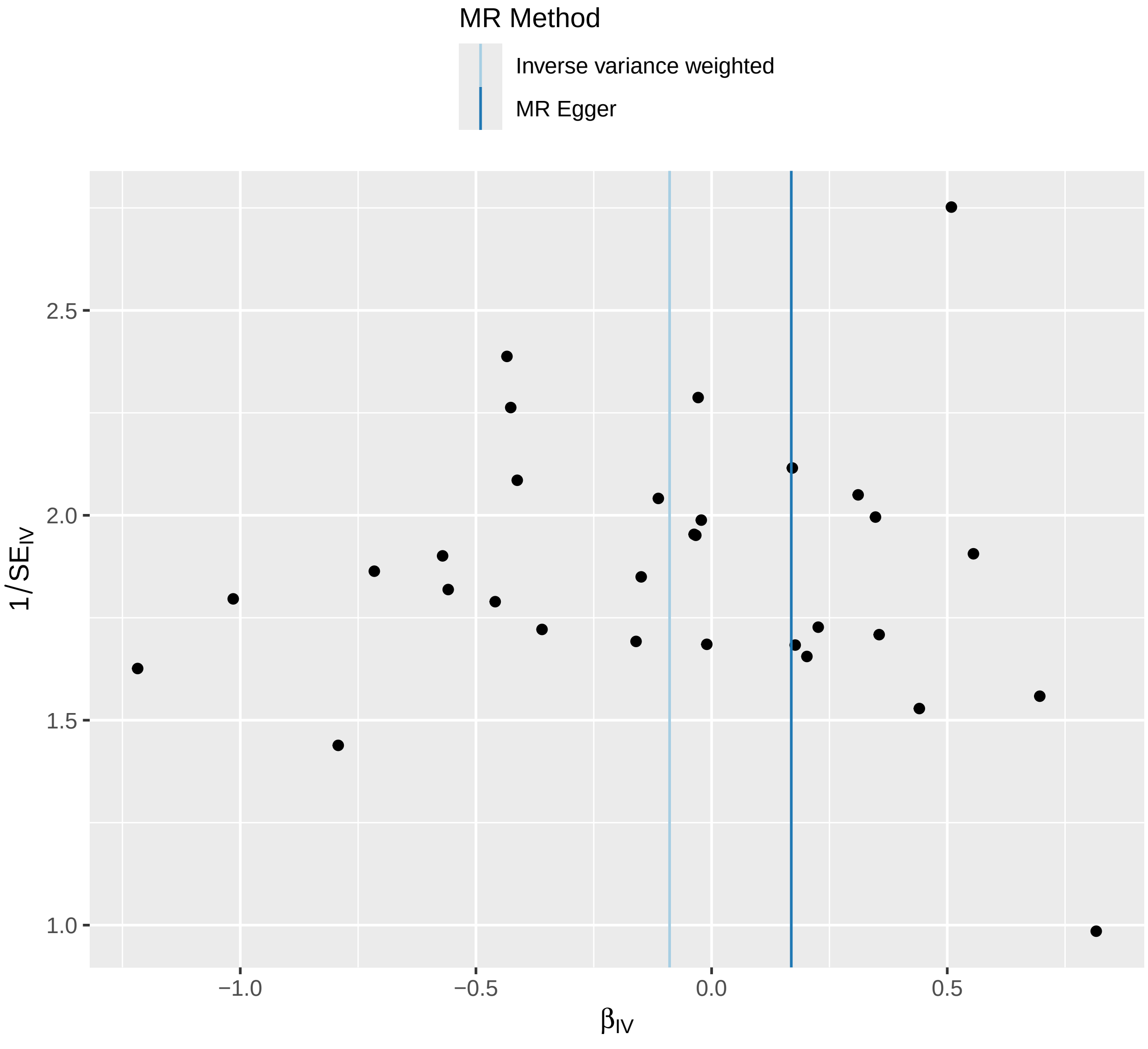

MR Method
Inverse variance weighted
MR Egger
2.5
2.0
1.5
1.0
1/SE_IV
−1.0
−0.5
0.0
0.5
β_IV

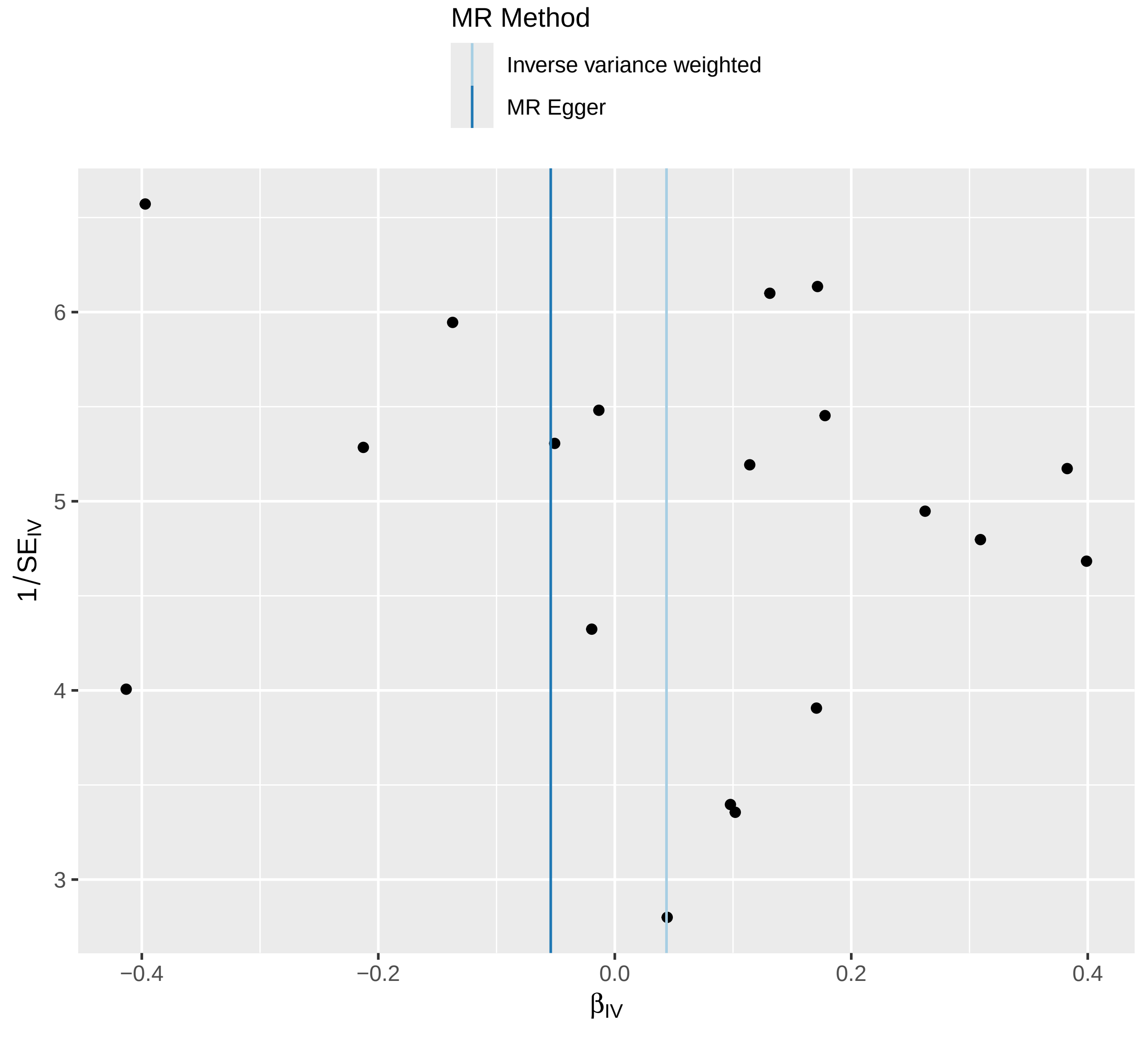
MR Method
Inverse variance weighted
MR Egger
6
5
4
3
1/SE_IV
−0.4
−0.2
0.0
0.2
0.4
β_IV

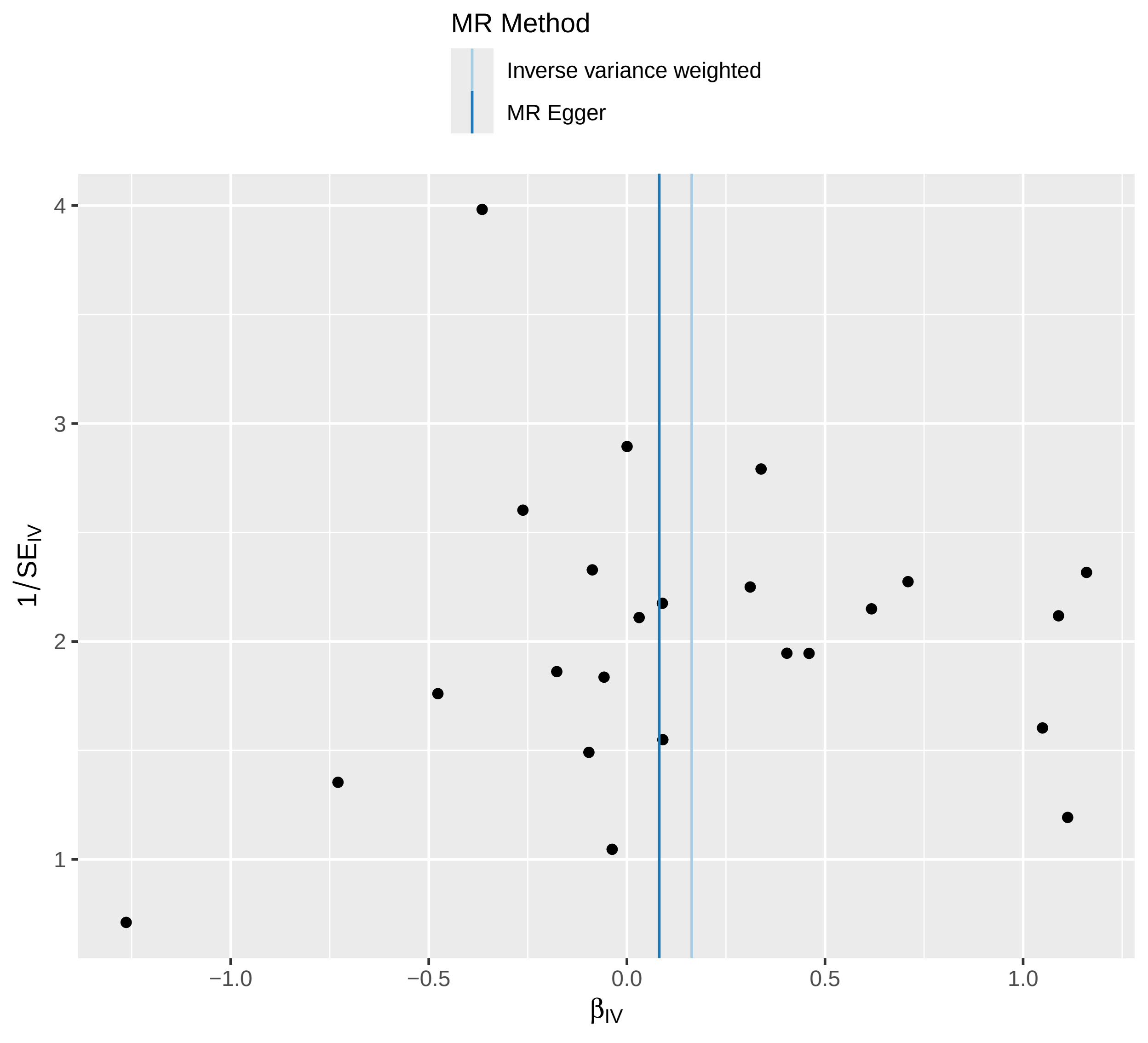

MR Method
Inverse variance weighted
MR Egger
4
3
2
1
1/SE_IV
−1.0
−0.5
0.0
0.5
1.0
β_IV

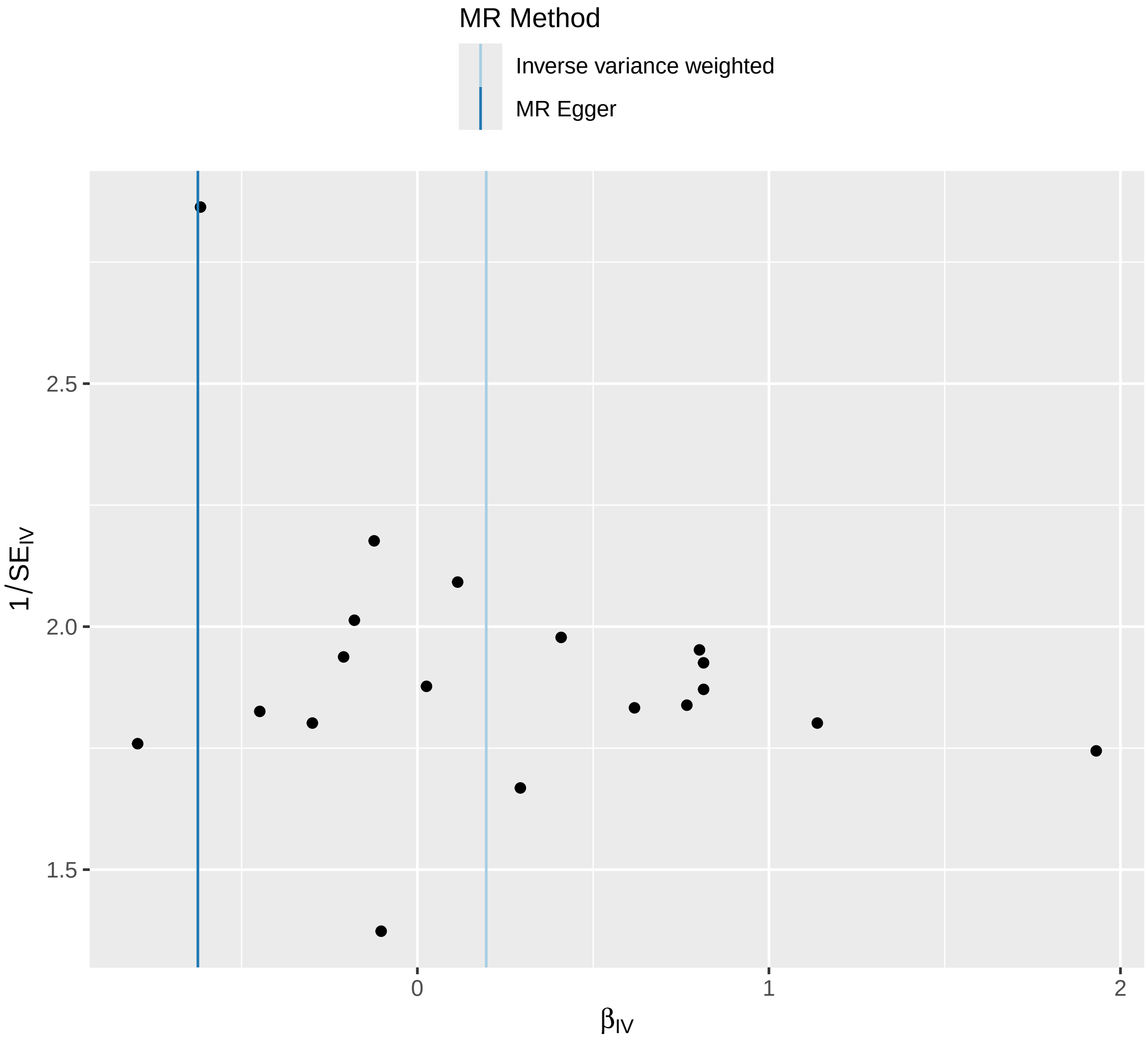
MR Method
Inverse variance weighted
MR Egger
2.5
2.0
1.5
1/SE_IV
0
1
2
β_IV

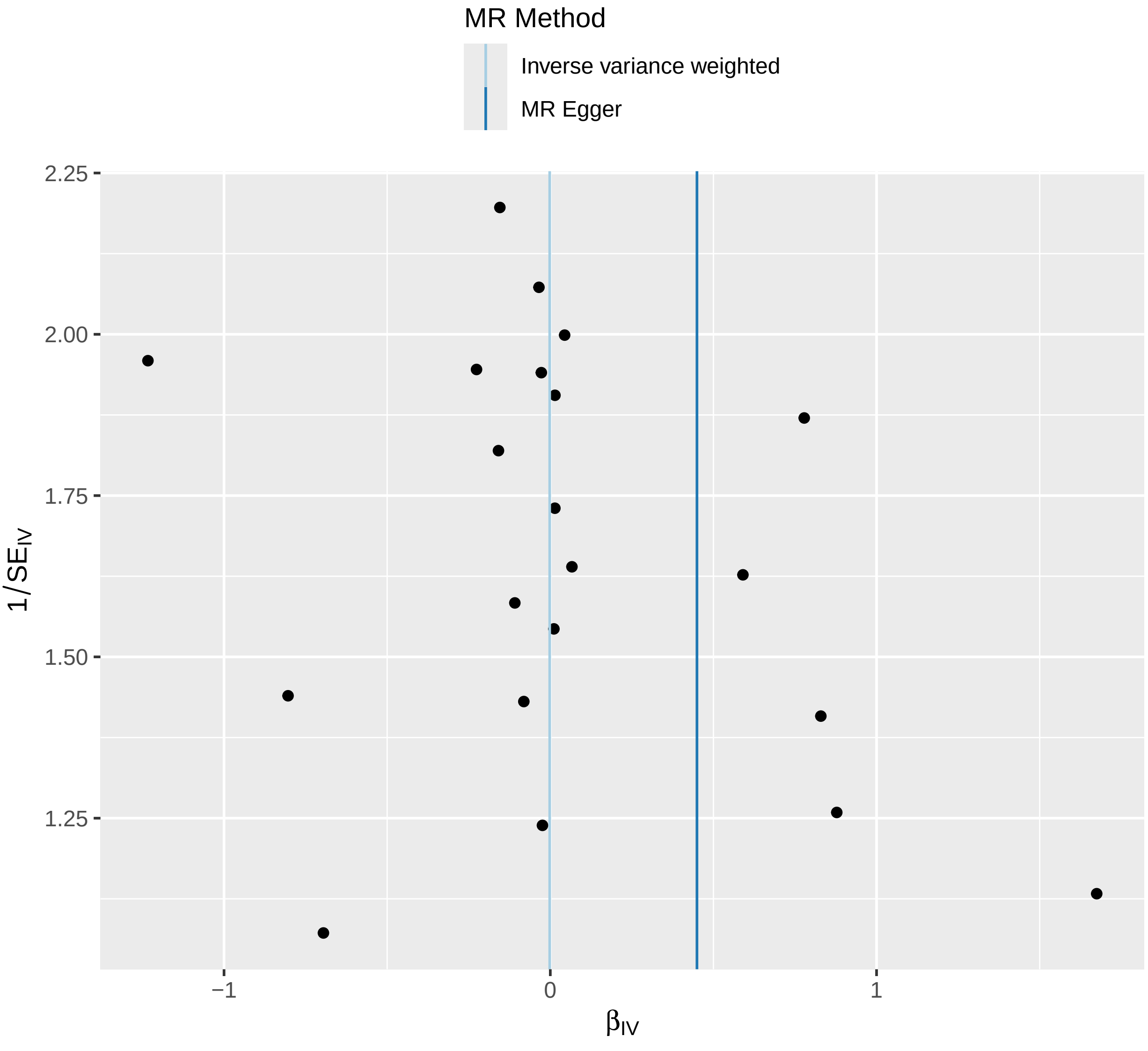
MR Method
Inverse variance weighted
MR Egger
2.25
2.00
1.75
1.50
1.25
1/SE$_{IV}$
−1
0
1
β$_{IV}$

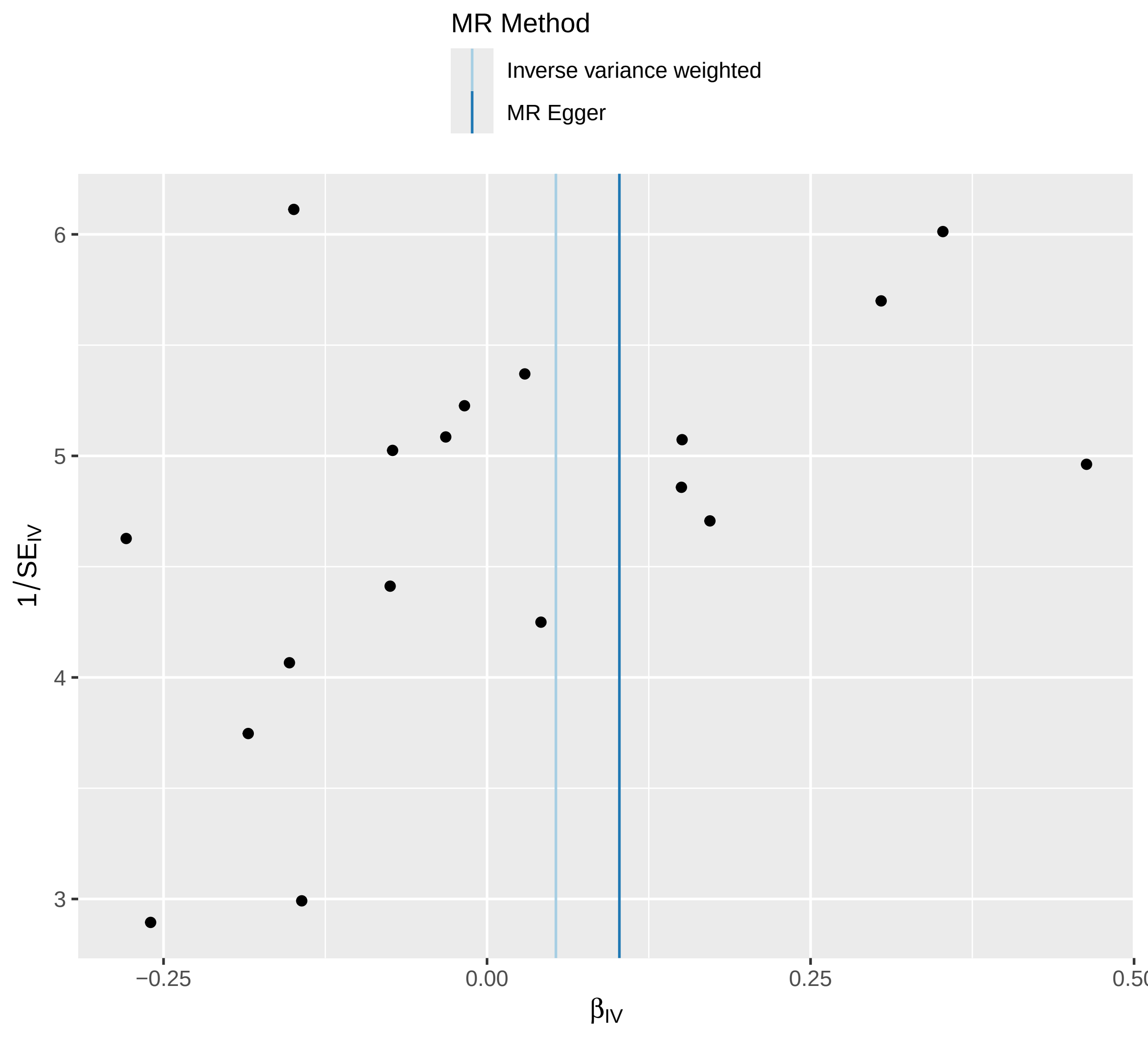
MR Method
Inverse variance weighted
MR Egger
6
5
4
3
1/SE$_{IV}$
−0.25
0.00
0.25
0.50
β$_{IV}$

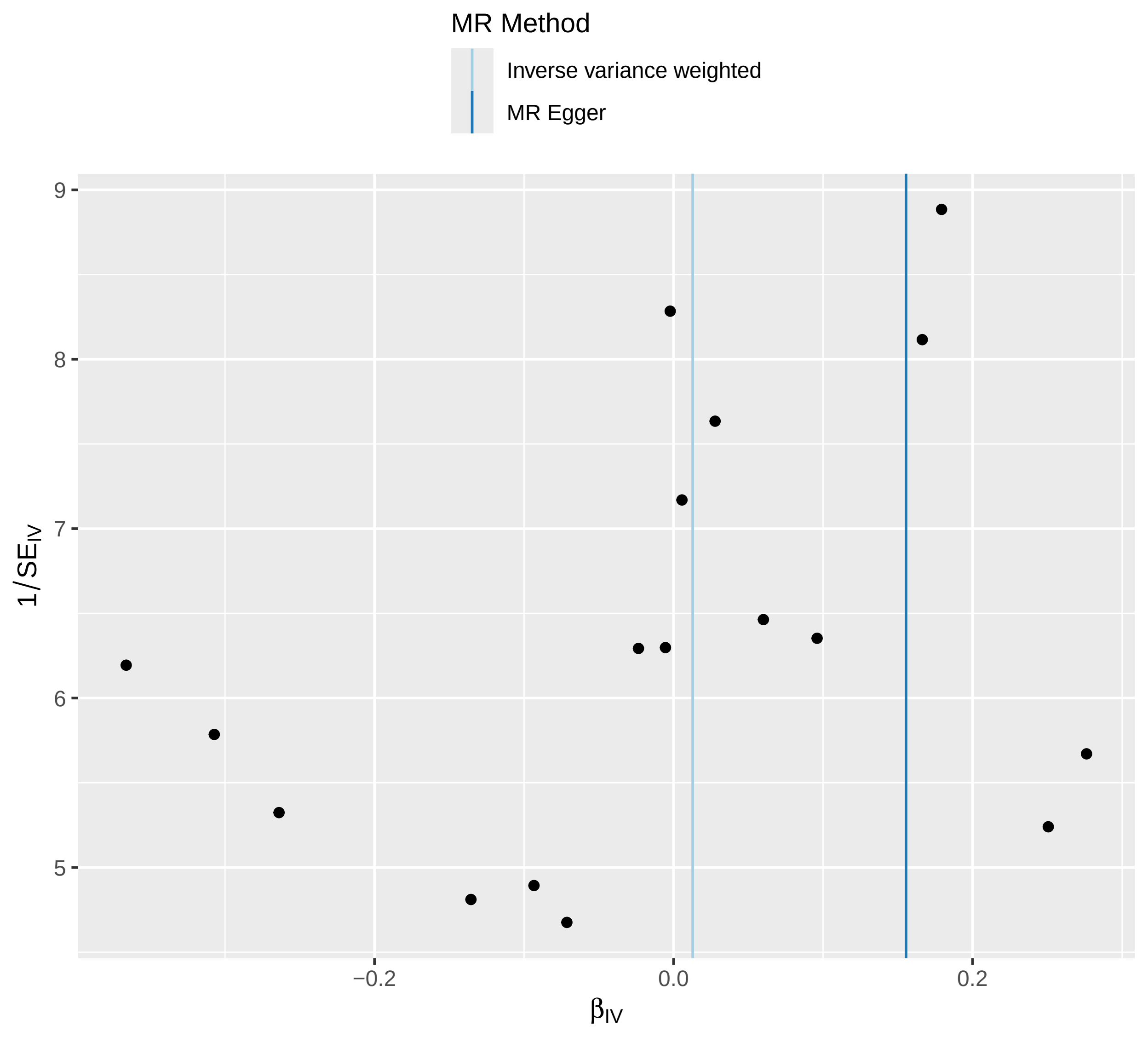
MR Method
Inverse variance weighted
MR Egger
9
8
7
6
5
1/SE$_{IV}$
−0.2
0.0
0.2
β$_{IV}$

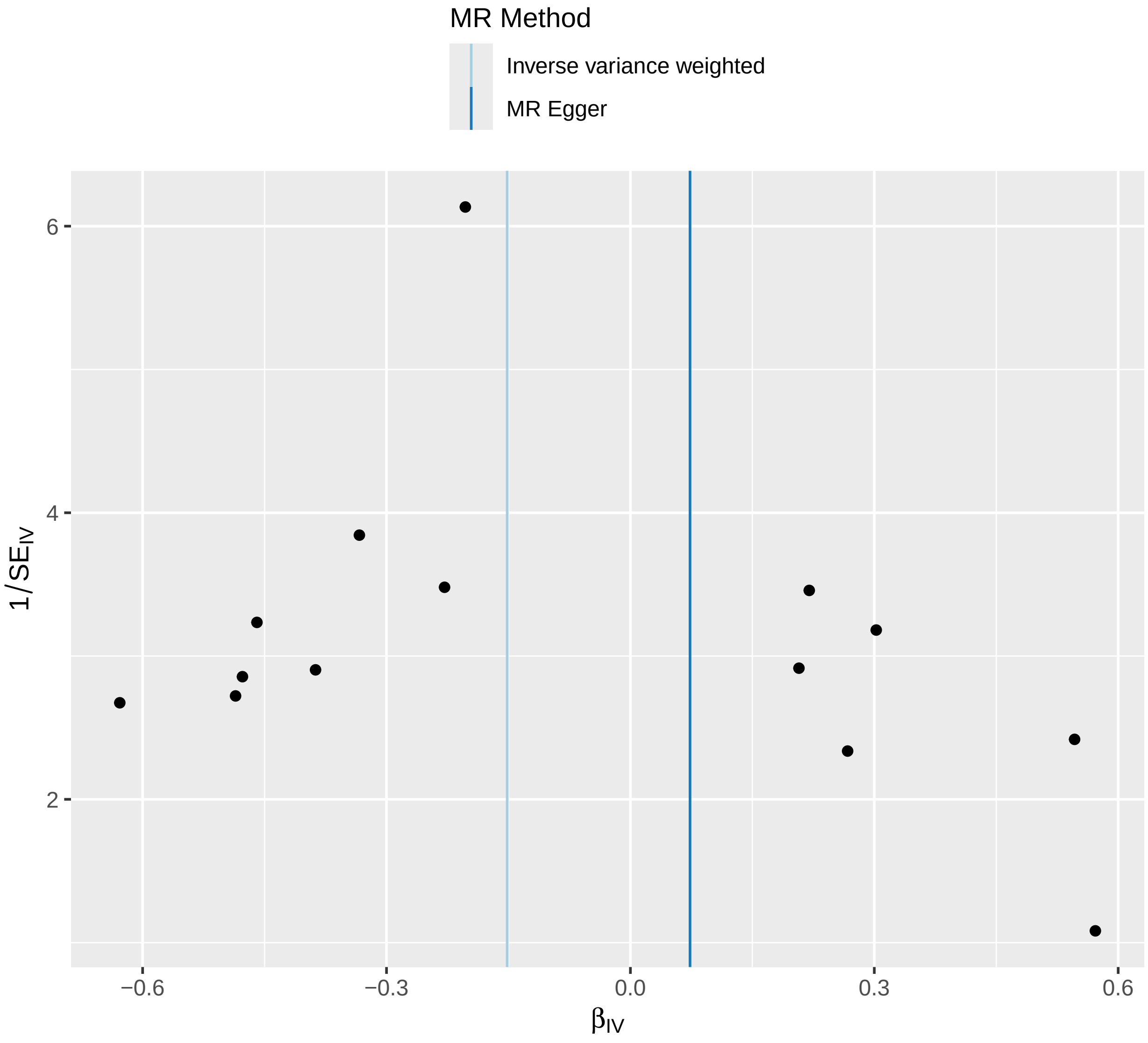
MR Method
Inverse variance weighted
MR Egger
6
4
2
1/SE$_{IV}$
−0.6
−0.3
0.0
0.3
0.6
β$_{IV}$

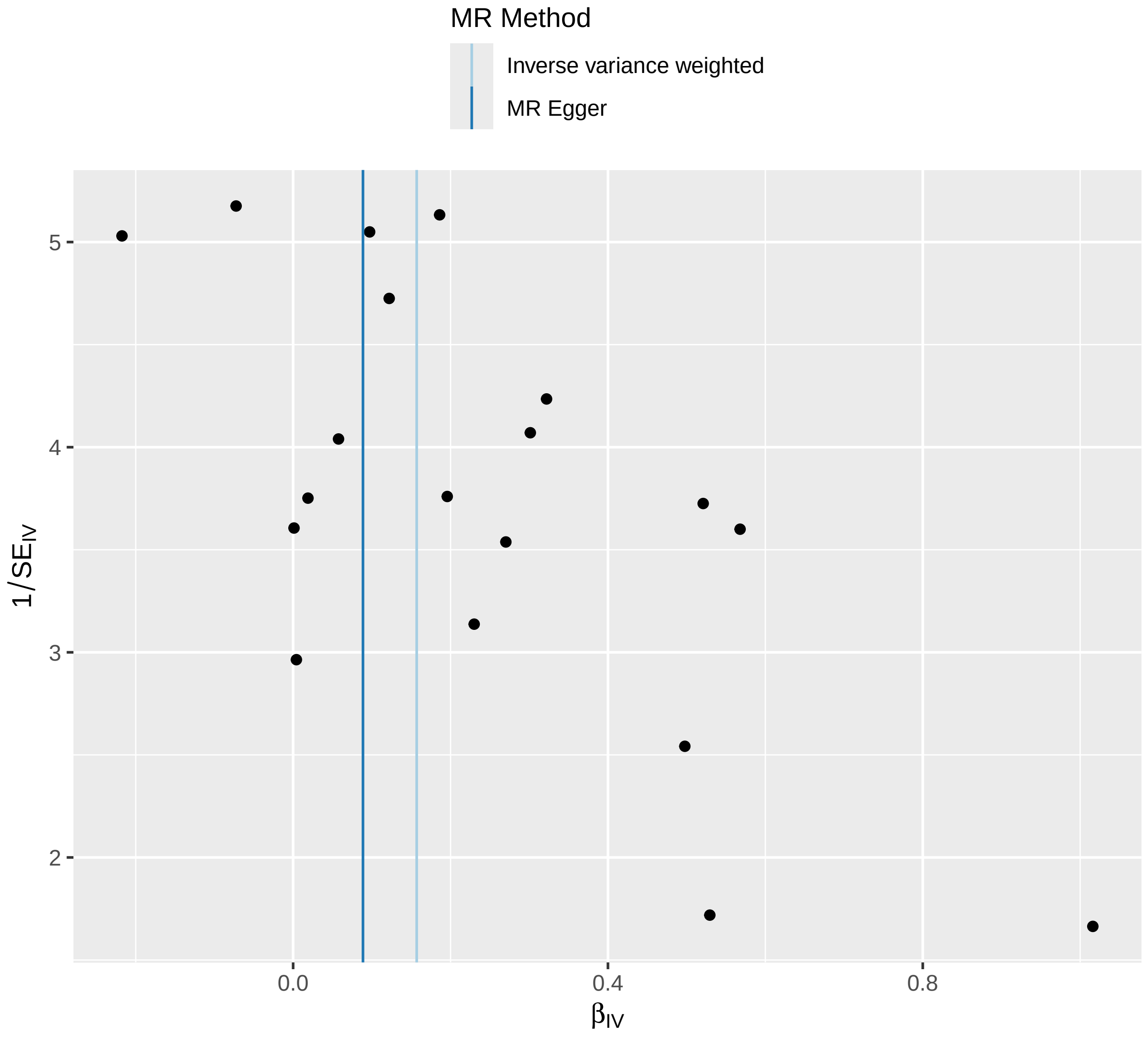
MR Method
Inverse variance weighted
MR Egger
5
4
3
2
1/SE$_{IV}$
0.0
0.4
0.8
β$_{IV}$

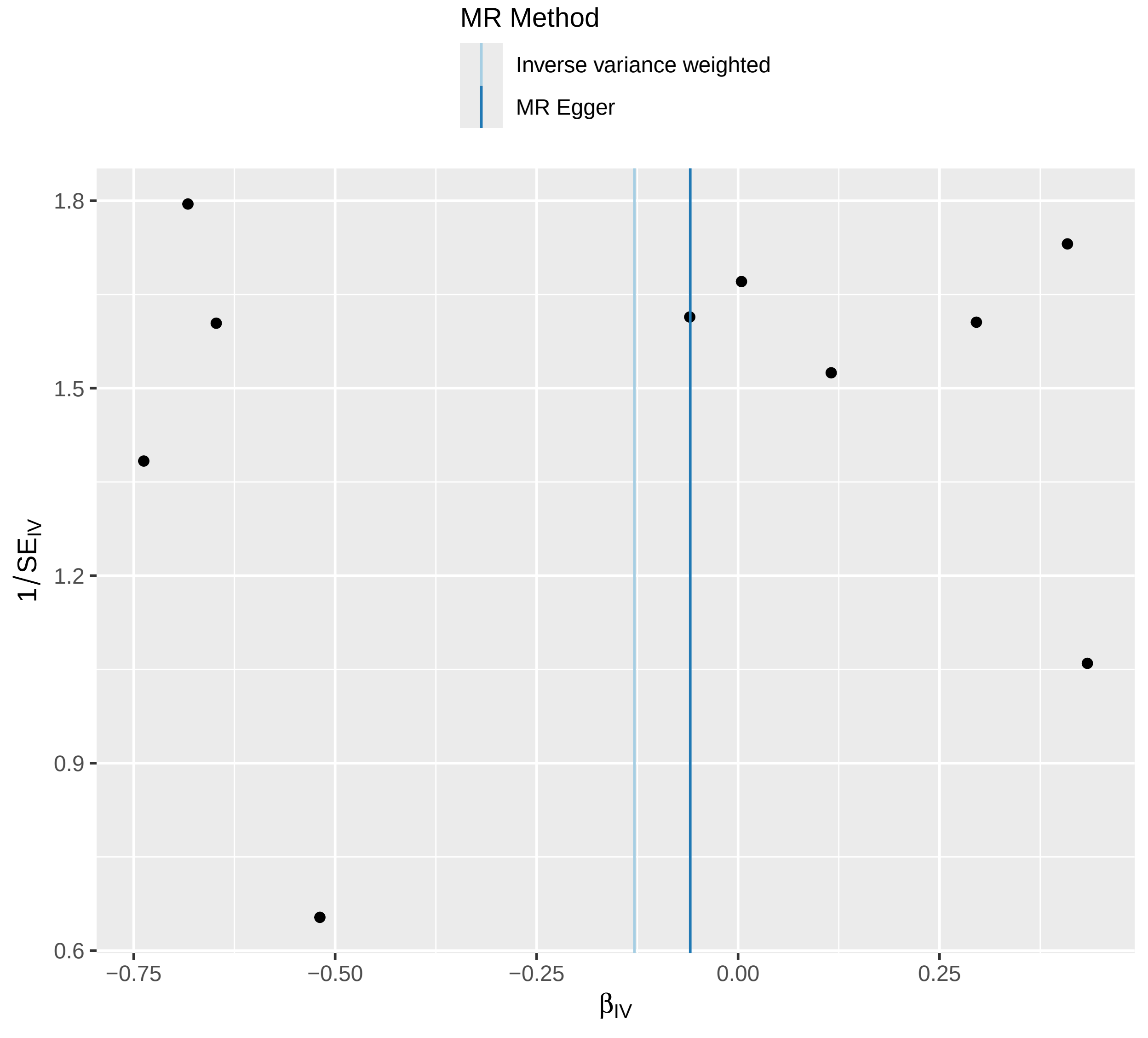

MR Method
Inverse variance weighted
MR Egger
1.8
1.5
1.2
0.9
0.6
$1/SE_{IV}$
−0.75
−0.50
−0.25
0.00
0.25
β_{IV}

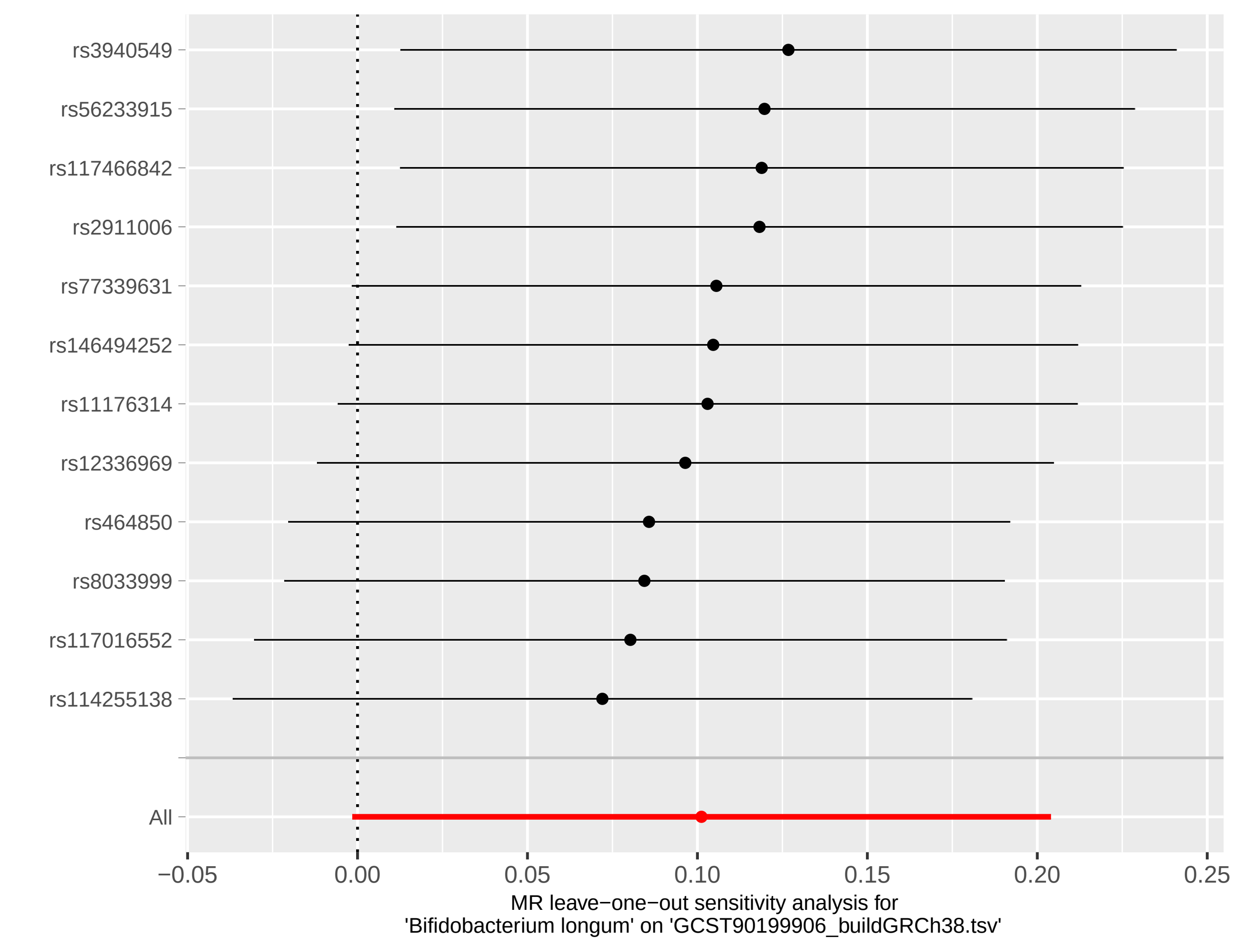
rs3940549
rs56233915
rs117466842
rs2911006
rs77339631
rs146494252
rs11176314
rs12336969
rs464850
rs8033999
rs117016552
rs114255138
All
−0.05
0.00
0.05
0.10
0.15
0.20
0.25
MR leave−one−out sensitivity analysis for
'Bifidobacterium longum' on 'GCST90199906_buildGRCh38.tsv'

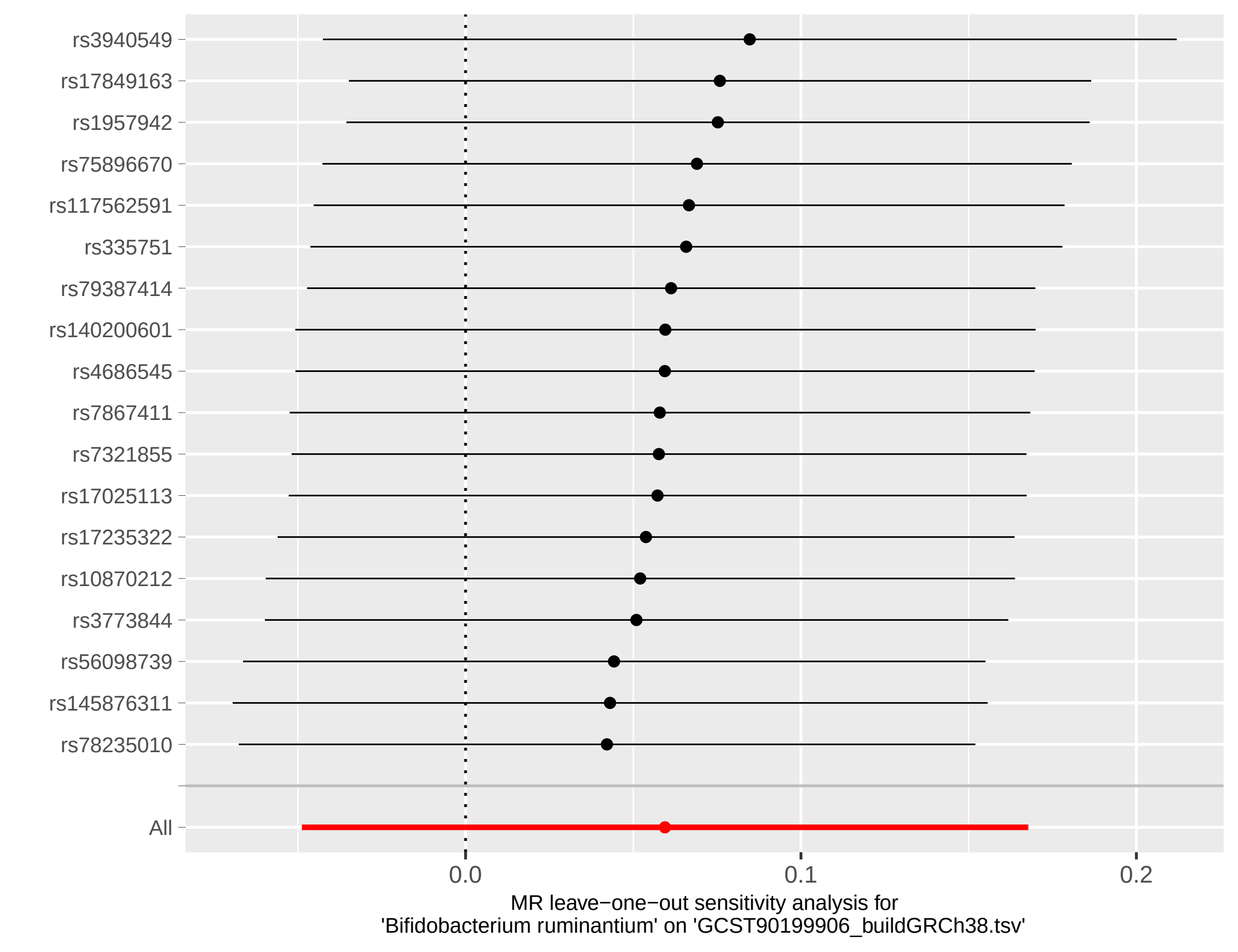

rs3940549
rs17849163
rs1957942
rs75896670
rs117562591
rs335751
rs79387414
rs140200601
rs4686545
rs7867411
rs7321855
rs17025113
rs17235322
rs10870212
rs3773844
rs56098739
rs145876311
rs78235010
All
0.0
0.1
0.2
MR leave−one−out sensitivity analysis for
'Bifidobacterium ruminantium' on 'GCST90199906_buildGRCh38.tsv'

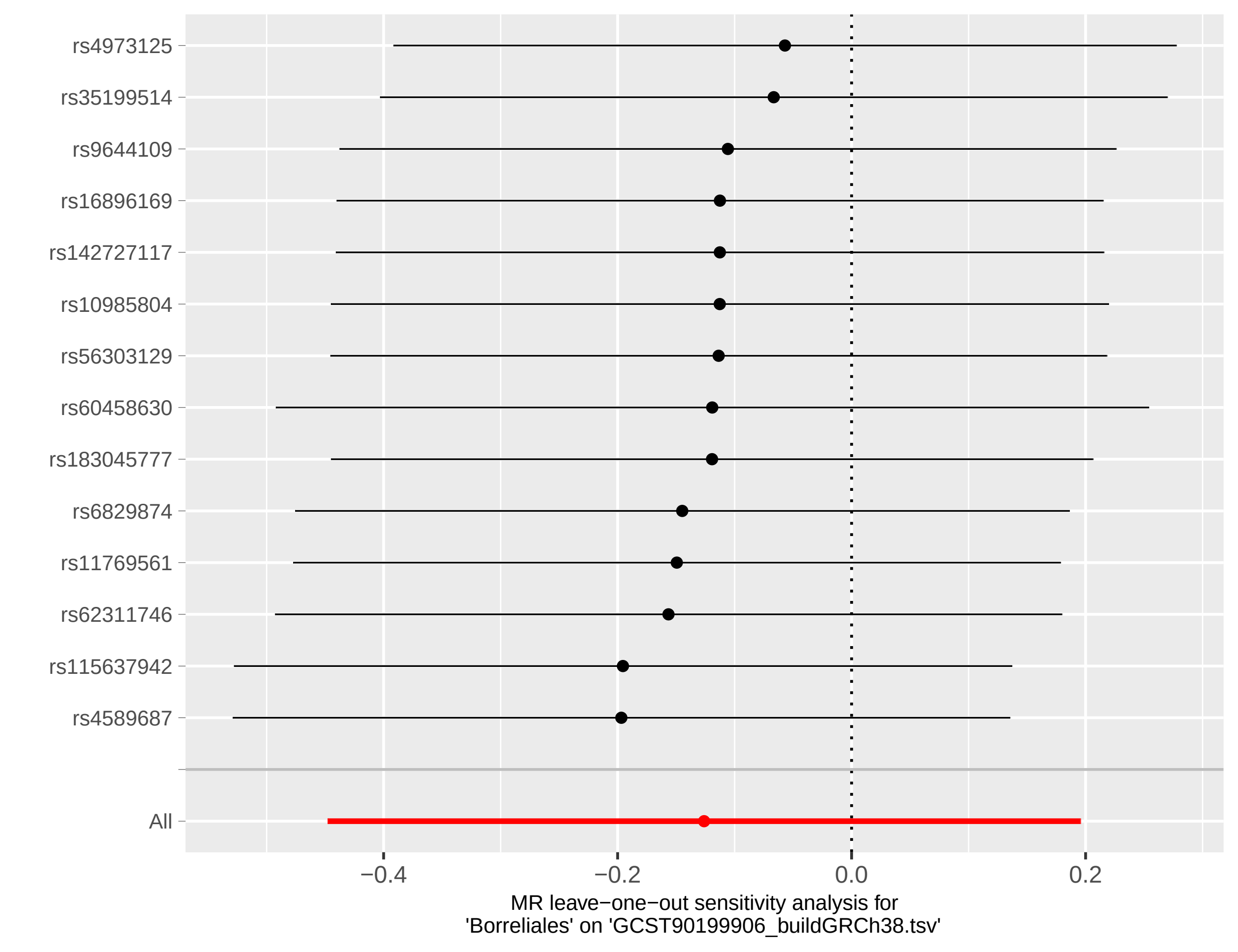

rs4973125
rs35199514
rs9644109
rs16896169
rs142727117
rs10985804
rs56303129
rs60458630
rs183045777
rs6829874
rs11769561
rs62311746
rs115637942
rs4589687
All
−0.4
−0.2
0.0
0.2
MR leave−one−out sensitivity analysis for
'Borreliales' on 'GCST90199906_buildGRCh38.tsv'

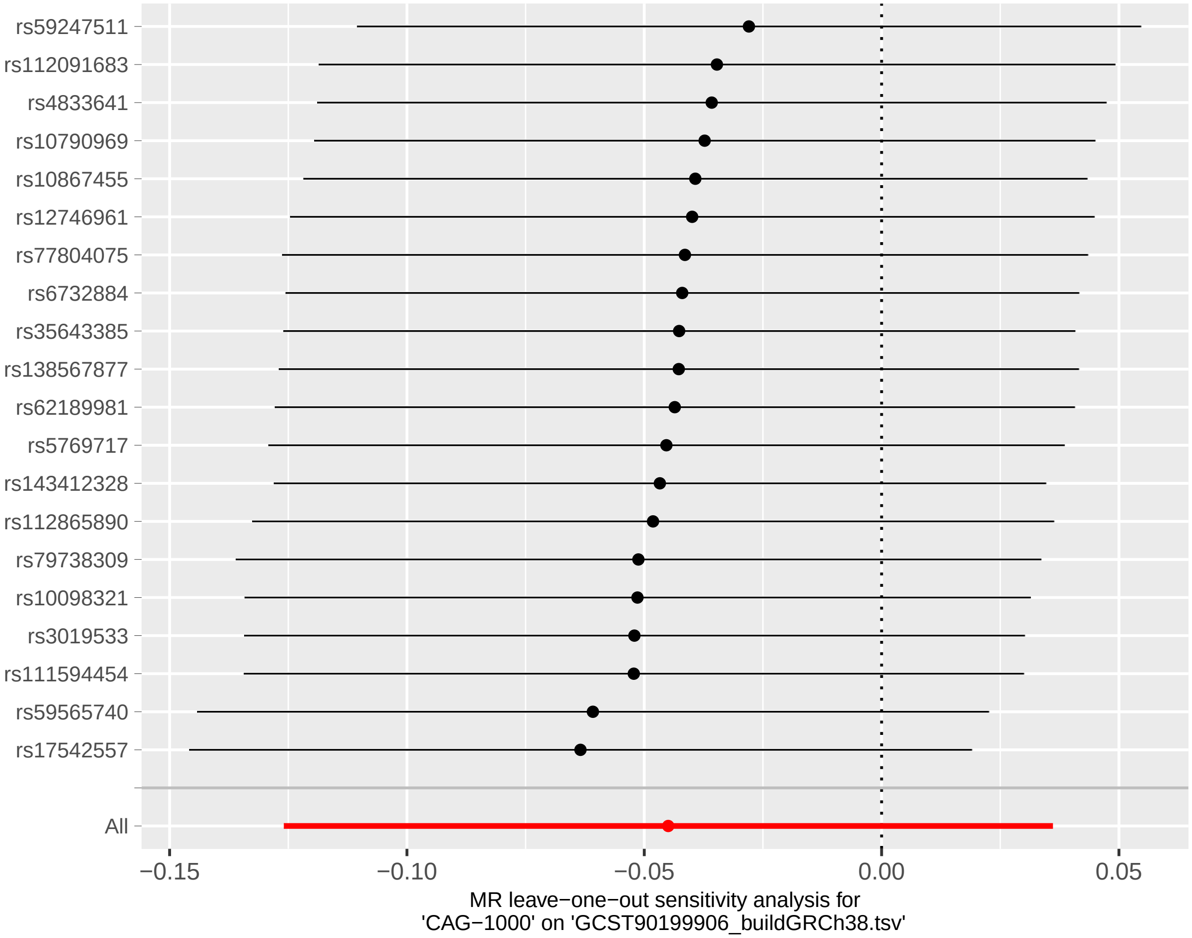
rs59247511
rs112091683
rs4833641
rs10790969
rs10867455
rs12746961
rs77804075
rs6732884
rs35643385
rs138567877
rs62189981
rs5769717
rs143412328
rs112865890
rs79738309
rs10098321
rs3019533
rs111594454
rs59565740
rs17542557
All
−0.15
−0.10
−0.05
0.00
0.05
MR leave−one−out sensitivity analysis for
'CAG−1000' on 'GCST90199906_buildGRCh38.tsv'

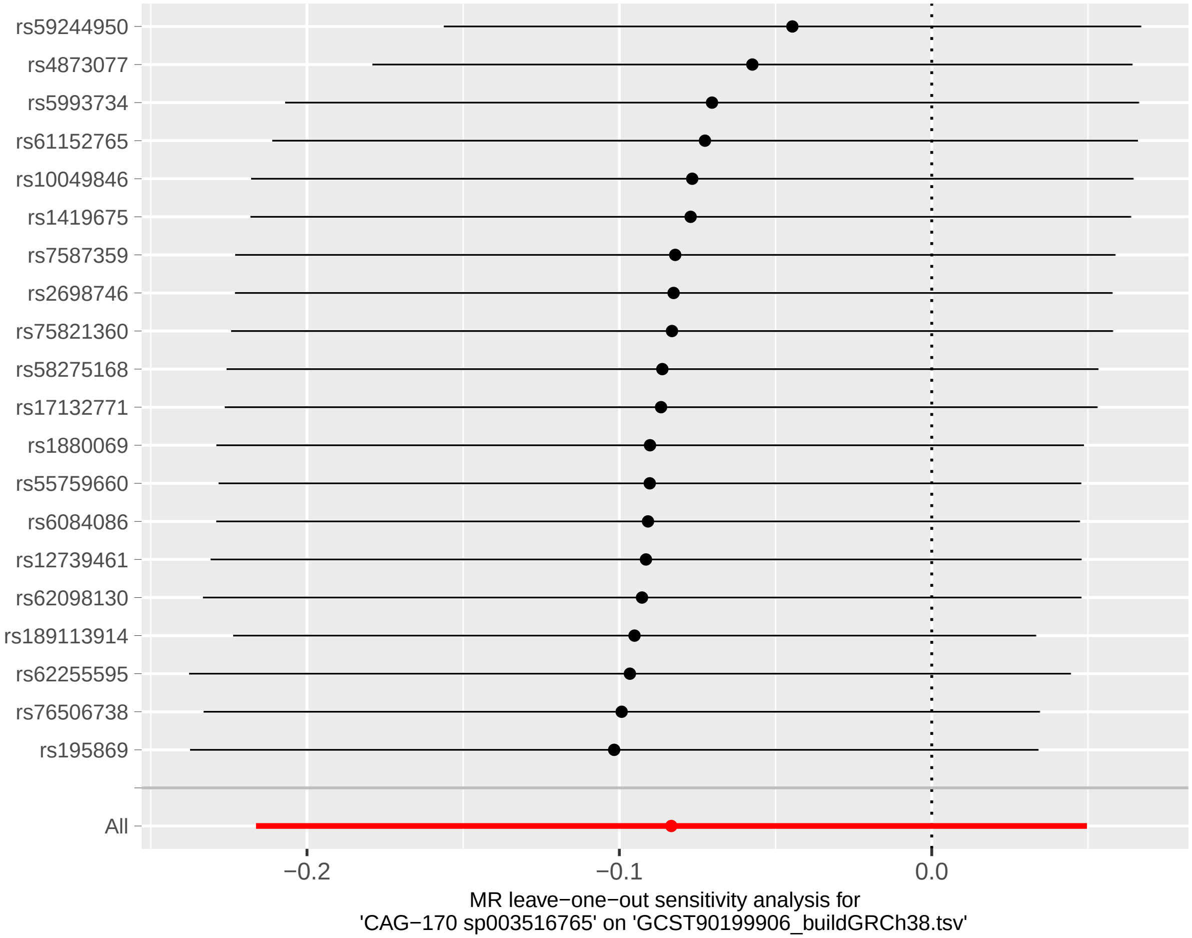
rs59244950
rs4873077
rs5993734
rs61152765
rs10049846
rs1419675
rs7587359
rs2698746
rs75821360
rs58275168
rs17132771
rs1880069
rs55759660
rs6084086
rs12739461
rs62098130
rs189113914
rs62255595
rs76506738
rs195869
All
−0.2
−0.1
0.0
MR leave−one−out sensitivity analysis for
'CAG−170 sp003516765' on 'GCST90199906_buildGRCh38.tsv'

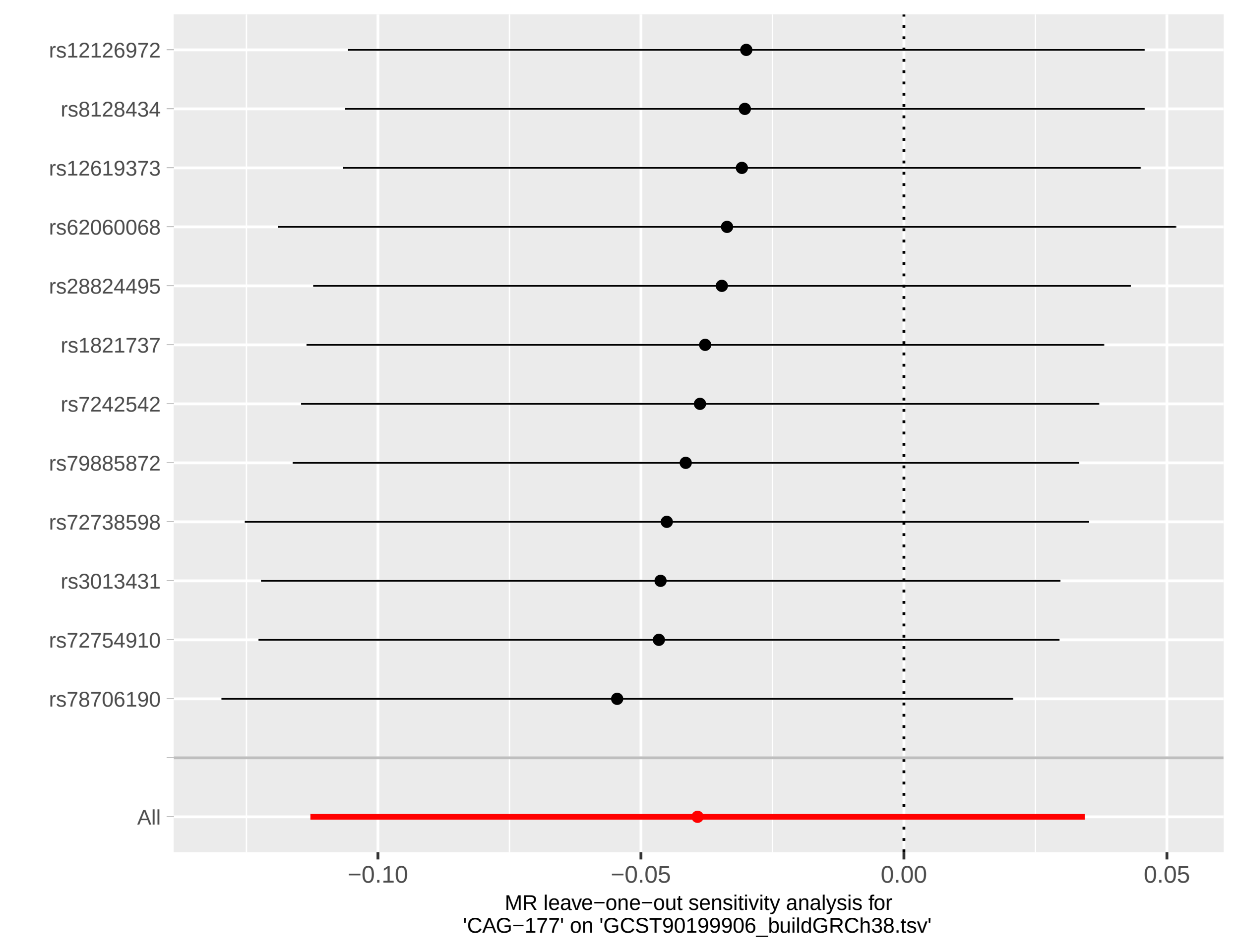
rs12126972
rs8128434
rs12619373
rs62060068
rs28824495
rs1821737
rs7242542
rs79885872
rs72738598
rs3013431
rs72754910
rs78706190
All
−0.10
−0.05
0.00
0.05
MR leave−one−out sensitivity analysis for
'CAG−177' on 'GCST90199906_buildGRCh38.tsv'

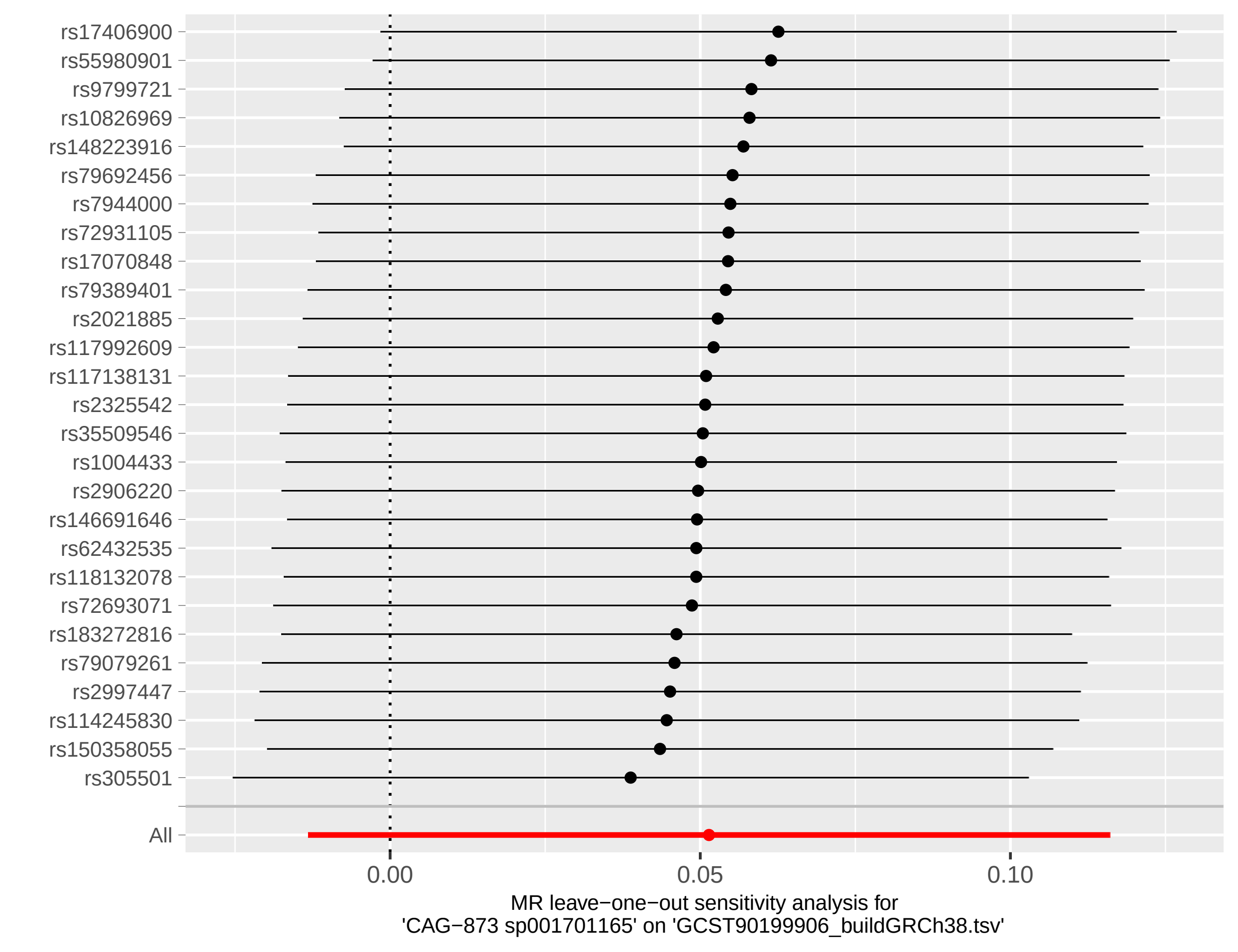
rs17406900
rs55980901
rs9799721
rs10826969
rs148223916
rs79692456
rs7944000
rs72931105
rs17070848
rs79389401
rs2021885
rs117992609
rs117138131
rs2325542
rs35509546
rs1004433
rs2906220
rs146691646
rs62432535
rs118132078
rs72693071
rs183272816
rs79079261
rs2997447
rs114245830
rs150358055
rs305501
All
0.00
0.05
0.10
MR leave−one−out sensitivity analysis for
'CAG−873 sp001701165' on 'GCST90199906_buildGRCh38.tsv'

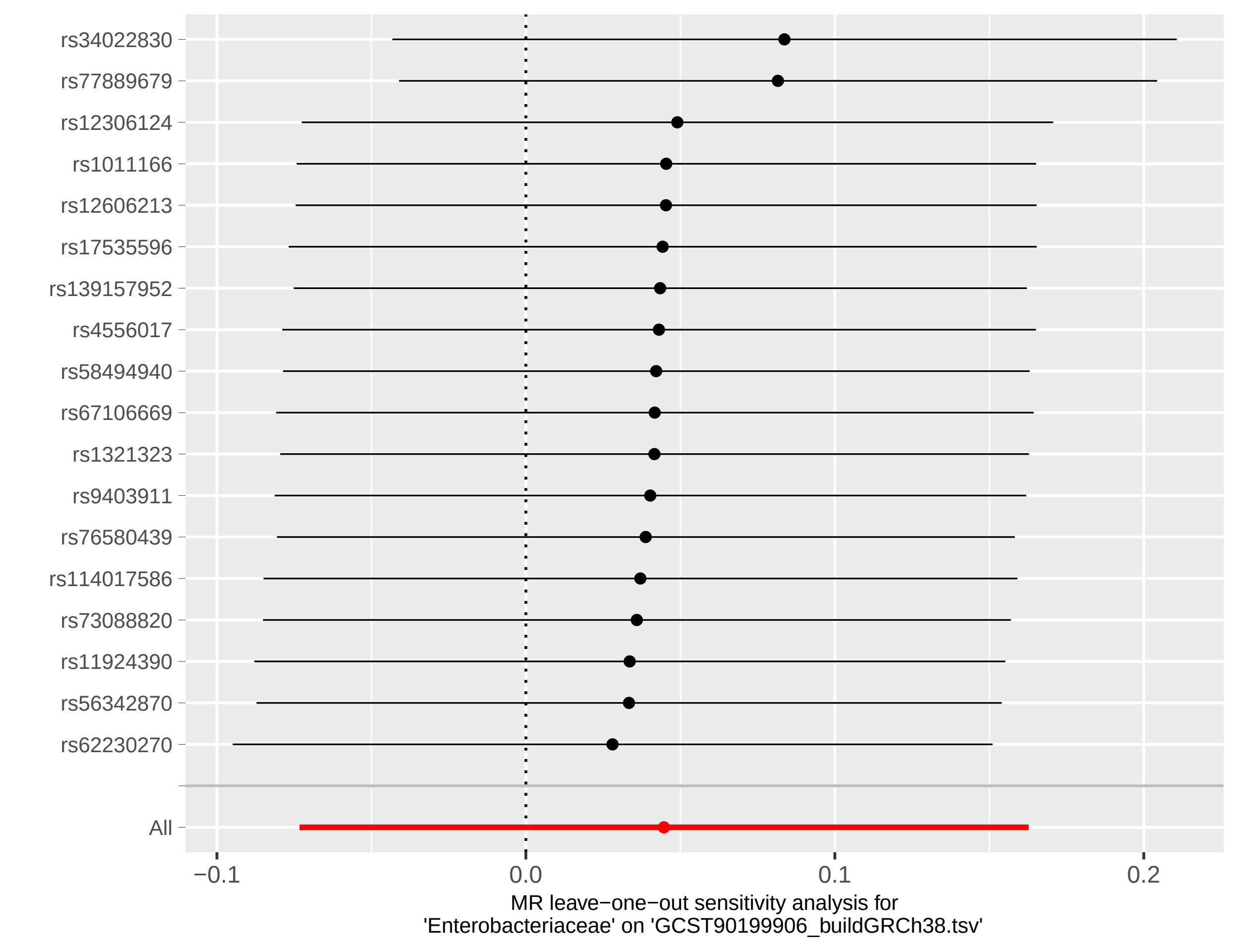

rs34022830
rs77889679
rs12306124
rs1011166
rs12606213
rs17535596
rs139157952
rs4556017
rs58494940
rs67106669
rs1321323
rs9403911
rs76580439
rs114017586
rs73088820
rs11924390
rs56342870
rs62230270
All
−0.1
0.0
0.1
0.2
MR leave−one−out sensitivity analysis for
'Enterobacteriaceae' on 'GCST90199906_buildGRCh38.tsv'

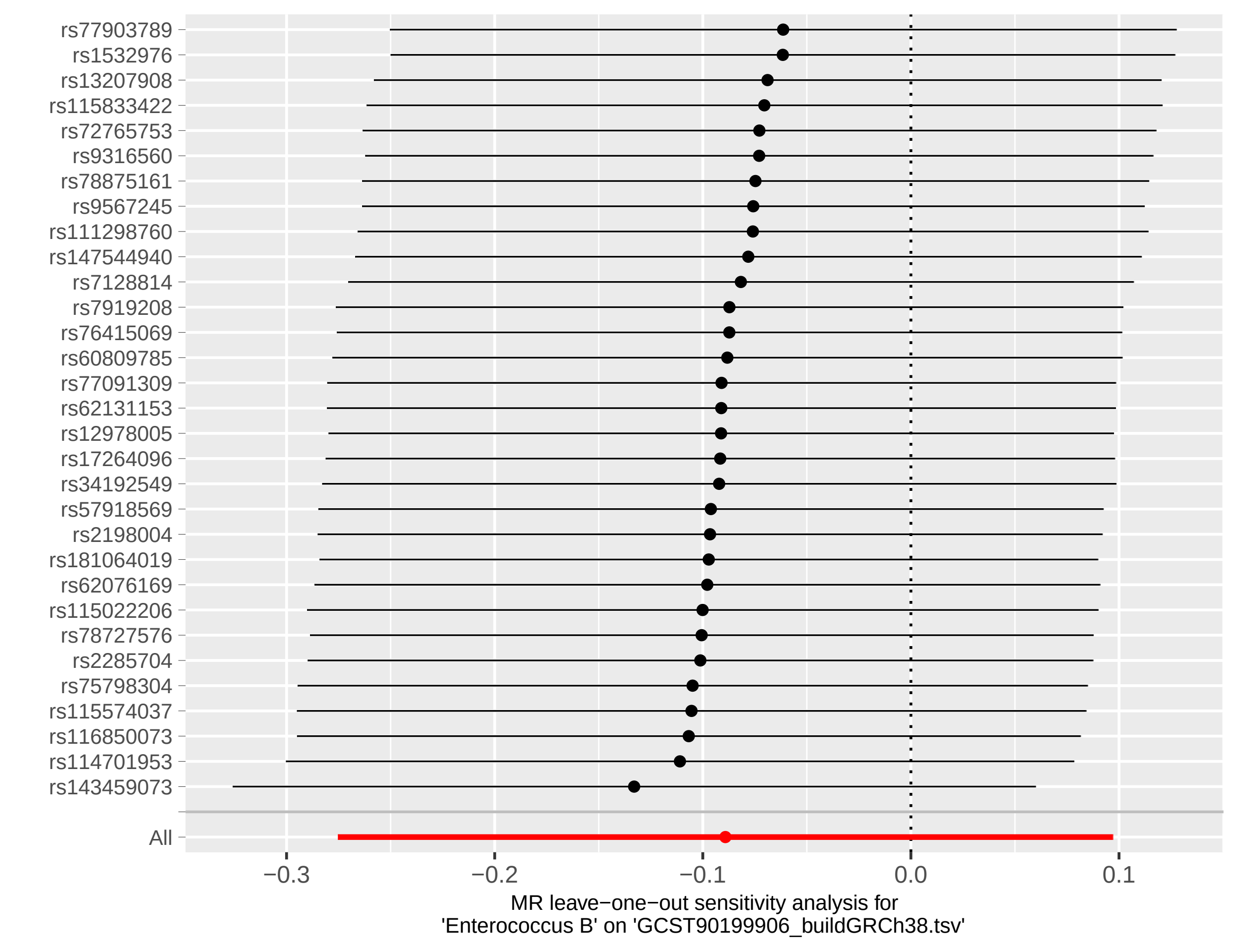

rs77903789
rs1532976
rs13207908
rs115833422
rs72765753
rs9316560
rs78875161
rs9567245
rs111298760
rs147544940
rs7128814
rs7919208
rs76415069
rs60809785
rs77091309
rs62131153
rs12978005
rs17264096
rs34192549
rs57918569
rs2198004
rs181064019
rs62076169
rs115022206
rs78727576
rs2285704
rs75798304
rs115574037
rs116850073
rs114701953
rs143459073
All
−0.3
−0.2
−0.1
0.0
0.1
MR leave−one−out sensitivity analysis for
'Enterococcus B' on 'GCST90199906_buildGRCh38.tsv'

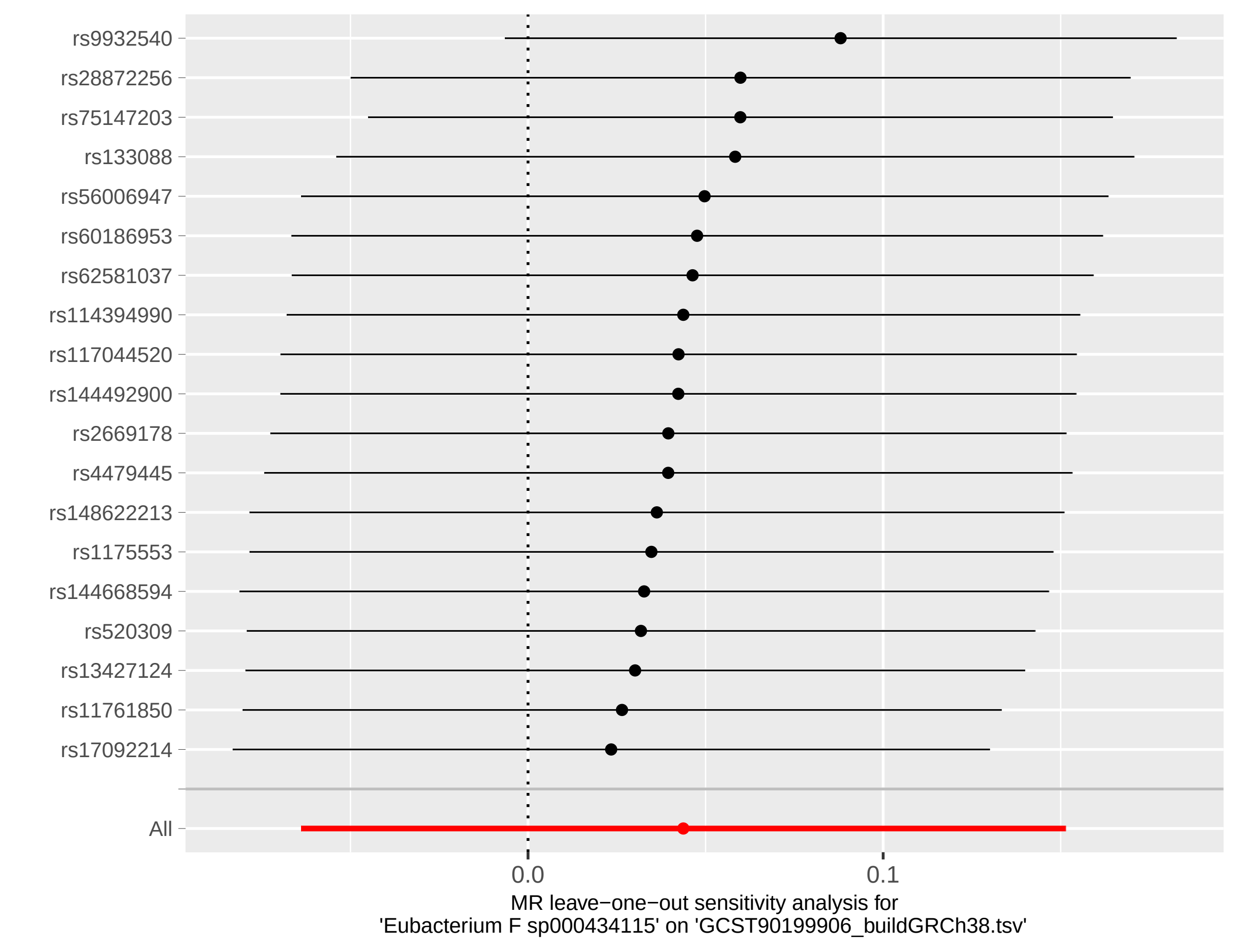
rs9932540
rs28872256
rs75147203
rs133088
rs56006947
rs60186953
rs62581037
rs114394990
rs117044520
rs144492900
rs2669178
rs4479445
rs148622213
rs1175553
rs144668594
rs520309
rs13427124
rs11761850
rs17092214
All
0.0
0.1
MR leave−one−out sensitivity analysis for
'Eubacterium F sp000434115' on 'GCST90199906_buildGRCh38.tsv'

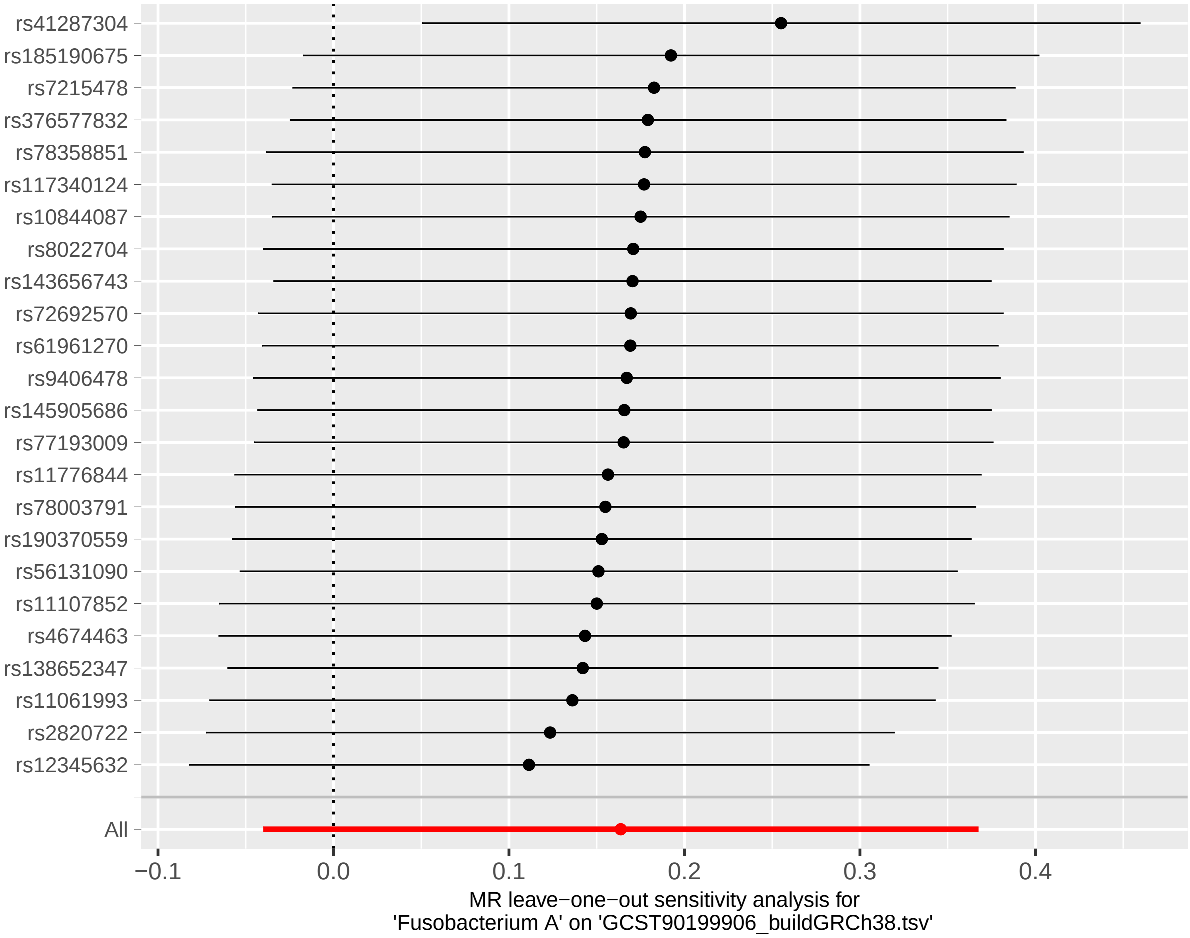

rs41287304
rs185190675
rs7215478
rs376577832
rs78358851
rs117340124
rs10844087
rs8022704
rs143656743
rs72692570
rs61961270
rs9406478
rs145905686
rs77193009
rs11776844
rs78003791
rs190370559
rs56131090
rs11107852
rs4674463
rs138652347
rs11061993
rs2820722
rs12345632
All
−0.1
0.0
0.1
0.2
0.3
0.4
MR leave−one−out sensitivity analysis for
'Fusobacterium A' on 'GCST90199906_buildGRCh38.tsv'

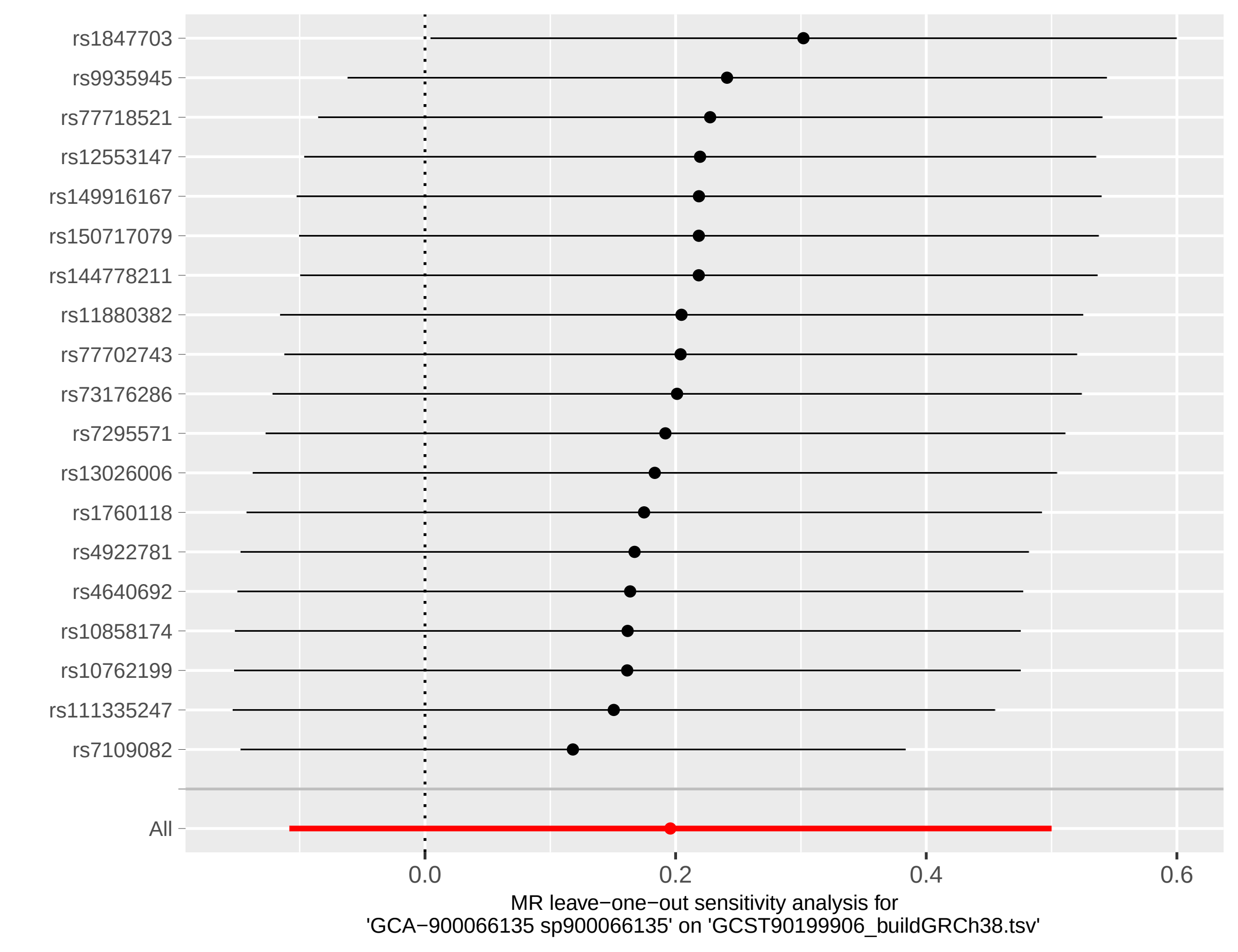
rs1847703
rs9935945
rs77718521
rs12553147
rs149916167
rs150717079
rs144778211
rs11880382
rs77702743
rs73176286
rs7295571
rs13026006
rs1760118
rs4922781
rs4640692
rs10858174
rs10762199
rs111335247
rs7109082
All
0.0
0.2
0.4
0.6
MR leave−one−out sensitivity analysis for
'GCA−900066135 sp900066135' on 'GCST90199906_buildGRCh38.tsv'

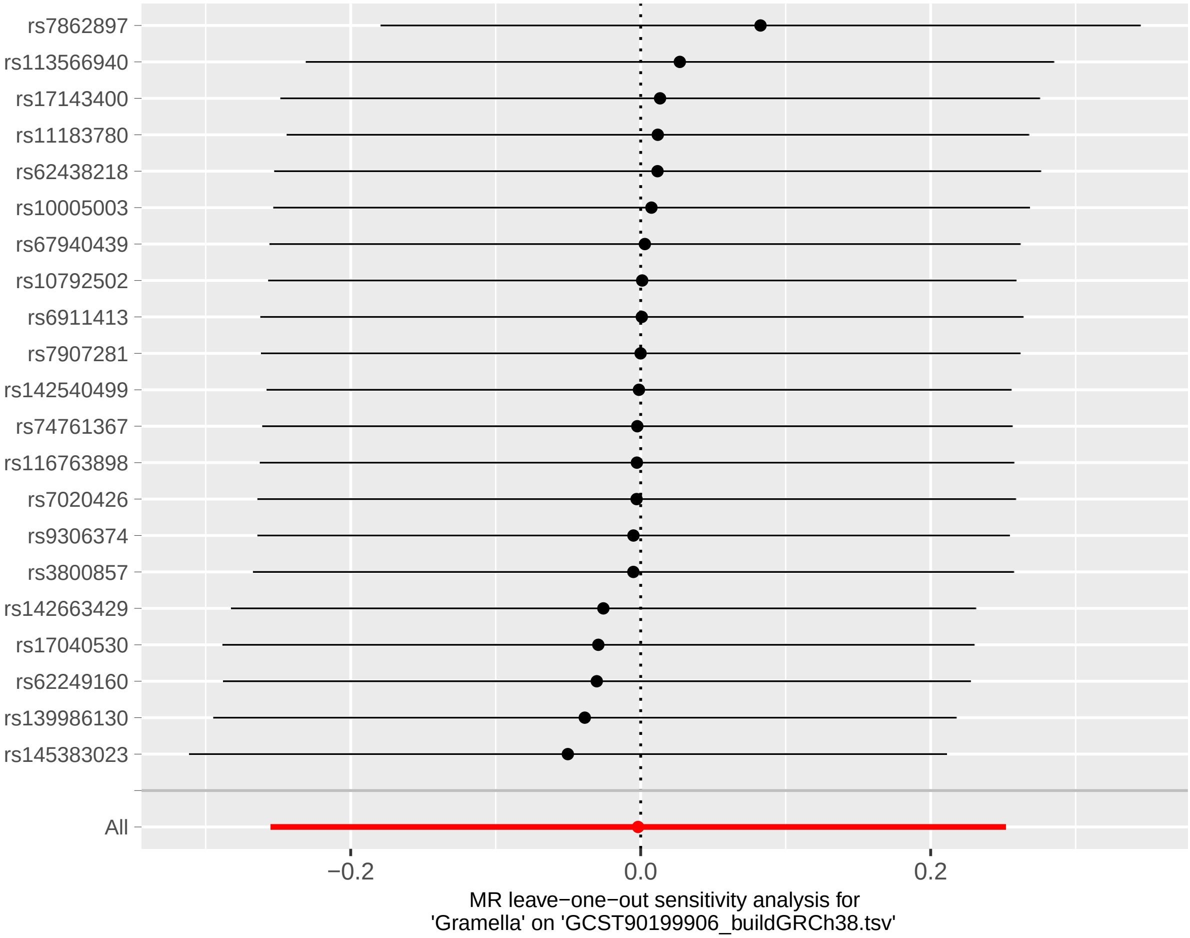
rs7862897
rs113566940
rs17143400
rs11183780
rs62438218
rs10005003
rs67940439
rs10792502
rs6911413
rs7907281
rs142540499
rs74761367
rs116763898
rs7020426
rs9306374
rs3800857
rs142663429
rs17040530
rs62249160
rs139986130
rs145383023
All
−0.2
0.0
0.2
MR leave−one−out sensitivity analysis for
'Gramella' on 'GCST90199906_buildGRCh38.tsv'

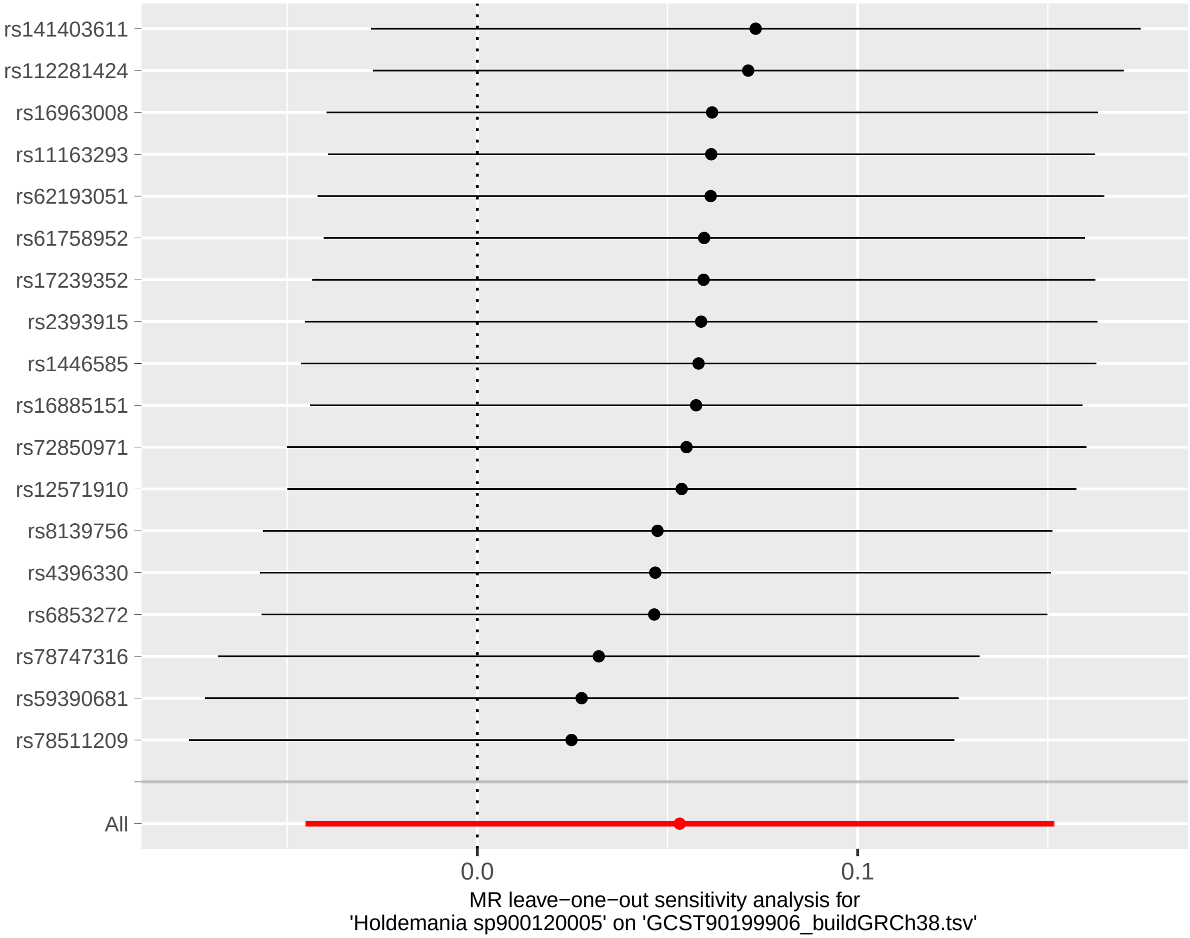

rs141403611
rs112281424
rs16963008
rs11163293
rs62193051
rs61758952
rs17239352
rs2393915
rs1446585
rs16885151
rs72850971
rs12571910
rs8139756
rs4396330
rs6853272
rs78747316
rs59390681
rs78511209
All
0.0
0.1
MR leave−one−out sensitivity analysis for
'Holdemania sp900120005' on 'GCST90199906_buildGRCh38.tsv'

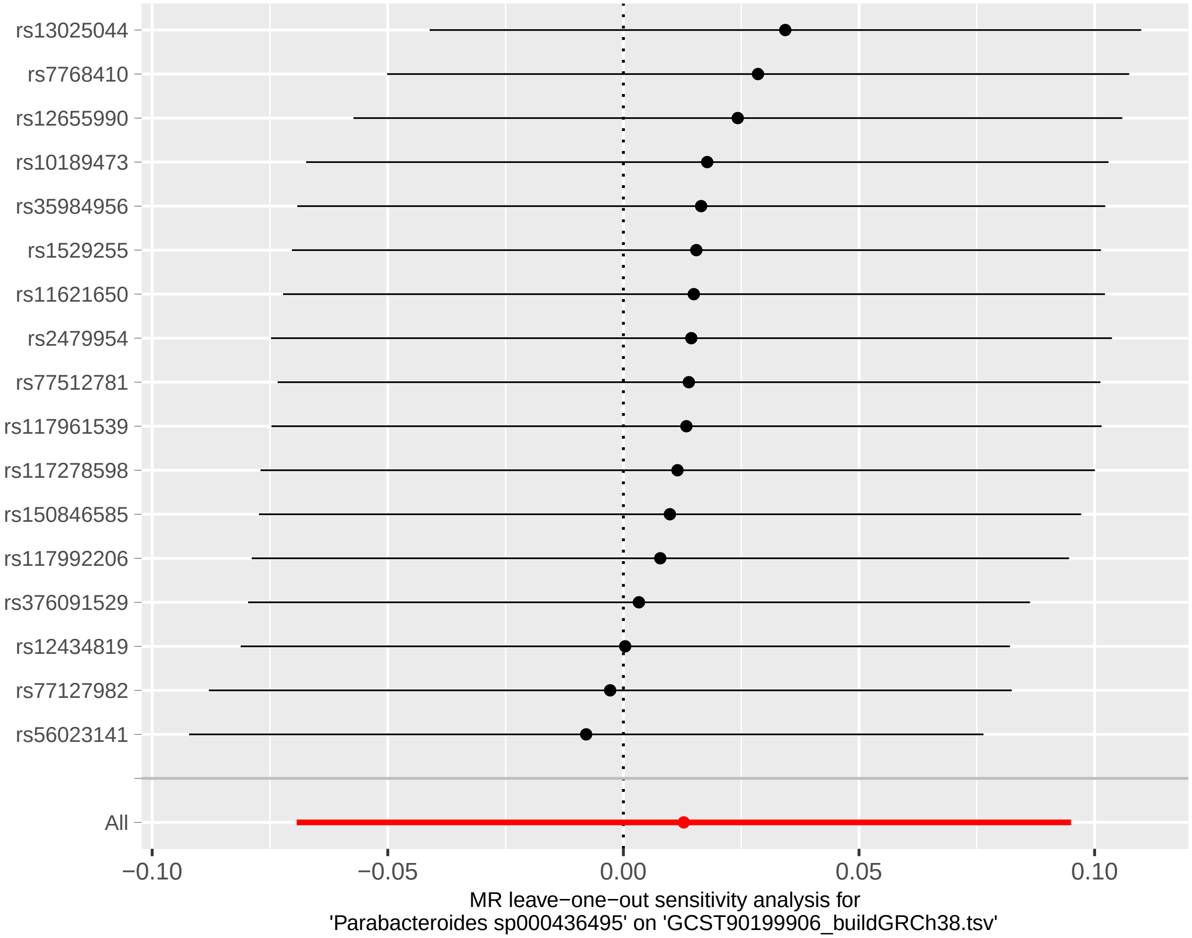
rs13025044
rs7768410
rs12655990
rs10189473
rs35984956
rs1529255
rs11621650
rs2479954
rs77512781
rs117961539
rs117278598
rs150846585
rs117992206
rs376091529
rs12434819
rs77127982
rs56023141
All
−0.10
−0.05
0.00
0.05
0.10
MR leave−one−out sensitivity analysis for
'Parabacteroides sp000436495' on 'GCST90199906_buildGRCh38.tsv'

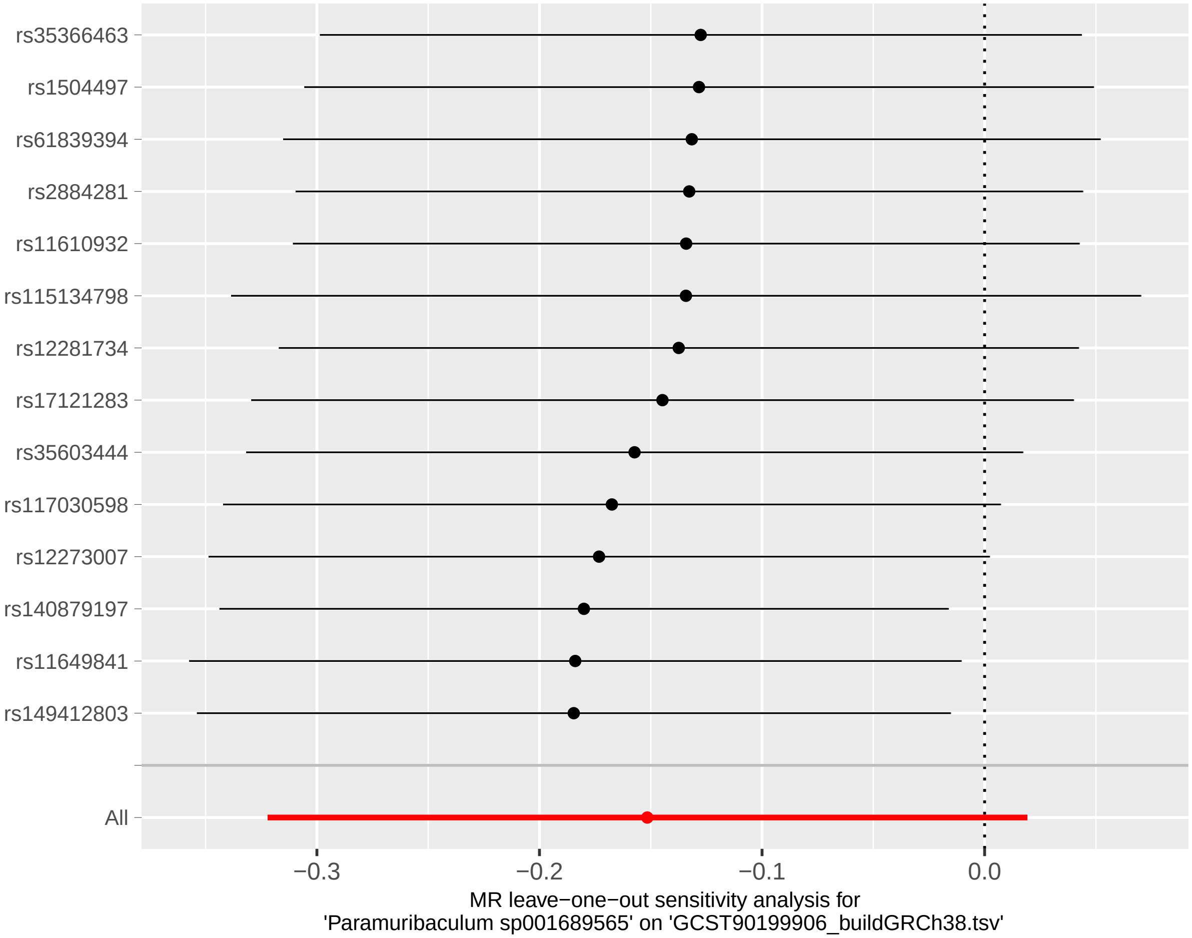

rs35366463
rs1504497
rs61839394
rs2884281
rs11610932
rs115134798
rs12281734
rs17121283
rs35603444
rs117030598
rs12273007
rs140879197
rs11649841
rs149412803
All
−0.3
−0.2
−0.1
0.0
MR leave−one−out sensitivity analysis for
'Paramuribaculum sp001689565' on 'GCST90199906_buildGRCh38.tsv'

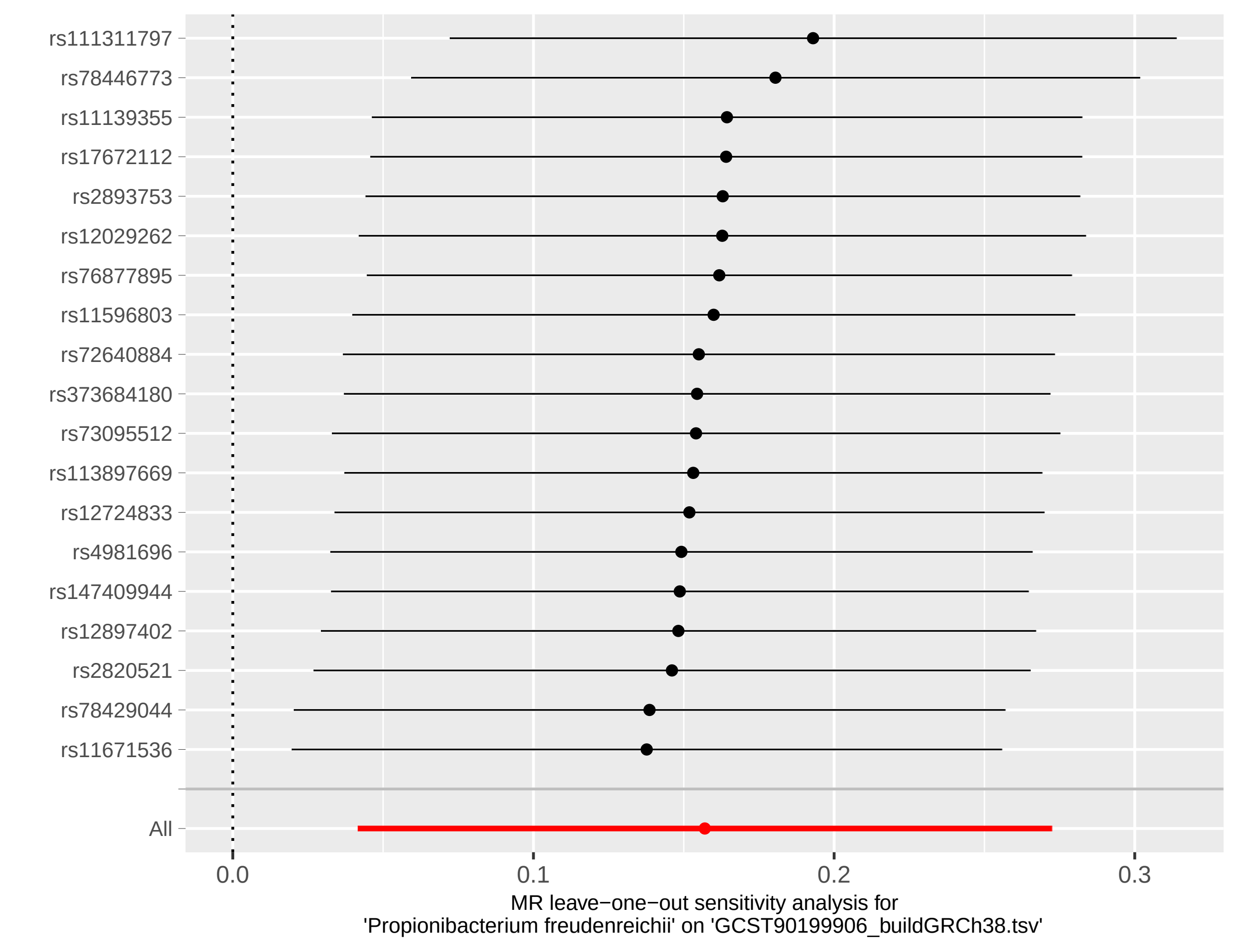

rs111311797
rs78446773
rs11139355
rs17672112
rs2893753
rs12029262
rs76877895
rs11596803
rs72640884
rs373684180
rs73095512
rs113897669
rs12724833
rs4981696
rs147409944
rs12897402
rs2820521
rs78429044
rs11671536
All
0.0
0.1
0.2
0.3
MR leave−one−out sensitivity analysis for
'Propionibacterium freudenreichii' on 'GCST90199906_buildGRCh38.tsv'

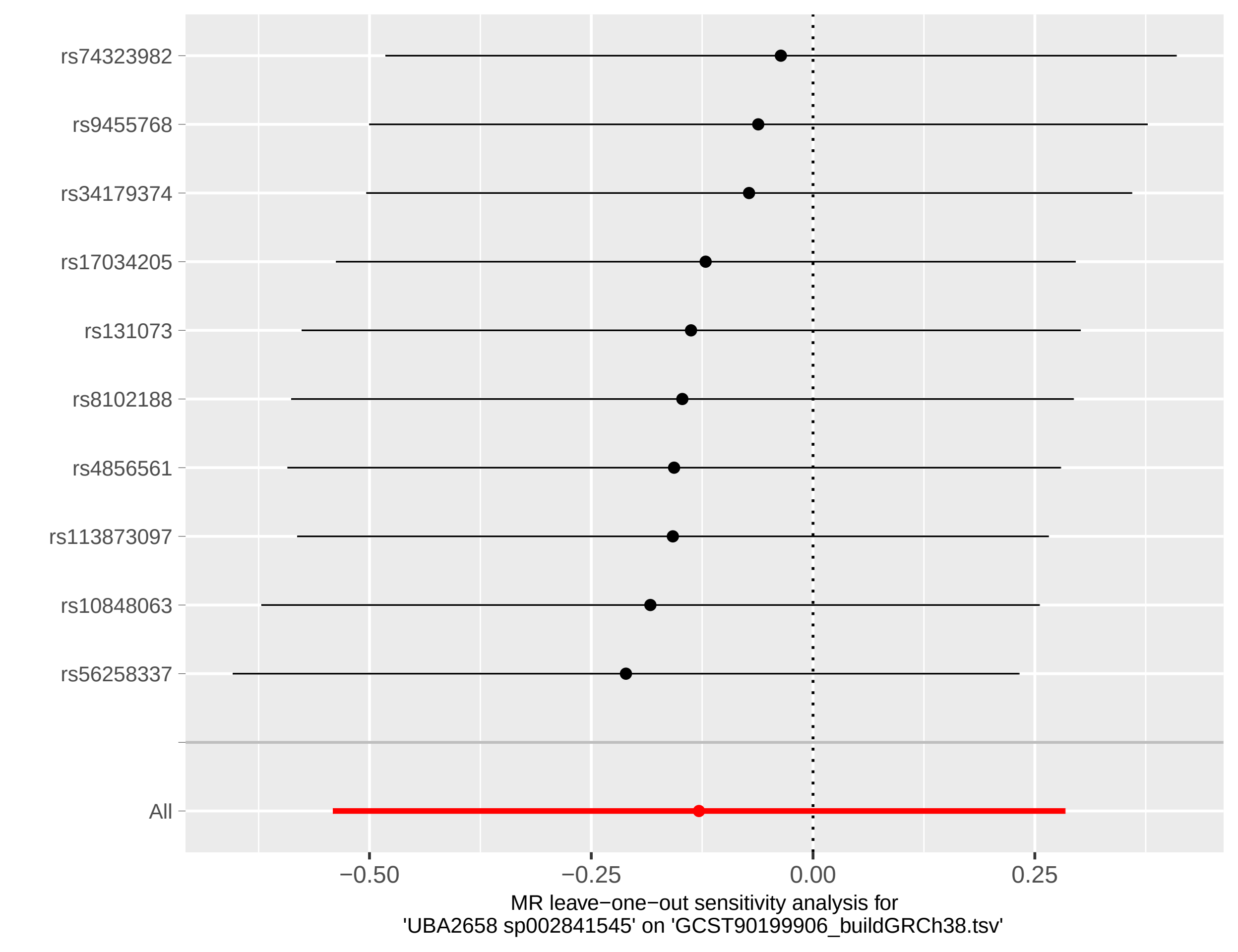
rs74323982
rs9455768
rs34179374
rs17034205
rs131073
rs8102188
rs4856561
rs113873097
rs10848063
rs56258337
All
−0.50
−0.25
0.00
0.25
MR leave−one−out sensitivity analysis for
'UBA2658 sp002841545' on 'GCST90199906_buildGRCh38.tsv'

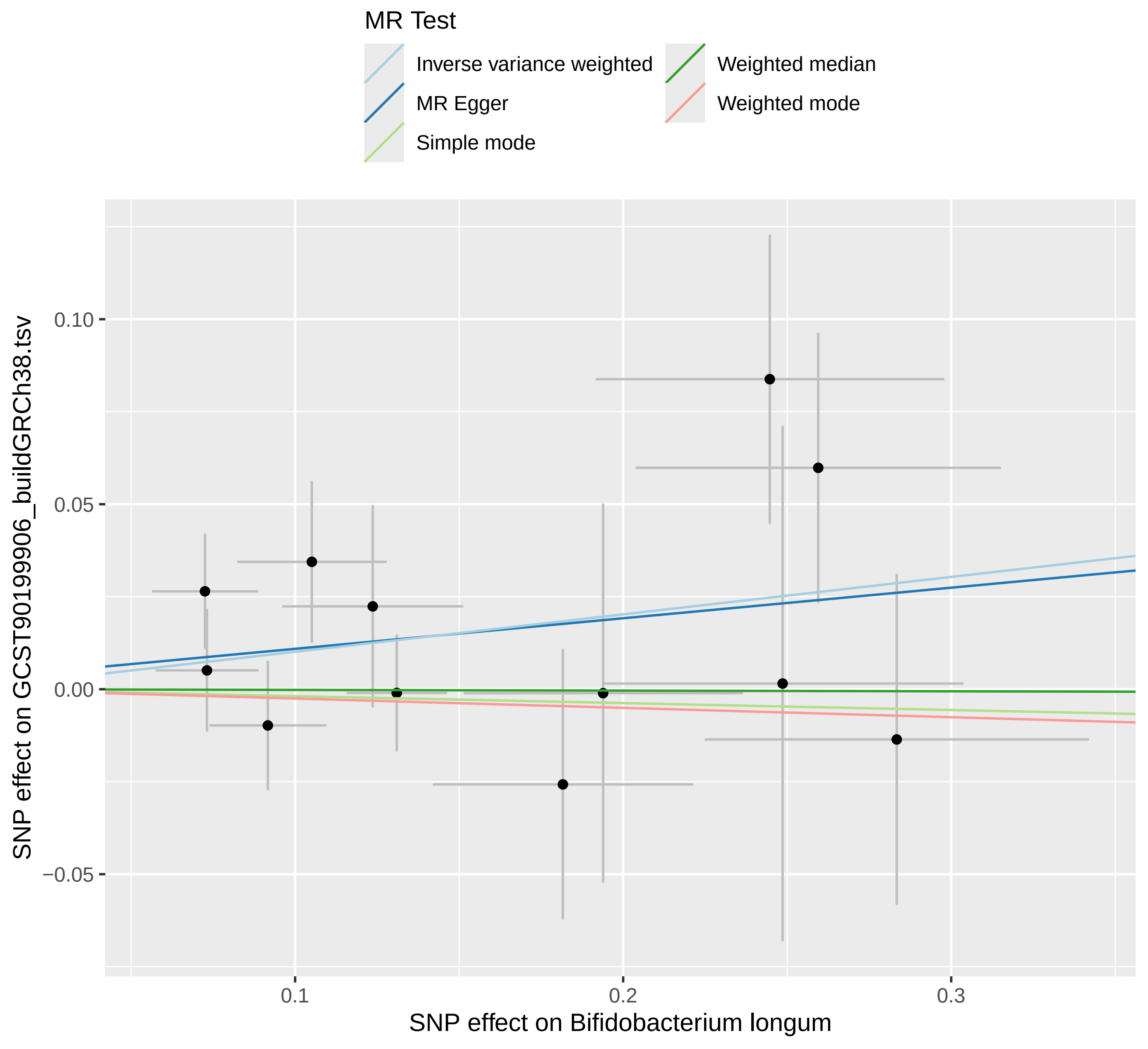
MR Test
Inverse variance weighted
MR Egger
Simple mode
Weighted median
Weighted mode
SNP effect on GCST90199906_buildGRCh38.tsv
0.10
0.05
0.00
−0.05
0.1
0.2
0.3
SNP effect on Bifidobacterium longum

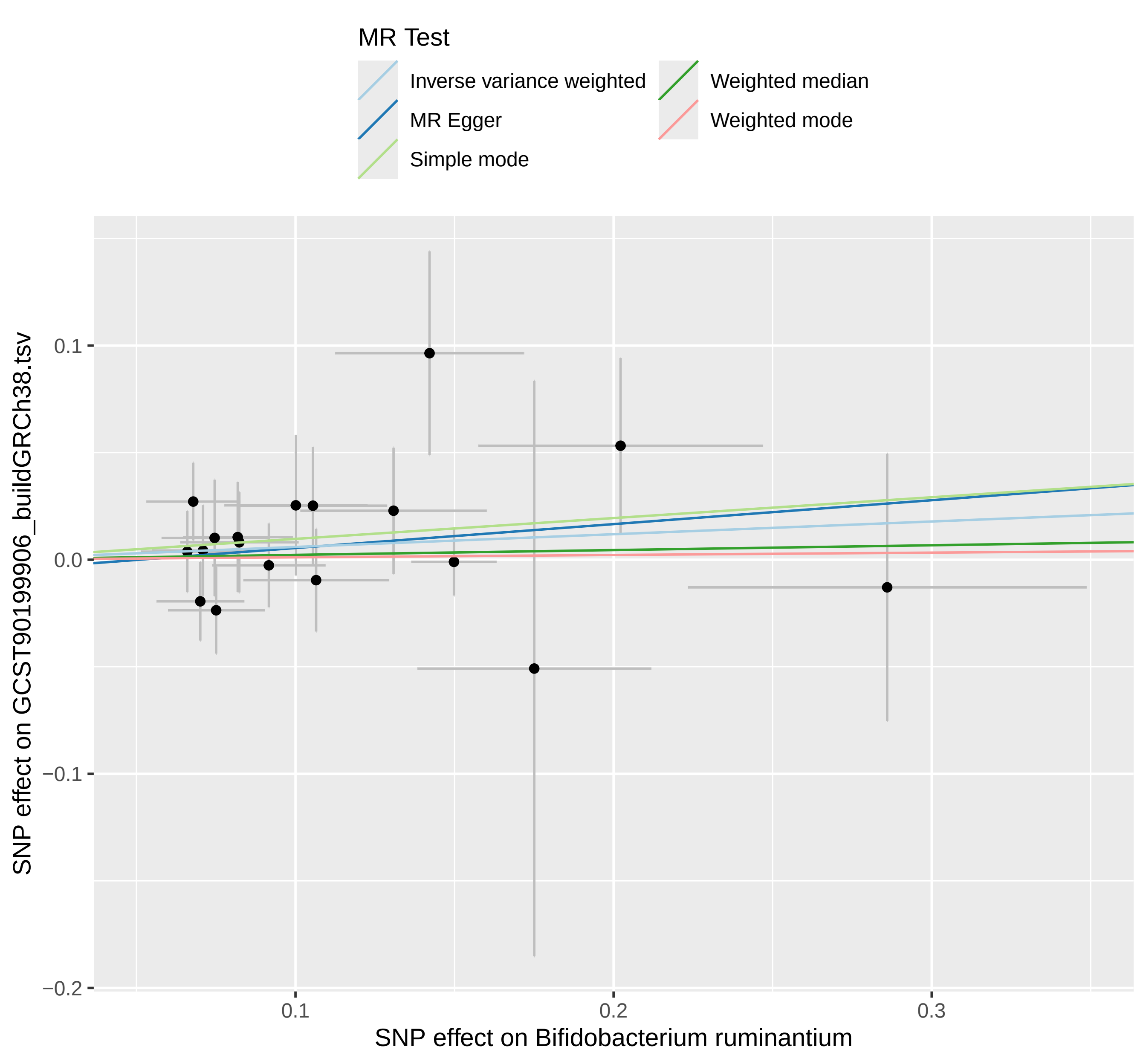
MR Test
Inverse variance weighted
MR Egger
Simple mode
Weighted median
Weighted mode
0.1
0.0
−0.1
−0.2
0.1
0.2
0.3
SNP effect on GCST90199906_buildGRCh38.tsv
SNP effect on Bifidobacterium ruminantium

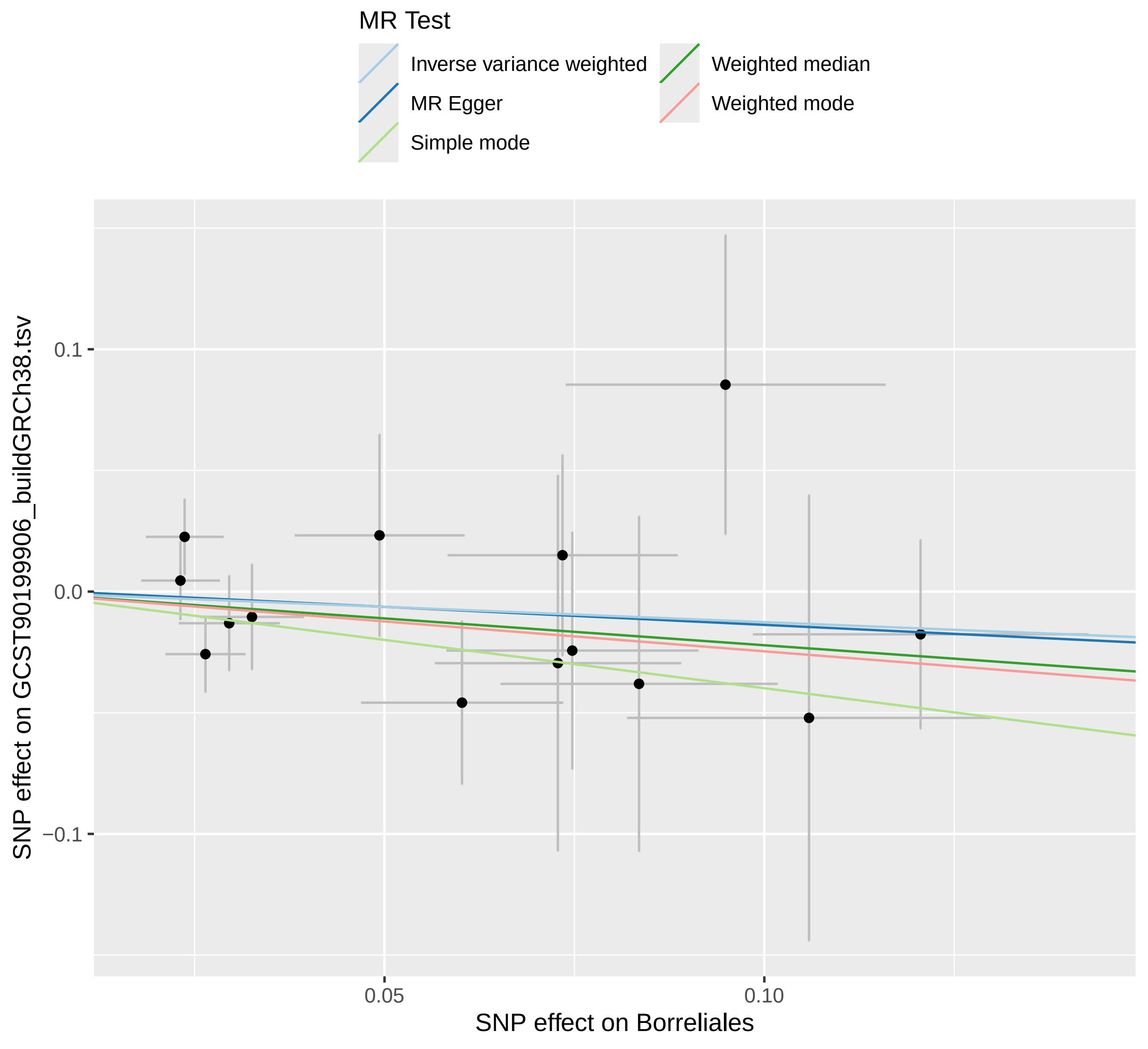
MR Test
Inverse variance weighted
MR Egger
Simple mode
Weighted median
Weighted mode
0.1
0.0
−0.1
0.05
0.10
SNP effect on GCST90199906_buildGRCh38.tsv
SNP effect on Borreliales

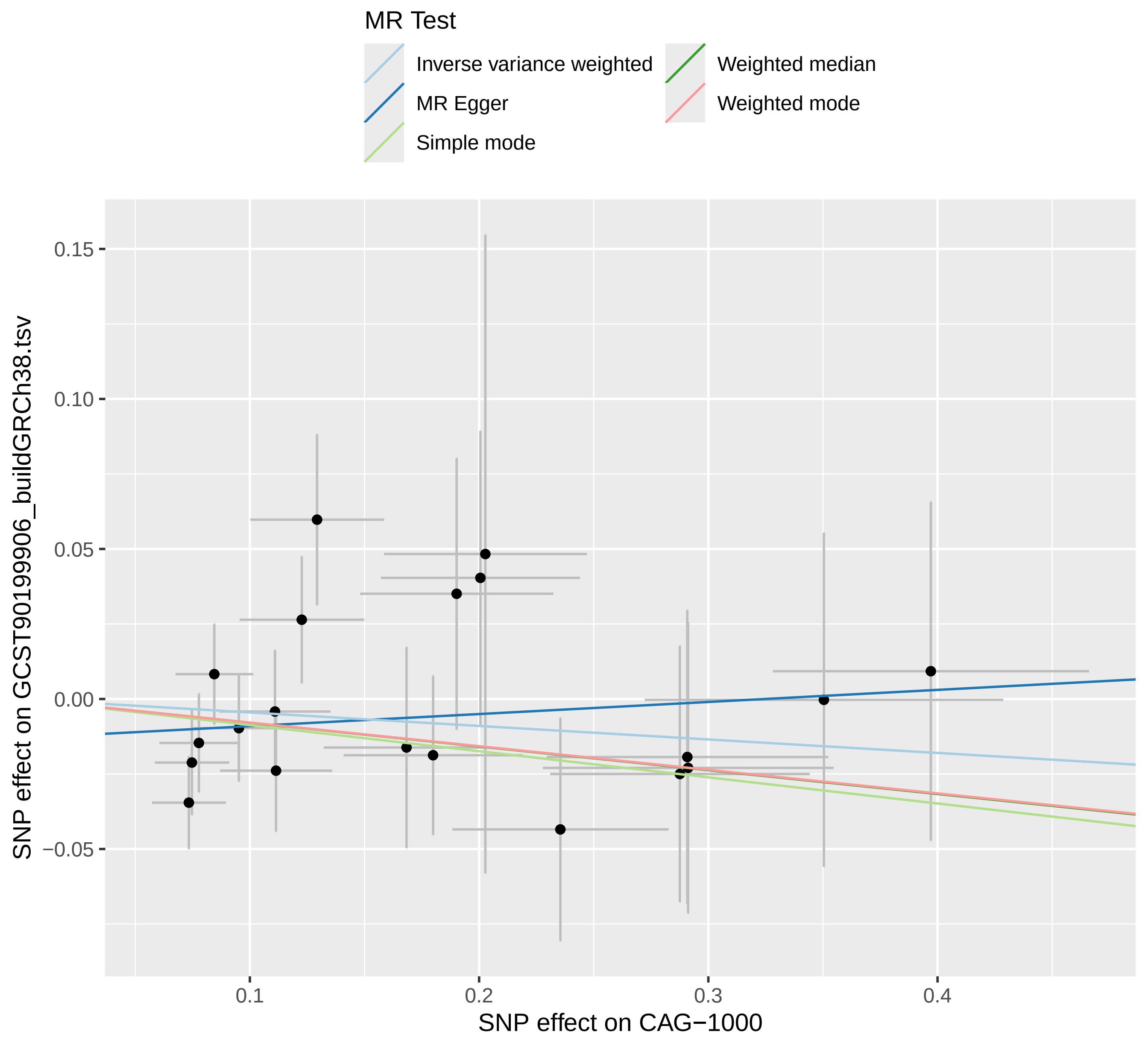
MR Test
Inverse variance weighted
MR Egger
Simple mode
Weighted median
Weighted mode
0.15
0.10
0.05
0.00
−0.05
SNP effect on GCST90199906_buildGRCh38.tsv
0.1
0.2
0.3
0.4
SNP effect on CAG−1000

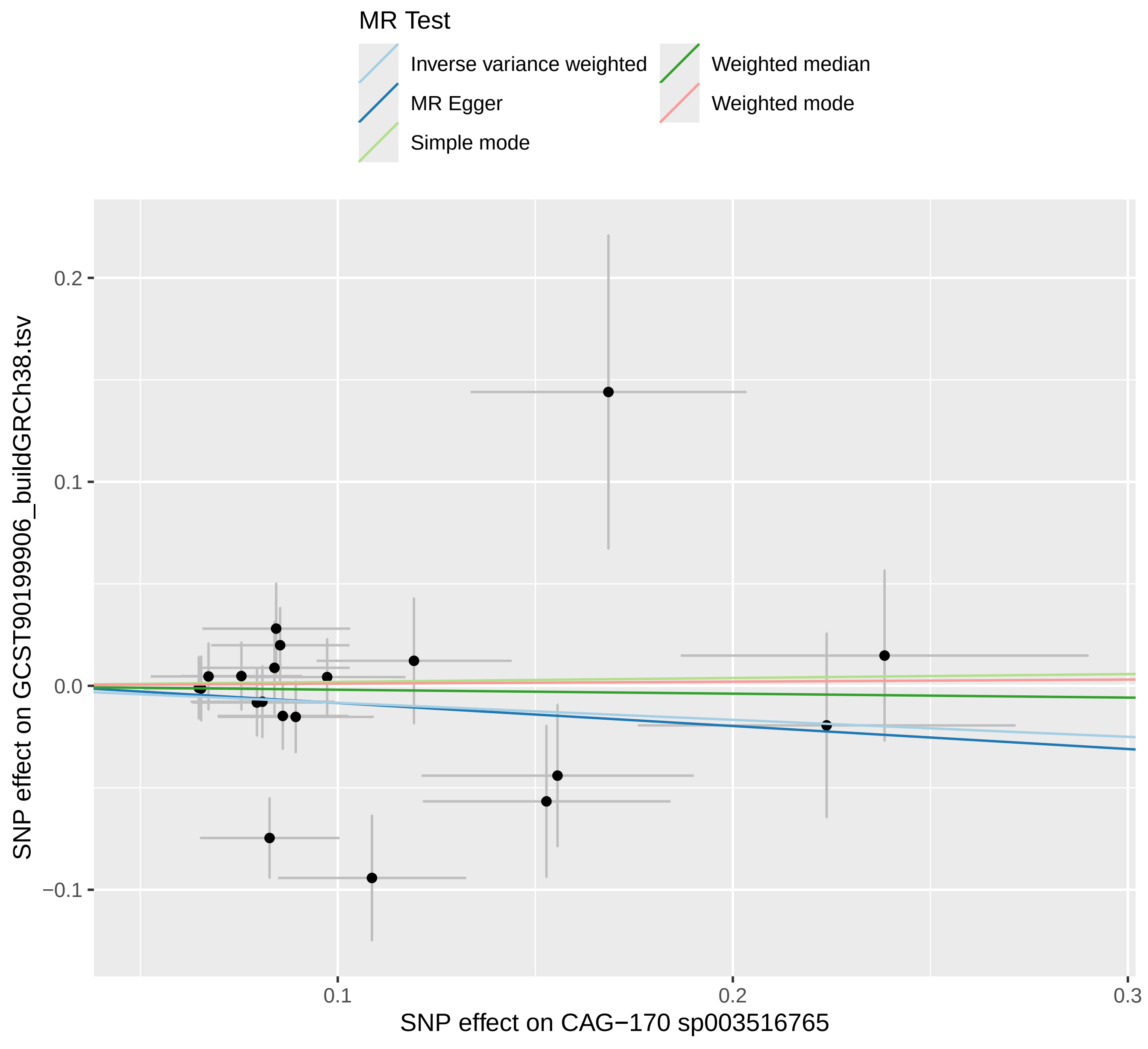
MR Test
Inverse variance weighted
MR Egger
Simple mode
Weighted median
Weighted mode
0.2
0.1
0.0
−0.1
0.1
0.2
0.3
SNP effect on GCST90199906_buildGRCh38.tsv
SNP effect on CAG−170 sp003516765

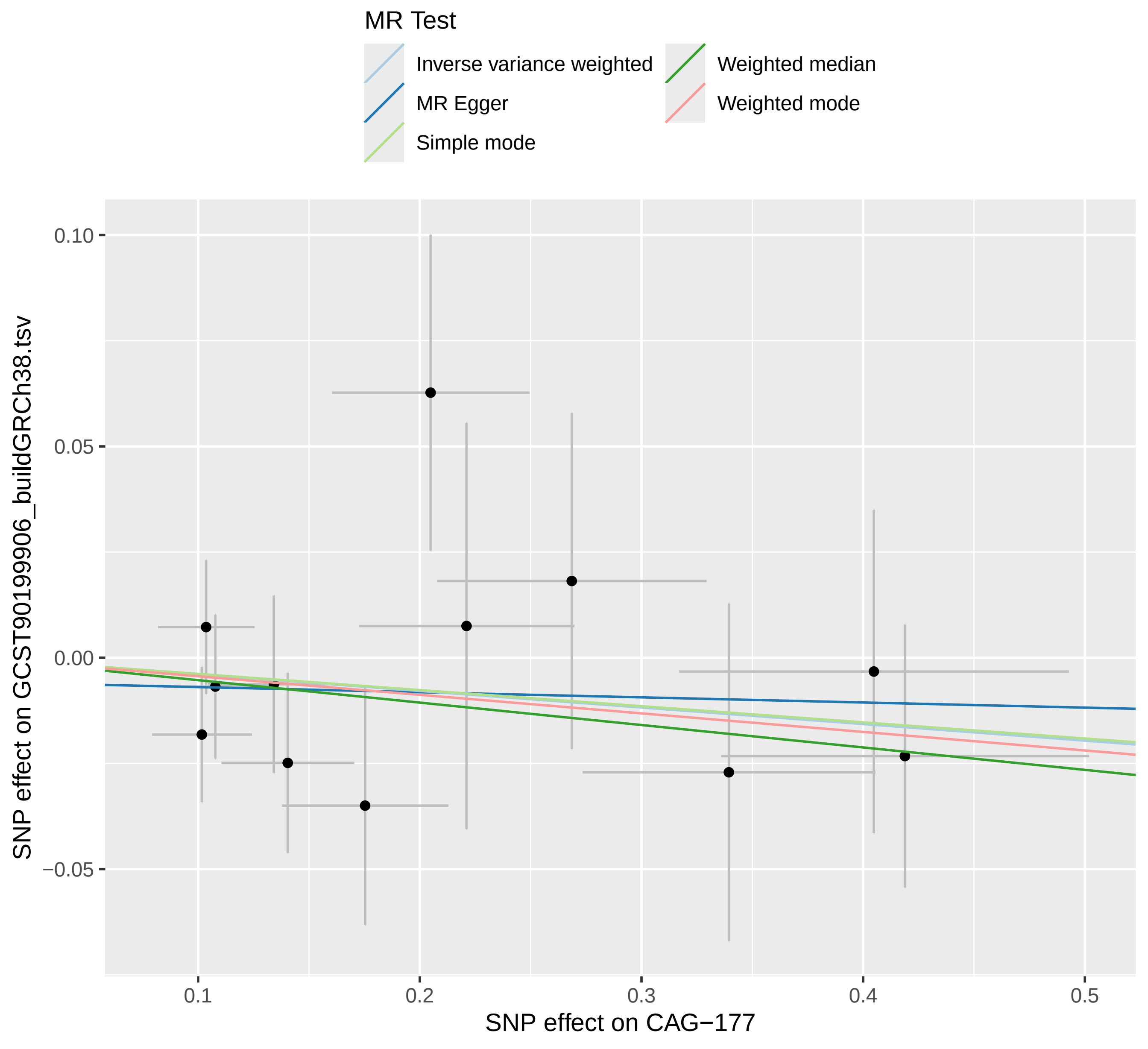

MR Test
Inverse variance weighted
MR Egger
Simple mode
Weighted median
Weighted mode
0.10
0.05
0.00
−0.05
0.1
0.2
0.3
0.4
0.5
SNP effect on CAG−177
SNP effect on GCST90199906_buildGRCh38.tsv

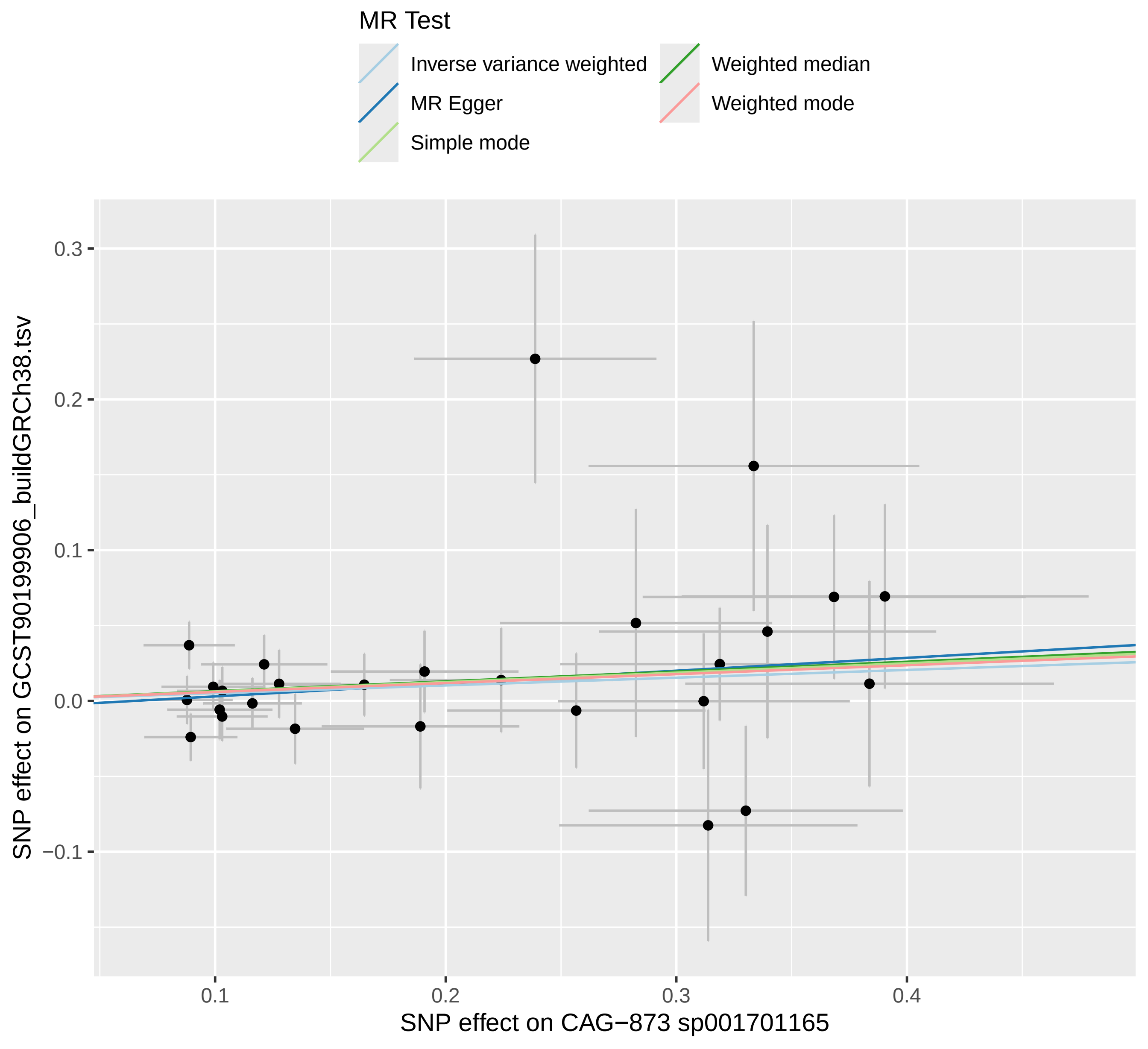

MR Test
Inverse variance weighted
MR Egger
Simple mode
Weighted median
Weighted mode
0.3
0.2
0.1
0.0
−0.1
0.1
0.2
0.3
0.4
SNP effect on GCST90199906_buildGRCh38.tsv
SNP effect on CAG−873 sp001701165

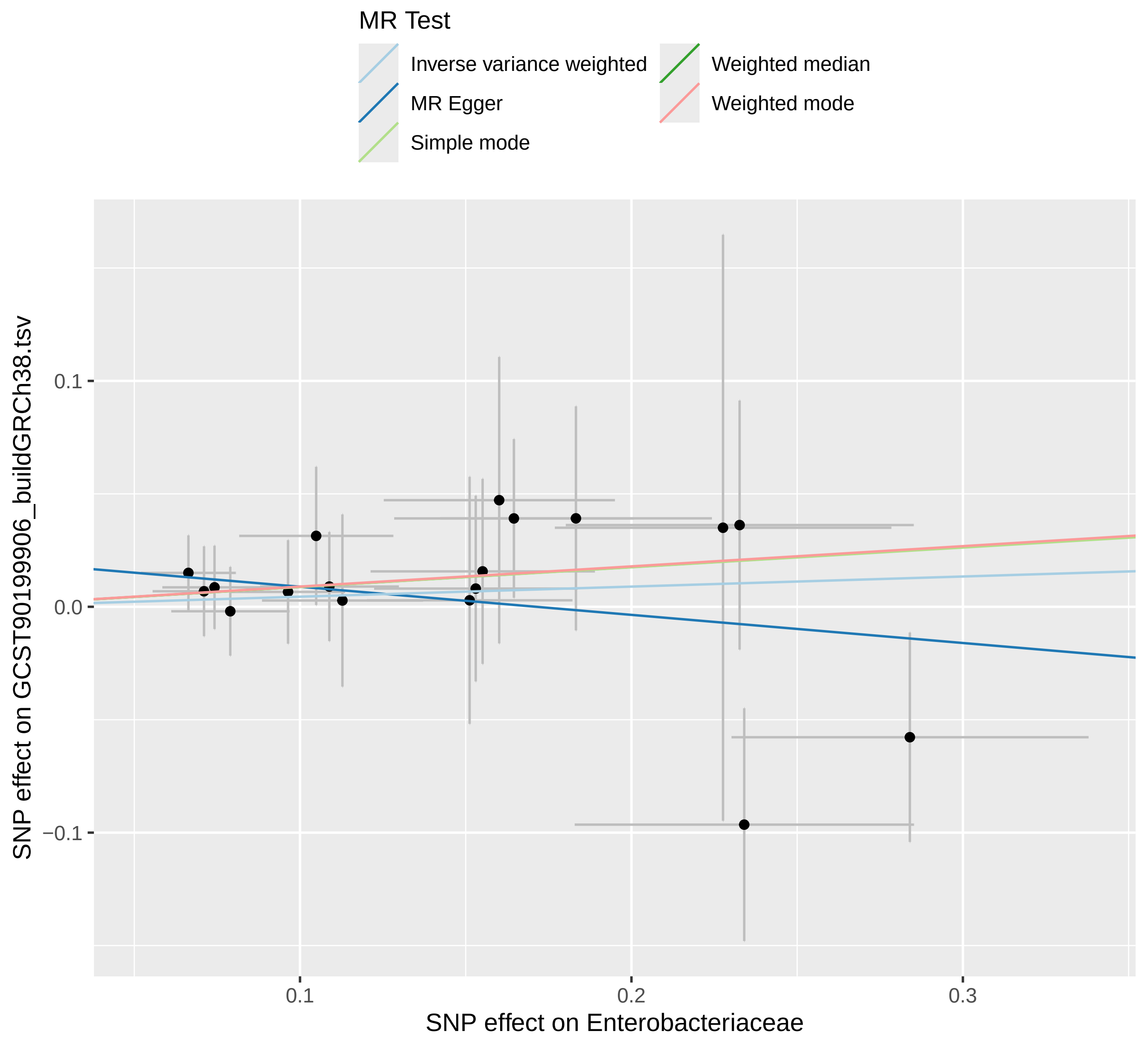

MR Test
Inverse variance weighted
MR Egger
Simple mode
Weighted median
Weighted mode
SNP effect on GCST90199906_buildGRCh38.tsv
0.1
0.0
−0.1
0.1
0.2
0.3
SNP effect on Enterobacteriaceae

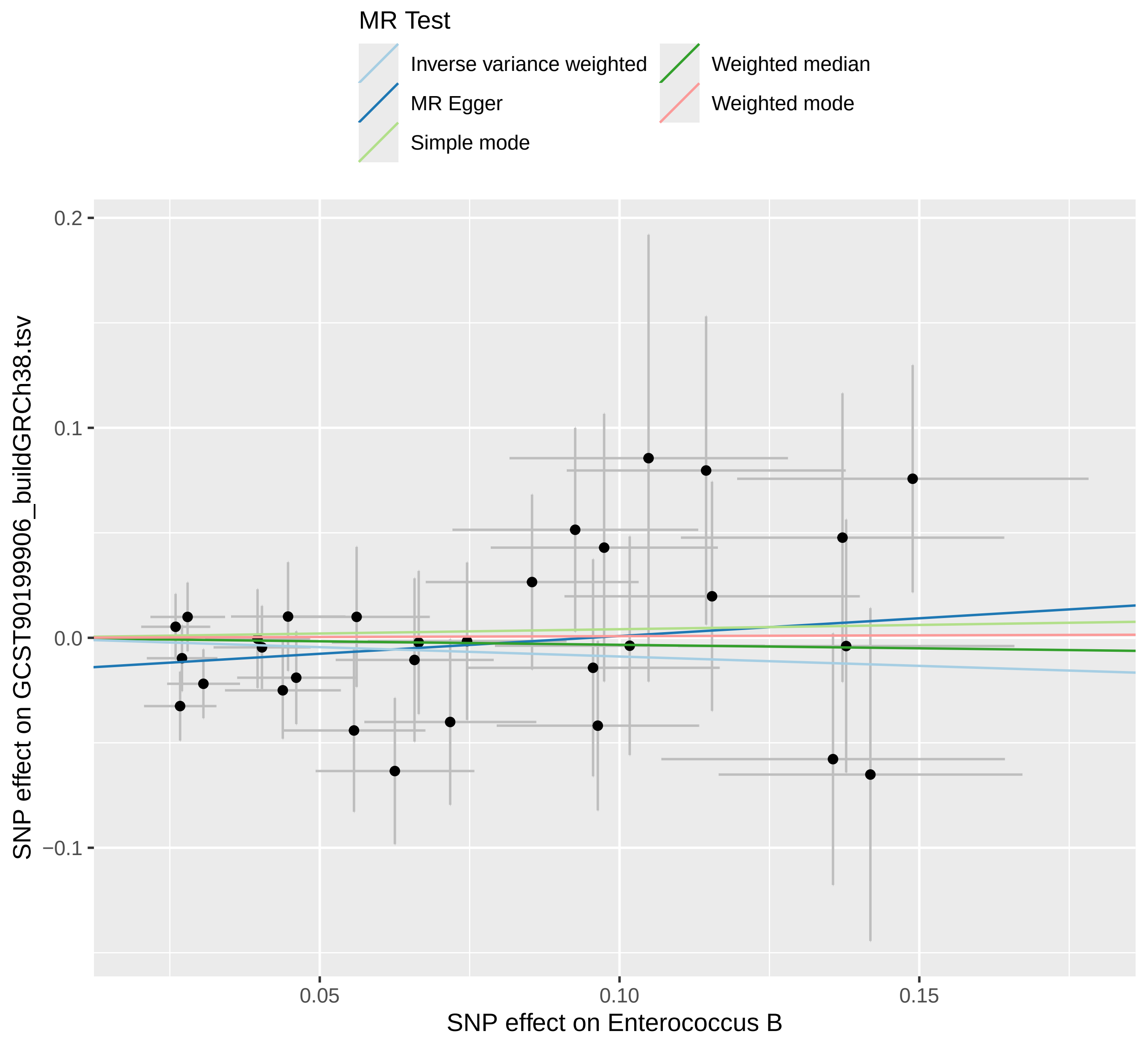
MR Test
Inverse variance weighted
MR Egger
Simple mode
Weighted median
Weighted mode
0.2
0.1
0.0
−0.1
0.05
0.10
0.15
SNP effect on GCST90199906_buildGRCh38.tsv
SNP effect on Enterococcus B

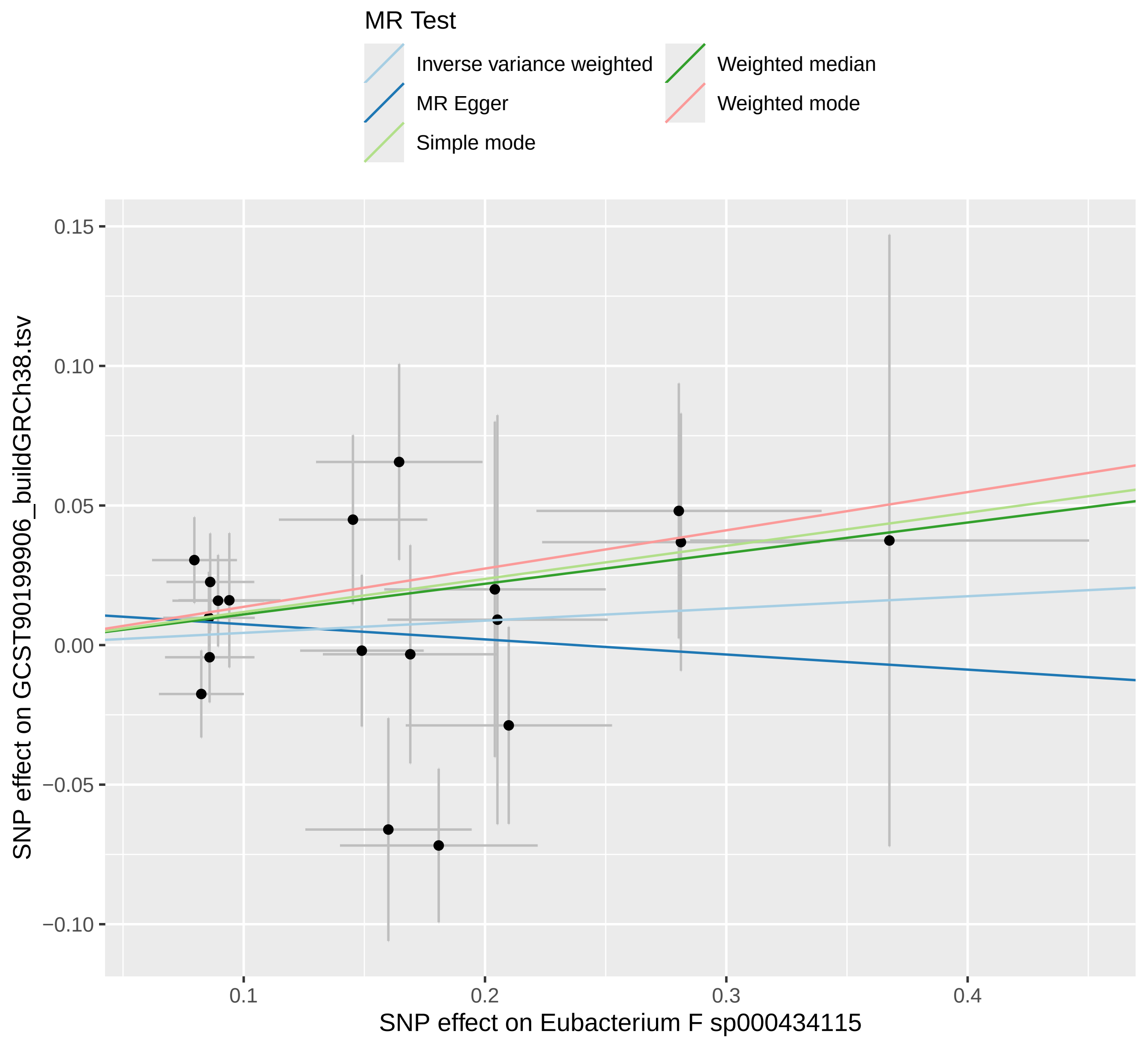

MR Test
Inverse variance weighted
MR Egger
Simple mode
Weighted median
Weighted mode
0.15
0.10
0.05
0.00
−0.05
−0.10
SNP effect on GCST90199906_buildGRCh38.tsv
0.1
0.2
0.3
0.4
SNP effect on Eubacterium F sp000434115

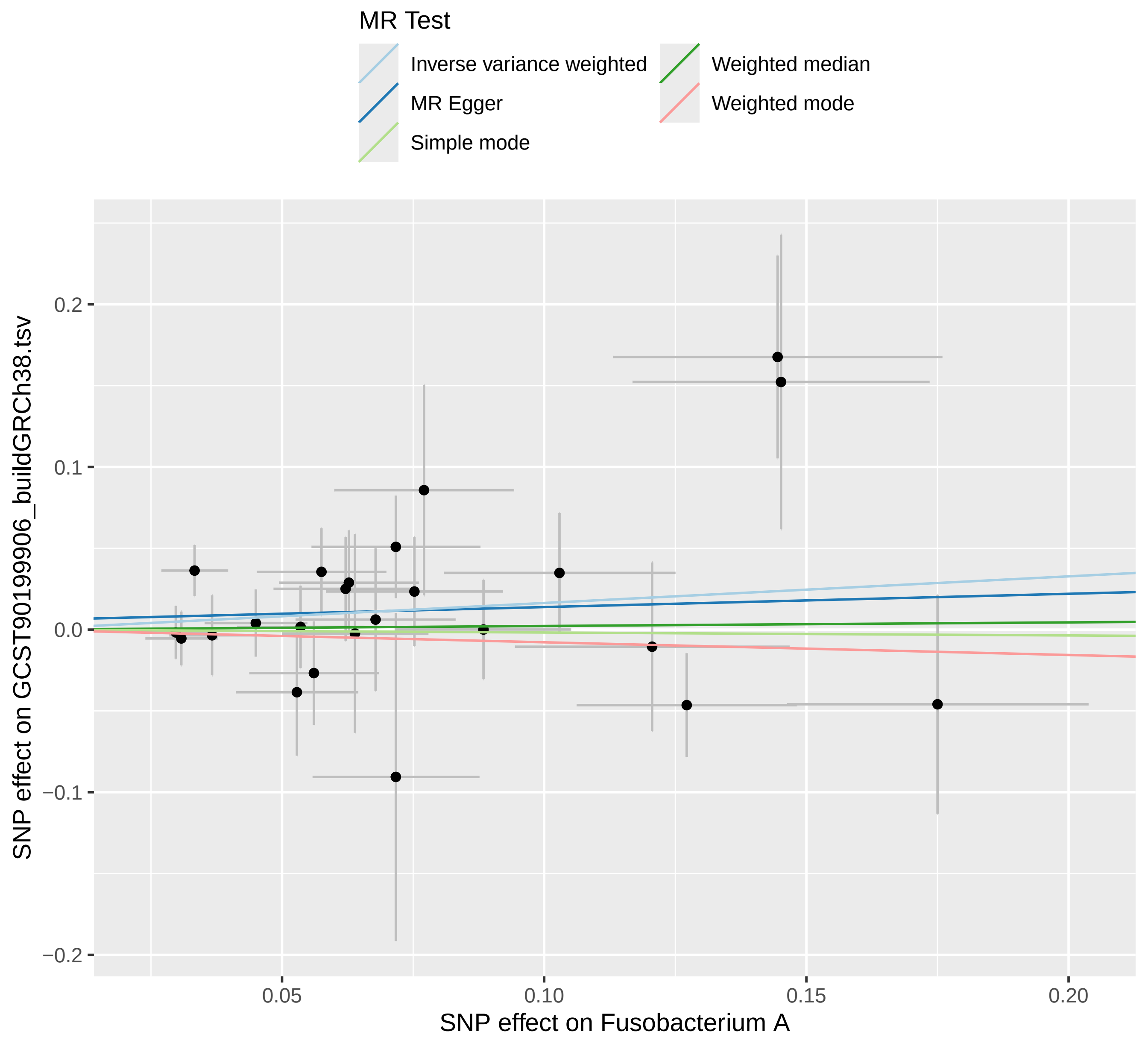

MR Test
Inverse variance weighted
MR Egger
Simple mode
Weighted median
Weighted mode
0.2
0.1
0.0
−0.1
−0.2
0.05
0.10
0.15
0.20
SNP effect on GCST90199906_buildGRCh38.tsv
SNP effect on Fusobacterium A

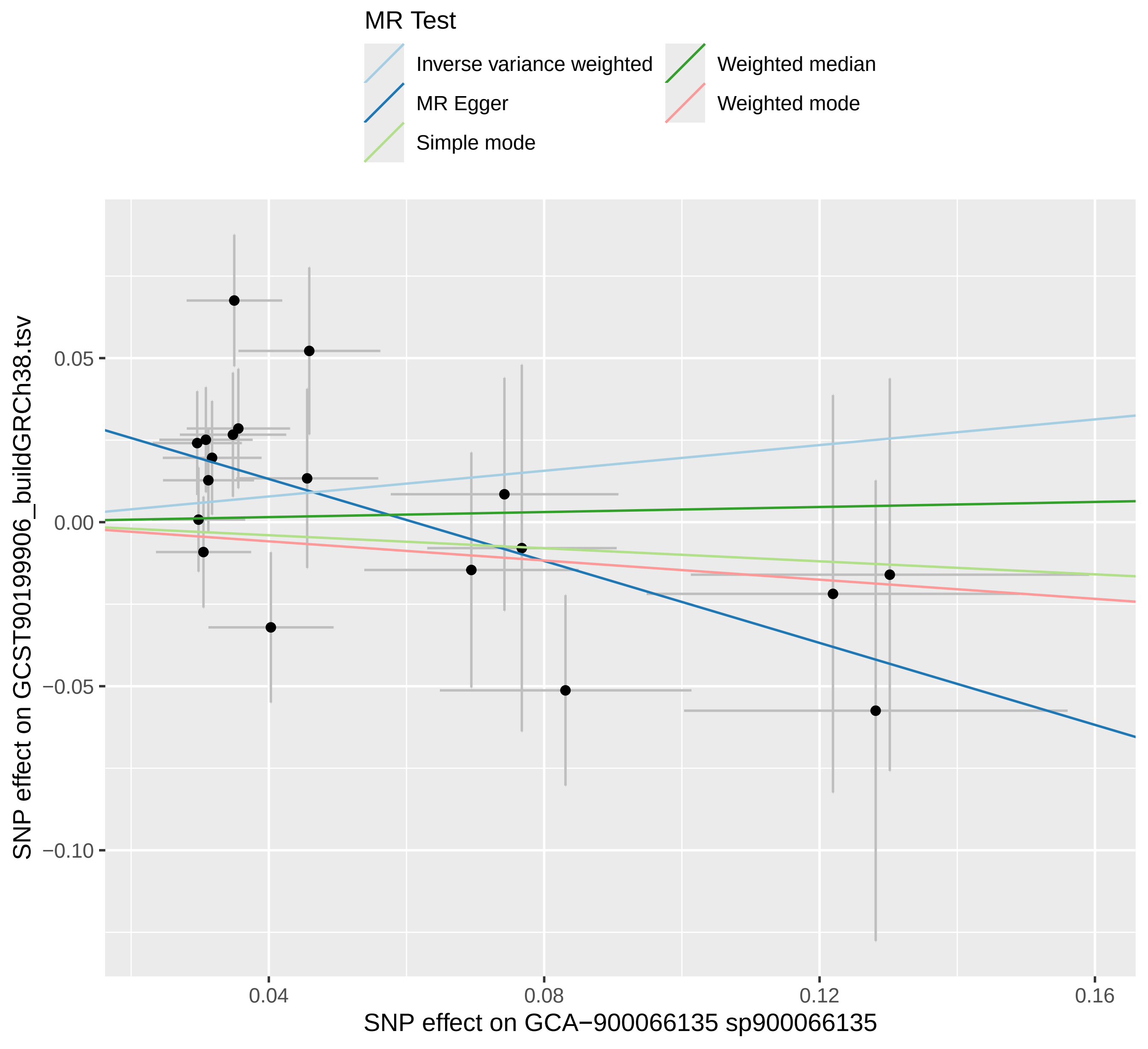
MR Test
Inverse variance weighted
MR Egger
Simple mode
Weighted median
Weighted mode
0.05
0.00
−0.05
−0.10
0.04
0.08
0.12
0.16
SNP effect on GCST90199906_buildGRCh38.tsv
SNP effect on GCA−900066135 sp900066135

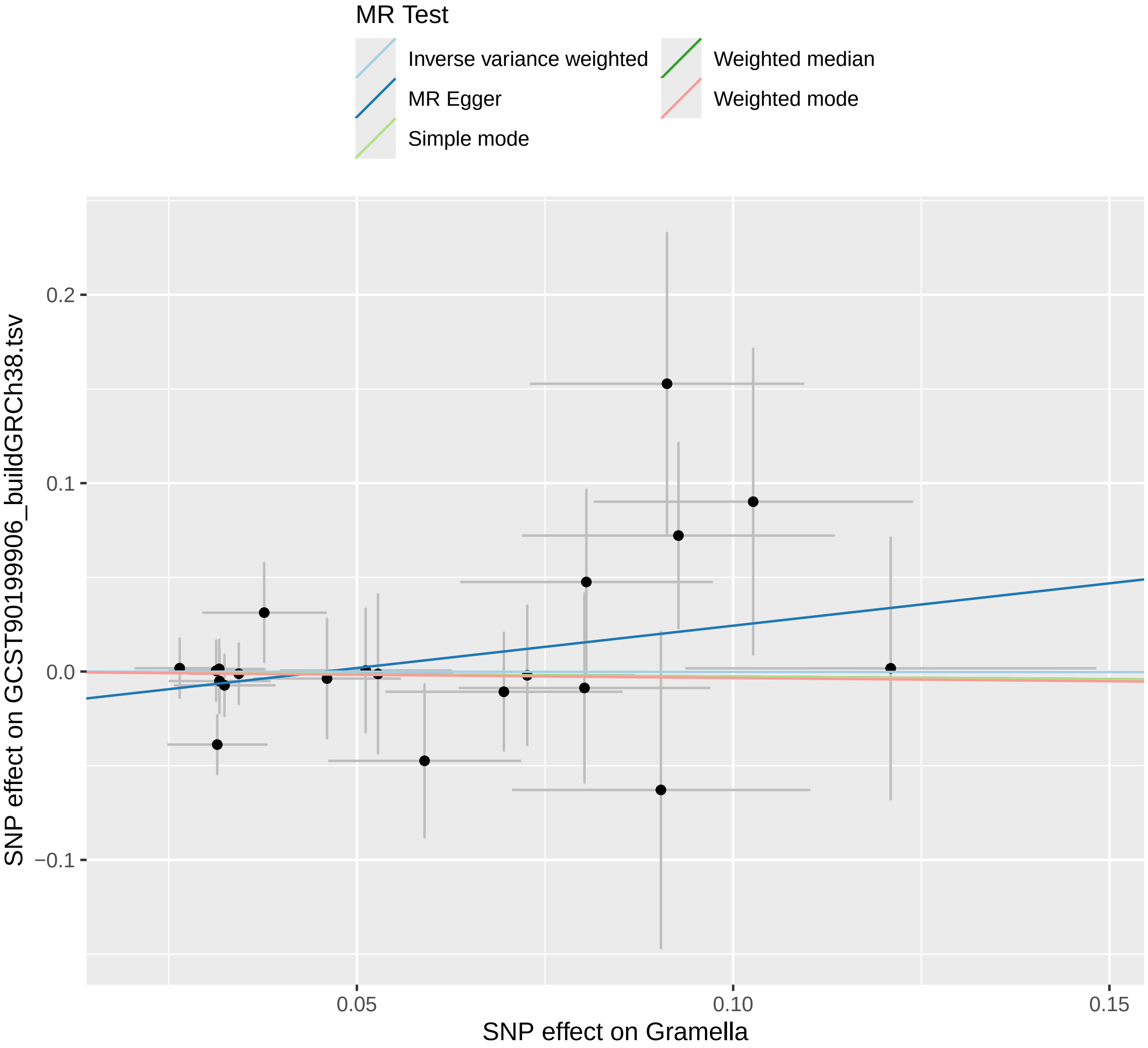
MR Test
Inverse variance weighted
MR Egger
Simple mode
Weighted median
Weighted mode
0.2
0.1
0.0
−0.1
0.05
0.10
0.15
SNP effect on GCST90199906_buildGRCh38.tsv
SNP effect on Gramella

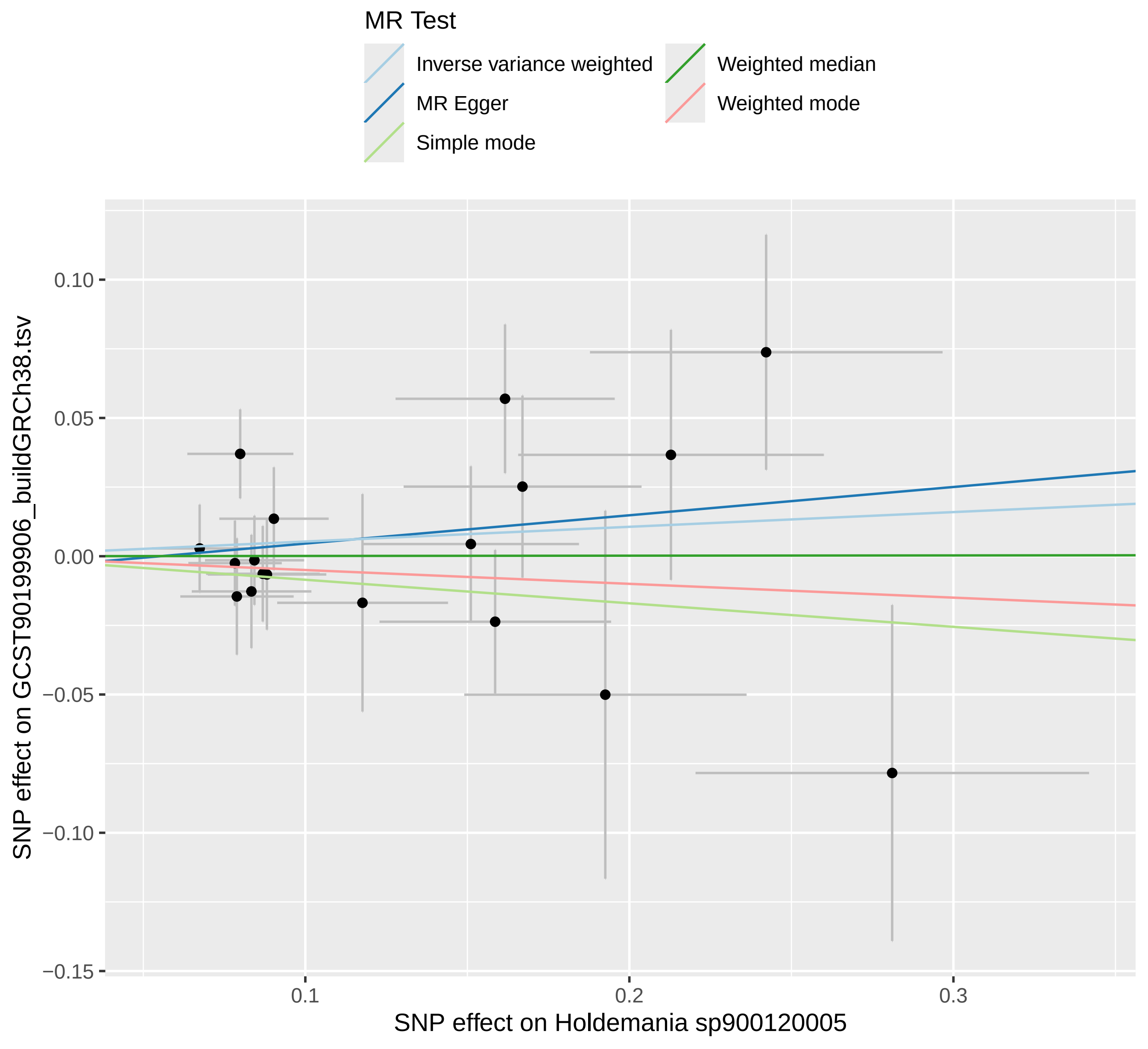
MR Test
Inverse variance weighted
MR Egger
Simple mode
Weighted median
Weighted mode
SNP effect on GCST90199906_buildGRCh38.tsv
0.10
0.05
0.00
−0.05
−0.10
−0.15
0.1
0.2
0.3
SNP effect on Holdemania sp900120005

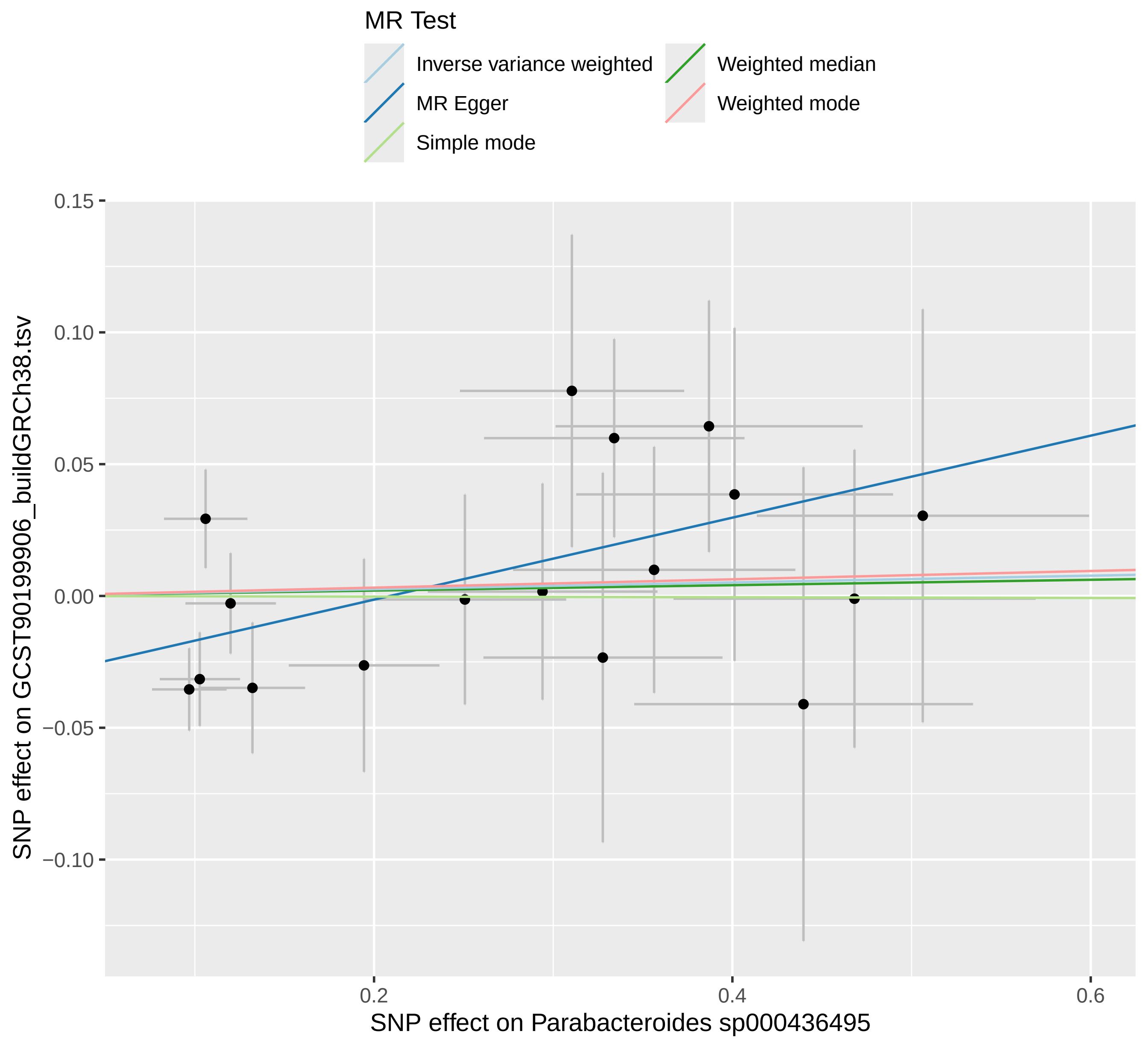
MR Test
Inverse variance weighted
MR Egger
Simple mode
Weighted median
Weighted mode
0.15
0.10
0.05
0.00
−0.05
−0.10
0.2
0.4
0.6
SNP effect on GCST90199906_buildGRCh38.tsv
SNP effect on Parabacteroides sp000436495

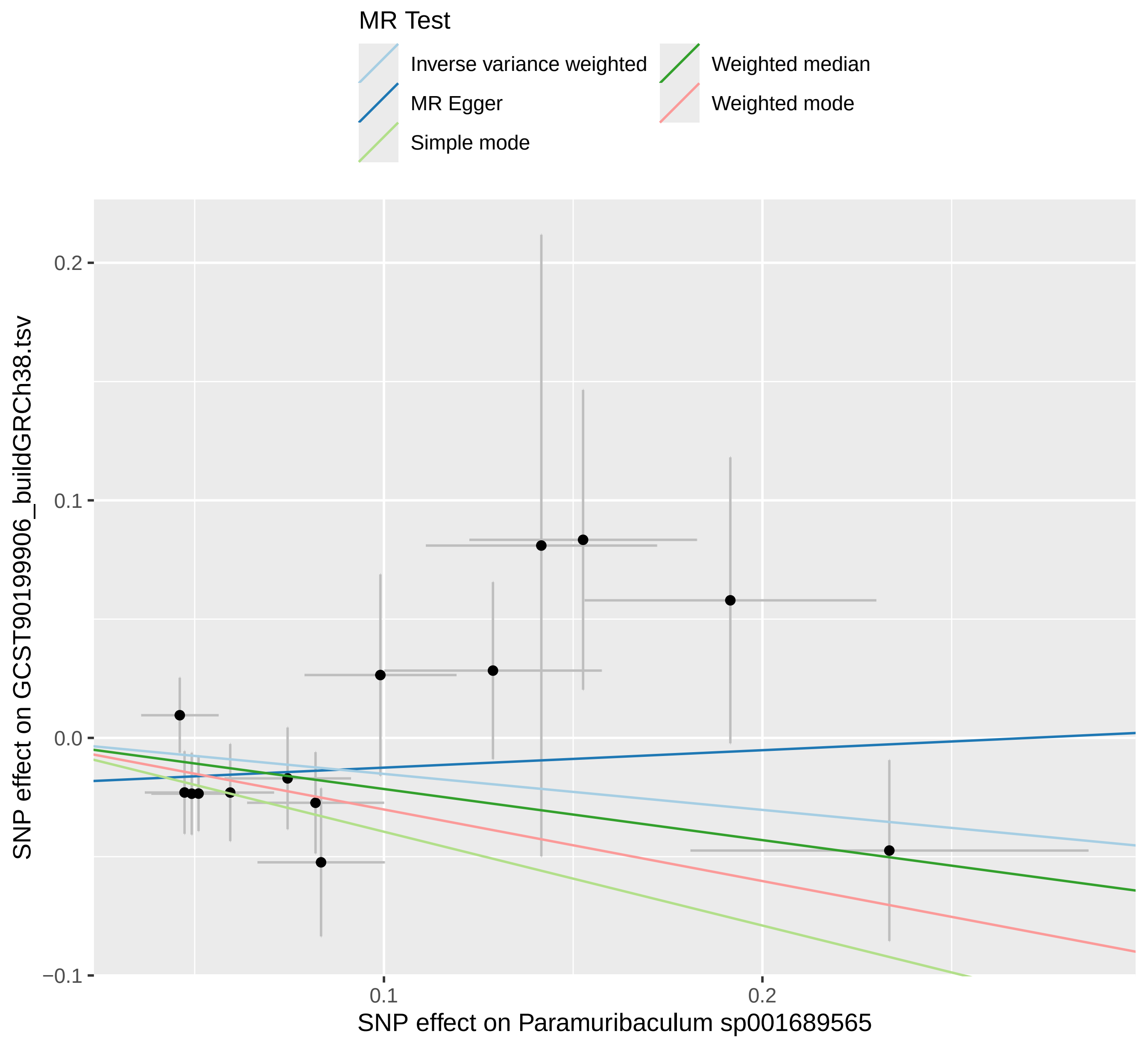
MR Test
Inverse variance weighted
MR Egger
Simple mode
Weighted median
Weighted mode
0.2
0.1
0.0
−0.1
0.1
0.2
SNP effect on GCST90199906_buildGRCh38.tsv
SNP effect on Paramuribaculum sp001689565

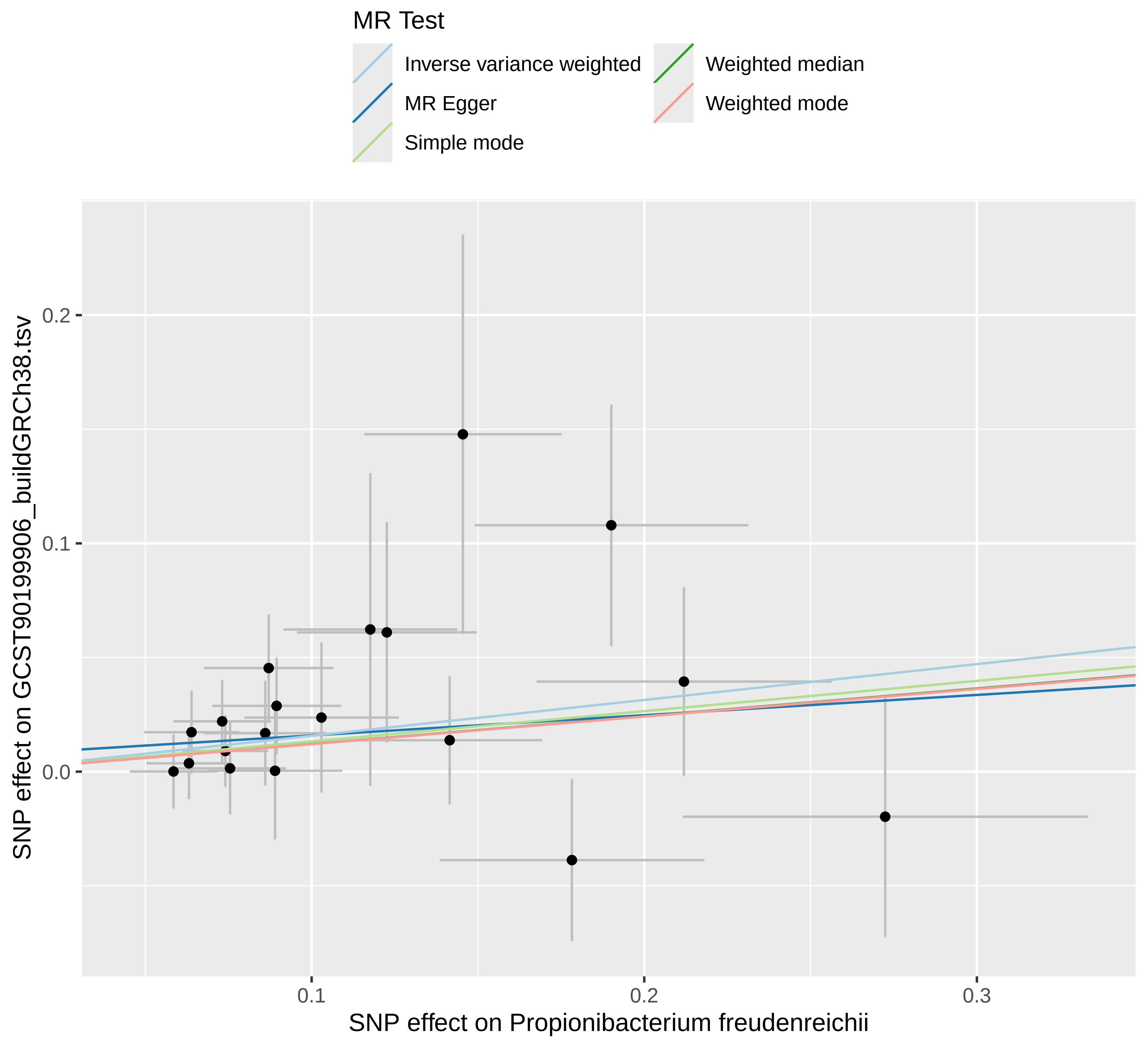
MR Test
Inverse variance weighted
MR Egger
Simple mode
Weighted median
Weighted mode
0.2
0.1
0.0
0.1
0.2
0.3
SNP effect on GCST90199906_buildGRCh38.tsv
SNP effect on Propionibacterium freudenreichii

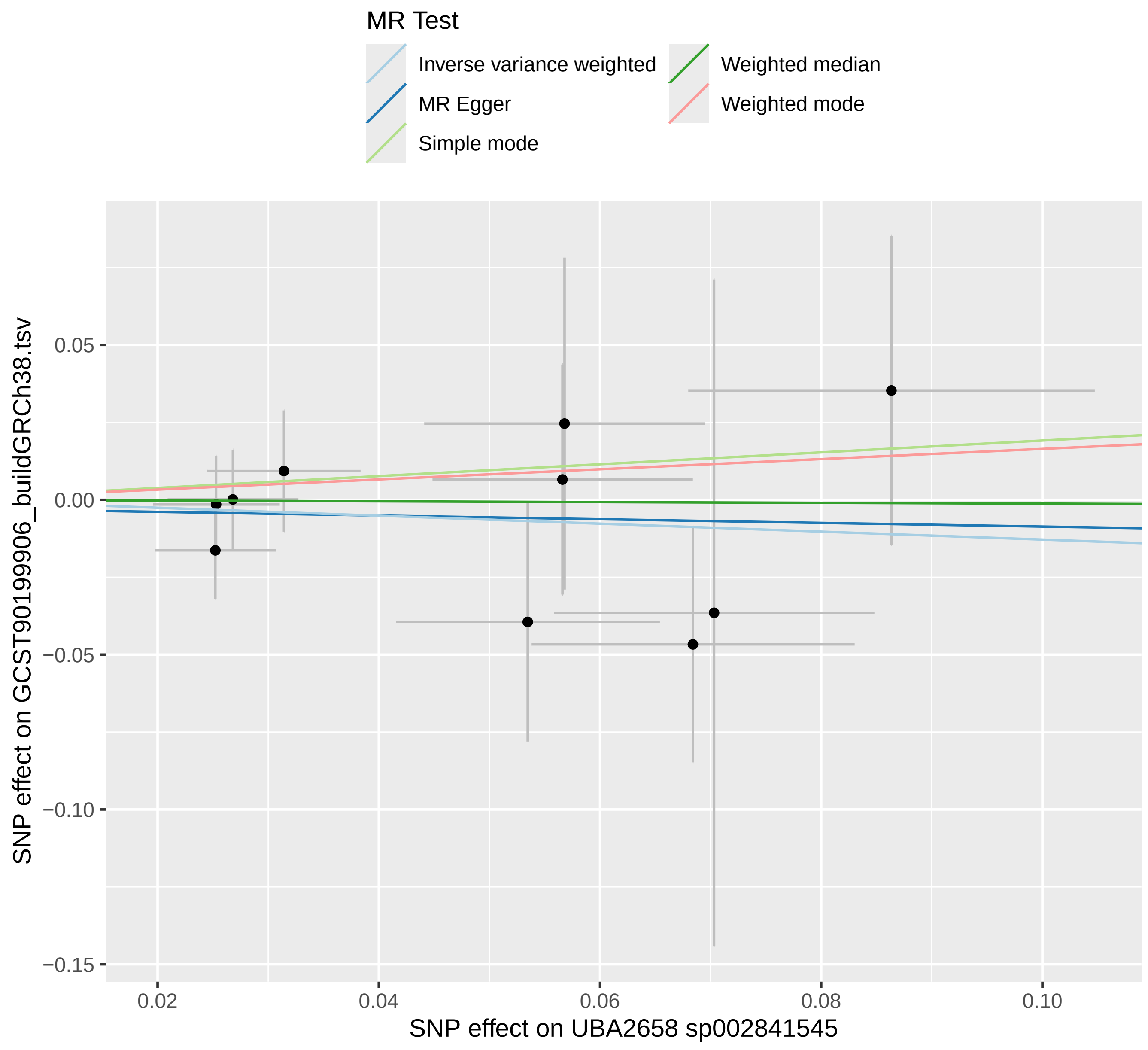
MR Test
Inverse variance weighted
MR Egger
Simple mode
Weighted median
Weighted mode
0.05
0.00
−0.05
−0.10
−0.15
0.02
0.04
0.06
0.08
0.10
SNP effect on GCST90199906_buildGRCh38.tsv
SNP effect on UBA2658 sp002841545

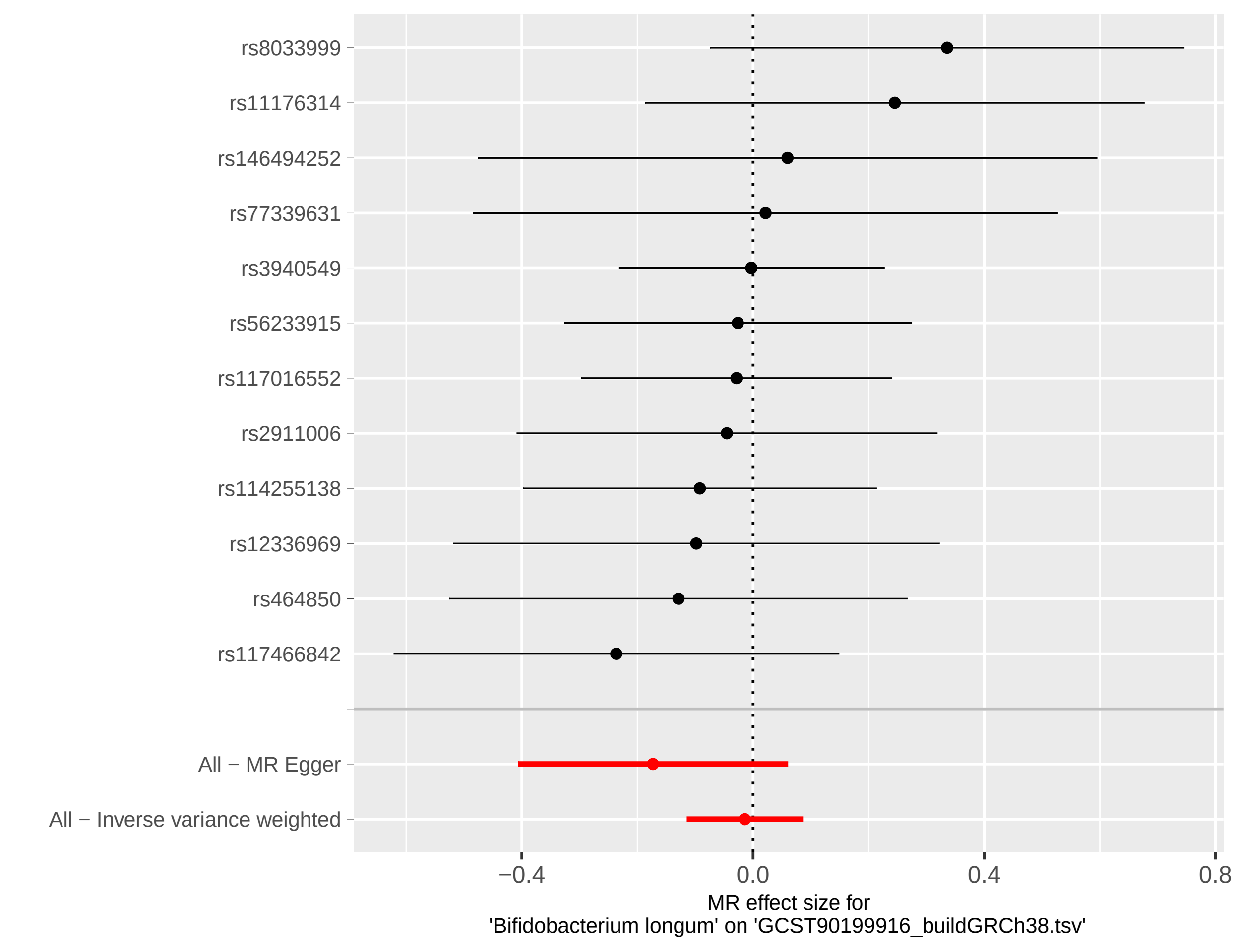
rs8033999
rs11176314
rs146494252
rs77339631
rs3940549
rs56233915
rs117016552
rs2911006
rs114255138
rs12336969
rs464850
rs117466842
All − MR Egger
All − Inverse variance weighted
−0.4
0.0
0.4
0.8
MR effect size for
'Bifidobacterium longum' on 'GCST90199916_buildGRCh38.tsv'

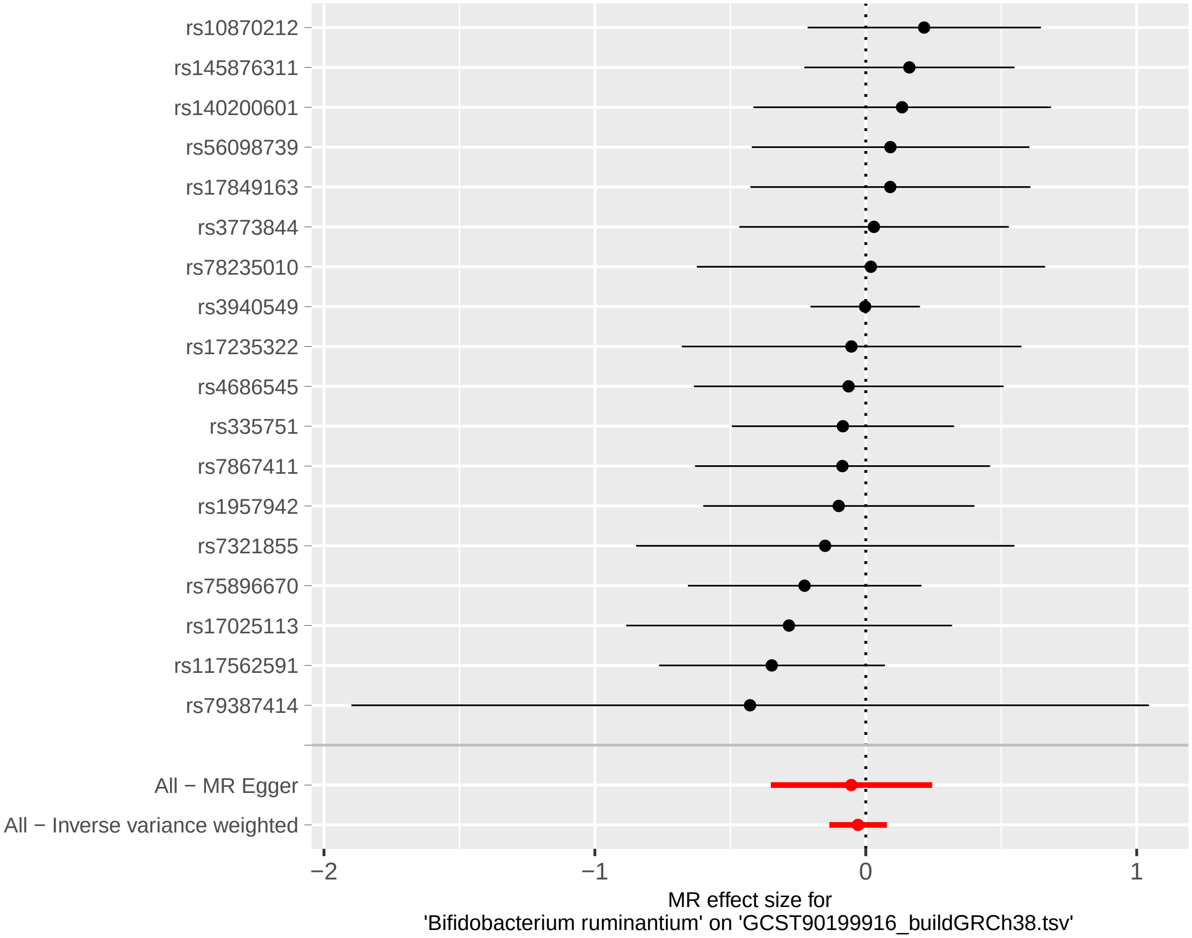
rs10870212
rs145876311
rs140200601
rs56098739
rs17849163
rs3773844
rs78235010
rs3940549
rs17235322
rs4686545
rs335751
rs7867411
rs1957942
rs7321855
rs75896670
rs17025113
rs117562591
rs79387414
All − MR Egger
All − Inverse variance weighted
−2
−1
0
1
MR effect size for
'Bifidobacterium ruminantium' on 'GCST90199916_buildGRCh38.tsv'

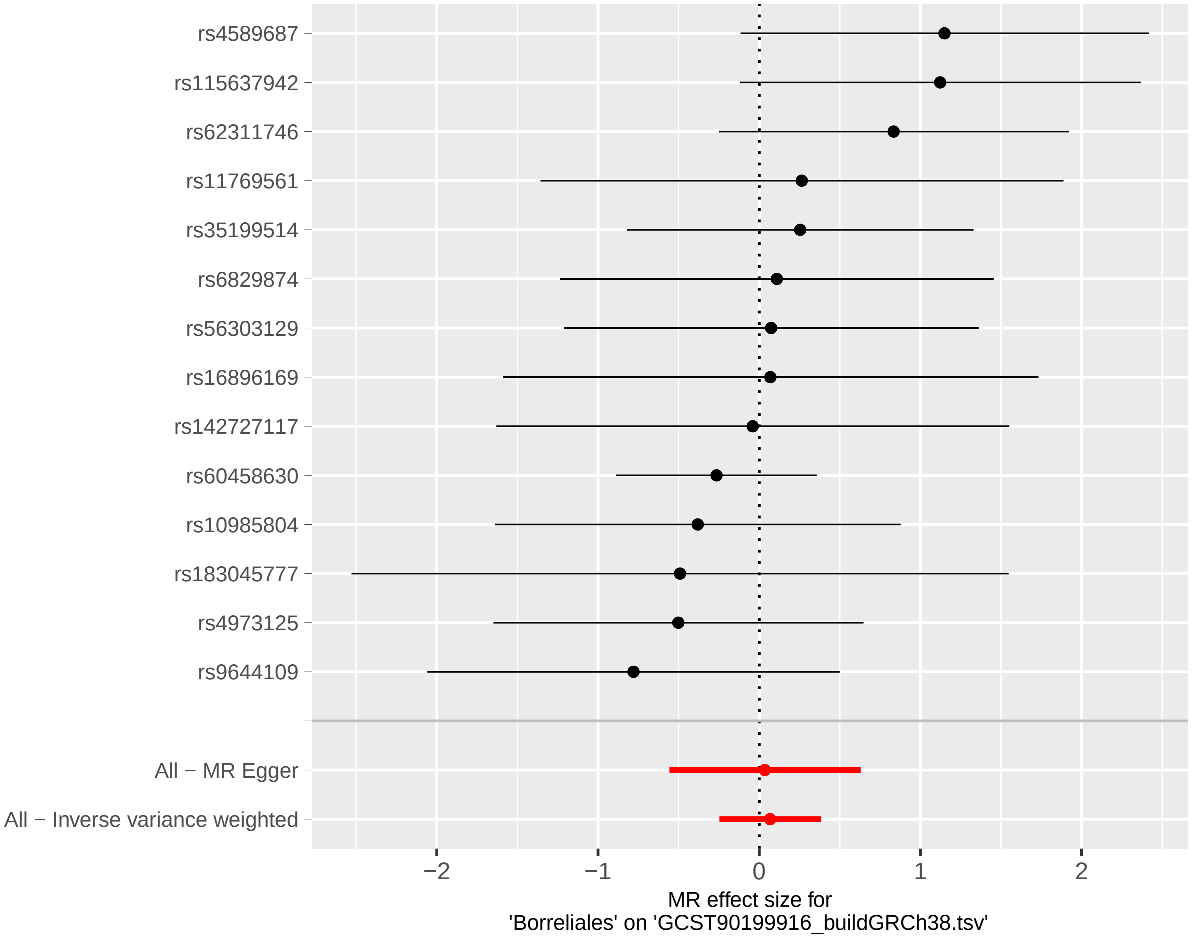

rs4589687
rs115637942
rs62311746
rs11769561
rs35199514
rs6829874
rs56303129
rs16896169
rs142727117
rs60458630
rs10985804
rs183045777
rs4973125
rs9644109
All − MR Egger
All − Inverse variance weighted
−2
−1
0
1
2
MR effect size for
'Borreliales' on 'GCST90199916_buildGRCh38.tsv'

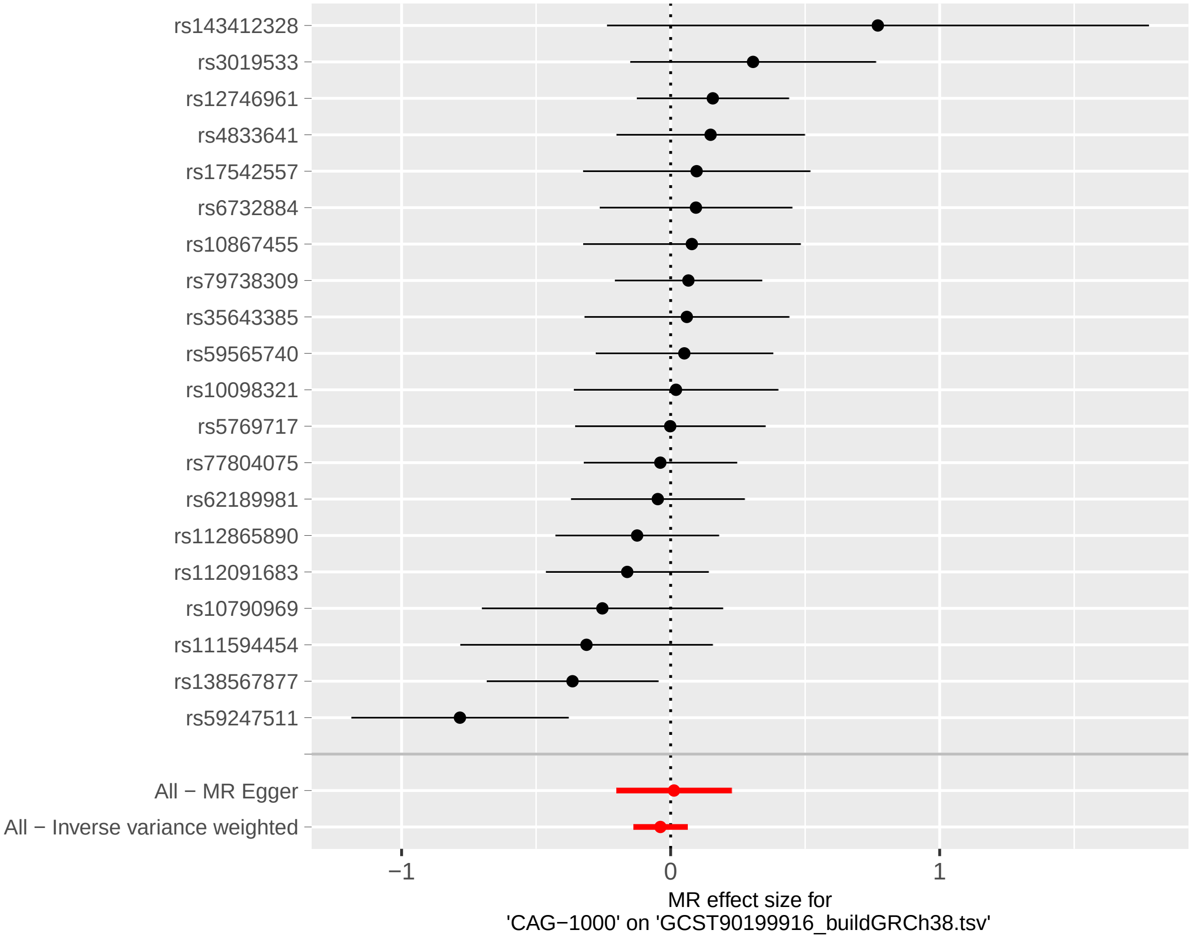
rs143412328
rs3019533
rs12746961
rs4833641
rs17542557
rs6732884
rs10867455
rs79738309
rs35643385
rs59565740
rs10098321
rs5769717
rs77804075
rs62189981
rs112865890
rs112091683
rs10790969
rs111594454
rs138567877
rs59247511
All − MR Egger
All − Inverse variance weighted
−1
0
1
MR effect size for
'CAG−1000' on 'GCST90199916_buildGRCh38.tsv'

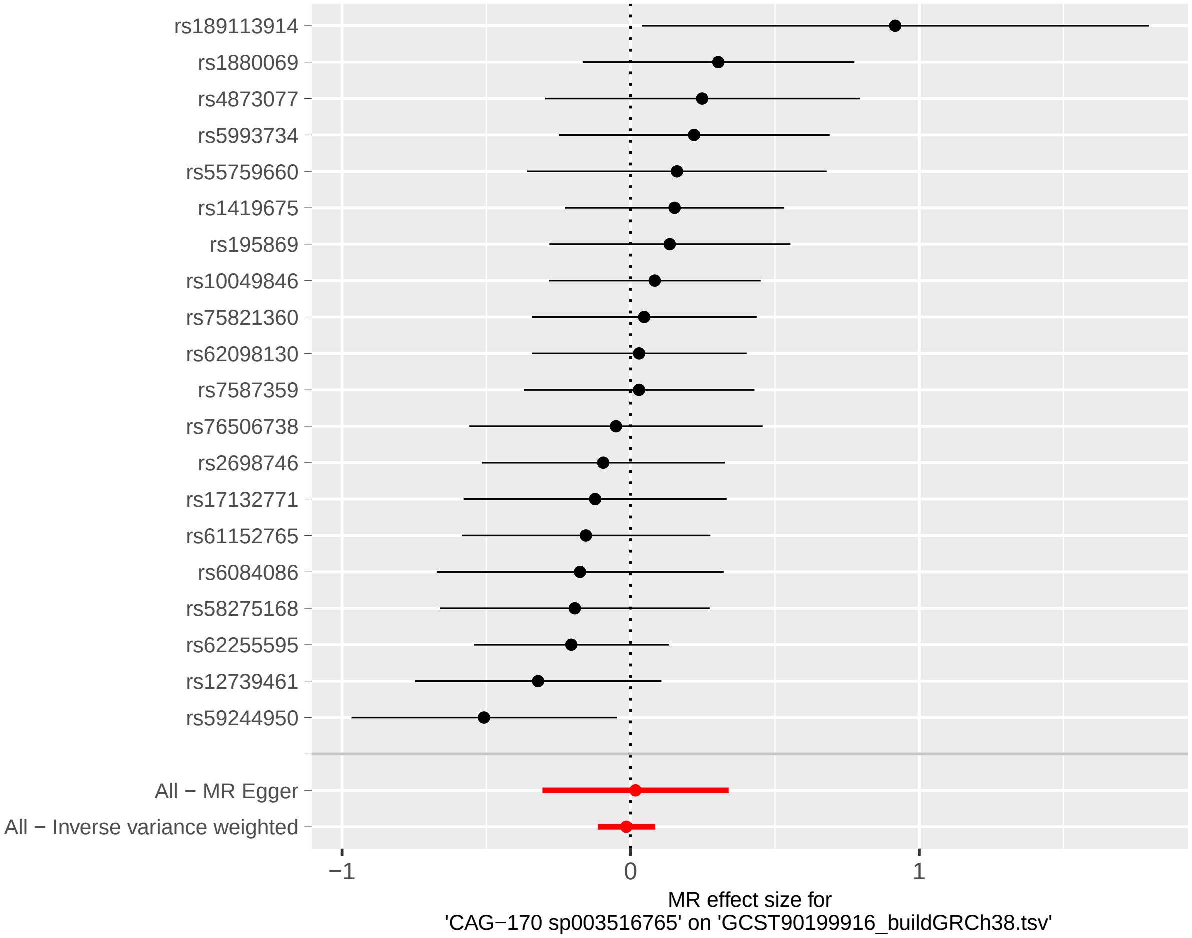
rs189113914
rs1880069
rs4873077
rs5993734
rs55759660
rs1419675
rs195869
rs10049846
rs75821360
rs62098130
rs7587359
rs76506738
rs2698746
rs17132771
rs61152765
rs6084086
rs58275168
rs62255595
rs12739461
rs59244950
All − MR Egger
All − Inverse variance weighted
−1
0
1
MR effect size for
'CAG−170 sp003516765' on 'GCST90199916_buildGRCh38.tsv'

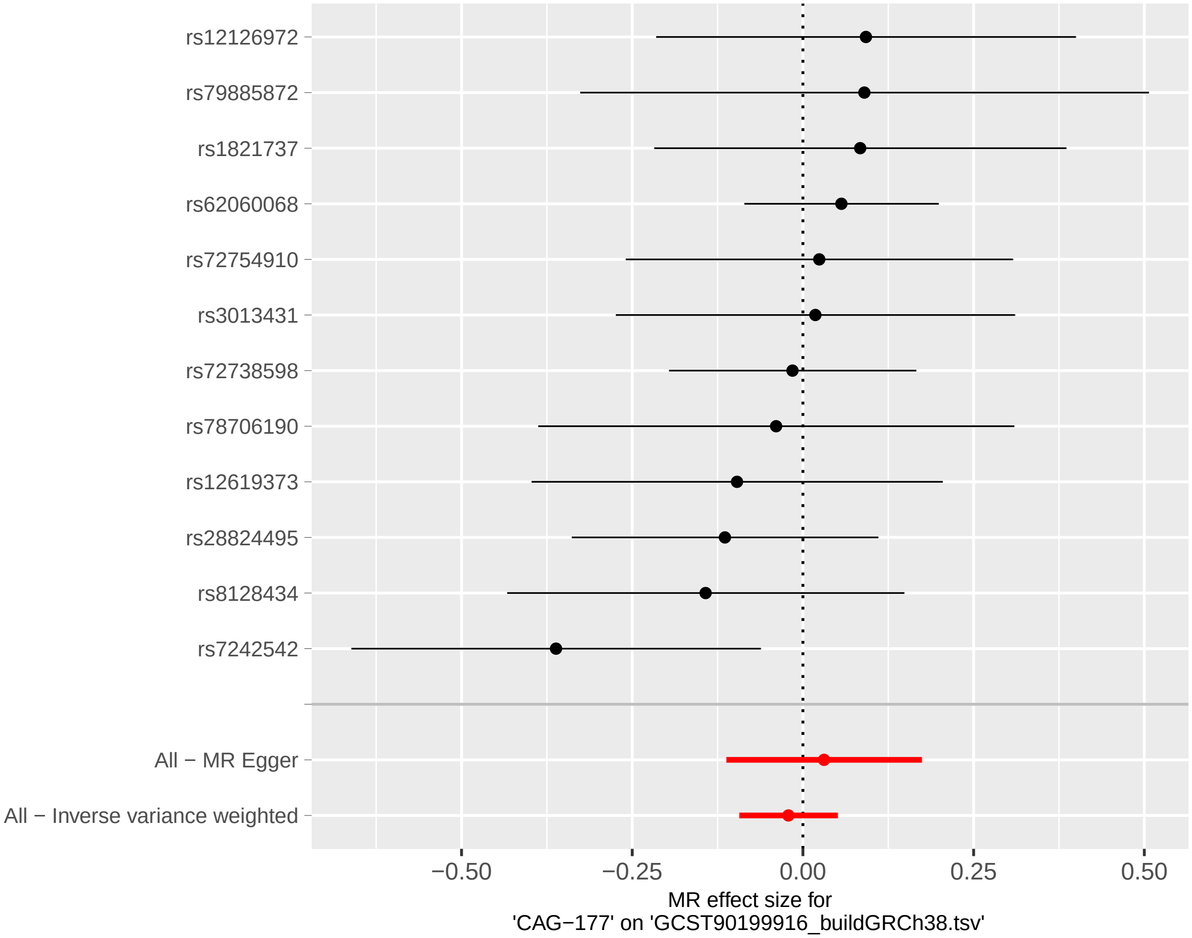

rs12126972
rs79885872
rs1821737
rs62060068
rs72754910
rs3013431
rs72738598
rs78706190
rs12619373
rs28824495
rs8128434
rs7242542
All − MR Egger
All − Inverse variance weighted
−0.50
−0.25
0.00
0.25
0.50
MR effect size for
'CAG−177' on 'GCST90199916_buildGRCh38.tsv'

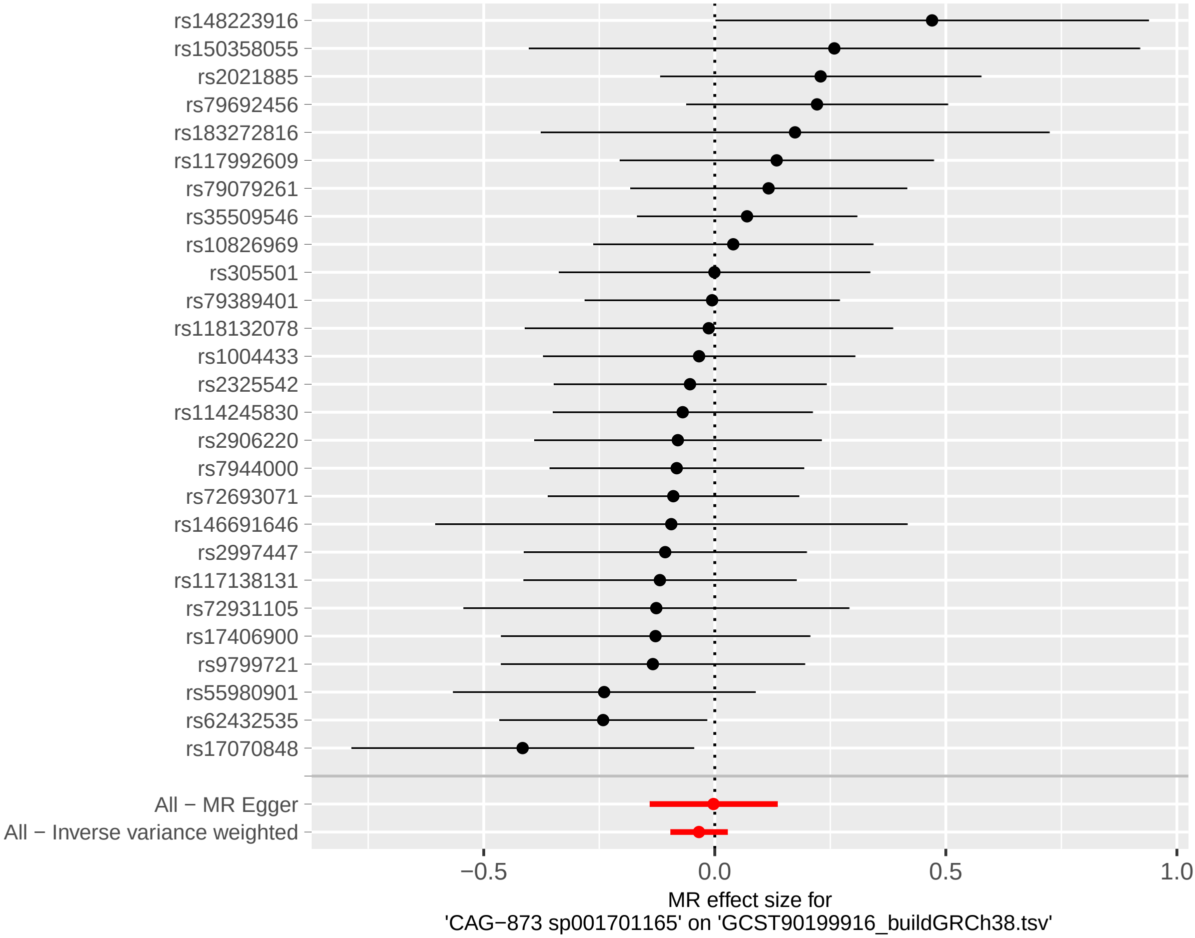
rs148223916
rs150358055
rs2021885
rs79692456
rs183272816
rs117992609
rs79079261
rs35509546
rs10826969
rs305501
rs79389401
rs118132078
rs1004433
rs2325542
rs114245830
rs2906220
rs7944000
rs72693071
rs146691646
rs2997447
rs117138131
rs72931105
rs17406900
rs9799721
rs55980901
rs62432535
rs17070848
All − MR Egger
All − Inverse variance weighted
−0.5
0.0
0.5
1.0
MR effect size for
'CAG−873 sp001701165' on 'GCST90199916_buildGRCh38.tsv'

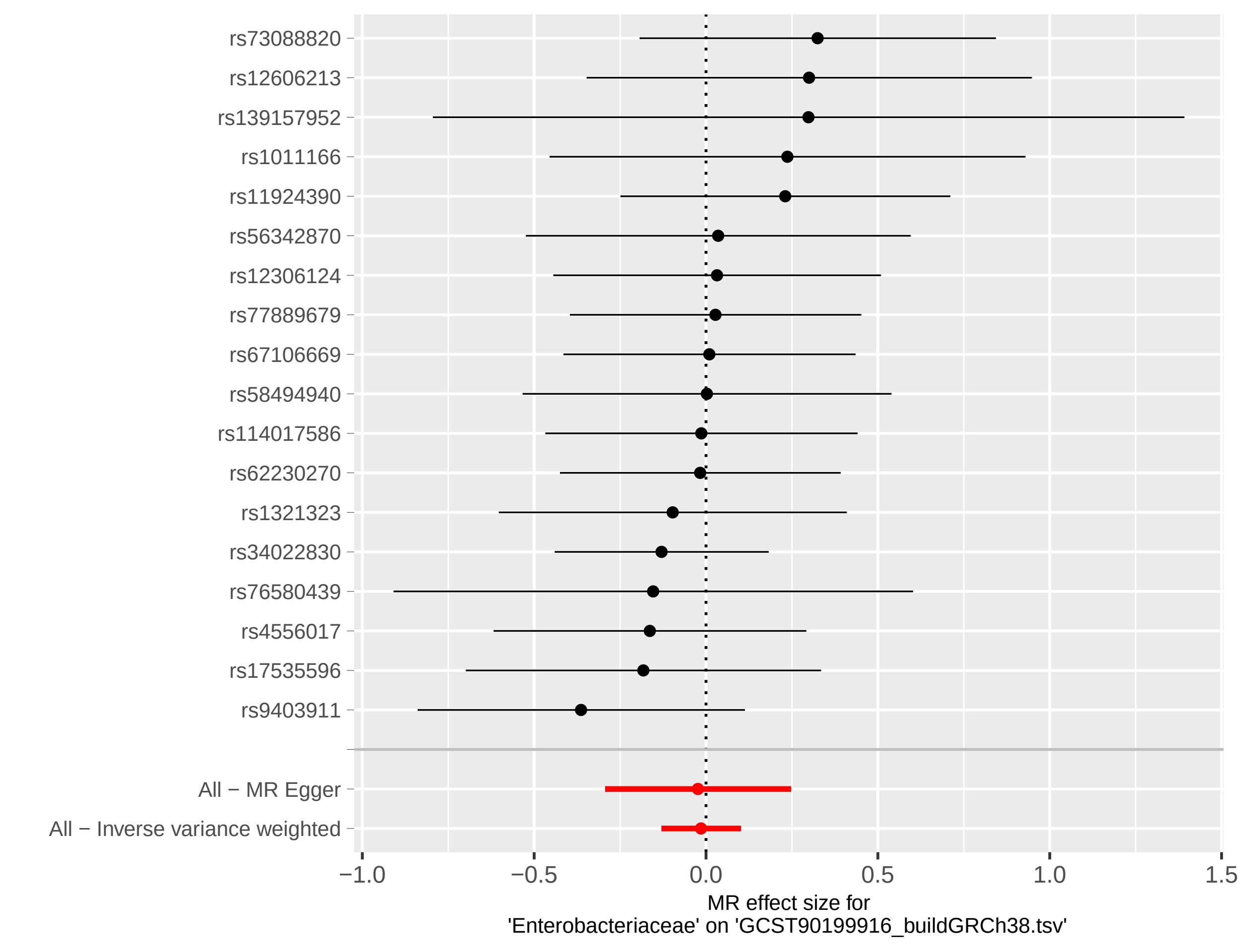

rs73088820
rs12606213
rs139157952
rs1011166
rs11924390
rs56342870
rs12306124
rs77889679
rs67106669
rs58494940
rs114017586
rs62230270
rs1321323
rs34022830
rs76580439
rs4556017
rs17535596
rs9403911
All − MR Egger
All − Inverse variance weighted
−1.0
−0.5
0.0
0.5
1.0
1.5
MR effect size for
'Enterobacteriaceae' on 'GCST90199916_buildGRCh38.tsv'

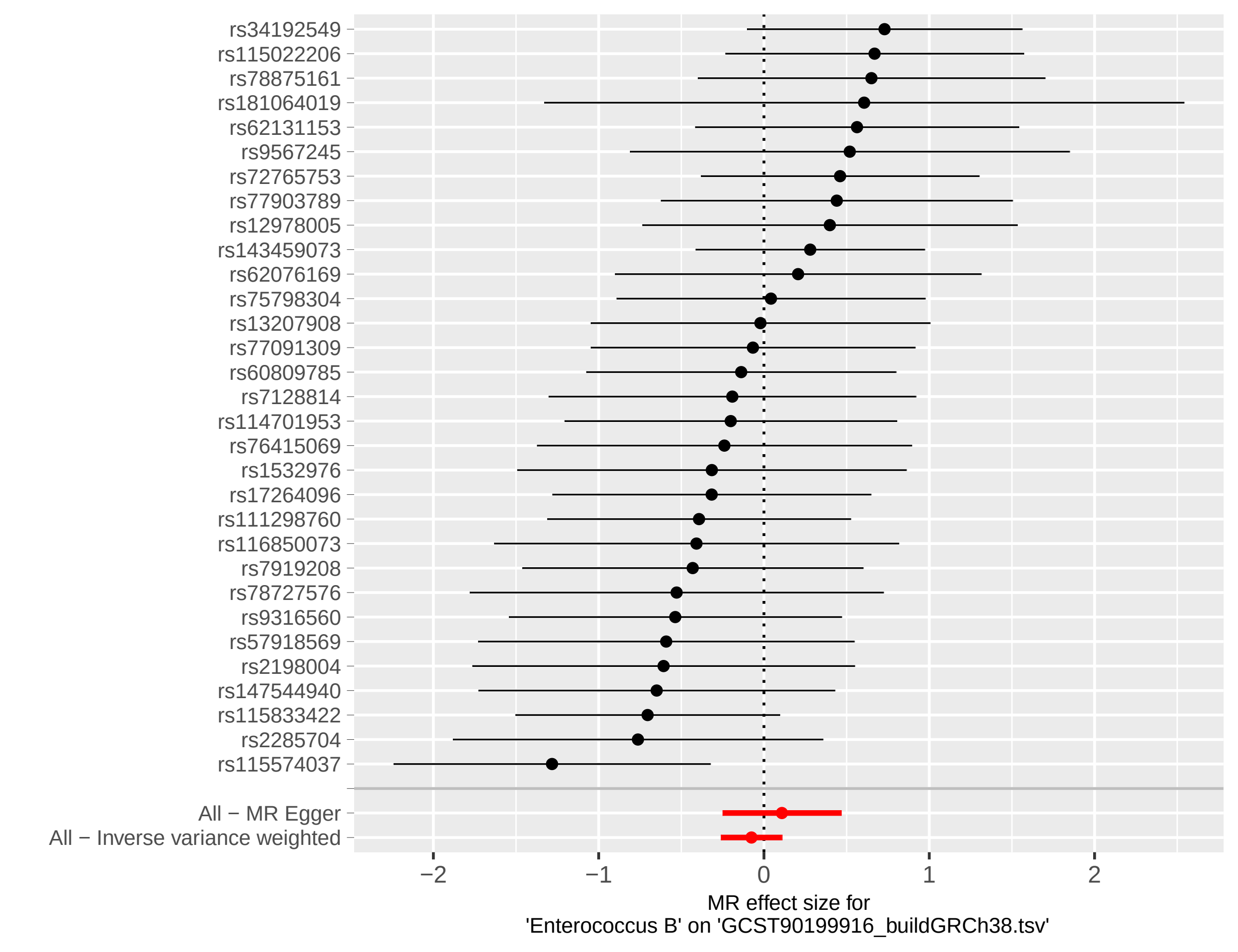

rs34192549
rs115022206
rs78875161
rs181064019
rs62131153
rs9567245
rs72765753
rs77903789
rs12978005
rs143459073
rs62076169
rs75798304
rs13207908
rs77091309
rs60809785
rs7128814
rs114701953
rs76415069
rs1532976
rs17264096
rs111298760
rs116850073
rs7919208
rs78727576
rs9316560
rs57918569
rs2198004
rs147544940
rs115833422
rs2285704
rs115574037
All − MR Egger
All − Inverse variance weighted
−2
−1
0
1
2
MR effect size for
'Enterococcus B' on 'GCST90199916_buildGRCh38.tsv'

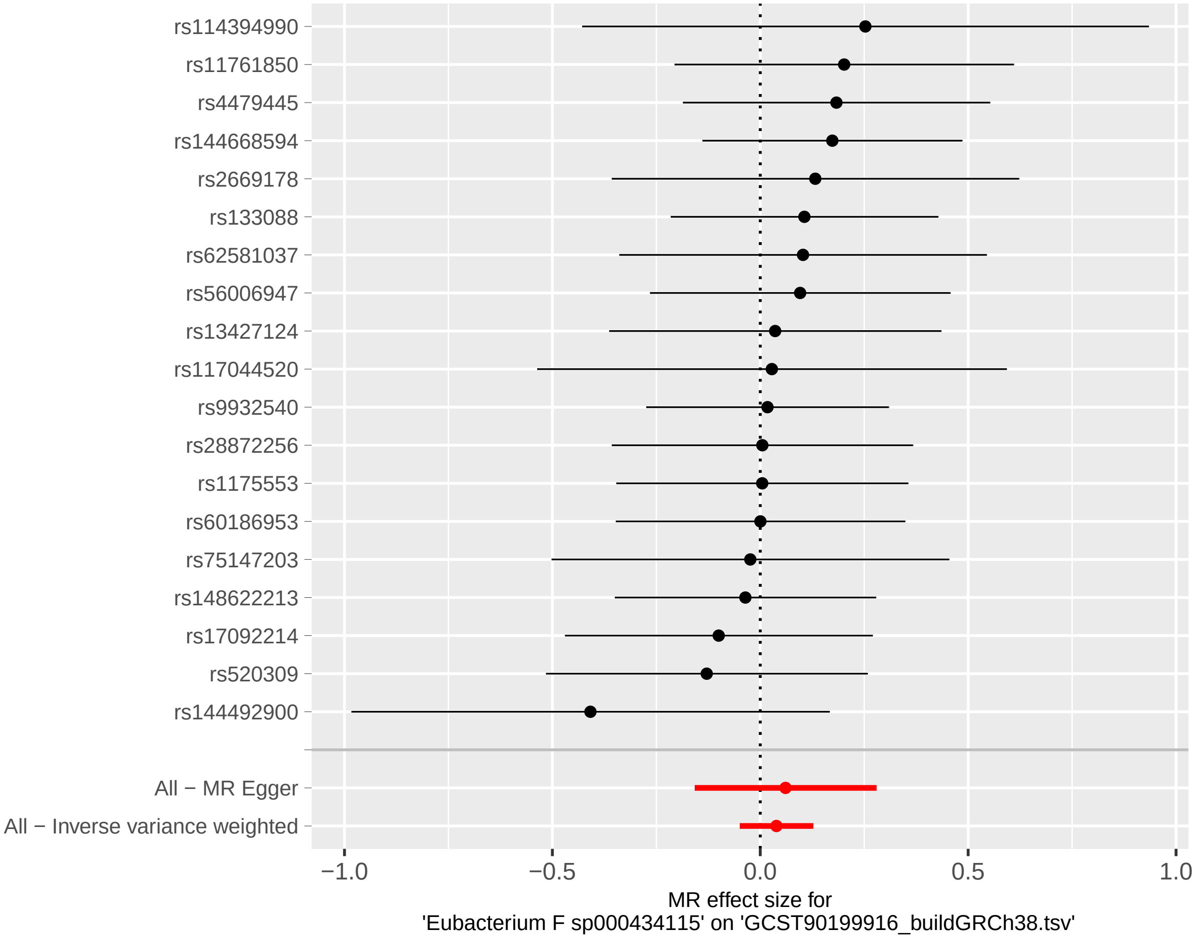
rs114394990
rs11761850
rs4479445
rs144668594
rs2669178
rs133088
rs62581037
rs56006947
rs13427124
rs117044520
rs9932540
rs28872256
rs1175553
rs60186953
rs75147203
rs148622213
rs17092214
rs520309
rs144492900
All − MR Egger
All − Inverse variance weighted
−1.0
−0.5
0.0
0.5
1.0
MR effect size for
'Eubacterium F sp000434115' on 'GCST90199916_buildGRCh38.tsv'

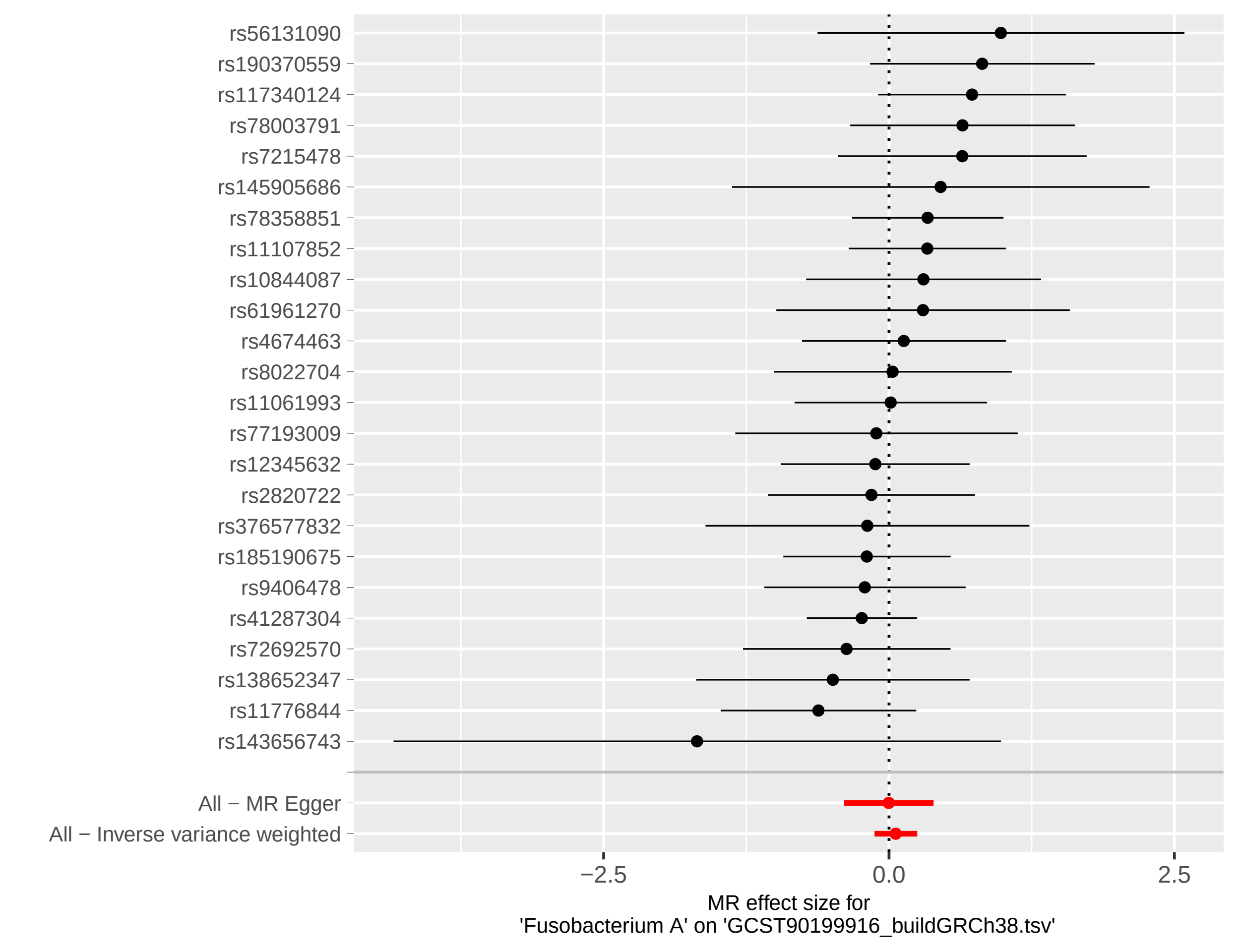
rs56131090
rs190370559
rs117340124
rs78003791
rs7215478
rs145905686
rs78358851
rs11107852
rs10844087
rs61961270
rs4674463
rs8022704
rs11061993
rs77193009
rs12345632
rs2820722
rs376577832
rs185190675
rs9406478
rs41287304
rs72692570
rs138652347
rs11776844
rs143656743
All − MR Egger
All − Inverse variance weighted
−2.5
0.0
2.5
MR effect size for
'Fusobacterium A' on 'GCST90199916_buildGRCh38.tsv'

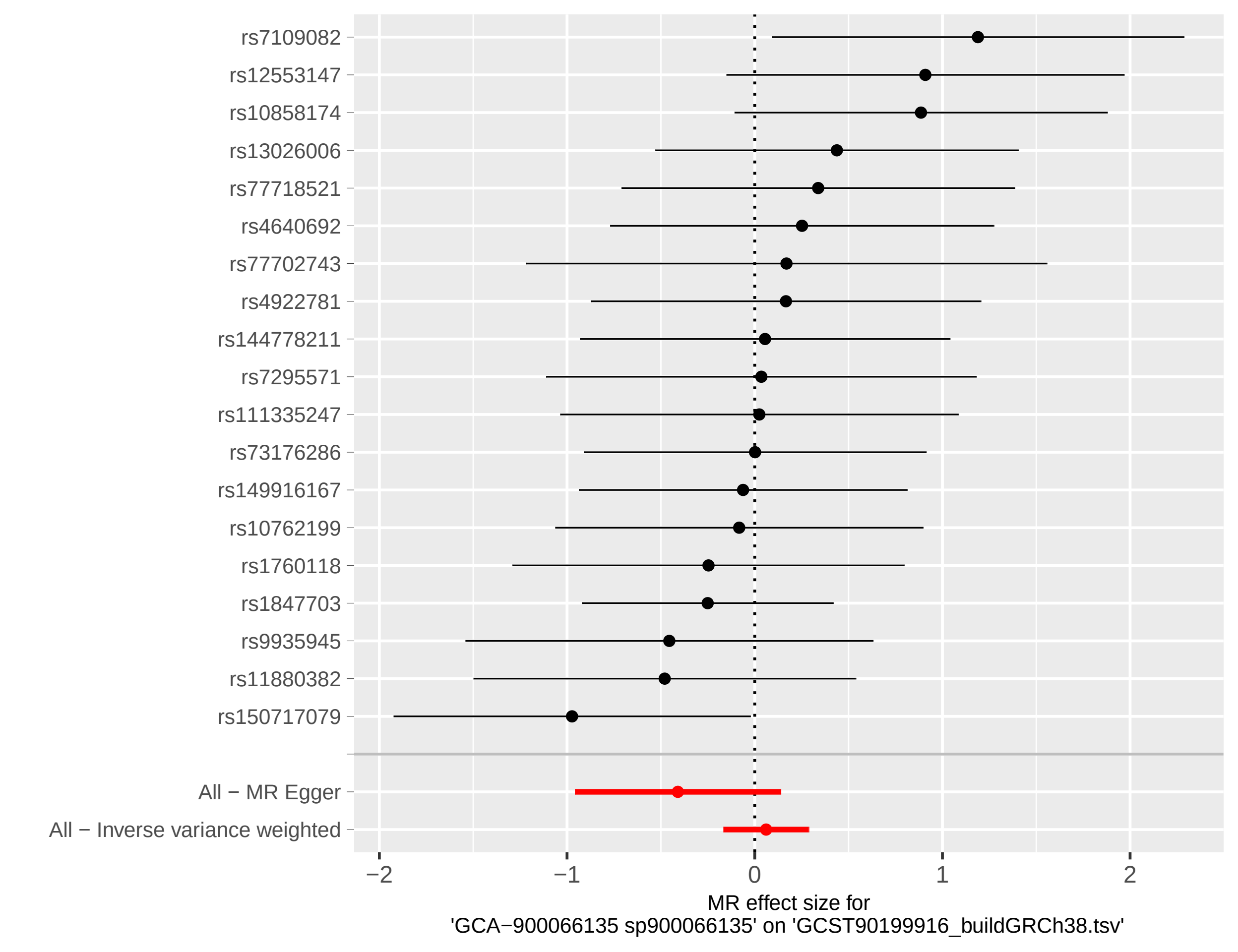

rs7109082
rs12553147
rs10858174
rs13026006
rs77718521
rs4640692
rs77702743
rs4922781
rs144778211
rs7295571
rs111335247
rs73176286
rs149916167
rs10762199
rs1760118
rs1847703
rs9935945
rs11880382
rs150717079
All − MR Egger
All − Inverse variance weighted
−2
−1
0
1
2
MR effect size for
'GCA−900066135 sp900066135' on 'GCST90199916_buildGRCh38.tsv'

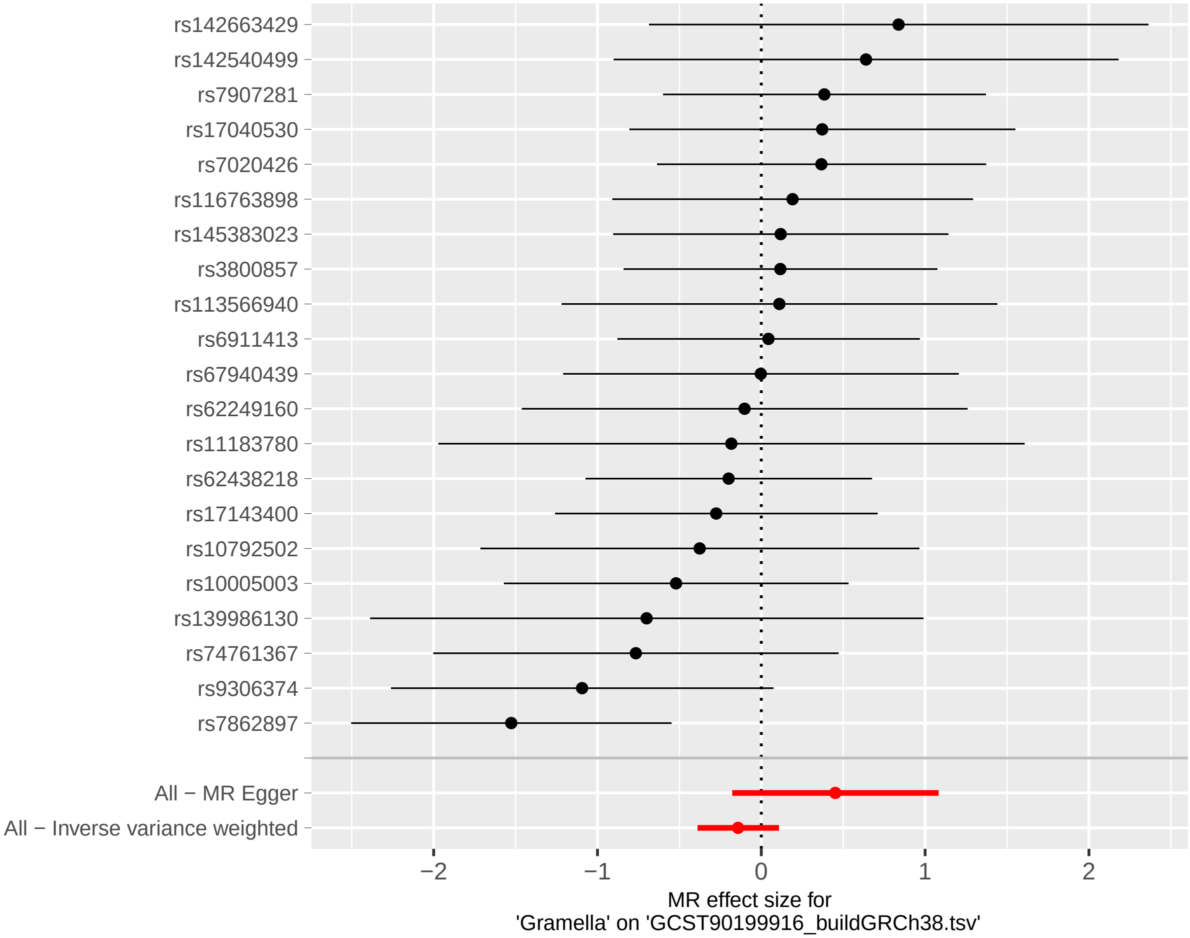

rs142663429
rs142540499
rs7907281
rs17040530
rs7020426
rs116763898
rs145383023
rs3800857
rs113566940
rs6911413
rs67940439
rs62249160
rs11183780
rs62438218
rs17143400
rs10792502
rs10005003
rs139986130
rs74761367
rs9306374
rs7862897
All − MR Egger
All − Inverse variance weighted
−2
−1
0
1
2
MR effect size for
'Gramella' on 'GCST90199916_buildGRCh38.tsv'

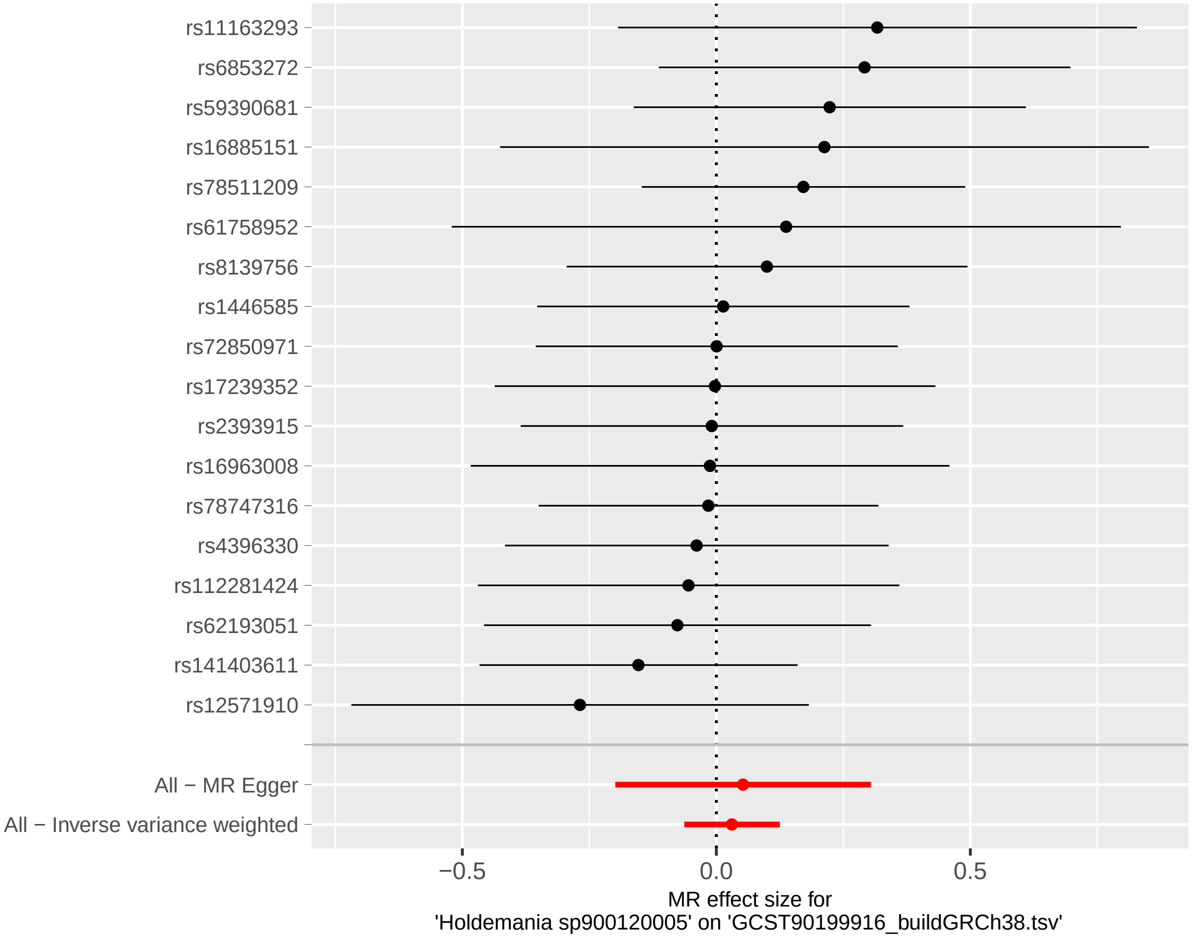
rs11163293
rs6853272
rs59390681
rs16885151
rs78511209
rs61758952
rs8139756
rs1446585
rs72850971
rs17239352
rs2393915
rs16963008
rs78747316
rs4396330
rs112281424
rs62193051
rs141403611
rs12571910
All − MR Egger
All − Inverse variance weighted
−0.5
0.0
0.5
MR effect size for
'Holdemania sp900120005' on 'GCST90199916_buildGRCh38.tsv'

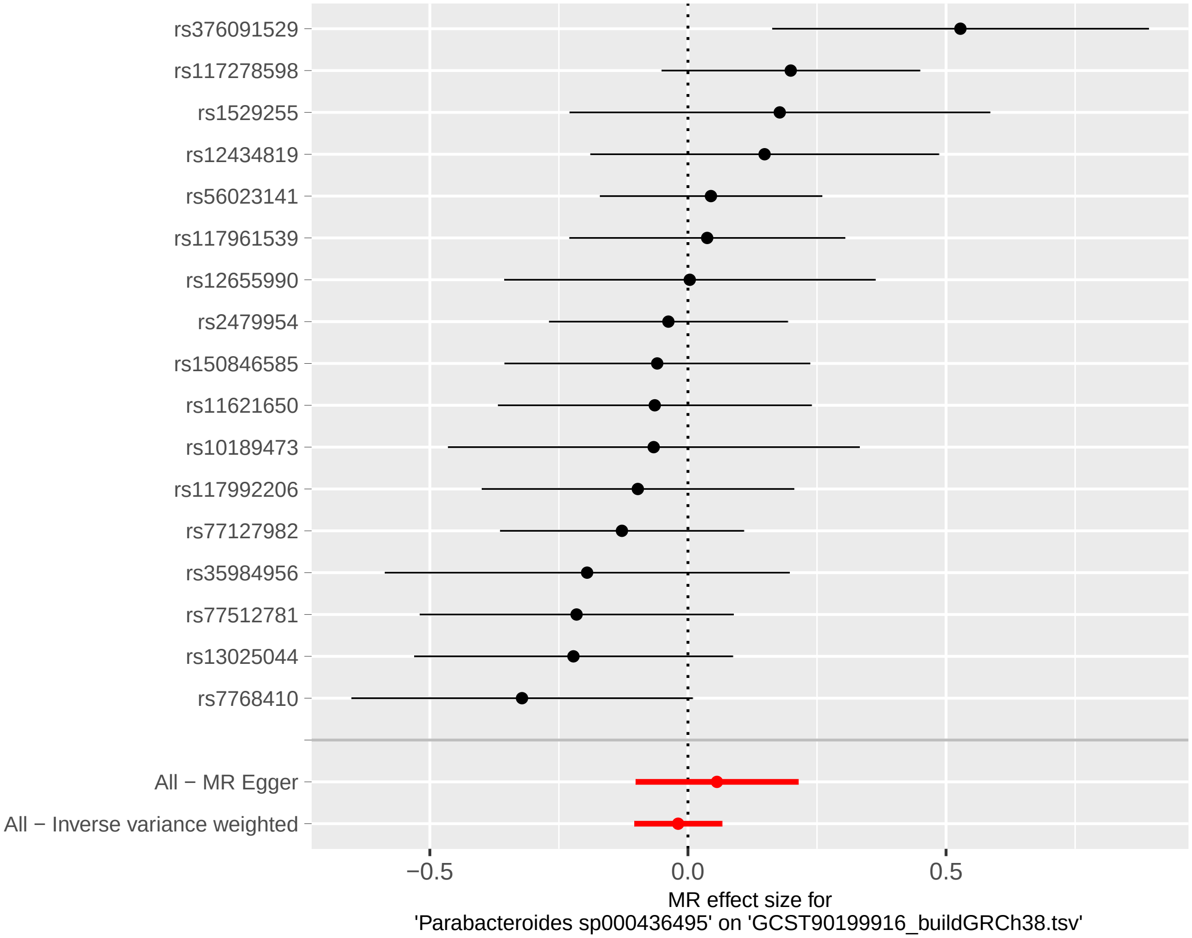

rs376091529
rs117278598
rs1529255
rs12434819
rs56023141
rs117961539
rs12655990
rs2479954
rs150846585
rs11621650
rs10189473
rs117992206
rs77127982
rs35984956
rs77512781
rs13025044
rs7768410
All − MR Egger
All − Inverse variance weighted
−0.5
0.0
0.5
MR effect size for
'Parabacteroides sp000436495' on 'GCST90199916_buildGRCh38.tsv'

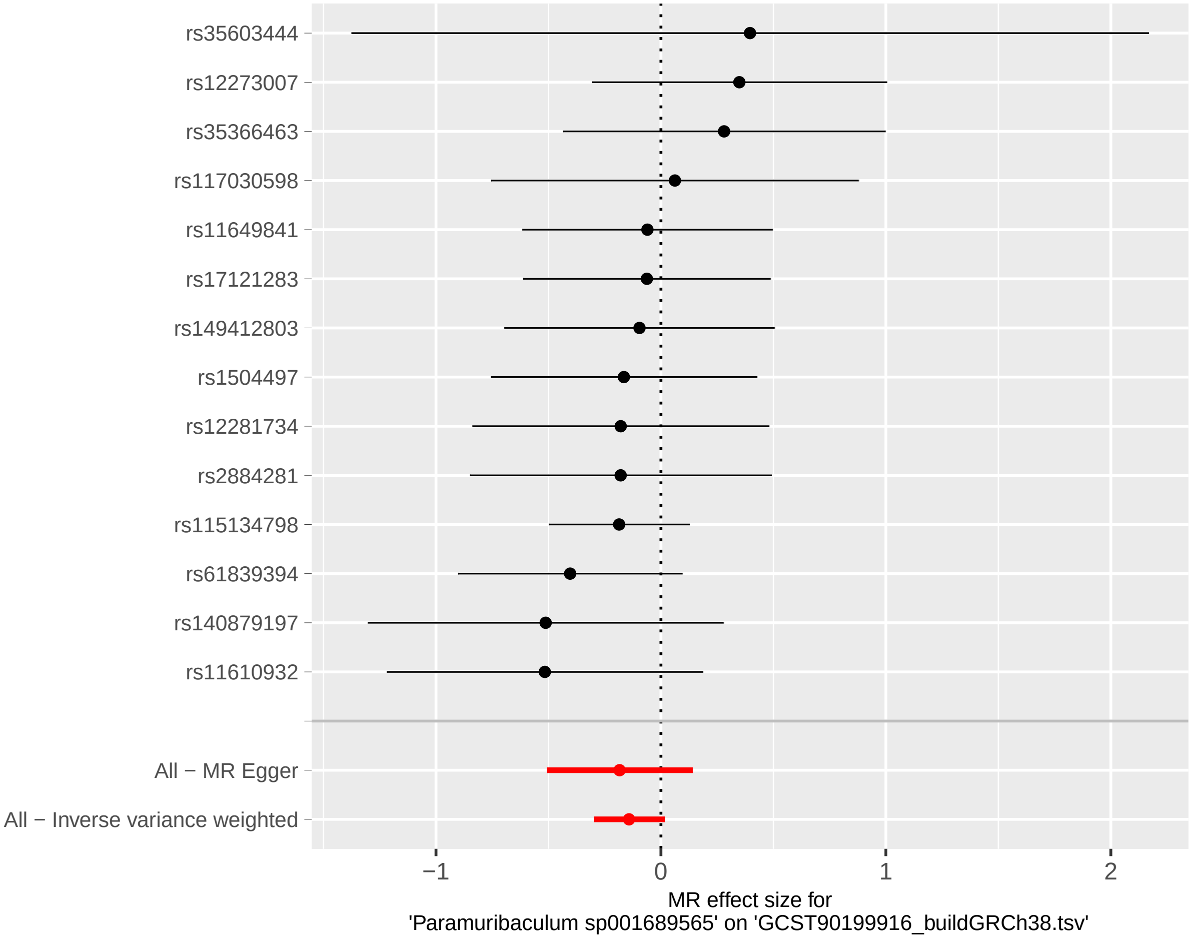
rs35603444
rs12273007
rs35366463
rs117030598
rs11649841
rs17121283
rs149412803
rs1504497
rs12281734
rs2884281
rs115134798
rs61839394
rs140879197
rs11610932
All − MR Egger
All − Inverse variance weighted
−1
0
1
2
MR effect size for
'Paramuribaculum sp001689565' on 'GCST90199916_buildGRCh38.tsv'

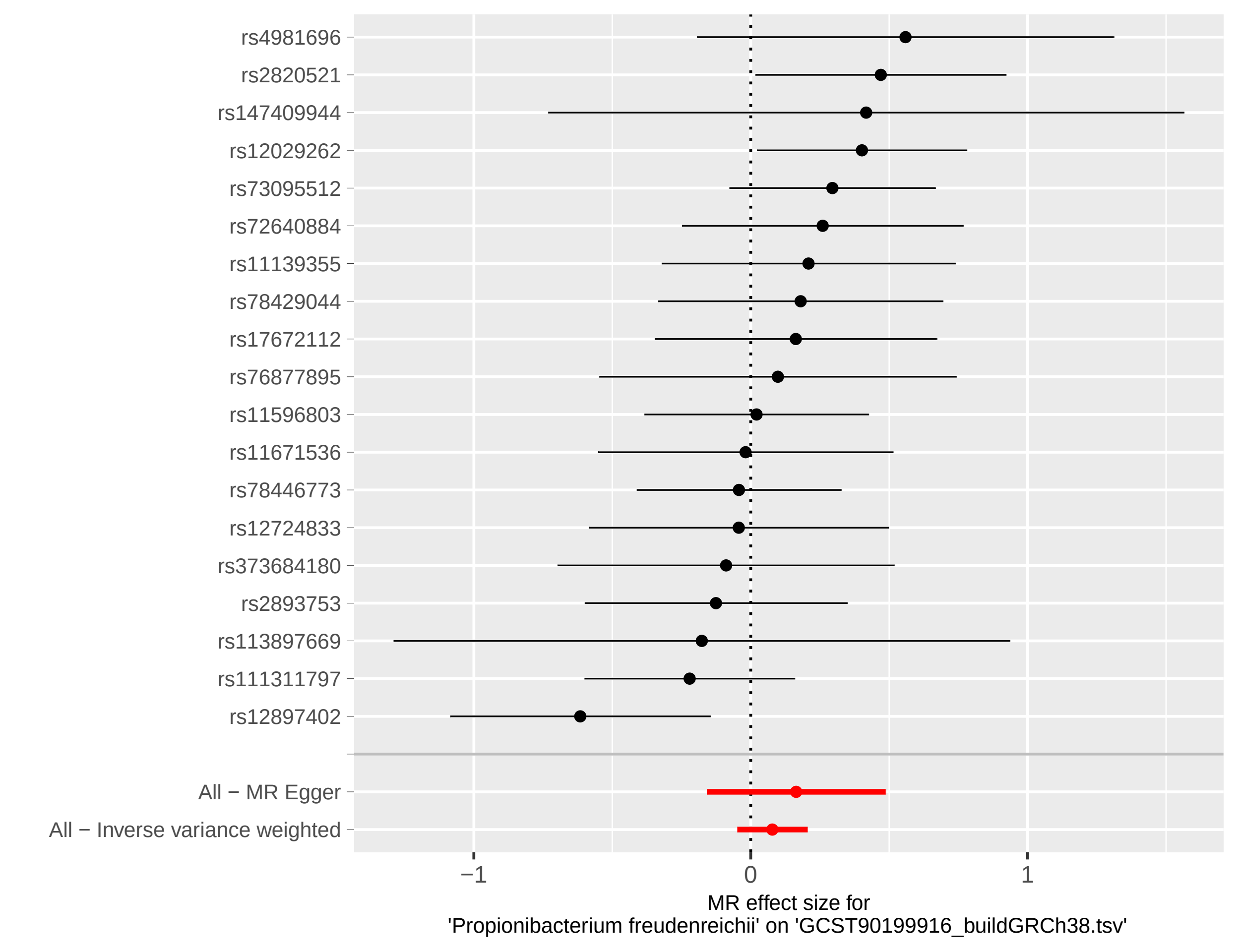
rs4981696
rs2820521
rs147409944
rs12029262
rs73095512
rs72640884
rs11139355
rs78429044
rs17672112
rs76877895
rs11596803
rs11671536
rs78446773
rs12724833
rs373684180
rs2893753
rs113897669
rs111311797
rs12897402
All − MR Egger
All − Inverse variance weighted
−1
0
1
MR effect size for
'Propionibacterium freudenreichii' on 'GCST90199916_buildGRCh38.tsv'

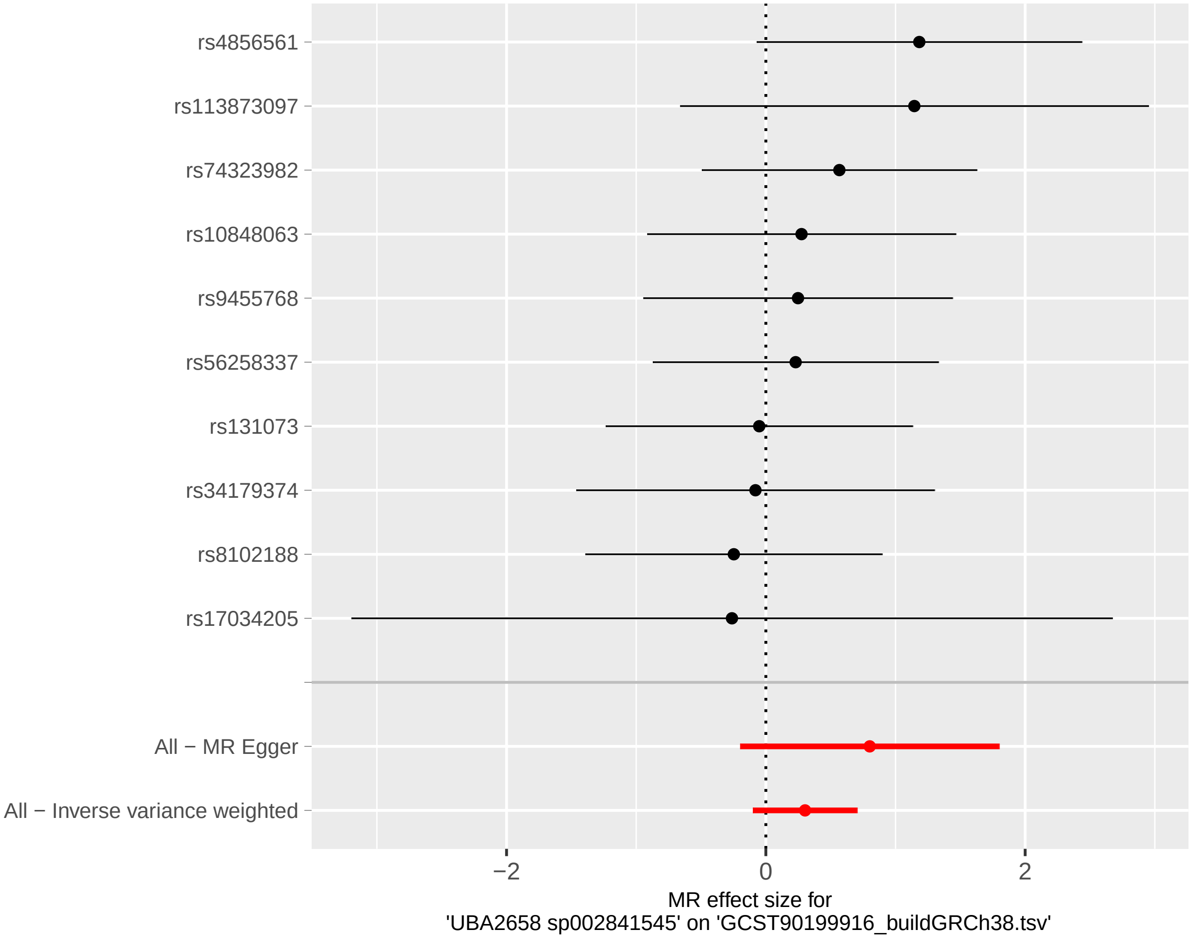

rs4856561
rs113873097
rs74323982
rs10848063
rs9455768
rs56258337
rs131073
rs34179374
rs8102188
rs17034205
All − MR Egger
All − Inverse variance weighted
−2
0
2
MR effect size for
'UBA2658 sp002841545' on 'GCST90199916_buildGRCh38.tsv'

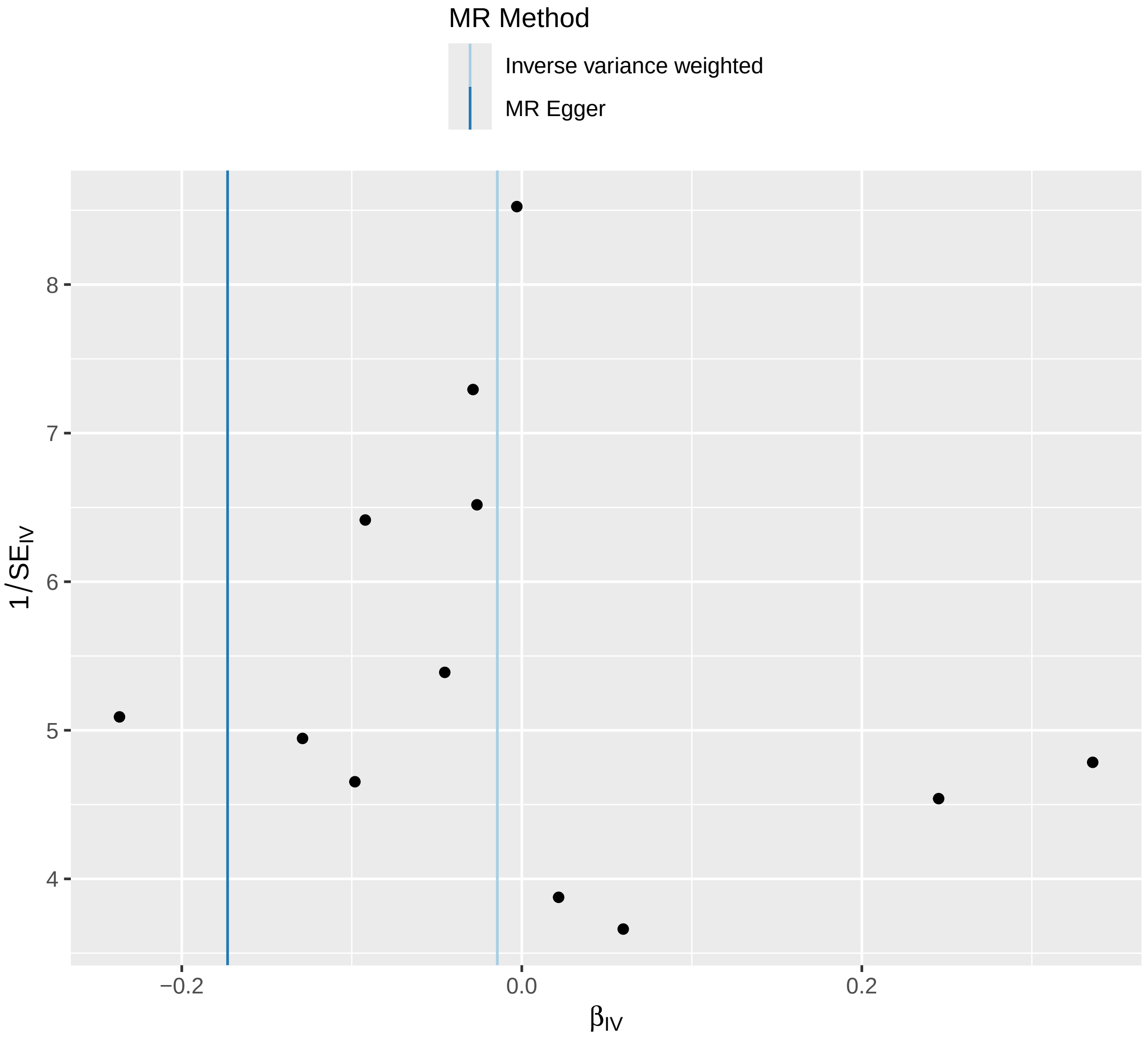
MR Method
Inverse variance weighted
MR Egger
8
7
6
5
4
1/SE$_{IV}$
−0.2
0.0
0.2
β$_{IV}$

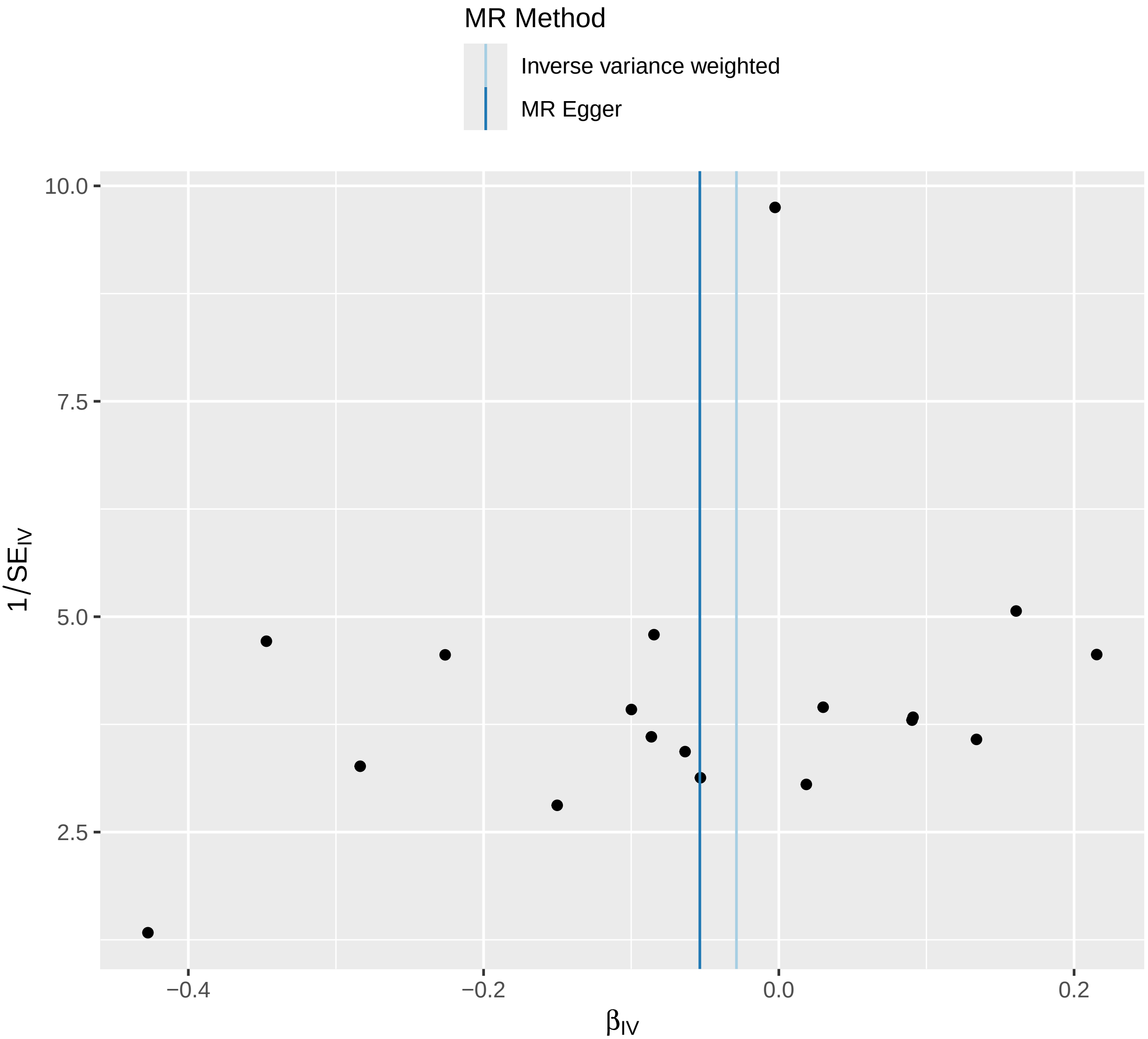

MR Method
Inverse variance weighted
MR Egger
10.0
7.5
5.0
2.5
1/SE_IV
−0.4
−0.2
0.0
0.2
β_IV

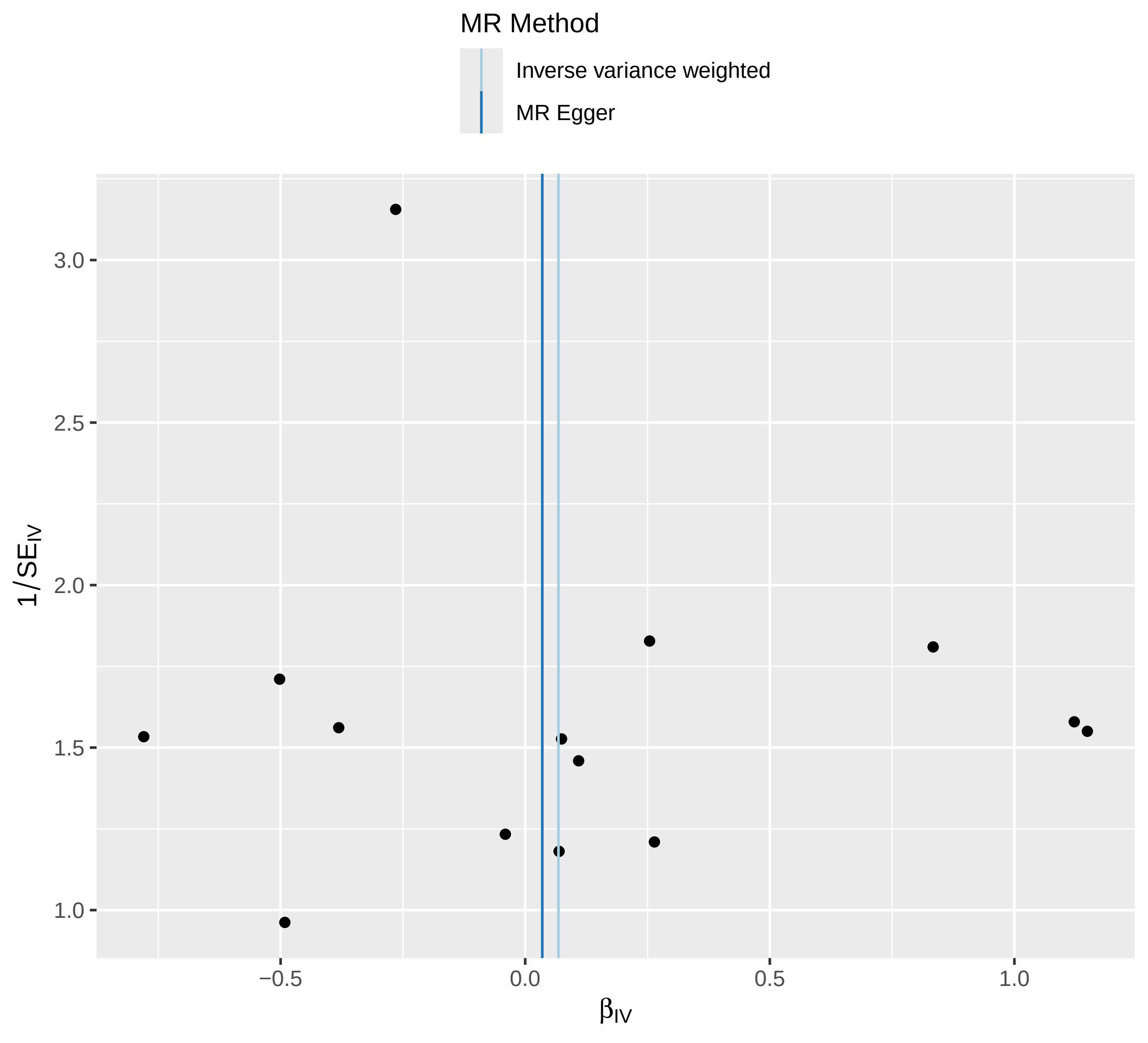
MR Method
Inverse variance weighted
MR Egger
3.0
2.5
2.0
1.5
1.0
−0.5
0.0
0.5
1.0
$1/SE_{IV}$
β_{IV}

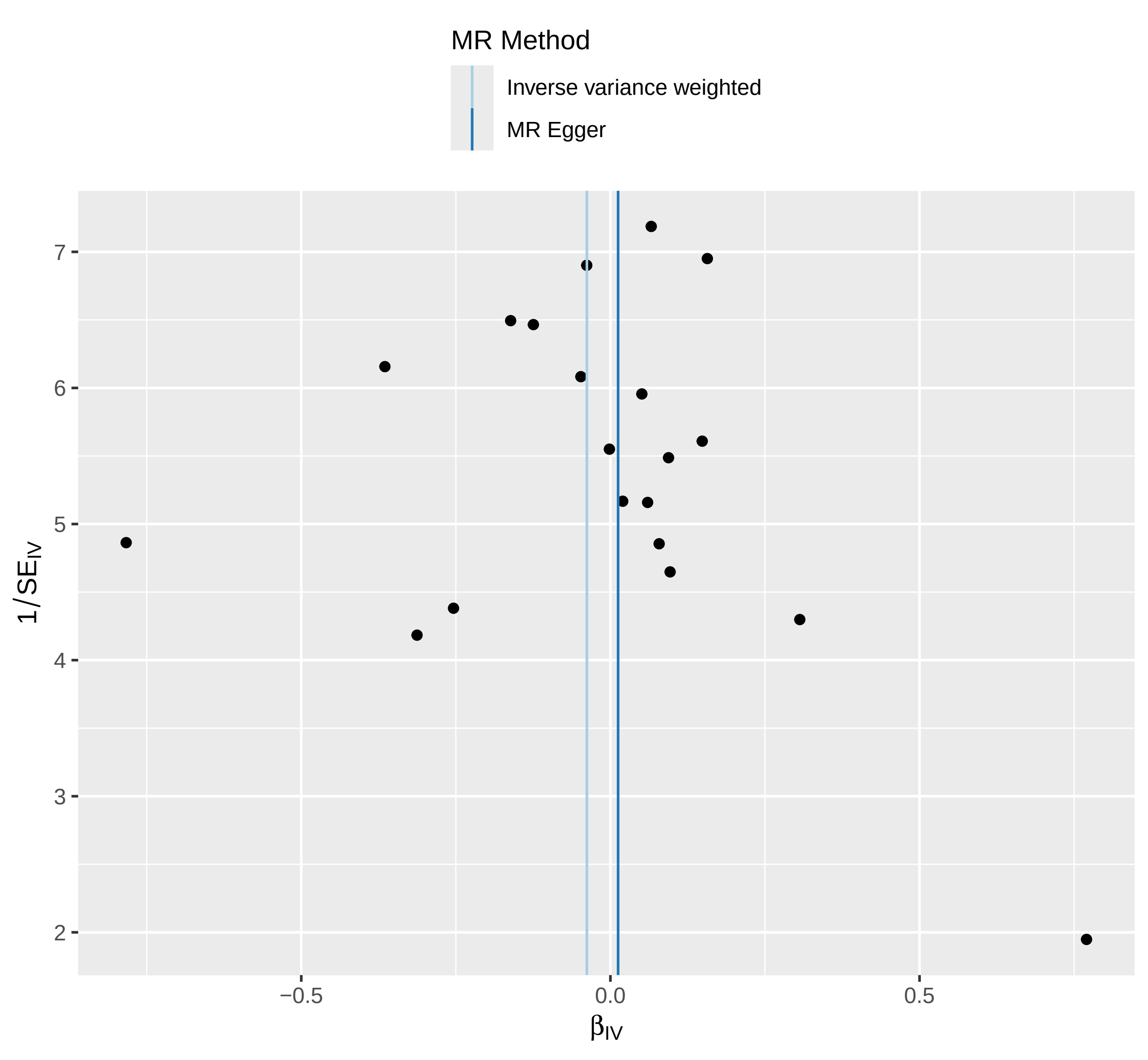
MR Method
Inverse variance weighted
MR Egger
1/SE_IV
7
6
5
4
3
2
−0.5
0.0
0.5
β_IV

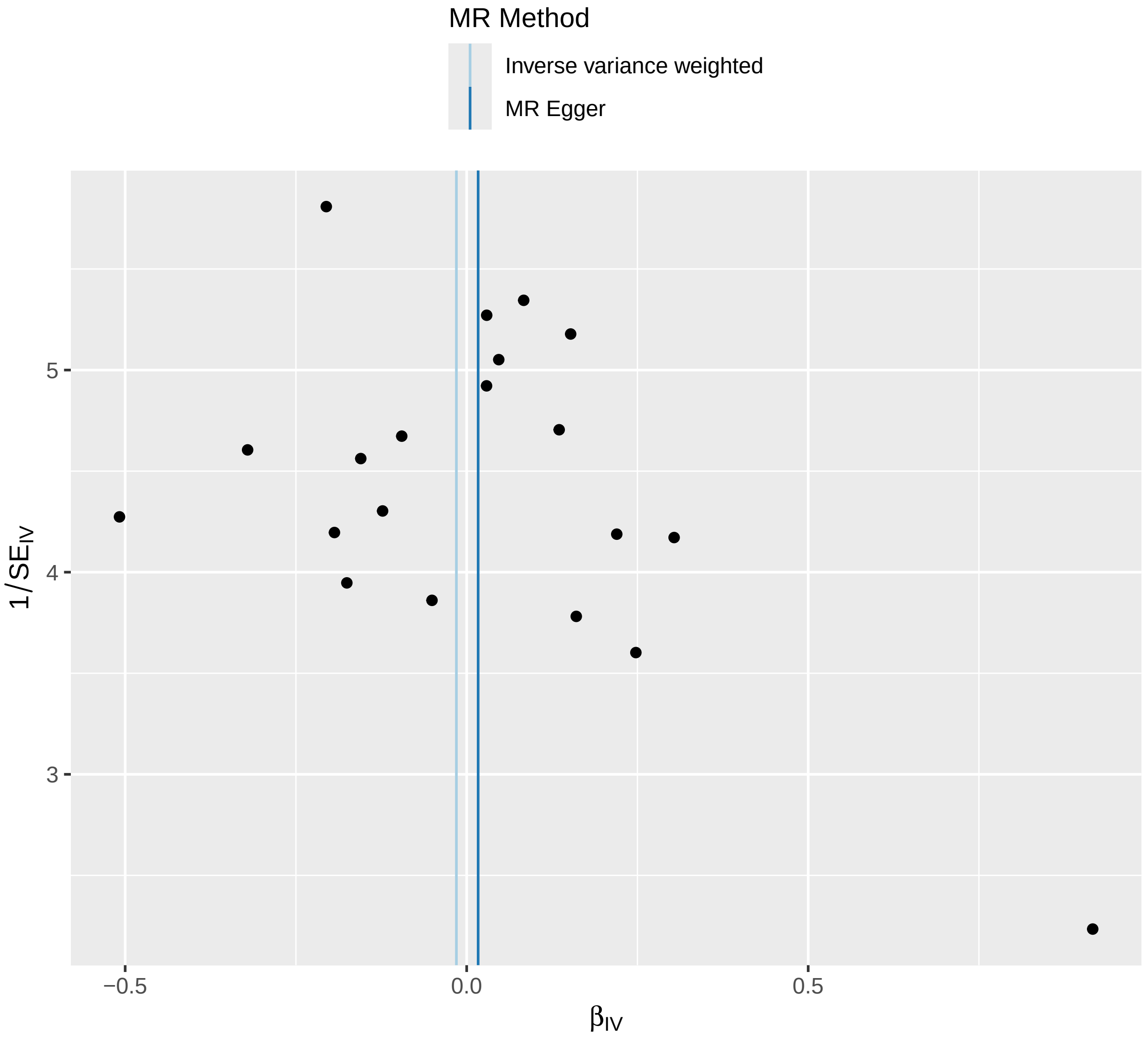
MR Method
Inverse variance weighted
MR Egger
1/SE$_{IV}$
5
4
3
−0.5
0.0
0.5
β$_{IV}$

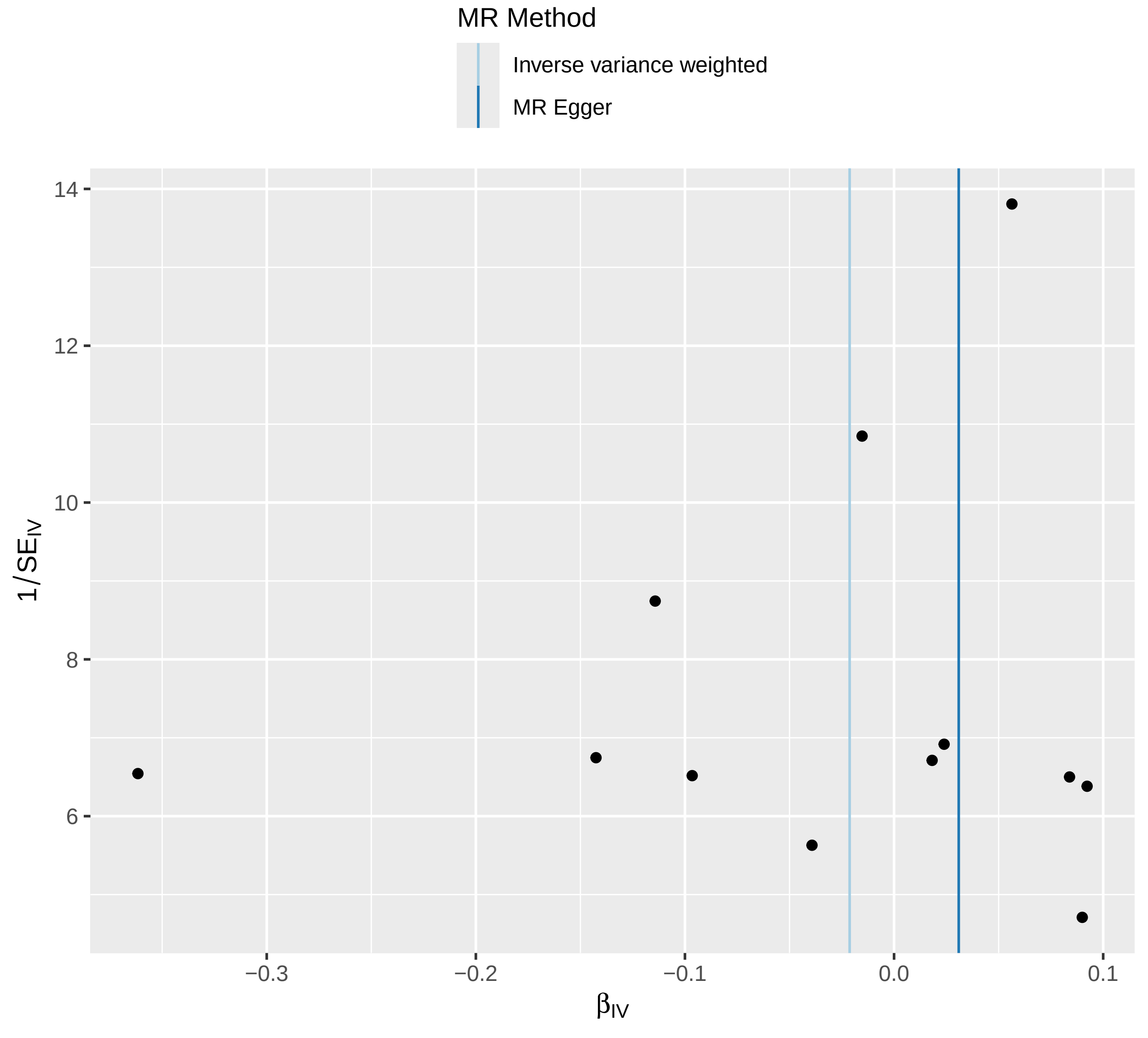
MR Method
Inverse variance weighted
MR Egger
14
12
10
8
6
1/SE_IV
−0.3
−0.2
−0.1
0.0
0.1
β_IV

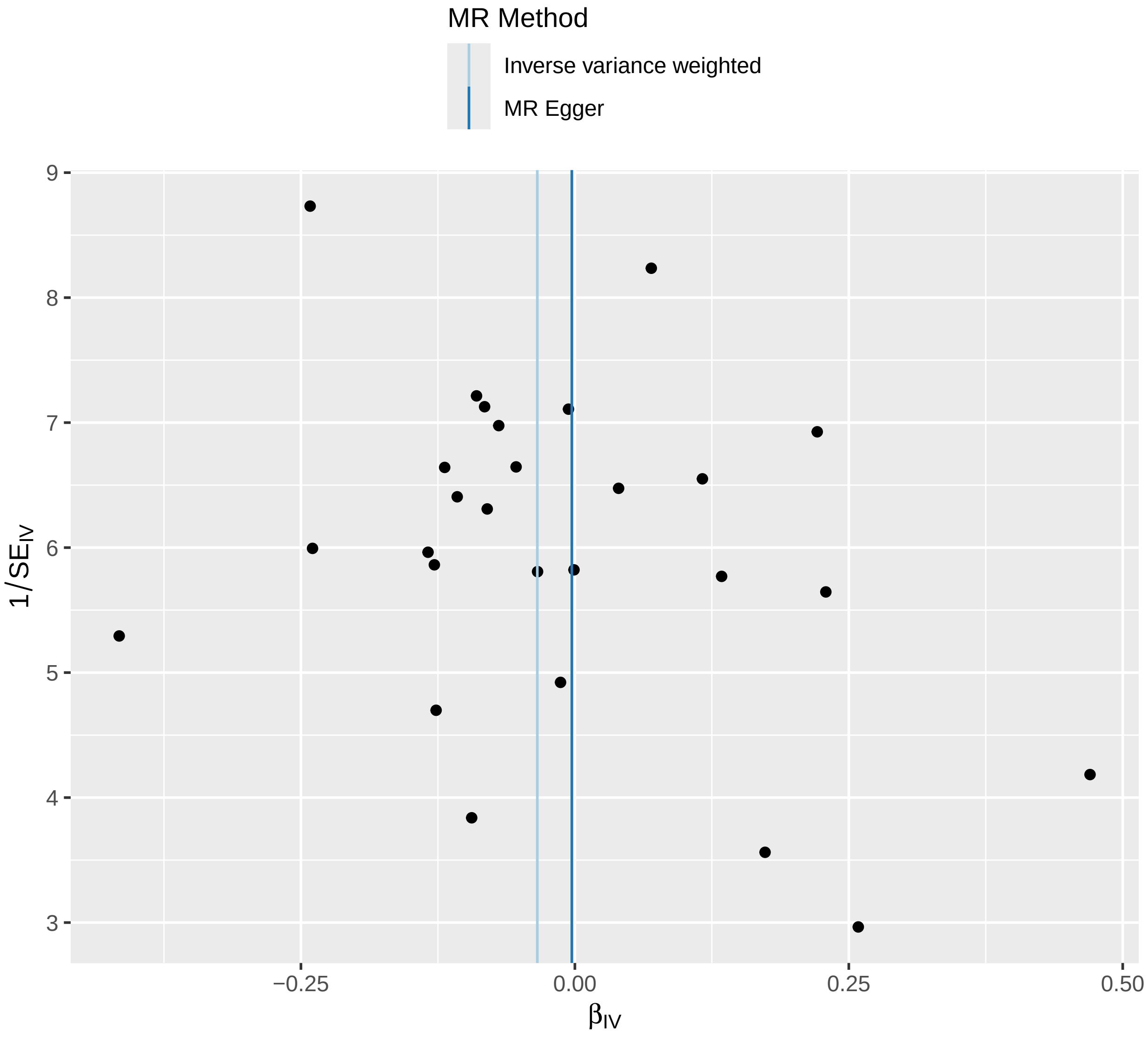
MR Method
Inverse variance weighted
MR Egger
1/SE_IV
9
8
7
6
5
4
3
−0.25
0.00
0.25
0.50
β_IV

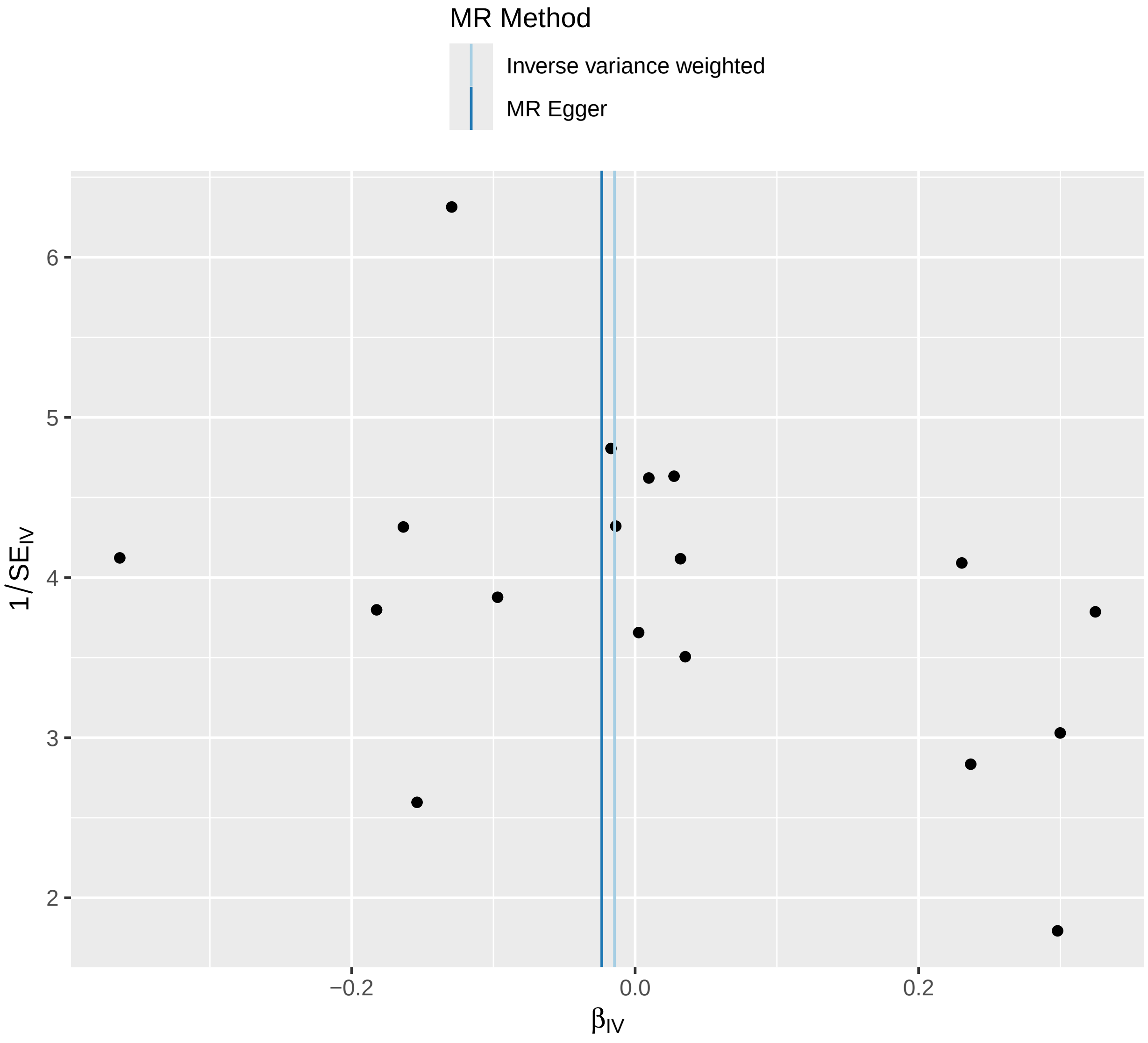
MR Method
Inverse variance weighted
MR Egger
1/SE_IV
6
5
4
3
2
−0.2
0.0
0.2
β_IV

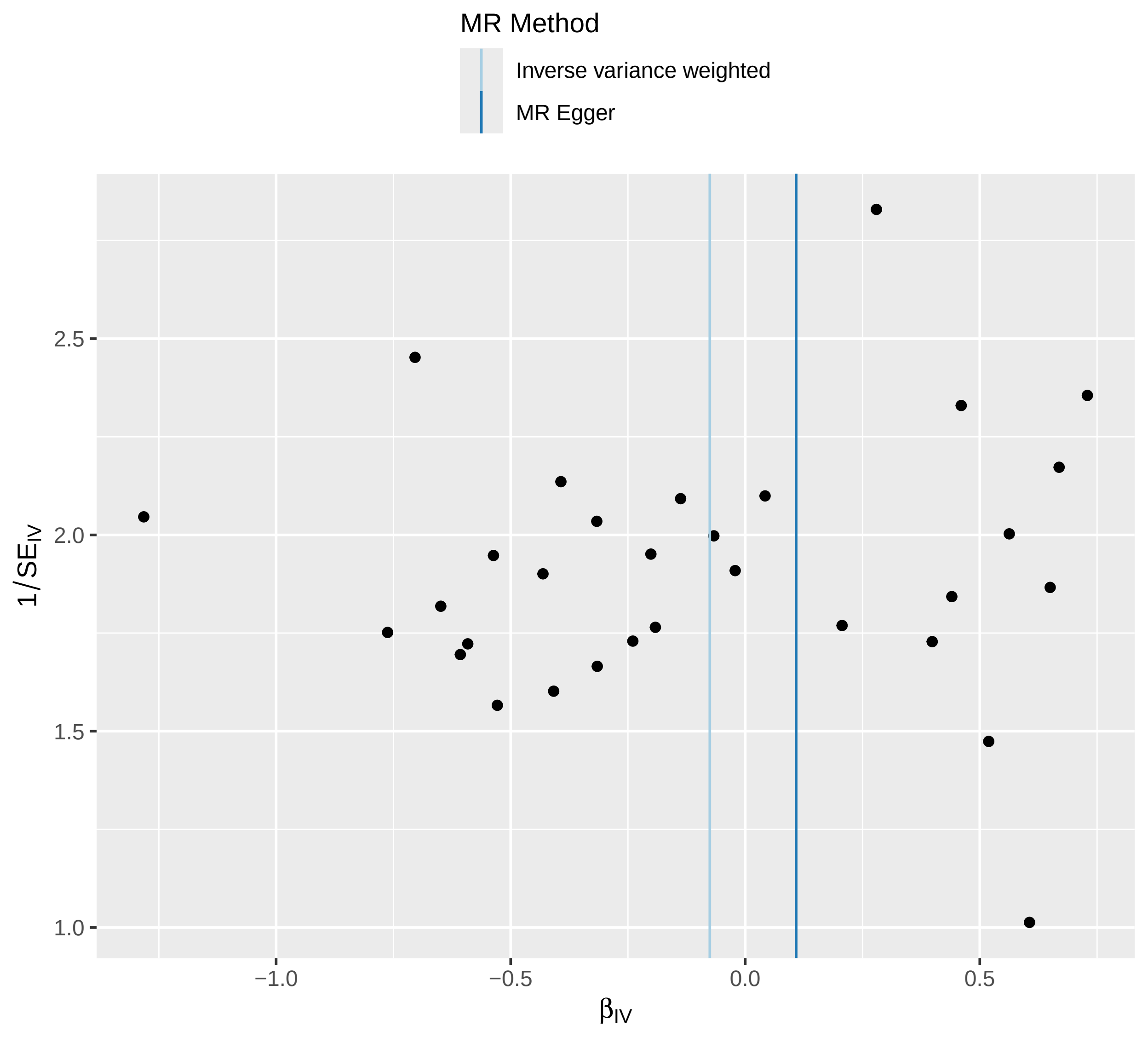

MR Method
Inverse variance weighted
MR Egger
2.5
2.0
1.5
1.0
1/SE_IV
−1.0
−0.5
0.0
0.5
β_IV

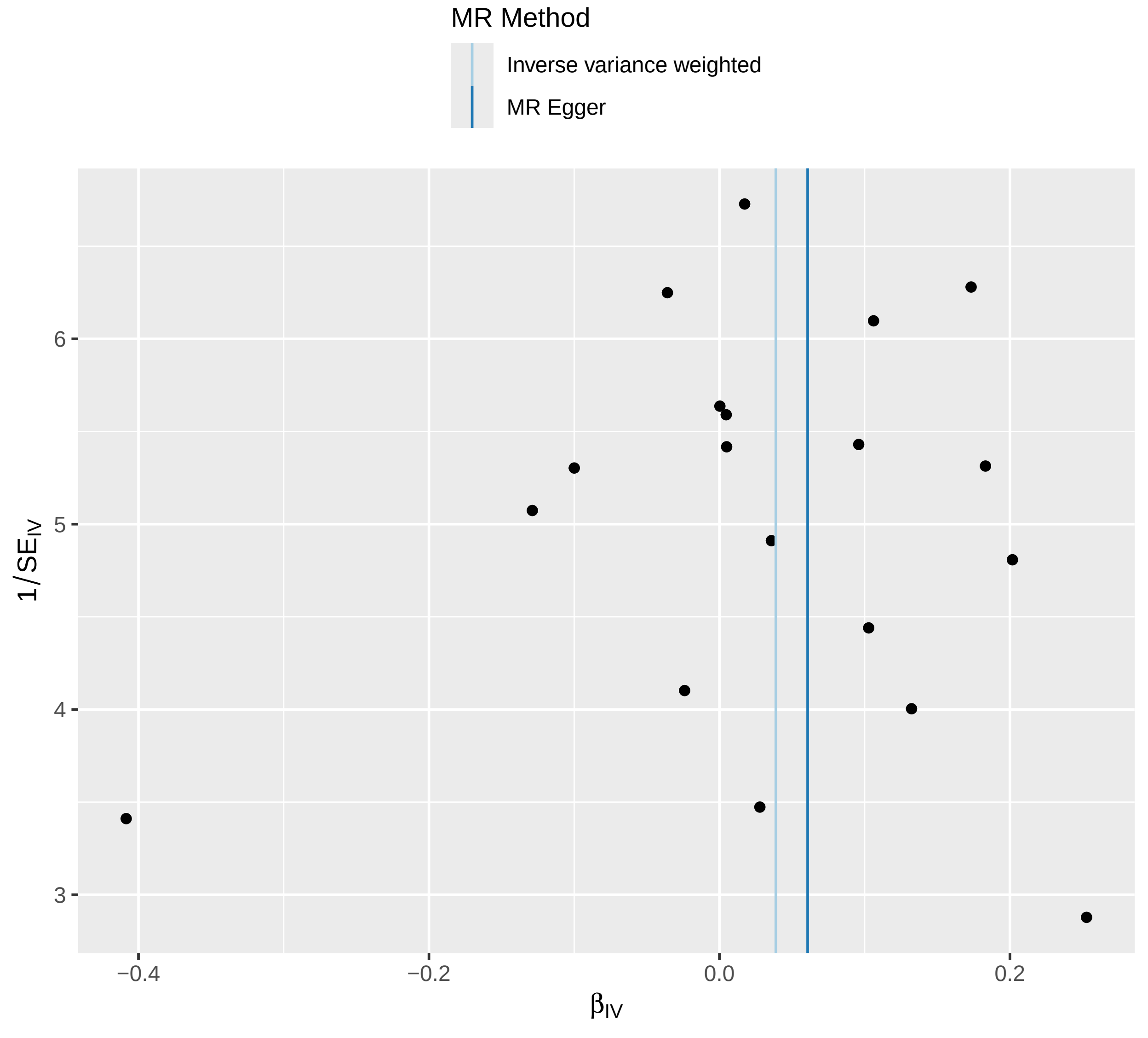

MR Method
Inverse variance weighted
MR Egger
1/SE$_{IV}$
6
5
4
3
−0.4
−0.2
0.0
0.2
β$_{IV}$

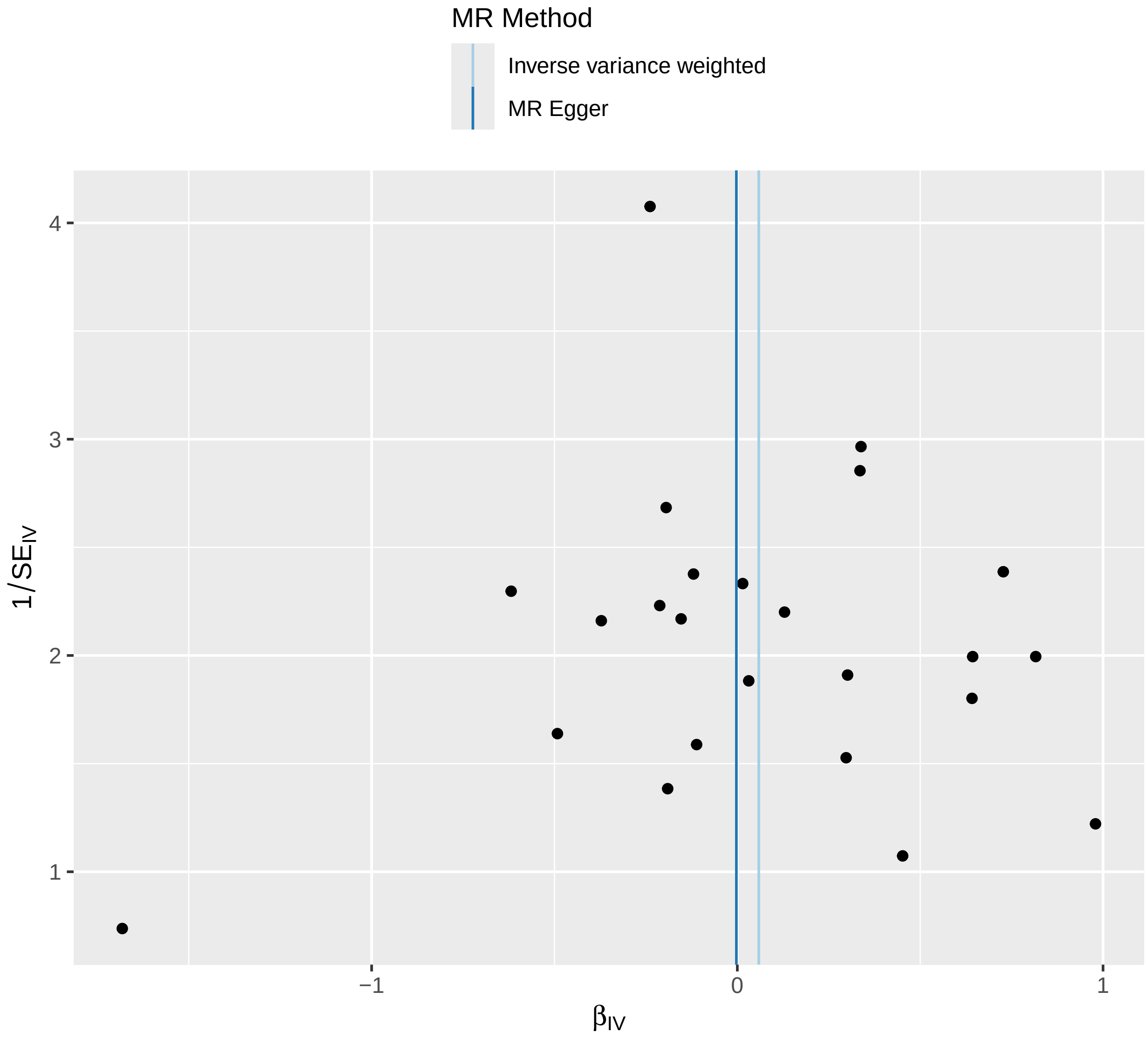

MR Method
Inverse variance weighted
MR Egger
4
3
2
1
1/SE$_{IV}$
−1
0
1
β$_{IV}$

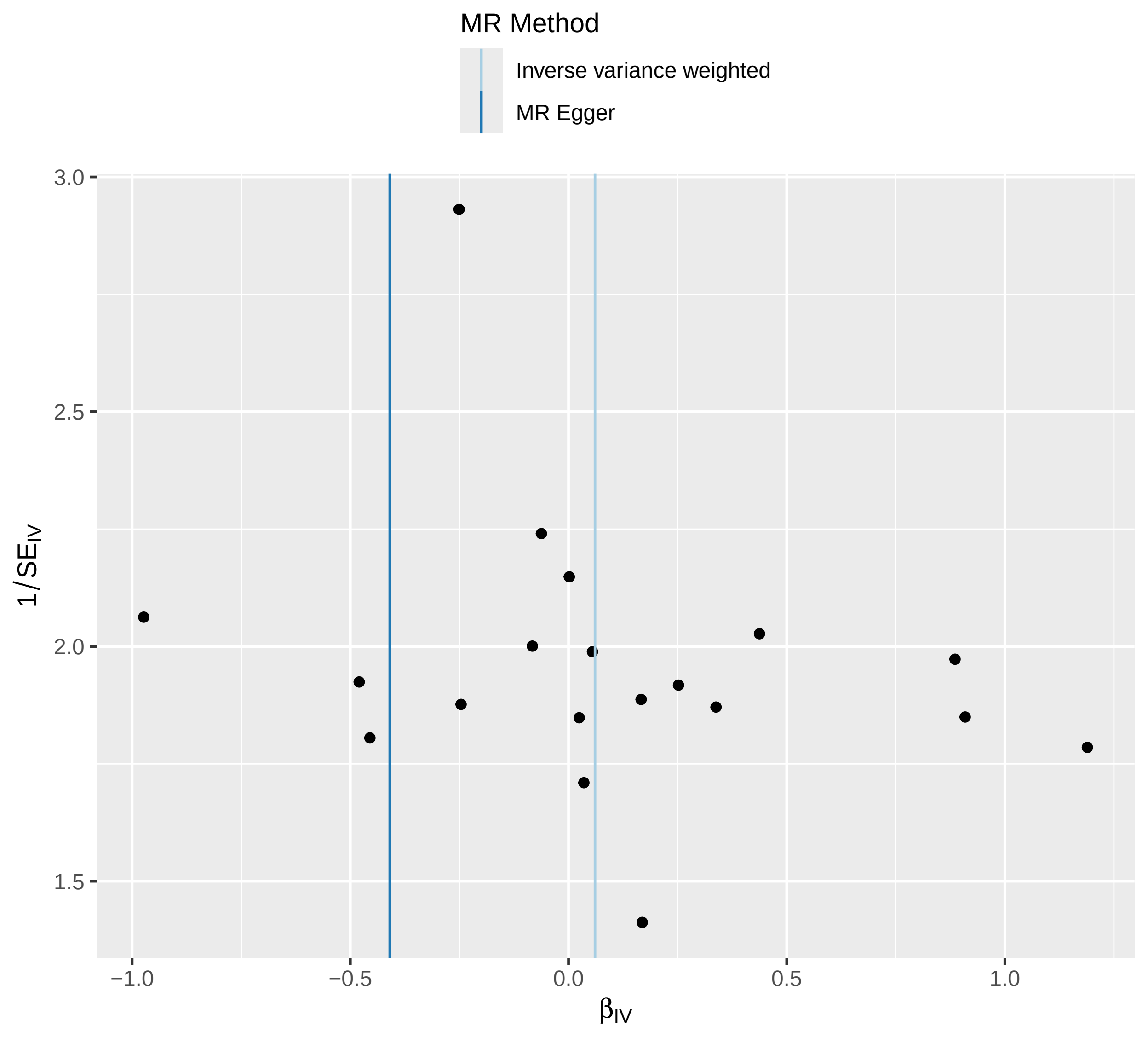

MR Method
Inverse variance weighted
MR Egger
3.0
2.5
2.0
1.5
1/SE_IV
−1.0
−0.5
0.0
0.5
1.0
β_IV

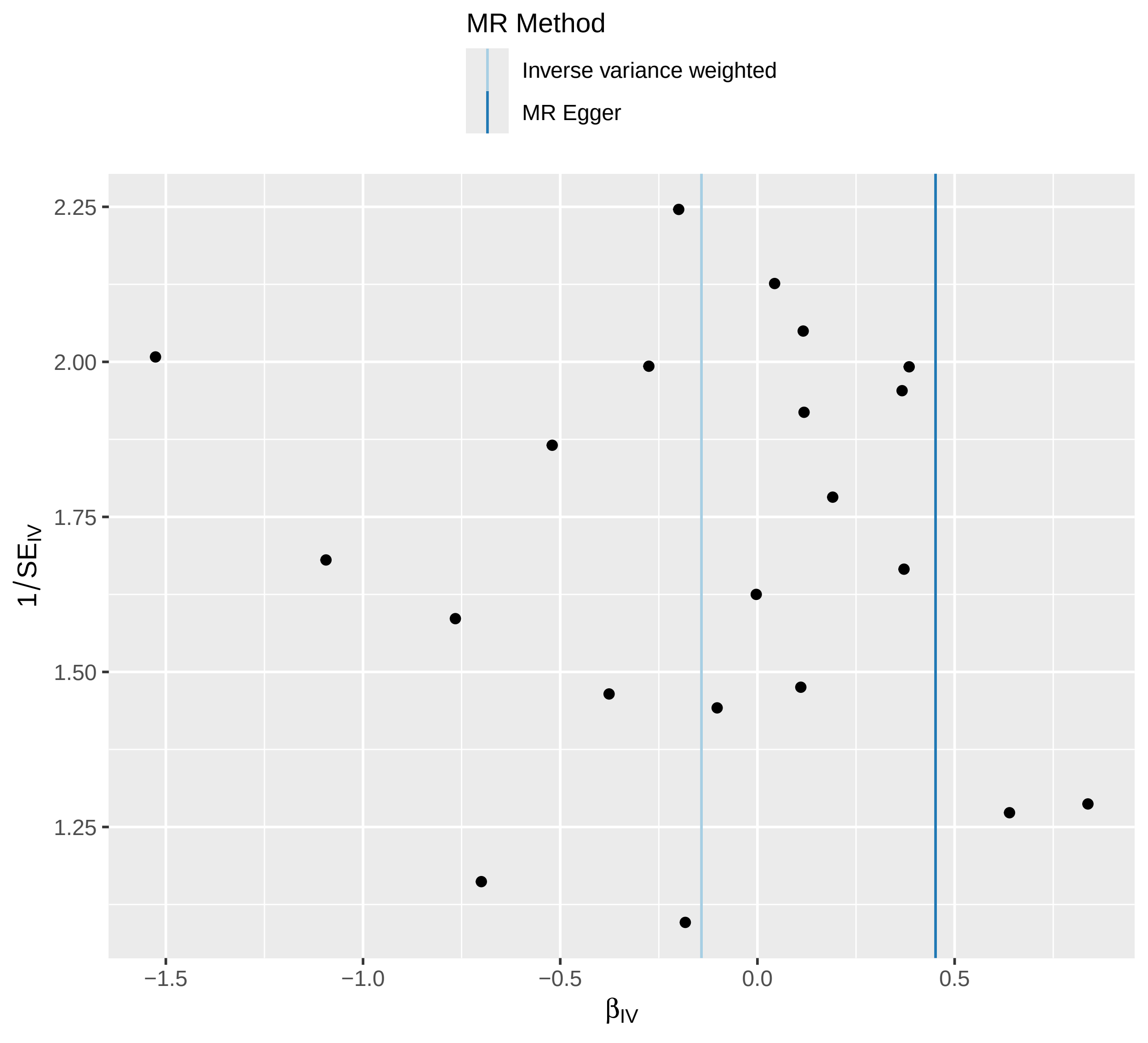

MR Method
Inverse variance weighted
MR Egger
2.25
2.00
1.75
1.50
1.25
1/SE_IV
−1.5
−1.0
−0.5
0.0
0.5
β_IV

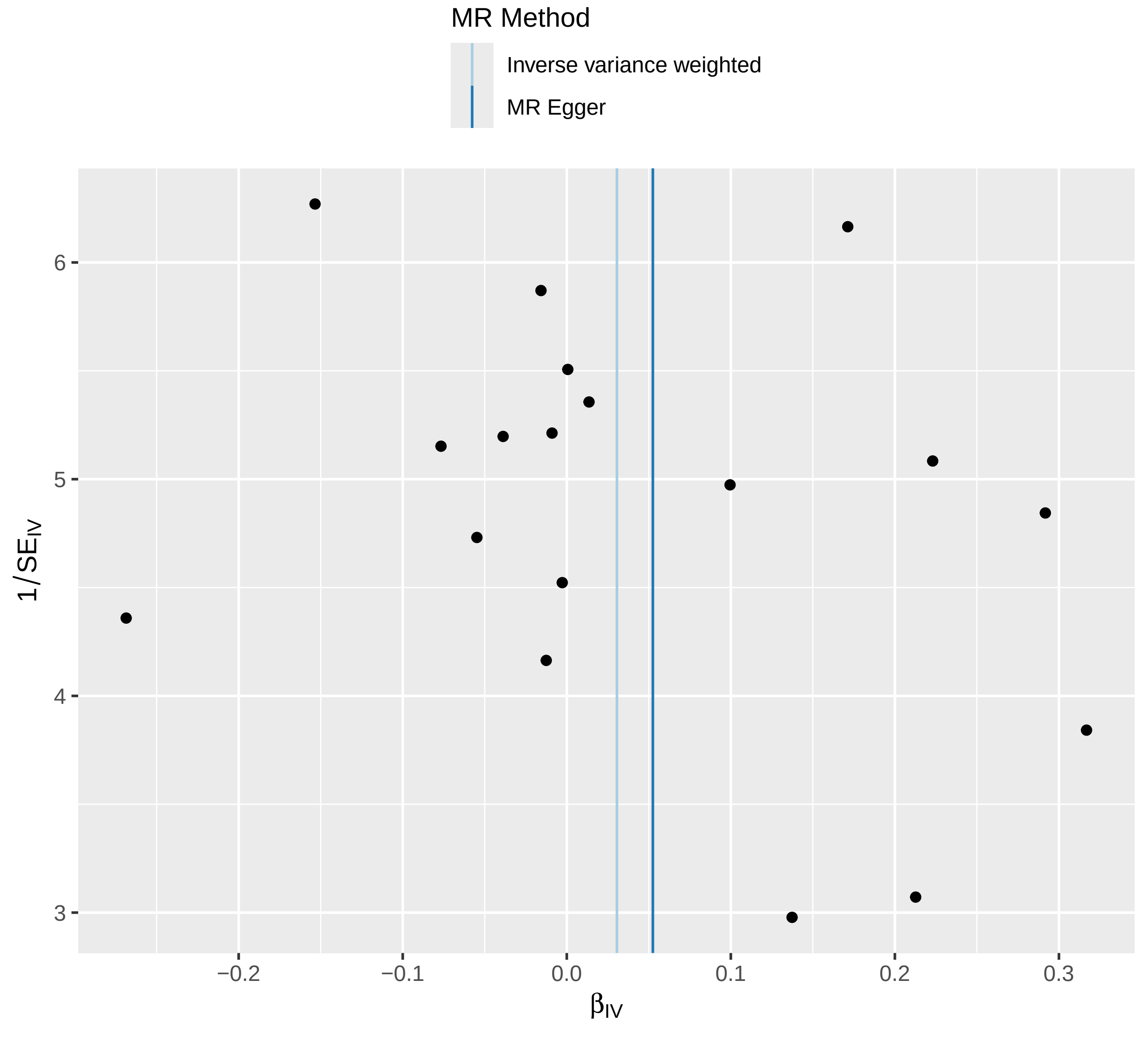

MR Method
Inverse variance weighted
MR Egger
6
5
4
3
1/SE_IV
−0.2
−0.1
0.0
0.1
0.2
0.3
β_IV

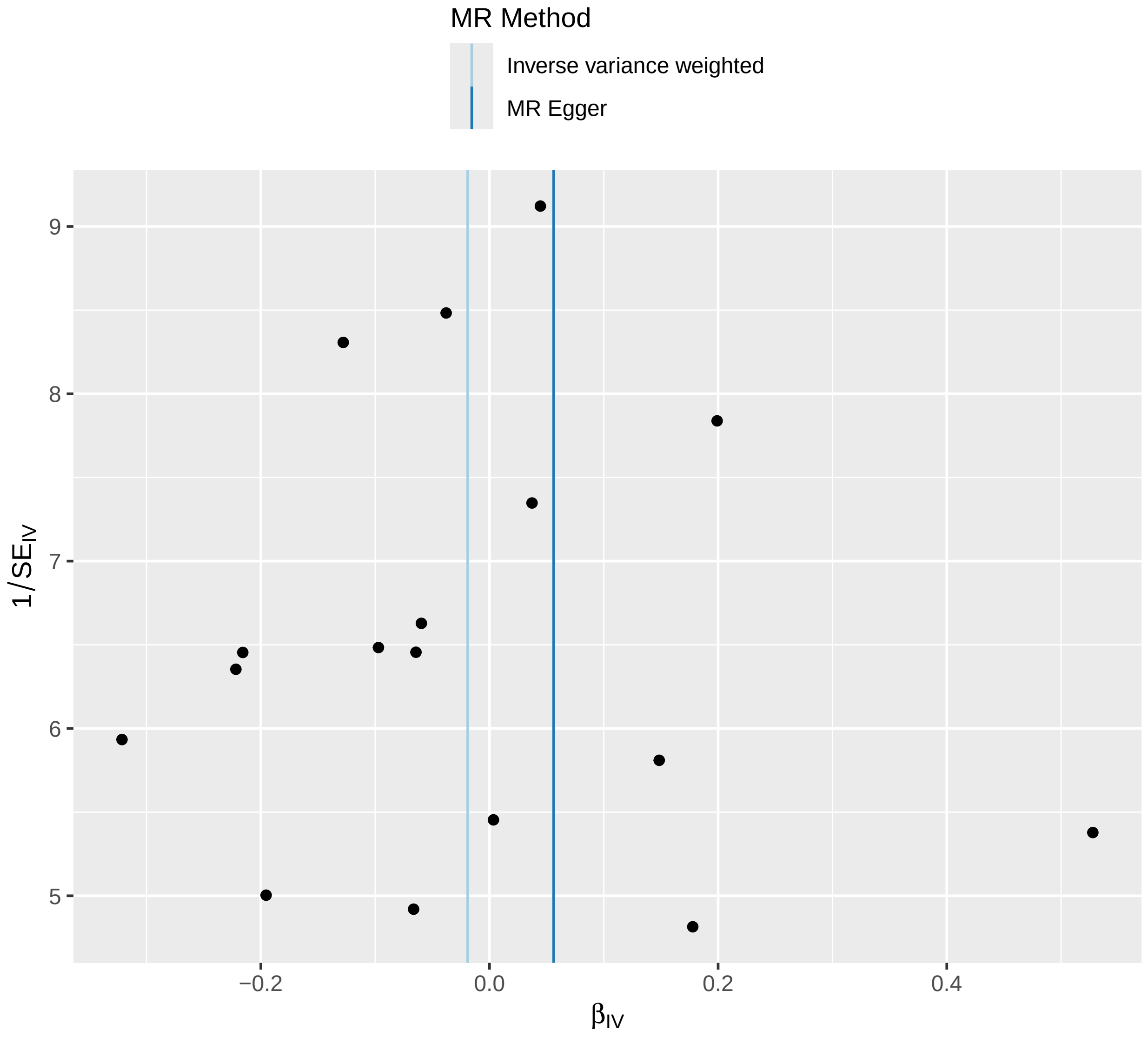
MR Method
Inverse variance weighted
MR Egger
9
8
7
6
5
1/SE$_{IV}$
−0.2
0.0
0.2
0.4
β$_{IV}$

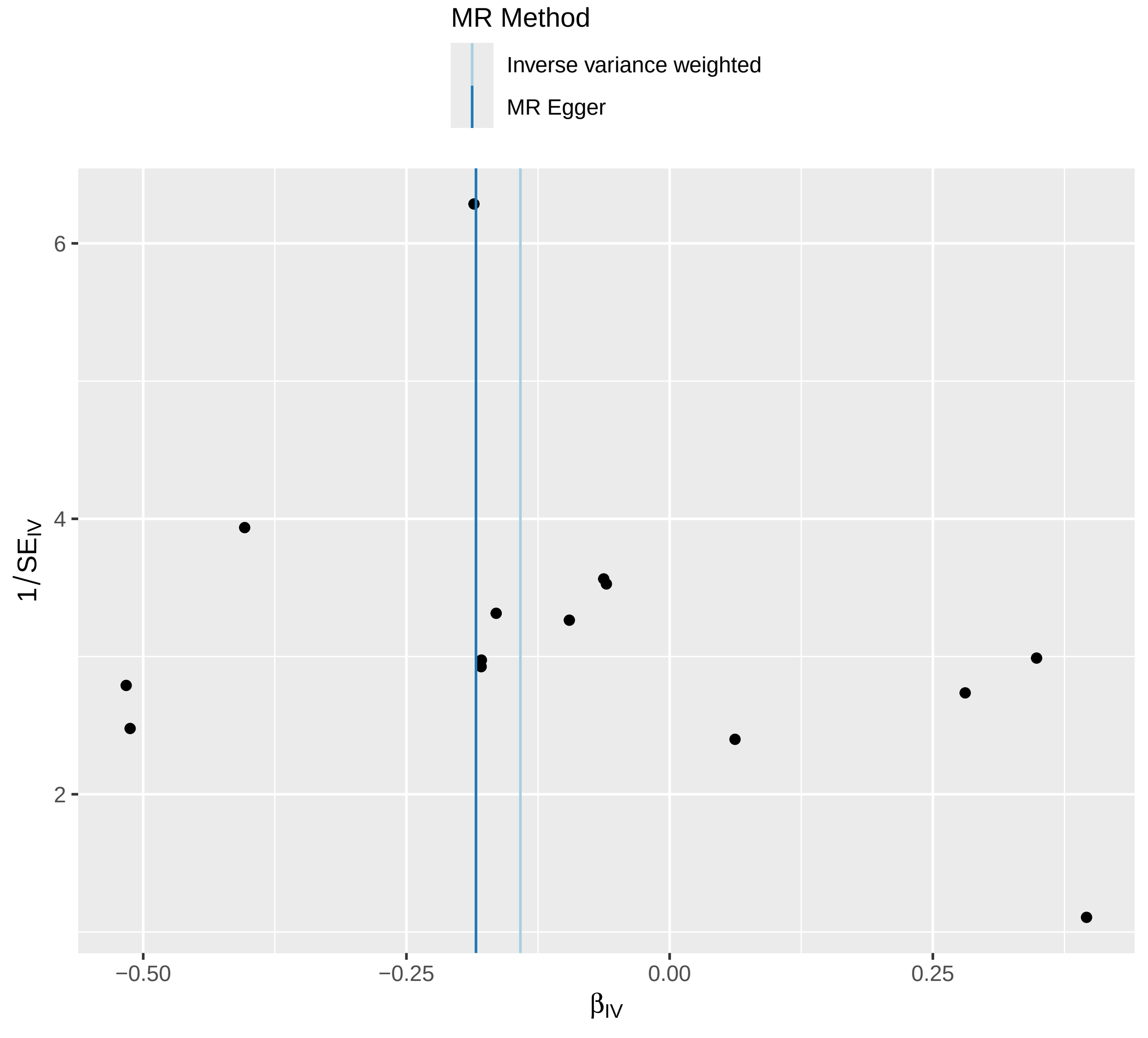

MR Method
Inverse variance weighted
MR Egger
6
4
2
1/SE_IV
−0.50
−0.25
0.00
0.25
β_IV

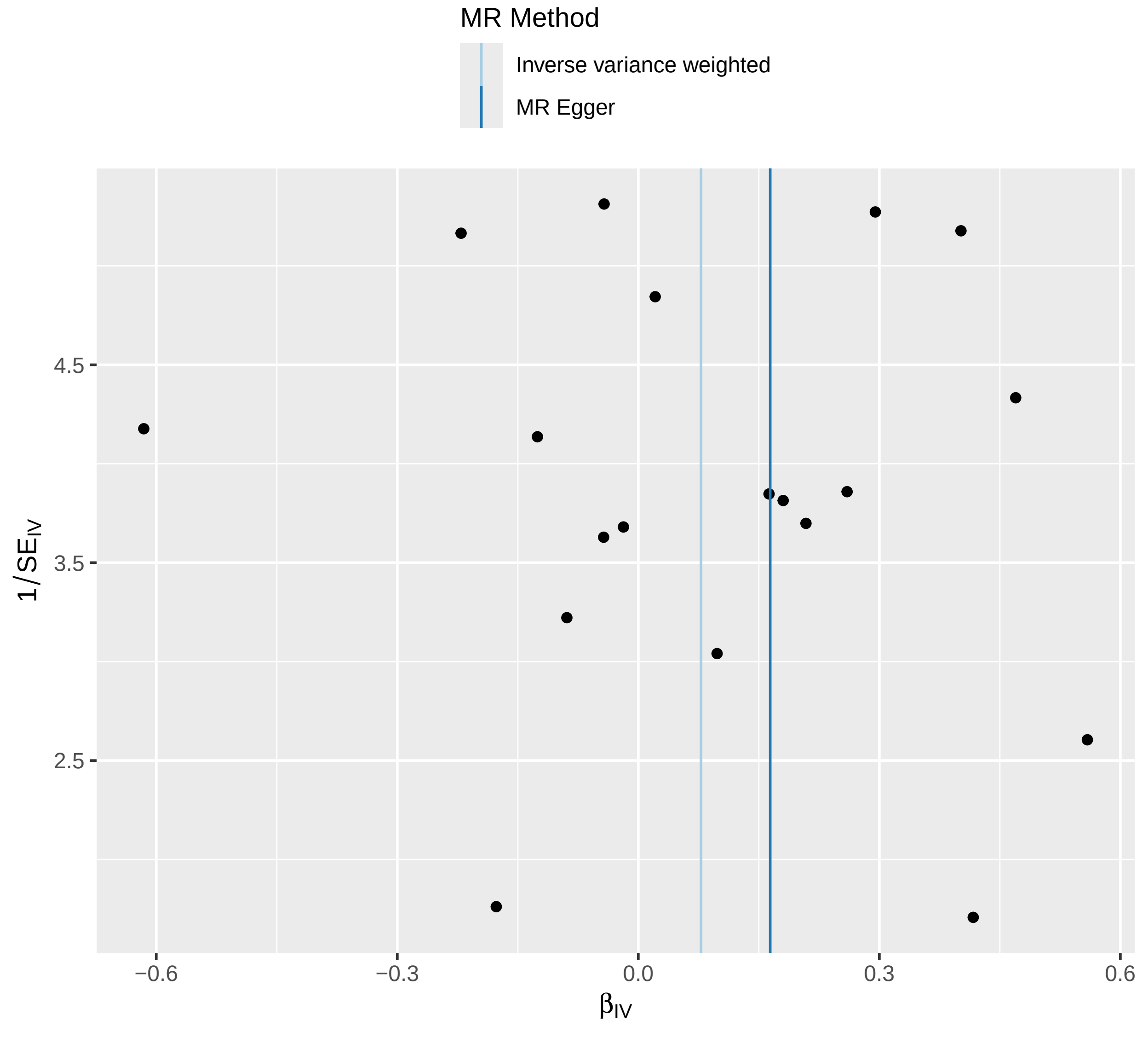
MR Method
Inverse variance weighted
MR Egger
4.5
3.5
2.5
1/SE_IV
−0.6
−0.3
0.0
0.3
0.6
β_IV

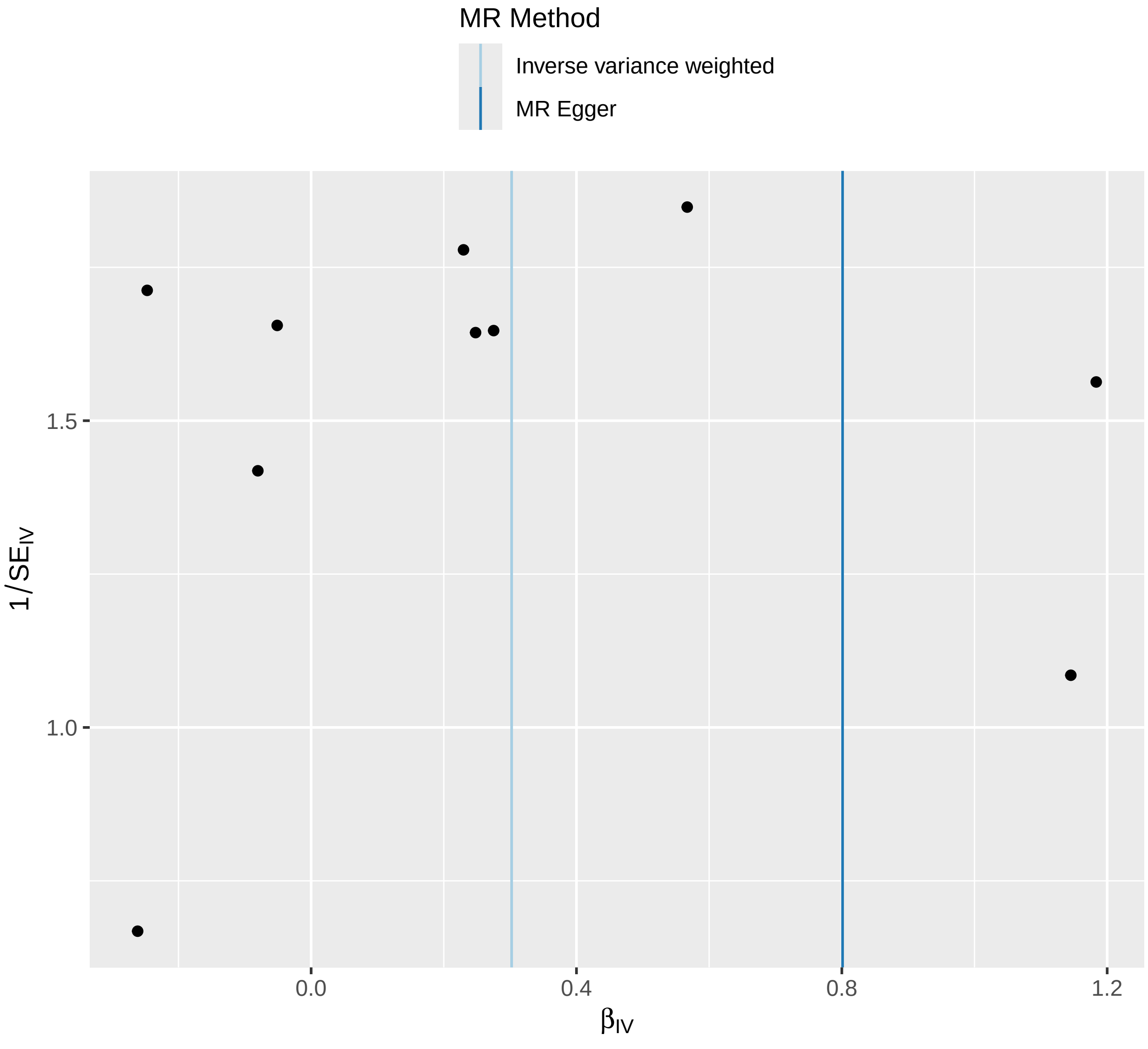

MR Method
Inverse variance weighted
MR Egger
1/SE_IV
1.5
1.0
0.0
0.4
0.8
1.2
β_IV

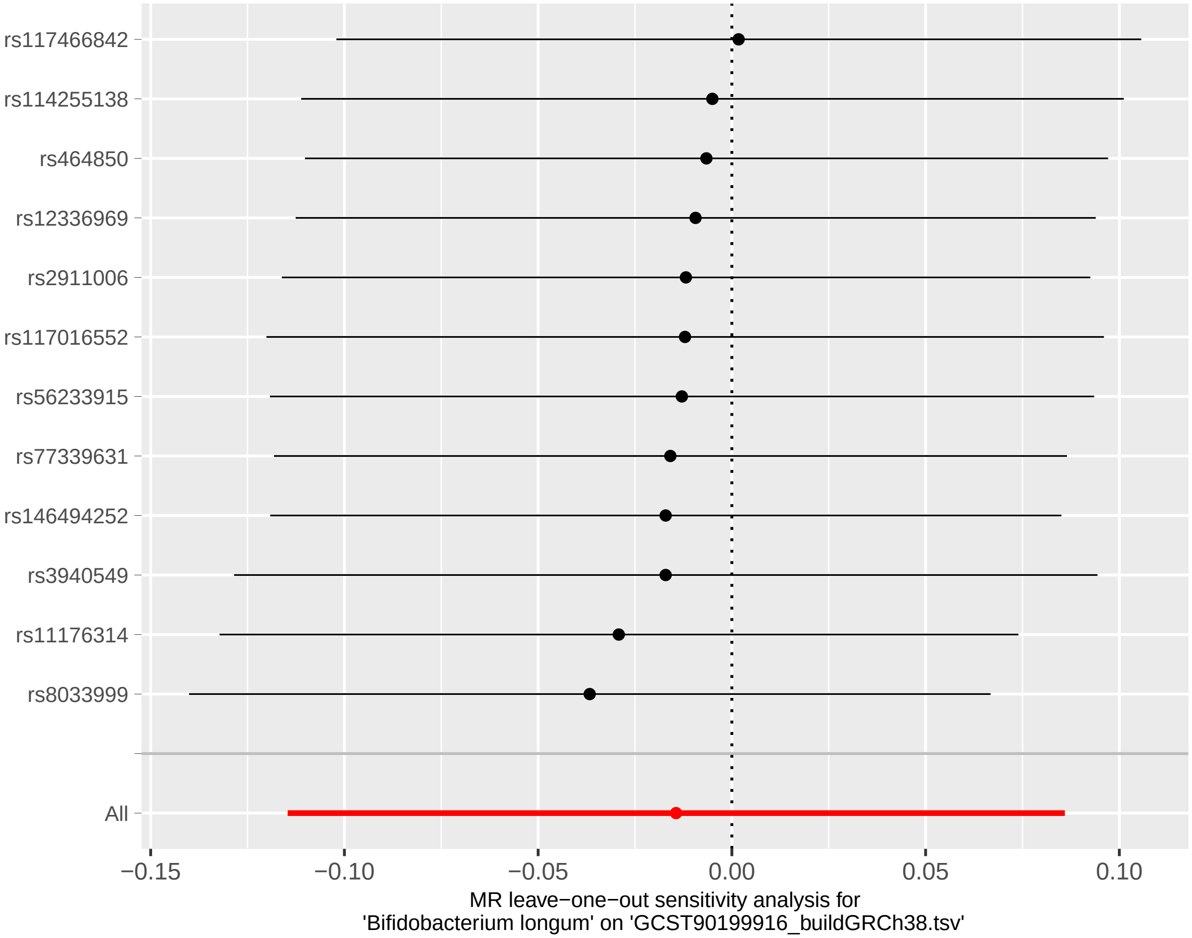

rs117466842
rs114255138
rs464850
rs12336969
rs2911006
rs117016552
rs56233915
rs77339631
rs146494252
rs3940549
rs11176314
rs8033999
All
−0.15
−0.10
−0.05
0.00
0.05
0.10
MR leave−one−out sensitivity analysis for
'Bifidobacterium longum' on 'GCST90199916_buildGRCh38.tsv'

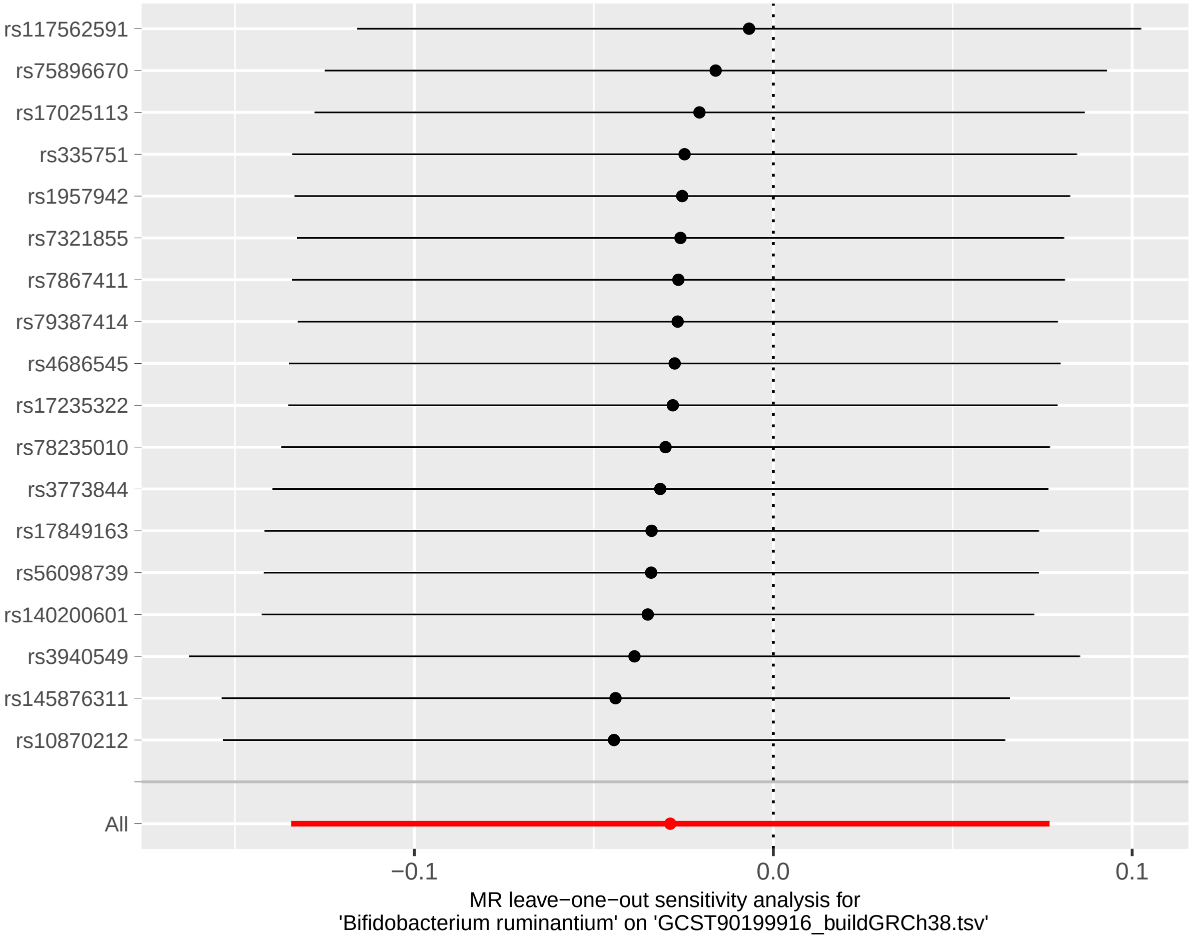

rs117562591
rs75896670
rs17025113
rs335751
rs1957942
rs7321855
rs7867411
rs79387414
rs4686545
rs17235322
rs78235010
rs3773844
rs17849163
rs56098739
rs140200601
rs3940549
rs145876311
rs10870212
All
−0.1
0.0
0.1
MR leave−one−out sensitivity analysis for
'Bifidobacterium ruminantium' on 'GCST90199916_buildGRCh38.tsv'

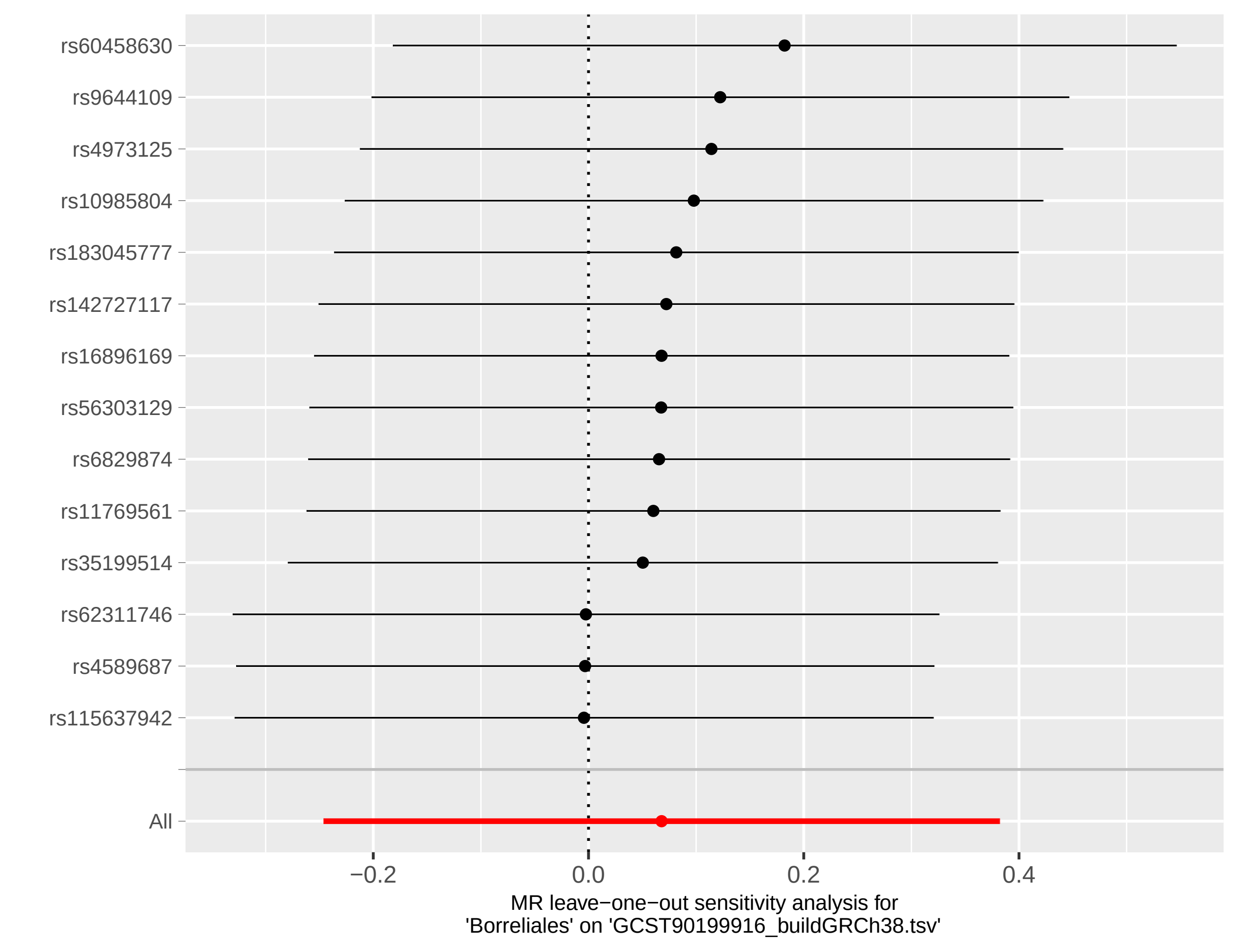

rs60458630
rs9644109
rs4973125
rs10985804
rs183045777
rs142727117
rs16896169
rs56303129
rs6829874
rs11769561
rs35199514
rs62311746
rs4589687
rs115637942
All
−0.2
0.0
0.2
0.4
MR leave−one−out sensitivity analysis for
'Borreliales' on 'GCST90199916_buildGRCh38.tsv'

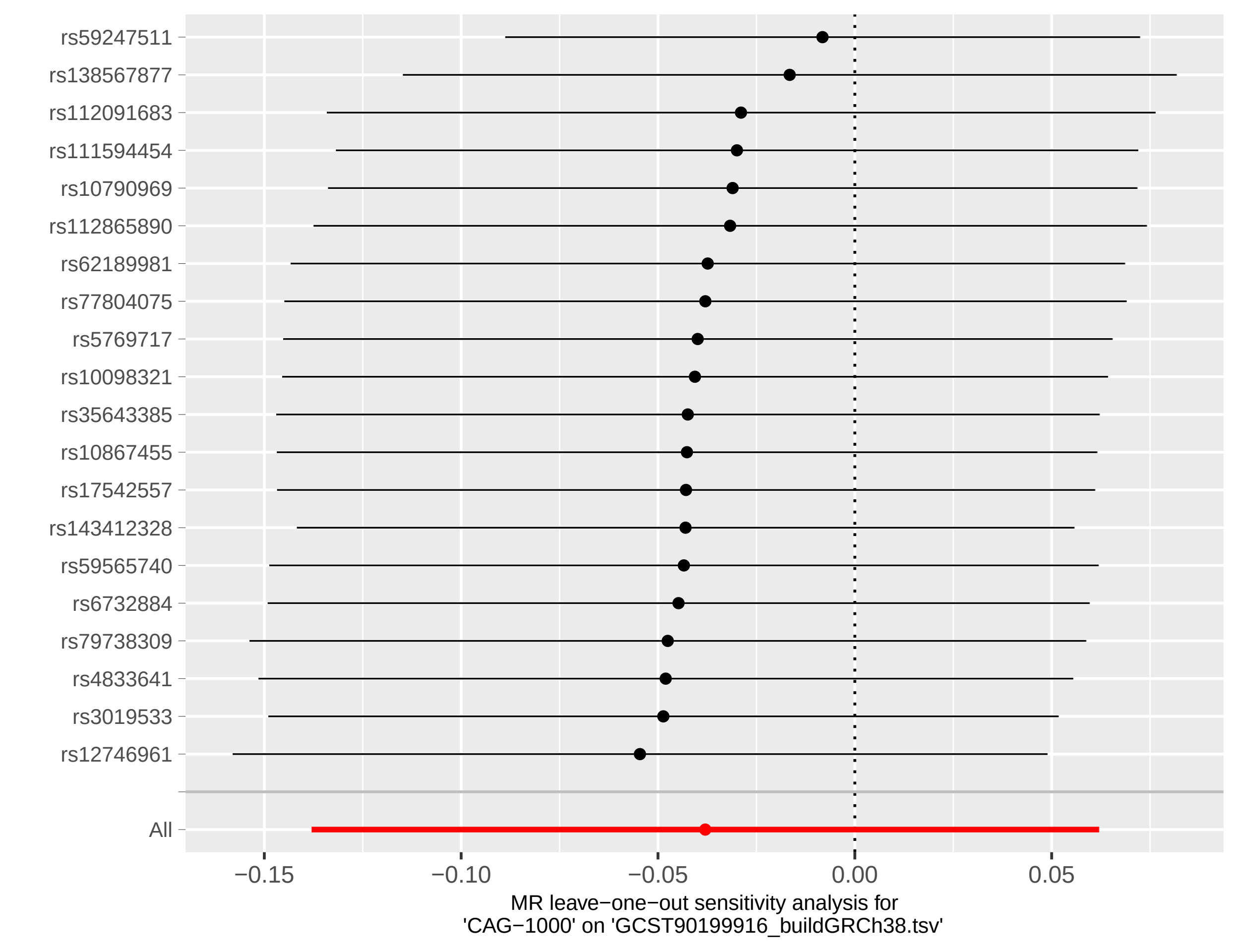
rs59247511
rs138567877
rs112091683
rs111594454
rs10790969
rs112865890
rs62189981
rs77804075
rs5769717
rs10098321
rs35643385
rs10867455
rs17542557
rs143412328
rs59565740
rs6732884
rs79738309
rs4833641
rs3019533
rs12746961
All
−0.15
−0.10
−0.05
0.00
0.05
MR leave−one−out sensitivity analysis for
'CAG−1000' on 'GCST90199916_buildGRCh38.tsv'

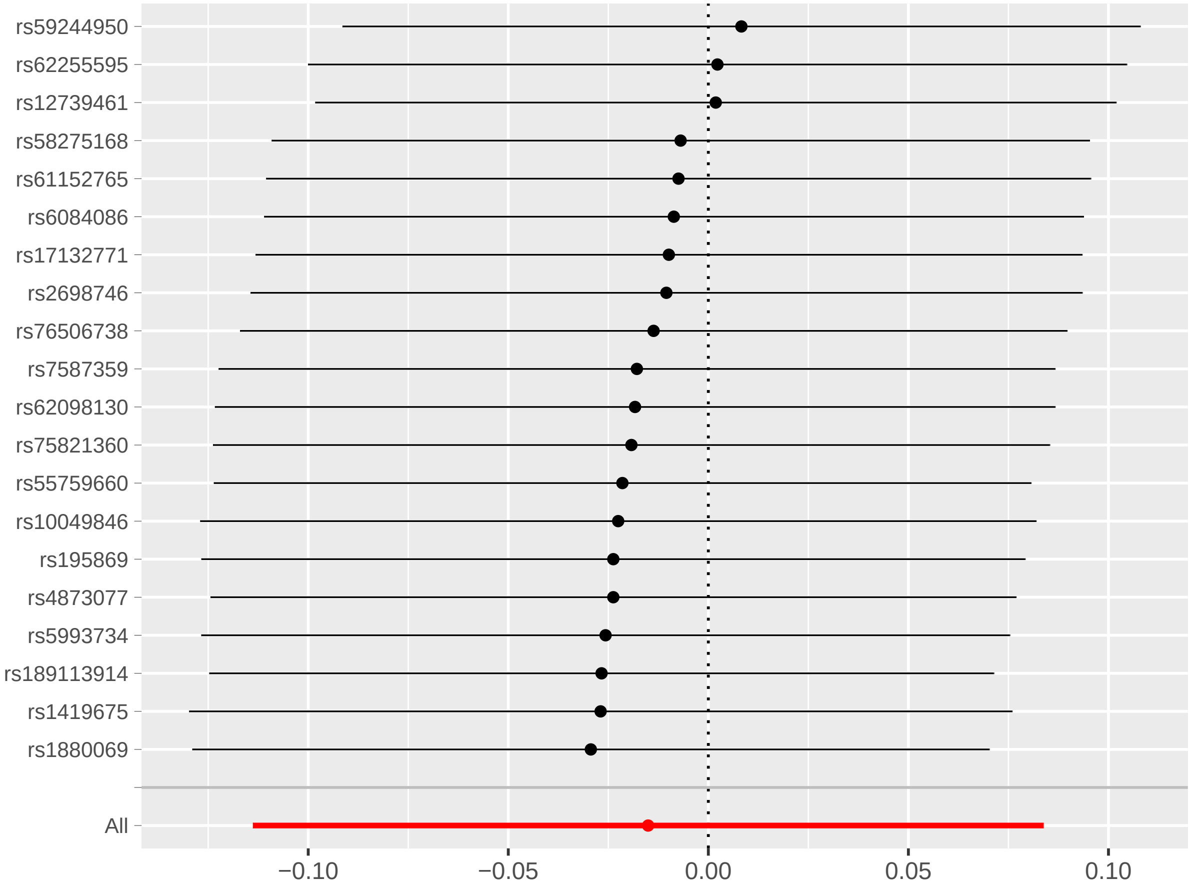

MR leave−one−out sensitivity analysis for
'CAG−170 sp003516765' on 'GCST90199916_buildGRCh38.tsv'

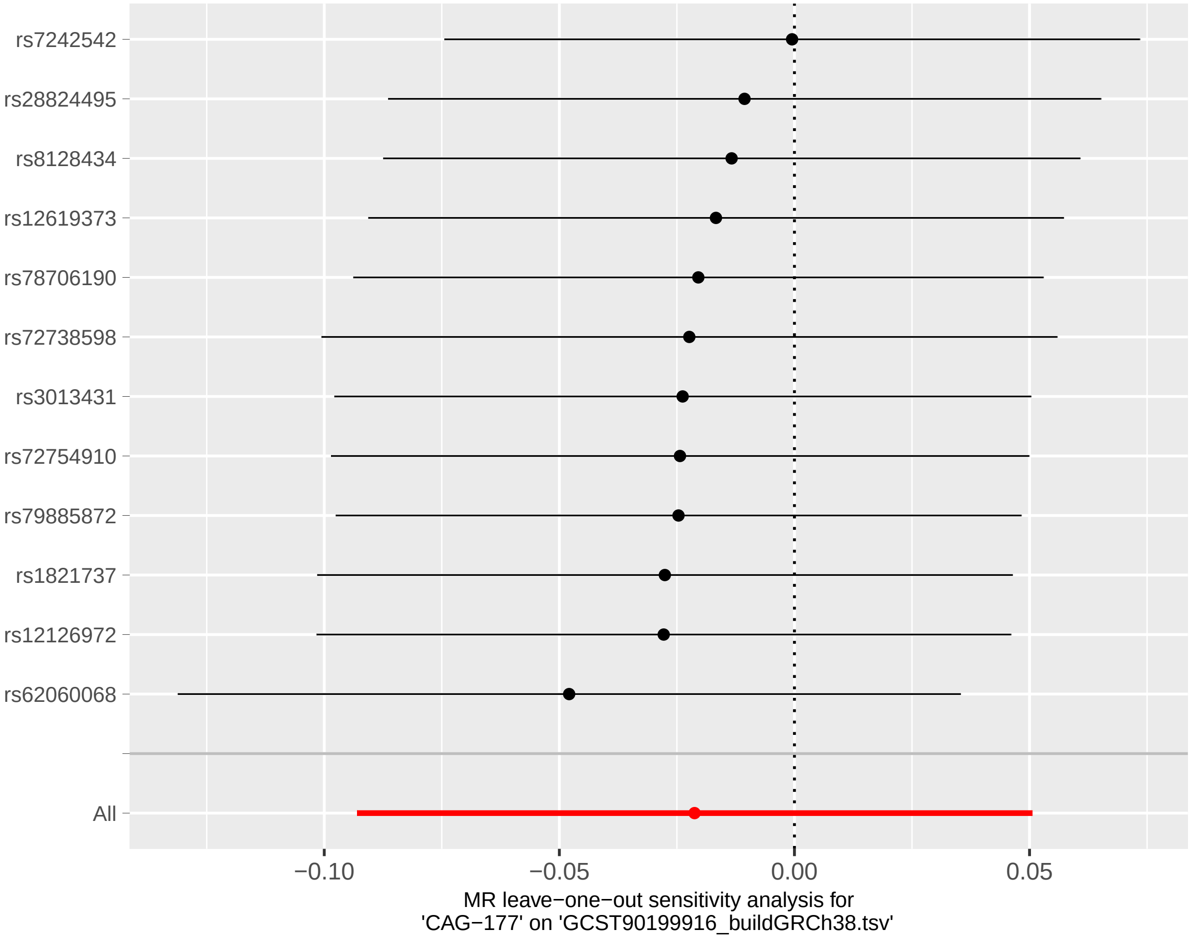
rs7242542
rs28824495
rs8128434
rs12619373
rs78706190
rs72738598
rs3013431
rs72754910
rs79885872
rs1821737
rs12126972
rs62060068
All
−0.10
−0.05
0.00
0.05
MR leave−one−out sensitivity analysis for
'CAG−177' on 'GCST90199916_buildGRCh38.tsv'

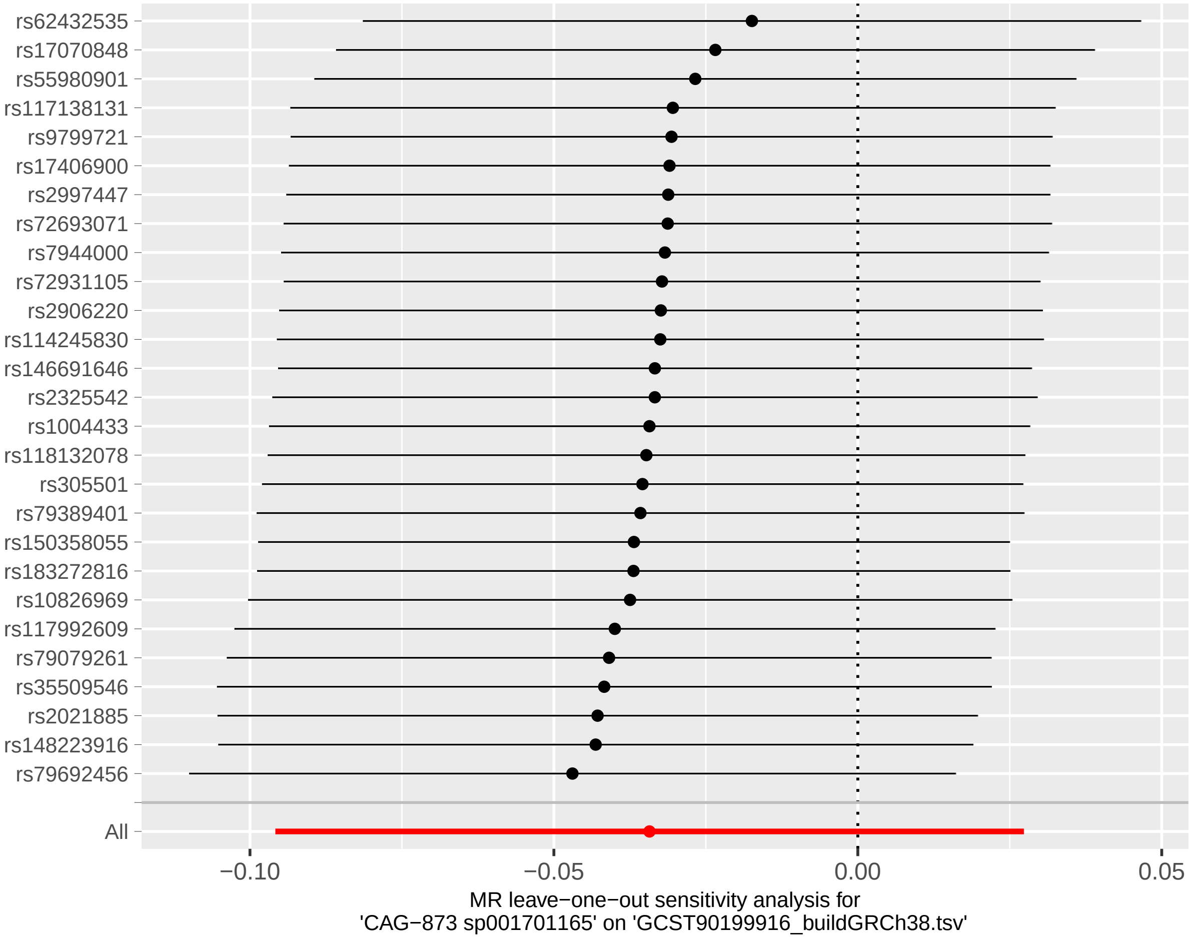

rs62432535
rs17070848
rs55980901
rs117138131
rs9799721
rs17406900
rs2997447
rs72693071
rs7944000
rs72931105
rs2906220
rs114245830
rs146691646
rs2325542
rs1004433
rs118132078
rs305501
rs79389401
rs150358055
rs183272816
rs10826969
rs117992609
rs79079261
rs35509546
rs2021885
rs148223916
rs79692456
All
−0.10
−0.05
0.00
0.05
MR leave−one−out sensitivity analysis for
'CAG−873 sp001701165' on 'GCST90199916_buildGRCh38.tsv'

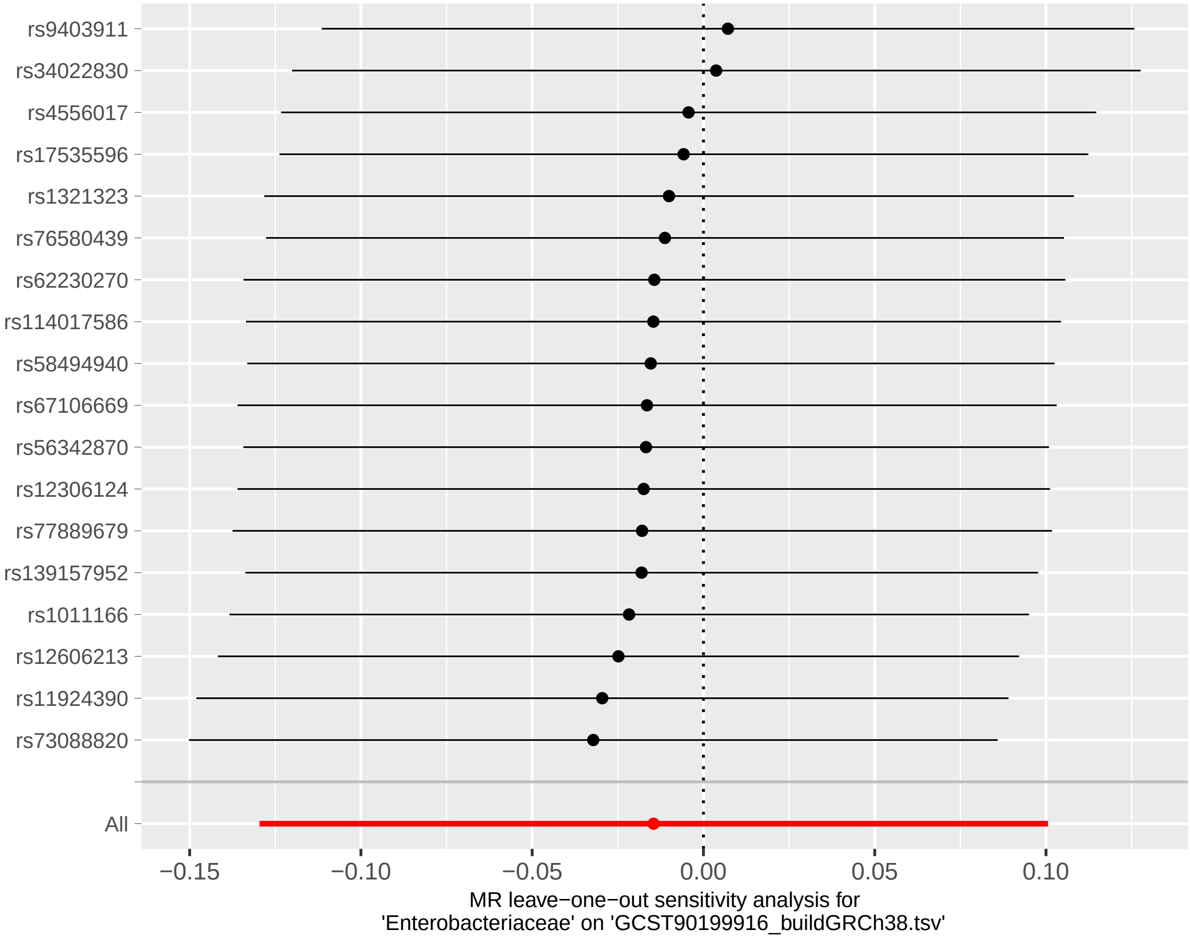

rs9403911
rs34022830
rs4556017
rs17535596
rs1321323
rs76580439
rs62230270
rs114017586
rs58494940
rs67106669
rs56342870
rs12306124
rs77889679
rs139157952
rs1011166
rs12606213
rs11924390
rs73088820
All
−0.15
−0.10
−0.05
0.00
0.05
0.10
MR leave−one−out sensitivity analysis for
'Enterobacteriaceae' on 'GCST90199916_buildGRCh38.tsv'

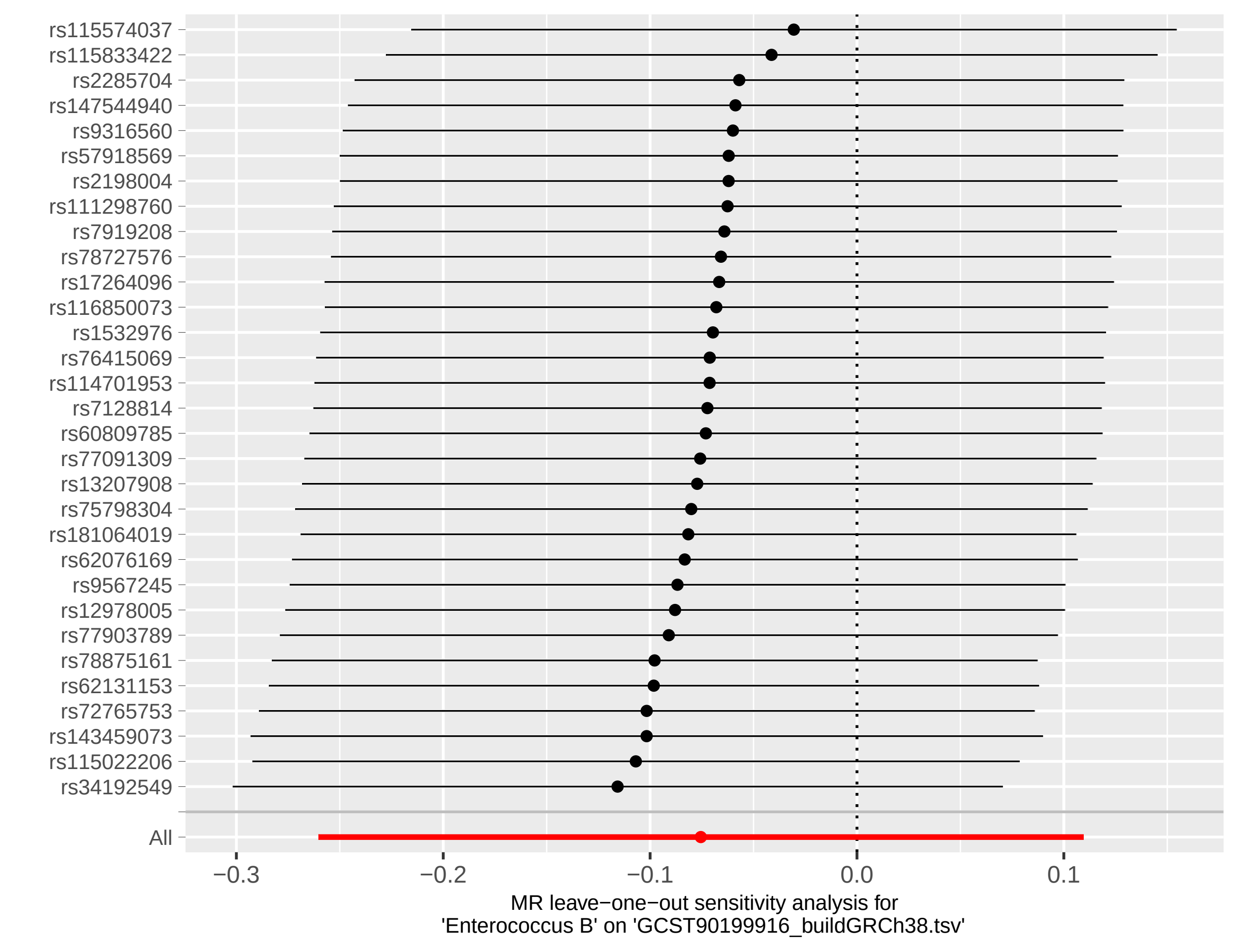
rs115574037
rs115833422
rs2285704
rs147544940
rs9316560
rs57918569
rs2198004
rs111298760
rs7919208
rs78727576
rs17264096
rs116850073
rs1532976
rs76415069
rs114701953
rs7128814
rs60809785
rs77091309
rs13207908
rs75798304
rs181064019
rs62076169
rs9567245
rs12978005
rs77903789
rs78875161
rs62131153
rs72765753
rs143459073
rs115022206
rs34192549
All
−0.3
−0.2
−0.1
0.0
0.1
MR leave−one−out sensitivity analysis for
'Enterococcus B' on 'GCST90199916_buildGRCh38.tsv'

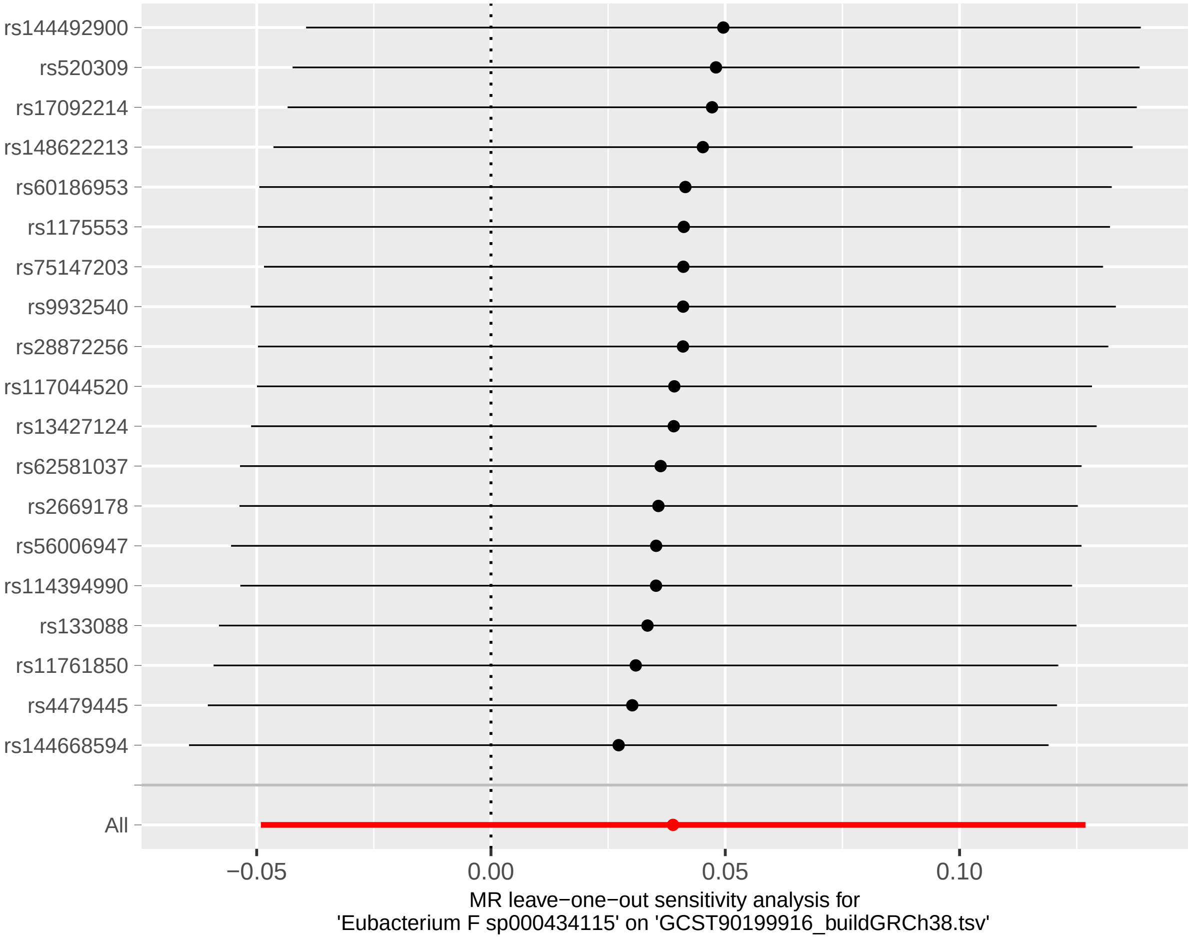

rs144492900
rs520309
rs17092214
rs148622213
rs60186953
rs1175553
rs75147203
rs9932540
rs28872256
rs117044520
rs13427124
rs62581037
rs2669178
rs56006947
rs114394990
rs133088
rs11761850
rs4479445
rs144668594
All
−0.05
0.00
0.05
0.10
MR leave−one−out sensitivity analysis for
'Eubacterium F sp000434115' on 'GCST90199916_buildGRCh38.tsv'

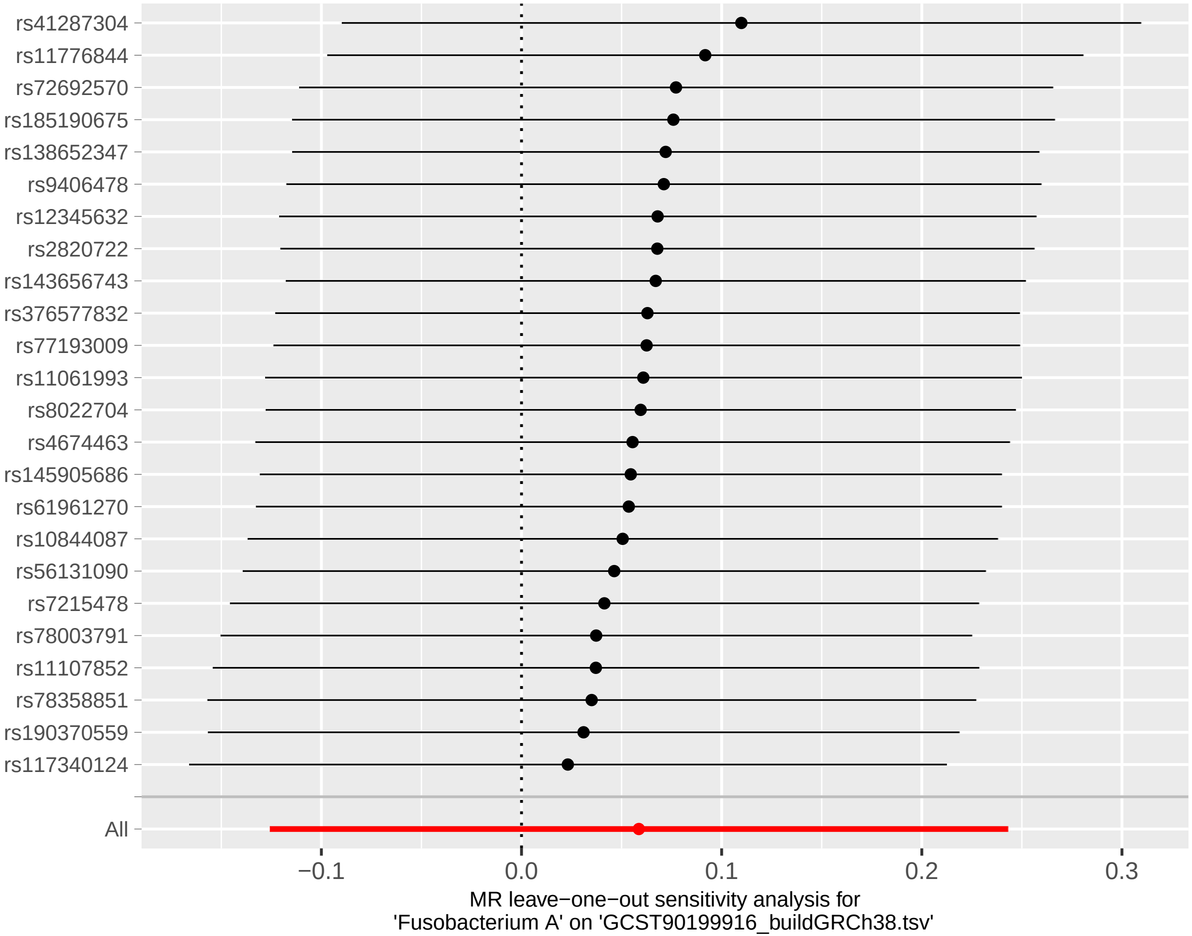

rs41287304
rs11776844
rs72692570
rs185190675
rs138652347
rs9406478
rs12345632
rs2820722
rs143656743
rs376577832
rs77193009
rs11061993
rs8022704
rs4674463
rs145905686
rs61961270
rs10844087
rs56131090
rs7215478
rs78003791
rs11107852
rs78358851
rs190370559
rs117340124
All
−0.1
0.0
0.1
0.2
0.3
MR leave−one−out sensitivity analysis for
'Fusobacterium A' on 'GCST90199916_buildGRCh38.tsv'

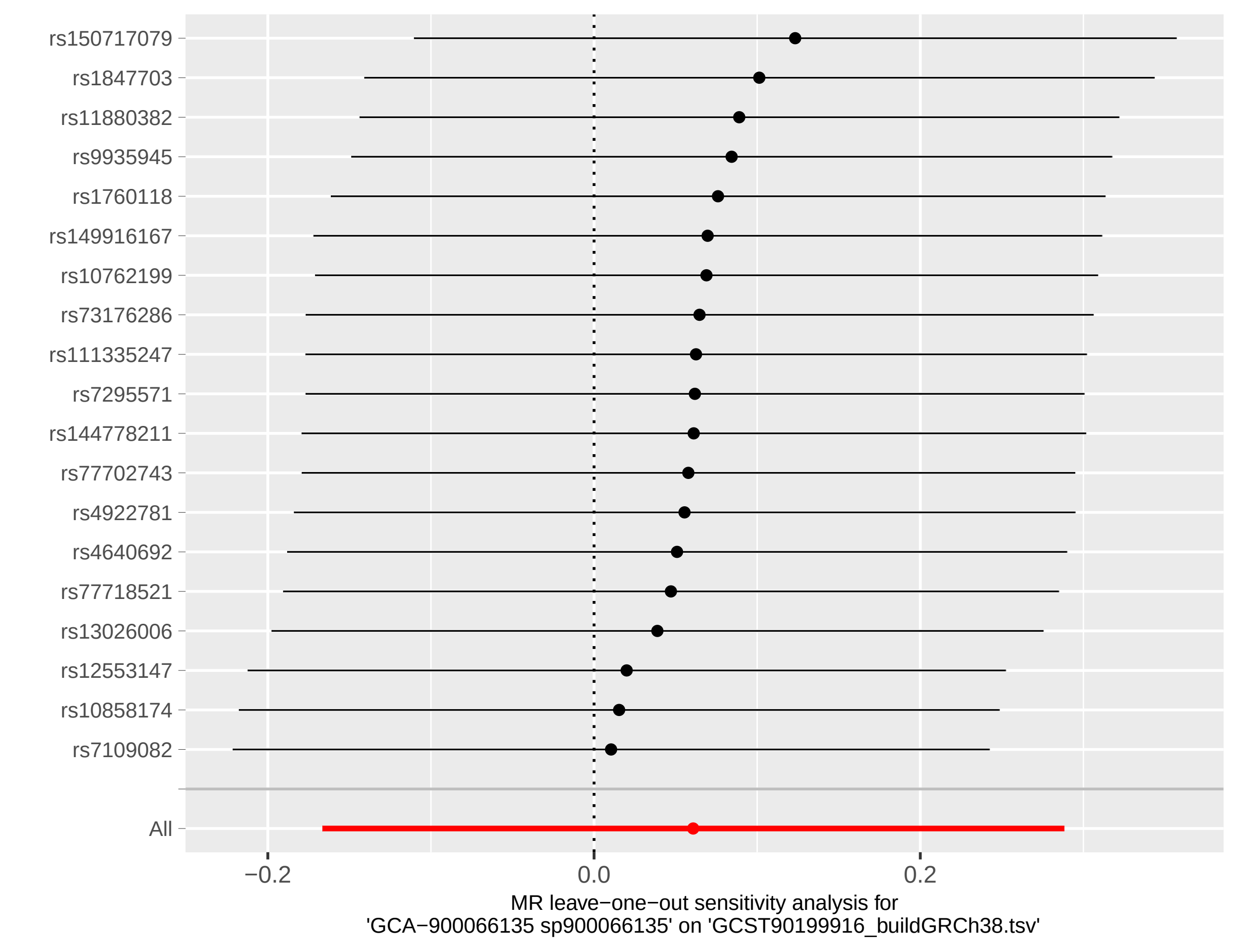

rs150717079
rs1847703
rs11880382
rs9935945
rs1760118
rs149916167
rs10762199
rs73176286
rs111335247
rs7295571
rs144778211
rs77702743
rs4922781
rs4640692
rs77718521
rs13026006
rs12553147
rs10858174
rs7109082
All
−0.2
0.0
0.2
MR leave−one−out sensitivity analysis for
'GCA−900066135 sp900066135' on 'GCST90199916_buildGRCh38.tsv'

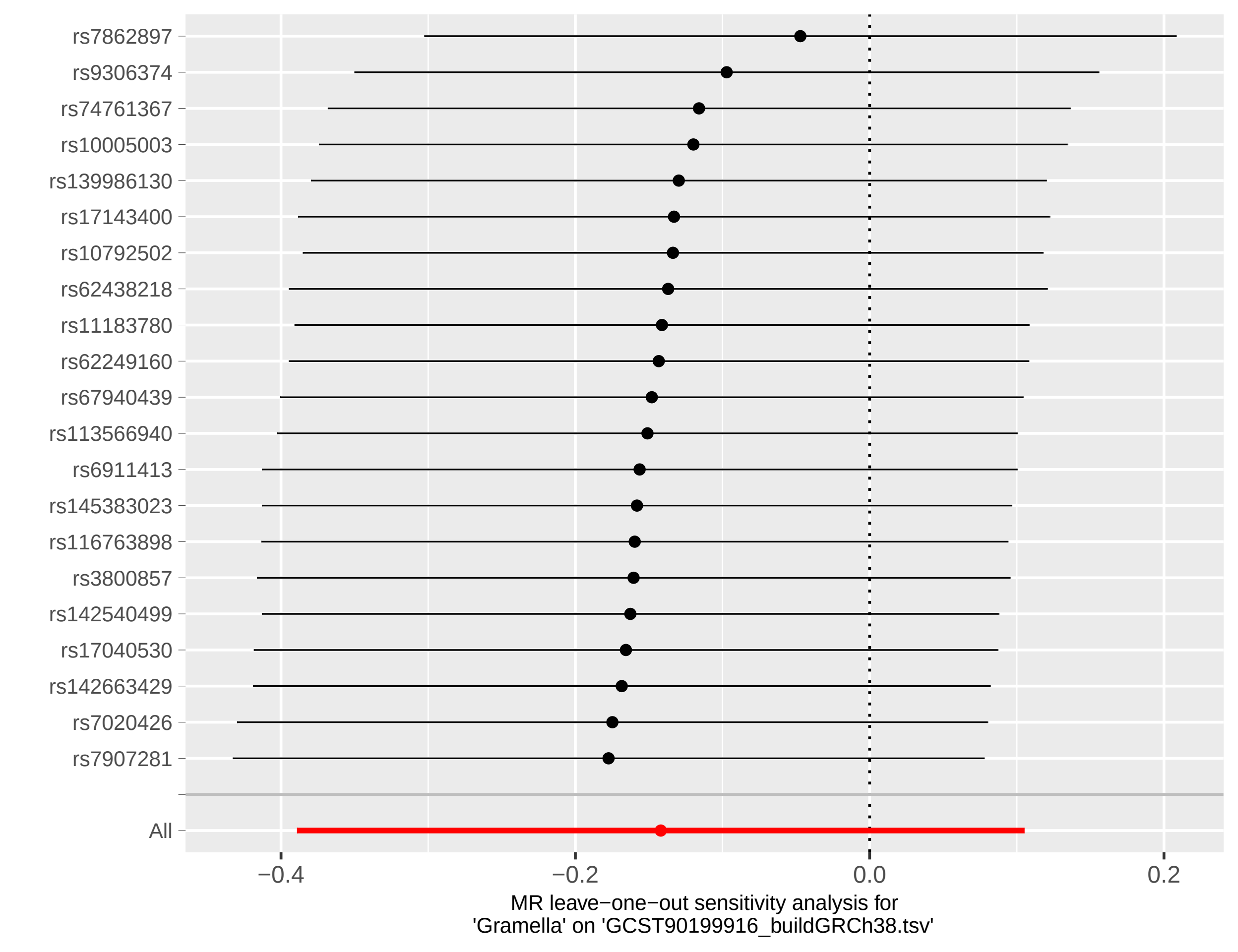
rs7862897
rs9306374
rs74761367
rs10005003
rs139986130
rs17143400
rs10792502
rs62438218
rs11183780
rs62249160
rs67940439
rs113566940
rs6911413
rs145383023
rs116763898
rs3800857
rs142540499
rs17040530
rs142663429
rs7020426
rs7907281
All
−0.4
−0.2
0.0
0.2
MR leave−one−out sensitivity analysis for
'Gramella' on 'GCST90199916_buildGRCh38.tsv'

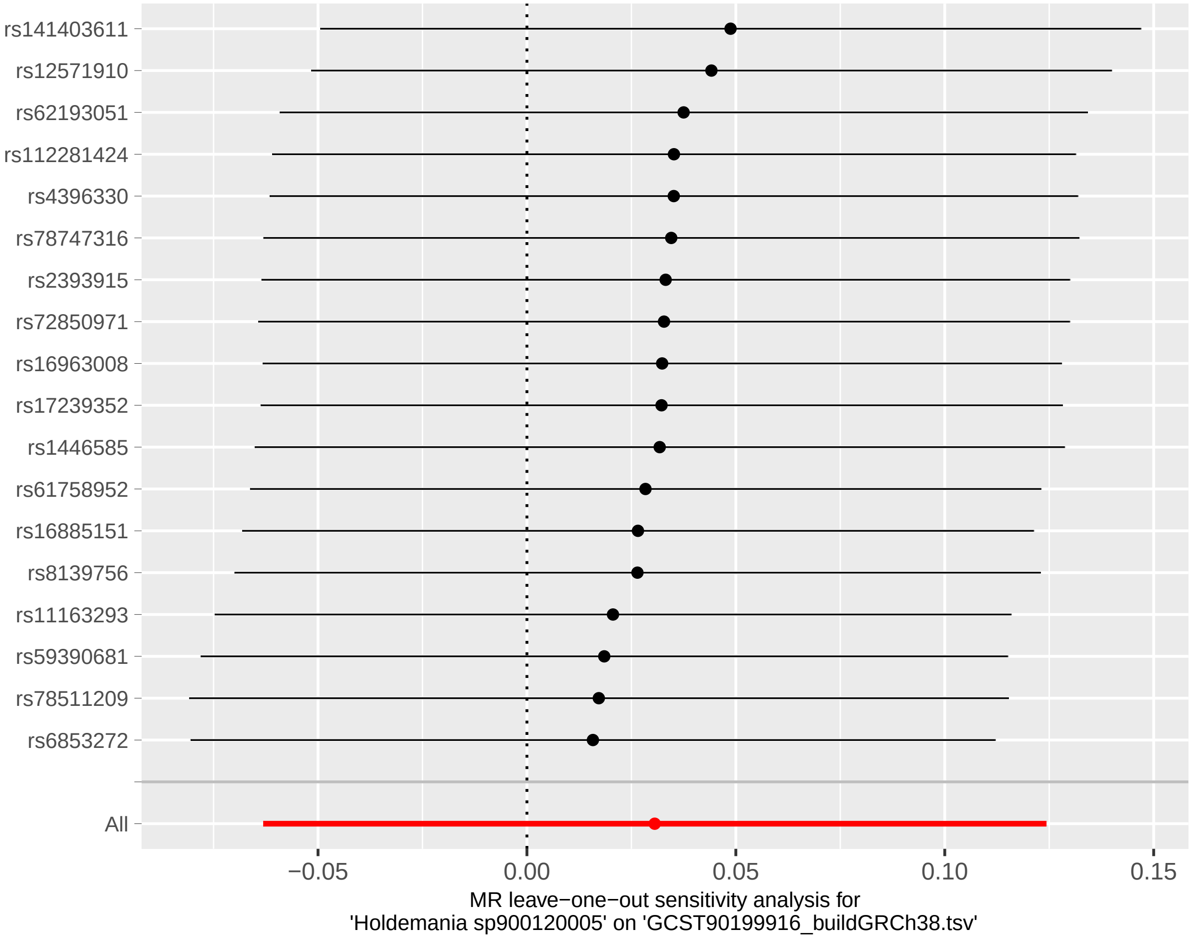
rs141403611
rs12571910
rs62193051
rs112281424
rs4396330
rs78747316
rs2393915
rs72850971
rs16963008
rs17239352
rs1446585
rs61758952
rs16885151
rs8139756
rs11163293
rs59390681
rs78511209
rs6853272
All
−0.05
0.00
0.05
0.10
0.15
MR leave−one−out sensitivity analysis for
'Holdemania sp900120005' on 'GCST90199916_buildGRCh38.tsv'

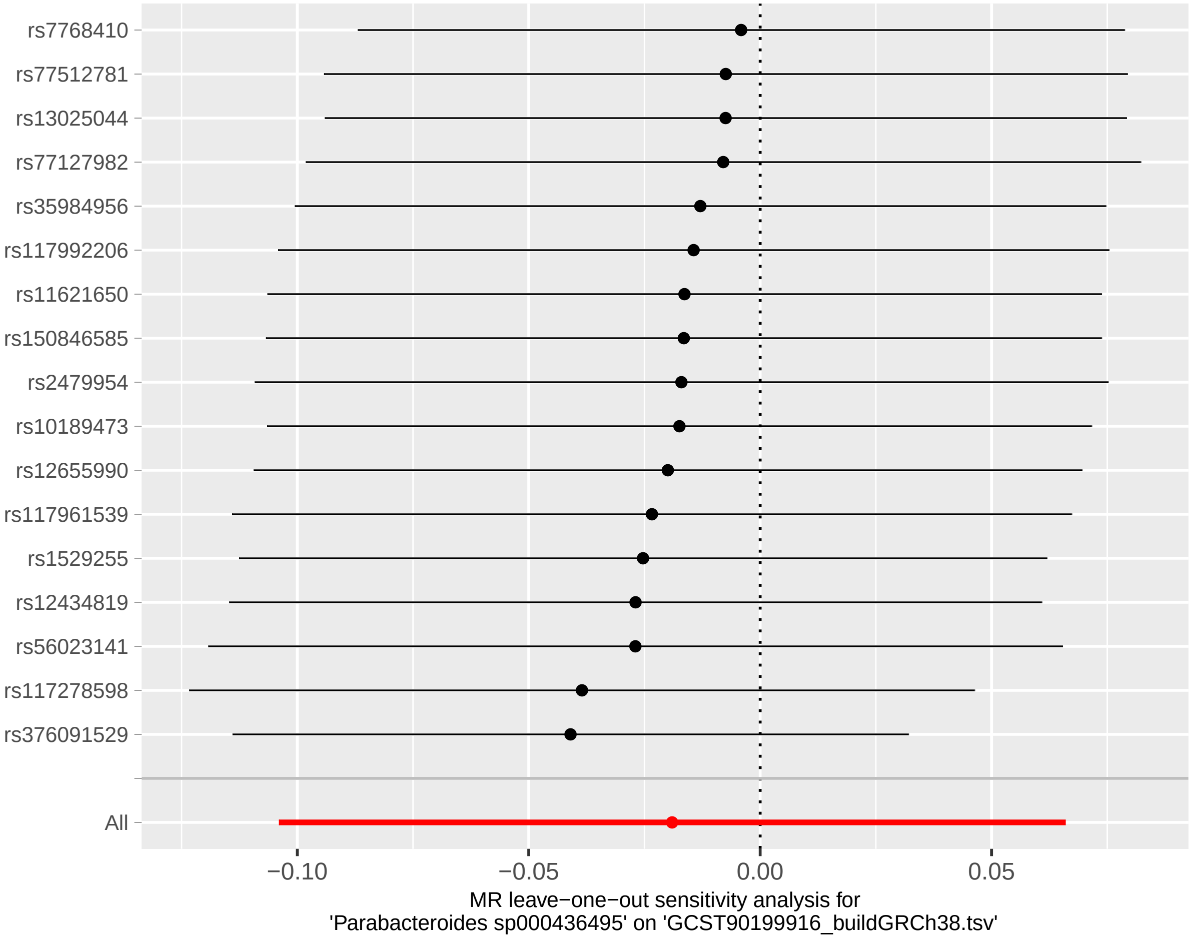

rs7768410
rs77512781
rs13025044
rs77127982
rs35984956
rs117992206
rs11621650
rs150846585
rs2479954
rs10189473
rs12655990
rs117961539
rs1529255
rs12434819
rs56023141
rs117278598
rs376091529
All
−0.10
−0.05
0.00
0.05
MR leave−one−out sensitivity analysis for
'Parabacteroides sp000436495' on 'GCST90199916_buildGRCh38.tsv'

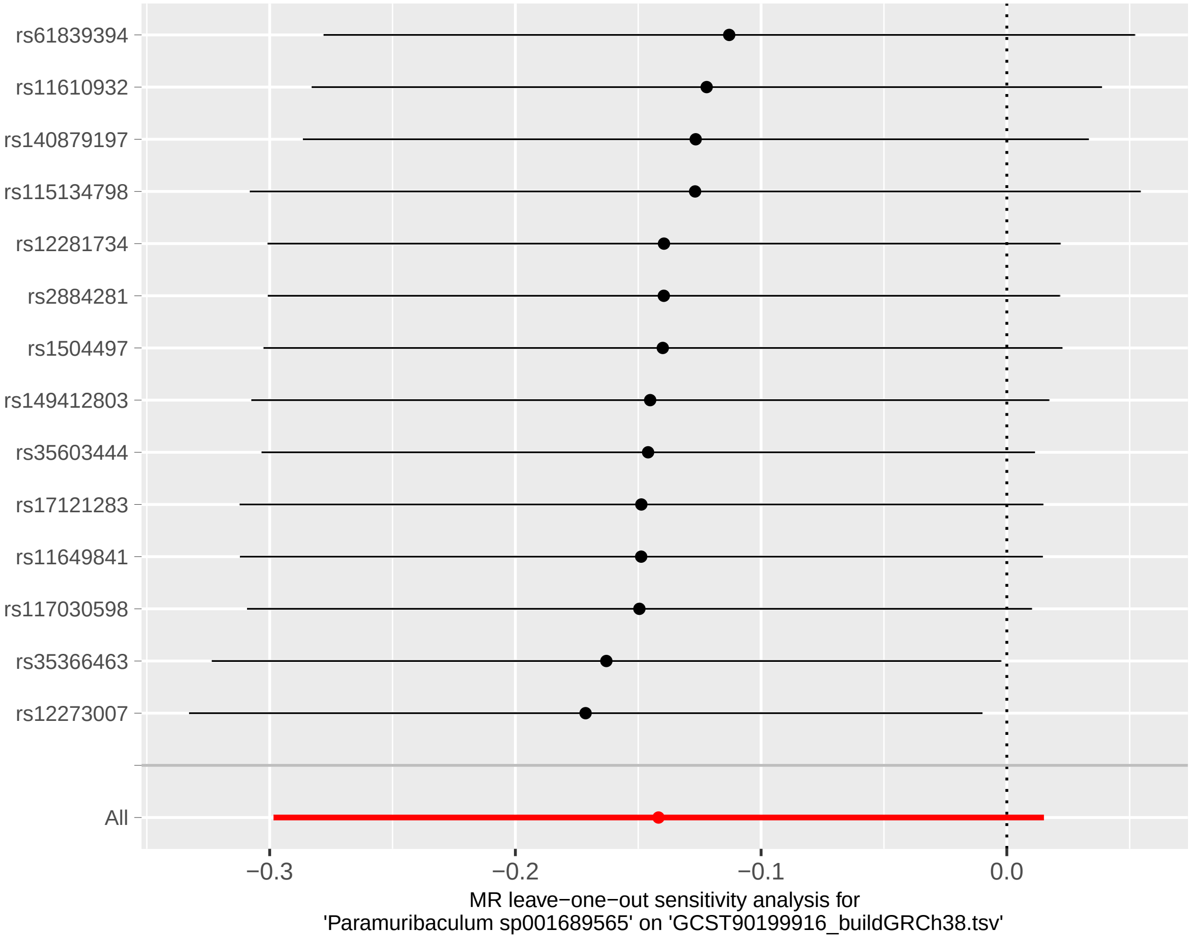
rs61839394
rs11610932
rs140879197
rs115134798
rs12281734
rs2884281
rs1504497
rs149412803
rs35603444
rs17121283
rs11649841
rs117030598
rs35366463
rs12273007
All
−0.3
−0.2
−0.1
0.0
MR leave−one−out sensitivity analysis for
'Paramuribaculum sp001689565' on 'GCST90199916_buildGRCh38.tsv'

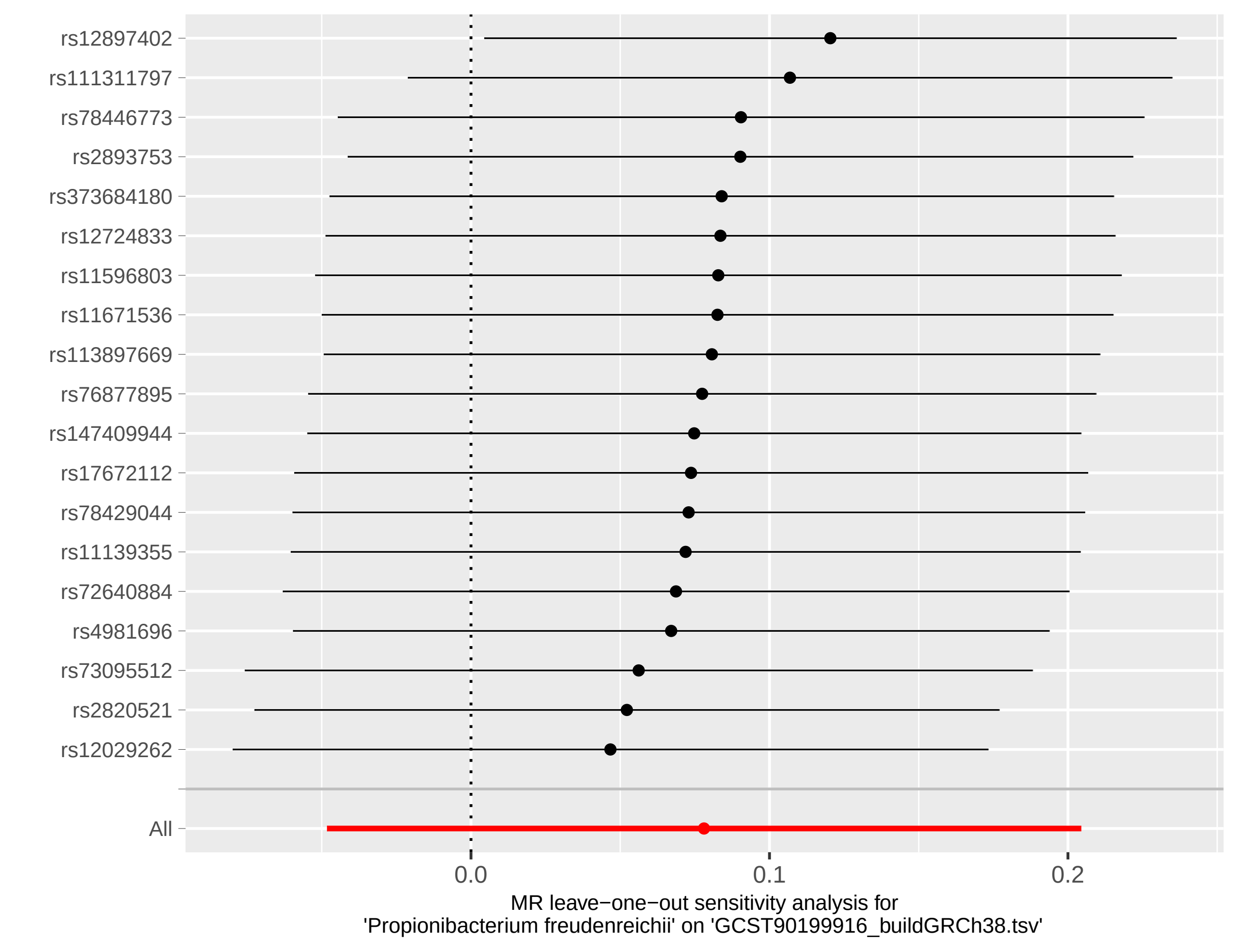
rs12897402
rs111311797
rs78446773
rs2893753
rs373684180
rs12724833
rs11596803
rs11671536
rs113897669
rs76877895
rs147409944
rs17672112
rs78429044
rs11139355
rs72640884
rs4981696
rs73095512
rs2820521
rs12029262
All
0.0
0.1
0.2
MR leave−one−out sensitivity analysis for
'Propionibacterium freudenreichii' on 'GCST90199916_buildGRCh38.tsv'

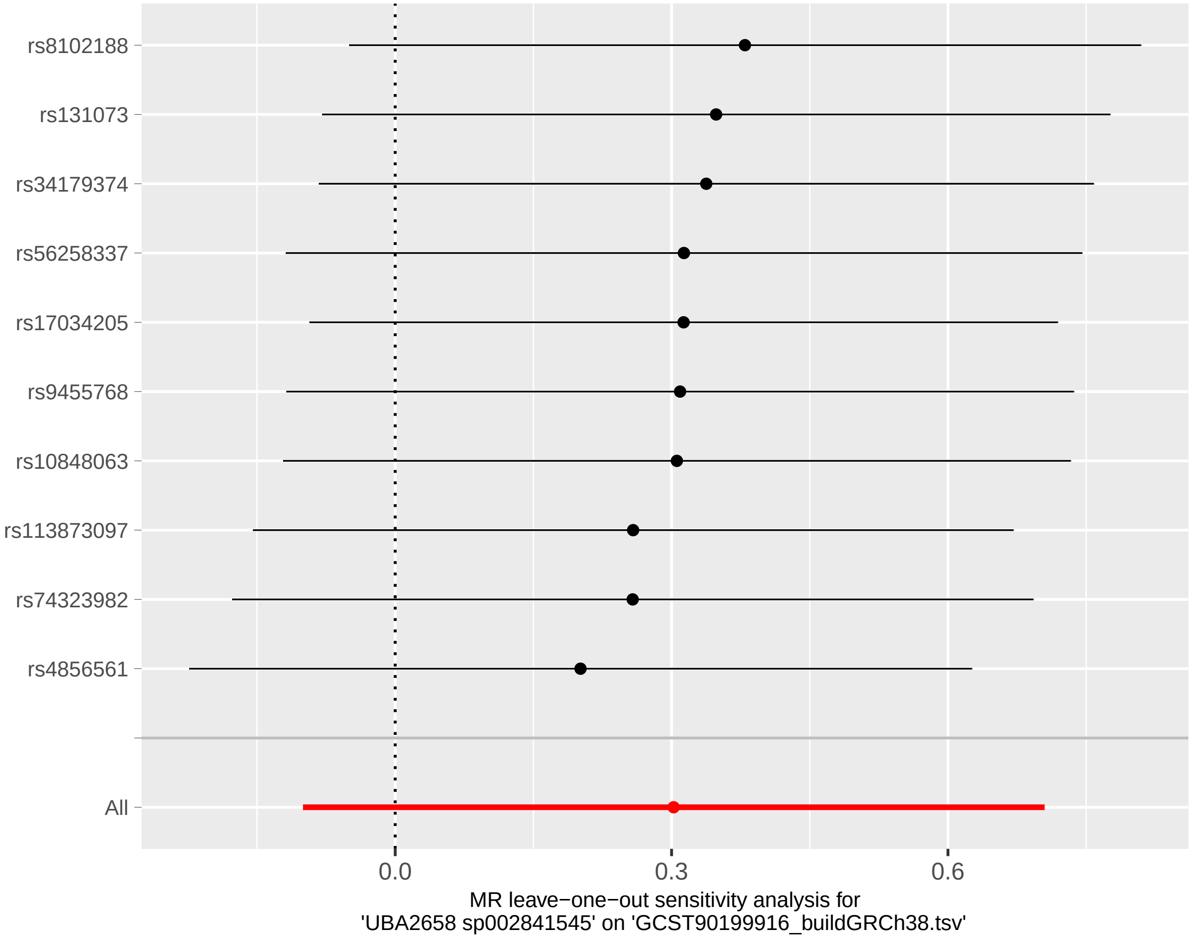
rs8102188
rs131073
rs34179374
rs56258337
rs17034205
rs9455768
rs10848063
rs113873097
rs74323982
rs4856561
All
0.0
0.3
0.6
MR leave−one−out sensitivity analysis for
'UBA2658 sp002841545' on 'GCST90199916_buildGRCh38.tsv'

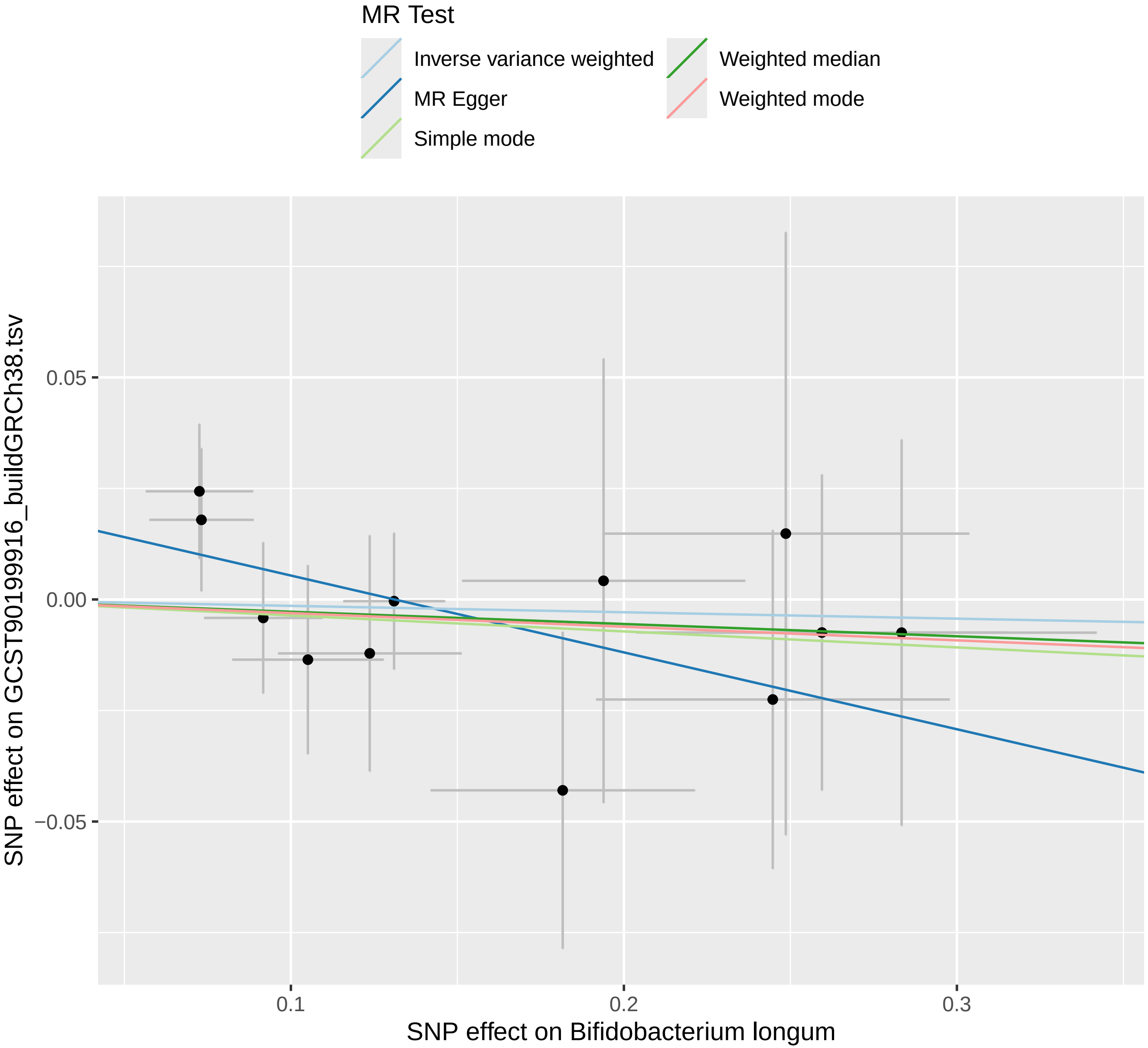
MR Test
Inverse variance weighted
MR Egger
Simple mode
Weighted median
Weighted mode
0.05
0.00
−0.05
0.1
0.2
0.3
SNP effect on GCST90199916_buildGRCh38.tsv
SNP effect on Bifidobacterium longum

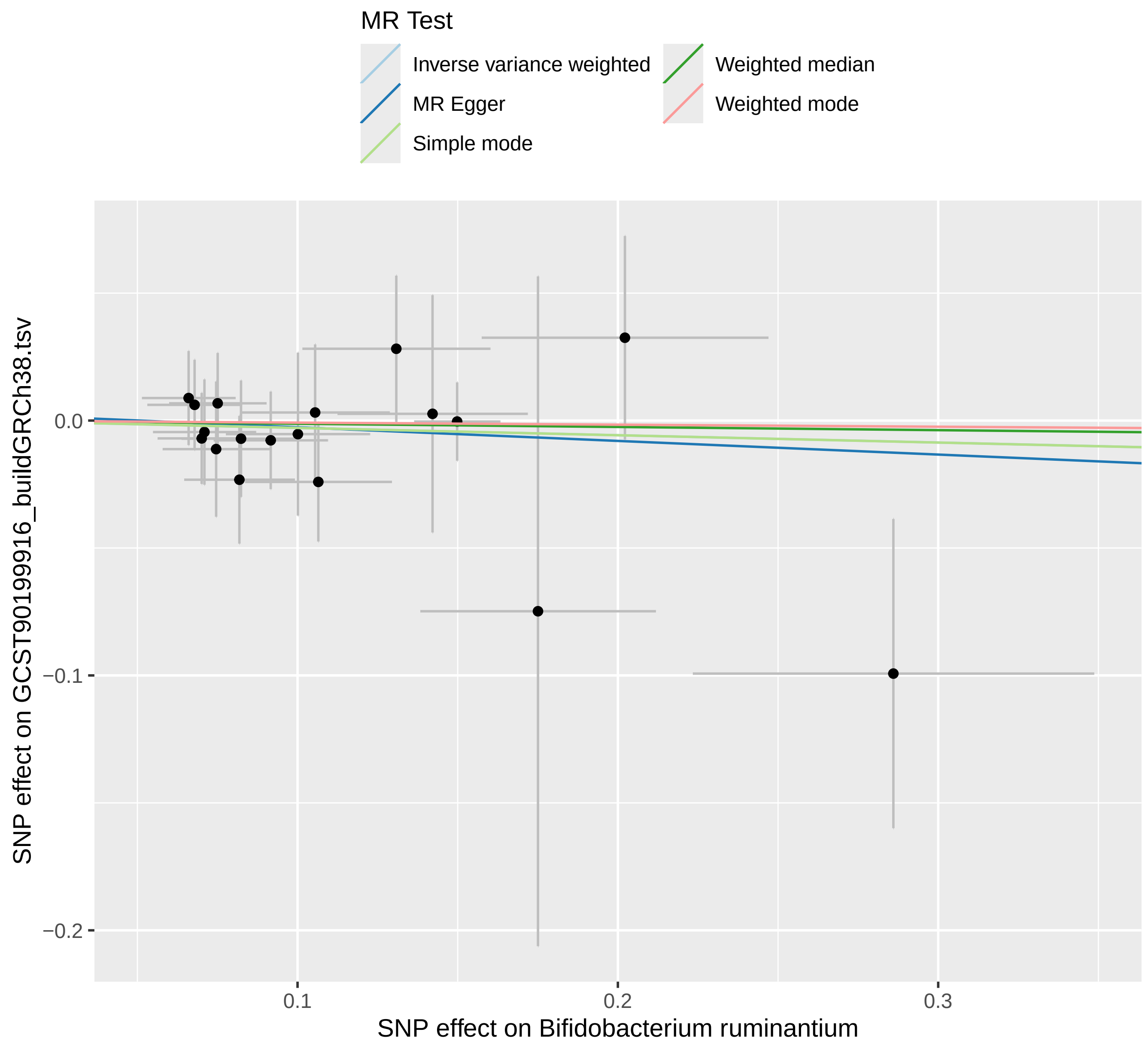

MR Test
Inverse variance weighted
MR Egger
Simple mode
Weighted median
Weighted mode
SNP effect on GCST90199916_buildGRCh38.tsv
0.0
−0.1
−0.2
0.1
0.2
0.3
SNP effect on Bifidobacterium ruminantium

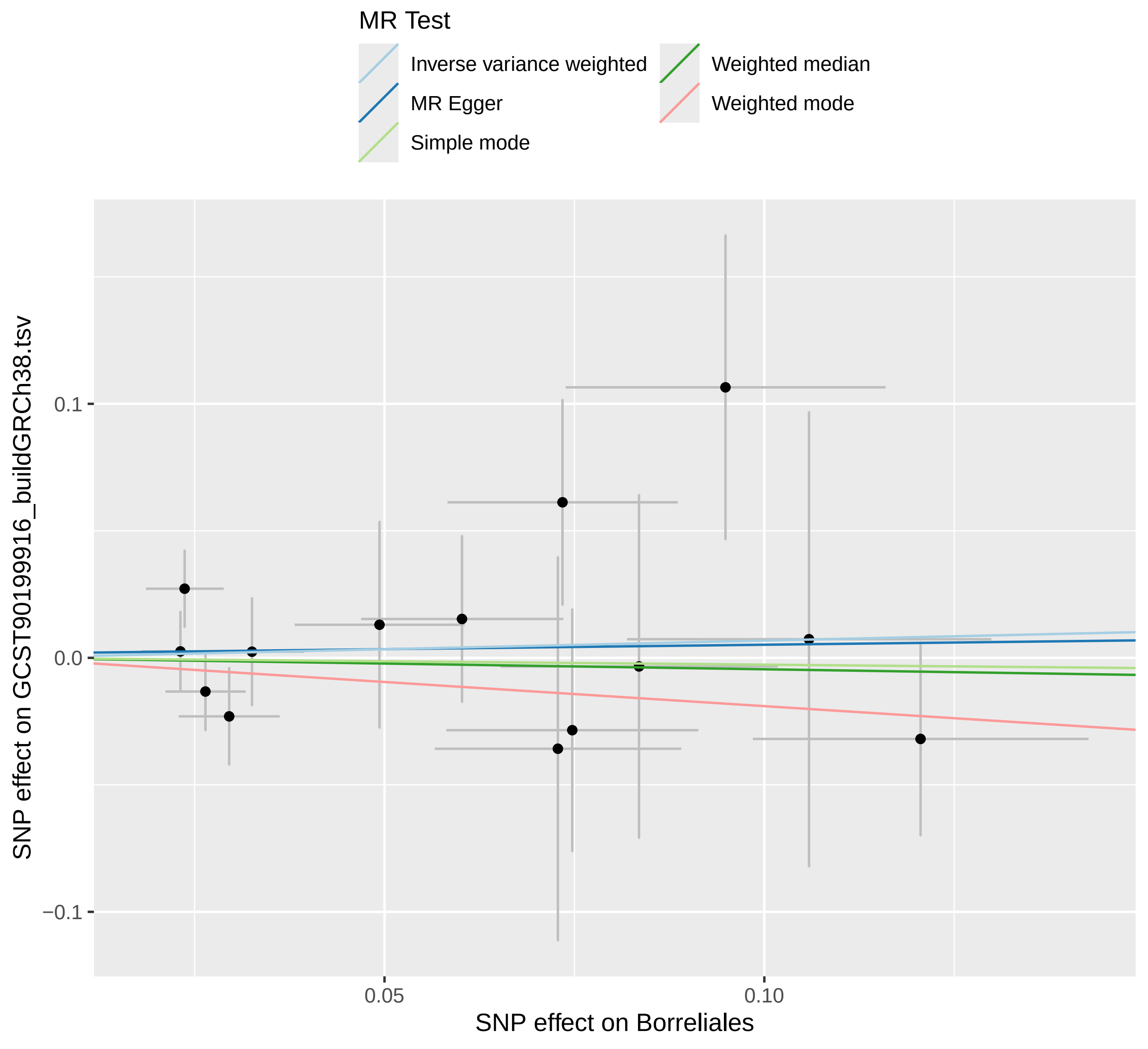
MR Test
Inverse variance weighted
MR Egger
Simple mode
Weighted median
Weighted mode
SNP effect on GCST90199916_buildGRCh38.tsv
0.1
0.0
−0.1
0.05
0.10
SNP effect on Borreliales

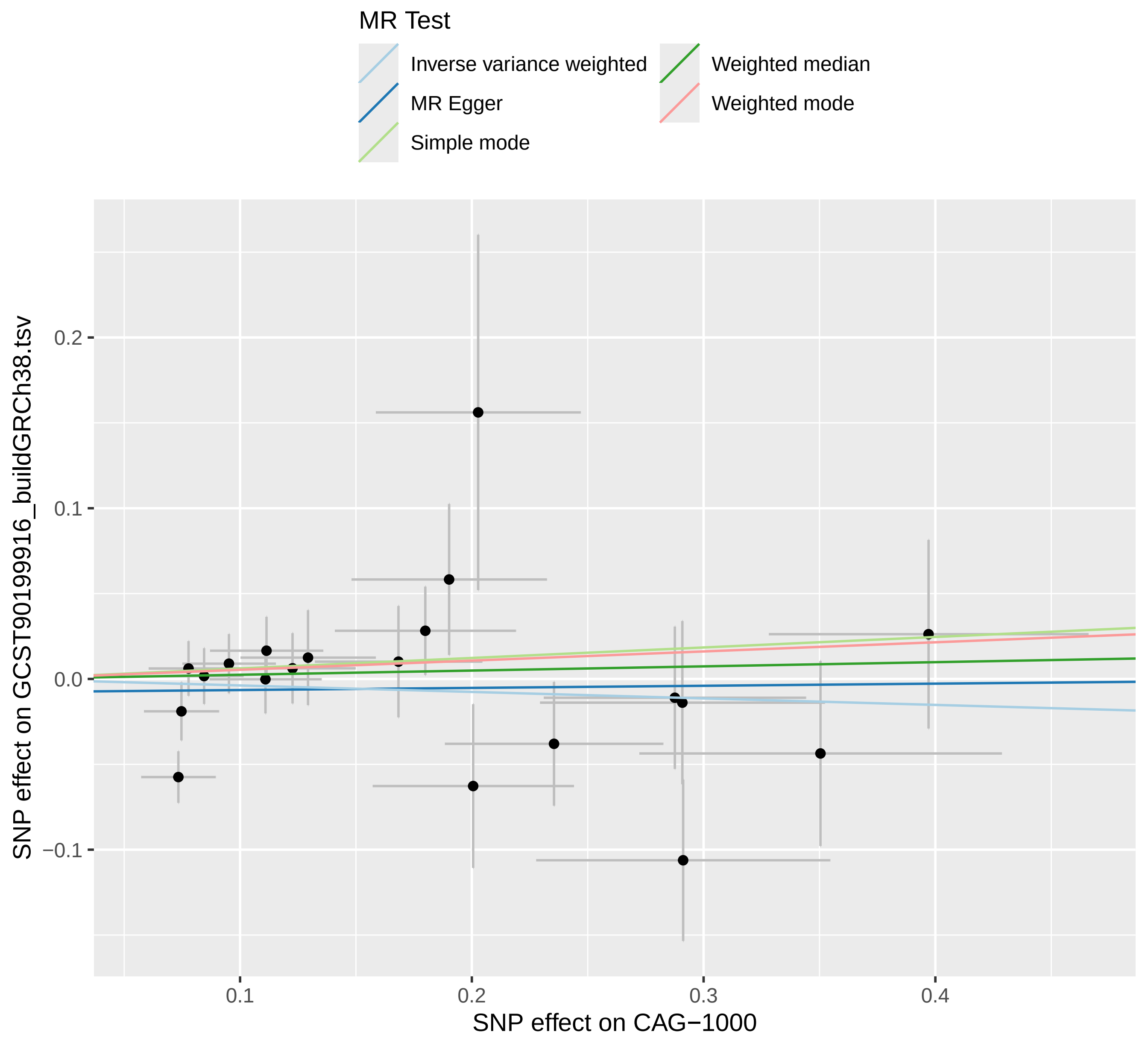

MR Test
Inverse variance weighted
MR Egger
Simple mode
Weighted median
Weighted mode
SNP effect on GCST90199916_buildGRCh38.tsv
0.2
0.1
0.0
−0.1
0.1
0.2
0.3
0.4
SNP effect on CAG−1000

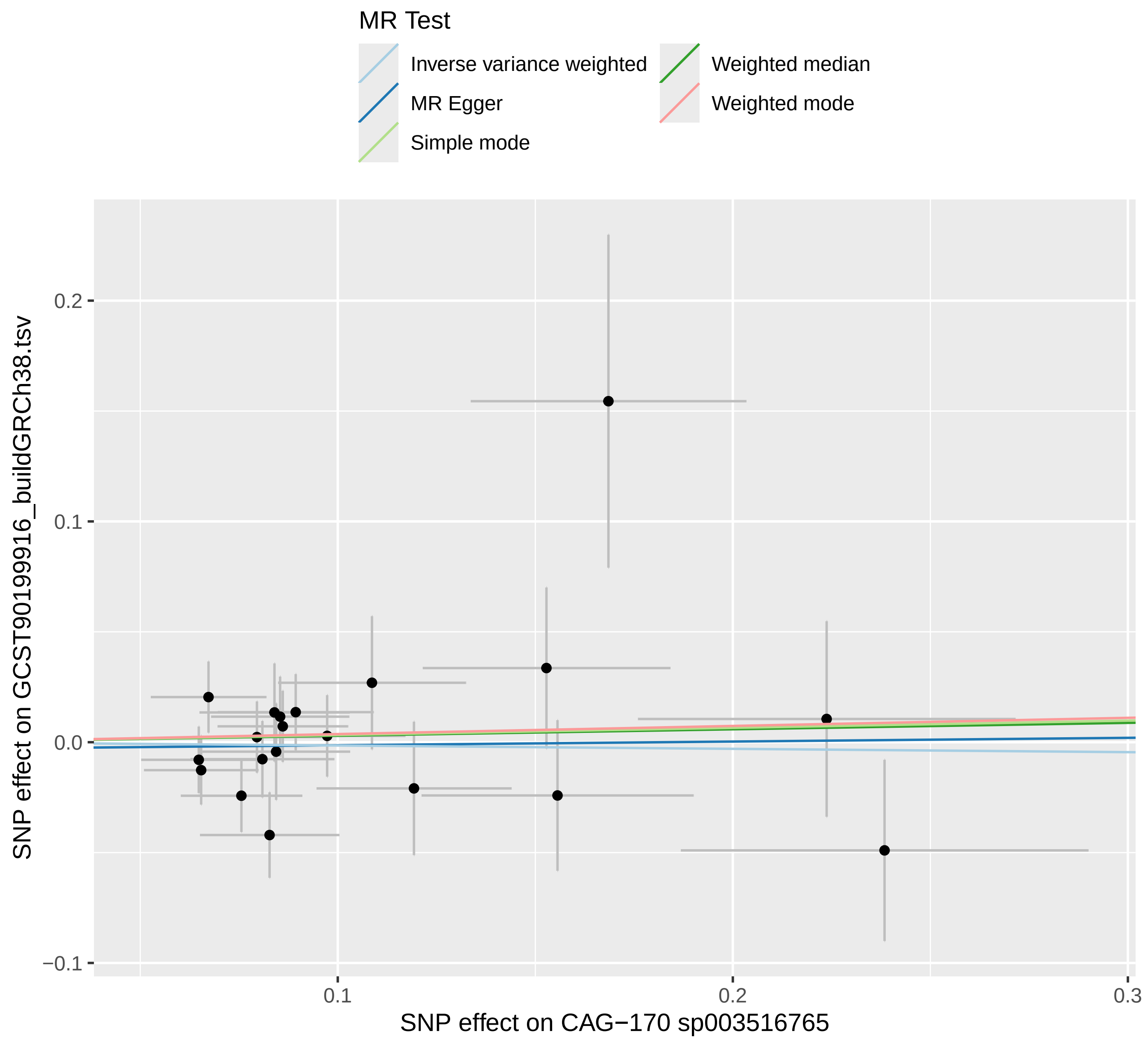
MR Test
Inverse variance weighted
MR Egger
Simple mode
Weighted median
Weighted mode
SNP effect on GCST90199916_buildGRCh38.tsv
0.2
0.1
0.0
−0.1
0.1
0.2
0.3
SNP effect on CAG−170 sp003516765

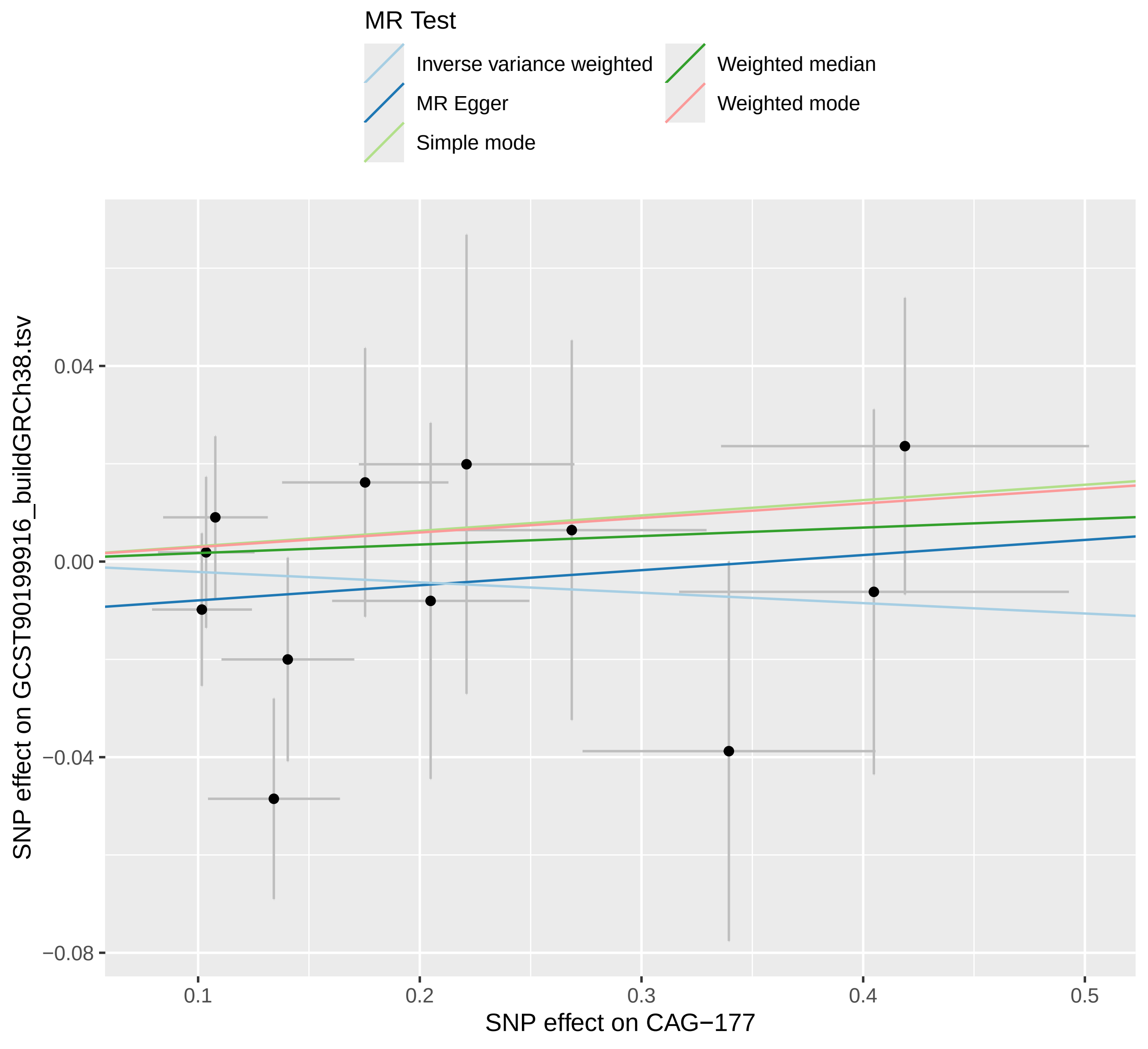

MR Test
Inverse variance weighted
MR Egger
Simple mode
Weighted median
Weighted mode
0.04
0.00
−0.04
−0.08
0.1
0.2
0.3
0.4
0.5
SNP effect on GCST90199916_buildGRCh38.tsv
SNP effect on CAG−177

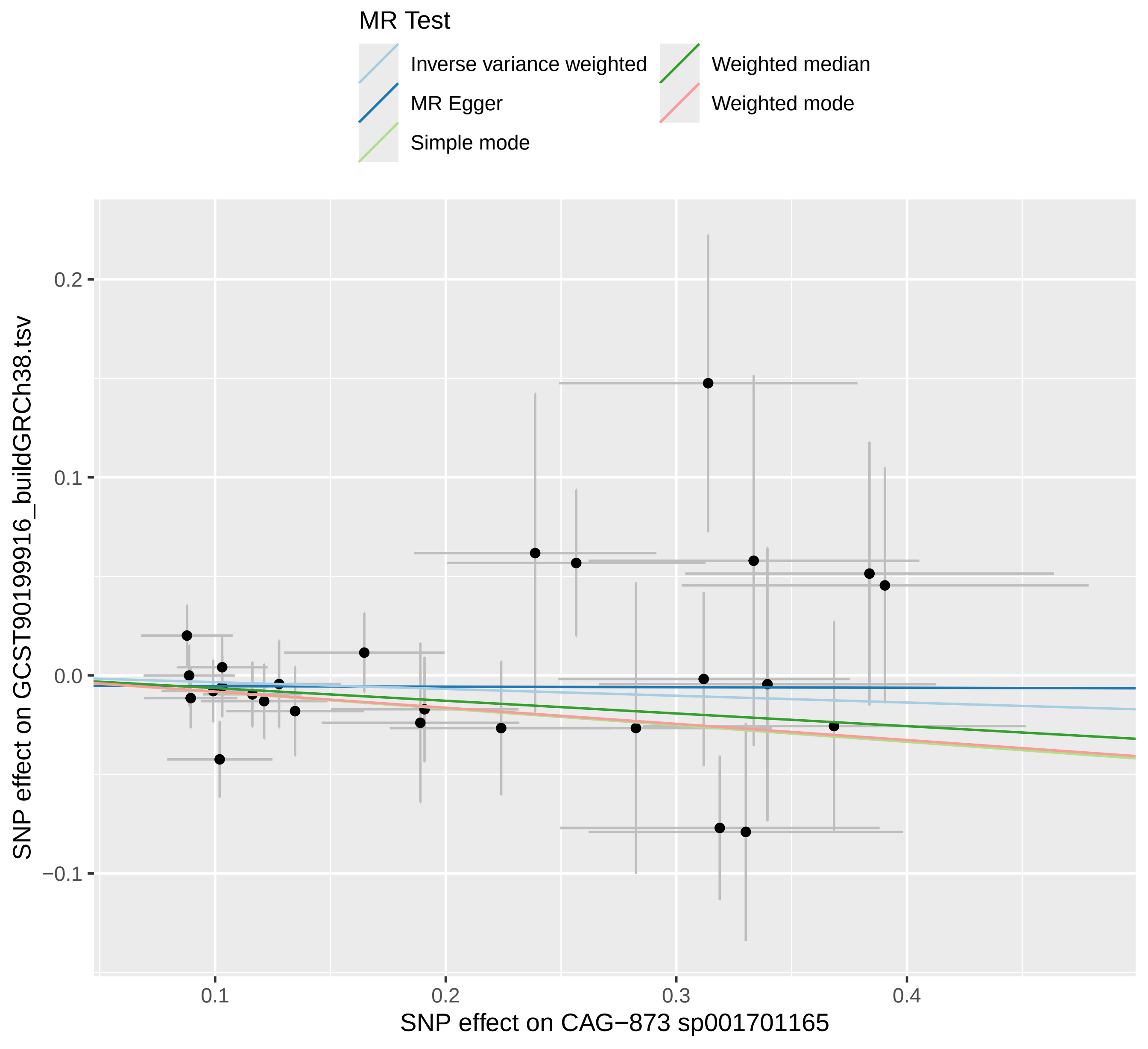
MR Test
Inverse variance weighted
MR Egger
Simple mode
Weighted median
Weighted mode
SNP effect on GCST90199916_buildGRCh38.tsv
0.2
0.1
0.0
−0.1
0.1
0.2
0.3
0.4
SNP effect on CAG−873 sp001701165

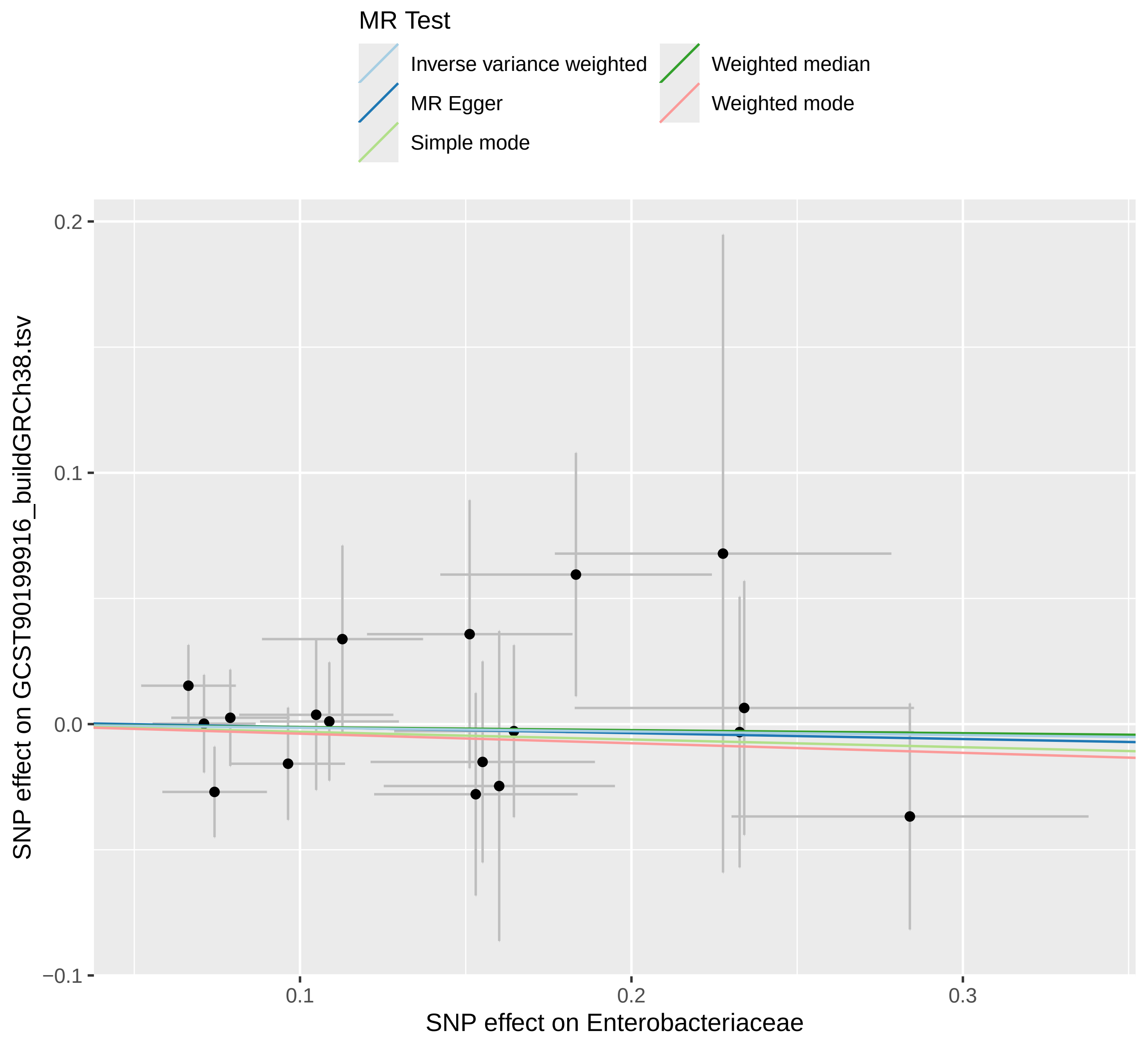
MR Test
Inverse variance weighted
MR Egger
Simple mode
Weighted median
Weighted mode
0.2
0.1
0.0
−0.1
0.1
0.2
0.3
SNP effect on GCST90199916_buildGRCh38.tsv
SNP effect on Enterobacteriaceae

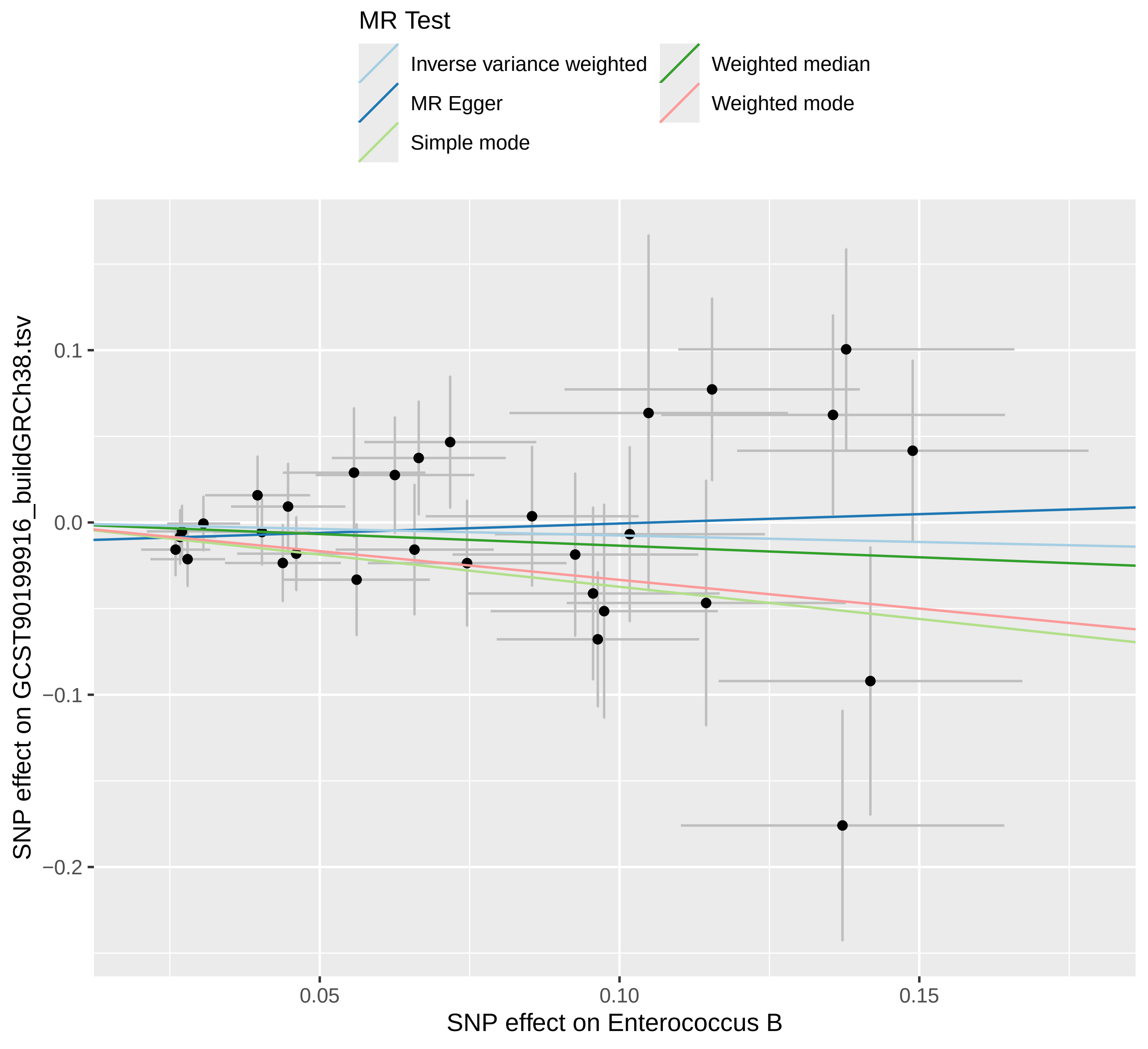
MR Test
Inverse variance weighted
MR Egger
Simple mode
Weighted median
Weighted mode
SNP effect on GCST90199916_buildGRCh38.tsv
0.1
0.0
−0.1
−0.2
0.05
0.10
0.15
SNP effect on Enterococcus B

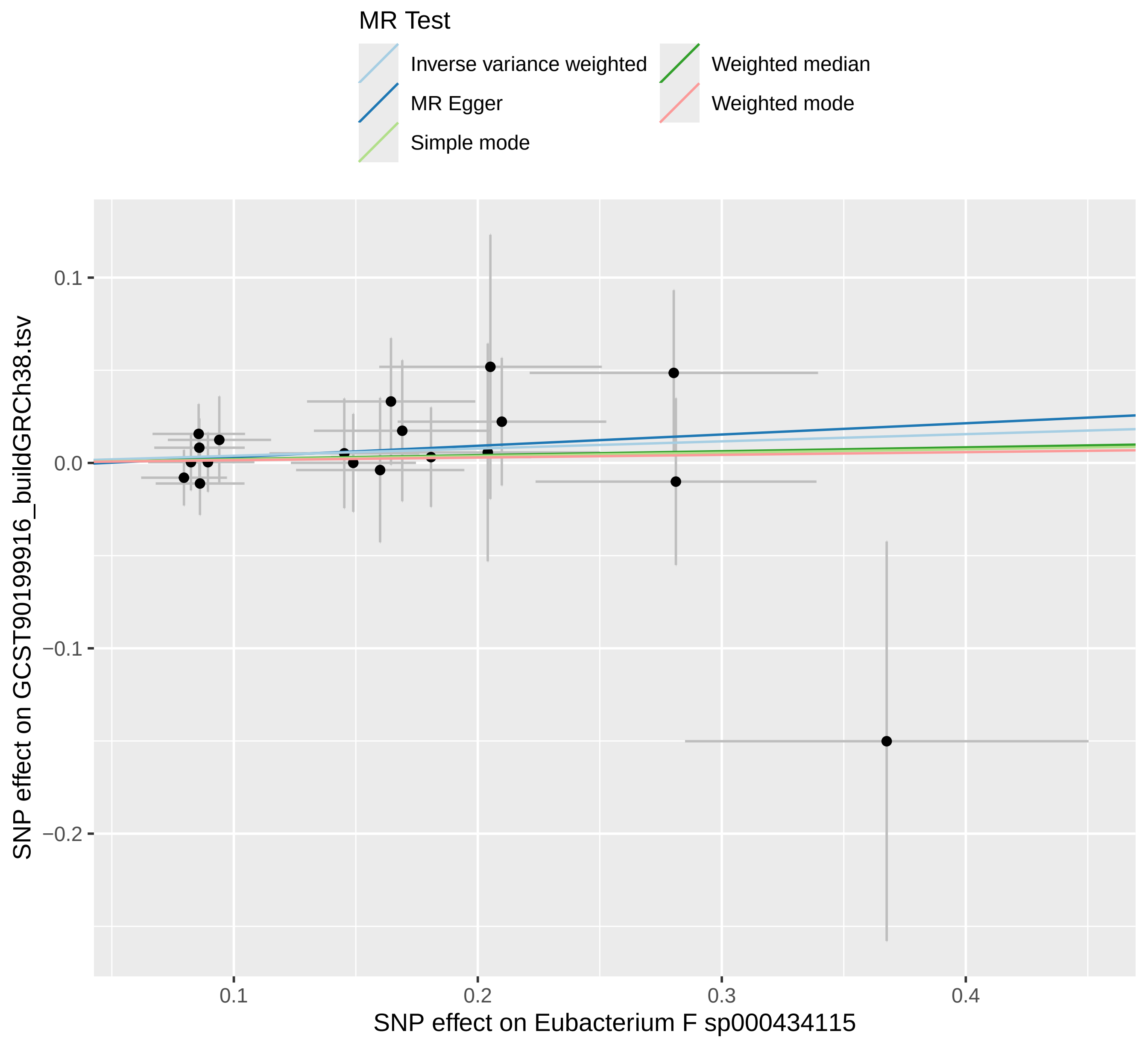
MR Test
Inverse variance weighted
MR Egger
Simple mode
Weighted median
Weighted mode
0.1
0.0
−0.1
−0.2
0.1
0.2
0.3
0.4
SNP effect on GCST90199916_buildGRCh38.tsv
SNP effect on Eubacterium F sp000434115

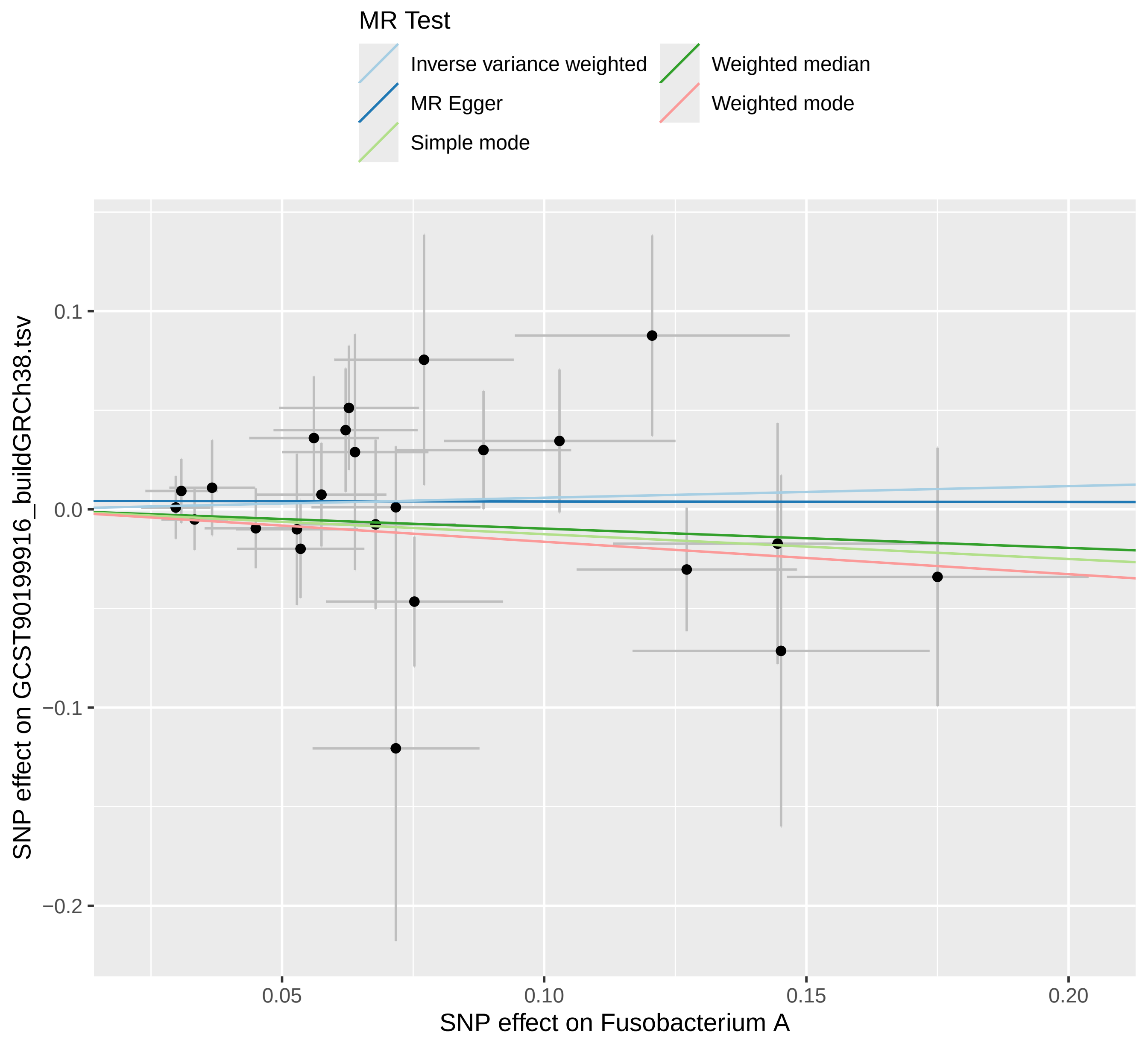
MR Test
Inverse variance weighted
MR Egger
Simple mode
Weighted median
Weighted mode
0.1
0.0
−0.1
−0.2
0.05
0.10
0.15
0.20
SNP effect on GCST90199916_buildGRCh38.tsv
SNP effect on Fusobacterium A

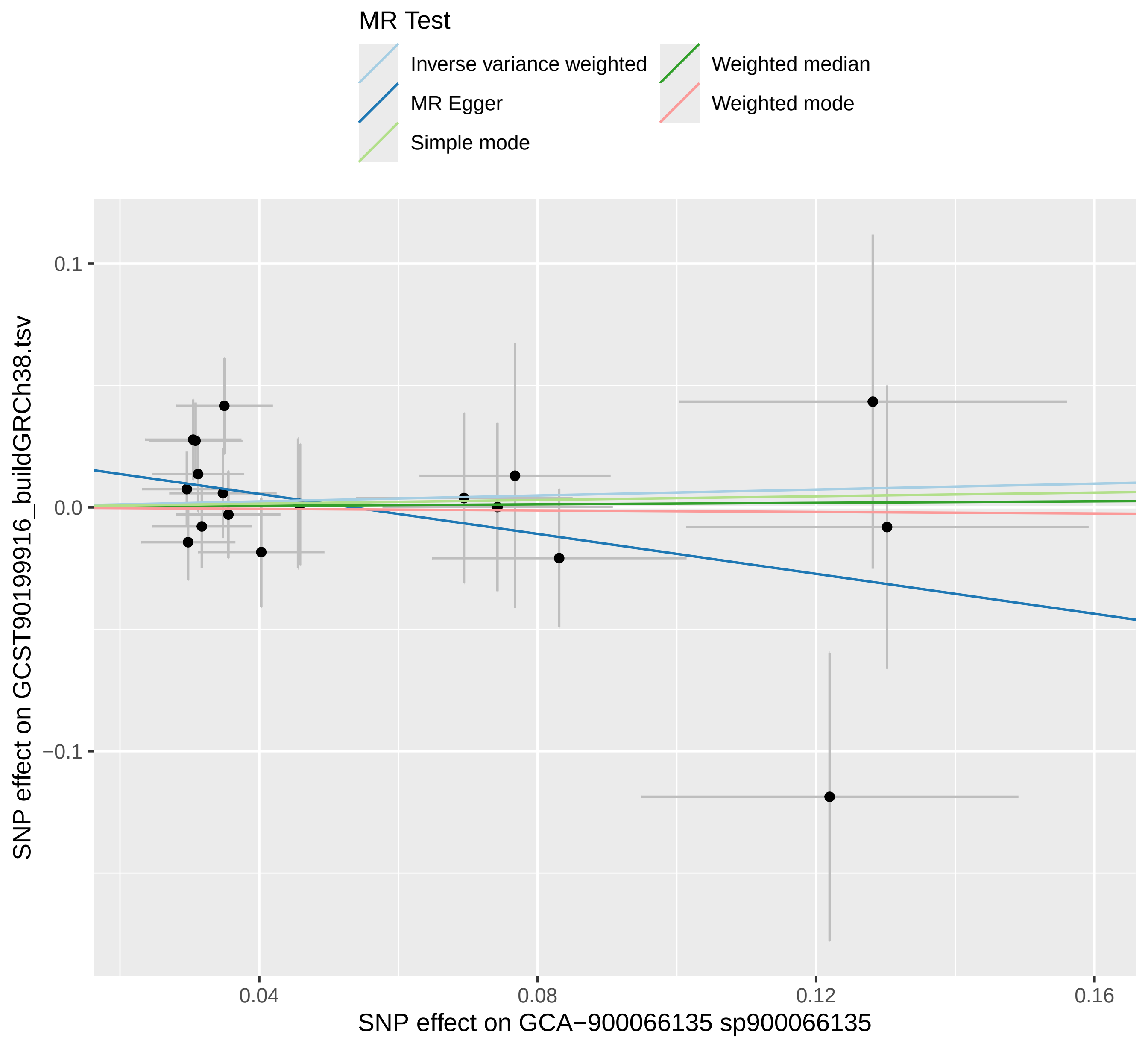

MR Test
Inverse variance weighted
MR Egger
Simple mode
Weighted median
Weighted mode
0.1
0.0
−0.1
0.04
0.08
0.12
0.16
SNP effect on GCST90199916_buildGRCh38.tsv
SNP effect on GCA−900066135 sp900066135

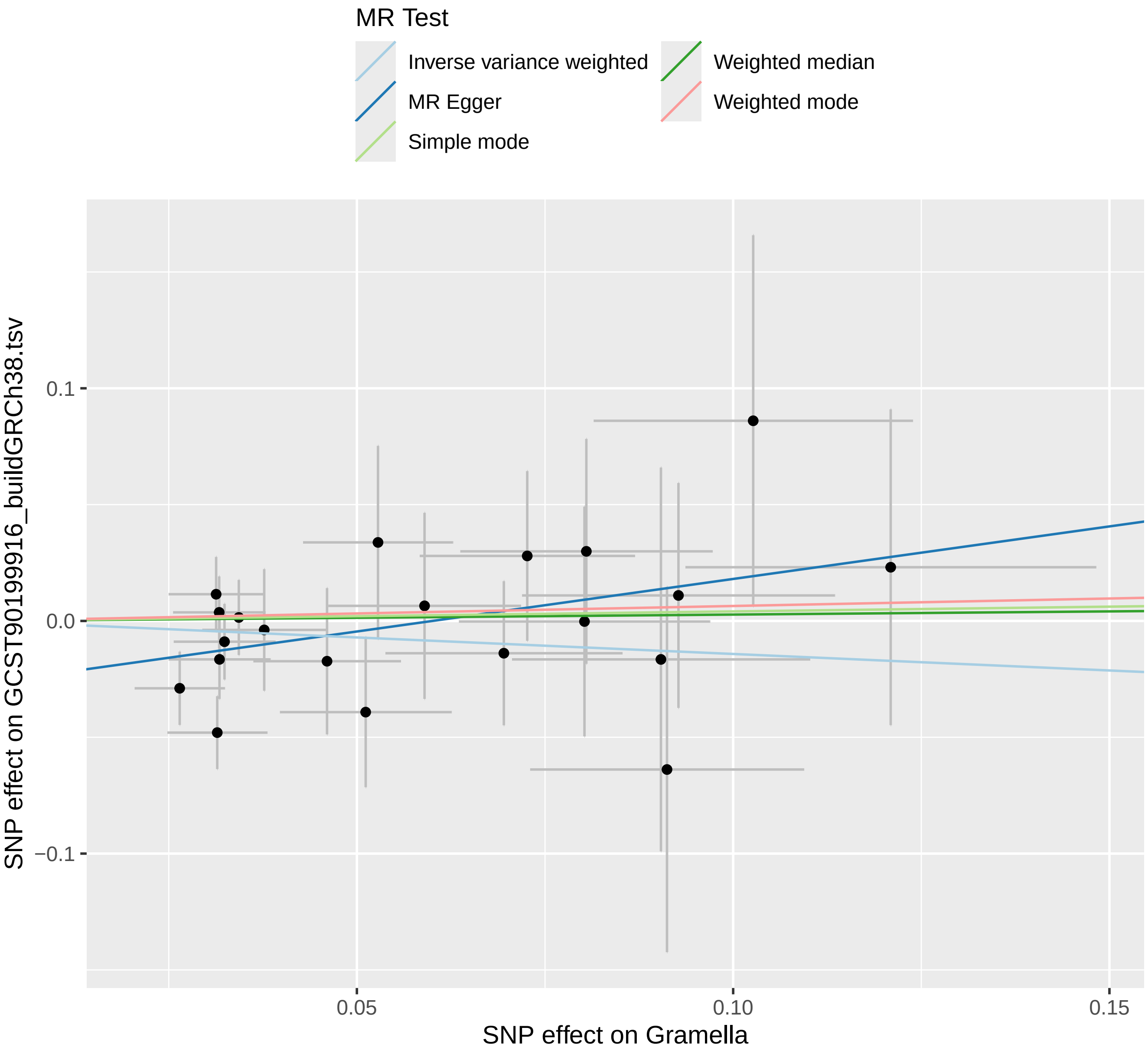
MR Test
Inverse variance weighted
MR Egger
Simple mode
Weighted median
Weighted mode
0.1
0.0
−0.1
0.05
0.10
0.15
SNP effect on GCST90199916_buildGRCh38.tsv
SNP effect on Gramella

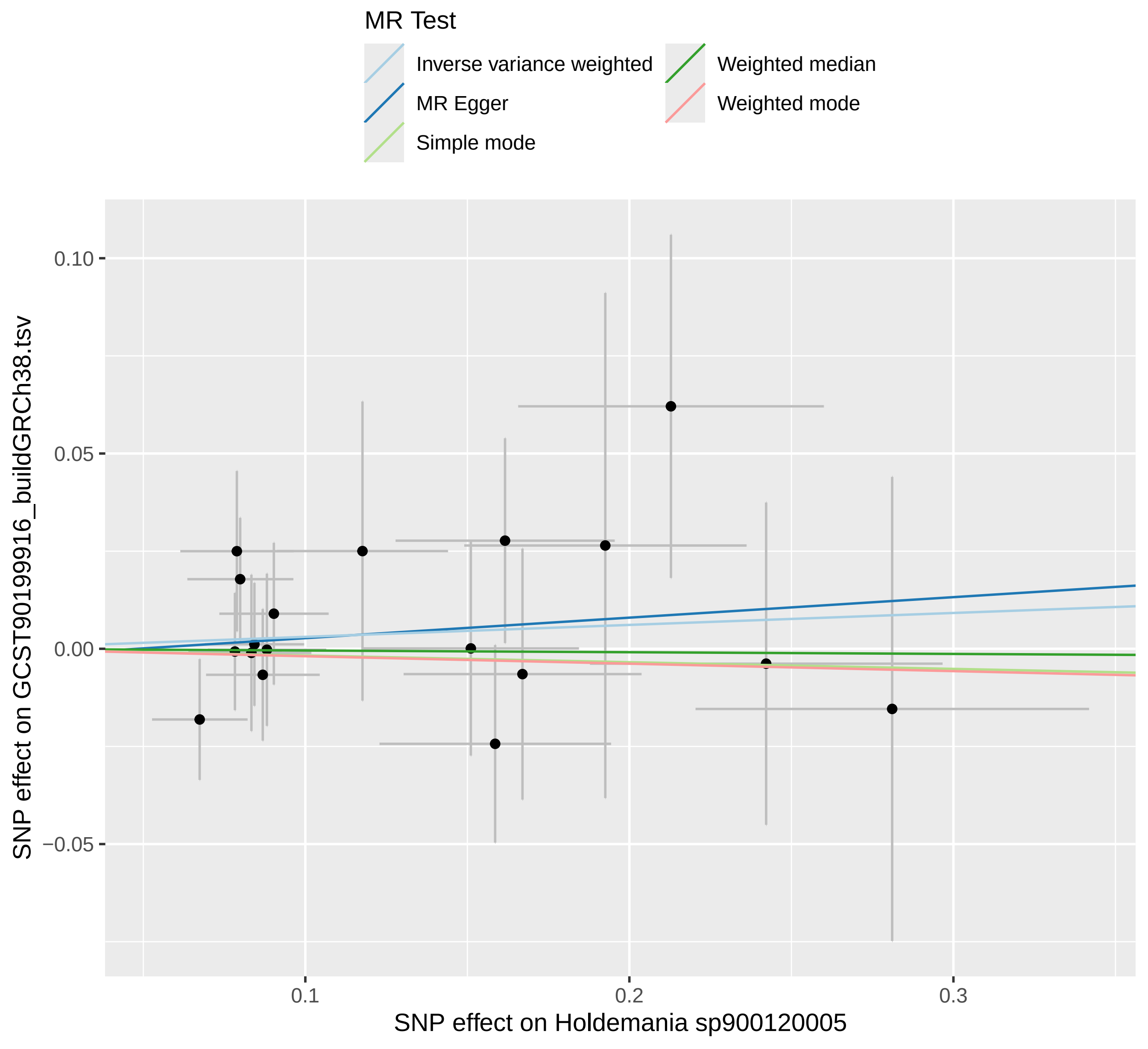

MR Test
Inverse variance weighted
MR Egger
Simple mode
Weighted median
Weighted mode
0.10
0.05
0.00
−0.05
0.1
0.2
0.3
SNP effect on GCST90199916_buildGRCh38.tsv
SNP effect on Holdemania sp900120005

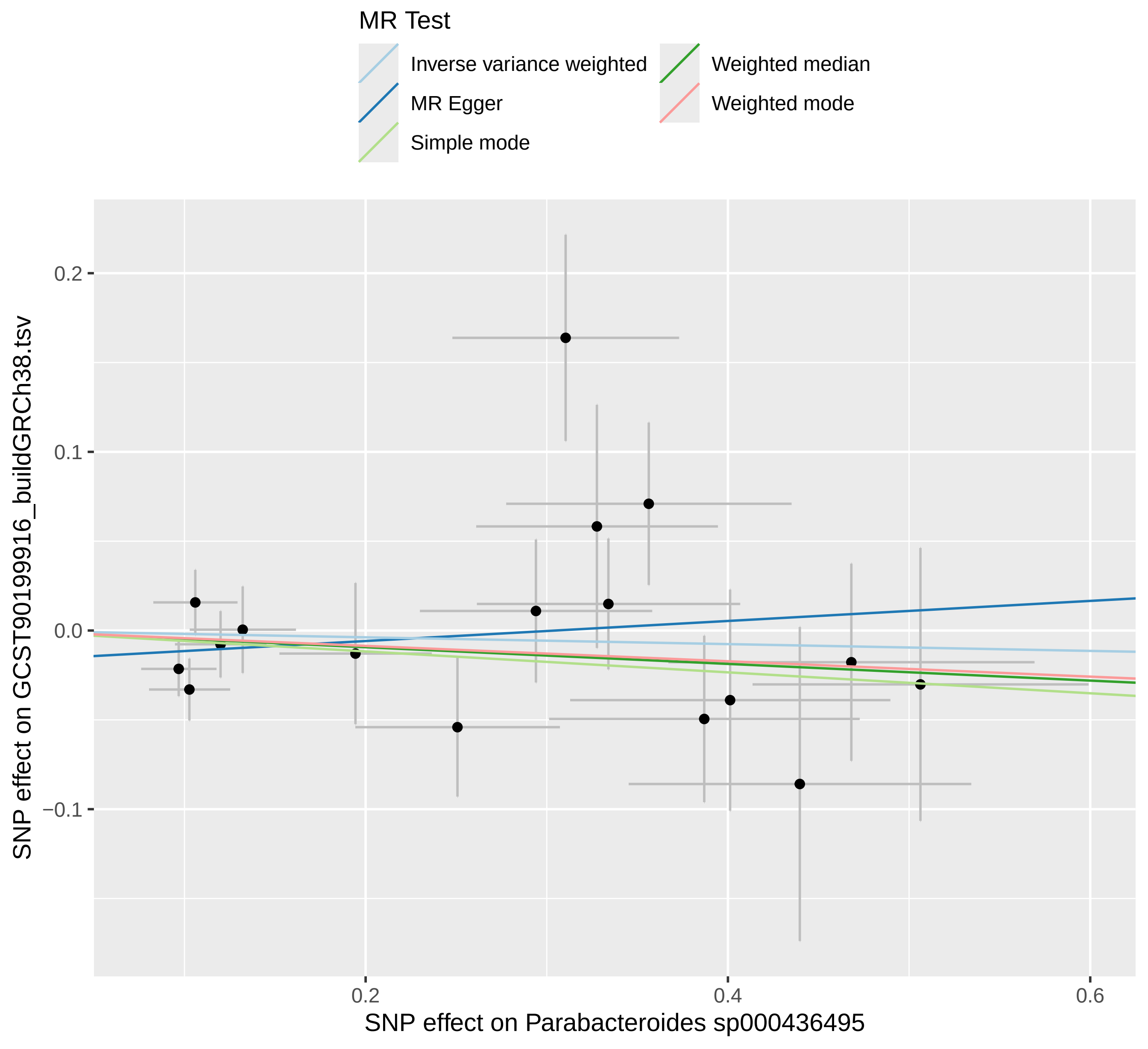

MR Test
Inverse variance weighted
MR Egger
Simple mode
Weighted median
Weighted mode
0.2
0.1
0.0
−0.1
0.2
0.4
0.6
SNP effect on GCST90199916_buildGRCh38.tsv
SNP effect on Parabacteroides sp000436495

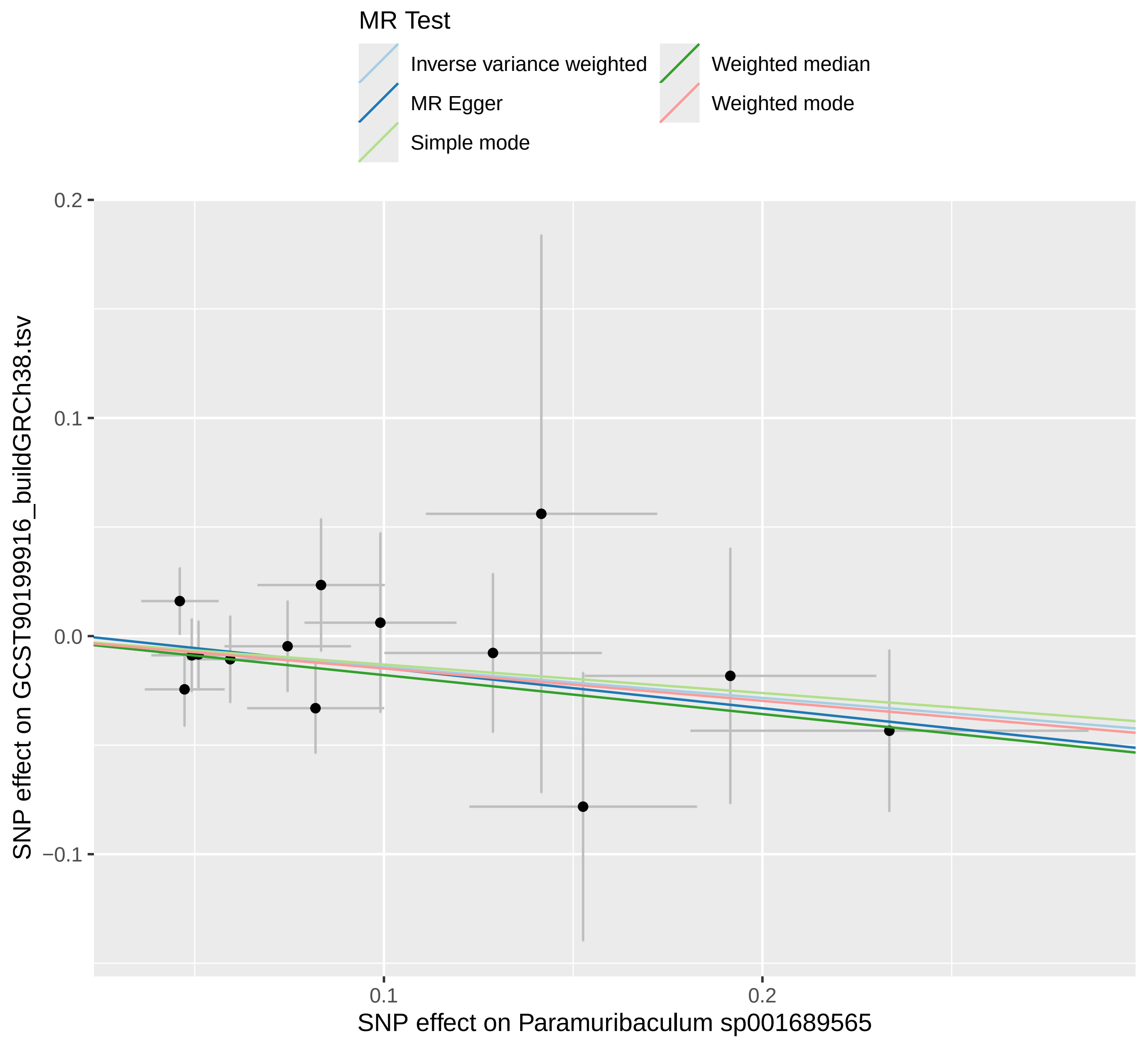

MR Test
Inverse variance weighted
MR Egger
Simple mode
Weighted median
Weighted mode
0.2
0.1
0.0
−0.1
0.1
0.2
SNP effect on GCST90199916_buildGRCh38.tsv
SNP effect on Paramuribaculum sp001689565

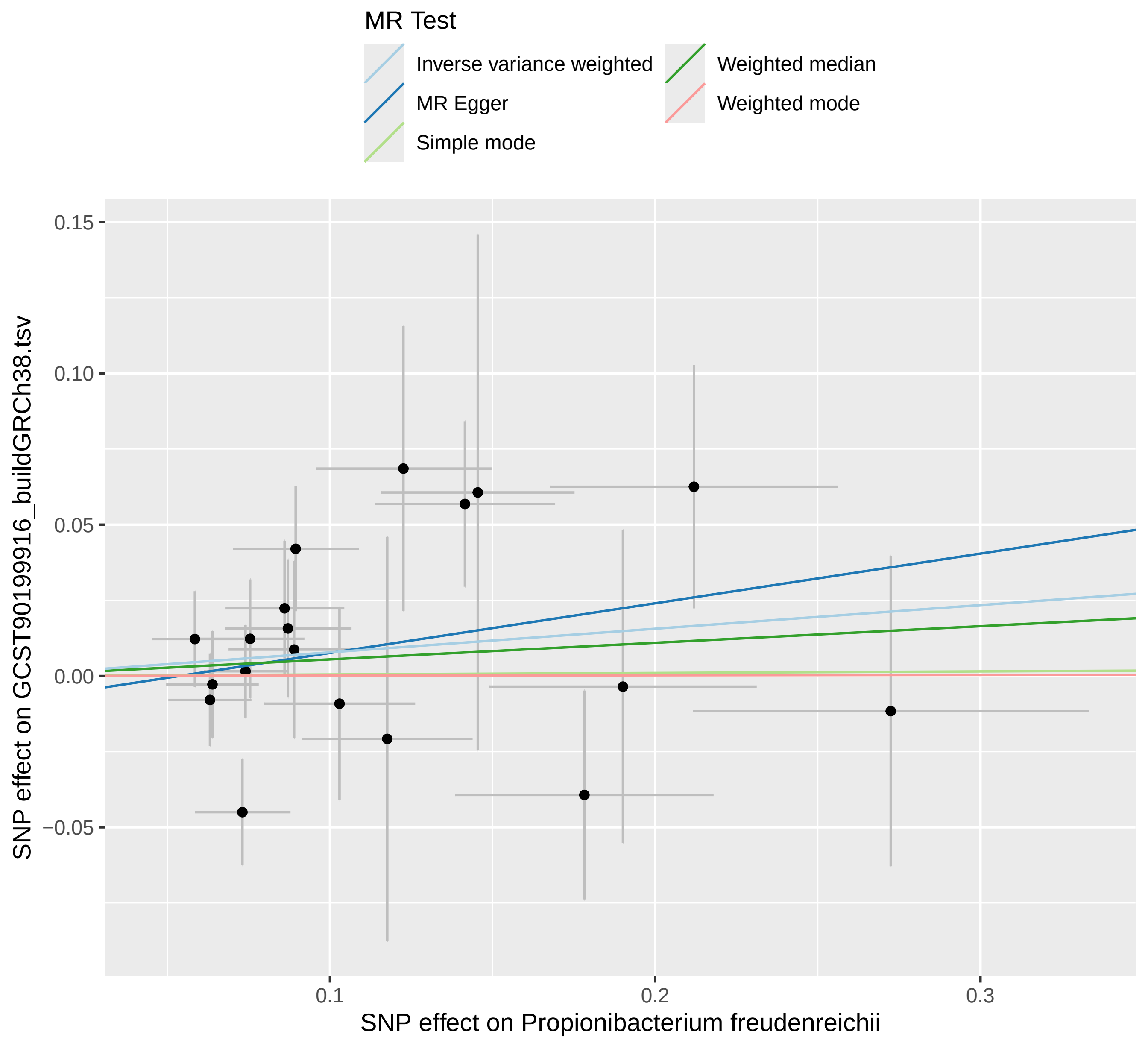
MR Test
Inverse variance weighted
MR Egger
Simple mode
Weighted median
Weighted mode
0.15
0.10
0.05
0.00
−0.05
0.1
0.2
0.3
SNP effect on GCST90199916_buildGRCh38.tsv
SNP effect on Propionibacterium freudenreichii

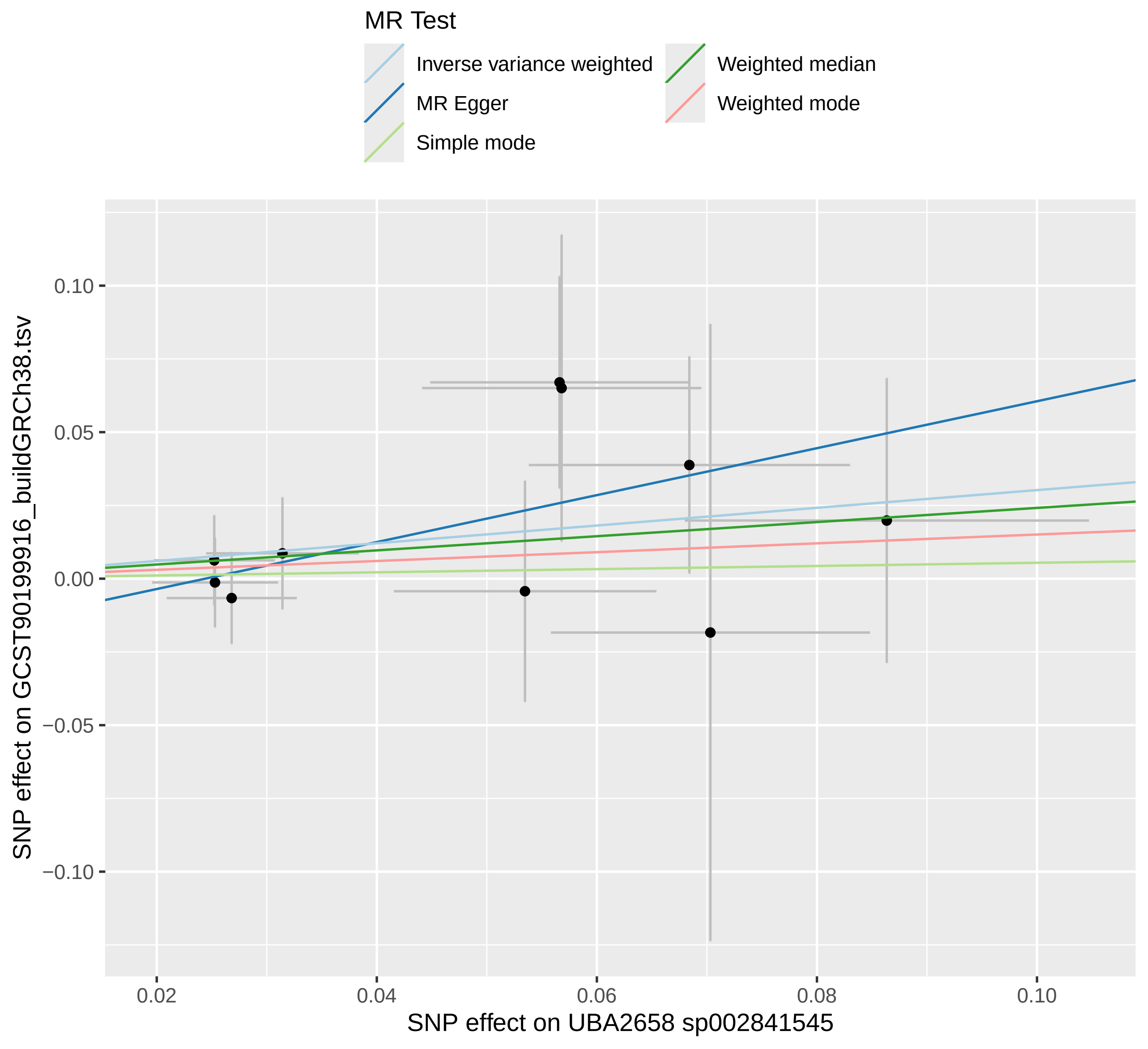

MR Test
Inverse variance weighted
MR Egger
Simple mode
Weighted median
Weighted mode
0.10
0.05
0.00
−0.05
−0.10
SNP effect on GCST90199916_buildGRCh38.tsv
0.02
0.04
0.06
0.08
0.10
SNP effect on UBA2658 sp002841545

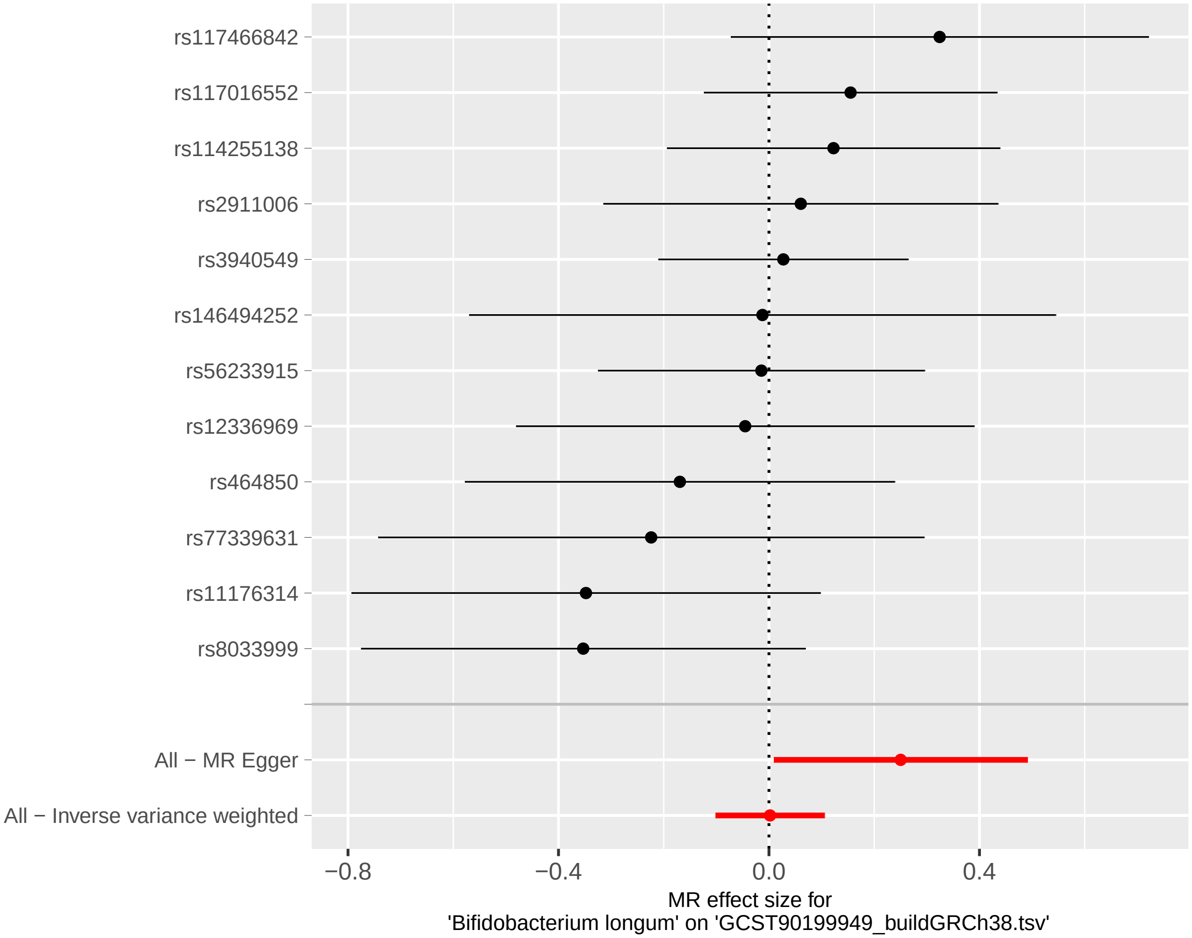
rs117466842
rs117016552
rs114255138
rs2911006
rs3940549
rs146494252
rs56233915
rs12336969
rs464850
rs77339631
rs11176314
rs8033999
All − MR Egger
All − Inverse variance weighted
−0.8
−0.4
0.0
0.4
MR effect size for
'Bifidobacterium longum' on 'GCST90199949_buildGRCh38.tsv'

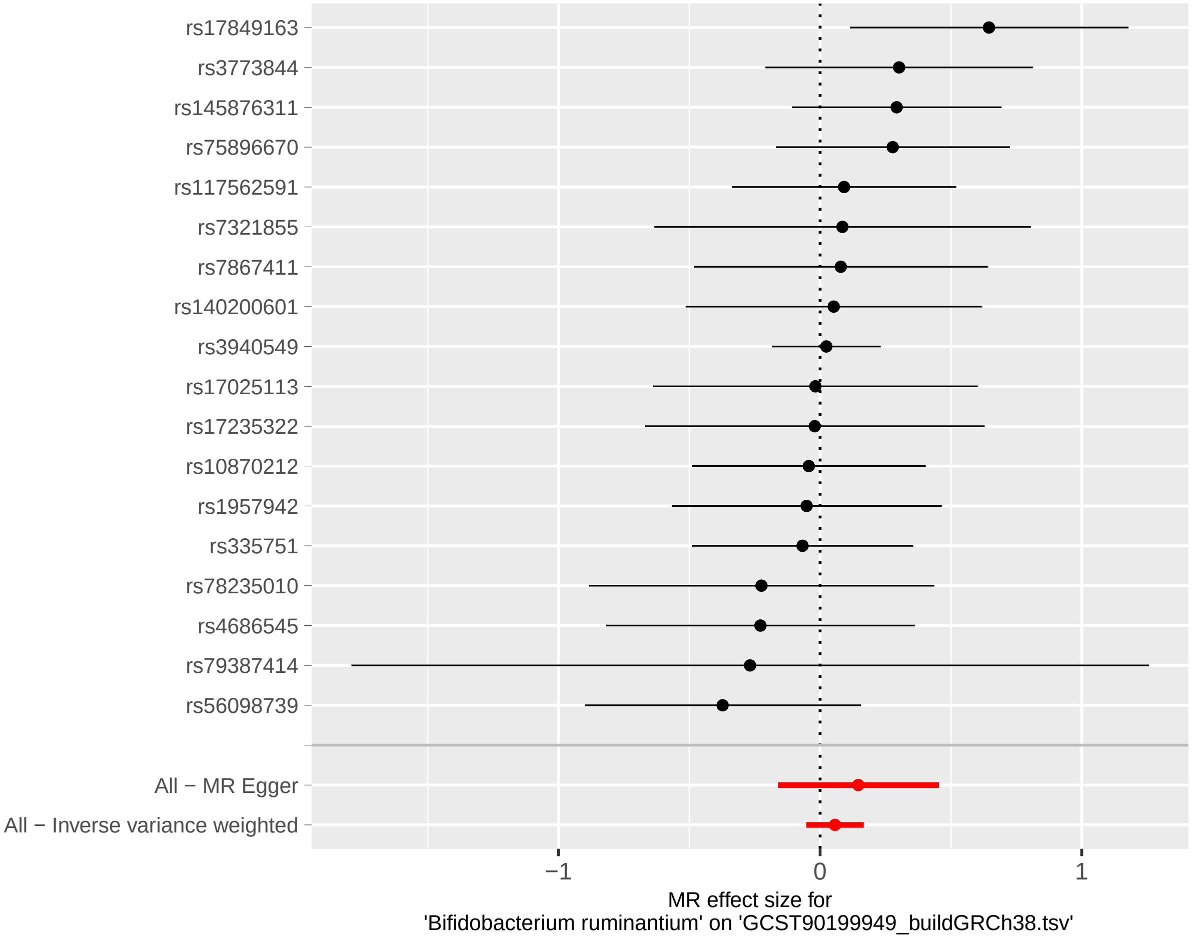
rs17849163
rs3773844
rs145876311
rs75896670
rs117562591
rs7321855
rs7867411
rs140200601
rs3940549
rs17025113
rs17235322
rs10870212
rs1957942
rs335751
rs78235010
rs4686545
rs79387414
rs56098739
All − MR Egger
All − Inverse variance weighted
−1
0
1
MR effect size for
'Bifidobacterium ruminantium' on 'GCST90199949_buildGRCh38.tsv'

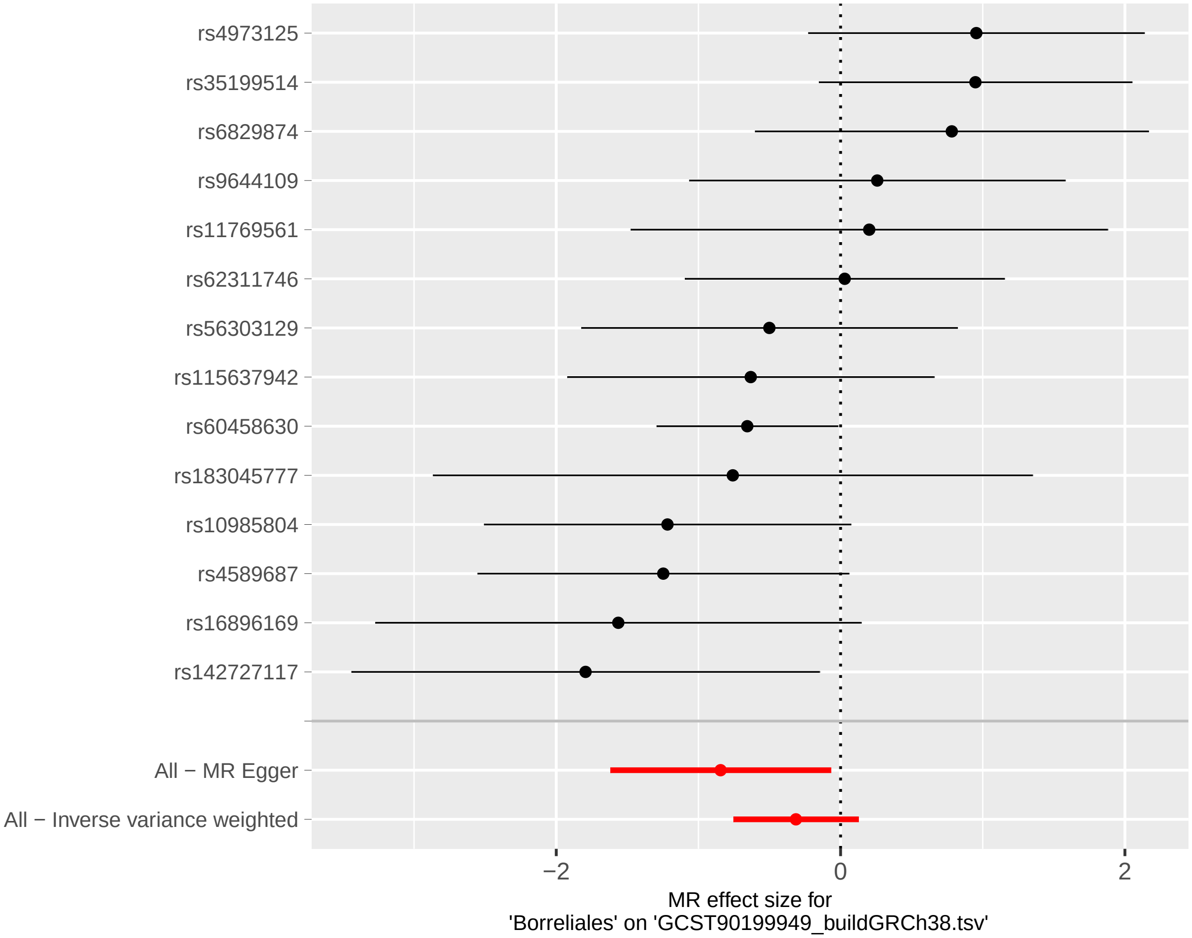

rs4973125
rs35199514
rs6829874
rs9644109
rs11769561
rs62311746
rs56303129
rs115637942
rs60458630
rs183045777
rs10985804
rs4589687
rs16896169
rs142727117
All − MR Egger
All − Inverse variance weighted
−2
0
2
MR effect size for
'Borreliales' on 'GCST90199949_buildGRCh38.tsv'

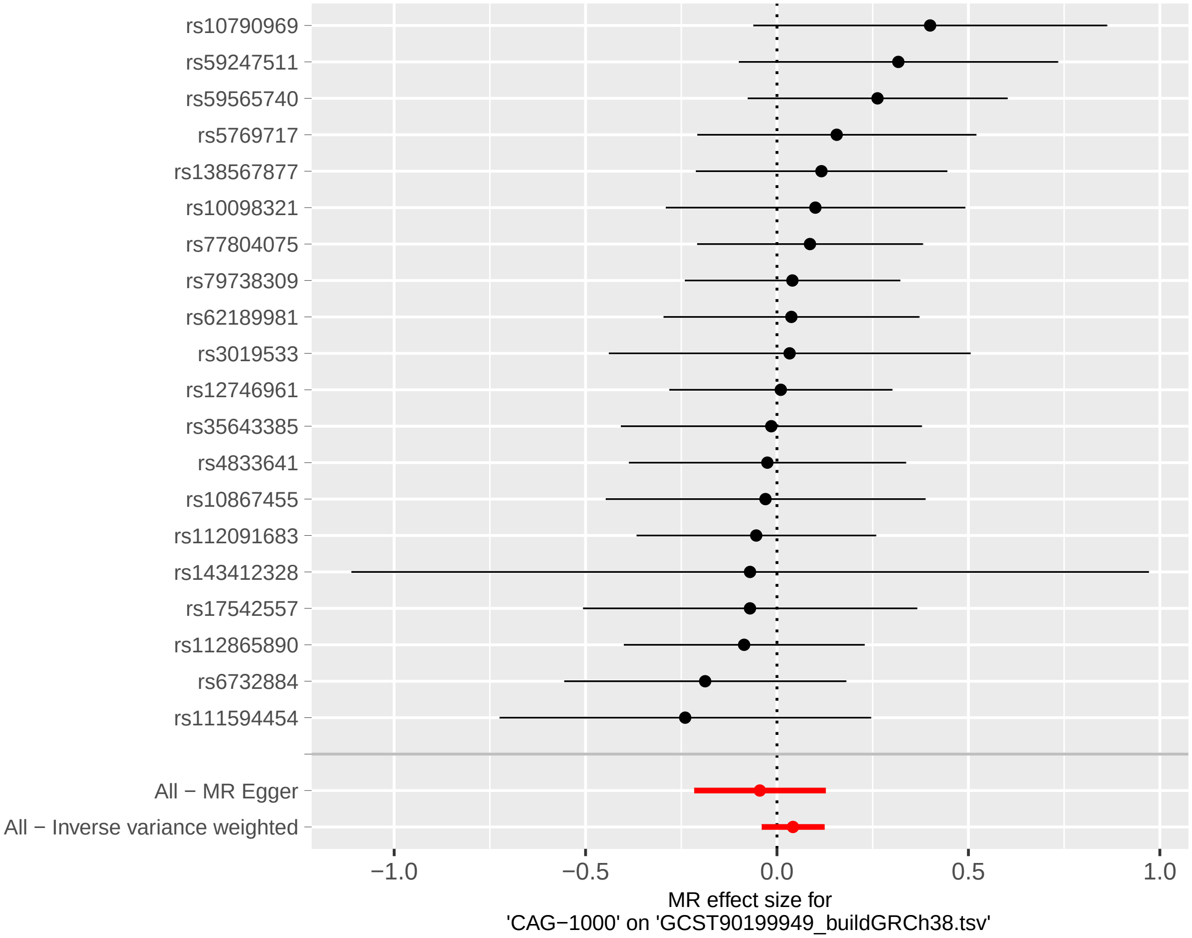
rs10790969
rs59247511
rs59565740
rs5769717
rs138567877
rs10098321
rs77804075
rs79738309
rs62189981
rs3019533
rs12746961
rs35643385
rs4833641
rs10867455
rs112091683
rs143412328
rs17542557
rs112865890
rs6732884
rs111594454
All − MR Egger
All − Inverse variance weighted
−1.0
−0.5
0.0
0.5
1.0
MR effect size for
'CAG−1000' on 'GCST90199949_buildGRCh38.tsv'

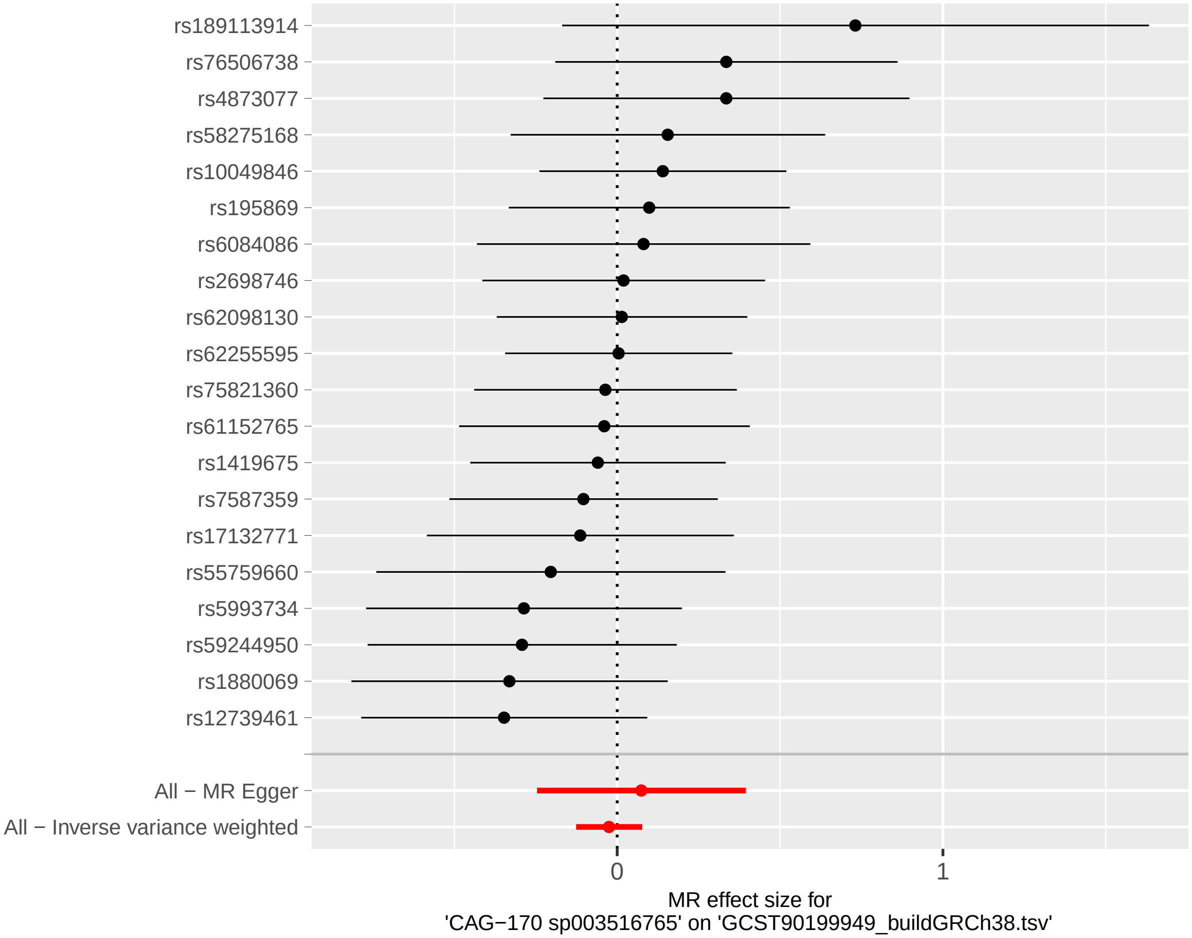

rs189113914
rs76506738
rs4873077
rs58275168
rs10049846
rs195869
rs6084086
rs2698746
rs62098130
rs62255595
rs75821360
rs61152765
rs1419675
rs7587359
rs17132771
rs55759660
rs5993734
rs59244950
rs1880069
rs12739461
All − MR Egger
All − Inverse variance weighted
0
1
MR effect size for
'CAG−170 sp003516765' on 'GCST90199949_buildGRCh38.tsv'

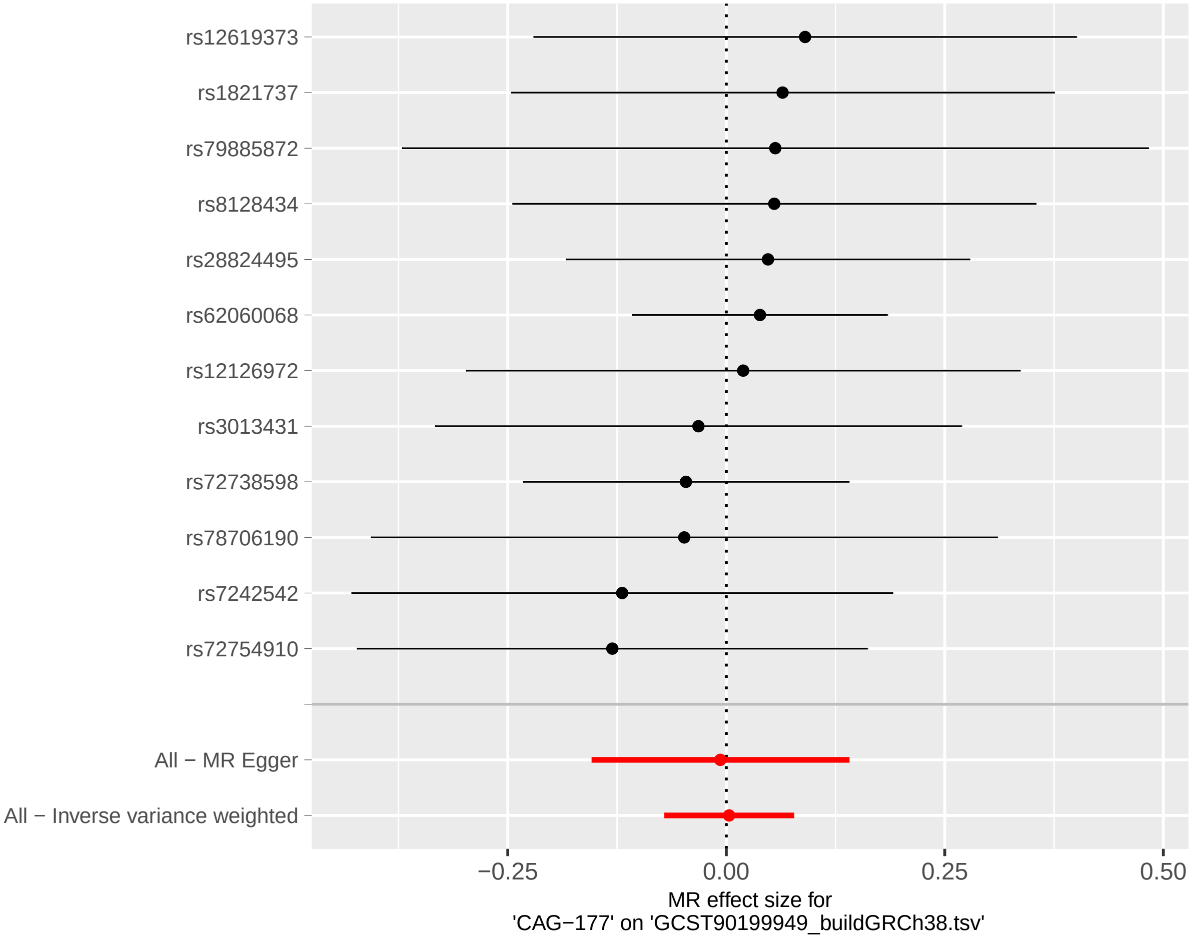
rs12619373
rs1821737
rs79885872
rs8128434
rs28824495
rs62060068
rs12126972
rs3013431
rs72738598
rs78706190
rs7242542
rs72754910
All − MR Egger
All − Inverse variance weighted
−0.25
0.00
0.25
0.50
MR effect size for
'CAG−177' on 'GCST90199949_buildGRCh38.tsv'

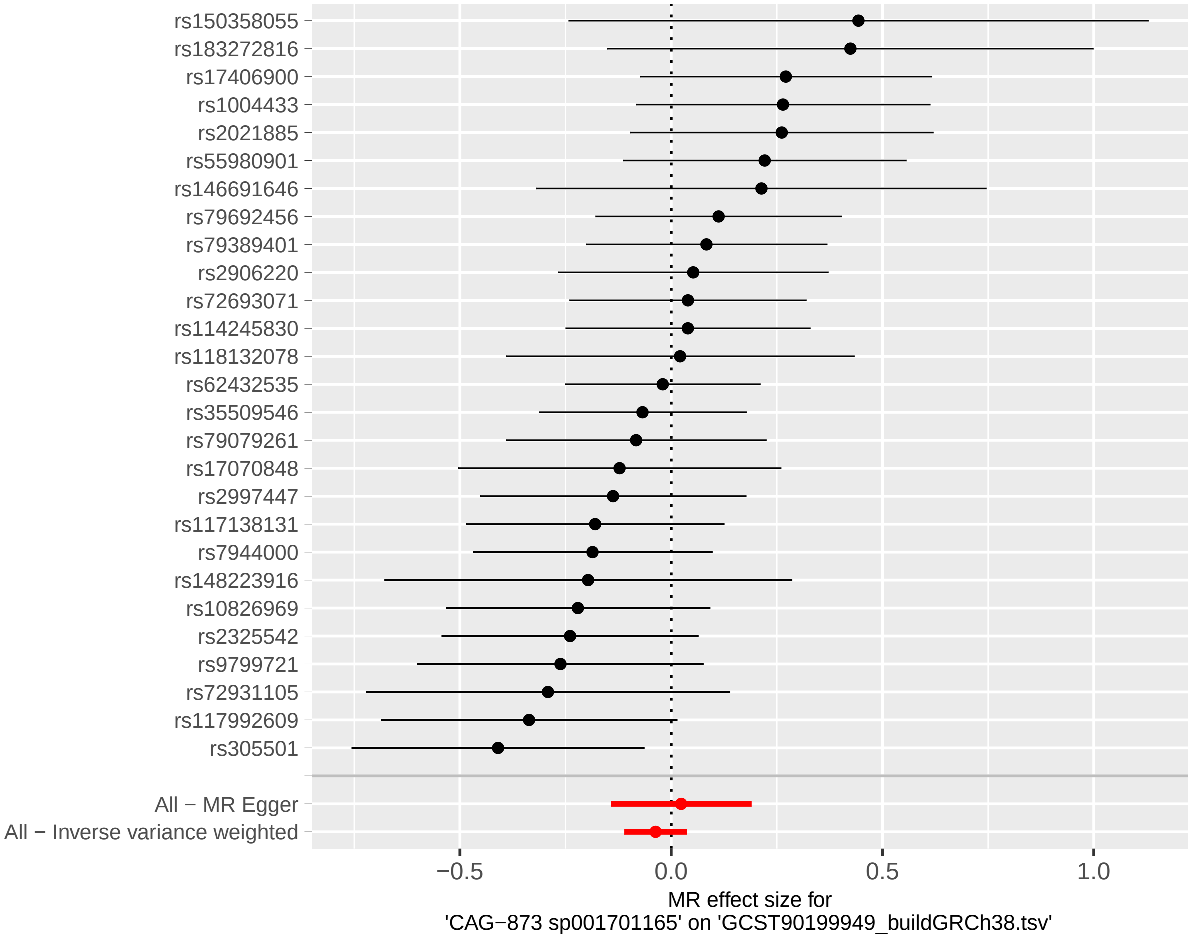
rs150358055
rs183272816
rs17406900
rs1004433
rs2021885
rs55980901
rs146691646
rs79692456
rs79389401
rs2906220
rs72693071
rs114245830
rs118132078
rs62432535
rs35509546
rs79079261
rs17070848
rs2997447
rs117138131
rs7944000
rs148223916
rs10826969
rs2325542
rs9799721
rs72931105
rs117992609
rs305501
All − MR Egger
All − Inverse variance weighted
−0.5
0.0
0.5
1.0
MR effect size for
'CAG−873 sp001701165' on 'GCST90199949_buildGRCh38.tsv'

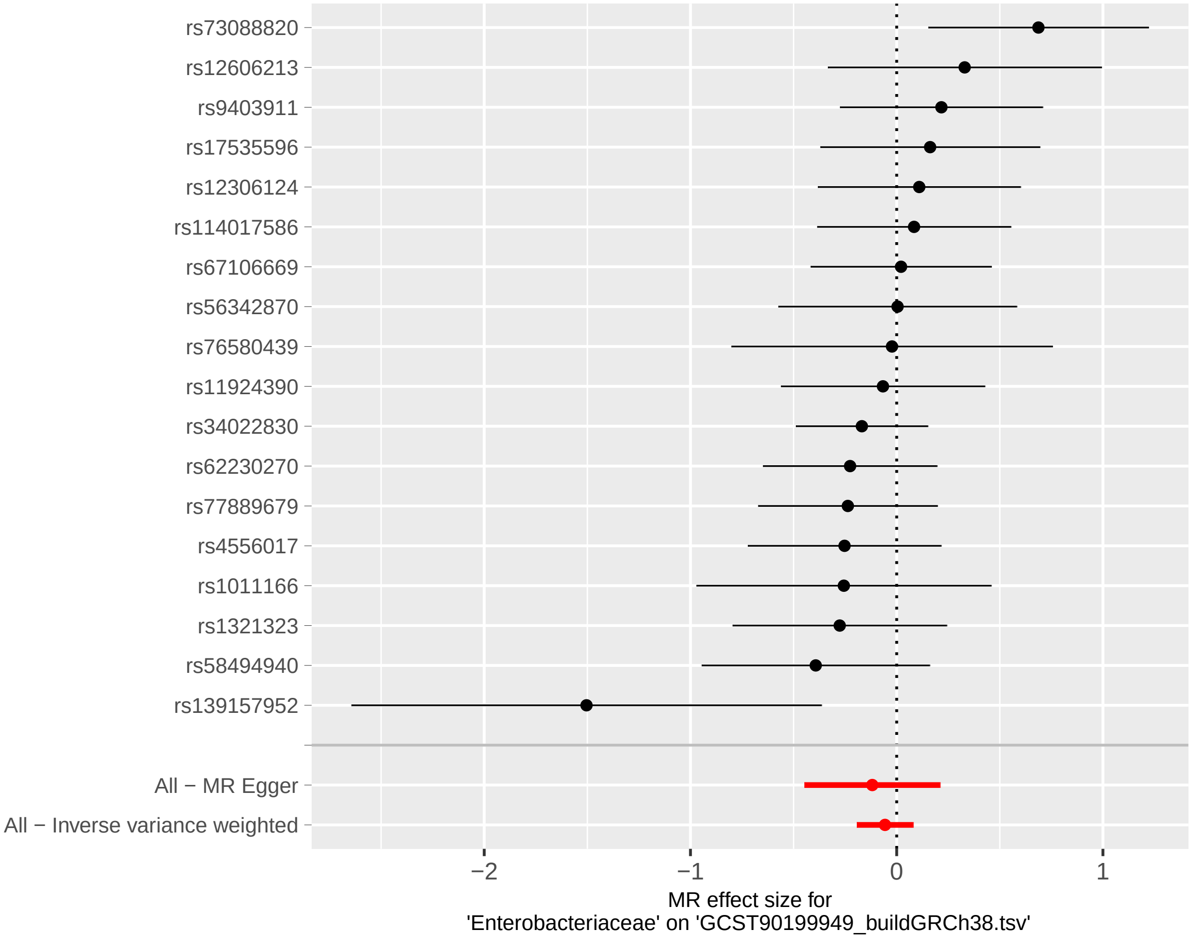

rs73088820
rs12606213
rs9403911
rs17535596
rs12306124
rs114017586
rs67106669
rs56342870
rs76580439
rs11924390
rs34022830
rs62230270
rs77889679
rs4556017
rs1011166
rs1321323
rs58494940
rs139157952
All − MR Egger
All − Inverse variance weighted
−2
−1
0
1
MR effect size for
'Enterobacteriaceae' on 'GCST90199949_buildGRCh38.tsv'

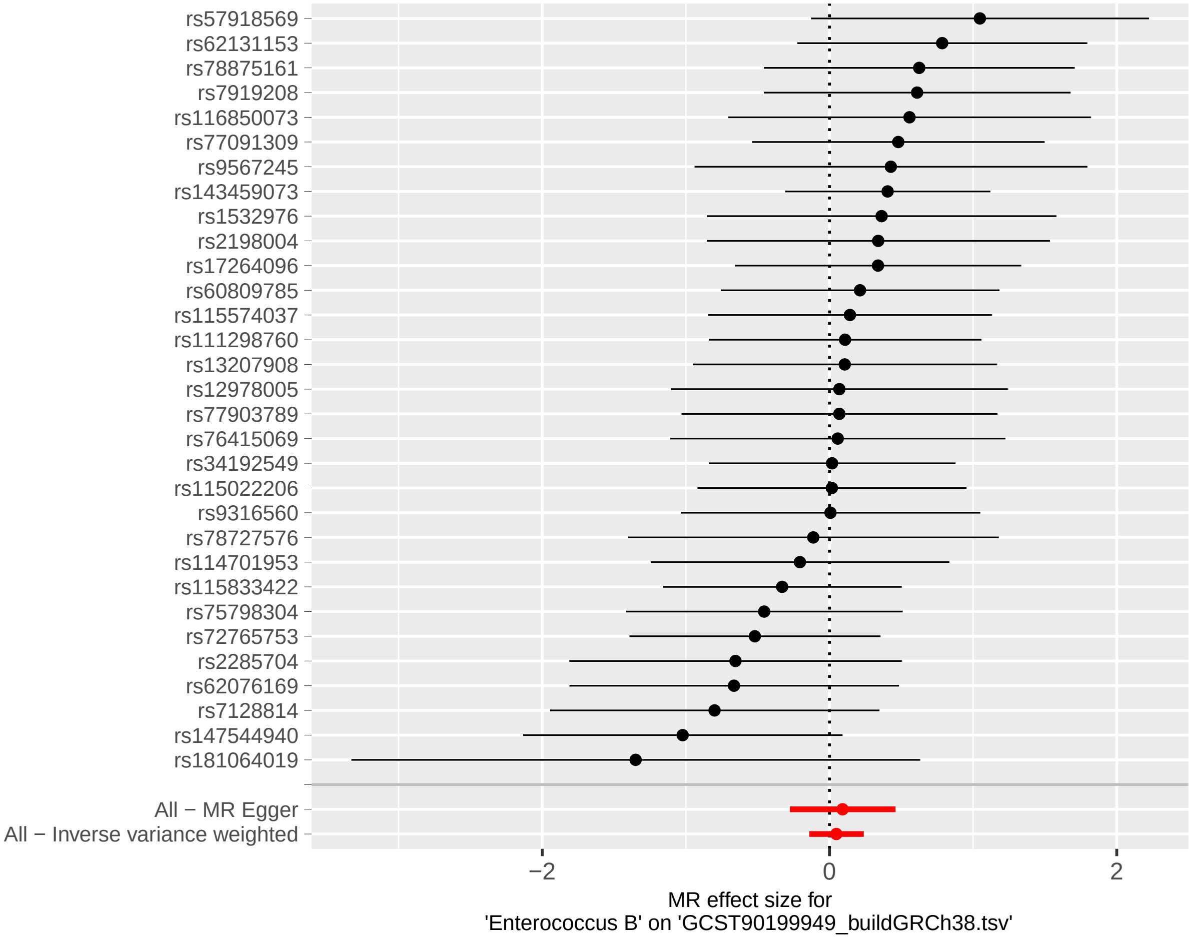
rs57918569
rs62131153
rs78875161
rs7919208
rs116850073
rs77091309
rs9567245
rs143459073
rs1532976
rs2198004
rs17264096
rs60809785
rs115574037
rs111298760
rs13207908
rs12978005
rs77903789
rs76415069
rs34192549
rs115022206
rs9316560
rs78727576
rs114701953
rs115833422
rs75798304
rs72765753
rs2285704
rs62076169
rs7128814
rs147544940
rs181064019
All − MR Egger
All − Inverse variance weighted
−2
0
2
MR effect size for
'Enterococcus B' on 'GCST90199949_buildGRCh38.tsv'

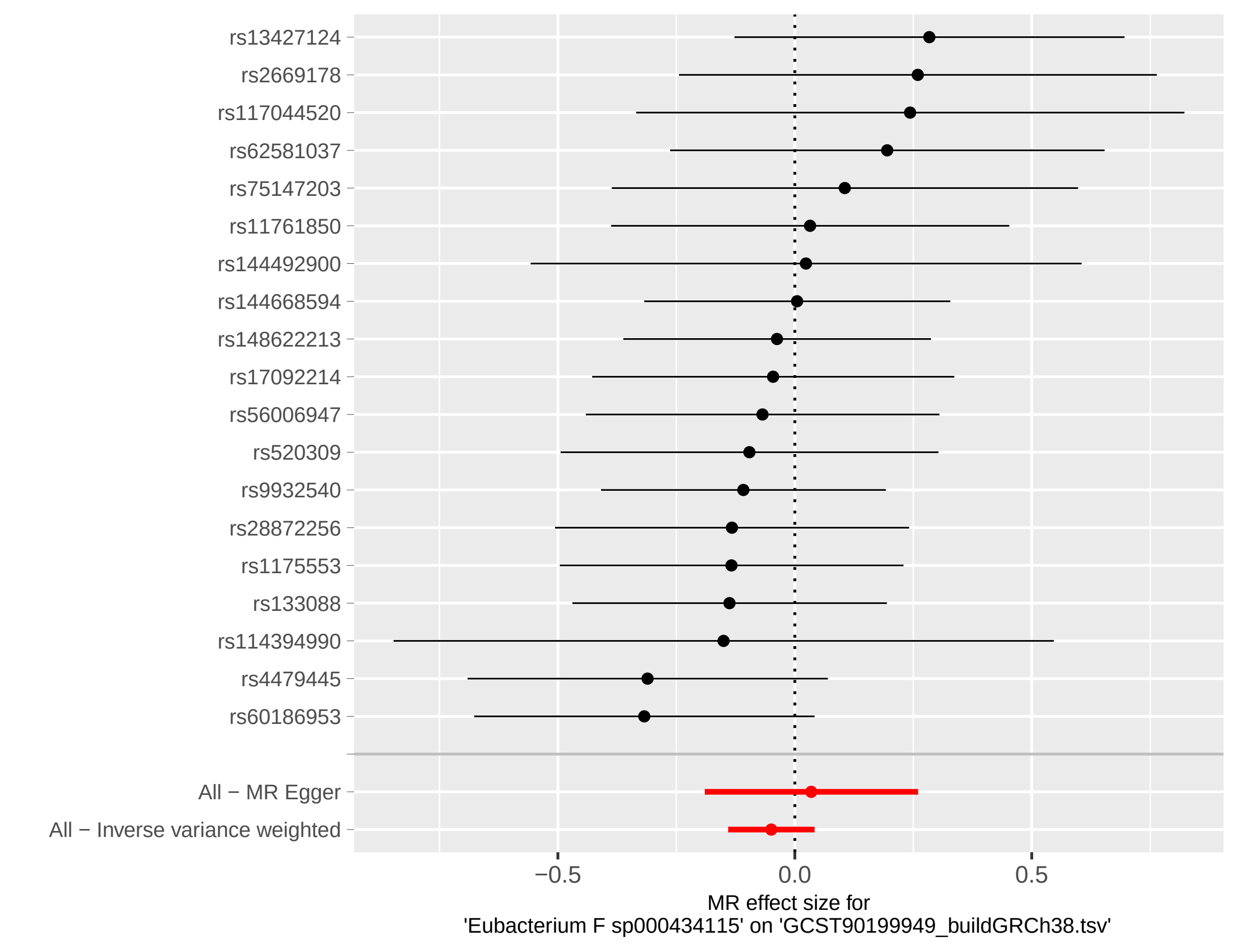
rs13427124
rs2669178
rs117044520
rs62581037
rs75147203
rs11761850
rs144492900
rs144668594
rs148622213
rs17092214
rs56006947
rs520309
rs9932540
rs28872256
rs1175553
rs133088
rs114394990
rs4479445
rs60186953
All − MR Egger
All − Inverse variance weighted
−0.5
0.0
0.5
MR effect size for
'Eubacterium F sp000434115' on 'GCST90199949_buildGRCh38.tsv'

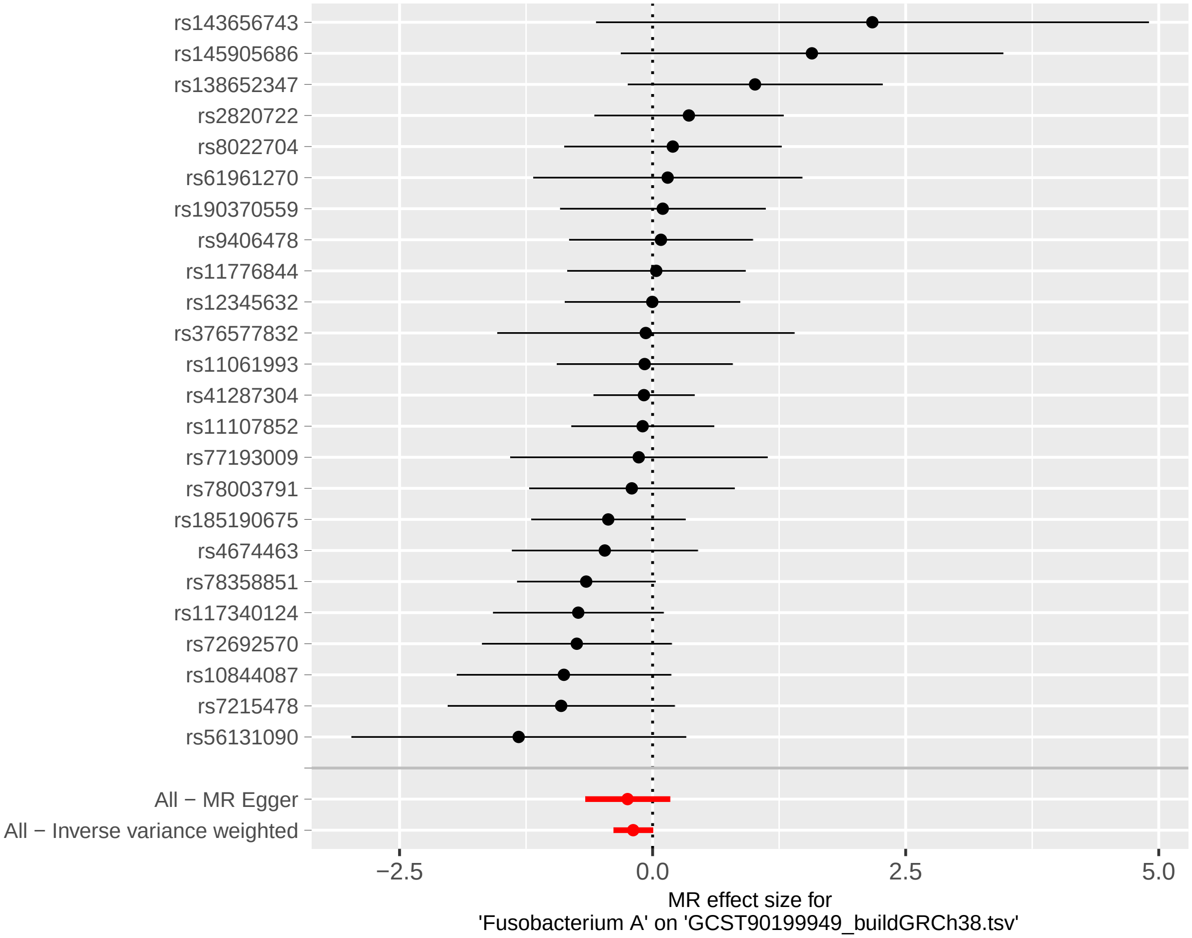
rs143656743
rs145905686
rs138652347
rs2820722
rs8022704
rs61961270
rs190370559
rs9406478
rs11776844
rs12345632
rs376577832
rs11061993
rs41287304
rs11107852
rs77193009
rs78003791
rs185190675
rs4674463
rs78358851
rs117340124
rs72692570
rs10844087
rs7215478
rs56131090
All − MR Egger
All − Inverse variance weighted
−2.5
0.0
2.5
5.0
MR effect size for
'Fusobacterium A' on 'GCST90199949_buildGRCh38.tsv'

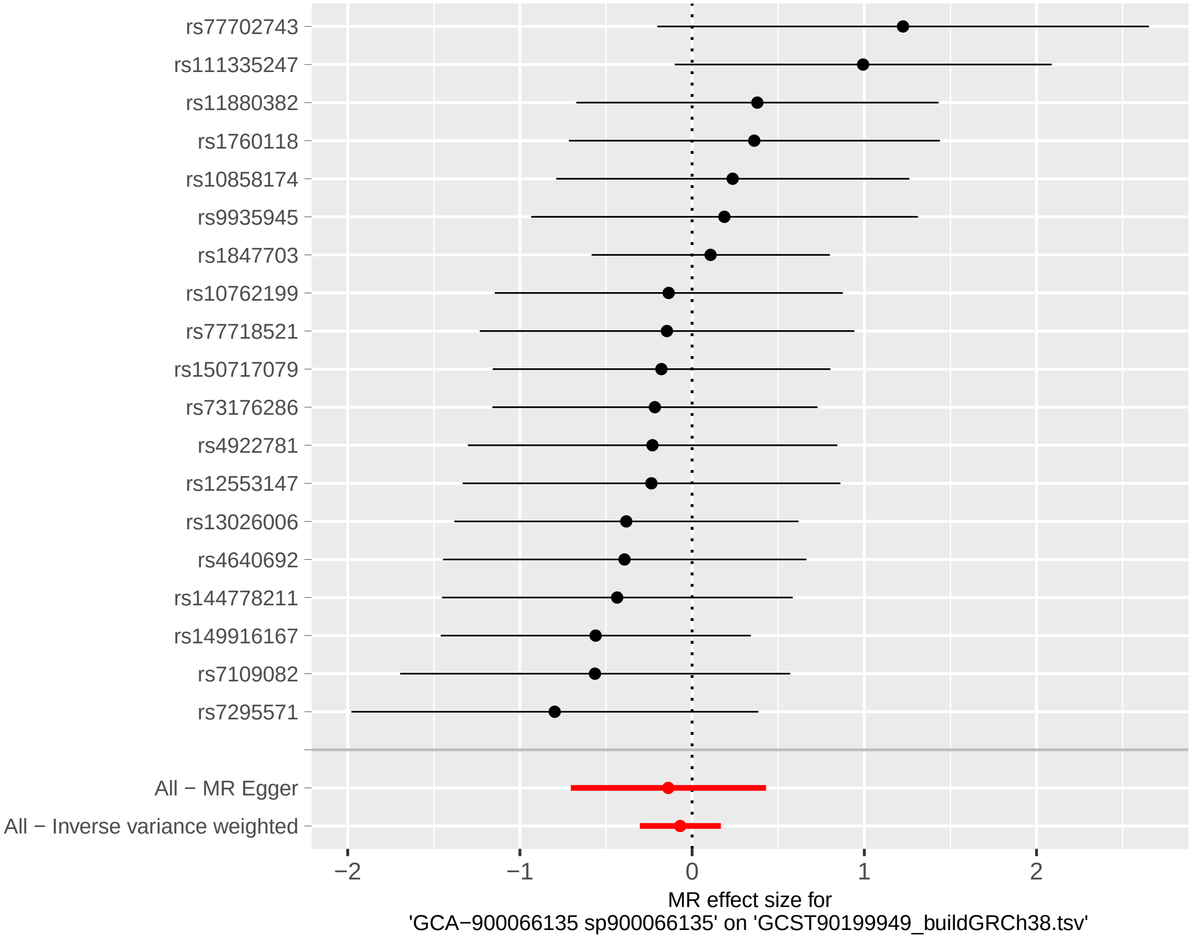

rs77702743
rs111335247
rs11880382
rs1760118
rs10858174
rs9935945
rs1847703
rs10762199
rs77718521
rs150717079
rs73176286
rs4922781
rs12553147
rs13026006
rs4640692
rs144778211
rs149916167
rs7109082
rs7295571
All − MR Egger
All − Inverse variance weighted
−2
−1
0
1
2
MR effect size for
'GCA−900066135 sp900066135' on 'GCST90199949_buildGRCh38.tsv'

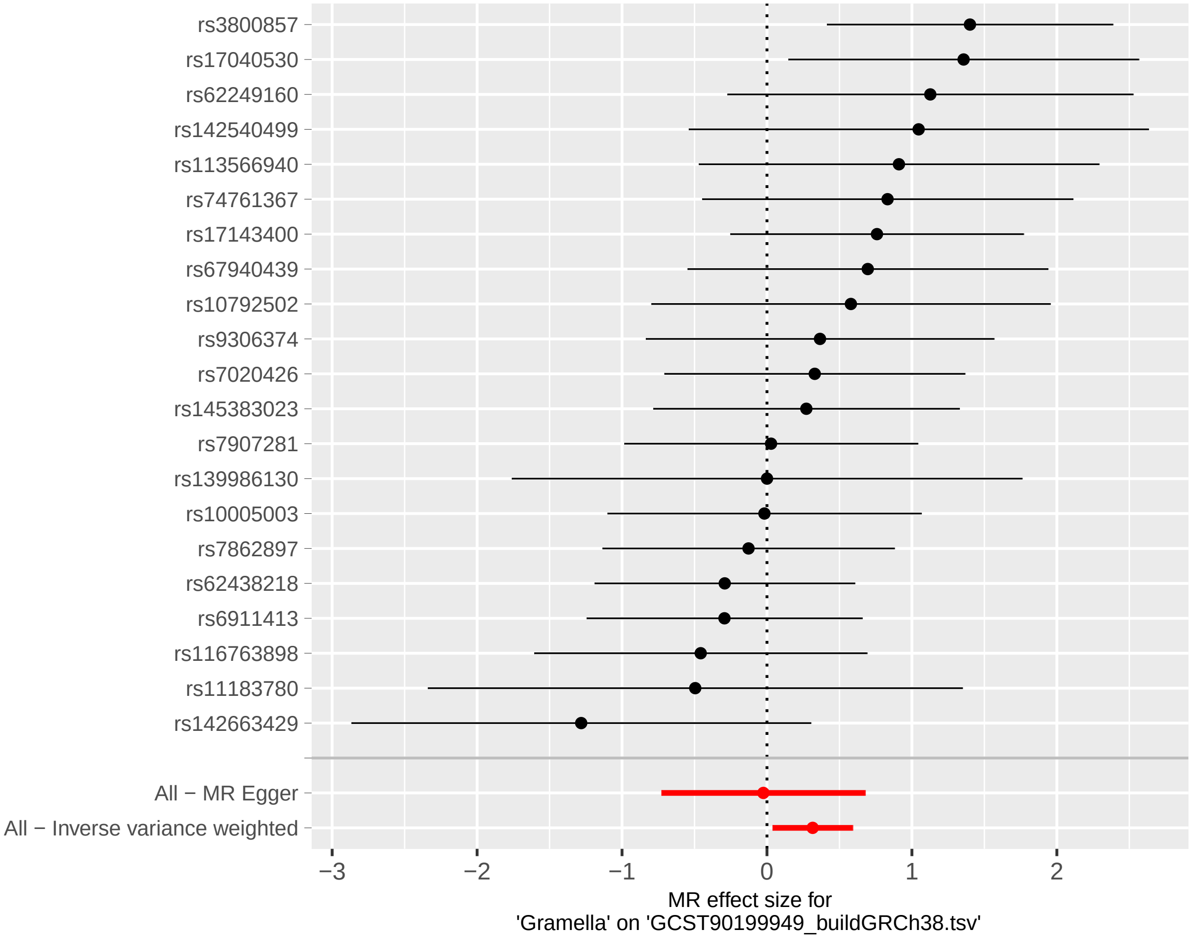

rs3800857
rs17040530
rs62249160
rs142540499
rs113566940
rs74761367
rs17143400
rs67940439
rs10792502
rs9306374
rs7020426
rs145383023
rs7907281
rs139986130
rs10005003
rs7862897
rs62438218
rs6911413
rs116763898
rs11183780
rs142663429
All − MR Egger
All − Inverse variance weighted
−3
−2
−1
0
1
2
MR effect size for
'Gramella' on 'GCST90199949_buildGRCh38.tsv'

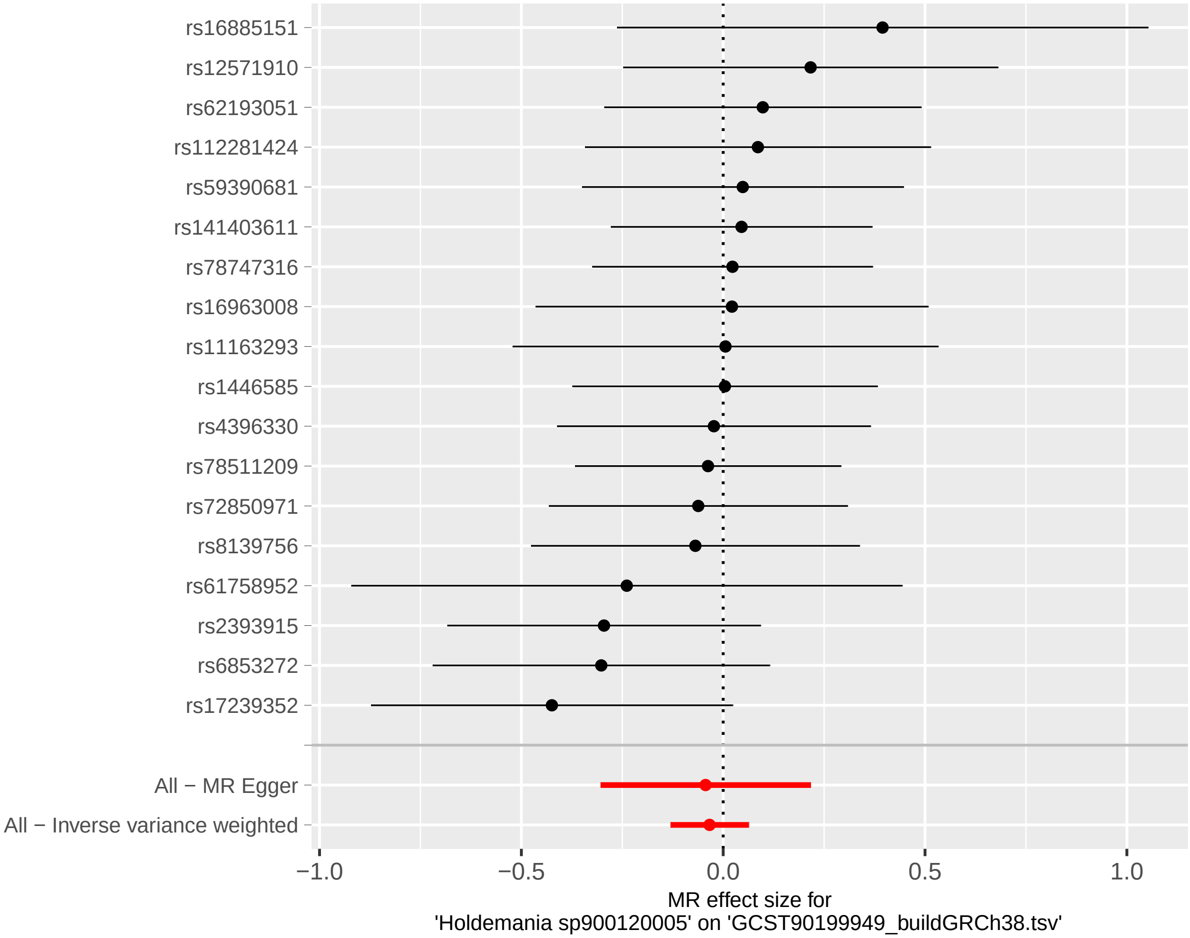
rs16885151
rs12571910
rs62193051
rs112281424
rs59390681
rs141403611
rs78747316
rs16963008
rs11163293
rs1446585
rs4396330
rs78511209
rs72850971
rs8139756
rs61758952
rs2393915
rs6853272
rs17239352
All − MR Egger
All − Inverse variance weighted
−1.0
−0.5
0.0
0.5
1.0
MR effect size for
'Holdemania sp900120005' on 'GCST90199949_buildGRCh38.tsv'

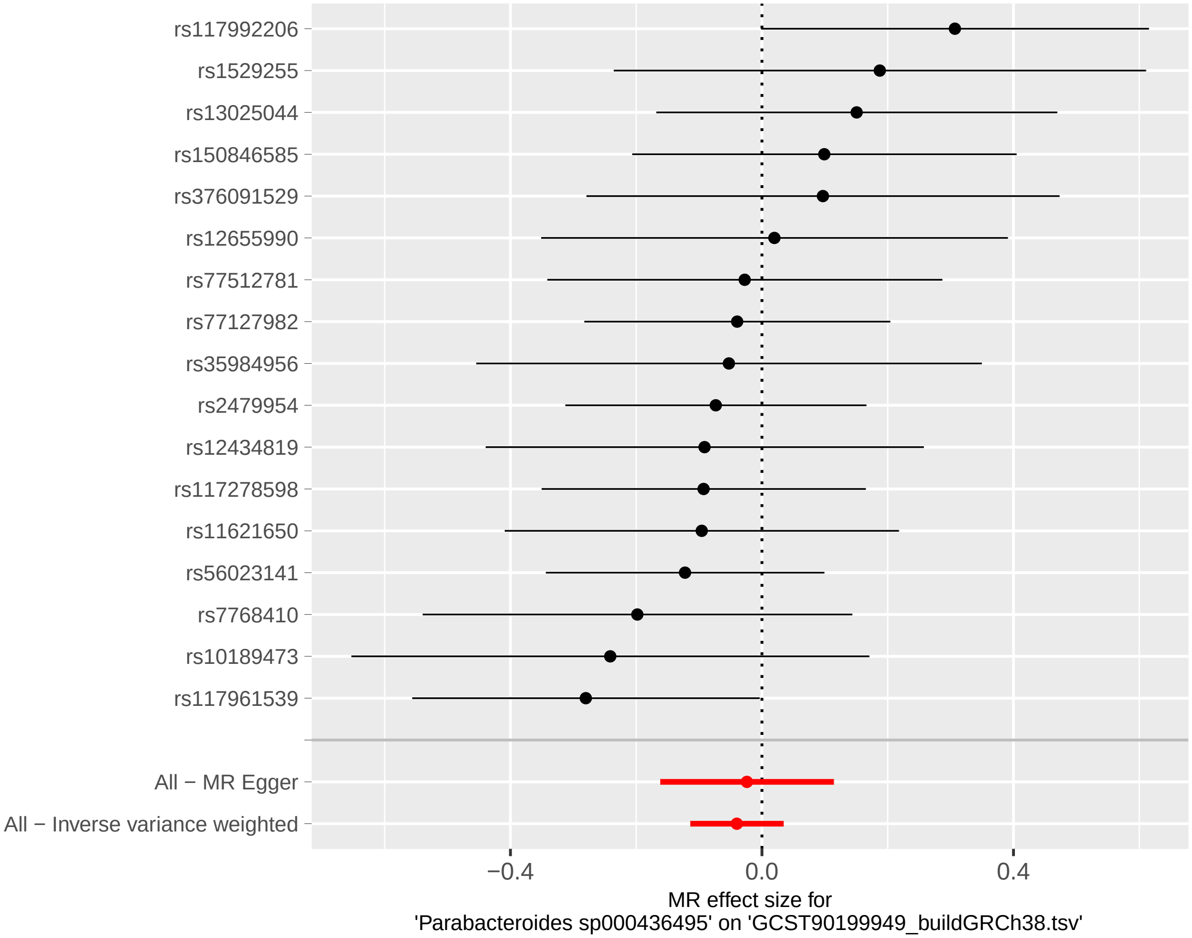

rs117992206
rs1529255
rs13025044
rs150846585
rs376091529
rs12655990
rs77512781
rs77127982
rs35984956
rs2479954
rs12434819
rs117278598
rs11621650
rs56023141
rs7768410
rs10189473
rs117961539
All − MR Egger
All − Inverse variance weighted
−0.4
0.0
0.4
MR effect size for
'Parabacteroides sp000436495' on 'GCST90199949_buildGRCh38.tsv'

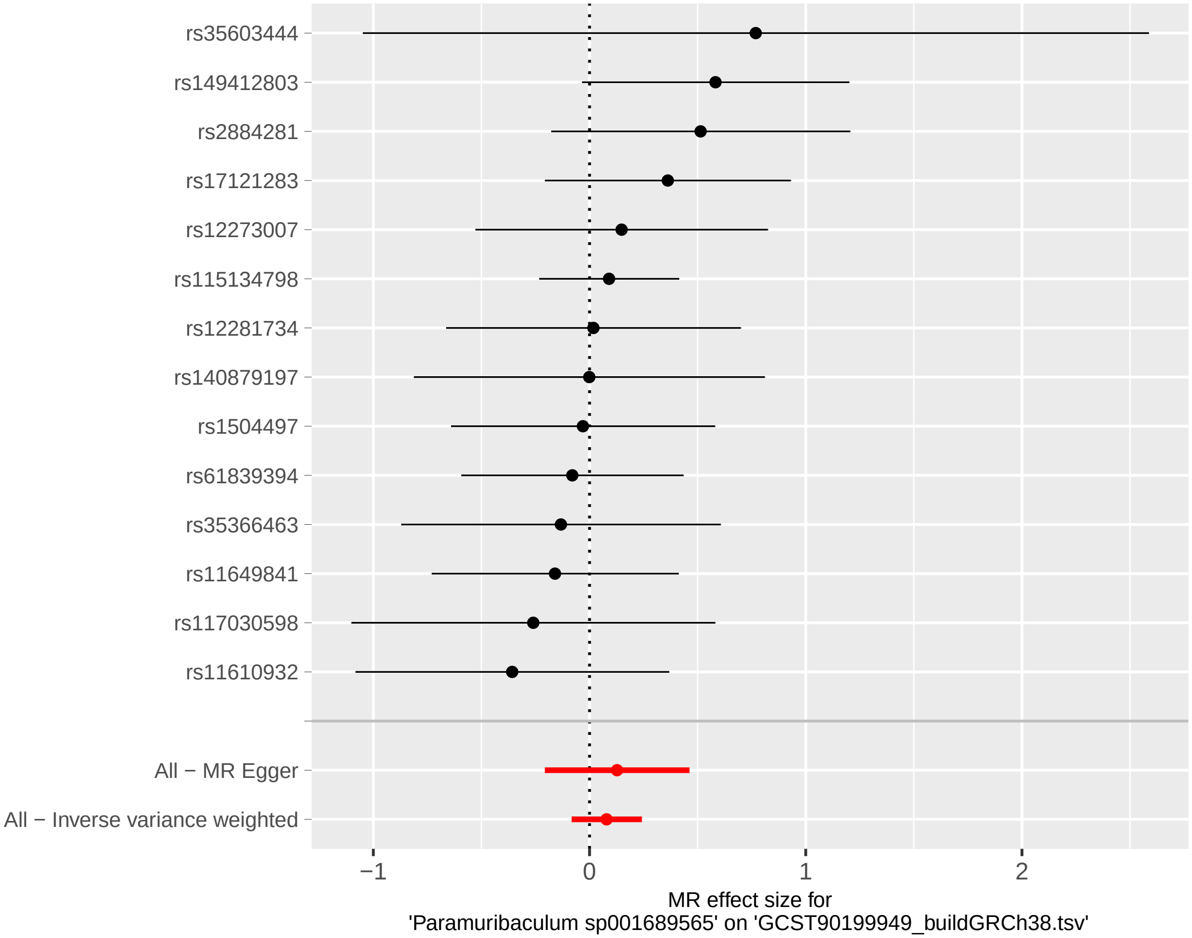

rs35603444
rs149412803
rs2884281
rs17121283
rs12273007
rs115134798
rs12281734
rs140879197
rs1504497
rs61839394
rs35366463
rs11649841
rs117030598
rs11610932
All − MR Egger
All − Inverse variance weighted
−1
0
1
2
MR effect size for
'Paramuribaculum sp001689565' on 'GCST90199949_buildGRCh38.tsv'

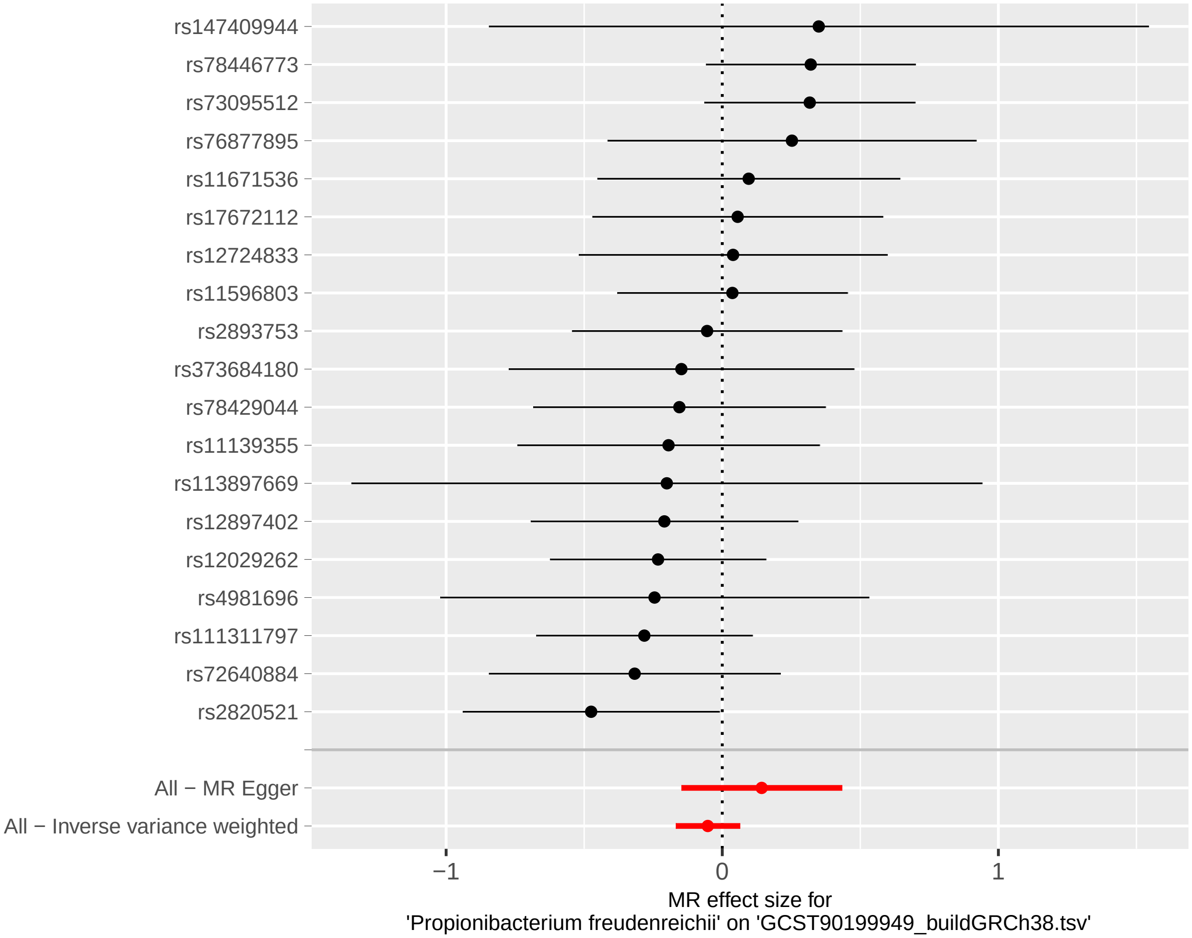
rs147409944
rs78446773
rs73095512
rs76877895
rs11671536
rs17672112
rs12724833
rs11596803
rs2893753
rs373684180
rs78429044
rs11139355
rs113897669
rs12897402
rs12029262
rs4981696
rs111311797
rs72640884
rs2820521
All − MR Egger
All − Inverse variance weighted
−1
0
1
MR effect size for
'Propionibacterium freudenreichii' on 'GCST90199949_buildGRCh38.tsv'

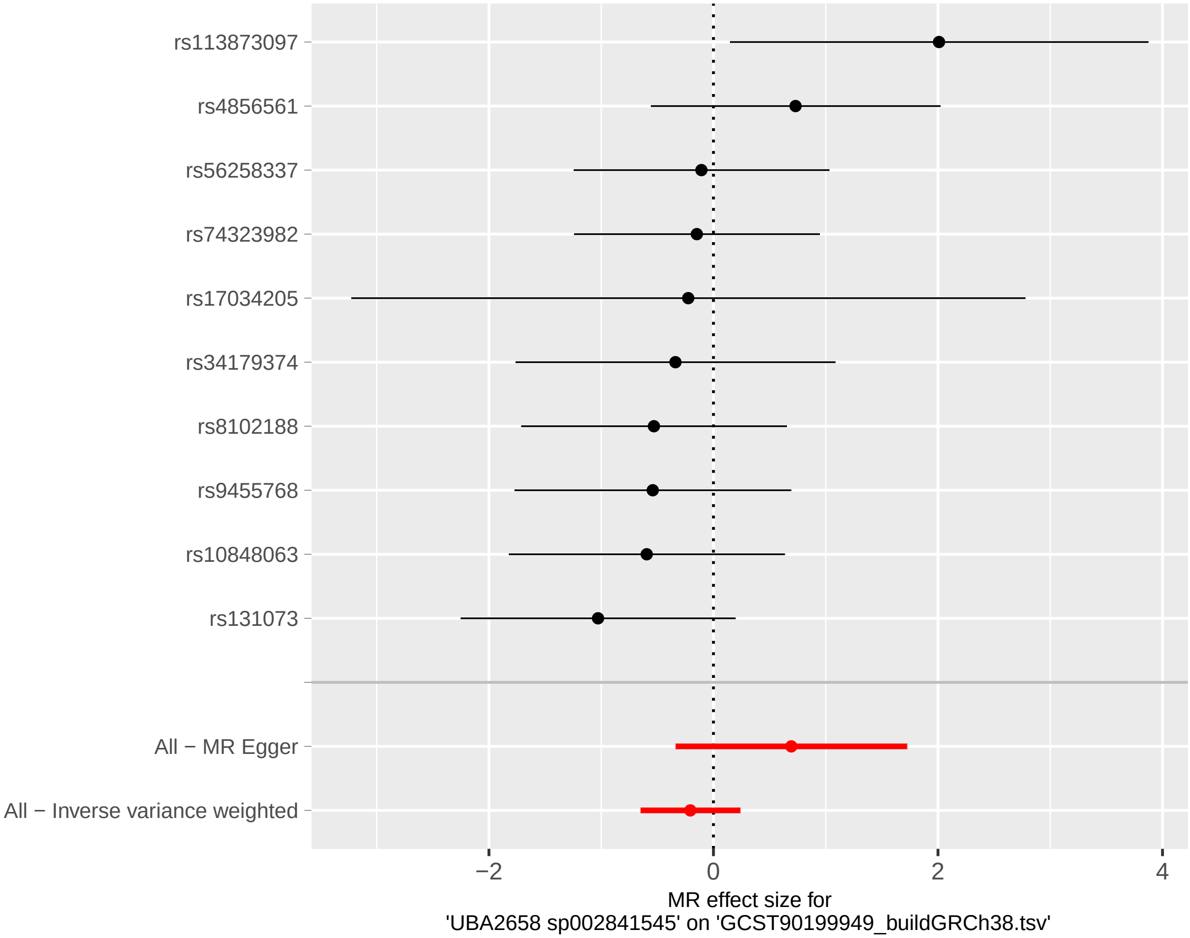

rs113873097
rs4856561
rs56258337
rs74323982
rs17034205
rs34179374
rs8102188
rs9455768
rs10848063
rs131073
All − MR Egger
All − Inverse variance weighted
−2
0
2
4
MR effect size for
'UBA2658 sp002841545' on 'GCST90199949_buildGRCh38.tsv'

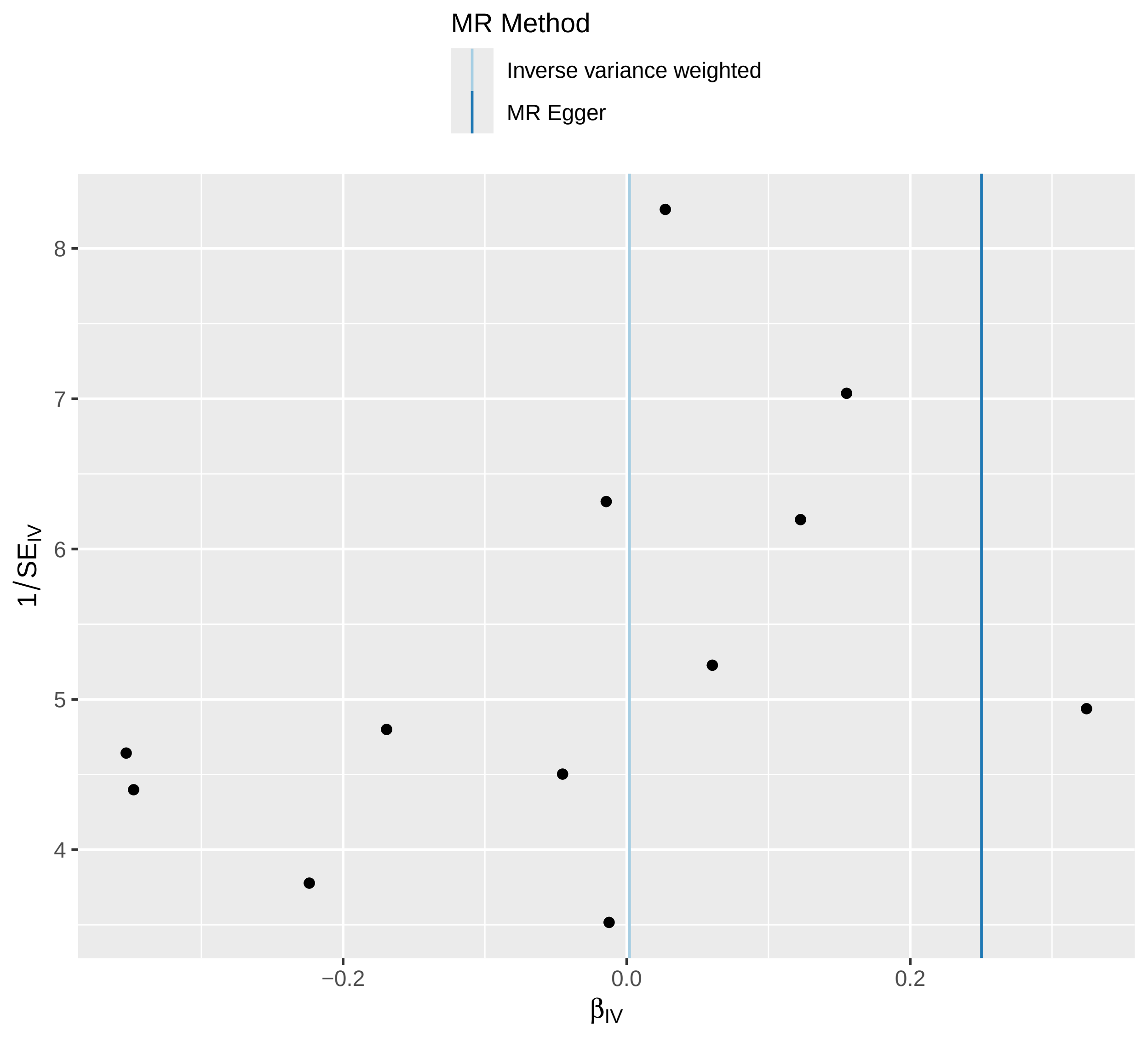
MR Method
Inverse variance weighted
MR Egger
8
7
6
5
4
1/SE_IV
−0.2
0.0
0.2
β_IV

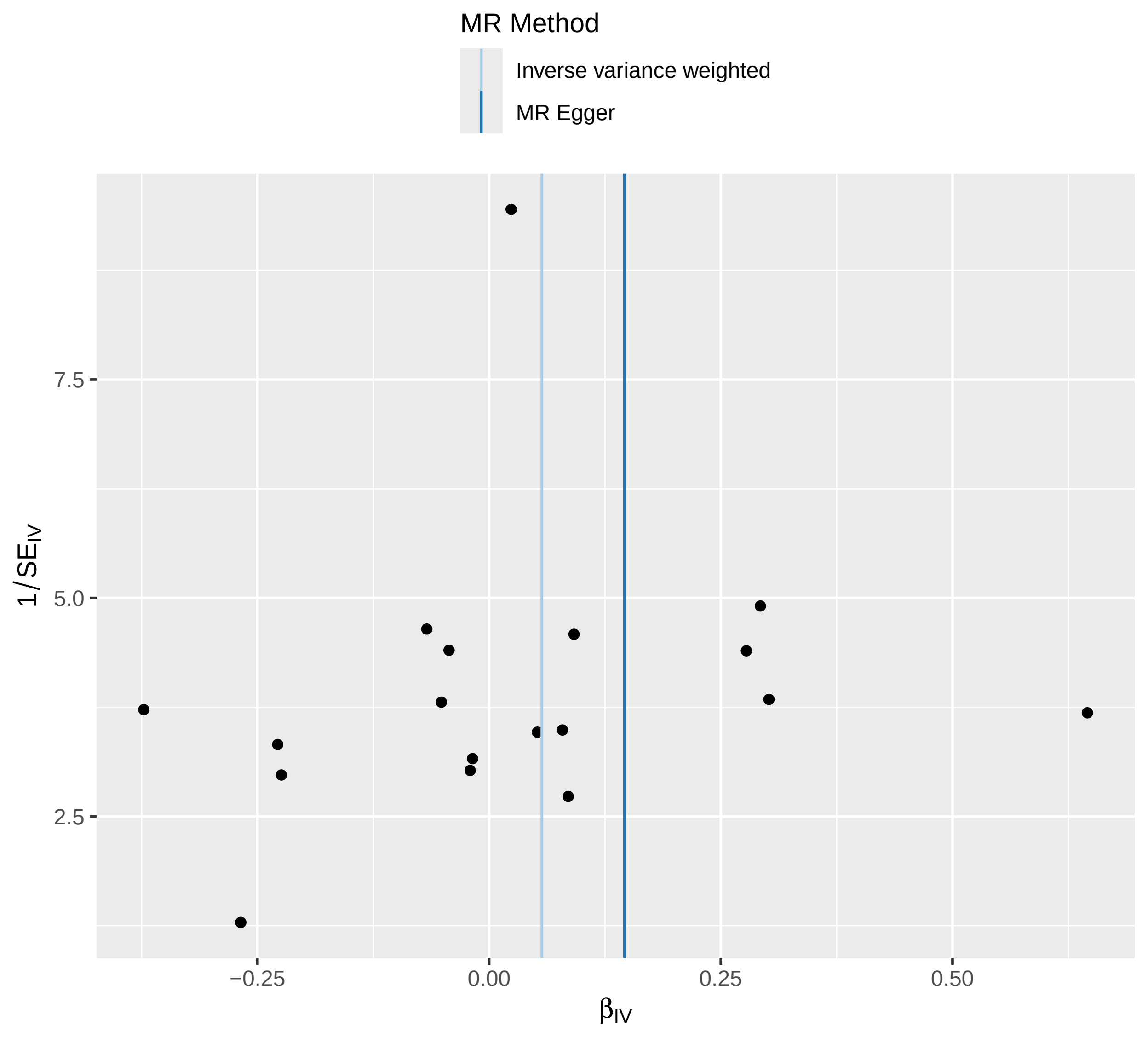
MR Method
Inverse variance weighted
MR Egger
7.5
5.0
2.5
1/SE_IV
−0.25
0.00
0.25
0.50
β_IV

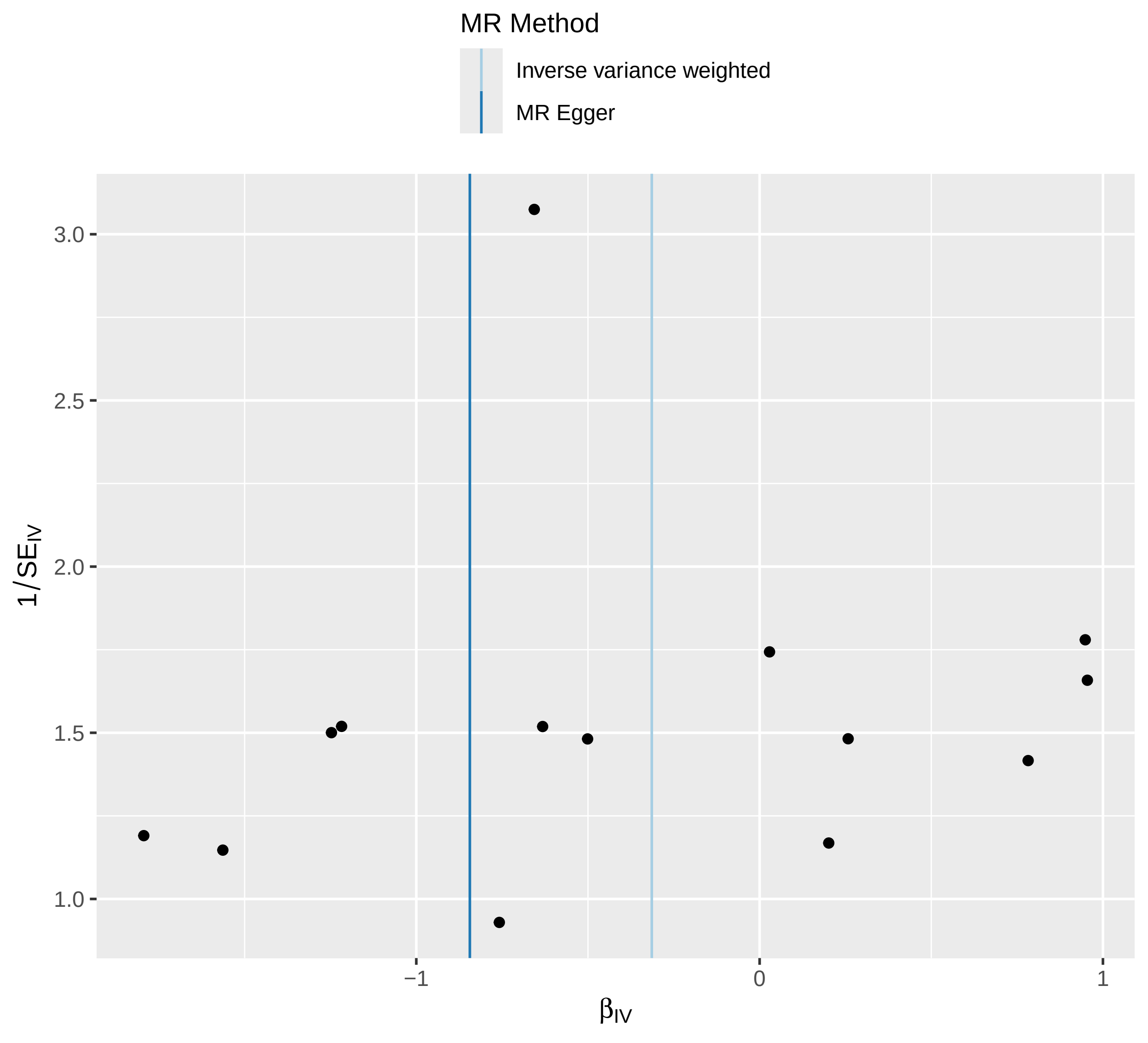
MR Method
Inverse variance weighted
MR Egger
3.0
2.5
2.0
1.5
1.0
1/SE_IV
−1
0
1
β_IV

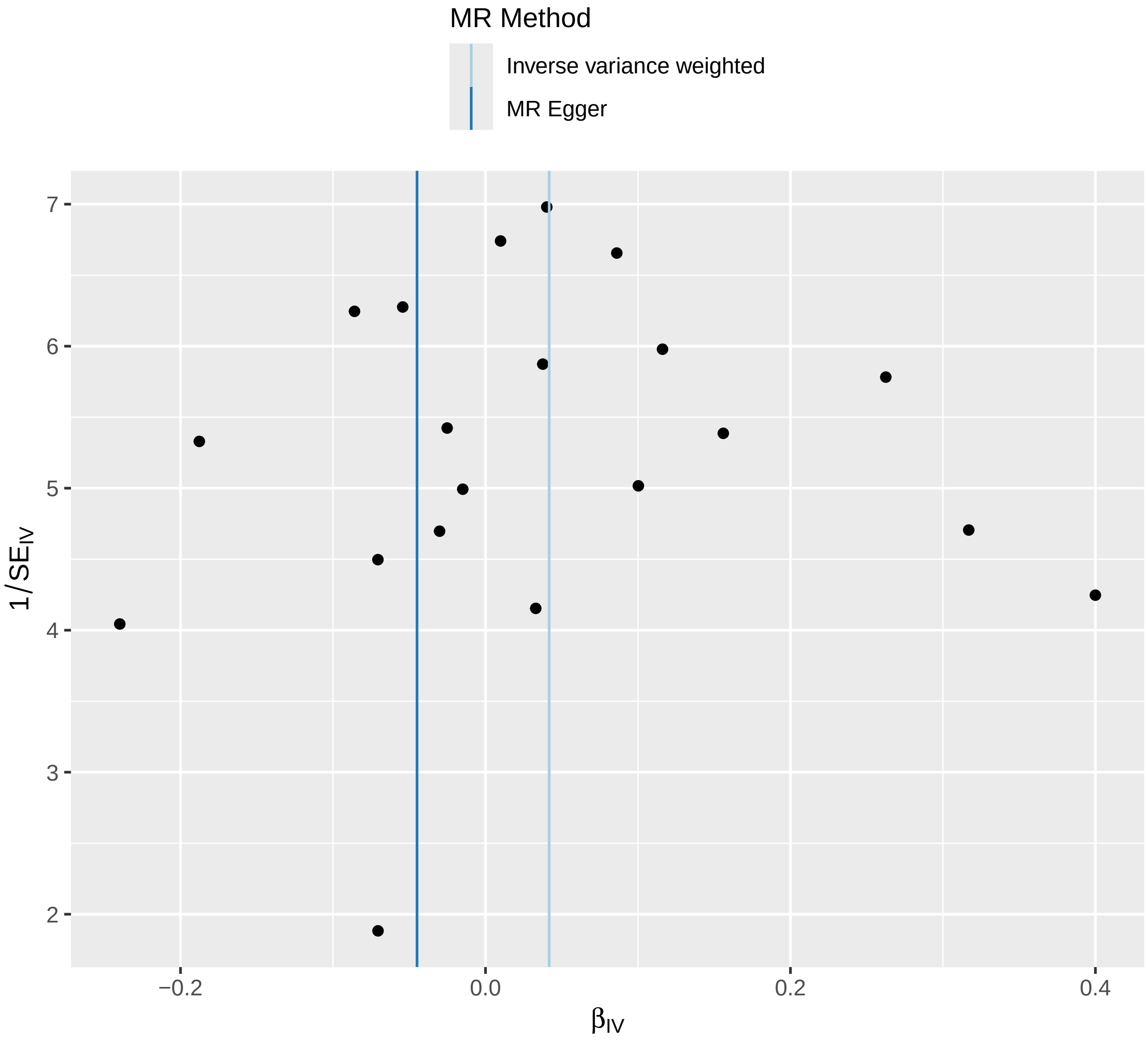

MR Method
Inverse variance weighted
MR Egger
7
6
5
4
3
2
1/SE_IV
−0.2
0.0
0.2
0.4
β_IV

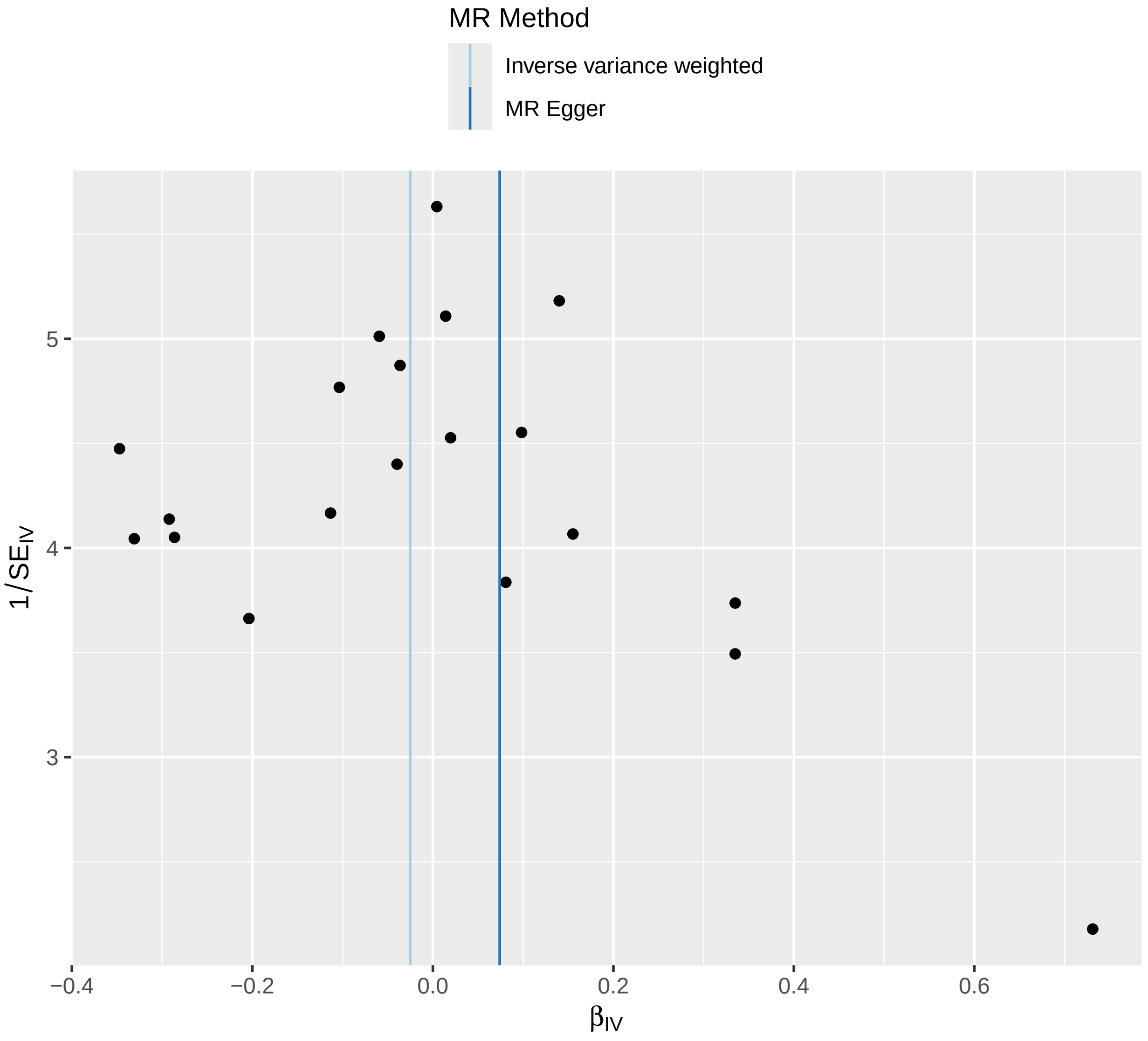

MR Method
Inverse variance weighted
MR Egger
5
4
3
1/SE_IV
−0.4
−0.2
0.0
0.2
0.4
0.6
β_IV

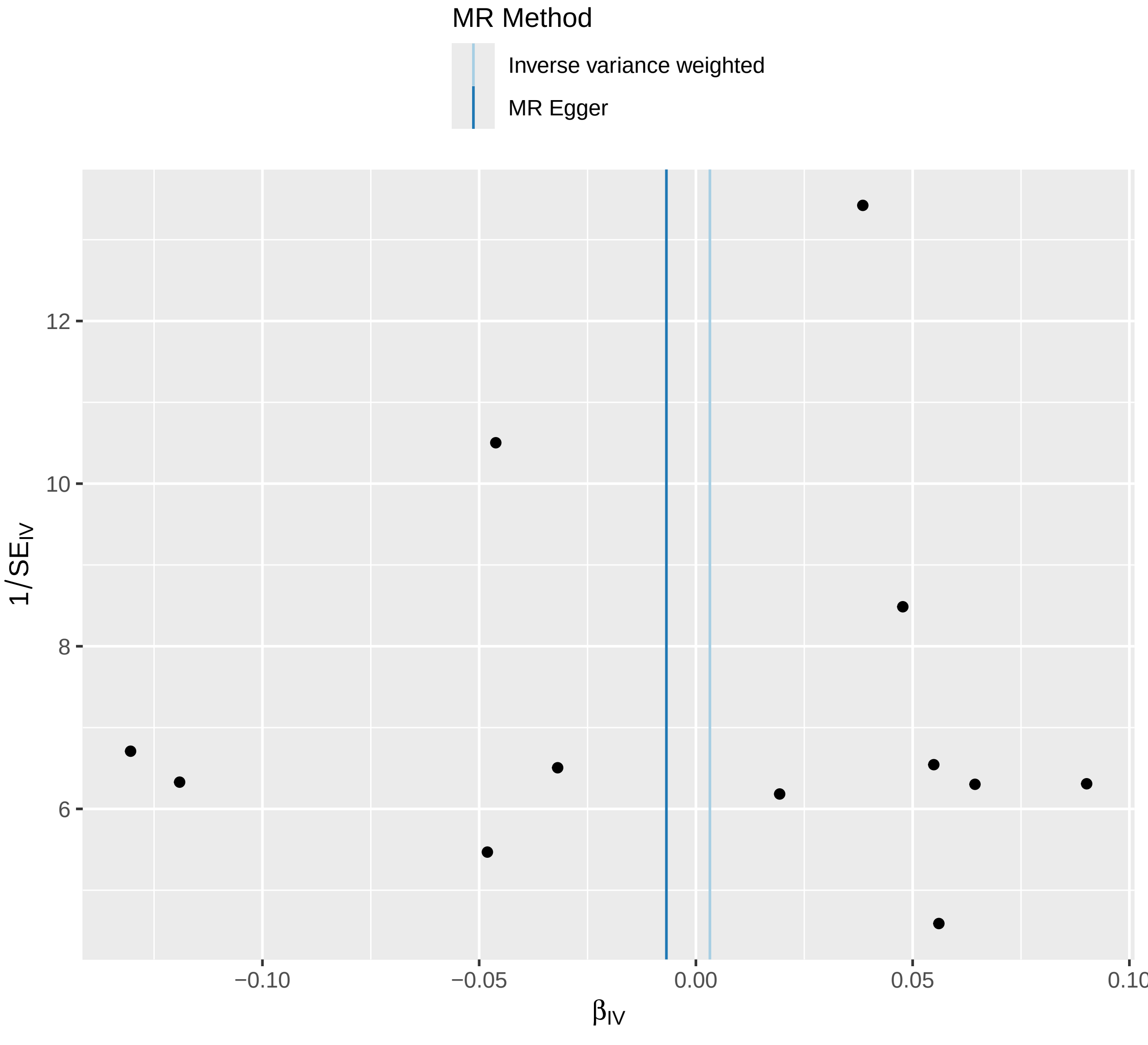
MR Method
Inverse variance weighted
MR Egger
12
10
8
6
$1/SE_{IV}$
−0.10
−0.05
0.00
0.05
0.10
β_{IV}

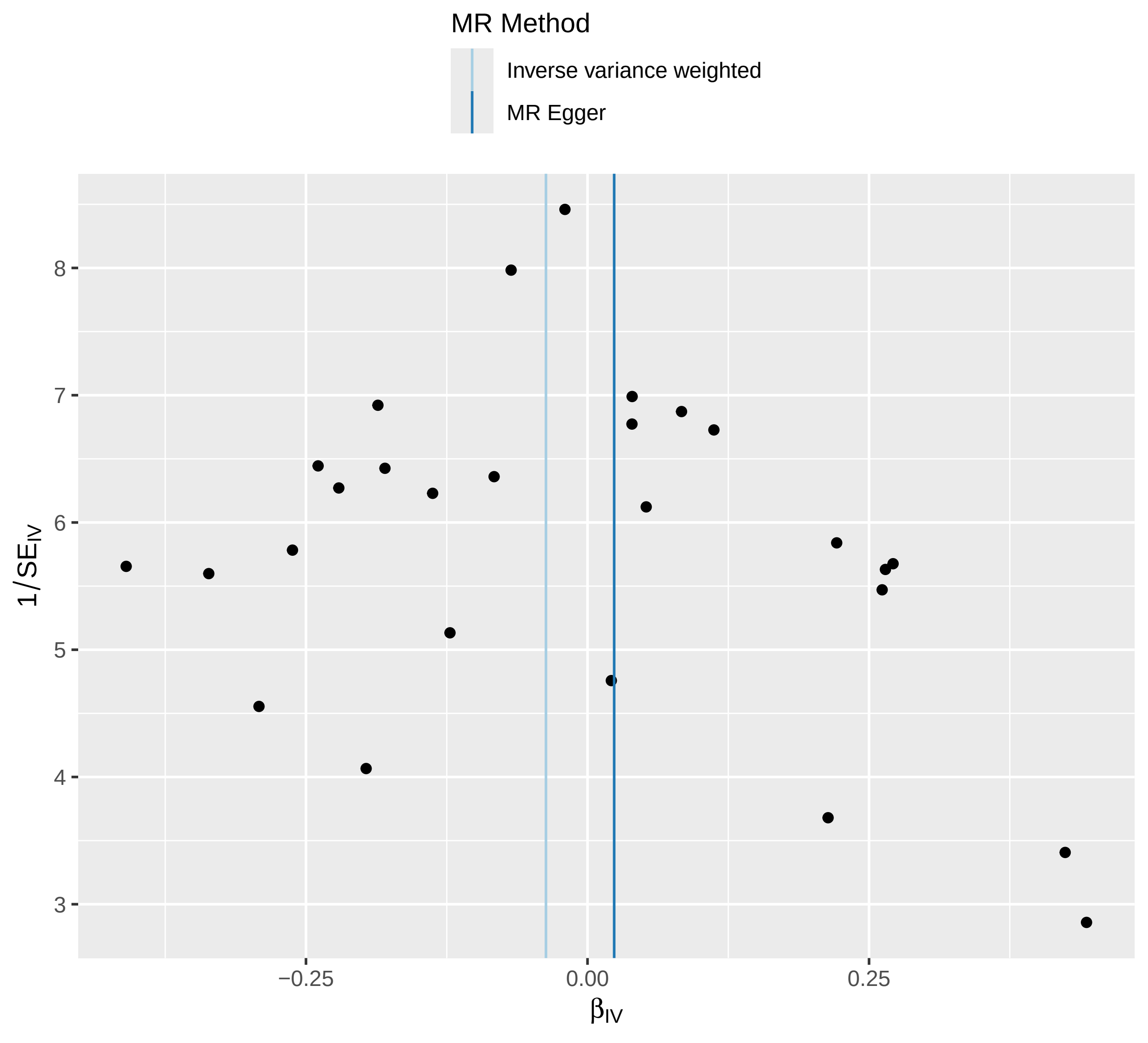

MR Method
Inverse variance weighted
MR Egger
1/SE_IV
8
7
6
5
4
3
−0.25
0.00
0.25
β_IV

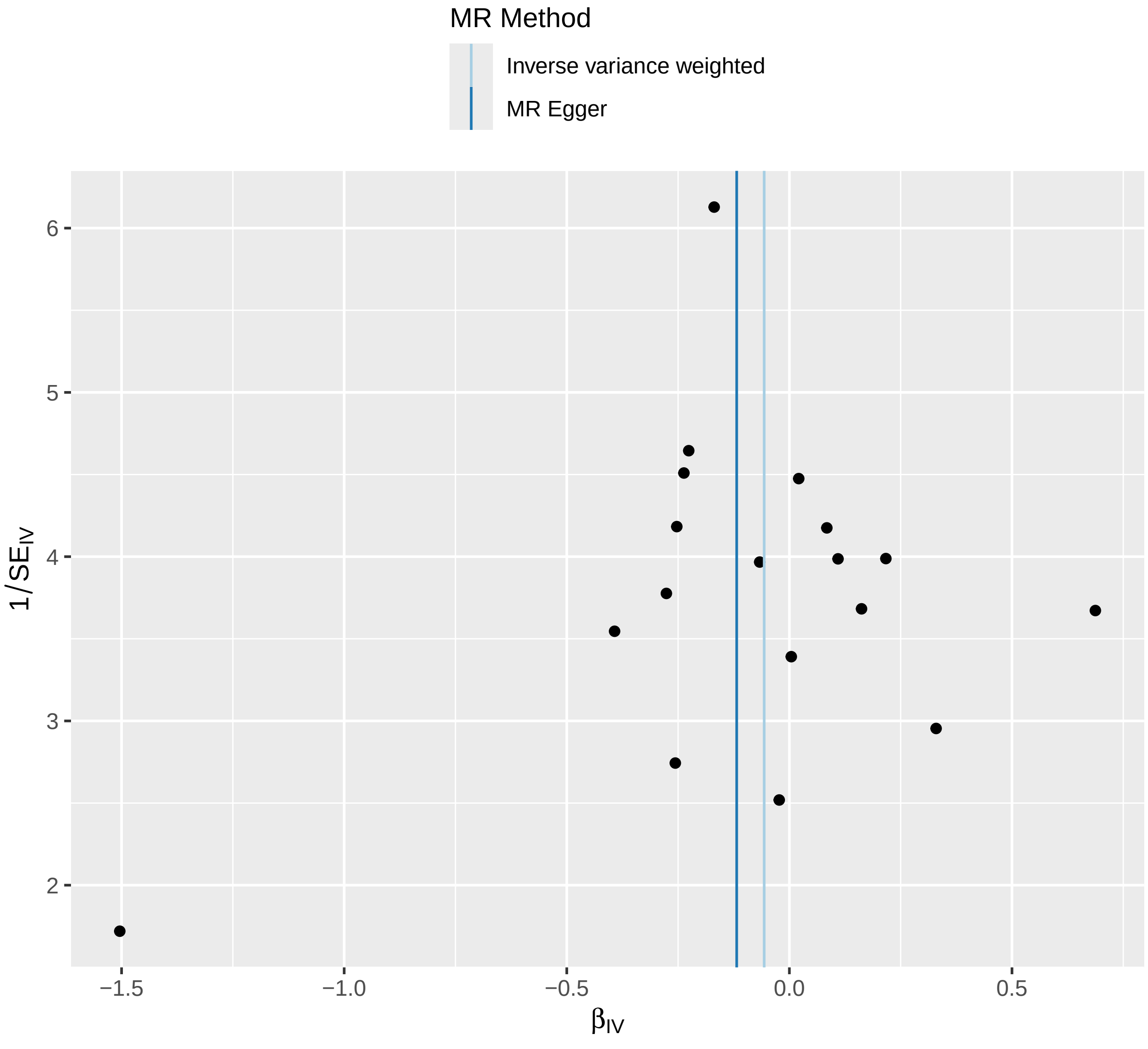
MR Method
Inverse variance weighted
MR Egger
6
5
4
3
2
1/SE_IV
−1.5
−1.0
−0.5
0.0
0.5
β_IV

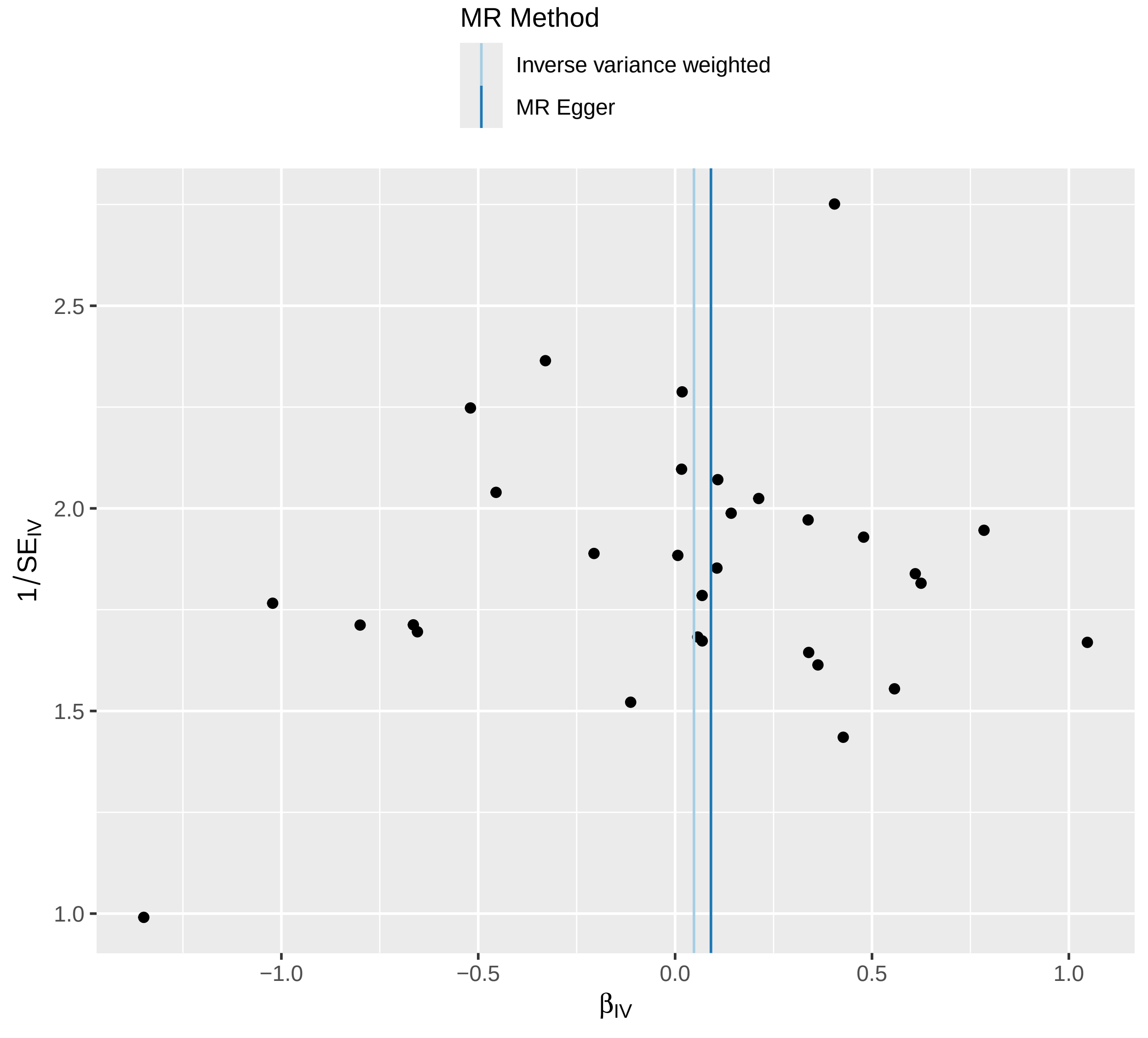
MR Method
Inverse variance weighted
MR Egger
2.5
2.0
1.5
1.0
$1/SE_{IV}$
−1.0
−0.5
0.0
0.5
1.0
β_{IV}

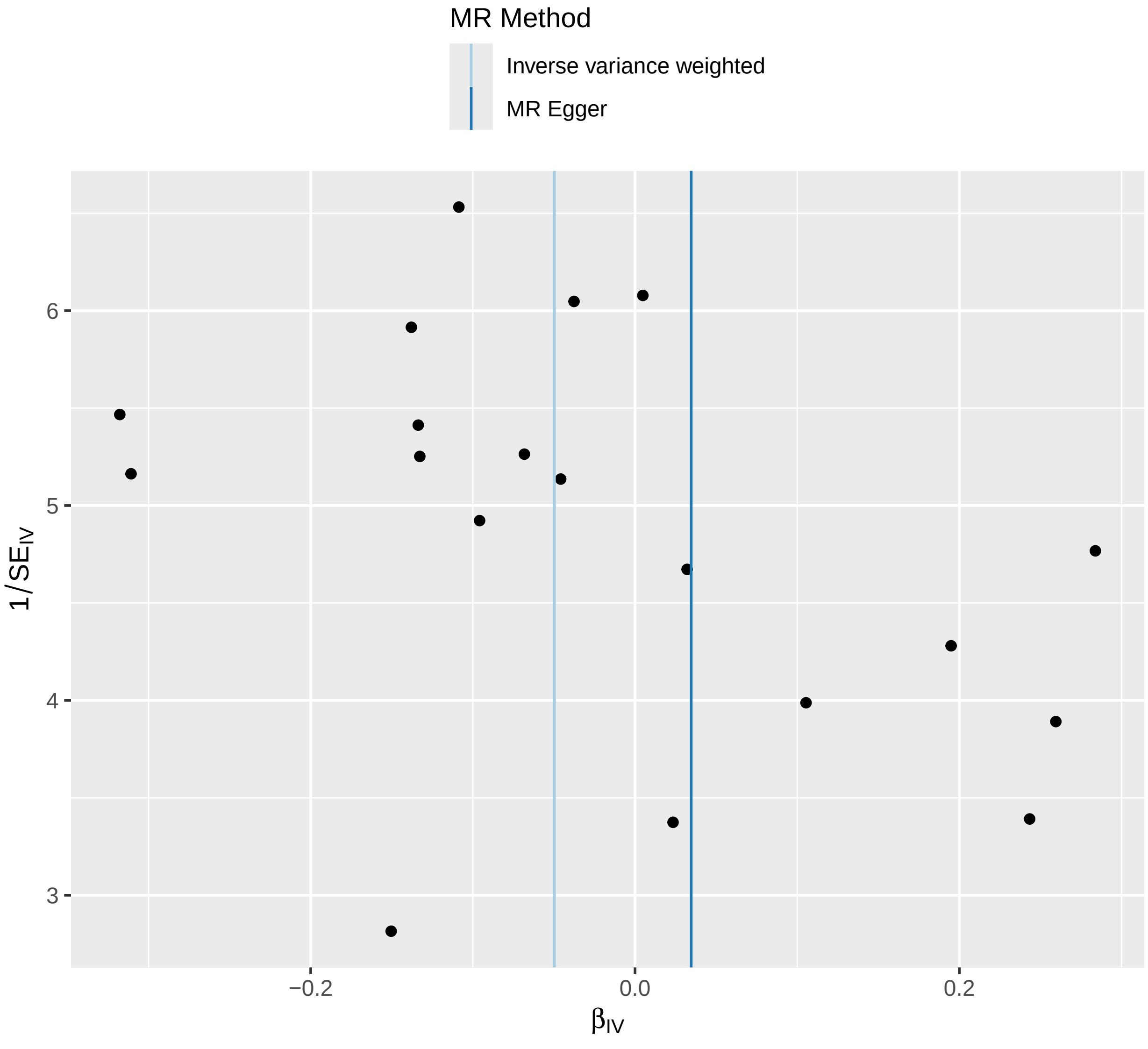
MR Method
Inverse variance weighted
MR Egger
1/SE$_{IV}$
6
5
4
3
−0.2
0.0
0.2
β$_{IV}$

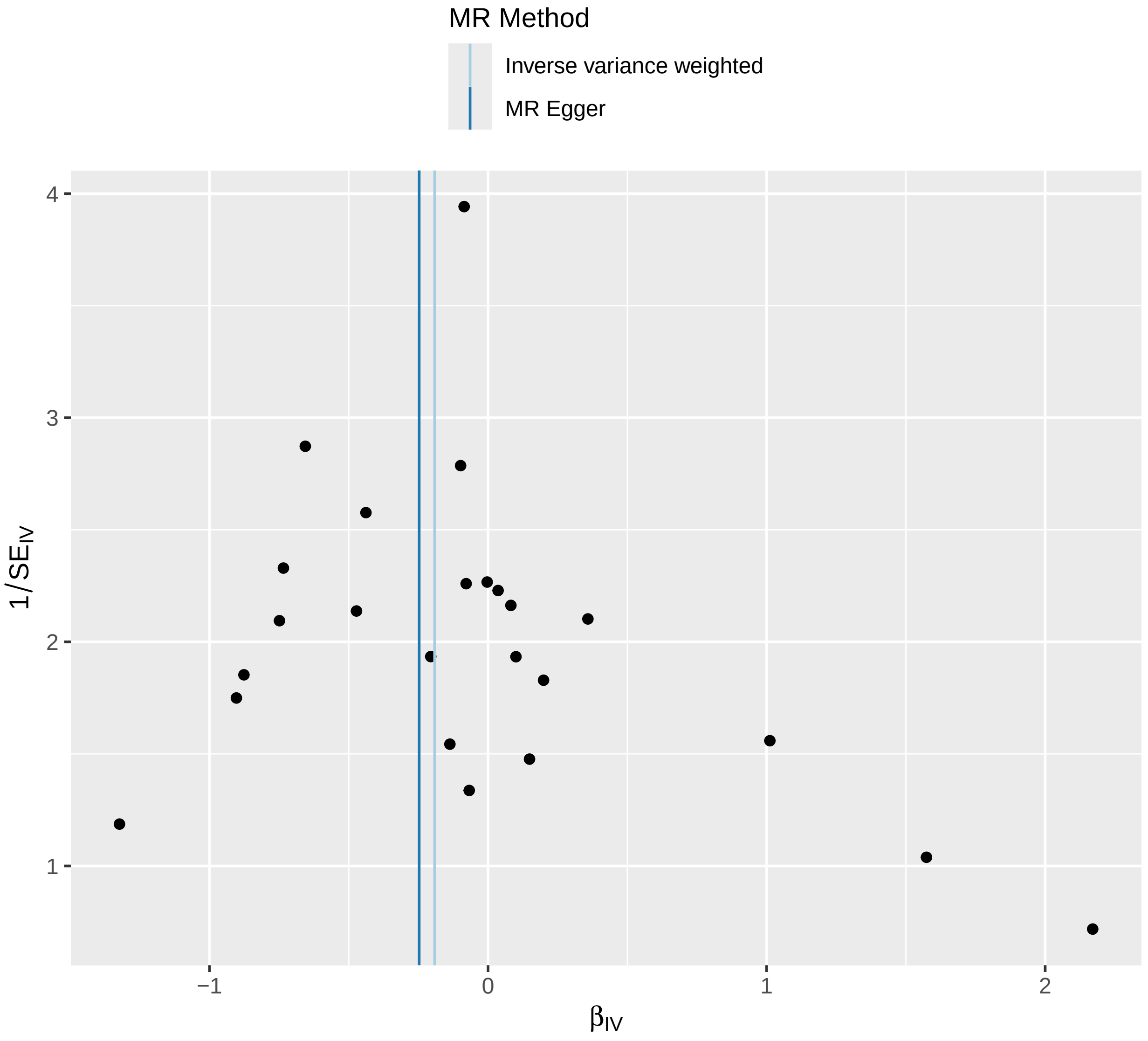
MR Method
Inverse variance weighted
MR Egger
4
3
2
1
−1
0
1
2
$1/SE_{IV}$
β_{IV}

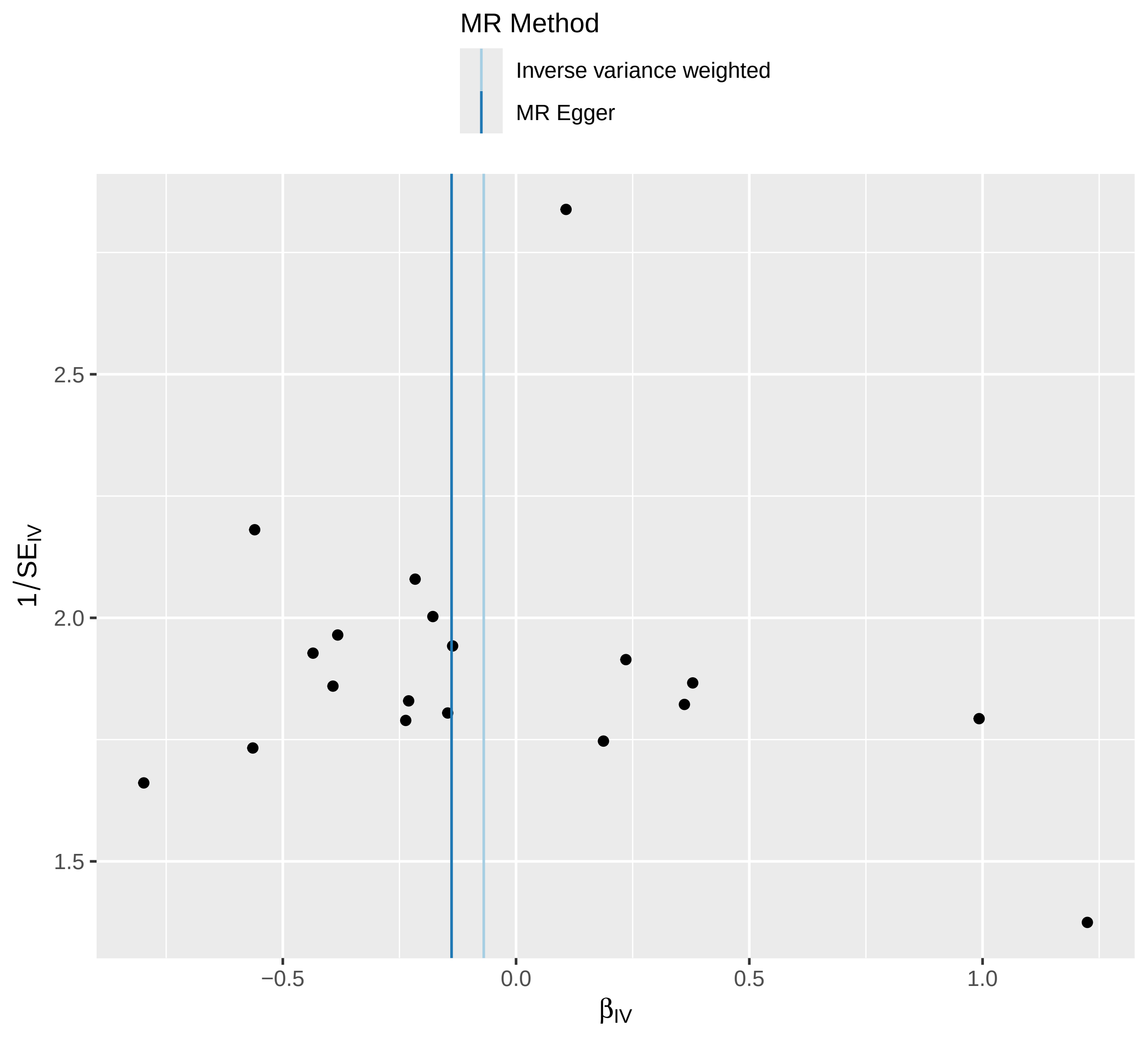
MR Method
Inverse variance weighted
MR Egger
2.5
2.0
1.5
$1/SE_{IV}$
−0.5
0.0
0.5
1.0
β_{IV}

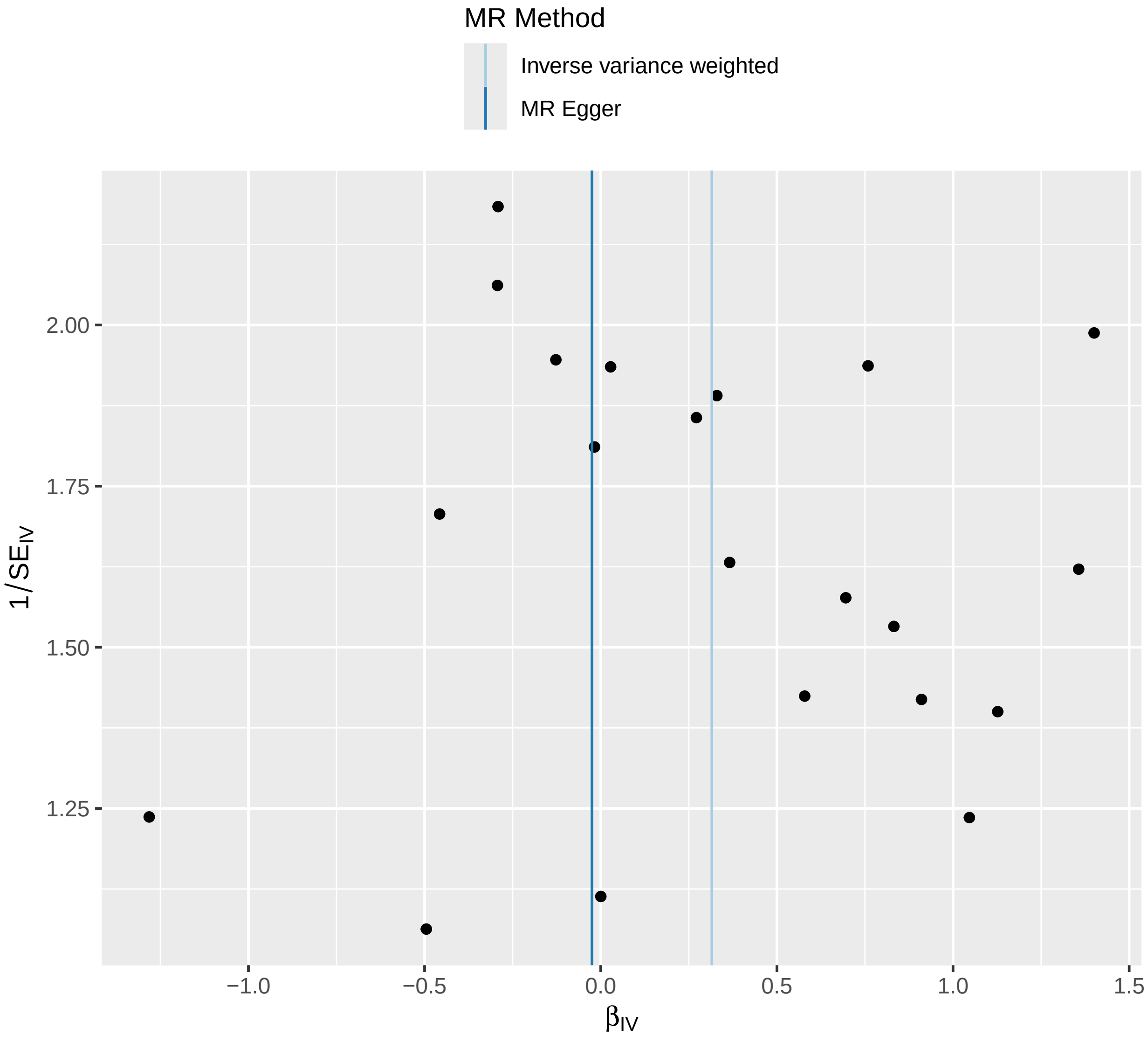
MR Method
Inverse variance weighted
MR Egger
2.00
1.75
1.50
1.25
1/SE_IV
−1.0
−0.5
0.0
0.5
1.0
1.5
β_IV

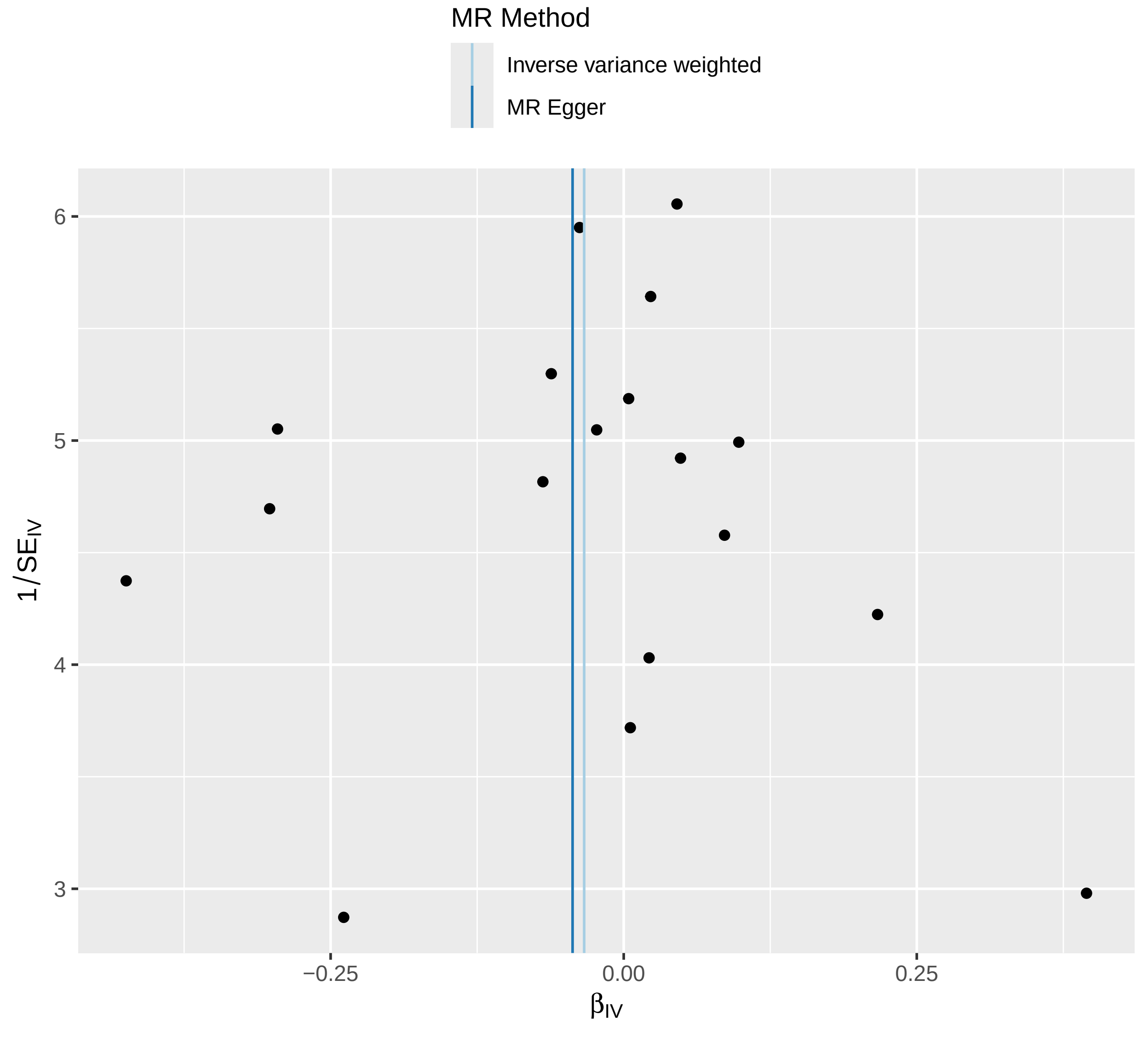

MR Method
Inverse variance weighted
MR Egger
6
5
4
3
1/SE_IV
−0.25
0.00
0.25
β_IV

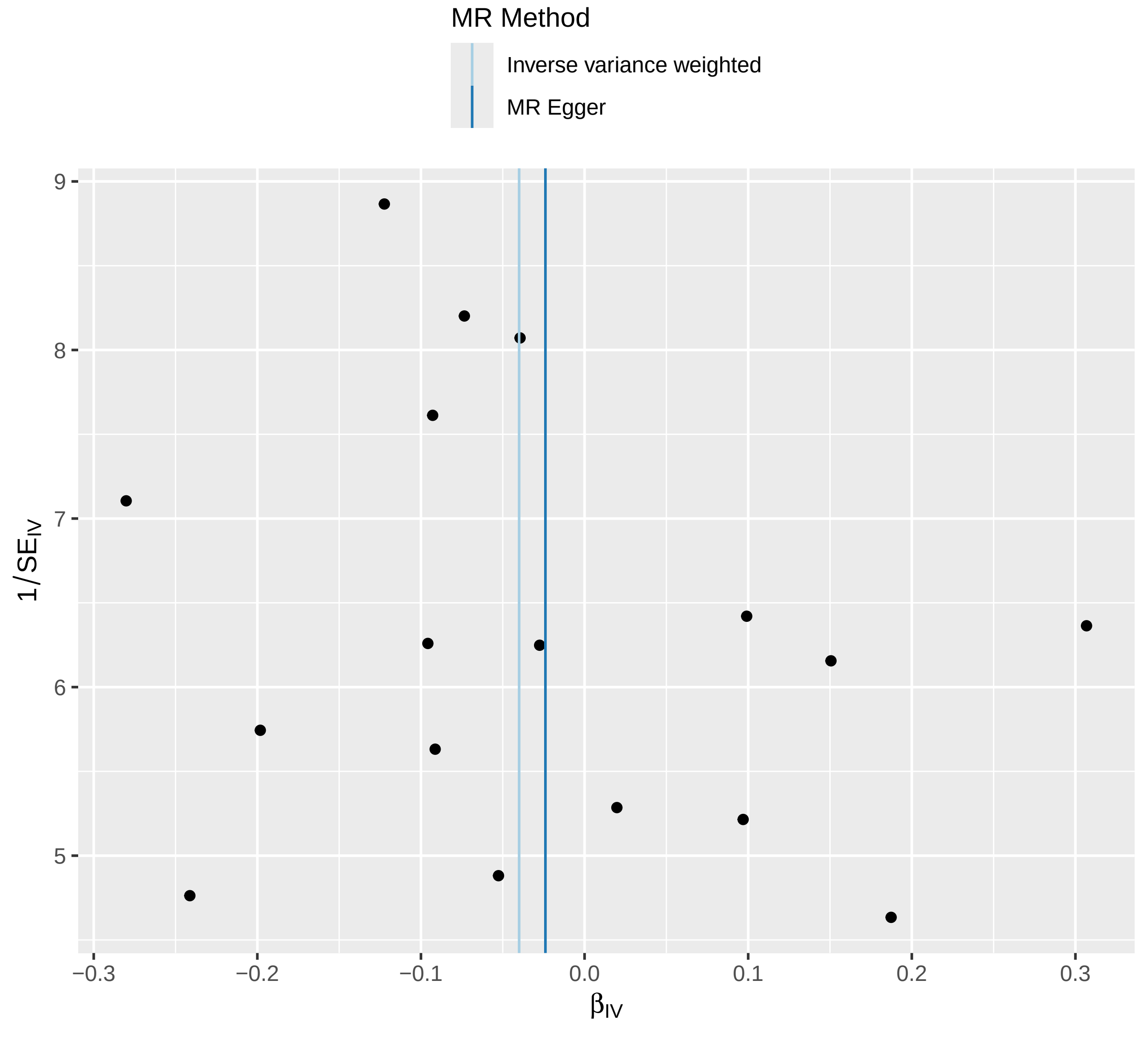
MR Method
Inverse variance weighted
MR Egger
9
8
7
6
5
1/SE_IV
−0.3
−0.2
−0.1
0.0
0.1
0.2
0.3
β_IV

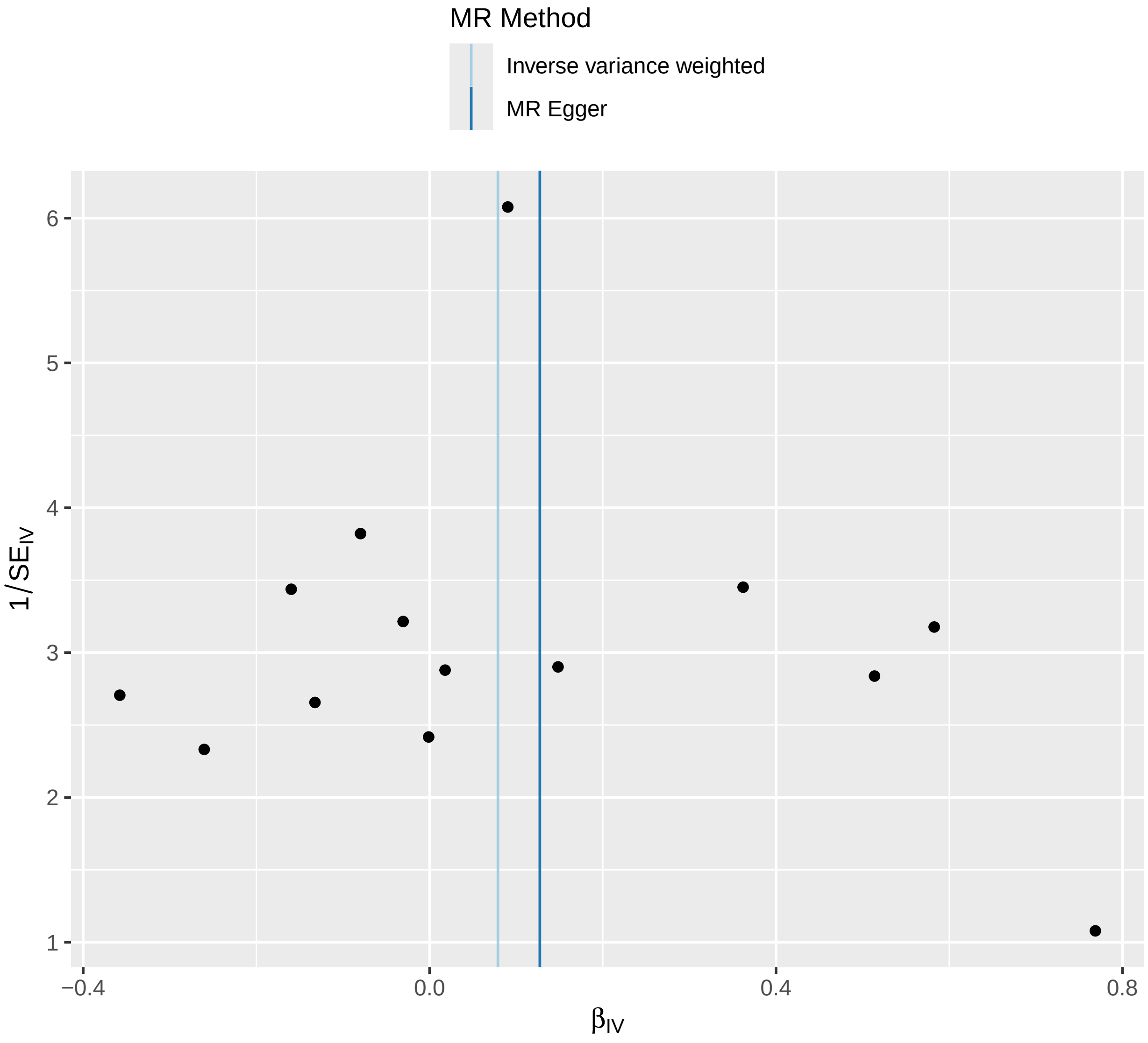
MR Method
Inverse variance weighted
MR Egger
1/SE_{IV}
β_{IV}
6
5
4
3
2
1
−0.4
0.0
0.4
0.8

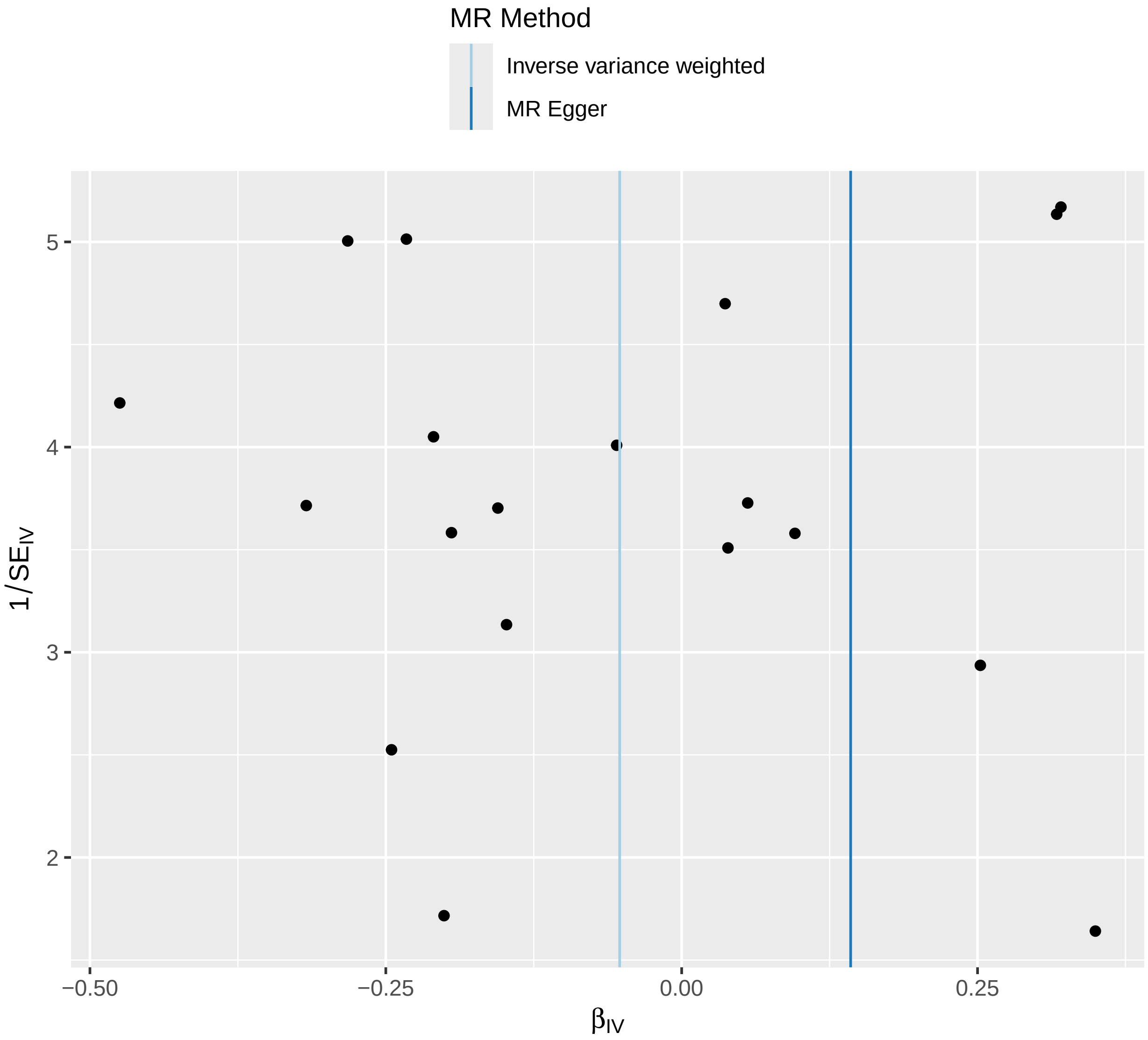

MR Method
Inverse variance weighted
MR Egger
5
4
3
2
1/SE_IV
−0.50
−0.25
0.00
0.25
β_IV

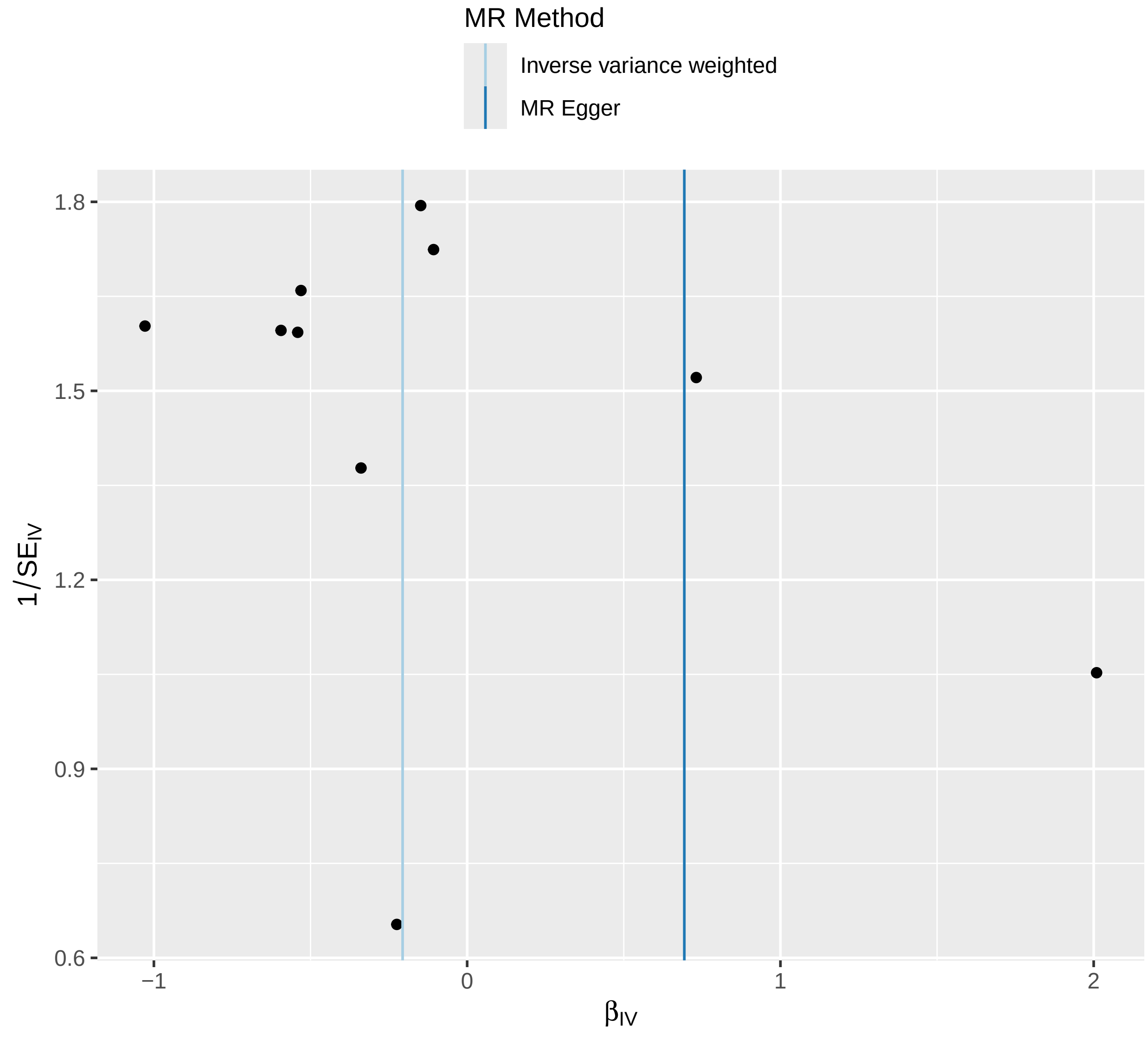
MR Method
Inverse variance weighted
MR Egger
1.8
1.5
1.2
0.9
0.6
$1/SE_{IV}$
−1
0
1
2
β_{IV}

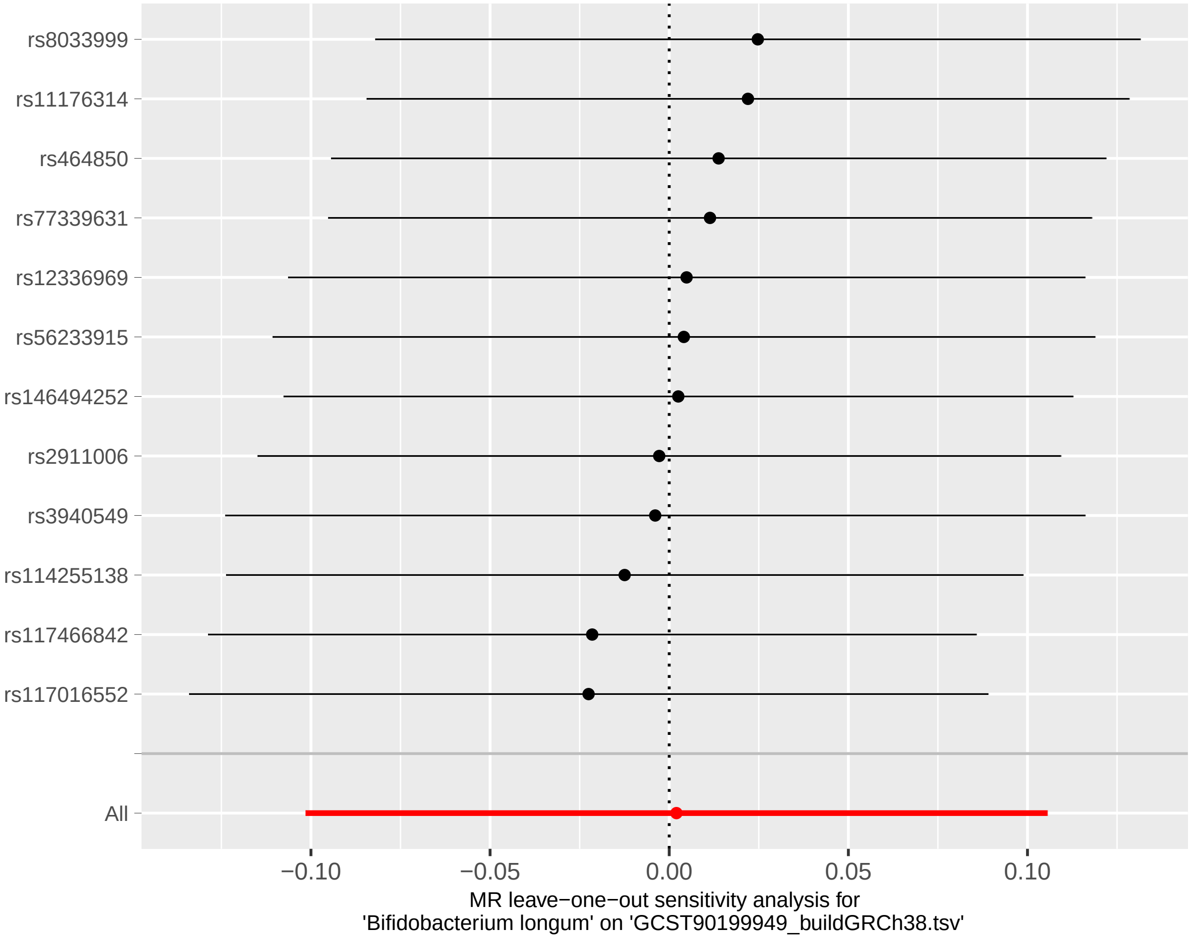
rs8033999
rs11176314
rs464850
rs77339631
rs12336969
rs56233915
rs146494252
rs2911006
rs3940549
rs114255138
rs117466842
rs117016552
All
−0.10
−0.05
0.00
0.05
0.10
MR leave−one−out sensitivity analysis for
'Bifidobacterium longum' on 'GCST90199949_buildGRCh38.tsv'

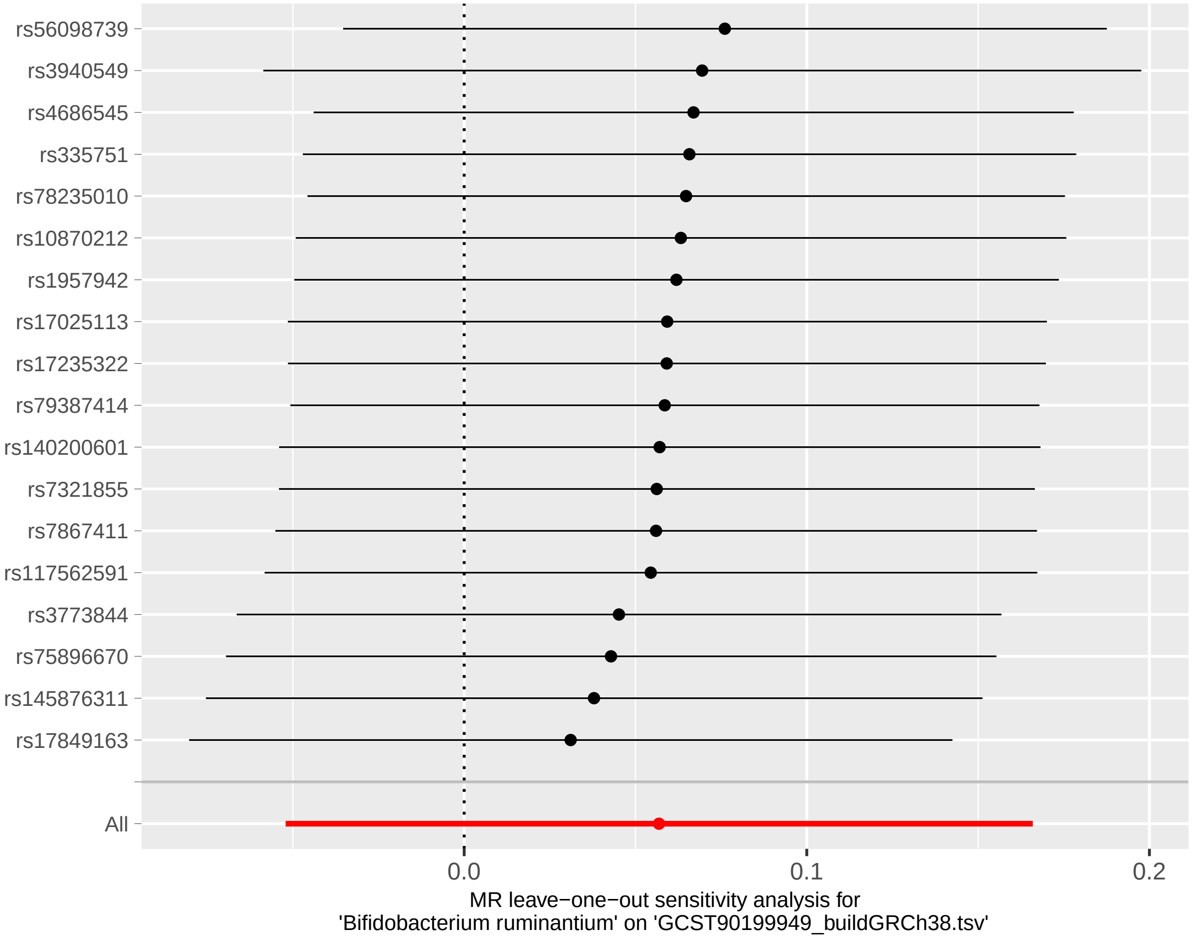

rs56098739
rs3940549
rs4686545
rs335751
rs78235010
rs10870212
rs1957942
rs17025113
rs17235322
rs79387414
rs140200601
rs7321855
rs7867411
rs117562591
rs3773844
rs75896670
rs145876311
rs17849163
All
0.0
0.1
0.2
MR leave−one−out sensitivity analysis for
'Bifidobacterium ruminantium' on 'GCST90199949_buildGRCh38.tsv'

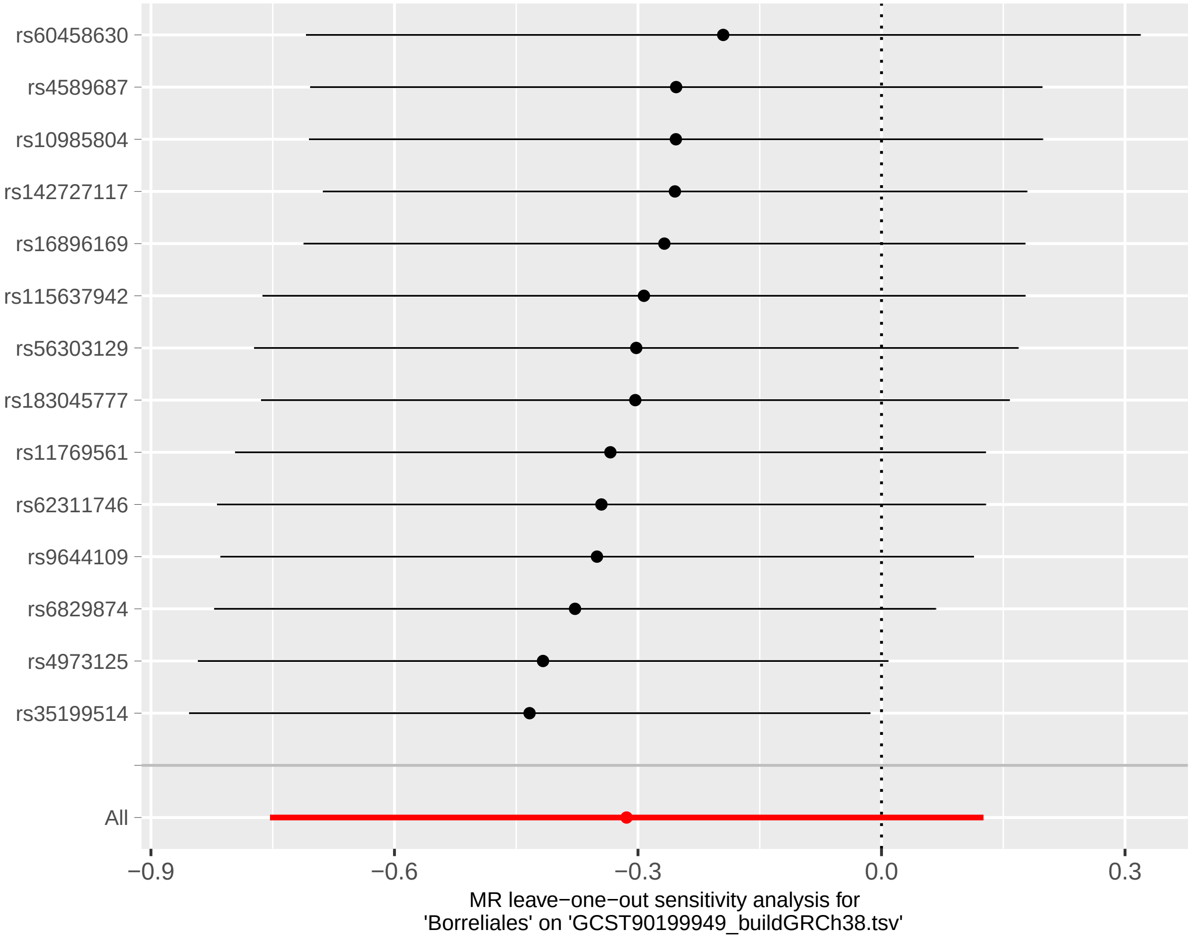

rs60458630
rs4589687
rs10985804
rs142727117
rs16896169
rs115637942
rs56303129
rs183045777
rs11769561
rs62311746
rs9644109
rs6829874
rs4973125
rs35199514
All
−0.9
−0.6
−0.3
0.0
0.3
MR leave−one−out sensitivity analysis for
'Borreliales' on 'GCST90199949_buildGRCh38.tsv'

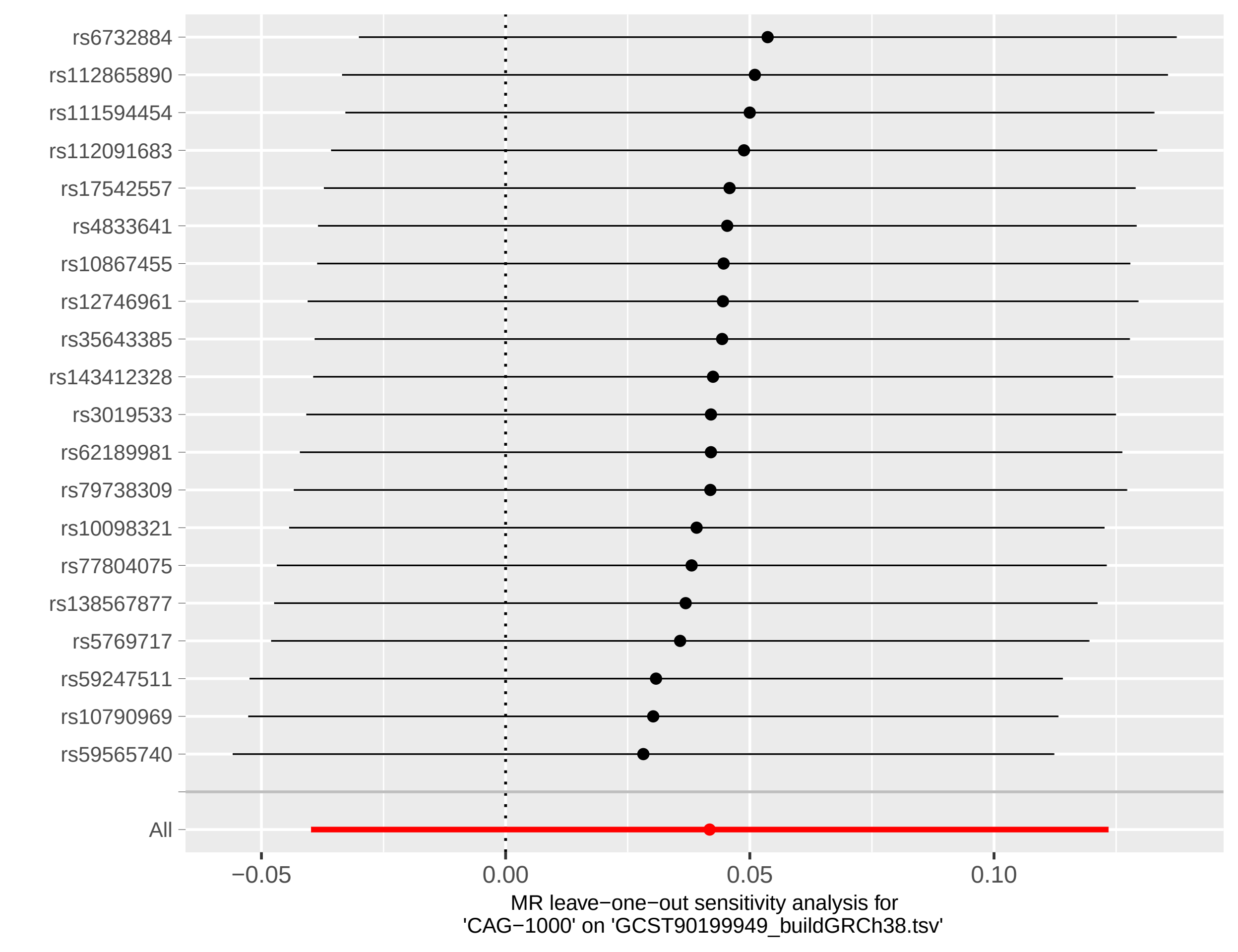

rs6732884
rs112865890
rs111594454
rs112091683
rs17542557
rs4833641
rs10867455
rs12746961
rs35643385
rs143412328
rs3019533
rs62189981
rs79738309
rs10098321
rs77804075
rs138567877
rs5769717
rs59247511
rs10790969
rs59565740
All
−0.05
0.00
0.05
0.10
MR leave−one−out sensitivity analysis for
'CAG−1000' on 'GCST90199949_buildGRCh38.tsv'

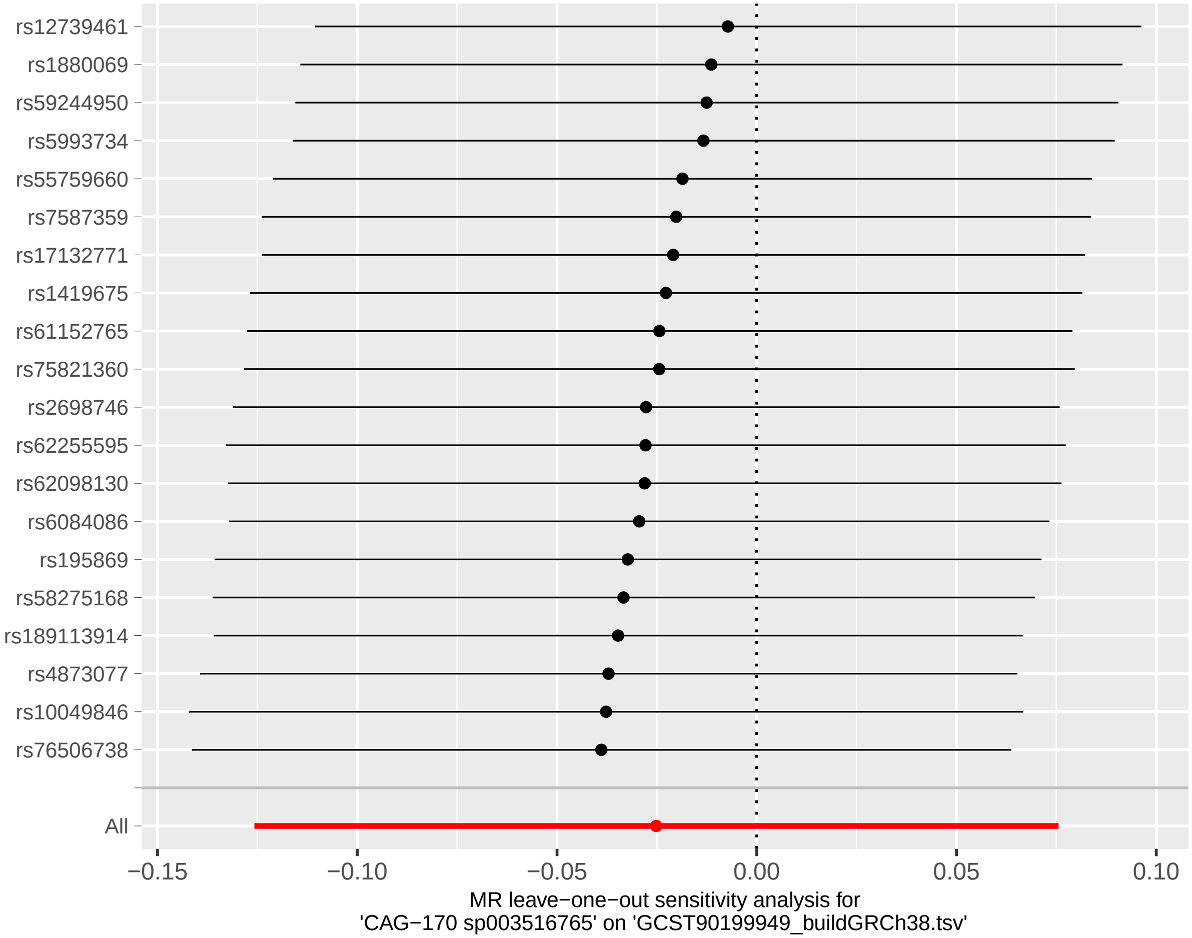

rs12739461
rs1880069
rs59244950
rs5993734
rs55759660
rs7587359
rs17132771
rs1419675
rs61152765
rs75821360
rs2698746
rs62255595
rs62098130
rs6084086
rs195869
rs58275168
rs189113914
rs4873077
rs10049846
rs76506738
All
−0.15
−0.10
−0.05
0.00
0.05
0.10
MR leave−one−out sensitivity analysis for
'CAG−170 sp003516765' on 'GCST90199949_buildGRCh38.tsv'

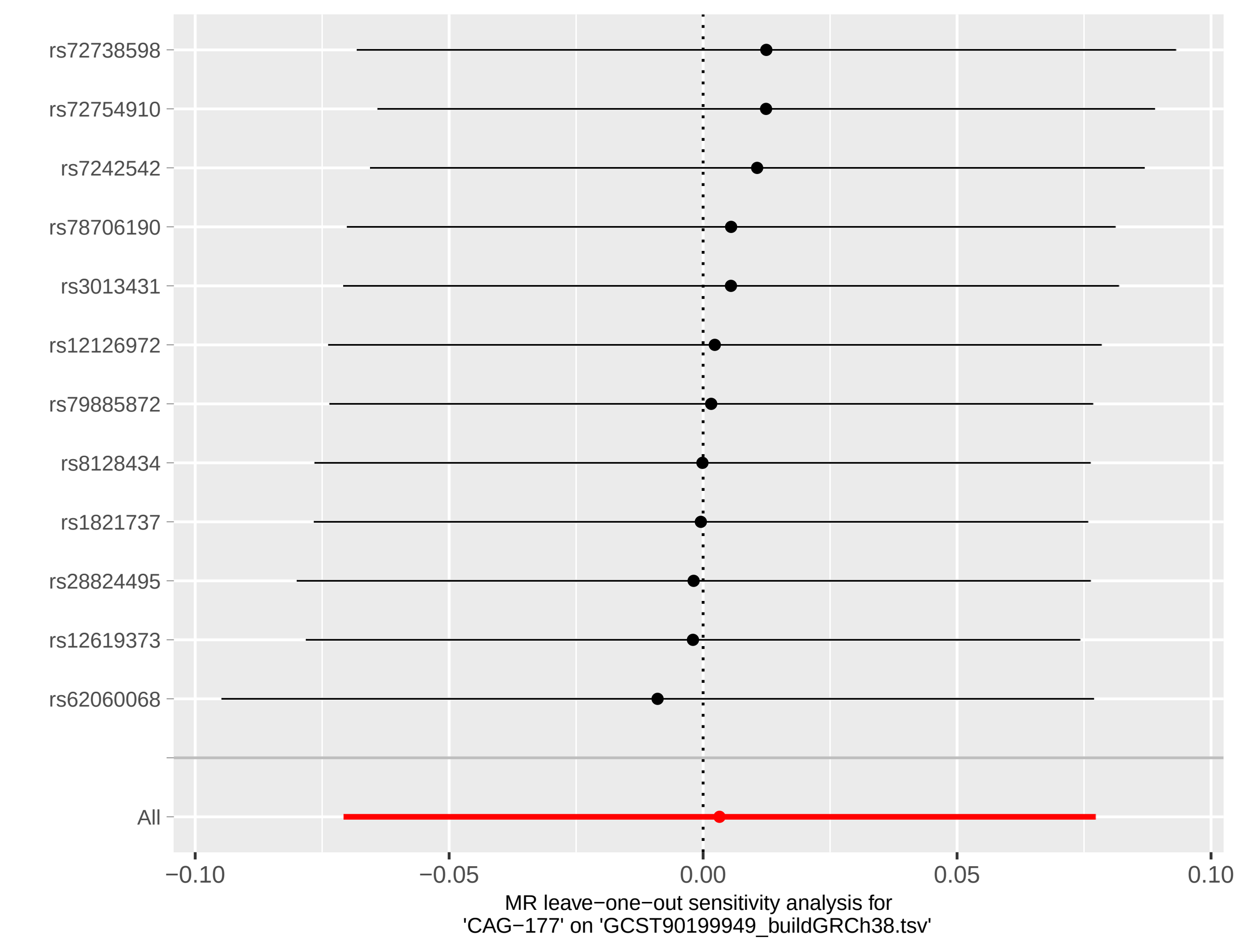
rs72738598
rs72754910
rs7242542
rs78706190
rs3013431
rs12126972
rs79885872
rs8128434
rs1821737
rs28824495
rs12619373
rs62060068
All
−0.10
−0.05
0.00
0.05
0.10
MR leave−one−out sensitivity analysis for
'CAG−177' on 'GCST90199949_buildGRCh38.tsv'

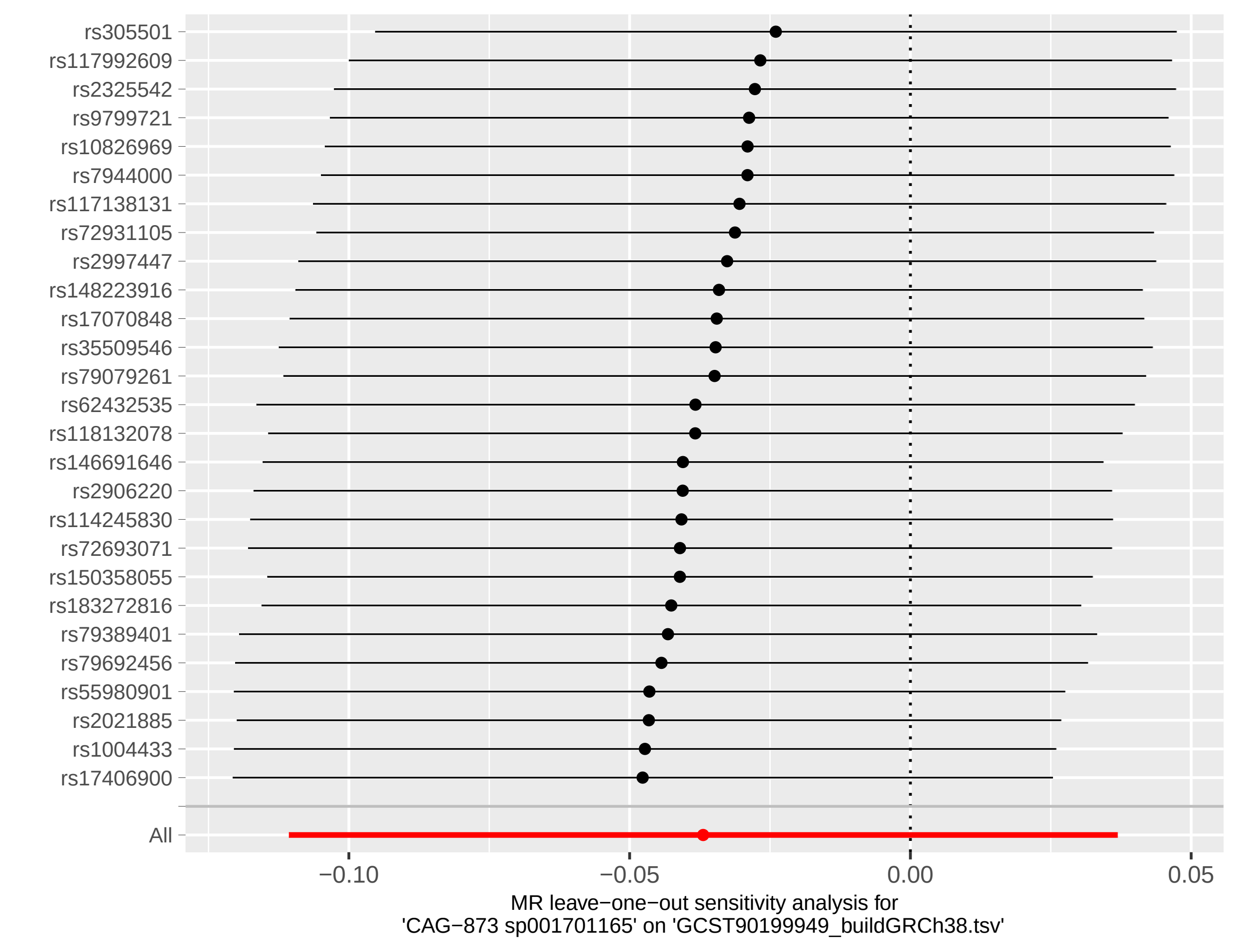
rs305501
rs117992609
rs2325542
rs9799721
rs10826969
rs7944000
rs117138131
rs72931105
rs2997447
rs148223916
rs17070848
rs35509546
rs79079261
rs62432535
rs118132078
rs146691646
rs2906220
rs114245830
rs72693071
rs150358055
rs183272816
rs79389401
rs79692456
rs55980901
rs2021885
rs1004433
rs17406900
All
−0.10
−0.05
0.00
0.05
MR leave−one−out sensitivity analysis for
'CAG−873 sp001701165' on 'GCST90199949_buildGRCh38.tsv'

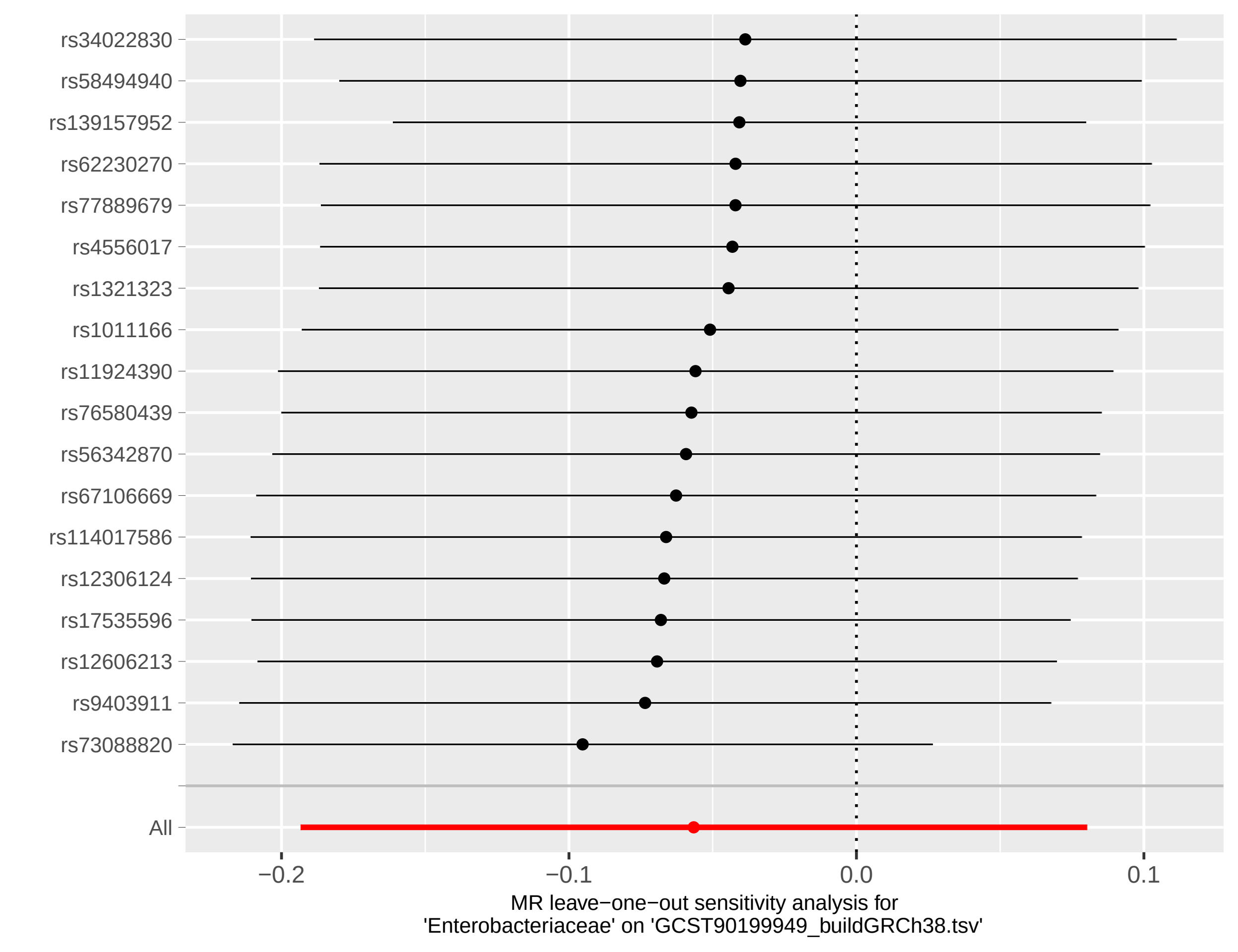
rs34022830
rs58494940
rs139157952
rs62230270
rs77889679
rs4556017
rs1321323
rs1011166
rs11924390
rs76580439
rs56342870
rs67106669
rs114017586
rs12306124
rs17535596
rs12606213
rs9403911
rs73088820
All
−0.2
−0.1
0.0
0.1
MR leave−one−out sensitivity analysis for
'Enterobacteriaceae' on 'GCST90199949_buildGRCh38.tsv'

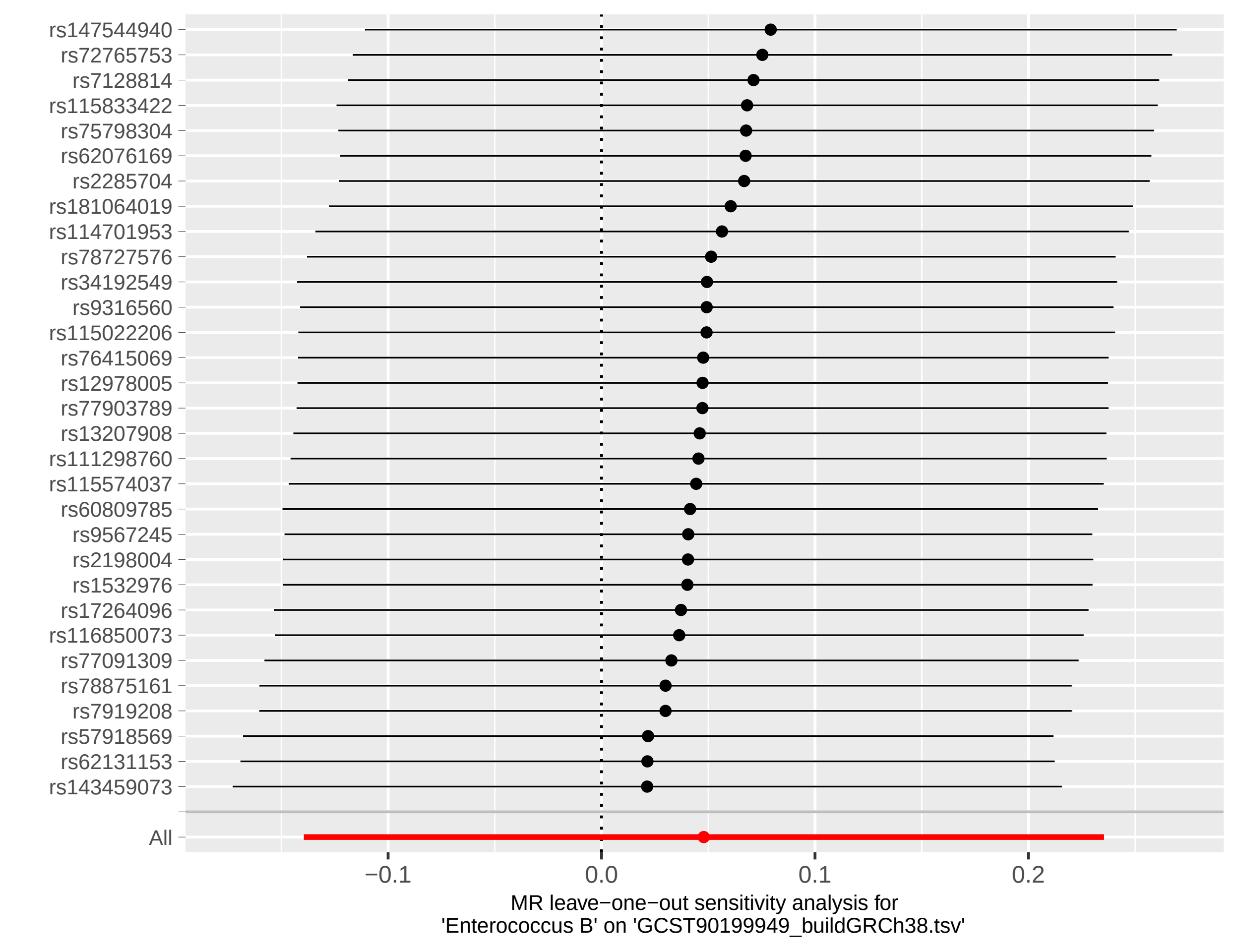

rs147544940
rs72765753
rs7128814
rs115833422
rs75798304
rs62076169
rs2285704
rs181064019
rs114701953
rs78727576
rs34192549
rs9316560
rs115022206
rs76415069
rs12978005
rs77903789
rs13207908
rs111298760
rs115574037
rs60809785
rs9567245
rs2198004
rs1532976
rs17264096
rs116850073
rs77091309
rs78875161
rs7919208
rs57918569
rs62131153
rs143459073
All
−0.1
0.0
0.1
0.2
MR leave−one−out sensitivity analysis for
'Enterococcus B' on 'GCST90199949_buildGRCh38.tsv'

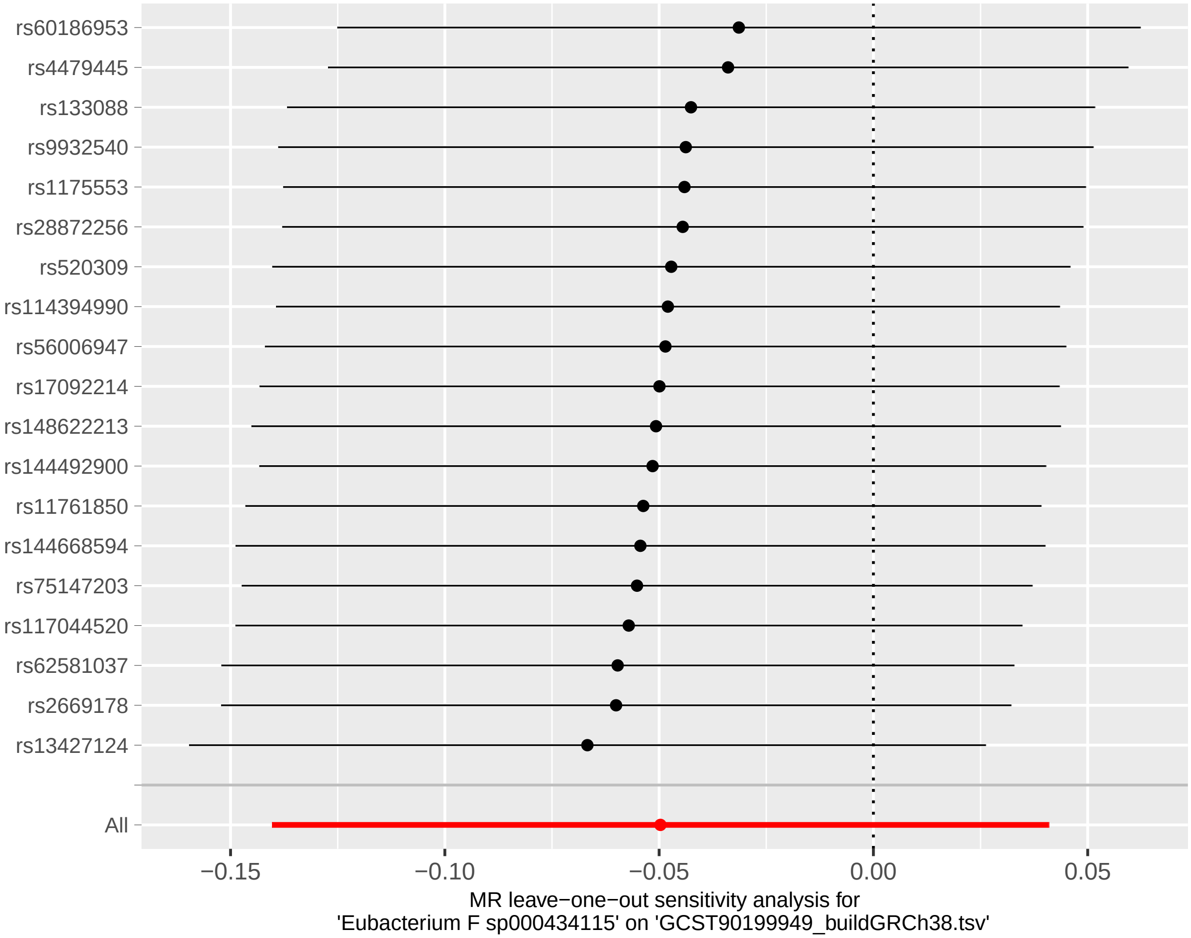

rs60186953
rs4479445
rs133088
rs9932540
rs1175553
rs28872256
rs520309
rs114394990
rs56006947
rs17092214
rs148622213
rs144492900
rs11761850
rs144668594
rs75147203
rs117044520
rs62581037
rs2669178
rs13427124
All
−0.15
−0.10
−0.05
0.00
0.05
MR leave−one−out sensitivity analysis for
'Eubacterium F sp000434115' on 'GCST90199949_buildGRCh38.tsv'

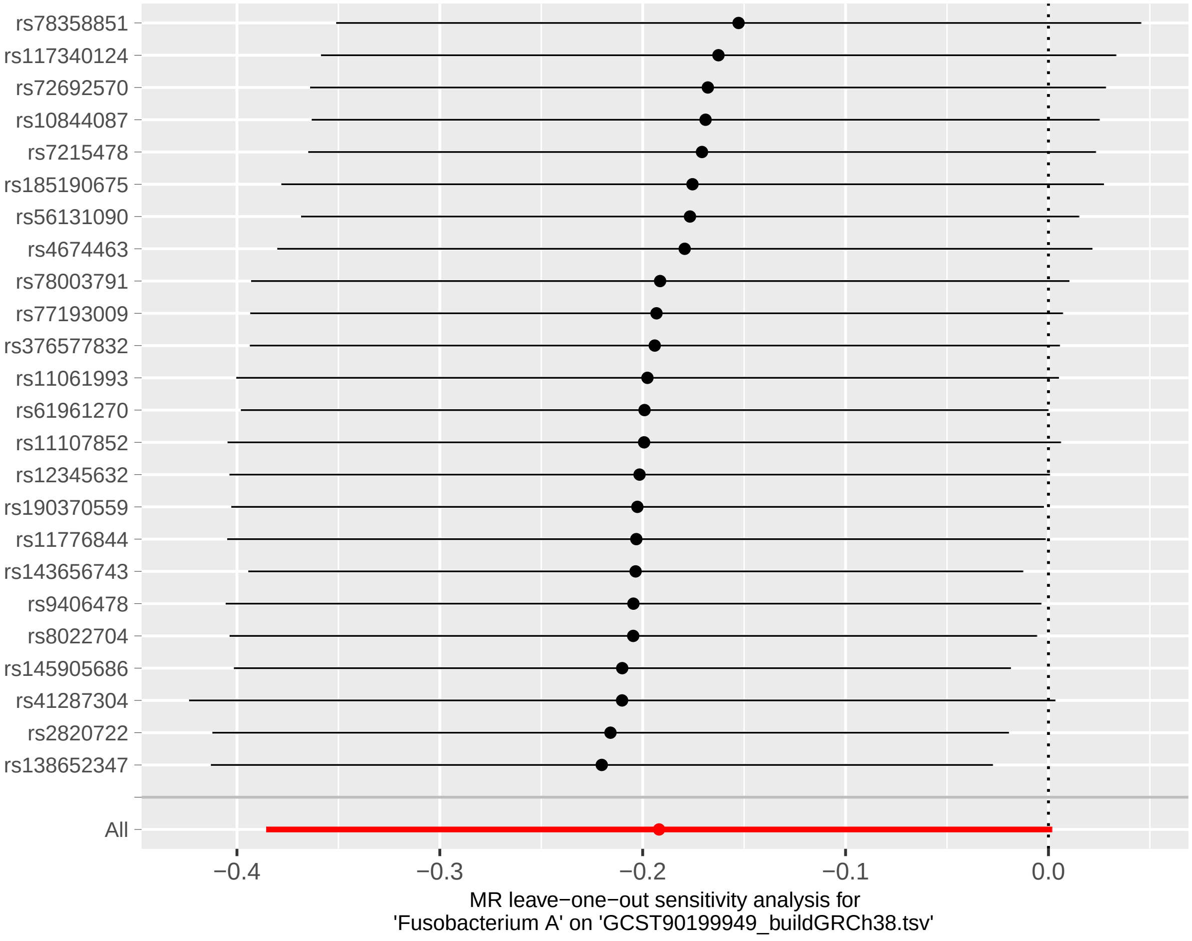

rs78358851
rs117340124
rs72692570
rs10844087
rs7215478
rs185190675
rs56131090
rs4674463
rs78003791
rs77193009
rs376577832
rs11061993
rs61961270
rs11107852
rs12345632
rs190370559
rs11776844
rs143656743
rs9406478
rs8022704
rs145905686
rs41287304
rs2820722
rs138652347
All
−0.4
−0.3
−0.2
−0.1
0.0
MR leave−one−out sensitivity analysis for
'Fusobacterium A' on 'GCST90199949_buildGRCh38.tsv'

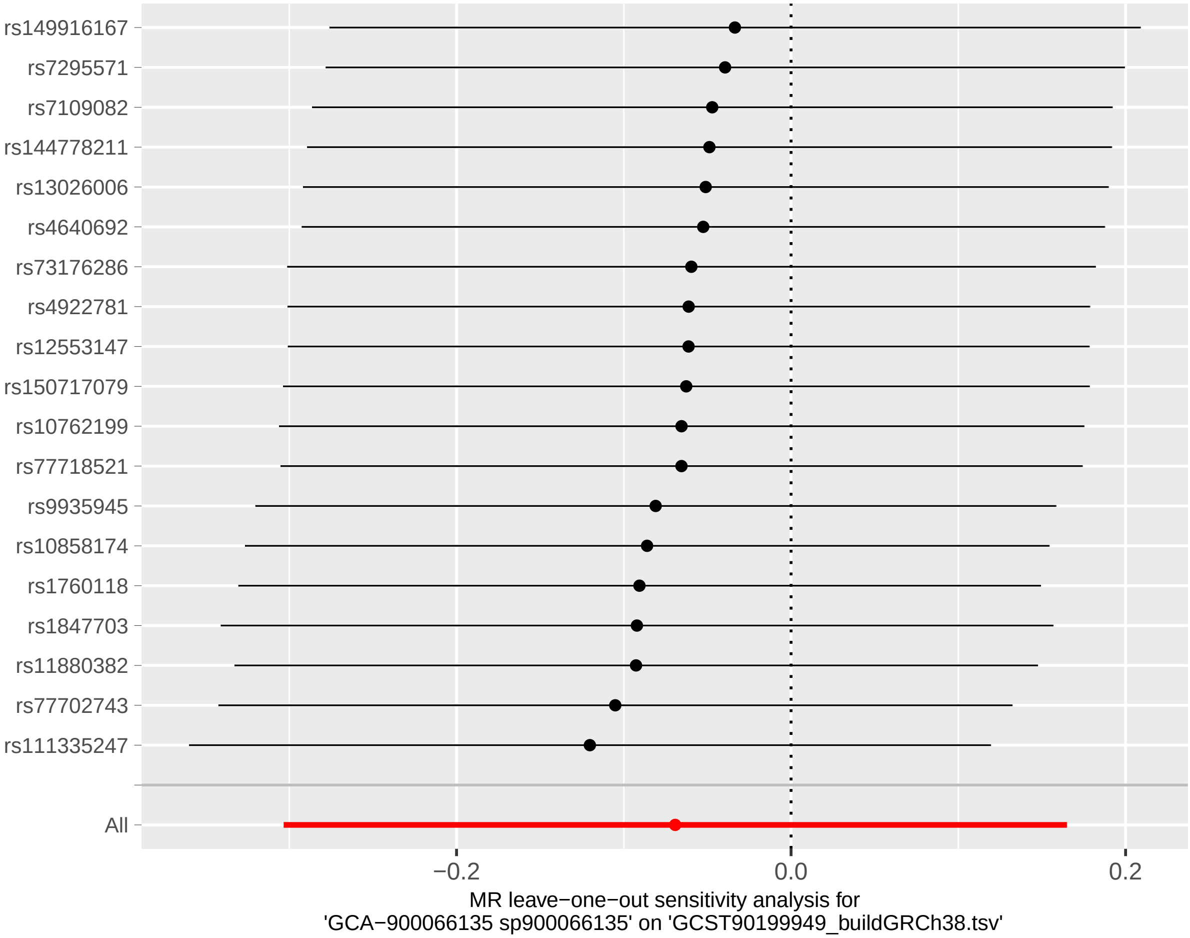

rs149916167
rs7295571
rs7109082
rs144778211
rs13026006
rs4640692
rs73176286
rs4922781
rs12553147
rs150717079
rs10762199
rs77718521
rs9935945
rs10858174
rs1760118
rs1847703
rs11880382
rs77702743
rs111335247
All
−0.2
0.0
0.2
MR leave−one−out sensitivity analysis for
'GCA−900066135 sp900066135' on 'GCST90199949_buildGRCh38.tsv'

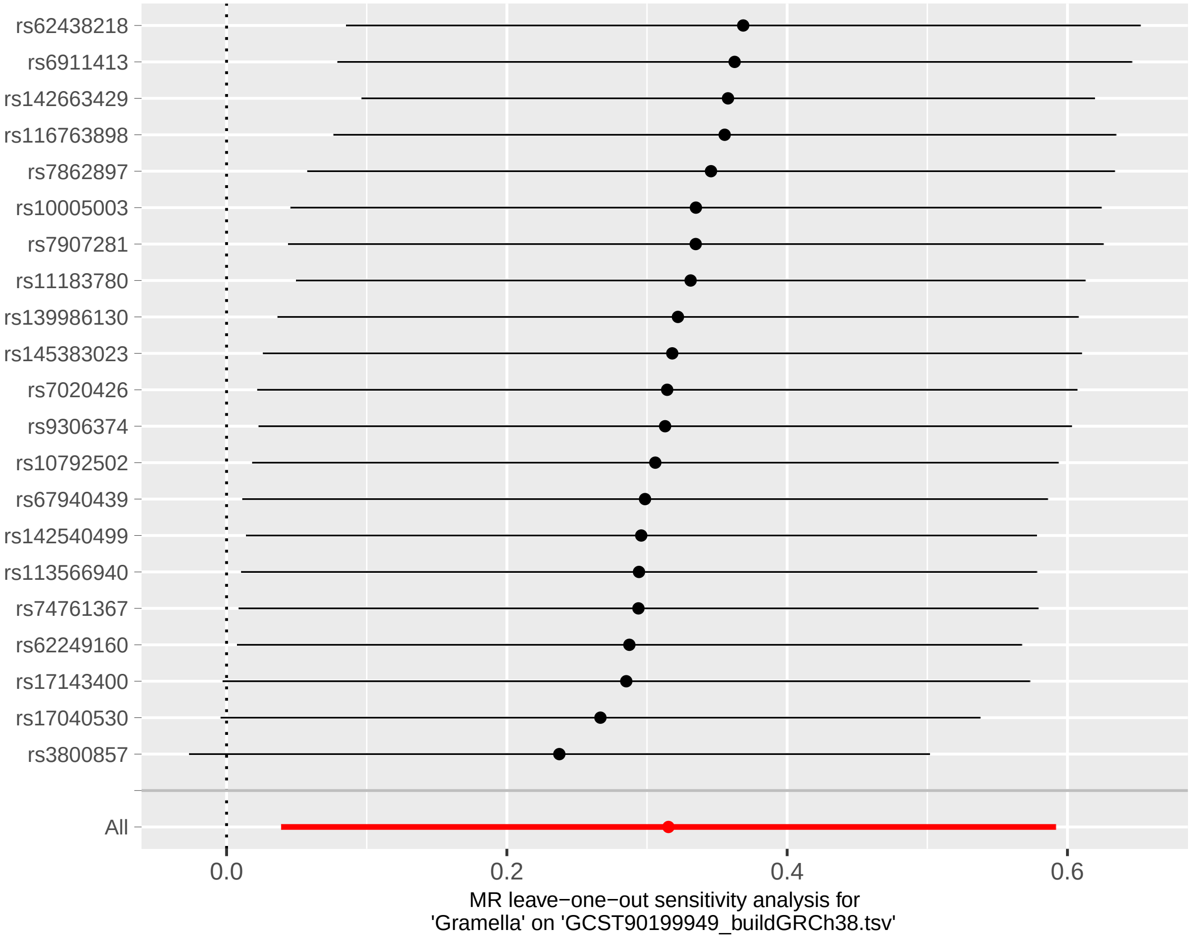
rs62438218
rs6911413
rs142663429
rs116763898
rs7862897
rs10005003
rs7907281
rs11183780
rs139986130
rs145383023
rs7020426
rs9306374
rs10792502
rs67940439
rs142540499
rs113566940
rs74761367
rs62249160
rs17143400
rs17040530
rs3800857
All
0.0
0.2
0.4
0.6
MR leave−one−out sensitivity analysis for
'Gramella' on 'GCST90199949_buildGRCh38.tsv'

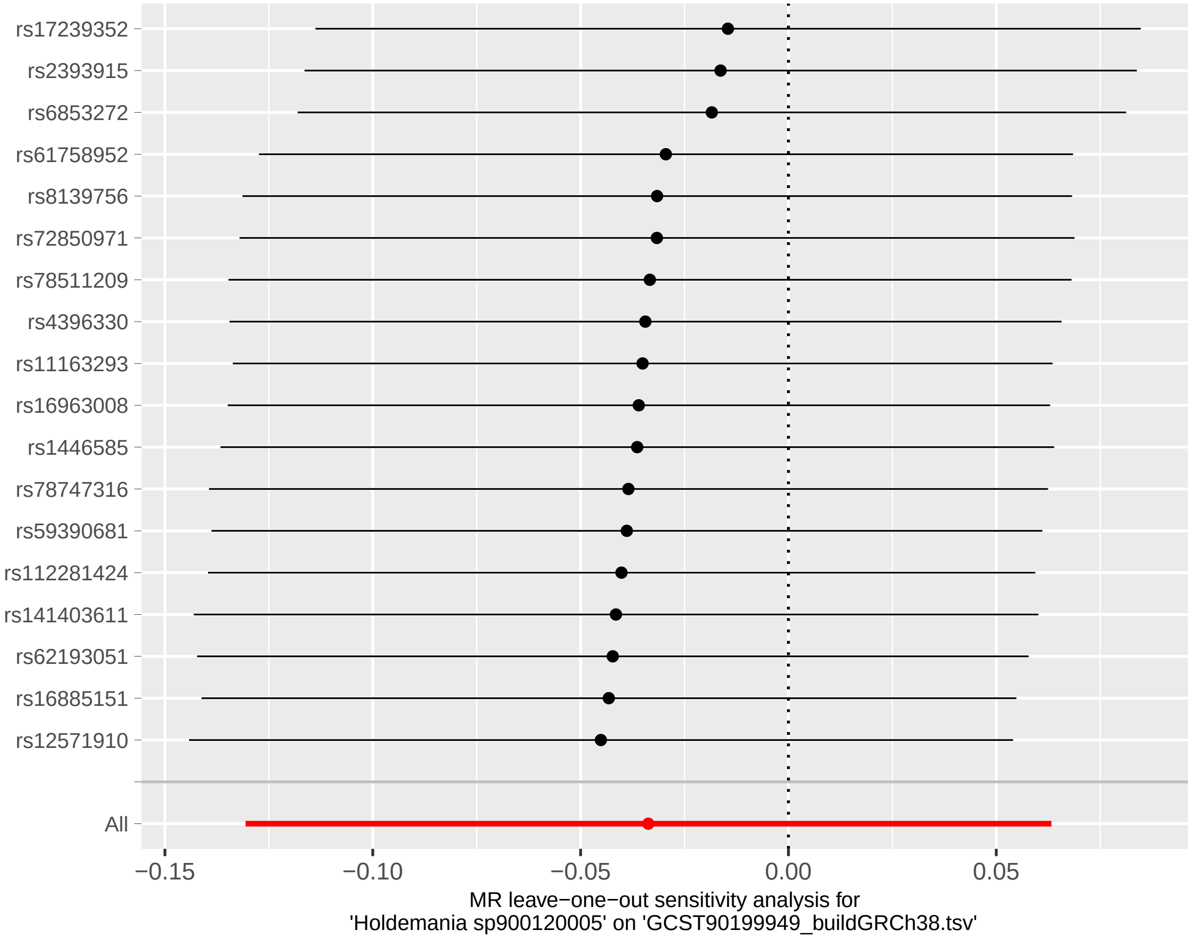

rs17239352
rs2393915
rs6853272
rs61758952
rs8139756
rs72850971
rs78511209
rs4396330
rs11163293
rs16963008
rs1446585
rs78747316
rs59390681
rs112281424
rs141403611
rs62193051
rs16885151
rs12571910
All
−0.15
−0.10
−0.05
0.00
0.05
MR leave−one−out sensitivity analysis for
'Holdemania sp900120005' on 'GCST90199949_buildGRCh38.tsv'

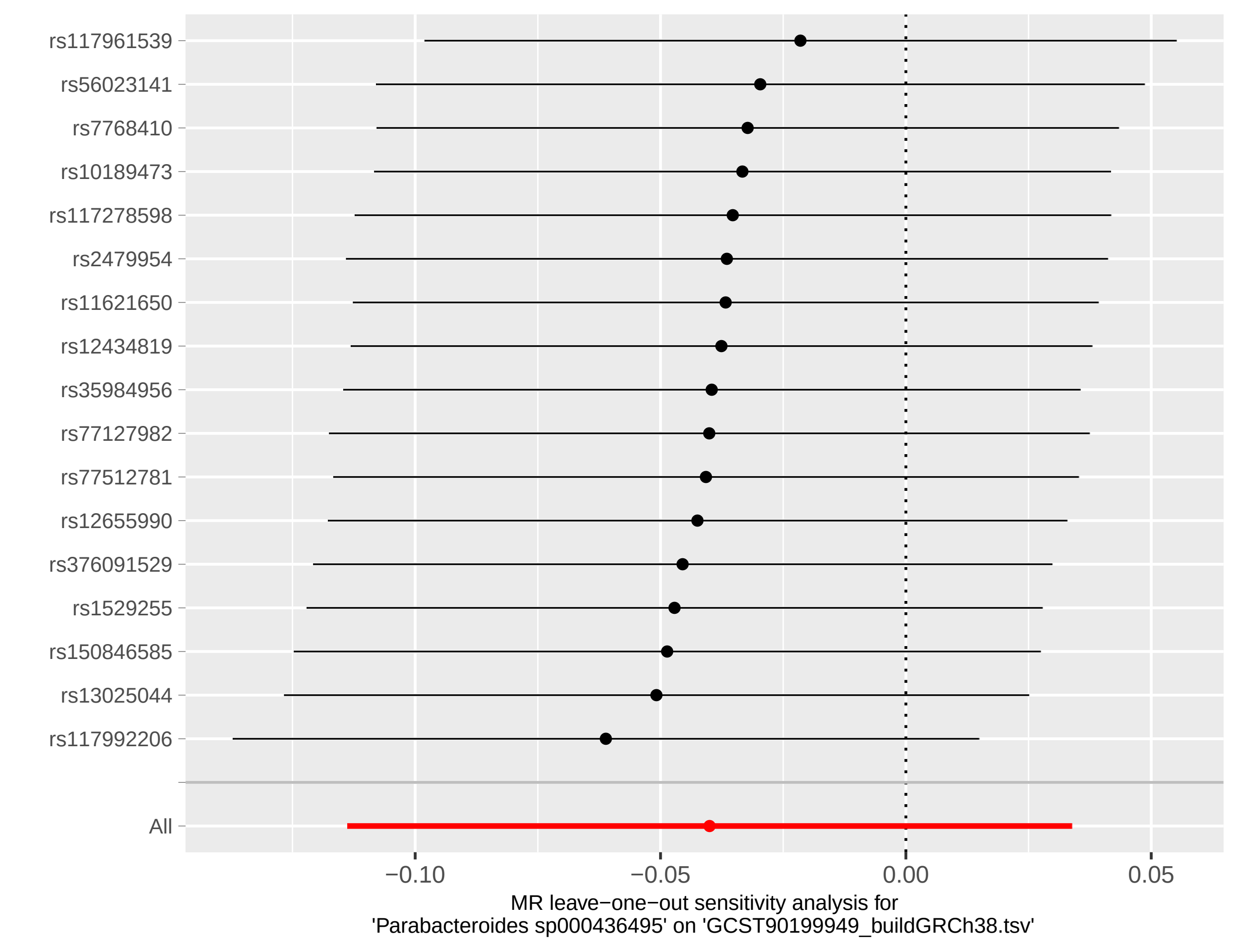

rs117961539
rs56023141
rs7768410
rs10189473
rs117278598
rs2479954
rs11621650
rs12434819
rs35984956
rs77127982
rs77512781
rs12655990
rs376091529
rs1529255
rs150846585
rs13025044
rs117992206
All
−0.10
−0.05
0.00
0.05
MR leave−one−out sensitivity analysis for
'Parabacteroides sp000436495' on 'GCST90199949_buildGRCh38.tsv'

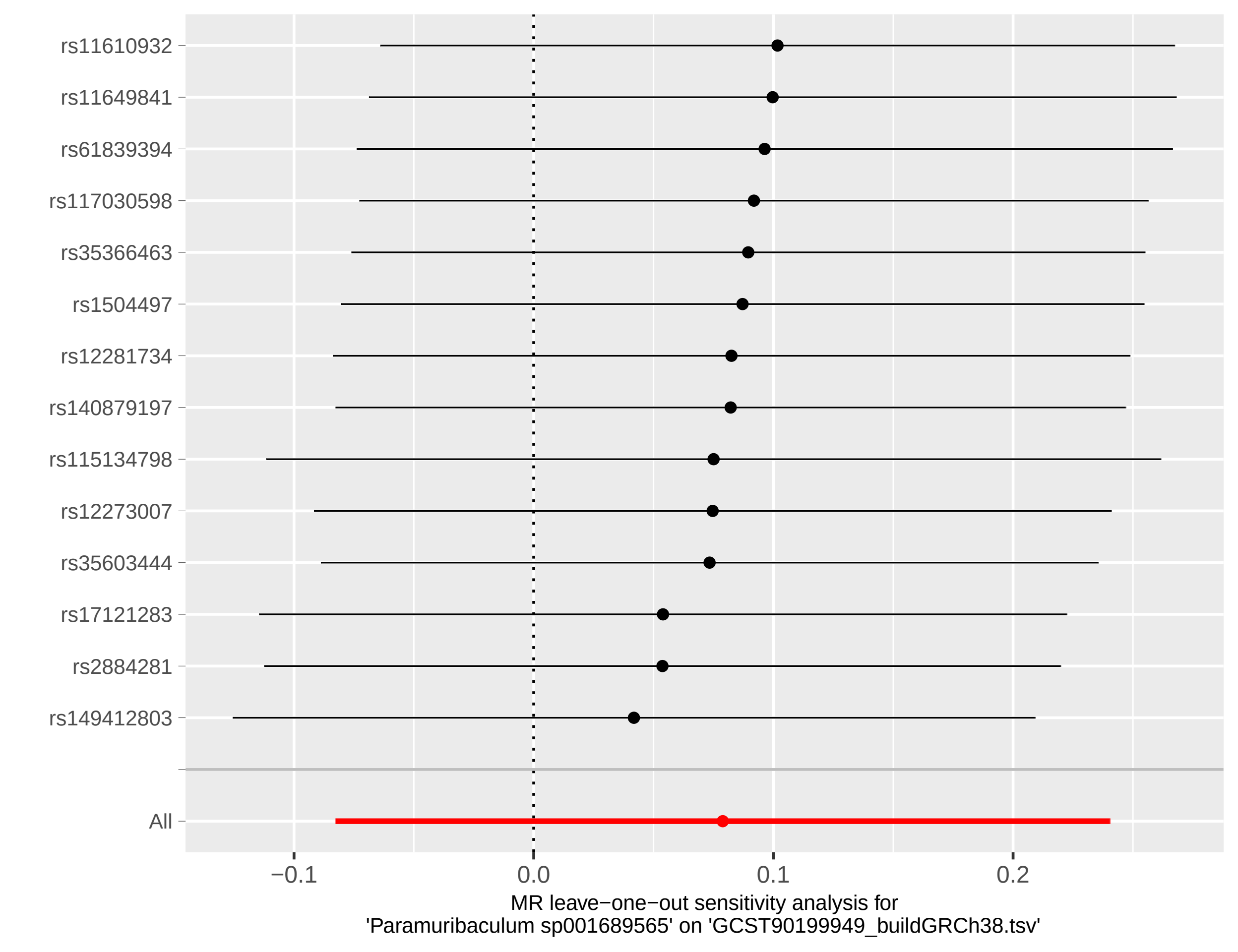

rs11610932
rs11649841
rs61839394
rs117030598
rs35366463
rs1504497
rs12281734
rs140879197
rs115134798
rs12273007
rs35603444
rs17121283
rs2884281
rs149412803
All
−0.1
0.0
0.1
0.2
MR leave−one−out sensitivity analysis for
'Paramuribaculum sp001689565' on 'GCST90199949_buildGRCh38.tsv'

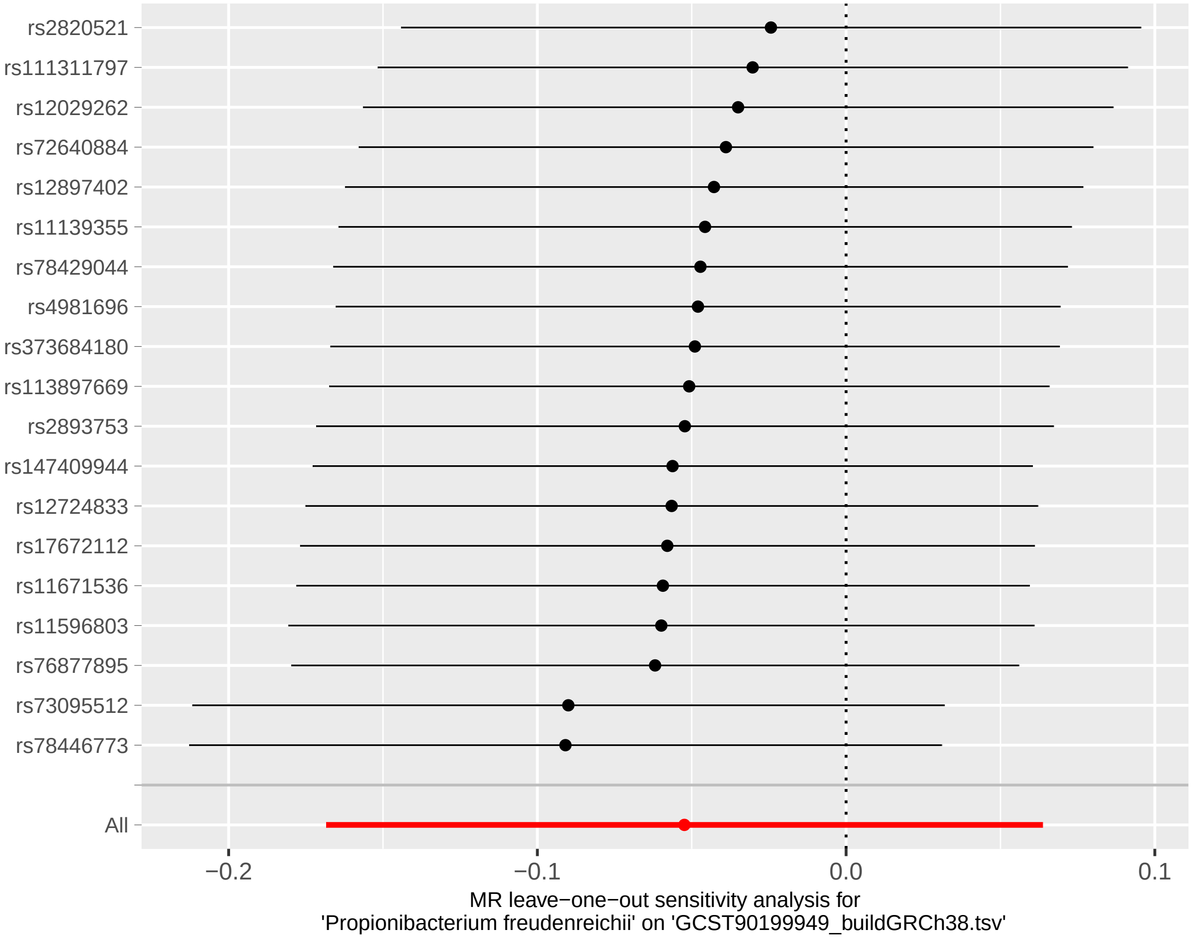
rs2820521
rs111311797
rs12029262
rs72640884
rs12897402
rs11139355
rs78429044
rs4981696
rs373684180
rs113897669
rs2893753
rs147409944
rs12724833
rs17672112
rs11671536
rs11596803
rs76877895
rs73095512
rs78446773
All
−0.2
−0.1
0.0
0.1
MR leave−one−out sensitivity analysis for
'Propionibacterium freudenreichii' on 'GCST90199949_buildGRCh38.tsv'

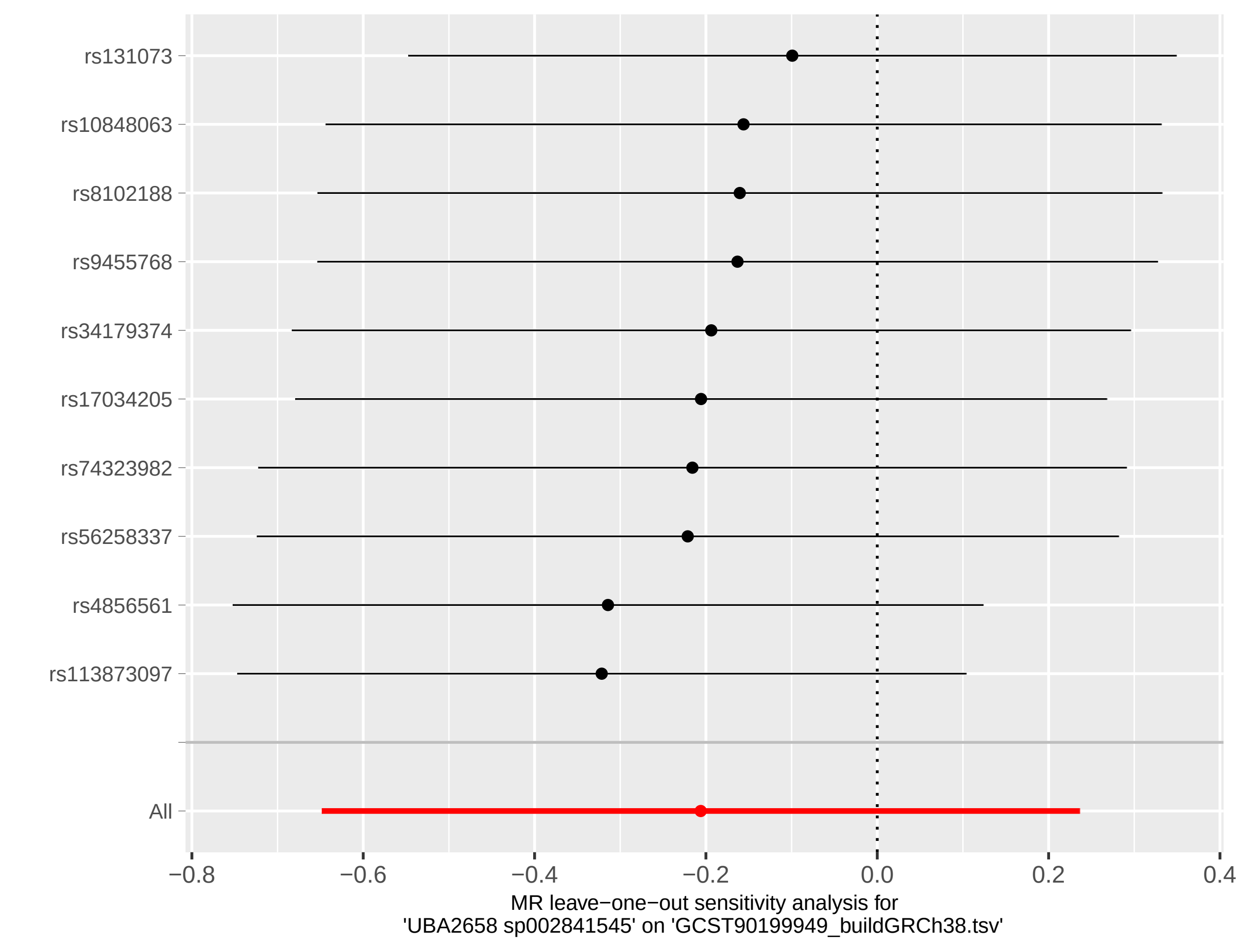
rs131073
rs10848063
rs8102188
rs9455768
rs34179374
rs17034205
rs74323982
rs56258337
rs4856561
rs113873097
All
−0.8
−0.6
−0.4
−0.2
0.0
0.2
0.4
MR leave−one−out sensitivity analysis for
'UBA2658 sp002841545' on 'GCST90199949_buildGRCh38.tsv'

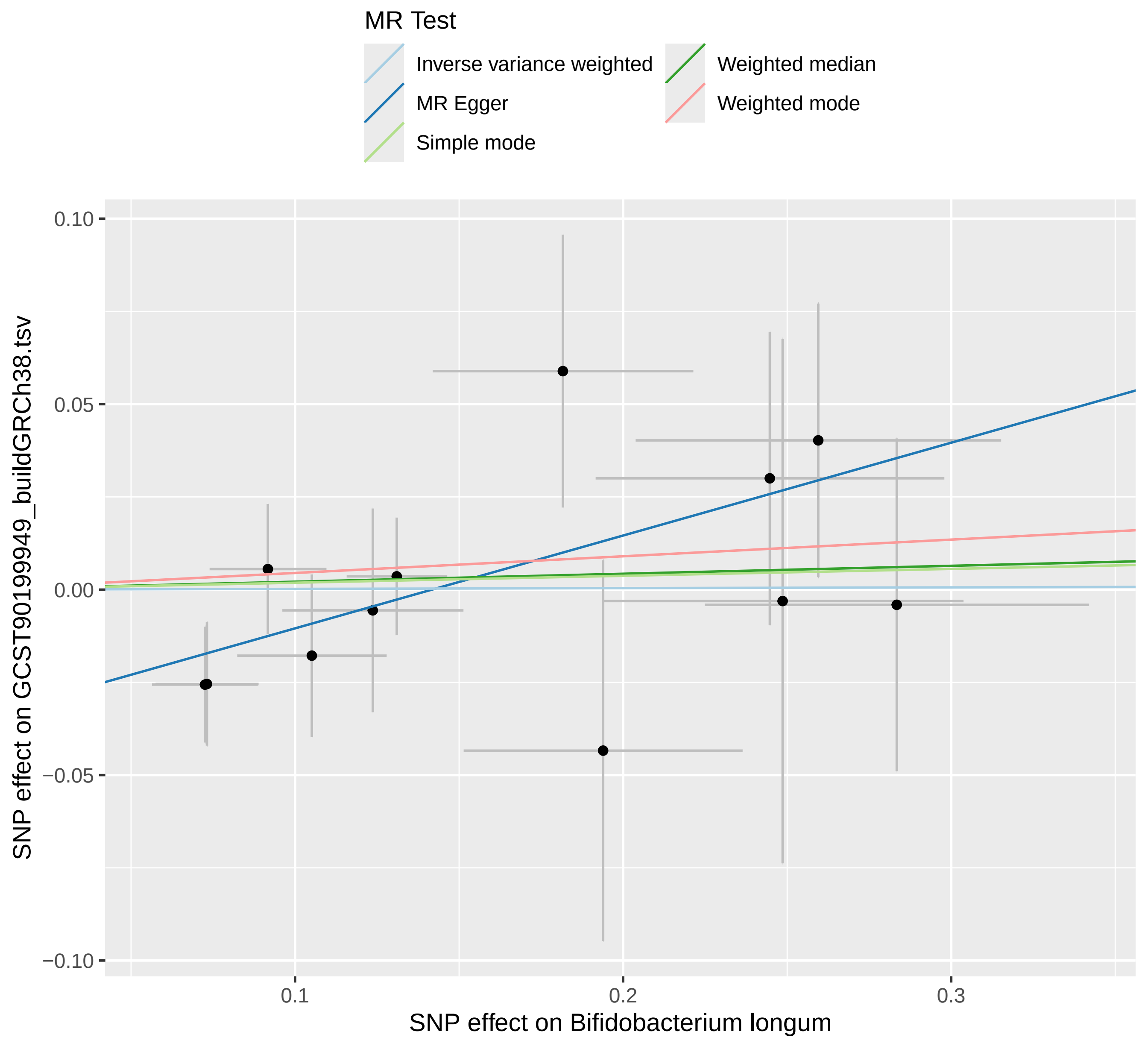
MR Test
Inverse variance weighted
MR Egger
Simple mode
Weighted median
Weighted mode
0.10
0.05
0.00
−0.05
−0.10
0.1
0.2
0.3
SNP effect on GCST90199949_buildGRCh38.tsv
SNP effect on Bifidobacterium longum

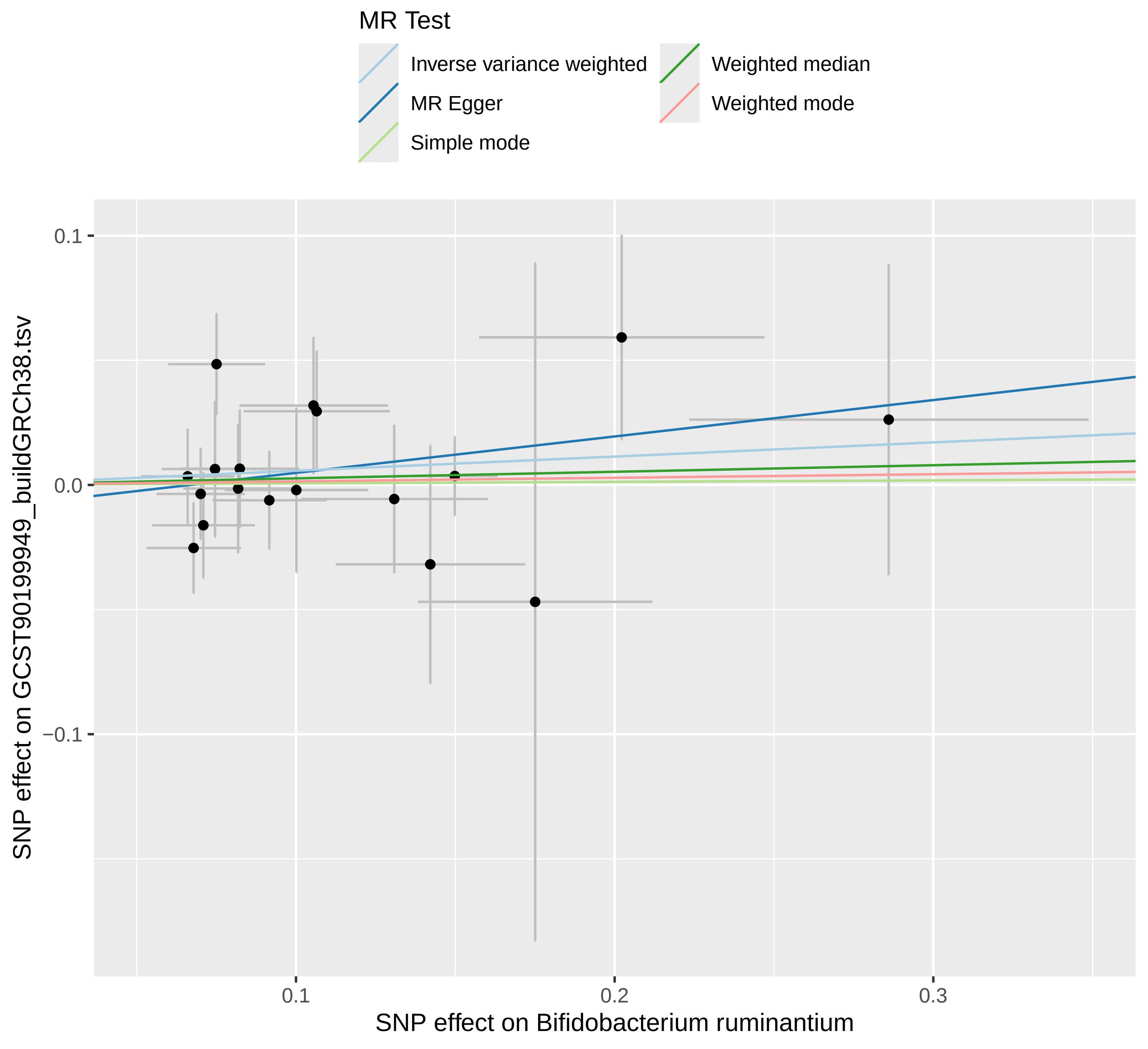

MR Test
Inverse variance weighted
MR Egger
Simple mode
Weighted median
Weighted mode
0.1
0.0
−0.1
0.1
0.2
0.3
SNP effect on GCST90199949_buildGRCh38.tsv
SNP effect on Bifidobacterium ruminantium

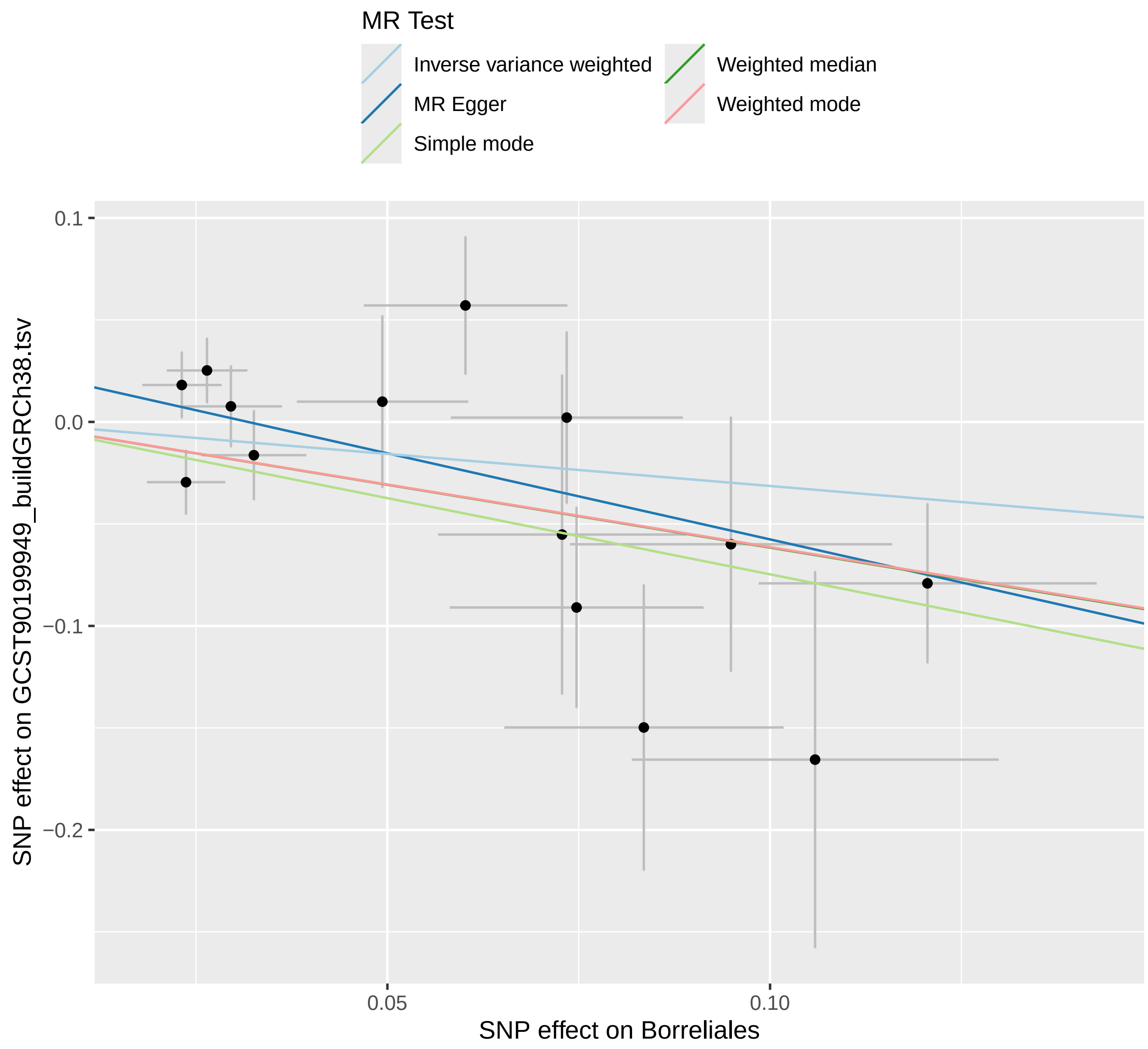
MR Test
Inverse variance weighted
MR Egger
Simple mode
Weighted median
Weighted mode
0.1
0.0
−0.1
−0.2
0.05
0.10
SNP effect on GCST90199949_buildGRCh38.tsv
SNP effect on Borreliales

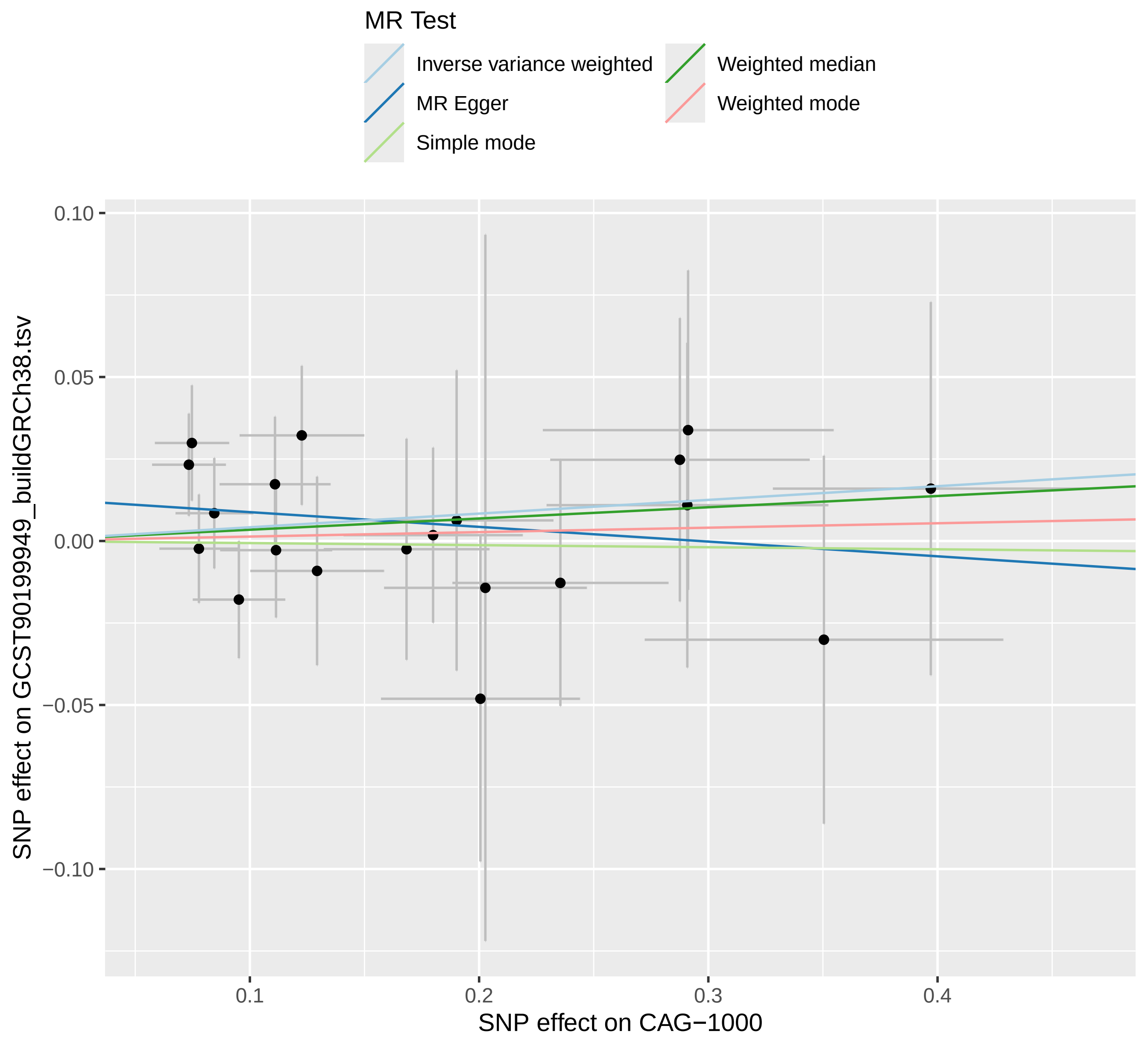
MR Test
Inverse variance weighted
MR Egger
Simple mode
Weighted median
Weighted mode
0.10
0.05
0.00
−0.05
−0.10
SNP effect on GCST90199949_buildGRCh38.tsv
0.1
0.2
0.3
0.4
SNP effect on CAG−1000

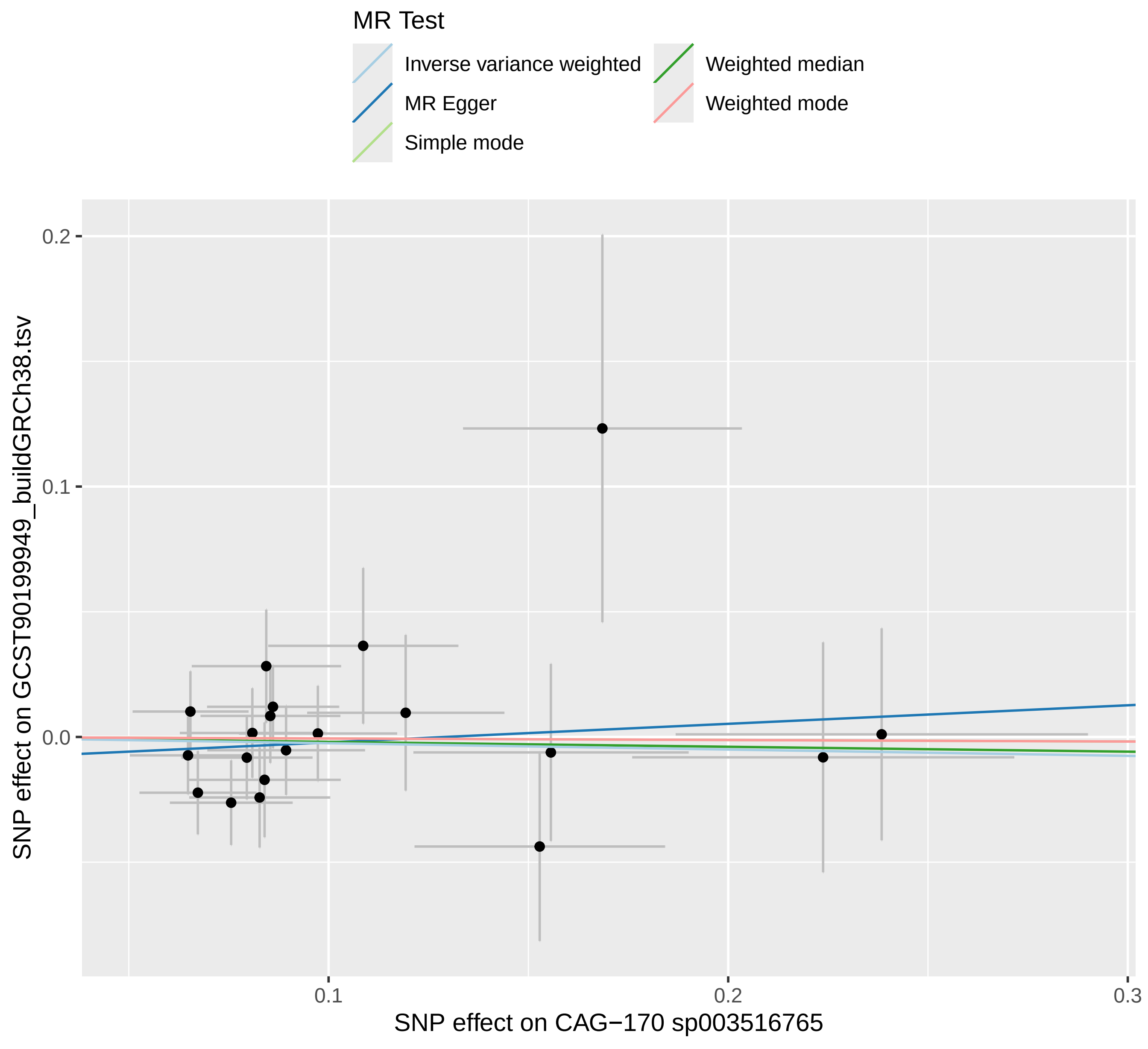
MR Test
Inverse variance weighted
MR Egger
Simple mode
Weighted median
Weighted mode
0.2
0.1
0.0
0.1
0.2
0.3
SNP effect on CAG−170 sp003516765
SNP effect on GCST90199949_buildGRCh38.tsv

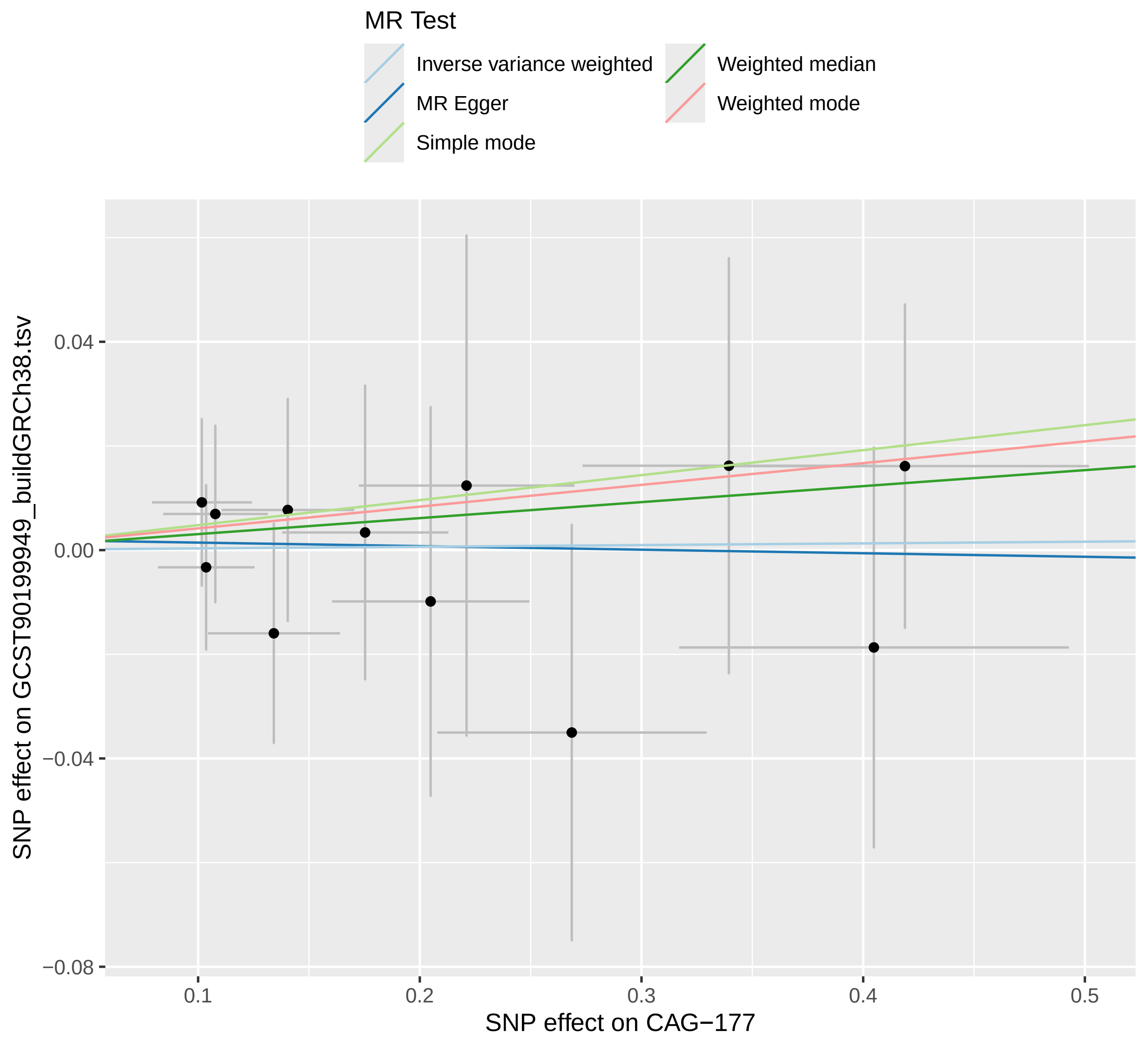

MR Test
Inverse variance weighted
MR Egger
Simple mode
Weighted median
Weighted mode
SNP effect on GCST90199949_buildGRCh38.tsv
0.04
0.00
−0.04
−0.08
0.1
0.2
0.3
0.4
0.5
SNP effect on CAG−177

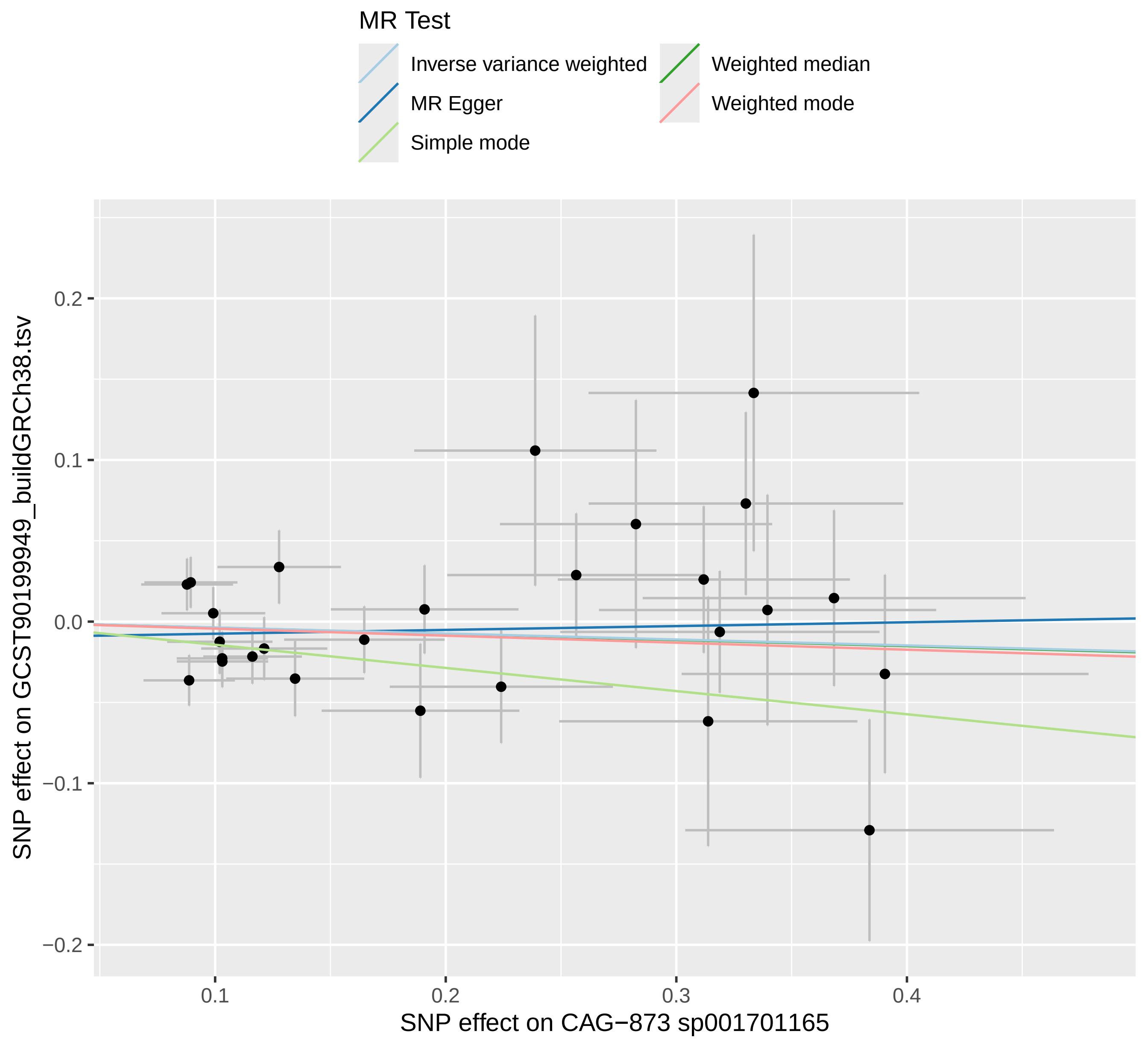

MR Test
Inverse variance weighted
MR Egger
Simple mode
Weighted median
Weighted mode
0.2
0.1
0.0
−0.1
−0.2
SNP effect on GCST90199949_buildGRCh38.tsv
0.1
0.2
0.3
0.4
SNP effect on CAG−873 sp001701165

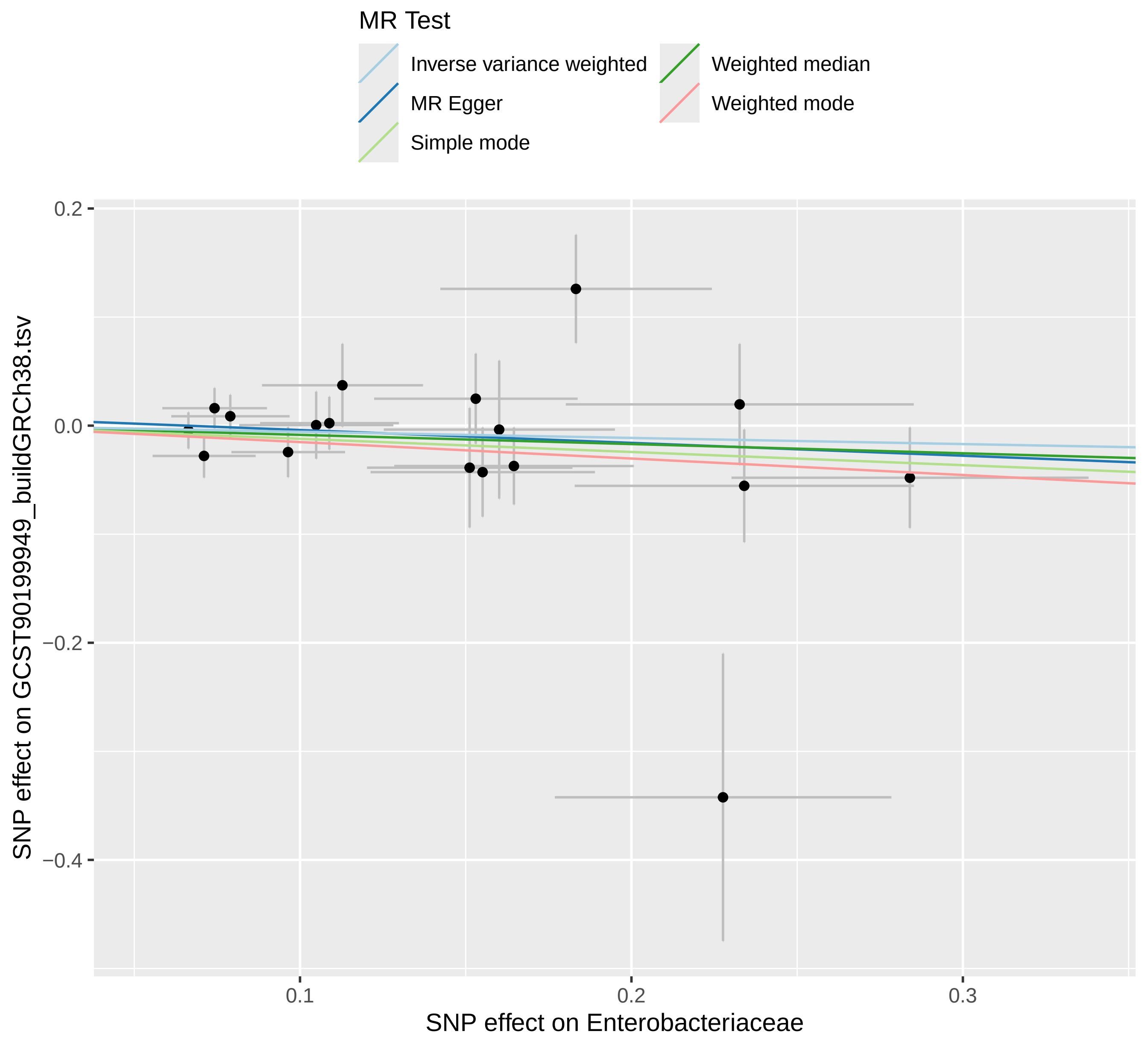

MR Test
Inverse variance weighted
MR Egger
Simple mode
Weighted median
Weighted mode
0.2
0.0
−0.2
−0.4
0.1
0.2
0.3
SNP effect on GCST90199949_buildGRCh38.tsv
SNP effect on Enterobacteriaceae

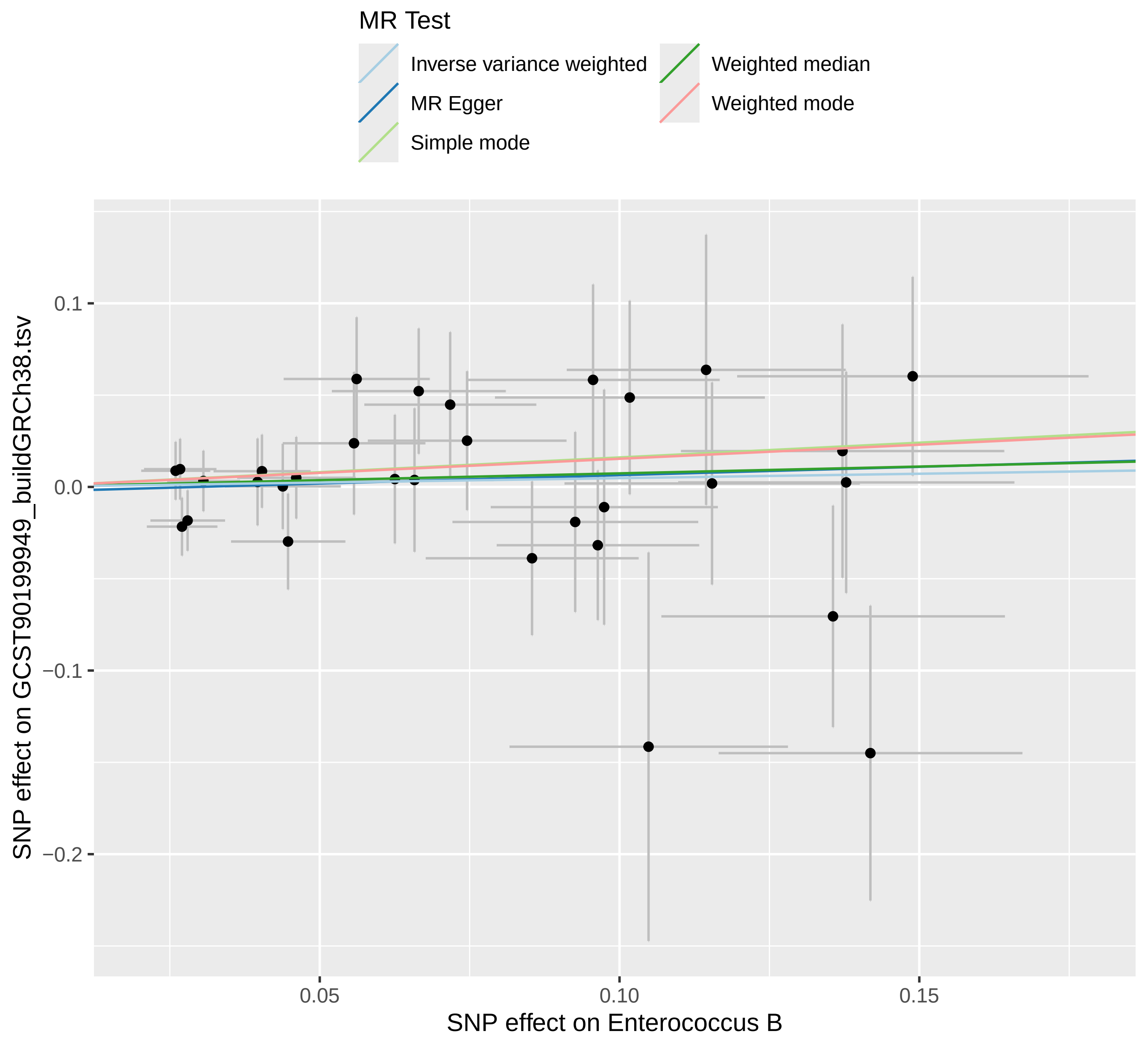

MR Test
Inverse variance weighted
MR Egger
Simple mode
Weighted median
Weighted mode
SNP effect on GCST90199949_buildGRCh38.tsv
0.1
0.0
−0.1
−0.2
0.05
0.10
0.15
SNP effect on Enterococcus B

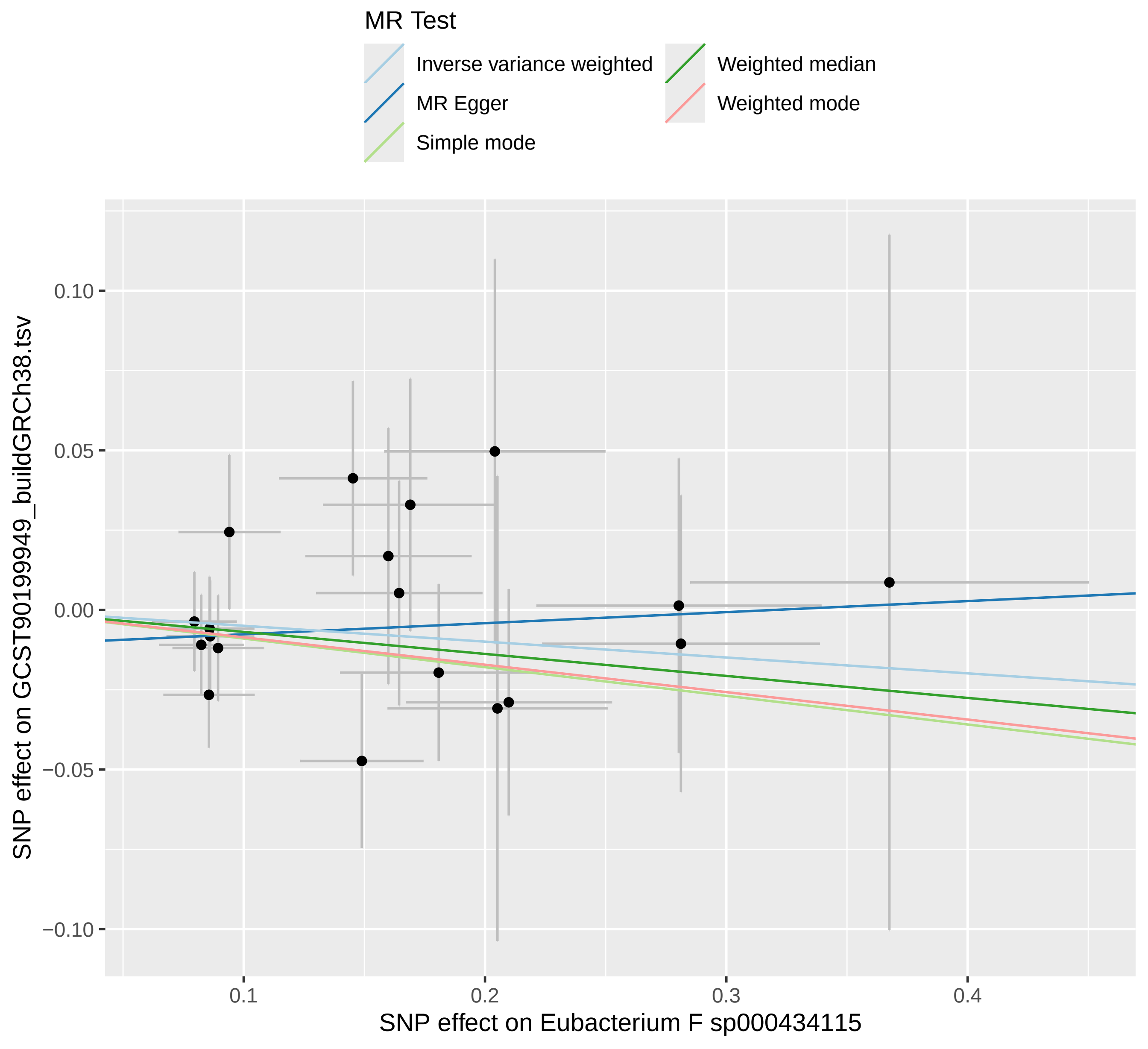

MR Test
Inverse variance weighted
MR Egger
Simple mode
Weighted median
Weighted mode
0.10
0.05
0.00
−0.05
−0.10
0.1
0.2
0.3
0.4
SNP effect on GCST90199949_buildGRCh38.tsv
SNP effect on Eubacterium F sp000434115

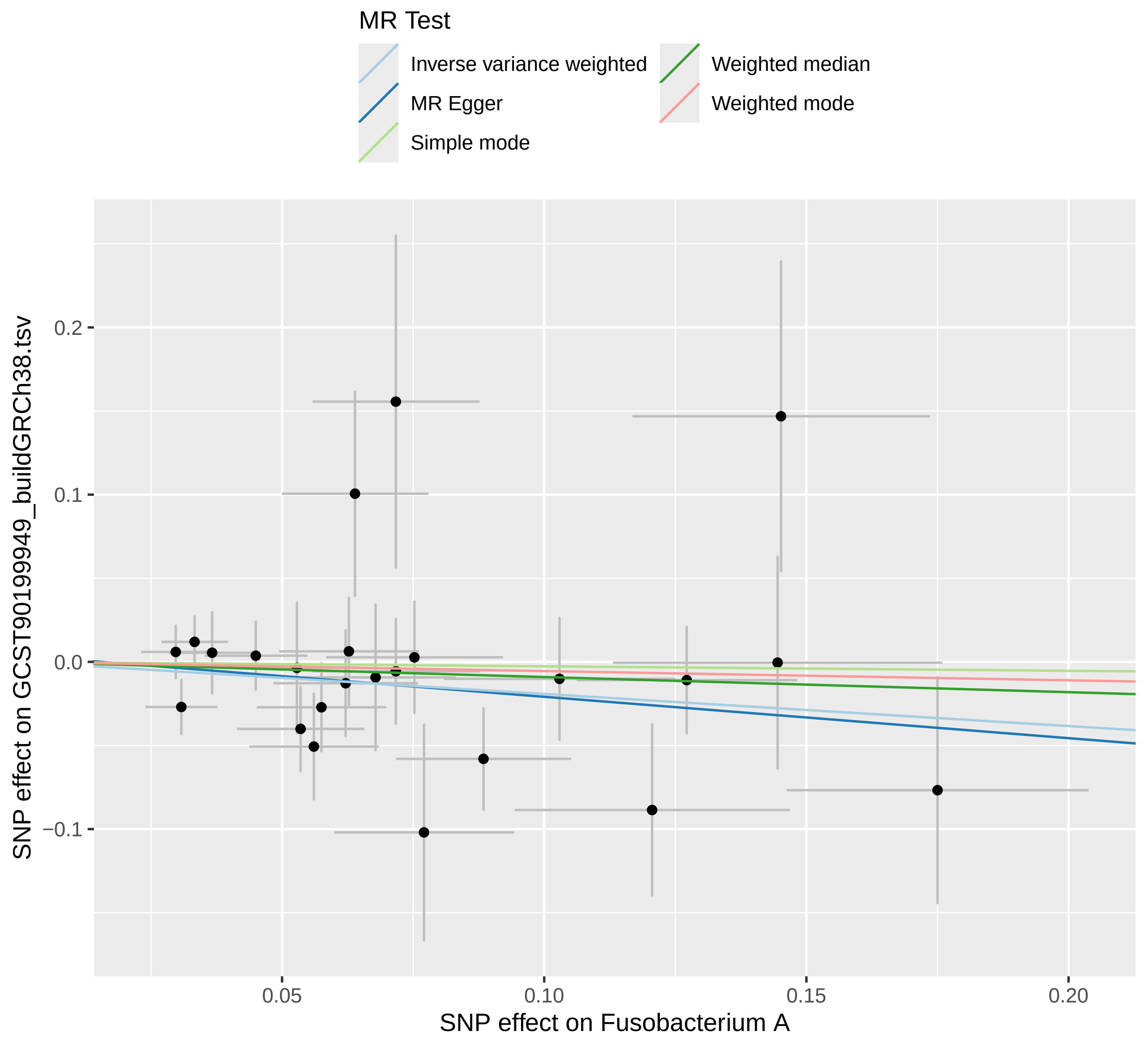
MR Test
Inverse variance weighted
MR Egger
Simple mode
Weighted median
Weighted mode
SNP effect on GCST90199949_buildGRCh38.tsv
0.2
0.1
0.0
−0.1
0.05
0.10
0.15
0.20
SNP effect on Fusobacterium A

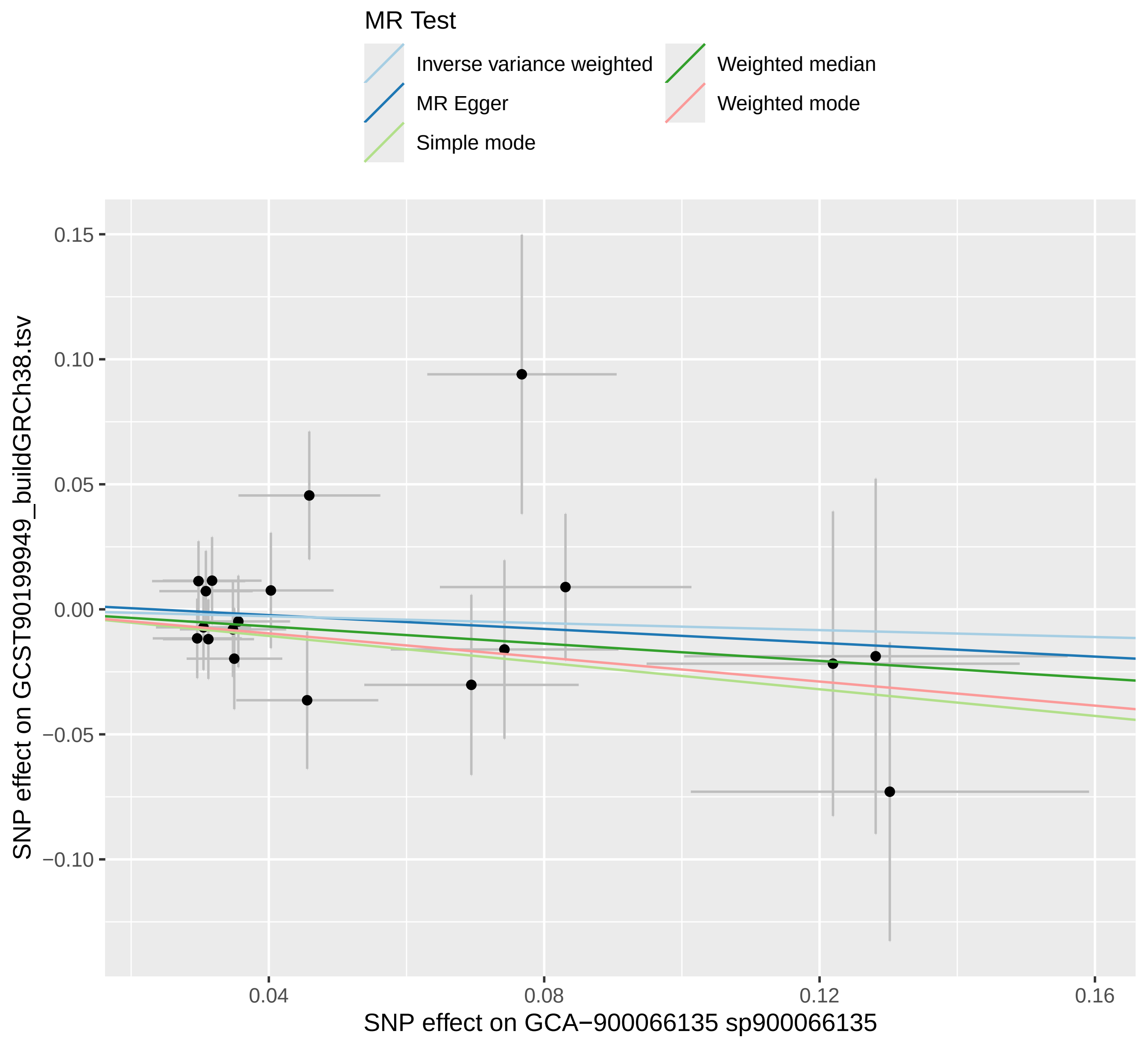

MR Test
Inverse variance weighted
MR Egger
Simple mode
Weighted median
Weighted mode
0.15
0.10
0.05
0.00
−0.05
−0.10
SNP effect on GCST90199949_buildGRCh38.tsv
0.04
0.08
0.12
0.16
SNP effect on GCA−900066135 sp900066135

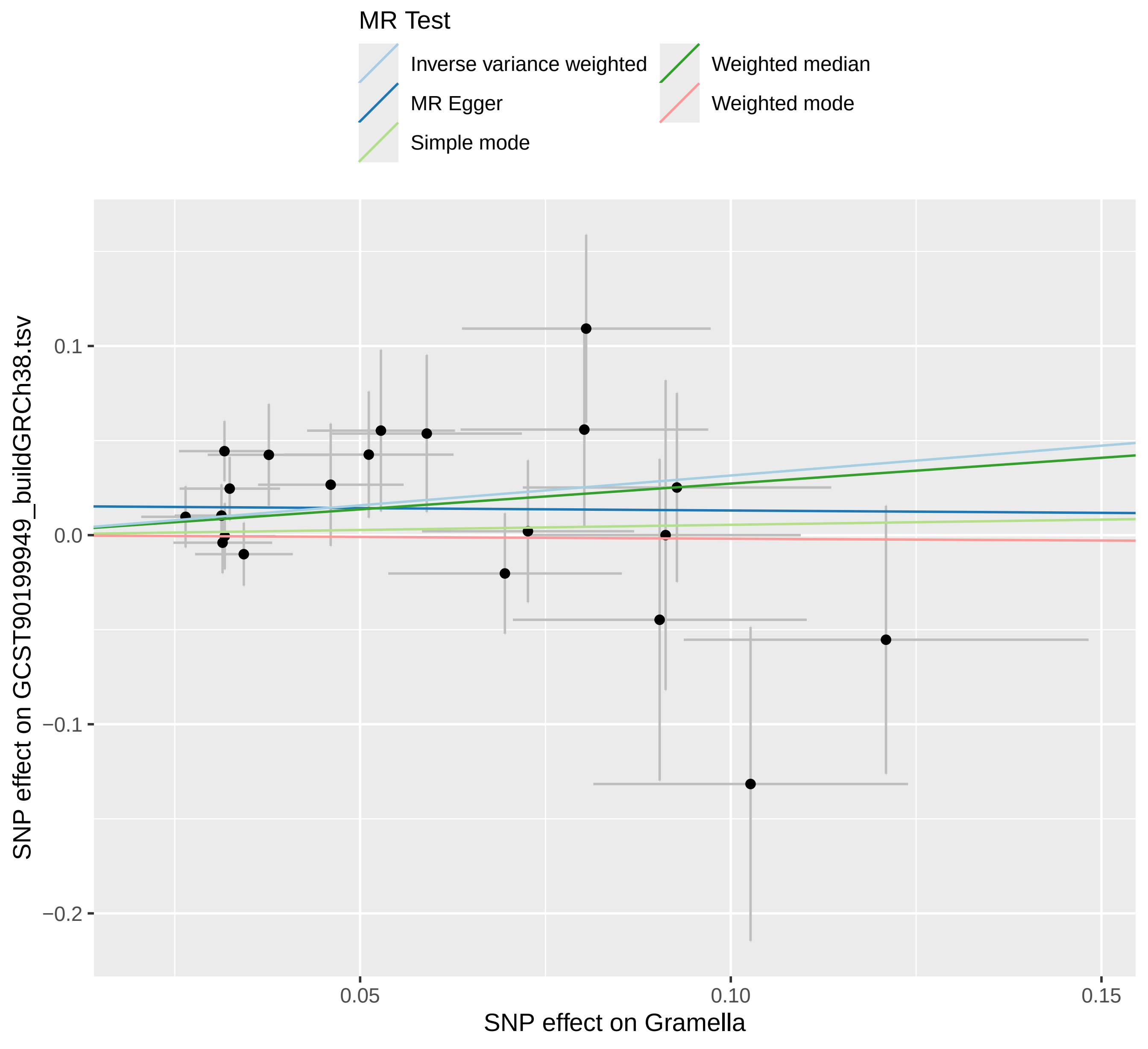
MR Test
Inverse variance weighted
MR Egger
Simple mode
Weighted median
Weighted mode
0.1
0.0
−0.1
−0.2
0.05
0.10
0.15
SNP effect on GCST90199949_buildGRCh38.tsv
SNP effect on Gramella

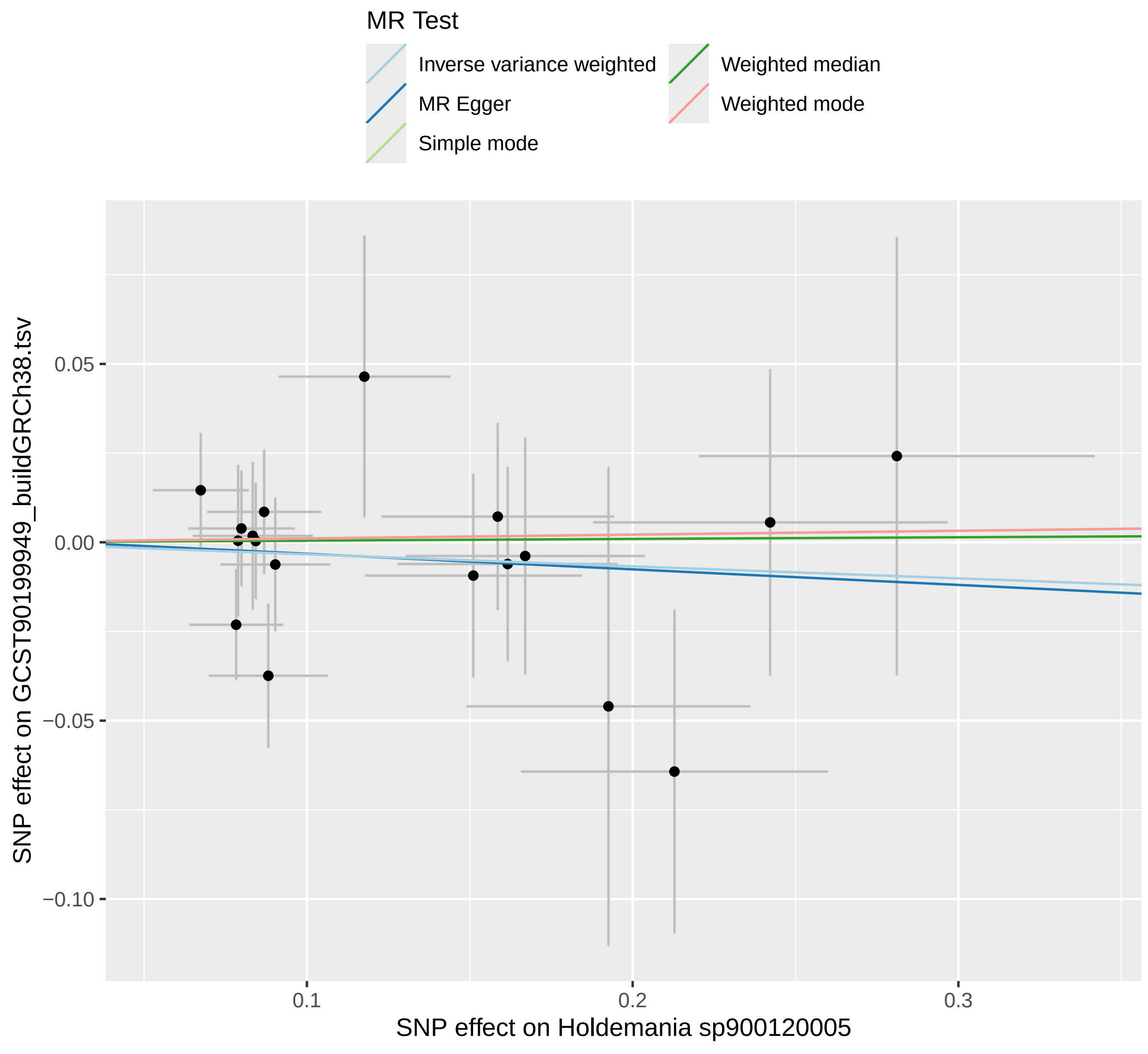

MR Test
Inverse variance weighted
MR Egger
Simple mode
Weighted median
Weighted mode
0.05
0.00
−0.05
−0.10
0.1
0.2
0.3
SNP effect on GCST90199949_buildGRCh38.tsv
SNP effect on Holdemania sp900120005

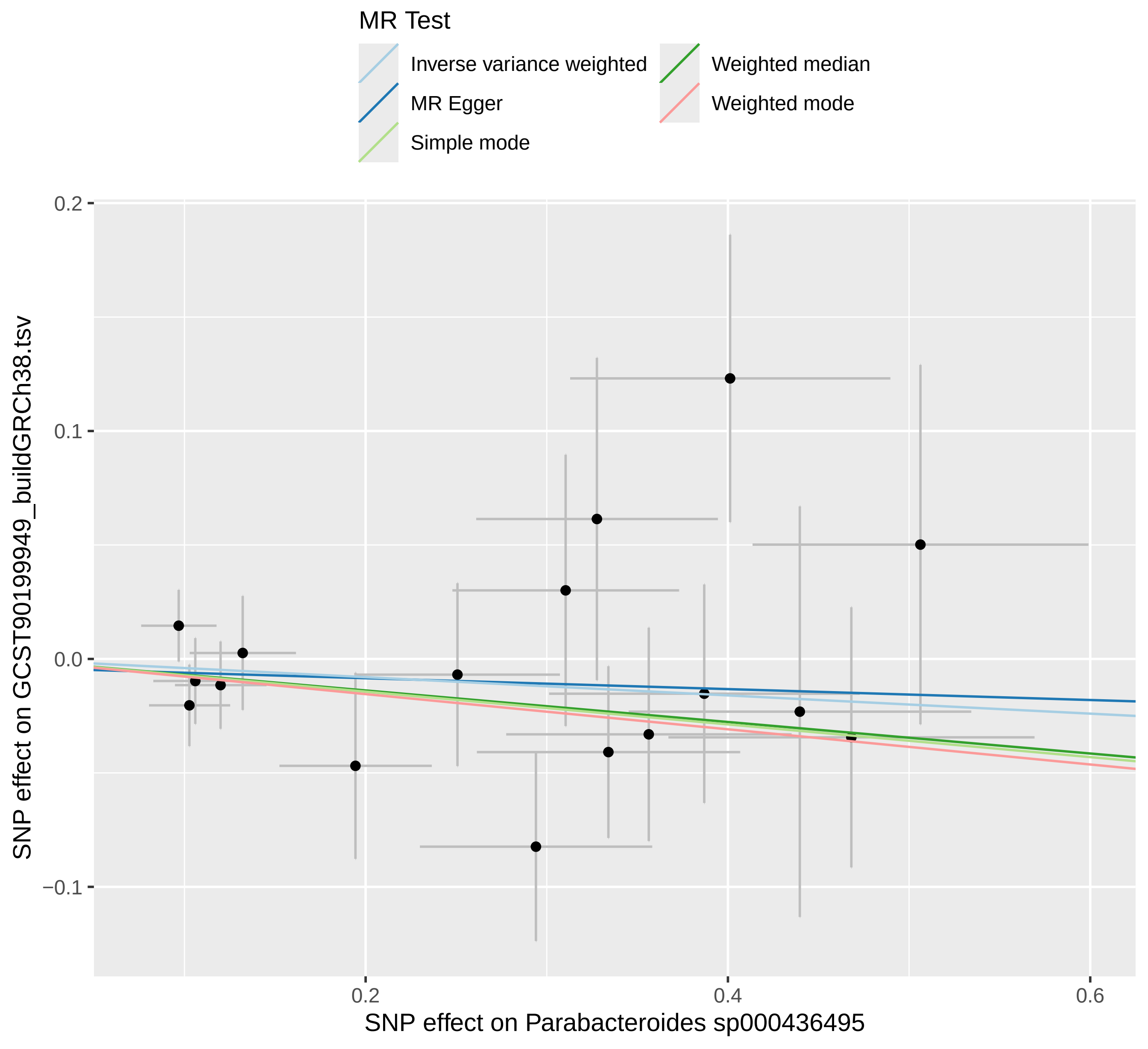
MR Test
Inverse variance weighted
MR Egger
Simple mode
Weighted median
Weighted mode
0.2
0.1
0.0
−0.1
0.2
0.4
0.6
SNP effect on GCST90199949_buildGRCh38.tsv
SNP effect on Parabacteroides sp000436495

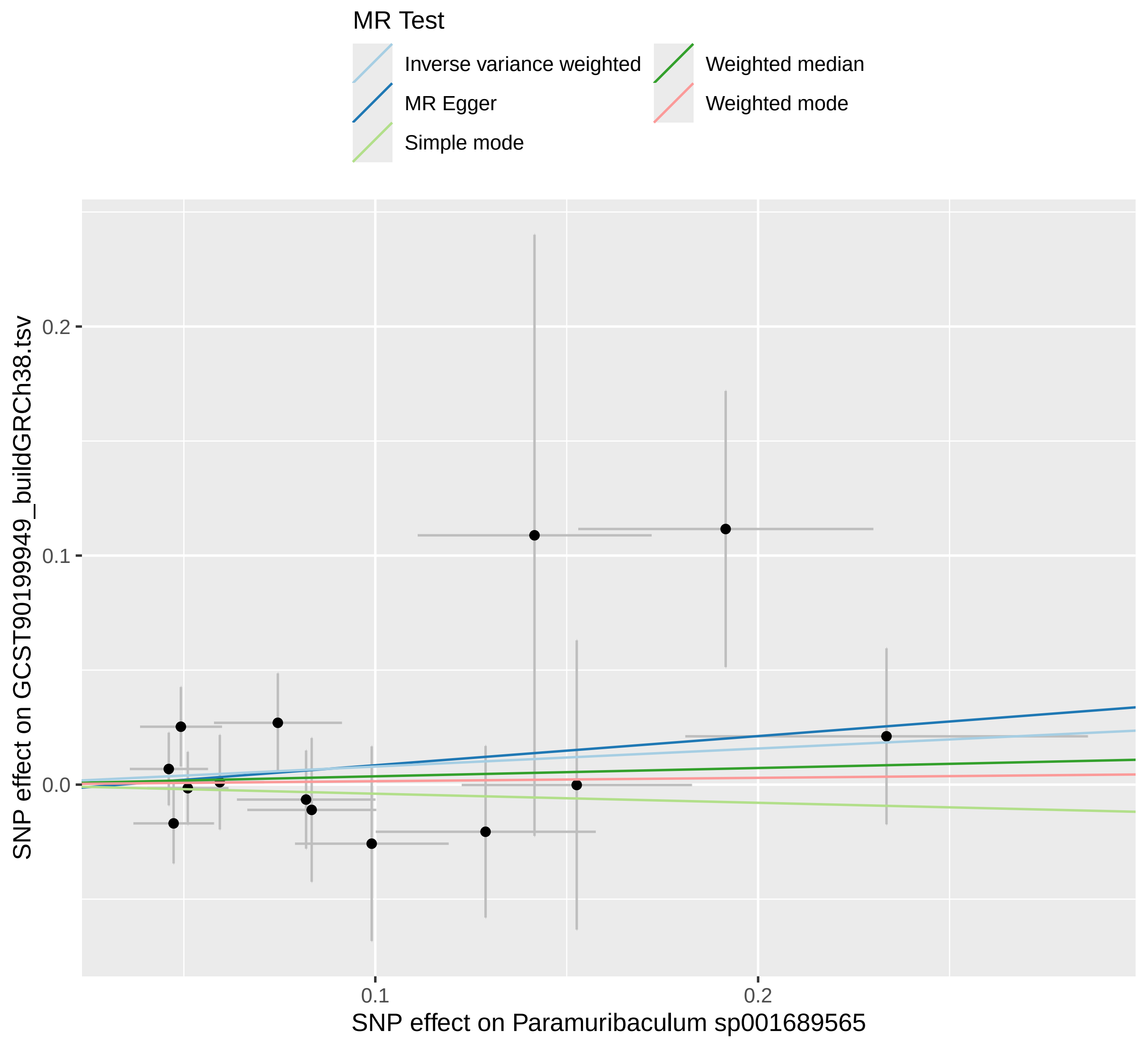

MR Test
Inverse variance weighted
MR Egger
Simple mode
Weighted median
Weighted mode
SNP effect on GCST90199949_buildGRCh38.tsv
0.2
0.1
0.0
0.1
0.2
SNP effect on Paramuribaculum sp001689565

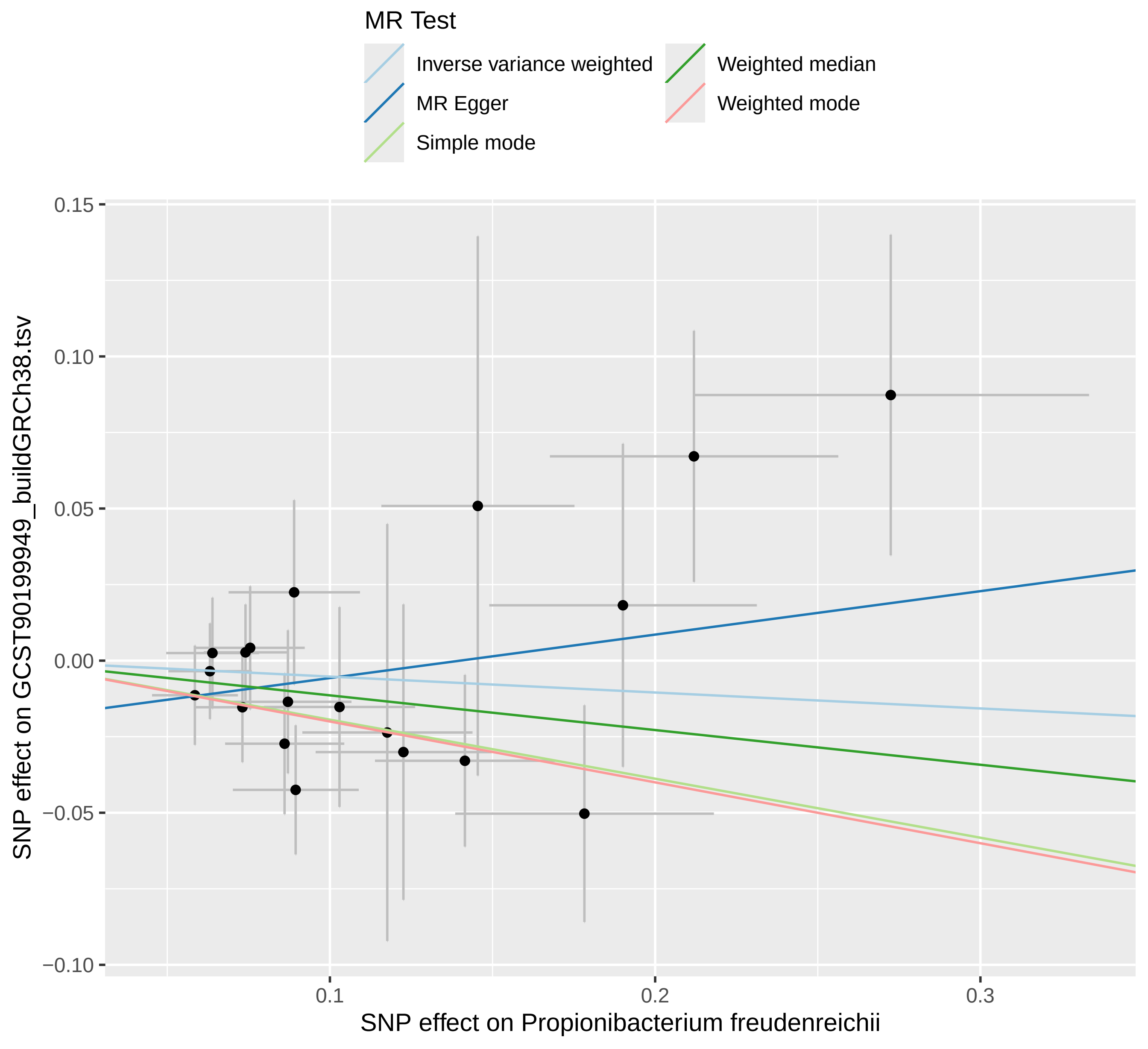

MR Test
Inverse variance weighted
MR Egger
Simple mode
Weighted median
Weighted mode
0.15
0.10
0.05
0.00
−0.05
−0.10
SNP effect on GCST90199949_buildGRCh38.tsv
0.1
0.2
0.3
SNP effect on Propionibacterium freudenreichii

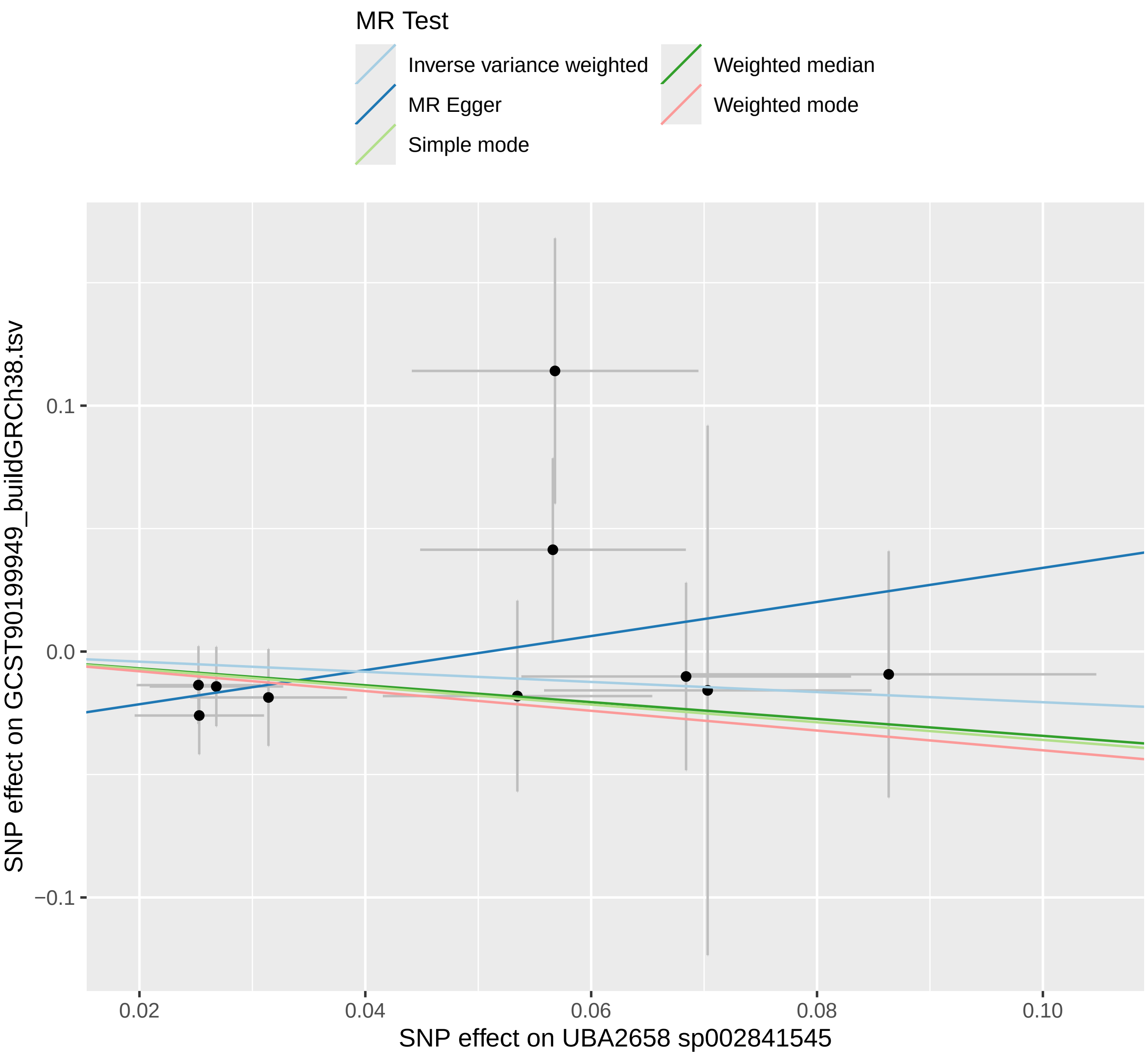

MR Test
Inverse variance weighted
MR Egger
Simple mode
Weighted median
Weighted mode
0.1
0.0
−0.1
0.02
0.04
0.06
0.08
0.10
SNP effect on GCST90199949_buildGRCh38.tsv
SNP effect on UBA2658 sp002841545

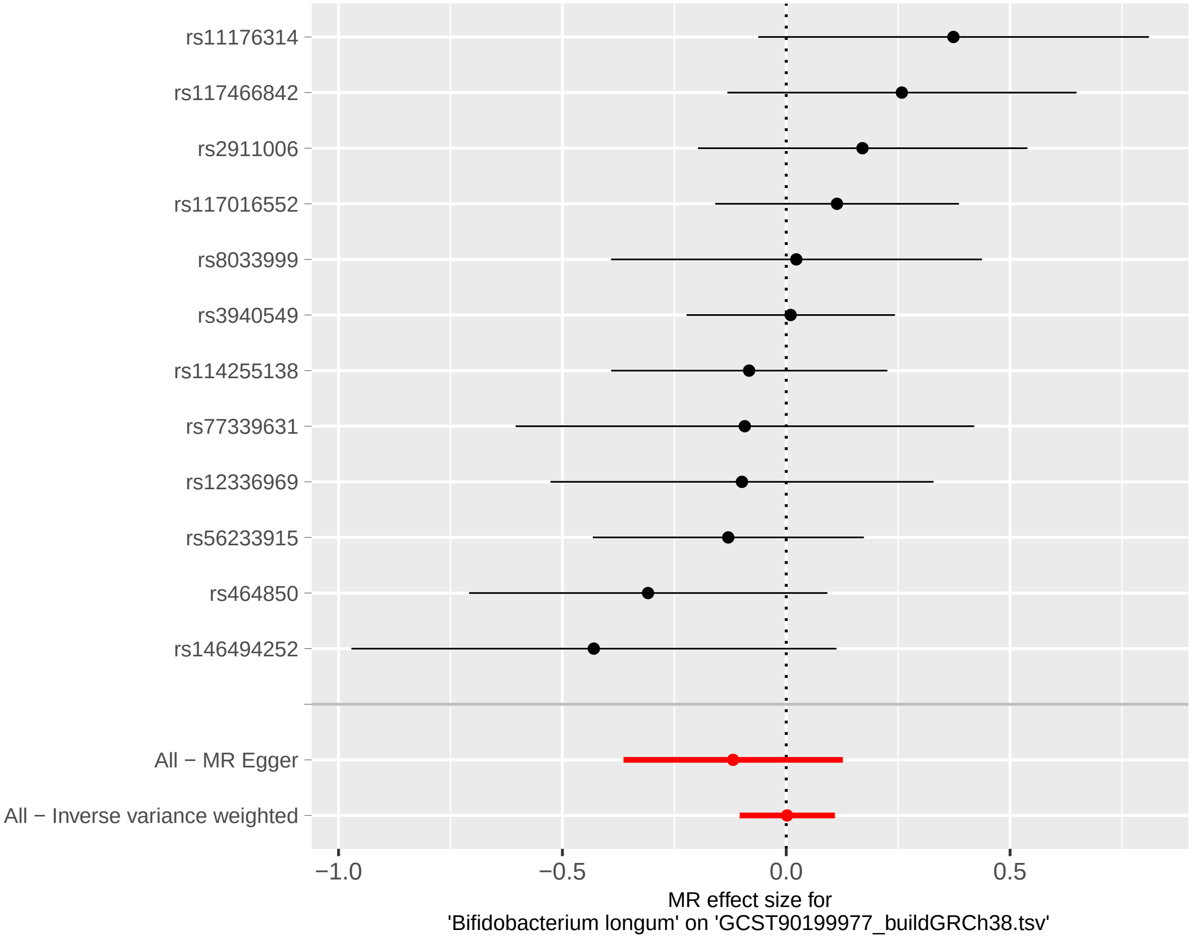
rs11176314
rs117466842
rs2911006
rs117016552
rs8033999
rs3940549
rs114255138
rs77339631
rs12336969
rs56233915
rs464850
rs146494252
All − MR Egger
All − Inverse variance weighted
−1.0
−0.5
0.0
0.5
MR effect size for
'Bifidobacterium longum' on 'GCST90199977_buildGRCh38.tsv'

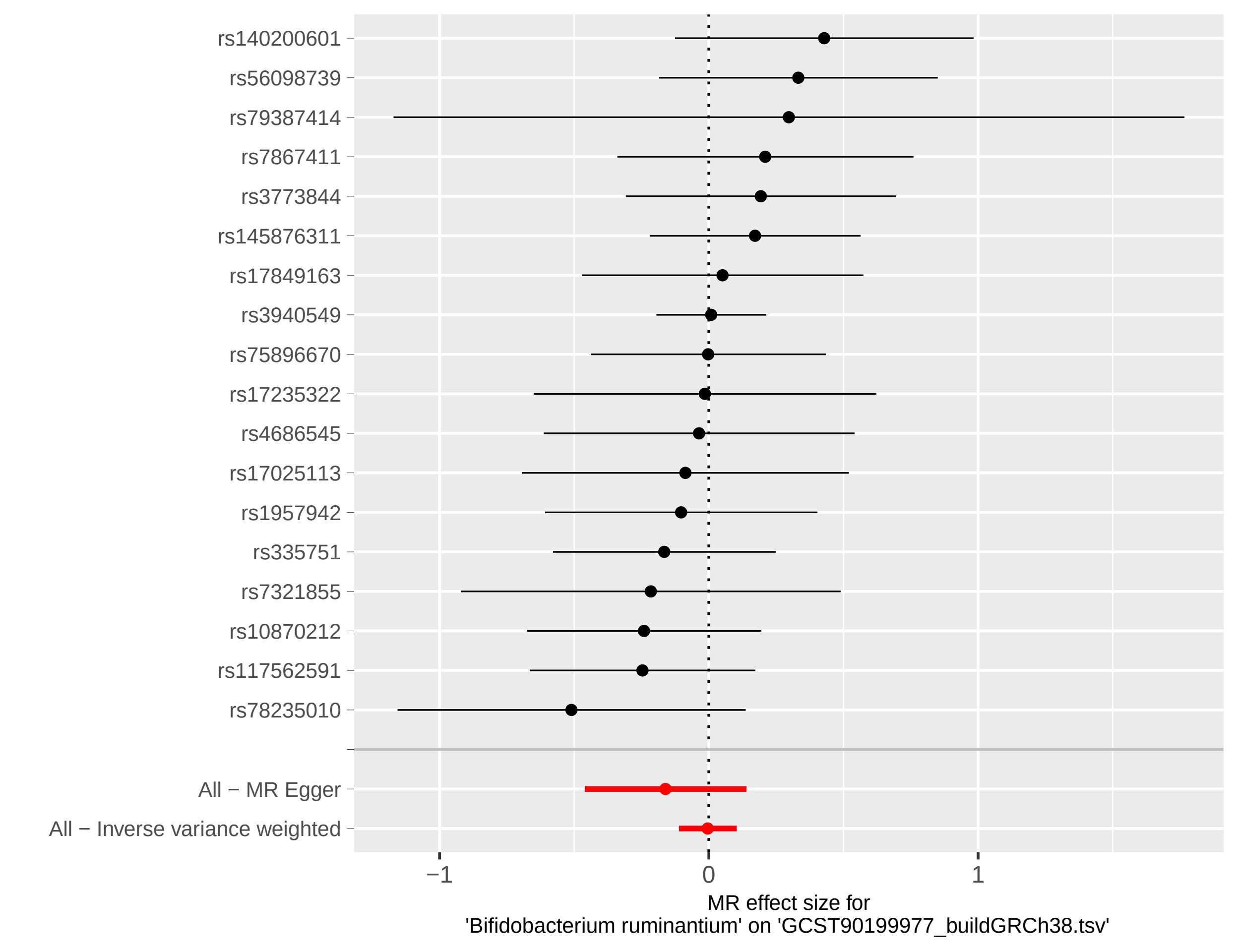
rs140200601
rs56098739
rs79387414
rs7867411
rs3773844
rs145876311
rs17849163
rs3940549
rs75896670
rs17235322
rs4686545
rs17025113
rs1957942
rs335751
rs7321855
rs10870212
rs117562591
rs78235010
All − MR Egger
All − Inverse variance weighted
−1
0
1
MR effect size for
'Bifidobacterium ruminantium' on 'GCST90199977_buildGRCh38.tsv'

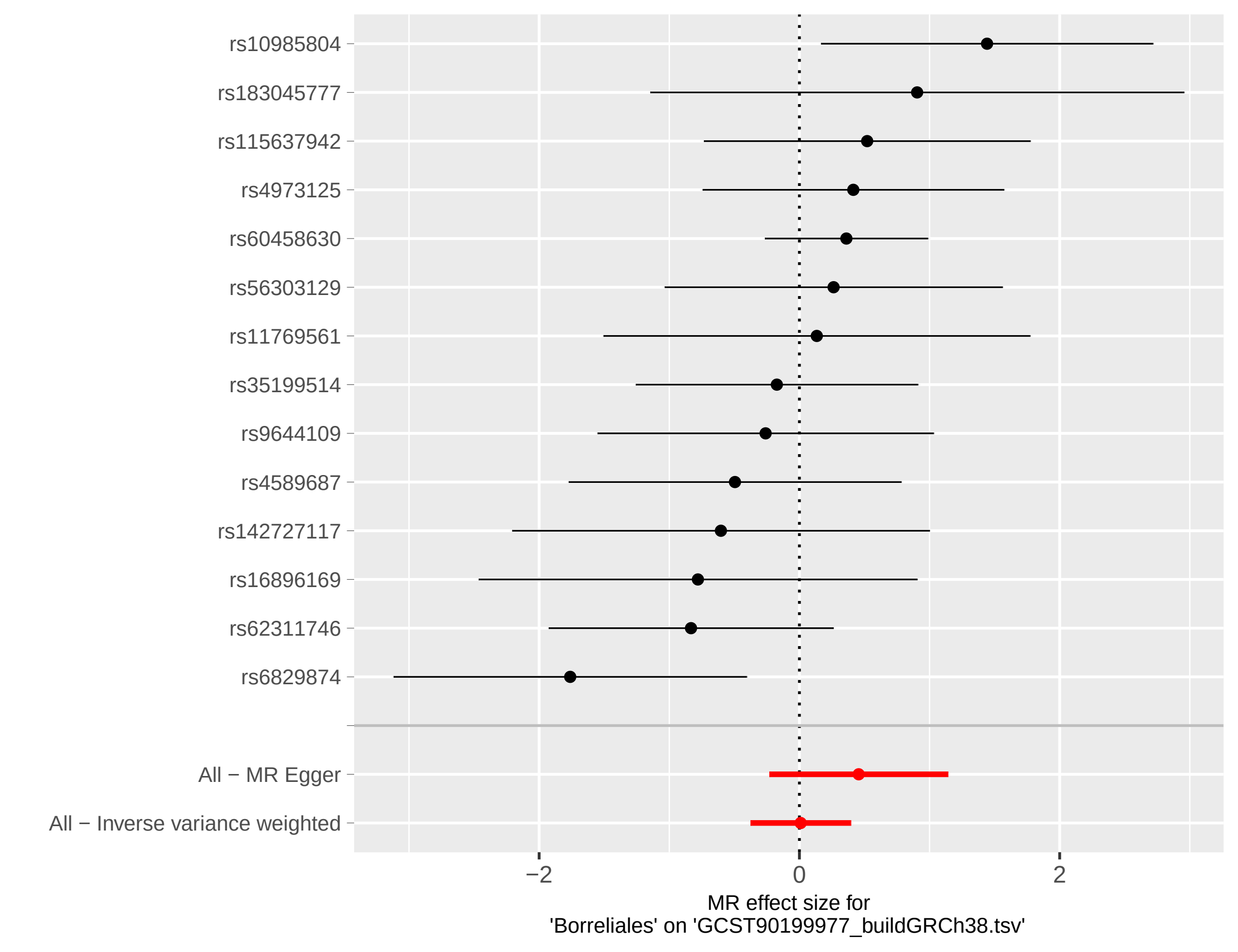
rs10985804
rs183045777
rs115637942
rs4973125
rs60458630
rs56303129
rs11769561
rs35199514
rs9644109
rs4589687
rs142727117
rs16896169
rs62311746
rs6829874
All − MR Egger
All − Inverse variance weighted
−2
0
2
MR effect size for
'Borreliales' on 'GCST90199977_buildGRCh38.tsv'

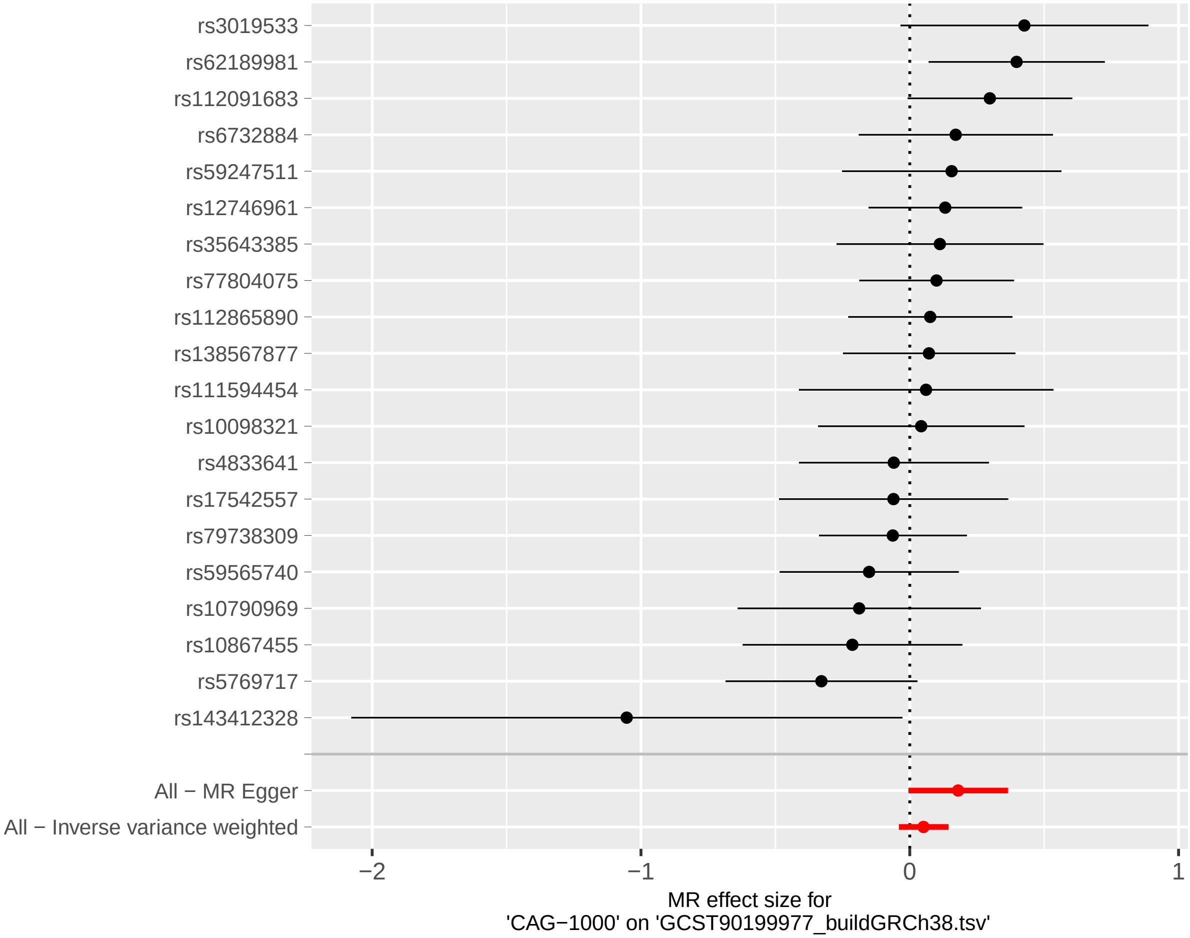
rs3019533
rs62189981
rs112091683
rs6732884
rs59247511
rs12746961
rs35643385
rs77804075
rs112865890
rs138567877
rs111594454
rs10098321
rs4833641
rs17542557
rs79738309
rs59565740
rs10790969
rs10867455
rs5769717
rs143412328
All − MR Egger
All − Inverse variance weighted
−2
−1
0
1
MR effect size for
'CAG−1000' on 'GCST90199977_buildGRCh38.tsv'

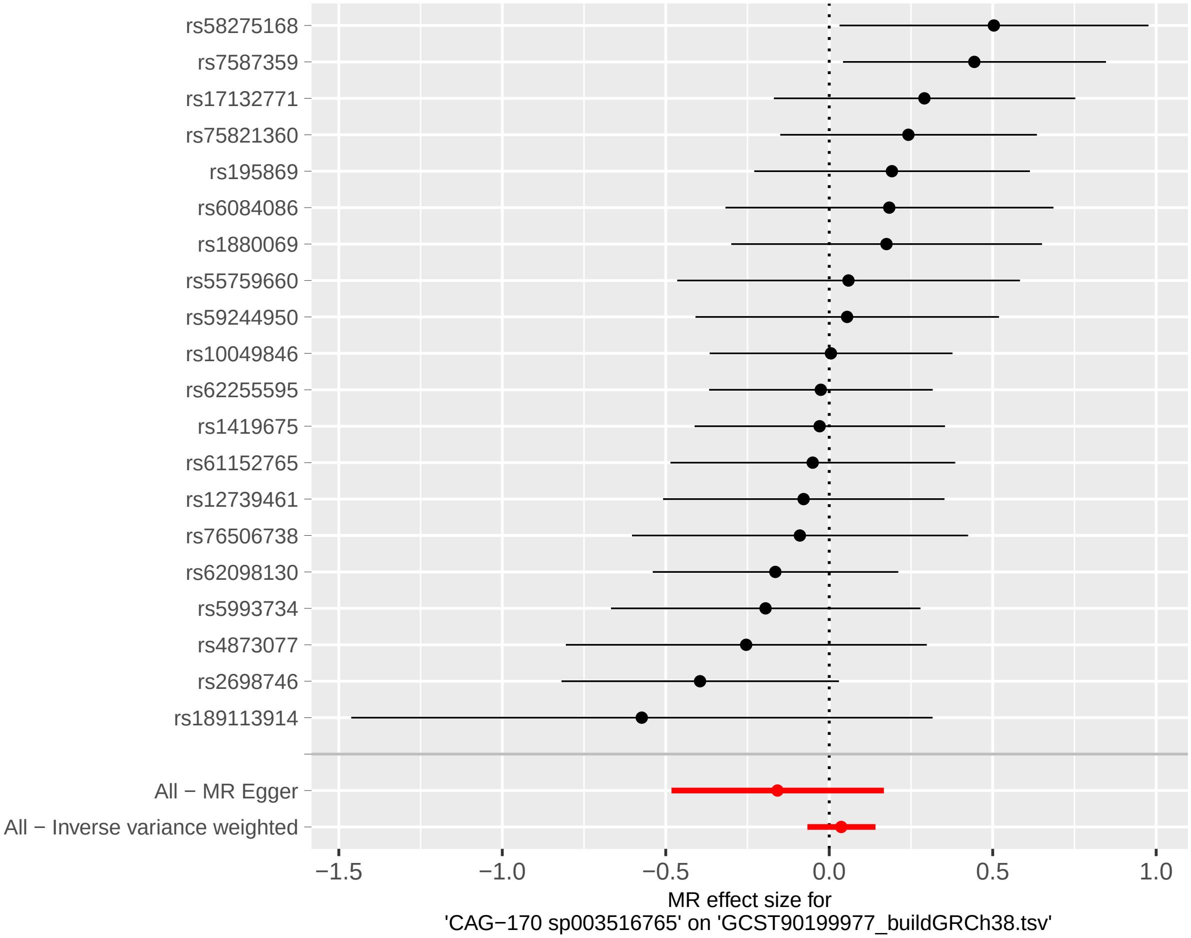

rs58275168
rs7587359
rs17132771
rs75821360
rs195869
rs6084086
rs1880069
rs55759660
rs59244950
rs10049846
rs62255595
rs1419675
rs61152765
rs12739461
rs76506738
rs62098130
rs5993734
rs4873077
rs2698746
rs189113914
All − MR Egger
All − Inverse variance weighted
−1.5
−1.0
−0.5
0.0
0.5
1.0
MR effect size for
'CAG−170 sp003516765' on 'GCST90199977_buildGRCh38.tsv'

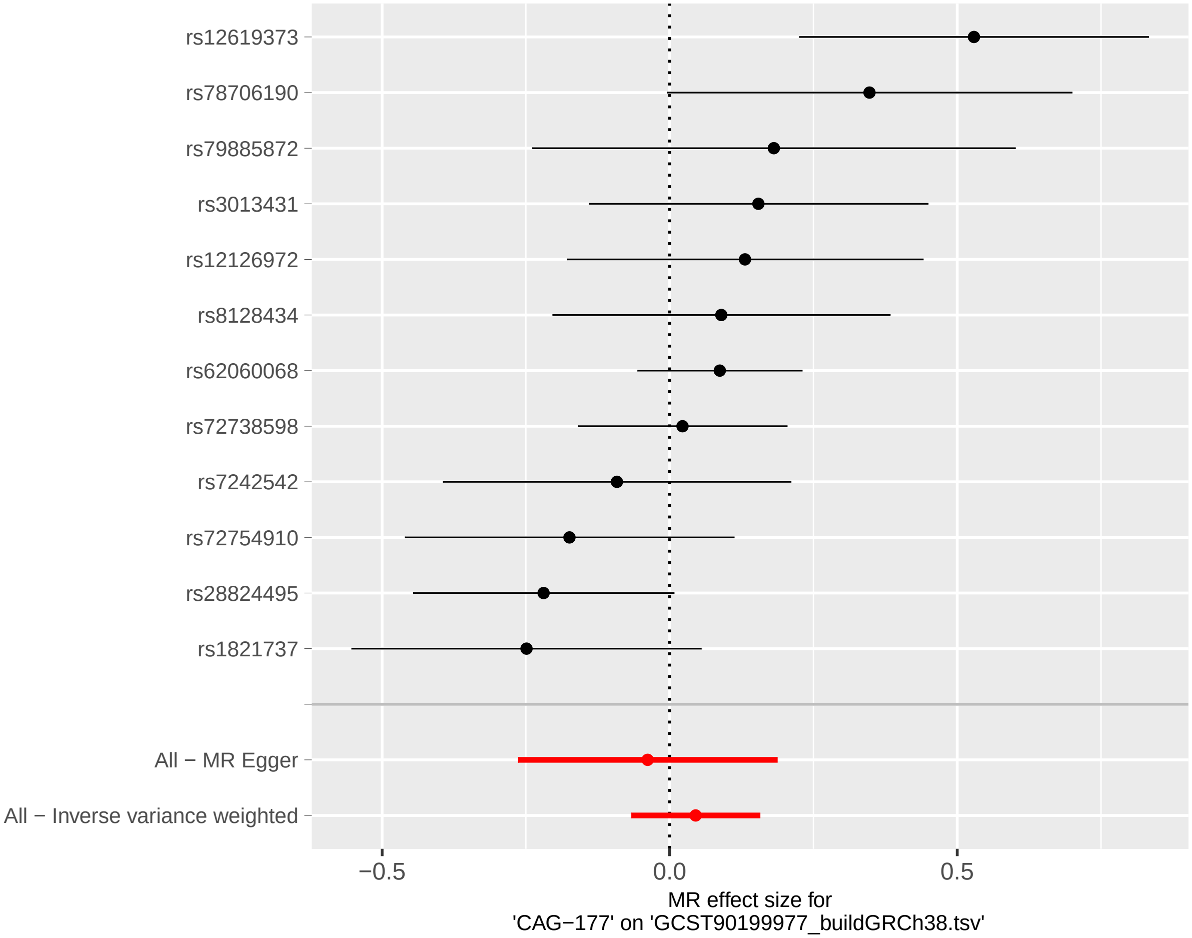

rs12619373
rs78706190
rs79885872
rs3013431
rs12126972
rs8128434
rs62060068
rs72738598
rs7242542
rs72754910
rs28824495
rs1821737
All − MR Egger
All − Inverse variance weighted
−0.5
0.0
0.5
MR effect size for
'CAG−177' on 'GCST90199977_buildGRCh38.tsv'

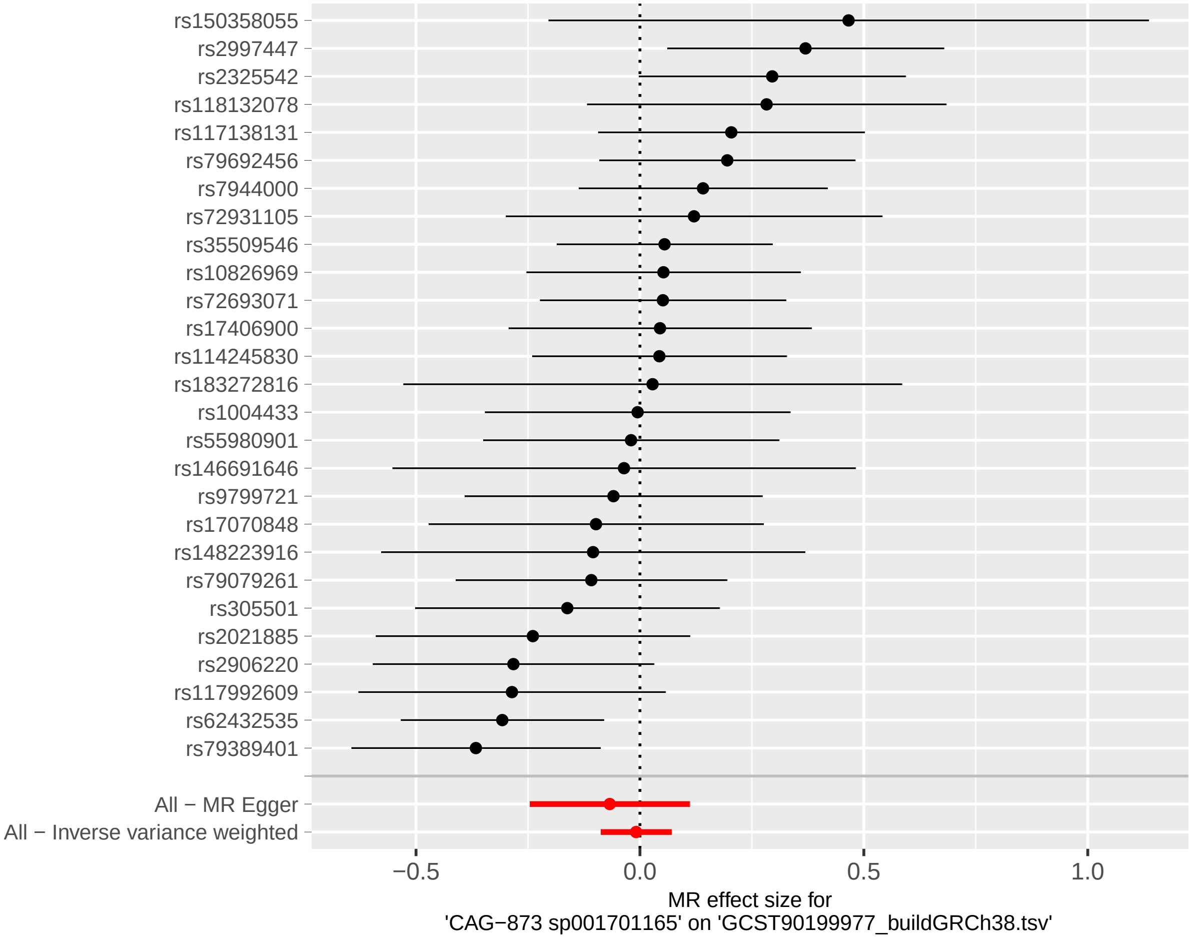

rs150358055
rs2997447
rs2325542
rs118132078
rs117138131
rs79692456
rs7944000
rs72931105
rs35509546
rs10826969
rs72693071
rs17406900
rs114245830
rs183272816
rs1004433
rs55980901
rs146691646
rs9799721
rs17070848
rs148223916
rs79079261
rs305501
rs2021885
rs2906220
rs117992609
rs62432535
rs79389401
All − MR Egger
All − Inverse variance weighted
−0.5
0.0
0.5
1.0
MR effect size for
'CAG−873 sp001701165' on 'GCST90199977_buildGRCh38.tsv'

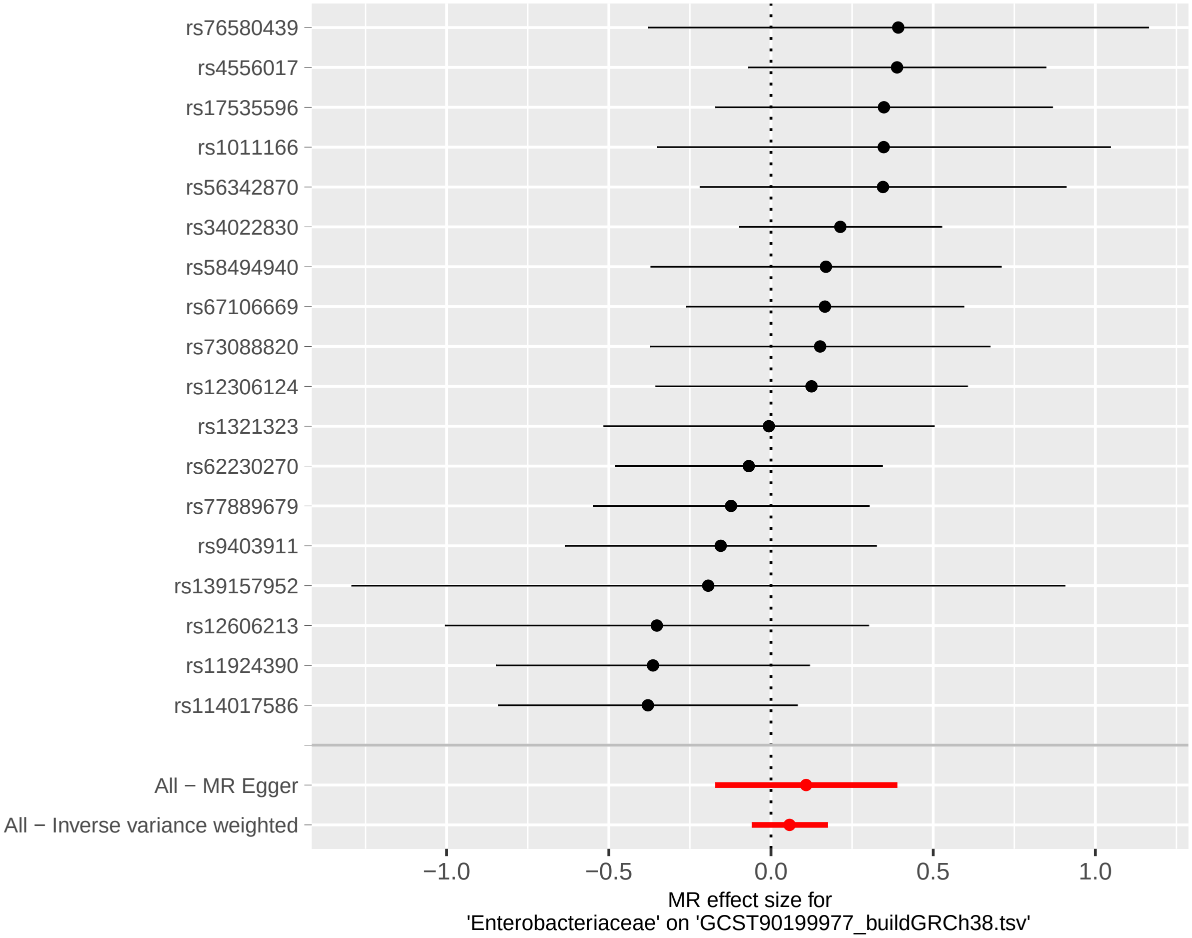
rs76580439
rs4556017
rs17535596
rs1011166
rs56342870
rs34022830
rs58494940
rs67106669
rs73088820
rs12306124
rs1321323
rs62230270
rs77889679
rs9403911
rs139157952
rs12606213
rs11924390
rs114017586
All − MR Egger
All − Inverse variance weighted
−1.0
−0.5
0.0
0.5
1.0
MR effect size for
'Enterobacteriaceae' on 'GCST90199977_buildGRCh38.tsv'

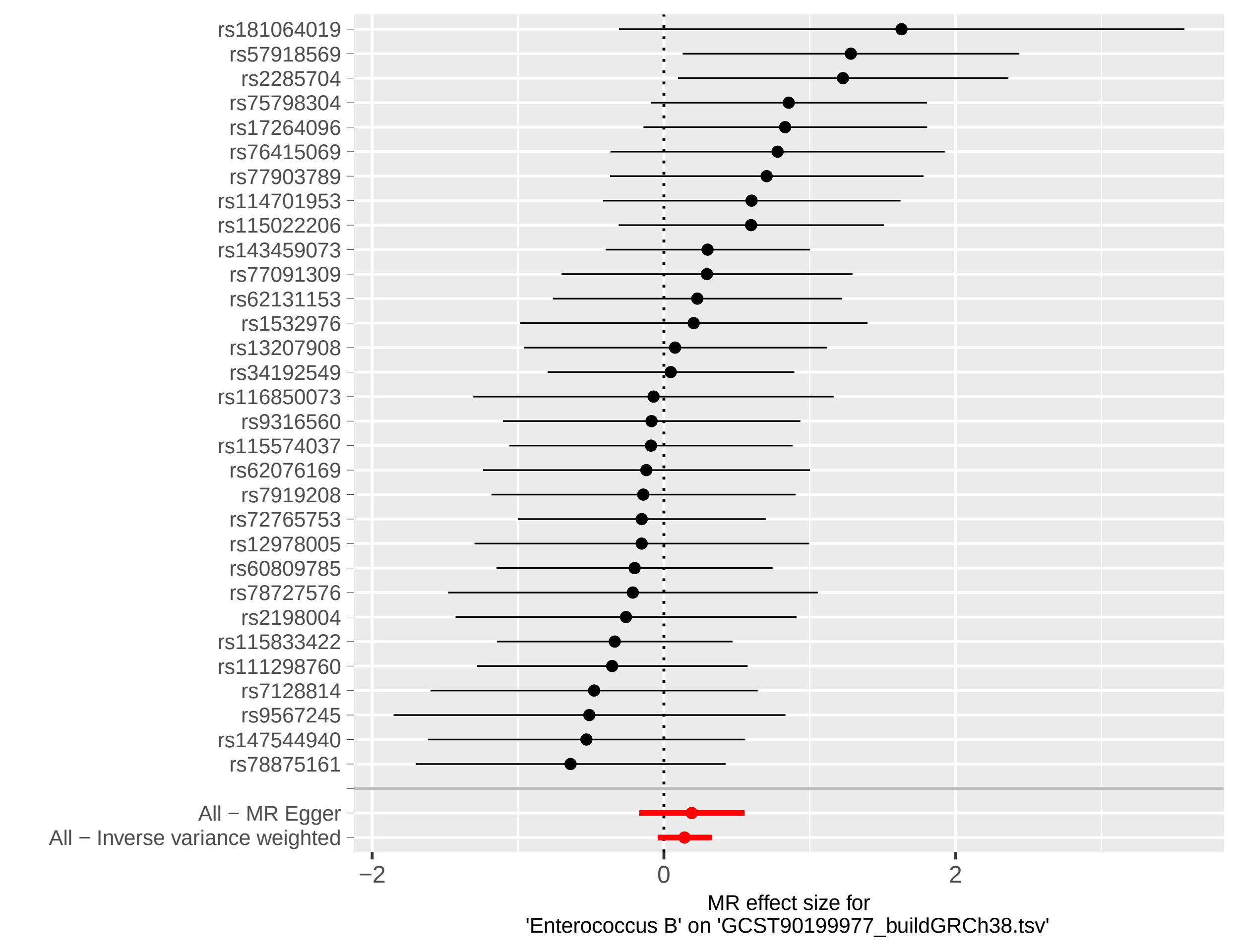
rs181064019
rs57918569
rs2285704
rs75798304
rs17264096
rs76415069
rs77903789
rs114701953
rs115022206
rs143459073
rs77091309
rs62131153
rs1532976
rs13207908
rs34192549
rs116850073
rs9316560
rs115574037
rs62076169
rs7919208
rs72765753
rs12978005
rs60809785
rs78727576
rs2198004
rs115833422
rs111298760
rs7128814
rs9567245
rs147544940
rs78875161
All − MR Egger
All − Inverse variance weighted
−2
0
2
MR effect size for
'Enterococcus B' on 'GCST90199977_buildGRCh38.tsv'

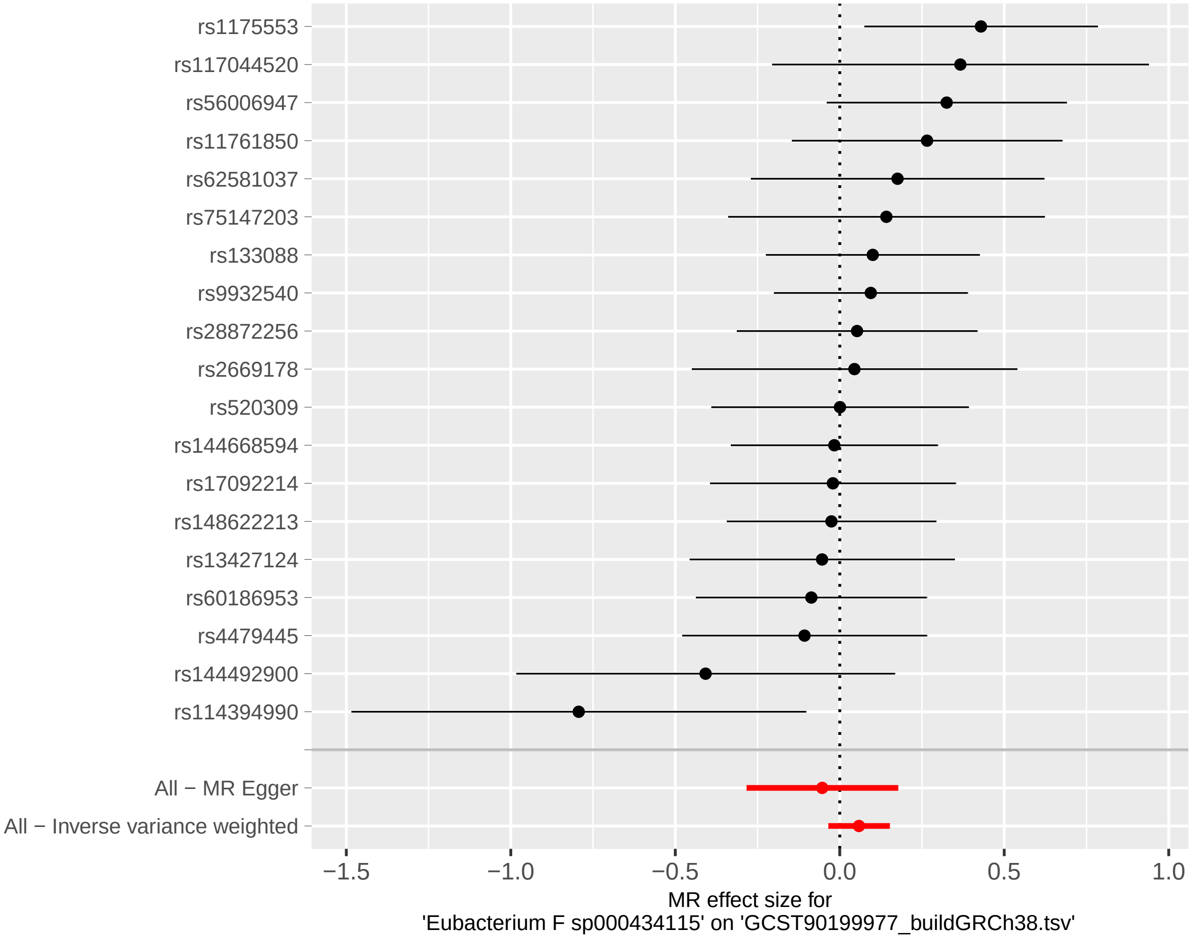
rs1175553
rs117044520
rs56006947
rs11761850
rs62581037
rs75147203
rs133088
rs9932540
rs28872256
rs2669178
rs520309
rs144668594
rs17092214
rs148622213
rs13427124
rs60186953
rs4479445
rs144492900
rs114394990
All − MR Egger
All − Inverse variance weighted
−1.5
−1.0
−0.5
0.0
0.5
1.0
MR effect size for
'Eubacterium F sp000434115' on 'GCST90199977_buildGRCh38.tsv'

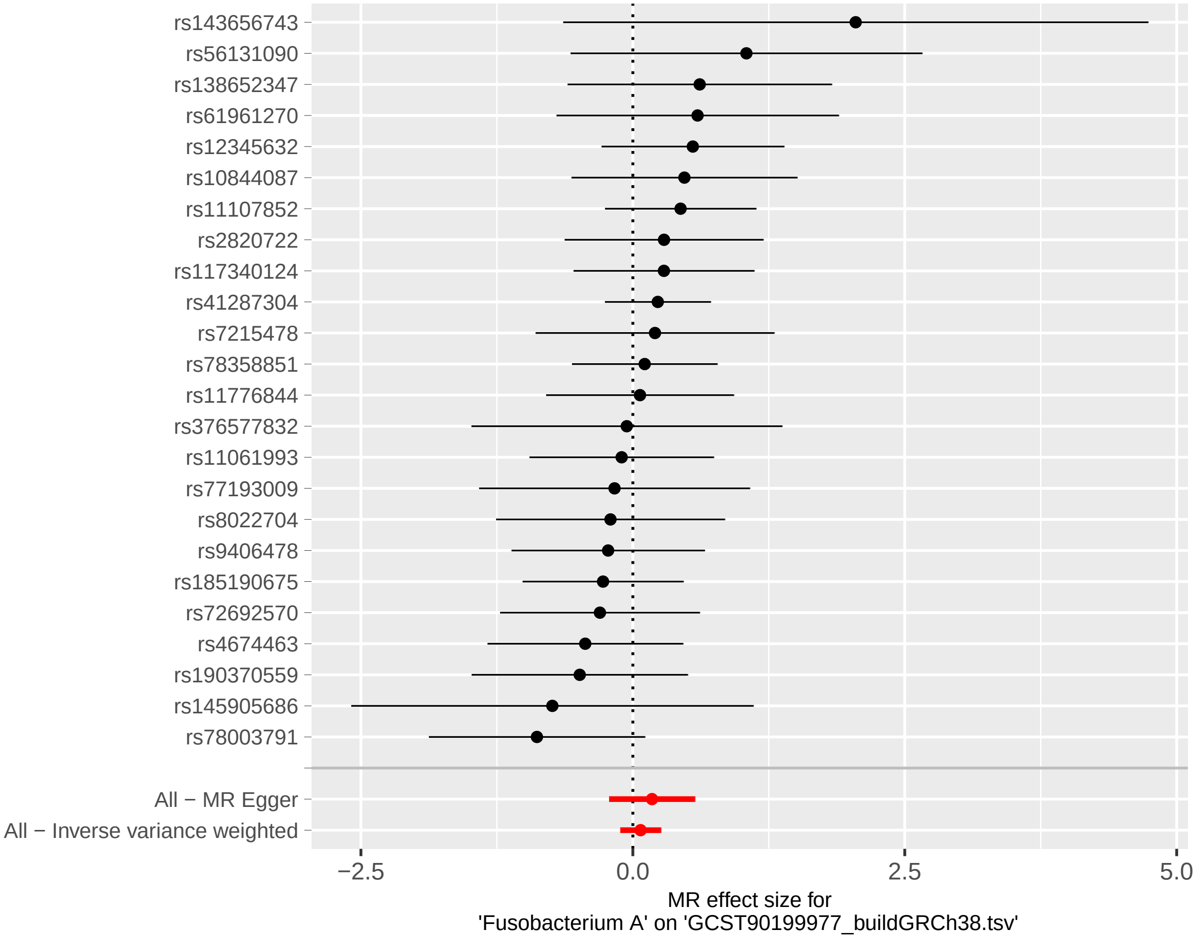
rs143656743
rs56131090
rs138652347
rs61961270
rs12345632
rs10844087
rs11107852
rs2820722
rs117340124
rs41287304
rs7215478
rs78358851
rs11776844
rs376577832
rs11061993
rs77193009
rs8022704
rs9406478
rs185190675
rs72692570
rs4674463
rs190370559
rs145905686
rs78003791
All − MR Egger
All − Inverse variance weighted
−2.5
0.0
2.5
5.0
MR effect size for
'Fusobacterium A' on 'GCST90199977_buildGRCh38.tsv'

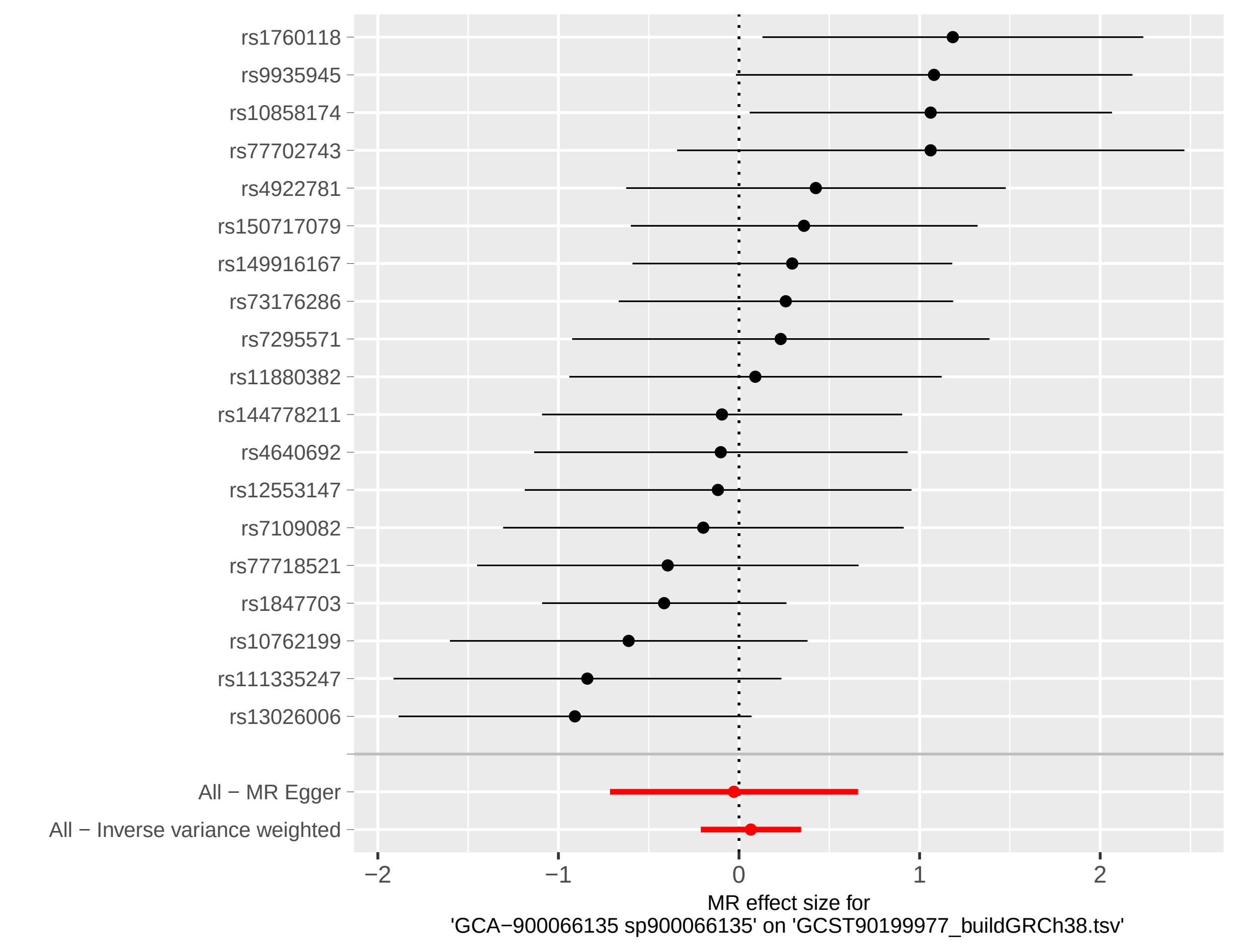

rs1760118
rs9935945
rs10858174
rs77702743
rs4922781
rs150717079
rs149916167
rs73176286
rs7295571
rs11880382
rs144778211
rs4640692
rs12553147
rs7109082
rs77718521
rs1847703
rs10762199
rs111335247
rs13026006
All − MR Egger
All − Inverse variance weighted
−2
−1
0
1
2
MR effect size for
'GCA−900066135 sp900066135' on 'GCST90199977_buildGRCh38.tsv'

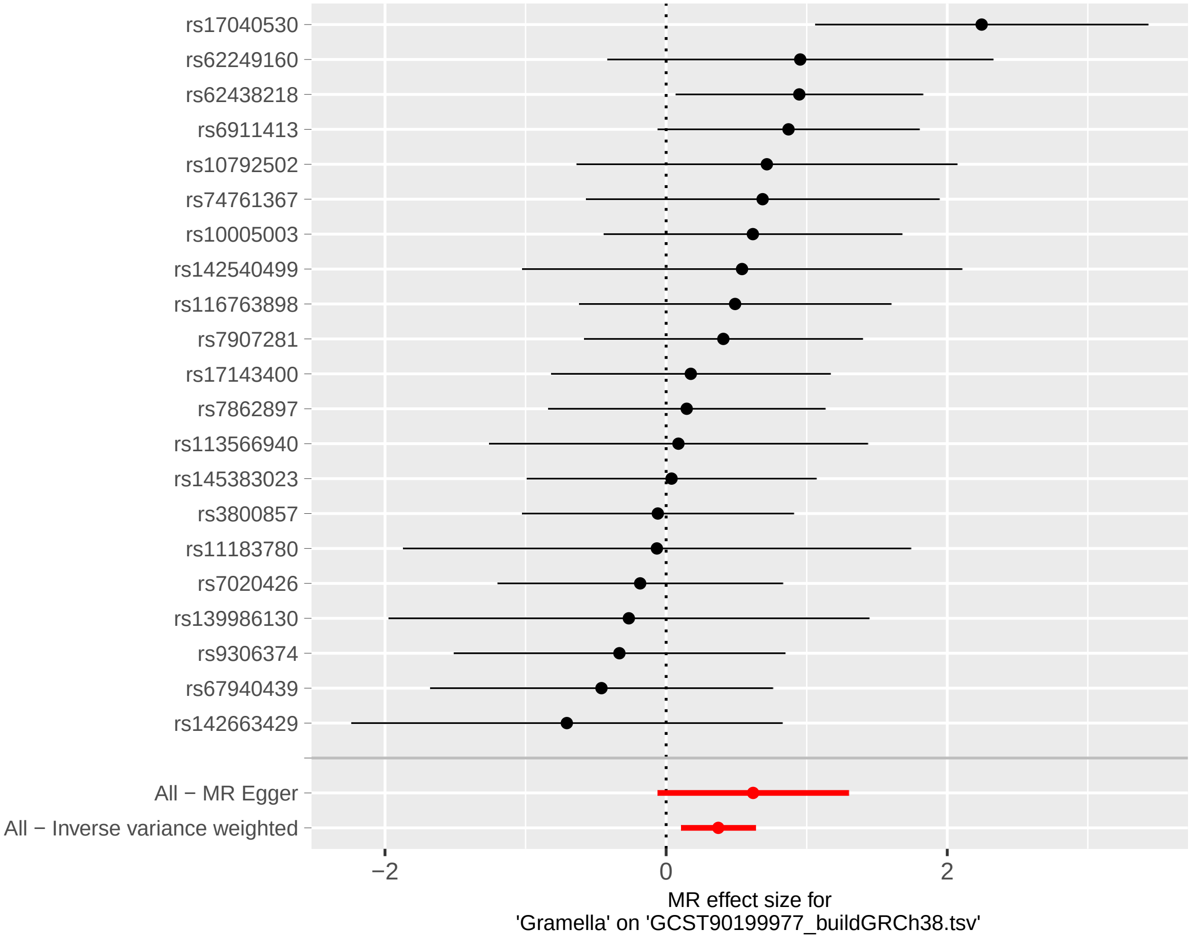
rs17040530
rs62249160
rs62438218
rs6911413
rs10792502
rs74761367
rs10005003
rs142540499
rs116763898
rs7907281
rs17143400
rs7862897
rs113566940
rs145383023
rs3800857
rs11183780
rs7020426
rs139986130
rs9306374
rs67940439
rs142663429
All − MR Egger
All − Inverse variance weighted
−2
0
2
MR effect size for
'Gramella' on 'GCST90199977_buildGRCh38.tsv'

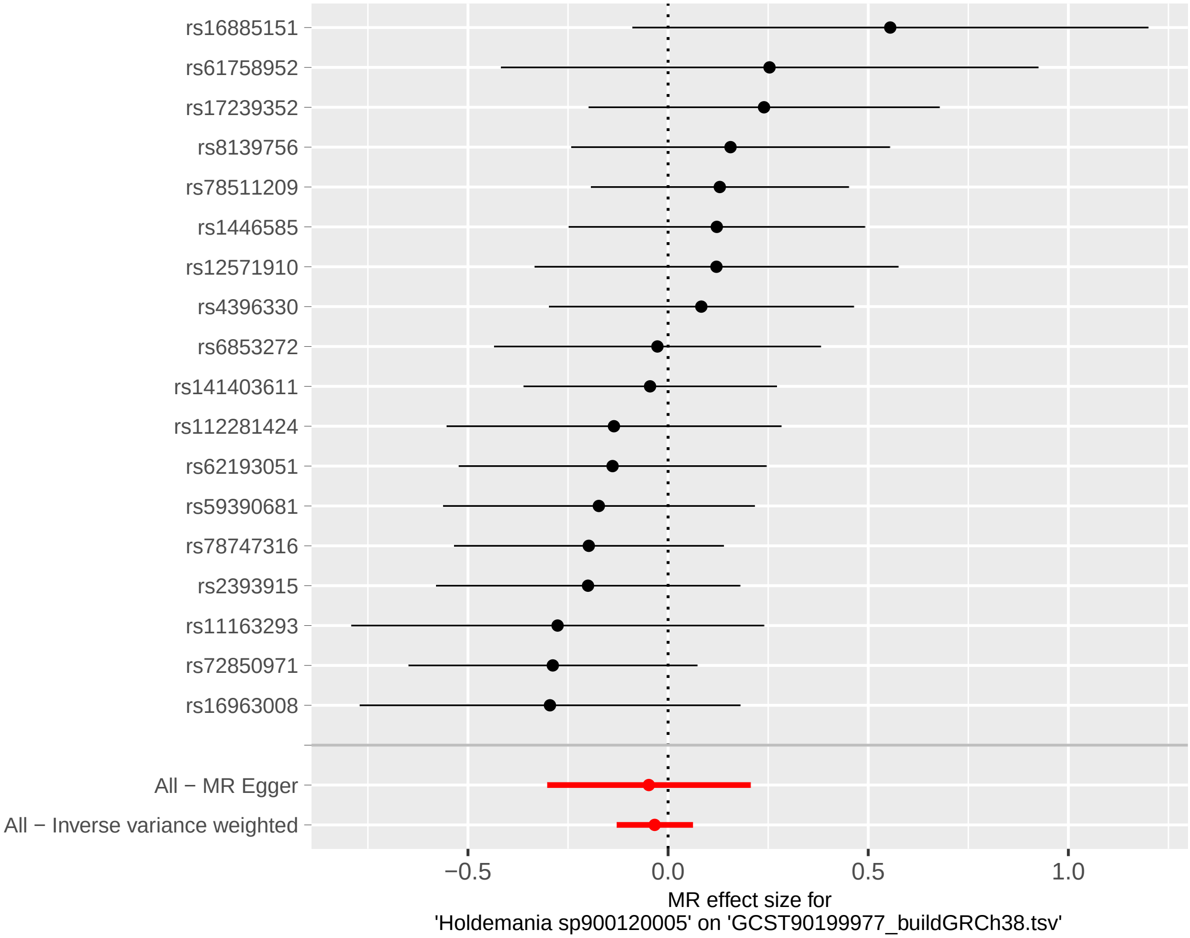
rs16885151
rs61758952
rs17239352
rs8139756
rs78511209
rs1446585
rs12571910
rs4396330
rs6853272
rs141403611
rs112281424
rs62193051
rs59390681
rs78747316
rs2393915
rs11163293
rs72850971
rs16963008
All − MR Egger
All − Inverse variance weighted
−0.5
0.0
0.5
1.0
MR effect size for
'Holdemania sp900120005' on 'GCST90199977_buildGRCh38.tsv'

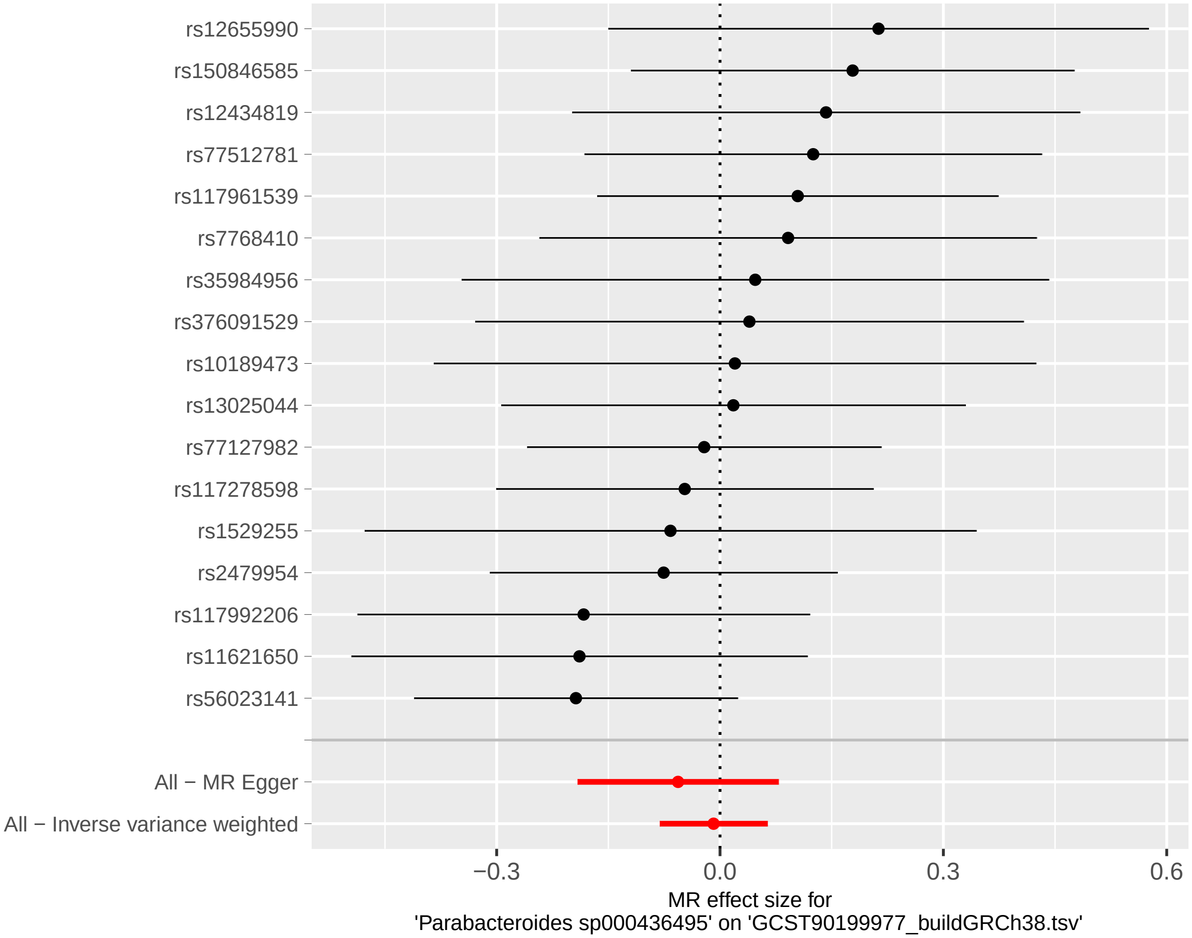
rs12655990
rs150846585
rs12434819
rs77512781
rs117961539
rs7768410
rs35984956
rs376091529
rs10189473
rs13025044
rs77127982
rs117278598
rs1529255
rs2479954
rs117992206
rs11621650
rs56023141
All − MR Egger
All − Inverse variance weighted
−0.3
0.0
0.3
0.6
MR effect size for
'Parabacteroides sp000436495' on 'GCST90199977_buildGRCh38.tsv'

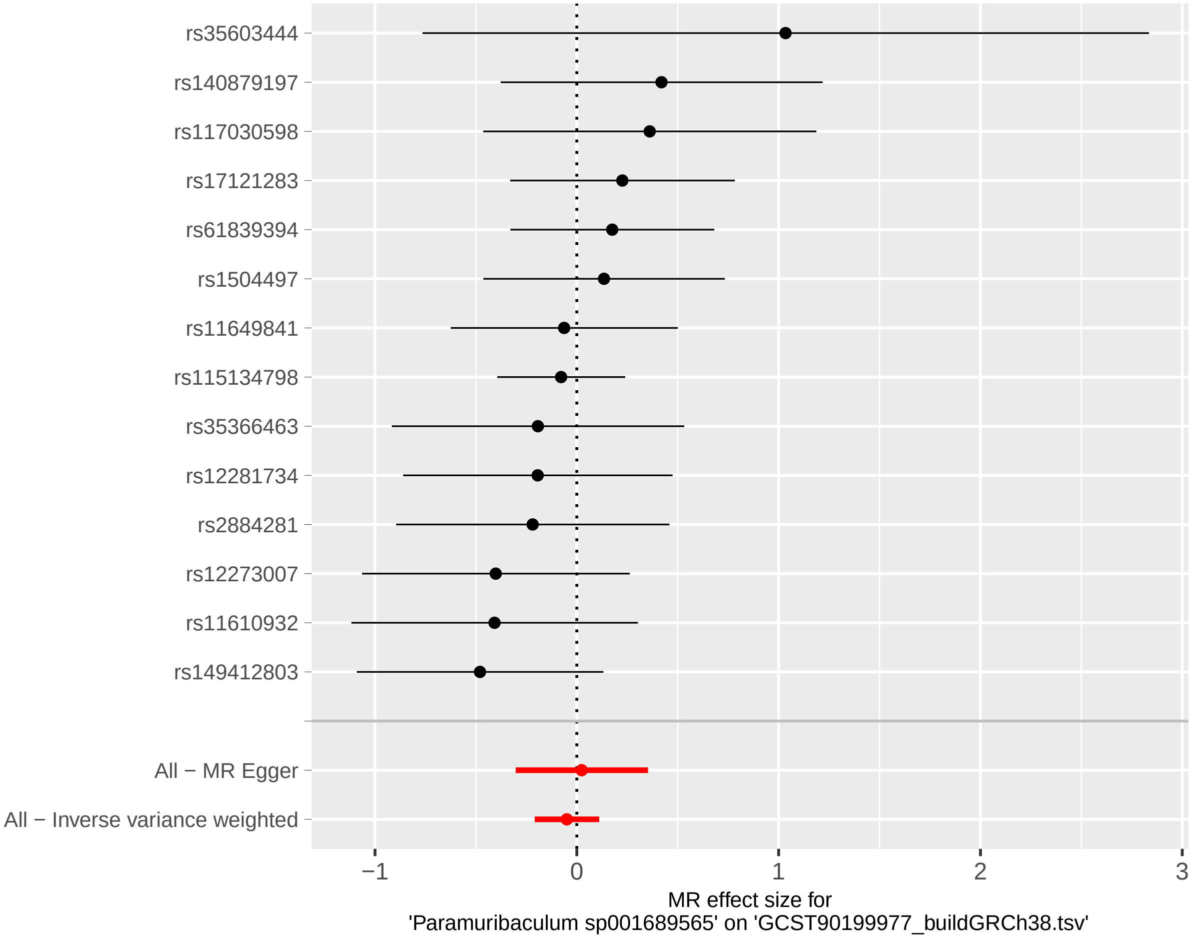

rs35603444
rs140879197
rs117030598
rs17121283
rs61839394
rs1504497
rs11649841
rs115134798
rs35366463
rs12281734
rs2884281
rs12273007
rs11610932
rs149412803
All − MR Egger
All − Inverse variance weighted
−1
0
1
2
3
MR effect size for
'Paramuribaculum sp001689565' on 'GCST90199977_buildGRCh38.tsv'

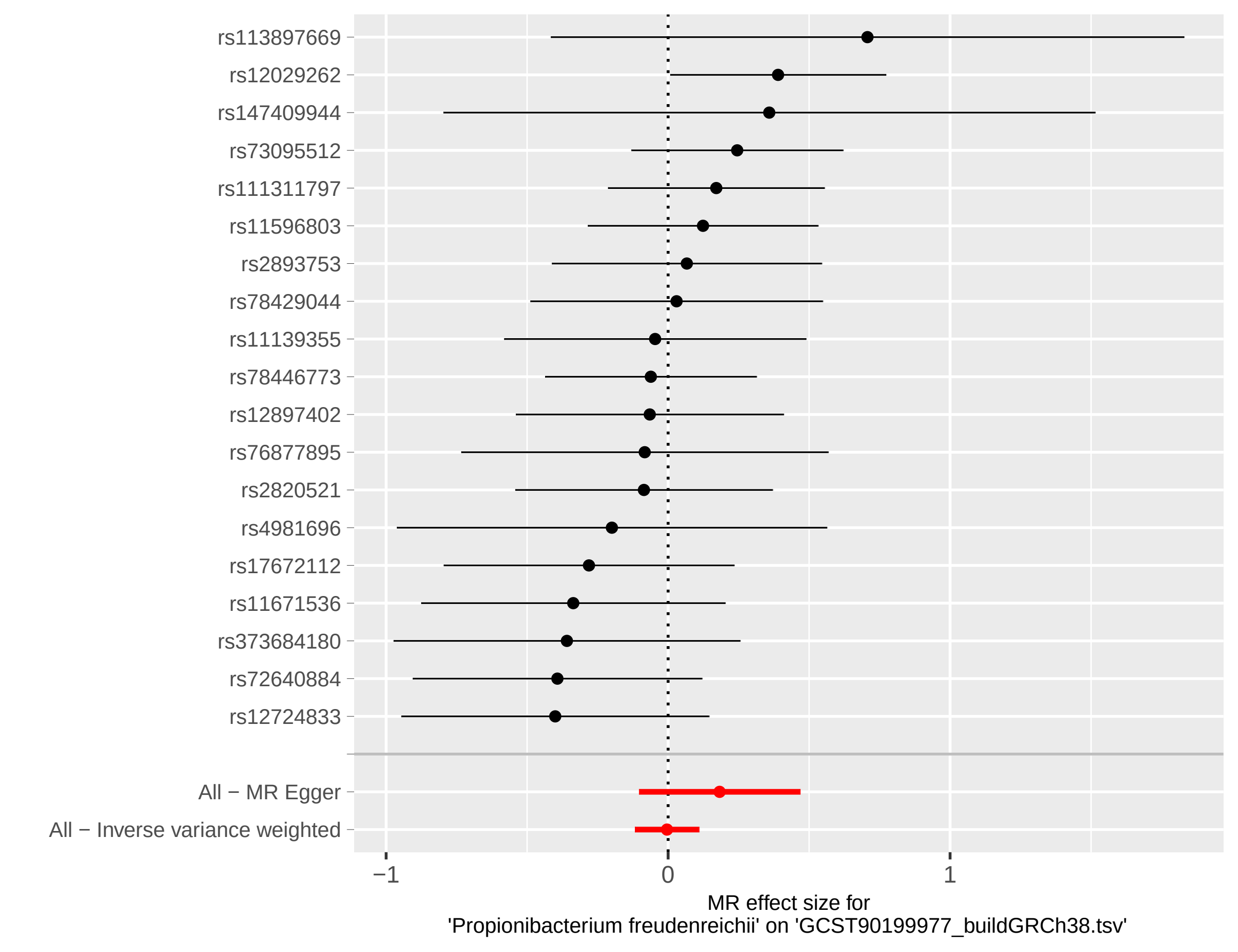
rs113897669
rs12029262
rs147409944
rs73095512
rs111311797
rs11596803
rs2893753
rs78429044
rs11139355
rs78446773
rs12897402
rs76877895
rs2820521
rs4981696
rs17672112
rs11671536
rs373684180
rs72640884
rs12724833
All − MR Egger
All − Inverse variance weighted
−1
0
1
MR effect size for
'Propionibacterium freudenreichii' on 'GCST90199977_buildGRCh38.tsv'

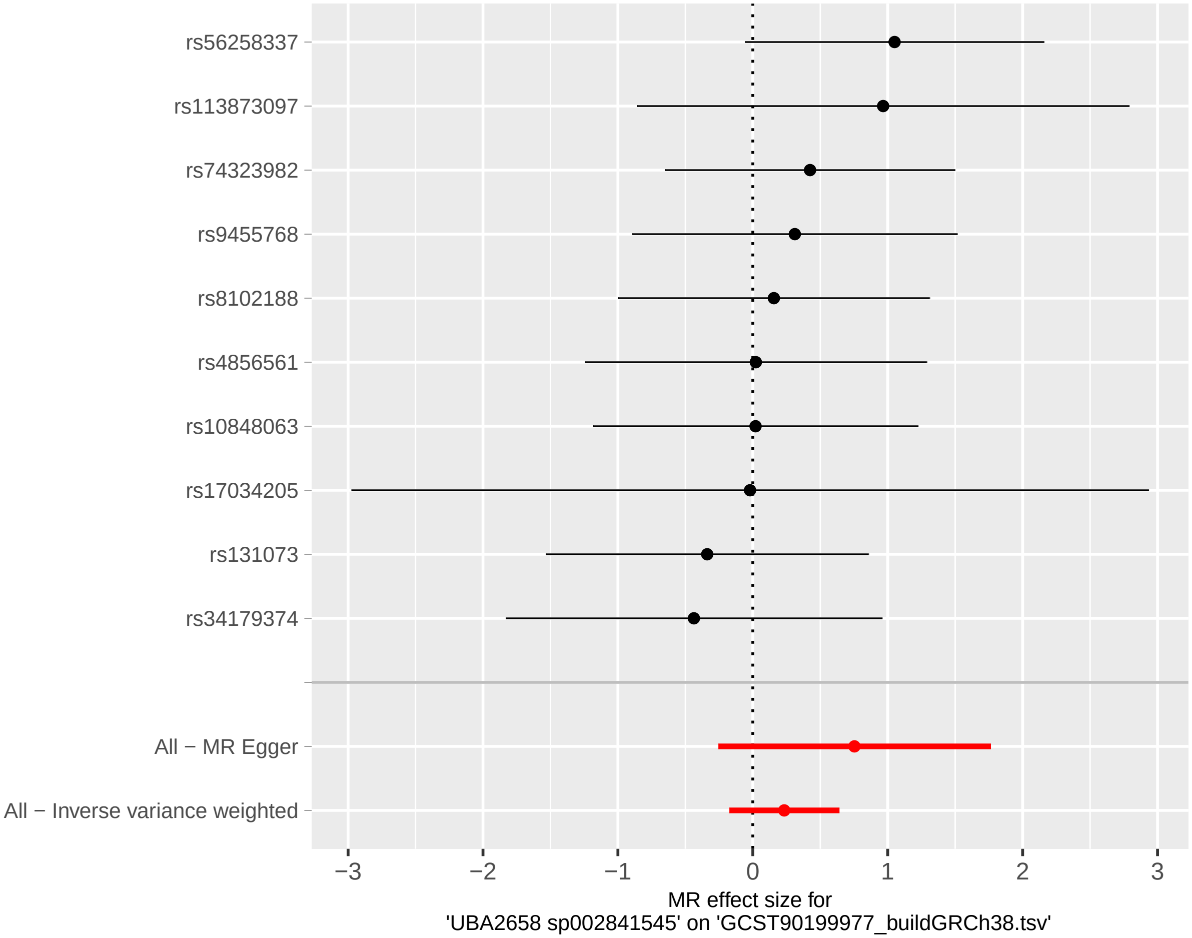
rs56258337
rs113873097
rs74323982
rs9455768
rs8102188
rs4856561
rs10848063
rs17034205
rs131073
rs34179374
All − MR Egger
All − Inverse variance weighted
−3
−2
−1
0
1
2
3
MR effect size for
'UBA2658 sp002841545' on 'GCST90199977_buildGRCh38.tsv'

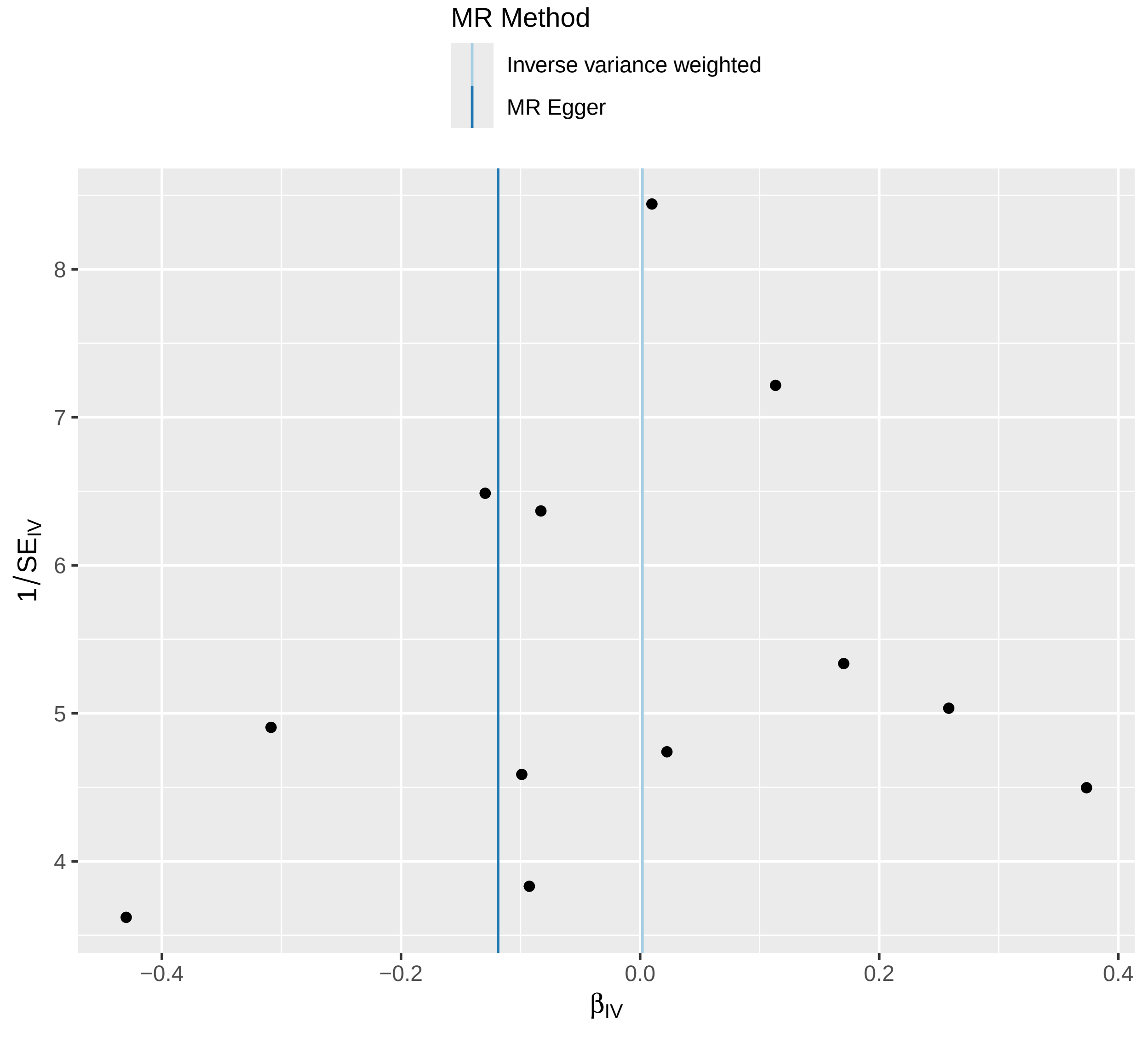

MR Method
Inverse variance weighted
MR Egger
8
7
6
5
4
1/SE_IV
−0.4
−0.2
0.0
0.2
0.4
β_IV

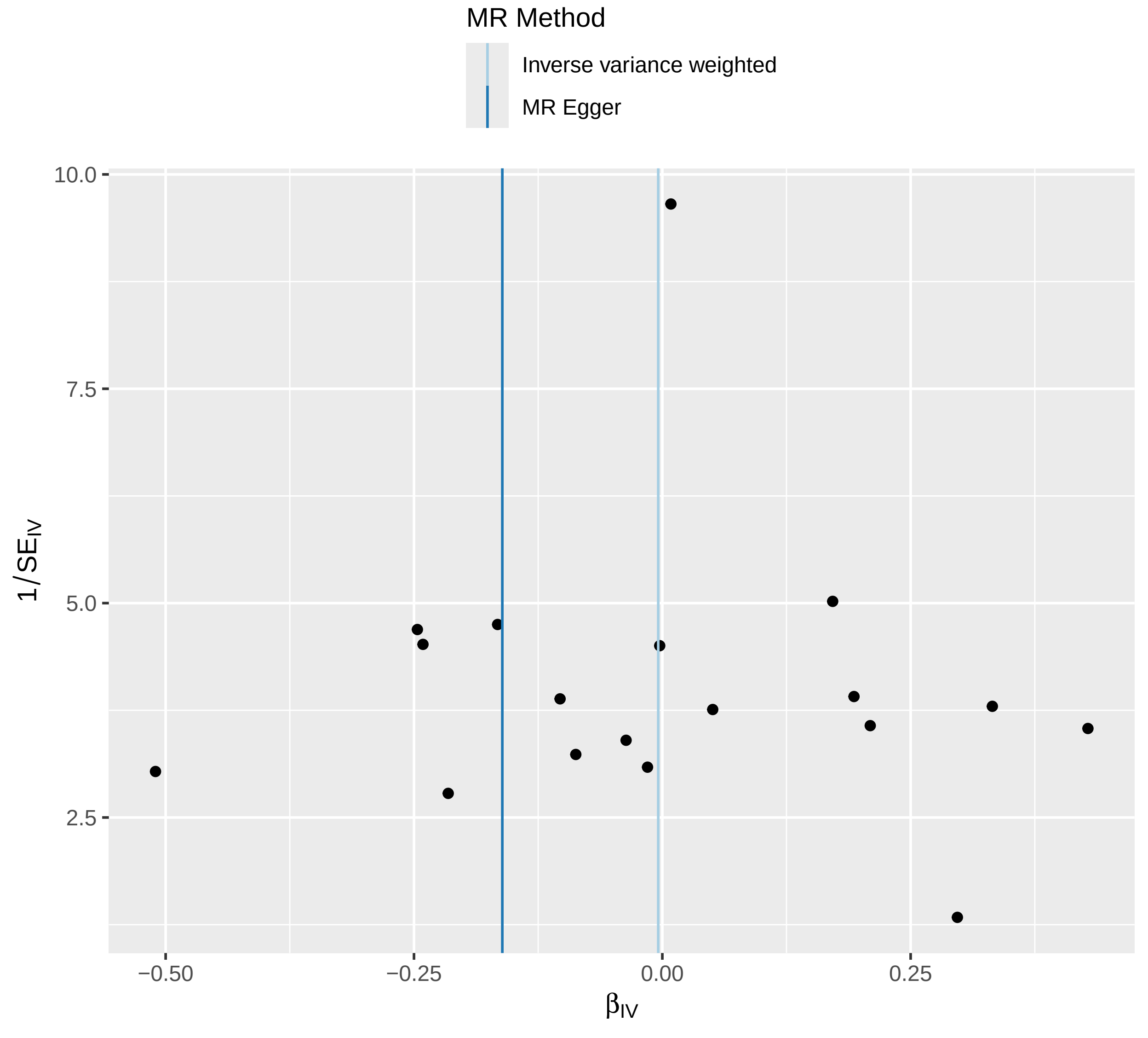
MR Method
Inverse variance weighted
MR Egger
10.0
7.5
5.0
2.5
1/SE$_{IV}$
−0.50
−0.25
0.00
0.25
β$_{IV}$

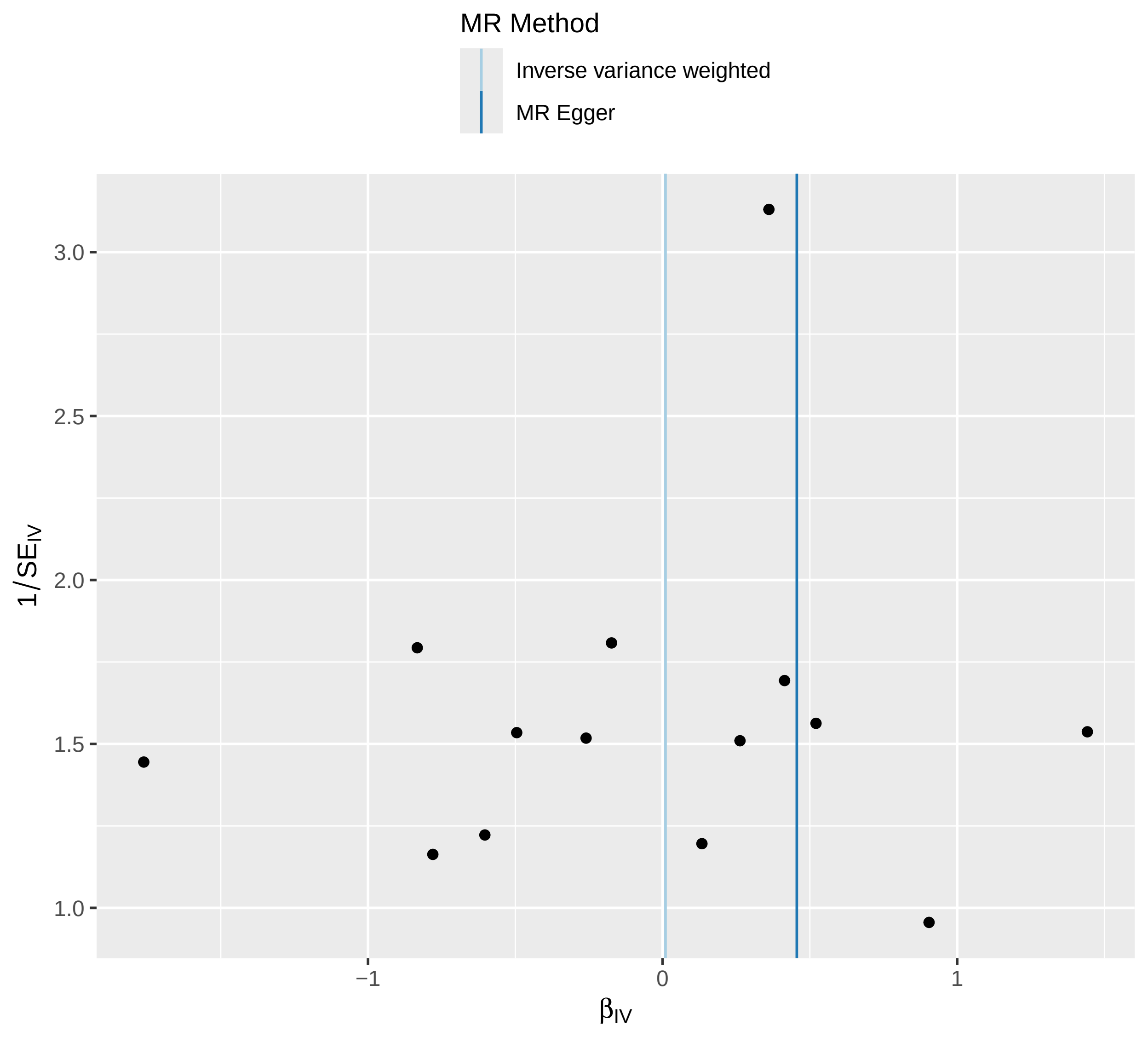

MR Method
Inverse variance weighted
MR Egger
3.0
2.5
2.0
1.5
1.0
$1/SE_{IV}$
−1
0
1
β_{IV}

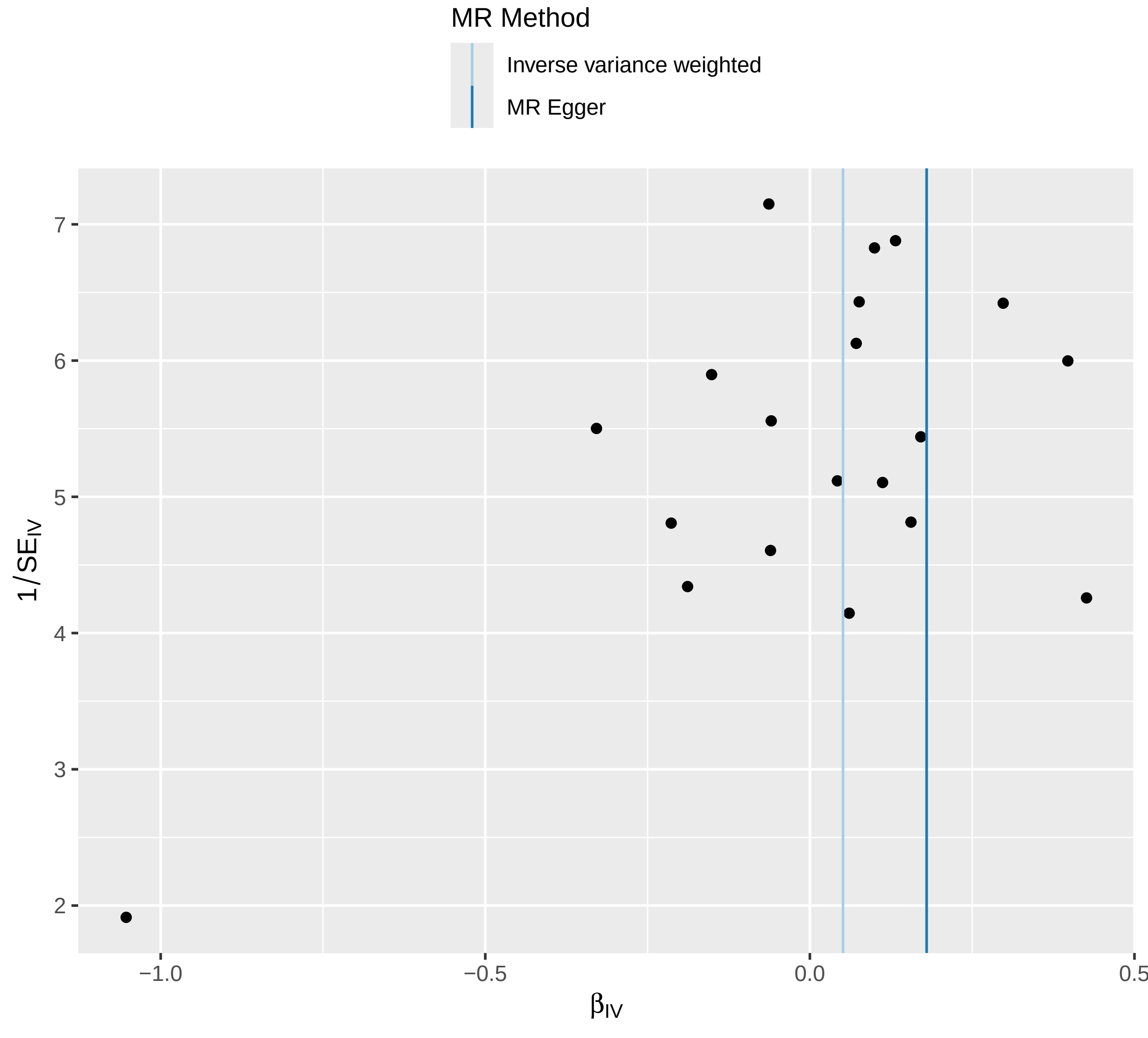
MR Method
Inverse variance weighted
MR Egger
1/SE_IV
7
6
5
4
3
2
−1.0
−0.5
0.0
0.5
β_IV

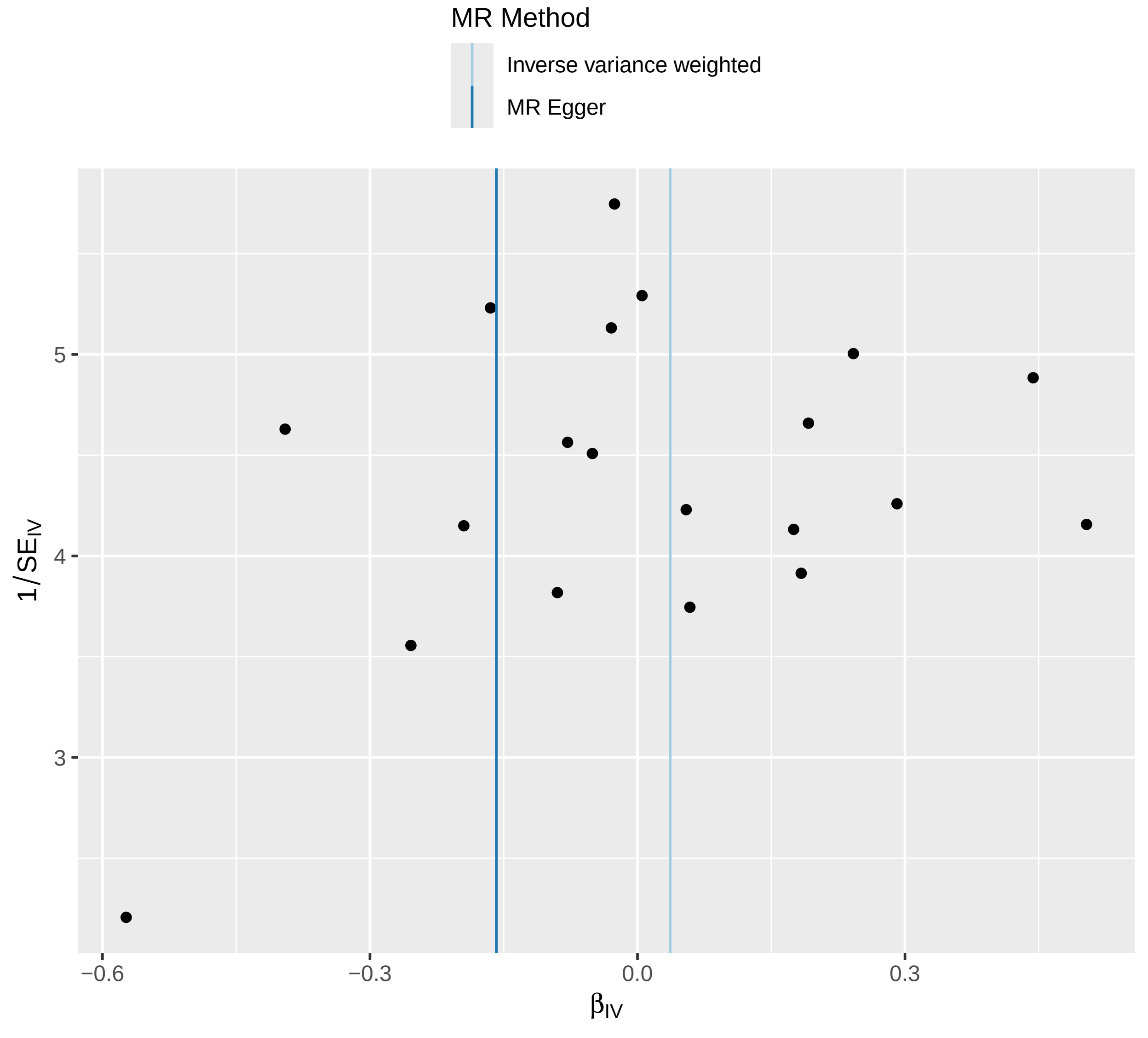

MR Method
Inverse variance weighted
MR Egger
5
4
3
1/SE_IV
−0.6
−0.3
0.0
0.3
β_IV

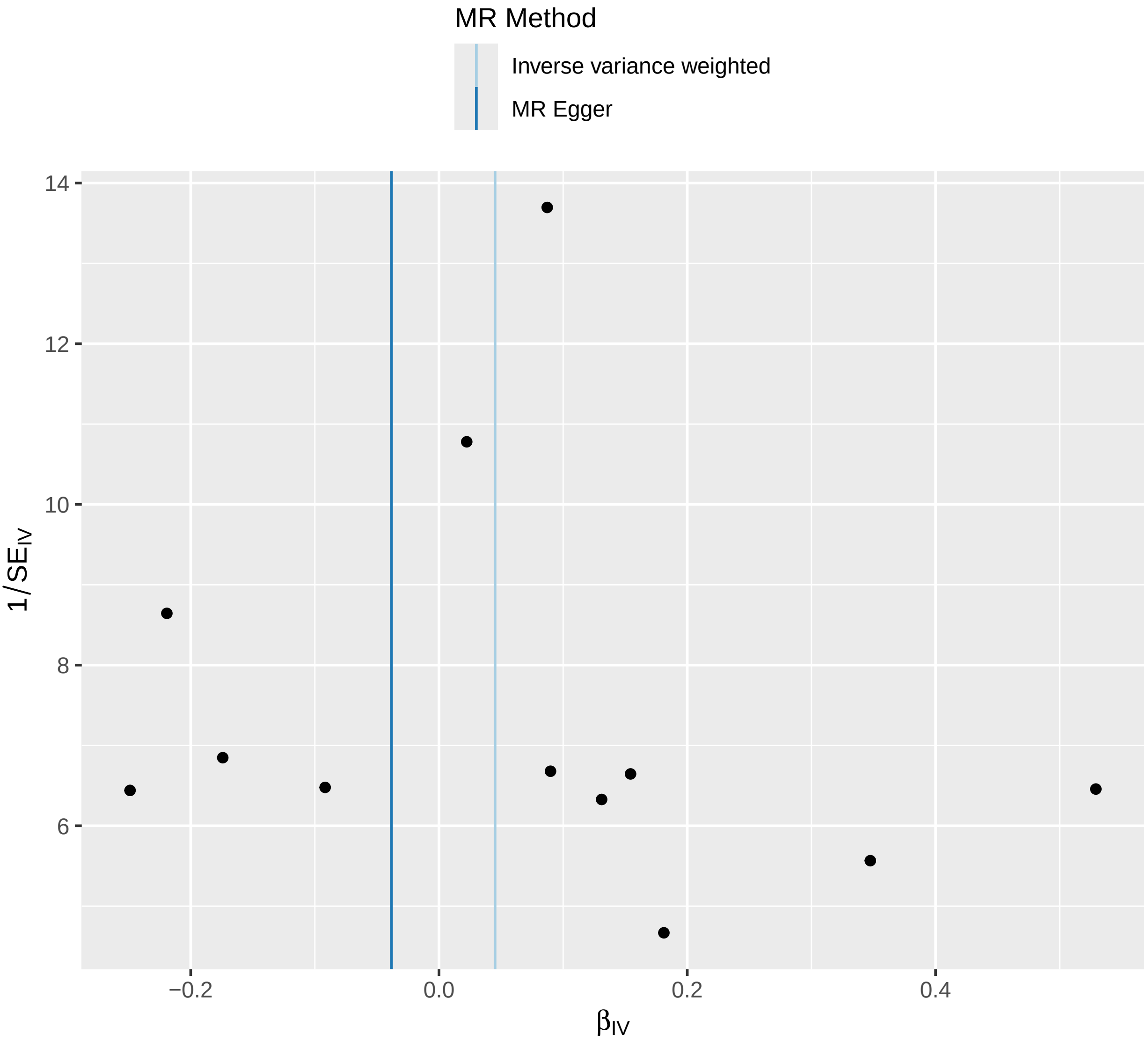
MR Method
Inverse variance weighted
MR Egger
14
12
10
8
6
1/SE_IV
−0.2
0.0
0.2
0.4
β_IV

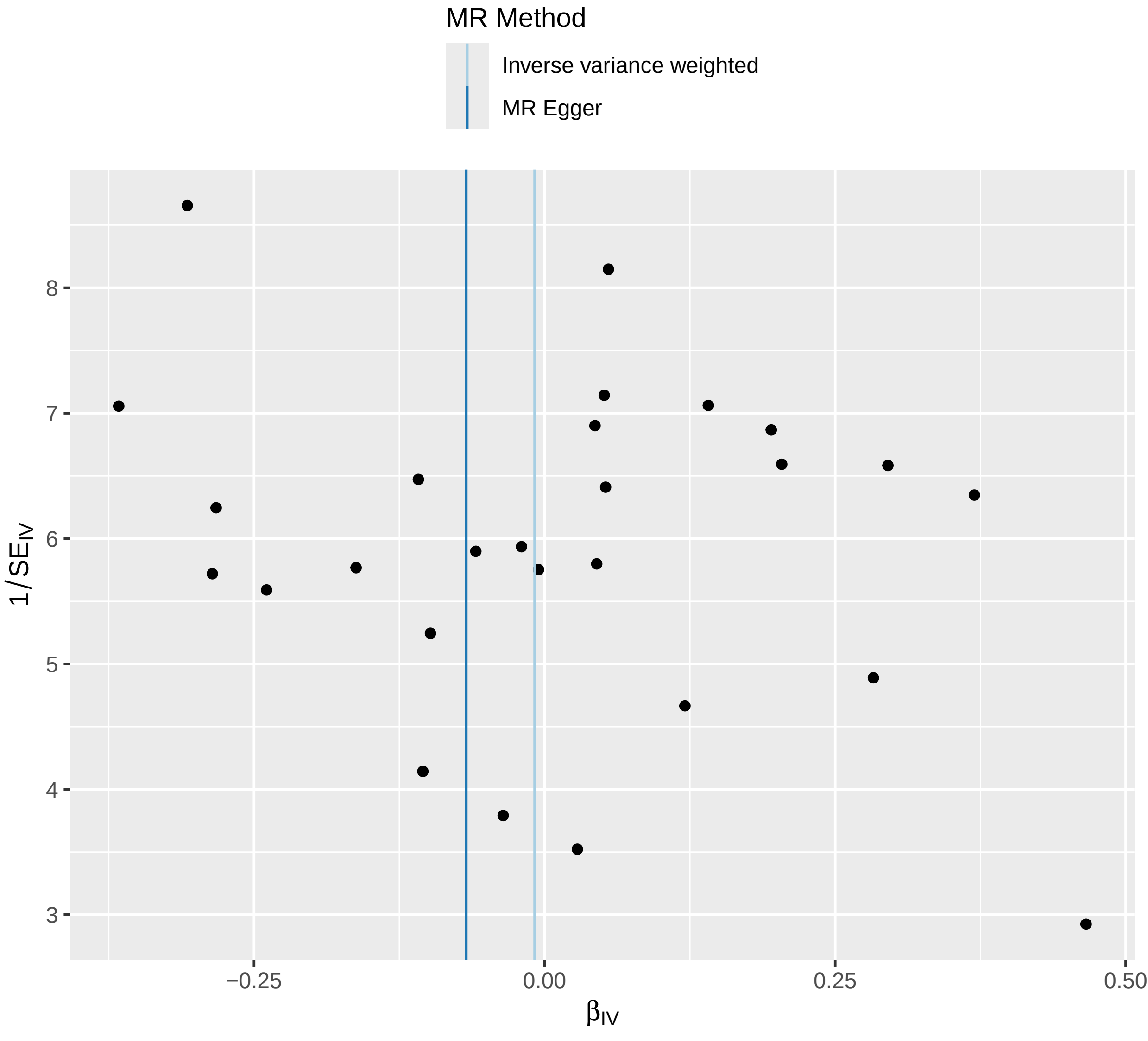
MR Method
Inverse variance weighted
MR Egger
8
7
6
5
4
3
1/SE_IV
−0.25
0.00
0.25
0.50
β_IV

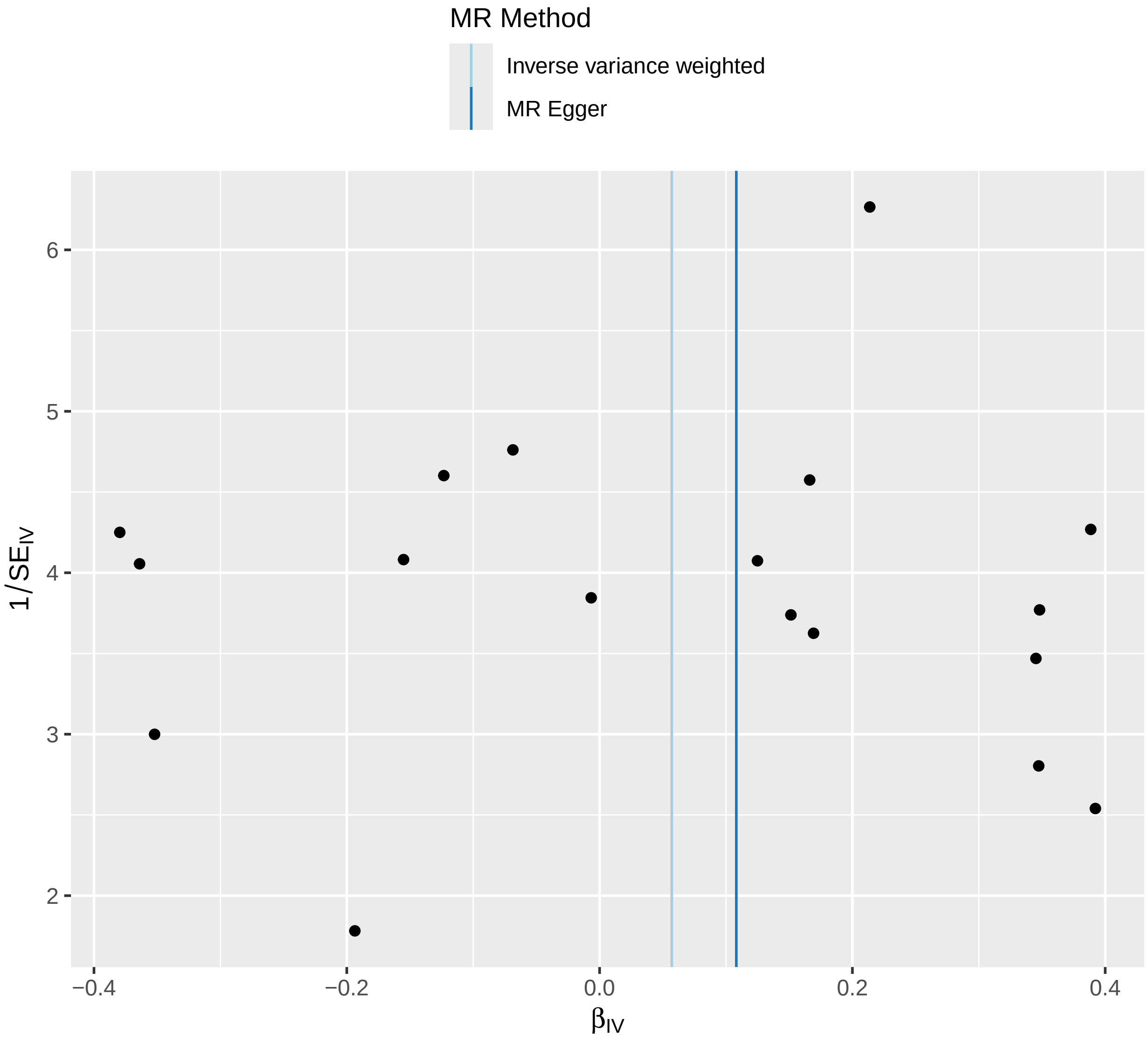
MR Method
Inverse variance weighted
MR Egger
6
5
4
3
2
1/SE_IV
−0.4
−0.2
0.0
0.2
0.4
β_IV

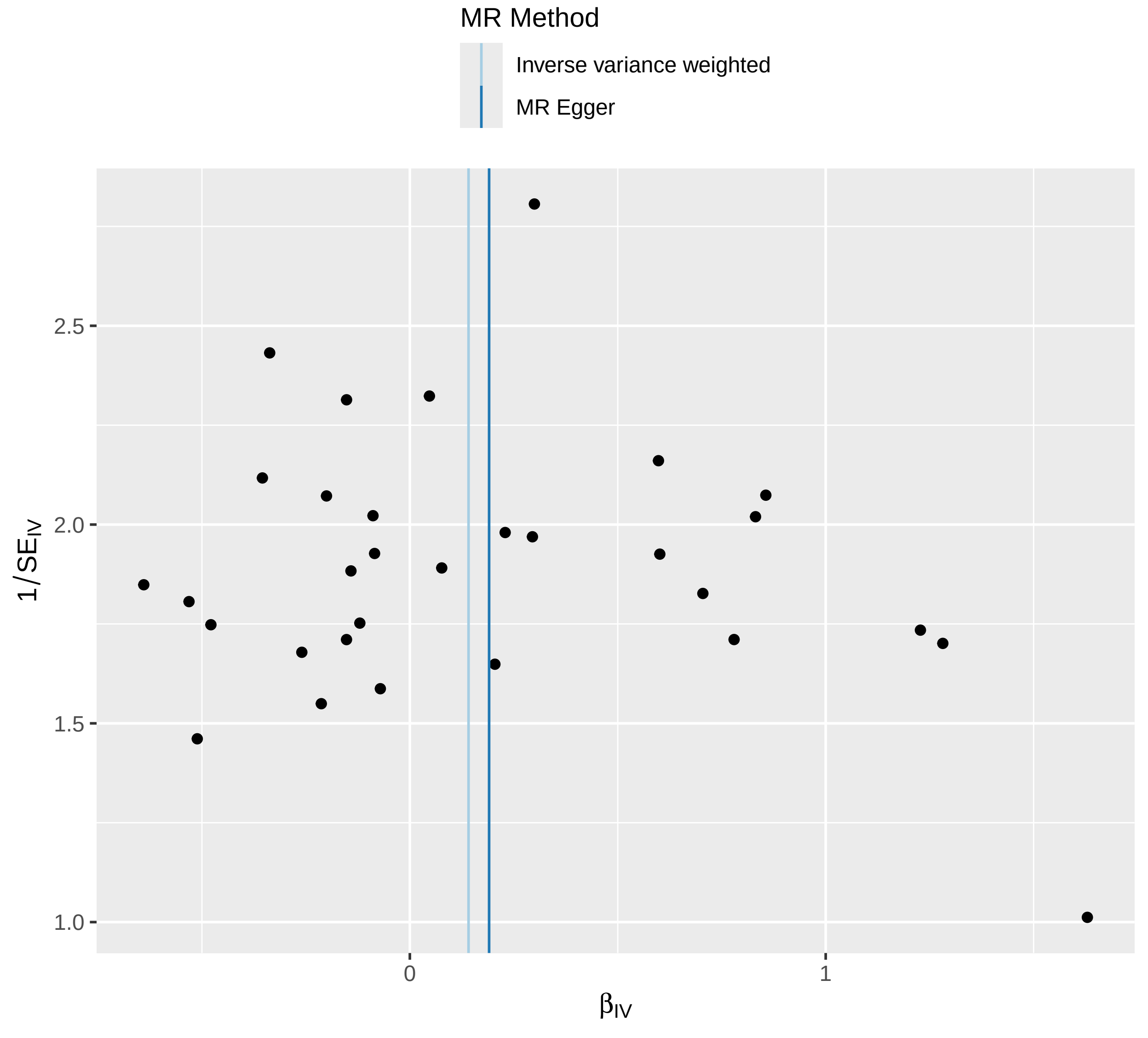
MR Method
Inverse variance weighted
MR Egger
2.5
2.0
1.5
1.0
1/SE_IV
0
1
β_IV

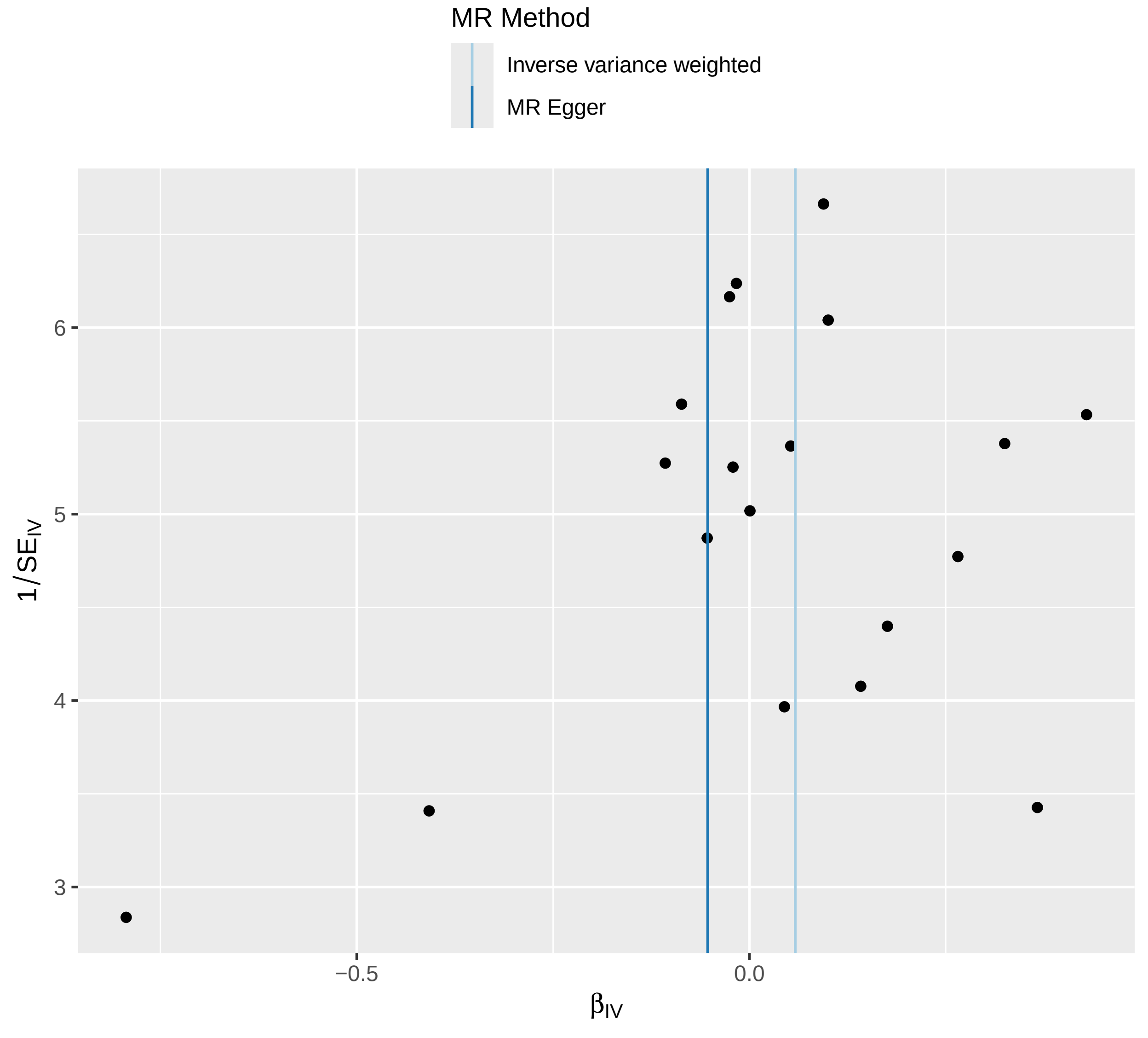
MR Method
Inverse variance weighted
MR Egger
6
5
4
3
1/SE_IV
−0.5
0.0
β_IV

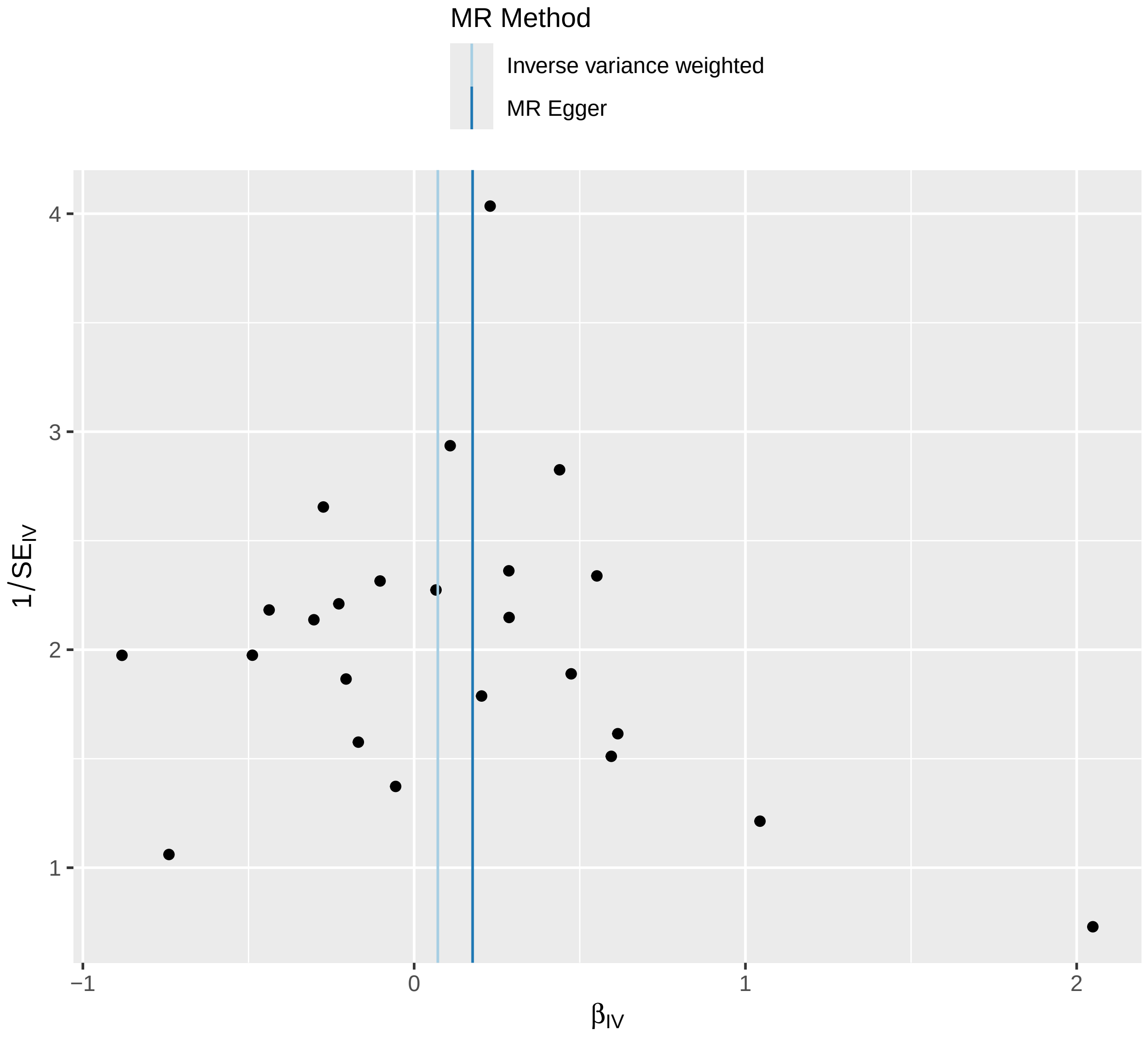
MR Method
Inverse variance weighted
MR Egger
1/SE$_{IV}$
4
3
2
1
−1
0
1
2
β$_{IV}$

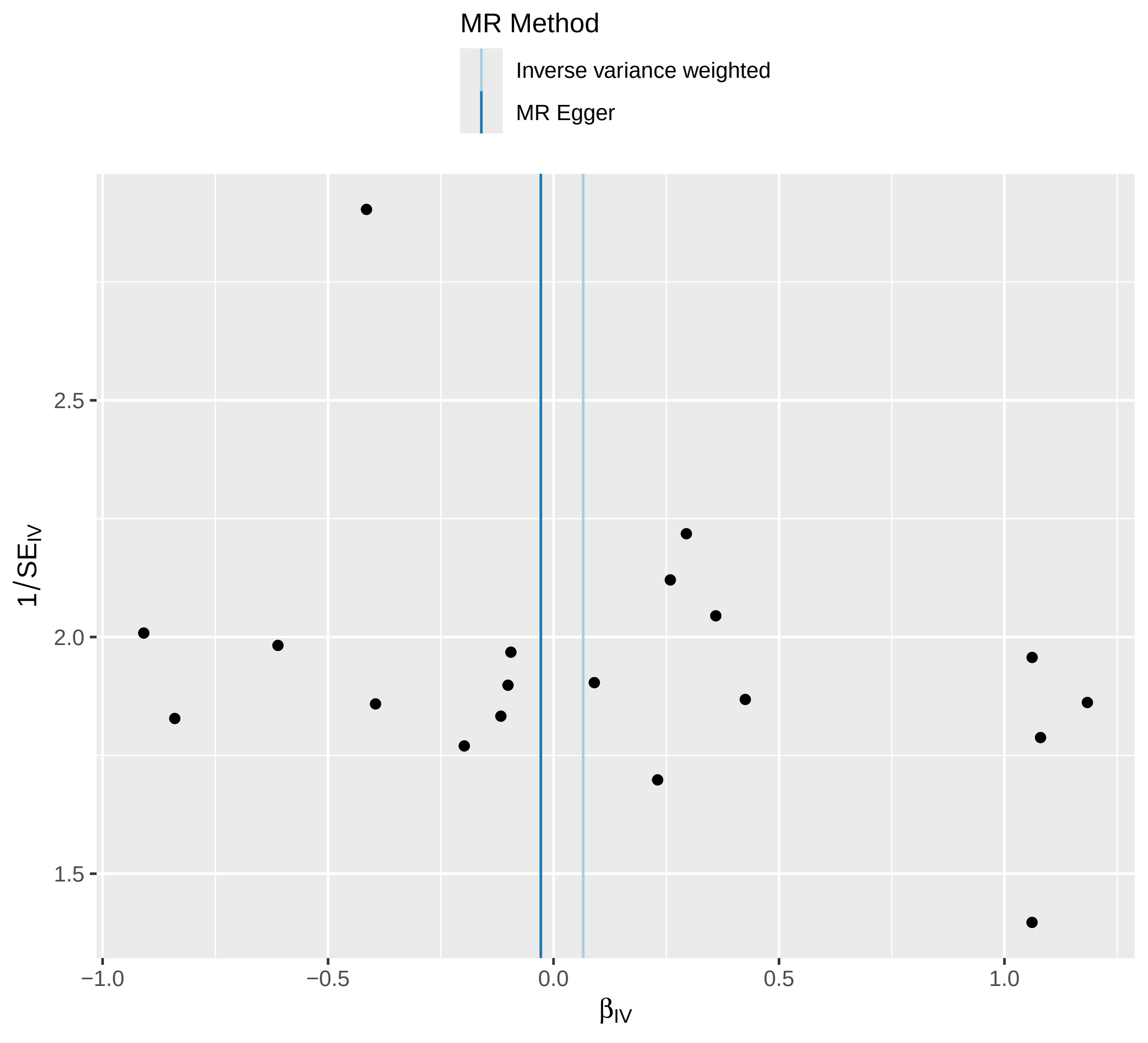

MR Method
Inverse variance weighted
MR Egger
2.5
2.0
1.5
$1/SE_{IV}$
−1.0
−0.5
0.0
0.5
1.0
β_{IV}

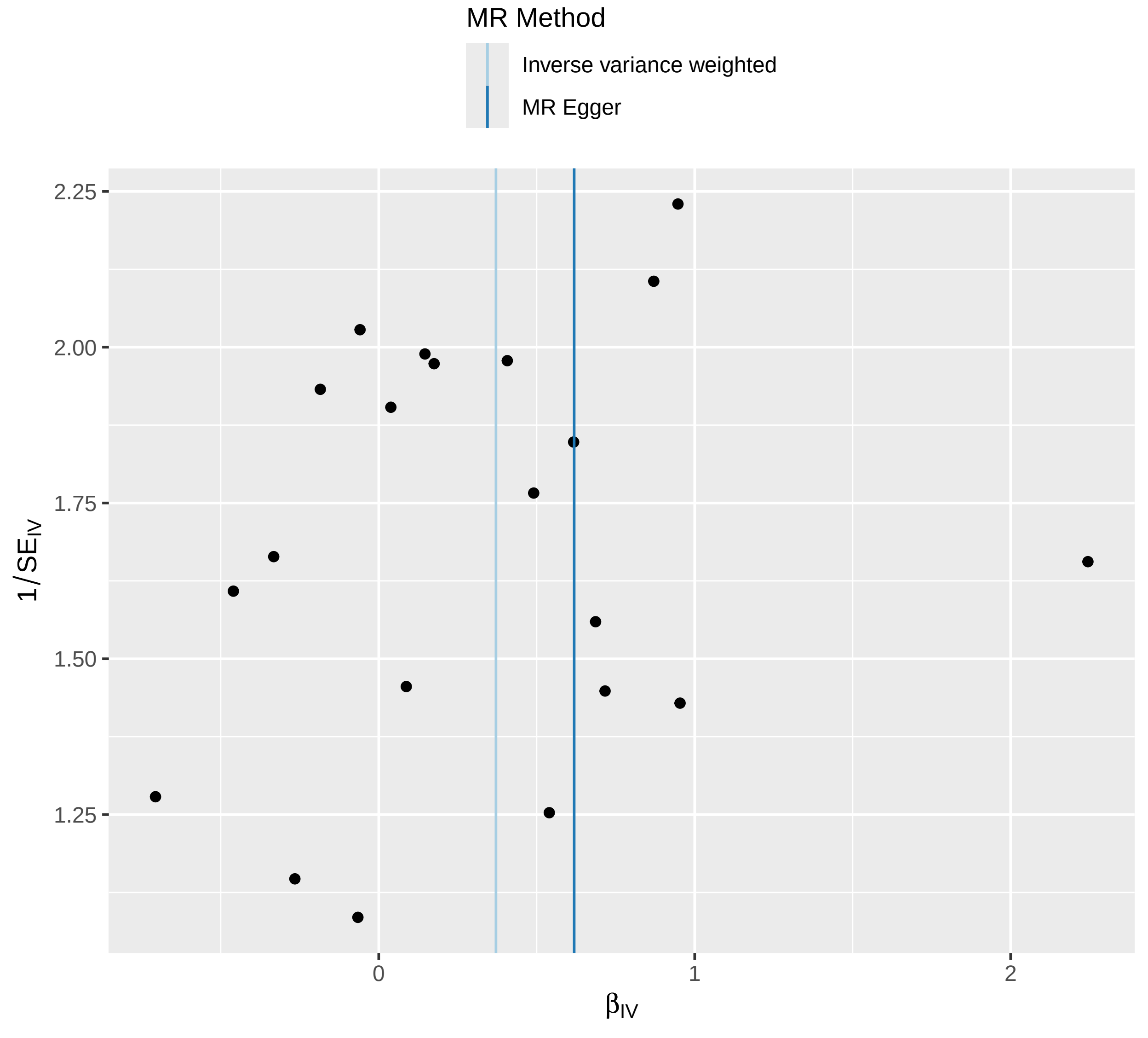

MR Method
Inverse variance weighted
MR Egger
2.25
2.00
1.75
1.50
1.25
1/SE_IV
0
1
2
β_IV

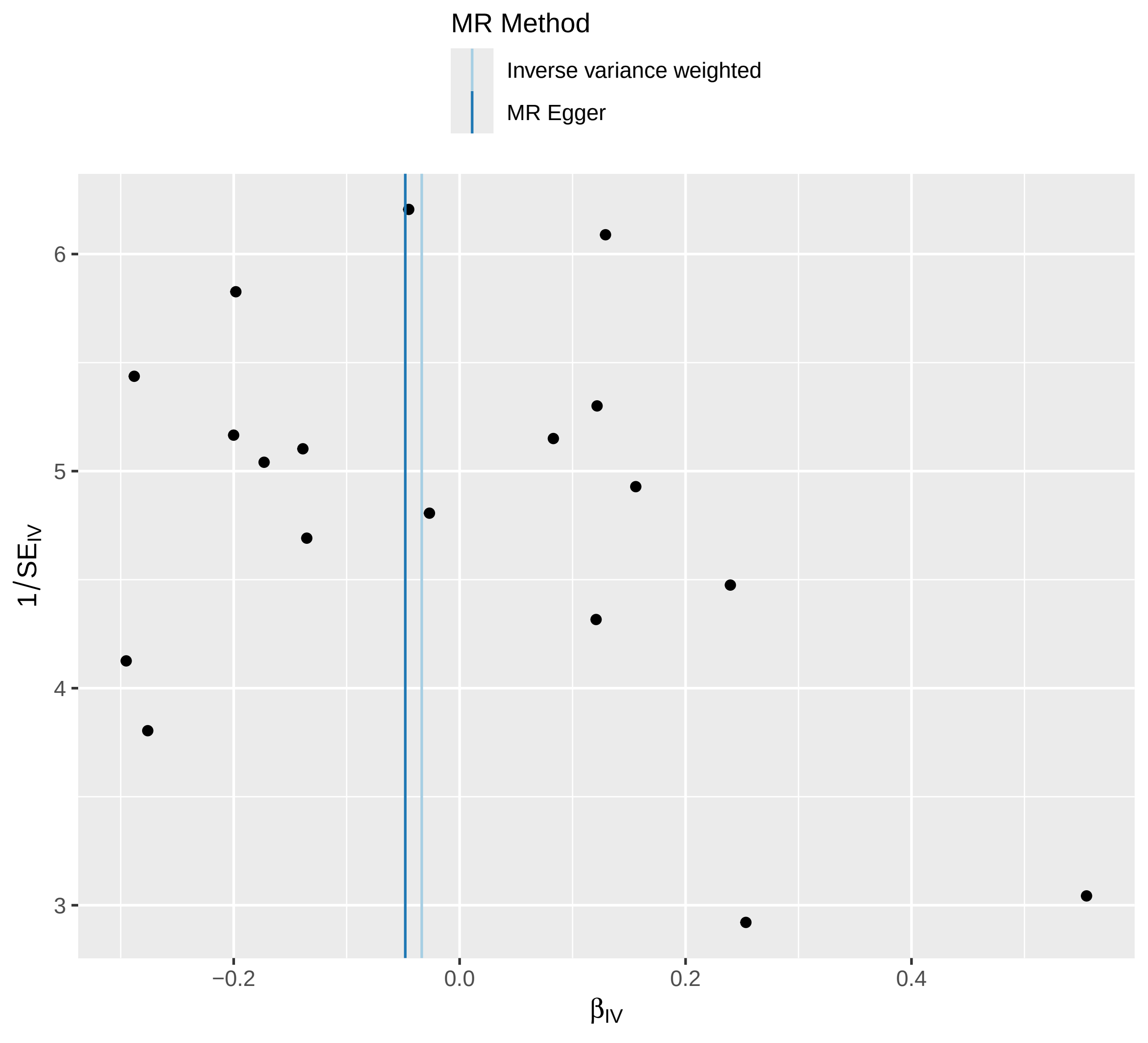
MR Method
Inverse variance weighted
MR Egger
6
5
4
3
1/SE$_{IV}$
−0.2
0.0
0.2
0.4
β$_{IV}$

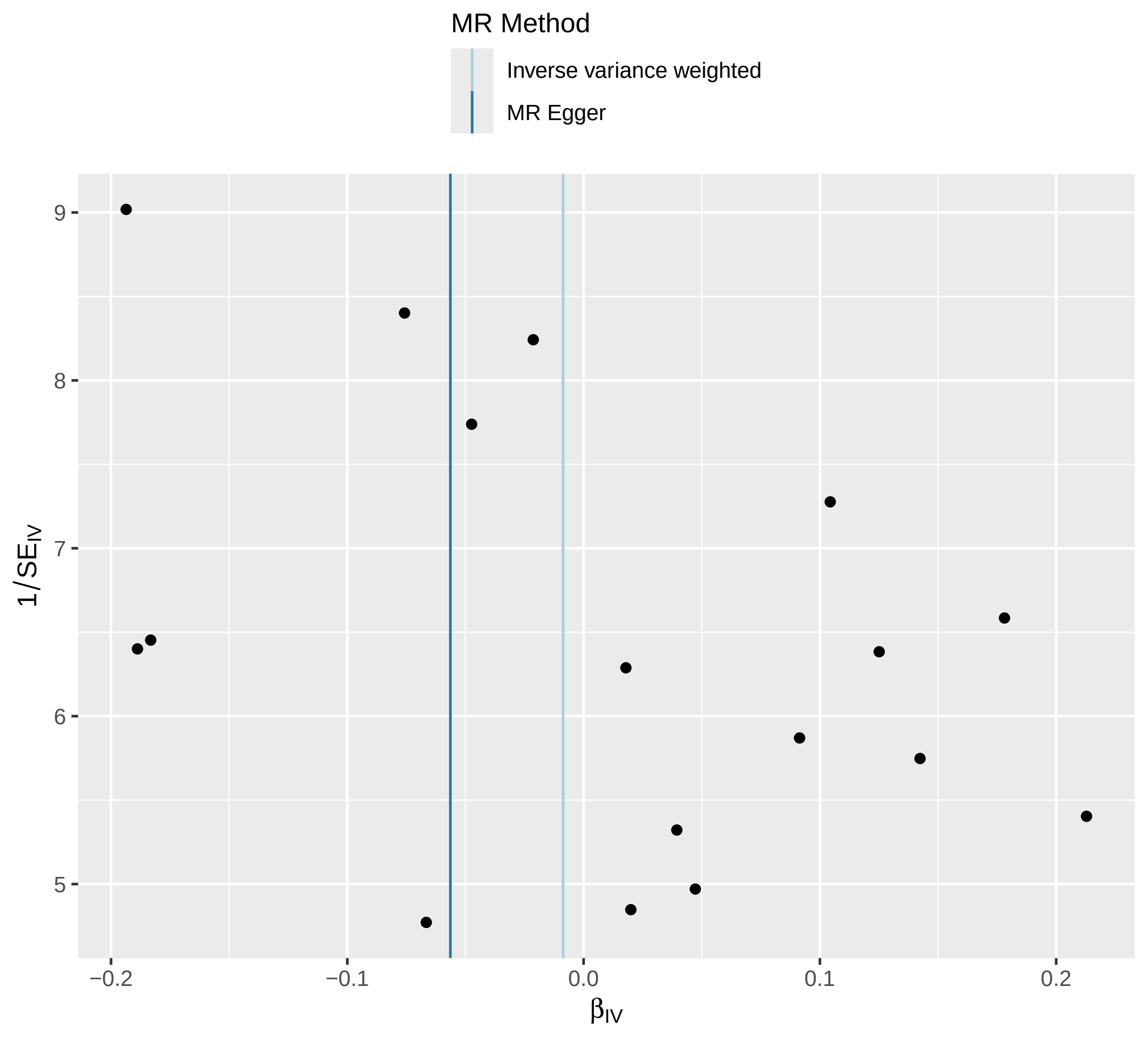
MR Method
Inverse variance weighted
MR Egger
9
8
7
6
5
1/SE_IV
−0.2
−0.1
0.0
0.1
0.2
β_IV

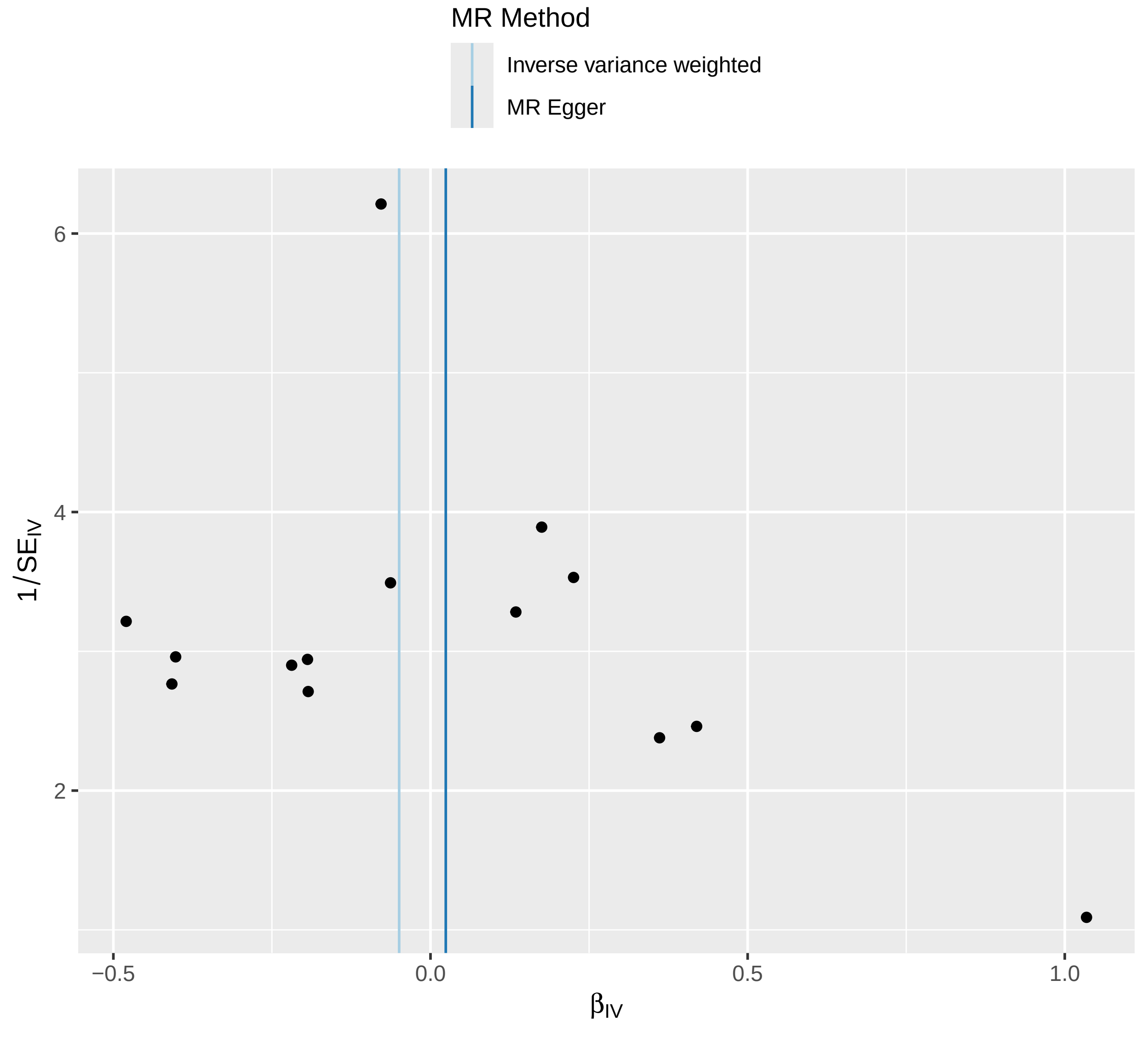
MR Method
Inverse variance weighted
MR Egger
6
4
2
1/SE$_{IV}$
−0.5
0.0
0.5
1.0
β$_{IV}$

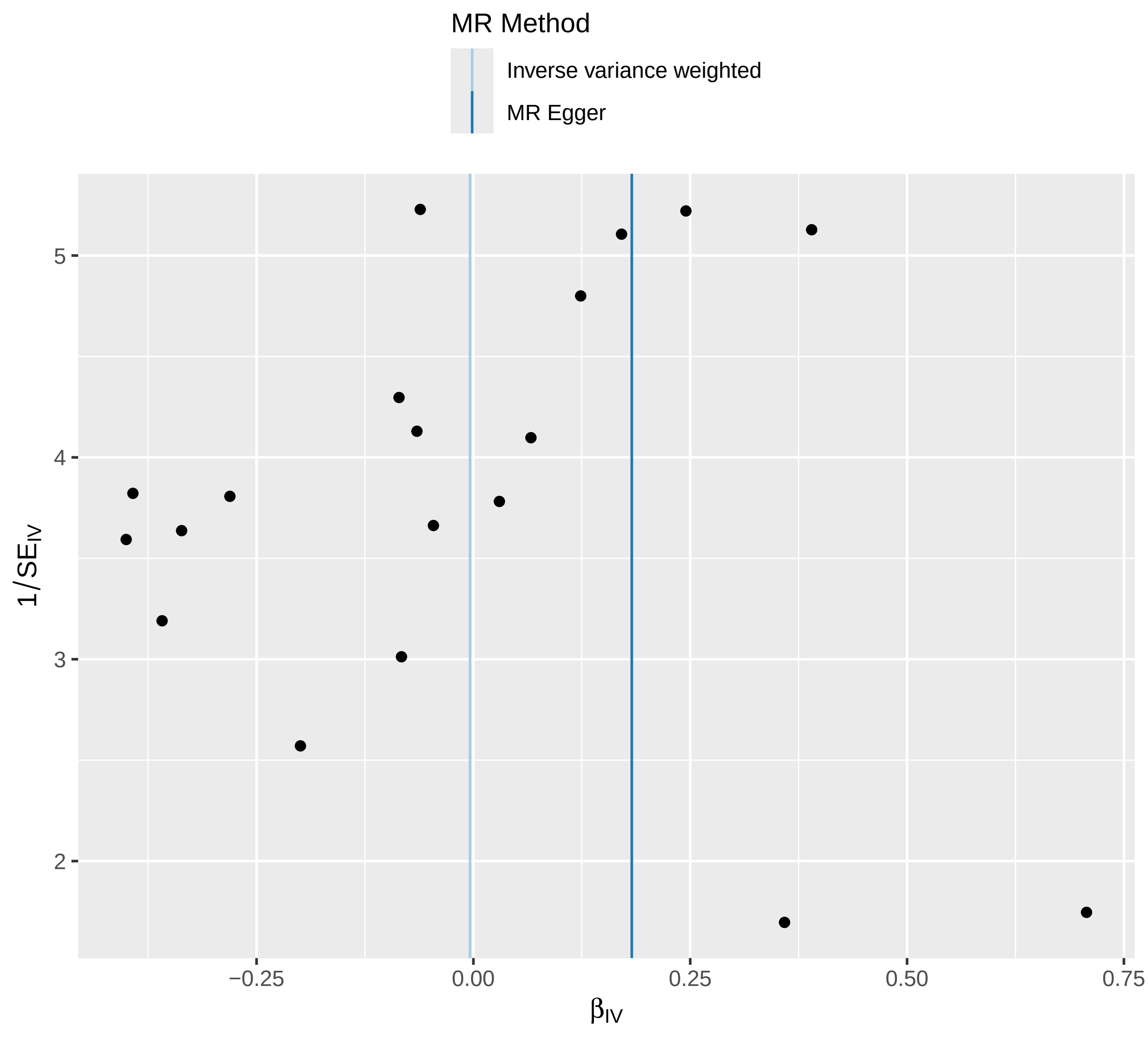

MR Method
Inverse variance weighted
MR Egger
5
4
3
2
1/SE_IV
−0.25
0.00
0.25
0.50
0.75
β_IV

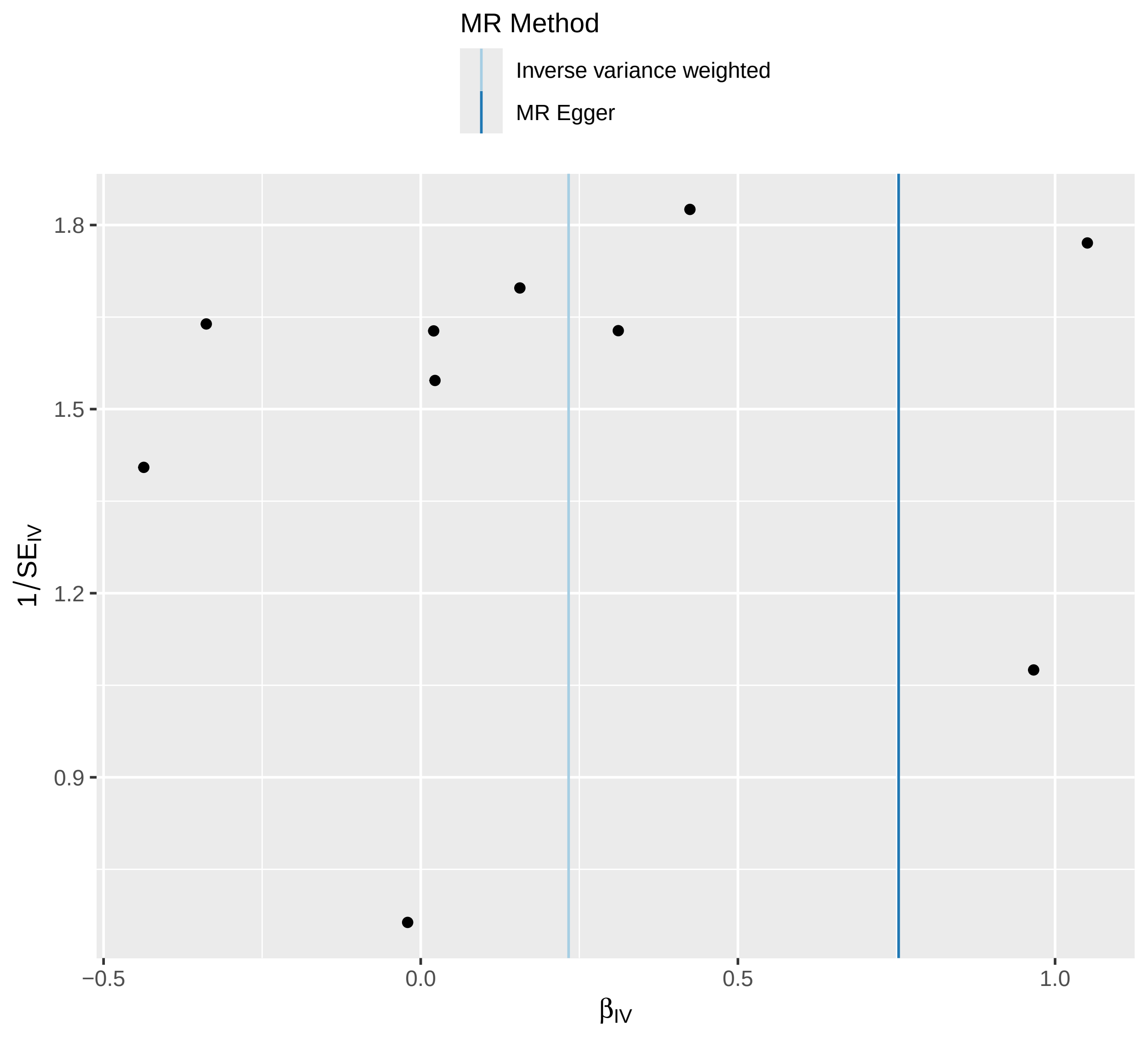
MR Method
Inverse variance weighted
MR Egger
1.8
1.5
1.2
0.9
1/SE_IV
−0.5
0.0
0.5
1.0
β_IV

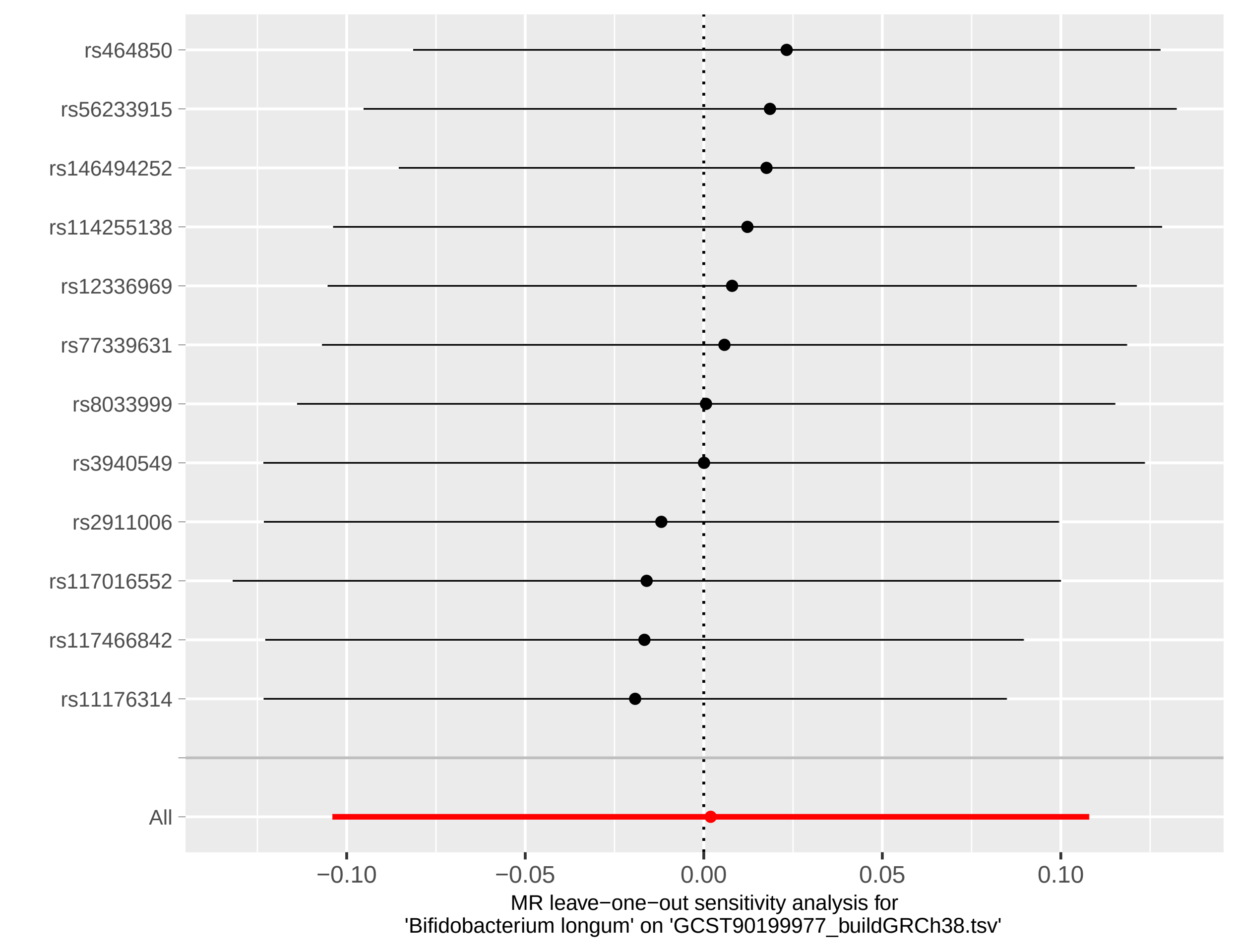

rs464850
rs56233915
rs146494252
rs114255138
rs12336969
rs77339631
rs8033999
rs3940549
rs2911006
rs117016552
rs117466842
rs11176314
All
−0.10
−0.05
0.00
0.05
0.10
MR leave−one−out sensitivity analysis for
'Bifidobacterium longum' on 'GCST90199977_buildGRCh38.tsv'

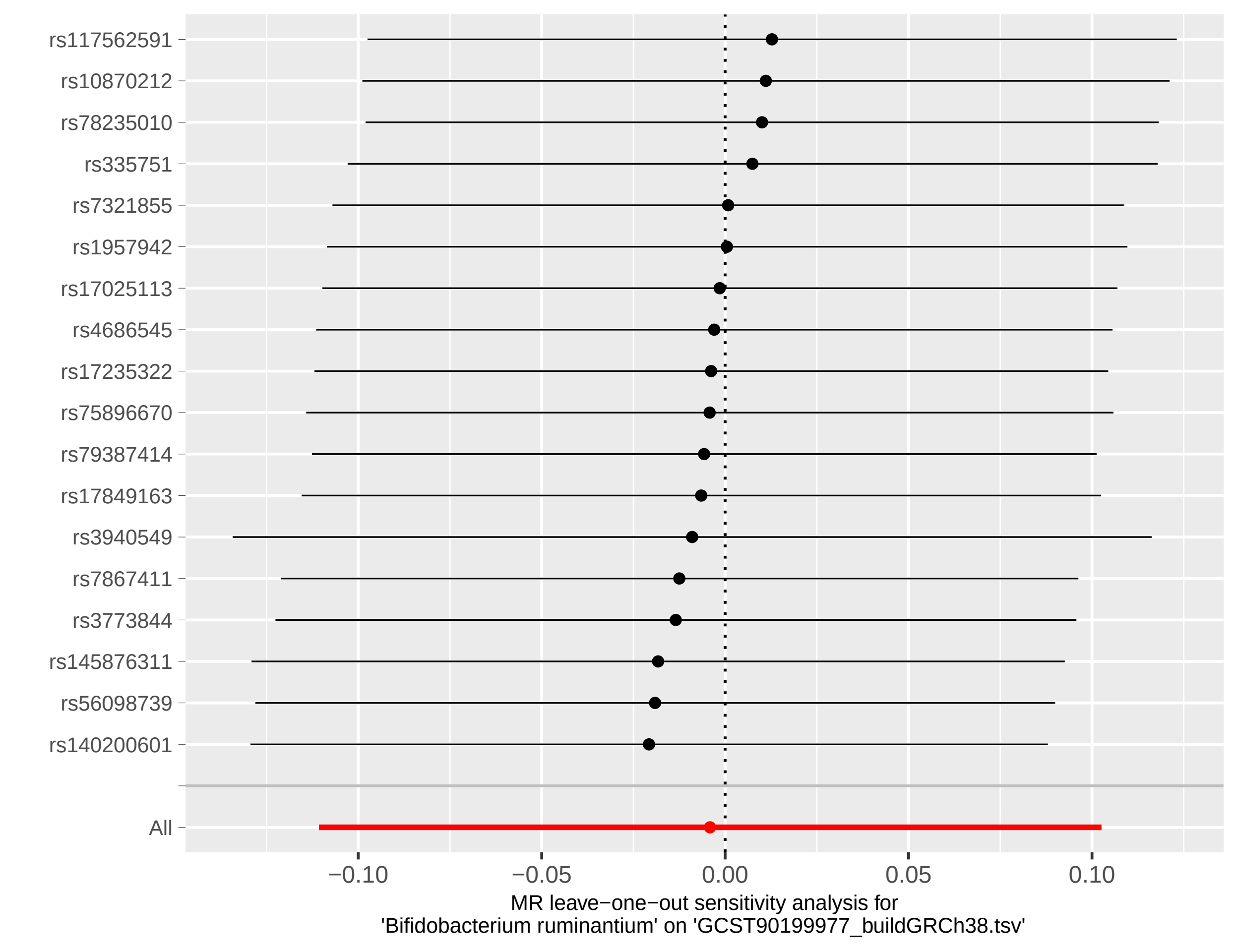

rs117562591
rs10870212
rs78235010
rs335751
rs7321855
rs1957942
rs17025113
rs4686545
rs17235322
rs75896670
rs79387414
rs17849163
rs3940549
rs7867411
rs3773844
rs145876311
rs56098739
rs140200601
All
−0.10
−0.05
0.00
0.05
0.10
MR leave−one−out sensitivity analysis for
'Bifidobacterium ruminantium' on 'GCST90199977_buildGRCh38.tsv'

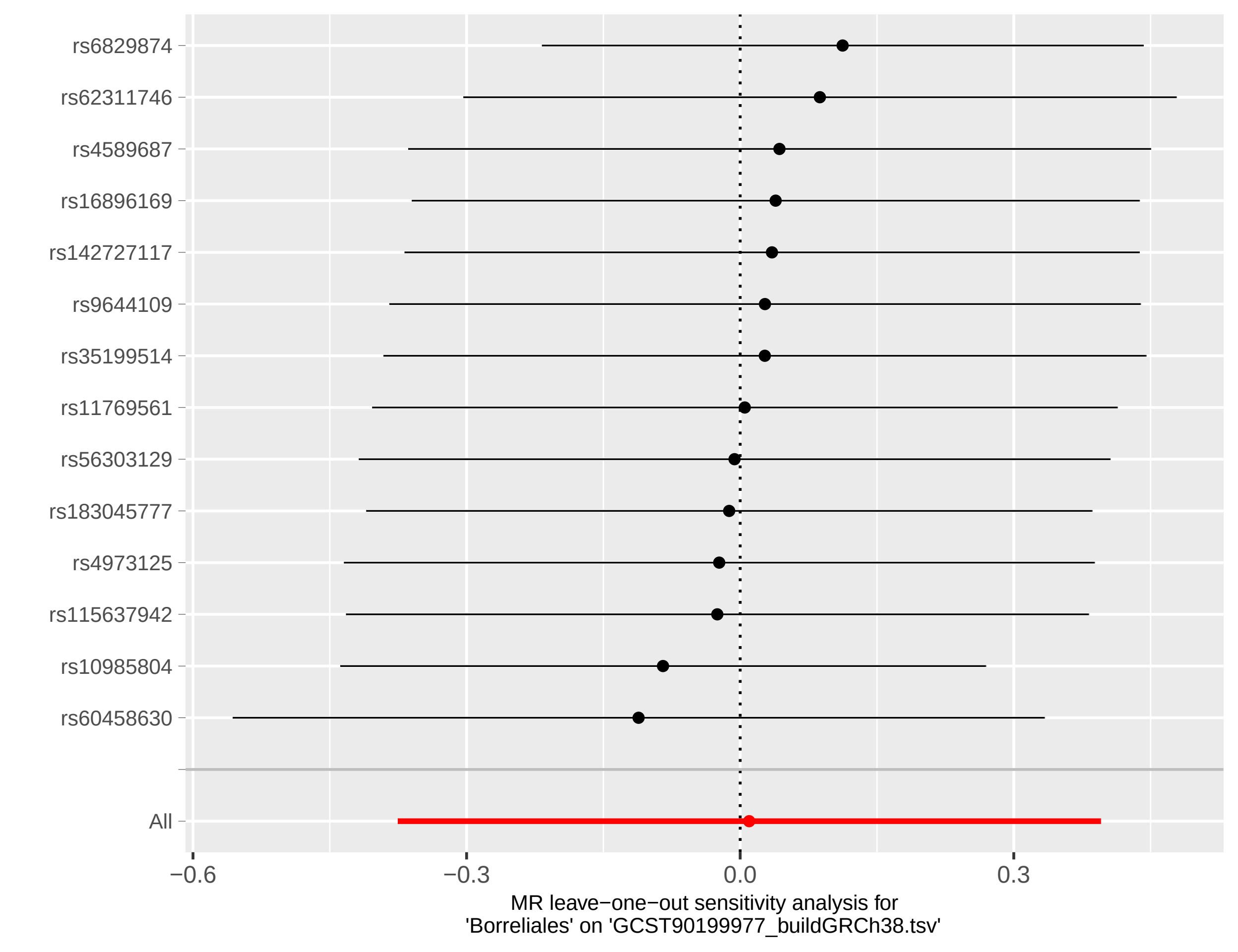

rs6829874
rs62311746
rs4589687
rs16896169
rs142727117
rs9644109
rs35199514
rs11769561
rs56303129
rs183045777
rs4973125
rs115637942
rs10985804
rs60458630
All
−0.6
−0.3
0.0
0.3
MR leave−one−out sensitivity analysis for
'Borreliales' on 'GCST90199977_buildGRCh38.tsv'

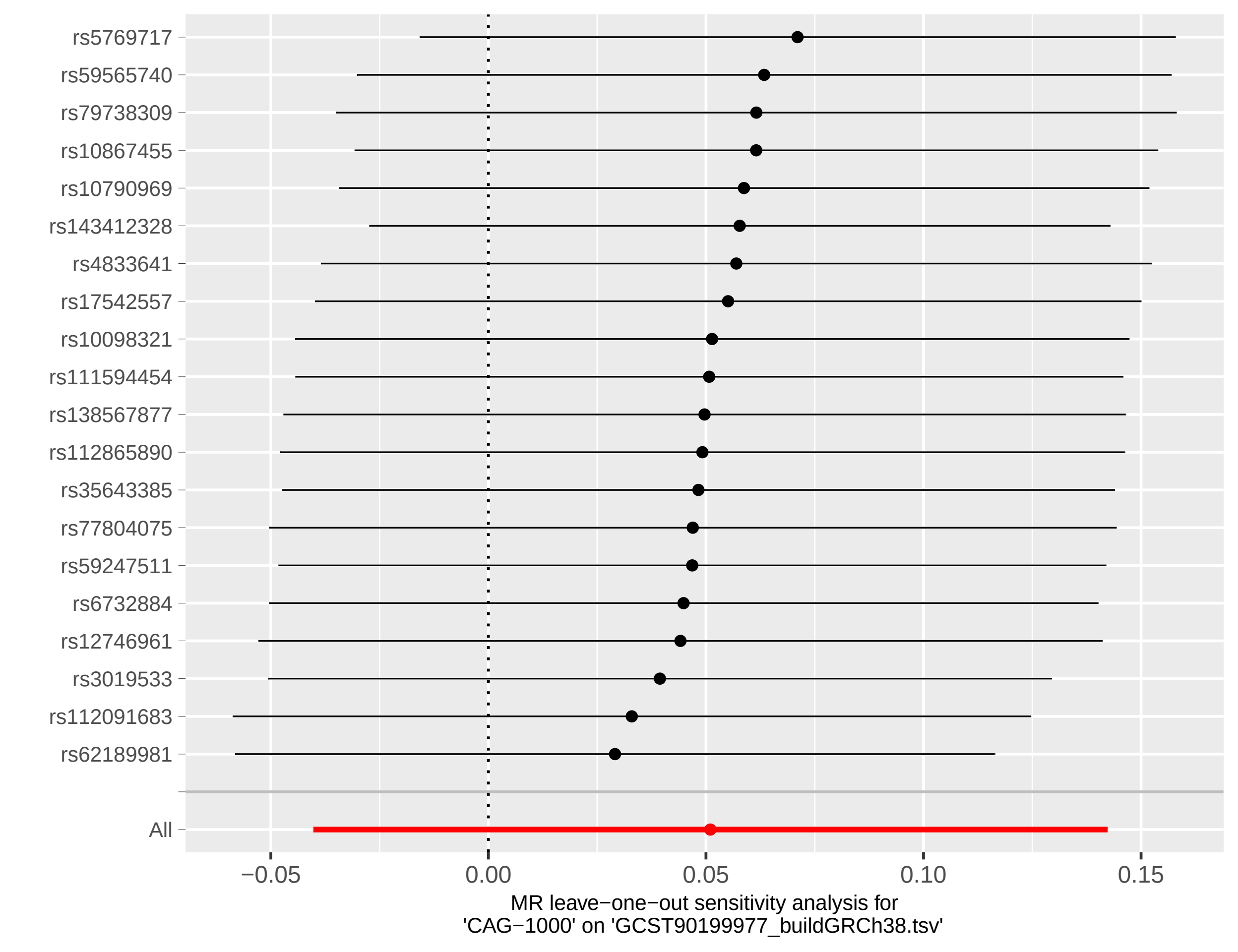
rs5769717
rs59565740
rs79738309
rs10867455
rs10790969
rs143412328
rs4833641
rs17542557
rs10098321
rs111594454
rs138567877
rs112865890
rs35643385
rs77804075
rs59247511
rs6732884
rs12746961
rs3019533
rs112091683
rs62189981
All
−0.05
0.00
0.05
0.10
0.15
MR leave−one−out sensitivity analysis for
'CAG−1000' on 'GCST90199977_buildGRCh38.tsv'

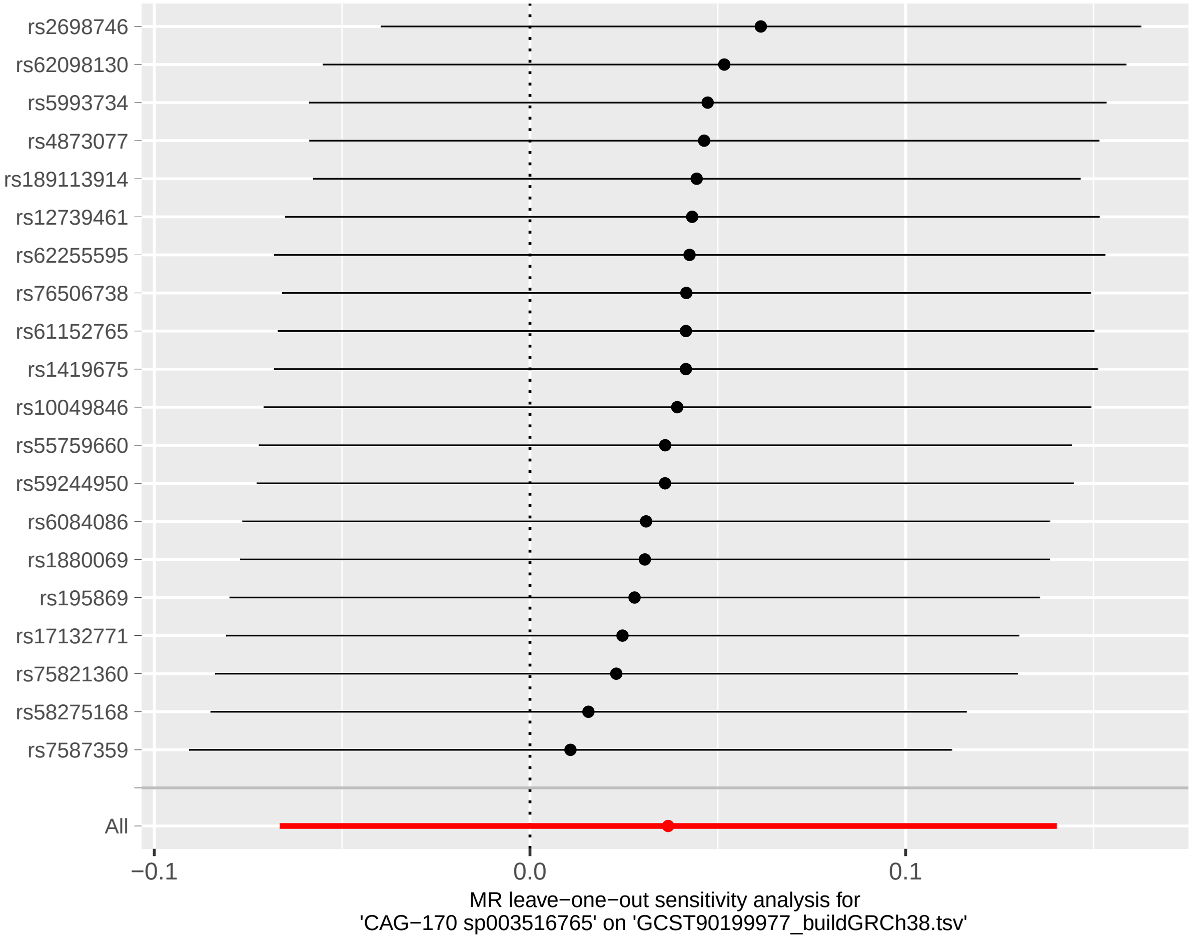
rs2698746
rs62098130
rs5993734
rs4873077
rs189113914
rs12739461
rs62255595
rs76506738
rs61152765
rs1419675
rs10049846
rs55759660
rs59244950
rs6084086
rs1880069
rs195869
rs17132771
rs75821360
rs58275168
rs7587359
All
−0.1
0.0
0.1
MR leave−one−out sensitivity analysis for
'CAG−170 sp003516765' on 'GCST90199977_buildGRCh38.tsv'

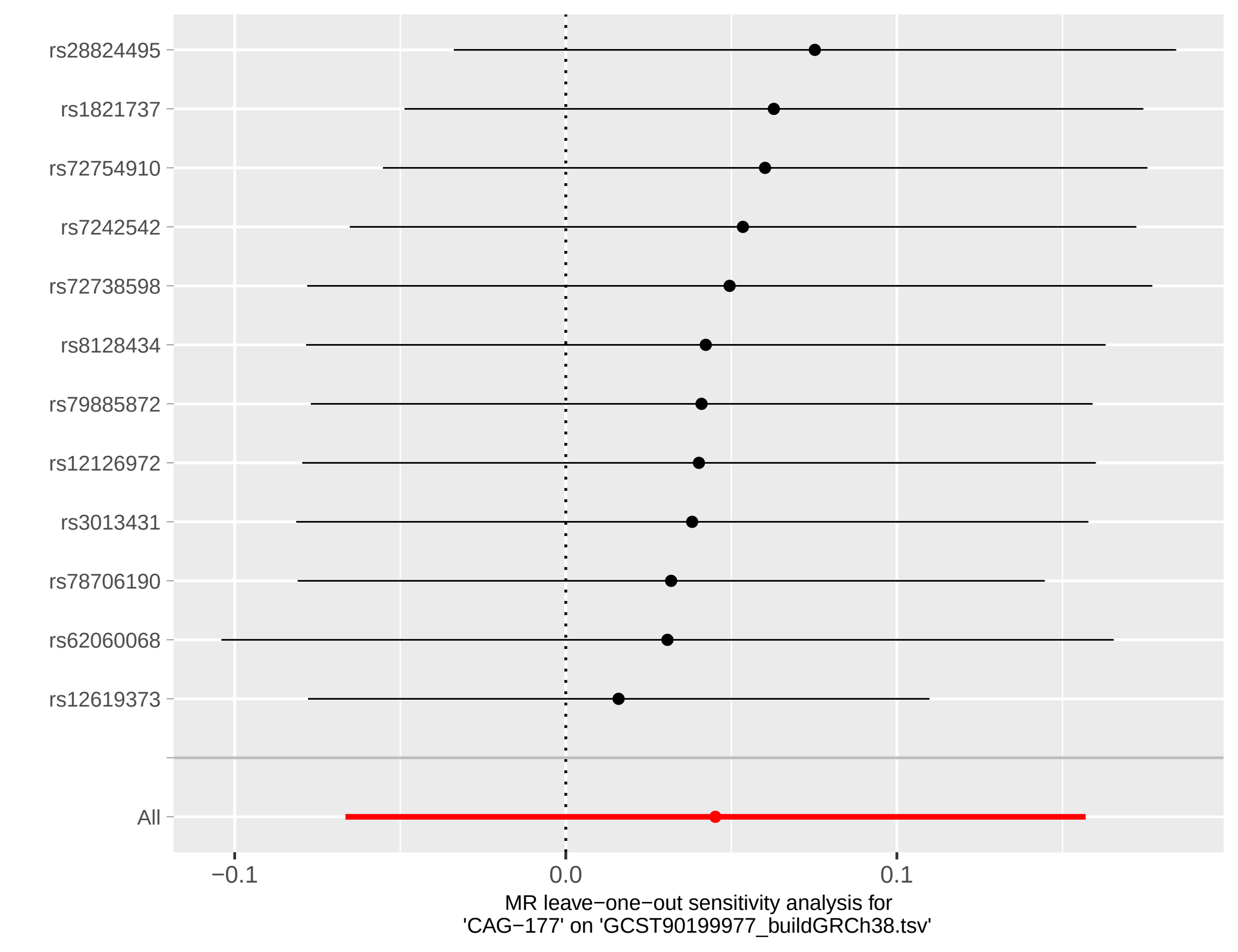

rs28824495
rs1821737
rs72754910
rs7242542
rs72738598
rs8128434
rs79885872
rs12126972
rs3013431
rs78706190
rs62060068
rs12619373
All
−0.1
0.0
0.1
MR leave−one−out sensitivity analysis for
'CAG−177' on 'GCST90199977_buildGRCh38.tsv'

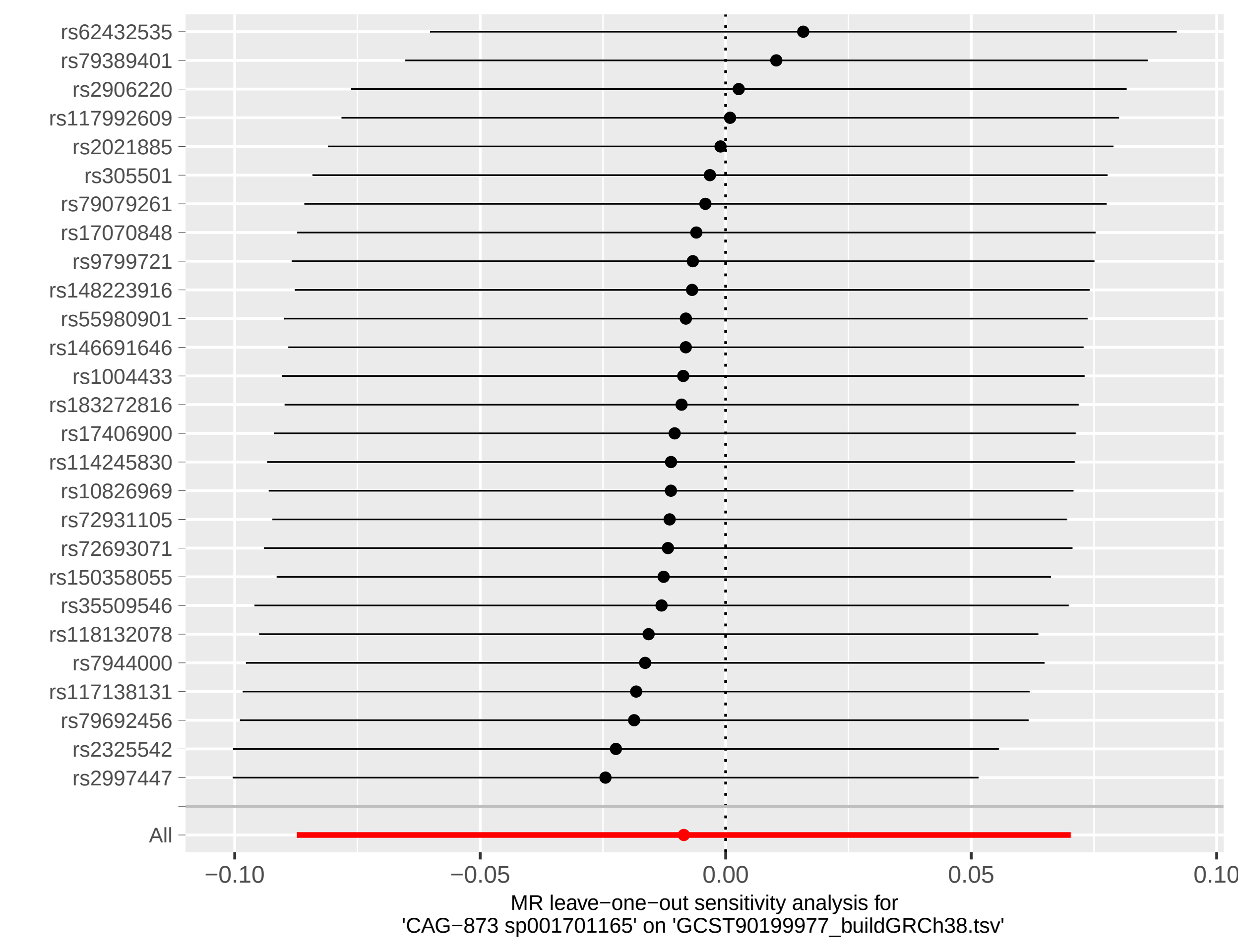

rs62432535
rs79389401
rs2906220
rs117992609
rs2021885
rs305501
rs79079261
rs17070848
rs9799721
rs148223916
rs55980901
rs146691646
rs1004433
rs183272816
rs17406900
rs114245830
rs10826969
rs72931105
rs72693071
rs150358055
rs35509546
rs118132078
rs7944000
rs117138131
rs79692456
rs2325542
rs2997447
All
−0.10
−0.05
0.00
0.05
0.10
MR leave−one−out sensitivity analysis for
'CAG−873 sp001701165' on 'GCST90199977_buildGRCh38.tsv'

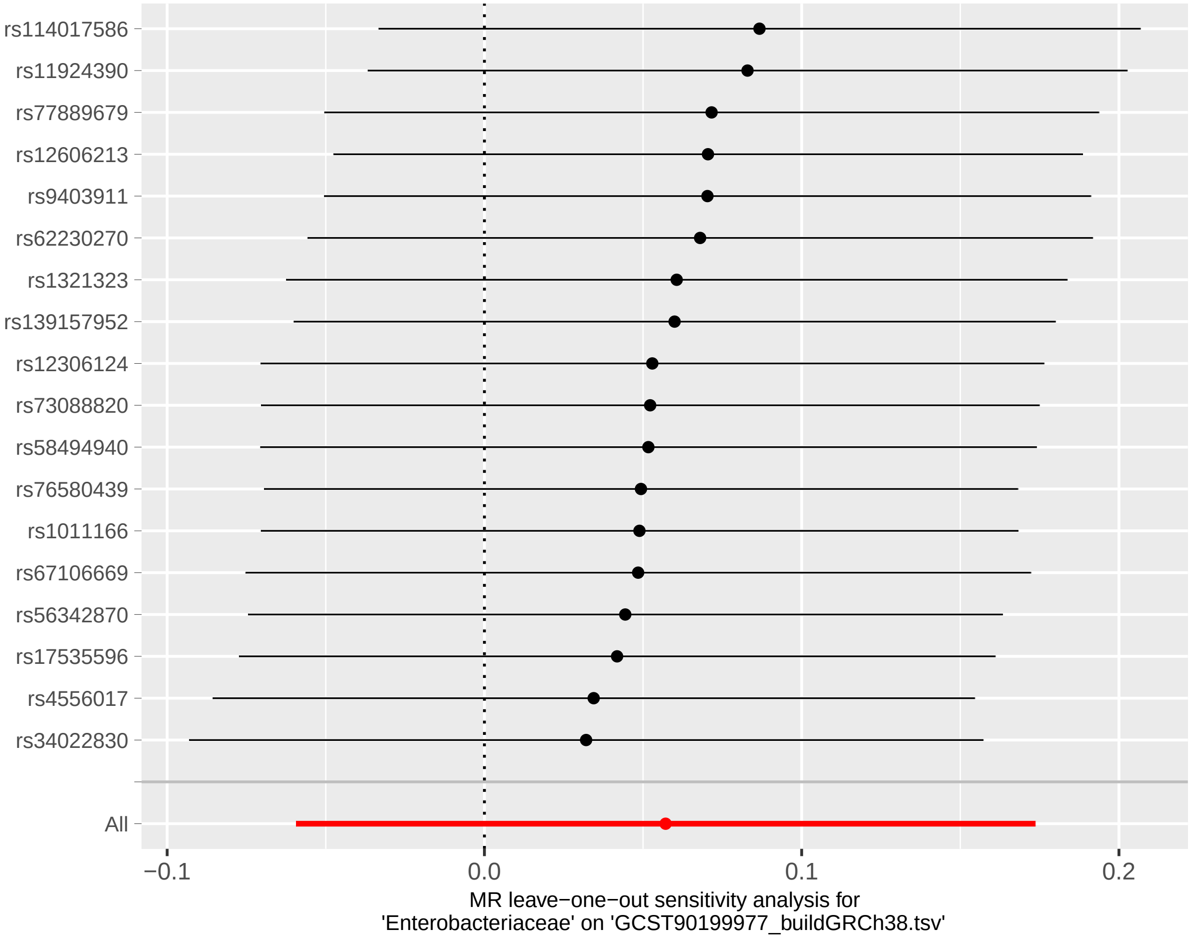
rs114017586
rs11924390
rs77889679
rs12606213
rs9403911
rs62230270
rs1321323
rs139157952
rs12306124
rs73088820
rs58494940
rs76580439
rs1011166
rs67106669
rs56342870
rs17535596
rs4556017
rs34022830
All
−0.1
0.0
0.1
0.2
MR leave−one−out sensitivity analysis for
'Enterobacteriaceae' on 'GCST90199977_buildGRCh38.tsv'

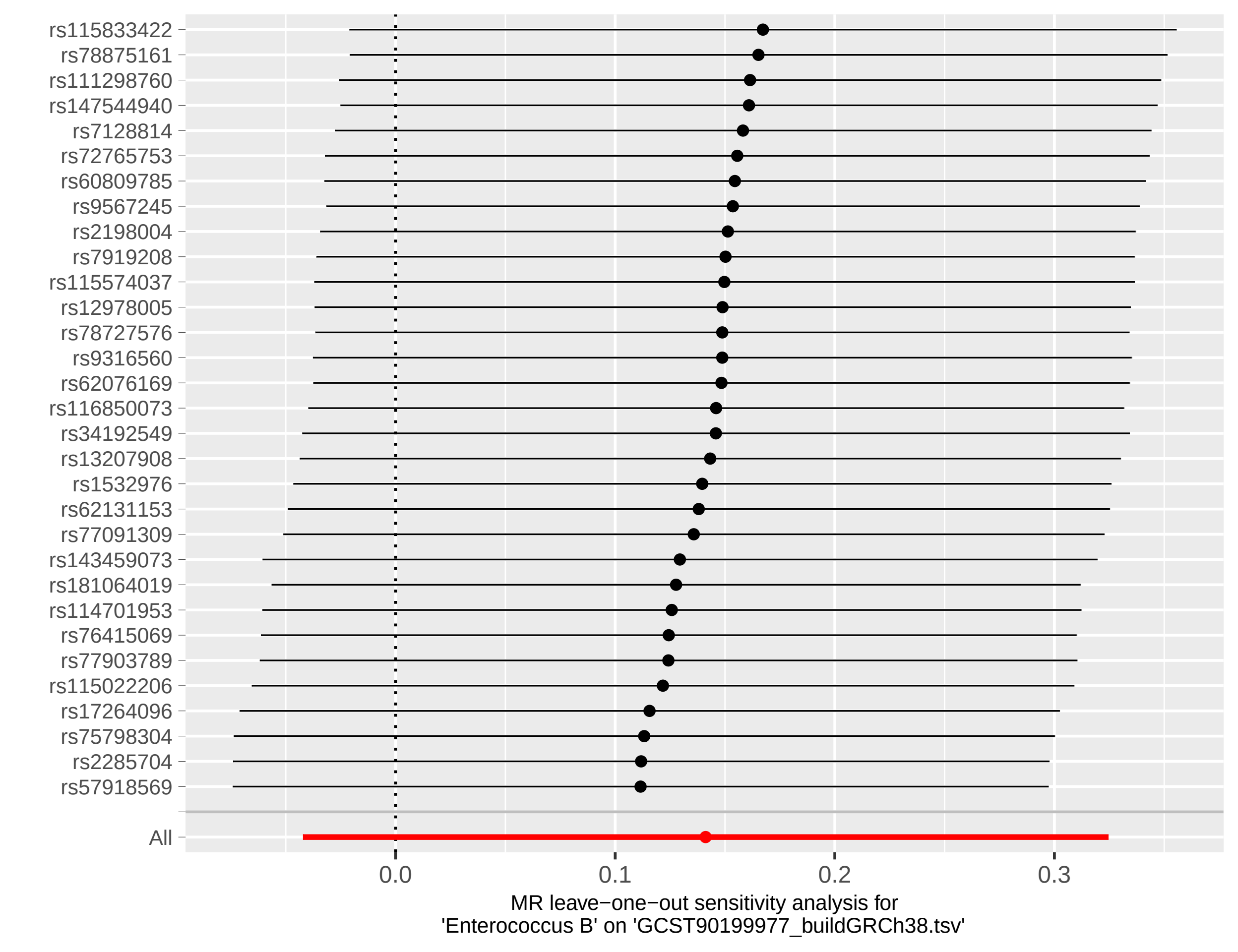

rs115833422
rs78875161
rs111298760
rs147544940
rs7128814
rs72765753
rs60809785
rs9567245
rs2198004
rs7919208
rs115574037
rs12978005
rs78727576
rs9316560
rs62076169
rs116850073
rs34192549
rs13207908
rs1532976
rs62131153
rs77091309
rs143459073
rs181064019
rs114701953
rs76415069
rs77903789
rs115022206
rs17264096
rs75798304
rs2285704
rs57918569
All
0.0
0.1
0.2
0.3
MR leave−one−out sensitivity analysis for
'Enterococcus B' on 'GCST90199977_buildGRCh38.tsv'

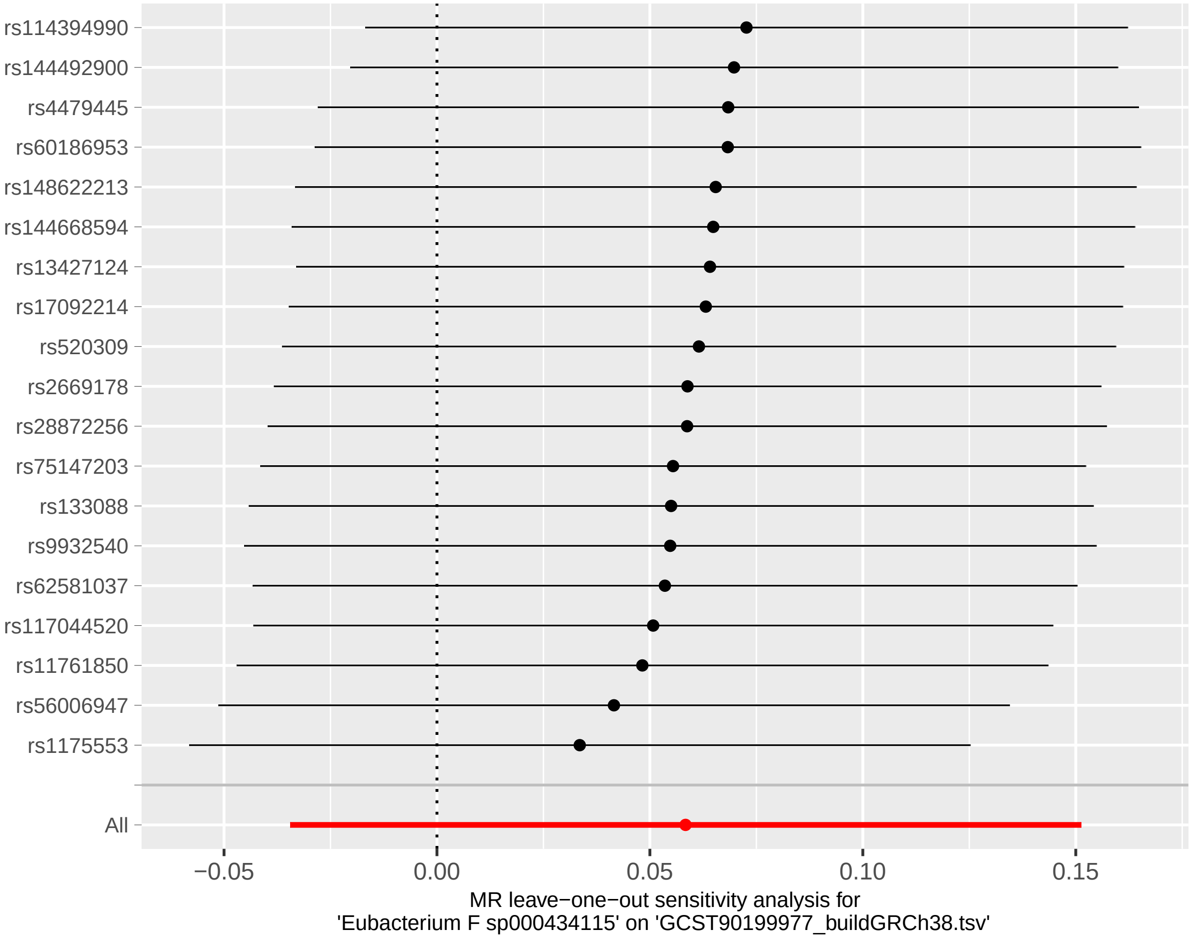
rs114394990
rs144492900
rs4479445
rs60186953
rs148622213
rs144668594
rs13427124
rs17092214
rs520309
rs2669178
rs28872256
rs75147203
rs133088
rs9932540
rs62581037
rs117044520
rs11761850
rs56006947
rs1175553
All
−0.05
0.00
0.05
0.10
0.15
MR leave−one−out sensitivity analysis for
'Eubacterium F sp000434115' on 'GCST90199977_buildGRCh38.tsv'

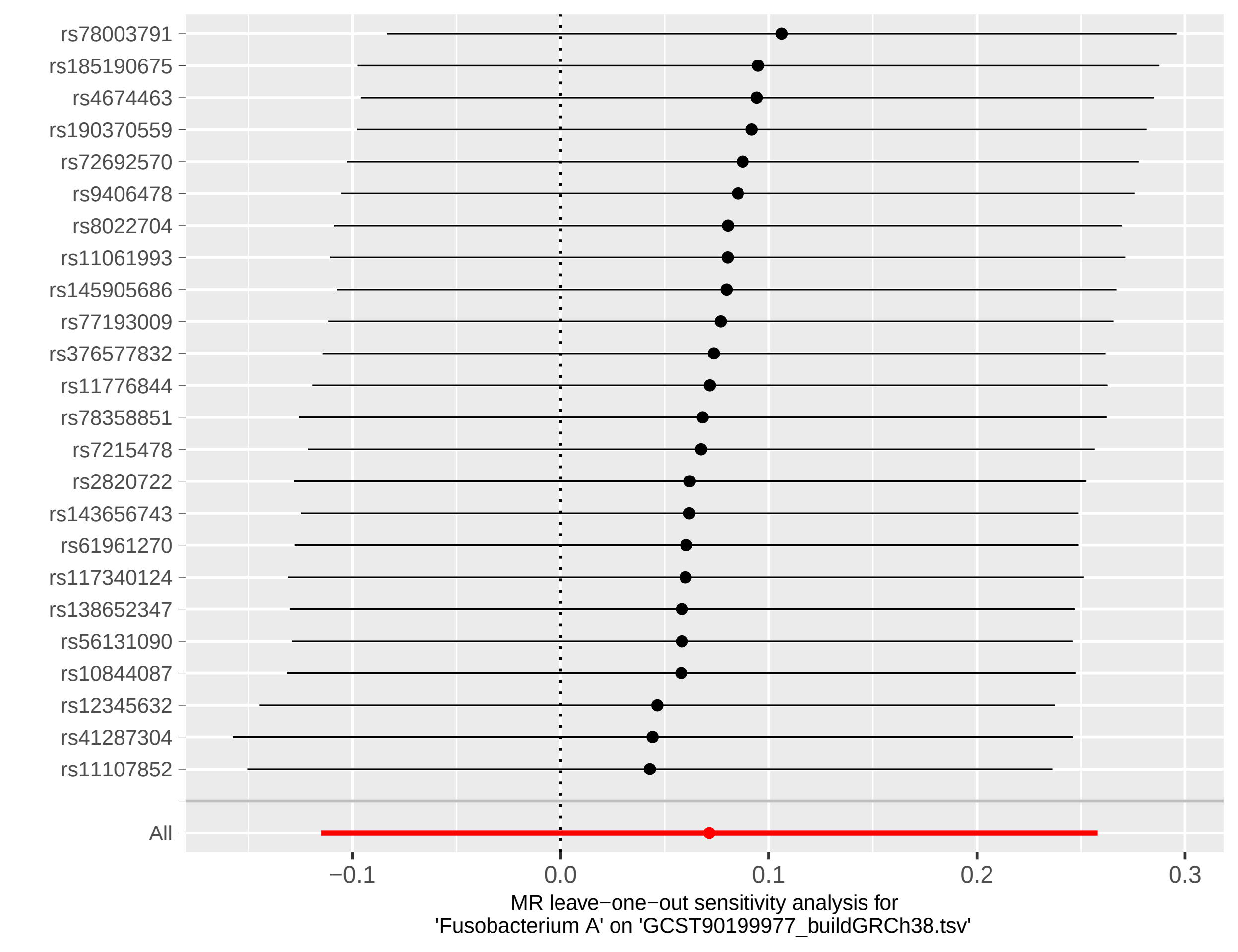
rs78003791
rs185190675
rs4674463
rs190370559
rs72692570
rs9406478
rs8022704
rs11061993
rs145905686
rs77193009
rs376577832
rs11776844
rs78358851
rs7215478
rs2820722
rs143656743
rs61961270
rs117340124
rs138652347
rs56131090
rs10844087
rs12345632
rs41287304
rs11107852
All
−0.1
0.0
0.1
0.2
0.3
MR leave−one−out sensitivity analysis for
'Fusobacterium A' on 'GCST90199977_buildGRCh38.tsv'

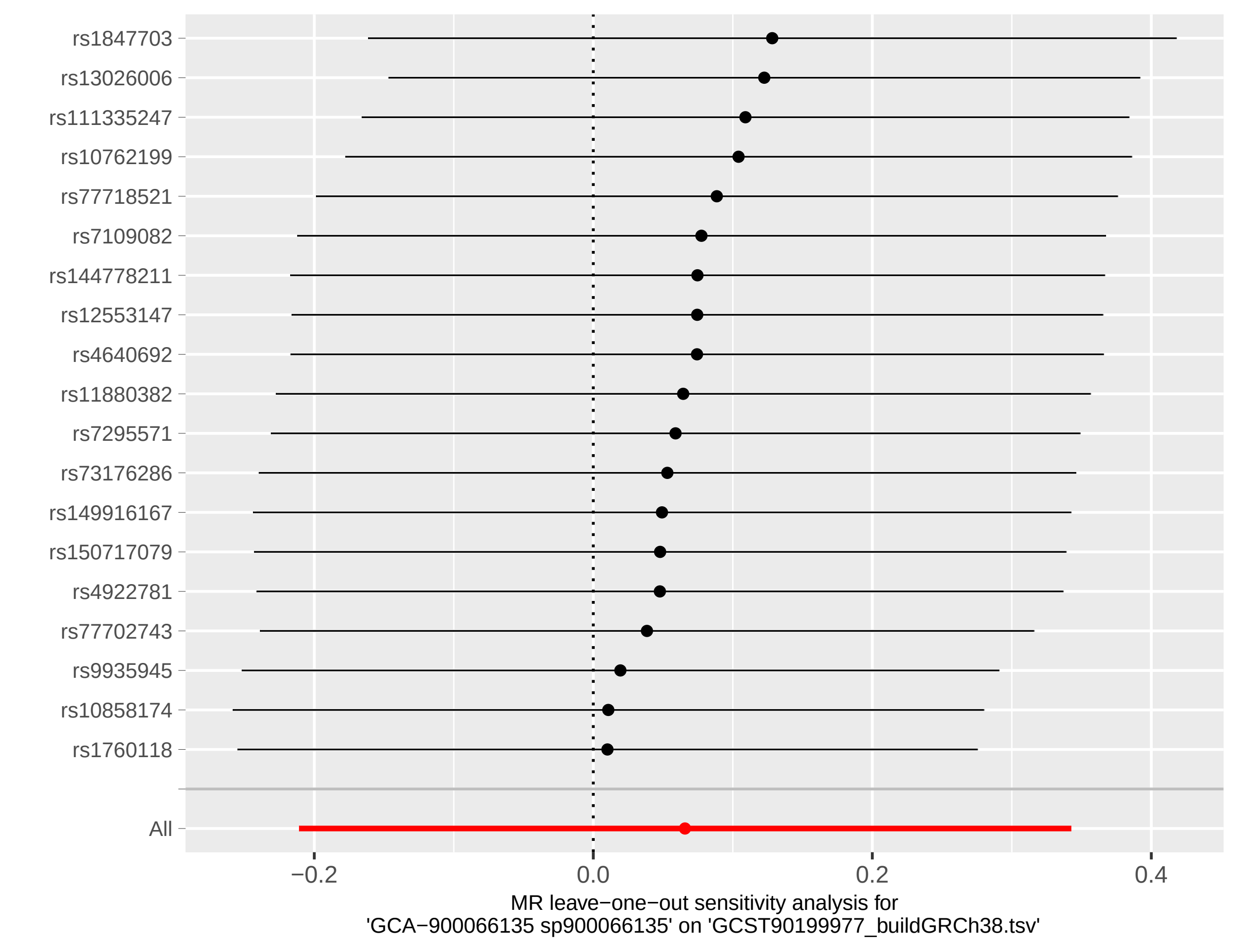

rs1847703
rs13026006
rs111335247
rs10762199
rs77718521
rs7109082
rs144778211
rs12553147
rs4640692
rs11880382
rs7295571
rs73176286
rs149916167
rs150717079
rs4922781
rs77702743
rs9935945
rs10858174
rs1760118
All
−0.2
0.0
0.2
0.4
MR leave−one−out sensitivity analysis for
'GCA−900066135 sp900066135' on 'GCST90199977_buildGRCh38.tsv'

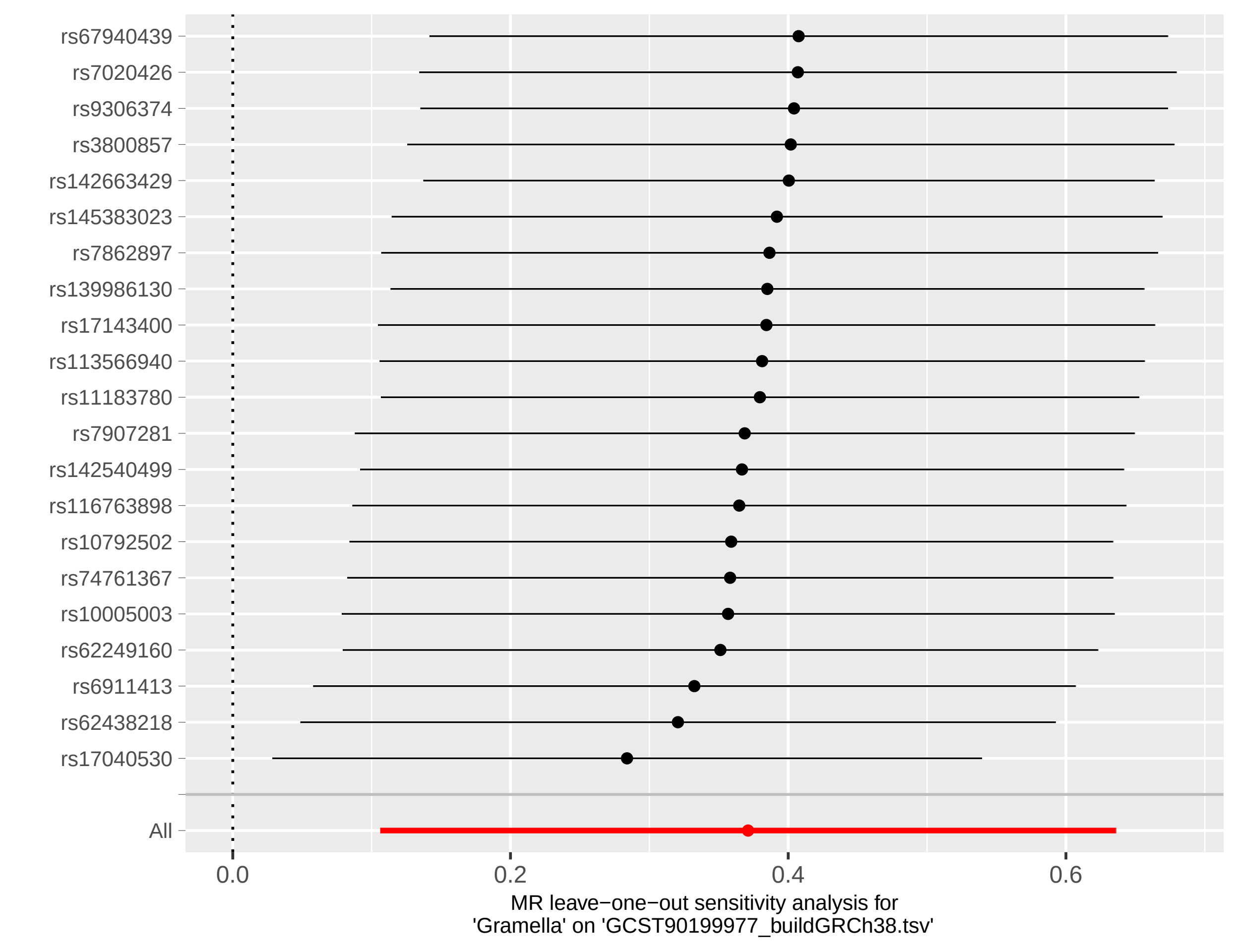

rs67940439
rs7020426
rs9306374
rs3800857
rs142663429
rs145383023
rs7862897
rs139986130
rs17143400
rs113566940
rs11183780
rs7907281
rs142540499
rs116763898
rs10792502
rs74761367
rs10005003
rs62249160
rs6911413
rs62438218
rs17040530
All
0.0
0.2
0.4
0.6
MR leave−one−out sensitivity analysis for
'Gramella' on 'GCST90199977_buildGRCh38.tsv'

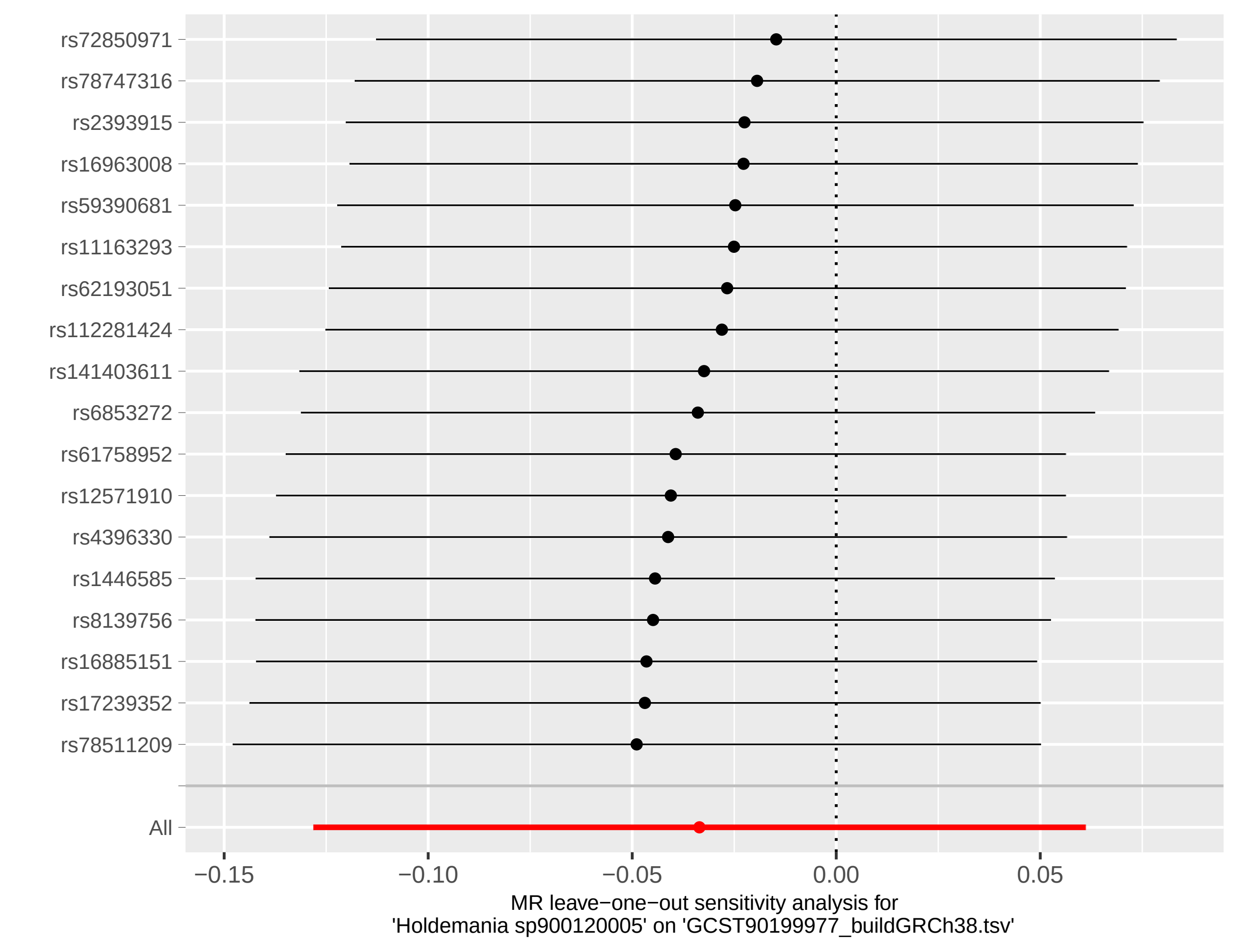

rs72850971
rs78747316
rs2393915
rs16963008
rs59390681
rs11163293
rs62193051
rs112281424
rs141403611
rs6853272
rs61758952
rs12571910
rs4396330
rs1446585
rs8139756
rs16885151
rs17239352
rs78511209
All
−0.15
−0.10
−0.05
0.00
0.05
MR leave−one−out sensitivity analysis for
'Holdemania sp900120005' on 'GCST90199977_buildGRCh38.tsv'

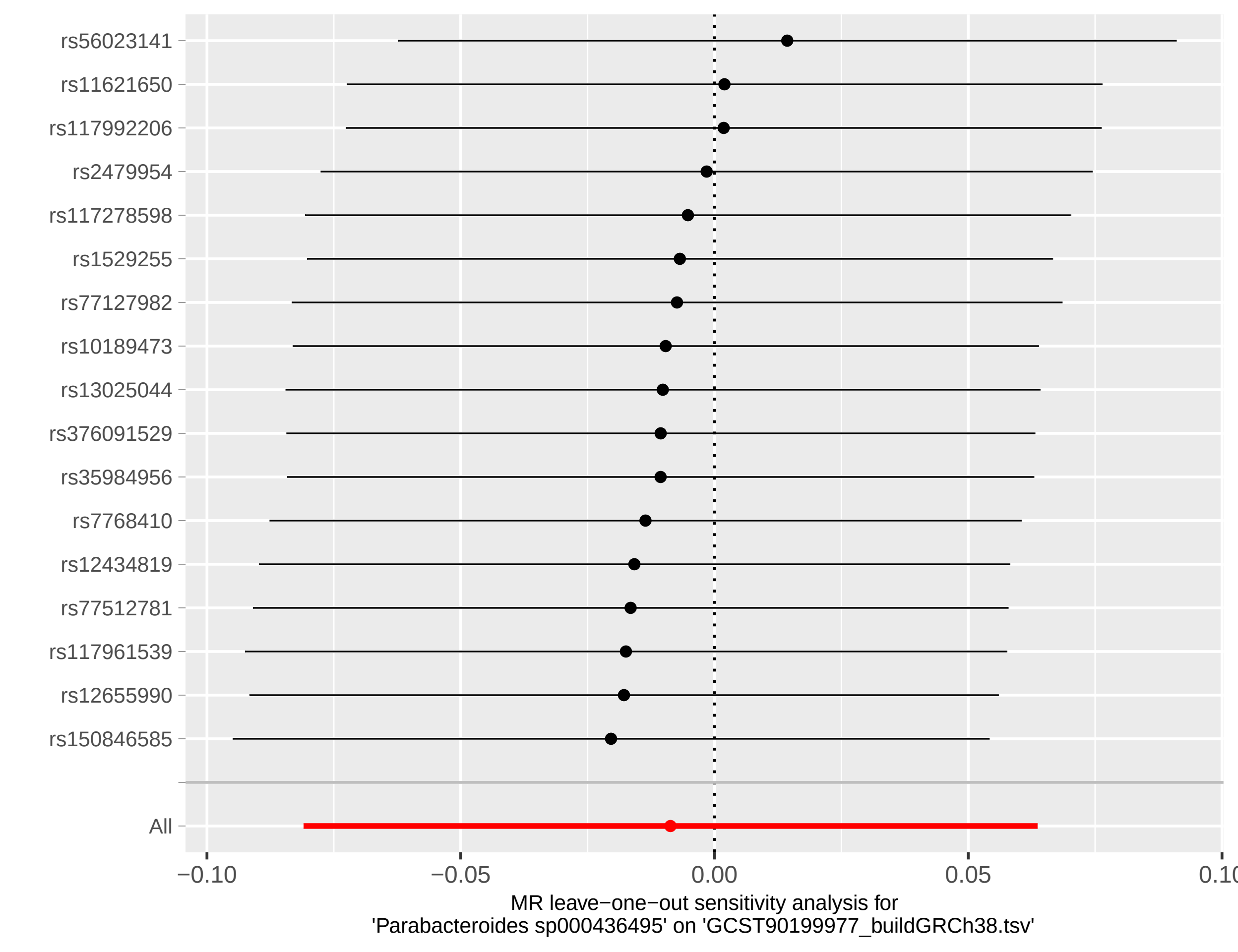
rs56023141
rs11621650
rs117992206
rs2479954
rs117278598
rs1529255
rs77127982
rs10189473
rs13025044
rs376091529
rs35984956
rs7768410
rs12434819
rs77512781
rs117961539
rs12655990
rs150846585
All
−0.10
−0.05
0.00
0.05
0.10
MR leave−one−out sensitivity analysis for
'Parabacteroides sp000436495' on 'GCST90199977_buildGRCh38.tsv'

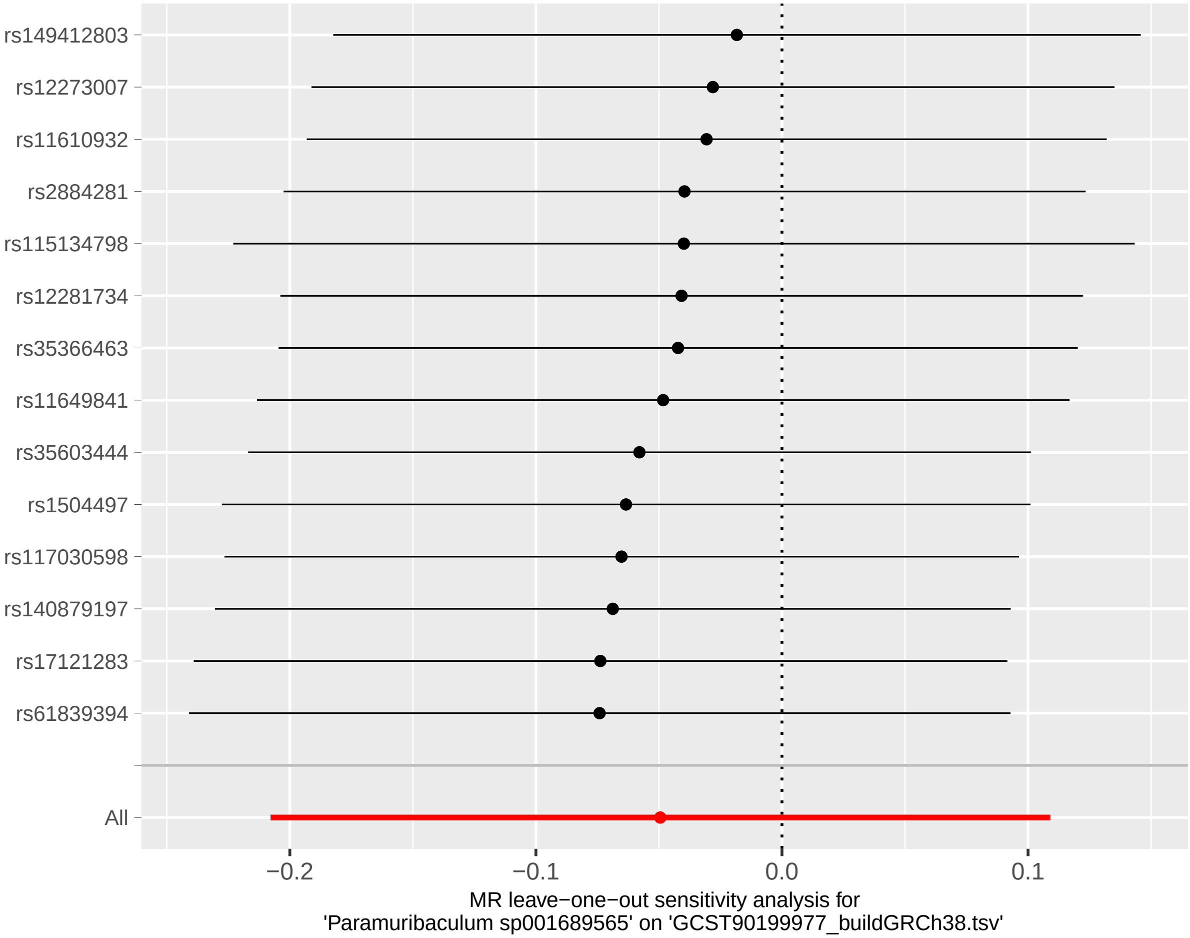

rs149412803
rs12273007
rs11610932
rs2884281
rs115134798
rs12281734
rs35366463
rs11649841
rs35603444
rs1504497
rs117030598
rs140879197
rs17121283
rs61839394
All
−0.2
−0.1
0.0
0.1
MR leave−one−out sensitivity analysis for
'Paramuribaculum sp001689565' on 'GCST90199977_buildGRCh38.tsv'

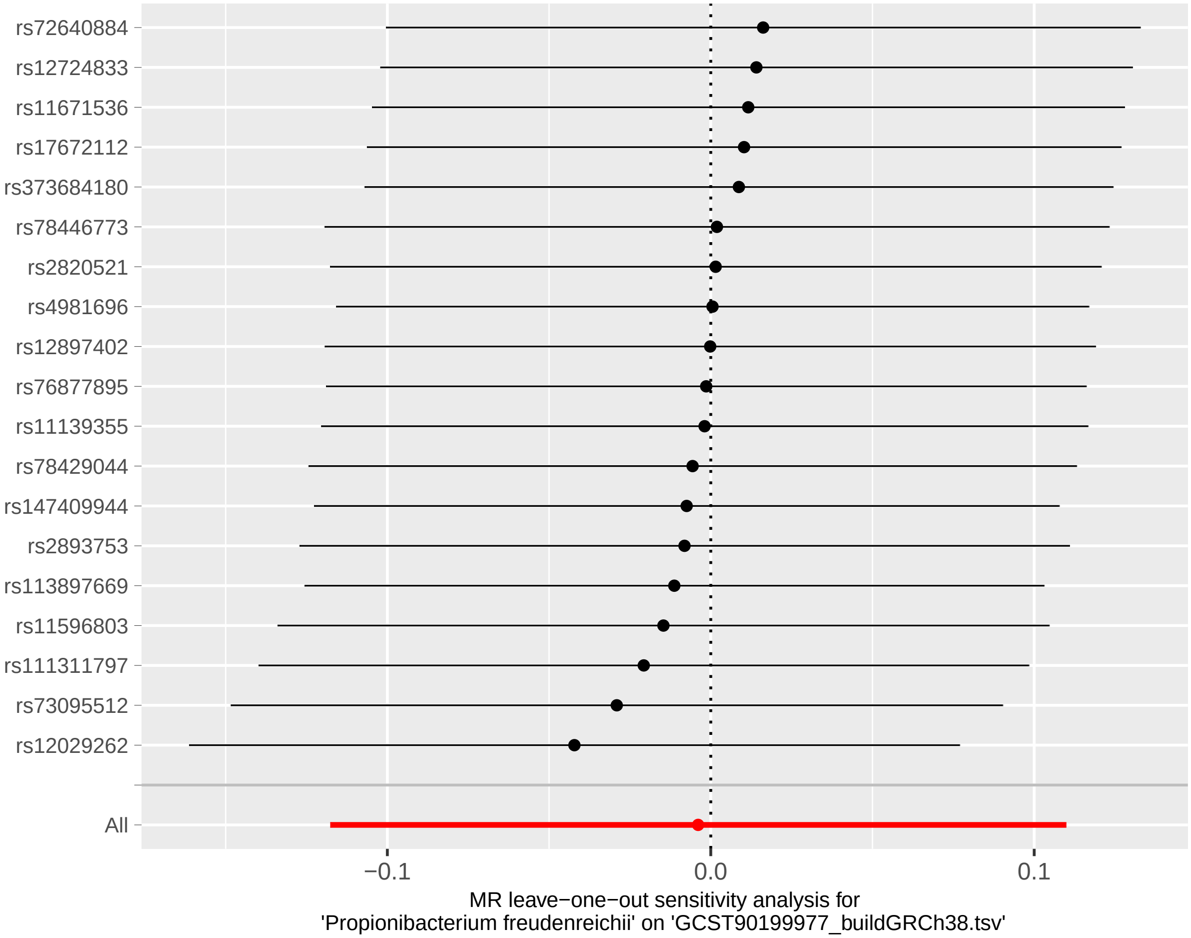
rs72640884
rs12724833
rs11671536
rs17672112
rs373684180
rs78446773
rs2820521
rs4981696
rs12897402
rs76877895
rs11139355
rs78429044
rs147409944
rs2893753
rs113897669
rs11596803
rs111311797
rs73095512
rs12029262
All
−0.1
0.0
0.1
MR leave−one−out sensitivity analysis for
'Propionibacterium freudenreichii' on 'GCST90199977_buildGRCh38.tsv'

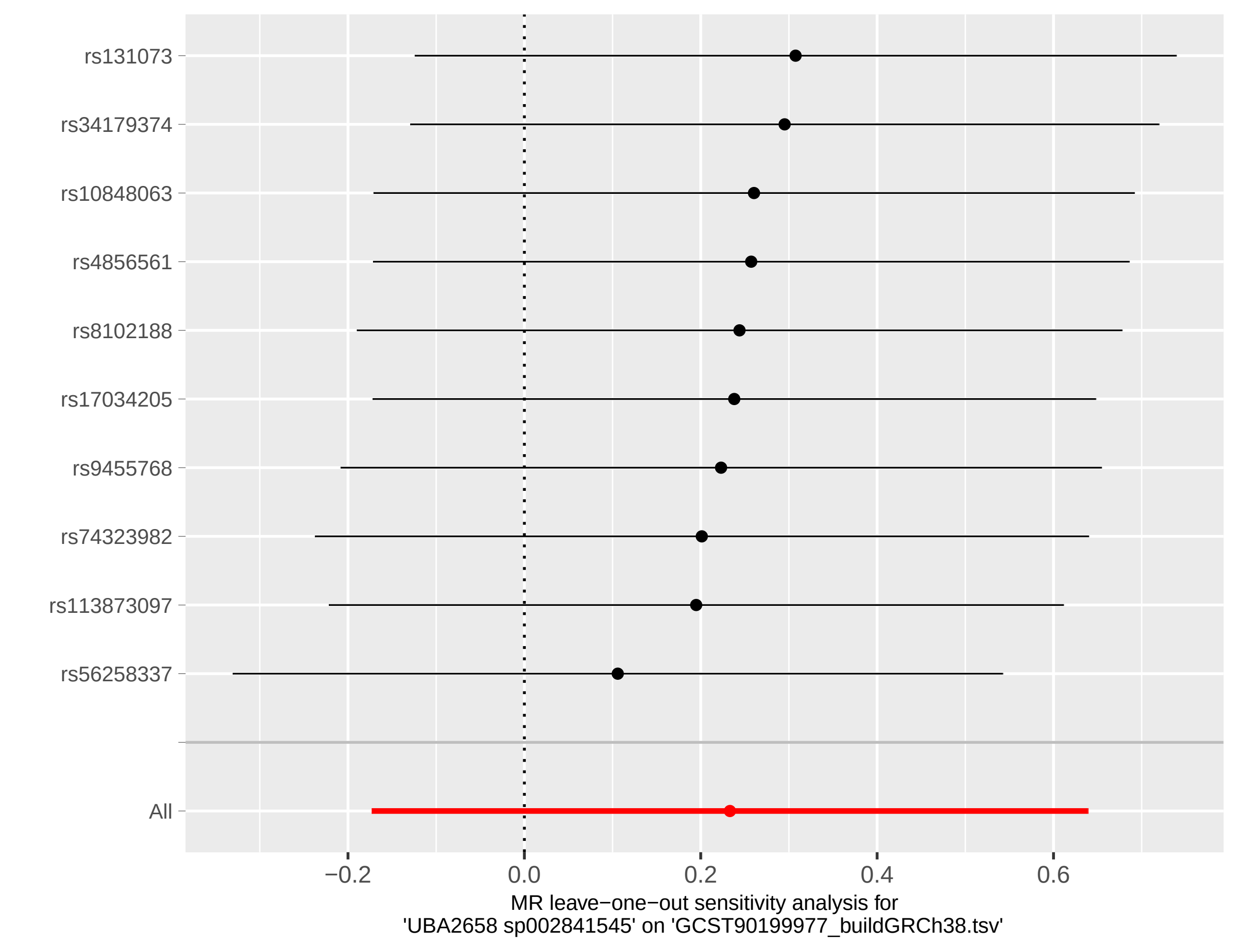

rs131073
rs34179374
rs10848063
rs4856561
rs8102188
rs17034205
rs9455768
rs74323982
rs113873097
rs56258337
All
−0.2
0.0
0.2
0.4
0.6
MR leave−one−out sensitivity analysis for
'UBA2658 sp002841545' on 'GCST90199977_buildGRCh38.tsv'

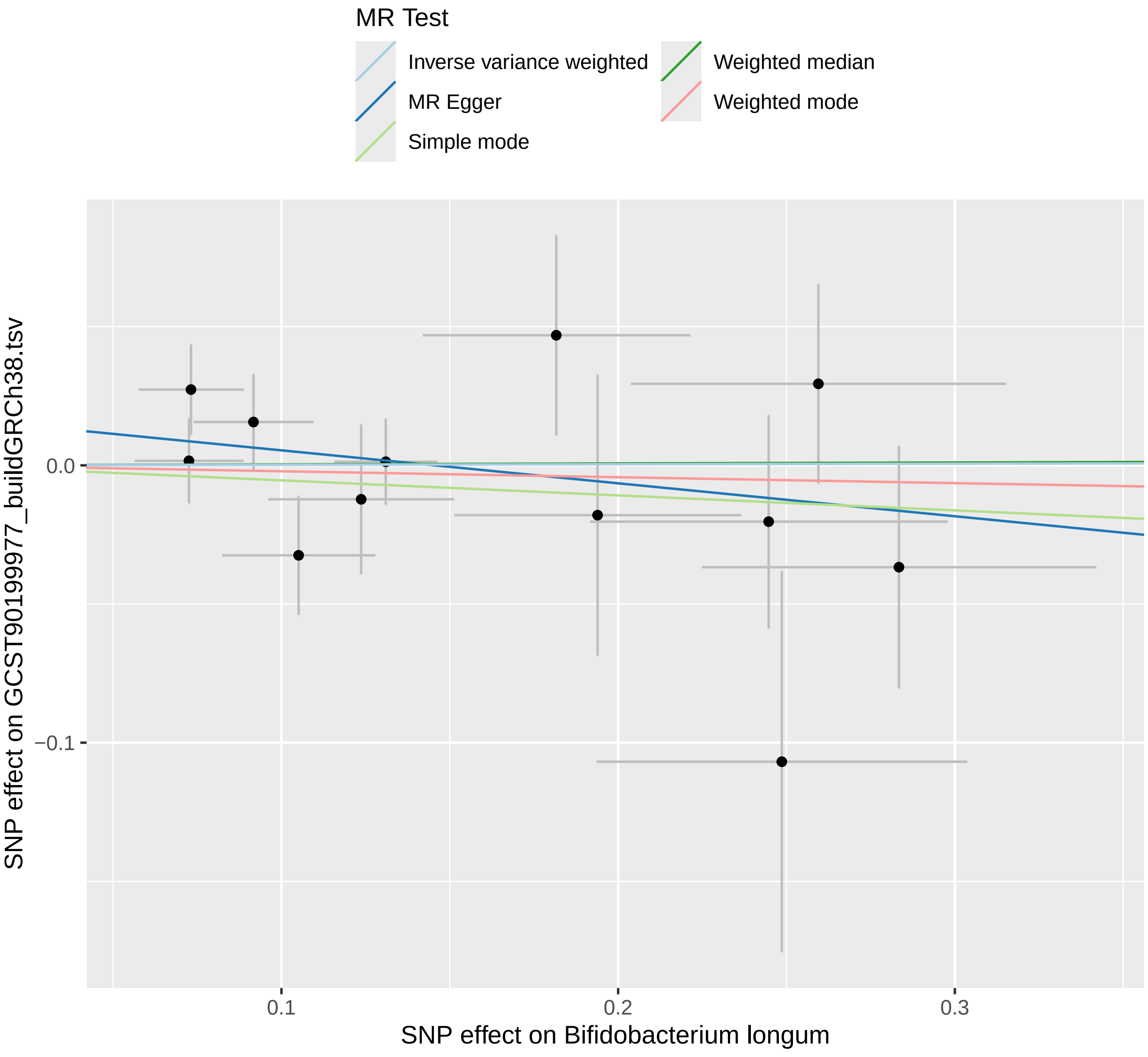
MR Test
Inverse variance weighted
MR Egger
Simple mode
Weighted median
Weighted mode
0.0
−0.1
0.1
0.2
0.3
SNP effect on GCST90199977_buildGRCh38.tsv
SNP effect on Bifidobacterium longum

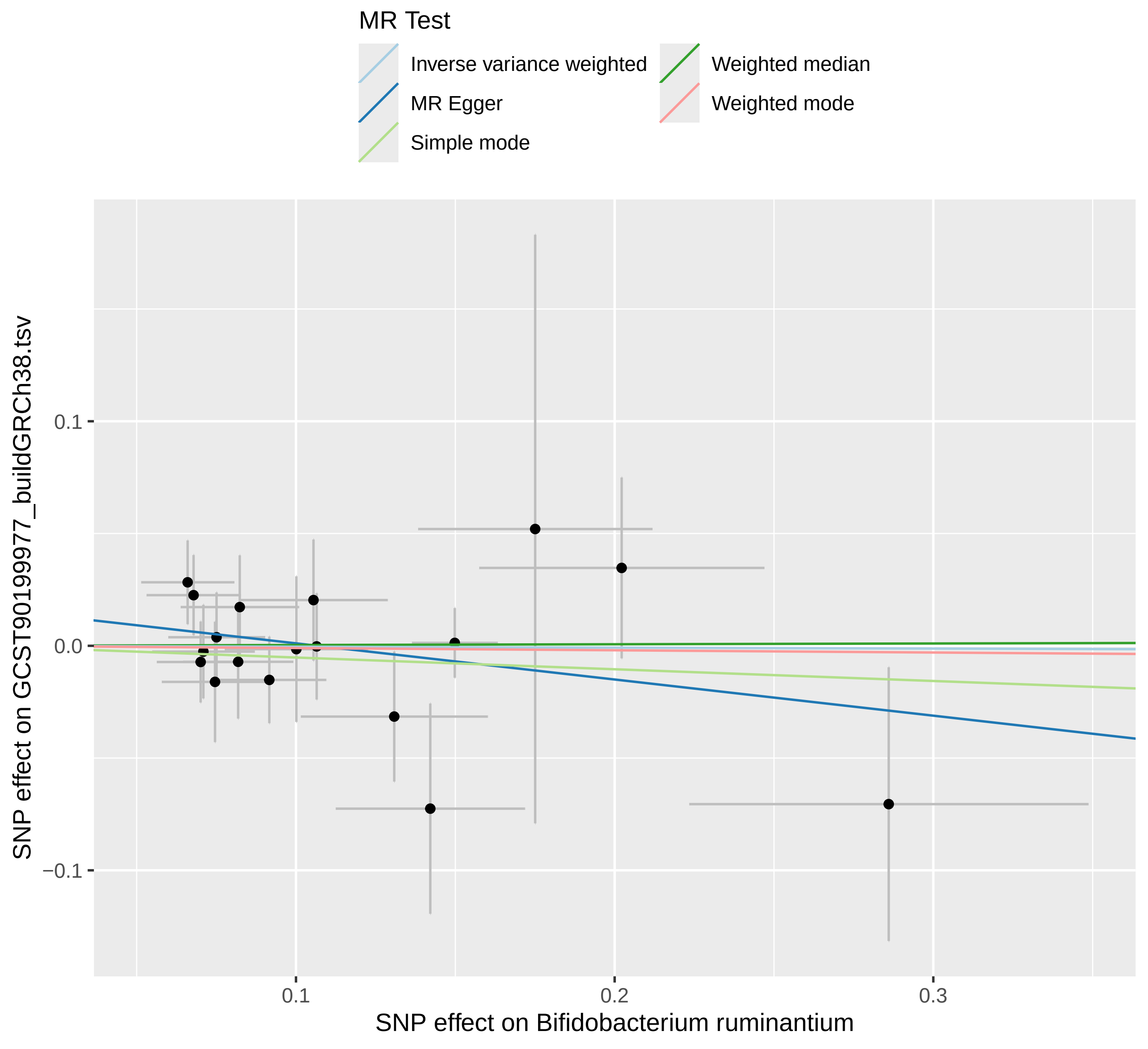
MR Test
Inverse variance weighted
MR Egger
Simple mode
Weighted median
Weighted mode
SNP effect on GCST90199977_buildGRCh38.tsv
0.1
0.0
−0.1
0.1
0.2
0.3
SNP effect on Bifidobacterium ruminantium

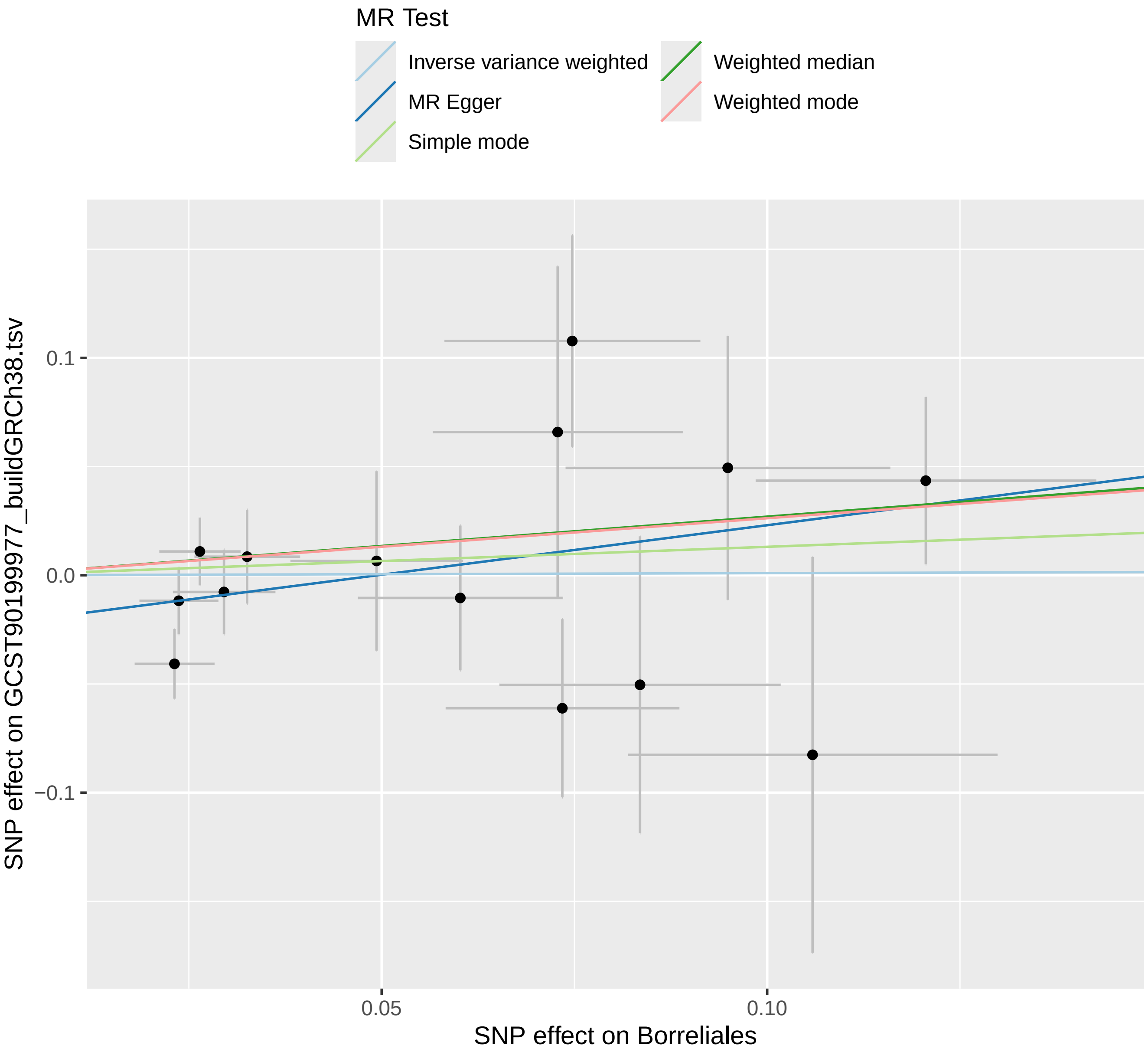
MR Test
Inverse variance weighted
MR Egger
Simple mode
Weighted median
Weighted mode
0.1
0.0
−0.1
0.05
0.10
SNP effect on GCST90199977_buildGRCh38.tsv
SNP effect on Borreliales

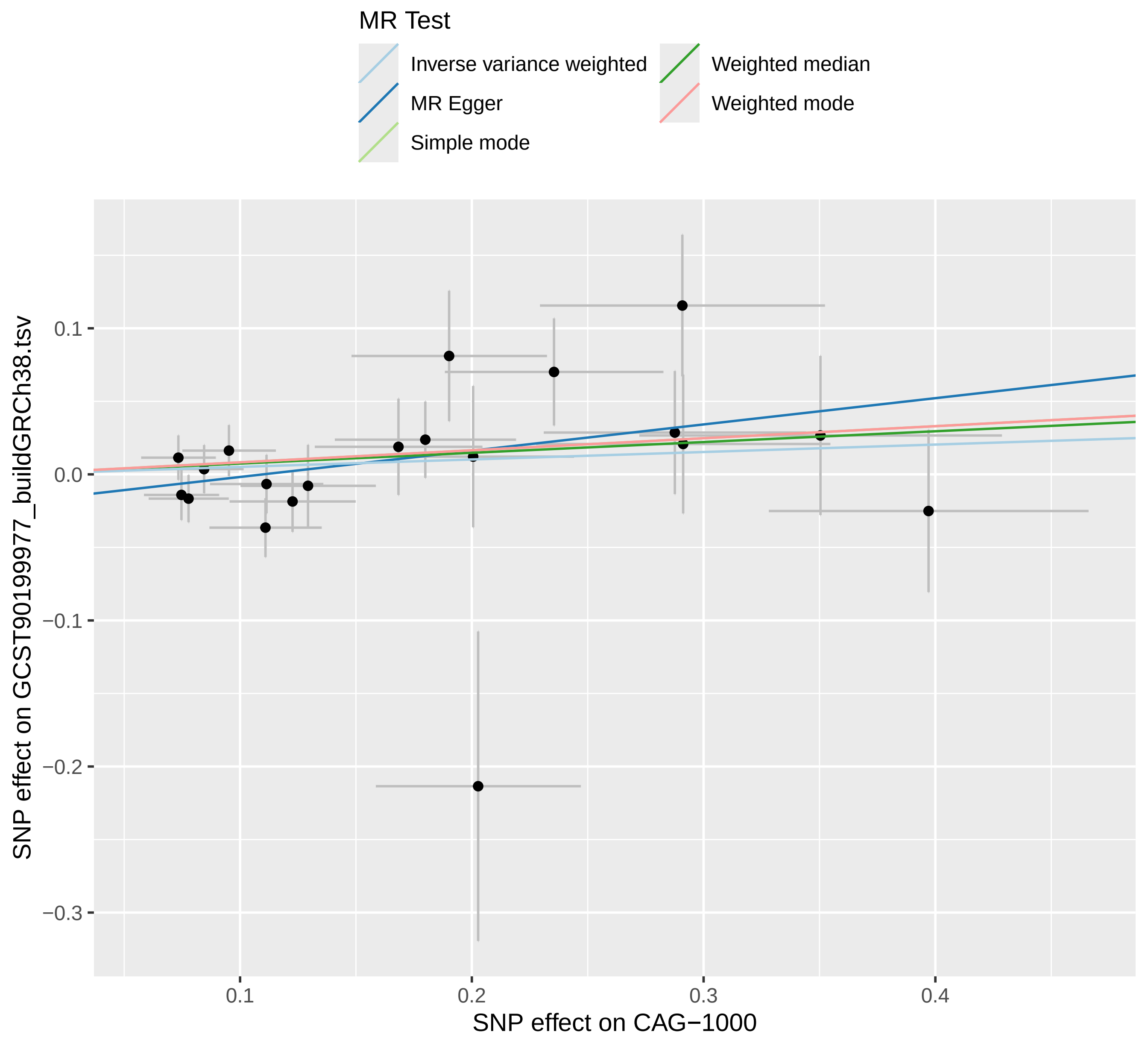
MR Test
Inverse variance weighted
MR Egger
Simple mode
Weighted median
Weighted mode
0.1
0.0
−0.1
−0.2
−0.3
0.1
0.2
0.3
0.4
SNP effect on CAG−1000
SNP effect on GCST90199977_buildGRCh38.tsv

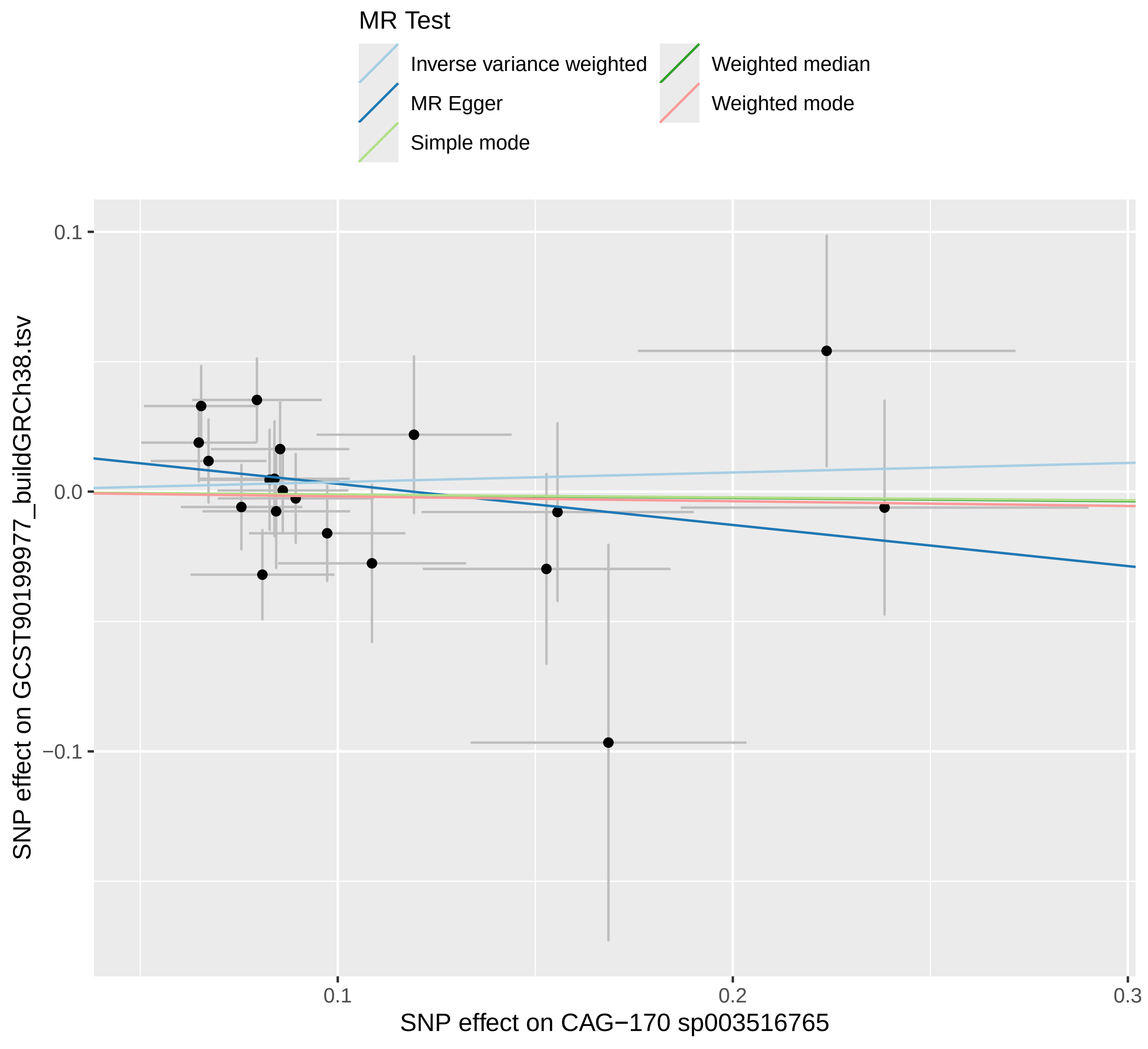
MR Test
Inverse variance weighted
MR Egger
Simple mode
Weighted median
Weighted mode
0.1
0.0
−0.1
SNP effect on GCST90199977_buildGRCh38.tsv
0.1
0.2
0.3
SNP effect on CAG−170 sp003516765

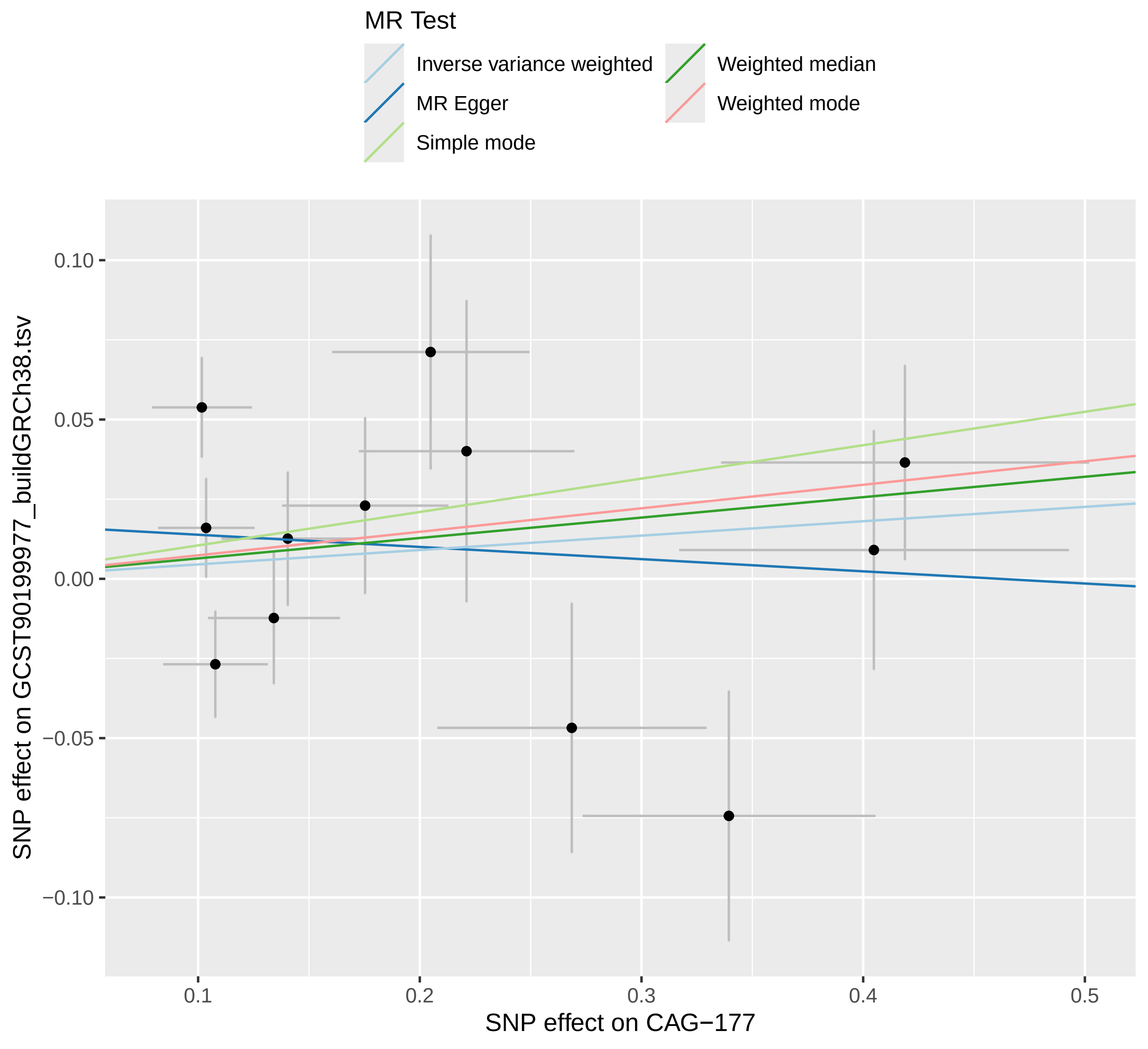
MR Test
Inverse variance weighted
MR Egger
Simple mode
Weighted median
Weighted mode
0.10
0.05
0.00
−0.05
−0.10
SNP effect on GCST90199977_buildGRCh38.tsv
0.1
0.2
0.3
0.4
0.5
SNP effect on CAG−177

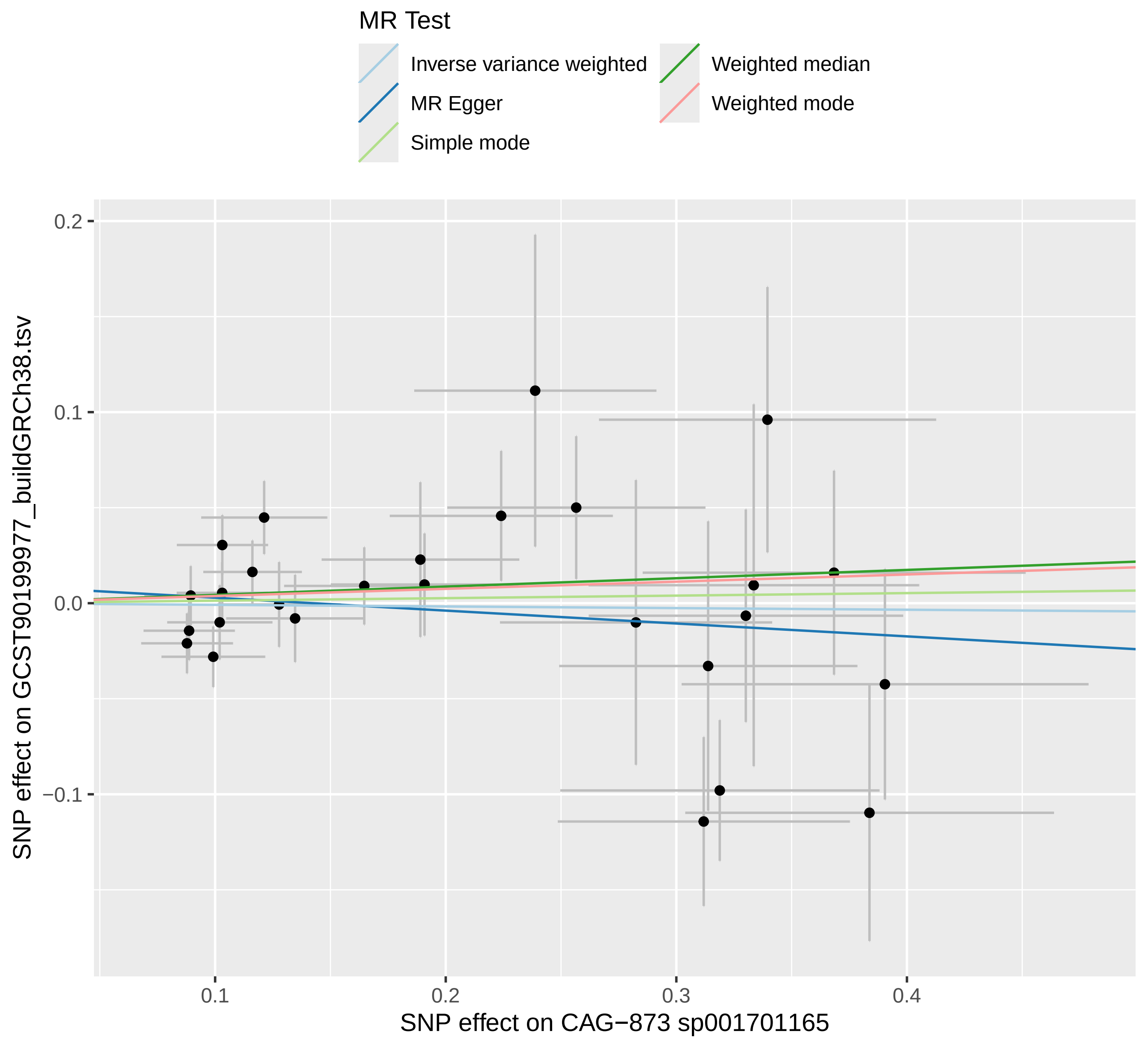
MR Test
Inverse variance weighted
MR Egger
Simple mode
Weighted median
Weighted mode
SNP effect on GCST90199977_buildGRCh38.tsv
0.2
0.1
0.0
−0.1
0.1
0.2
0.3
0.4
SNP effect on CAG−873 sp001701165

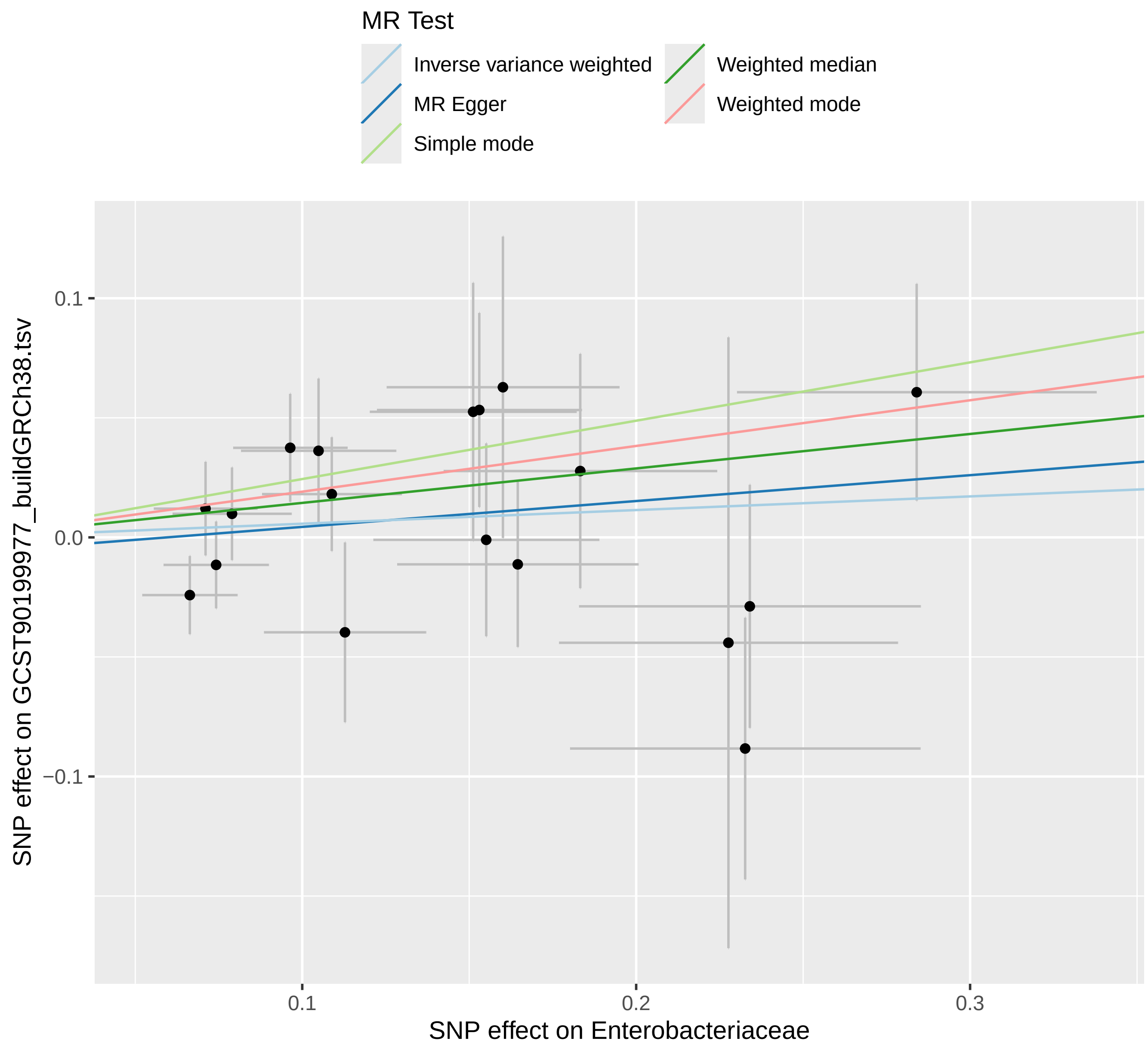

MR Test
Inverse variance weighted
MR Egger
Simple mode
Weighted median
Weighted mode
0.1
0.0
−0.1
SNP effect on GCST90199977_buildGRCh38.tsv
0.1
0.2
0.3
SNP effect on Enterobacteriaceae

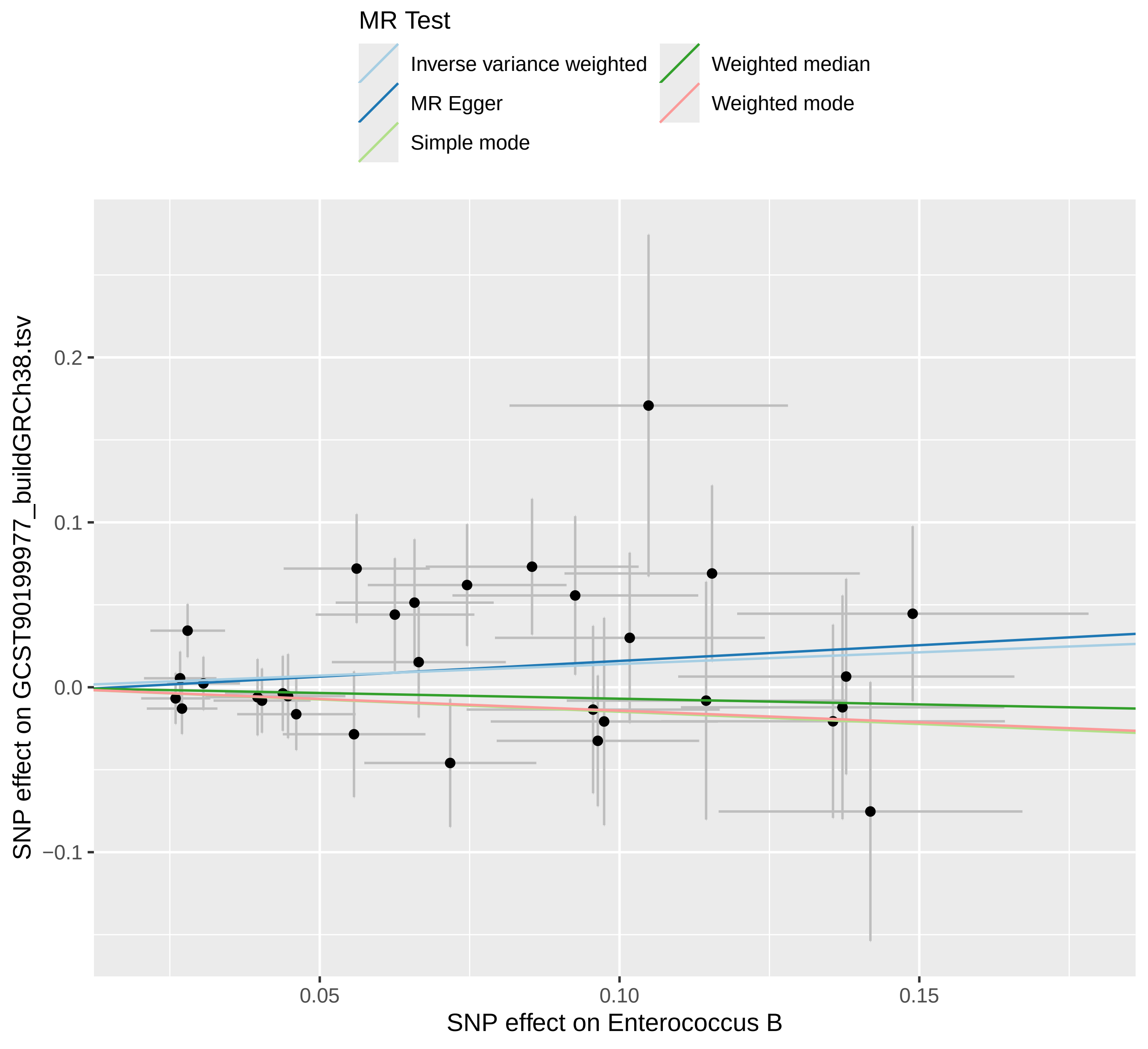
MR Test
Inverse variance weighted
MR Egger
Simple mode
Weighted median
Weighted mode
SNP effect on GCST90199977_buildGRCh38.tsv
0.2
0.1
0.0
−0.1
0.05
0.10
0.15
SNP effect on Enterococcus B

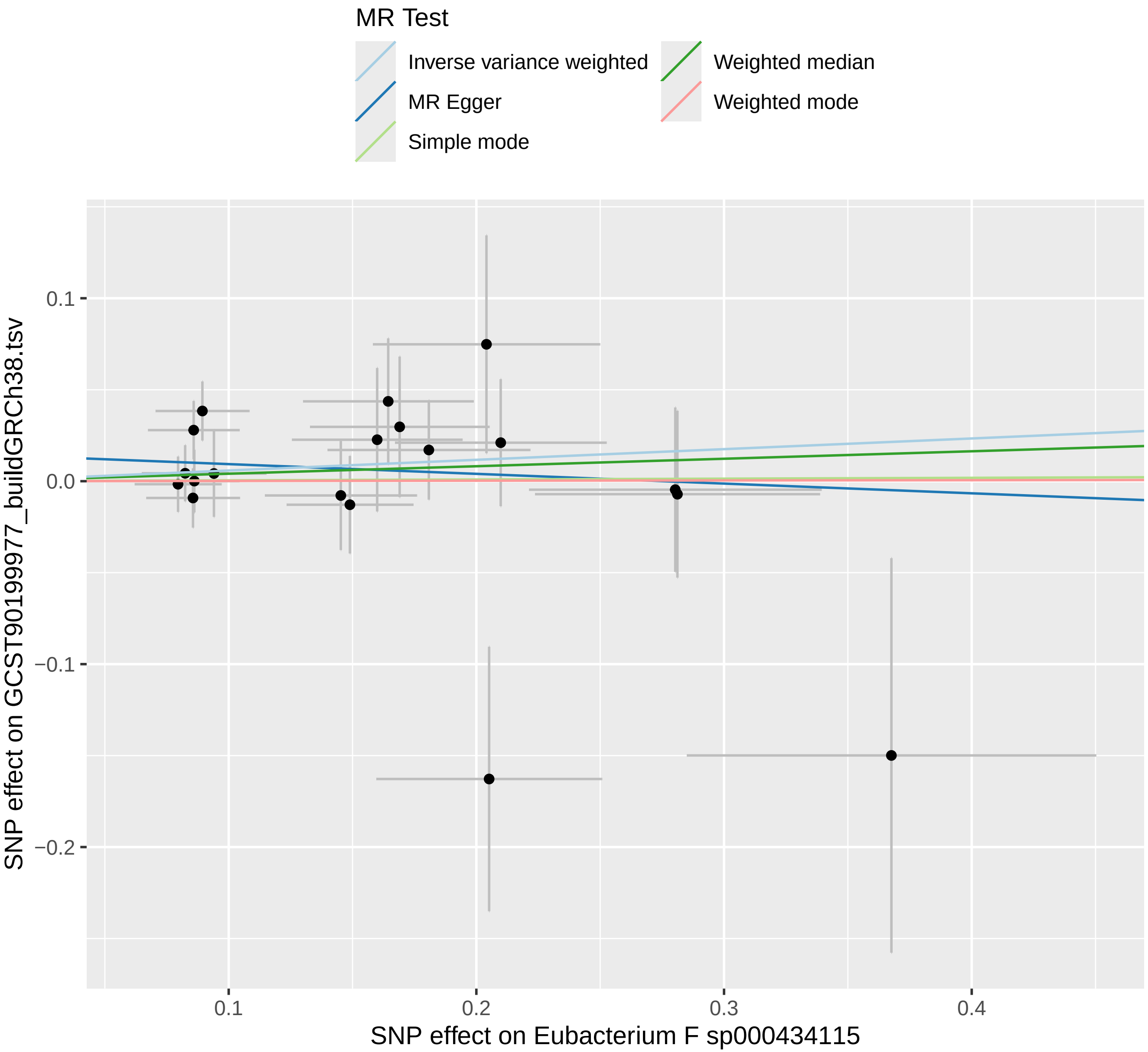
MR Test
Inverse variance weighted
MR Egger
Simple mode
Weighted median
Weighted mode
0.1
0.0
−0.1
−0.2
SNP effect on GCST90199977_buildGRCh38.tsv
0.1
0.2
0.3
0.4
SNP effect on Eubacterium F sp000434115

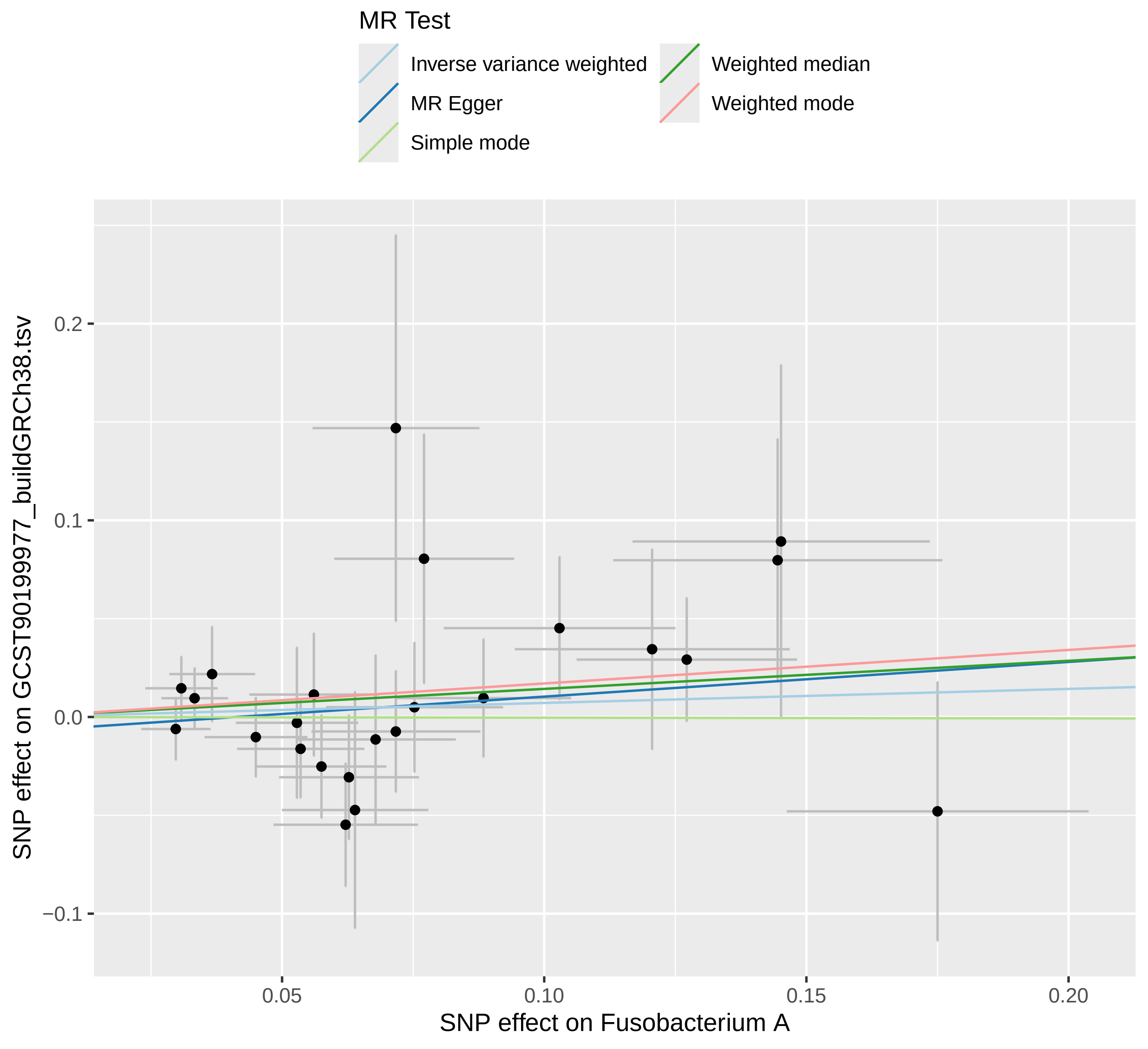

MR Test
Inverse variance weighted
MR Egger
Simple mode
Weighted median
Weighted mode
SNP effect on GCST90199977_buildGRCh38.tsv
0.2
0.1
0.0
−0.1
0.05
0.10
0.15
0.20
SNP effect on Fusobacterium A

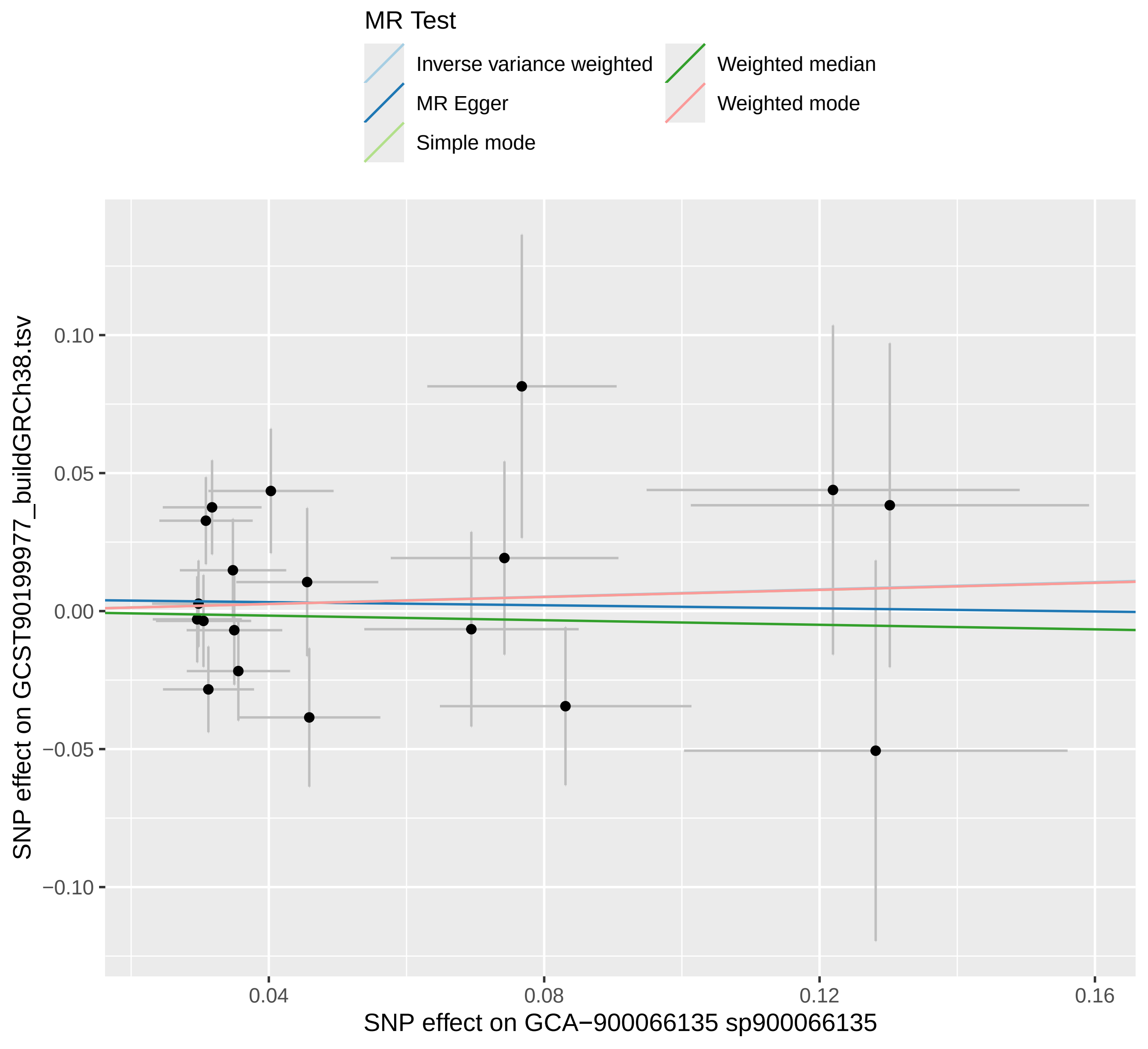

MR Test
Inverse variance weighted
MR Egger
Simple mode
Weighted median
Weighted mode
0.10
0.05
0.00
−0.05
−0.10
0.04
0.08
0.12
0.16
SNP effect on GCST90199977_buildGRCh38.tsv
SNP effect on GCA−900066135 sp900066135

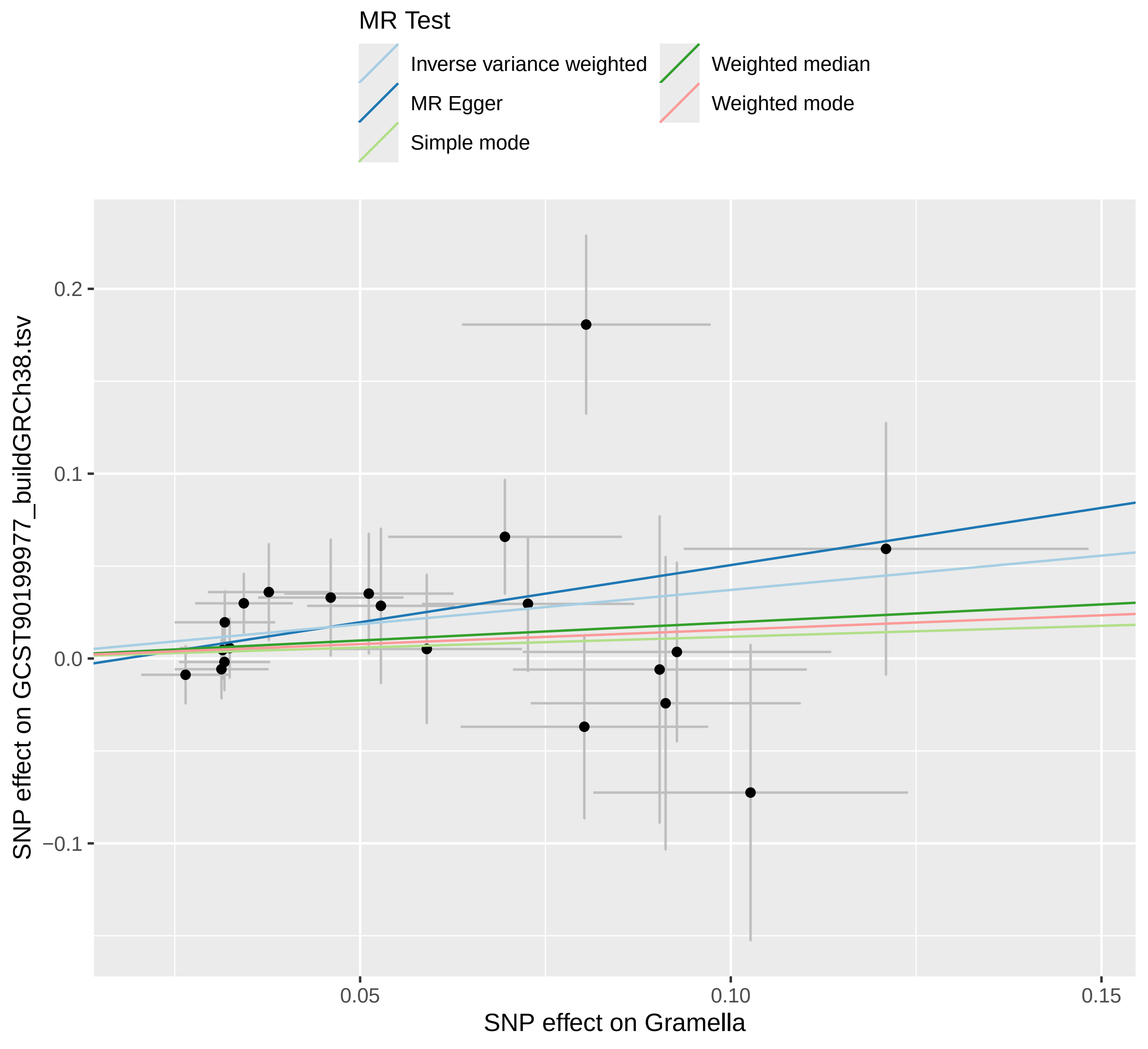
MR Test
Inverse variance weighted
MR Egger
Simple mode
Weighted median
Weighted mode
SNP effect on GCST90199977_buildGRCh38.tsv
0.2
0.1
0.0
−0.1
0.05
0.10
0.15
SNP effect on Gramella

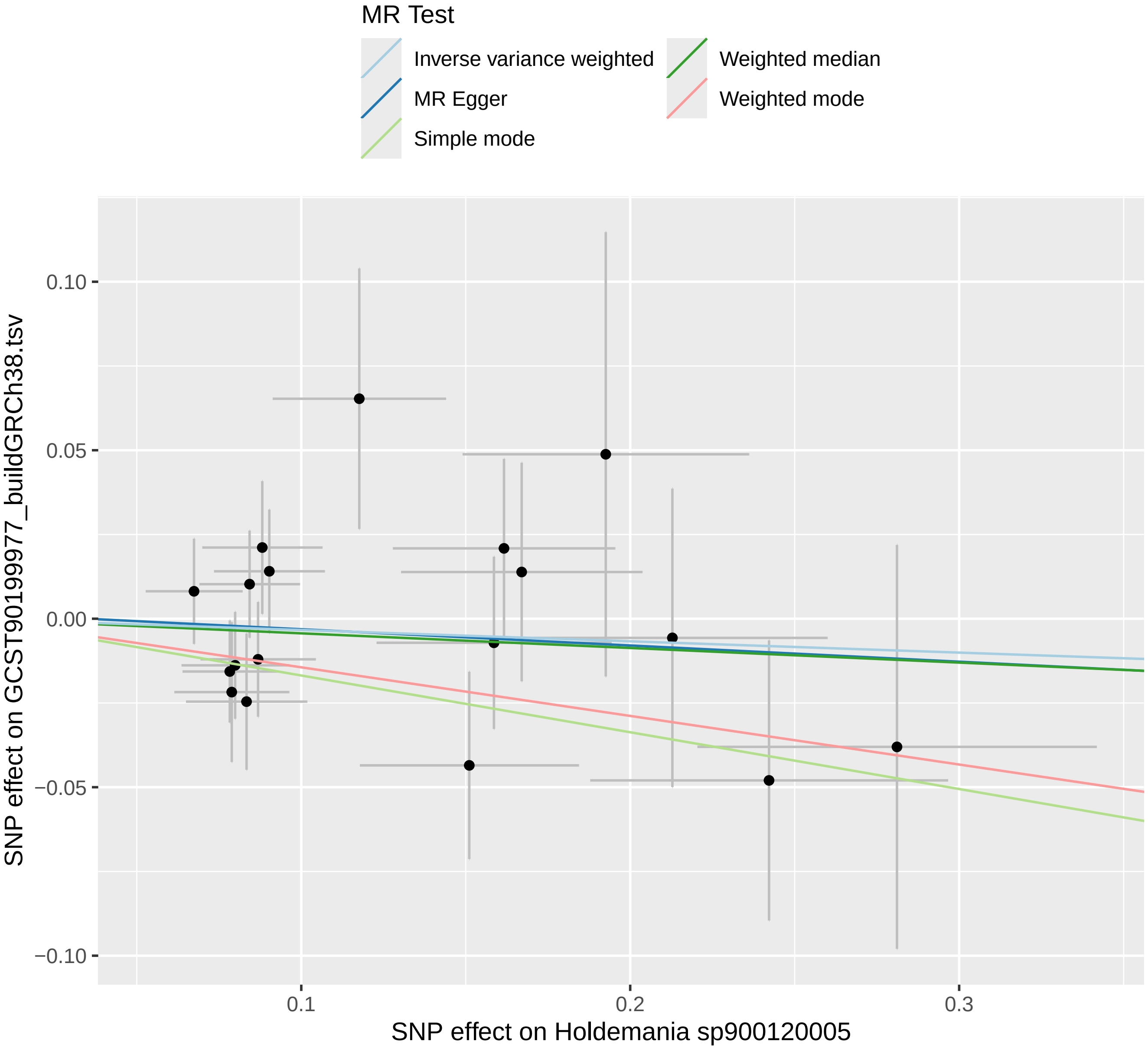
MR Test
Inverse variance weighted
MR Egger
Simple mode
Weighted median
Weighted mode
0.10
0.05
0.00
−0.05
−0.10
0.1
0.2
0.3
SNP effect on GCST90199977_buildGRCh38.tsv
SNP effect on Holdemania sp900120005

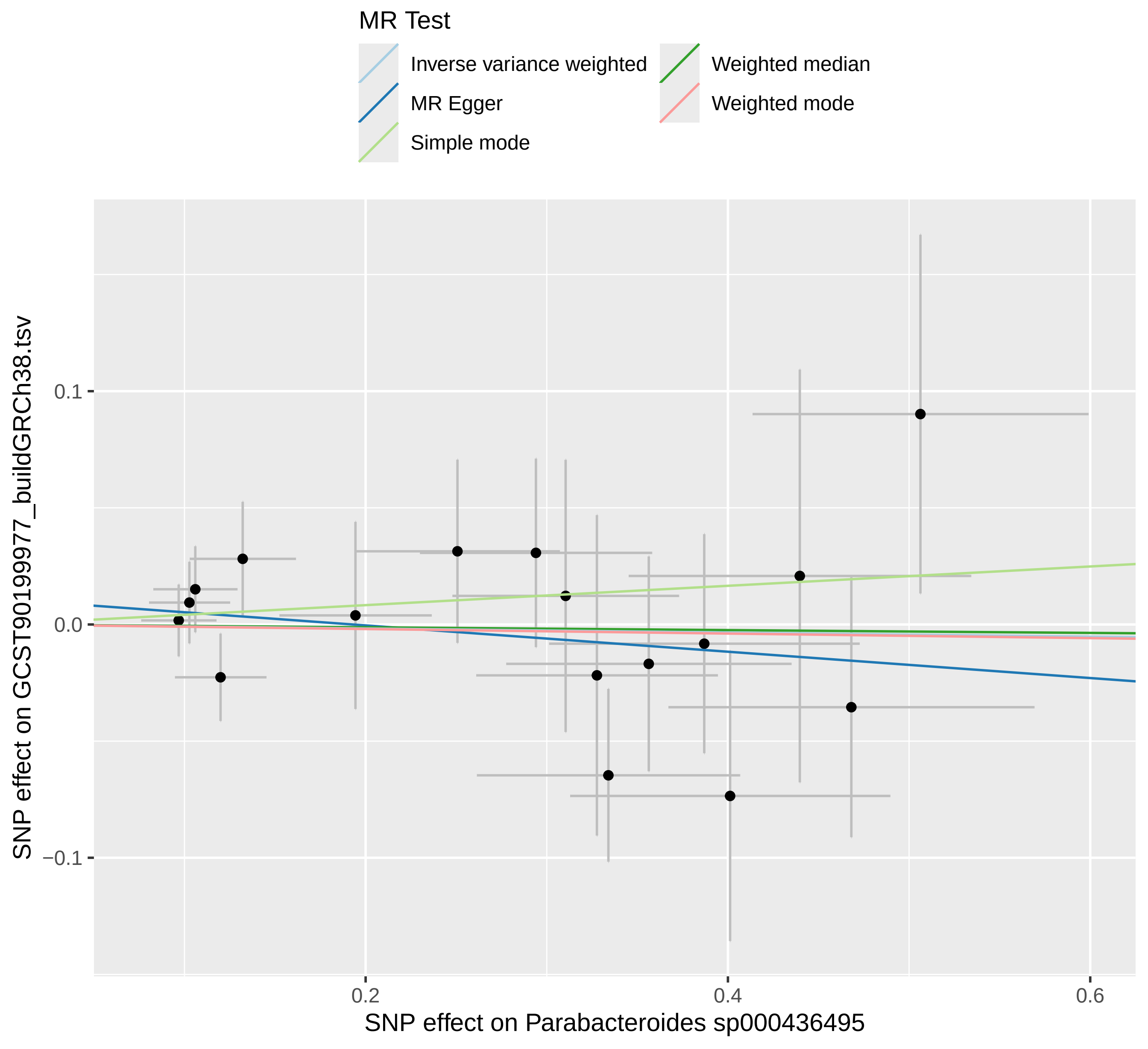

MR Test
Inverse variance weighted
MR Egger
Simple mode
Weighted median
Weighted mode
0.1
0.0
−0.1
0.2
0.4
0.6
SNP effect on GCST90199977_buildGRCh38.tsv
SNP effect on Parabacteroides sp000436495

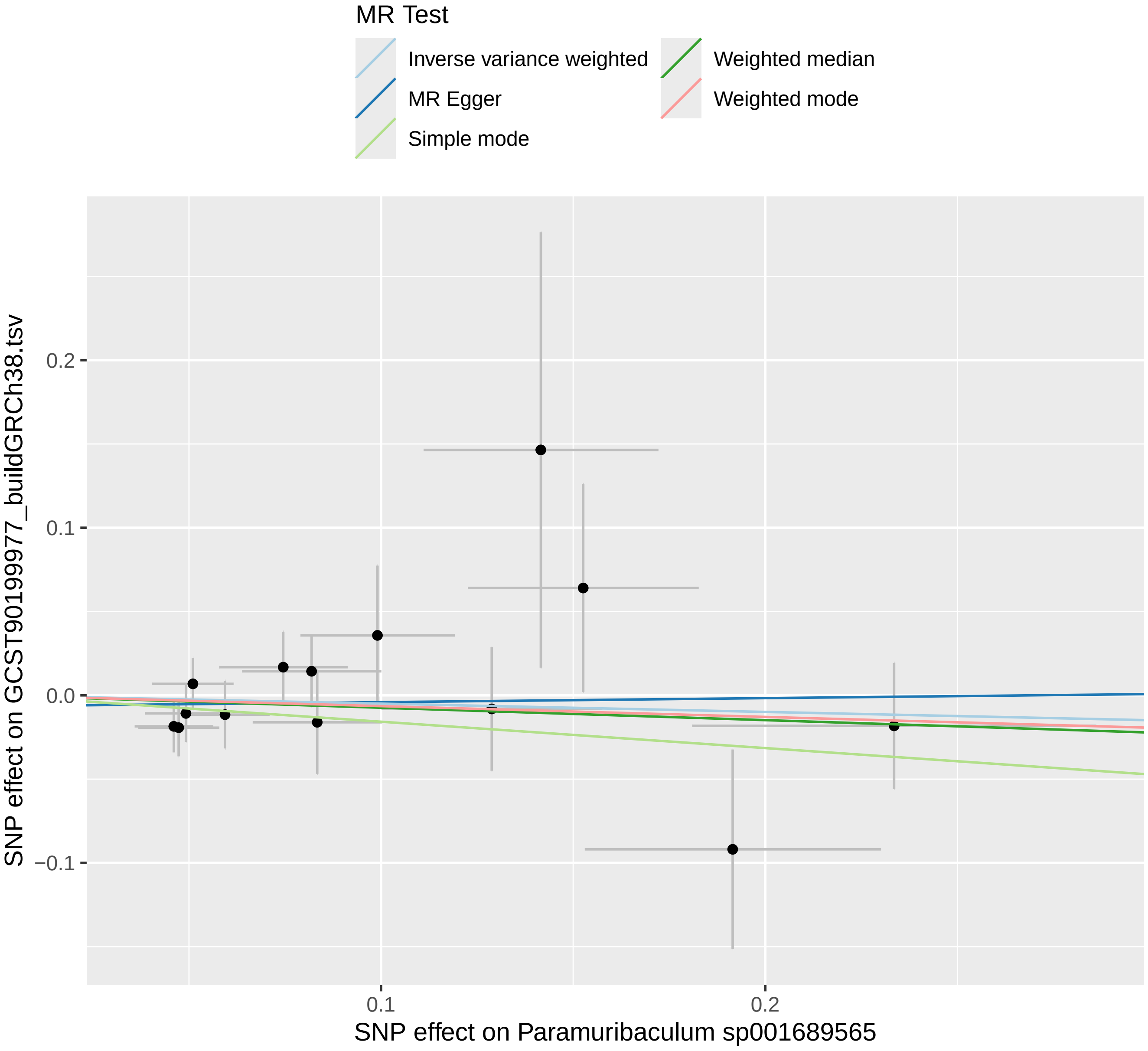
MR Test
Inverse variance weighted
MR Egger
Simple mode
Weighted median
Weighted mode
0.2
0.1
0.0
−0.1
0.1
0.2
SNP effect on GCST90199977_buildGRCh38.tsv
SNP effect on Paramuribaculum sp001689565

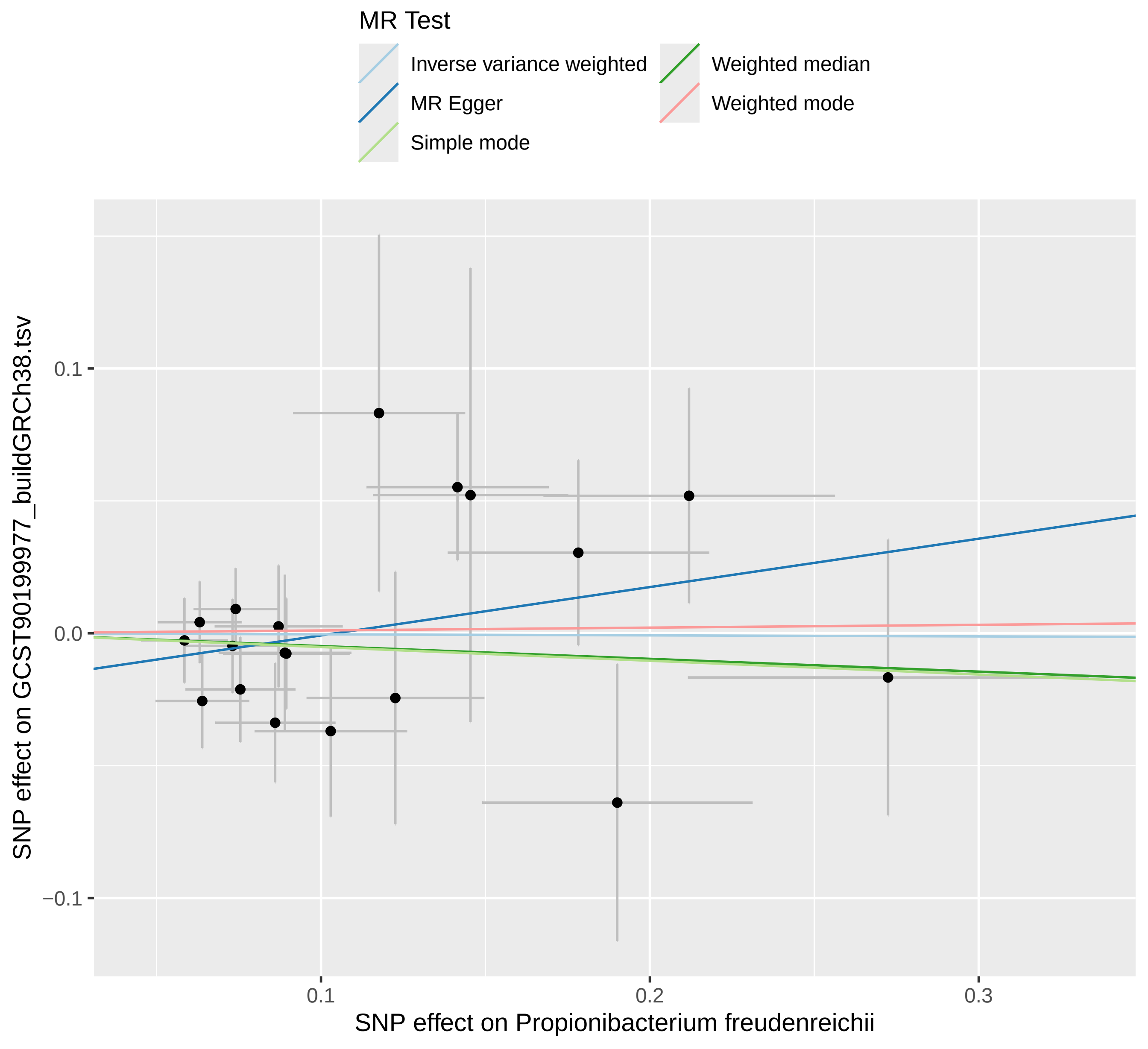

MR Test
Inverse variance weighted
MR Egger
Simple mode
Weighted median
Weighted mode
SNP effect on GCST90199977_buildGRCh38.tsv
0.1
0.0
−0.1
0.1
0.2
0.3
SNP effect on Propionibacterium freudenreichii

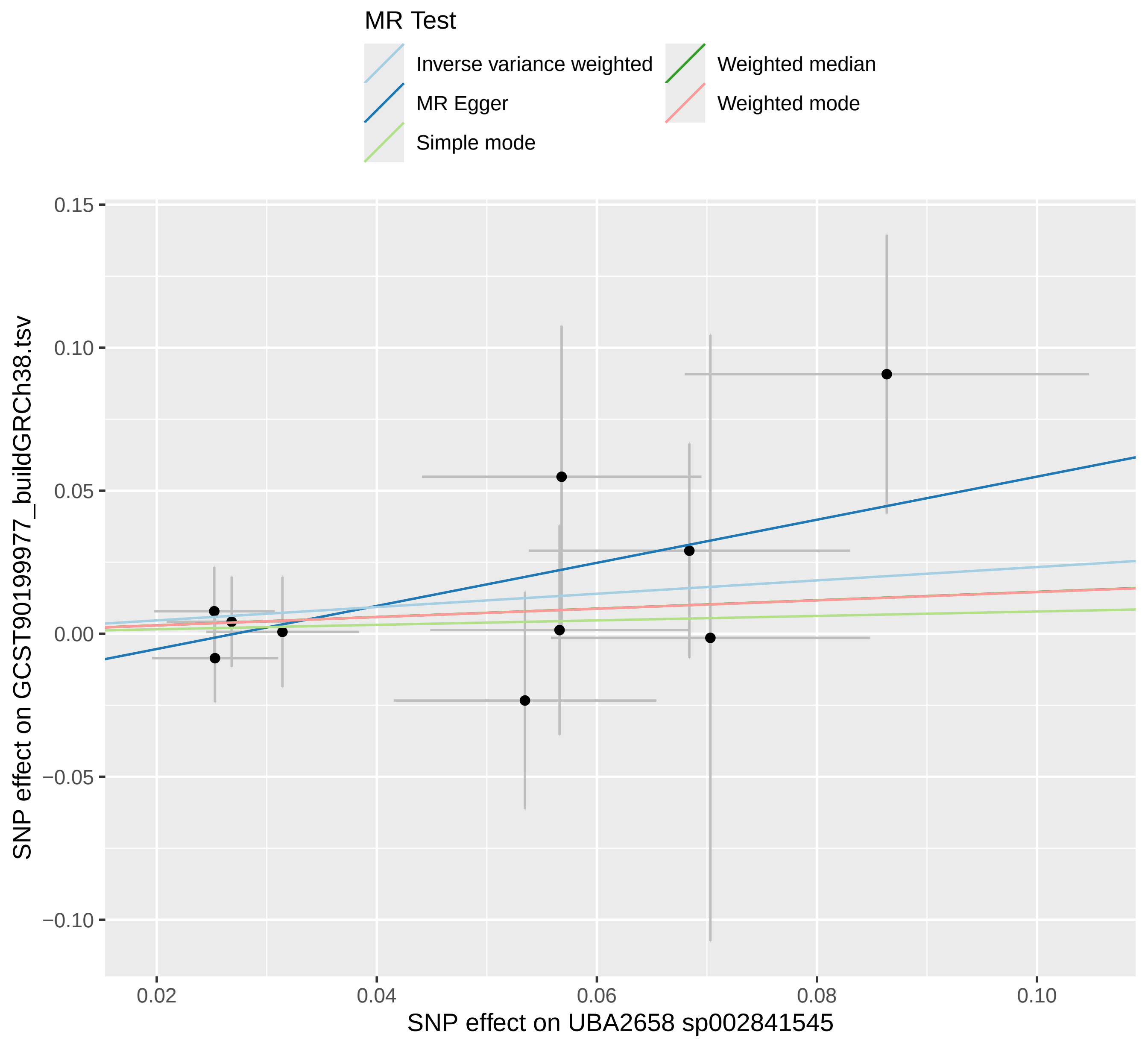

MR Test
Inverse variance weighted
MR Egger
Simple mode
Weighted median
Weighted mode
0.15
0.10
0.05
0.00
−0.05
−0.10
0.02
0.04
0.06
0.08
0.10
SNP effect on GCST90199977_buildGRCh38.tsv
SNP effect on UBA2658 sp002841545

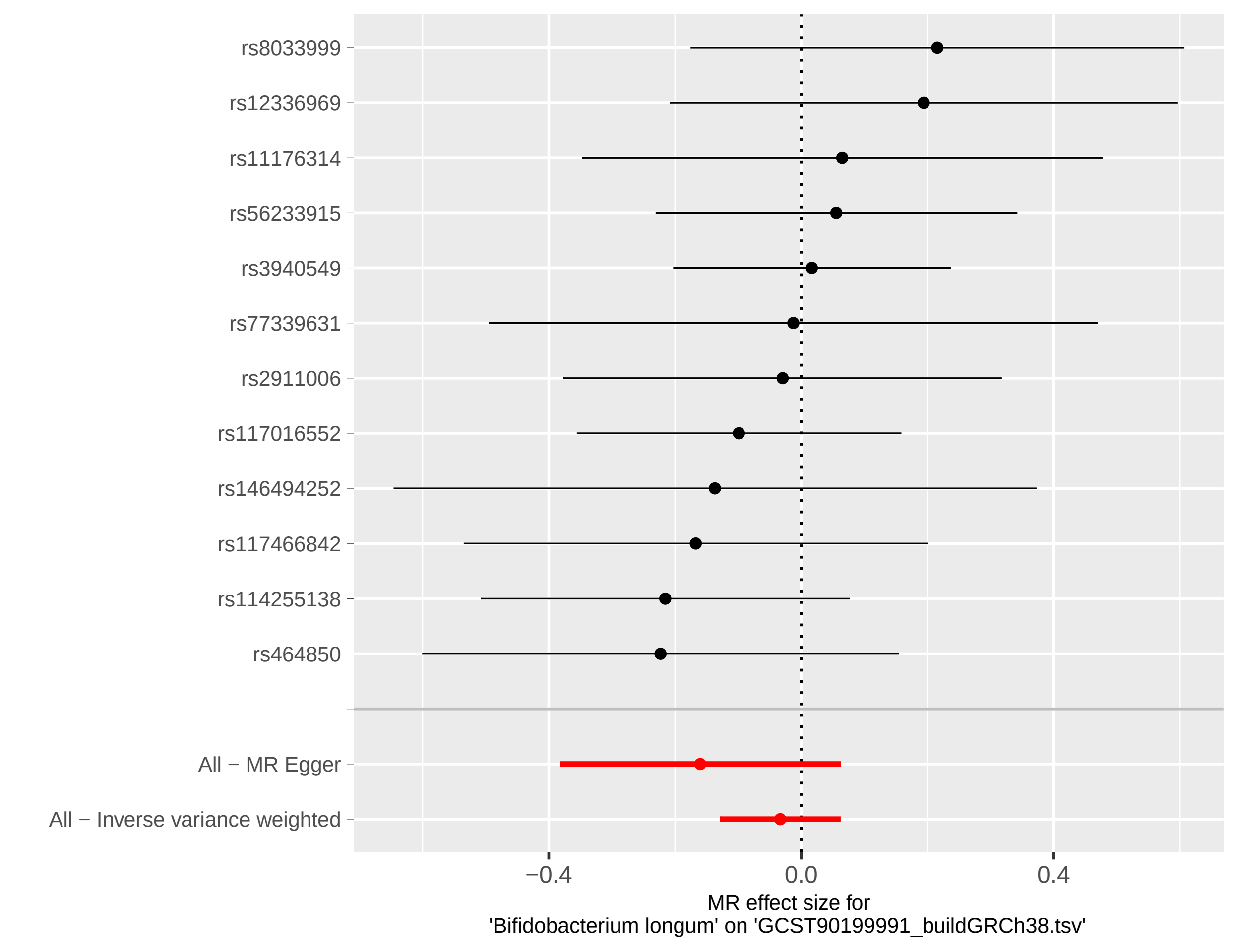
rs8033999
rs12336969
rs11176314
rs56233915
rs3940549
rs77339631
rs2911006
rs117016552
rs146494252
rs117466842
rs114255138
rs464850
All − MR Egger
All − Inverse variance weighted
−0.4
0.0
0.4
MR effect size for
'Bifidobacterium longum' on 'GCST90199991_buildGRCh38.tsv'

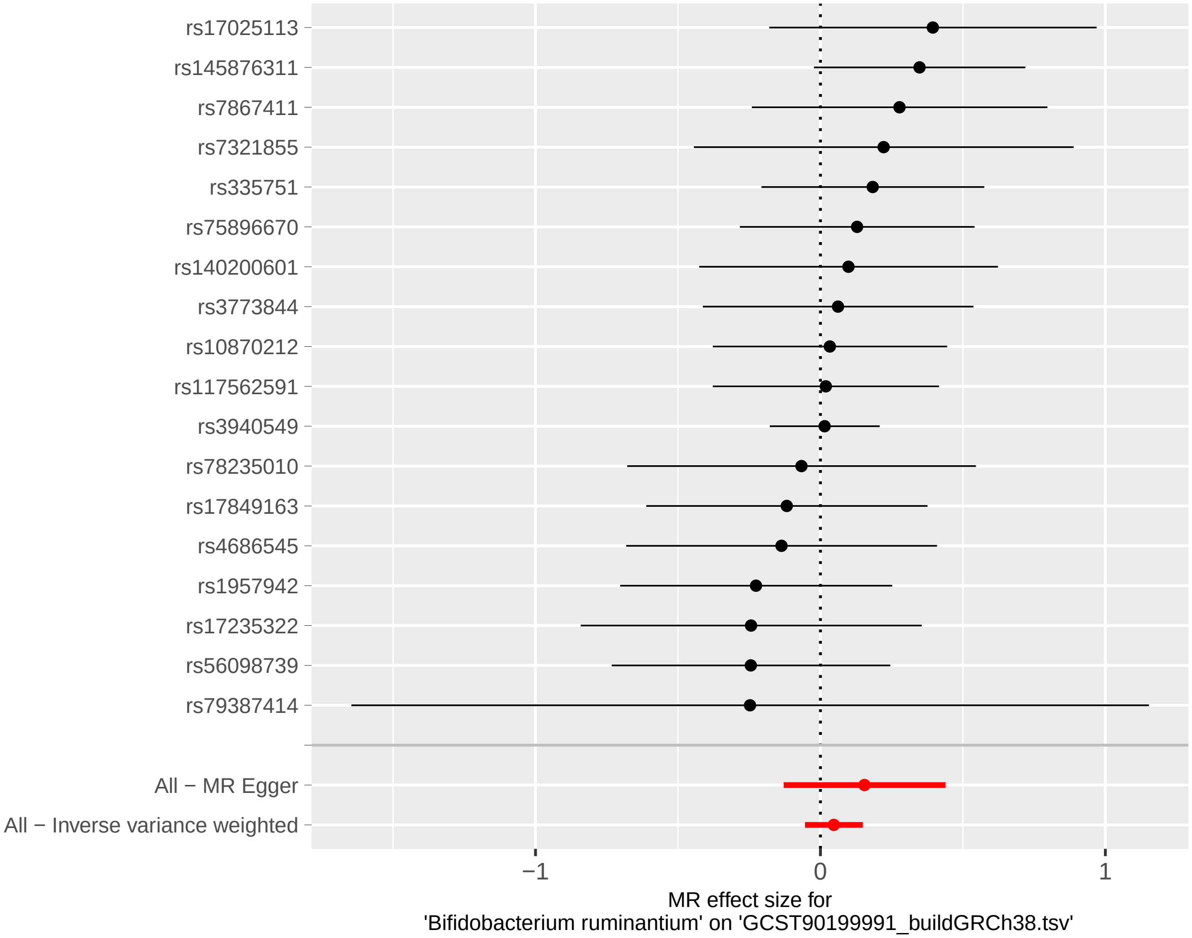
rs17025113
rs145876311
rs7867411
rs7321855
rs335751
rs75896670
rs140200601
rs3773844
rs10870212
rs117562591
rs3940549
rs78235010
rs17849163
rs4686545
rs1957942
rs17235322
rs56098739
rs79387414
All − MR Egger
All − Inverse variance weighted
−1
0
1
MR effect size for
'Bifidobacterium ruminantium' on 'GCST90199991_buildGRCh38.tsv'

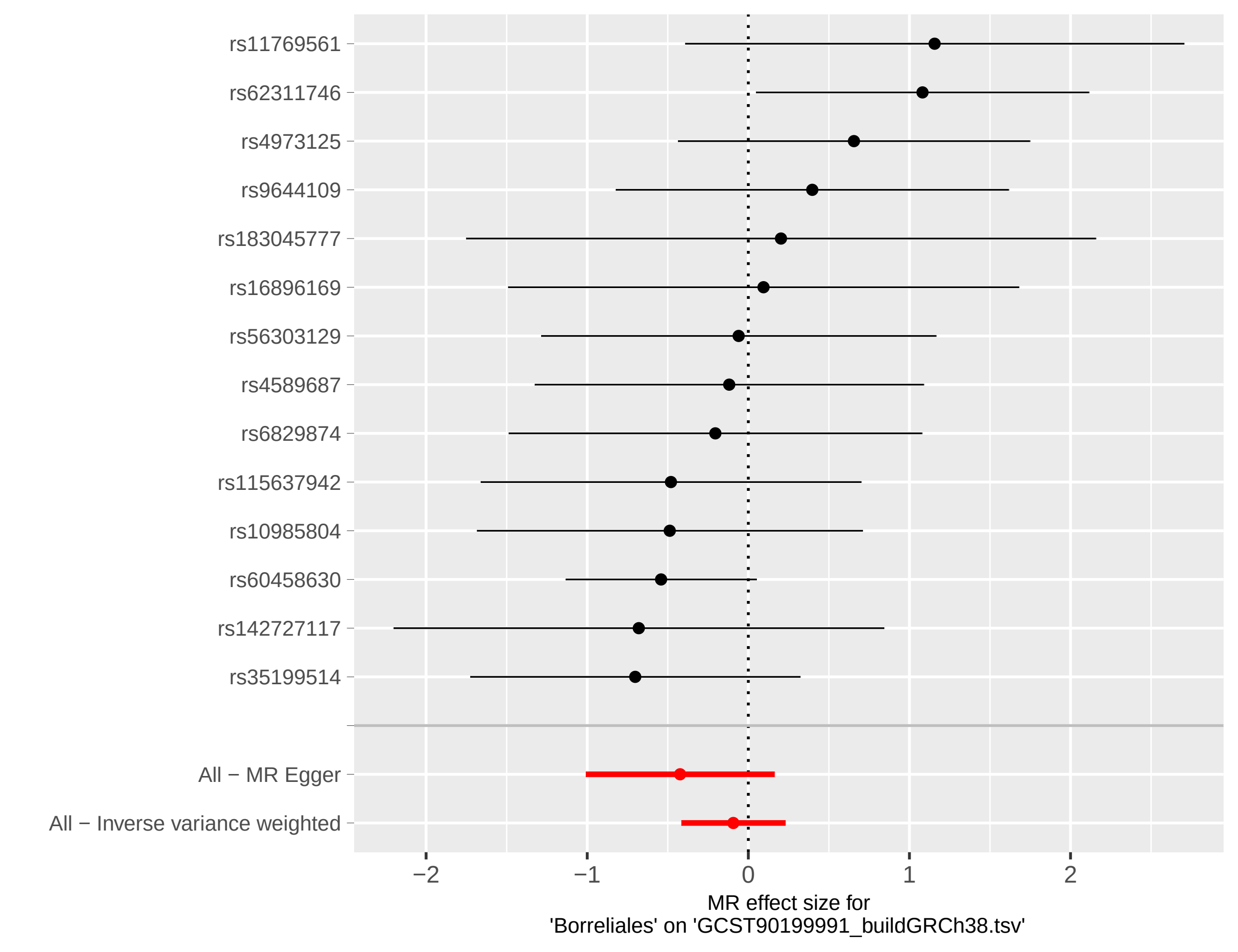
rs11769561
rs62311746
rs4973125
rs9644109
rs183045777
rs16896169
rs56303129
rs4589687
rs6829874
rs115637942
rs10985804
rs60458630
rs142727117
rs35199514
All − MR Egger
All − Inverse variance weighted
−2
−1
0
1
2
MR effect size for
'Borreliales' on 'GCST90199991_buildGRCh38.tsv'

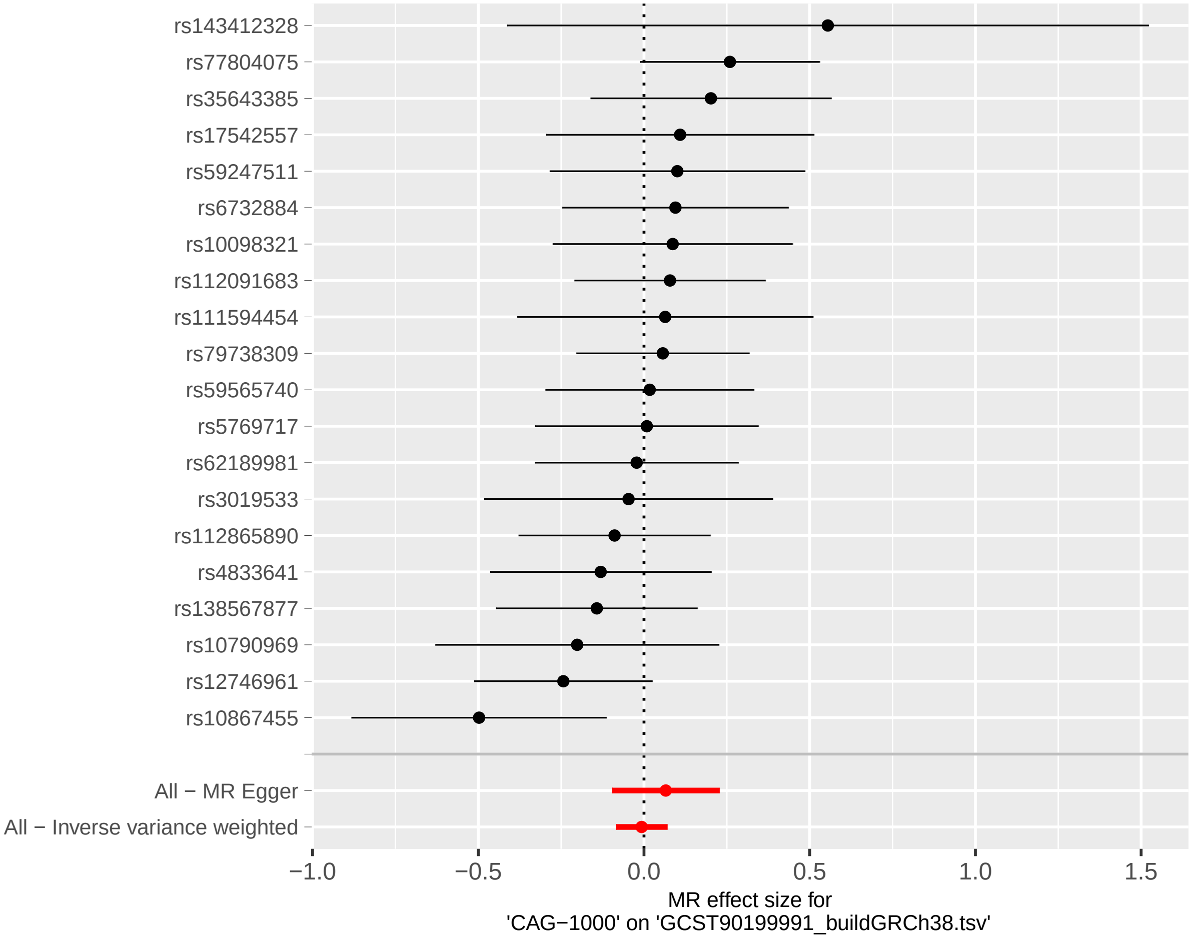

rs143412328
rs77804075
rs35643385
rs17542557
rs59247511
rs6732884
rs10098321
rs112091683
rs111594454
rs79738309
rs59565740
rs5769717
rs62189981
rs3019533
rs112865890
rs4833641
rs138567877
rs10790969
rs12746961
rs10867455
All − MR Egger
All − Inverse variance weighted
−1.0
−0.5
0.0
0.5
1.0
1.5
MR effect size for
'CAG−1000' on 'GCST90199991_buildGRCh38.tsv'

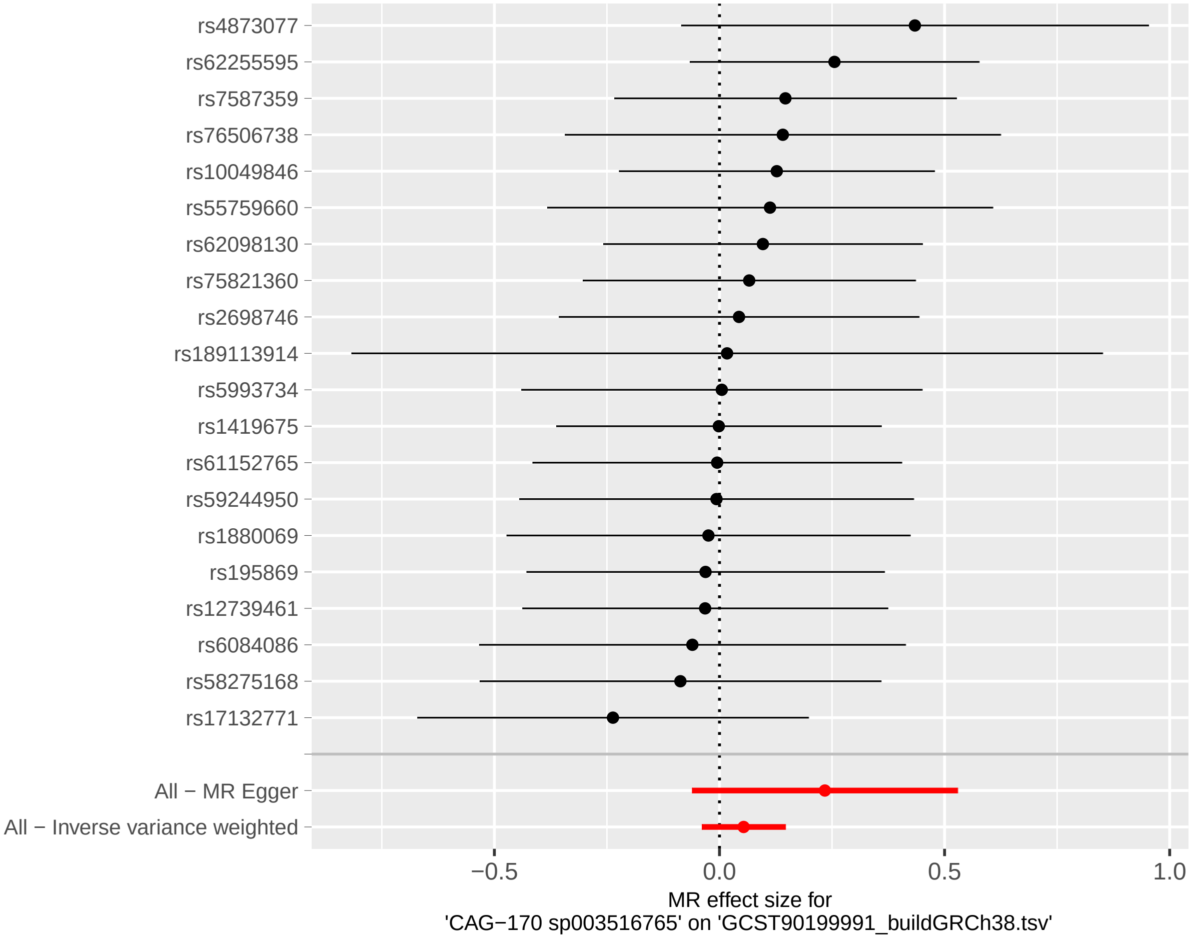
rs4873077
rs62255595
rs7587359
rs76506738
rs10049846
rs55759660
rs62098130
rs75821360
rs2698746
rs189113914
rs5993734
rs1419675
rs61152765
rs59244950
rs1880069
rs195869
rs12739461
rs6084086
rs58275168
rs17132771
All − MR Egger
All − Inverse variance weighted
−0.5
0.0
0.5
1.0
MR effect size for
'CAG−170 sp003516765' on 'GCST90199991_buildGRCh38.tsv'

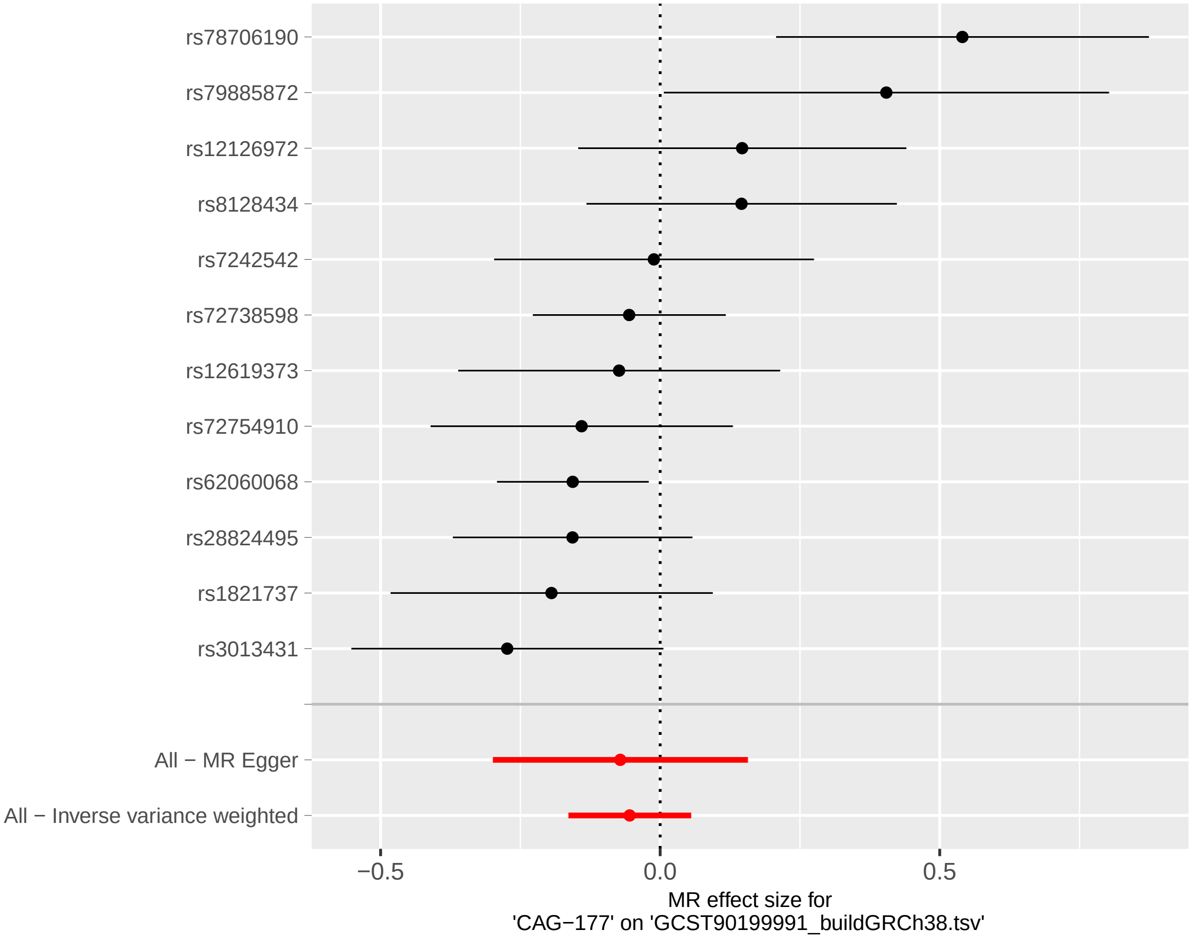

rs78706190
rs79885872
rs12126972
rs8128434
rs7242542
rs72738598
rs12619373
rs72754910
rs62060068
rs28824495
rs1821737
rs3013431
All − MR Egger
All − Inverse variance weighted
−0.5
0.0
0.5
MR effect size for
'CAG−177' on 'GCST90199991_buildGRCh38.tsv'

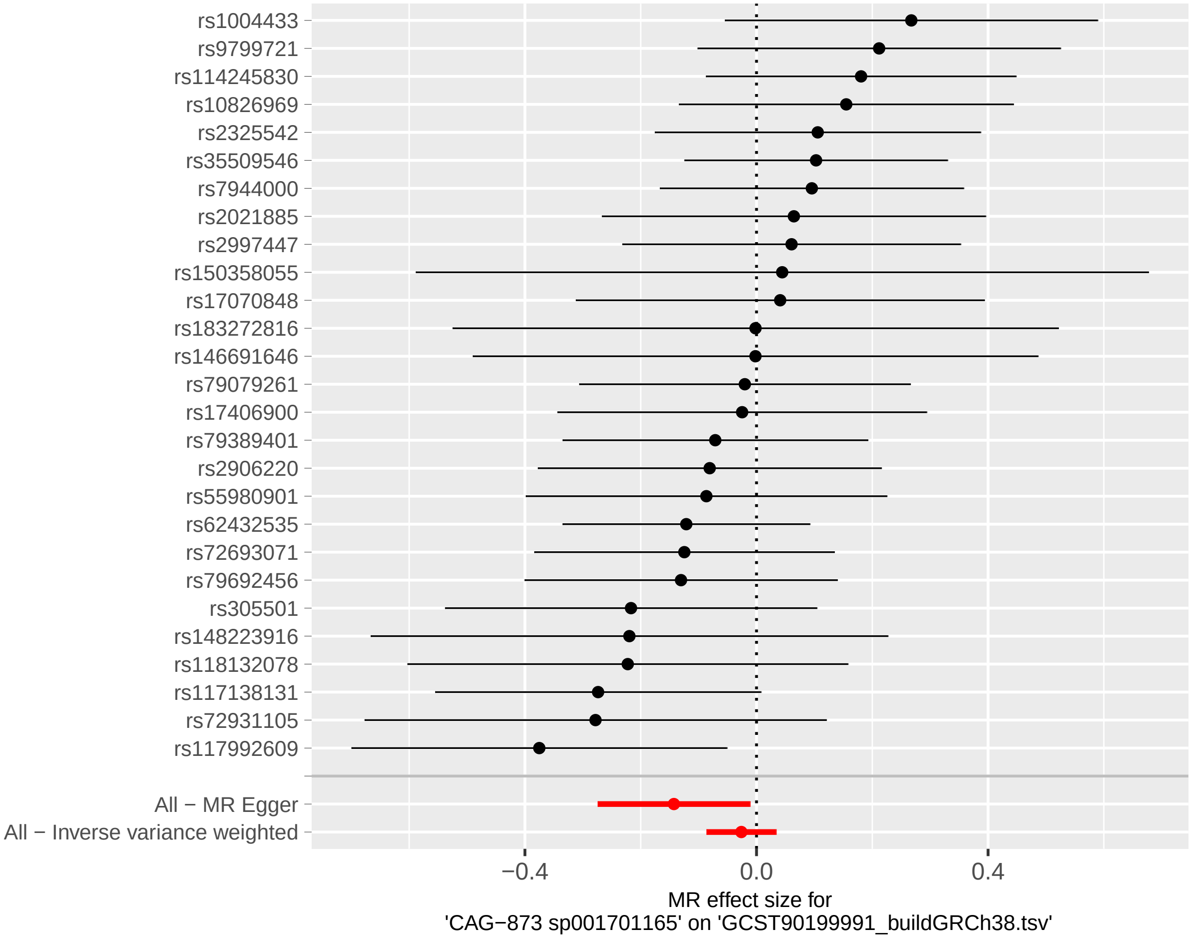

rs1004433
rs9799721
rs114245830
rs10826969
rs2325542
rs35509546
rs7944000
rs2021885
rs2997447
rs150358055
rs17070848
rs183272816
rs146691646
rs79079261
rs17406900
rs79389401
rs2906220
rs55980901
rs62432535
rs72693071
rs79692456
rs305501
rs148223916
rs118132078
rs117138131
rs72931105
rs117992609
All − MR Egger
All − Inverse variance weighted
−0.4
0.0
0.4
MR effect size for
'CAG−873 sp001701165' on 'GCST90199991_buildGRCh38.tsv'

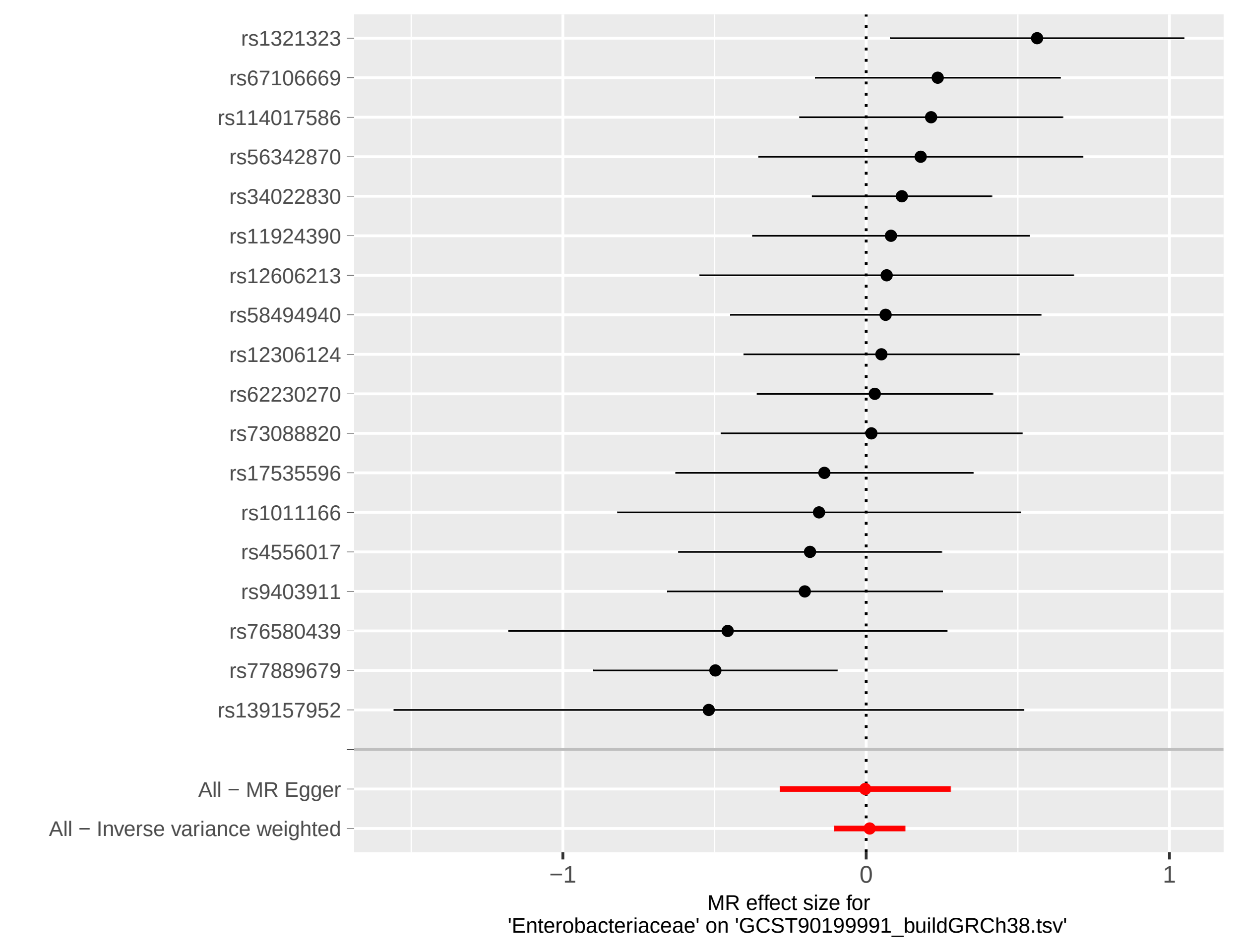
rs1321323
rs67106669
rs114017586
rs56342870
rs34022830
rs11924390
rs12606213
rs58494940
rs12306124
rs62230270
rs73088820
rs17535596
rs1011166
rs4556017
rs9403911
rs76580439
rs77889679
rs139157952
All − MR Egger
All − Inverse variance weighted
−1
0
1
MR effect size for
'Enterobacteriaceae' on 'GCST90199991_buildGRCh38.tsv'

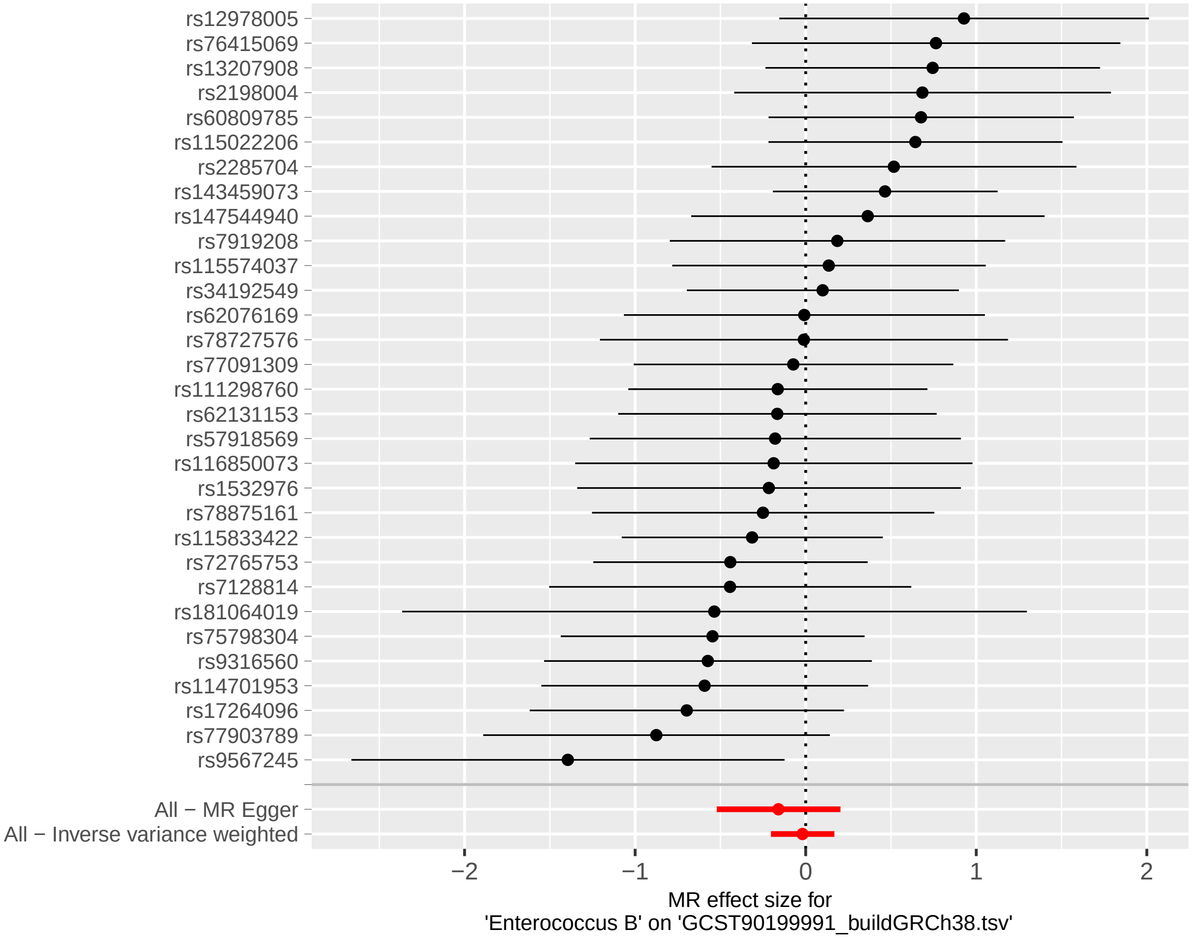
rs12978005
rs76415069
rs13207908
rs2198004
rs60809785
rs115022206
rs2285704
rs143459073
rs147544940
rs7919208
rs115574037
rs34192549
rs62076169
rs78727576
rs77091309
rs111298760
rs62131153
rs57918569
rs116850073
rs1532976
rs78875161
rs115833422
rs72765753
rs7128814
rs181064019
rs75798304
rs9316560
rs114701953
rs17264096
rs77903789
rs9567245
All − MR Egger
All − Inverse variance weighted
−2
−1
0
1
2
MR effect size for
'Enterococcus B' on 'GCST90199991_buildGRCh38.tsv'

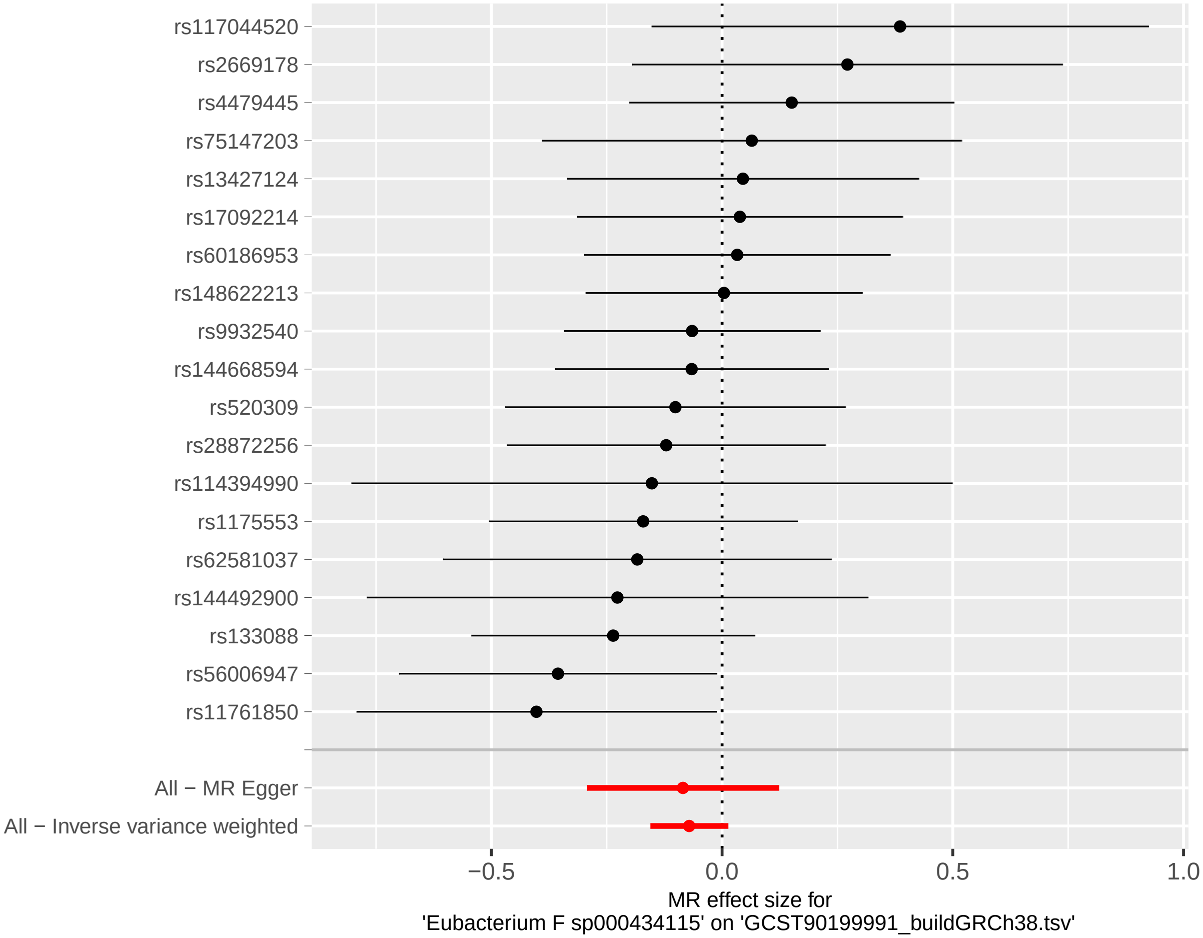
rs117044520
rs2669178
rs4479445
rs75147203
rs13427124
rs17092214
rs60186953
rs148622213
rs9932540
rs144668594
rs520309
rs28872256
rs114394990
rs1175553
rs62581037
rs144492900
rs133088
rs56006947
rs11761850
All − MR Egger
All − Inverse variance weighted
−0.5
0.0
0.5
1.0
MR effect size for
'Eubacterium F sp000434115' on 'GCST90199991_buildGRCh38.tsv'

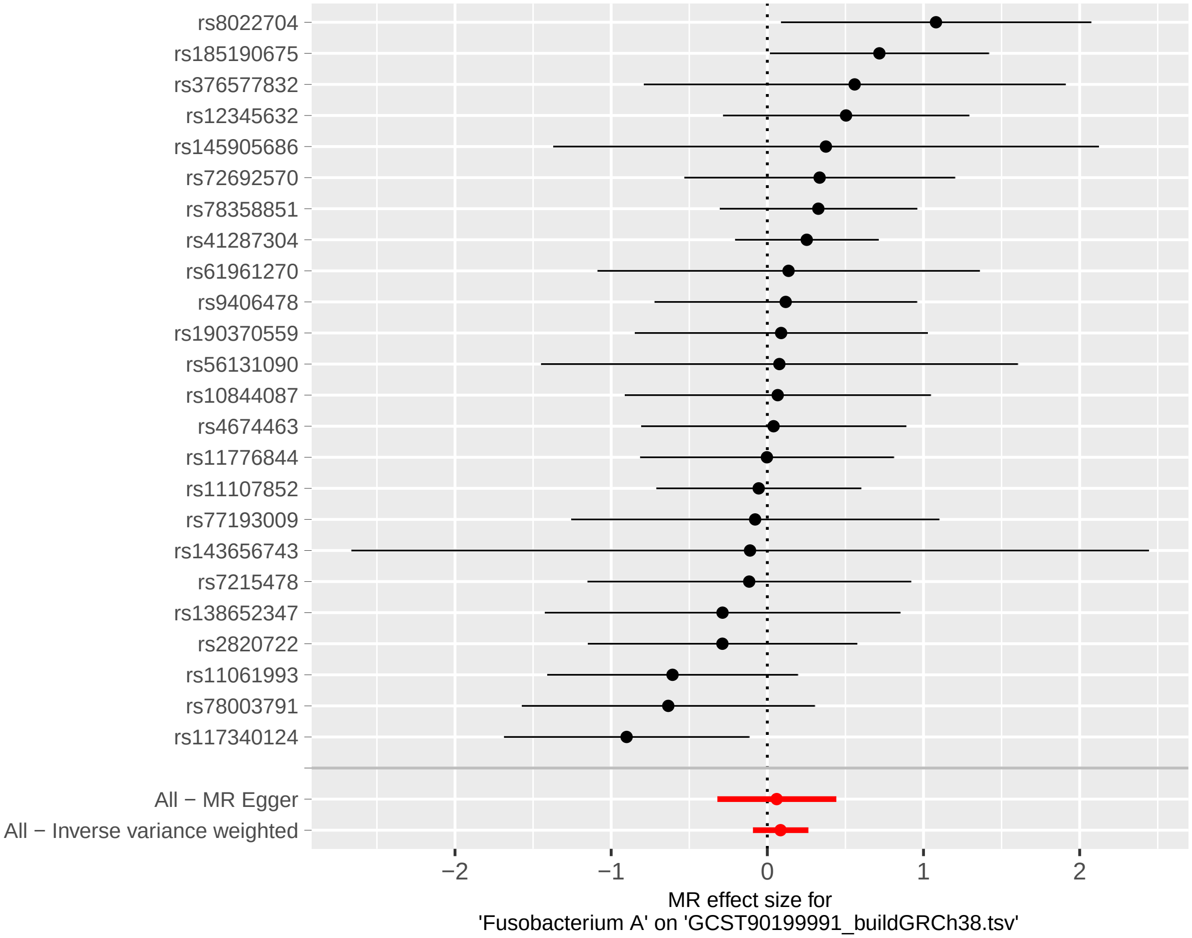
rs8022704
rs185190675
rs376577832
rs12345632
rs145905686
rs72692570
rs78358851
rs41287304
rs61961270
rs9406478
rs190370559
rs56131090
rs10844087
rs4674463
rs11776844
rs11107852
rs77193009
rs143656743
rs7215478
rs138652347
rs2820722
rs11061993
rs78003791
rs117340124
All − MR Egger
All − Inverse variance weighted
−2
−1
0
1
2
MR effect size for
'Fusobacterium A' on 'GCST90199991_buildGRCh38.tsv'

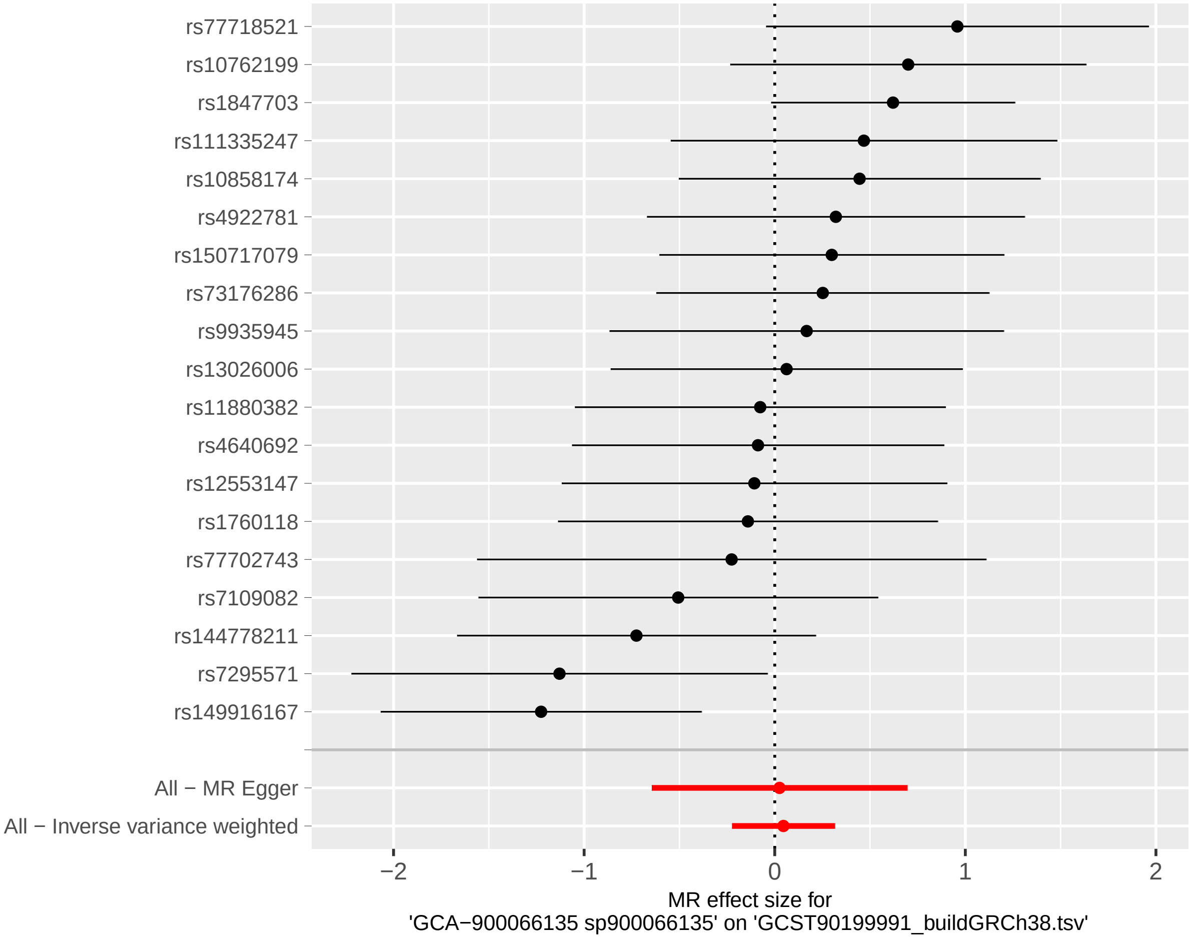

rs77718521
rs10762199
rs1847703
rs111335247
rs10858174
rs4922781
rs150717079
rs73176286
rs9935945
rs13026006
rs11880382
rs4640692
rs12553147
rs1760118
rs77702743
rs7109082
rs144778211
rs7295571
rs149916167
All − MR Egger
All − Inverse variance weighted
−2
−1
0
1
2
MR effect size for
'GCA−900066135 sp900066135' on 'GCST90199991_buildGRCh38.tsv'

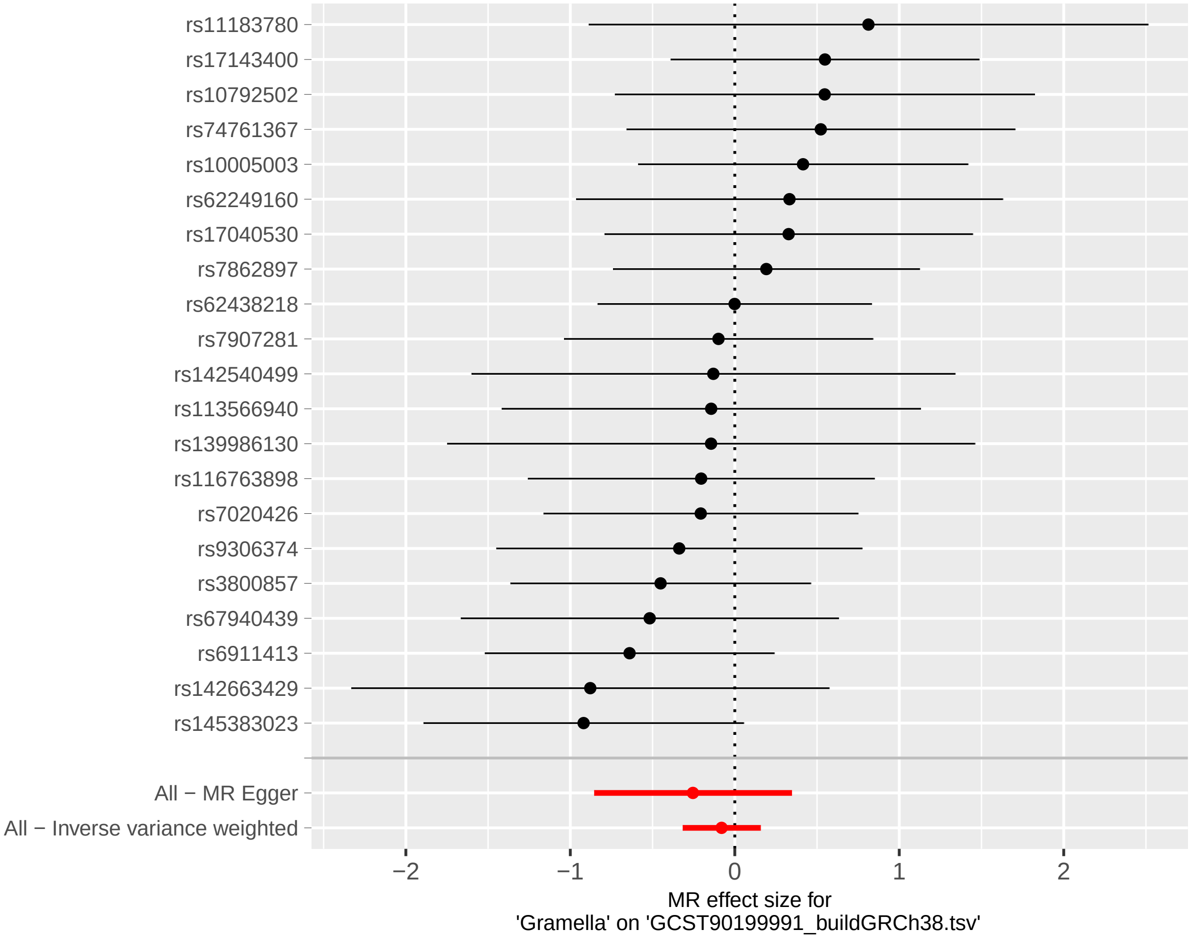
rs11183780
rs17143400
rs10792502
rs74761367
rs10005003
rs62249160
rs17040530
rs7862897
rs62438218
rs7907281
rs142540499
rs113566940
rs139986130
rs116763898
rs7020426
rs9306374
rs3800857
rs67940439
rs6911413
rs142663429
rs145383023
All − MR Egger
All − Inverse variance weighted
−2
−1
0
1
2
MR effect size for
'Gramella' on 'GCST90199991_buildGRCh38.tsv'

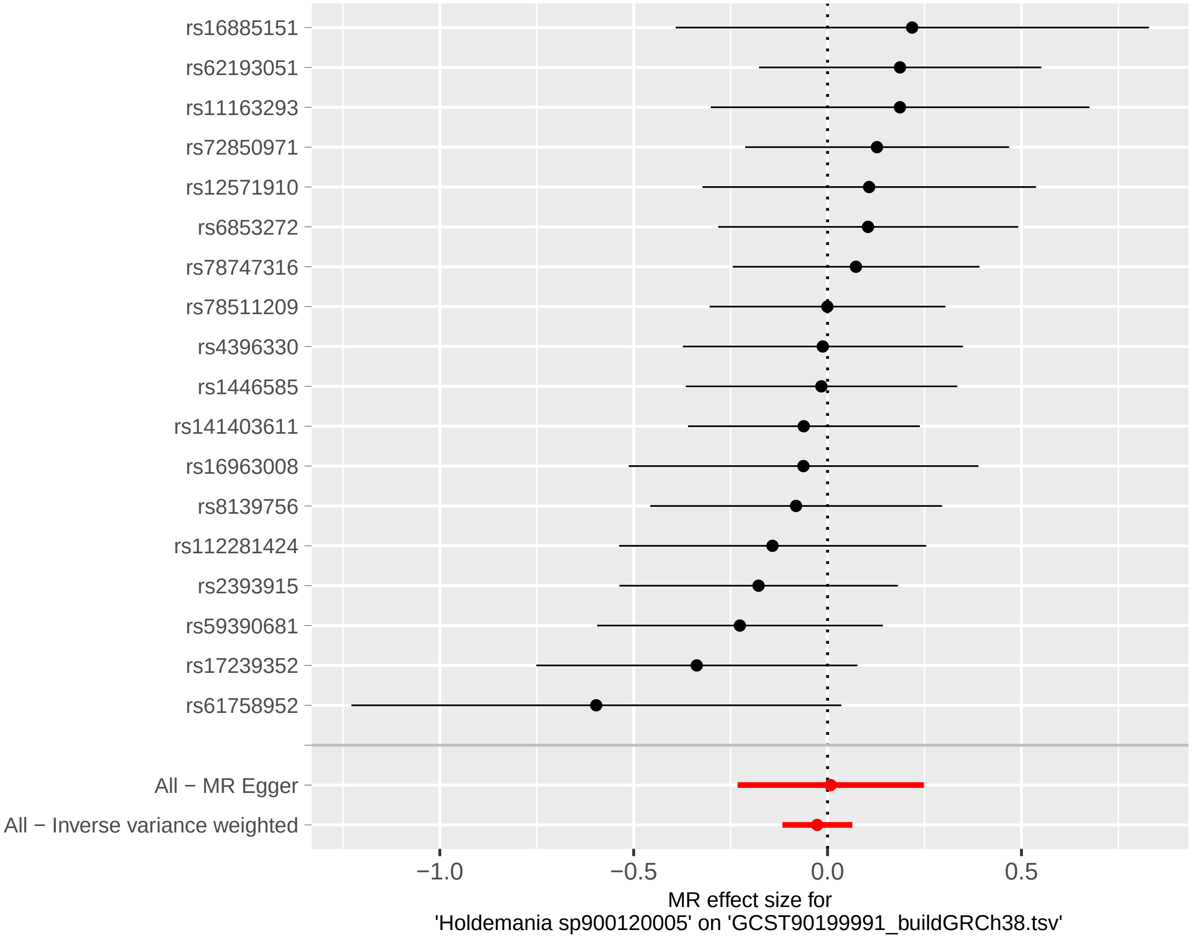

rs16885151
rs62193051
rs11163293
rs72850971
rs12571910
rs6853272
rs78747316
rs78511209
rs4396330
rs1446585
rs141403611
rs16963008
rs8139756
rs112281424
rs2393915
rs59390681
rs17239352
rs61758952
All − MR Egger
All − Inverse variance weighted
−1.0
−0.5
0.0
0.5
MR effect size for
'Holdemania sp900120005' on 'GCST90199991_buildGRCh38.tsv'

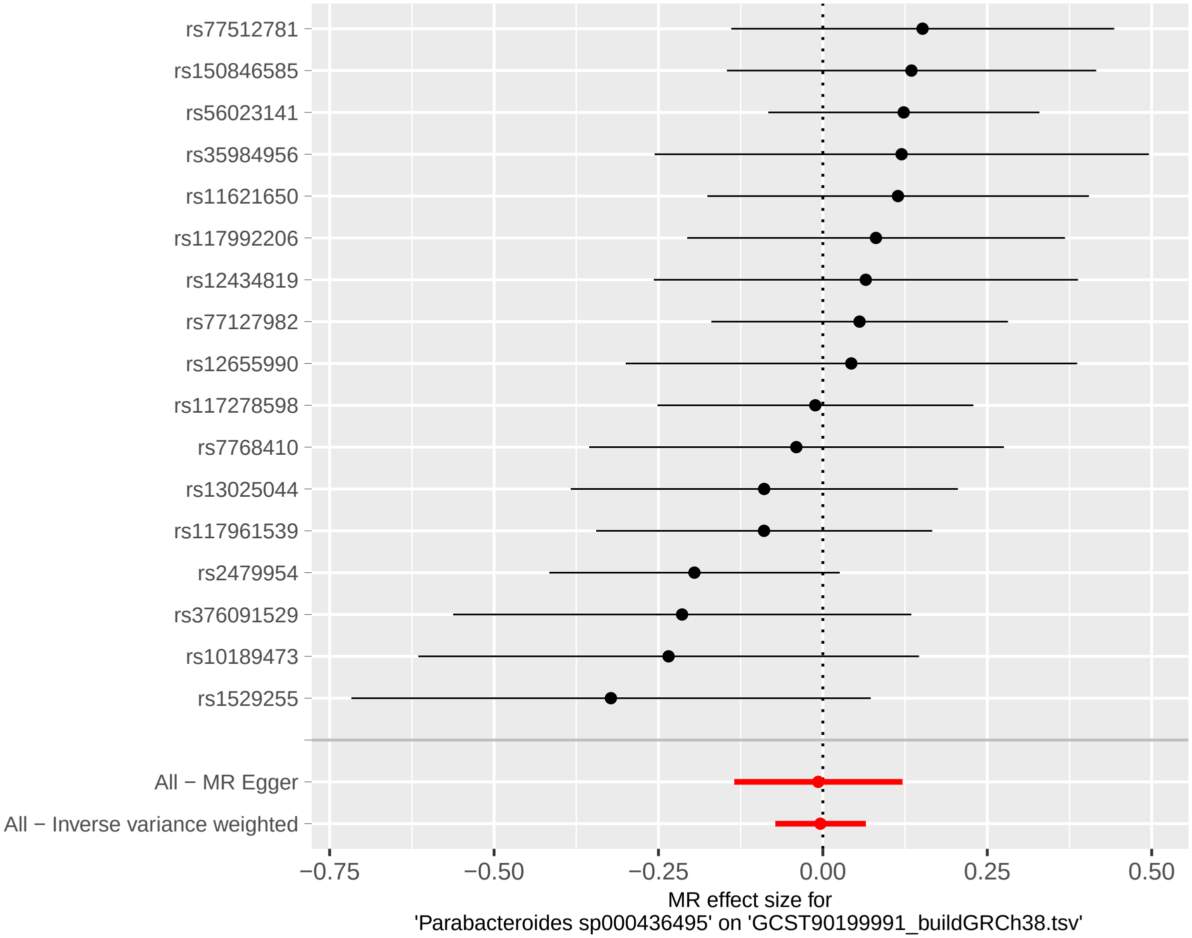

rs77512781
rs150846585
rs56023141
rs35984956
rs11621650
rs117992206
rs12434819
rs77127982
rs12655990
rs117278598
rs7768410
rs13025044
rs117961539
rs2479954
rs376091529
rs10189473
rs1529255
All − MR Egger
All − Inverse variance weighted
−0.75
−0.50
−0.25
0.00
0.25
0.50
MR effect size for
'Parabacteroides sp000436495' on 'GCST90199991_buildGRCh38.tsv'

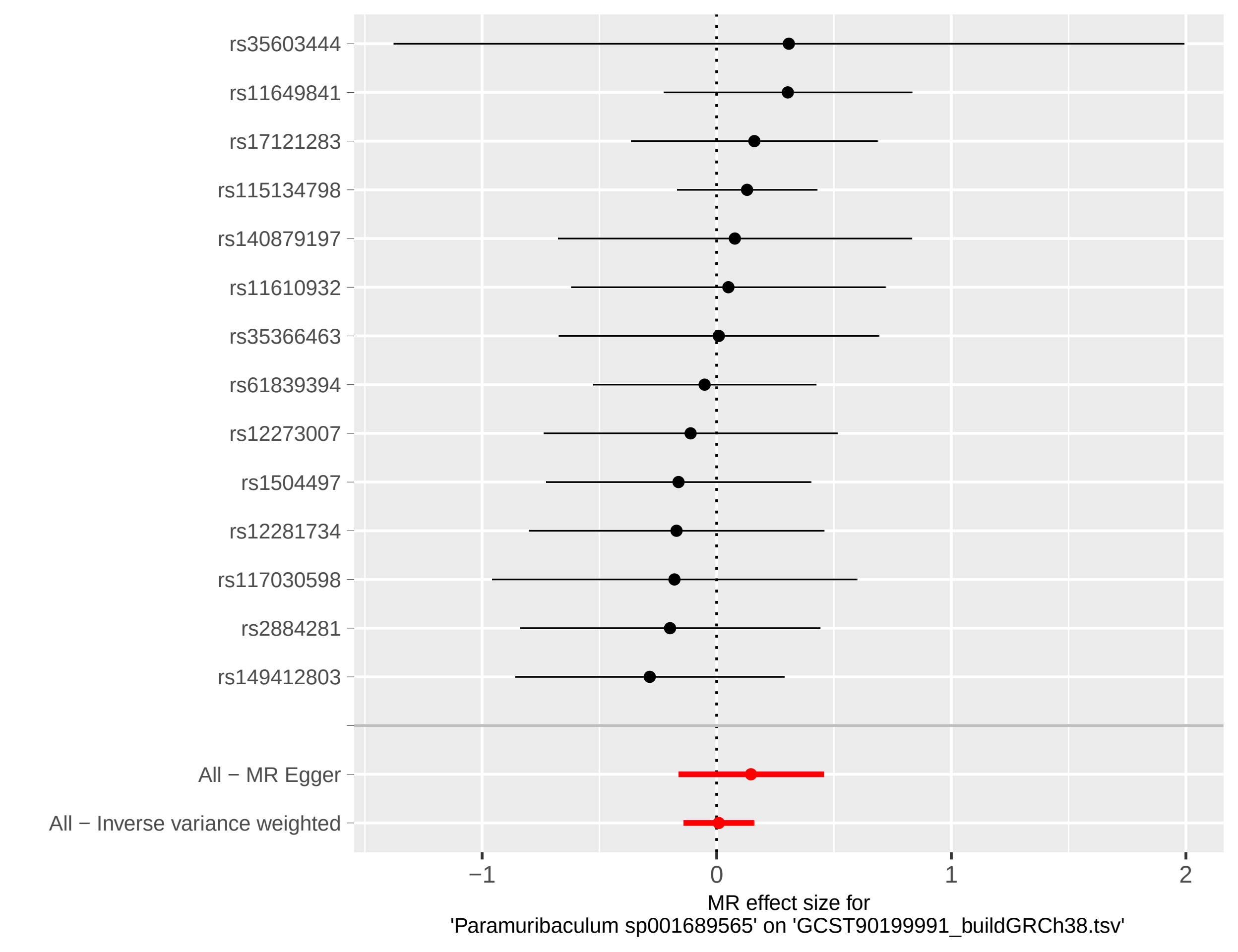
rs35603444
rs11649841
rs17121283
rs115134798
rs140879197
rs11610932
rs35366463
rs61839394
rs12273007
rs1504497
rs12281734
rs117030598
rs2884281
rs149412803
All − MR Egger
All − Inverse variance weighted
−1
0
1
2
MR effect size for
'Paramuribaculum sp001689565' on 'GCST90199991_buildGRCh38.tsv'

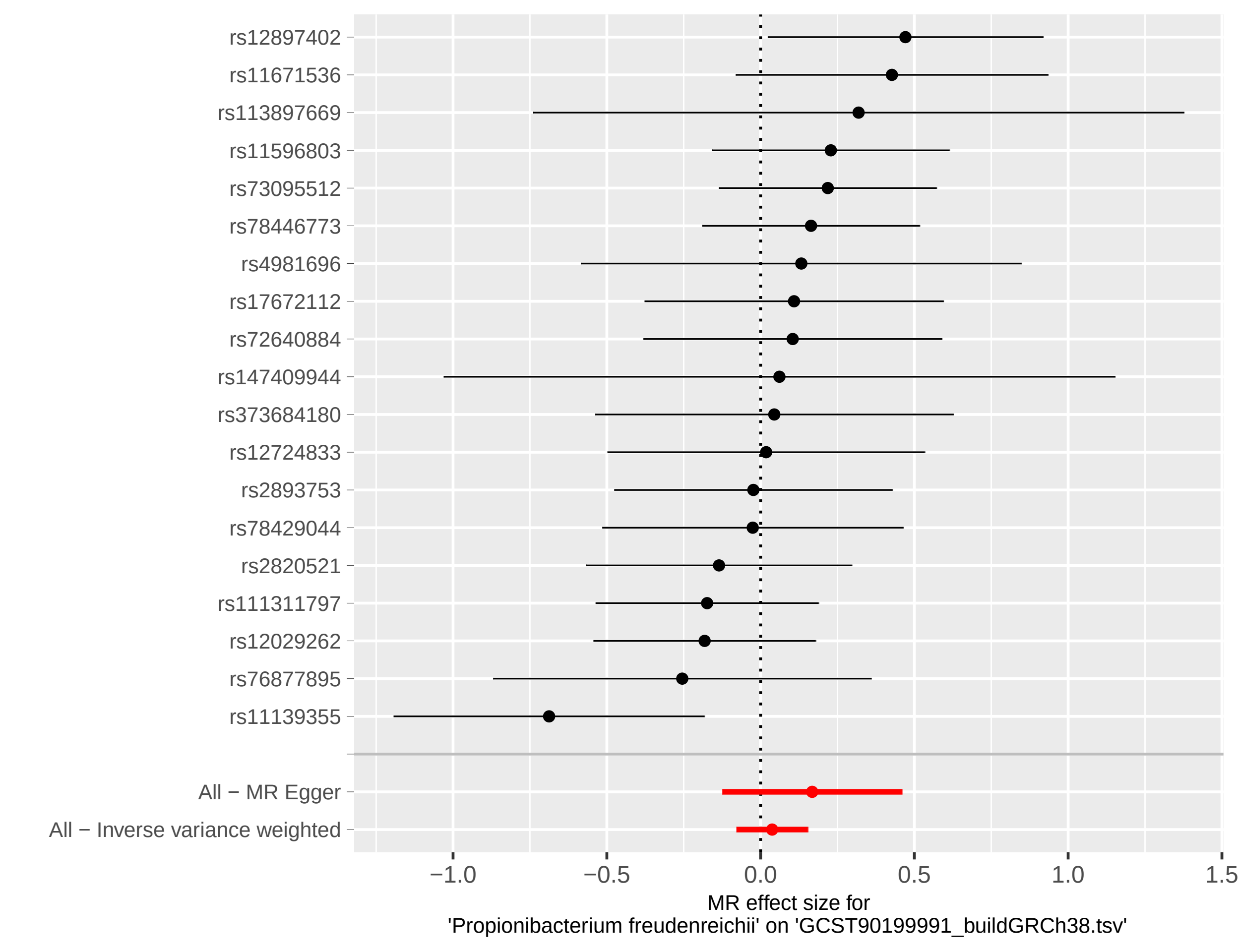
rs12897402
rs11671536
rs113897669
rs11596803
rs73095512
rs78446773
rs4981696
rs17672112
rs72640884
rs147409944
rs373684180
rs12724833
rs2893753
rs78429044
rs2820521
rs111311797
rs12029262
rs76877895
rs11139355
All − MR Egger
All − Inverse variance weighted
−1.0
−0.5
0.0
0.5
1.0
1.5
MR effect size for
'Propionibacterium freudenreichii' on 'GCST90199991_buildGRCh38.tsv'

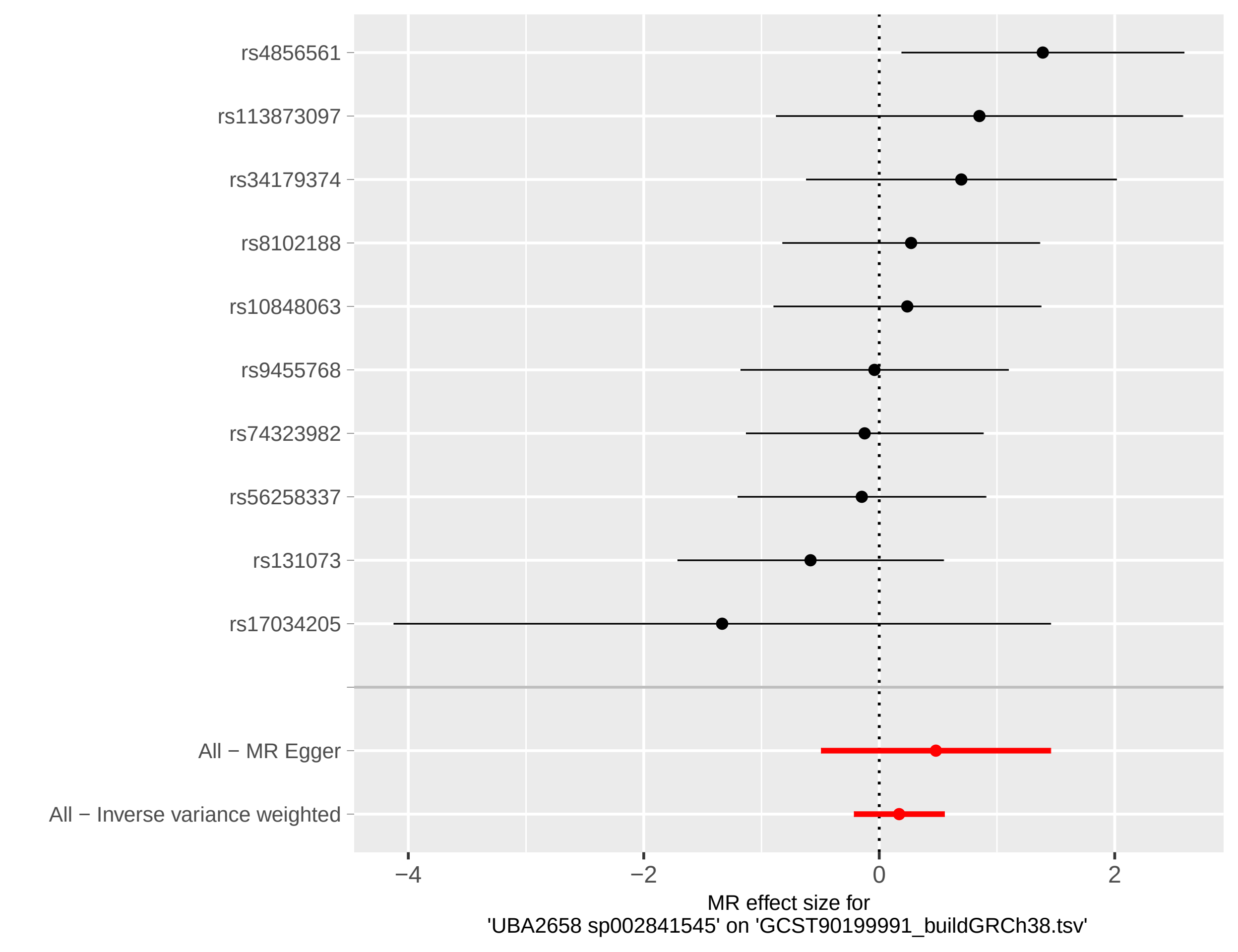

rs4856561
rs113873097
rs34179374
rs8102188
rs10848063
rs9455768
rs74323982
rs56258337
rs131073
rs17034205
All − MR Egger
All − Inverse variance weighted
−4
−2
0
2
MR effect size for
'UBA2658 sp002841545' on 'GCST90199991_buildGRCh38.tsv'

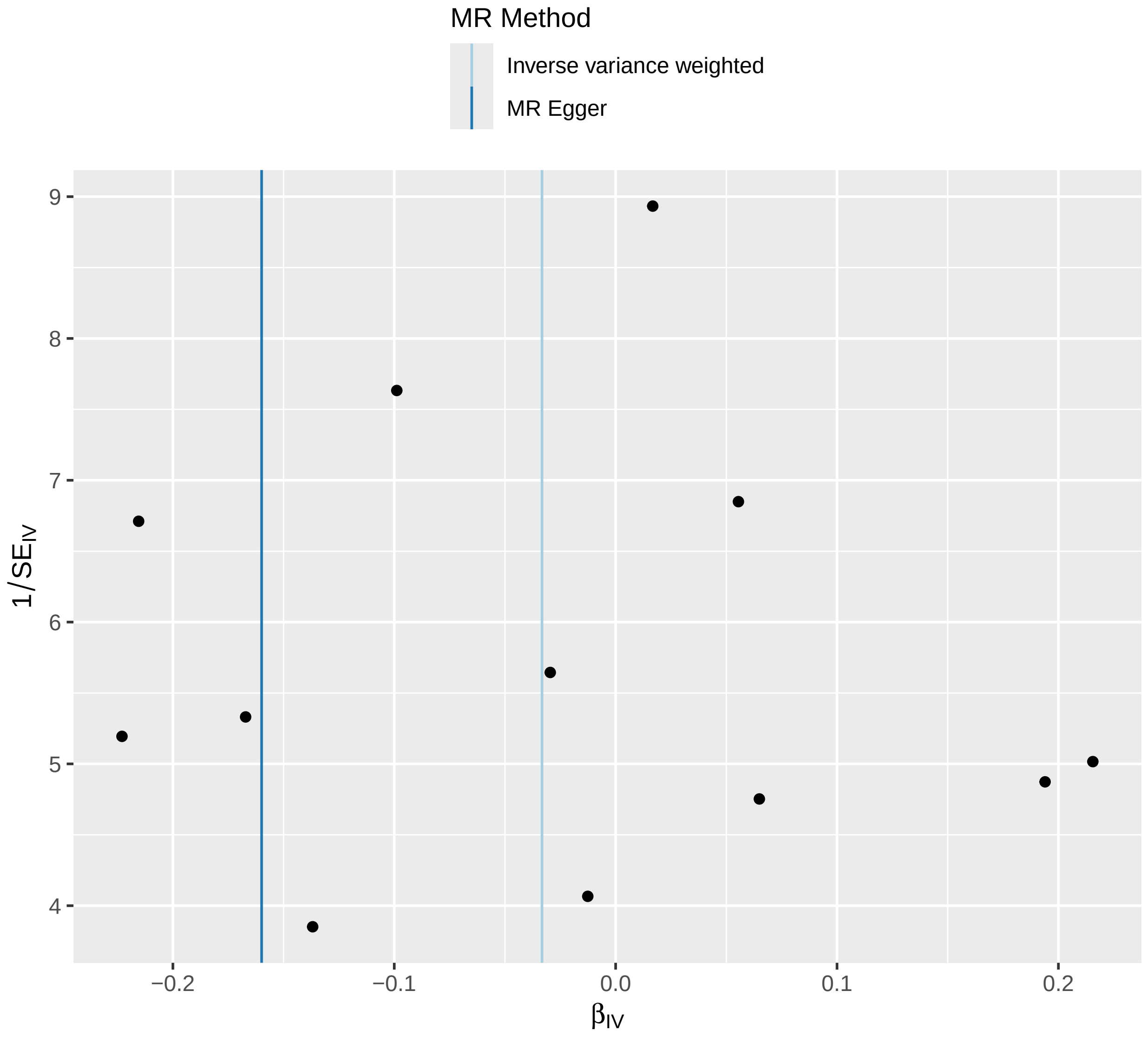
MR Method
Inverse variance weighted
MR Egger
9
8
7
6
5
4
1/SE_IV
−0.2
−0.1
0.0
0.1
0.2
β_IV

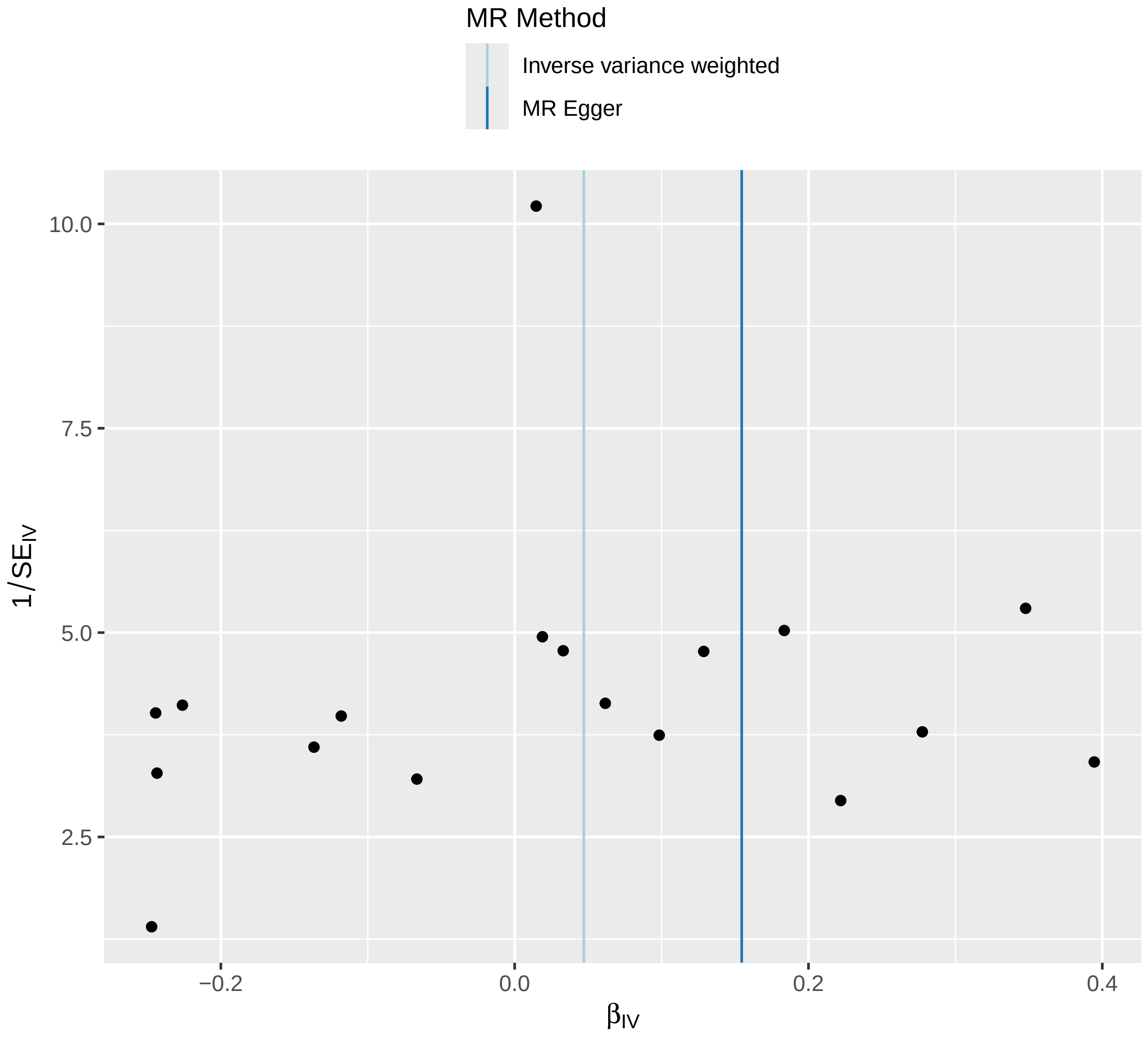
MR Method
Inverse variance weighted
MR Egger
$1/SE_{IV}$
10.0
7.5
5.0
2.5
−0.2
0.0
0.2
0.4
β_{IV}

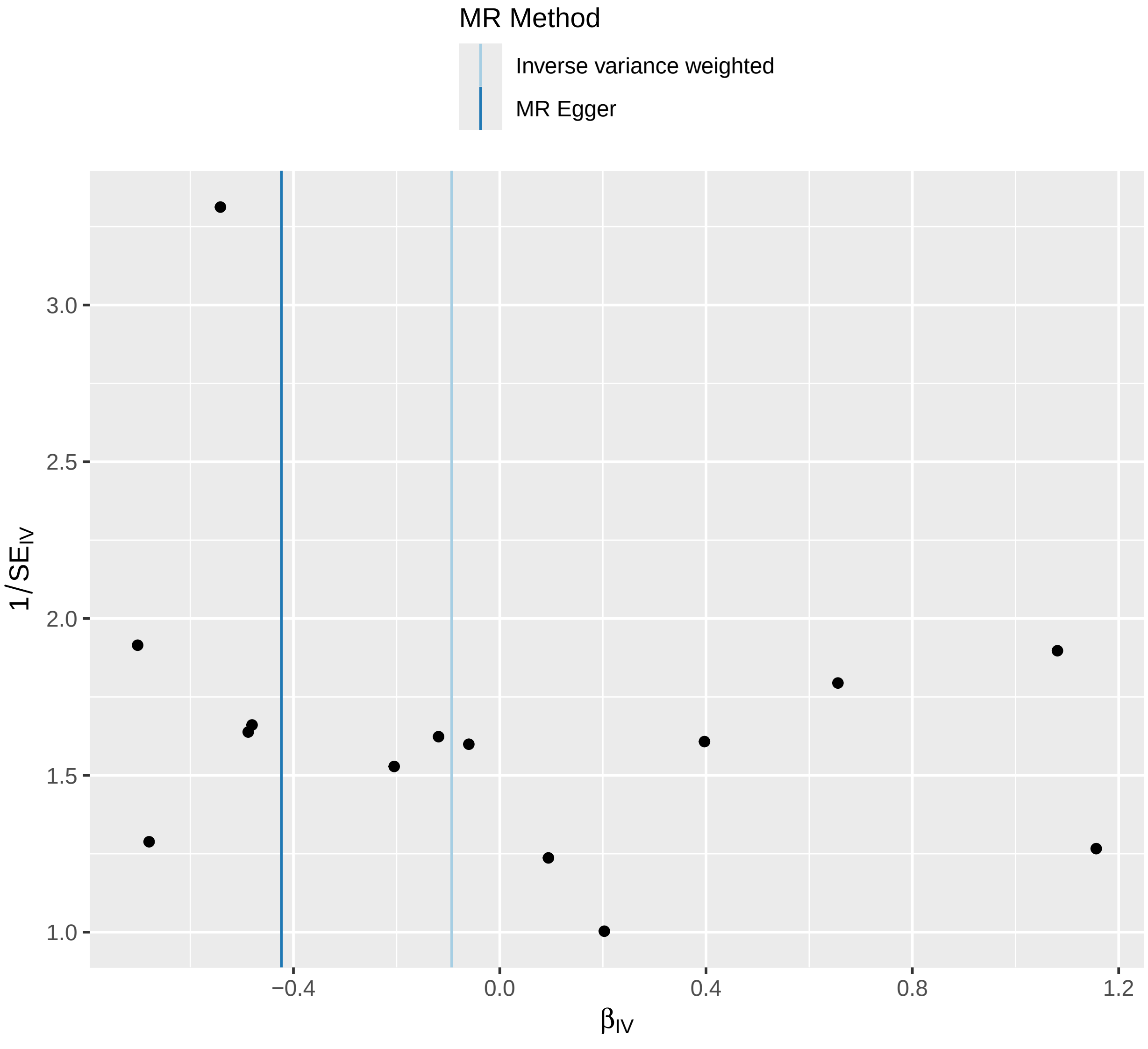

MR Method
Inverse variance weighted
MR Egger
3.0
2.5
2.0
1.5
1.0
1/SE_IV
−0.4
0.0
0.4
0.8
1.2
β_IV

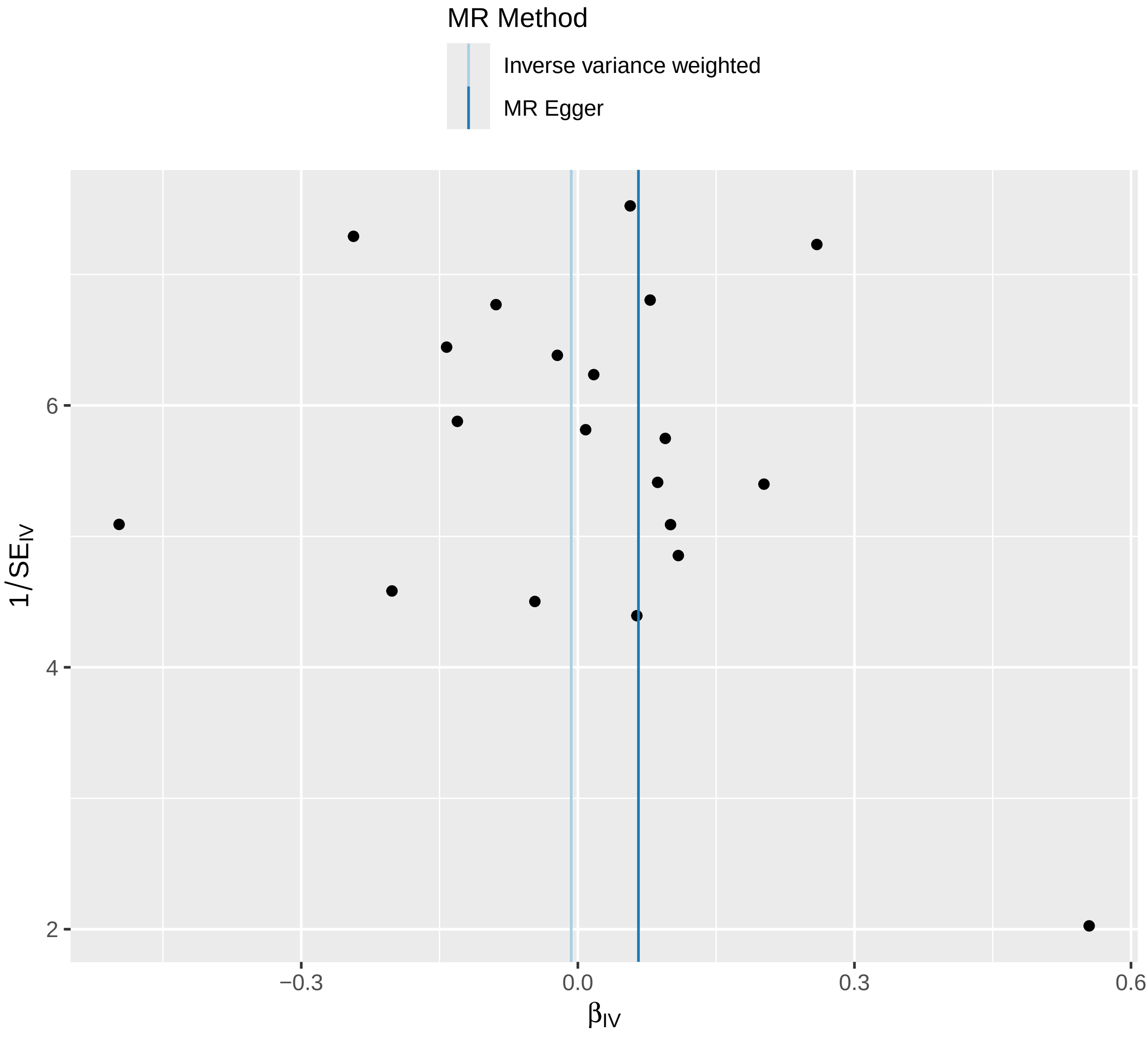
MR Method
Inverse variance weighted
MR Egger
1/SE_IV
6
4
2
−0.3
0.0
0.3
0.6
β_IV

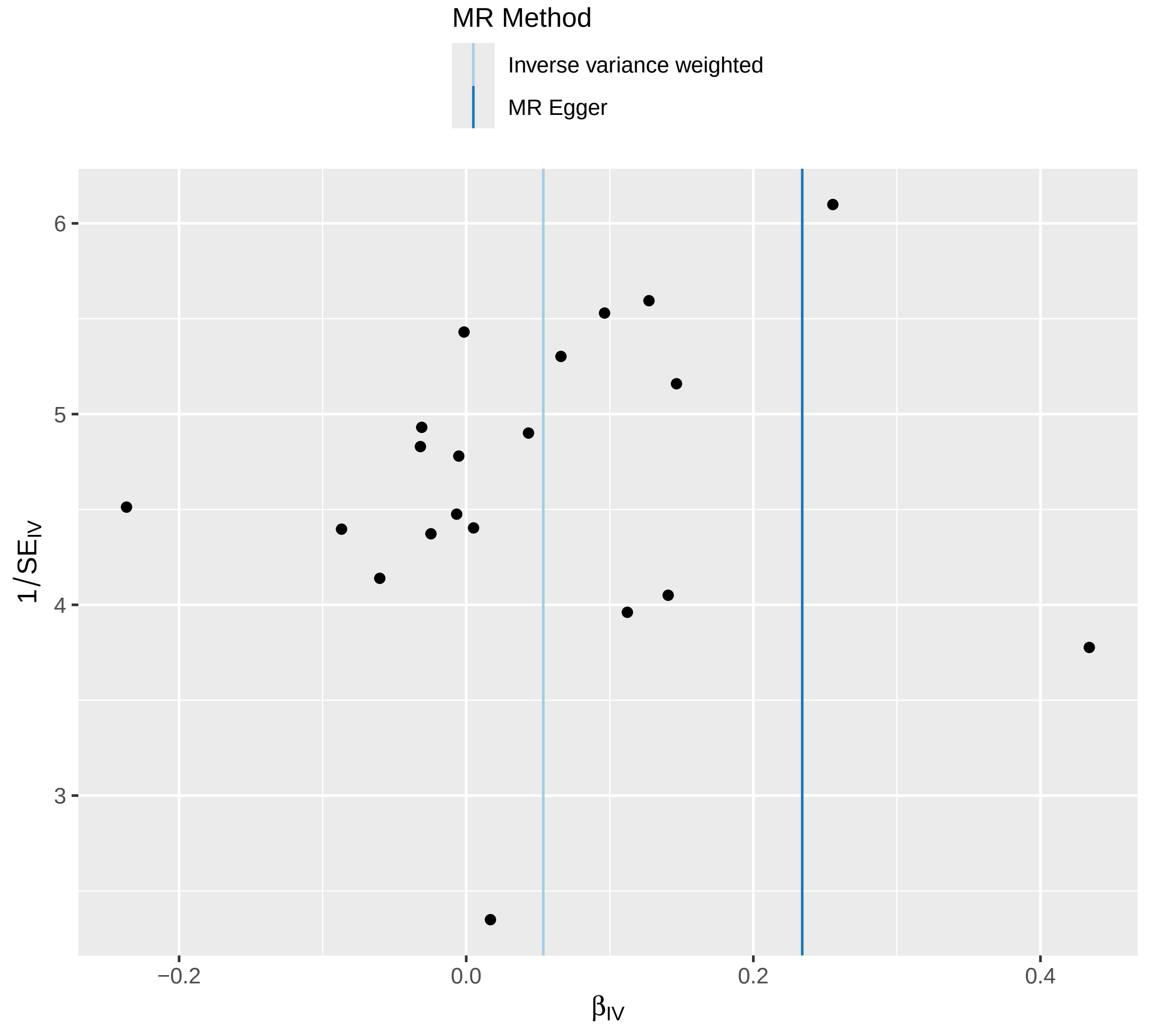
MR Method
Inverse variance weighted
MR Egger
6
5
4
3
−0.2
0.0
0.2
0.4
$1/SE_{IV}$
β_{IV}

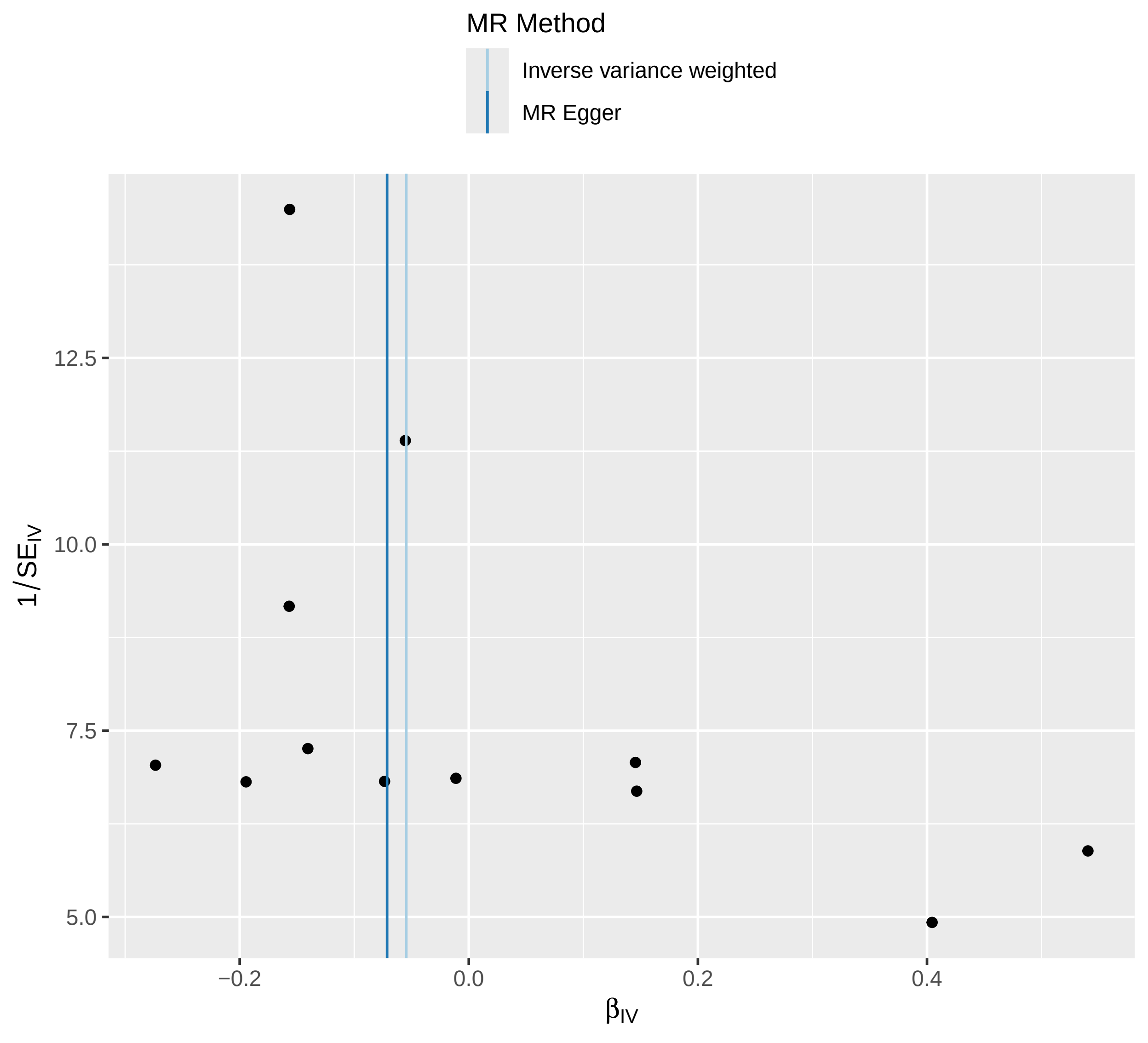
MR Method
Inverse variance weighted
MR Egger
12.5
10.0
7.5
5.0
$1/SE_{IV}$
−0.2
0.0
0.2
0.4
β_{IV}

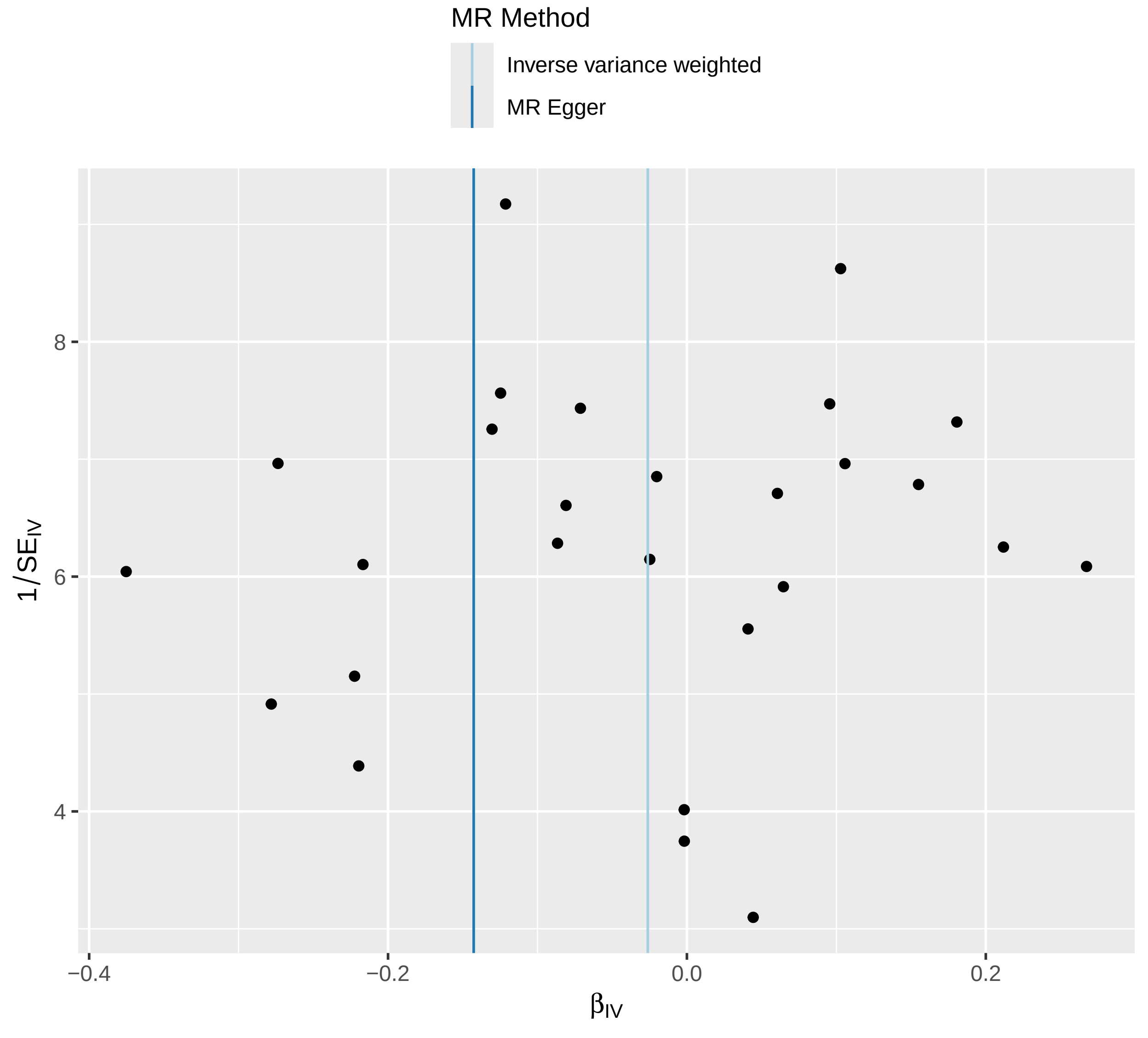
MR Method
Inverse variance weighted
MR Egger
8
6
4
1/SE$_{IV}$
−0.4
−0.2
0.0
0.2
β$_{IV}$

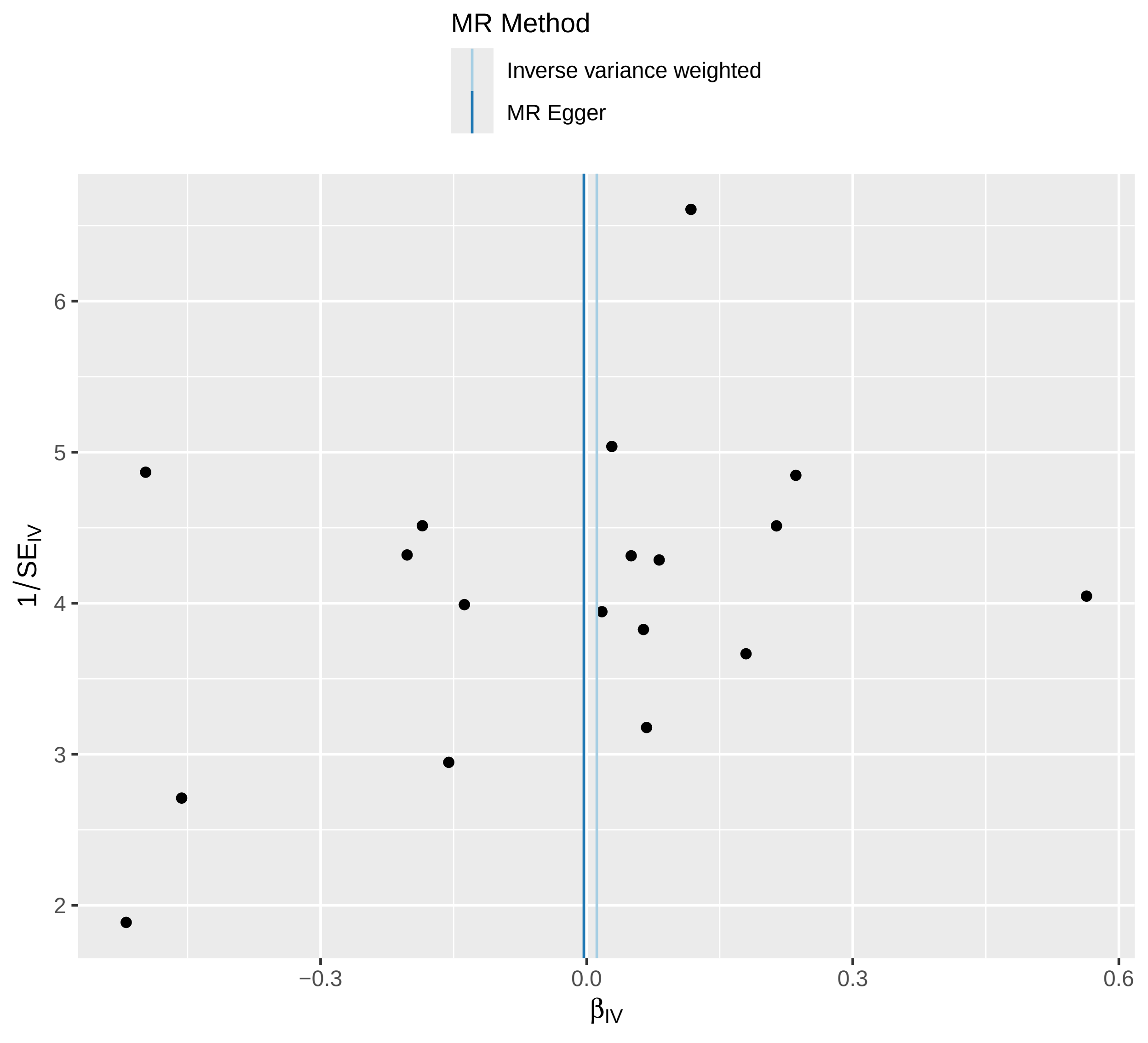
MR Method
Inverse variance weighted
MR Egger
6
5
4
3
2
1/SE_IV
−0.3
0.0
0.3
0.6
β_IV

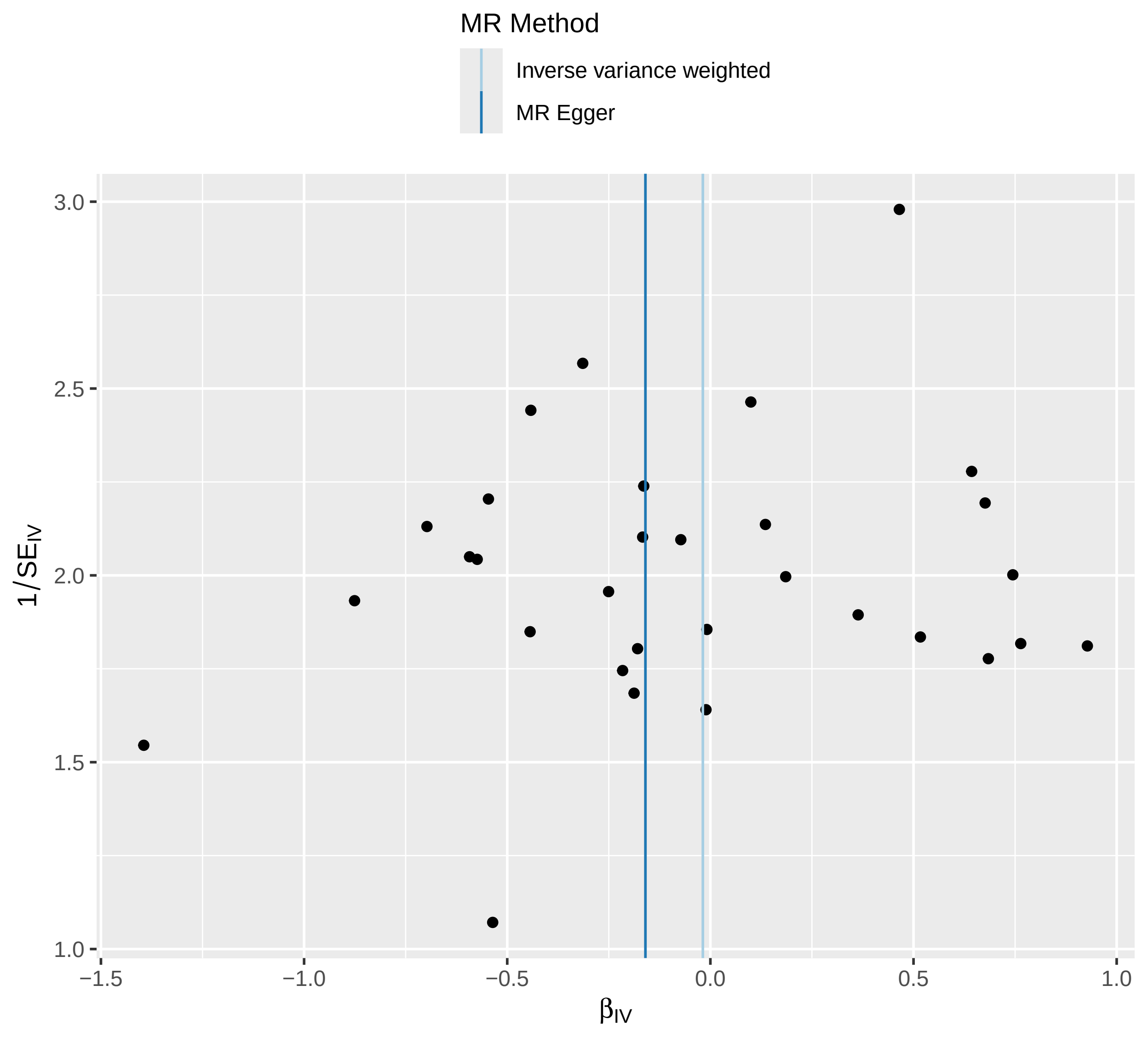
MR Method
Inverse variance weighted
MR Egger
$1/SE_{IV}$
3.0
2.5
2.0
1.5
1.0
−1.5
−1.0
−0.5
0.0
0.5
1.0
β_{IV}

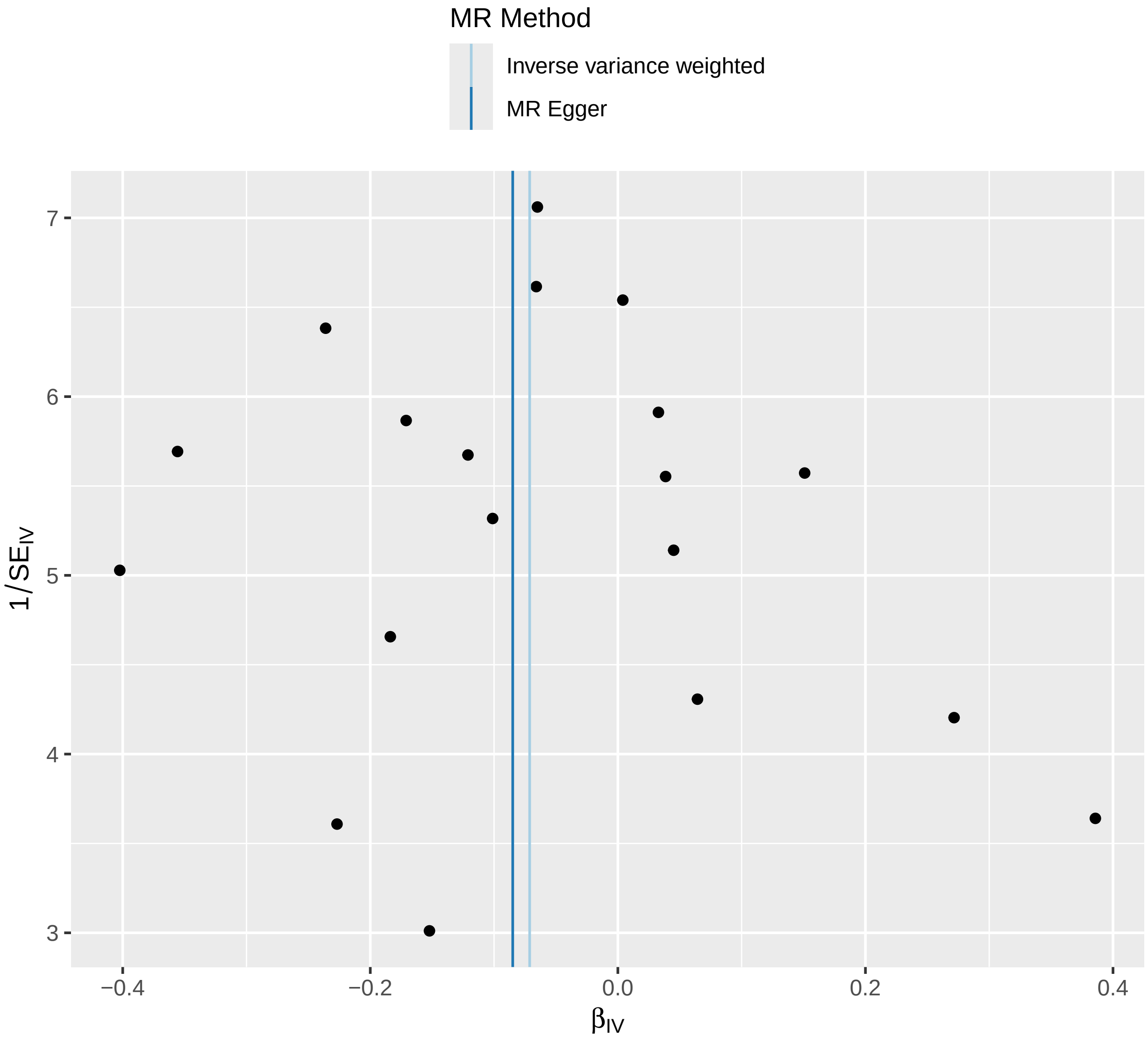
MR Method
Inverse variance weighted
MR Egger
$1/SE_{IV}$
7
6
5
4
3
−0.4
−0.2
0.0
0.2
0.4
β_{IV}

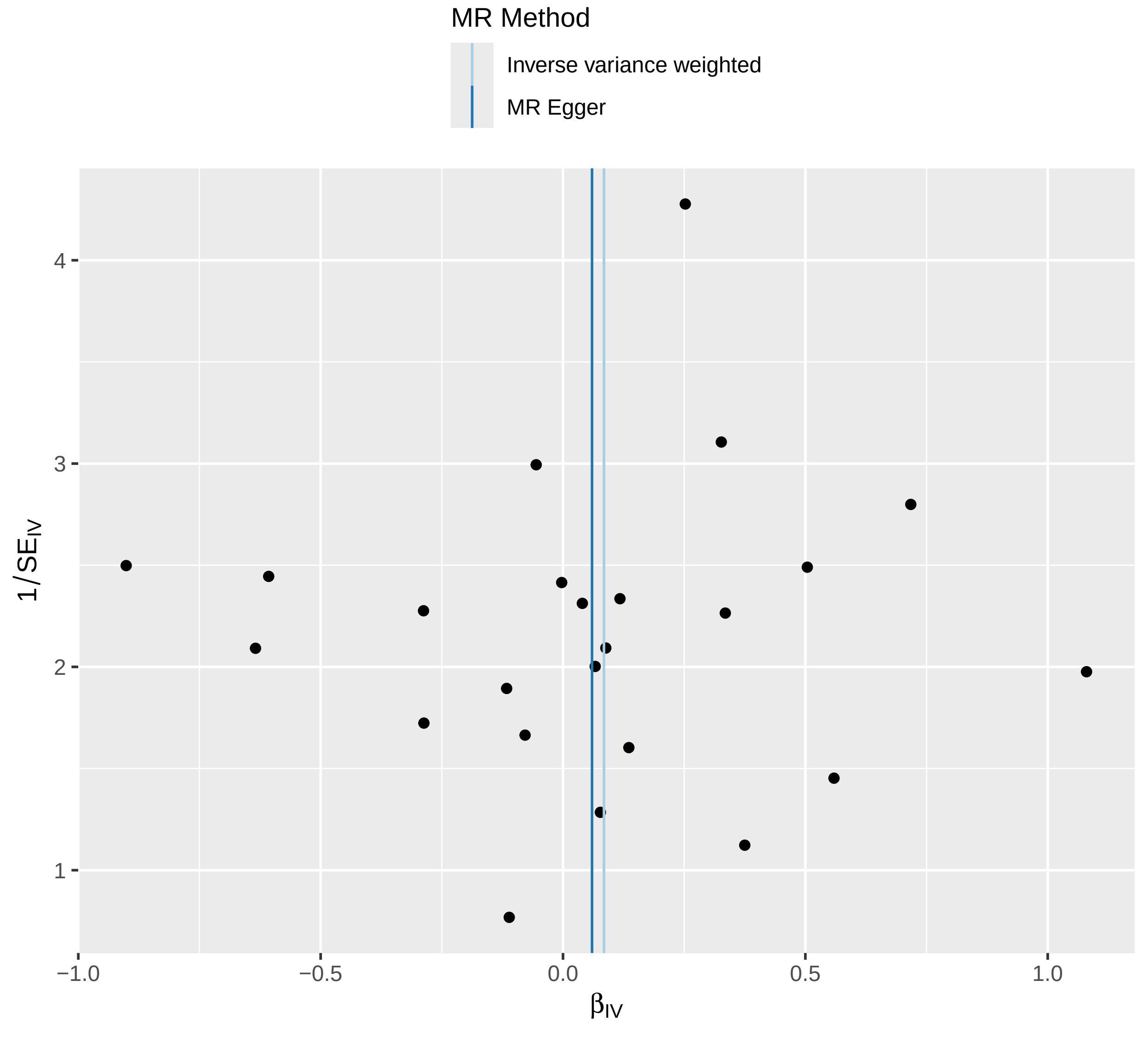
MR Method
Inverse variance weighted
MR Egger
1/SE_IV
4
3
2
1
−1.0
−0.5
0.0
0.5
1.0
β_IV

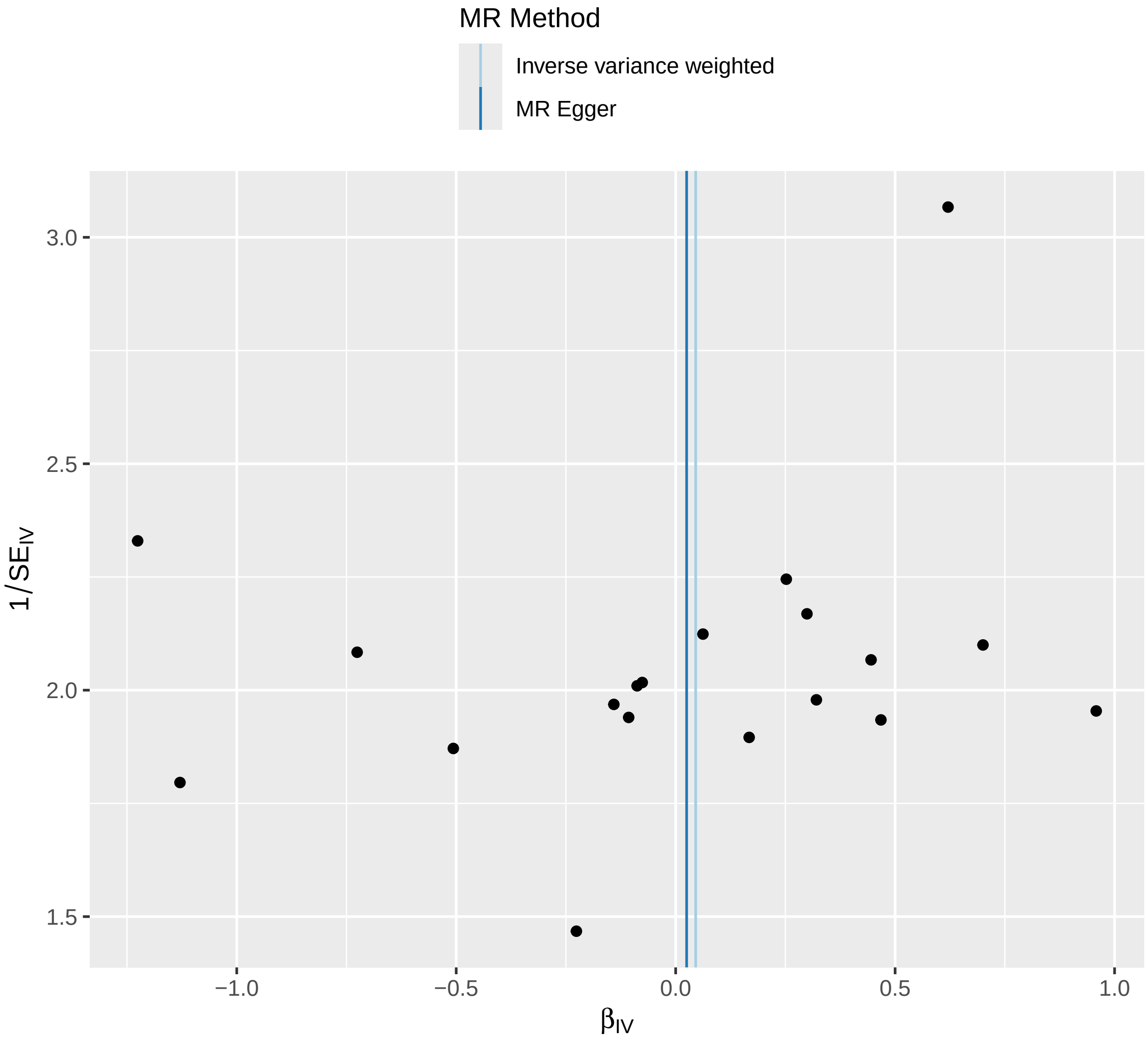
MR Method
Inverse variance weighted
MR Egger
3.0
2.5
2.0
1.5
1/SE_IV
−1.0
−0.5
0.0
0.5
1.0
β_IV

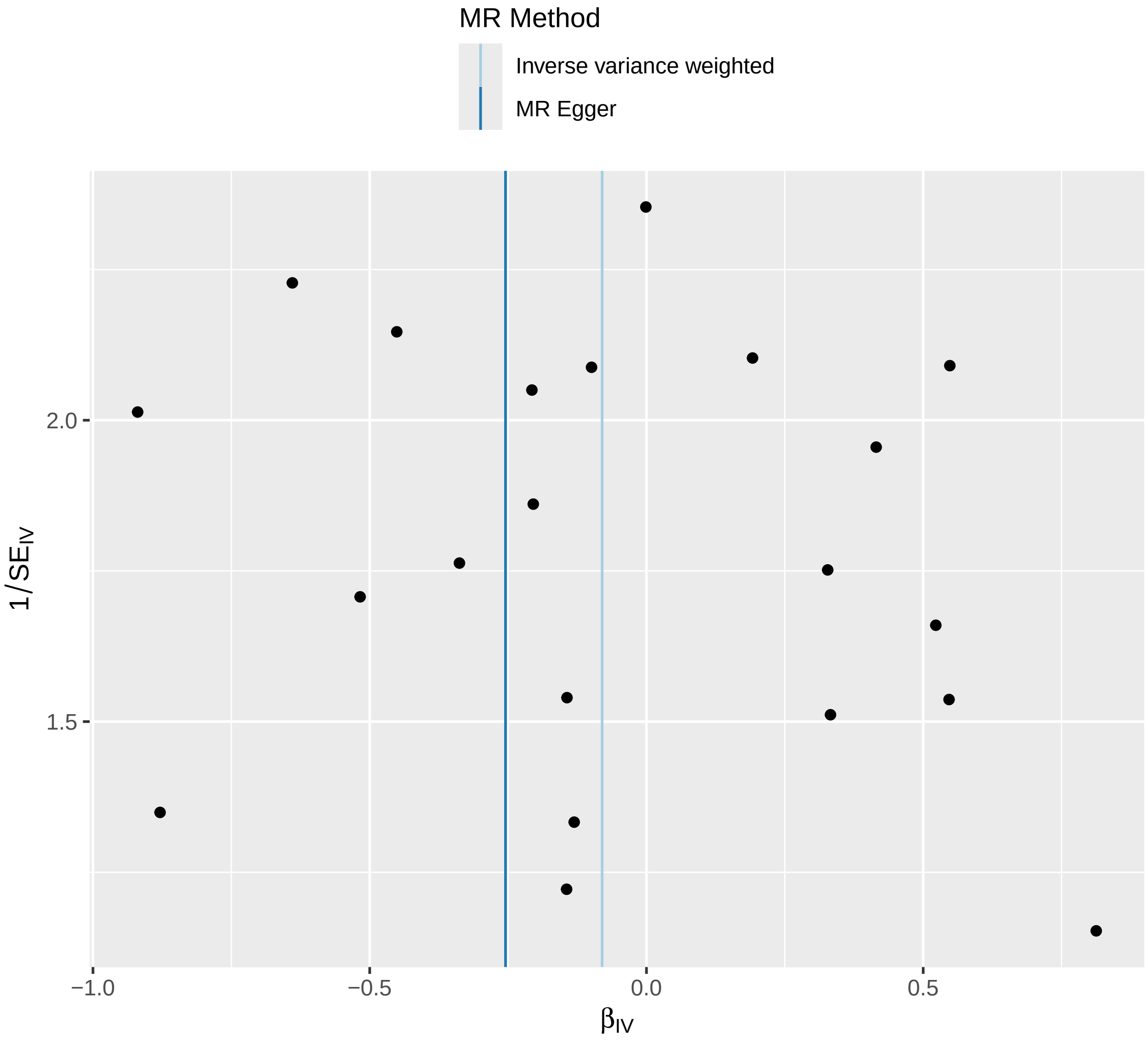

MR Method
Inverse variance weighted
MR Egger
2.0
1.5
1/SE$_{IV}$
−1.0
−0.5
0.0
0.5
β$_{IV}$

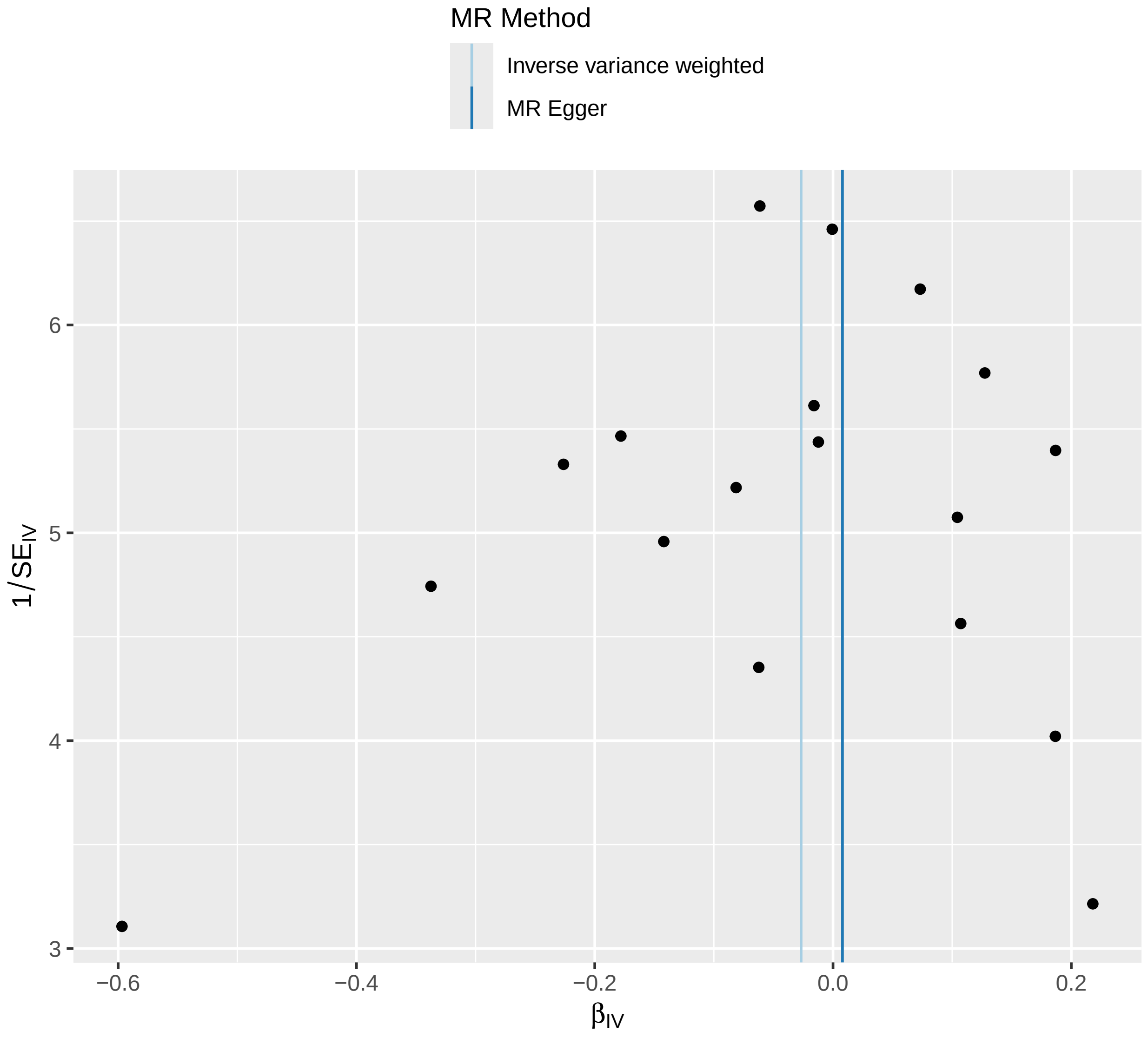

MR Method
Inverse variance weighted
MR Egger
6
5
4
3
1/SE$_{IV}$
−0.6
−0.4
−0.2
0.0
0.2
β_{IV}

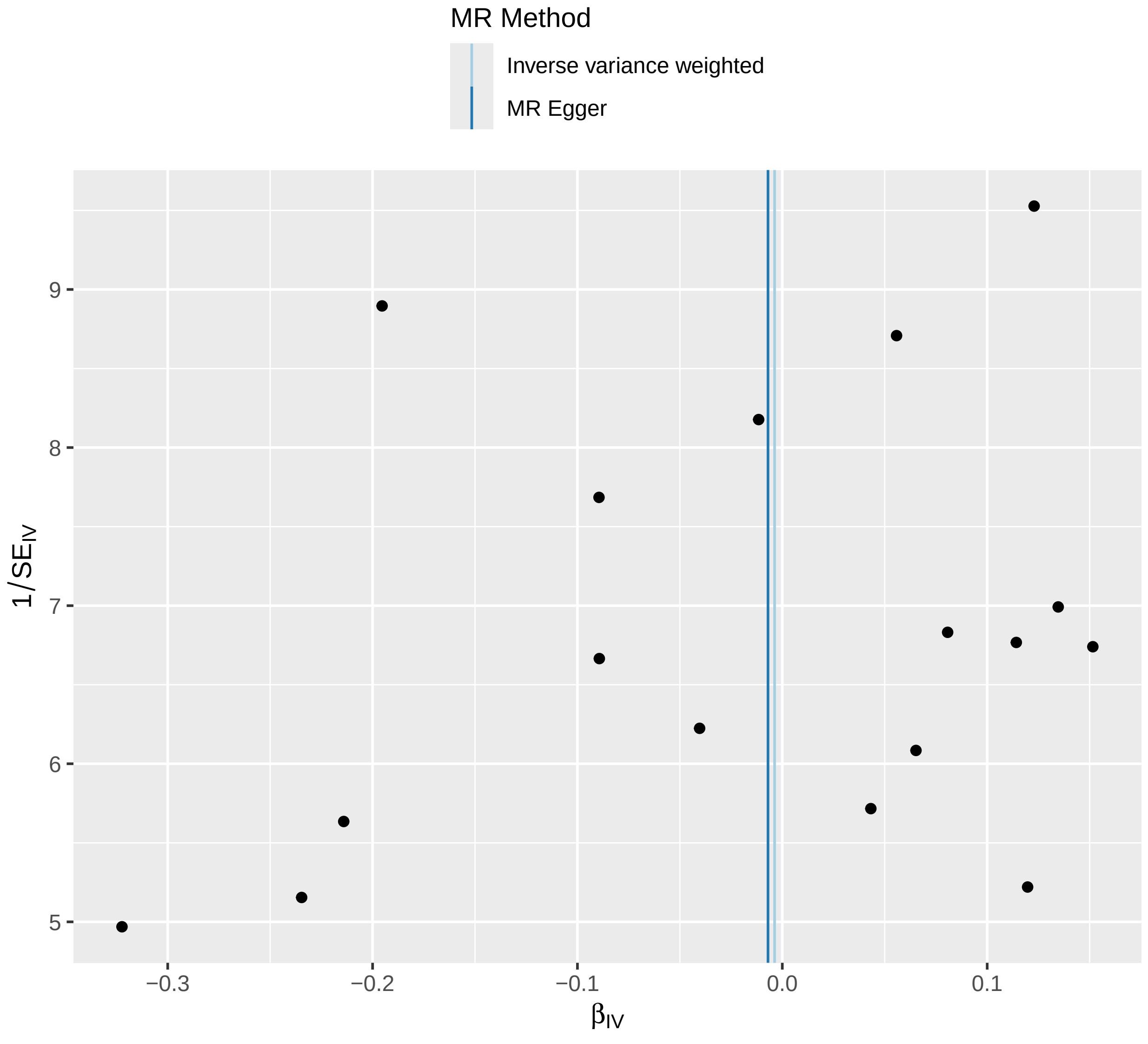
MR Method
Inverse variance weighted
MR Egger
1/SE_IV
β_IV
9
8
7
6
5
−0.3
−0.2
−0.1
0.0
0.1

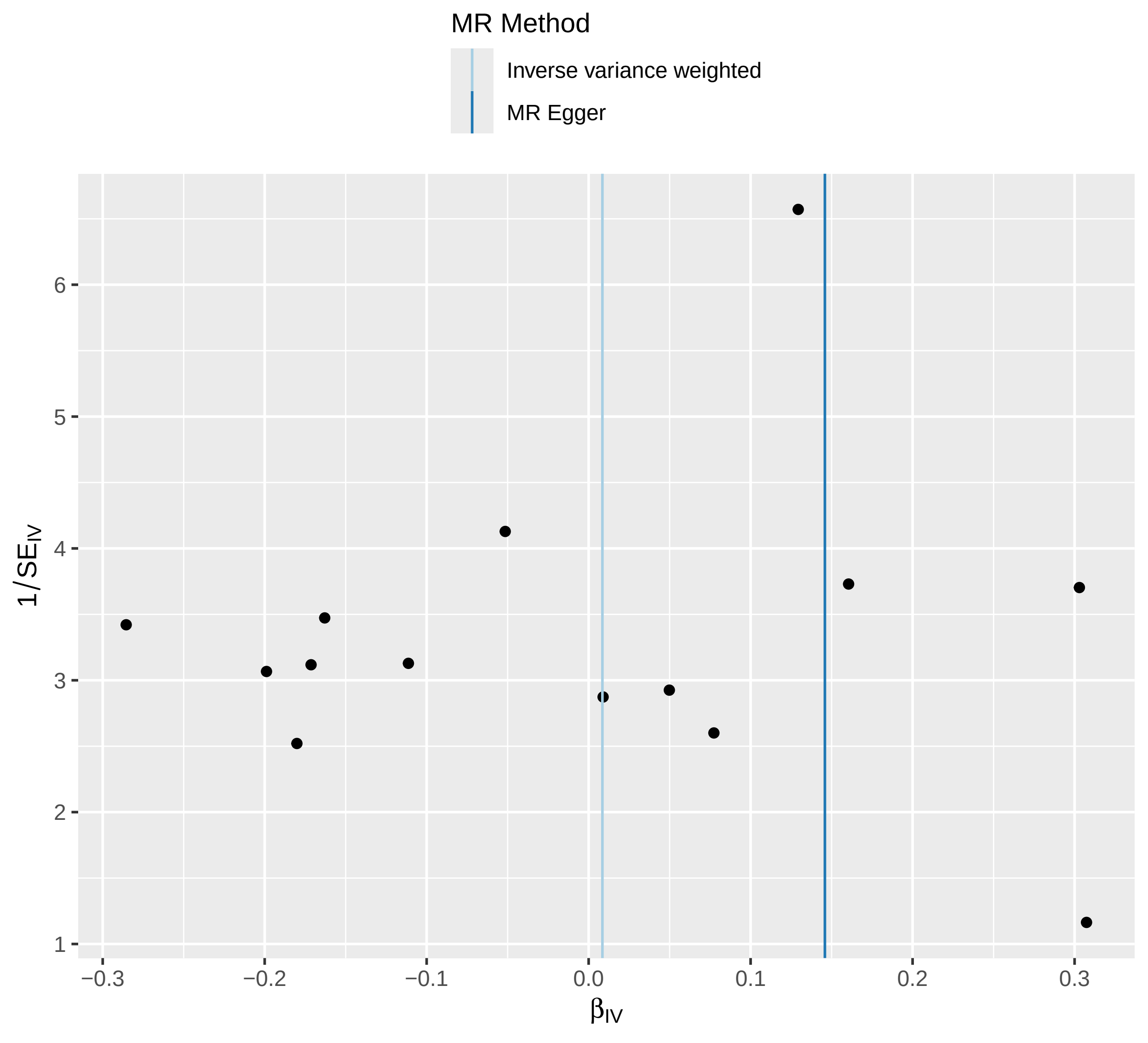

MR Method
Inverse variance weighted
MR Egger
6
5
4
3
2
1
1/SE_IV
−0.3
−0.2
−0.1
0.0
0.1
0.2
0.3
β_IV

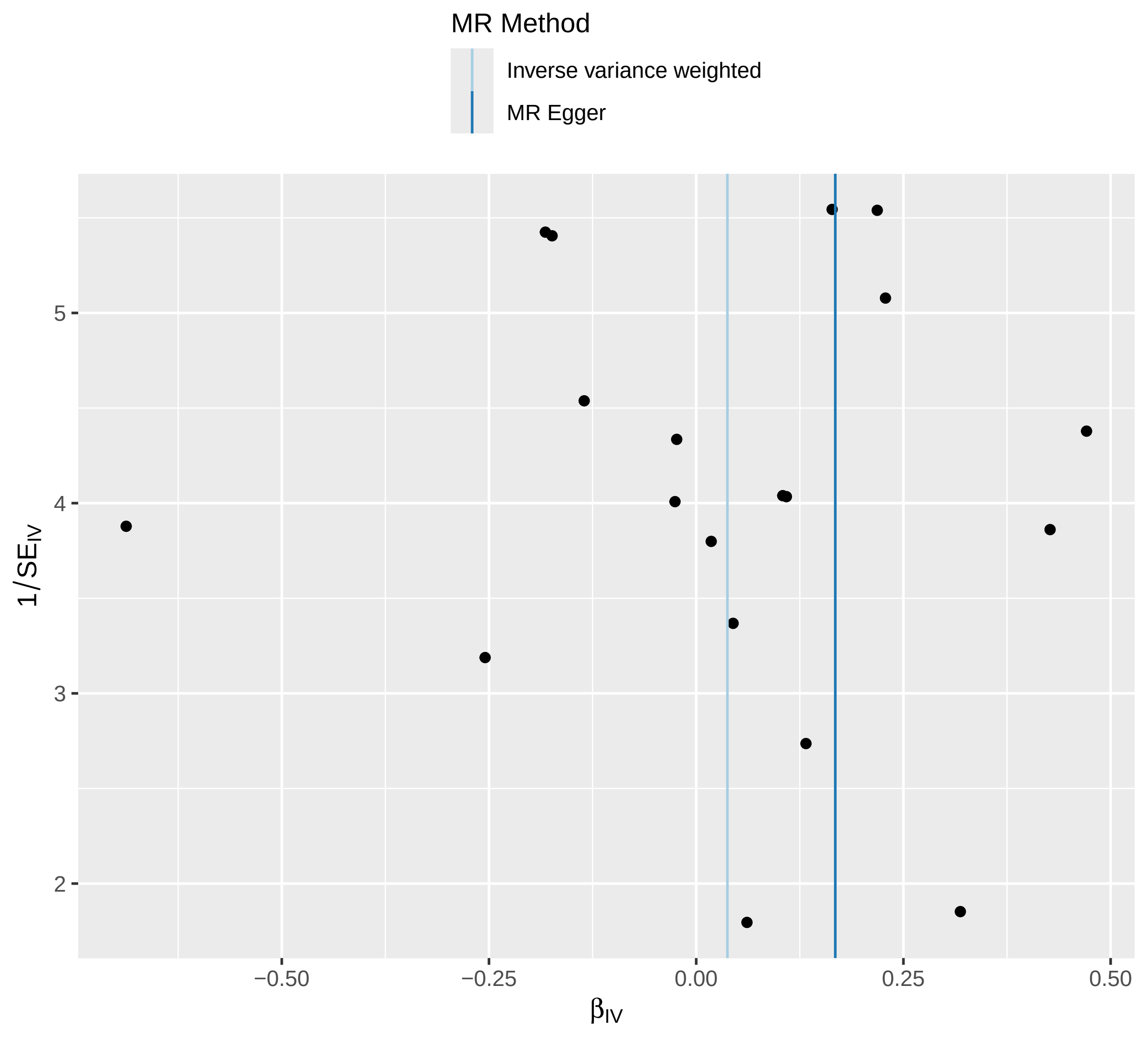
MR Method
Inverse variance weighted
MR Egger
1/SE_IV
β_IV
−0.50
−0.25
0.00
0.25
0.50
2
3
4
5

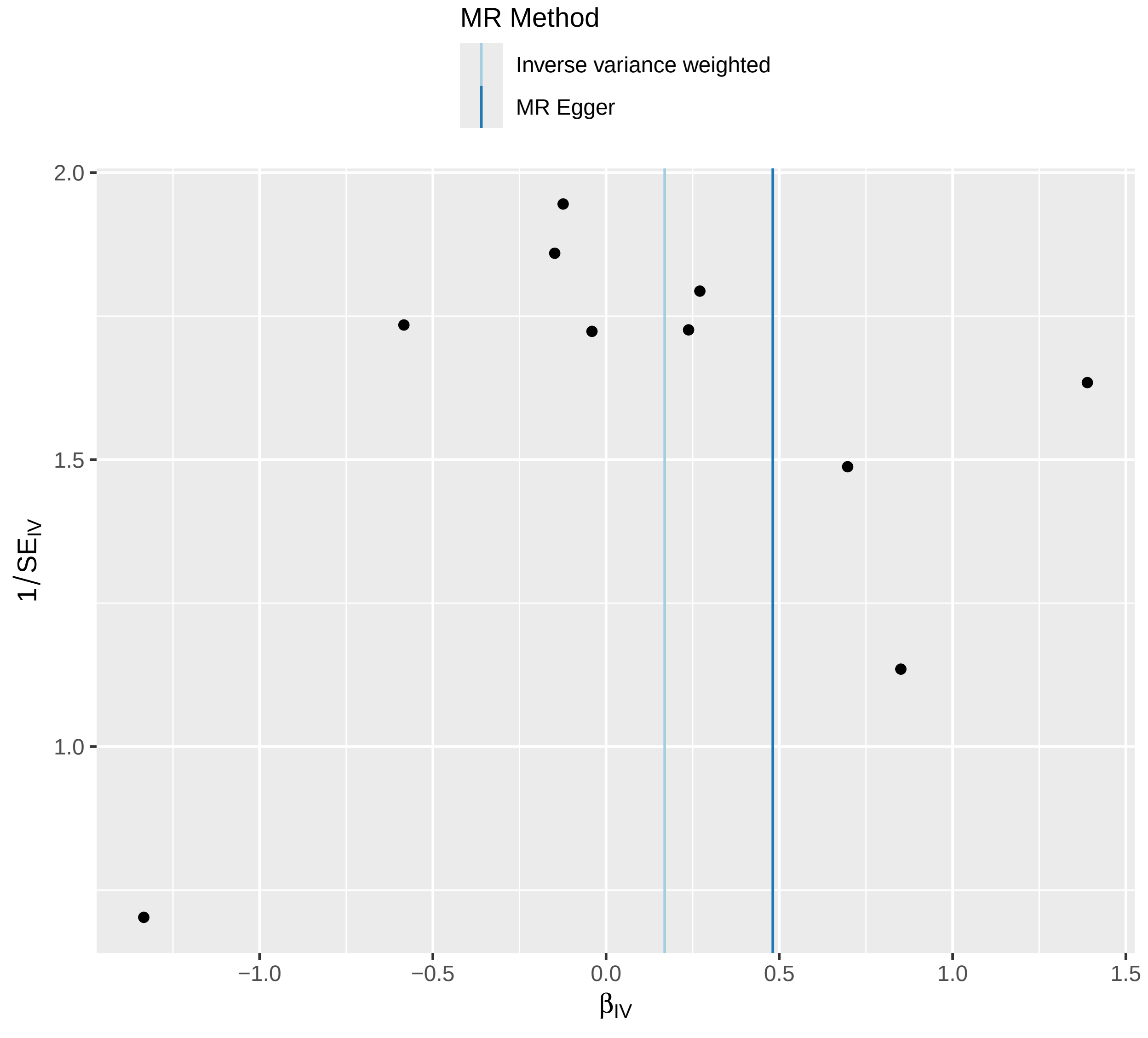
MR Method
Inverse variance weighted
MR Egger
2.0
1.5
1.0
1/SE_IV
−1.0
−0.5
0.0
0.5
1.0
1.5
β_IV

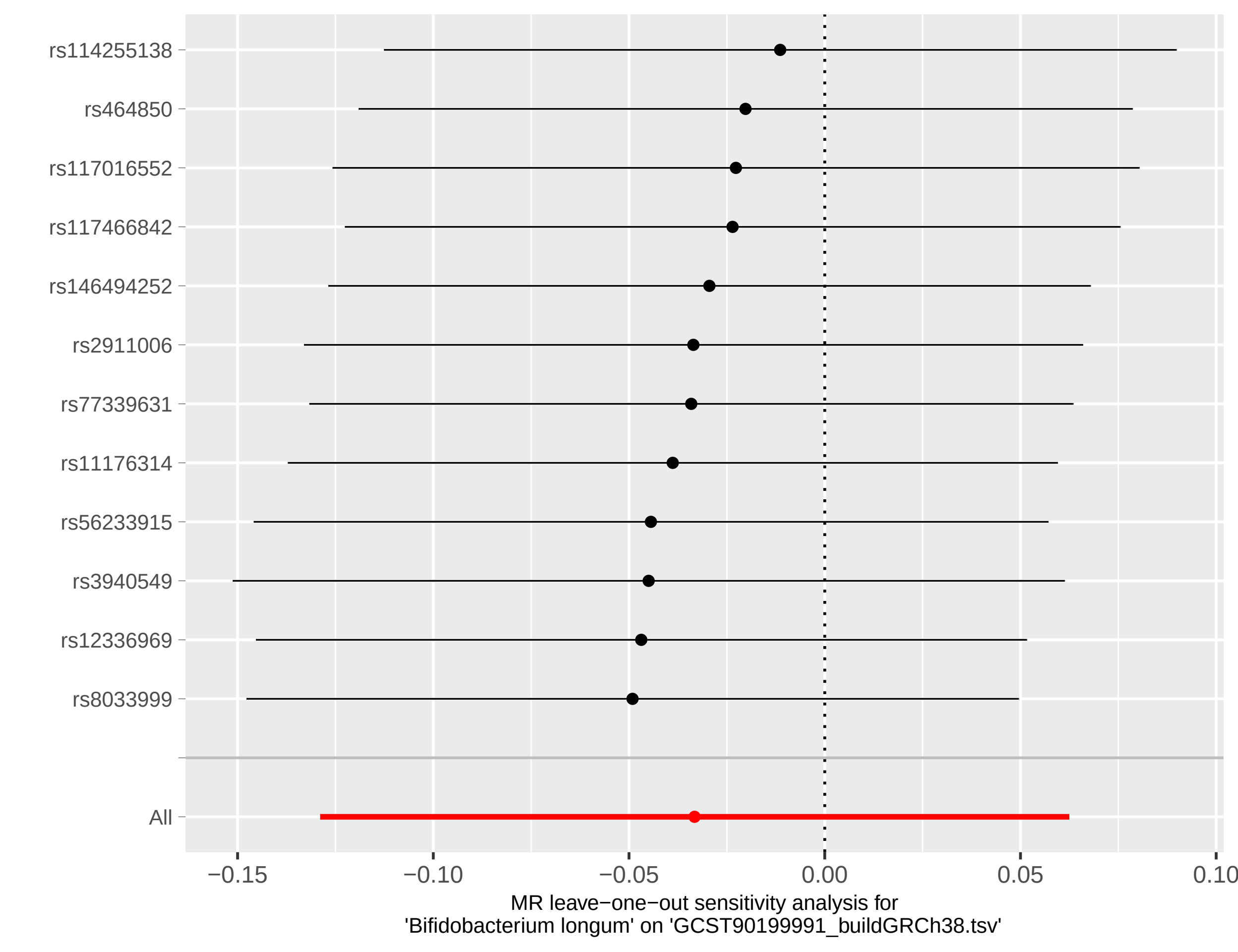

rs114255138
rs464850
rs117016552
rs117466842
rs146494252
rs2911006
rs77339631
rs11176314
rs56233915
rs3940549
rs12336969
rs8033999
All
−0.15
−0.10
−0.05
0.00
0.05
0.10
MR leave−one−out sensitivity analysis for
'Bifidobacterium longum' on 'GCST90199991_buildGRCh38.tsv'

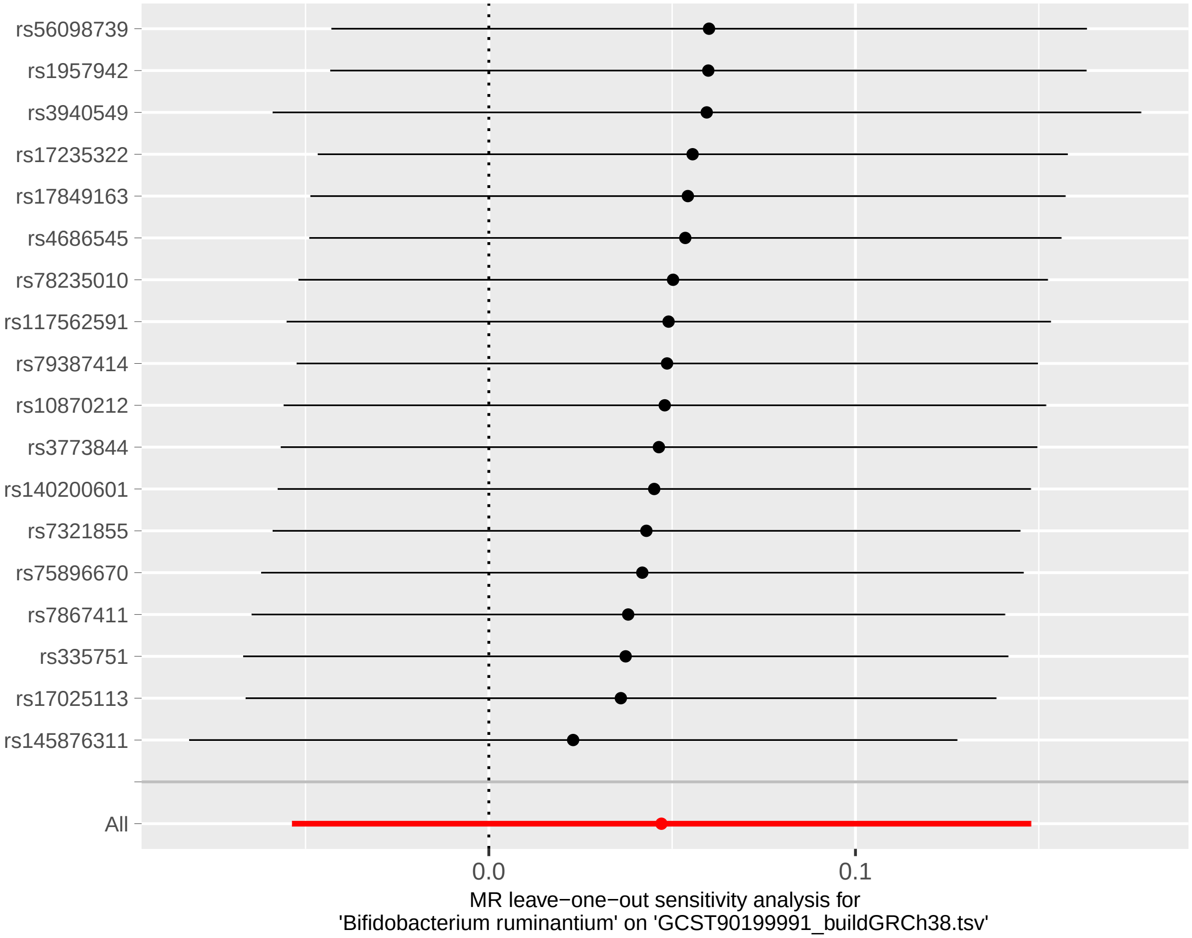
rs56098739
rs1957942
rs3940549
rs17235322
rs17849163
rs4686545
rs78235010
rs117562591
rs79387414
rs10870212
rs3773844
rs140200601
rs7321855
rs75896670
rs7867411
rs335751
rs17025113
rs145876311
All
0.0
0.1
MR leave−one−out sensitivity analysis for
'Bifidobacterium ruminantium' on 'GCST90199991_buildGRCh38.tsv'

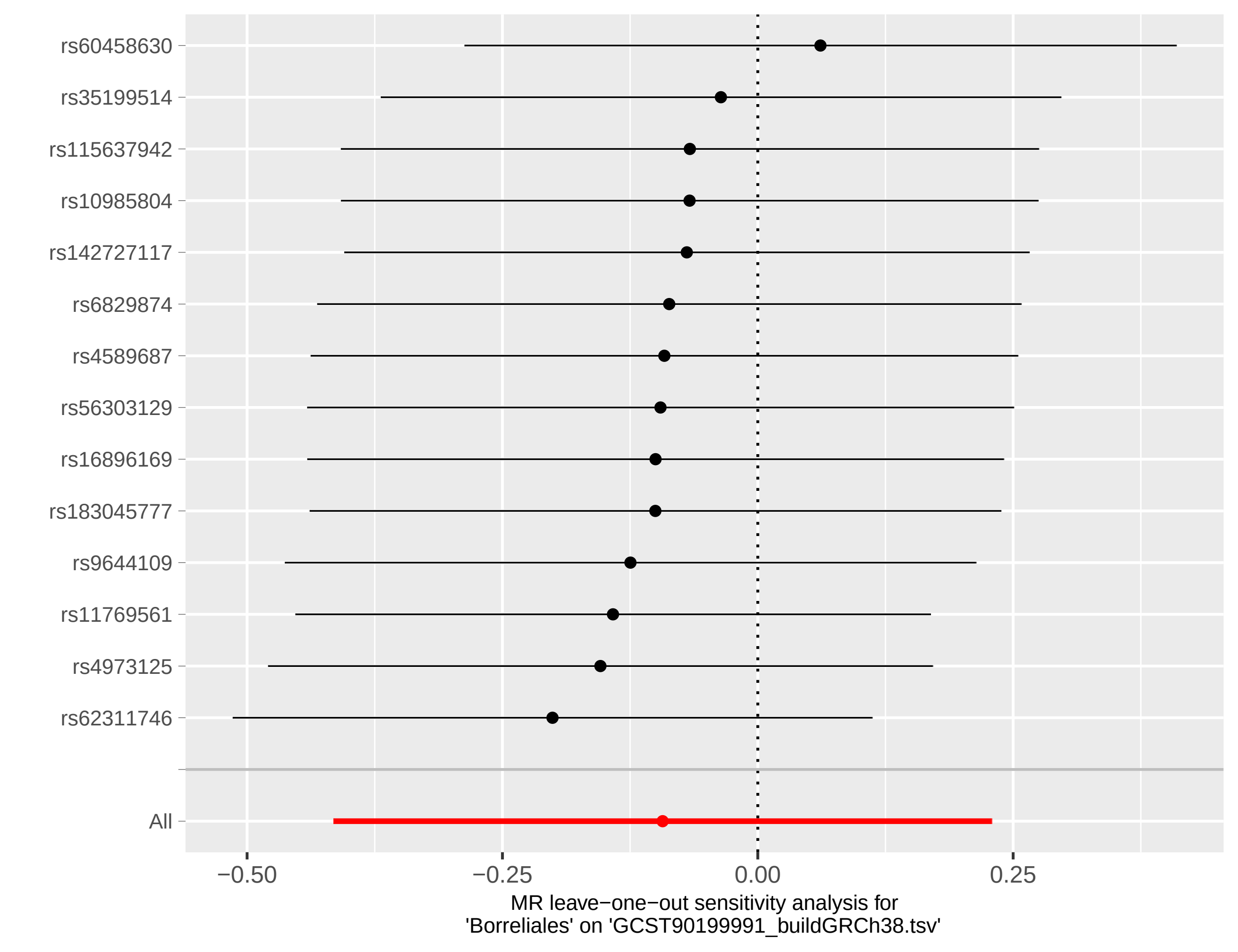

rs60458630
rs35199514
rs115637942
rs10985804
rs142727117
rs6829874
rs4589687
rs56303129
rs16896169
rs183045777
rs9644109
rs11769561
rs4973125
rs62311746
All
−0.50
−0.25
0.00
0.25
MR leave−one−out sensitivity analysis for
'Borreliales' on 'GCST90199991_buildGRCh38.tsv'

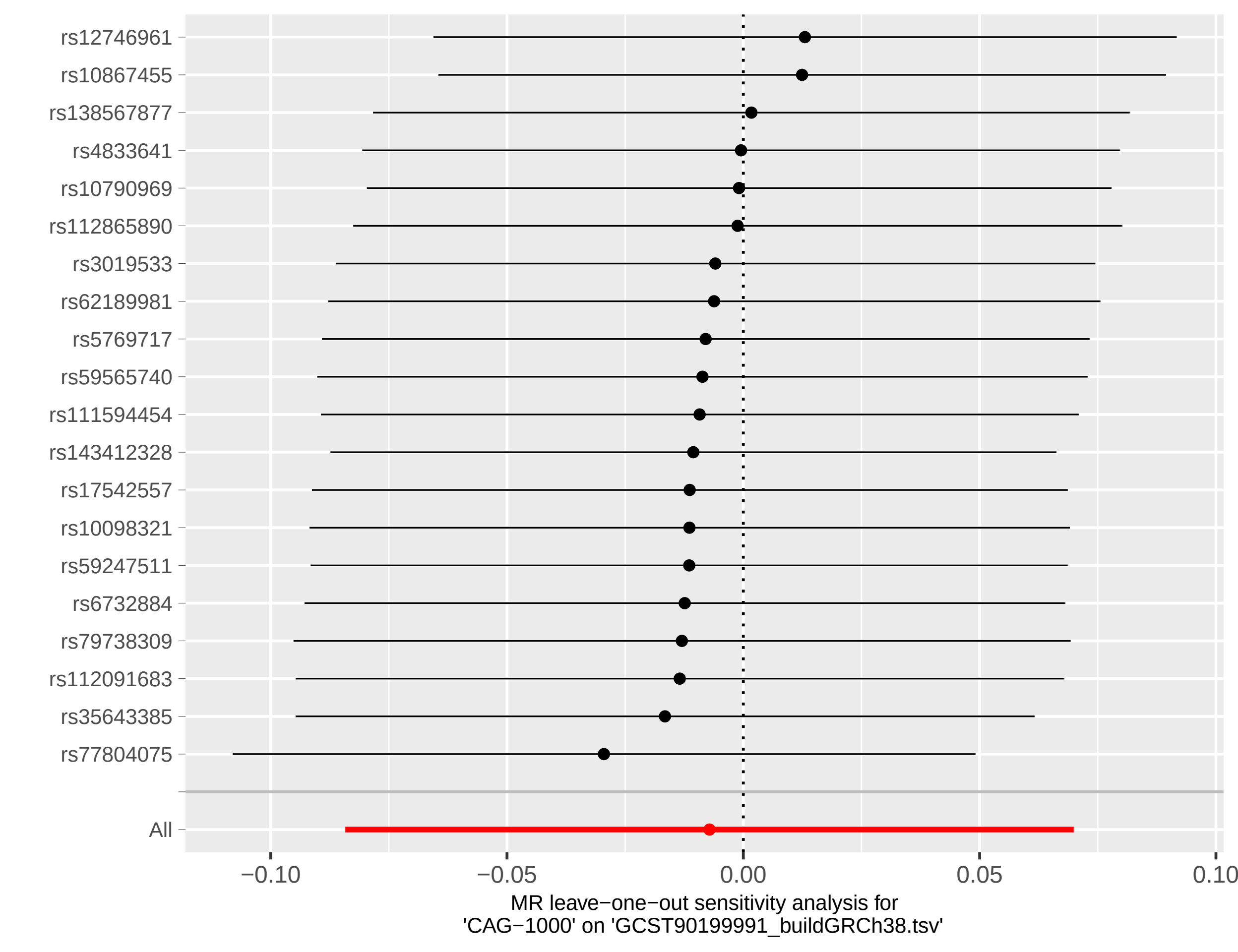
rs12746961
rs10867455
rs138567877
rs4833641
rs10790969
rs112865890
rs3019533
rs62189981
rs5769717
rs59565740
rs111594454
rs143412328
rs17542557
rs10098321
rs59247511
rs6732884
rs79738309
rs112091683
rs35643385
rs77804075
All
−0.10
−0.05
0.00
0.05
0.10
MR leave−one−out sensitivity analysis for
'CAG−1000' on 'GCST90199991_buildGRCh38.tsv'

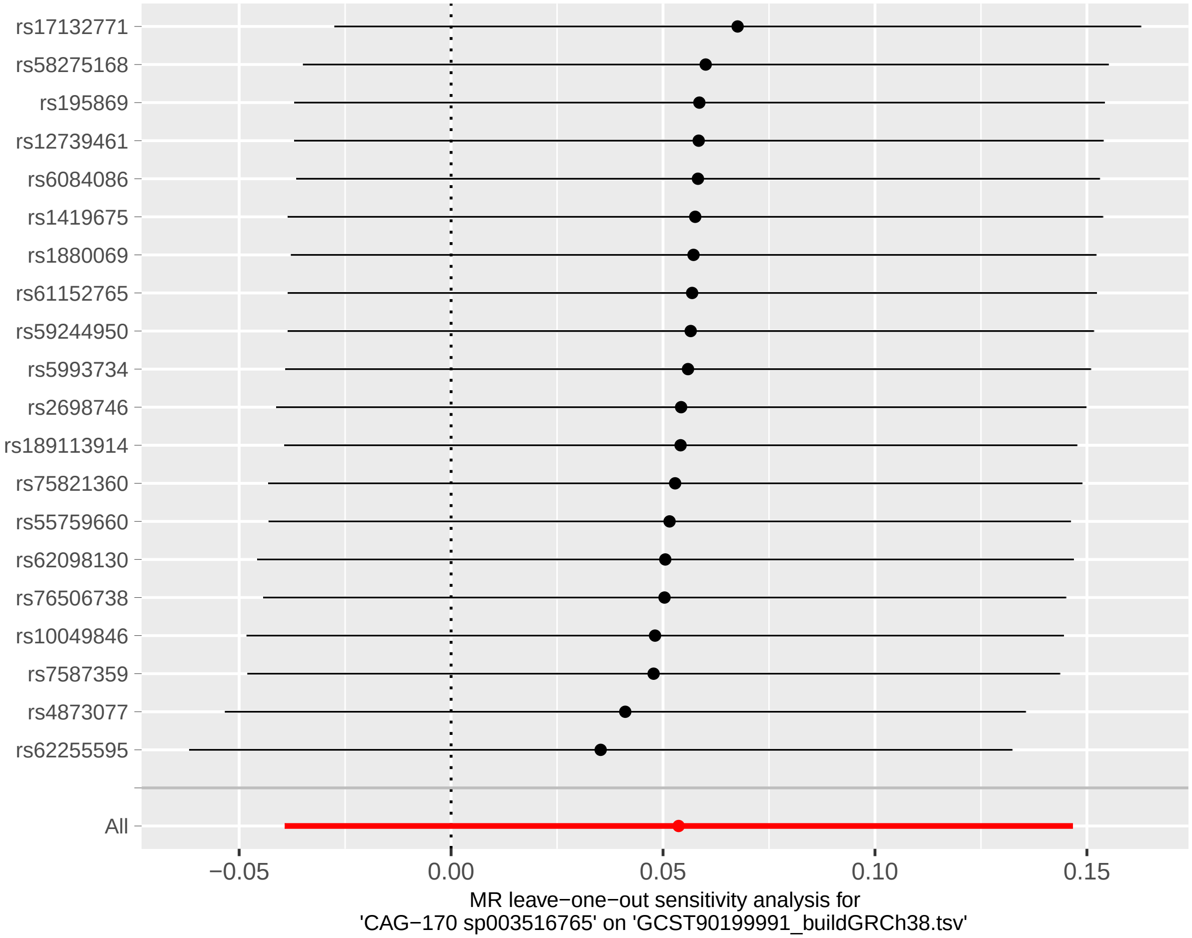
rs17132771
rs58275168
rs195869
rs12739461
rs6084086
rs1419675
rs1880069
rs61152765
rs59244950
rs5993734
rs2698746
rs189113914
rs75821360
rs55759660
rs62098130
rs76506738
rs10049846
rs7587359
rs4873077
rs62255595
All
−0.05
0.00
0.05
0.10
0.15
MR leave−one−out sensitivity analysis for
'CAG−170 sp003516765' on 'GCST90199991_buildGRCh38.tsv'

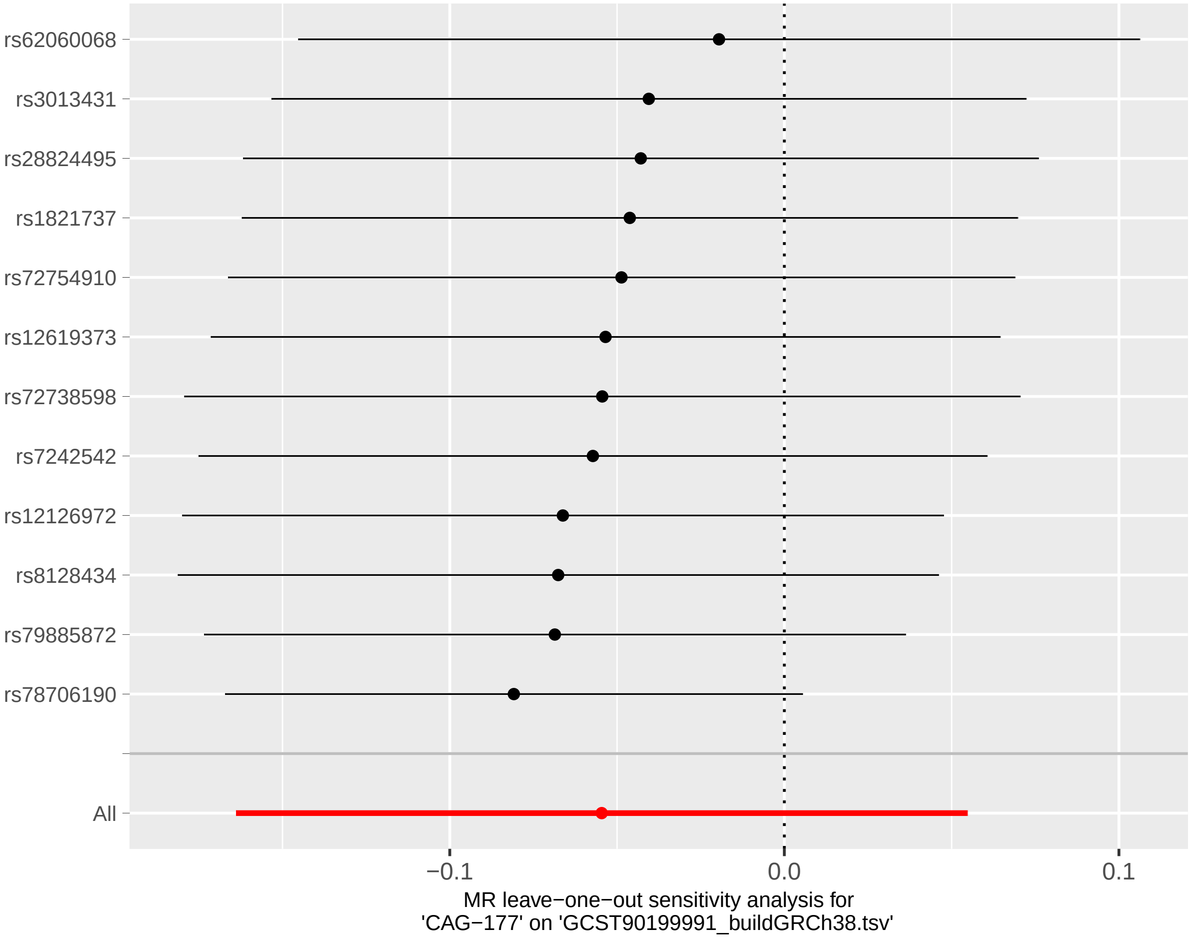

rs62060068
rs3013431
rs28824495
rs1821737
rs72754910
rs12619373
rs72738598
rs7242542
rs12126972
rs8128434
rs79885872
rs78706190
All
−0.1
0.0
0.1
MR leave−one−out sensitivity analysis for
'CAG−177' on 'GCST90199991_buildGRCh38.tsv'

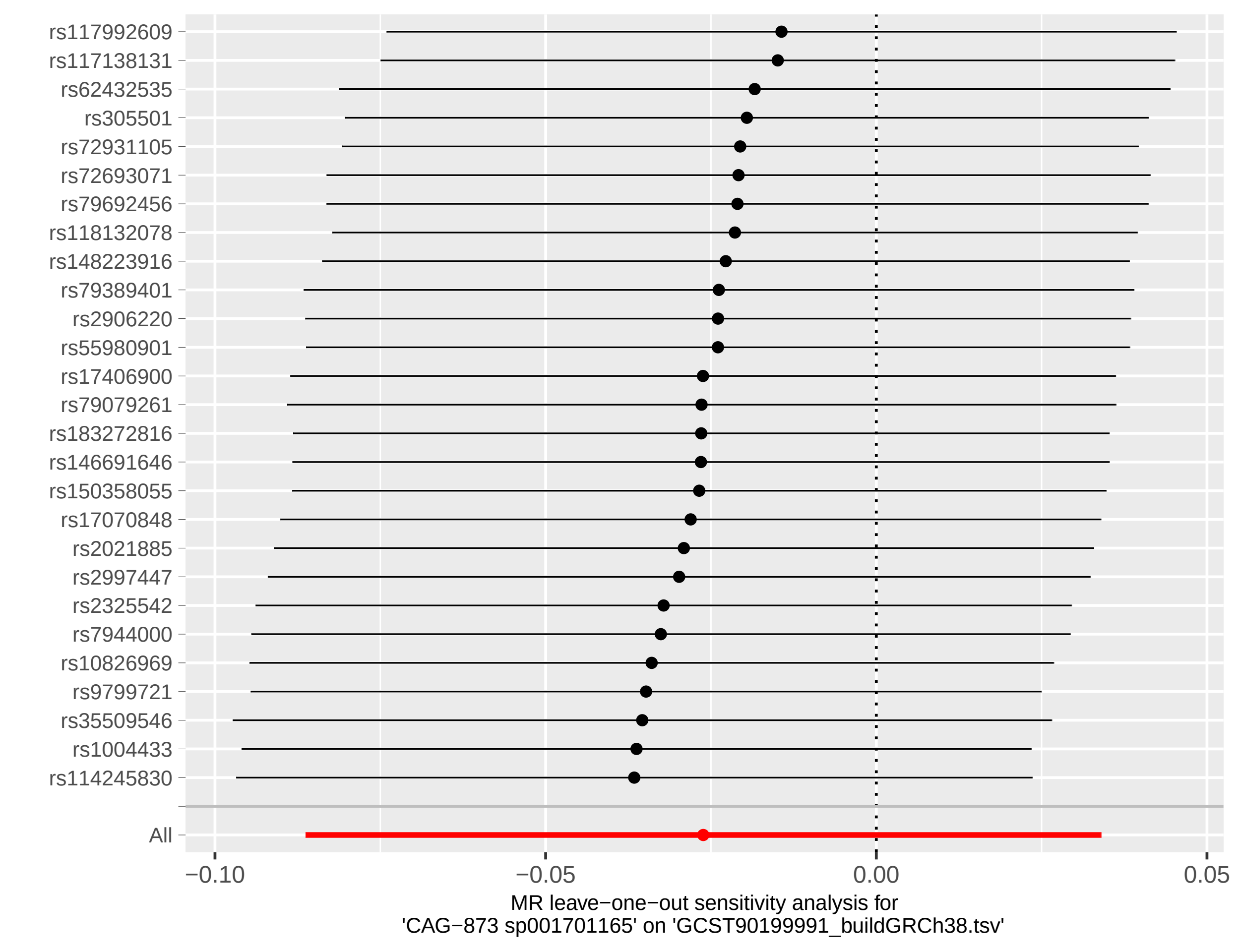

rs117992609
rs117138131
rs62432535
rs305501
rs72931105
rs72693071
rs79692456
rs118132078
rs148223916
rs79389401
rs2906220
rs55980901
rs17406900
rs79079261
rs183272816
rs146691646
rs150358055
rs17070848
rs2021885
rs2997447
rs2325542
rs7944000
rs10826969
rs9799721
rs35509546
rs1004433
rs114245830
All
−0.10
−0.05
0.00
0.05
MR leave−one−out sensitivity analysis for
'CAG−873 sp001701165' on 'GCST90199991_buildGRCh38.tsv'

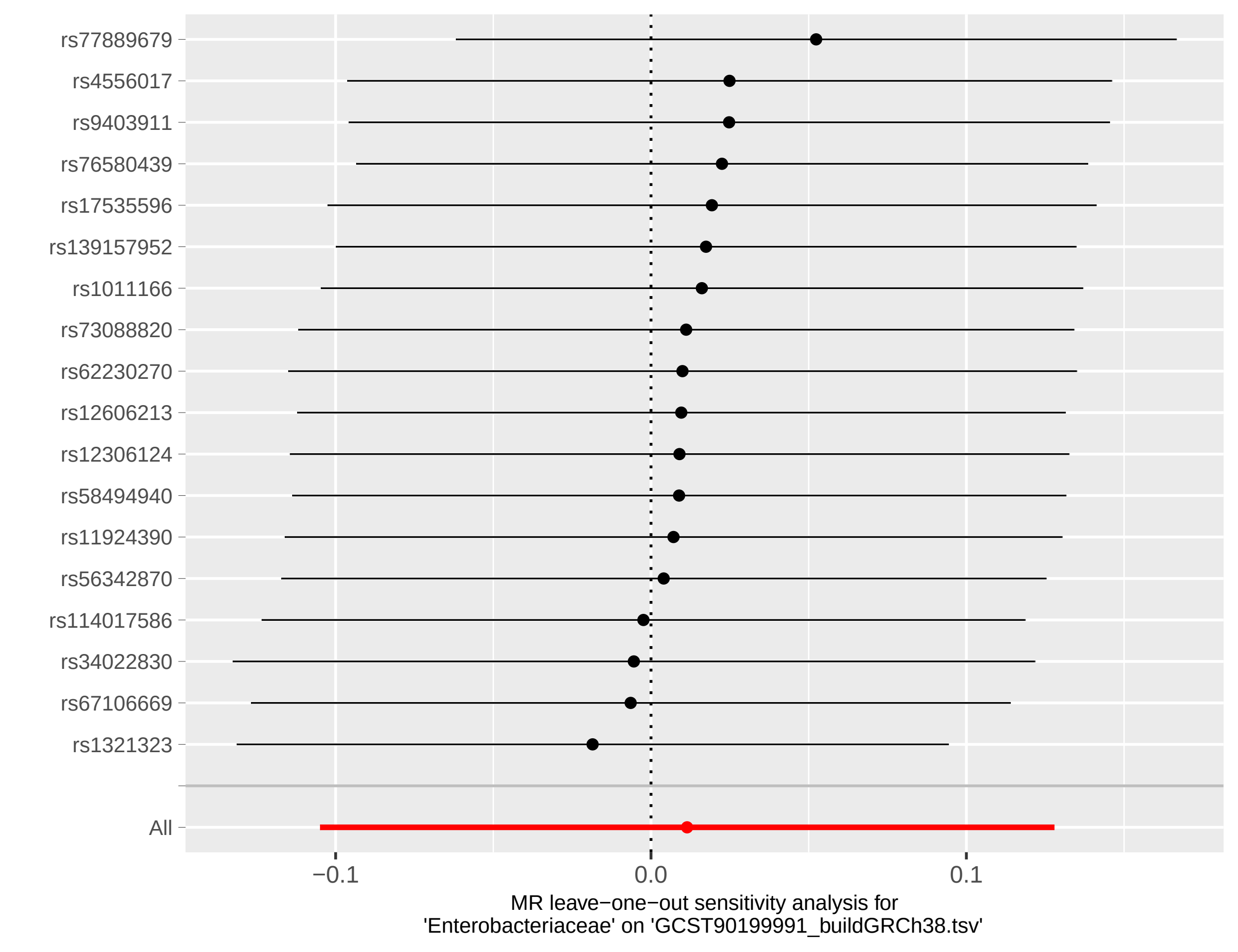

rs77889679
rs4556017
rs9403911
rs76580439
rs17535596
rs139157952
rs1011166
rs73088820
rs62230270
rs12606213
rs12306124
rs58494940
rs11924390
rs56342870
rs114017586
rs34022830
rs67106669
rs1321323
All
−0.1
0.0
0.1
MR leave−one−out sensitivity analysis for
'Enterobacteriaceae' on 'GCST90199991_buildGRCh38.tsv'

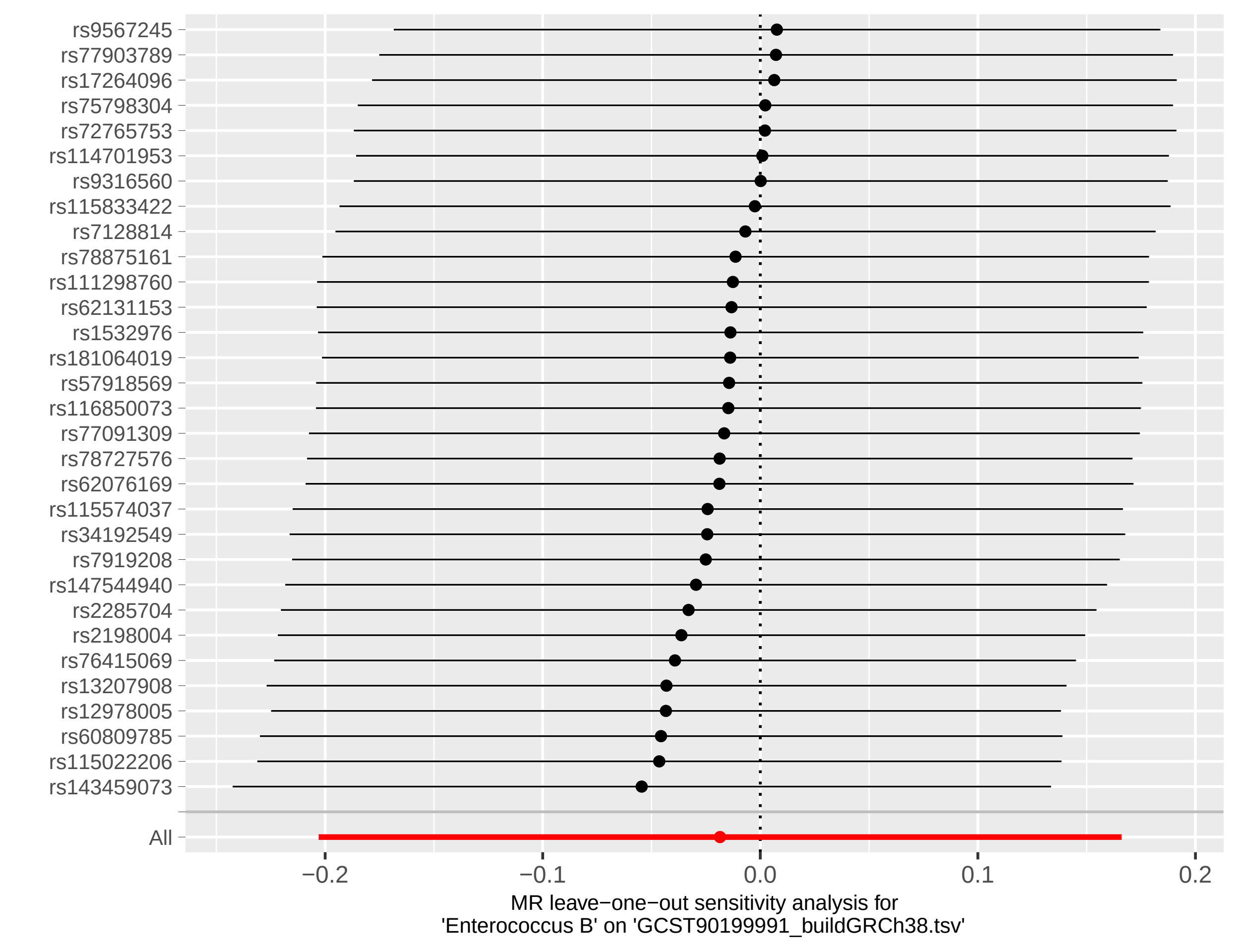

rs9567245
rs77903789
rs17264096
rs75798304
rs72765753
rs114701953
rs9316560
rs115833422
rs7128814
rs78875161
rs111298760
rs62131153
rs1532976
rs181064019
rs57918569
rs116850073
rs77091309
rs78727576
rs62076169
rs115574037
rs34192549
rs7919208
rs147544940
rs2285704
rs2198004
rs76415069
rs13207908
rs12978005
rs60809785
rs115022206
rs143459073
All
−0.2
−0.1
0.0
0.1
0.2
MR leave−one−out sensitivity analysis for
'Enterococcus B' on 'GCST90199991_buildGRCh38.tsv'

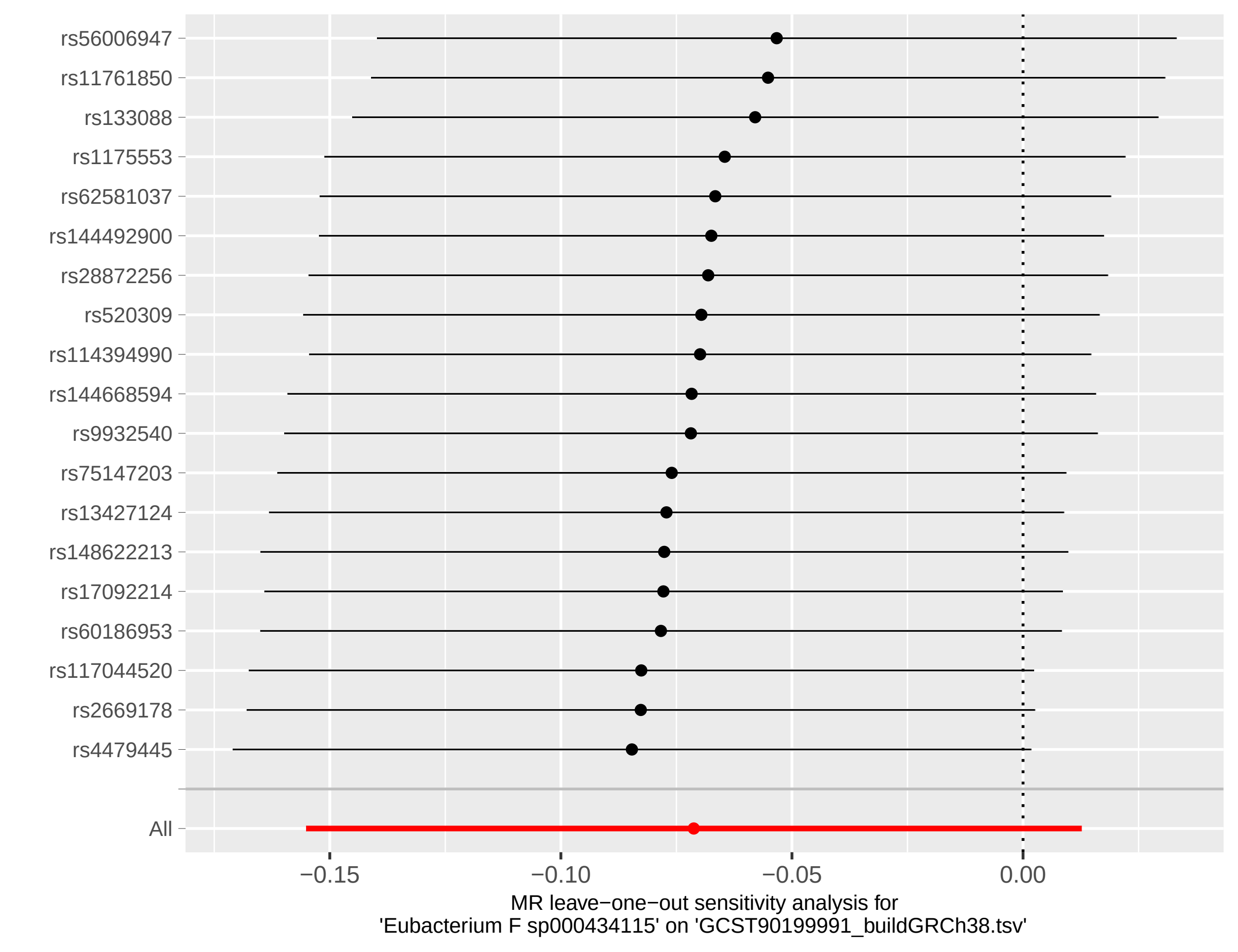

rs56006947
rs11761850
rs133088
rs1175553
rs62581037
rs144492900
rs28872256
rs520309
rs114394990
rs144668594
rs9932540
rs75147203
rs13427124
rs148622213
rs17092214
rs60186953
rs117044520
rs2669178
rs4479445
All
−0.15
−0.10
−0.05
0.00
MR leave−one−out sensitivity analysis for
'Eubacterium F sp000434115' on 'GCST90199991_buildGRCh38.tsv'

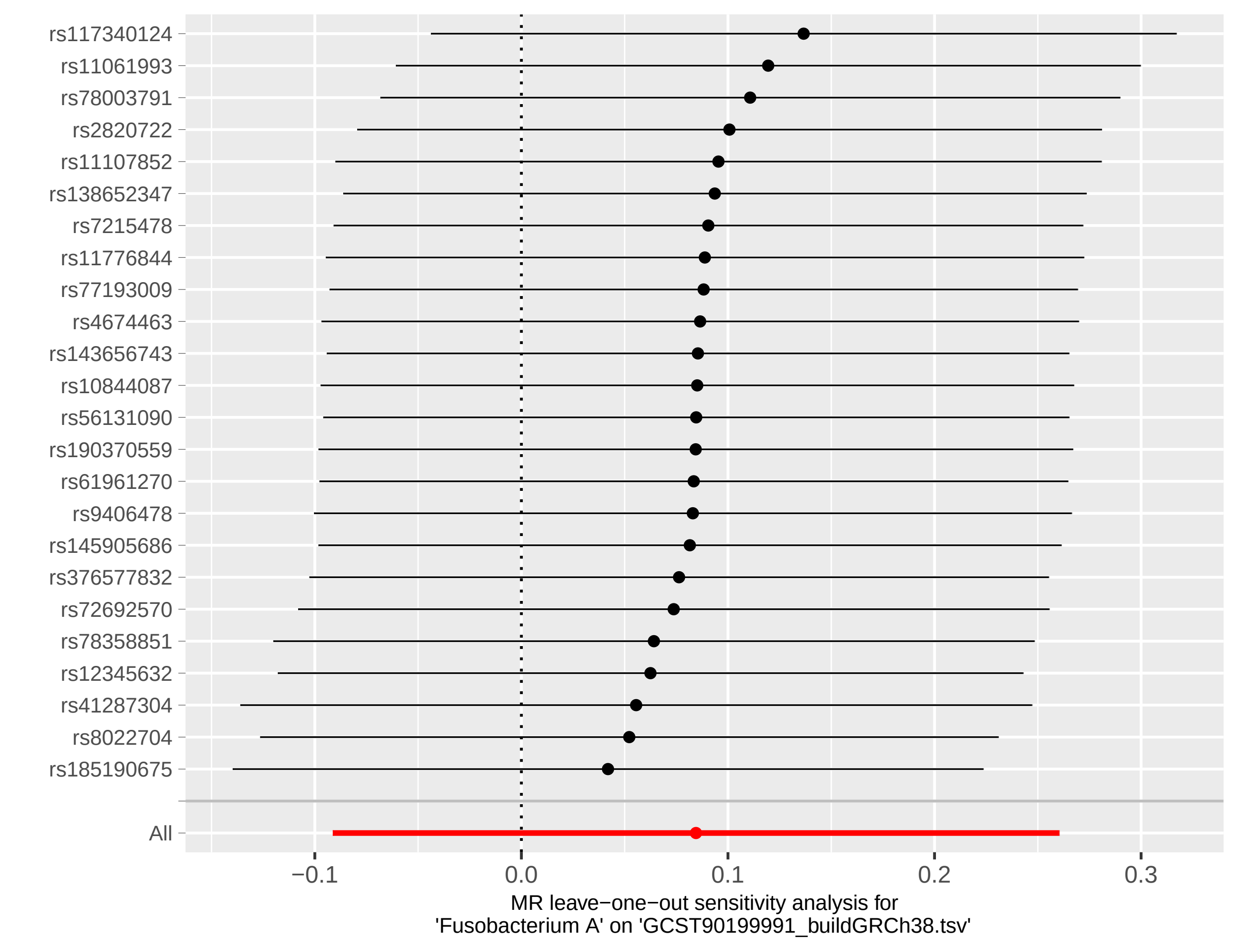

rs117340124
rs11061993
rs78003791
rs2820722
rs11107852
rs138652347
rs7215478
rs11776844
rs77193009
rs4674463
rs143656743
rs10844087
rs56131090
rs190370559
rs61961270
rs9406478
rs145905686
rs376577832
rs72692570
rs78358851
rs12345632
rs41287304
rs8022704
rs185190675
All
−0.1
0.0
0.1
0.2
0.3
MR leave−one−out sensitivity analysis for
'Fusobacterium A' on 'GCST90199991_buildGRCh38.tsv'

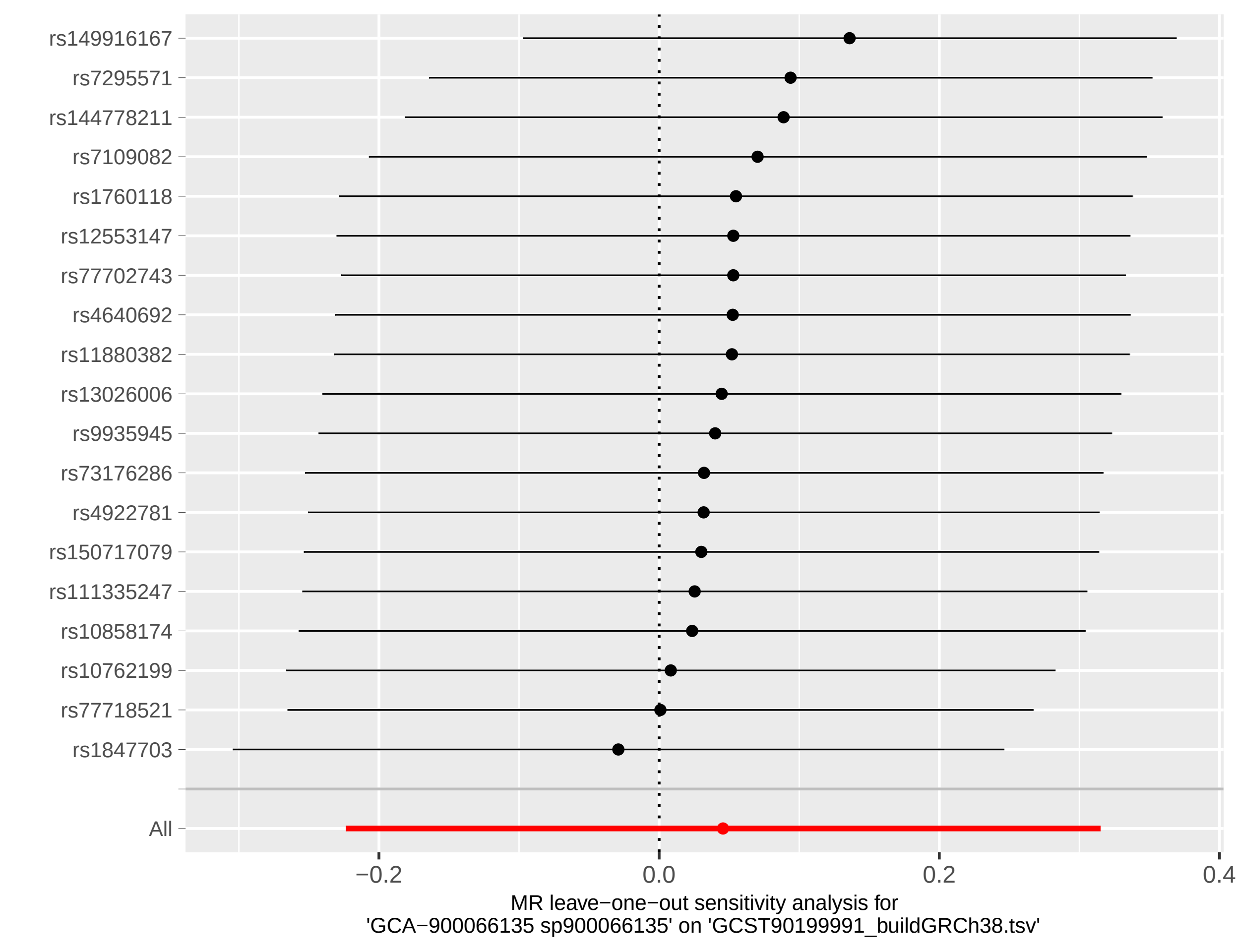
rs149916167
rs7295571
rs144778211
rs7109082
rs1760118
rs12553147
rs77702743
rs4640692
rs11880382
rs13026006
rs9935945
rs73176286
rs4922781
rs150717079
rs111335247
rs10858174
rs10762199
rs77718521
rs1847703
All
−0.2
0.0
0.2
0.4
MR leave−one−out sensitivity analysis for
'GCA−900066135 sp900066135' on 'GCST90199991_buildGRCh38.tsv'

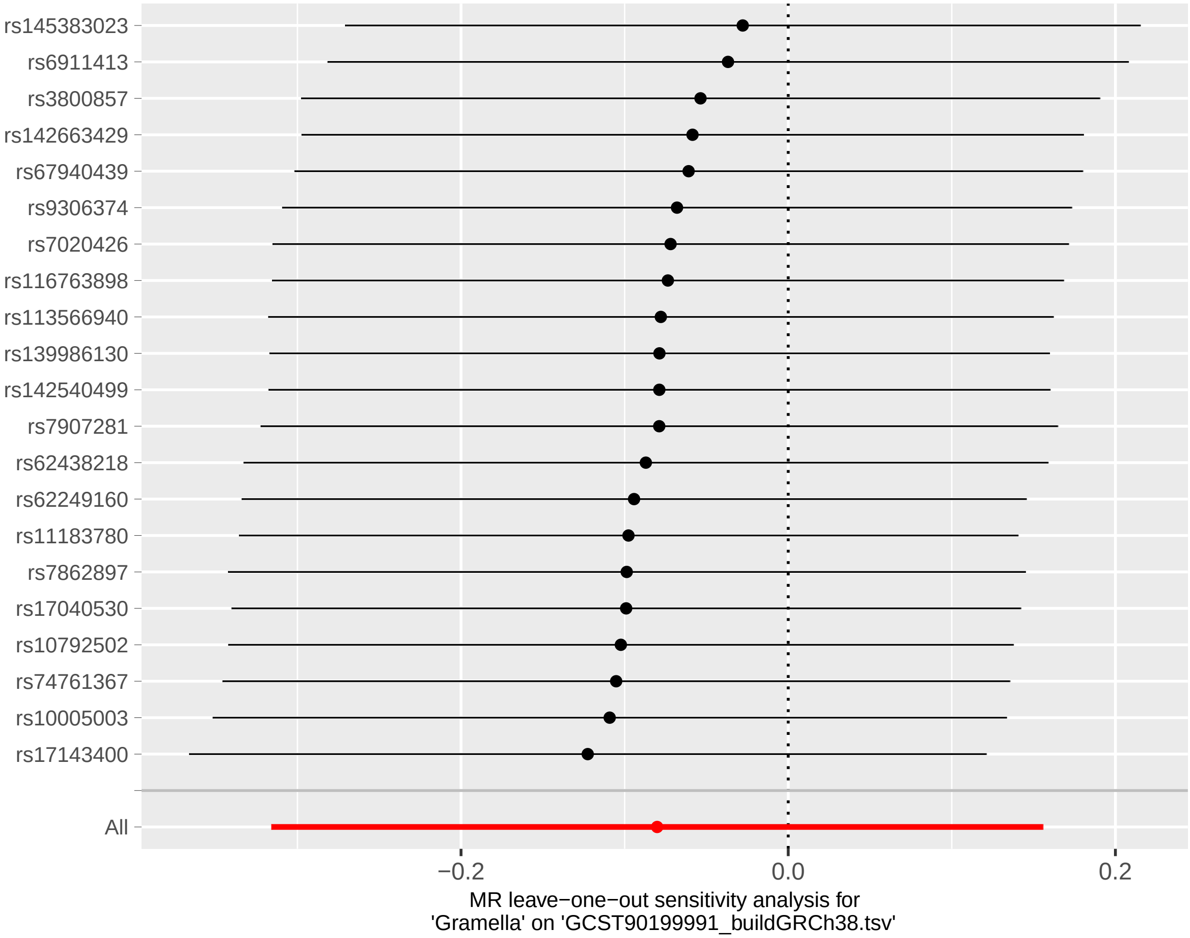

rs145383023
rs6911413
rs3800857
rs142663429
rs67940439
rs9306374
rs7020426
rs116763898
rs113566940
rs139986130
rs142540499
rs7907281
rs62438218
rs62249160
rs11183780
rs7862897
rs17040530
rs10792502
rs74761367
rs10005003
rs17143400
All
−0.2
0.0
0.2
MR leave−one−out sensitivity analysis for
'Gramella' on 'GCST90199991_buildGRCh38.tsv'

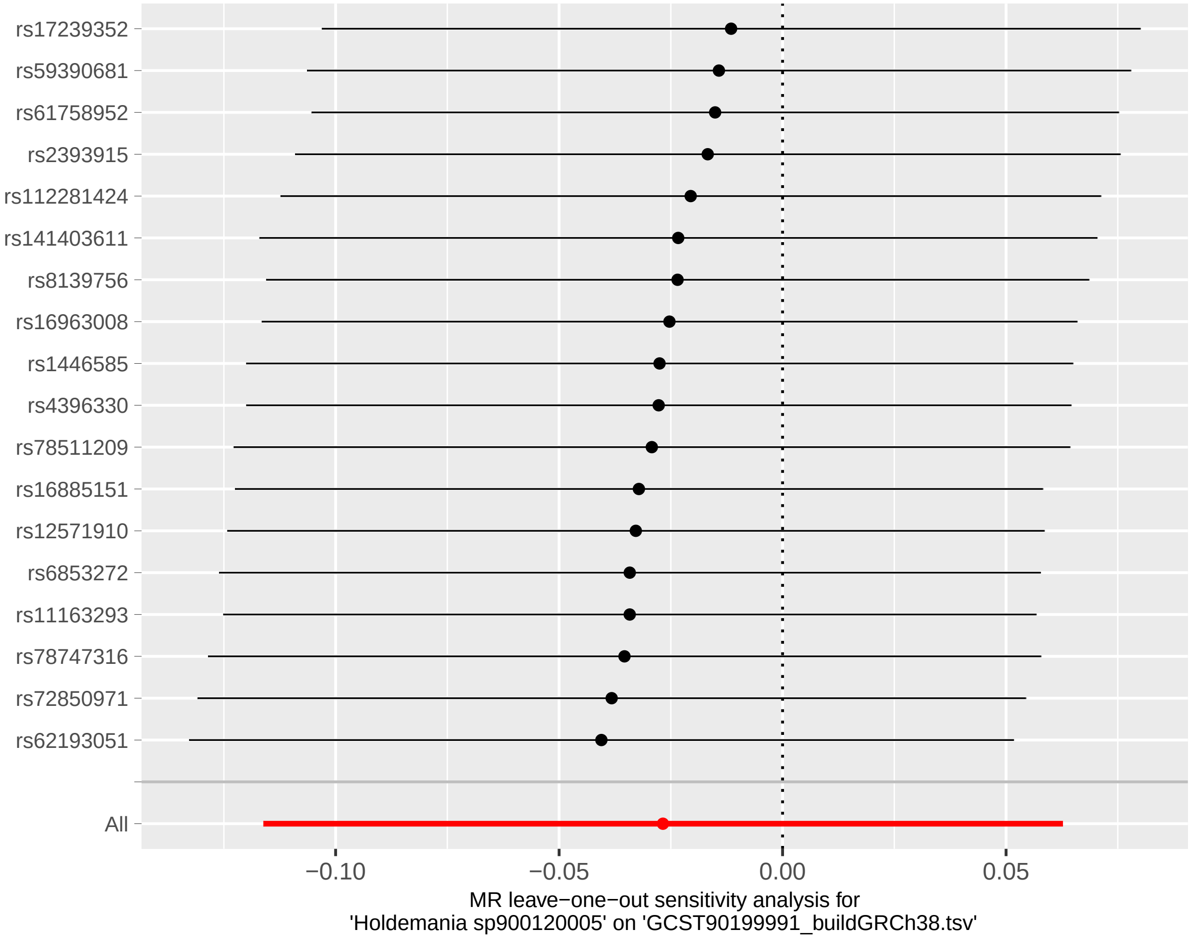
rs17239352
rs59390681
rs61758952
rs2393915
rs112281424
rs141403611
rs8139756
rs16963008
rs1446585
rs4396330
rs78511209
rs16885151
rs12571910
rs6853272
rs11163293
rs78747316
rs72850971
rs62193051
All
−0.10
−0.05
0.00
0.05
MR leave−one−out sensitivity analysis for
'Holdemania sp900120005' on 'GCST90199991_buildGRCh38.tsv'

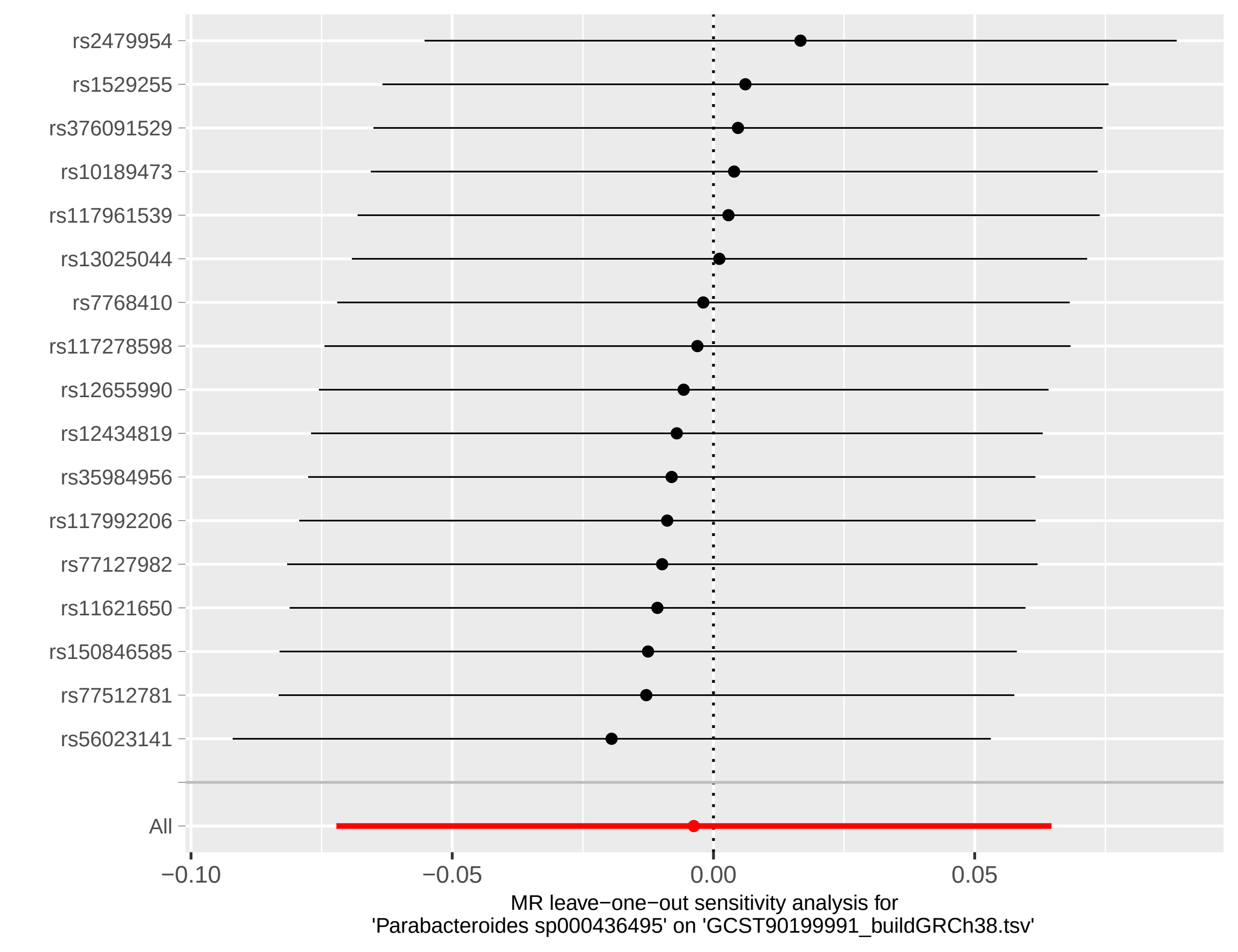
rs2479954
rs1529255
rs376091529
rs10189473
rs117961539
rs13025044
rs7768410
rs117278598
rs12655990
rs12434819
rs35984956
rs117992206
rs77127982
rs11621650
rs150846585
rs77512781
rs56023141
All
−0.10
−0.05
0.00
0.05
MR leave−one−out sensitivity analysis for
'Parabacteroides sp000436495' on 'GCST90199991_buildGRCh38.tsv'

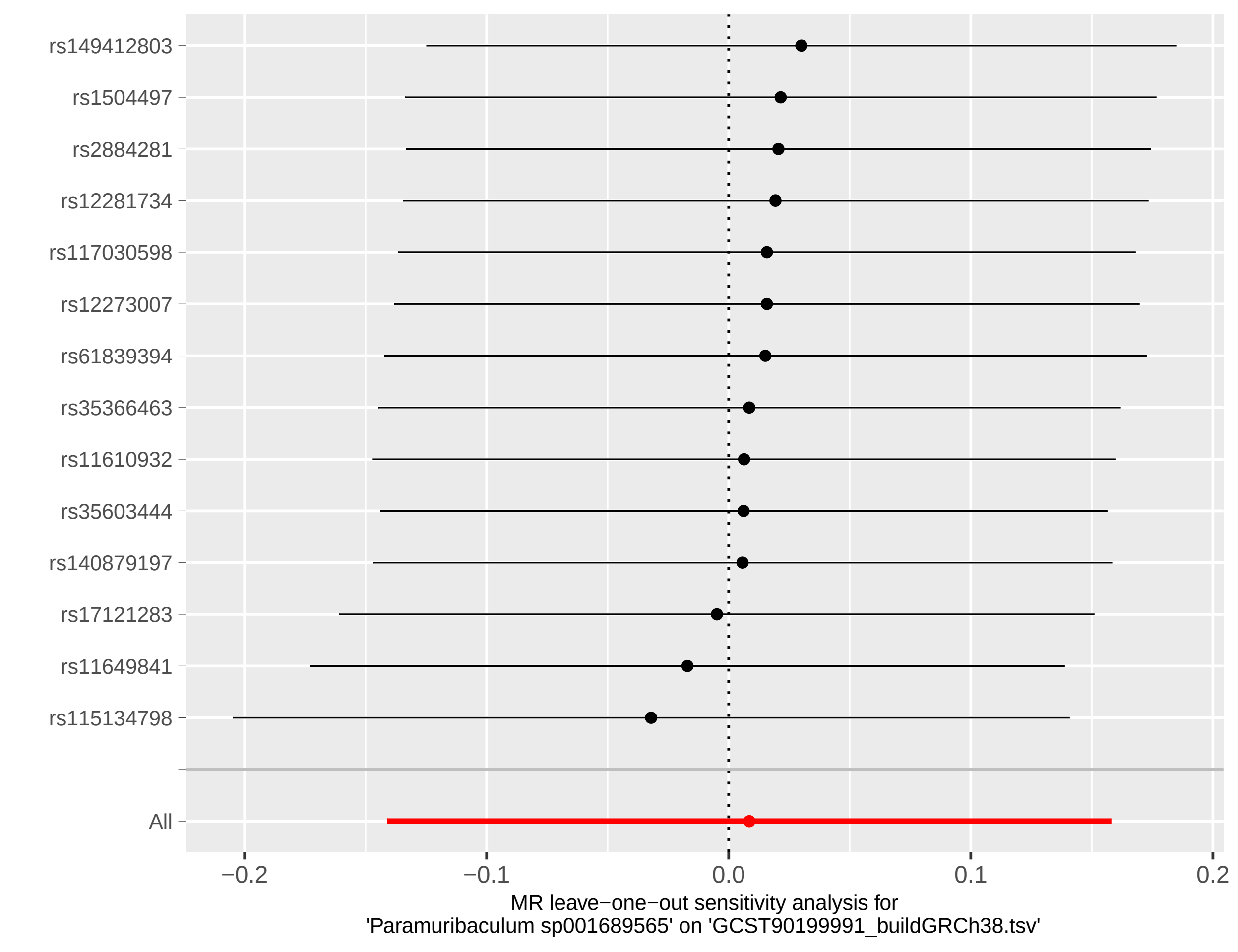
rs149412803
rs1504497
rs2884281
rs12281734
rs117030598
rs12273007
rs61839394
rs35366463
rs11610932
rs35603444
rs140879197
rs17121283
rs11649841
rs115134798
All
−0.2
−0.1
0.0
0.1
0.2
MR leave−one−out sensitivity analysis for
'Paramuribaculum sp001689565' on 'GCST90199991_buildGRCh38.tsv'

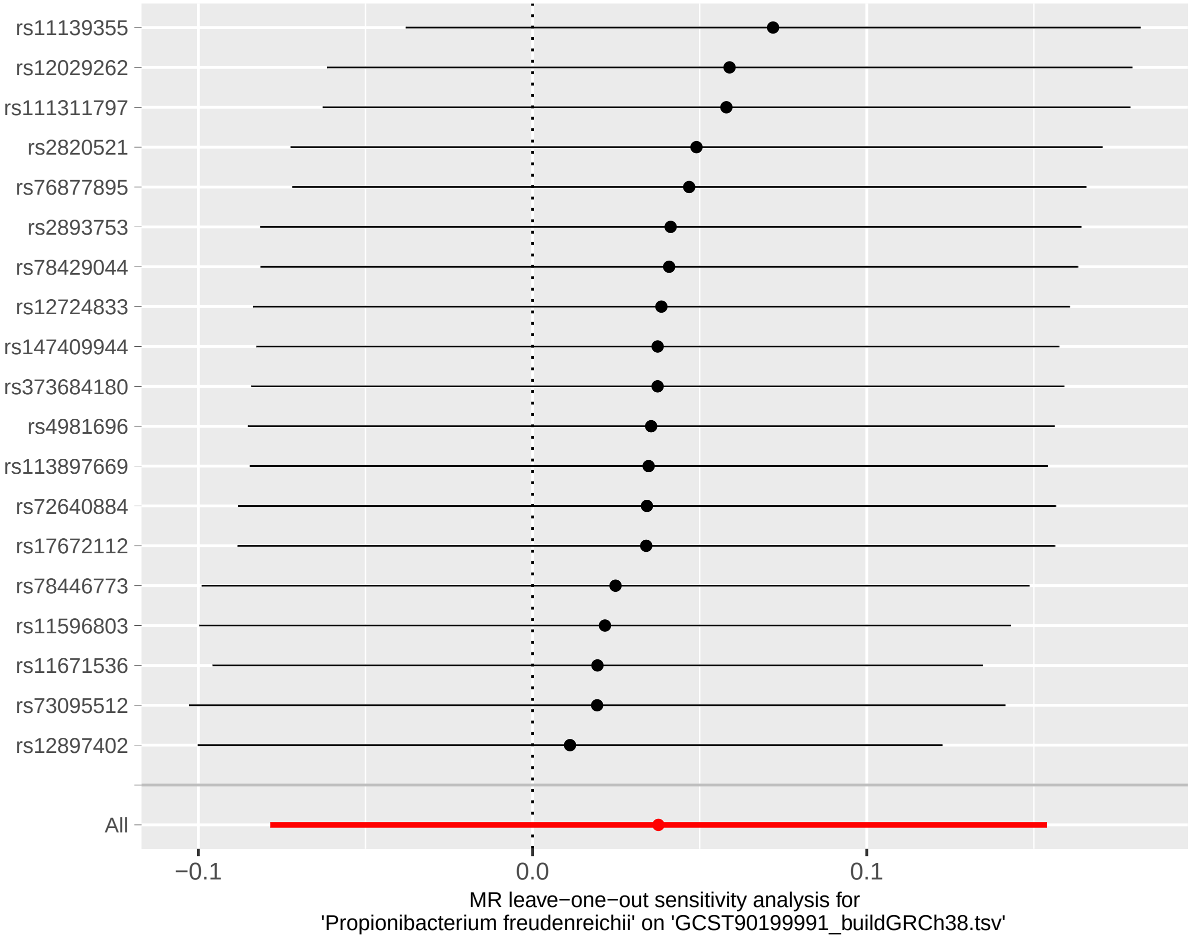
rs11139355
rs12029262
rs111311797
rs2820521
rs76877895
rs2893753
rs78429044
rs12724833
rs147409944
rs373684180
rs4981696
rs113897669
rs72640884
rs17672112
rs78446773
rs11596803
rs11671536
rs73095512
rs12897402
All
−0.1
0.0
0.1
MR leave−one−out sensitivity analysis for
'Propionibacterium freudenreichii' on 'GCST90199991_buildGRCh38.tsv'

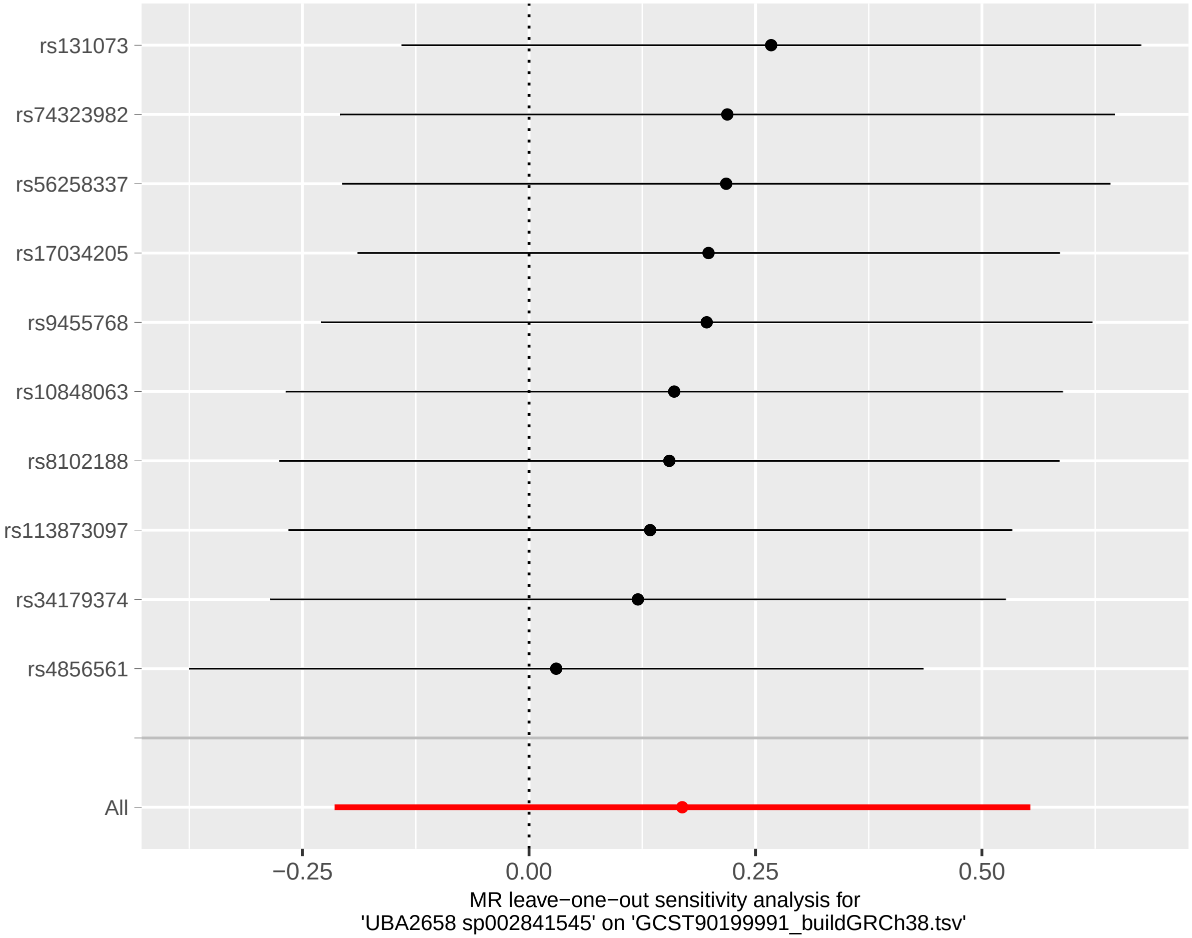

rs131073
rs74323982
rs56258337
rs17034205
rs9455768
rs10848063
rs8102188
rs113873097
rs34179374
rs4856561
All
−0.25
0.00
0.25
0.50
MR leave−one−out sensitivity analysis for
'UBA2658 sp002841545' on 'GCST90199991_buildGRCh38.tsv'

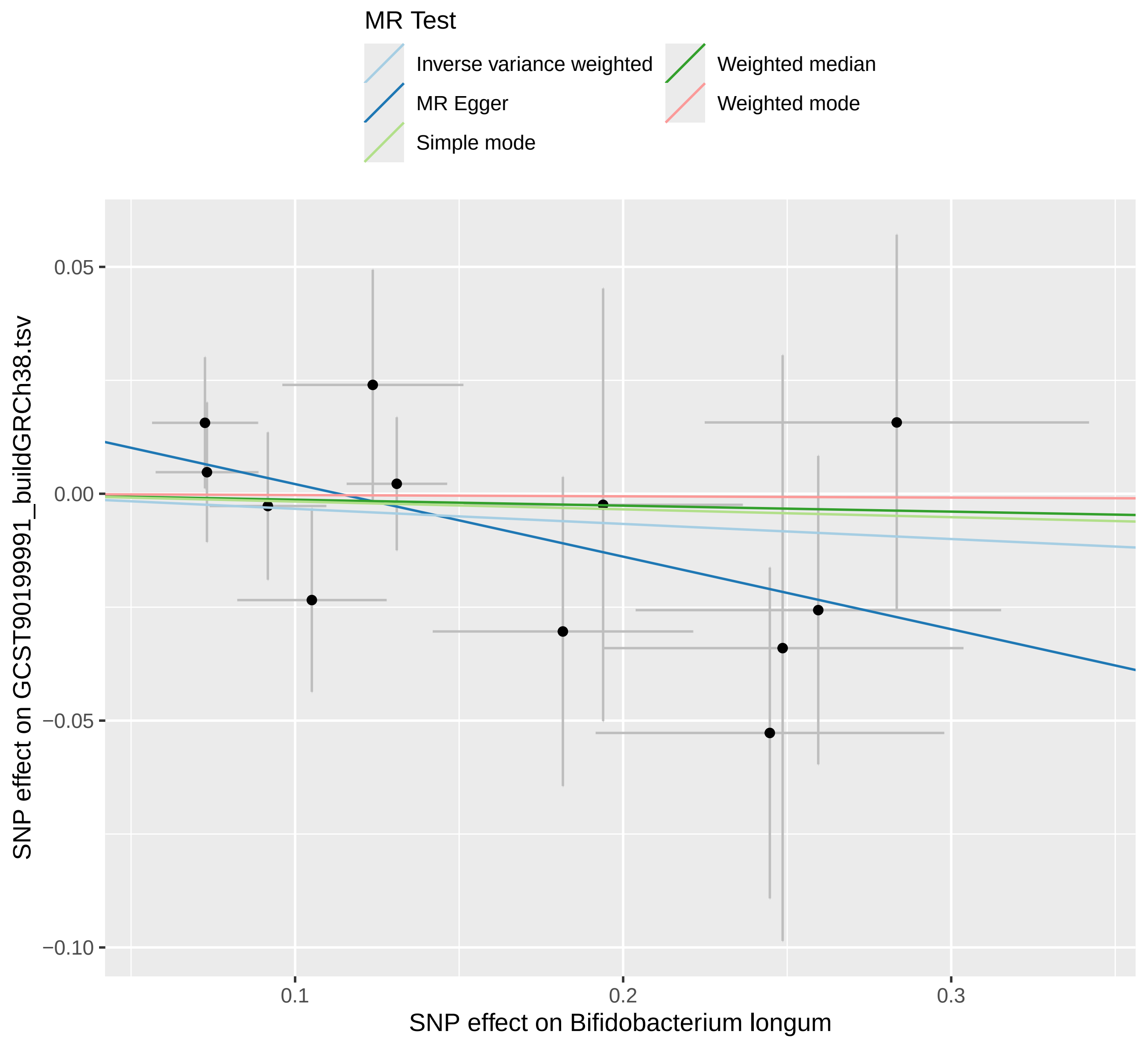
MR Test
Inverse variance weighted
MR Egger
Simple mode
Weighted median
Weighted mode
0.05
0.00
−0.05
−0.10
0.1
0.2
0.3
SNP effect on GCST90199991_buildGRCh38.tsv
SNP effect on Bifidobacterium longum

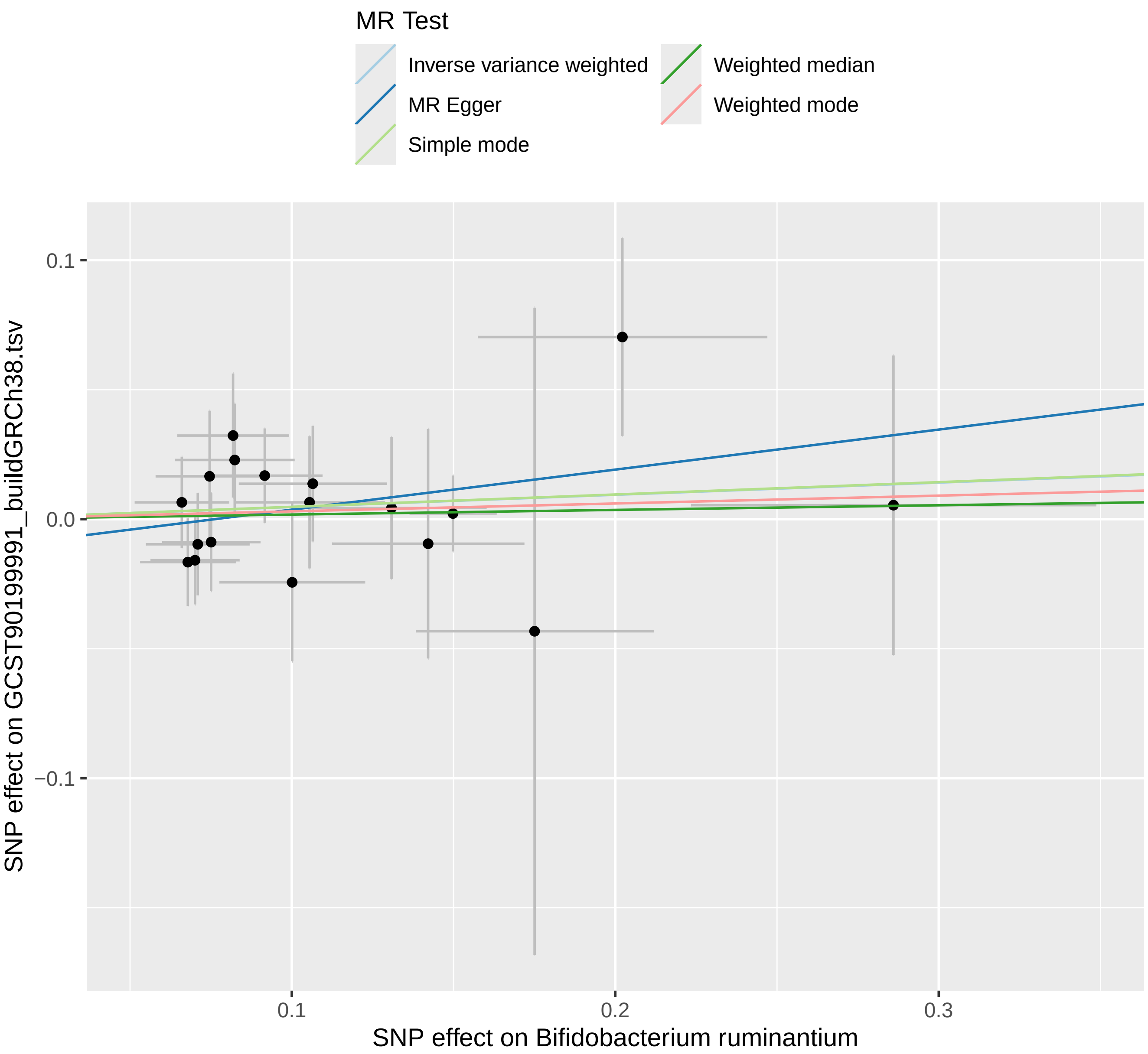
MR Test
Inverse variance weighted
MR Egger
Simple mode
Weighted median
Weighted mode
0.1
0.0
−0.1
SNP effect on GCST90199991_buildGRCh38.tsv
0.1
0.2
0.3
SNP effect on Bifidobacterium ruminantium

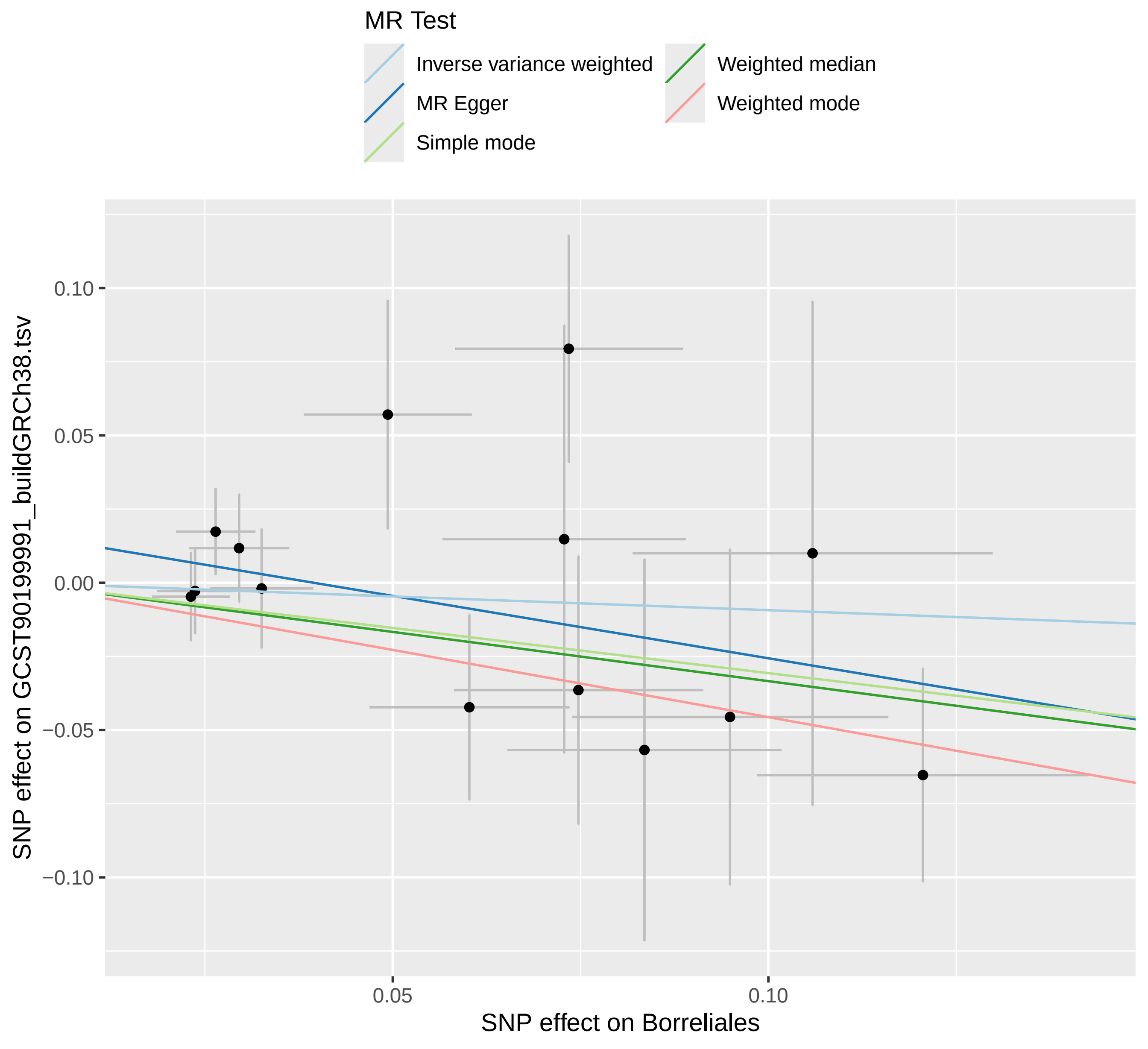

MR Test
Inverse variance weighted
MR Egger
Simple mode
Weighted median
Weighted mode
0.10
0.05
0.00
−0.05
−0.10
0.05
0.10
SNP effect on GCST90199991_buildGRCh38.tsv
SNP effect on Borreliales

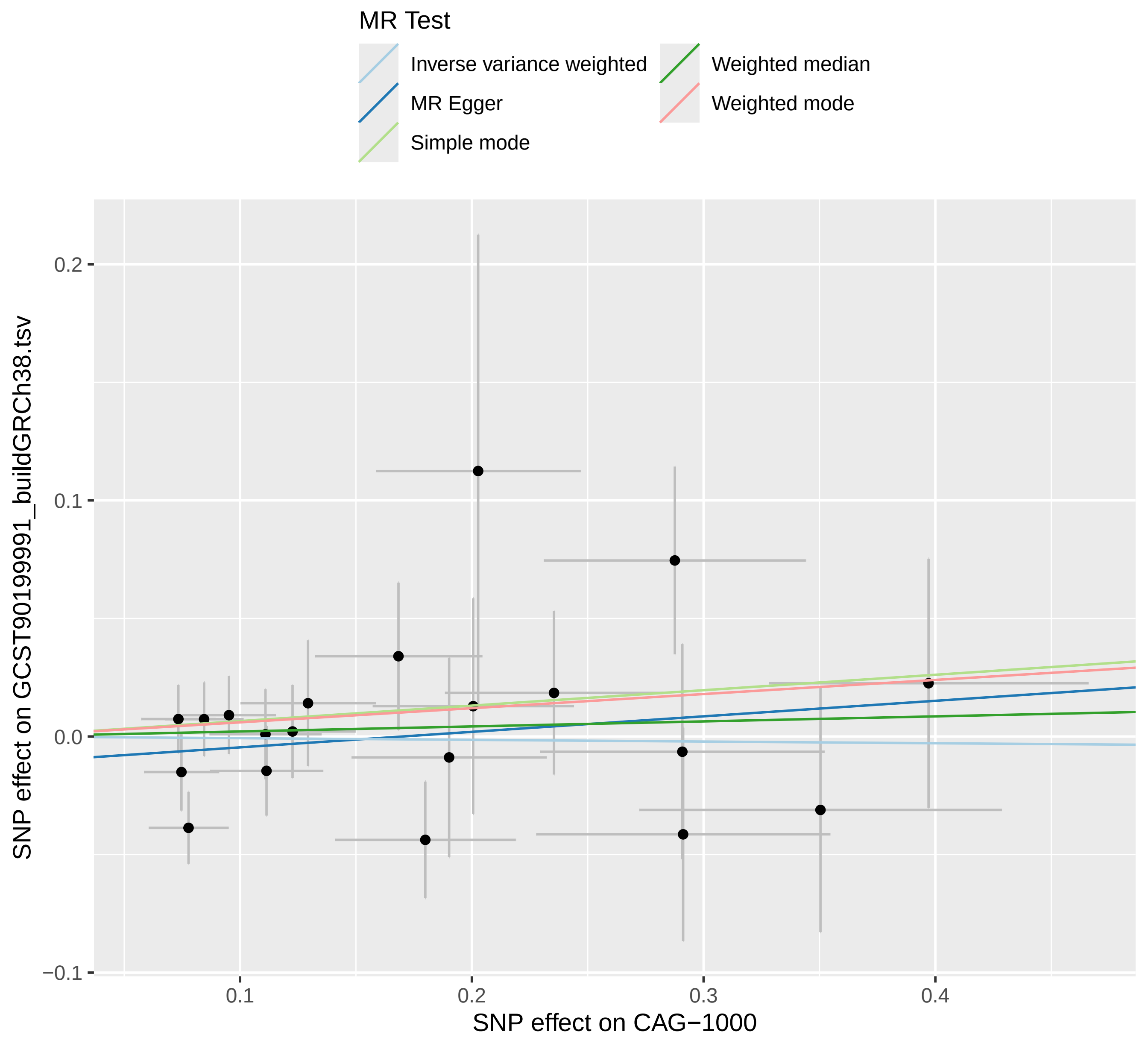

MR Test
Inverse variance weighted
MR Egger
Simple mode
Weighted median
Weighted mode
0.2
0.1
0.0
−0.1
0.1
0.2
0.3
0.4
SNP effect on GCST90199991_buildGRCh38.tsv
SNP effect on CAG−1000

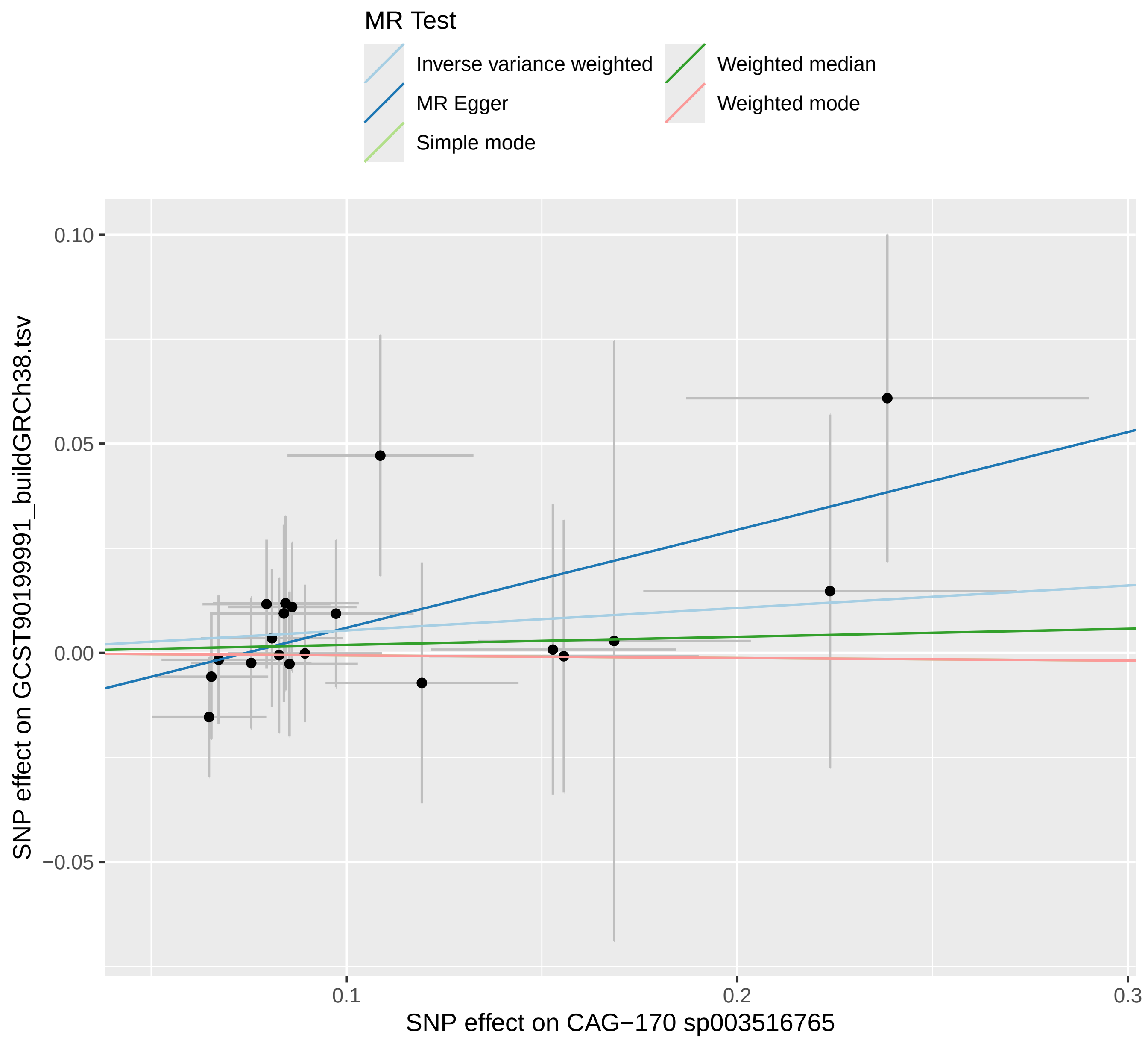

MR Test
Inverse variance weighted
MR Egger
Simple mode
Weighted median
Weighted mode
SNP effect on GCST90199991_buildGRCh38.tsv
0.10
0.05
0.00
−0.05
0.1
0.2
0.3
SNP effect on CAG−170 sp003516765

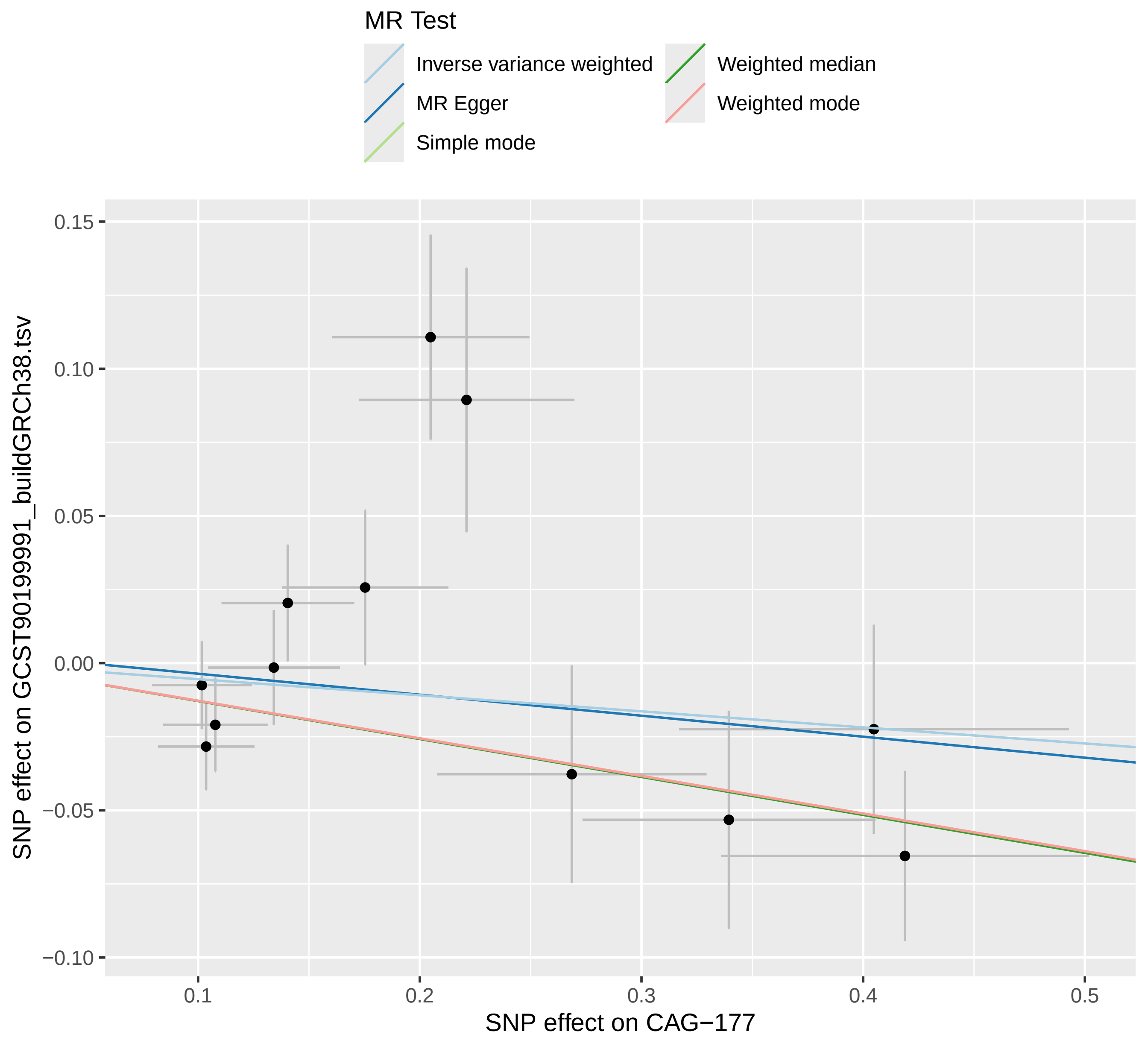
MR Test
Inverse variance weighted
MR Egger
Simple mode
Weighted median
Weighted mode
SNP effect on GCST90199991_buildGRCh38.tsv
0.15
0.10
0.05
0.00
−0.05
−0.10
0.1
0.2
0.3
0.4
0.5
SNP effect on CAG−177

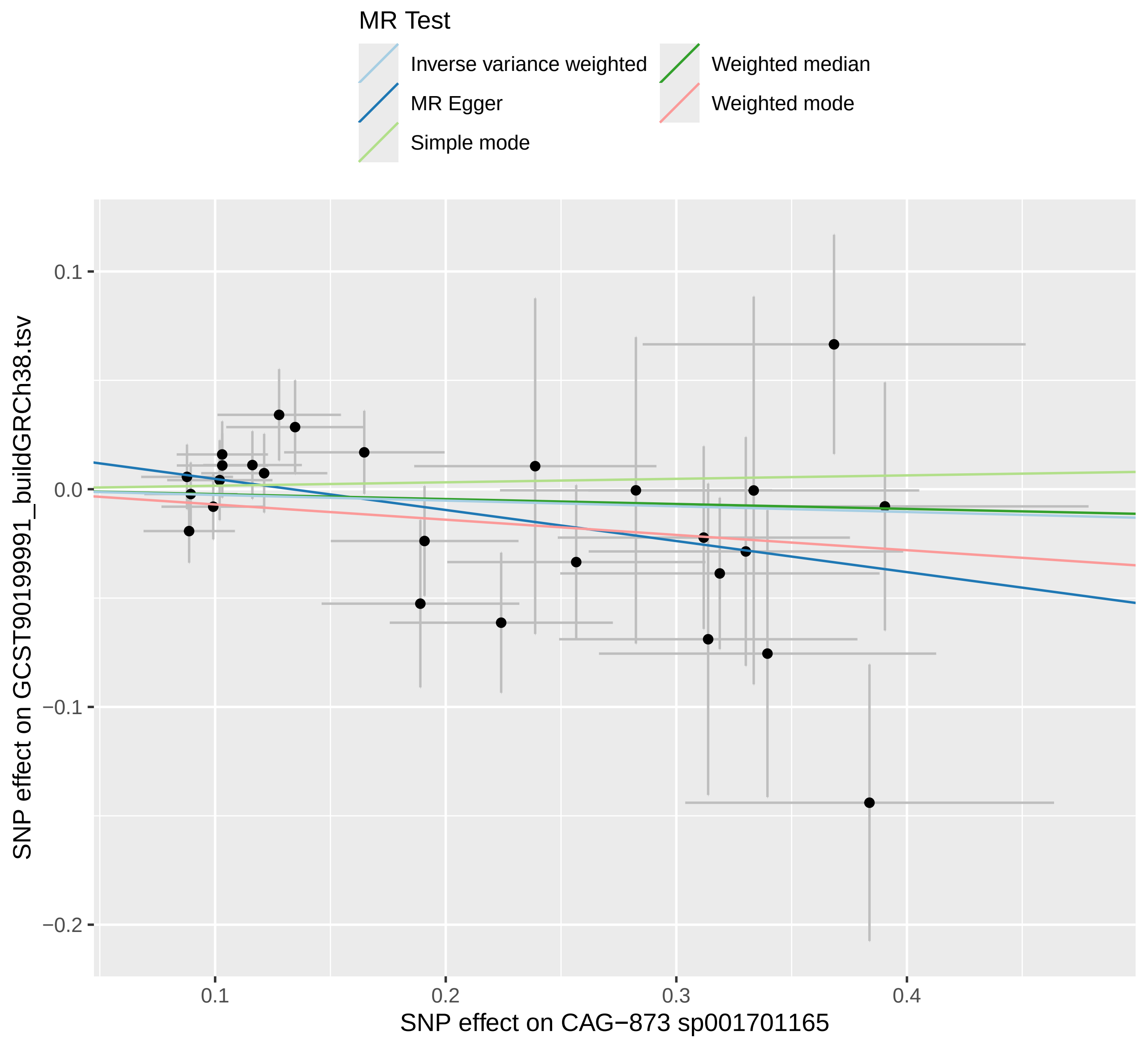

MR Test
Inverse variance weighted
MR Egger
Simple mode
Weighted median
Weighted mode
0.1
0.0
−0.1
−0.2
0.1
0.2
0.3
0.4
SNP effect on GCST90199991_buildGRCh38.tsv
SNP effect on CAG−873 sp001701165

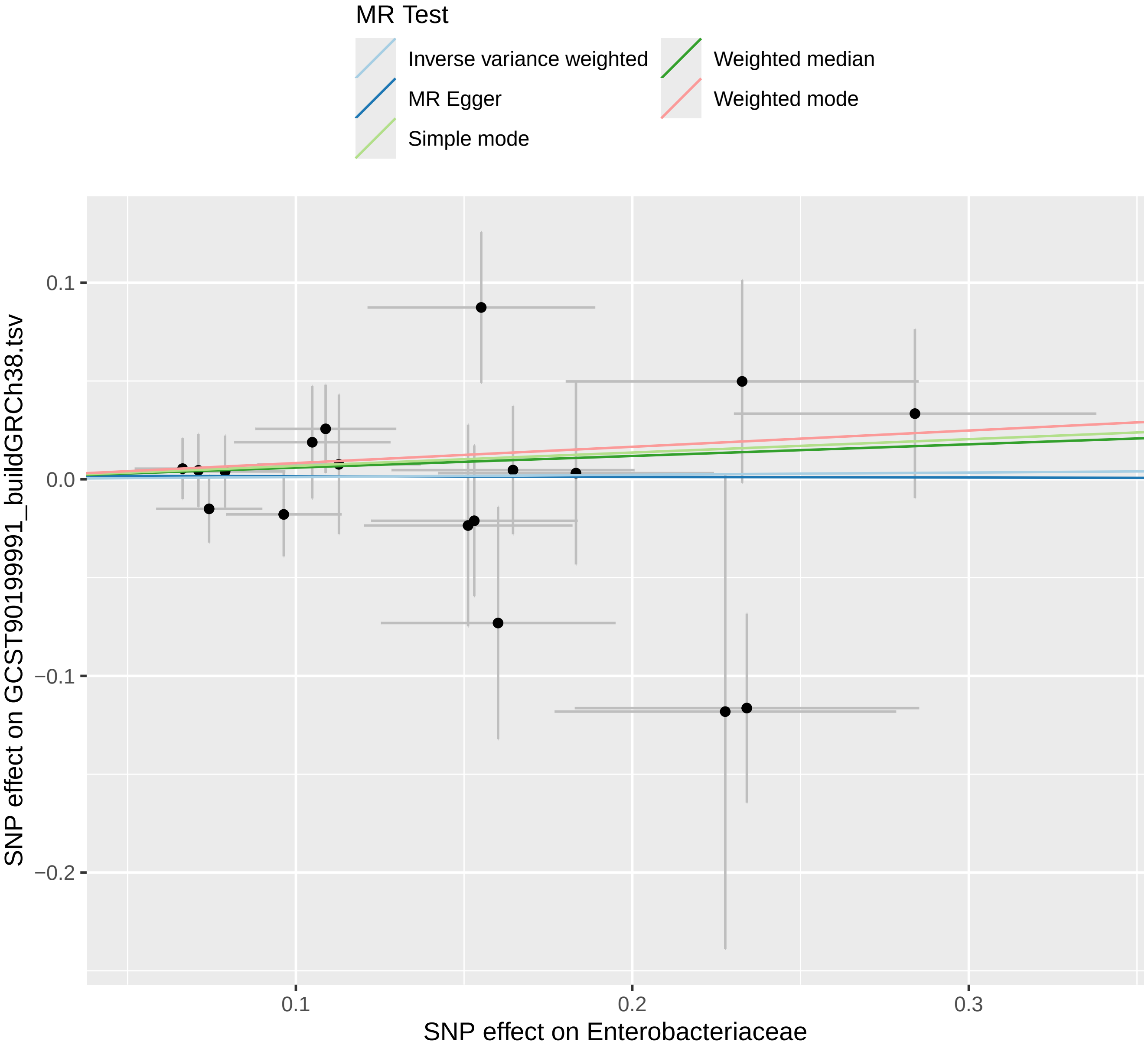

MR Test
Inverse variance weighted
MR Egger
Simple mode
Weighted median
Weighted mode
0.1
0.0
−0.1
−0.2
SNP effect on GCST90199991_buildGRCh38.tsv
0.1
0.2
0.3
SNP effect on Enterobacteriaceae

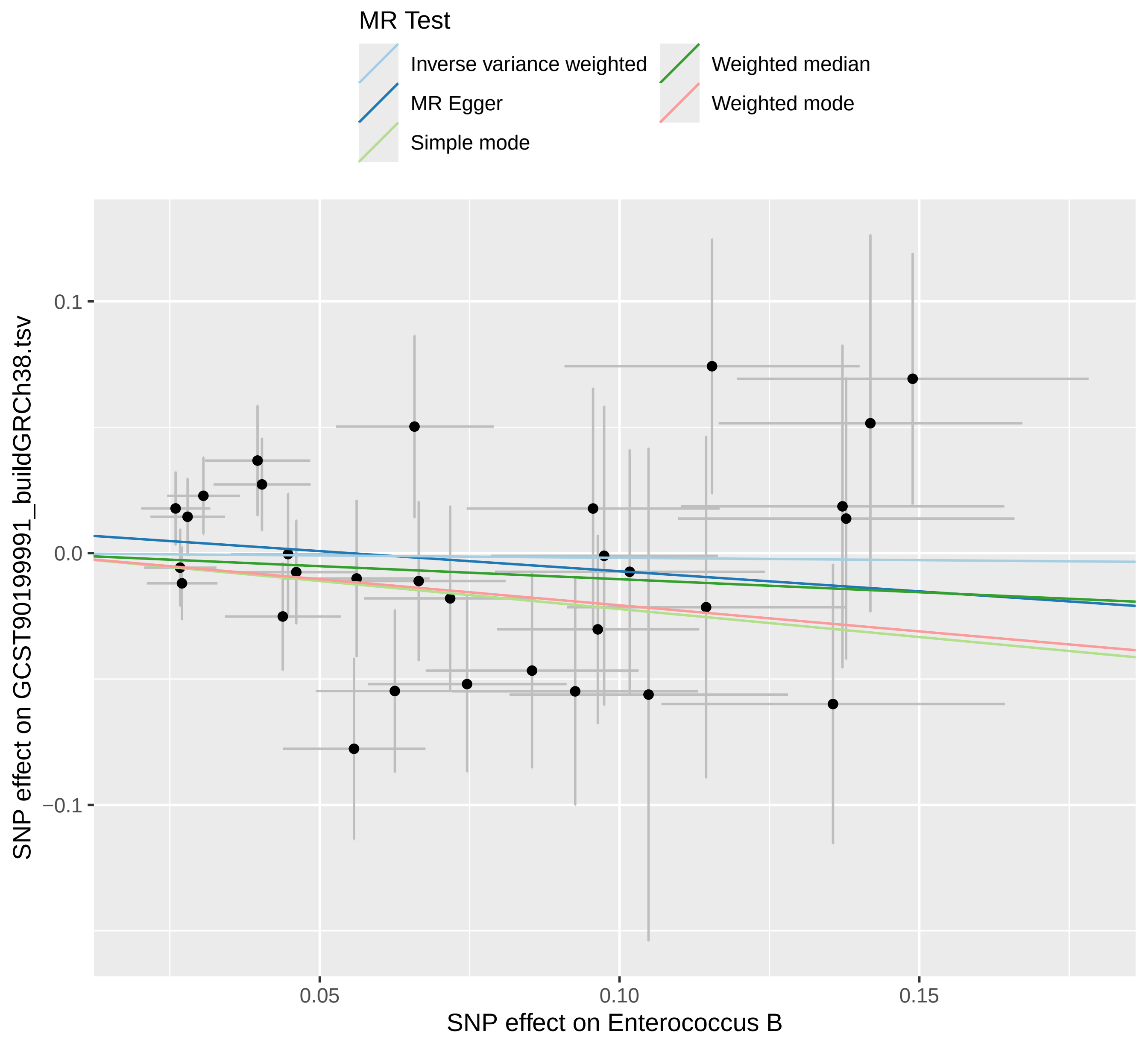
MR Test
Inverse variance weighted
MR Egger
Simple mode
Weighted median
Weighted mode
0.1
0.0
−0.1
0.05
0.10
0.15
SNP effect on GCST90199991_buildGRCh38.tsv
SNP effect on Enterococcus B

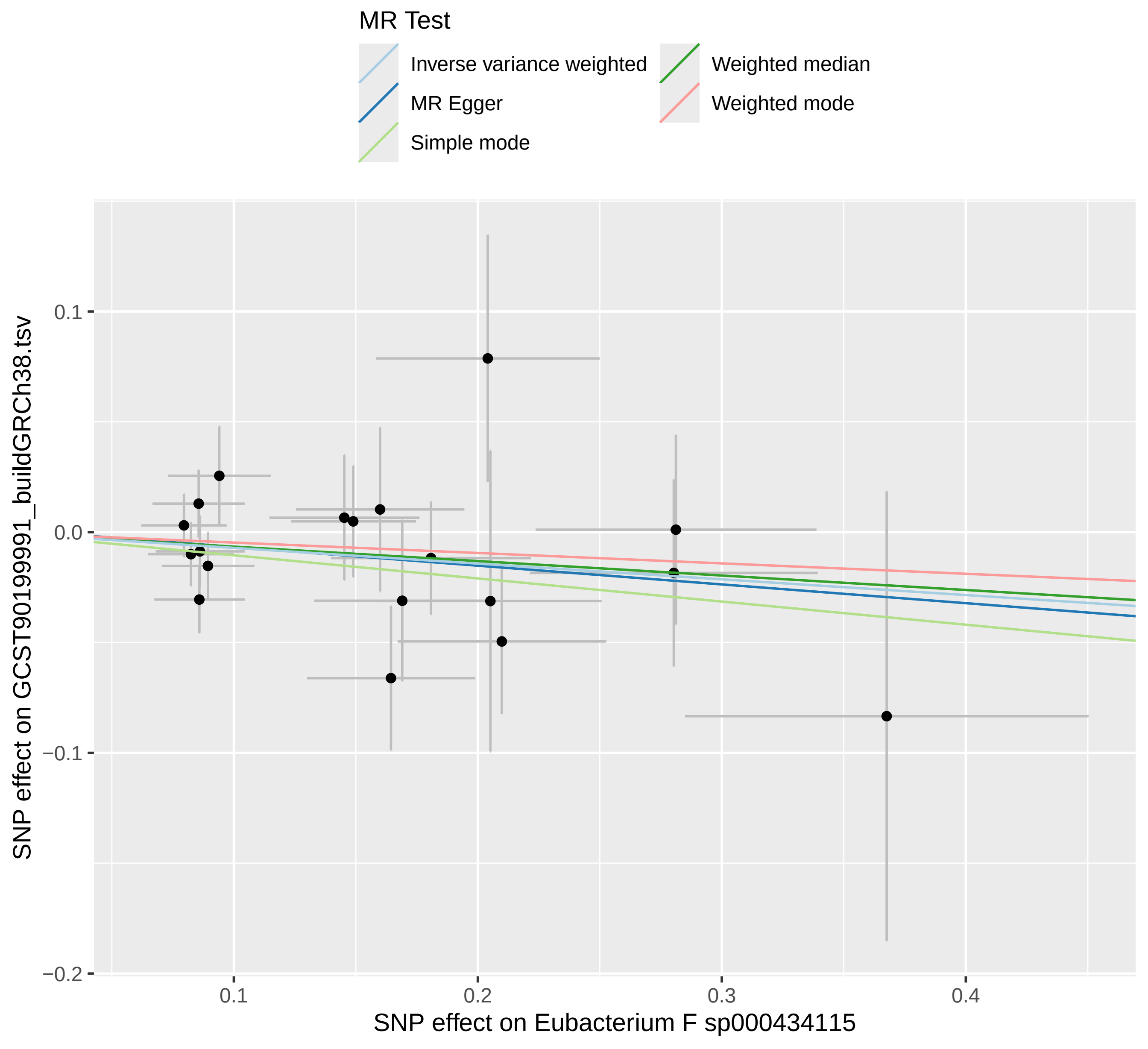

MR Test
Inverse variance weighted
MR Egger
Simple mode
Weighted median
Weighted mode
SNP effect on GCST90199991_buildGRCh38.tsv
SNP effect on Eubacterium F sp000434115
0.1
0.0
−0.1
−0.2
0.1
0.2
0.3
0.4

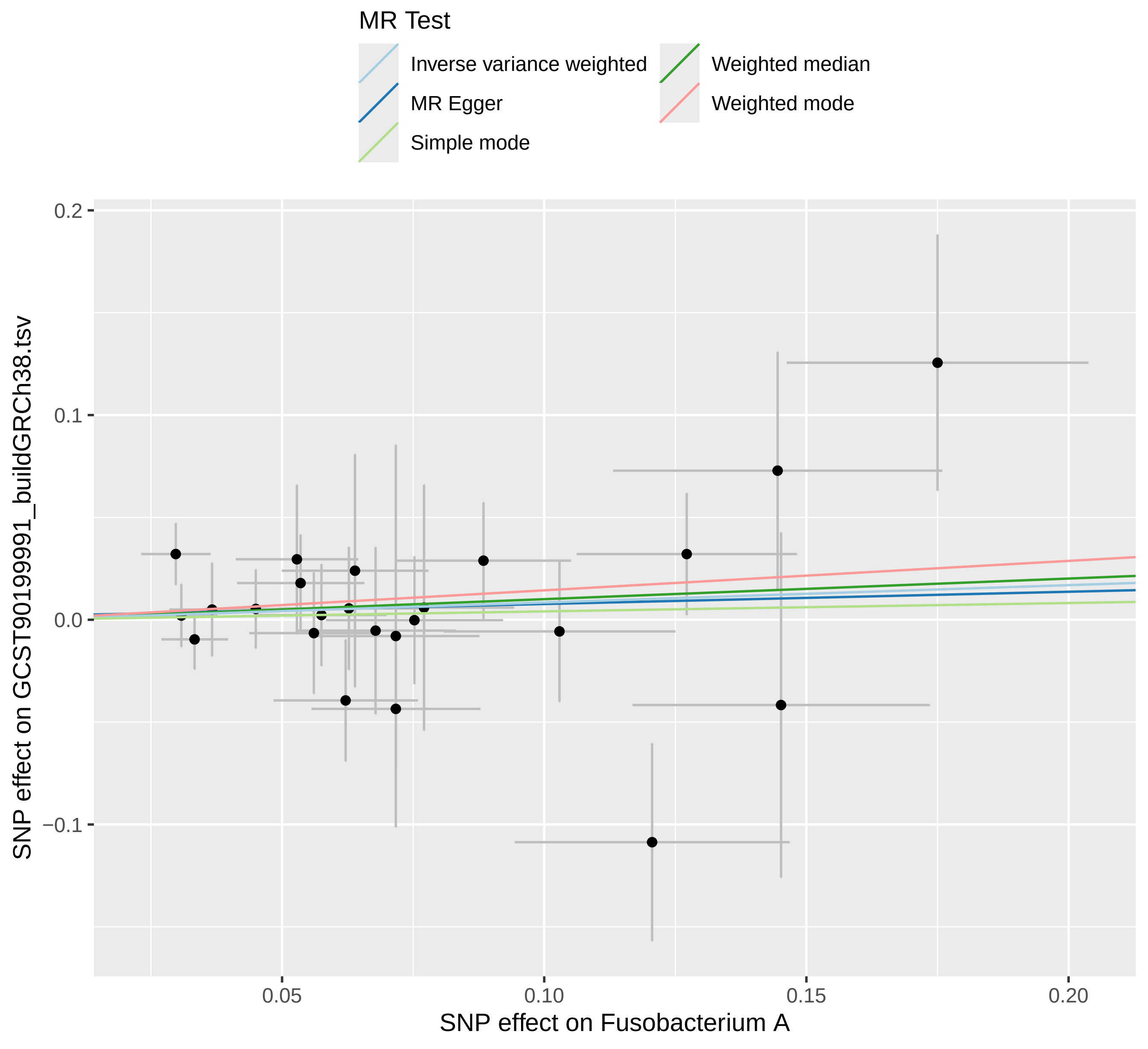
MR Test
Inverse variance weighted
MR Egger
Simple mode
Weighted median
Weighted mode
0.2
0.1
0.0
−0.1
0.05
0.10
0.15
0.20
SNP effect on GCST90199991_buildGRCh38.tsv
SNP effect on Fusobacterium A

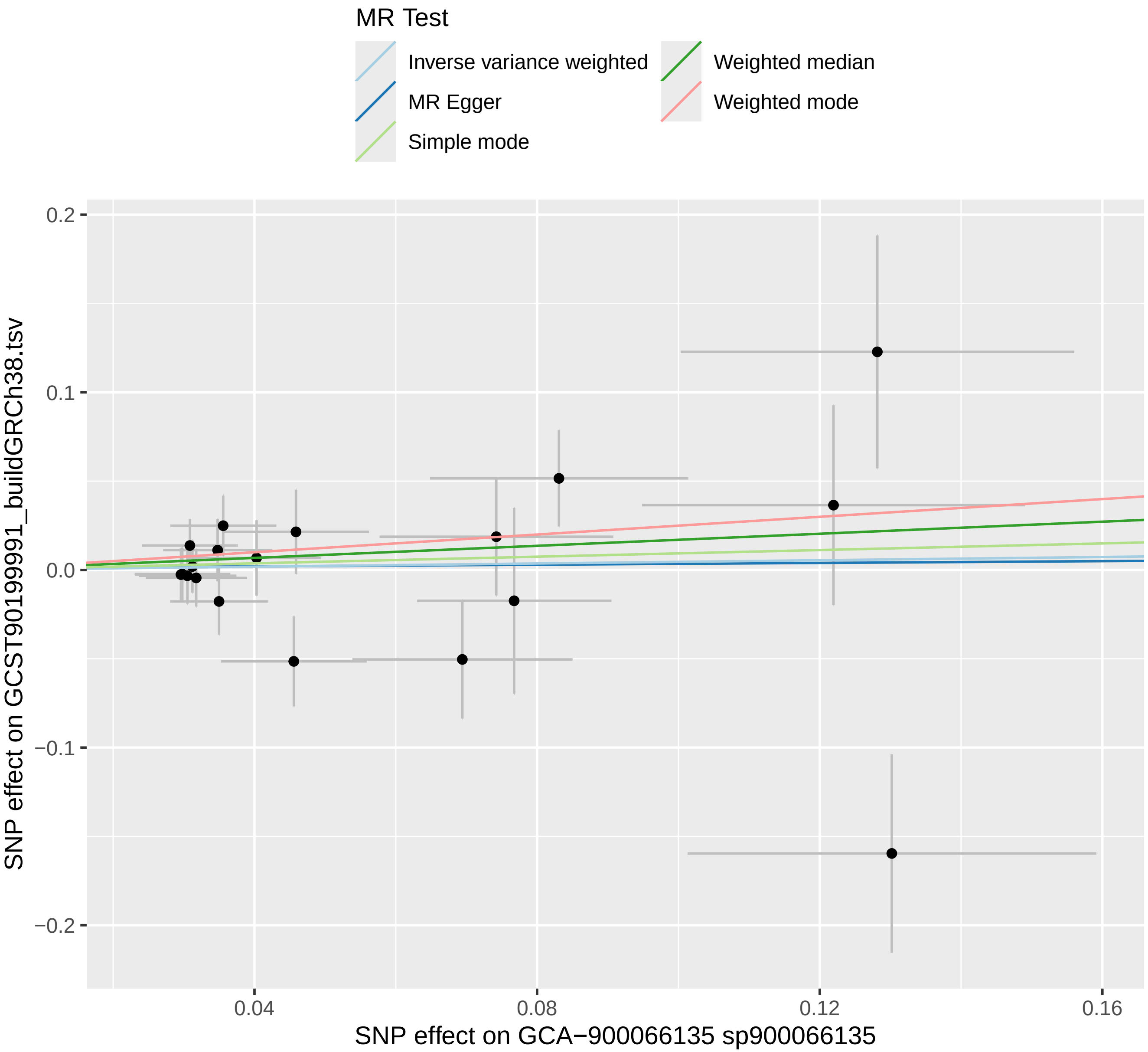

MR Test
Inverse variance weighted
MR Egger
Simple mode
Weighted median
Weighted mode
SNP effect on GCST90199991_buildGRCh38.tsv
0.2
0.1
0.0
−0.1
−0.2
0.04
0.08
0.12
0.16
SNP effect on GCA−900066135 sp900066135

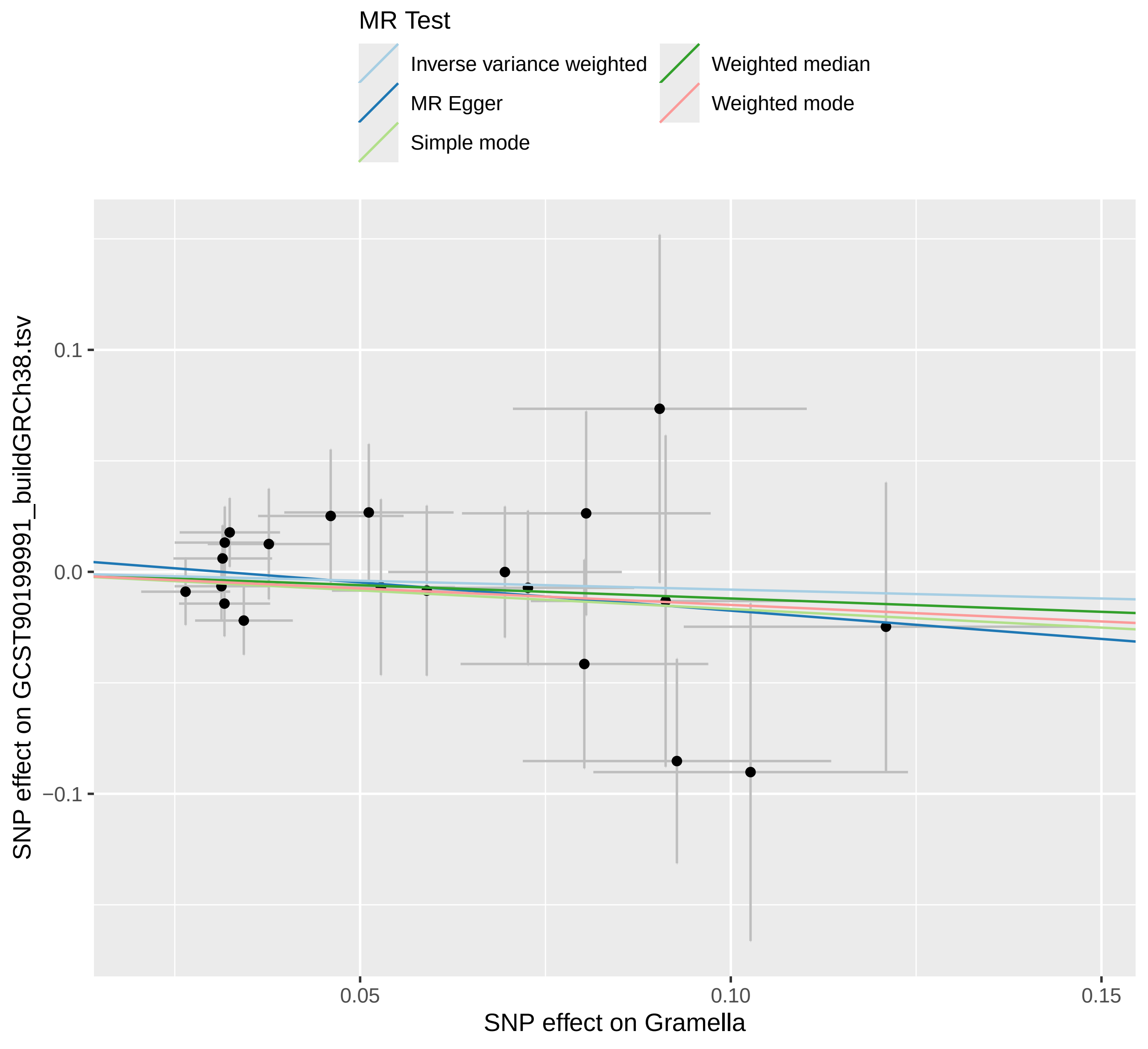

MR Test
Inverse variance weighted
MR Egger
Simple mode
Weighted median
Weighted mode
0.1
0.0
−0.1
0.05
0.10
0.15
SNP effect on GCST90199991_buildGRCh38.tsv
SNP effect on Gramella

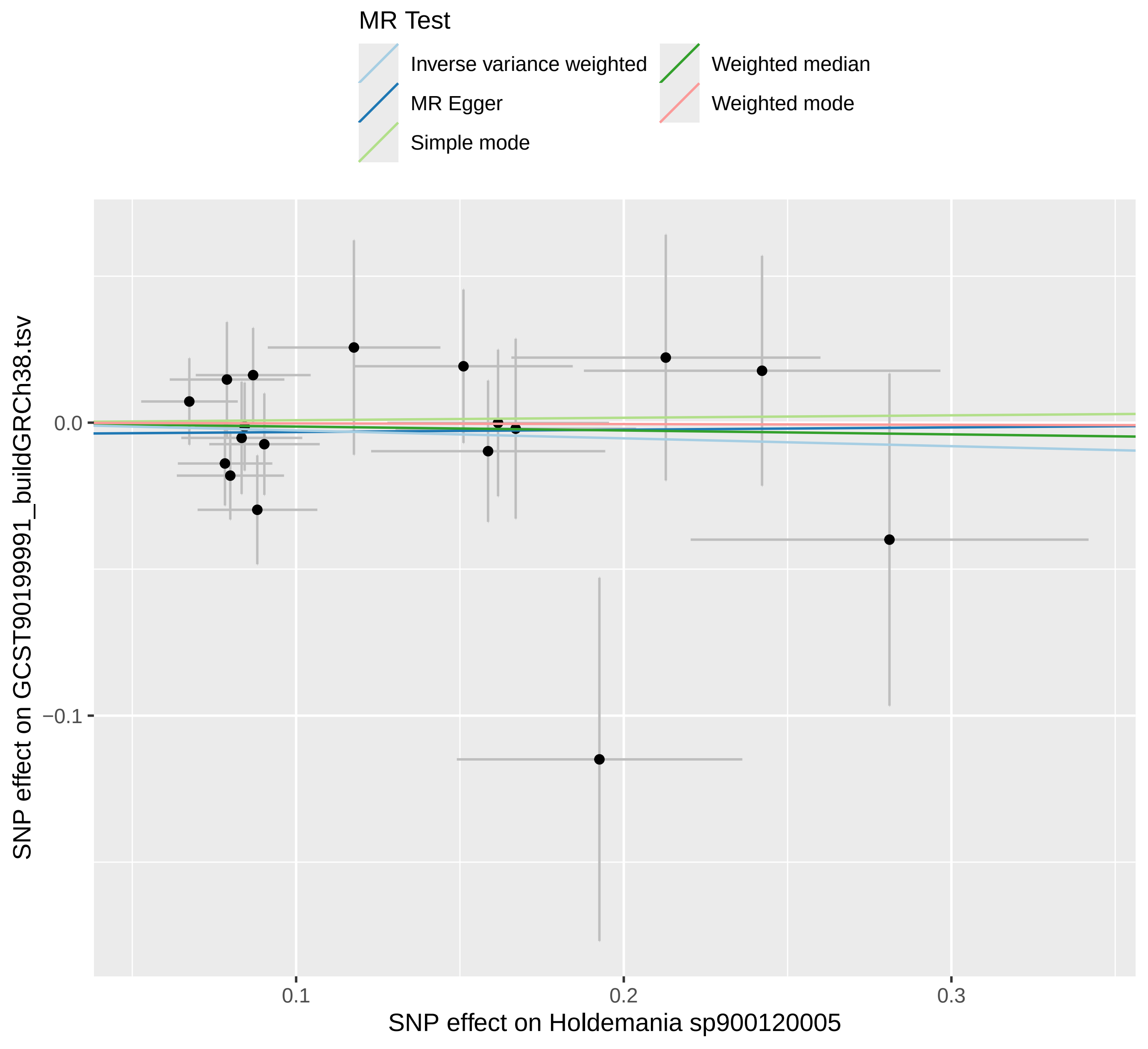

MR Test
Inverse variance weighted
MR Egger
Simple mode
Weighted median
Weighted mode
SNP effect on GCST90199991_buildGRCh38.tsv
0.0
−0.1
0.1
0.2
0.3
SNP effect on Holdemania sp900120005

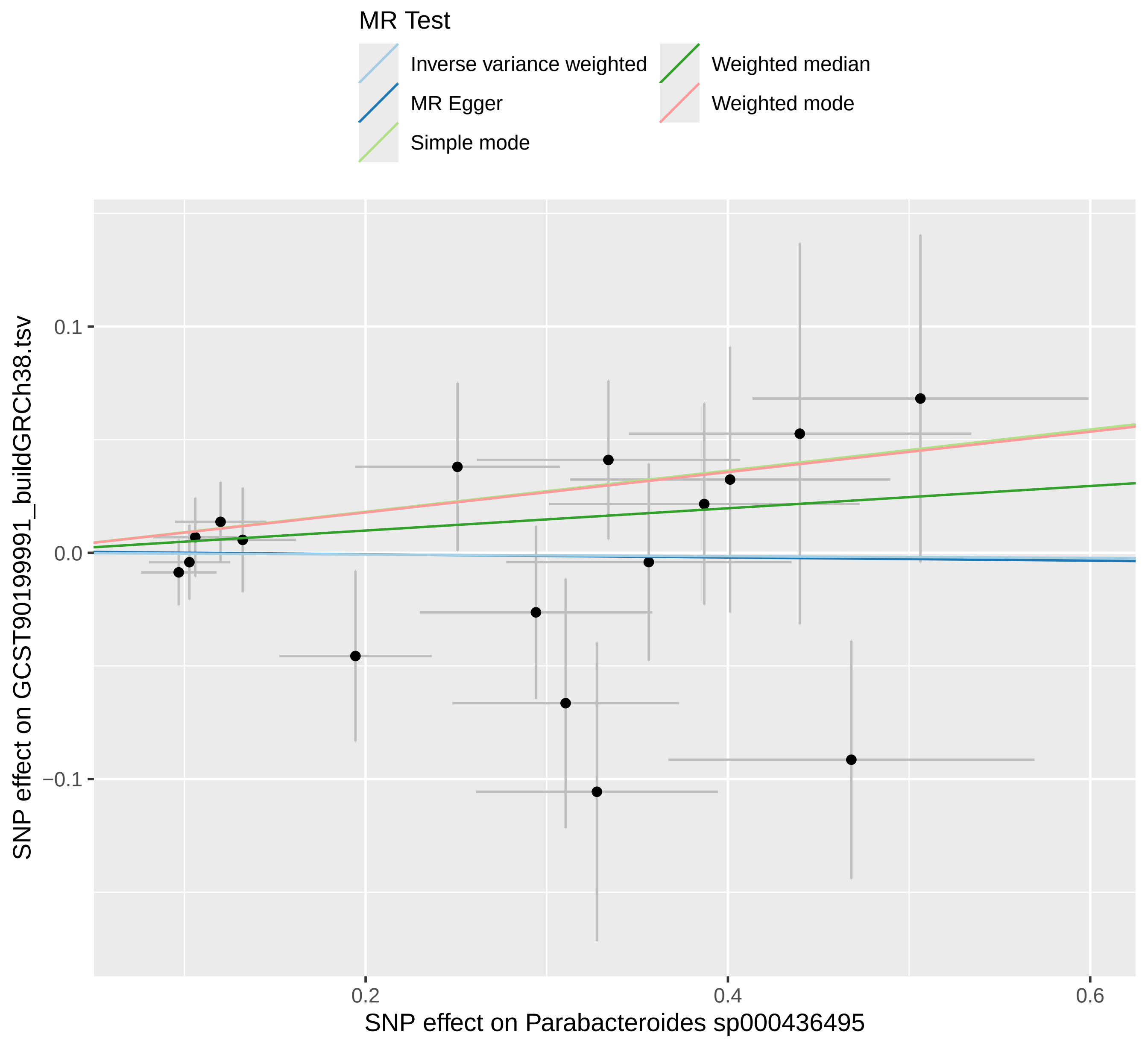

MR Test
Inverse variance weighted
MR Egger
Simple mode
Weighted median
Weighted mode
0.1
0.0
−0.1
0.2
0.4
0.6
SNP effect on GCST90199991_buildGRCh38.tsv
SNP effect on Parabacteroides sp000436495

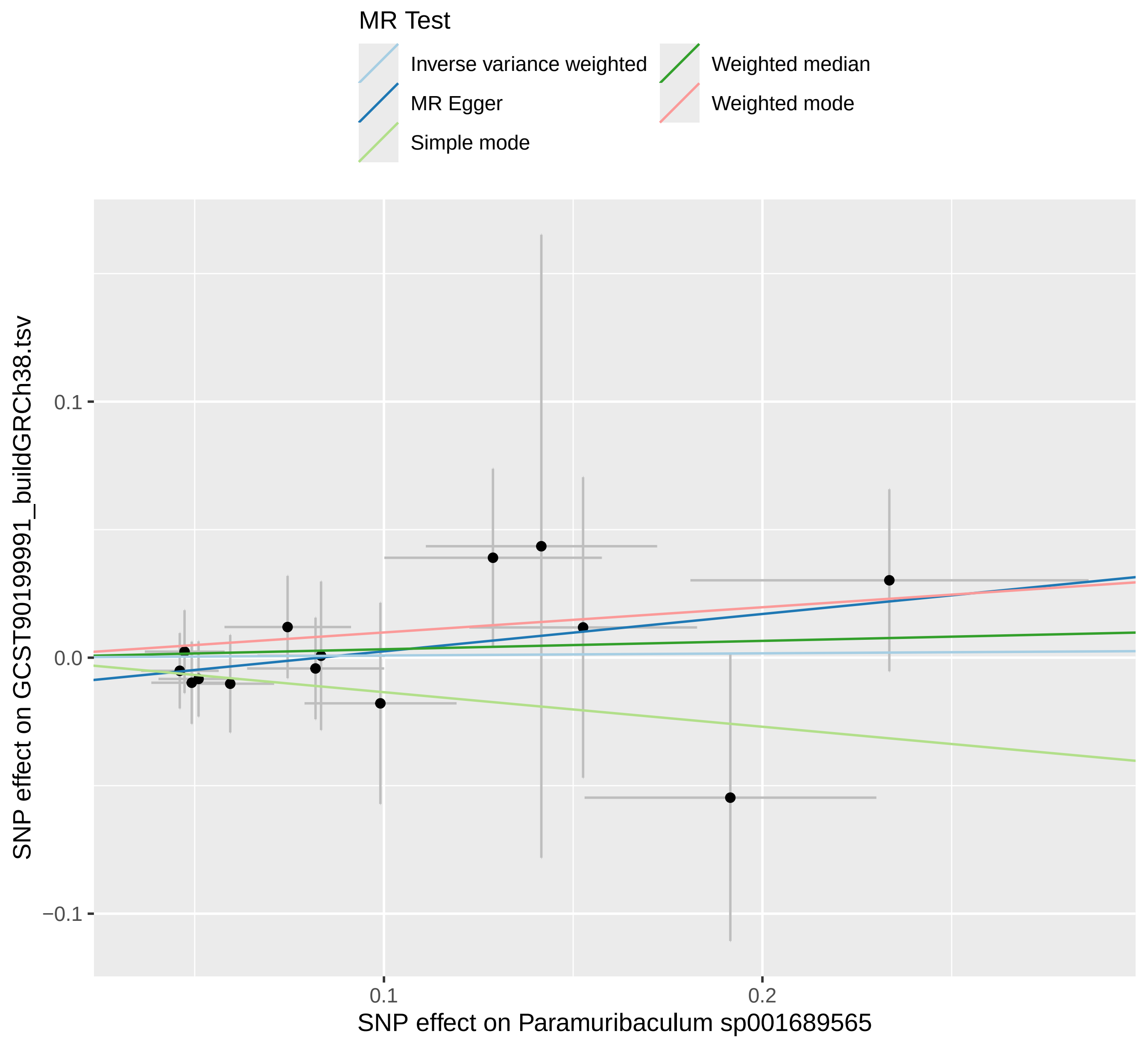
MR Test
Inverse variance weighted
MR Egger
Simple mode
Weighted median
Weighted mode
SNP effect on GCST90199991_buildGRCh38.tsv
0.1
0.0
−0.1
0.1
0.2
SNP effect on Paramuribaculum sp001689565

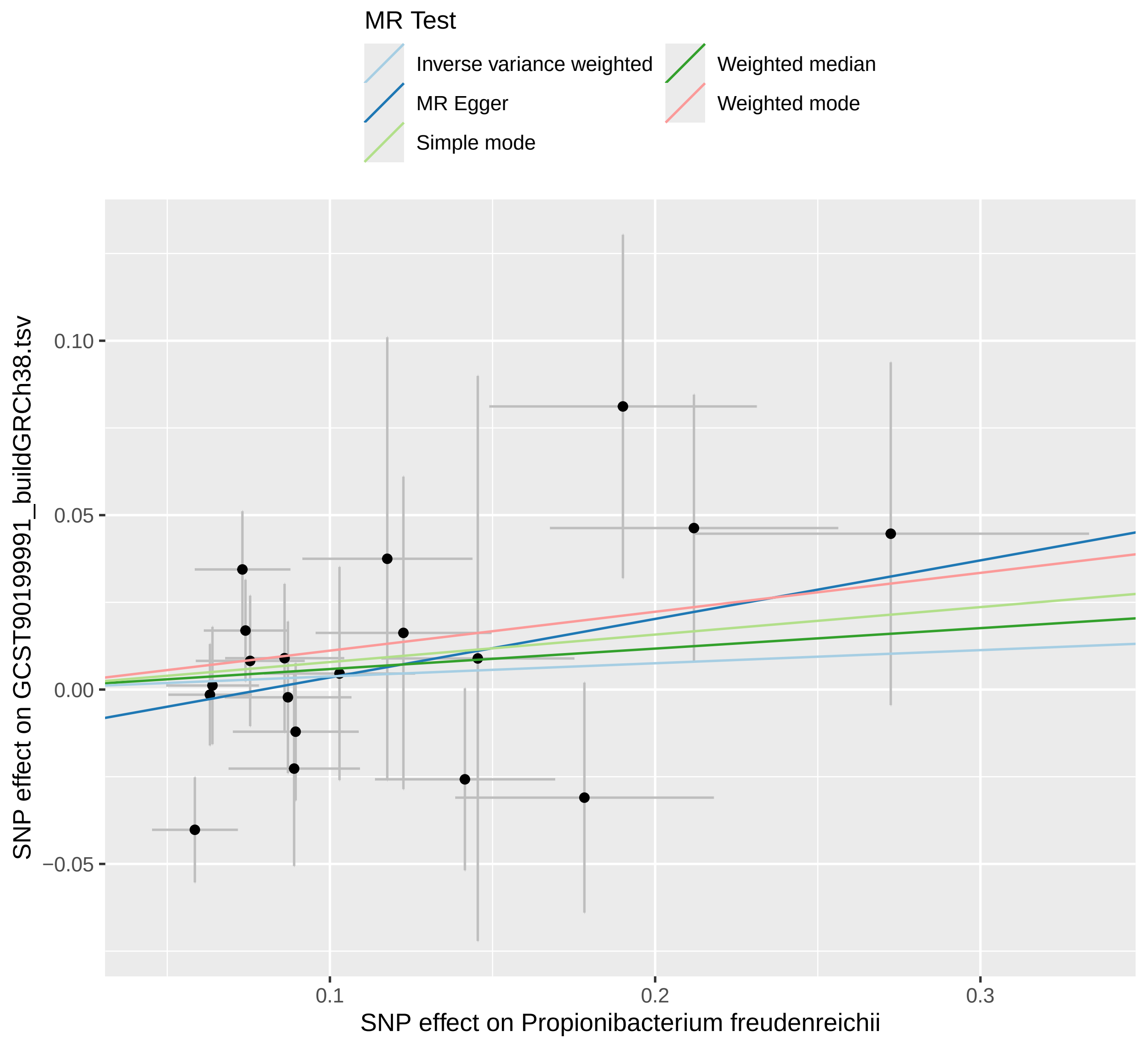
MR Test
Inverse variance weighted
MR Egger
Simple mode
Weighted median
Weighted mode
0.10
0.05
0.00
−0.05
0.1
0.2
0.3
SNP effect on GCST90199991_buildGRCh38.tsv
SNP effect on Propionibacterium freudenreichii

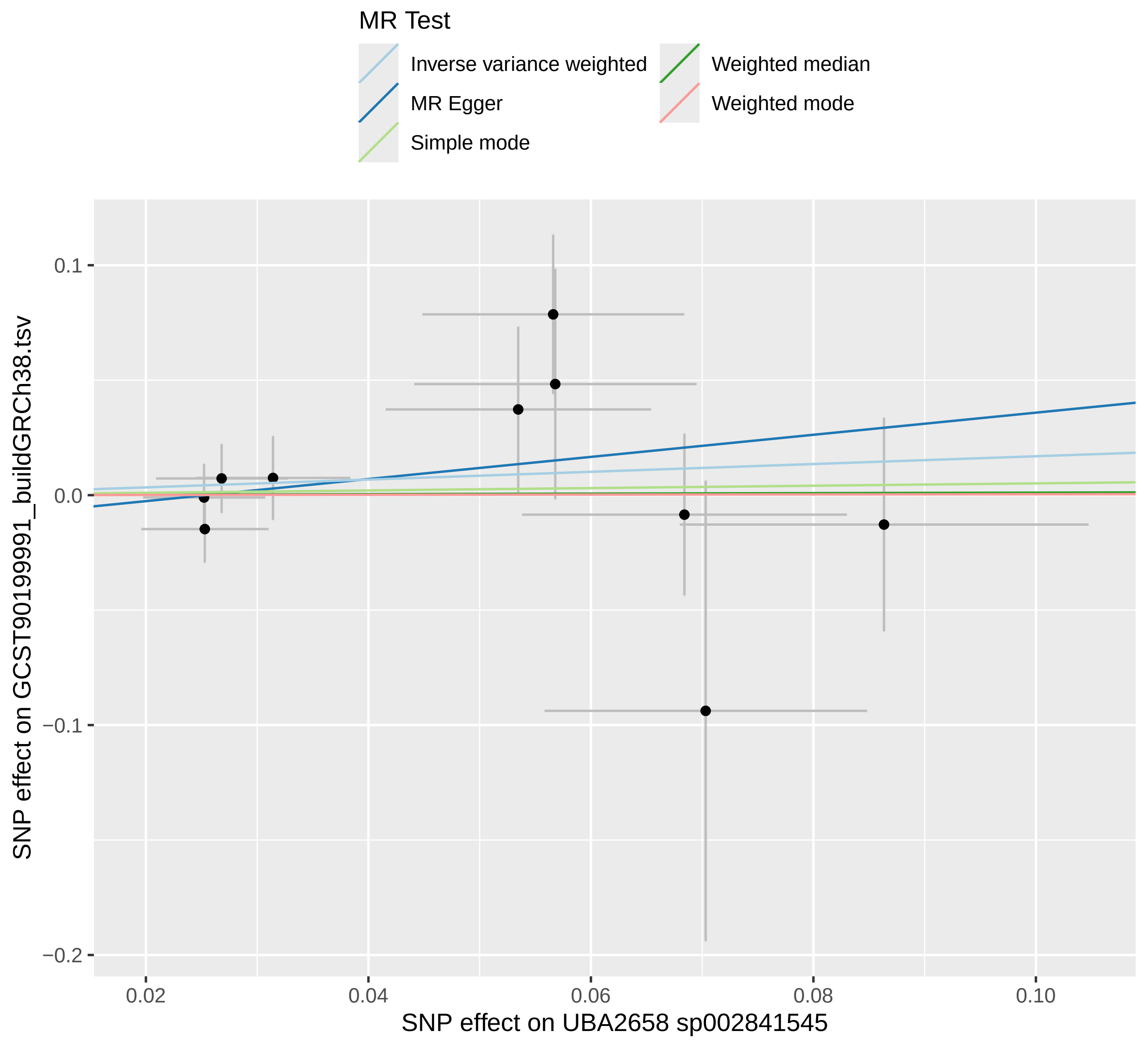

MR Test
Inverse variance weighted
MR Egger
Simple mode
Weighted median
Weighted mode
0.1
0.0
−0.1
−0.2
0.02
0.04
0.06
0.08
0.10
SNP effect on GCST90199991_buildGRCh38.tsv
SNP effect on UBA2658 sp002841545

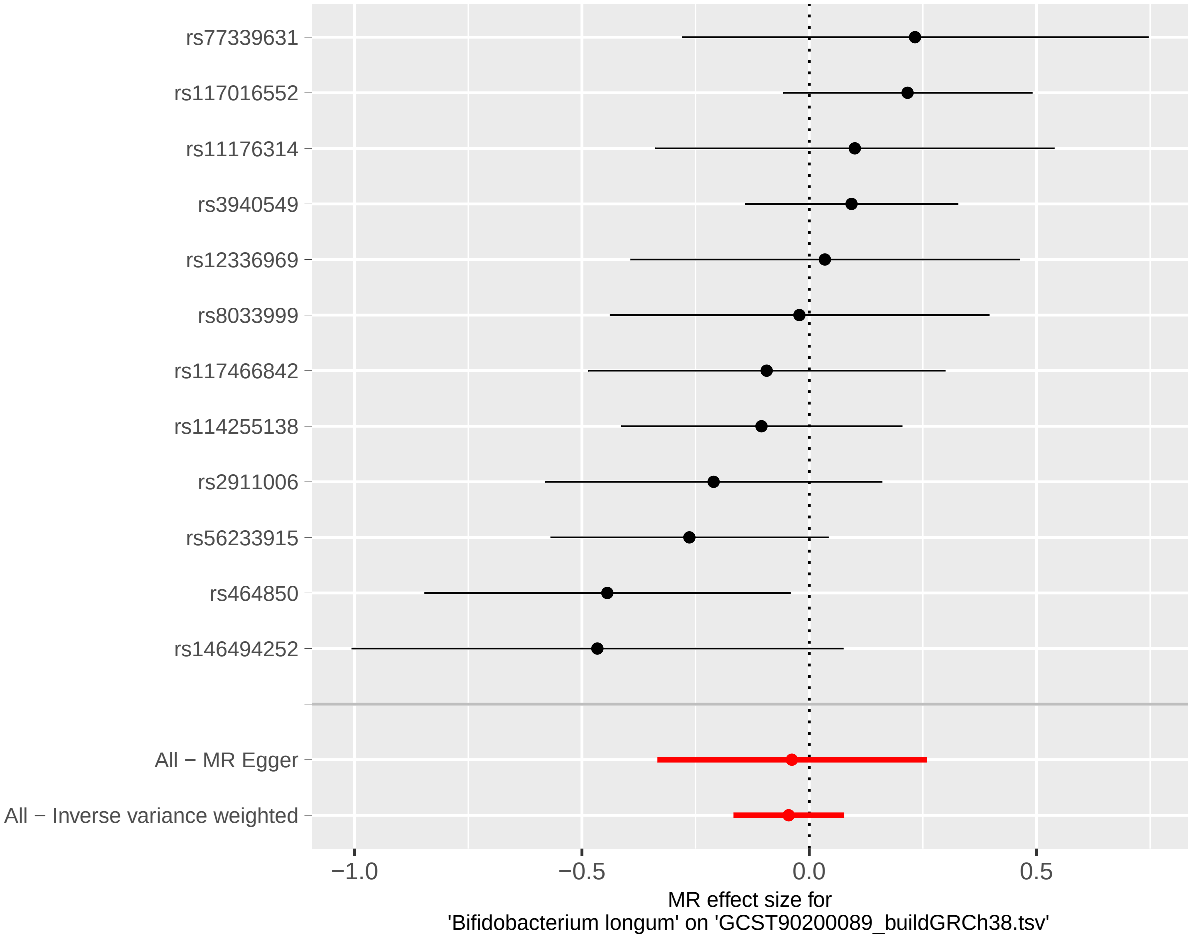
rs77339631
rs117016552
rs11176314
rs3940549
rs12336969
rs8033999
rs117466842
rs114255138
rs2911006
rs56233915
rs464850
rs146494252
All − MR Egger
All − Inverse variance weighted
−1.0
−0.5
0.0
0.5
MR effect size for
'Bifidobacterium longum' on 'GCST90200089_buildGRCh38.tsv'

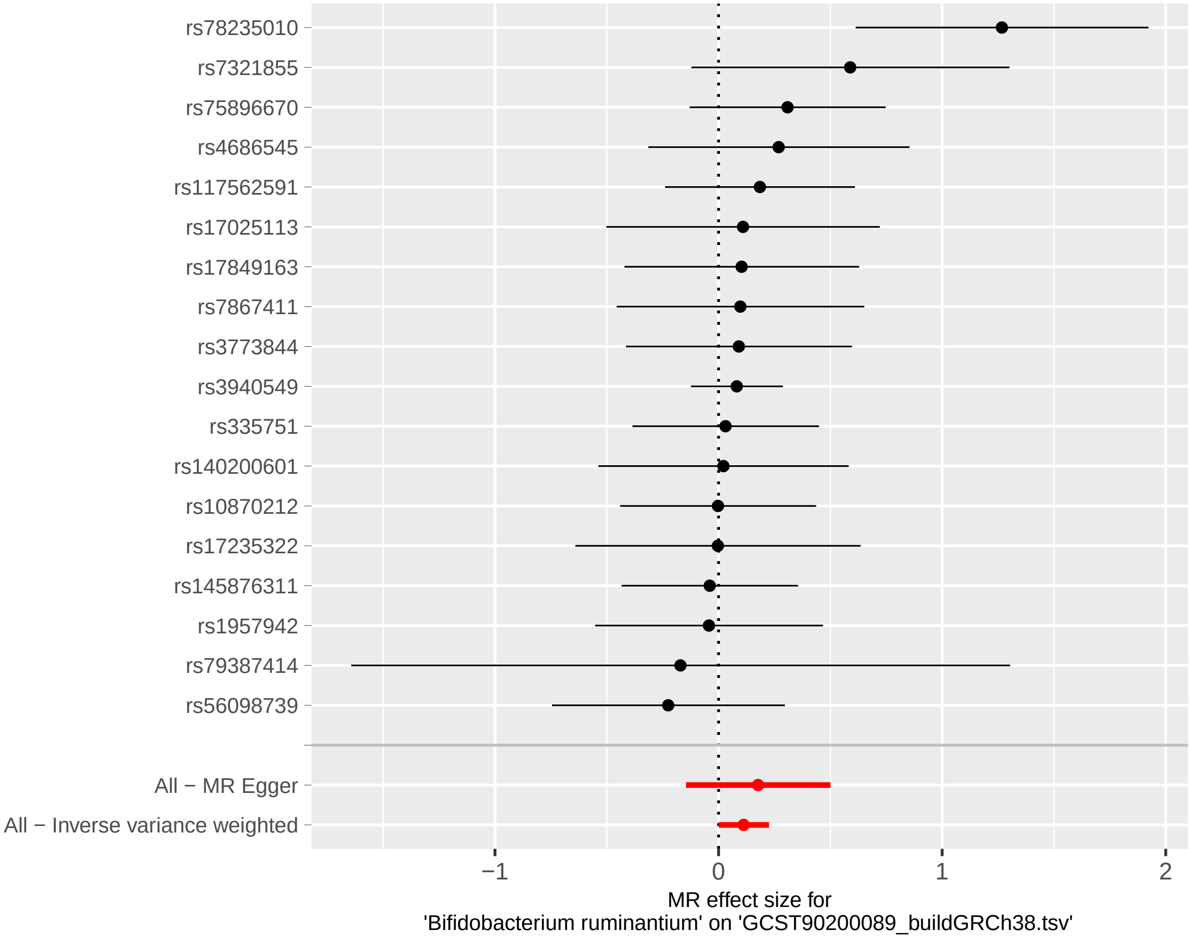
rs78235010
rs7321855
rs75896670
rs4686545
rs117562591
rs17025113
rs17849163
rs7867411
rs3773844
rs3940549
rs335751
rs140200601
rs10870212
rs17235322
rs145876311
rs1957942
rs79387414
rs56098739
All − MR Egger
All − Inverse variance weighted
−1
0
1
2
MR effect size for
'Bifidobacterium ruminantium' on 'GCST90200089_buildGRCh38.tsv'

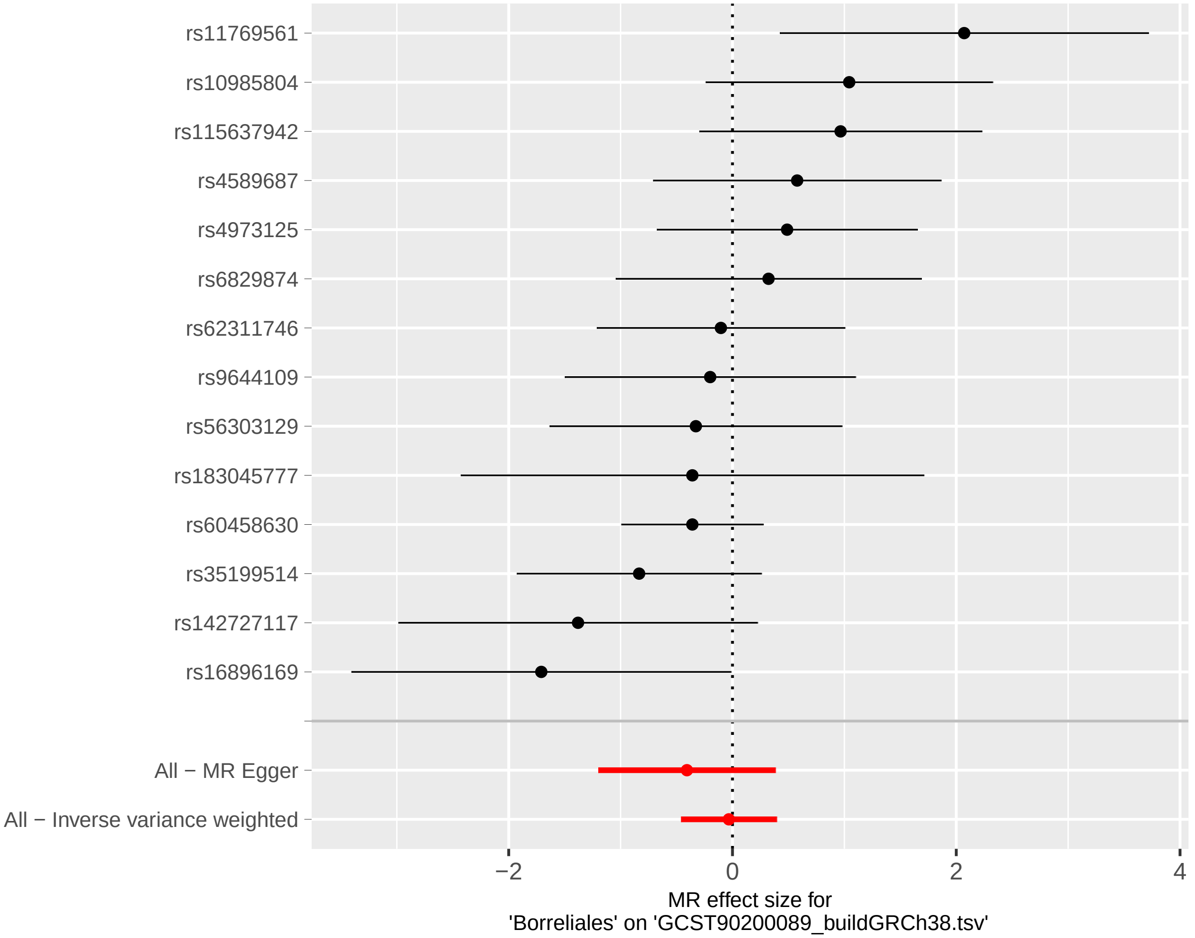
rs11769561
rs10985804
rs115637942
rs4589687
rs4973125
rs6829874
rs62311746
rs9644109
rs56303129
rs183045777
rs60458630
rs35199514
rs142727117
rs16896169
All − MR Egger
All − Inverse variance weighted
−2
0
2
4
MR effect size for
'Borreliales' on 'GCST90200089_buildGRCh38.tsv'

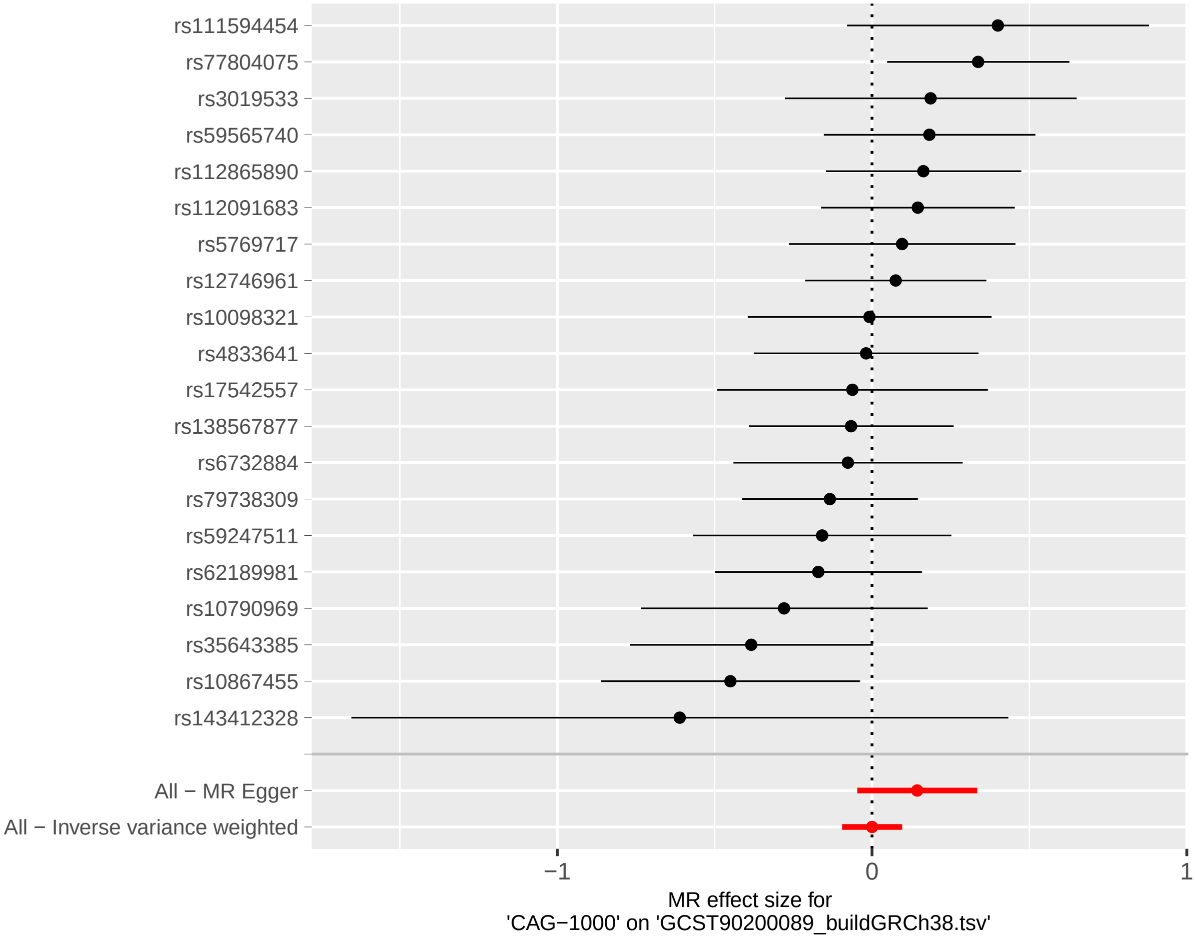
rs111594454
rs77804075
rs3019533
rs59565740
rs112865890
rs112091683
rs5769717
rs12746961
rs10098321
rs4833641
rs17542557
rs138567877
rs6732884
rs79738309
rs59247511
rs62189981
rs10790969
rs35643385
rs10867455
rs143412328
All − MR Egger
All − Inverse variance weighted
−1
0
1
MR effect size for
'CAG−1000' on 'GCST90200089_buildGRCh38.tsv'

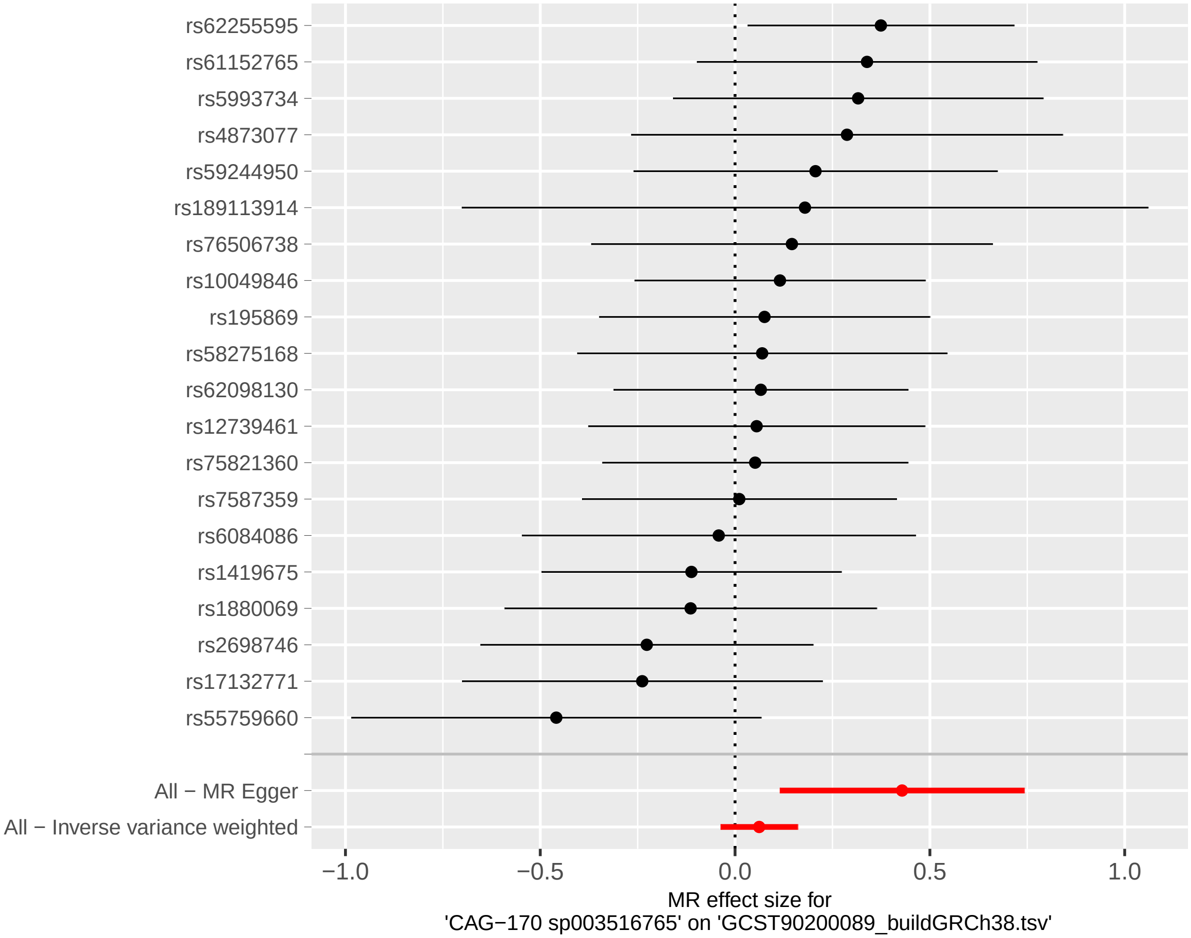

rs62255595
rs61152765
rs5993734
rs4873077
rs59244950
rs189113914
rs76506738
rs10049846
rs195869
rs58275168
rs62098130
rs12739461
rs75821360
rs7587359
rs6084086
rs1419675
rs1880069
rs2698746
rs17132771
rs55759660
All − MR Egger
All − Inverse variance weighted
−1.0
−0.5
0.0
0.5
1.0
MR effect size for
'CAG−170 sp003516765' on 'GCST90200089_buildGRCh38.tsv'

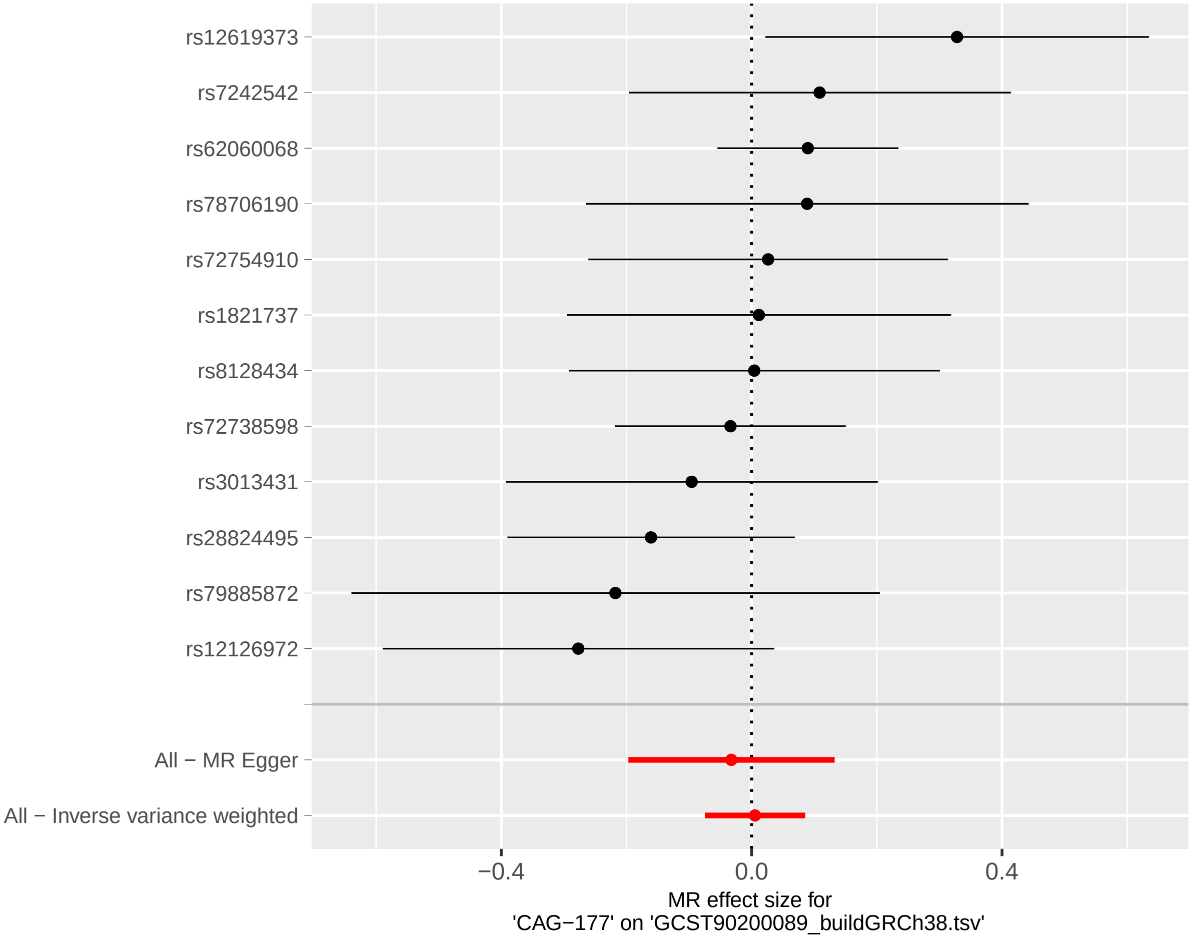
rs12619373
rs7242542
rs62060068
rs78706190
rs72754910
rs1821737
rs8128434
rs72738598
rs3013431
rs28824495
rs79885872
rs12126972
All − MR Egger
All − Inverse variance weighted
−0.4
0.0
0.4
MR effect size for
'CAG−177' on 'GCST90200089_buildGRCh38.tsv'

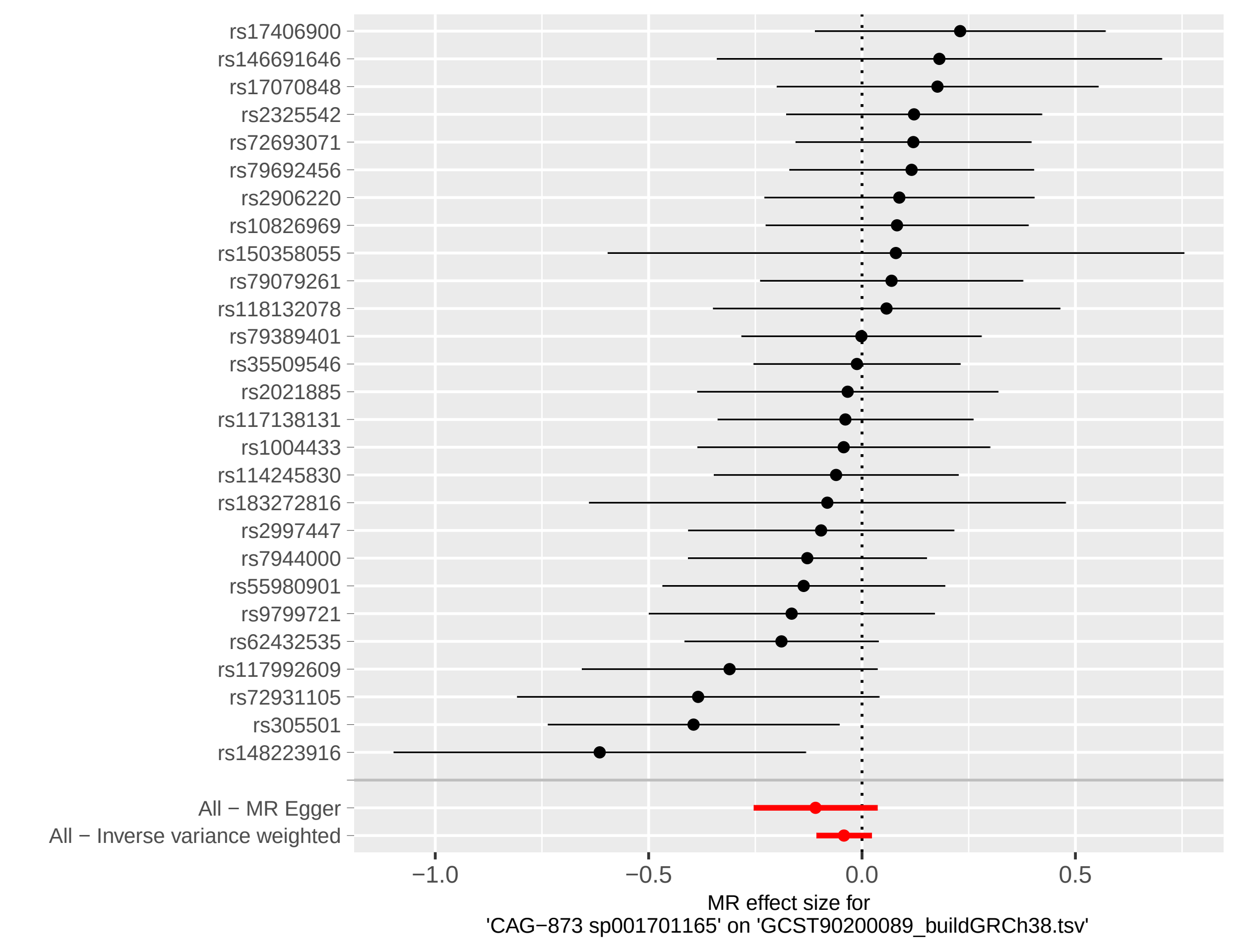

rs17406900
rs146691646
rs17070848
rs2325542
rs72693071
rs79692456
rs2906220
rs10826969
rs150358055
rs79079261
rs118132078
rs79389401
rs35509546
rs2021885
rs117138131
rs1004433
rs114245830
rs183272816
rs2997447
rs7944000
rs55980901
rs9799721
rs62432535
rs117992609
rs72931105
rs305501
rs148223916
All − MR Egger
All − Inverse variance weighted
−1.0
−0.5
0.0
0.5
MR effect size for
'CAG−873 sp001701165' on 'GCST90200089_buildGRCh38.tsv'

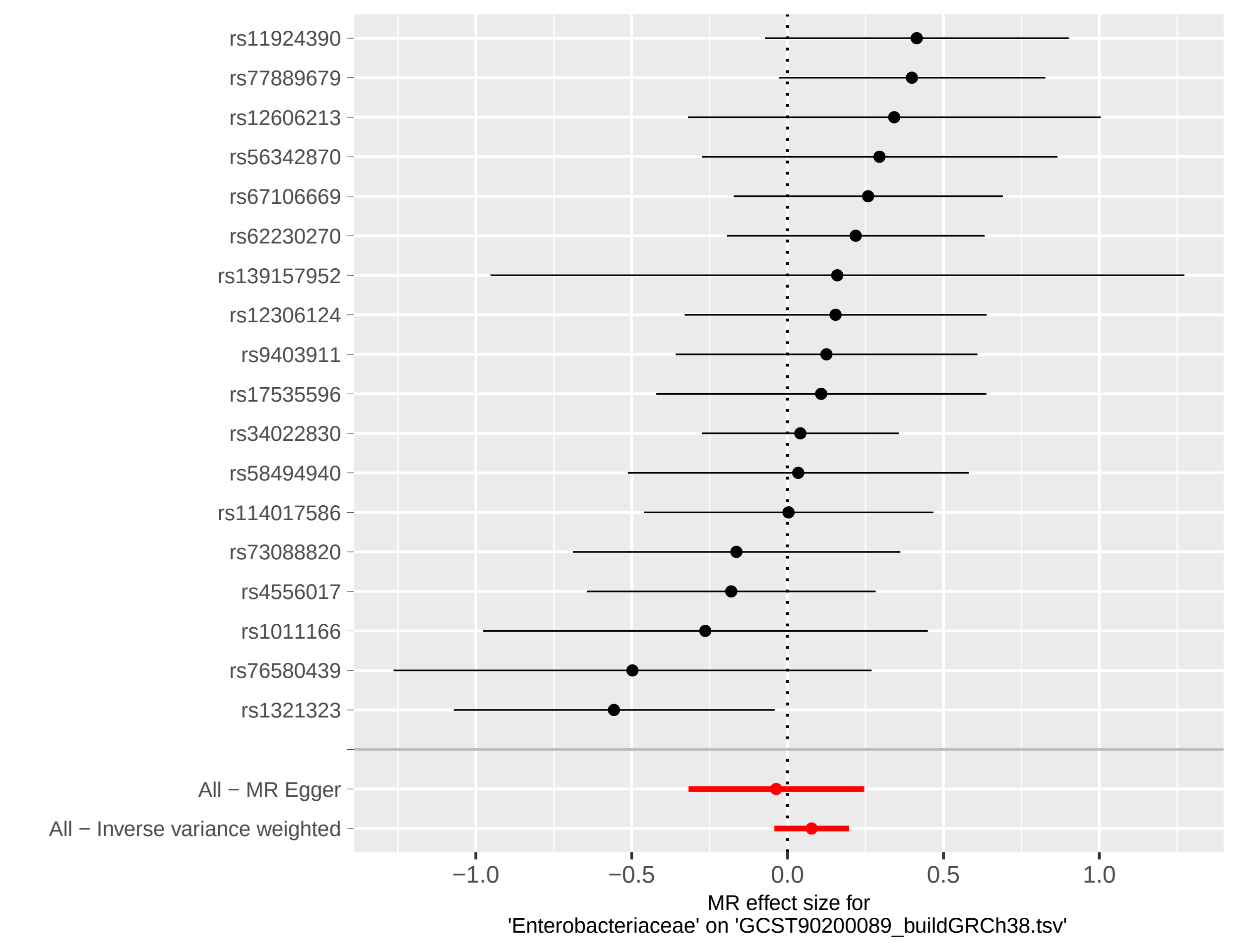

rs11924390
rs77889679
rs12606213
rs56342870
rs67106669
rs62230270
rs139157952
rs12306124
rs9403911
rs17535596
rs34022830
rs58494940
rs114017586
rs73088820
rs4556017
rs1011166
rs76580439
rs1321323
All − MR Egger
All − Inverse variance weighted
−1.0
−0.5
0.0
0.5
1.0
MR effect size for
'Enterobacteriaceae' on 'GCST90200089_buildGRCh38.tsv'

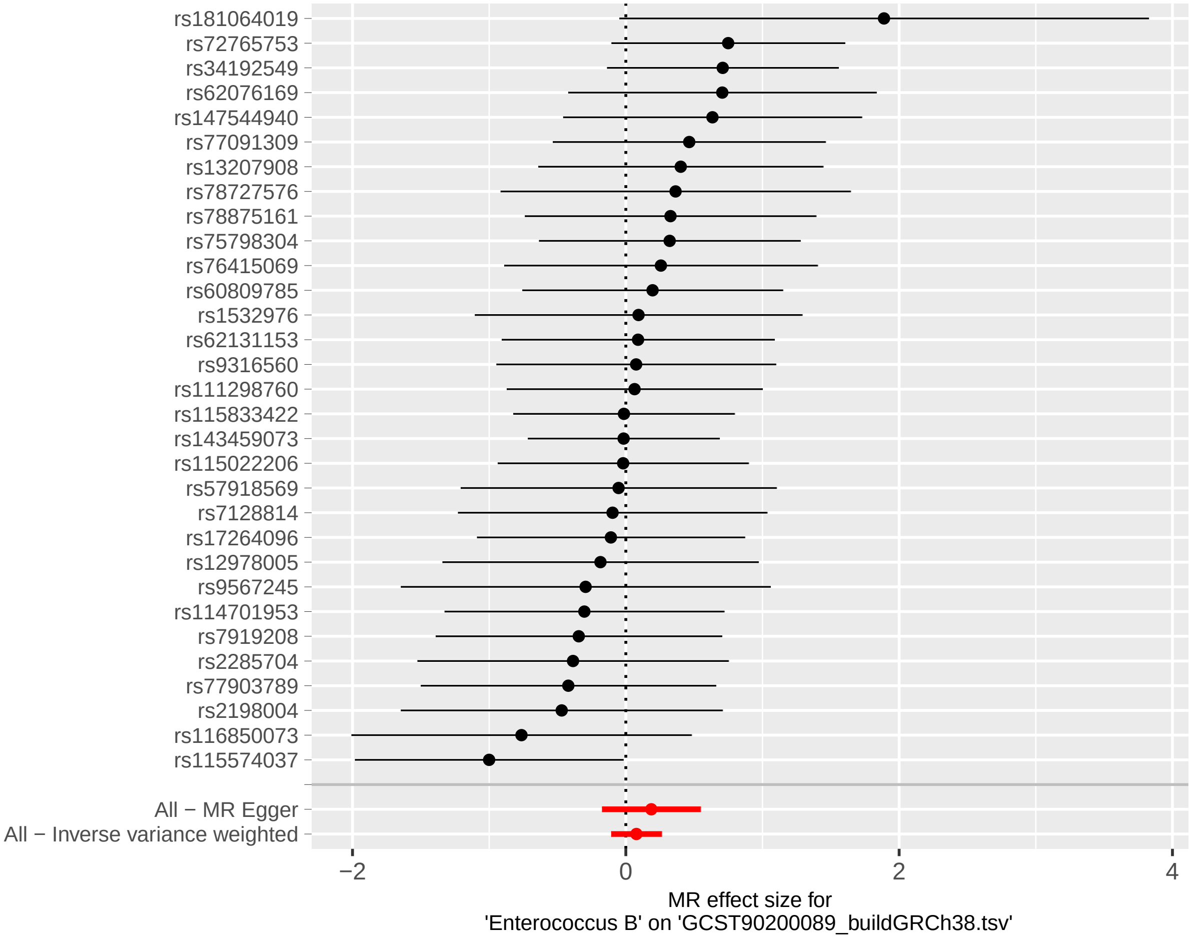

rs181064019
rs72765753
rs34192549
rs62076169
rs147544940
rs77091309
rs13207908
rs78727576
rs78875161
rs75798304
rs76415069
rs60809785
rs1532976
rs62131153
rs9316560
rs111298760
rs115833422
rs143459073
rs115022206
rs57918569
rs7128814
rs17264096
rs12978005
rs9567245
rs114701953
rs7919208
rs2285704
rs77903789
rs2198004
rs116850073
rs115574037
All − MR Egger
All − Inverse variance weighted
−2
0
2
4
MR effect size for
'Enterococcus B' on 'GCST90200089_buildGRCh38.tsv'

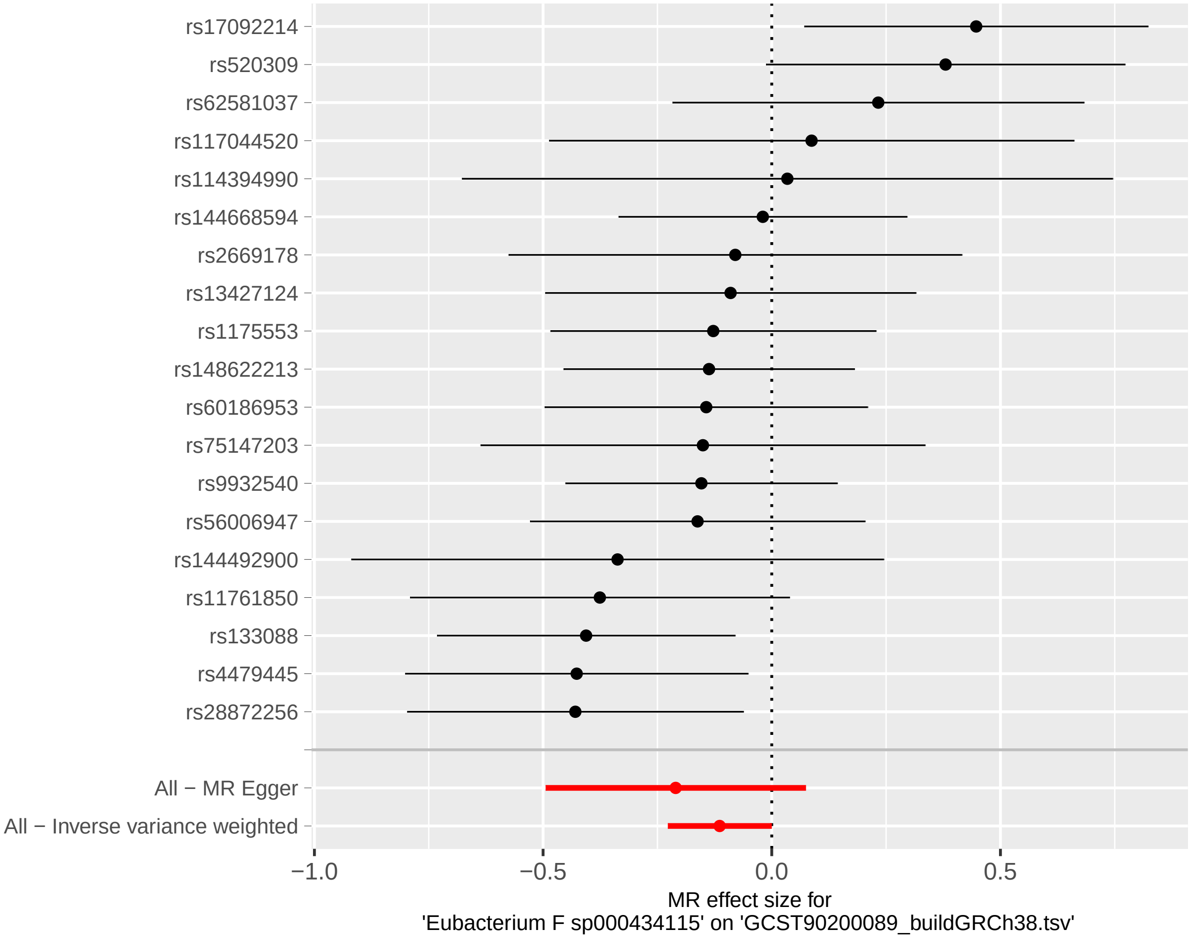
rs17092214
rs520309
rs62581037
rs117044520
rs114394990
rs144668594
rs2669178
rs13427124
rs1175553
rs148622213
rs60186953
rs75147203
rs9932540
rs56006947
rs144492900
rs11761850
rs133088
rs4479445
rs28872256
All − MR Egger
All − Inverse variance weighted
−1.0
−0.5
0.0
0.5
MR effect size for
'Eubacterium F sp000434115' on 'GCST90200089_buildGRCh38.tsv'

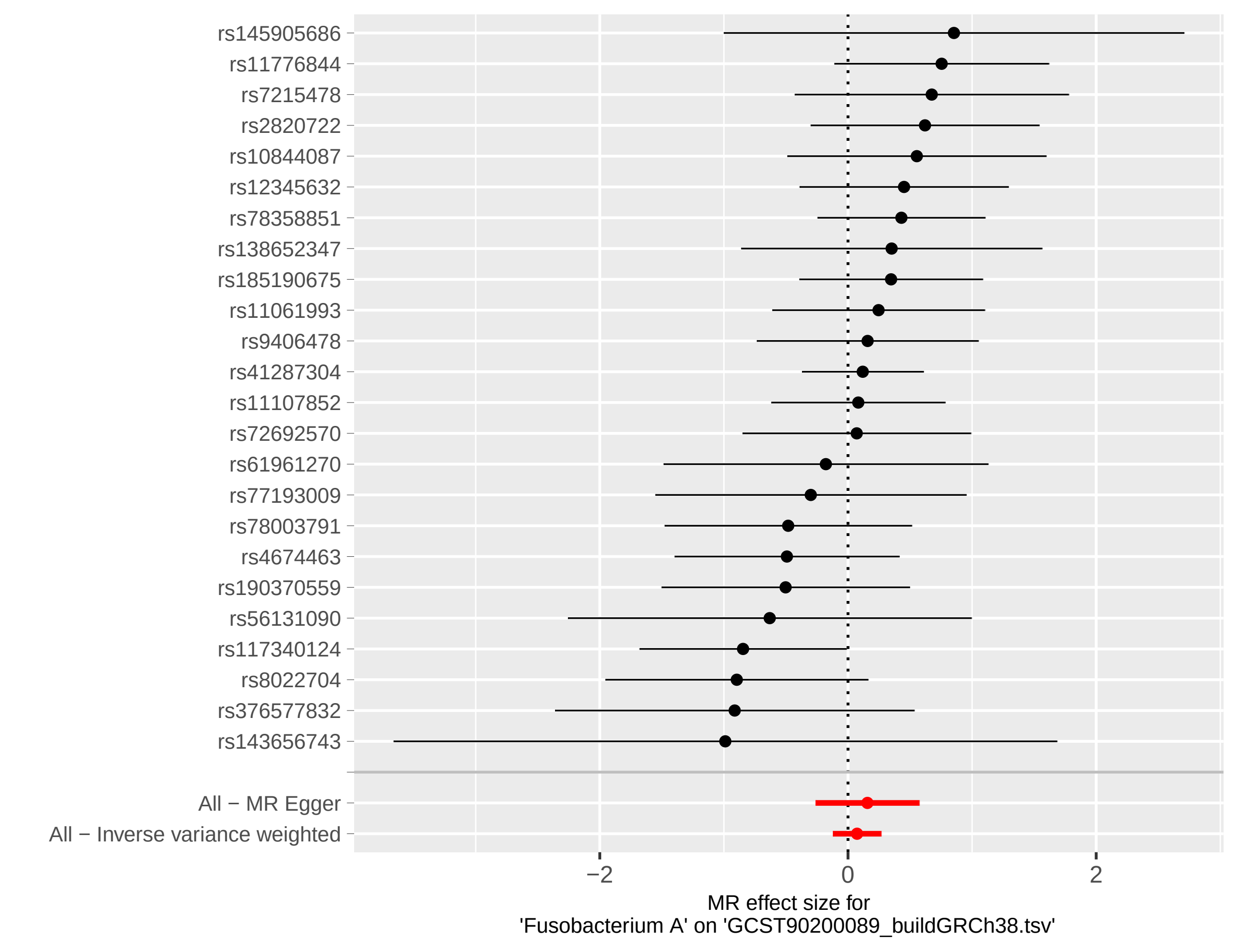

rs145905686
rs11776844
rs7215478
rs2820722
rs10844087
rs12345632
rs78358851
rs138652347
rs185190675
rs11061993
rs9406478
rs41287304
rs11107852
rs72692570
rs61961270
rs77193009
rs78003791
rs4674463
rs190370559
rs56131090
rs117340124
rs8022704
rs376577832
rs143656743
All − MR Egger
All − Inverse variance weighted
−2
0
2
MR effect size for
'Fusobacterium A' on 'GCST90200089_buildGRCh38.tsv'

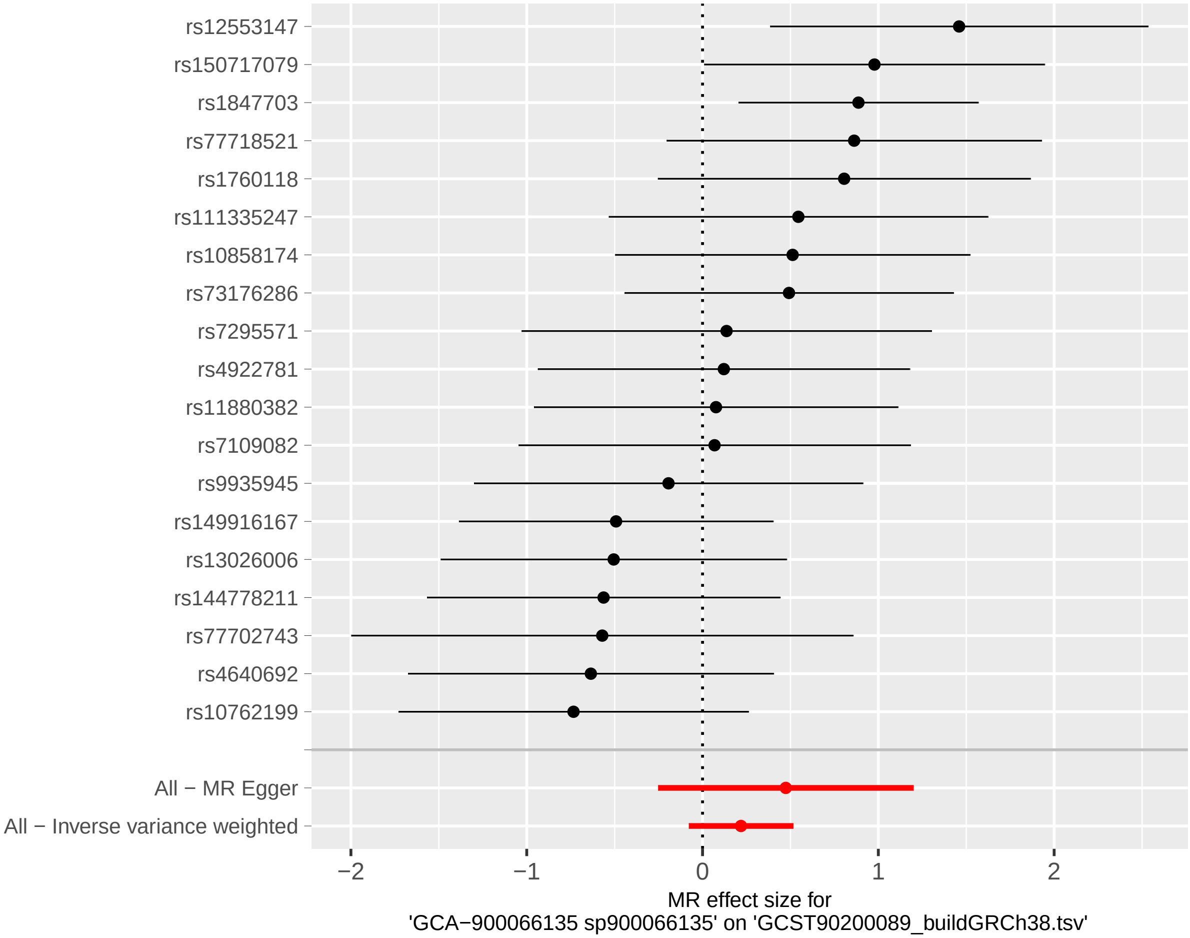
rs12553147
rs150717079
rs1847703
rs77718521
rs1760118
rs111335247
rs10858174
rs73176286
rs7295571
rs4922781
rs11880382
rs7109082
rs9935945
rs149916167
rs13026006
rs144778211
rs77702743
rs4640692
rs10762199
All − MR Egger
All − Inverse variance weighted
−2
−1
0
1
2
MR effect size for
'GCA−900066135 sp900066135' on 'GCST90200089_buildGRCh38.tsv'

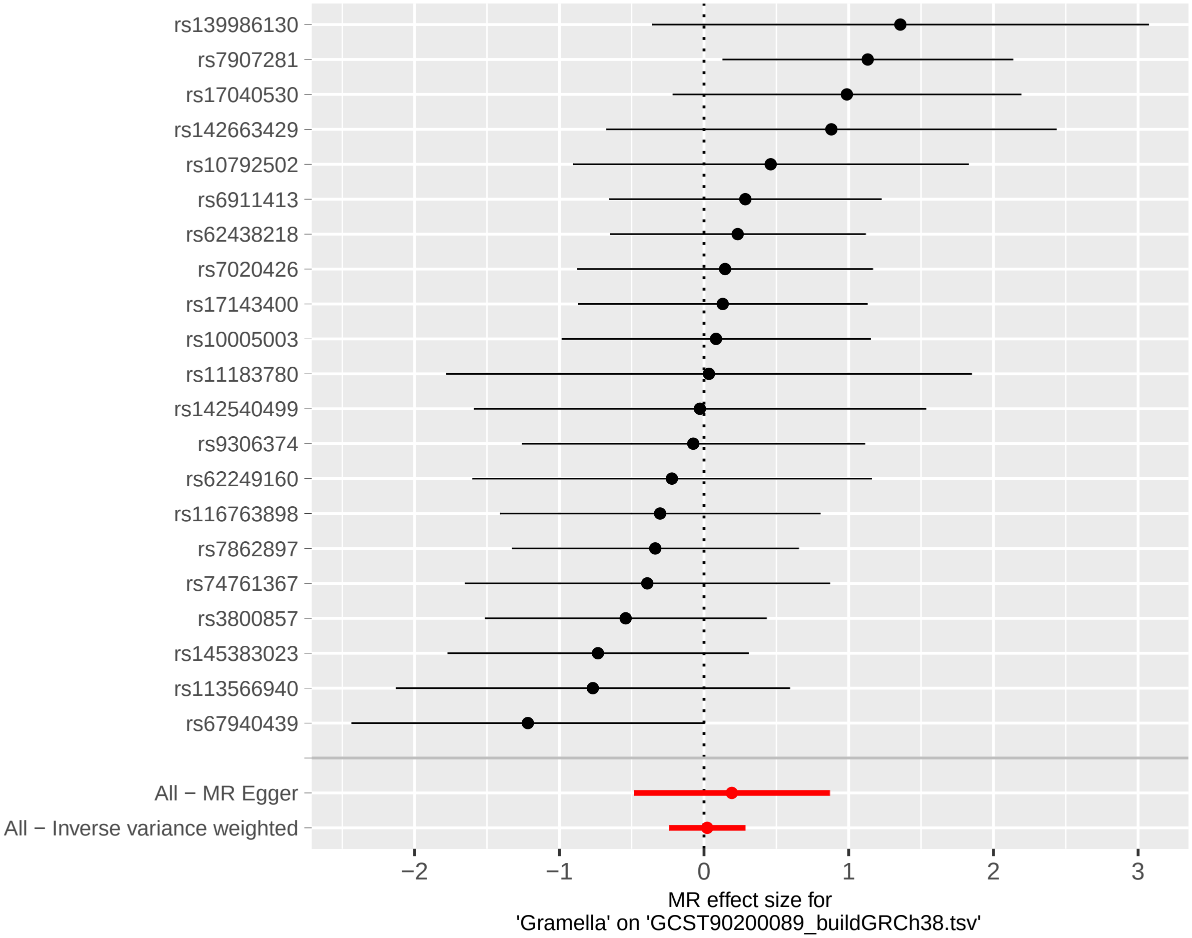
rs139986130
rs7907281
rs17040530
rs142663429
rs10792502
rs6911413
rs62438218
rs7020426
rs17143400
rs10005003
rs11183780
rs142540499
rs9306374
rs62249160
rs116763898
rs7862897
rs74761367
rs3800857
rs145383023
rs113566940
rs67940439
All − MR Egger
All − Inverse variance weighted
−2
−1
0
1
2
3
MR effect size for
'Gramella' on 'GCST90200089_buildGRCh38.tsv'

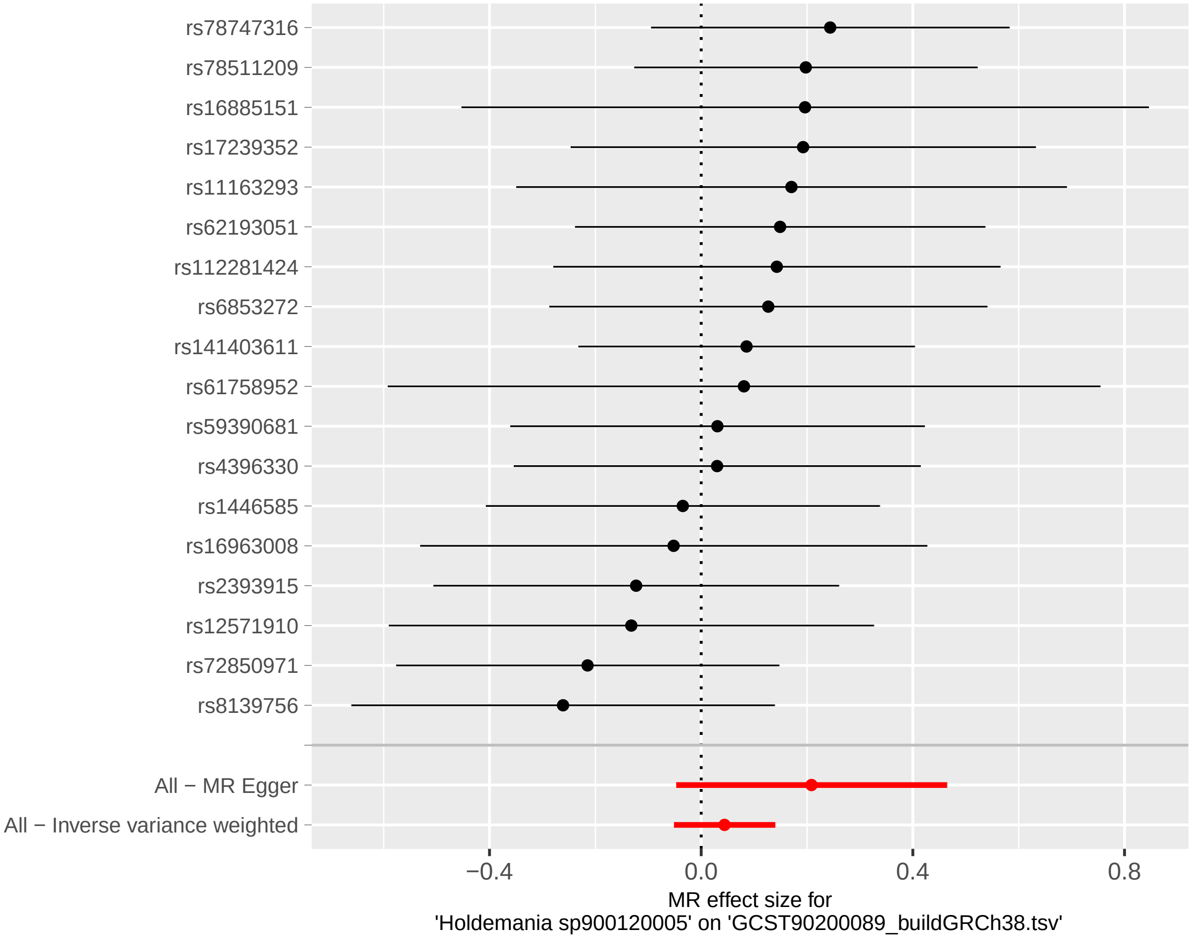
rs78747316
rs78511209
rs16885151
rs17239352
rs11163293
rs62193051
rs112281424
rs6853272
rs141403611
rs61758952
rs59390681
rs4396330
rs1446585
rs16963008
rs2393915
rs12571910
rs72850971
rs8139756
All − MR Egger
All − Inverse variance weighted
−0.4
0.0
0.4
0.8
MR effect size for
'Holdemania sp900120005' on 'GCST90200089_buildGRCh38.tsv'

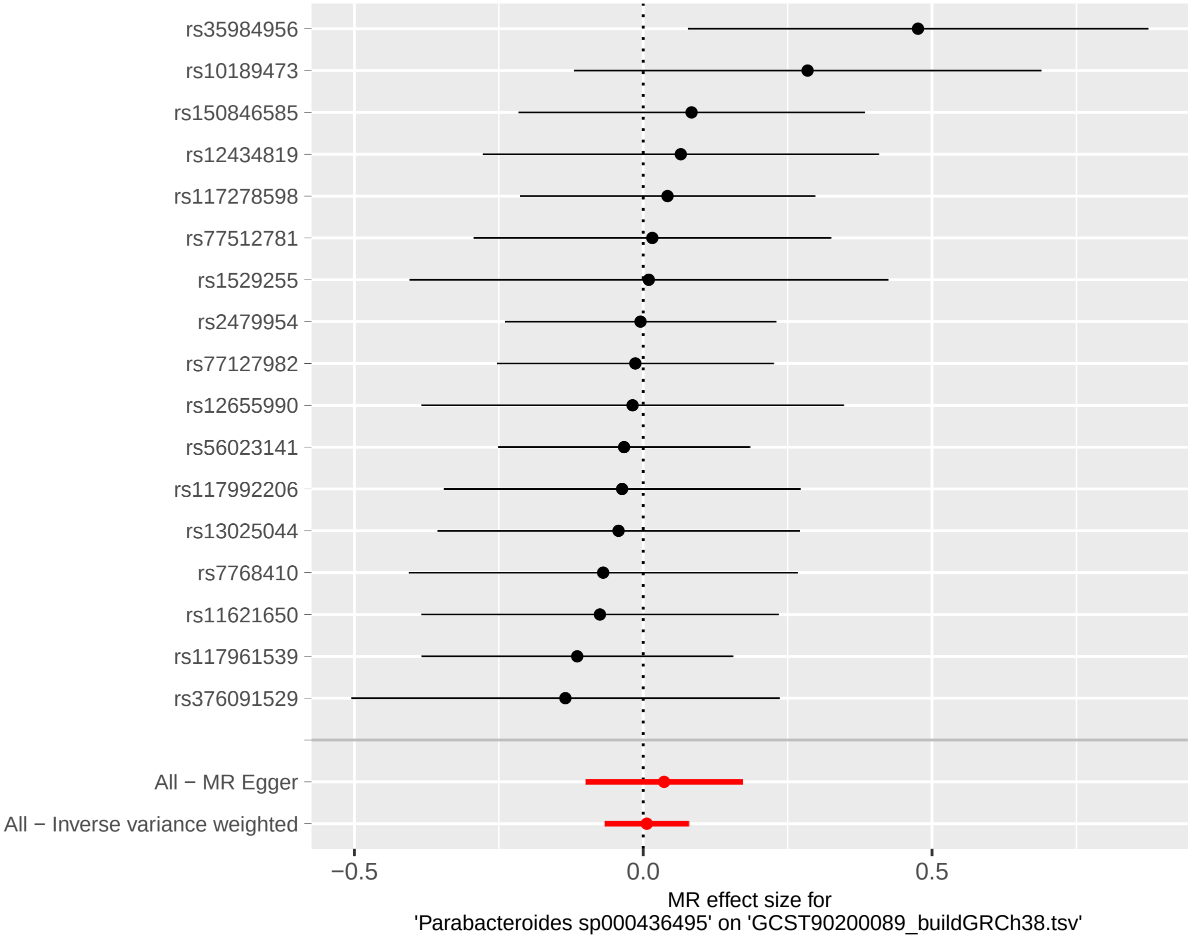
rs35984956
rs10189473
rs150846585
rs12434819
rs117278598
rs77512781
rs1529255
rs2479954
rs77127982
rs12655990
rs56023141
rs117992206
rs13025044
rs7768410
rs11621650
rs117961539
rs376091529
All − MR Egger
All − Inverse variance weighted
−0.5
0.0
0.5
MR effect size for
'Parabacteroides sp000436495' on 'GCST90200089_buildGRCh38.tsv'

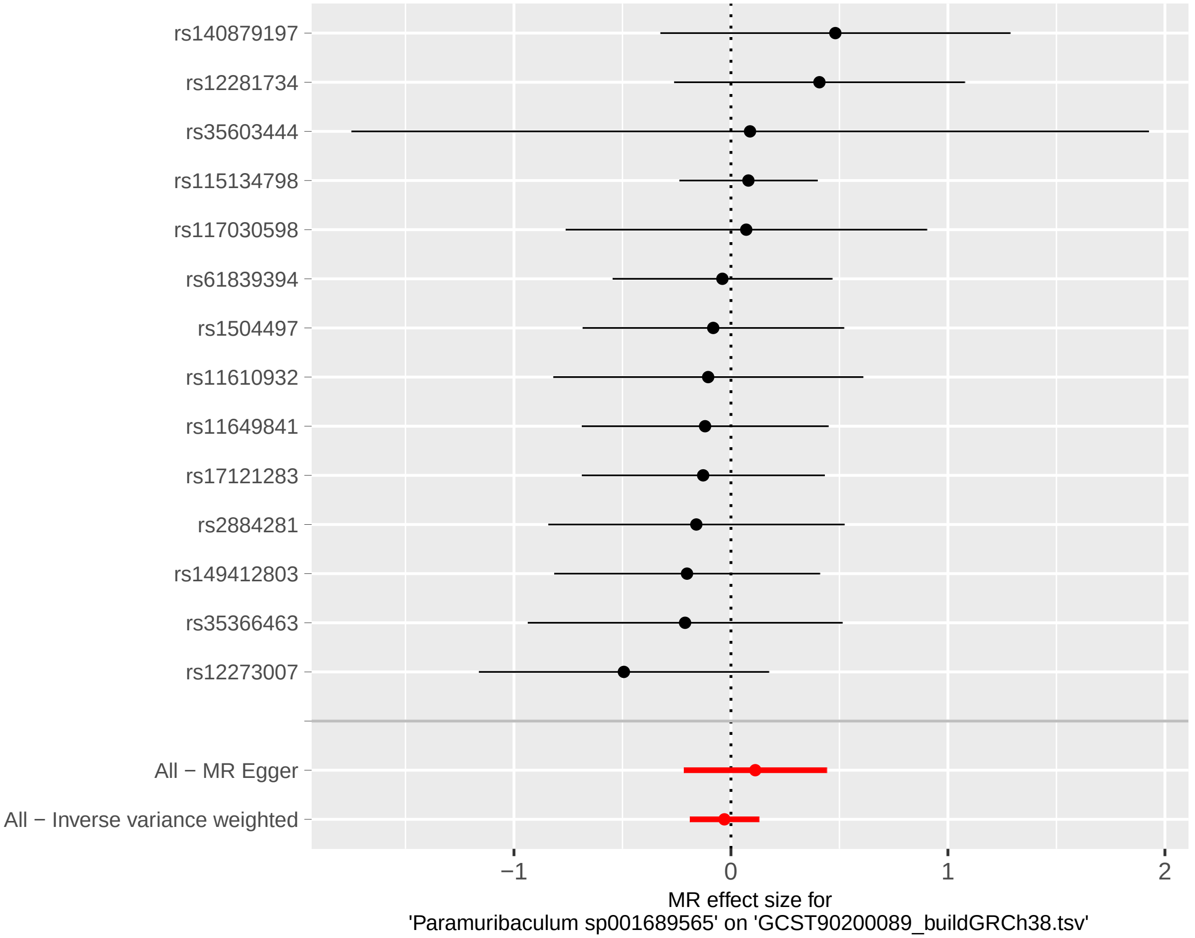
rs140879197
rs12281734
rs35603444
rs115134798
rs117030598
rs61839394
rs1504497
rs11610932
rs11649841
rs17121283
rs2884281
rs149412803
rs35366463
rs12273007
All − MR Egger
All − Inverse variance weighted
−1
0
1
2
MR effect size for
'Paramuribaculum sp001689565' on 'GCST90200089_buildGRCh38.tsv'

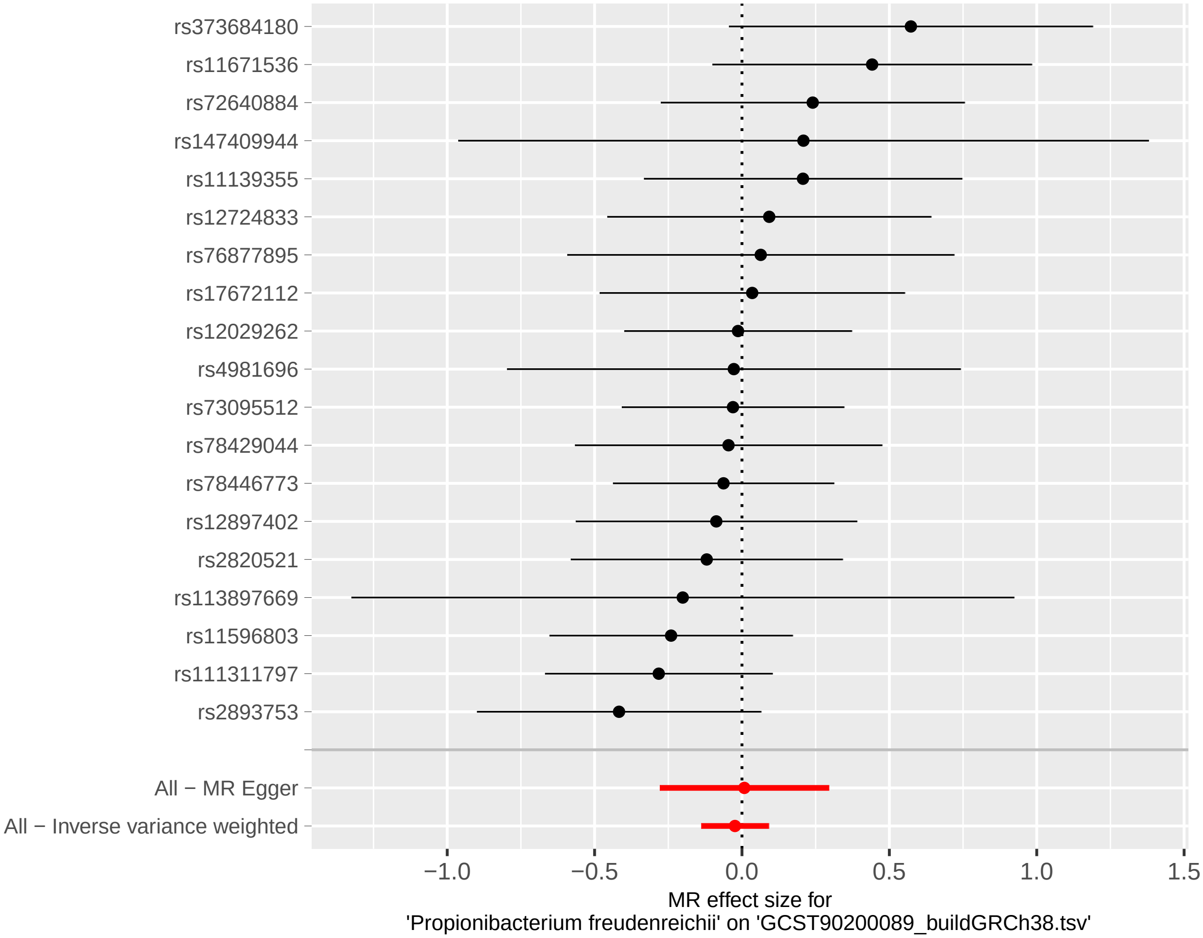
rs373684180
rs11671536
rs72640884
rs147409944
rs11139355
rs12724833
rs76877895
rs17672112
rs12029262
rs4981696
rs73095512
rs78429044
rs78446773
rs12897402
rs2820521
rs113897669
rs11596803
rs111311797
rs2893753
All − MR Egger
All − Inverse variance weighted
−1.0
−0.5
0.0
0.5
1.0
1.5
MR effect size for
'Propionibacterium freudenreichii' on 'GCST90200089_buildGRCh38.tsv'

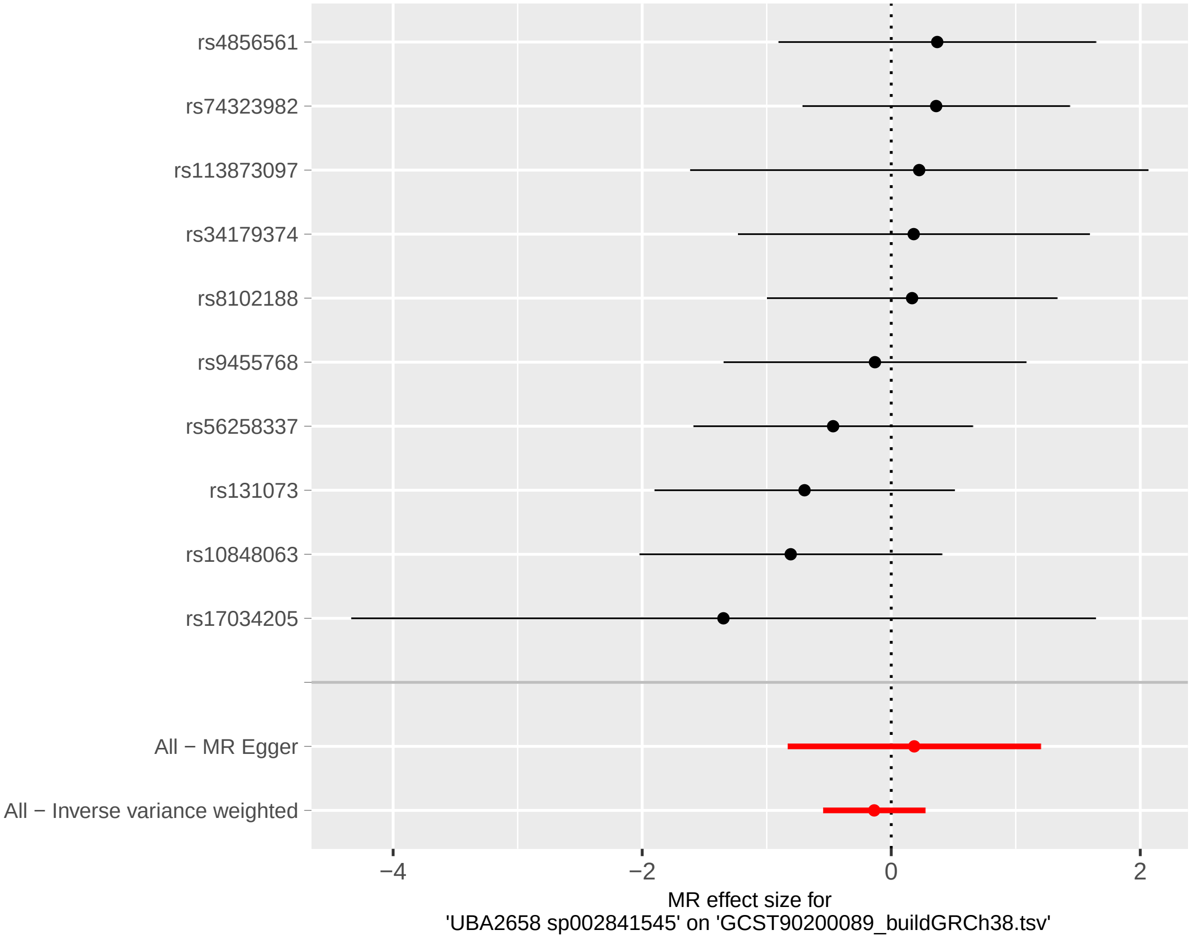
rs4856561
rs74323982
rs113873097
rs34179374
rs8102188
rs9455768
rs56258337
rs131073
rs10848063
rs17034205
All − MR Egger
All − Inverse variance weighted
−4
−2
0
2
MR effect size for
'UBA2658 sp002841545' on 'GCST90200089_buildGRCh38.tsv'

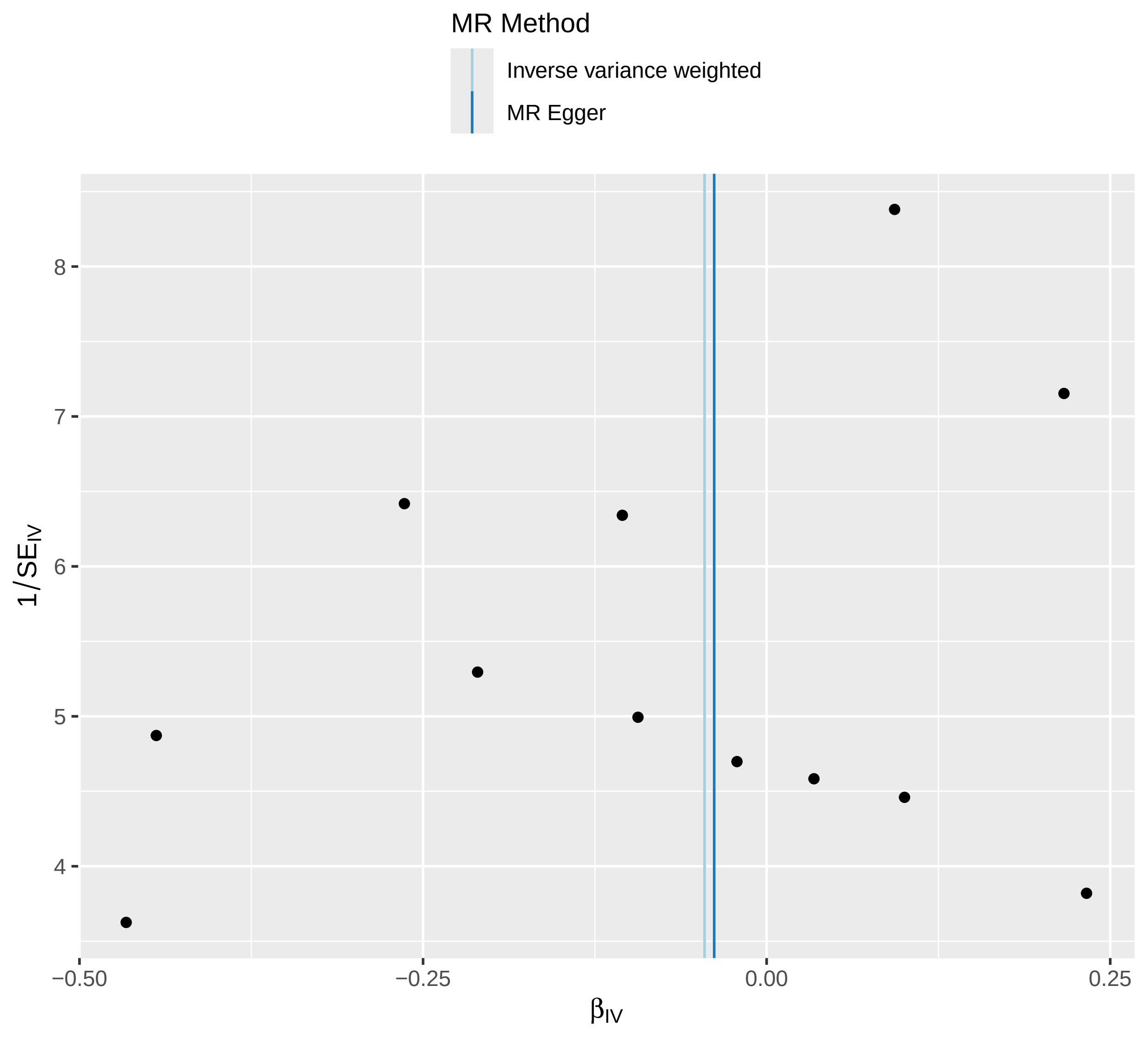

MR Method
Inverse variance weighted
MR Egger
8
7
6
5
4
1/SE_IV
−0.50
−0.25
0.00
0.25
β_IV

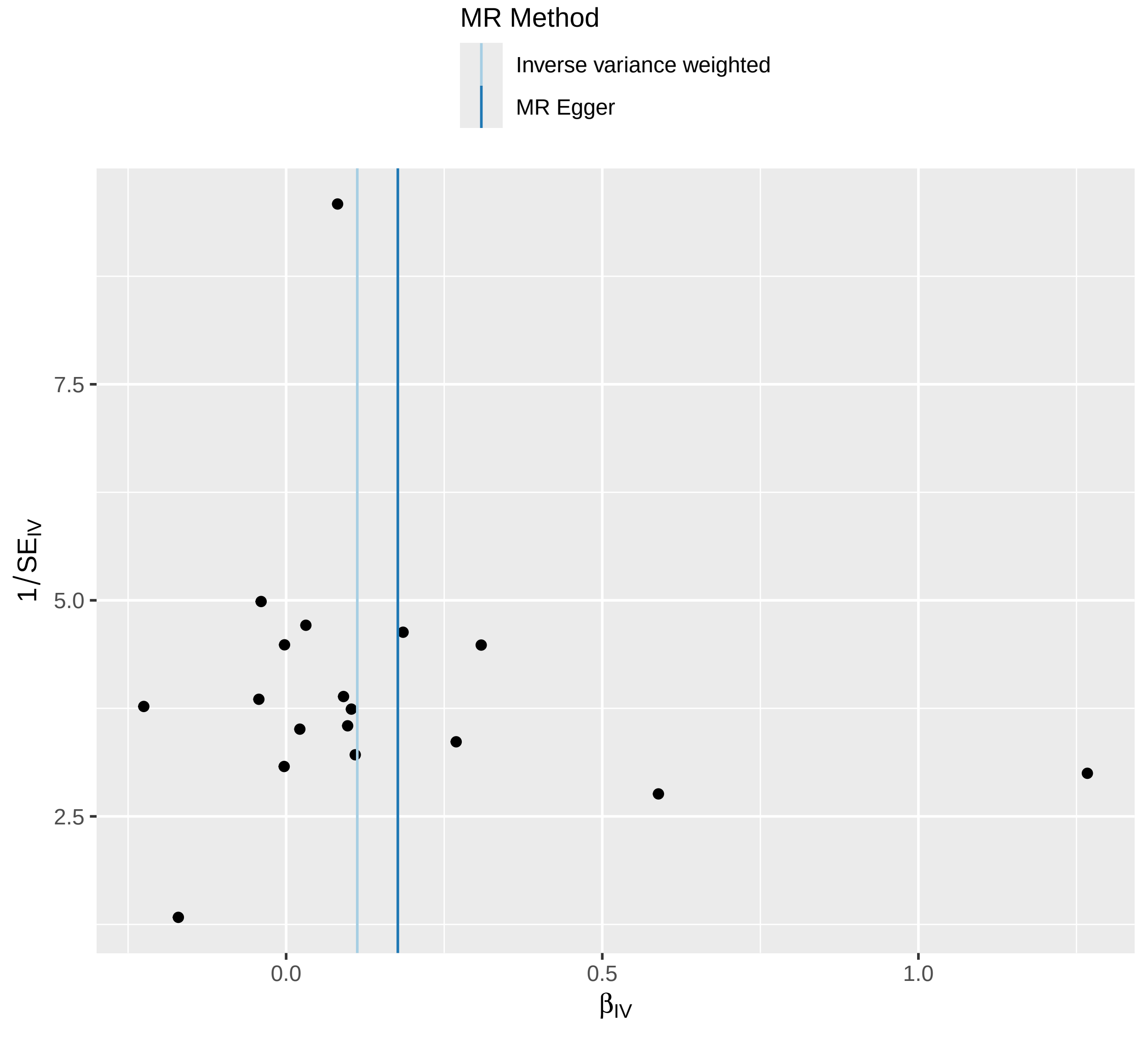
MR Method
Inverse variance weighted
MR Egger
7.5
5.0
2.5
1/SE$_{IV}$
0.0
0.5
1.0
β$_{IV}$

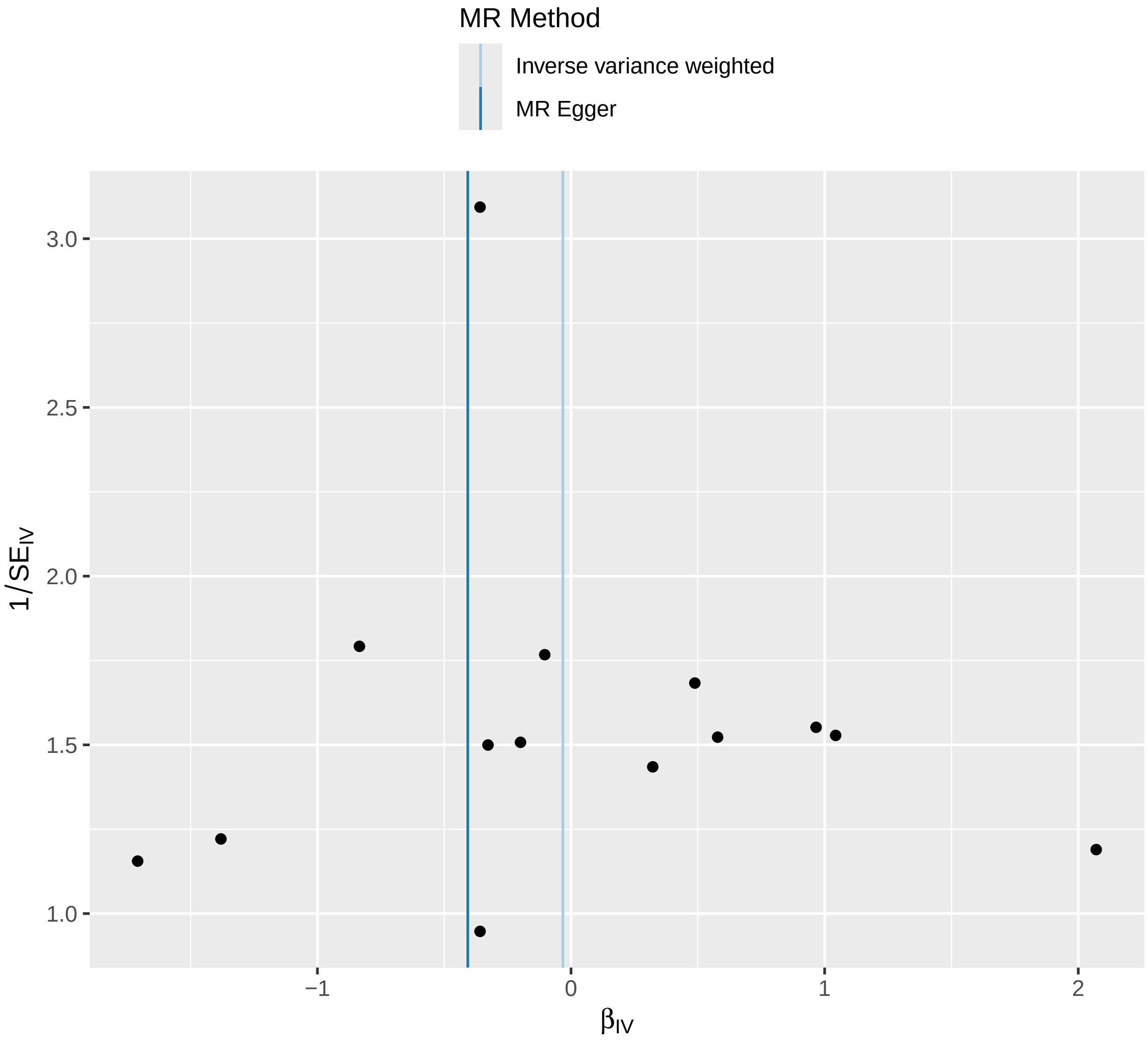

MR Method
Inverse variance weighted
MR Egger
3.0
2.5
2.0
1.5
1.0
$1/SE_{IV}$
−1
0
1
2
β_{IV}

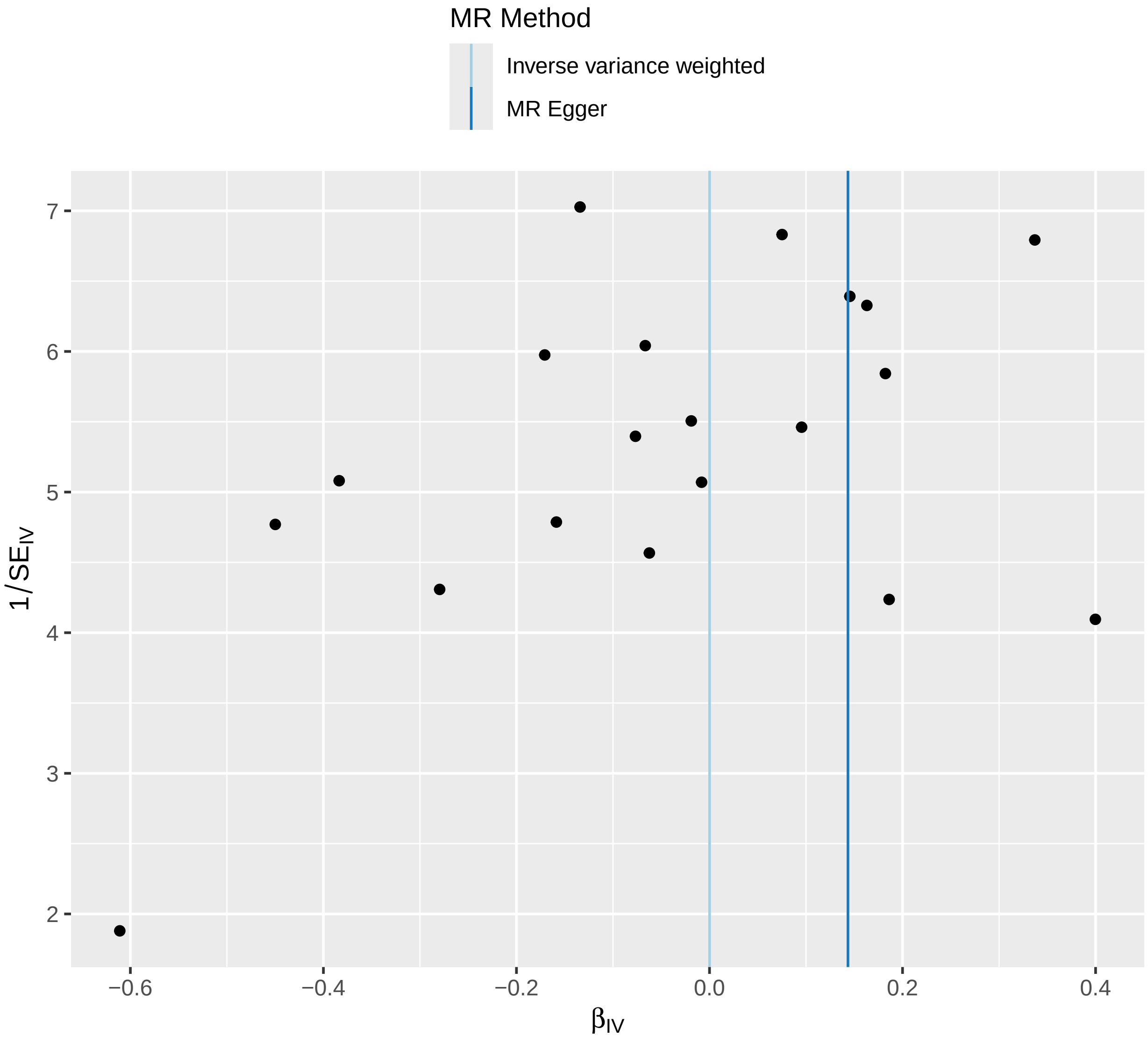

MR Method
Inverse variance weighted
MR Egger
1/SE$_{IV}$
β_{IV}
7
6
5
4
3
2
−0.6
−0.4
−0.2
0.0
0.2
0.4

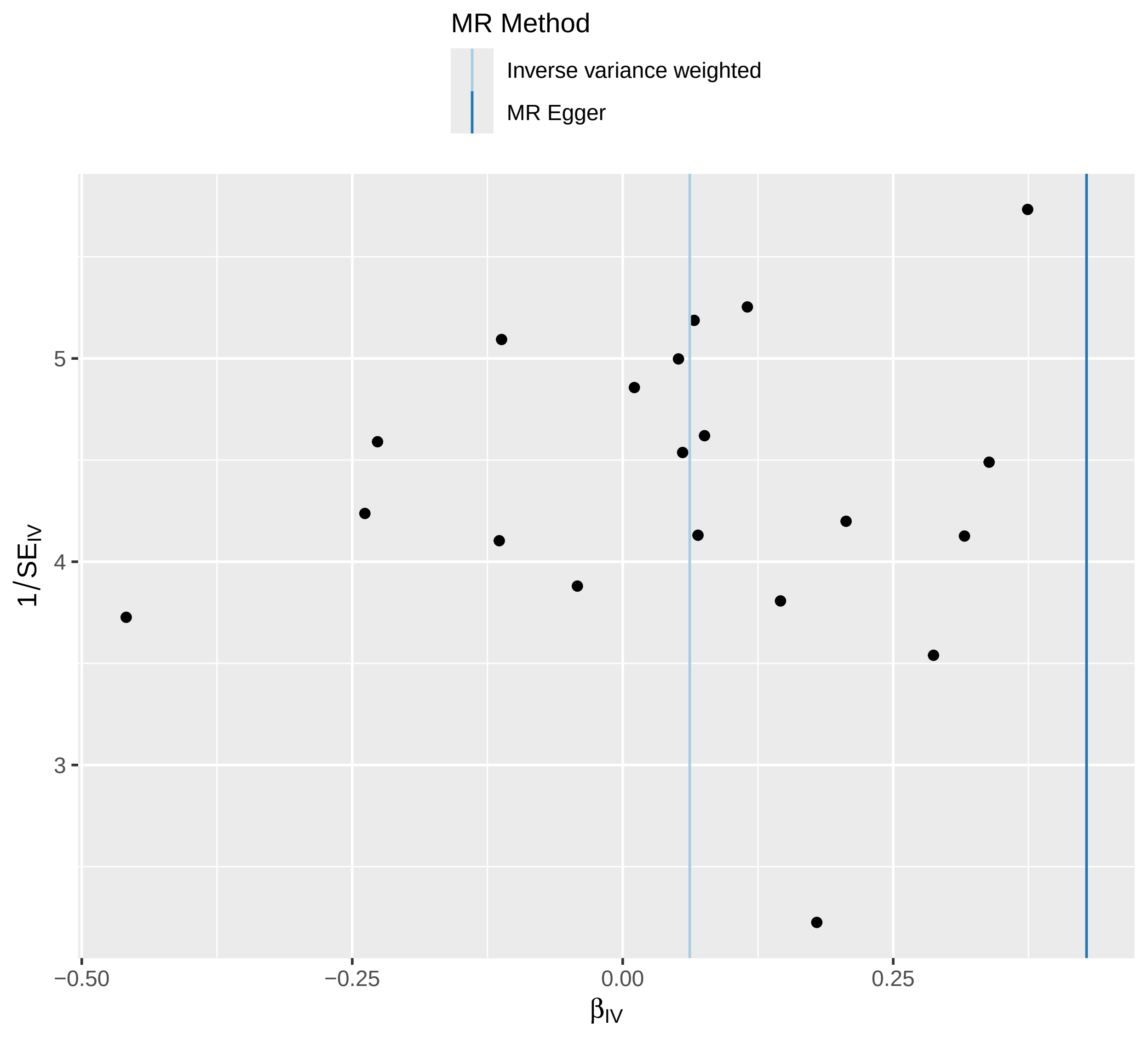

MR Method
Inverse variance weighted
MR Egger
5
4
3
1/SE_IV
−0.50
−0.25
0.00
0.25
β_IV

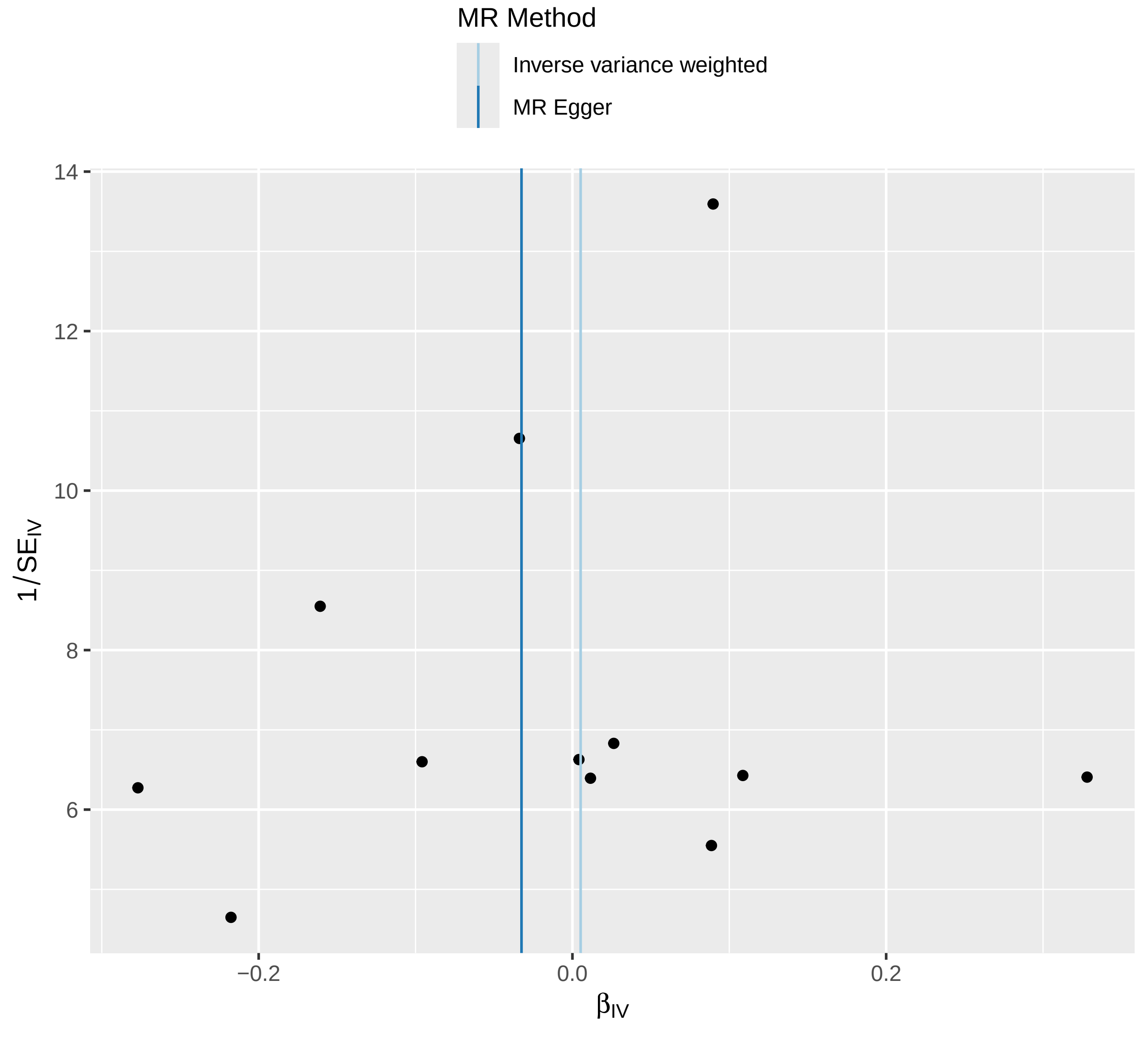
MR Method
Inverse variance weighted
MR Egger
14
12
10
8
6
1/SE$_{IV}$
−0.2
0.0
0.2
β_{IV}

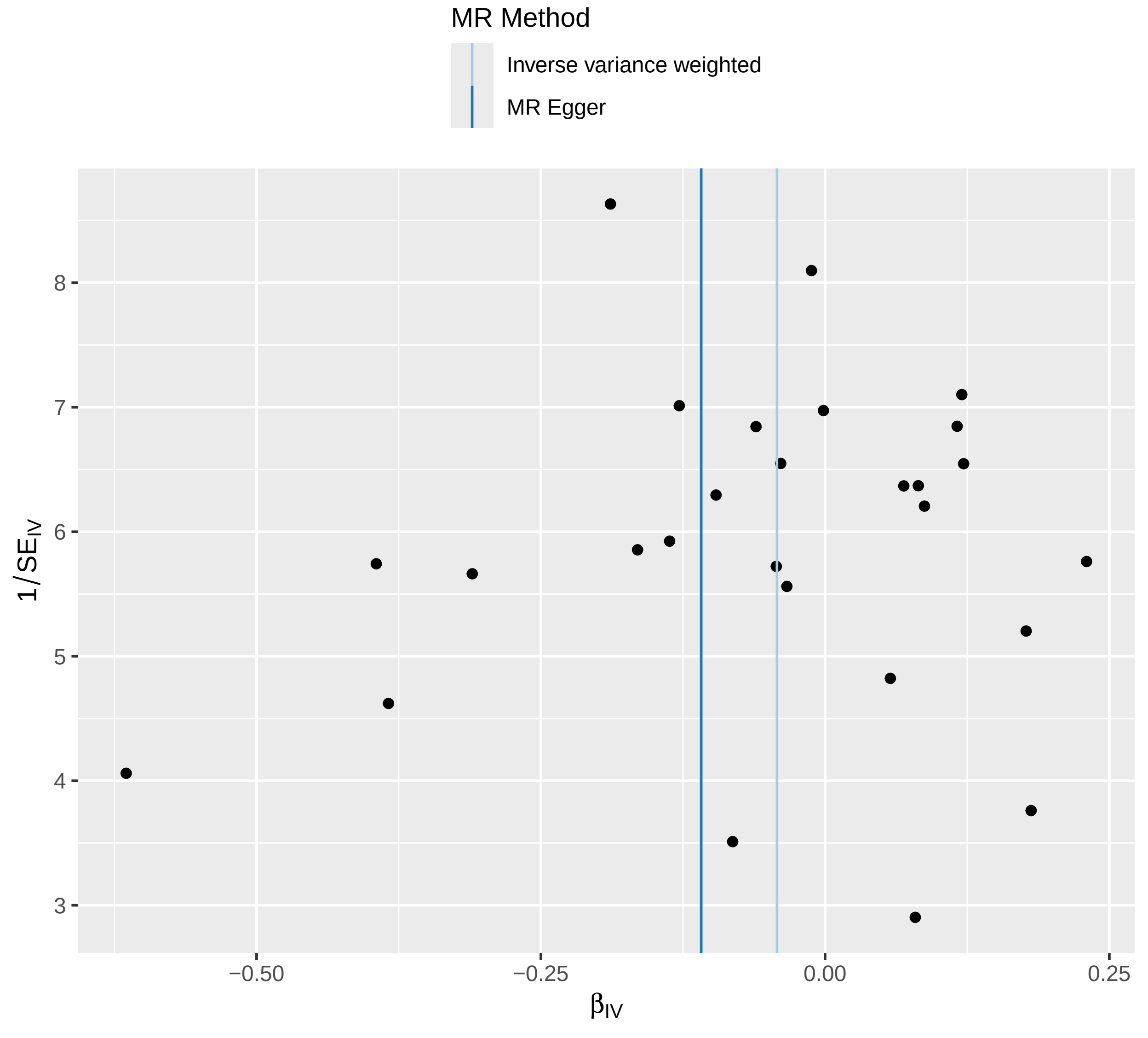
MR Method
Inverse variance weighted
MR Egger
1/SE_IV
β_IV
8
7
6
5
4
3
−0.50
−0.25
0.00
0.25

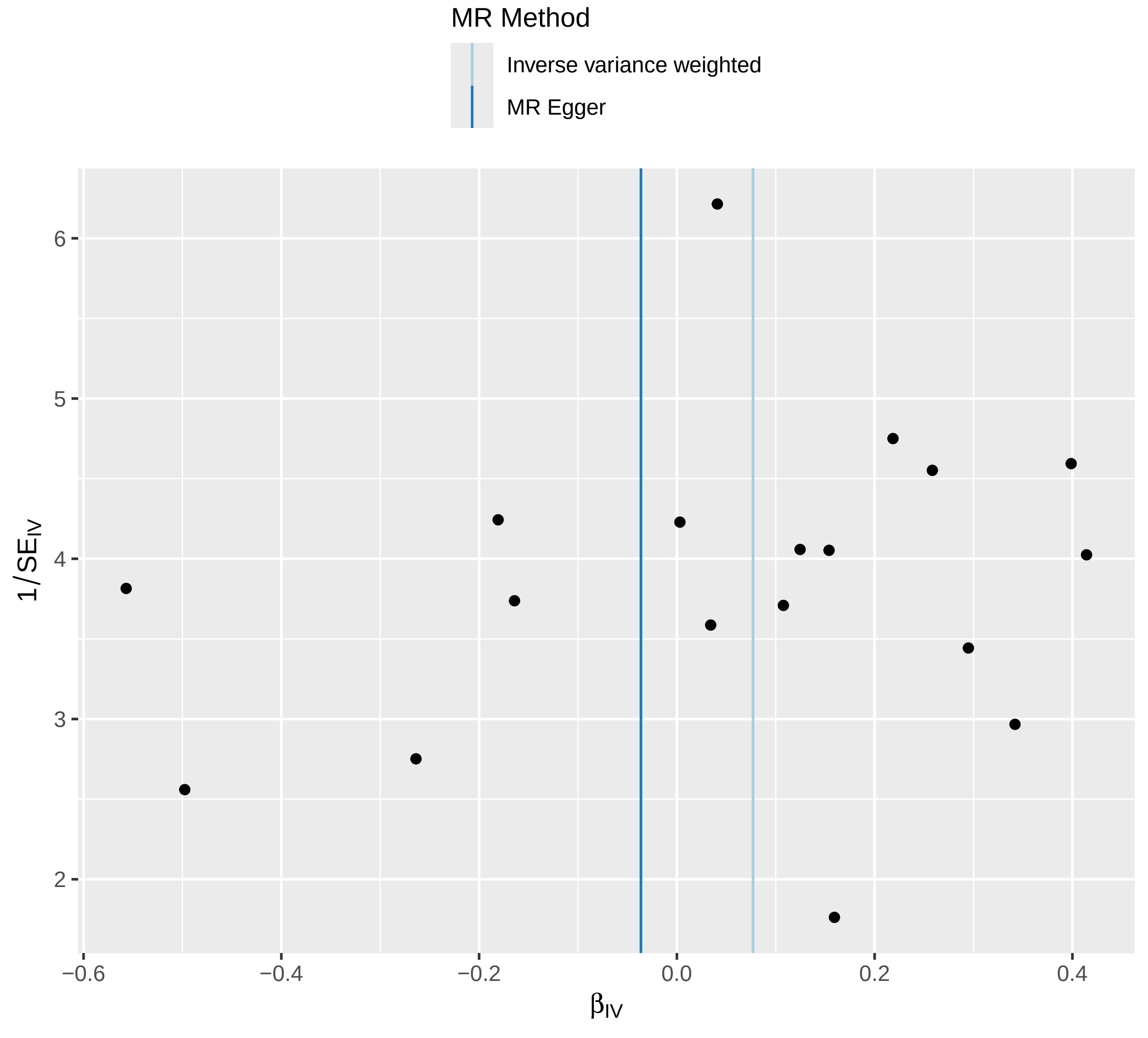

MR Method
Inverse variance weighted
MR Egger
6
5
4
3
2
1/SE_IV
−0.6
−0.4
−0.2
0.0
0.2
0.4
β_IV

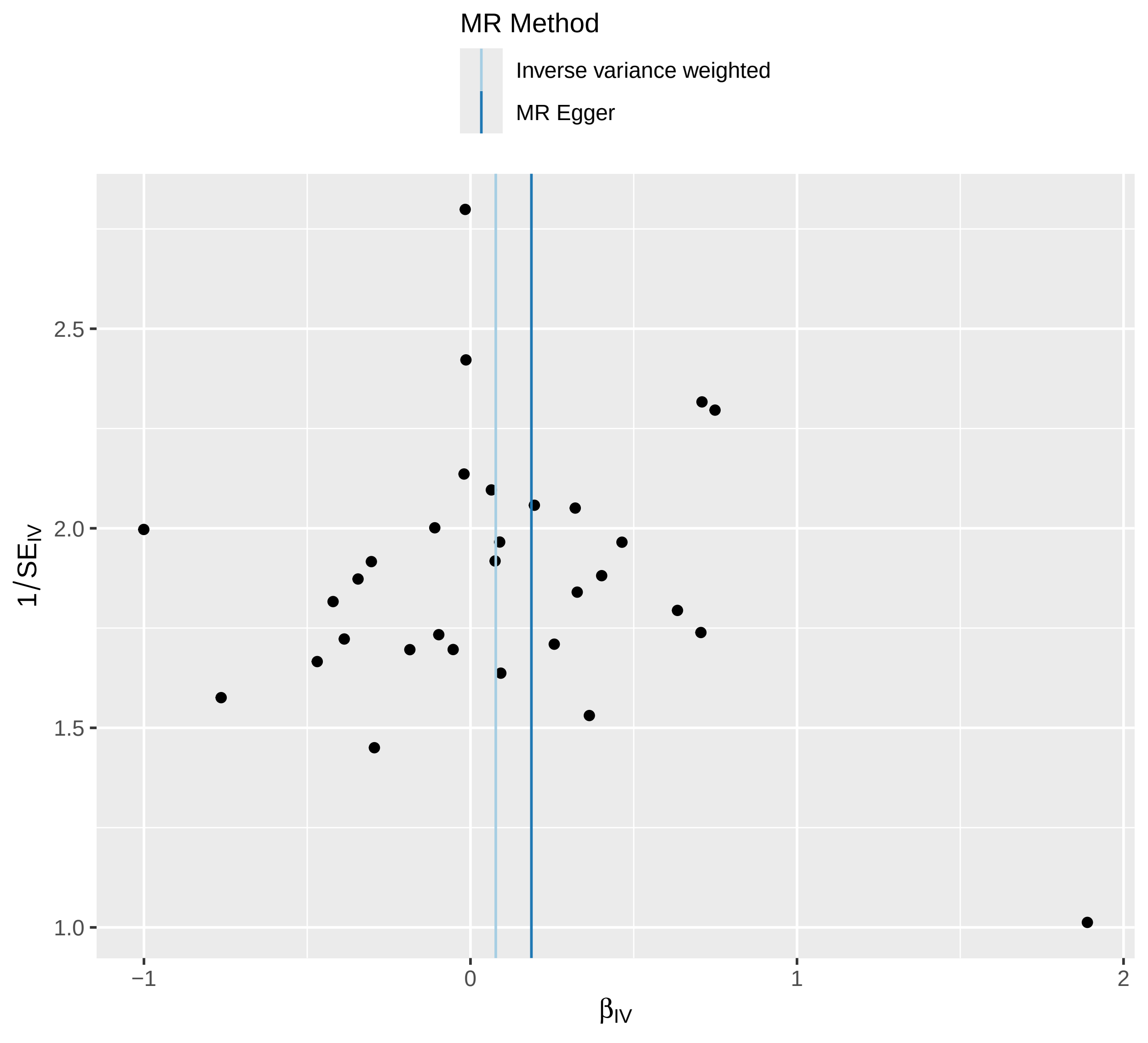

MR Method
Inverse variance weighted
MR Egger
2.5
2.0
1.5
1.0
1/SE_IV
−1
0
1
2
β_IV

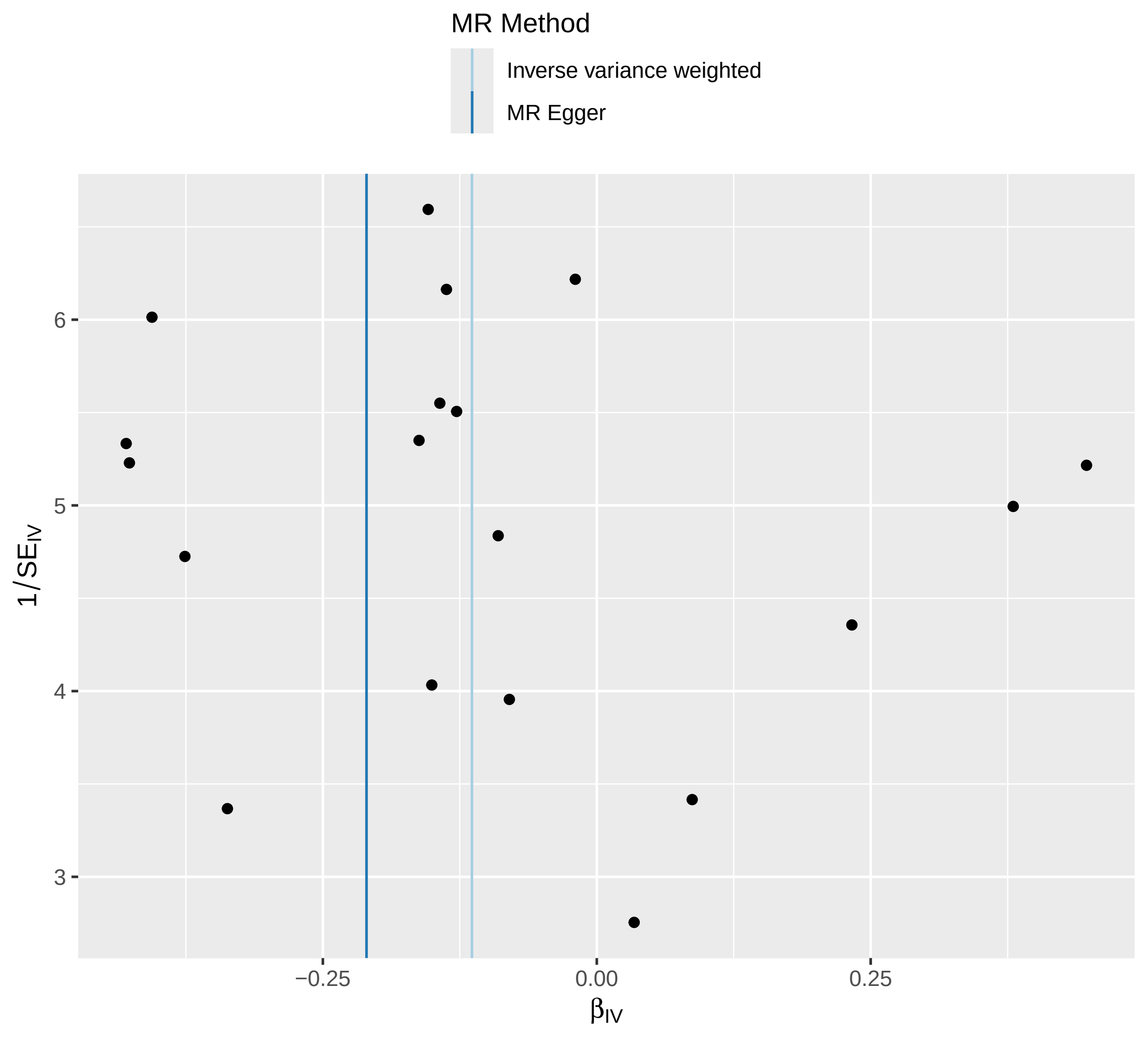

MR Method
Inverse variance weighted
MR Egger
6
5
4
3
1/SE_IV
−0.25
0.00
0.25
β_IV

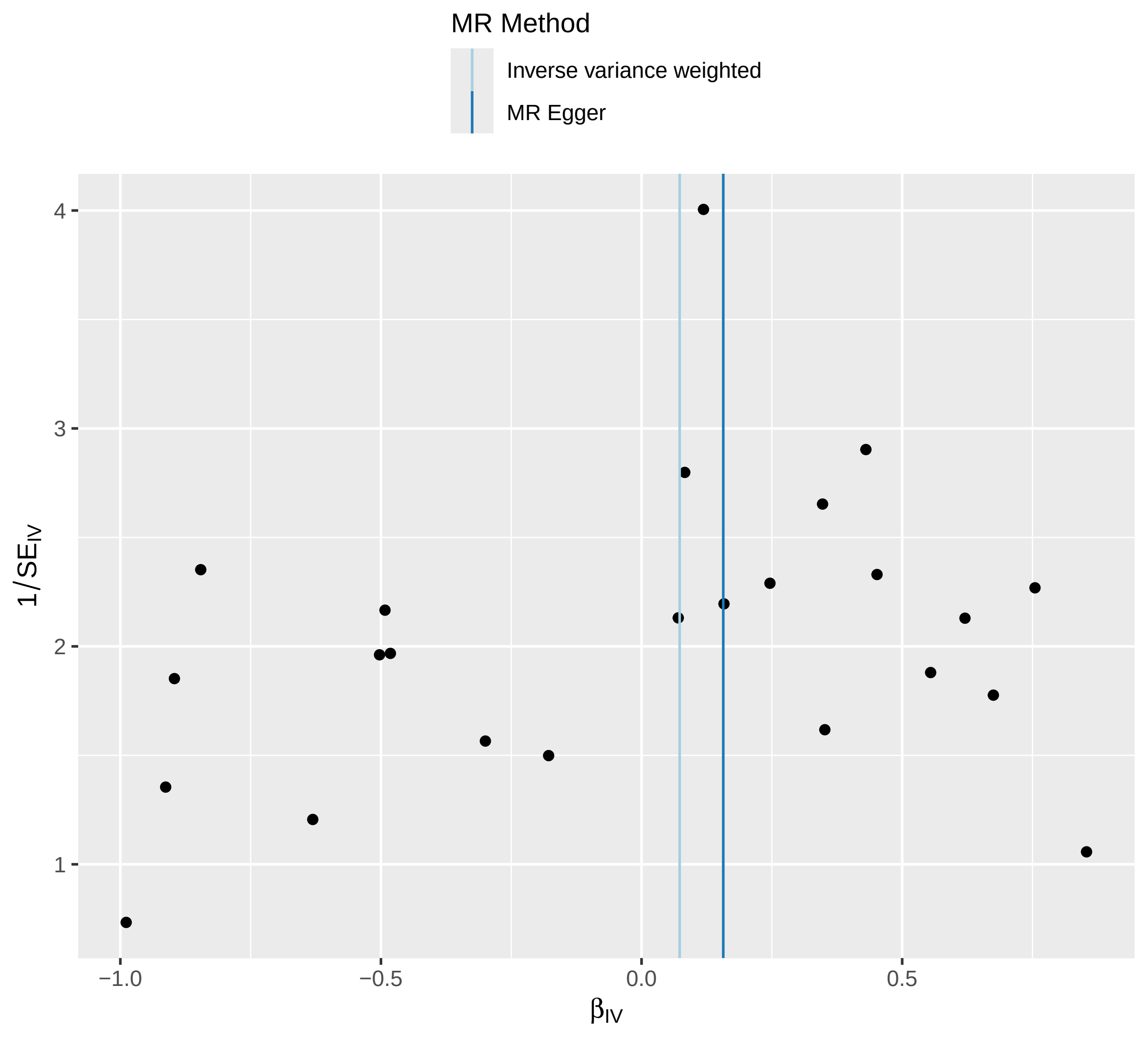

MR Method
Inverse variance weighted
MR Egger
4
3
2
1
$1/SE_{IV}$
−1.0
−0.5
0.0
0.5
β_{IV}

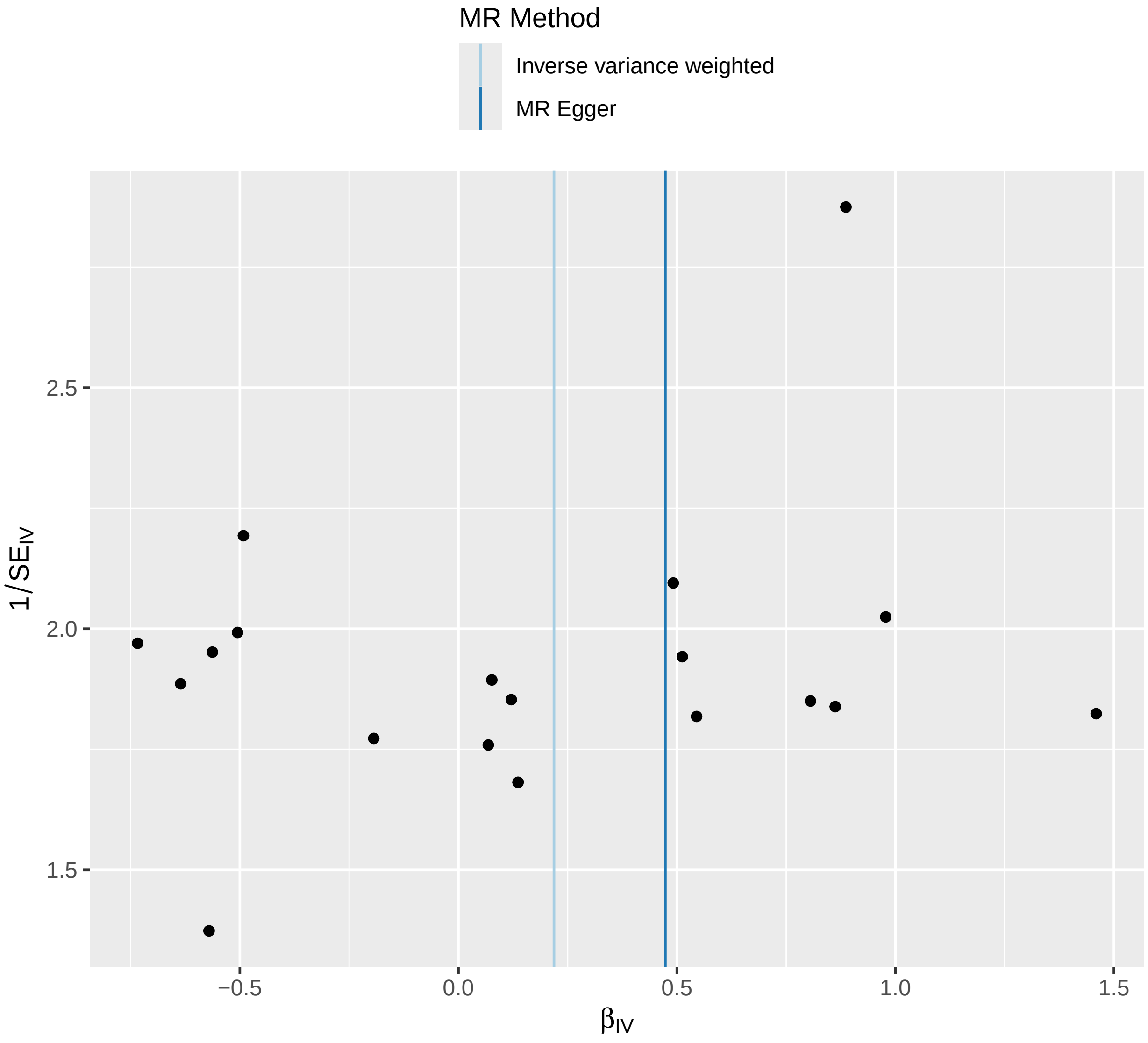

MR Method
Inverse variance weighted
MR Egger
2.5
2.0
1.5
$1/SE_{IV}$
−0.5
0.0
0.5
1.0
1.5
β_{IV}

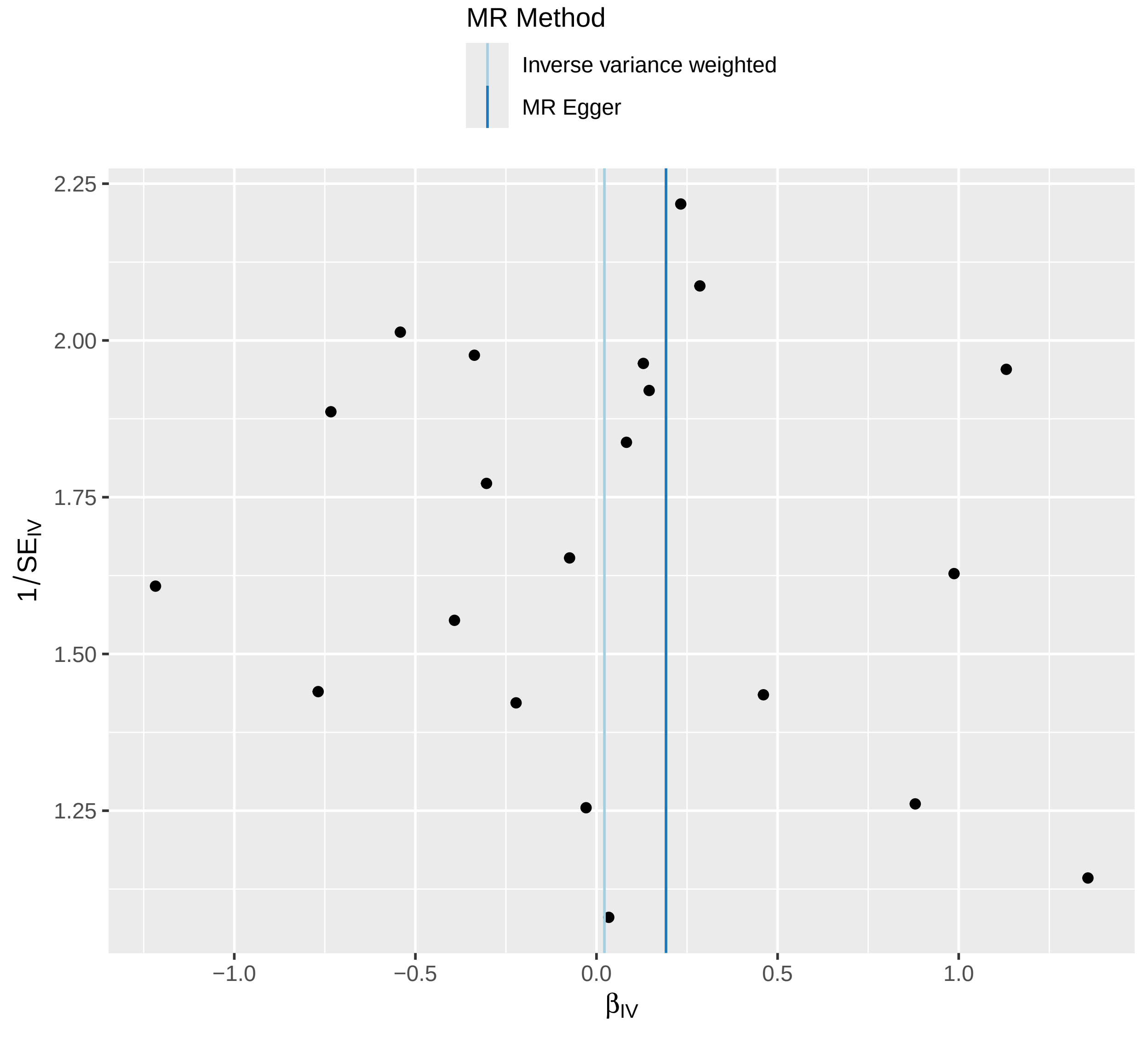

MR Method
Inverse variance weighted
MR Egger
2.25
2.00
1.75
1.50
1.25
1/SE$_{IV}$
−1.0
−0.5
0.0
0.5
1.0
β$_{IV}$

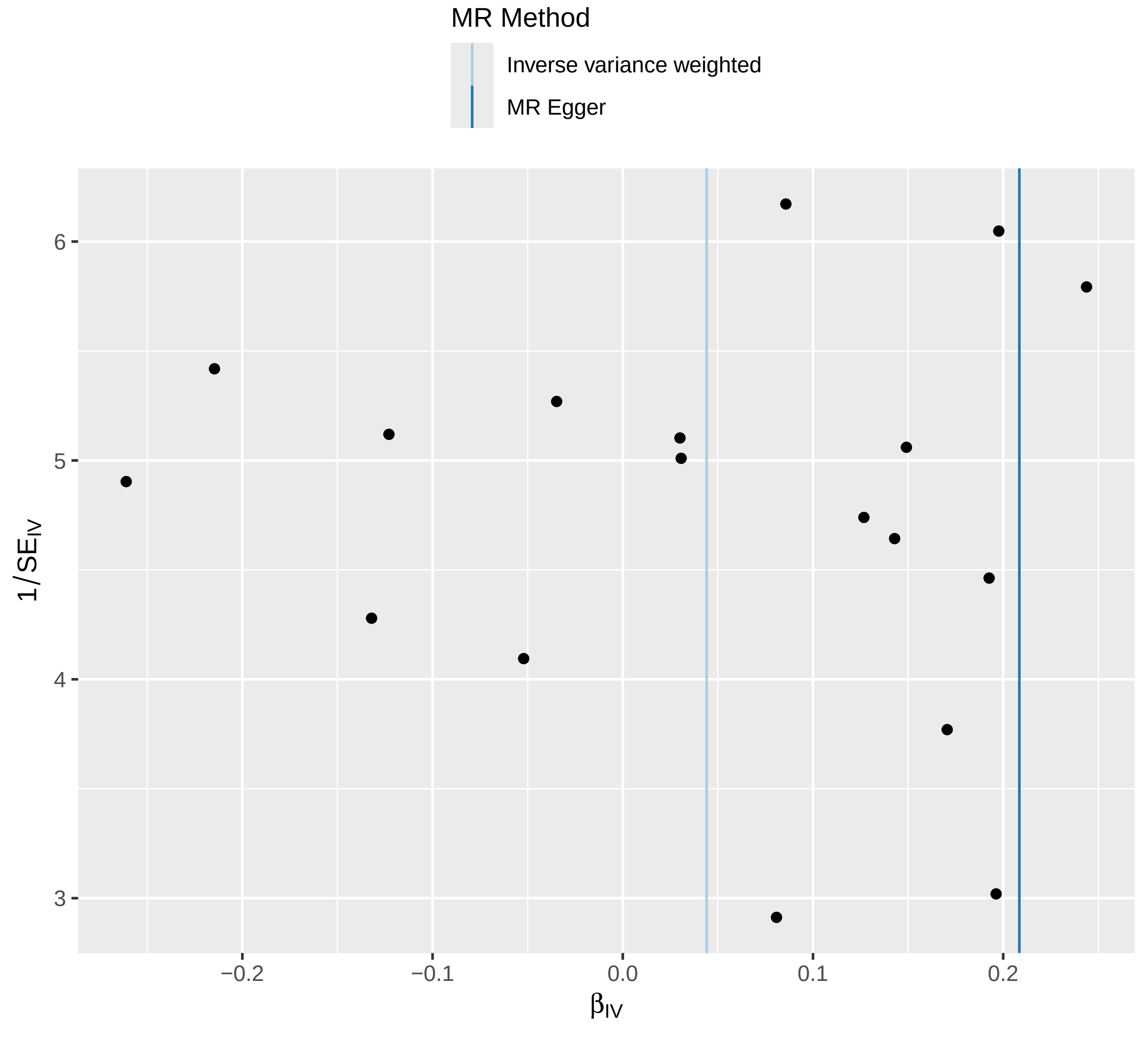
MR Method
Inverse variance weighted
MR Egger
6
5
4
3
1/SE_IV
−0.2
−0.1
0.0
0.1
0.2
β_IV

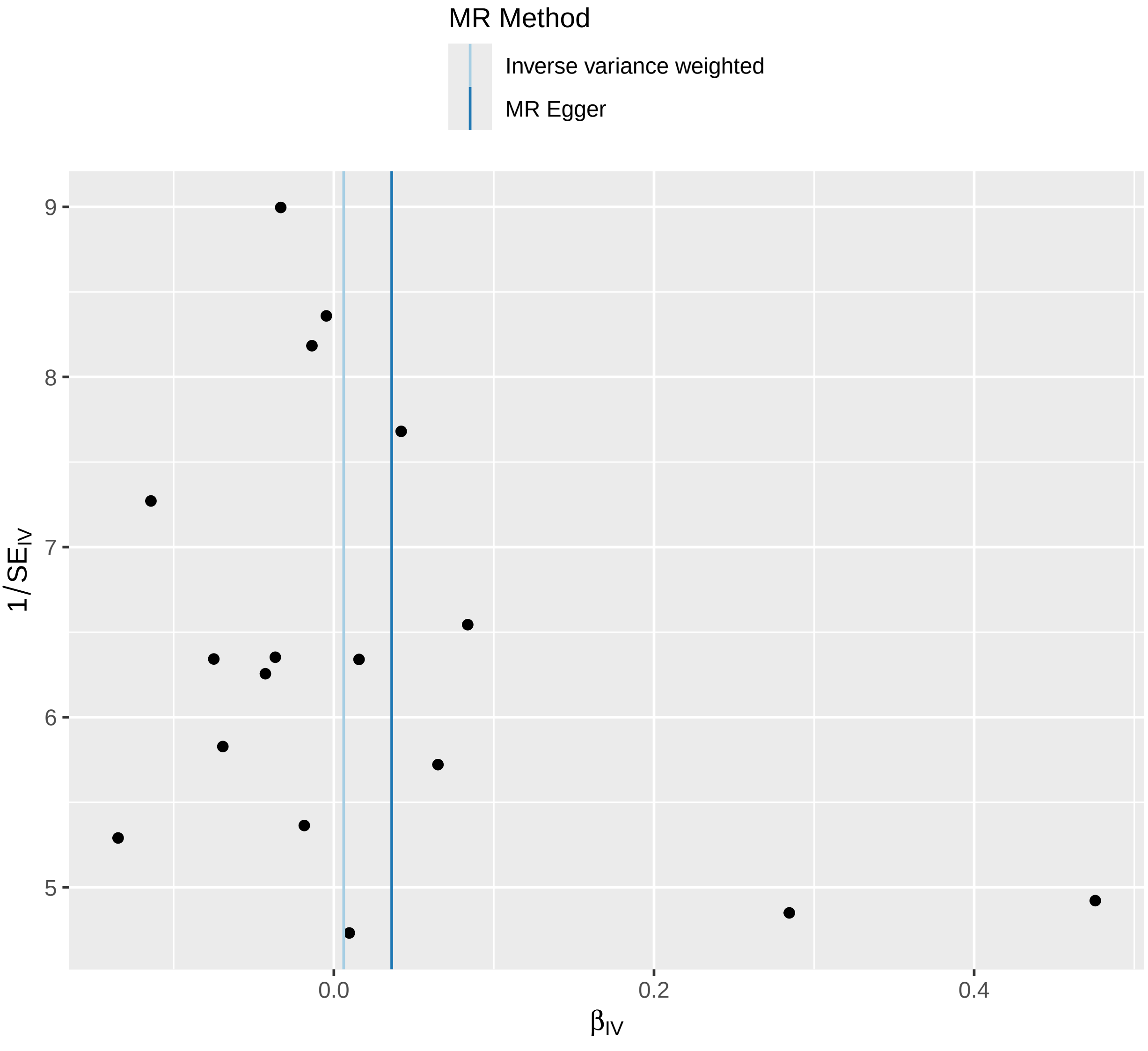

MR Method
Inverse variance weighted
MR Egger
9
8
7
6
5
1/SE_IV
0.0
0.2
0.4
β_IV

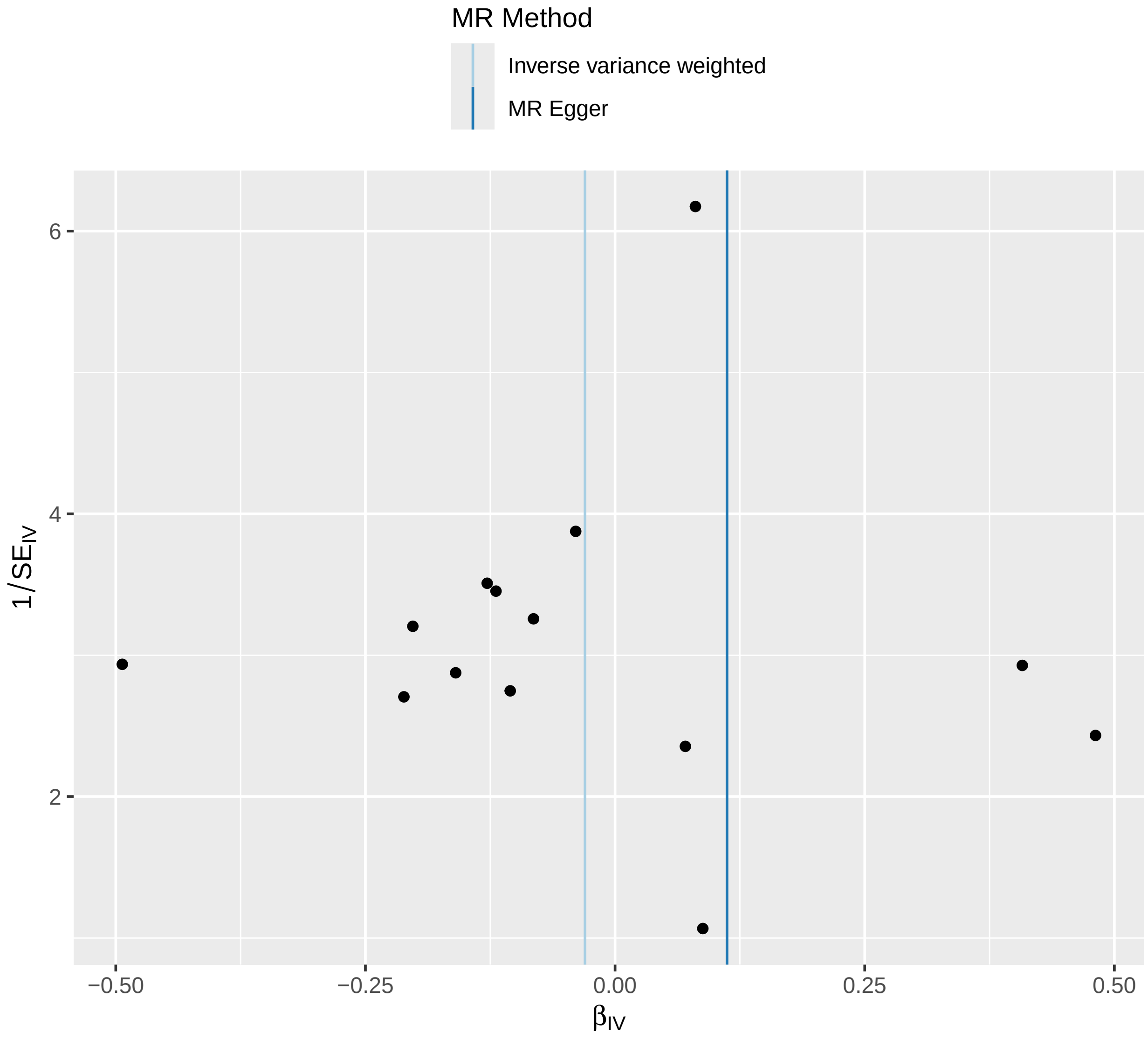

MR Method
Inverse variance weighted
MR Egger
6
4
2
1/SE_IV
−0.50
−0.25
0.00
0.25
0.50
β_IV

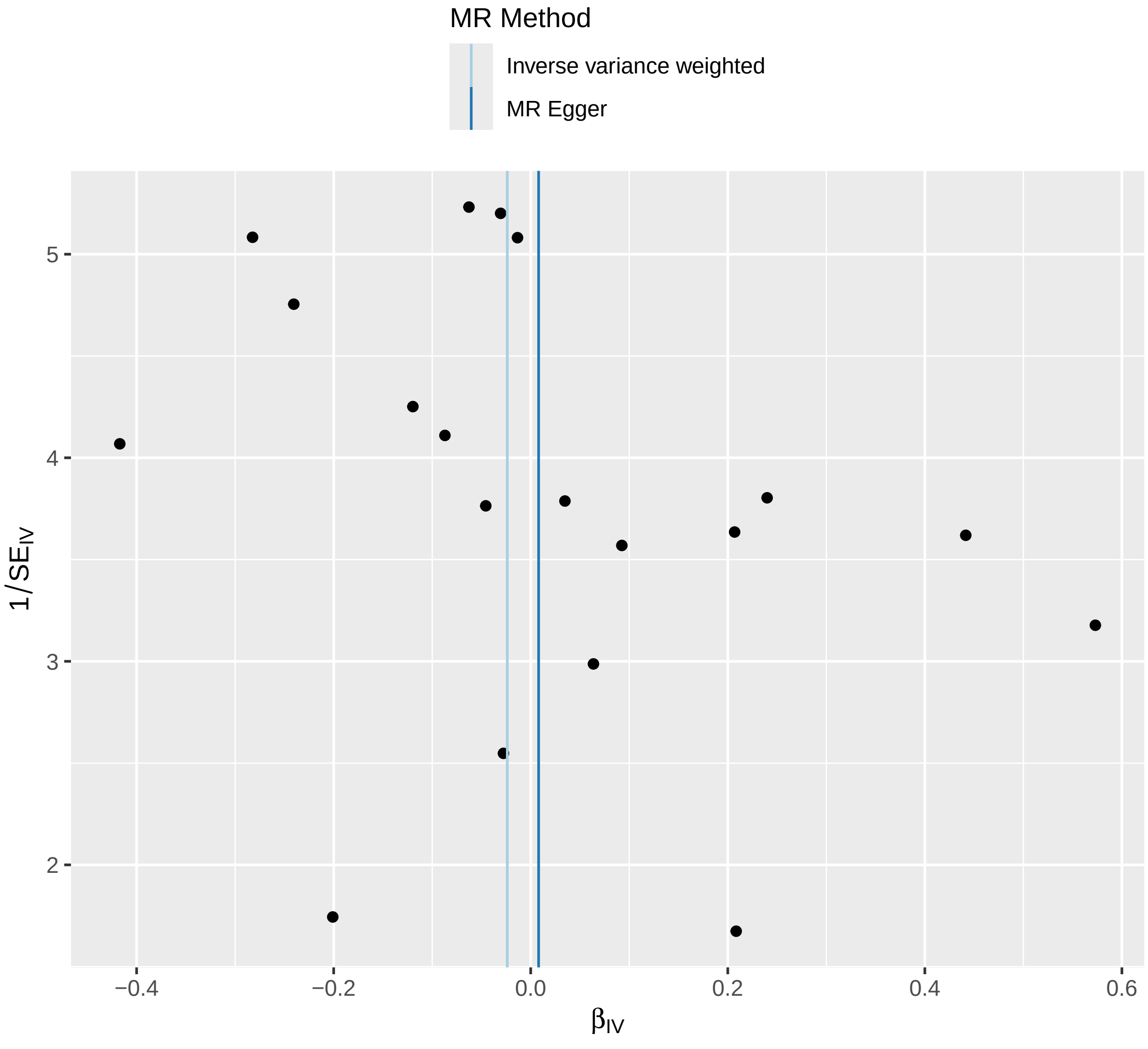

MR Method
Inverse variance weighted
MR Egger
5
4
3
2
−0.4
−0.2
0.0
0.2
0.4
0.6
$1/SE_{IV}$
β_{IV}

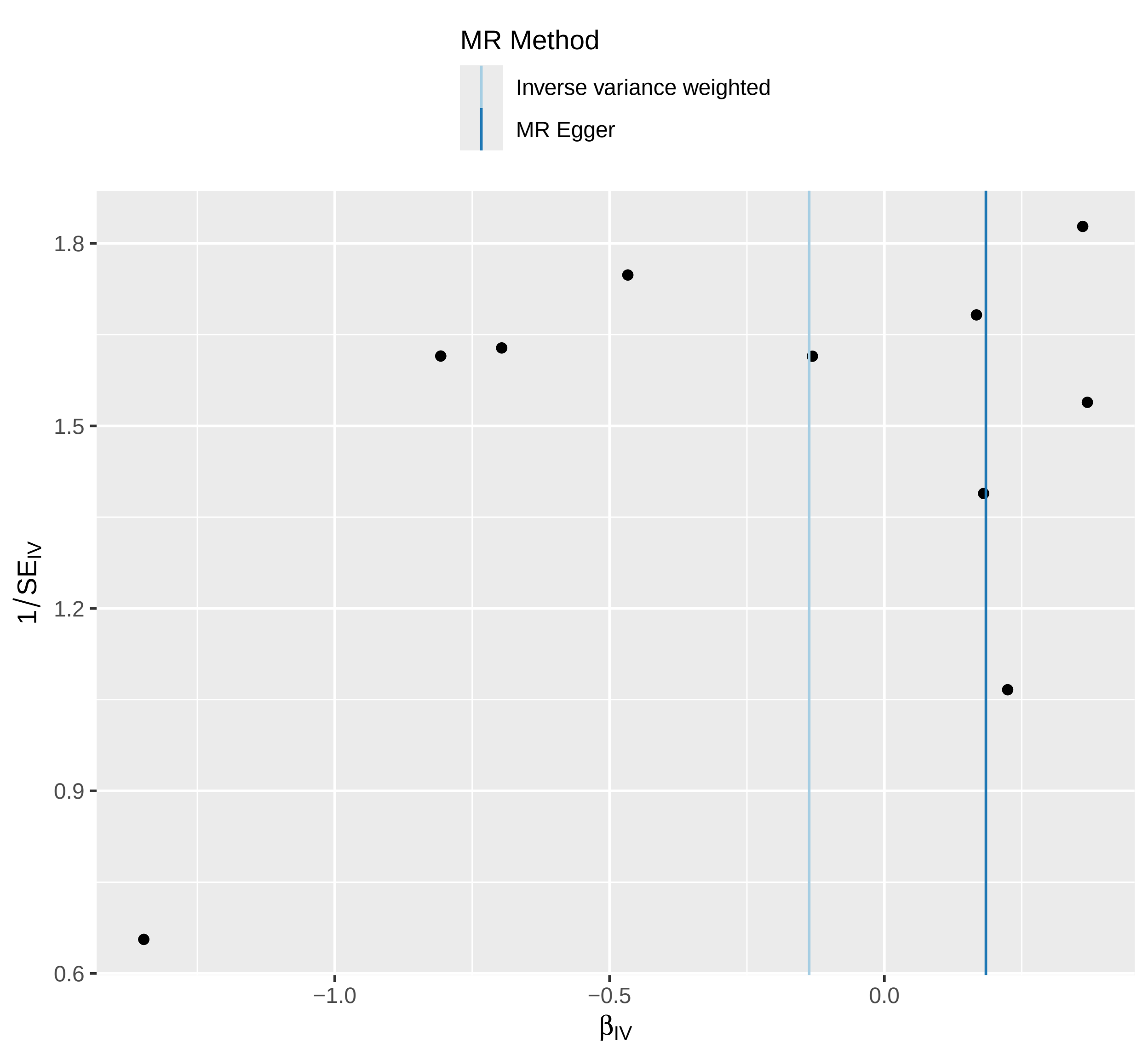

MR Method
Inverse variance weighted
MR Egger
1.8
1.5
1.2
0.9
0.6
−1.0
−0.5
0.0
1/SE_IV
β_IV

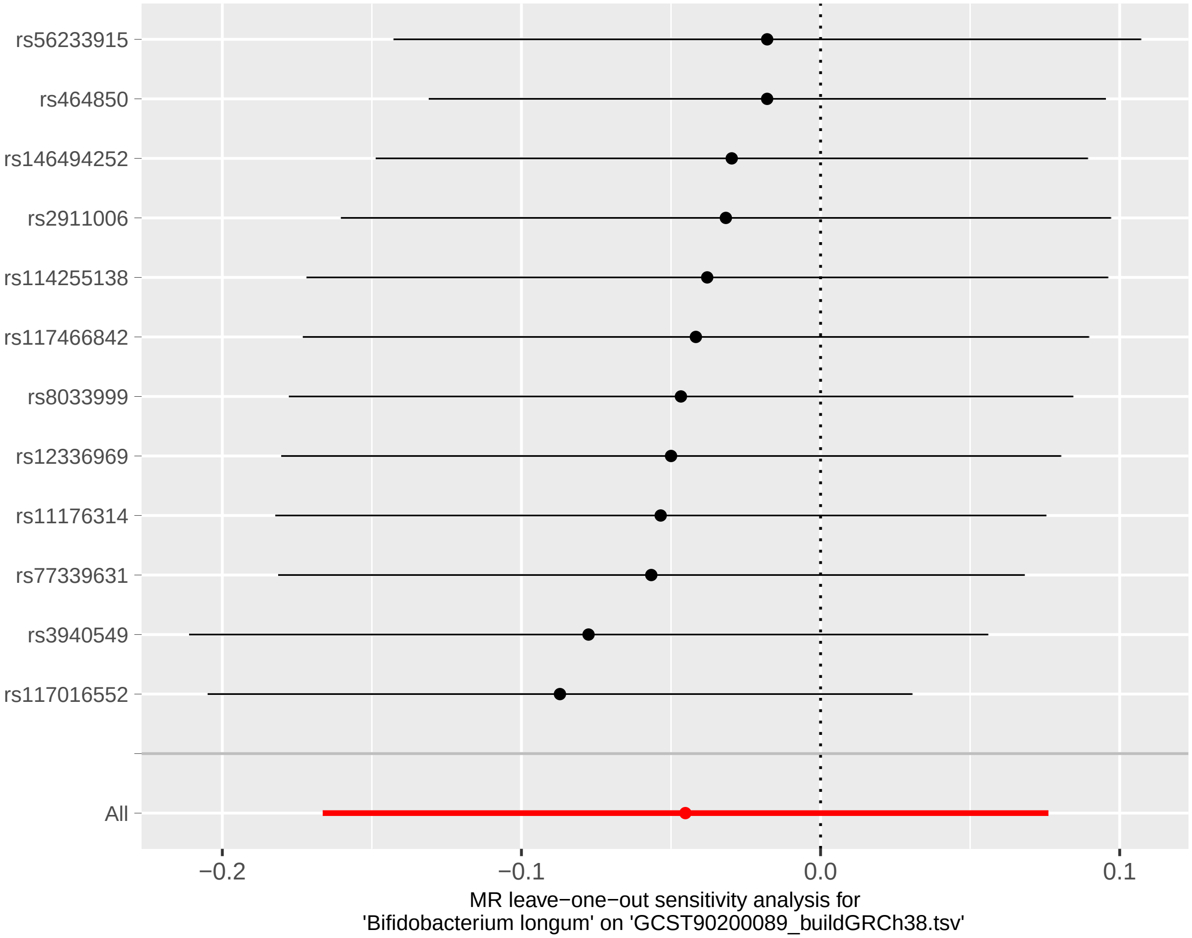
rs56233915
rs464850
rs146494252
rs2911006
rs114255138
rs117466842
rs8033999
rs12336969
rs11176314
rs77339631
rs3940549
rs117016552
All
−0.2
−0.1
0.0
0.1
MR leave−one−out sensitivity analysis for
'Bifidobacterium longum' on 'GCST90200089_buildGRCh38.tsv'

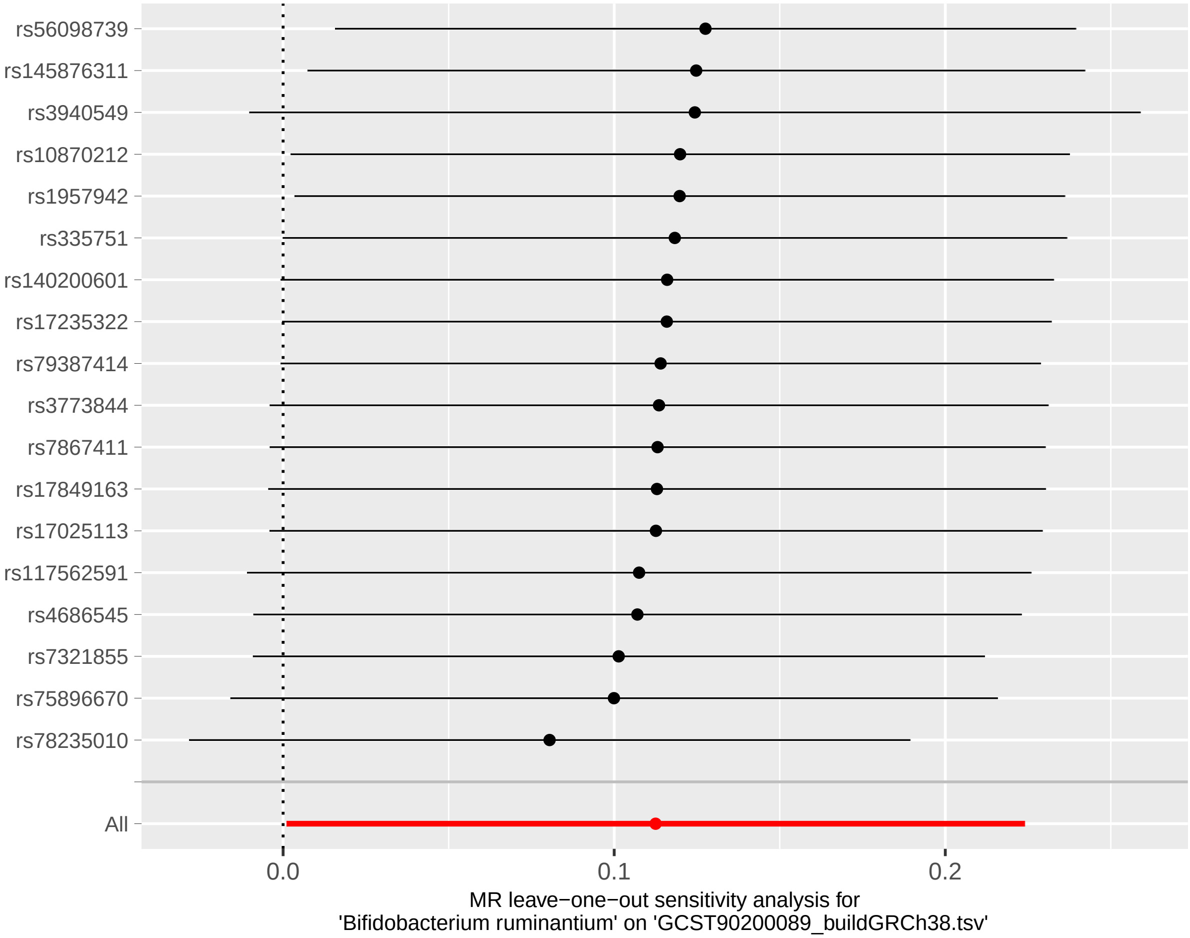

rs56098739
rs145876311
rs3940549
rs10870212
rs1957942
rs335751
rs140200601
rs17235322
rs79387414
rs3773844
rs7867411
rs17849163
rs17025113
rs117562591
rs4686545
rs7321855
rs75896670
rs78235010
All
0.0
0.1
0.2
MR leave−one−out sensitivity analysis for
'Bifidobacterium ruminantium' on 'GCST90200089_buildGRCh38.tsv'

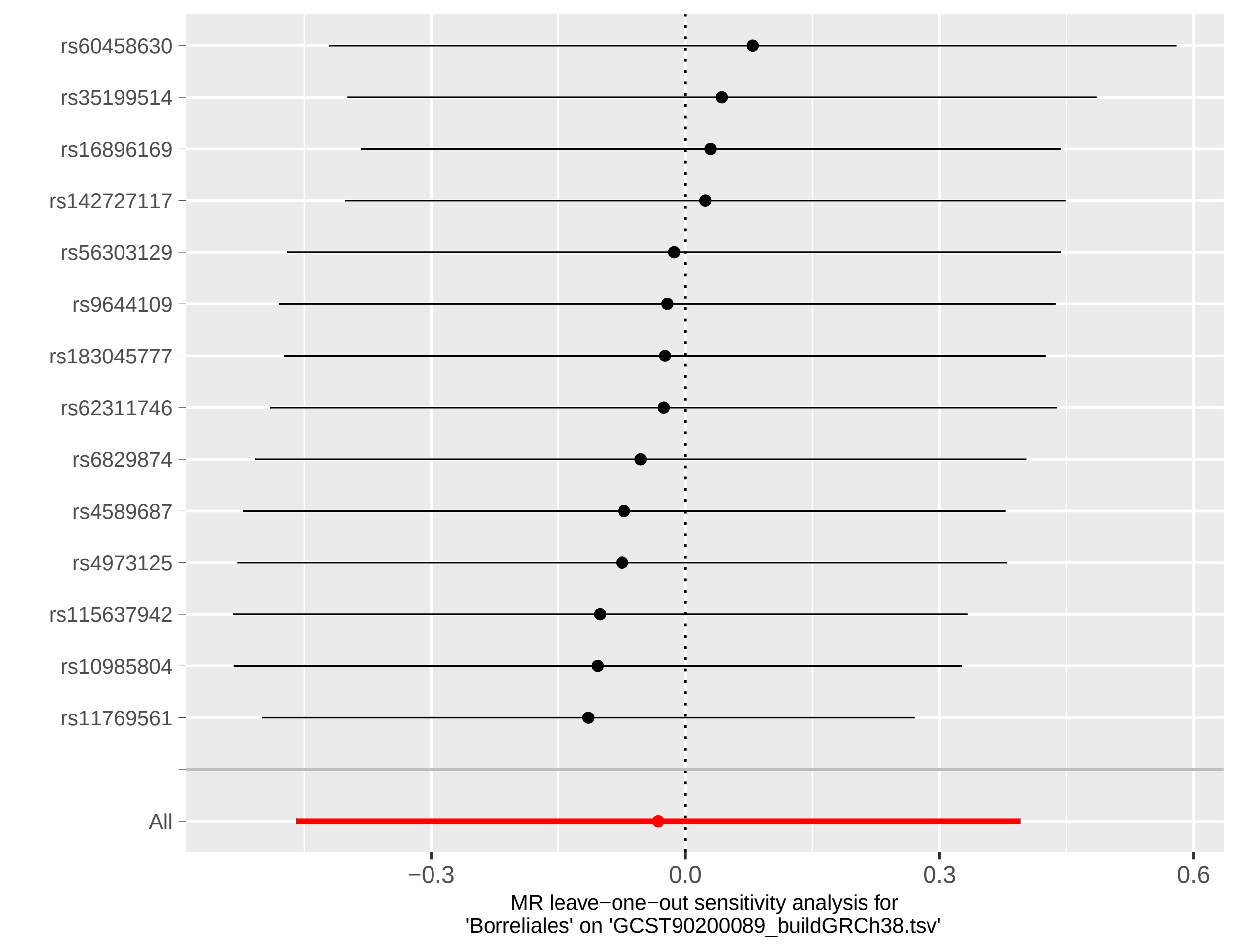

rs60458630
rs35199514
rs16896169
rs142727117
rs56303129
rs9644109
rs183045777
rs62311746
rs6829874
rs4589687
rs4973125
rs115637942
rs10985804
rs11769561
All
−0.3
0.0
0.3
0.6
MR leave−one−out sensitivity analysis for
'Borreliales' on 'GCST90200089_buildGRCh38.tsv'

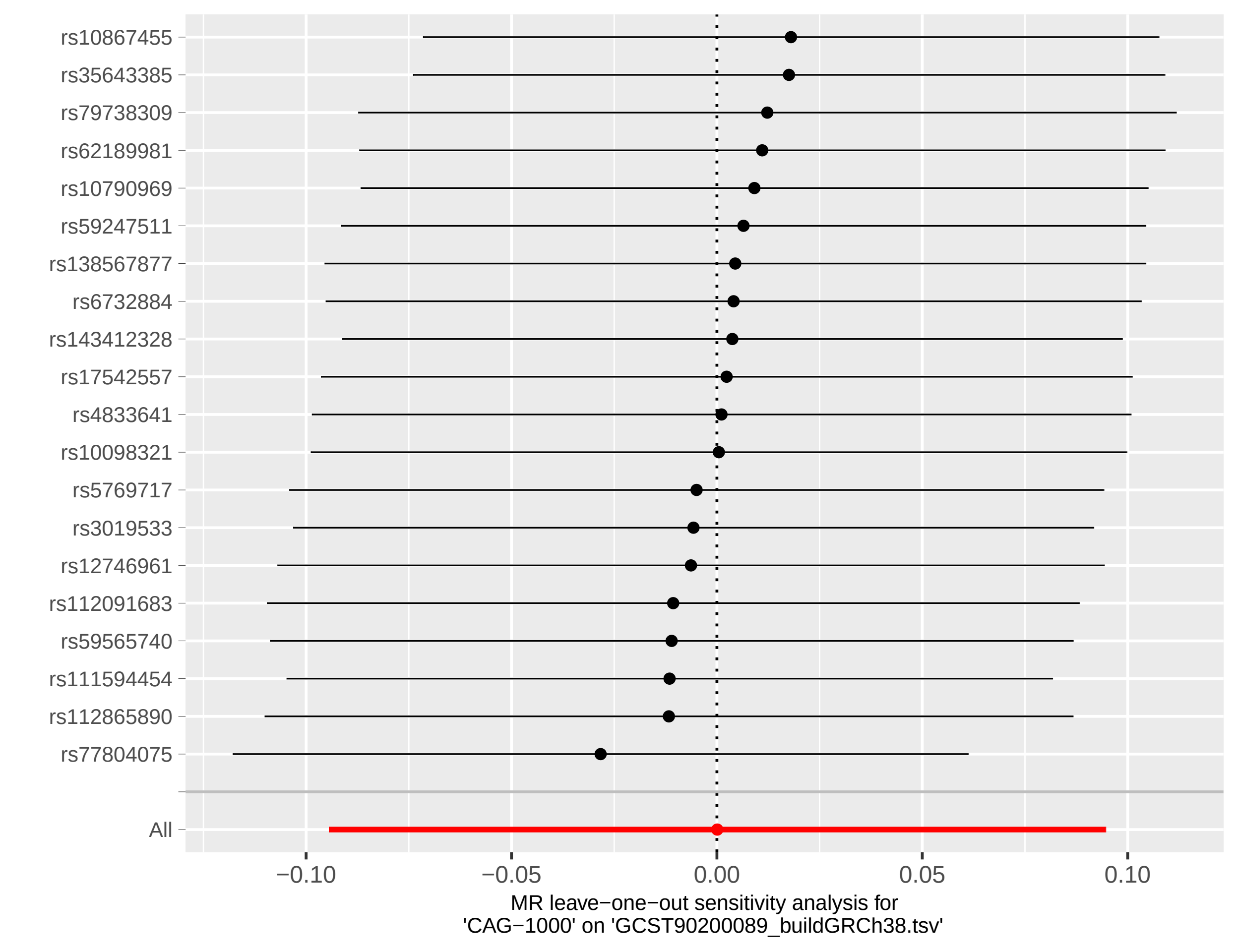
rs10867455
rs35643385
rs79738309
rs62189981
rs10790969
rs59247511
rs138567877
rs6732884
rs143412328
rs17542557
rs4833641
rs10098321
rs5769717
rs3019533
rs12746961
rs112091683
rs59565740
rs111594454
rs112865890
rs77804075
All
−0.10
−0.05
0.00
0.05
0.10
MR leave−one−out sensitivity analysis for
'CAG−1000' on 'GCST90200089_buildGRCh38.tsv'

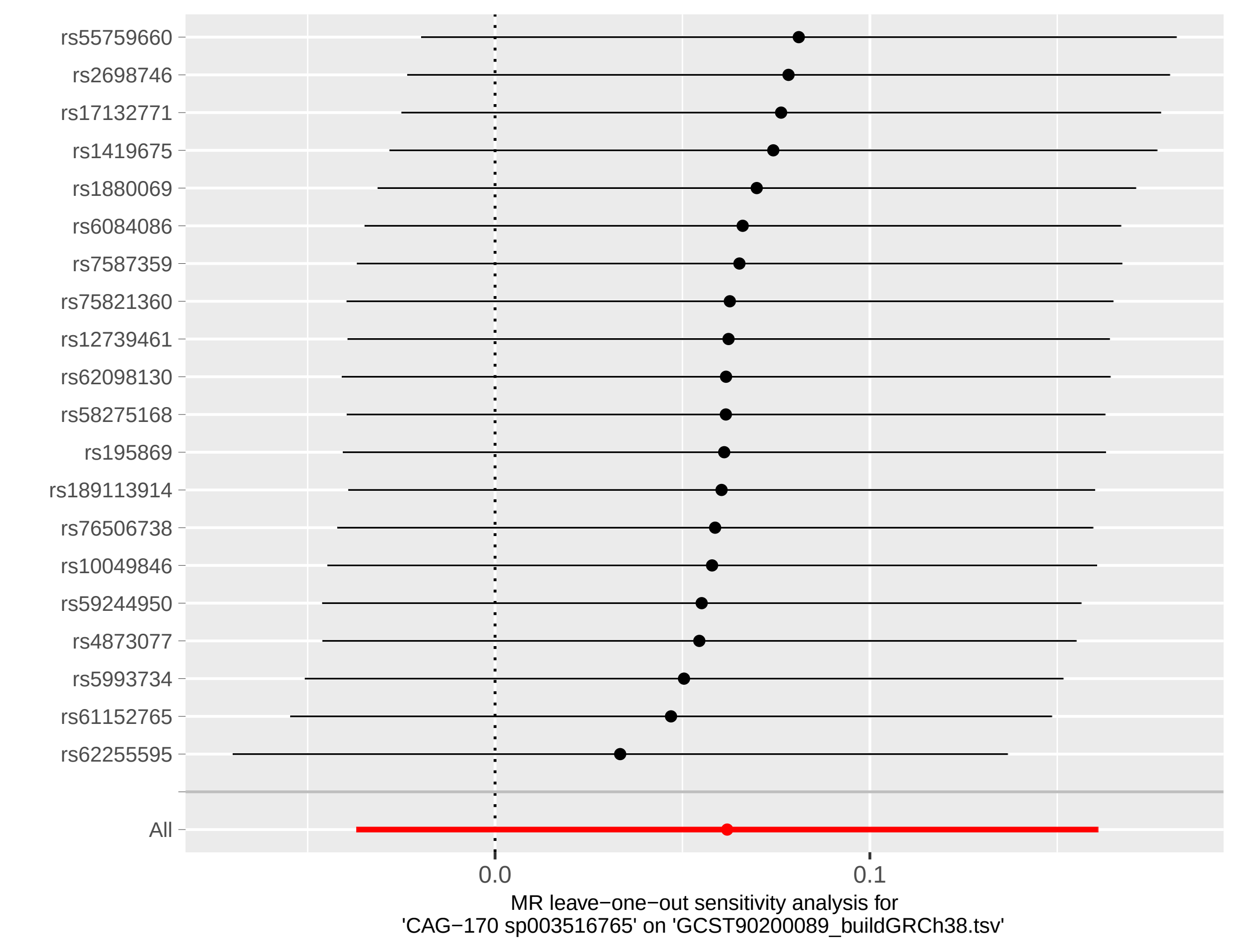
rs55759660
rs2698746
rs17132771
rs1419675
rs1880069
rs6084086
rs7587359
rs75821360
rs12739461
rs62098130
rs58275168
rs195869
rs189113914
rs76506738
rs10049846
rs59244950
rs4873077
rs5993734
rs61152765
rs62255595
All
0.0
0.1
MR leave−one−out sensitivity analysis for
'CAG−170 sp003516765' on 'GCST90200089_buildGRCh38.tsv'

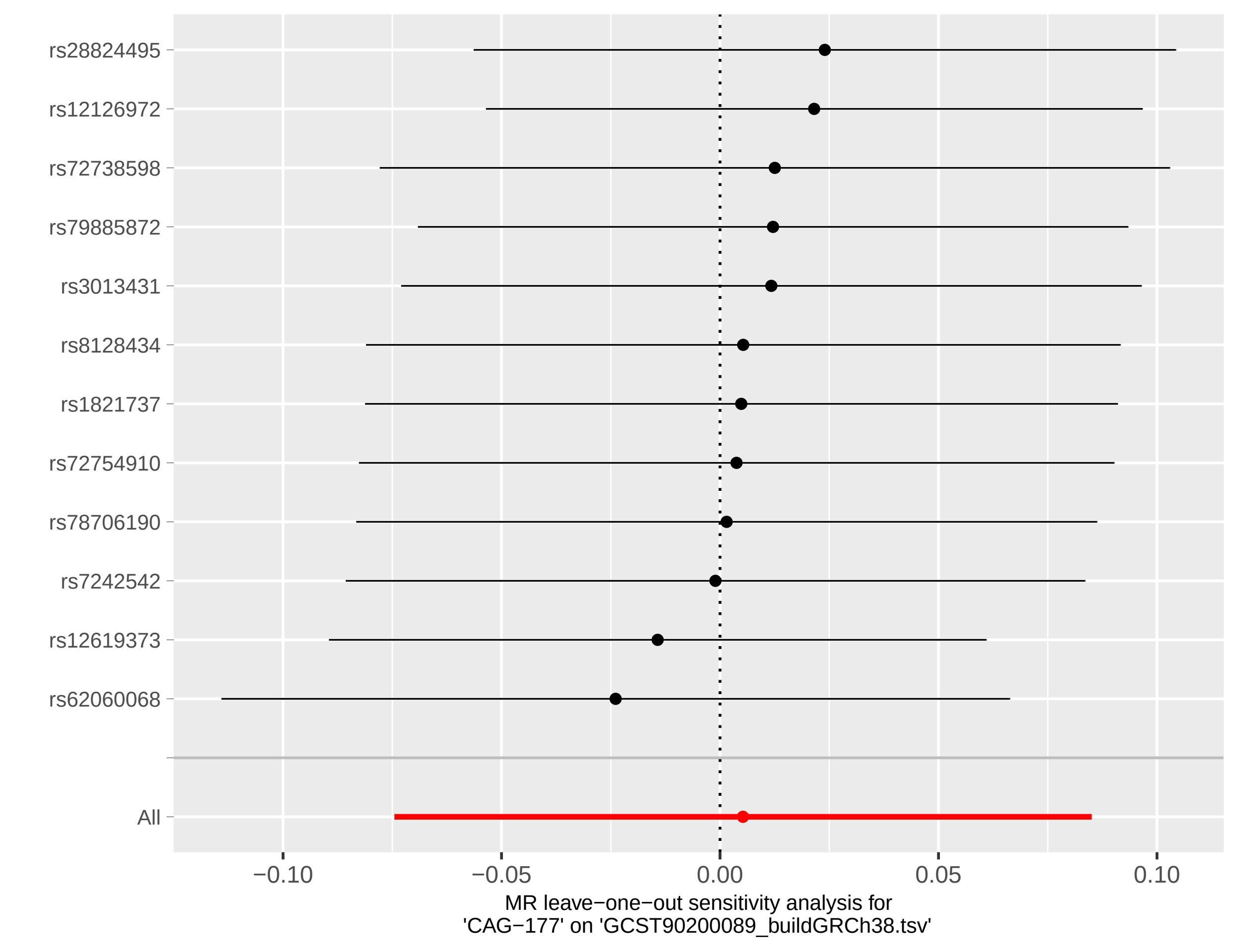
rs28824495
rs12126972
rs72738598
rs79885872
rs3013431
rs8128434
rs1821737
rs72754910
rs78706190
rs7242542
rs12619373
rs62060068
All
−0.10
−0.05
0.00
0.05
0.10
MR leave−one−out sensitivity analysis for
'CAG−177' on 'GCST90200089_buildGRCh38.tsv'

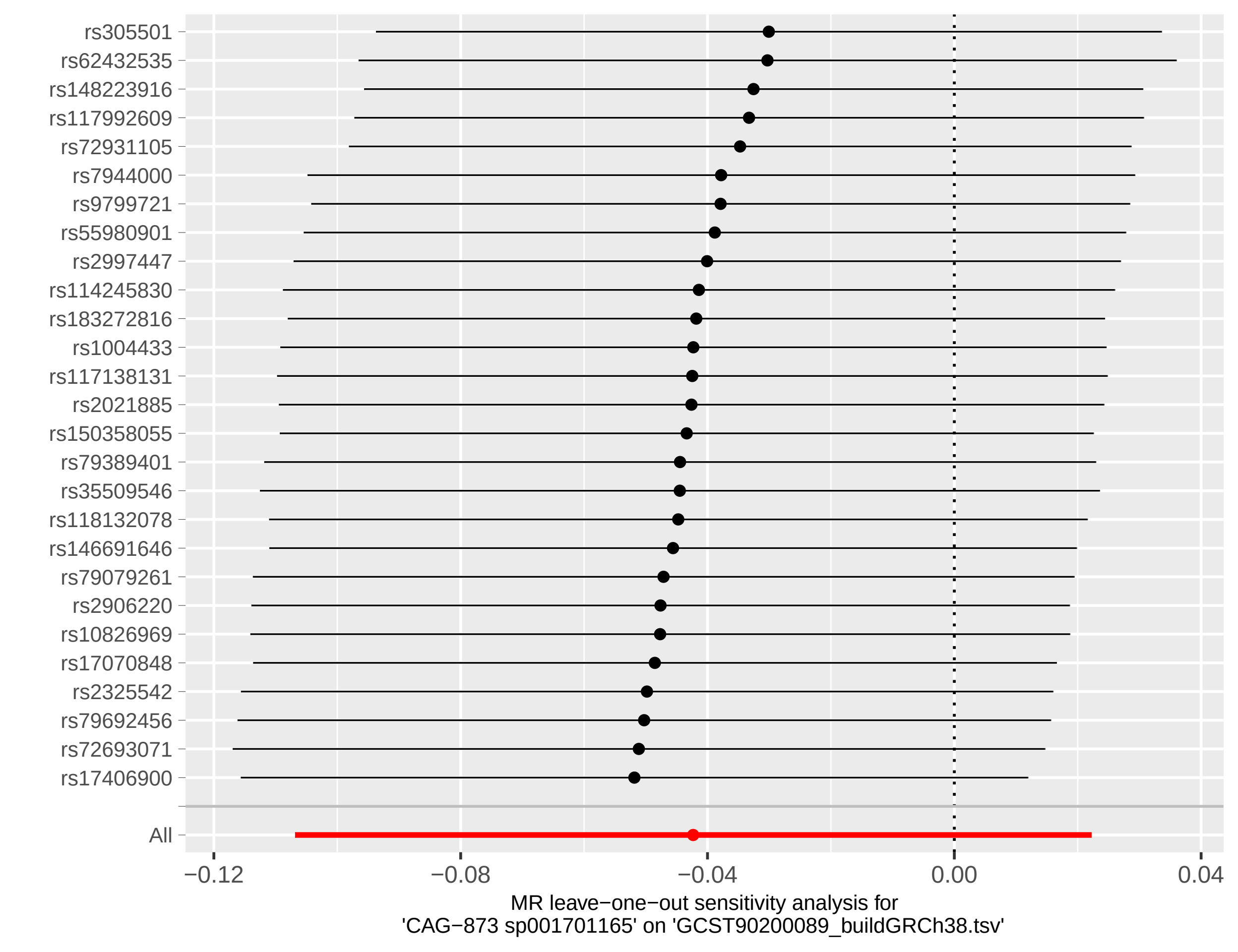
rs305501
rs62432535
rs148223916
rs117992609
rs72931105
rs7944000
rs9799721
rs55980901
rs2997447
rs114245830
rs183272816
rs1004433
rs117138131
rs2021885
rs150358055
rs79389401
rs35509546
rs118132078
rs146691646
rs79079261
rs2906220
rs10826969
rs17070848
rs2325542
rs79692456
rs72693071
rs17406900
All
−0.12
−0.08
−0.04
0.00
0.04
MR leave−one−out sensitivity analysis for
'CAG−873 sp001701165' on 'GCST90200089_buildGRCh38.tsv'

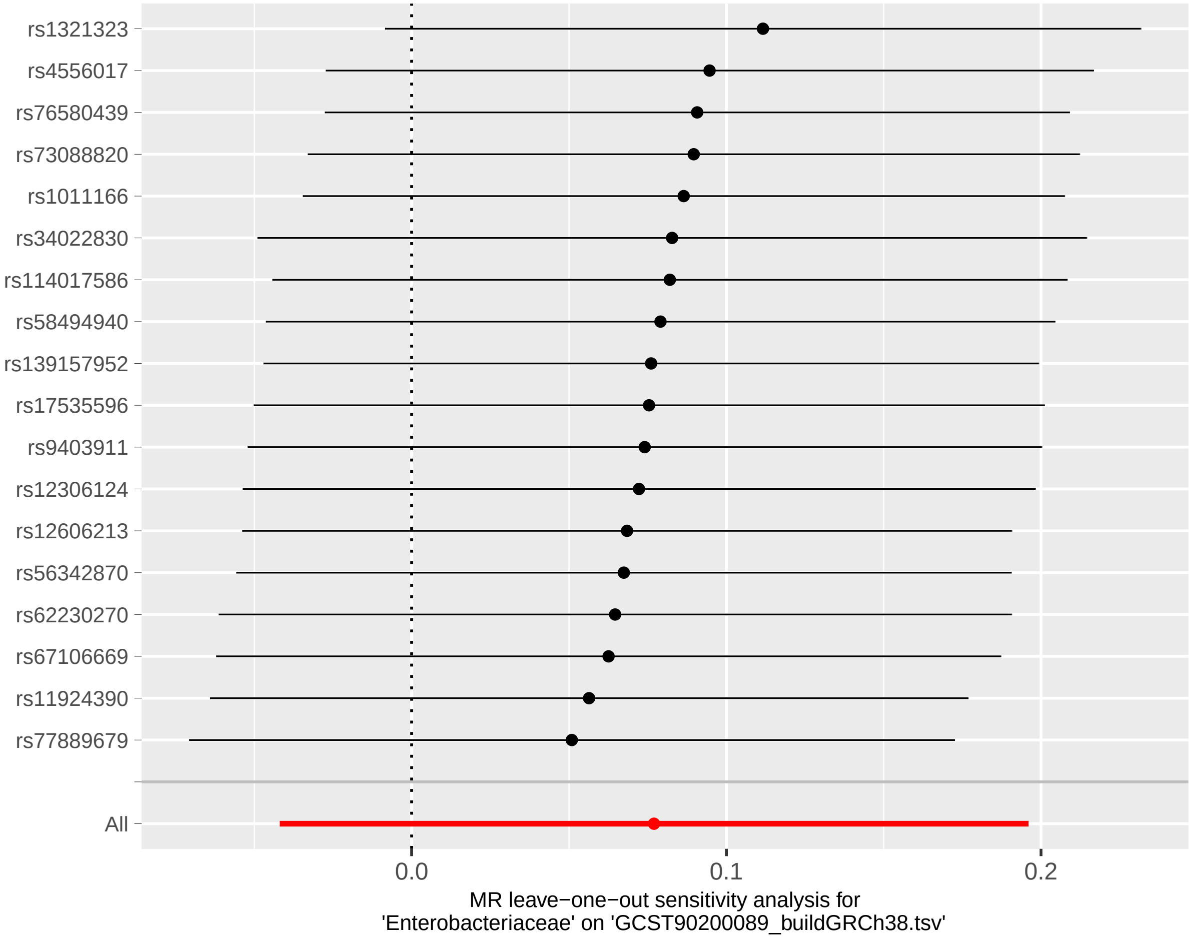
rs1321323
rs4556017
rs76580439
rs73088820
rs1011166
rs34022830
rs114017586
rs58494940
rs139157952
rs17535596
rs9403911
rs12306124
rs12606213
rs56342870
rs62230270
rs67106669
rs11924390
rs77889679
All
0.0
0.1
0.2
MR leave−one−out sensitivity analysis for
'Enterobacteriaceae' on 'GCST90200089_buildGRCh38.tsv'

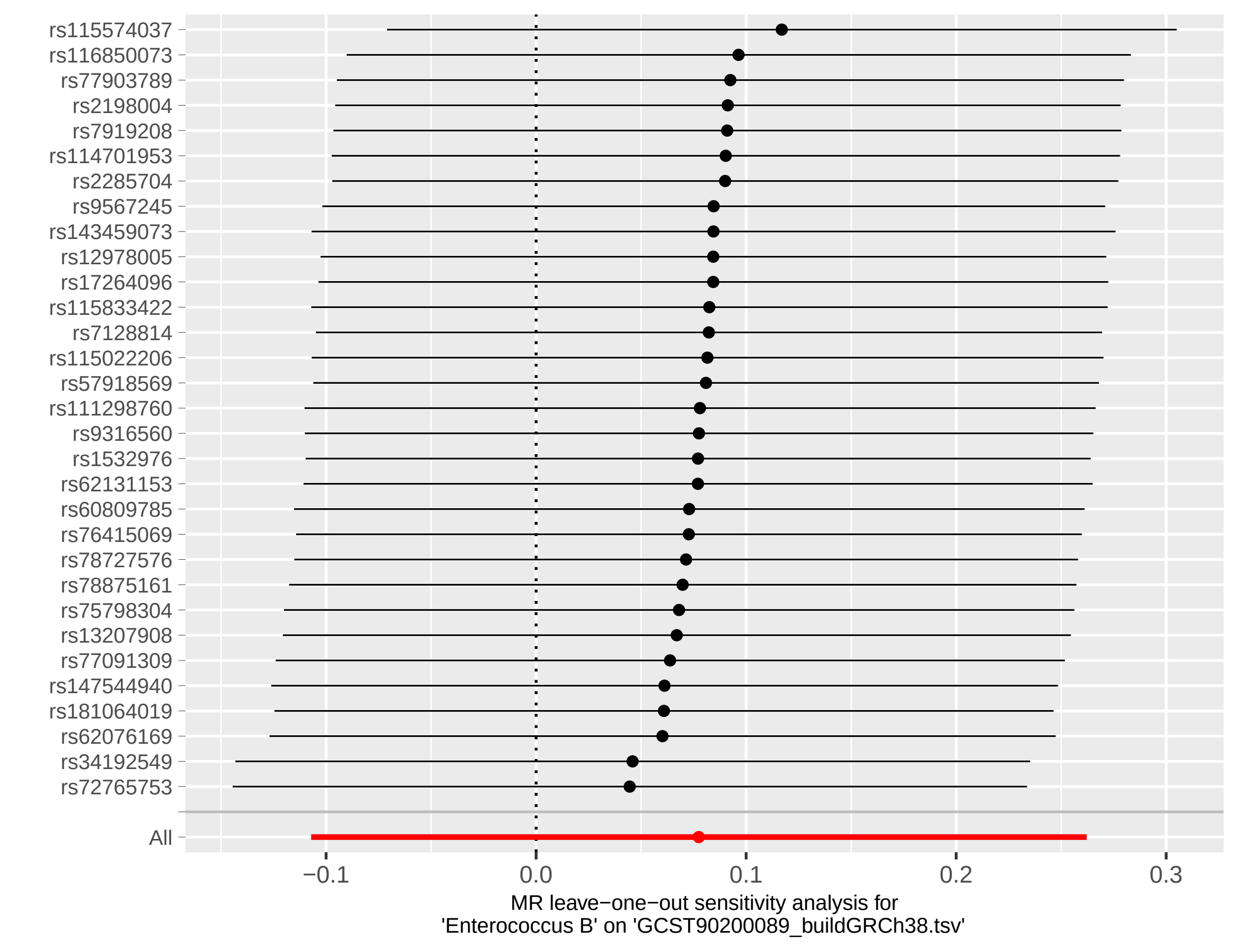
rs115574037
rs116850073
rs77903789
rs2198004
rs7919208
rs114701953
rs2285704
rs9567245
rs143459073
rs12978005
rs17264096
rs115833422
rs7128814
rs115022206
rs57918569
rs111298760
rs9316560
rs1532976
rs62131153
rs60809785
rs76415069
rs78727576
rs78875161
rs75798304
rs13207908
rs77091309
rs147544940
rs181064019
rs62076169
rs34192549
rs72765753
All
−0.1
0.0
0.1
0.2
0.3
MR leave−one−out sensitivity analysis for
'Enterococcus B' on 'GCST90200089_buildGRCh38.tsv'

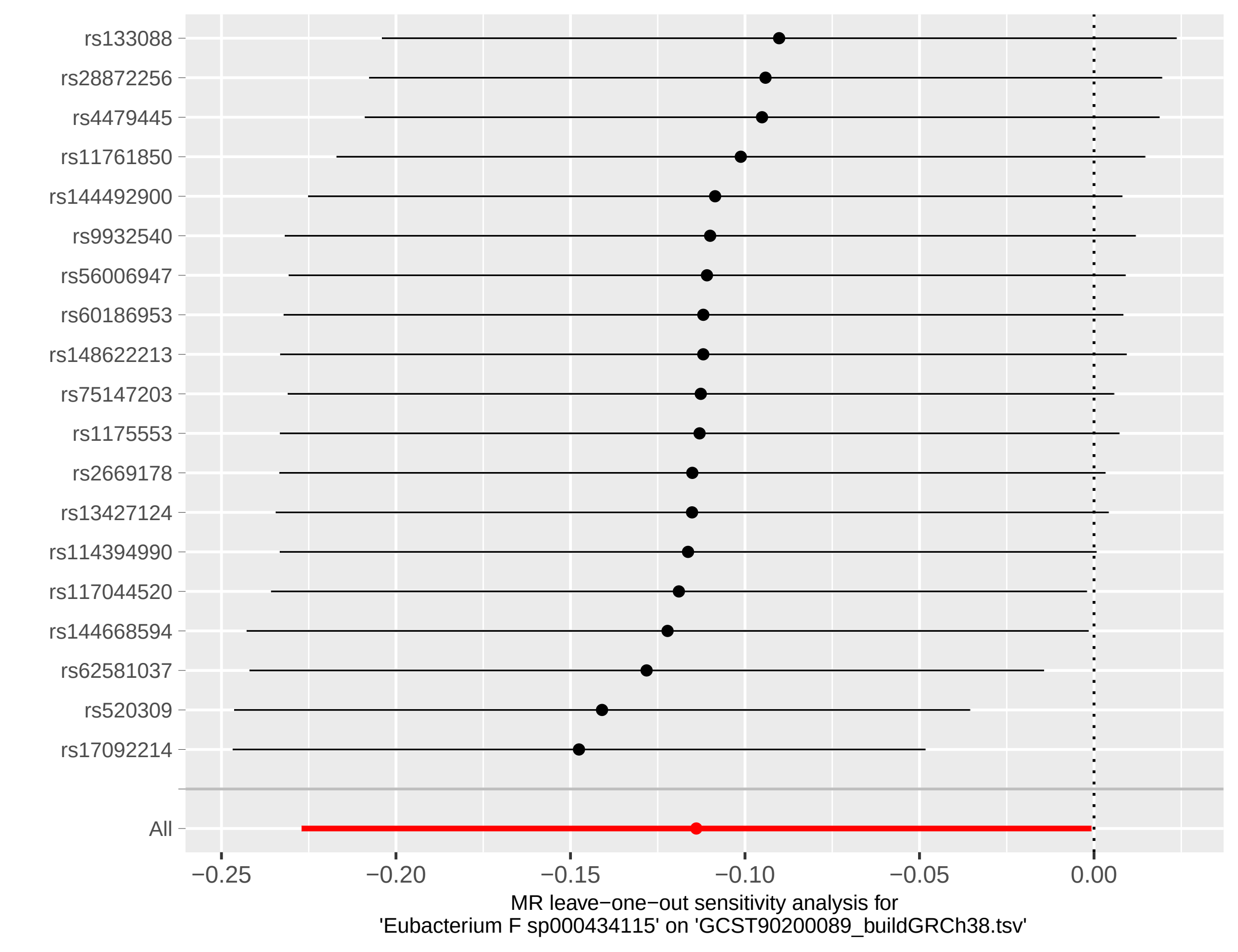

rs133088
rs28872256
rs4479445
rs11761850
rs144492900
rs9932540
rs56006947
rs60186953
rs148622213
rs75147203
rs1175553
rs2669178
rs13427124
rs114394990
rs117044520
rs144668594
rs62581037
rs520309
rs17092214
All
−0.25
−0.20
−0.15
−0.10
−0.05
0.00
MR leave−one−out sensitivity analysis for
'Eubacterium F sp000434115' on 'GCST90200089_buildGRCh38.tsv'

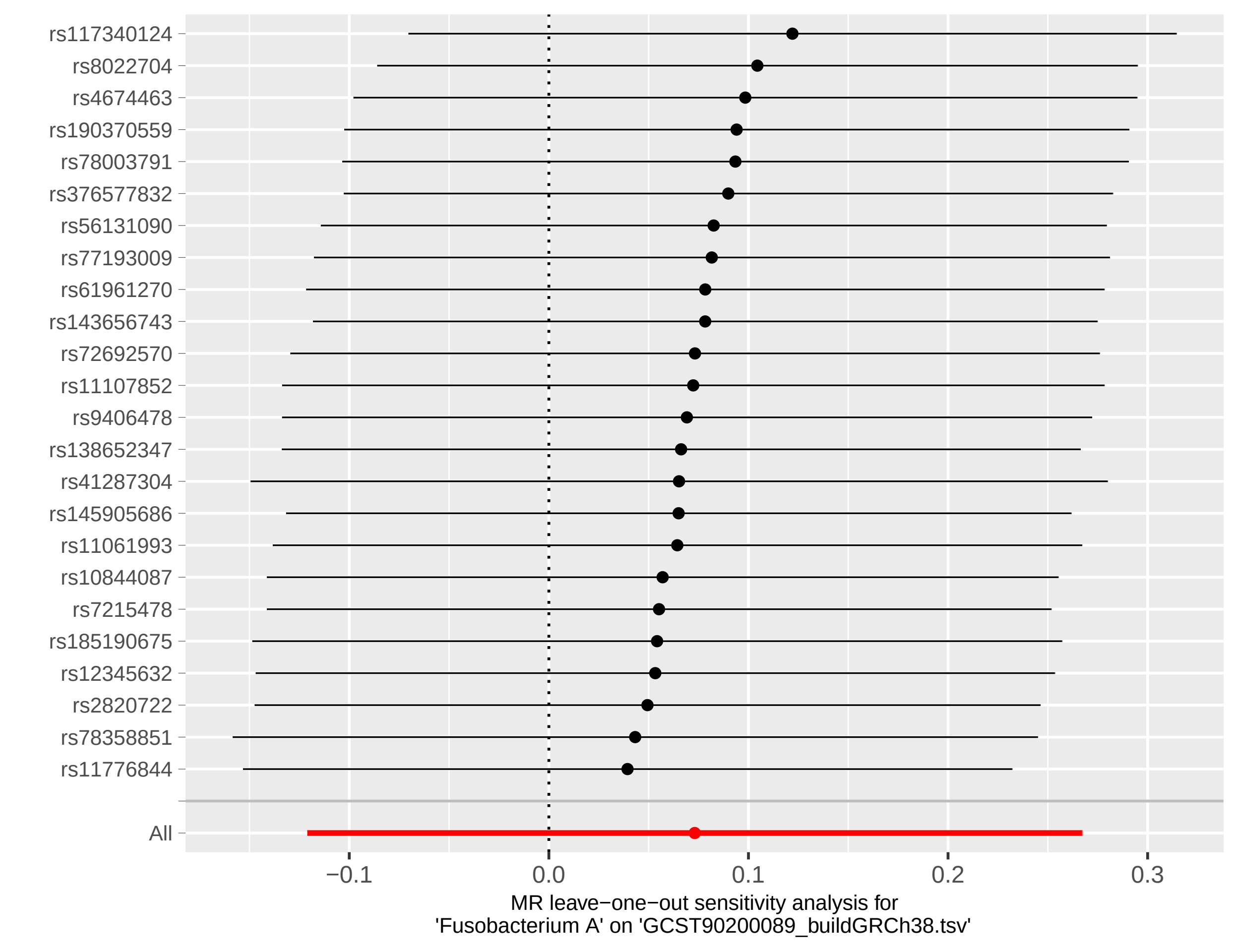
rs117340124
rs8022704
rs4674463
rs190370559
rs78003791
rs376577832
rs56131090
rs77193009
rs61961270
rs143656743
rs72692570
rs11107852
rs9406478
rs138652347
rs41287304
rs145905686
rs11061993
rs10844087
rs7215478
rs185190675
rs12345632
rs2820722
rs78358851
rs11776844
All
−0.1
0.0
0.1
0.2
0.3
MR leave−one−out sensitivity analysis for
'Fusobacterium A' on 'GCST90200089_buildGRCh38.tsv'

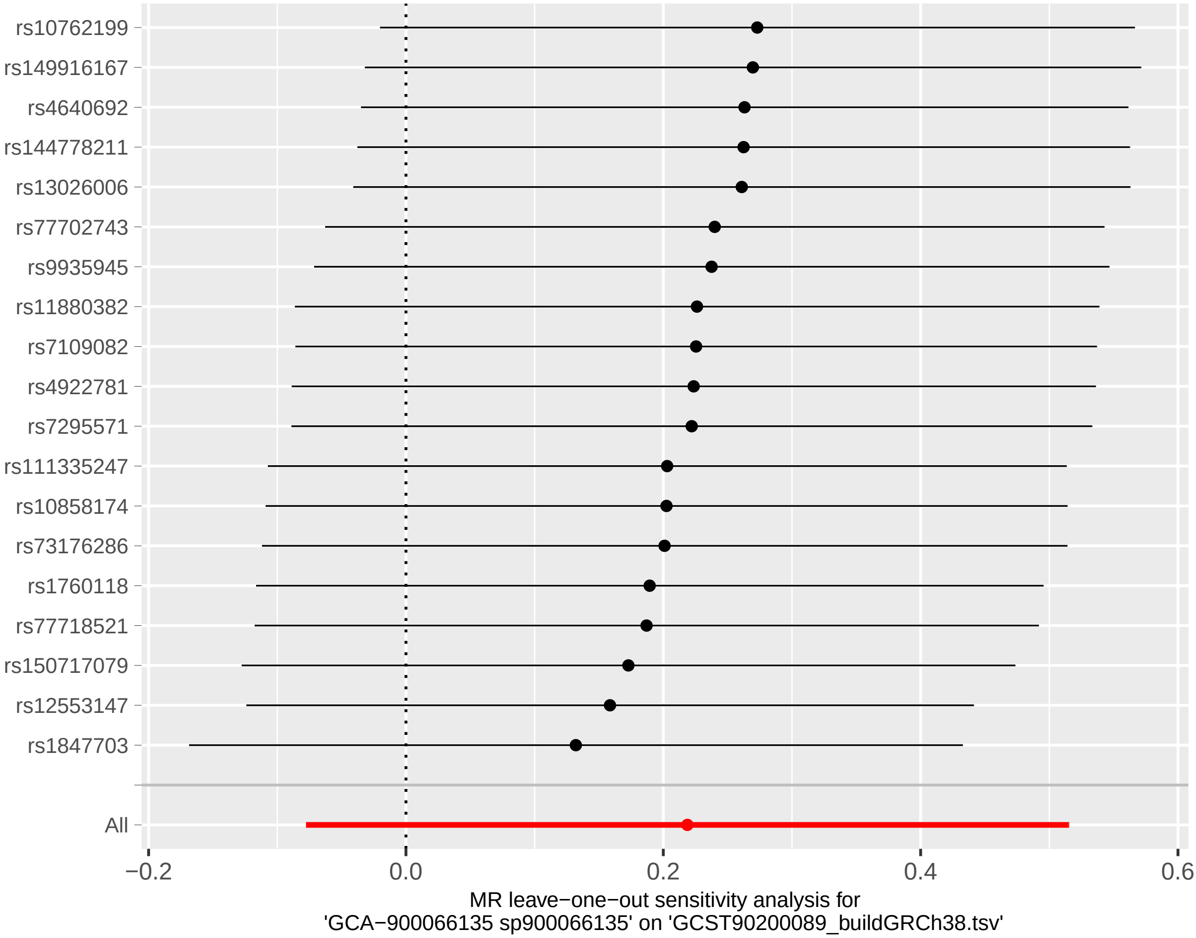
rs10762199
rs149916167
rs4640692
rs144778211
rs13026006
rs77702743
rs9935945
rs11880382
rs7109082
rs4922781
rs7295571
rs111335247
rs10858174
rs73176286
rs1760118
rs77718521
rs150717079
rs12553147
rs1847703
All
−0.2
0.0
0.2
0.4
0.6
MR leave−one−out sensitivity analysis for
'GCA−900066135 sp900066135' on 'GCST90200089_buildGRCh38.tsv'

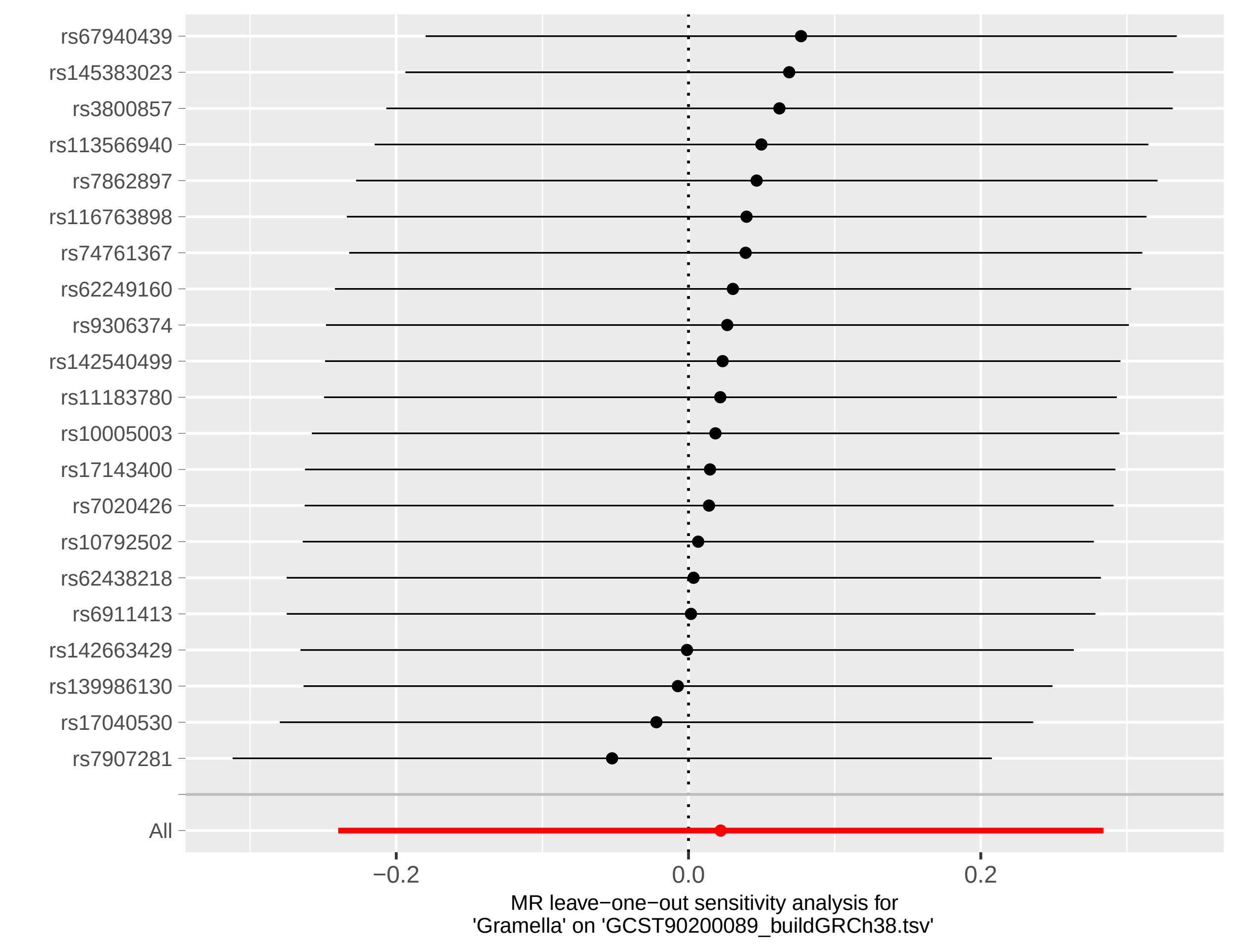

rs67940439
rs145383023
rs3800857
rs113566940
rs7862897
rs116763898
rs74761367
rs62249160
rs9306374
rs142540499
rs11183780
rs10005003
rs17143400
rs7020426
rs10792502
rs62438218
rs6911413
rs142663429
rs139986130
rs17040530
rs7907281
All
−0.2
0.0
0.2
MR leave−one−out sensitivity analysis for
'Gramella' on 'GCST90200089_buildGRCh38.tsv'

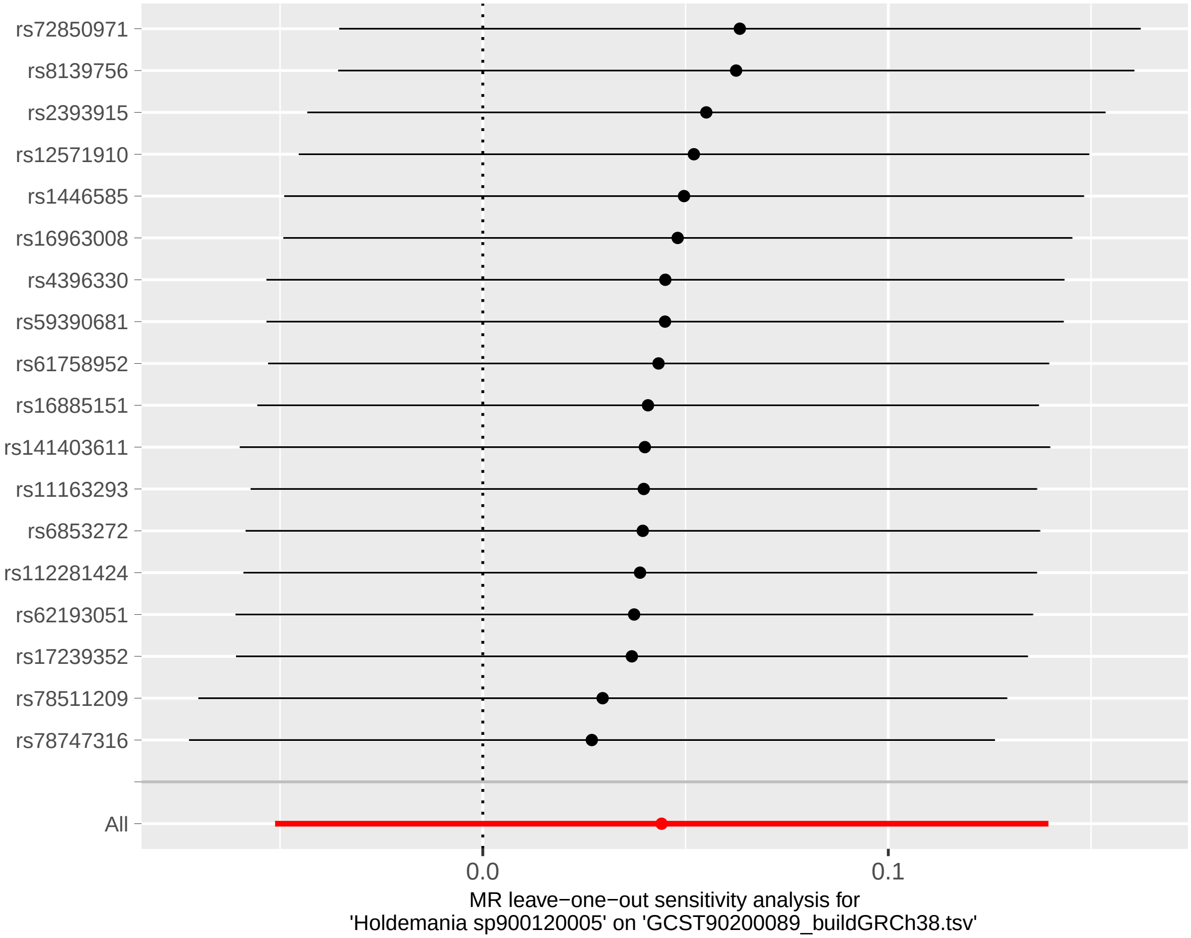

rs72850971
rs8139756
rs2393915
rs12571910
rs1446585
rs16963008
rs4396330
rs59390681
rs61758952
rs16885151
rs141403611
rs11163293
rs6853272
rs112281424
rs62193051
rs17239352
rs78511209
rs78747316
All
0.0
0.1
MR leave−one−out sensitivity analysis for
'Holdemania sp900120005' on 'GCST90200089_buildGRCh38.tsv'

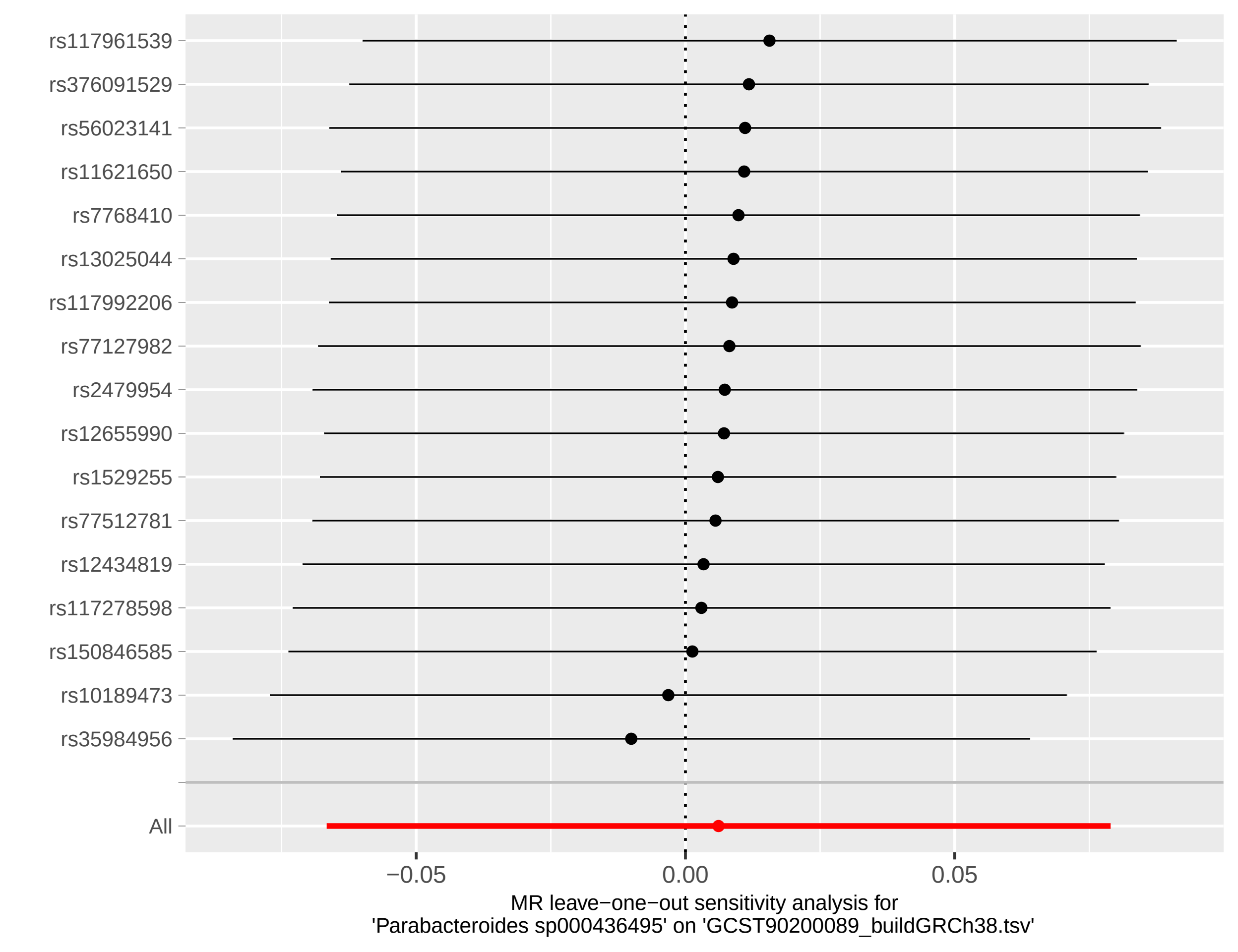
rs117961539
rs376091529
rs56023141
rs11621650
rs7768410
rs13025044
rs117992206
rs77127982
rs2479954
rs12655990
rs1529255
rs77512781
rs12434819
rs117278598
rs150846585
rs10189473
rs35984956
All
−0.05
0.00
0.05
MR leave−one−out sensitivity analysis for
'Parabacteroides sp000436495' on 'GCST90200089_buildGRCh38.tsv'

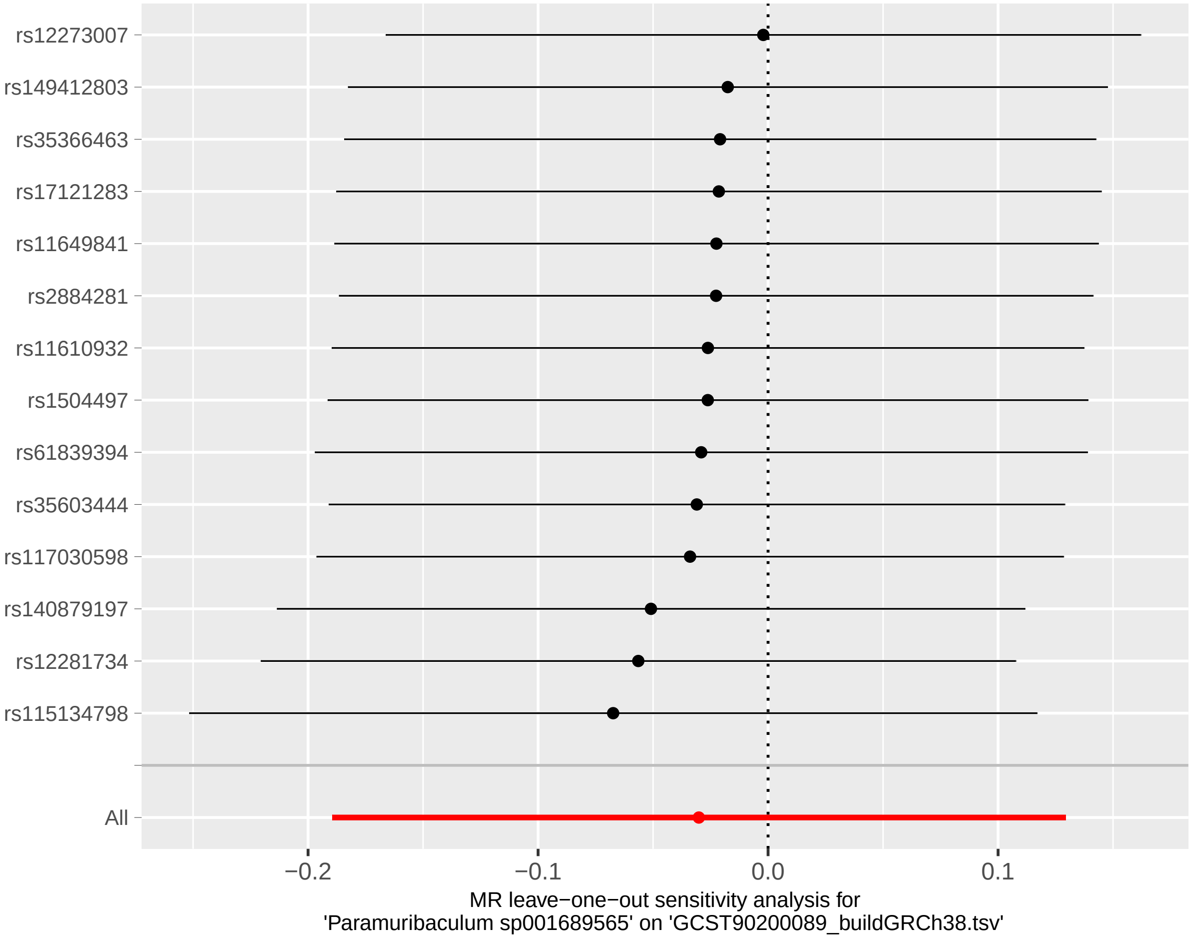
rs12273007
rs149412803
rs35366463
rs17121283
rs11649841
rs2884281
rs11610932
rs1504497
rs61839394
rs35603444
rs117030598
rs140879197
rs12281734
rs115134798
All
−0.2
−0.1
0.0
0.1
MR leave−one−out sensitivity analysis for
'Paramuribaculum sp001689565' on 'GCST90200089_buildGRCh38.tsv'

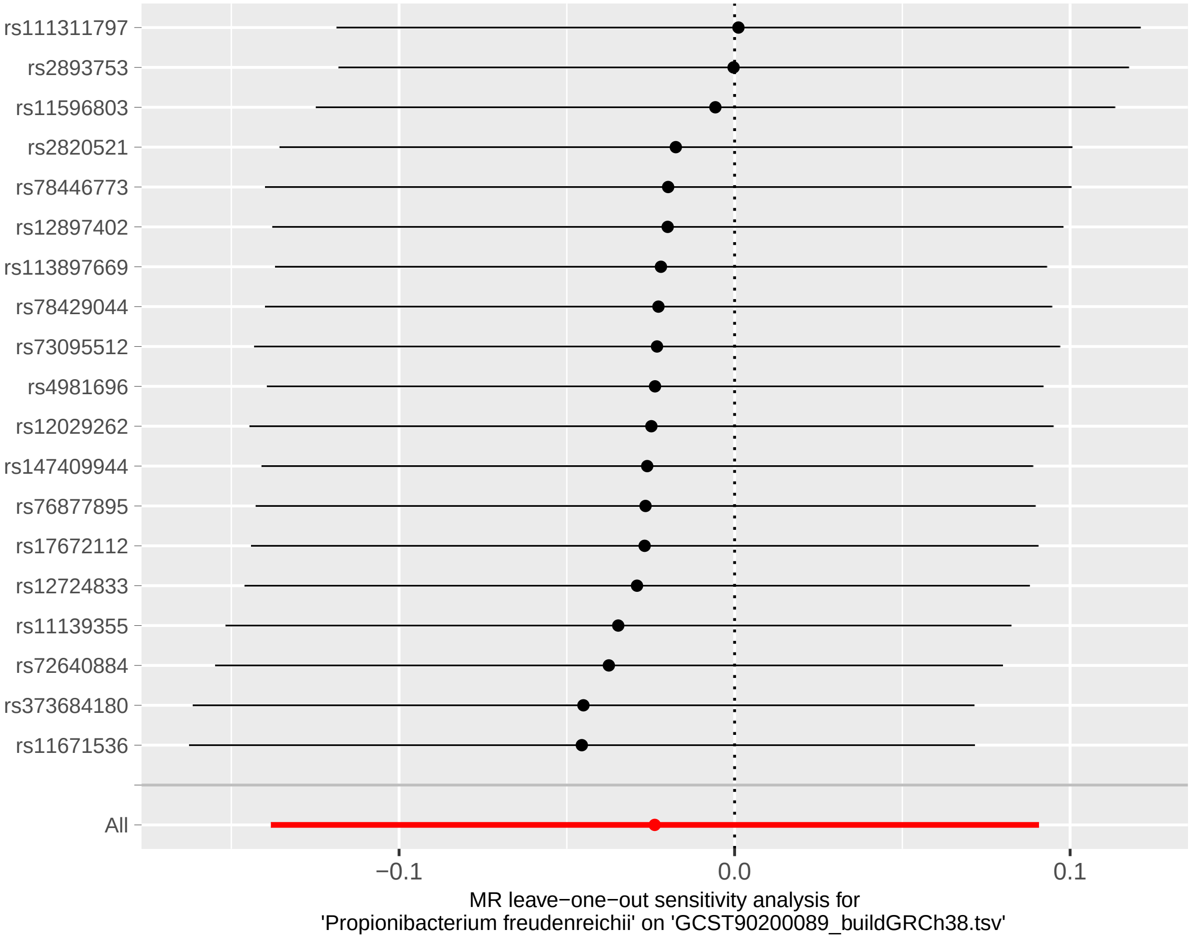
rs111311797
rs2893753
rs11596803
rs2820521
rs78446773
rs12897402
rs113897669
rs78429044
rs73095512
rs4981696
rs12029262
rs147409944
rs76877895
rs17672112
rs12724833
rs11139355
rs72640884
rs373684180
rs11671536
All
−0.1
0.0
0.1
MR leave−one−out sensitivity analysis for
'Propionibacterium freudenreichii' on 'GCST90200089_buildGRCh38.tsv'

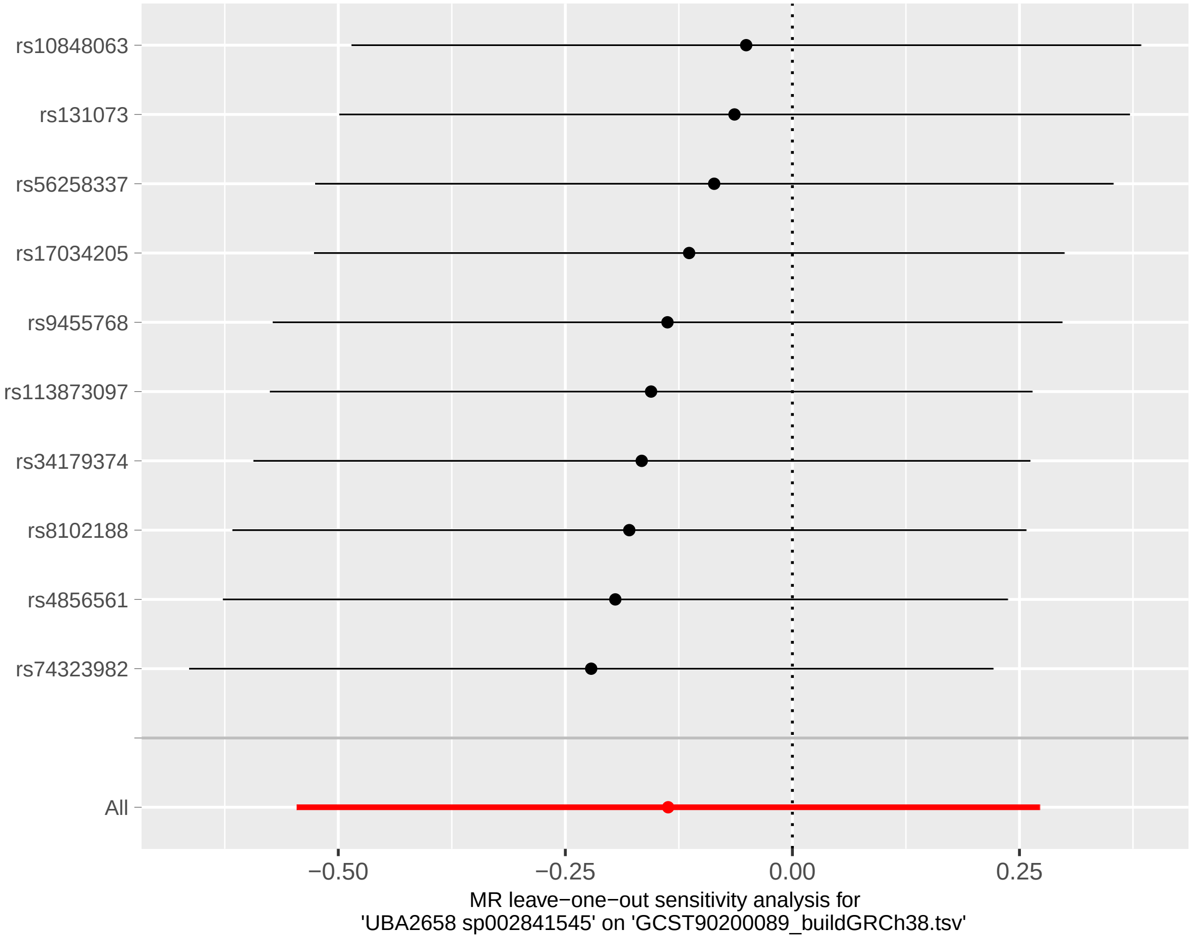

rs10848063
rs131073
rs56258337
rs17034205
rs9455768
rs113873097
rs34179374
rs8102188
rs4856561
rs74323982
All
−0.50
−0.25
0.00
0.25
MR leave−one−out sensitivity analysis for
'UBA2658 sp002841545' on 'GCST90200089_buildGRCh38.tsv'

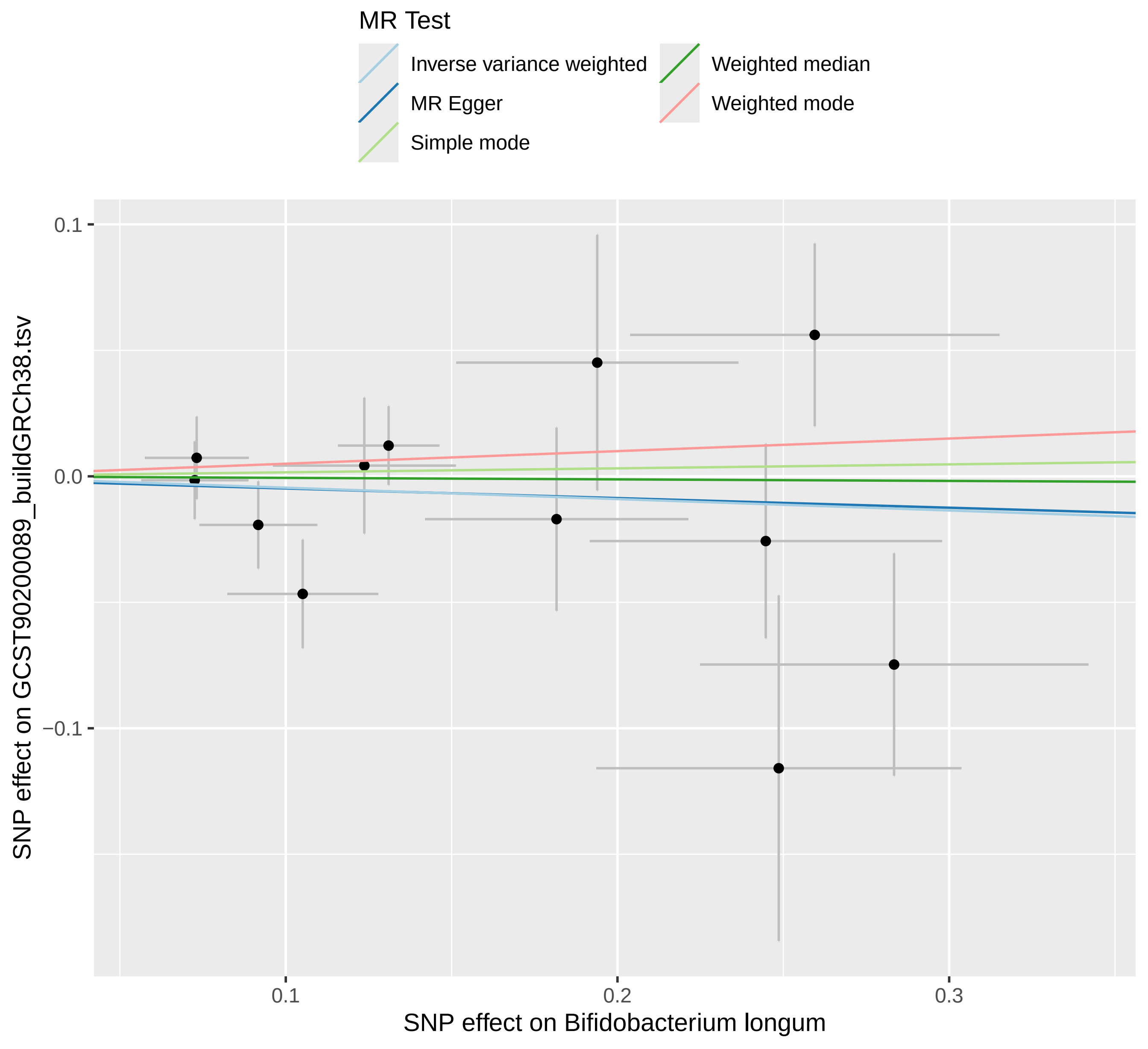
MR Test
Inverse variance weighted
MR Egger
Simple mode
Weighted median
Weighted mode
0.1
0.0
−0.1
0.1
0.2
0.3
SNP effect on GCST90200089_buildGRCh38.tsv
SNP effect on Bifidobacterium longum

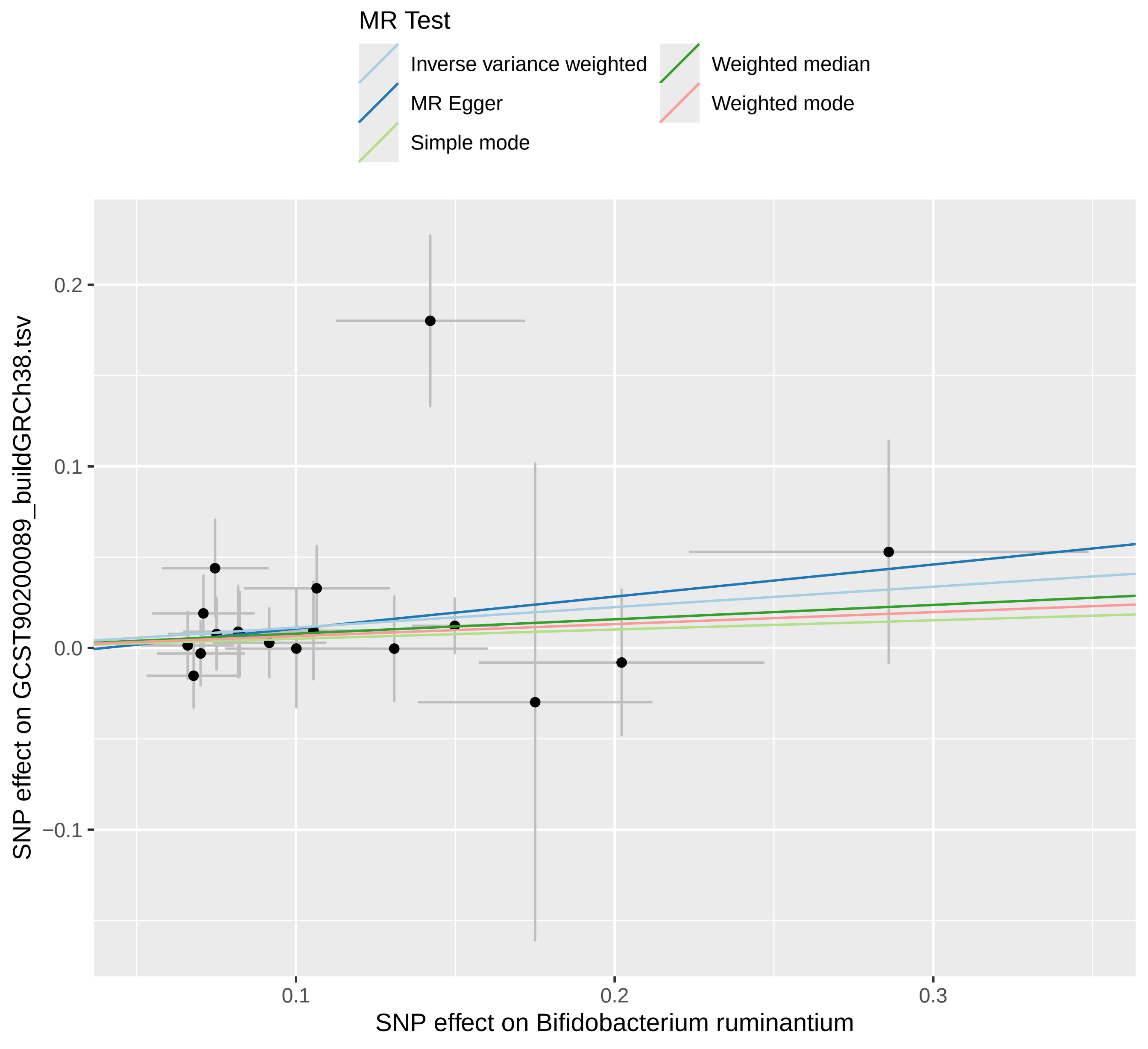

MR Test
Inverse variance weighted
MR Egger
Simple mode
Weighted median
Weighted mode
0.2
0.1
0.0
−0.1
0.1
0.2
0.3
SNP effect on GCST90200089_buildGRCh38.tsv
SNP effect on Bifidobacterium ruminantium

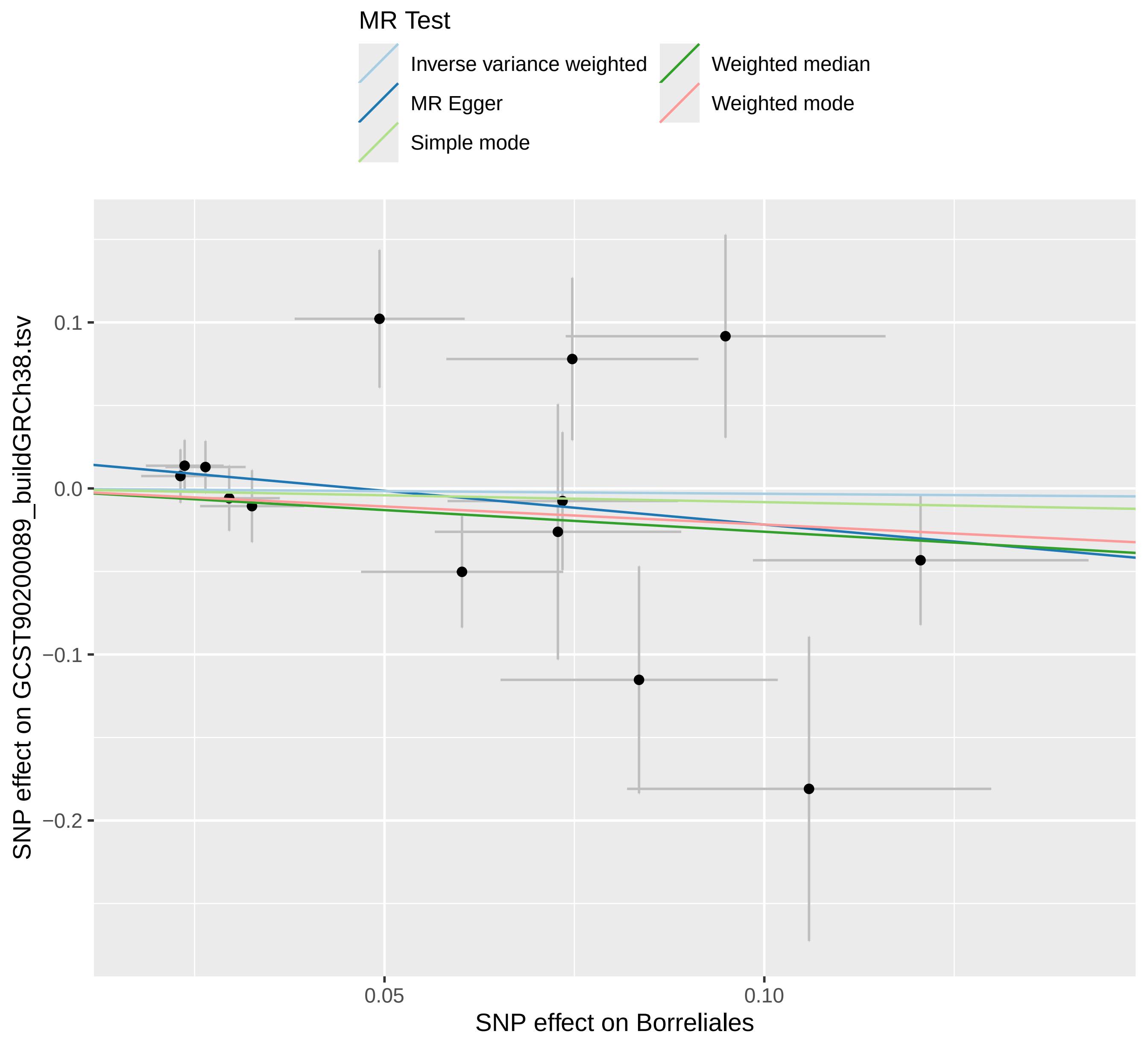
MR Test
Inverse variance weighted
MR Egger
Simple mode
Weighted median
Weighted mode
SNP effect on GCST90200089_buildGRCh38.tsv
0.1
0.0
−0.1
−0.2
0.05
0.10
SNP effect on Borreliales

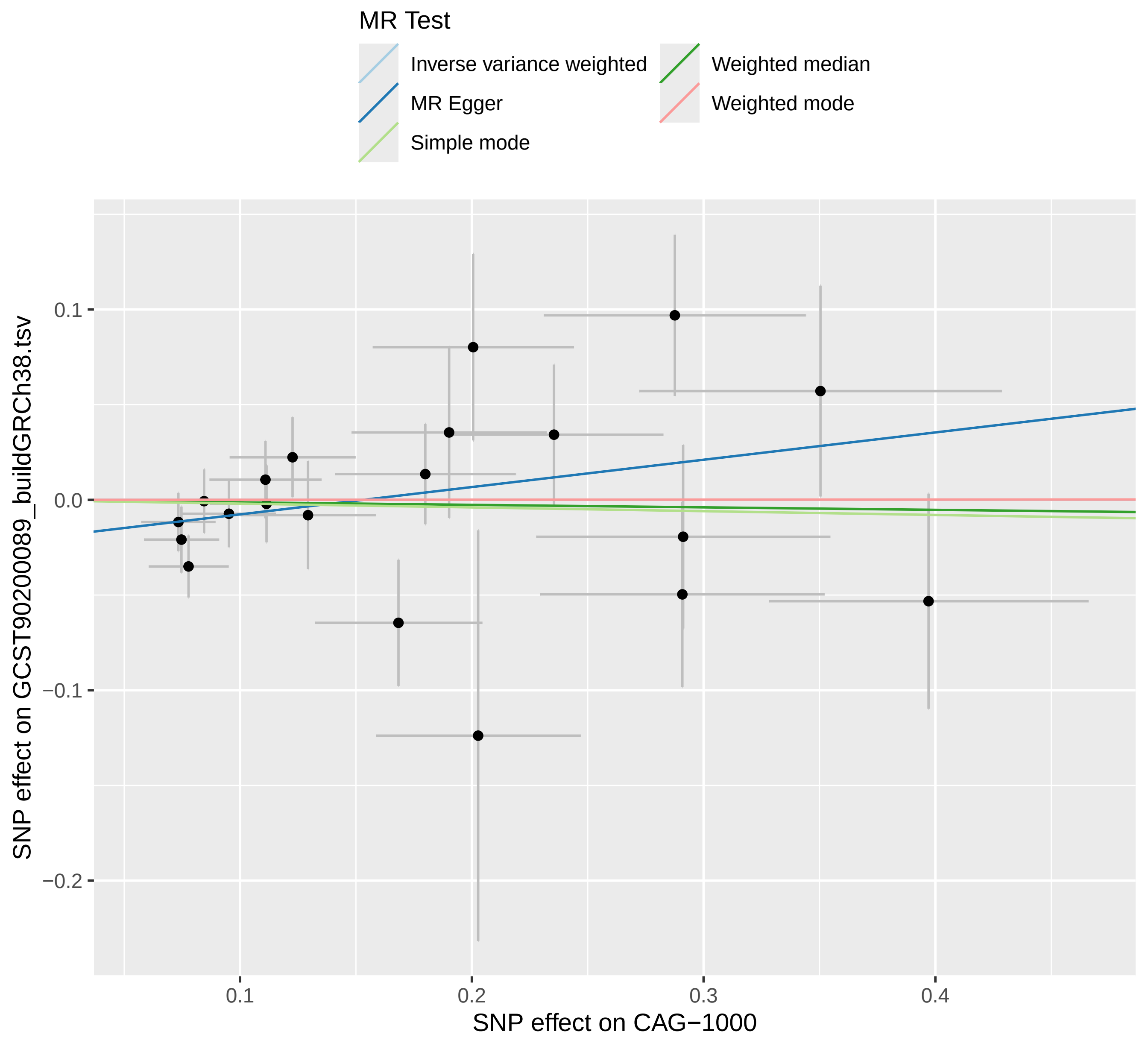
MR Test
Inverse variance weighted
MR Egger
Simple mode
Weighted median
Weighted mode
0.1
0.0
−0.1
−0.2
0.1
0.2
0.3
0.4
SNP effect on GCST90200089_buildGRCh38.tsv
SNP effect on CAG−1000

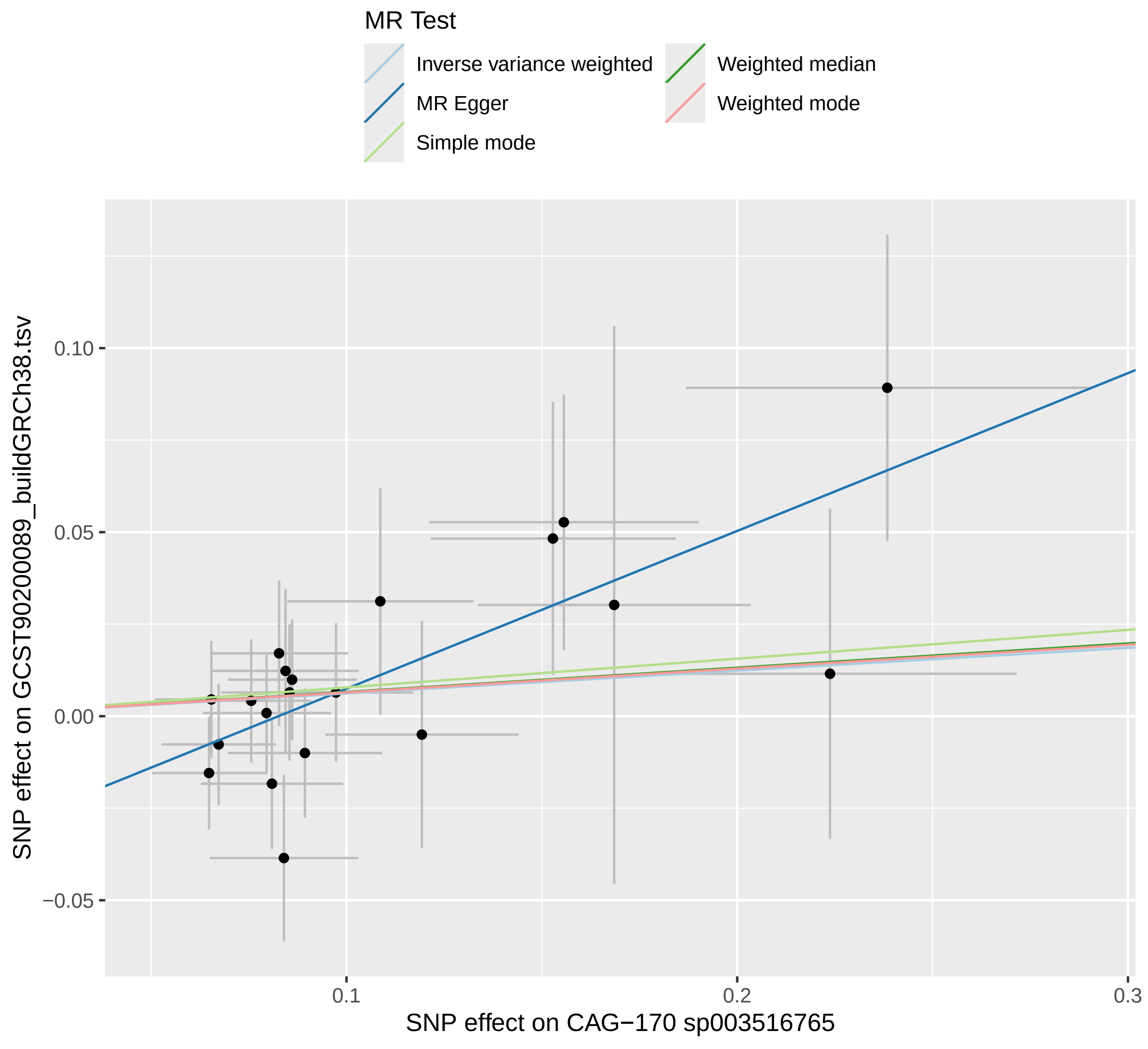

MR Test
Inverse variance weighted
MR Egger
Simple mode
Weighted median
Weighted mode
SNP effect on GCST90200089_buildGRCh38.tsv
0.10
0.05
0.00
−0.05
0.1
0.2
0.3
SNP effect on CAG−170 sp003516765

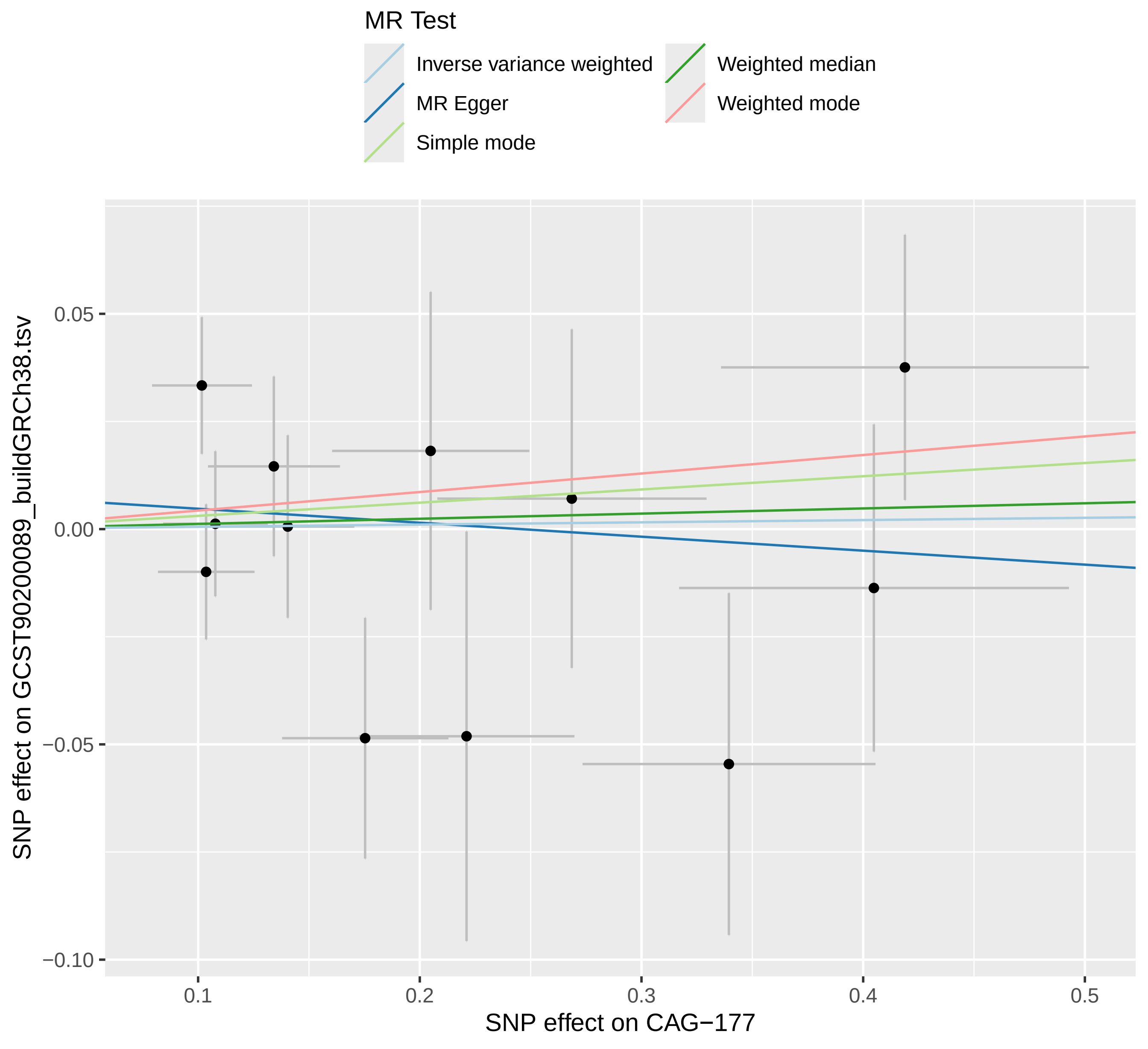
MR Test
Inverse variance weighted
MR Egger
Simple mode
Weighted median
Weighted mode
0.05
0.00
−0.05
−0.10
0.1
0.2
0.3
0.4
0.5
SNP effect on GCST90200089_buildGRCh38.tsv
SNP effect on CAG−177

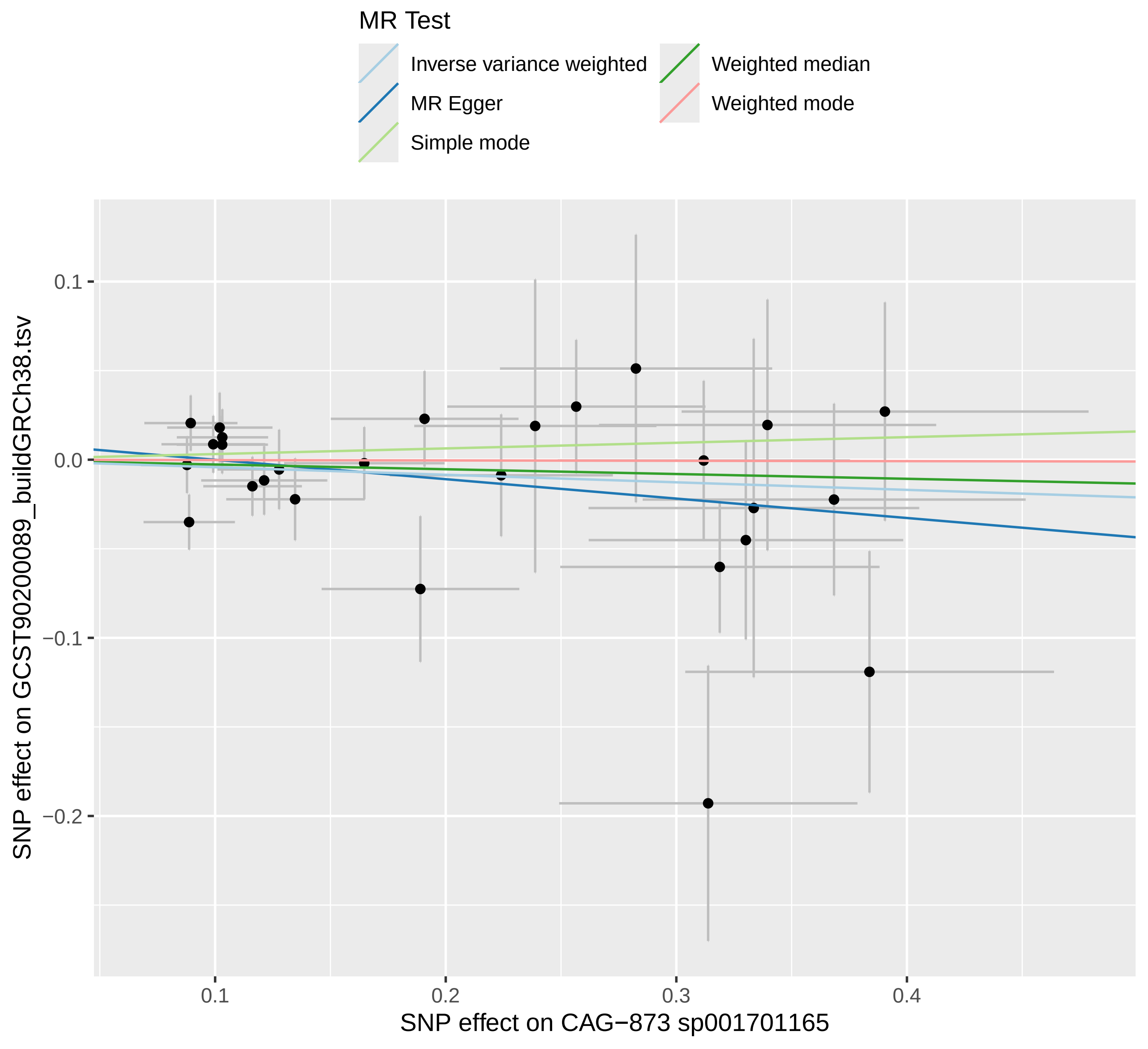
MR Test
Inverse variance weighted
MR Egger
Simple mode
Weighted median
Weighted mode
0.1
0.0
−0.1
−0.2
0.1
0.2
0.3
0.4
SNP effect on GCST90200089_buildGRCh38.tsv
SNP effect on CAG−873 sp001701165

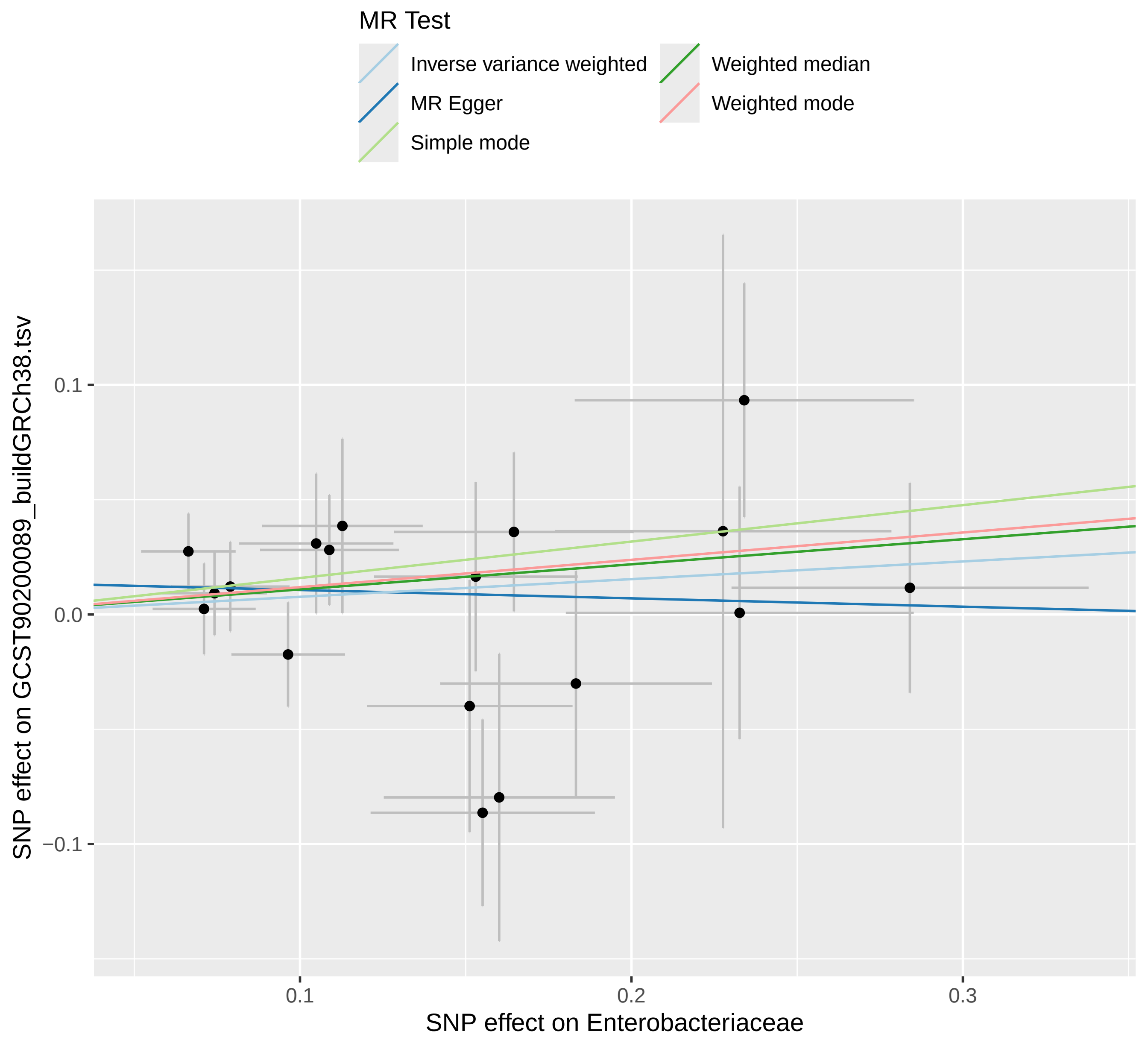
MR Test
Inverse variance weighted
MR Egger
Simple mode
Weighted median
Weighted mode
SNP effect on GCST90200089_buildGRCh38.tsv
0.1
0.0
−0.1
0.1
0.2
0.3
SNP effect on Enterobacteriaceae

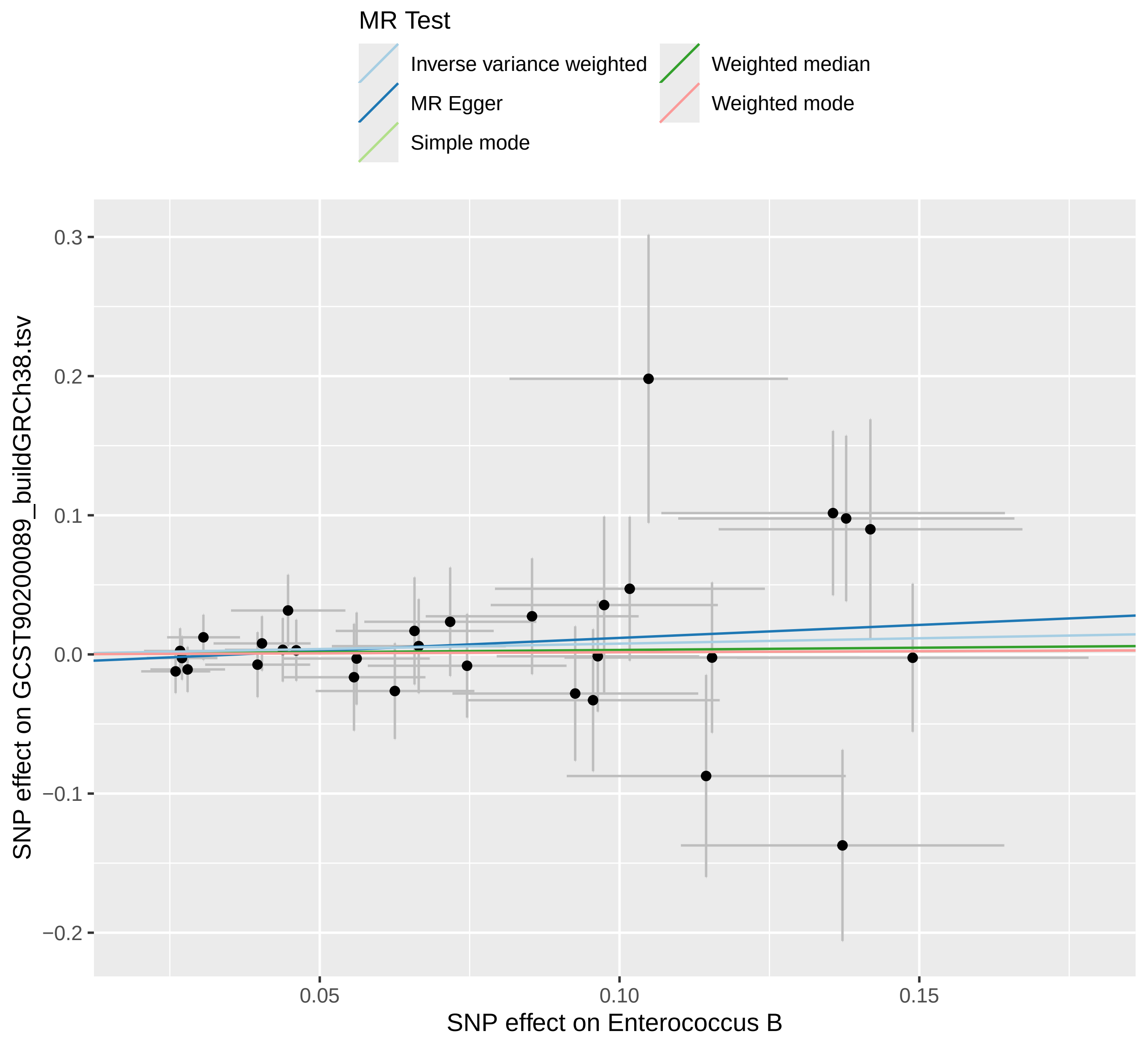
MR Test
Inverse variance weighted
MR Egger
Simple mode
Weighted median
Weighted mode
0.3
0.2
0.1
0.0
−0.1
−0.2
0.05
0.10
0.15
SNP effect on GCST90200089_buildGRCh38.tsv
SNP effect on Enterococcus B

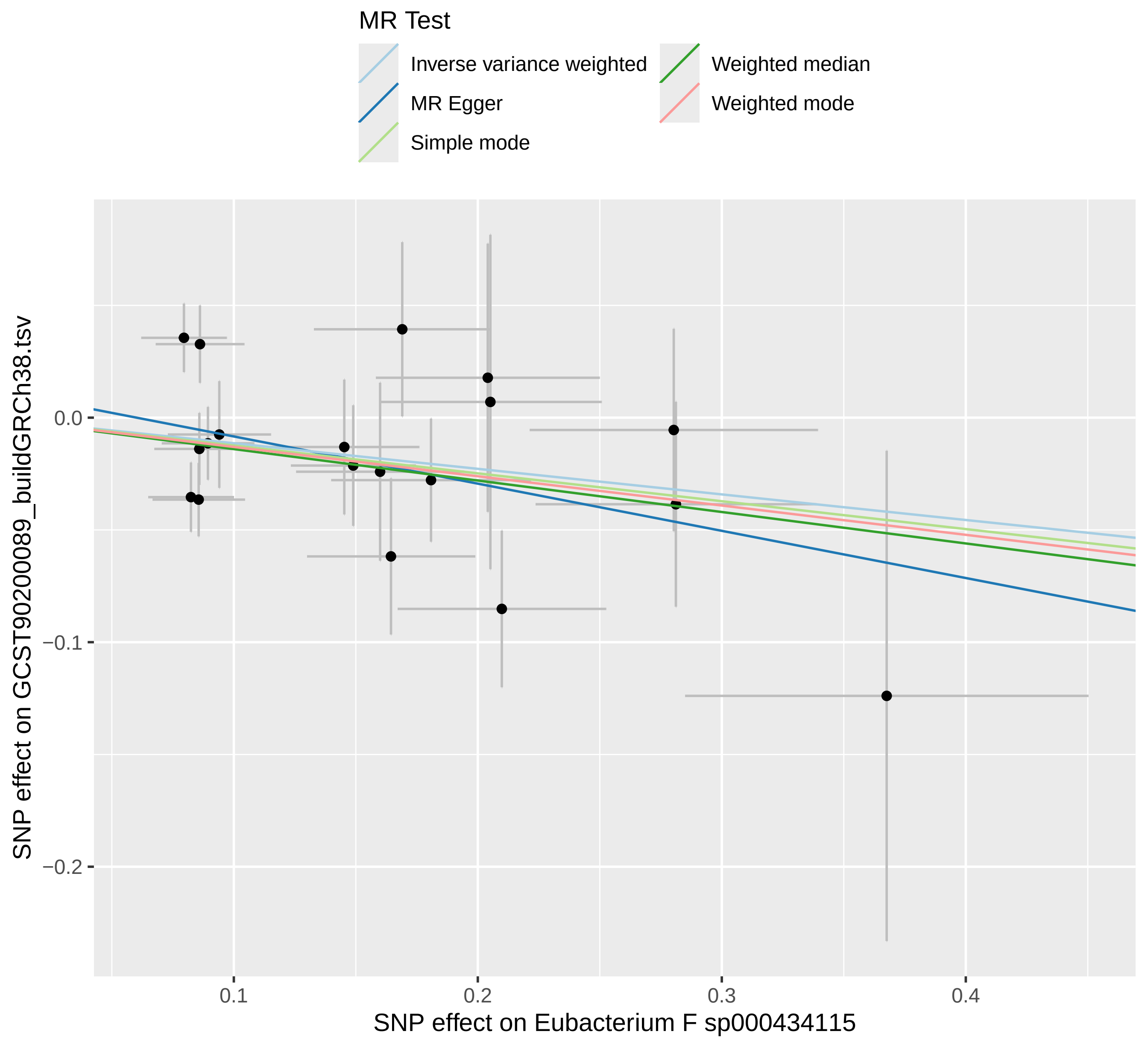
MR Test
Inverse variance weighted
MR Egger
Simple mode
Weighted median
Weighted mode
SNP effect on GCST90200089_buildGRCh38.tsv
0.0
−0.1
−0.2
0.1
0.2
0.3
0.4
SNP effect on Eubacterium F sp000434115

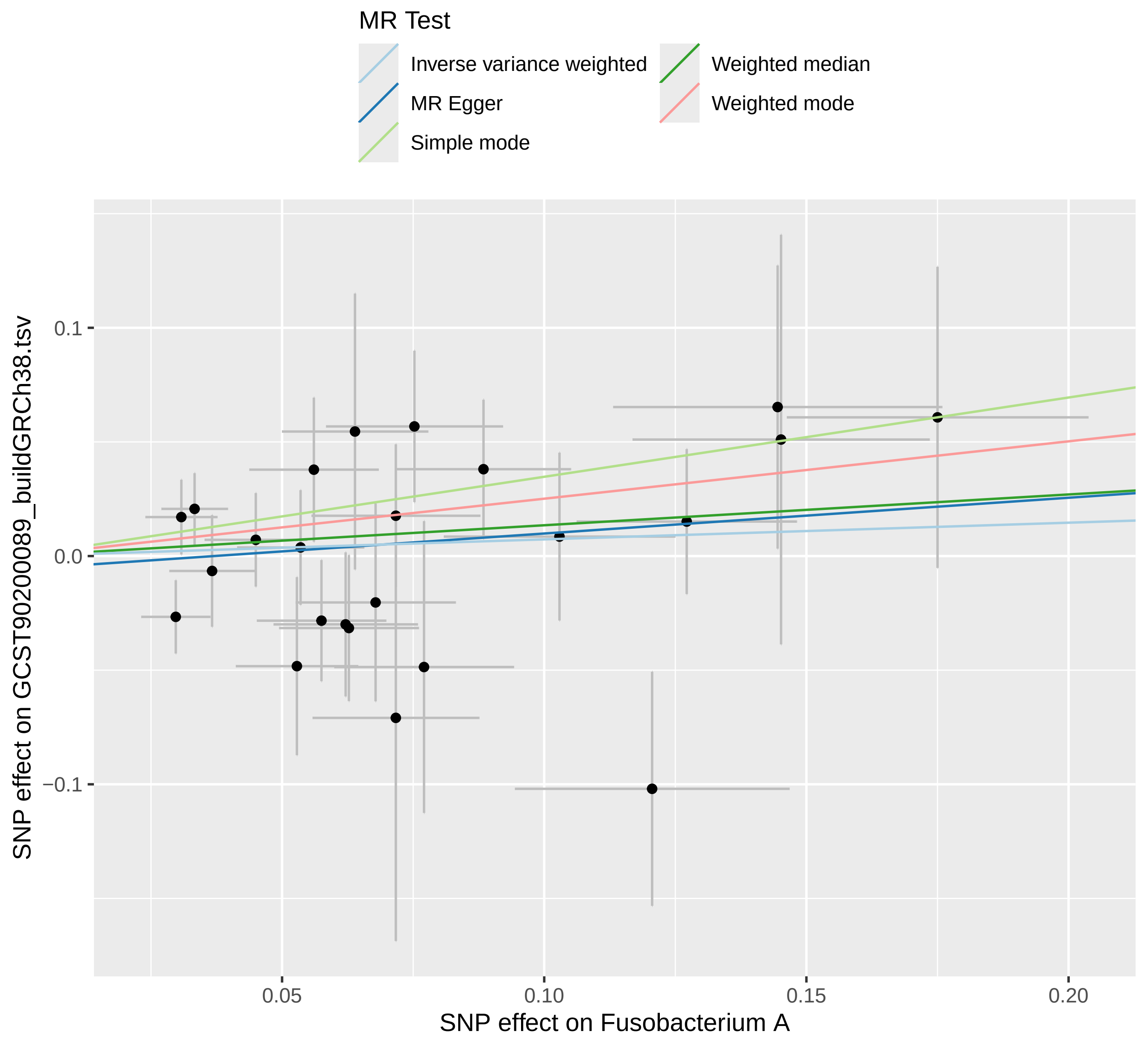
MR Test
Inverse variance weighted
MR Egger
Simple mode
Weighted median
Weighted mode
0.1
0.0
−0.1
0.05
0.10
0.15
0.20
SNP effect on GCST90200089_buildGRCh38.tsv
SNP effect on Fusobacterium A

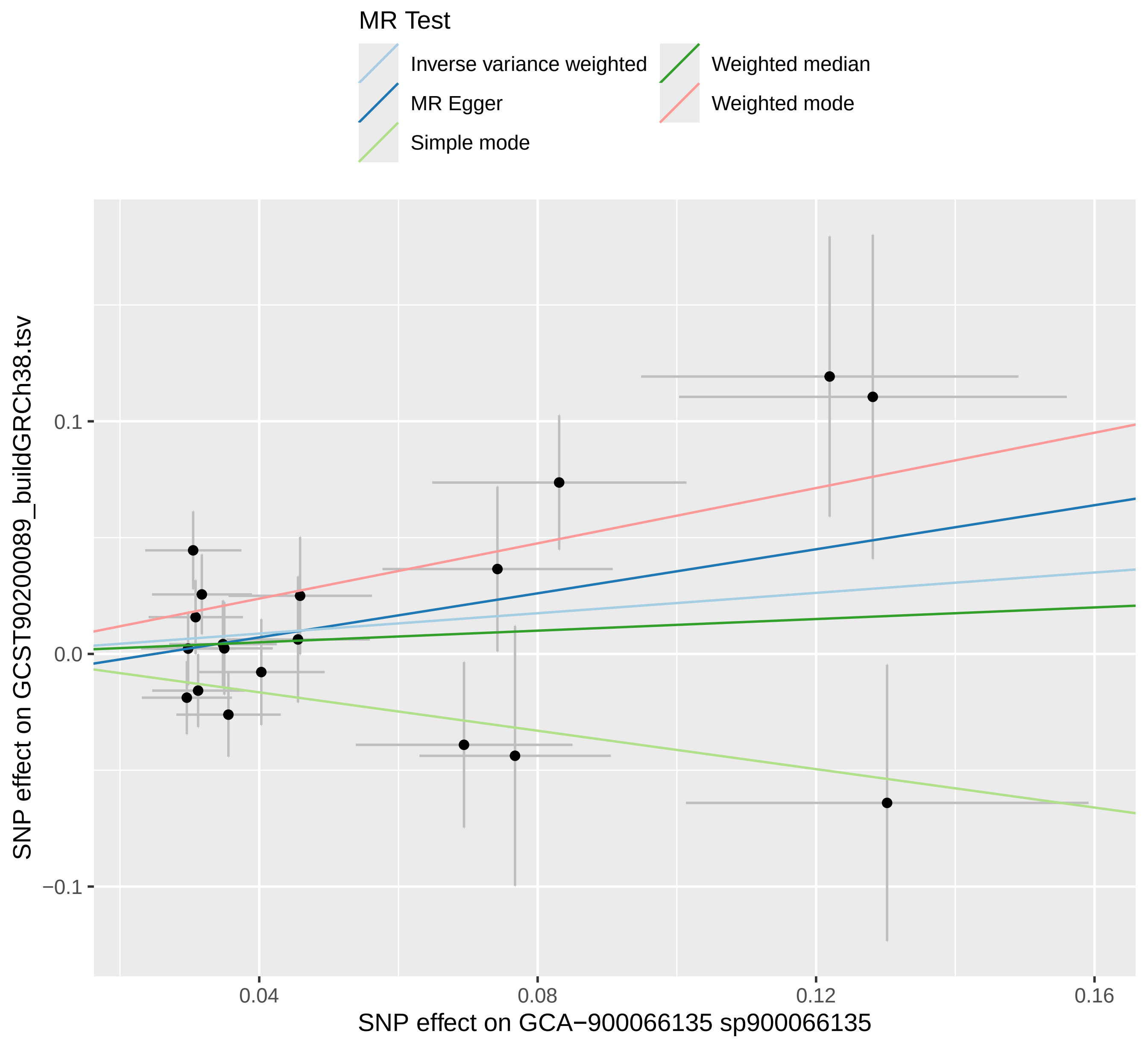
MR Test
Inverse variance weighted
MR Egger
Simple mode
Weighted median
Weighted mode
0.1
0.0
−0.1
0.04
0.08
0.12
0.16
SNP effect on GCST90200089_buildGRCh38.tsv
SNP effect on GCA−900066135 sp900066135

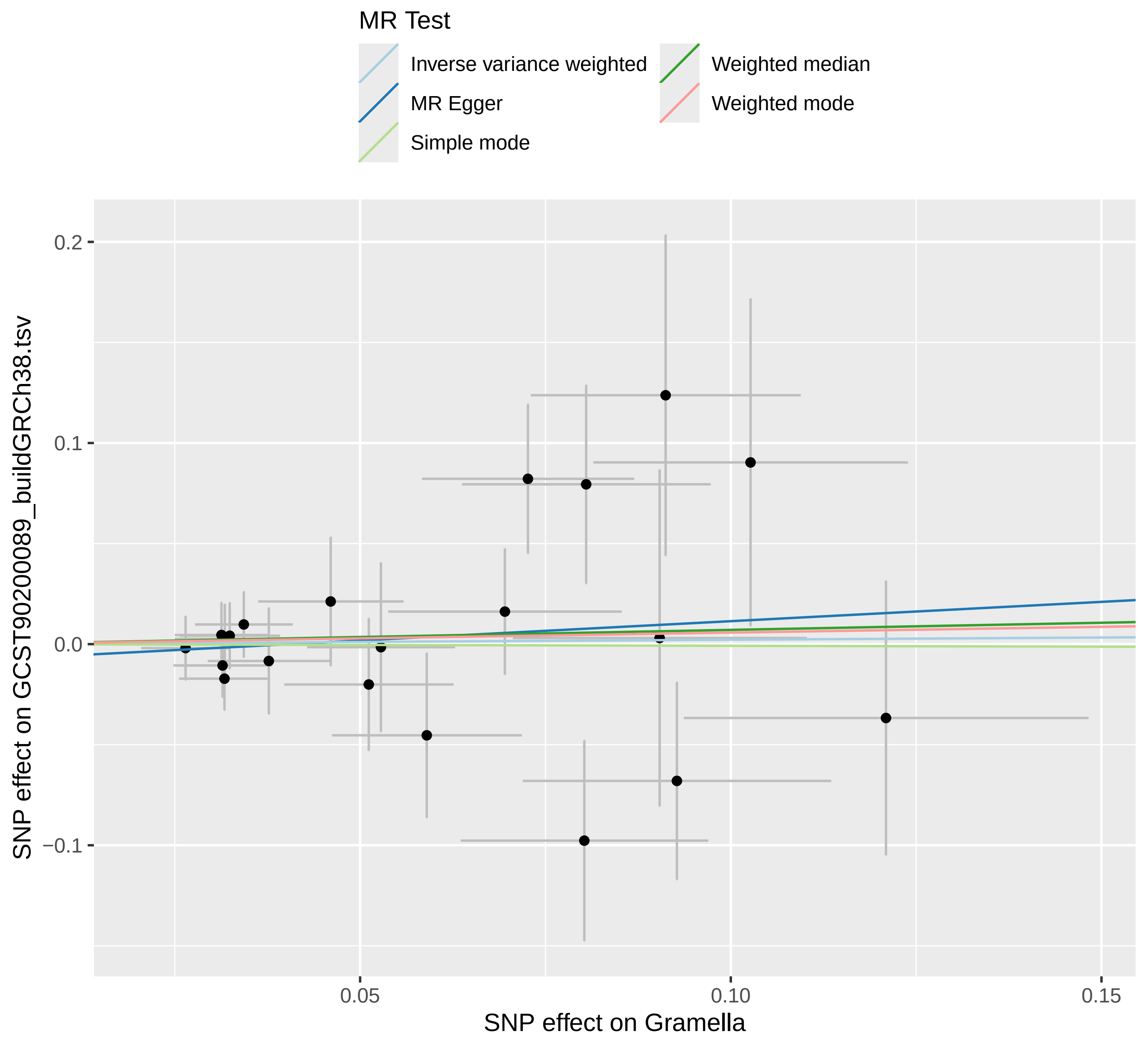
MR Test
Inverse variance weighted
MR Egger
Simple mode
Weighted median
Weighted mode
0.2
0.1
0.0
−0.1
0.05
0.10
0.15
SNP effect on GCST90200089_buildGRCh38.tsv
SNP effect on Gramella

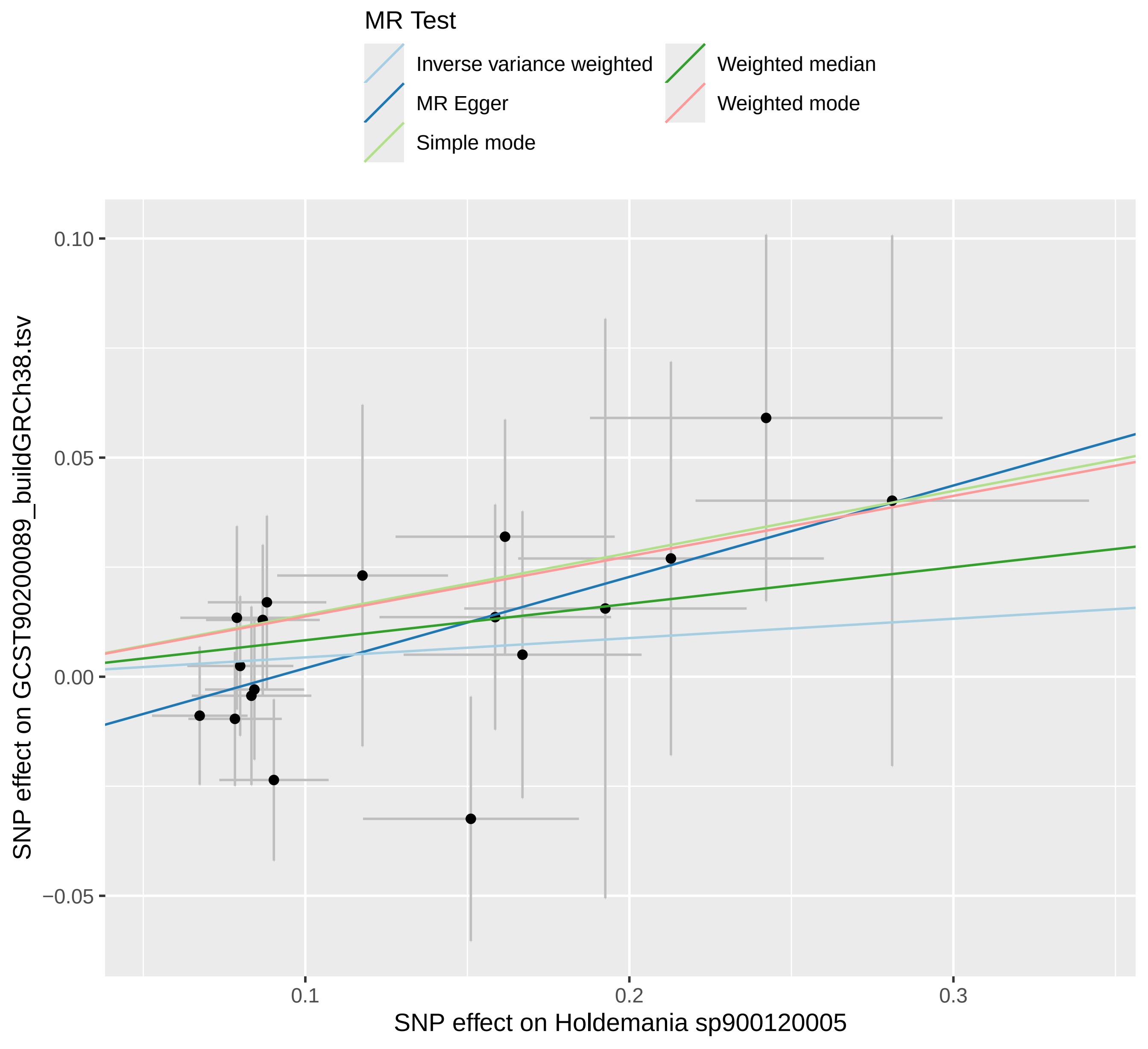

MR Test
Inverse variance weighted
MR Egger
Simple mode
Weighted median
Weighted mode
0.10
0.05
0.00
−0.05
0.1
0.2
0.3
SNP effect on GCST90200089_buildGRCh38.tsv
SNP effect on Holdemania sp900120005

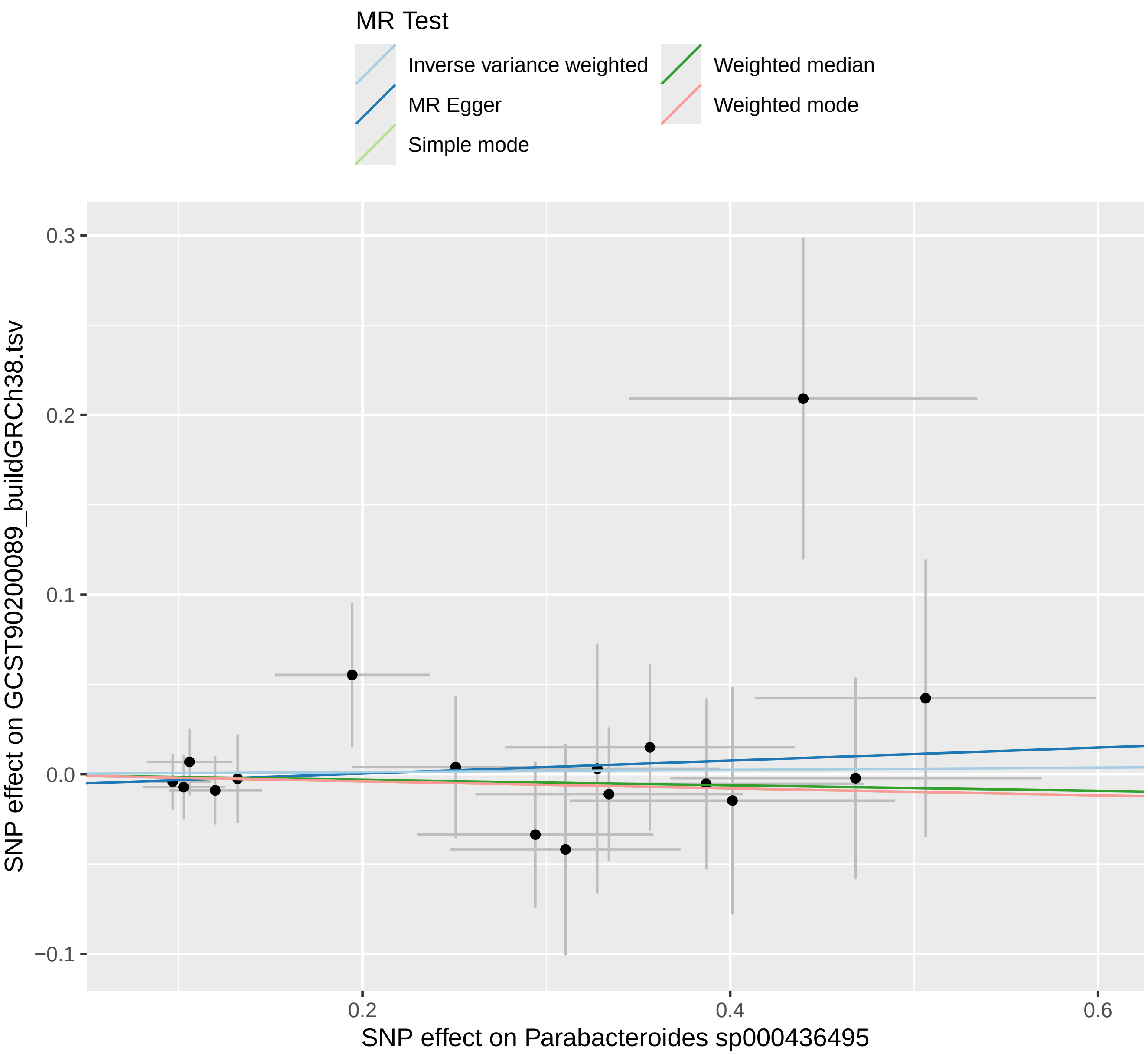
MR Test
Inverse variance weighted
MR Egger
Simple mode
Weighted median
Weighted mode
SNP effect on GCST90200089_buildGRCh38.tsv
0.3
0.2
0.1
0.0
−0.1
0.2
0.4
0.6
SNP effect on Parabacteroides sp000436495

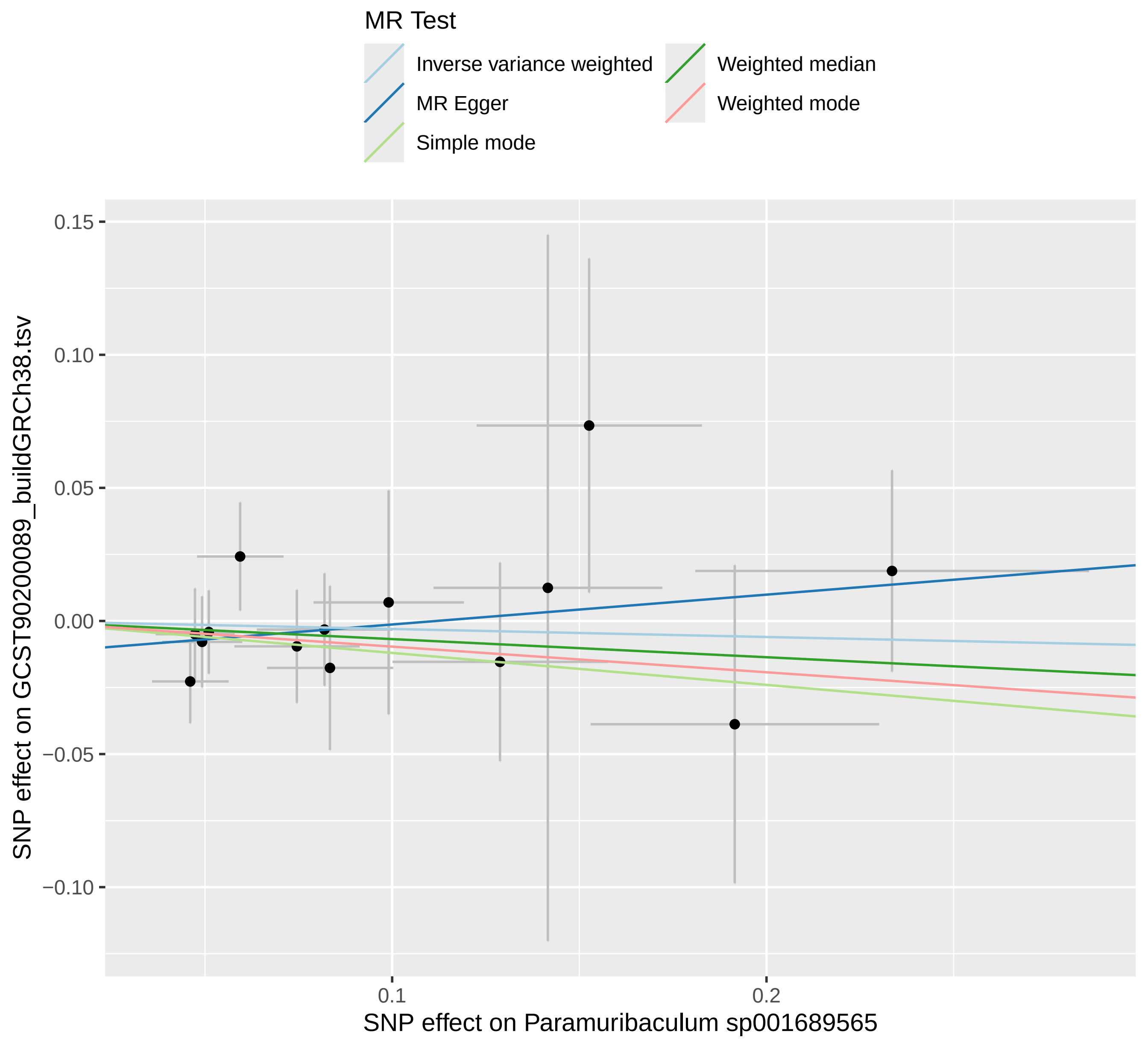
MR Test
Inverse variance weighted
MR Egger
Simple mode
Weighted median
Weighted mode
0.15
0.10
0.05
0.00
−0.05
−0.10
SNP effect on GCST90200089_buildGRCh38.tsv
0.1
0.2
SNP effect on Paramuribaculum sp001689565

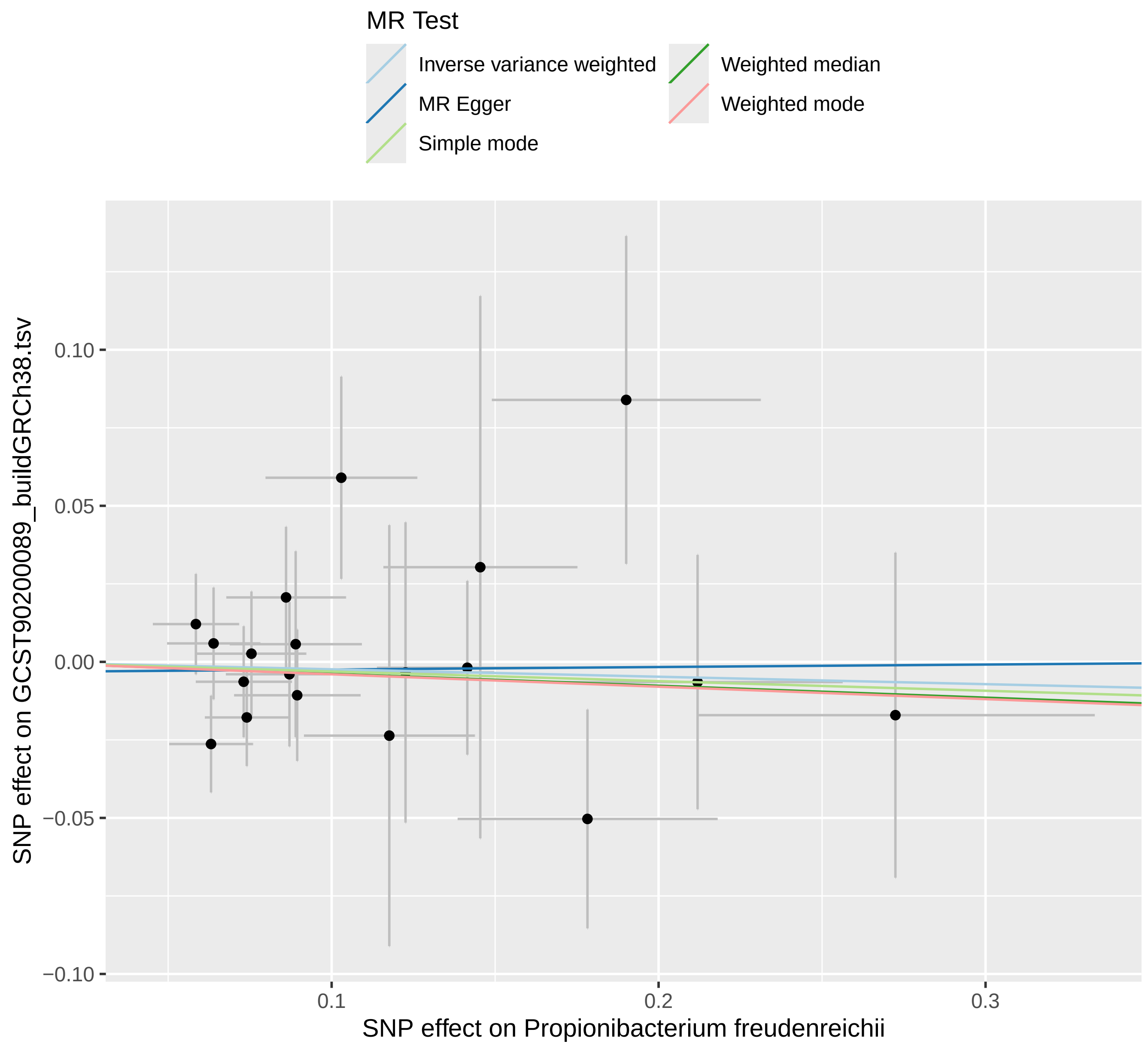
MR Test
Inverse variance weighted
MR Egger
Simple mode
Weighted median
Weighted mode
SNP effect on GCST90200089_buildGRCh38.tsv
0.10
0.05
0.00
−0.05
−0.10
0.1
0.2
0.3
SNP effect on Propionibacterium freudenreichii

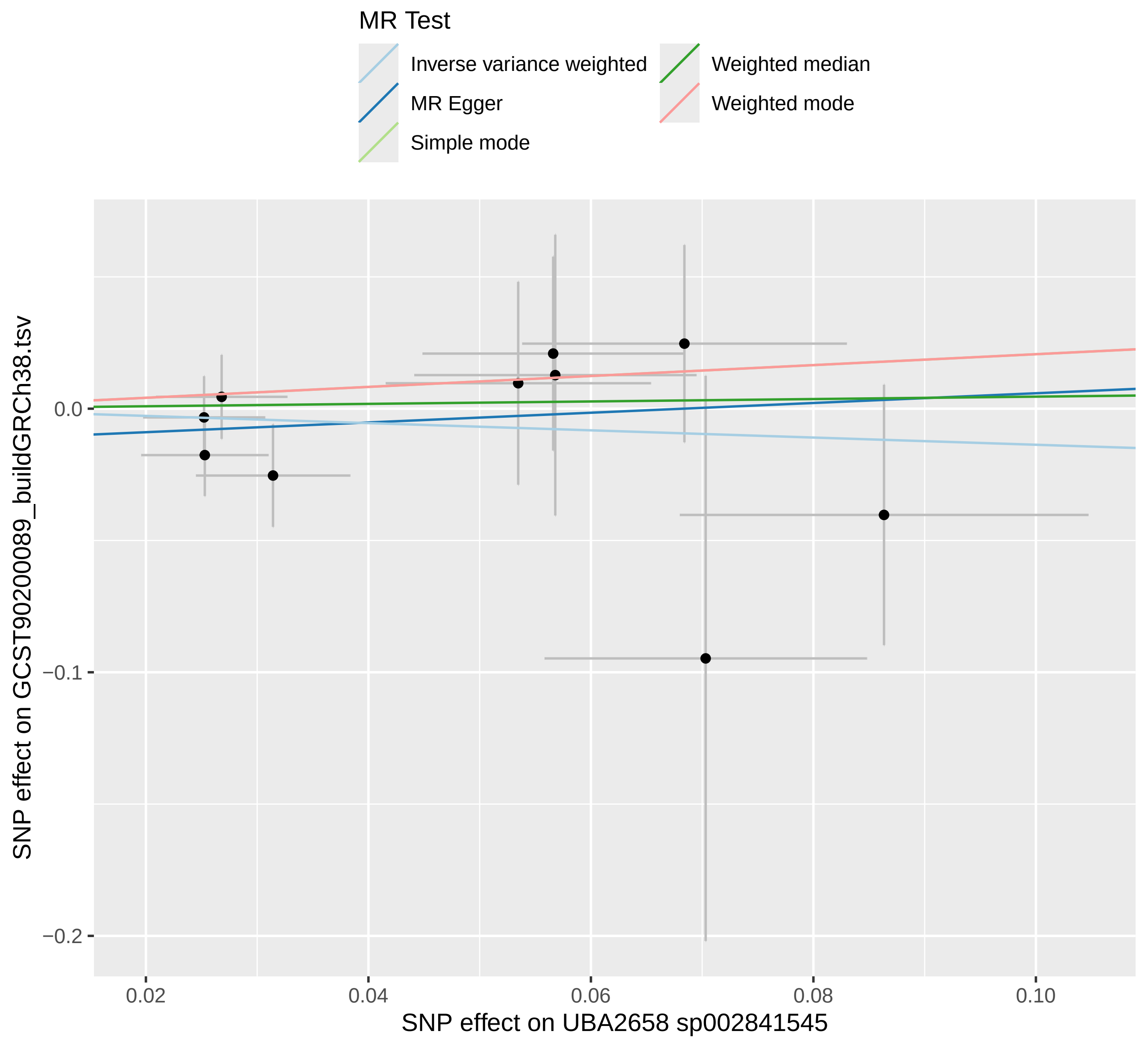
MR Test
Inverse variance weighted
MR Egger
Simple mode
Weighted median
Weighted mode
SNP effect on GCST90200089_buildGRCh38.tsv
0.0
−0.1
−0.2
0.02
0.04
0.06
0.08
0.10
SNP effect on UBA2658 sp002841545

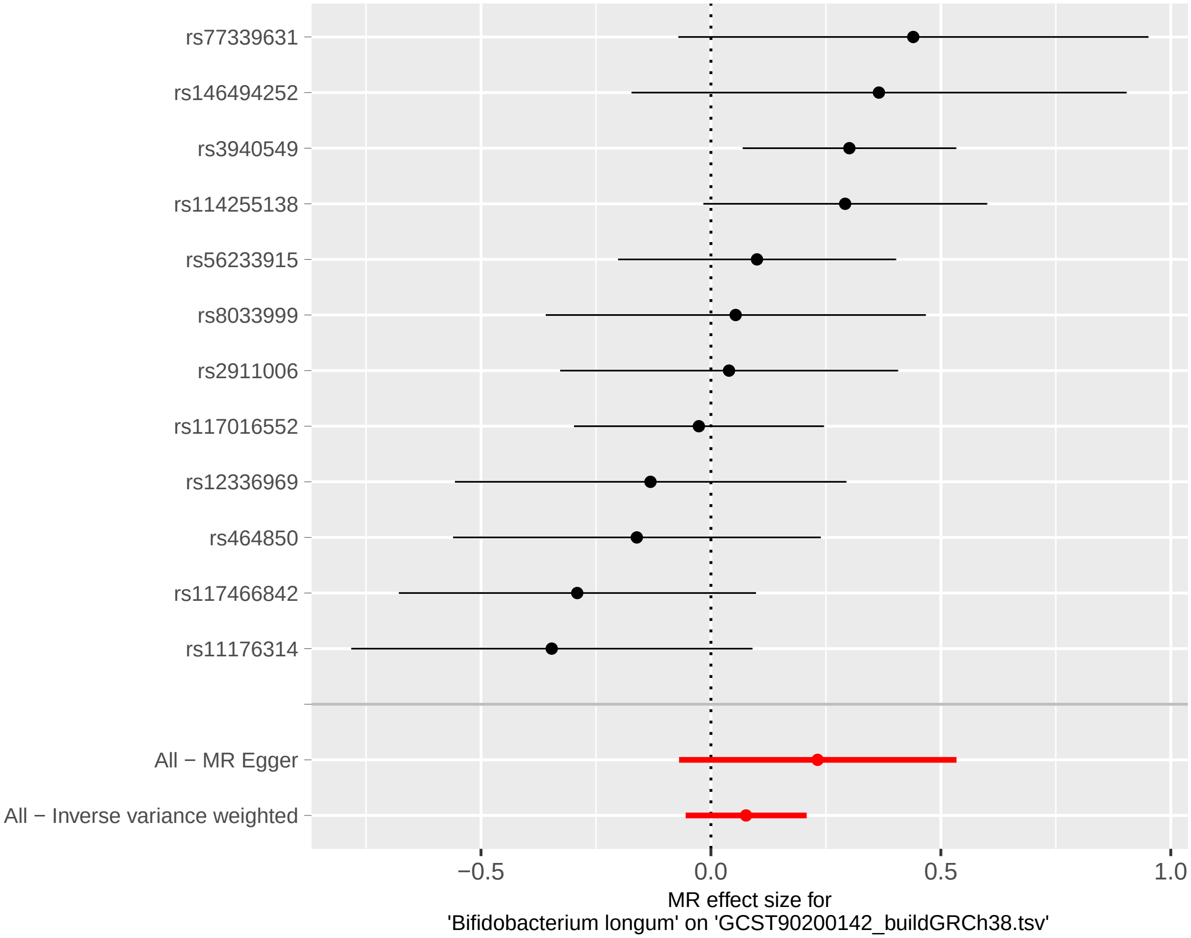
rs77339631
rs146494252
rs3940549
rs114255138
rs56233915
rs8033999
rs2911006
rs117016552
rs12336969
rs464850
rs117466842
rs11176314
All − MR Egger
All − Inverse variance weighted
−0.5
0.0
0.5
1.0
MR effect size for
'Bifidobacterium longum' on 'GCST90200142_buildGRCh38.tsv'

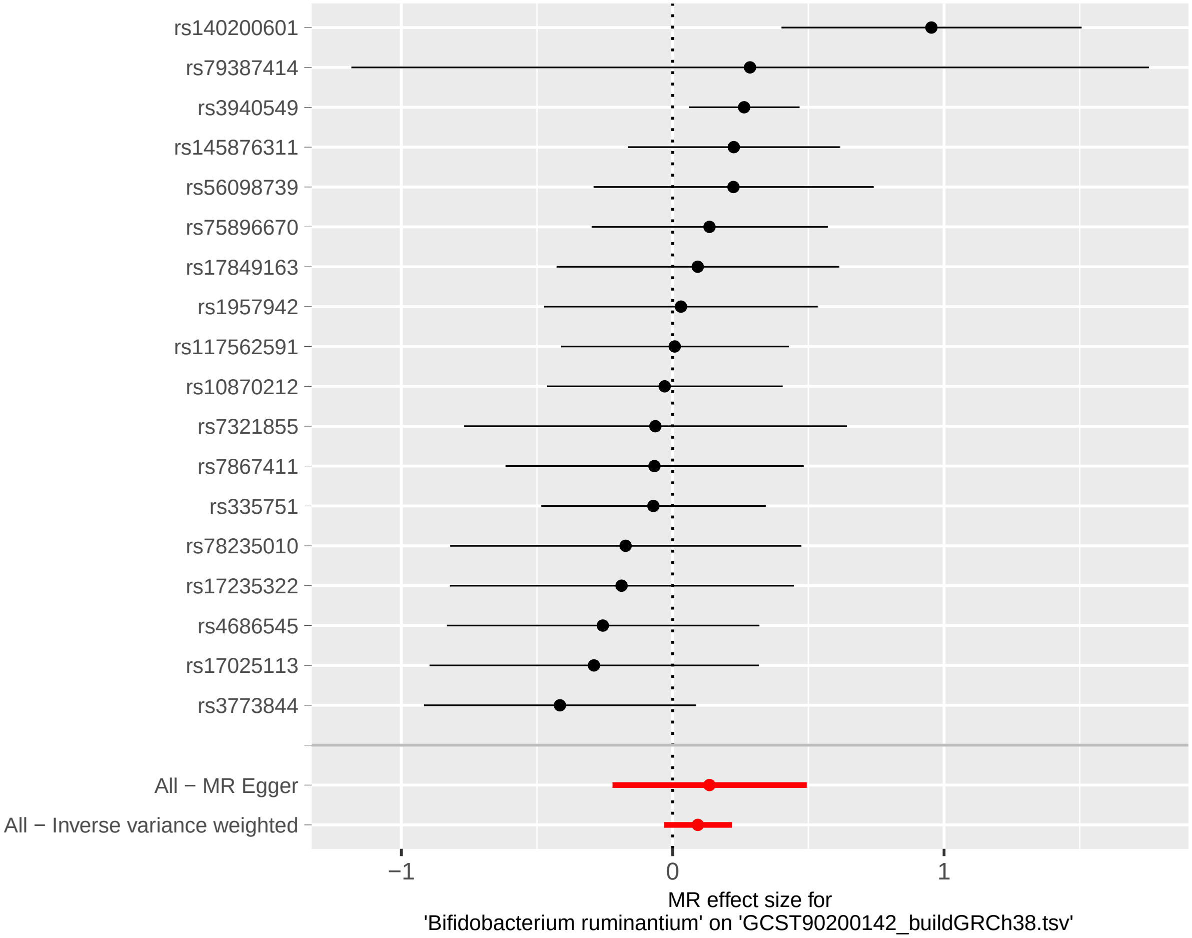

rs140200601
rs79387414
rs3940549
rs145876311
rs56098739
rs75896670
rs17849163
rs1957942
rs117562591
rs10870212
rs7321855
rs7867411
rs335751
rs78235010
rs17235322
rs4686545
rs17025113
rs3773844
All − MR Egger
All − Inverse variance weighted
−1
0
1
MR effect size for
'Bifidobacterium ruminantium' on 'GCST90200142_buildGRCh38.tsv'

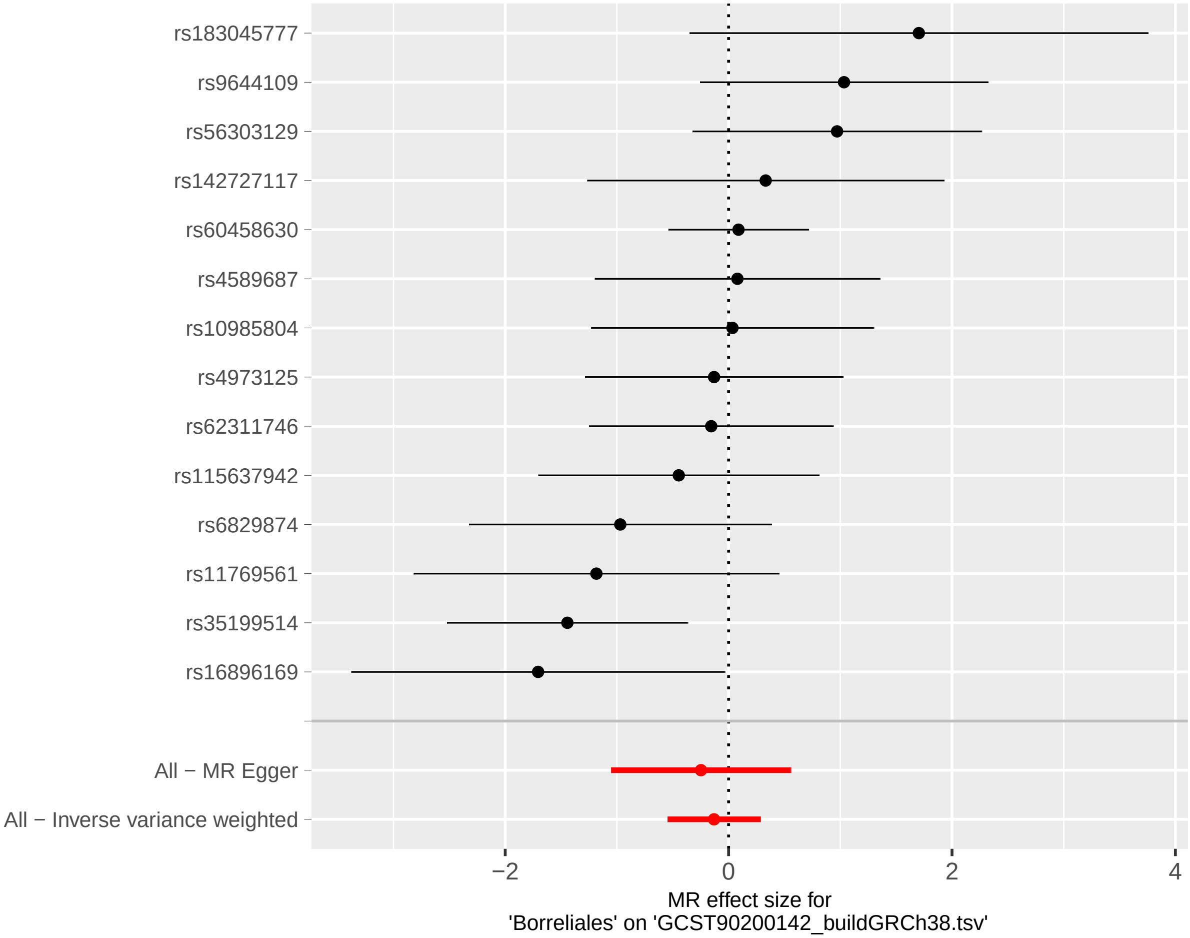
rs183045777
rs9644109
rs56303129
rs142727117
rs60458630
rs4589687
rs10985804
rs4973125
rs62311746
rs115637942
rs6829874
rs11769561
rs35199514
rs16896169
All − MR Egger
All − Inverse variance weighted
−2
0
2
4
MR effect size for
'Borreliales' on 'GCST90200142_buildGRCh38.tsv'

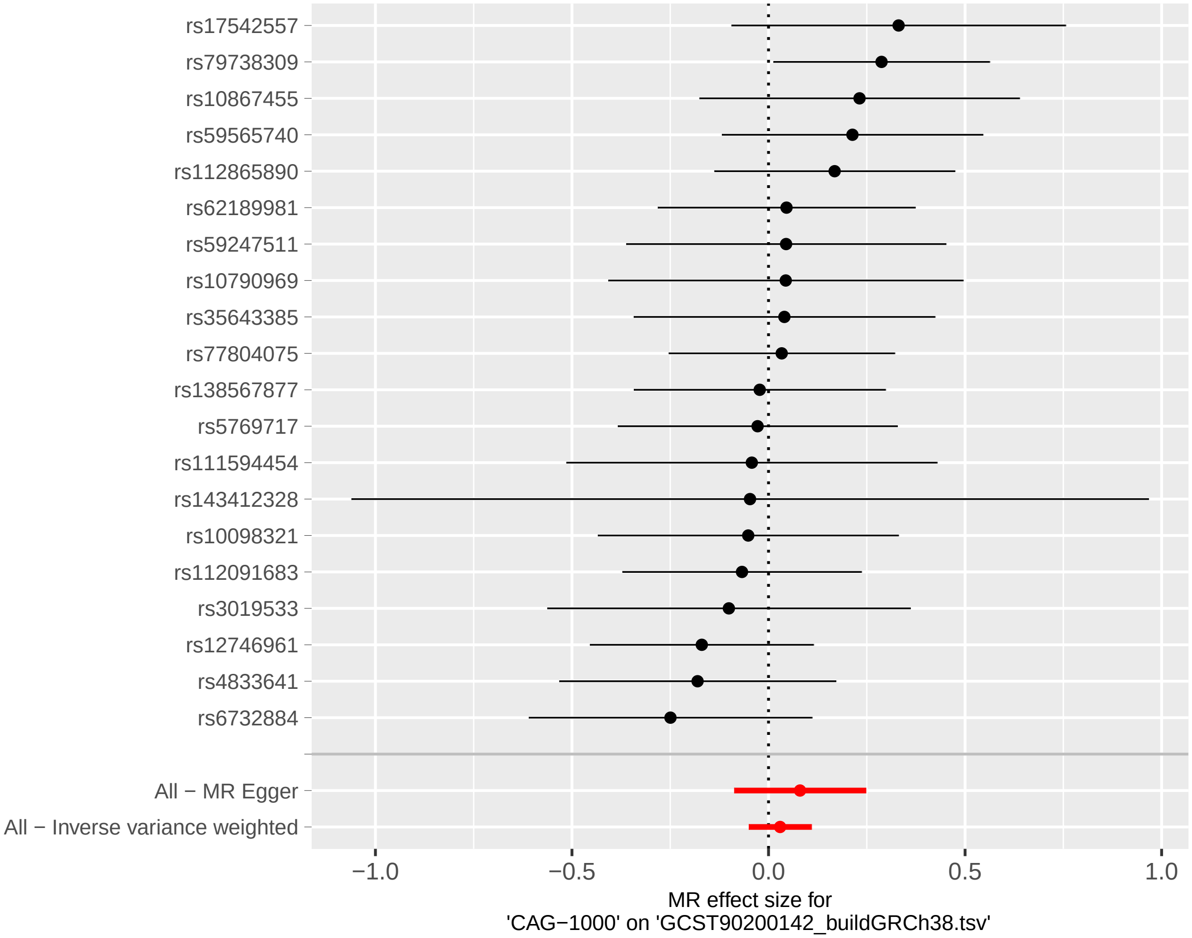
rs17542557
rs79738309
rs10867455
rs59565740
rs112865890
rs62189981
rs59247511
rs10790969
rs35643385
rs77804075
rs138567877
rs5769717
rs111594454
rs143412328
rs10098321
rs112091683
rs3019533
rs12746961
rs4833641
rs6732884
All − MR Egger
All − Inverse variance weighted
−1.0
−0.5
0.0
0.5
1.0
MR effect size for
'CAG−1000' on 'GCST90200142_buildGRCh38.tsv'

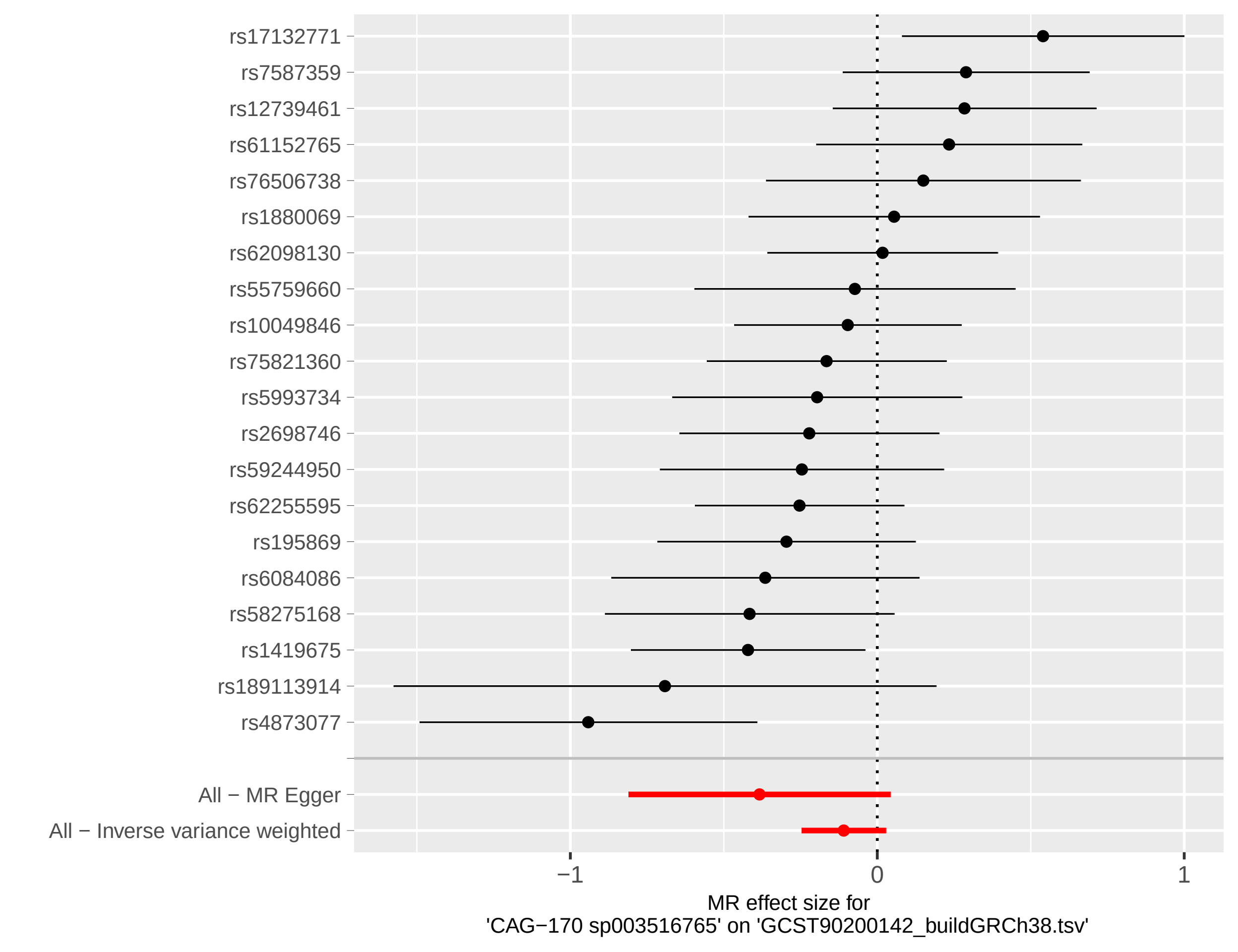

rs17132771
rs7587359
rs12739461
rs61152765
rs76506738
rs1880069
rs62098130
rs55759660
rs10049846
rs75821360
rs5993734
rs2698746
rs59244950
rs62255595
rs195869
rs6084086
rs58275168
rs1419675
rs189113914
rs4873077
All − MR Egger
All − Inverse variance weighted
−1
0
1
MR effect size for
'CAG−170 sp003516765' on 'GCST90200142_buildGRCh38.tsv'

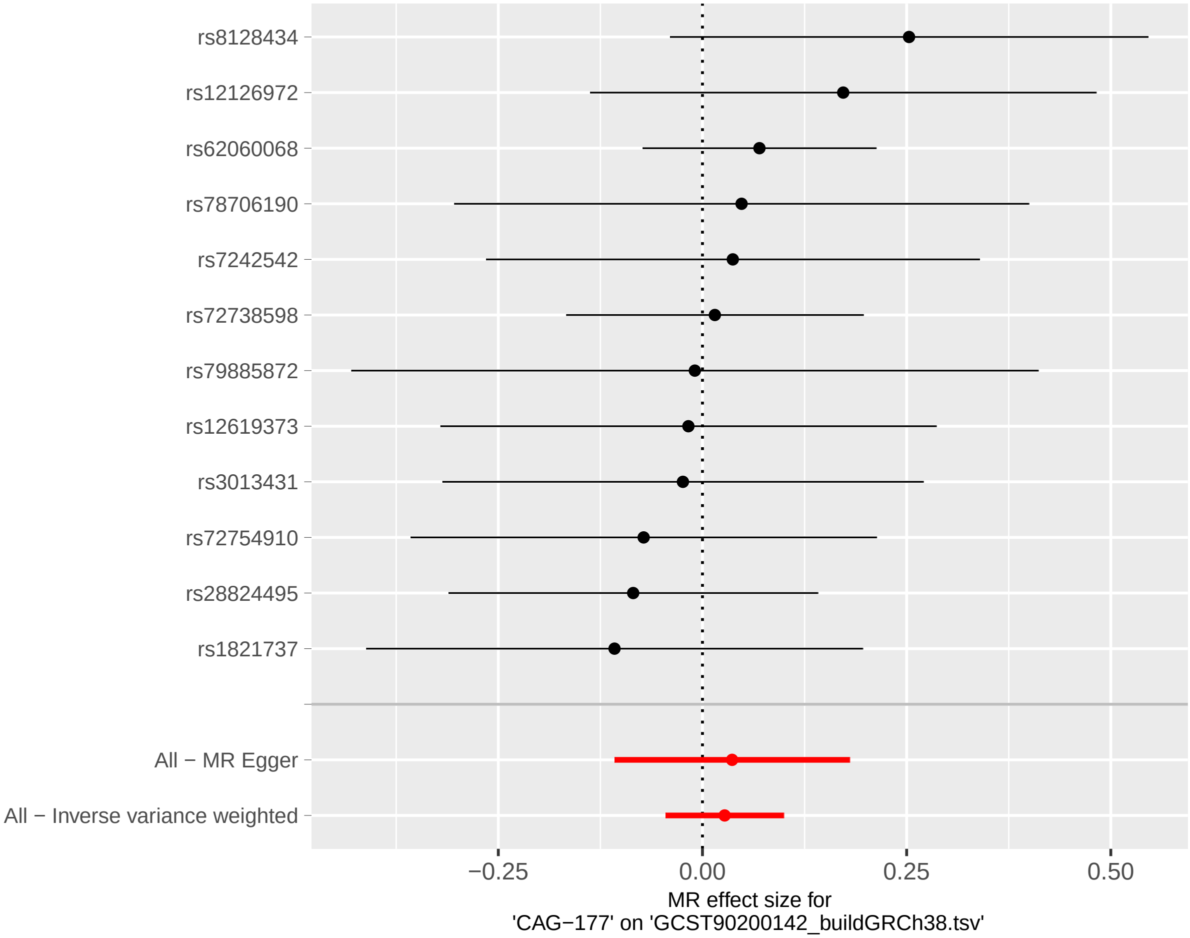
rs8128434
rs12126972
rs62060068
rs78706190
rs7242542
rs72738598
rs79885872
rs12619373
rs3013431
rs72754910
rs28824495
rs1821737
All − MR Egger
All − Inverse variance weighted
−0.25
0.00
0.25
0.50
MR effect size for
'CAG−177' on 'GCST90200142_buildGRCh38.tsv'

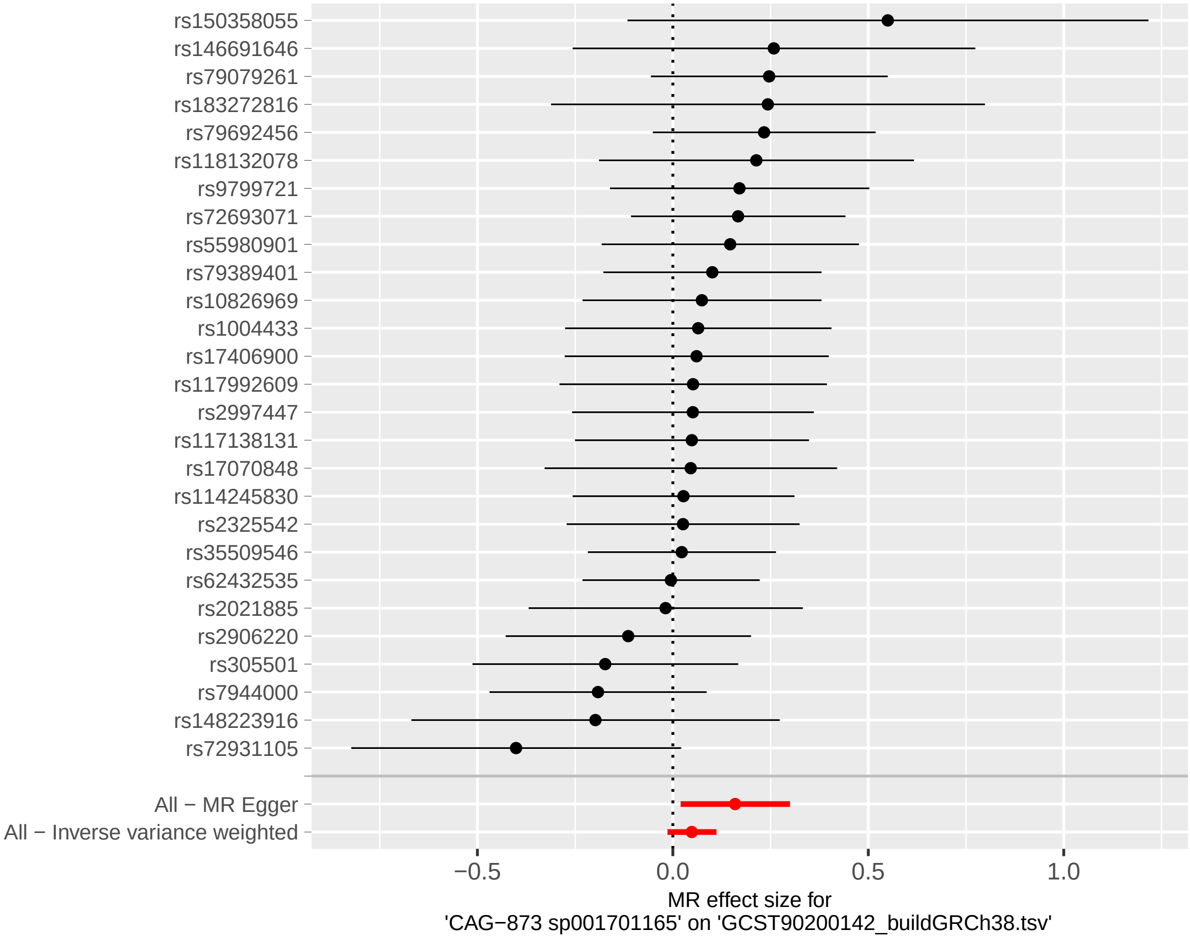
rs150358055
rs146691646
rs79079261
rs183272816
rs79692456
rs118132078
rs9799721
rs72693071
rs55980901
rs79389401
rs10826969
rs1004433
rs17406900
rs117992609
rs2997447
rs117138131
rs17070848
rs114245830
rs2325542
rs35509546
rs62432535
rs2021885
rs2906220
rs305501
rs7944000
rs148223916
rs72931105
All − MR Egger
All − Inverse variance weighted
−0.5
0.0
0.5
1.0
MR effect size for
'CAG−873 sp001701165' on 'GCST90200142_buildGRCh38.tsv'

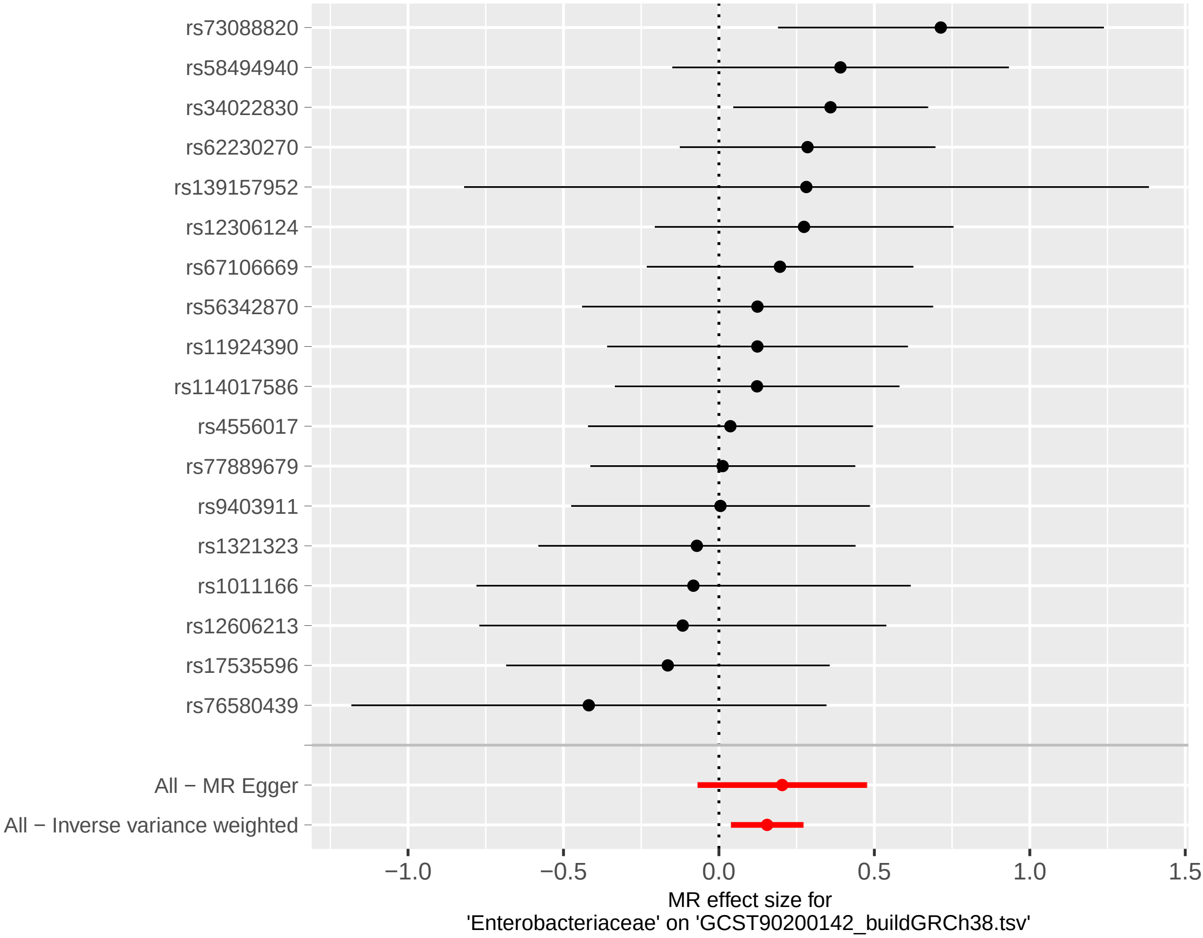
rs73088820
rs58494940
rs34022830
rs62230270
rs139157952
rs12306124
rs67106669
rs56342870
rs11924390
rs114017586
rs4556017
rs77889679
rs9403911
rs1321323
rs1011166
rs12606213
rs17535596
rs76580439
All − MR Egger
All − Inverse variance weighted
−1.0
−0.5
0.0
0.5
1.0
1.5
MR effect size for
'Enterobacteriaceae' on 'GCST90200142_buildGRCh38.tsv'

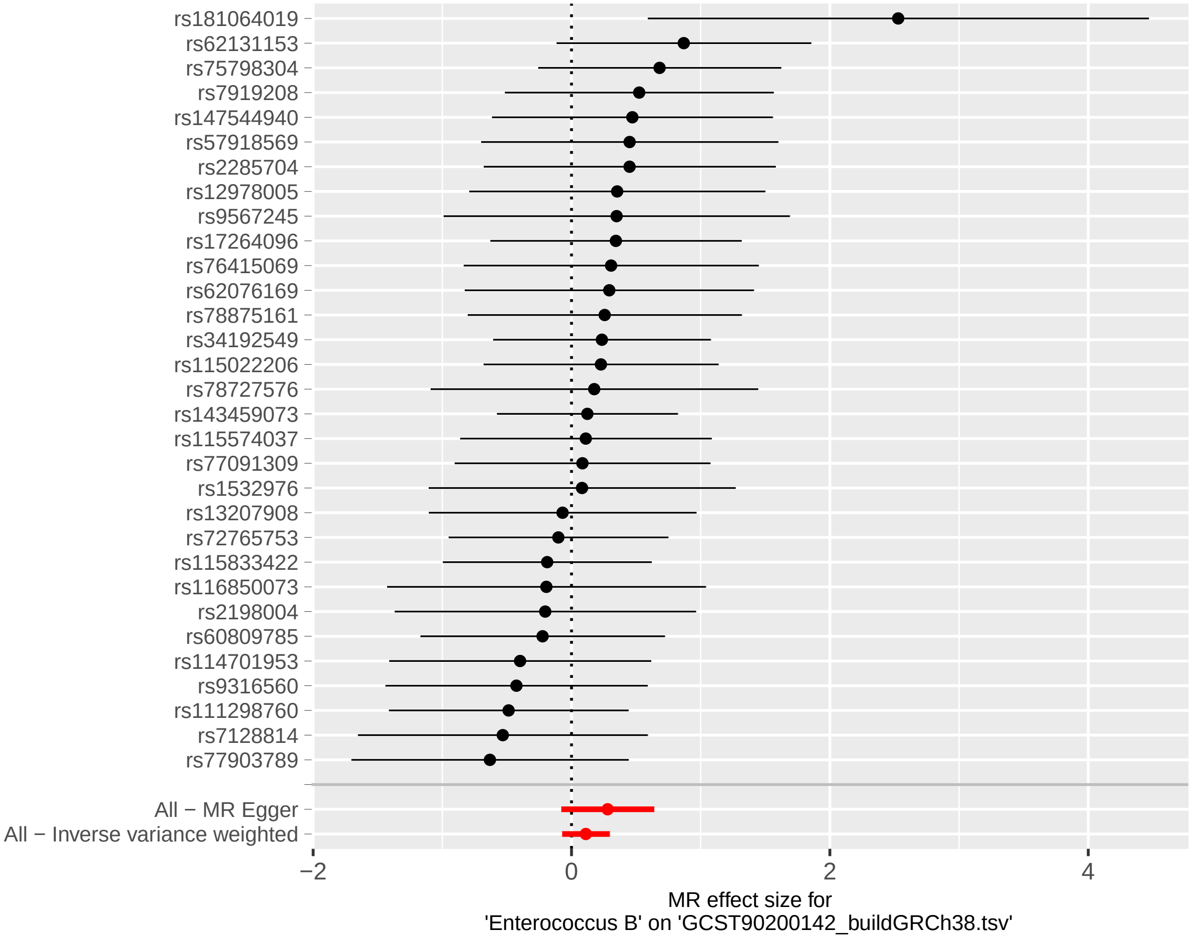
rs181064019
rs62131153
rs75798304
rs7919208
rs147544940
rs57918569
rs2285704
rs12978005
rs9567245
rs17264096
rs76415069
rs62076169
rs78875161
rs34192549
rs115022206
rs78727576
rs143459073
rs115574037
rs77091309
rs1532976
rs13207908
rs72765753
rs115833422
rs116850073
rs2198004
rs60809785
rs114701953
rs9316560
rs111298760
rs7128814
rs77903789
All − MR Egger
All − Inverse variance weighted
−2
0
2
4
MR effect size for
'Enterococcus B' on 'GCST90200142_buildGRCh38.tsv'

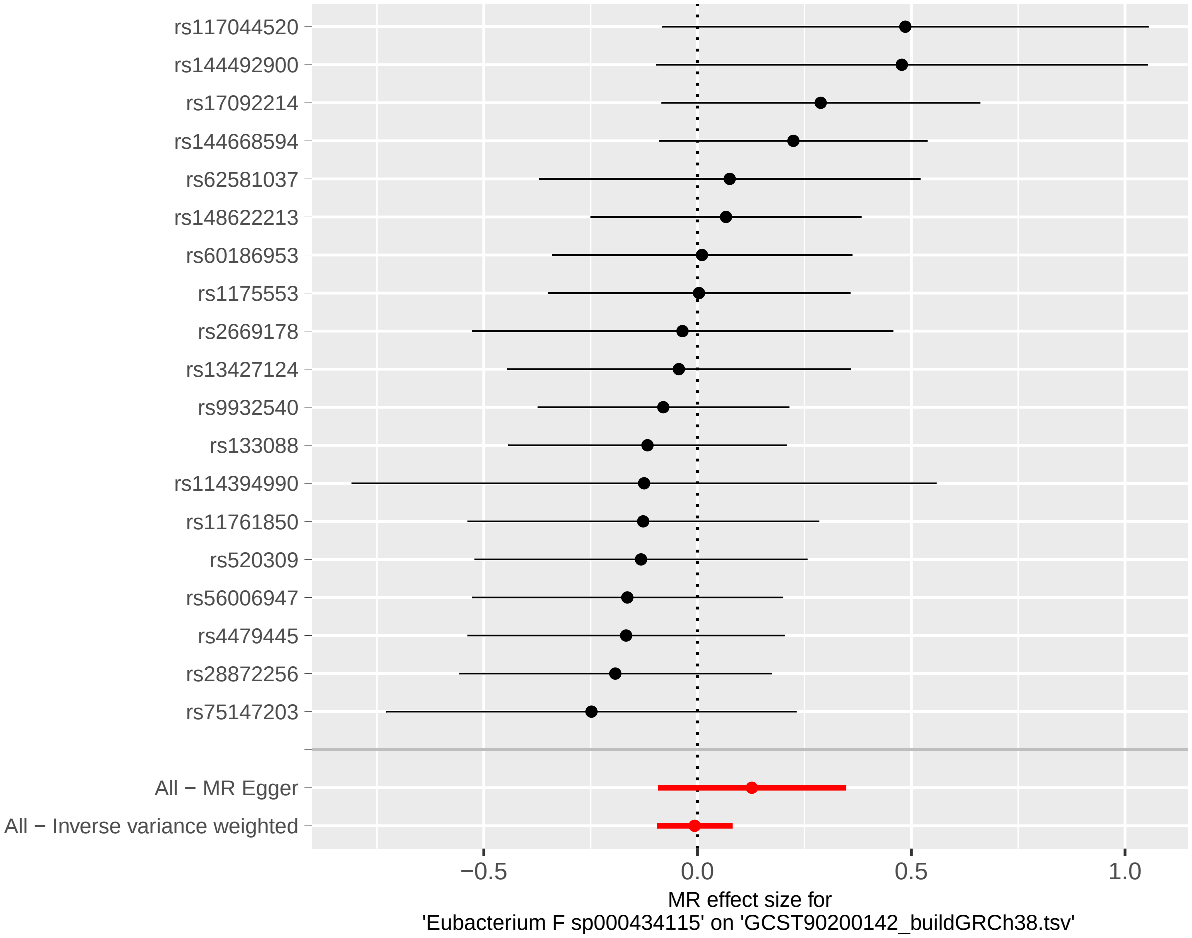
rs117044520
rs144492900
rs17092214
rs144668594
rs62581037
rs148622213
rs60186953
rs1175553
rs2669178
rs13427124
rs9932540
rs133088
rs114394990
rs11761850
rs520309
rs56006947
rs4479445
rs28872256
rs75147203
All − MR Egger
All − Inverse variance weighted
−0.5
0.0
0.5
1.0
MR effect size for
'Eubacterium F sp000434115' on 'GCST90200142_buildGRCh38.tsv'

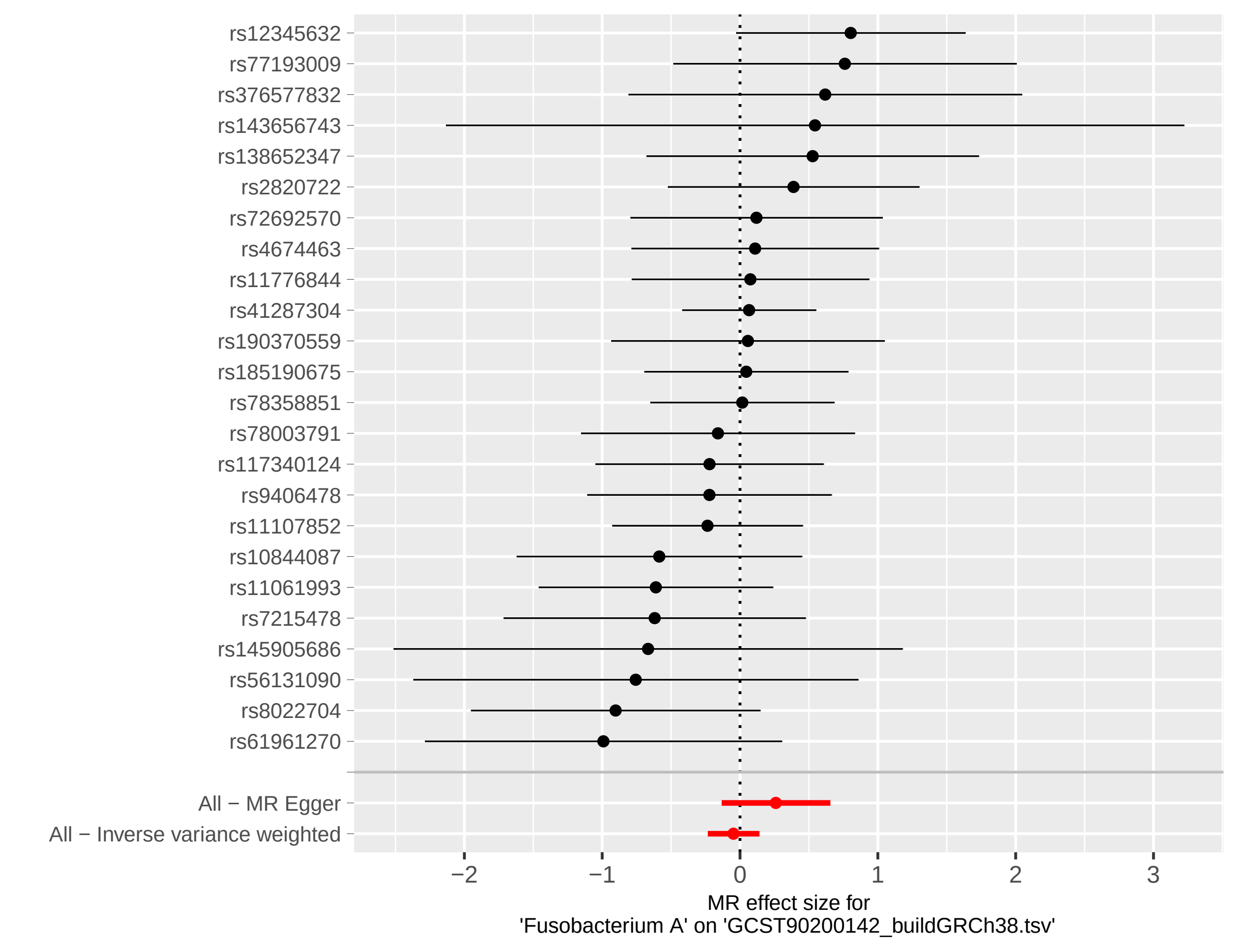
rs12345632
rs77193009
rs376577832
rs143656743
rs138652347
rs2820722
rs72692570
rs4674463
rs11776844
rs41287304
rs190370559
rs185190675
rs78358851
rs78003791
rs117340124
rs9406478
rs11107852
rs10844087
rs11061993
rs7215478
rs145905686
rs56131090
rs8022704
rs61961270
All − MR Egger
All − Inverse variance weighted
−2
−1
0
1
2
3
MR effect size for
'Fusobacterium A' on 'GCST90200142_buildGRCh38.tsv'

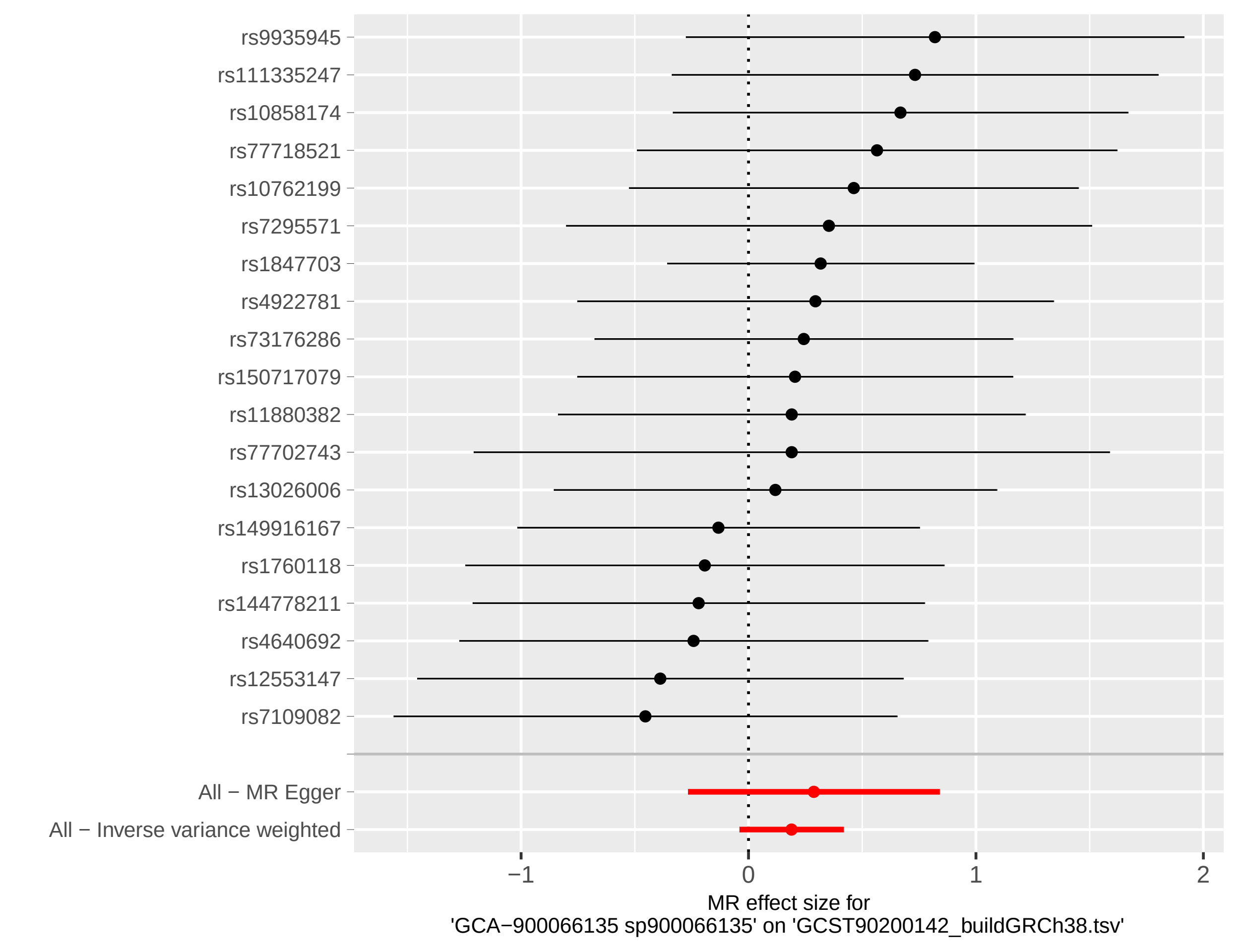

rs9935945
rs111335247
rs10858174
rs77718521
rs10762199
rs7295571
rs1847703
rs4922781
rs73176286
rs150717079
rs11880382
rs77702743
rs13026006
rs149916167
rs1760118
rs144778211
rs4640692
rs12553147
rs7109082
All − MR Egger
All − Inverse variance weighted
−1
0
1
2
MR effect size for
'GCA−900066135 sp900066135' on 'GCST90200142_buildGRCh38.tsv'

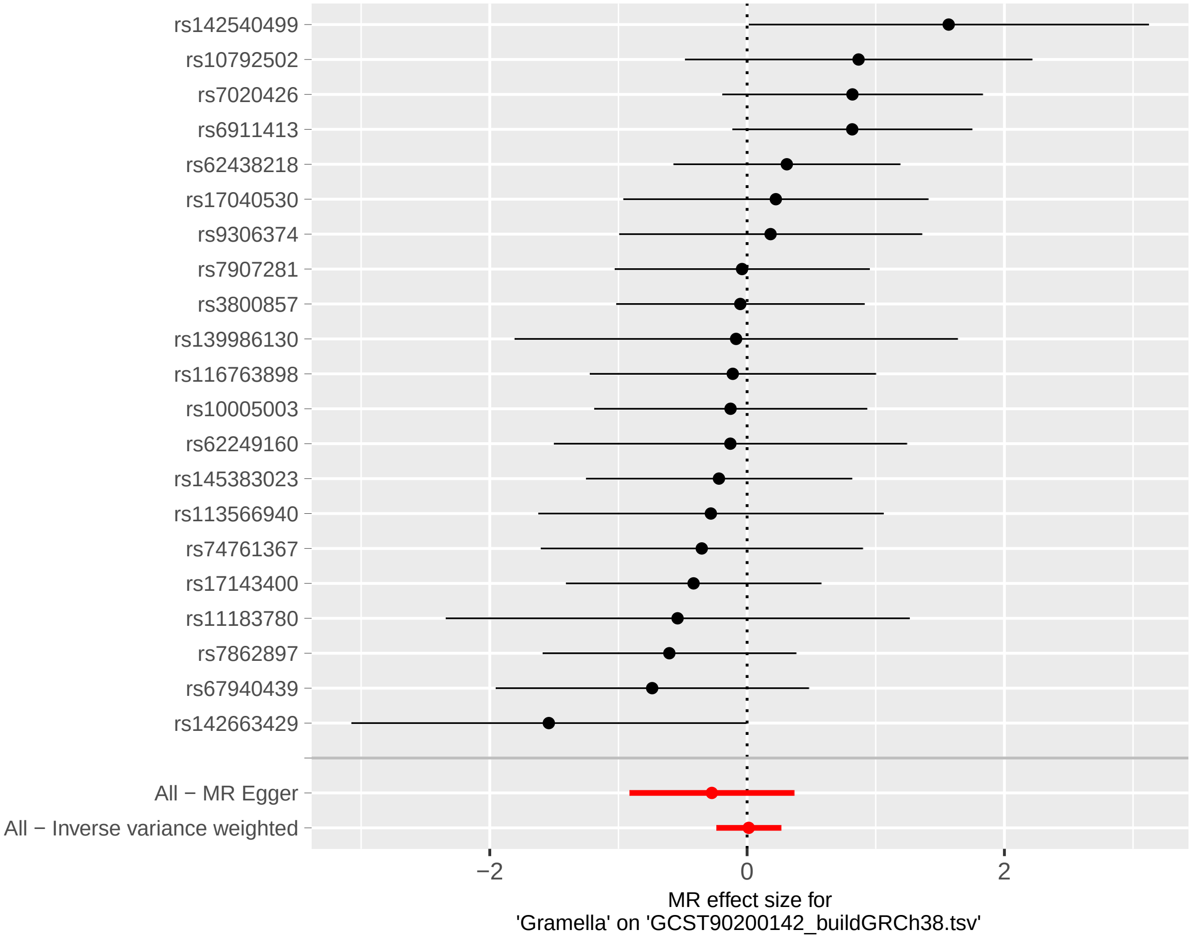

rs142540499
rs10792502
rs7020426
rs6911413
rs62438218
rs17040530
rs9306374
rs7907281
rs3800857
rs139986130
rs116763898
rs10005003
rs62249160
rs145383023
rs113566940
rs74761367
rs17143400
rs11183780
rs7862897
rs67940439
rs142663429
All − MR Egger
All − Inverse variance weighted
−2
0
2
MR effect size for
'Gramella' on 'GCST90200142_buildGRCh38.tsv'

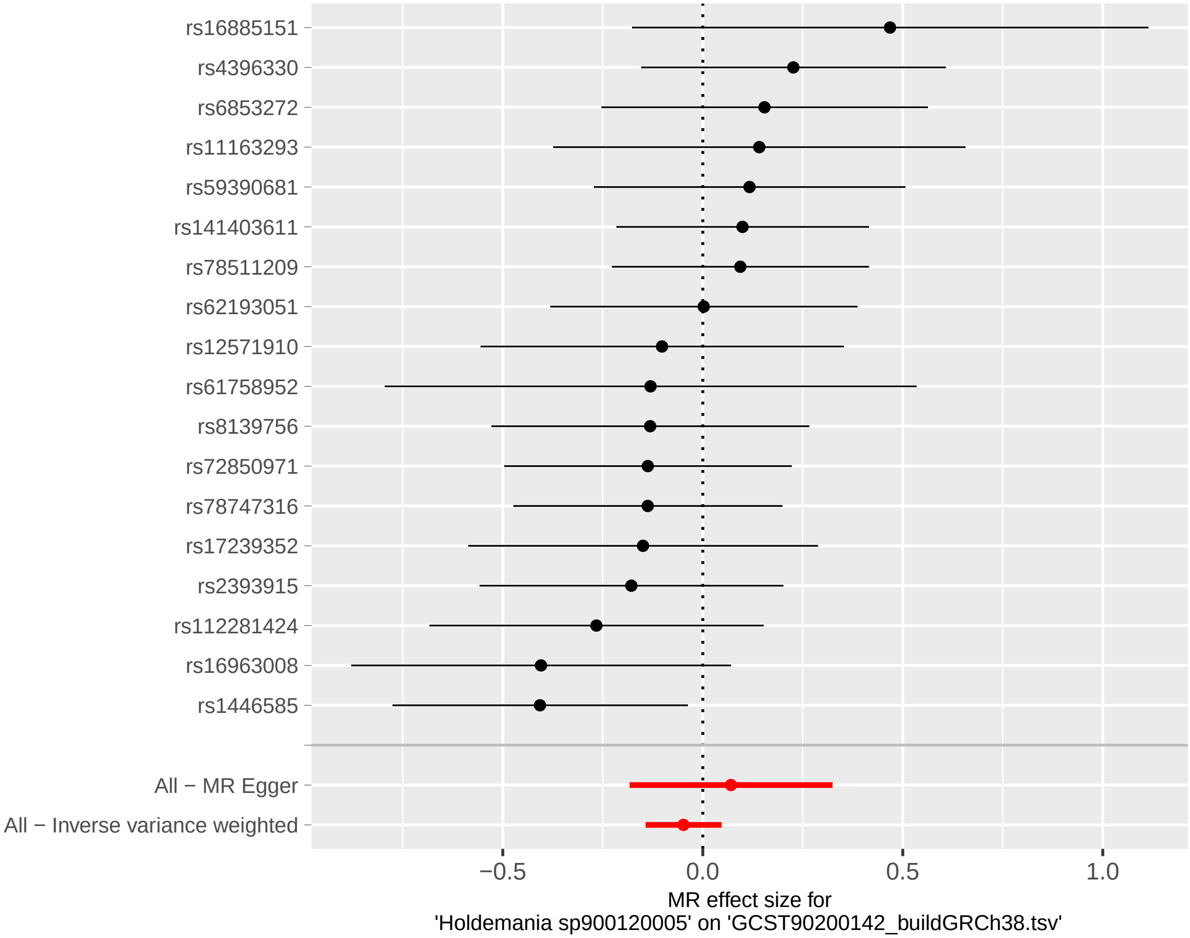
rs16885151
rs4396330
rs6853272
rs11163293
rs59390681
rs141403611
rs78511209
rs62193051
rs12571910
rs61758952
rs8139756
rs72850971
rs78747316
rs17239352
rs2393915
rs112281424
rs16963008
rs1446585
All − MR Egger
All − Inverse variance weighted
−0.5
0.0
0.5
1.0
MR effect size for
'Holdemania sp900120005' on 'GCST90200142_buildGRCh38.tsv'

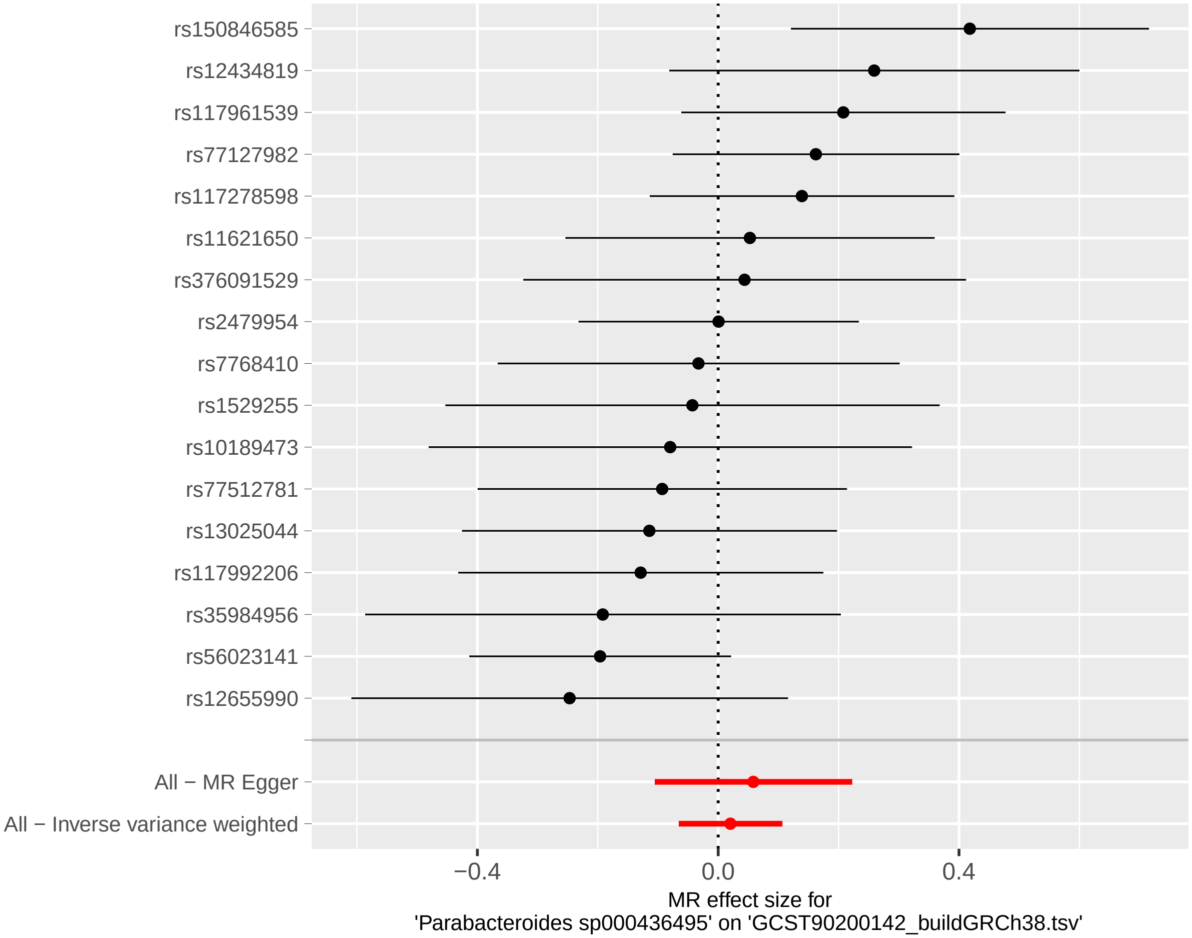

rs150846585
rs12434819
rs117961539
rs77127982
rs117278598
rs11621650
rs376091529
rs2479954
rs7768410
rs1529255
rs10189473
rs77512781
rs13025044
rs117992206
rs35984956
rs56023141
rs12655990
All − MR Egger
All − Inverse variance weighted
−0.4
0.0
0.4
MR effect size for
'Parabacteroides sp000436495' on 'GCST90200142_buildGRCh38.tsv'

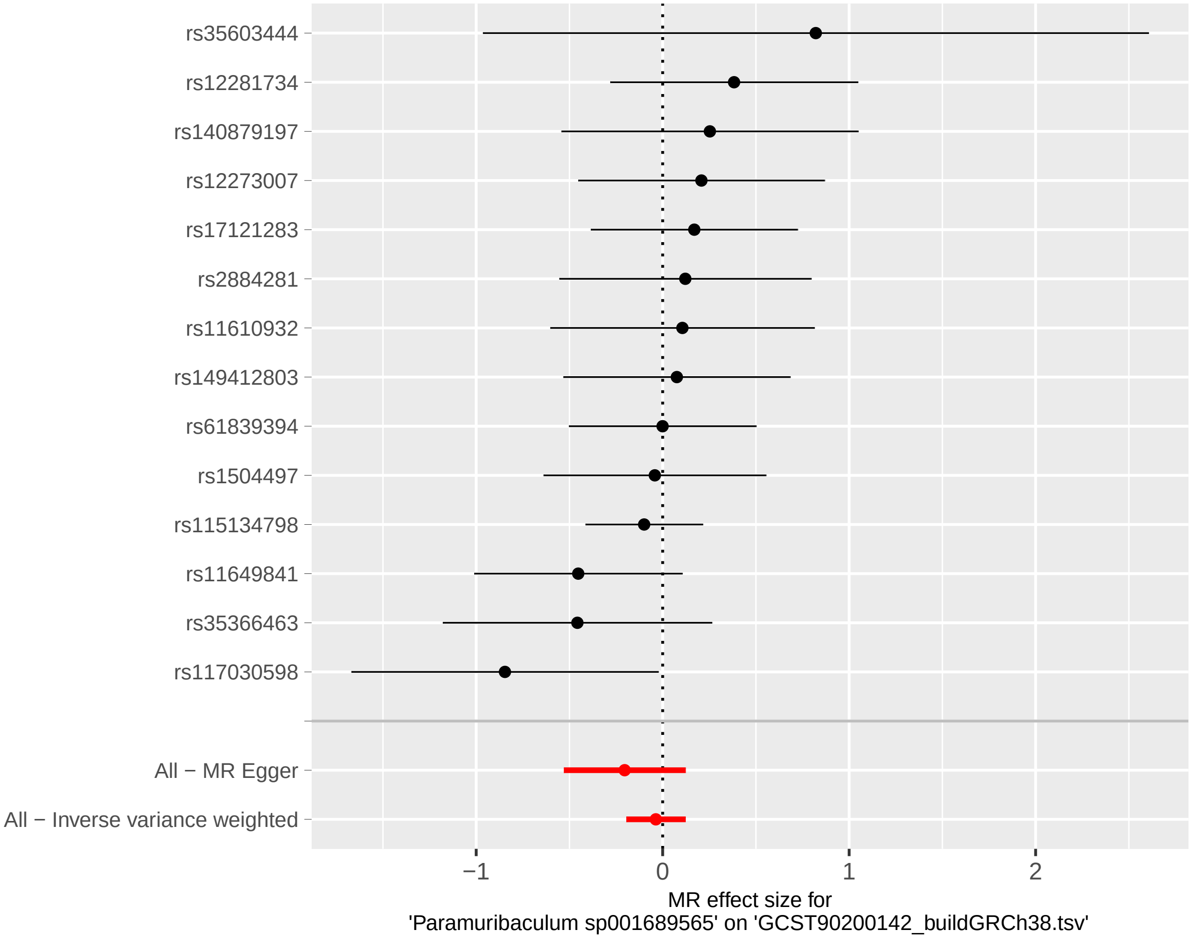
rs35603444
rs12281734
rs140879197
rs12273007
rs17121283
rs2884281
rs11610932
rs149412803
rs61839394
rs1504497
rs115134798
rs11649841
rs35366463
rs117030598
All − MR Egger
All − Inverse variance weighted
−1
0
1
2
MR effect size for
'Paramuribaculum sp001689565' on 'GCST90200142_buildGRCh38.tsv'

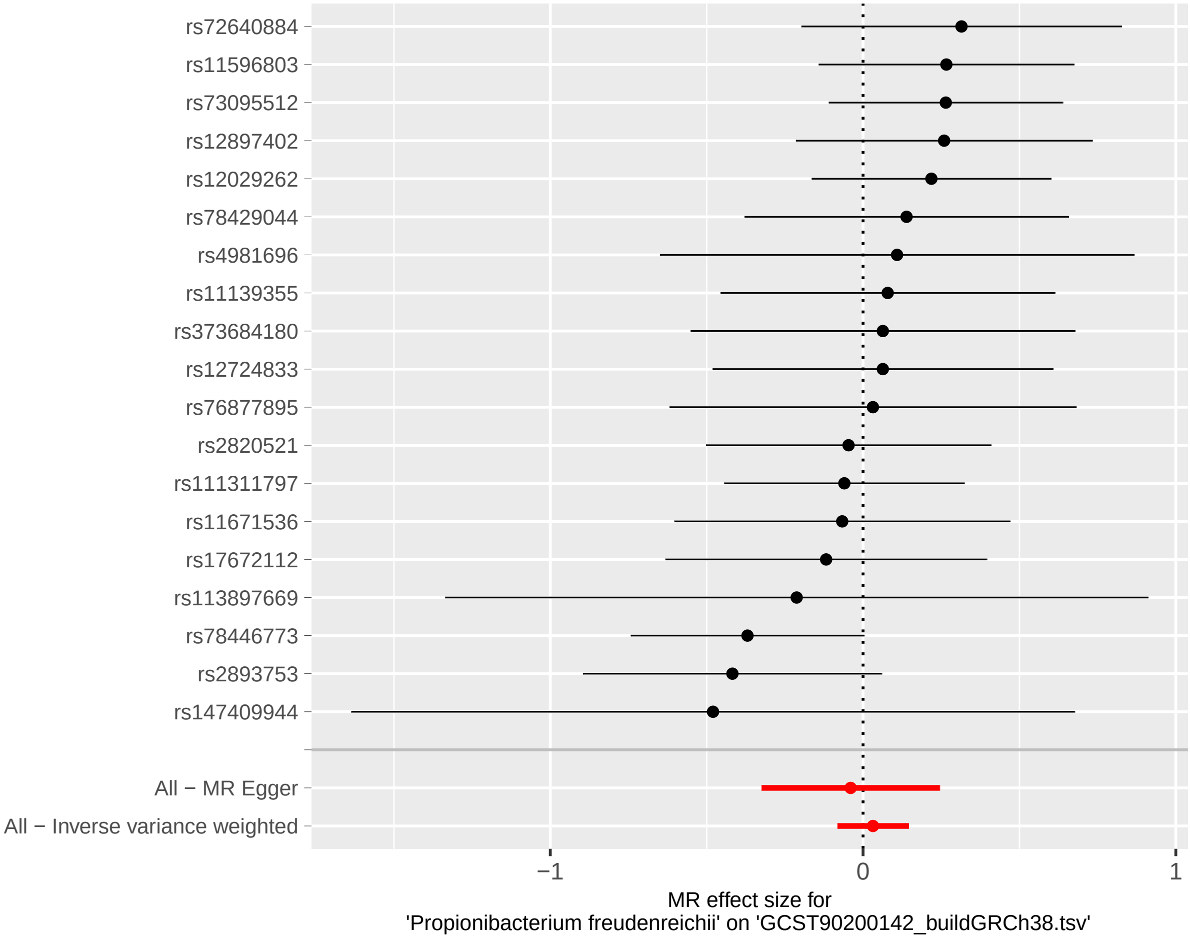
rs72640884
rs11596803
rs73095512
rs12897402
rs12029262
rs78429044
rs4981696
rs11139355
rs373684180
rs12724833
rs76877895
rs2820521
rs111311797
rs11671536
rs17672112
rs113897669
rs78446773
rs2893753
rs147409944
All − MR Egger
All − Inverse variance weighted
−1
0
1
MR effect size for
'Propionibacterium freudenreichii' on 'GCST90200142_buildGRCh38.tsv'

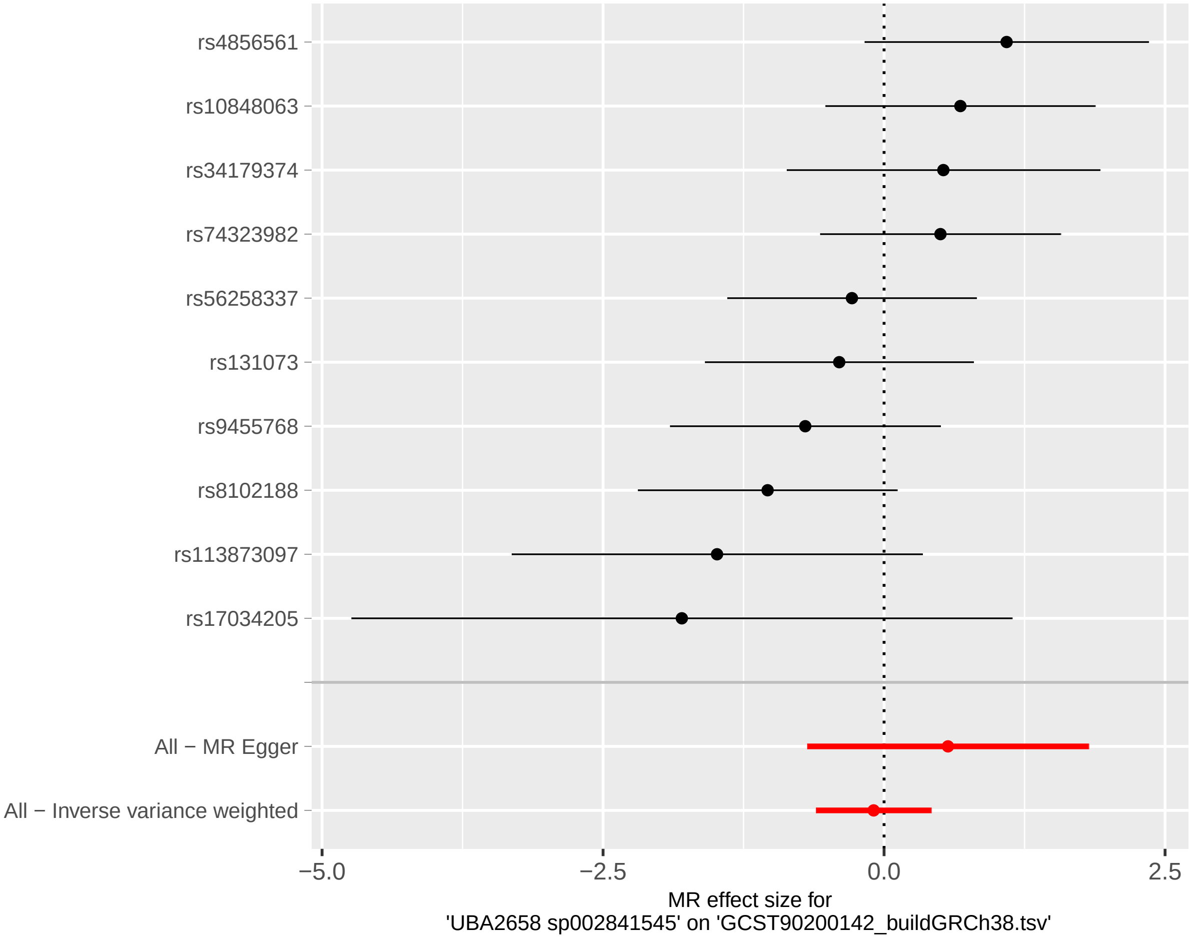
rs4856561
rs10848063
rs34179374
rs74323982
rs56258337
rs131073
rs9455768
rs8102188
rs113873097
rs17034205
All − MR Egger
All − Inverse variance weighted
−5.0
−2.5
0.0
2.5
MR effect size for
'UBA2658 sp002841545' on 'GCST90200142_buildGRCh38.tsv'

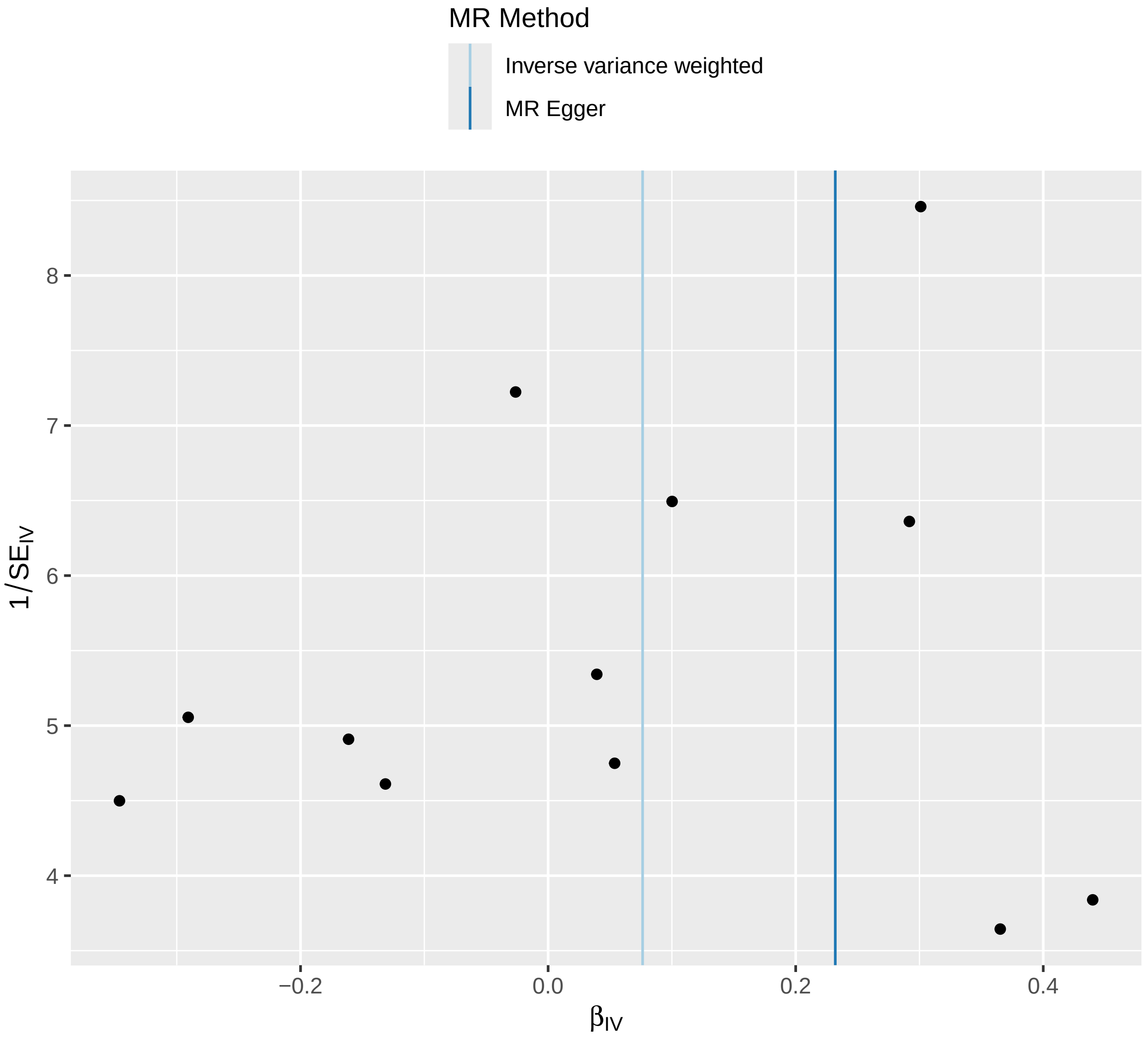

MR Method
Inverse variance weighted
MR Egger
8
7
6
5
4
1/SE_IV
−0.2
0.0
0.2
0.4
β_IV

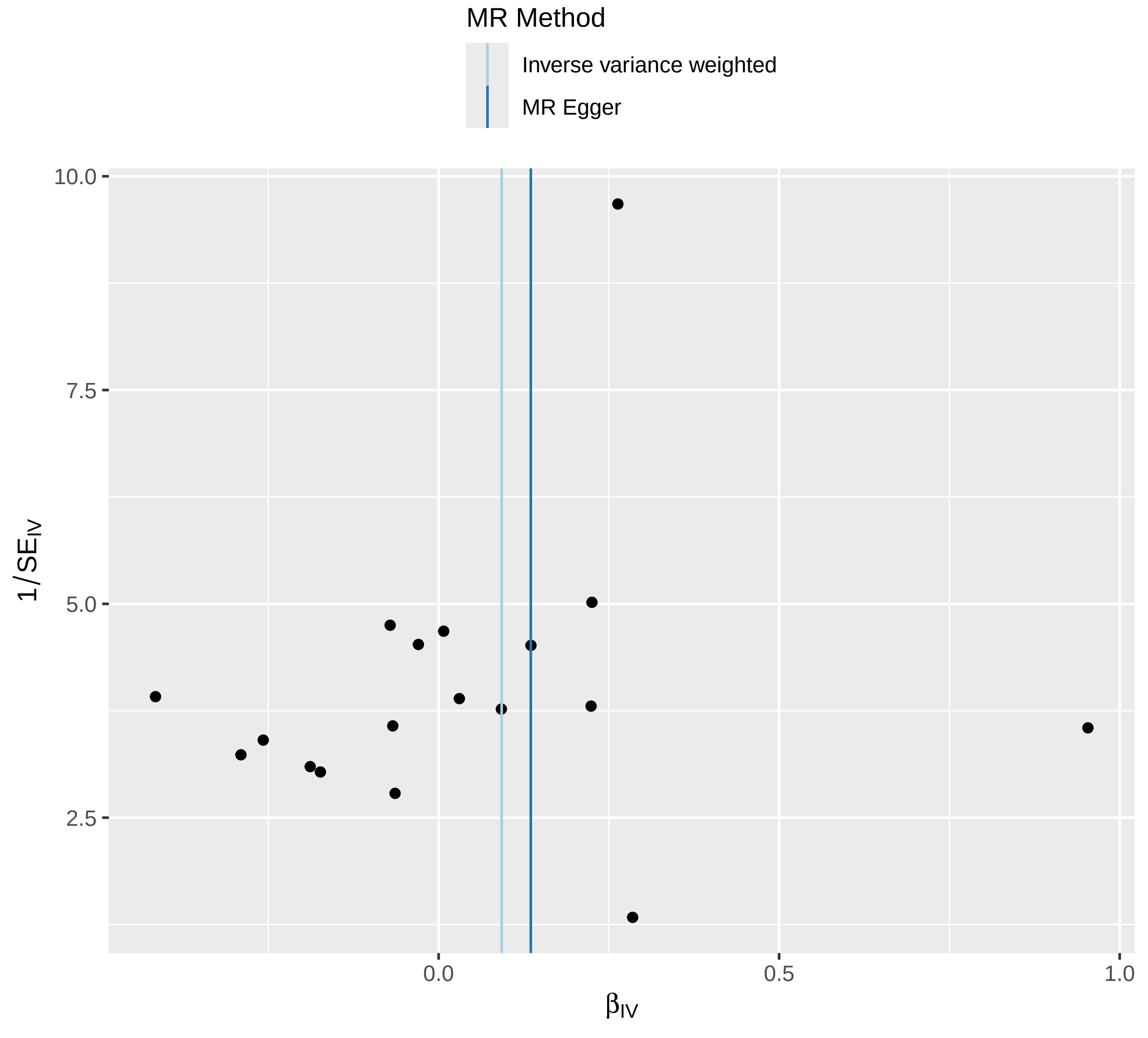
MR Method
Inverse variance weighted
MR Egger
10.0
7.5
5.0
2.5
1/SE_IV
0.0
0.5
1.0
β_IV

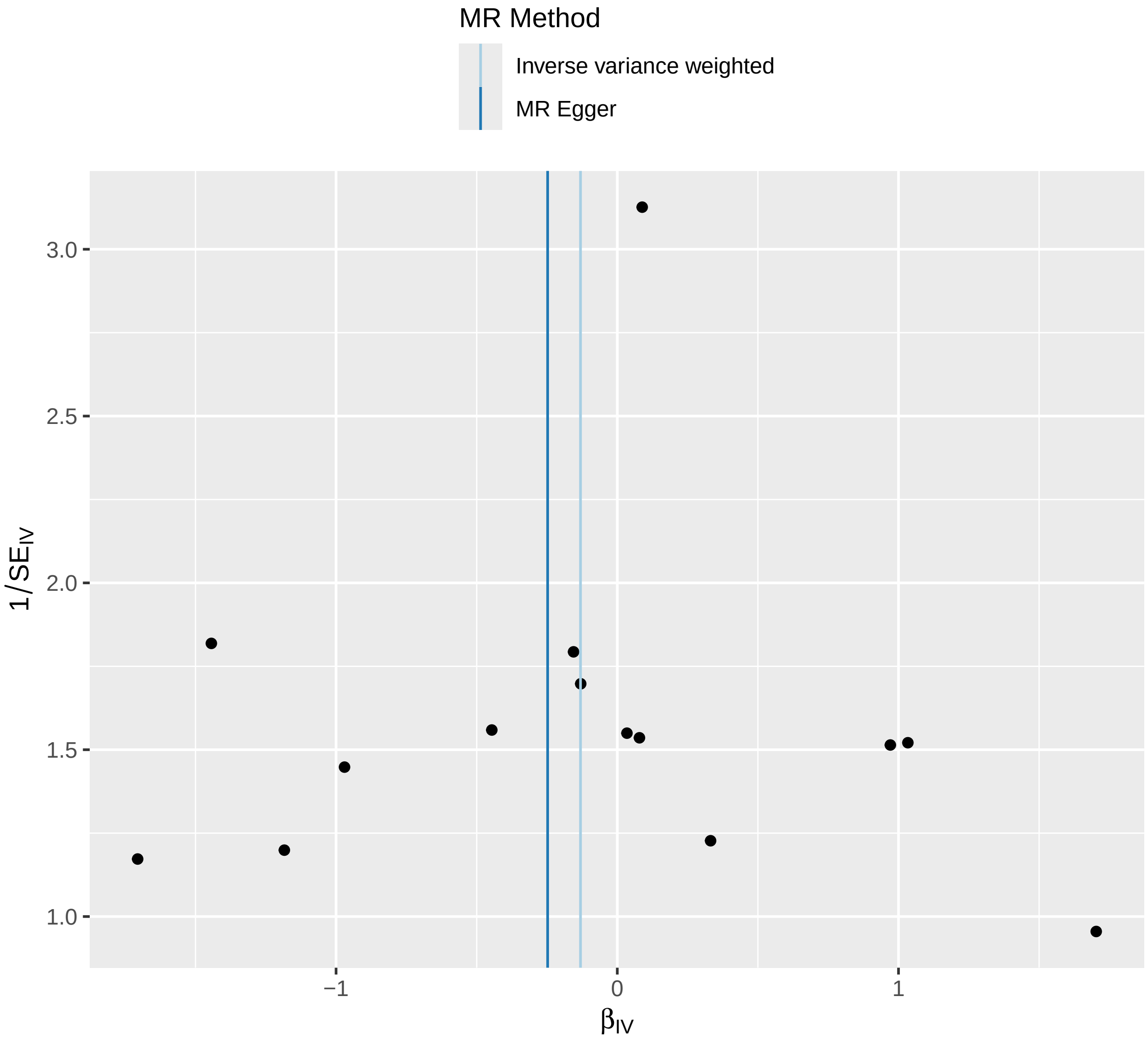
MR Method
Inverse variance weighted
MR Egger
3.0
2.5
2.0
1.5
1.0
$1/SE_{IV}$
−1
0
1
β_{IV}

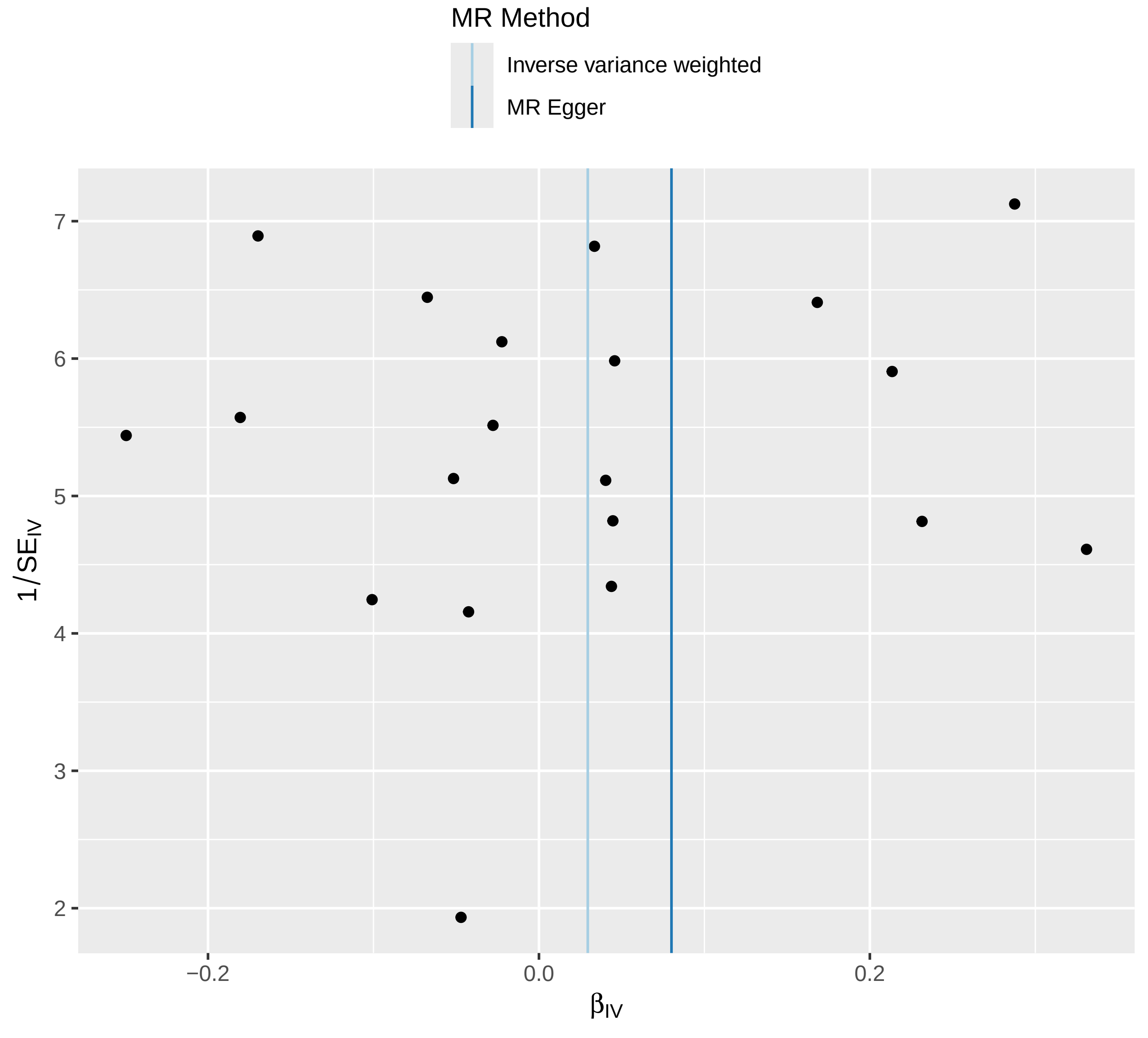
MR Method
Inverse variance weighted
MR Egger
$1/SE_{IV}$
7
6
5
4
3
2
−0.2
0.0
0.2
β_{IV}

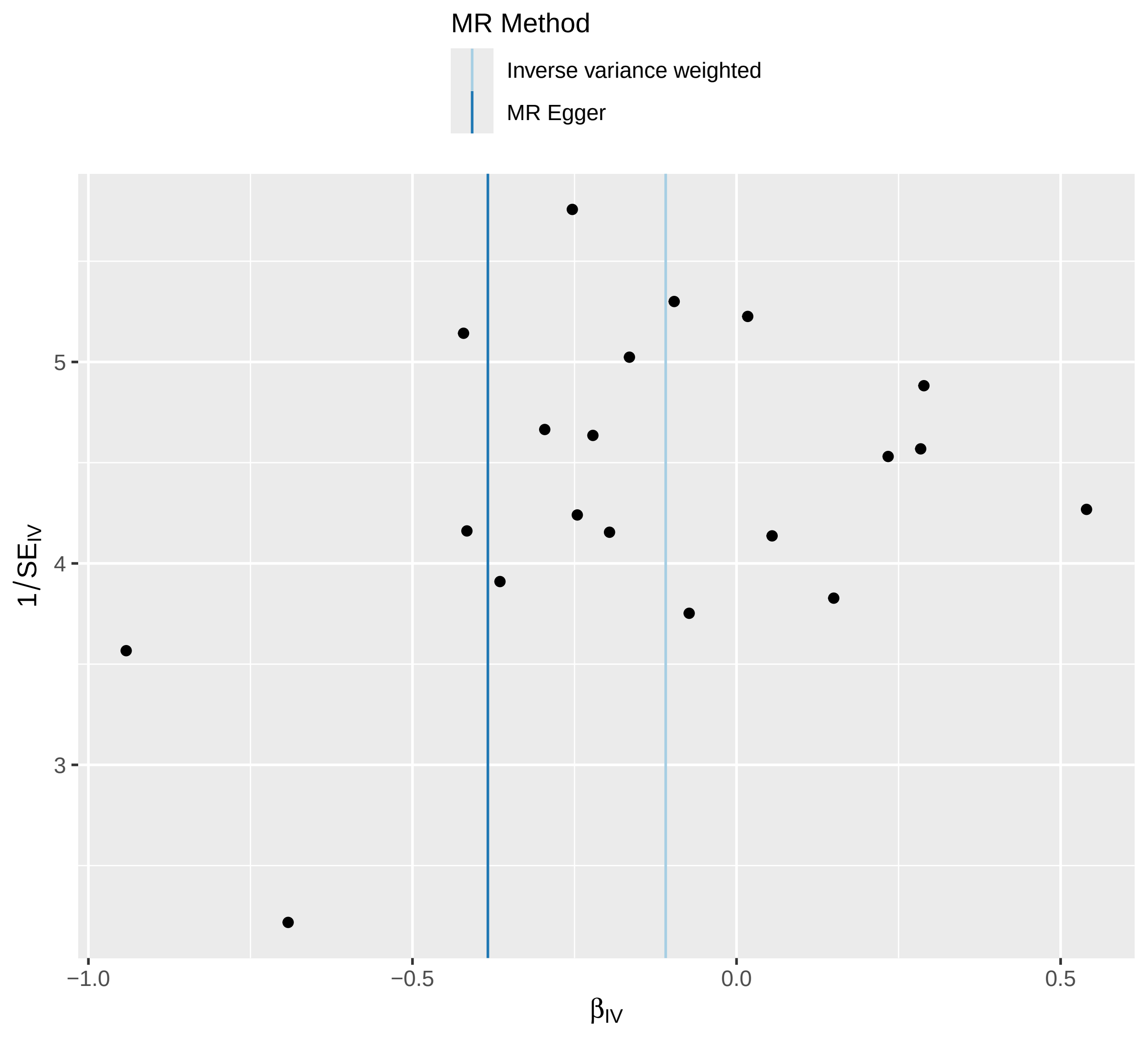

MR Method
Inverse variance weighted
MR Egger
5
4
3
1/SE$_{IV}$
−1.0
−0.5
0.0
0.5
β$_{IV}$

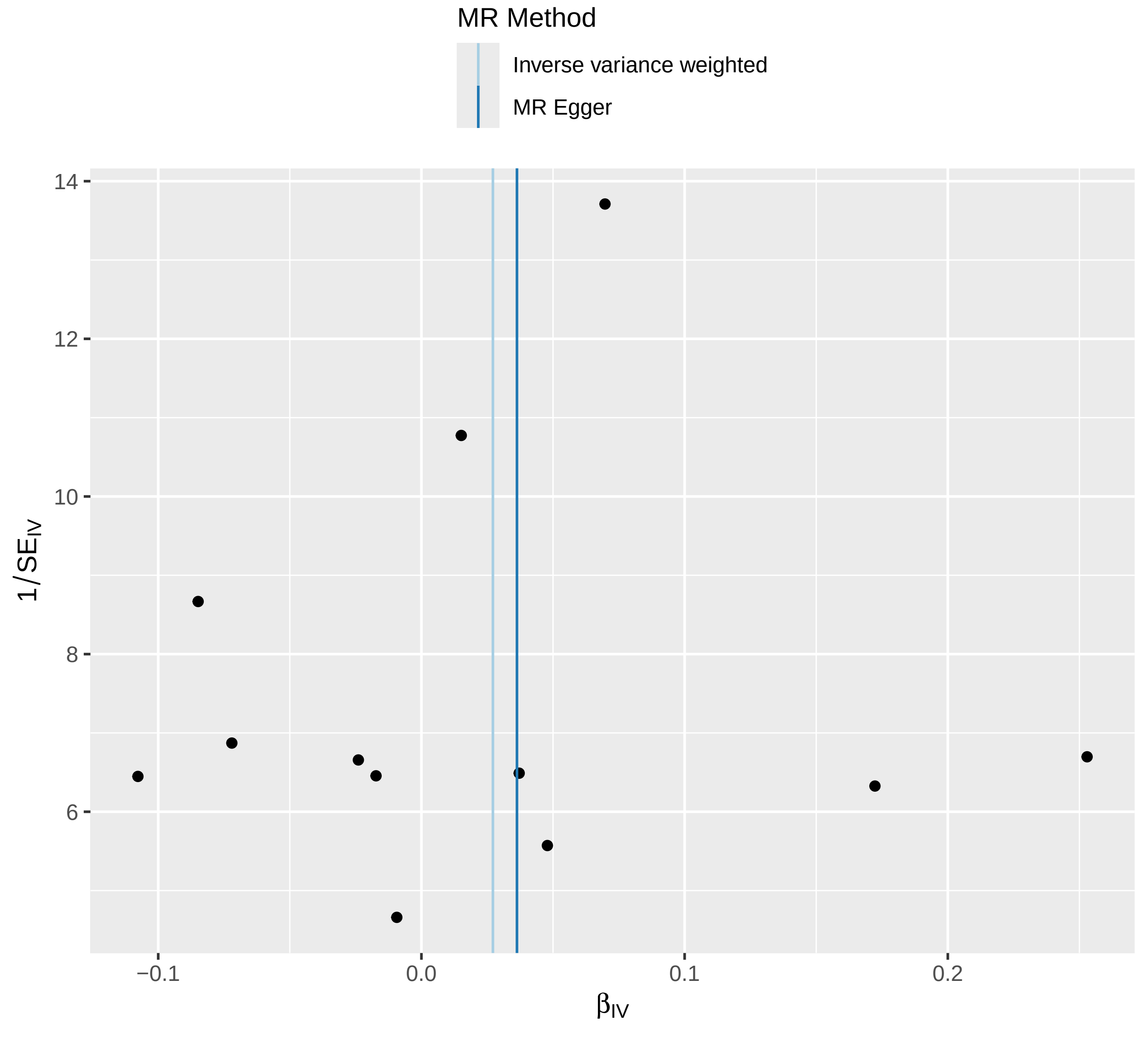

MR Method
Inverse variance weighted
MR Egger
14
12
10
8
6
1/SE$_{IV}$
−0.1
0.0
0.1
0.2
β$_{IV}$

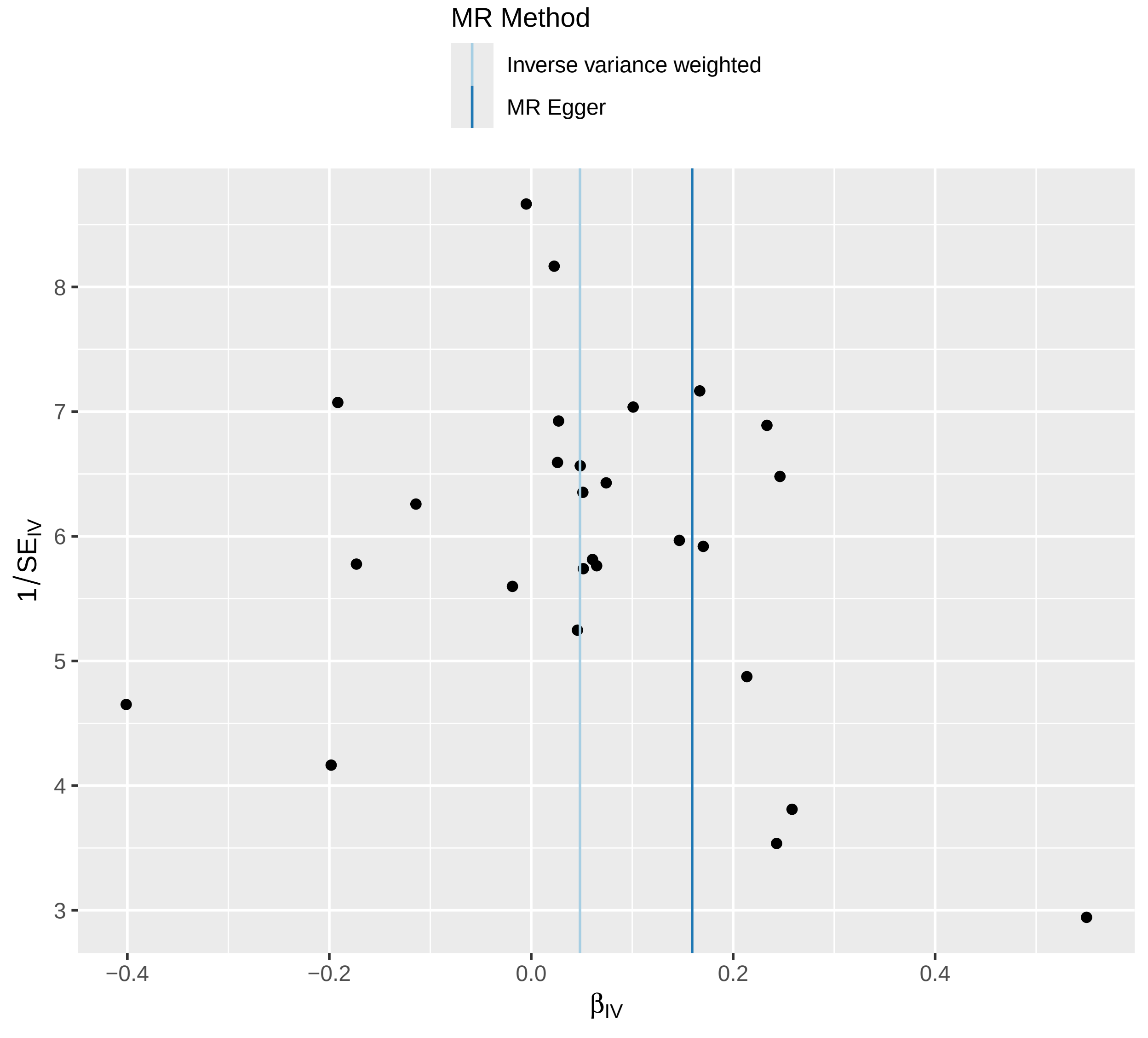
MR Method
Inverse variance weighted
MR Egger
1/SE_IV
8
7
6
5
4
3
−0.4
−0.2
0.0
0.2
0.4
β_IV

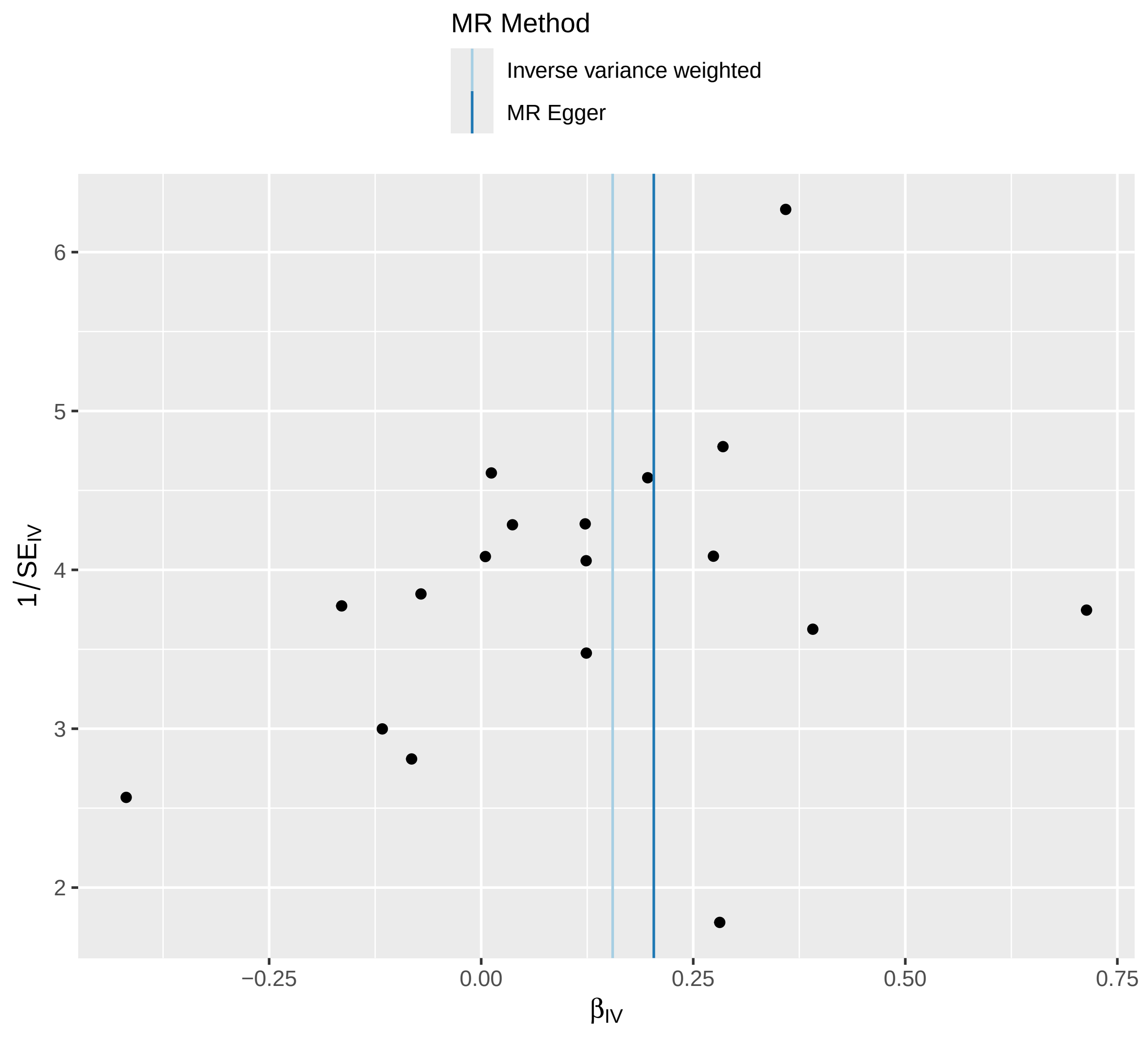

MR Method
Inverse variance weighted
MR Egger
6
5
4
3
2
$1/SE_{IV}$
−0.25
0.00
0.25
0.50
0.75
β_{IV}

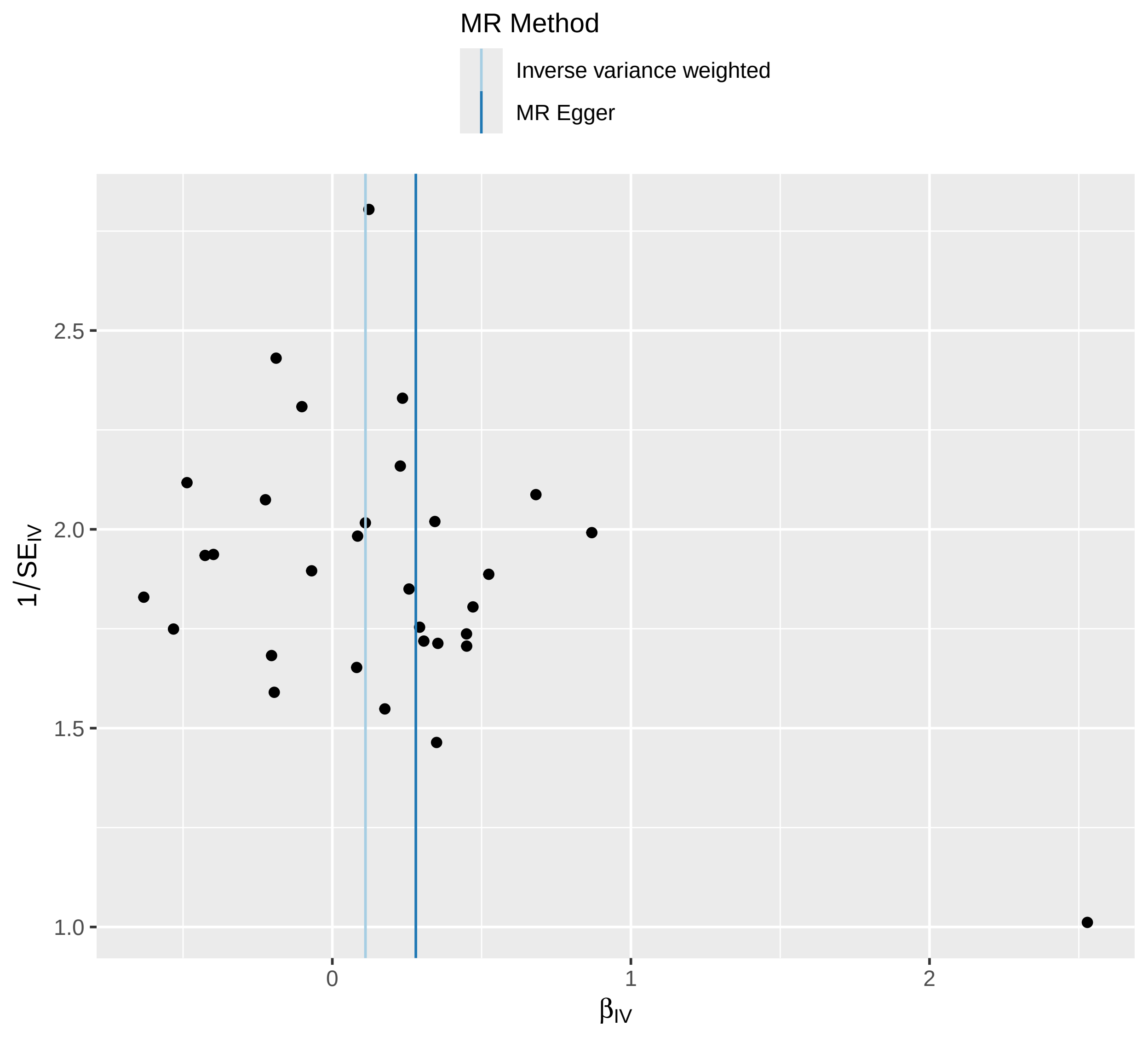
MR Method
Inverse variance weighted
MR Egger
2.5
2.0
1.5
1.0
$1/SE_{IV}$
0
1
2
β_{IV}

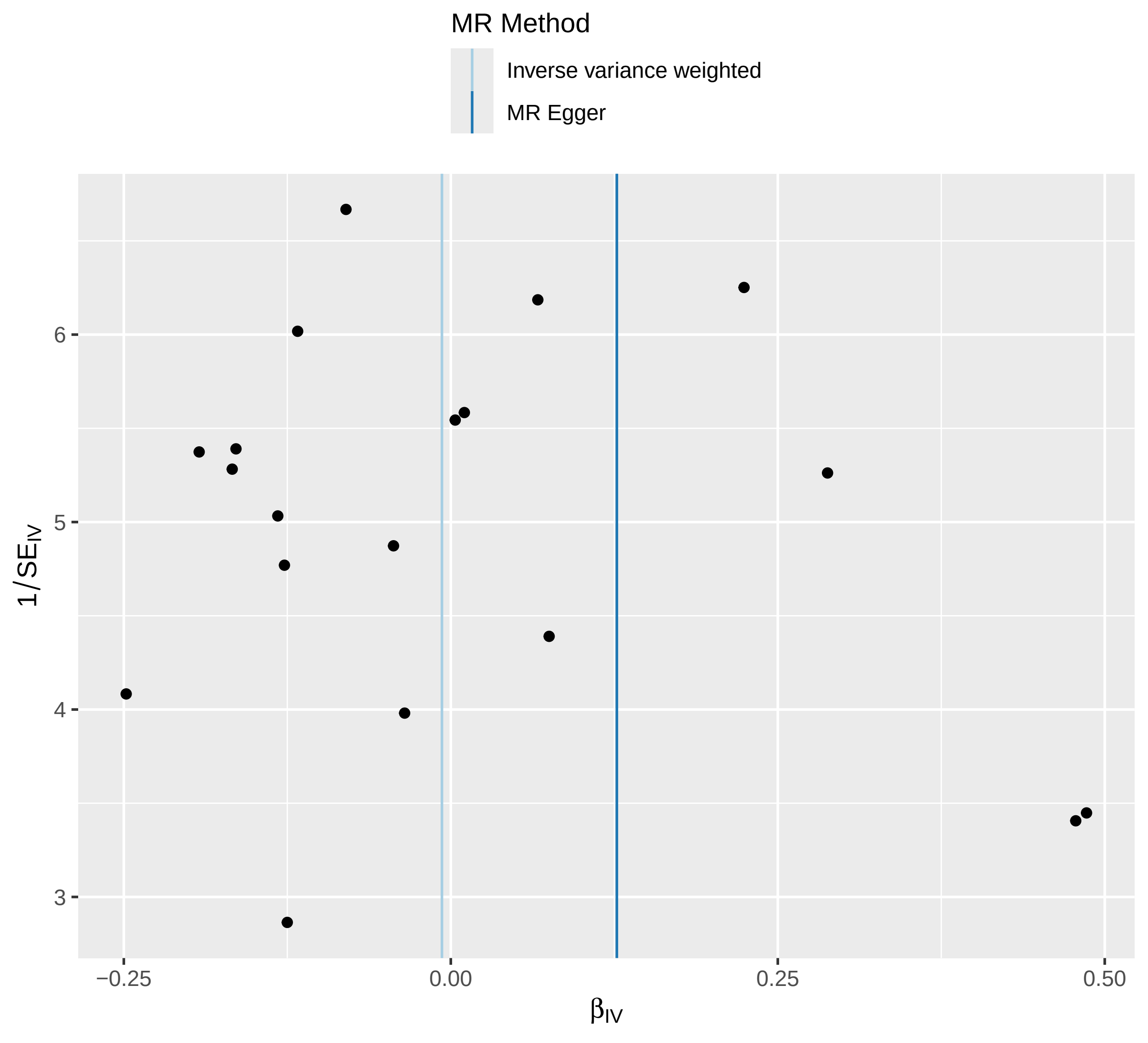

MR Method
Inverse variance weighted
MR Egger
6
5
4
3
$1/SE_{IV}$
−0.25
0.00
0.25
0.50
β_{IV}

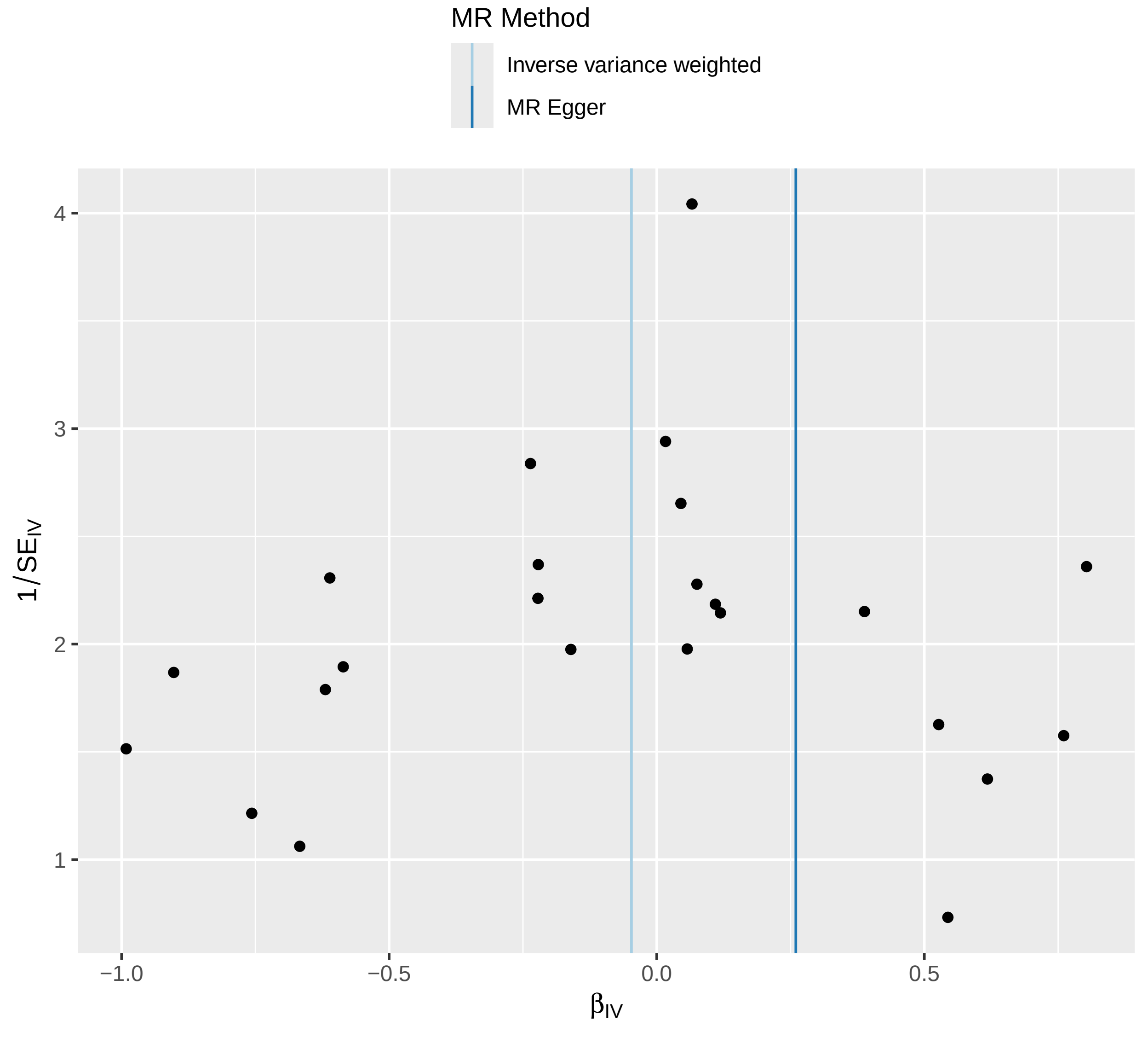
MR Method
Inverse variance weighted
MR Egger
1/SE_{IV}
4
3
2
1
−1.0
−0.5
0.0
0.5
β_{IV}

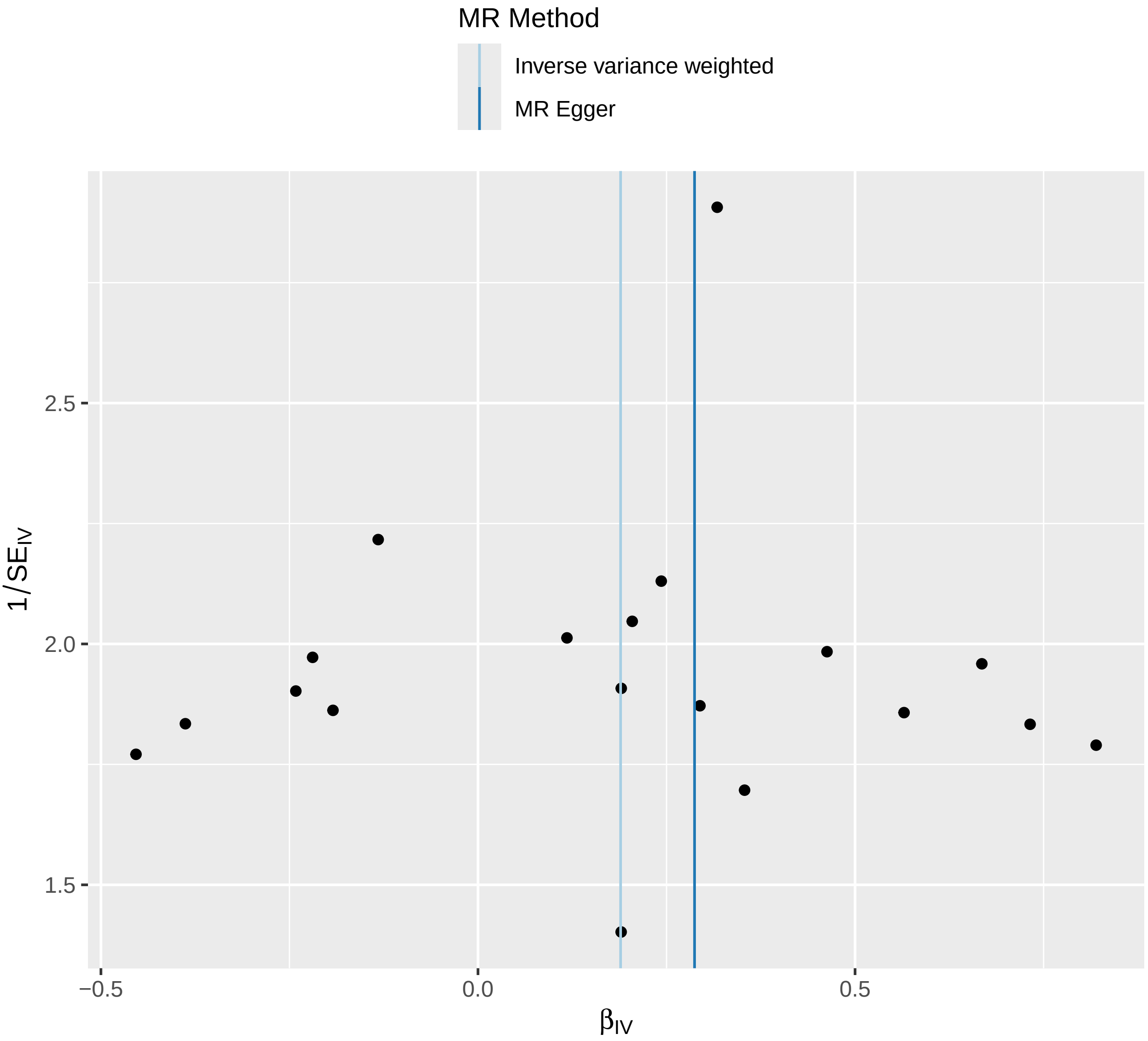
MR Method
Inverse variance weighted
MR Egger
2.5
2.0
1.5
1/SE_IV
−0.5
0.0
0.5
β_IV

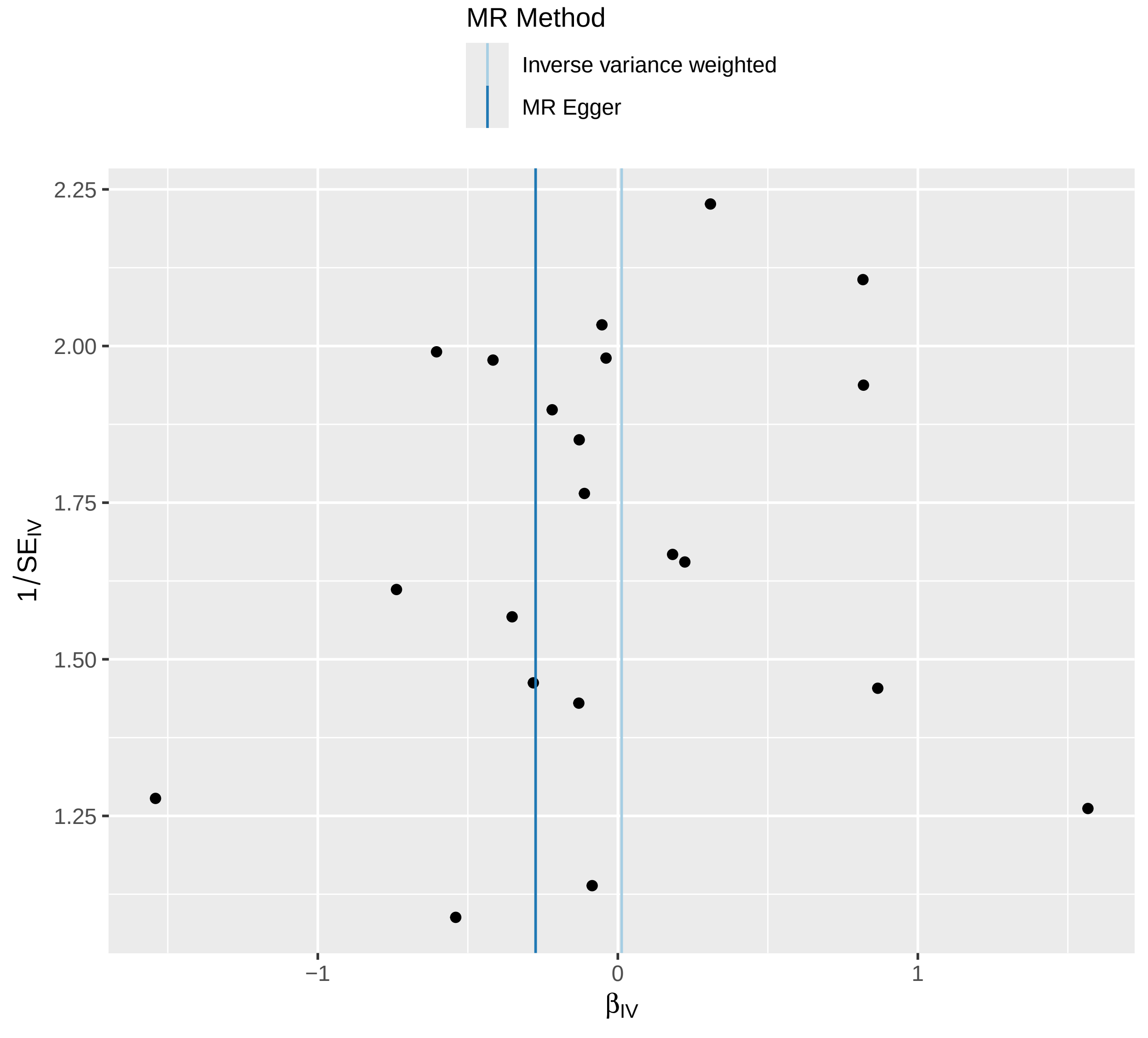
MR Method
Inverse variance weighted
MR Egger
2.25
2.00
1.75
1.50
1.25
1/SE$_{IV}$
−1
0
1
β$_{IV}$

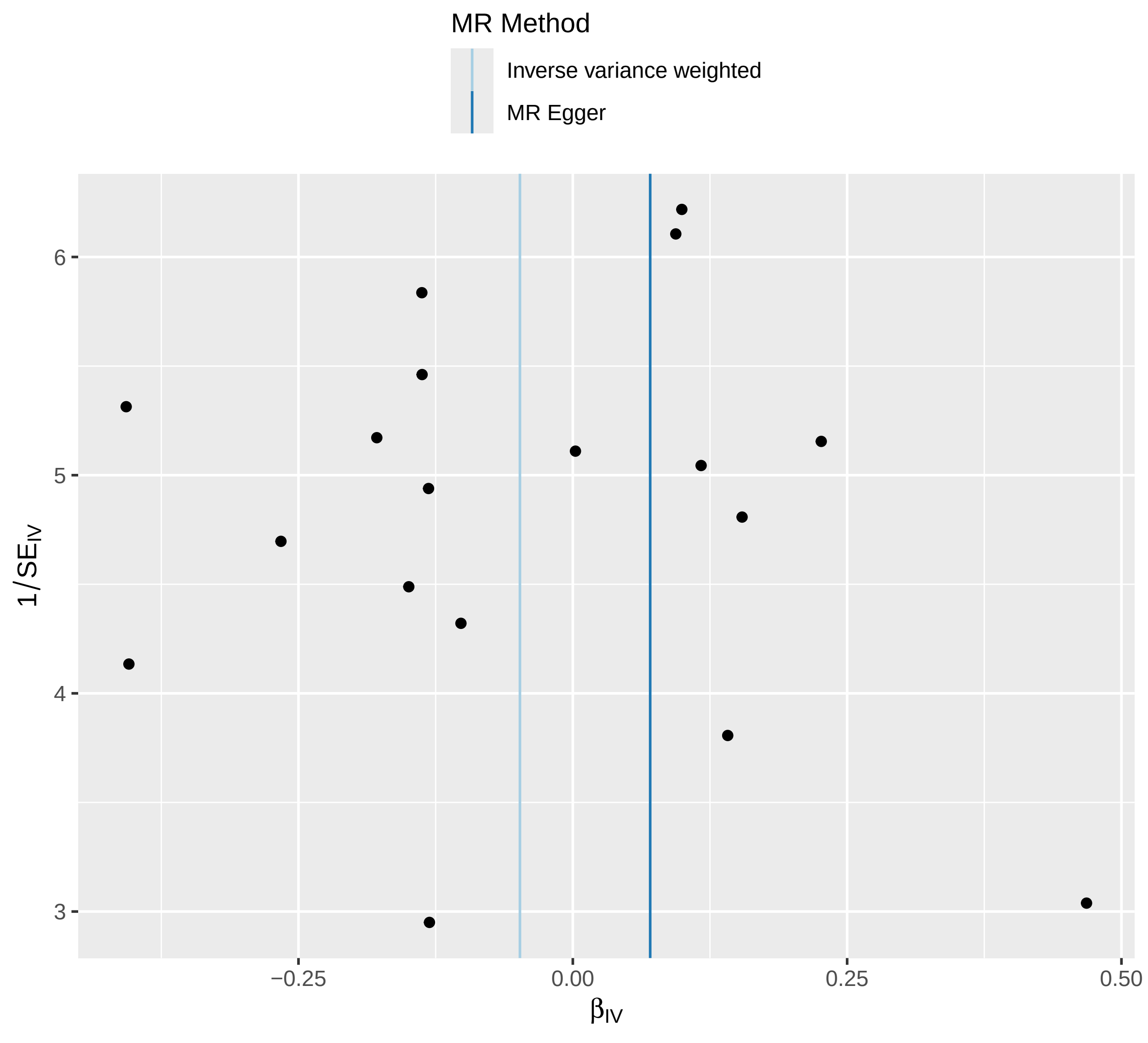
MR Method
Inverse variance weighted
MR Egger
6
5
4
3
1/SE$_{IV}$
−0.25
0.00
0.25
0.50
β$_{IV}$

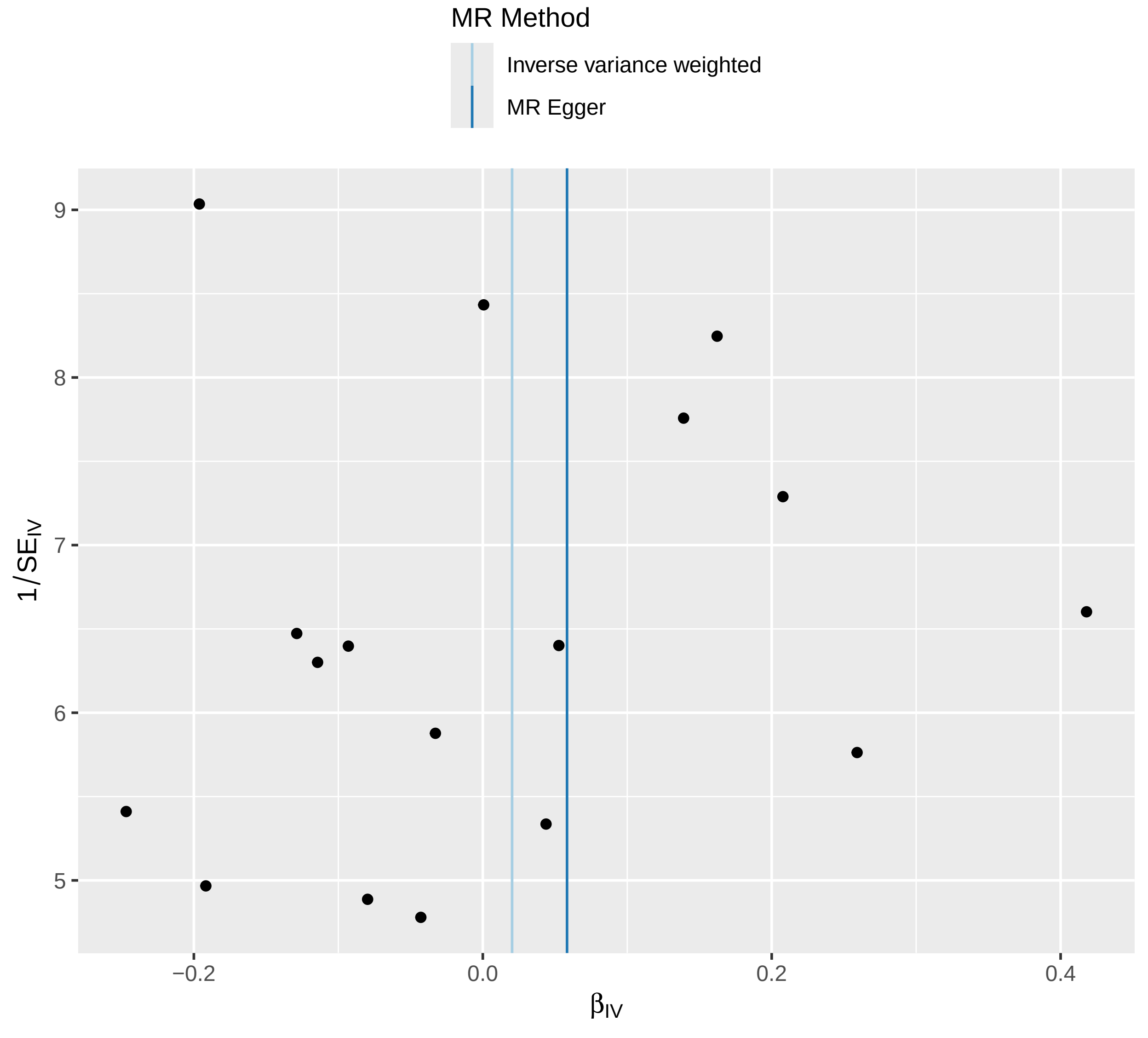

MR Method
Inverse variance weighted
MR Egger
9
8
7
6
5
1/SE_IV
−0.2
0.0
0.2
0.4
β_IV

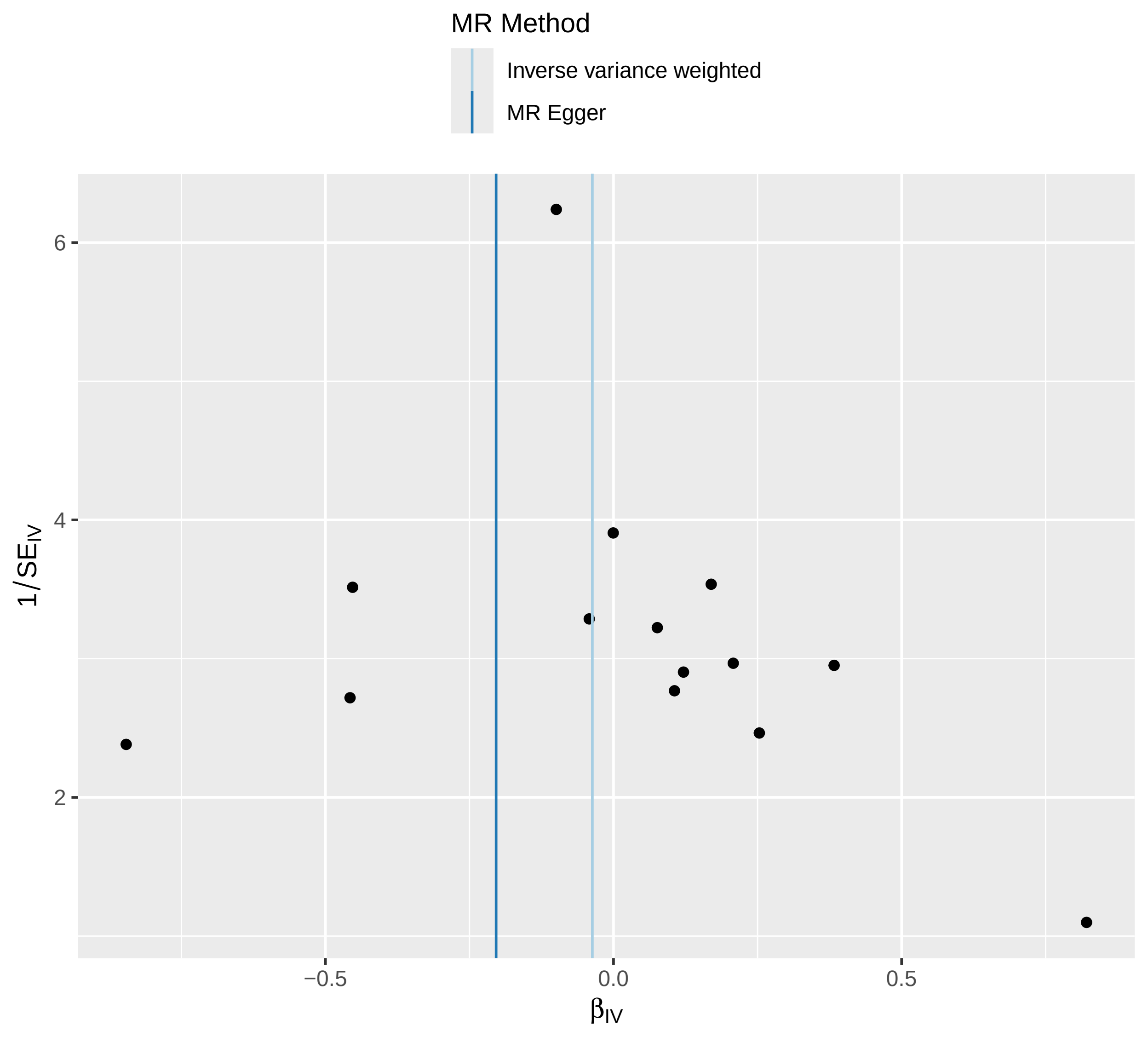
MR Method
Inverse variance weighted
MR Egger
6
4
2
1/SE_IV
−0.5
0.0
0.5
β_IV

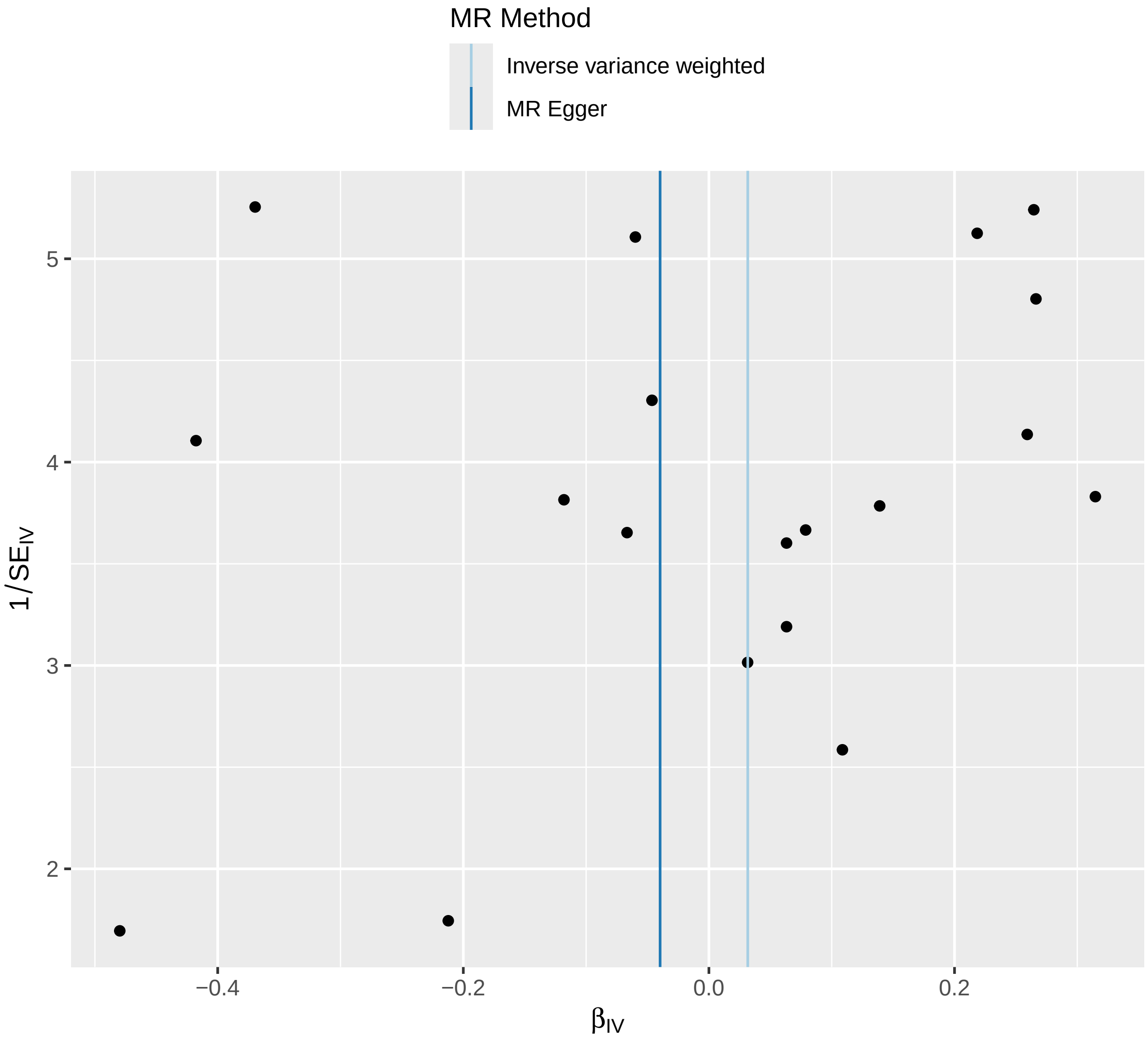

MR Method
Inverse variance weighted
MR Egger
5
4
3
2
1/SE$_{IV}$
−0.4
−0.2
0.0
0.2
β$_{IV}$

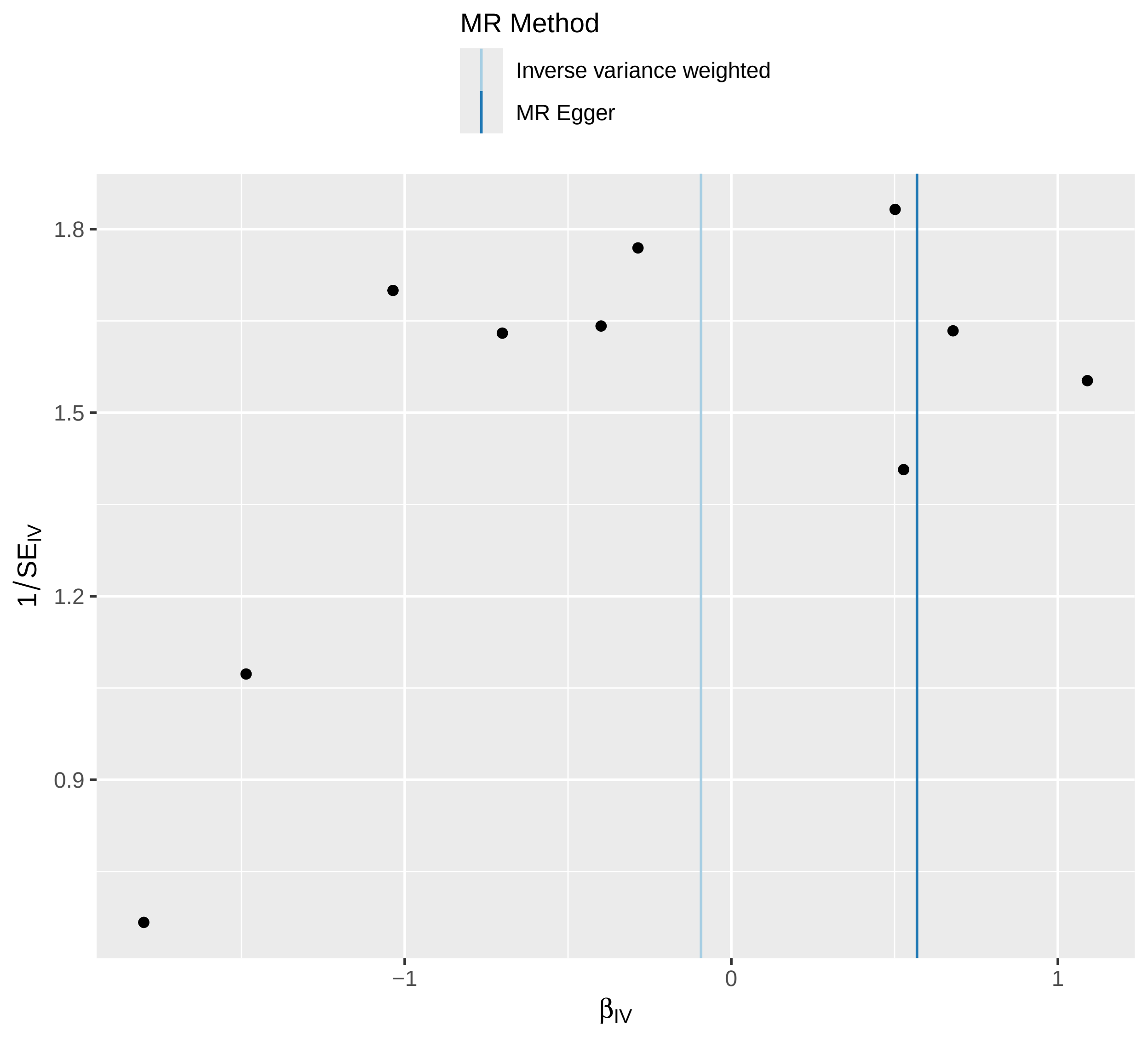
MR Method
Inverse variance weighted
MR Egger
1.8
1.5
1.2
0.9
1/SE$_{IV}$
−1
0
1
β$_{IV}$

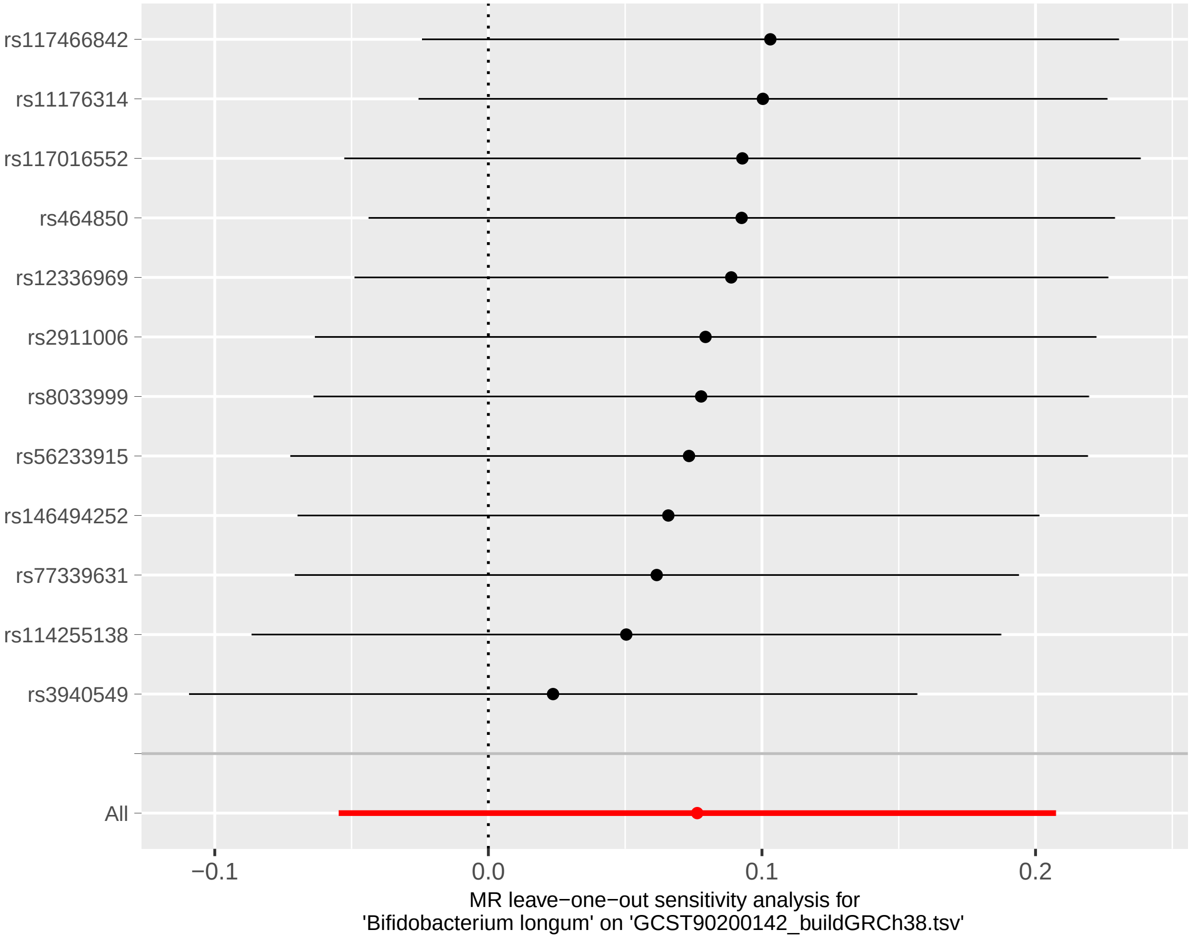
rs117466842
rs11176314
rs117016552
rs464850
rs12336969
rs2911006
rs8033999
rs56233915
rs146494252
rs77339631
rs114255138
rs3940549
All
−0.1
0.0
0.1
0.2
MR leave−one−out sensitivity analysis for
'Bifidobacterium longum' on 'GCST90200142_buildGRCh38.tsv'

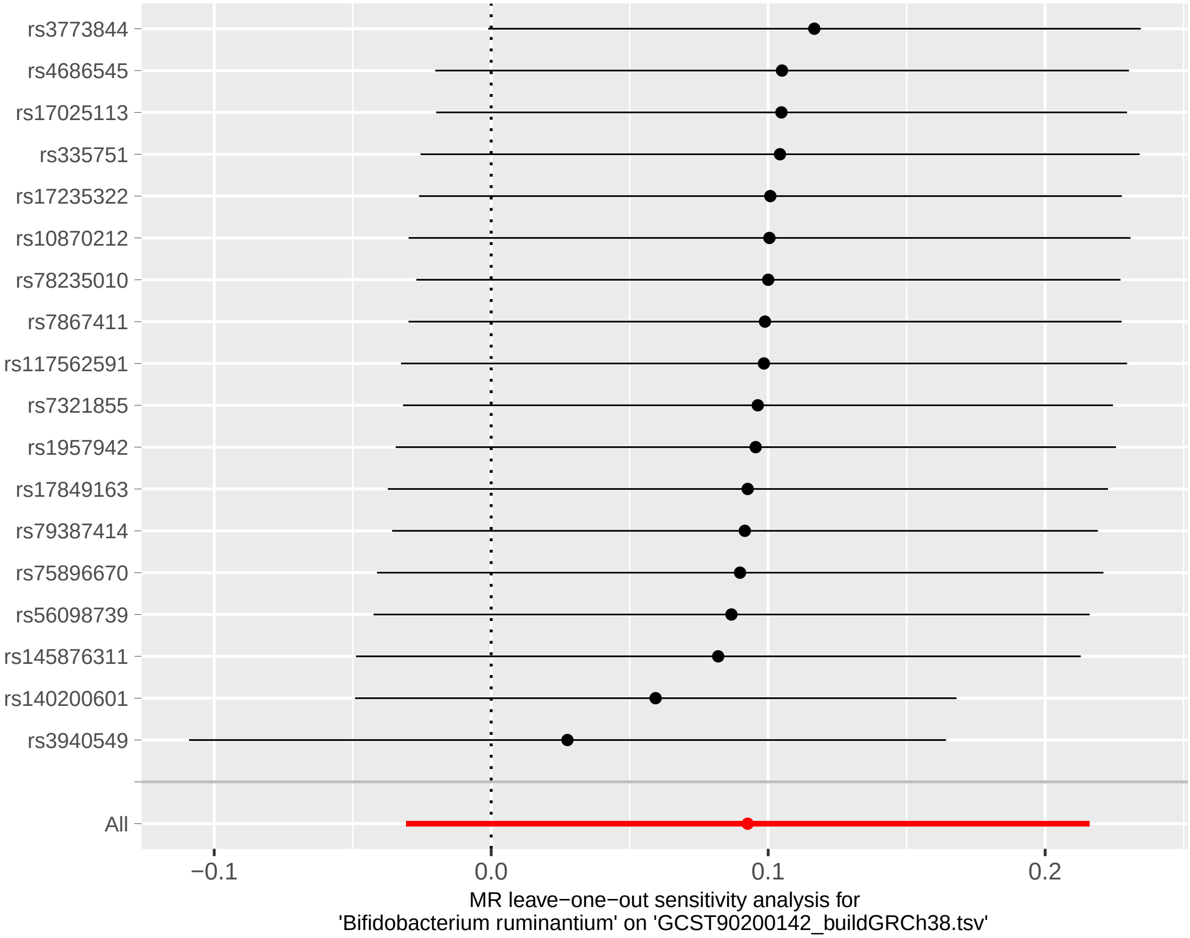

rs3773844
rs4686545
rs17025113
rs335751
rs17235322
rs10870212
rs78235010
rs7867411
rs117562591
rs7321855
rs1957942
rs17849163
rs79387414
rs75896670
rs56098739
rs145876311
rs140200601
rs3940549
All
−0.1
0.0
0.1
0.2
MR leave−one−out sensitivity analysis for
'Bifidobacterium ruminantium' on 'GCST90200142_buildGRCh38.tsv'

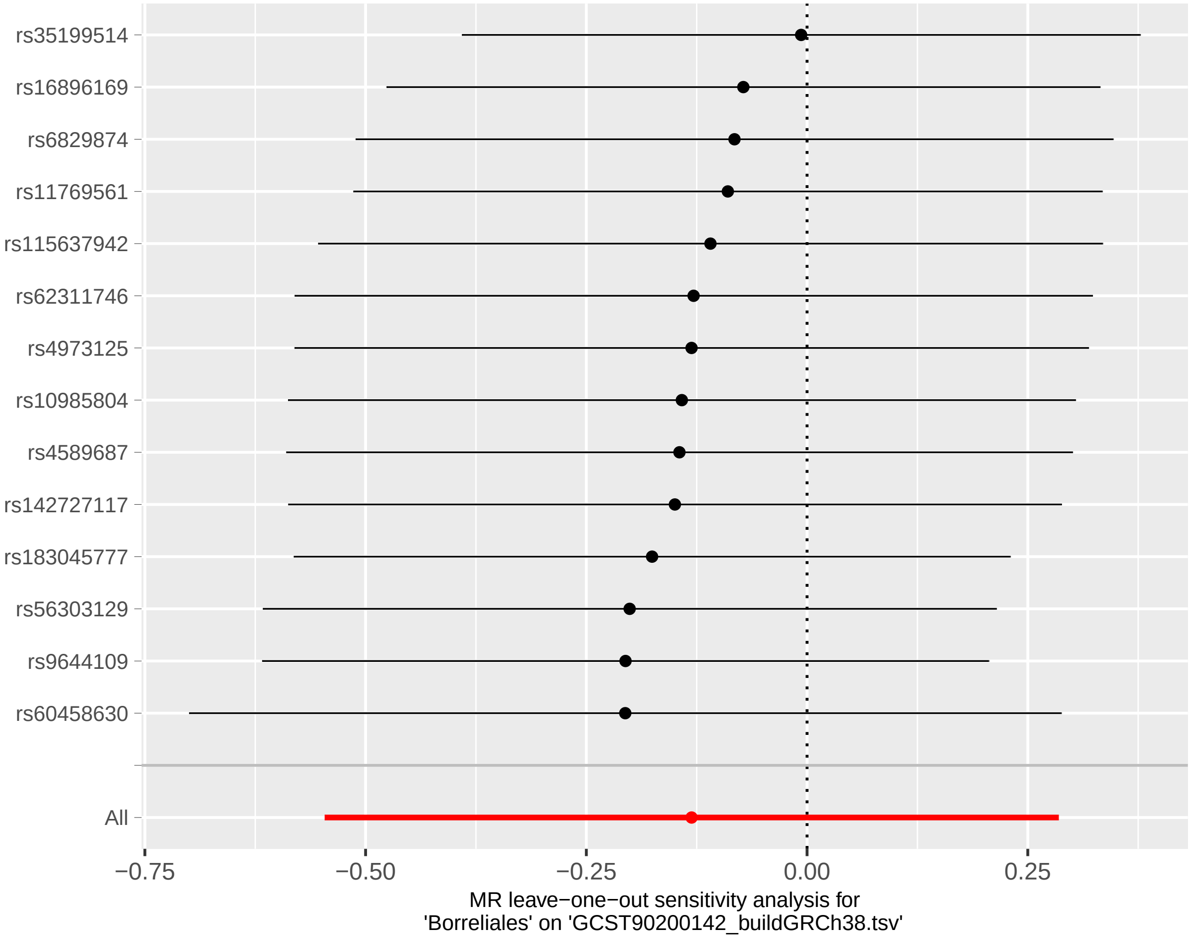

rs35199514
rs16896169
rs6829874
rs11769561
rs115637942
rs62311746
rs4973125
rs10985804
rs4589687
rs142727117
rs183045777
rs56303129
rs9644109
rs60458630
All
−0.75
−0.50
−0.25
0.00
0.25
MR leave−one−out sensitivity analysis for
'Borreliales' on 'GCST90200142_buildGRCh38.tsv'

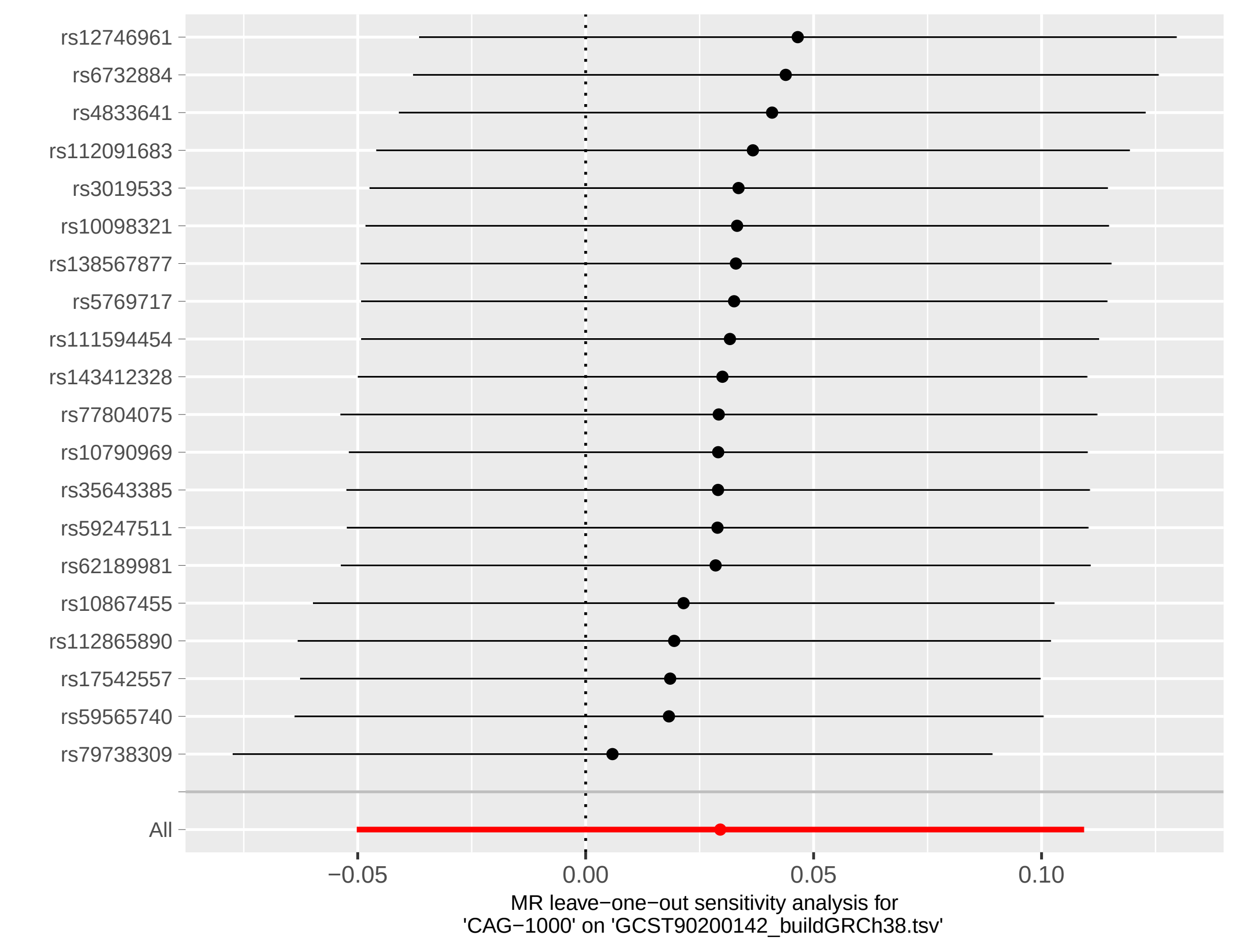

rs12746961
rs6732884
rs4833641
rs112091683
rs3019533
rs10098321
rs138567877
rs5769717
rs111594454
rs143412328
rs77804075
rs10790969
rs35643385
rs59247511
rs62189981
rs10867455
rs112865890
rs17542557
rs59565740
rs79738309
All
−0.05
0.00
0.05
0.10
MR leave−one−out sensitivity analysis for
'CAG−1000' on 'GCST90200142_buildGRCh38.tsv'

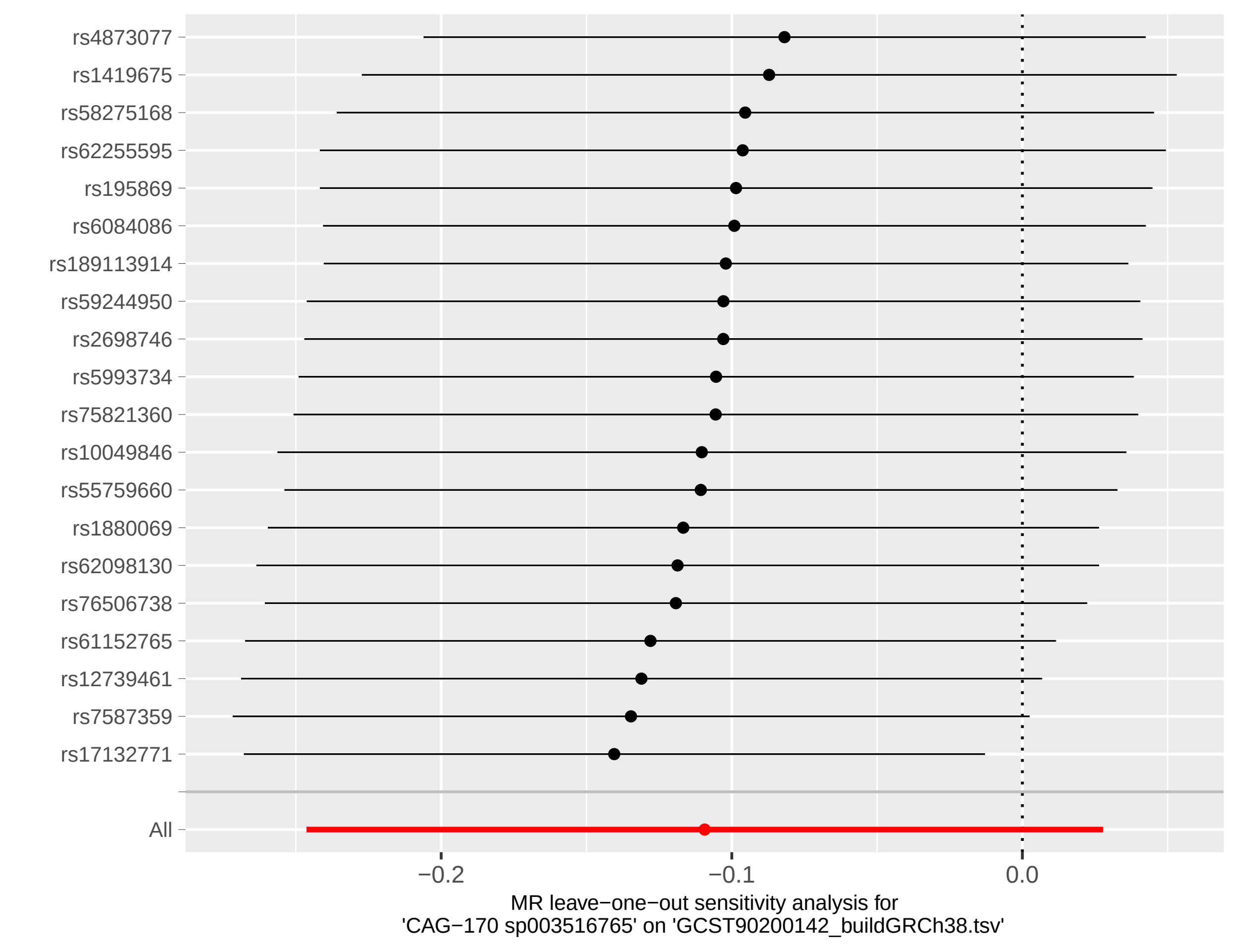
rs4873077
rs1419675
rs58275168
rs62255595
rs195869
rs6084086
rs189113914
rs59244950
rs2698746
rs5993734
rs75821360
rs10049846
rs55759660
rs1880069
rs62098130
rs76506738
rs61152765
rs12739461
rs7587359
rs17132771
All
−0.2
−0.1
0.0
MR leave−one−out sensitivity analysis for
'CAG−170 sp003516765' on 'GCST90200142_buildGRCh38.tsv'

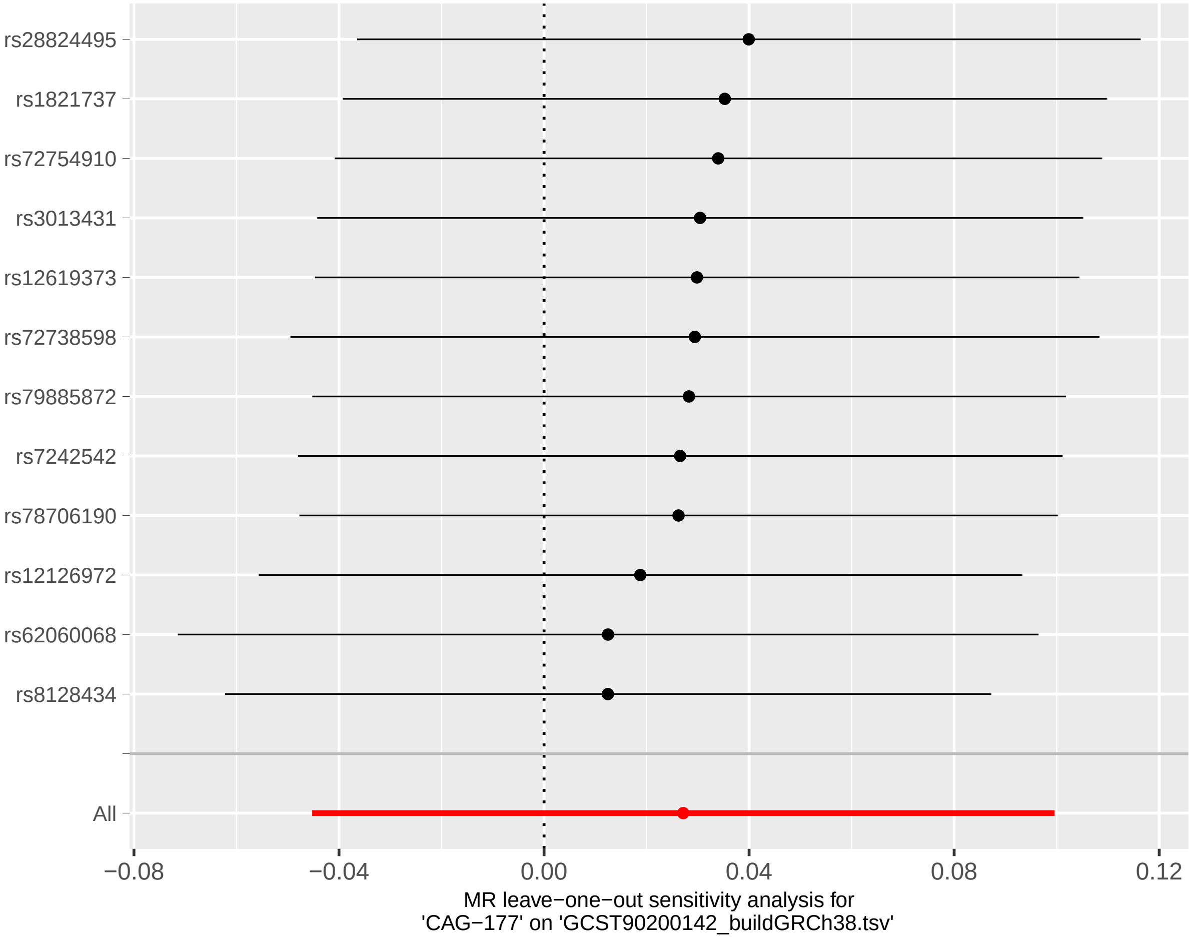

rs28824495
rs1821737
rs72754910
rs3013431
rs12619373
rs72738598
rs79885872
rs7242542
rs78706190
rs12126972
rs62060068
rs8128434
All
−0.08
−0.04
0.00
0.04
0.08
0.12
MR leave−one−out sensitivity analysis for
'CAG−177' on 'GCST90200142_buildGRCh38.tsv'

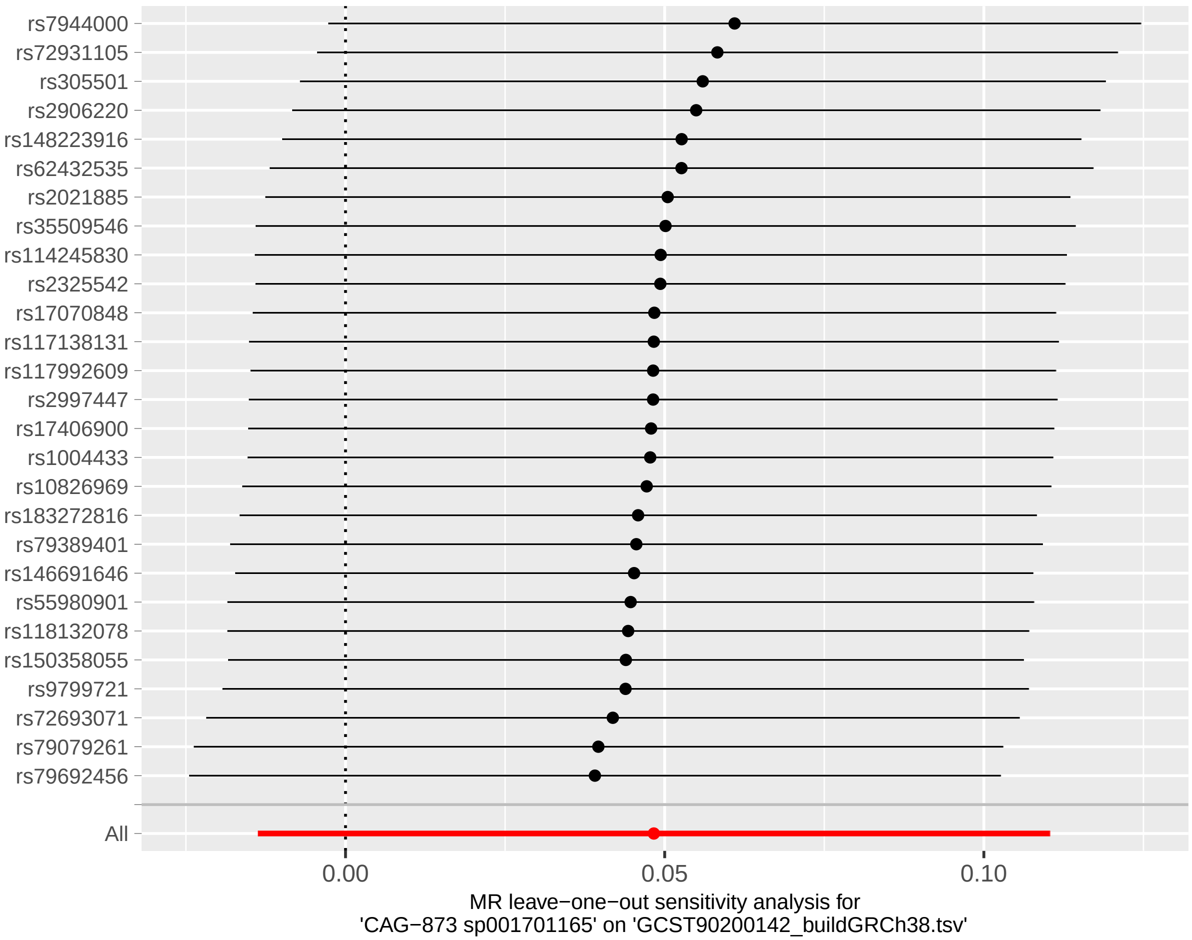

rs7944000
rs72931105
rs305501
rs2906220
rs148223916
rs62432535
rs2021885
rs35509546
rs114245830
rs2325542
rs17070848
rs117138131
rs117992609
rs2997447
rs17406900
rs1004433
rs10826969
rs183272816
rs79389401
rs146691646
rs55980901
rs118132078
rs150358055
rs9799721
rs72693071
rs79079261
rs79692456
All
0.00
0.05
0.10
MR leave−one−out sensitivity analysis for
'CAG−873 sp001701165' on 'GCST90200142_buildGRCh38.tsv'

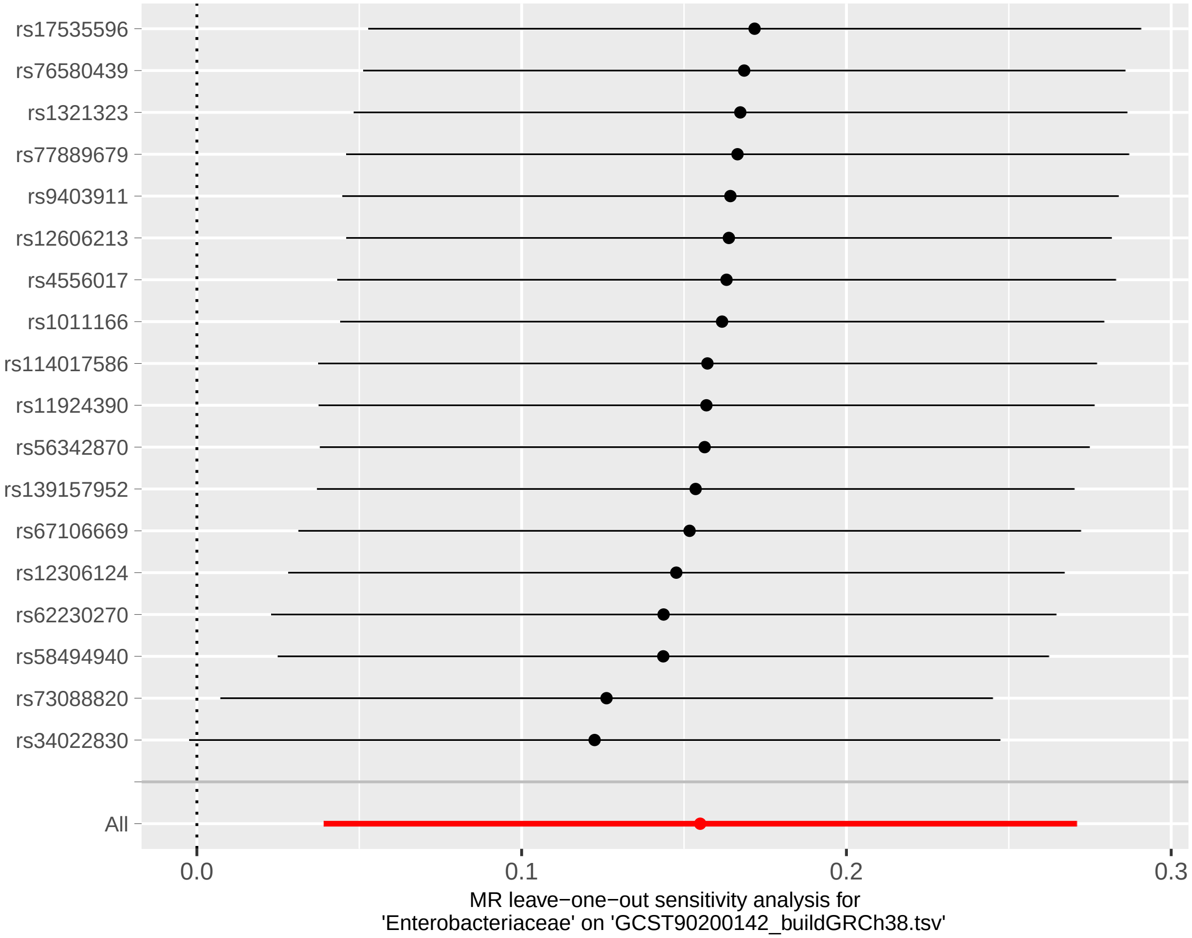
rs17535596
rs76580439
rs1321323
rs77889679
rs9403911
rs12606213
rs4556017
rs1011166
rs114017586
rs11924390
rs56342870
rs139157952
rs67106669
rs12306124
rs62230270
rs58494940
rs73088820
rs34022830
All
0.0
0.1
0.2
0.3
MR leave−one−out sensitivity analysis for
'Enterobacteriaceae' on 'GCST90200142_buildGRCh38.tsv'

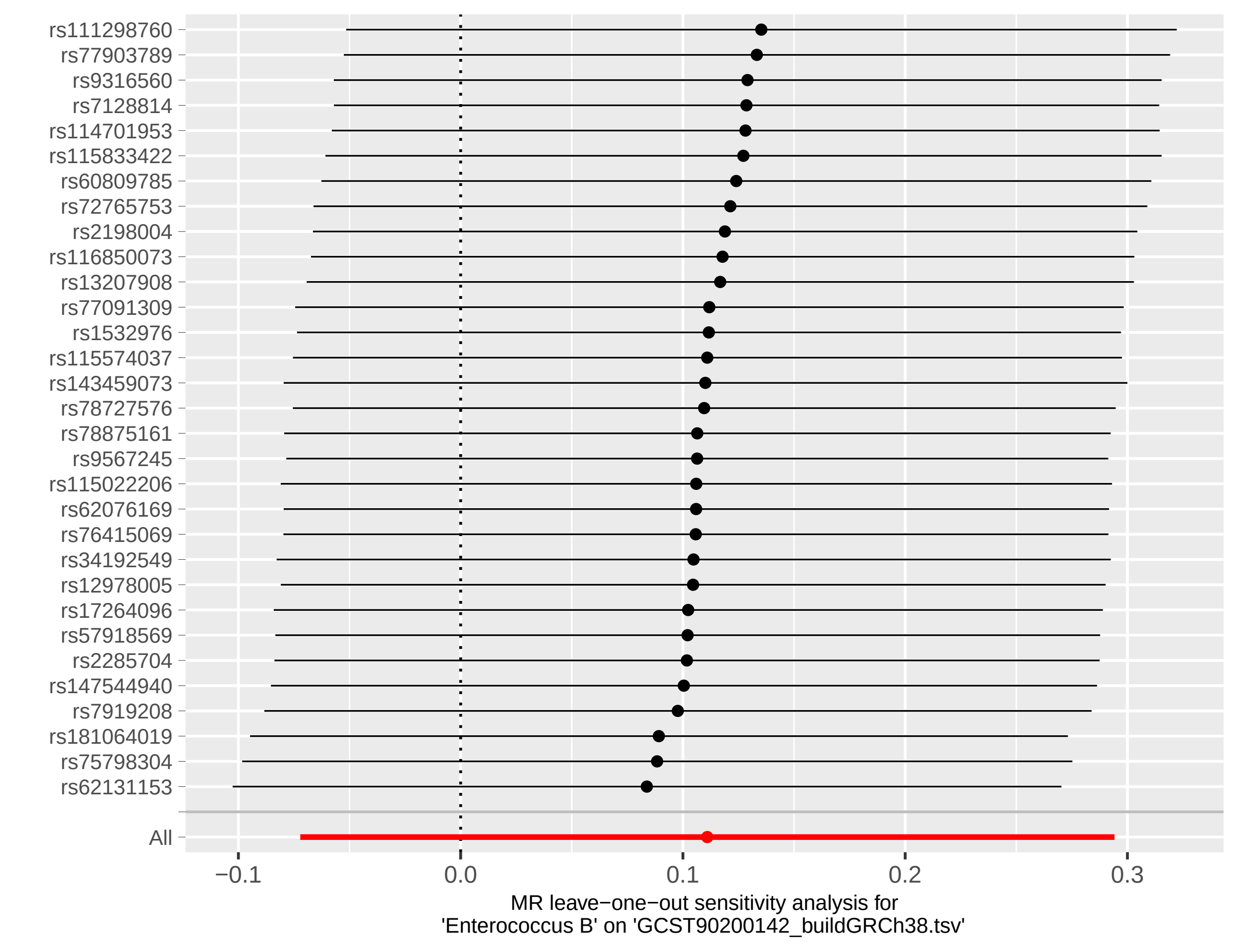

rs111298760
rs77903789
rs9316560
rs7128814
rs114701953
rs115833422
rs60809785
rs72765753
rs2198004
rs116850073
rs13207908
rs77091309
rs1532976
rs115574037
rs143459073
rs78727576
rs78875161
rs9567245
rs115022206
rs62076169
rs76415069
rs34192549
rs12978005
rs17264096
rs57918569
rs2285704
rs147544940
rs7919208
rs181064019
rs75798304
rs62131153
All
−0.1
0.0
0.1
0.2
0.3
MR leave−one−out sensitivity analysis for
'Enterococcus B' on 'GCST90200142_buildGRCh38.tsv'

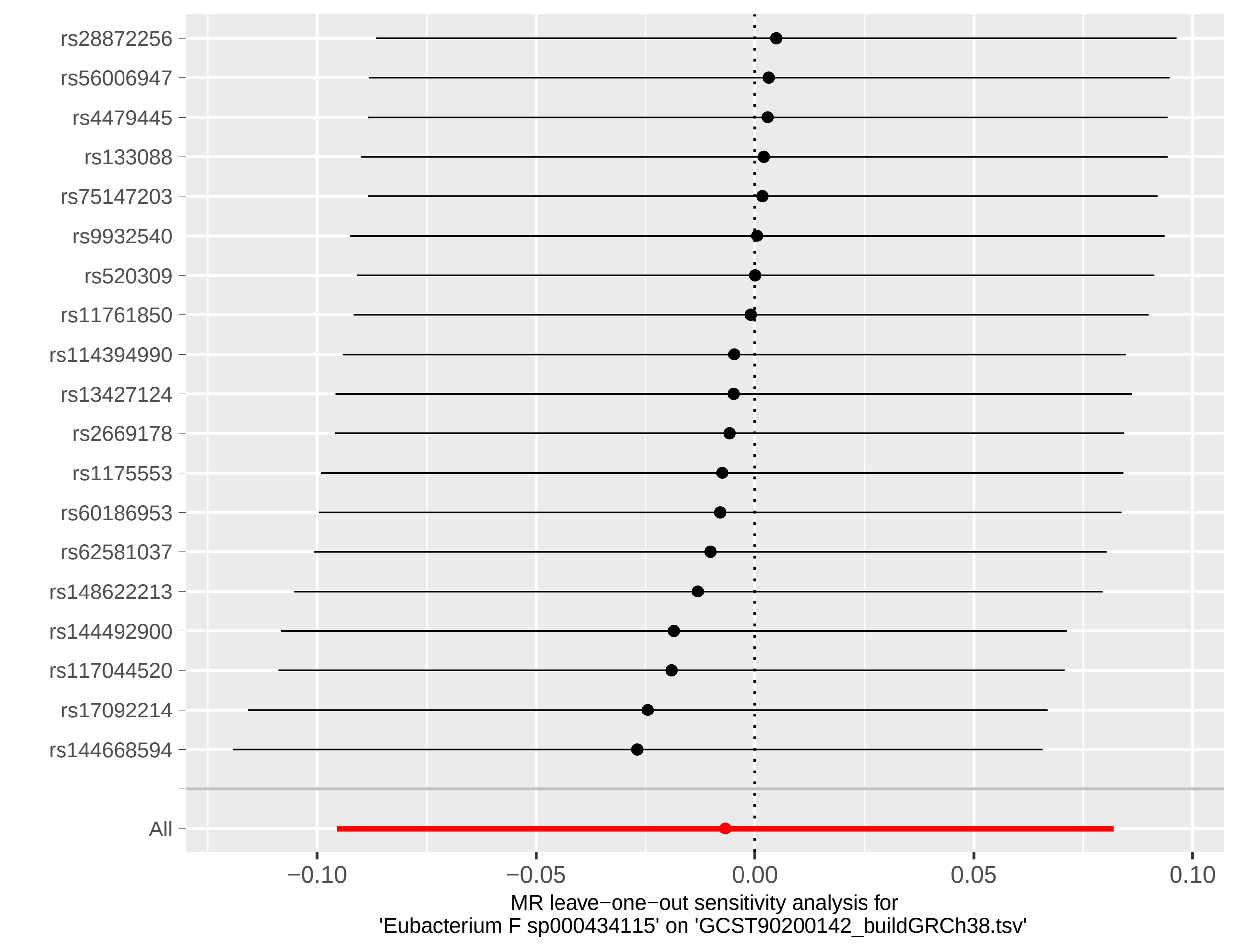
rs28872256
rs56006947
rs4479445
rs133088
rs75147203
rs9932540
rs520309
rs11761850
rs114394990
rs13427124
rs2669178
rs1175553
rs60186953
rs62581037
rs148622213
rs144492900
rs117044520
rs17092214
rs144668594
All
−0.10
−0.05
0.00
0.05
0.10
MR leave−one−out sensitivity analysis for
'Eubacterium F sp000434115' on 'GCST90200142_buildGRCh38.tsv'

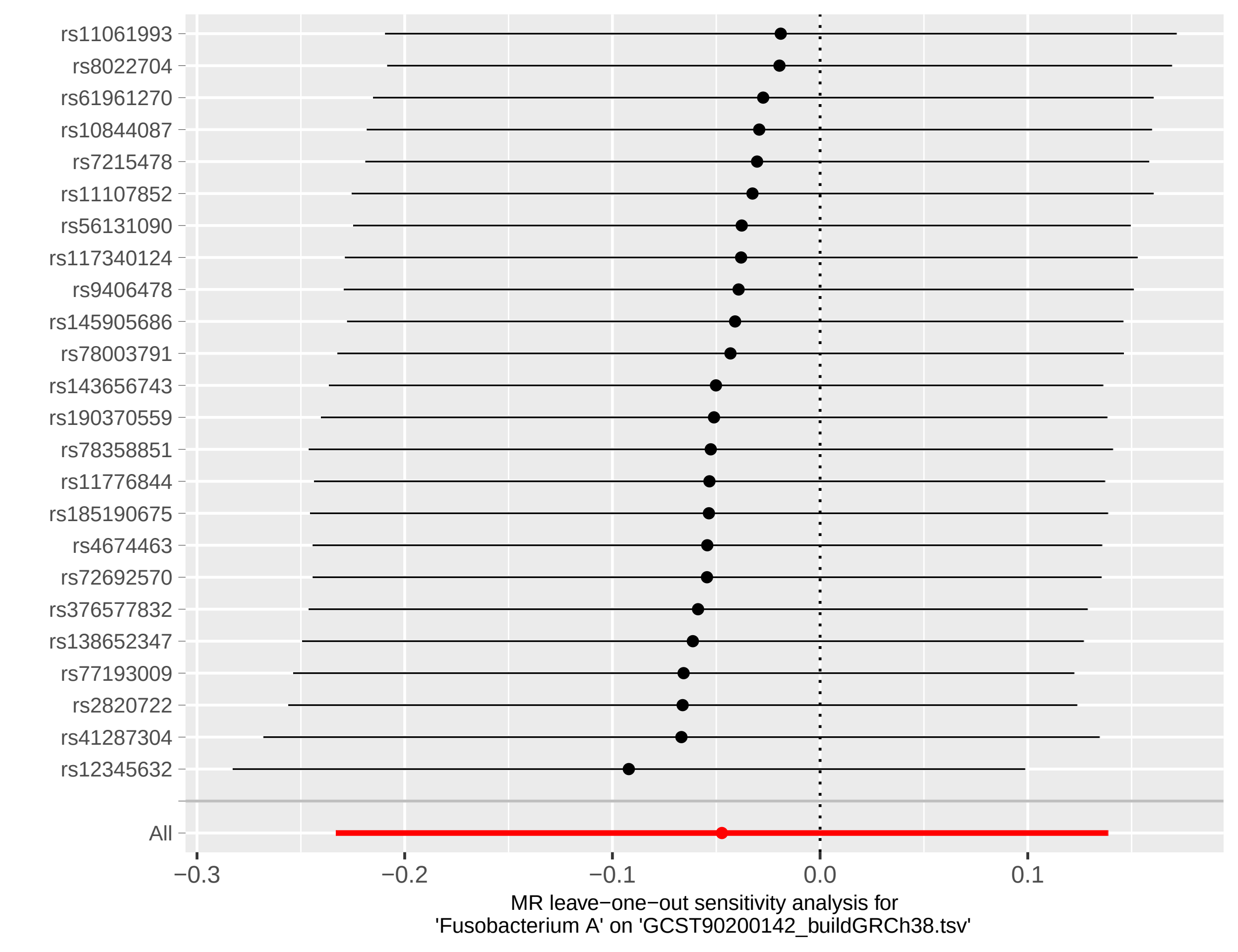
rs11061993
rs8022704
rs61961270
rs10844087
rs7215478
rs11107852
rs56131090
rs117340124
rs9406478
rs145905686
rs78003791
rs143656743
rs190370559
rs78358851
rs11776844
rs185190675
rs4674463
rs72692570
rs376577832
rs138652347
rs77193009
rs2820722
rs41287304
rs12345632
All
−0.3
−0.2
−0.1
0.0
0.1
MR leave−one−out sensitivity analysis for
'Fusobacterium A' on 'GCST90200142_buildGRCh38.tsv'

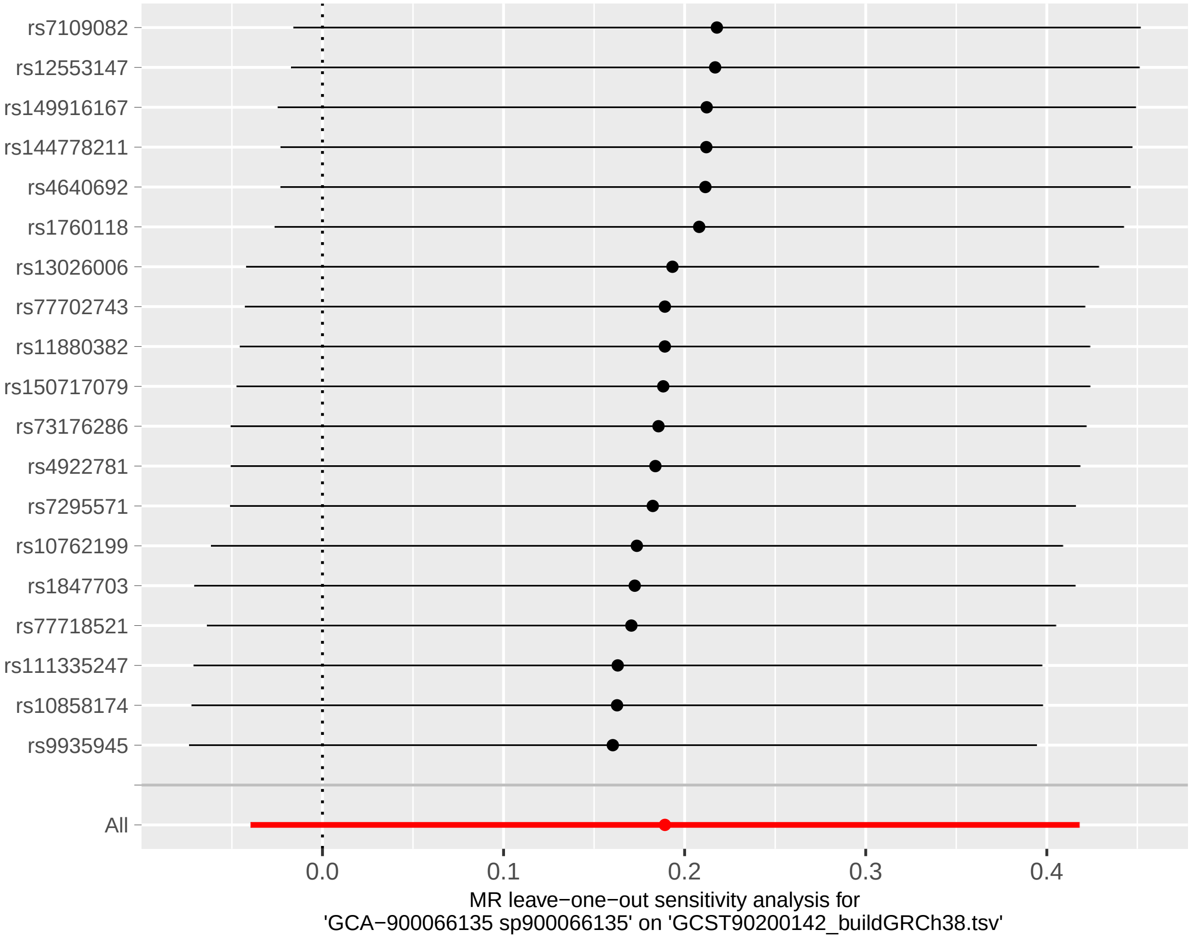

rs7109082
rs12553147
rs149916167
rs144778211
rs4640692
rs1760118
rs13026006
rs77702743
rs11880382
rs150717079
rs73176286
rs4922781
rs7295571
rs10762199
rs1847703
rs77718521
rs111335247
rs10858174
rs9935945
All
0.0
0.1
0.2
0.3
0.4
MR leave−one−out sensitivity analysis for
'GCA−900066135 sp900066135' on 'GCST90200142_buildGRCh38.tsv'

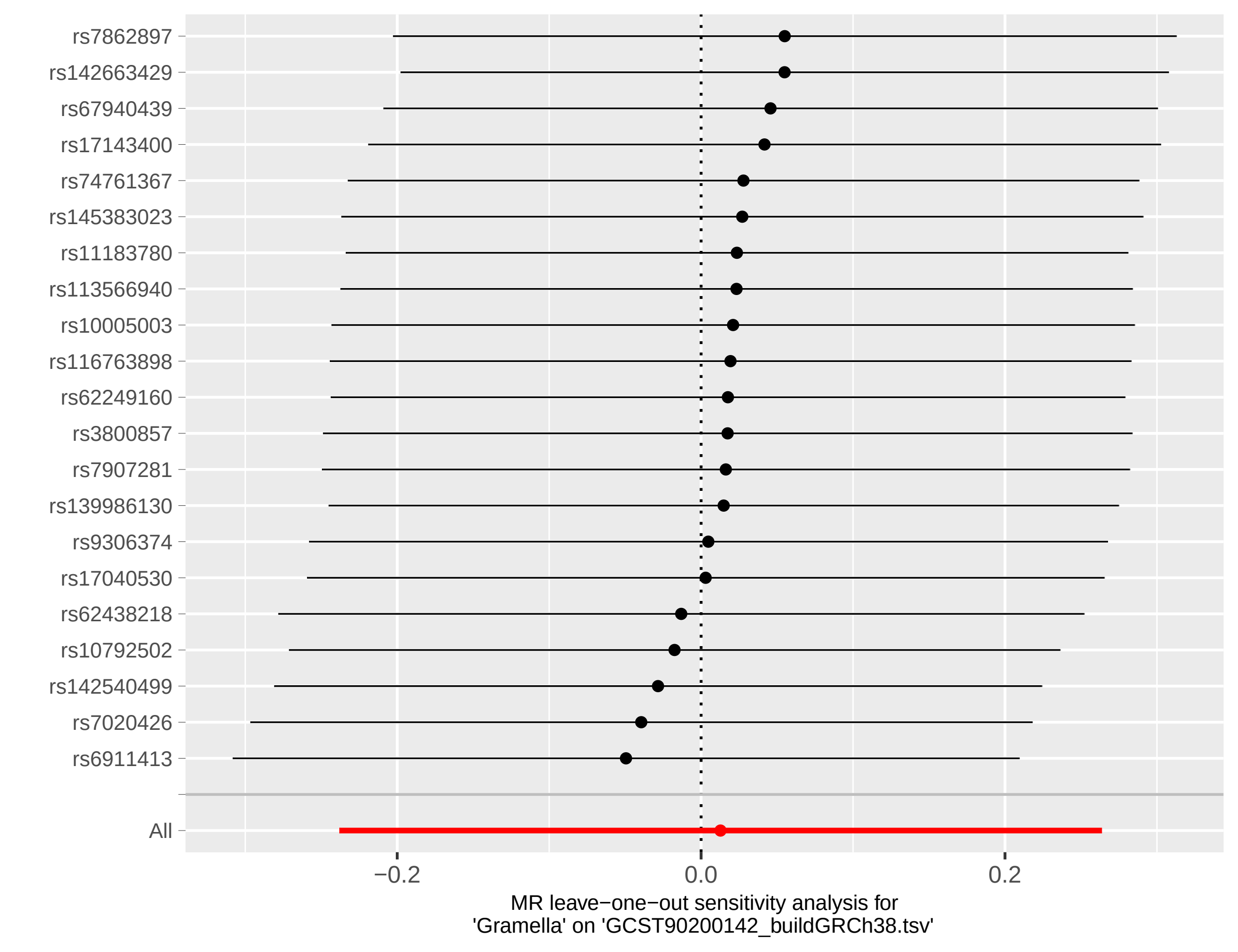
rs7862897
rs142663429
rs67940439
rs17143400
rs74761367
rs145383023
rs11183780
rs113566940
rs10005003
rs116763898
rs62249160
rs3800857
rs7907281
rs139986130
rs9306374
rs17040530
rs62438218
rs10792502
rs142540499
rs7020426
rs6911413
All
−0.2
0.0
0.2
MR leave−one−out sensitivity analysis for
'Gramella' on 'GCST90200142_buildGRCh38.tsv'

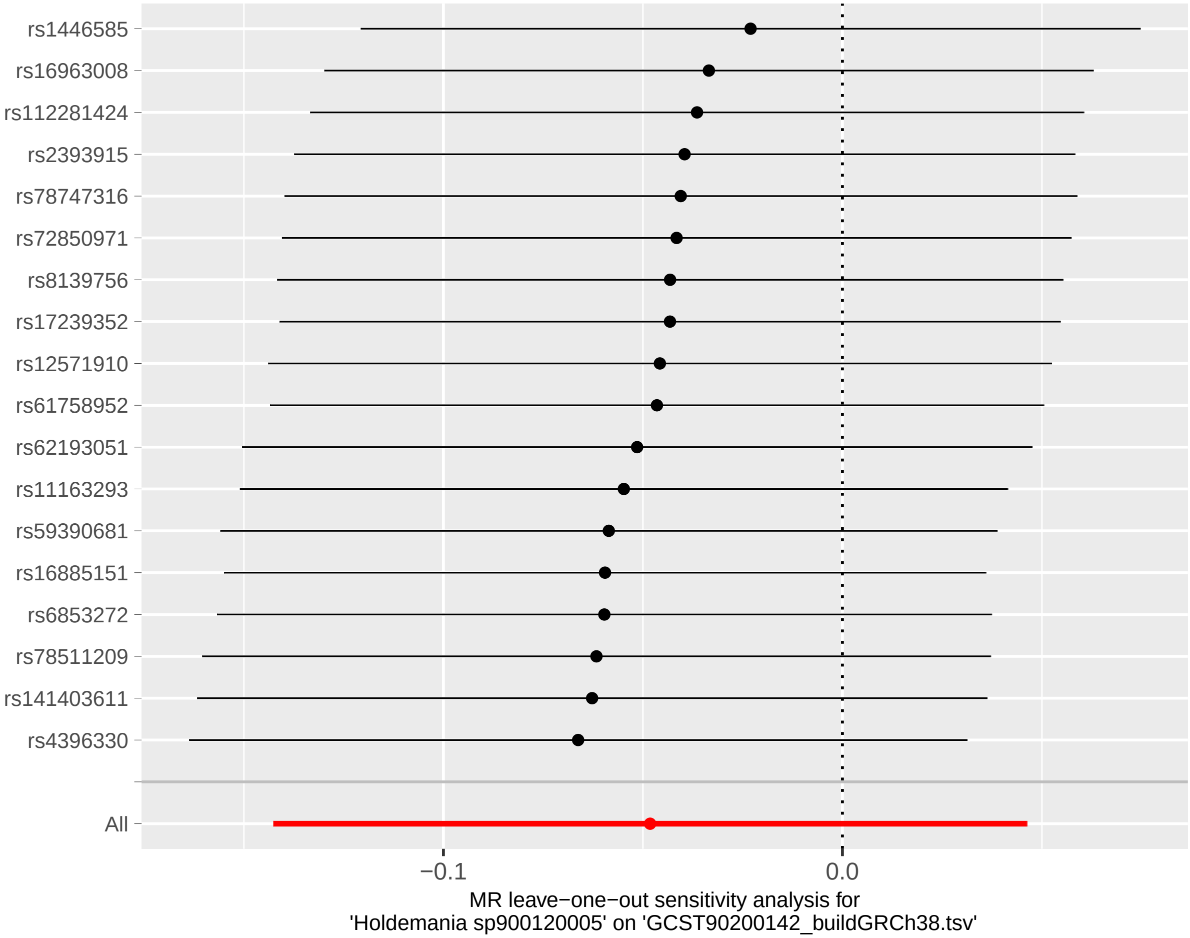

rs1446585
rs16963008
rs112281424
rs2393915
rs78747316
rs72850971
rs8139756
rs17239352
rs12571910
rs61758952
rs62193051
rs11163293
rs59390681
rs16885151
rs6853272
rs78511209
rs141403611
rs4396330
All
−0.1
0.0
MR leave−one−out sensitivity analysis for
'Holdemania sp900120005' on 'GCST90200142_buildGRCh38.tsv'

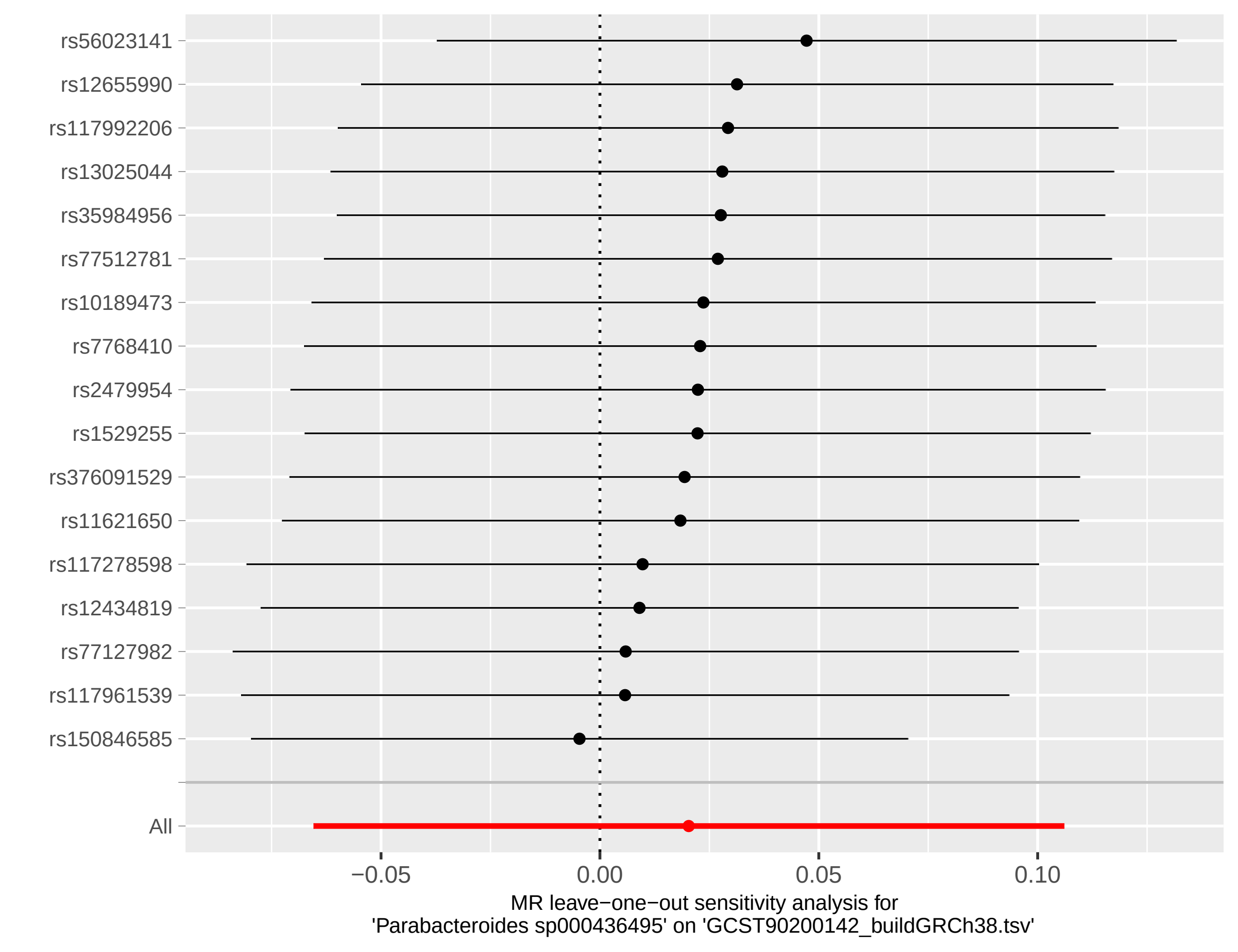
rs56023141
rs12655990
rs117992206
rs13025044
rs35984956
rs77512781
rs10189473
rs7768410
rs2479954
rs1529255
rs376091529
rs11621650
rs117278598
rs12434819
rs77127982
rs117961539
rs150846585
All
−0.05
0.00
0.05
0.10
MR leave−one−out sensitivity analysis for
'Parabacteroides sp000436495' on 'GCST90200142_buildGRCh38.tsv'

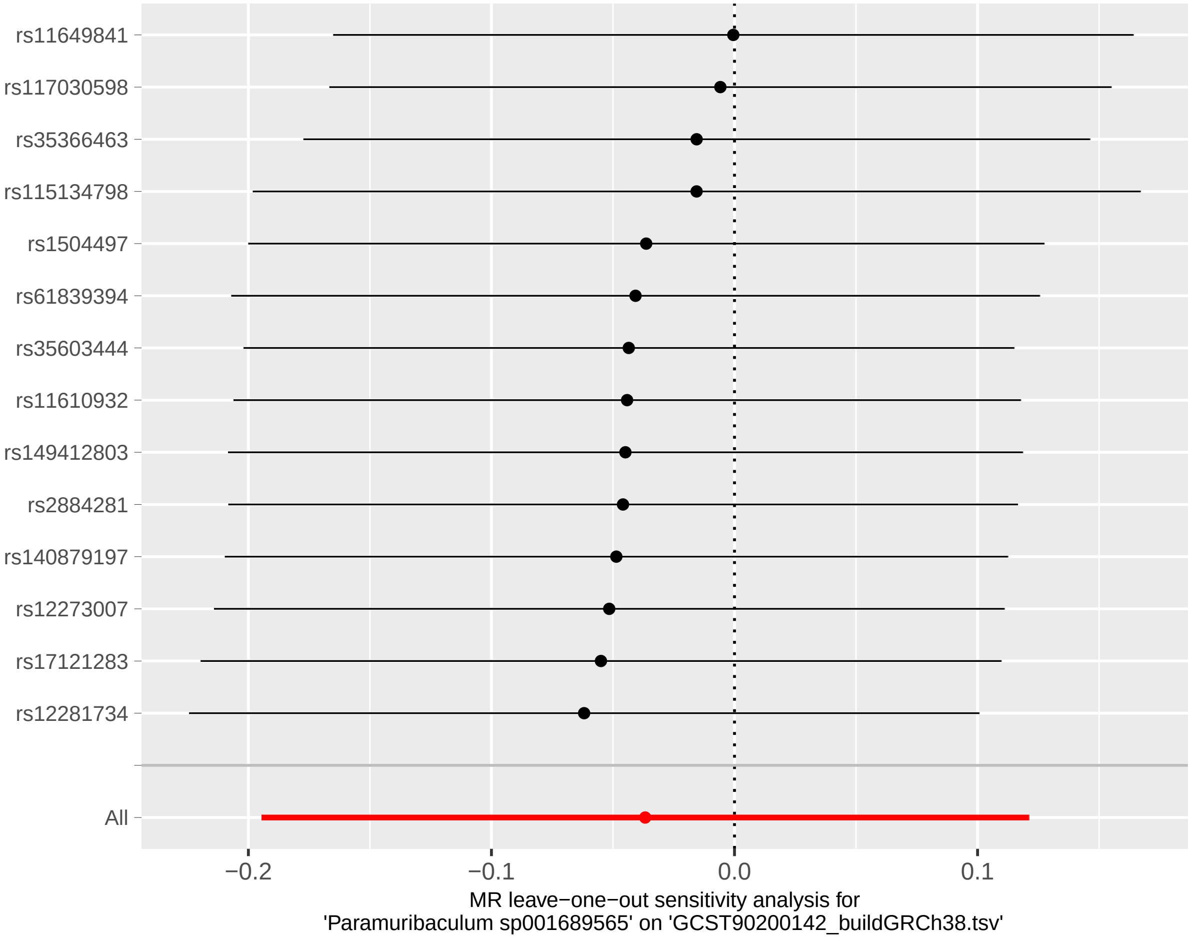
rs11649841
rs117030598
rs35366463
rs115134798
rs1504497
rs61839394
rs35603444
rs11610932
rs149412803
rs2884281
rs140879197
rs12273007
rs17121283
rs12281734
All
−0.2
−0.1
0.0
0.1
MR leave−one−out sensitivity analysis for
'Paramuribaculum sp001689565' on 'GCST90200142_buildGRCh38.tsv'

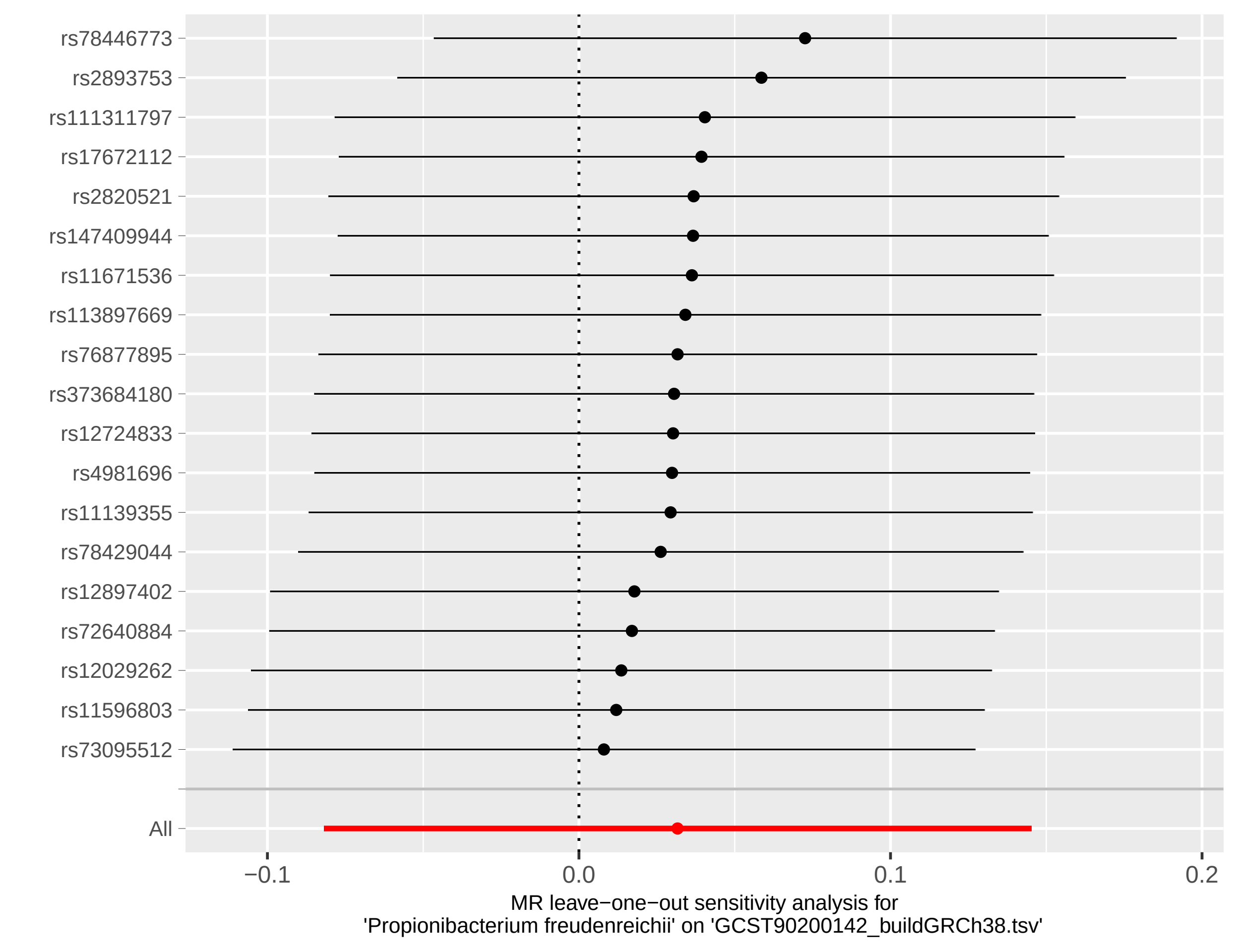
rs78446773
rs2893753
rs111311797
rs17672112
rs2820521
rs147409944
rs11671536
rs113897669
rs76877895
rs373684180
rs12724833
rs4981696
rs11139355
rs78429044
rs12897402
rs72640884
rs12029262
rs11596803
rs73095512
All
−0.1
0.0
0.1
0.2
MR leave−one−out sensitivity analysis for
'Propionibacterium freudenreichii' on 'GCST90200142_buildGRCh38.tsv'

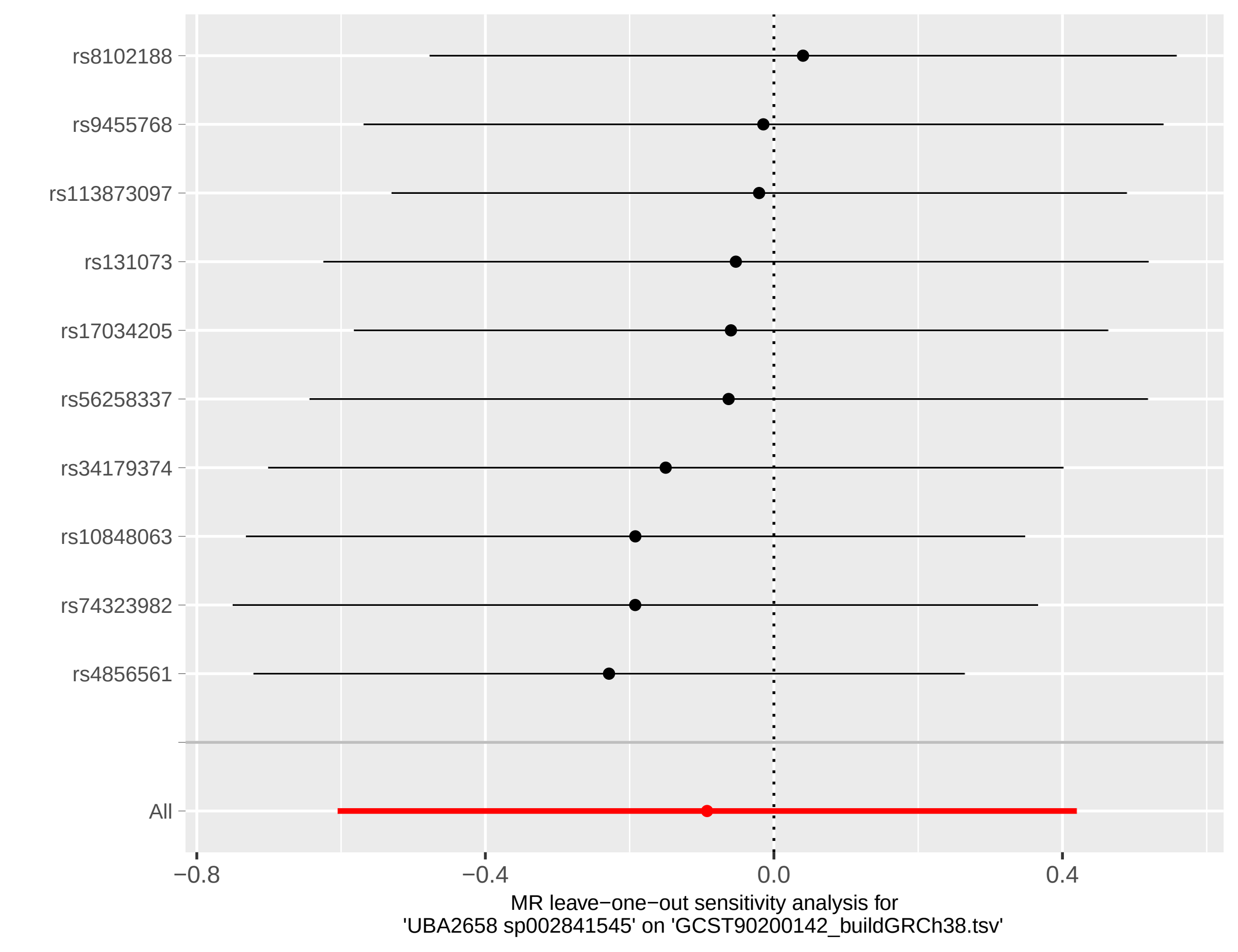

rs8102188
rs9455768
rs113873097
rs131073
rs17034205
rs56258337
rs34179374
rs10848063
rs74323982
rs4856561
All
−0.8
−0.4
0.0
0.4
MR leave−one−out sensitivity analysis for
'UBA2658 sp002841545' on 'GCST90200142_buildGRCh38.tsv'

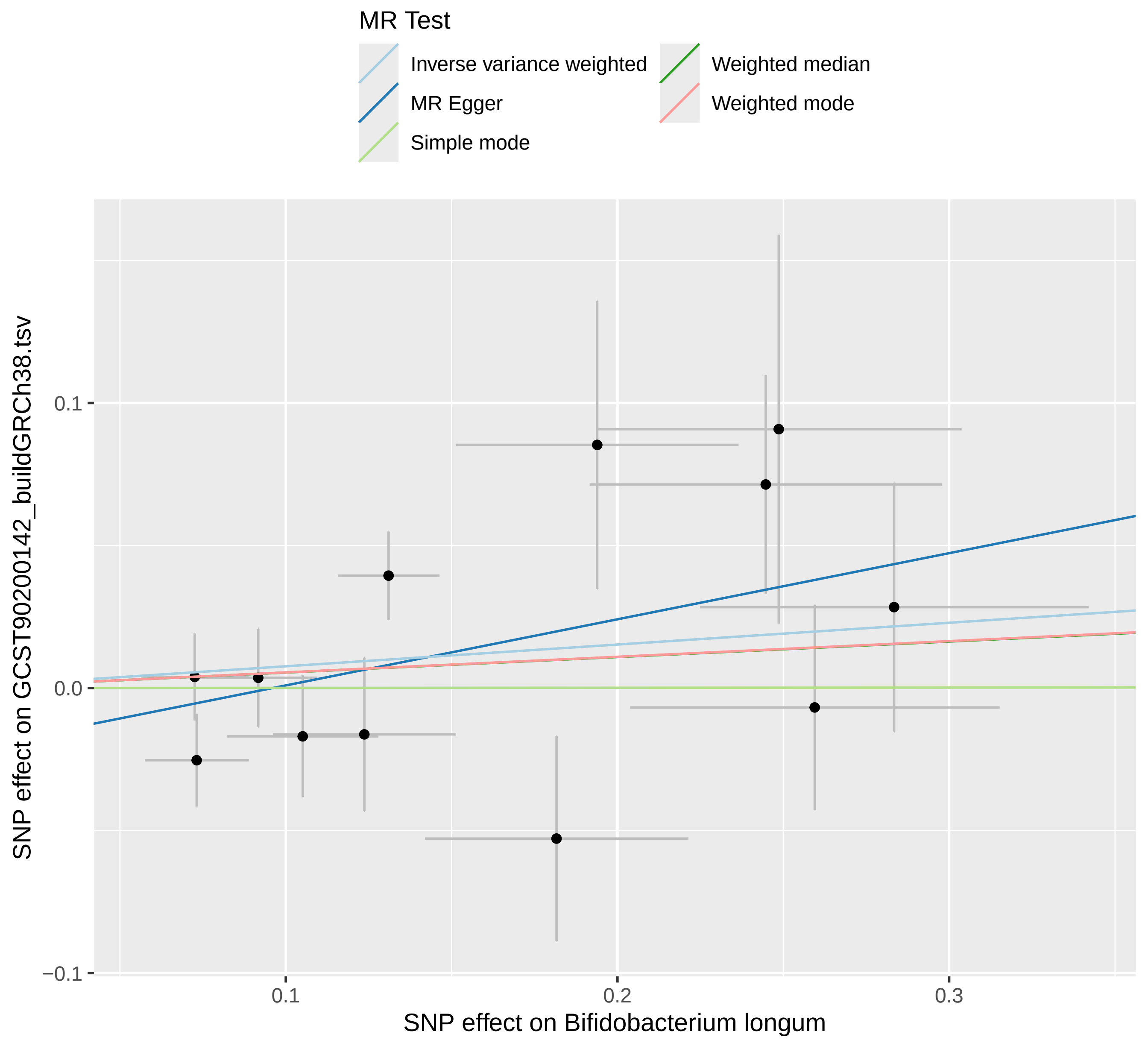

MR Test
Inverse variance weighted
MR Egger
Simple mode
Weighted median
Weighted mode
SNP effect on GCST90200142_buildGRCh38.tsv
0.1
0.0
−0.1
0.1
0.2
0.3
SNP effect on Bifidobacterium longum

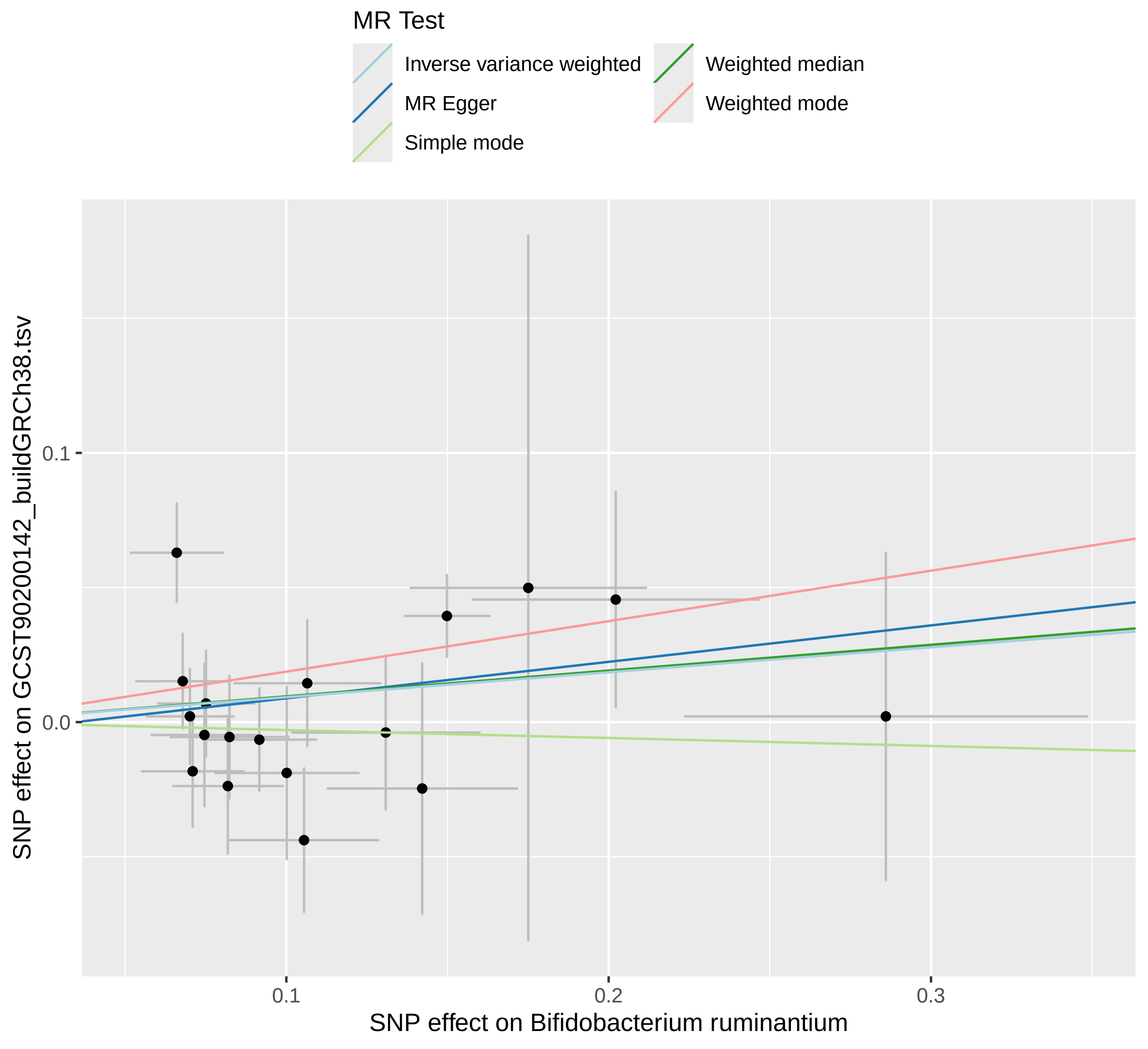

MR Test
Inverse variance weighted
MR Egger
Simple mode
Weighted median
Weighted mode
0.1
0.0
0.1
0.2
0.3
SNP effect on GCST90200142_buildGRCh38.tsv
SNP effect on Bifidobacterium ruminantium

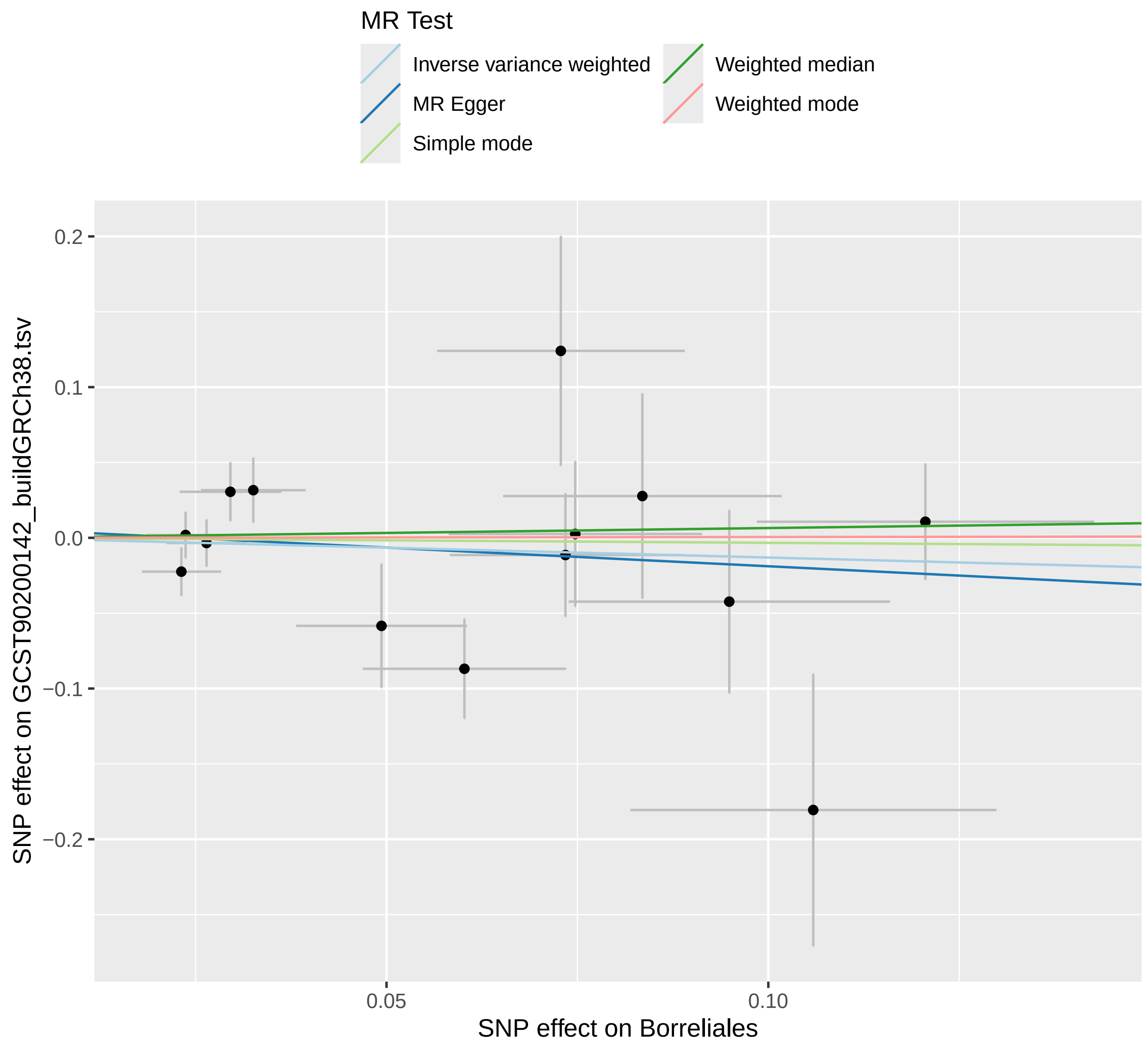

MR Test
Inverse variance weighted
MR Egger
Simple mode
Weighted median
Weighted mode
0.2
0.1
0.0
−0.1
−0.2
0.05
0.10
SNP effect on GCST90200142_buildGRCh38.tsv
SNP effect on Borreliales

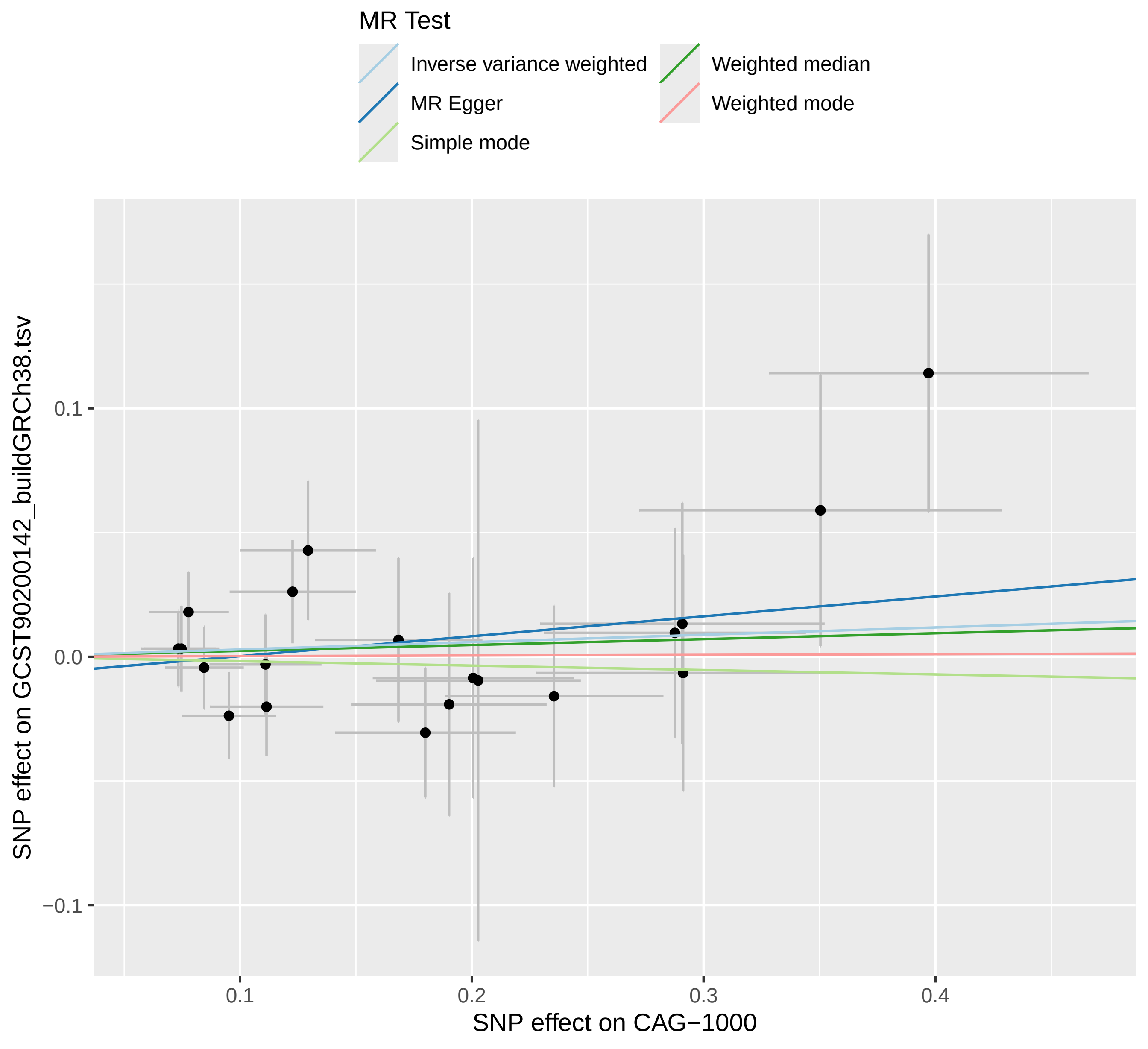
MR Test
Inverse variance weighted
MR Egger
Simple mode
Weighted median
Weighted mode
SNP effect on GCST90200142_buildGRCh38.tsv
0.1
0.0
−0.1
0.1
0.2
0.3
0.4
SNP effect on CAG−1000

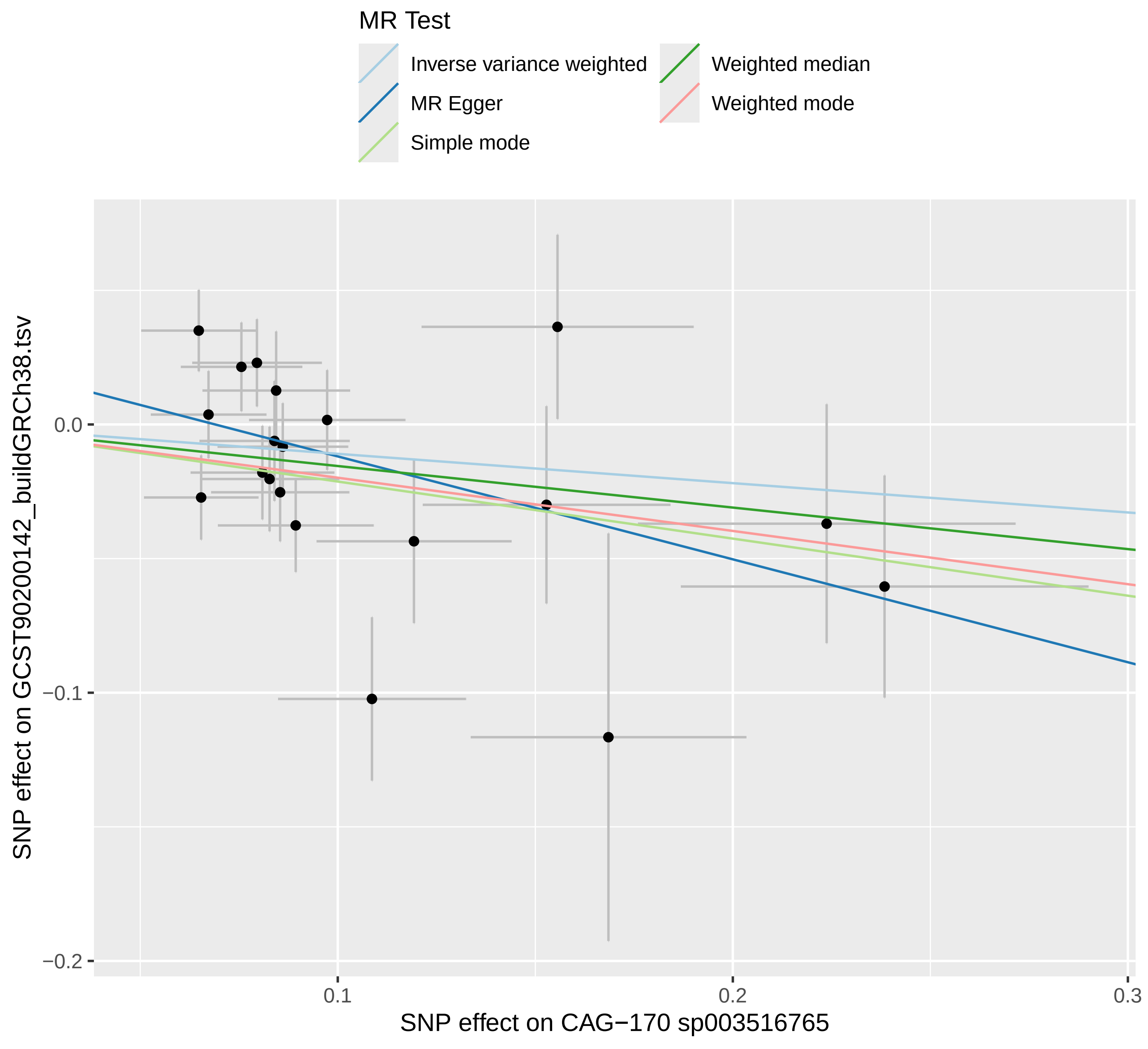

MR Test
Inverse variance weighted
MR Egger
Simple mode
Weighted median
Weighted mode
0.0
−0.1
−0.2
0.1
0.2
0.3
SNP effect on GCST90200142_buildGRCh38.tsv
SNP effect on CAG−170 sp003516765

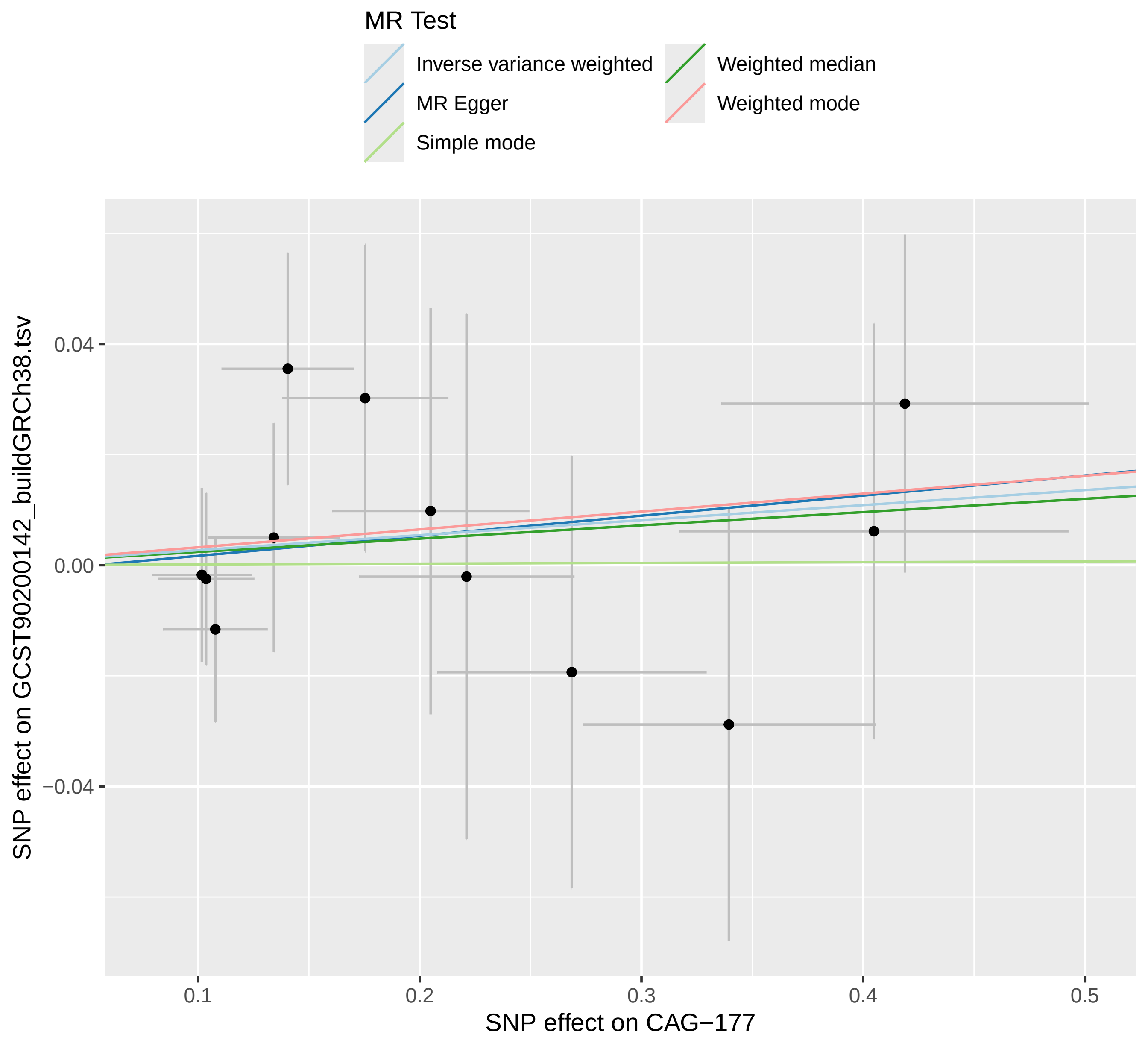

MR Test
Inverse variance weighted
MR Egger
Simple mode
Weighted median
Weighted mode
0.04
0.00
−0.04
0.1
0.2
0.3
0.4
0.5
SNP effect on GCST90200142_buildGRCh38.tsv
SNP effect on CAG−177

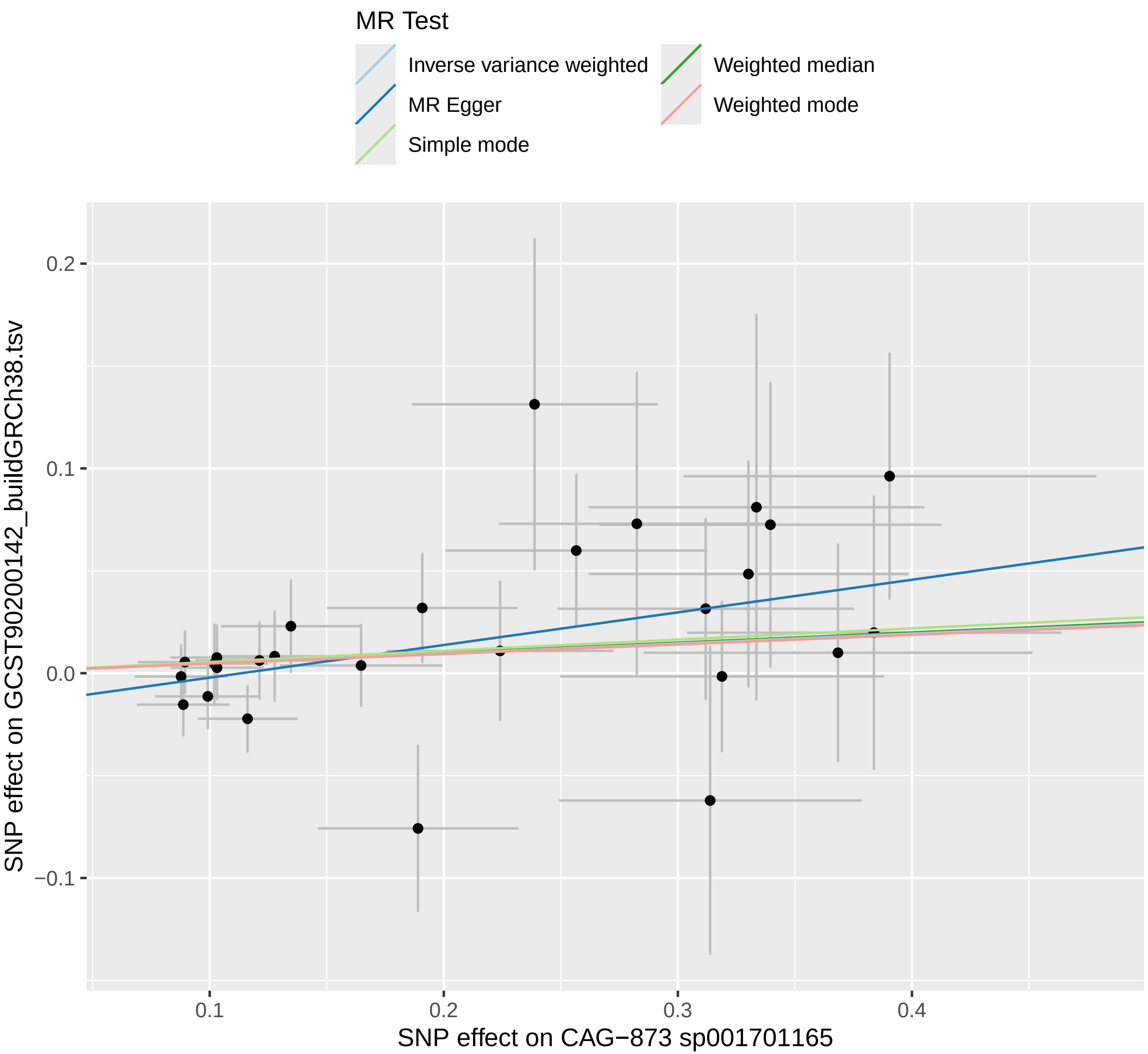
MR Test
Inverse variance weighted
MR Egger
Simple mode
Weighted median
Weighted mode
0.2
0.1
0.0
−0.1
0.1
0.2
0.3
0.4
SNP effect on GCST90200142_buildGRCh38.tsv
SNP effect on CAG−873 sp001701165

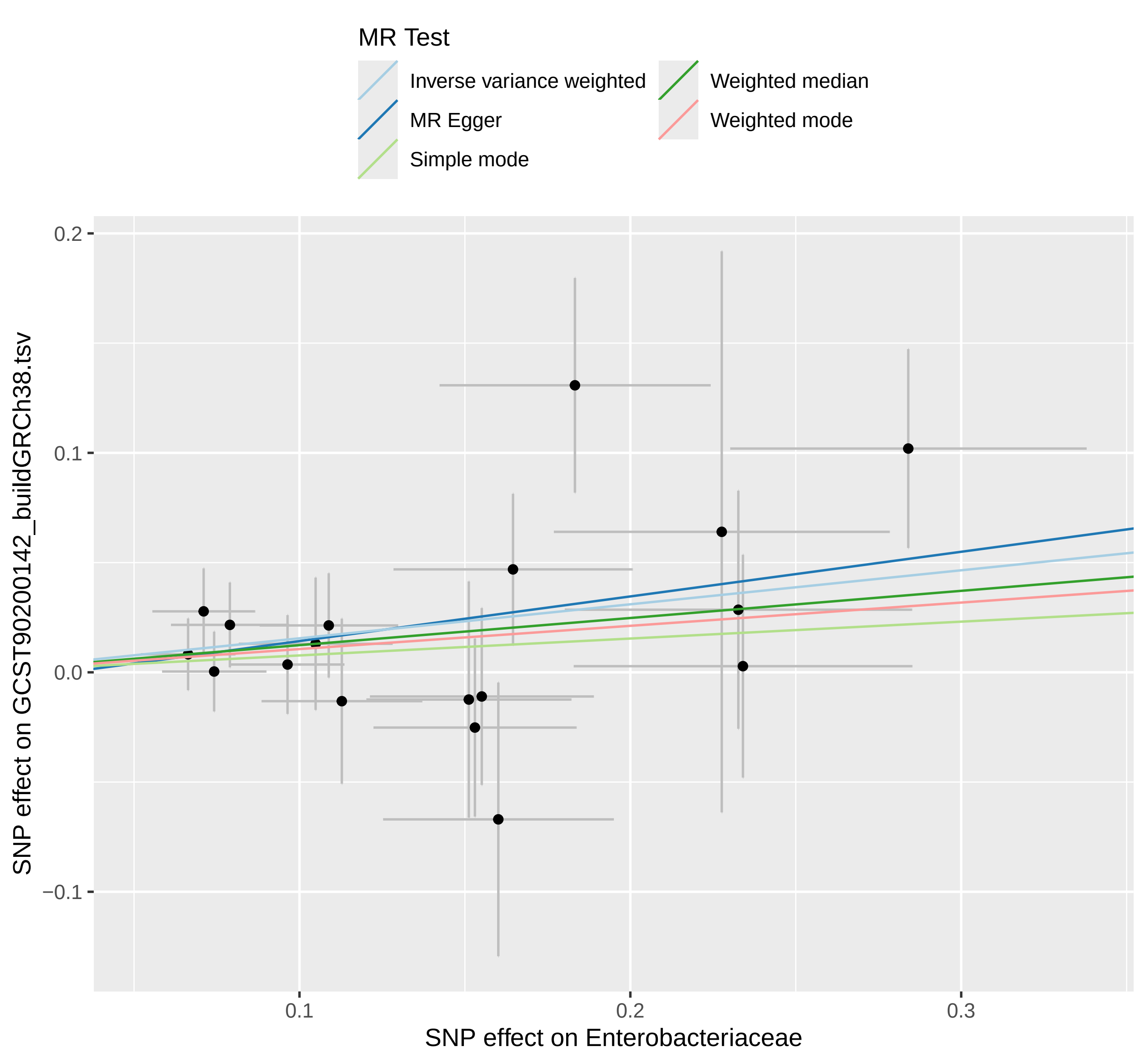
MR Test
Inverse variance weighted
MR Egger
Simple mode
Weighted median
Weighted mode
SNP effect on GCST90200142_buildGRCh38.tsv
0.2
0.1
0.0
−0.1
0.1
0.2
0.3
SNP effect on Enterobacteriaceae

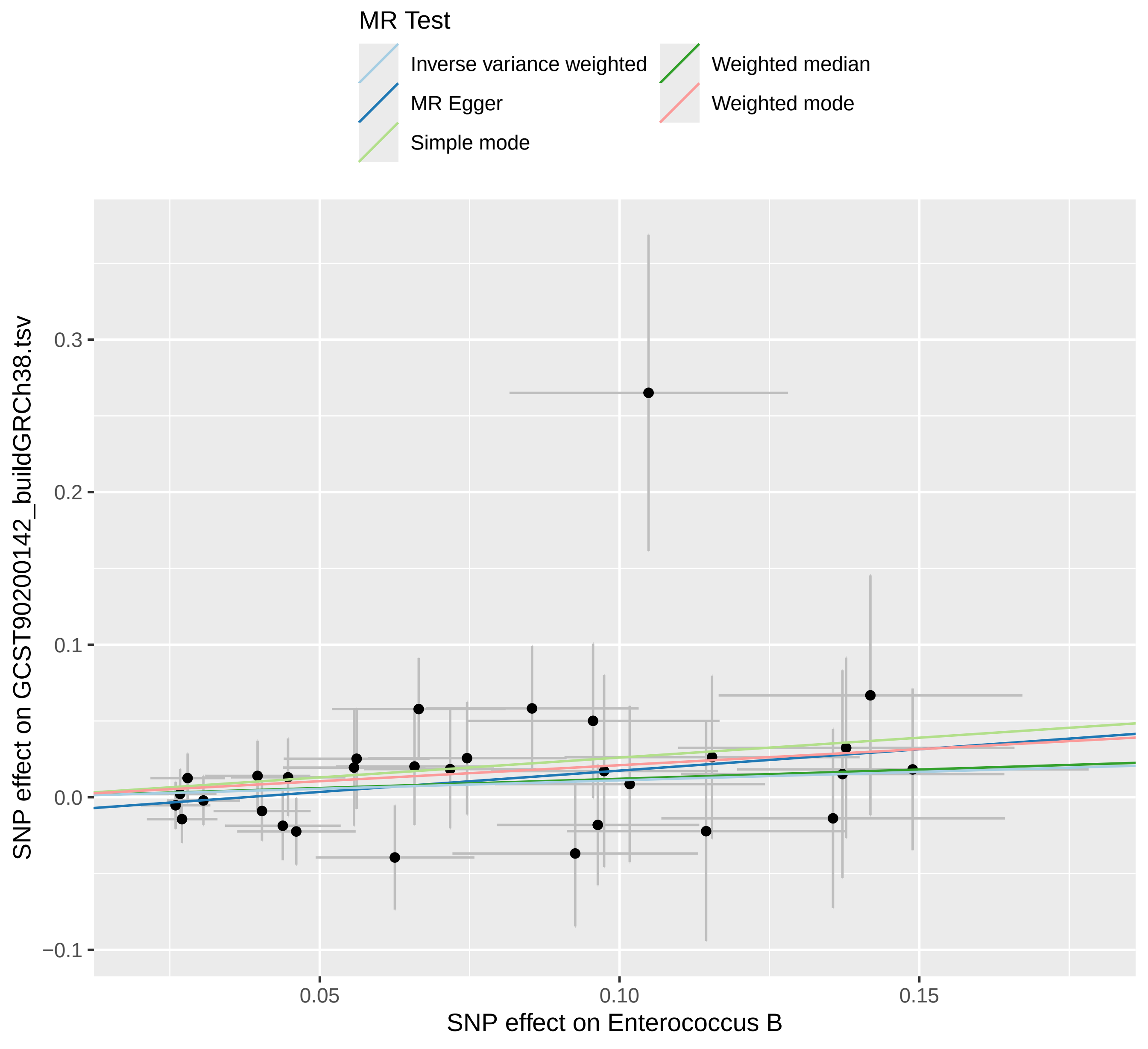

MR Test
Inverse variance weighted
MR Egger
Simple mode
Weighted median
Weighted mode
0.3
0.2
0.1
0.0
−0.1
0.05
0.10
0.15
SNP effect on GCST90200142_buildGRCh38.tsv
SNP effect on Enterococcus B

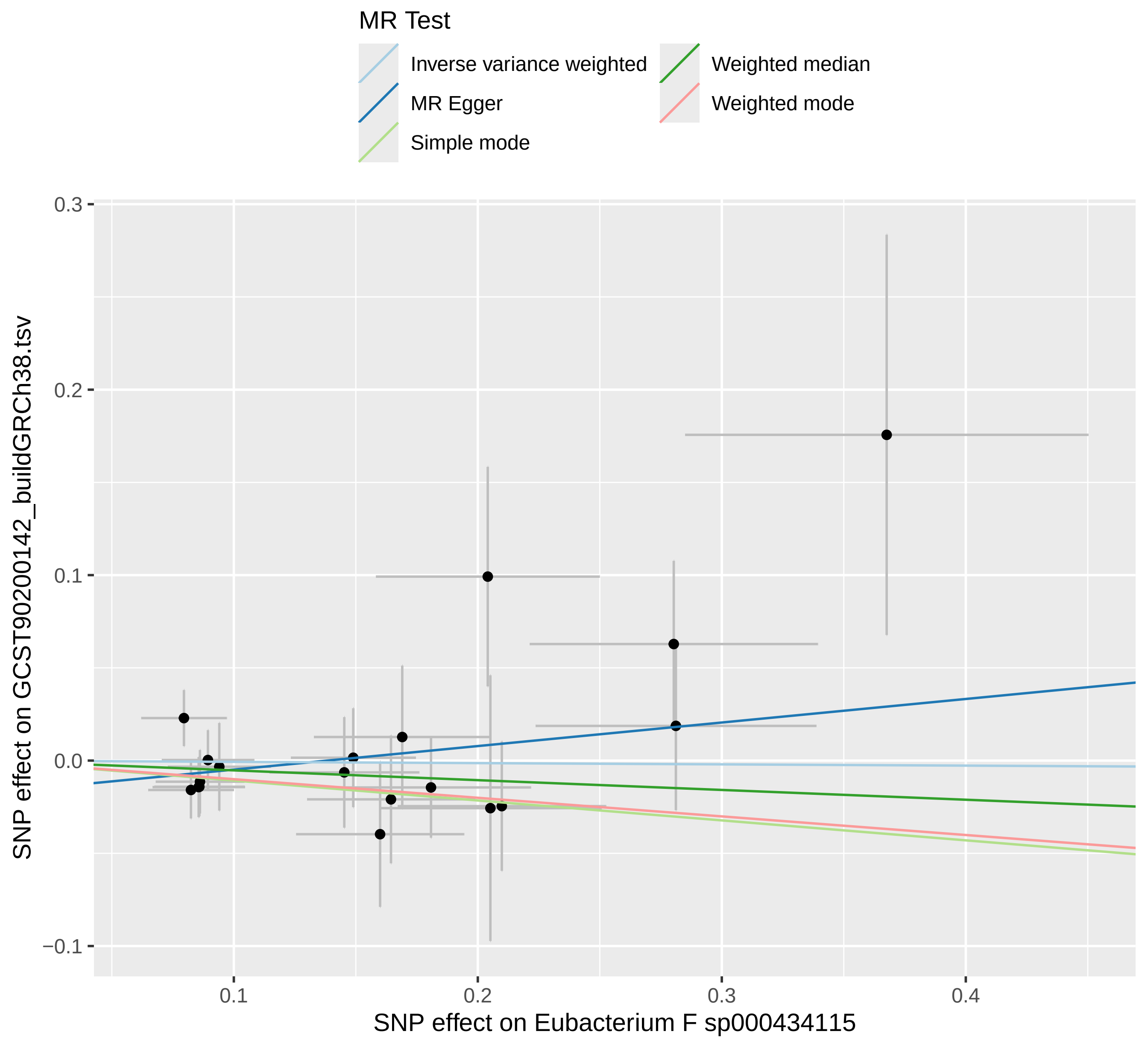
MR Test
Inverse variance weighted
MR Egger
Simple mode
Weighted median
Weighted mode
0.3
0.2
0.1
0.0
−0.1
0.1
0.2
0.3
0.4
SNP effect on GCST90200142_buildGRCh38.tsv
SNP effect on Eubacterium F sp000434115

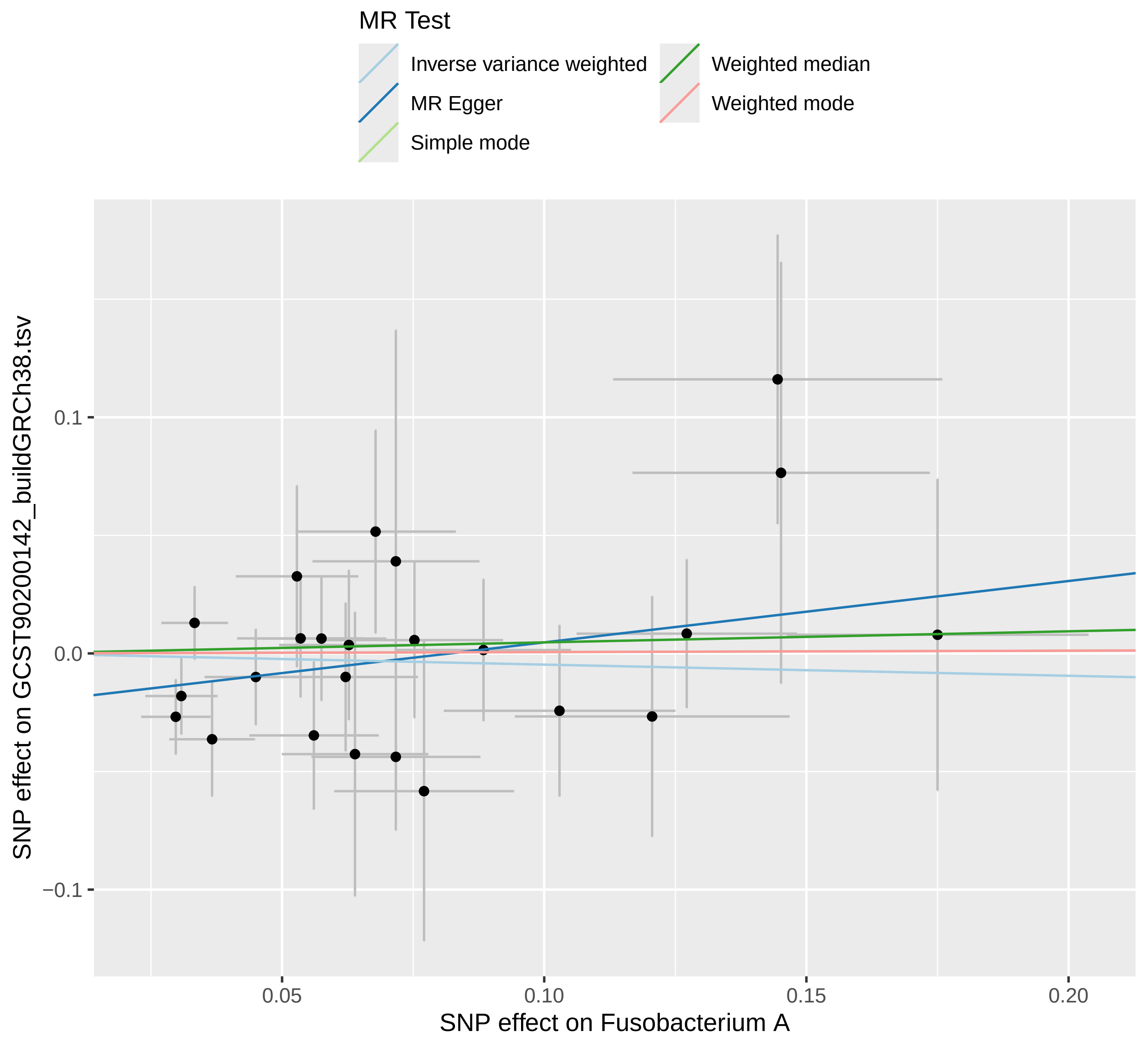
MR Test
Inverse variance weighted
MR Egger
Simple mode
Weighted median
Weighted mode
SNP effect on GCST90200142_buildGRCh38.tsv
0.1
0.0
−0.1
0.05
0.10
0.15
0.20
SNP effect on Fusobacterium A

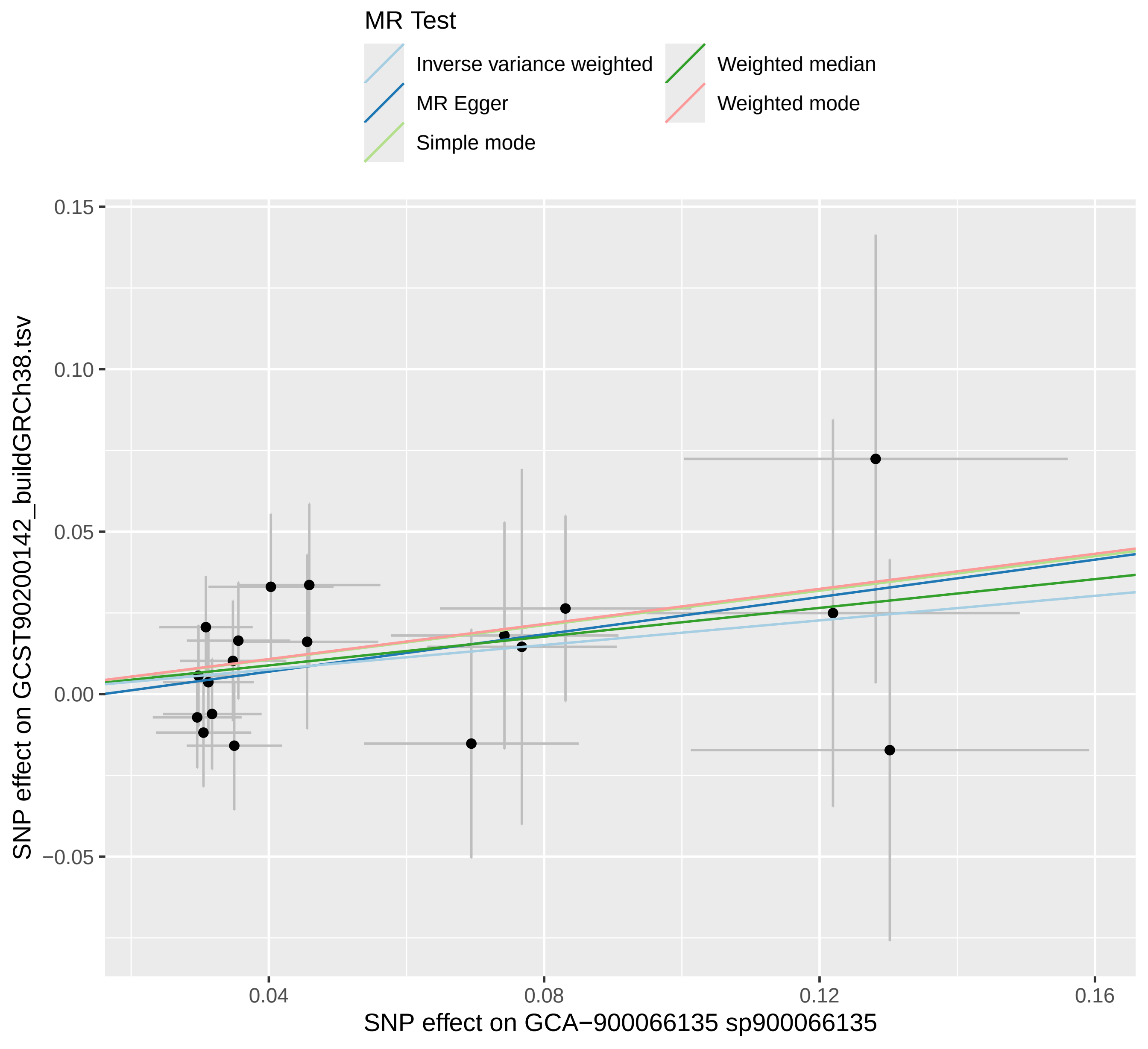
MR Test
Inverse variance weighted
MR Egger
Simple mode
Weighted median
Weighted mode
0.15
0.10
0.05
0.00
−0.05
0.04
0.08
0.12
0.16
SNP effect on GCST90200142_buildGRCh38.tsv
SNP effect on GCA−900066135 sp900066135

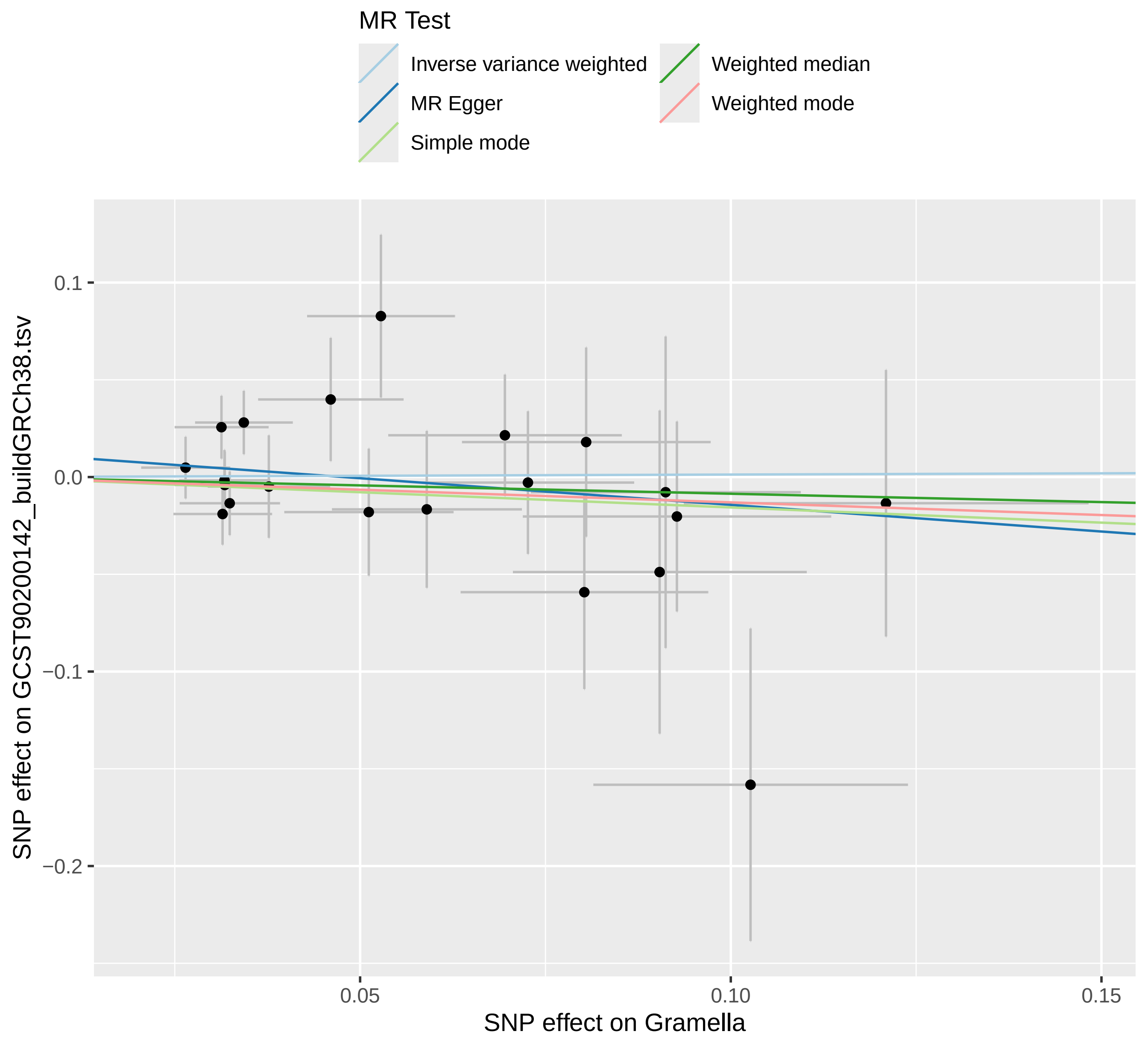
MR Test
Inverse variance weighted
MR Egger
Simple mode
Weighted median
Weighted mode
0.1
0.0
−0.1
−0.2
SNP effect on GCST90200142_buildGRCh38.tsv
0.05
0.10
0.15
SNP effect on Gramella

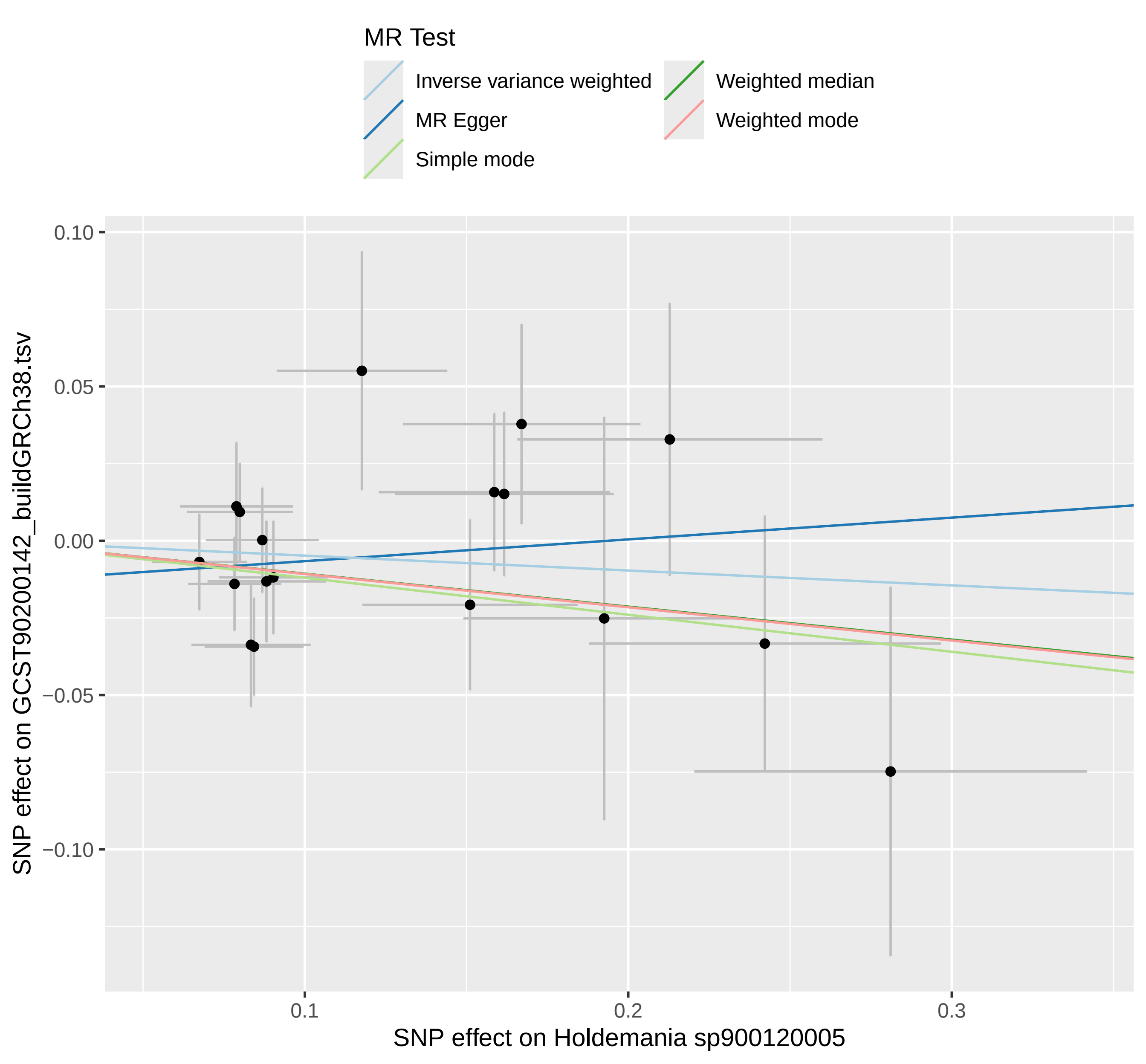

MR Test
Inverse variance weighted
MR Egger
Simple mode
Weighted median
Weighted mode
0.10
0.05
0.00
−0.05
−0.10
0.1
0.2
0.3
SNP effect on GCST90200142_buildGRCh38.tsv
SNP effect on Holdemania sp900120005

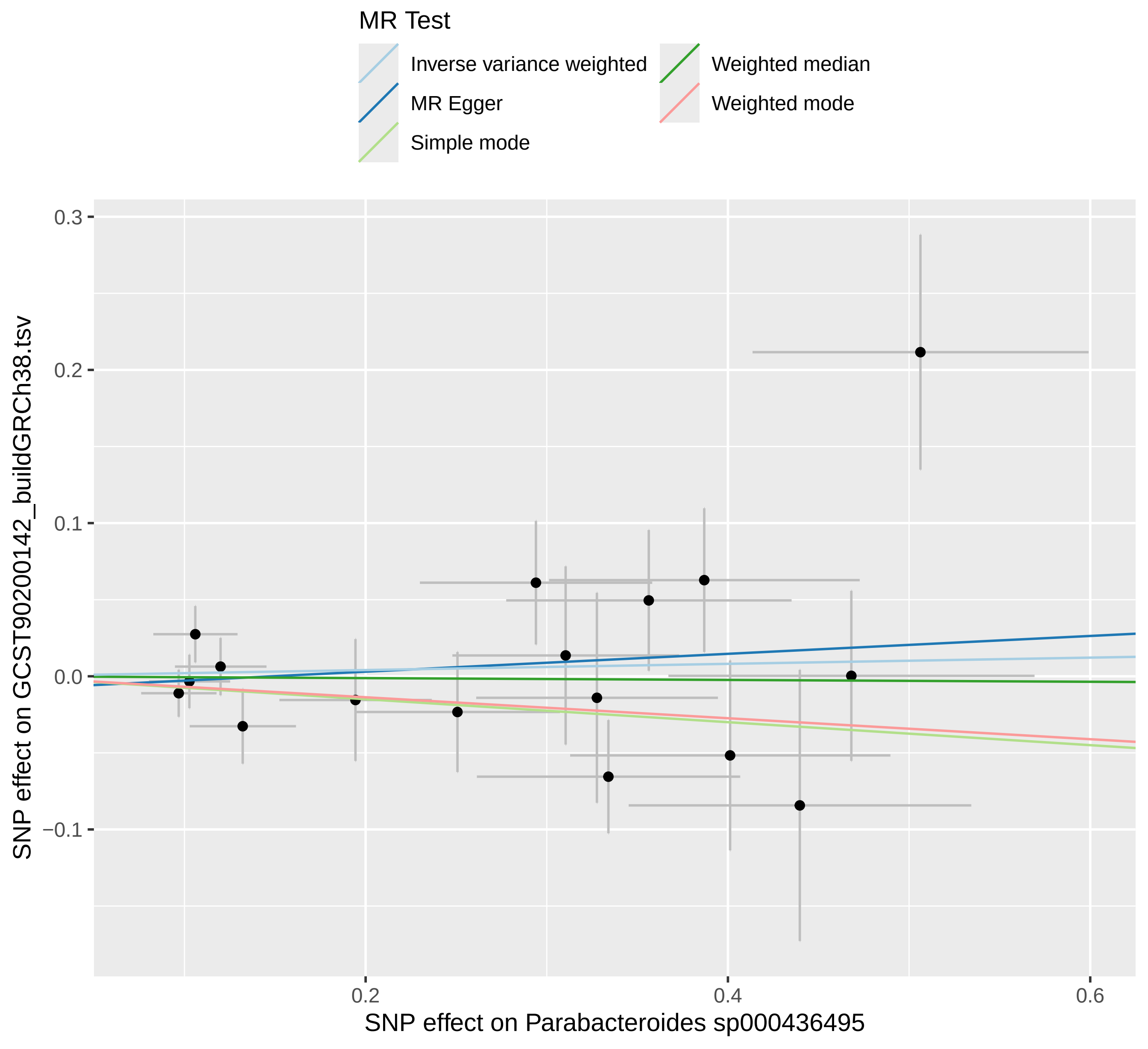

MR Test
Inverse variance weighted
MR Egger
Simple mode
Weighted median
Weighted mode
0.3
0.2
0.1
0.0
−0.1
0.2
0.4
0.6
SNP effect on GCST90200142_buildGRCh38.tsv
SNP effect on Parabacteroides sp000436495

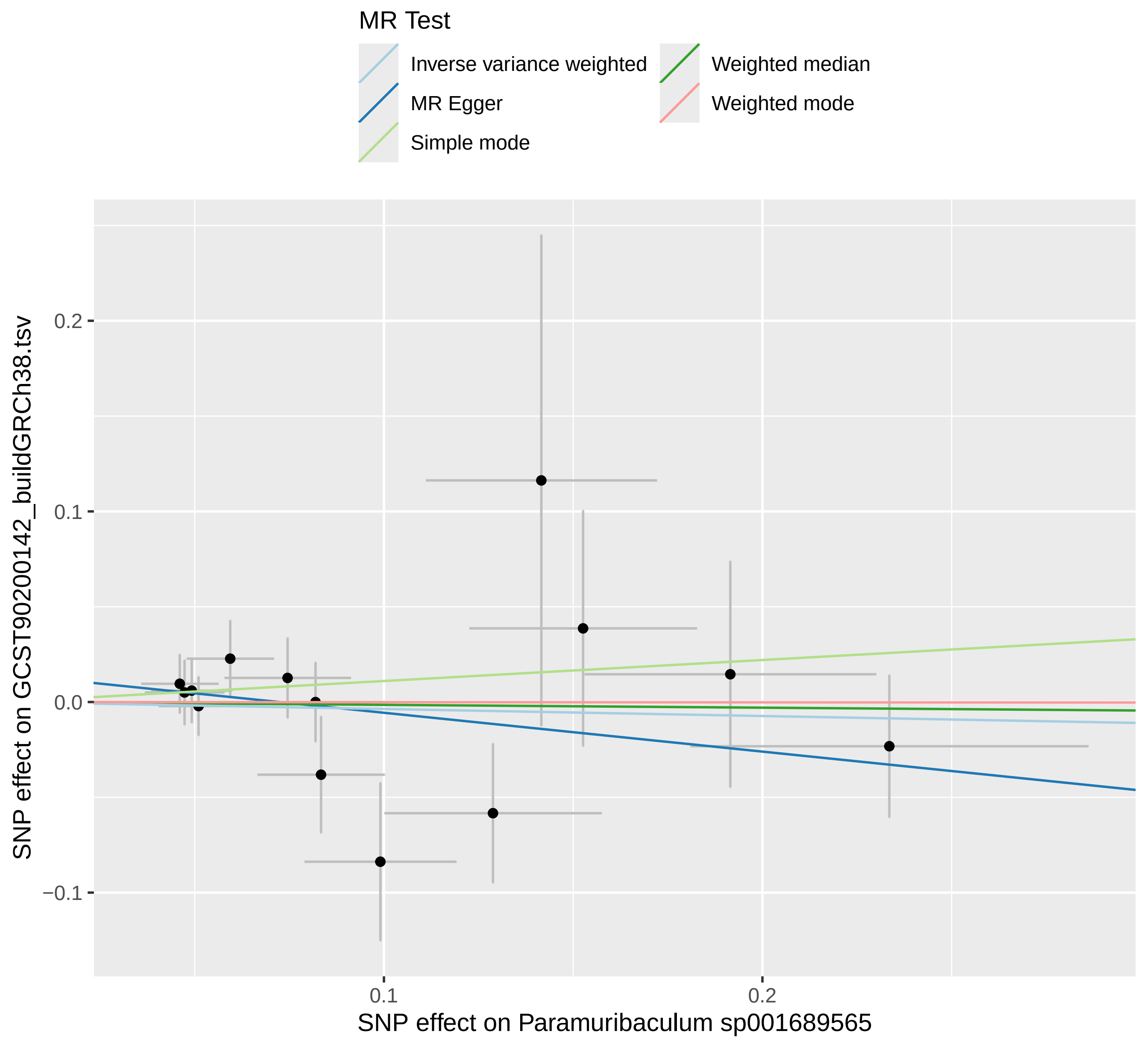

MR Test
Inverse variance weighted
MR Egger
Simple mode
Weighted median
Weighted mode
0.2
0.1
0.0
−0.1
0.1
0.2
SNP effect on GCST90200142_buildGRCh38.tsv
SNP effect on Paramuribaculum sp001689565

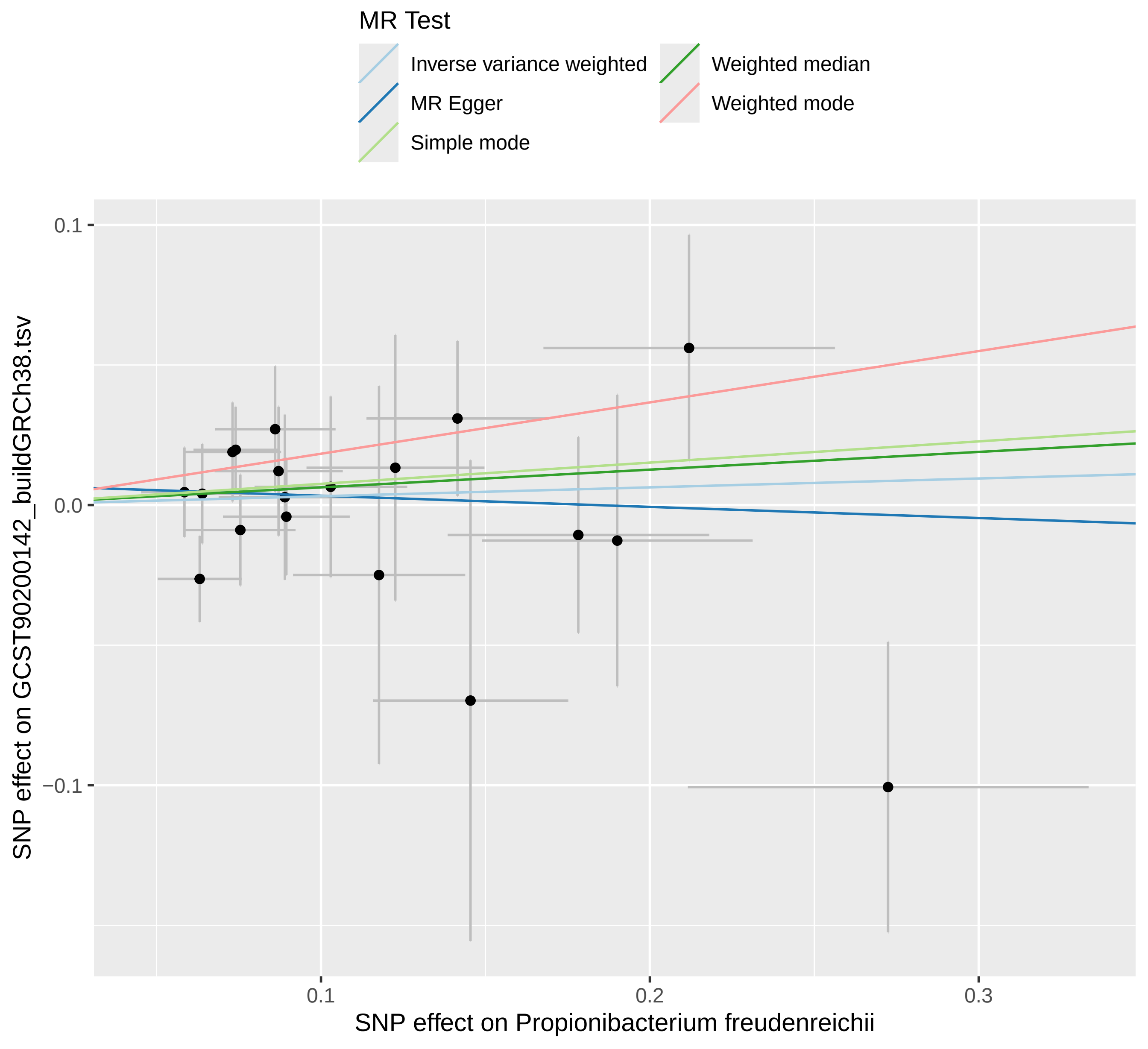

MR Test
Inverse variance weighted
MR Egger
Simple mode
Weighted median
Weighted mode
0.1
0.0
−0.1
0.1
0.2
0.3
SNP effect on GCST90200142_buildGRCh38.tsv
SNP effect on Propionibacterium freudenreichii

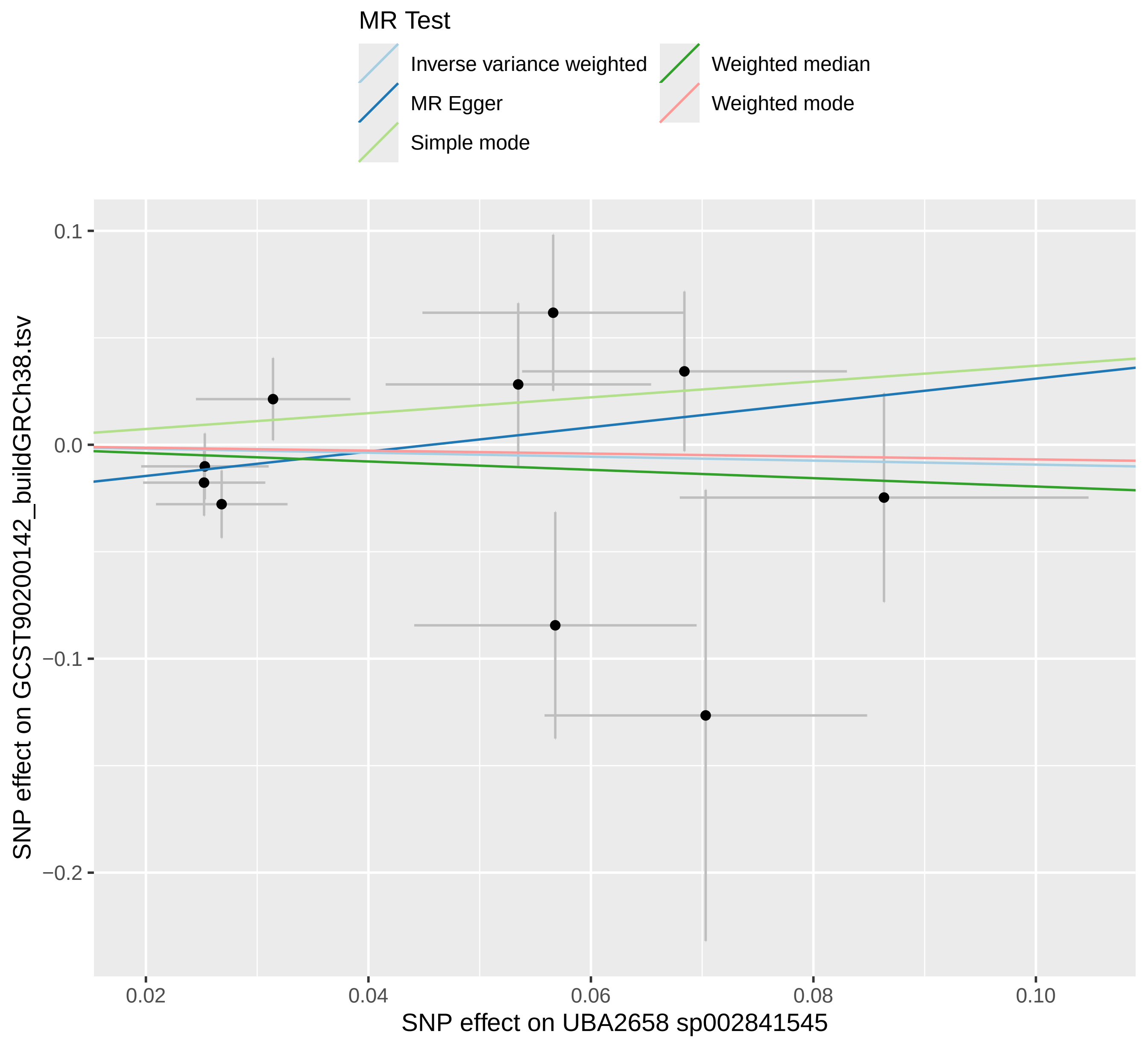

MR Test
Inverse variance weighted
MR Egger
Simple mode
Weighted median
Weighted mode
0.1
0.0
−0.1
−0.2
0.02
0.04
0.06
0.08
0.10
SNP effect on GCST90200142_buildGRCh38.tsv
SNP effect on UBA2658 sp002841545

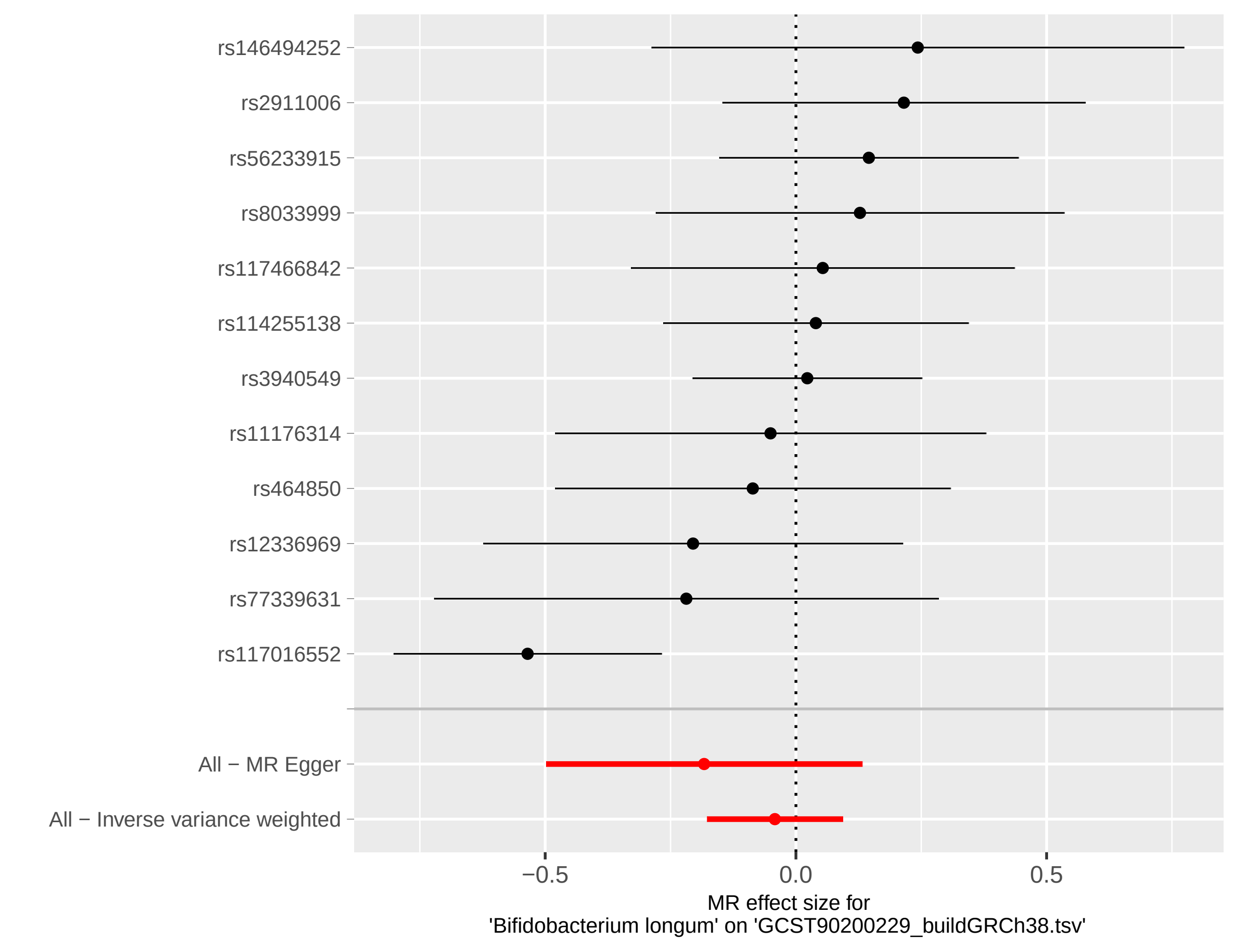

rs146494252
rs2911006
rs56233915
rs8033999
rs117466842
rs114255138
rs3940549
rs11176314
rs464850
rs12336969
rs77339631
rs117016552
All − MR Egger
All − Inverse variance weighted
−0.5
0.0
0.5
MR effect size for
'Bifidobacterium longum' on 'GCST90200229_buildGRCh38.tsv'

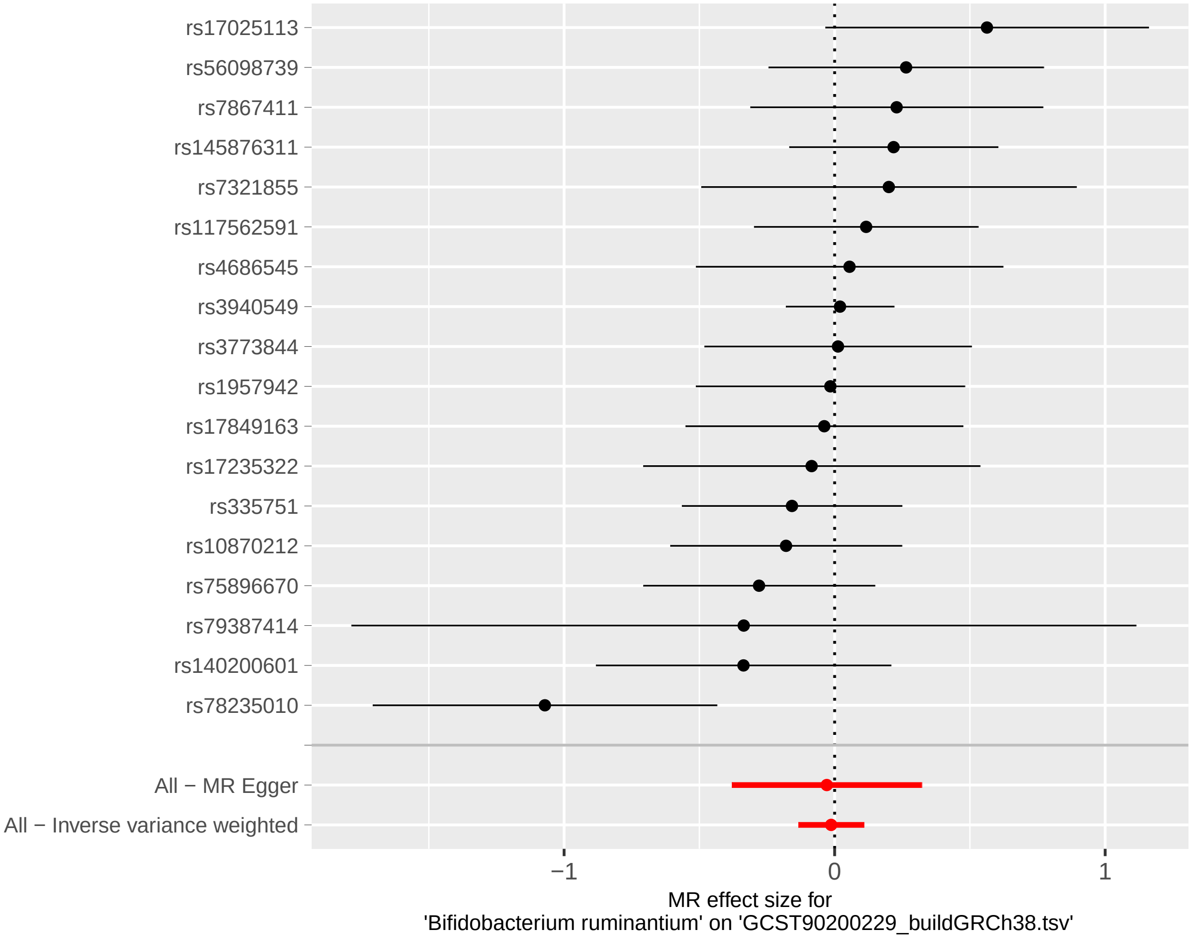

rs17025113
rs56098739
rs7867411
rs145876311
rs7321855
rs117562591
rs4686545
rs3940549
rs3773844
rs1957942
rs17849163
rs17235322
rs335751
rs10870212
rs75896670
rs79387414
rs140200601
rs78235010
All − MR Egger
All − Inverse variance weighted
−1
0
1
MR effect size for
'Bifidobacterium ruminantium' on 'GCST90200229_buildGRCh38.tsv'

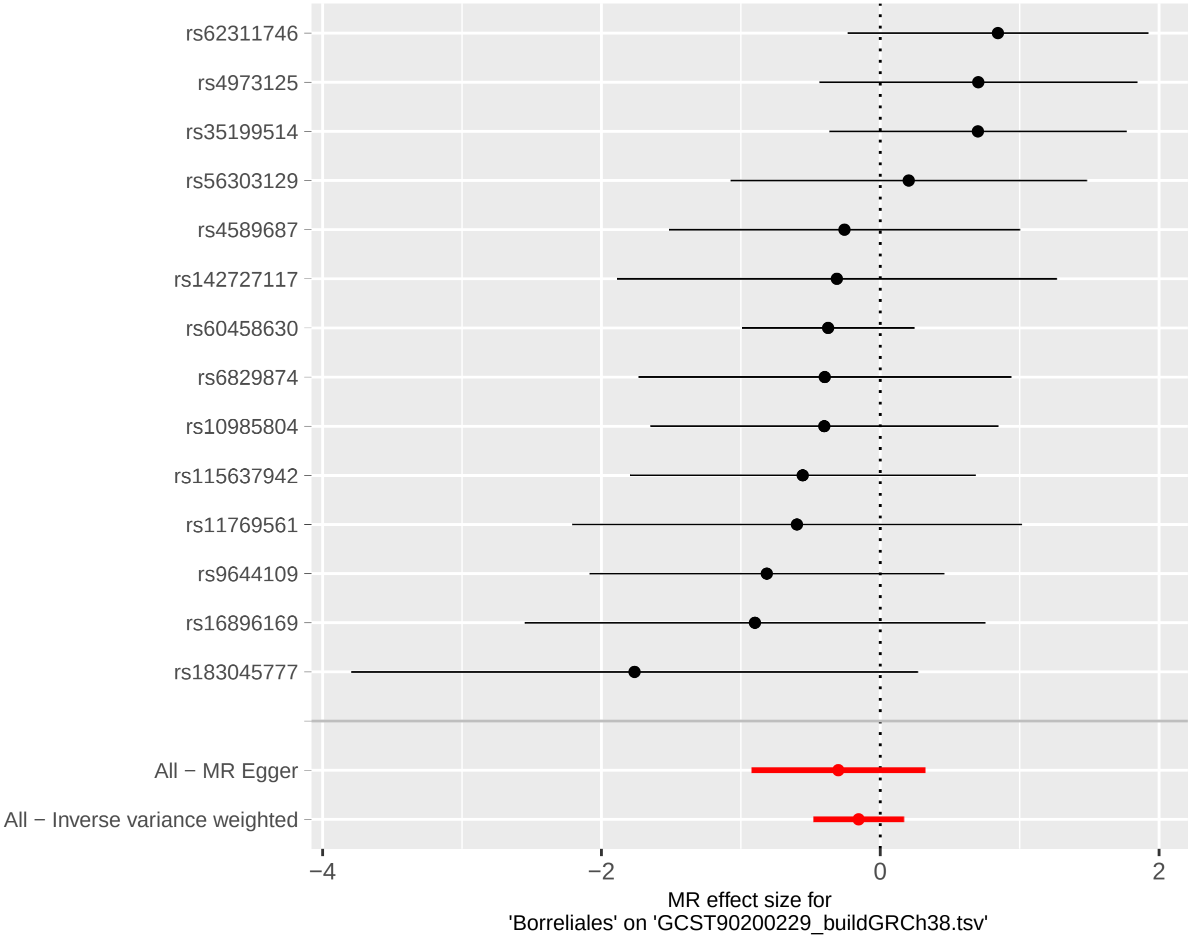
rs62311746
rs4973125
rs35199514
rs56303129
rs4589687
rs142727117
rs60458630
rs6829874
rs10985804
rs115637942
rs11769561
rs9644109
rs16896169
rs183045777
All − MR Egger
All − Inverse variance weighted
−4
−2
0
2
MR effect size for
'Borreliales' on 'GCST90200229_buildGRCh38.tsv'

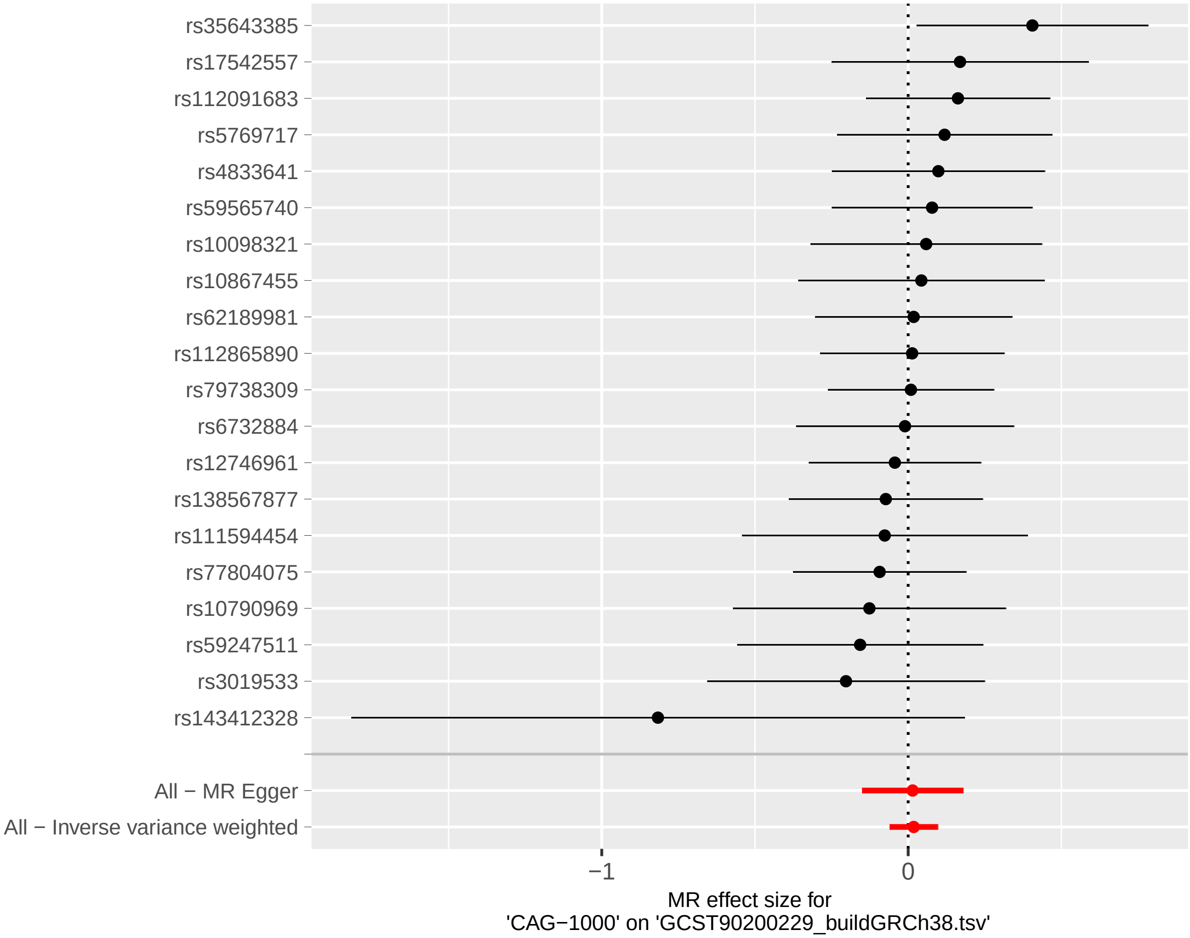
rs35643385
rs17542557
rs112091683
rs5769717
rs4833641
rs59565740
rs10098321
rs10867455
rs62189981
rs112865890
rs79738309
rs6732884
rs12746961
rs138567877
rs111594454
rs77804075
rs10790969
rs59247511
rs3019533
rs143412328
All − MR Egger
All − Inverse variance weighted
−1
0
MR effect size for
'CAG−1000' on 'GCST90200229_buildGRCh38.tsv'

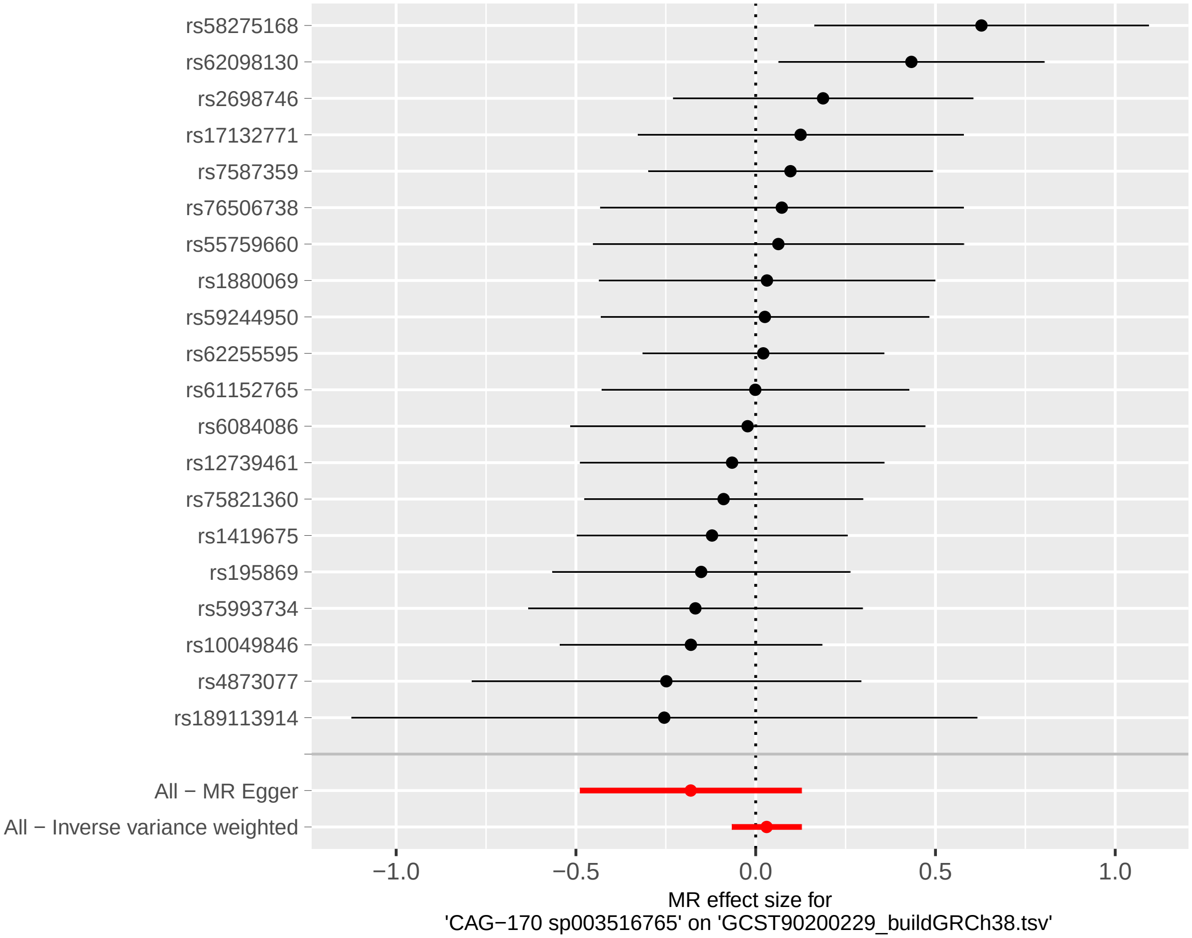
rs58275168
rs62098130
rs2698746
rs17132771
rs7587359
rs76506738
rs55759660
rs1880069
rs59244950
rs62255595
rs61152765
rs6084086
rs12739461
rs75821360
rs1419675
rs195869
rs5993734
rs10049846
rs4873077
rs189113914
All − MR Egger
All − Inverse variance weighted
−1.0
−0.5
0.0
0.5
1.0
MR effect size for
'CAG−170 sp003516765' on 'GCST90200229_buildGRCh38.tsv'

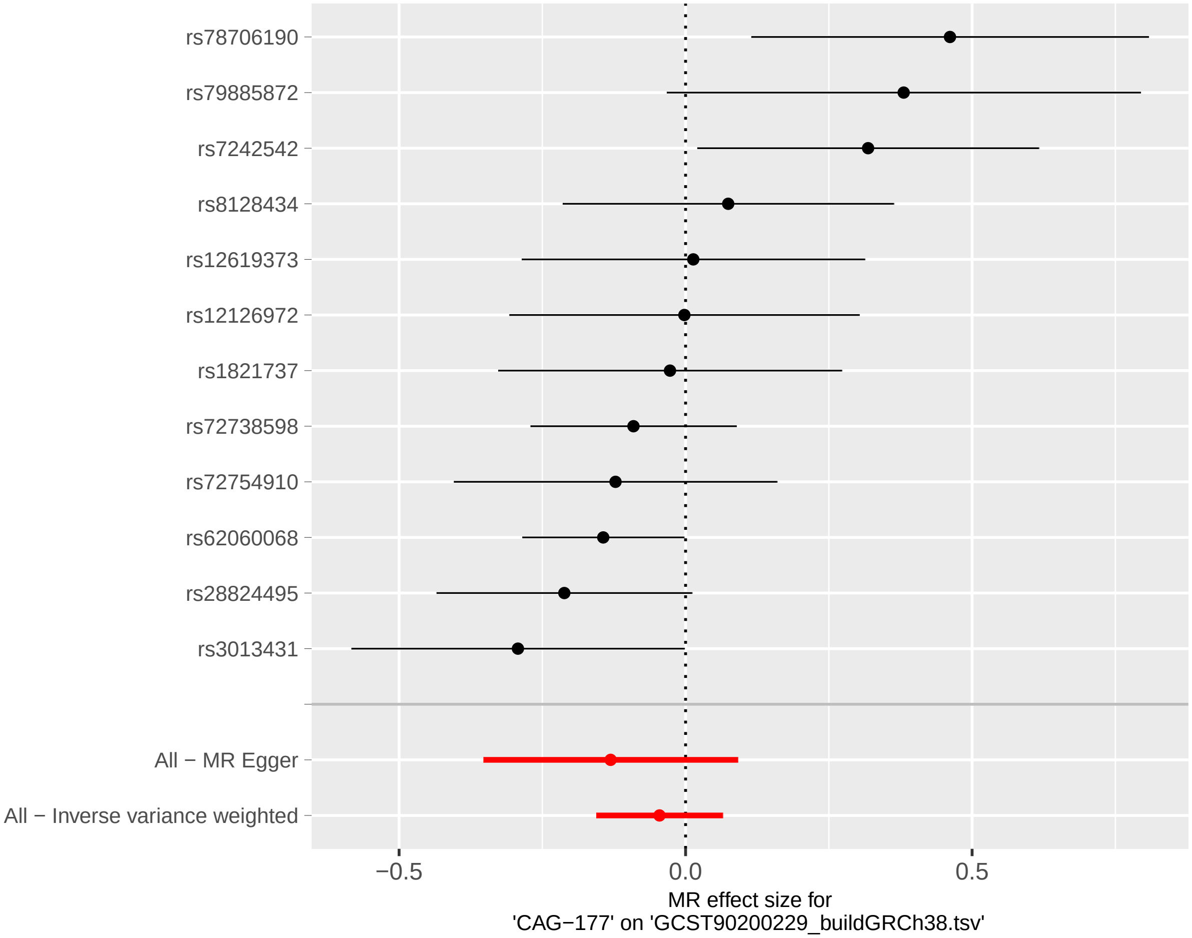
rs78706190
rs79885872
rs7242542
rs8128434
rs12619373
rs12126972
rs1821737
rs72738598
rs72754910
rs62060068
rs28824495
rs3013431
All − MR Egger
All − Inverse variance weighted
−0.5
0.0
0.5
MR effect size for
'CAG−177' on 'GCST90200229_buildGRCh38.tsv'

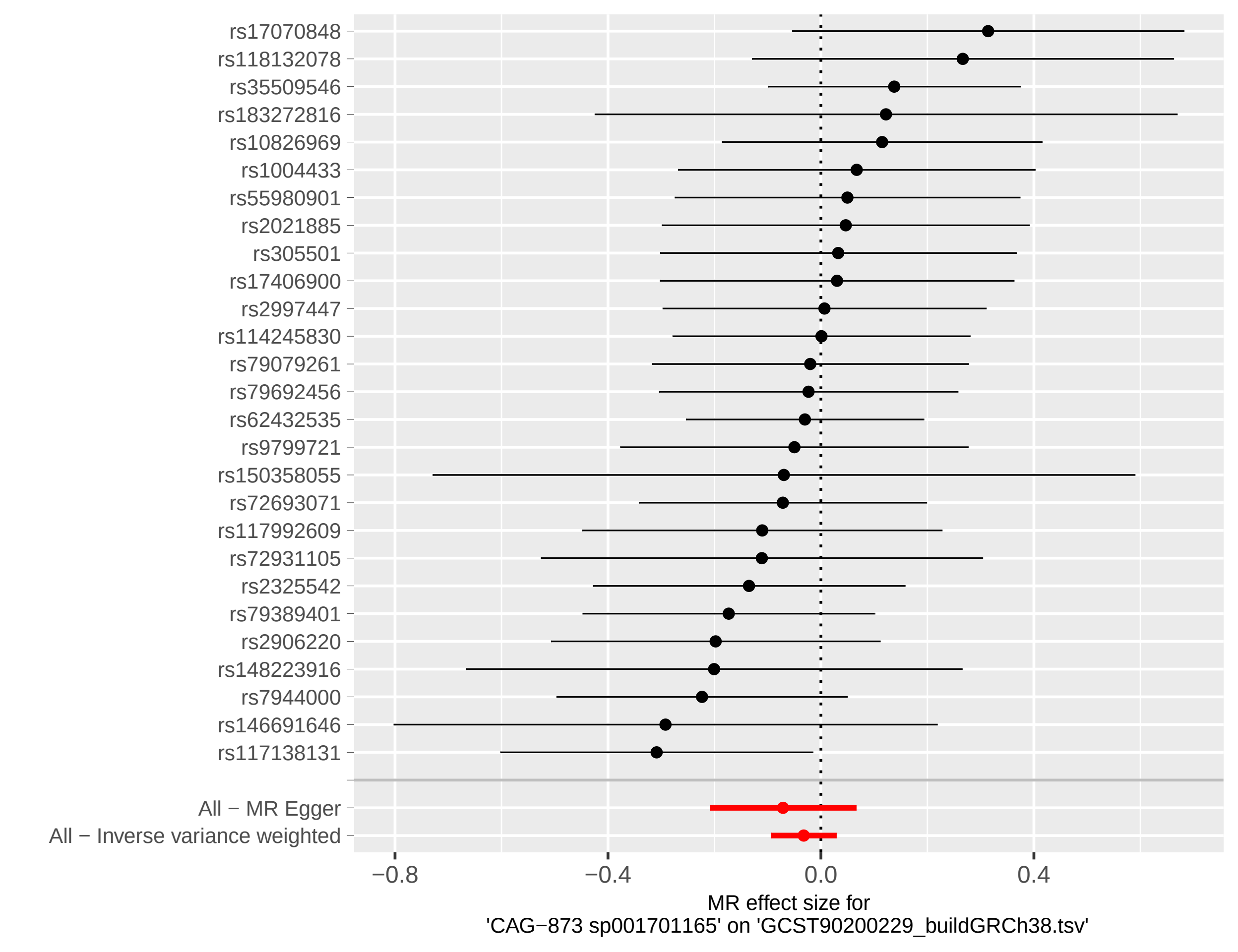

rs17070848
rs118132078
rs35509546
rs183272816
rs10826969
rs1004433
rs55980901
rs2021885
rs305501
rs17406900
rs2997447
rs114245830
rs79079261
rs79692456
rs62432535
rs9799721
rs150358055
rs72693071
rs117992609
rs72931105
rs2325542
rs79389401
rs2906220
rs148223916
rs7944000
rs146691646
rs117138131
All − MR Egger
All − Inverse variance weighted
−0.8
−0.4
0.0
0.4
MR effect size for
'CAG−873 sp001701165' on 'GCST90200229_buildGRCh38.tsv'

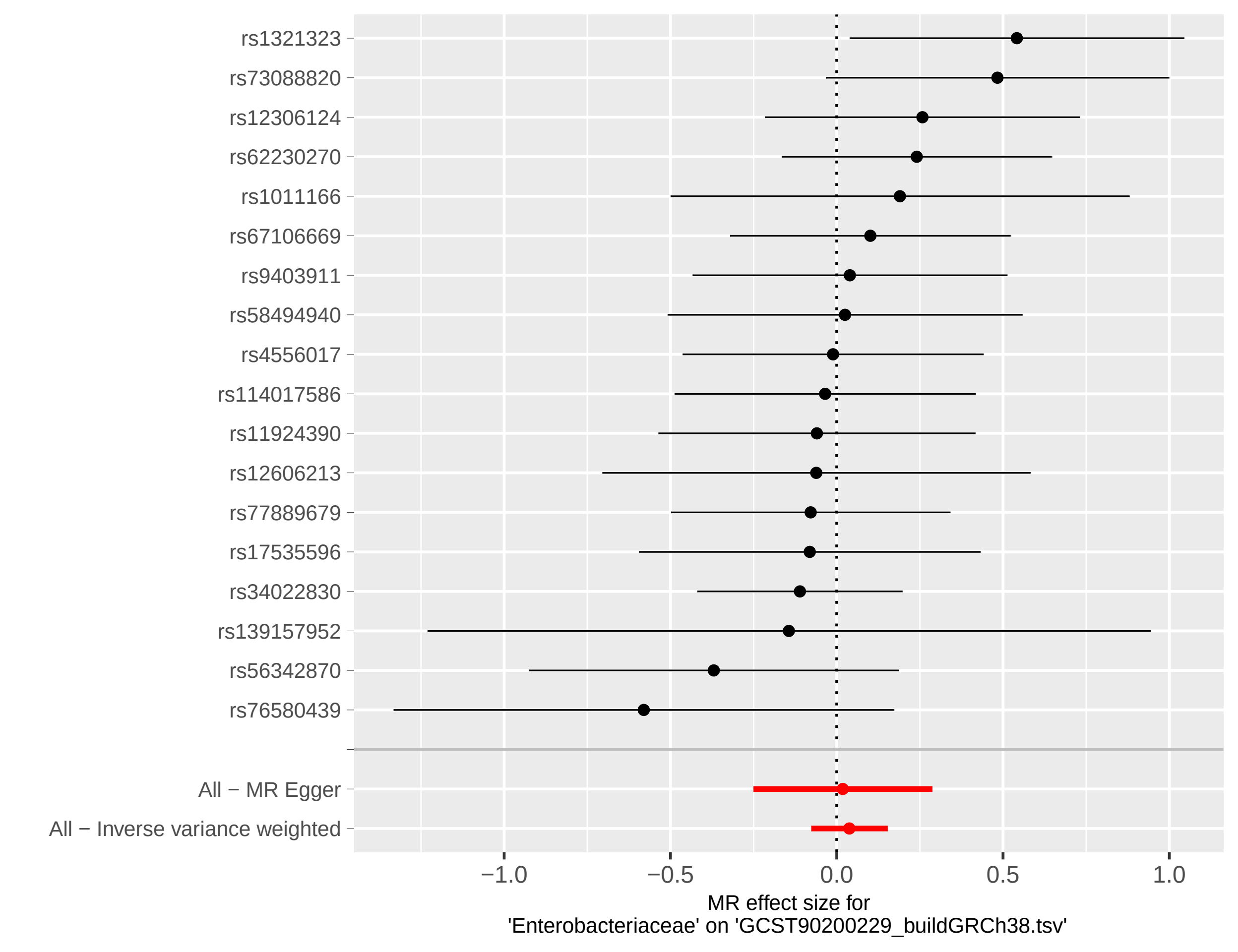

rs1321323
rs73088820
rs12306124
rs62230270
rs1011166
rs67106669
rs9403911
rs58494940
rs4556017
rs114017586
rs11924390
rs12606213
rs77889679
rs17535596
rs34022830
rs139157952
rs56342870
rs76580439
All − MR Egger
All − Inverse variance weighted
−1.0
−0.5
0.0
0.5
1.0
MR effect size for
'Enterobacteriaceae' on 'GCST90200229_buildGRCh38.tsv'

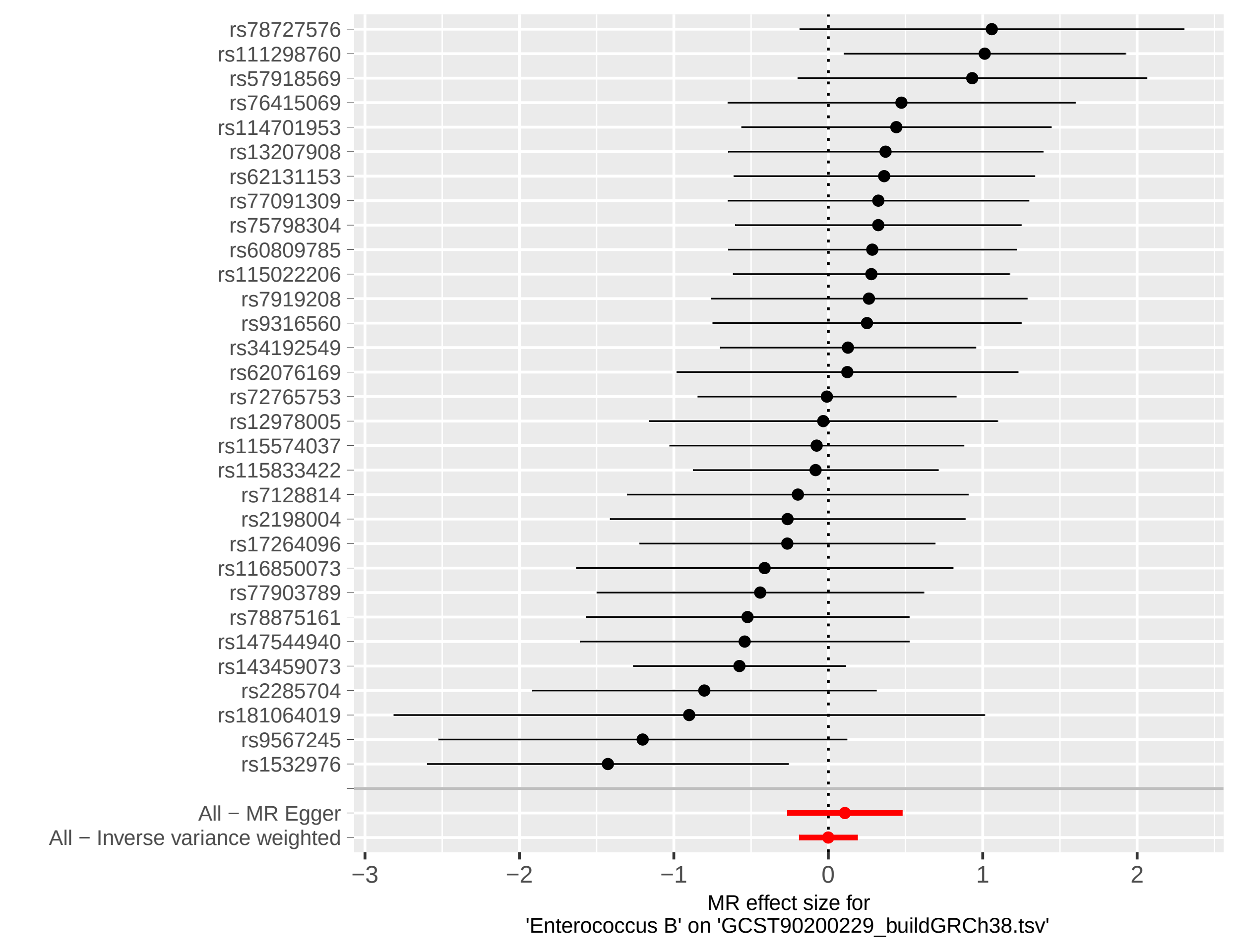
rs78727576
rs111298760
rs57918569
rs76415069
rs114701953
rs13207908
rs62131153
rs77091309
rs75798304
rs60809785
rs115022206
rs7919208
rs9316560
rs34192549
rs62076169
rs72765753
rs12978005
rs115574037
rs115833422
rs7128814
rs2198004
rs17264096
rs116850073
rs77903789
rs78875161
rs147544940
rs143459073
rs2285704
rs181064019
rs9567245
rs1532976
All − MR Egger
All − Inverse variance weighted
−3
−2
−1
0
1
2
MR effect size for
'Enterococcus B' on 'GCST90200229_buildGRCh38.tsv'

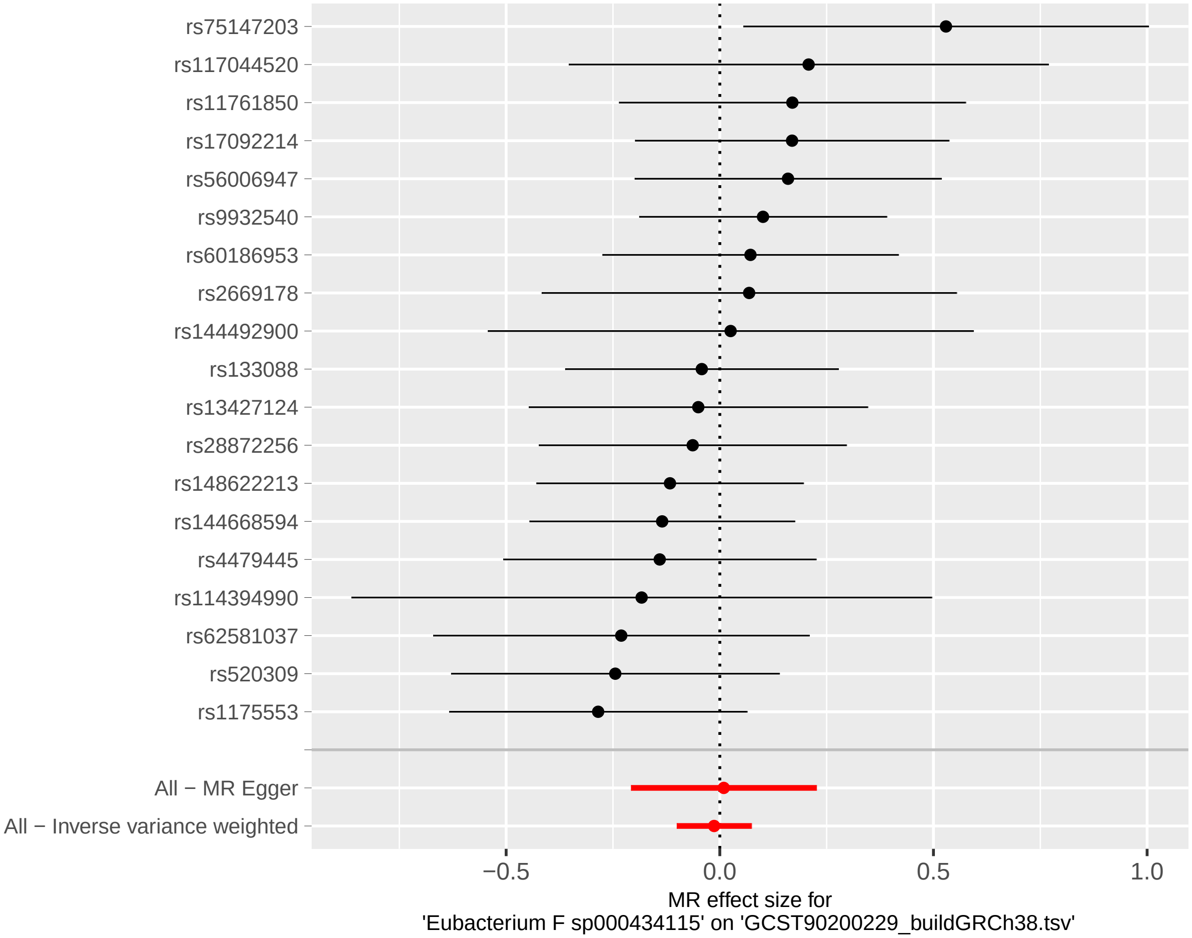

rs75147203
rs117044520
rs11761850
rs17092214
rs56006947
rs9932540
rs60186953
rs2669178
rs144492900
rs133088
rs13427124
rs28872256
rs148622213
rs144668594
rs4479445
rs114394990
rs62581037
rs520309
rs1175553
All − MR Egger
All − Inverse variance weighted
−0.5
0.0
0.5
1.0
MR effect size for
'Eubacterium F sp000434115' on 'GCST90200229_buildGRCh38.tsv'

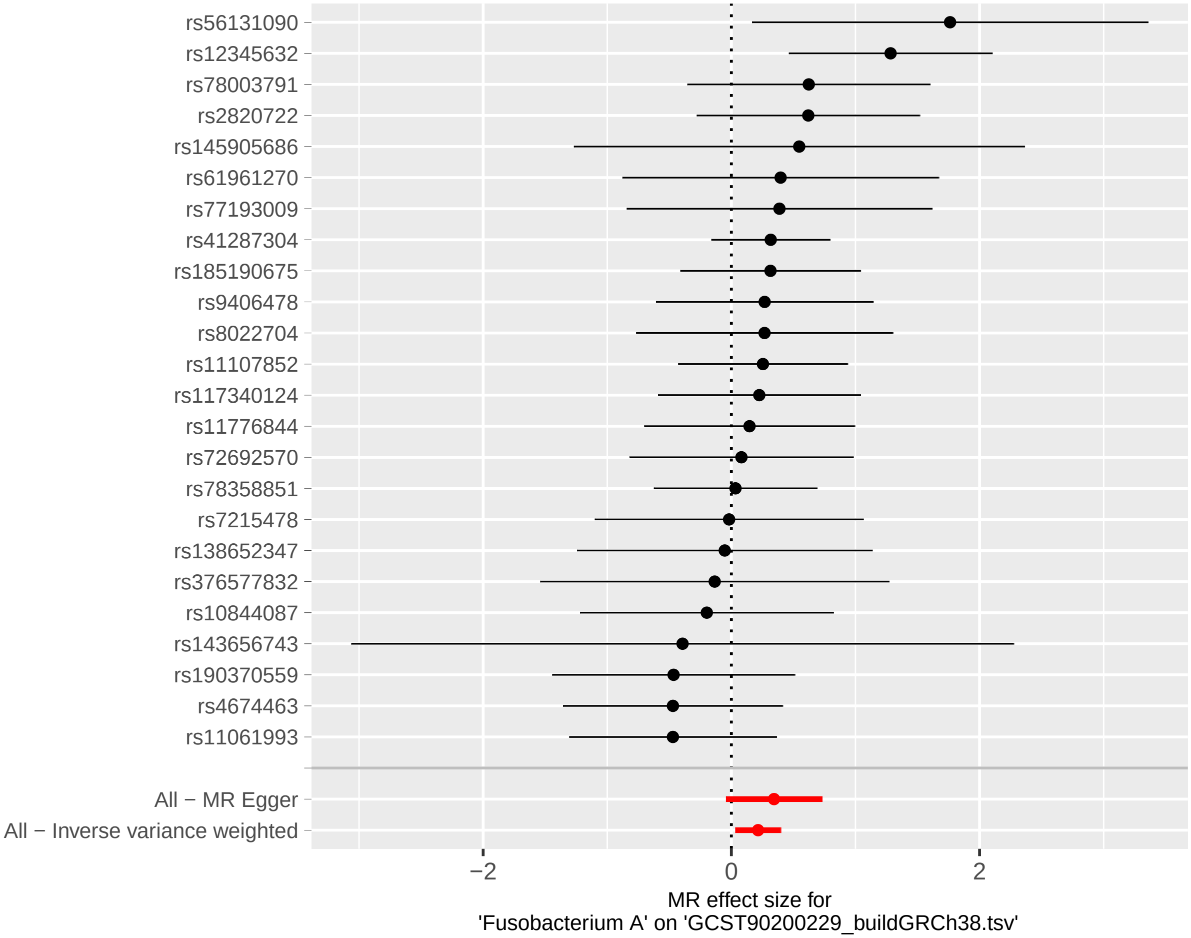
rs56131090
rs12345632
rs78003791
rs2820722
rs145905686
rs61961270
rs77193009
rs41287304
rs185190675
rs9406478
rs8022704
rs11107852
rs117340124
rs11776844
rs72692570
rs78358851
rs7215478
rs138652347
rs376577832
rs10844087
rs143656743
rs190370559
rs4674463
rs11061993
All − MR Egger
All − Inverse variance weighted
−2
0
2
MR effect size for
'Fusobacterium A' on 'GCST90200229_buildGRCh38.tsv'

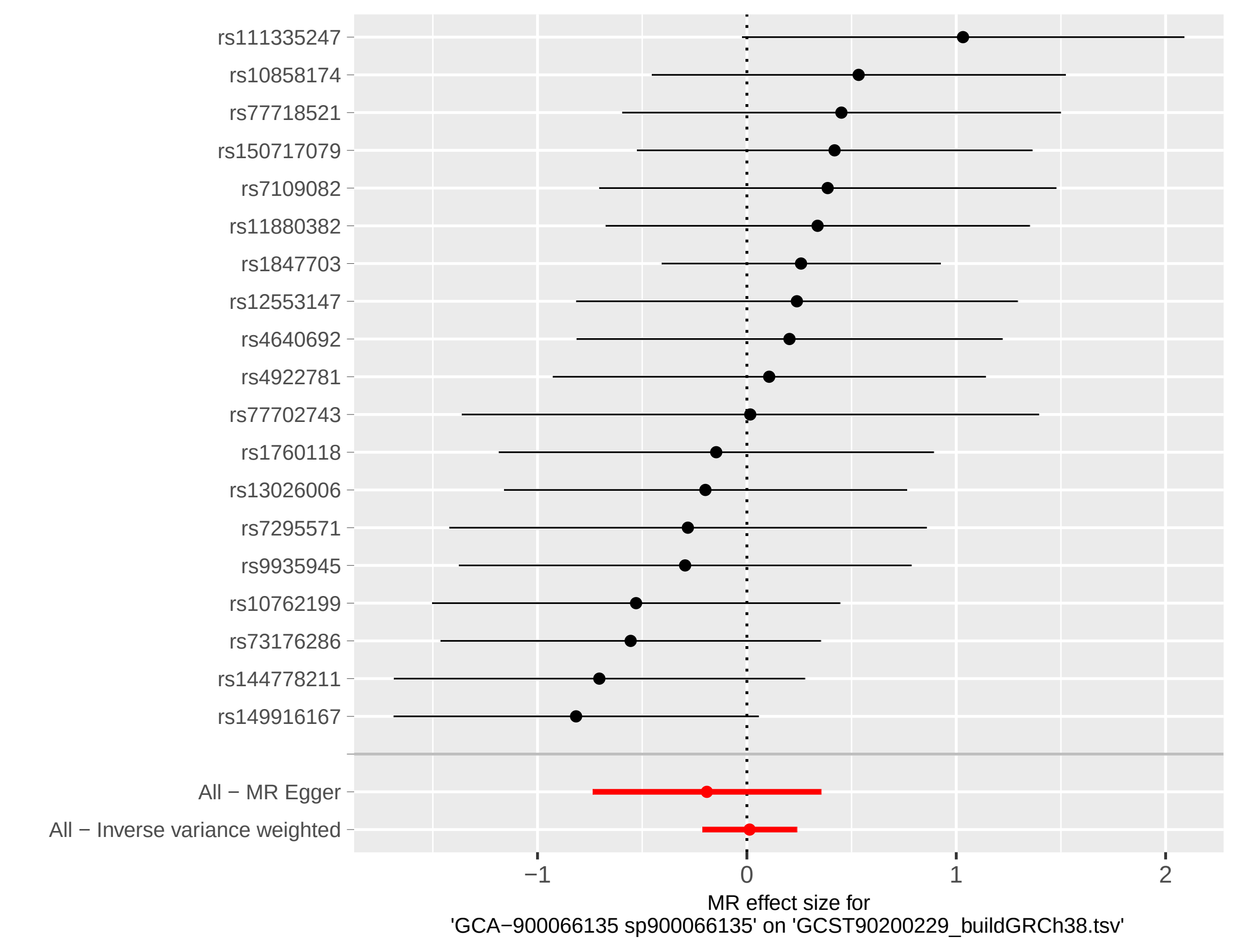
rs111335247
rs10858174
rs77718521
rs150717079
rs7109082
rs11880382
rs1847703
rs12553147
rs4640692
rs4922781
rs77702743
rs1760118
rs13026006
rs7295571
rs9935945
rs10762199
rs73176286
rs144778211
rs149916167
All − MR Egger
All − Inverse variance weighted
−1
0
1
2
MR effect size for
'GCA−900066135 sp900066135' on 'GCST90200229_buildGRCh38.tsv'

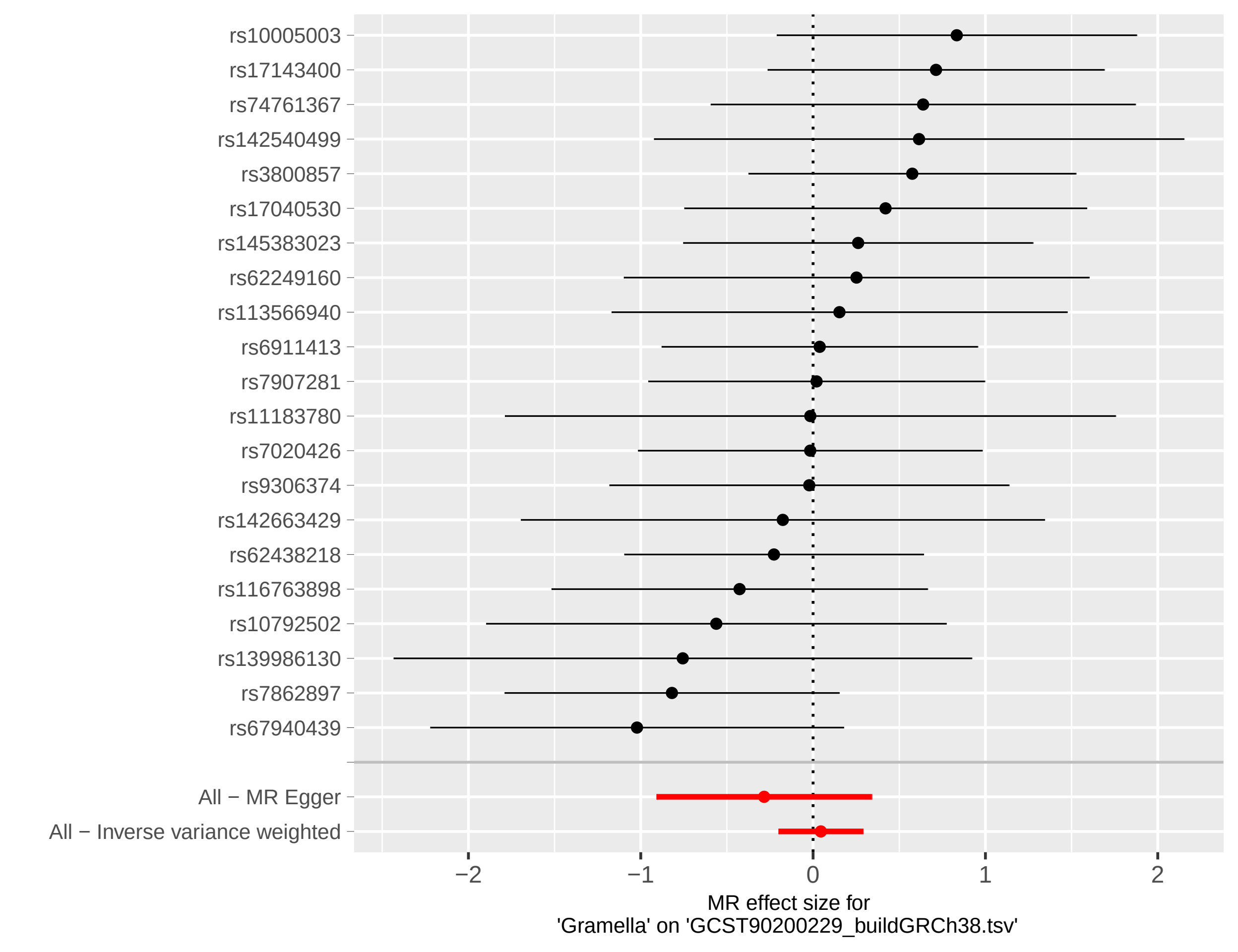
rs10005003
rs17143400
rs74761367
rs142540499
rs3800857
rs17040530
rs145383023
rs62249160
rs113566940
rs6911413
rs7907281
rs11183780
rs7020426
rs9306374
rs142663429
rs62438218
rs116763898
rs10792502
rs139986130
rs7862897
rs67940439
All − MR Egger
All − Inverse variance weighted
−2
−1
0
1
2
MR effect size for
'Gramella' on 'GCST90200229_buildGRCh38.tsv'

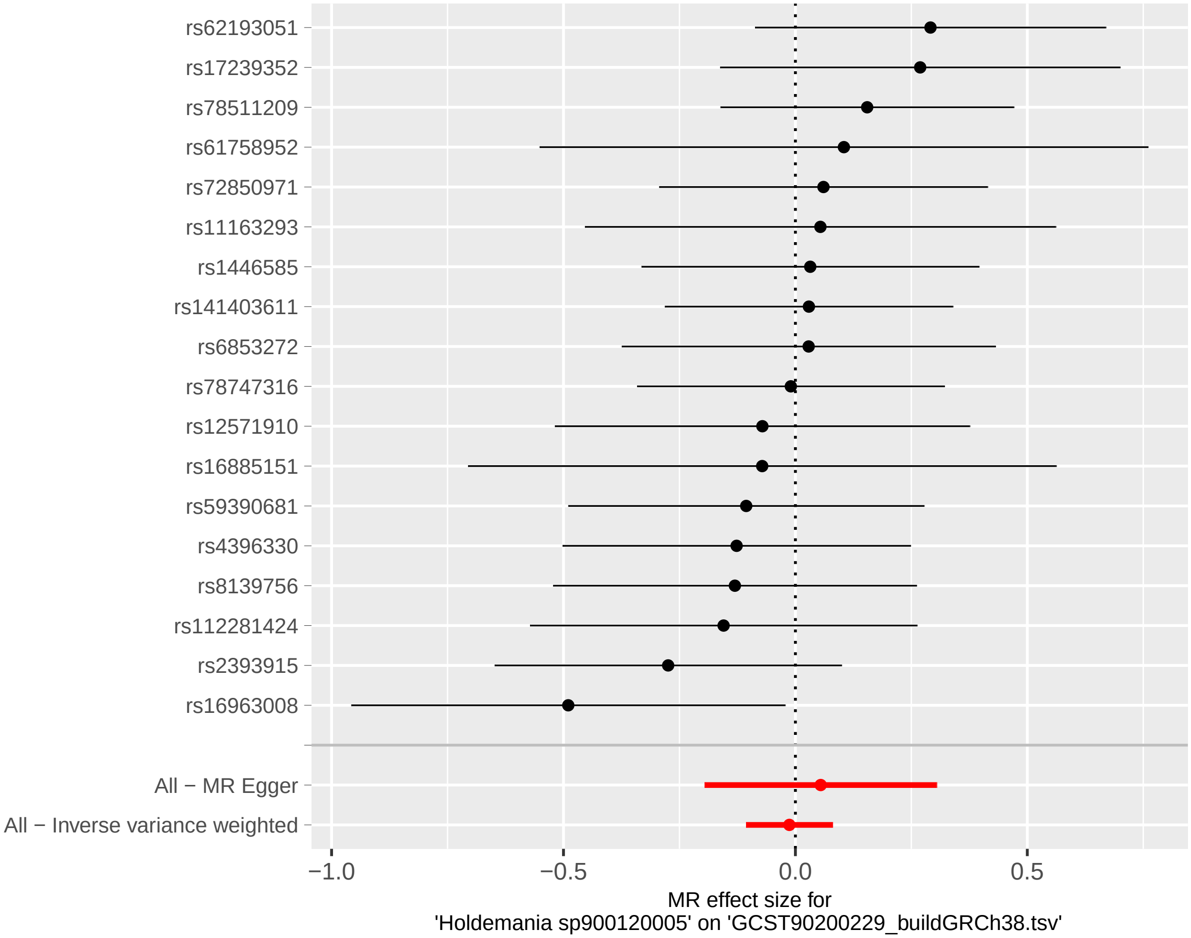
rs62193051
rs17239352
rs78511209
rs61758952
rs72850971
rs11163293
rs1446585
rs141403611
rs6853272
rs78747316
rs12571910
rs16885151
rs59390681
rs4396330
rs8139756
rs112281424
rs2393915
rs16963008
All − MR Egger
All − Inverse variance weighted
−1.0
−0.5
0.0
0.5
MR effect size for
'Holdemania sp900120005' on 'GCST90200229_buildGRCh38.tsv'

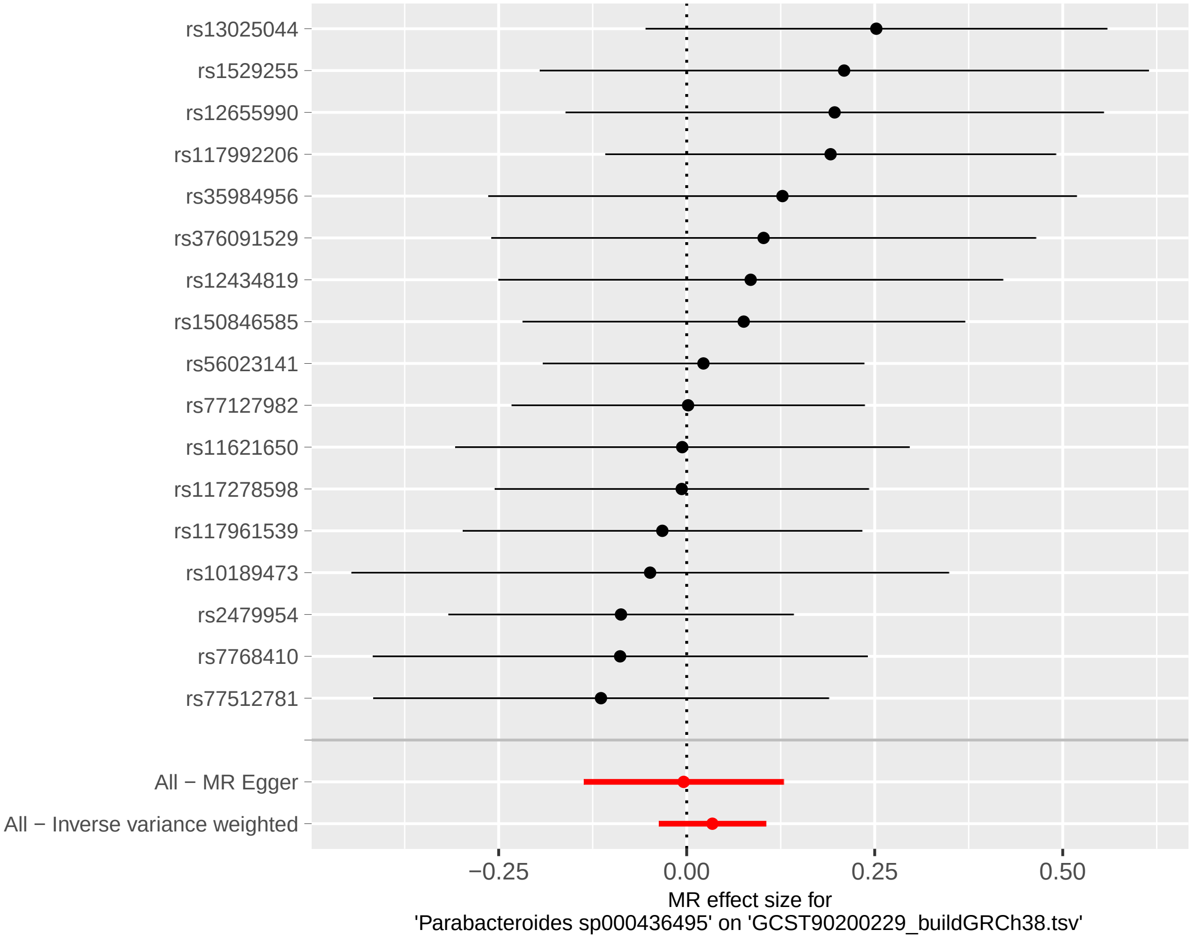
rs13025044
rs1529255
rs12655990
rs117992206
rs35984956
rs376091529
rs12434819
rs150846585
rs56023141
rs77127982
rs11621650
rs117278598
rs117961539
rs10189473
rs2479954
rs7768410
rs77512781
All − MR Egger
All − Inverse variance weighted
−0.25
0.00
0.25
0.50
MR effect size for
'Parabacteroides sp000436495' on 'GCST90200229_buildGRCh38.tsv'

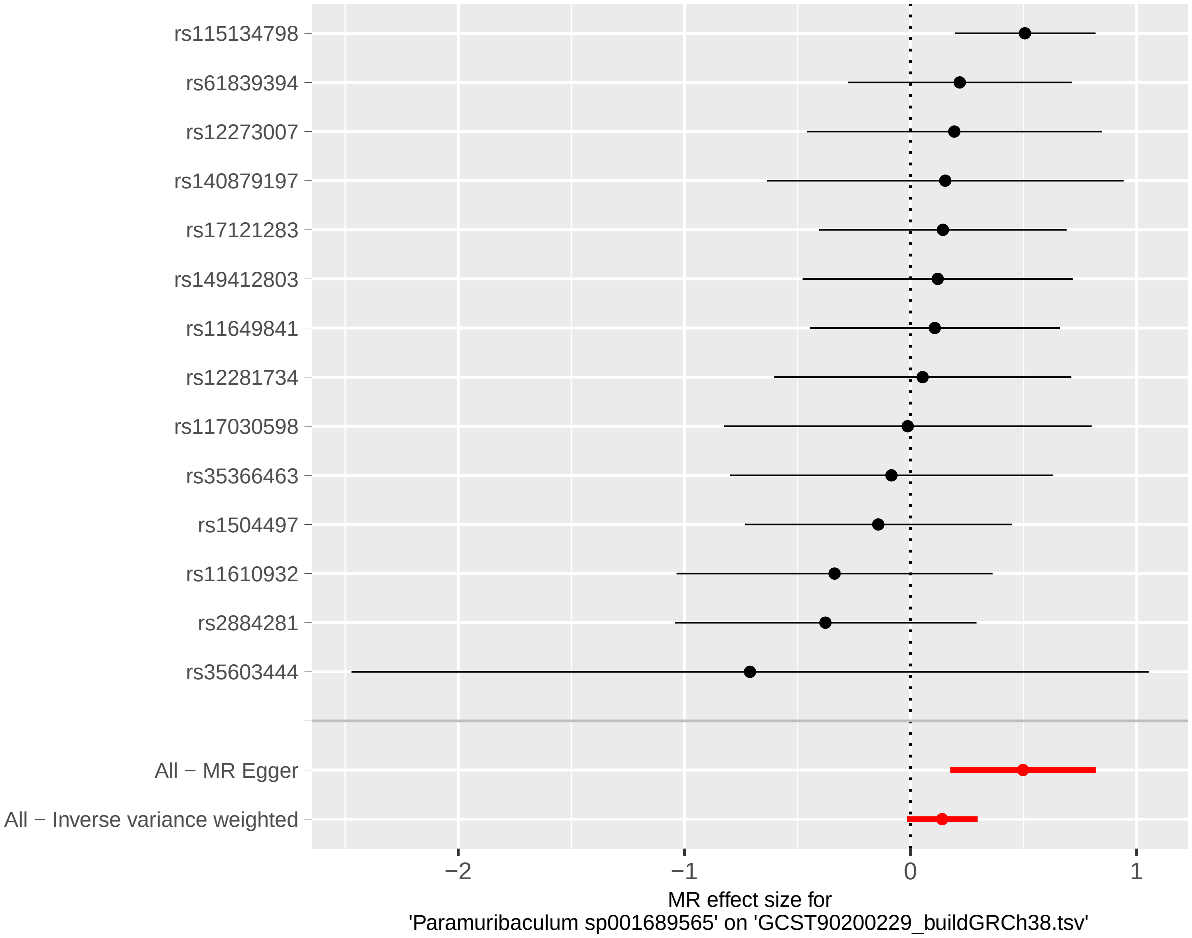
rs115134798
rs61839394
rs12273007
rs140879197
rs17121283
rs149412803
rs11649841
rs12281734
rs117030598
rs35366463
rs1504497
rs11610932
rs2884281
rs35603444
All − MR Egger
All − Inverse variance weighted
−2
−1
0
1
MR effect size for
'Paramuribaculum sp001689565' on 'GCST90200229_buildGRCh38.tsv'

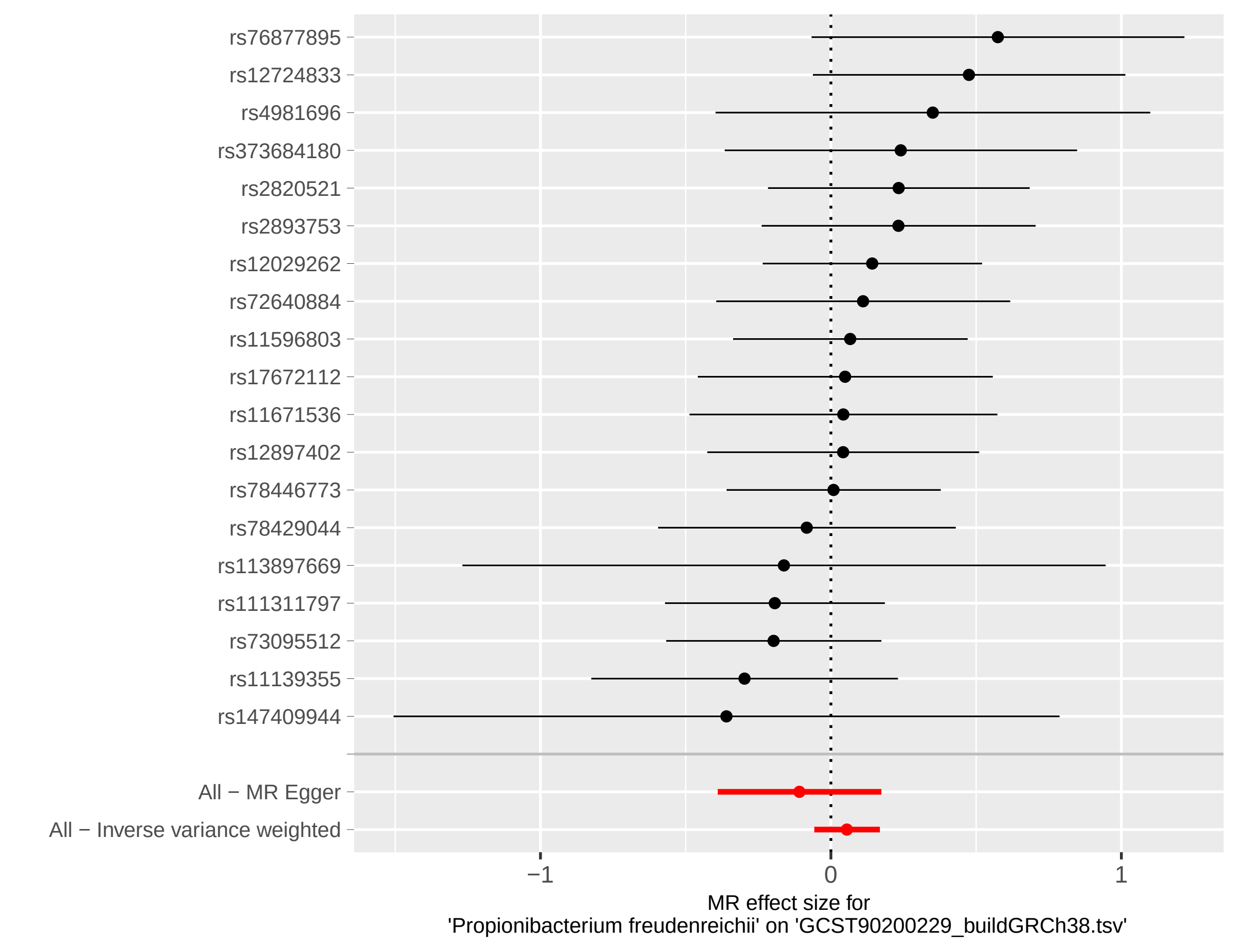

rs76877895
rs12724833
rs4981696
rs373684180
rs2820521
rs2893753
rs12029262
rs72640884
rs11596803
rs17672112
rs11671536
rs12897402
rs78446773
rs78429044
rs113897669
rs111311797
rs73095512
rs11139355
rs147409944
All − MR Egger
All − Inverse variance weighted
−1
0
1
MR effect size for
'Propionibacterium freudenreichii' on 'GCST90200229_buildGRCh38.tsv'

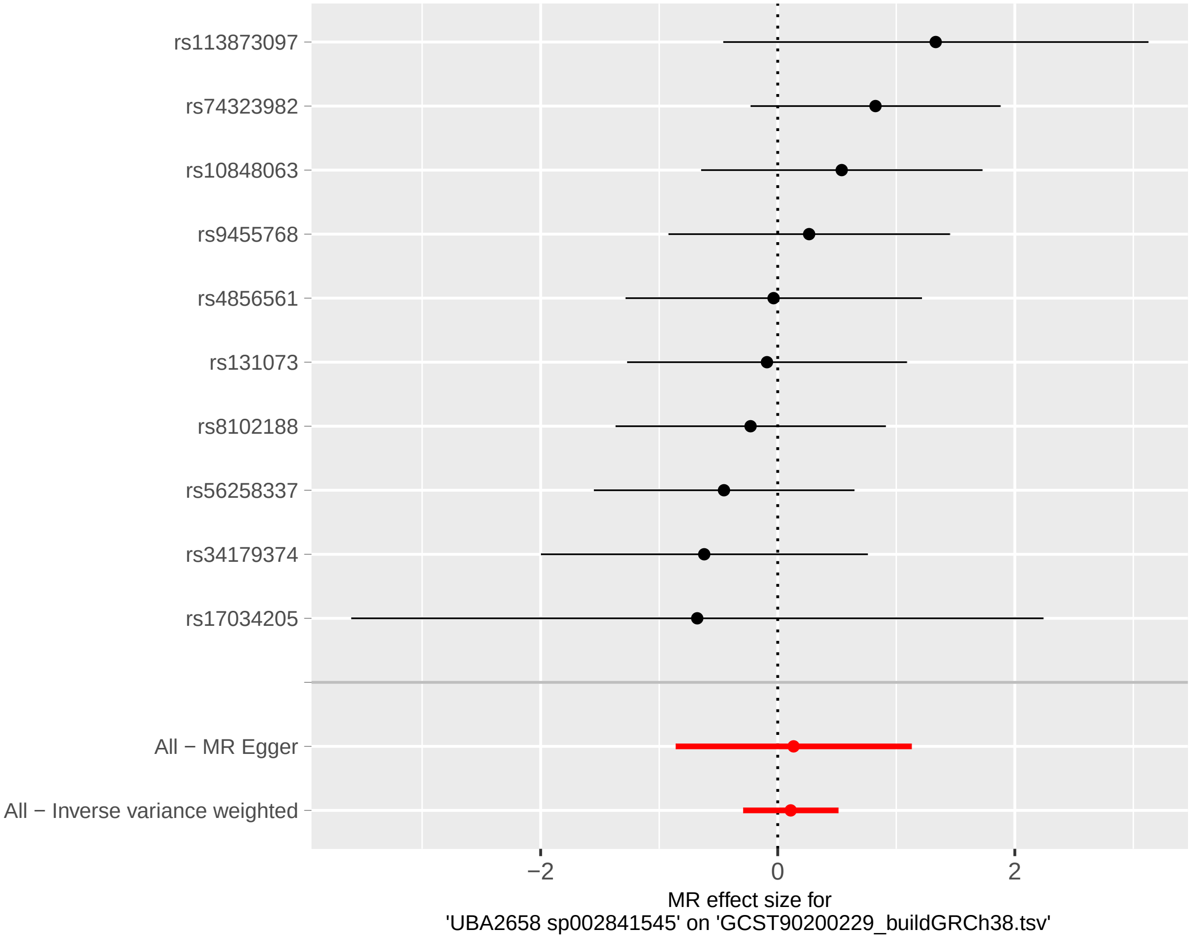

rs113873097
rs74323982
rs10848063
rs9455768
rs4856561
rs131073
rs8102188
rs56258337
rs34179374
rs17034205
All − MR Egger
All − Inverse variance weighted
−2
0
2
MR effect size for
'UBA2658 sp002841545' on 'GCST90200229_buildGRCh38.tsv'

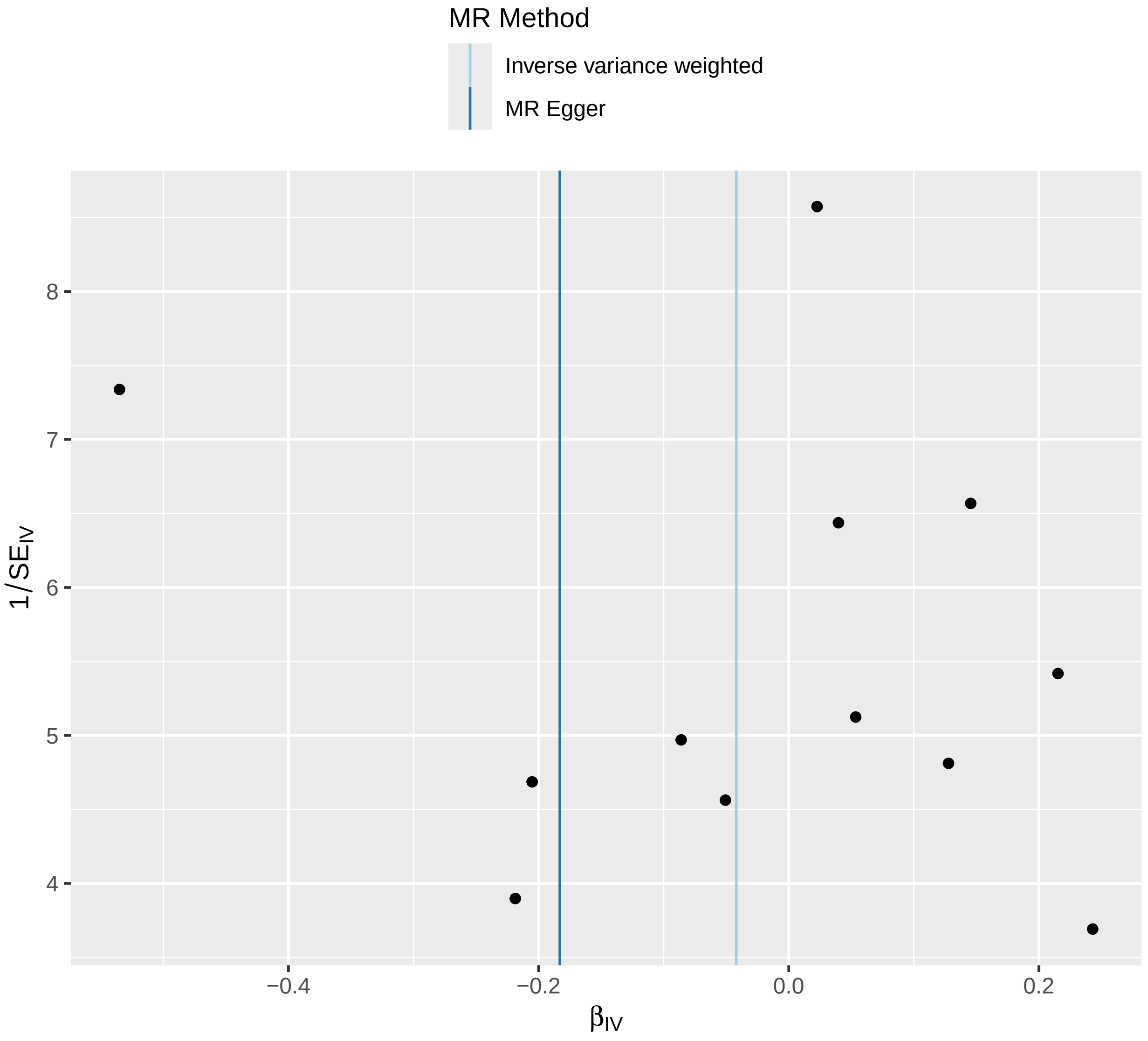
MR Method
Inverse variance weighted
MR Egger
8
7
6
5
4
1/SE$_{IV}$
−0.4
−0.2
0.0
0.2
β$_{IV}$

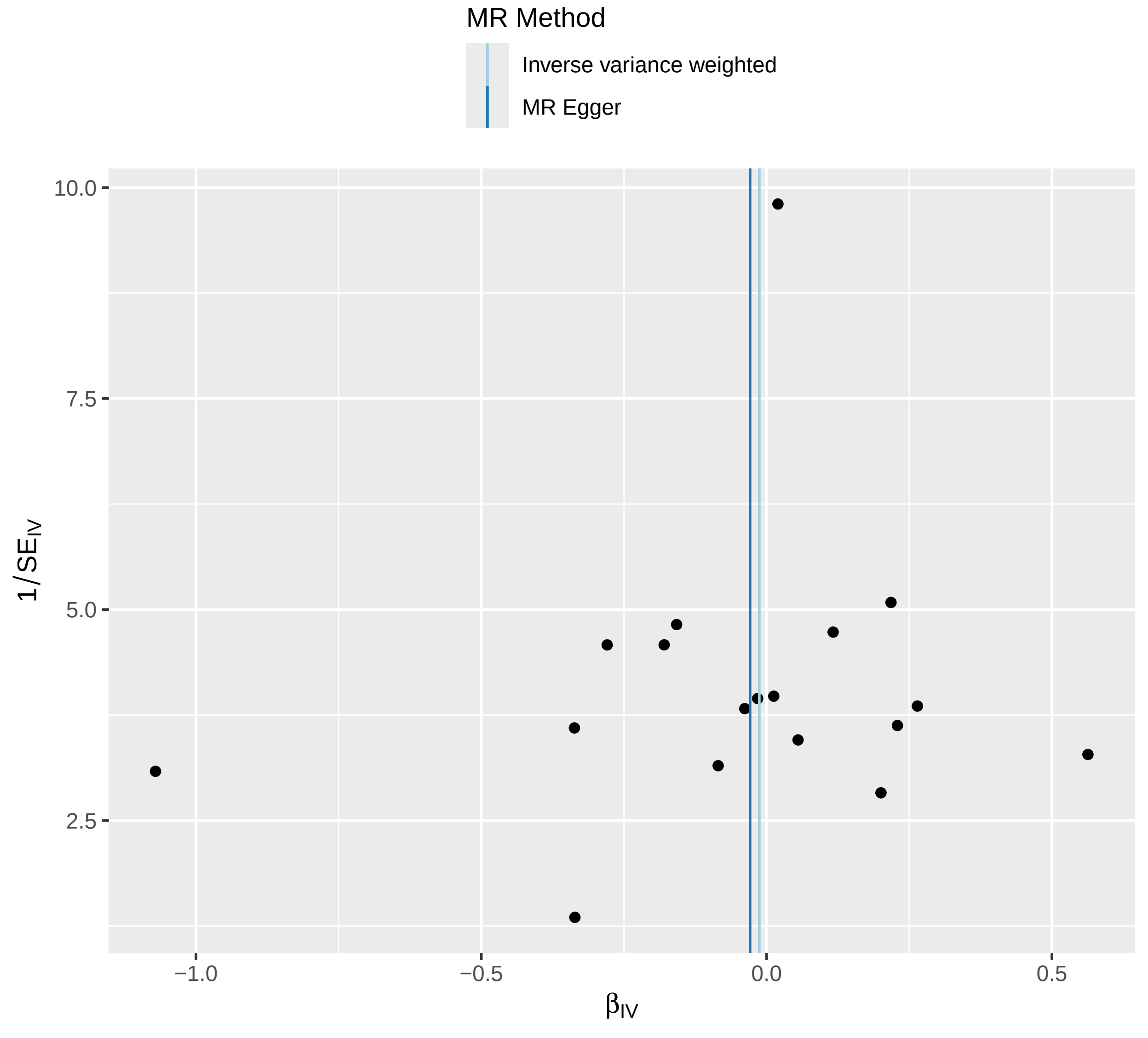
MR Method
Inverse variance weighted
MR Egger
10.0
7.5
5.0
2.5
1/SE$_{IV}$
−1.0
−0.5
0.0
0.5
β$_{IV}$

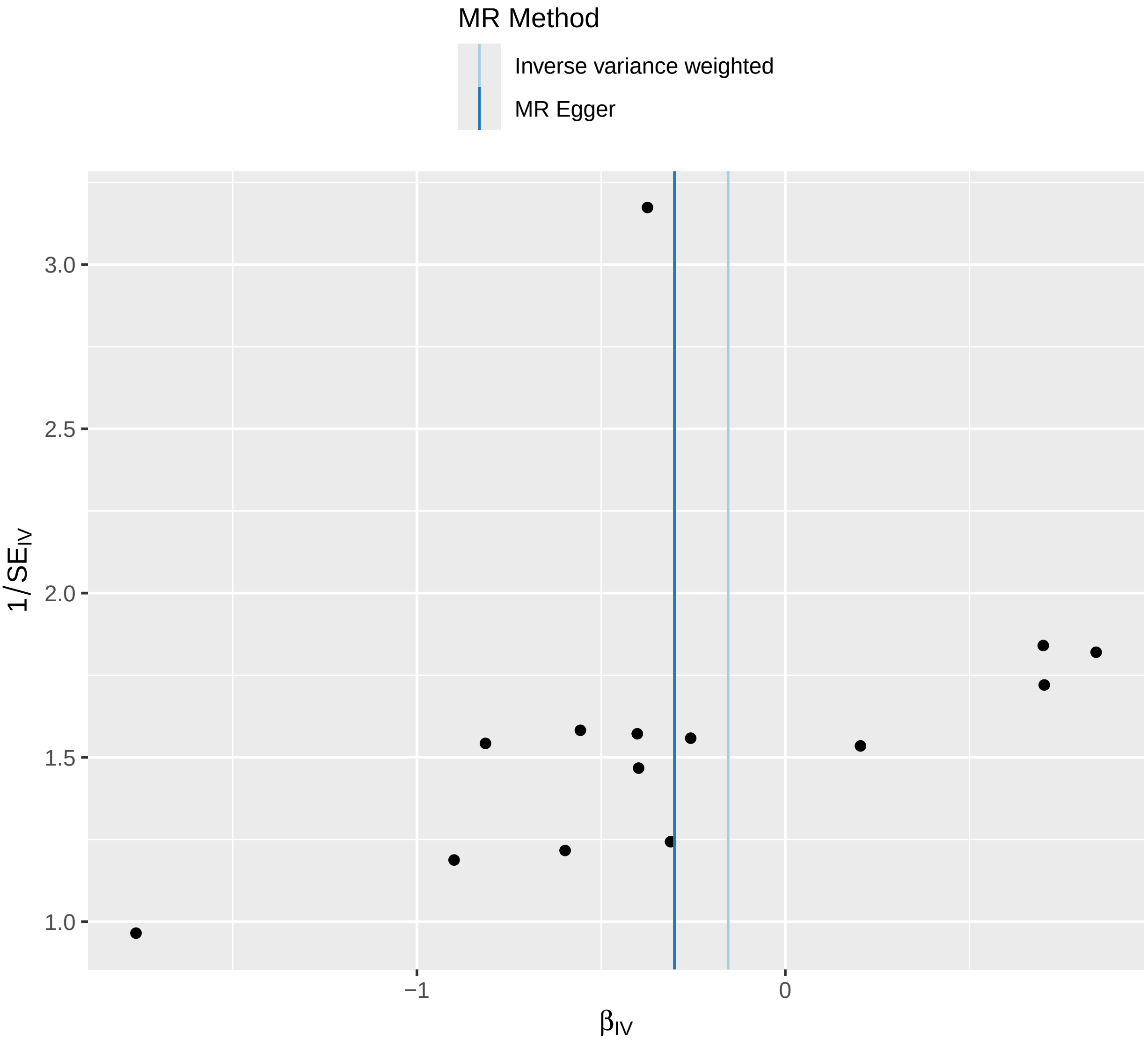

MR Method
Inverse variance weighted
MR Egger
3.0
2.5
2.0
1.5
1.0
1/SE_IV
−1
0
β_IV

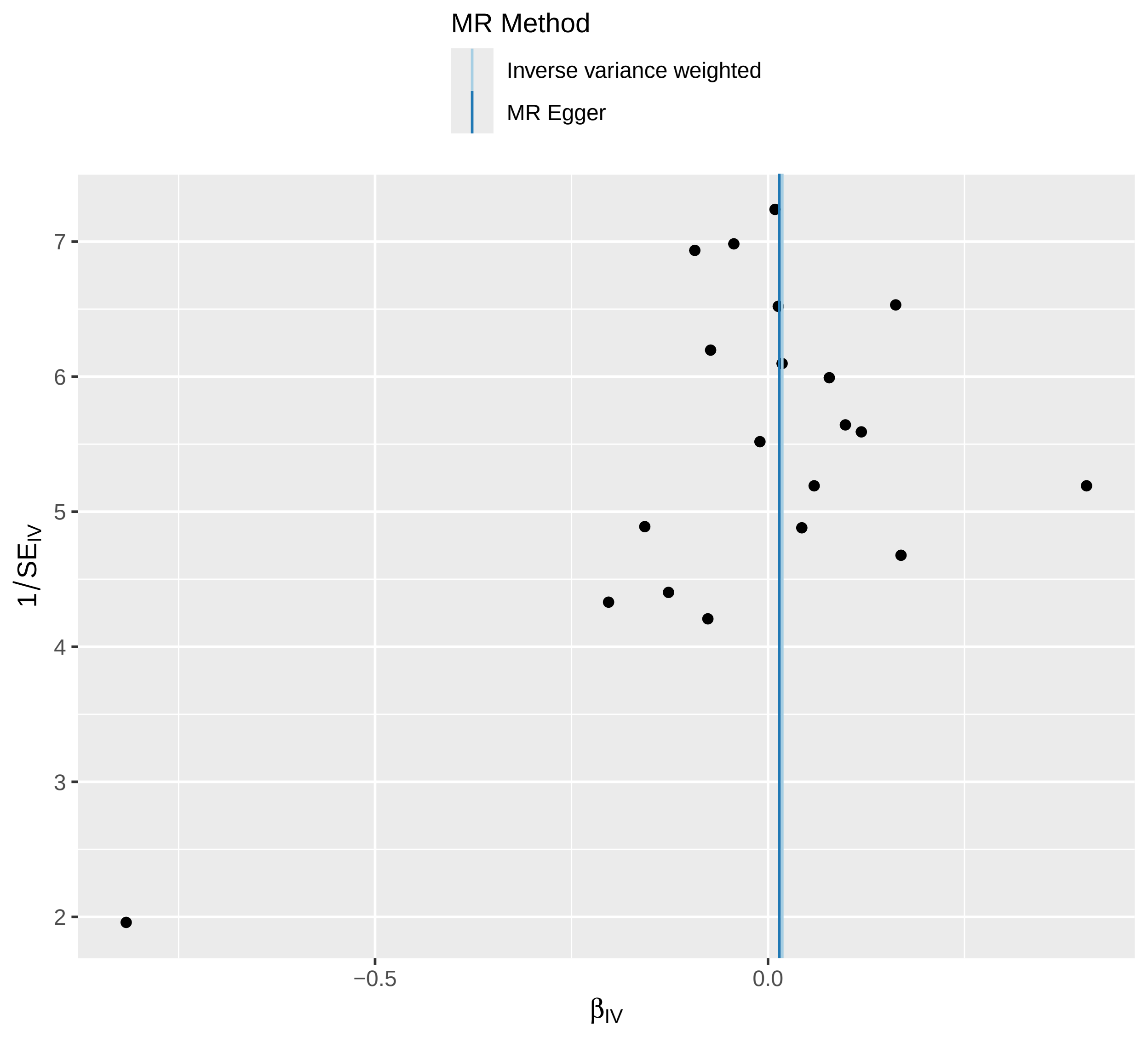
MR Method
Inverse variance weighted
MR Egger
7
6
5
4
3
2
$1/SE_{IV}$
−0.5
0.0
β_{IV}

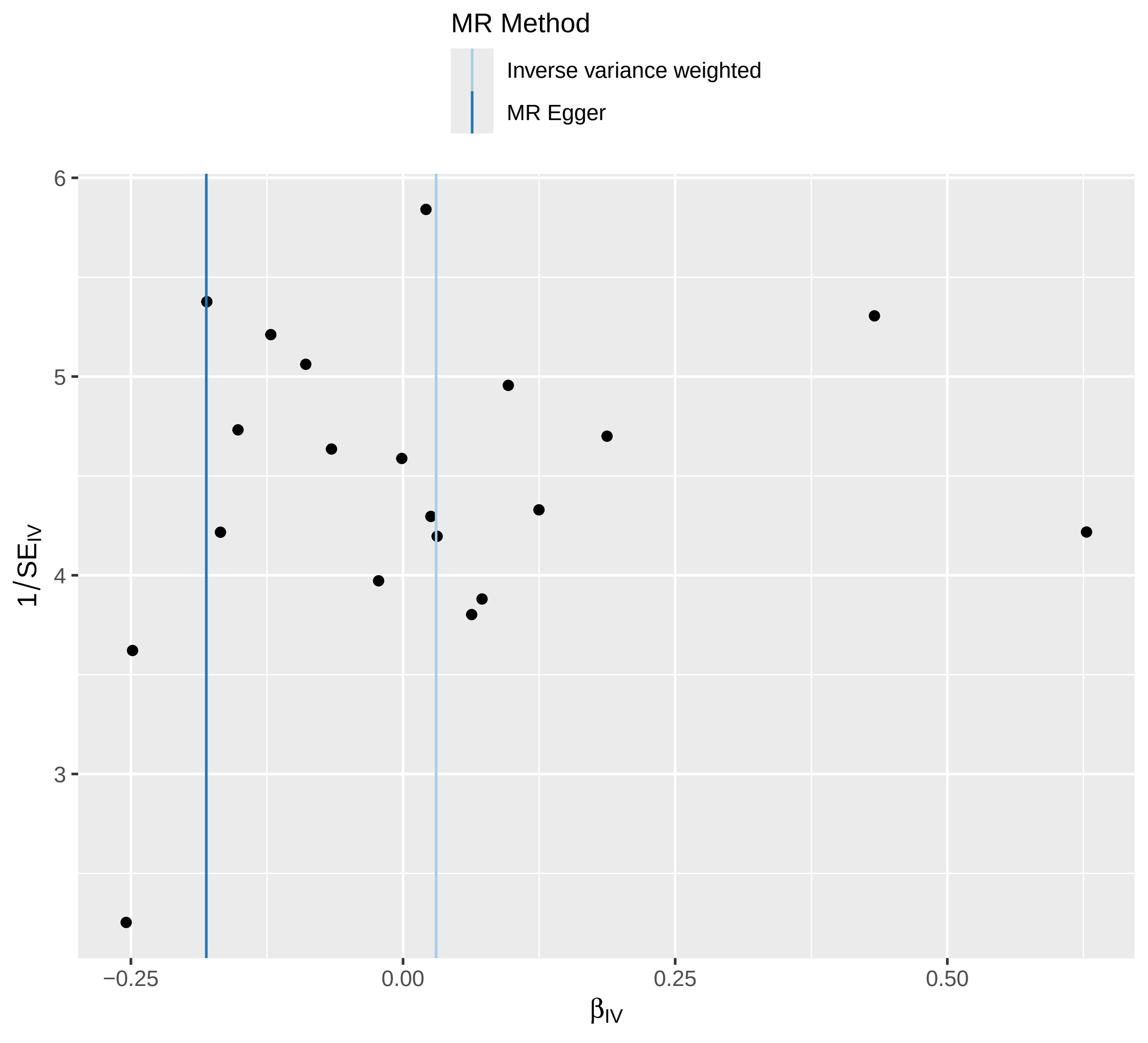

MR Method
Inverse variance weighted
MR Egger
6
5
4
3
1/SE_IV
−0.25
0.00
0.25
0.50
β_IV

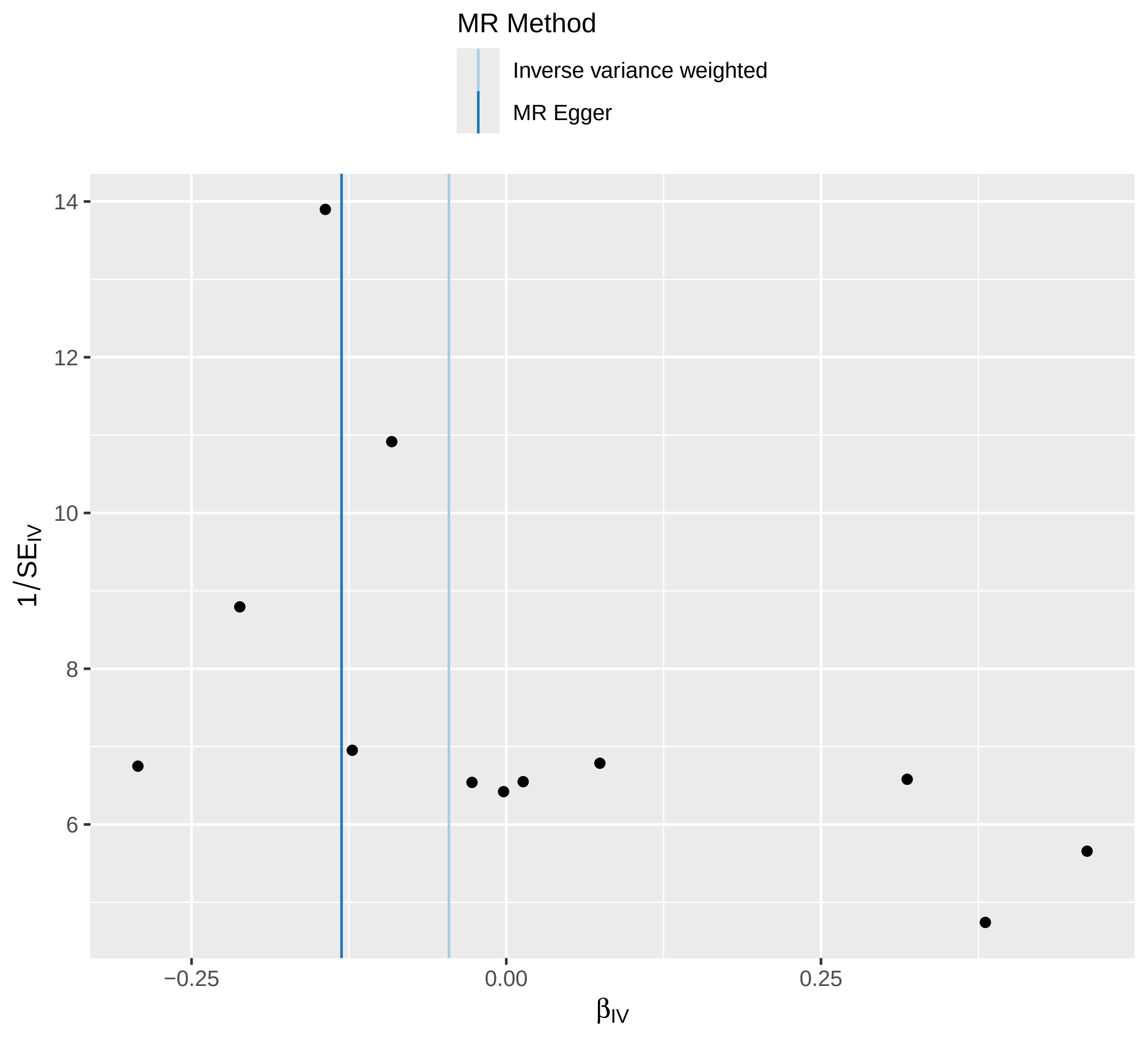

MR Method
Inverse variance weighted
MR Egger
14
12
10
8
6
1/SE_IV
−0.25
0.00
0.25
β_IV

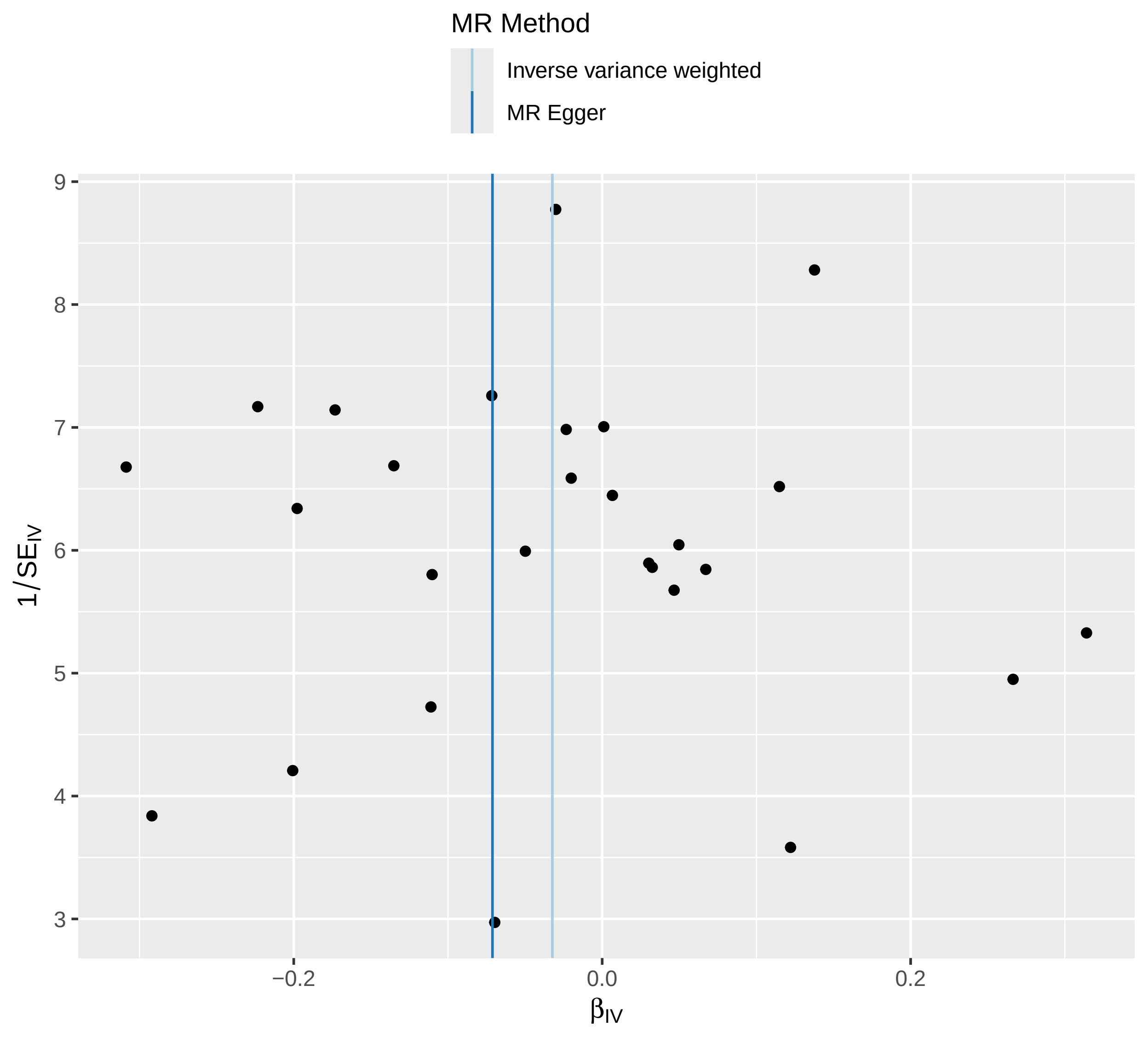

MR Method
Inverse variance weighted
MR Egger
9
8
7
6
5
4
3
1/SE_IV
−0.2
0.0
0.2
β_IV

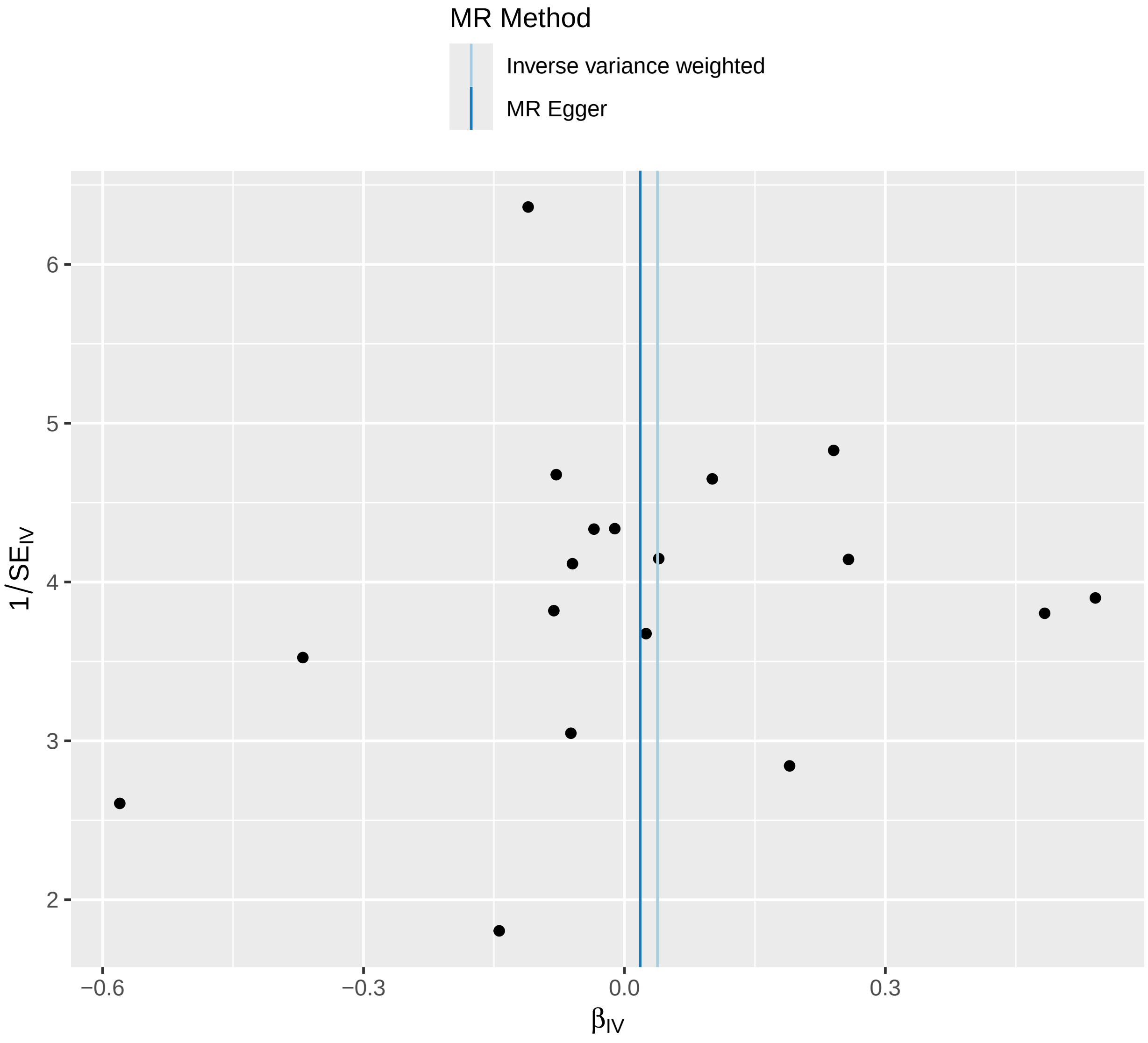
MR Method
Inverse variance weighted
MR Egger
6
5
4
3
2
1/SE_IV
−0.6
−0.3
0.0
0.3
β_IV

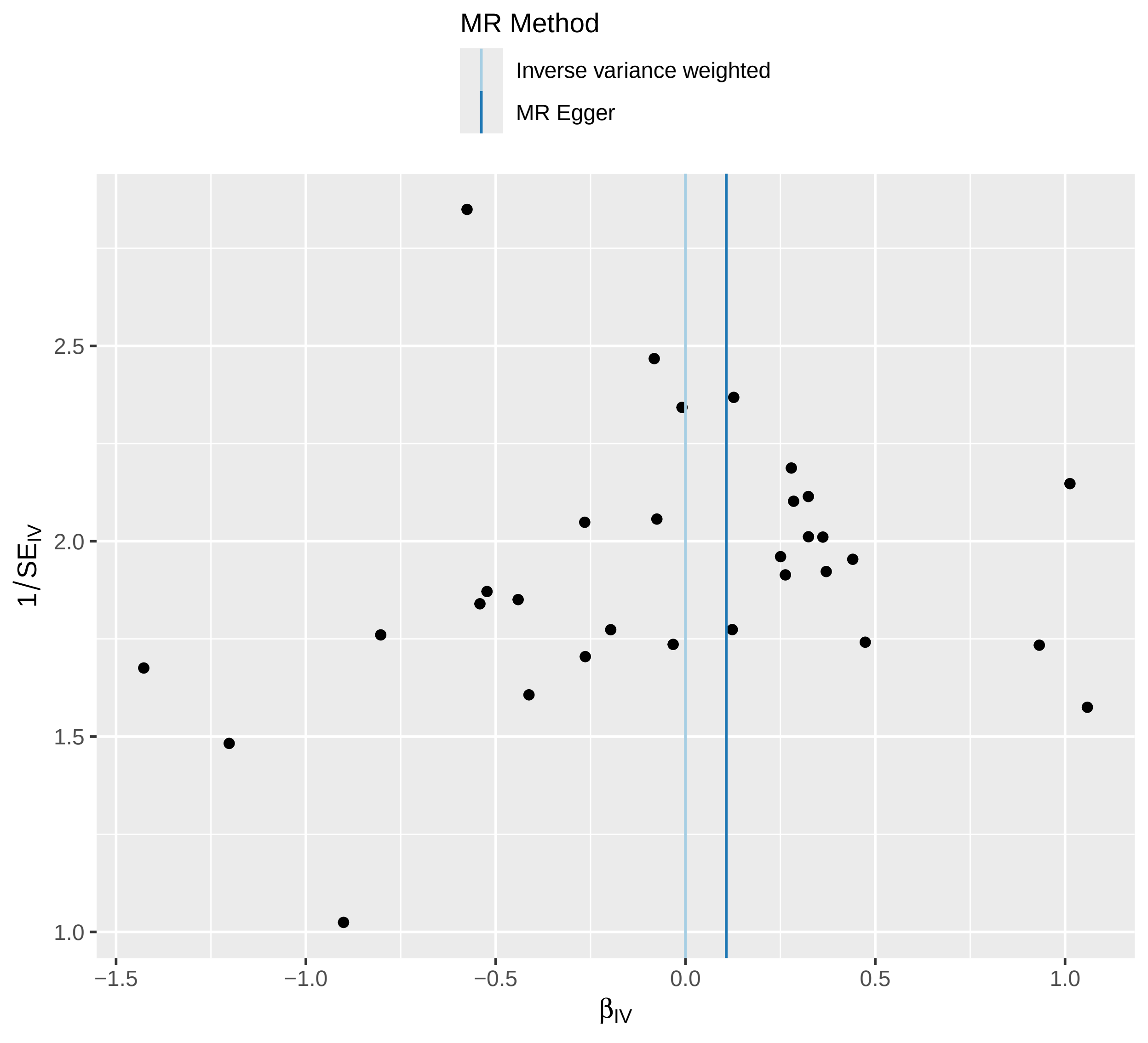

MR Method
Inverse variance weighted
MR Egger
1/SE_IV
2.5
2.0
1.5
1.0
−1.5
−1.0
−0.5
0.0
0.5
1.0
β_IV

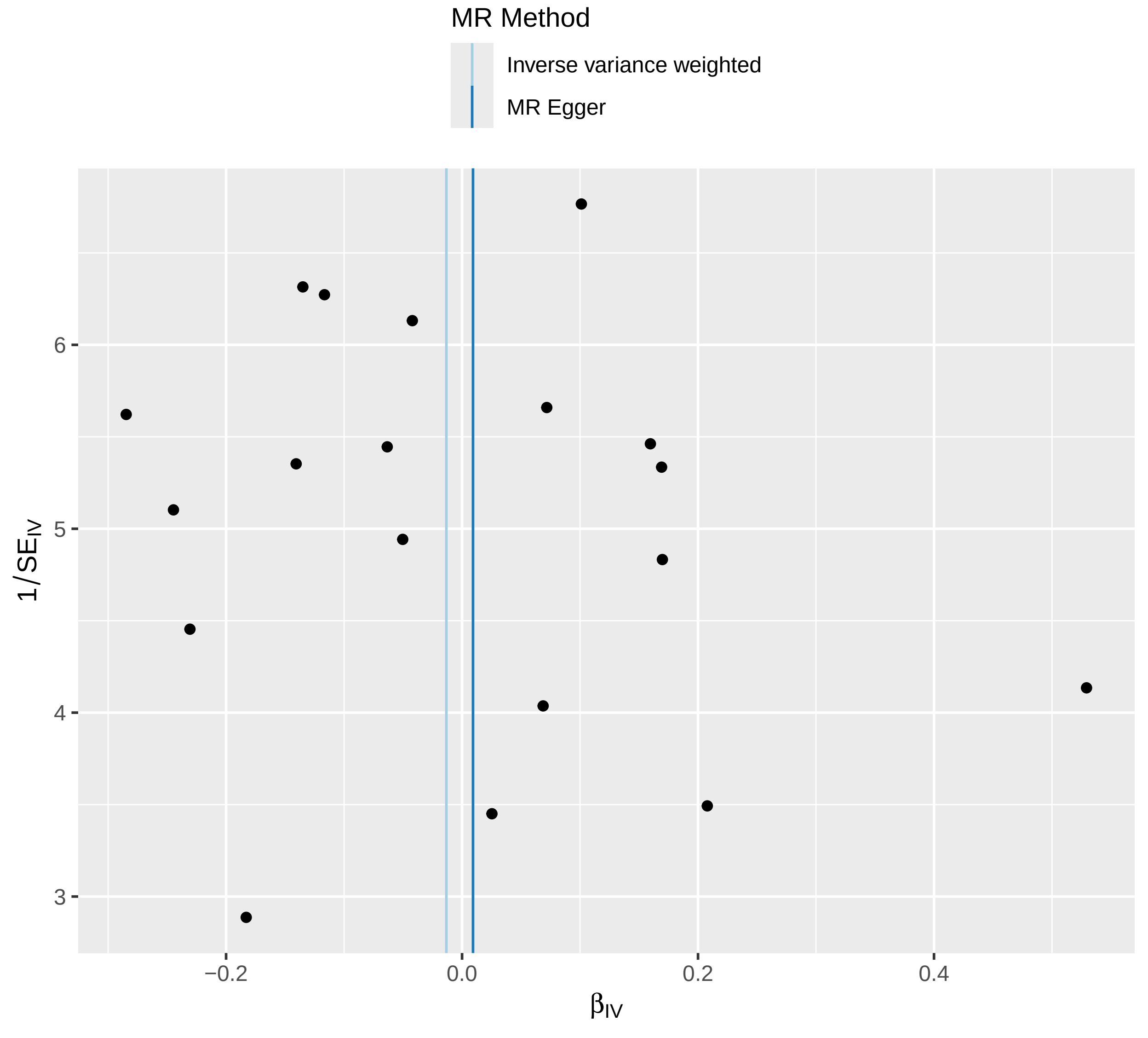

MR Method
Inverse variance weighted
MR Egger
6
5
4
3
1/SE$_{IV}$
−0.2
0.0
0.2
0.4
β$_{IV}$

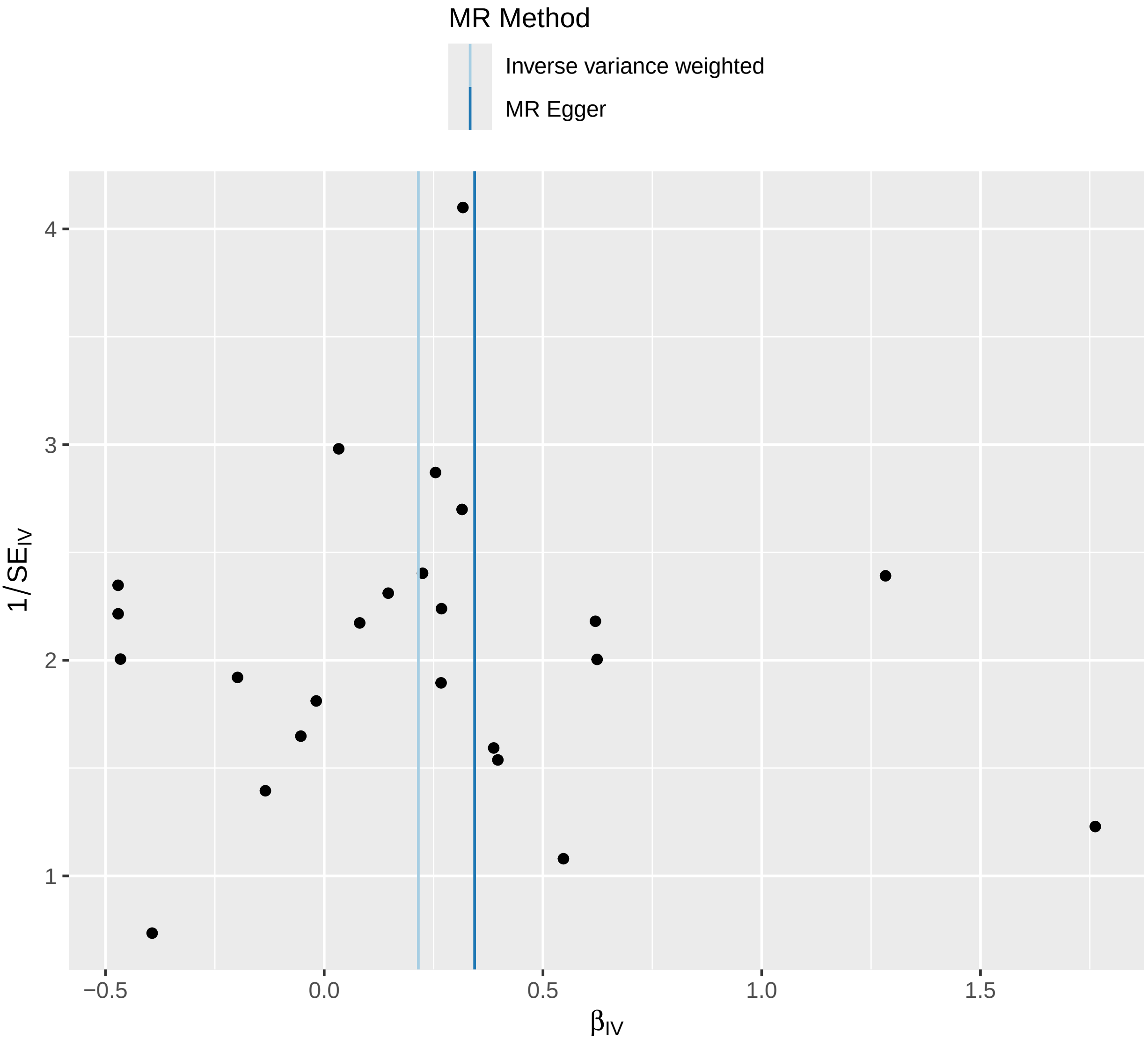
MR Method
Inverse variance weighted
MR Egger
1/SE_IV
β_IV
4
3
2
1
−0.5
0.0
0.5
1.0
1.5

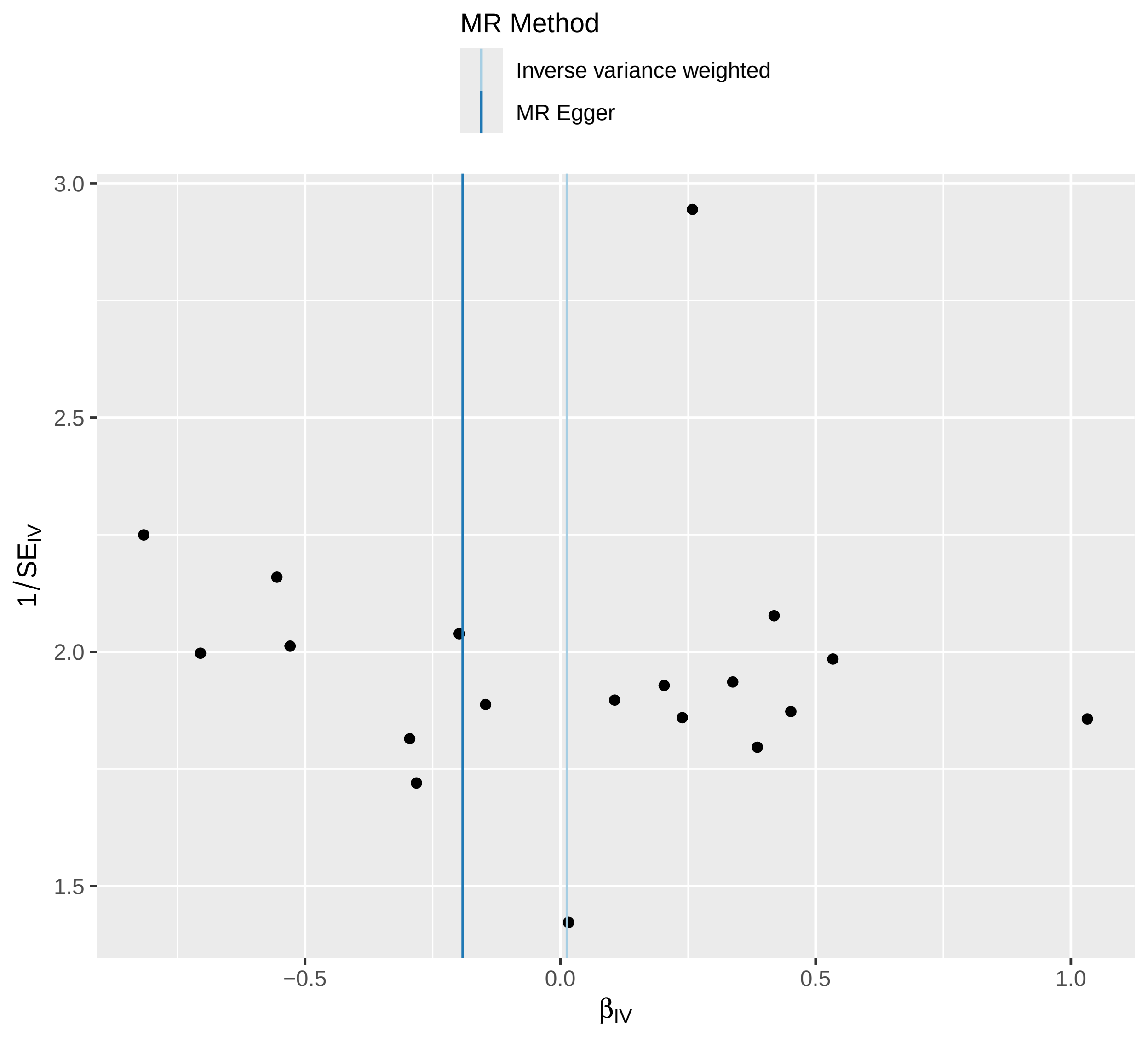
MR Method
Inverse variance weighted
MR Egger
3.0
2.5
2.0
1.5
−0.5
0.0
0.5
1.0
$1/SE_{IV}$
β_{IV}

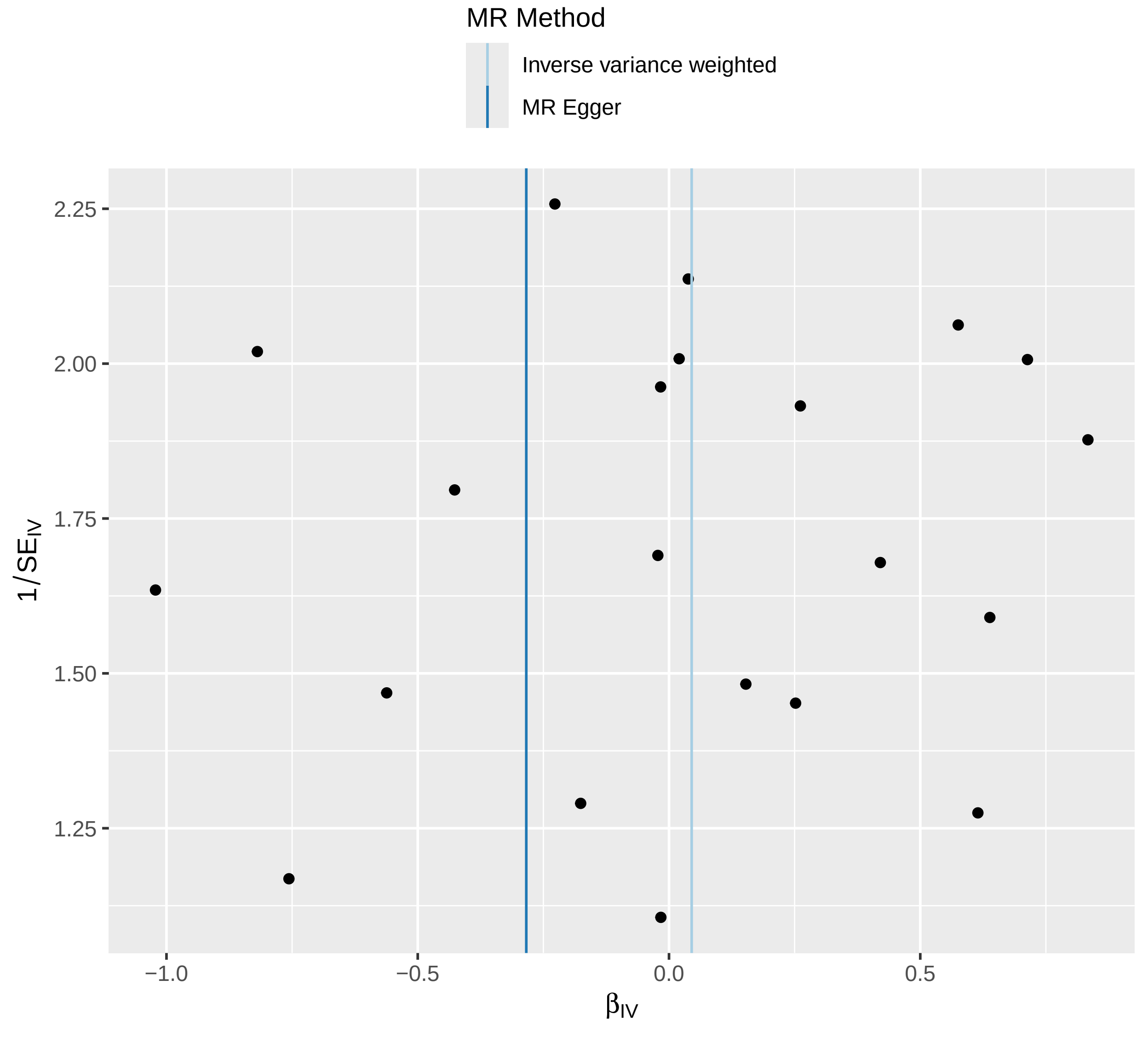
MR Method
Inverse variance weighted
MR Egger
2.25
2.00
1.75
1.50
1.25
1/SE_IV
−1.0
−0.5
0.0
0.5
β_IV

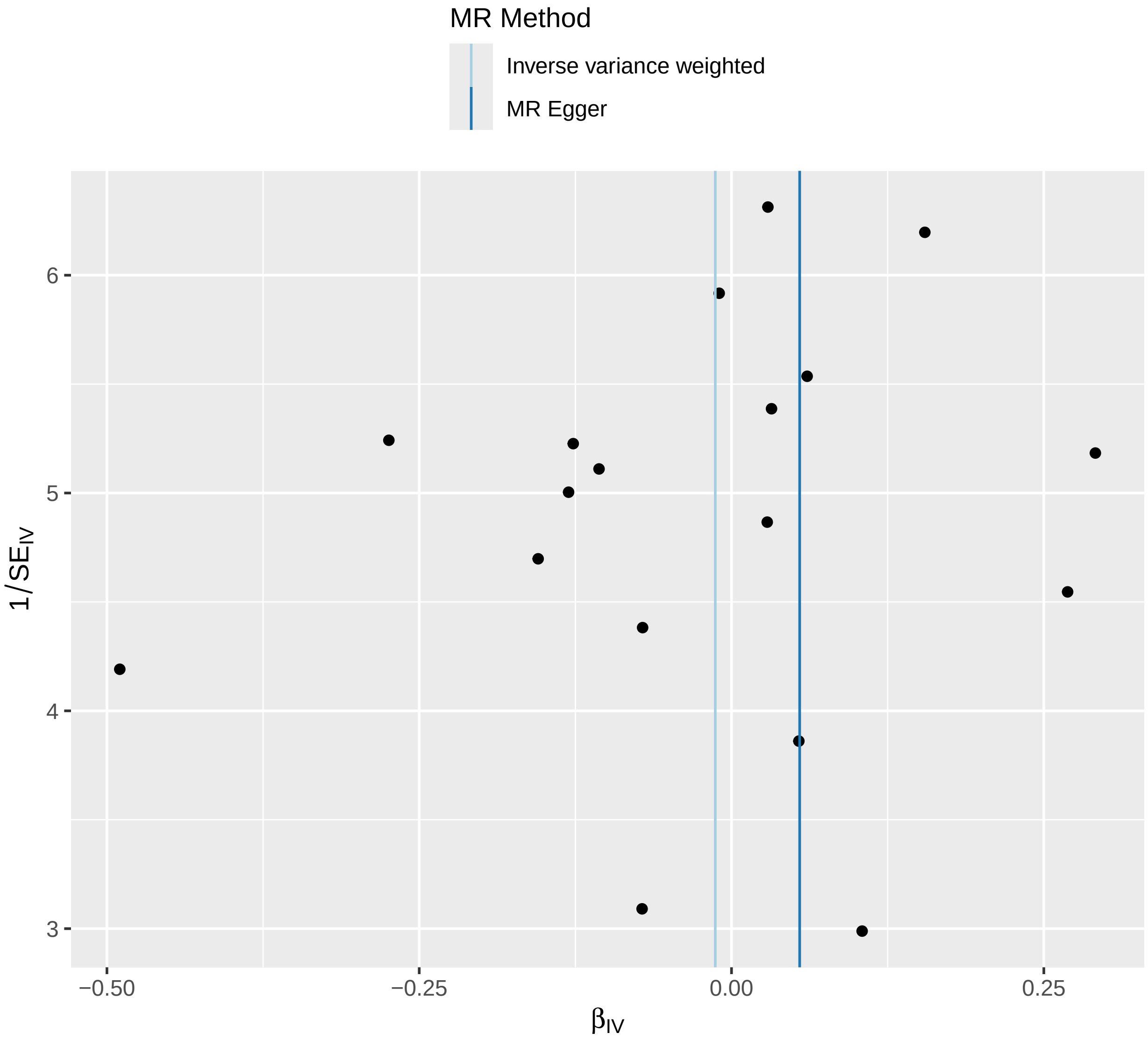

MR Method
Inverse variance weighted
MR Egger
6
5
4
3
1/SE_IV
−0.50
−0.25
0.00
0.25
β_IV

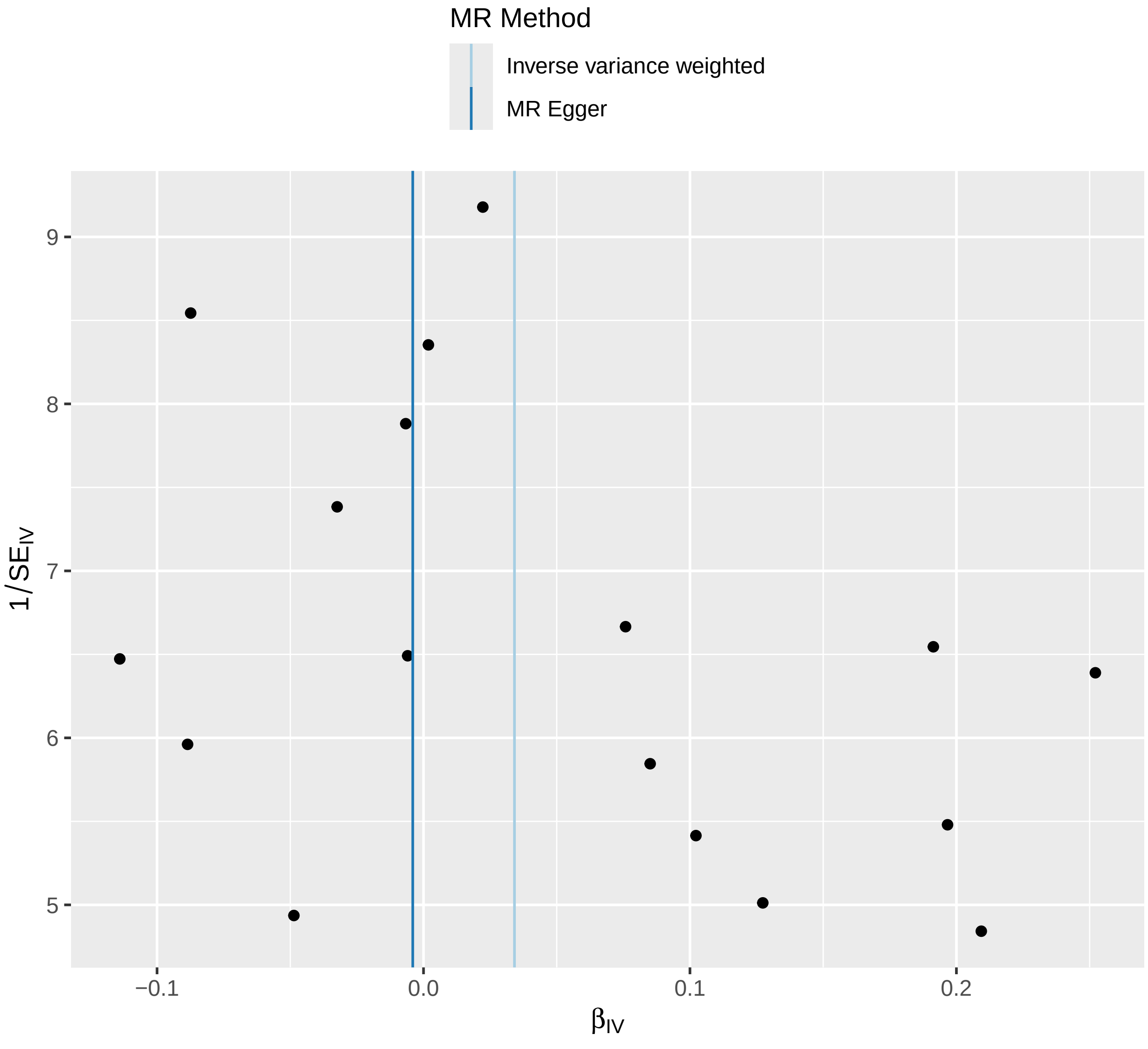
MR Method
Inverse variance weighted
MR Egger
9
8
7
6
5
$1/SE_{IV}$
−0.1
0.0
0.1
0.2
β_{IV}

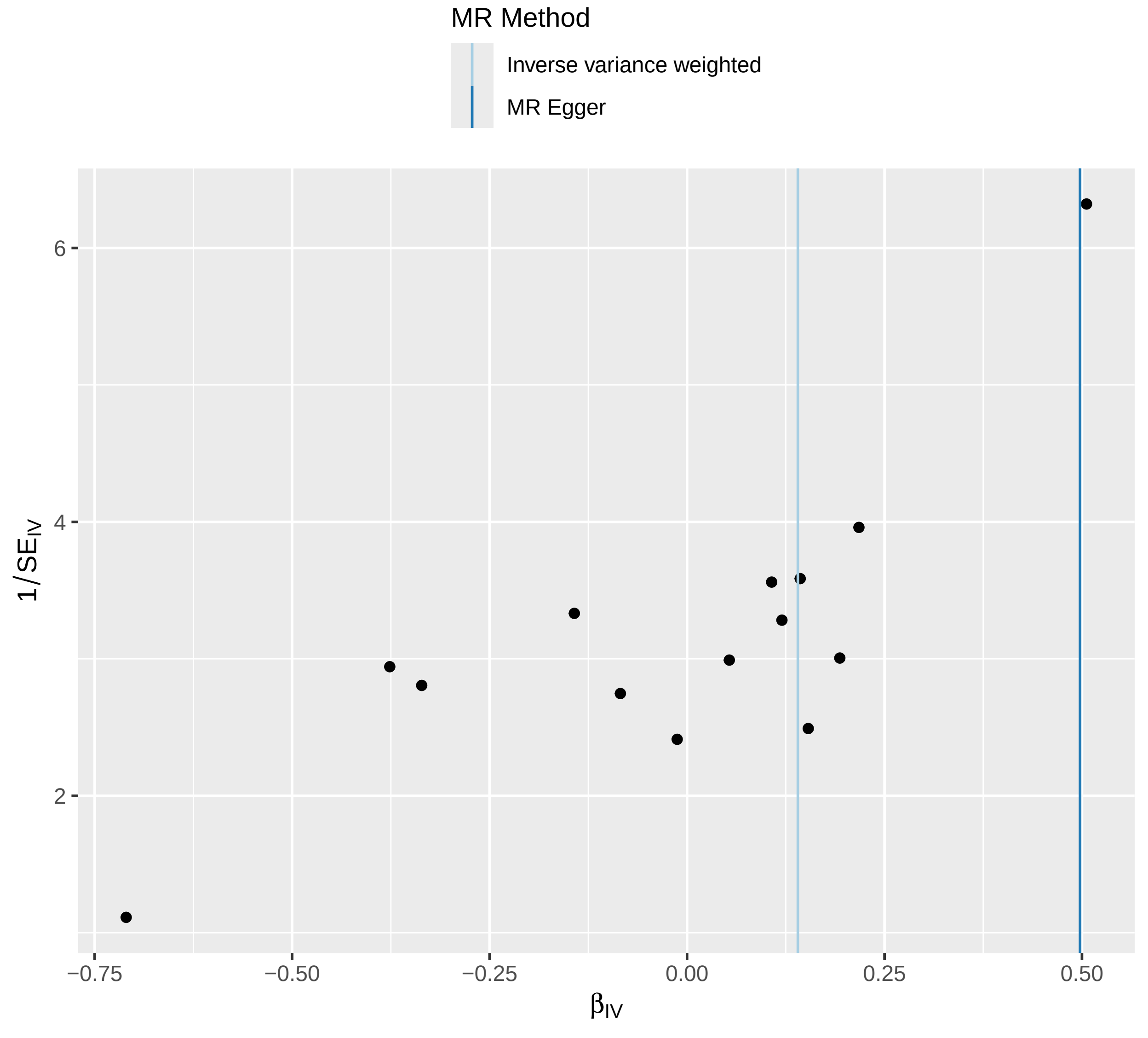

MR Method
Inverse variance weighted
MR Egger
6
4
2
$1/SE_{IV}$
−0.75
−0.50
−0.25
0.00
0.25
0.50
β_{IV}

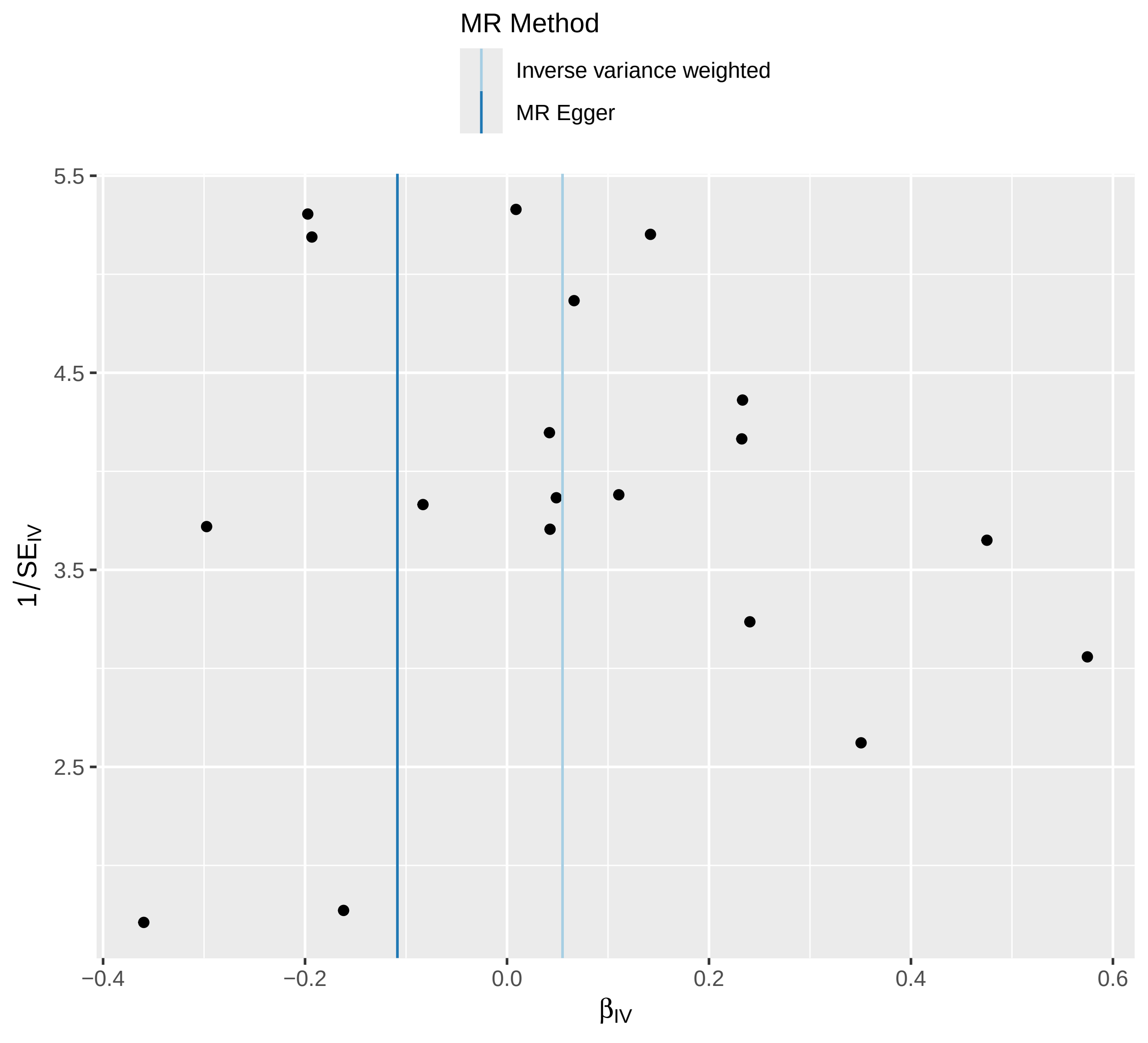

MR Method
Inverse variance weighted
MR Egger
5.5
4.5
3.5
2.5
1/SE$_{IV}$
−0.4
−0.2
0.0
0.2
0.4
0.6
β$_{IV}$

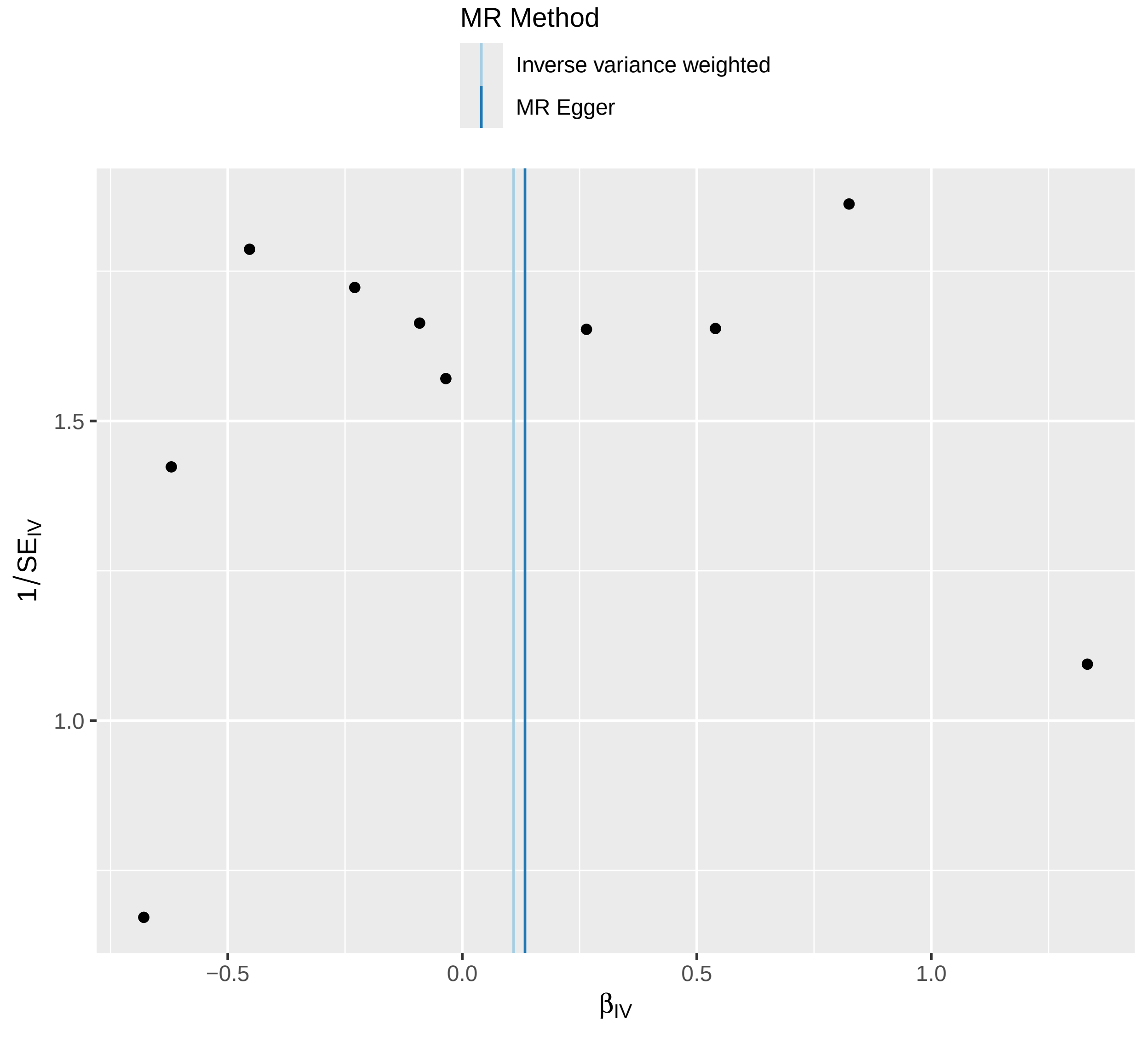

MR Method
Inverse variance weighted
MR Egger
1.5
1.0
1/SE_IV
−0.5
0.0
0.5
1.0
β_IV

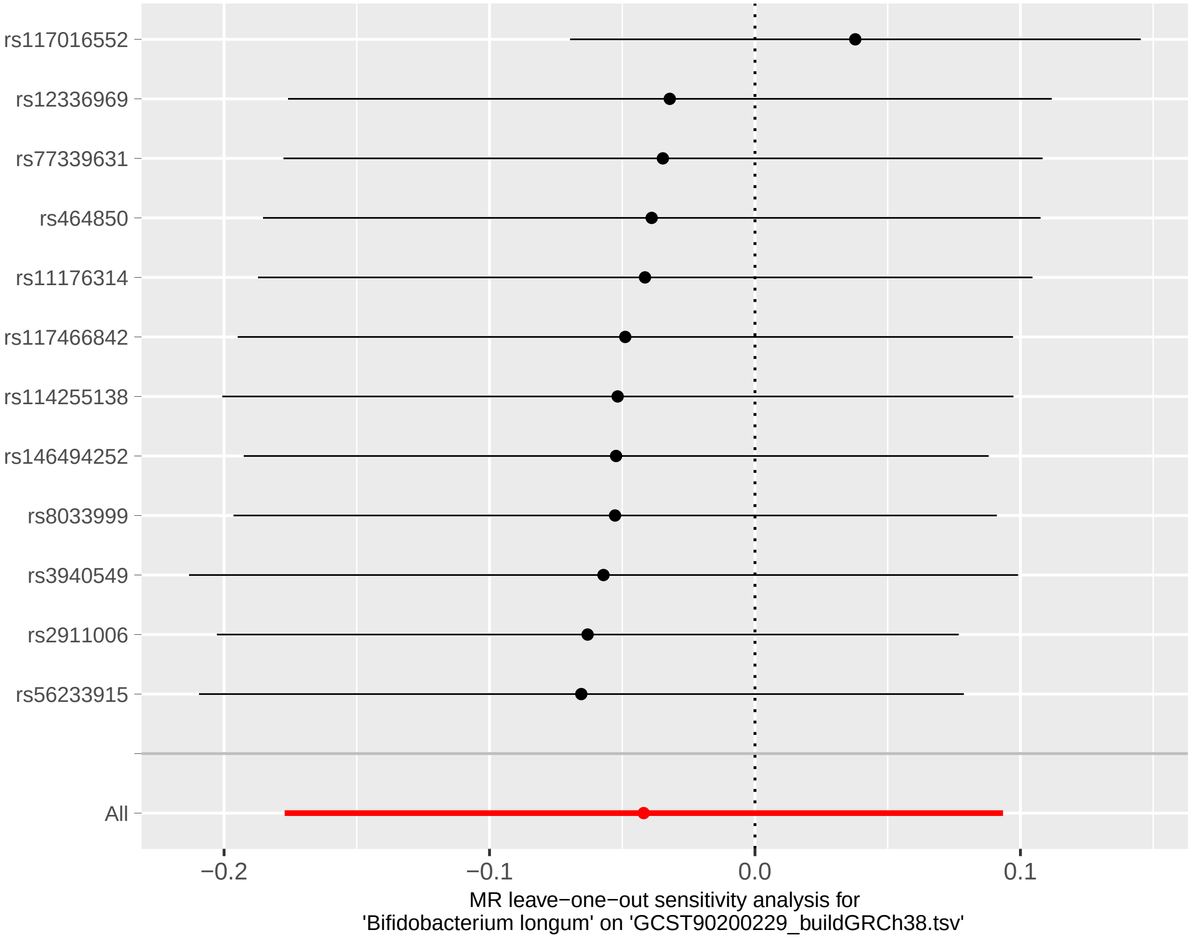

rs117016552
rs12336969
rs77339631
rs464850
rs11176314
rs117466842
rs114255138
rs146494252
rs8033999
rs3940549
rs2911006
rs56233915
All
−0.2
−0.1
0.0
0.1
MR leave−one−out sensitivity analysis for
'Bifidobacterium longum' on 'GCST90200229_buildGRCh38.tsv'

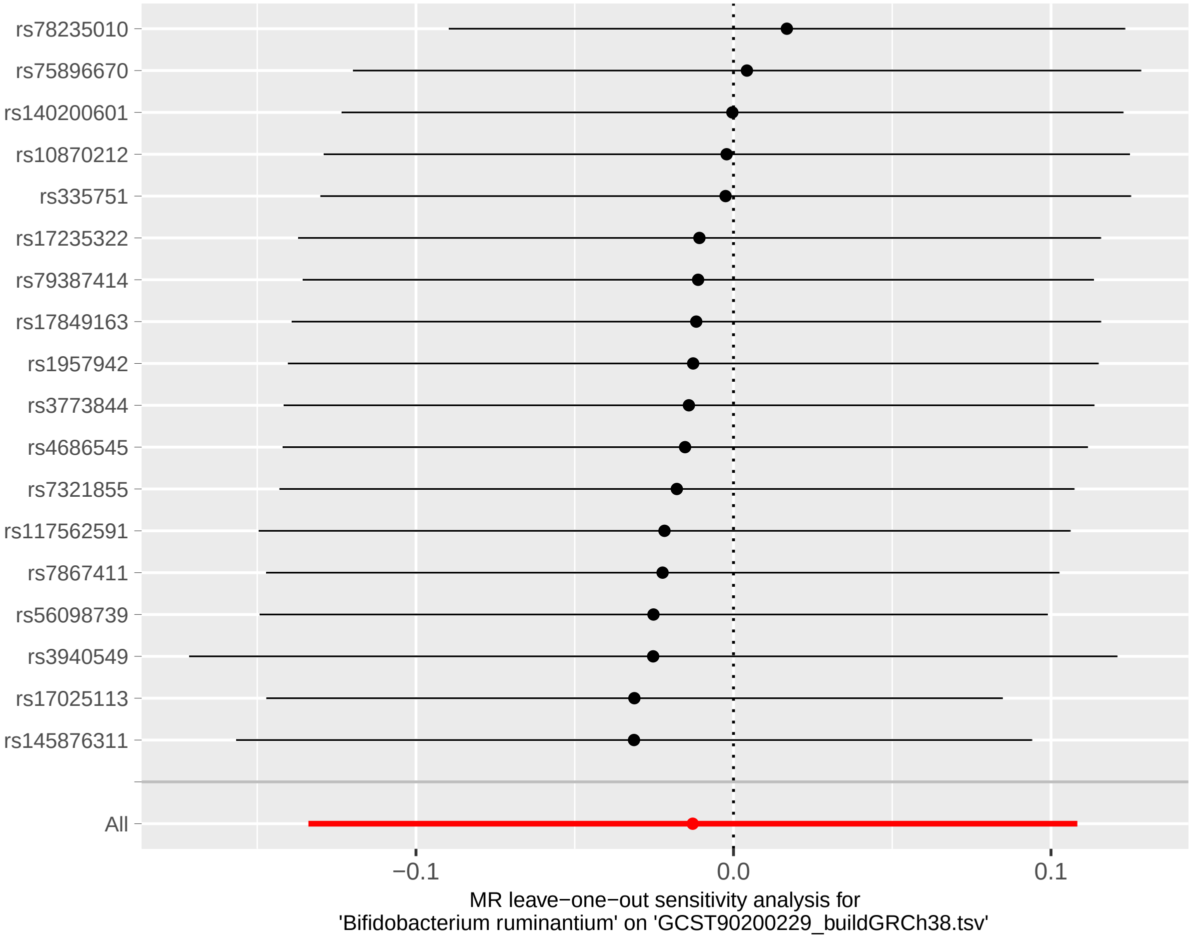
rs78235010
rs75896670
rs140200601
rs10870212
rs335751
rs17235322
rs79387414
rs17849163
rs1957942
rs3773844
rs4686545
rs7321855
rs117562591
rs7867411
rs56098739
rs3940549
rs17025113
rs145876311
All
−0.1
0.0
0.1
MR leave−one−out sensitivity analysis for
'Bifidobacterium ruminantium' on 'GCST90200229_buildGRCh38.tsv'

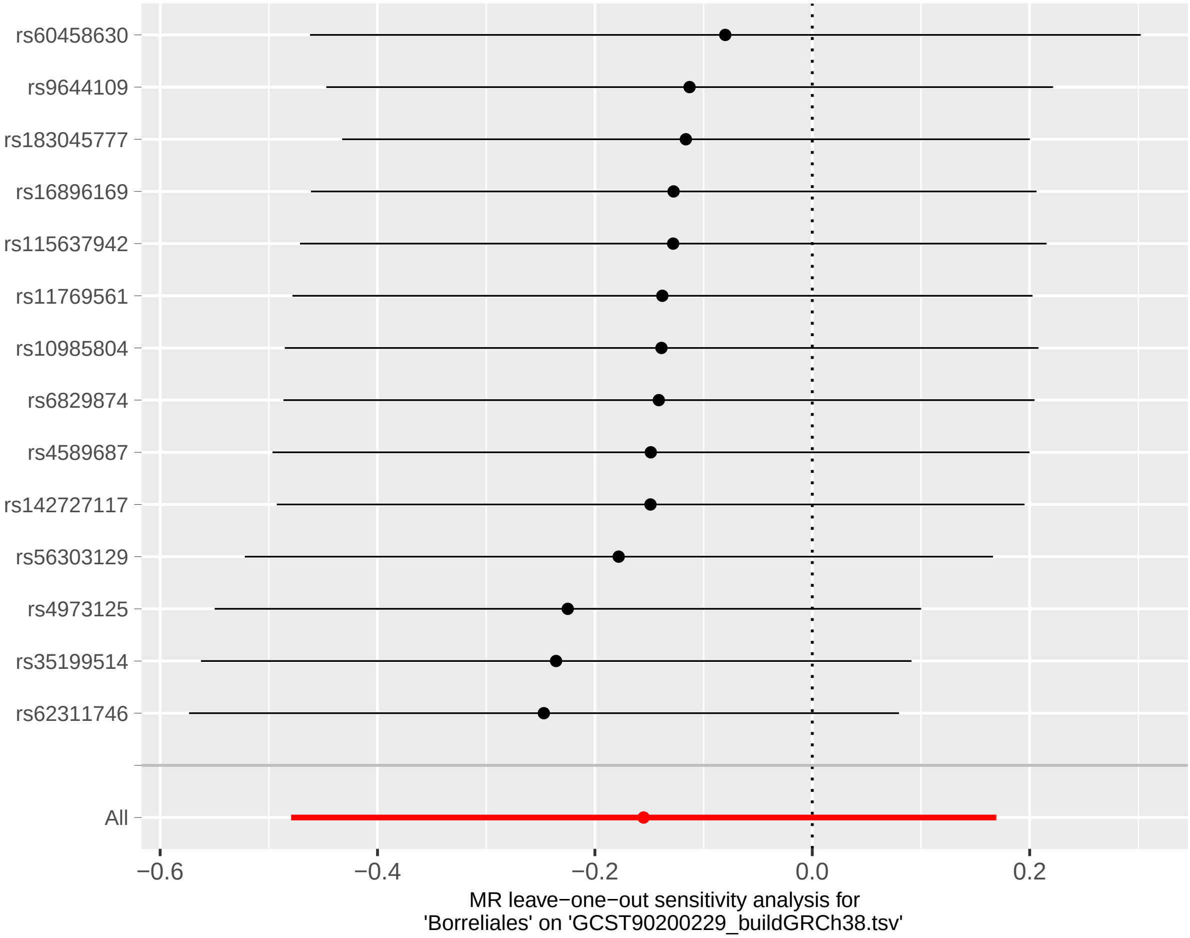

rs60458630
rs9644109
rs183045777
rs16896169
rs115637942
rs11769561
rs10985804
rs6829874
rs4589687
rs142727117
rs56303129
rs4973125
rs35199514
rs62311746
All
−0.6
−0.4
−0.2
0.0
0.2
MR leave−one−out sensitivity analysis for
'Borreliales' on 'GCST90200229_buildGRCh38.tsv'

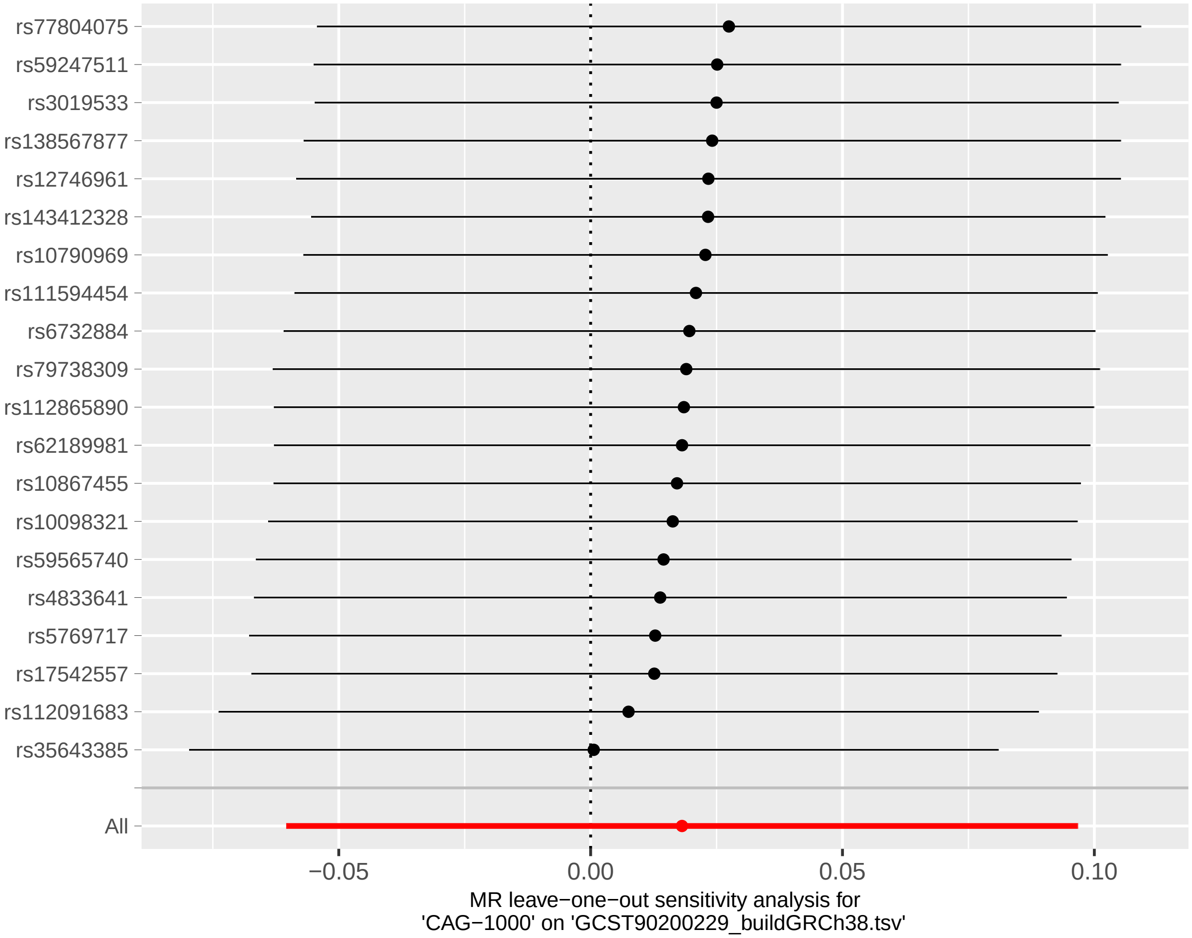

rs77804075
rs59247511
rs3019533
rs138567877
rs12746961
rs143412328
rs10790969
rs111594454
rs6732884
rs79738309
rs112865890
rs62189981
rs10867455
rs10098321
rs59565740
rs4833641
rs5769717
rs17542557
rs112091683
rs35643385
All
−0.05
0.00
0.05
0.10
MR leave−one−out sensitivity analysis for
'CAG−1000' on 'GCST90200229_buildGRCh38.tsv'

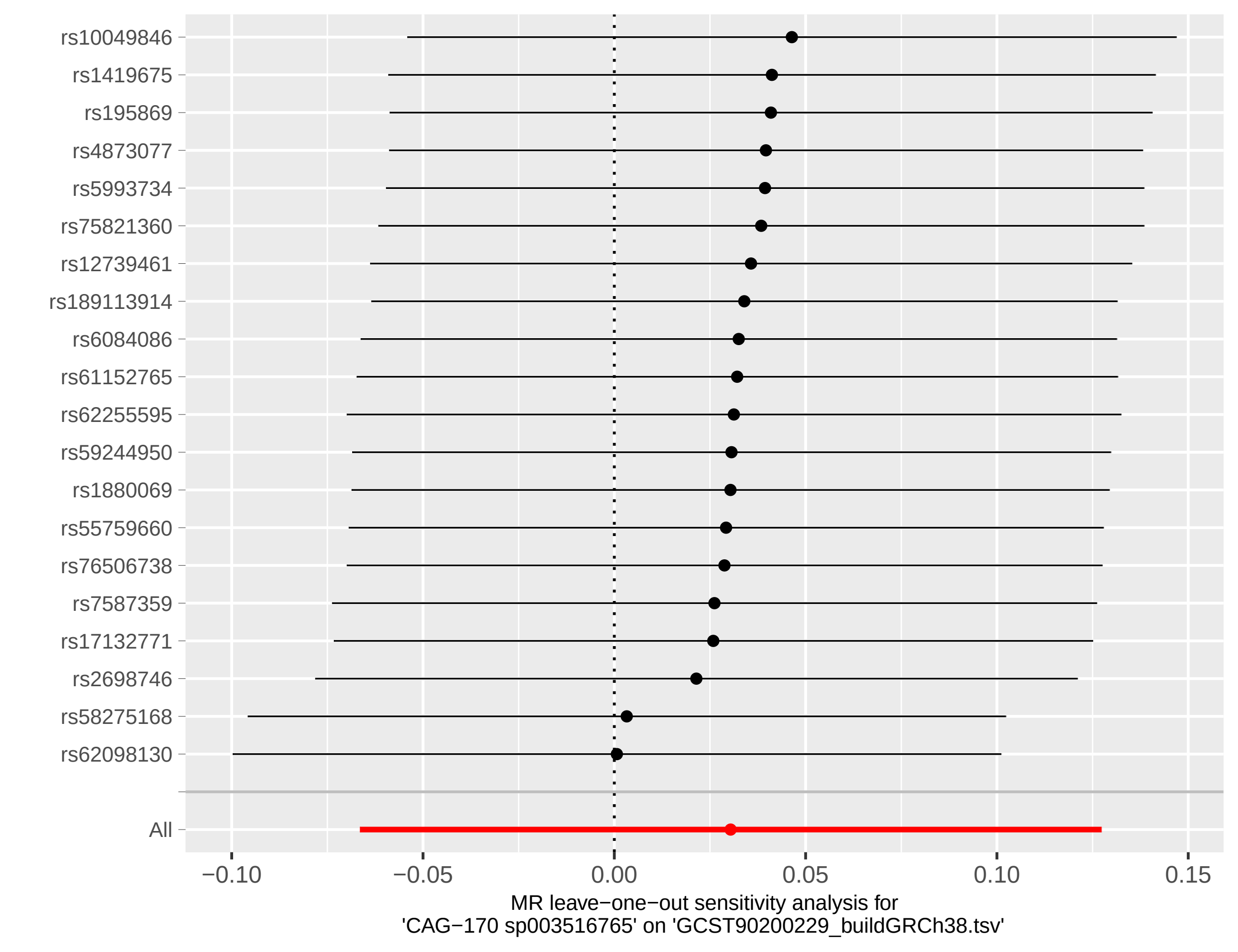

rs10049846
rs1419675
rs195869
rs4873077
rs5993734
rs75821360
rs12739461
rs189113914
rs6084086
rs61152765
rs62255595
rs59244950
rs1880069
rs55759660
rs76506738
rs7587359
rs17132771
rs2698746
rs58275168
rs62098130
All
−0.10
−0.05
0.00
0.05
0.10
0.15
MR leave−one−out sensitivity analysis for
'CAG−170 sp003516765' on 'GCST90200229_buildGRCh38.tsv'

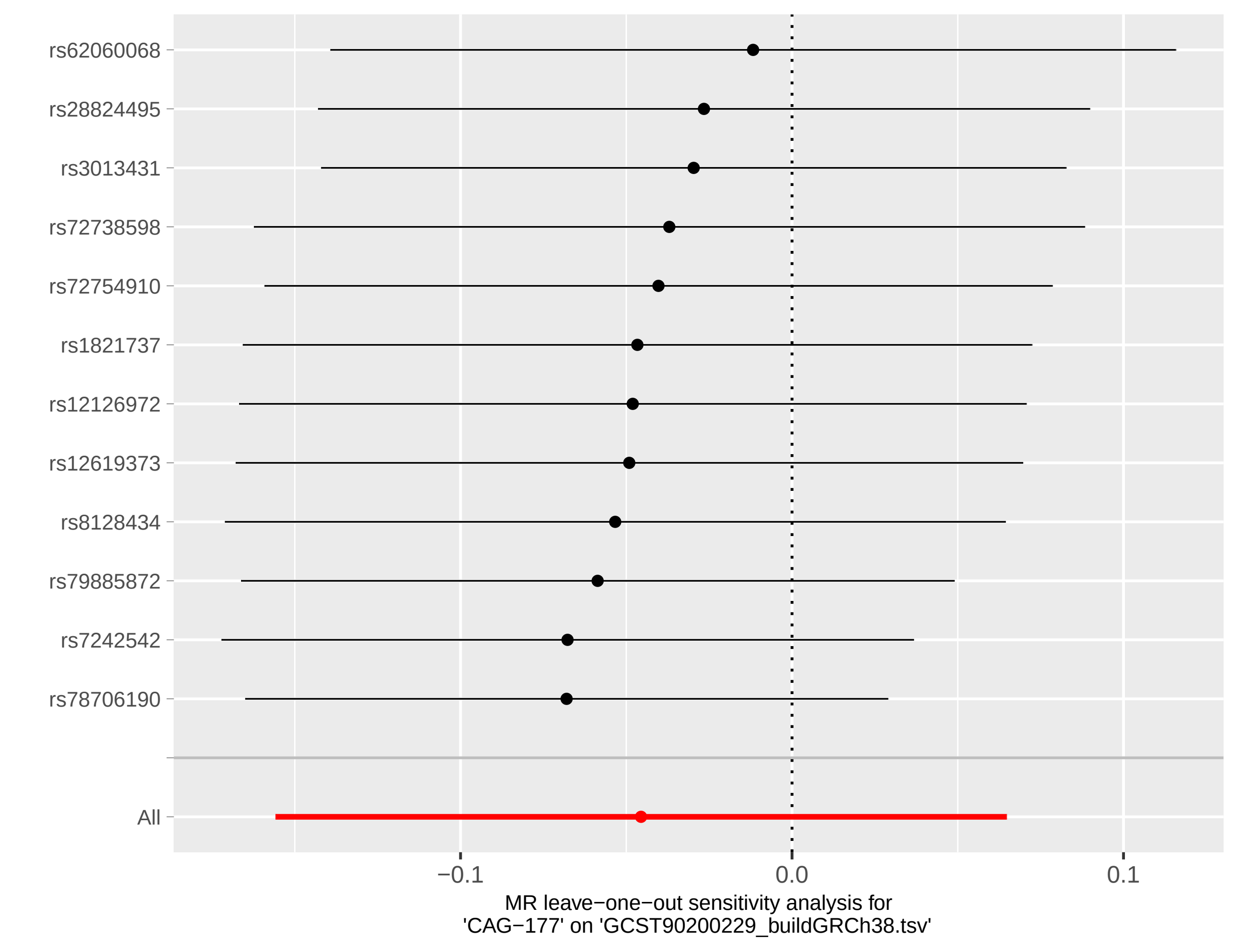

rs62060068
rs28824495
rs3013431
rs72738598
rs72754910
rs1821737
rs12126972
rs12619373
rs8128434
rs79885872
rs7242542
rs78706190
All
−0.1
0.0
0.1
MR leave−one−out sensitivity analysis for
'CAG−177' on 'GCST90200229_buildGRCh38.tsv'

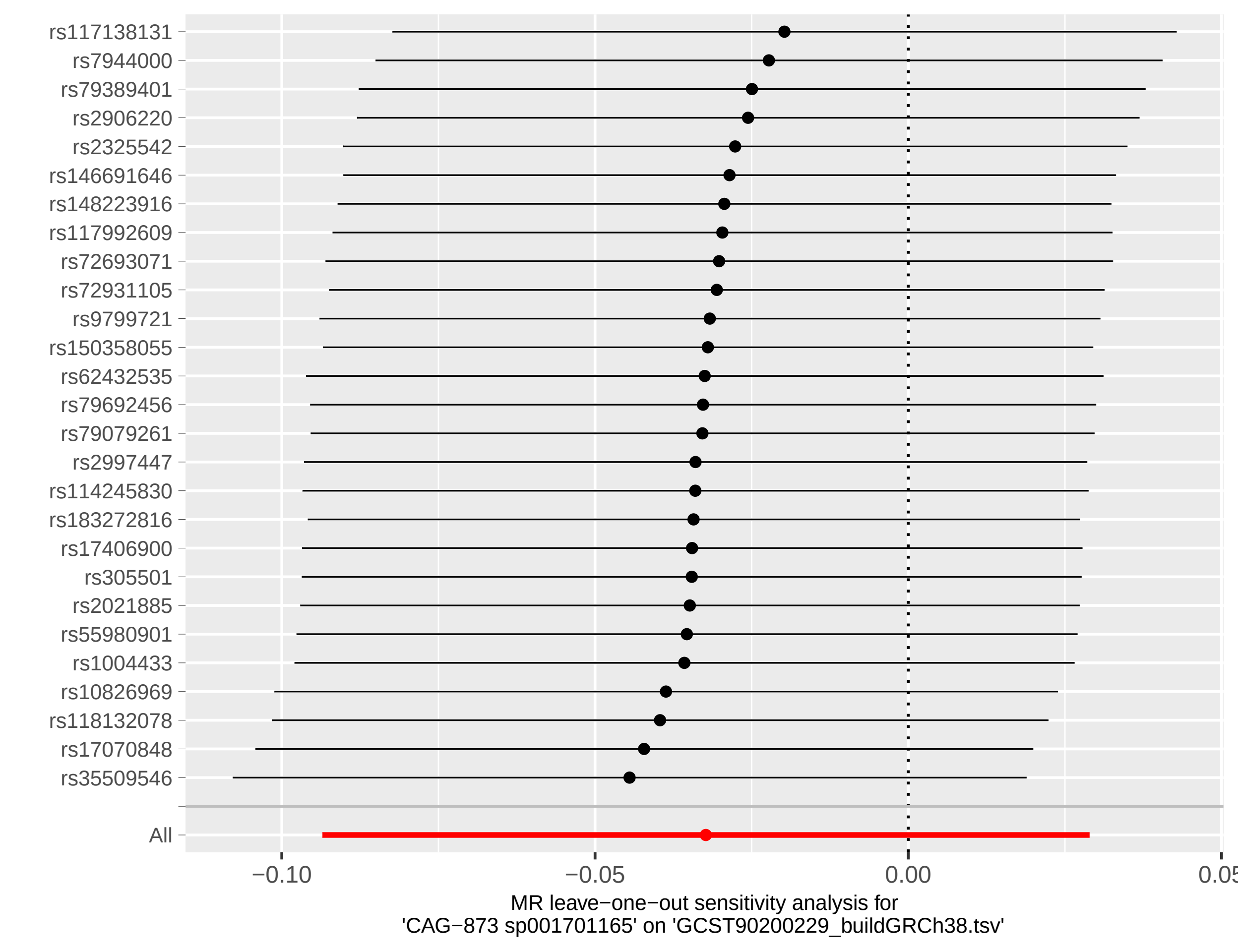
rs117138131
rs7944000
rs79389401
rs2906220
rs2325542
rs146691646
rs148223916
rs117992609
rs72693071
rs72931105
rs9799721
rs150358055
rs62432535
rs79692456
rs79079261
rs2997447
rs114245830
rs183272816
rs17406900
rs305501
rs2021885
rs55980901
rs1004433
rs10826969
rs118132078
rs17070848
rs35509546
All
−0.10
−0.05
0.00
MR leave−one−out sensitivity analysis for
'CAG−873 sp001701165' on 'GCST90200229_buildGRCh38.tsv'

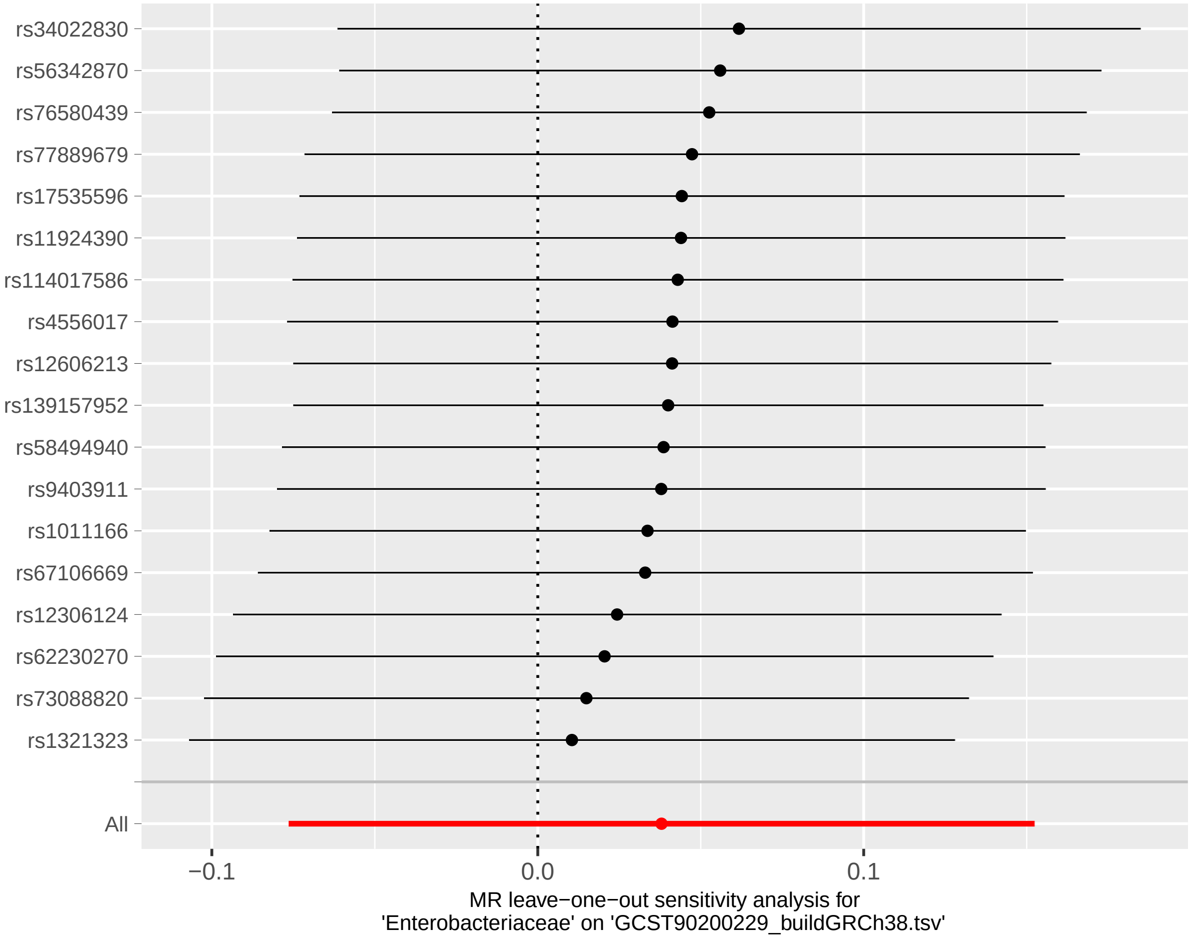
rs34022830
rs56342870
rs76580439
rs77889679
rs17535596
rs11924390
rs114017586
rs4556017
rs12606213
rs139157952
rs58494940
rs9403911
rs1011166
rs67106669
rs12306124
rs62230270
rs73088820
rs1321323
All
−0.1
0.0
0.1
MR leave−one−out sensitivity analysis for
'Enterobacteriaceae' on 'GCST90200229_buildGRCh38.tsv'

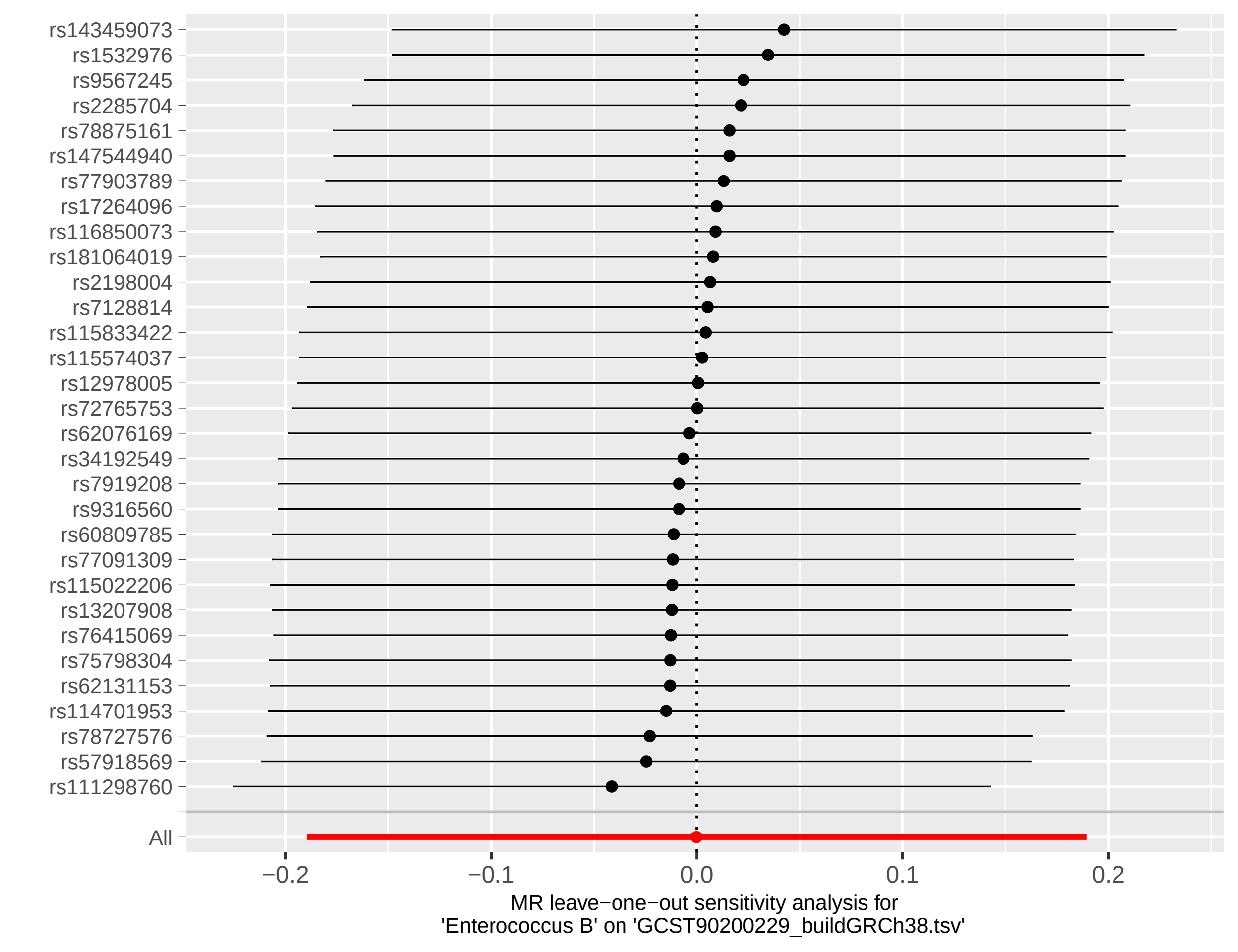
rs143459073
rs1532976
rs9567245
rs2285704
rs78875161
rs147544940
rs77903789
rs17264096
rs116850073
rs181064019
rs2198004
rs7128814
rs115833422
rs115574037
rs12978005
rs72765753
rs62076169
rs34192549
rs7919208
rs9316560
rs60809785
rs77091309
rs115022206
rs13207908
rs76415069
rs75798304
rs62131153
rs114701953
rs78727576
rs57918569
rs111298760
All
−0.2
−0.1
0.0
0.1
0.2
MR leave−one−out sensitivity analysis for
'Enterococcus B' on 'GCST90200229_buildGRCh38.tsv'

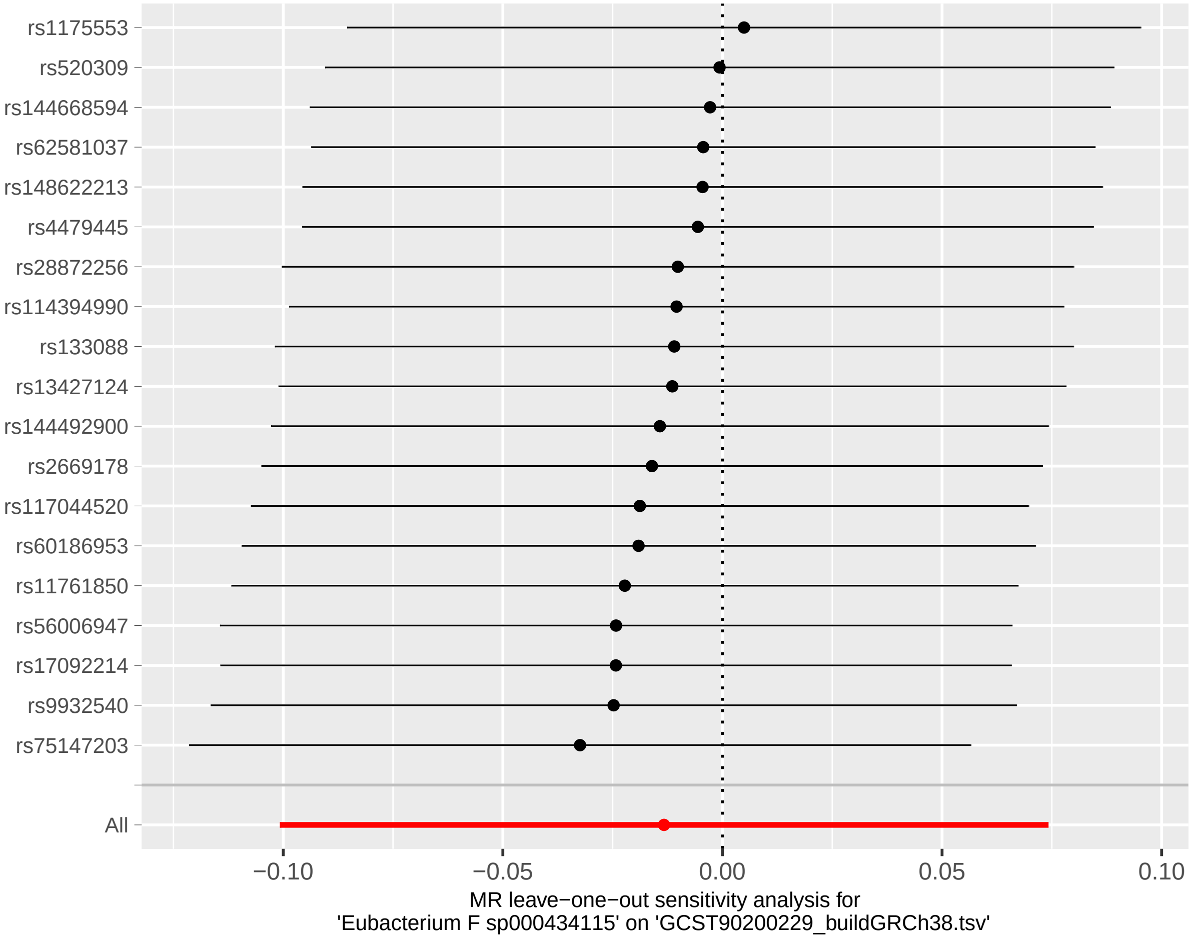

rs1175553
rs520309
rs144668594
rs62581037
rs148622213
rs4479445
rs28872256
rs114394990
rs133088
rs13427124
rs144492900
rs2669178
rs117044520
rs60186953
rs11761850
rs56006947
rs17092214
rs9932540
rs75147203
All
−0.10
−0.05
0.00
0.05
0.10
MR leave−one−out sensitivity analysis for
'Eubacterium F sp000434115' on 'GCST90200229_buildGRCh38.tsv'

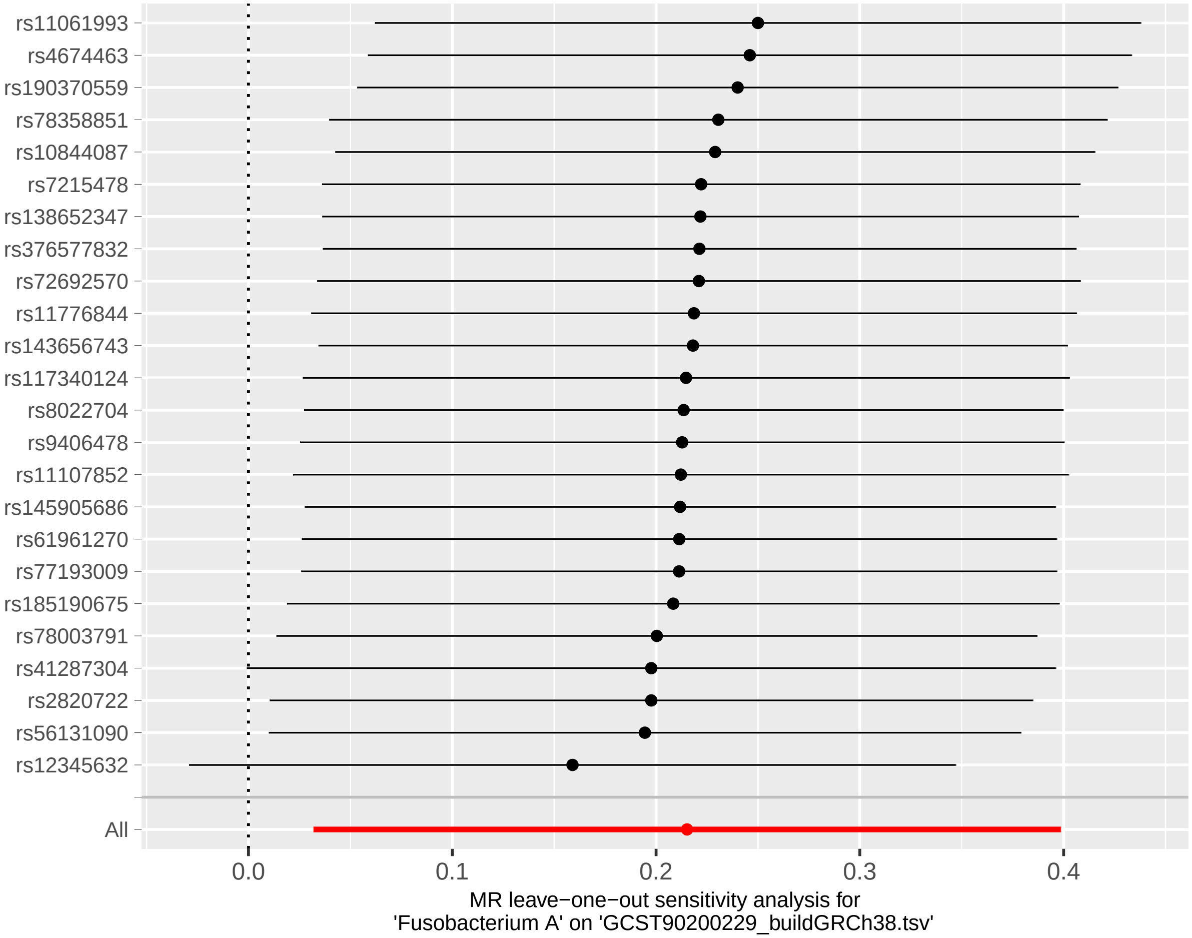
rs11061993
rs4674463
rs190370559
rs78358851
rs10844087
rs7215478
rs138652347
rs376577832
rs72692570
rs11776844
rs143656743
rs117340124
rs8022704
rs9406478
rs11107852
rs145905686
rs61961270
rs77193009
rs185190675
rs78003791
rs41287304
rs2820722
rs56131090
rs12345632
All
0.0
0.1
0.2
0.3
0.4
MR leave−one−out sensitivity analysis for
'Fusobacterium A' on 'GCST90200229_buildGRCh38.tsv'

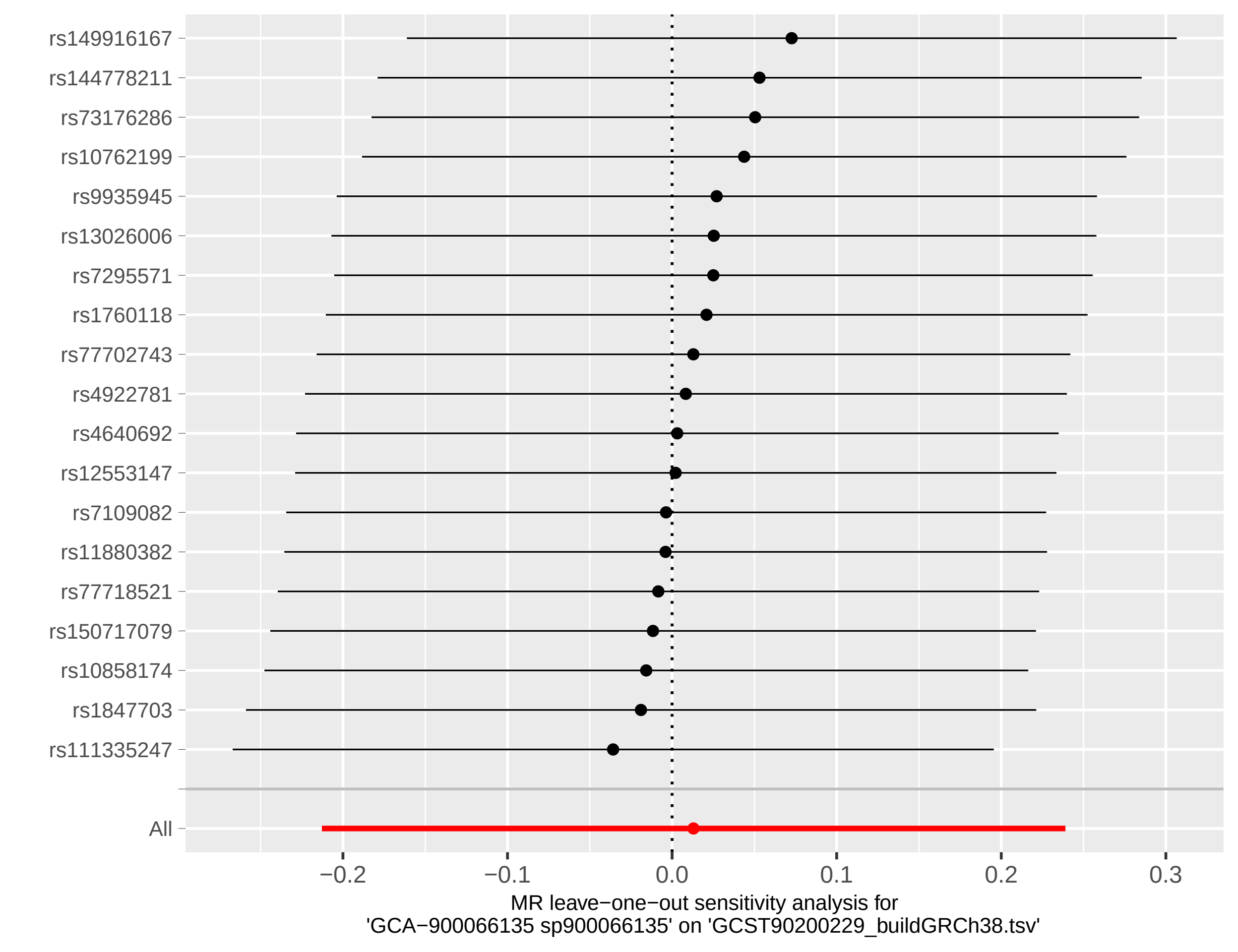

rs149916167
rs144778211
rs73176286
rs10762199
rs9935945
rs13026006
rs7295571
rs1760118
rs77702743
rs4922781
rs4640692
rs12553147
rs7109082
rs11880382
rs77718521
rs150717079
rs10858174
rs1847703
rs111335247
All
−0.2
−0.1
0.0
0.1
0.2
0.3
MR leave−one−out sensitivity analysis for
'GCA−900066135 sp900066135' on 'GCST90200229_buildGRCh38.tsv'

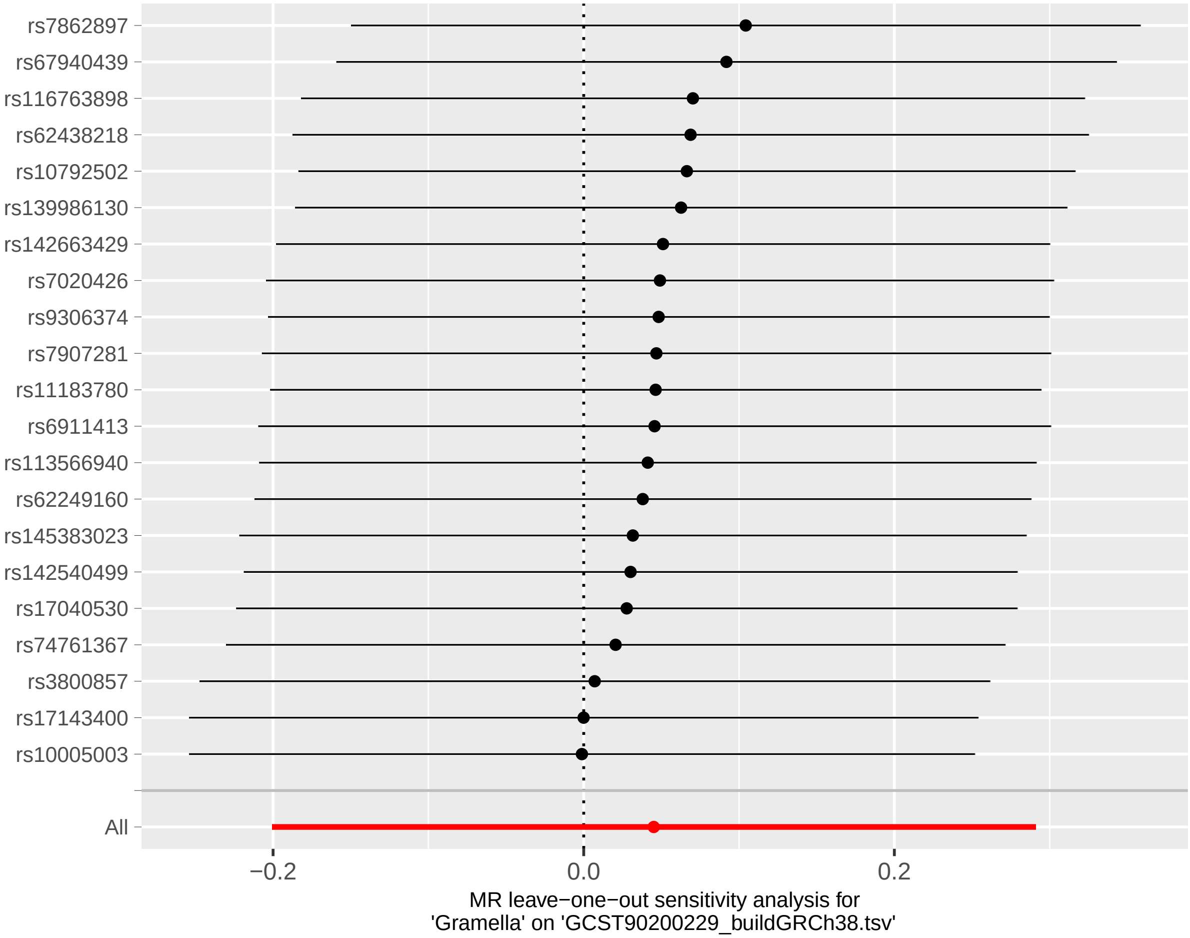

rs7862897
rs67940439
rs116763898
rs62438218
rs10792502
rs139986130
rs142663429
rs7020426
rs9306374
rs7907281
rs11183780
rs6911413
rs113566940
rs62249160
rs145383023
rs142540499
rs17040530
rs74761367
rs3800857
rs17143400
rs10005003
All
−0.2
0.0
0.2
MR leave−one−out sensitivity analysis for
'Gramella' on 'GCST90200229_buildGRCh38.tsv'

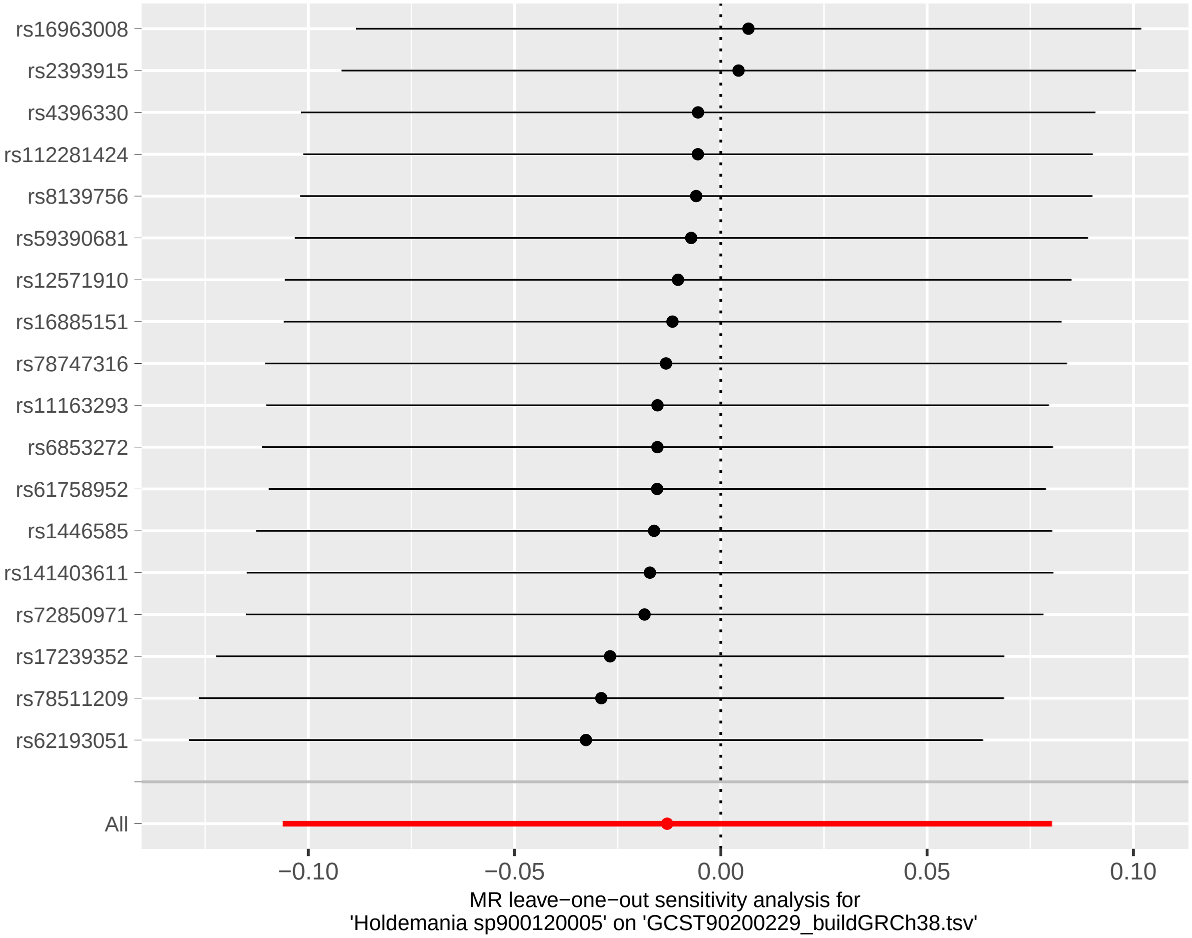

rs16963008
rs2393915
rs4396330
rs112281424
rs8139756
rs59390681
rs12571910
rs16885151
rs78747316
rs11163293
rs6853272
rs61758952
rs1446585
rs141403611
rs72850971
rs17239352
rs78511209
rs62193051
All
−0.10
−0.05
0.00
0.05
0.10
MR leave−one−out sensitivity analysis for
'Holdemania sp900120005' on 'GCST90200229_buildGRCh38.tsv'

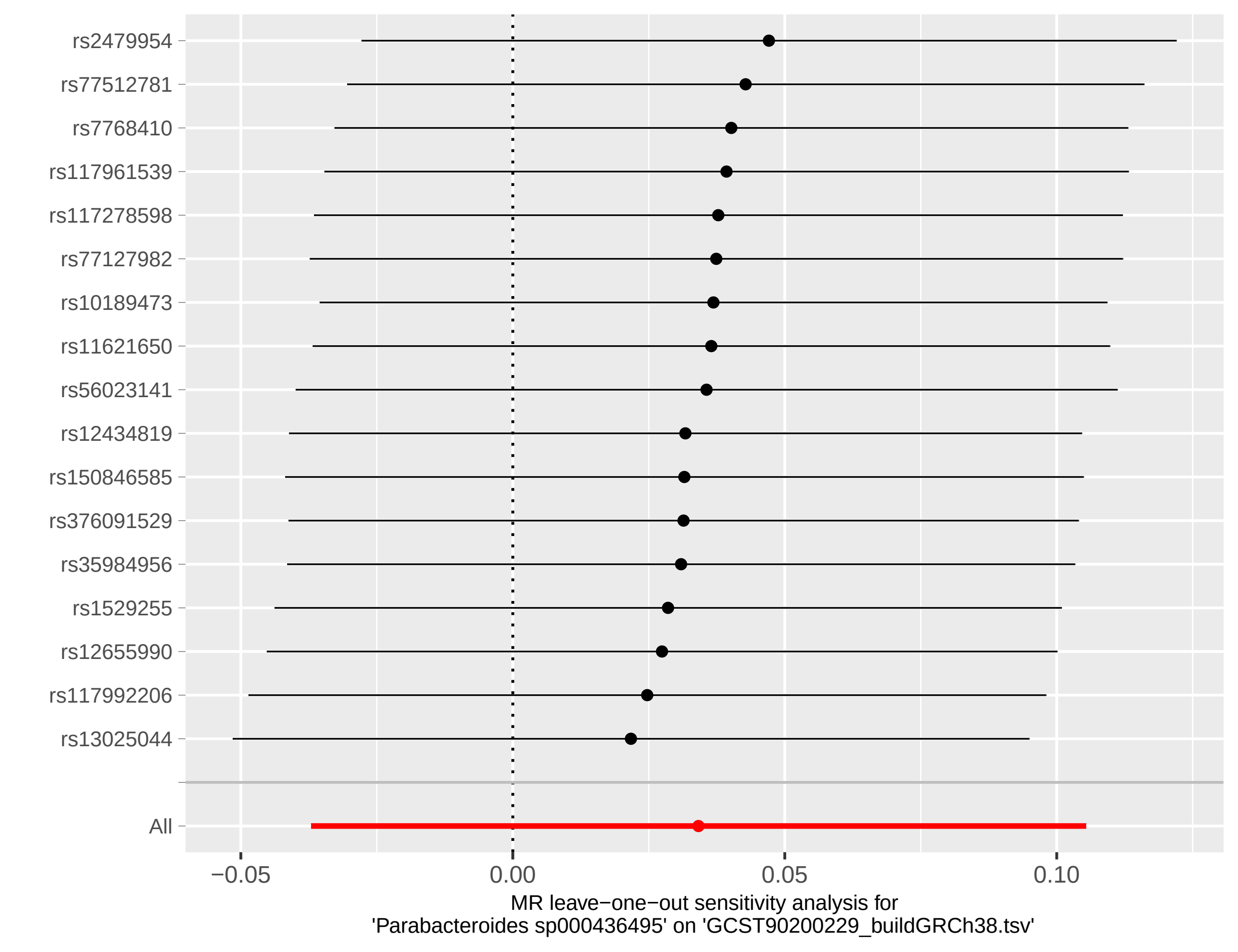
rs2479954
rs77512781
rs7768410
rs117961539
rs117278598
rs77127982
rs10189473
rs11621650
rs56023141
rs12434819
rs150846585
rs376091529
rs35984956
rs1529255
rs12655990
rs117992206
rs13025044
All
−0.05
0.00
0.05
0.10
MR leave−one−out sensitivity analysis for
'Parabacteroides sp000436495' on 'GCST90200229_buildGRCh38.tsv'

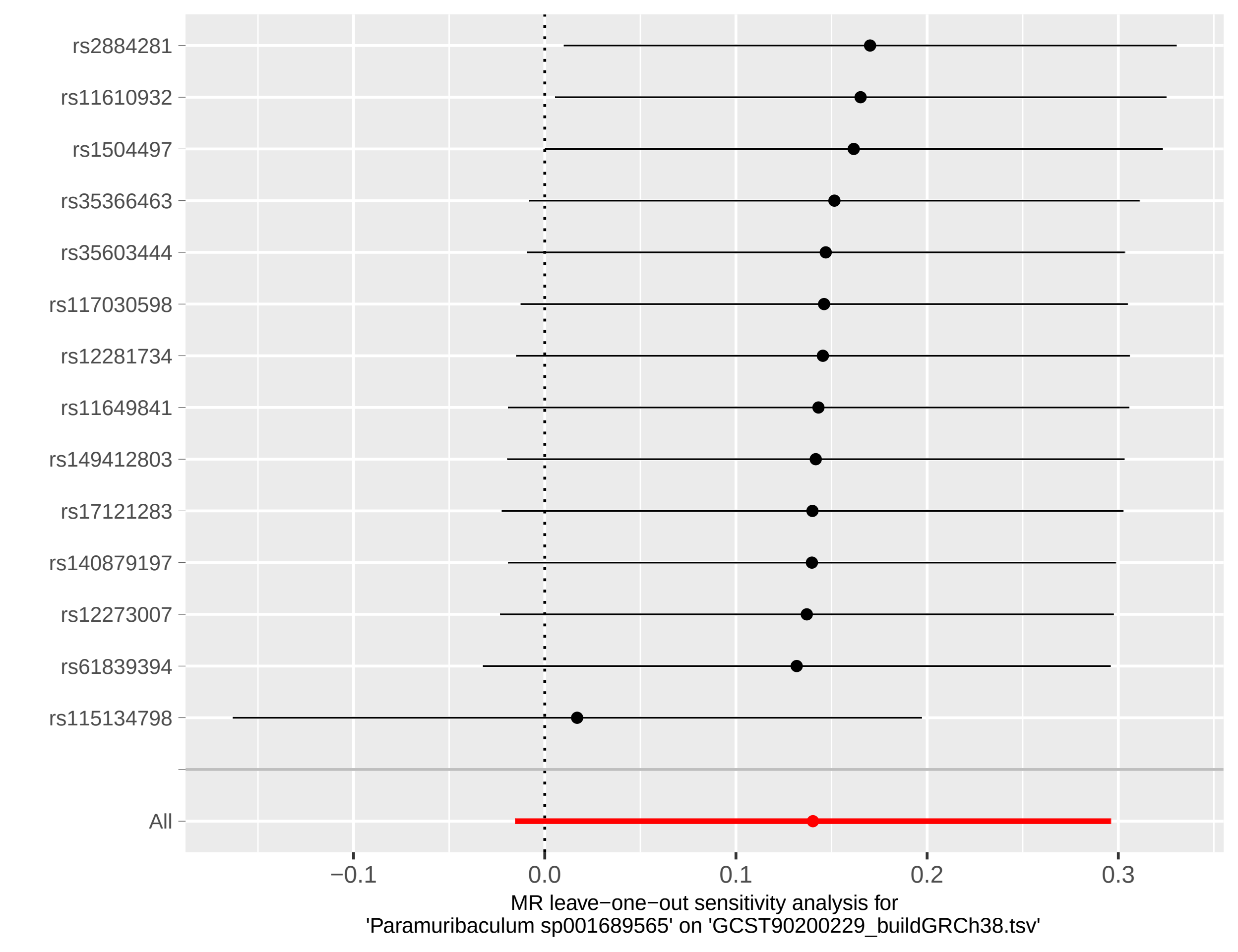

rs2884281
rs11610932
rs1504497
rs35366463
rs35603444
rs117030598
rs12281734
rs11649841
rs149412803
rs17121283
rs140879197
rs12273007
rs61839394
rs115134798
All
−0.1
0.0
0.1
0.2
0.3
MR leave−one−out sensitivity analysis for
'Paramuribaculum sp001689565' on 'GCST90200229_buildGRCh38.tsv'

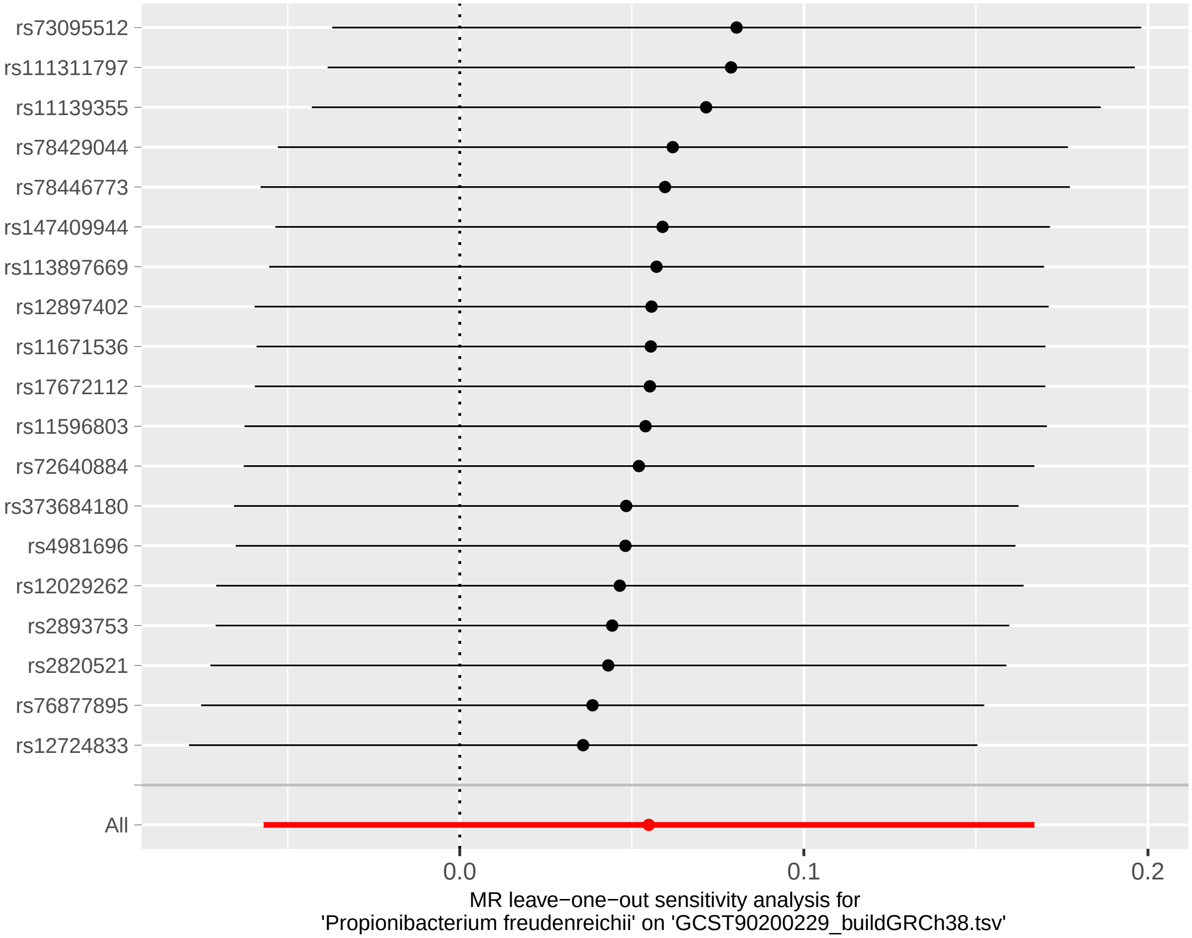
rs73095512
rs111311797
rs11139355
rs78429044
rs78446773
rs147409944
rs113897669
rs12897402
rs11671536
rs17672112
rs11596803
rs72640884
rs373684180
rs4981696
rs12029262
rs2893753
rs2820521
rs76877895
rs12724833
All
0.0
0.1
0.2
MR leave−one−out sensitivity analysis for
'Propionibacterium freudenreichii' on 'GCST90200229_buildGRCh38.tsv'

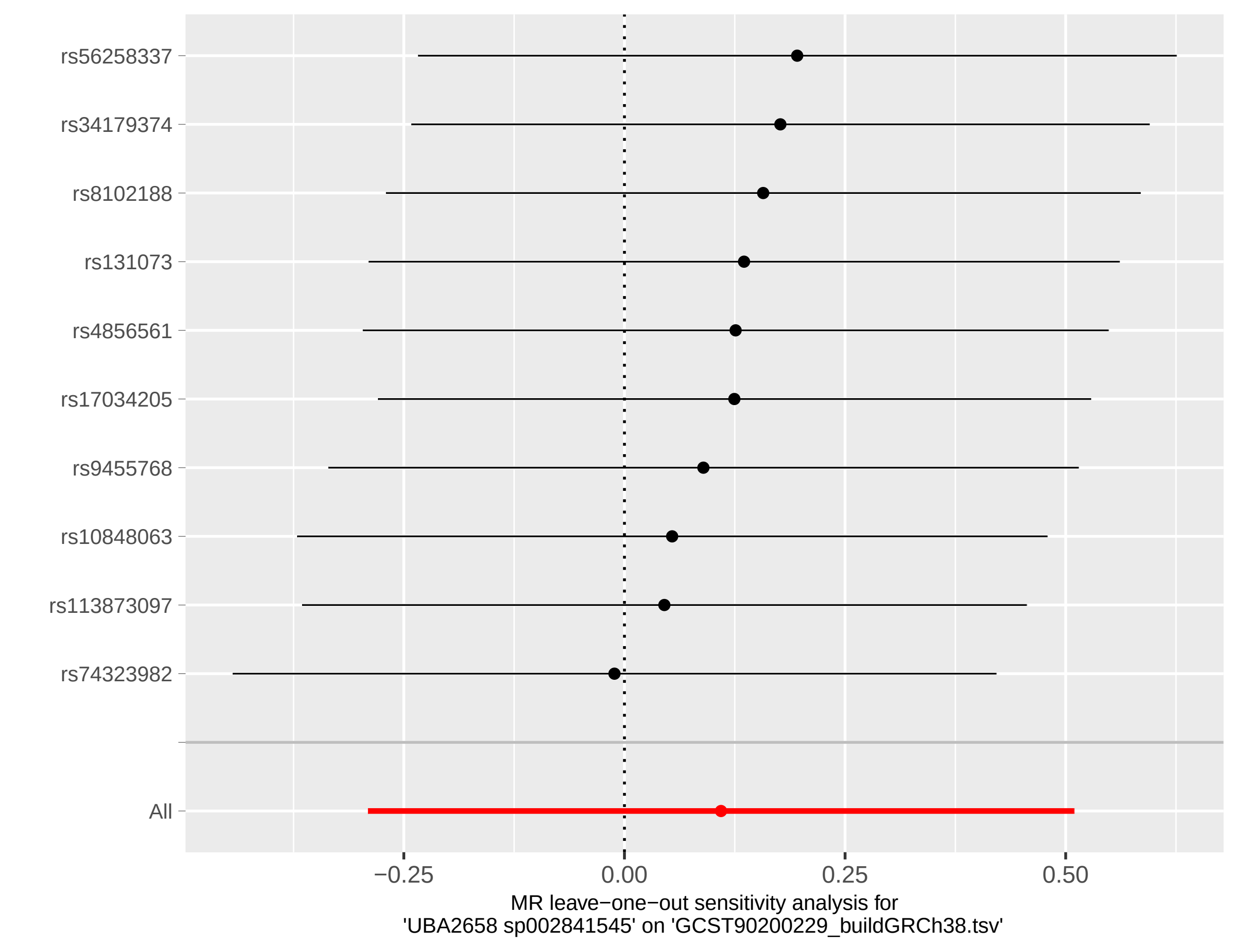

rs56258337
rs34179374
rs8102188
rs131073
rs4856561
rs17034205
rs9455768
rs10848063
rs113873097
rs74323982
All
−0.25
0.00
0.25
0.50
MR leave−one−out sensitivity analysis for
'UBA2658 sp002841545' on 'GCST90200229_buildGRCh38.tsv'

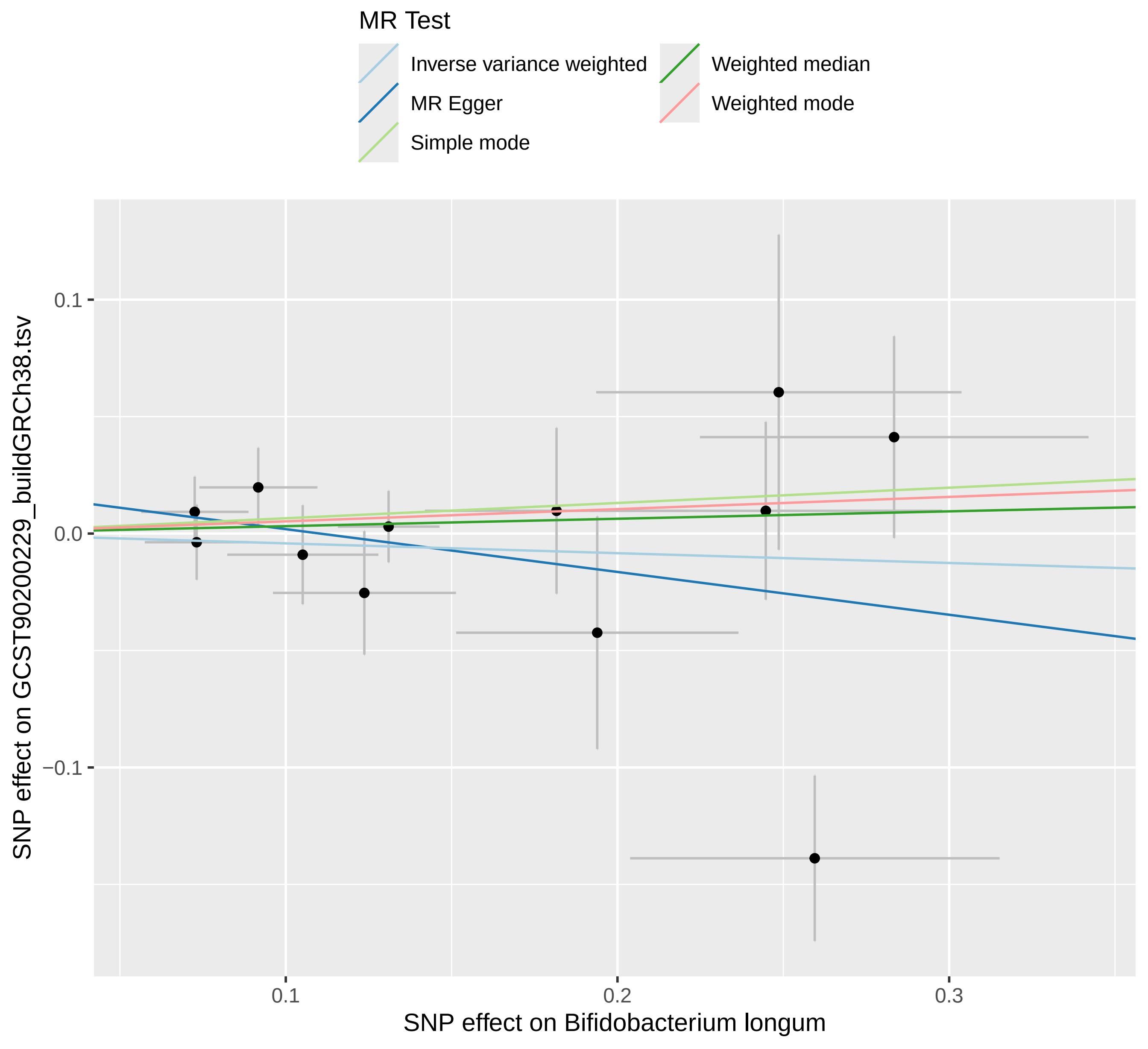
MR Test
Inverse variance weighted
MR Egger
Simple mode
Weighted median
Weighted mode
0.1
0.0
−0.1
0.1
0.2
0.3
SNP effect on GCST90200229_buildGRCh38.tsv
SNP effect on Bifidobacterium longum

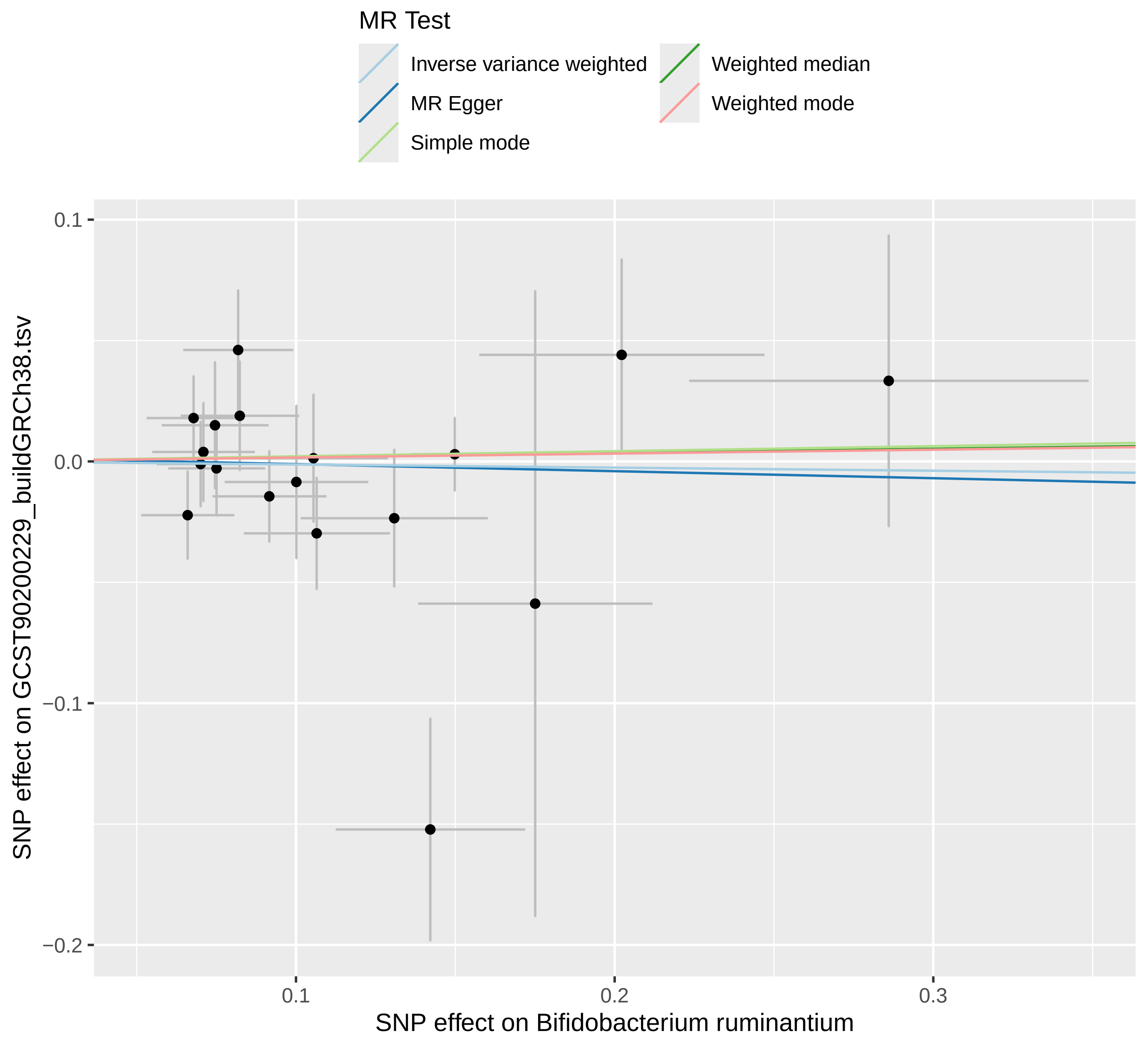
MR Test
Inverse variance weighted
MR Egger
Simple mode
Weighted median
Weighted mode
0.1
0.0
−0.1
−0.2
0.1
0.2
0.3
SNP effect on GCST90200229_buildGRCh38.tsv
SNP effect on Bifidobacterium ruminantium

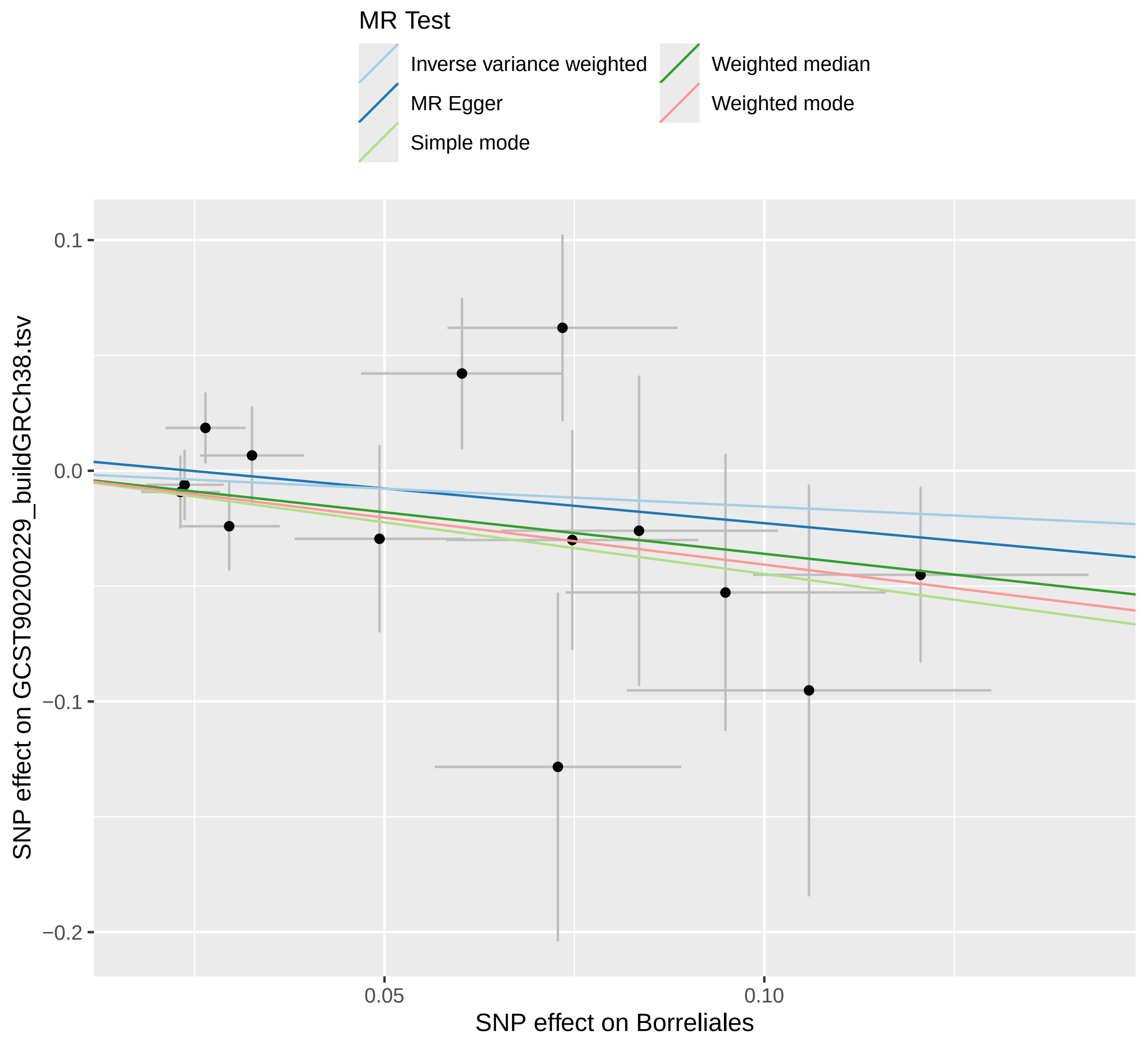
MR Test
Inverse variance weighted
MR Egger
Simple mode
Weighted median
Weighted mode
SNP effect on GCST90200229_buildGRCh38.tsv
0.1
0.0
−0.1
−0.2
0.05
0.10
SNP effect on Borreliales

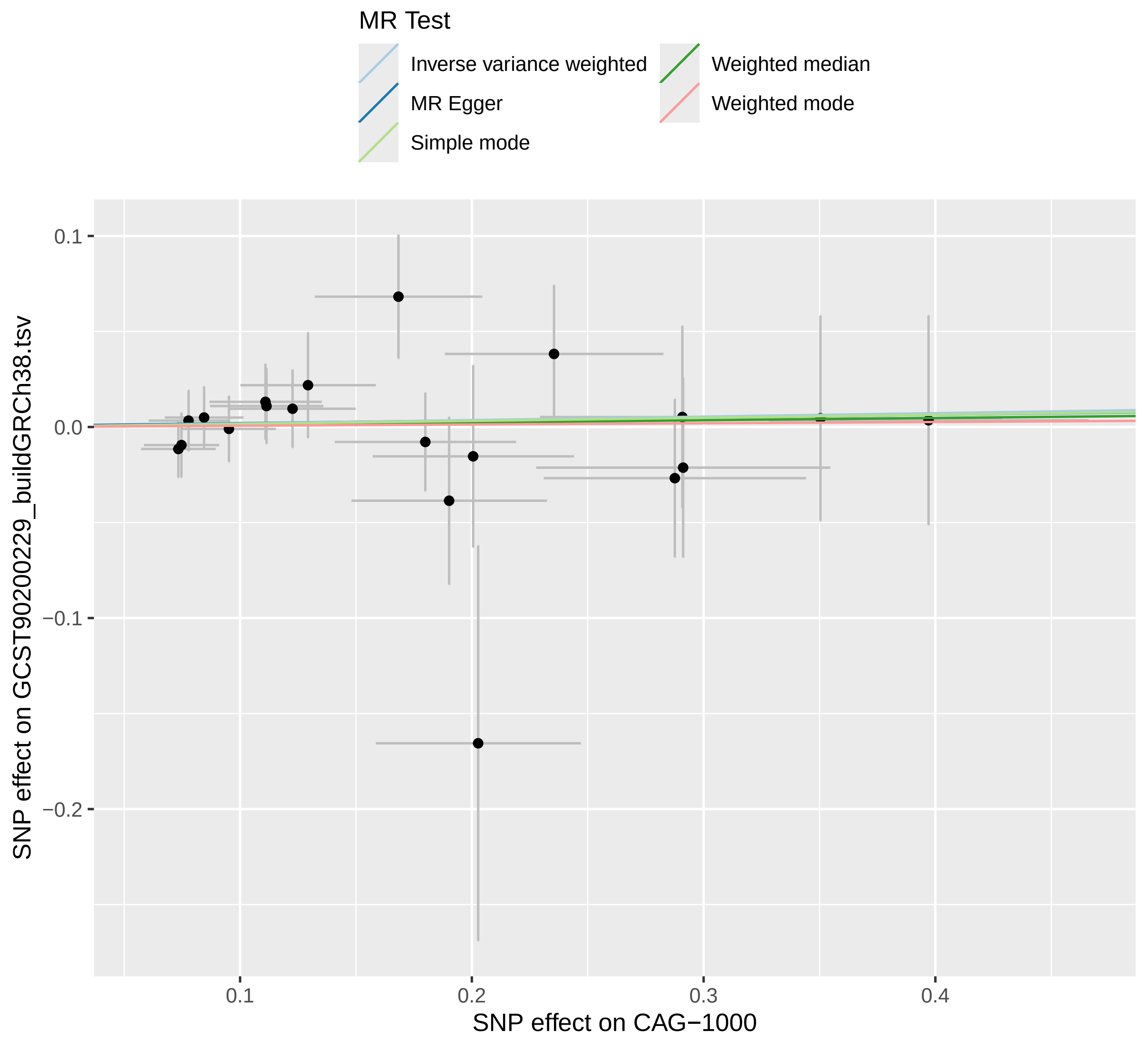
MR Test
Inverse variance weighted
MR Egger
Simple mode
Weighted median
Weighted mode
0.1
0.0
−0.1
−0.2
0.1
0.2
0.3
0.4
SNP effect on GCST90200229_buildGRCh38.tsv
SNP effect on CAG−1000

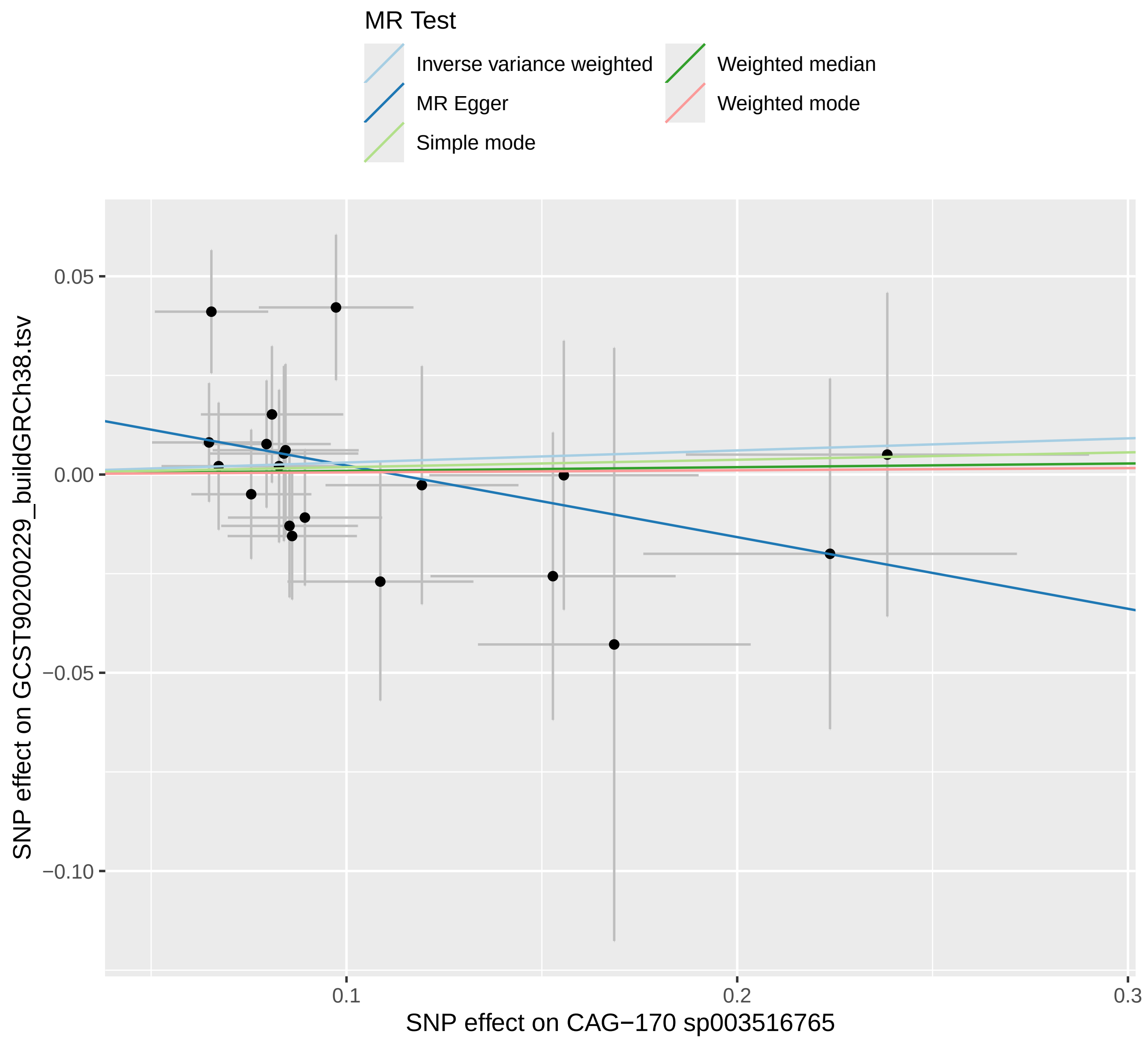
MR Test
Inverse variance weighted
MR Egger
Simple mode
Weighted median
Weighted mode
0.05
0.00
−0.05
−0.10
0.1
0.2
0.3
SNP effect on GCST90200229_buildGRCh38.tsv
SNP effect on CAG−170 sp003516765

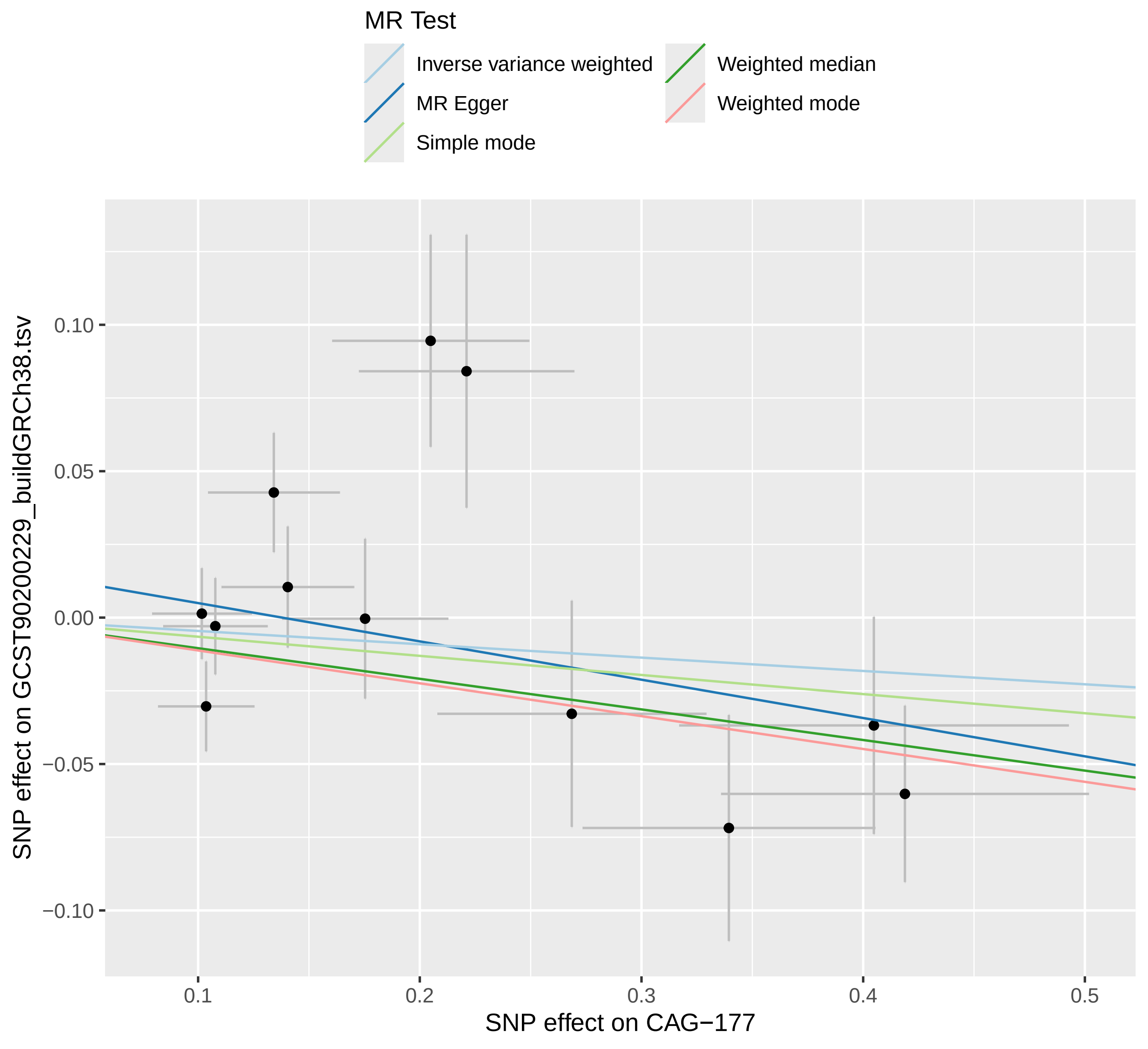

MR Test
Inverse variance weighted
MR Egger
Simple mode
Weighted median
Weighted mode
0.10
0.05
0.00
−0.05
−0.10
SNP effect on GCST90200229_buildGRCh38.tsv
0.1
0.2
0.3
0.4
0.5
SNP effect on CAG−177

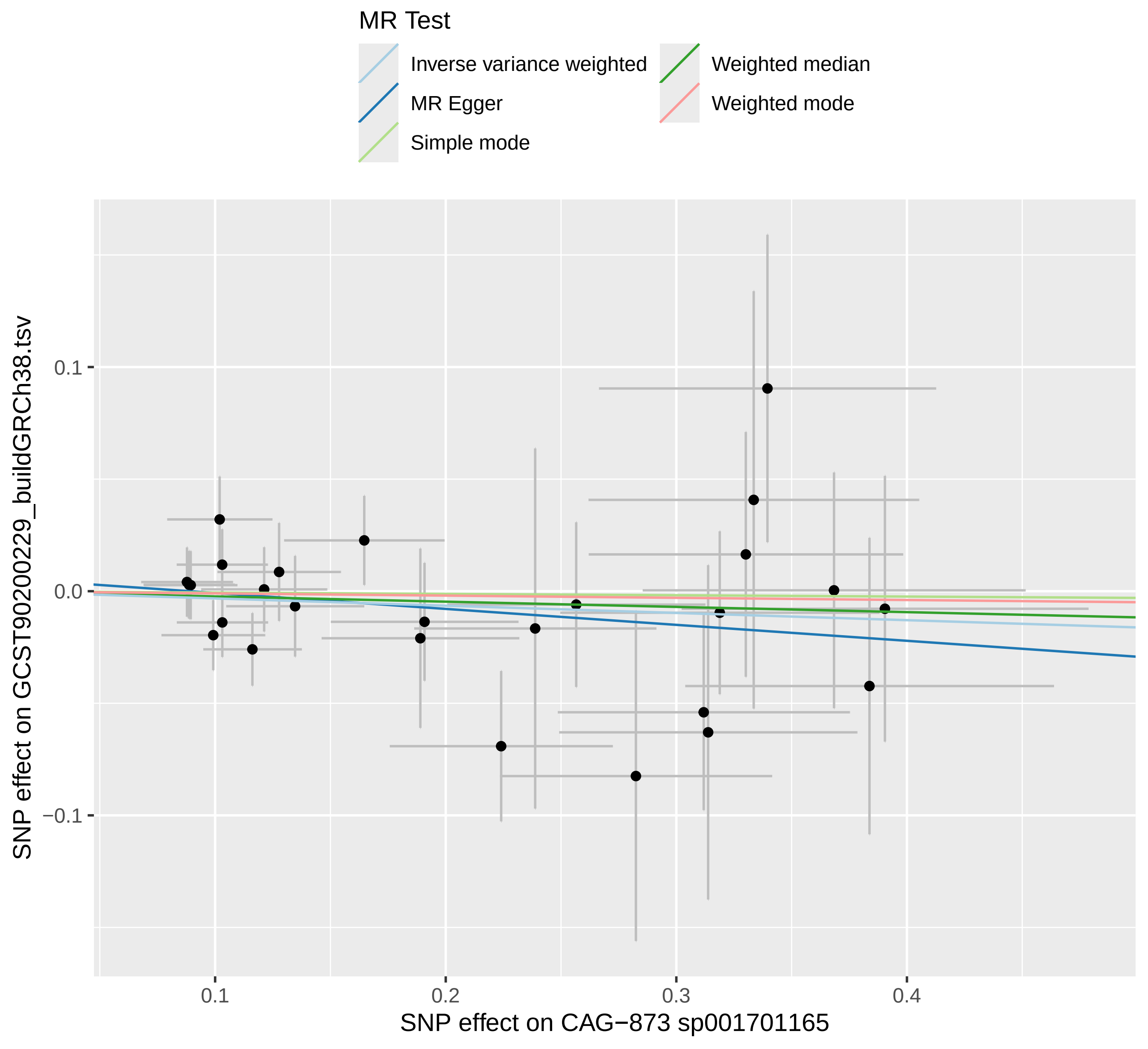
MR Test
Inverse variance weighted
MR Egger
Simple mode
Weighted median
Weighted mode
0.1
0.0
−0.1
0.1
0.2
0.3
0.4
SNP effect on GCST90200229_buildGRCh38.tsv
SNP effect on CAG−873 sp001701165

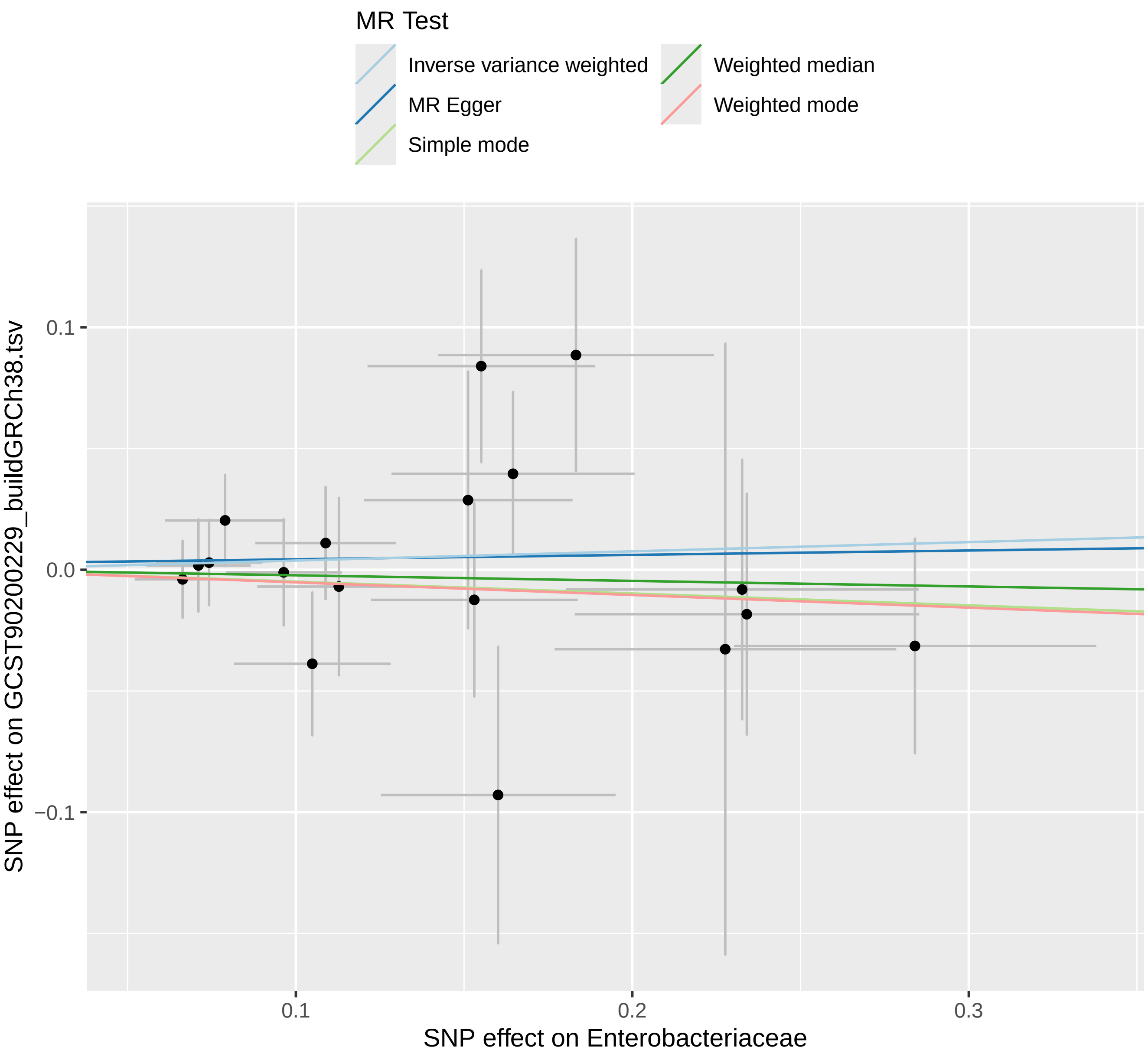

MR Test
Inverse variance weighted
MR Egger
Simple mode
Weighted median
Weighted mode
0.1
0.0
−0.1
0.1
0.2
0.3
SNP effect on GCST90200229_buildGRCh38.tsv
SNP effect on Enterobacteriaceae

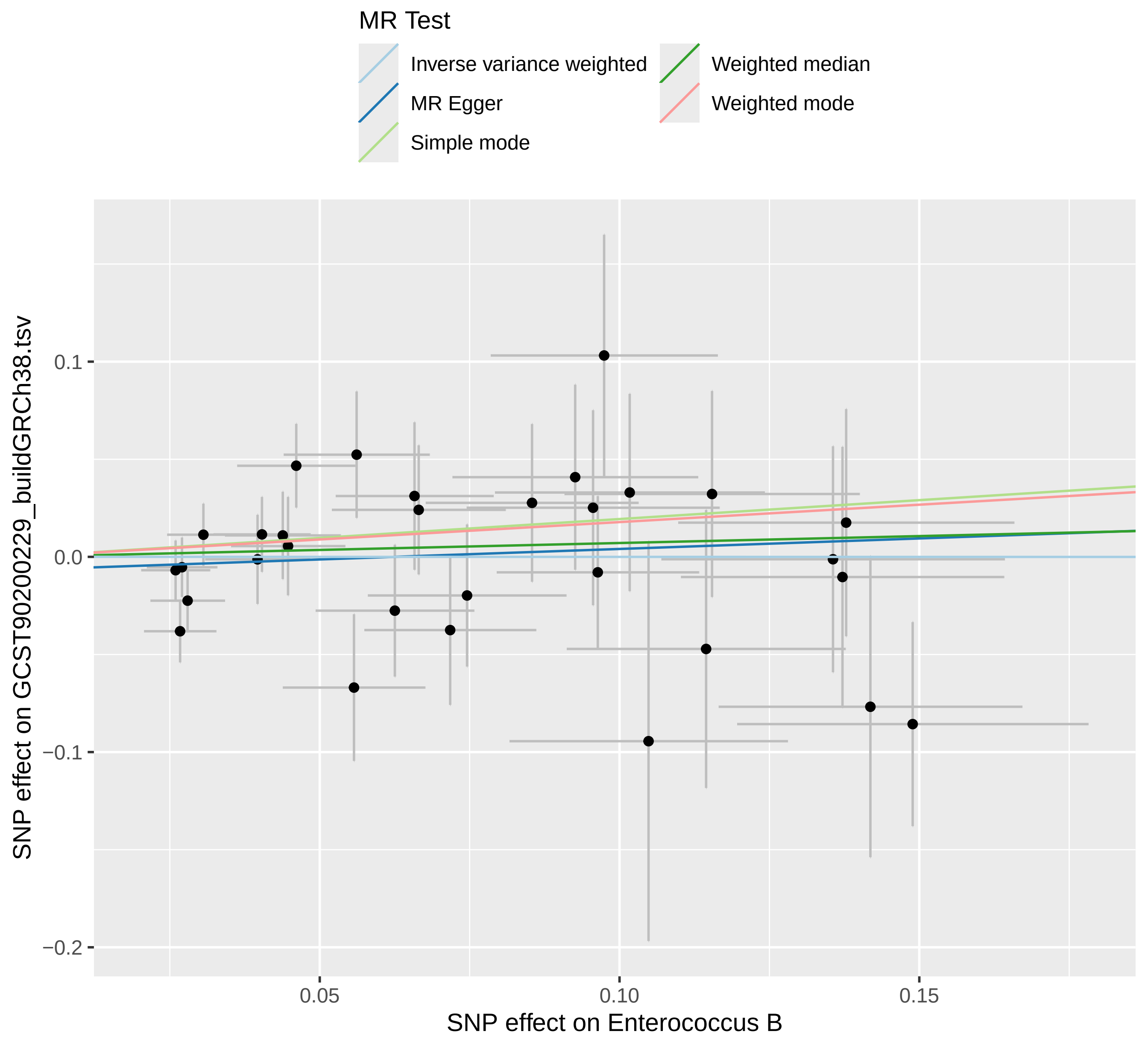
MR Test
Inverse variance weighted
MR Egger
Simple mode
Weighted median
Weighted mode
0.1
0.0
−0.1
−0.2
0.05
0.10
0.15
SNP effect on GCST90200229_buildGRCh38.tsv
SNP effect on Enterococcus B

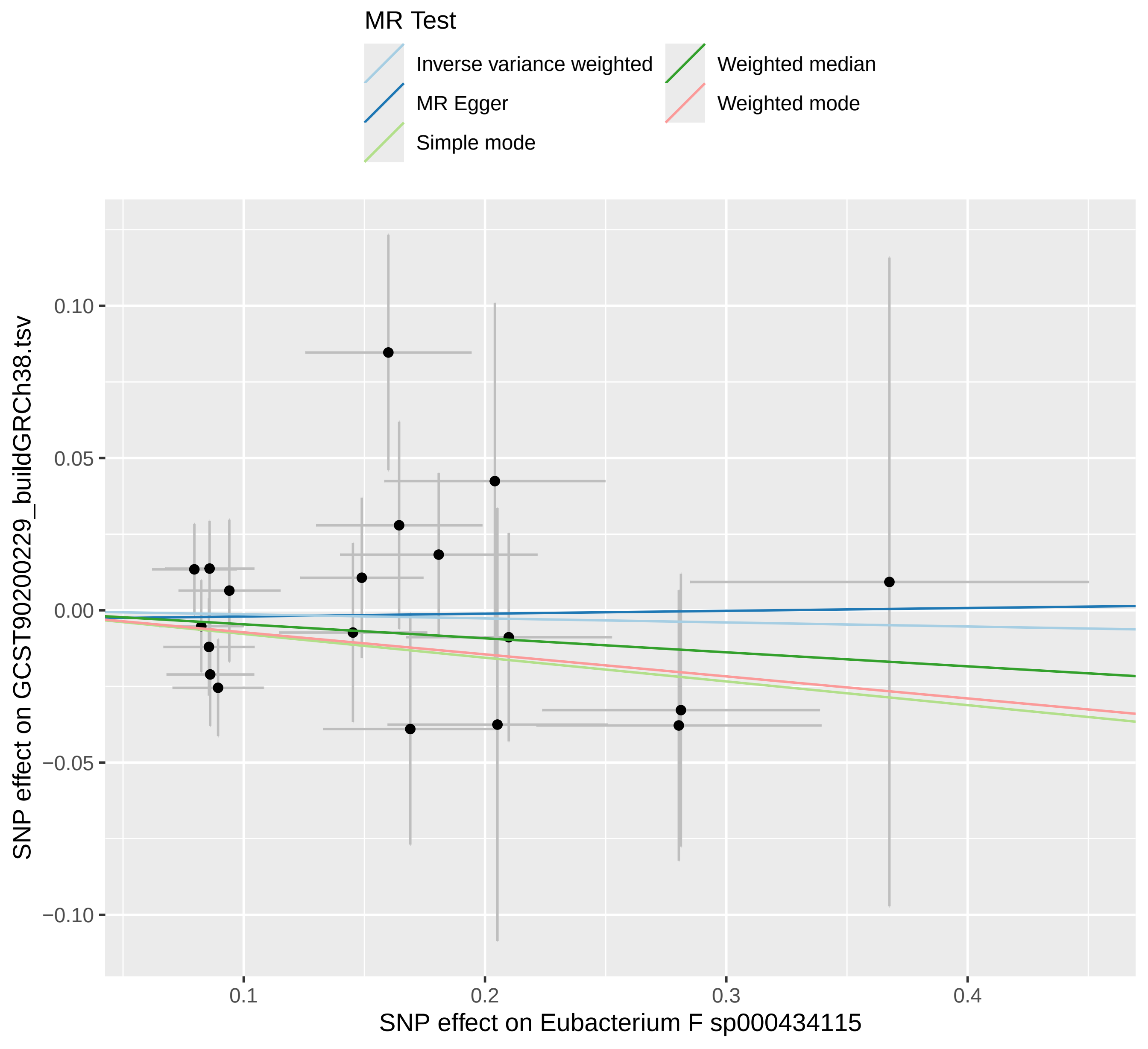
MR Test
Inverse variance weighted
MR Egger
Simple mode
Weighted median
Weighted mode
SNP effect on GCST90200229_buildGRCh38.tsv
0.10
0.05
0.00
−0.05
−0.10
0.1
0.2
0.3
0.4
SNP effect on Eubacterium F sp000434115

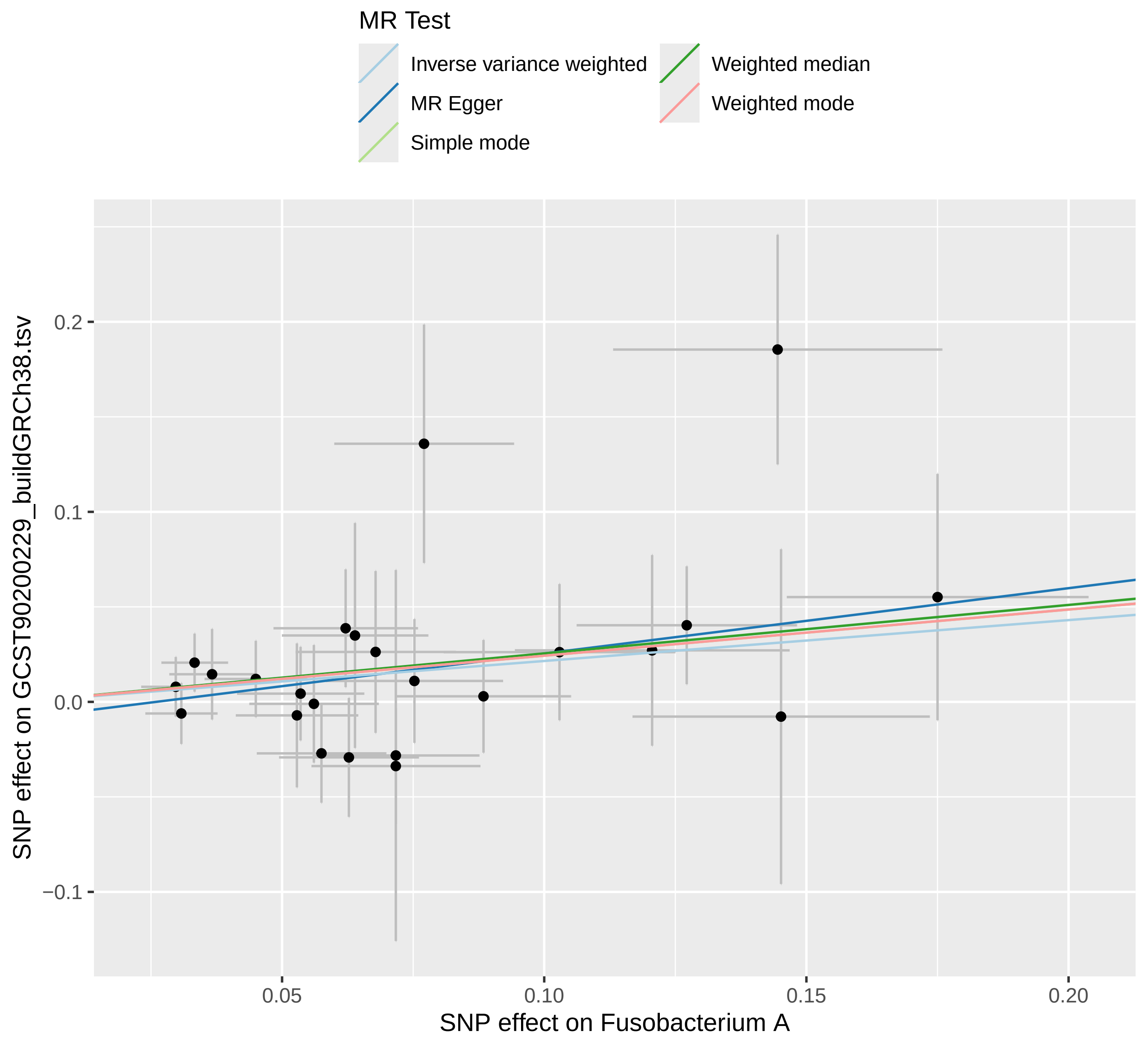

MR Test
Inverse variance weighted
MR Egger
Simple mode
Weighted median
Weighted mode
SNP effect on GCST90200229_buildGRCh38.tsv
0.2
0.1
0.0
−0.1
0.05
0.10
0.15
0.20
SNP effect on Fusobacterium A

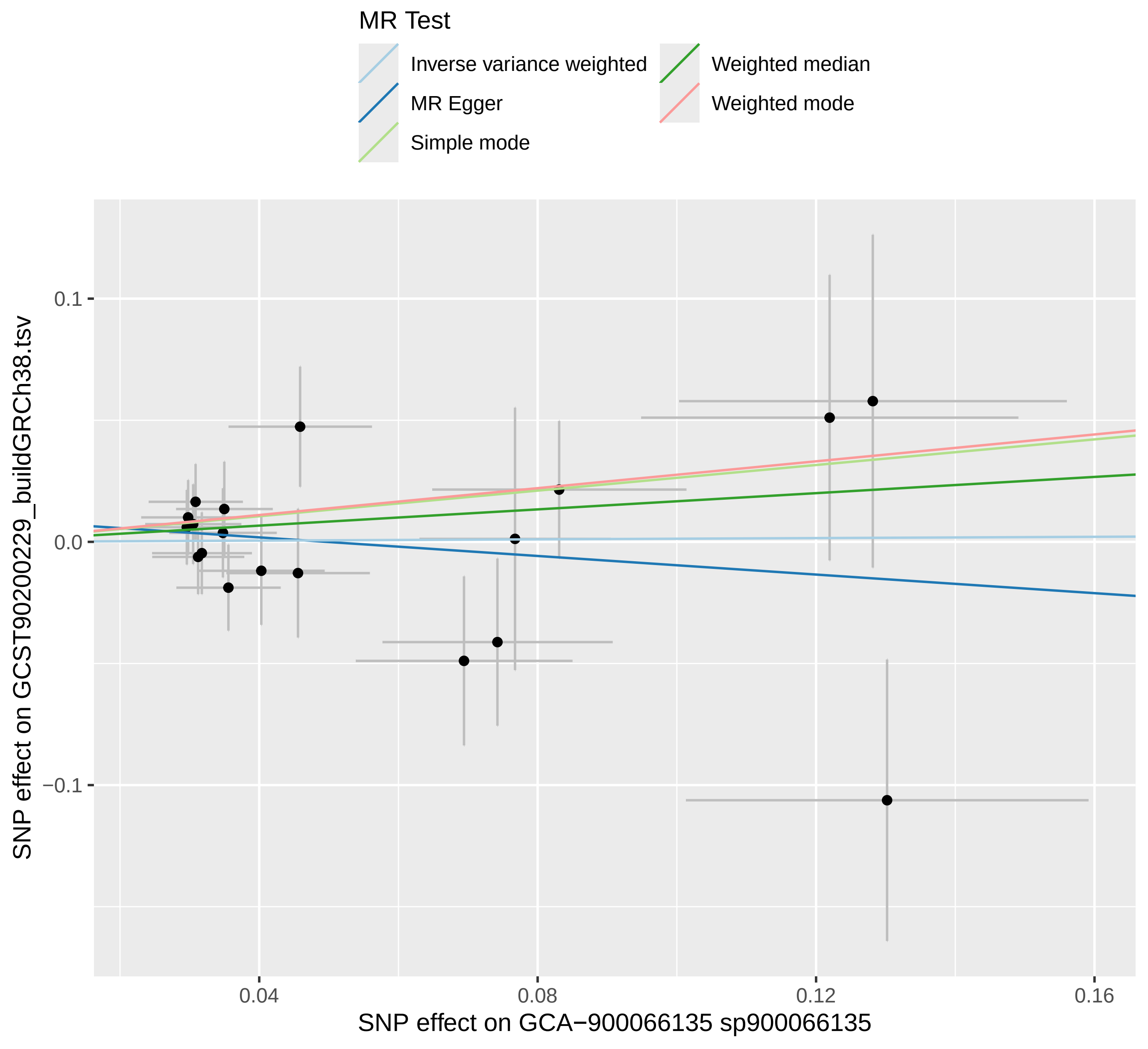
MR Test
Inverse variance weighted
MR Egger
Simple mode
Weighted median
Weighted mode
0.1
0.0
−0.1
0.04
0.08
0.12
0.16
SNP effect on GCST90200229_buildGRCh38.tsv
SNP effect on GCA−900066135 sp900066135

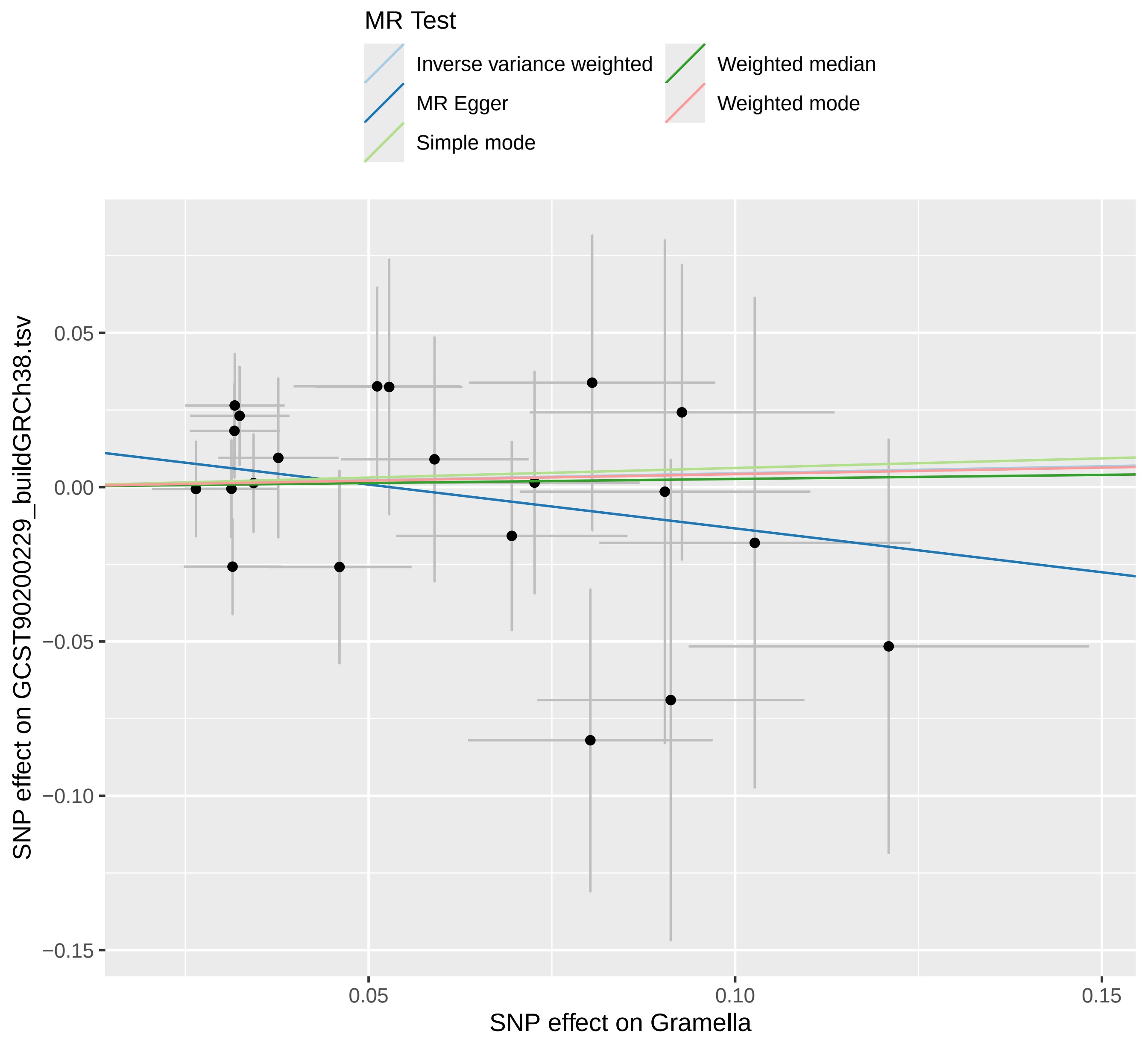
MR Test
Inverse variance weighted
MR Egger
Simple mode
Weighted median
Weighted mode
0.05
0.00
−0.05
−0.10
−0.15
SNP effect on GCST90200229_buildGRCh38.tsv
0.05
0.10
0.15
SNP effect on Gramella

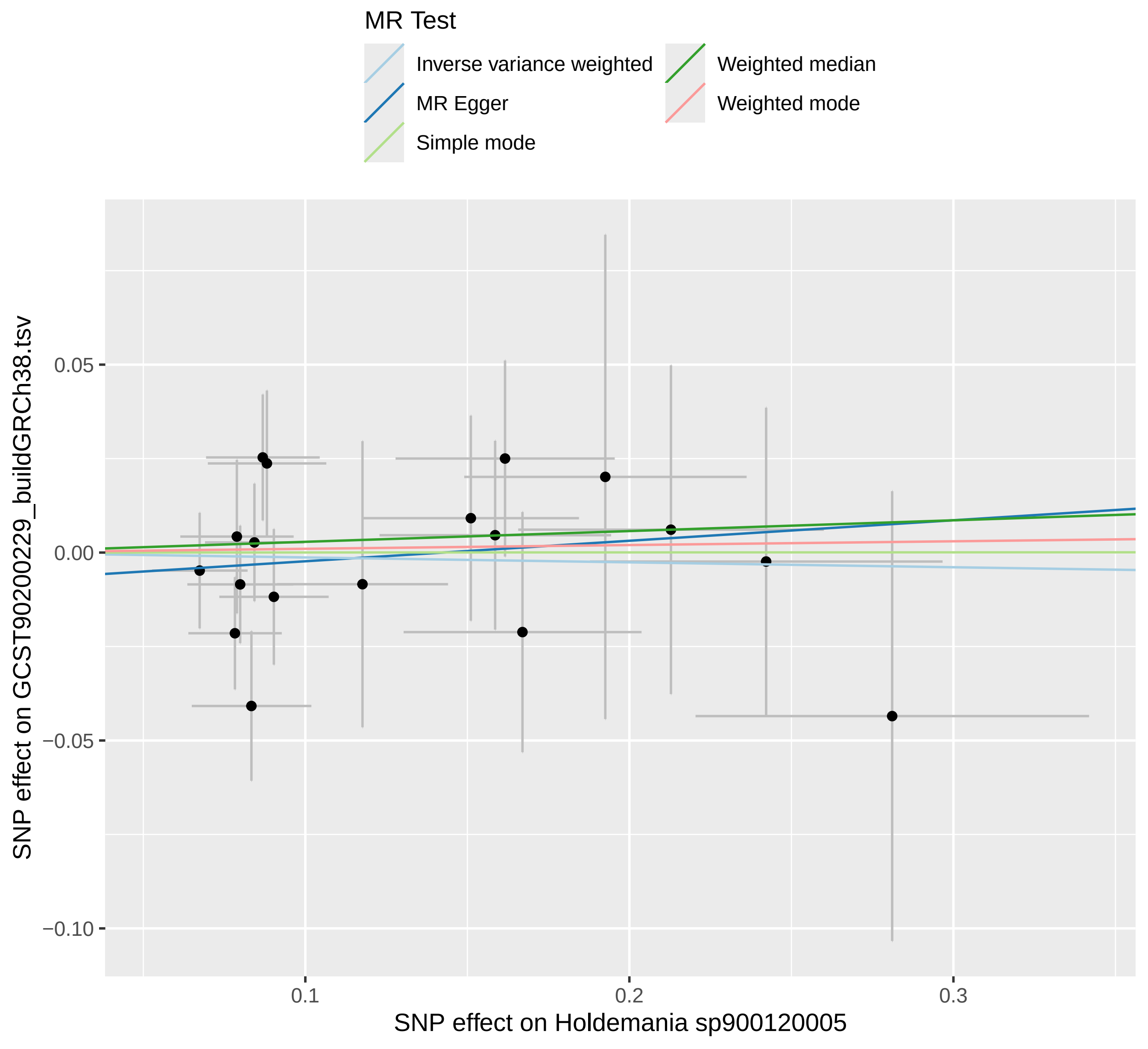

MR Test
Inverse variance weighted
MR Egger
Simple mode
Weighted median
Weighted mode
0.05
0.00
−0.05
−0.10
0.1
0.2
0.3
SNP effect on GCST90200229_buildGRCh38.tsv
SNP effect on Holdemania sp900120005

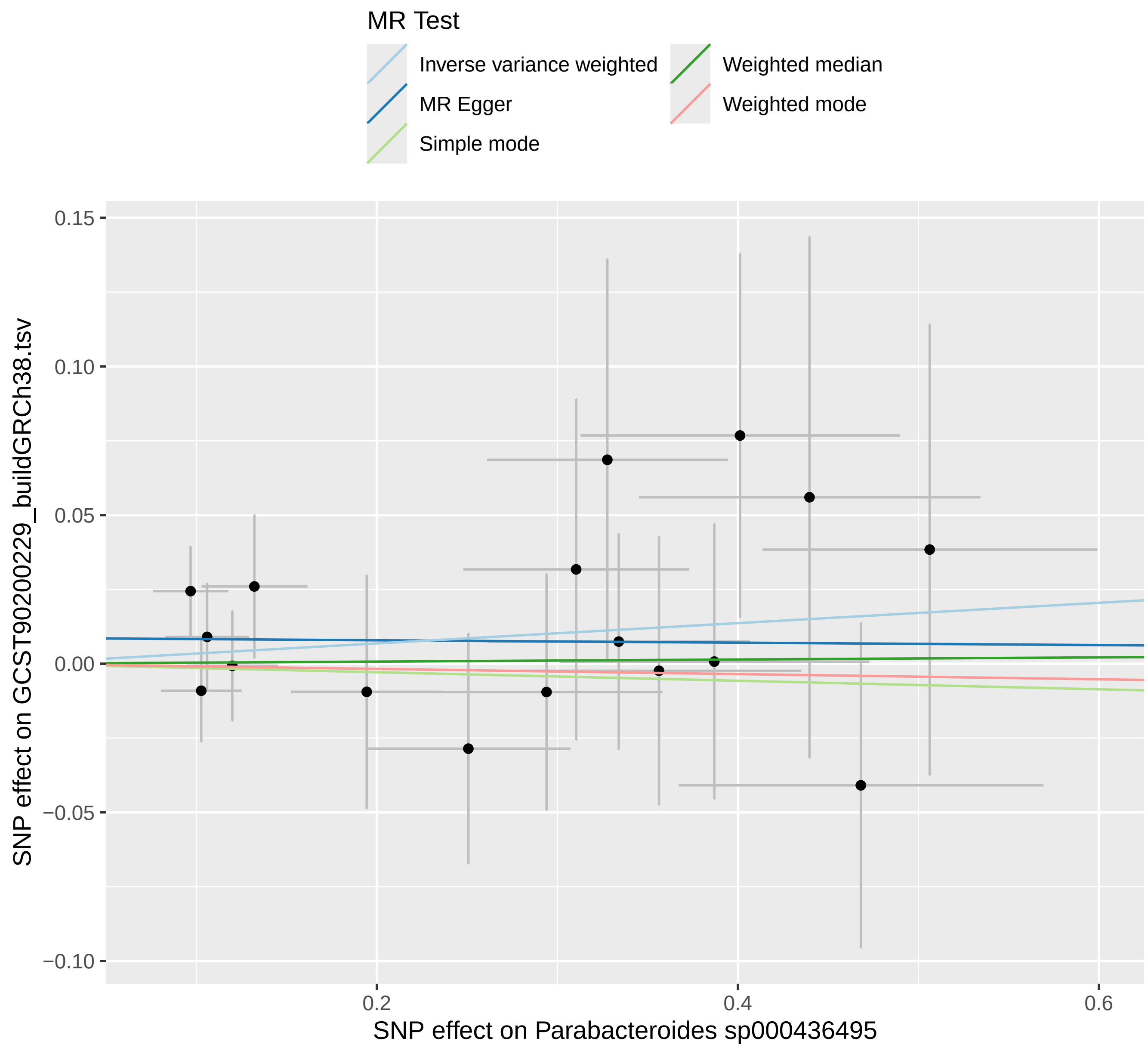

MR Test
Inverse variance weighted
MR Egger
Simple mode
Weighted median
Weighted mode
0.15
0.10
0.05
0.00
−0.05
−0.10
0.2
0.4
0.6
SNP effect on GCST90200229_buildGRCh38.tsv
SNP effect on Parabacteroides sp000436495

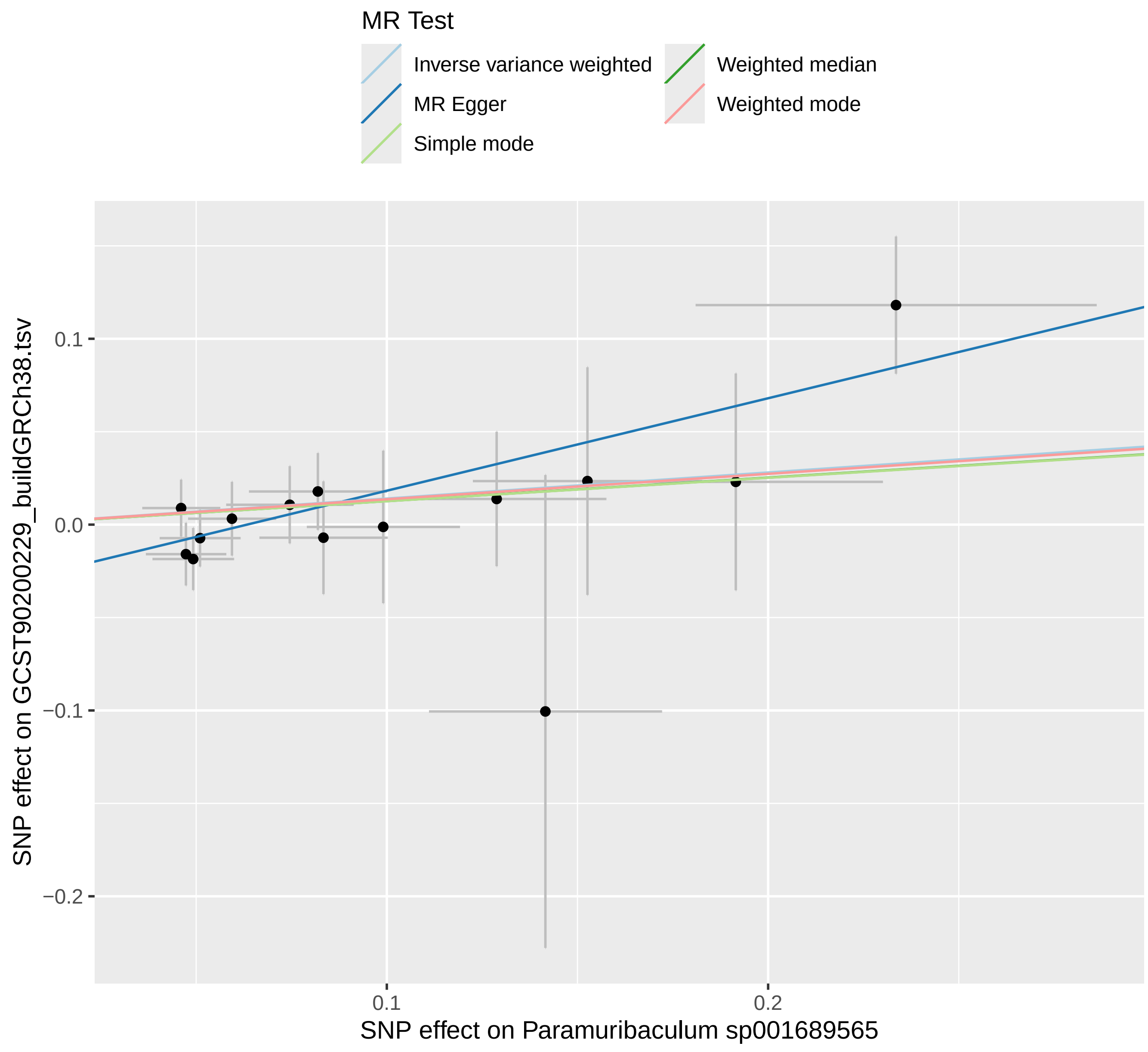
MR Test
Inverse variance weighted
MR Egger
Simple mode
Weighted median
Weighted mode
0.1
0.0
−0.1
−0.2
0.1
0.2
SNP effect on GCST90200229_buildGRCh38.tsv
SNP effect on Paramuribaculum sp001689565

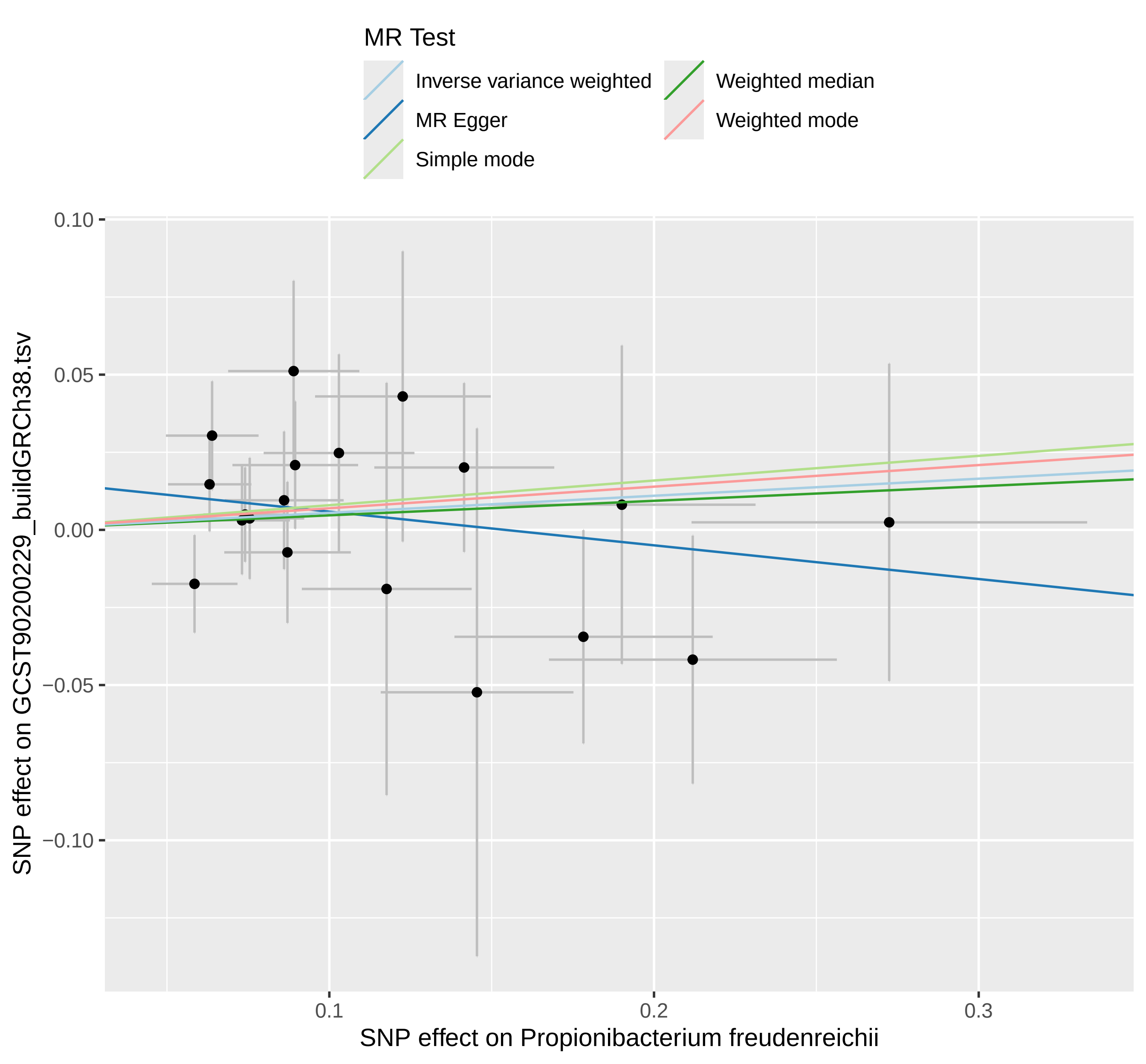
MR Test
Inverse variance weighted
MR Egger
Simple mode
Weighted median
Weighted mode
0.10
0.05
0.00
−0.05
−0.10
0.1
0.2
0.3
SNP effect on GCST90200229_buildGRCh38.tsv
SNP effect on Propionibacterium freudenreichii

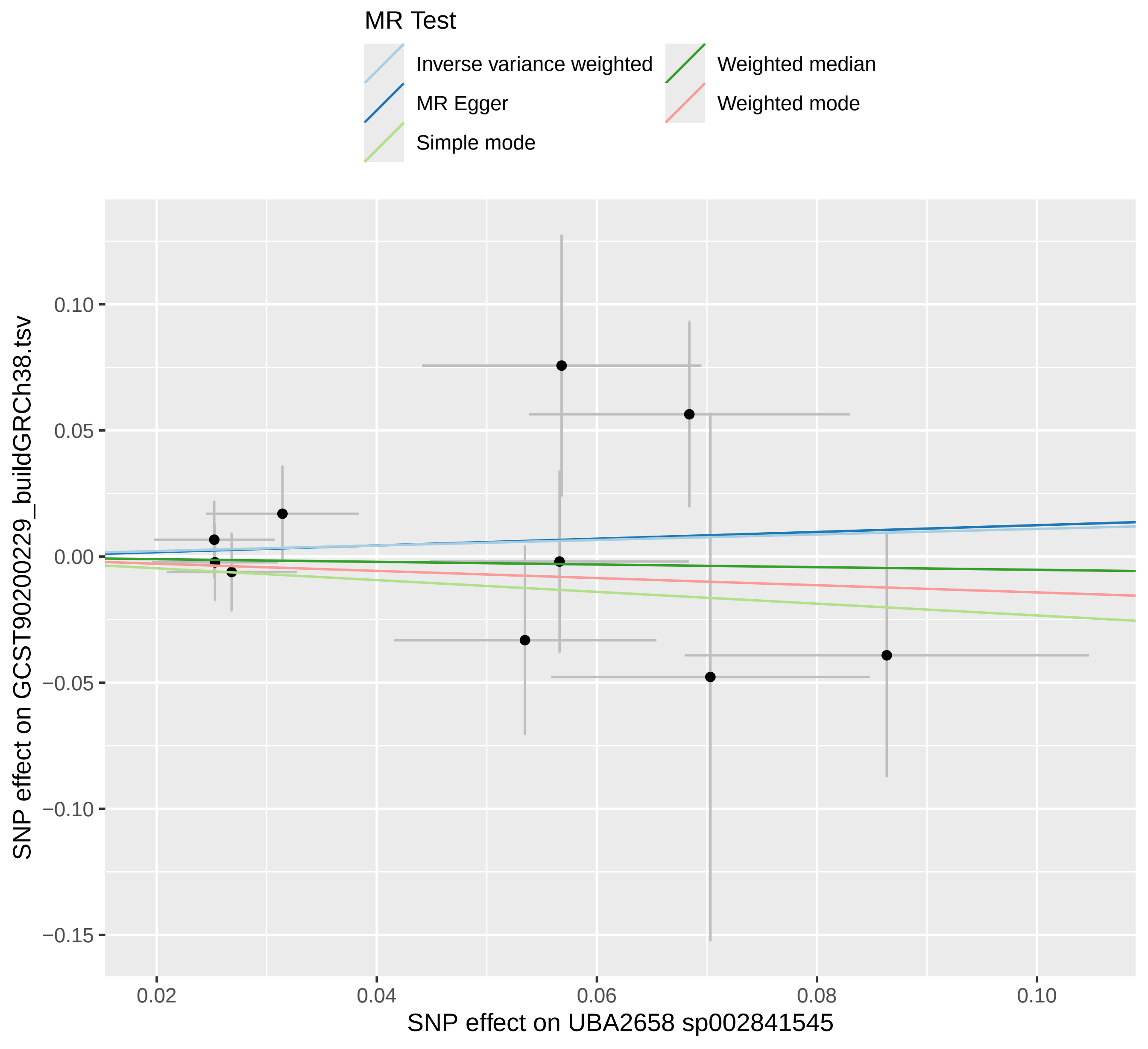
MR Test
Inverse variance weighted
MR Egger
Simple mode
Weighted median
Weighted mode
0.10
0.05
0.00
−0.05
−0.10
−0.15
SNP effect on GCST90200229_buildGRCh38.tsv
0.02
0.04
0.06
0.08
0.10
SNP effect on UBA2658 sp002841545

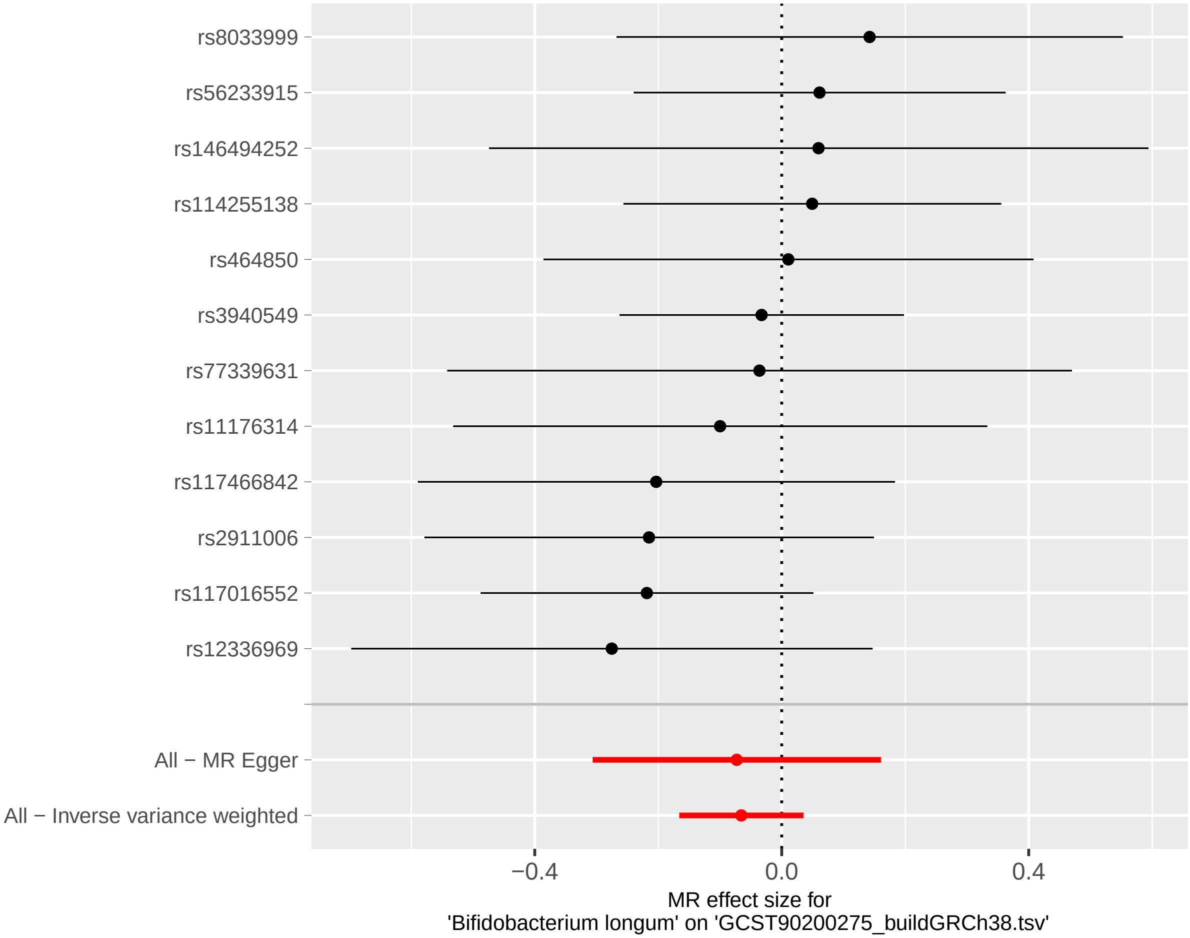

rs8033999
rs56233915
rs146494252
rs114255138
rs464850
rs3940549
rs77339631
rs11176314
rs117466842
rs2911006
rs117016552
rs12336969
All − MR Egger
All − Inverse variance weighted
−0.4
0.0
0.4
MR effect size for
'Bifidobacterium longum' on 'GCST90200275_buildGRCh38.tsv'

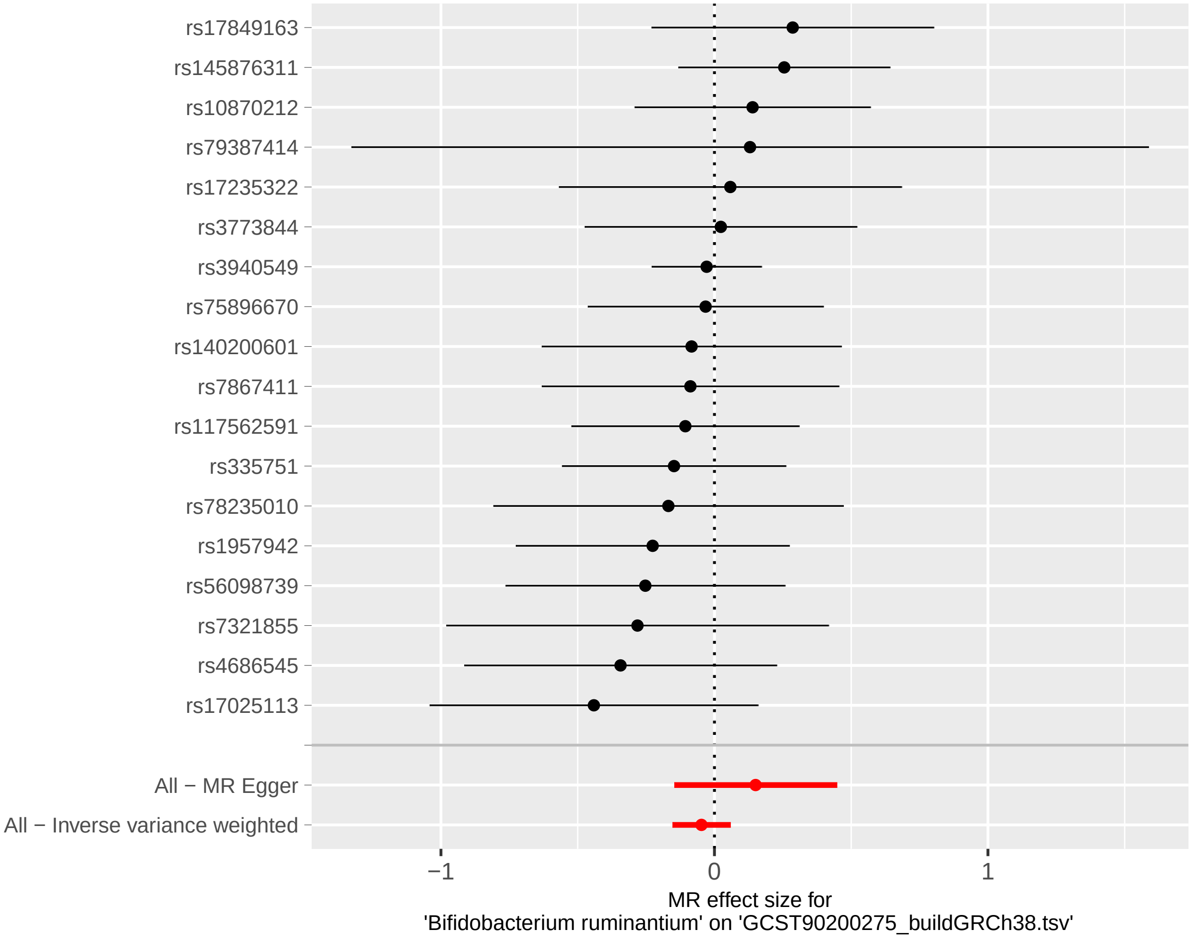
rs17849163
rs145876311
rs10870212
rs79387414
rs17235322
rs3773844
rs3940549
rs75896670
rs140200601
rs7867411
rs117562591
rs335751
rs78235010
rs1957942
rs56098739
rs7321855
rs4686545
rs17025113
All − MR Egger
All − Inverse variance weighted
−1
0
1
MR effect size for
'Bifidobacterium ruminantium' on 'GCST90200275_buildGRCh38.tsv'

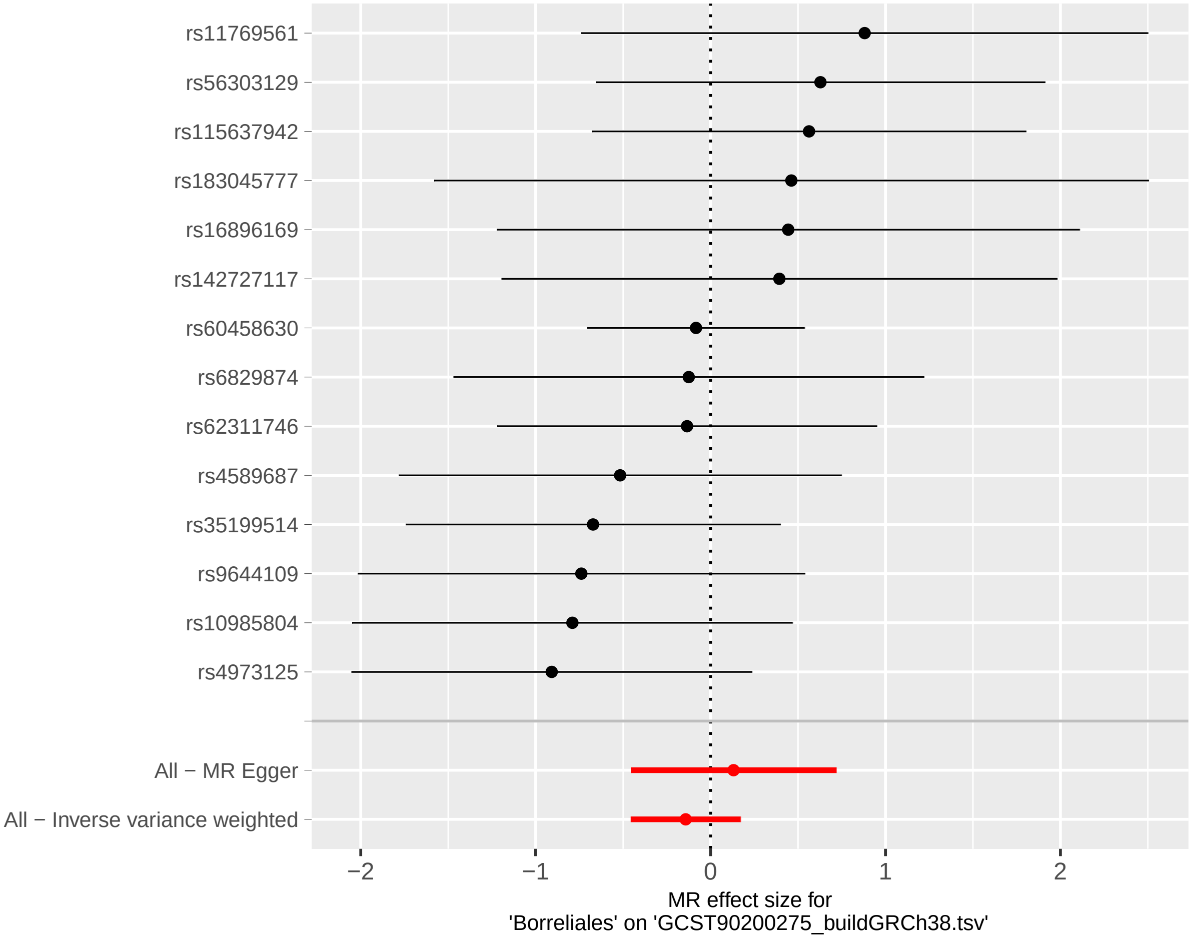
rs11769561
rs56303129
rs115637942
rs183045777
rs16896169
rs142727117
rs60458630
rs6829874
rs62311746
rs4589687
rs35199514
rs9644109
rs10985804
rs4973125
All − MR Egger
All − Inverse variance weighted
−2
−1
0
1
2
MR effect size for
'Borreliales' on 'GCST90200275_buildGRCh38.tsv'

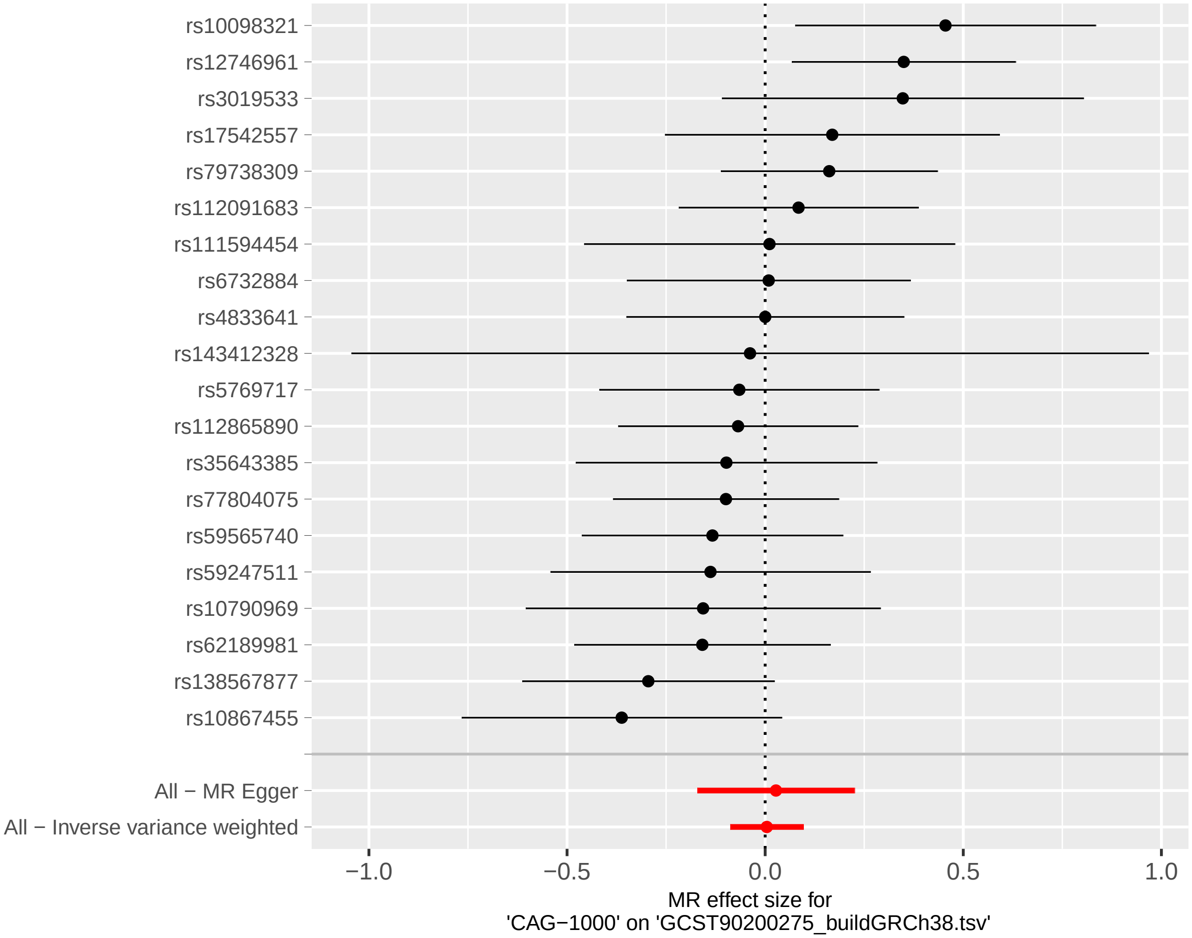

rs10098321
rs12746961
rs3019533
rs17542557
rs79738309
rs112091683
rs111594454
rs6732884
rs4833641
rs143412328
rs5769717
rs112865890
rs35643385
rs77804075
rs59565740
rs59247511
rs10790969
rs62189981
rs138567877
rs10867455
All − MR Egger
All − Inverse variance weighted
−1.0
−0.5
0.0
0.5
1.0
MR effect size for
'CAG−1000' on 'GCST90200275_buildGRCh38.tsv'

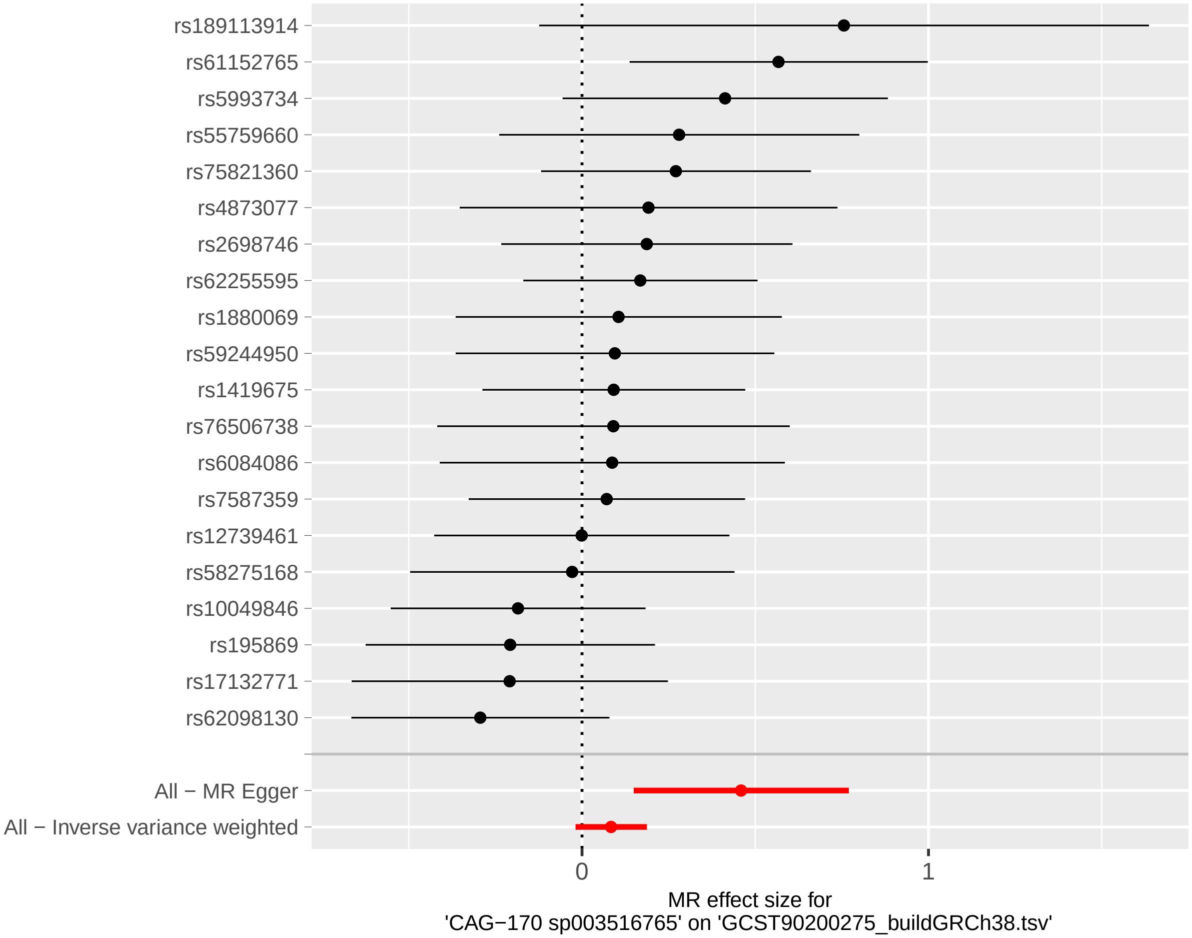
rs189113914
rs61152765
rs5993734
rs55759660
rs75821360
rs4873077
rs2698746
rs62255595
rs1880069
rs59244950
rs1419675
rs76506738
rs6084086
rs7587359
rs12739461
rs58275168
rs10049846
rs195869
rs17132771
rs62098130
All − MR Egger
All − Inverse variance weighted
0
1
MR effect size for
'CAG−170 sp003516765' on 'GCST90200275_buildGRCh38.tsv'

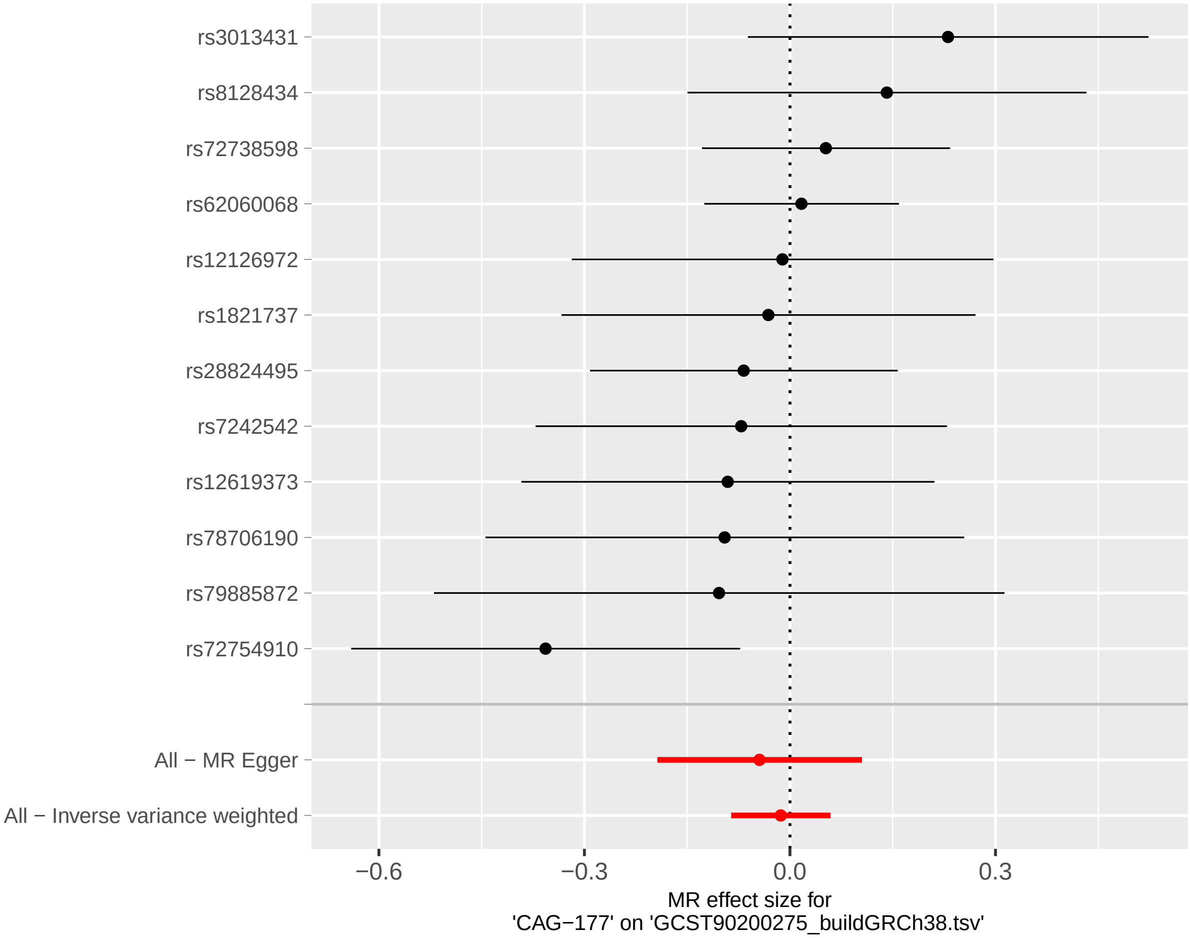

rs3013431
rs8128434
rs72738598
rs62060068
rs12126972
rs1821737
rs28824495
rs7242542
rs12619373
rs78706190
rs79885872
rs72754910
All − MR Egger
All − Inverse variance weighted
−0.6
−0.3
0.0
0.3
MR effect size for
'CAG−177' on 'GCST90200275_buildGRCh38.tsv'

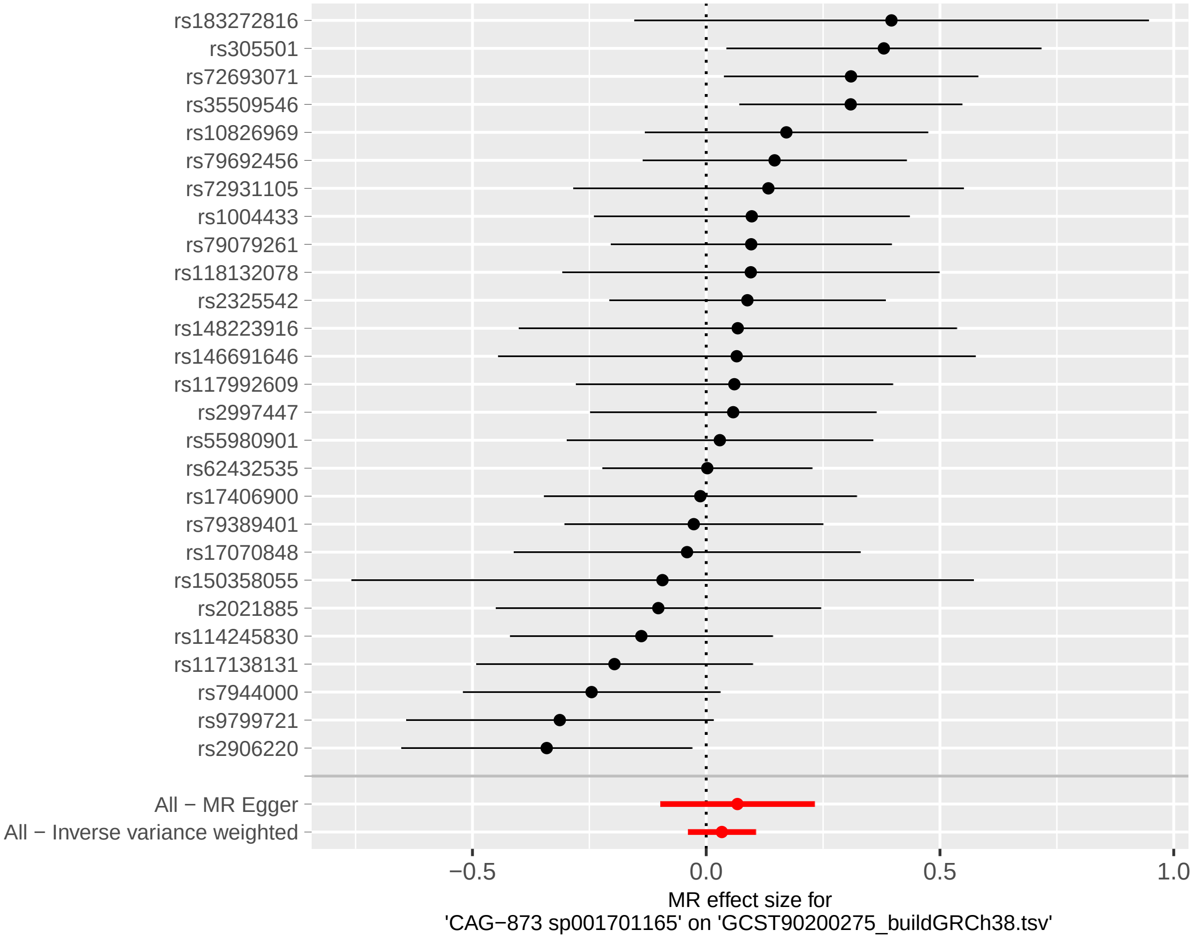

rs183272816
rs305501
rs72693071
rs35509546
rs10826969
rs79692456
rs72931105
rs1004433
rs79079261
rs118132078
rs2325542
rs148223916
rs146691646
rs117992609
rs2997447
rs55980901
rs62432535
rs17406900
rs79389401
rs17070848
rs150358055
rs2021885
rs114245830
rs117138131
rs7944000
rs9799721
rs2906220
All − MR Egger
All − Inverse variance weighted
−0.5
0.0
0.5
1.0
MR effect size for
'CAG−873 sp001701165' on 'GCST90200275_buildGRCh38.tsv'

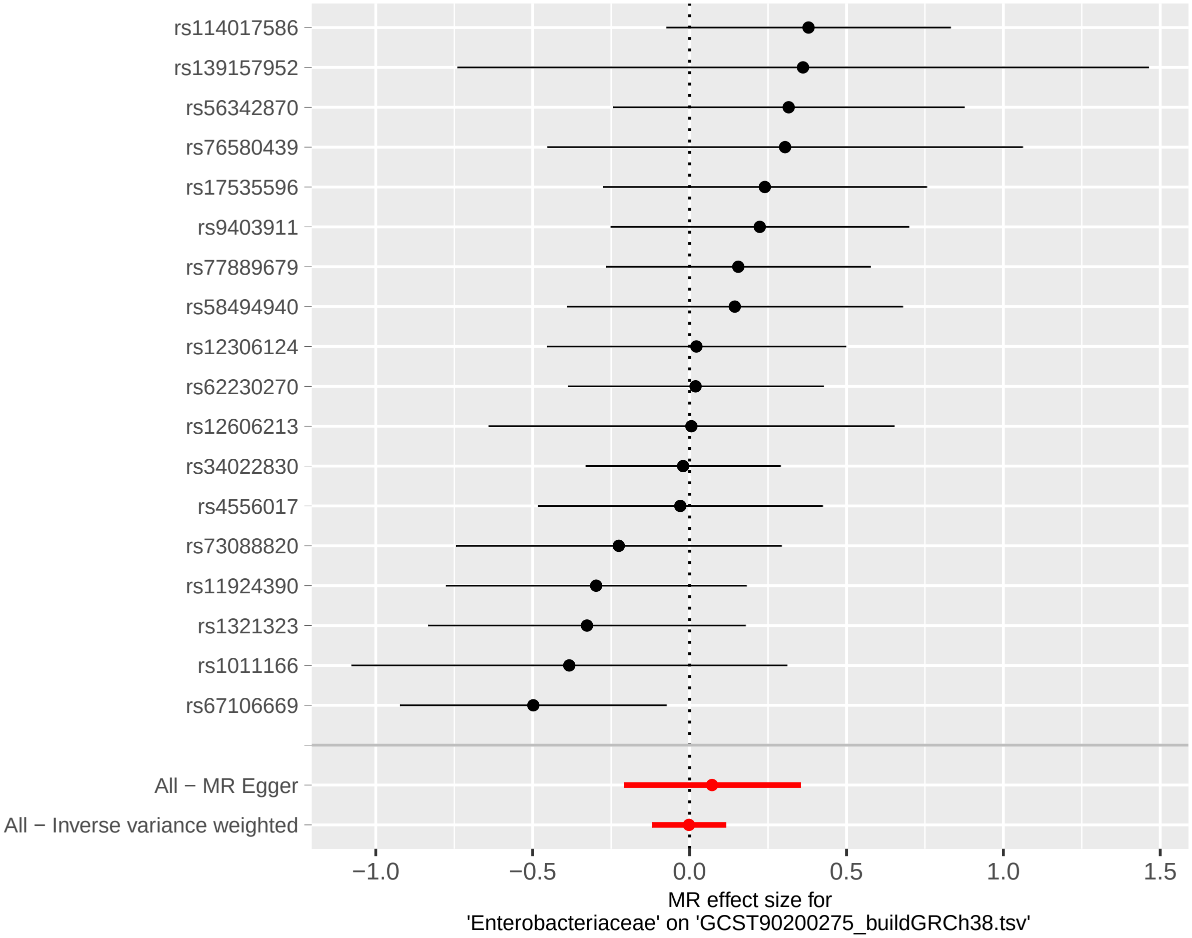

rs114017586
rs139157952
rs56342870
rs76580439
rs17535596
rs9403911
rs77889679
rs58494940
rs12306124
rs62230270
rs12606213
rs34022830
rs4556017
rs73088820
rs11924390
rs1321323
rs1011166
rs67106669
All − MR Egger
All − Inverse variance weighted
−1.0
−0.5
0.0
0.5
1.0
1.5
MR effect size for
'Enterobacteriaceae' on 'GCST90200275_buildGRCh38.tsv'

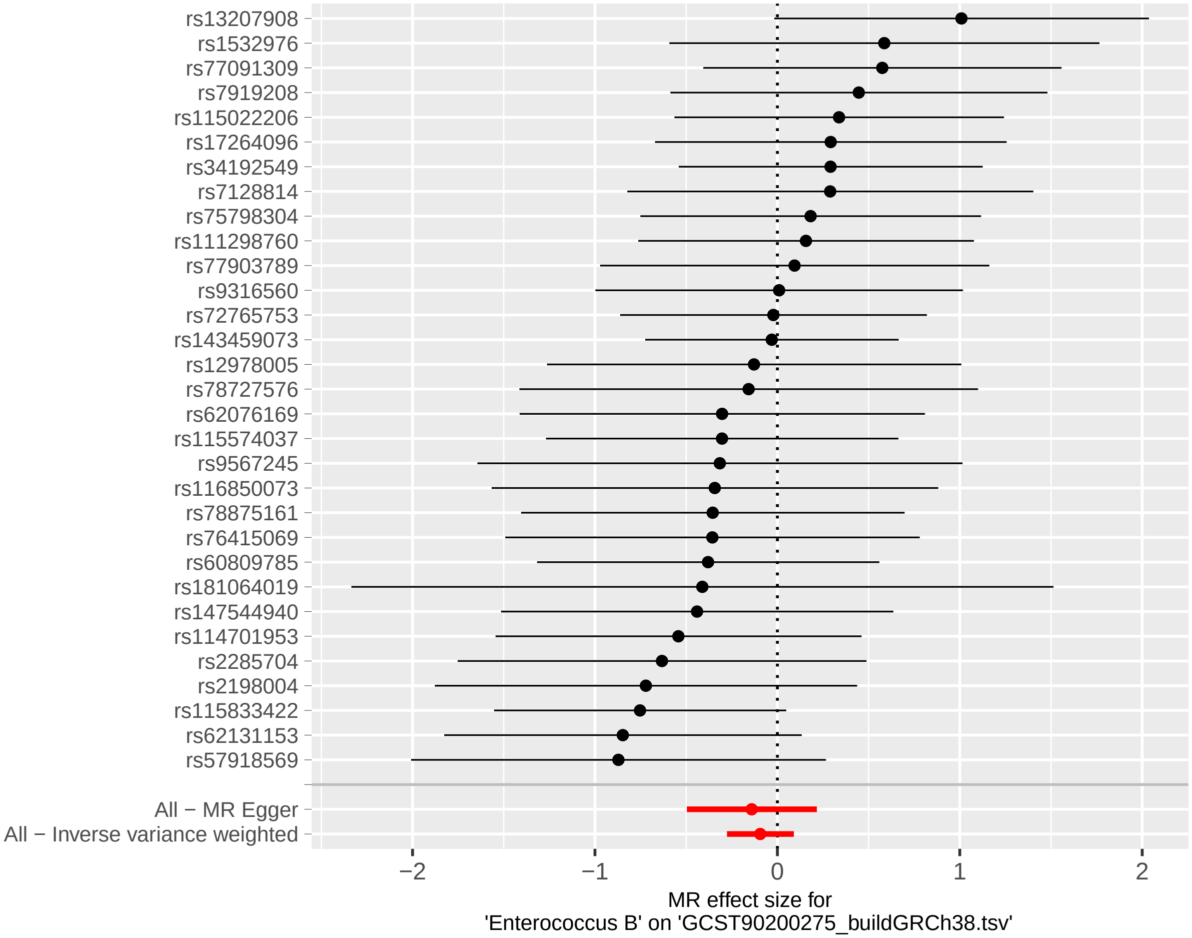
rs13207908
rs1532976
rs77091309
rs7919208
rs115022206
rs17264096
rs34192549
rs7128814
rs75798304
rs111298760
rs77903789
rs9316560
rs72765753
rs143459073
rs12978005
rs78727576
rs62076169
rs115574037
rs9567245
rs116850073
rs78875161
rs76415069
rs60809785
rs181064019
rs147544940
rs114701953
rs2285704
rs2198004
rs115833422
rs62131153
rs57918569
All − MR Egger
All − Inverse variance weighted
−2
−1
0
1
2
MR effect size for
'Enterococcus B' on 'GCST90200275_buildGRCh38.tsv'

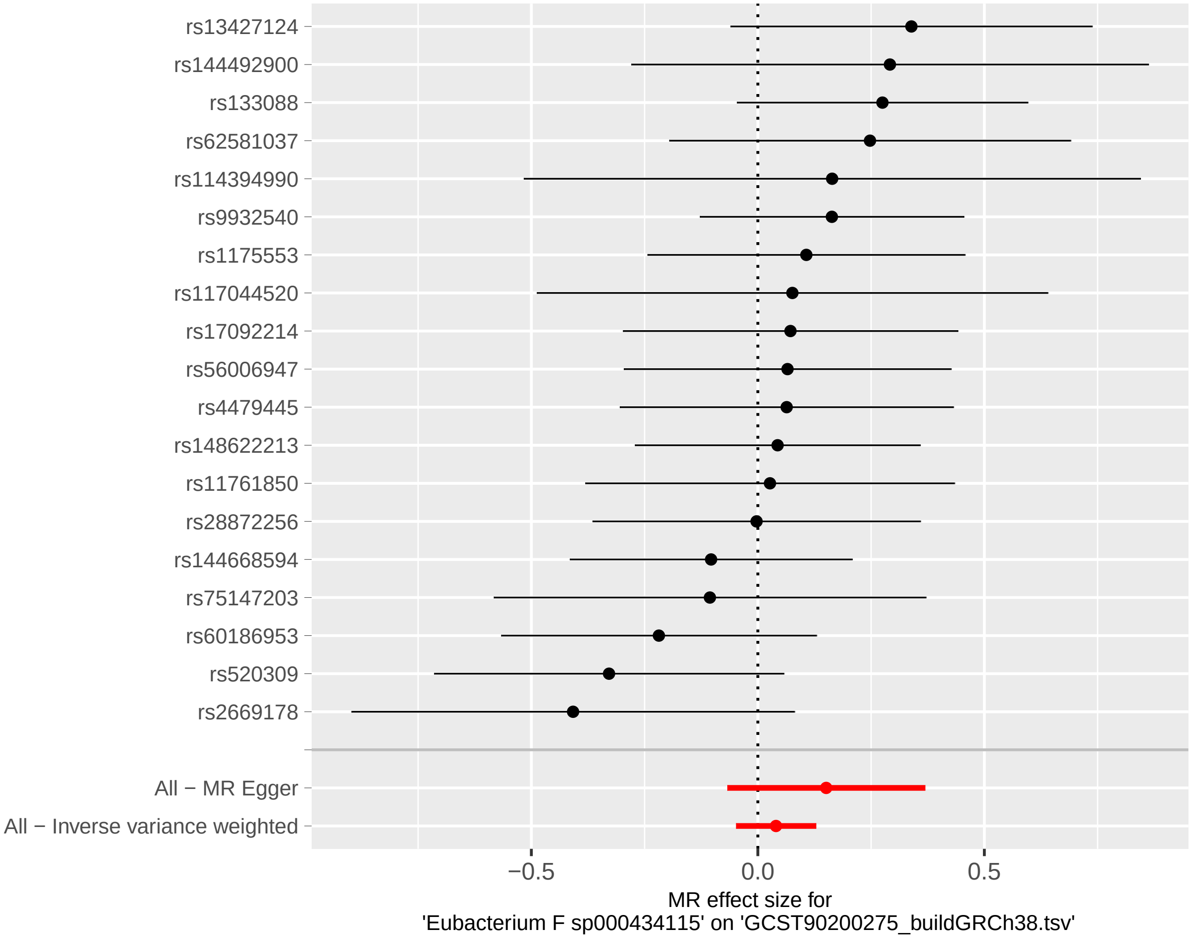

rs13427124
rs144492900
rs133088
rs62581037
rs114394990
rs9932540
rs1175553
rs117044520
rs17092214
rs56006947
rs4479445
rs148622213
rs11761850
rs28872256
rs144668594
rs75147203
rs60186953
rs520309
rs2669178
All − MR Egger
All − Inverse variance weighted
−0.5
0.0
0.5
MR effect size for
'Eubacterium F sp000434115' on 'GCST90200275_buildGRCh38.tsv'

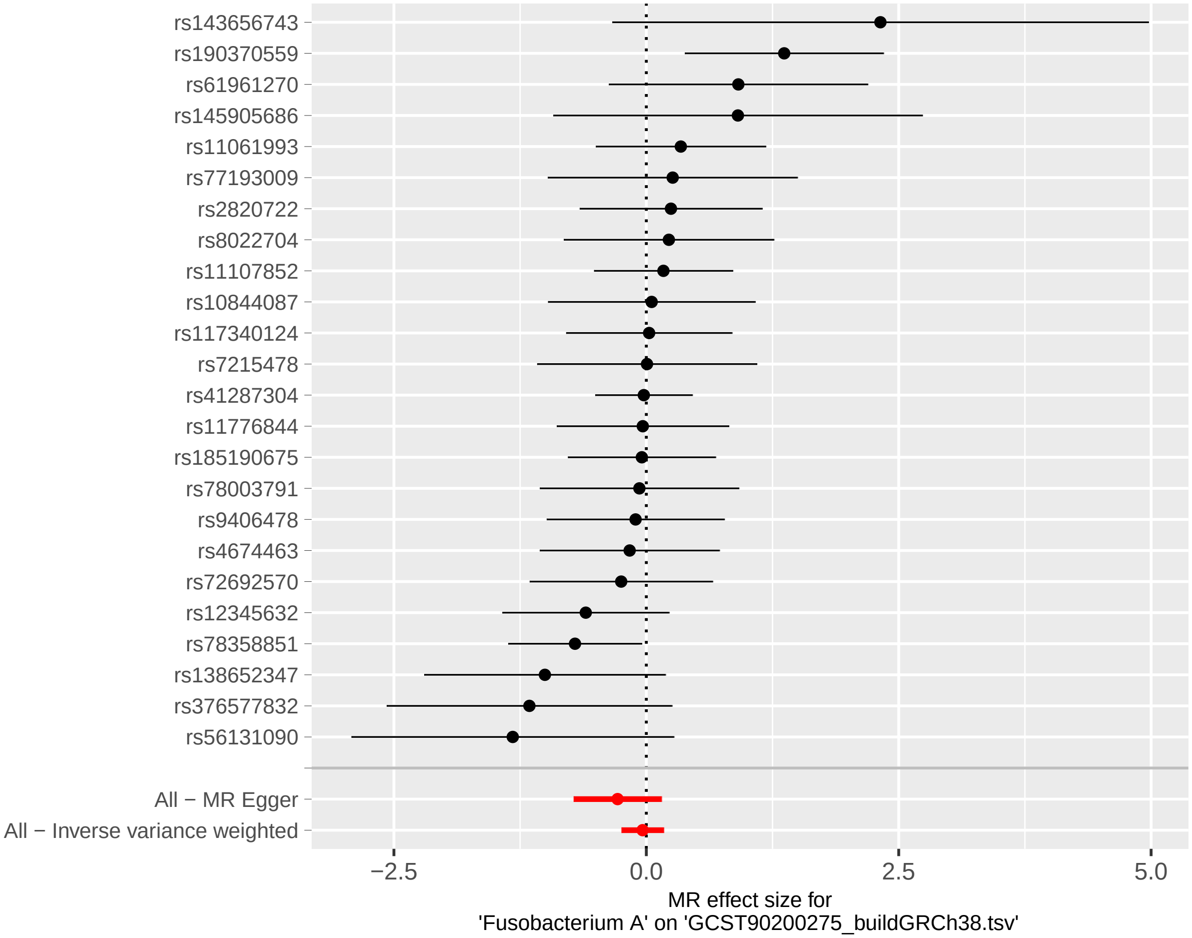
rs143656743
rs190370559
rs61961270
rs145905686
rs11061993
rs77193009
rs2820722
rs8022704
rs11107852
rs10844087
rs117340124
rs7215478
rs41287304
rs11776844
rs185190675
rs78003791
rs9406478
rs4674463
rs72692570
rs12345632
rs78358851
rs138652347
rs376577832
rs56131090
All − MR Egger
All − Inverse variance weighted
−2.5
0.0
2.5
5.0
MR effect size for
'Fusobacterium A' on 'GCST90200275_buildGRCh38.tsv'

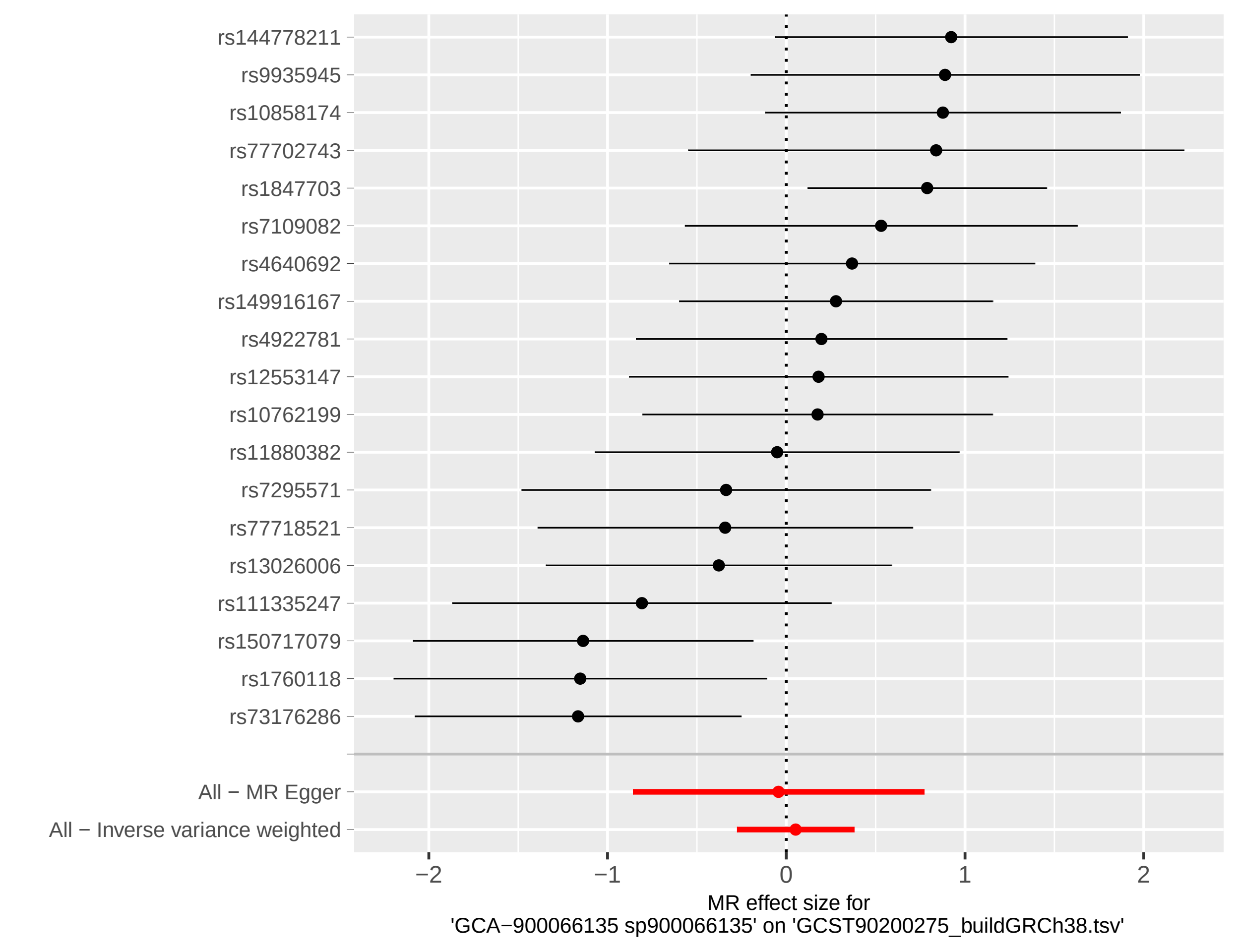

rs144778211
rs9935945
rs10858174
rs77702743
rs1847703
rs7109082
rs4640692
rs149916167
rs4922781
rs12553147
rs10762199
rs11880382
rs7295571
rs77718521
rs13026006
rs111335247
rs150717079
rs1760118
rs73176286
All − MR Egger
All − Inverse variance weighted
−2
−1
0
1
2
MR effect size for
'GCA−900066135 sp900066135' on 'GCST90200275_buildGRCh38.tsv'

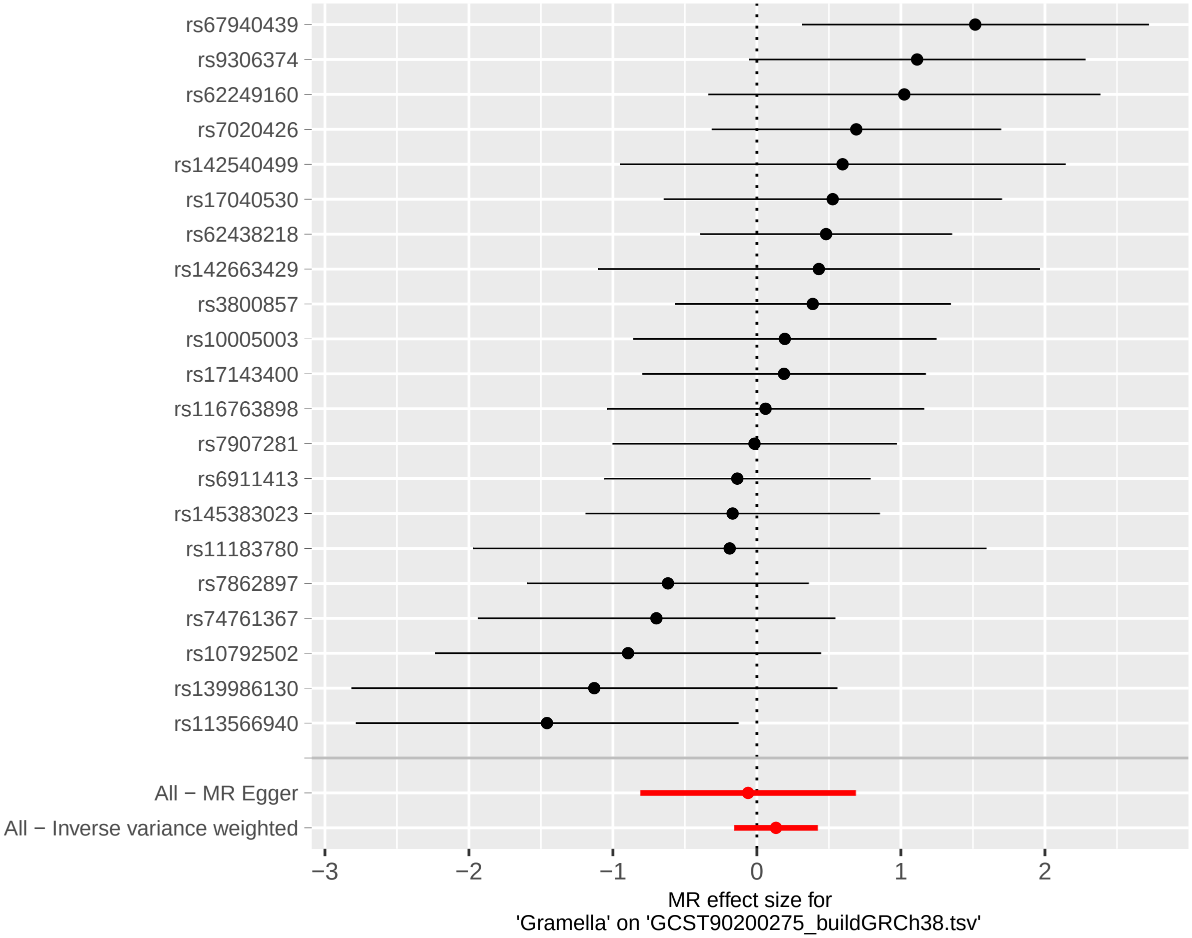

rs67940439
rs9306374
rs62249160
rs7020426
rs142540499
rs17040530
rs62438218
rs142663429
rs3800857
rs10005003
rs17143400
rs116763898
rs7907281
rs6911413
rs145383023
rs11183780
rs7862897
rs74761367
rs10792502
rs139986130
rs113566940
All − MR Egger
All − Inverse variance weighted
−3
−2
−1
0
1
2
MR effect size for
'Gramella' on 'GCST90200275_buildGRCh38.tsv'

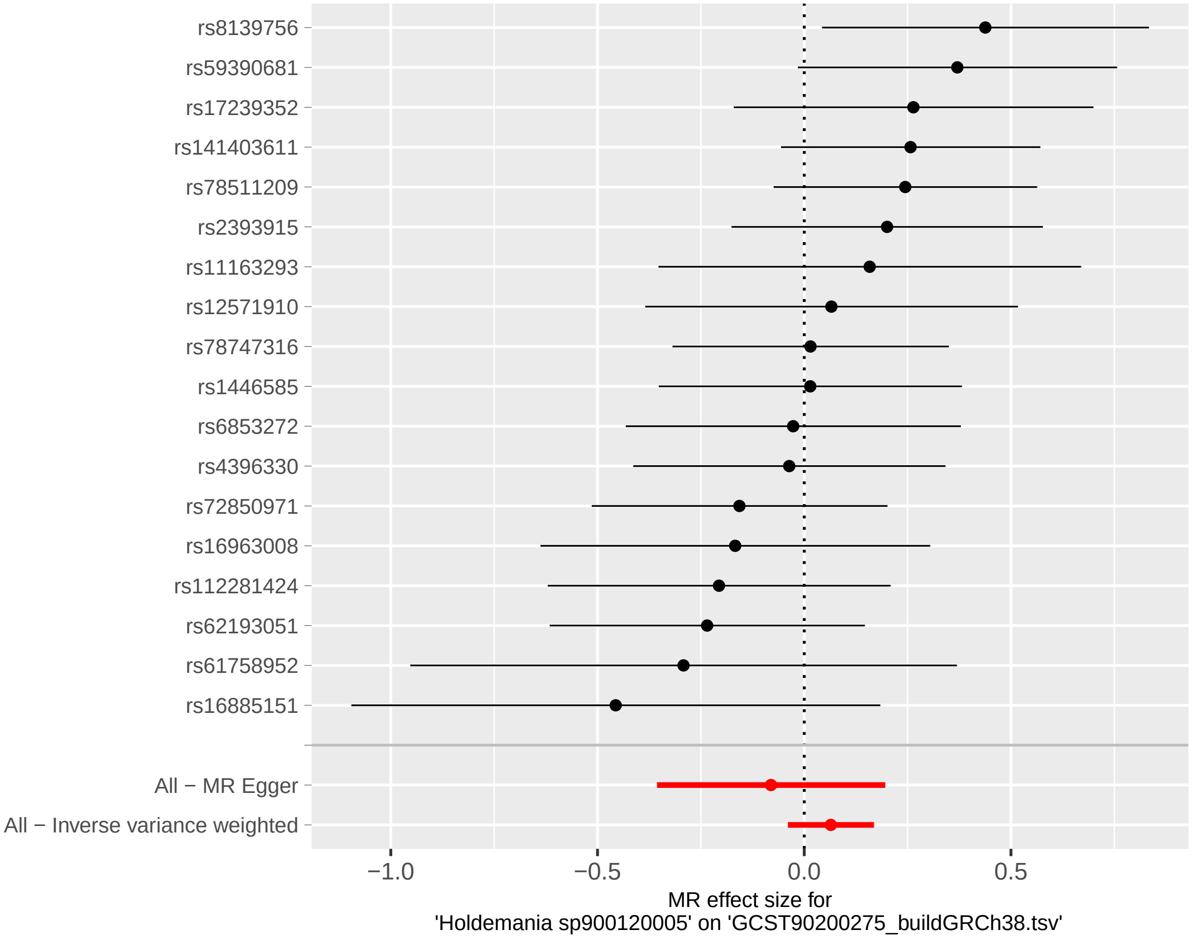
rs8139756
rs59390681
rs17239352
rs141403611
rs78511209
rs2393915
rs11163293
rs12571910
rs78747316
rs1446585
rs6853272
rs4396330
rs72850971
rs16963008
rs112281424
rs62193051
rs61758952
rs16885151
All − MR Egger
All − Inverse variance weighted
−1.0
−0.5
0.0
0.5
MR effect size for
'Holdemania sp900120005' on 'GCST90200275_buildGRCh38.tsv'

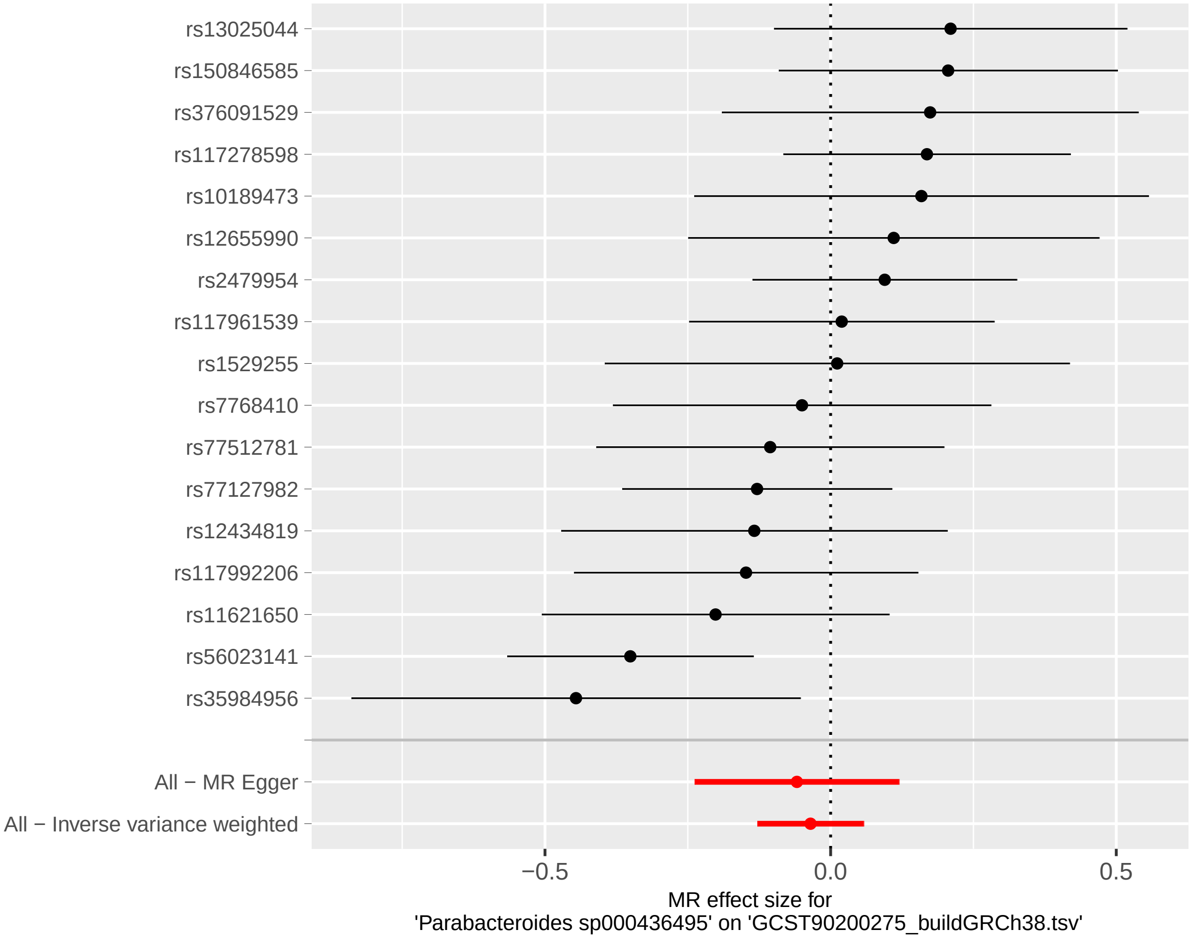
rs13025044
rs150846585
rs376091529
rs117278598
rs10189473
rs12655990
rs2479954
rs117961539
rs1529255
rs7768410
rs77512781
rs77127982
rs12434819
rs117992206
rs11621650
rs56023141
rs35984956
All − MR Egger
All − Inverse variance weighted
−0.5
0.0
0.5
MR effect size for
'Parabacteroides sp000436495' on 'GCST90200275_buildGRCh38.tsv'

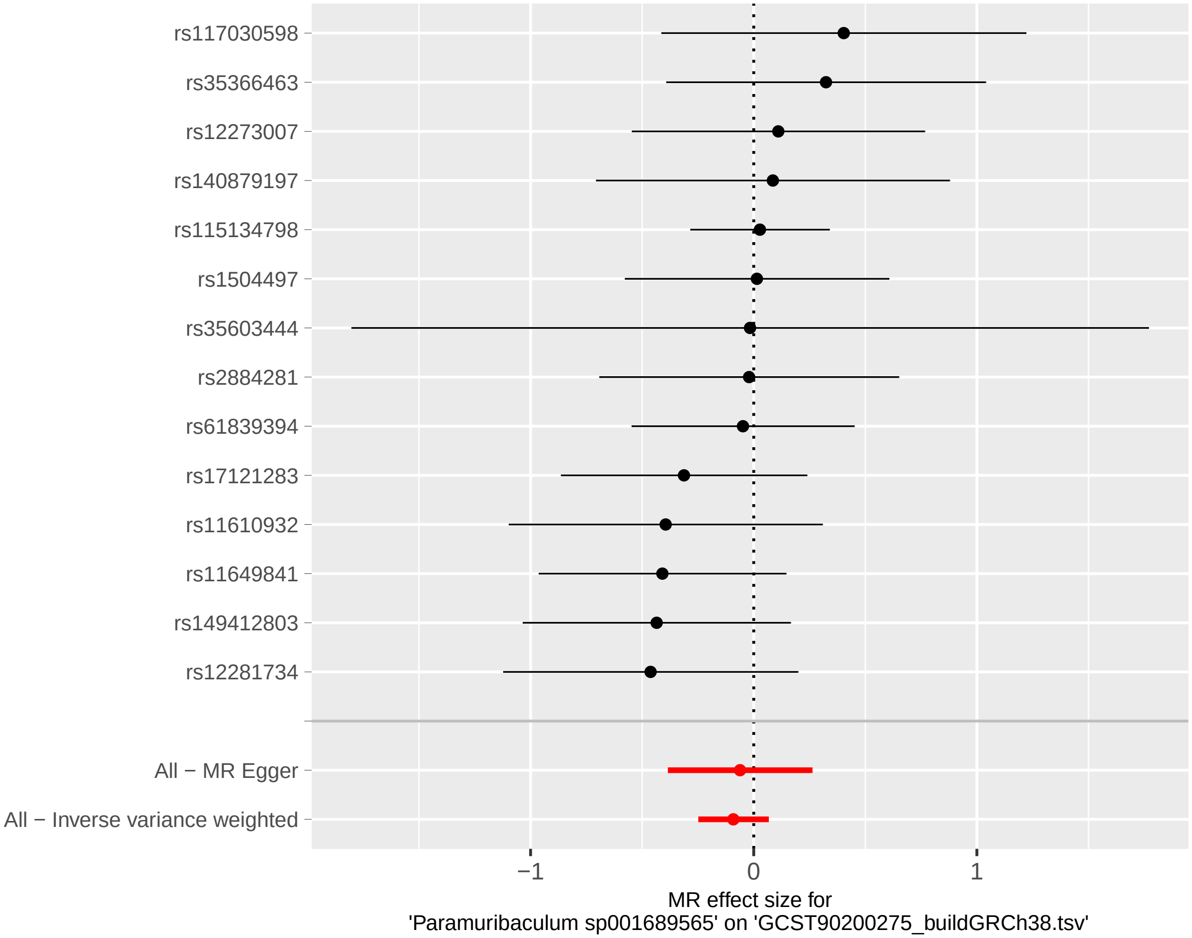

rs117030598
rs35366463
rs12273007
rs140879197
rs115134798
rs1504497
rs35603444
rs2884281
rs61839394
rs17121283
rs11610932
rs11649841
rs149412803
rs12281734
All − MR Egger
All − Inverse variance weighted
−1
0
1
MR effect size for
'Paramuribaculum sp001689565' on 'GCST90200275_buildGRCh38.tsv'

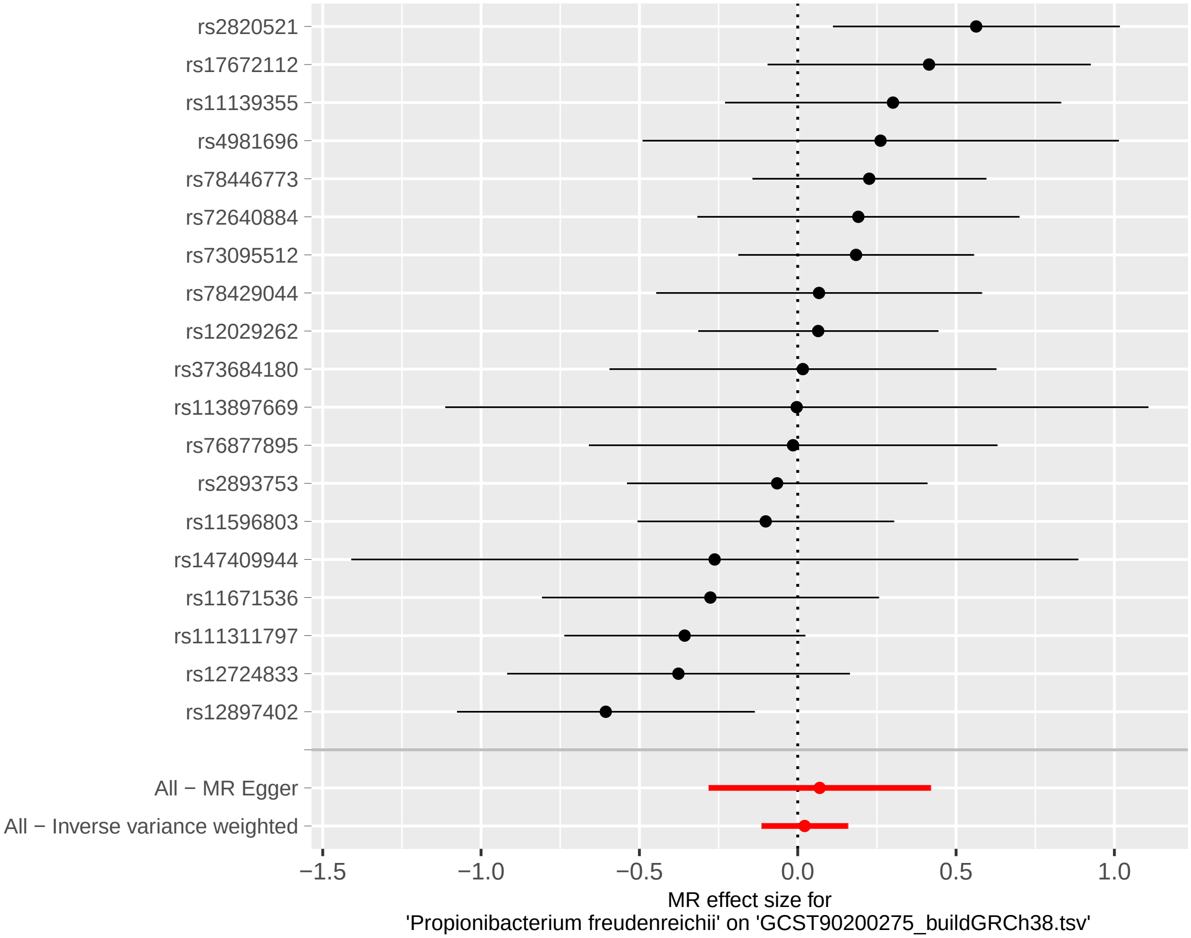
rs2820521
rs17672112
rs11139355
rs4981696
rs78446773
rs72640884
rs73095512
rs78429044
rs12029262
rs373684180
rs113897669
rs76877895
rs2893753
rs11596803
rs147409944
rs11671536
rs111311797
rs12724833
rs12897402
All − MR Egger
All − Inverse variance weighted
−1.5
−1.0
−0.5
0.0
0.5
1.0
MR effect size for
'Propionibacterium freudenreichii' on 'GCST90200275_buildGRCh38.tsv'

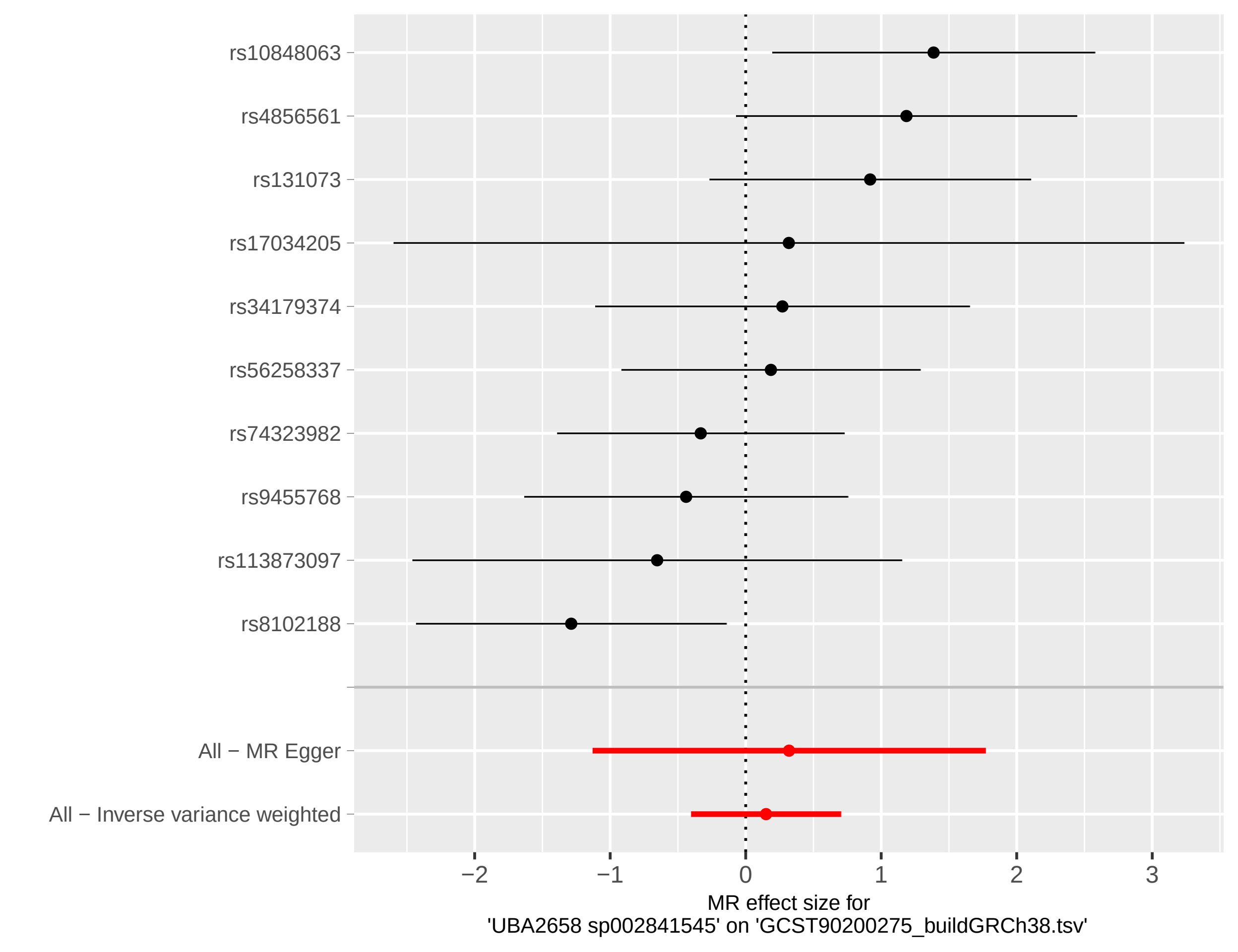

rs10848063
rs4856561
rs131073
rs17034205
rs34179374
rs56258337
rs74323982
rs9455768
rs113873097
rs8102188
All − MR Egger
All − Inverse variance weighted
−2
−1
0
1
2
3
MR effect size for
'UBA2658 sp002841545' on 'GCST90200275_buildGRCh38.tsv'

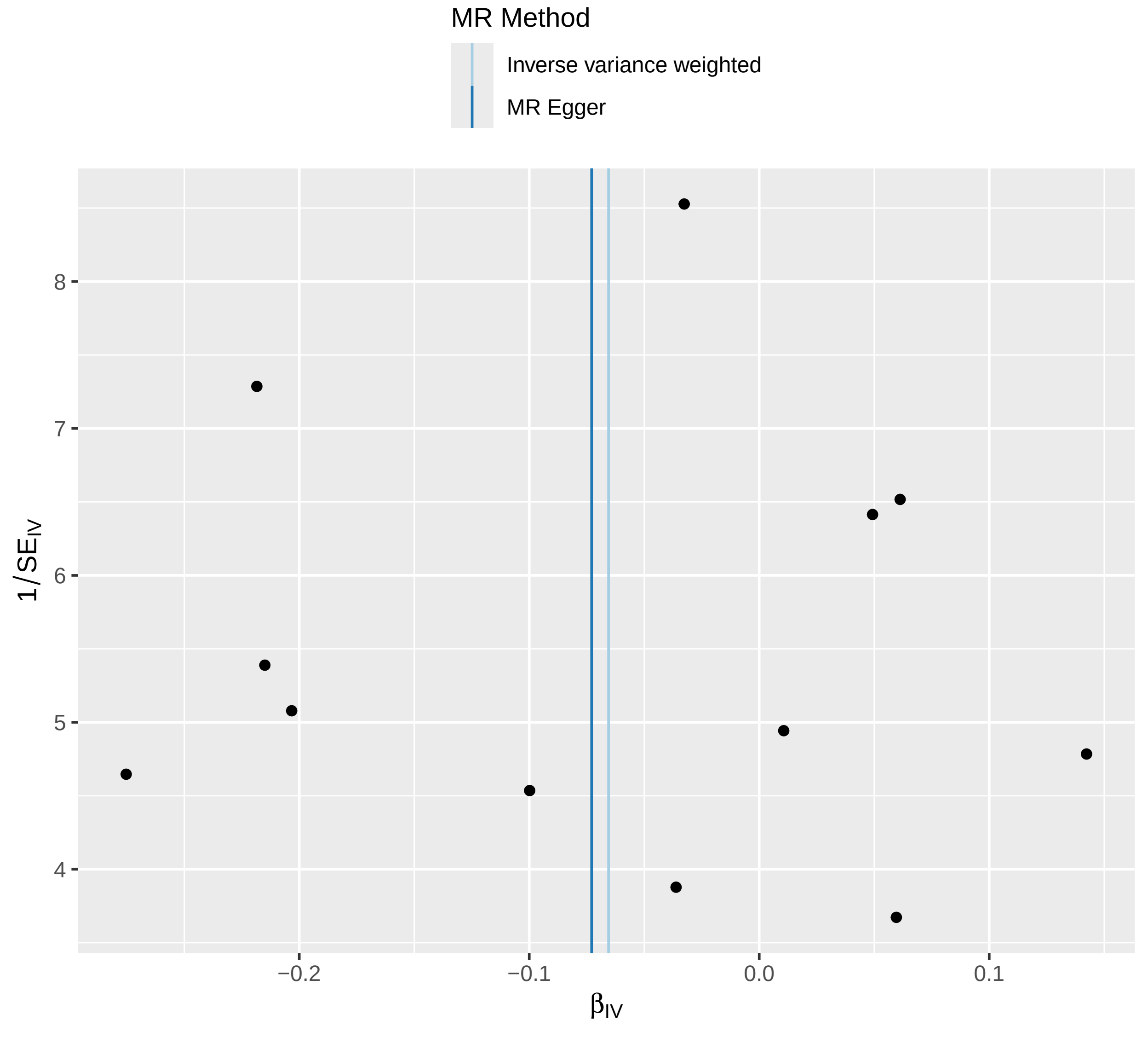
MR Method
Inverse variance weighted
MR Egger
8
7
6
5
4
1/SE_IV
−0.2
−0.1
0.0
0.1
β_IV

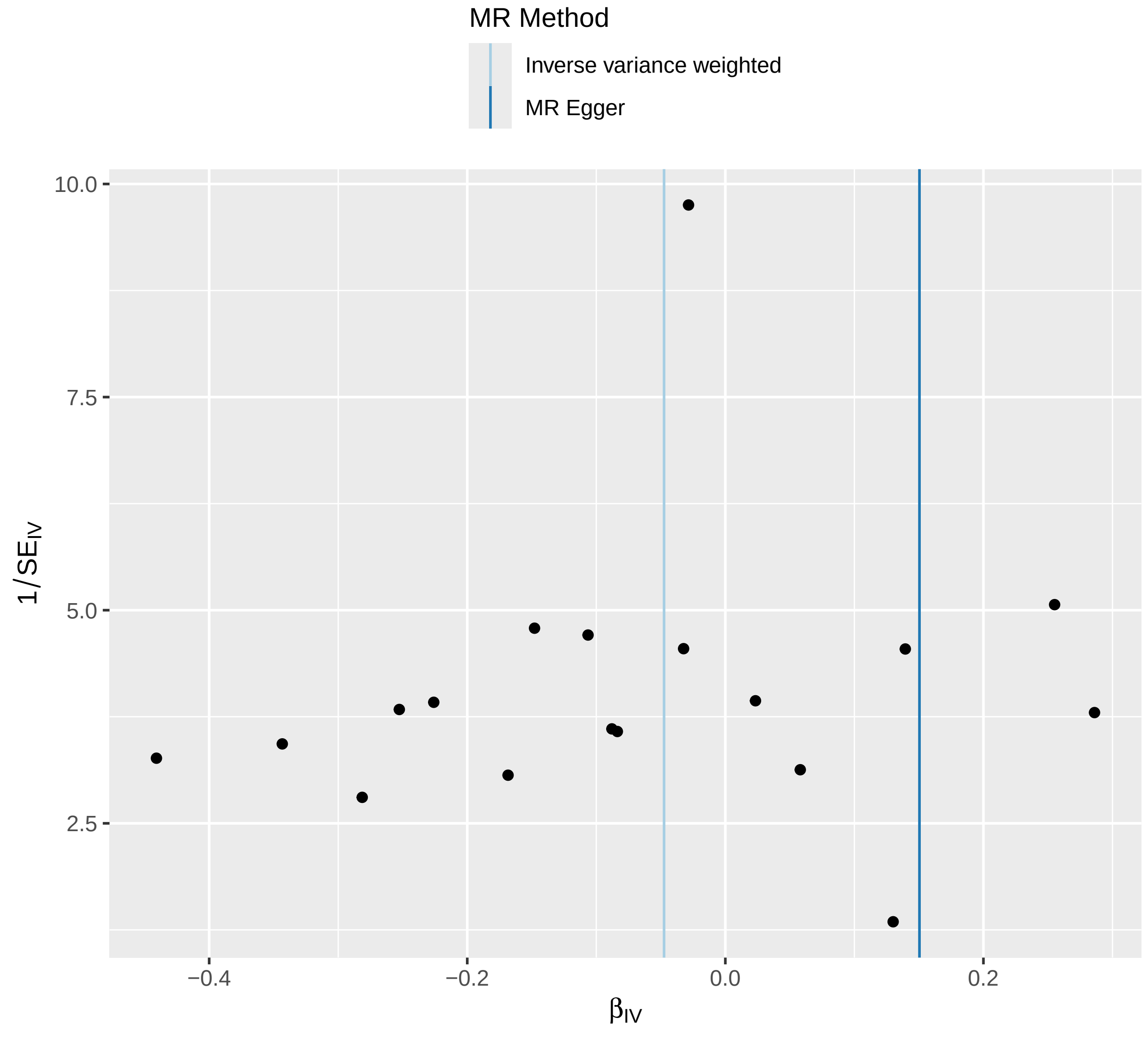
MR Method
Inverse variance weighted
MR Egger
10.0
7.5
5.0
2.5
1/SE$_{IV}$
−0.4
−0.2
0.0
0.2
β$_{IV}$

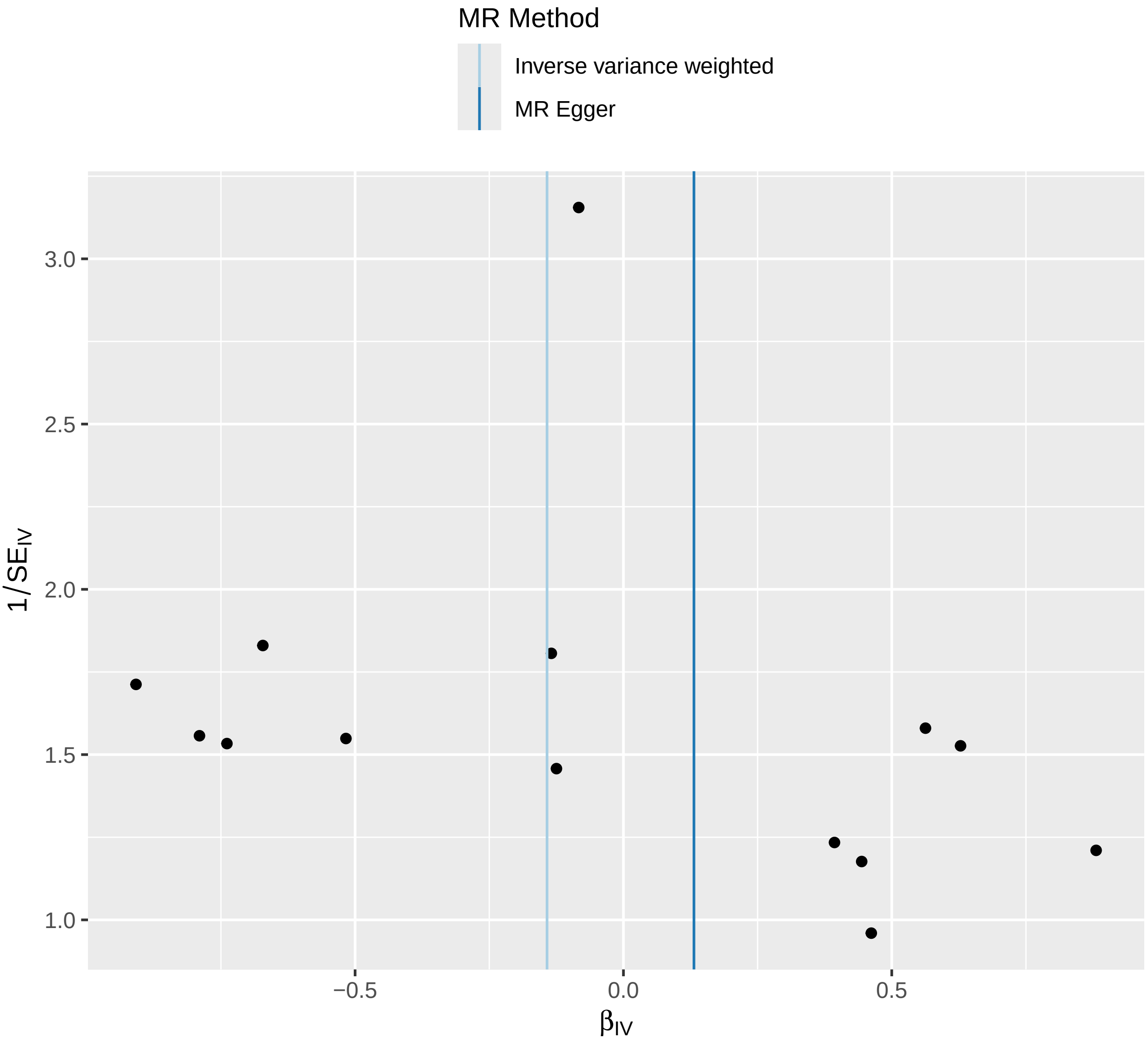
MR Method
Inverse variance weighted
MR Egger
3.0
2.5
2.0
1.5
1.0
$1/SE_{IV}$
−0.5
0.0
0.5
β_{IV}

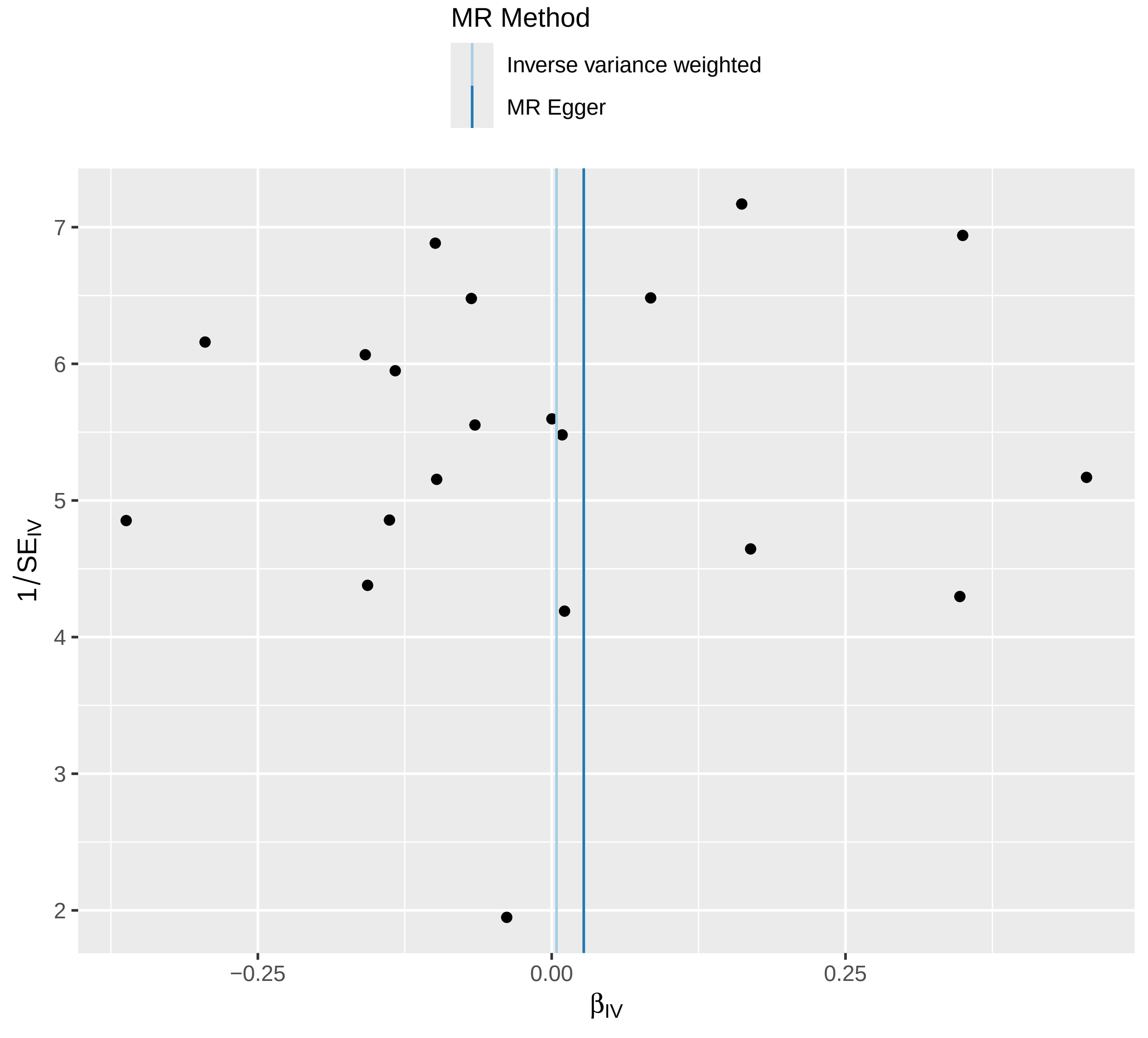
MR Method
Inverse variance weighted
MR Egger
7
6
5
4
3
2
$1/SE_{IV}$
−0.25
0.00
0.25
β_{IV}

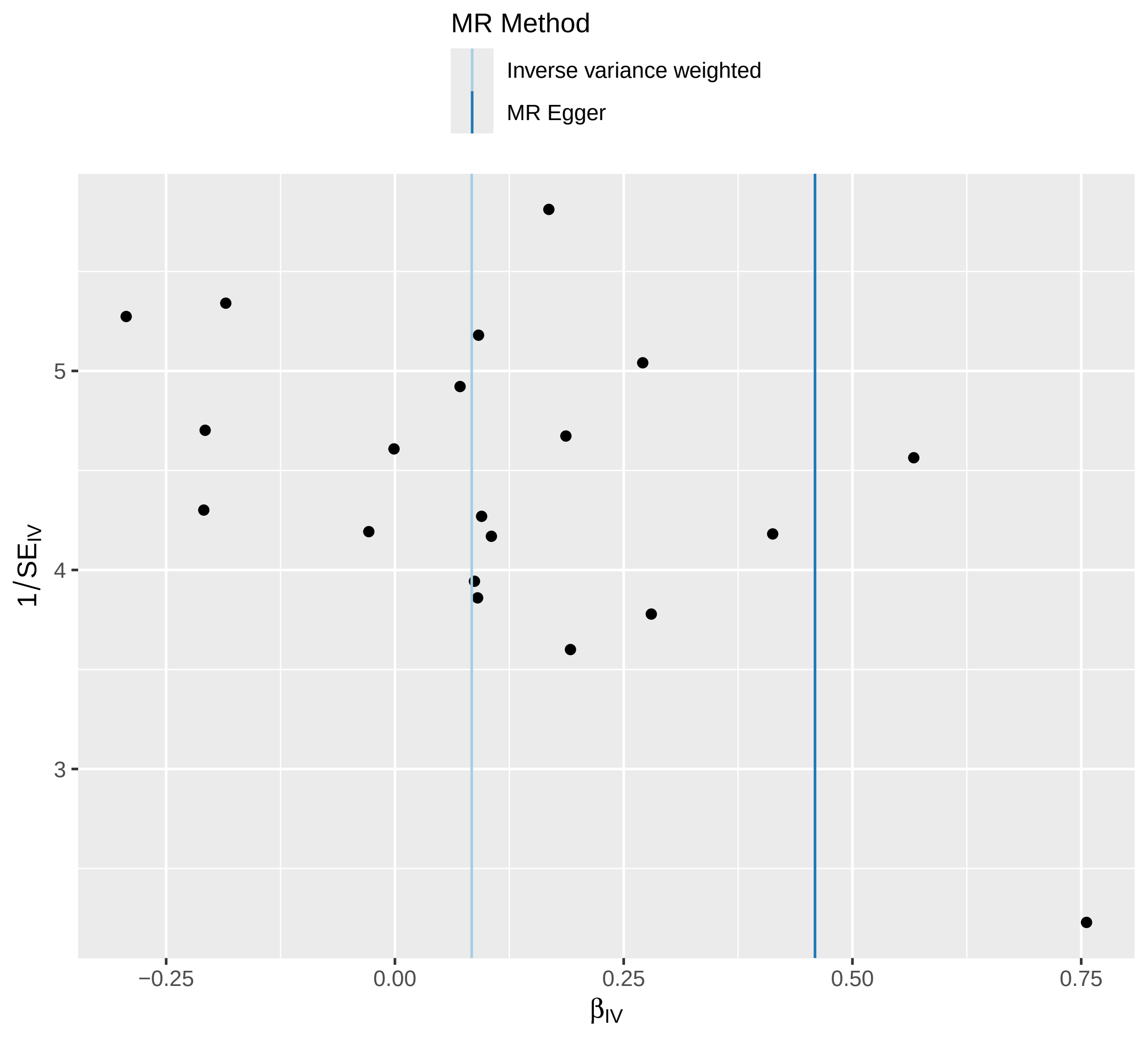

MR Method
Inverse variance weighted
MR Egger
5
4
3
1/SE_IV
−0.25
0.00
0.25
0.50
0.75
β_IV

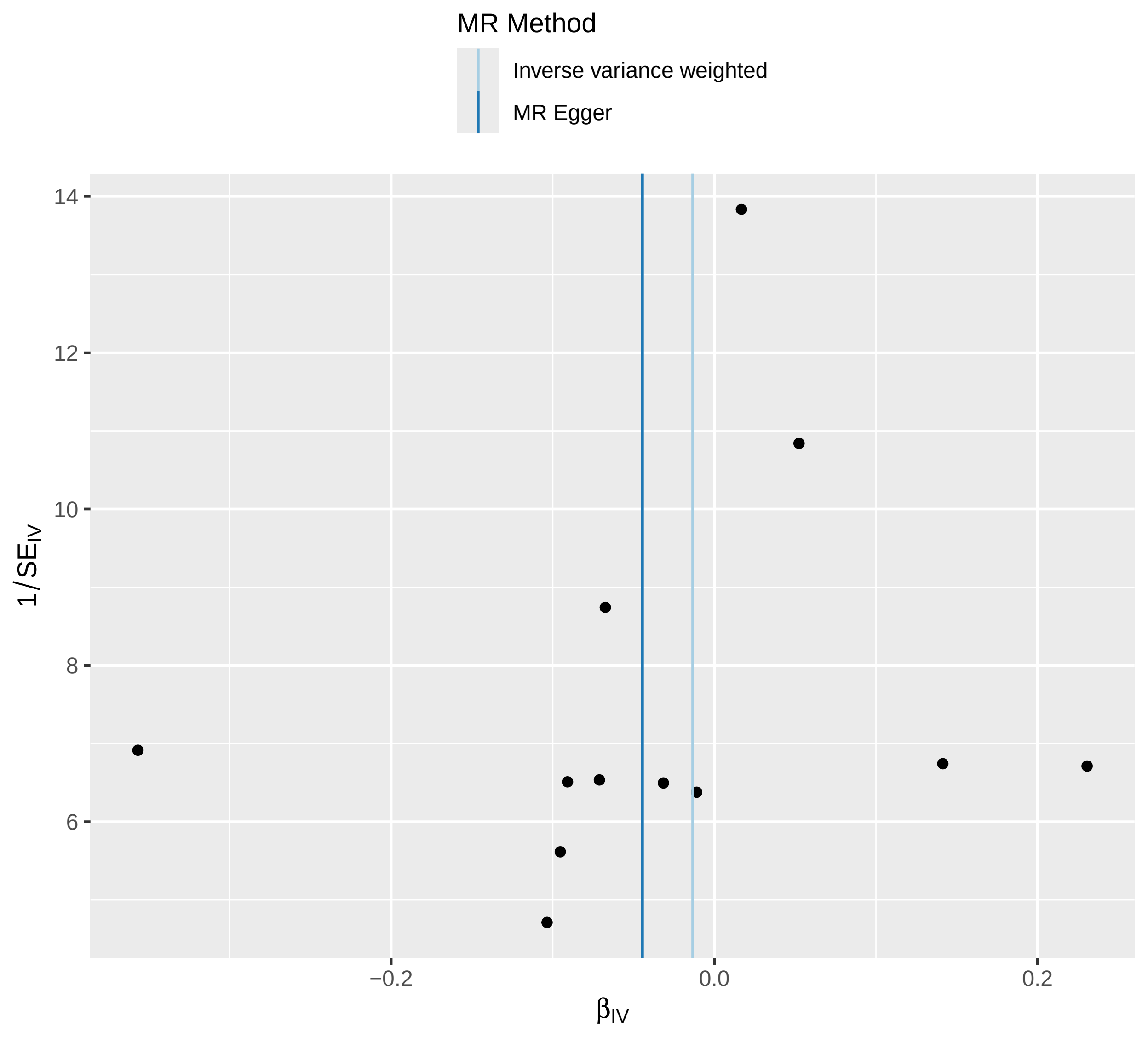
MR Method
Inverse variance weighted
MR Egger
14
12
10
8
6
$1/SE_{IV}$
−0.2
0.0
0.2
β_{IV}

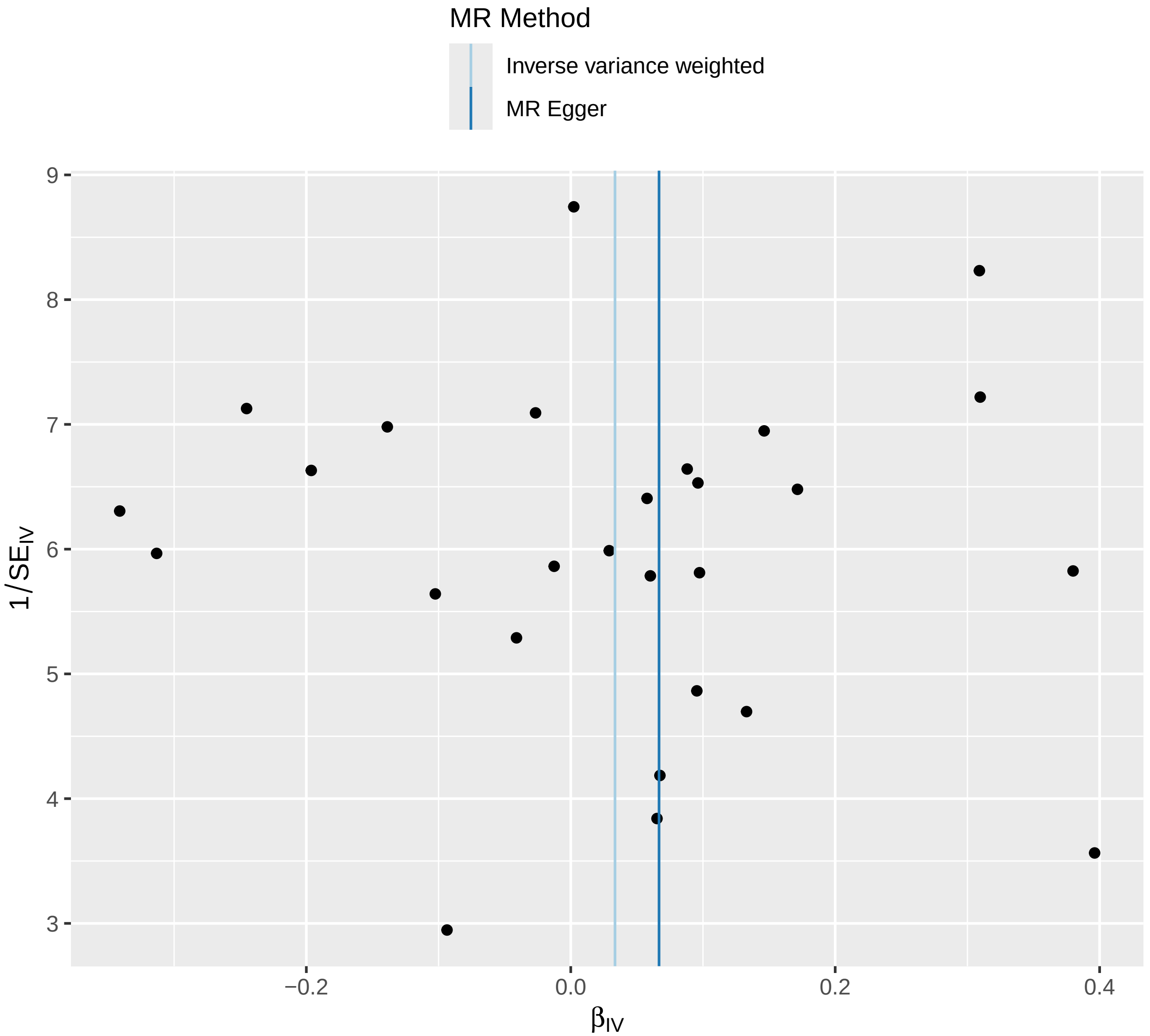

MR Method
Inverse variance weighted
MR Egger
9
8
7
6
5
4
3
1/SE_IV
−0.2
0.0
0.2
0.4
β_IV

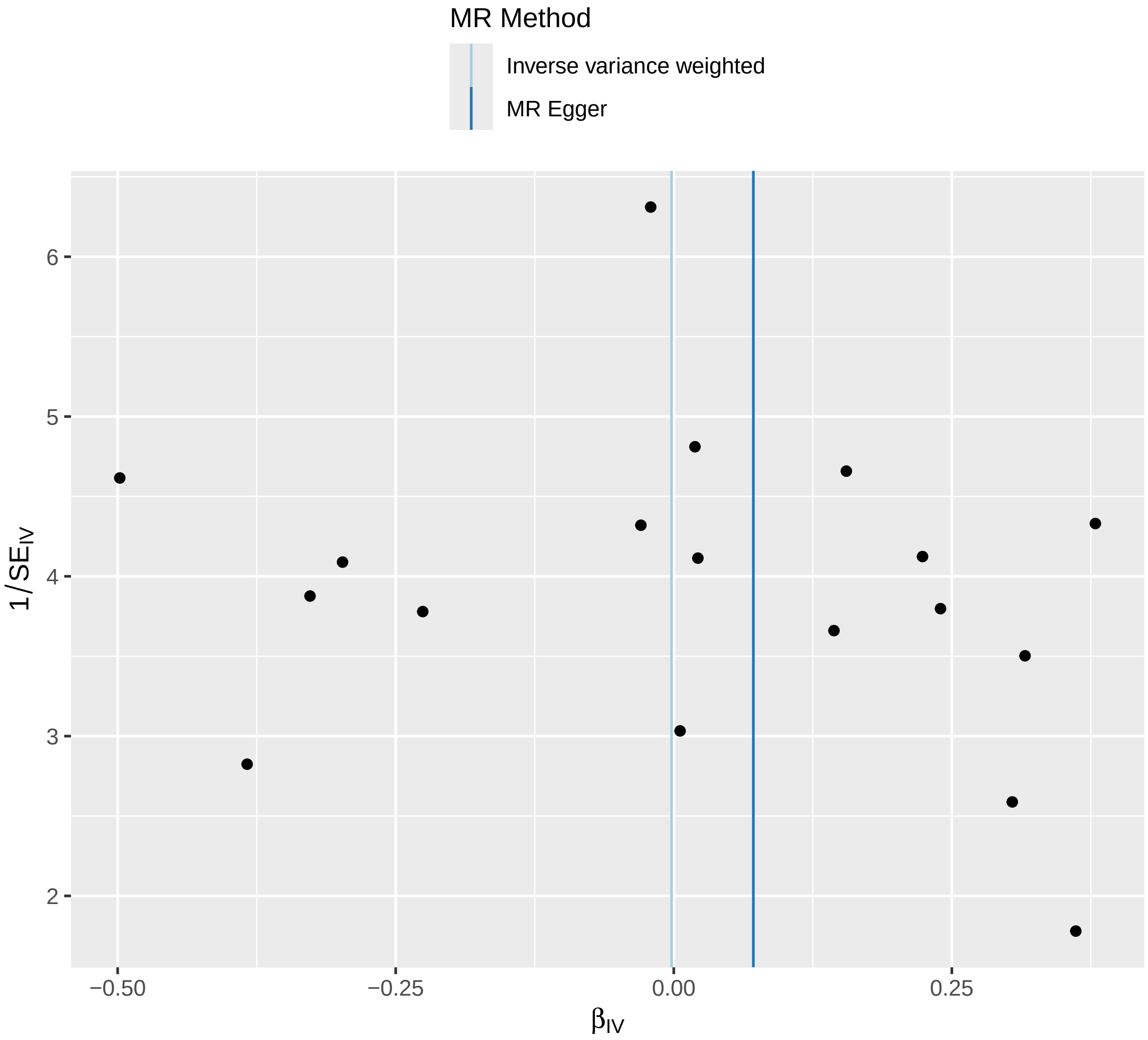

MR Method
Inverse variance weighted
MR Egger
1/SE_IV
6
5
4
3
2
β_IV
−0.50
−0.25
0.00
0.25

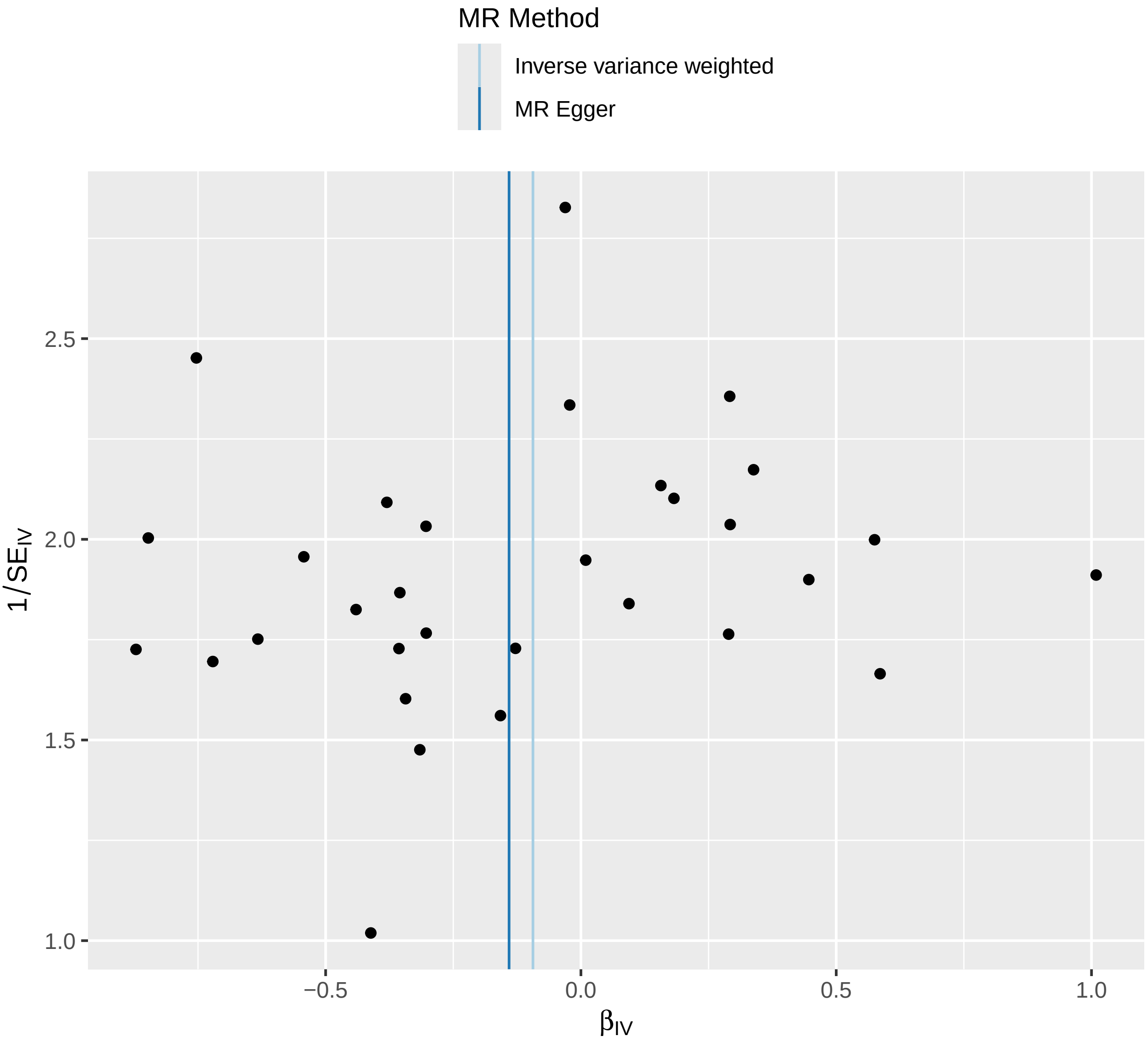

MR Method
Inverse variance weighted
MR Egger
2.5
2.0
1.5
1.0
1/SE$_{IV}$
−0.5
0.0
0.5
1.0
β$_{IV}$

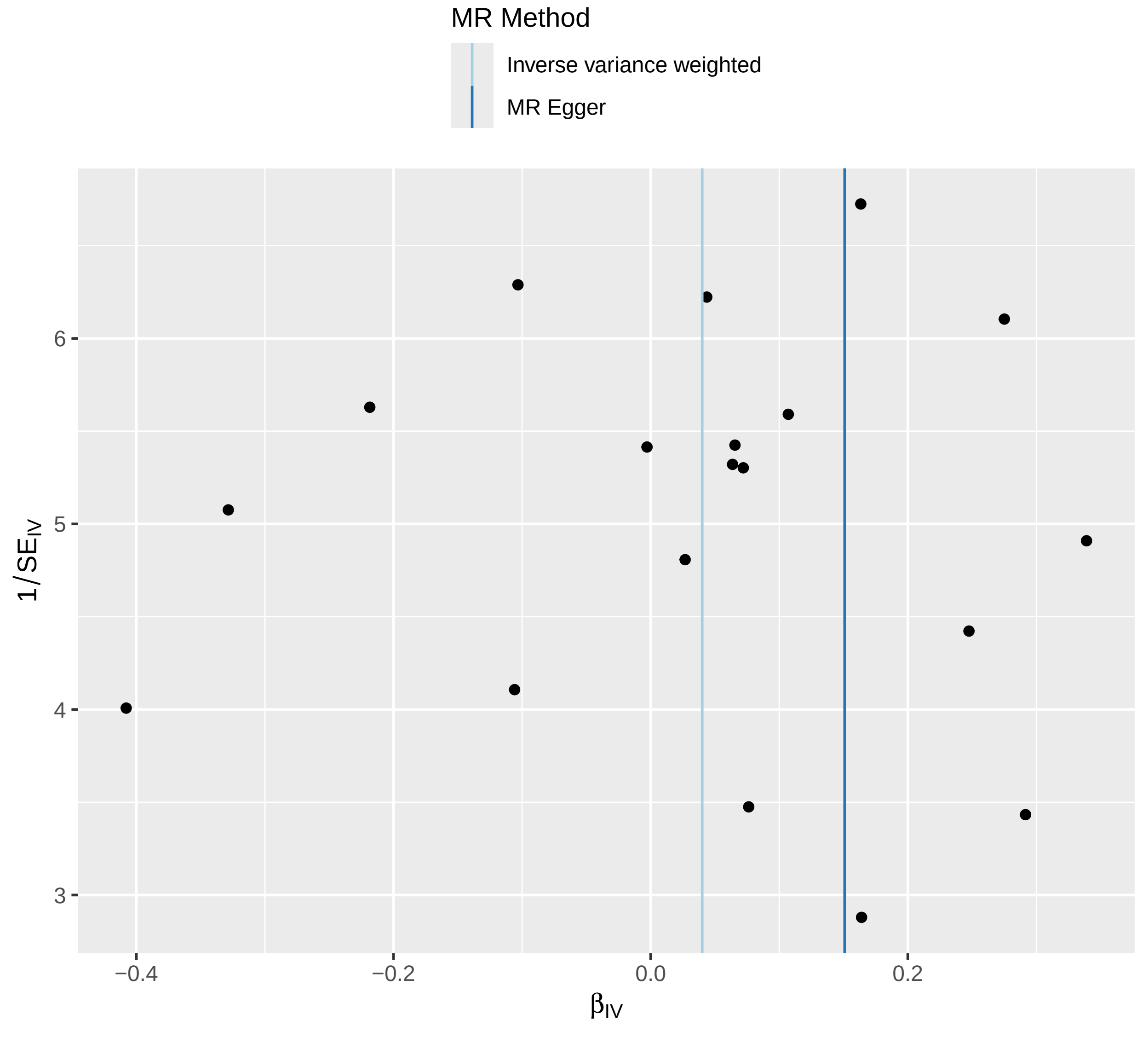
MR Method
Inverse variance weighted
MR Egger
6
5
4
3
1/SE$_{IV}$
−0.4
−0.2
0.0
0.2
β$_{IV}$

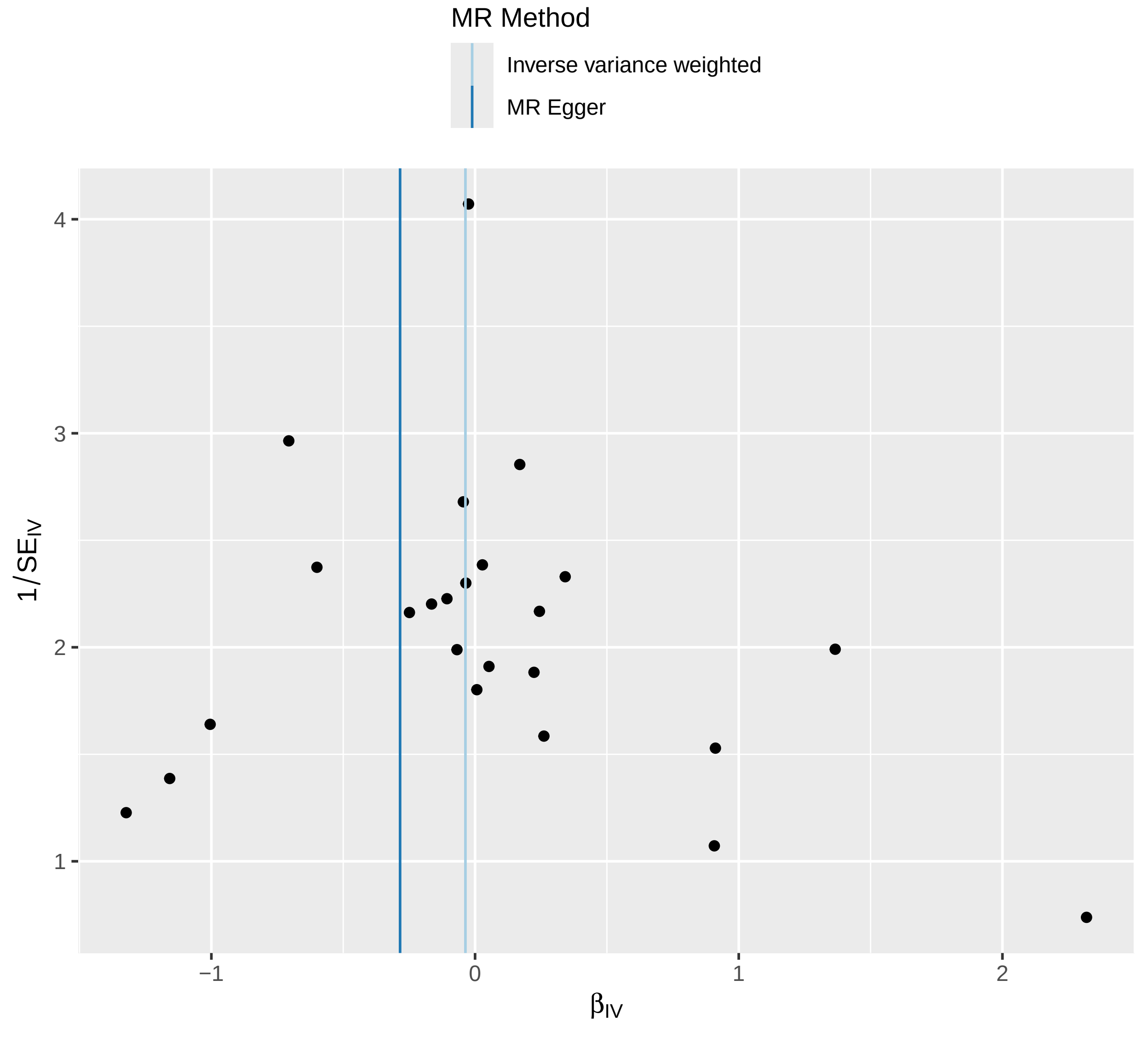

MR Method
Inverse variance weighted
MR Egger
4
3
2
1
1/SE$_{IV}$
−1
0
1
2
β$_{IV}$

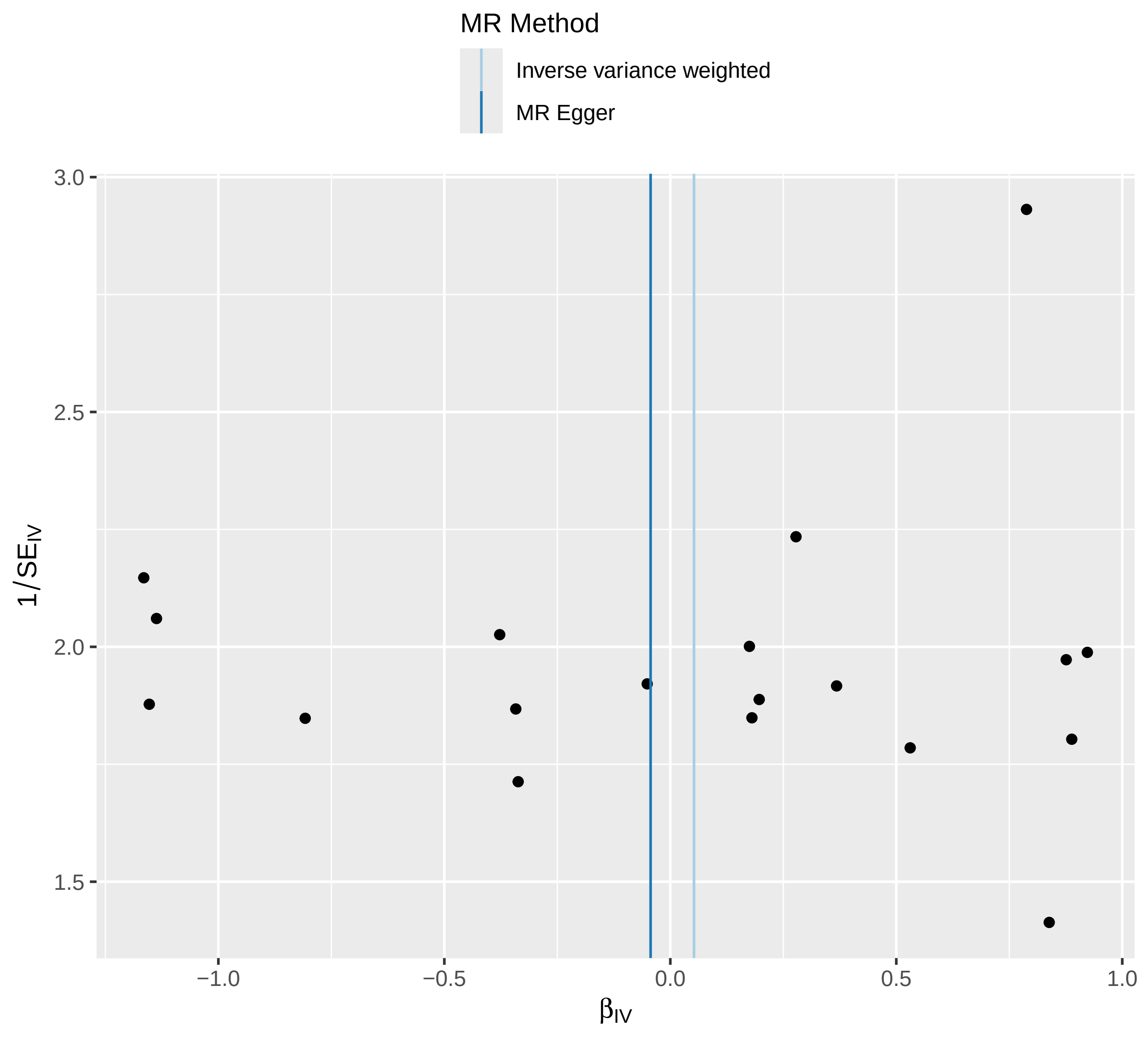
MR Method
Inverse variance weighted
MR Egger
3.0
2.5
2.0
1.5
$1/SE_{IV}$
−1.0
−0.5
0.0
0.5
1.0
β_{IV}

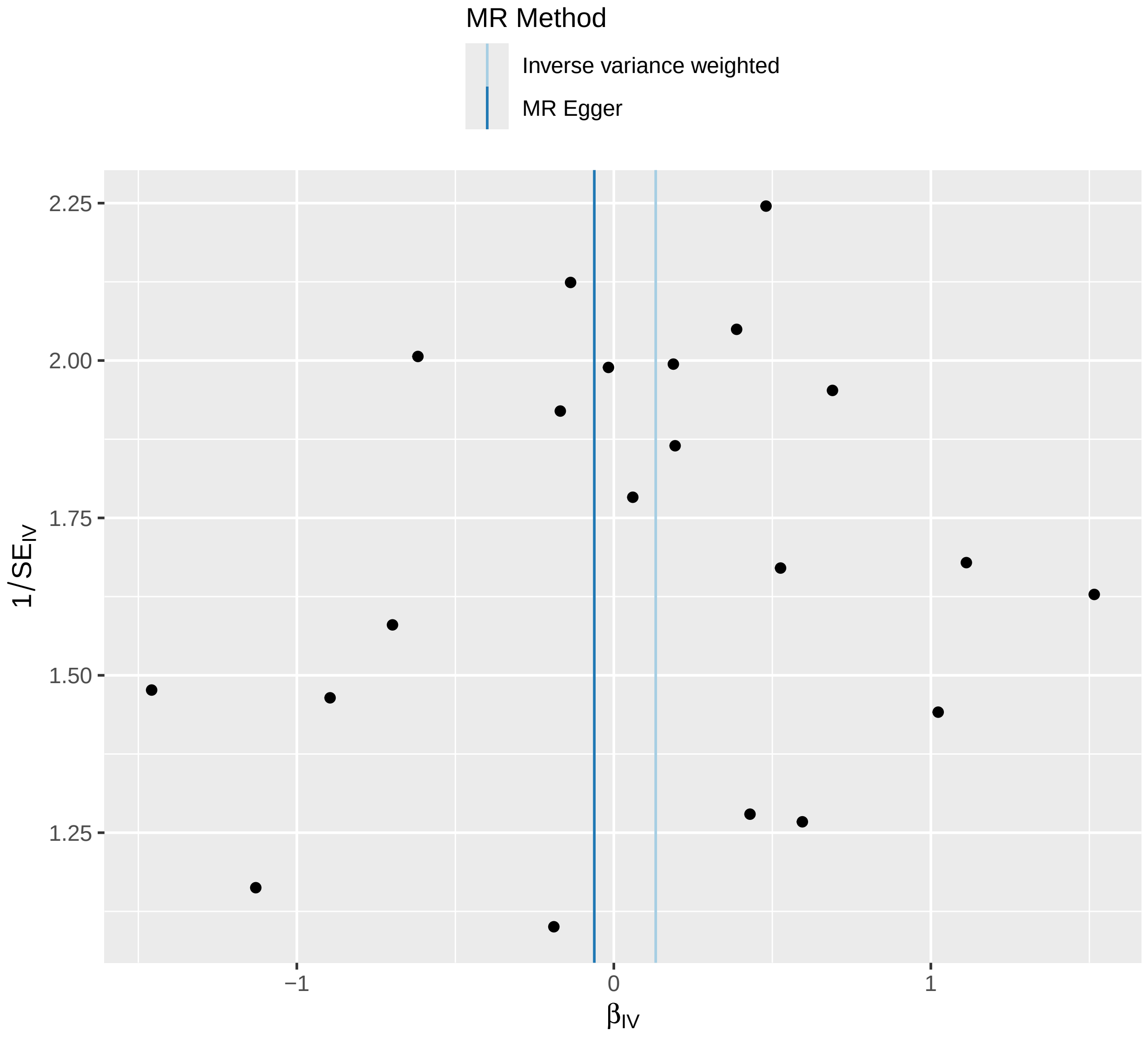

MR Method
Inverse variance weighted
MR Egger
2.25
2.00
1.75
1.50
1.25
1/SE_IV
−1
0
1
β_IV

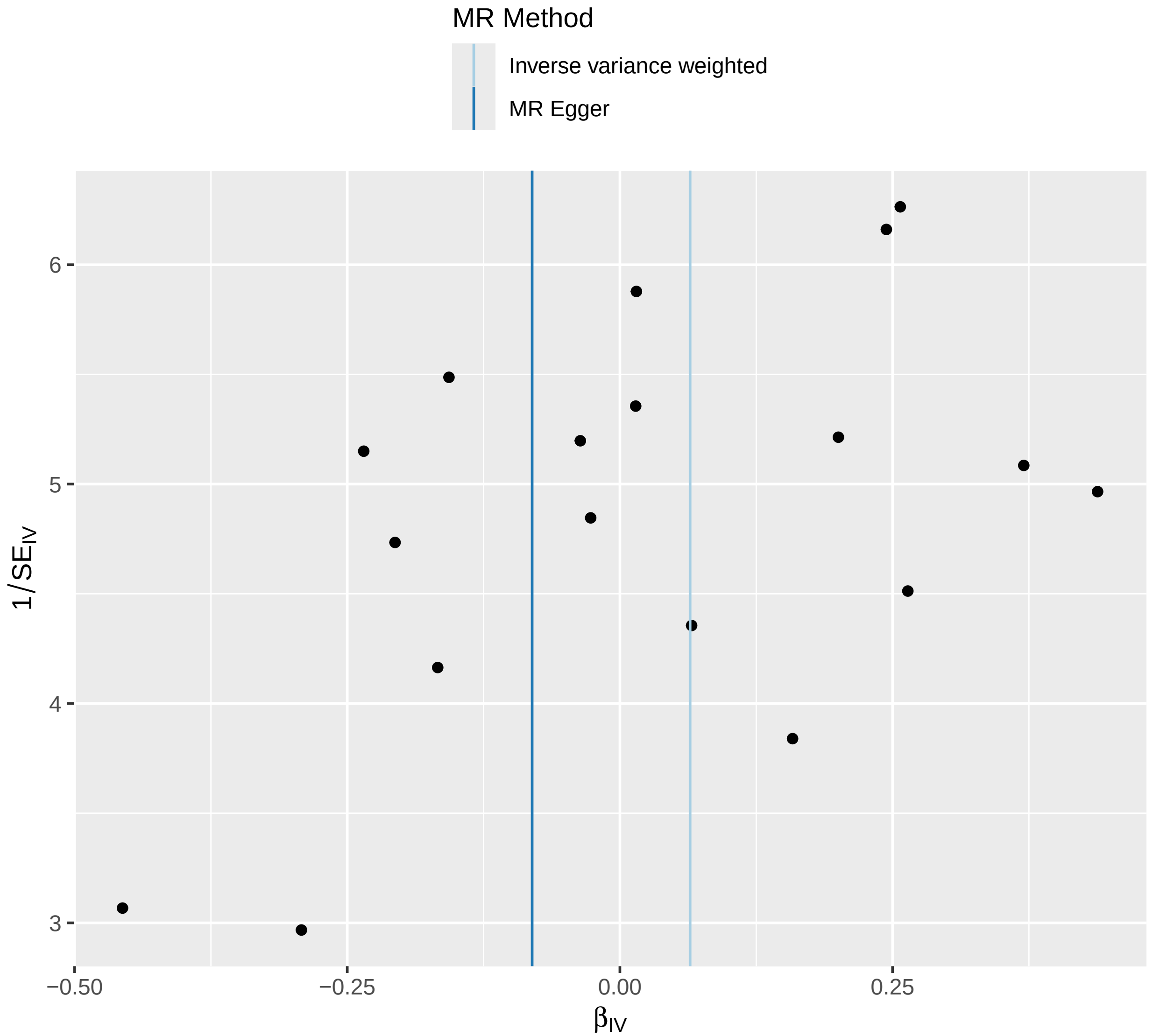

MR Method
Inverse variance weighted
MR Egger
6
5
4
3
1/SE$_{IV}$
−0.50
−0.25
0.00
0.25
β$_{IV}$

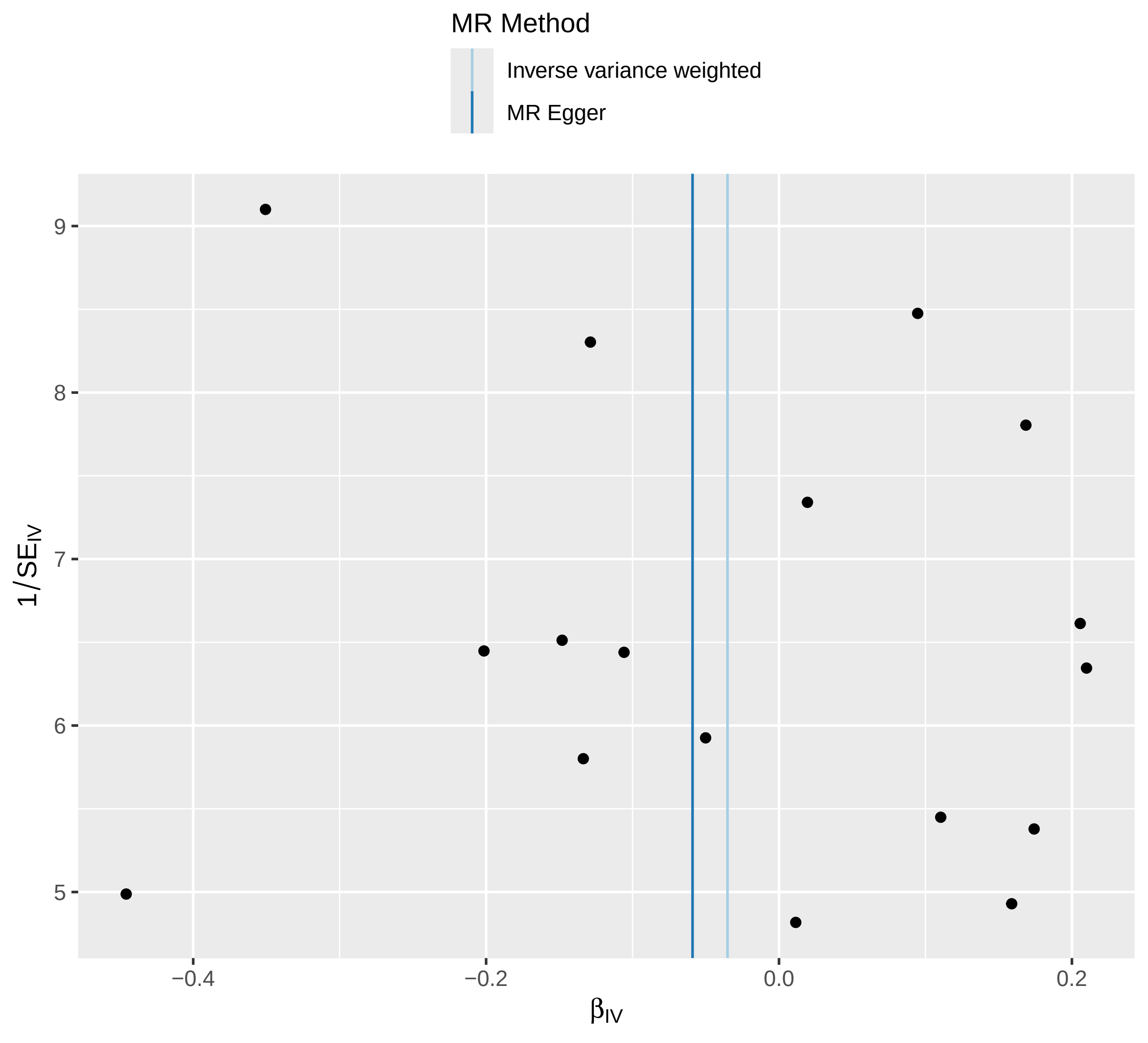

MR Method
Inverse variance weighted
MR Egger
9
8
7
6
5
1/SE_IV
−0.4
−0.2
0.0
0.2
β_IV

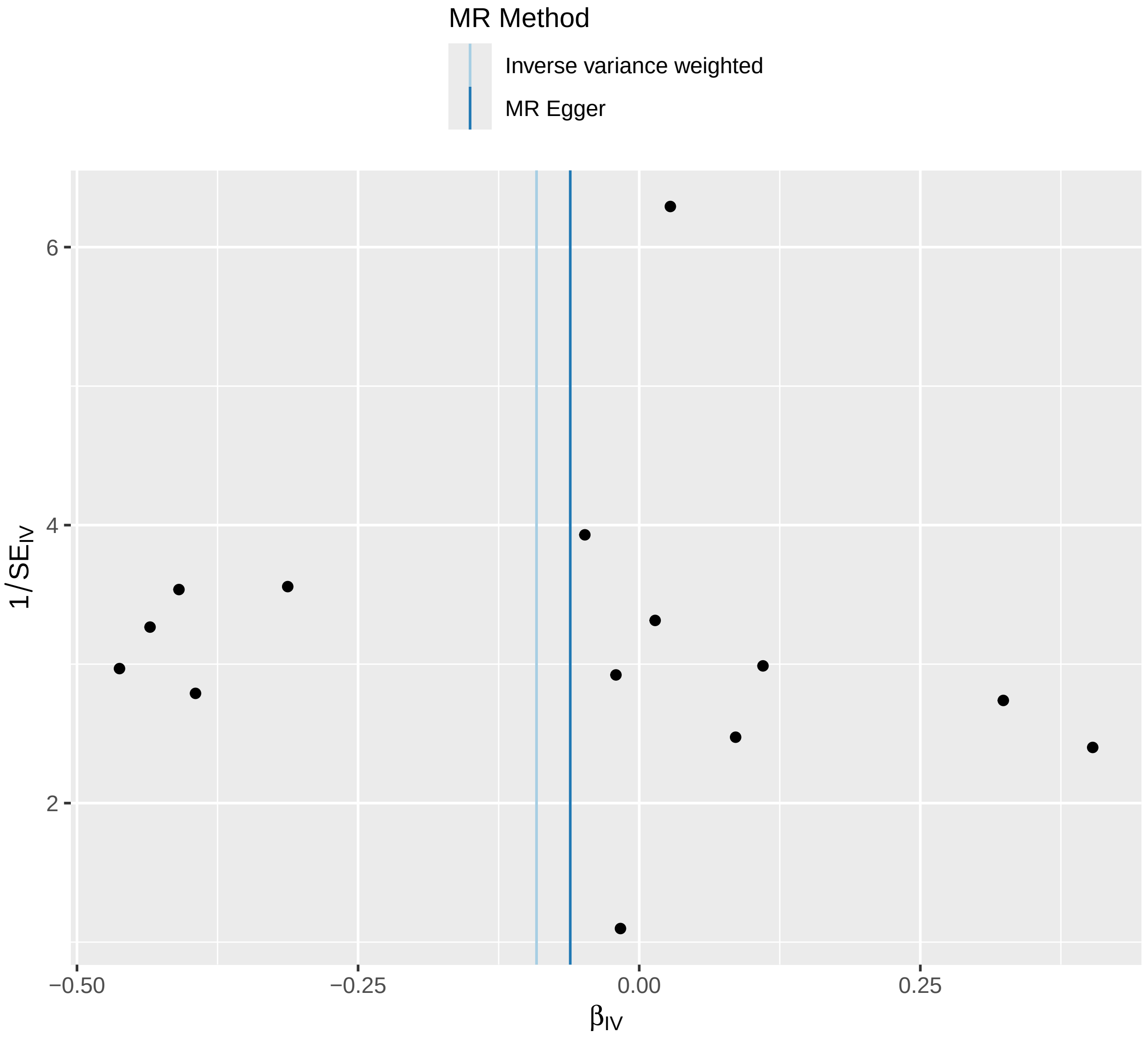
MR Method
Inverse variance weighted
MR Egger
6
4
2
1/SE$_{IV}$
−0.50
−0.25
0.00
0.25
β$_{IV}$

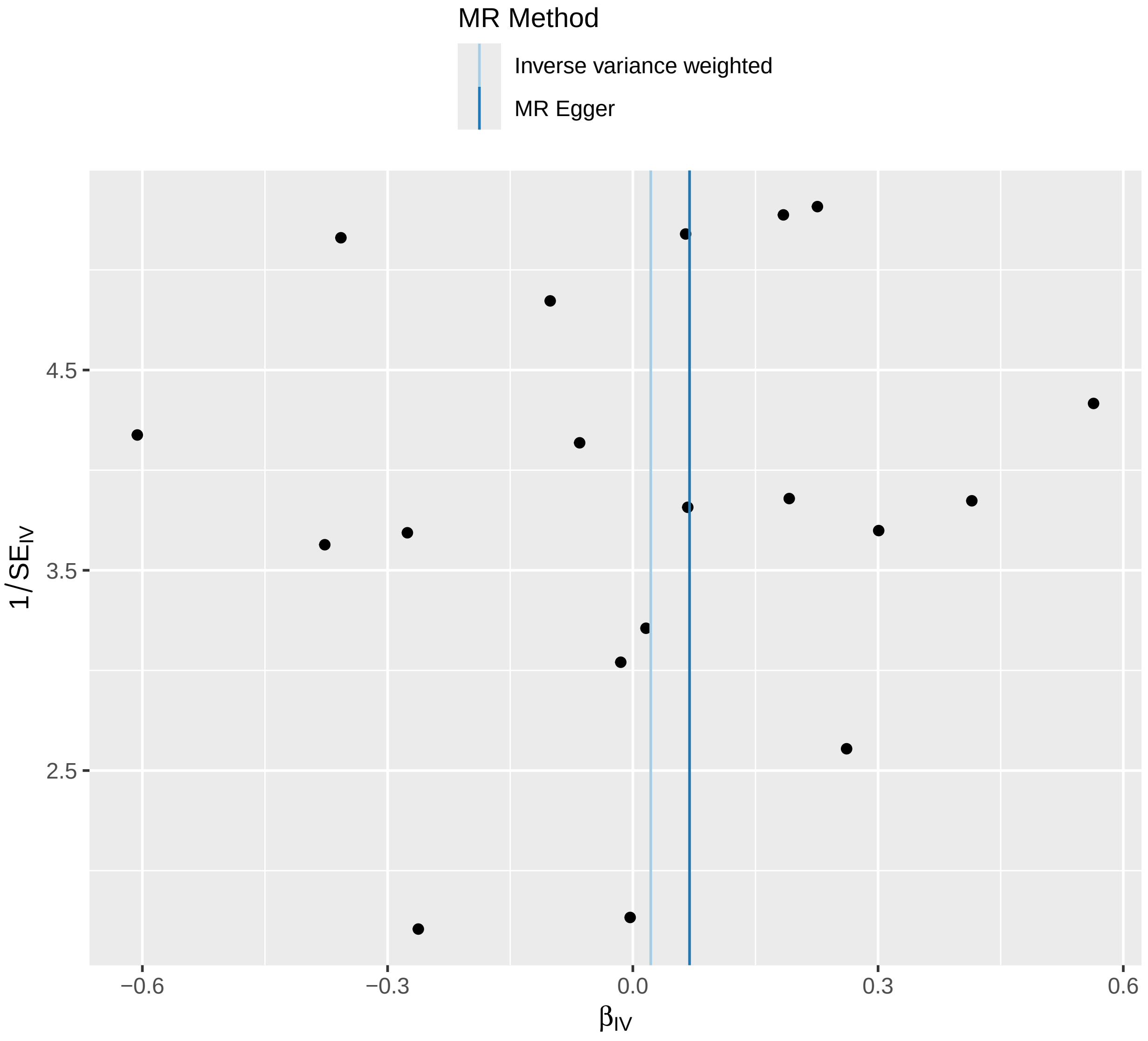

MR Method
Inverse variance weighted
MR Egger
4.5
3.5
2.5
1/SE$_{IV}$
−0.6
−0.3
0.0
0.3
0.6
β$_{IV}$

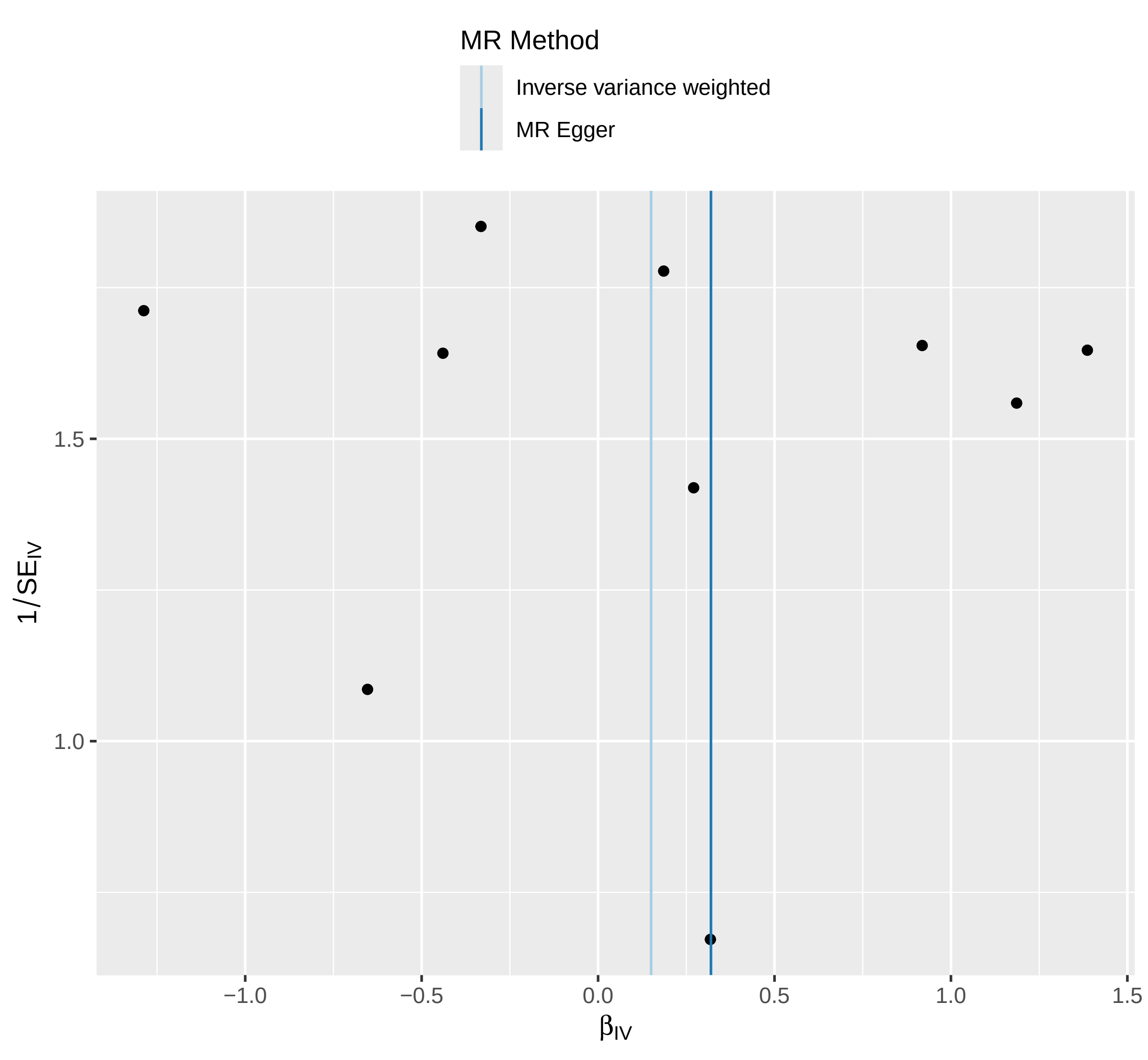

MR Method
Inverse variance weighted
MR Egger
1.5
1.0
1/SE$_{IV}$
−1.0
−0.5
0.0
0.5
1.0
1.5
β$_{IV}$

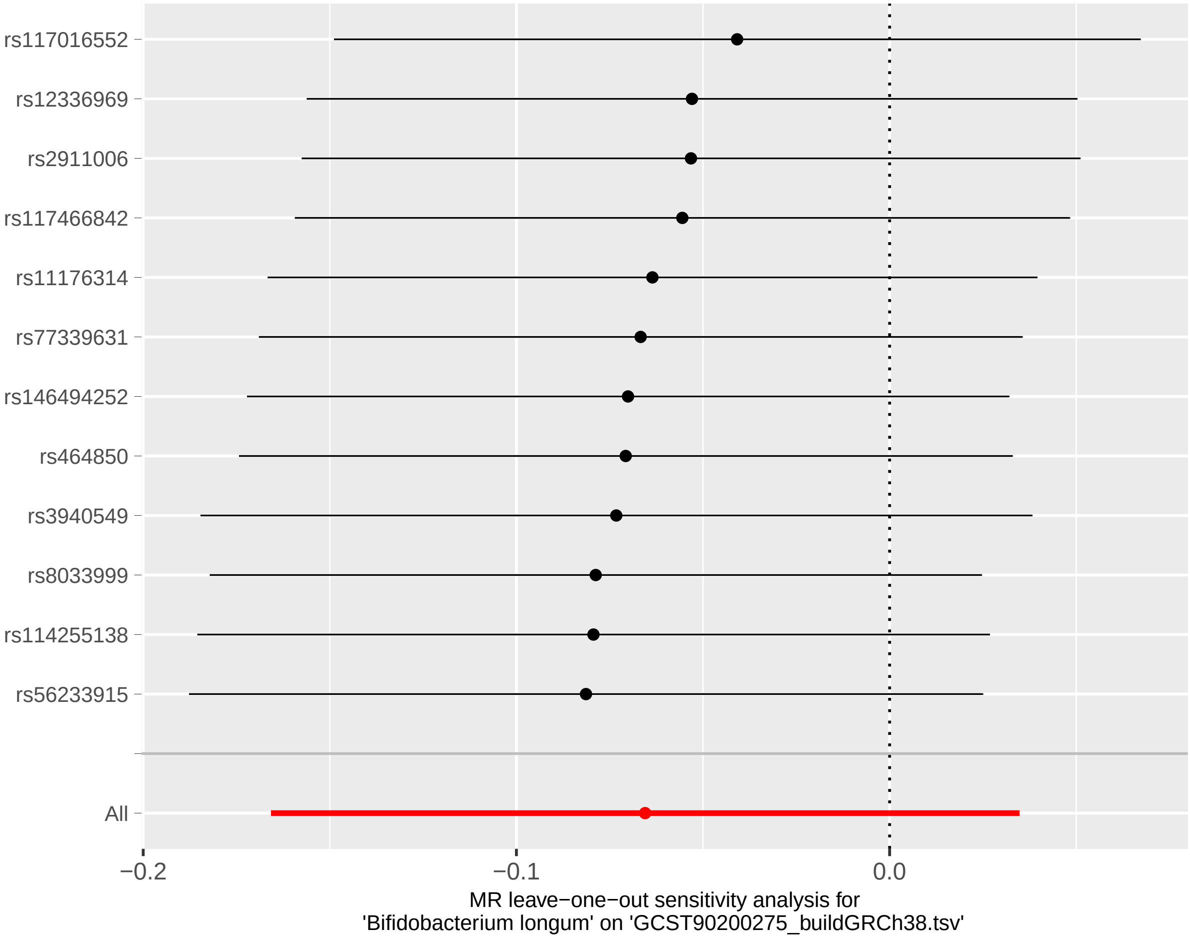

rs117016552
rs12336969
rs2911006
rs117466842
rs11176314
rs77339631
rs146494252
rs464850
rs3940549
rs8033999
rs114255138
rs56233915
All
−0.2
−0.1
0.0
MR leave−one−out sensitivity analysis for
'Bifidobacterium longum' on 'GCST90200275_buildGRCh38.tsv'

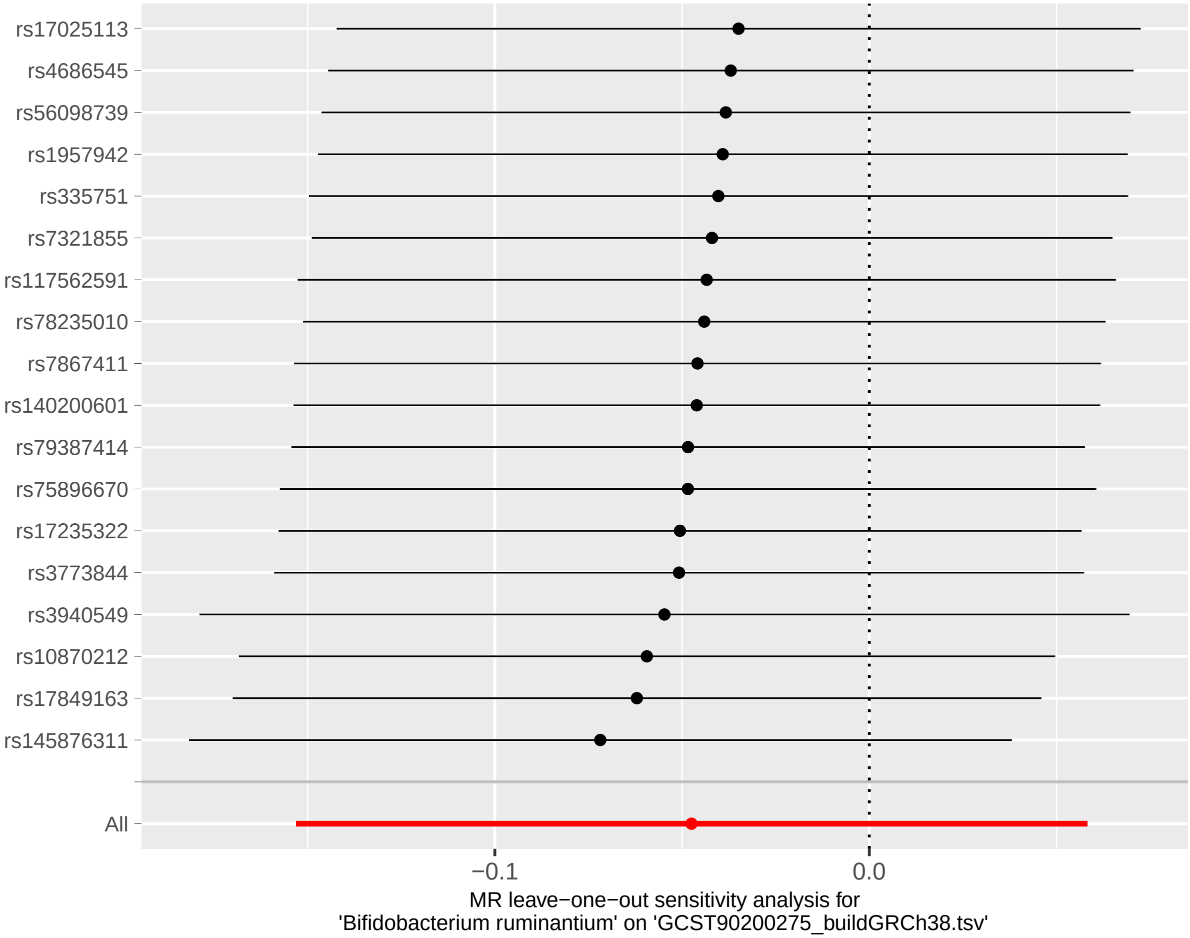
rs17025113
rs4686545
rs56098739
rs1957942
rs335751
rs7321855
rs117562591
rs78235010
rs7867411
rs140200601
rs79387414
rs75896670
rs17235322
rs3773844
rs3940549
rs10870212
rs17849163
rs145876311
All
−0.1
0.0
MR leave−one−out sensitivity analysis for
'Bifidobacterium ruminantium' on 'GCST90200275_buildGRCh38.tsv'

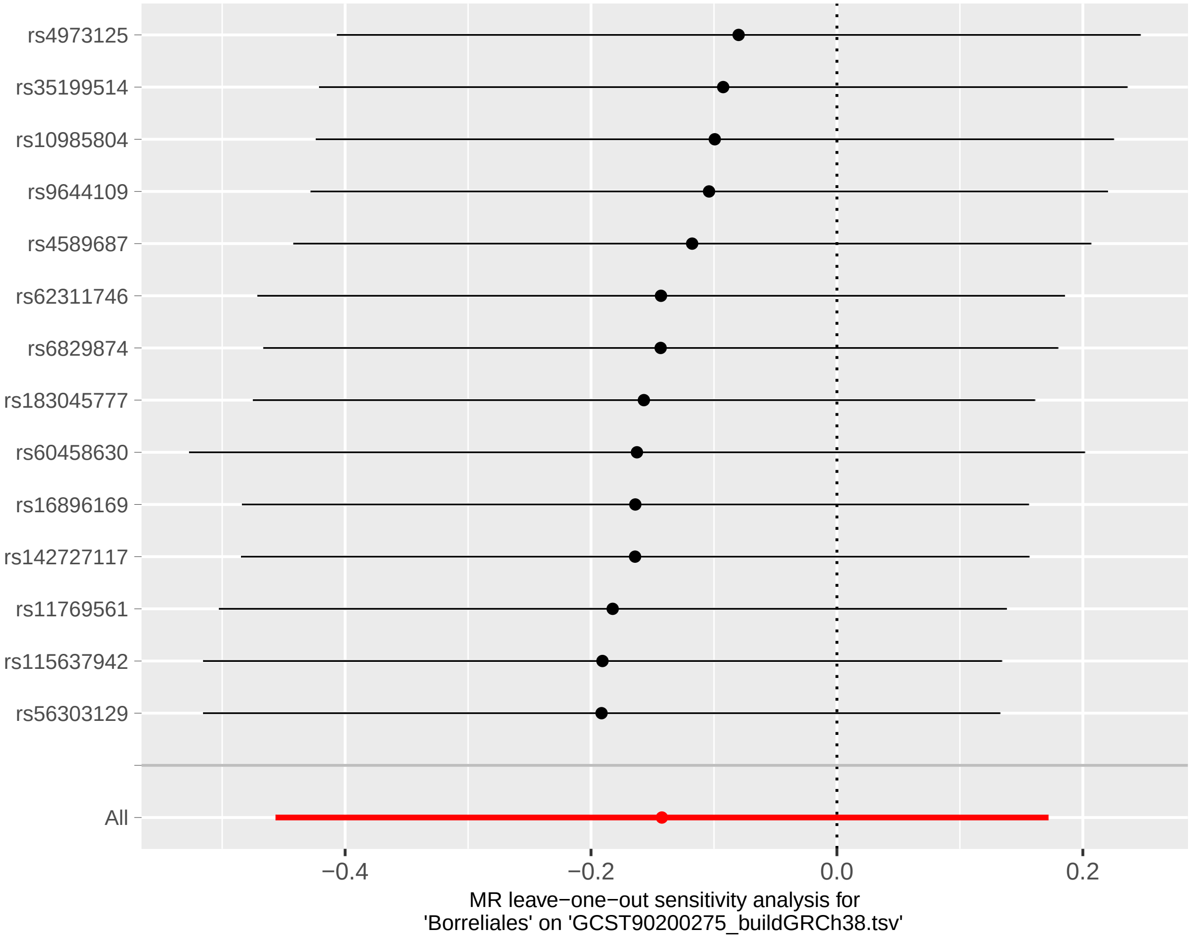
rs4973125
rs35199514
rs10985804
rs9644109
rs4589687
rs62311746
rs6829874
rs183045777
rs60458630
rs16896169
rs142727117
rs11769561
rs115637942
rs56303129
All
−0.4
−0.2
0.0
0.2
MR leave−one−out sensitivity analysis for
'Borreliales' on 'GCST90200275_buildGRCh38.tsv'

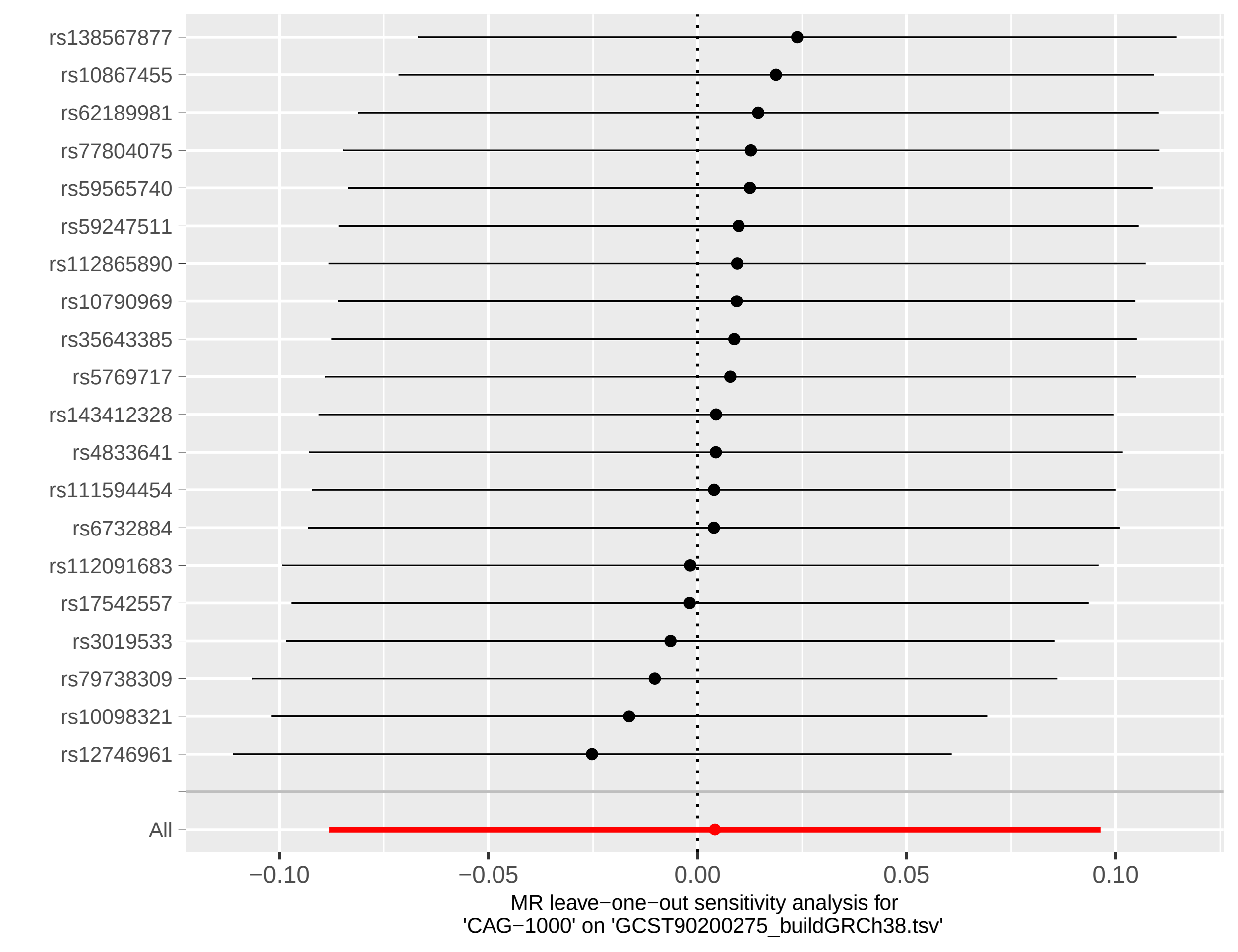
rs138567877
rs10867455
rs62189981
rs77804075
rs59565740
rs59247511
rs112865890
rs10790969
rs35643385
rs5769717
rs143412328
rs4833641
rs111594454
rs6732884
rs112091683
rs17542557
rs3019533
rs79738309
rs10098321
rs12746961
All
−0.10
−0.05
0.00
0.05
0.10
MR leave−one−out sensitivity analysis for
'CAG−1000' on 'GCST90200275_buildGRCh38.tsv'

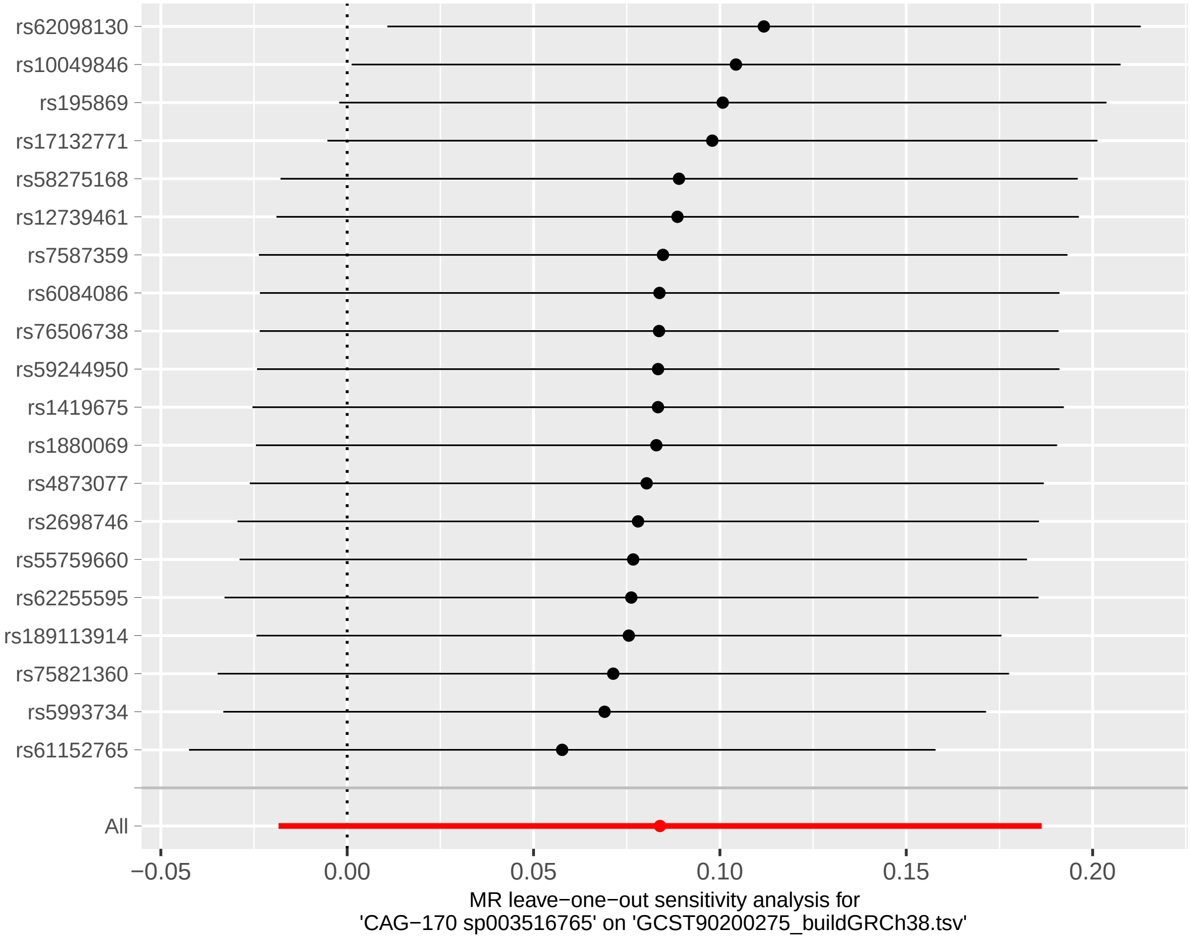
rs62098130
rs10049846
rs195869
rs17132771
rs58275168
rs12739461
rs7587359
rs6084086
rs76506738
rs59244950
rs1419675
rs1880069
rs4873077
rs2698746
rs55759660
rs62255595
rs189113914
rs75821360
rs5993734
rs61152765
All
−0.05
0.00
0.05
0.10
0.15
0.20
MR leave−one−out sensitivity analysis for
'CAG−170 sp003516765' on 'GCST90200275_buildGRCh38.tsv'

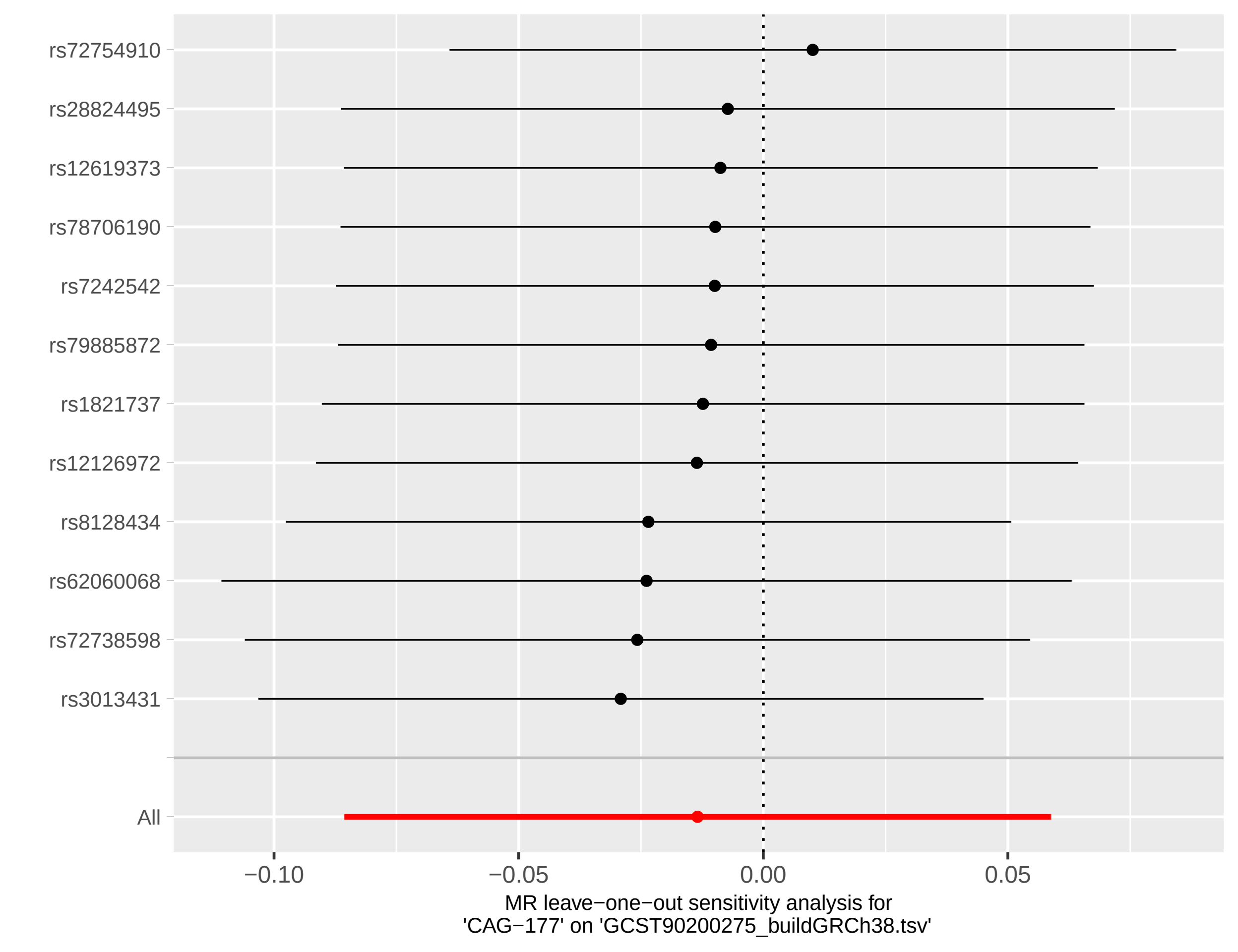
rs72754910
rs28824495
rs12619373
rs78706190
rs7242542
rs79885872
rs1821737
rs12126972
rs8128434
rs62060068
rs72738598
rs3013431
All
−0.10
−0.05
0.00
0.05
MR leave−one−out sensitivity analysis for
'CAG−177' on 'GCST90200275_buildGRCh38.tsv'

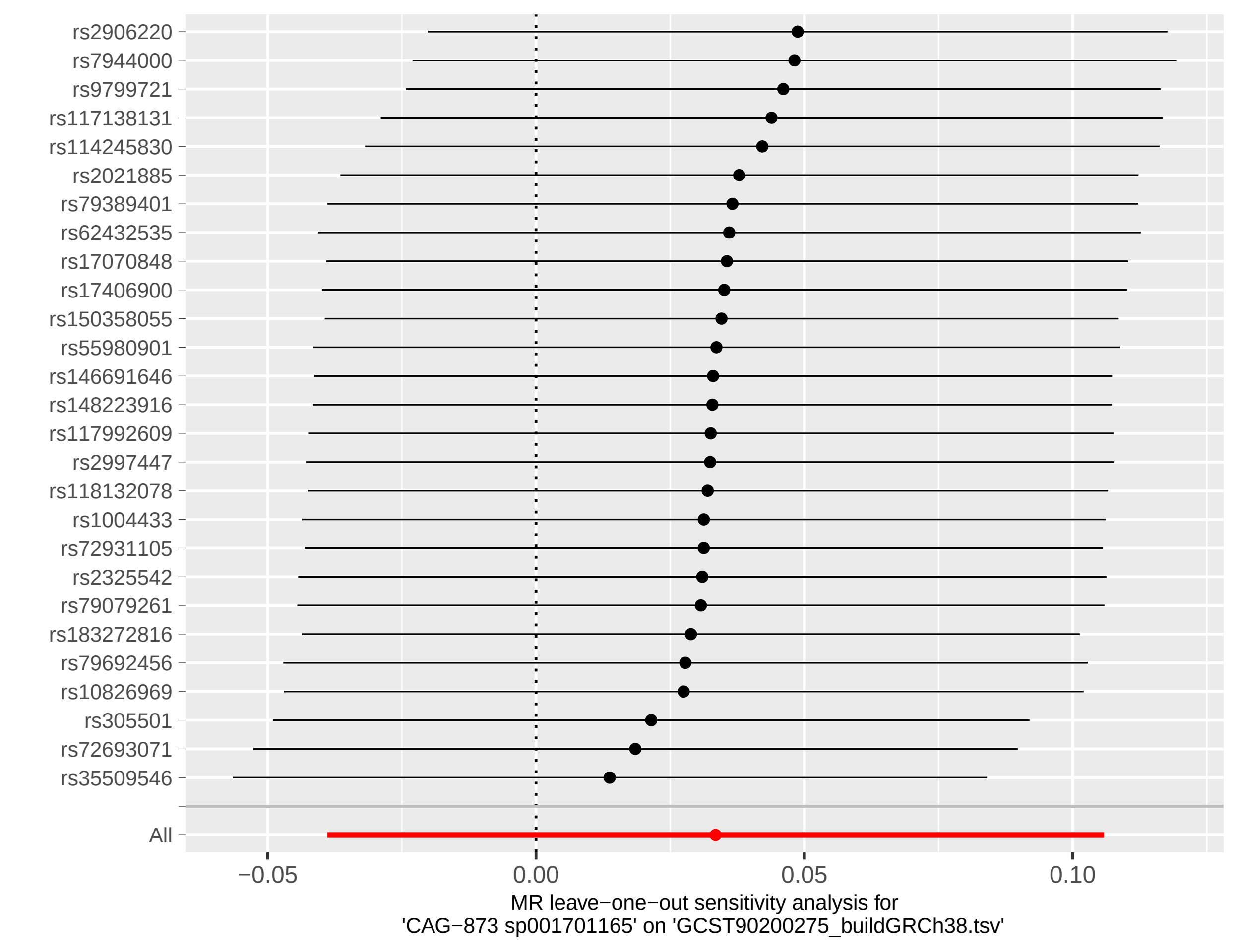
rs2906220
rs7944000
rs9799721
rs117138131
rs114245830
rs2021885
rs79389401
rs62432535
rs17070848
rs17406900
rs150358055
rs55980901
rs146691646
rs148223916
rs117992609
rs2997447
rs118132078
rs1004433
rs72931105
rs2325542
rs79079261
rs183272816
rs79692456
rs10826969
rs305501
rs72693071
rs35509546
All
−0.05
0.00
0.05
0.10
MR leave−one−out sensitivity analysis for
'CAG−873 sp001701165' on 'GCST90200275_buildGRCh38.tsv'

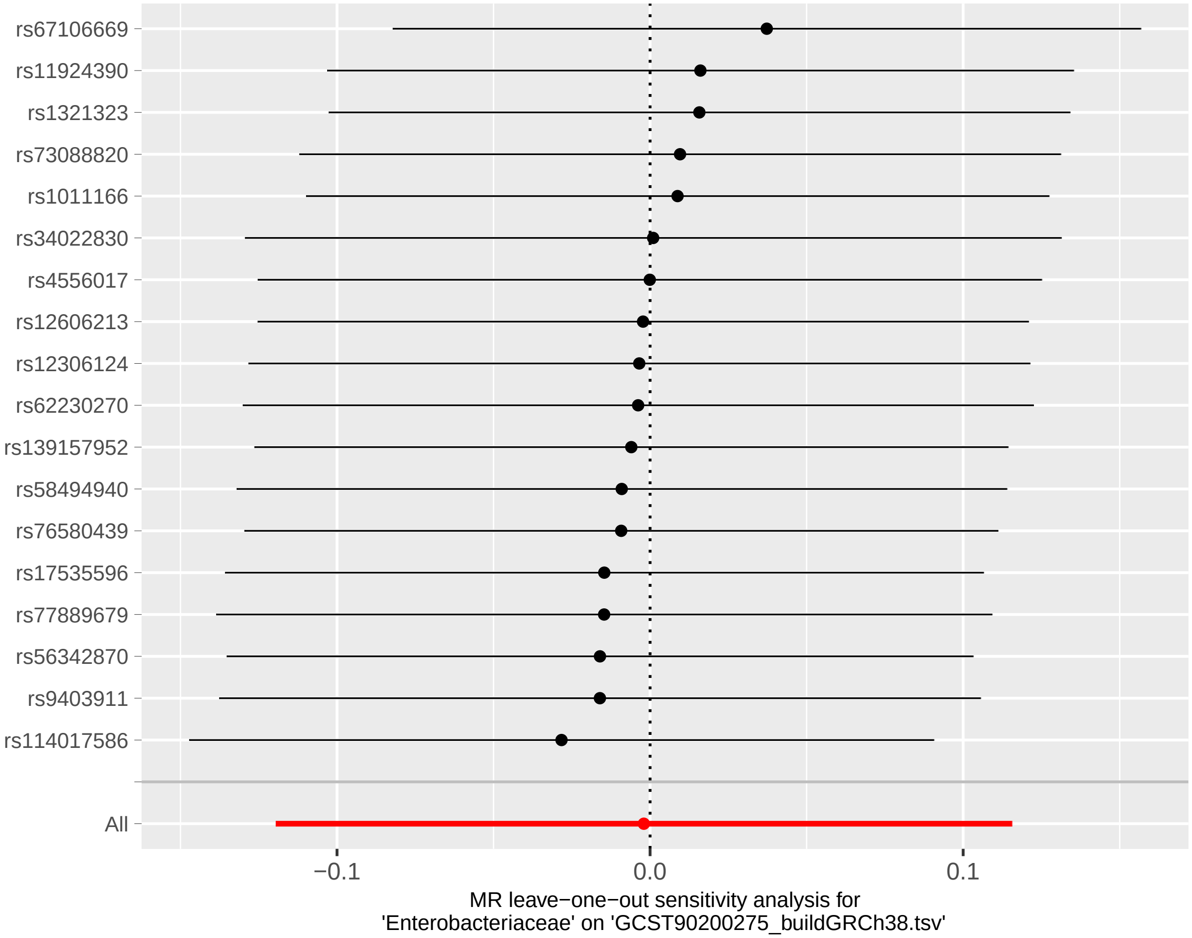
rs67106669
rs11924390
rs1321323
rs73088820
rs1011166
rs34022830
rs4556017
rs12606213
rs12306124
rs62230270
rs139157952
rs58494940
rs76580439
rs17535596
rs77889679
rs56342870
rs9403911
rs114017586
All
−0.1
0.0
0.1
MR leave−one−out sensitivity analysis for
'Enterobacteriaceae' on 'GCST90200275_buildGRCh38.tsv'

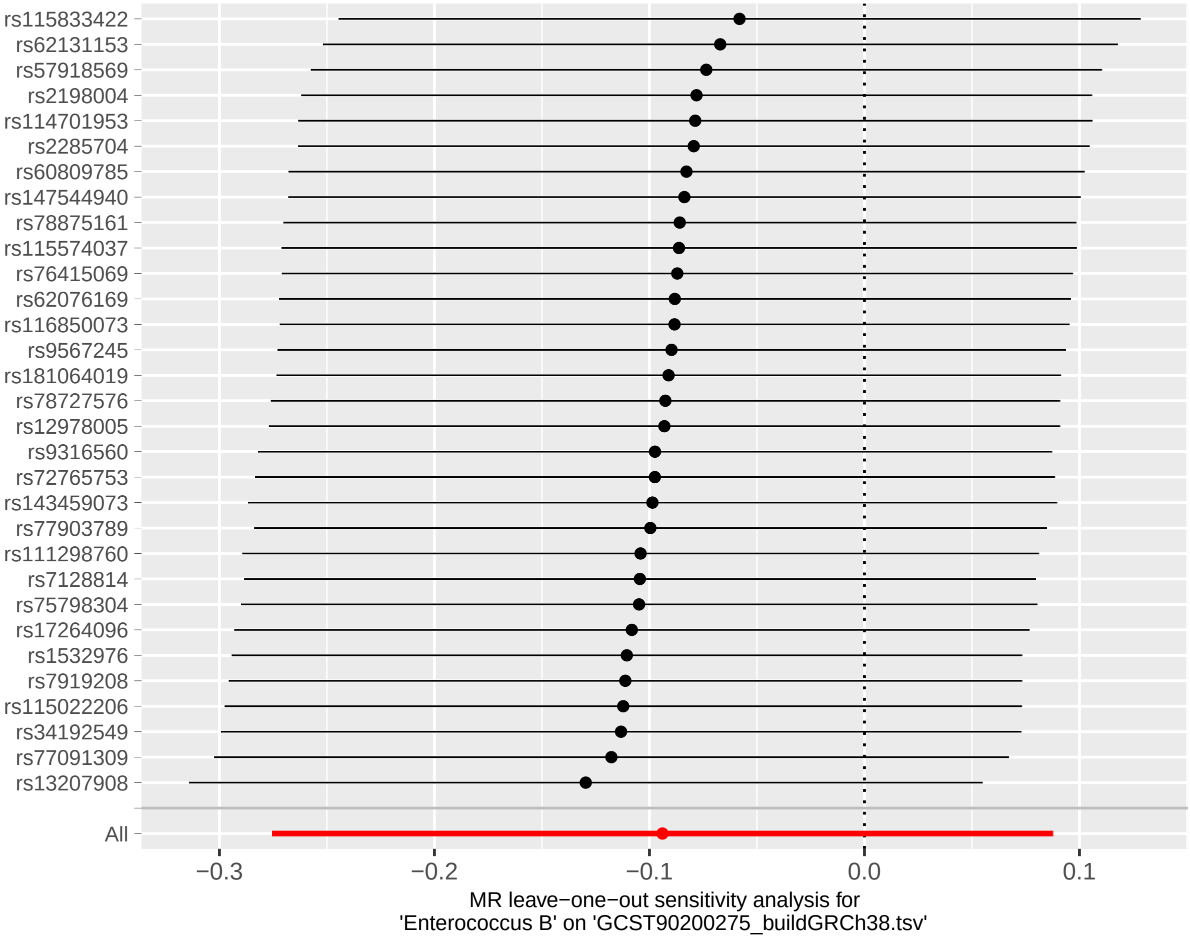
rs115833422
rs62131153
rs57918569
rs2198004
rs114701953
rs2285704
rs60809785
rs147544940
rs78875161
rs115574037
rs76415069
rs62076169
rs116850073
rs9567245
rs181064019
rs78727576
rs12978005
rs9316560
rs72765753
rs143459073
rs77903789
rs111298760
rs7128814
rs75798304
rs17264096
rs1532976
rs7919208
rs115022206
rs34192549
rs77091309
rs13207908
All
−0.3
−0.2
−0.1
0.0
0.1
MR leave−one−out sensitivity analysis for
'Enterococcus B' on 'GCST90200275_buildGRCh38.tsv'

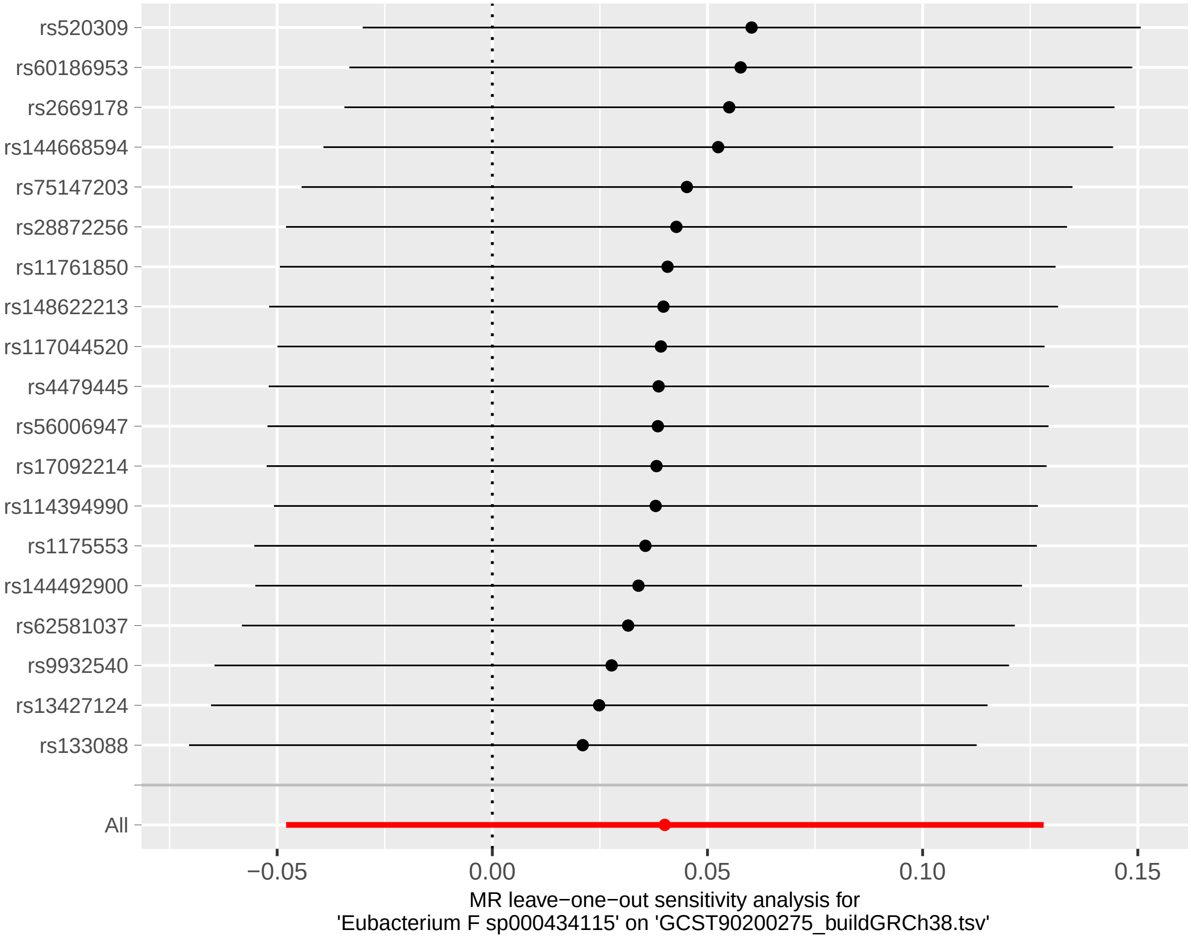

rs520309
rs60186953
rs2669178
rs144668594
rs75147203
rs28872256
rs11761850
rs148622213
rs117044520
rs4479445
rs56006947
rs17092214
rs114394990
rs1175553
rs144492900
rs62581037
rs9932540
rs13427124
rs133088
All
−0.05
0.00
0.05
0.10
0.15
MR leave−one−out sensitivity analysis for
'Eubacterium F sp000434115' on 'GCST90200275_buildGRCh38.tsv'

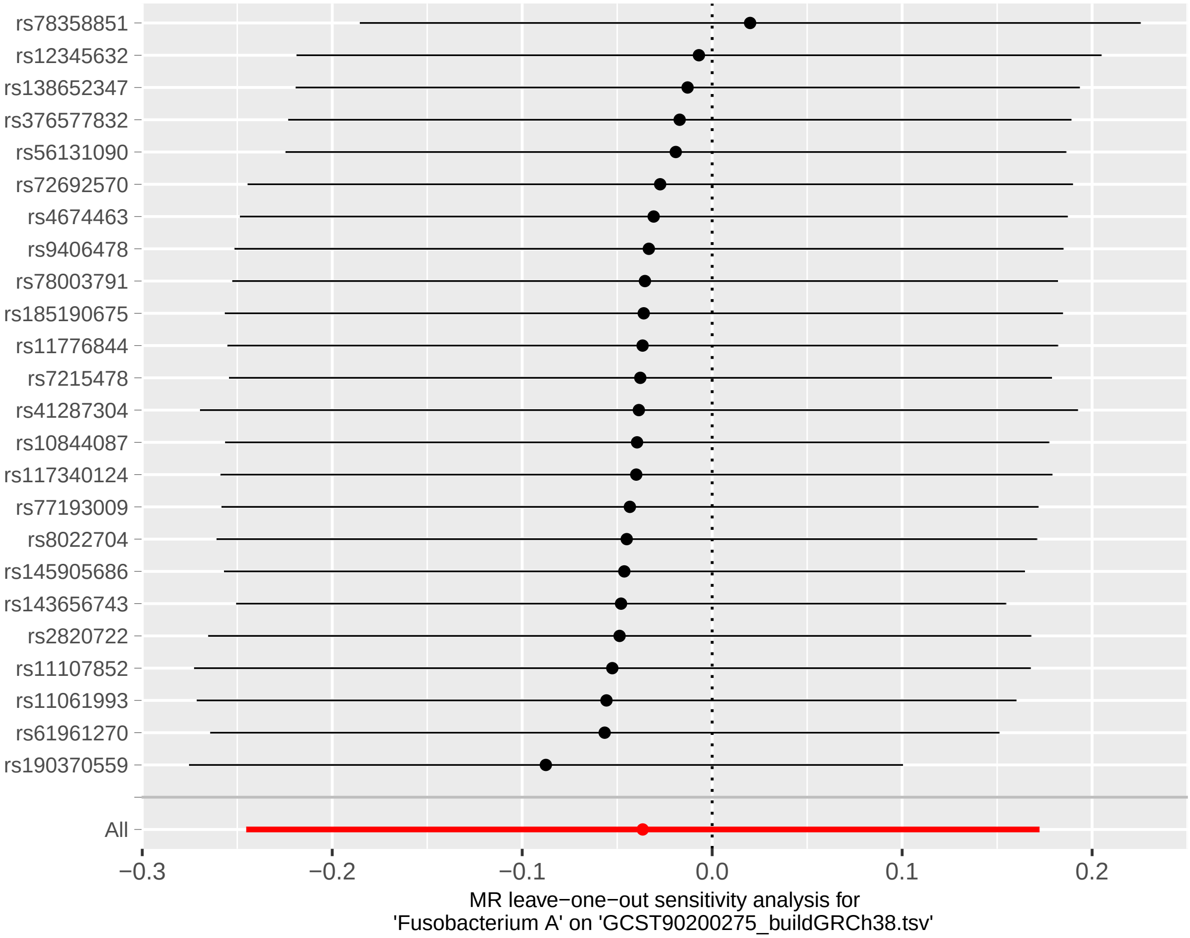
rs78358851
rs12345632
rs138652347
rs376577832
rs56131090
rs72692570
rs4674463
rs9406478
rs78003791
rs185190675
rs11776844
rs7215478
rs41287304
rs10844087
rs117340124
rs77193009
rs8022704
rs145905686
rs143656743
rs2820722
rs11107852
rs11061993
rs61961270
rs190370559
All
−0.3
−0.2
−0.1
0.0
0.1
0.2
MR leave−one−out sensitivity analysis for
'Fusobacterium A' on 'GCST90200275_buildGRCh38.tsv'

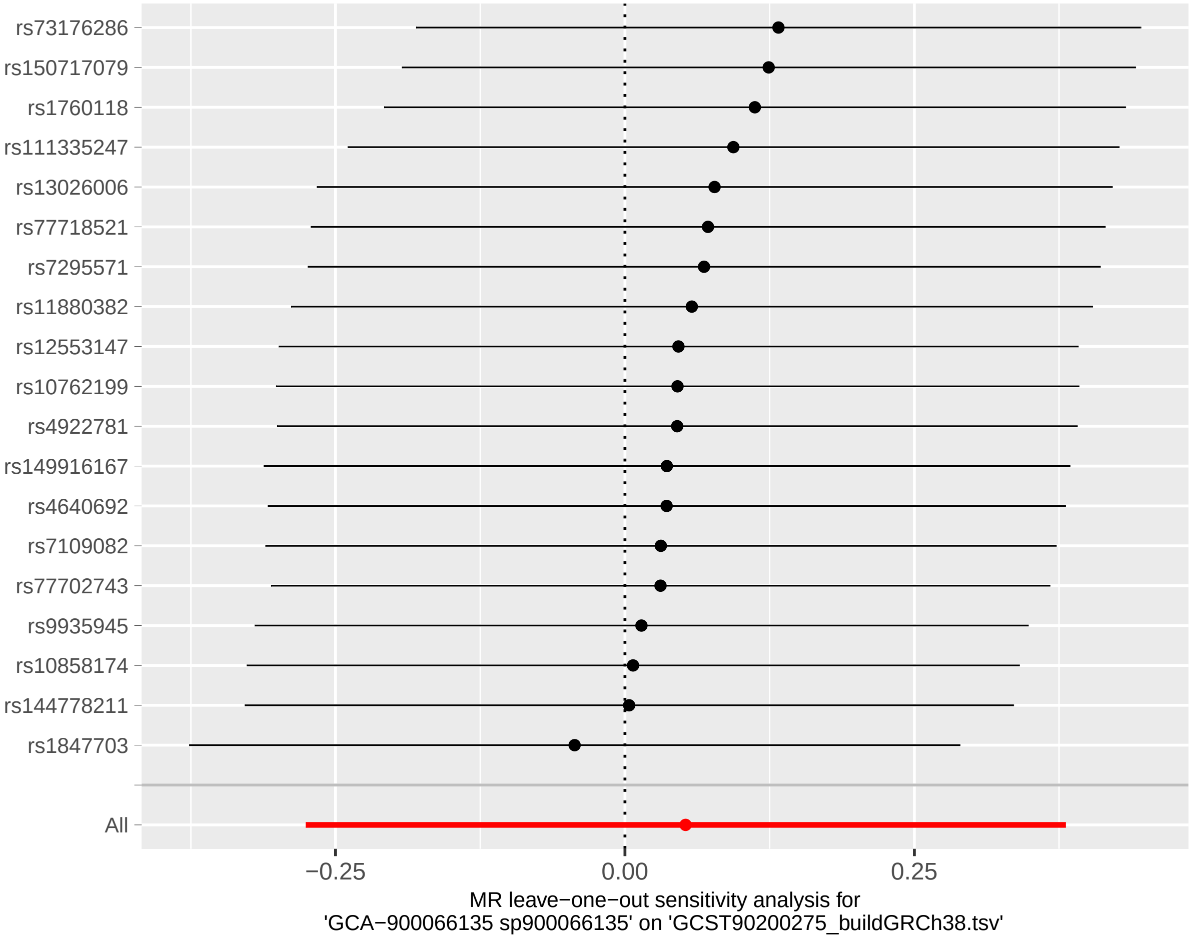

rs73176286
rs150717079
rs1760118
rs111335247
rs13026006
rs77718521
rs7295571
rs11880382
rs12553147
rs10762199
rs4922781
rs149916167
rs4640692
rs7109082
rs77702743
rs9935945
rs10858174
rs144778211
rs1847703
All
−0.25
0.00
0.25
MR leave−one−out sensitivity analysis for
'GCA−900066135 sp900066135' on 'GCST90200275_buildGRCh38.tsv'

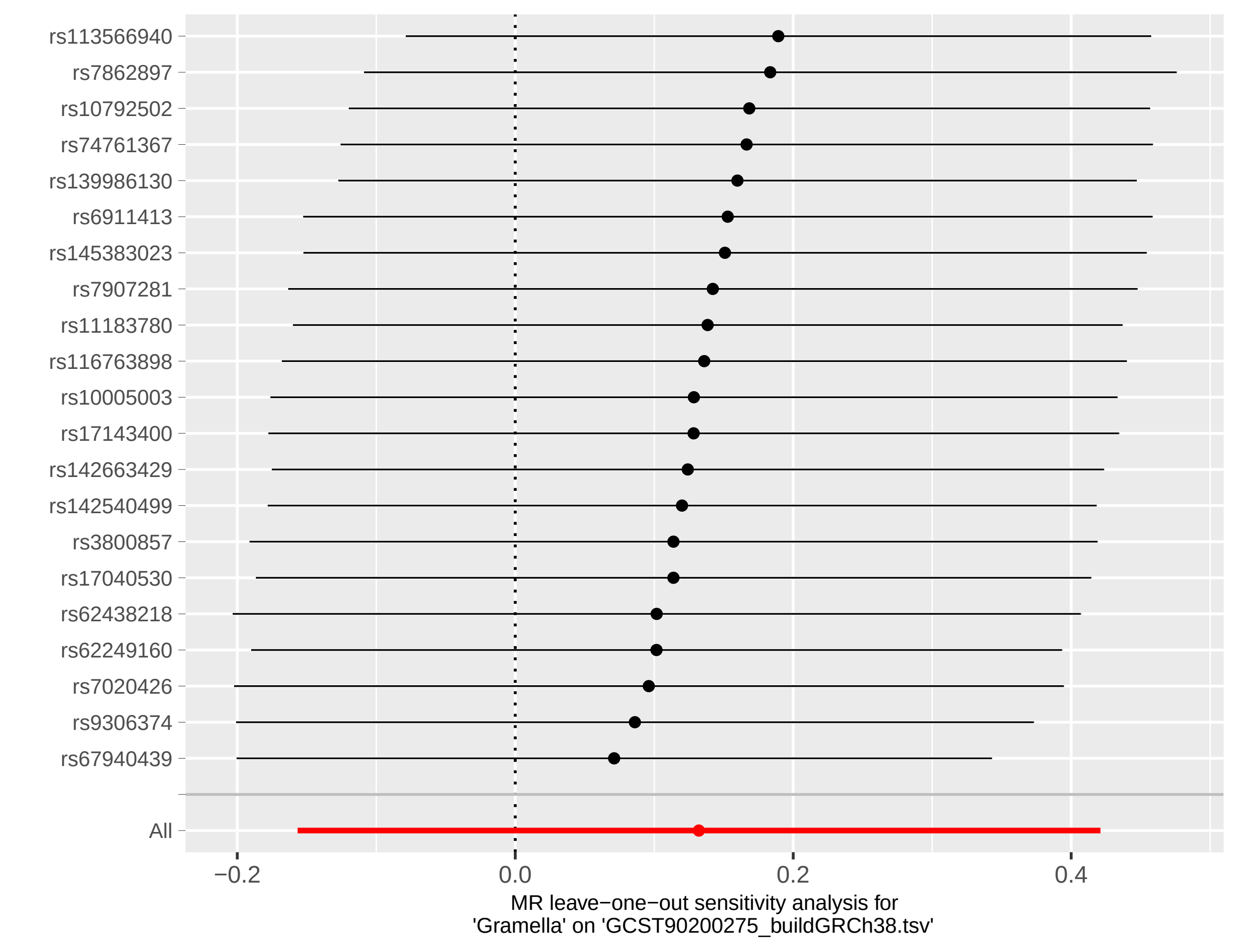
rs113566940
rs7862897
rs10792502
rs74761367
rs139986130
rs6911413
rs145383023
rs7907281
rs11183780
rs116763898
rs10005003
rs17143400
rs142663429
rs142540499
rs3800857
rs17040530
rs62438218
rs62249160
rs7020426
rs9306374
rs67940439
All
−0.2
0.0
0.2
0.4
MR leave−one−out sensitivity analysis for
'Gramella' on 'GCST90200275_buildGRCh38.tsv'

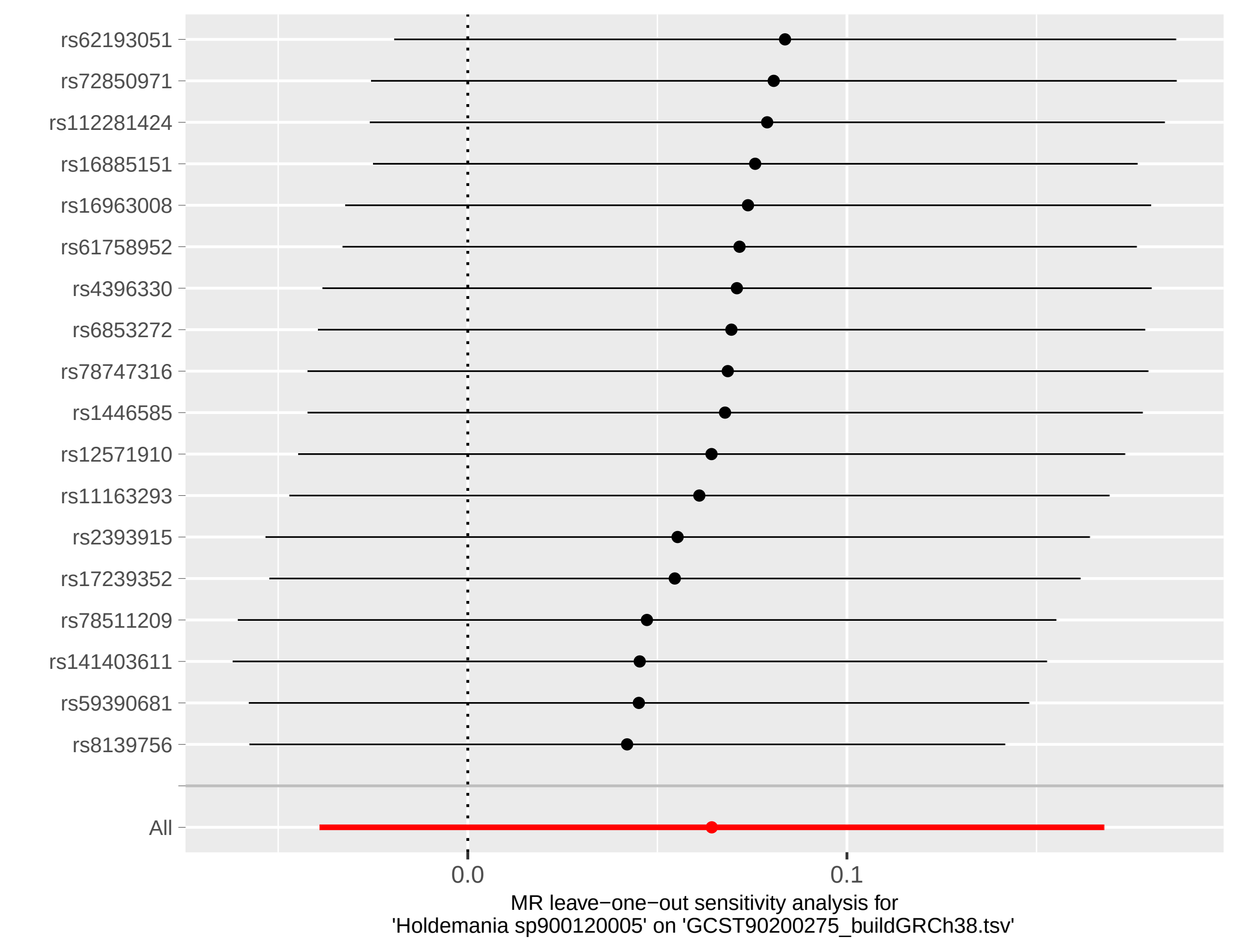
rs62193051
rs72850971
rs112281424
rs16885151
rs16963008
rs61758952
rs4396330
rs6853272
rs78747316
rs1446585
rs12571910
rs11163293
rs2393915
rs17239352
rs78511209
rs141403611
rs59390681
rs8139756
All
0.0
0.1
MR leave−one−out sensitivity analysis for
'Holdemania sp900120005' on 'GCST90200275_buildGRCh38.tsv'

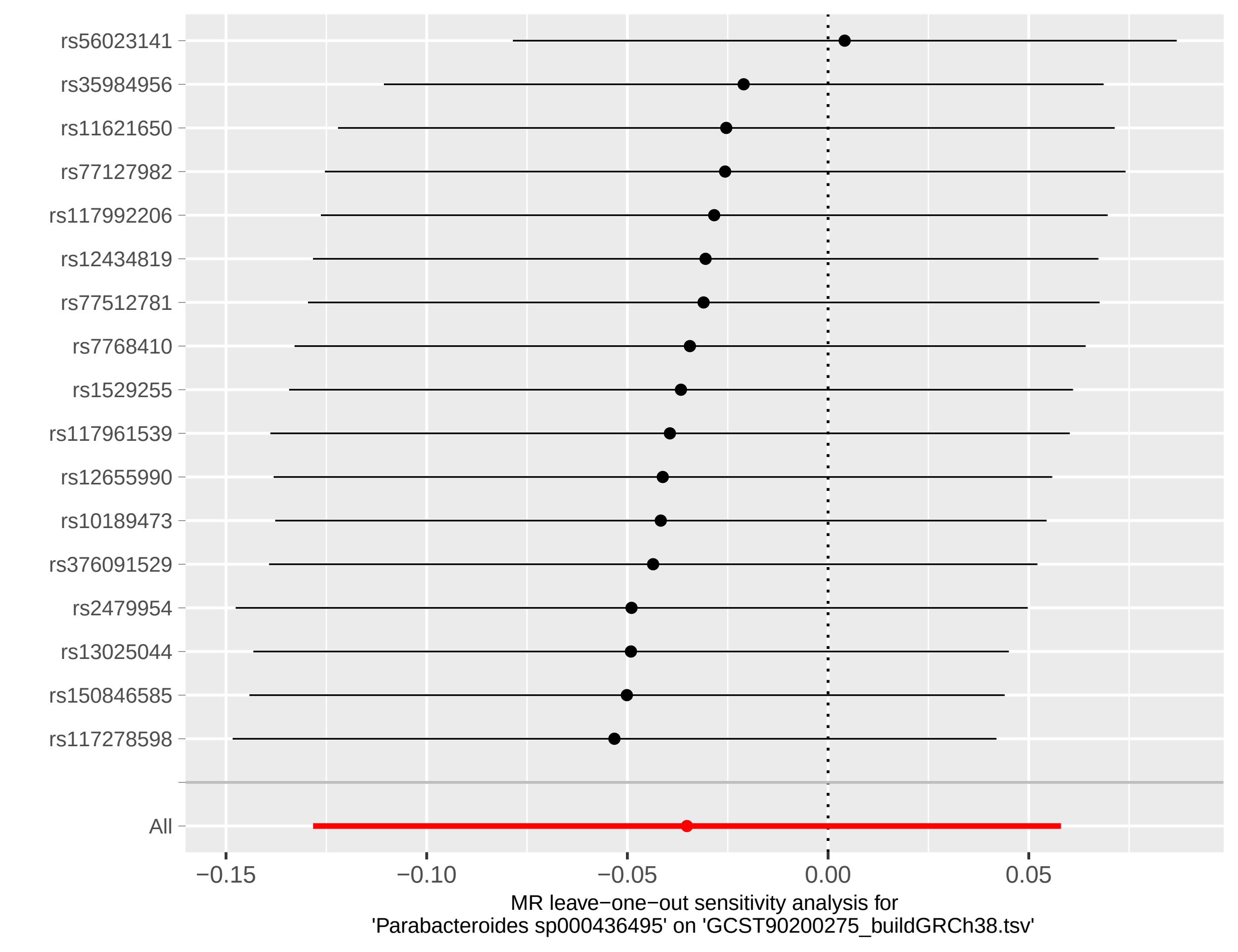
rs56023141
rs35984956
rs11621650
rs77127982
rs117992206
rs12434819
rs77512781
rs7768410
rs1529255
rs117961539
rs12655990
rs10189473
rs376091529
rs2479954
rs13025044
rs150846585
rs117278598
All
−0.15
−0.10
−0.05
0.00
0.05
MR leave−one−out sensitivity analysis for
'Parabacteroides sp000436495' on 'GCST90200275_buildGRCh38.tsv'

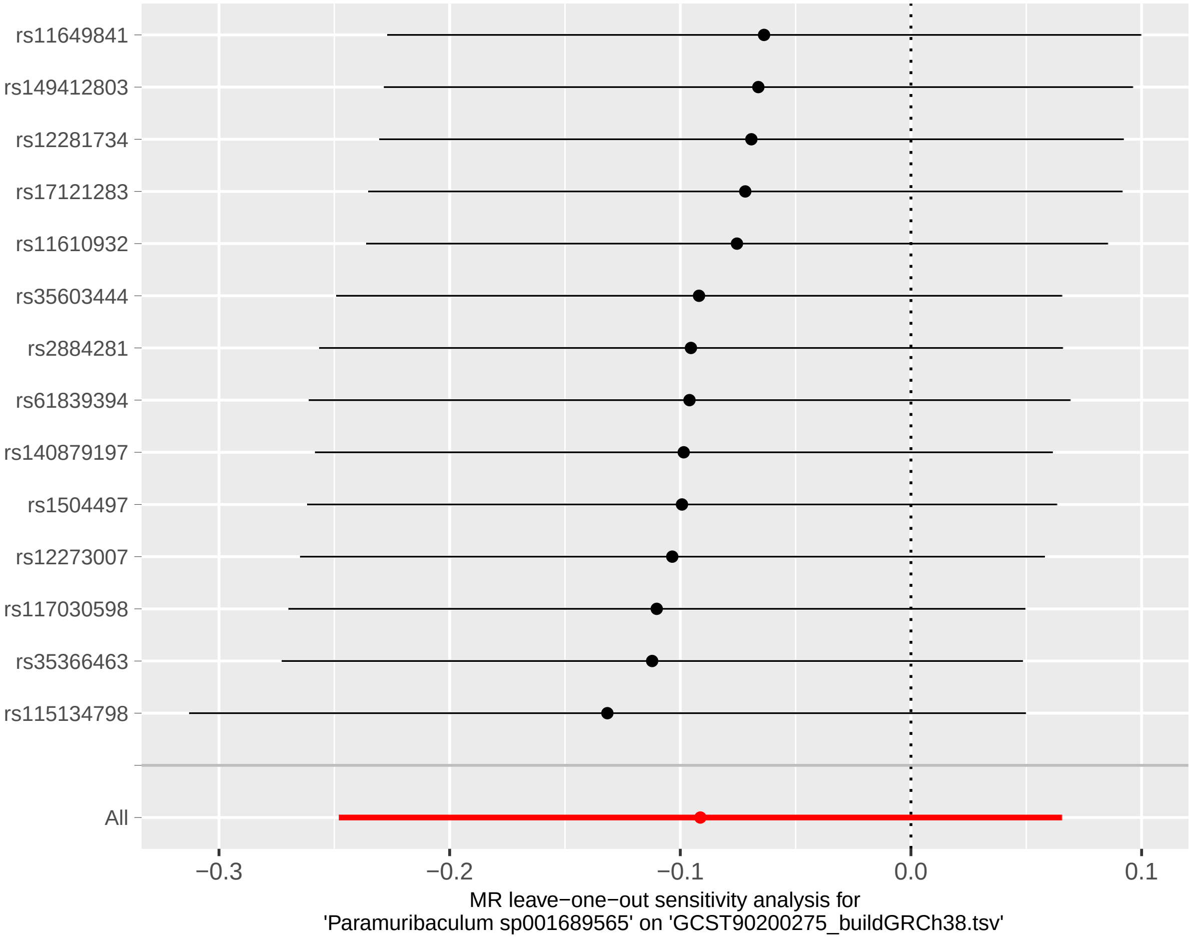

rs11649841
rs149412803
rs12281734
rs17121283
rs11610932
rs35603444
rs2884281
rs61839394
rs140879197
rs1504497
rs12273007
rs117030598
rs35366463
rs115134798
All
−0.3
−0.2
−0.1
0.0
0.1
MR leave−one−out sensitivity analysis for
'Paramuribaculum sp001689565' on 'GCST90200275_buildGRCh38.tsv'

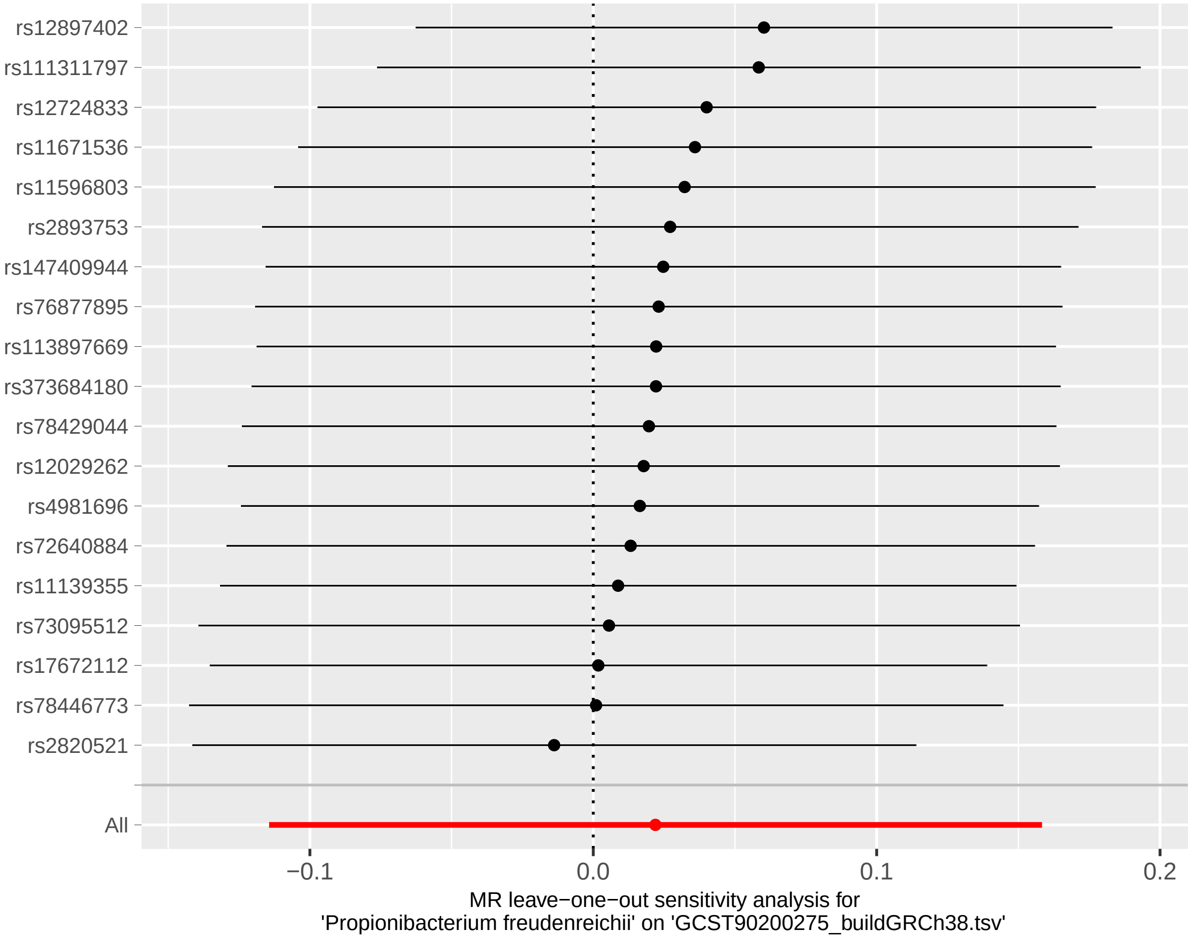
rs12897402
rs111311797
rs12724833
rs11671536
rs11596803
rs2893753
rs147409944
rs76877895
rs113897669
rs373684180
rs78429044
rs12029262
rs4981696
rs72640884
rs11139355
rs73095512
rs17672112
rs78446773
rs2820521
All
−0.1
0.0
0.1
0.2
MR leave−one−out sensitivity analysis for
'Propionibacterium freudenreichii' on 'GCST90200275_buildGRCh38.tsv'

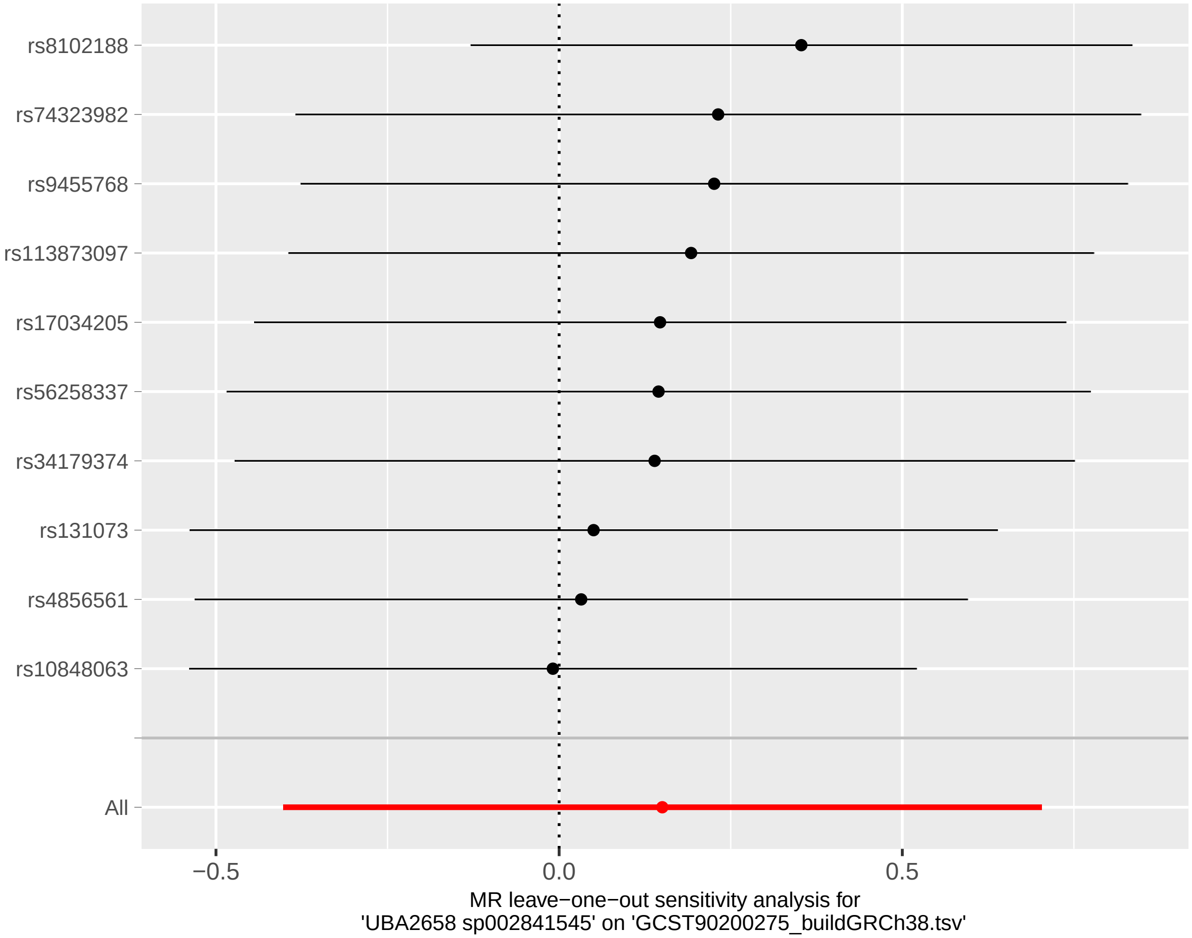

rs8102188
rs74323982
rs9455768
rs113873097
rs17034205
rs56258337
rs34179374
rs131073
rs4856561
rs10848063
All
−0.5
0.0
0.5
MR leave−one−out sensitivity analysis for
'UBA2658 sp002841545' on 'GCST90200275_buildGRCh38.tsv'

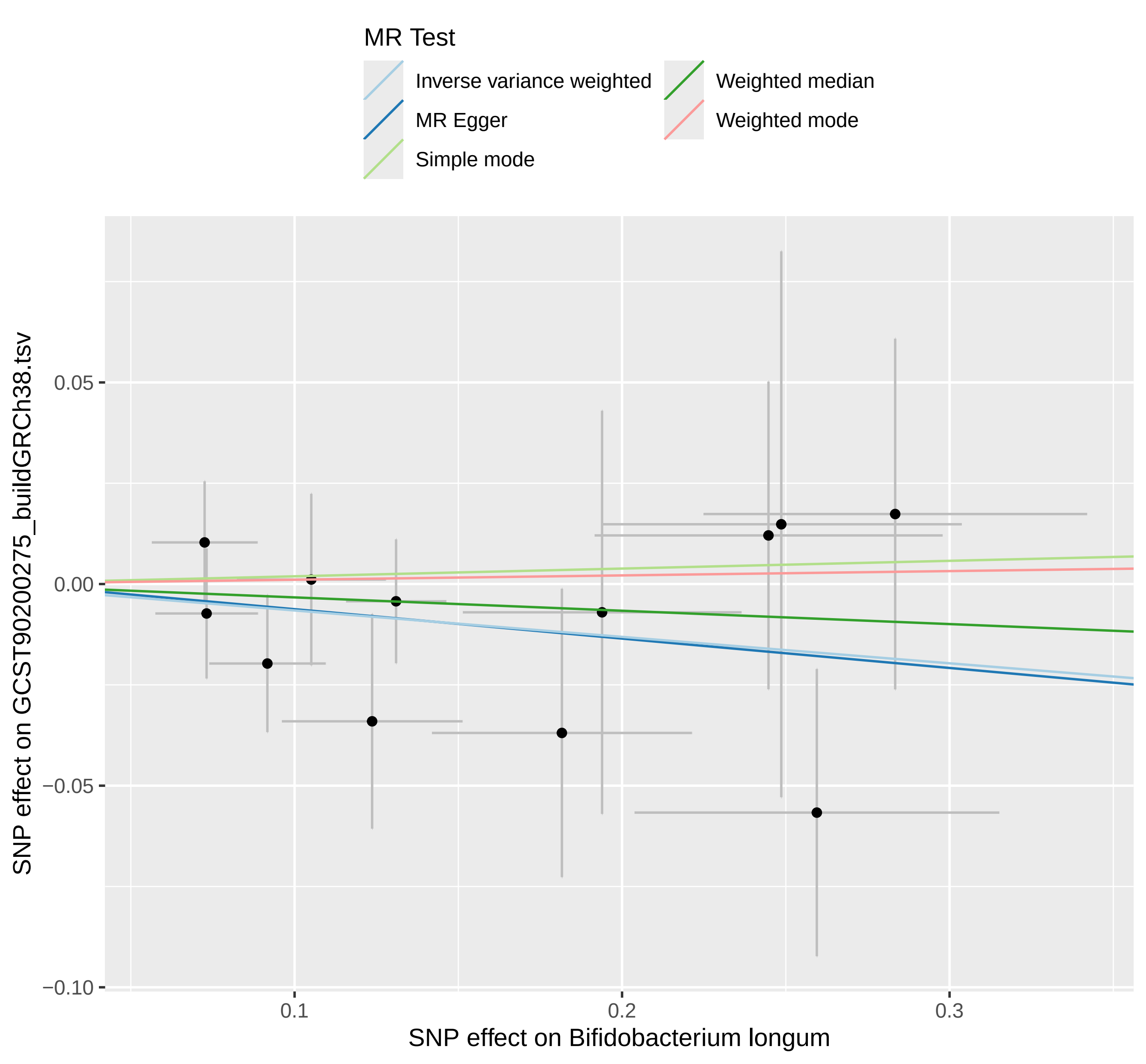

MR Test
Inverse variance weighted
MR Egger
Simple mode
Weighted median
Weighted mode
SNP effect on GCST90200275_buildGRCh38.tsv
0.05
0.00
−0.05
−0.10
0.1
0.2
0.3
SNP effect on Bifidobacterium longum

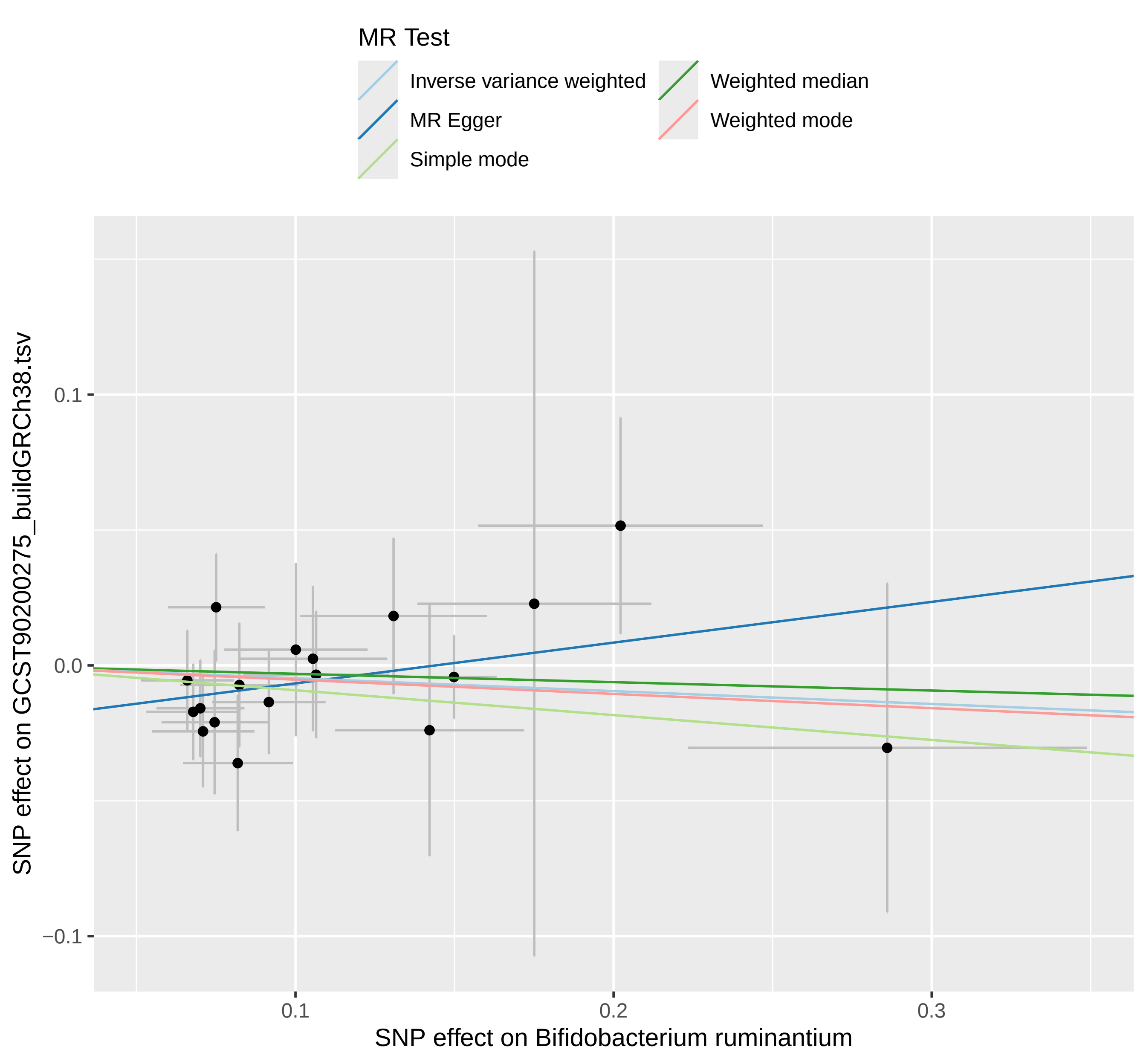

MR Test
Inverse variance weighted
MR Egger
Simple mode
Weighted median
Weighted mode
SNP effect on GCST90200275_buildGRCh38.tsv
0.1
0.0
−0.1
0.1
0.2
0.3
SNP effect on Bifidobacterium ruminantium

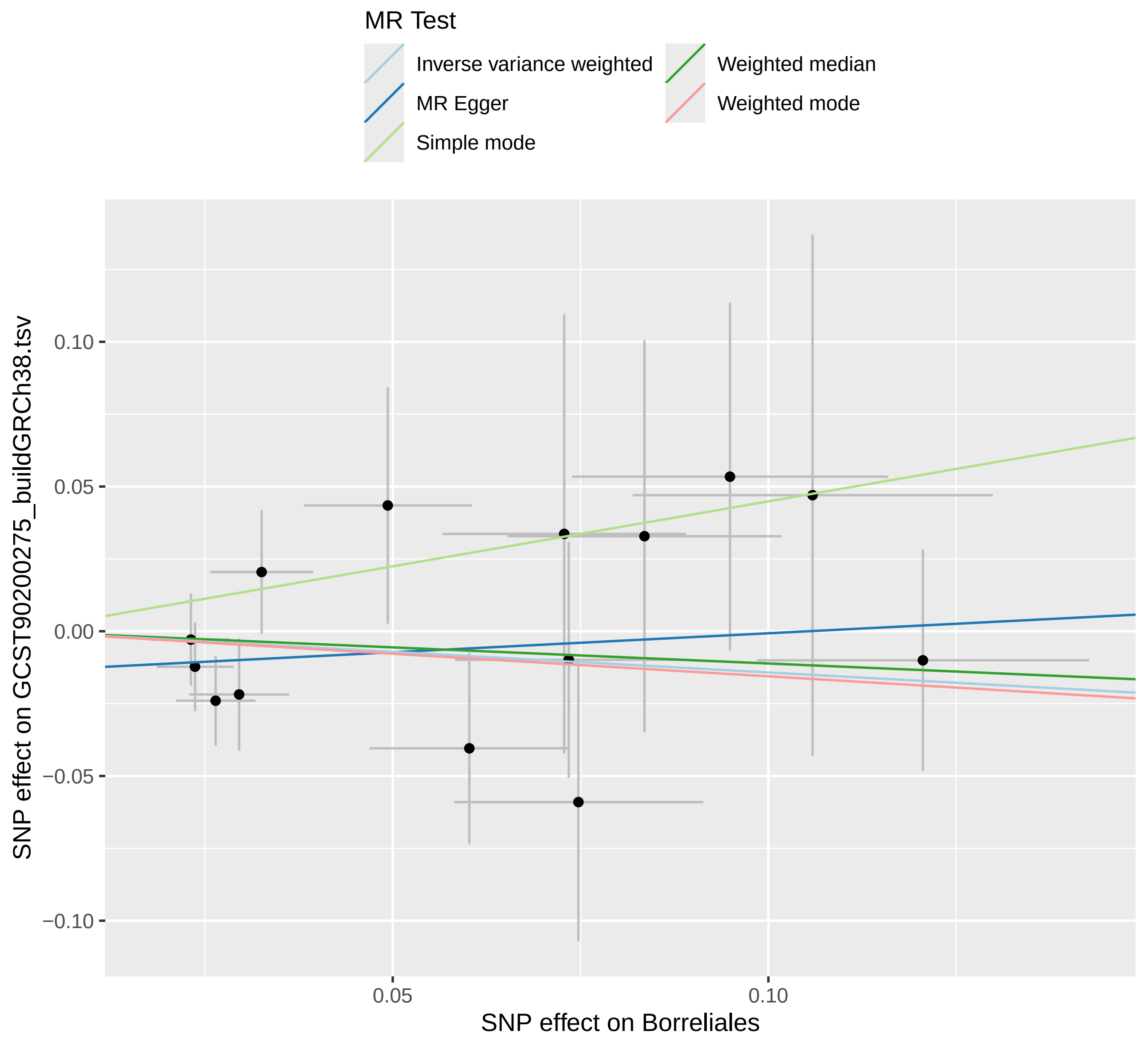

MR Test
Inverse variance weighted
MR Egger
Simple mode
Weighted median
Weighted mode
0.10
0.05
0.00
−0.05
−0.10
0.05
0.10
SNP effect on GCST90200275_buildGRCh38.tsv
SNP effect on Borreliales

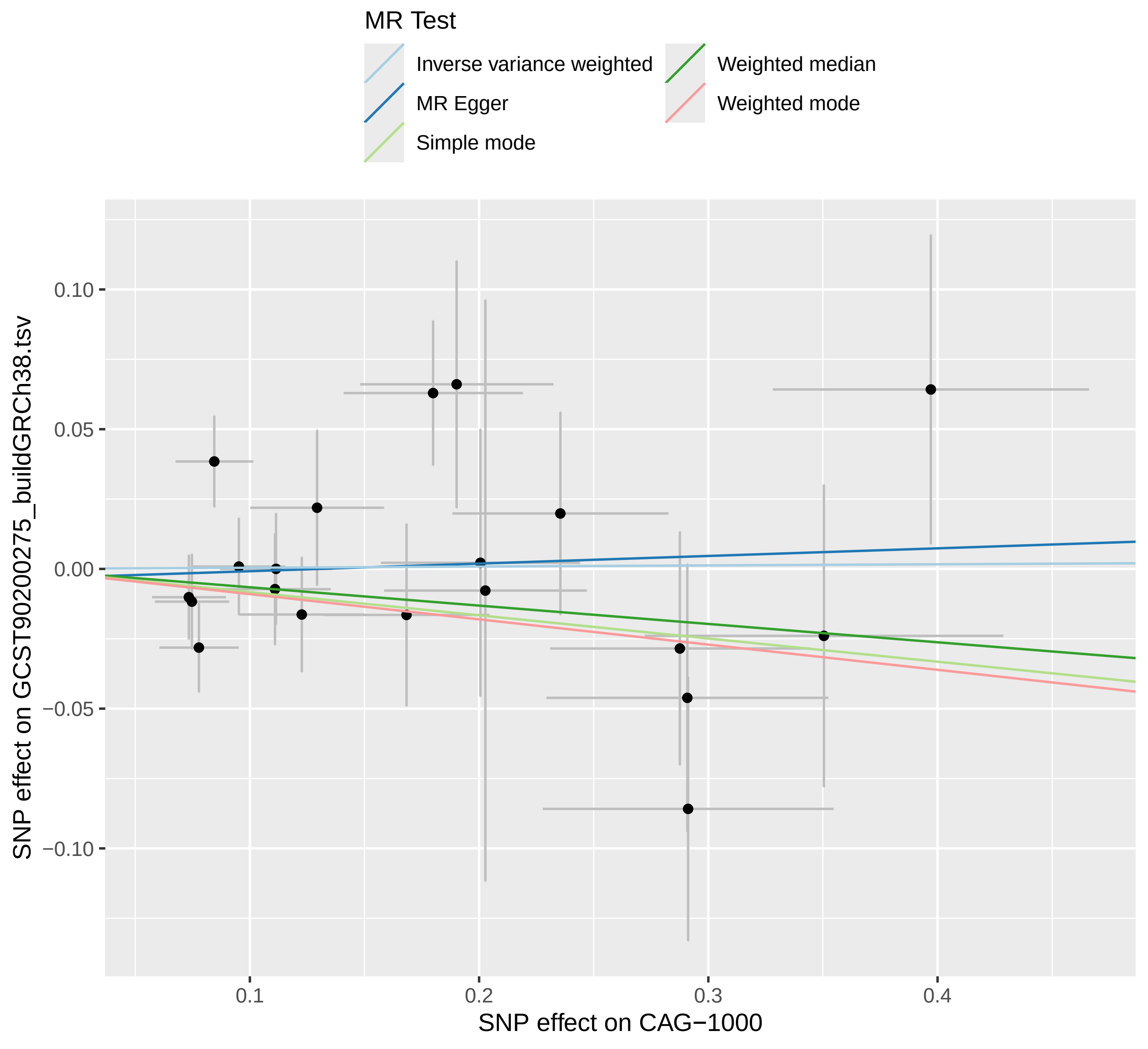

MR Test
Inverse variance weighted
MR Egger
Simple mode
Weighted median
Weighted mode
SNP effect on GCST90200275_buildGRCh38.tsv
0.10
0.05
0.00
−0.05
−0.10
0.1
0.2
0.3
0.4
SNP effect on CAG−1000

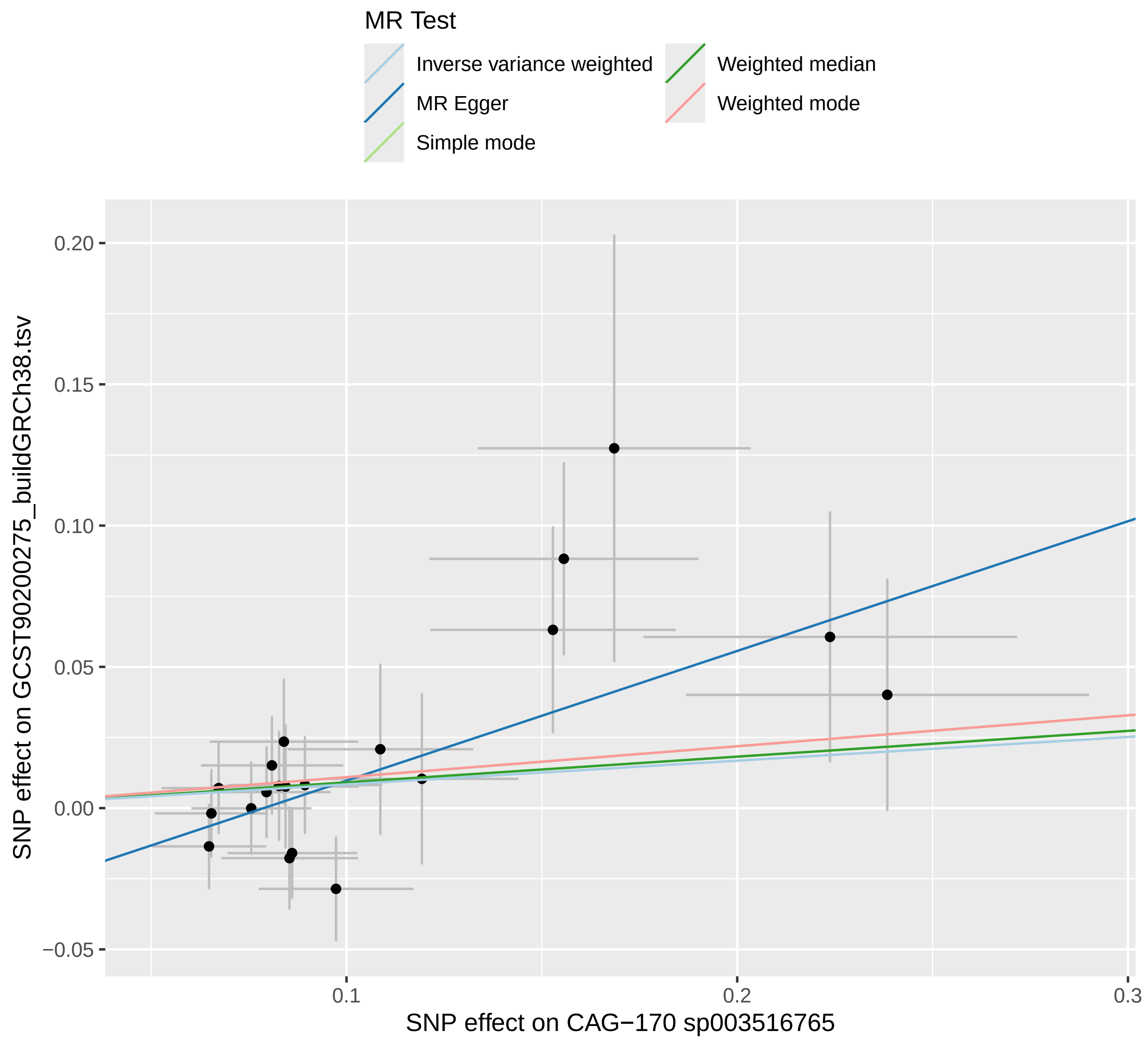

MR Test
Inverse variance weighted
MR Egger
Simple mode
Weighted median
Weighted mode
SNP effect on GCST90200275_buildGRCh38.tsv
0.20
0.15
0.10
0.05
0.00
−0.05
0.1
0.2
0.3
SNP effect on CAG−170 sp003516765

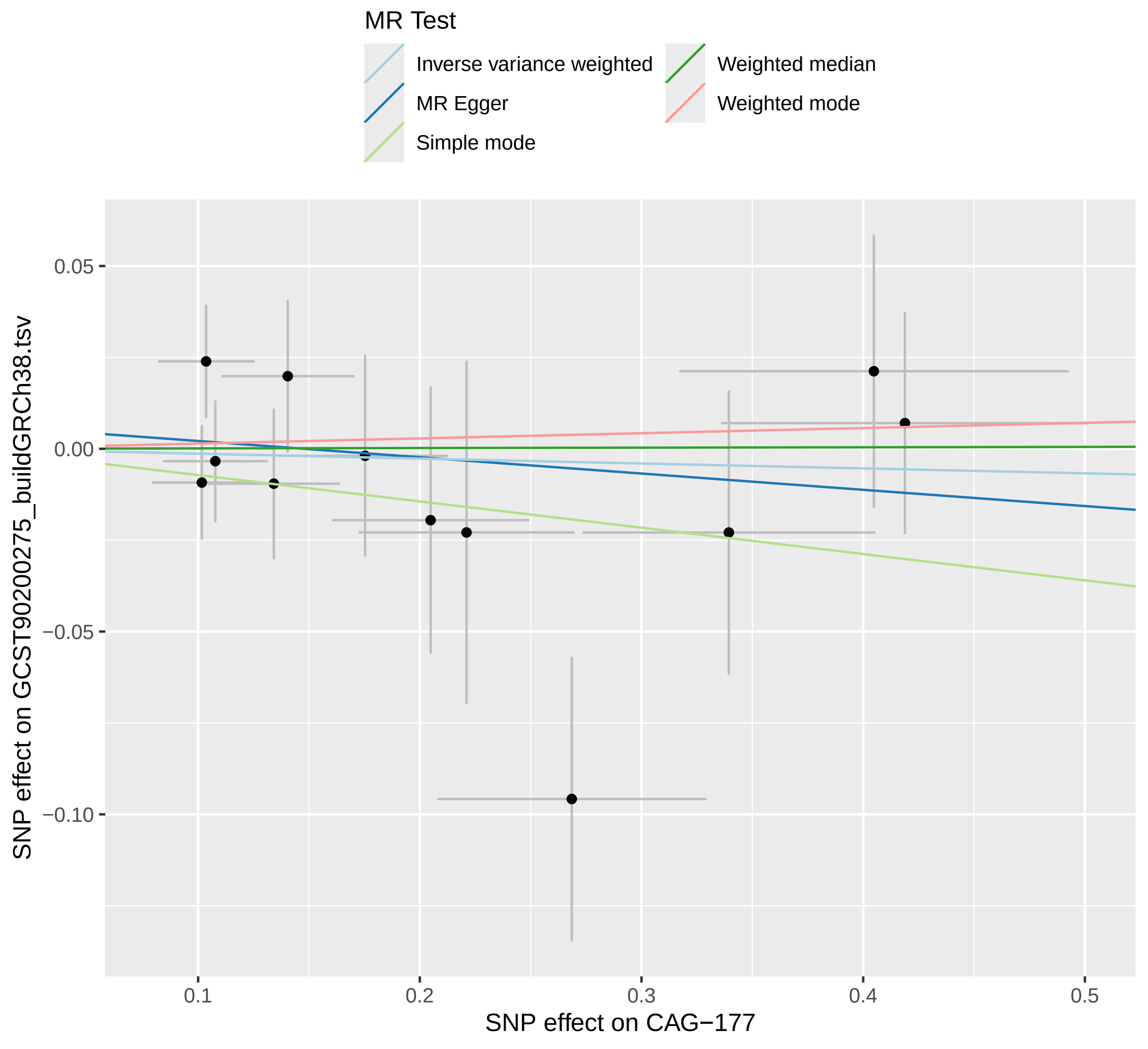
MR Test
Inverse variance weighted
MR Egger
Simple mode
Weighted median
Weighted mode
SNP effect on GCST90200275_buildGRCh38.tsv
0.05
0.00
−0.05
−0.10
0.1
0.2
0.3
0.4
0.5
SNP effect on CAG−177

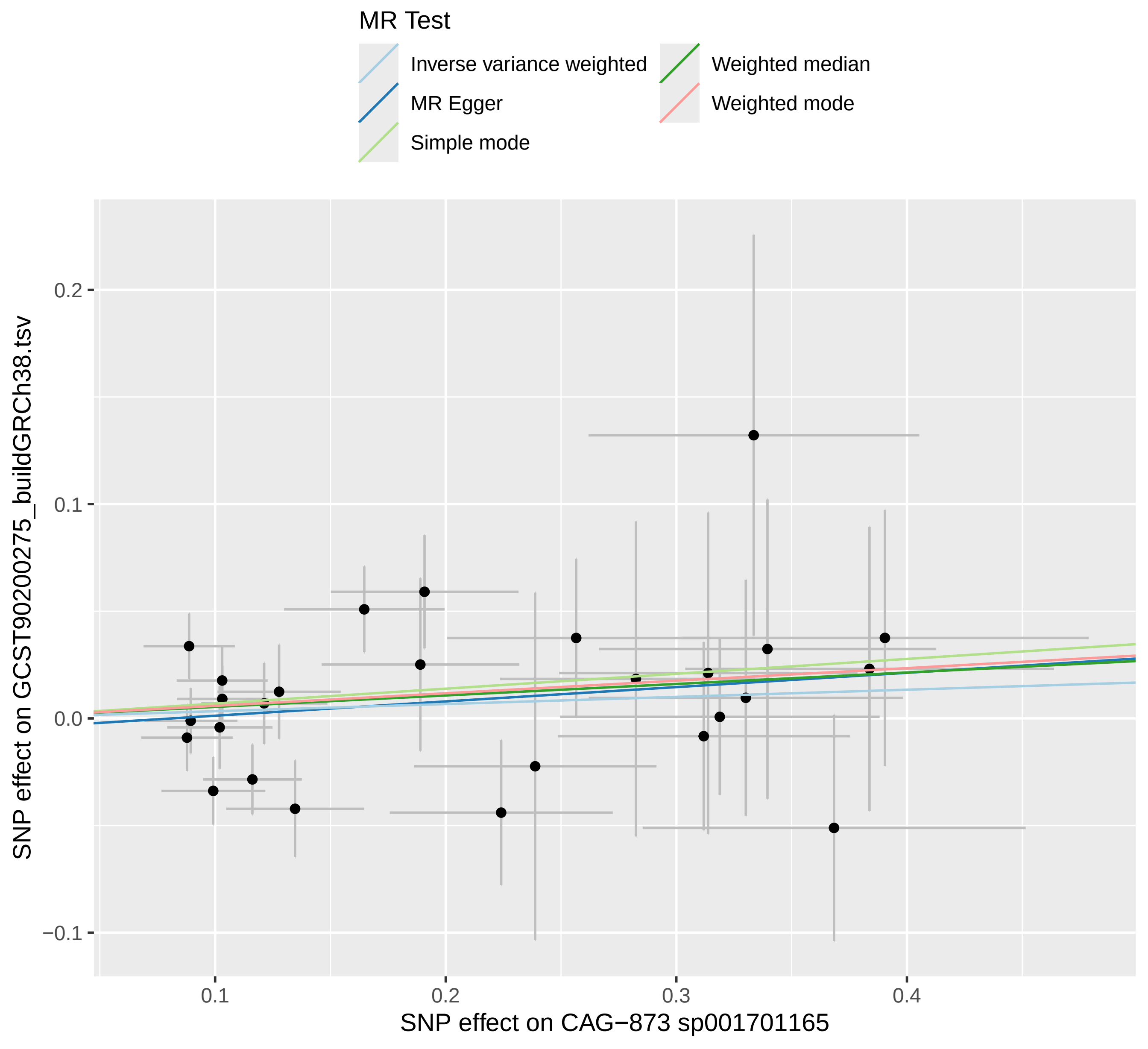
MR Test
Inverse variance weighted
MR Egger
Simple mode
Weighted median
Weighted mode
SNP effect on GCST90200275_buildGRCh38.tsv
0.2
0.1
0.0
−0.1
0.1
0.2
0.3
0.4
SNP effect on CAG−873 sp001701165

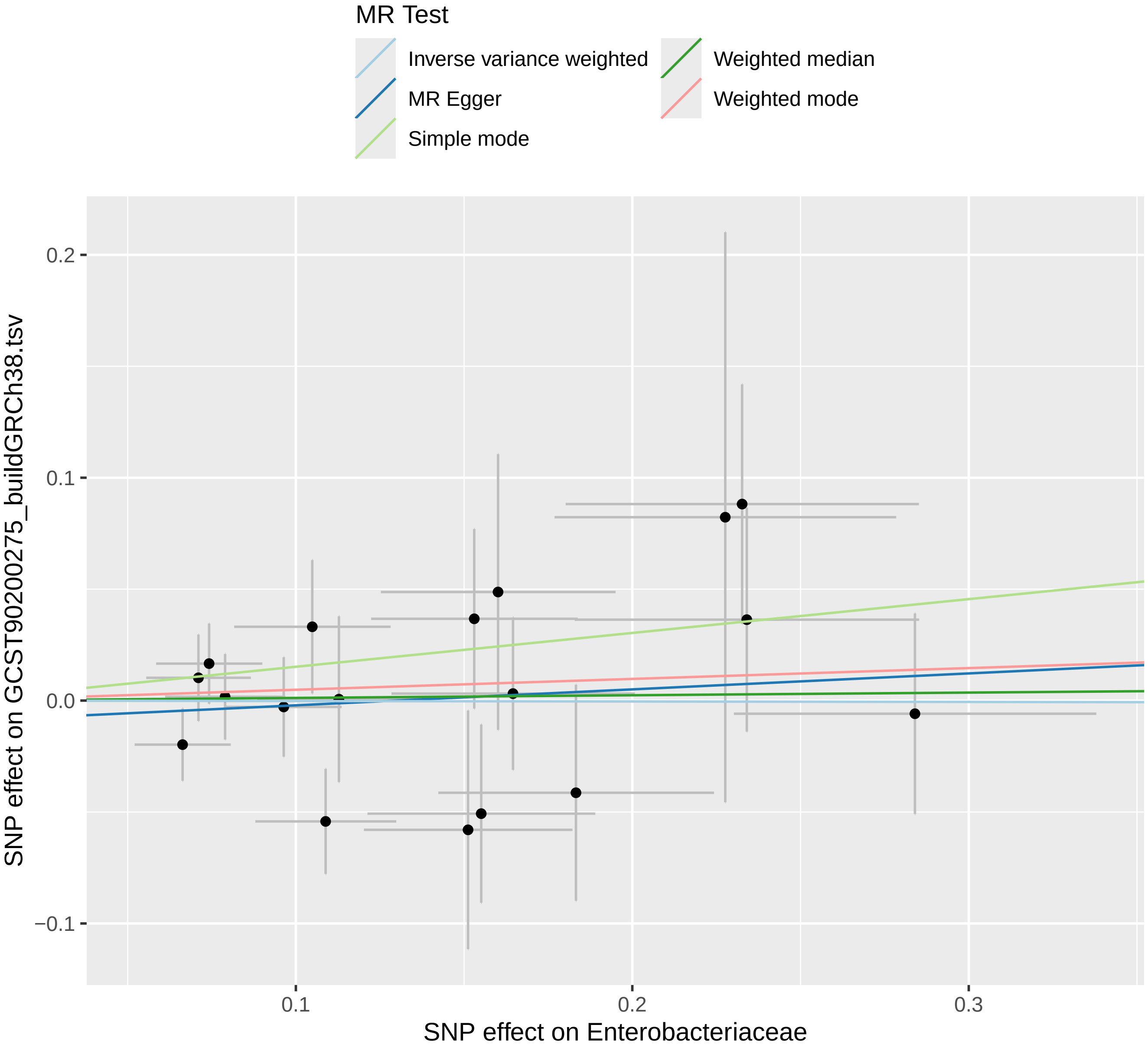
MR Test
Inverse variance weighted
MR Egger
Simple mode
Weighted median
Weighted mode
SNP effect on GCST90200275_buildGRCh38.tsv
0.2
0.1
0.0
−0.1
0.1
0.2
0.3
SNP effect on Enterobacteriaceae

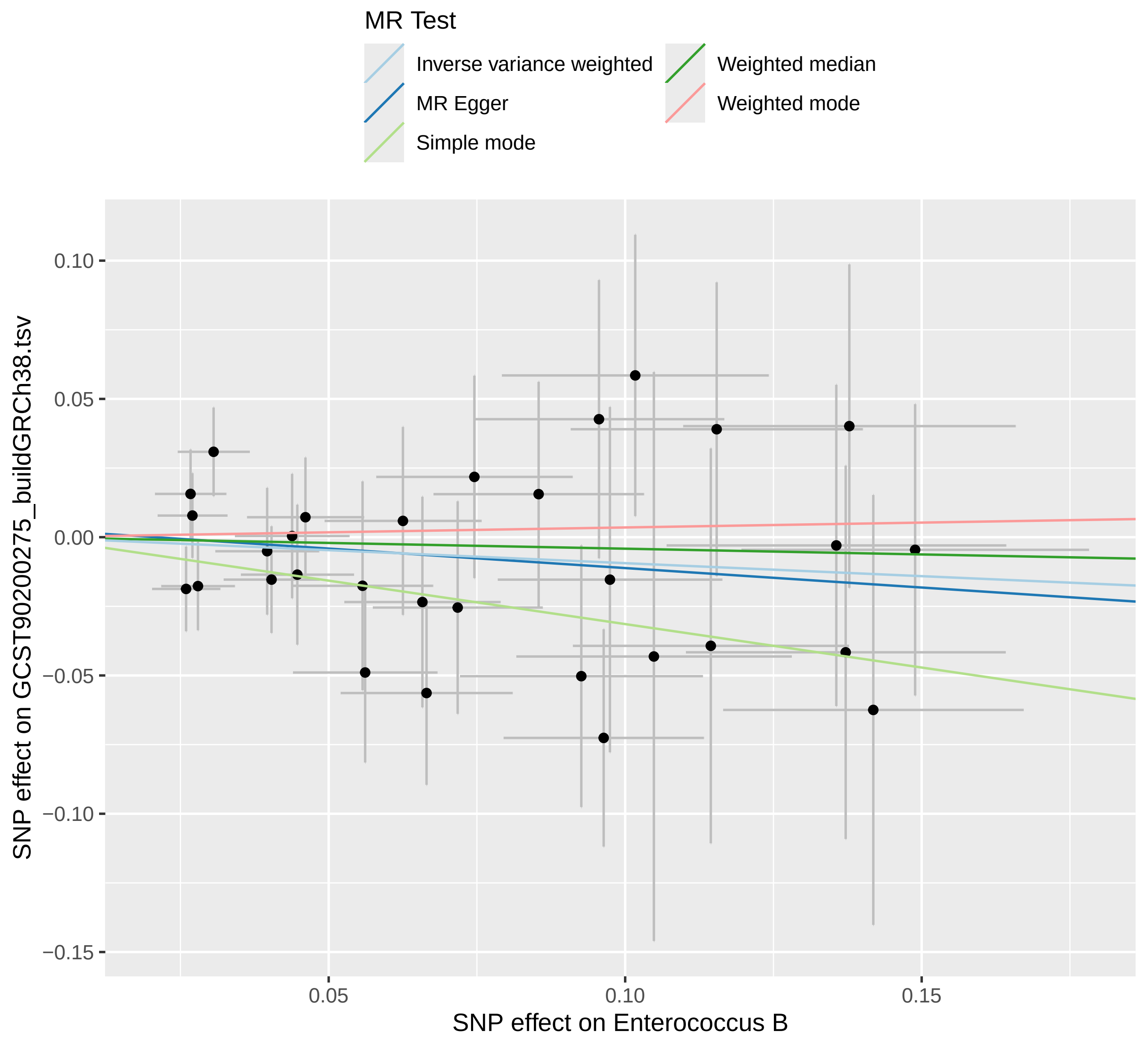

MR Test
Inverse variance weighted
MR Egger
Simple mode
Weighted median
Weighted mode
0.10
0.05
0.00
−0.05
−0.10
−0.15
SNP effect on GCST90200275_buildGRCh38.tsv
0.05
0.10
0.15
SNP effect on Enterococcus B

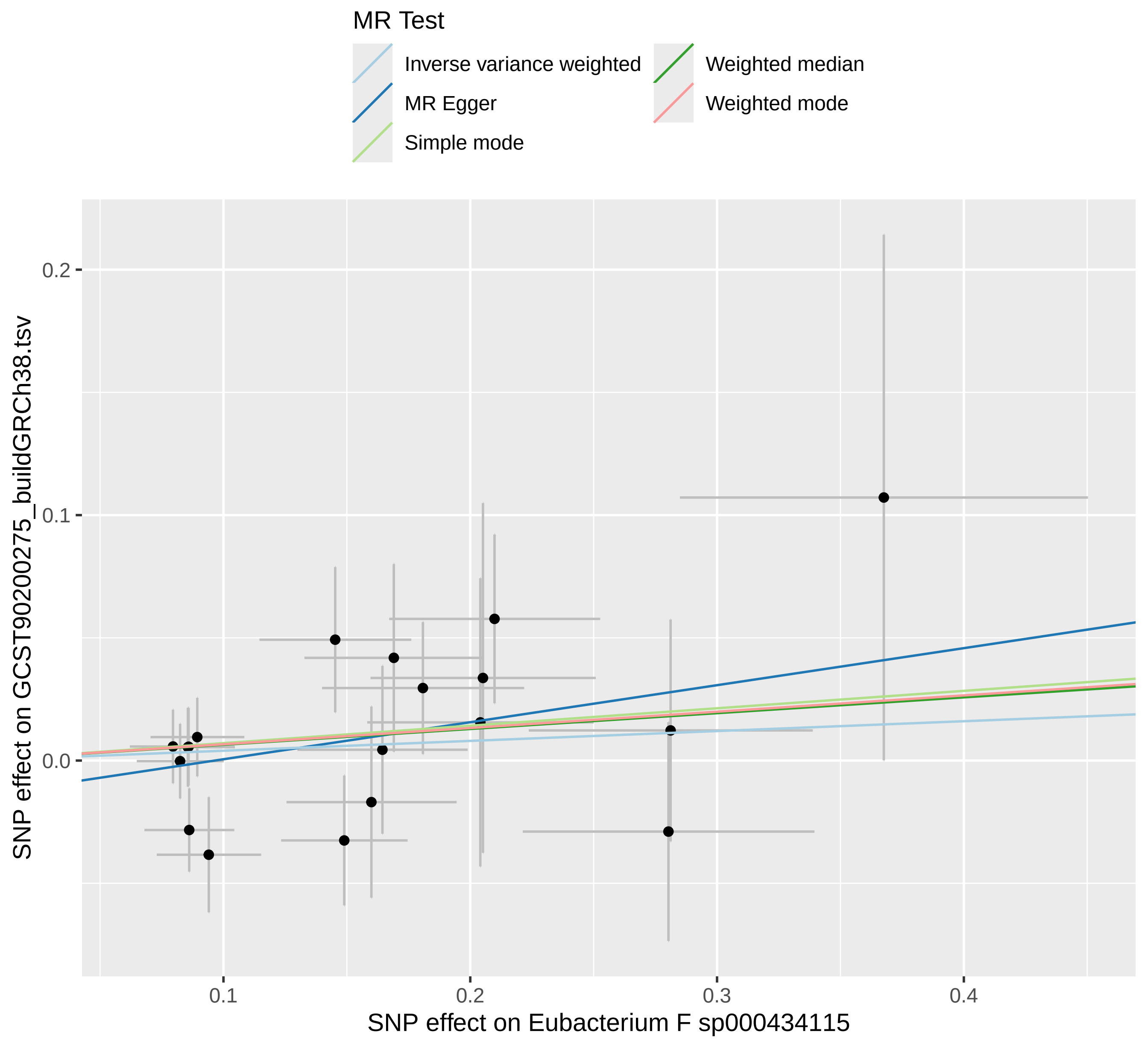
MR Test
Inverse variance weighted
MR Egger
Simple mode
Weighted median
Weighted mode
SNP effect on GCST90200275_buildGRCh38.tsv
0.2
0.1
0.0
0.1
0.2
0.3
0.4
SNP effect on Eubacterium F sp000434115

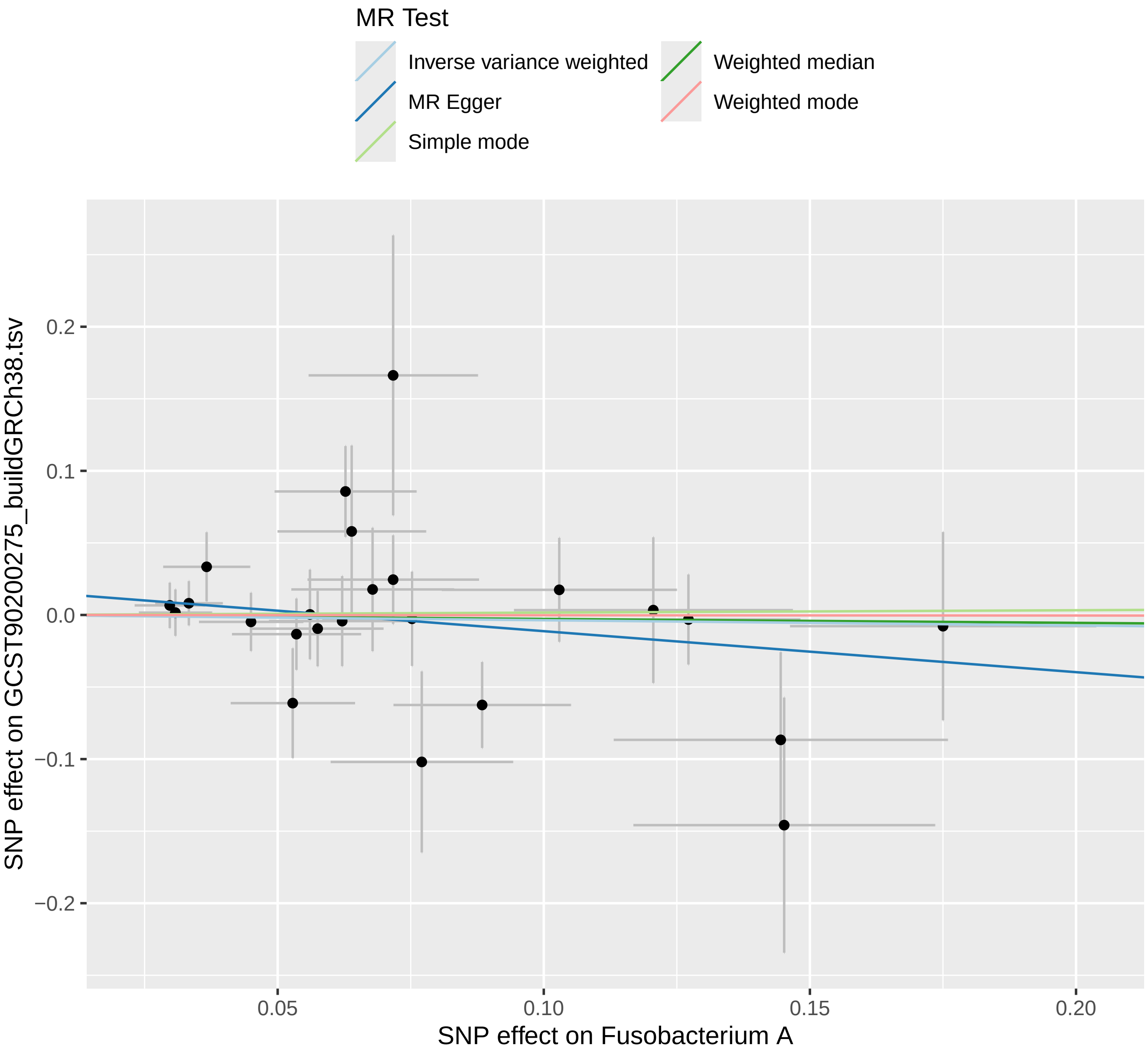
MR Test
Inverse variance weighted
MR Egger
Simple mode
Weighted median
Weighted mode
0.2
0.1
0.0
−0.1
−0.2
0.05
0.10
0.15
0.20
SNP effect on GCST90200275_buildGRCh38.tsv
SNP effect on Fusobacterium A

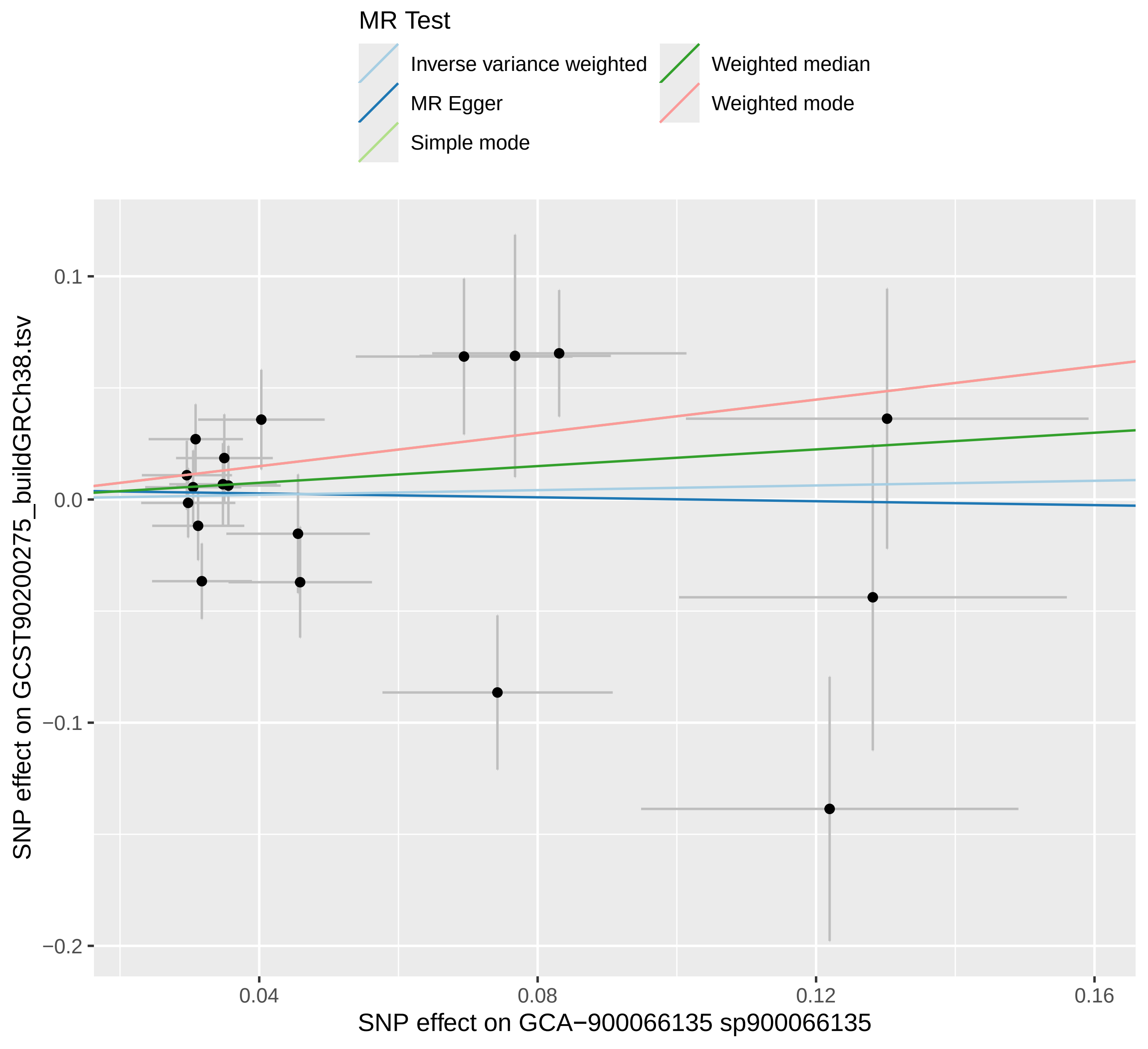
MR Test
Inverse variance weighted
MR Egger
Simple mode
Weighted median
Weighted mode
0.1
0.0
−0.1
−0.2
SNP effect on GCST90200275_buildGRCh38.tsv
0.04
0.08
0.12
0.16
SNP effect on GCA−900066135 sp900066135

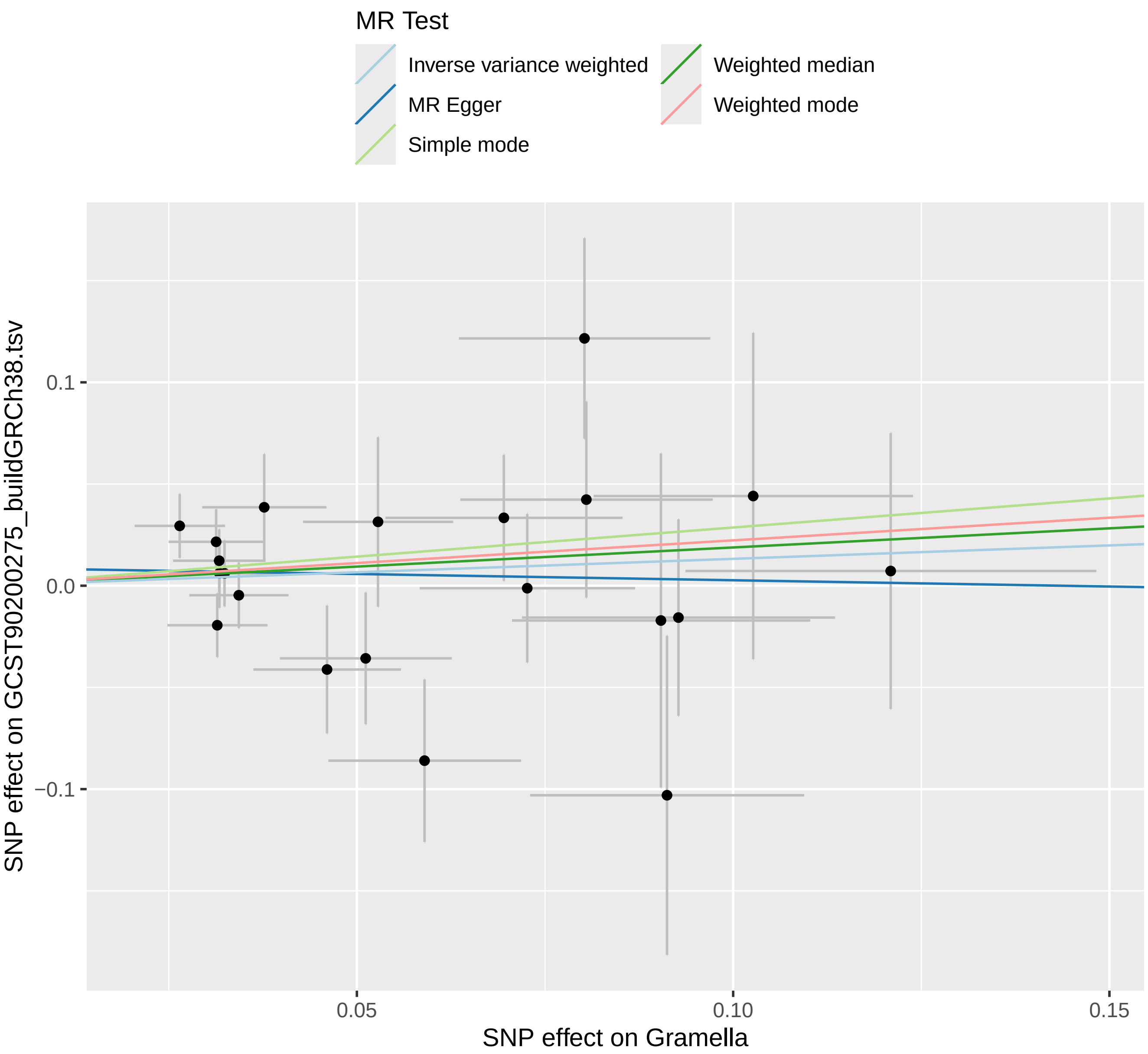
MR Test
Inverse variance weighted
MR Egger
Simple mode
Weighted median
Weighted mode
0.1
0.0
−0.1
0.05
0.10
0.15
SNP effect on GCST90200275_buildGRCh38.tsv
SNP effect on Gramella

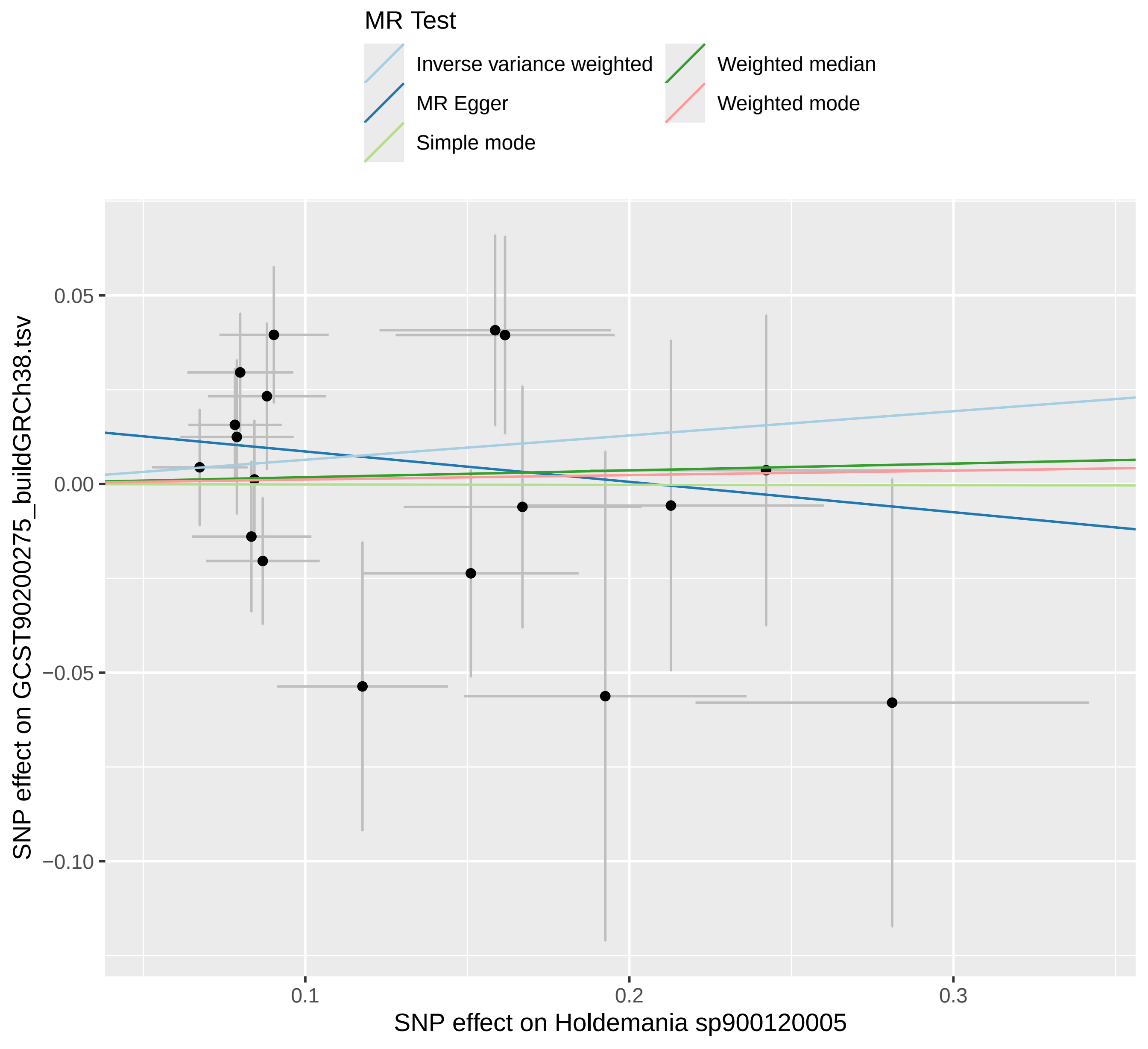

MR Test
Inverse variance weighted
MR Egger
Simple mode
Weighted median
Weighted mode
0.05
0.00
−0.05
−0.10
0.1
0.2
0.3
SNP effect on GCST90200275_buildGRCh38.tsv
SNP effect on Holdemania sp900120005

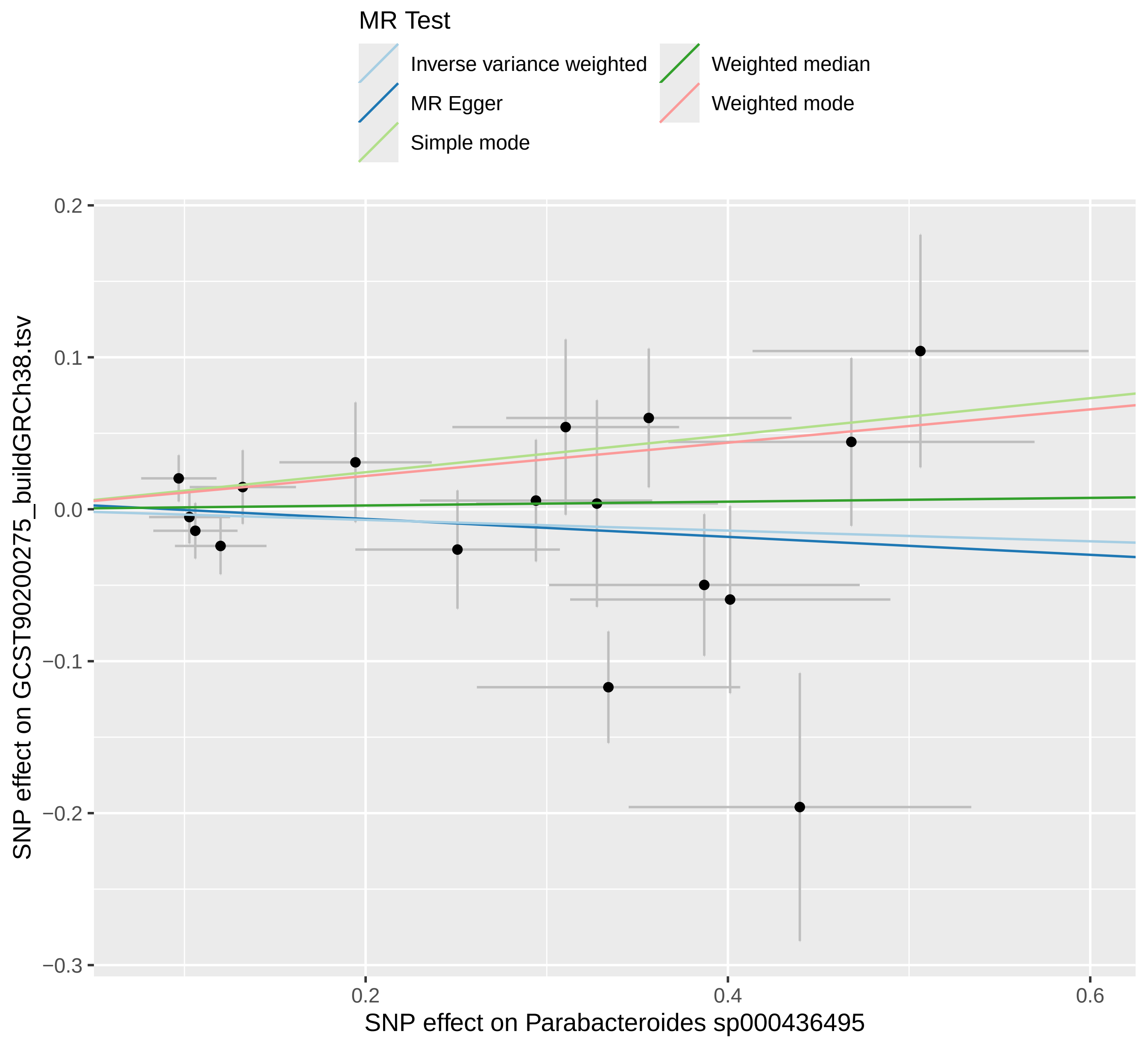
MR Test
Inverse variance weighted
MR Egger
Simple mode
Weighted median
Weighted mode
0.2
0.1
0.0
−0.1
−0.2
−0.3
SNP effect on GCST90200275_buildGRCh38.tsv
0.2
0.4
0.6
SNP effect on Parabacteroides sp000436495

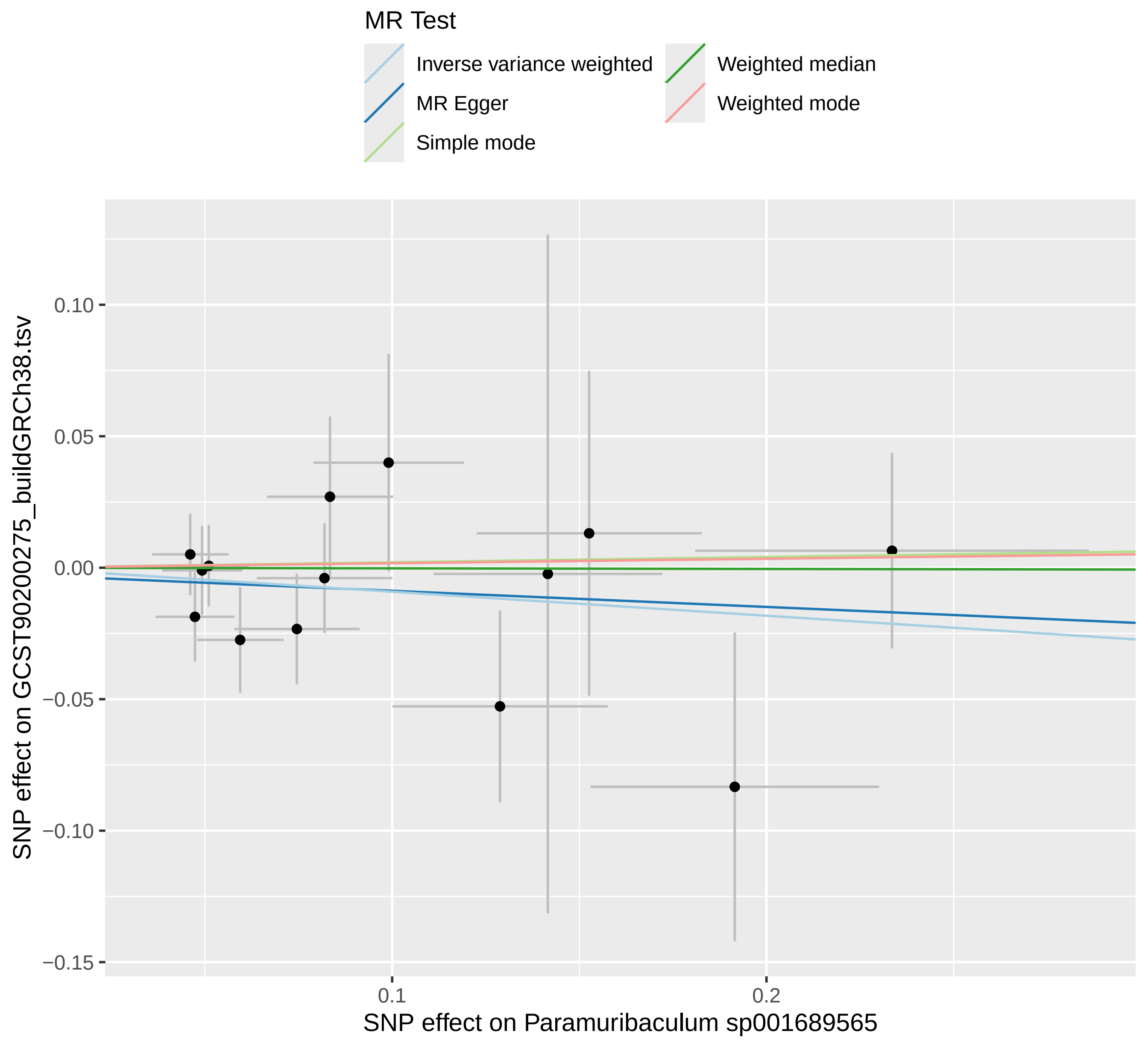
MR Test
Inverse variance weighted
MR Egger
Simple mode
Weighted median
Weighted mode
0.10
0.05
0.00
−0.05
−0.10
−0.15
0.1
0.2
SNP effect on GCST90200275_buildGRCh38.tsv
SNP effect on Paramuribaculum sp001689565

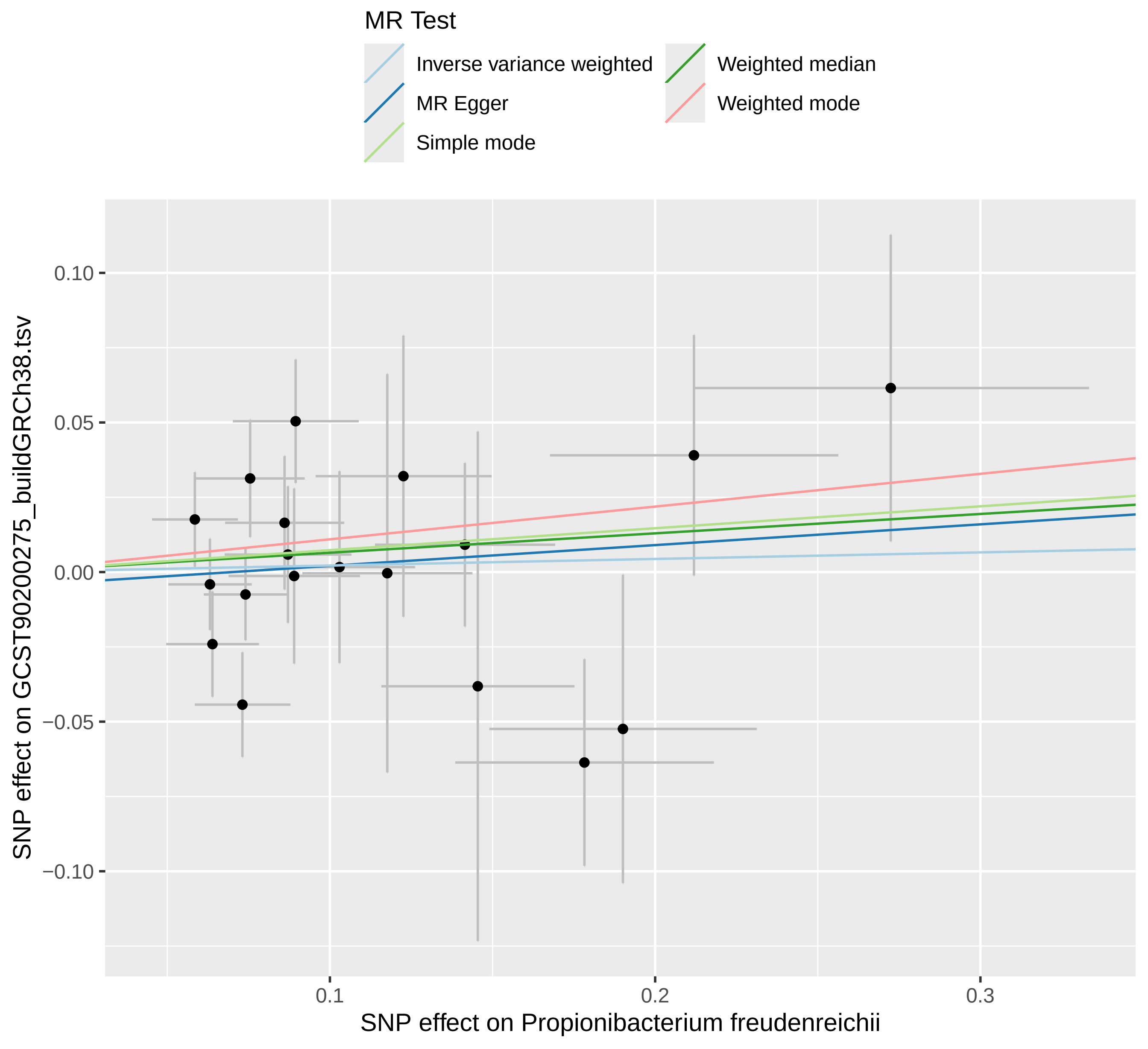

MR Test
Inverse variance weighted
MR Egger
Simple mode
Weighted median
Weighted mode
0.10
0.05
0.00
−0.05
−0.10
0.1
0.2
0.3
SNP effect on GCST90200275_buildGRCh38.tsv
SNP effect on Propionibacterium freudenreichii

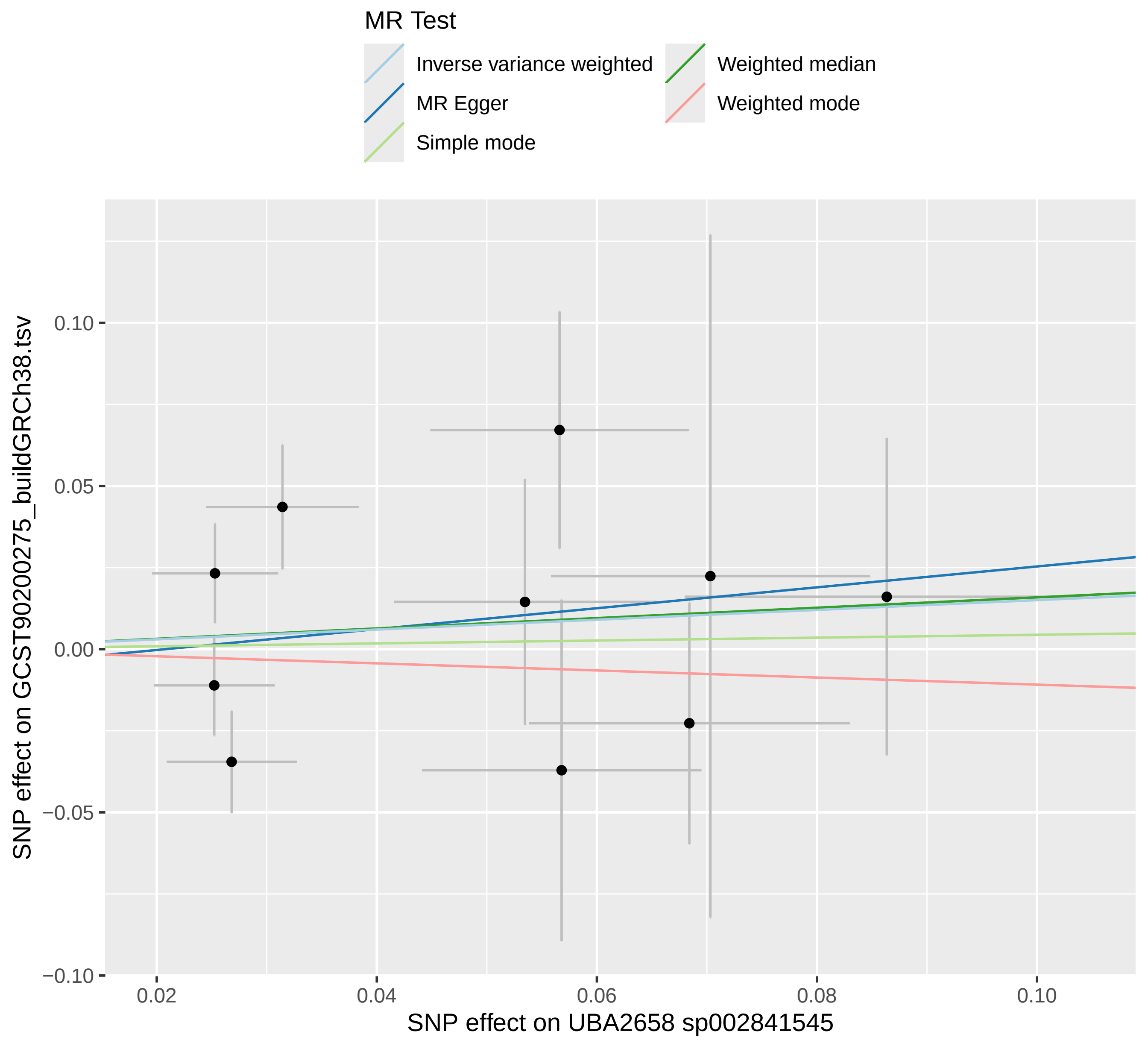
MR Test
Inverse variance weighted
MR Egger
Simple mode
Weighted median
Weighted mode
SNP effect on GCST90200275_buildGRCh38.tsv
0.10
0.05
0.00
−0.05
−0.10
0.02
0.04
0.06
0.08
0.10
SNP effect on UBA2658 sp002841545

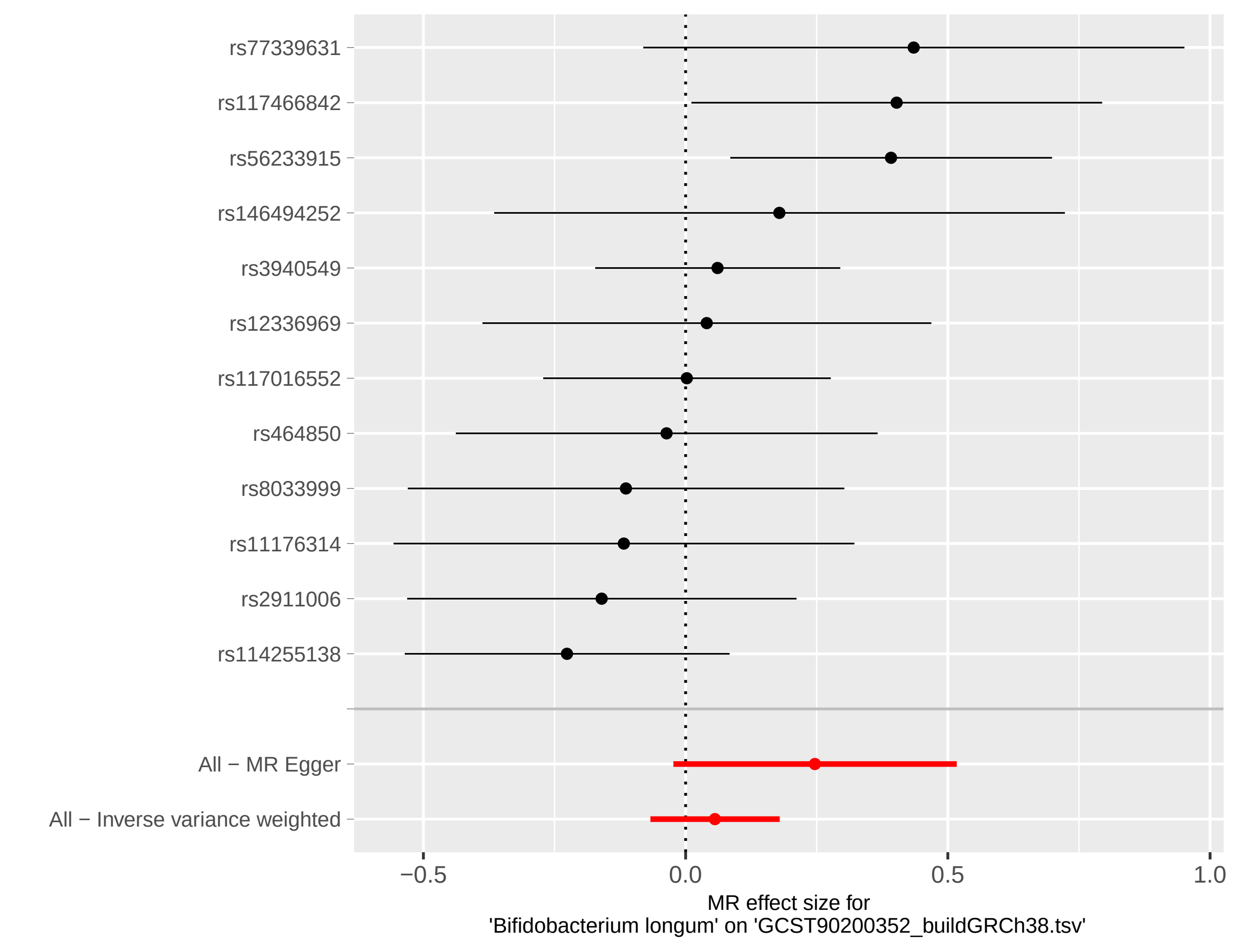
rs77339631
rs117466842
rs56233915
rs146494252
rs3940549
rs12336969
rs117016552
rs464850
rs8033999
rs11176314
rs2911006
rs114255138
All − MR Egger
All − Inverse variance weighted
−0.5
0.0
0.5
1.0
MR effect size for
'Bifidobacterium longum' on 'GCST90200352_buildGRCh38.tsv'

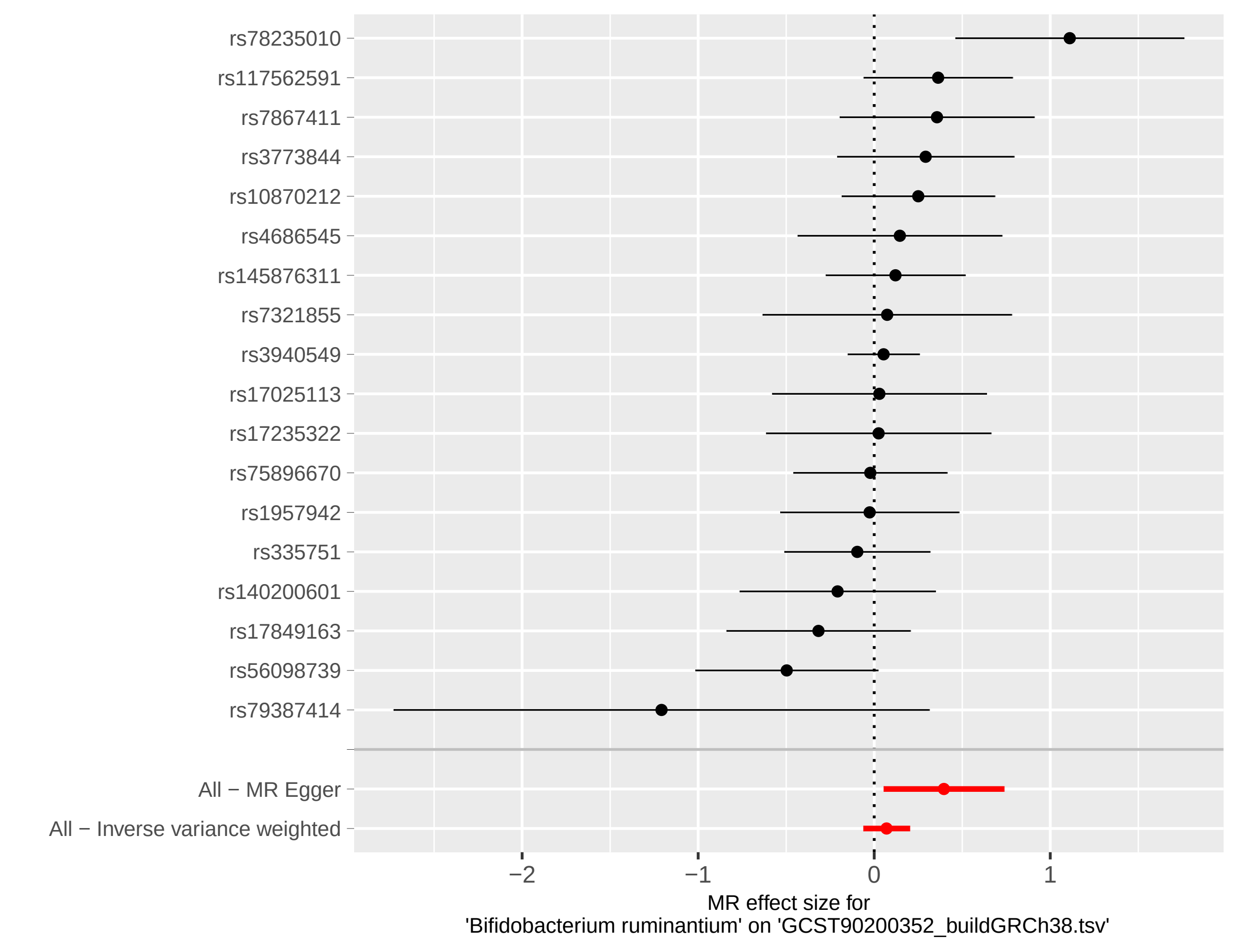
rs78235010
rs117562591
rs7867411
rs3773844
rs10870212
rs4686545
rs145876311
rs7321855
rs3940549
rs17025113
rs17235322
rs75896670
rs1957942
rs335751
rs140200601
rs17849163
rs56098739
rs79387414
All − MR Egger
All − Inverse variance weighted
−2
−1
0
1
MR effect size for
'Bifidobacterium ruminantium' on 'GCST90200352_buildGRCh38.tsv'

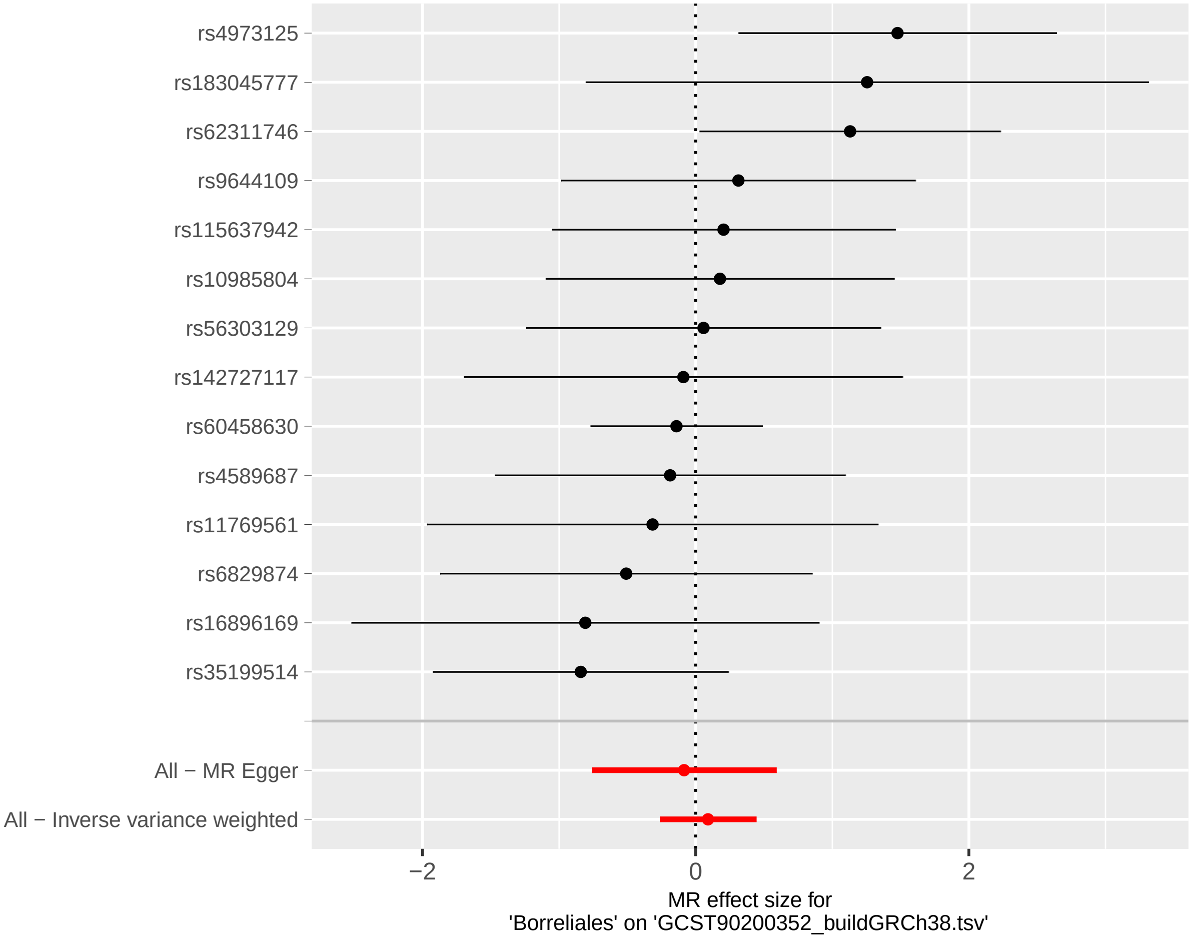
rs4973125
rs183045777
rs62311746
rs9644109
rs115637942
rs10985804
rs56303129
rs142727117
rs60458630
rs4589687
rs11769561
rs6829874
rs16896169
rs35199514
All − MR Egger
All − Inverse variance weighted
−2
0
2
MR effect size for
'Borreliales' on 'GCST90200352_buildGRCh38.tsv'

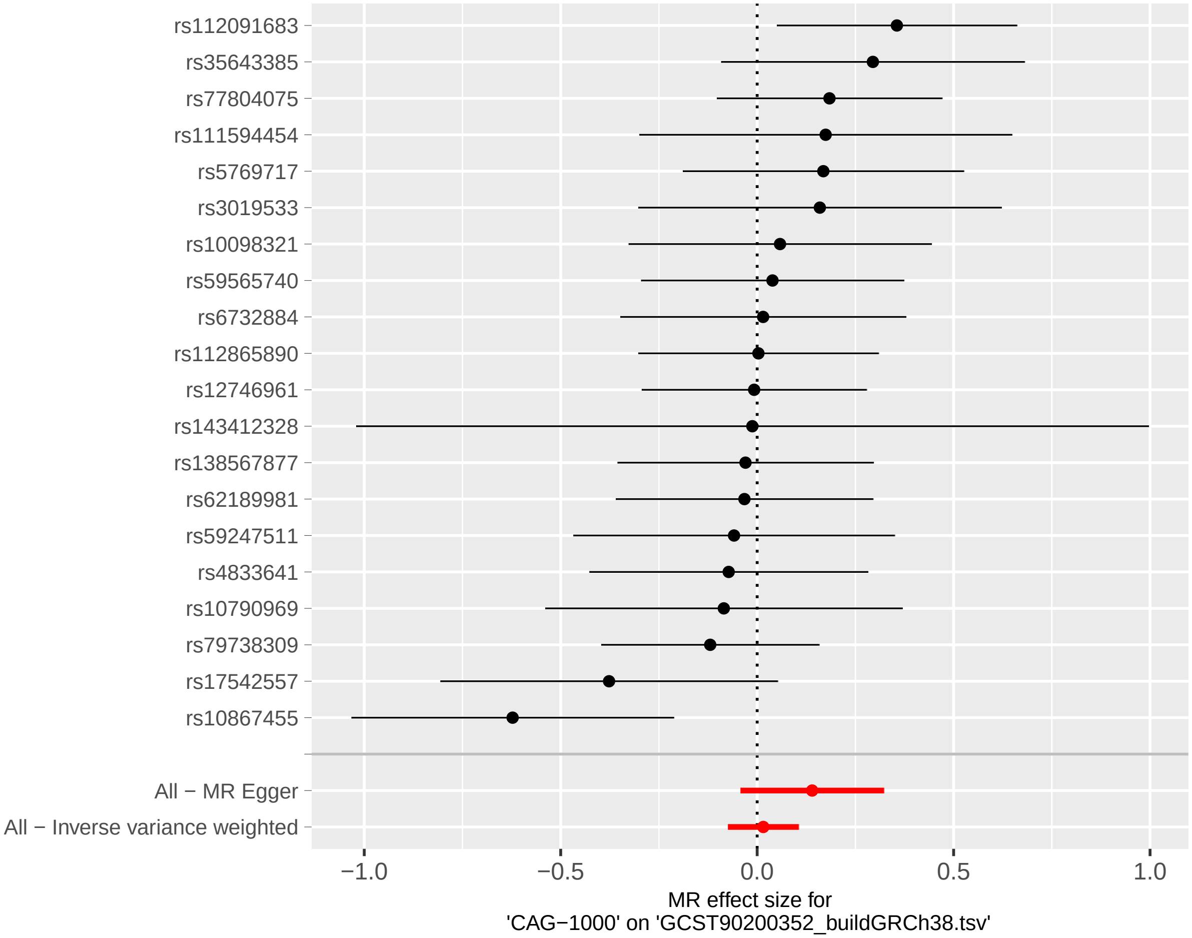
rs112091683
rs35643385
rs77804075
rs111594454
rs5769717
rs3019533
rs10098321
rs59565740
rs6732884
rs112865890
rs12746961
rs143412328
rs138567877
rs62189981
rs59247511
rs4833641
rs10790969
rs79738309
rs17542557
rs10867455
All − MR Egger
All − Inverse variance weighted
−1.0
−0.5
0.0
0.5
1.0
MR effect size for
'CAG−1000' on 'GCST90200352_buildGRCh38.tsv'

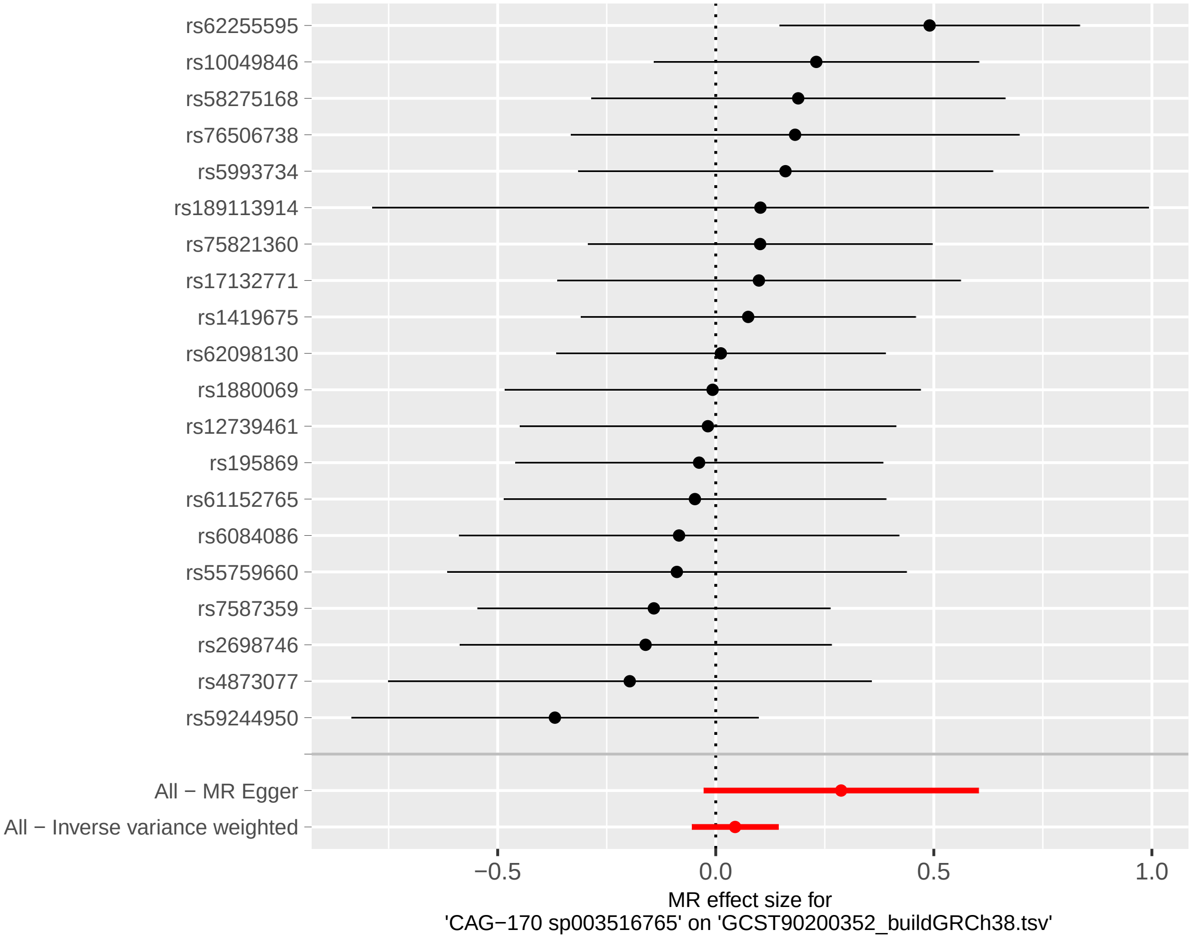
rs62255595
rs10049846
rs58275168
rs76506738
rs5993734
rs189113914
rs75821360
rs17132771
rs1419675
rs62098130
rs1880069
rs12739461
rs195869
rs61152765
rs6084086
rs55759660
rs7587359
rs2698746
rs4873077
rs59244950
All − MR Egger
All − Inverse variance weighted
−0.5
0.0
0.5
1.0
MR effect size for
'CAG−170 sp003516765' on 'GCST90200352_buildGRCh38.tsv'

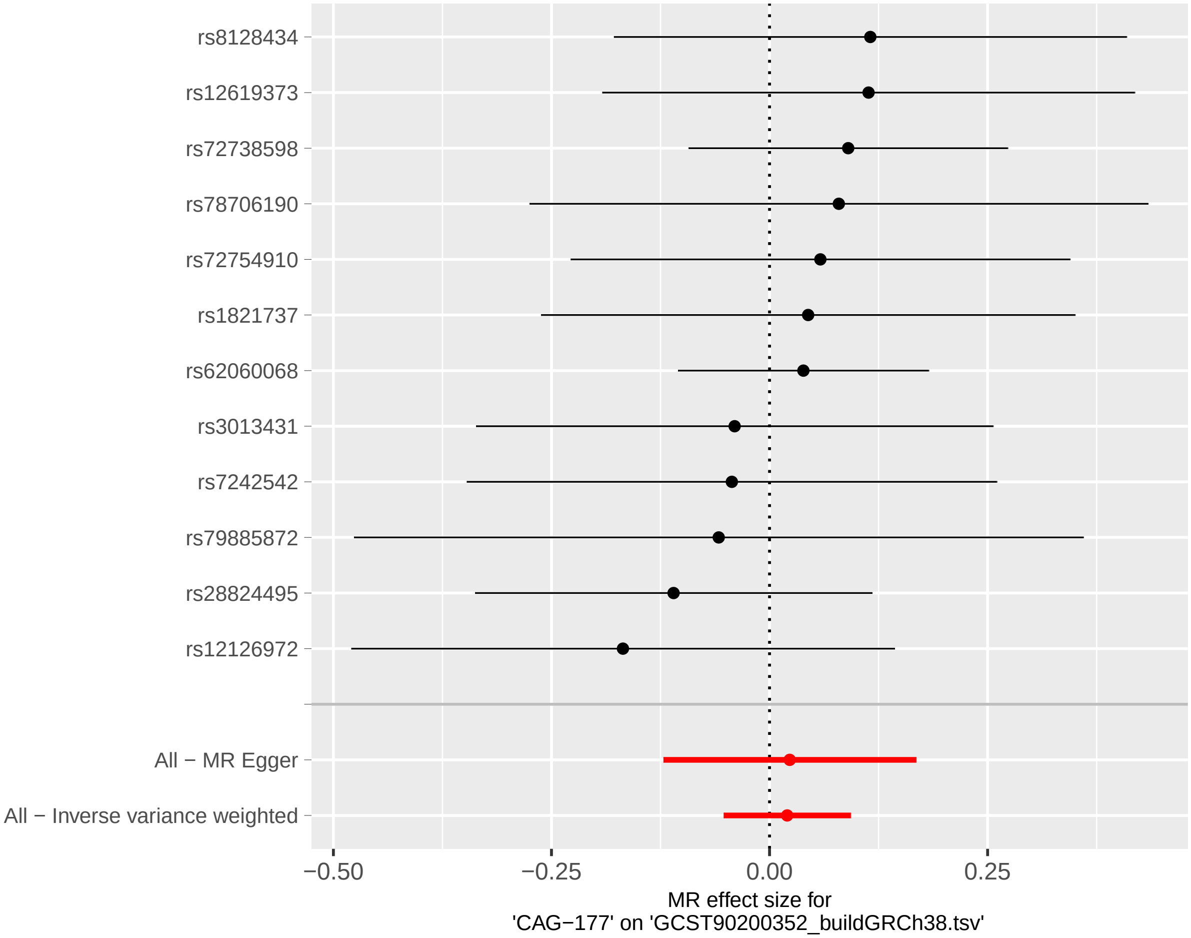

rs8128434
rs12619373
rs72738598
rs78706190
rs72754910
rs1821737
rs62060068
rs3013431
rs7242542
rs79885872
rs28824495
rs12126972
All − MR Egger
All − Inverse variance weighted
−0.50
−0.25
0.00
0.25
MR effect size for
'CAG−177' on 'GCST90200352_buildGRCh38.tsv'

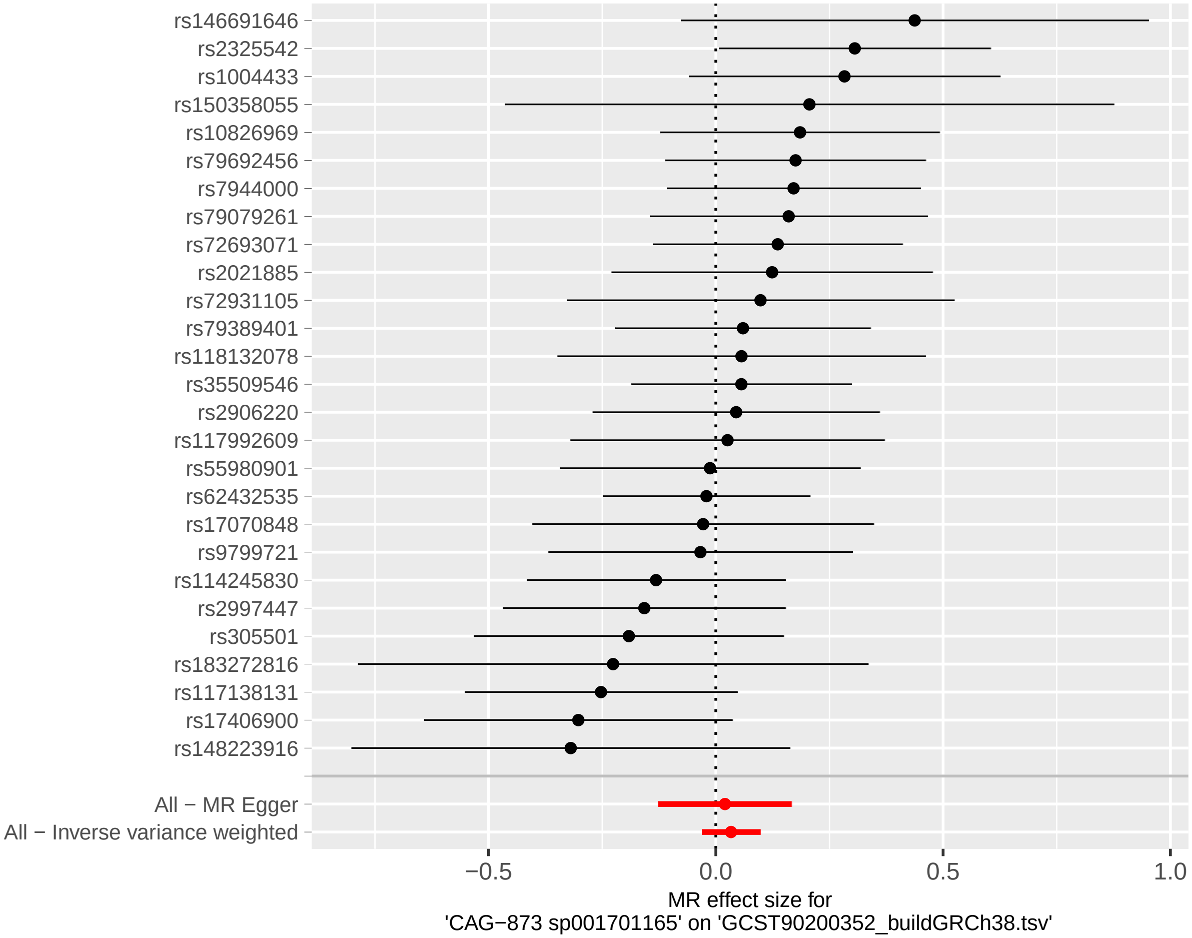

rs146691646
rs2325542
rs1004433
rs150358055
rs10826969
rs79692456
rs7944000
rs79079261
rs72693071
rs2021885
rs72931105
rs79389401
rs118132078
rs35509546
rs2906220
rs117992609
rs55980901
rs62432535
rs17070848
rs9799721
rs114245830
rs2997447
rs305501
rs183272816
rs117138131
rs17406900
rs148223916
All − MR Egger
All − Inverse variance weighted
−0.5
0.0
0.5
1.0
MR effect size for
'CAG−873 sp001701165' on 'GCST90200352_buildGRCh38.tsv'

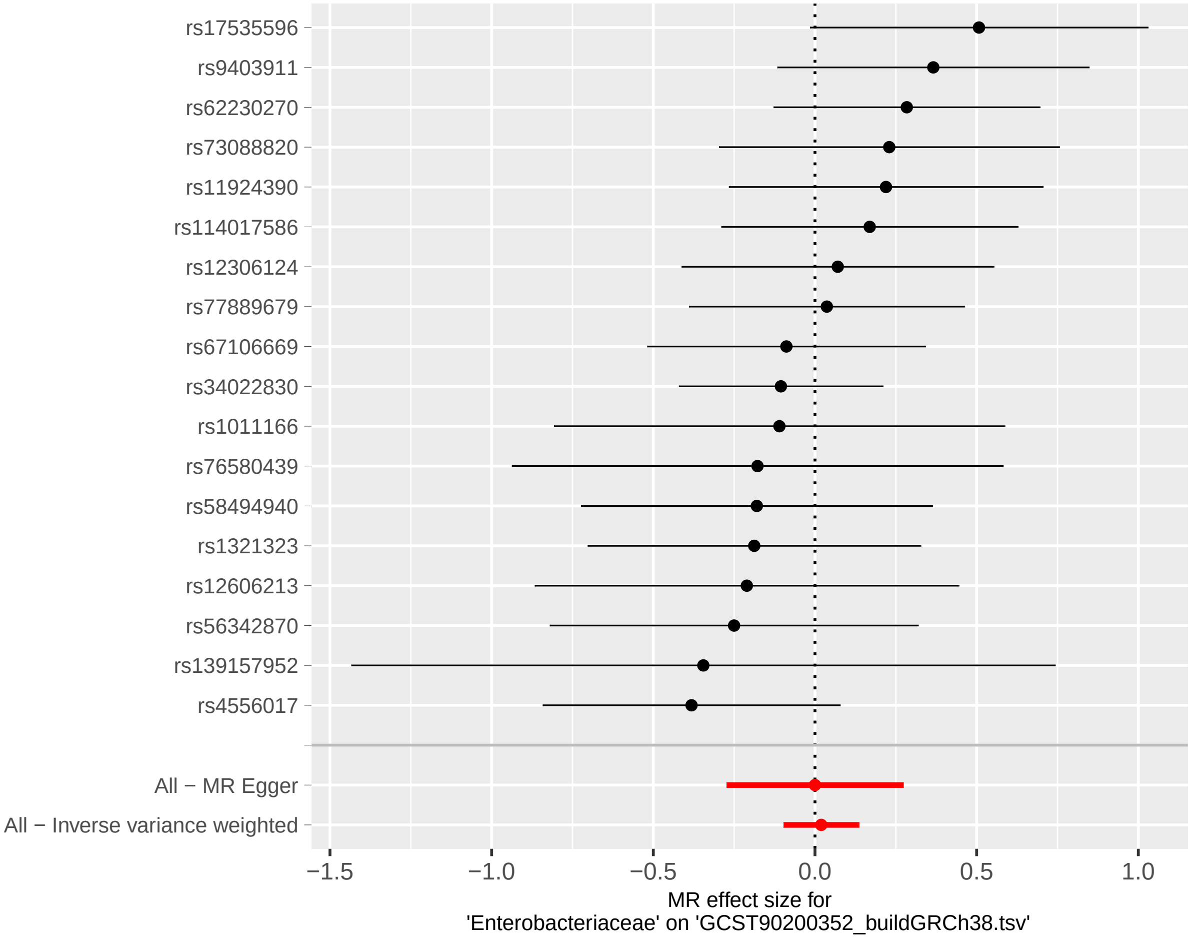

rs17535596
rs9403911
rs62230270
rs73088820
rs11924390
rs114017586
rs12306124
rs77889679
rs67106669
rs34022830
rs1011166
rs76580439
rs58494940
rs1321323
rs12606213
rs56342870
rs139157952
rs4556017
All − MR Egger
All − Inverse variance weighted
−1.5
−1.0
−0.5
0.0
0.5
1.0
MR effect size for
'Enterobacteriaceae' on 'GCST90200352_buildGRCh38.tsv'

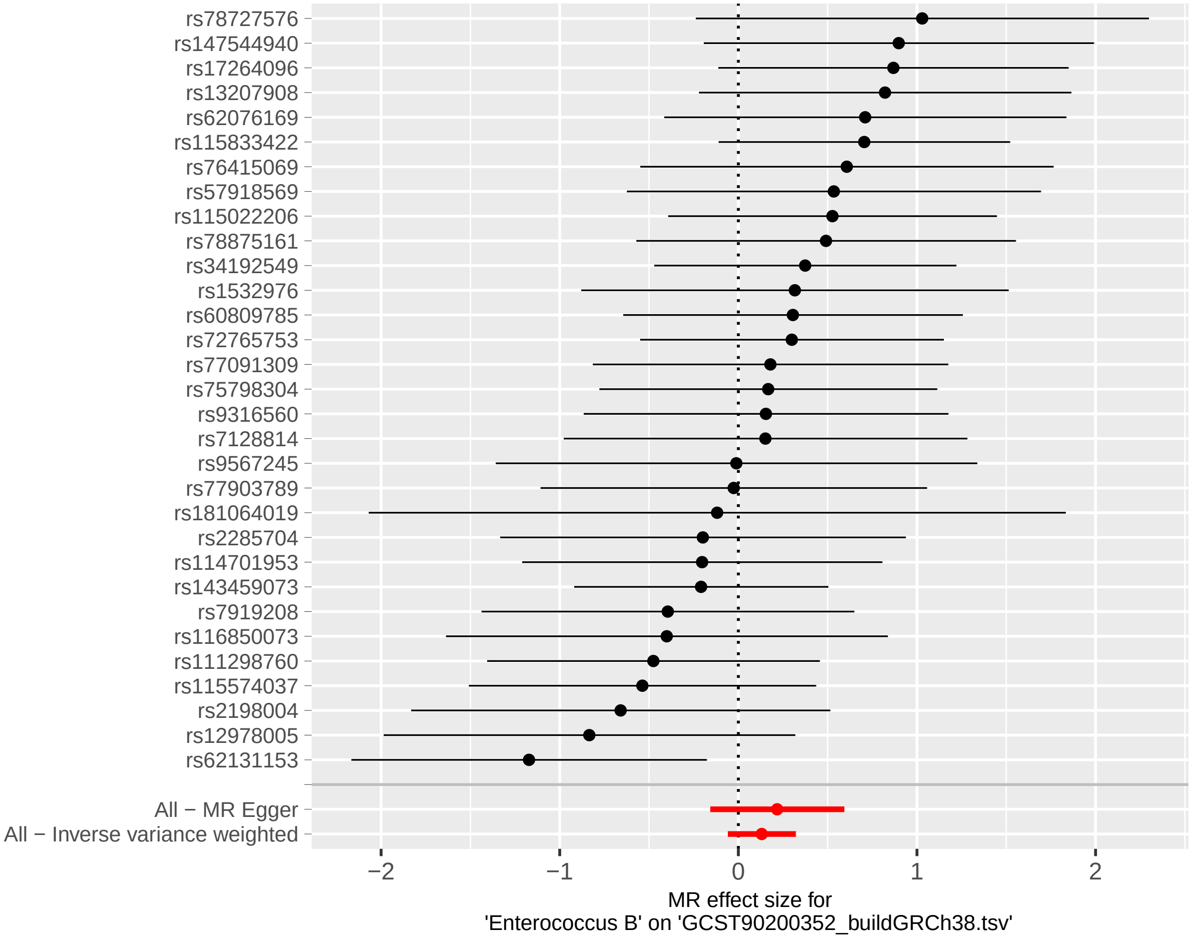
rs78727576
rs147544940
rs17264096
rs13207908
rs62076169
rs115833422
rs76415069
rs57918569
rs115022206
rs78875161
rs34192549
rs1532976
rs60809785
rs72765753
rs77091309
rs75798304
rs9316560
rs7128814
rs9567245
rs77903789
rs181064019
rs2285704
rs114701953
rs143459073
rs7919208
rs116850073
rs111298760
rs115574037
rs2198004
rs12978005
rs62131153
All − MR Egger
All − Inverse variance weighted
−2
−1
0
1
2
MR effect size for
'Enterococcus B' on 'GCST90200352_buildGRCh38.tsv'

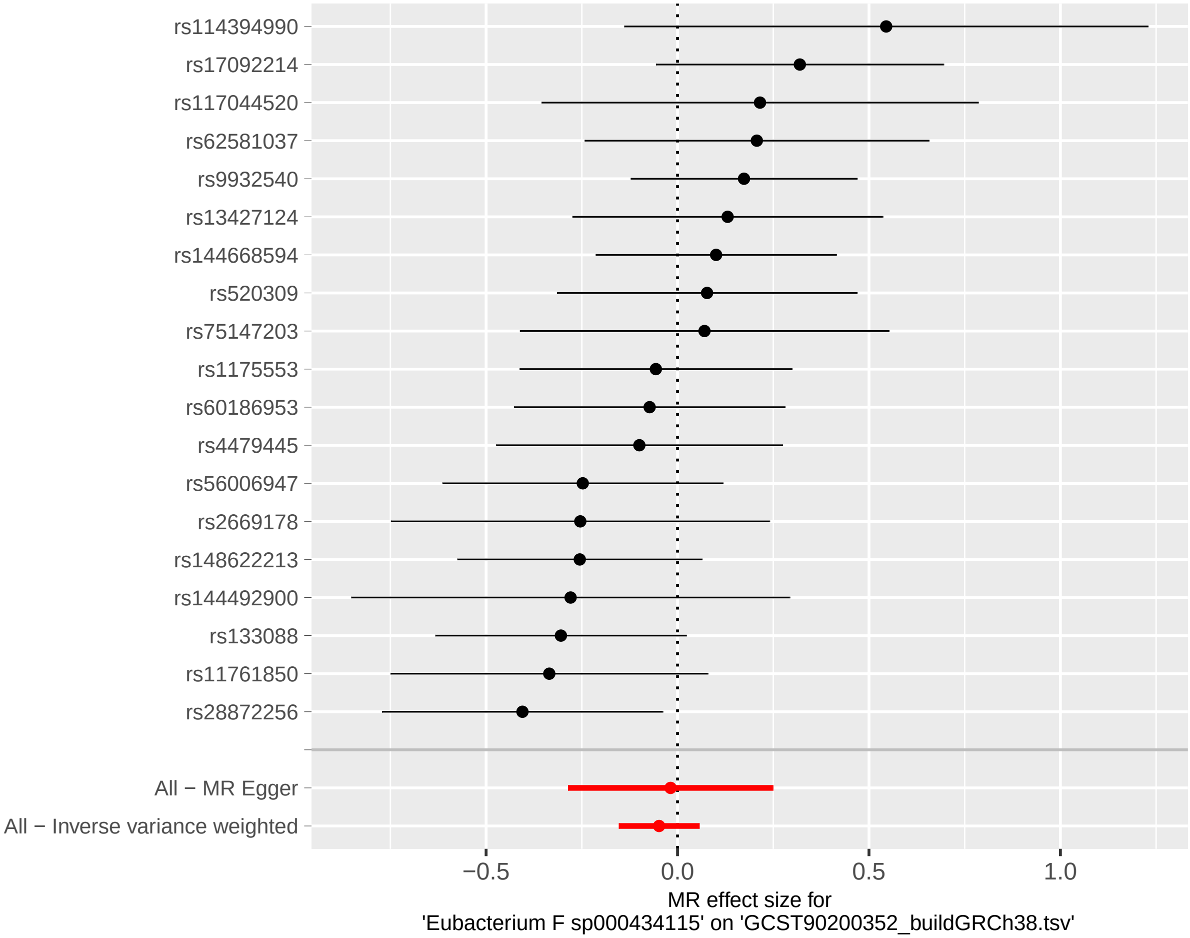
rs114394990
rs17092214
rs117044520
rs62581037
rs9932540
rs13427124
rs144668594
rs520309
rs75147203
rs1175553
rs60186953
rs4479445
rs56006947
rs2669178
rs148622213
rs144492900
rs133088
rs11761850
rs28872256
All − MR Egger
All − Inverse variance weighted
−0.5
0.0
0.5
1.0
MR effect size for
'Eubacterium F sp000434115' on 'GCST90200352_buildGRCh38.tsv'

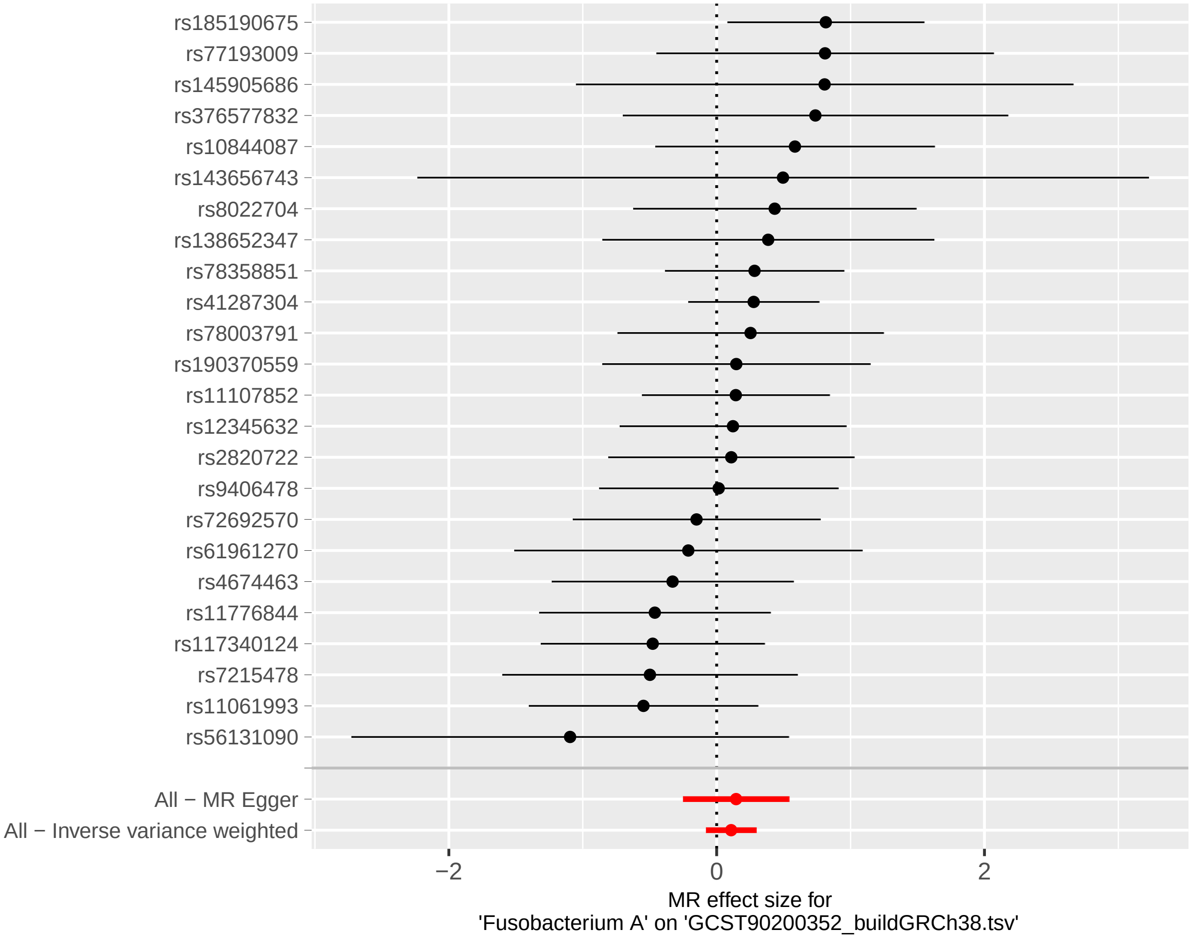

rs185190675
rs77193009
rs145905686
rs376577832
rs10844087
rs143656743
rs8022704
rs138652347
rs78358851
rs41287304
rs78003791
rs190370559
rs11107852
rs12345632
rs2820722
rs9406478
rs72692570
rs61961270
rs4674463
rs11776844
rs117340124
rs7215478
rs11061993
rs56131090
All − MR Egger
All − Inverse variance weighted
−2
0
2
MR effect size for
'Fusobacterium A' on 'GCST90200352_buildGRCh38.tsv'

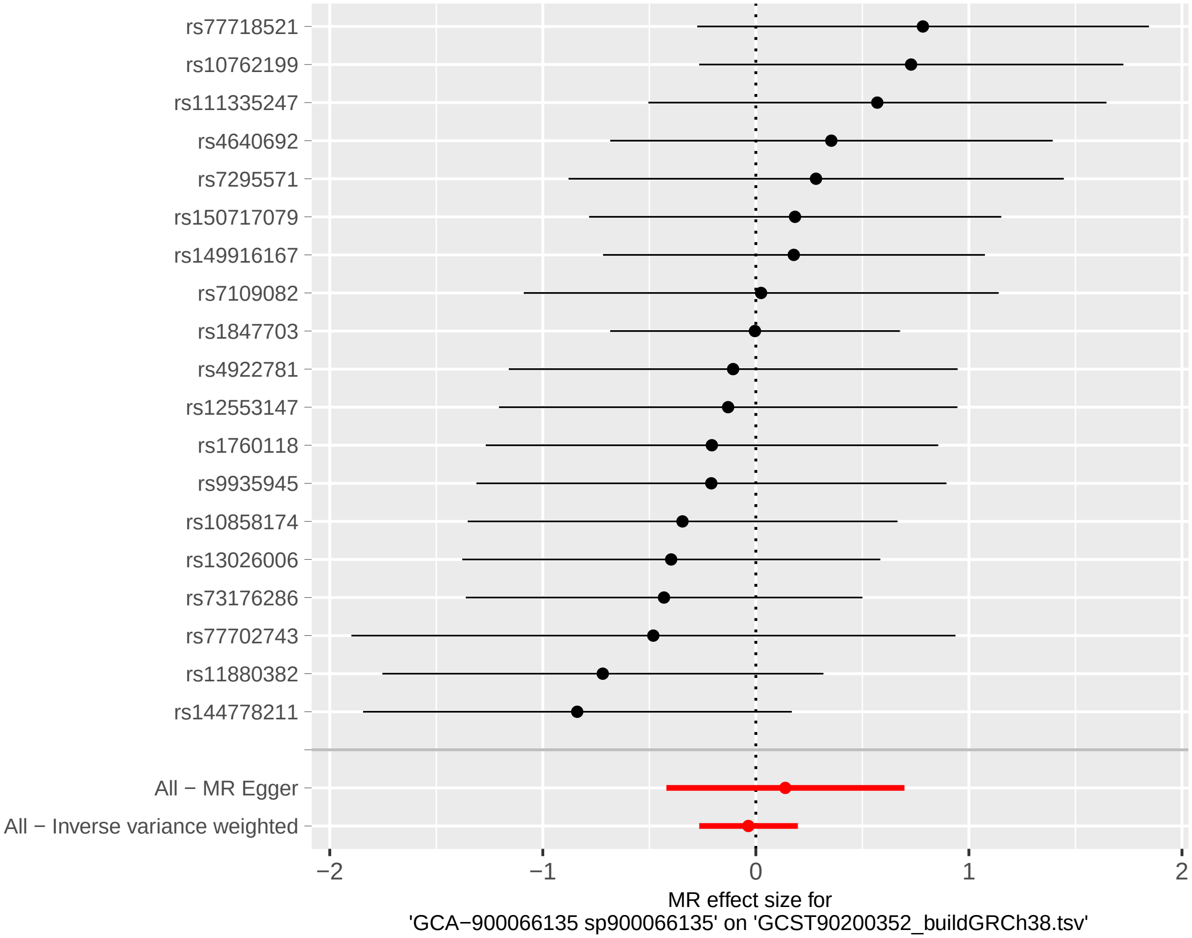
rs77718521
rs10762199
rs111335247
rs4640692
rs7295571
rs150717079
rs149916167
rs7109082
rs1847703
rs4922781
rs12553147
rs1760118
rs9935945
rs10858174
rs13026006
rs73176286
rs77702743
rs11880382
rs144778211
All − MR Egger
All − Inverse variance weighted
−2
−1
0
1
2
MR effect size for
'GCA−900066135 sp900066135' on 'GCST90200352_buildGRCh38.tsv'

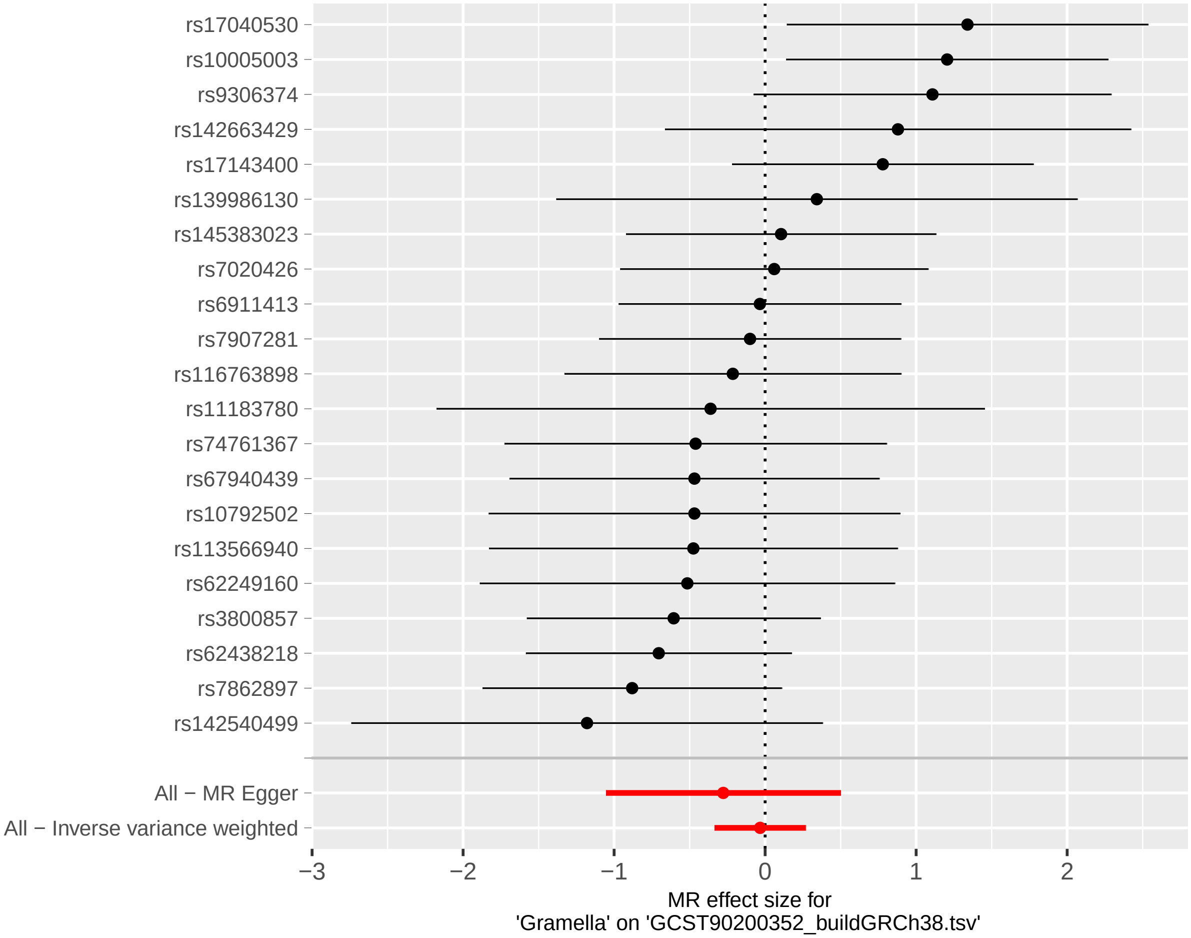

rs17040530
rs10005003
rs9306374
rs142663429
rs17143400
rs139986130
rs145383023
rs7020426
rs6911413
rs7907281
rs116763898
rs11183780
rs74761367
rs67940439
rs10792502
rs113566940
rs62249160
rs3800857
rs62438218
rs7862897
rs142540499
All − MR Egger
All − Inverse variance weighted
−3
−2
−1
0
1
2
MR effect size for
'Gramella' on 'GCST90200352_buildGRCh38.tsv'

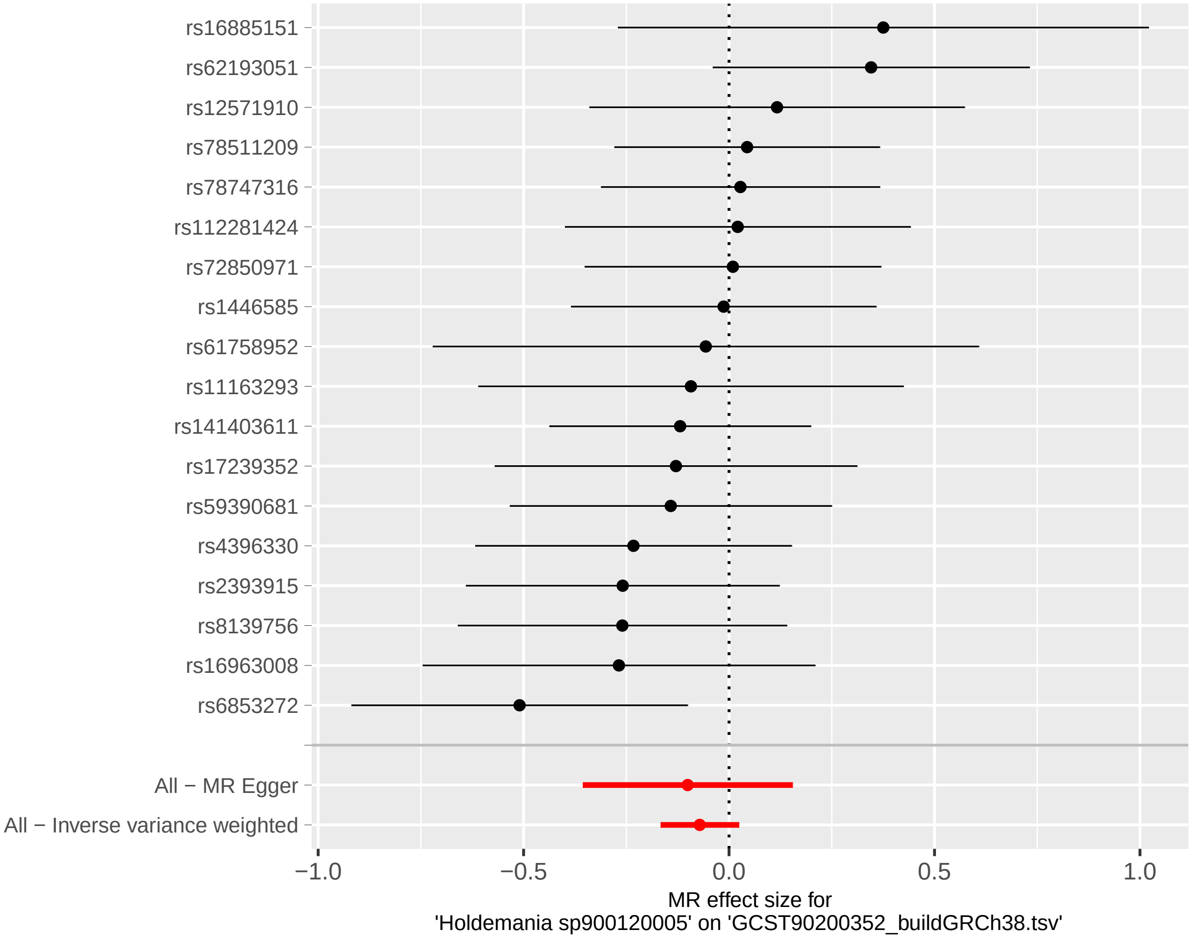
rs16885151
rs62193051
rs12571910
rs78511209
rs78747316
rs112281424
rs72850971
rs1446585
rs61758952
rs11163293
rs141403611
rs17239352
rs59390681
rs4396330
rs2393915
rs8139756
rs16963008
rs6853272
All − MR Egger
All − Inverse variance weighted
−1.0
−0.5
0.0
0.5
1.0
MR effect size for
'Holdemania sp900120005' on 'GCST90200352_buildGRCh38.tsv'

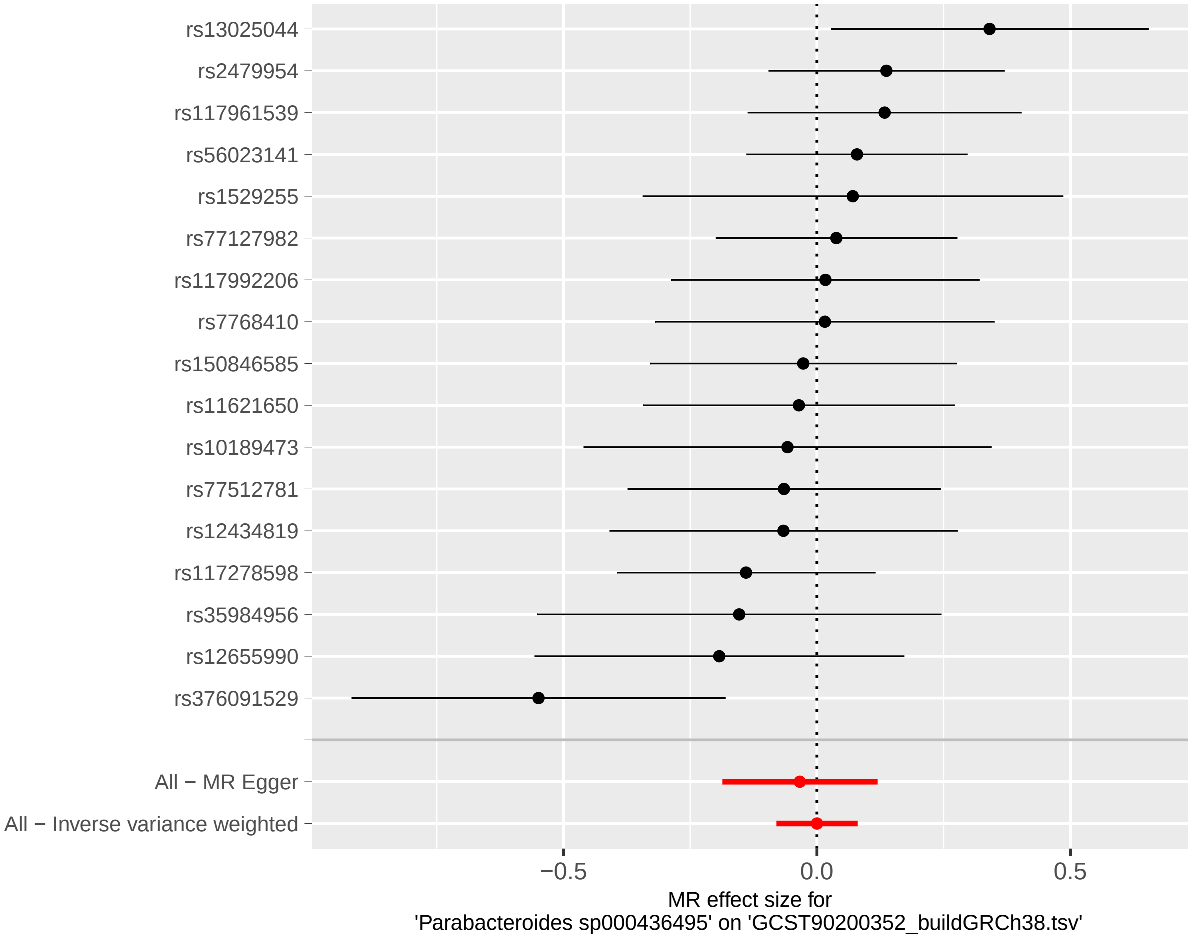

rs13025044
rs2479954
rs117961539
rs56023141
rs1529255
rs77127982
rs117992206
rs7768410
rs150846585
rs11621650
rs10189473
rs77512781
rs12434819
rs117278598
rs35984956
rs12655990
rs376091529
All − MR Egger
All − Inverse variance weighted
−0.5
0.0
0.5
MR effect size for
'Parabacteroides sp000436495' on 'GCST90200352_buildGRCh38.tsv'

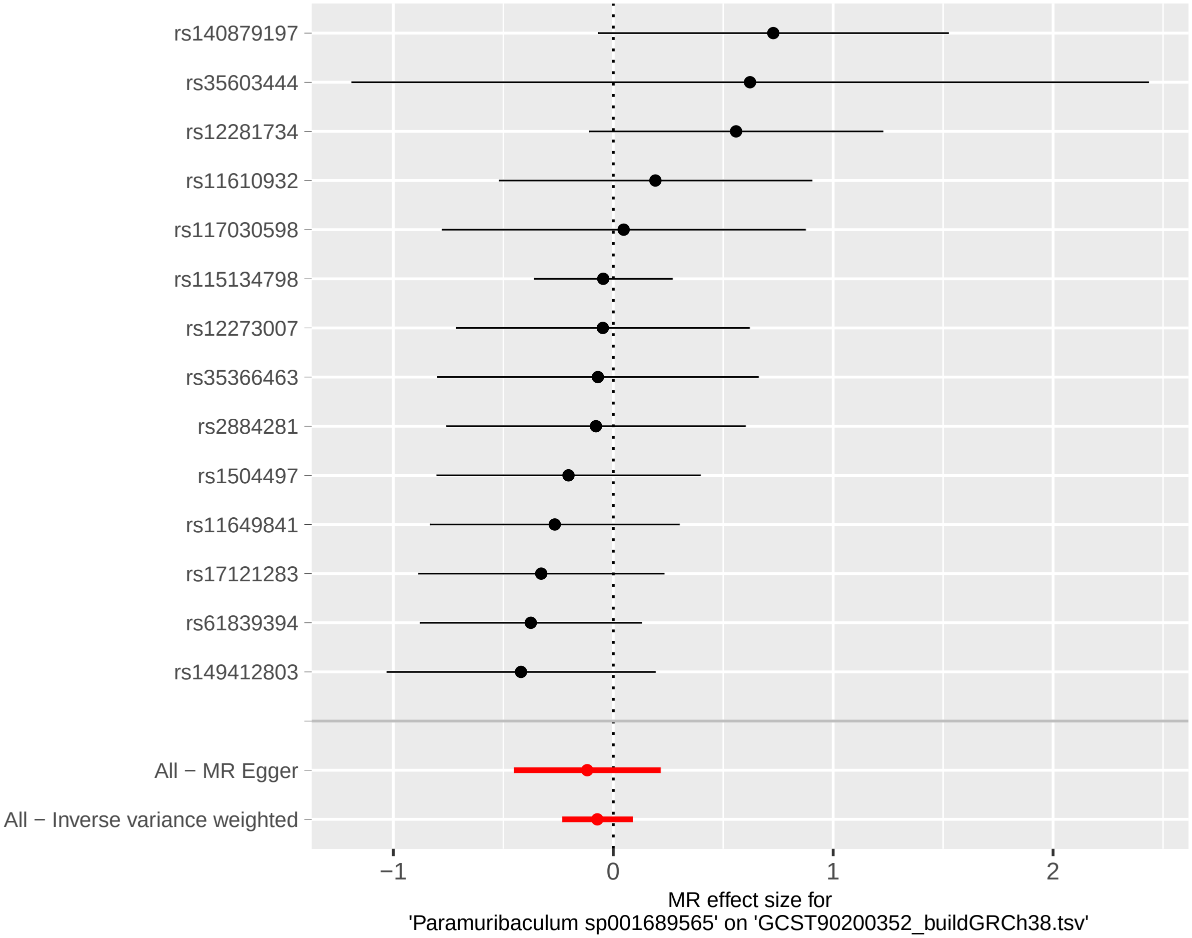

rs140879197
rs35603444
rs12281734
rs11610932
rs117030598
rs115134798
rs12273007
rs35366463
rs2884281
rs1504497
rs11649841
rs17121283
rs61839394
rs149412803
All − MR Egger
All − Inverse variance weighted
−1
0
1
2
MR effect size for
'Paramuribaculum sp001689565' on 'GCST90200352_buildGRCh38.tsv'

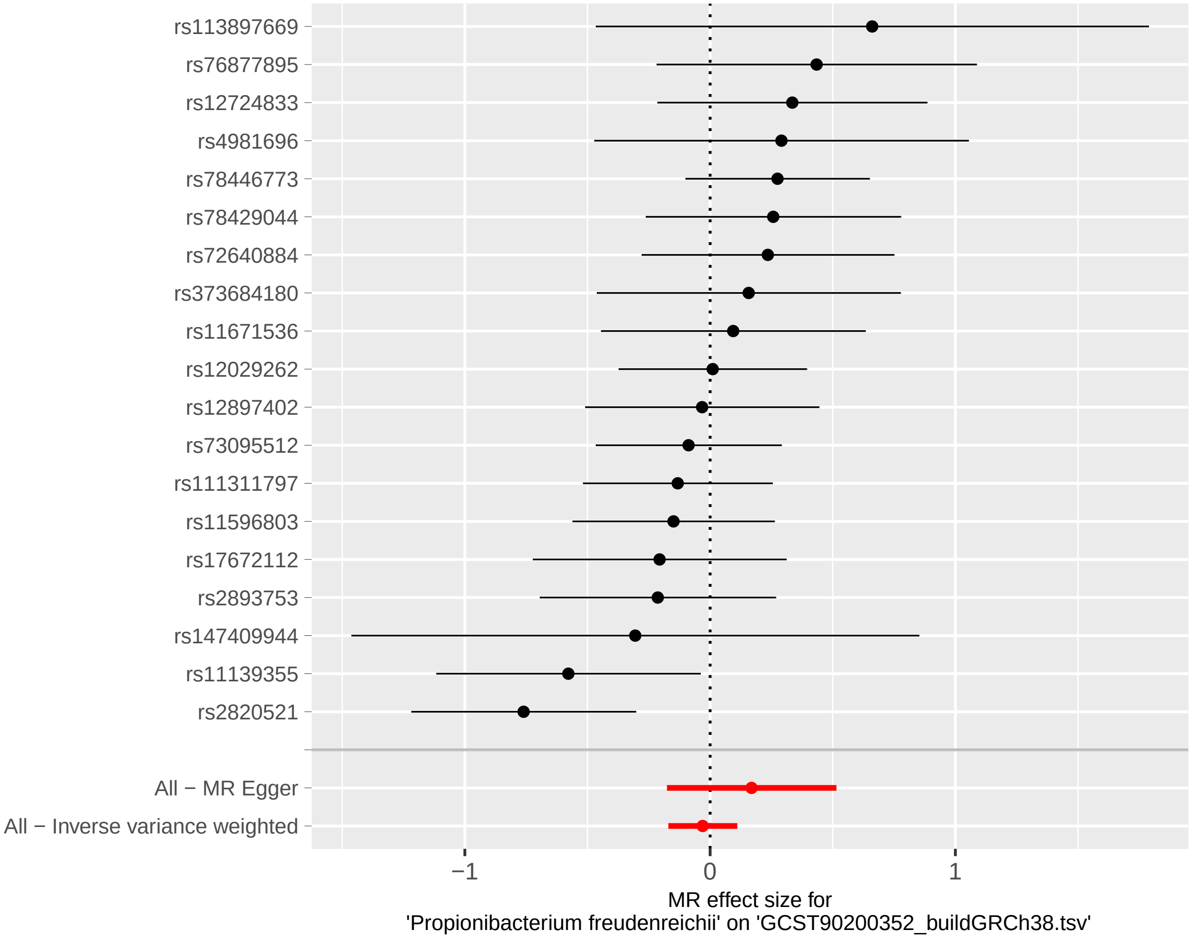

rs113897669
rs76877895
rs12724833
rs4981696
rs78446773
rs78429044
rs72640884
rs373684180
rs11671536
rs12029262
rs12897402
rs73095512
rs111311797
rs11596803
rs17672112
rs2893753
rs147409944
rs11139355
rs2820521
All − MR Egger
All − Inverse variance weighted
−1
0
1
MR effect size for
'Propionibacterium freudenreichii' on 'GCST90200352_buildGRCh38.tsv'

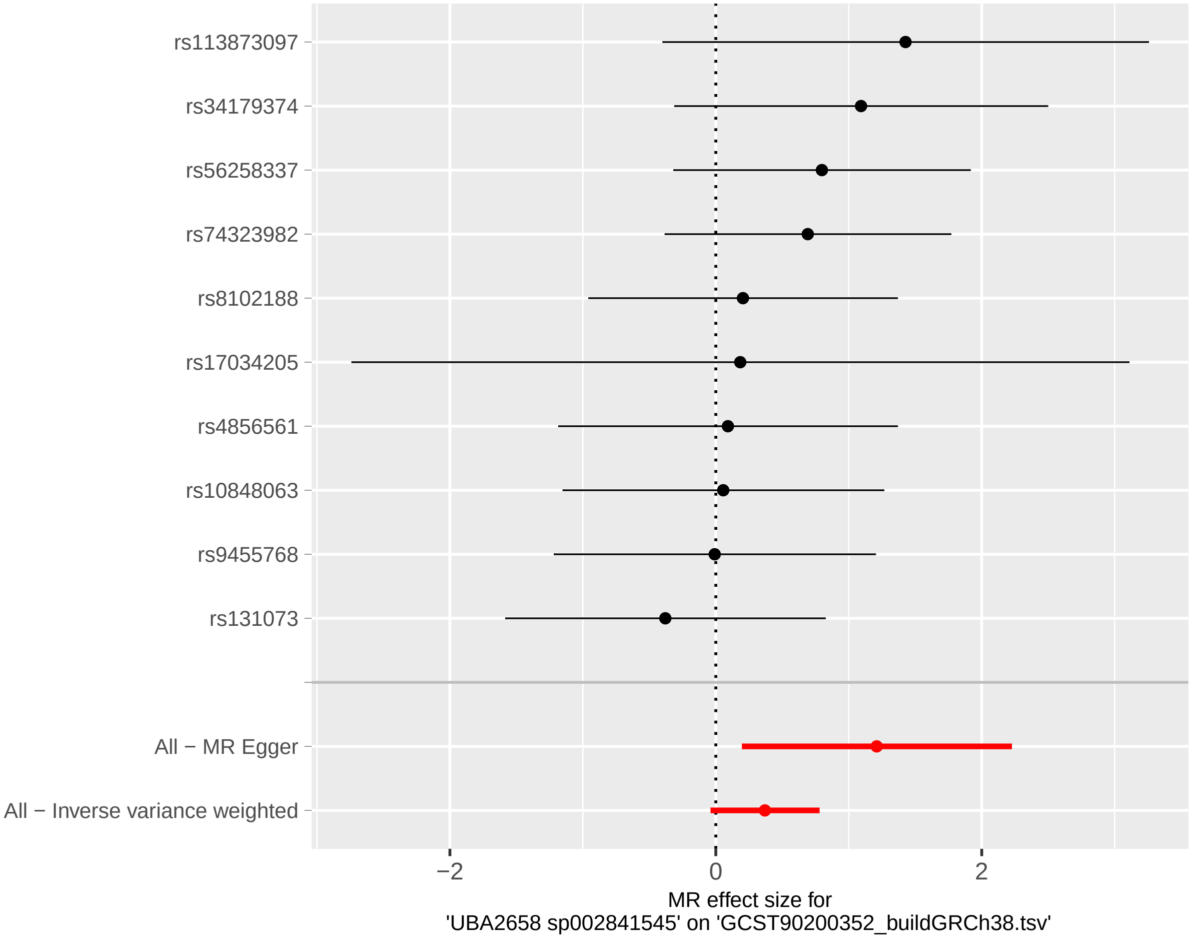
rs113873097
rs34179374
rs56258337
rs74323982
rs8102188
rs17034205
rs4856561
rs10848063
rs9455768
rs131073
All − MR Egger
All − Inverse variance weighted
−2
0
2
MR effect size for
'UBA2658 sp002841545' on 'GCST90200352_buildGRCh38.tsv'

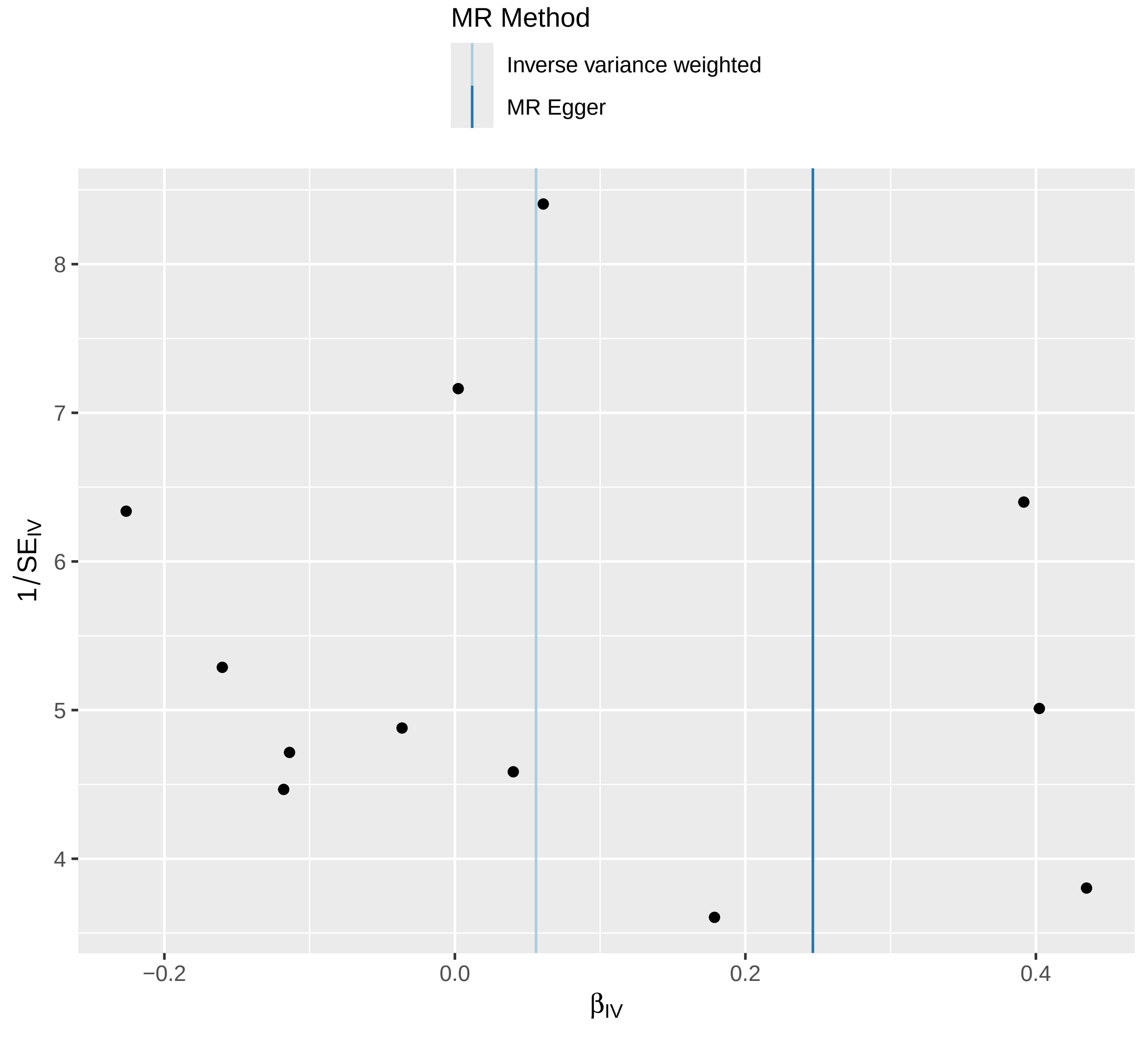
MR Method
Inverse variance weighted
MR Egger
8
7
6
5
4
1/SE_IV
−0.2
0.0
0.2
0.4
β_IV

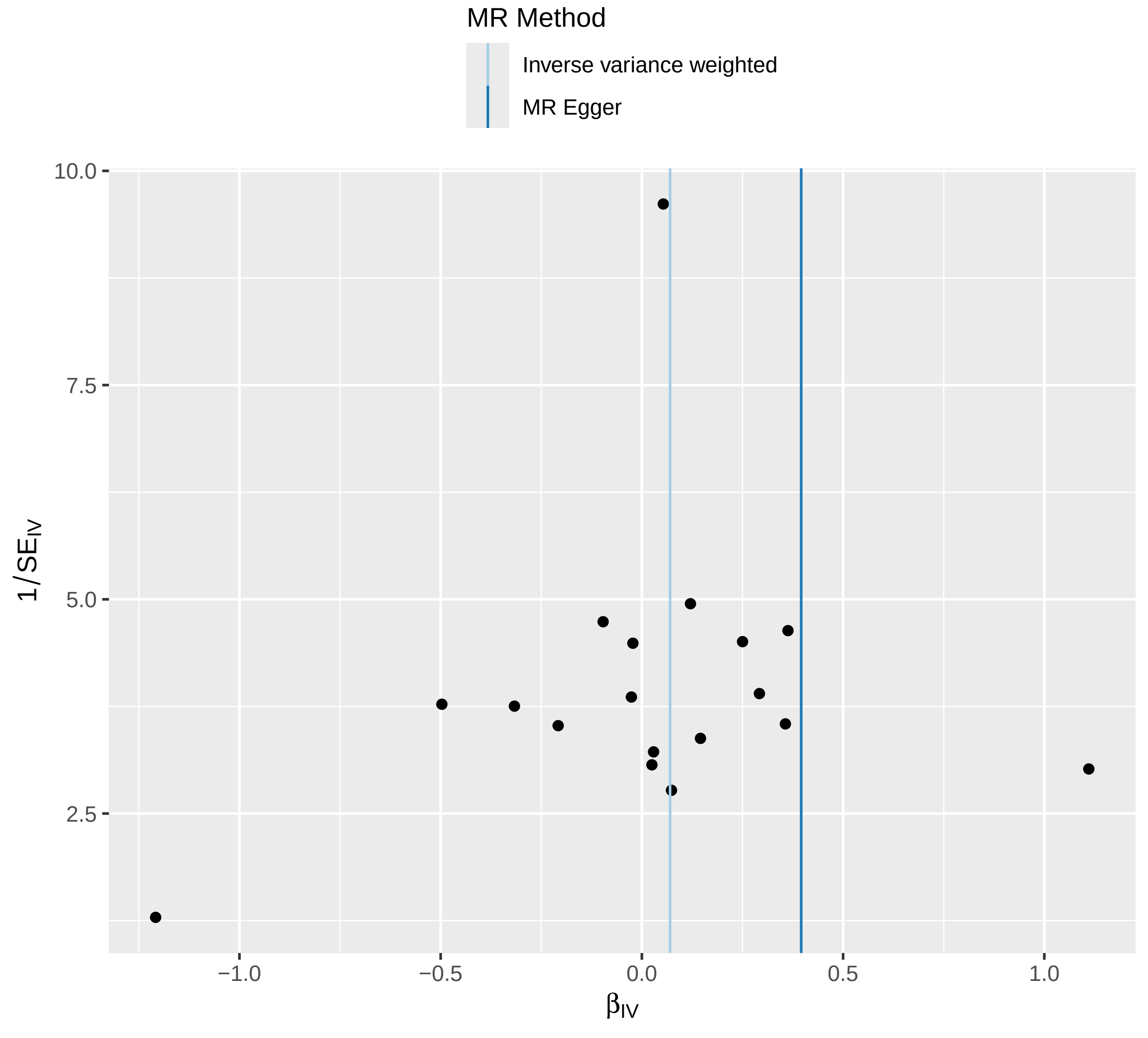

MR Method
Inverse variance weighted
MR Egger
10.0
7.5
5.0
2.5
1/SE_IV
−1.0
−0.5
0.0
0.5
1.0
β_IV

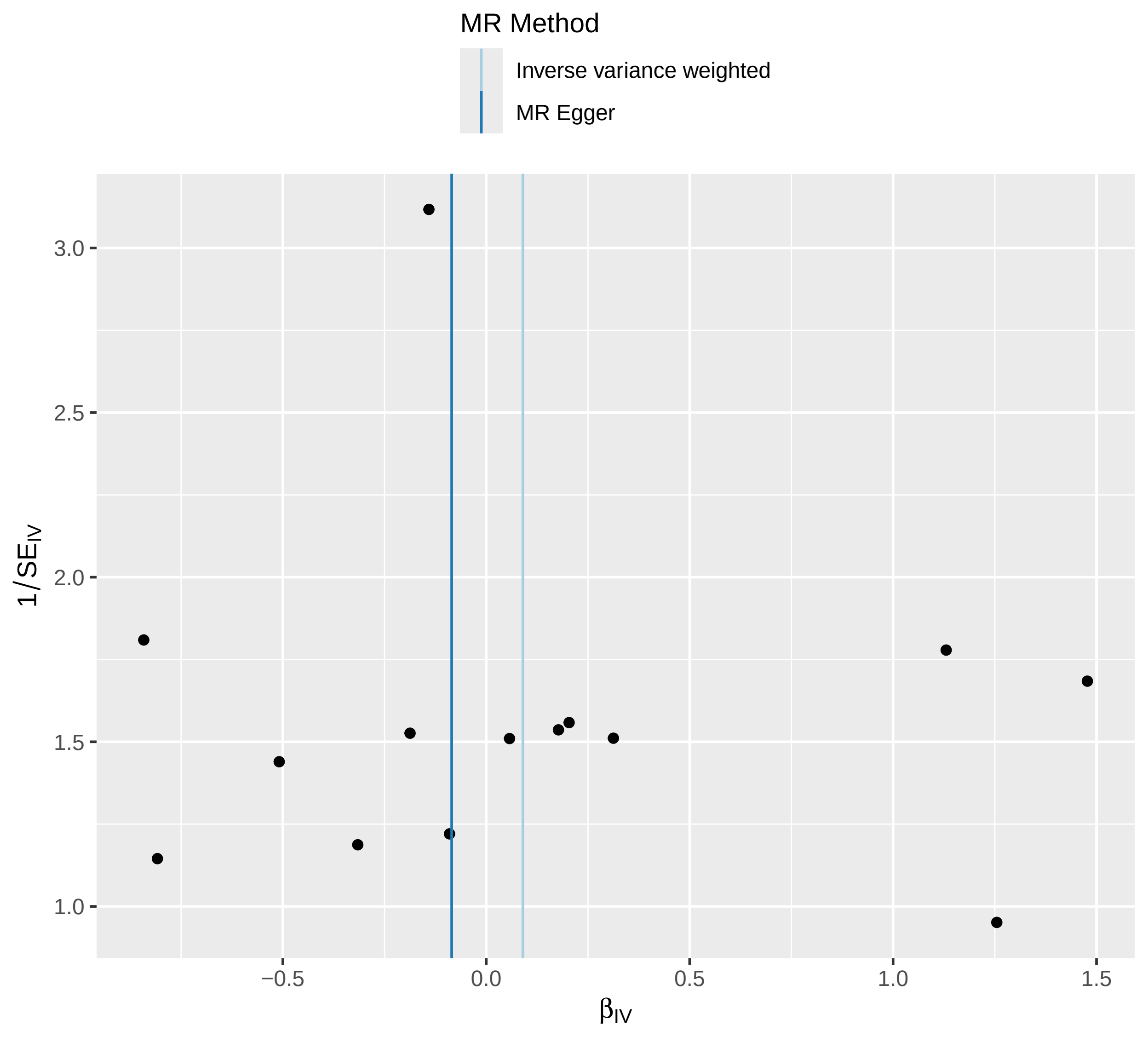
MR Method
Inverse variance weighted
MR Egger
1/SE_IV
β_IV
3.0
2.5
2.0
1.5
1.0
−0.5
0.0
0.5
1.0
1.5

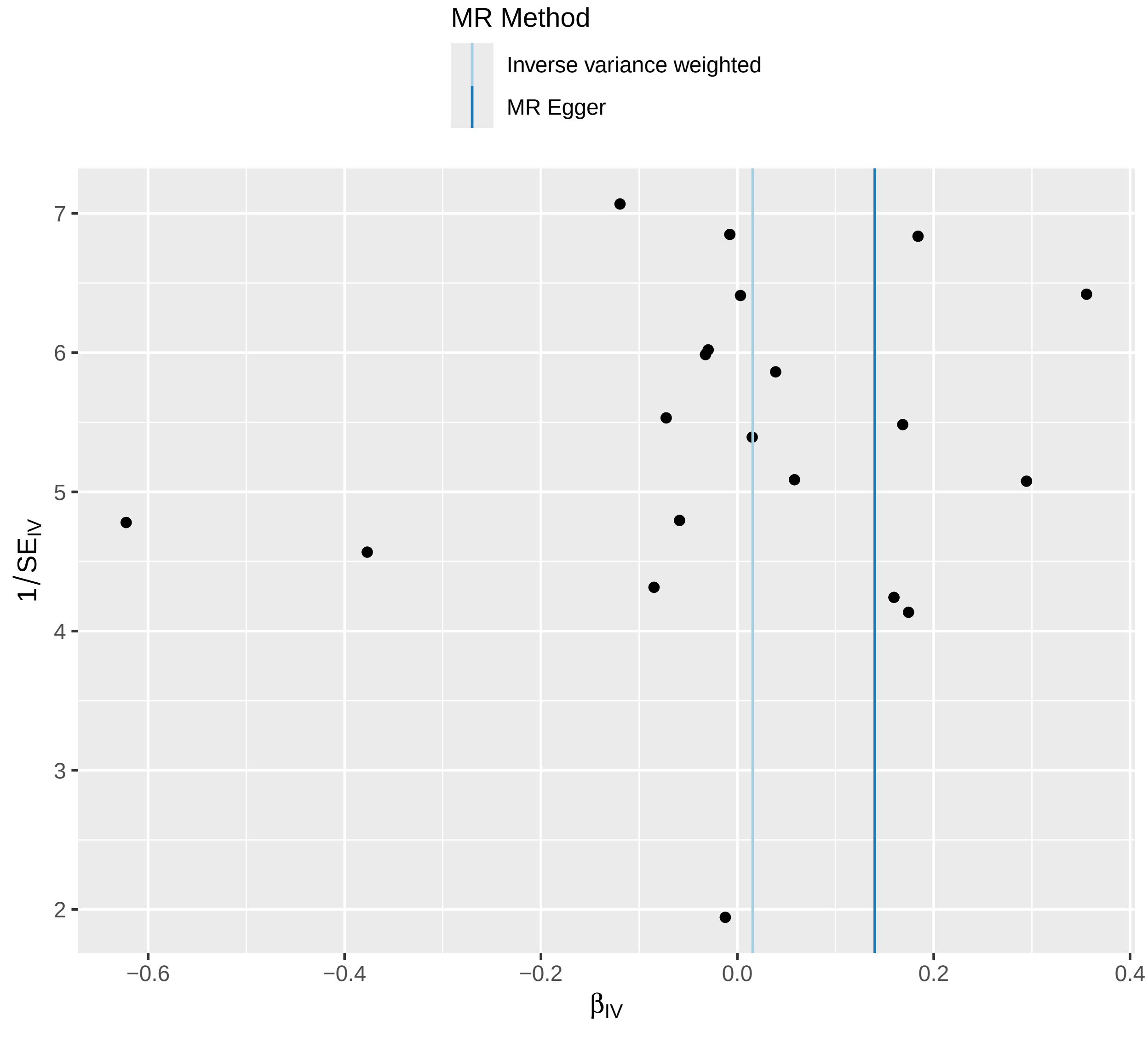

MR Method
Inverse variance weighted
MR Egger
1/SE$_{IV}$
7
6
5
4
3
2
−0.6
−0.4
−0.2
0.0
0.2
0.4
β$_{IV}$

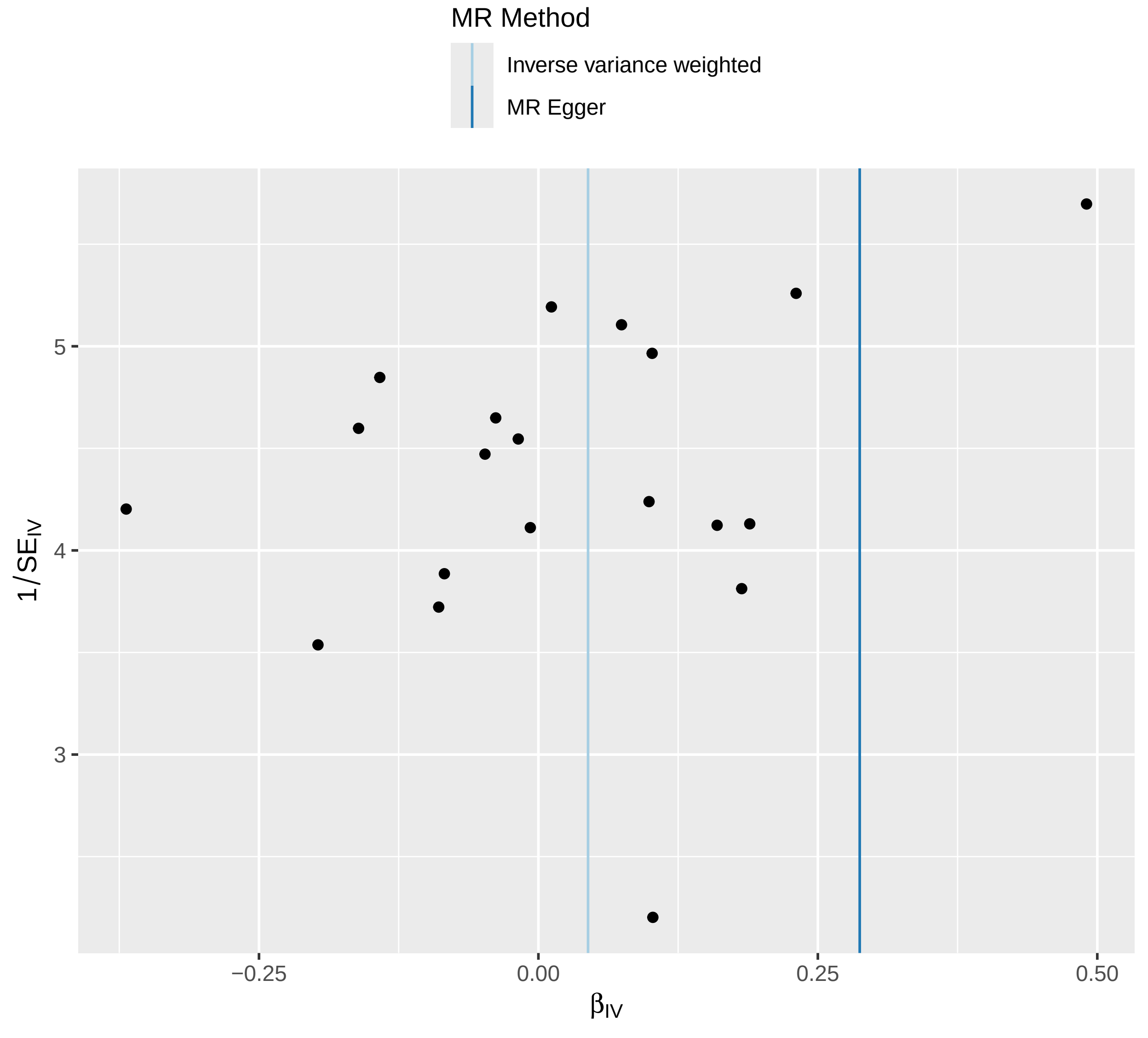
MR Method
Inverse variance weighted
MR Egger
5
4
3
1/SE_IV
−0.25
0.00
0.25
0.50
β_IV

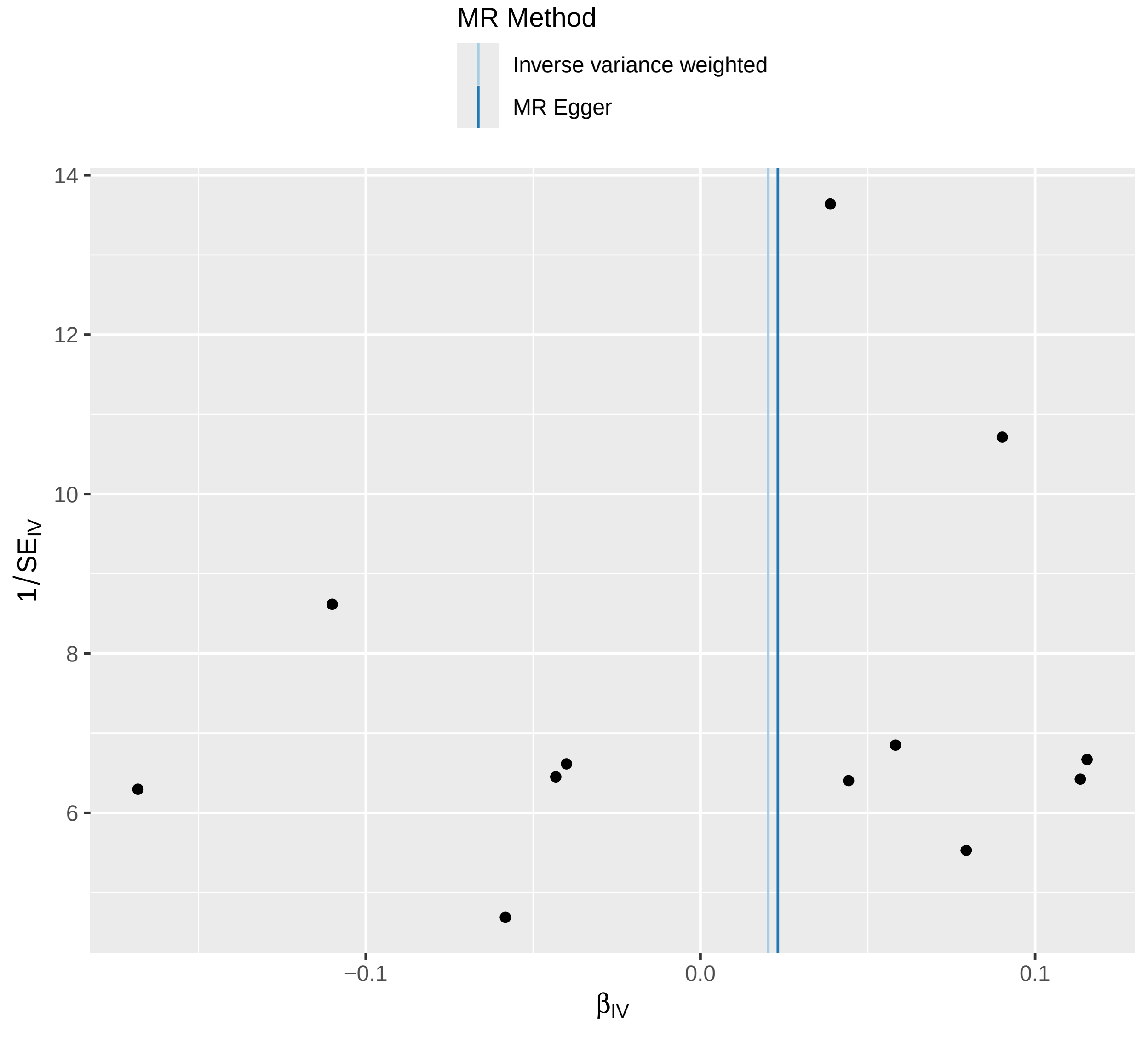

MR Method
Inverse variance weighted
MR Egger
14
12
10
8
6
1/SE_IV
−0.1
0.0
0.1
β_IV

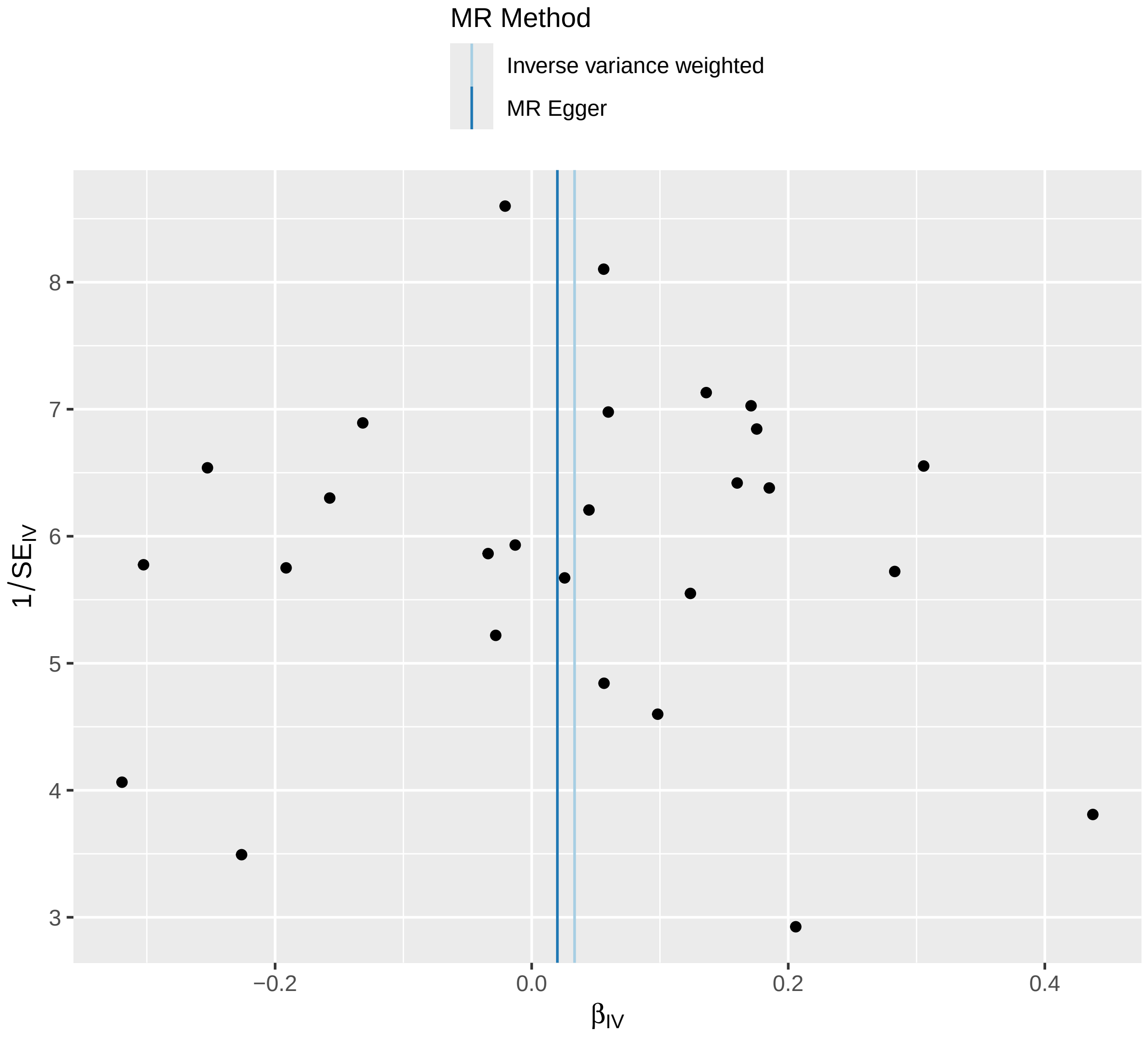

MR Method
Inverse variance weighted
MR Egger
1/SE_IV
8
7
6
5
4
3
−0.2
0.0
0.2
0.4
β_IV

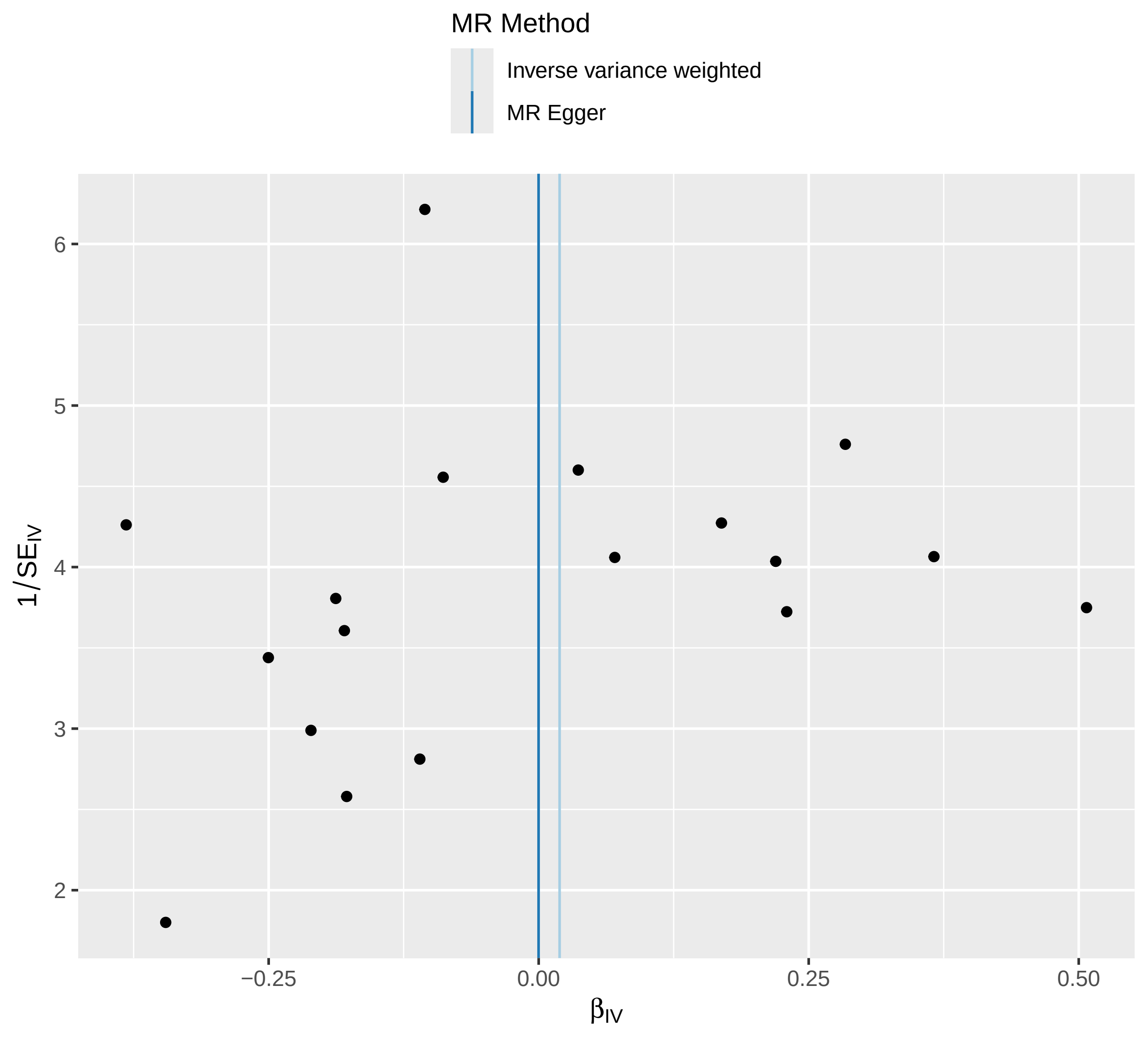

MR Method
Inverse variance weighted
MR Egger
6
5
4
3
2
1/SE_IV
−0.25
0.00
0.25
0.50
β_IV

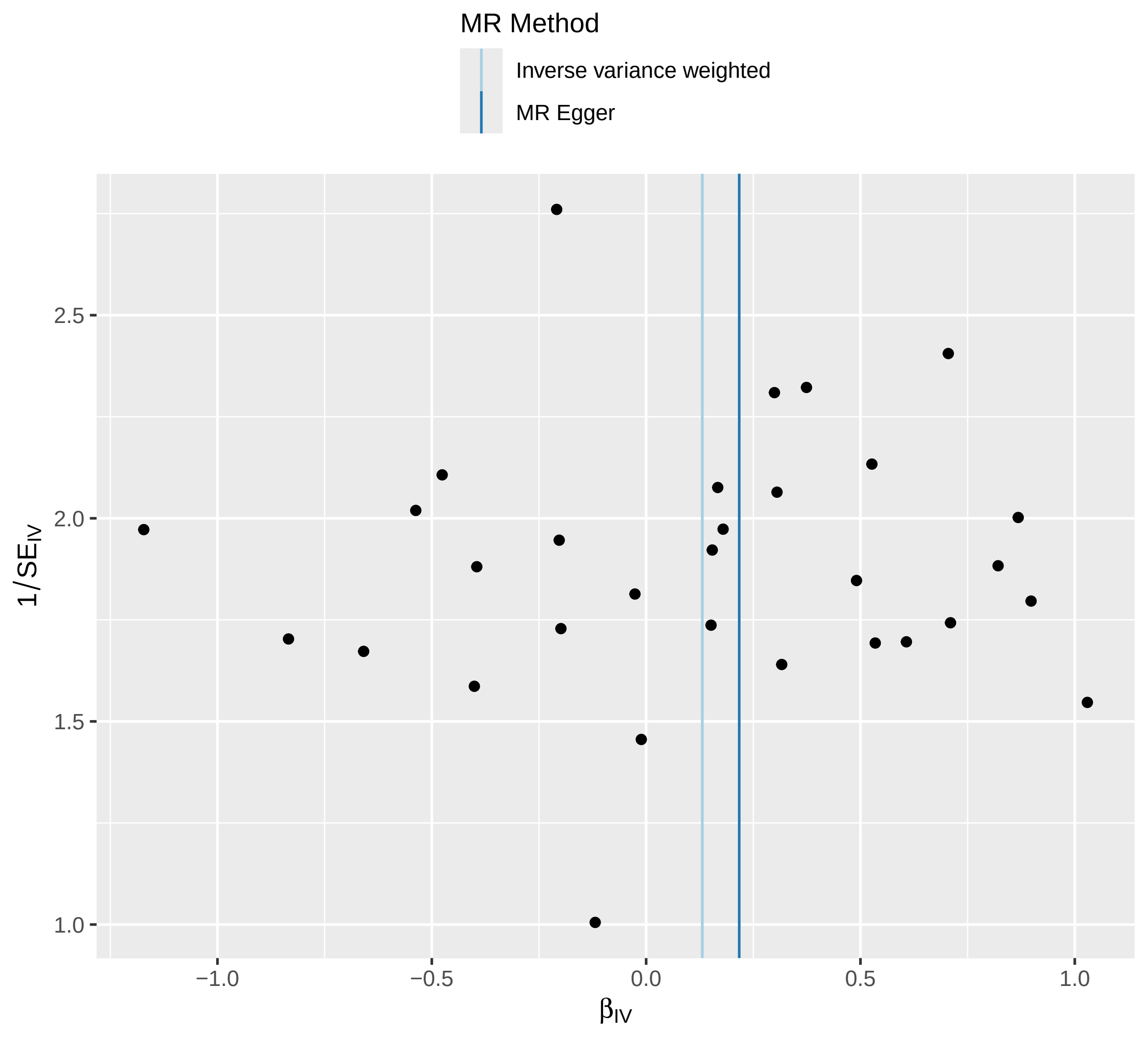

MR Method
Inverse variance weighted
MR Egger
2.5
2.0
1.5
1.0
$1/SE_{IV}$
−1.0
−0.5
0.0
0.5
1.0
β_{IV}

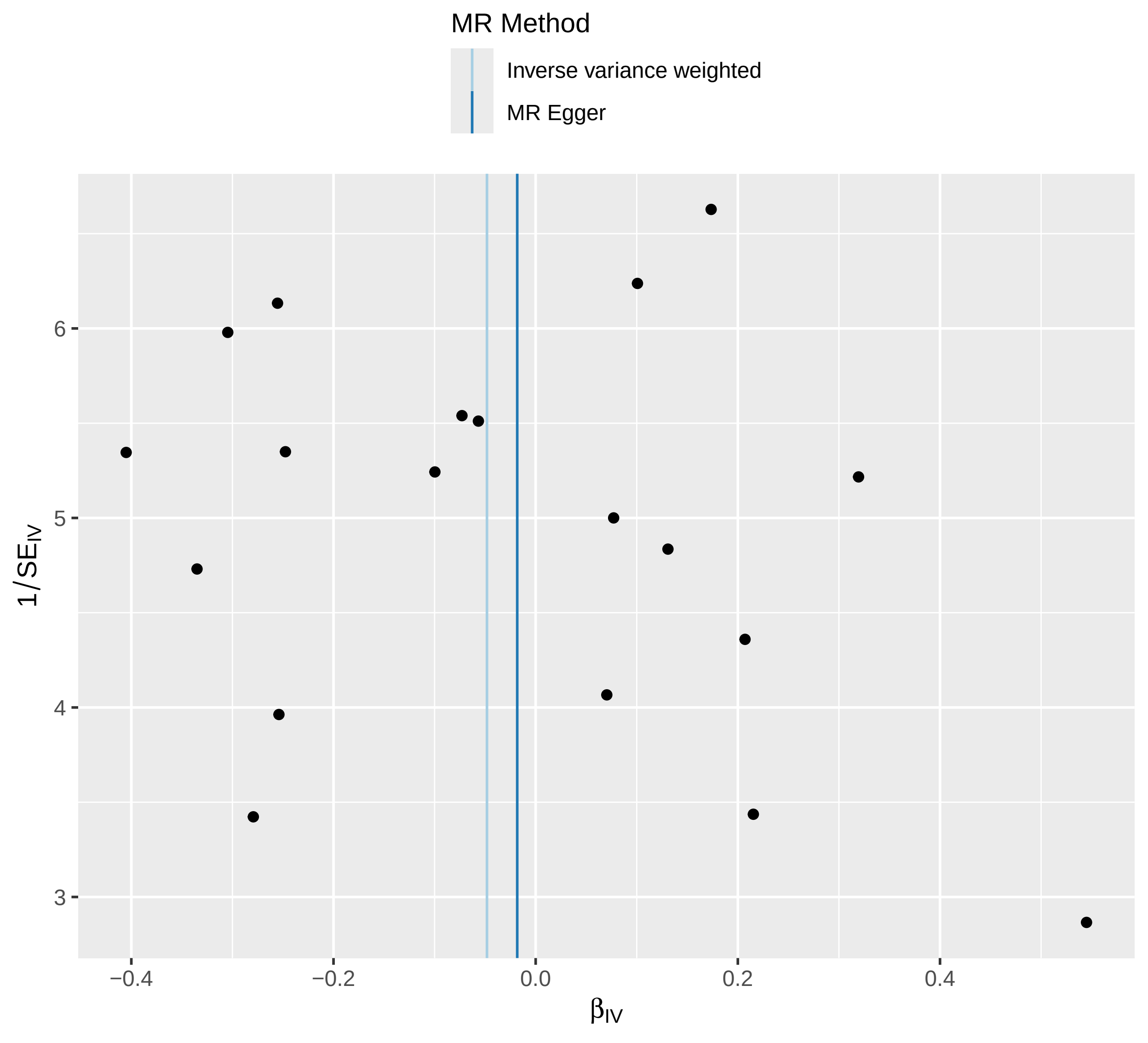

MR Method
Inverse variance weighted
MR Egger
6
5
4
3
1/SE_IV
−0.4
−0.2
0.0
0.2
0.4
β_IV

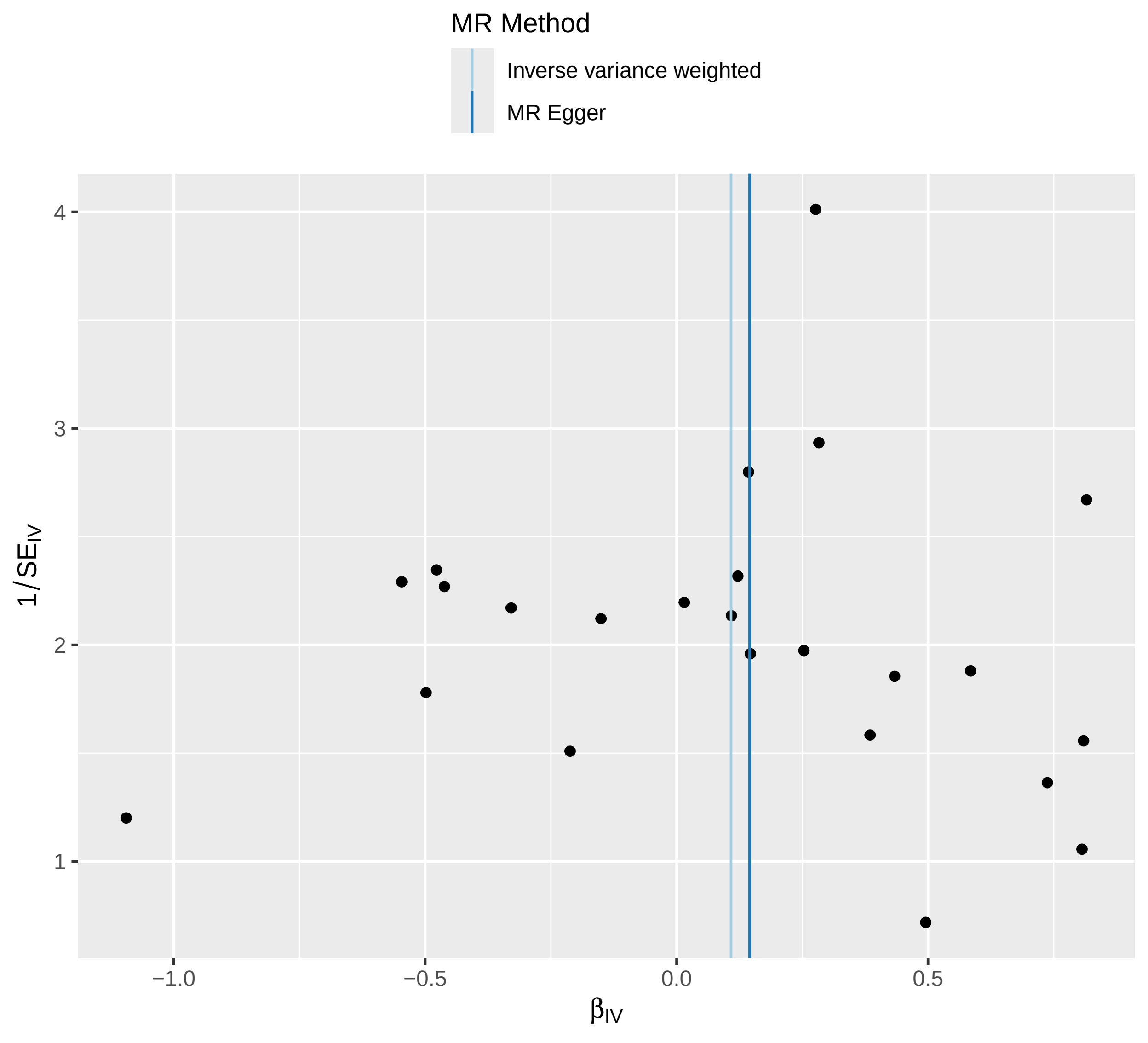

MR Method
Inverse variance weighted
MR Egger
4
3
2
1
1/SE_IV
−1.0
−0.5
0.0
0.5
β_IV

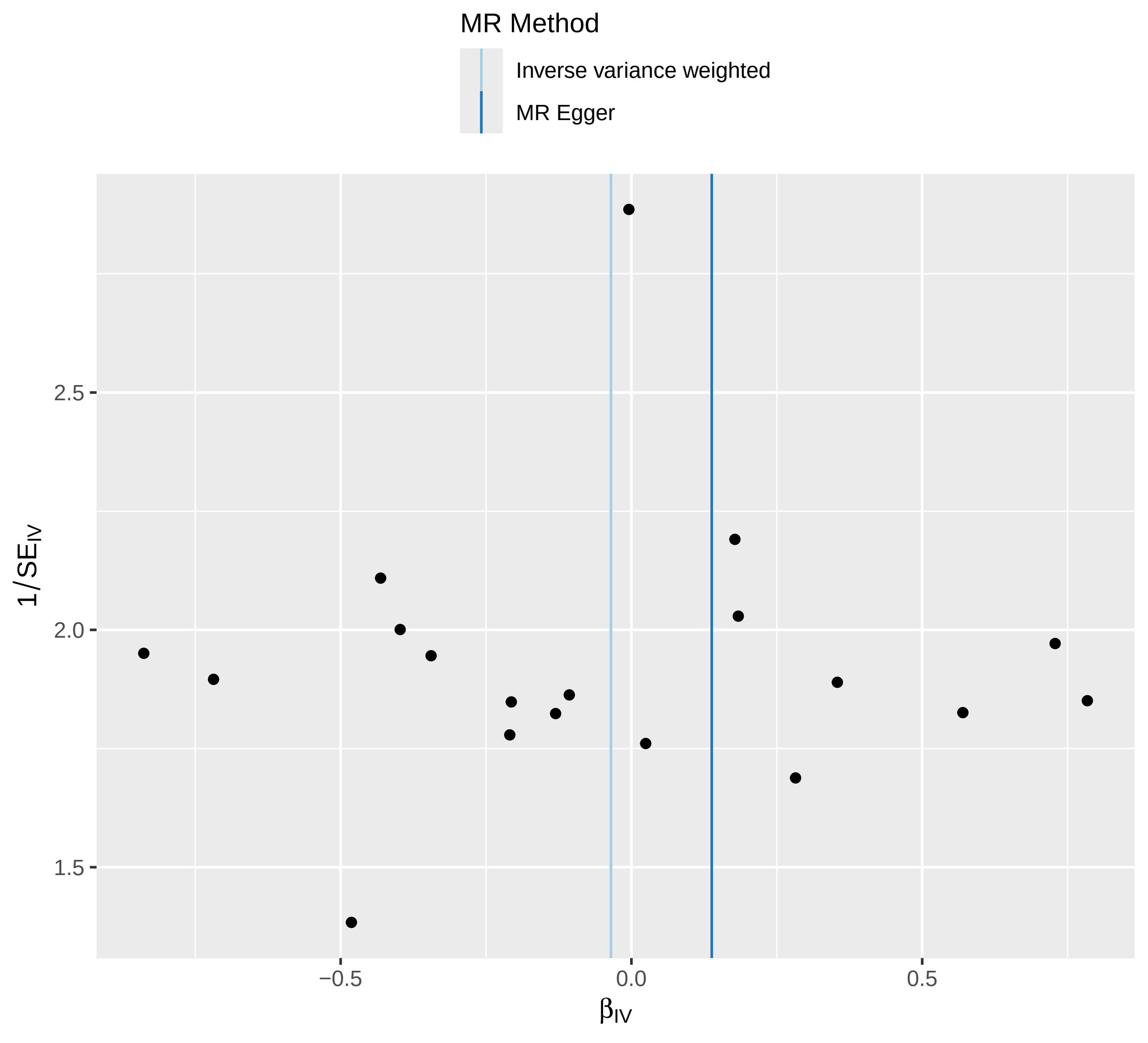
MR Method
Inverse variance weighted
MR Egger
2.5
2.0
1.5
1/SE$_{IV}$
−0.5
0.0
0.5
β$_{IV}$

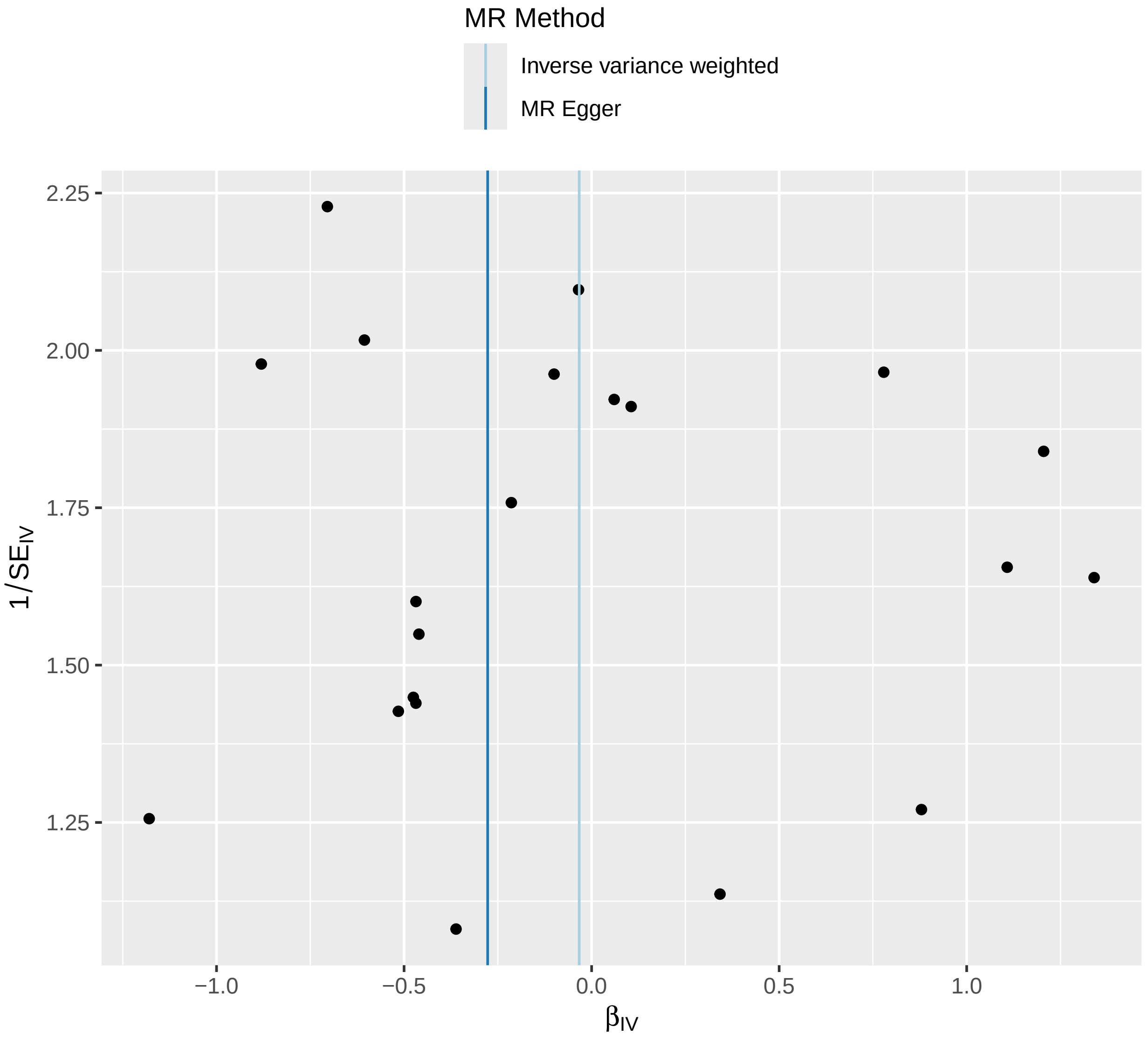

MR Method
Inverse variance weighted
MR Egger
2.25
2.00
1.75
1.50
1.25
1/SE_IV
−1.0
−0.5
0.0
0.5
1.0
β_IV

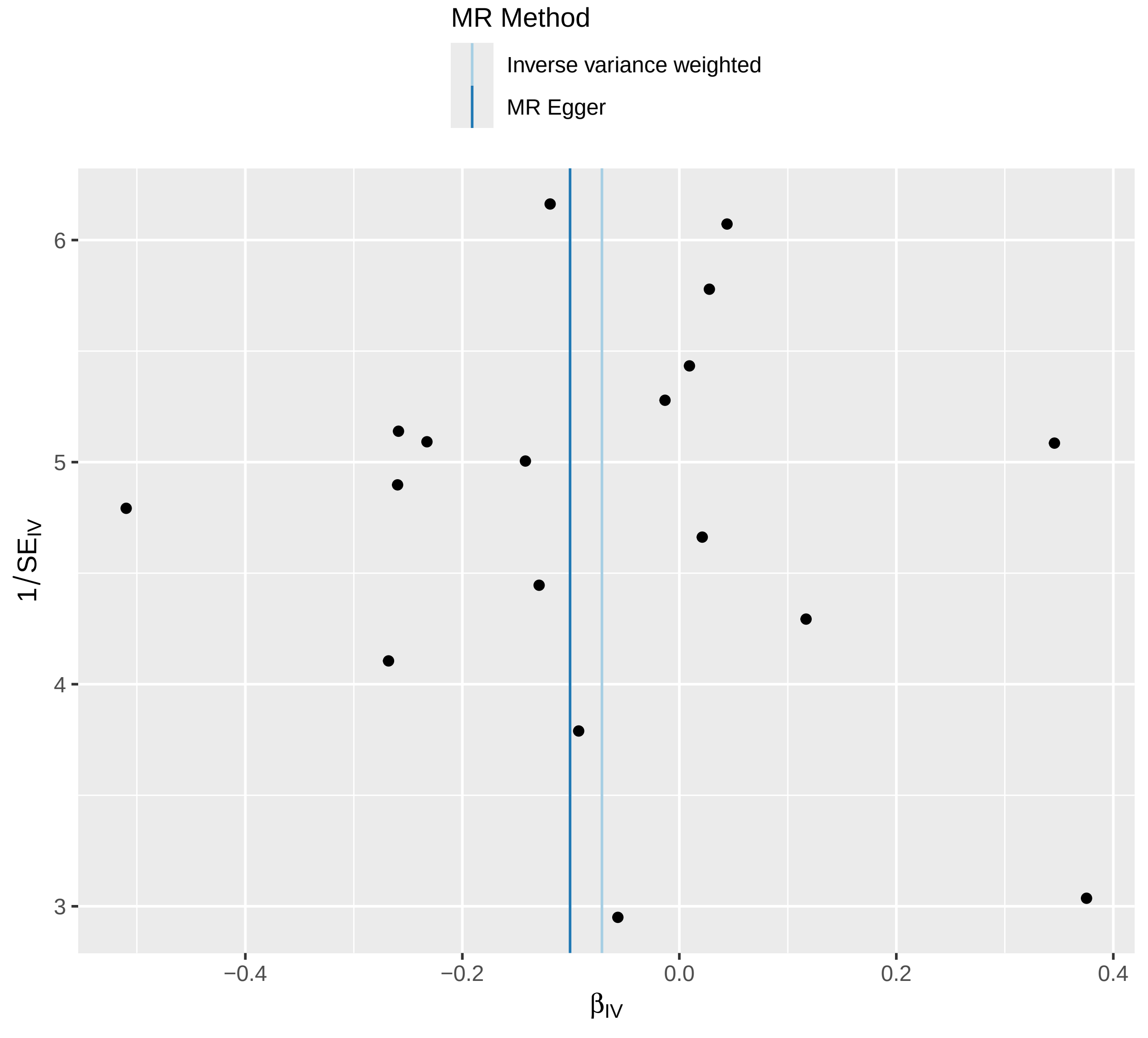

MR Method
Inverse variance weighted
MR Egger
6
5
4
3
−0.4
−0.2
0.0
0.2
0.4
$1/SE_{IV}$
β_{IV}

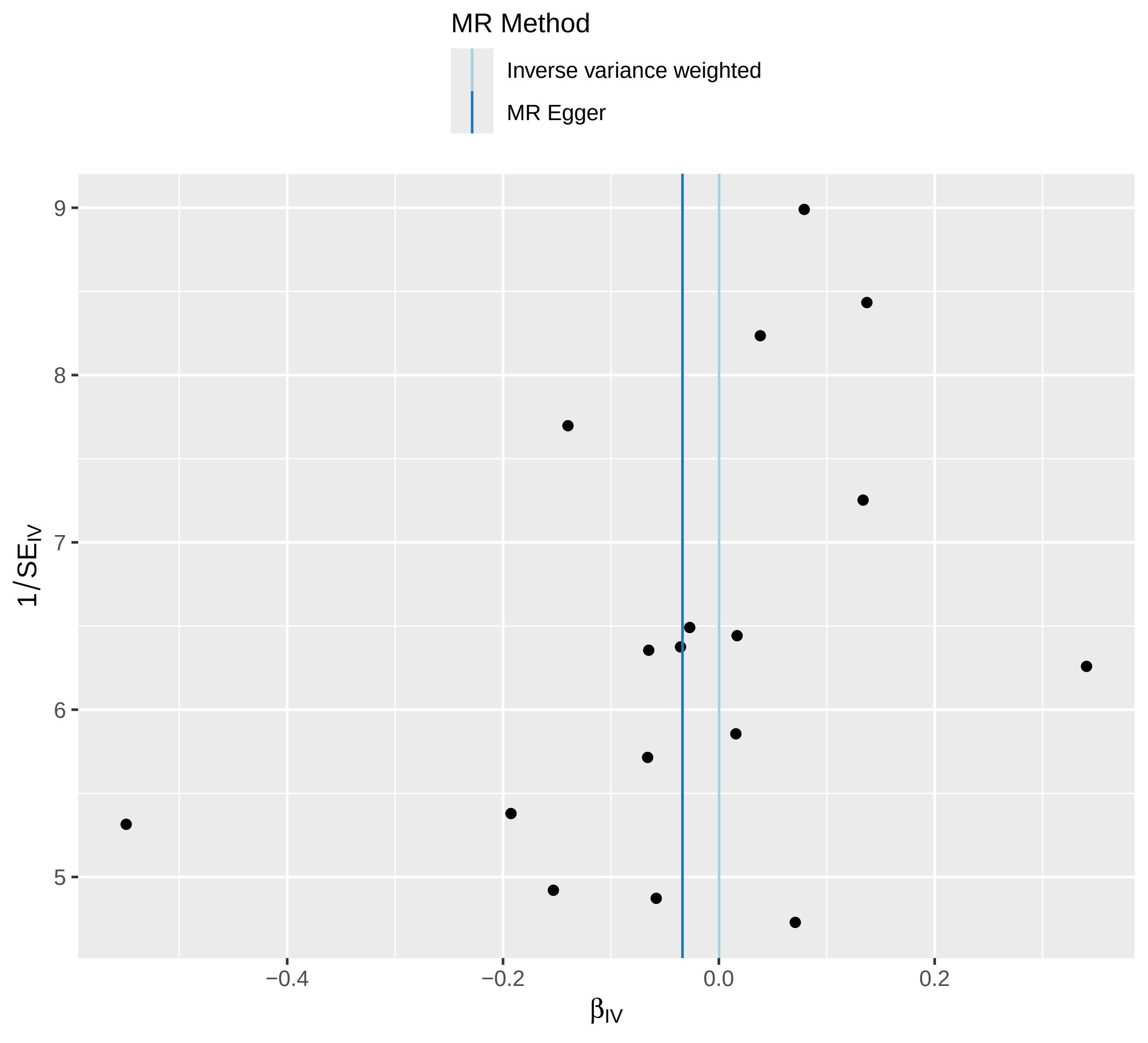
MR Method
Inverse variance weighted
MR Egger
9
8
7
6
5
1/SE_IV
−0.4
−0.2
0.0
0.2
β_IV

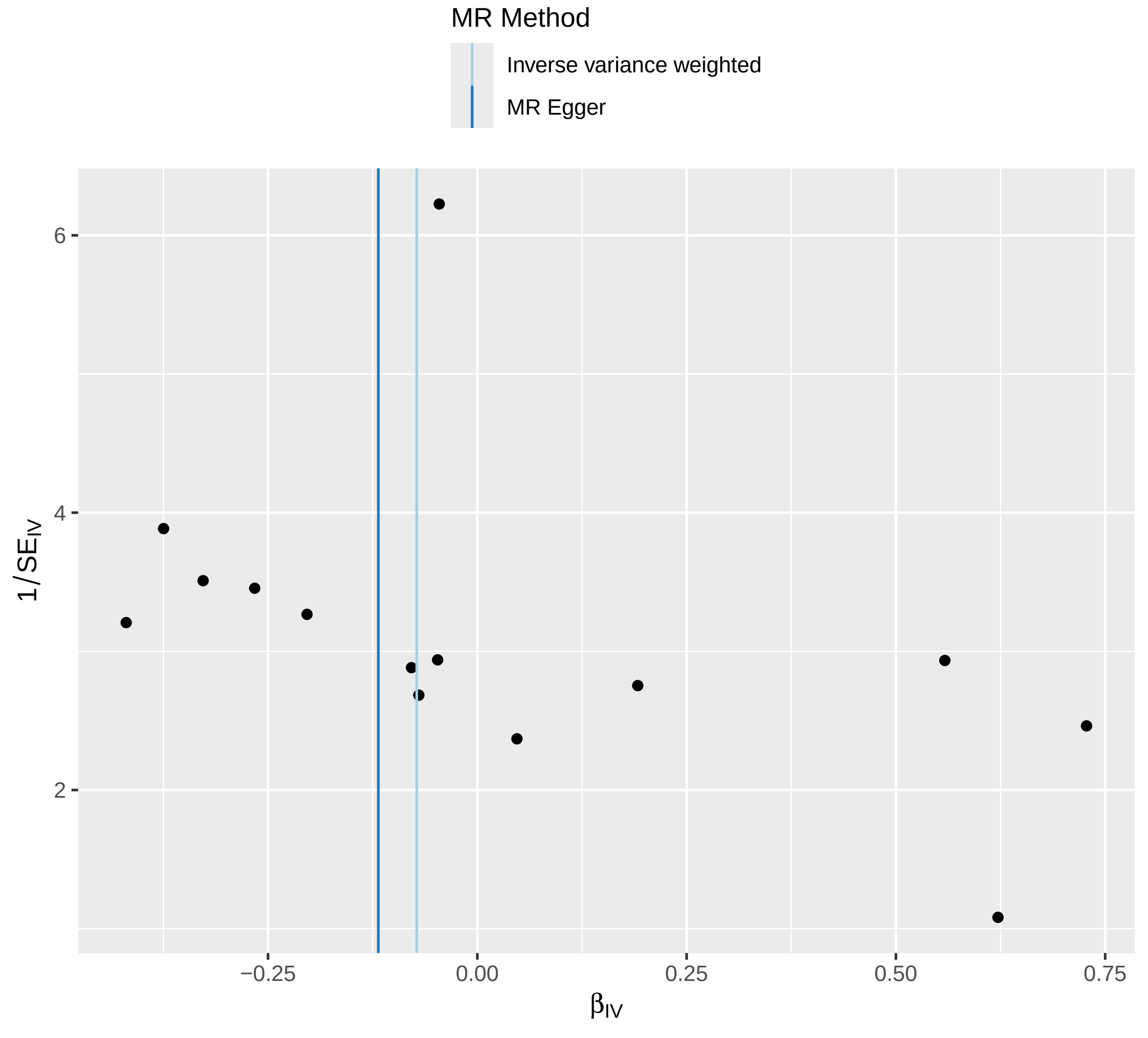
MR Method
Inverse variance weighted
MR Egger
6
4
2
$1/SE_{IV}$
−0.25
0.00
0.25
0.50
0.75
β_{IV}

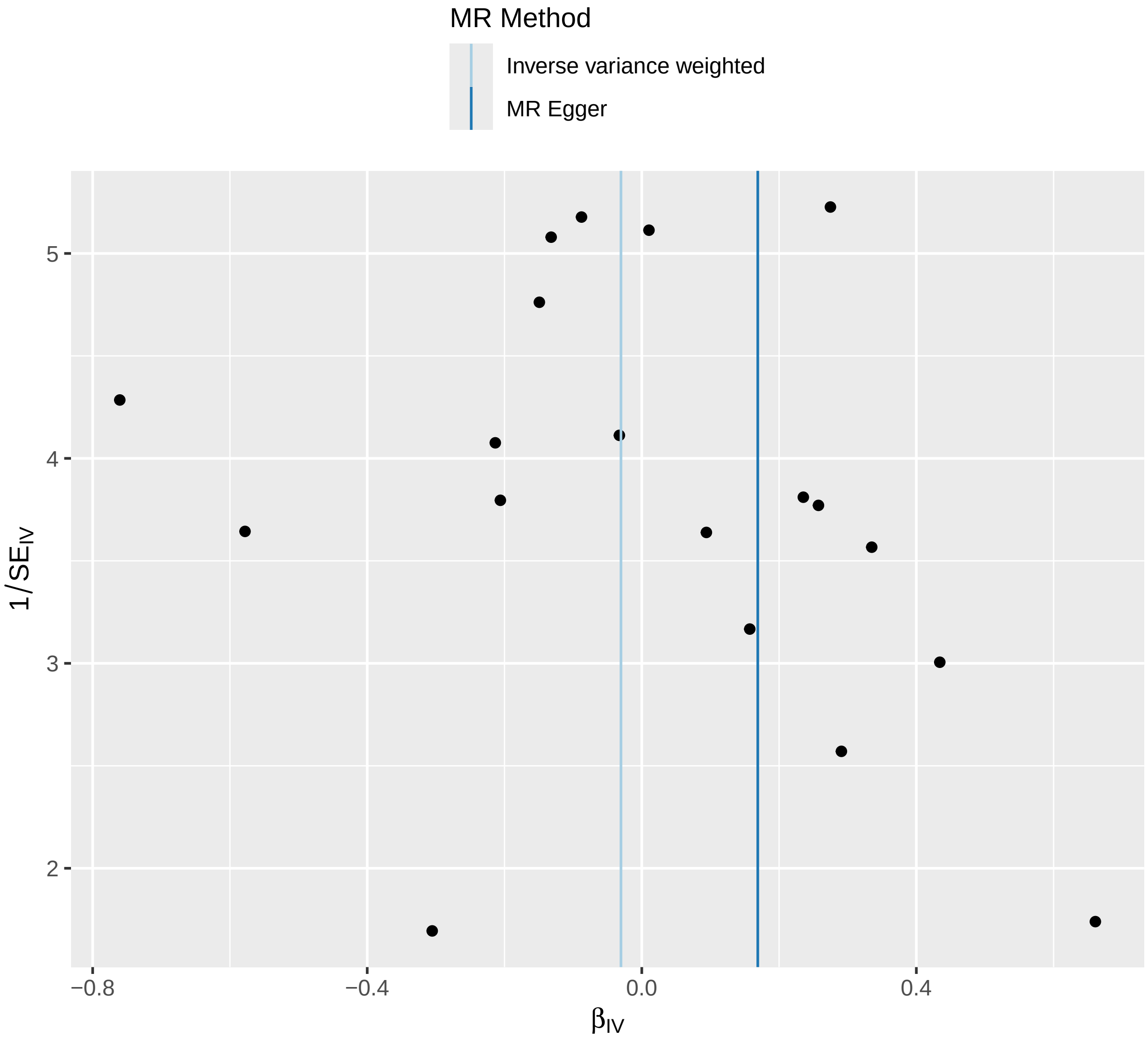
MR Method
Inverse variance weighted
MR Egger
1/SE_IV
β_IV
5
4
3
2
−0.8
−0.4
0.0
0.4

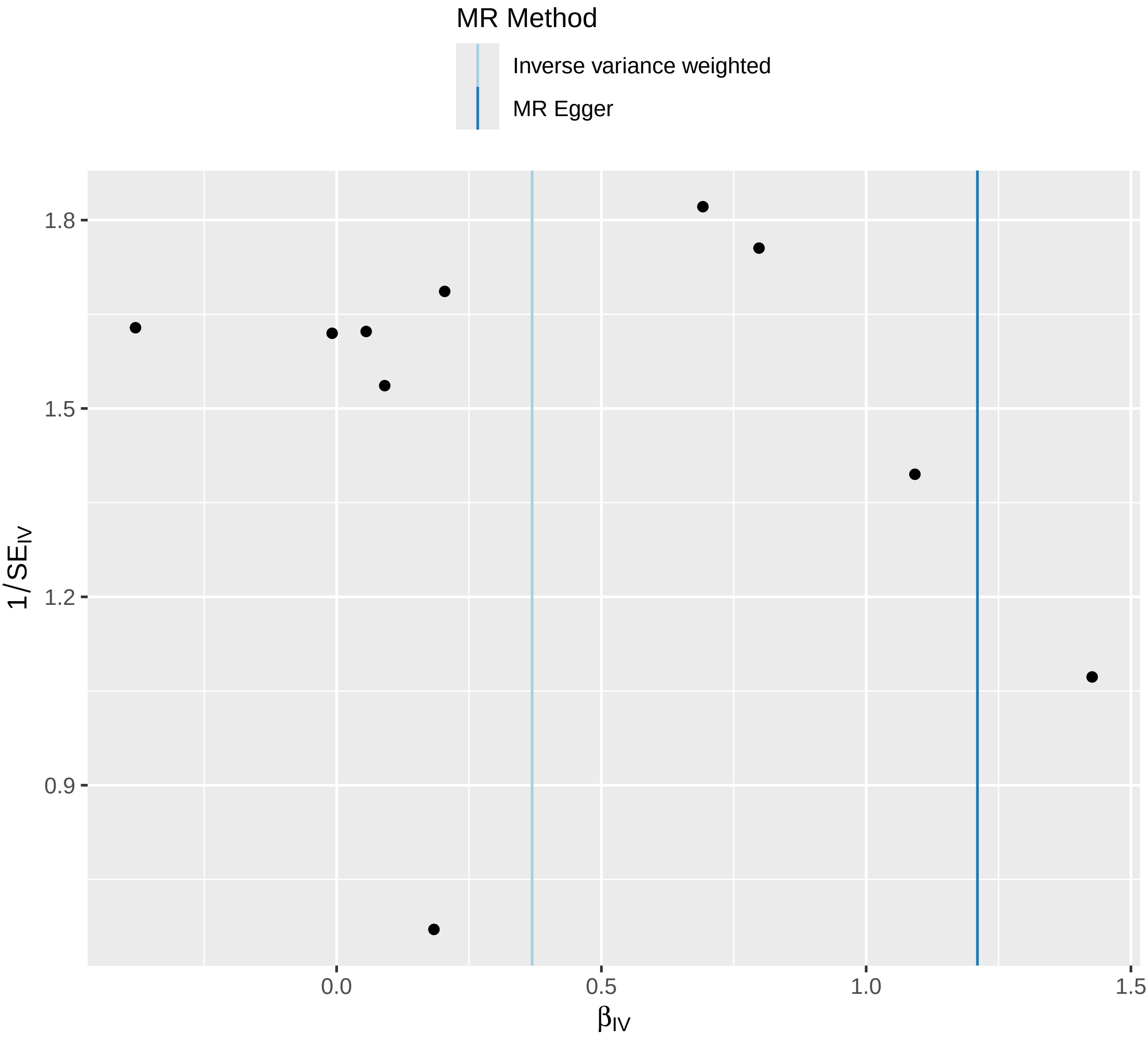

MR Method
Inverse variance weighted
MR Egger
1.8
1.5
1.2
0.9
$1/SE_{IV}$
0.0
0.5
1.0
1.5
β_{IV}

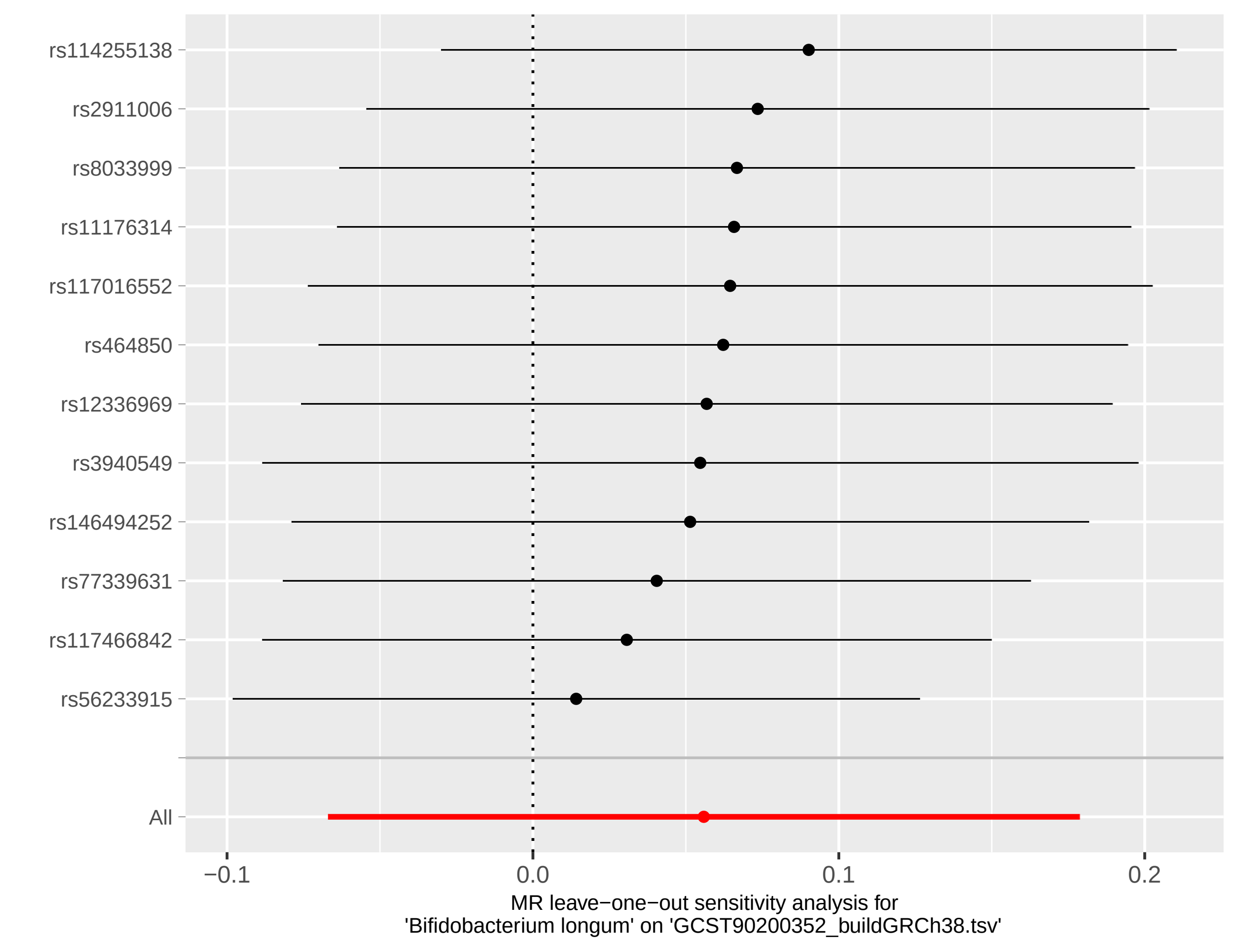

rs114255138
rs2911006
rs8033999
rs11176314
rs117016552
rs464850
rs12336969
rs3940549
rs146494252
rs77339631
rs117466842
rs56233915
All
−0.1
0.0
0.1
0.2
MR leave−one−out sensitivity analysis for
'Bifidobacterium longum' on 'GCST90200352_buildGRCh38.tsv'

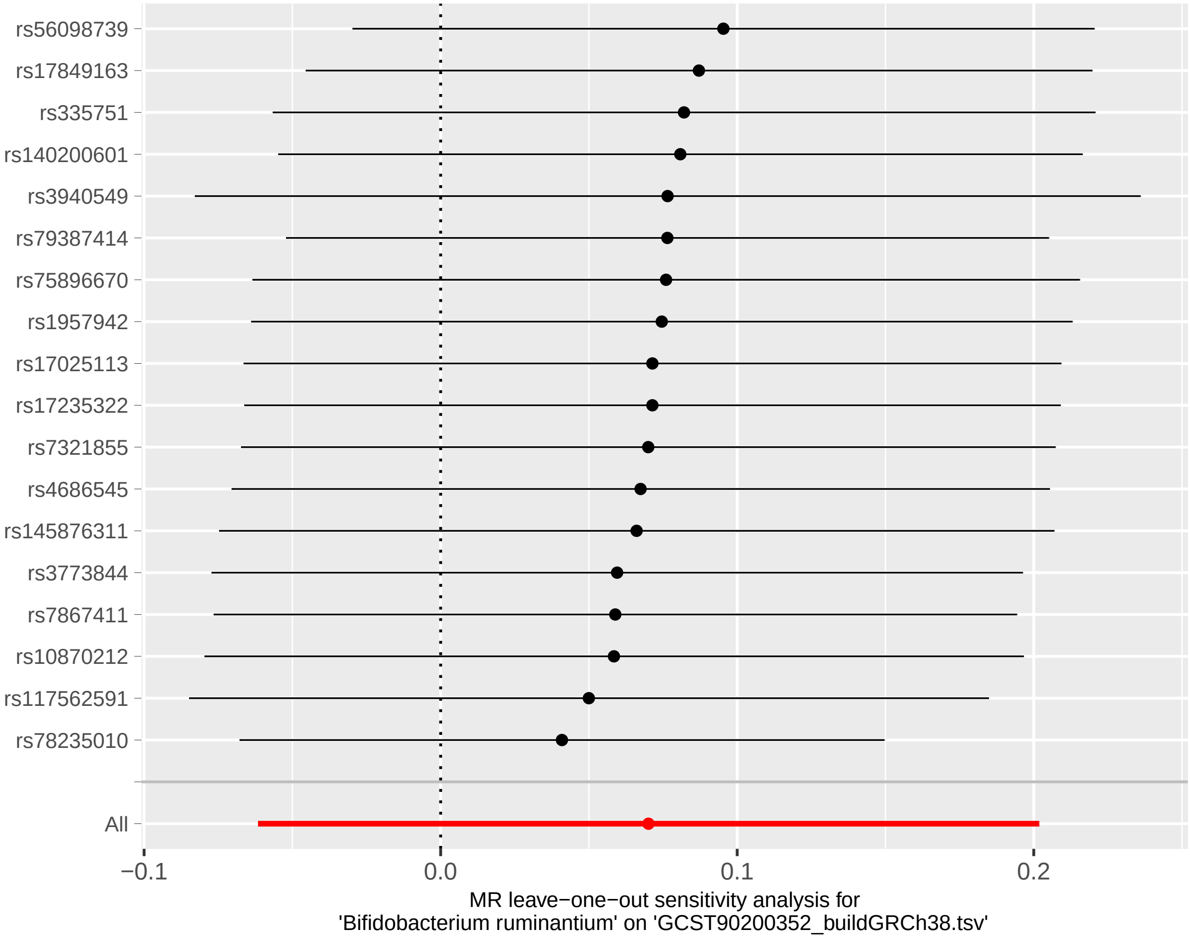

rs56098739
rs17849163
rs335751
rs140200601
rs3940549
rs79387414
rs75896670
rs1957942
rs17025113
rs17235322
rs7321855
rs4686545
rs145876311
rs3773844
rs7867411
rs10870212
rs117562591
rs78235010
All
−0.1
0.0
0.1
0.2
MR leave−one−out sensitivity analysis for
'Bifidobacterium ruminantium' on 'GCST90200352_buildGRCh38.tsv'

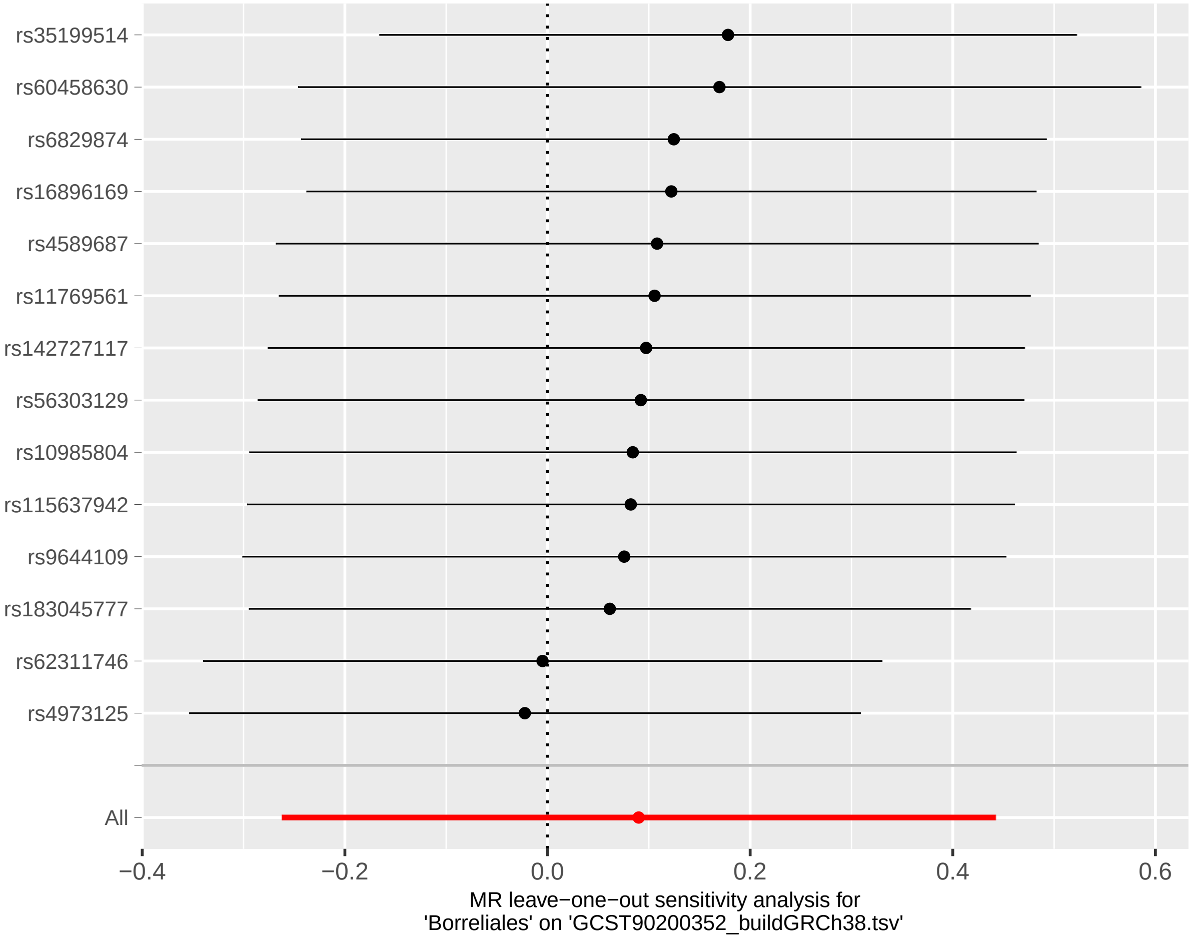
rs35199514
rs60458630
rs6829874
rs16896169
rs4589687
rs11769561
rs142727117
rs56303129
rs10985804
rs115637942
rs9644109
rs183045777
rs62311746
rs4973125
All
−0.4
−0.2
0.0
0.2
0.4
0.6
MR leave−one−out sensitivity analysis for
'Borreliales' on 'GCST90200352_buildGRCh38.tsv'

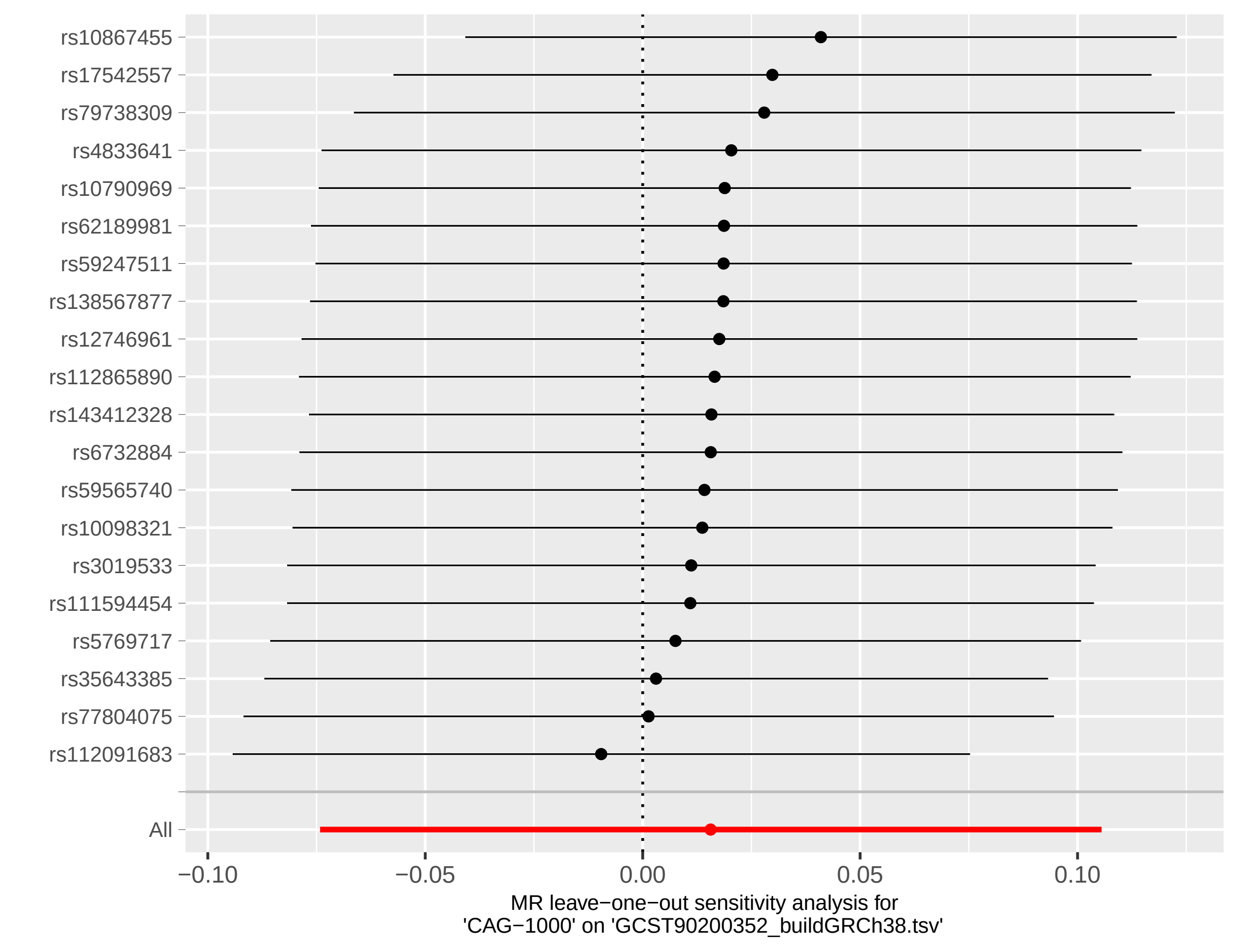

rs10867455
rs17542557
rs79738309
rs4833641
rs10790969
rs62189981
rs59247511
rs138567877
rs12746961
rs112865890
rs143412328
rs6732884
rs59565740
rs10098321
rs3019533
rs111594454
rs5769717
rs35643385
rs77804075
rs112091683
All
−0.10
−0.05
0.00
0.05
0.10
MR leave−one−out sensitivity analysis for
'CAG−1000' on 'GCST90200352_buildGRCh38.tsv'

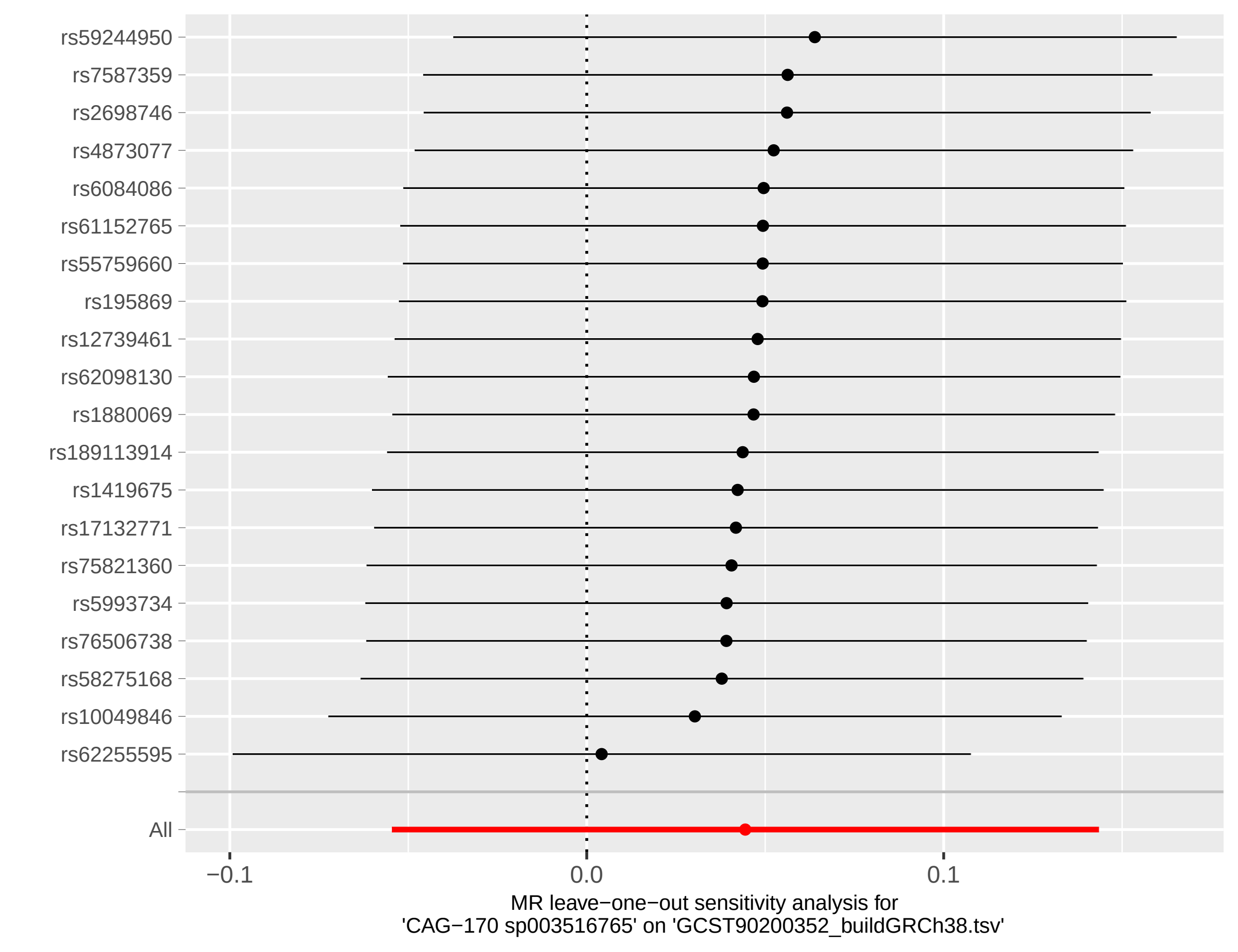

rs59244950
rs7587359
rs2698746
rs4873077
rs6084086
rs61152765
rs55759660
rs195869
rs12739461
rs62098130
rs1880069
rs189113914
rs1419675
rs17132771
rs75821360
rs5993734
rs76506738
rs58275168
rs10049846
rs62255595
All
−0.1
0.0
0.1
MR leave−one−out sensitivity analysis for
'CAG−170 sp003516765' on 'GCST90200352_buildGRCh38.tsv'

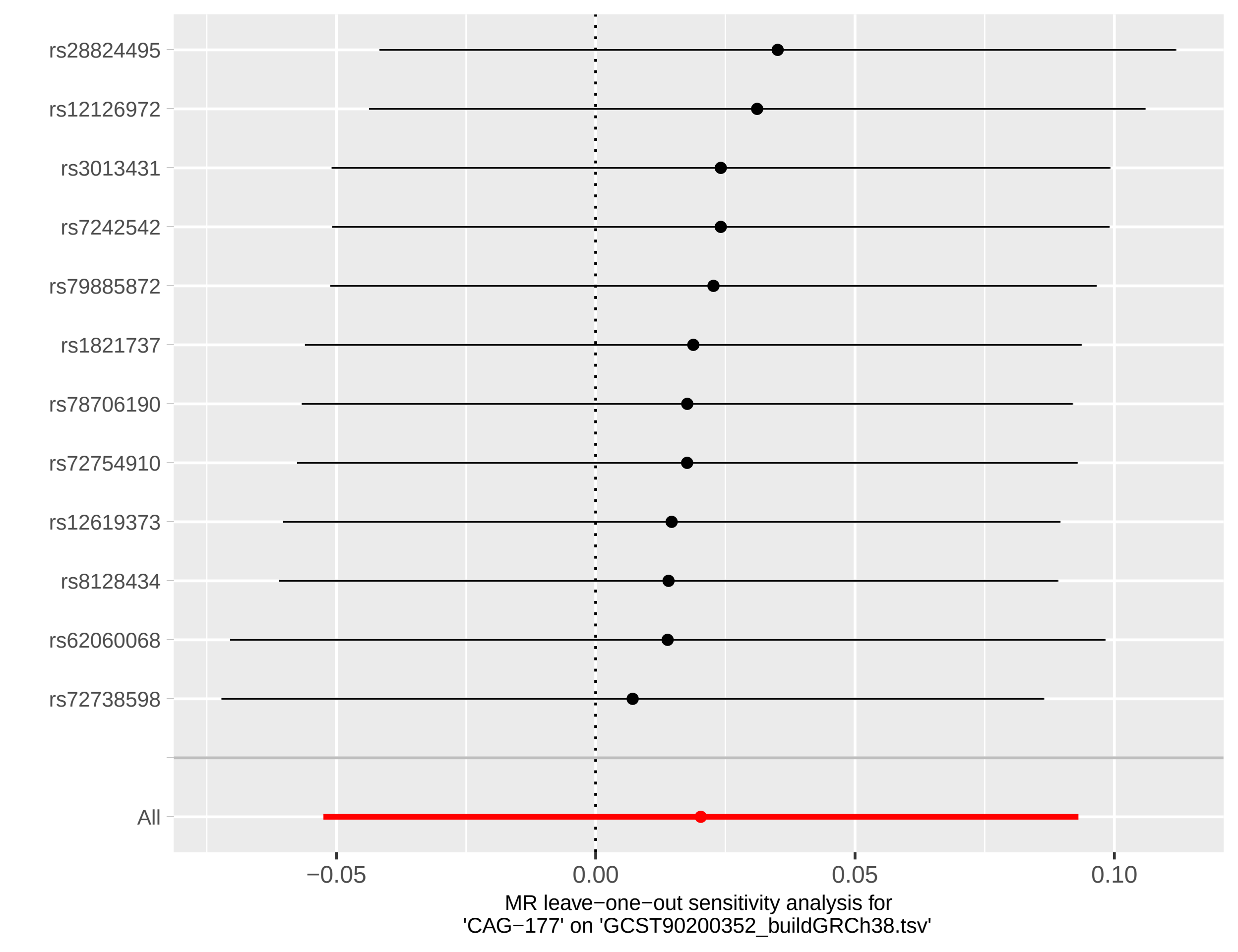
rs28824495
rs12126972
rs3013431
rs7242542
rs79885872
rs1821737
rs78706190
rs72754910
rs12619373
rs8128434
rs62060068
rs72738598
All
−0.05
0.00
0.05
0.10
MR leave−one−out sensitivity analysis for
'CAG−177' on 'GCST90200352_buildGRCh38.tsv'

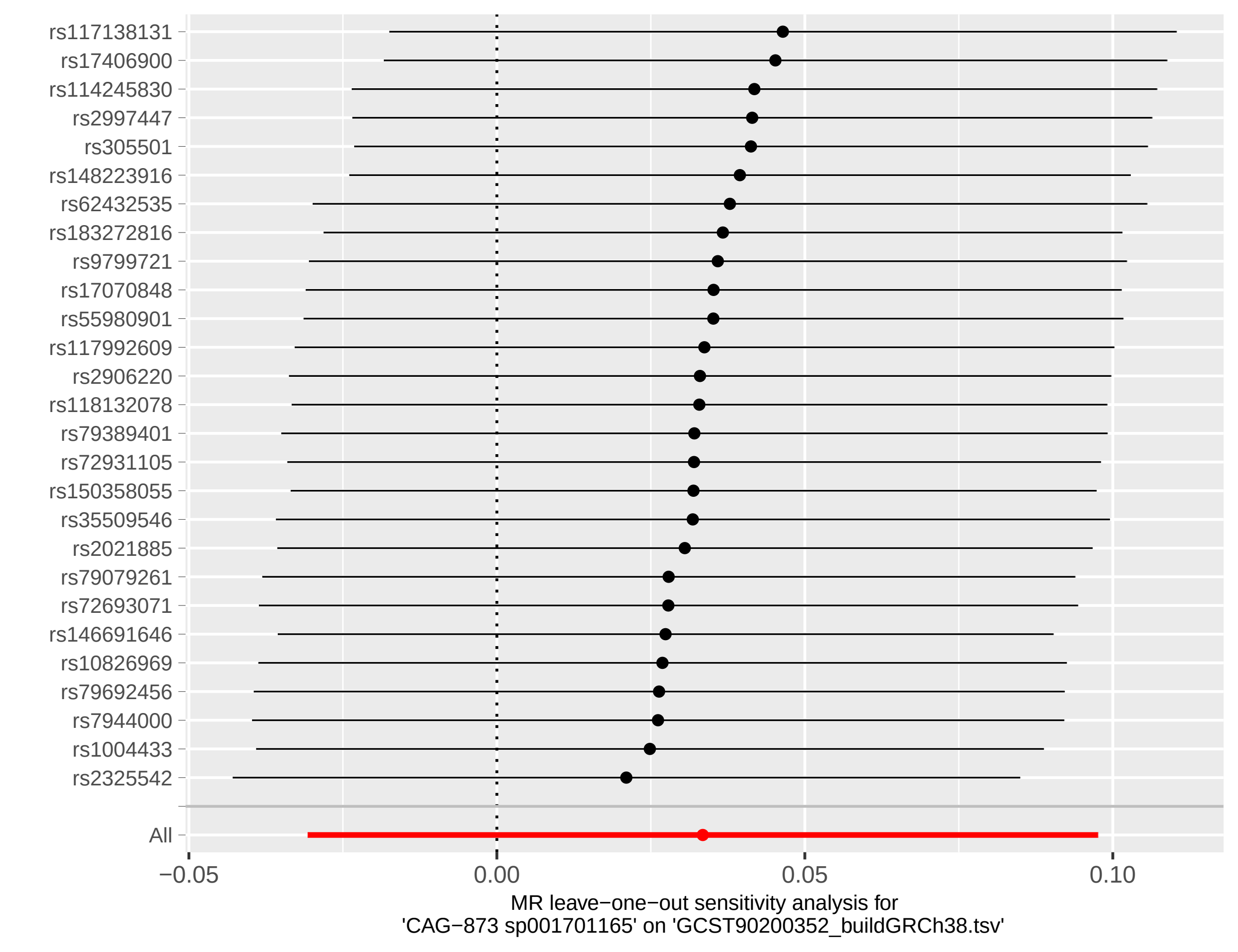
rs117138131
rs17406900
rs114245830
rs2997447
rs305501
rs148223916
rs62432535
rs183272816
rs9799721
rs17070848
rs55980901
rs117992609
rs2906220
rs118132078
rs79389401
rs72931105
rs150358055
rs35509546
rs2021885
rs79079261
rs72693071
rs146691646
rs10826969
rs79692456
rs7944000
rs1004433
rs2325542
All
−0.05
0.00
0.05
0.10
MR leave−one−out sensitivity analysis for
'CAG−873 sp001701165' on 'GCST90200352_buildGRCh38.tsv'

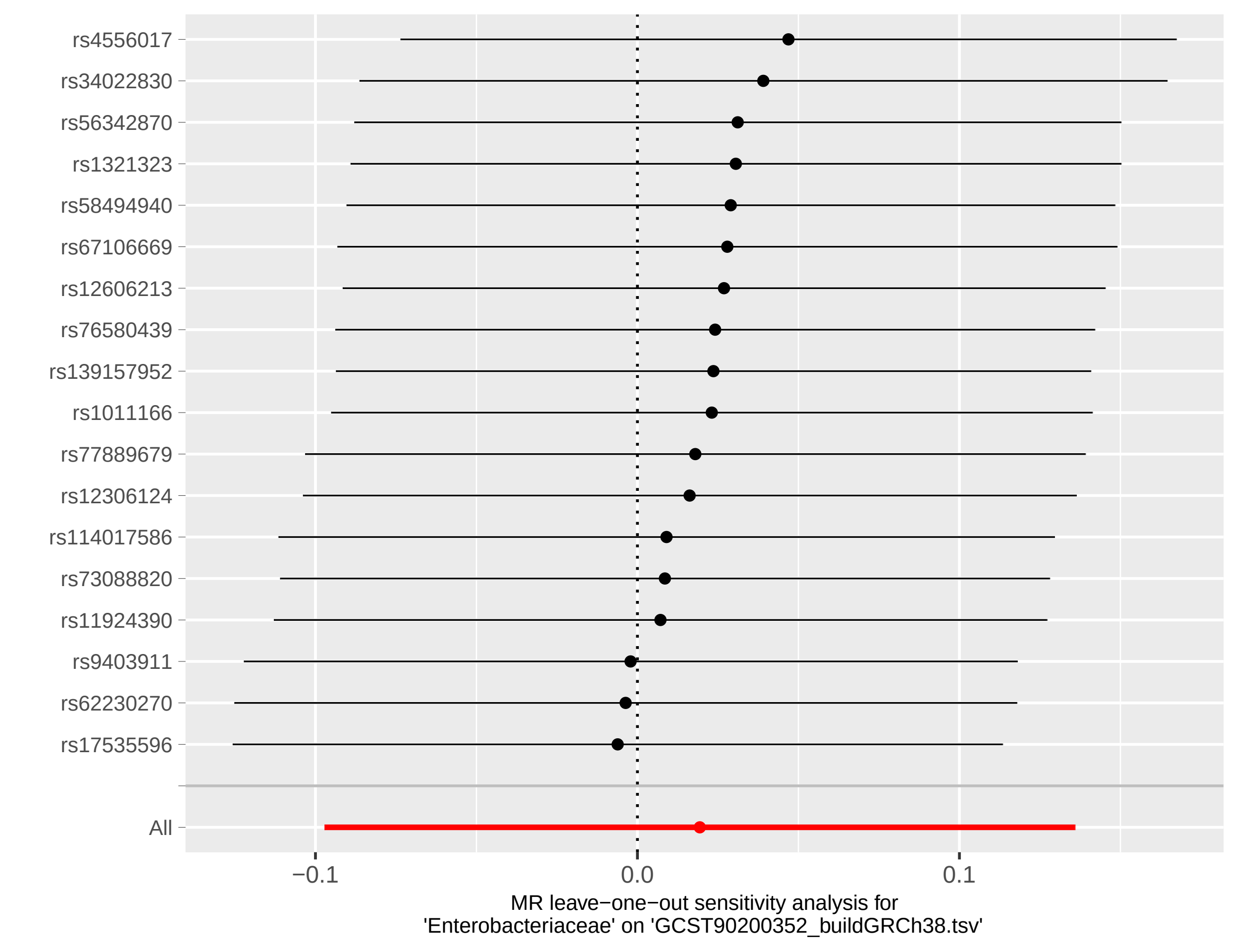
rs4556017
rs34022830
rs56342870
rs1321323
rs58494940
rs67106669
rs12606213
rs76580439
rs139157952
rs1011166
rs77889679
rs12306124
rs114017586
rs73088820
rs11924390
rs9403911
rs62230270
rs17535596
All
−0.1
0.0
0.1
MR leave−one−out sensitivity analysis for
'Enterobacteriaceae' on 'GCST90200352_buildGRCh38.tsv'

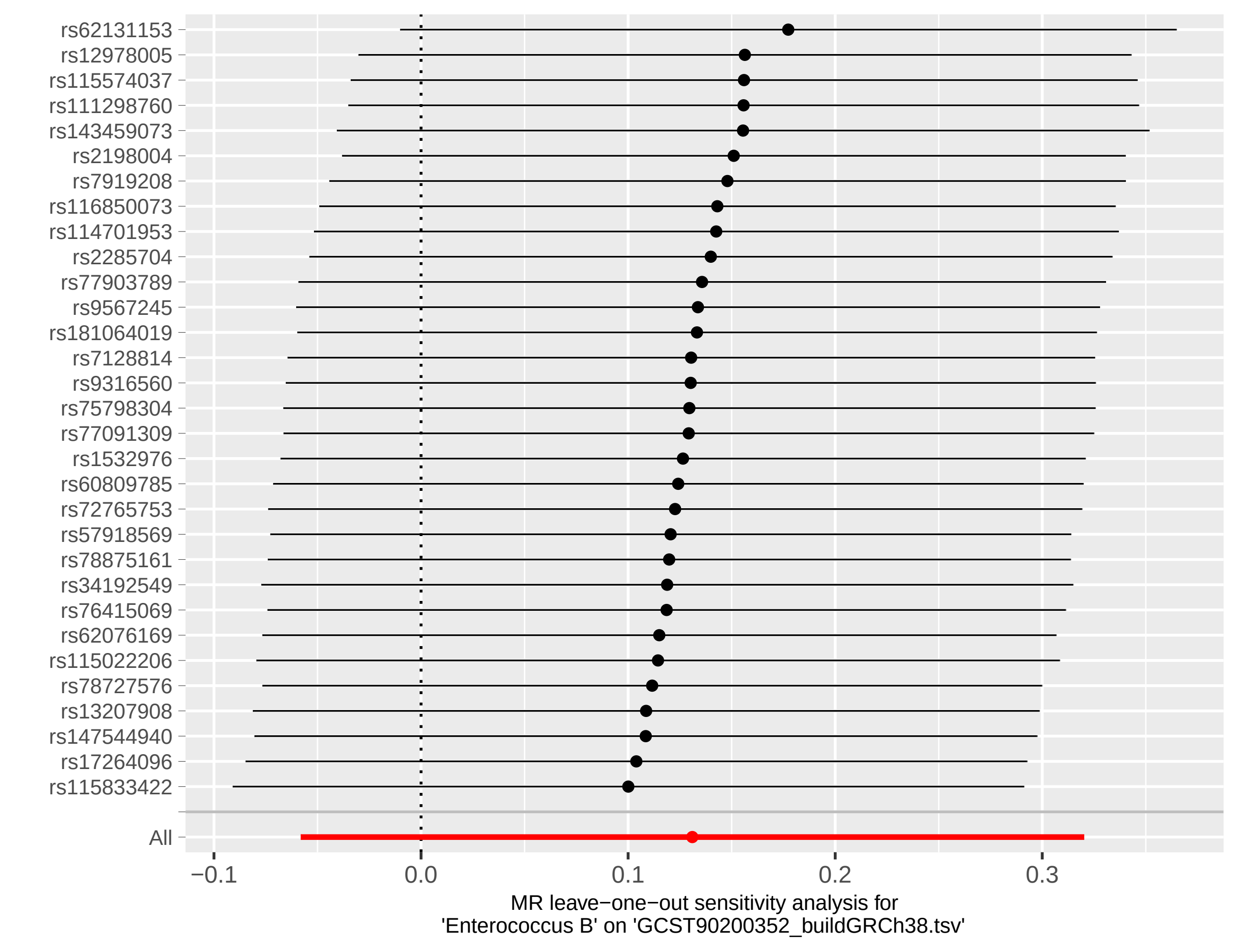
rs62131153
rs12978005
rs115574037
rs111298760
rs143459073
rs2198004
rs7919208
rs116850073
rs114701953
rs2285704
rs77903789
rs9567245
rs181064019
rs7128814
rs9316560
rs75798304
rs77091309
rs1532976
rs60809785
rs72765753
rs57918569
rs78875161
rs34192549
rs76415069
rs62076169
rs115022206
rs78727576
rs13207908
rs147544940
rs17264096
rs115833422
All
−0.1
0.0
0.1
0.2
0.3
MR leave−one−out sensitivity analysis for
'Enterococcus B' on 'GCST90200352_buildGRCh38.tsv'

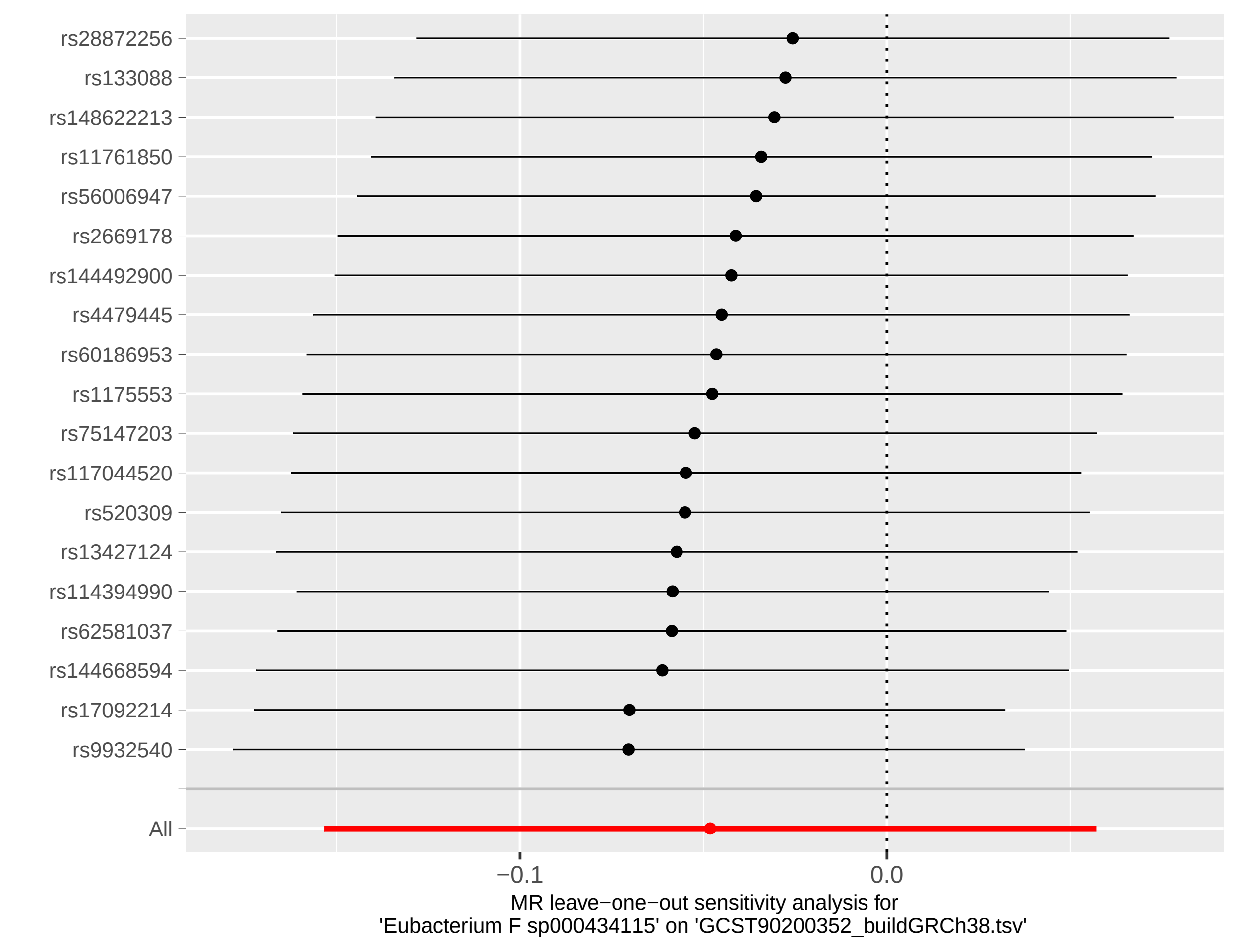
rs28872256
rs133088
rs148622213
rs11761850
rs56006947
rs2669178
rs144492900
rs4479445
rs60186953
rs1175553
rs75147203
rs117044520
rs520309
rs13427124
rs114394990
rs62581037
rs144668594
rs17092214
rs9932540
All
−0.1
0.0
MR leave−one−out sensitivity analysis for
'Eubacterium F sp000434115' on 'GCST90200352_buildGRCh38.tsv'

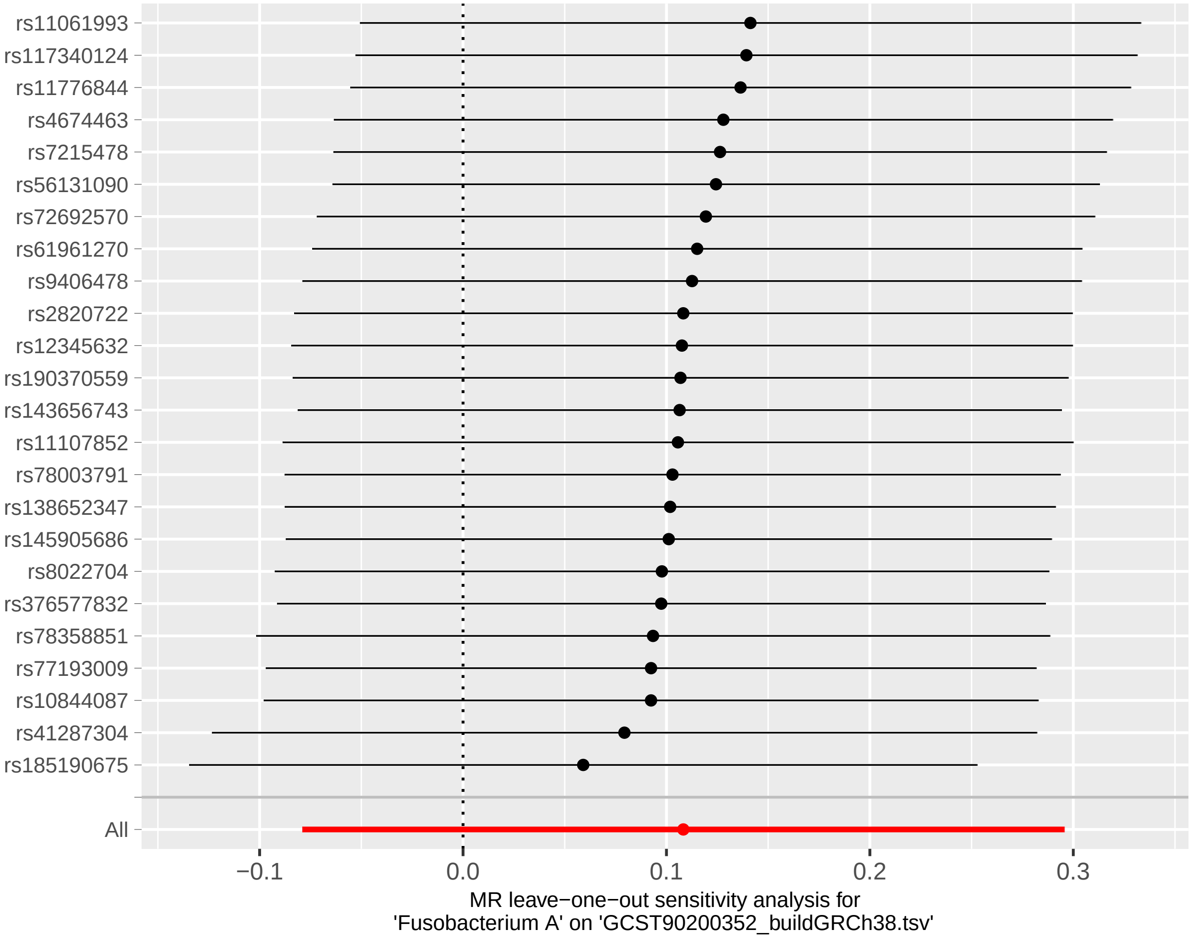
rs11061993
rs117340124
rs11776844
rs4674463
rs7215478
rs56131090
rs72692570
rs61961270
rs9406478
rs2820722
rs12345632
rs190370559
rs143656743
rs11107852
rs78003791
rs138652347
rs145905686
rs8022704
rs376577832
rs78358851
rs77193009
rs10844087
rs41287304
rs185190675
All
−0.1
0.0
0.1
0.2
0.3
MR leave−one−out sensitivity analysis for
'Fusobacterium A' on 'GCST90200352_buildGRCh38.tsv'

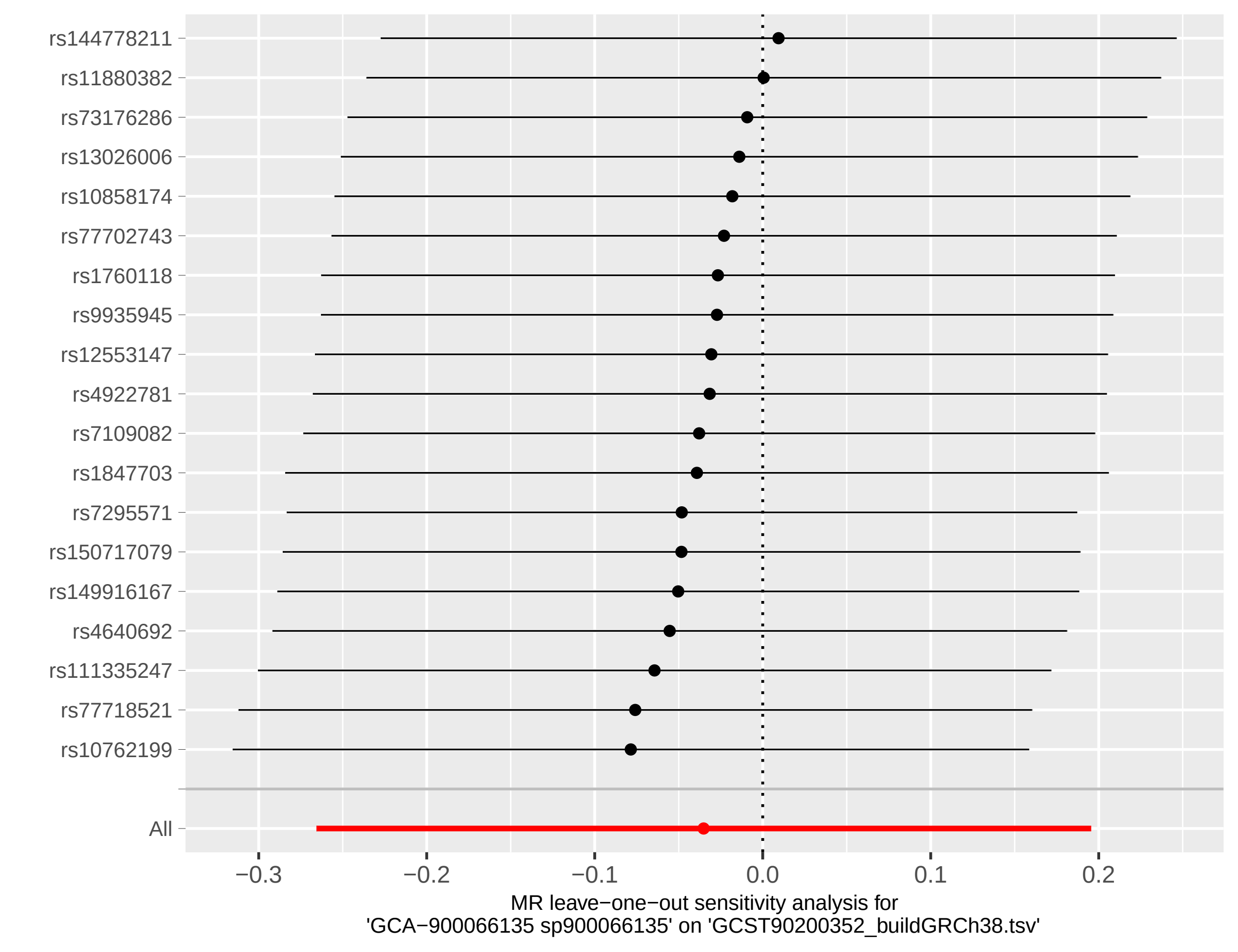
rs144778211
rs11880382
rs73176286
rs13026006
rs10858174
rs77702743
rs1760118
rs9935945
rs12553147
rs4922781
rs7109082
rs1847703
rs7295571
rs150717079
rs149916167
rs4640692
rs111335247
rs77718521
rs10762199
All
−0.3
−0.2
−0.1
0.0
0.1
0.2
MR leave−one−out sensitivity analysis for
'GCA−900066135 sp900066135' on 'GCST90200352_buildGRCh38.tsv'

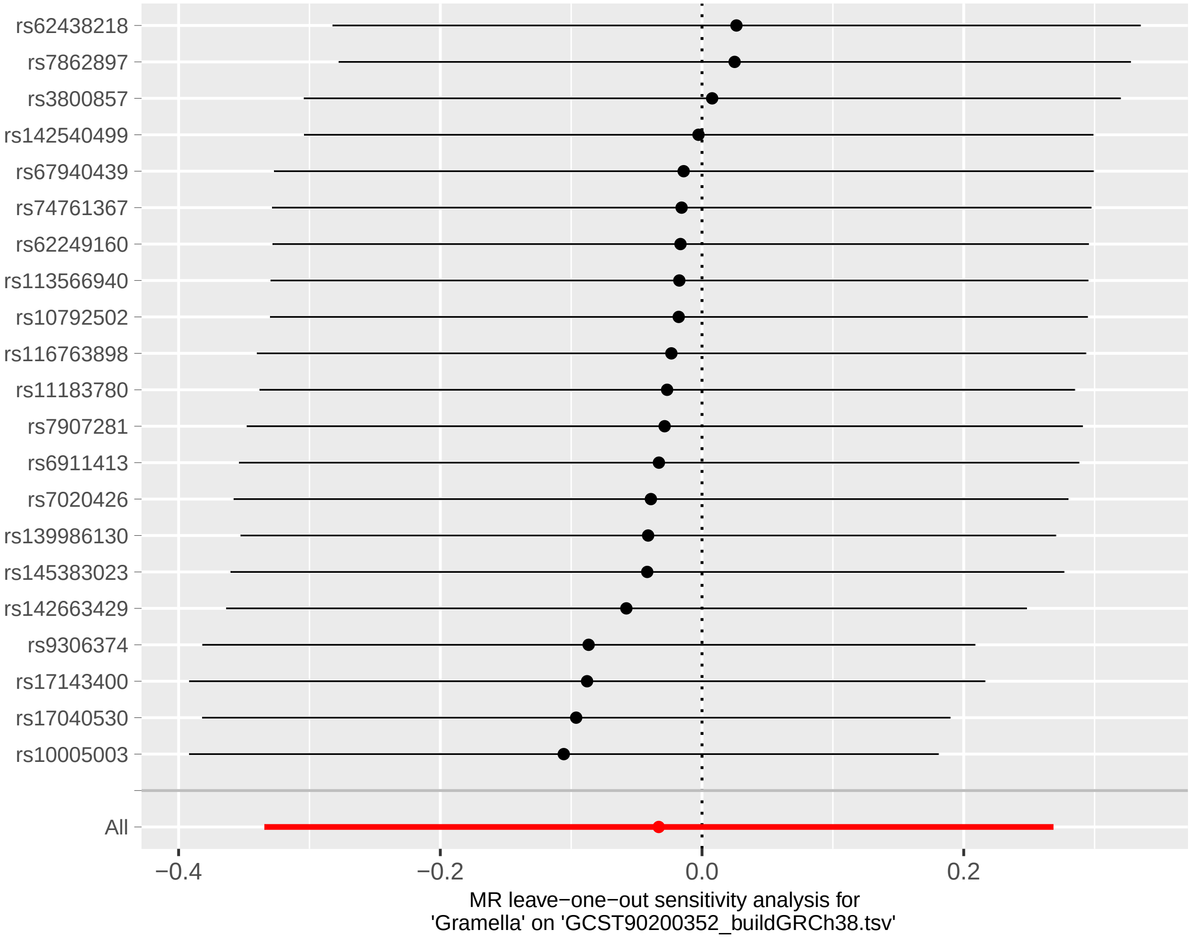
rs62438218
rs7862897
rs3800857
rs142540499
rs67940439
rs74761367
rs62249160
rs113566940
rs10792502
rs116763898
rs11183780
rs7907281
rs6911413
rs7020426
rs139986130
rs145383023
rs142663429
rs9306374
rs17143400
rs17040530
rs10005003
All
−0.4
−0.2
0.0
0.2
MR leave−one−out sensitivity analysis for
'Gramella' on 'GCST90200352_buildGRCh38.tsv'

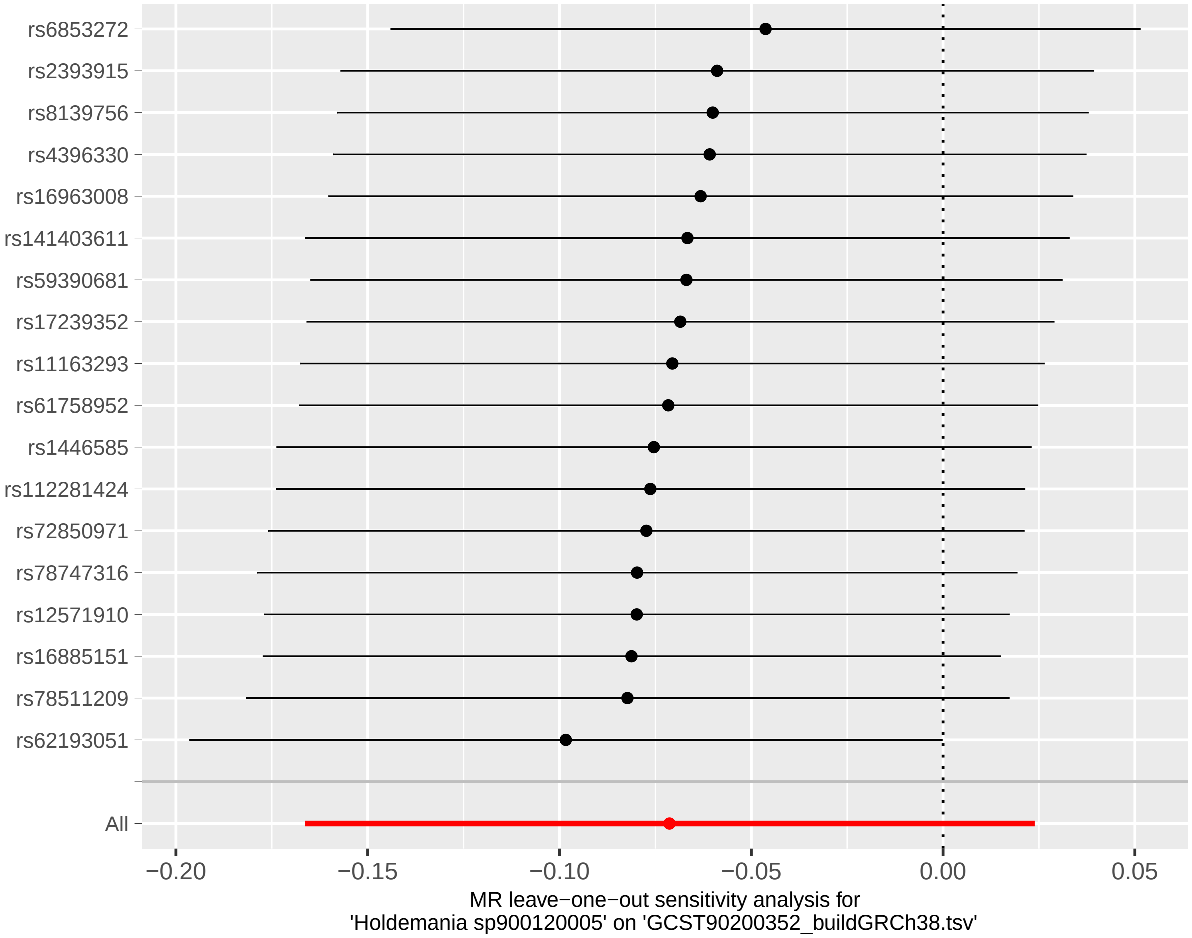

rs6853272
rs2393915
rs8139756
rs4396330
rs16963008
rs141403611
rs59390681
rs17239352
rs11163293
rs61758952
rs1446585
rs112281424
rs72850971
rs78747316
rs12571910
rs16885151
rs78511209
rs62193051
All
−0.20
−0.15
−0.10
−0.05
0.00
0.05
MR leave−one−out sensitivity analysis for
'Holdemania sp900120005' on 'GCST90200352_buildGRCh38.tsv'

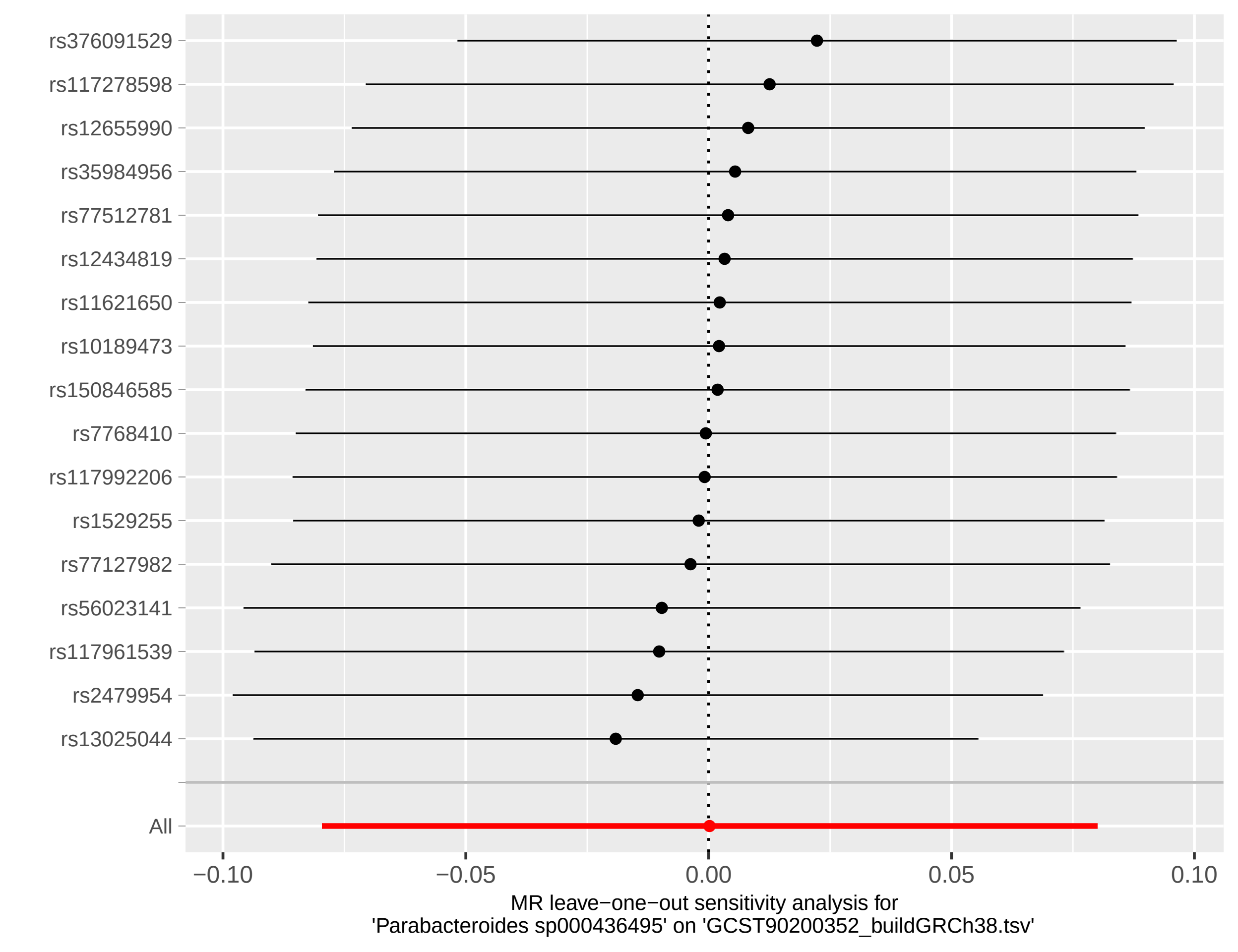

rs376091529
rs117278598
rs12655990
rs35984956
rs77512781
rs12434819
rs11621650
rs10189473
rs150846585
rs7768410
rs117992206
rs1529255
rs77127982
rs56023141
rs117961539
rs2479954
rs13025044
All
−0.10
−0.05
0.00
0.05
0.10
MR leave−one−out sensitivity analysis for
'Parabacteroides sp000436495' on 'GCST90200352_buildGRCh38.tsv'

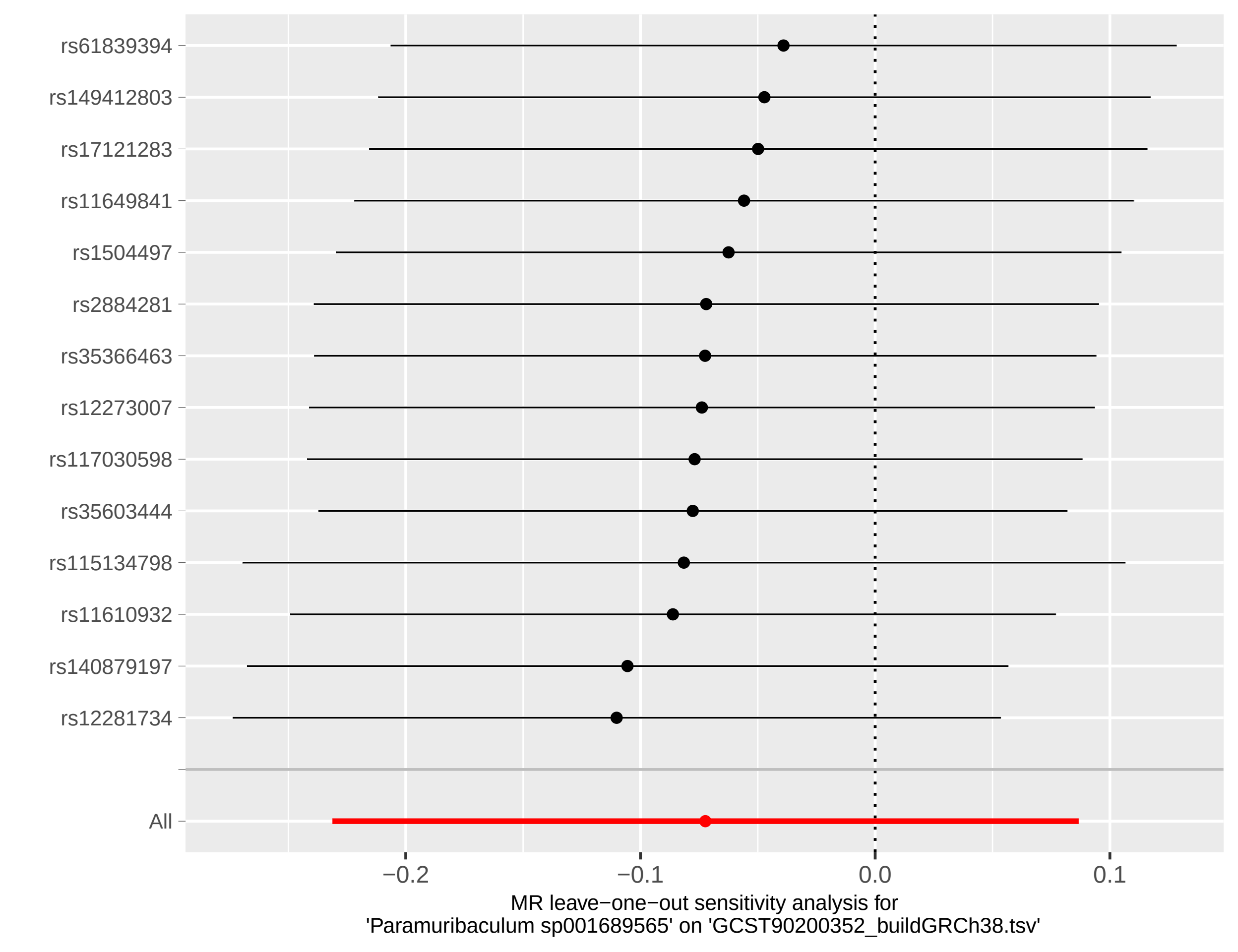

rs61839394
rs149412803
rs17121283
rs11649841
rs1504497
rs2884281
rs35366463
rs12273007
rs117030598
rs35603444
rs115134798
rs11610932
rs140879197
rs12281734
All
−0.2
−0.1
0.0
0.1
MR leave−one−out sensitivity analysis for
'Paramuribaculum sp001689565' on 'GCST90200352_buildGRCh38.tsv'

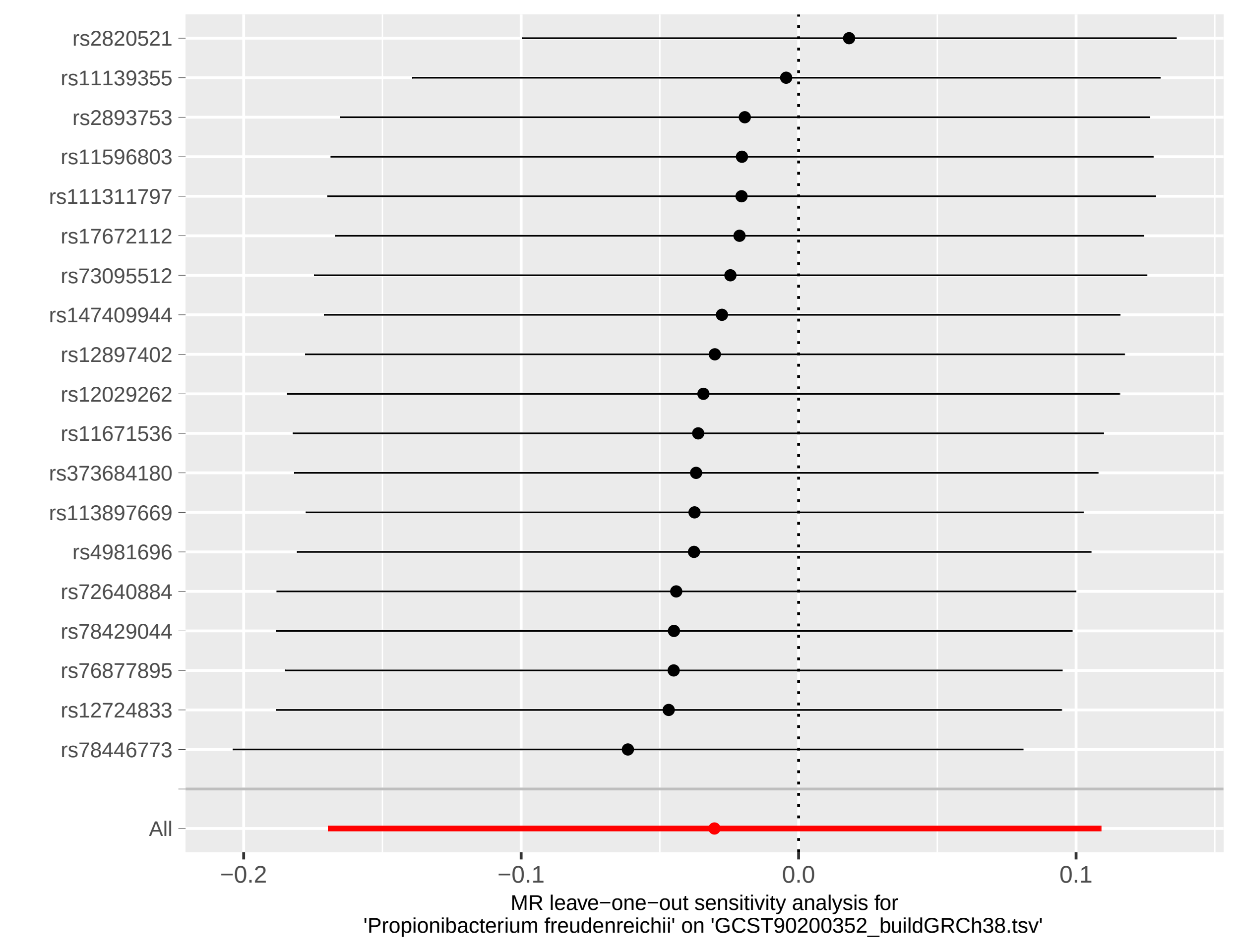
rs2820521
rs11139355
rs2893753
rs11596803
rs111311797
rs17672112
rs73095512
rs147409944
rs12897402
rs12029262
rs11671536
rs373684180
rs113897669
rs4981696
rs72640884
rs78429044
rs76877895
rs12724833
rs78446773
All
−0.2
−0.1
0.0
0.1
MR leave−one−out sensitivity analysis for
'Propionibacterium freudenreichii' on 'GCST90200352_buildGRCh38.tsv'

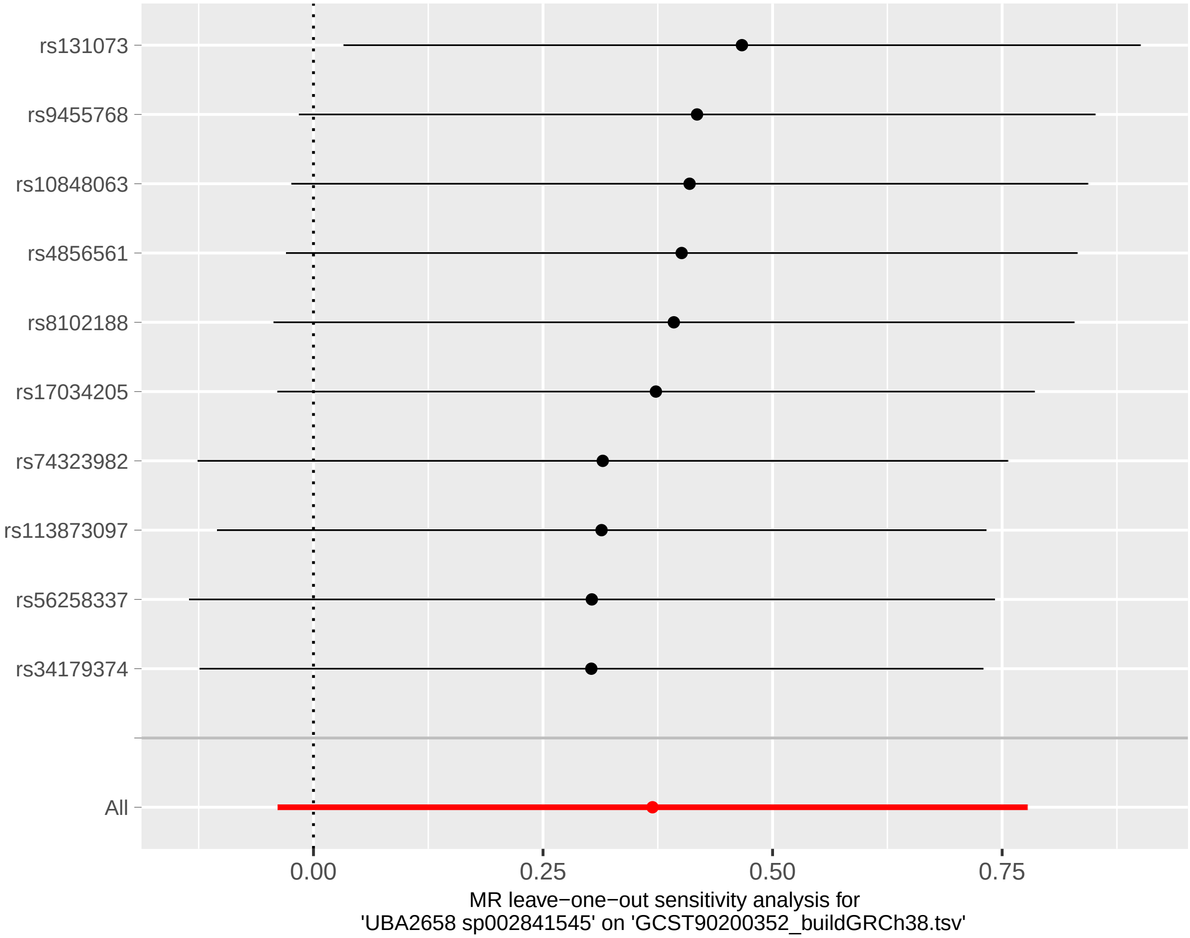

rs131073
rs9455768
rs10848063
rs4856561
rs8102188
rs17034205
rs74323982
rs113873097
rs56258337
rs34179374
All
0.00
0.25
0.50
0.75
MR leave−one−out sensitivity analysis for
'UBA2658 sp002841545' on 'GCST90200352_buildGRCh38.tsv'

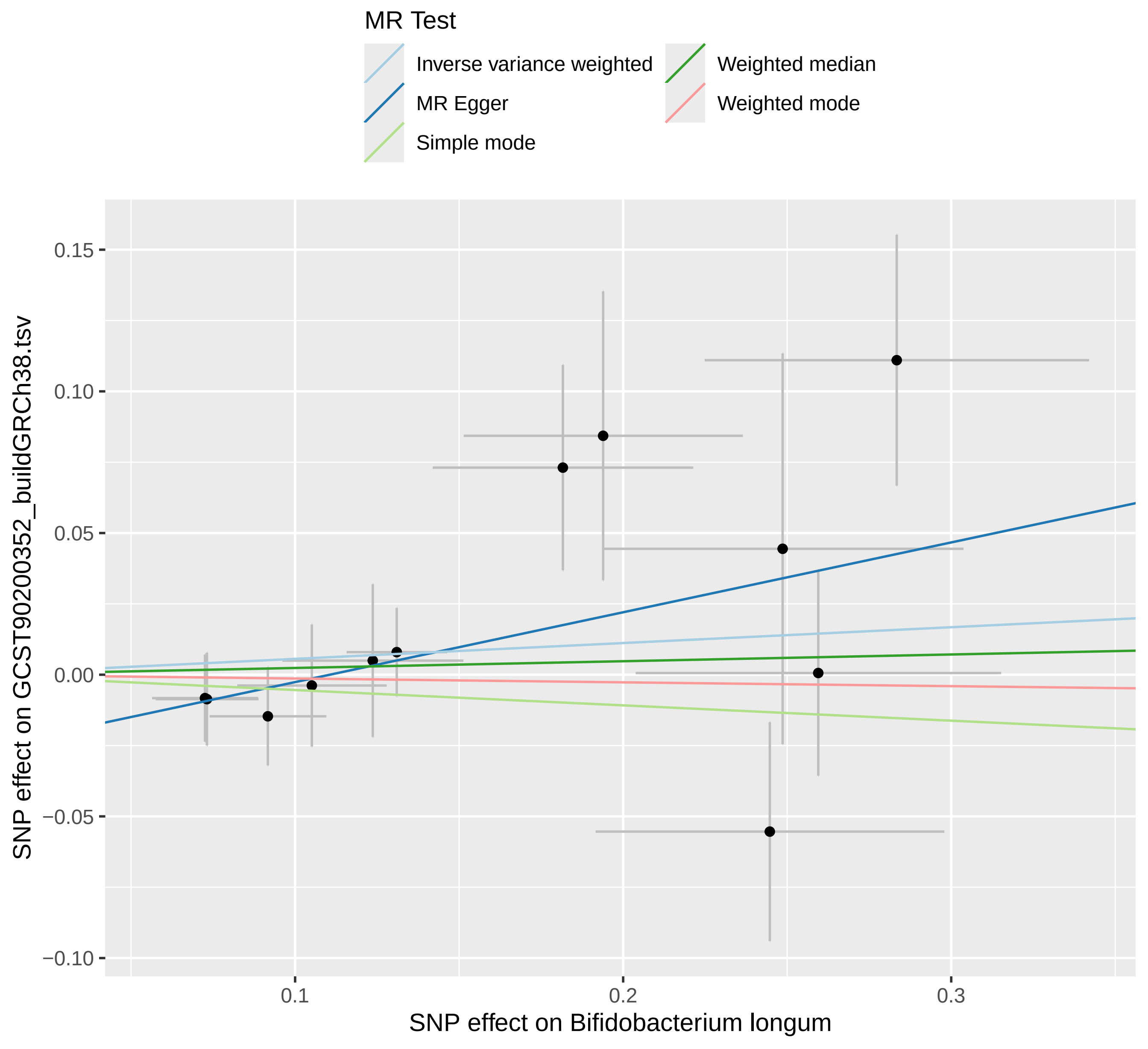

MR Test
Inverse variance weighted
MR Egger
Simple mode
Weighted median
Weighted mode
SNP effect on GCST90200352_buildGRCh38.tsv
0.15
0.10
0.05
0.00
−0.05
−0.10
0.1
0.2
0.3
SNP effect on Bifidobacterium longum

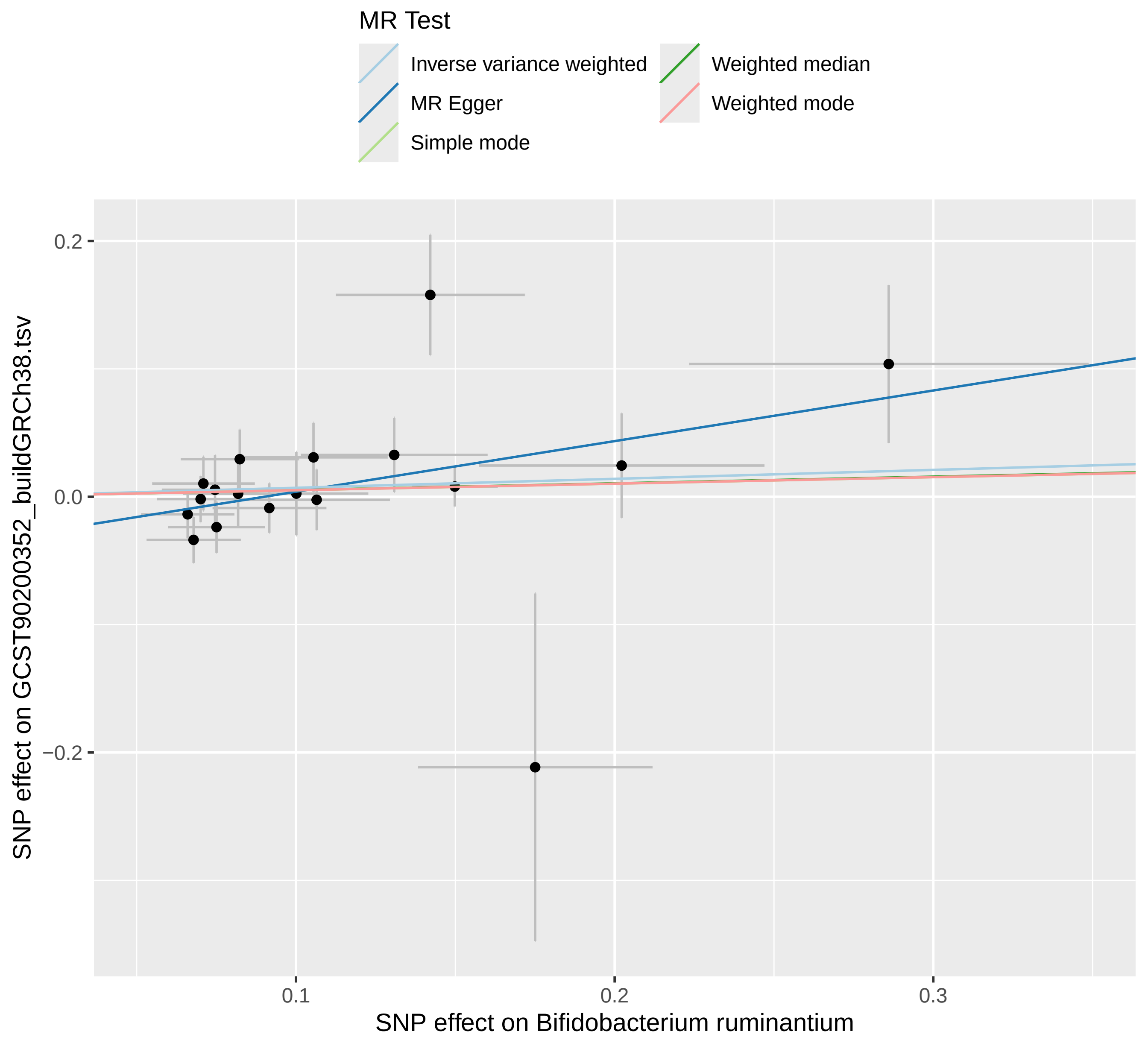
MR Test
Inverse variance weighted
MR Egger
Simple mode
Weighted median
Weighted mode
0.2
0.0
−0.2
0.1
0.2
0.3
SNP effect on GCST90200352_buildGRCh38.tsv
SNP effect on Bifidobacterium ruminantium

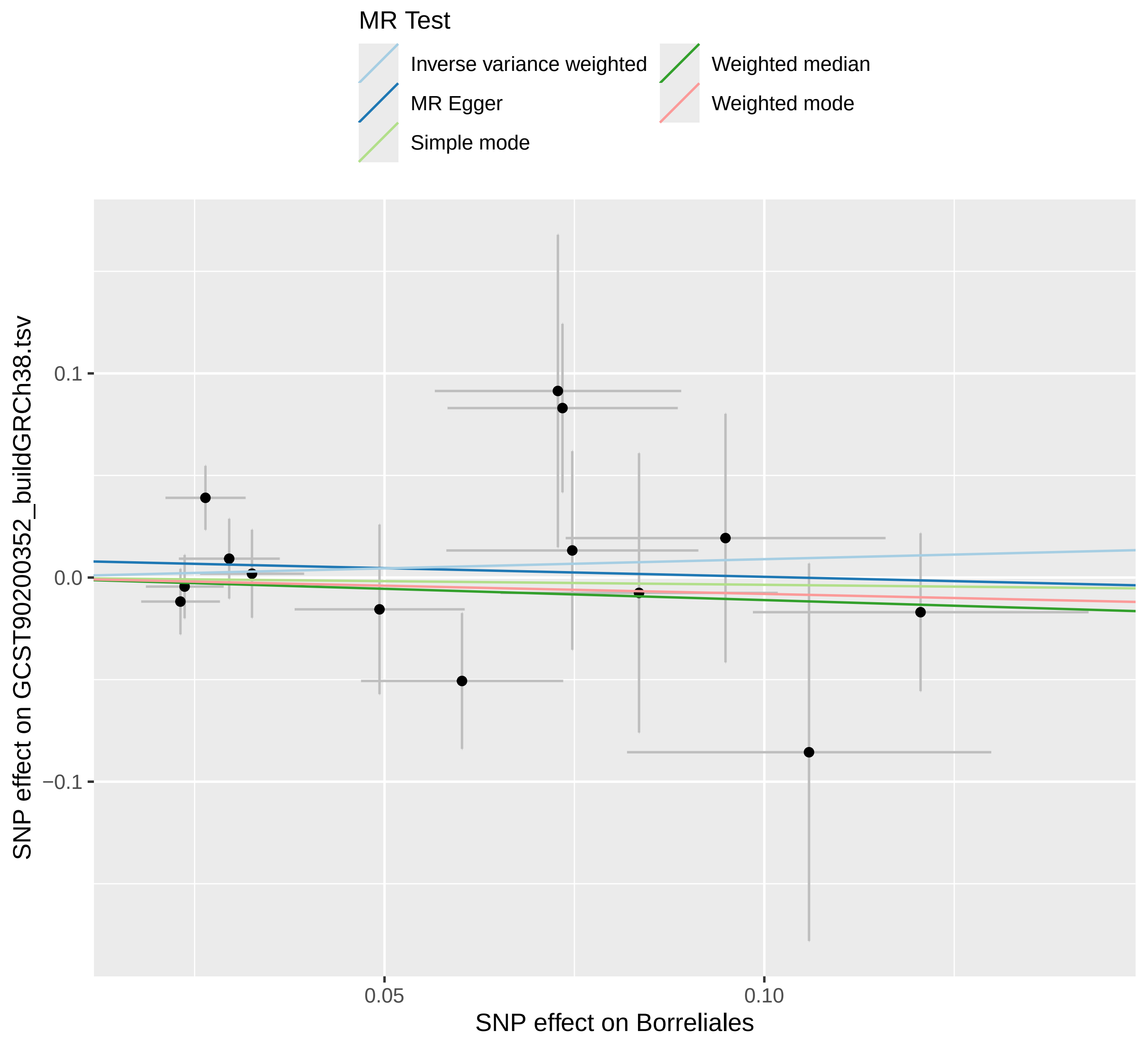

MR Test
Inverse variance weighted
MR Egger
Simple mode
Weighted median
Weighted mode
0.1
0.0
−0.1
0.05
0.10
SNP effect on GCST90200352_buildGRCh38.tsv
SNP effect on Borreliales

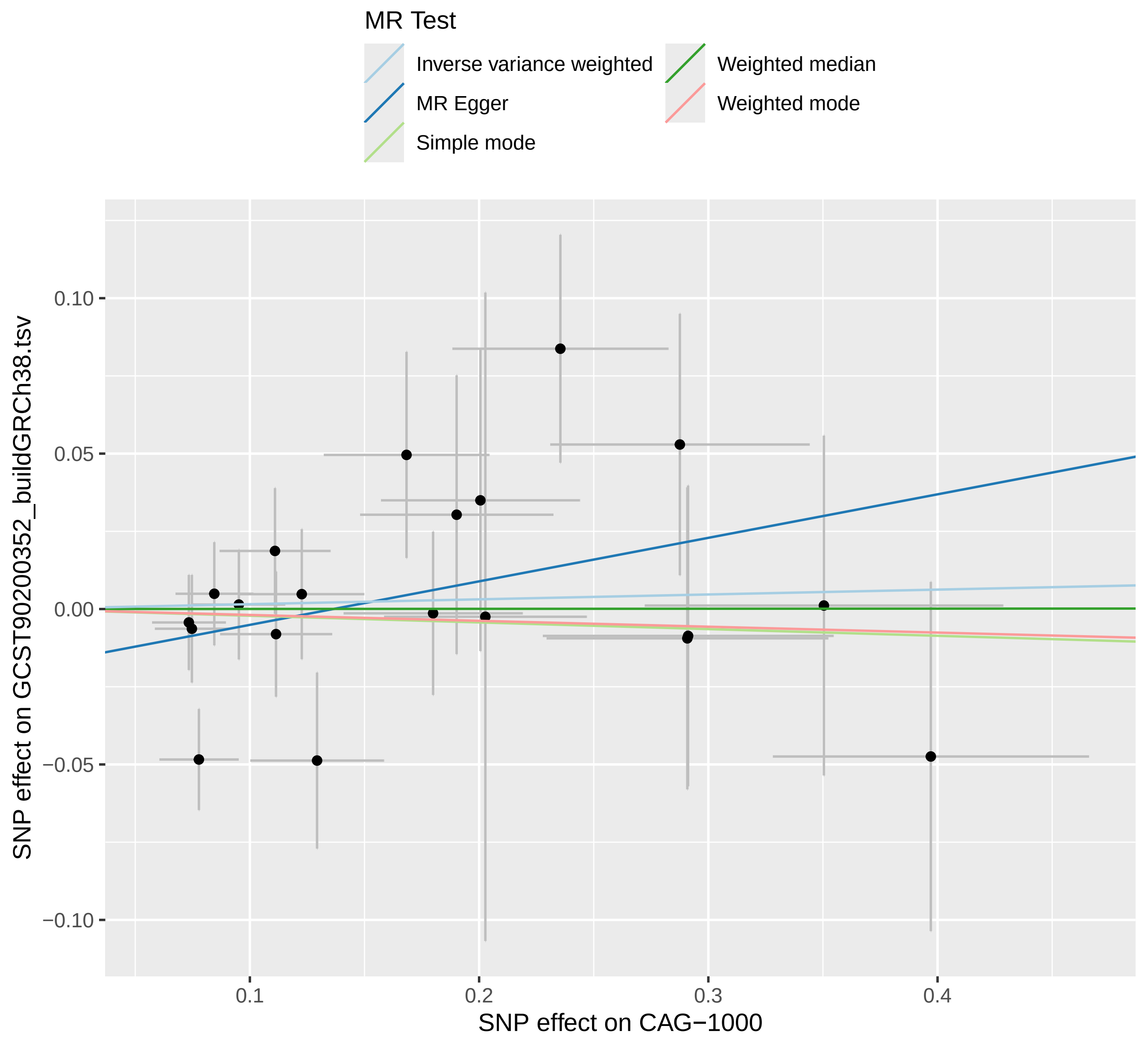

MR Test
Inverse variance weighted
MR Egger
Simple mode
Weighted median
Weighted mode
SNP effect on GCST90200352_buildGRCh38.tsv
0.10
0.05
0.00
−0.05
−0.10
0.1
0.2
0.3
0.4
SNP effect on CAG−1000

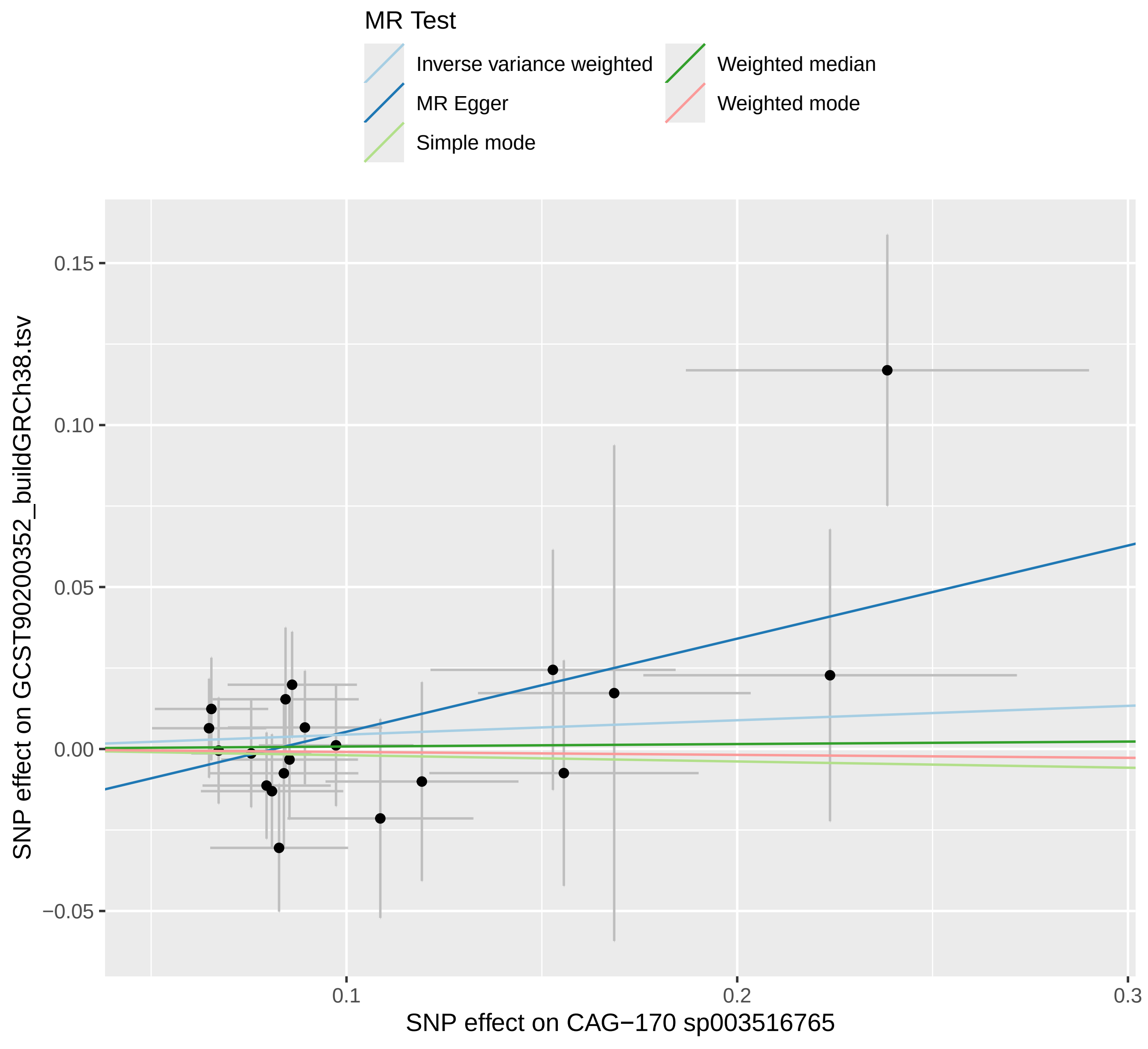
MR Test
Inverse variance weighted
MR Egger
Simple mode
Weighted median
Weighted mode
0.15
0.10
0.05
0.00
−0.05
0.1
0.2
0.3
SNP effect on GCST90200352_buildGRCh38.tsv
SNP effect on CAG−170 sp003516765

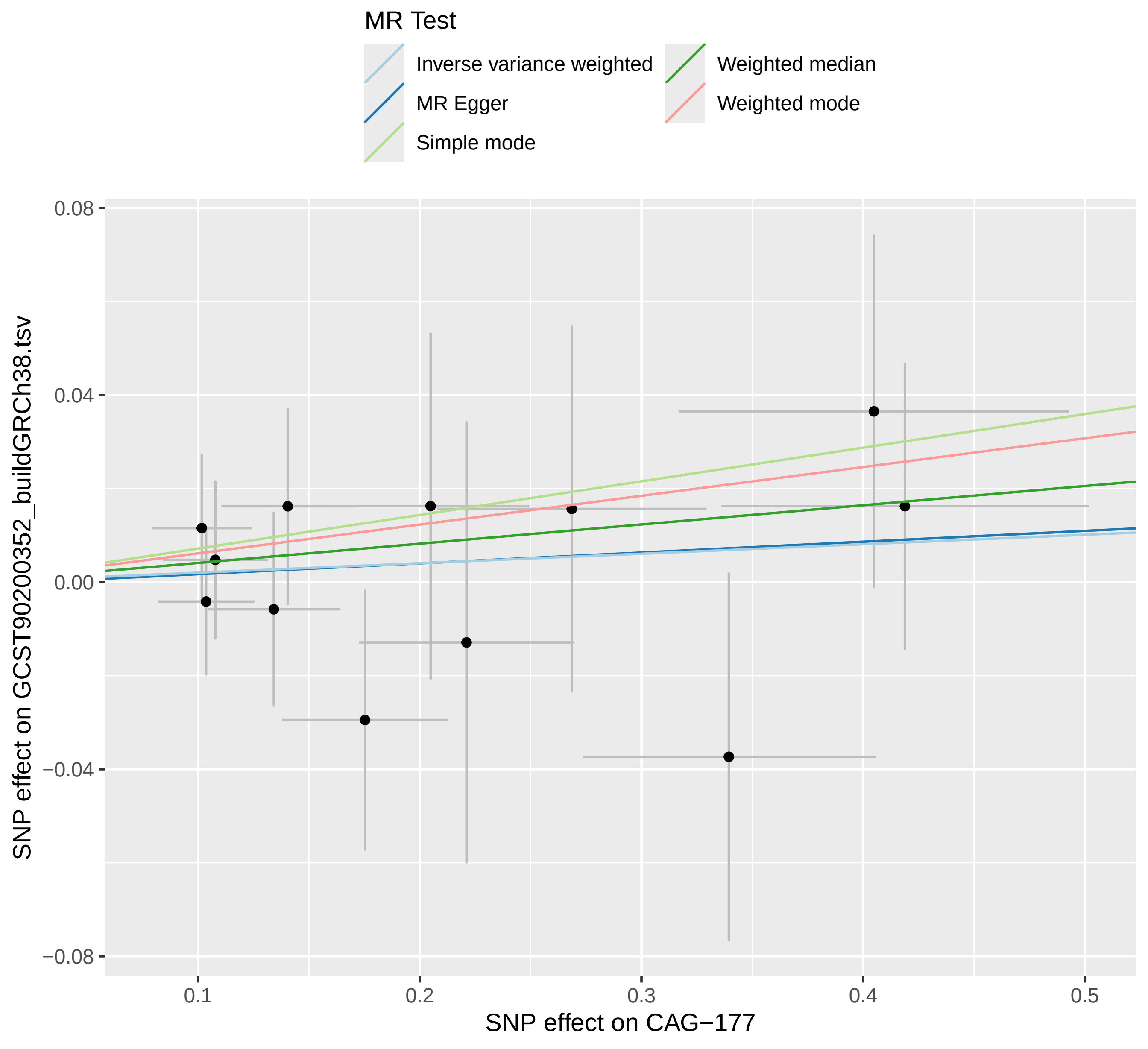
MR Test
Inverse variance weighted
MR Egger
Simple mode
Weighted median
Weighted mode
0.08
0.04
0.00
−0.04
−0.08
SNP effect on GCST90200352_buildGRCh38.tsv
0.1
0.2
0.3
0.4
0.5
SNP effect on CAG−177

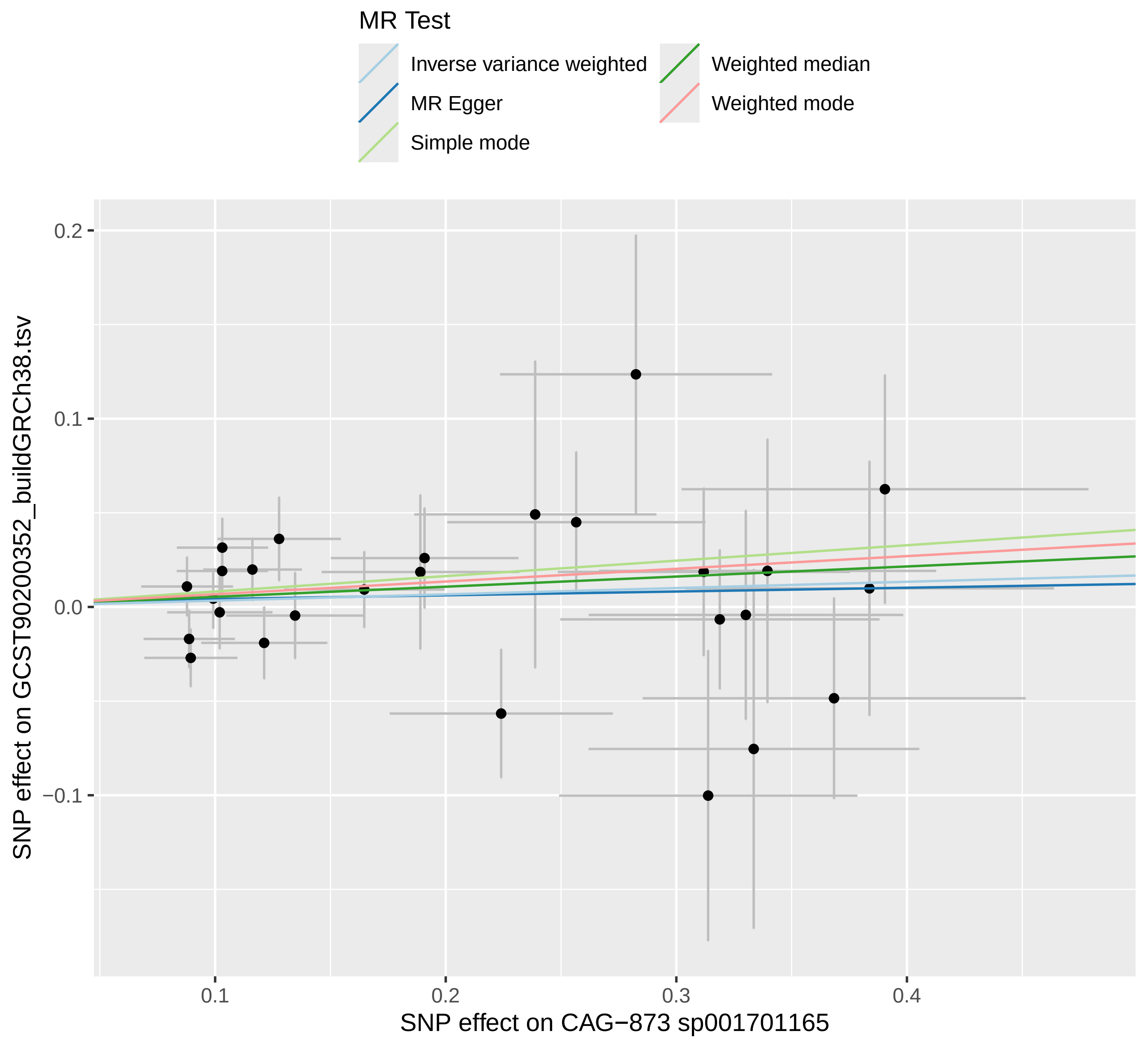

MR Test
Inverse variance weighted
MR Egger
Simple mode
Weighted median
Weighted mode
0.2
0.1
0.0
−0.1
0.1
0.2
0.3
0.4
SNP effect on GCST90200352_buildGRCh38.tsv
SNP effect on CAG−873 sp001701165

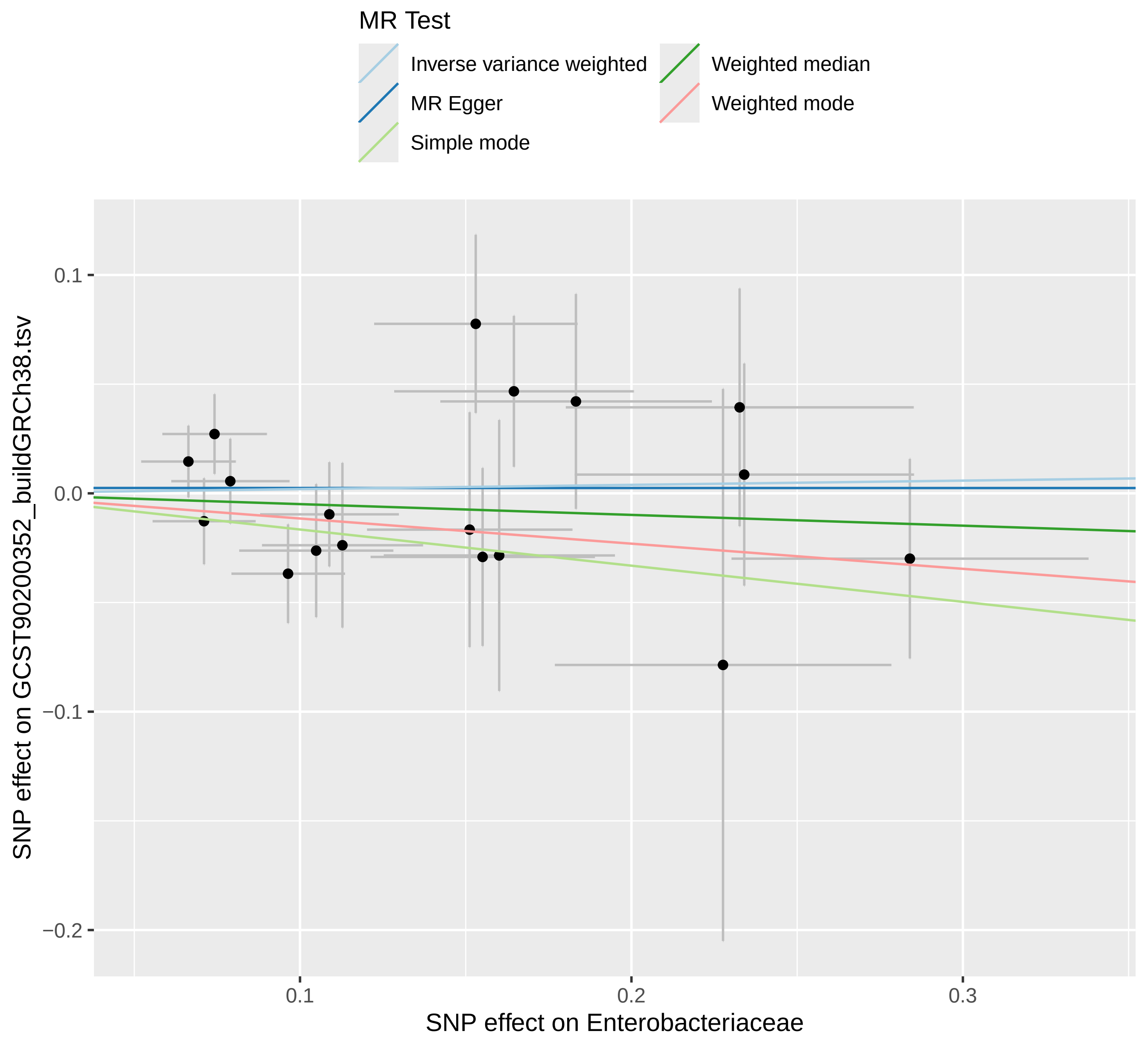

MR Test
Inverse variance weighted
MR Egger
Simple mode
Weighted median
Weighted mode
0.1
0.0
−0.1
−0.2
0.1
0.2
0.3
SNP effect on GCST90200352_buildGRCh38.tsv
SNP effect on Enterobacteriaceae

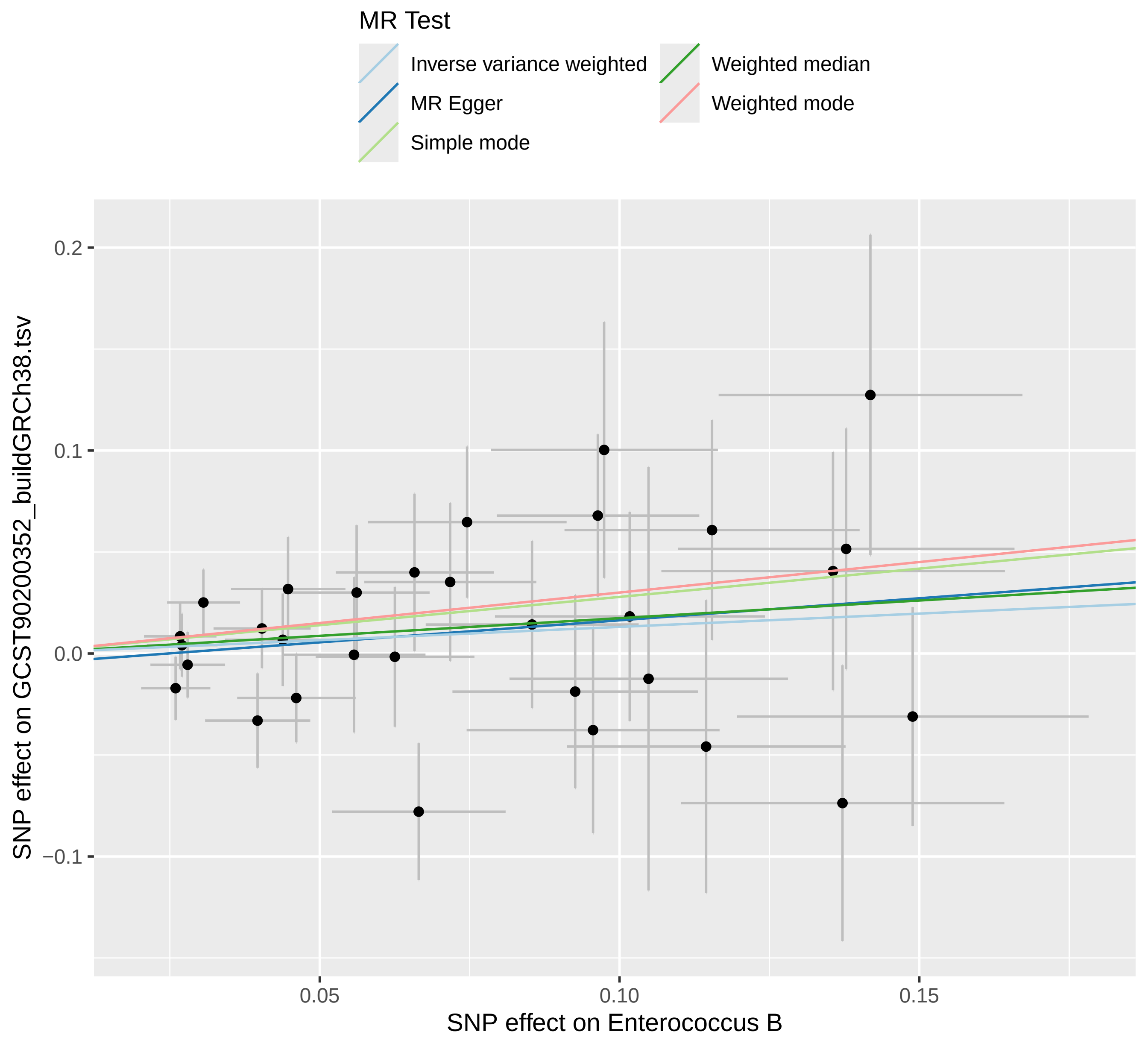
MR Test
Inverse variance weighted
MR Egger
Simple mode
Weighted median
Weighted mode
0.2
0.1
0.0
−0.1
0.05
0.10
0.15
SNP effect on GCST90200352_buildGRCh38.tsv
SNP effect on Enterococcus B

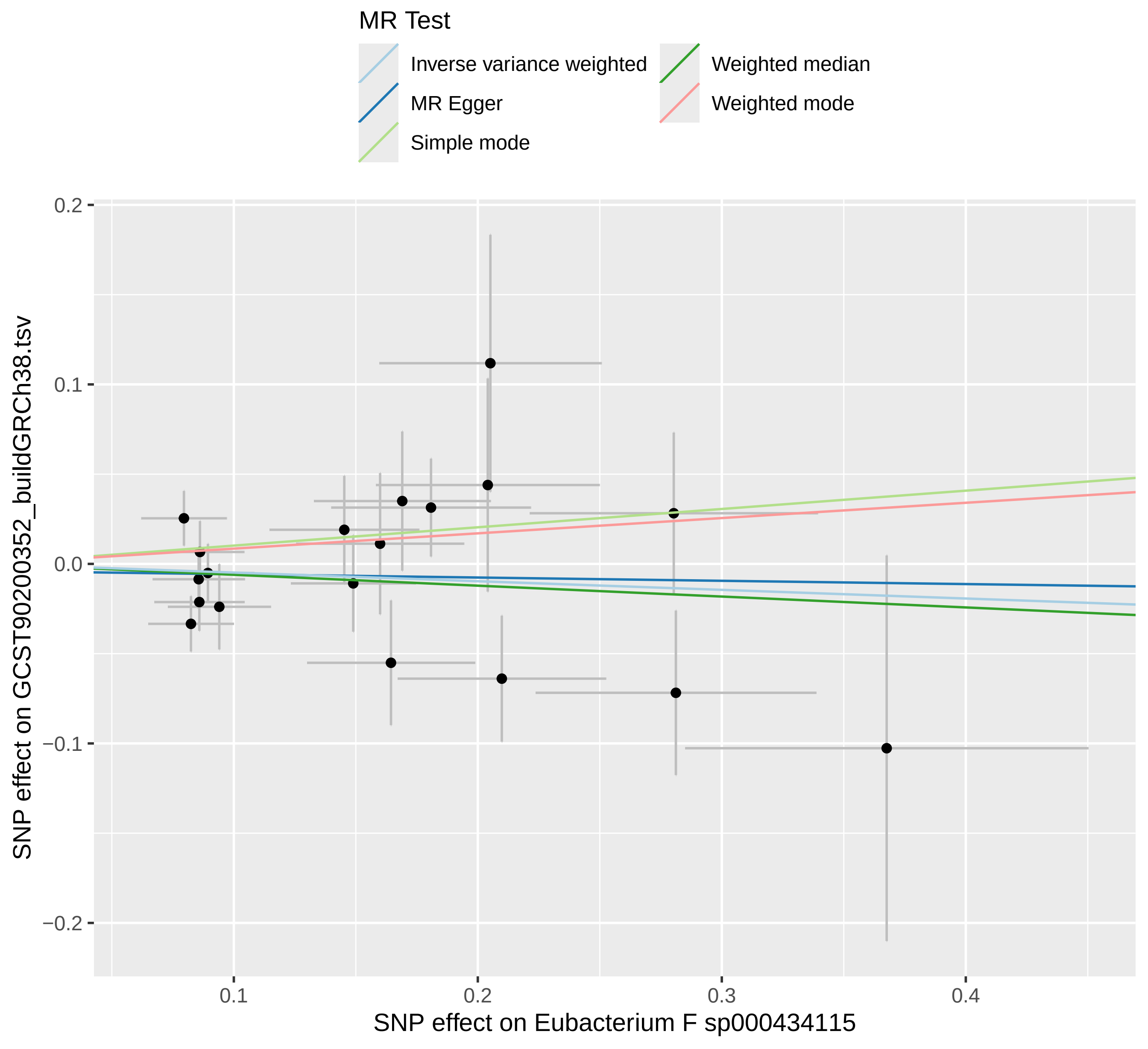
MR Test
Inverse variance weighted
MR Egger
Simple mode
Weighted median
Weighted mode
0.2
0.1
0.0
−0.1
−0.2
0.1
0.2
0.3
0.4
SNP effect on GCST90200352_buildGRCh38.tsv
SNP effect on Eubacterium F sp000434115

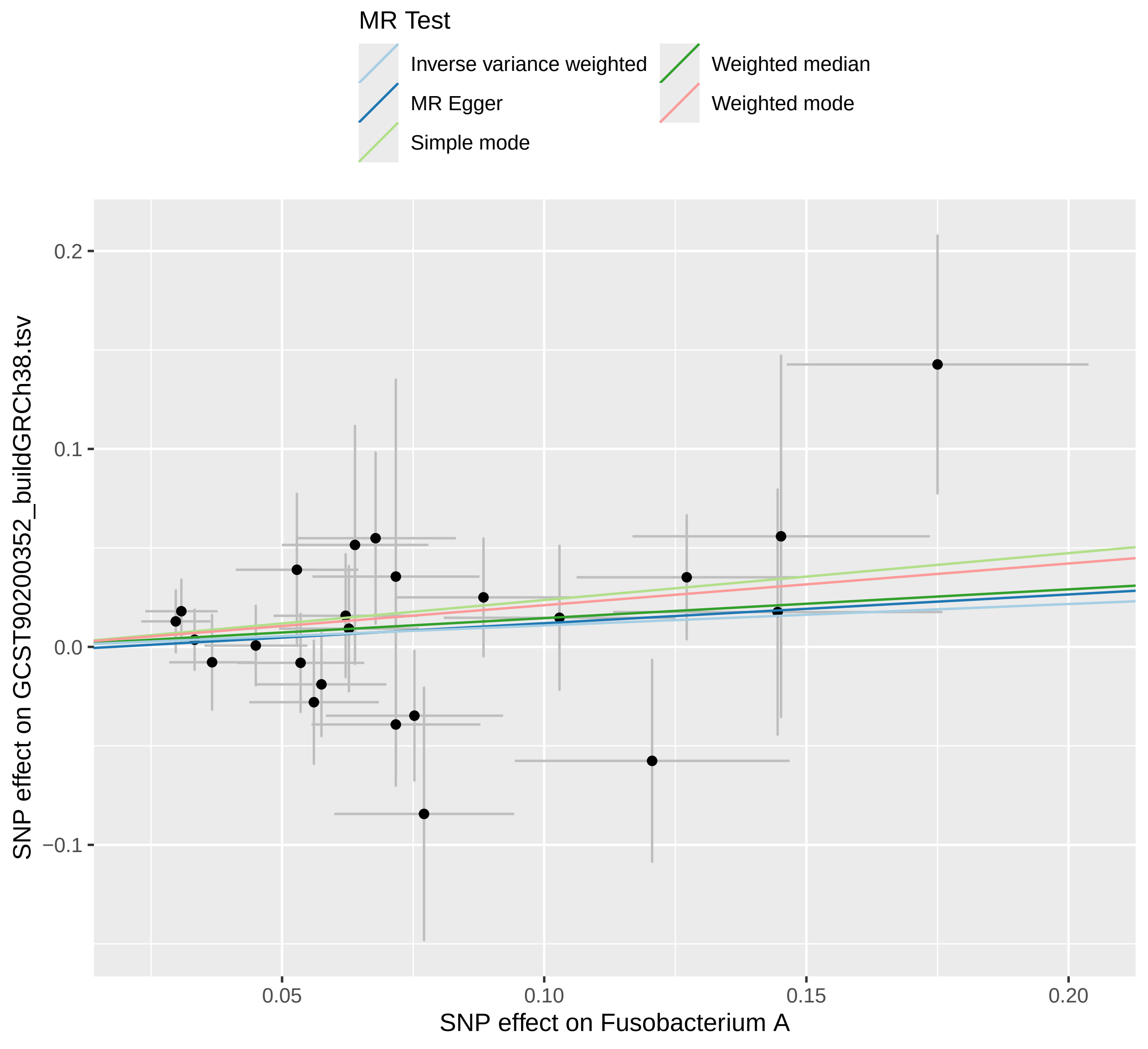
MR Test
Inverse variance weighted
MR Egger
Simple mode
Weighted median
Weighted mode
SNP effect on GCST90200352_buildGRCh38.tsv
0.2
0.1
0.0
−0.1
0.05
0.10
0.15
0.20
SNP effect on Fusobacterium A

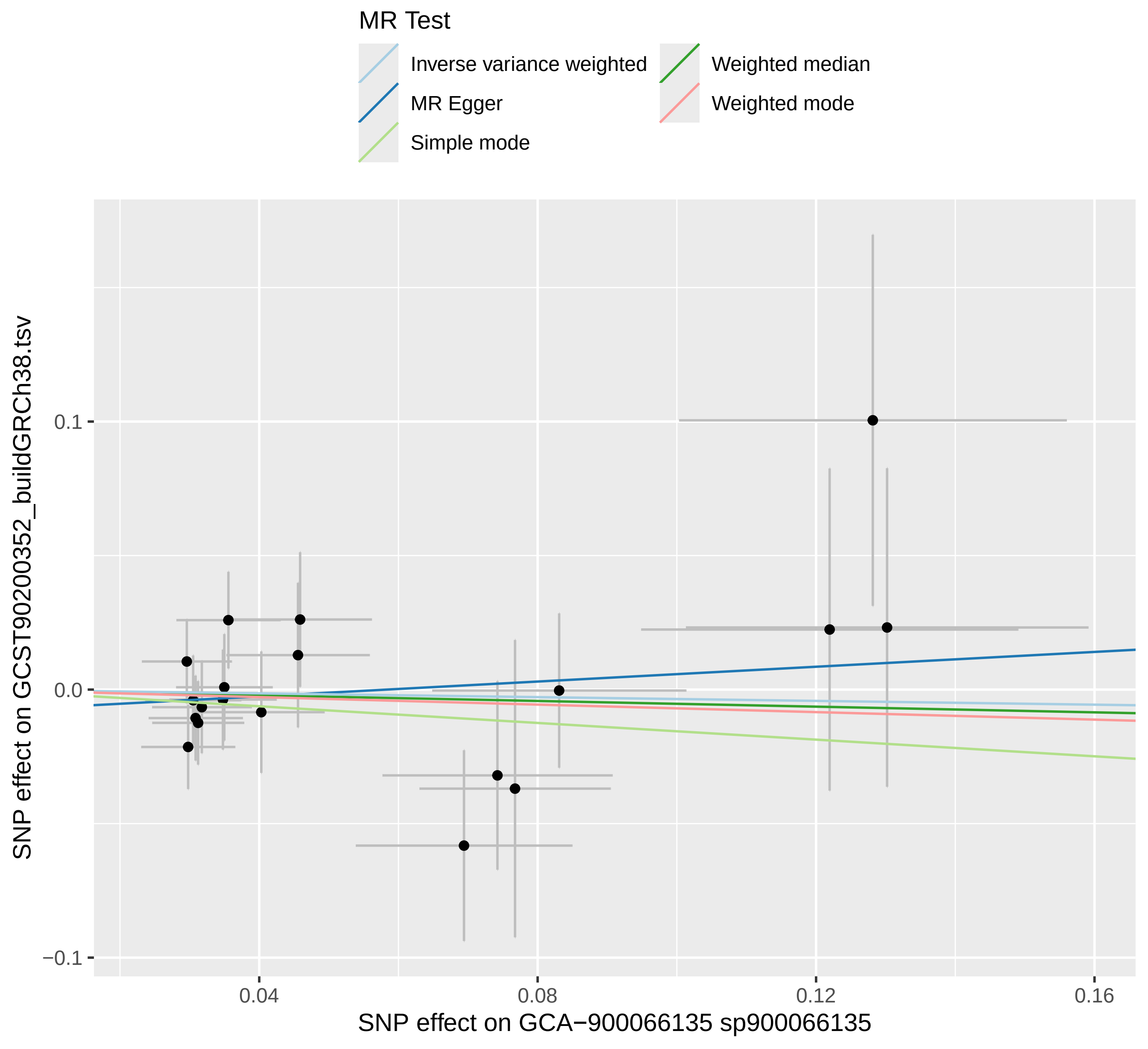
MR Test
Inverse variance weighted
MR Egger
Simple mode
Weighted median
Weighted mode
SNP effect on GCST90200352_buildGRCh38.tsv
0.1
0.0
−0.1
0.04
0.08
0.12
0.16
SNP effect on GCA−900066135 sp900066135

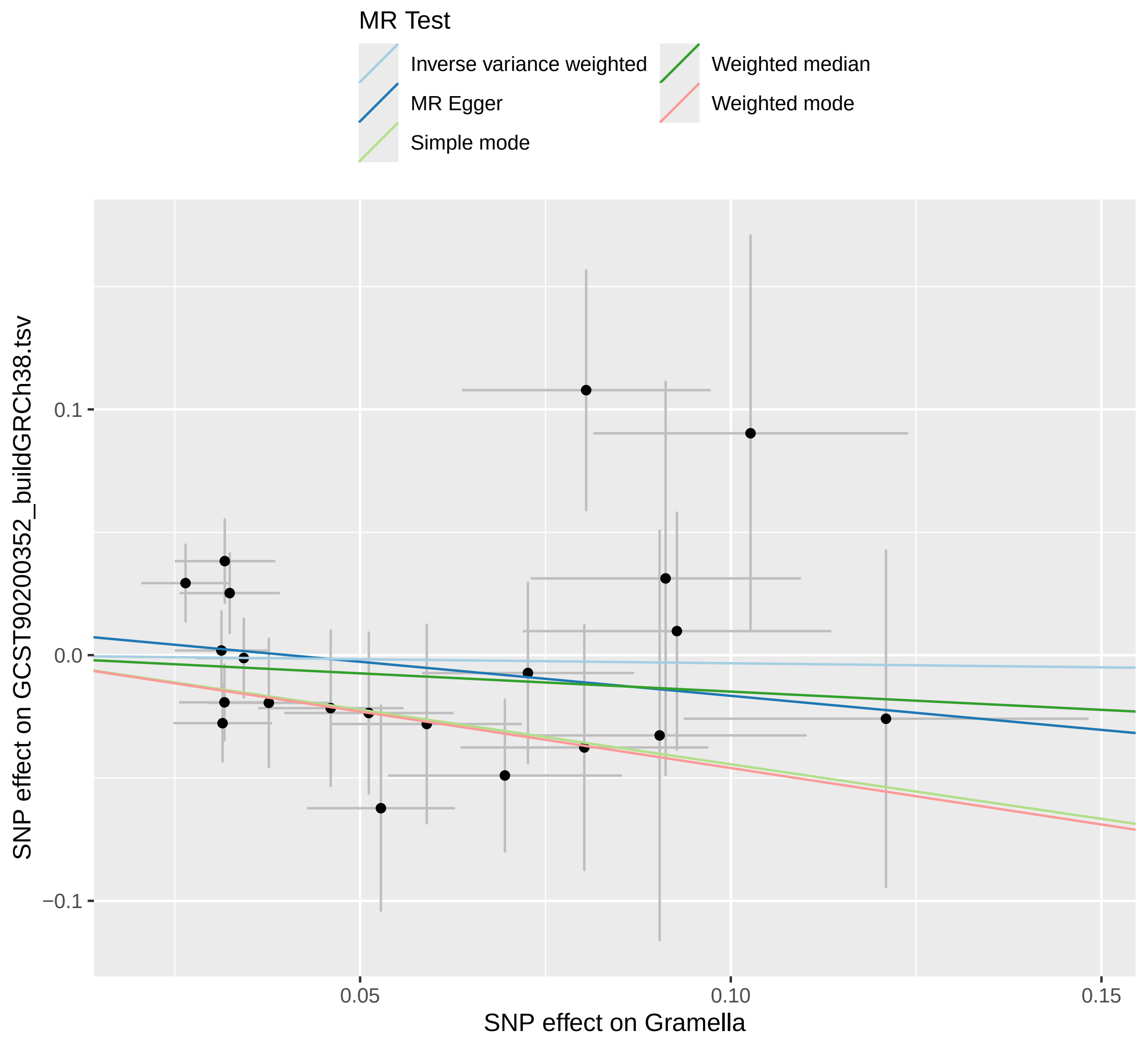
MR Test
Inverse variance weighted
MR Egger
Simple mode
Weighted median
Weighted mode
0.1
0.0
−0.1
0.05
0.10
0.15
SNP effect on GCST90200352_buildGRCh38.tsv
SNP effect on Gramella

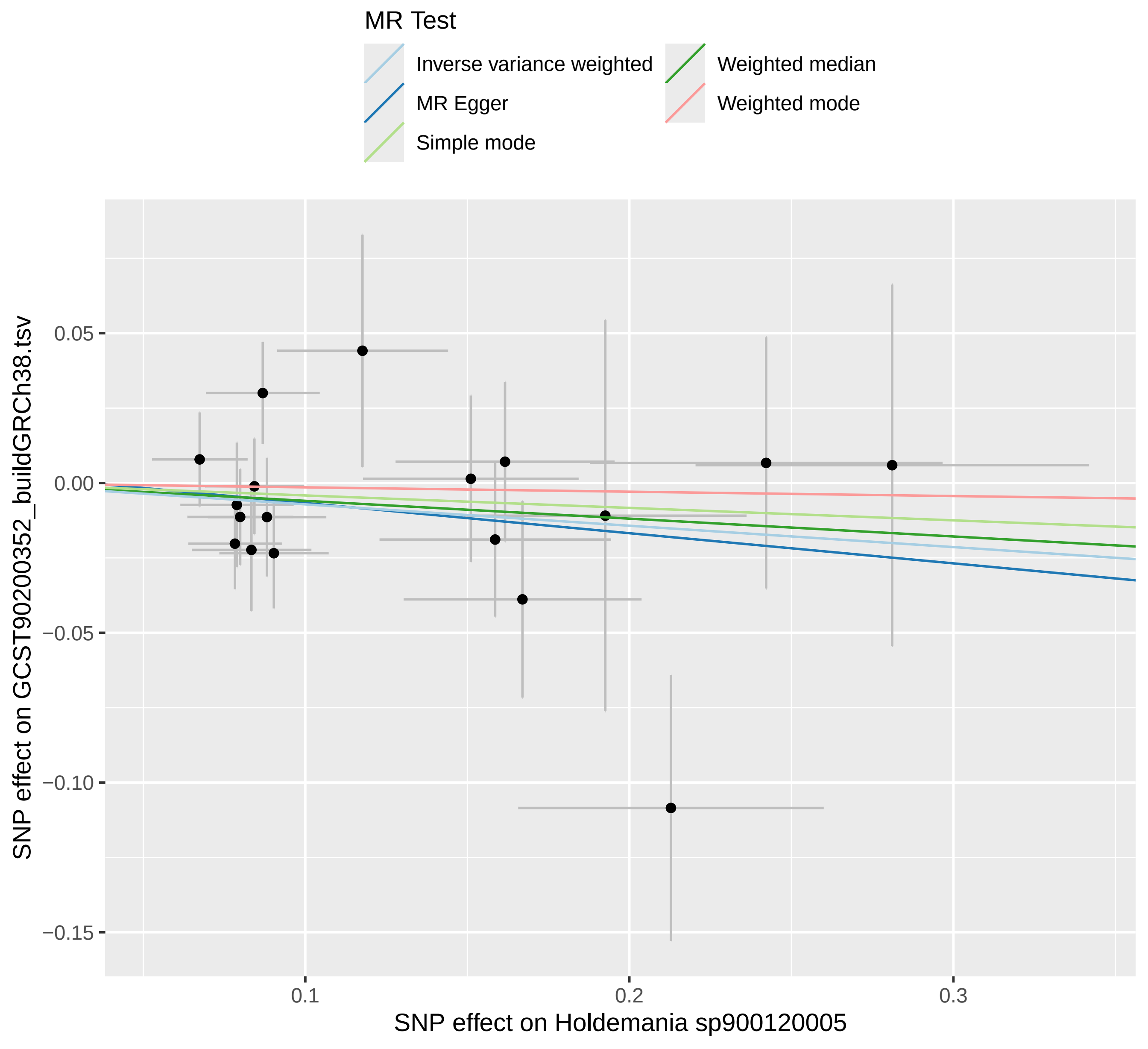
MR Test
Inverse variance weighted
MR Egger
Simple mode
Weighted median
Weighted mode
0.05
0.00
−0.05
−0.10
−0.15
0.1
0.2
0.3
SNP effect on GCST90200352_buildGRCh38.tsv
SNP effect on Holdemania sp900120005

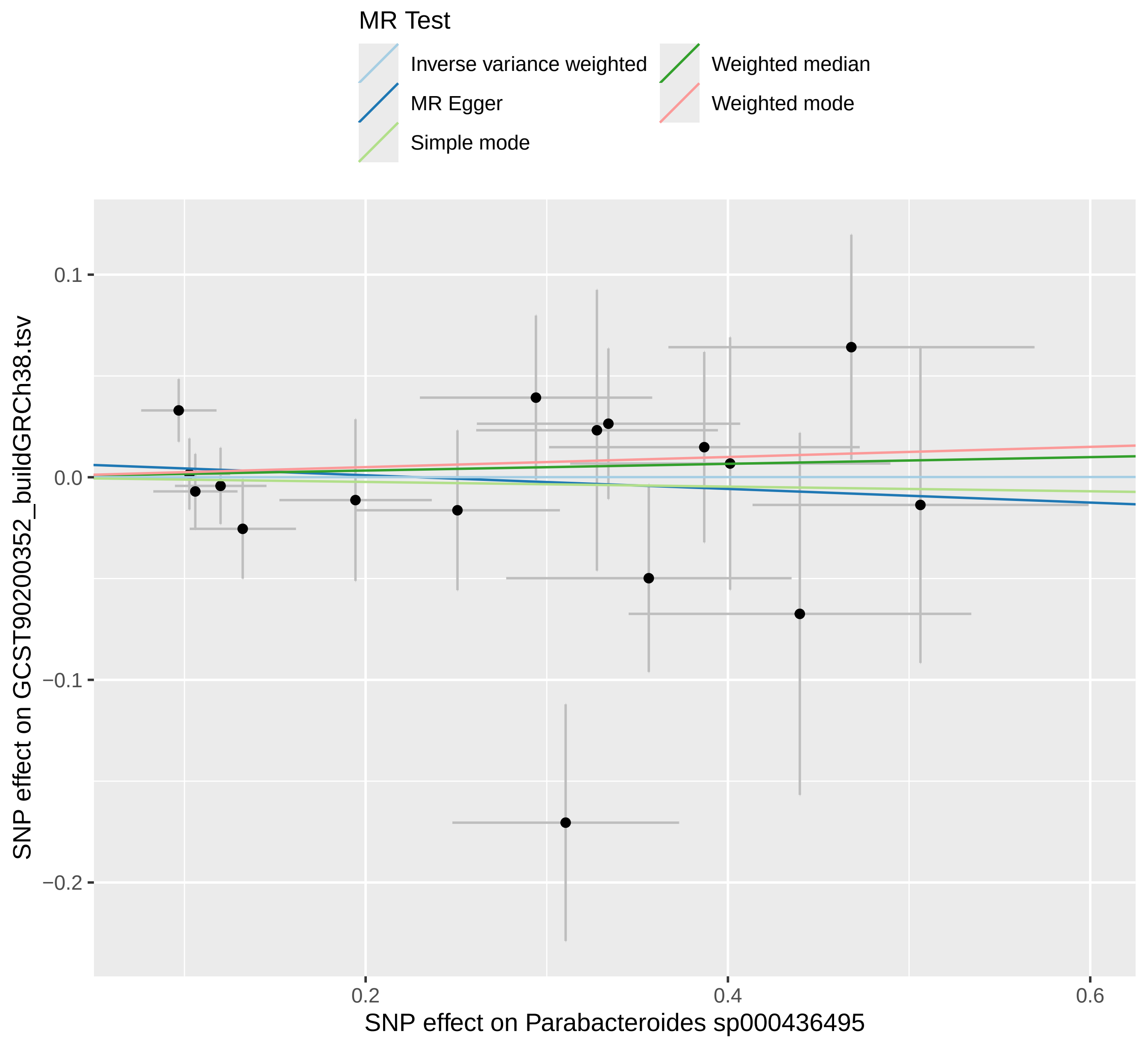
MR Test
Inverse variance weighted
MR Egger
Simple mode
Weighted median
Weighted mode
0.1
0.0
−0.1
−0.2
0.2
0.4
0.6
SNP effect on GCST90200352_buildGRCh38.tsv
SNP effect on Parabacteroides sp000436495

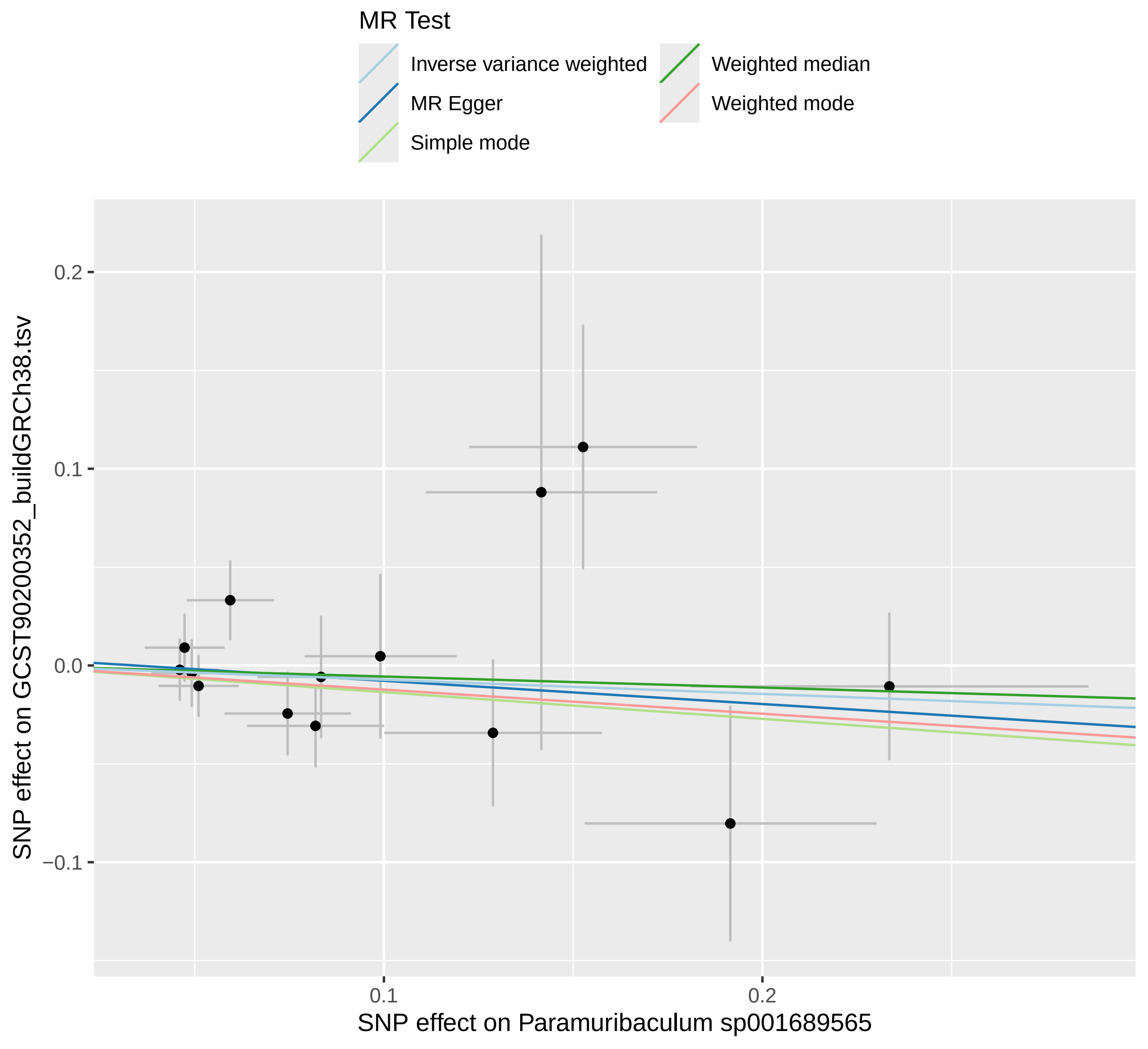
MR Test
Inverse variance weighted
MR Egger
Simple mode
Weighted median
Weighted mode
0.2
0.1
0.0
−0.1
0.1
0.2
SNP effect on GCST90200352_buildGRCh38.tsv
SNP effect on Paramuribaculum sp001689565

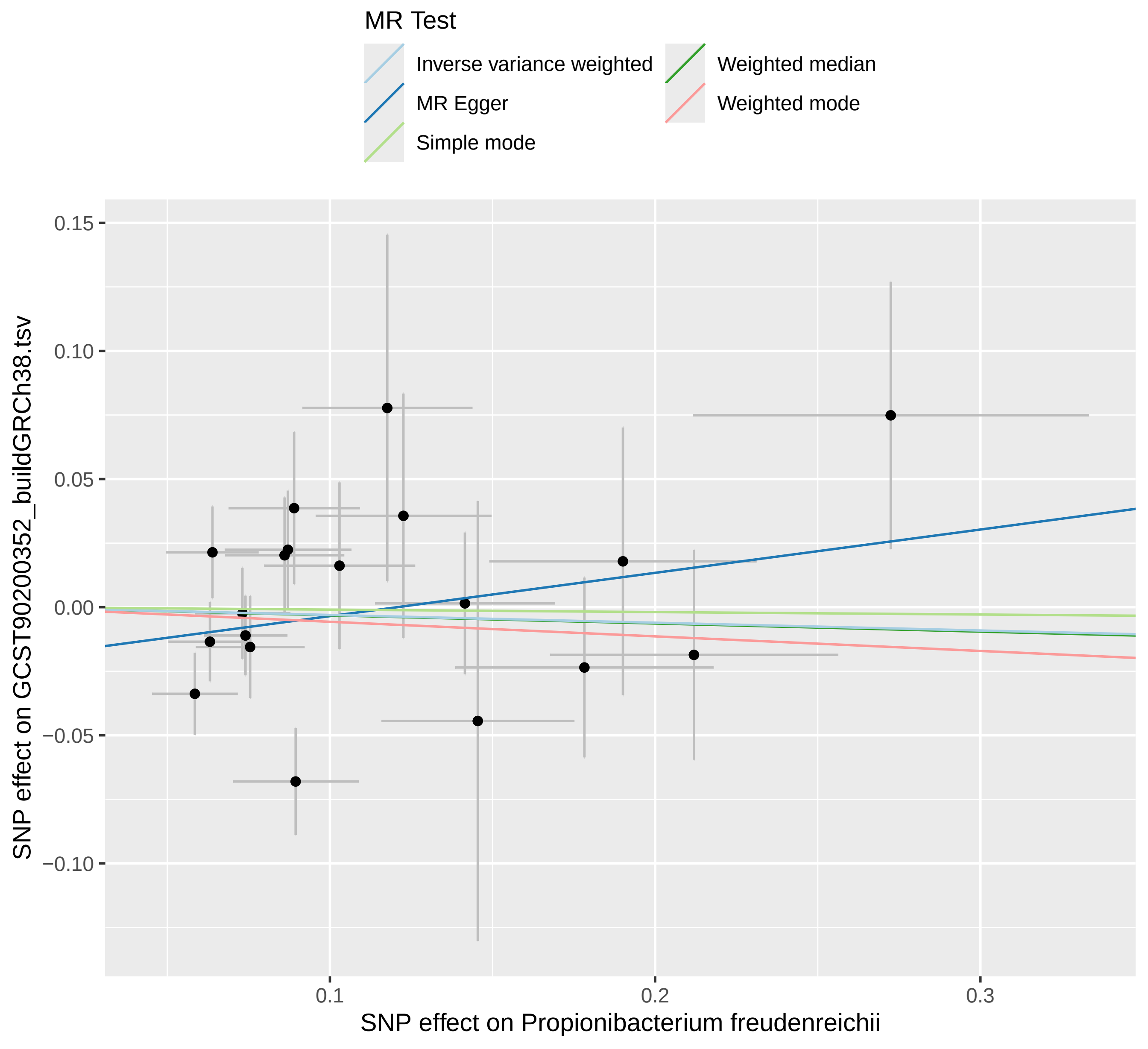

MR Test
Inverse variance weighted
MR Egger
Simple mode
Weighted median
Weighted mode
0.15
0.10
0.05
0.00
−0.05
−0.10
0.1
0.2
0.3
SNP effect on GCST90200352_buildGRCh38.tsv
SNP effect on Propionibacterium freudenreichii

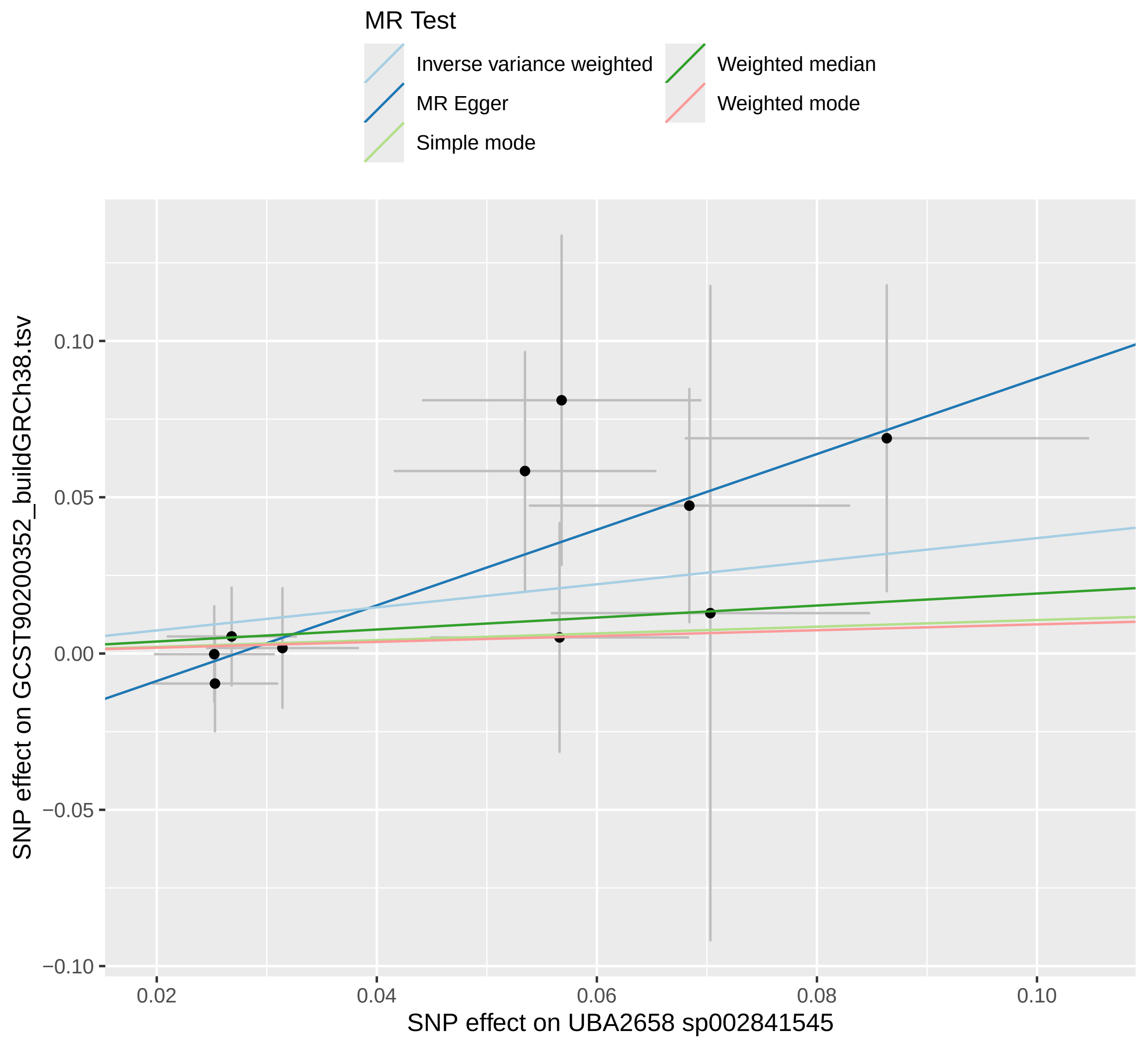

MR Test
Inverse variance weighted
MR Egger
Simple mode
Weighted median
Weighted mode
0.10
0.05
0.00
−0.05
−0.10
0.02
0.04
0.06
0.08
0.10
SNP effect on GCST90200352_buildGRCh38.tsv
SNP effect on UBA2658 sp002841545

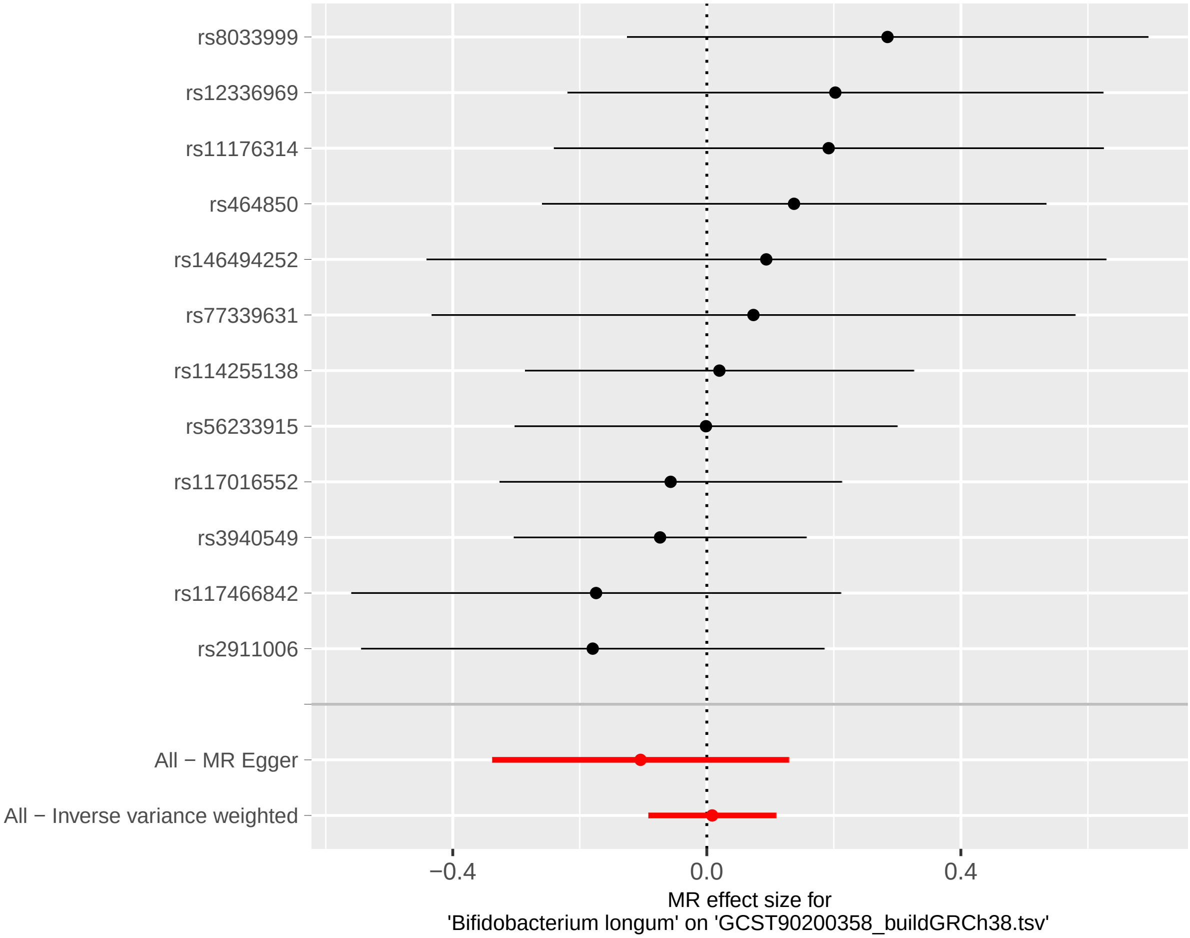
rs8033999
rs12336969
rs11176314
rs464850
rs146494252
rs77339631
rs114255138
rs56233915
rs117016552
rs3940549
rs117466842
rs2911006
All − MR Egger
All − Inverse variance weighted
−0.4
0.0
0.4
MR effect size for
'Bifidobacterium longum' on 'GCST90200358_buildGRCh38.tsv'

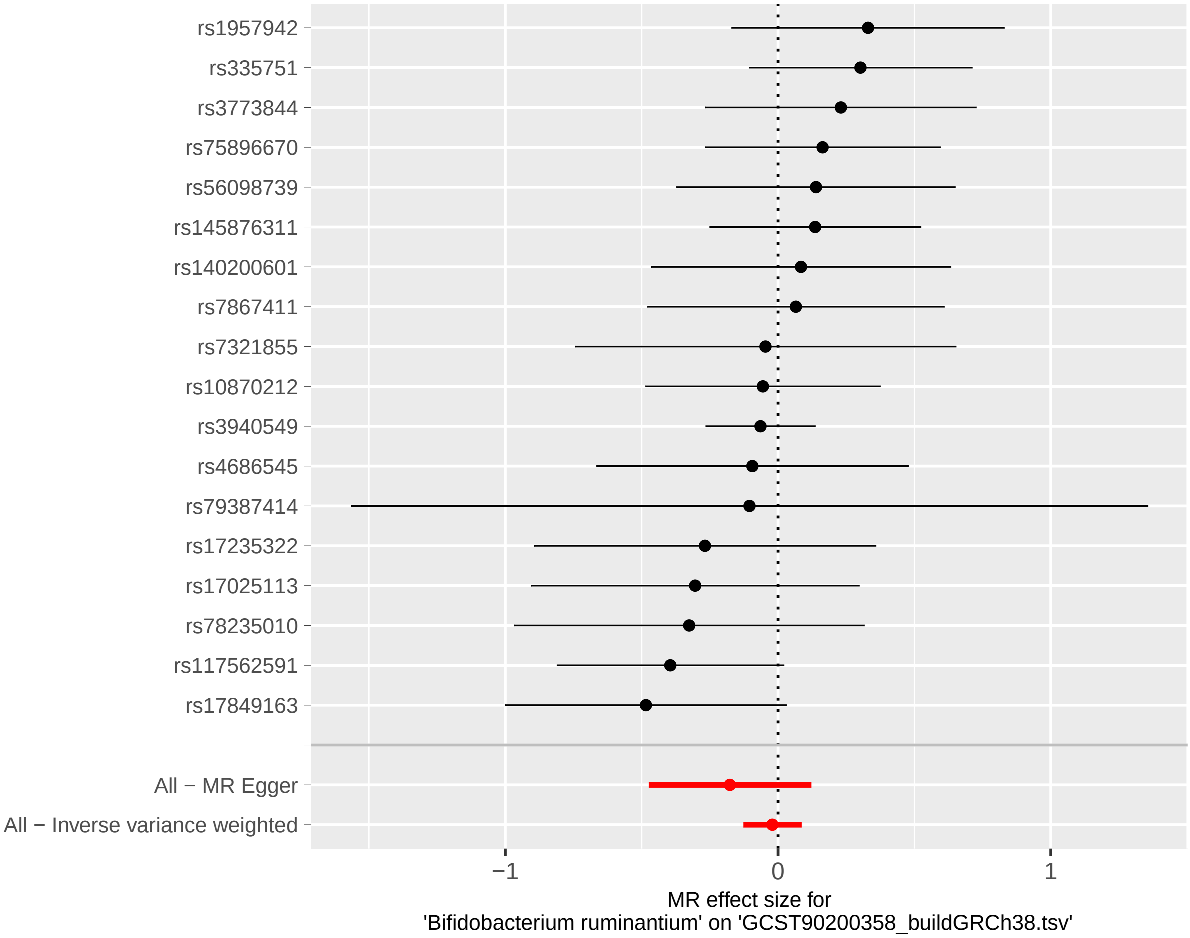
rs1957942
rs335751
rs3773844
rs75896670
rs56098739
rs145876311
rs140200601
rs7867411
rs7321855
rs10870212
rs3940549
rs4686545
rs79387414
rs17235322
rs17025113
rs78235010
rs117562591
rs17849163
All − MR Egger
All − Inverse variance weighted
−1
0
1
MR effect size for
'Bifidobacterium ruminantium' on 'GCST90200358_buildGRCh38.tsv'

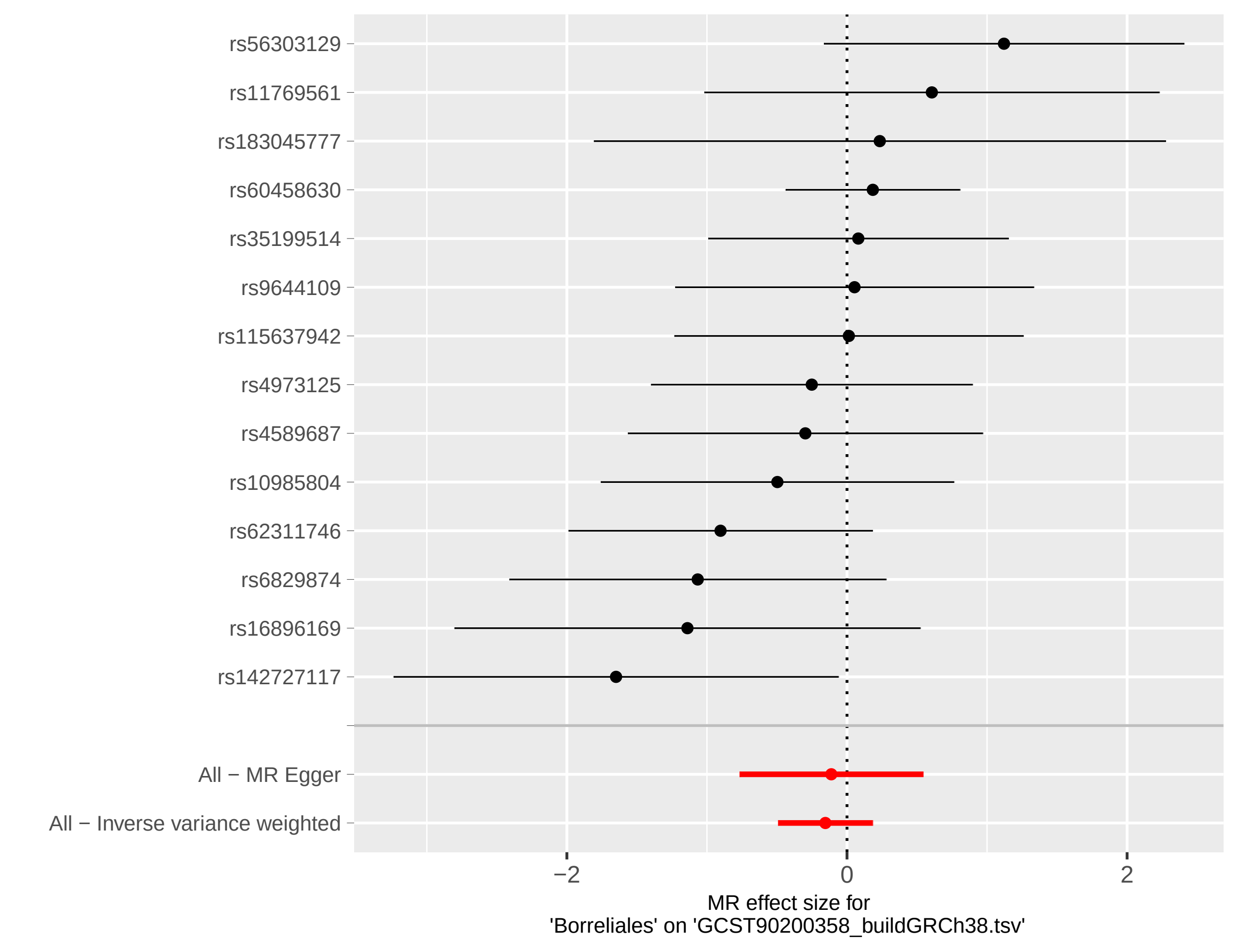

rs56303129
rs11769561
rs183045777
rs60458630
rs35199514
rs9644109
rs115637942
rs4973125
rs4589687
rs10985804
rs62311746
rs6829874
rs16896169
rs142727117
All − MR Egger
All − Inverse variance weighted
−2
0
2
MR effect size for
'Borreliales' on 'GCST90200358_buildGRCh38.tsv'

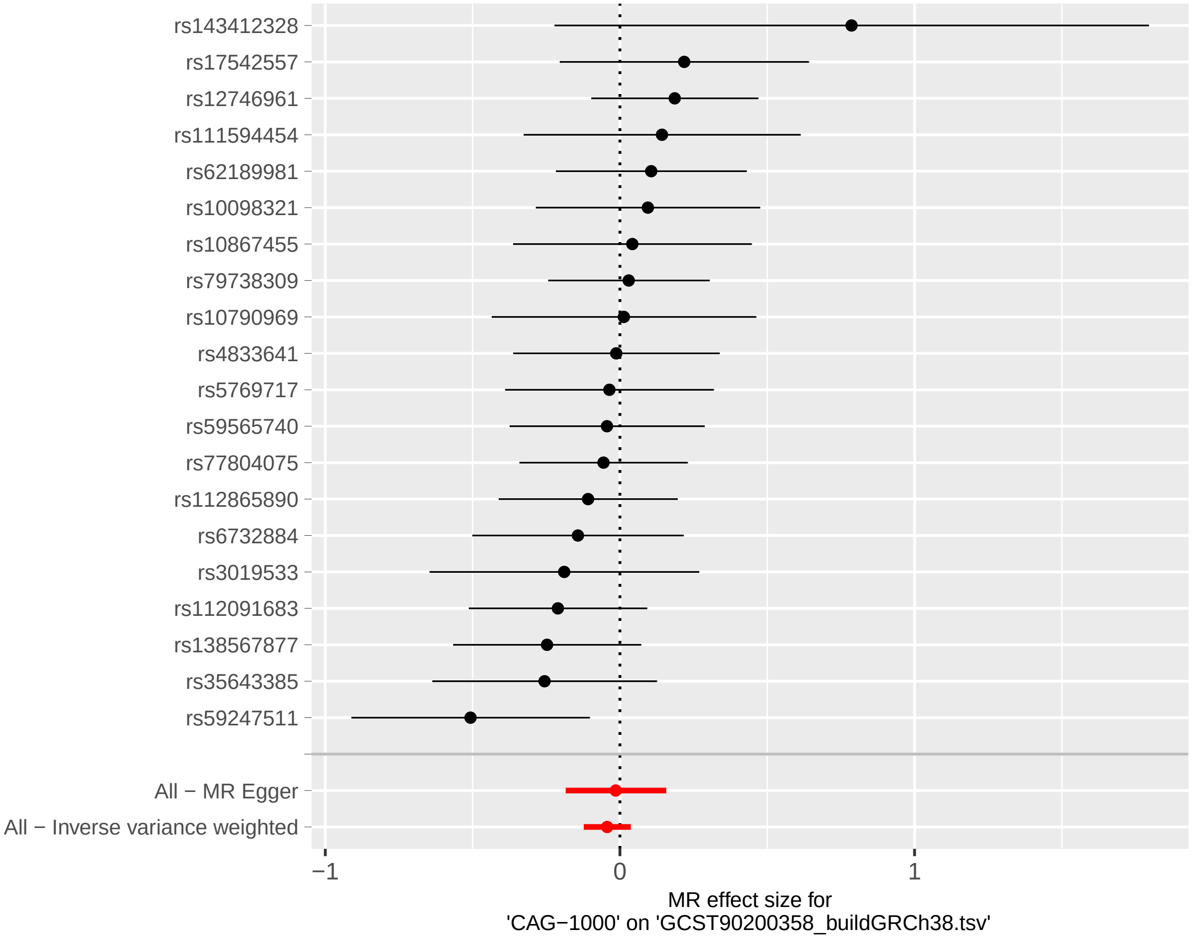
rs143412328
rs17542557
rs12746961
rs111594454
rs62189981
rs10098321
rs10867455
rs79738309
rs10790969
rs4833641
rs5769717
rs59565740
rs77804075
rs112865890
rs6732884
rs3019533
rs112091683
rs138567877
rs35643385
rs59247511
All − MR Egger
All − Inverse variance weighted
−1
0
1
MR effect size for
'CAG−1000' on 'GCST90200358_buildGRCh38.tsv'

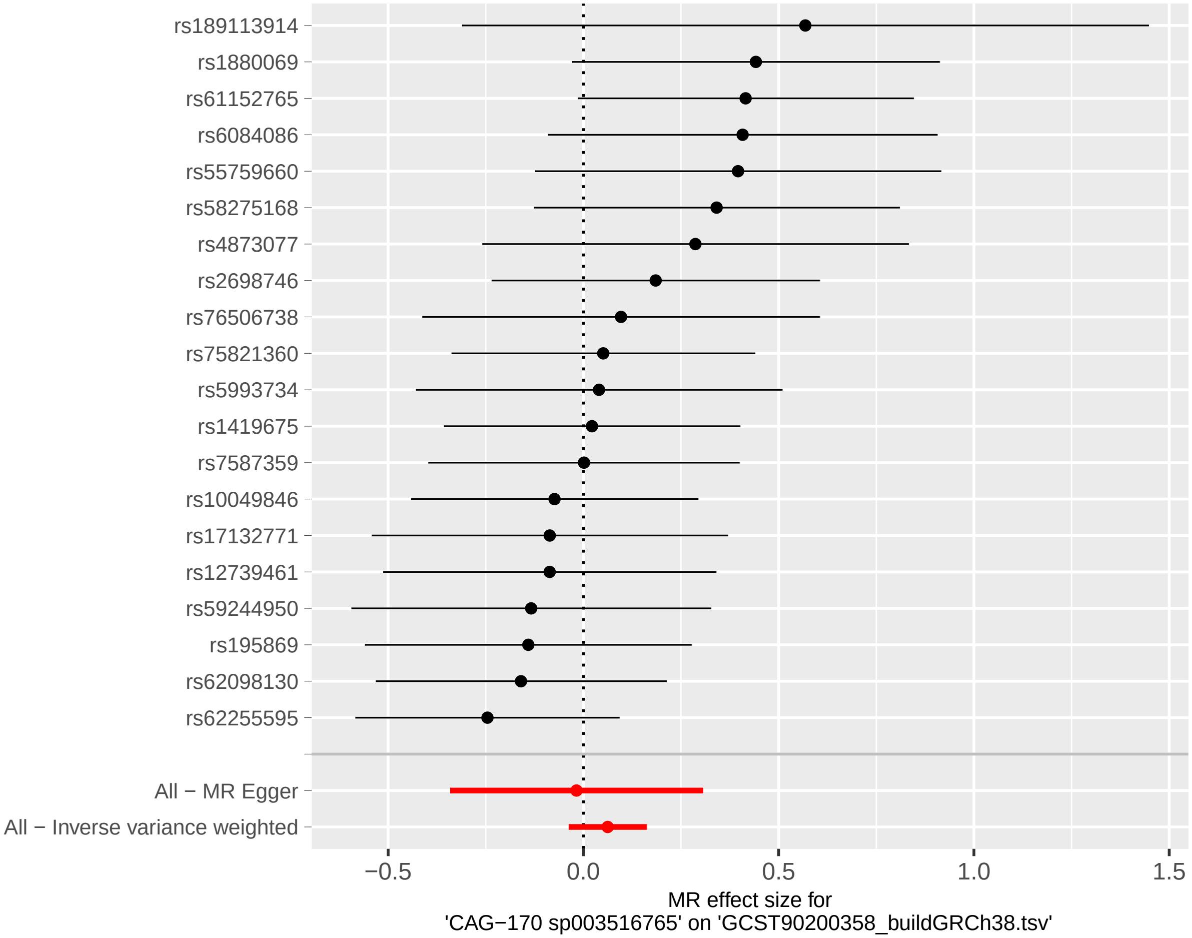

rs189113914
rs1880069
rs61152765
rs6084086
rs55759660
rs58275168
rs4873077
rs2698746
rs76506738
rs75821360
rs5993734
rs1419675
rs7587359
rs10049846
rs17132771
rs12739461
rs59244950
rs195869
rs62098130
rs62255595
All − MR Egger
All − Inverse variance weighted
−0.5
0.0
0.5
1.0
1.5
MR effect size for
'CAG−170 sp003516765' on 'GCST90200358_buildGRCh38.tsv'

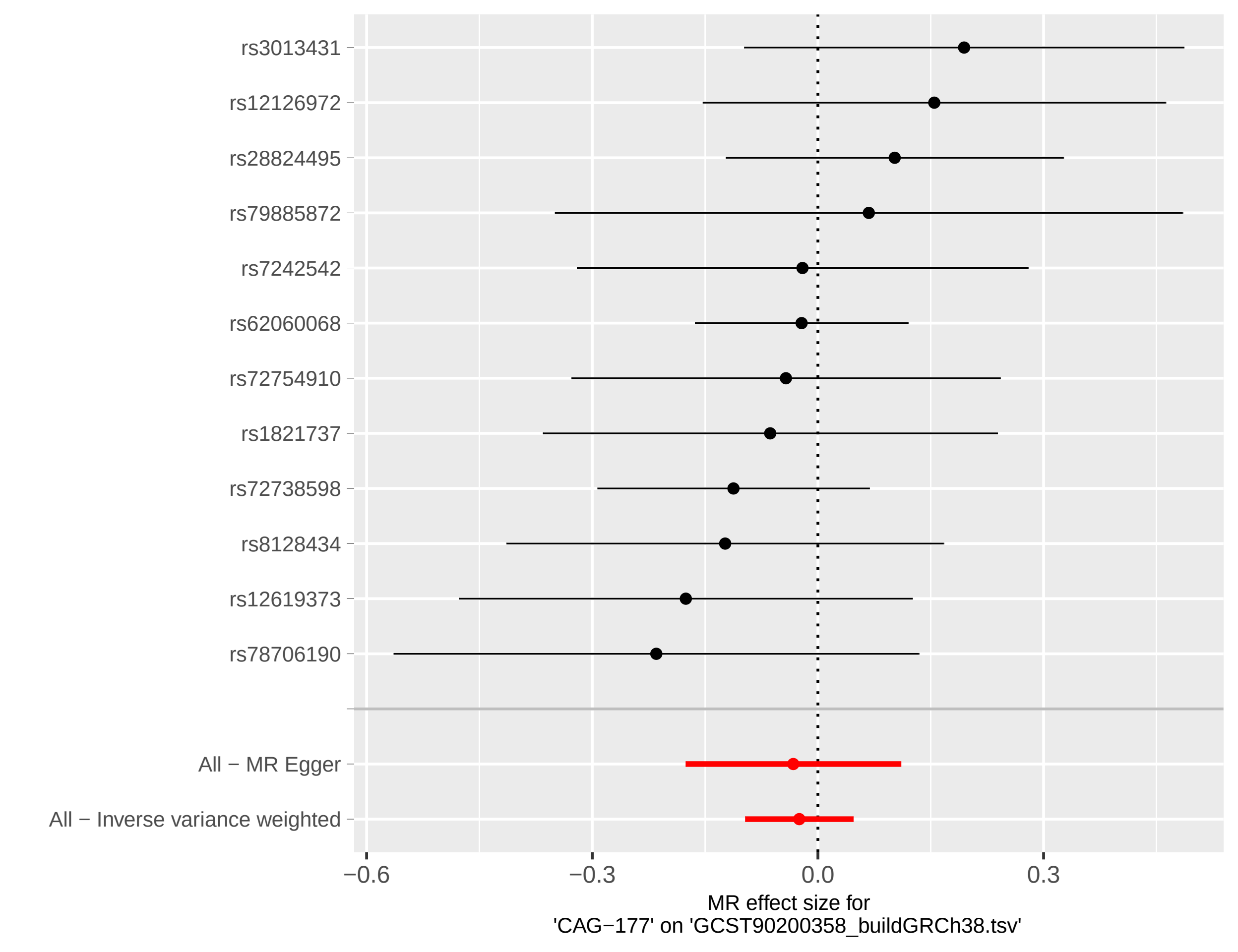
rs3013431
rs12126972
rs28824495
rs79885872
rs7242542
rs62060068
rs72754910
rs1821737
rs72738598
rs8128434
rs12619373
rs78706190
All − MR Egger
All − Inverse variance weighted
−0.6
−0.3
0.0
0.3
MR effect size for
'CAG−177' on 'GCST90200358_buildGRCh38.tsv'

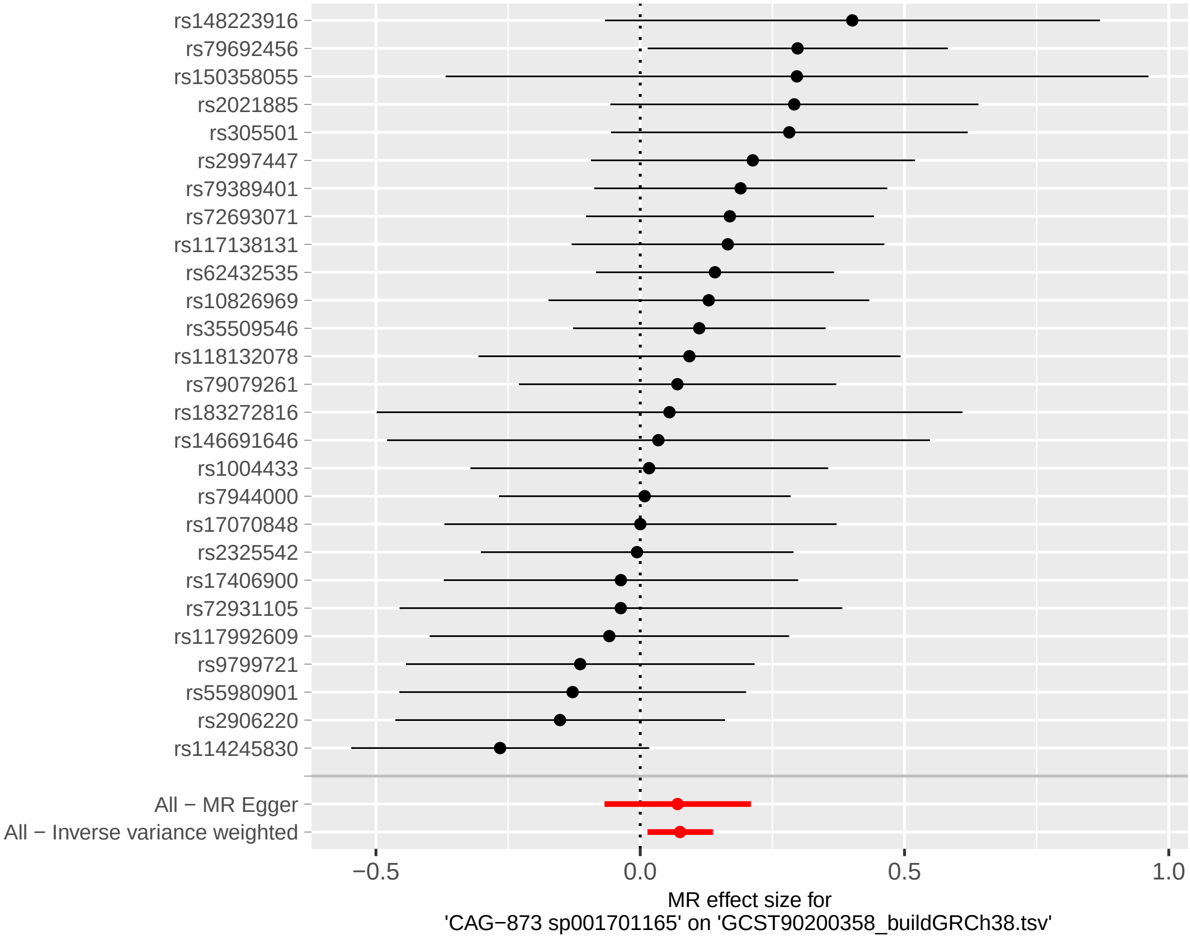

rs148223916
rs79692456
rs150358055
rs2021885
rs305501
rs2997447
rs79389401
rs72693071
rs117138131
rs62432535
rs10826969
rs35509546
rs118132078
rs79079261
rs183272816
rs146691646
rs1004433
rs7944000
rs17070848
rs2325542
rs17406900
rs72931105
rs117992609
rs9799721
rs55980901
rs2906220
rs114245830
All − MR Egger
All − Inverse variance weighted
−0.5
0.0
0.5
1.0
MR effect size for
'CAG−873 sp001701165' on 'GCST90200358_buildGRCh38.tsv'

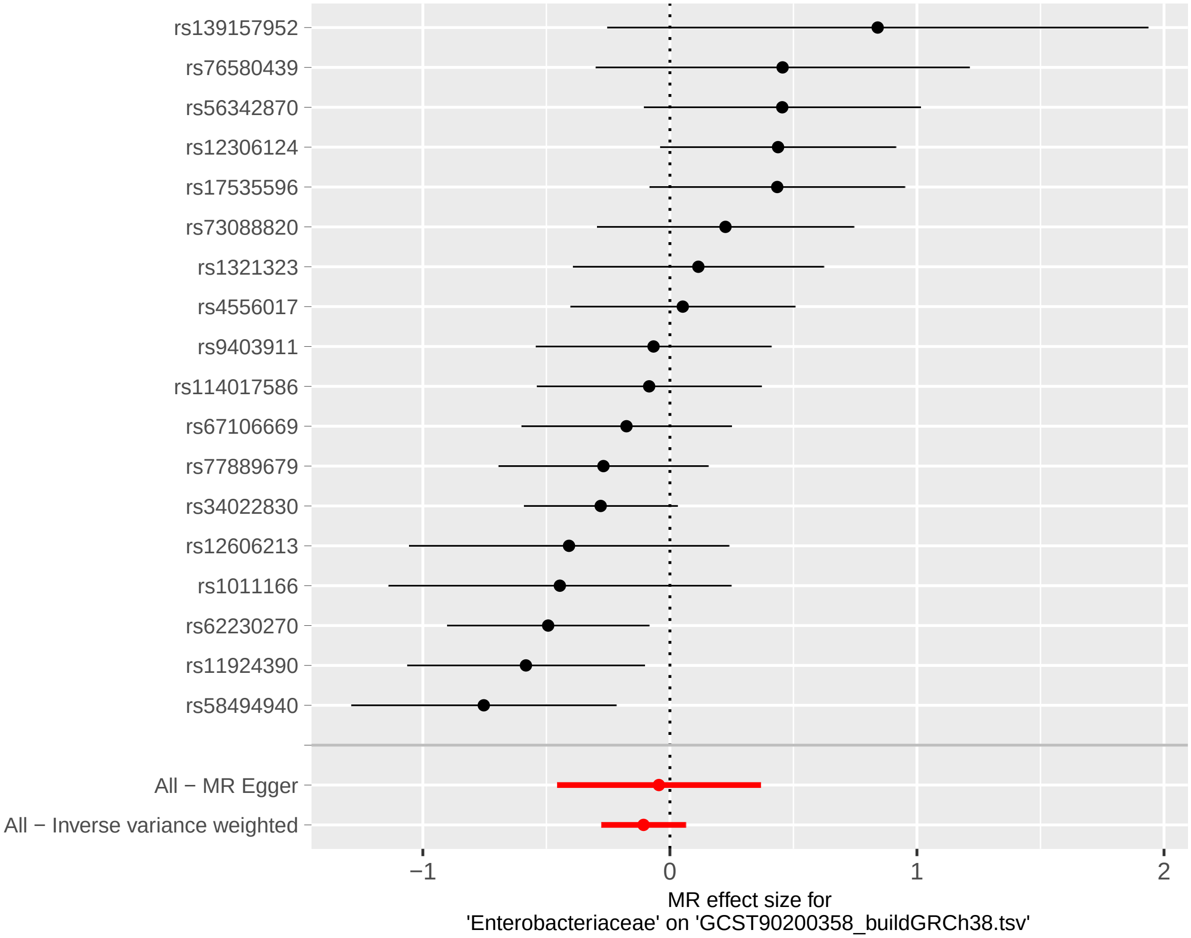

rs139157952
rs76580439
rs56342870
rs12306124
rs17535596
rs73088820
rs1321323
rs4556017
rs9403911
rs114017586
rs67106669
rs77889679
rs34022830
rs12606213
rs1011166
rs62230270
rs11924390
rs58494940
All − MR Egger
All − Inverse variance weighted
−1
0
1
2
MR effect size for
'Enterobacteriaceae' on 'GCST90200358_buildGRCh38.tsv'

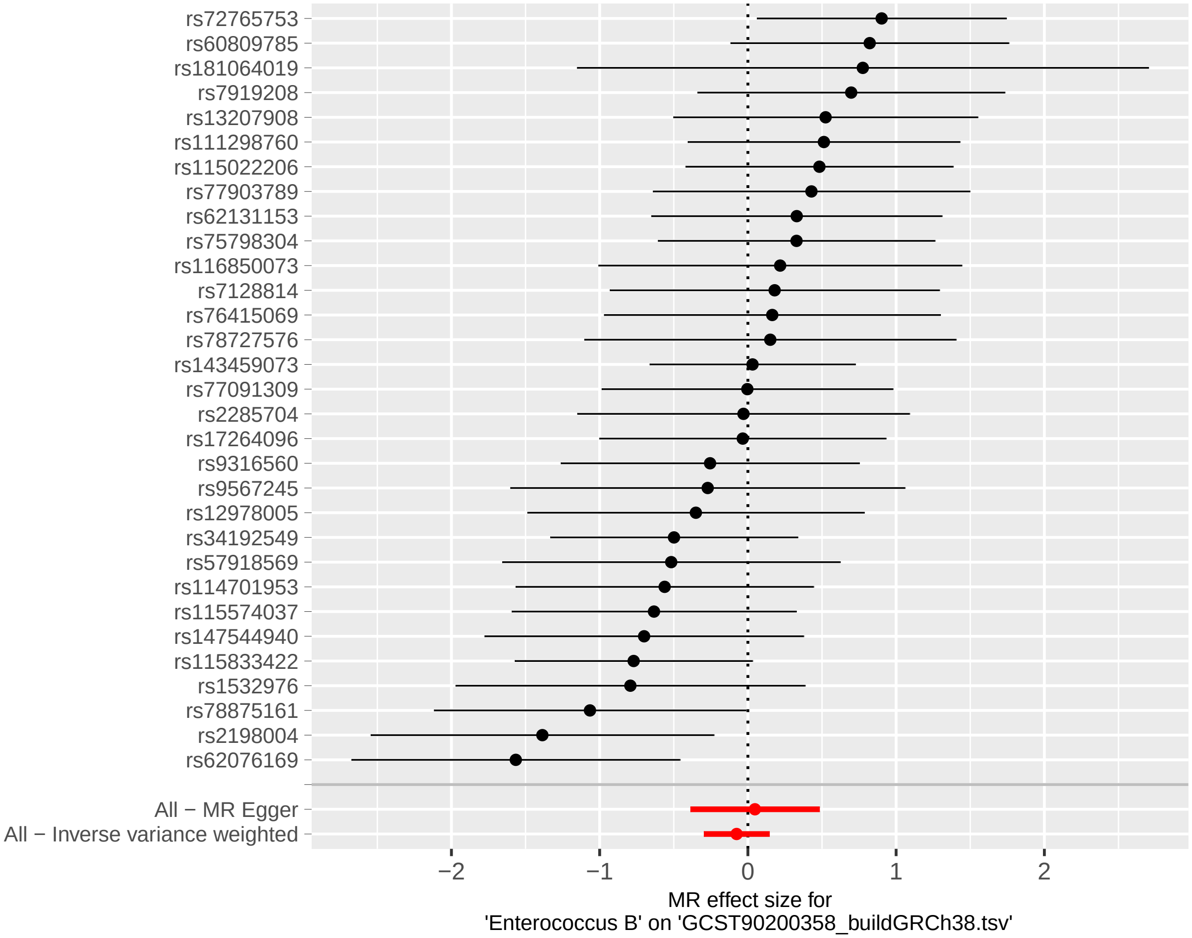
rs72765753
rs60809785
rs181064019
rs7919208
rs13207908
rs111298760
rs115022206
rs77903789
rs62131153
rs75798304
rs116850073
rs7128814
rs76415069
rs78727576
rs143459073
rs77091309
rs2285704
rs17264096
rs9316560
rs9567245
rs12978005
rs34192549
rs57918569
rs114701953
rs115574037
rs147544940
rs115833422
rs1532976
rs78875161
rs2198004
rs62076169
All − MR Egger
All − Inverse variance weighted
−2
−1
0
1
2
MR effect size for
'Enterococcus B' on 'GCST90200358_buildGRCh38.tsv'

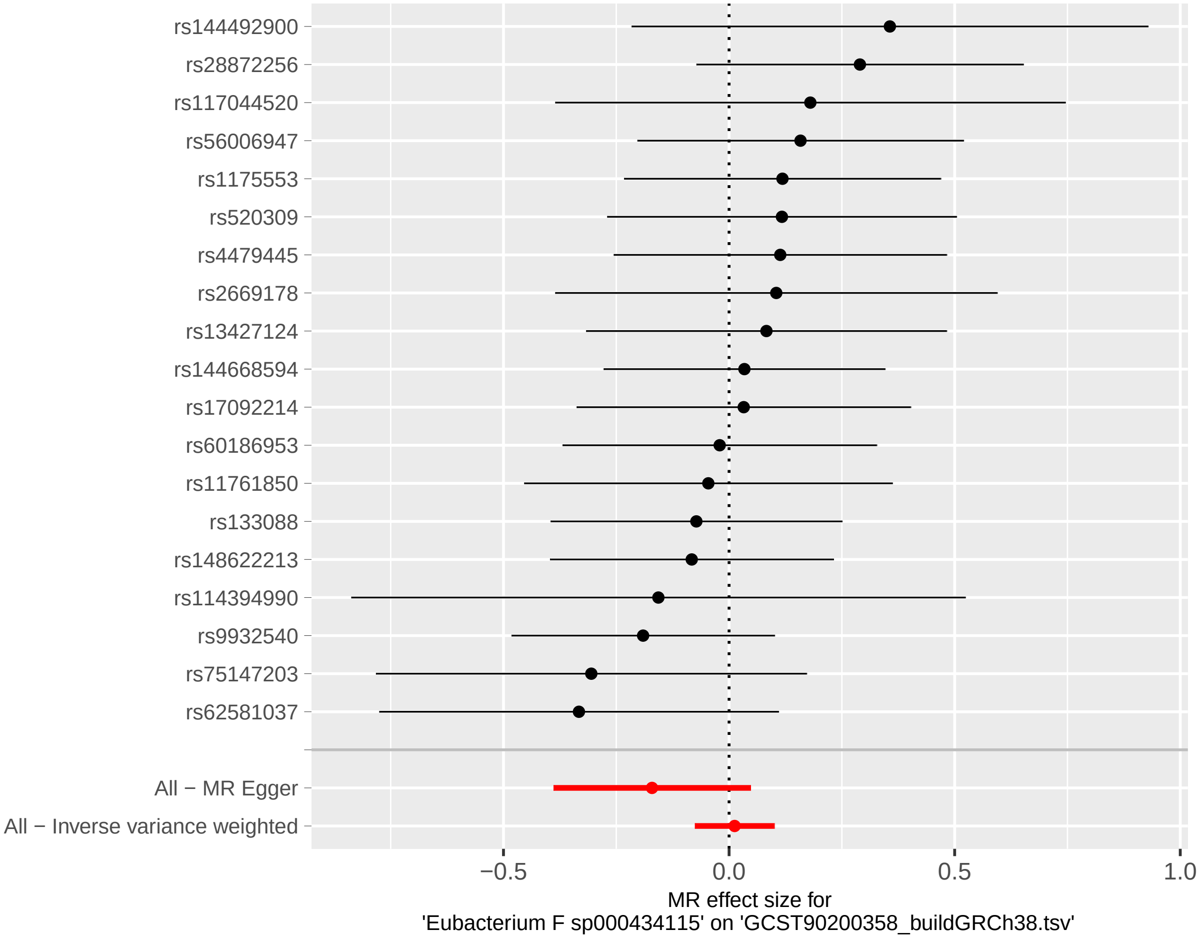
rs144492900
rs28872256
rs117044520
rs56006947
rs1175553
rs520309
rs4479445
rs2669178
rs13427124
rs144668594
rs17092214
rs60186953
rs11761850
rs133088
rs148622213
rs114394990
rs9932540
rs75147203
rs62581037
All − MR Egger
All − Inverse variance weighted
−0.5
0.0
0.5
1.0
MR effect size for
'Eubacterium F sp000434115' on 'GCST90200358_buildGRCh38.tsv'

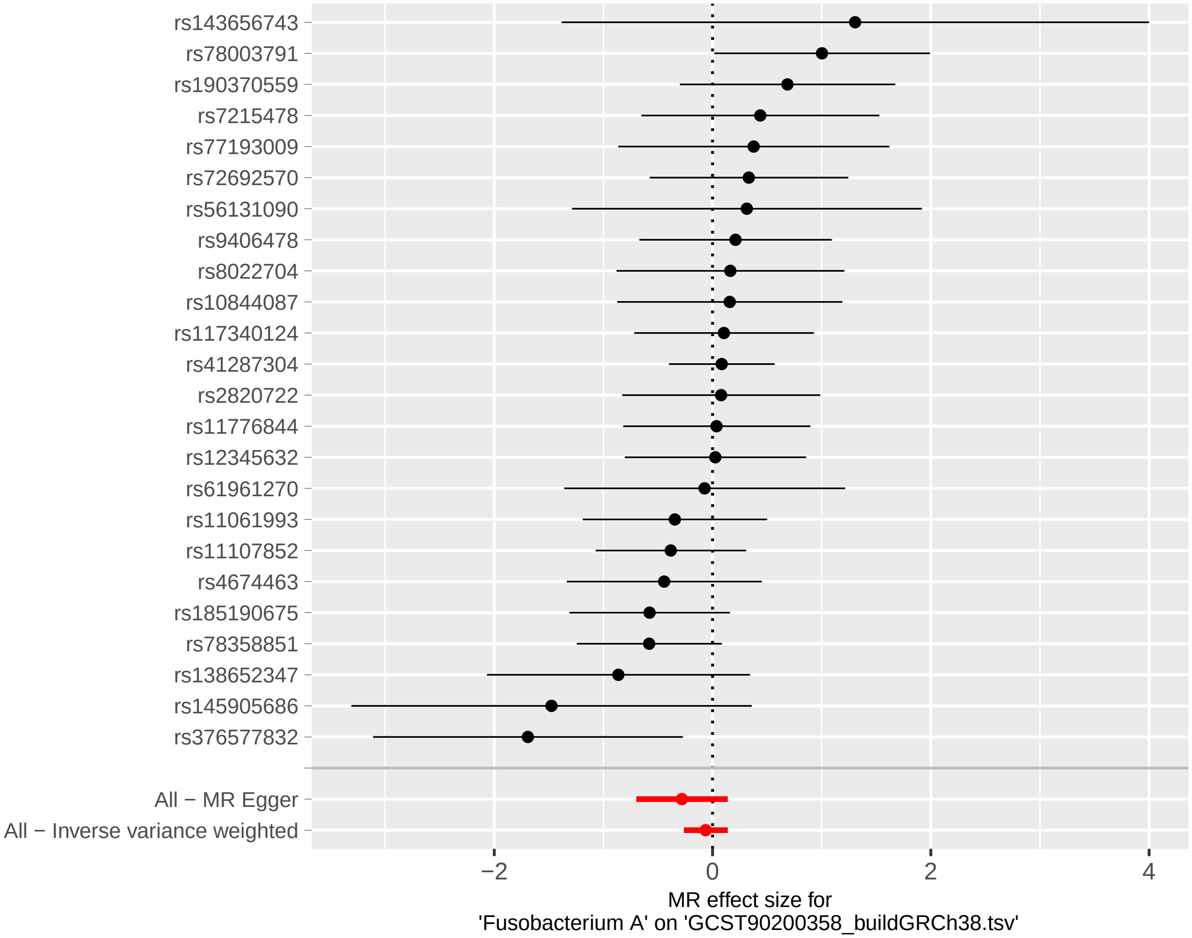

rs143656743
rs78003791
rs190370559
rs7215478
rs77193009
rs72692570
rs56131090
rs9406478
rs8022704
rs10844087
rs117340124
rs41287304
rs2820722
rs11776844
rs12345632
rs61961270
rs11061993
rs11107852
rs4674463
rs185190675
rs78358851
rs138652347
rs145905686
rs376577832
All − MR Egger
All − Inverse variance weighted
−2
0
2
4
MR effect size for
'Fusobacterium A' on 'GCST90200358_buildGRCh38.tsv'

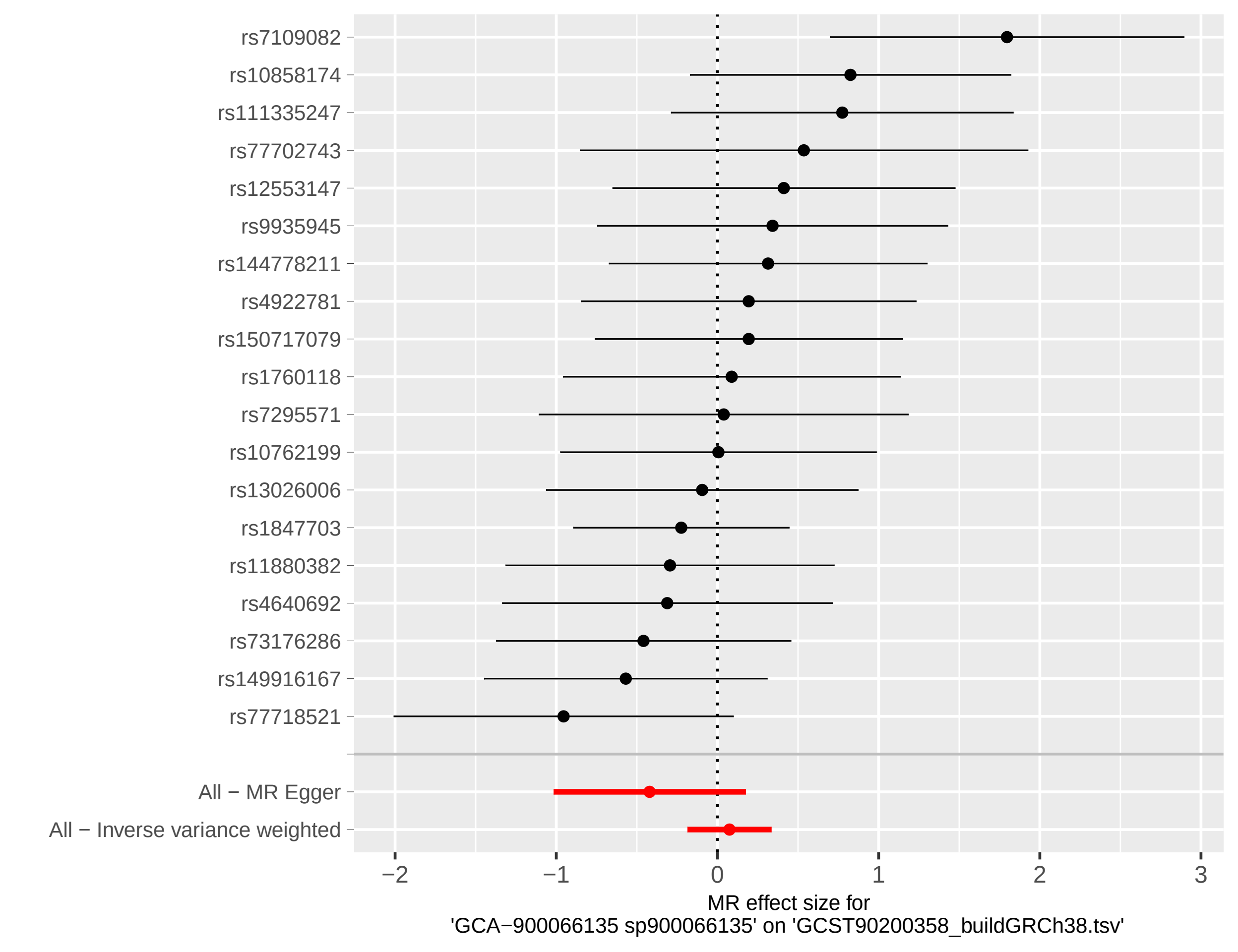

rs7109082
rs10858174
rs111335247
rs77702743
rs12553147
rs9935945
rs144778211
rs4922781
rs150717079
rs1760118
rs7295571
rs10762199
rs13026006
rs1847703
rs11880382
rs4640692
rs73176286
rs149916167
rs77718521
All − MR Egger
All − Inverse variance weighted
−2
−1
0
1
2
3
MR effect size for
'GCA−900066135 sp900066135' on 'GCST90200358_buildGRCh38.tsv'

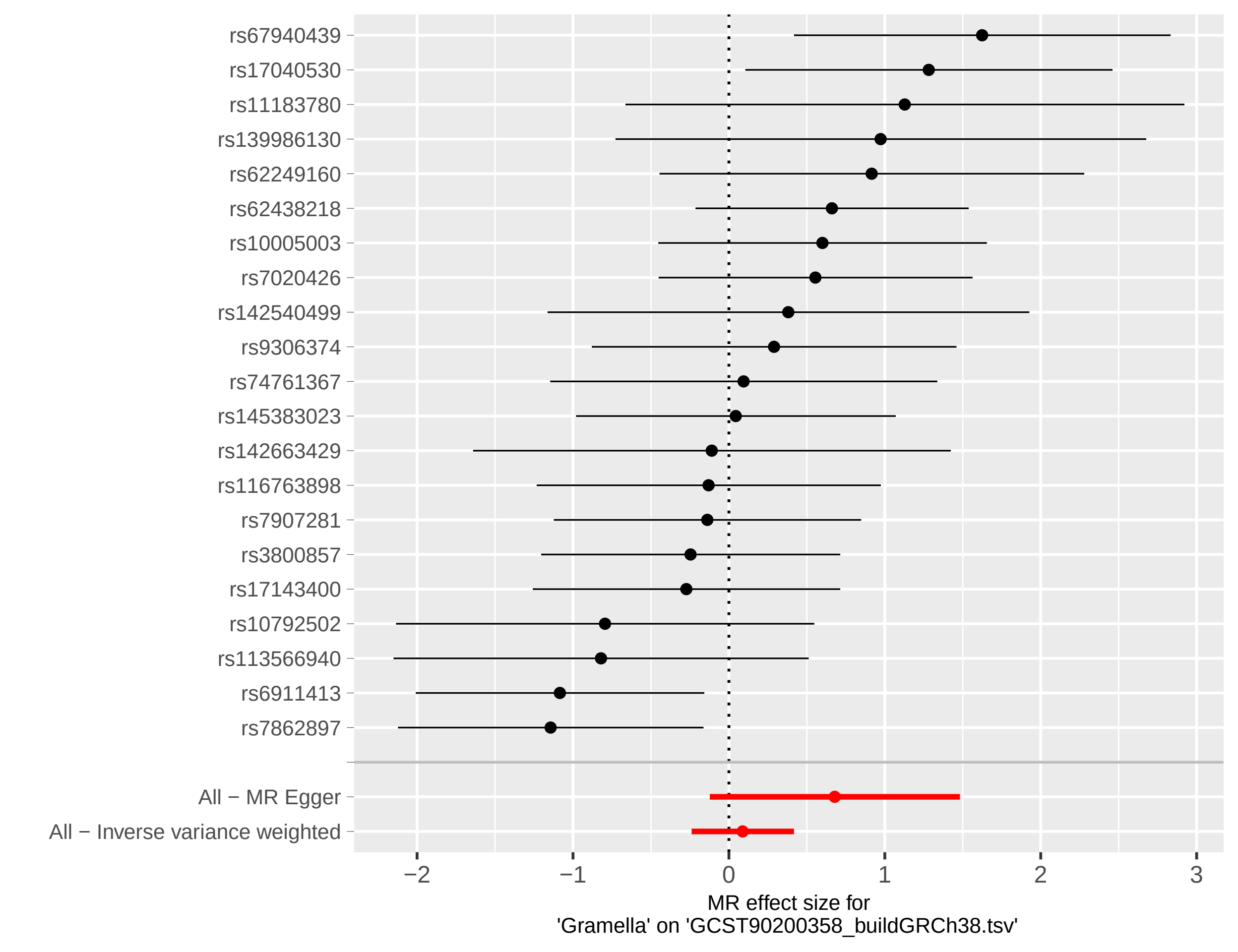

rs67940439
rs17040530
rs11183780
rs139986130
rs62249160
rs62438218
rs10005003
rs7020426
rs142540499
rs9306374
rs74761367
rs145383023
rs142663429
rs116763898
rs7907281
rs3800857
rs17143400
rs10792502
rs113566940
rs6911413
rs7862897
All − MR Egger
All − Inverse variance weighted
−2
−1
0
1
2
3
MR effect size for
'Gramella' on 'GCST90200358_buildGRCh38.tsv'

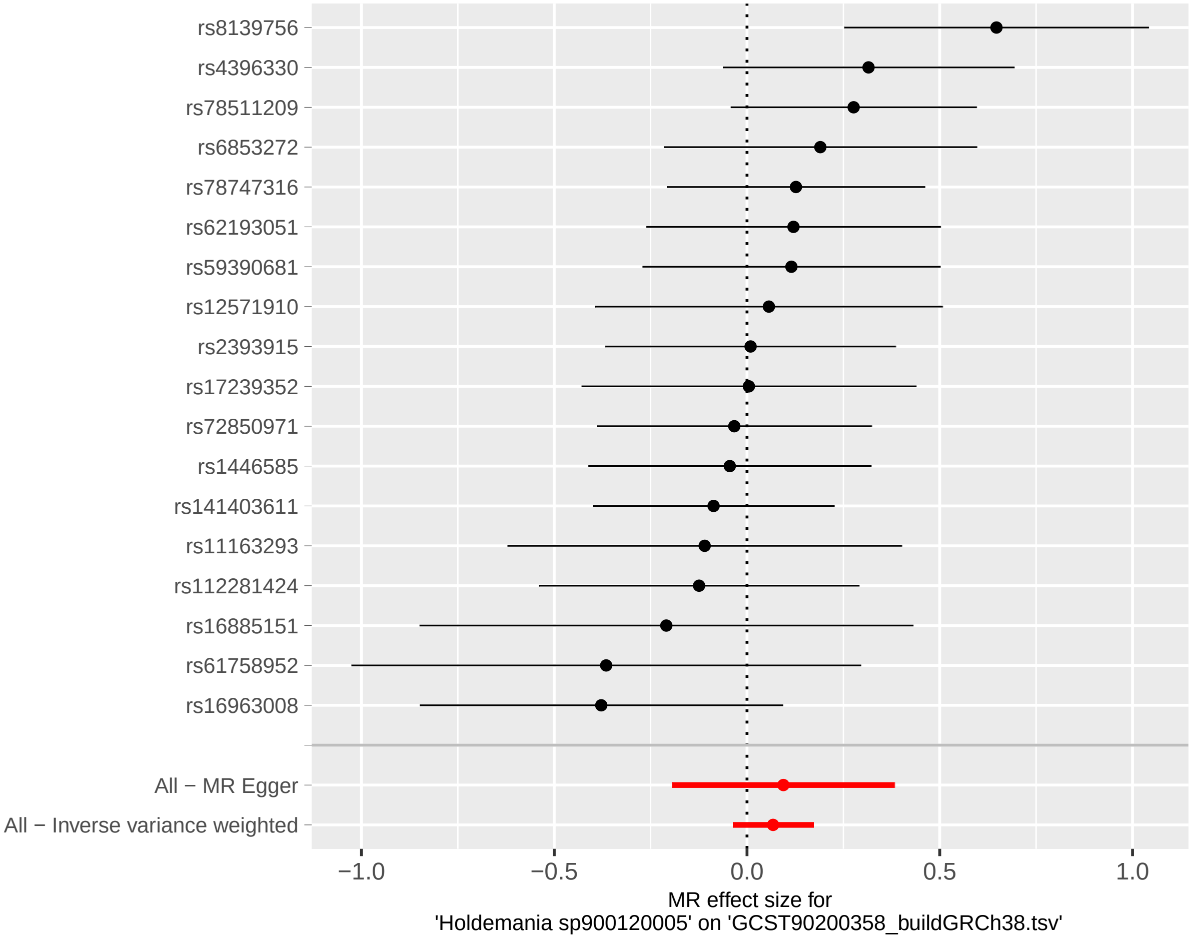
rs8139756
rs4396330
rs78511209
rs6853272
rs78747316
rs62193051
rs59390681
rs12571910
rs2393915
rs17239352
rs72850971
rs1446585
rs141403611
rs11163293
rs112281424
rs16885151
rs61758952
rs16963008
All − MR Egger
All − Inverse variance weighted
−1.0
−0.5
0.0
0.5
1.0
MR effect size for
'Holdemania sp900120005' on 'GCST90200358_buildGRCh38.tsv'

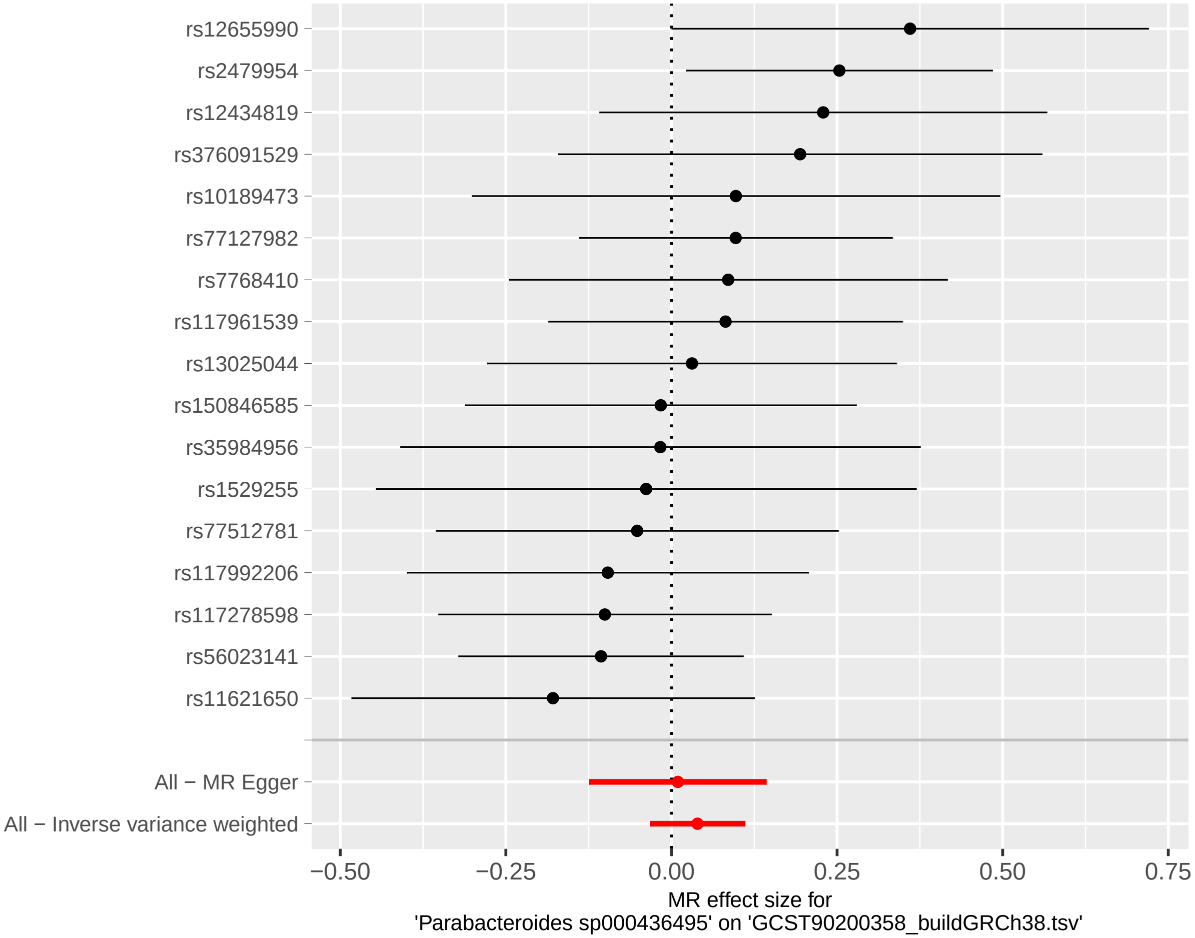

rs12655990
rs2479954
rs12434819
rs376091529
rs10189473
rs77127982
rs7768410
rs117961539
rs13025044
rs150846585
rs35984956
rs1529255
rs77512781
rs117992206
rs117278598
rs56023141
rs11621650
All − MR Egger
All − Inverse variance weighted
−0.50
−0.25
0.00
0.25
0.50
0.75
MR effect size for
'Parabacteroides sp000436495' on 'GCST90200358_buildGRCh38.tsv'

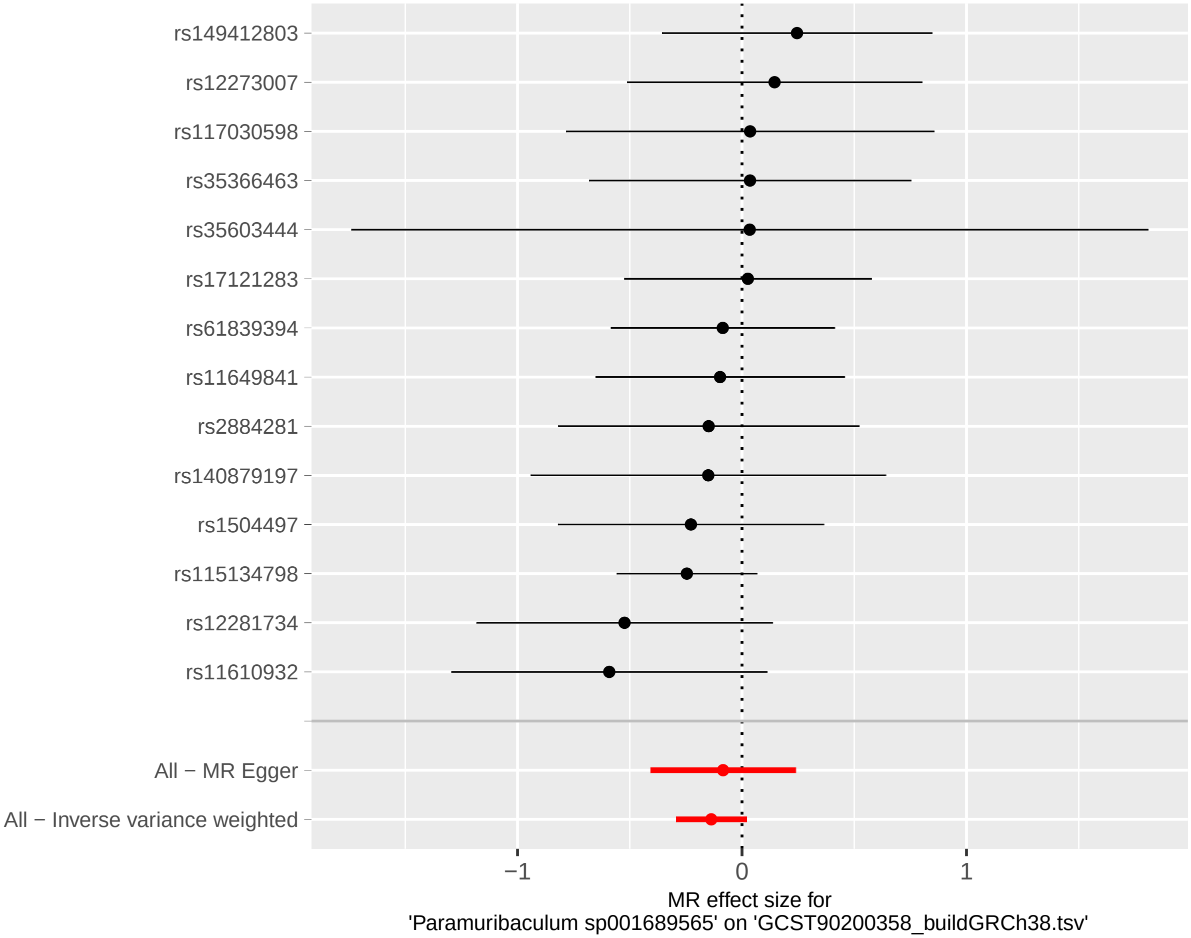

rs149412803
rs12273007
rs117030598
rs35366463
rs35603444
rs17121283
rs61839394
rs11649841
rs2884281
rs140879197
rs1504497
rs115134798
rs12281734
rs11610932
All − MR Egger
All − Inverse variance weighted
−1
0
1
MR effect size for
'Paramuribaculum sp001689565' on 'GCST90200358_buildGRCh38.tsv'

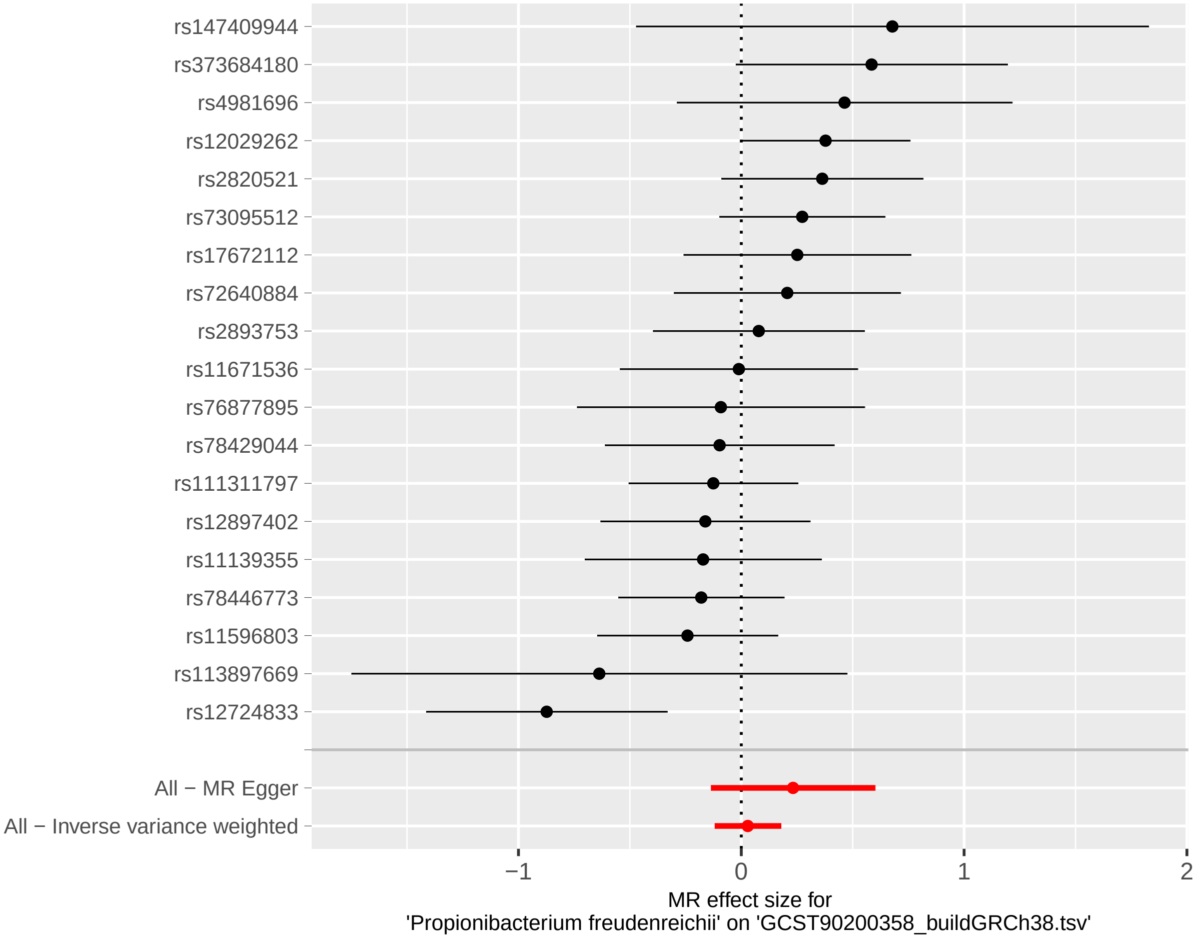

rs147409944
rs373684180
rs4981696
rs12029262
rs2820521
rs73095512
rs17672112
rs72640884
rs2893753
rs11671536
rs76877895
rs78429044
rs111311797
rs12897402
rs11139355
rs78446773
rs11596803
rs113897669
rs12724833
All − MR Egger
All − Inverse variance weighted
−1
0
1
2
MR effect size for
'Propionibacterium freudenreichii' on 'GCST90200358_buildGRCh38.tsv'

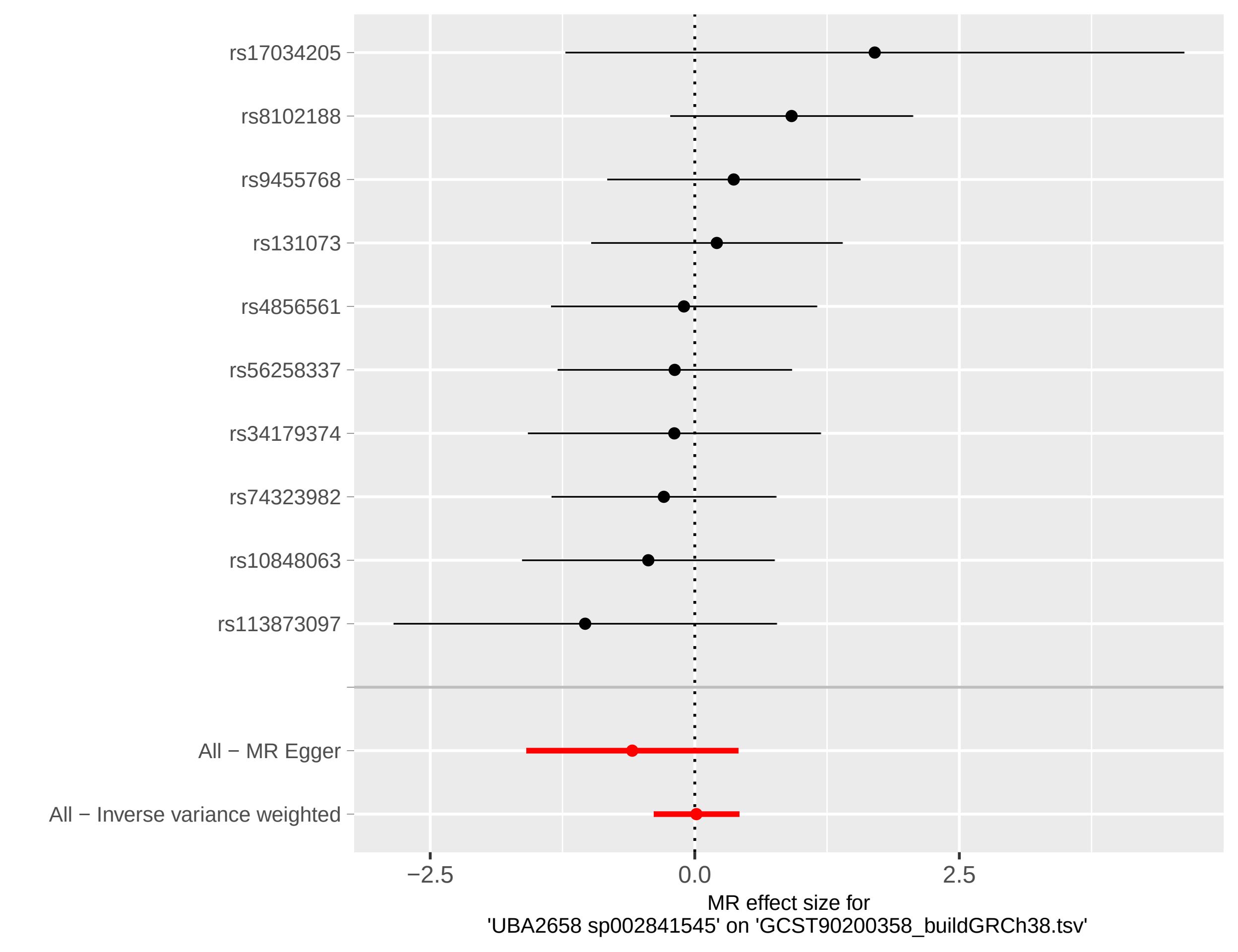

rs17034205
rs8102188
rs9455768
rs131073
rs4856561
rs56258337
rs34179374
rs74323982
rs10848063
rs113873097
All − MR Egger
All − Inverse variance weighted
−2.5
0.0
2.5
MR effect size for
'UBA2658 sp002841545' on 'GCST90200358_buildGRCh38.tsv'

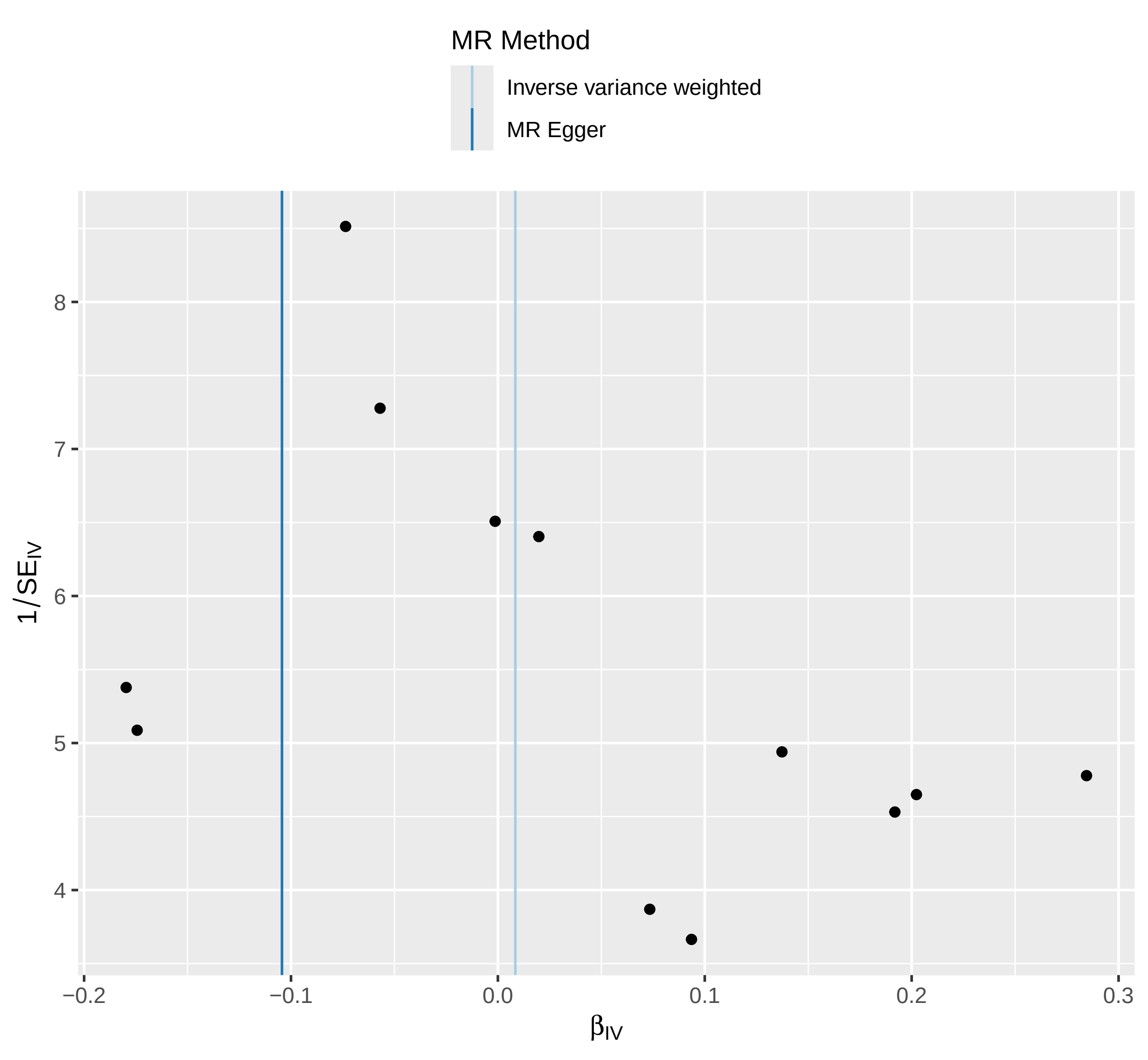

MR Method
Inverse variance weighted
MR Egger
1/SE_IV
8
7
6
5
4
−0.2
−0.1
0.0
0.1
0.2
0.3
β_IV

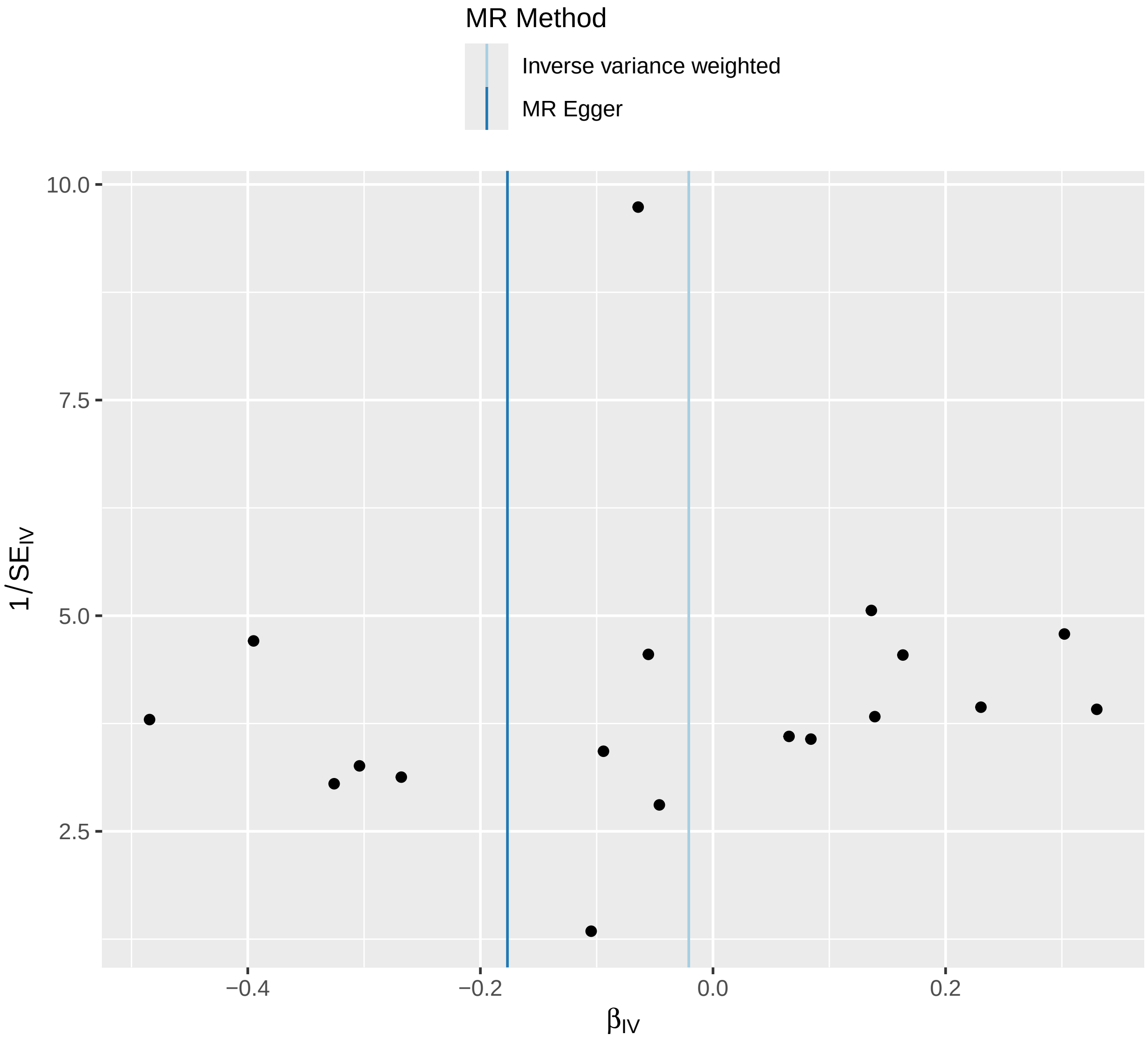
MR Method
Inverse variance weighted
MR Egger
10.0
7.5
5.0
2.5
$1/SE_{IV}$
−0.4
−0.2
0.0
0.2
β_{IV}

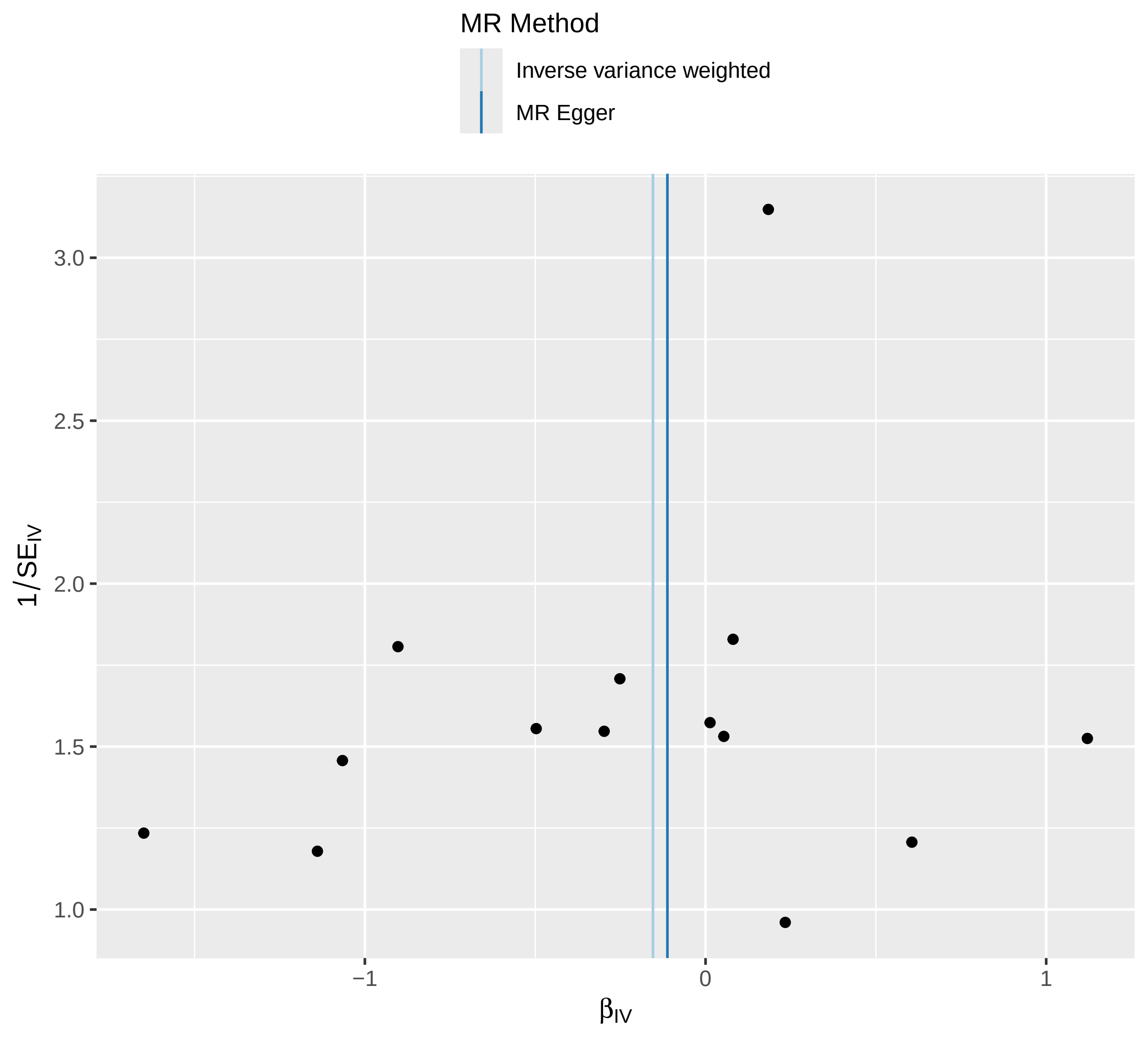

MR Method
Inverse variance weighted
MR Egger
3.0
2.5
2.0
1.5
1.0
1/SE_IV
−1
0
1
β_IV

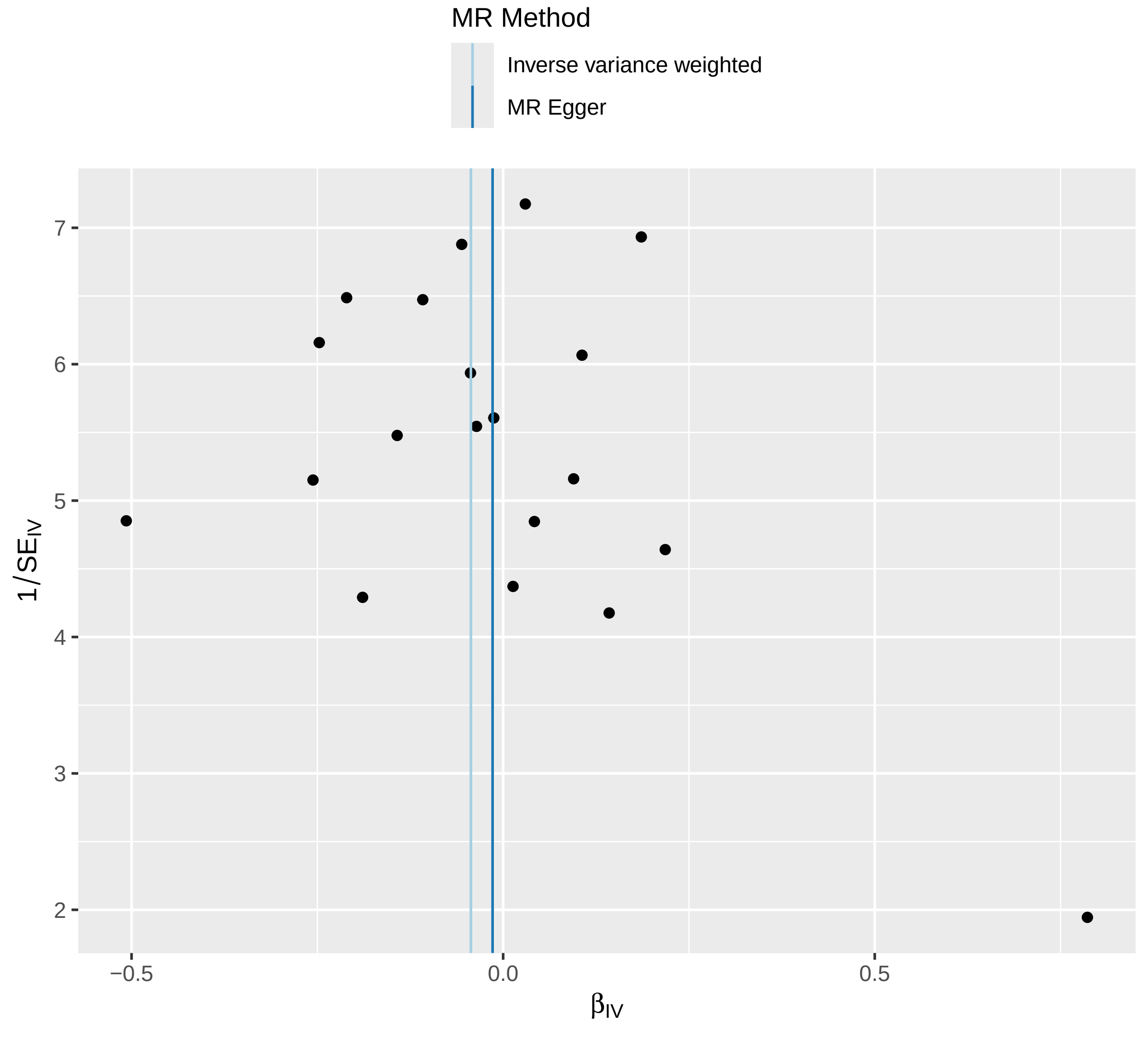

MR Method
Inverse variance weighted
MR Egger
7
6
5
4
3
2
1/SE_IV
−0.5
0.0
0.5
β_IV

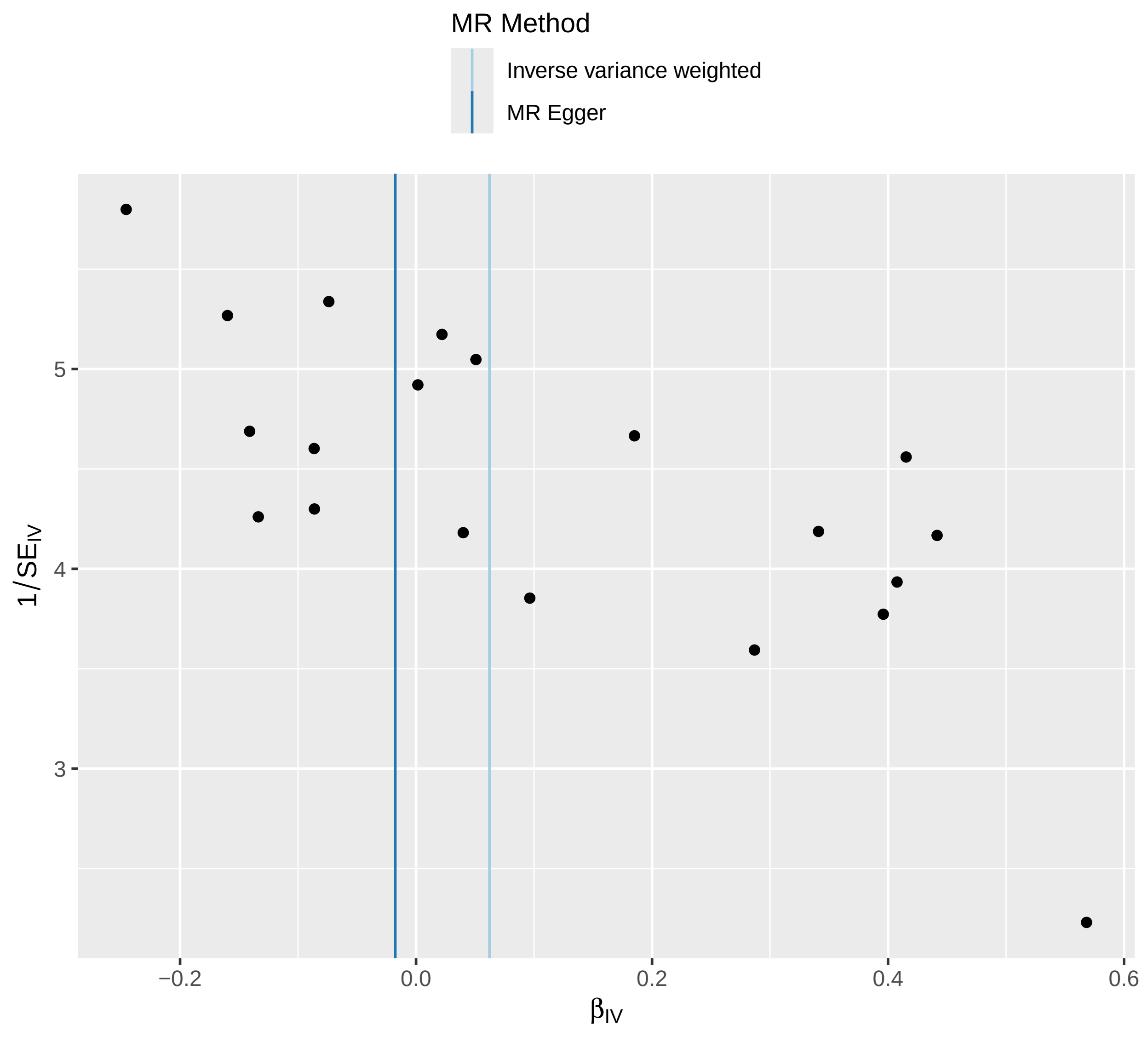
MR Method
Inverse variance weighted
MR Egger
5
4
3
1/SE_IV
−0.2
0.0
0.2
0.4
0.6
β_IV

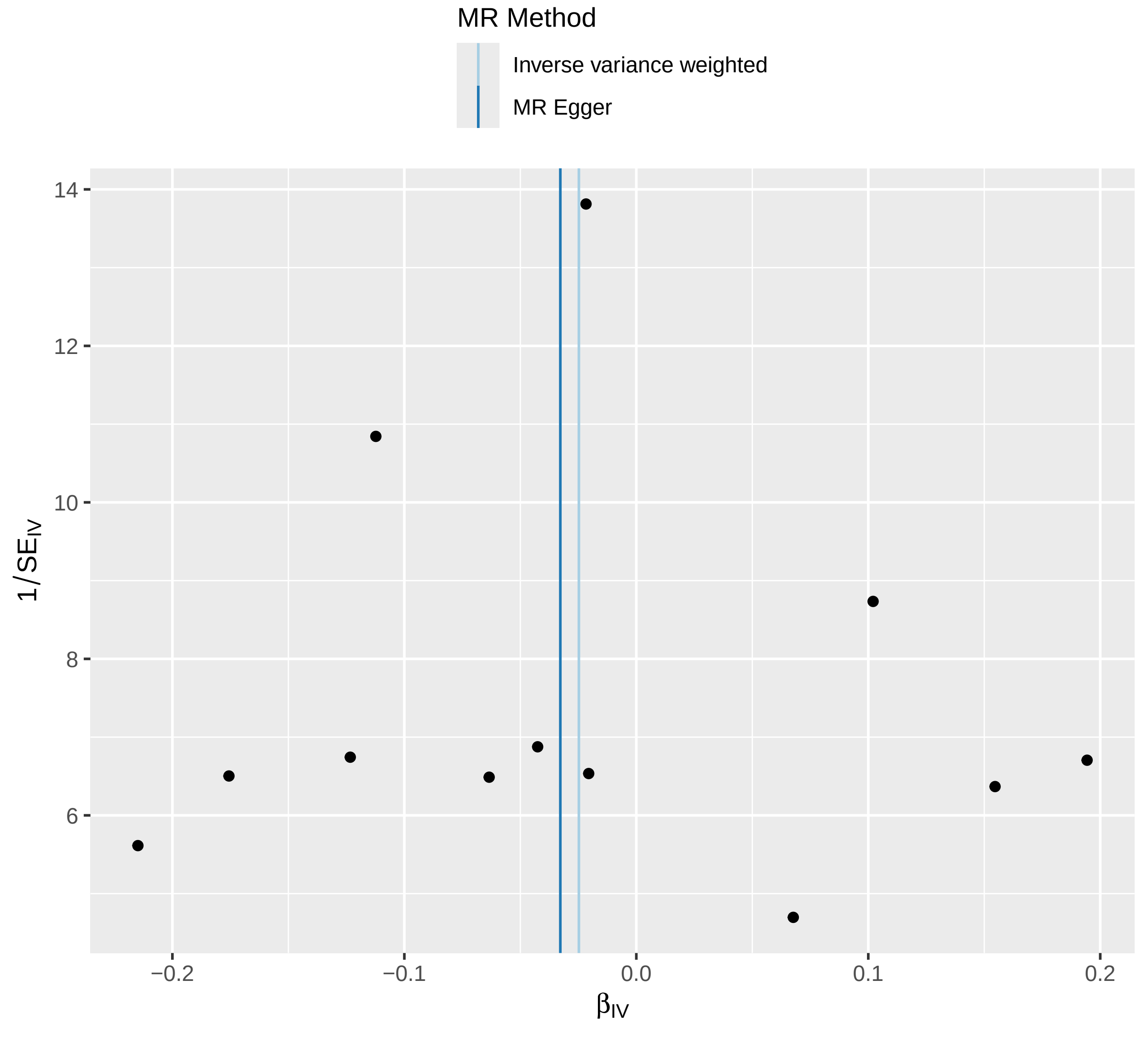

MR Method
Inverse variance weighted
MR Egger
14
12
10
8
6
1/SE_IV
−0.2
−0.1
0.0
0.1
0.2
β_IV

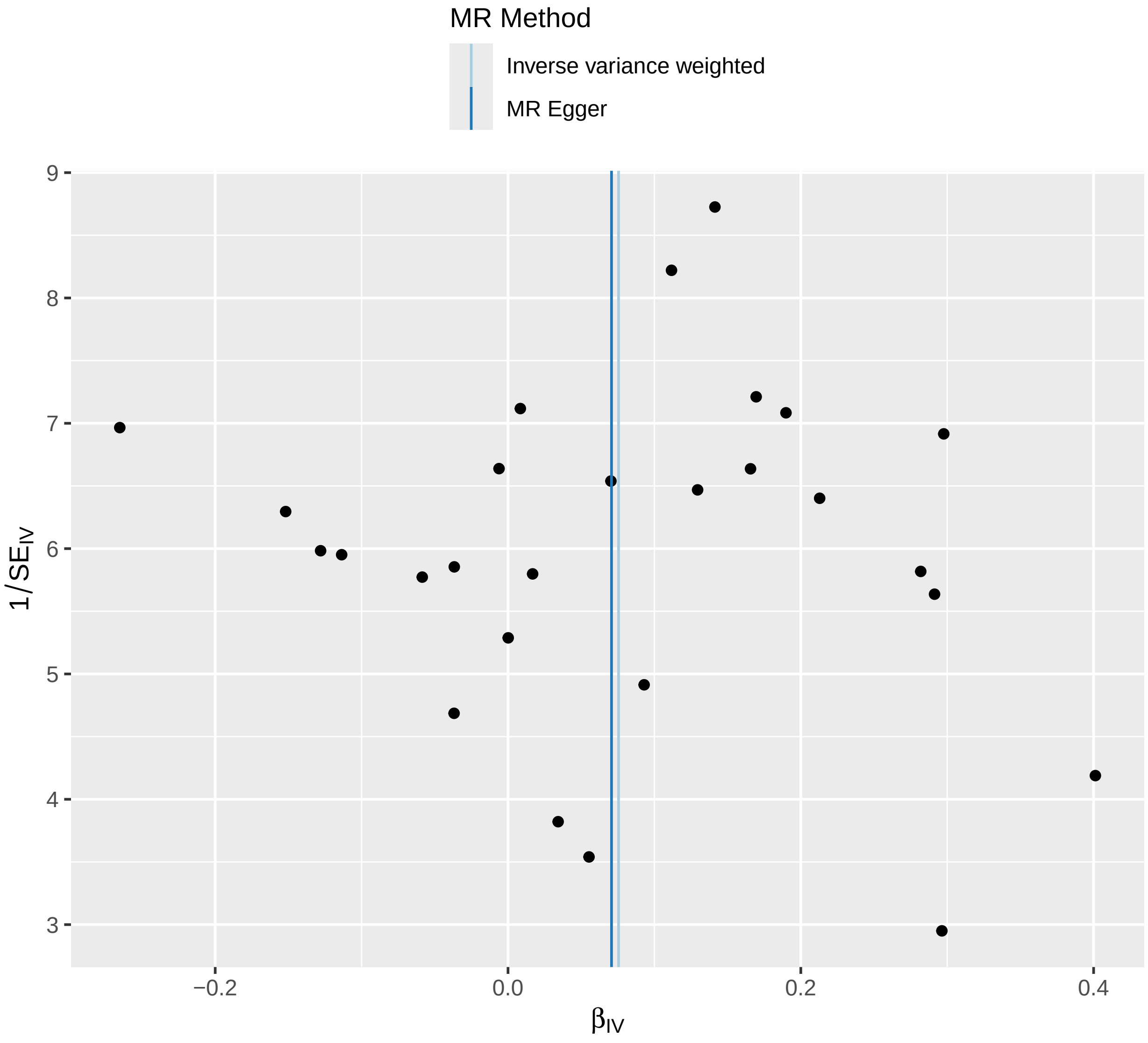
MR Method
Inverse variance weighted
MR Egger
9
8
7
6
5
4
3
1/SE_IV
−0.2
0.0
0.2
0.4
β_IV

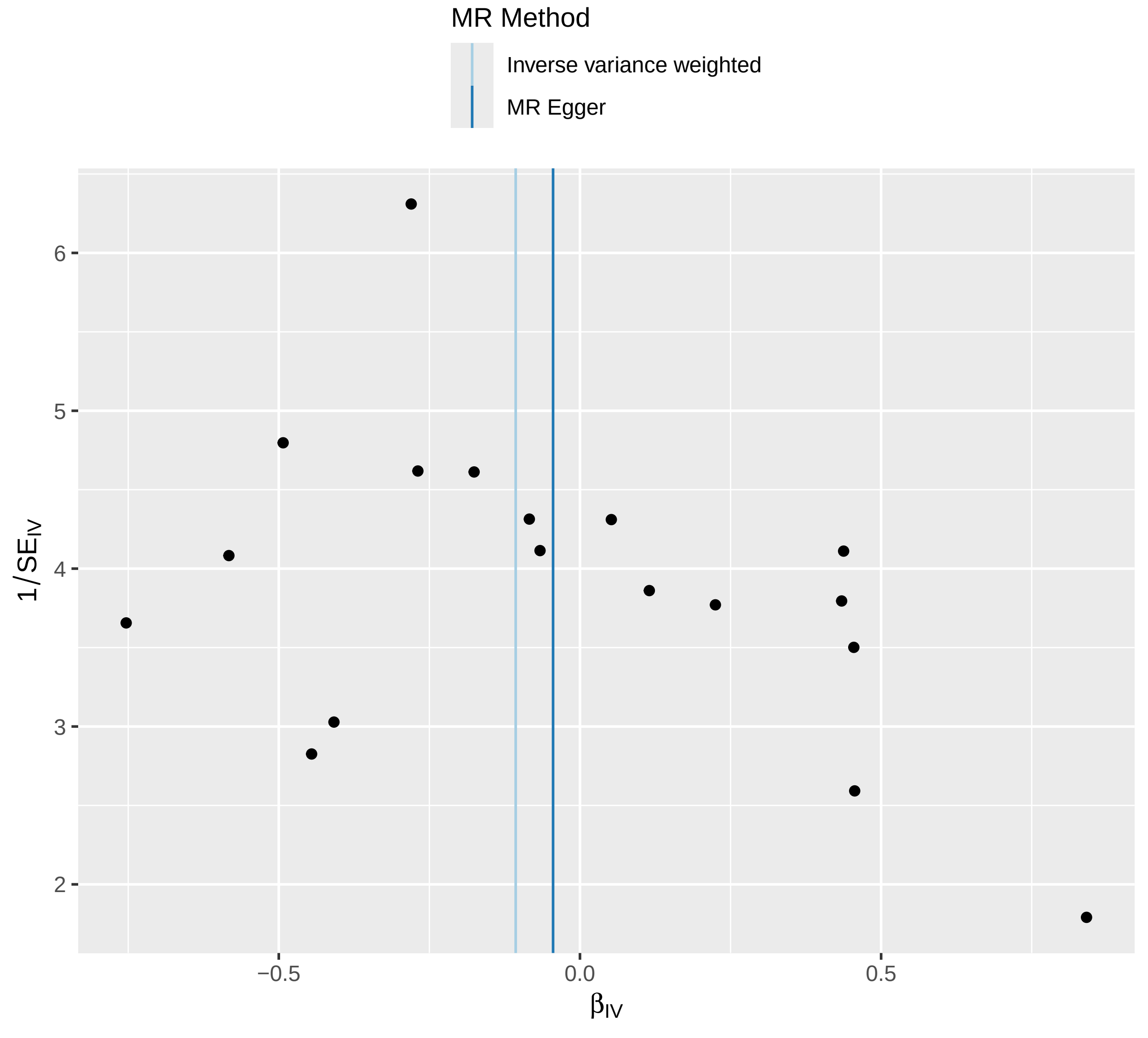
MR Method
Inverse variance weighted
MR Egger
6
5
4
3
2
−0.5
0.0
0.5
$1/SE_{IV}$
β_{IV}

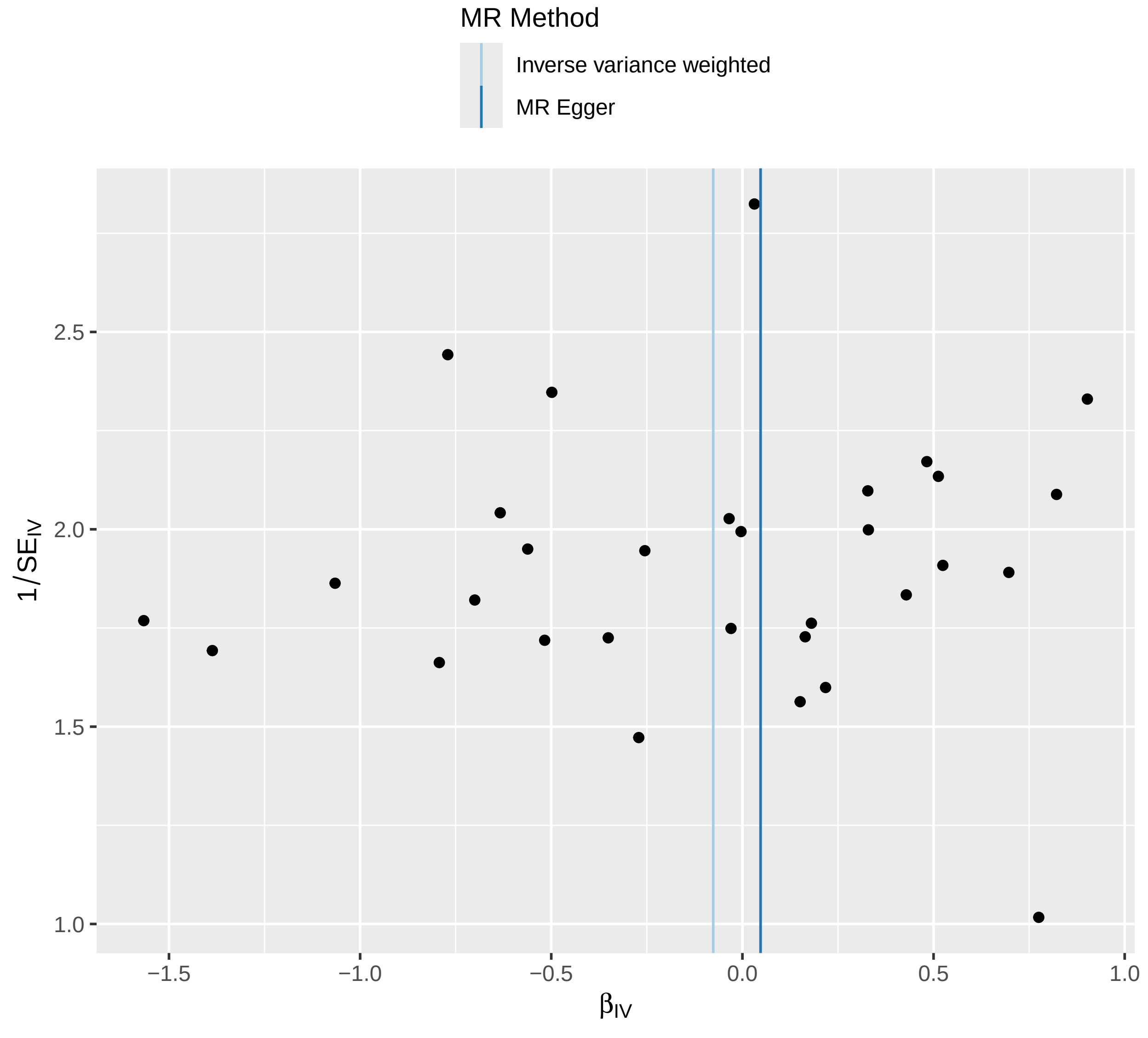
MR Method
Inverse variance weighted
MR Egger
2.5
2.0
1.5
1.0
1/SE$_{IV}$
−1.5
−1.0
−0.5
0.0
0.5
1.0
β$_{IV}$

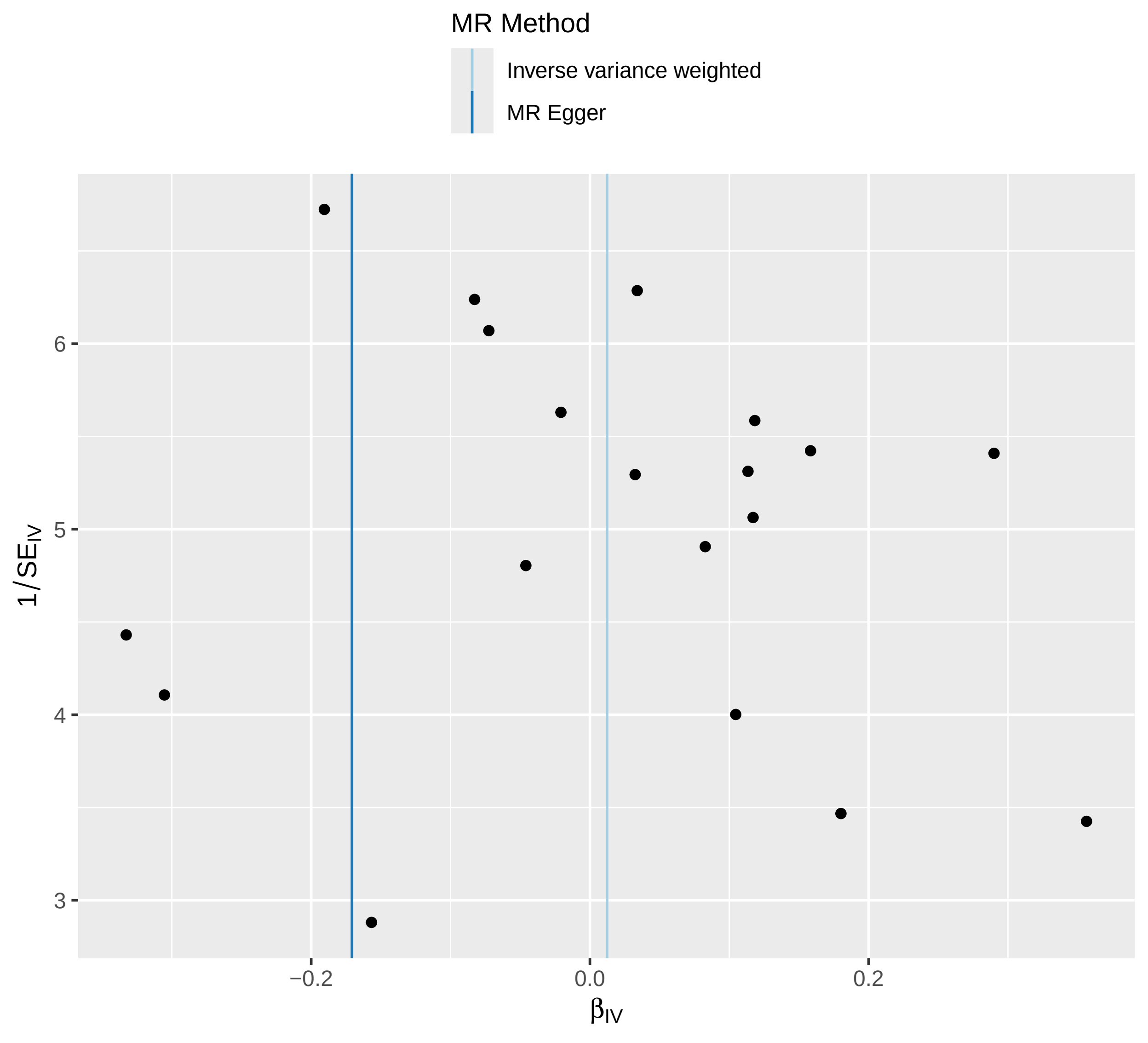
MR Method
Inverse variance weighted
MR Egger
1/SE$_{IV}$
6
5
4
3
−0.2
0.0
0.2
β$_{IV}$

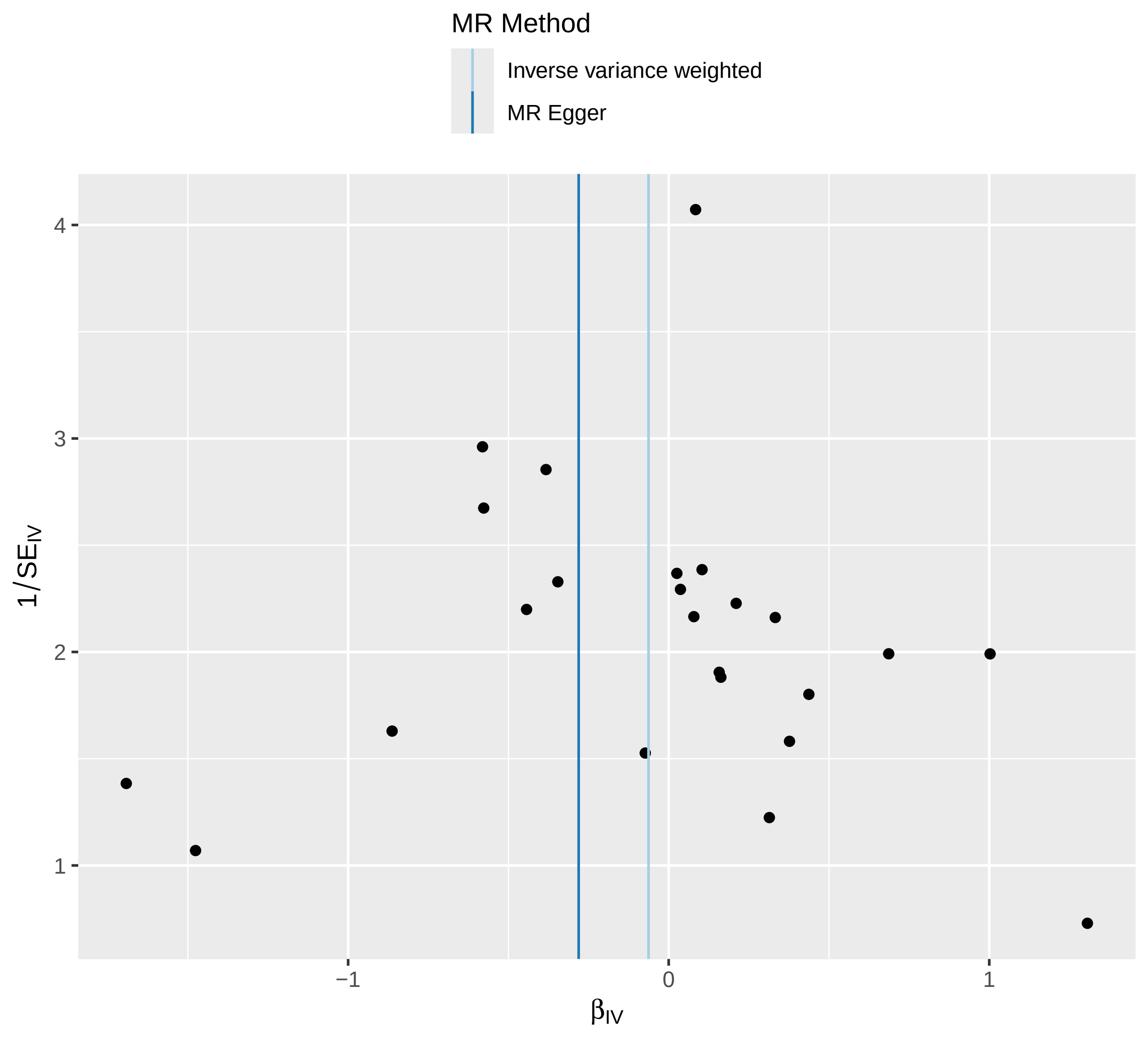

MR Method
Inverse variance weighted
MR Egger
4
3
2
1
−1
0
1
1/SE$_{IV}$
β$_{IV}$

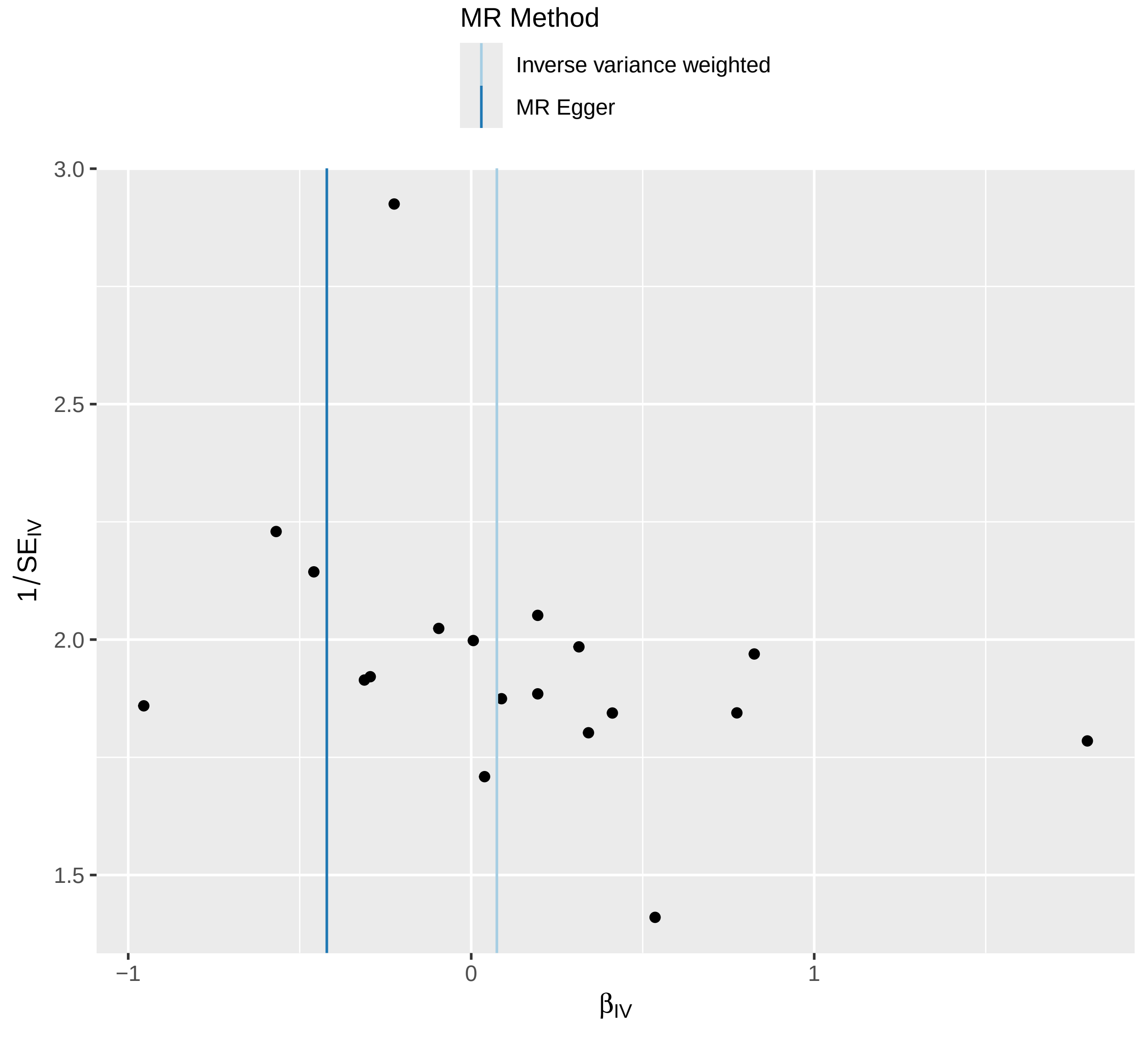

MR Method
Inverse variance weighted
MR Egger
3.0
2.5
2.0
1.5
1/SE$_{IV}$
−1
0
1
β$_{IV}$

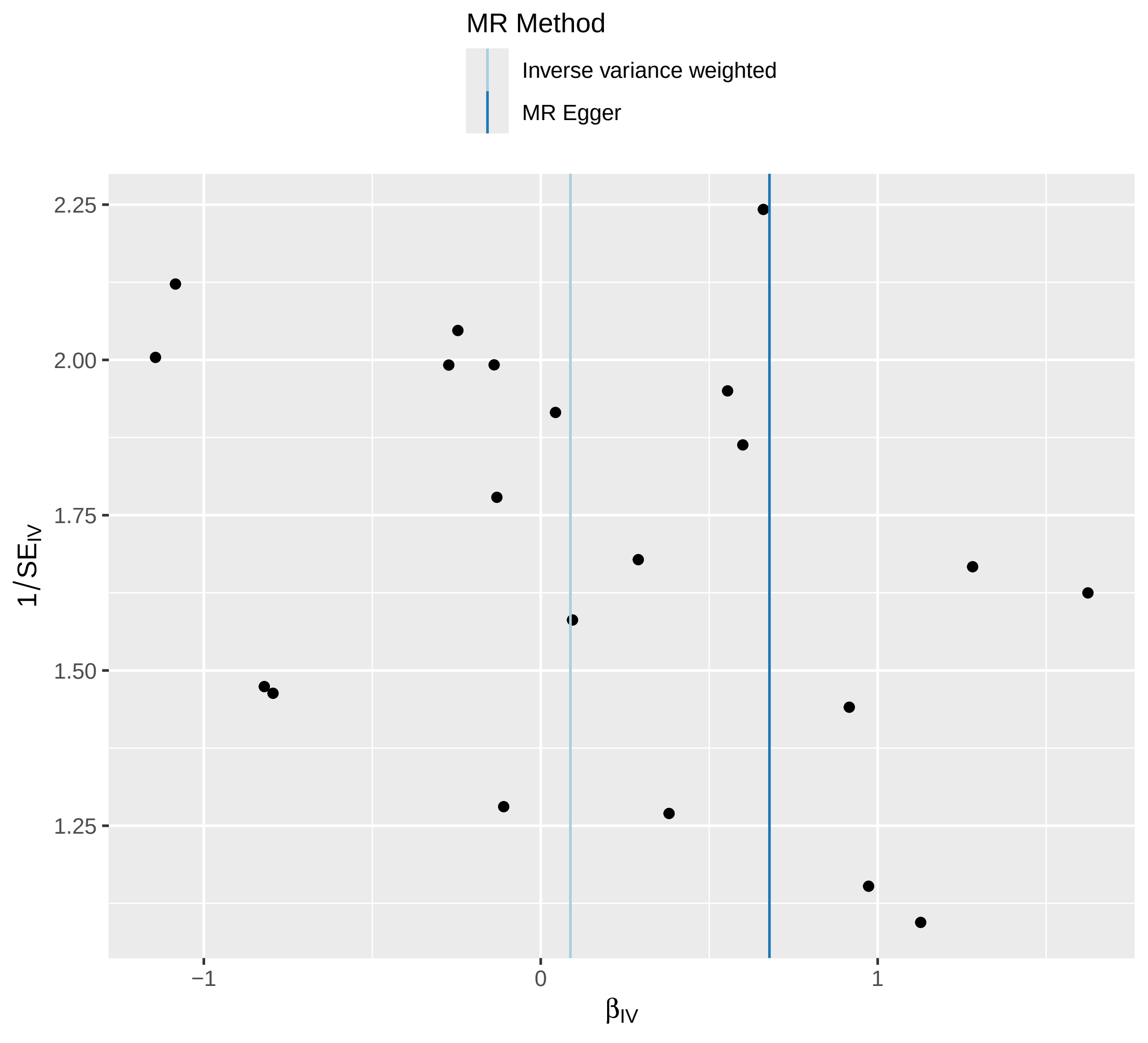
MR Method
Inverse variance weighted
MR Egger
2.25
2.00
1.75
1.50
1.25
1/SE_IV
−1
0
1
β_IV

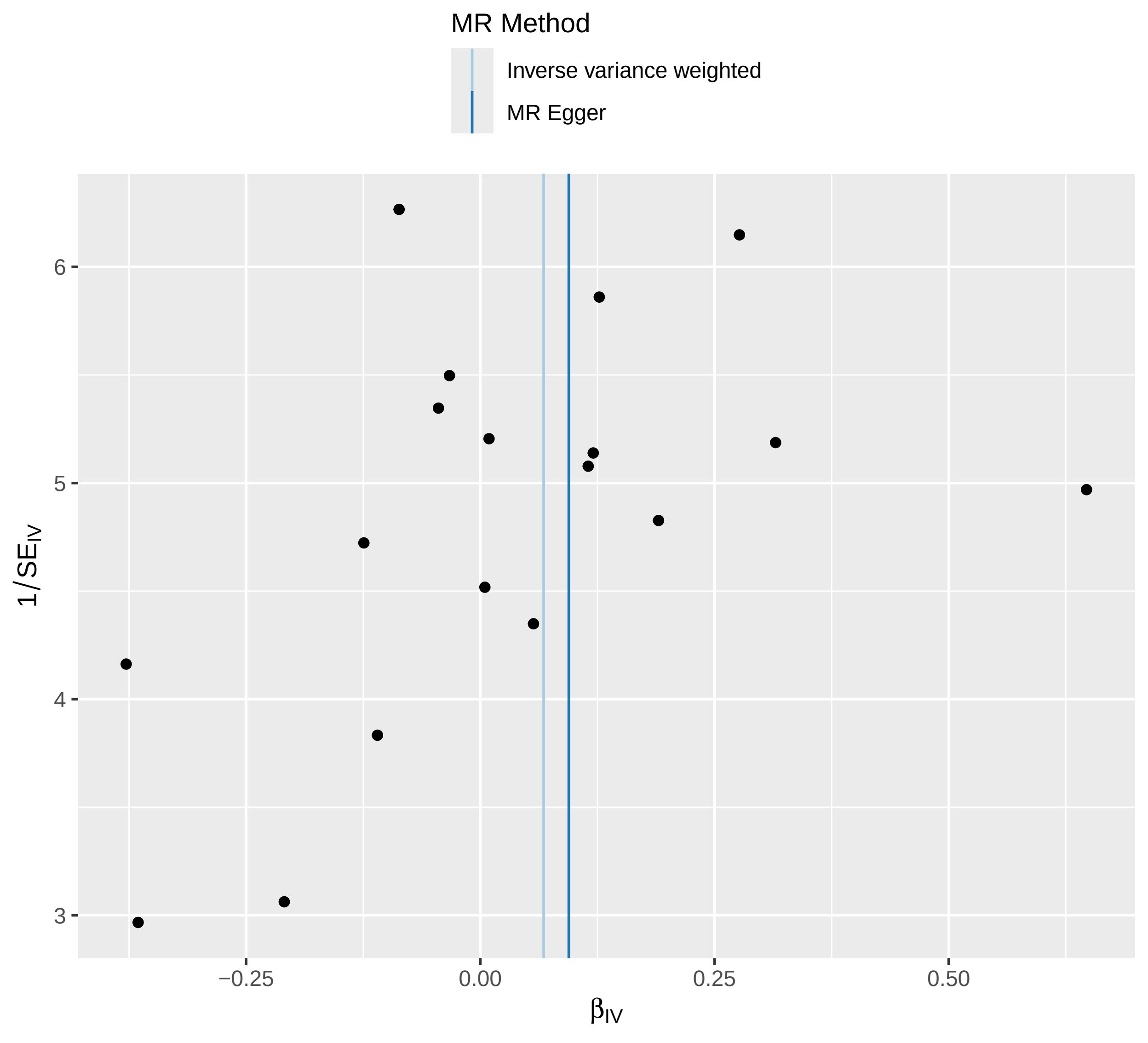
MR Method
Inverse variance weighted
MR Egger
6
5
4
3
$1/SE_{IV}$
−0.25
0.00
0.25
0.50
β_{IV}

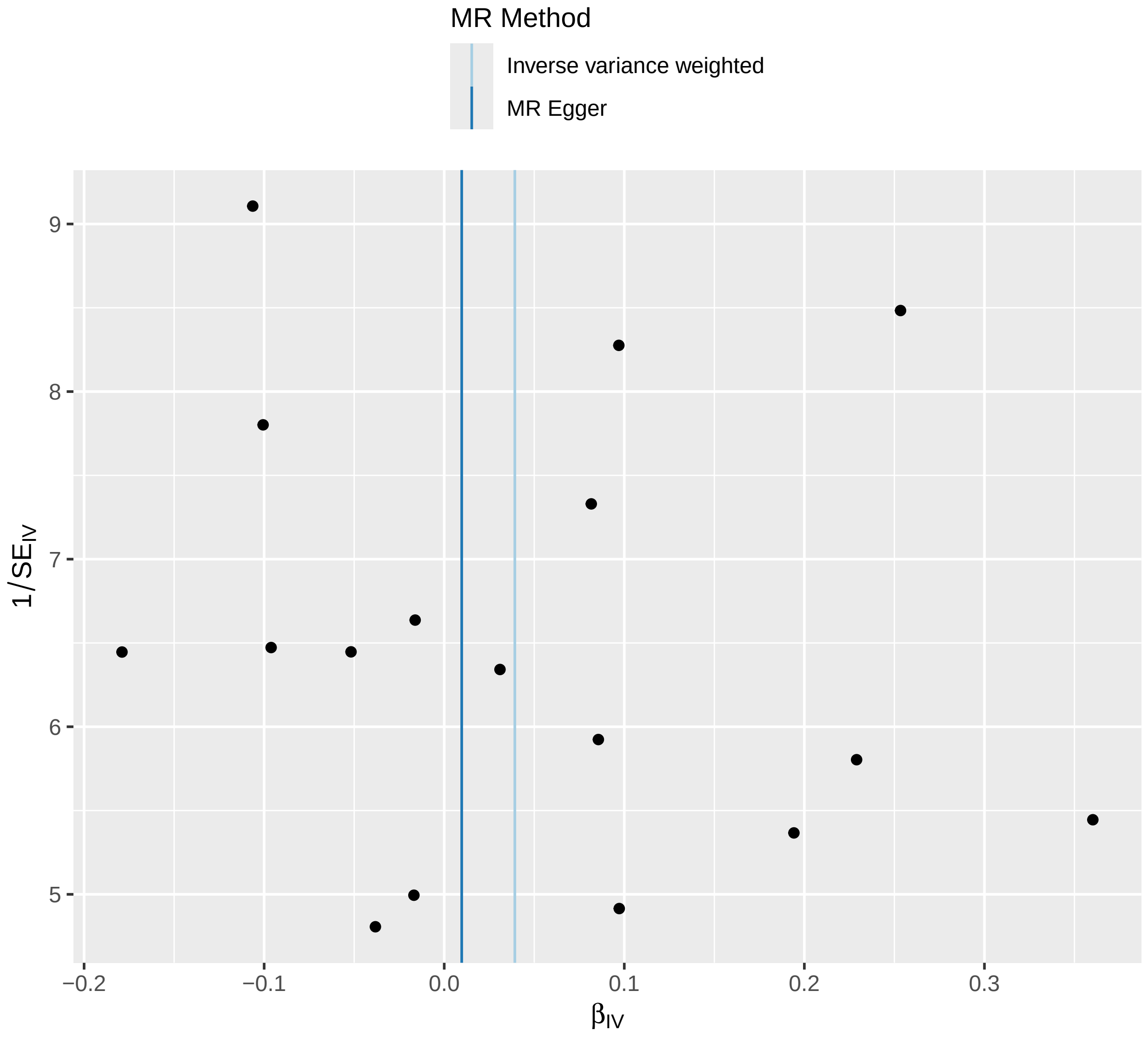
MR Method
Inverse variance weighted
MR Egger
9
8
7
6
5
1/SE_IV
−0.2
−0.1
0.0
0.1
0.2
0.3
β_IV

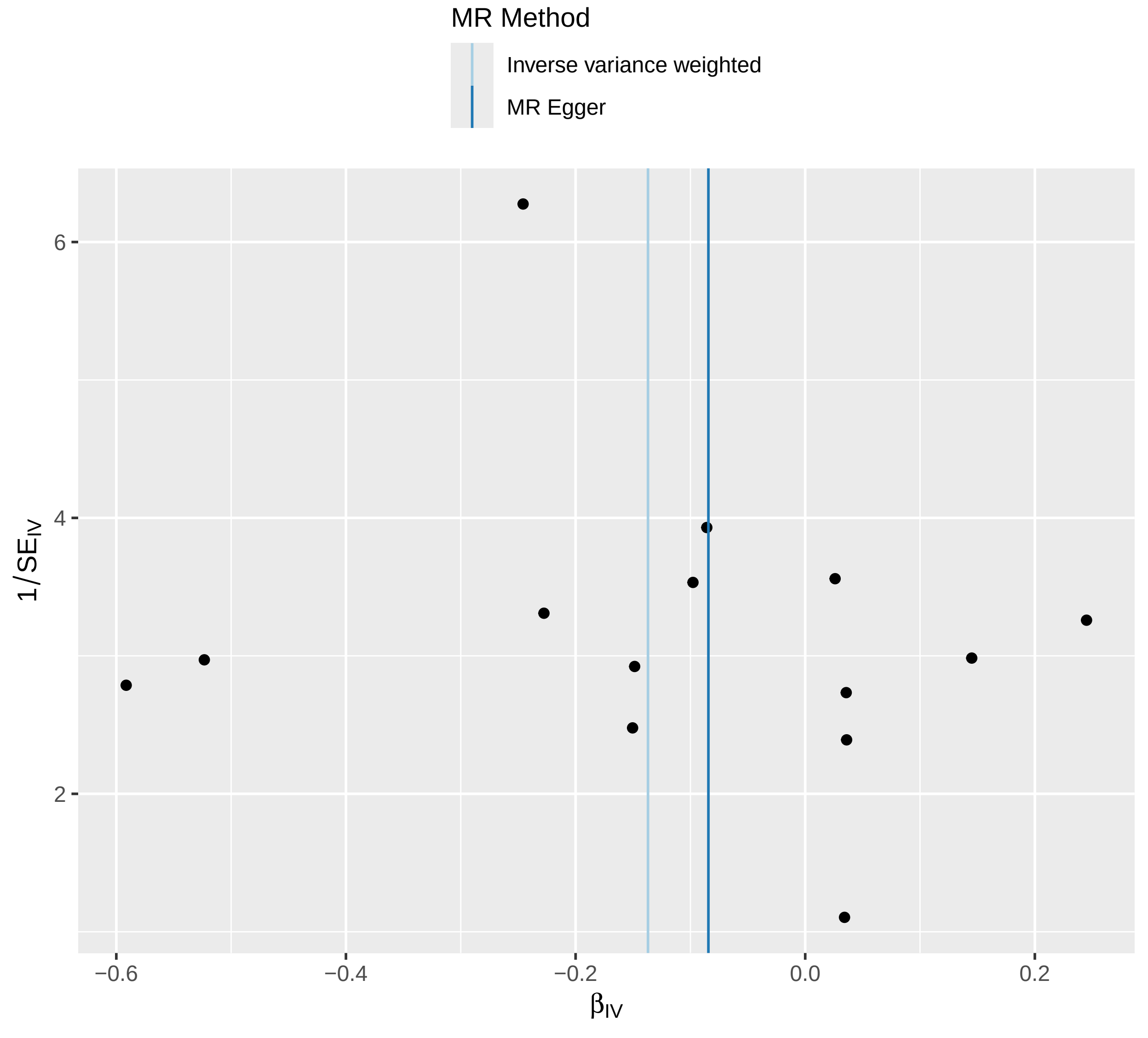

MR Method
Inverse variance weighted
MR Egger
6
4
2
$1/SE_{IV}$
−0.6
−0.4
−0.2
0.0
0.2
β_{IV}

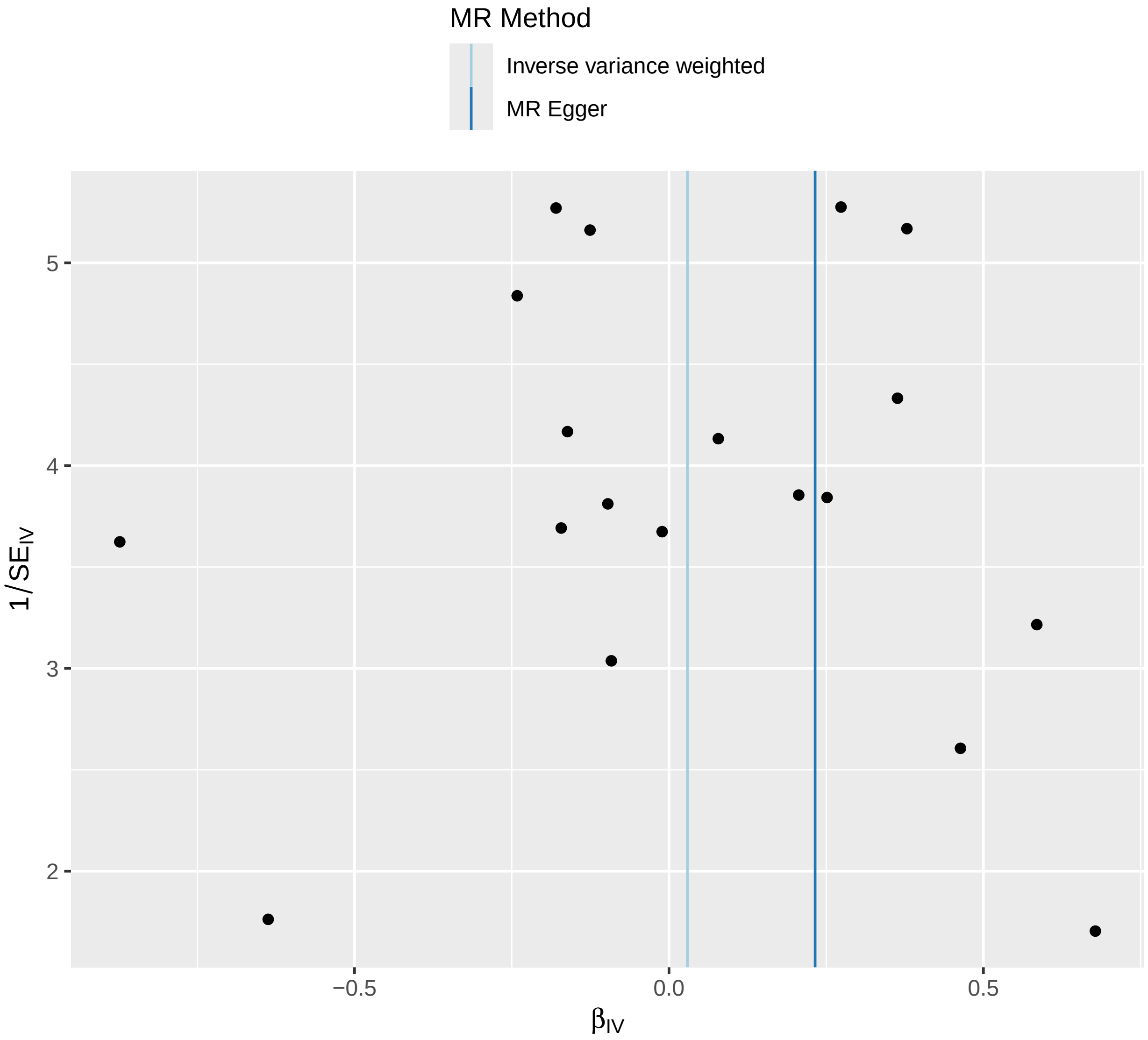
MR Method
Inverse variance weighted
MR Egger
5
4
3
2
1/SE$_{IV}$
−0.5
0.0
0.5
β$_{IV}$

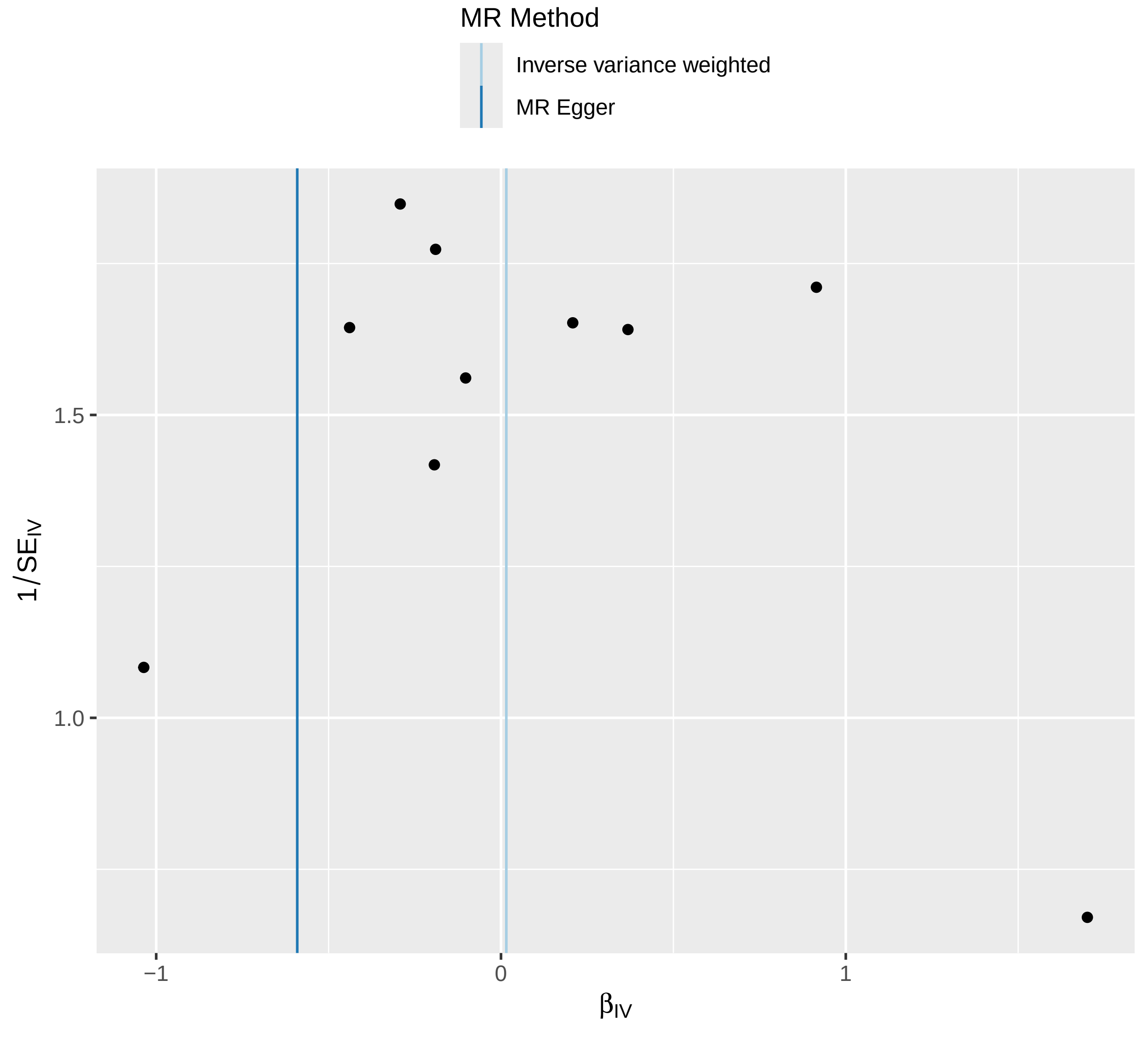

MR Method
Inverse variance weighted
MR Egger
1.5
1.0
−1
0
1
1/SE_IV
β_IV

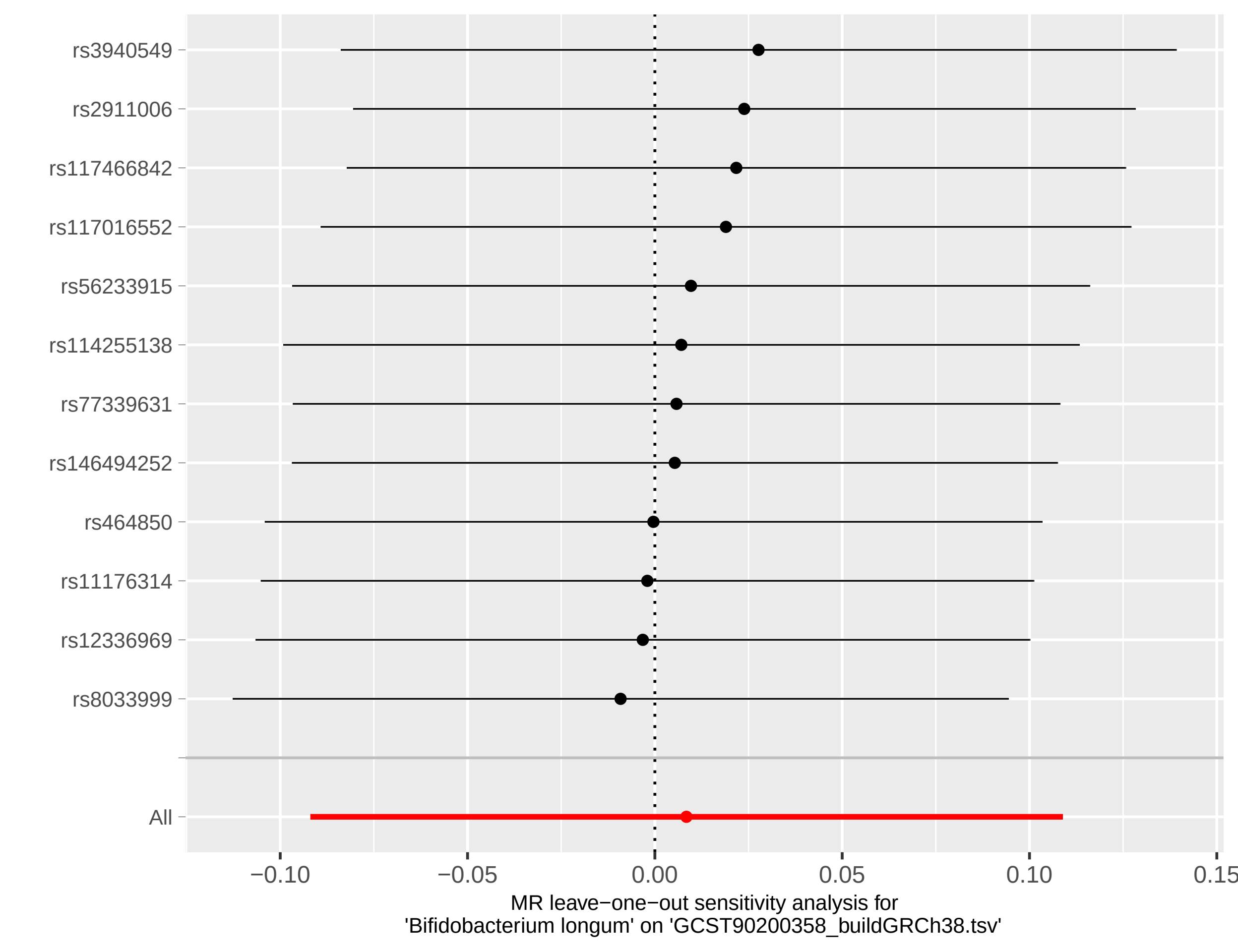

rs3940549
rs2911006
rs117466842
rs117016552
rs56233915
rs114255138
rs77339631
rs146494252
rs464850
rs11176314
rs12336969
rs8033999
All
−0.10
−0.05
0.00
0.05
0.10
0.15
MR leave−one−out sensitivity analysis for
'Bifidobacterium longum' on 'GCST90200358_buildGRCh38.tsv'

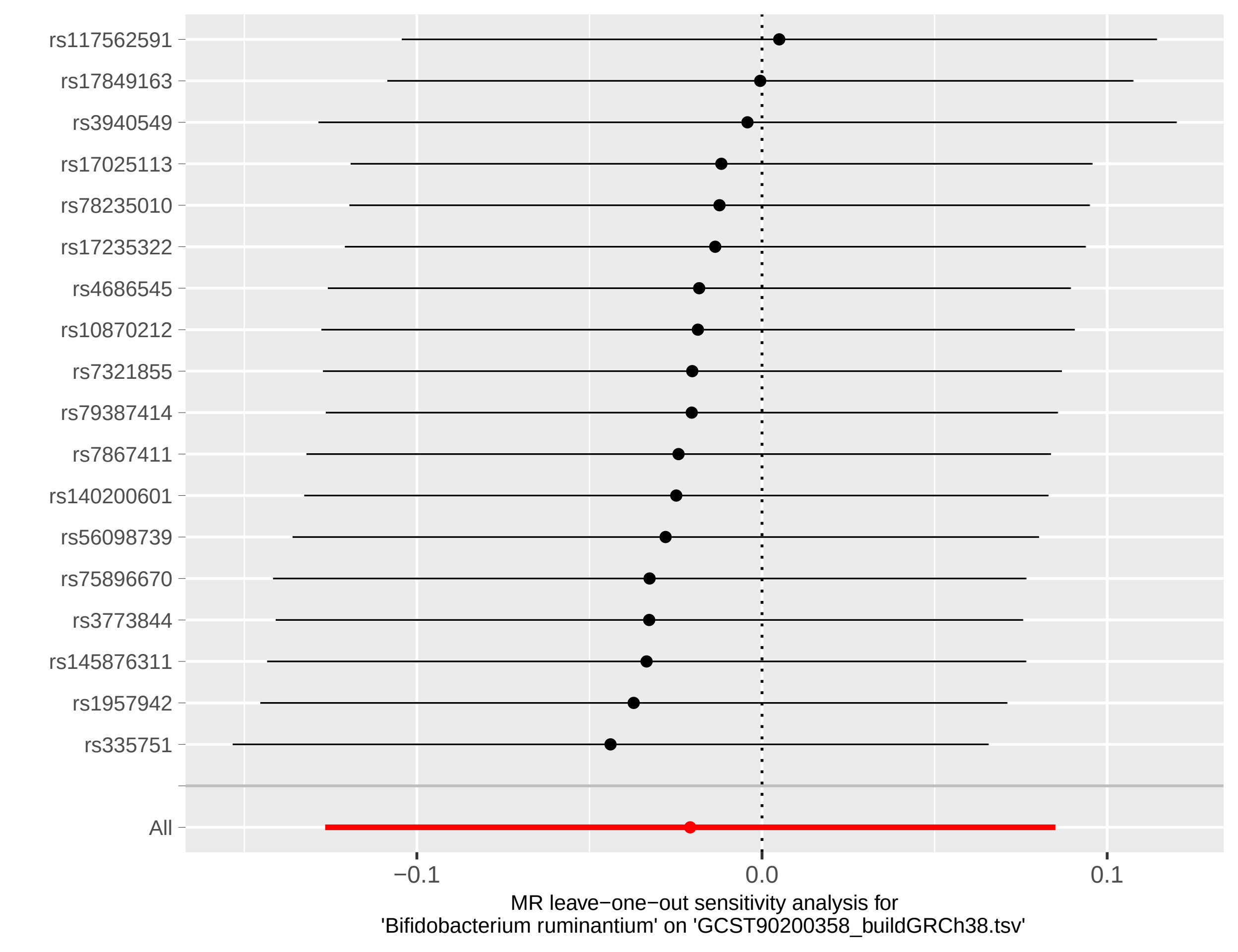
rs117562591
rs17849163
rs3940549
rs17025113
rs78235010
rs17235322
rs4686545
rs10870212
rs7321855
rs79387414
rs7867411
rs140200601
rs56098739
rs75896670
rs3773844
rs145876311
rs1957942
rs335751
All
−0.1
0.0
0.1
MR leave−one−out sensitivity analysis for
'Bifidobacterium ruminantium' on 'GCST90200358_buildGRCh38.tsv'

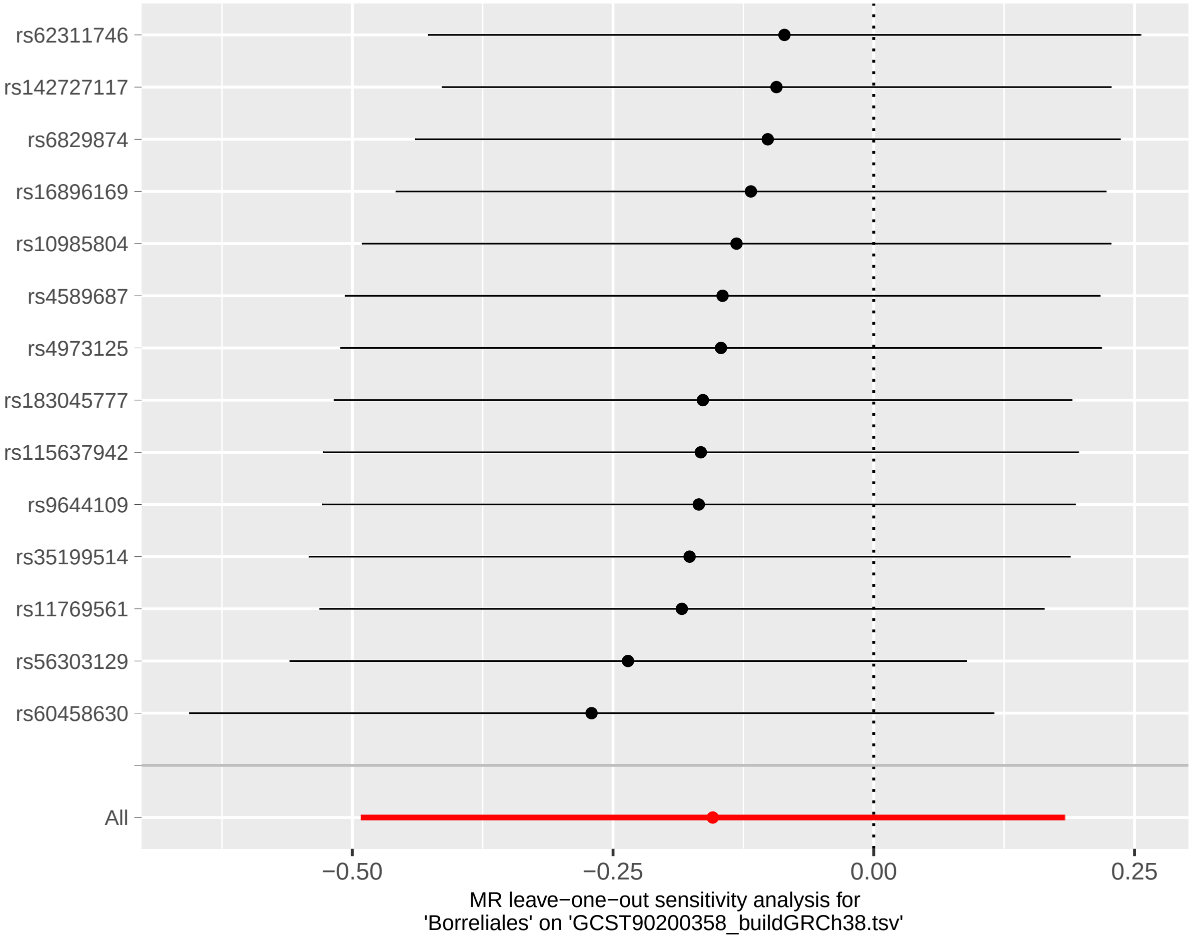

rs62311746
rs142727117
rs6829874
rs16896169
rs10985804
rs4589687
rs4973125
rs183045777
rs115637942
rs9644109
rs35199514
rs11769561
rs56303129
rs60458630
All
−0.50
−0.25
0.00
0.25
MR leave−one−out sensitivity analysis for
'Borreliales' on 'GCST90200358_buildGRCh38.tsv'

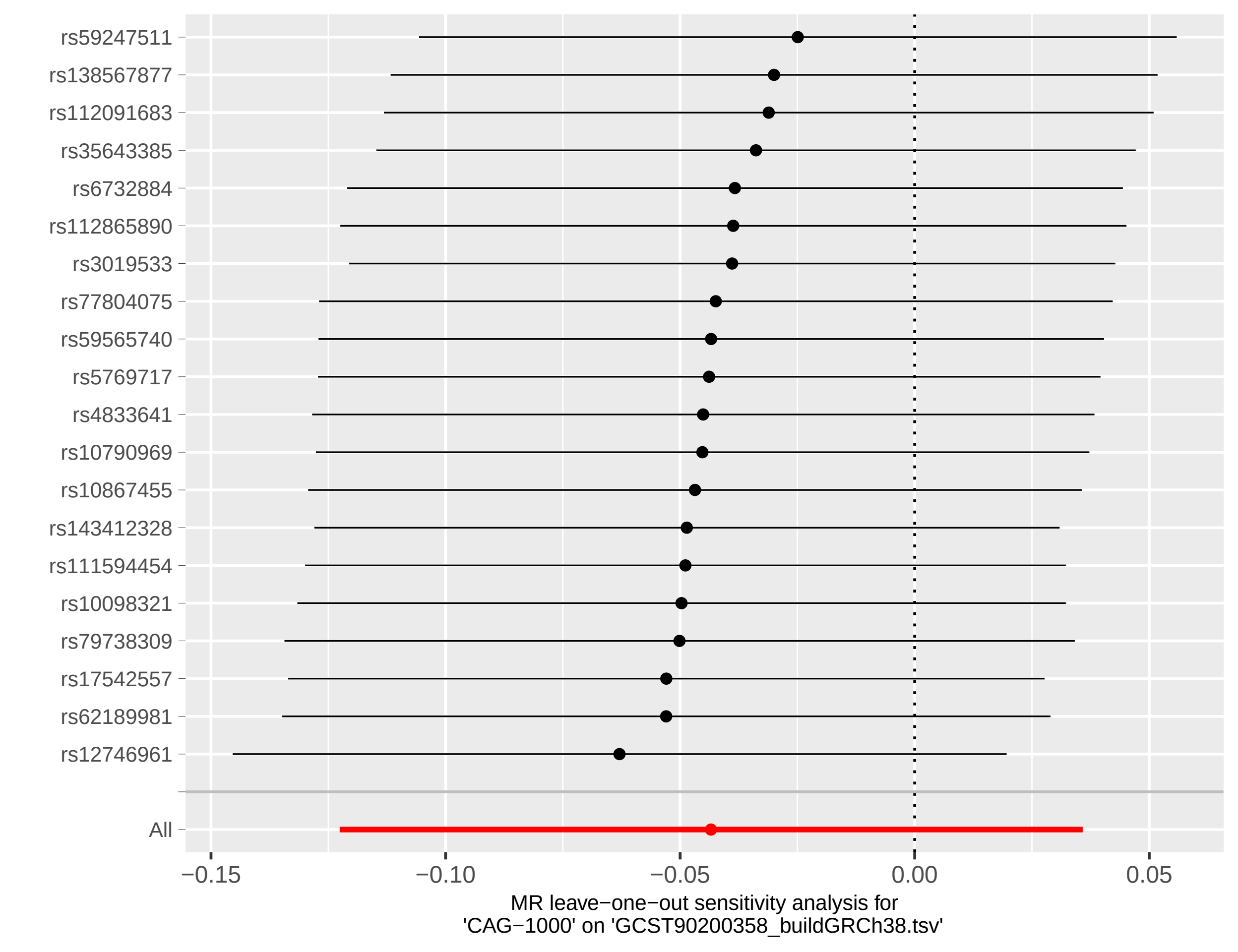

rs59247511
rs138567877
rs112091683
rs35643385
rs6732884
rs112865890
rs3019533
rs77804075
rs59565740
rs5769717
rs4833641
rs10790969
rs10867455
rs143412328
rs111594454
rs10098321
rs79738309
rs17542557
rs62189981
rs12746961
All
−0.15
−0.10
−0.05
0.00
0.05
MR leave−one−out sensitivity analysis for
'CAG−1000' on 'GCST90200358_buildGRCh38.tsv'

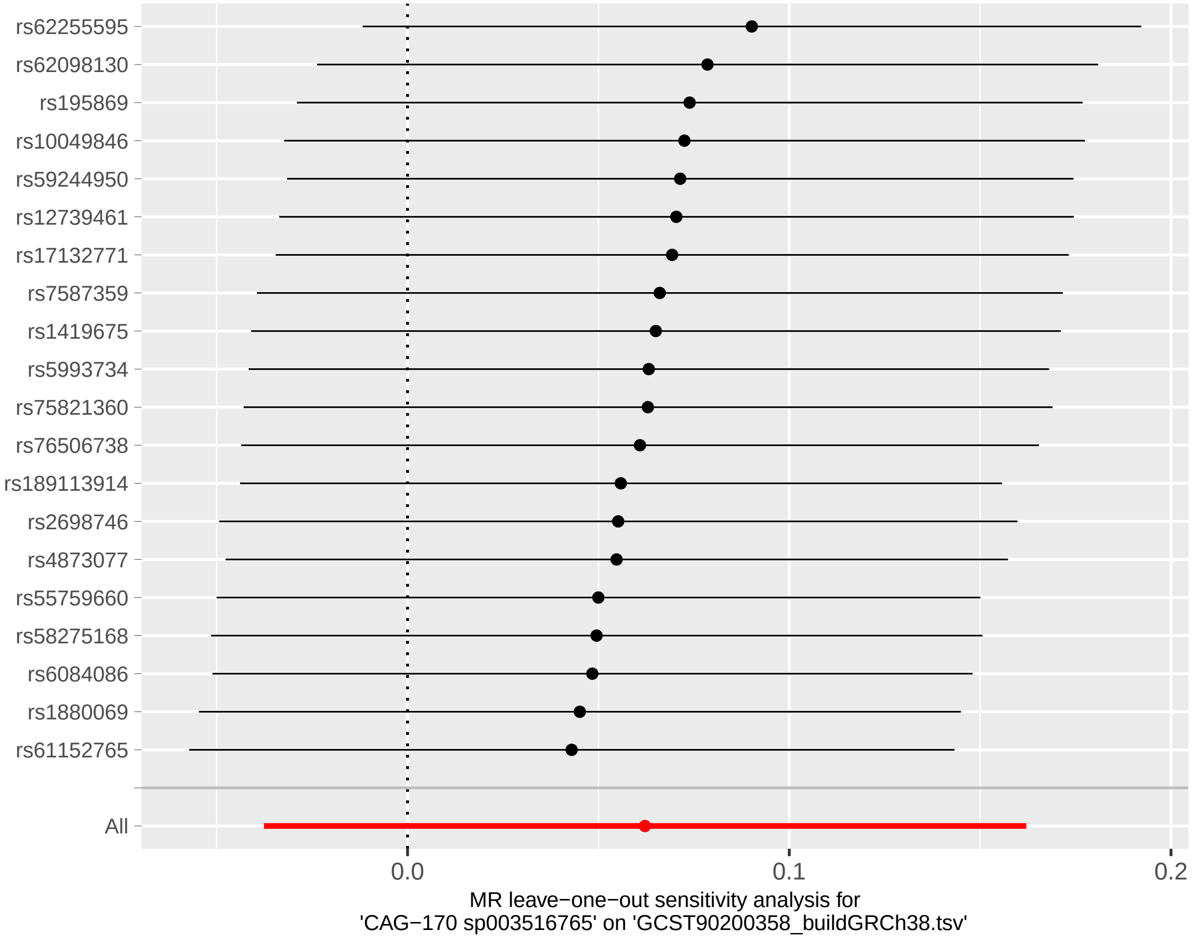

rs62255595
rs62098130
rs195869
rs10049846
rs59244950
rs12739461
rs17132771
rs7587359
rs1419675
rs5993734
rs75821360
rs76506738
rs189113914
rs2698746
rs4873077
rs55759660
rs58275168
rs6084086
rs1880069
rs61152765
All
0.0
0.1
0.2
MR leave−one−out sensitivity analysis for
'CAG−170 sp003516765' on 'GCST90200358_buildGRCh38.tsv'

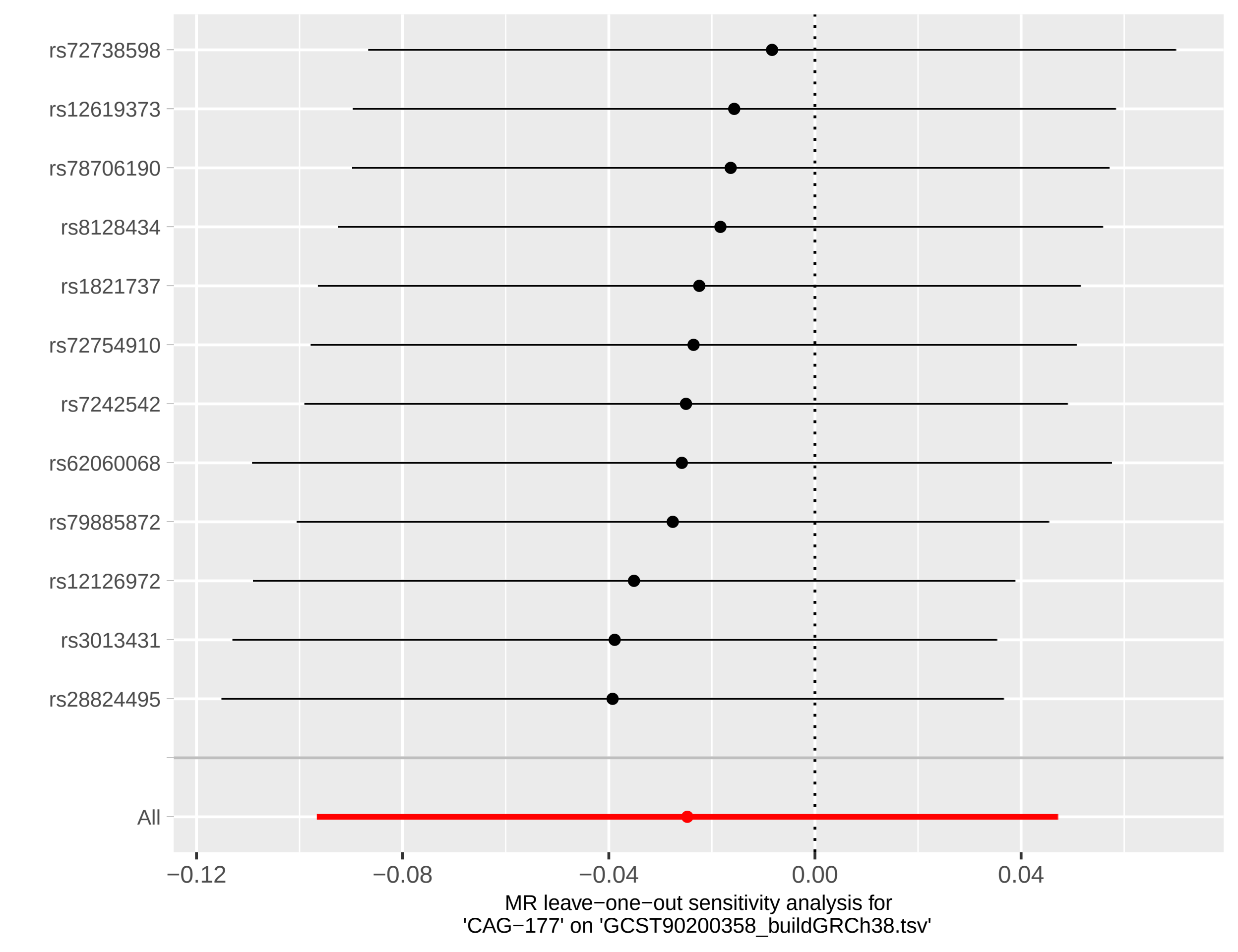
rs72738598
rs12619373
rs78706190
rs8128434
rs1821737
rs72754910
rs7242542
rs62060068
rs79885872
rs12126972
rs3013431
rs28824495
All
−0.12
−0.08
−0.04
0.00
0.04
MR leave−one−out sensitivity analysis for
'CAG−177' on 'GCST90200358_buildGRCh38.tsv'

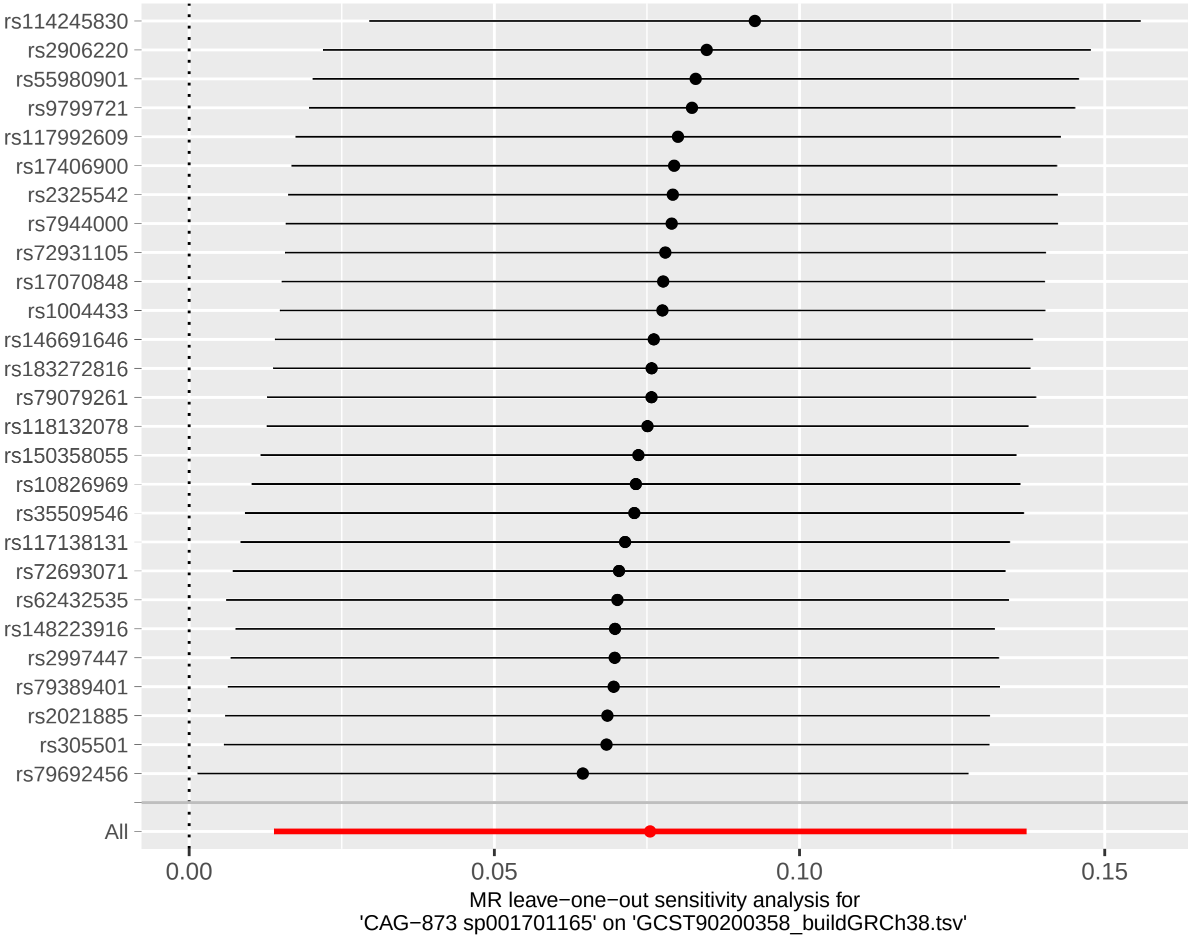

rs114245830
rs2906220
rs55980901
rs9799721
rs117992609
rs17406900
rs2325542
rs7944000
rs72931105
rs17070848
rs1004433
rs146691646
rs183272816
rs79079261
rs118132078
rs150358055
rs10826969
rs35509546
rs117138131
rs72693071
rs62432535
rs148223916
rs2997447
rs79389401
rs2021885
rs305501
rs79692456
All
0.00
0.05
0.10
0.15
MR leave−one−out sensitivity analysis for
'CAG−873 sp001701165' on 'GCST90200358_buildGRCh38.tsv'

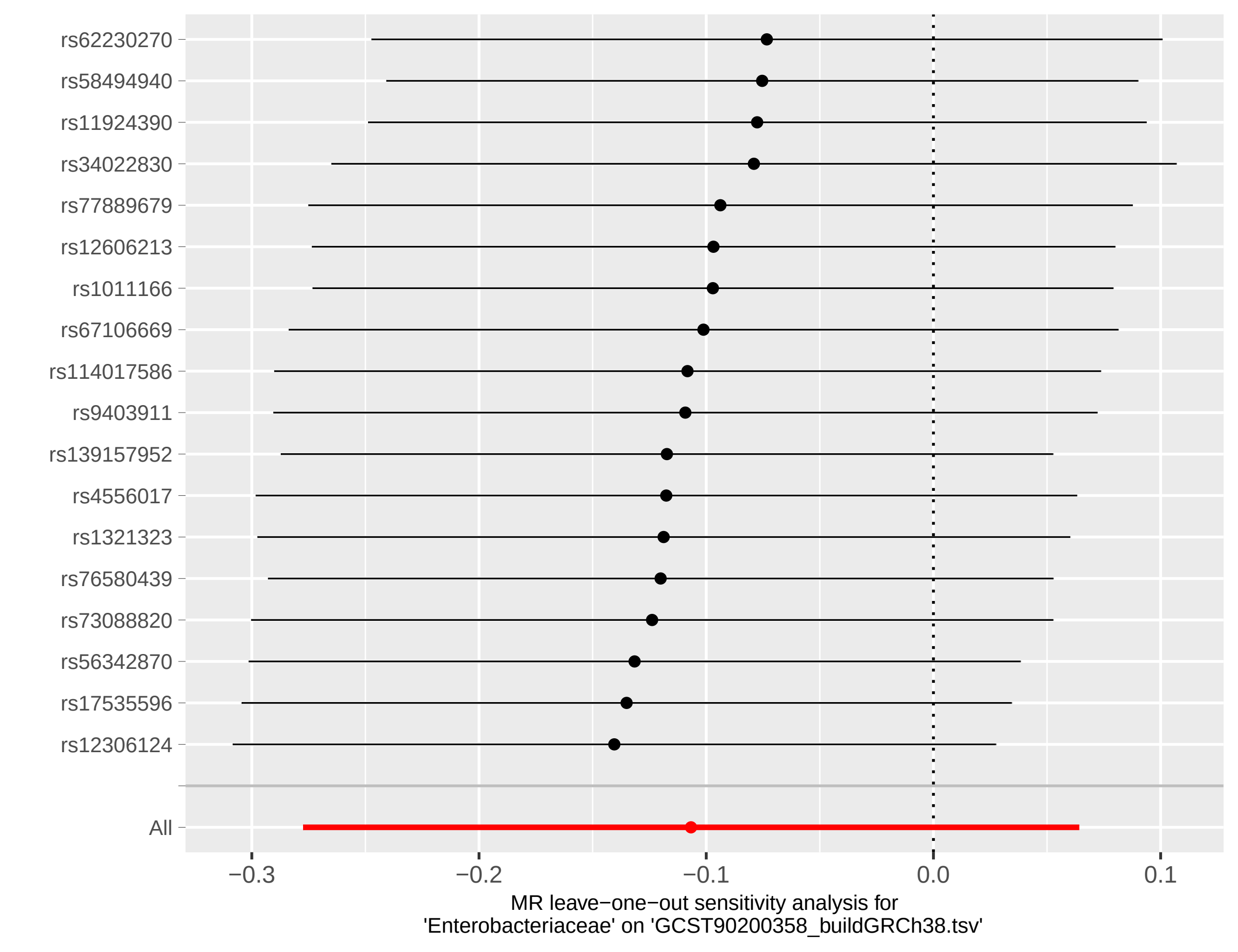
rs62230270
rs58494940
rs11924390
rs34022830
rs77889679
rs12606213
rs1011166
rs67106669
rs114017586
rs9403911
rs139157952
rs4556017
rs1321323
rs76580439
rs73088820
rs56342870
rs17535596
rs12306124
All
−0.3
−0.2
−0.1
0.0
0.1
MR leave−one−out sensitivity analysis for
'Enterobacteriaceae' on 'GCST90200358_buildGRCh38.tsv'

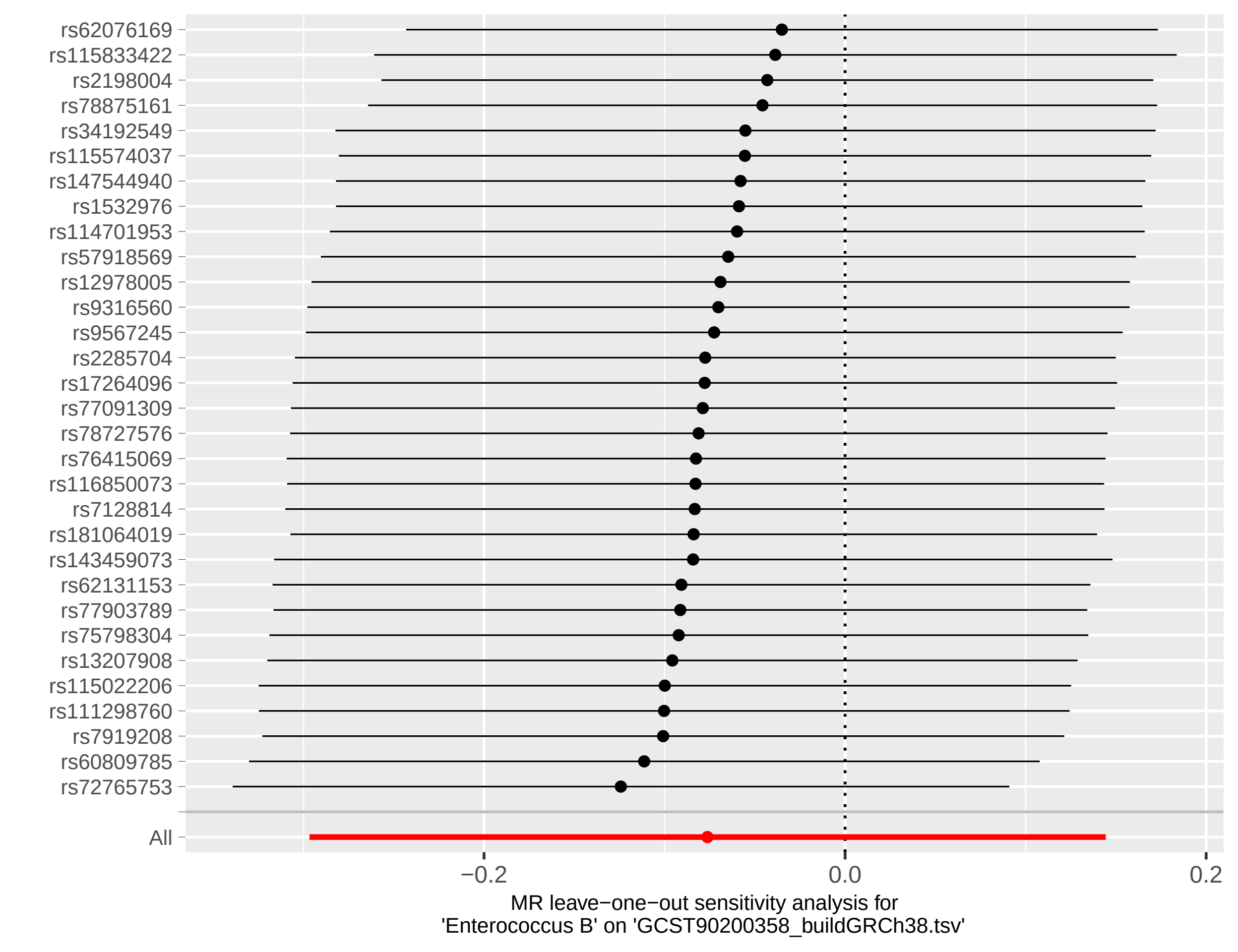
rs62076169
rs115833422
rs2198004
rs78875161
rs34192549
rs115574037
rs147544940
rs1532976
rs114701953
rs57918569
rs12978005
rs9316560
rs9567245
rs2285704
rs17264096
rs77091309
rs78727576
rs76415069
rs116850073
rs7128814
rs181064019
rs143459073
rs62131153
rs77903789
rs75798304
rs13207908
rs115022206
rs111298760
rs7919208
rs60809785
rs72765753
All
−0.2
0.0
0.2
MR leave−one−out sensitivity analysis for
'Enterococcus B' on 'GCST90200358_buildGRCh38.tsv'

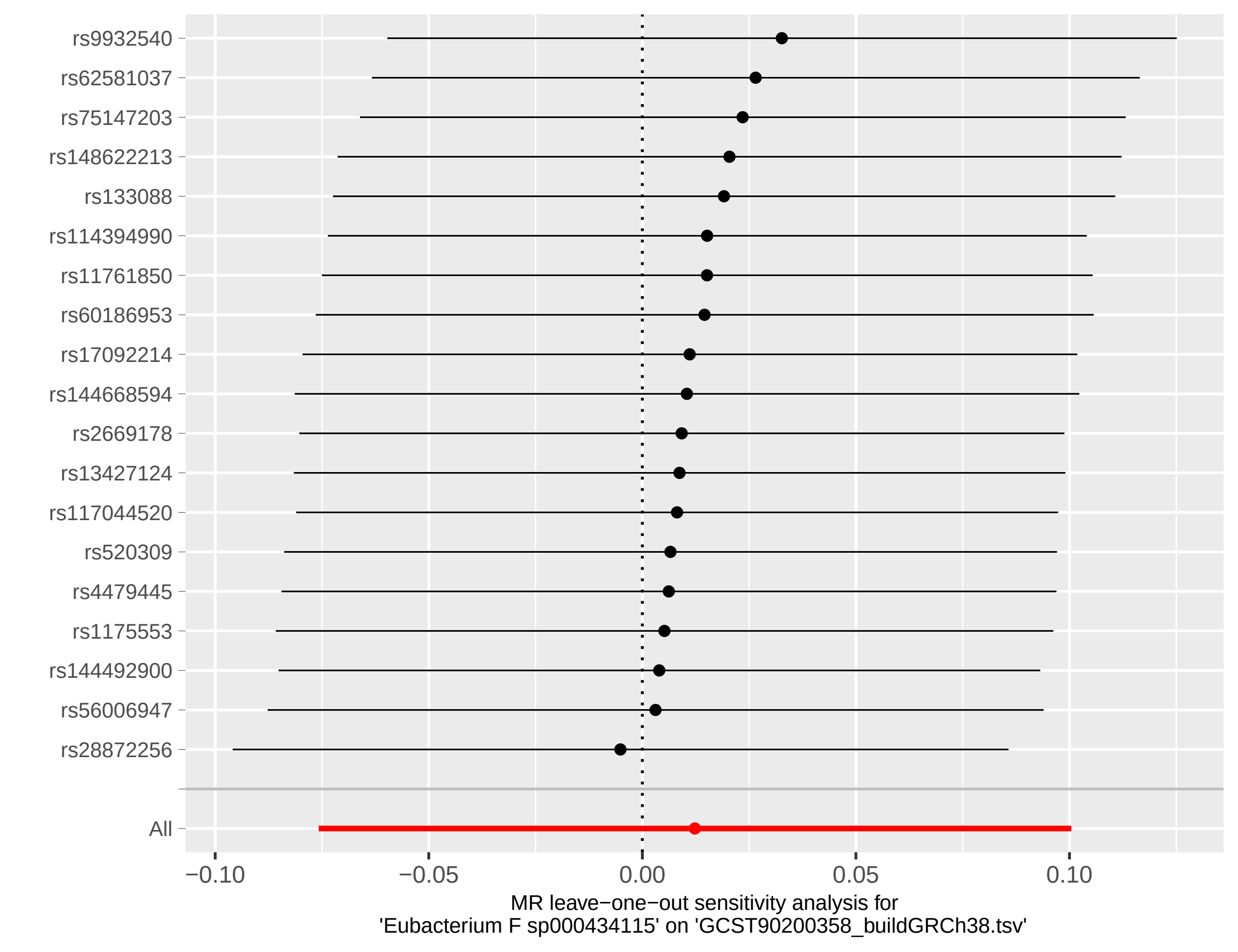

rs9932540
rs62581037
rs75147203
rs148622213
rs133088
rs114394990
rs11761850
rs60186953
rs17092214
rs144668594
rs2669178
rs13427124
rs117044520
rs520309
rs4479445
rs1175553
rs144492900
rs56006947
rs28872256
All
−0.10
−0.05
0.00
0.05
0.10
MR leave−one−out sensitivity analysis for
'Eubacterium F sp000434115' on 'GCST90200358_buildGRCh38.tsv'

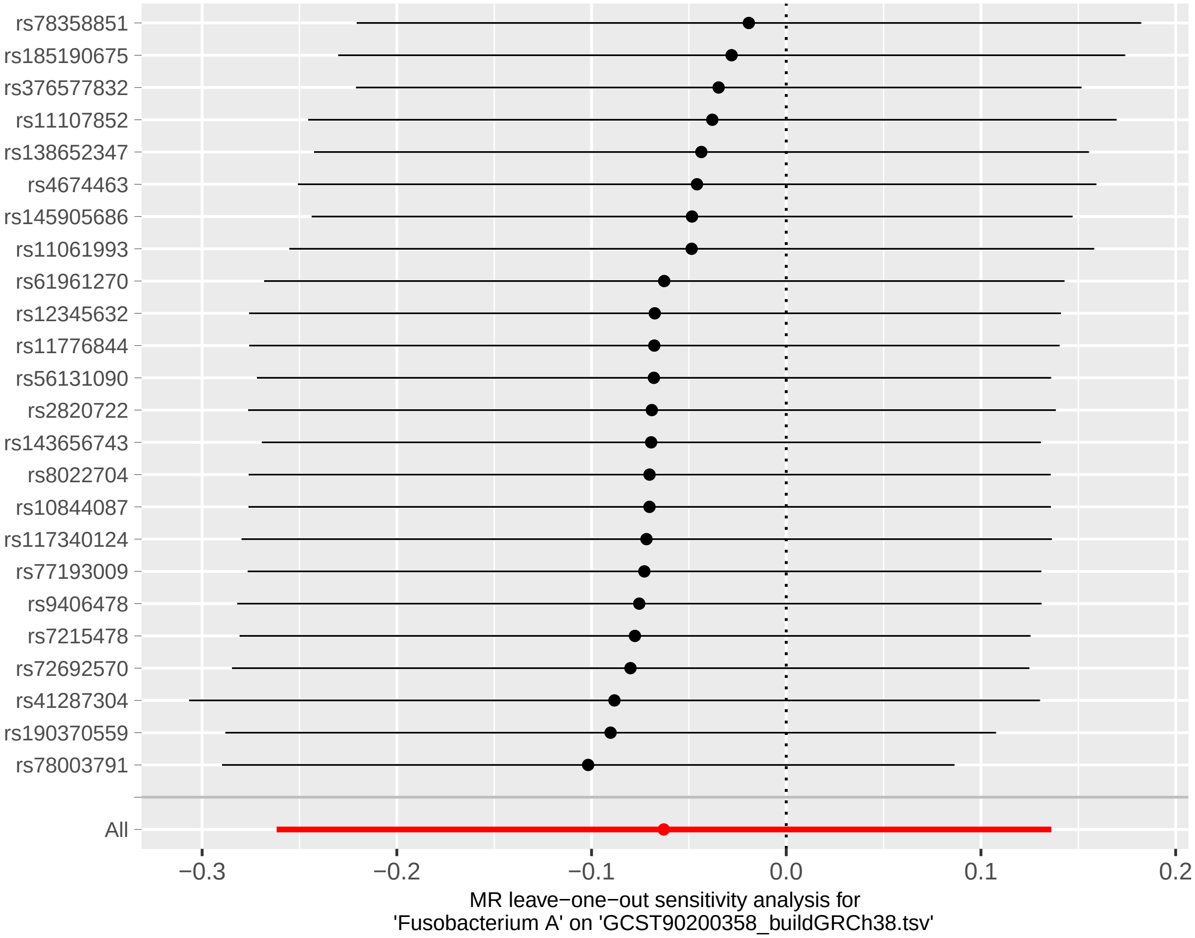
rs78358851
rs185190675
rs376577832
rs11107852
rs138652347
rs4674463
rs145905686
rs11061993
rs61961270
rs12345632
rs11776844
rs56131090
rs2820722
rs143656743
rs8022704
rs10844087
rs117340124
rs77193009
rs9406478
rs7215478
rs72692570
rs41287304
rs190370559
rs78003791
All
−0.3
−0.2
−0.1
0.0
0.1
0.2
MR leave−one−out sensitivity analysis for
'Fusobacterium A' on 'GCST90200358_buildGRCh38.tsv'

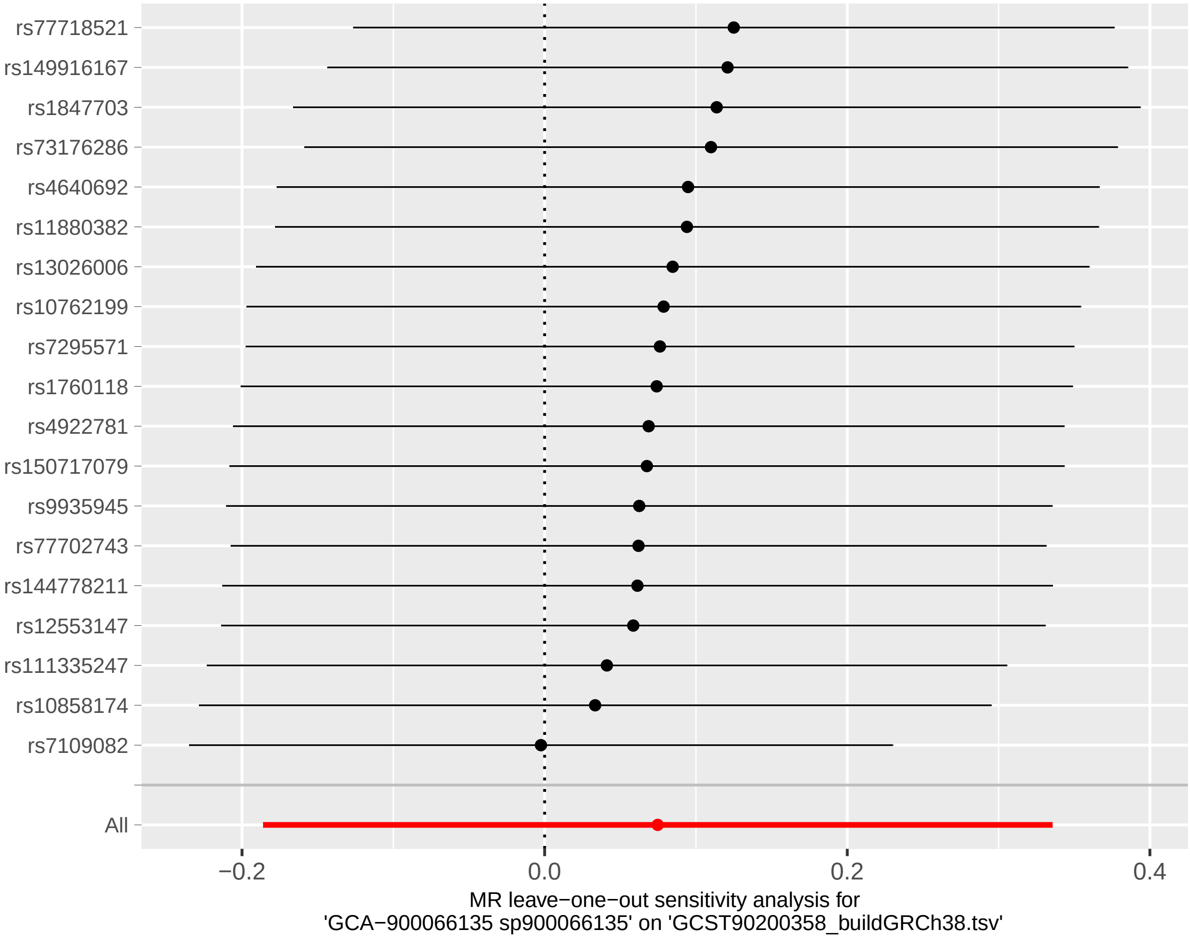

rs77718521
rs149916167
rs1847703
rs73176286
rs4640692
rs11880382
rs13026006
rs10762199
rs7295571
rs1760118
rs4922781
rs150717079
rs9935945
rs77702743
rs144778211
rs12553147
rs111335247
rs10858174
rs7109082
All
−0.2
0.0
0.2
0.4
MR leave−one−out sensitivity analysis for
'GCA−900066135 sp900066135' on 'GCST90200358_buildGRCh38.tsv'

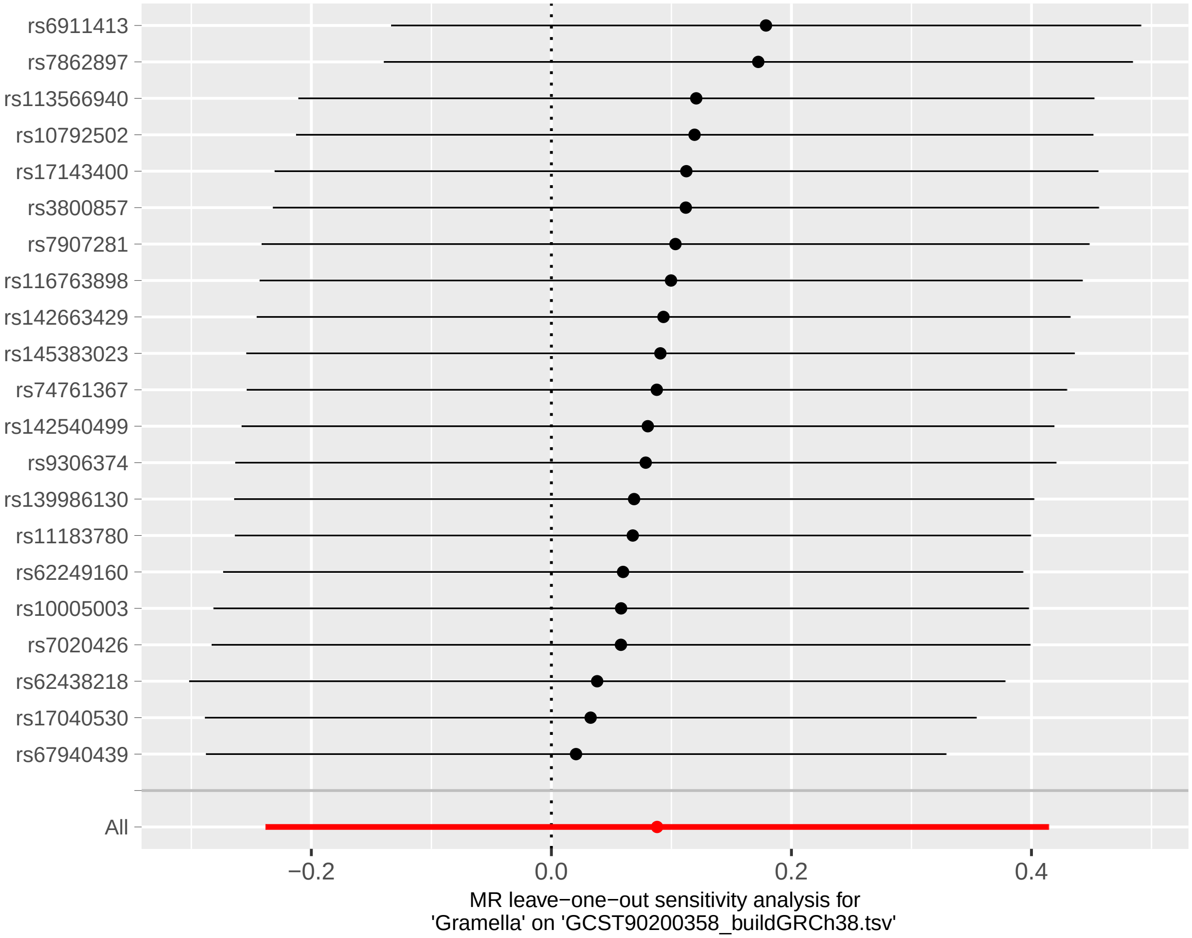

rs6911413
rs7862897
rs113566940
rs10792502
rs17143400
rs3800857
rs7907281
rs116763898
rs142663429
rs145383023
rs74761367
rs142540499
rs9306374
rs139986130
rs11183780
rs62249160
rs10005003
rs7020426
rs62438218
rs17040530
rs67940439
All
−0.2
0.0
0.2
0.4
MR leave−one−out sensitivity analysis for
'Gramella' on 'GCST90200358_buildGRCh38.tsv'

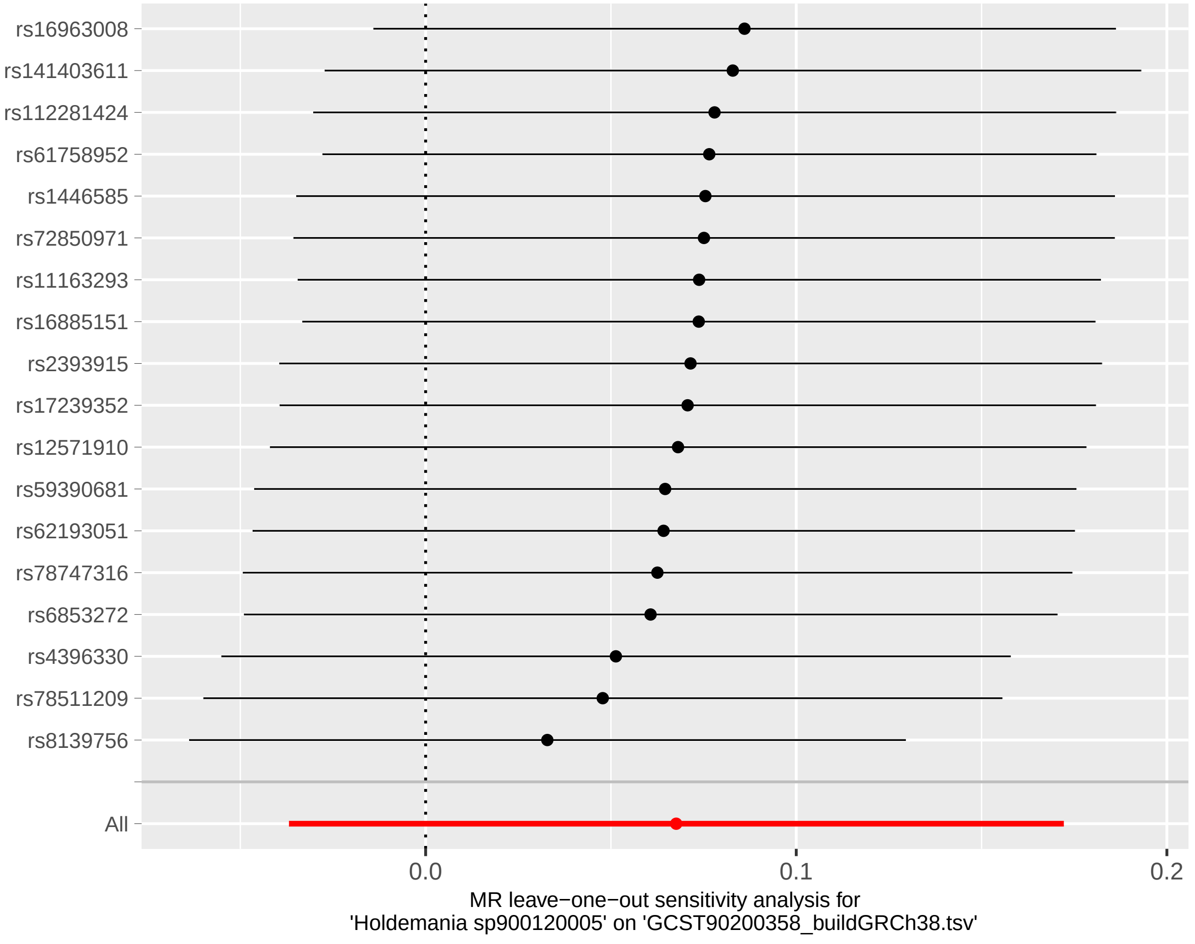
rs16963008
rs141403611
rs112281424
rs61758952
rs1446585
rs72850971
rs11163293
rs16885151
rs2393915
rs17239352
rs12571910
rs59390681
rs62193051
rs78747316
rs6853272
rs4396330
rs78511209
rs8139756
All
0.0
0.1
0.2
MR leave−one−out sensitivity analysis for
'Holdemania sp900120005' on 'GCST90200358_buildGRCh38.tsv'

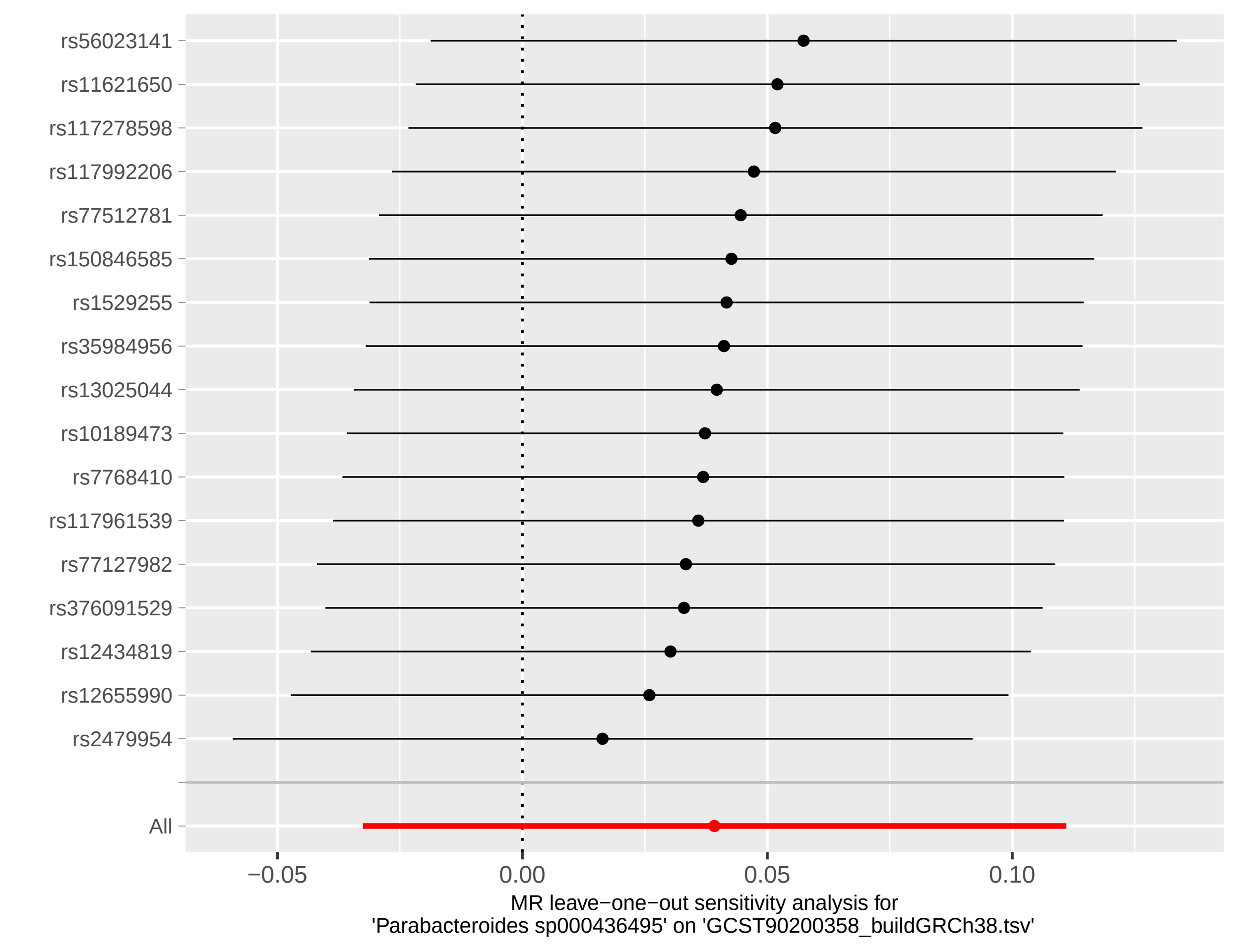

rs56023141
rs11621650
rs117278598
rs117992206
rs77512781
rs150846585
rs1529255
rs35984956
rs13025044
rs10189473
rs7768410
rs117961539
rs77127982
rs376091529
rs12434819
rs12655990
rs2479954
All
−0.05
0.00
0.05
0.10
MR leave−one−out sensitivity analysis for
'Parabacteroides sp000436495' on 'GCST90200358_buildGRCh38.tsv'

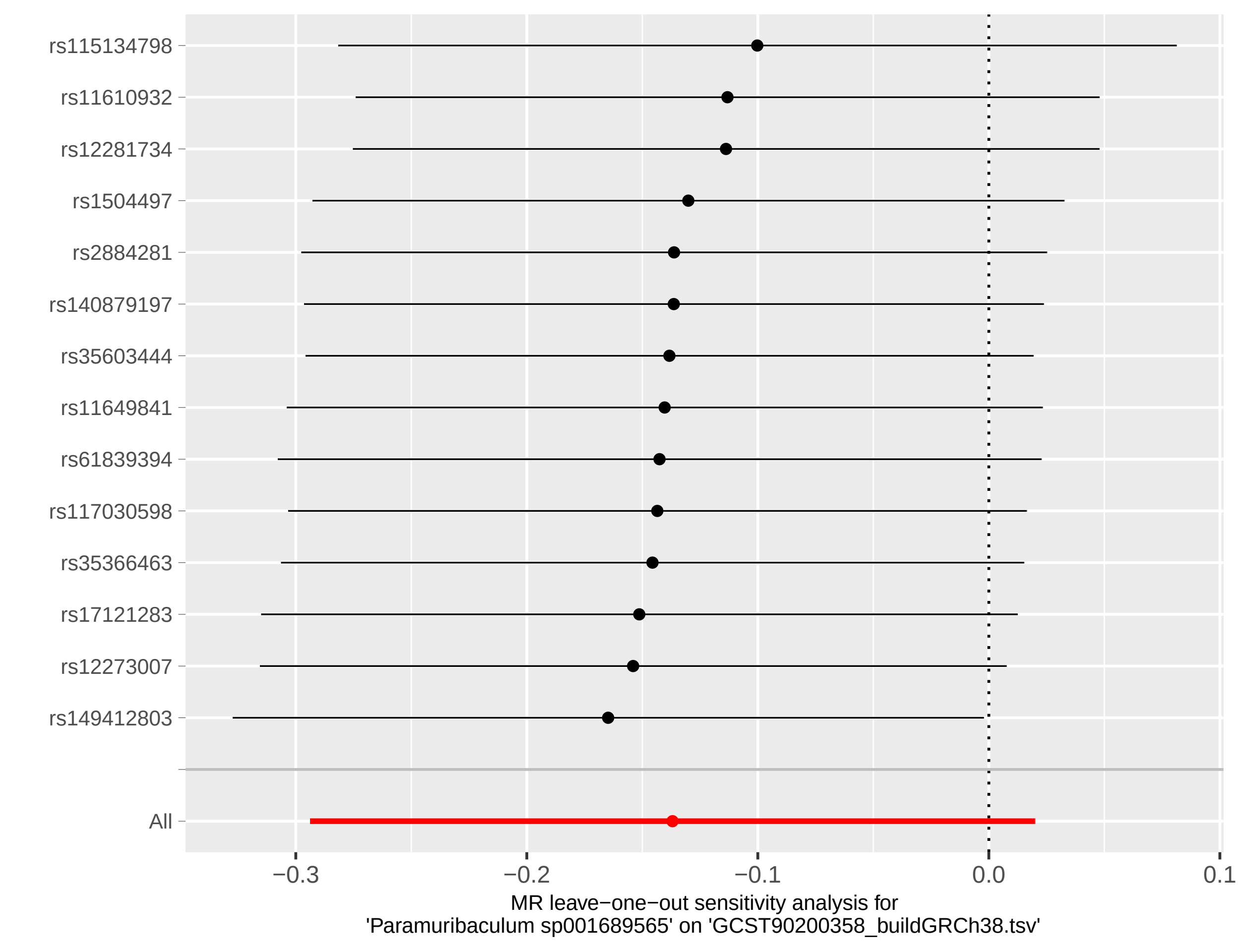

rs115134798
rs11610932
rs12281734
rs1504497
rs2884281
rs140879197
rs35603444
rs11649841
rs61839394
rs117030598
rs35366463
rs17121283
rs12273007
rs149412803
All
−0.3
−0.2
−0.1
0.0
0.1
MR leave−one−out sensitivity analysis for
'Paramuribaculum sp001689565' on 'GCST90200358_buildGRCh38.tsv'

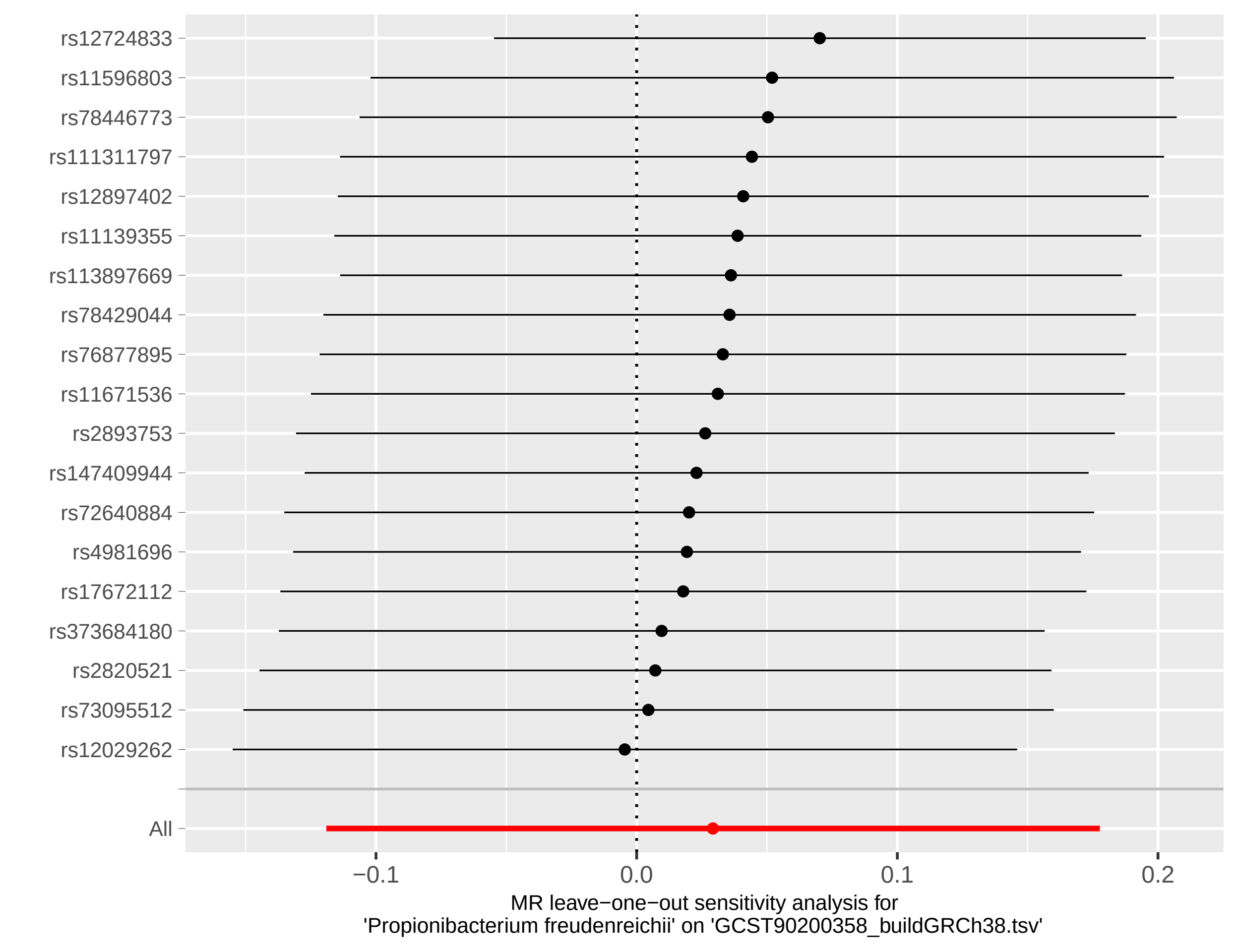
rs12724833
rs11596803
rs78446773
rs111311797
rs12897402
rs11139355
rs113897669
rs78429044
rs76877895
rs11671536
rs2893753
rs147409944
rs72640884
rs4981696
rs17672112
rs373684180
rs2820521
rs73095512
rs12029262
All
−0.1
0.0
0.1
0.2
MR leave−one−out sensitivity analysis for
'Propionibacterium freudenreichii' on 'GCST90200358_buildGRCh38.tsv'

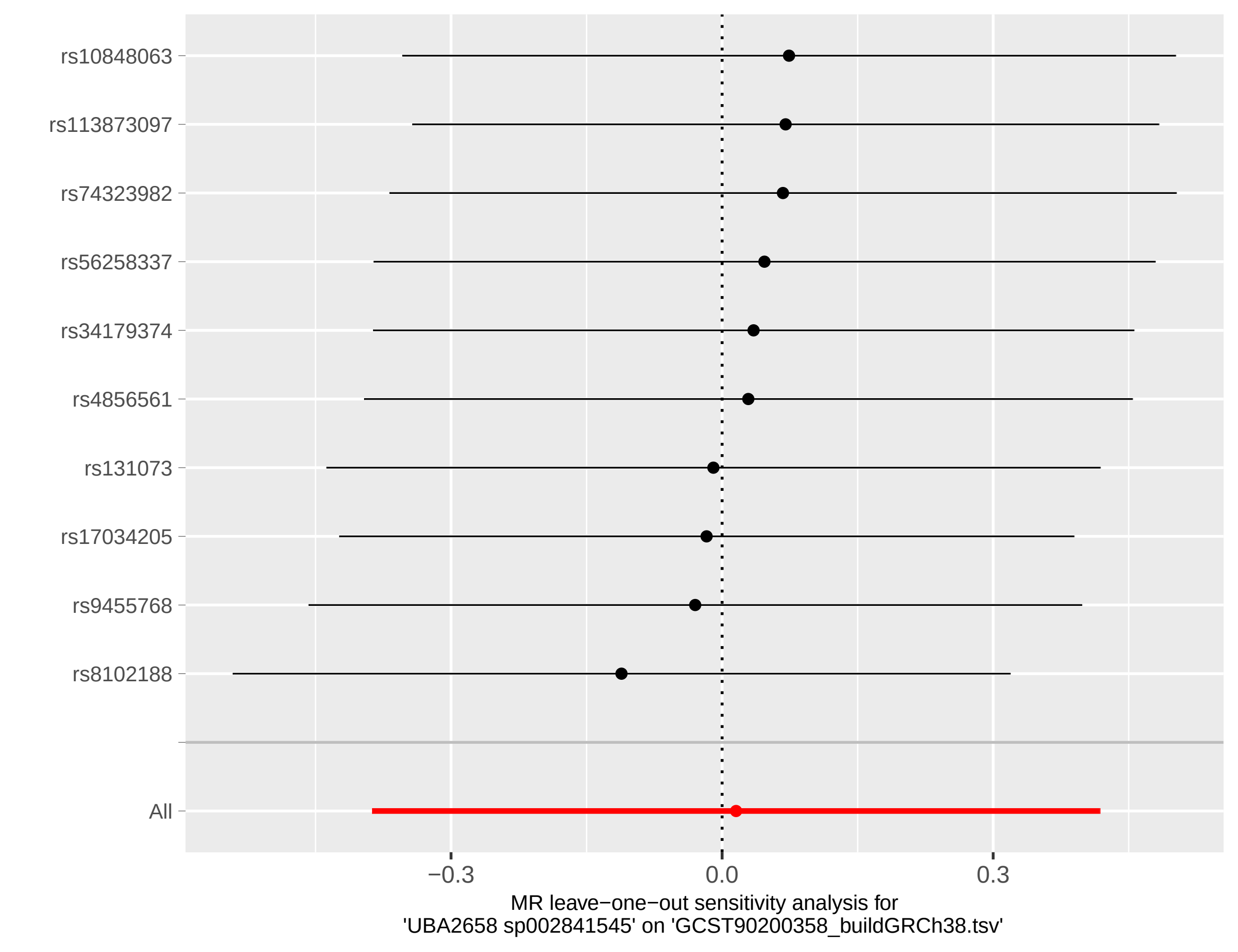

rs10848063
rs113873097
rs74323982
rs56258337
rs34179374
rs4856561
rs131073
rs17034205
rs9455768
rs8102188
All
−0.3
0.0
0.3
MR leave−one−out sensitivity analysis for
'UBA2658 sp002841545' on 'GCST90200358_buildGRCh38.tsv'

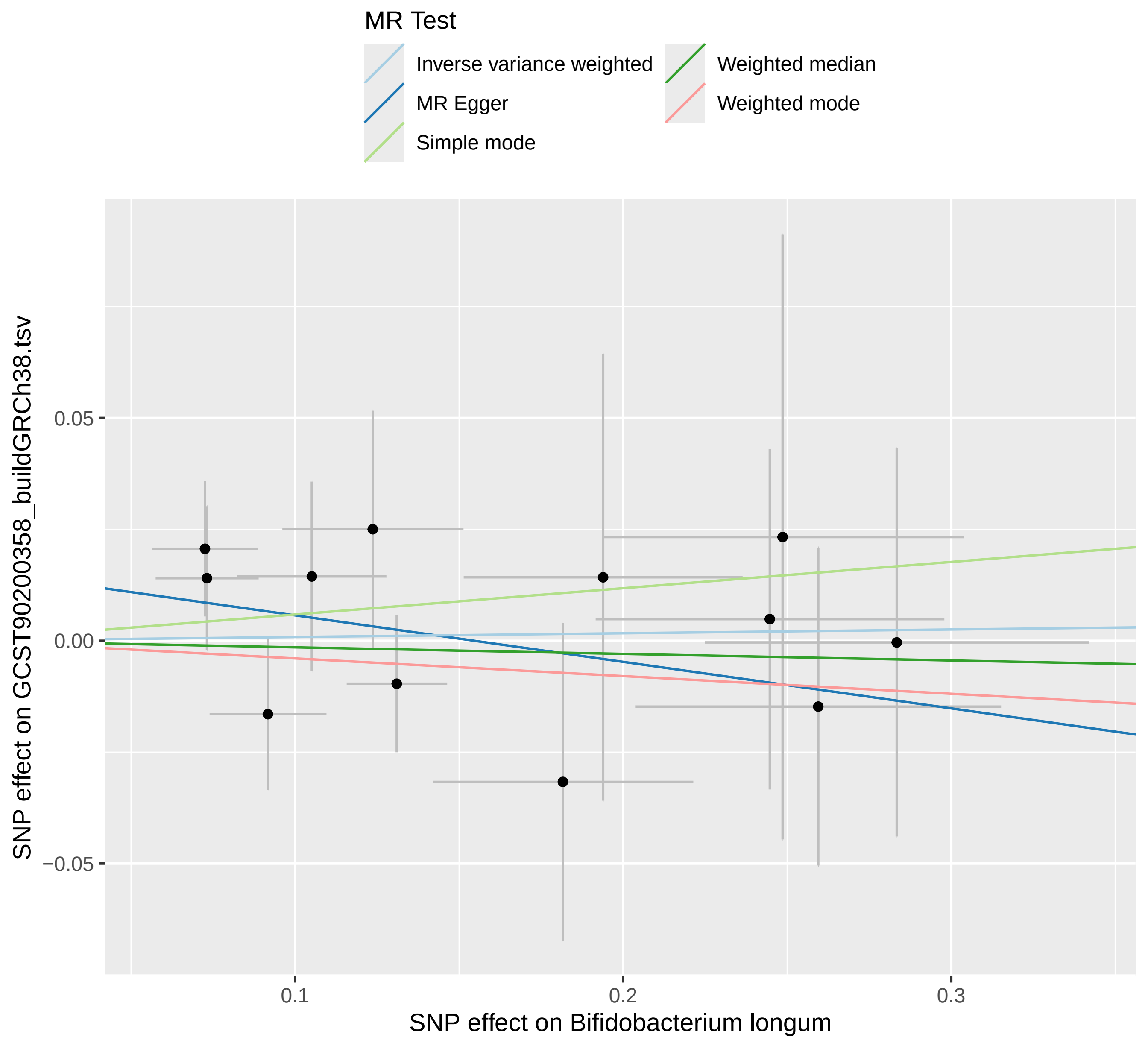
MR Test
Inverse variance weighted
MR Egger
Simple mode
Weighted median
Weighted mode
SNP effect on GCST90200358_buildGRCh38.tsv
0.05
0.00
−0.05
0.1
0.2
0.3
SNP effect on Bifidobacterium longum

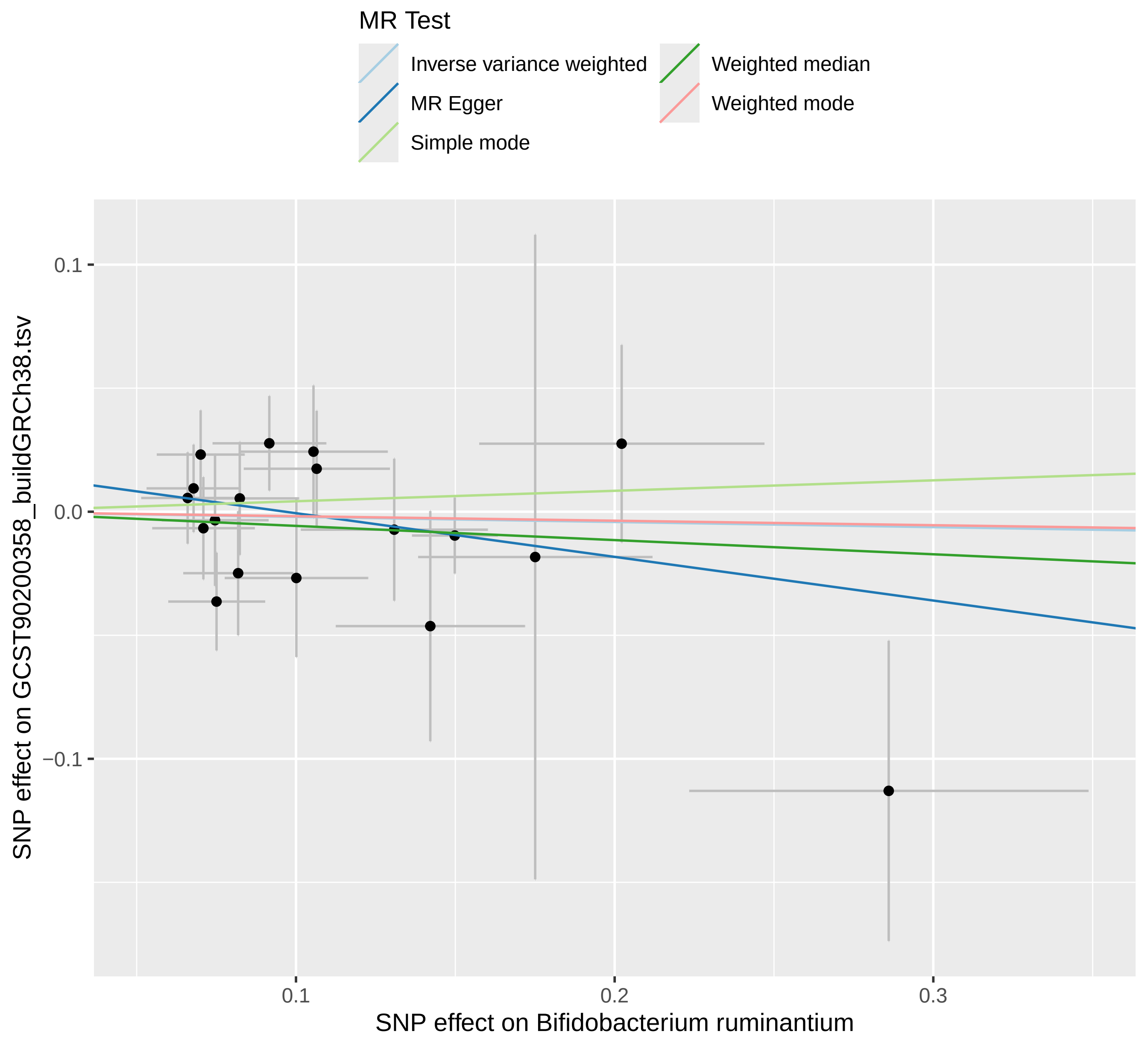
MR Test
Inverse variance weighted
MR Egger
Simple mode
Weighted median
Weighted mode
SNP effect on GCST90200358_buildGRCh38.tsv
0.1
0.0
−0.1
0.1
0.2
0.3
SNP effect on Bifidobacterium ruminantium

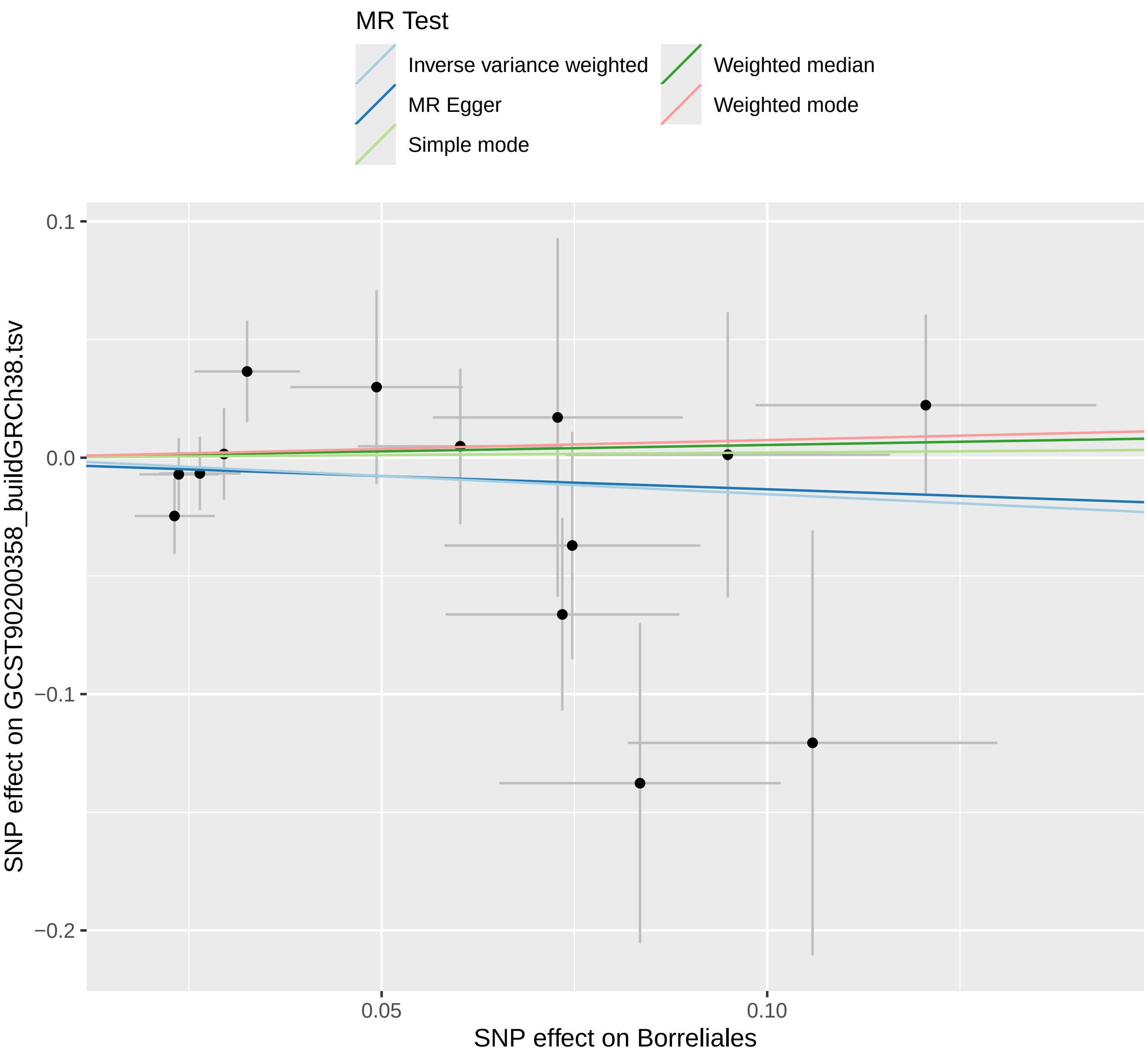
MR Test
Inverse variance weighted
MR Egger
Simple mode
Weighted median
Weighted mode
0.1
0.0
−0.1
−0.2
0.05
0.10
SNP effect on Borreliales
SNP effect on GCST90200358_buildGRCh38.tsv

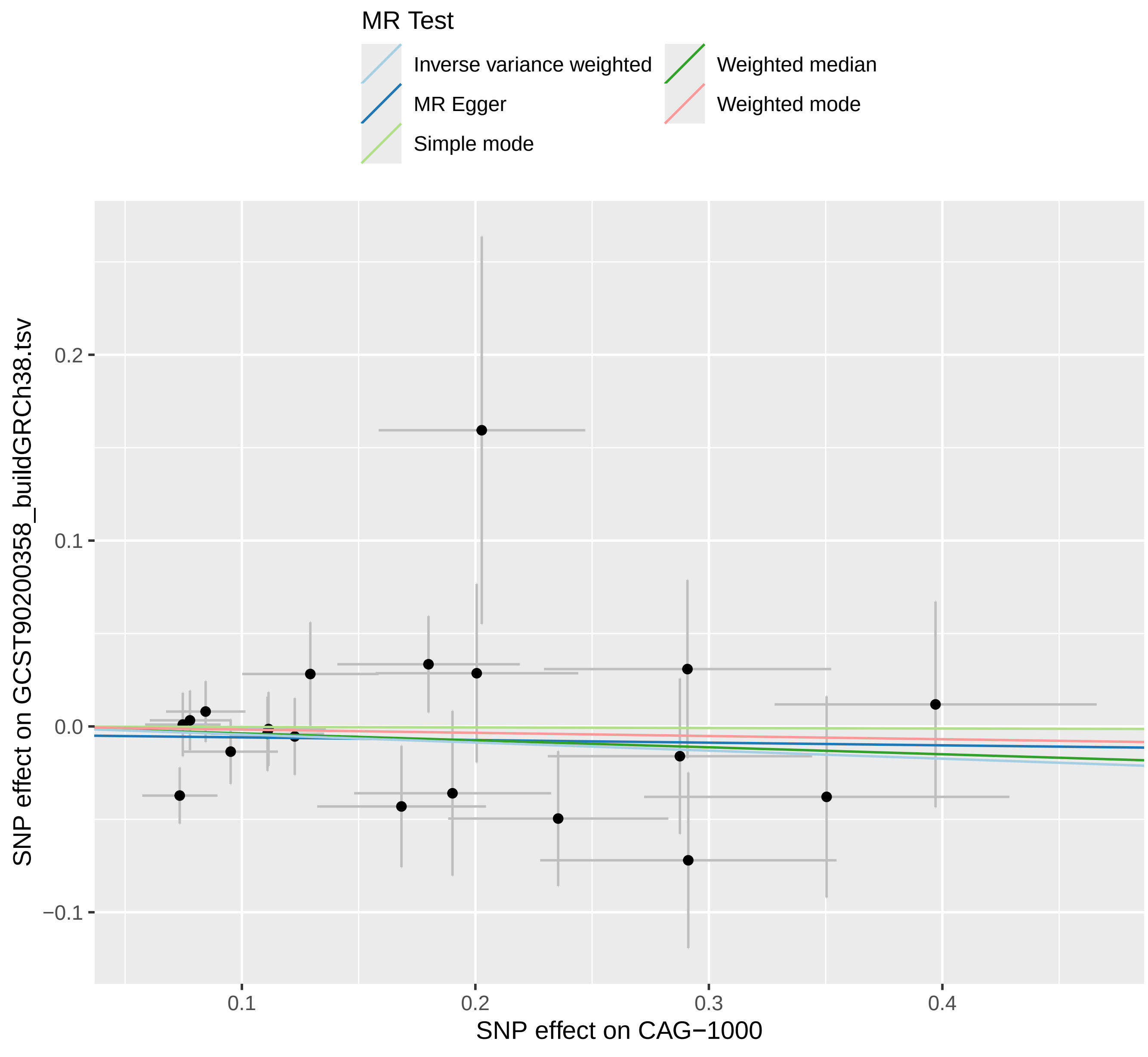

MR Test
Inverse variance weighted
MR Egger
Simple mode
Weighted median
Weighted mode
SNP effect on GCST90200358_buildGRCh38.tsv
0.2
0.1
0.0
−0.1
0.1
0.2
0.3
0.4
SNP effect on CAG−1000

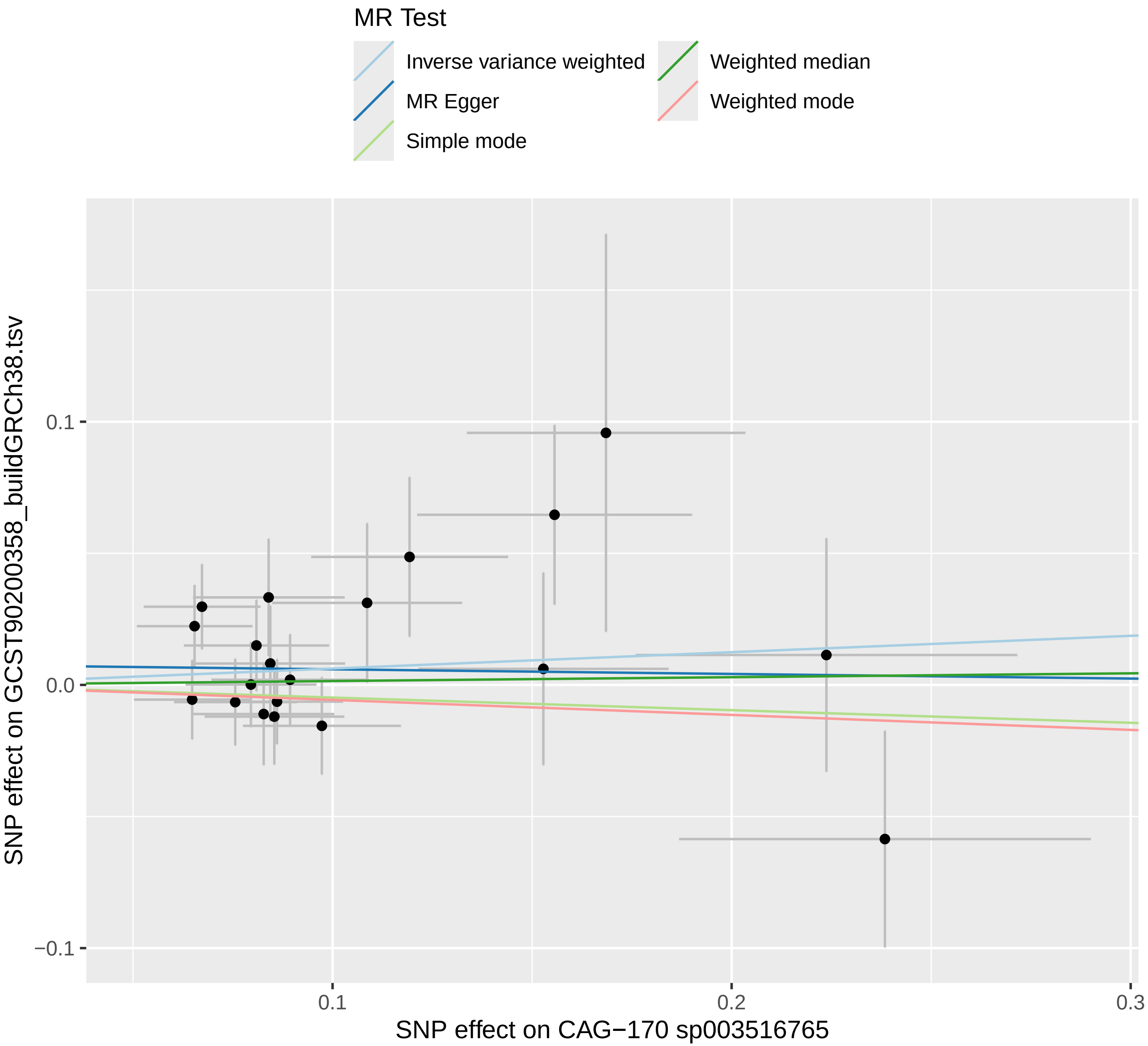
MR Test
Inverse variance weighted
MR Egger
Simple mode
Weighted median
Weighted mode
SNP effect on GCST90200358_buildGRCh38.tsv
0.1
0.0
−0.1
0.1
0.2
0.3
SNP effect on CAG−170 sp003516765

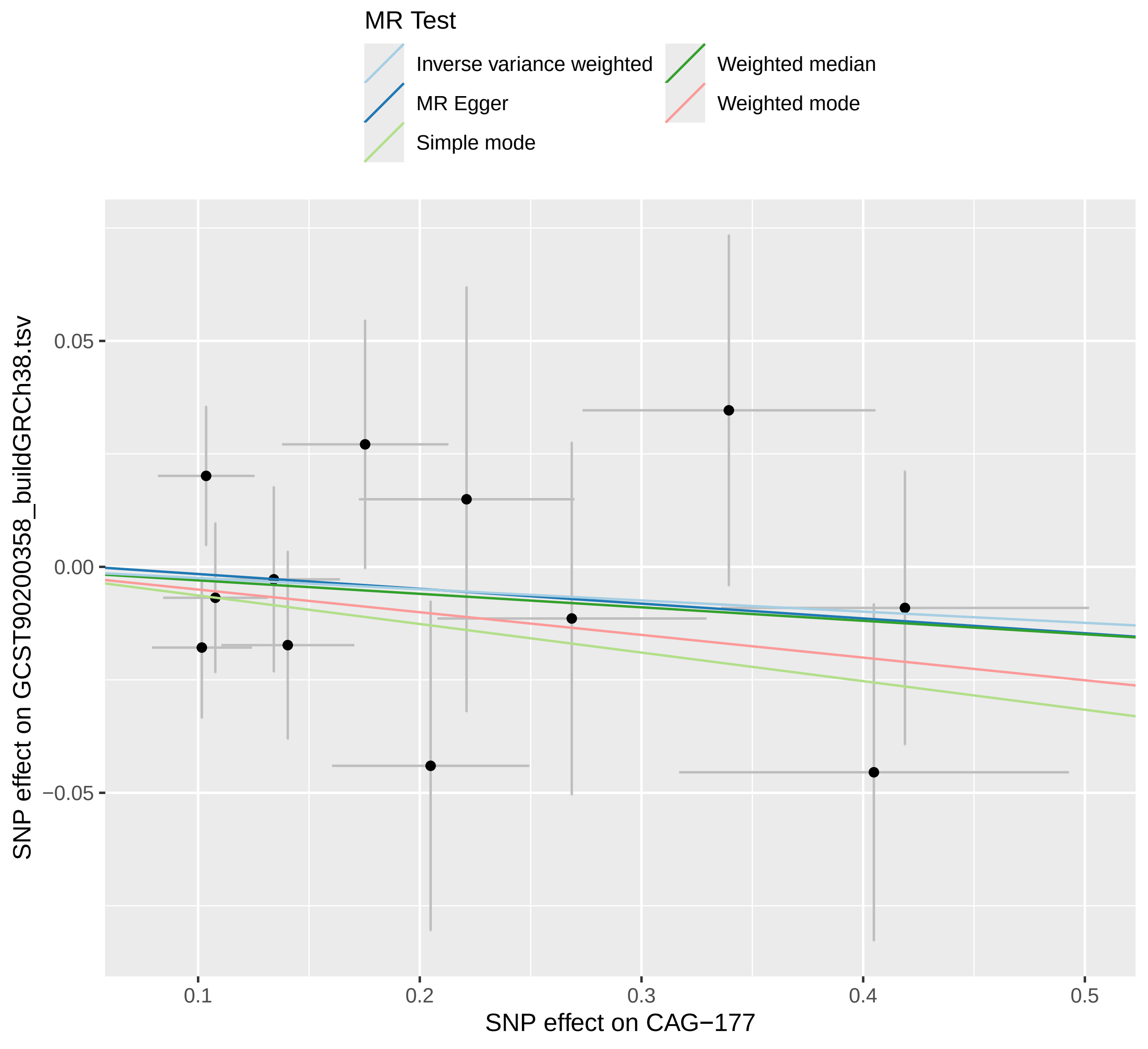
MR Test
Inverse variance weighted
MR Egger
Simple mode
Weighted median
Weighted mode
SNP effect on GCST90200358_buildGRCh38.tsv
0.05
0.00
−0.05
0.1
0.2
0.3
0.4
0.5
SNP effect on CAG−177

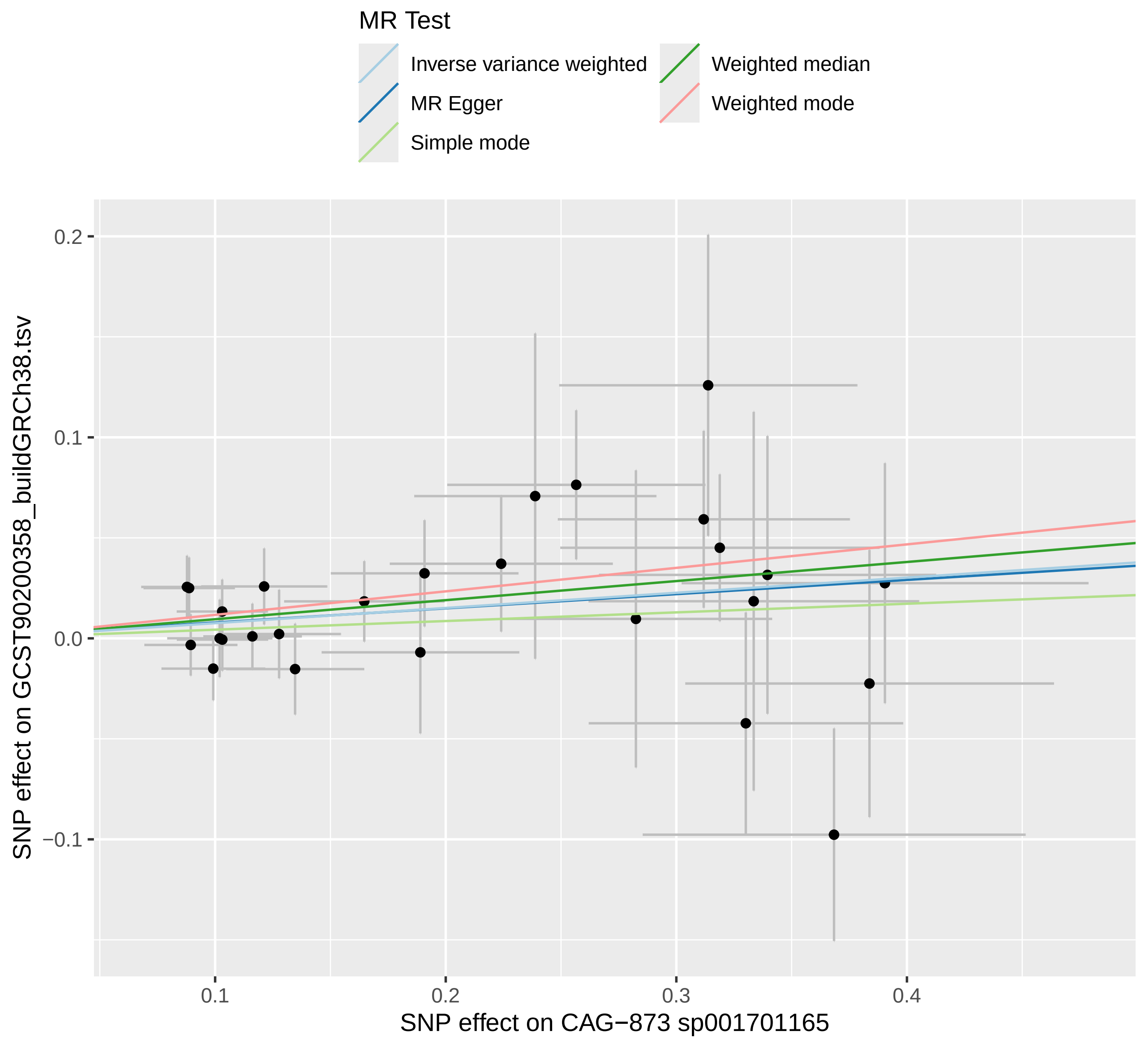
MR Test
Inverse variance weighted
MR Egger
Simple mode
Weighted median
Weighted mode
0.2
0.1
0.0
−0.1
0.1
0.2
0.3
0.4
SNP effect on GCST90020358_buildGRCh38.tsv
SNP effect on CAG−873 sp001701165

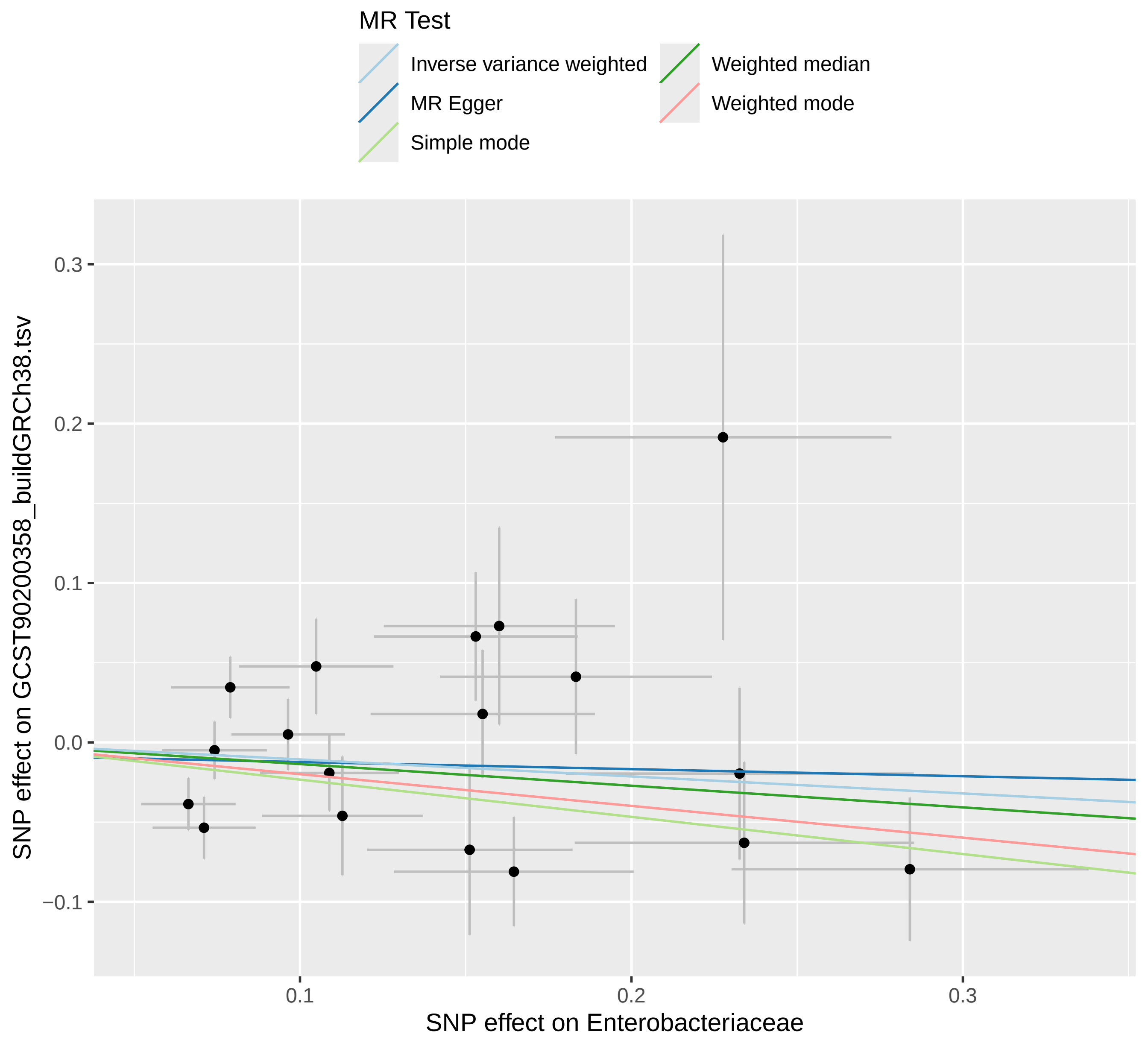

MR Test
Inverse variance weighted
MR Egger
Simple mode
Weighted median
Weighted mode
0.3
0.2
0.1
0.0
−0.1
0.1
0.2
0.3
SNP effect on GCST90200358_buildGRCh38.tsv
SNP effect on Enterobacteriaceae

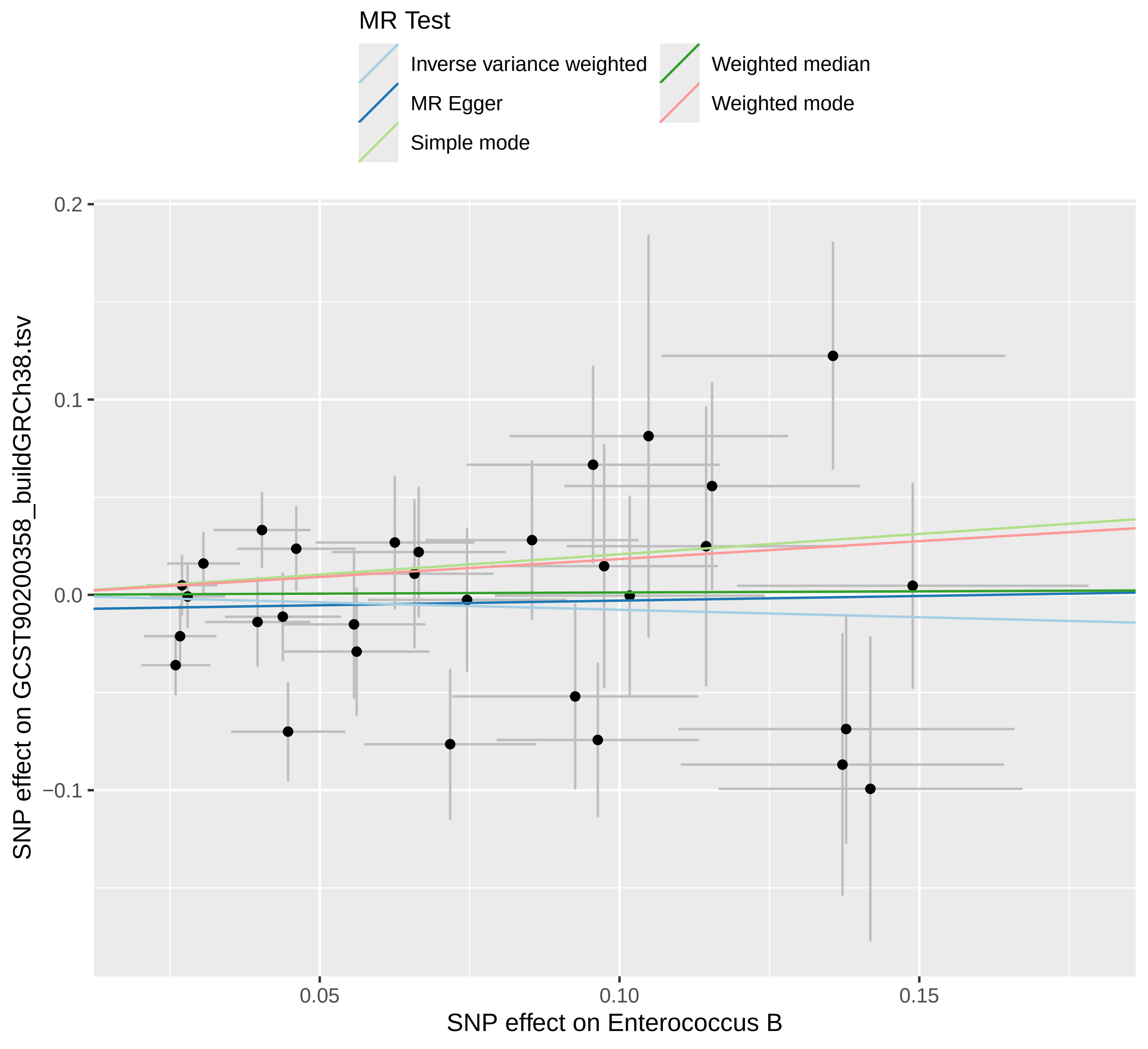
MR Test
Inverse variance weighted
MR Egger
Simple mode
Weighted median
Weighted mode
0.2
0.1
0.0
−0.1
0.05
0.10
0.15
SNP effect on GCST90200358_buildGRCh38.tsv
SNP effect on Enterococcus B

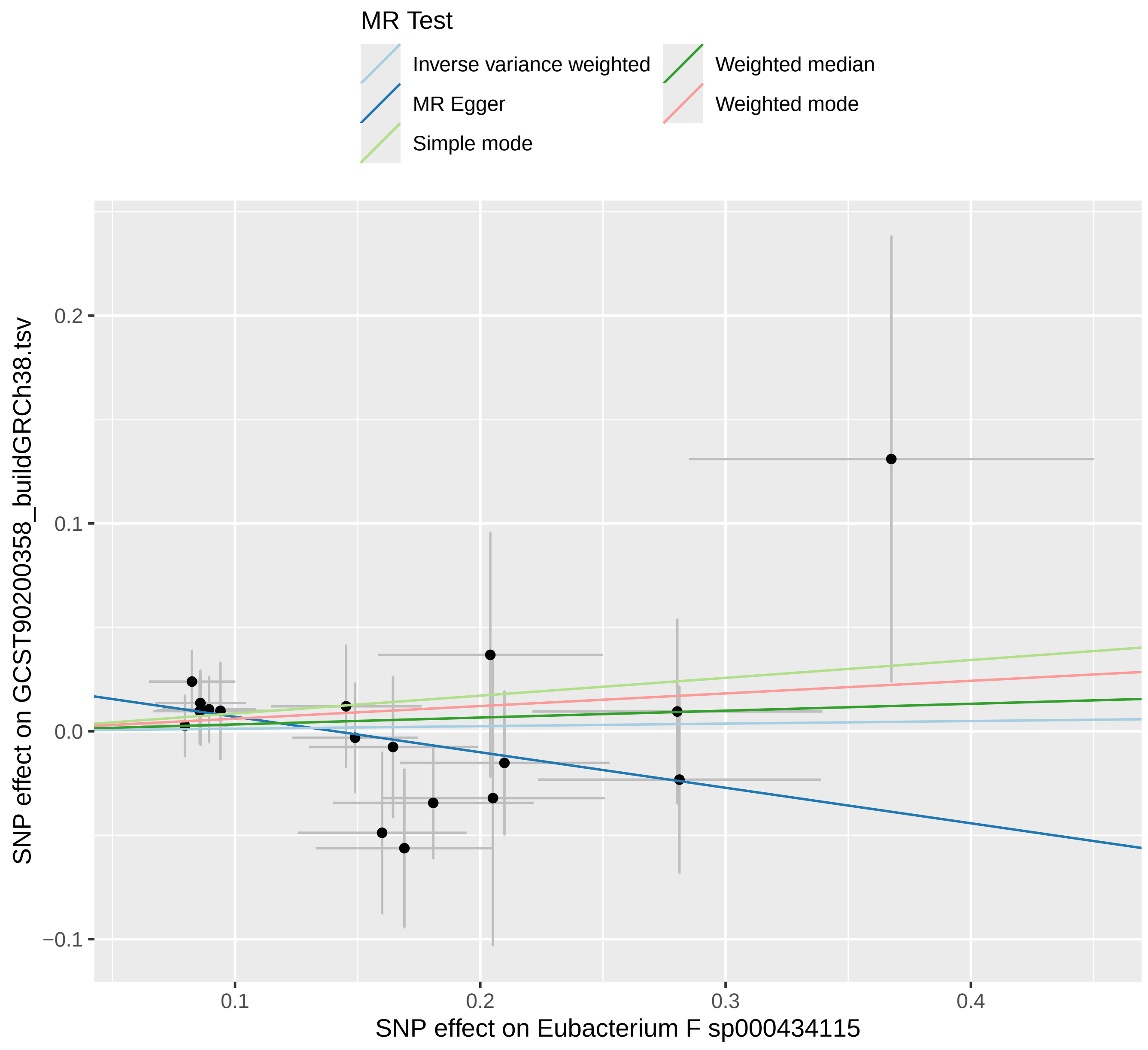

MR Test
Inverse variance weighted
MR Egger
Simple mode
Weighted median
Weighted mode
0.2
0.1
0.0
−0.1
SNP effect on GCST90200358_buildGRCh38.tsv
0.1
0.2
0.3
0.4
SNP effect on Eubacterium F sp000434115

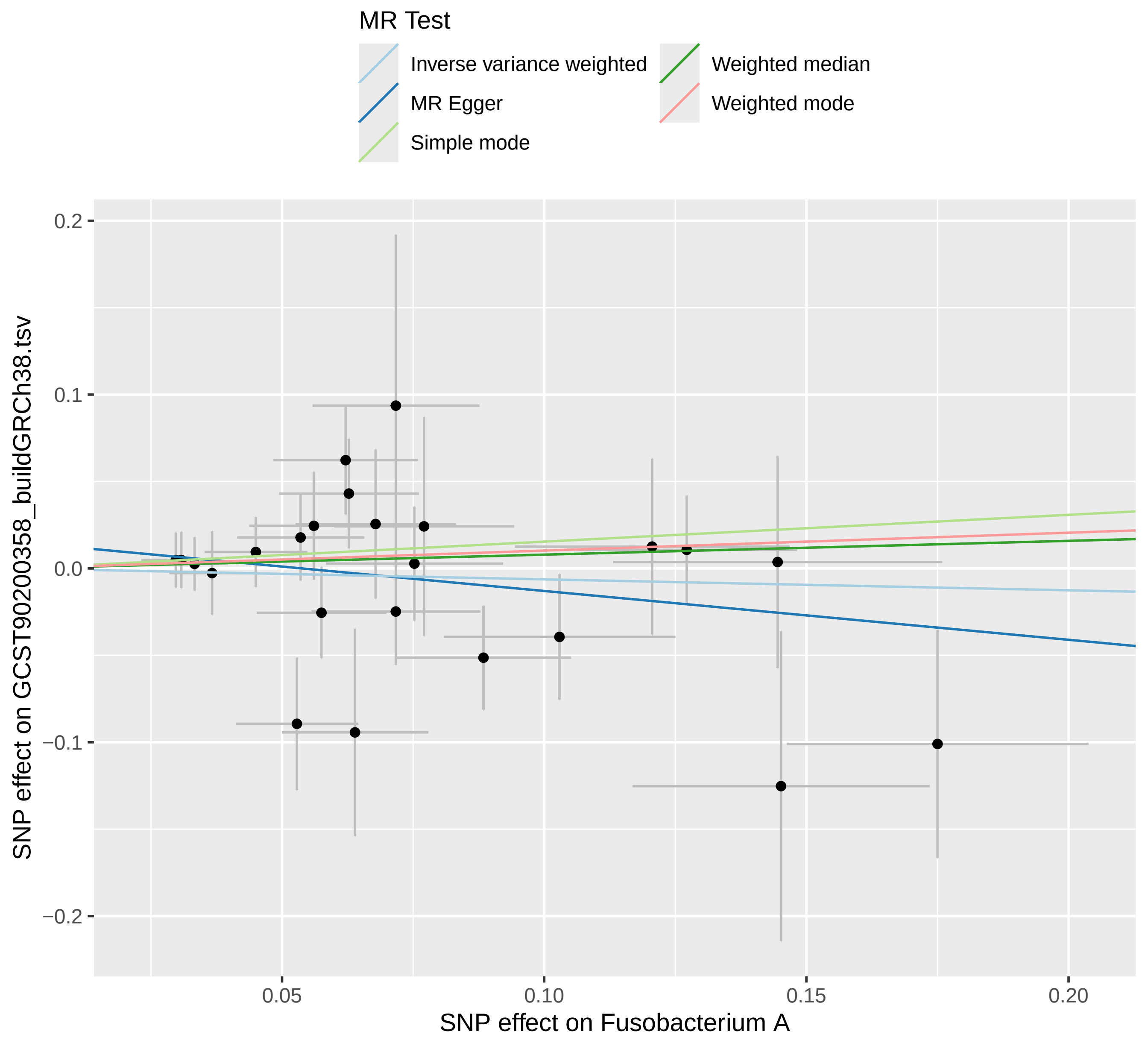
MR Test
Inverse variance weighted
MR Egger
Simple mode
Weighted median
Weighted mode
SNP effect on GCST90200358_buildGRCh38.tsv
0.2
0.1
0.0
−0.1
−0.2
0.05
0.10
0.15
0.20
SNP effect on Fusobacterium A

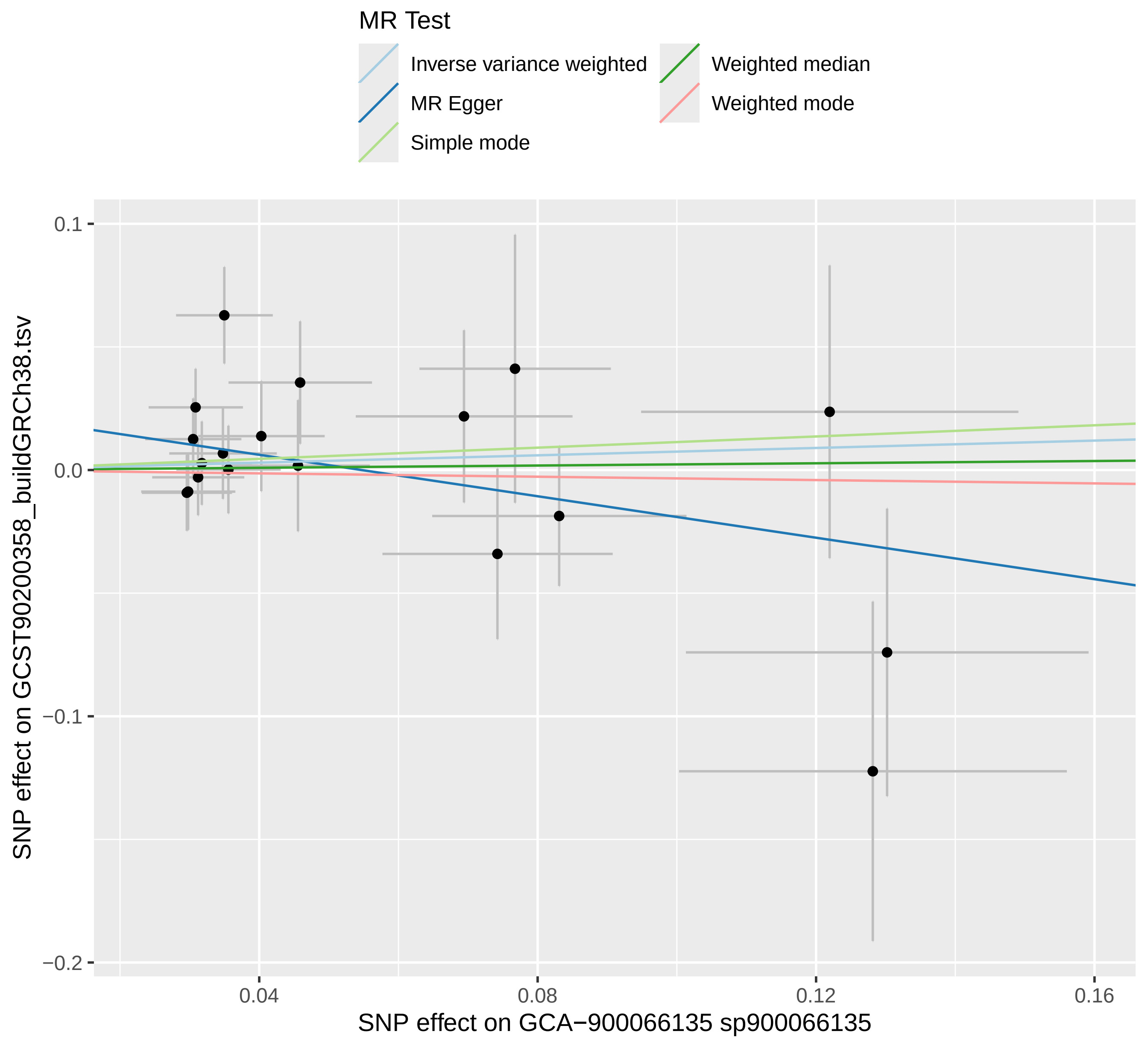
MR Test
Inverse variance weighted
MR Egger
Simple mode
Weighted median
Weighted mode
0.1
0.0
−0.1
−0.2
0.04
0.08
0.12
0.16
SNP effect on GCST90200358_buildGRCh38.tsv
SNP effect on GCA−900066135 sp900066135

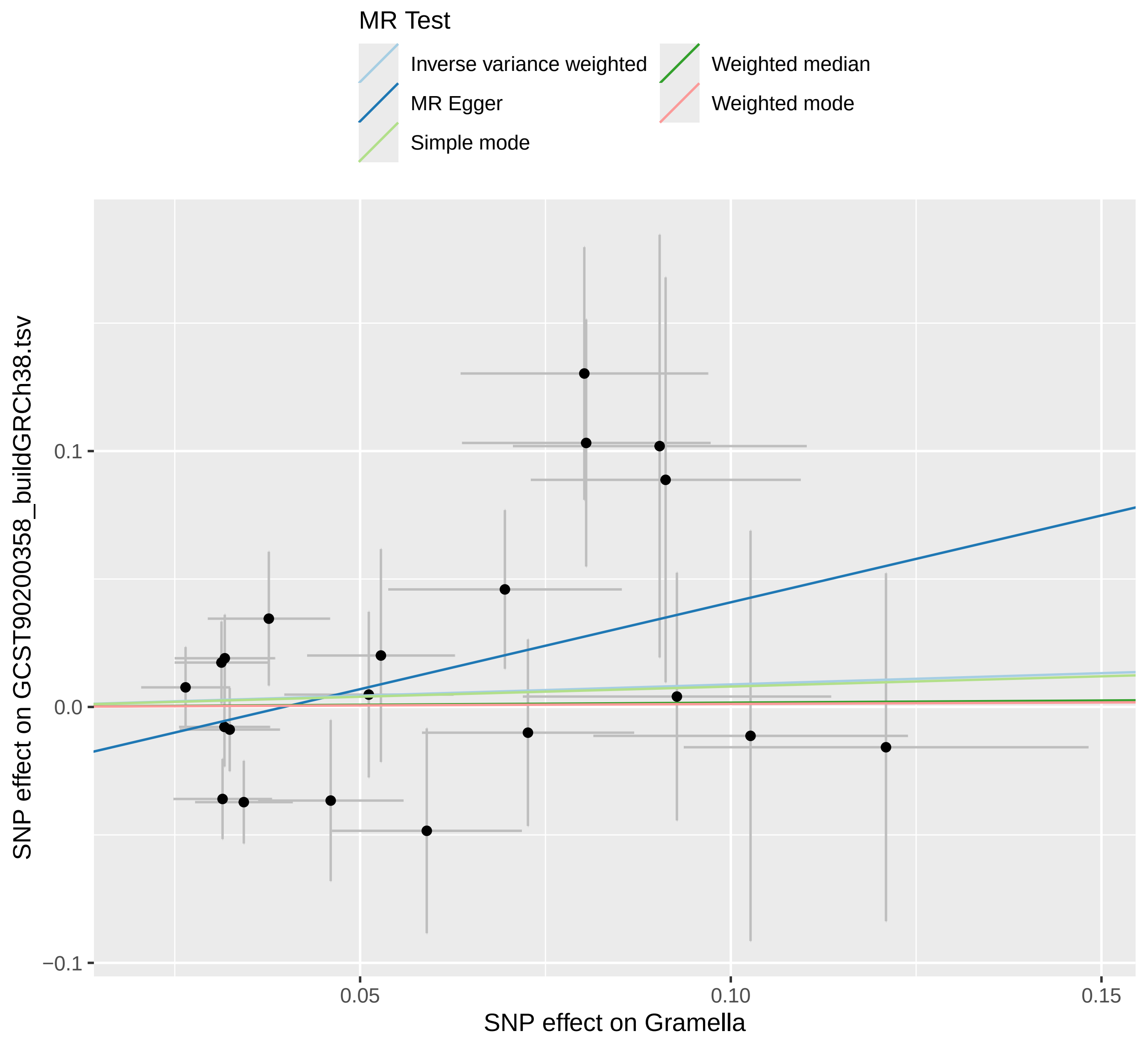

MR Test
Inverse variance weighted
MR Egger
Simple mode
Weighted median
Weighted mode
SNP effect on GCST90200358_buildGRCh38.tsv
0.1
0.0
−0.1
0.05
0.10
0.15
SNP effect on Gramella

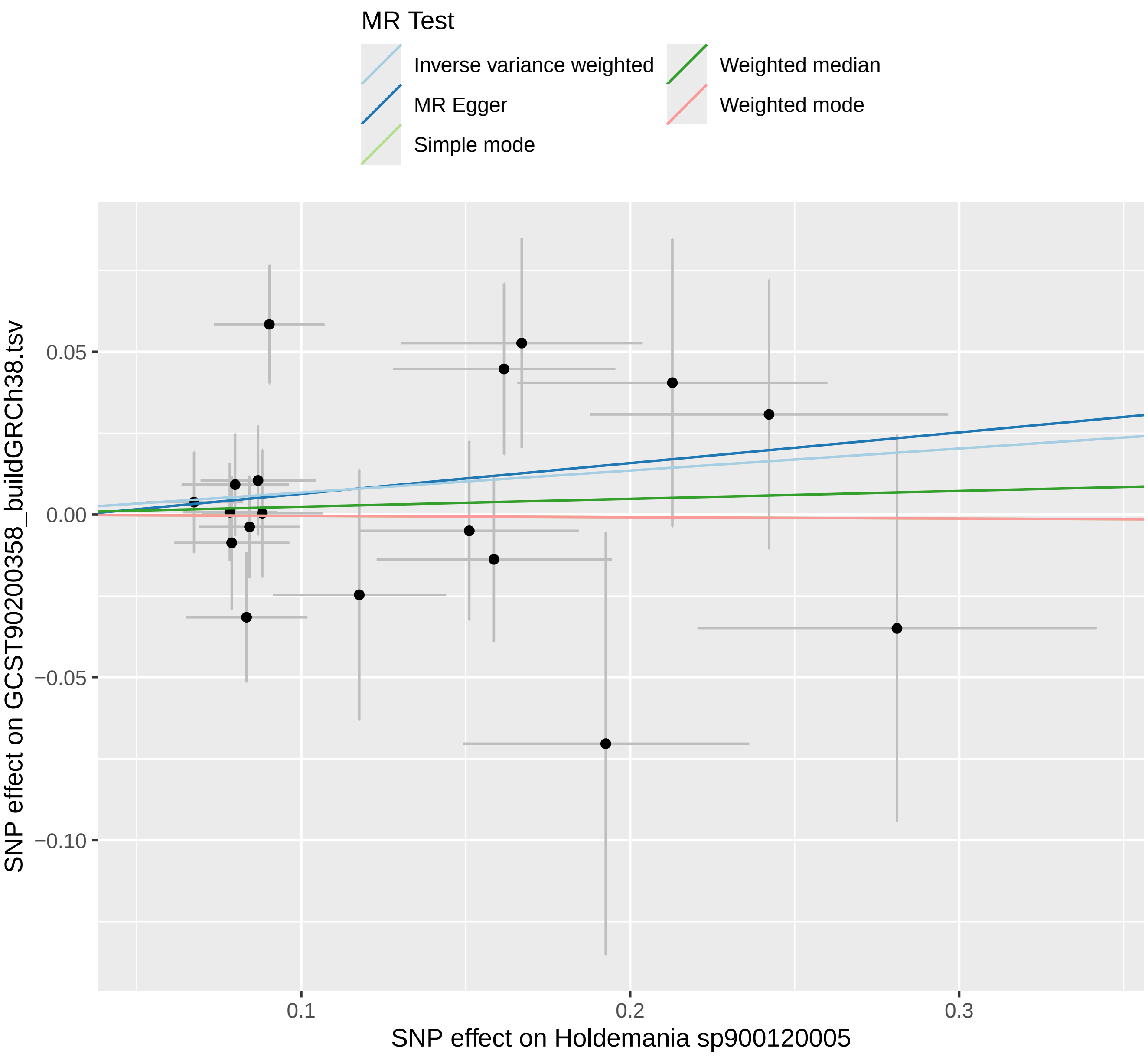
MR Test
Inverse variance weighted
MR Egger
Simple mode
Weighted median
Weighted mode
0.05
0.00
−0.05
−0.10
0.1
0.2
0.3
SNP effect on GCST90200358_buildGRCh38.tsv
SNP effect on Holdemania sp900120005

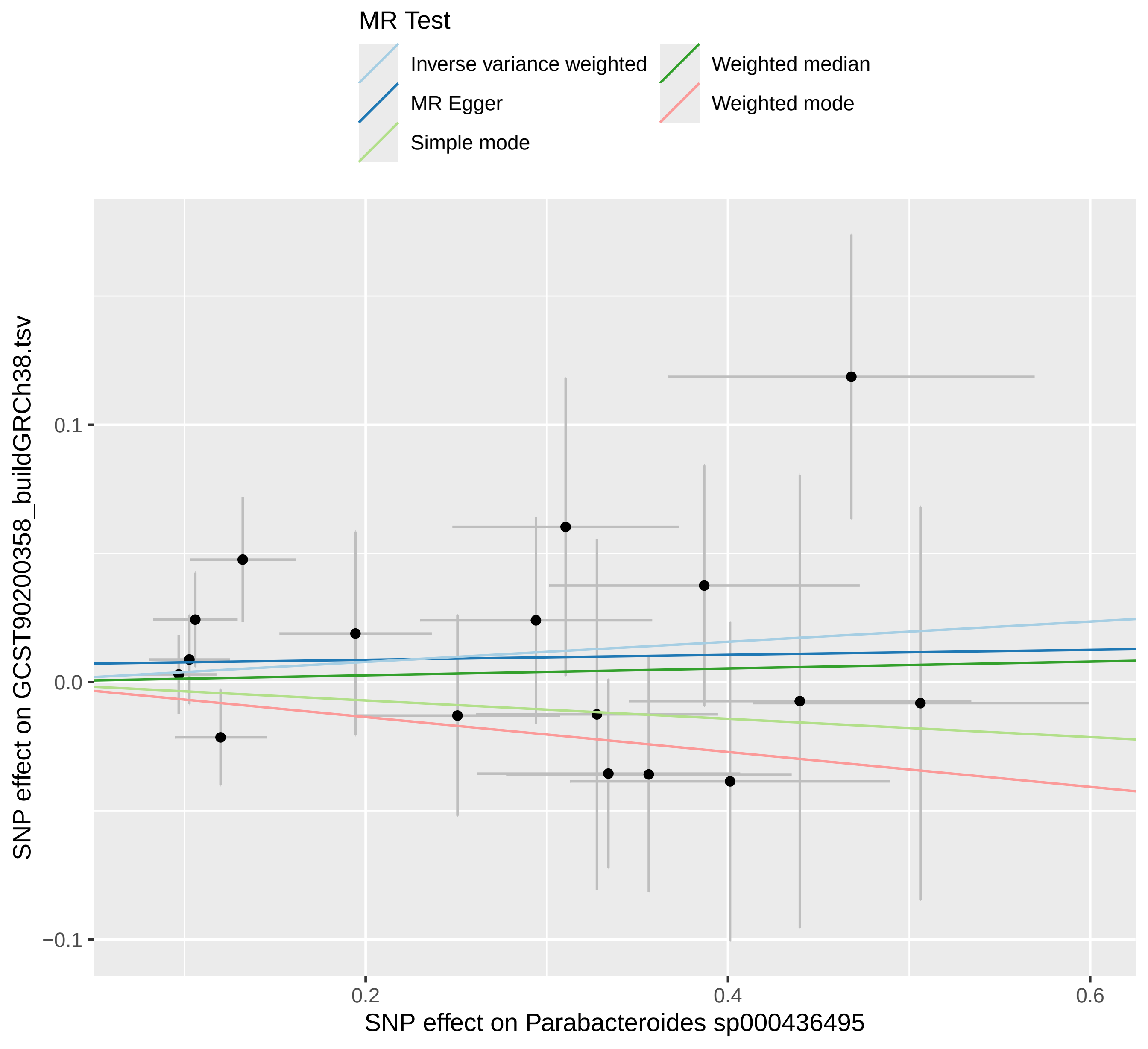
MR Test
Inverse variance weighted
MR Egger
Simple mode
Weighted median
Weighted mode
0.1
0.0
−0.1
0.2
0.4
0.6
SNP effect on GCST90200358_buildGRCh38.tsv
SNP effect on Parabacteroides sp000436495

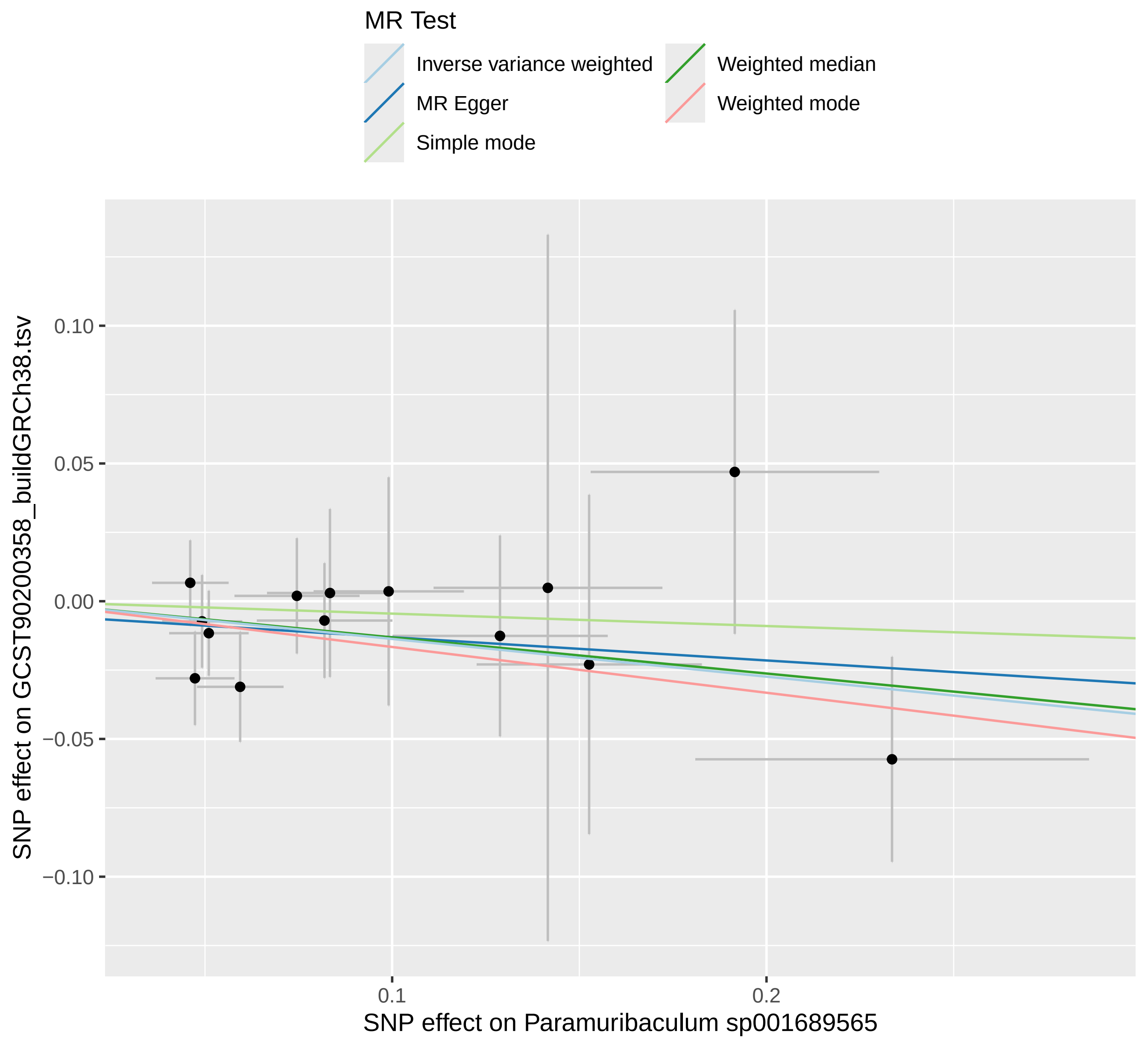
MR Test
Inverse variance weighted
MR Egger
Simple mode
Weighted median
Weighted mode
0.10
0.05
0.00
−0.05
−0.10
SNP effect on GCST90200358_buildGRCh38.tsv
0.1
0.2
SNP effect on Paramuribaculum sp001689565

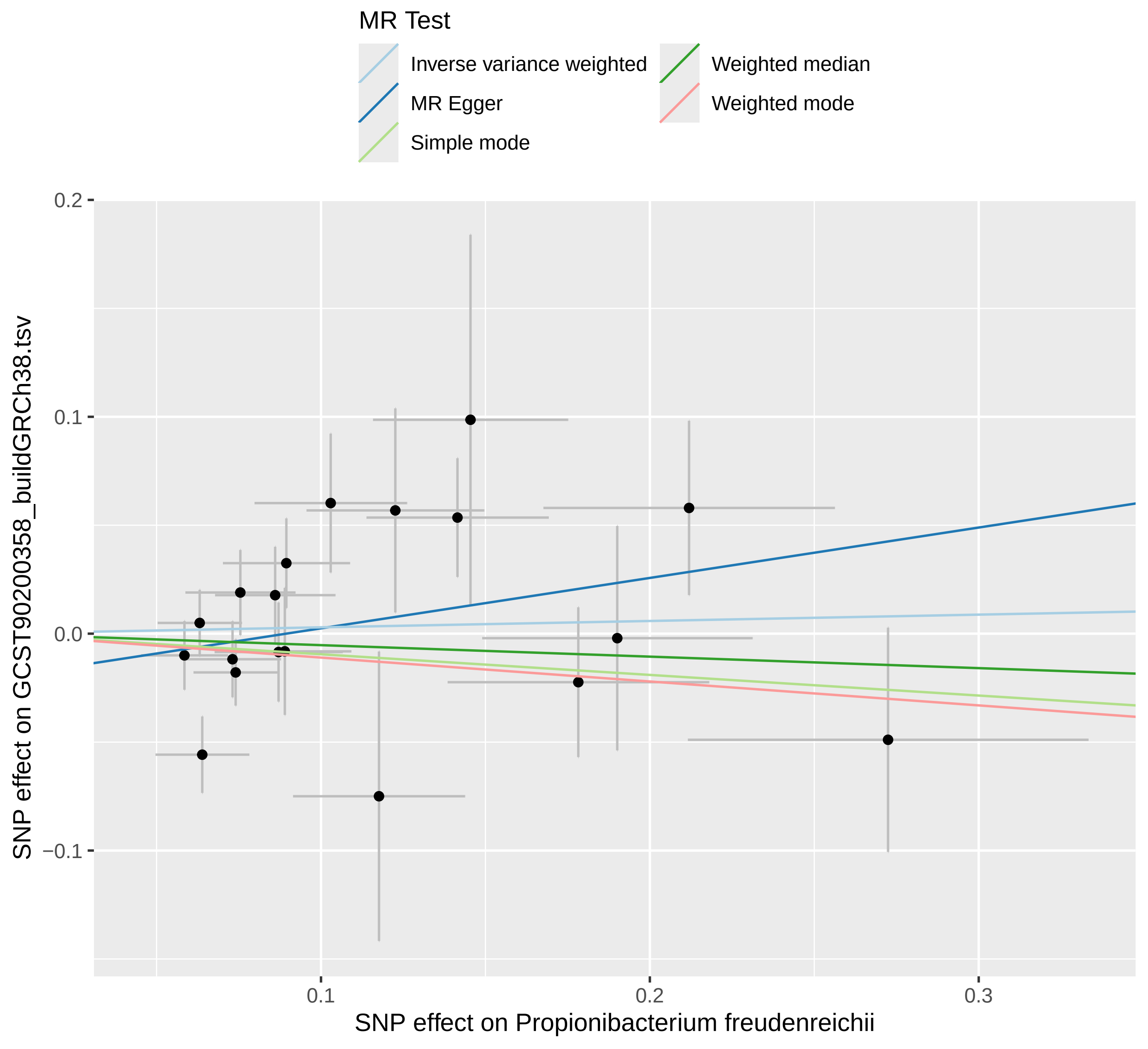

MR Test
Inverse variance weighted
MR Egger
Simple mode
Weighted median
Weighted mode
0.2
0.1
0.0
−0.1
0.1
0.2
0.3
SNP effect on GCST90200358_buildGRCh38.tsv
SNP effect on Propionibacterium freudenreichii

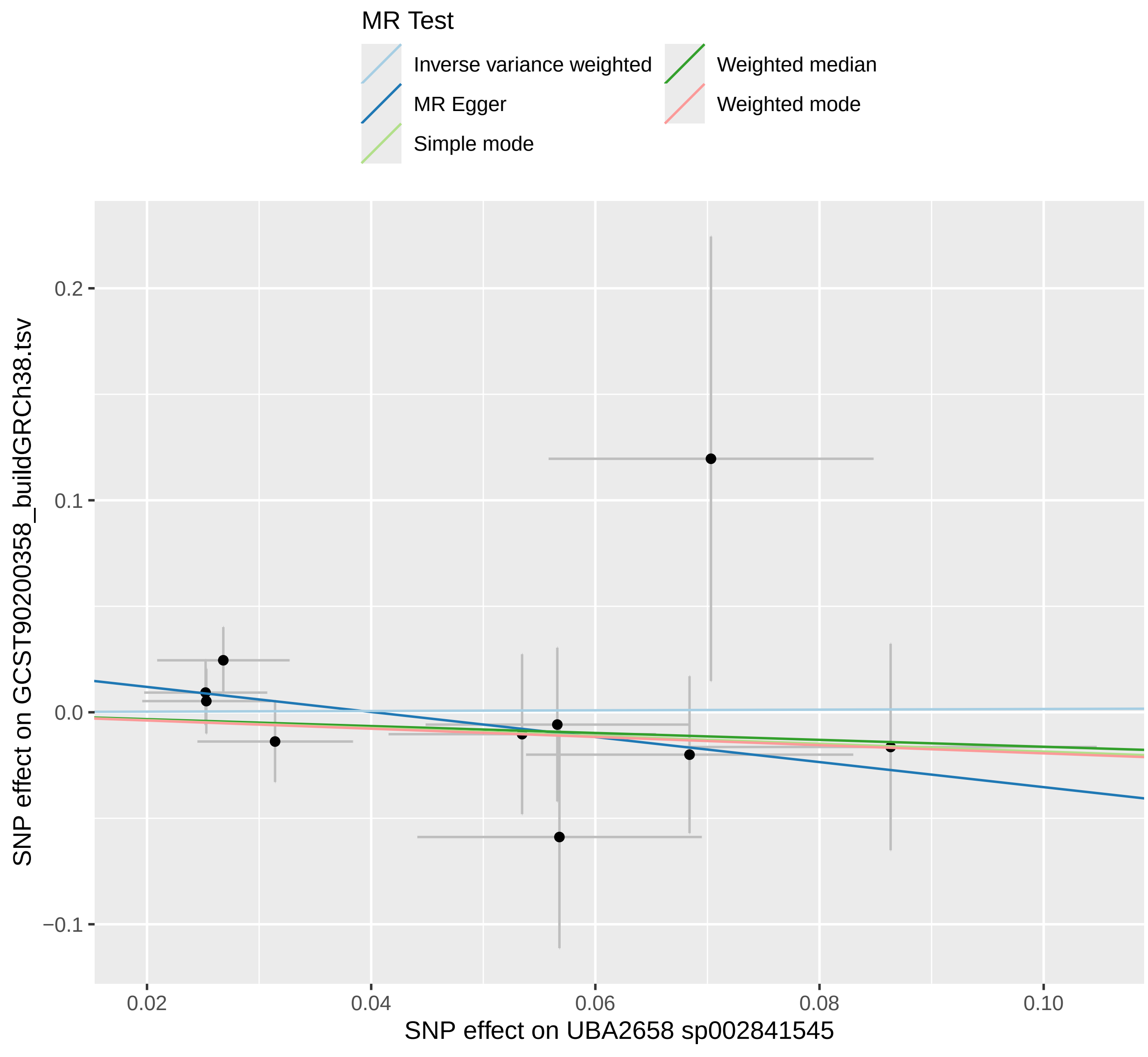
MR Test
Inverse variance weighted
MR Egger
Simple mode
Weighted median
Weighted mode
0.2
0.1
0.0
−0.1
0.02
0.04
0.06
0.08
0.10
SNP effect on GCST90200358_buildGRCh38.tsv
SNP effect on UBA2658 sp002841545

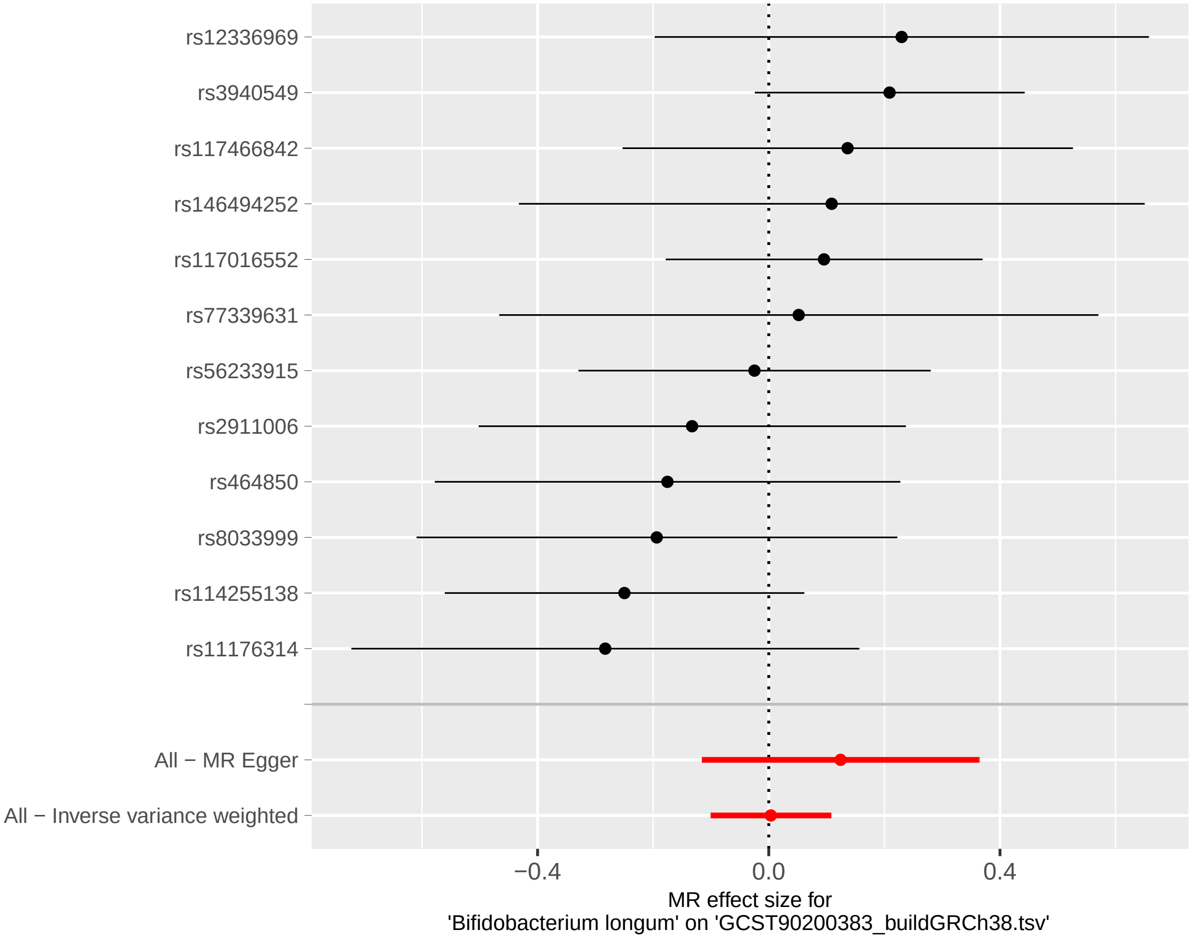
rs12336969
rs3940549
rs117466842
rs146494252
rs117016552
rs77339631
rs56233915
rs2911006
rs464850
rs8033999
rs114255138
rs11176314
All − MR Egger
All − Inverse variance weighted
−0.4
0.0
0.4
MR effect size for
'Bifidobacterium longum' on 'GCST90200383_buildGRCh38.tsv'

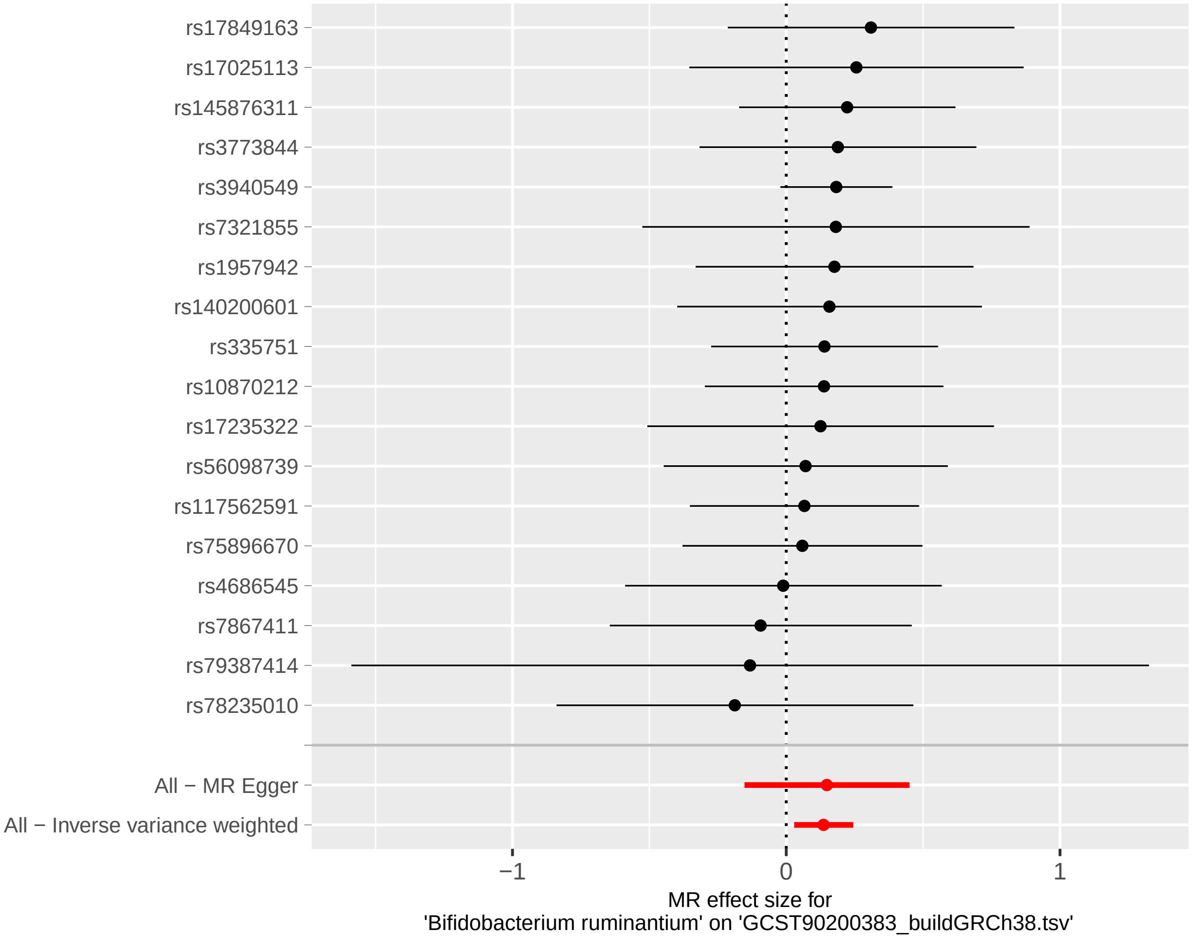

rs17849163
rs17025113
rs145876311
rs3773844
rs3940549
rs7321855
rs1957942
rs140200601
rs335751
rs10870212
rs17235322
rs56098739
rs117562591
rs75896670
rs4686545
rs7867411
rs79387414
rs78235010
All − MR Egger
All − Inverse variance weighted
−1
0
1
MR effect size for
'Bifidobacterium ruminantium' on 'GCST90200383_buildGRCh38.tsv'

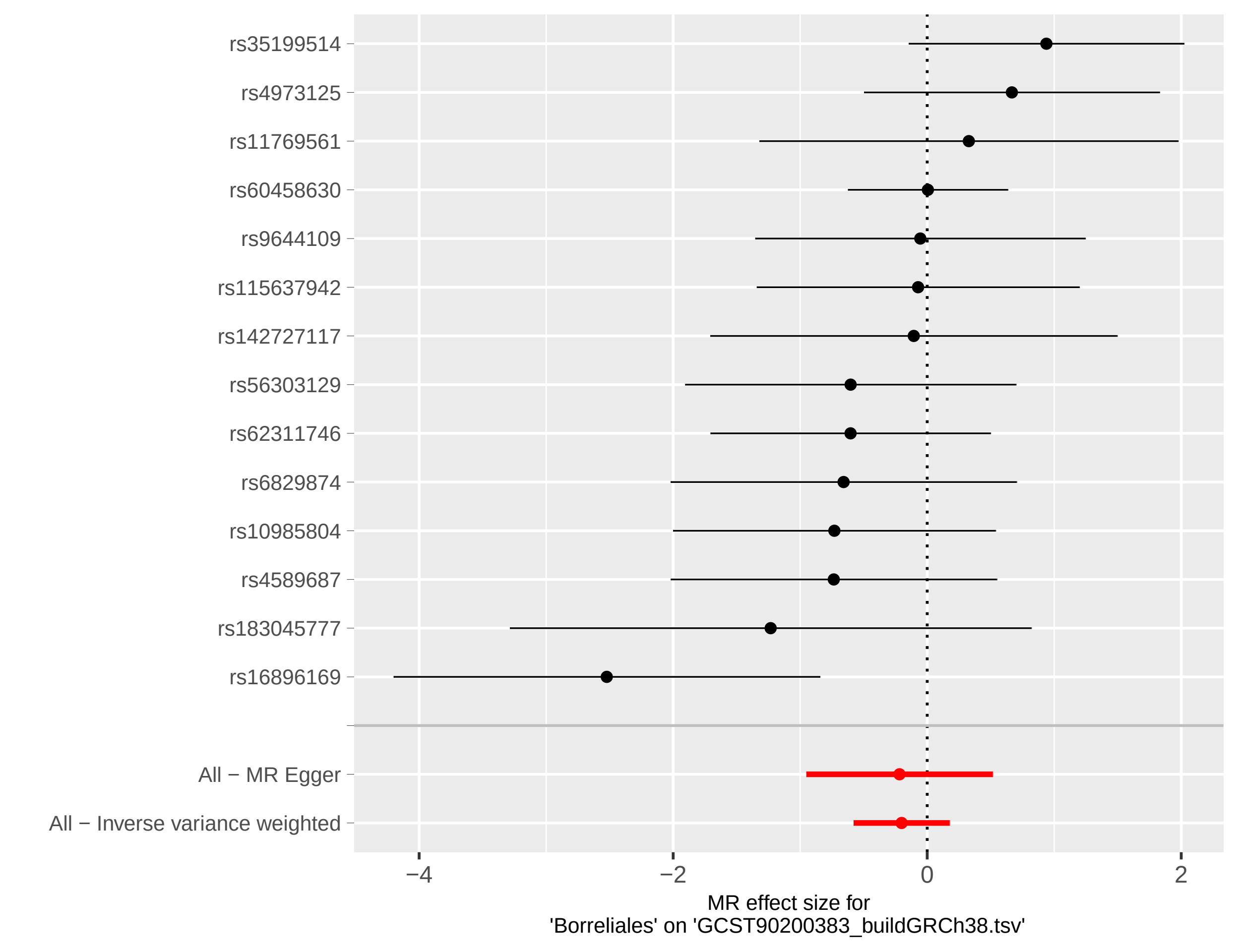
rs35199514
rs4973125
rs11769561
rs60458630
rs9644109
rs115637942
rs142727117
rs56303129
rs62311746
rs6829874
rs10985804
rs4589687
rs183045777
rs16896169
All − MR Egger
All − Inverse variance weighted
−4
−2
0
2
MR effect size for
'Borreliales' on 'GCST90200383_buildGRCh38.tsv'

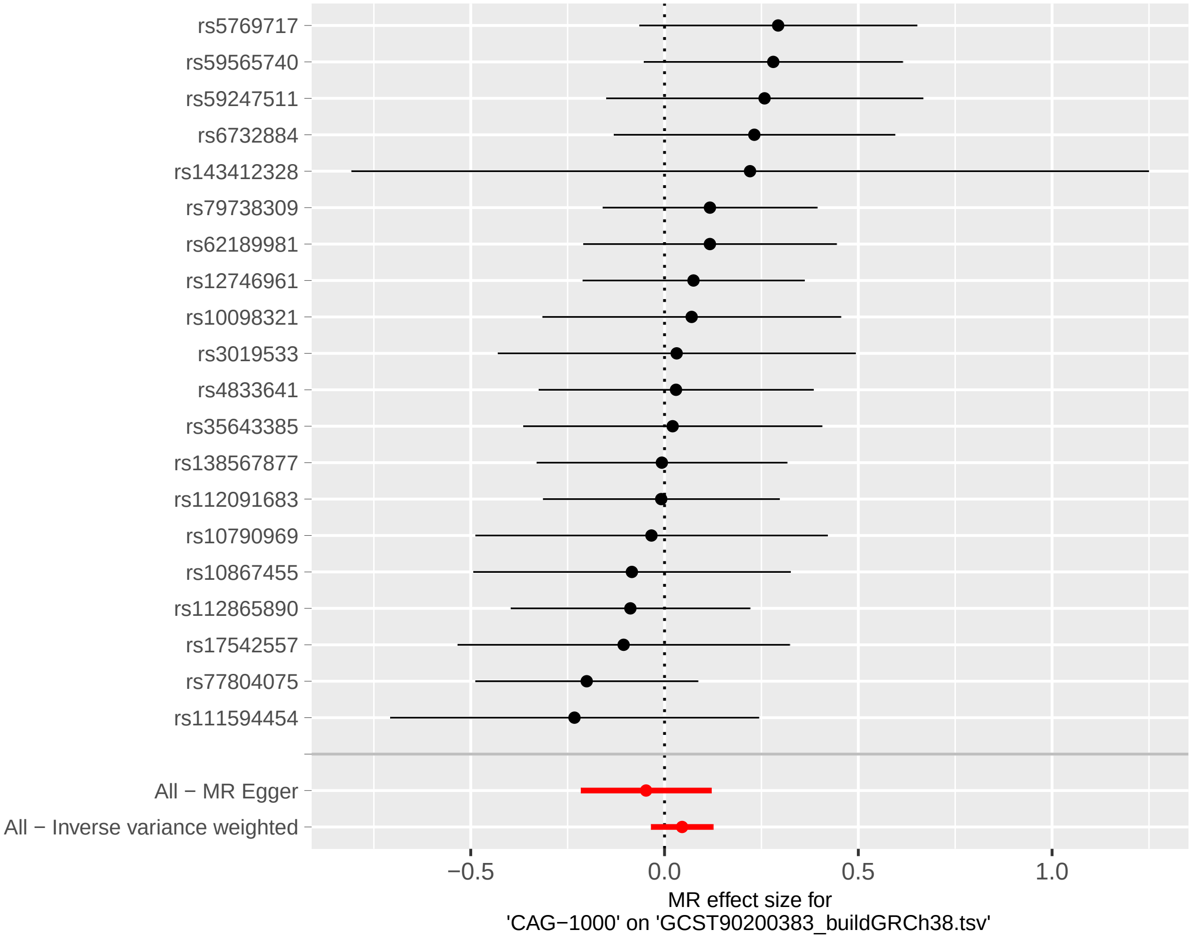

rs5769717
rs59565740
rs59247511
rs6732884
rs143412328
rs79738309
rs62189981
rs12746961
rs10098321
rs3019533
rs4833641
rs35643385
rs138567877
rs112091683
rs10790969
rs10867455
rs112865890
rs17542557
rs77804075
rs111594454
All − MR Egger
All − Inverse variance weighted
−0.5
0.0
0.5
1.0
MR effect size for
'CAG−1000' on 'GCST90200383_buildGRCh38.tsv'

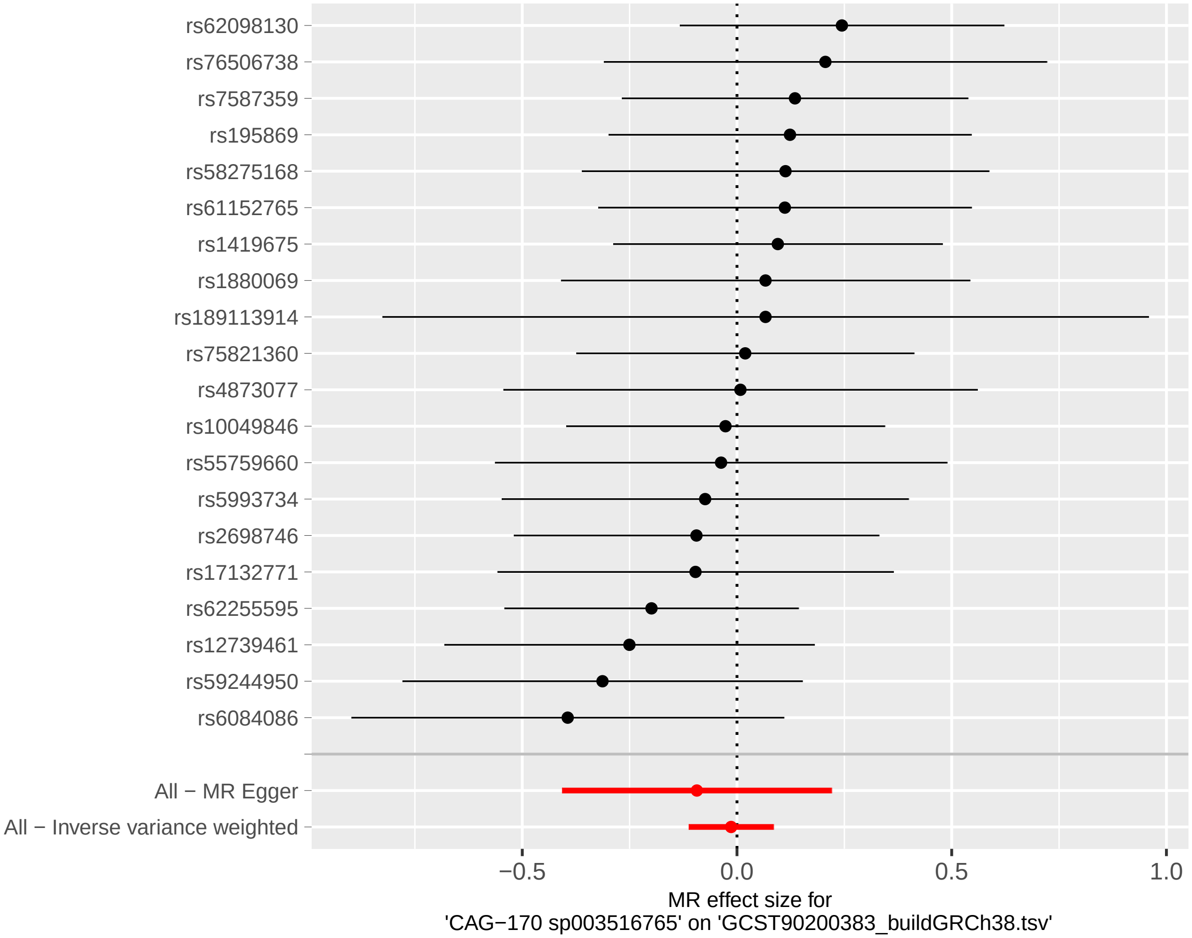

rs62098130
rs76506738
rs7587359
rs195869
rs58275168
rs61152765
rs1419675
rs1880069
rs189113914
rs75821360
rs4873077
rs10049846
rs55759660
rs5993734
rs2698746
rs17132771
rs62255595
rs12739461
rs59244950
rs6084086
All − MR Egger
All − Inverse variance weighted
−0.5
0.0
0.5
1.0
MR effect size for
'CAG−170 sp003516765' on 'GCST90200383_buildGRCh38.tsv'

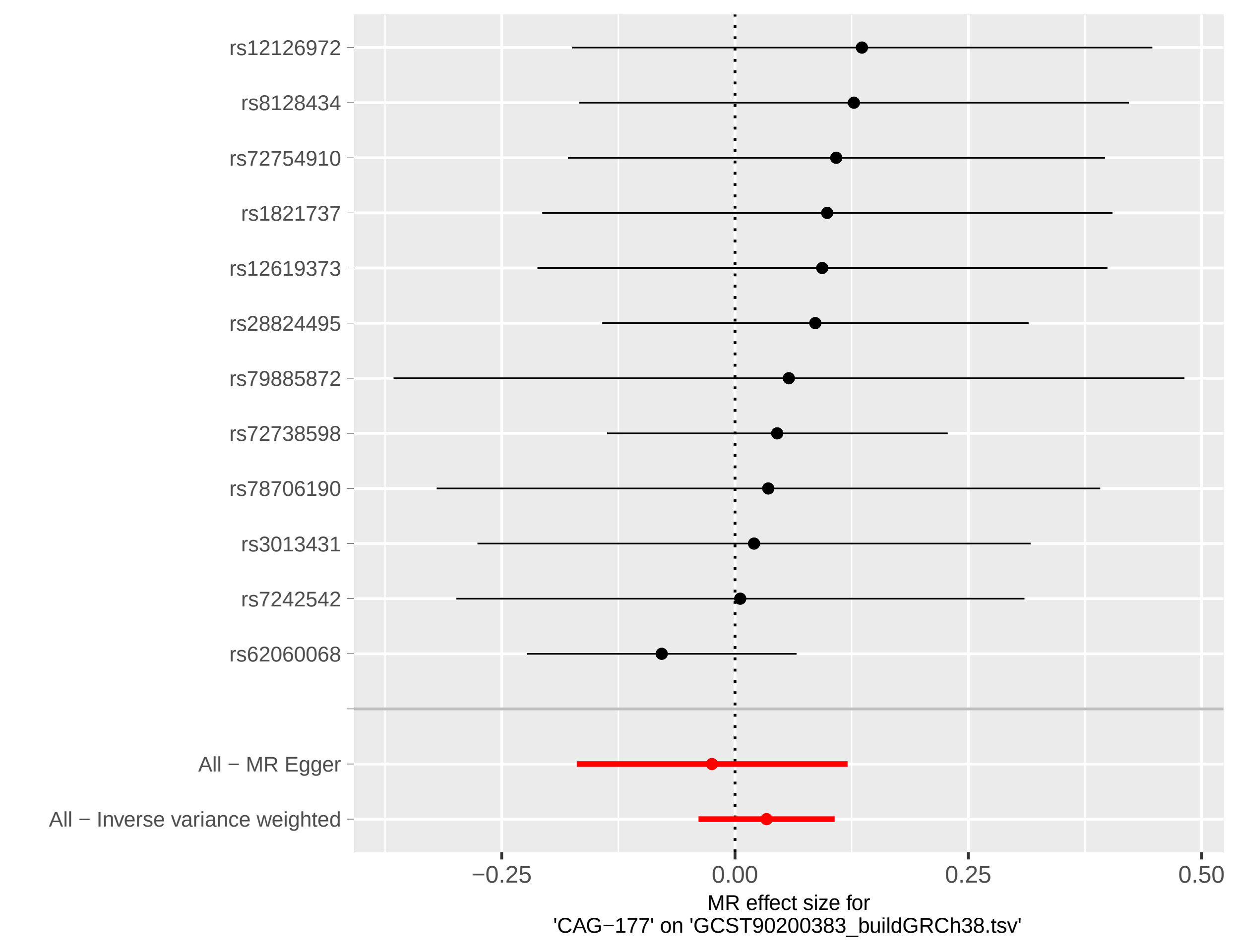

rs12126972
rs8128434
rs72754910
rs1821737
rs12619373
rs28824495
rs79885872
rs72738598
rs78706190
rs3013431
rs7242542
rs62060068
All − MR Egger
All − Inverse variance weighted
−0.25
0.00
0.25
0.50
MR effect size for
'CAG−177' on 'GCST90200383_buildGRCh38.tsv'

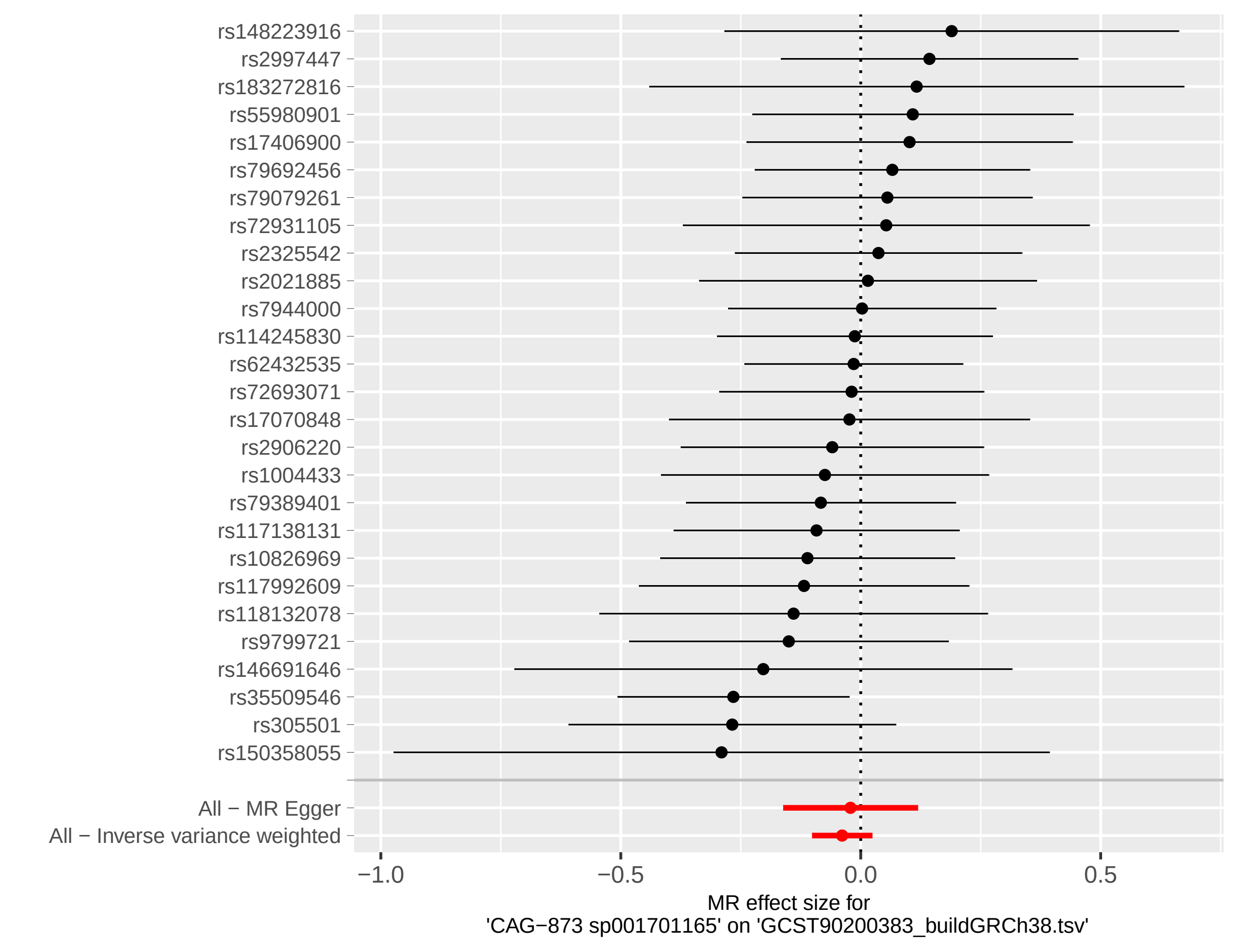
rs148223916
rs2997447
rs183272816
rs55980901
rs17406900
rs79692456
rs79079261
rs72931105
rs2325542
rs2021885
rs7944000
rs114245830
rs62432535
rs72693071
rs17070848
rs2906220
rs1004433
rs79389401
rs117138131
rs10826969
rs117992609
rs118132078
rs9799721
rs146691646
rs35509546
rs305501
rs150358055
All − MR Egger
All − Inverse variance weighted
−1.0
−0.5
0.0
0.5
MR effect size for
'CAG−873 sp001701165' on 'GCST90200383_buildGRCh38.tsv'

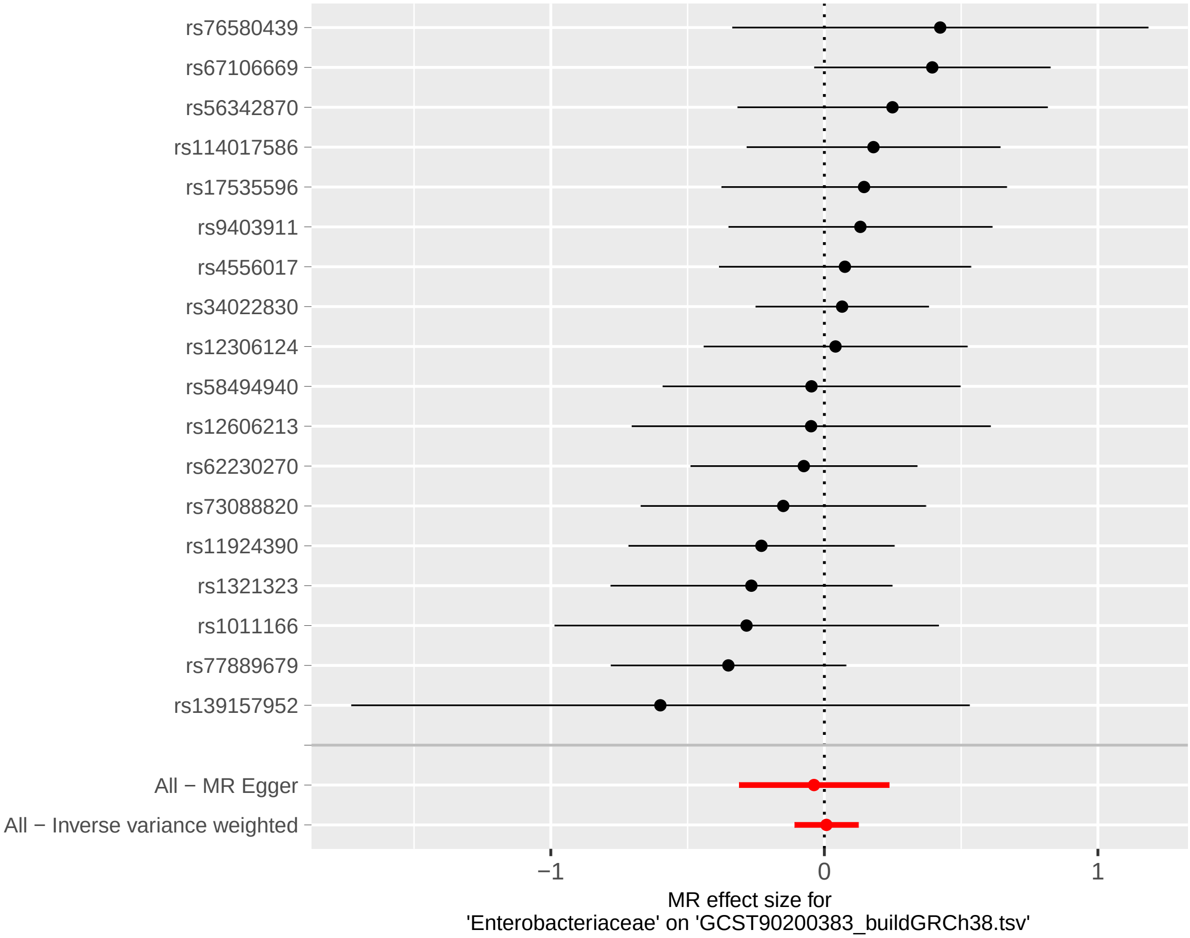

rs76580439
rs67106669
rs56342870
rs114017586
rs17535596
rs9403911
rs4556017
rs34022830
rs12306124
rs58494940
rs12606213
rs62230270
rs73088820
rs11924390
rs1321323
rs1011166
rs77889679
rs139157952
All − MR Egger
All − Inverse variance weighted
−1
0
1
MR effect size for
'Enterobacteriaceae' on 'GCST90200383_buildGRCh38.tsv'

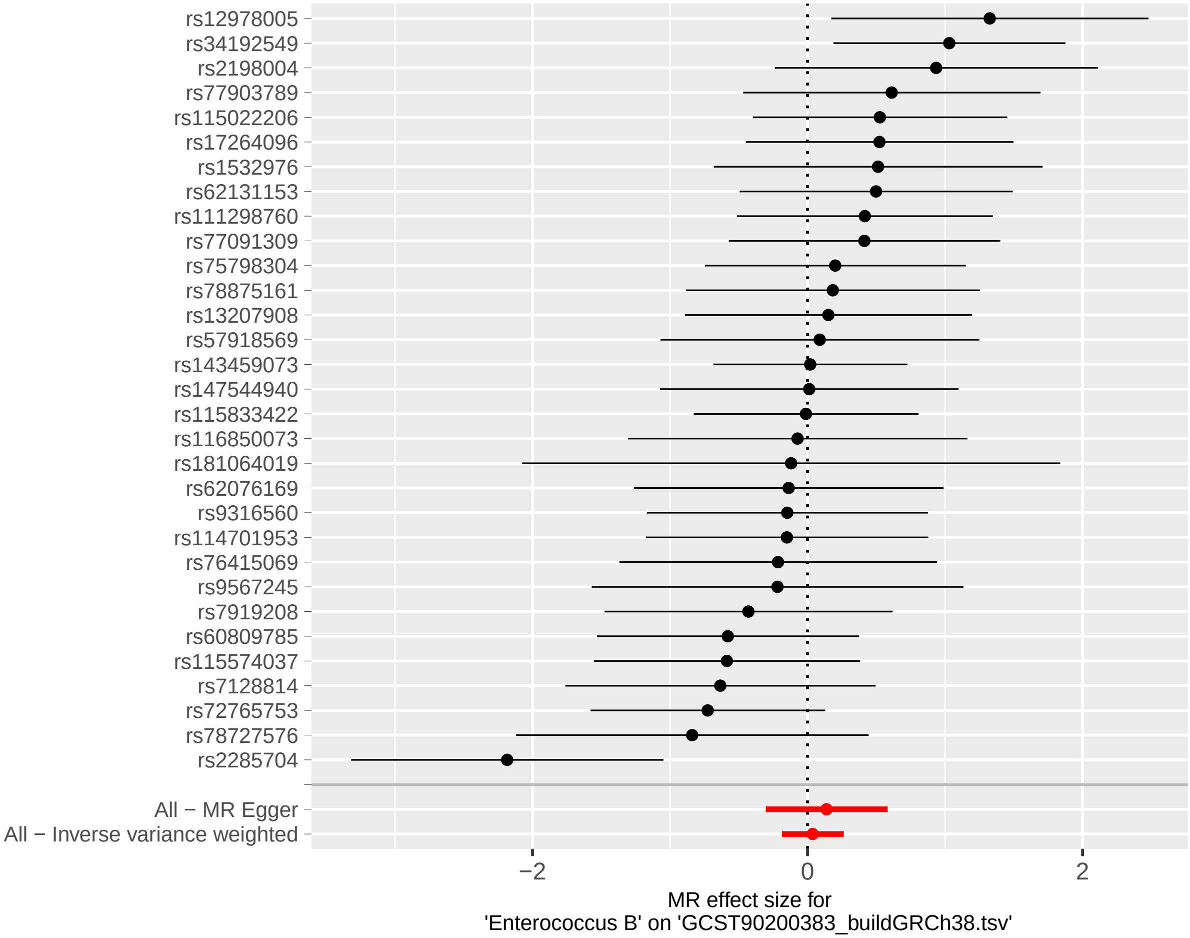
rs12978005
rs34192549
rs2198004
rs77903789
rs115022206
rs17264096
rs1532976
rs62131153
rs111298760
rs77091309
rs75798304
rs78875161
rs13207908
rs57918569
rs143459073
rs147544940
rs115833422
rs116850073
rs181064019
rs62076169
rs9316560
rs114701953
rs76415069
rs9567245
rs7919208
rs60809785
rs115574037
rs7128814
rs72765753
rs78727576
rs2285704
All − MR Egger
All − Inverse variance weighted
−2
0
2
MR effect size for
'Enterococcus B' on 'GCST90200383_buildGRCh38.tsv'

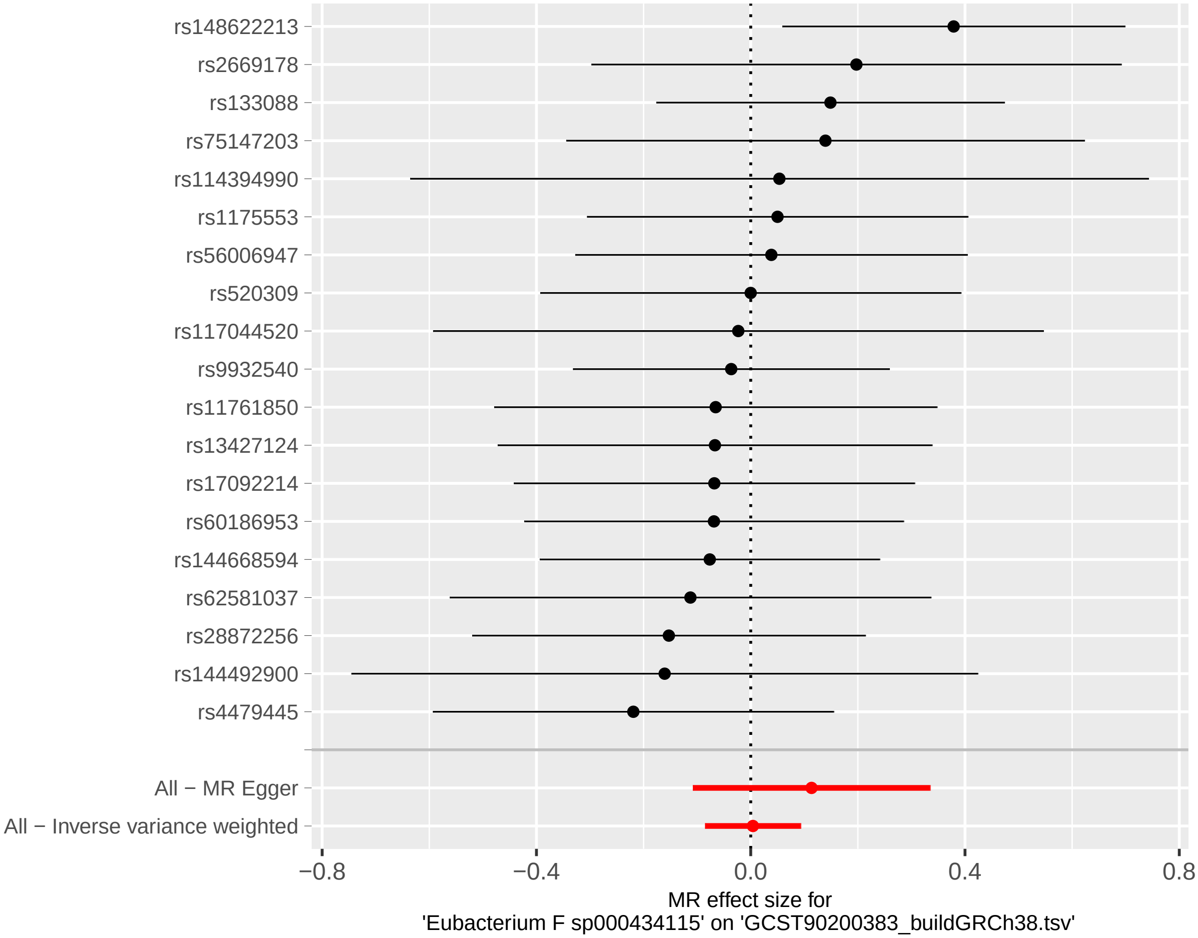
rs148622213
rs2669178
rs133088
rs75147203
rs114394990
rs1175553
rs56006947
rs520309
rs117044520
rs9932540
rs11761850
rs13427124
rs17092214
rs60186953
rs144668594
rs62581037
rs28872256
rs144492900
rs4479445
All − MR Egger
All − Inverse variance weighted
−0.8
−0.4
0.0
0.4
0.8
MR effect size for
'Eubacterium F sp000434115' on 'GCST90200383_buildGRCh38.tsv'

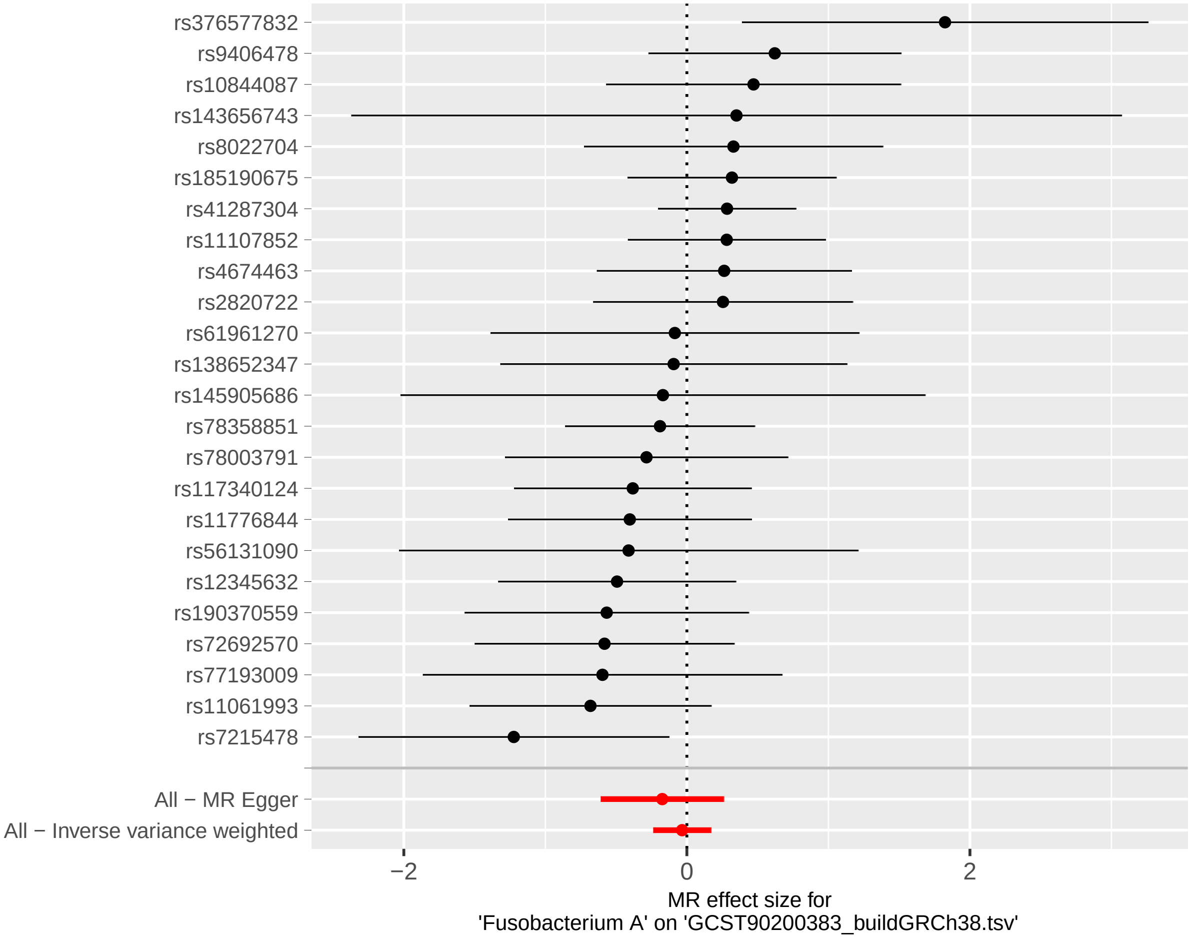
rs376577832
rs9406478
rs10844087
rs143656743
rs8022704
rs185190675
rs41287304
rs11107852
rs4674463
rs2820722
rs61961270
rs138652347
rs145905686
rs78358851
rs78003791
rs117340124
rs11776844
rs56131090
rs12345632
rs190370559
rs72692570
rs77193009
rs11061993
rs7215478
All − MR Egger
All − Inverse variance weighted
−2
0
2
MR effect size for
'Fusobacterium A' on 'GCST90200383_buildGRCh38.tsv'

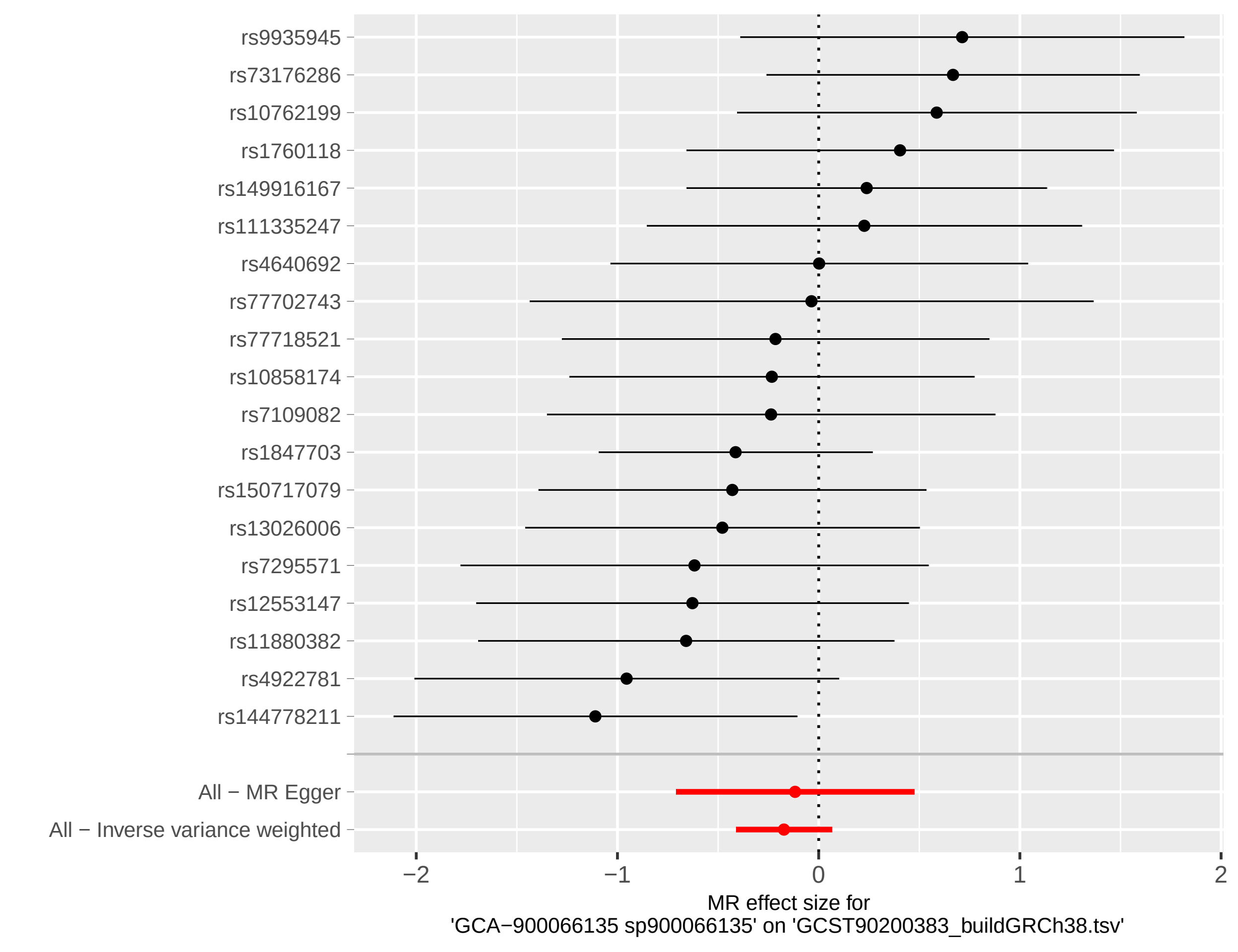
rs9935945
rs73176286
rs10762199
rs1760118
rs149916167
rs111335247
rs4640692
rs77702743
rs77718521
rs10858174
rs7109082
rs1847703
rs150717079
rs13026006
rs7295571
rs12553147
rs11880382
rs4922781
rs144778211
All − MR Egger
All − Inverse variance weighted
−2
−1
0
1
2
MR effect size for
'GCA−900066135 sp900066135' on 'GCST90200383_buildGRCh38.tsv'

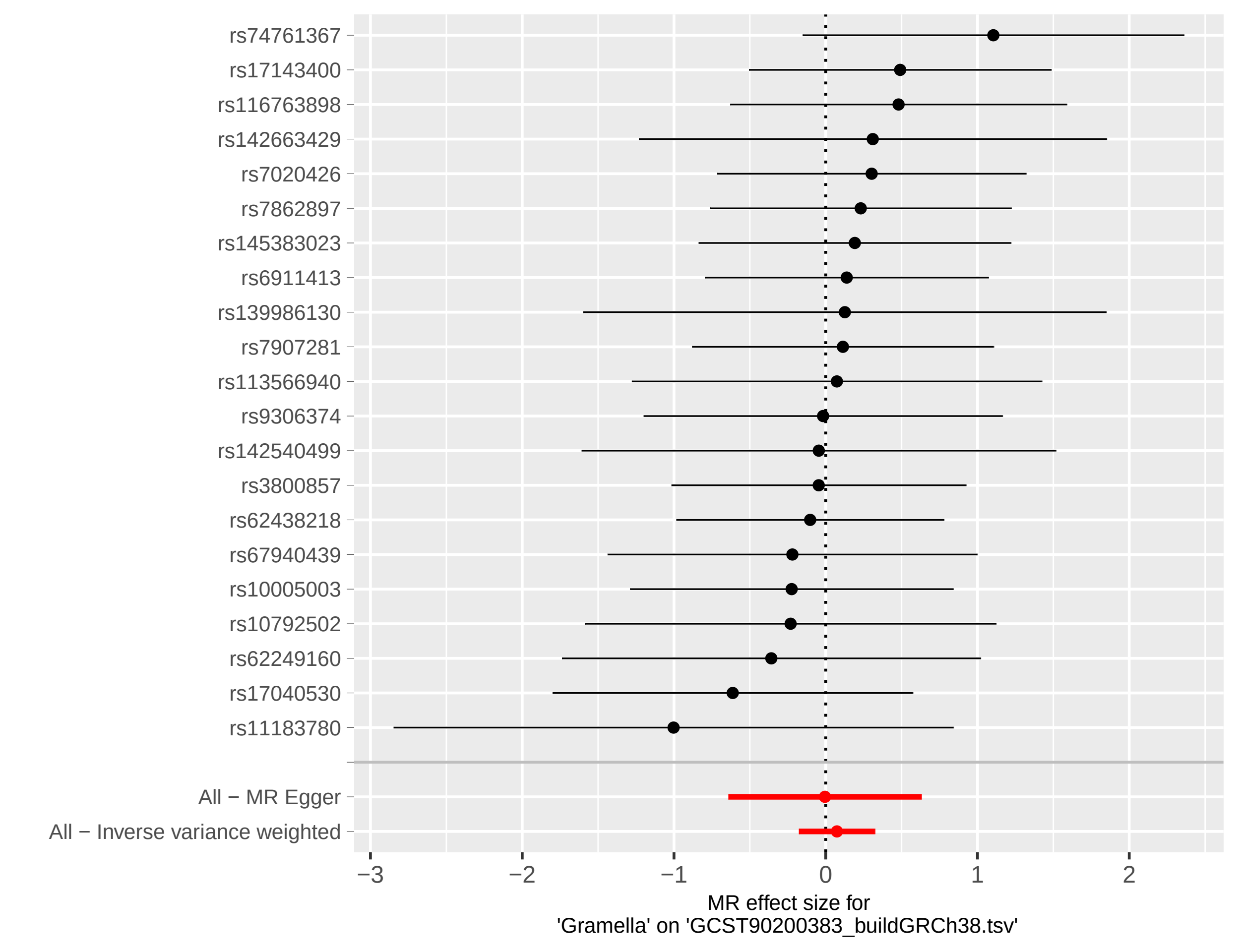

rs74761367
rs17143400
rs116763898
rs142663429
rs7020426
rs7862897
rs145383023
rs6911413
rs139986130
rs7907281
rs113566940
rs9306374
rs142540499
rs3800857
rs62438218
rs67940439
rs10005003
rs10792502
rs62249160
rs17040530
rs11183780
All − MR Egger
All − Inverse variance weighted
−3
−2
−1
0
1
2
MR effect size for
'Gramella' on 'GCST90200383_buildGRCh38.tsv'

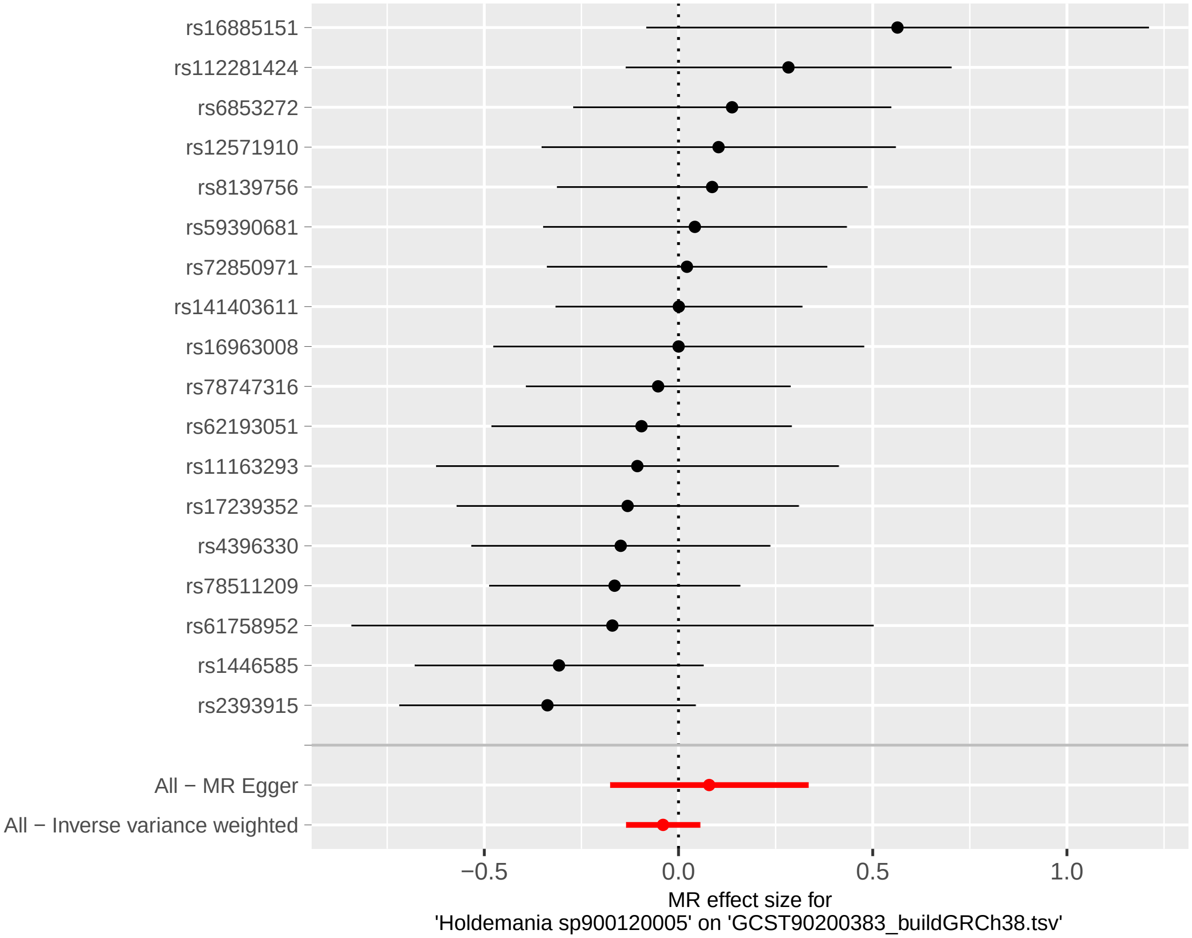

rs16885151
rs112281424
rs6853272
rs12571910
rs8139756
rs59390681
rs72850971
rs141403611
rs16963008
rs78747316
rs62193051
rs11163293
rs17239352
rs4396330
rs78511209
rs61758952
rs1446585
rs2393915
All − MR Egger
All − Inverse variance weighted
−0.5
0.0
0.5
1.0
MR effect size for
'Holdemania sp900120005' on 'GCST90200383_buildGRCh38.tsv'

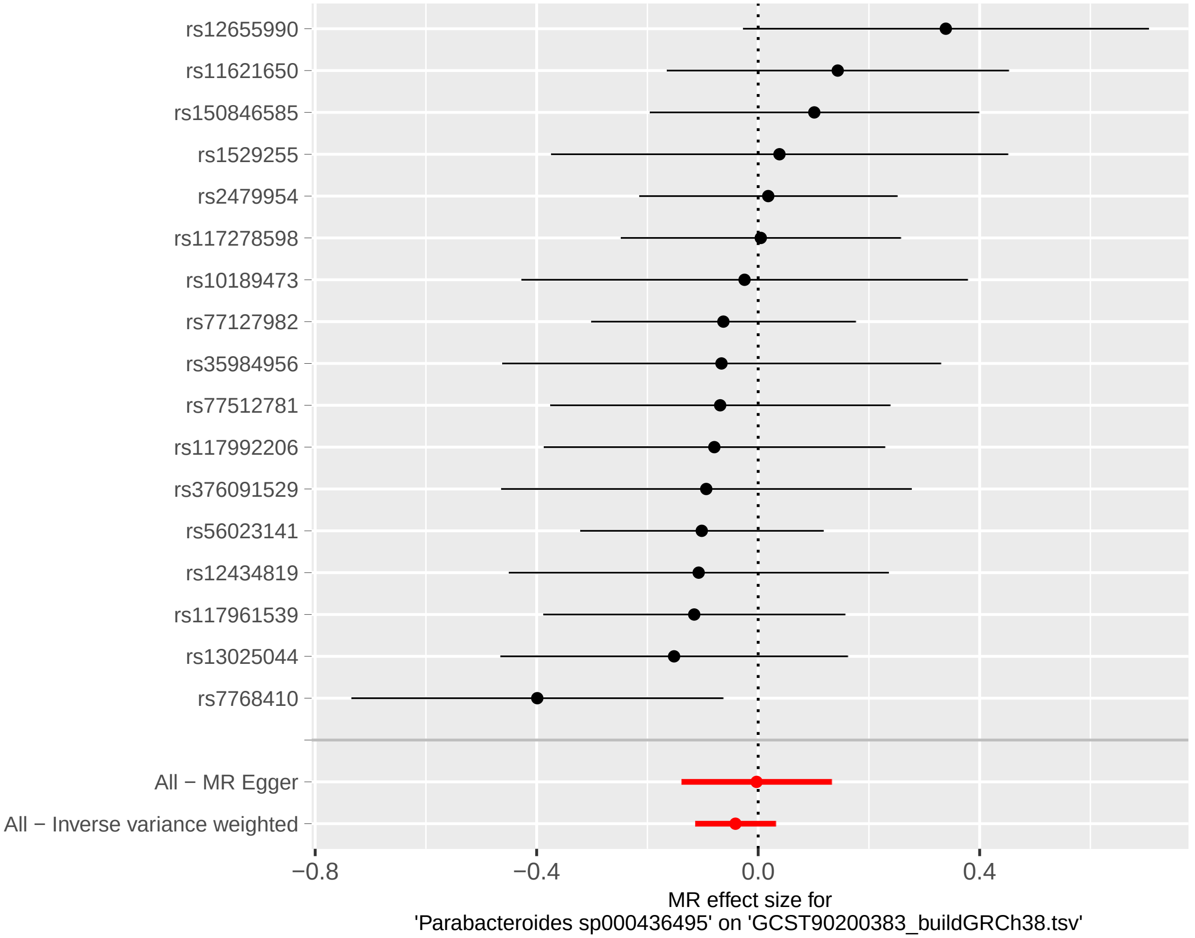
rs12655990
rs11621650
rs150846585
rs1529255
rs2479954
rs117278598
rs10189473
rs77127982
rs35984956
rs77512781
rs117992206
rs376091529
rs56023141
rs12434819
rs117961539
rs13025044
rs7768410
All − MR Egger
All − Inverse variance weighted
−0.8
−0.4
0.0
0.4
MR effect size for
'Parabacteroides sp000436495' on 'GCST90200383_buildGRCh38.tsv'

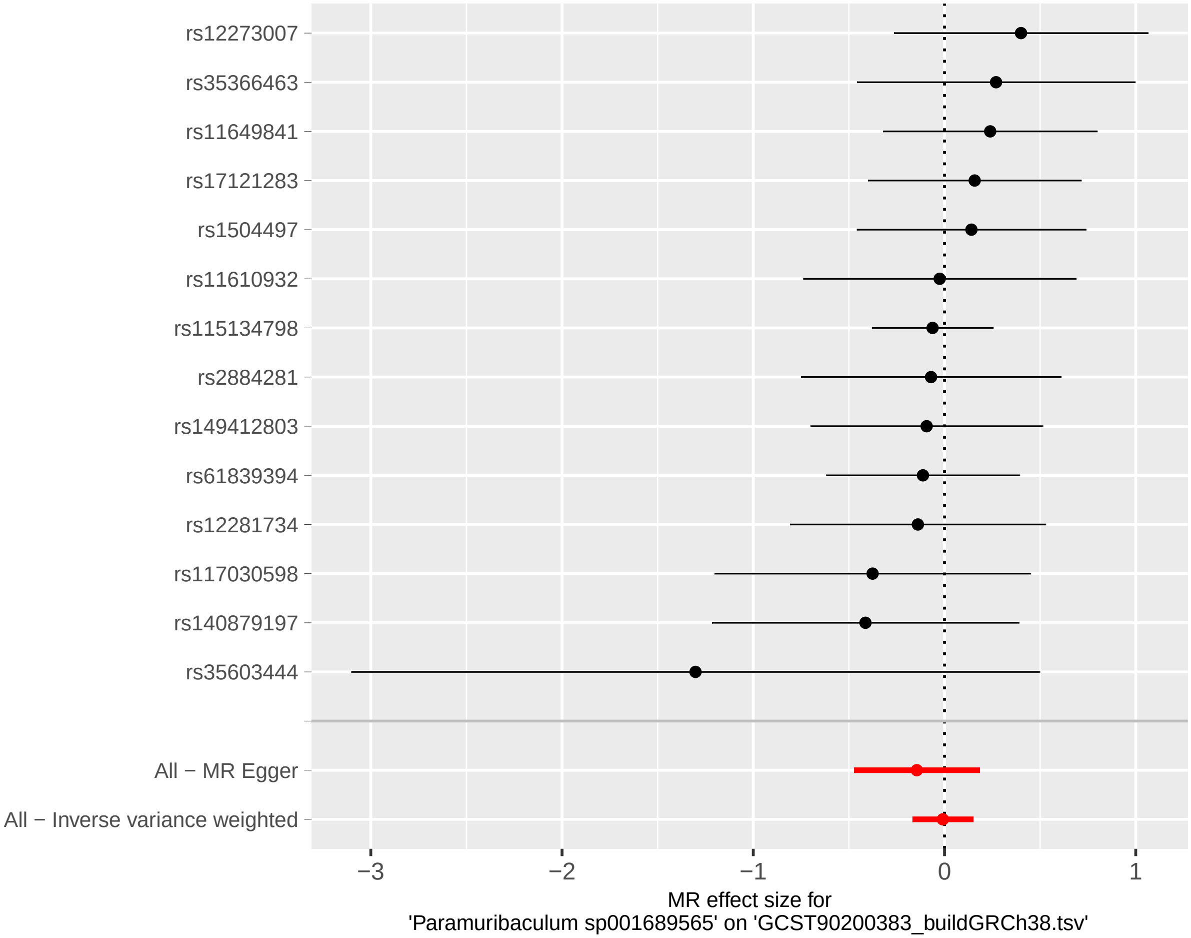

rs12273007
rs35366463
rs11649841
rs17121283
rs1504497
rs11610932
rs115134798
rs2884281
rs149412803
rs61839394
rs12281734
rs117030598
rs140879197
rs35603444
All − MR Egger
All − Inverse variance weighted
−3
−2
−1
0
1
MR effect size for
'Paramuribaculum sp001689565' on 'GCST90200383_buildGRCh38.tsv'

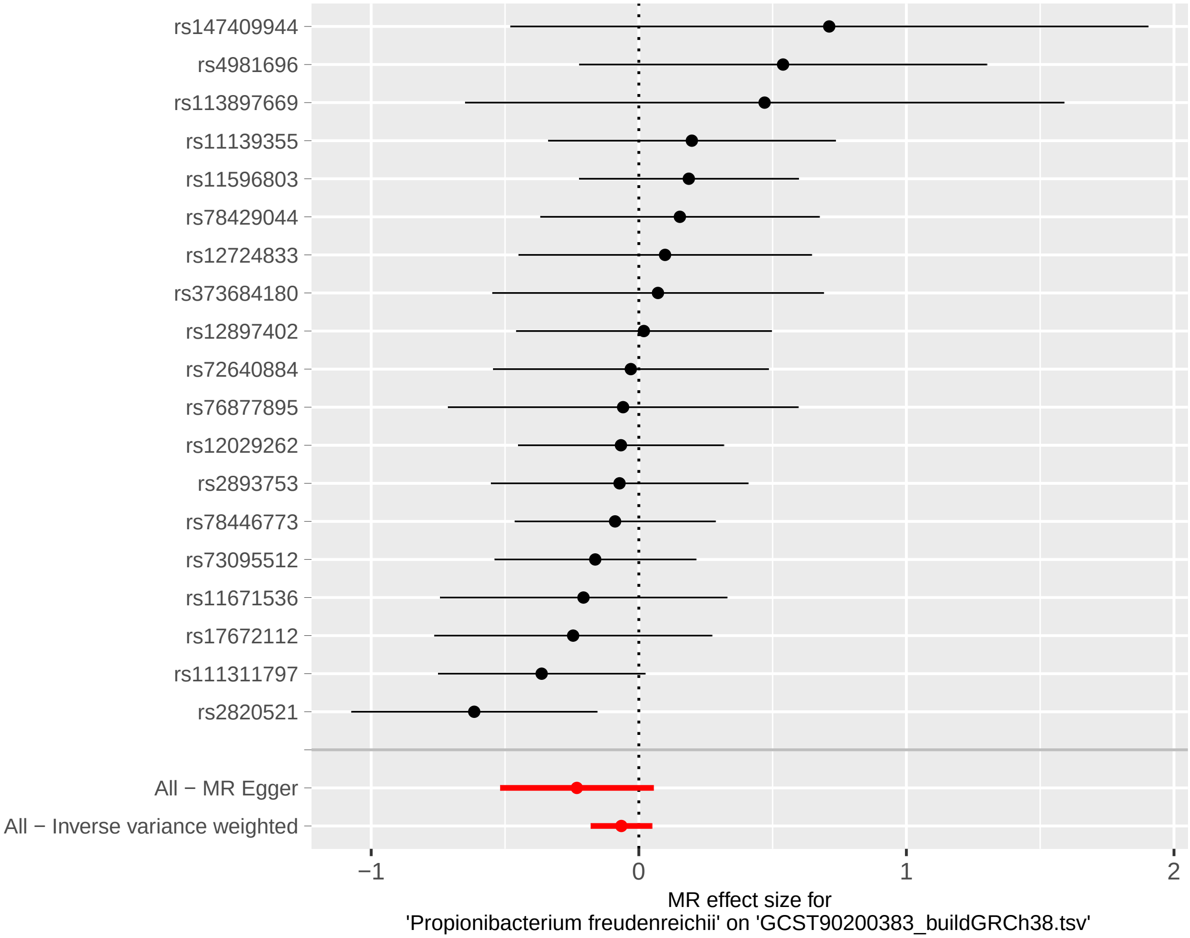
rs147409944
rs4981696
rs113897669
rs11139355
rs11596803
rs78429044
rs12724833
rs373684180
rs12897402
rs72640884
rs76877895
rs12029262
rs2893753
rs78446773
rs73095512
rs11671536
rs17672112
rs111311797
rs2820521
All − MR Egger
All − Inverse variance weighted
−1
0
1
2
MR effect size for
'Propionibacterium freudenreichii' on 'GCST90200383_buildGRCh38.tsv'

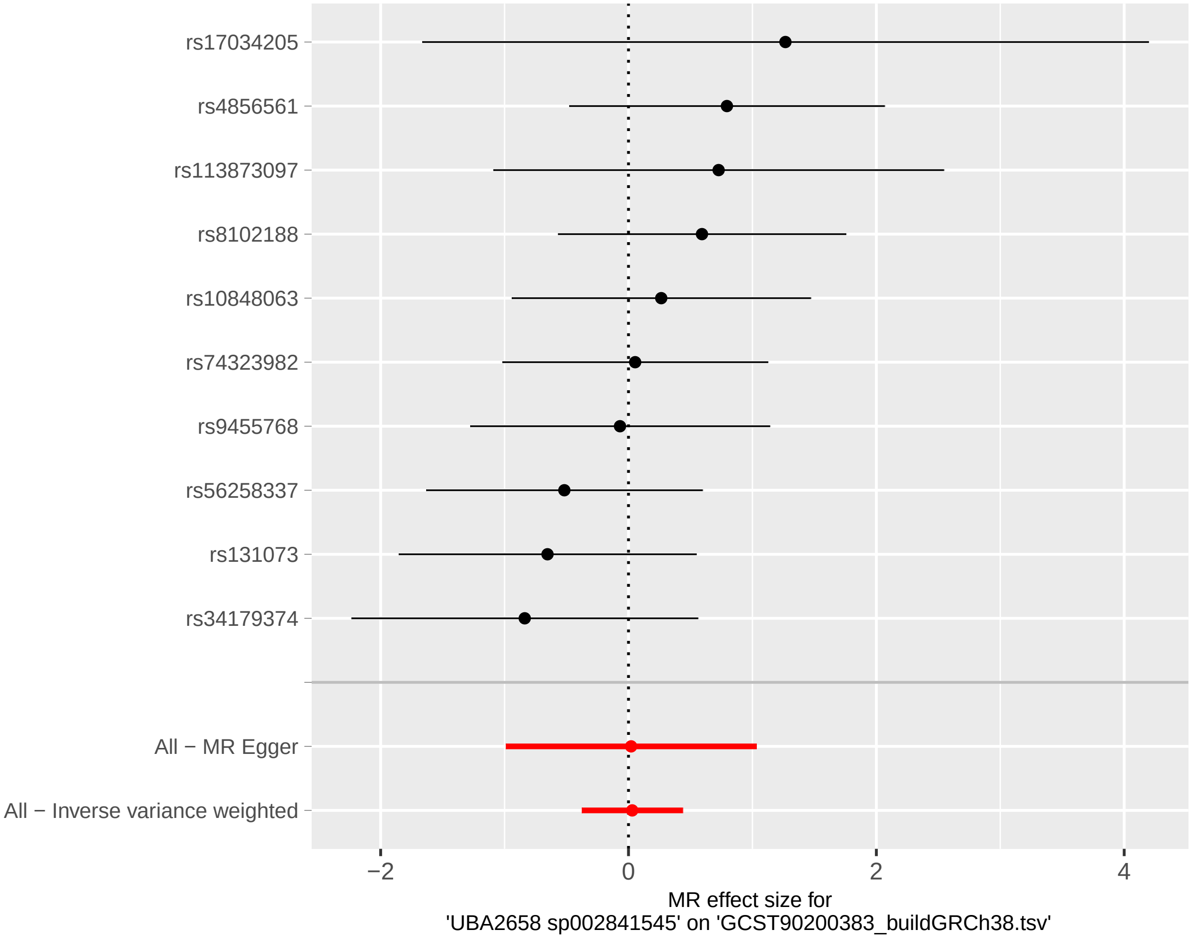
rs17034205
rs4856561
rs113873097
rs8102188
rs10848063
rs74323982
rs9455768
rs56258337
rs131073
rs34179374
All − MR Egger
All − Inverse variance weighted
−2
0
2
4
MR effect size for
'UBA2658 sp002841545' on 'GCST90200383_buildGRCh38.tsv'

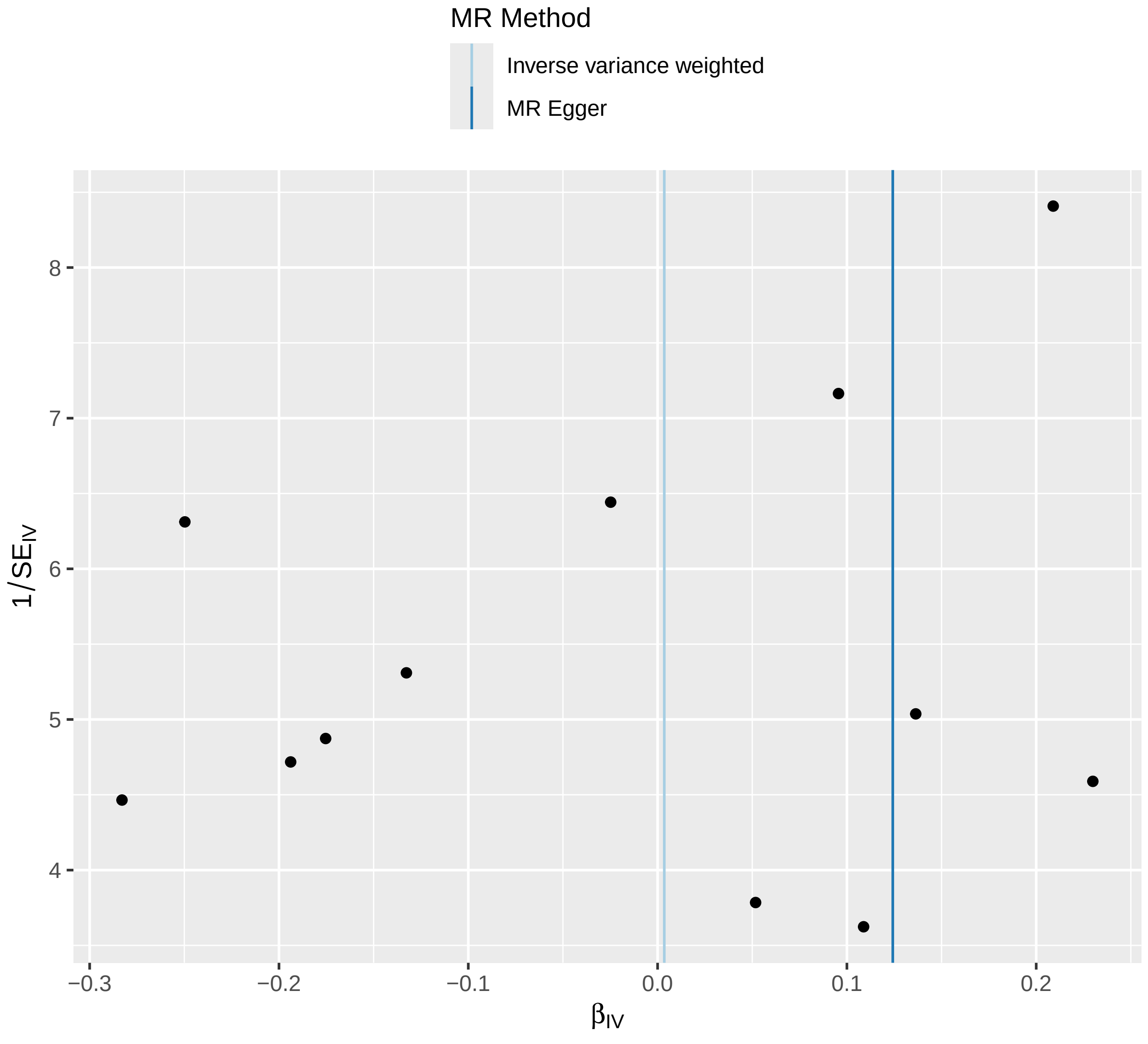

MR Method
Inverse variance weighted
MR Egger
8
7
6
5
4
1/SE_IV
−0.3
−0.2
−0.1
0.0
0.1
0.2
β_IV

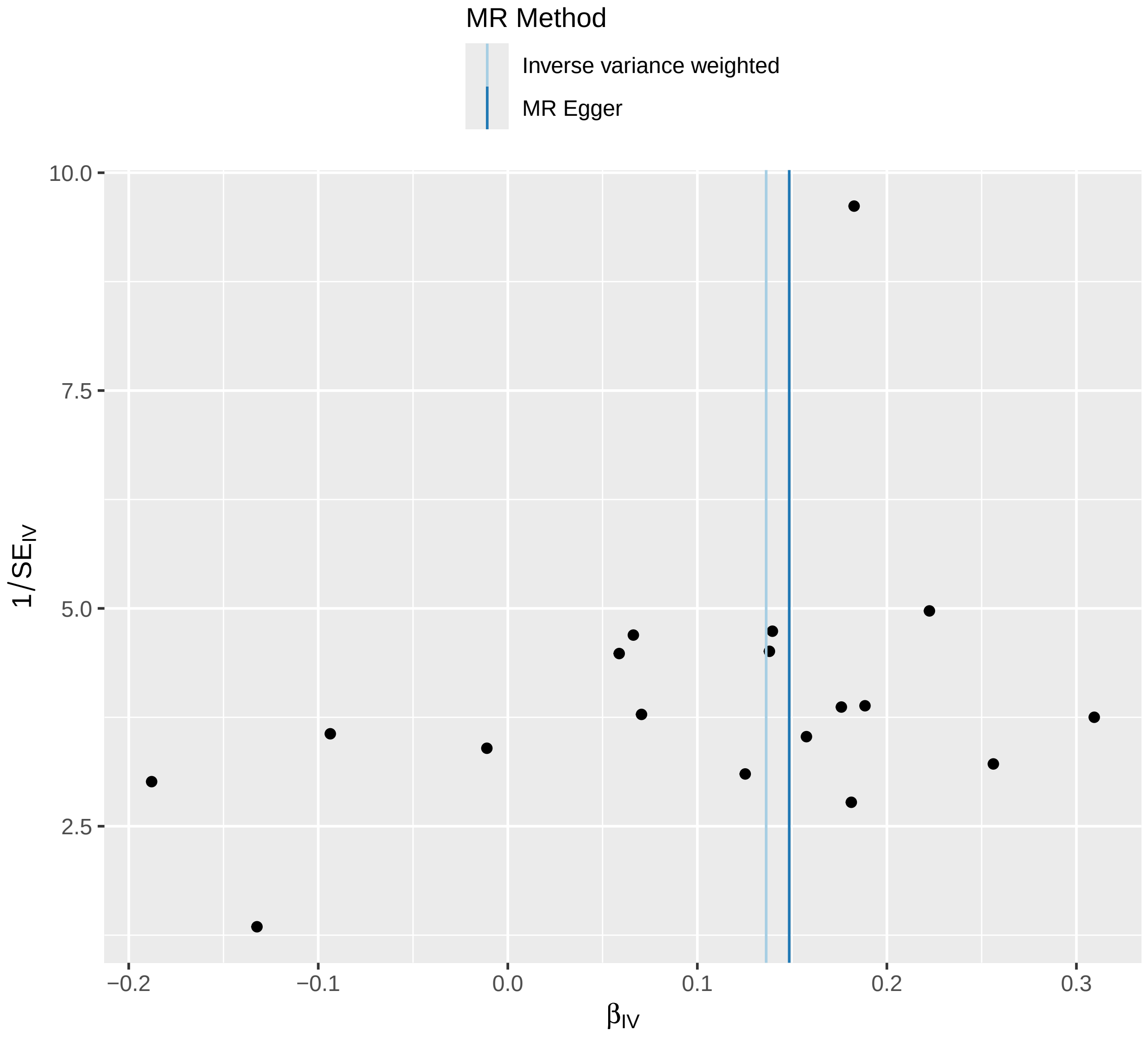

MR Method
Inverse variance weighted
MR Egger
10.0
7.5
5.0
2.5
1/SE$_{IV}$
−0.2
−0.1
0.0
0.1
0.2
0.3
β$_{IV}$

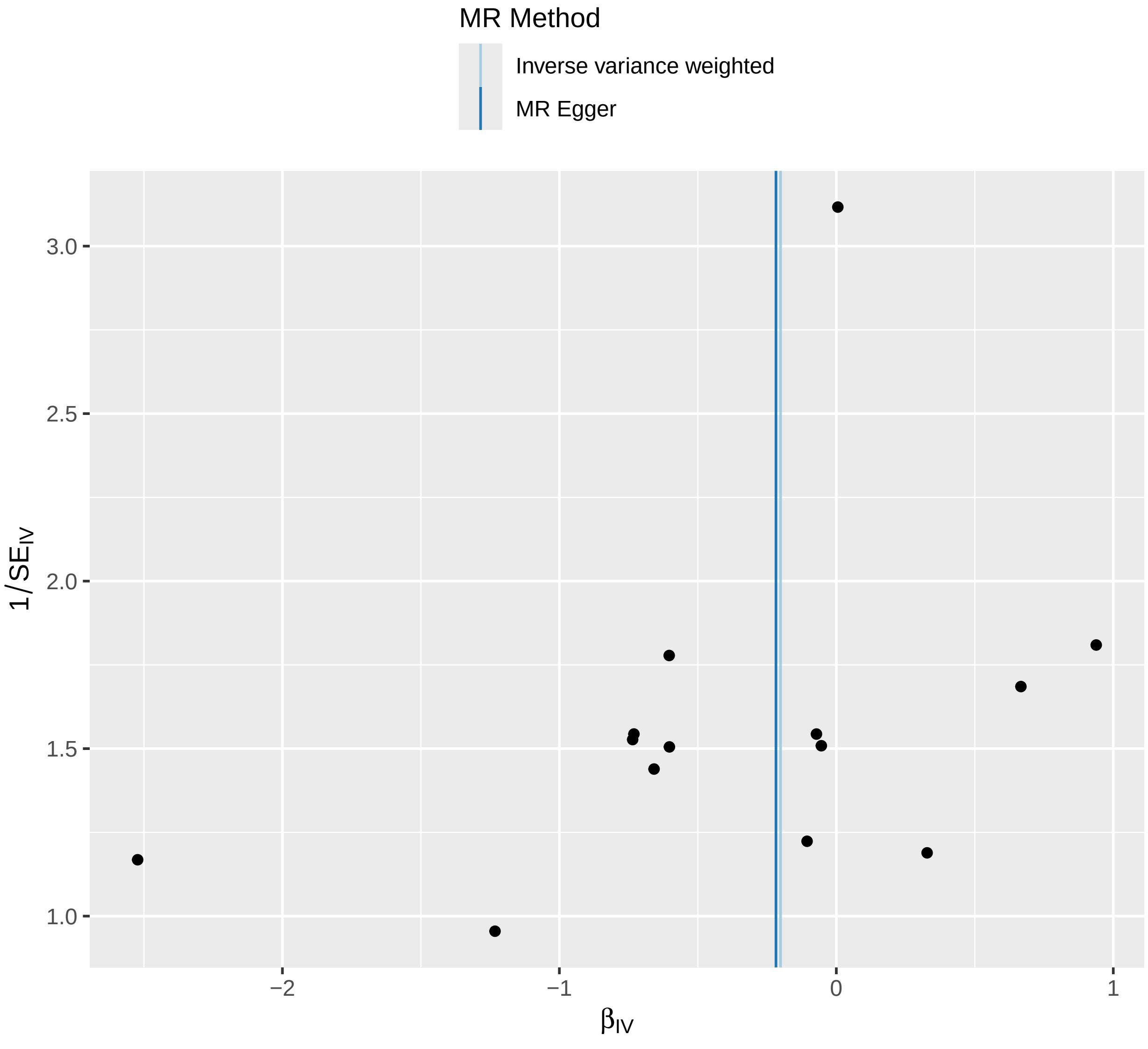

MR Method
Inverse variance weighted
MR Egger
3.0
2.5
2.0
1.5
1.0
1/SE_IV
−2
−1
0
1
β_IV

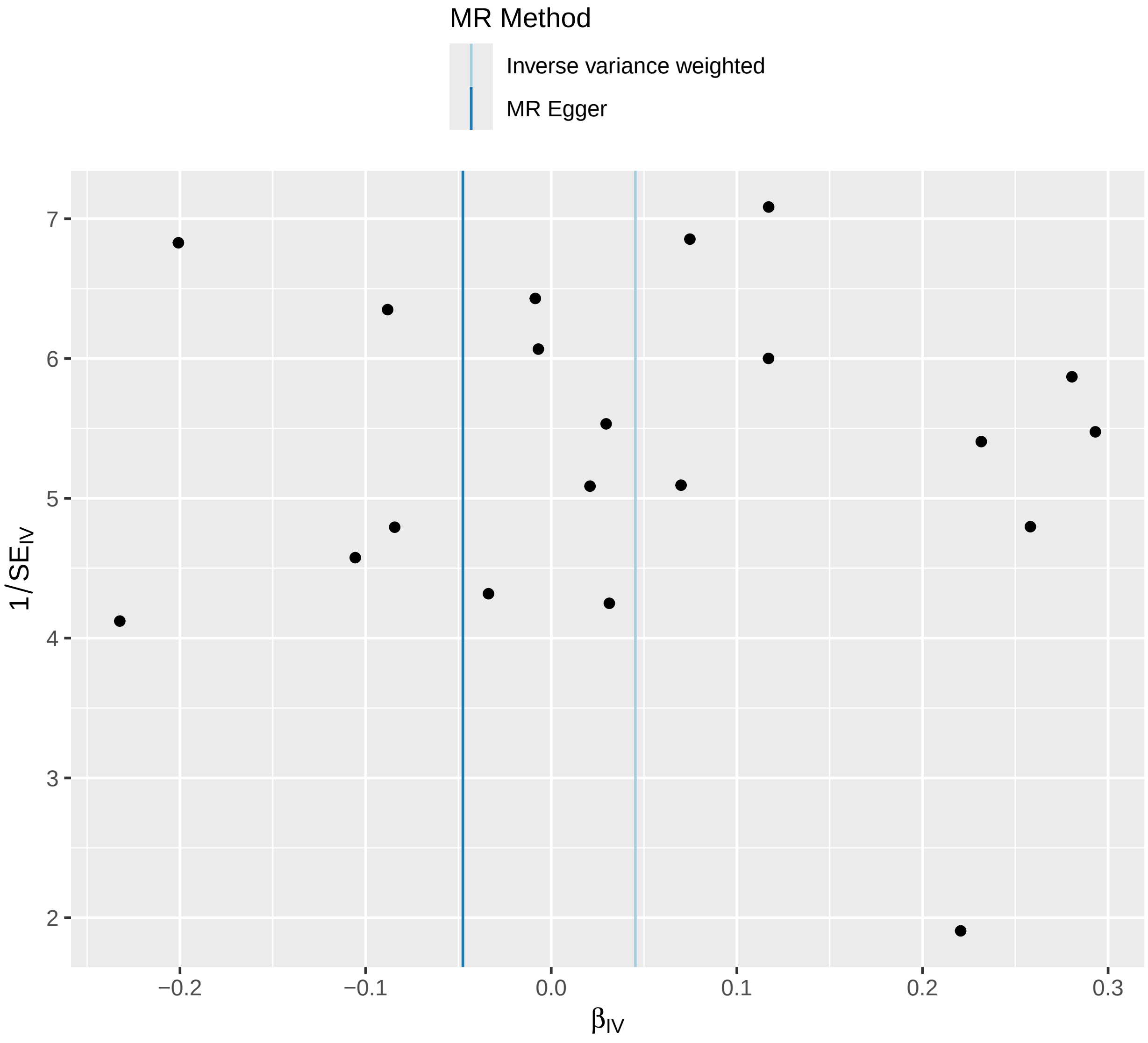
MR Method
Inverse variance weighted
MR Egger
7
6
5
4
3
2
1/SE_IV
−0.2
−0.1
0.0
0.1
0.2
0.3
β_IV

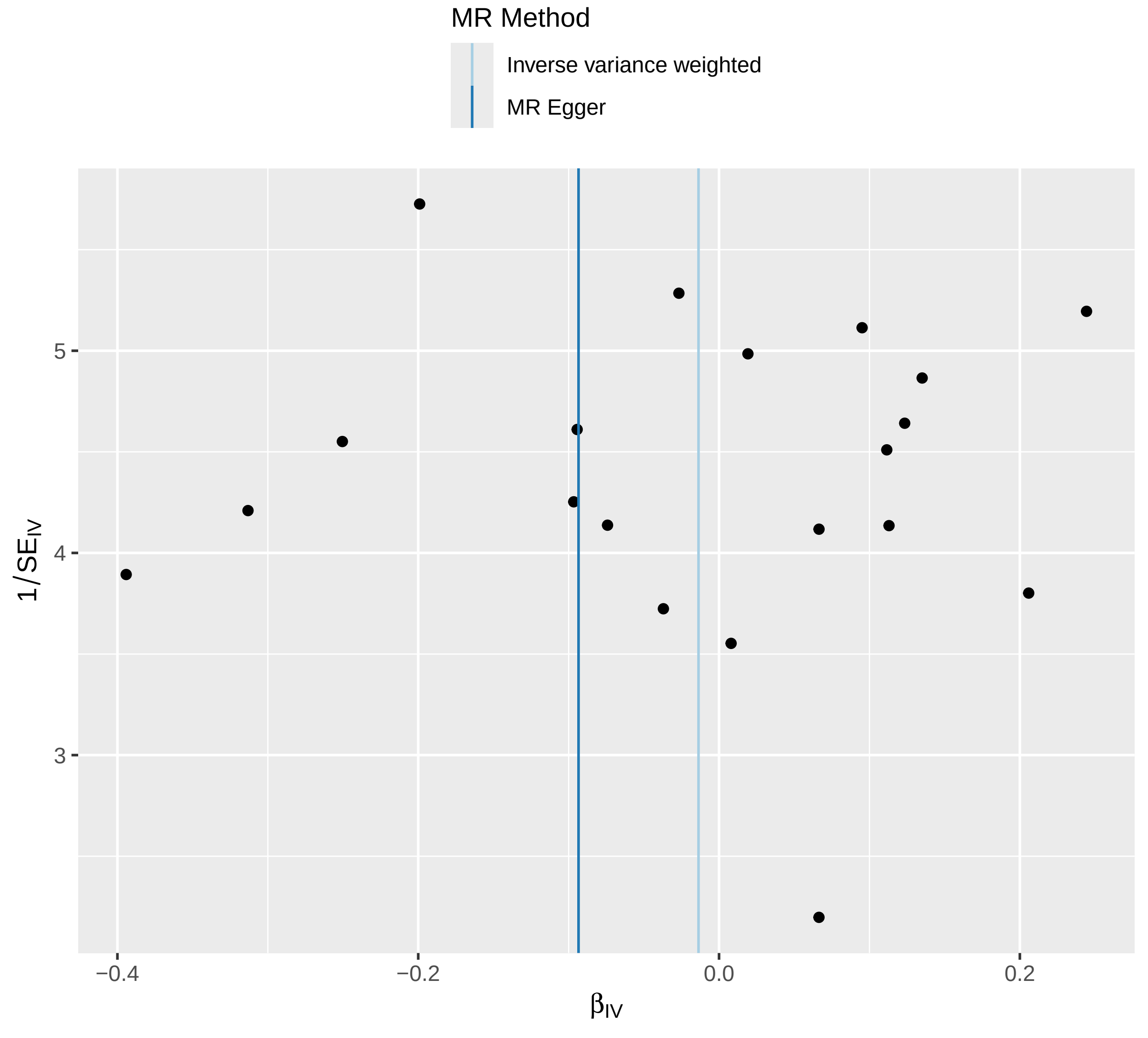
MR Method
Inverse variance weighted
MR Egger
1/SE$_{IV}$
5
4
3
−0.4
−0.2
0.0
0.2
β$_{IV}$

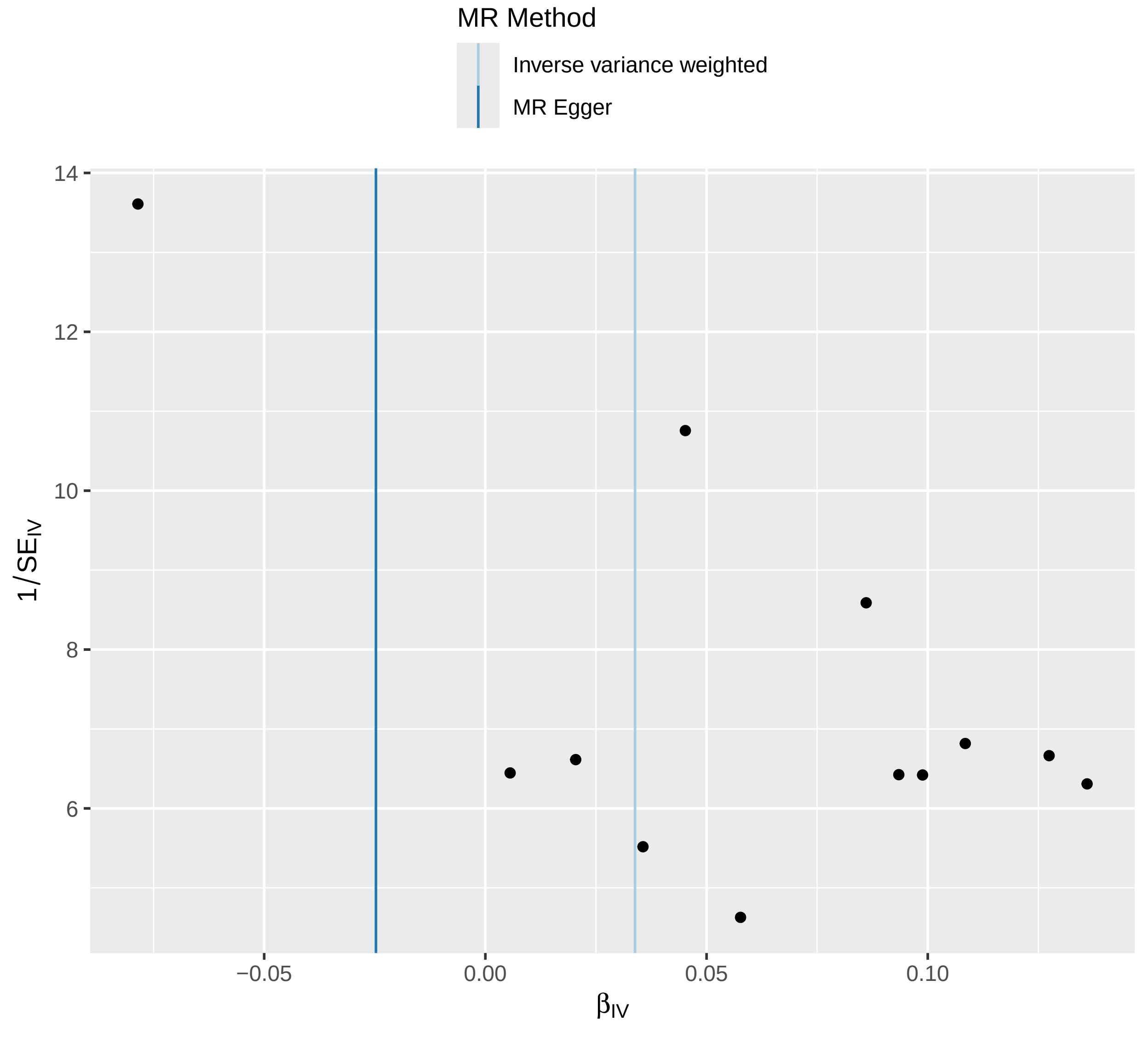
MR Method
Inverse variance weighted
MR Egger
14
12
10
8
6
$1/SE_{IV}$
−0.05
0.00
0.05
0.10
β_{IV}

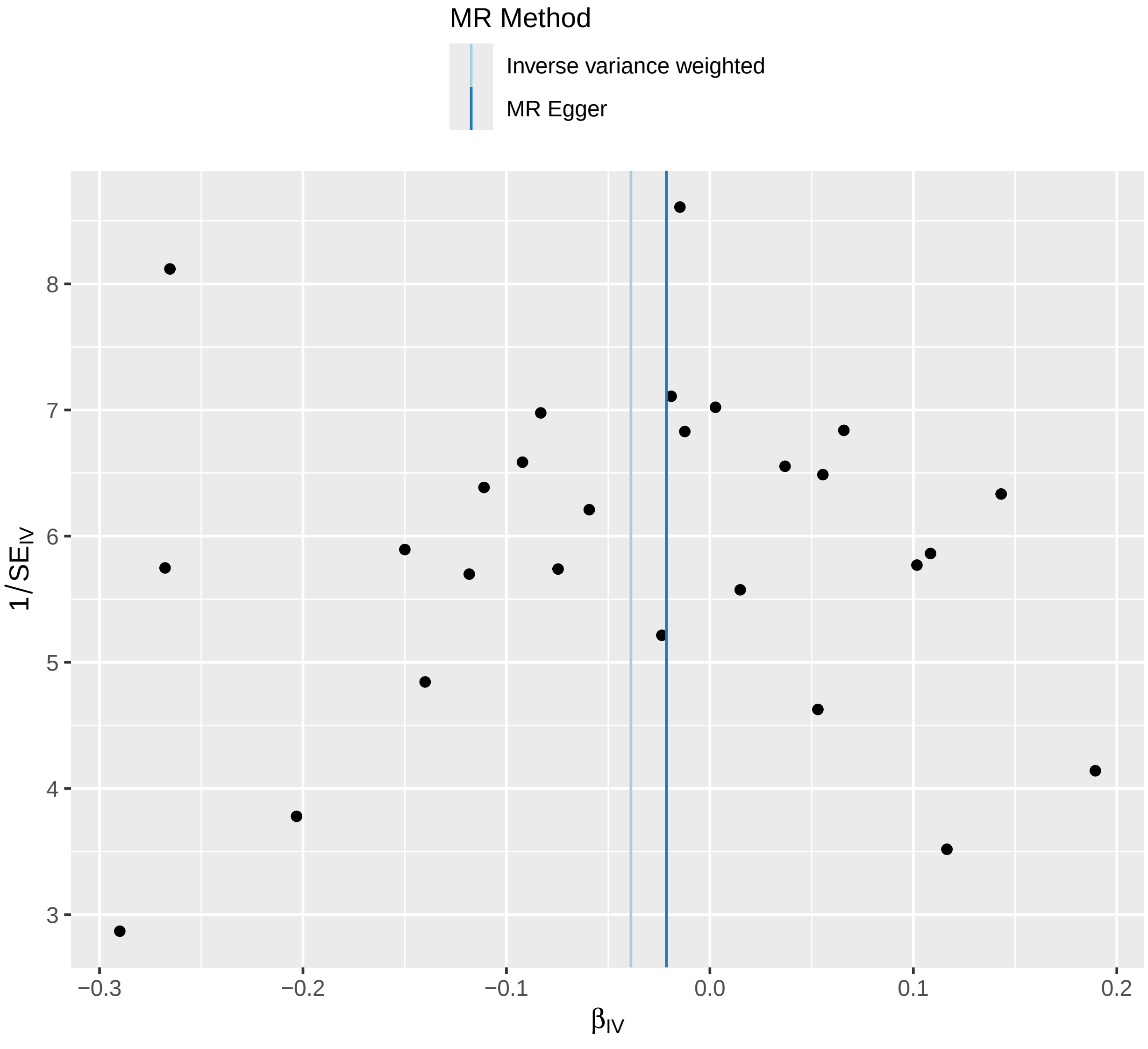
MR Method
Inverse variance weighted
MR Egger
1/SE$_{IV}$
8
7
6
5
4
3
−0.3
−0.2
−0.1
0.0
0.1
0.2
β$_{IV}$

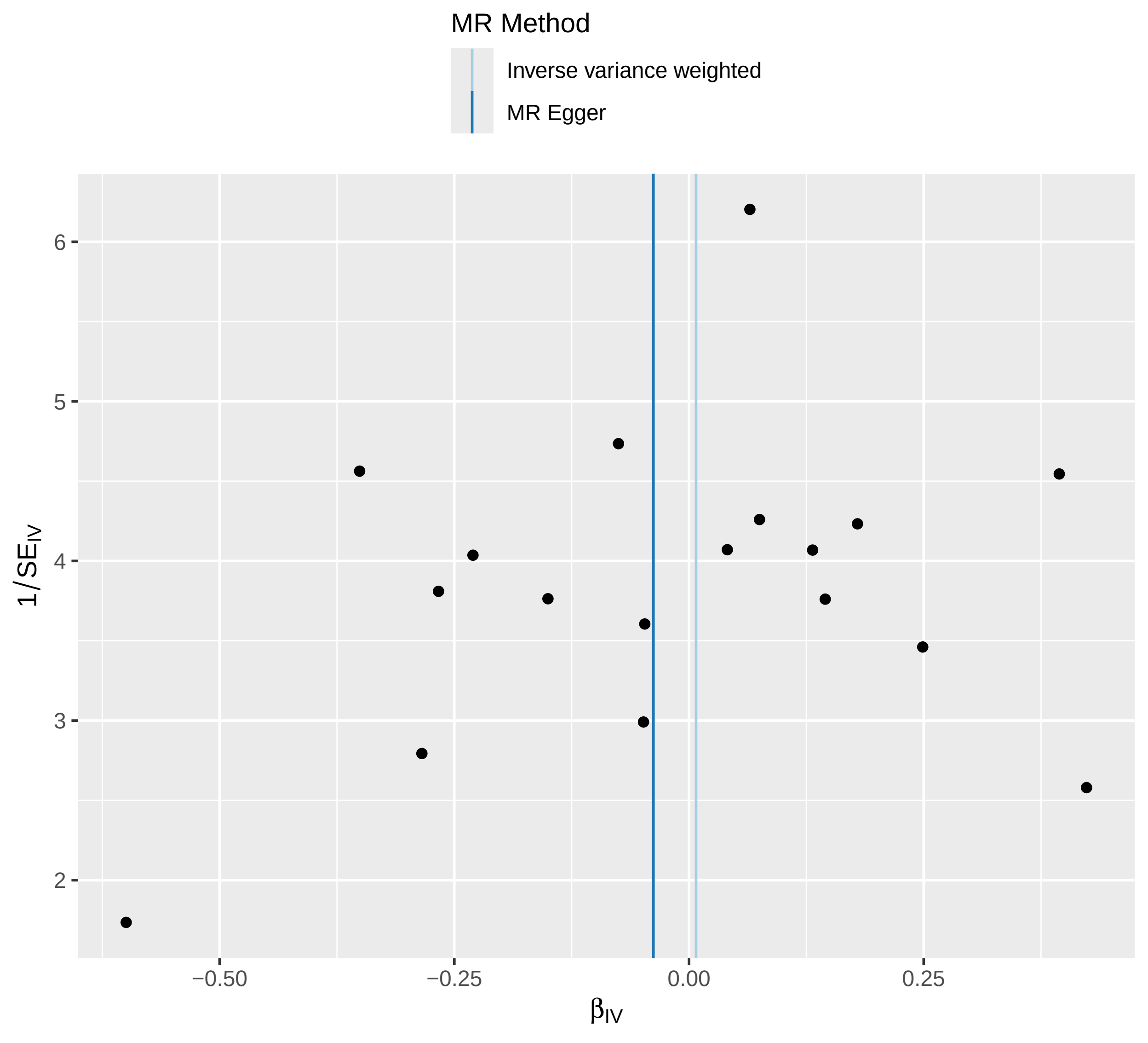
MR Method
Inverse variance weighted
MR Egger
6
5
4
3
2
$1/SE_{IV}$
−0.50
−0.25
0.00
0.25
β_{IV}

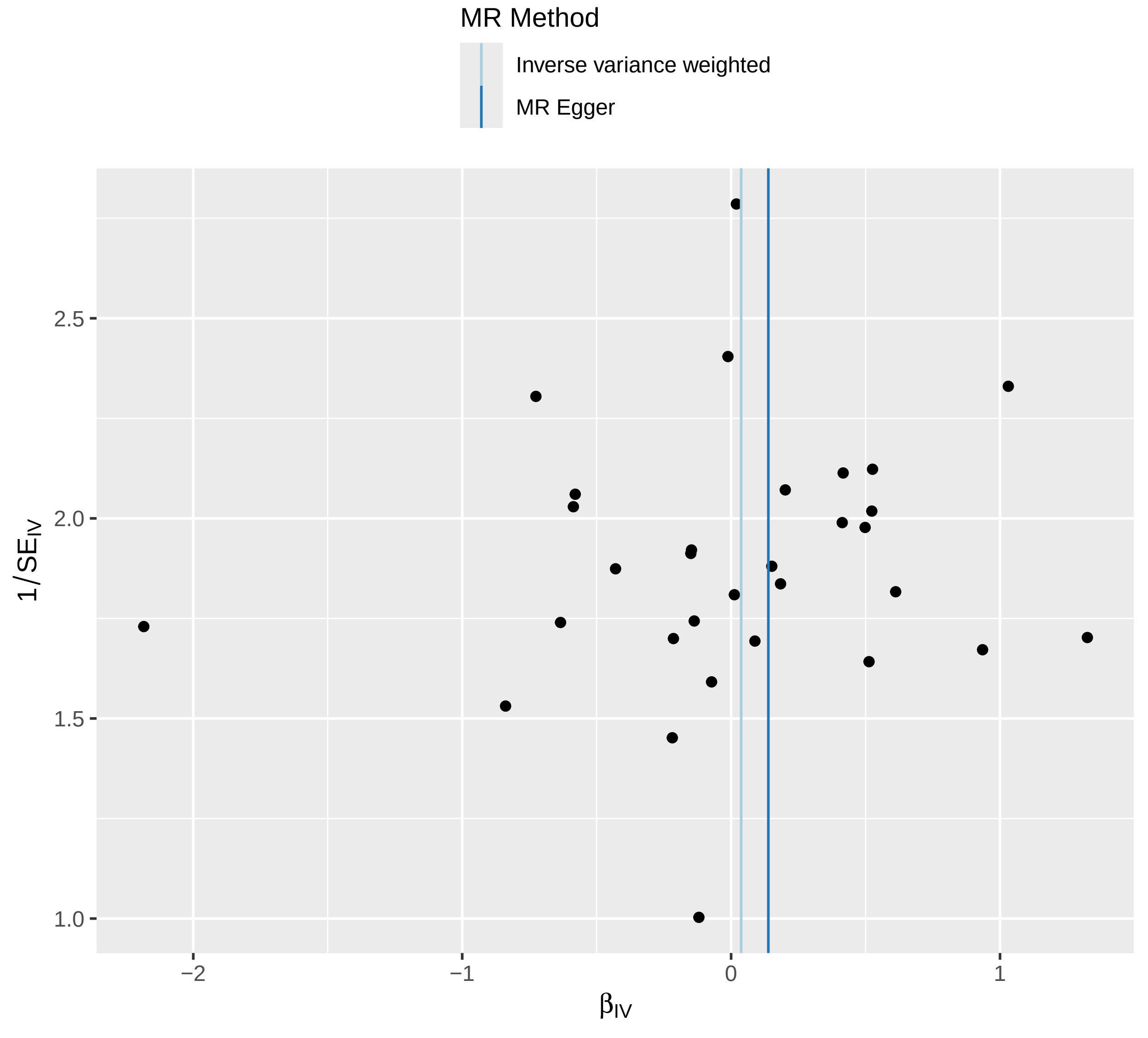
MR Method
Inverse variance weighted
MR Egger
2.5
2.0
1.5
1.0
$1/SE_{IV}$
−2
−1
0
1
β_{IV}

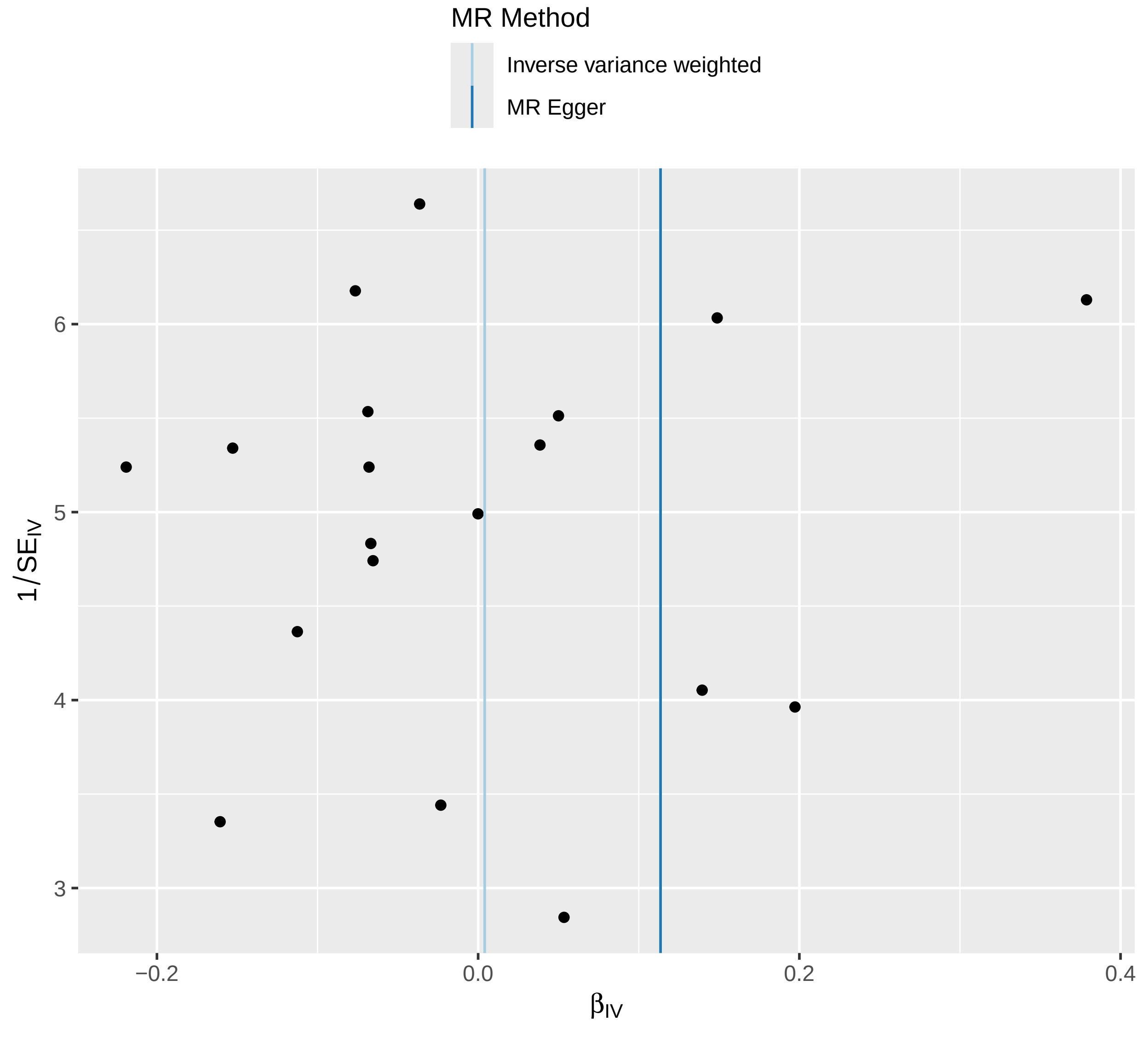
MR Method
Inverse variance weighted
MR Egger
6
5
4
3
1/SE$_{IV}$
−0.2
0.0
0.2
0.4
β$_{IV}$

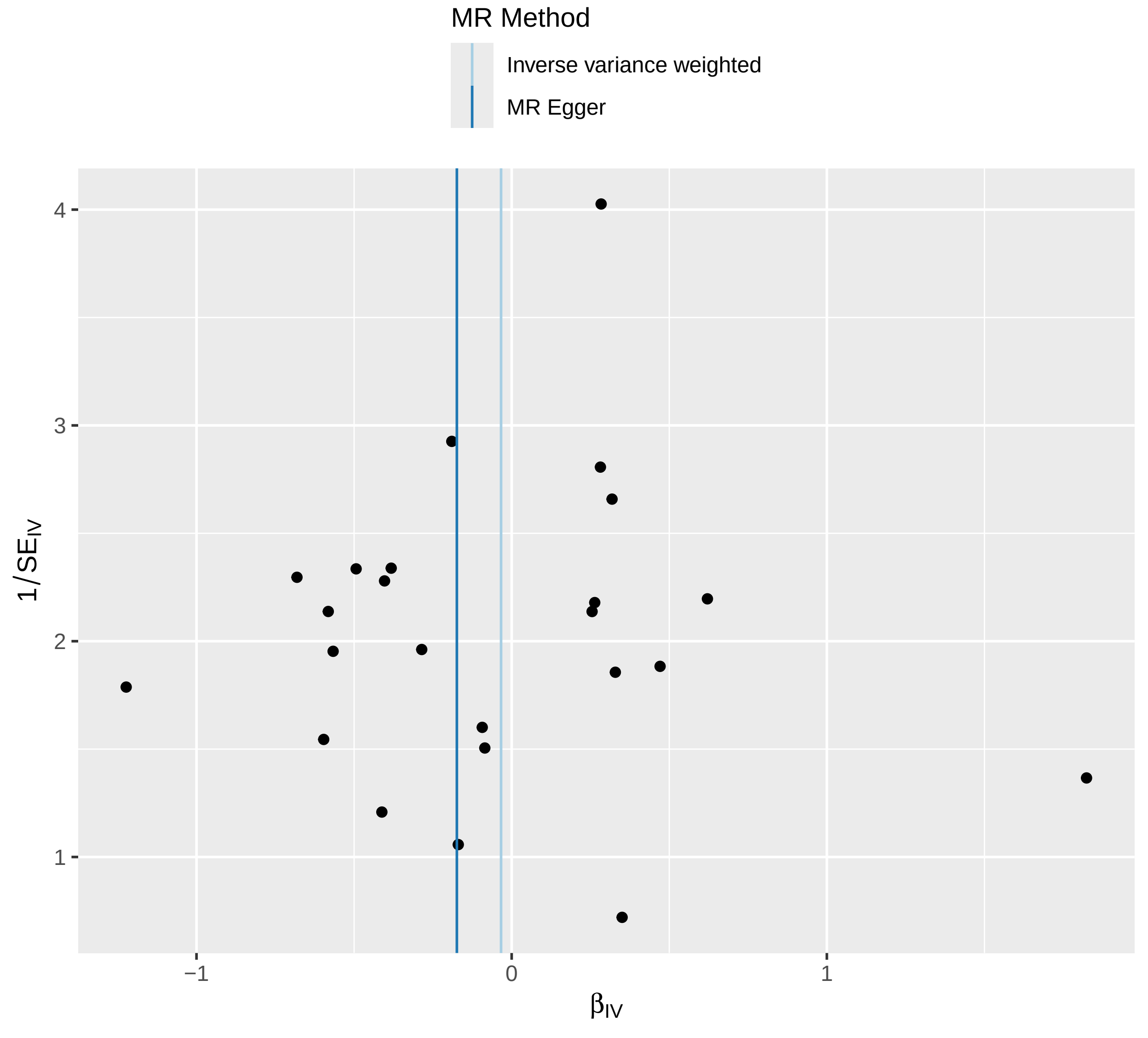
MR Method
Inverse variance weighted
MR Egger
$1/SE_{IV}$
4
3
2
1
−1
0
1
β_{IV}

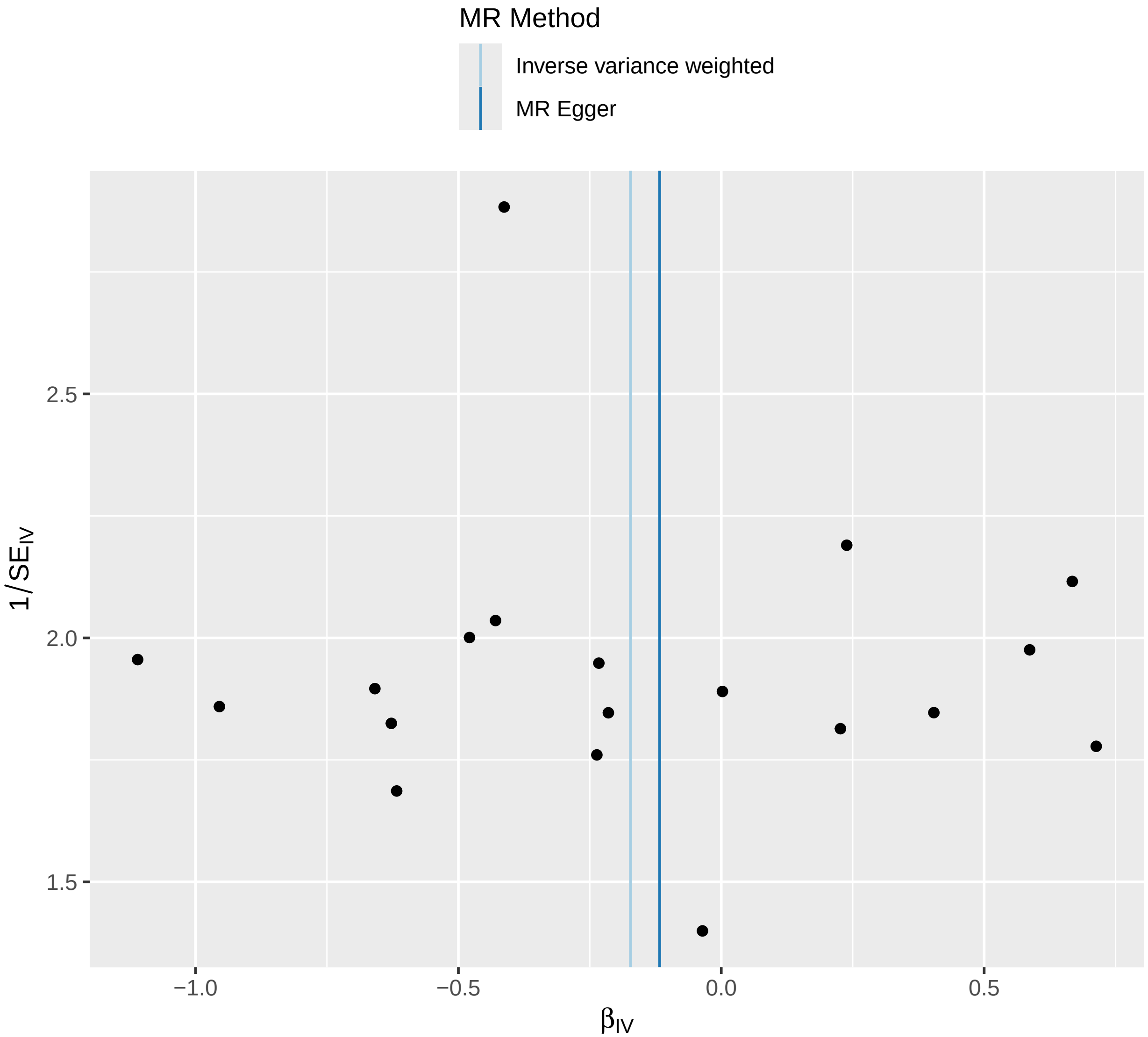
MR Method
Inverse variance weighted
MR Egger
2.5
2.0
1.5
−1.0
−0.5
0.0
0.5
$1/SE_{IV}$
β_{IV}

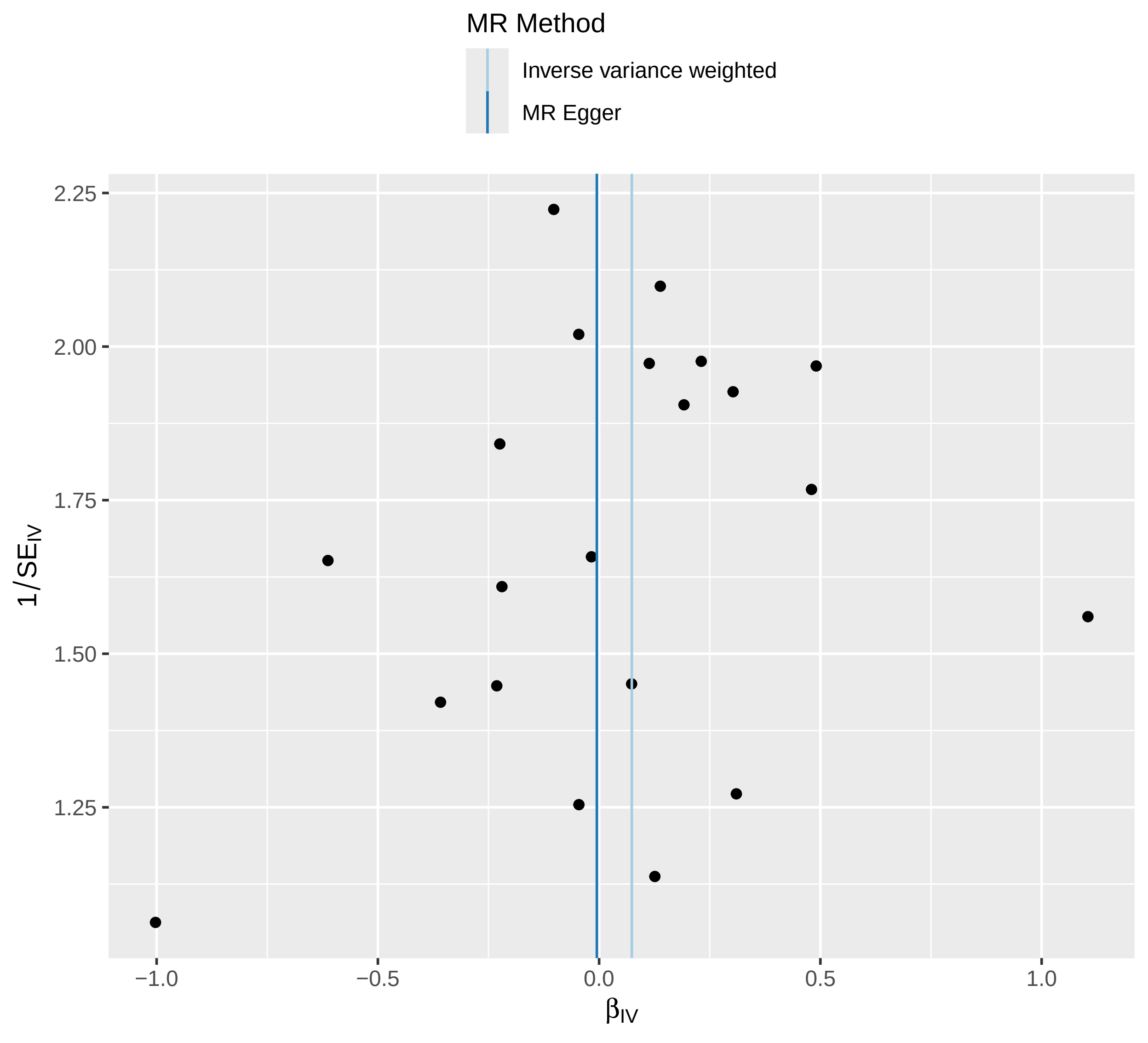
MR Method
Inverse variance weighted
MR Egger
2.25
2.00
1.75
1.50
1.25
1/SE_IV
−1.0
−0.5
0.0
0.5
1.0
β_IV

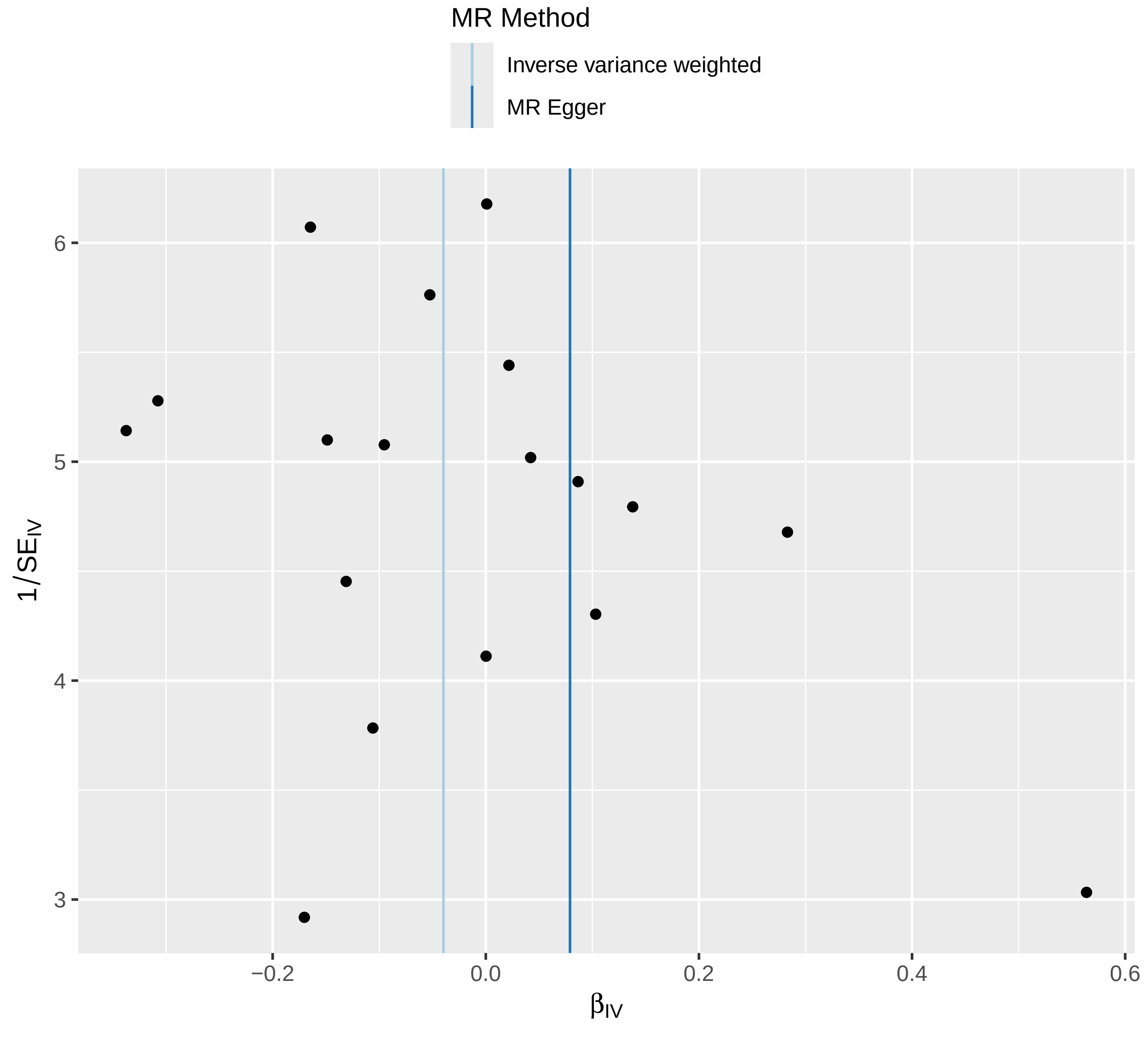
MR Method
Inverse variance weighted
MR Egger
1/SE$_{IV}$
6
5
4
3
−0.2
0.0
0.2
0.4
0.6
β$_{IV}$

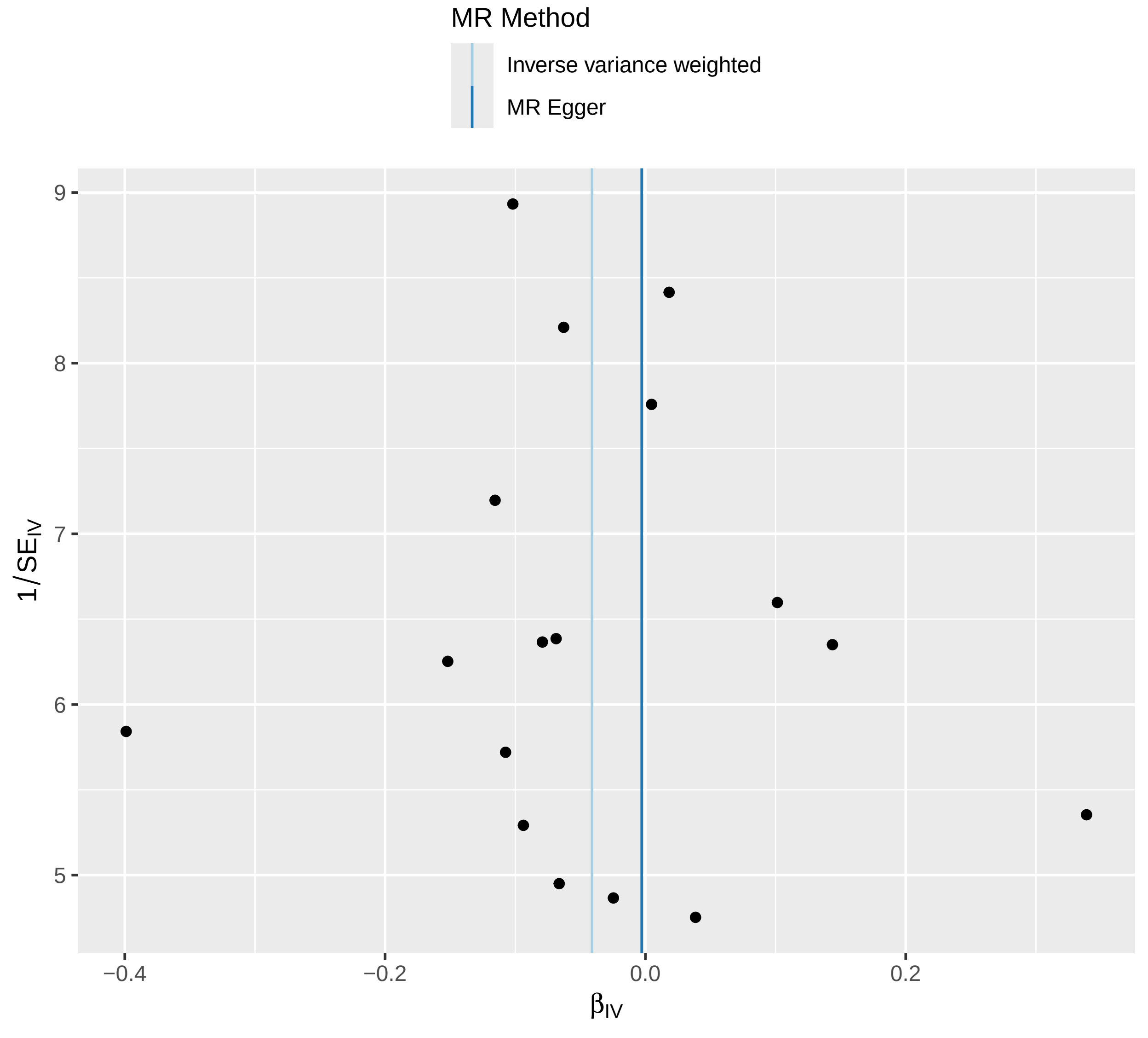
MR Method
Inverse variance weighted
MR Egger
$1/SE_{IV}$
9
8
7
6
5
−0.4
−0.2
0.0
0.2
β_{IV}

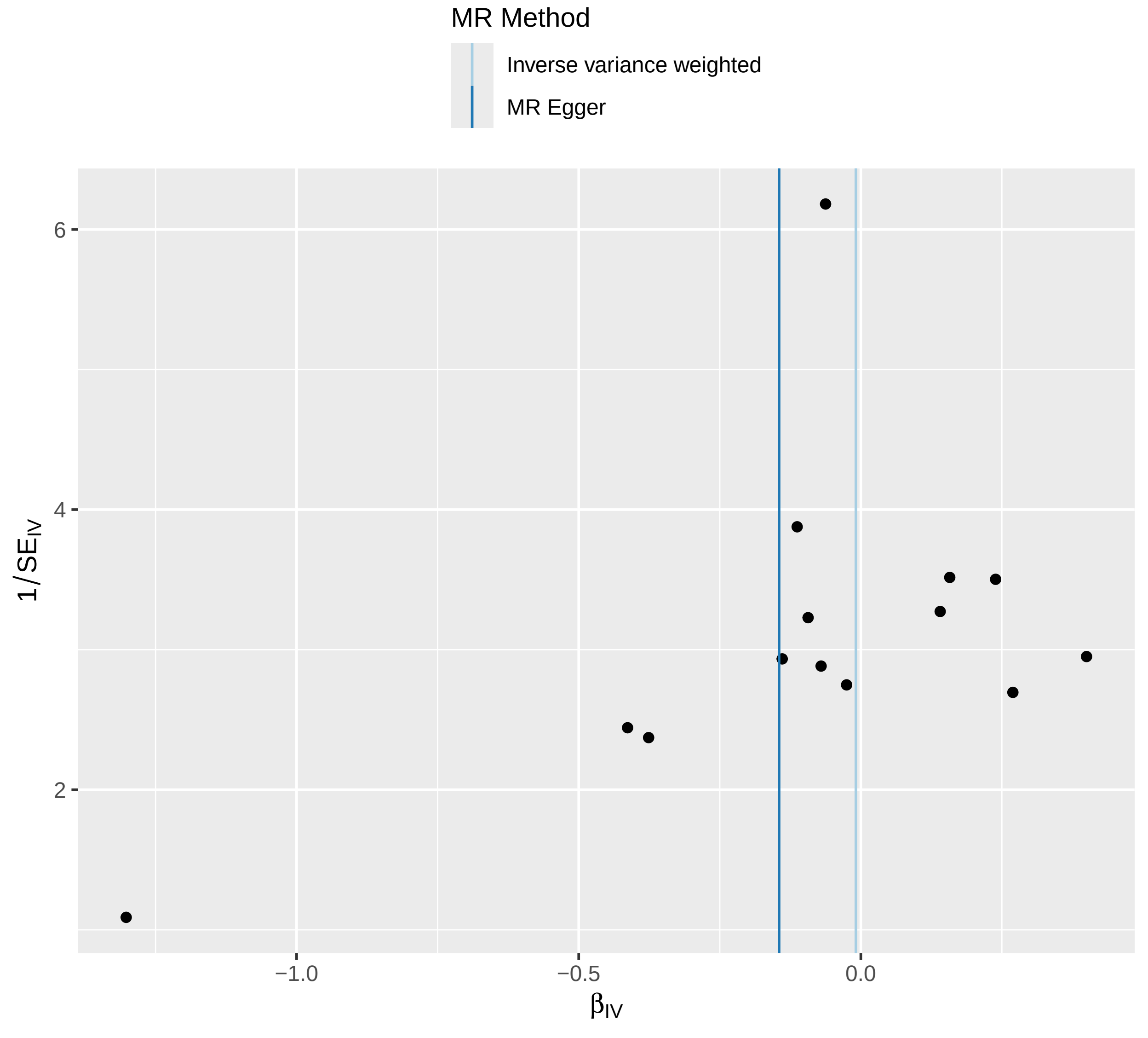

MR Method
Inverse variance weighted
MR Egger
6
4
2
−1.0
−0.5
0.0
$1/SE_{IV}$
β_{IV}

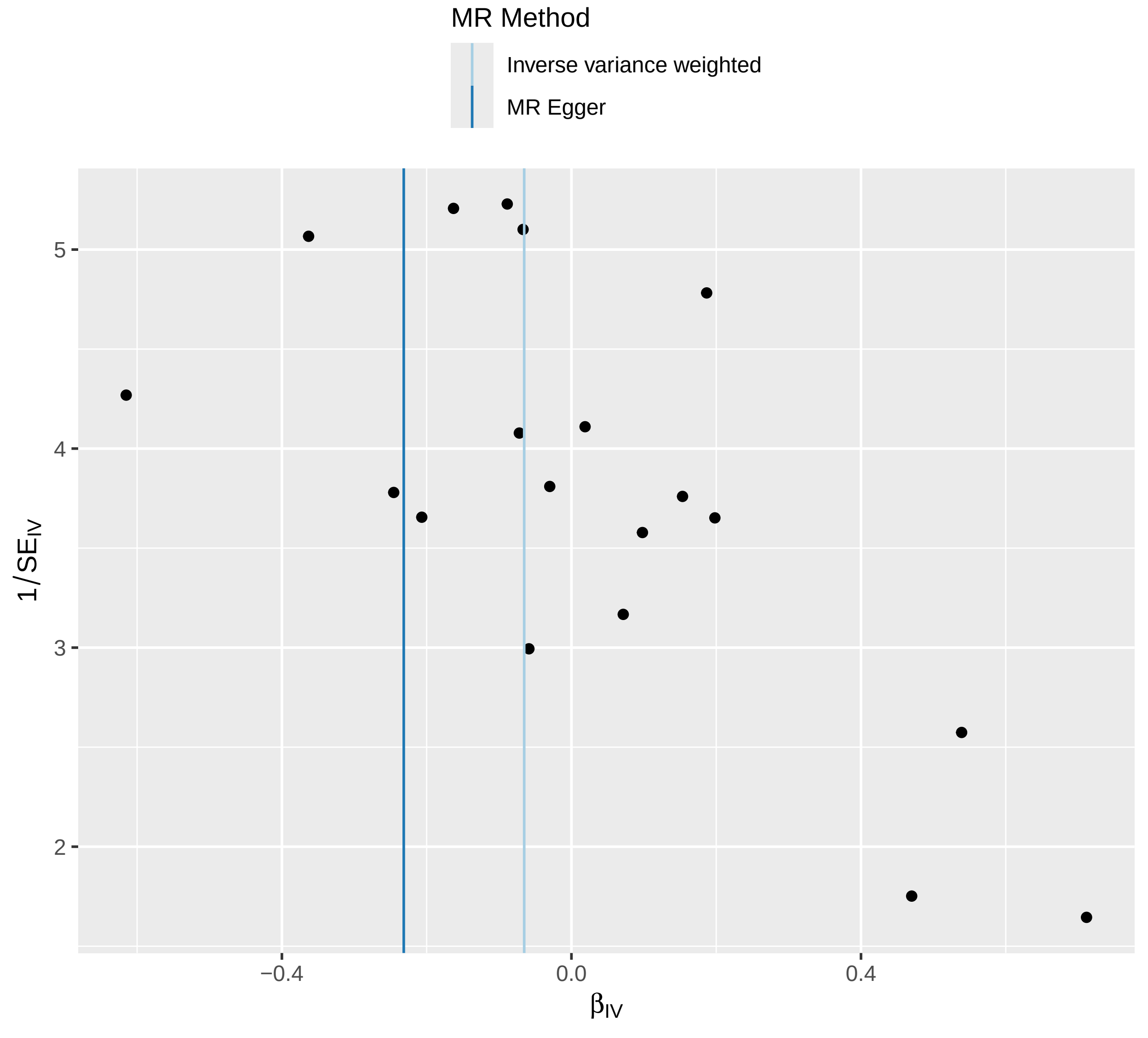

MR Method
Inverse variance weighted
MR Egger
5
4
3
2
1/SE_IV
−0.4
0.0
0.4
β_IV

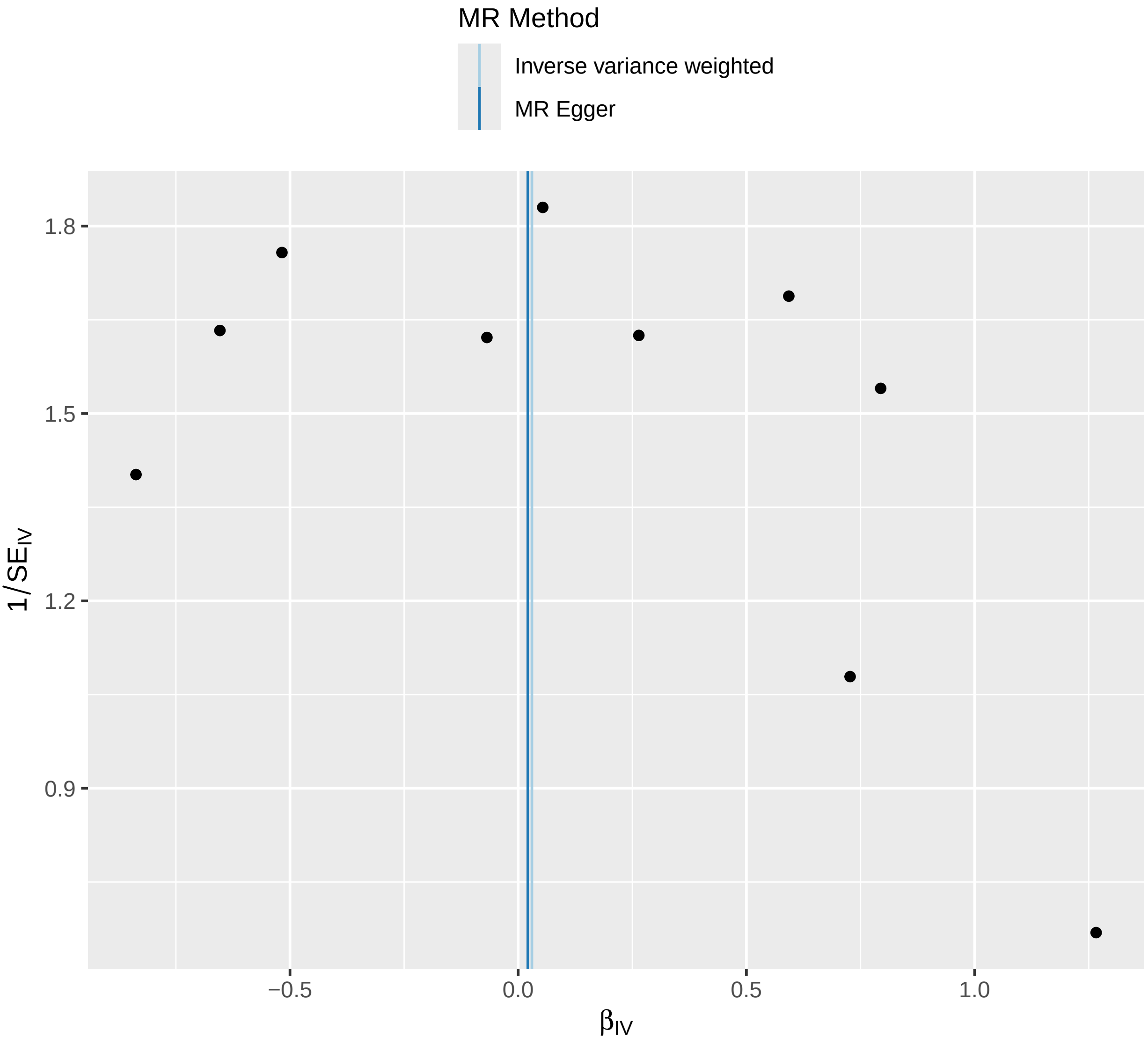
MR Method
Inverse variance weighted
MR Egger
1.8
1.5
1.2
0.9
$1/SE_{IV}$
−0.5
0.0
0.5
1.0
β_{IV}

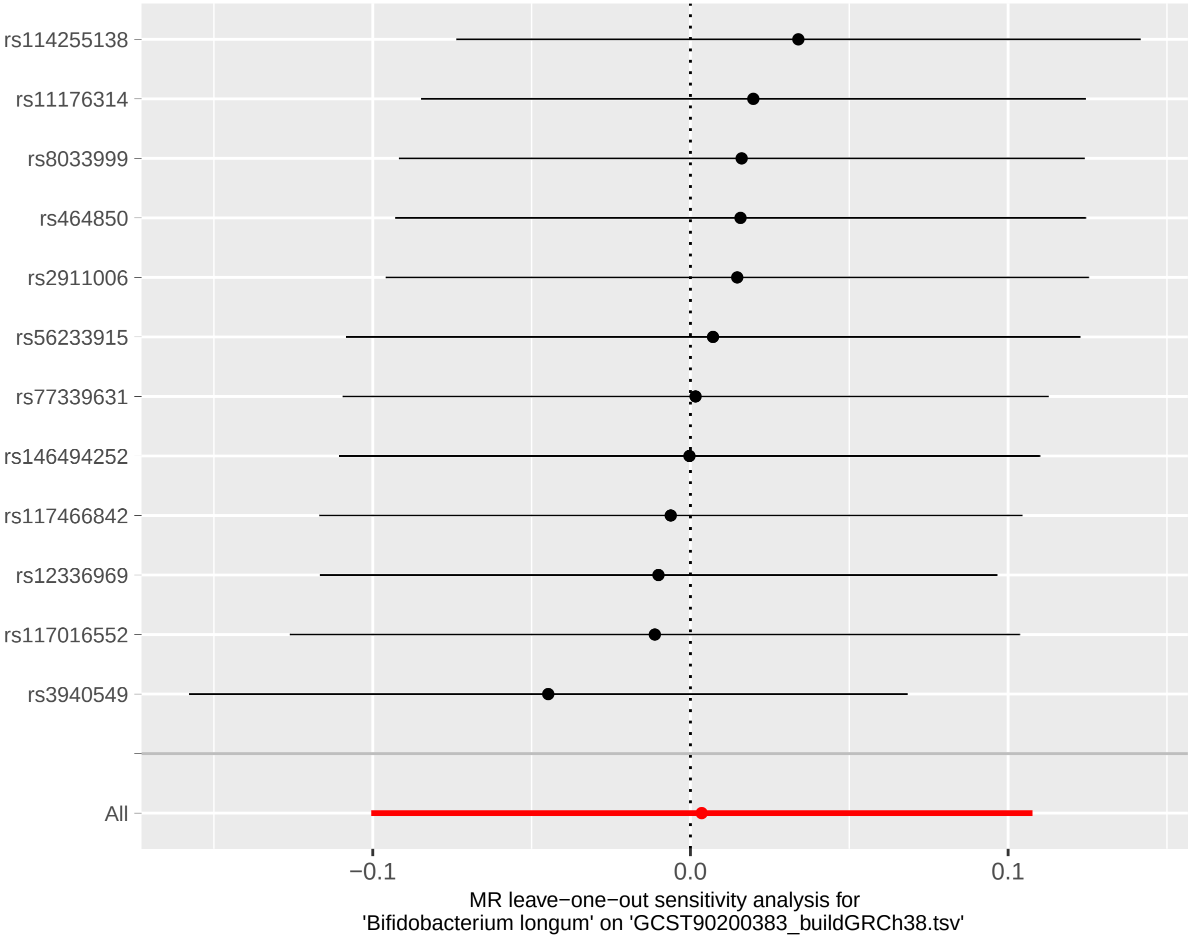
rs114255138
rs11176314
rs8033999
rs464850
rs2911006
rs56233915
rs77339631
rs146494252
rs117466842
rs12336969
rs117016552
rs3940549
All
−0.1
0.0
0.1
MR leave−one−out sensitivity analysis for
'Bifidobacterium longum' on 'GCST90200383_buildGRCh38.tsv'

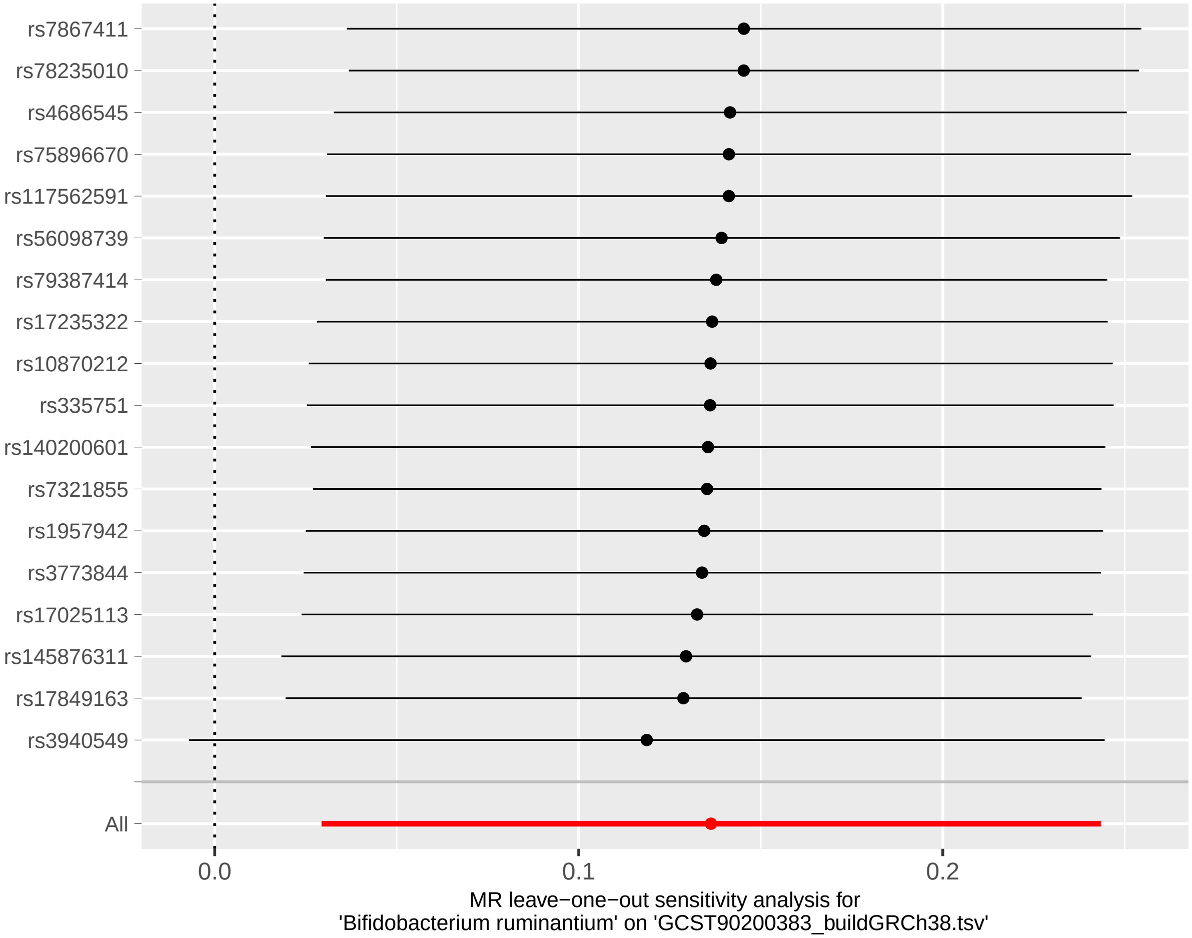
rs7867411
rs78235010
rs4686545
rs75896670
rs117562591
rs56098739
rs79387414
rs17235322
rs10870212
rs335751
rs140200601
rs7321855
rs1957942
rs3773844
rs17025113
rs145876311
rs17849163
rs3940549
All
0.0
0.1
0.2
MR leave−one−out sensitivity analysis for
'Bifidobacterium ruminantium' on 'GCST90200383_buildGRCh38.tsv'

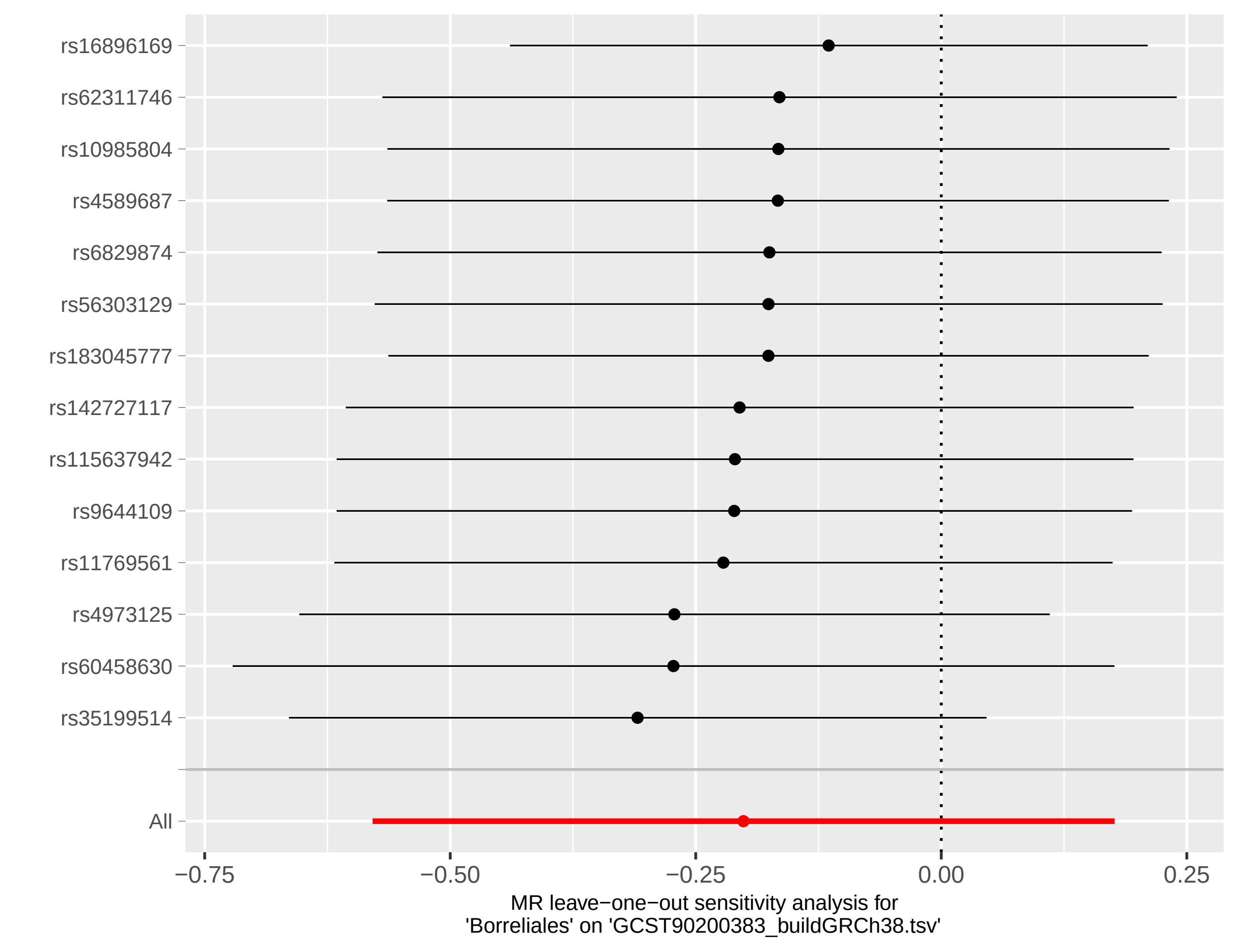

rs16896169
rs62311746
rs10985804
rs4589687
rs6829874
rs56303129
rs183045777
rs142727117
rs115637942
rs9644109
rs11769561
rs4973125
rs60458630
rs35199514
All
−0.75
−0.50
−0.25
0.00
0.25
MR leave−one−out sensitivity analysis for
'Borreliales' on 'GCST90200383_buildGRCh38.tsv'

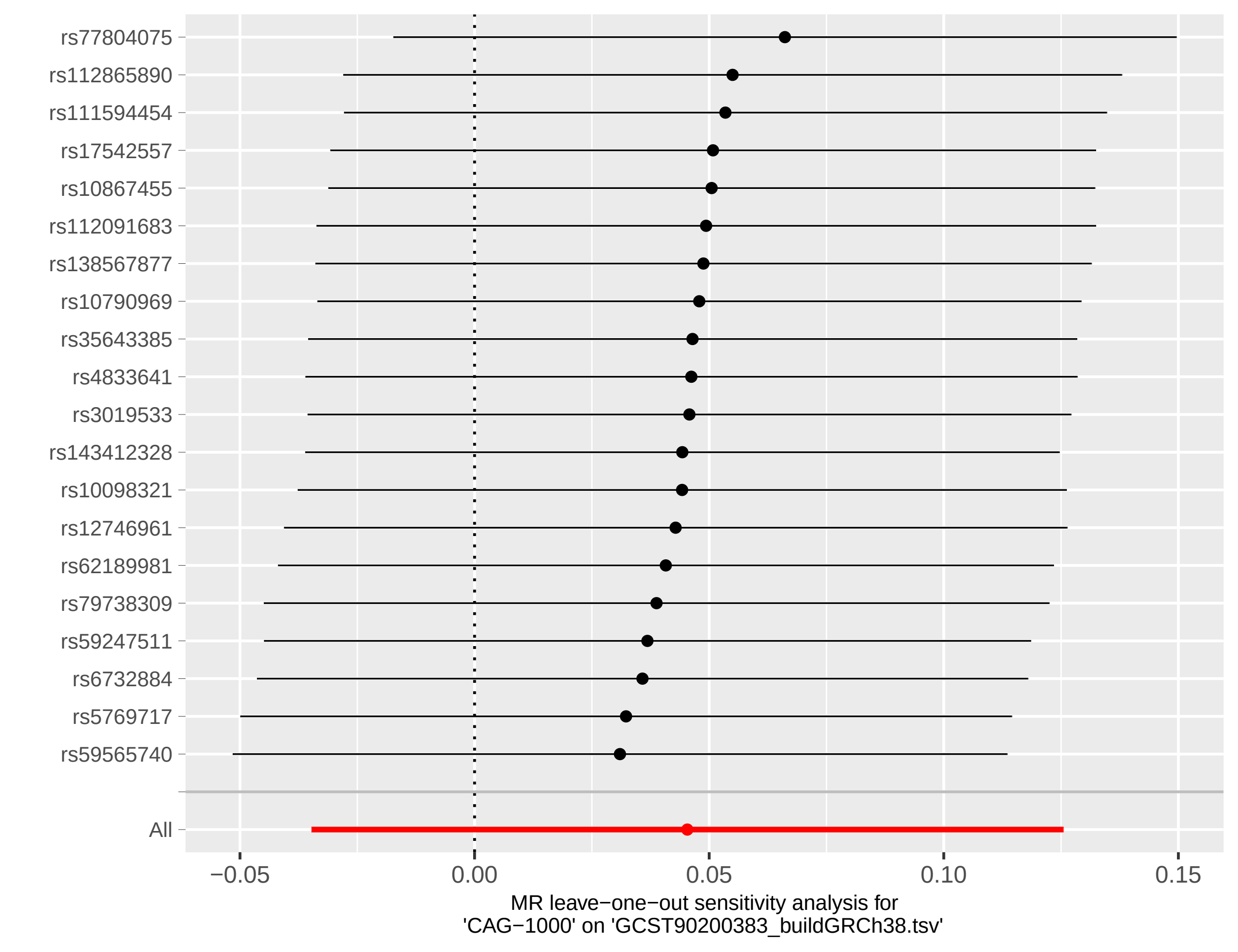

rs77804075
rs112865890
rs111594454
rs17542557
rs10867455
rs112091683
rs138567877
rs10790969
rs35643385
rs4833641
rs3019533
rs143412328
rs10098321
rs12746961
rs62189981
rs79738309
rs59247511
rs6732884
rs5769717
rs59565740
All
−0.05
0.00
0.05
0.10
0.15
MR leave−one−out sensitivity analysis for
'CAG−1000' on 'GCST90200383_buildGRCh38.tsv'

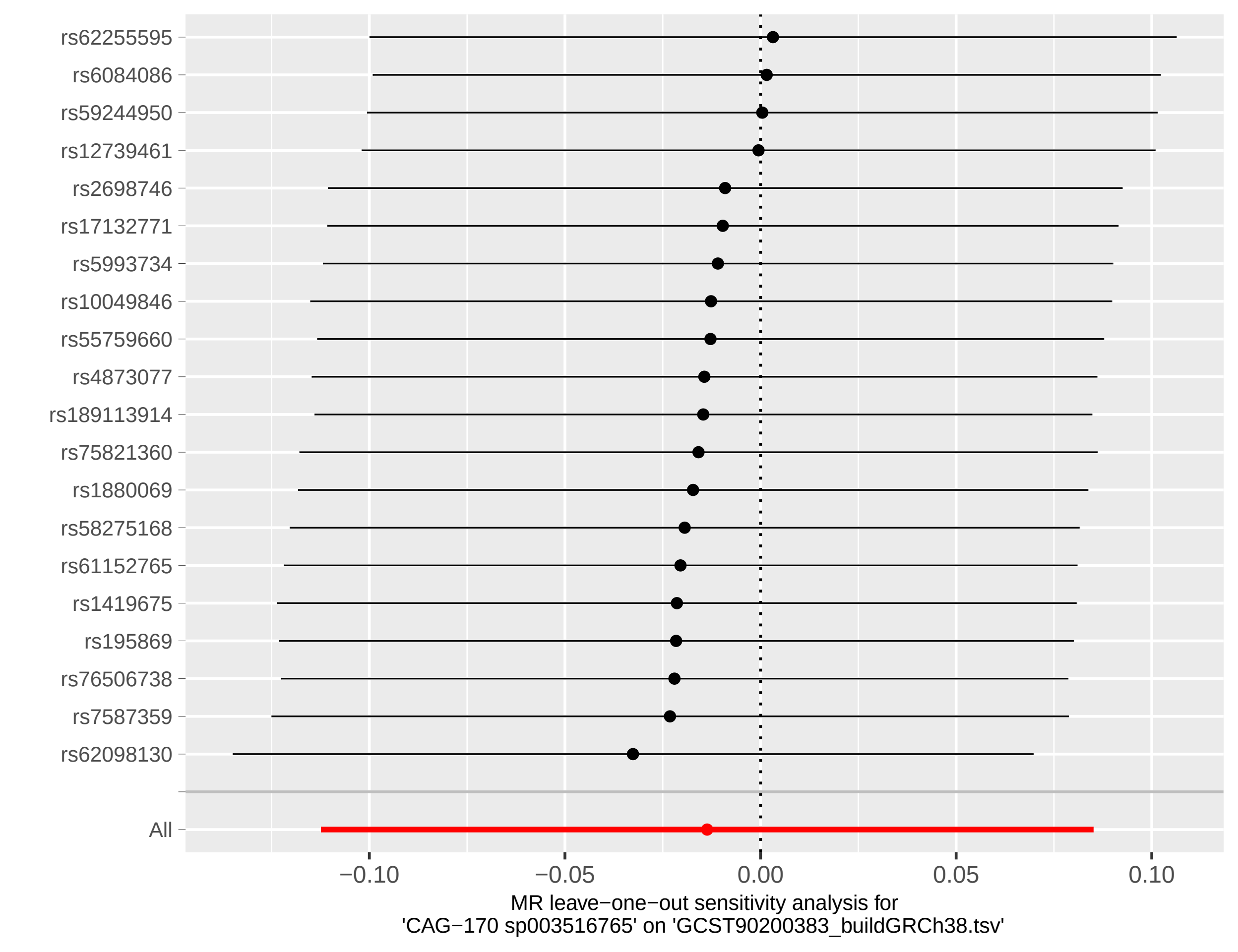

rs62255595
rs6084086
rs59244950
rs12739461
rs2698746
rs17132771
rs5993734
rs10049846
rs55759660
rs4873077
rs189113914
rs75821360
rs1880069
rs58275168
rs61152765
rs1419675
rs195869
rs76506738
rs7587359
rs62098130
All
−0.10
−0.05
0.00
0.05
0.10
MR leave−one−out sensitivity analysis for
'CAG−170 sp003516765' on 'GCST90200383_buildGRCh38.tsv'

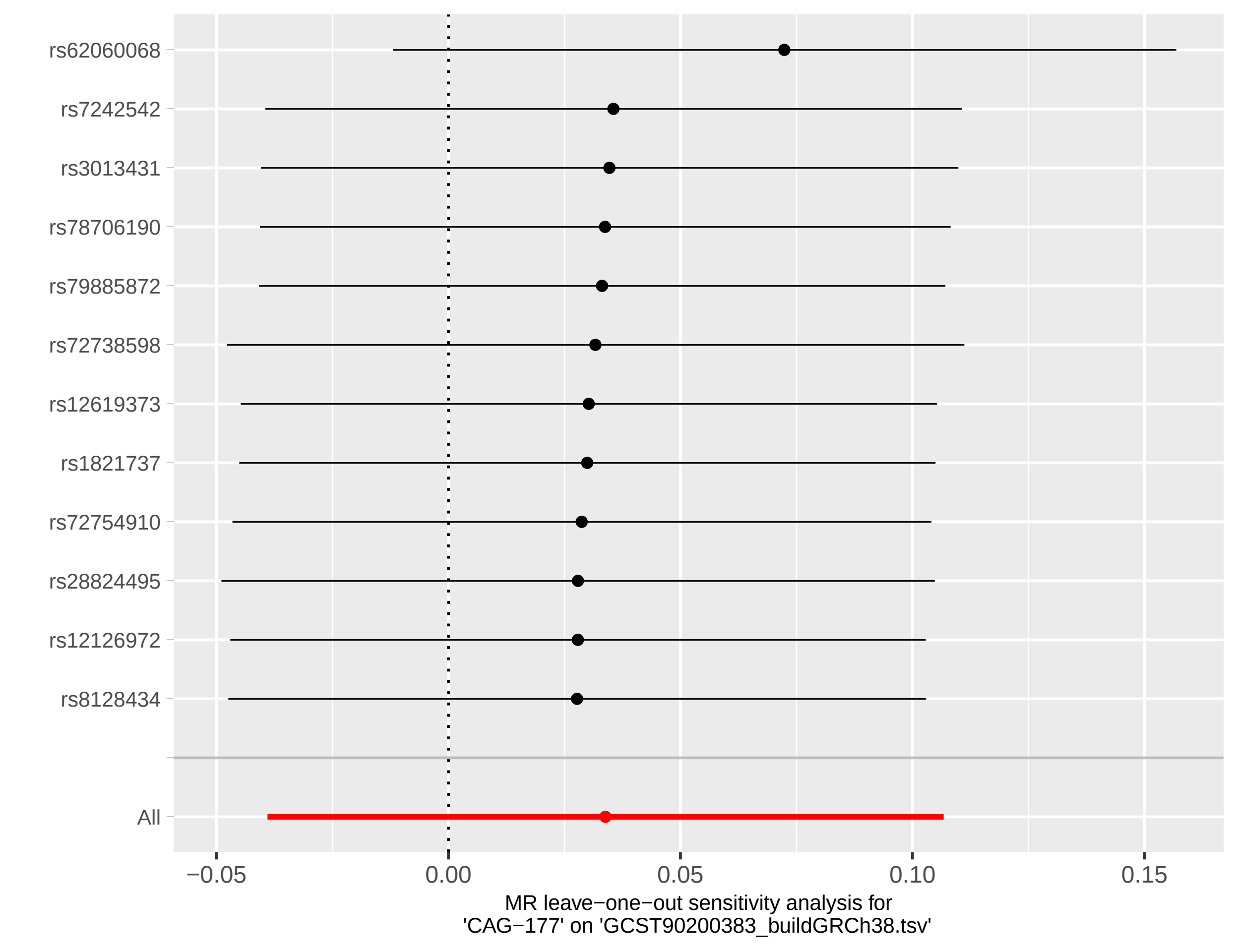

rs62060068
rs7242542
rs3013431
rs78706190
rs79885872
rs72738598
rs12619373
rs1821737
rs72754910
rs28824495
rs12126972
rs8128434
All
−0.05
0.00
0.05
0.10
0.15
MR leave−one−out sensitivity analysis for
'CAG−177' on 'GCST90200383_buildGRCh38.tsv'

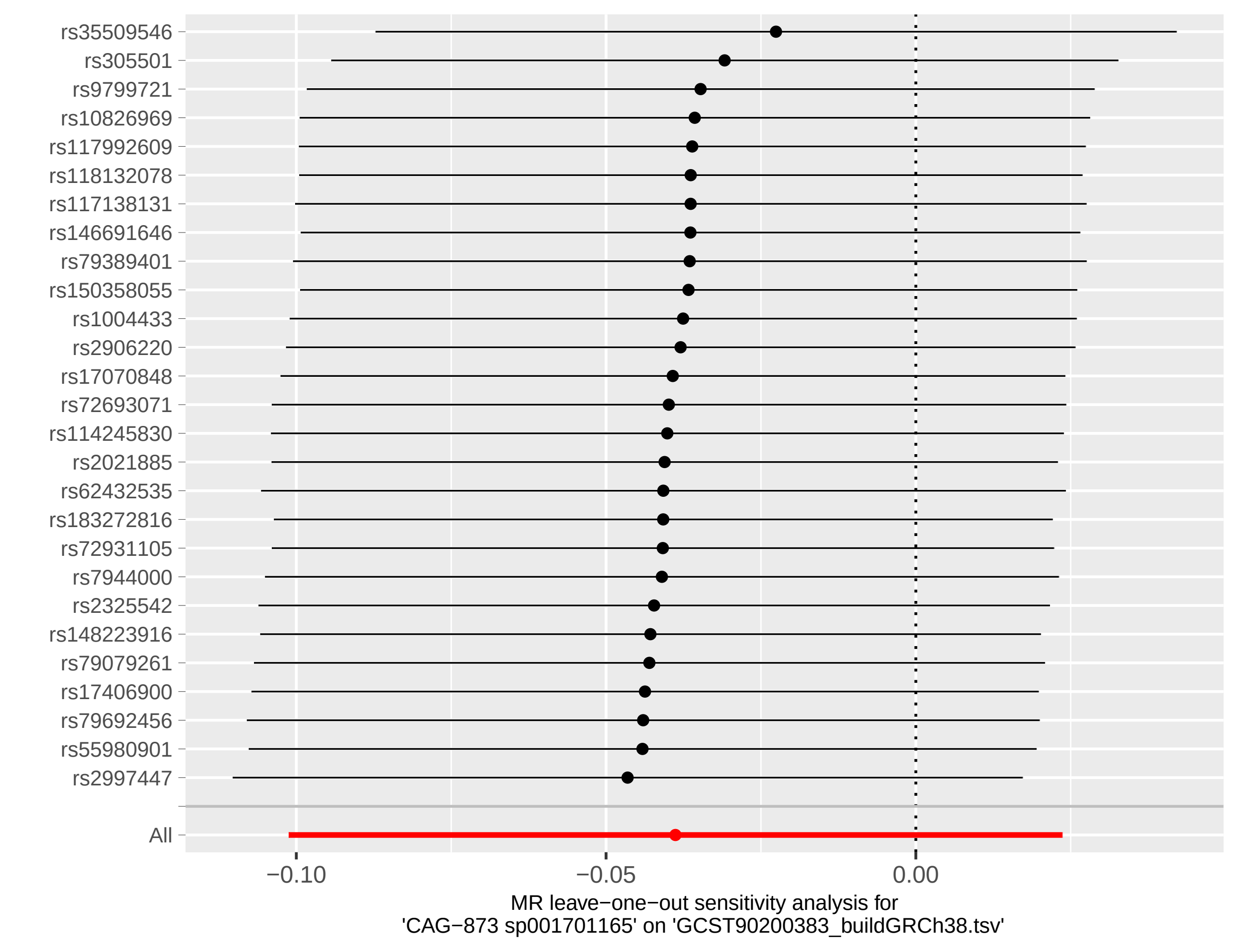

rs35509546
rs305501
rs9799721
rs10826969
rs117992609
rs118132078
rs117138131
rs146691646
rs79389401
rs150358055
rs1004433
rs2906220
rs17070848
rs72693071
rs114245830
rs2021885
rs62432535
rs183272816
rs72931105
rs7944000
rs2325542
rs148223916
rs79079261
rs17406900
rs79692456
rs55980901
rs2997447
All
−0.10
−0.05
0.00
MR leave−one−out sensitivity analysis for
'CAG−873 sp001701165' on 'GCST90200383_buildGRCh38.tsv'

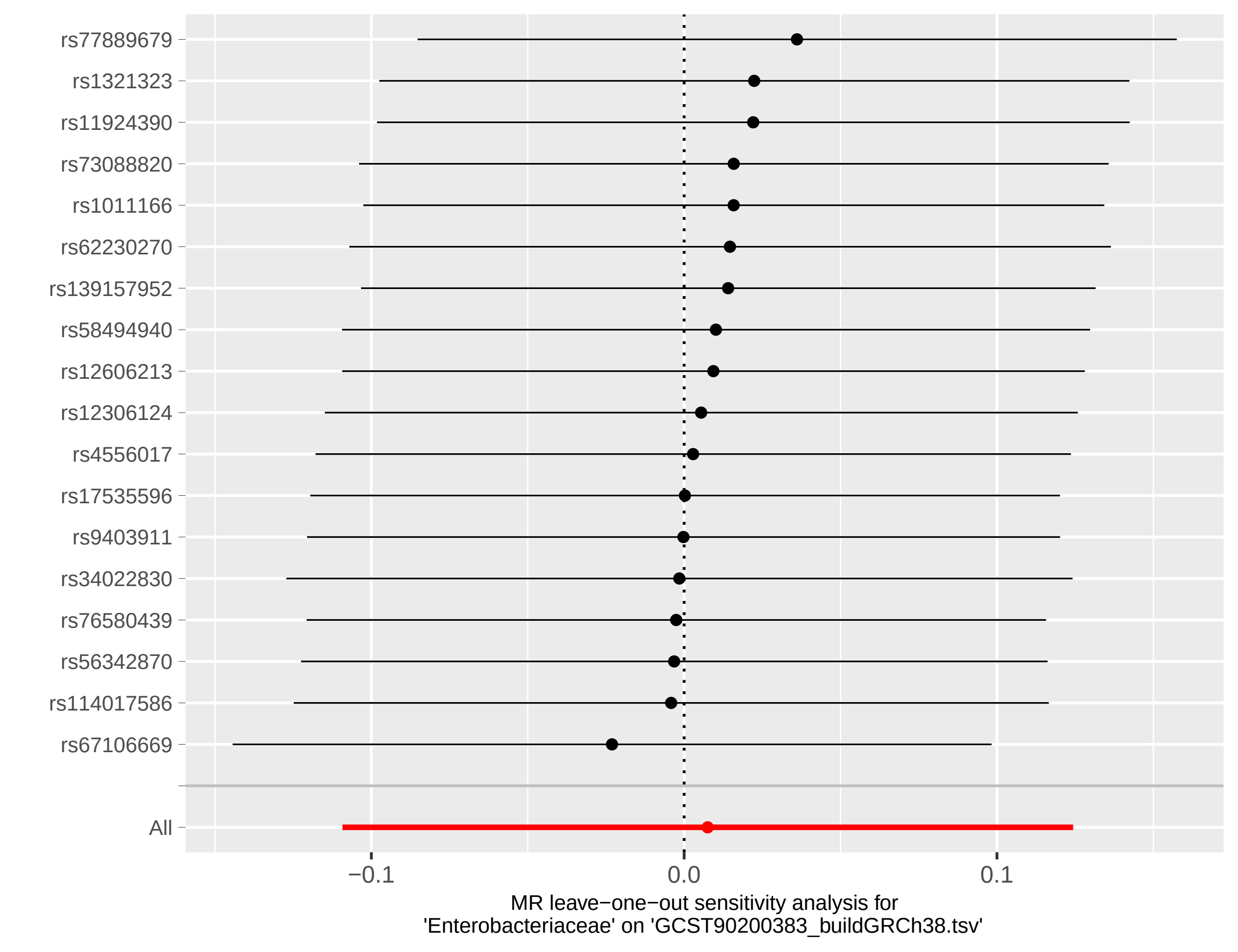

rs77889679
rs1321323
rs11924390
rs73088820
rs1011166
rs62230270
rs139157952
rs58494940
rs12606213
rs12306124
rs4556017
rs17535596
rs9403911
rs34022830
rs76580439
rs56342870
rs114017586
rs67106669
All
−0.1
0.0
0.1
MR leave−one−out sensitivity analysis for
'Enterobacteriaceae' on 'GCST90200383_buildGRCh38.tsv'

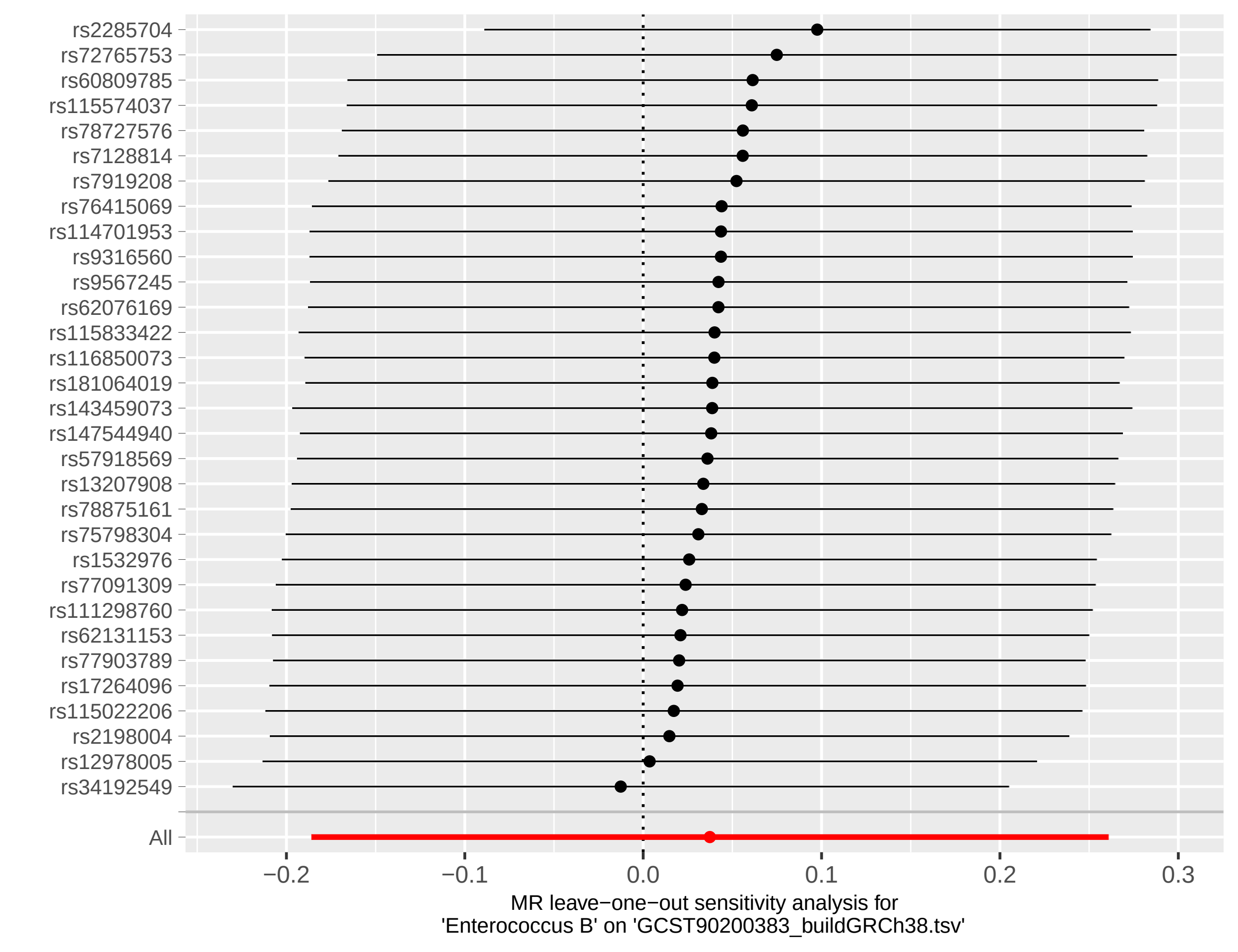
rs2285704
rs72765753
rs60809785
rs115574037
rs78727576
rs7128814
rs7919208
rs76415069
rs114701953
rs9316560
rs9567245
rs62076169
rs115833422
rs116850073
rs181064019
rs143459073
rs147544940
rs57918569
rs13207908
rs78875161
rs75798304
rs1532976
rs77091309
rs111298760
rs62131153
rs77903789
rs17264096
rs115022206
rs2198004
rs12978005
rs34192549
All
−0.2
−0.1
0.0
0.1
0.2
0.3
MR leave−one−out sensitivity analysis for
'Enterococcus B' on 'GCST90200383_buildGRCh38.tsv'

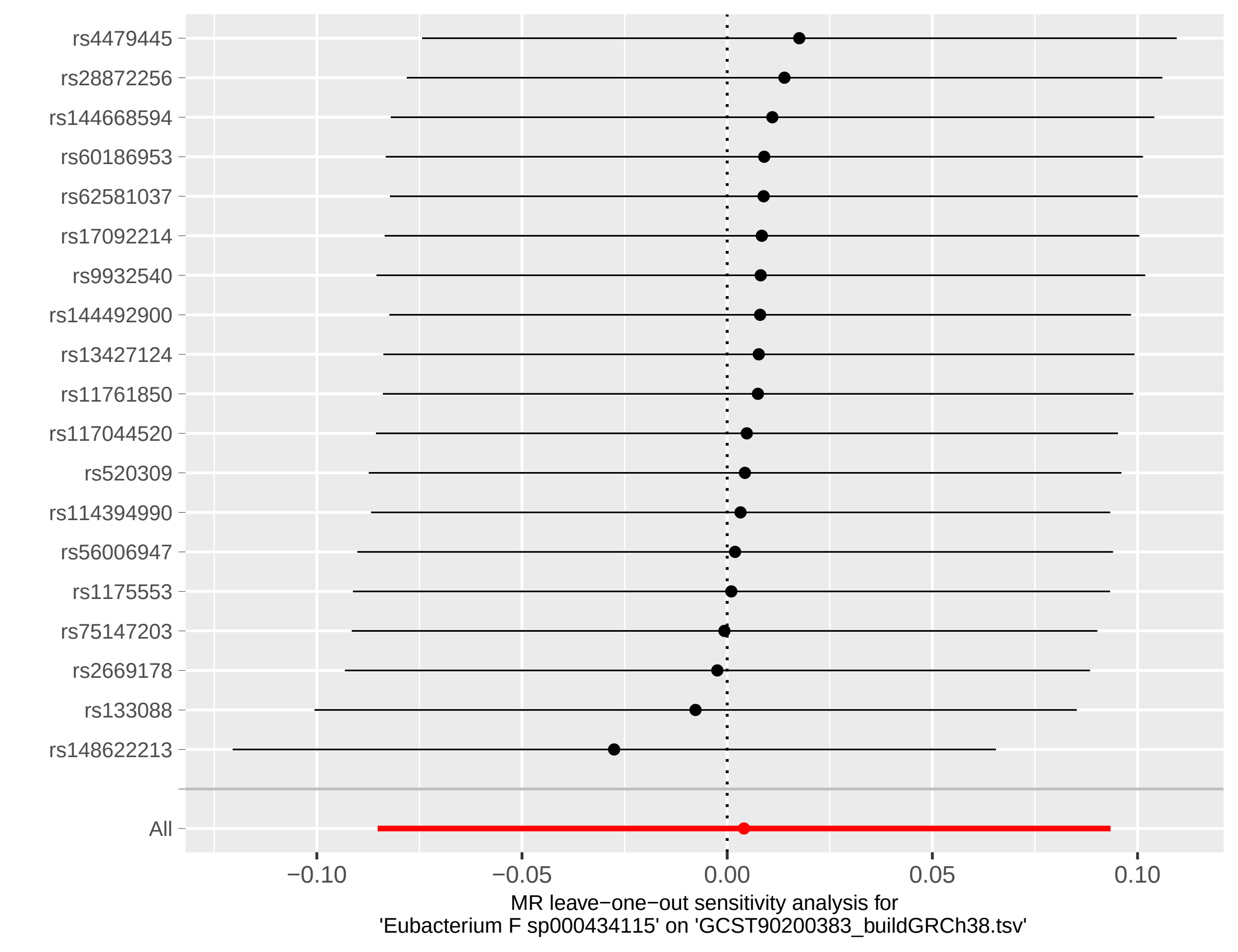

rs4479445
rs28872256
rs144668594
rs60186953
rs62581037
rs17092214
rs9932540
rs144492900
rs13427124
rs11761850
rs117044520
rs520309
rs114394990
rs56006947
rs1175553
rs75147203
rs2669178
rs133088
rs148622213
All
−0.10
−0.05
0.00
0.05
0.10
MR leave−one−out sensitivity analysis for
'Eubacterium F sp000434115' on 'GCST90200383_buildGRCh38.tsv'

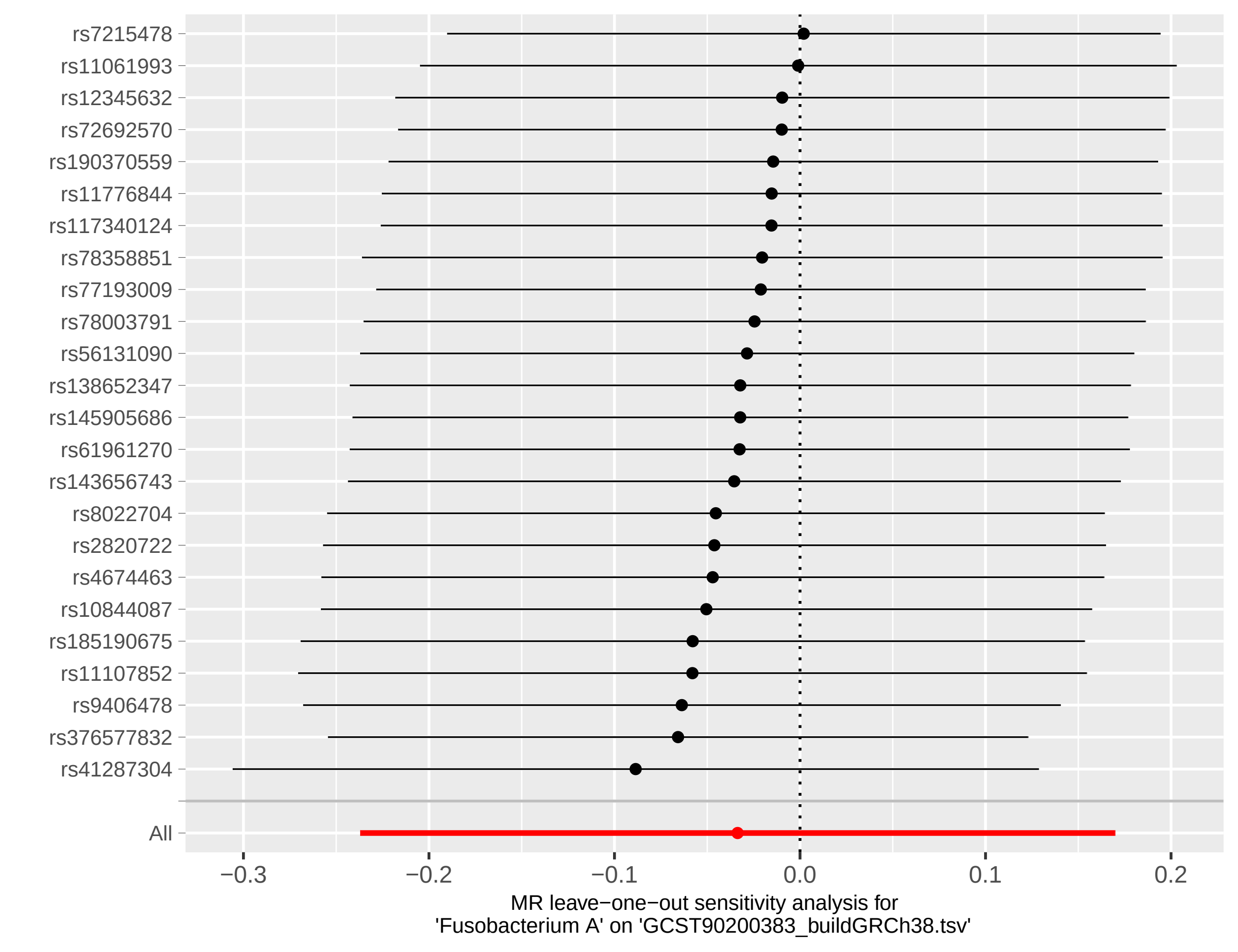

rs7215478
rs11061993
rs12345632
rs72692570
rs190370559
rs11776844
rs117340124
rs78358851
rs77193009
rs78003791
rs56131090
rs138652347
rs145905686
rs61961270
rs143656743
rs8022704
rs2820722
rs4674463
rs10844087
rs185190675
rs11107852
rs9406478
rs376577832
rs41287304
All
−0.3
−0.2
−0.1
0.0
0.1
0.2
MR leave−one−out sensitivity analysis for
'Fusobacterium A' on 'GCST90200383_buildGRCh38.tsv'

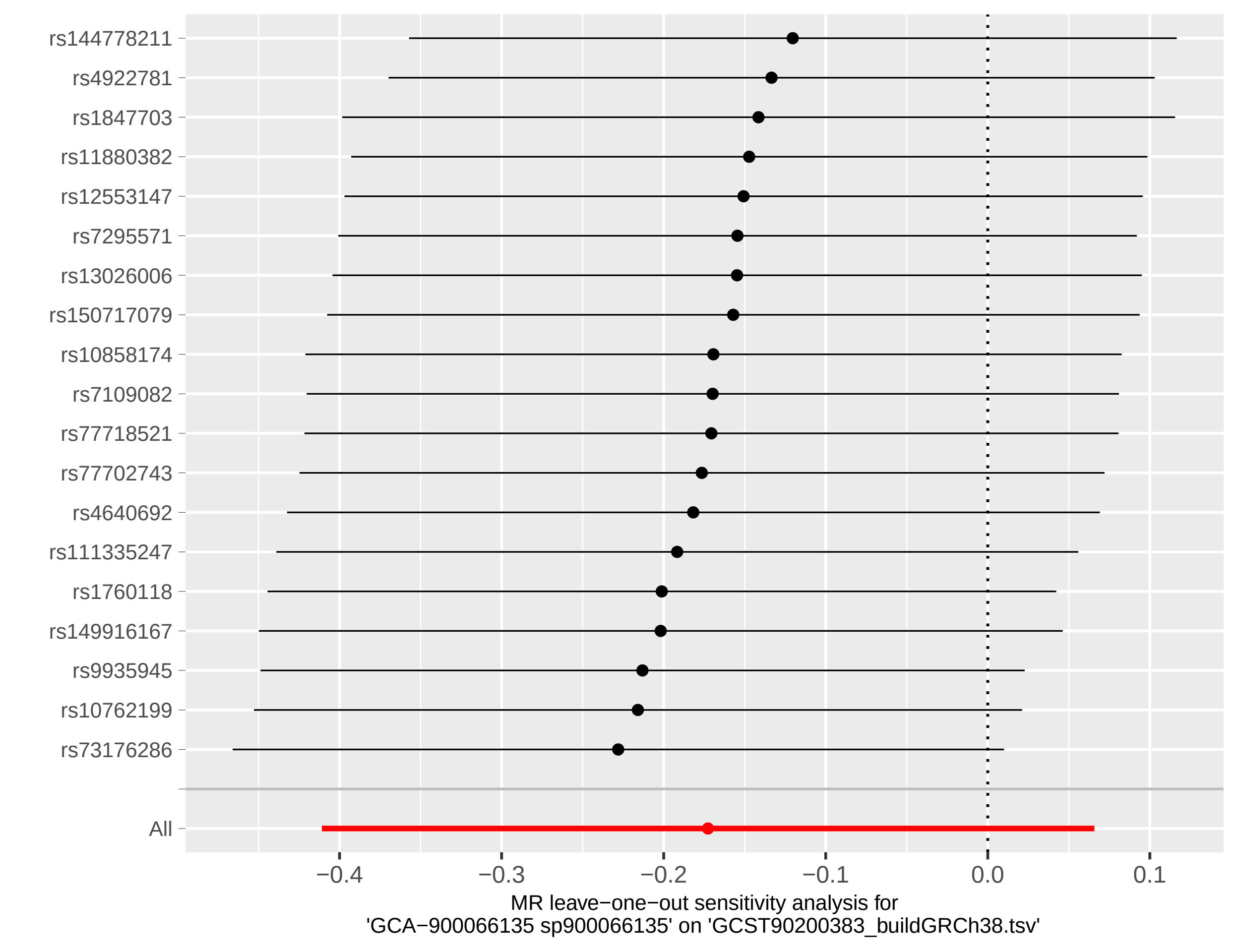
rs144778211
rs4922781
rs1847703
rs11880382
rs12553147
rs7295571
rs13026006
rs150717079
rs10858174
rs7109082
rs77718521
rs77702743
rs4640692
rs111335247
rs1760118
rs149916167
rs9935945
rs10762199
rs73176286
All
−0.4
−0.3
−0.2
−0.1
0.0
0.1
MR leave−one−out sensitivity analysis for
'GCA−900066135 sp900066135' on 'GCST90200383_buildGRCh38.tsv'

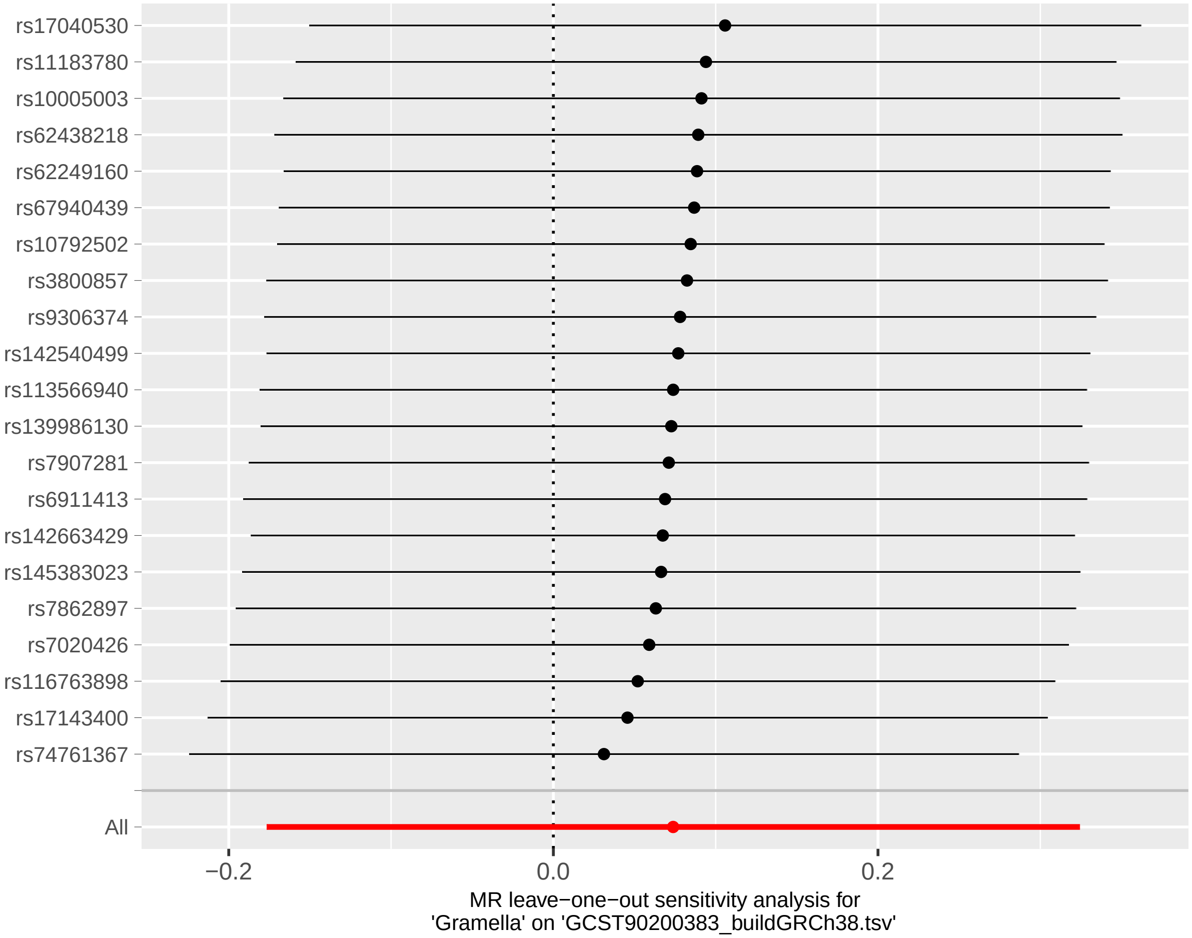

rs17040530
rs11183780
rs10005003
rs62438218
rs62249160
rs67940439
rs10792502
rs3800857
rs9306374
rs142540499
rs113566940
rs139986130
rs7907281
rs6911413
rs142663429
rs145383023
rs7862897
rs7020426
rs116763898
rs17143400
rs74761367
All
−0.2
0.0
0.2
MR leave−one−out sensitivity analysis for
'Gramella' on 'GCST90200383_buildGRCh38.tsv'

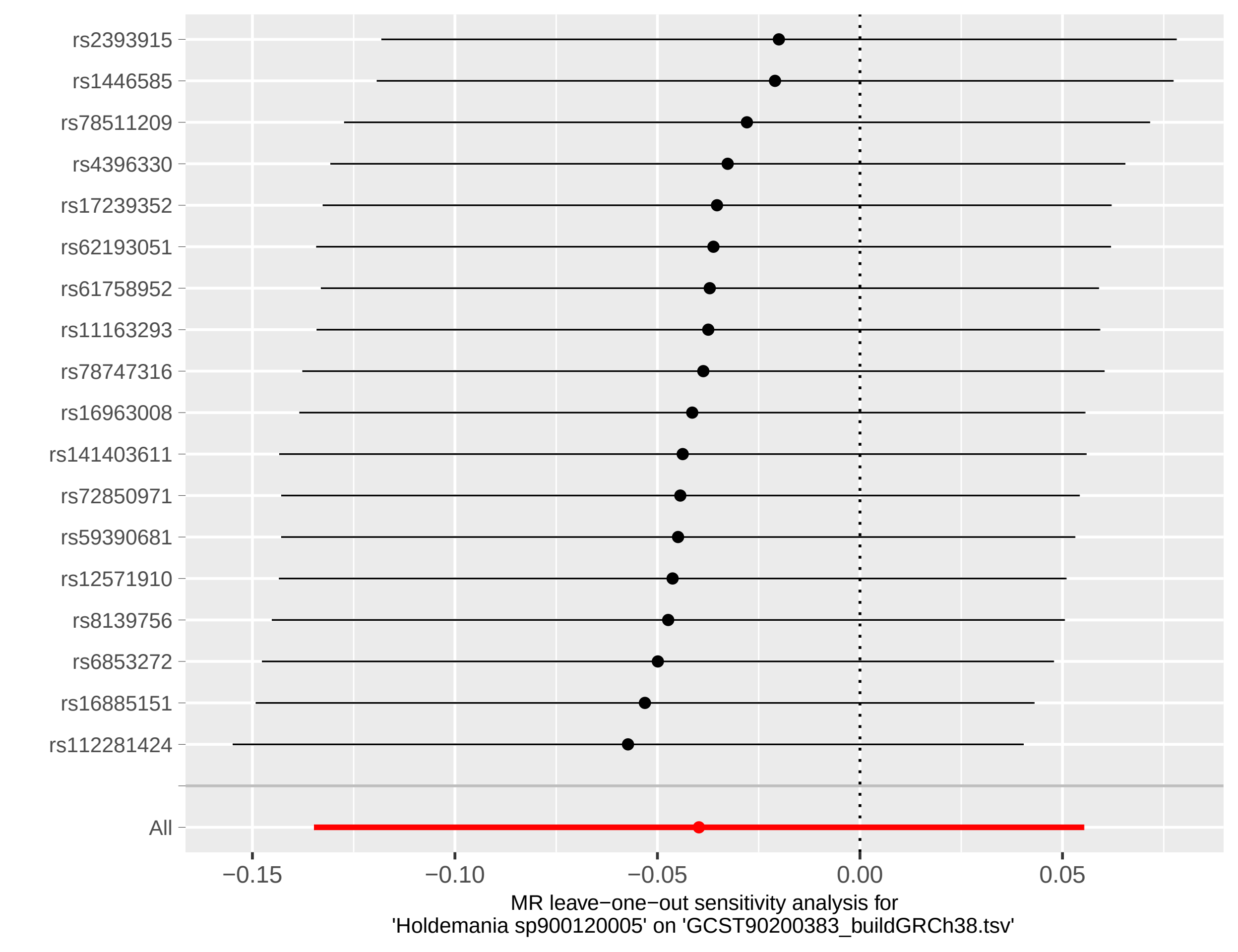

rs2393915
rs1446585
rs78511209
rs4396330
rs17239352
rs62193051
rs61758952
rs11163293
rs78747316
rs16963008
rs141403611
rs72850971
rs59390681
rs12571910
rs8139756
rs6853272
rs16885151
rs112281424
All
−0.15
−0.10
−0.05
0.00
0.05
MR leave−one−out sensitivity analysis for
'Holdemania sp900120005' on 'GCST90200383_buildGRCh38.tsv'

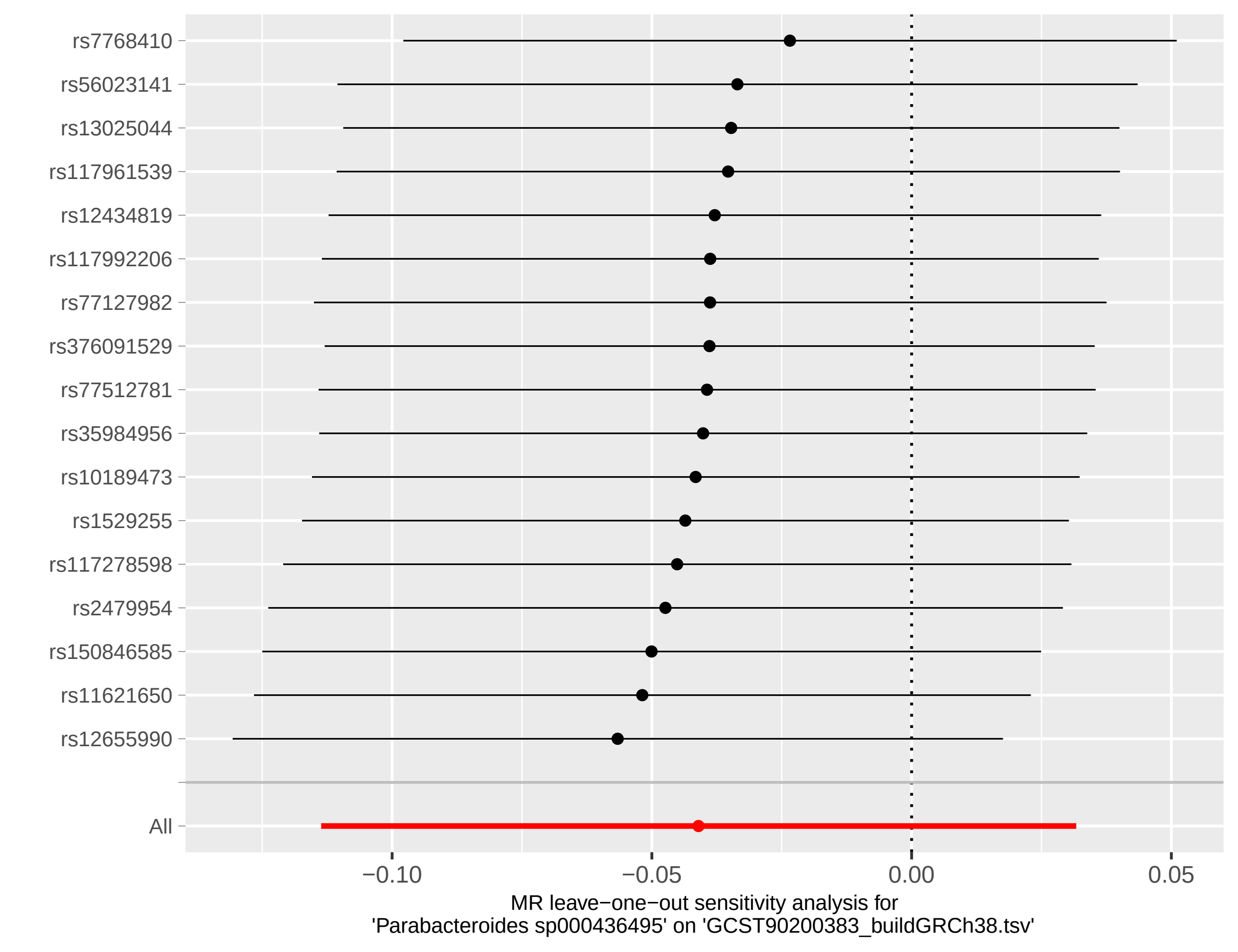

rs7768410
rs56023141
rs13025044
rs117961539
rs12434819
rs117992206
rs77127982
rs376091529
rs77512781
rs35984956
rs10189473
rs1529255
rs117278598
rs2479954
rs150846585
rs11621650
rs12655990
All
−0.10
−0.05
0.00
0.05
MR leave−one−out sensitivity analysis for
'Parabacteroides sp000436495' on 'GCST90200383_buildGRCh38.tsv'

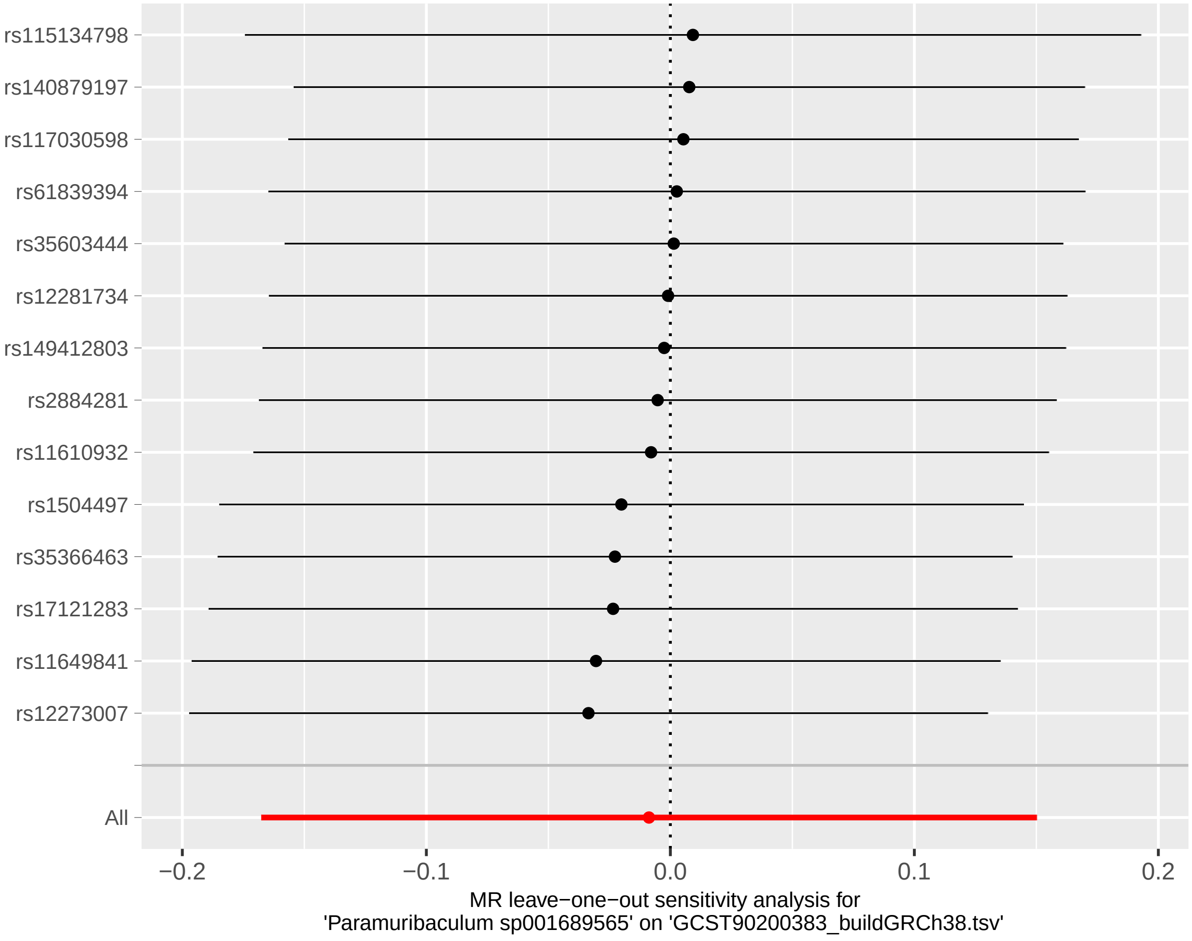

rs115134798
rs140879197
rs117030598
rs61839394
rs35603444
rs12281734
rs149412803
rs2884281
rs11610932
rs1504497
rs35366463
rs17121283
rs11649841
rs12273007
All
−0.2
−0.1
0.0
0.1
0.2
MR leave−one−out sensitivity analysis for
'Paramuribaculum sp001689565' on 'GCST90200383_buildGRCh38.tsv'

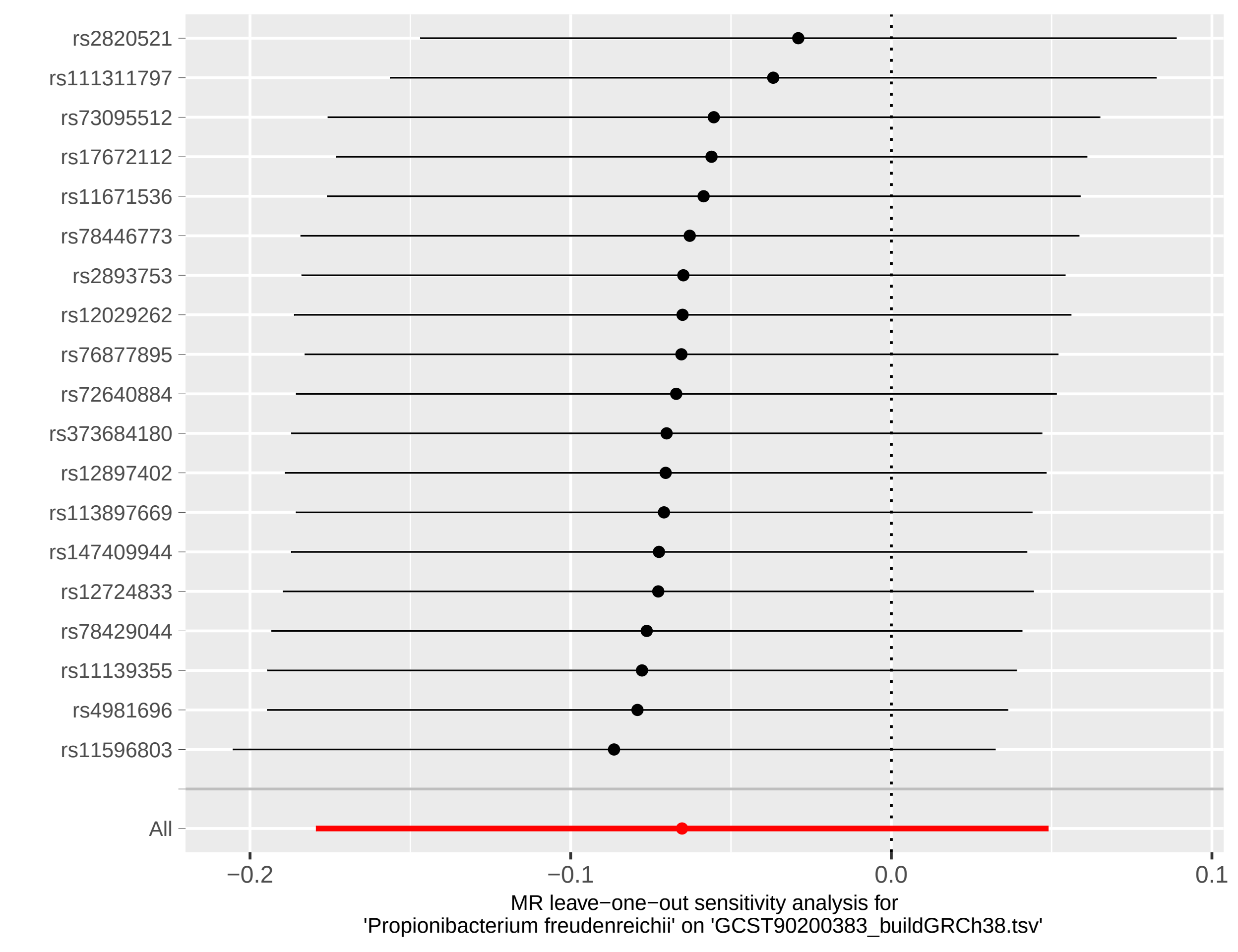
rs2820521
rs111311797
rs73095512
rs17672112
rs11671536
rs78446773
rs2893753
rs12029262
rs76877895
rs72640884
rs373684180
rs12897402
rs113897669
rs147409944
rs12724833
rs78429044
rs11139355
rs4981696
rs11596803
All
−0.2
−0.1
0.0
0.1
MR leave−one−out sensitivity analysis for
'Propionibacterium freudenreichii' on 'GCST90200383_buildGRCh38.tsv'

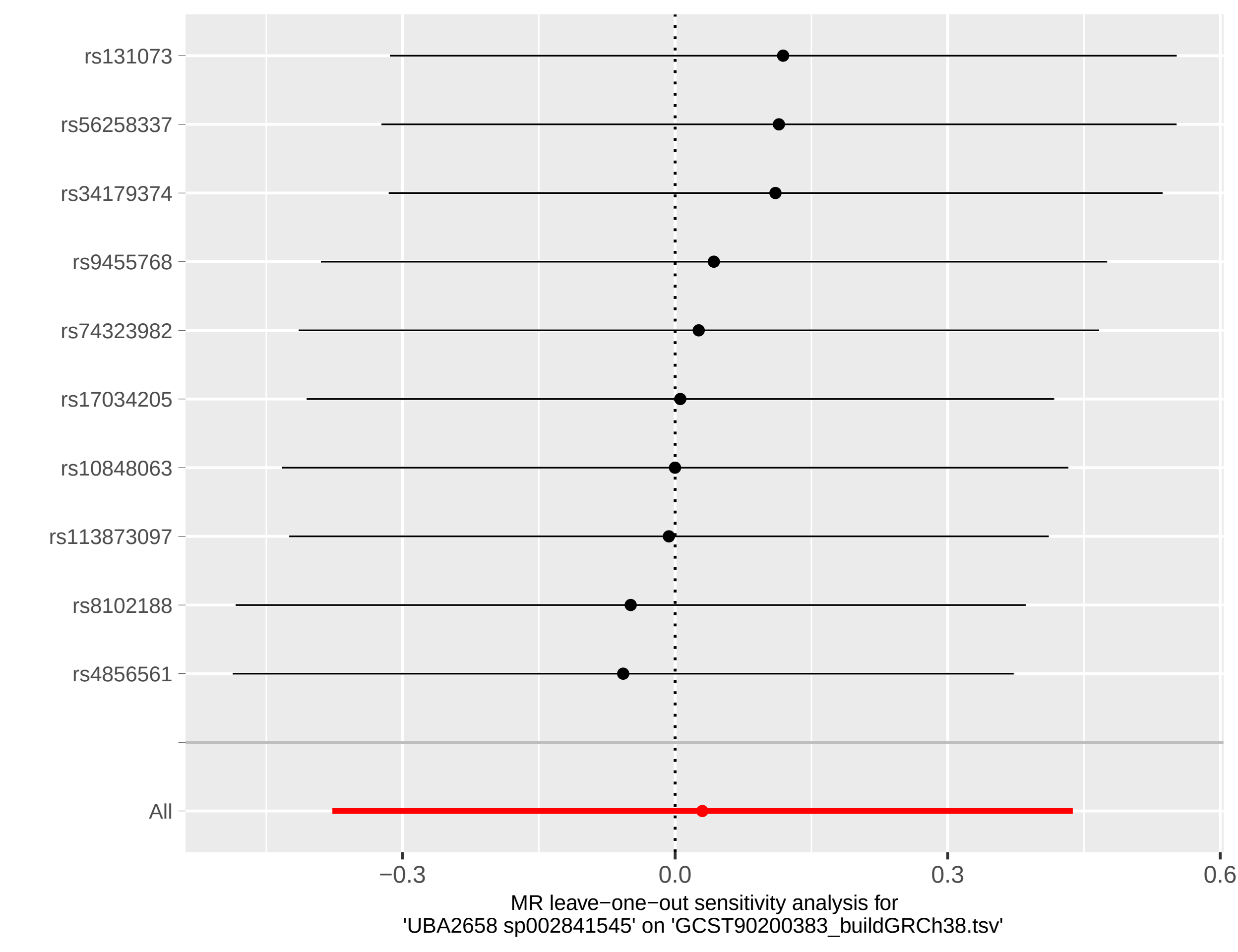

rs131073
rs56258337
rs34179374
rs9455768
rs74323982
rs17034205
rs10848063
rs113873097
rs8102188
rs4856561
All
−0.3
0.0
0.3
0.6
MR leave−one−out sensitivity analysis for
'UBA2658 sp002841545' on 'GCST90200383_buildGRCh38.tsv'

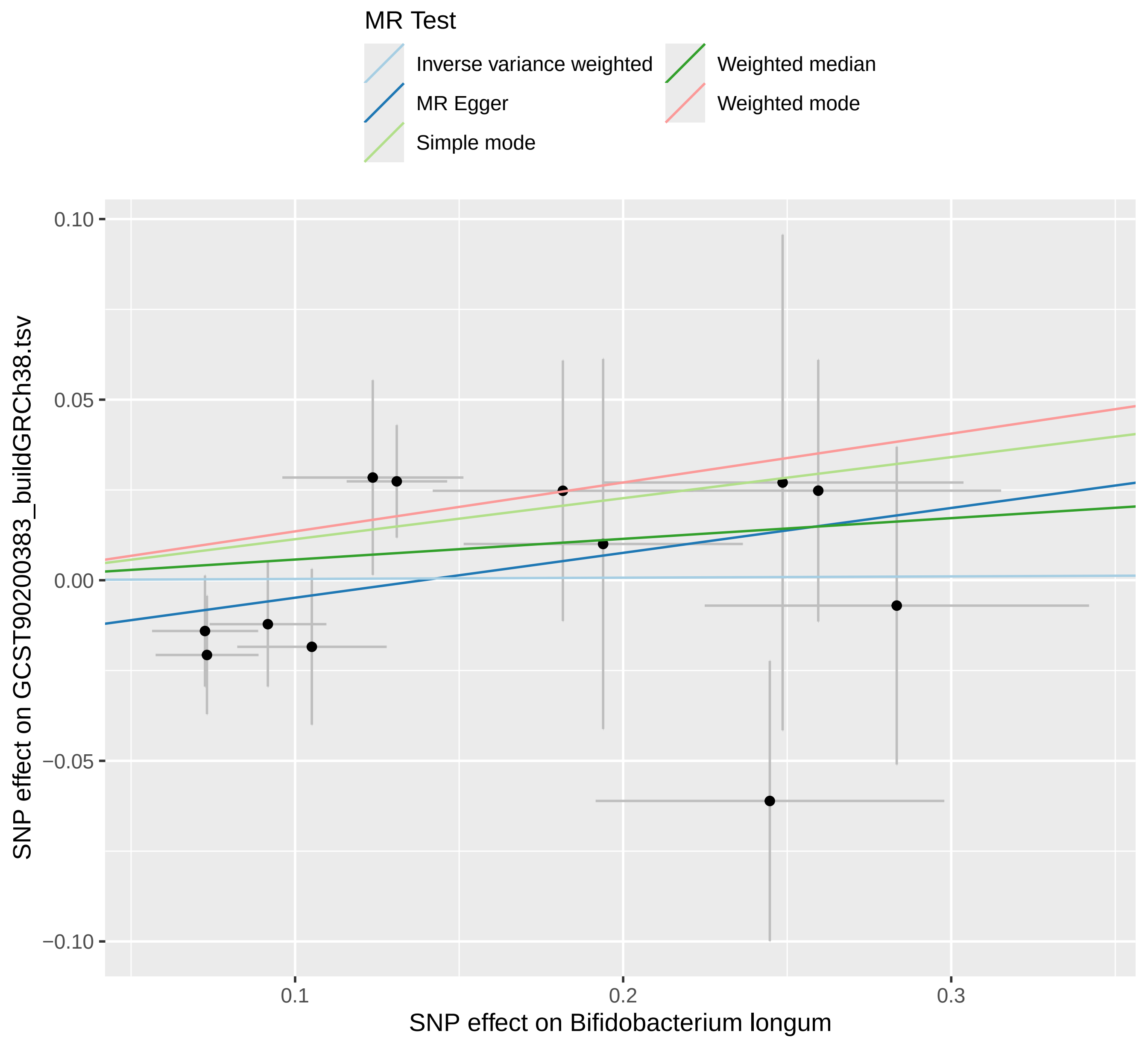

MR Test
Inverse variance weighted
MR Egger
Simple mode
Weighted median
Weighted mode
0.10
0.05
0.00
−0.05
−0.10
0.1
0.2
0.3
SNP effect on GCST90200383_buildGRCh38.tsv
SNP effect on Bifidobacterium longum

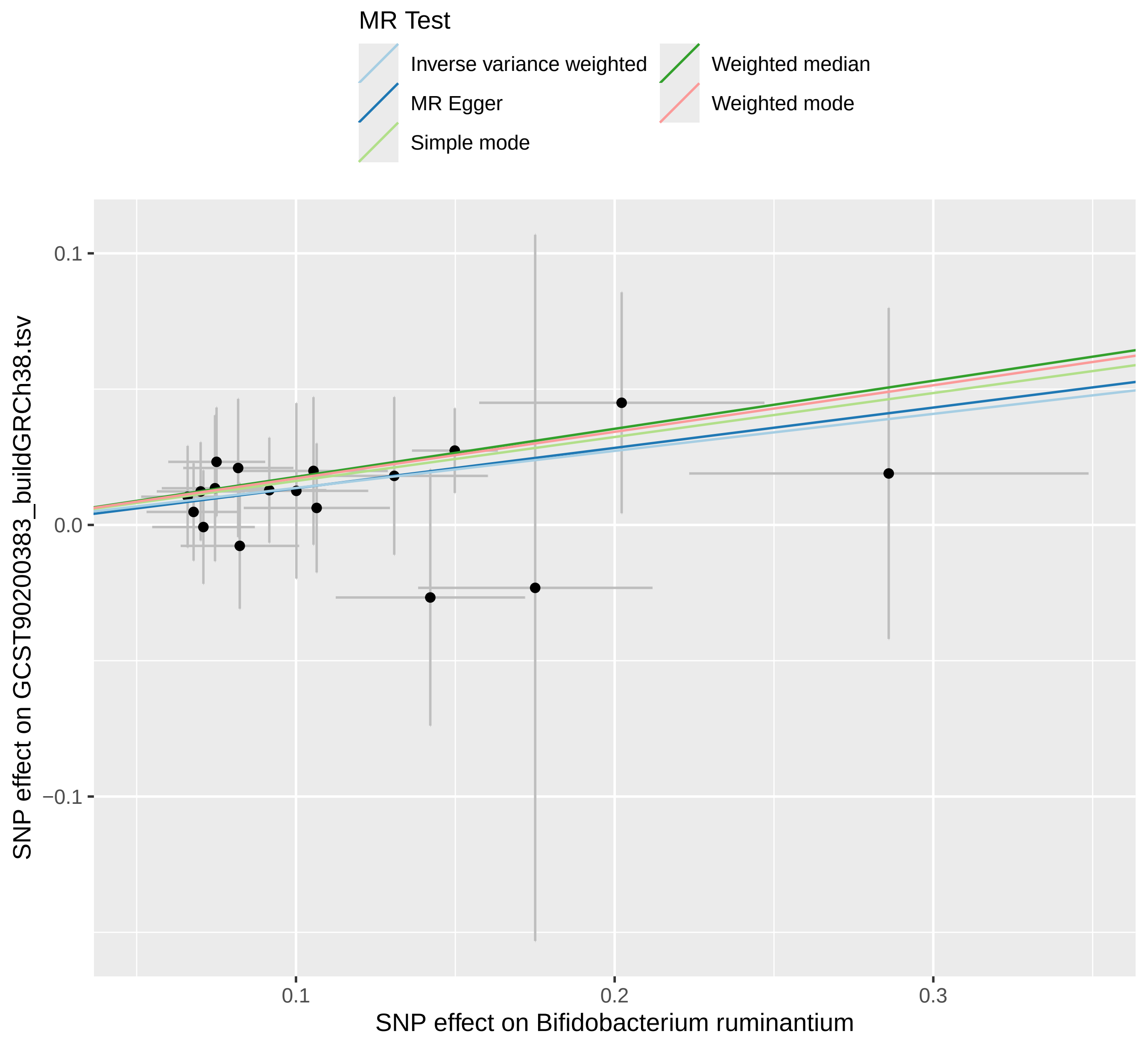

MR Test
Inverse variance weighted
MR Egger
Simple mode
Weighted median
Weighted mode
0.1
0.0
−0.1
0.1
0.2
0.3
SNP effect on GCST90200383_buildGRCh38.tsv
SNP effect on Bifidobacterium ruminantium

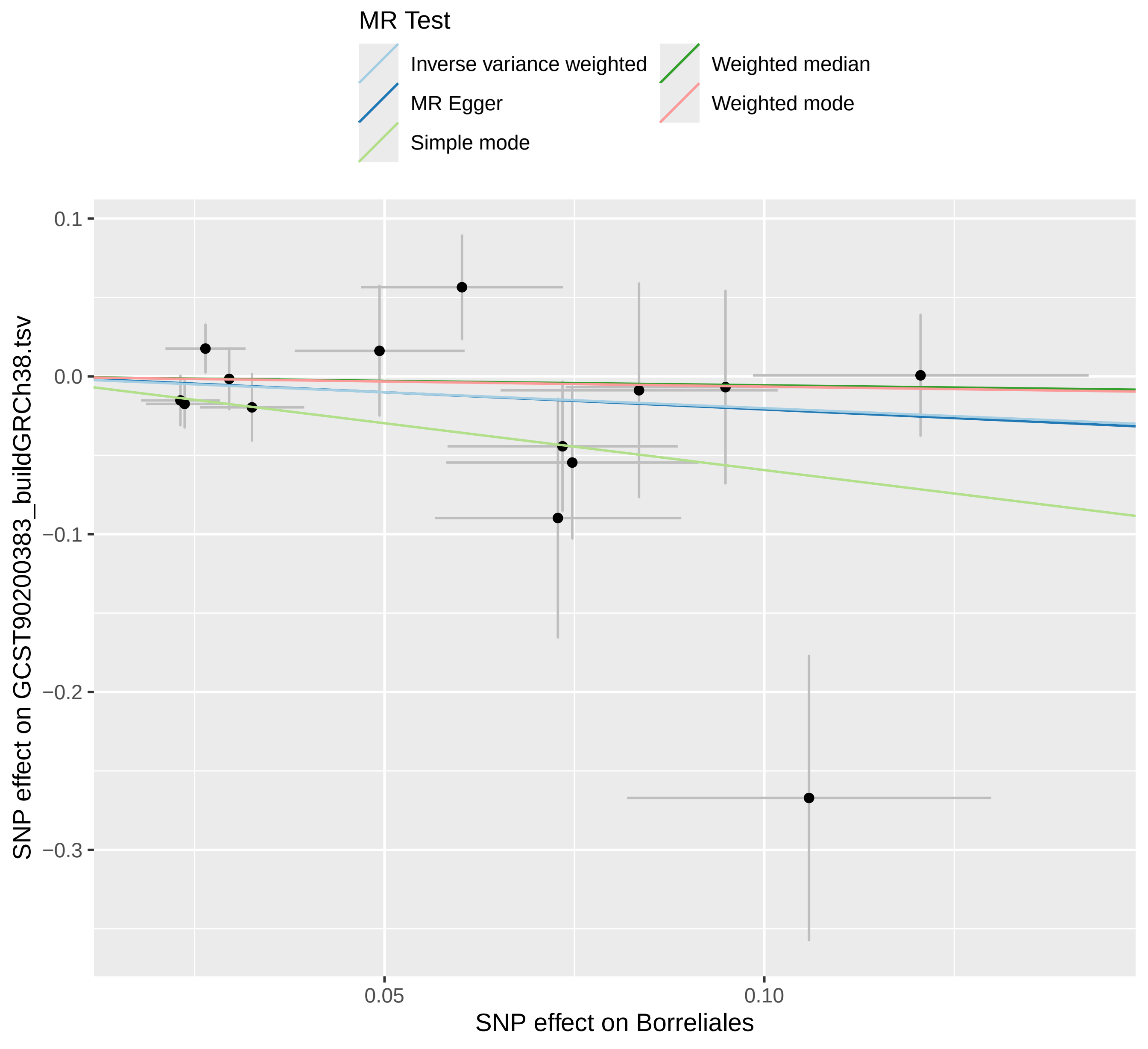

MR Test
Inverse variance weighted
MR Egger
Simple mode
Weighted median
Weighted mode
0.1
0.0
−0.1
−0.2
−0.3
SNP effect on GCST90200383_buildGRCh38.tsv
0.05
0.10
SNP effect on Borreliales

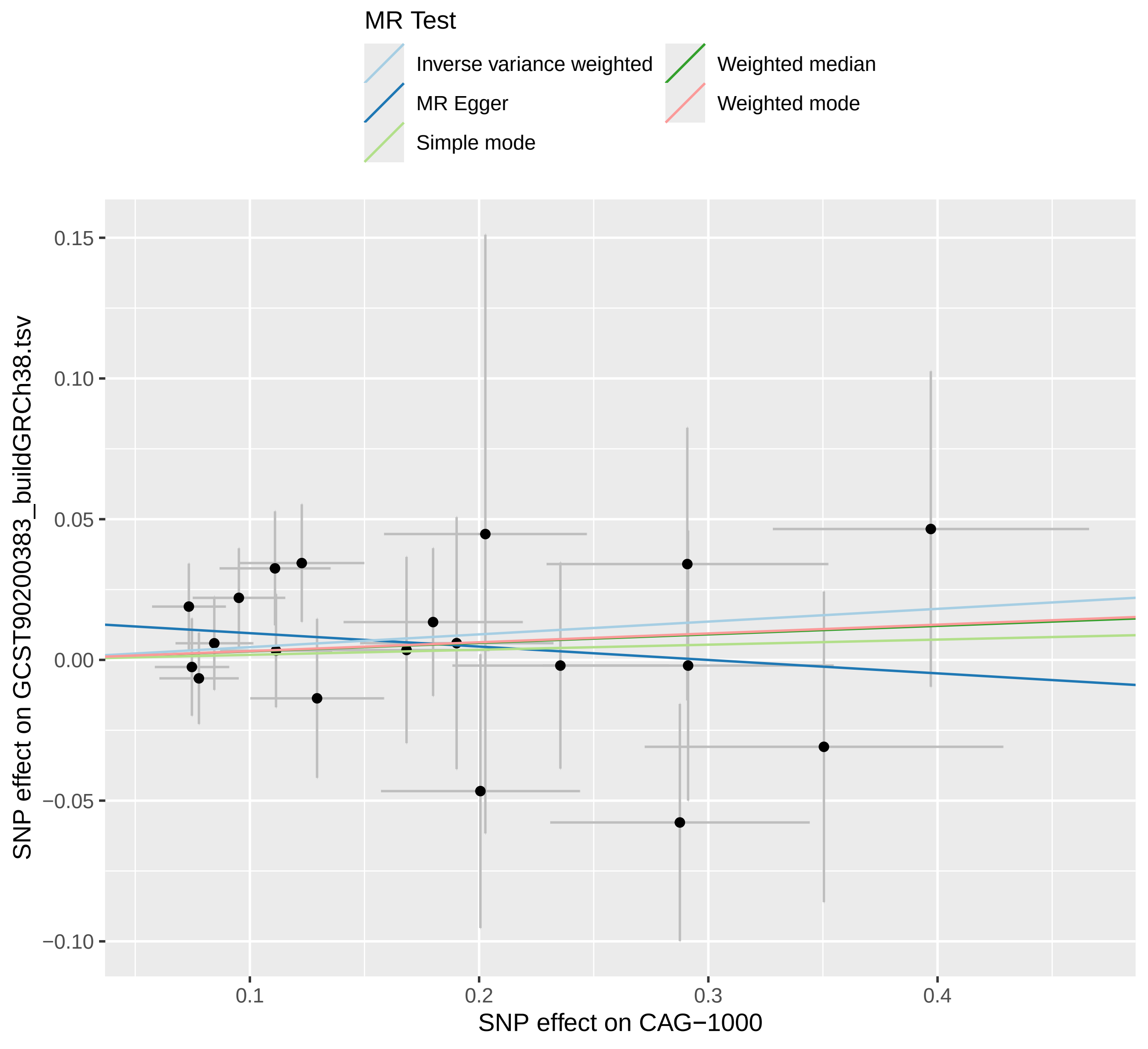
MR Test
Inverse variance weighted
MR Egger
Simple mode
Weighted median
Weighted mode
SNP effect on GCST90200383_buildGRCh38.tsv
0.15
0.10
0.05
0.00
−0.05
−0.10
0.1
0.2
0.3
0.4
SNP effect on CAG−1000

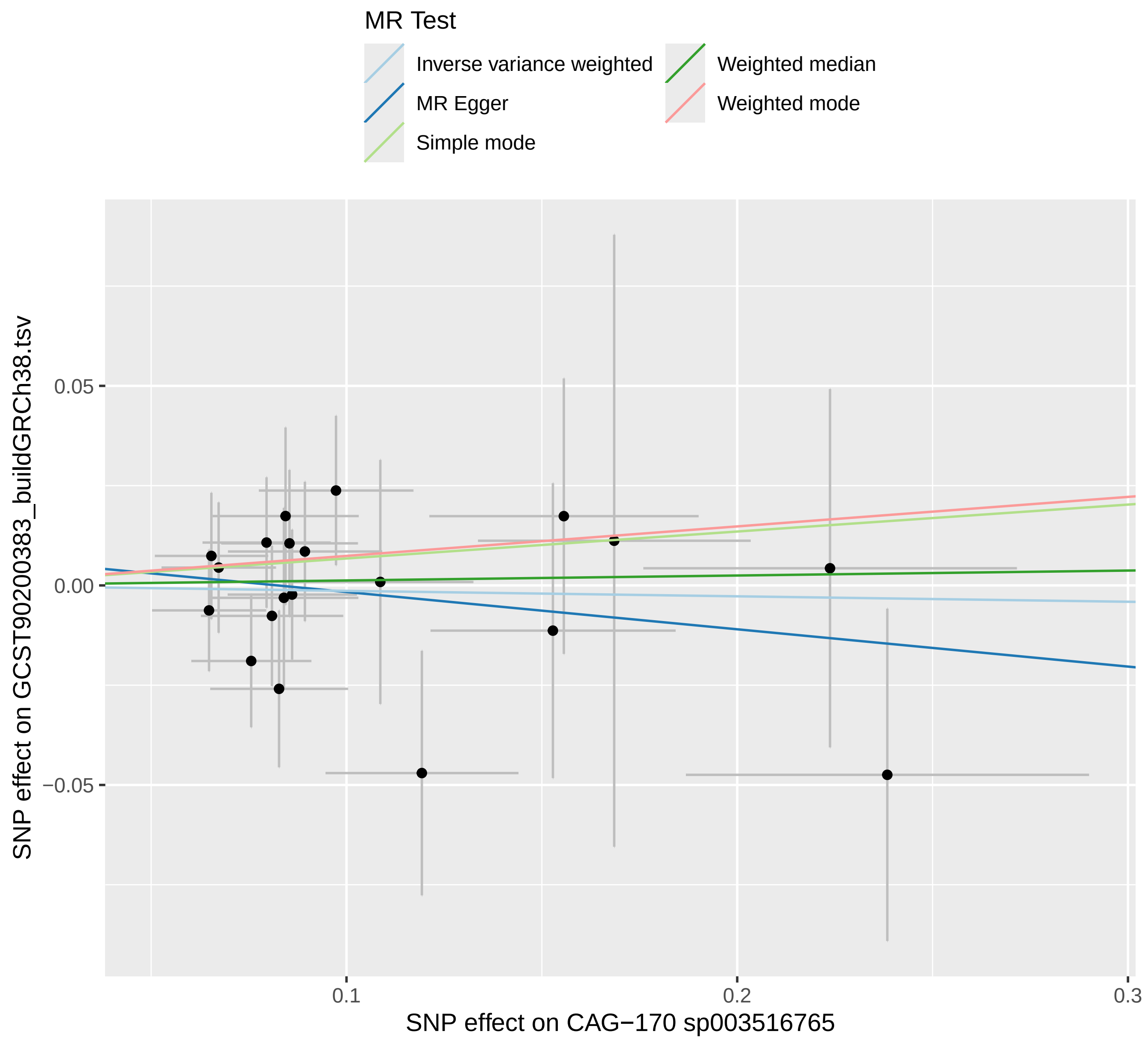
MR Test
Inverse variance weighted
MR Egger
Simple mode
Weighted median
Weighted mode
0.05
0.00
−0.05
0.1
0.2
0.3
SNP effect on GCST90200383_buildGRCh38.tsv
SNP effect on CAG−170 sp003516765

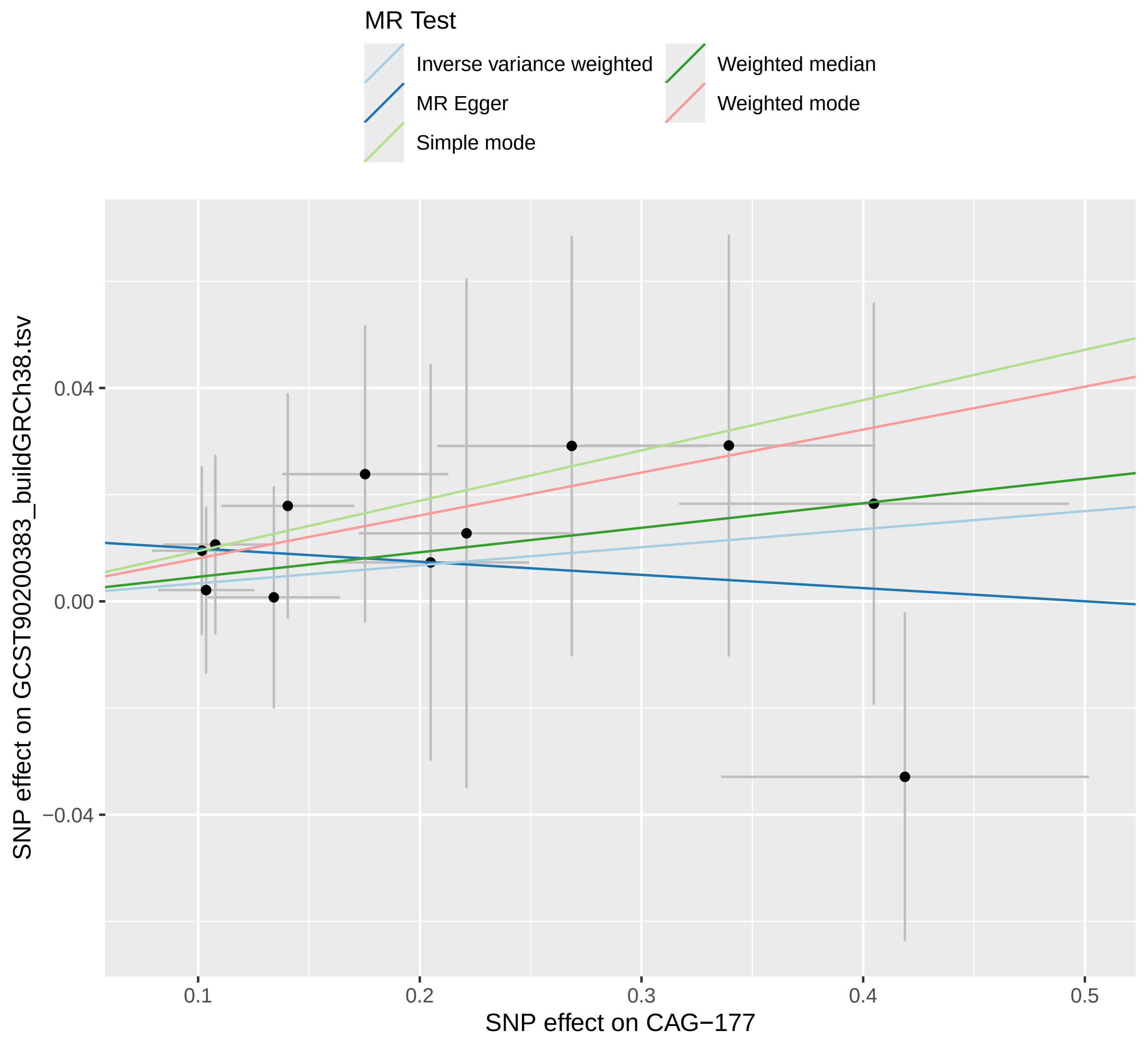

MR Test
Inverse variance weighted
MR Egger
Simple mode
Weighted median
Weighted mode
0.04
0.00
−0.04
0.1
0.2
0.3
0.4
0.5
SNP effect on GCST90200383_buildGRCh38.tsv
SNP effect on CAG−177

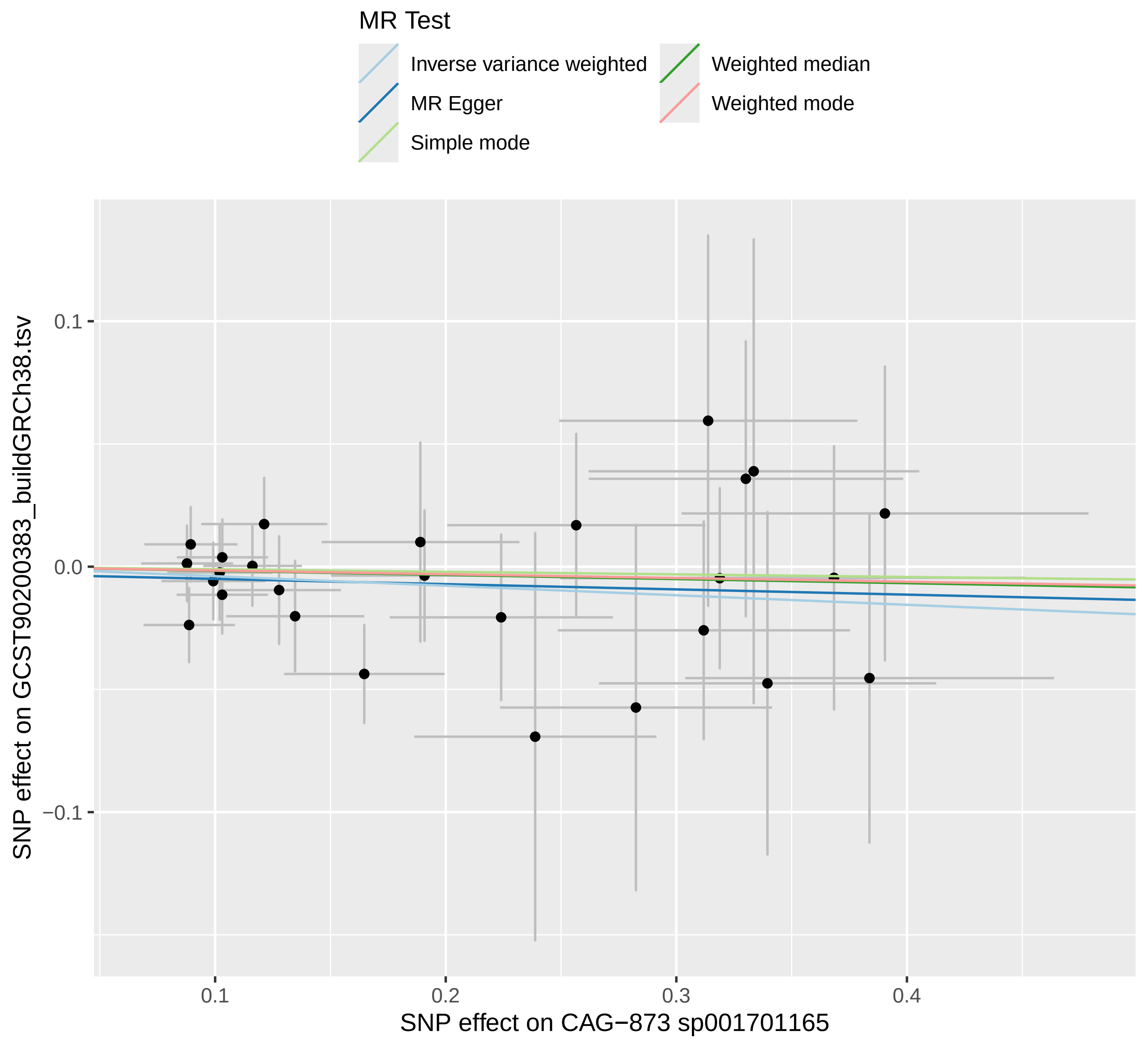
MR Test
Inverse variance weighted
MR Egger
Simple mode
Weighted median
Weighted mode
0.1
0.0
−0.1
0.1
0.2
0.3
0.4
SNP effect on GCST90200383_buildGRCh38.tsv
SNP effect on CAG−873 sp001701165

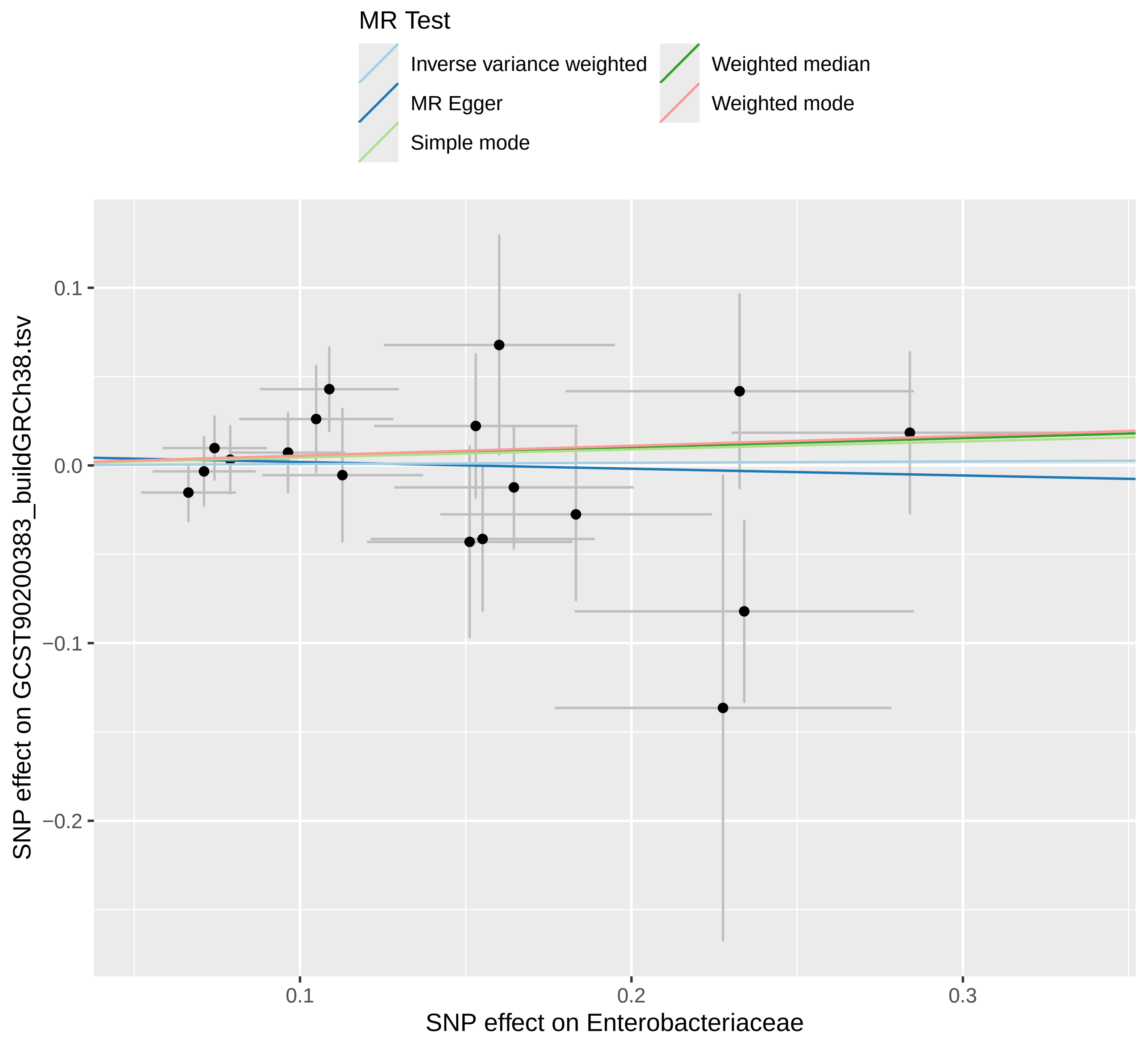
MR Test
Inverse variance weighted
MR Egger
Simple mode
Weighted median
Weighted mode
0.1
0.0
−0.1
−0.2
SNP effect on GCST90200383_buildGRCh38.tsv
0.1
0.2
0.3
SNP effect on Enterobacteriaceae

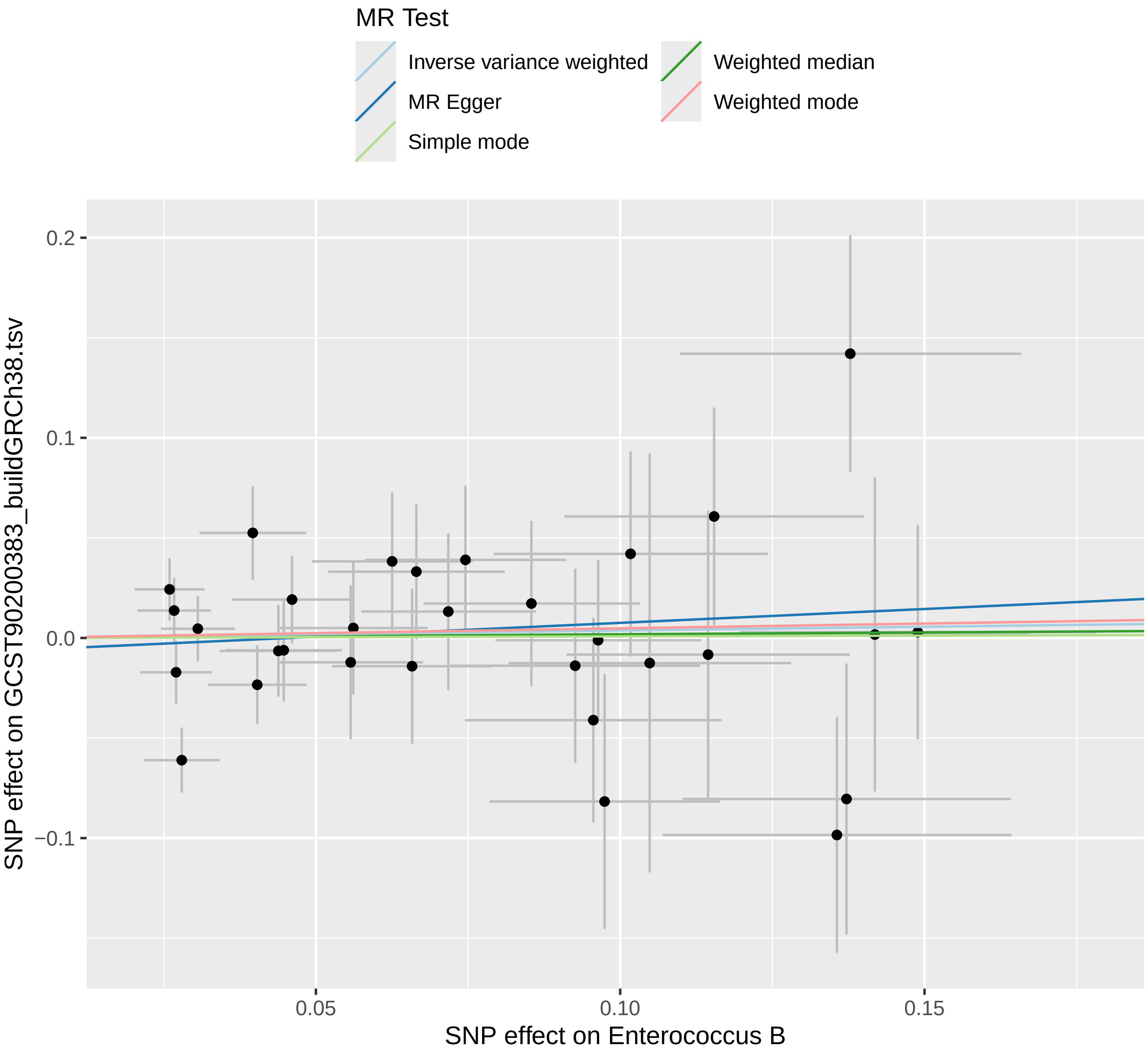
MR Test
Inverse variance weighted
MR Egger
Simple mode
Weighted median
Weighted mode
0.2
0.1
0.0
−0.1
0.05
0.10
0.15
SNP effect on GCST90200383_buildGRCh38.tsv
SNP effect on Enterococcus B

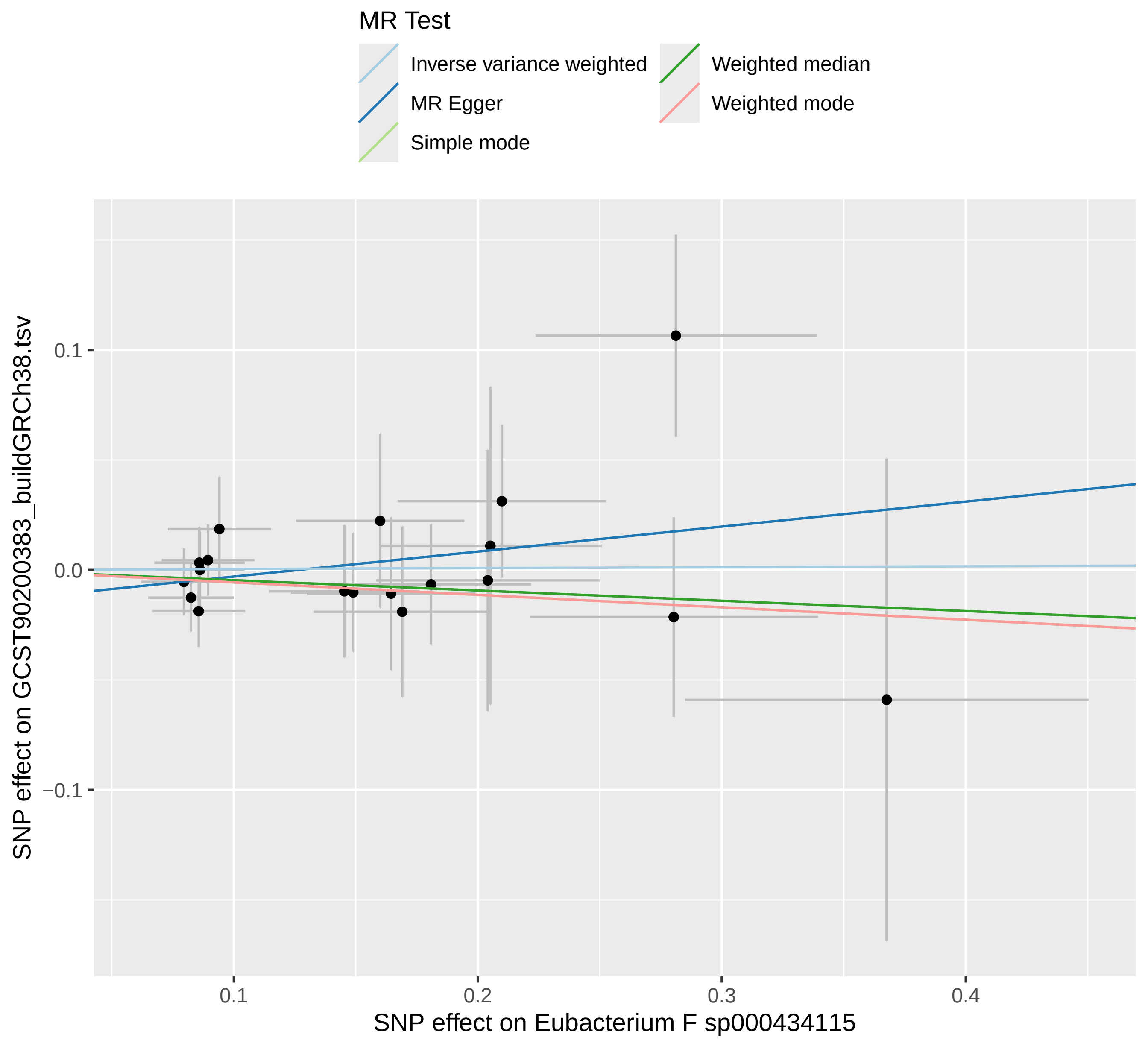

MR Test
Inverse variance weighted
MR Egger
Simple mode
Weighted median
Weighted mode
0.1
0.0
−0.1
0.1
0.2
0.3
0.4
SNP effect on GCST90200383_buildGRCh38.tsv
SNP effect on Eubacterium F sp000434115

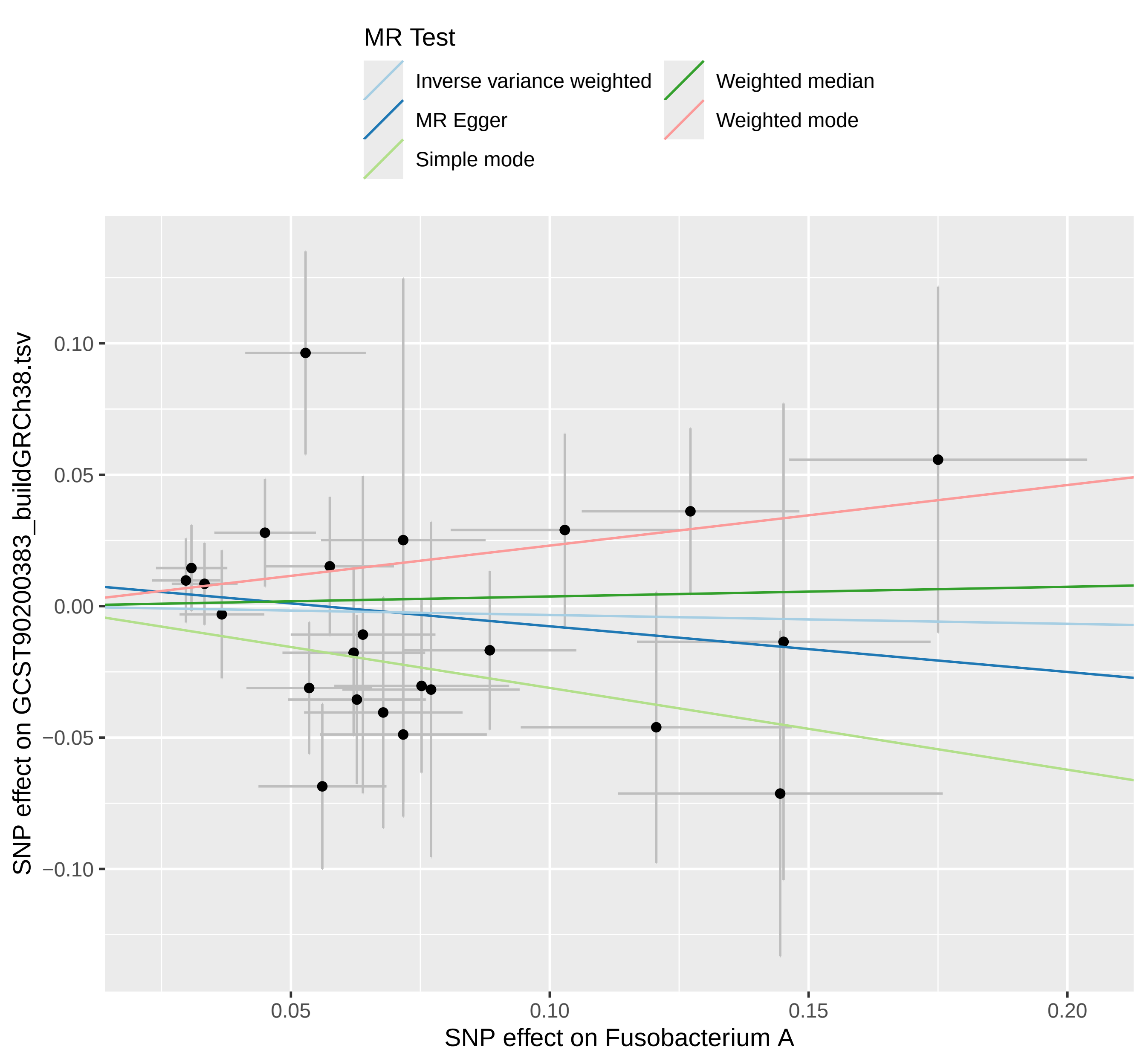

MR Test
Inverse variance weighted
MR Egger
Simple mode
Weighted median
Weighted mode
0.10
0.05
0.00
−0.05
−0.10
0.05
0.10
0.15
0.20
SNP effect on GCST90200383_buildGRCh38.tsv
SNP effect on Fusobacterium A

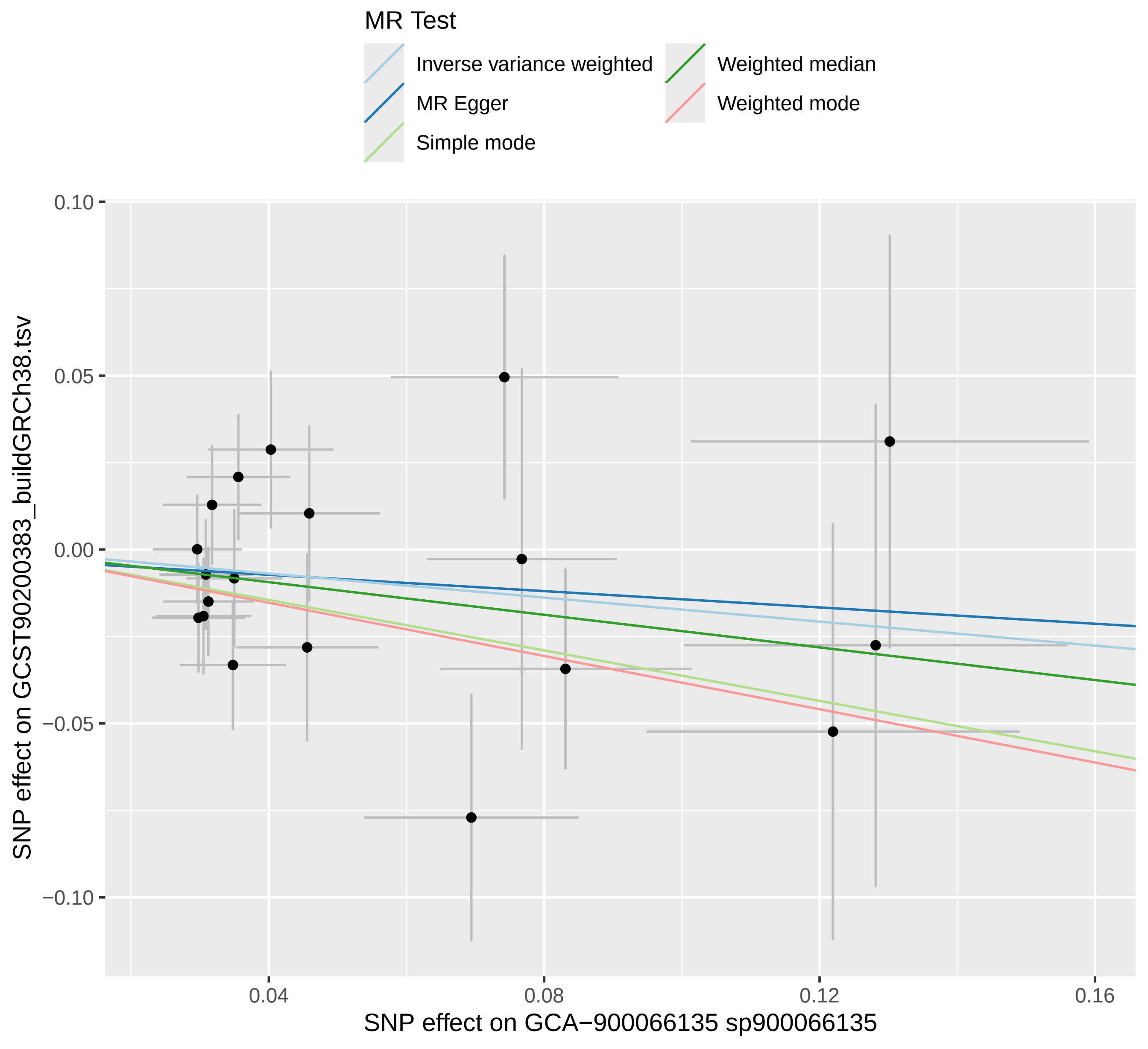

MR Test
Inverse variance weighted
MR Egger
Simple mode
Weighted median
Weighted mode
0.10
0.05
0.00
−0.05
−0.10
0.04
0.08
0.12
0.16
SNP effect on GCST90200383_buildGRCh38.tsv
SNP effect on GCA−900066135 sp900066135

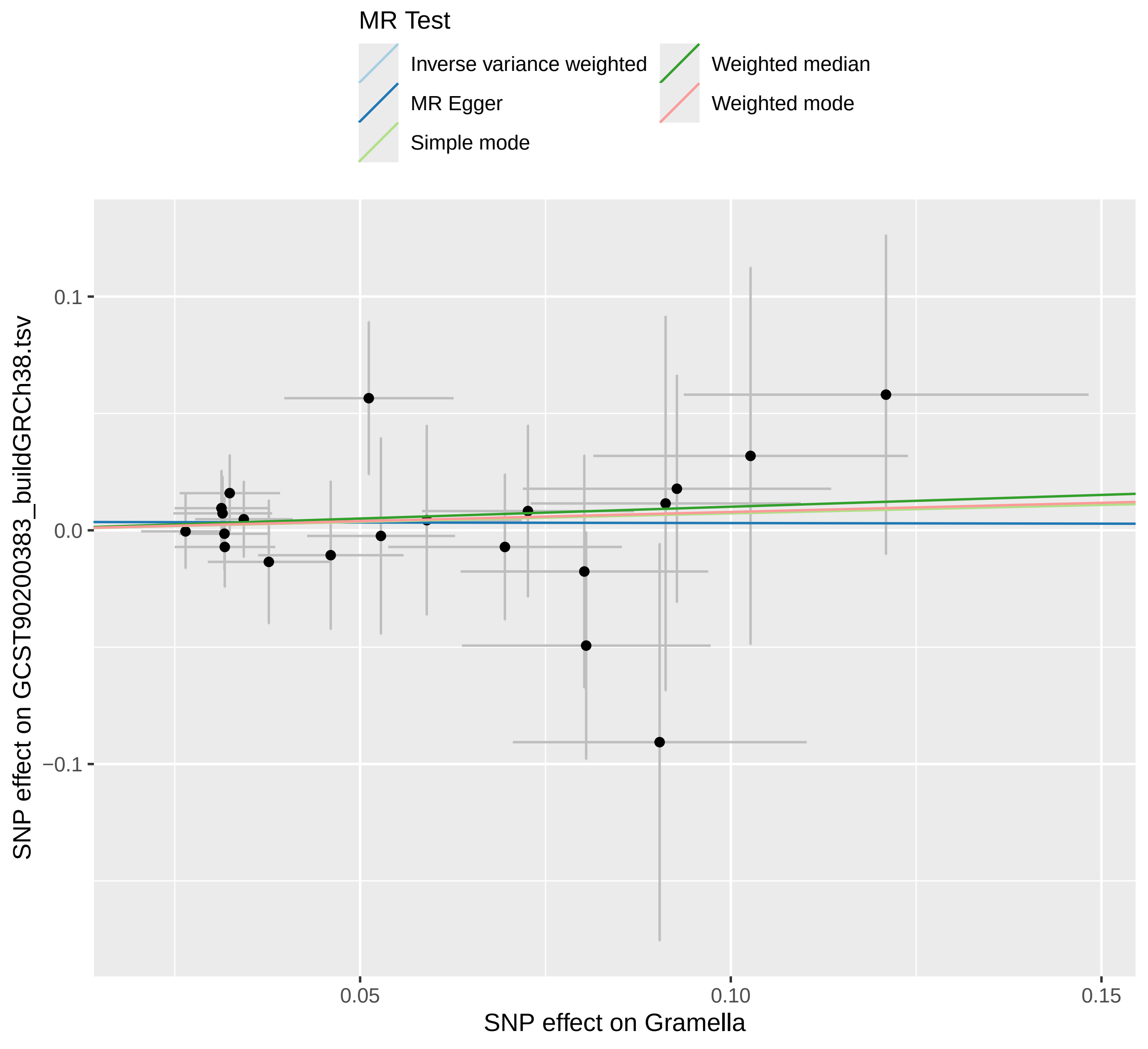

MR Test
Inverse variance weighted
MR Egger
Simple mode
Weighted median
Weighted mode
0.1
0.0
−0.1
0.05
0.10
0.15
SNP effect on GCST90200383_buildGRCh38.tsv
SNP effect on Gramella

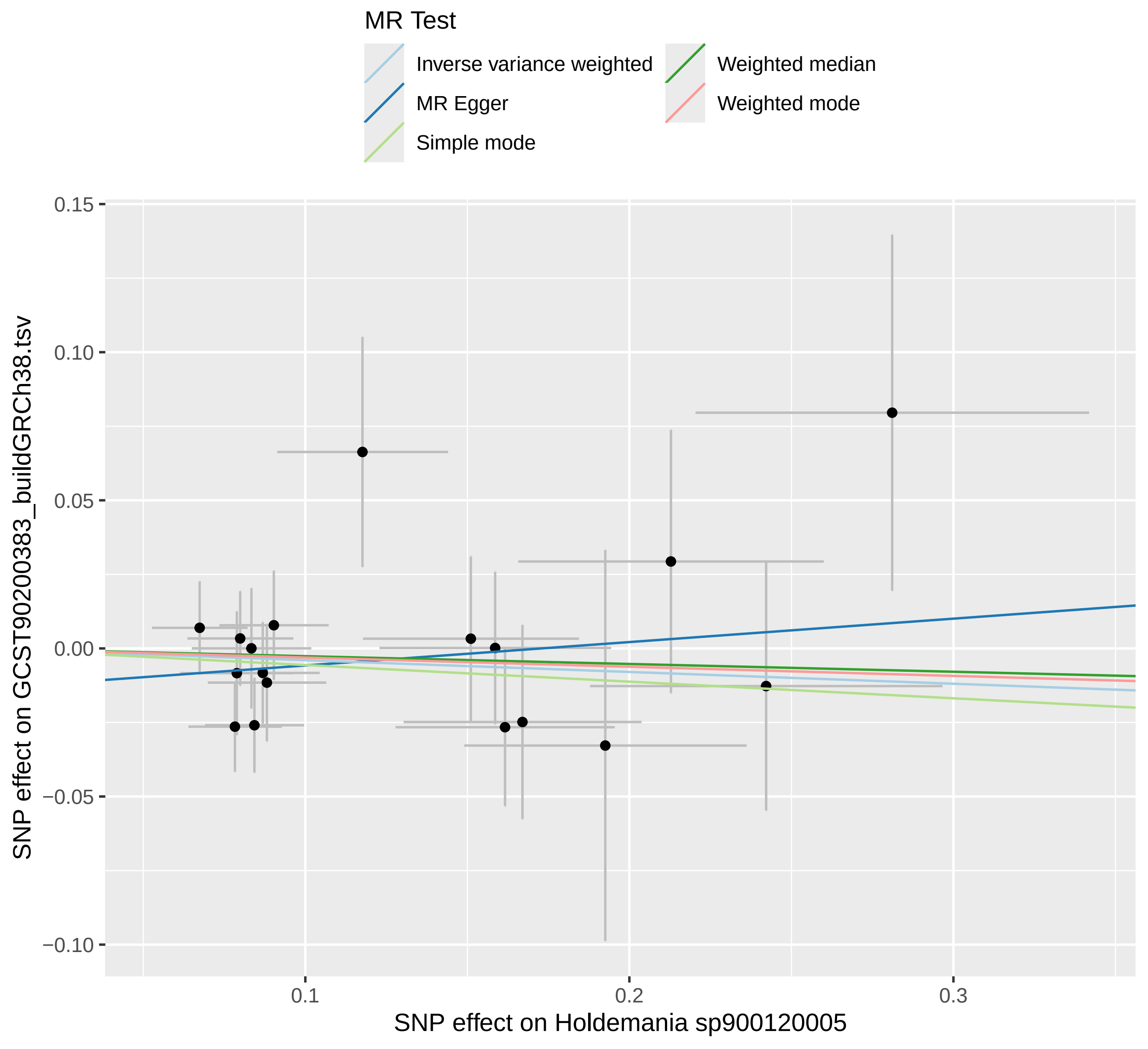
MR Test
Inverse variance weighted
MR Egger
Simple mode
Weighted median
Weighted mode
0.15
0.10
0.05
0.00
−0.05
−0.10
SNP effect on GCST90200383_buildGRCh38.tsv
0.1
0.2
0.3
SNP effect on Holdemania sp900120005

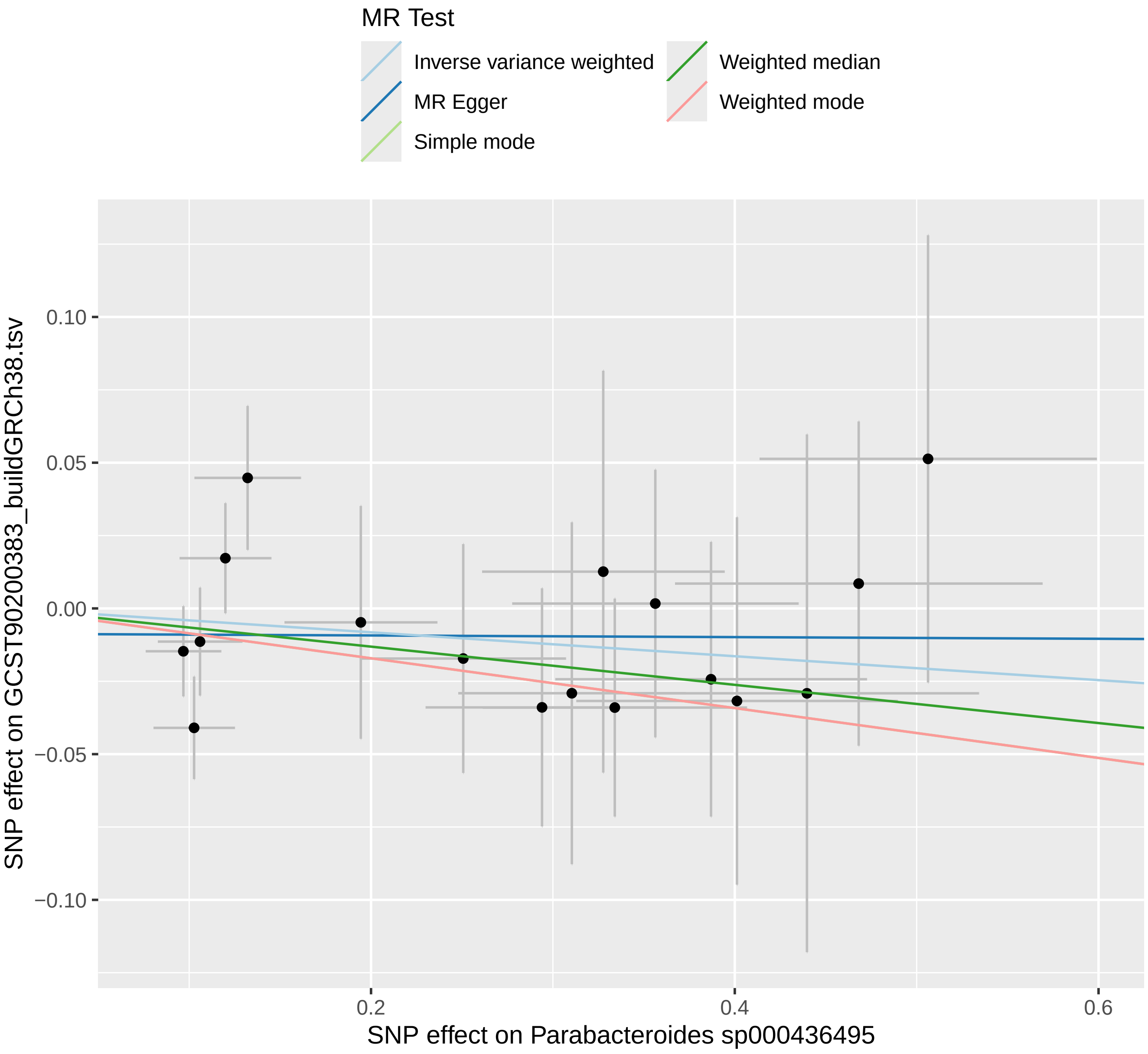
MR Test
Inverse variance weighted
MR Egger
Simple mode
Weighted median
Weighted mode
0.10
0.05
0.00
−0.05
−0.10
0.2
0.4
0.6
SNP effect on GCST90200383_buildGRCh38.tsv
SNP effect on Parabacteroides sp000436495

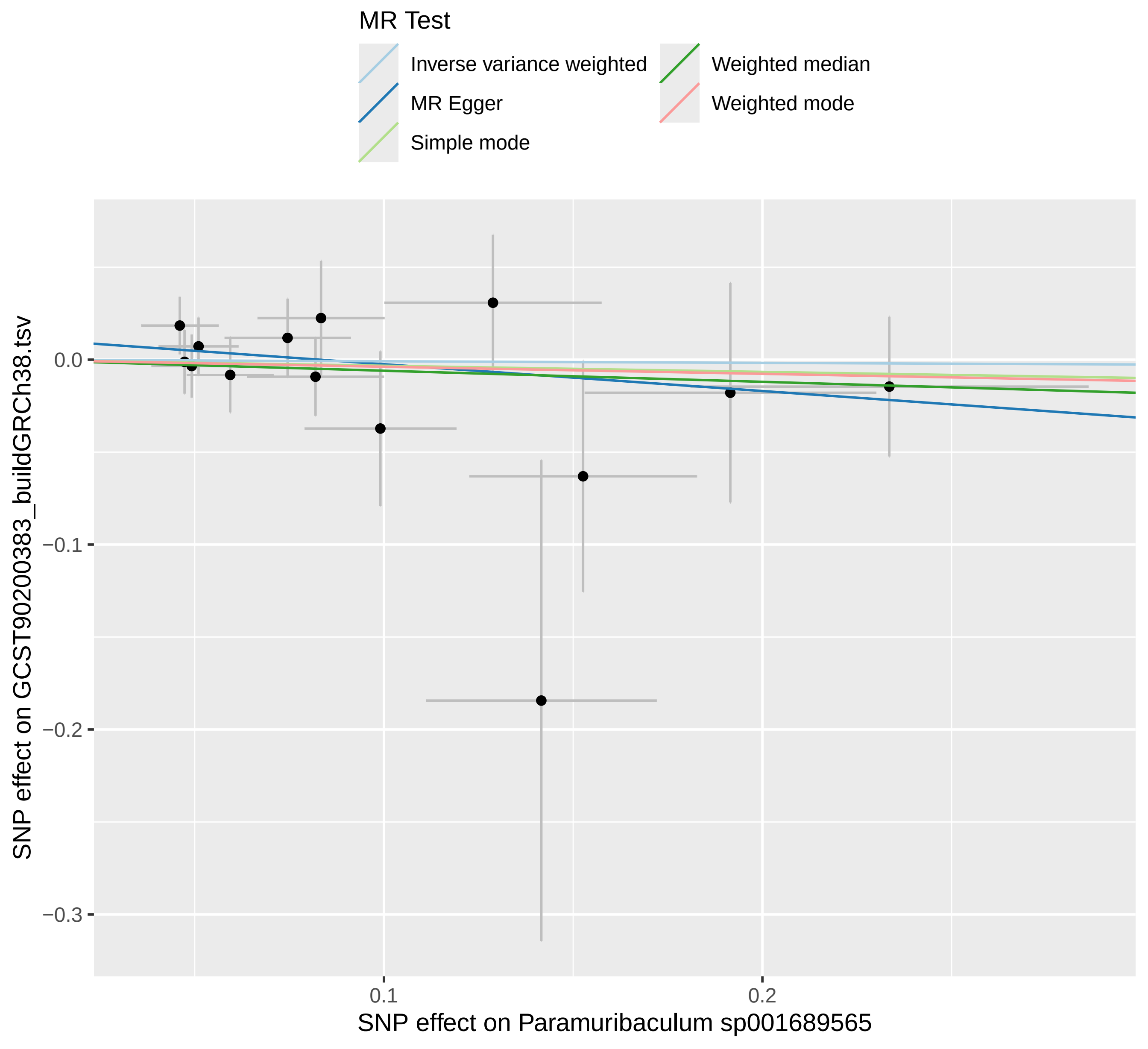
MR Test
Inverse variance weighted
MR Egger
Simple mode
Weighted median
Weighted mode
0.0
−0.1
−0.2
−0.3
0.1
0.2
SNP effect on GCST90200383_buildGRCh38.tsv
SNP effect on Paramuribaculum sp001689565

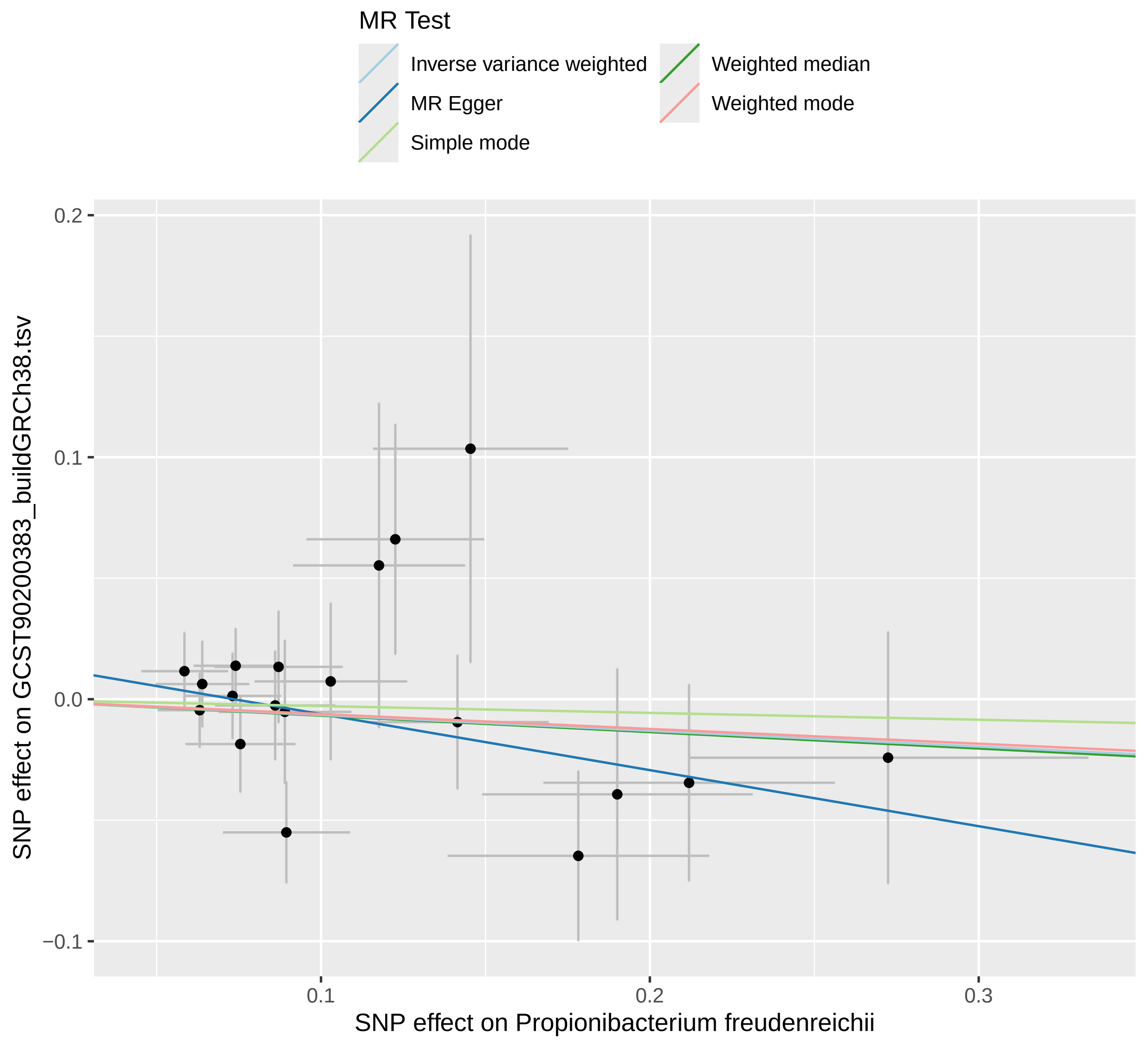
MR Test
Inverse variance weighted
MR Egger
Simple mode
Weighted median
Weighted mode
0.2
0.1
0.0
−0.1
0.1
0.2
0.3
SNP effect on GCST90200383_buildGRCh38.tsv
SNP effect on Propionibacterium freudenreichii

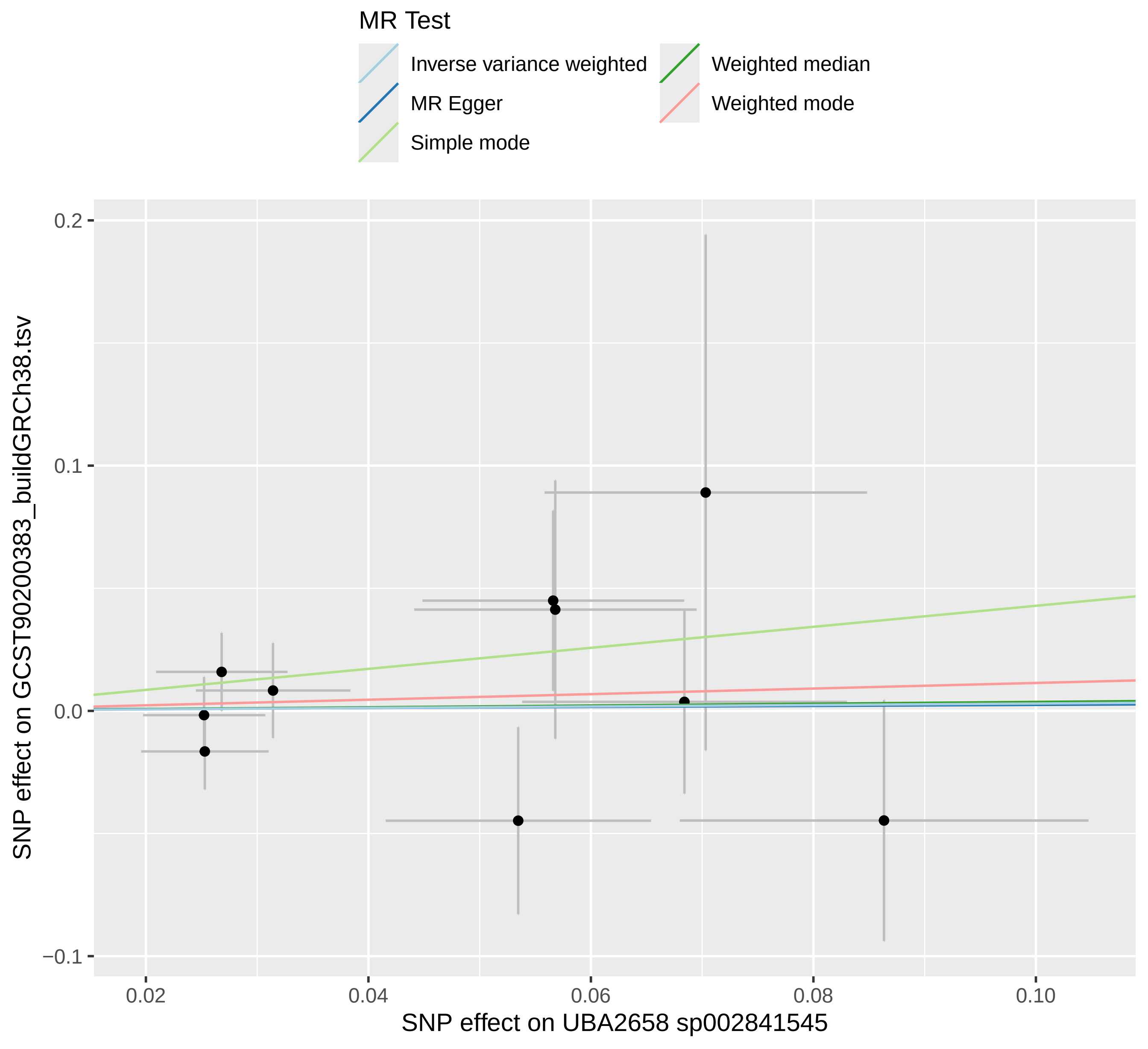
MR Test
Inverse variance weighted
MR Egger
Simple mode
Weighted median
Weighted mode
0.2
0.1
0.0
−0.1
0.02
0.04
0.06
0.08
0.10
SNP effect on GCST90200383_buildGRCh38.tsv
SNP effect on UBA2658 sp002841545

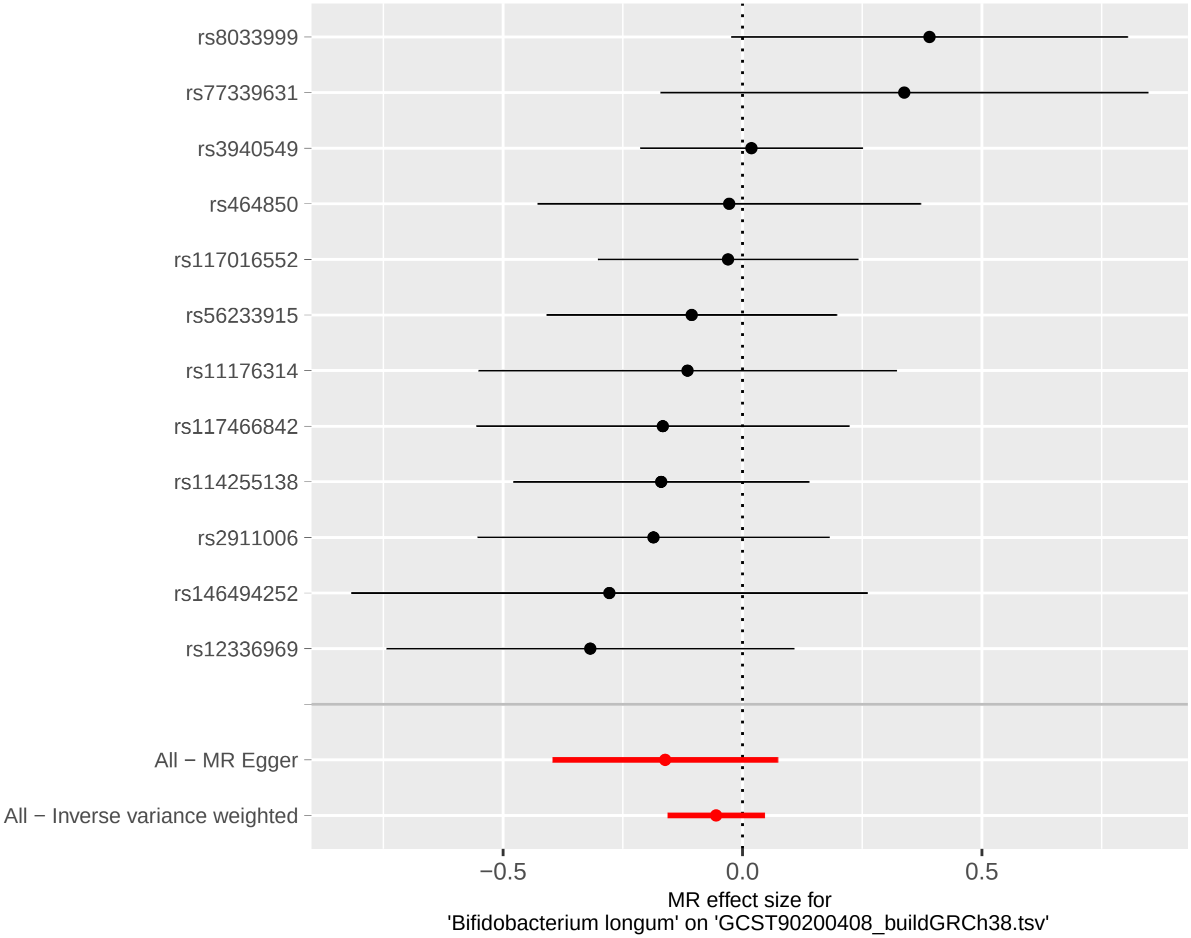

rs8033999
rs77339631
rs3940549
rs464850
rs117016552
rs56233915
rs11176314
rs117466842
rs114255138
rs2911006
rs146494252
rs12336969
All − MR Egger
All − Inverse variance weighted
−0.5
0.0
0.5
MR effect size for
'Bifidobacterium longum' on 'GCST90200408_buildGRCh38.tsv'

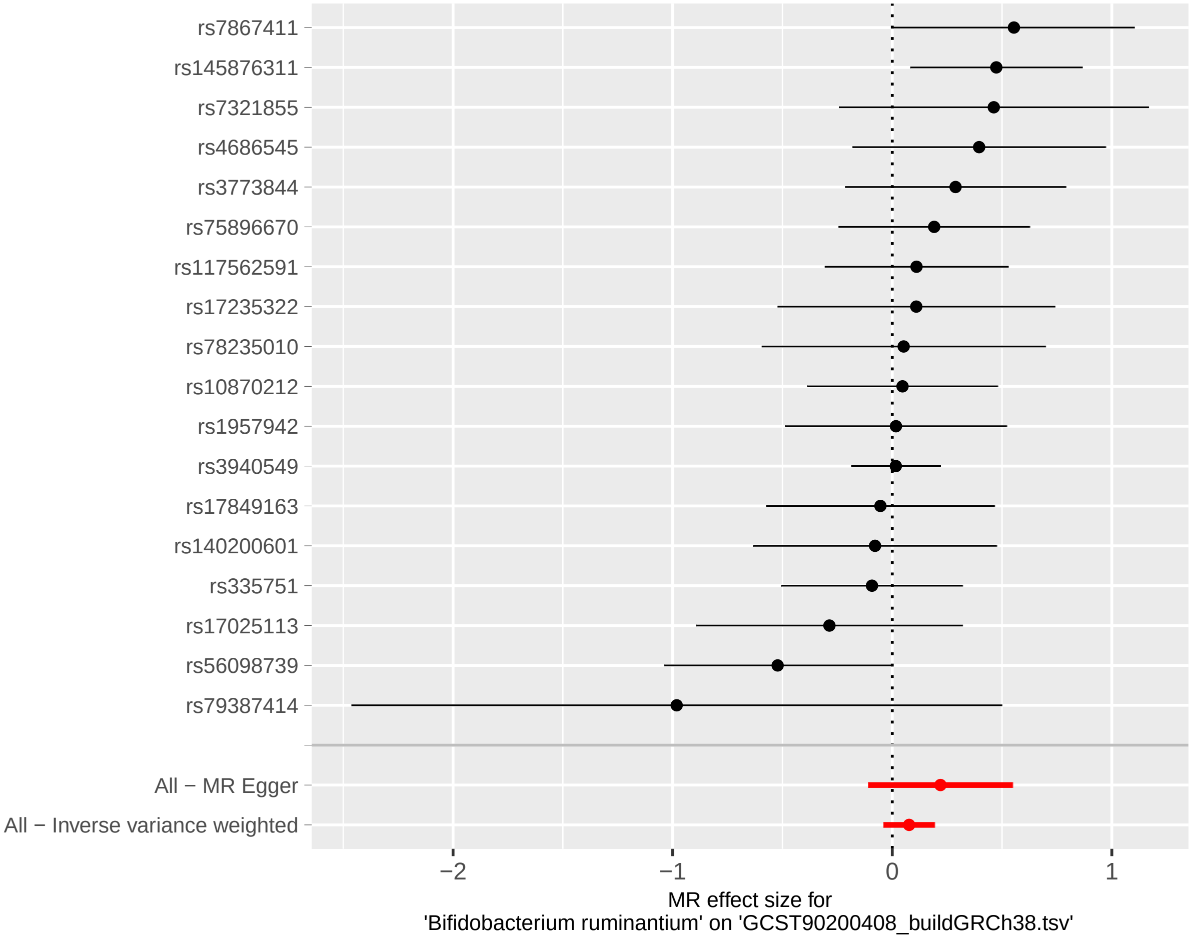
rs7867411
rs145876311
rs7321855
rs4686545
rs3773844
rs75896670
rs117562591
rs17235322
rs78235010
rs10870212
rs1957942
rs3940549
rs17849163
rs140200601
rs335751
rs17025113
rs56098739
rs79387414
All − MR Egger
All − Inverse variance weighted
−2
−1
0
1
MR effect size for
'Bifidobacterium ruminantium' on 'GCST90200408_buildGRCh38.tsv'

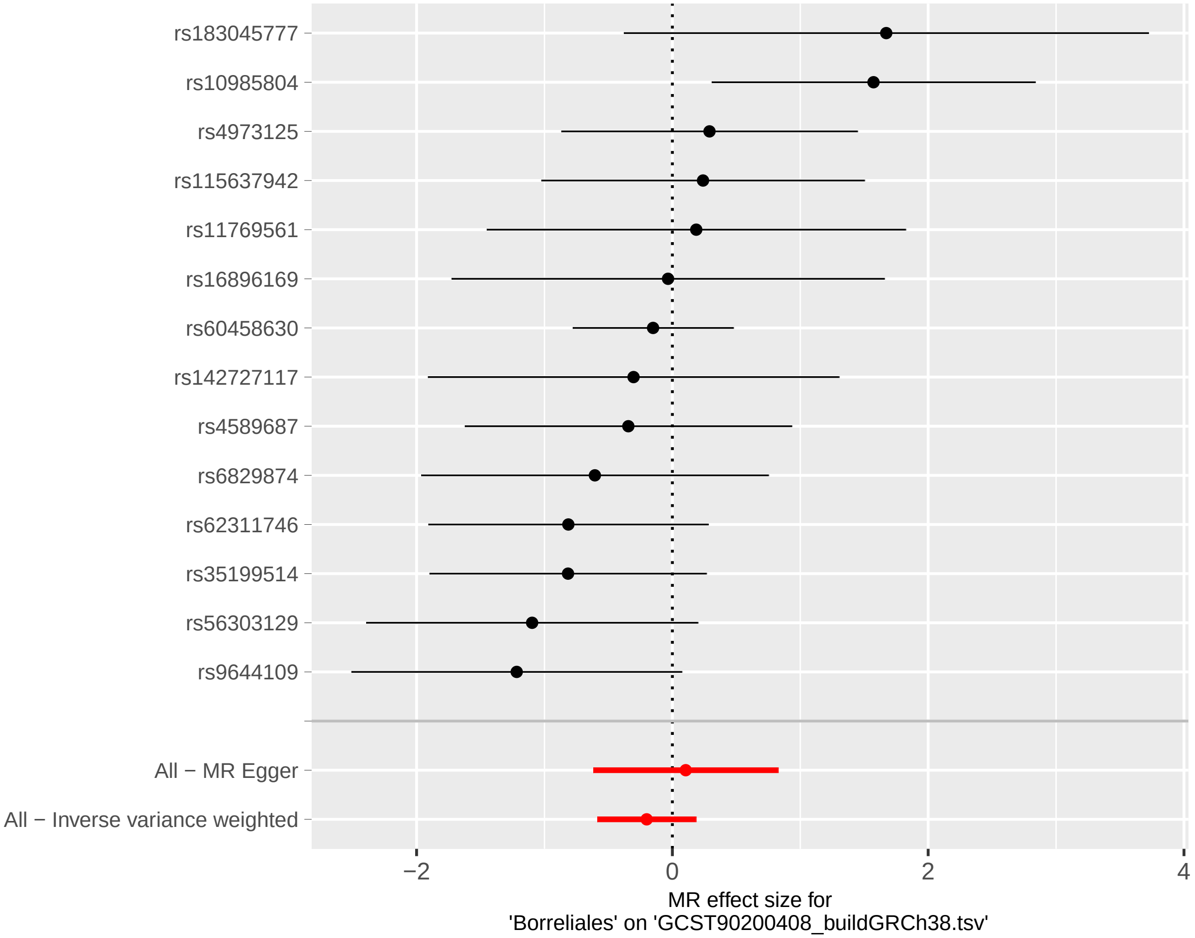
rs183045777
rs10985804
rs4973125
rs115637942
rs11769561
rs16896169
rs60458630
rs142727117
rs4589687
rs6829874
rs62311746
rs35199514
rs56303129
rs9644109
All − MR Egger
All − Inverse variance weighted
−2
0
2
4
MR effect size for
'Borreliales' on 'GCST90200408_buildGRCh38.tsv'

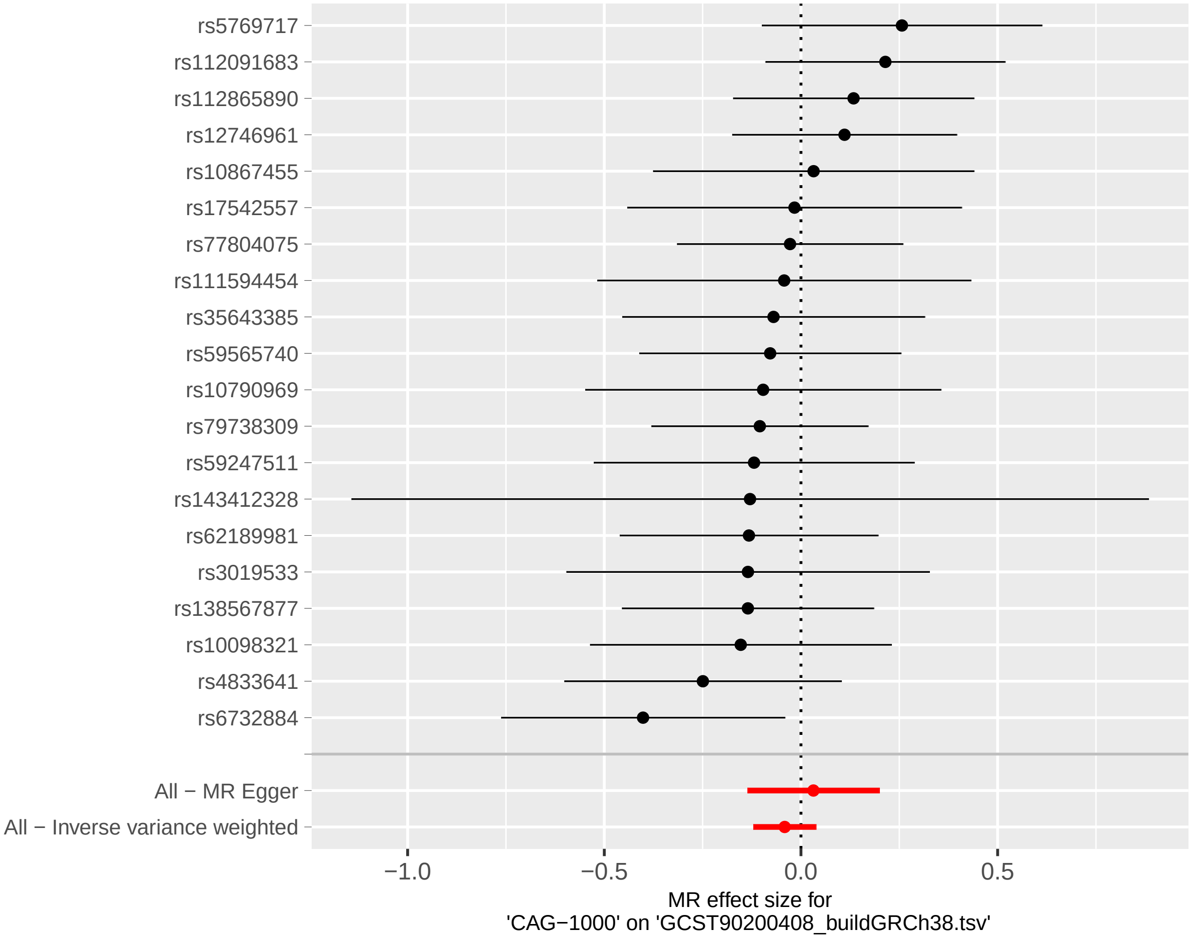

rs5769717
rs112091683
rs112865890
rs12746961
rs10867455
rs17542557
rs77804075
rs111594454
rs35643385
rs59565740
rs10790969
rs79738309
rs59247511
rs143412328
rs62189981
rs3019533
rs138567877
rs10098321
rs4833641
rs6732884
All − MR Egger
All − Inverse variance weighted
−1.0
−0.5
0.0
0.5
MR effect size for
'CAG−1000' on 'GCST90200408_buildGRCh38.tsv'

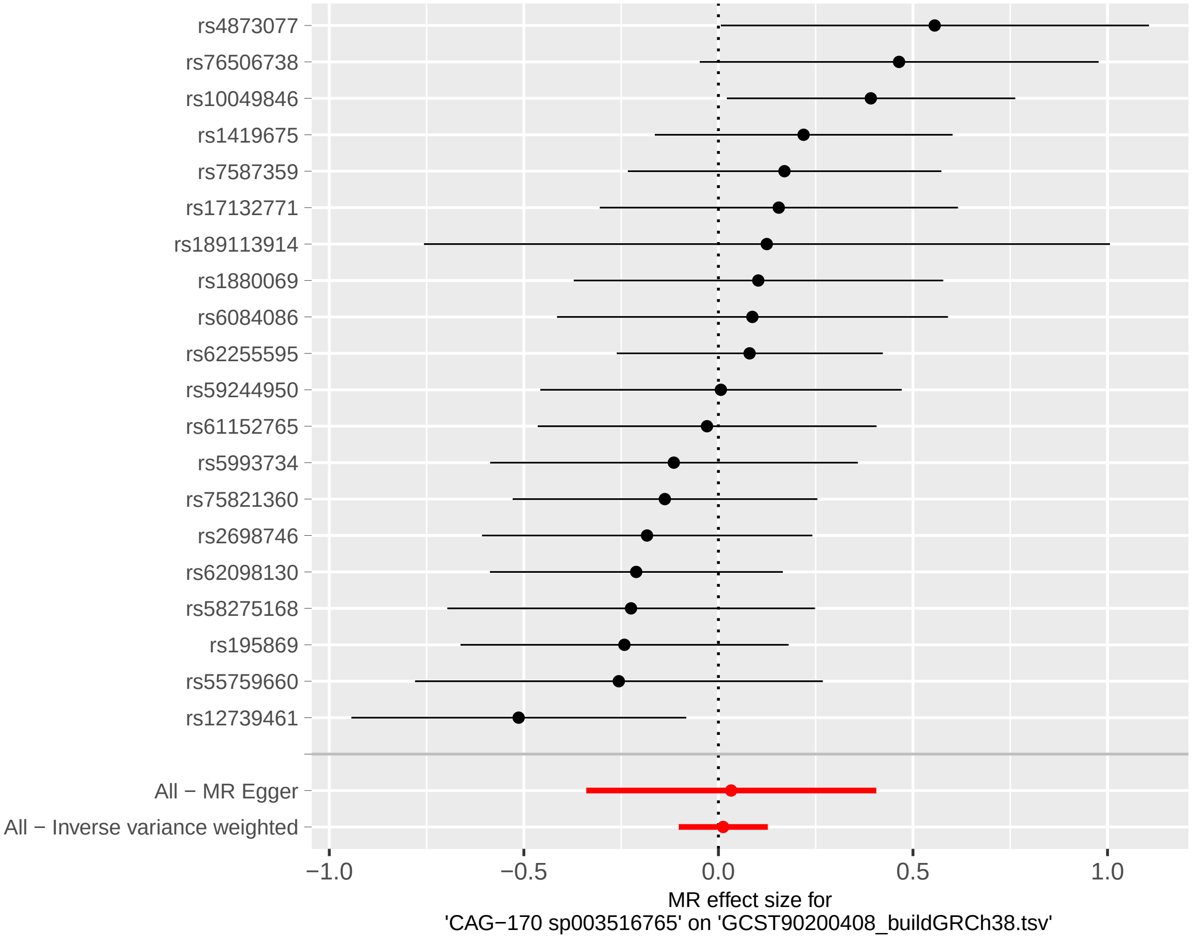
rs4873077
rs76506738
rs10049846
rs1419675
rs7587359
rs17132771
rs189113914
rs1880069
rs6084086
rs62255595
rs59244950
rs61152765
rs5993734
rs75821360
rs2698746
rs62098130
rs58275168
rs195869
rs55759660
rs12739461
All − MR Egger
All − Inverse variance weighted
−1.0
−0.5
0.0
0.5
1.0
MR effect size for
'CAG−170 sp003516765' on 'GCST90200408_buildGRCh38.tsv'

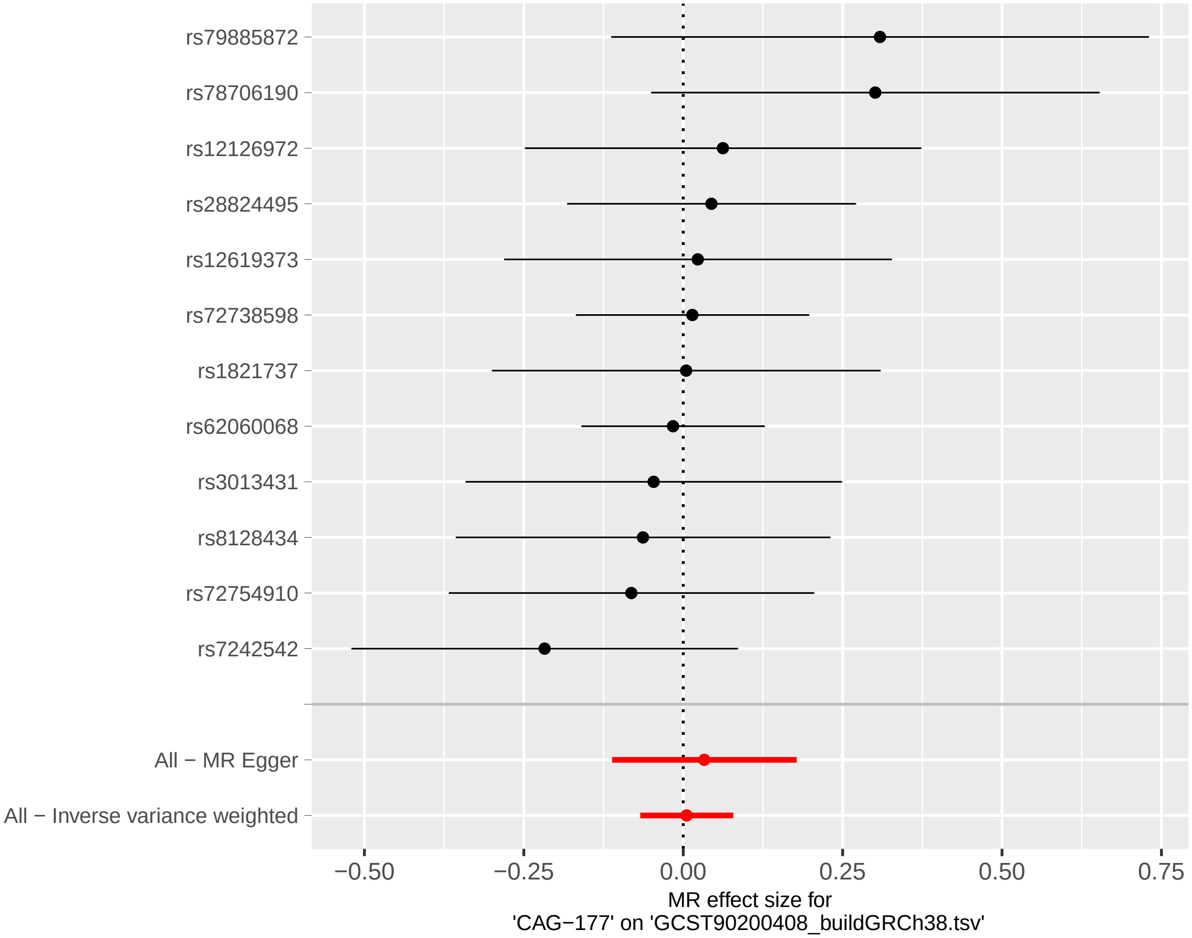

rs79885872
rs78706190
rs12126972
rs28824495
rs12619373
rs72738598
rs1821737
rs62060068
rs3013431
rs8128434
rs72754910
rs7242542
All − MR Egger
All − Inverse variance weighted
−0.50
−0.25
0.00
0.25
0.50
0.75
MR effect size for
'CAG−177' on 'GCST90200408_buildGRCh38.tsv'

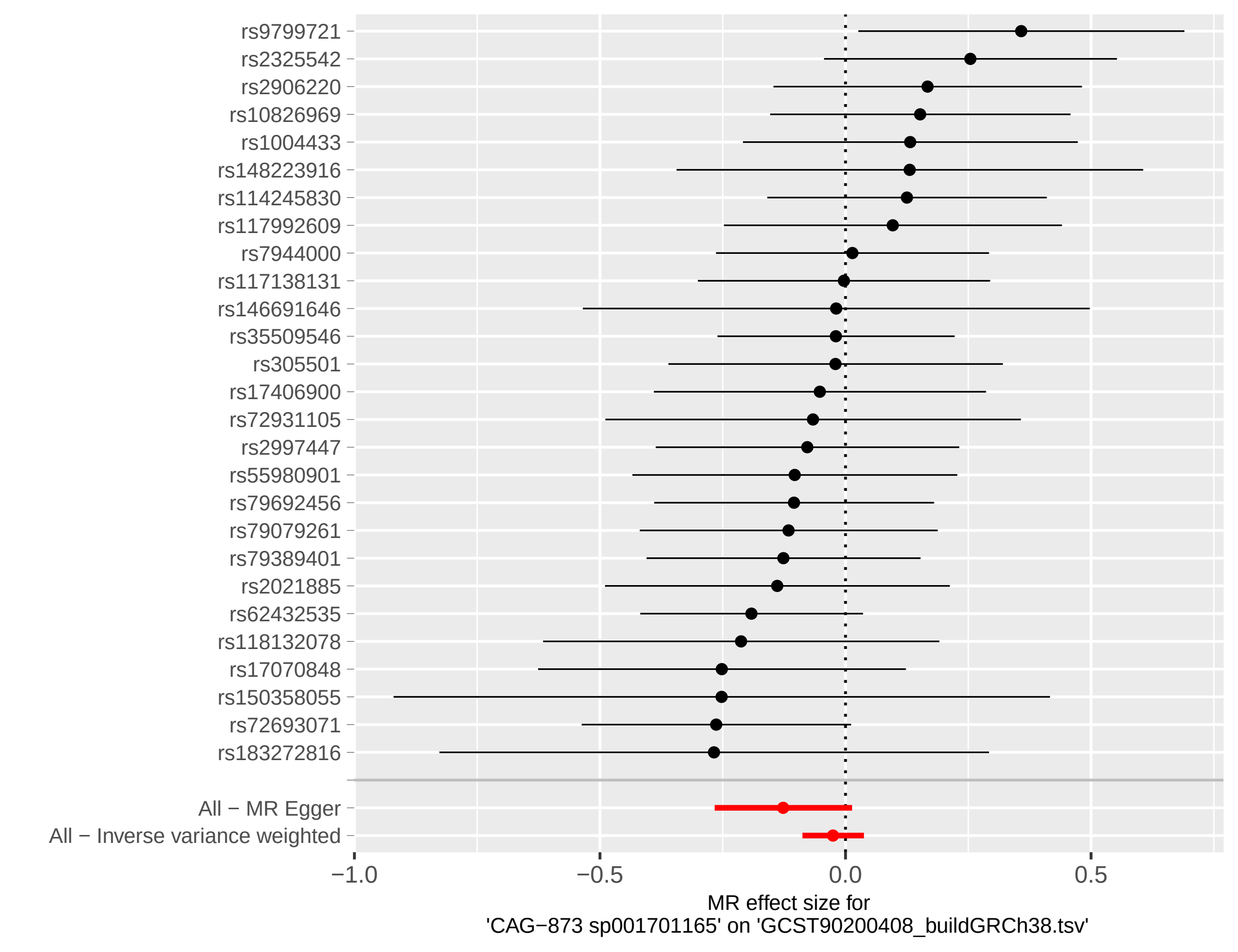

rs9799721
rs2325542
rs2906220
rs10826969
rs1004433
rs148223916
rs114245830
rs117992609
rs7944000
rs117138131
rs146691646
rs35509546
rs305501
rs17406900
rs72931105
rs2997447
rs55980901
rs79692456
rs79079261
rs79389401
rs2021885
rs62432535
rs118132078
rs17070848
rs150358055
rs72693071
rs183272816
All − MR Egger
All − Inverse variance weighted
−1.0
−0.5
0.0
0.5
MR effect size for
'CAG−873 sp001701165' on 'GCST90200408_buildGRCh38.tsv'

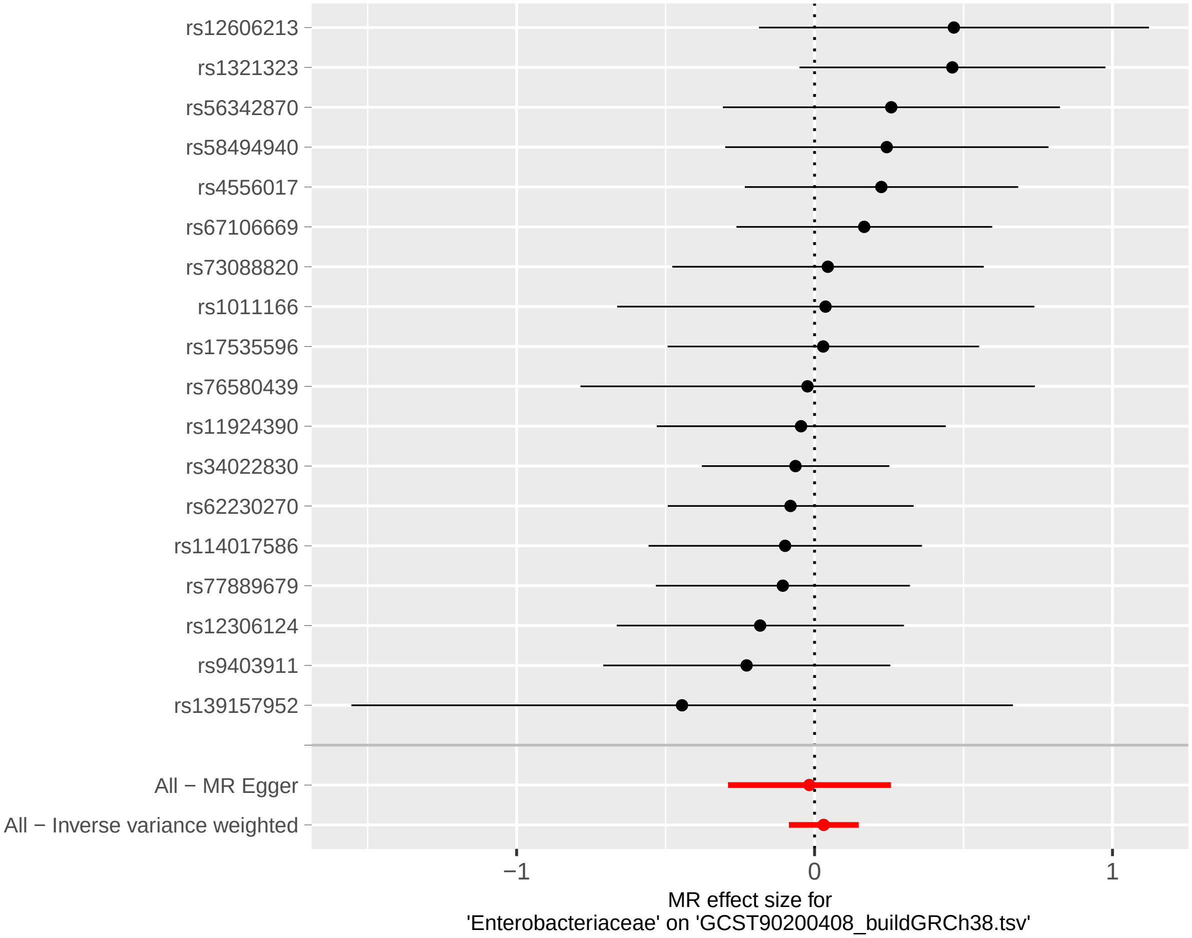
rs12606213
rs1321323
rs56342870
rs58494940
rs4556017
rs67106669
rs73088820
rs1011166
rs17535596
rs76580439
rs11924390
rs34022830
rs62230270
rs114017586
rs77889679
rs12306124
rs9403911
rs139157952
All − MR Egger
All − Inverse variance weighted
−1
0
1
MR effect size for
'Enterobacteriaceae' on 'GCST90200408_buildGRCh38.tsv'

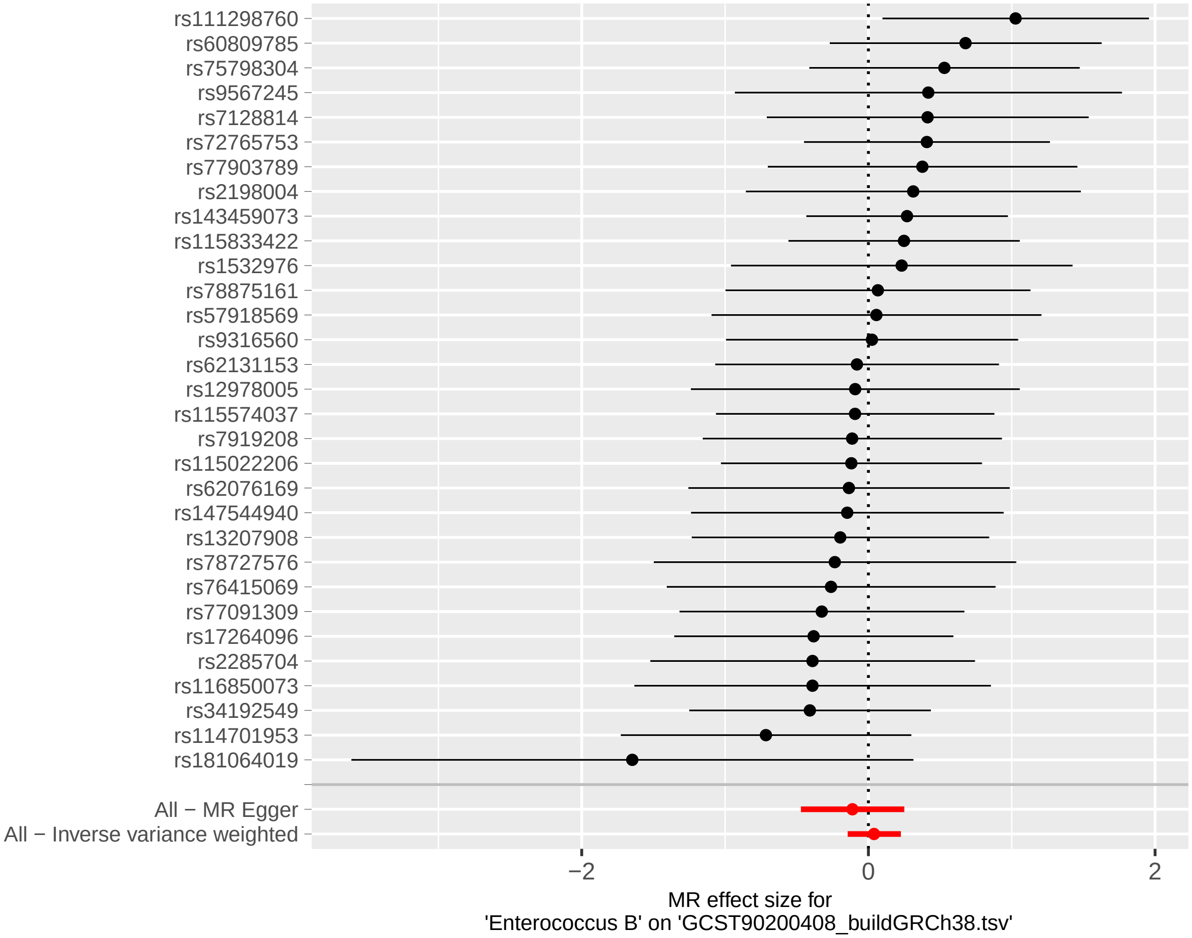
rs111298760
rs60809785
rs75798304
rs9567245
rs7128814
rs72765753
rs77903789
rs2198004
rs143459073
rs115833422
rs1532976
rs78875161
rs57918569
rs9316560
rs62131153
rs12978005
rs115574037
rs7919208
rs115022206
rs62076169
rs147544940
rs13207908
rs78727576
rs76415069
rs77091309
rs17264096
rs2285704
rs116850073
rs34192549
rs114701953
rs181064019
All − MR Egger
All − Inverse variance weighted
−2
0
2
MR effect size for
'Enterococcus B' on 'GCST90200408_buildGRCh38.tsv'

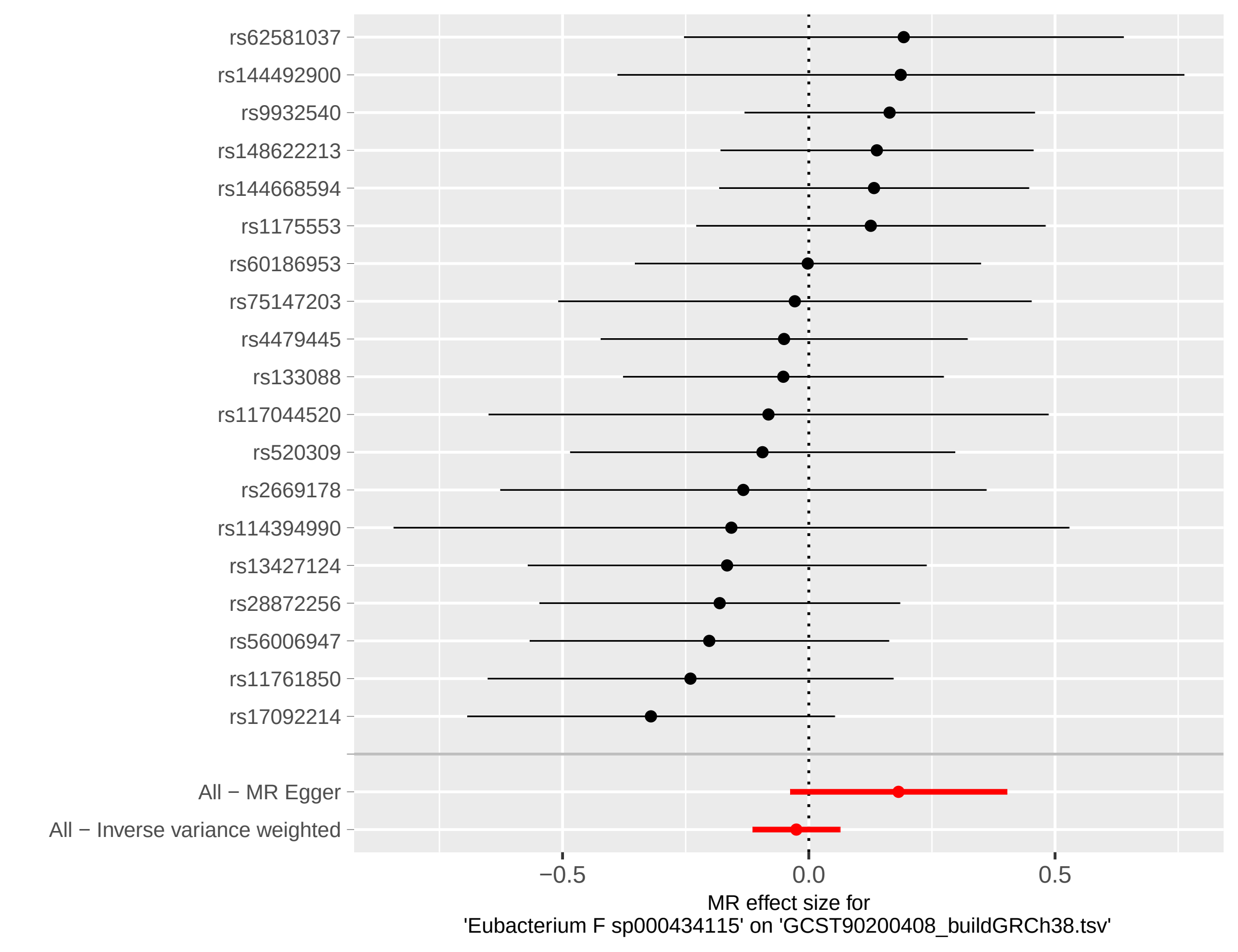

rs62581037
rs144492900
rs9932540
rs148622213
rs144668594
rs1175553
rs60186953
rs75147203
rs4479445
rs133088
rs117044520
rs520309
rs2669178
rs114394990
rs13427124
rs28872256
rs56006947
rs11761850
rs17092214
All − MR Egger
All − Inverse variance weighted
−0.5
0.0
0.5
MR effect size for
'Eubacterium F sp000434115' on 'GCST90200408_buildGRCh38.tsv'

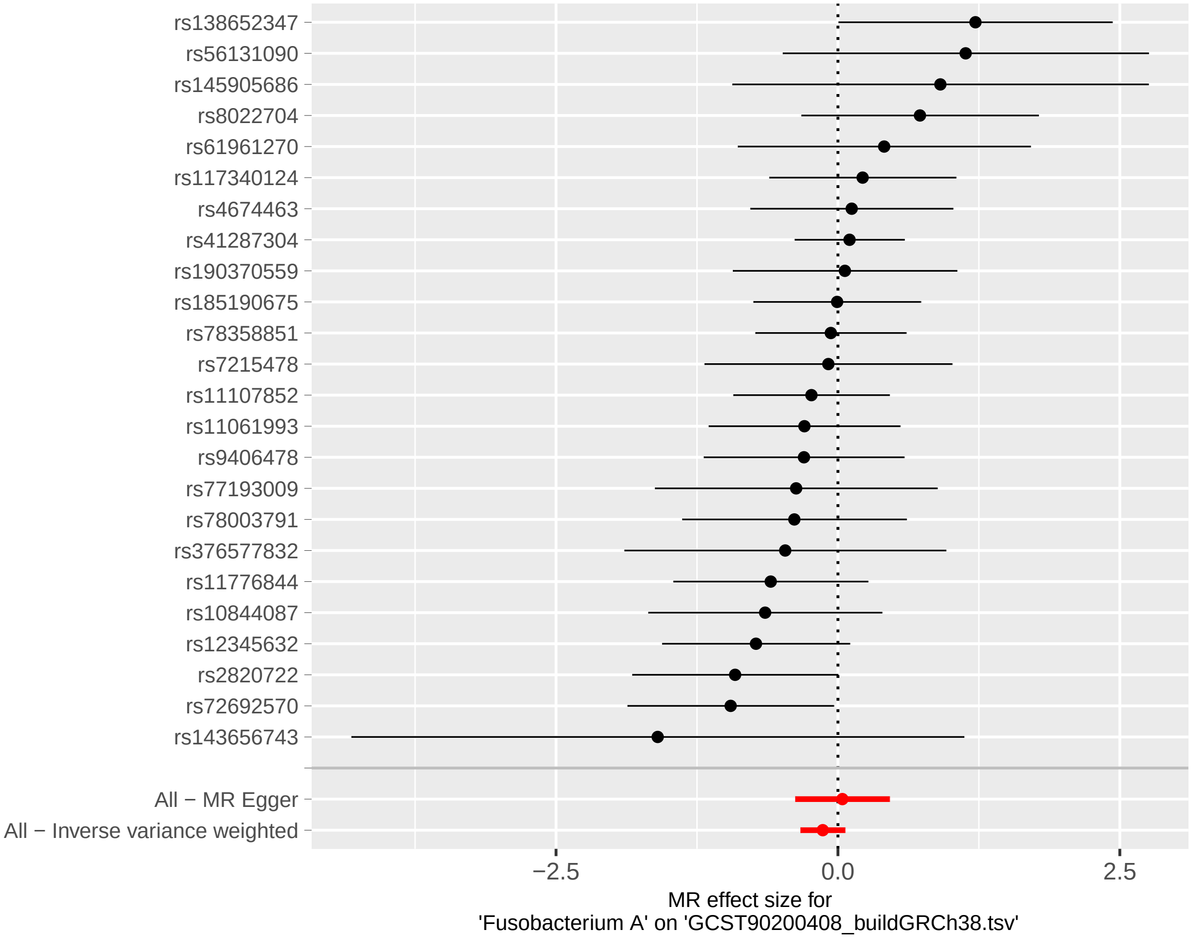
rs138652347
rs56131090
rs145905686
rs8022704
rs61961270
rs117340124
rs4674463
rs41287304
rs190370559
rs185190675
rs78358851
rs7215478
rs11107852
rs11061993
rs9406478
rs77193009
rs78003791
rs376577832
rs11776844
rs10844087
rs12345632
rs2820722
rs72692570
rs143656743
All − MR Egger
All − Inverse variance weighted
−2.5
0.0
2.5
MR effect size for
'Fusobacterium A' on 'GCST90200408_buildGRCh38.tsv'

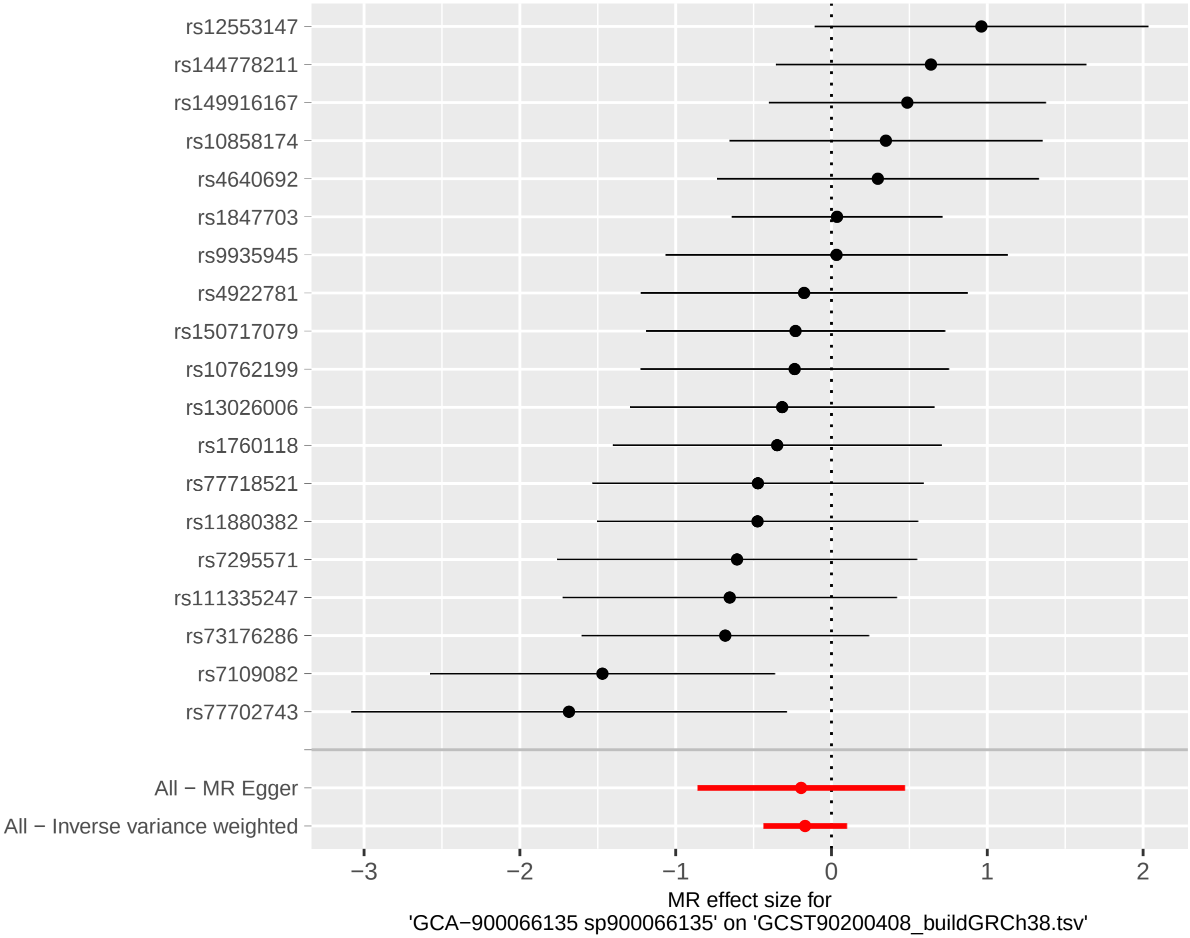

rs12553147
rs144778211
rs149916167
rs10858174
rs4640692
rs1847703
rs9935945
rs4922781
rs150717079
rs10762199
rs13026006
rs1760118
rs77718521
rs11880382
rs7295571
rs111335247
rs73176286
rs7109082
rs77702743
All − MR Egger
All − Inverse variance weighted
−3
−2
−1
0
1
2
MR effect size for
'GCA−900066135 sp900066135' on 'GCST90200408_buildGRCh38.tsv'

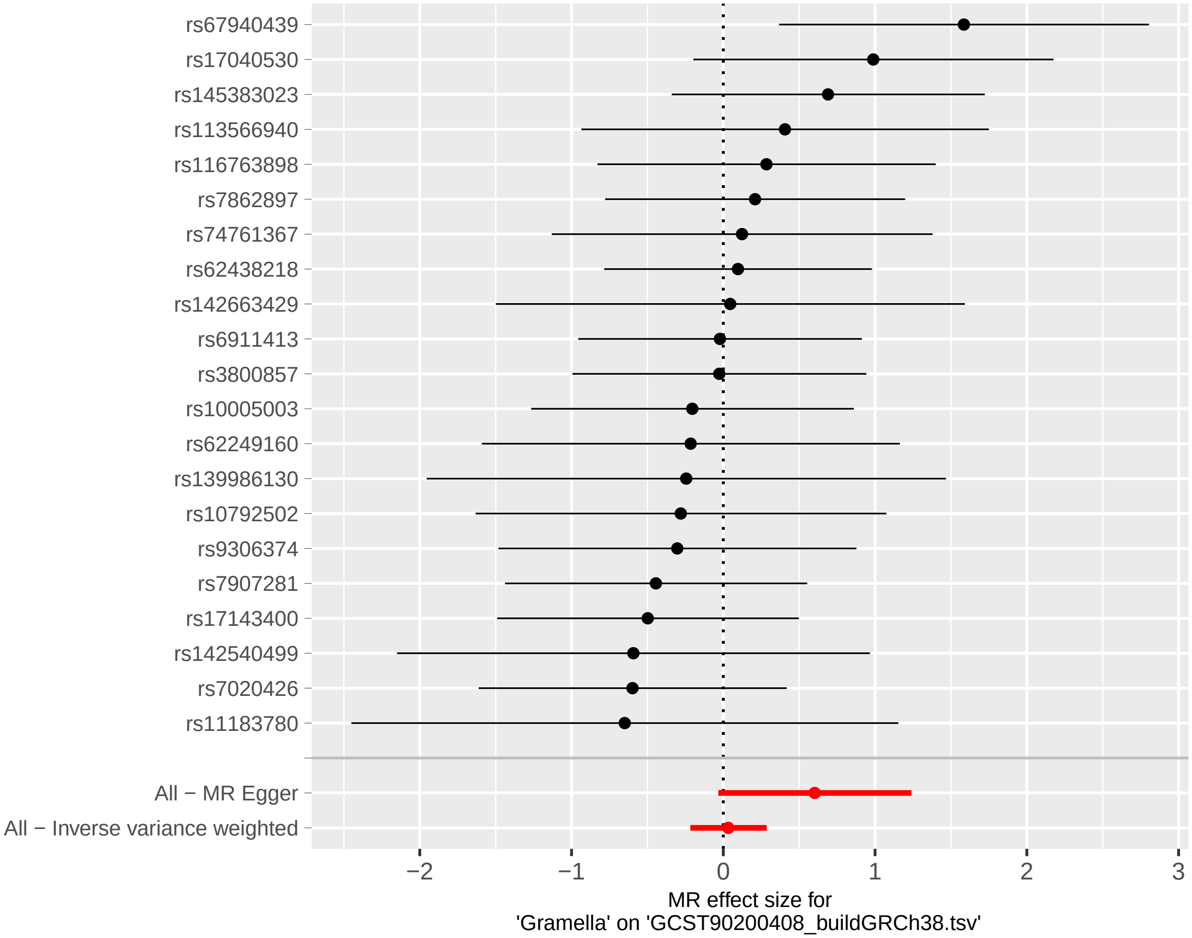
rs67940439
rs17040530
rs145383023
rs113566940
rs116763898
rs7862897
rs74761367
rs62438218
rs142663429
rs6911413
rs3800857
rs10005003
rs62249160
rs139986130
rs10792502
rs9306374
rs7907281
rs17143400
rs142540499
rs7020426
rs11183780
All − MR Egger
All − Inverse variance weighted
−2
−1
0
1
2
3
MR effect size for
'Gramella' on 'GCST90200408_buildGRCh38.tsv'

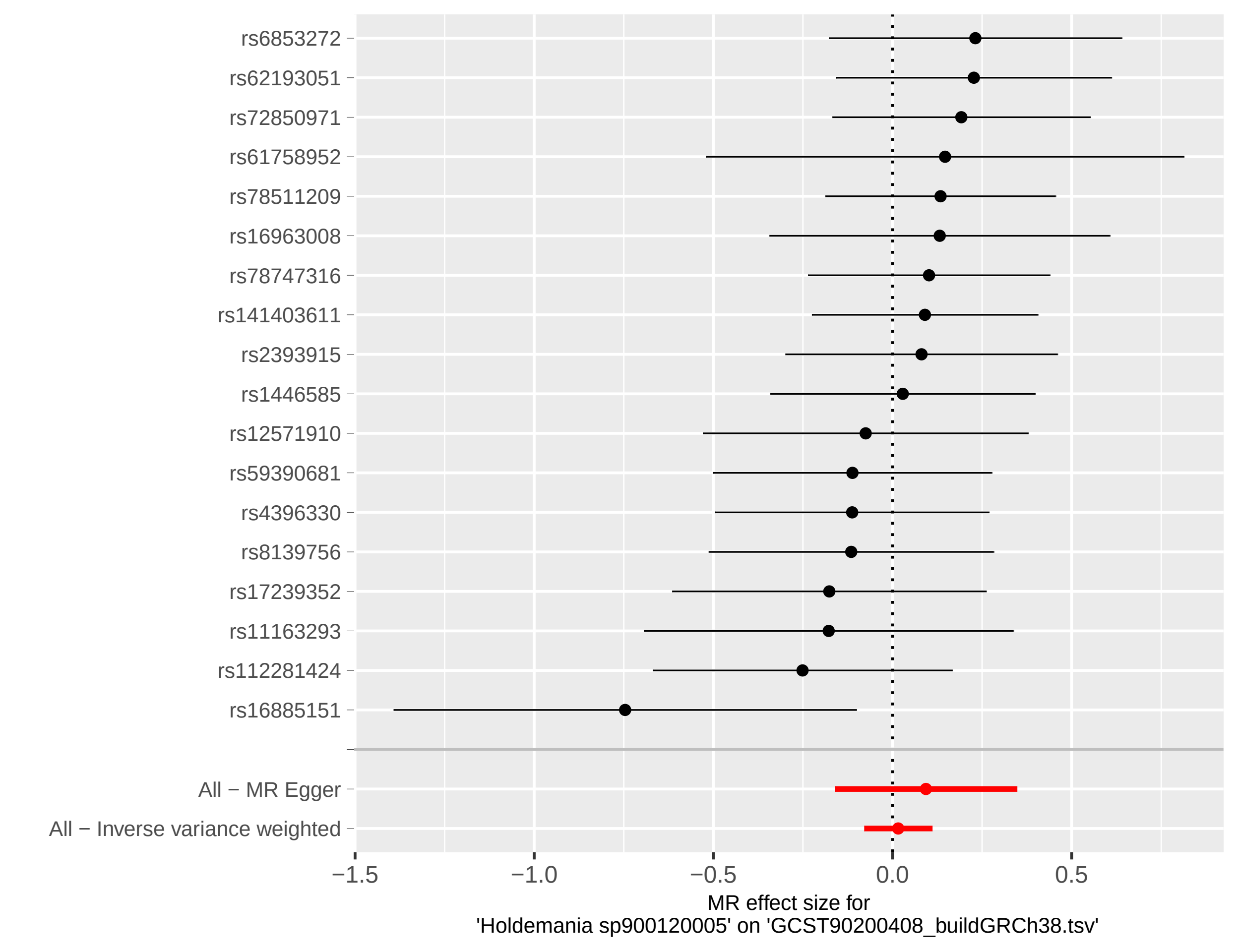
rs6853272
rs62193051
rs72850971
rs61758952
rs78511209
rs16963008
rs78747316
rs141403611
rs2393915
rs1446585
rs12571910
rs59390681
rs4396330
rs8139756
rs17239352
rs11163293
rs112281424
rs16885151
All − MR Egger
All − Inverse variance weighted
−1.5
−1.0
−0.5
0.0
0.5
MR effect size for
'Holdemania sp900120005' on 'GCST90200408_buildGRCh38.tsv'

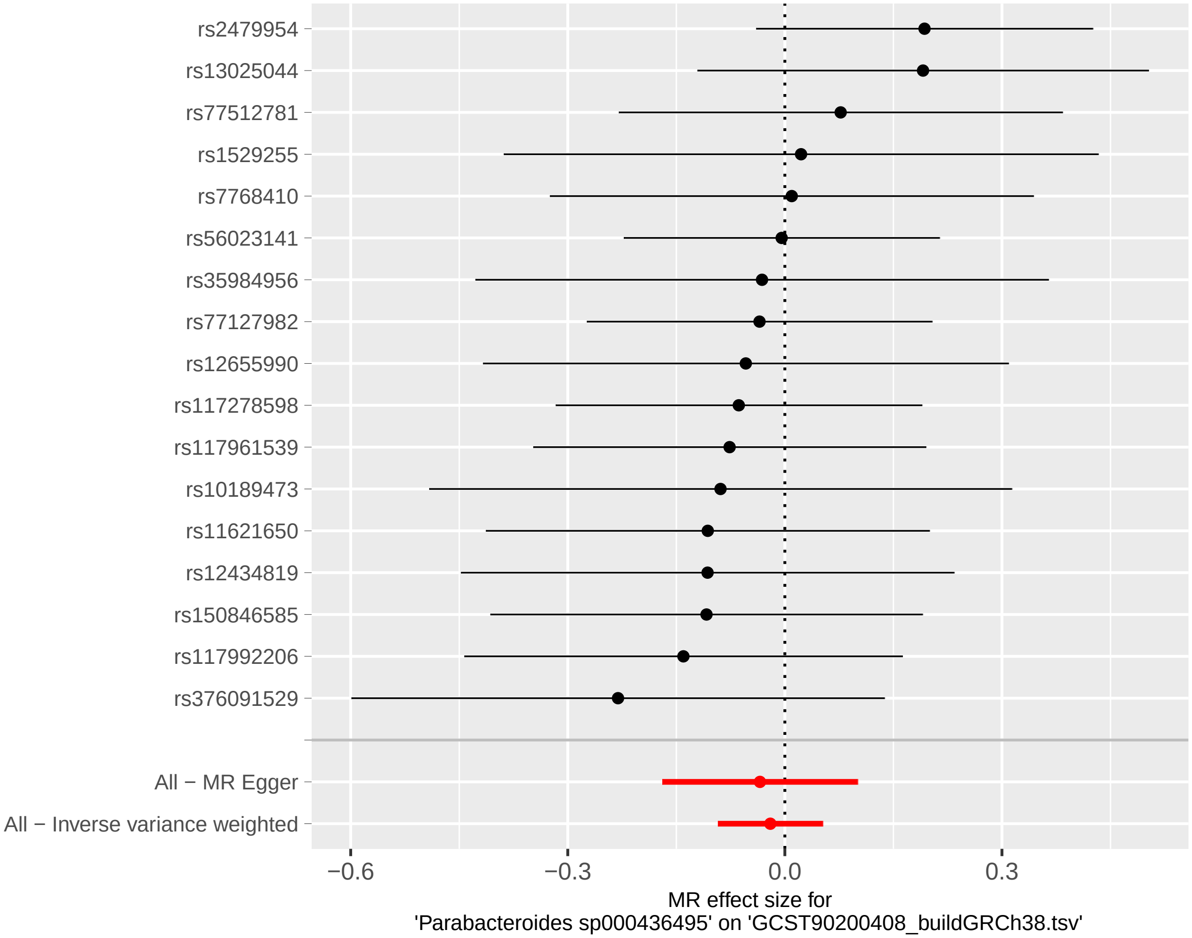
rs2479954
rs13025044
rs77512781
rs1529255
rs7768410
rs56023141
rs35984956
rs77127982
rs12655990
rs117278598
rs117961539
rs10189473
rs11621650
rs12434819
rs150846585
rs117992206
rs376091529
All − MR Egger
All − Inverse variance weighted
−0.6
−0.3
0.0
0.3
MR effect size for
'Parabacteroides sp000436495' on 'GCST90200408_buildGRCh38.tsv'

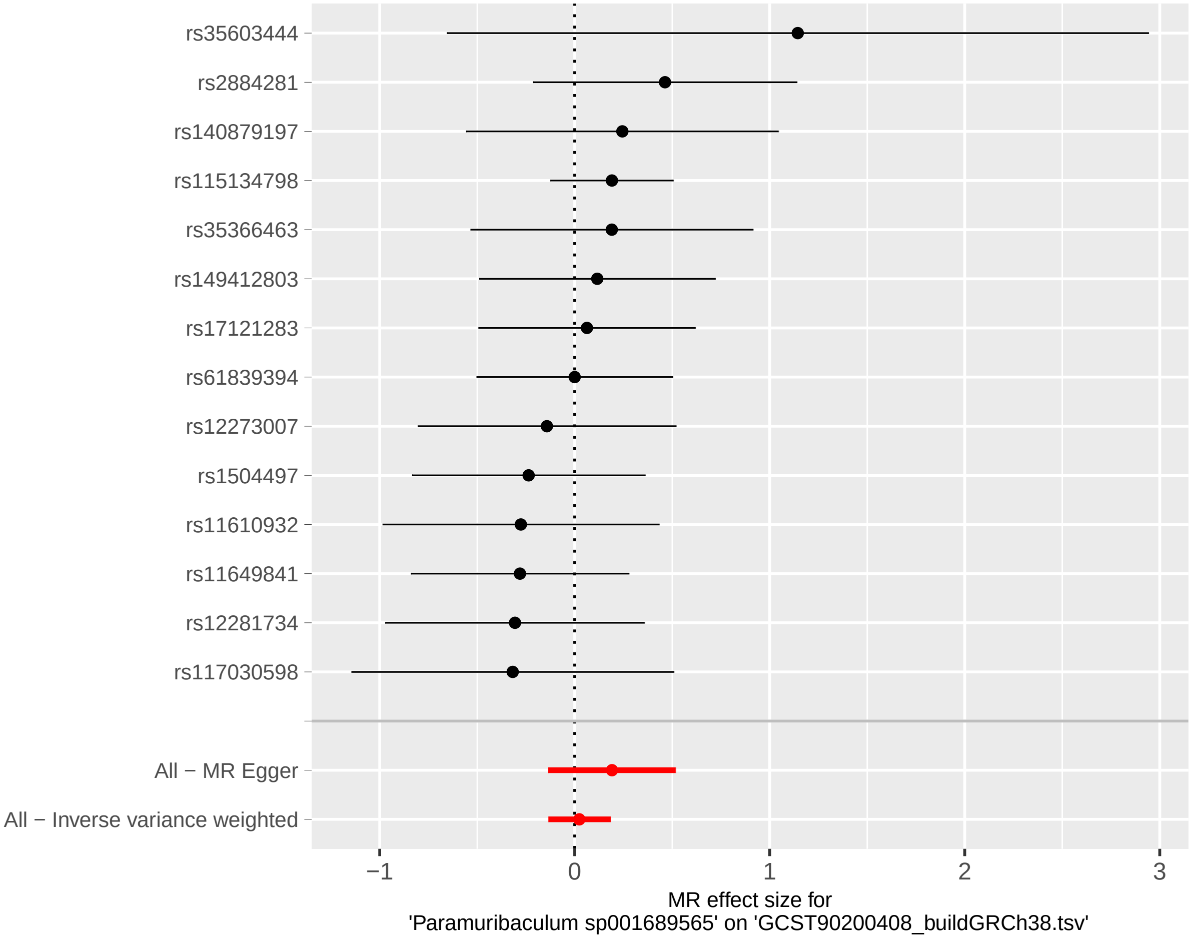
rs35603444
rs2884281
rs140879197
rs115134798
rs35366463
rs149412803
rs17121283
rs61839394
rs12273007
rs1504497
rs11610932
rs11649841
rs12281734
rs117030598
All − MR Egger
All − Inverse variance weighted
−1
0
1
2
3
MR effect size for
'Paramuribaculum sp001689565' on 'GCST90200408_buildGRCh38.tsv'

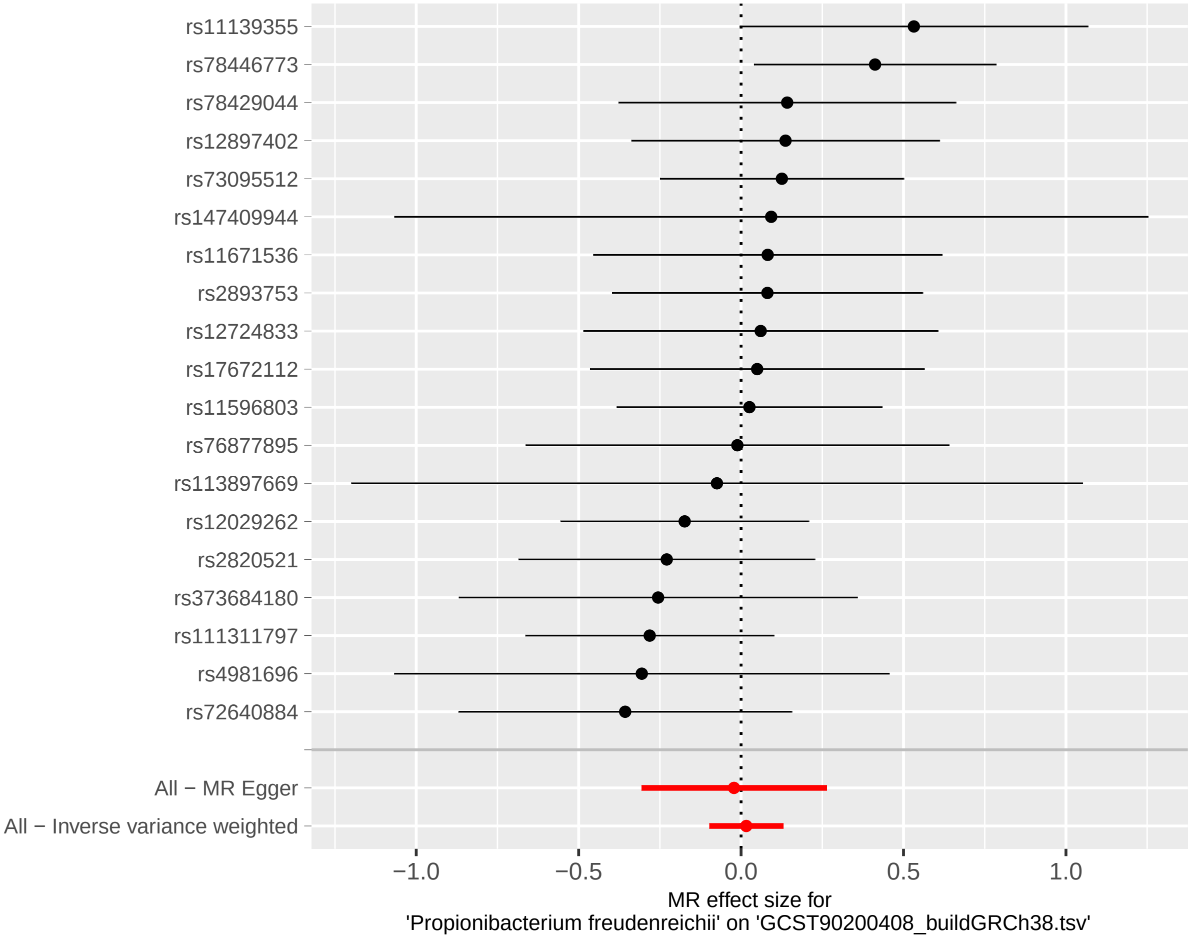
rs11139355
rs78446773
rs78429044
rs12897402
rs73095512
rs147409944
rs11671536
rs2893753
rs12724833
rs17672112
rs11596803
rs76877895
rs113897669
rs12029262
rs2820521
rs373684180
rs111311797
rs4981696
rs72640884
All − MR Egger
All − Inverse variance weighted
−1.0
−0.5
0.0
0.5
1.0
MR effect size for
'Propionibacterium freudenreichii' on 'GCST90200408_buildGRCh38.tsv'

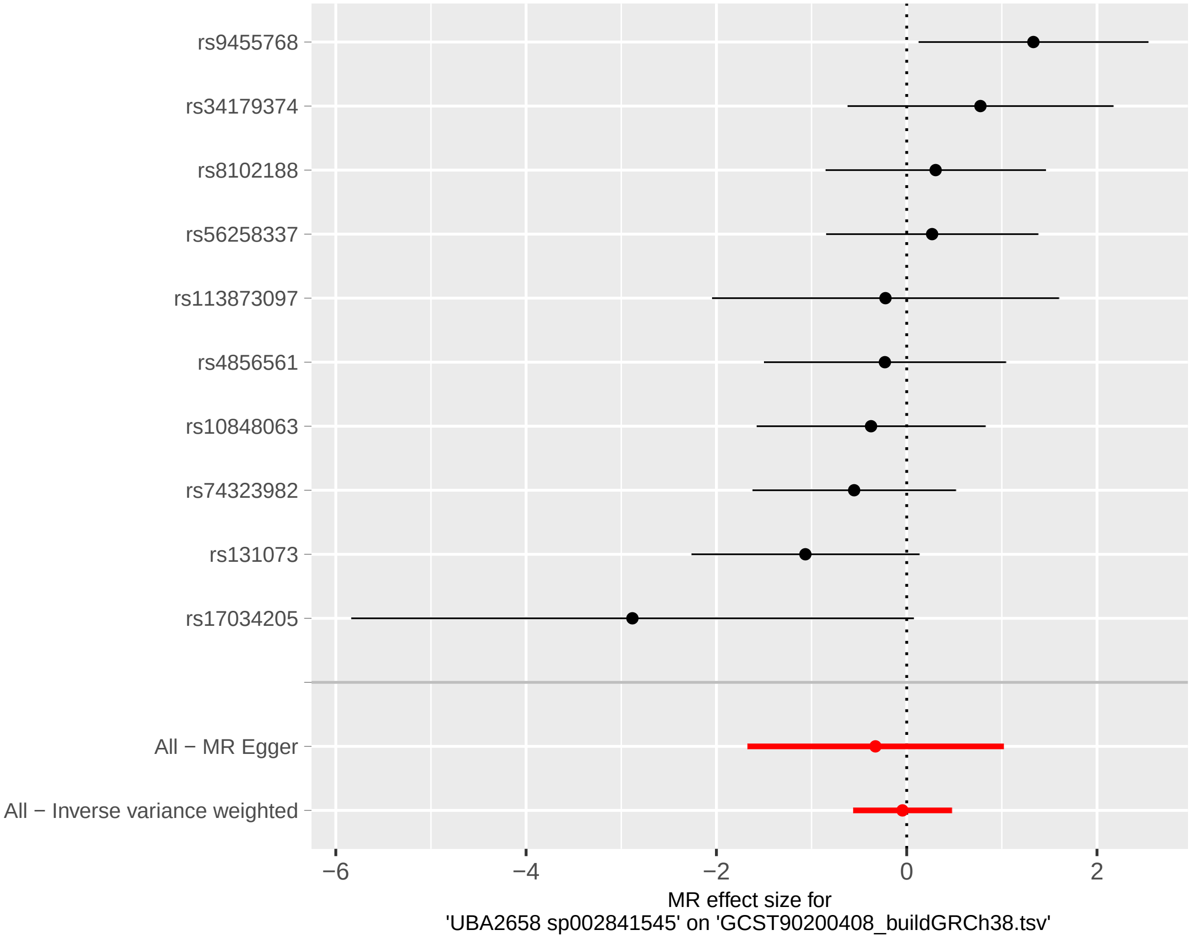
rs9455768
rs34179374
rs8102188
rs56258337
rs113873097
rs4856561
rs10848063
rs74323982
rs131073
rs17034205
All − MR Egger
All − Inverse variance weighted
−6
−4
−2
0
2
MR effect size for
'UBA2658 sp002841545' on 'GCST90200408_buildGRCh38.tsv'

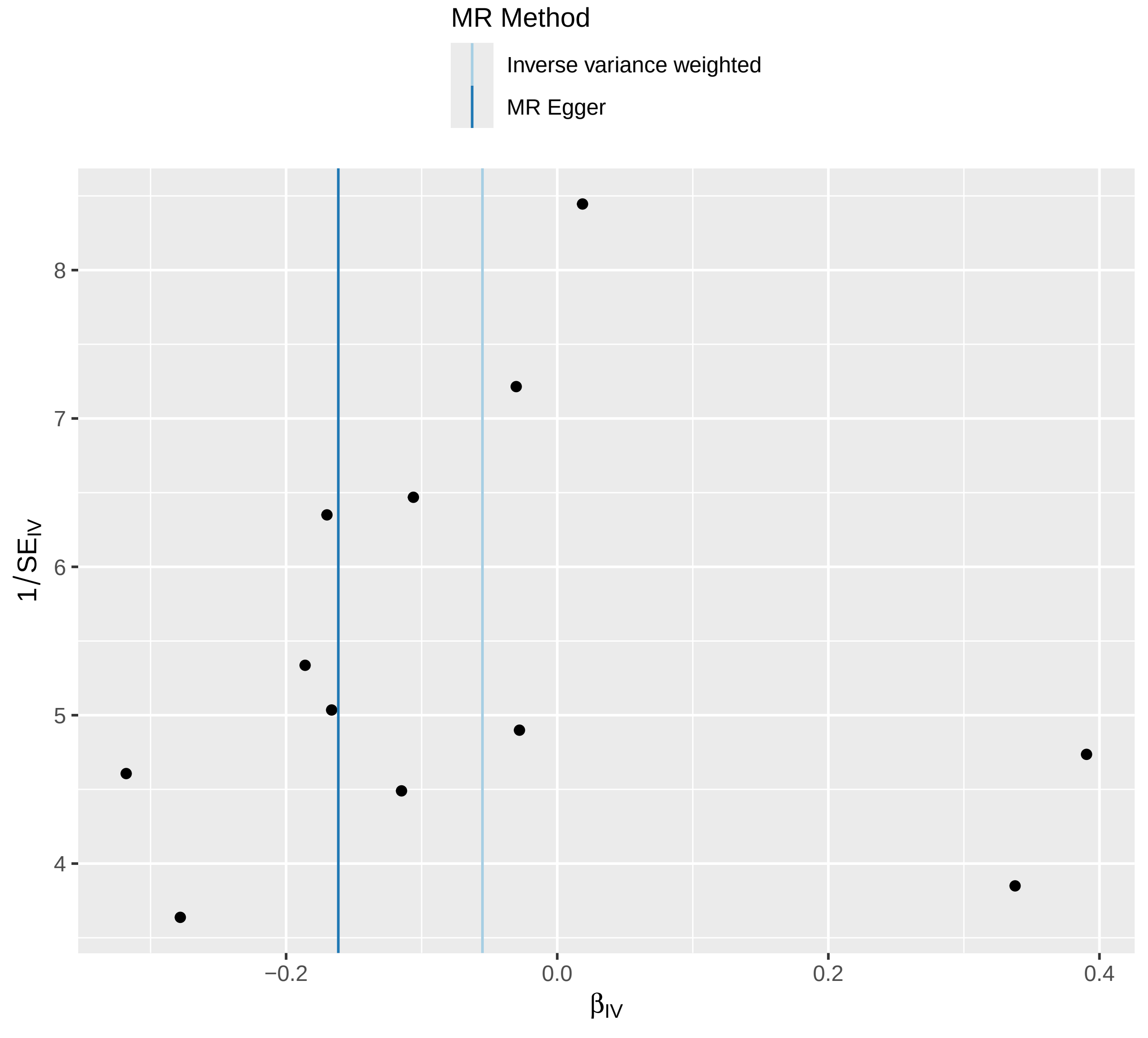
MR Method
Inverse variance weighted
MR Egger
1/SE$_{IV}$
8
7
6
5
4
−0.2
0.0
0.2
0.4
β$_{IV}$

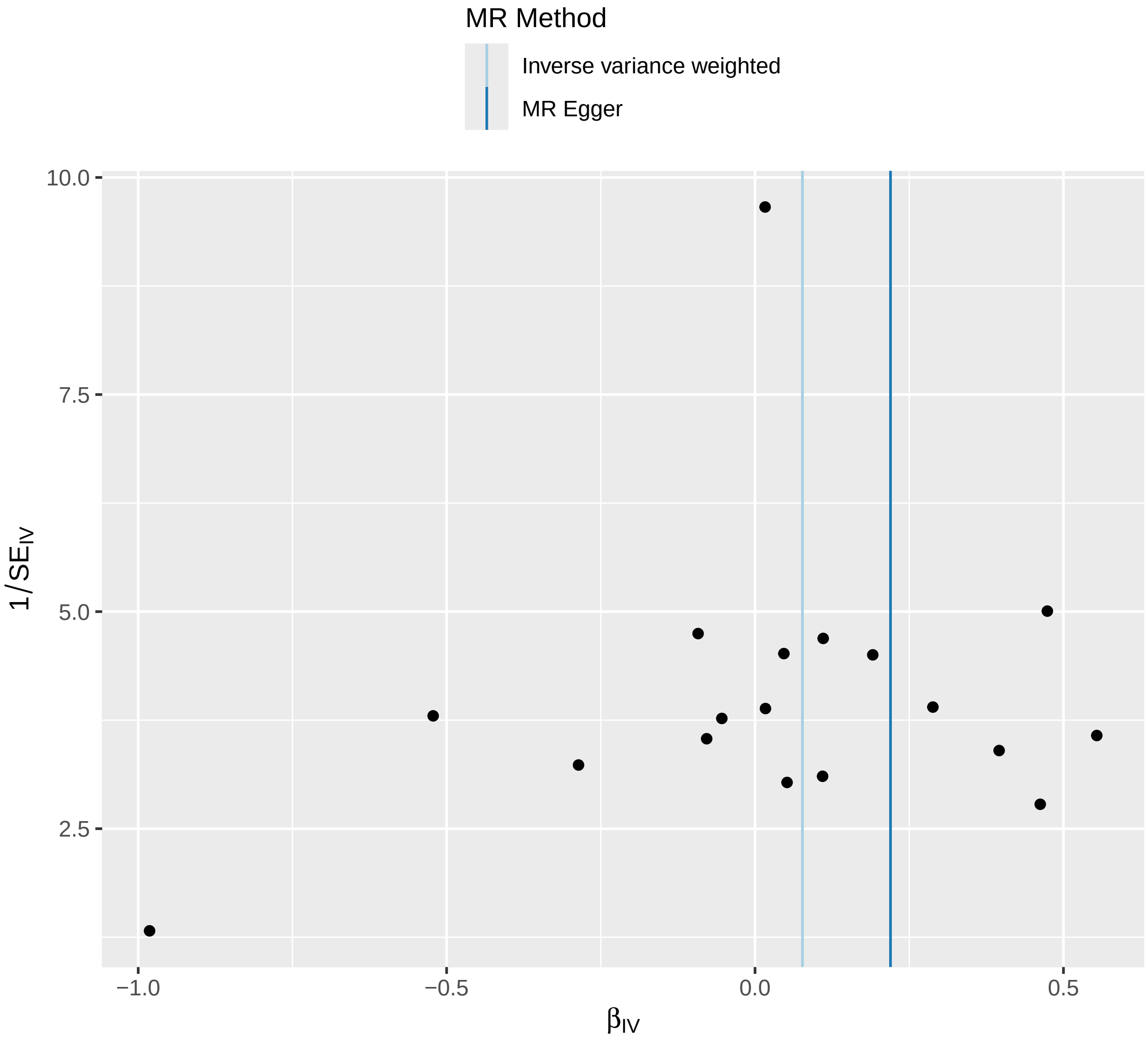
MR Method
Inverse variance weighted
MR Egger
10.0
7.5
5.0
2.5
1/SE$_{IV}$
−1.0
−0.5
0.0
0.5
β$_{IV}$

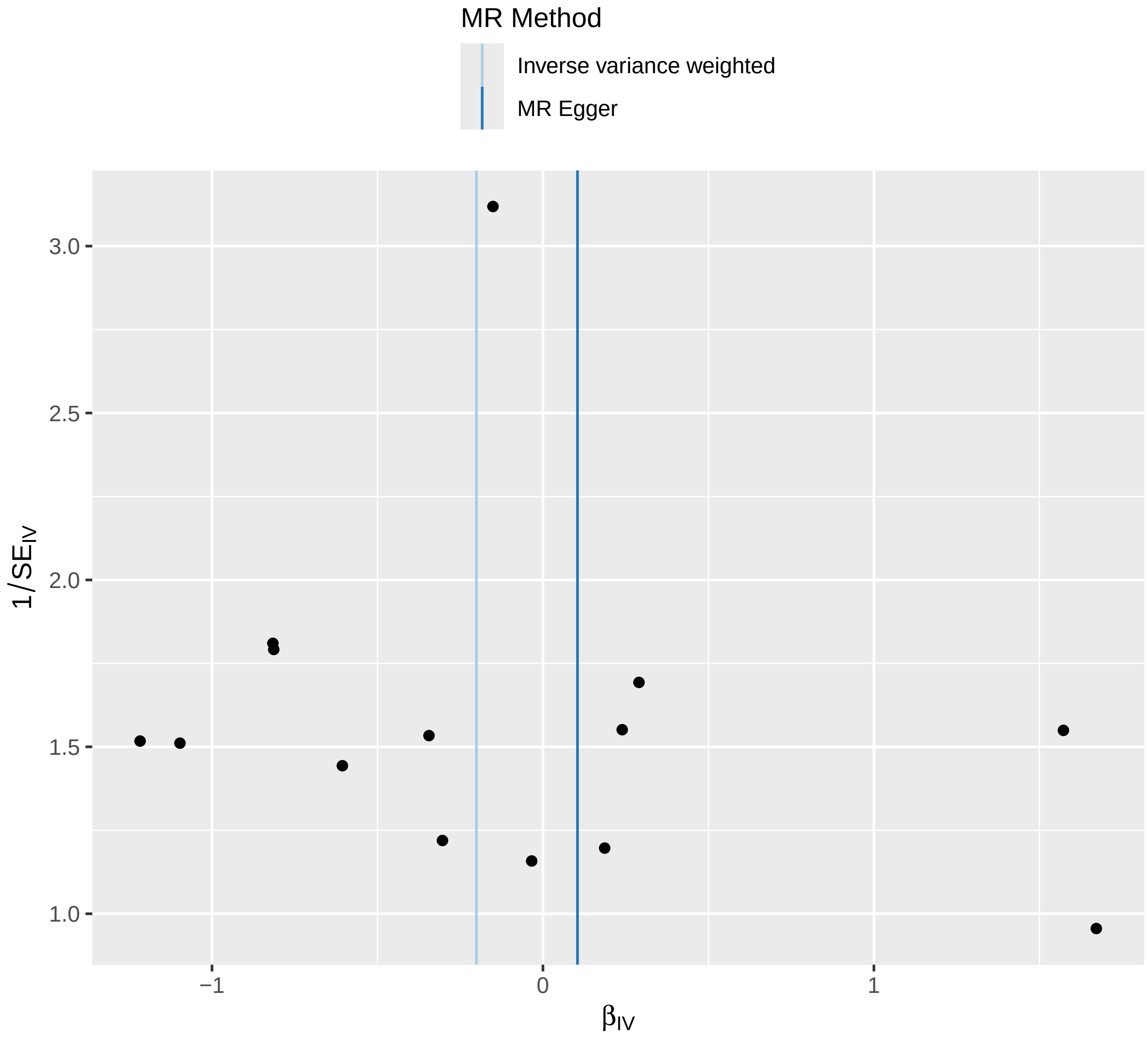

MR Method
Inverse variance weighted
MR Egger
3.0
2.5
2.0
1.5
1.0
1/SE_IV
−1
0
1
β_IV

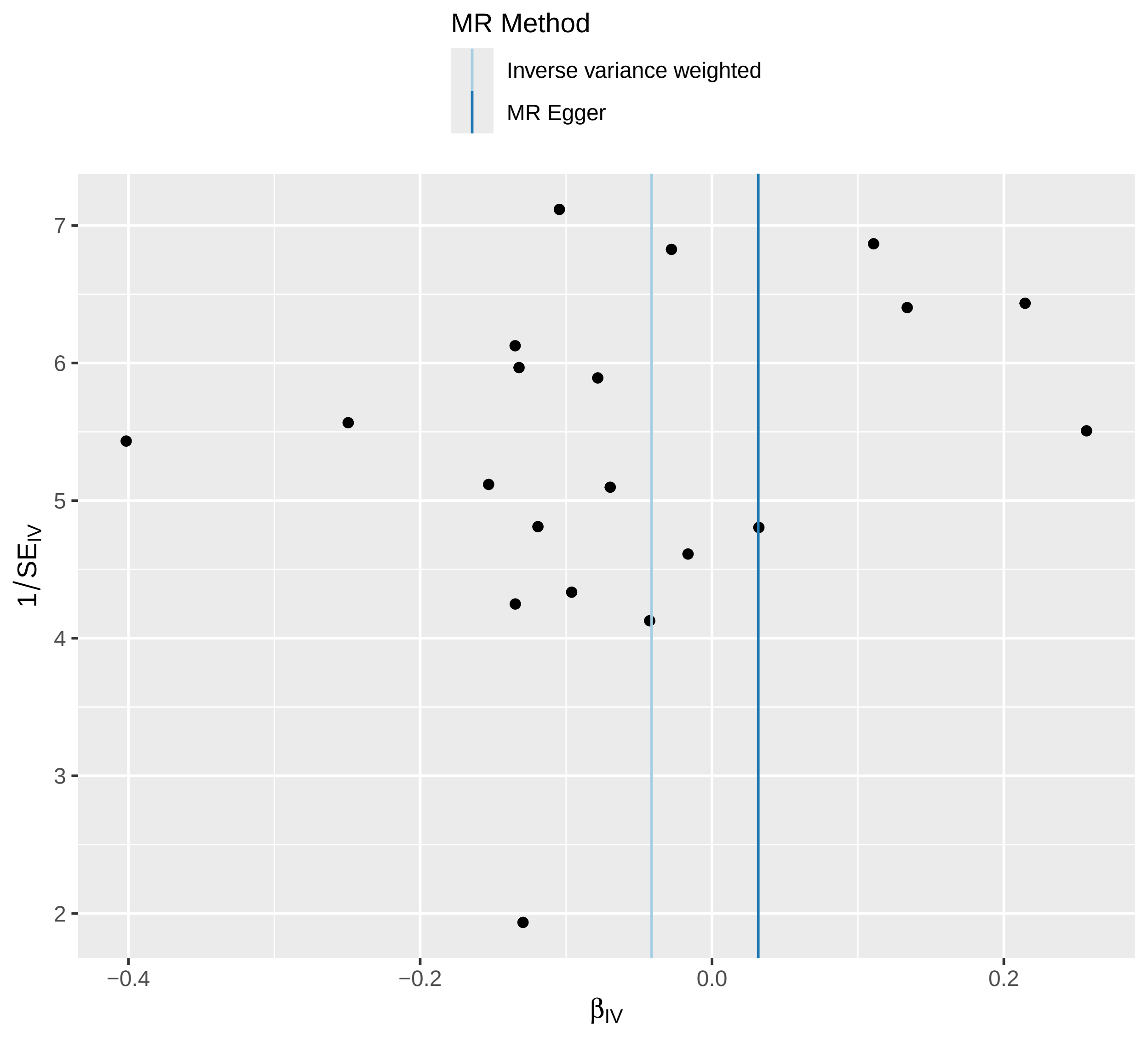

MR Method
Inverse variance weighted
MR Egger
7
6
5
4
3
2
1/SE$_{IV}$
−0.4
−0.2
0.0
0.2
β_{IV}

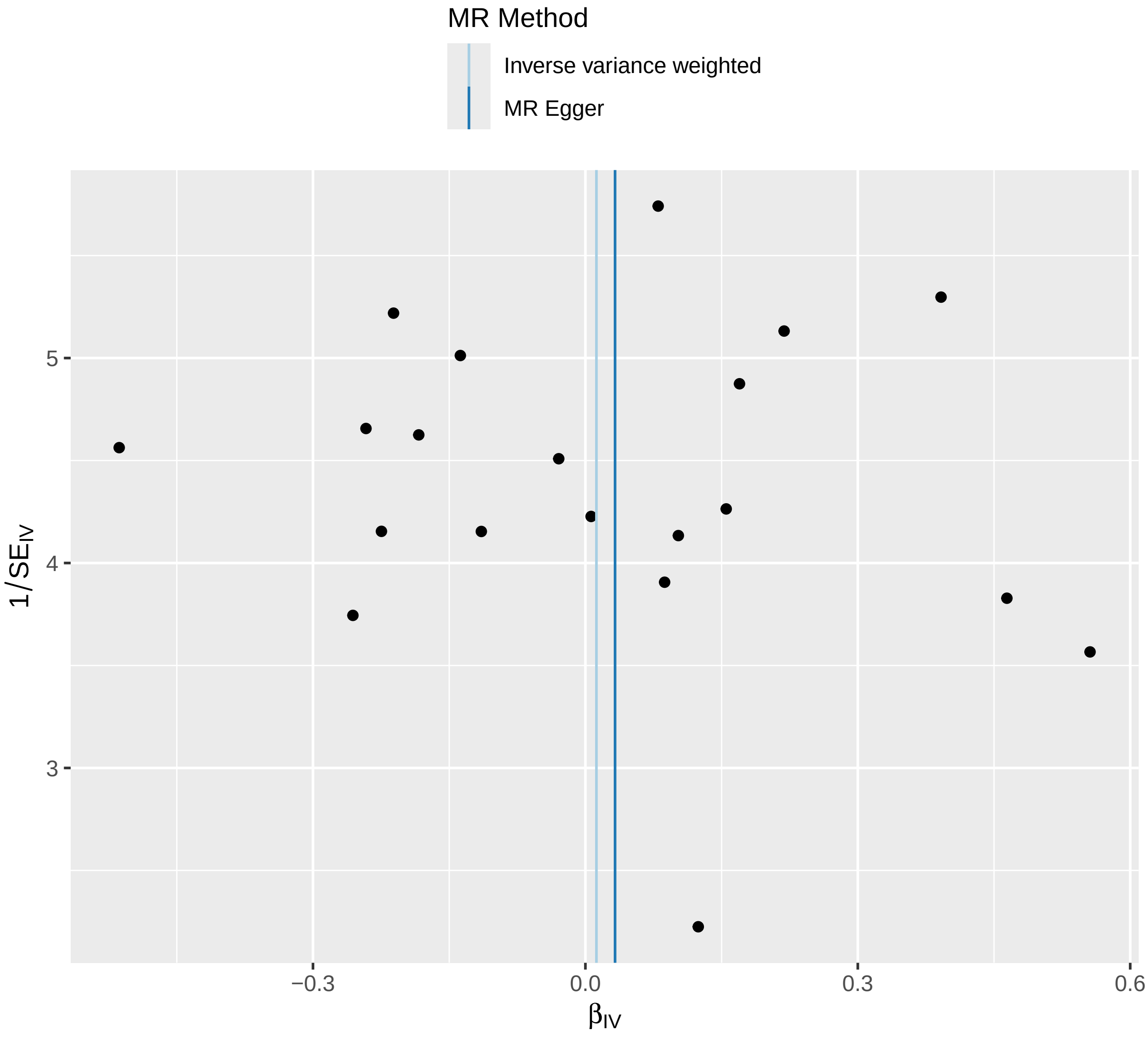

MR Method
Inverse variance weighted
MR Egger
5
4
3
1/SE$_{IV}$
−0.3
0.0
0.3
0.6
β$_{IV}$

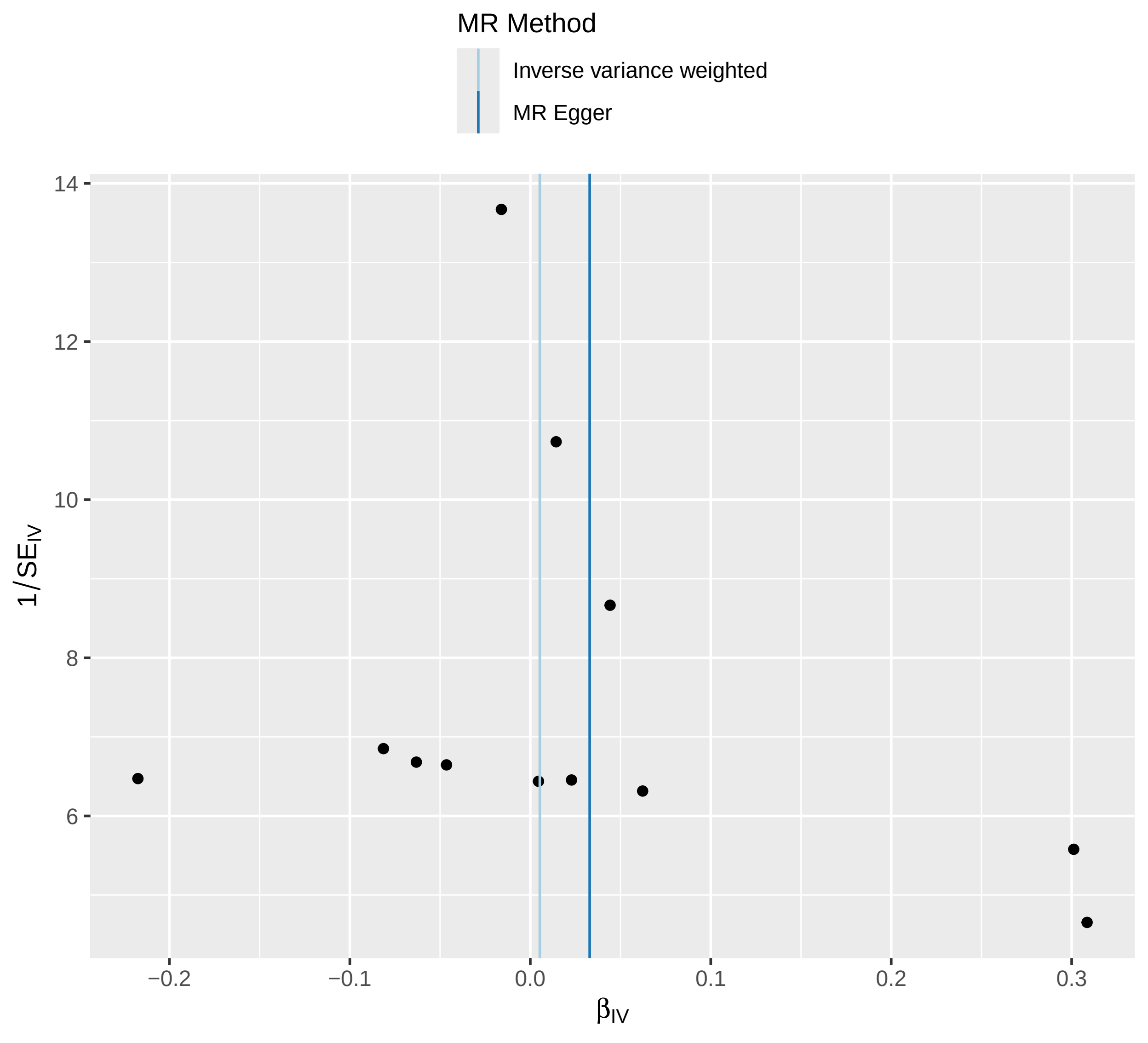

MR Method
Inverse variance weighted
MR Egger
14
12
10
8
6
1/SE$_{IV}$
−0.2
−0.1
0.0
0.1
0.2
0.3
β$_{IV}$

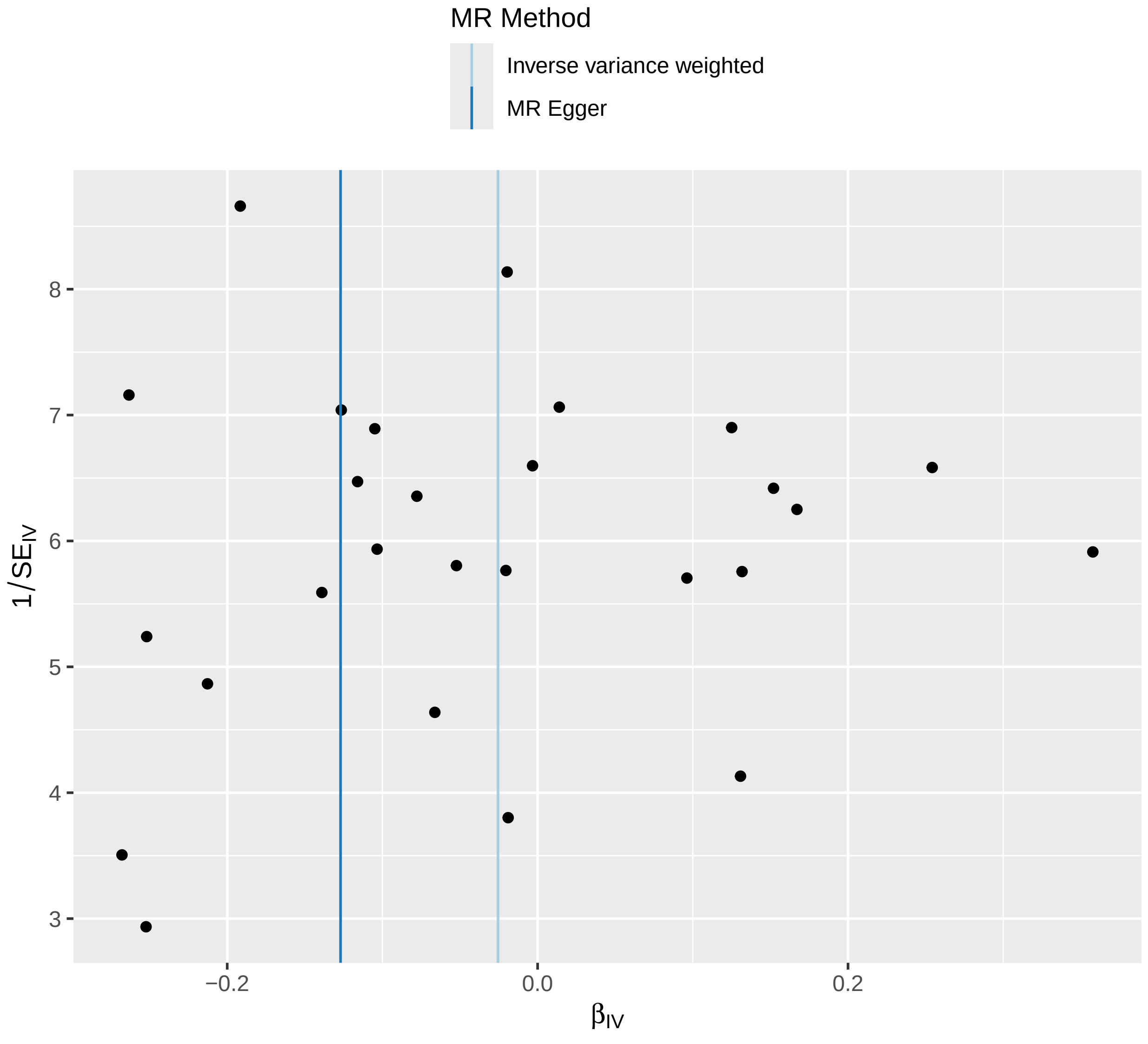

MR Method
Inverse variance weighted
MR Egger
1/SE_IV
8
7
6
5
4
3
−0.2
0.0
0.2
β_IV

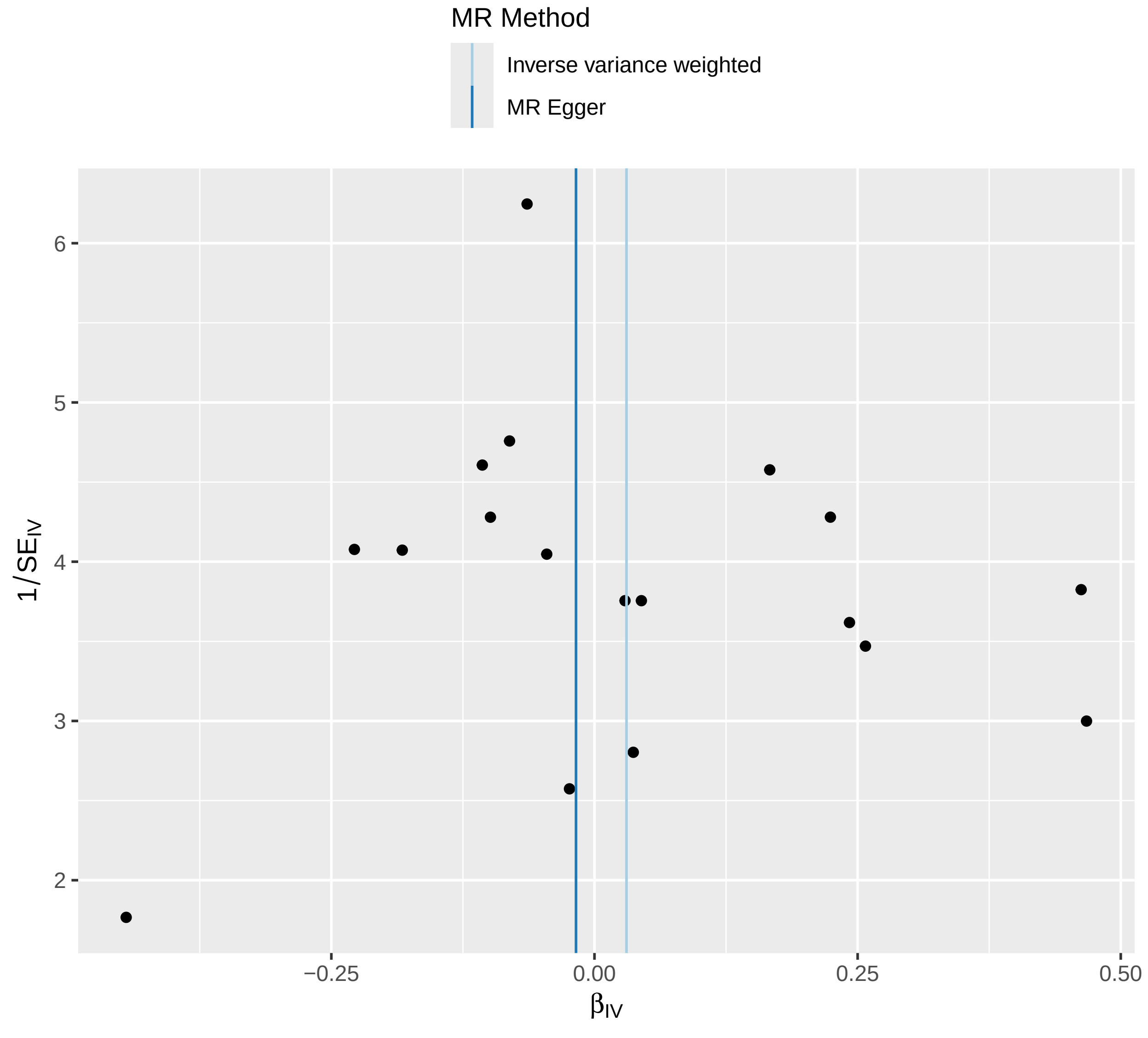
MR Method
Inverse variance weighted
MR Egger
1/SE$_{IV}$
6
5
4
3
2
−0.25
0.00
0.25
0.50
β_{IV}

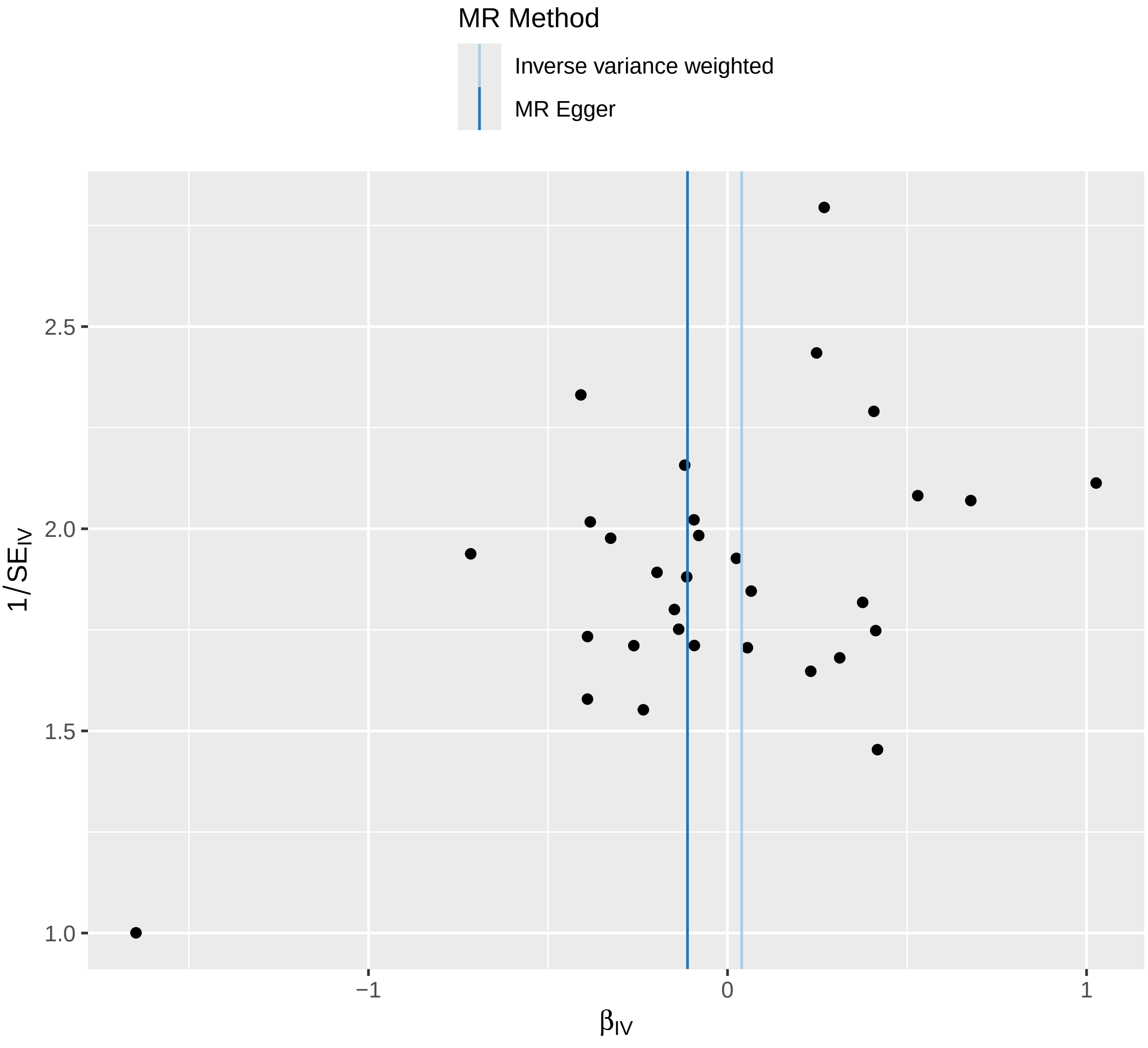
MR Method
Inverse variance weighted
MR Egger
2.5
2.0
1.5
1.0
$1/SE_{IV}$
−1
0
1
β_{IV}

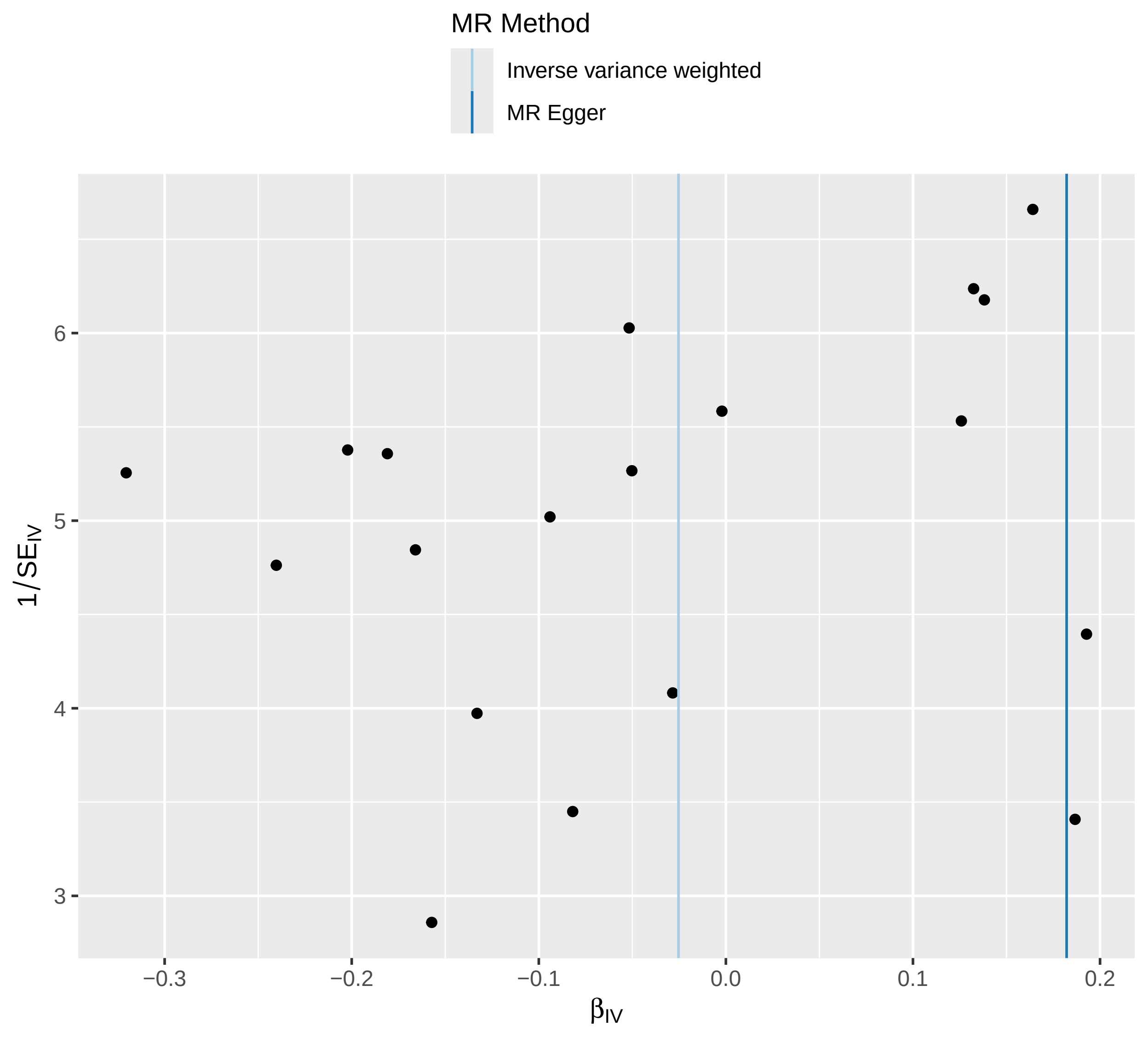
MR Method
Inverse variance weighted
MR Egger
1/SE_IV
β_IV
6
5
4
3
−0.3
−0.2
−0.1
0.0
0.1
0.2

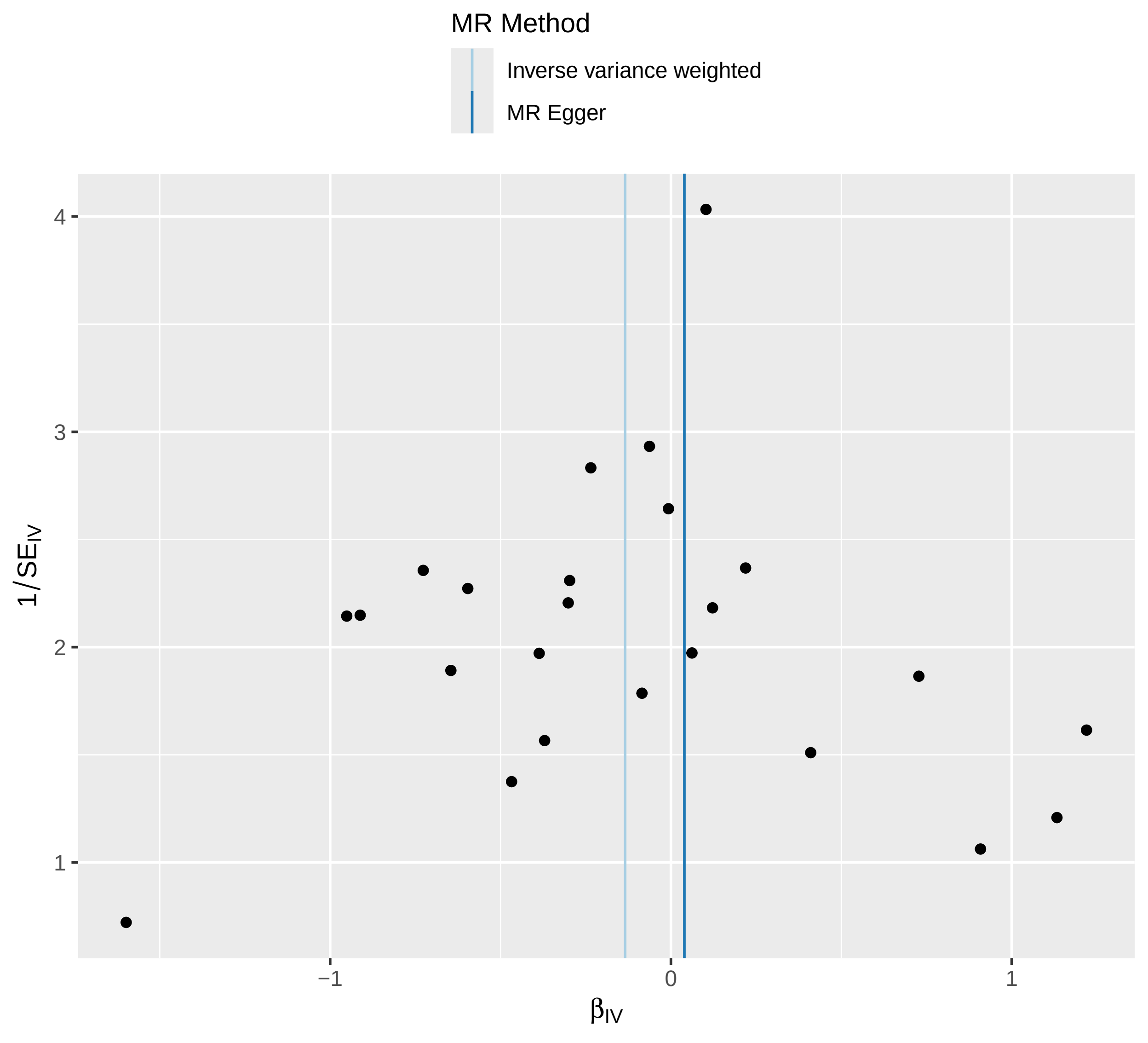
MR Method
Inverse variance weighted
MR Egger
1/SE$_{IV}$
4
3
2
1
−1
0
1
β$_{IV}$

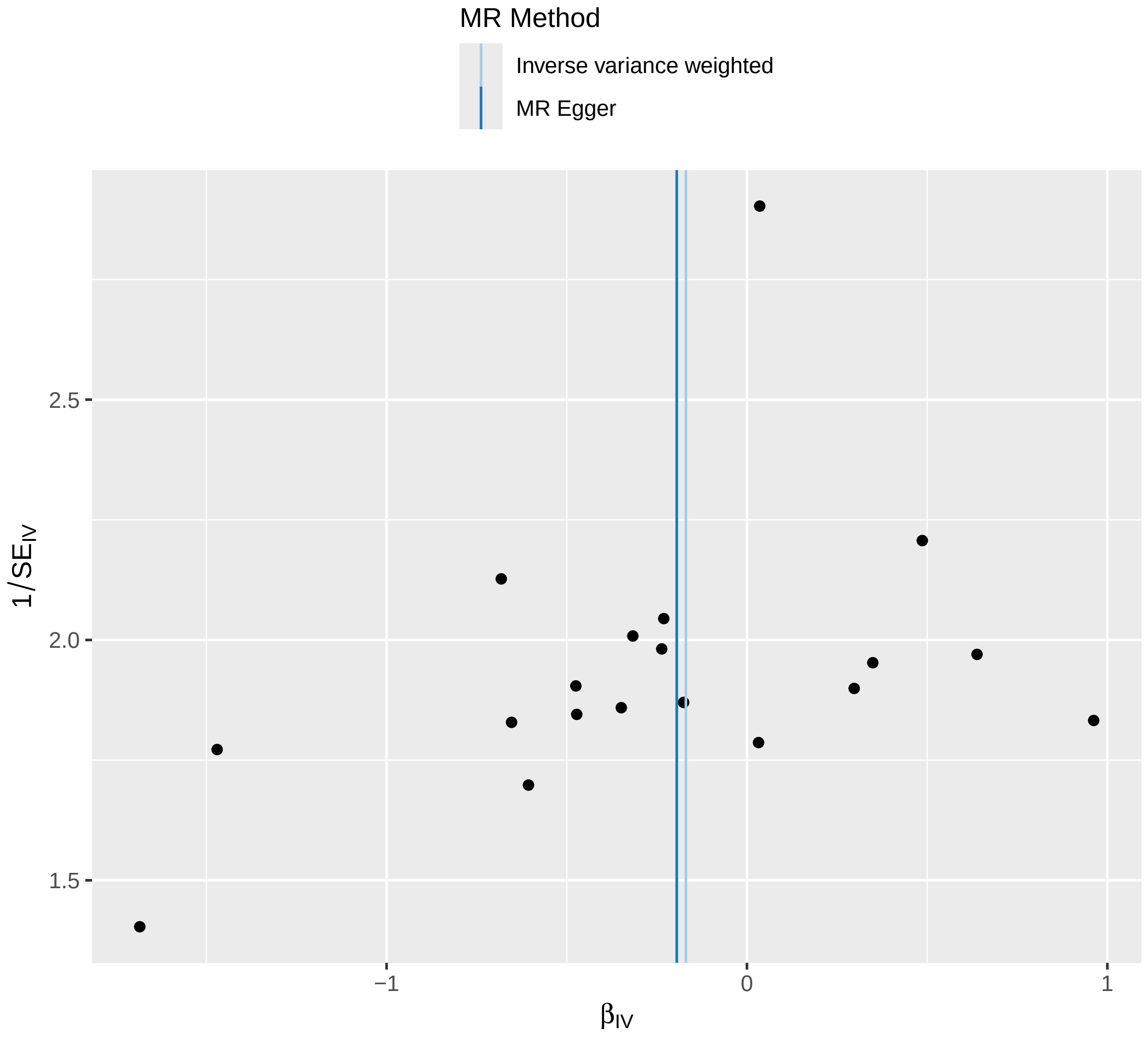
MR Method
Inverse variance weighted
MR Egger
2.5
2.0
1.5
1/SE_IV
−1
0
1
β_IV

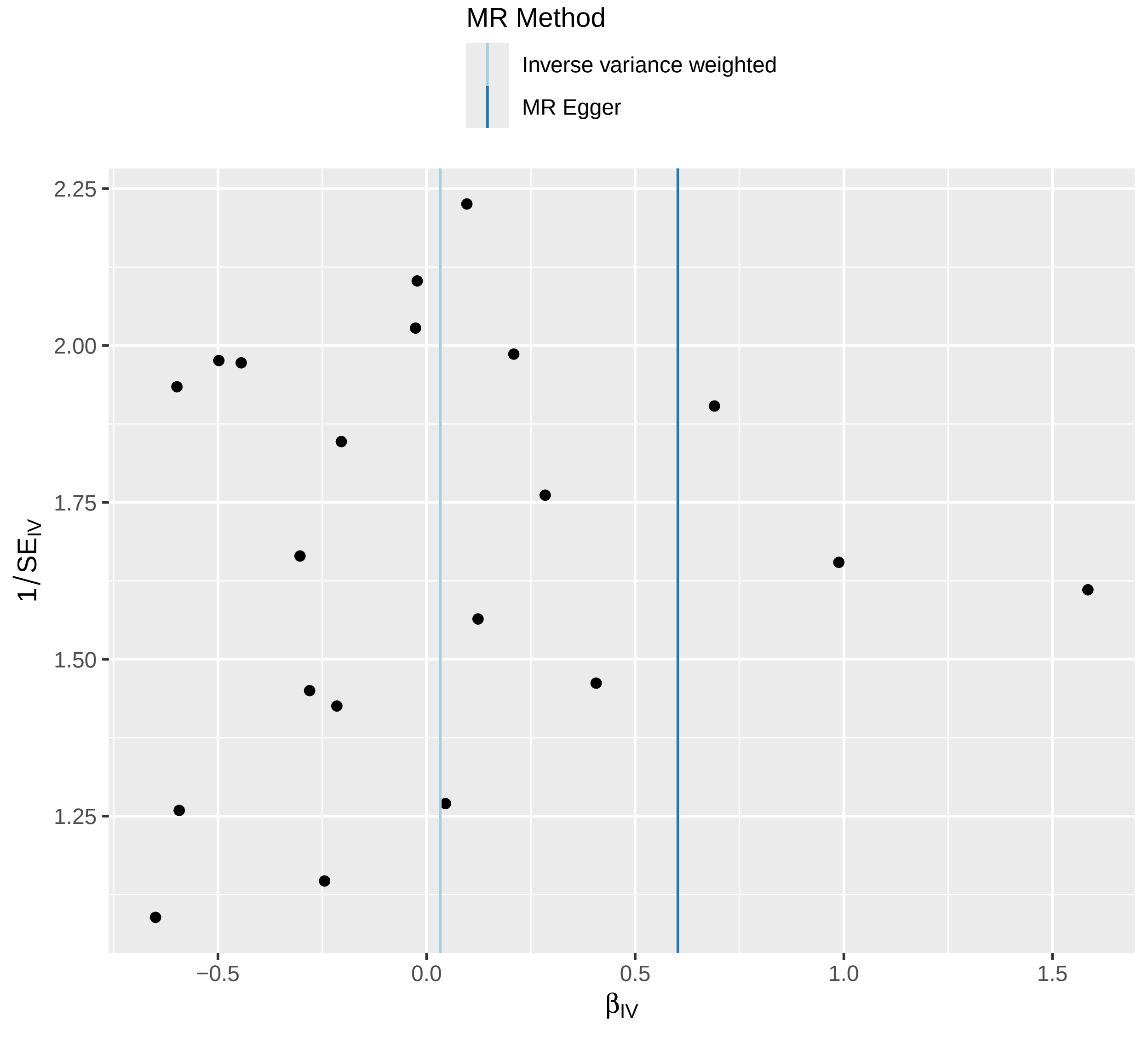

MR Method
Inverse variance weighted
MR Egger
2.25
2.00
1.75
1.50
1.25
1/SE$_{IV}$
−0.5
0.0
0.5
1.0
1.5
β$_{IV}$

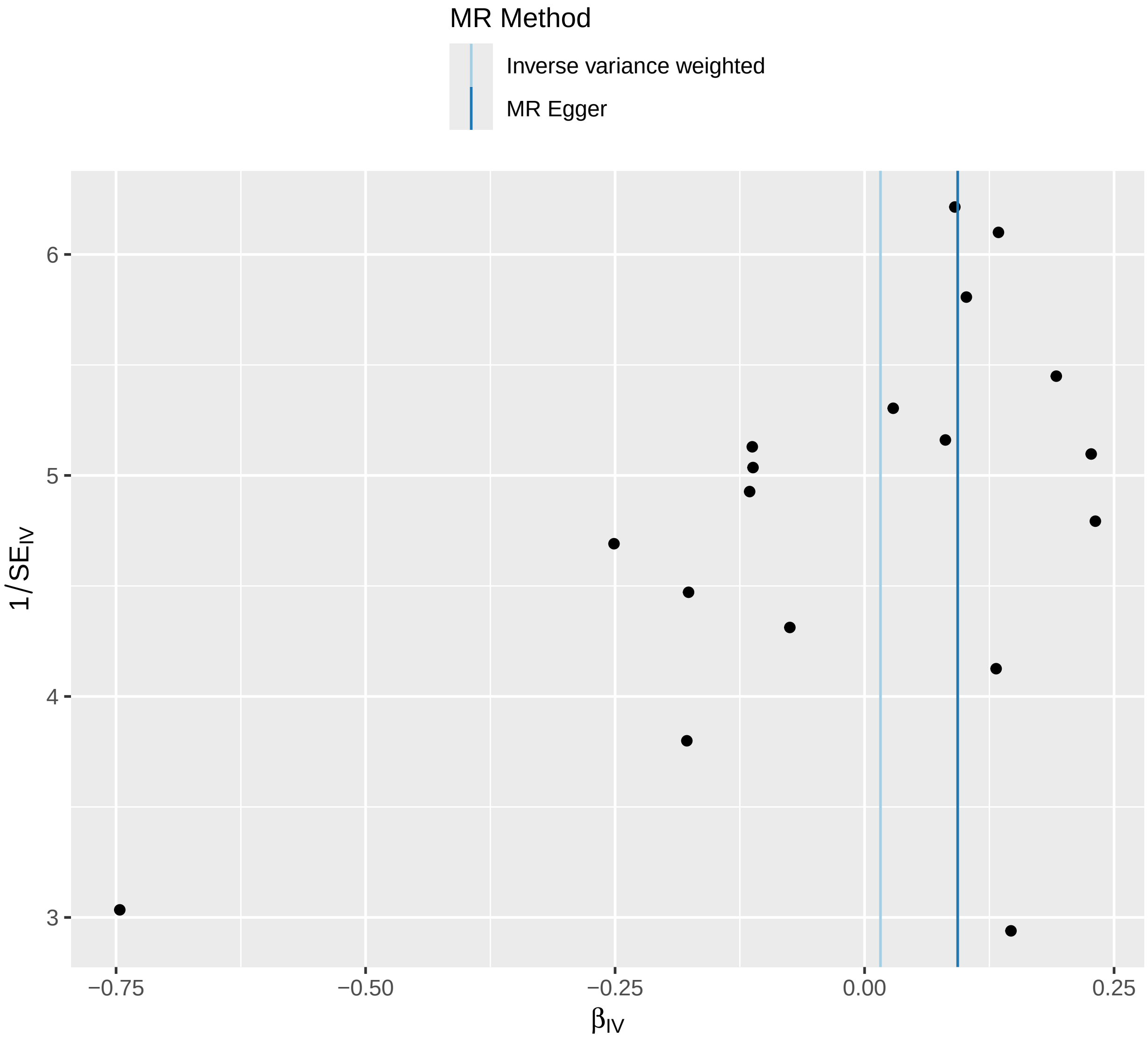

MR Method
Inverse variance weighted
MR Egger
1/SE$_{IV}$
6
5
4
3
−0.75
−0.50
−0.25
0.00
0.25
β$_{IV}$

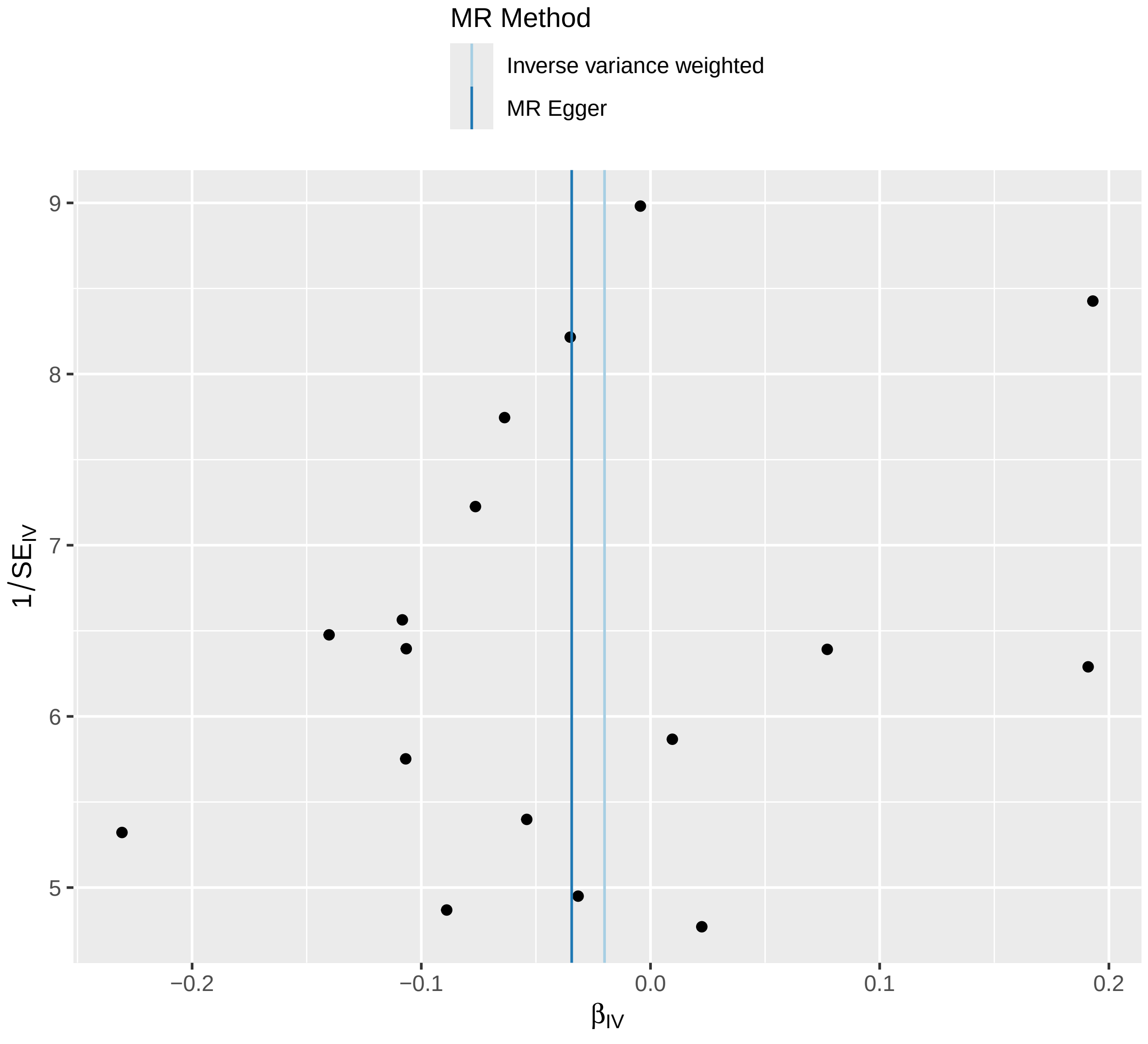

MR Method
Inverse variance weighted
MR Egger
9
8
7
6
5
$1/SE_{IV}$
−0.2
−0.1
0.0
0.1
0.2
β_{IV}

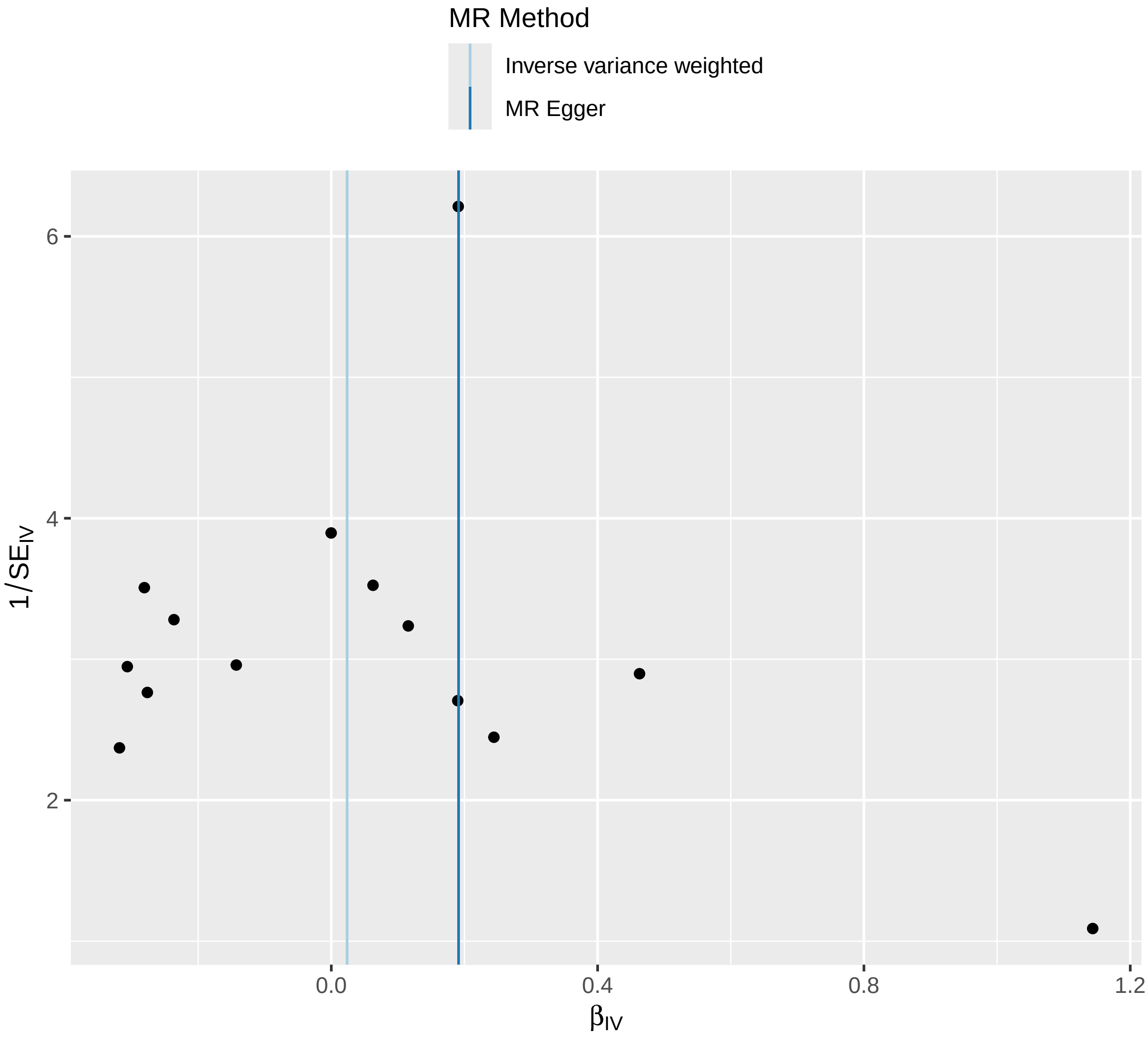

MR Method
Inverse variance weighted
MR Egger
6
4
2
1/SE_IV
0.0
0.4
0.8
1.2
β_IV

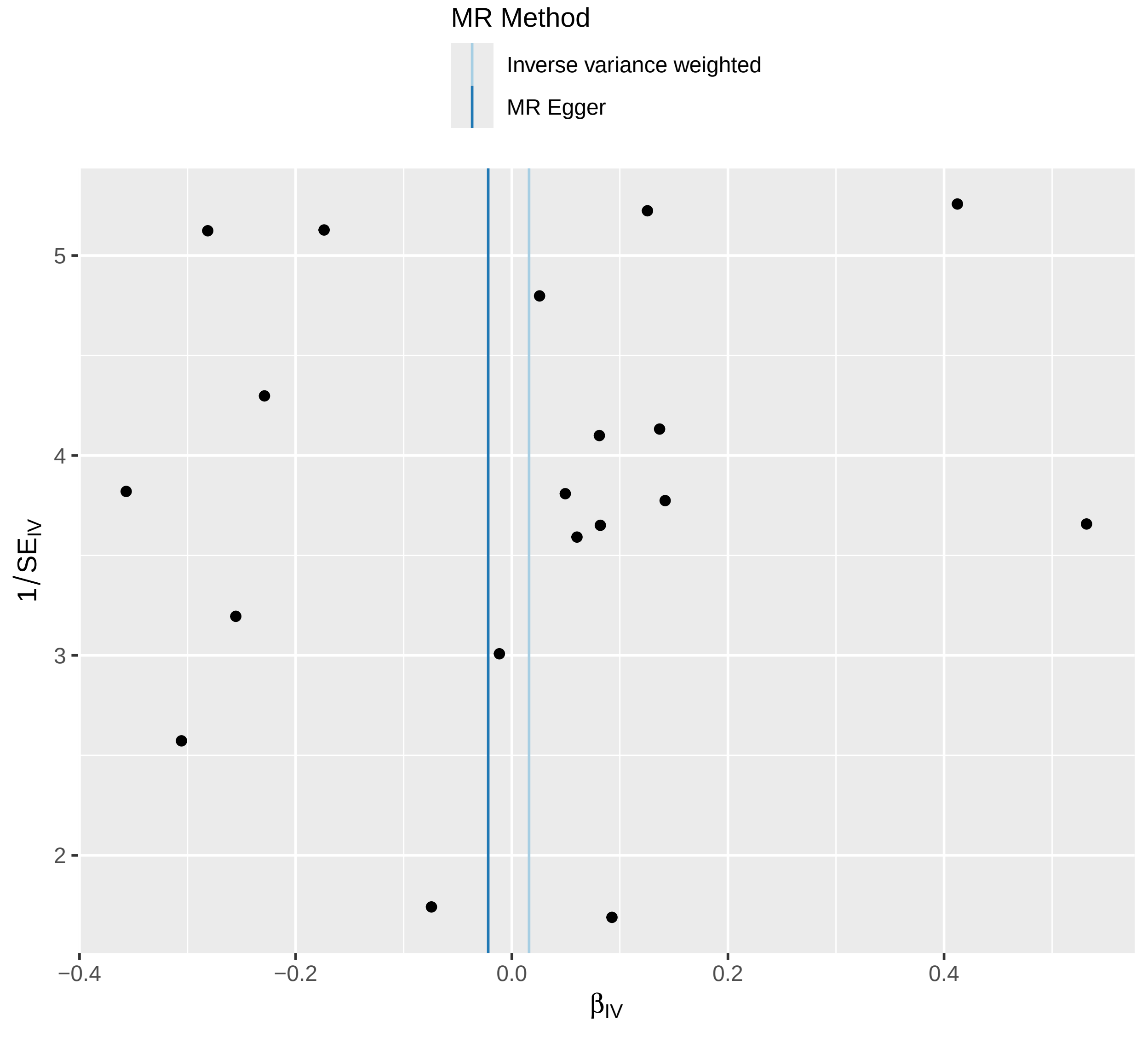
MR Method
Inverse variance weighted
MR Egger
1/SE$_{IV}$
5
4
3
2
−0.4
−0.2
0.0
0.2
0.4
β_{IV}

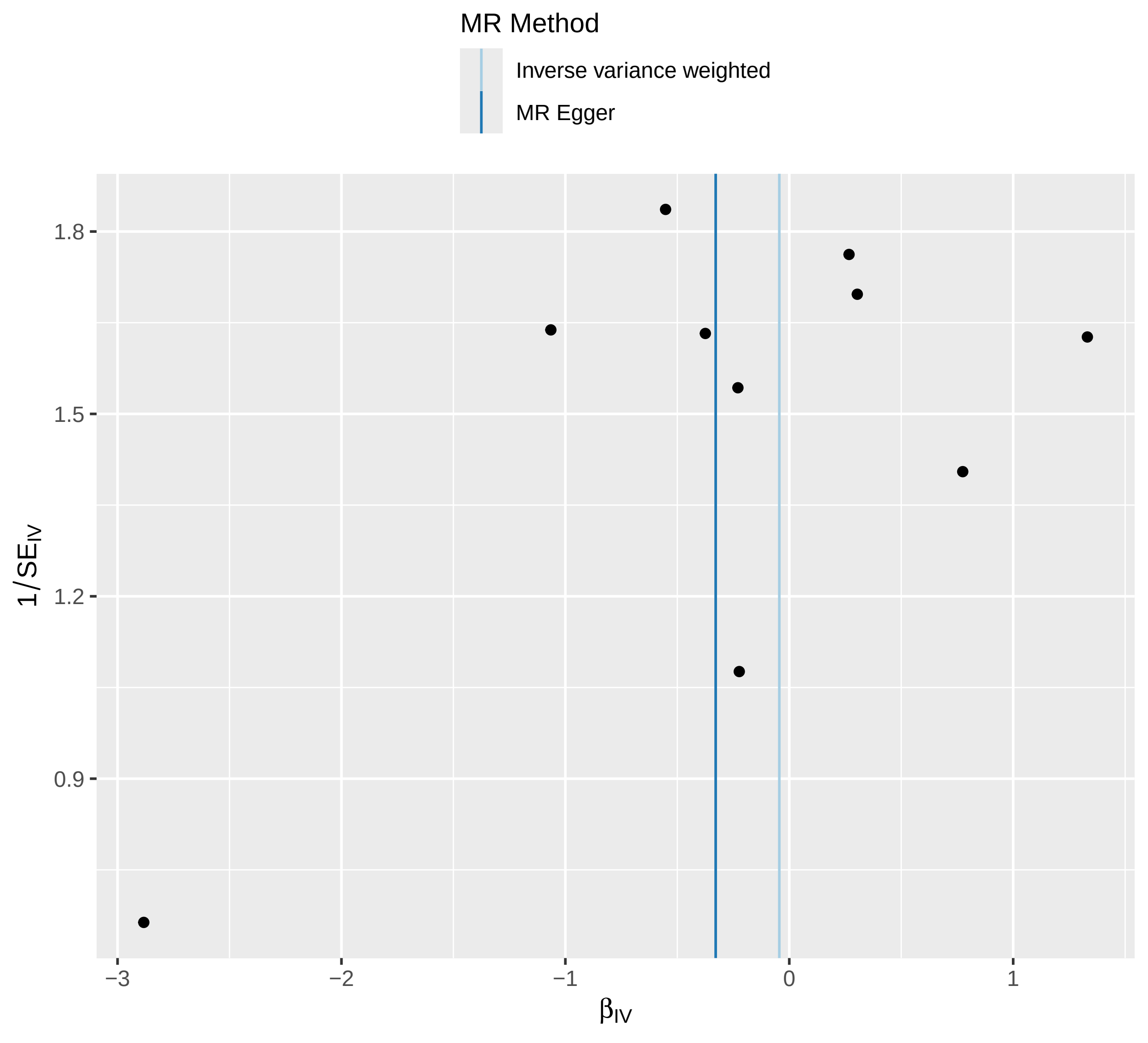
MR Method
Inverse variance weighted
MR Egger
1.8
1.5
1.2
0.9
−3
−2
−1
0
1
$1/SE_{IV}$
β_{IV}

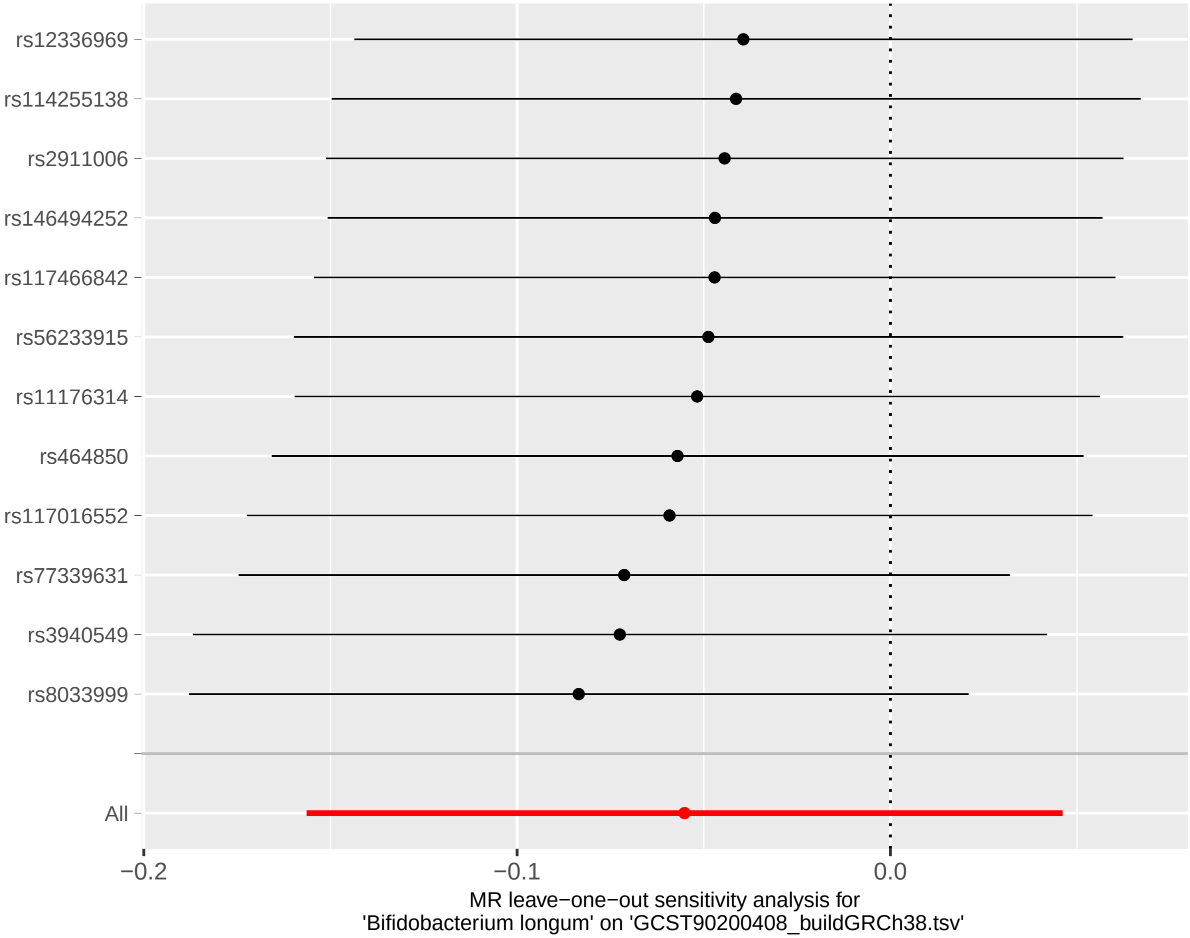
rs12336969
rs114255138
rs2911006
rs146494252
rs117466842
rs56233915
rs11176314
rs464850
rs117016552
rs77339631
rs3940549
rs8033999
All
−0.2
−0.1
0.0
MR leave−one−out sensitivity analysis for
'Bifidobacterium longum' on 'GCST90200408_buildGRCh38.tsv'

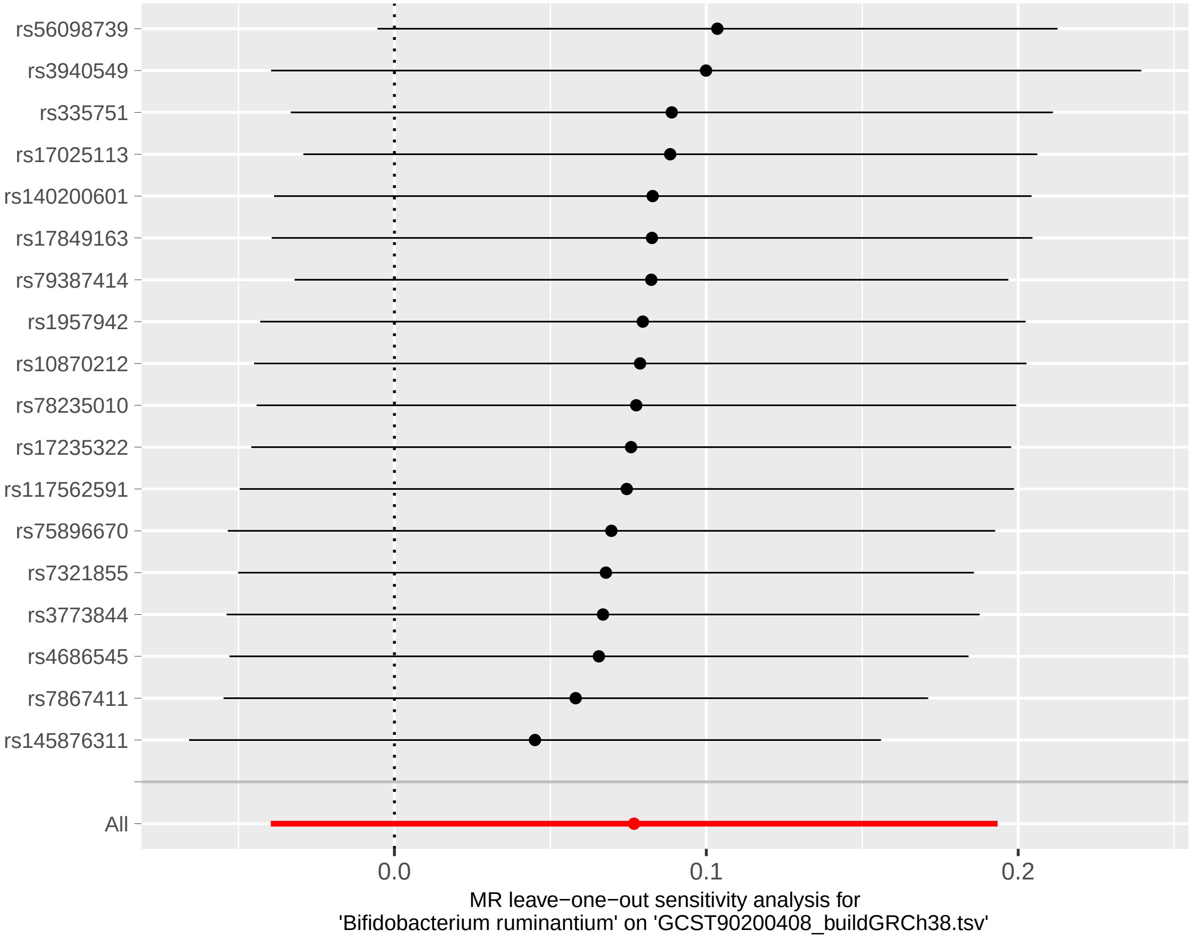
rs56098739
rs3940549
rs335751
rs17025113
rs140200601
rs17849163
rs79387414
rs1957942
rs10870212
rs78235010
rs17235322
rs117562591
rs75896670
rs7321855
rs3773844
rs4686545
rs7867411
rs145876311
All
0.0
0.1
0.2
MR leave−one−out sensitivity analysis for
'Bifidobacterium ruminantium' on 'GCST90200408_buildGRCh38.tsv'

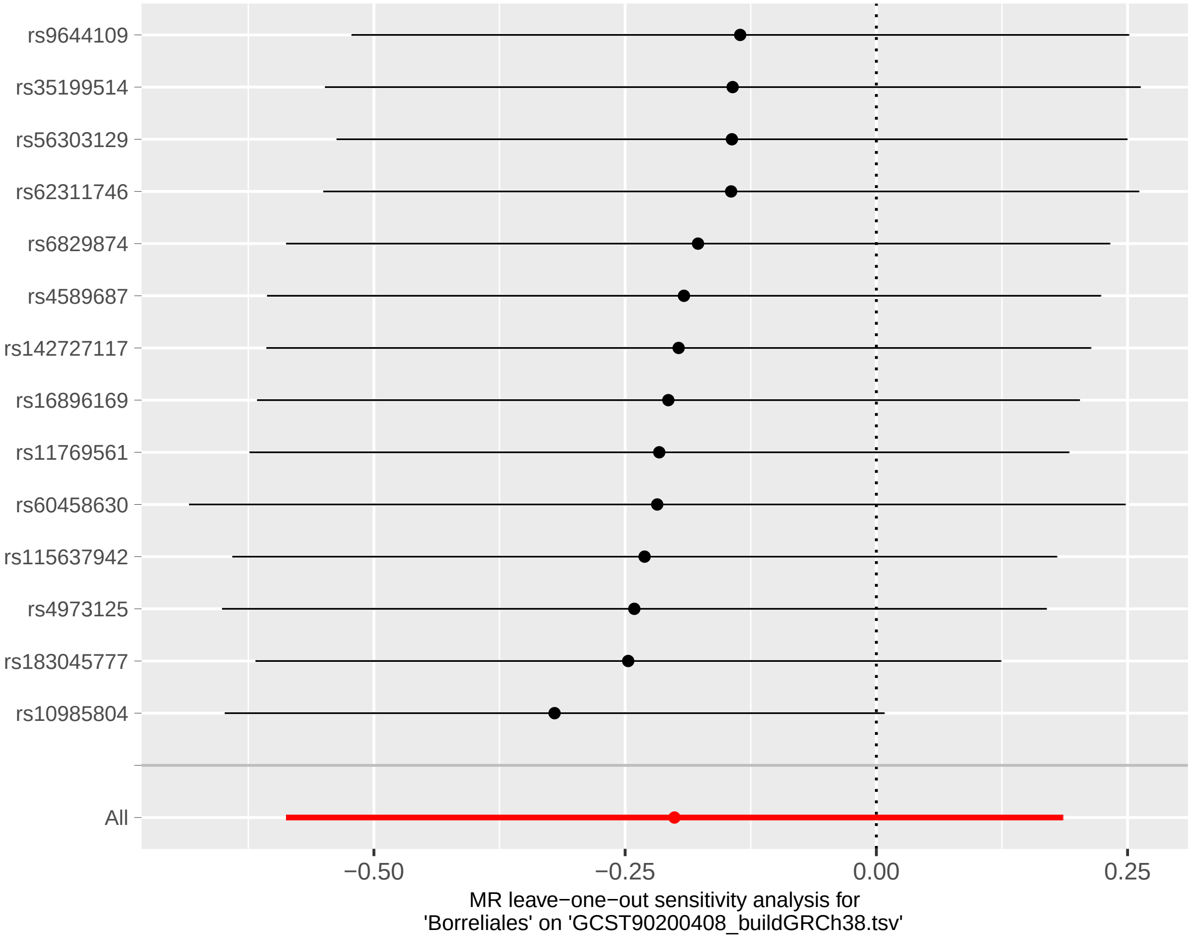

rs9644109
rs35199514
rs56303129
rs62311746
rs6829874
rs4589687
rs142727117
rs16896169
rs11769561
rs60458630
rs115637942
rs4973125
rs183045777
rs10985804
All
−0.50
−0.25
0.00
0.25
MR leave−one−out sensitivity analysis for
'Borreliales' on 'GCST90200408_buildGRCh38.tsv'

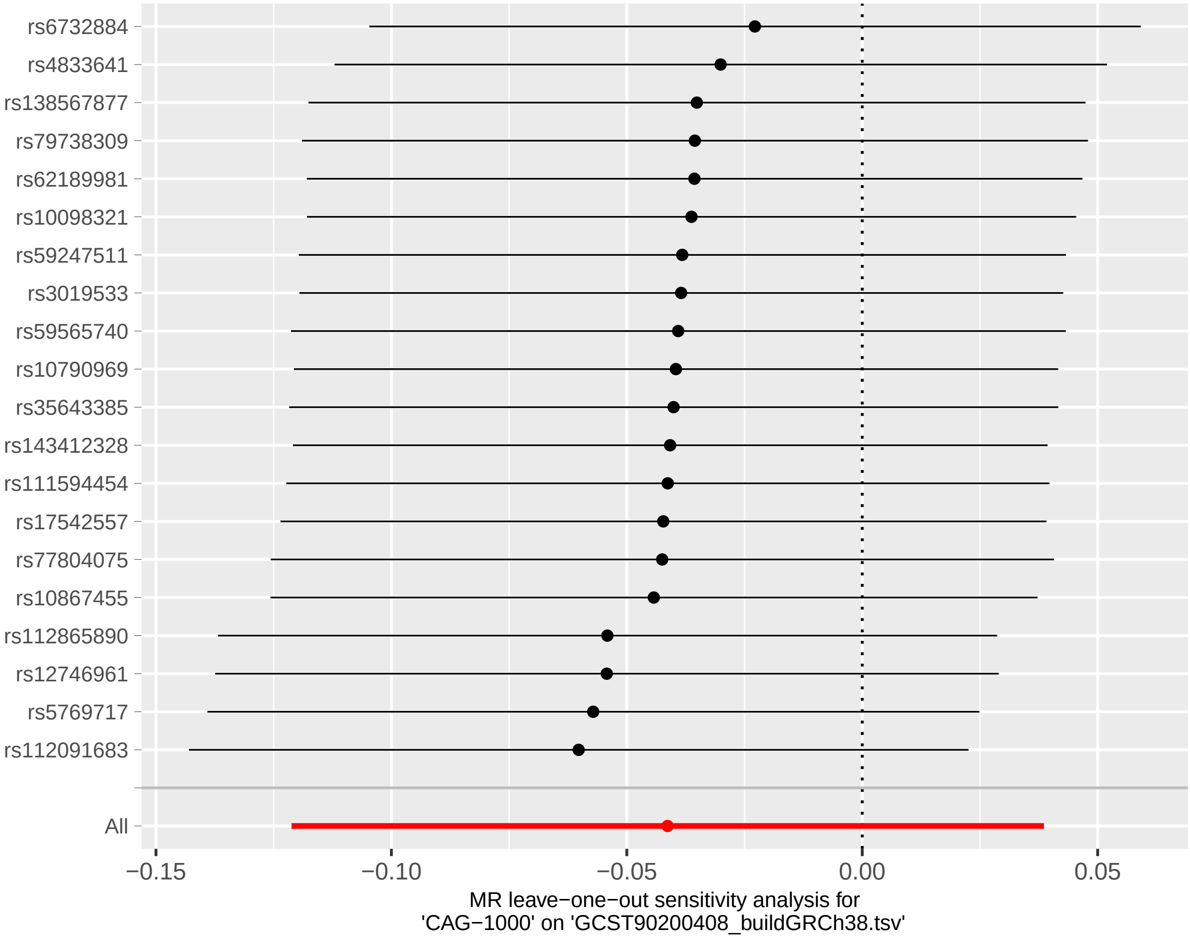

rs6732884
rs4833641
rs138567877
rs79738309
rs62189981
rs10098321
rs59247511
rs3019533
rs59565740
rs10790969
rs35643385
rs143412328
rs111594454
rs17542557
rs77804075
rs10867455
rs112865890
rs12746961
rs5769717
rs112091683
All
−0.15
−0.10
−0.05
0.00
0.05
MR leave−one−out sensitivity analysis for
'CAG−1000' on 'GCST90200408_buildGRCh38.tsv'

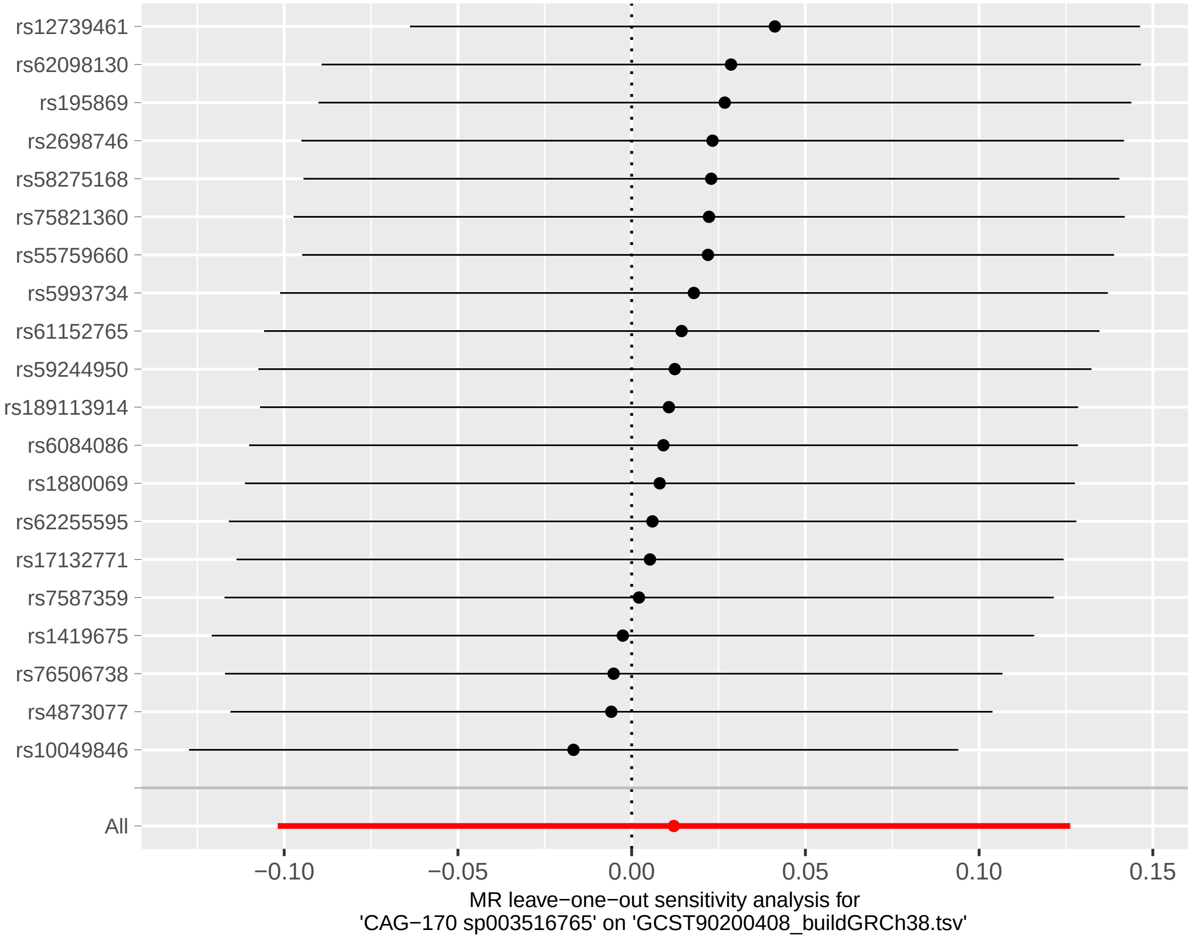

rs12739461
rs62098130
rs195869
rs2698746
rs58275168
rs75821360
rs55759660
rs5993734
rs61152765
rs59244950
rs189113914
rs6084086
rs1880069
rs62255595
rs17132771
rs7587359
rs1419675
rs76506738
rs4873077
rs10049846
All
−0.10
−0.05
0.00
0.05
0.10
0.15
MR leave−one−out sensitivity analysis for
'CAG−170 sp003516765' on 'GCST90200408_buildGRCh38.tsv'

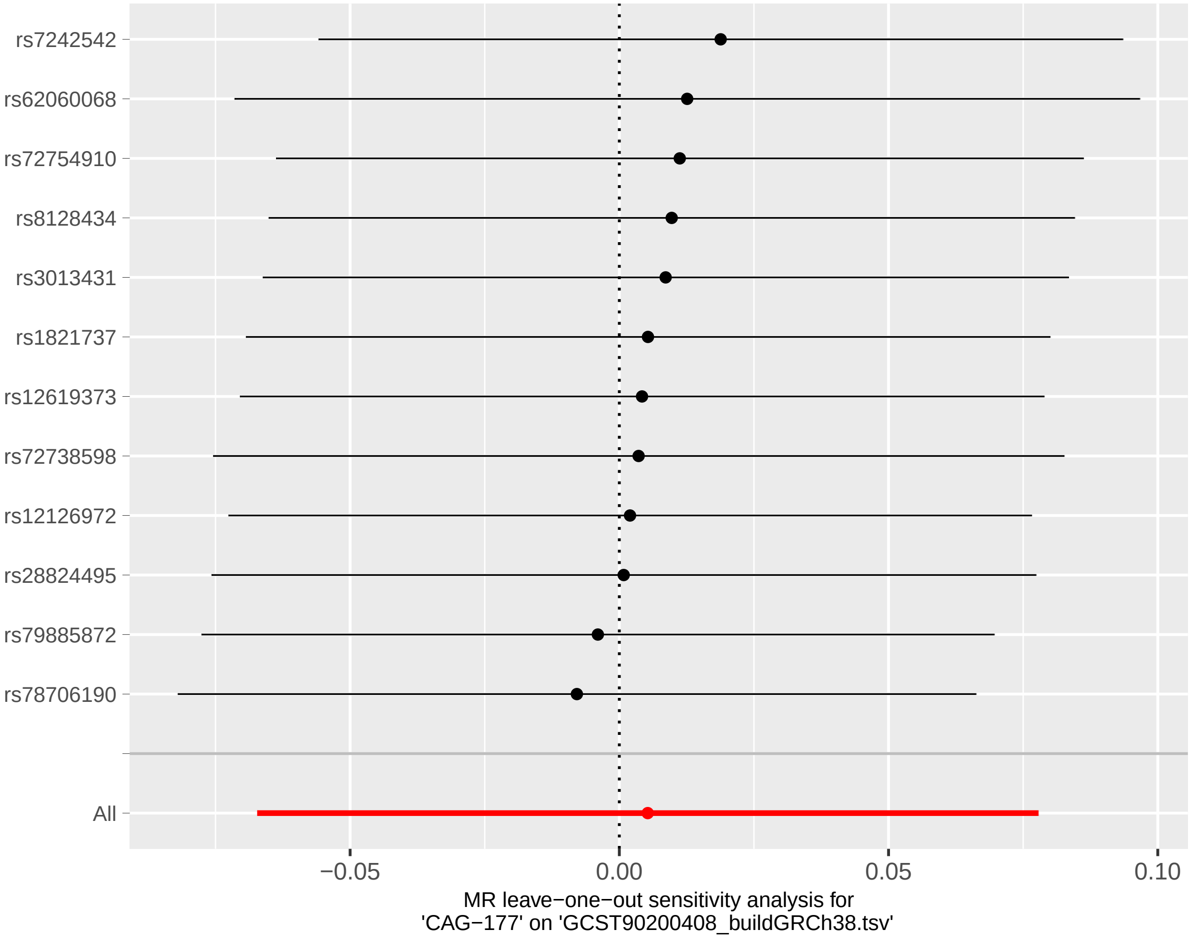
rs7242542
rs62060068
rs72754910
rs8128434
rs3013431
rs1821737
rs12619373
rs72738598
rs12126972
rs28824495
rs79885872
rs78706190
All
−0.05
0.00
0.05
0.10
MR leave−one−out sensitivity analysis for
'CAG−177' on 'GCST90200408_buildGRCh38.tsv'

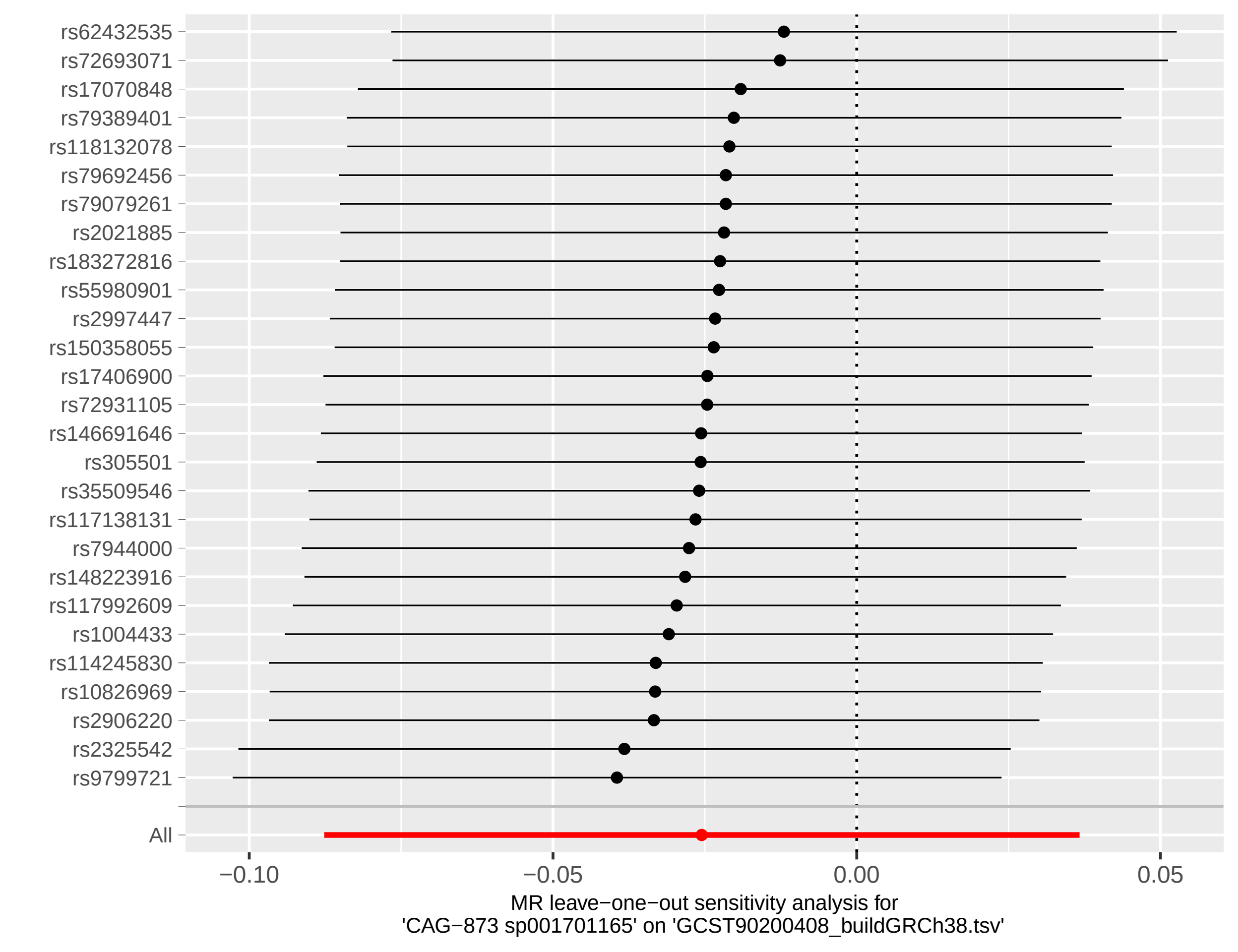

rs62432535
rs72693071
rs17070848
rs79389401
rs118132078
rs79692456
rs79079261
rs2021885
rs183272816
rs55980901
rs2997447
rs150358055
rs17406900
rs72931105
rs146691646
rs305501
rs35509546
rs117138131
rs7944000
rs148223916
rs117992609
rs1004433
rs114245830
rs10826969
rs2906220
rs2325542
rs9799721
All
−0.10
−0.05
0.00
0.05
MR leave−one−out sensitivity analysis for
'CAG−873 sp001701165' on 'GCST90200408_buildGRCh38.tsv'

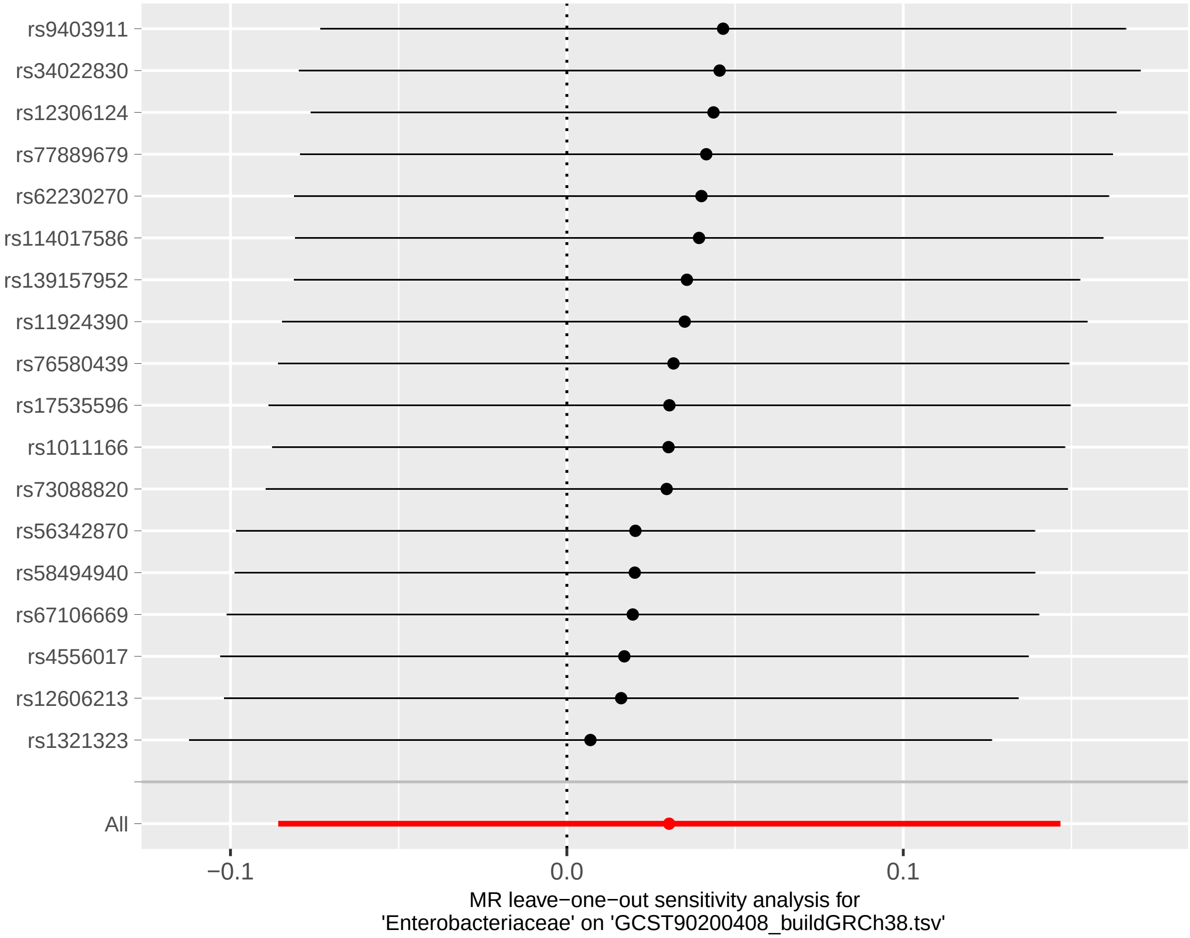
rs9403911
rs34022830
rs12306124
rs77889679
rs62230270
rs114017586
rs139157952
rs11924390
rs76580439
rs17535596
rs1011166
rs73088820
rs56342870
rs58494940
rs67106669
rs4556017
rs12606213
rs1321323
All
−0.1
0.0
0.1
MR leave−one−out sensitivity analysis for
'Enterobacteriaceae' on 'GCST90200408_buildGRCh38.tsv'

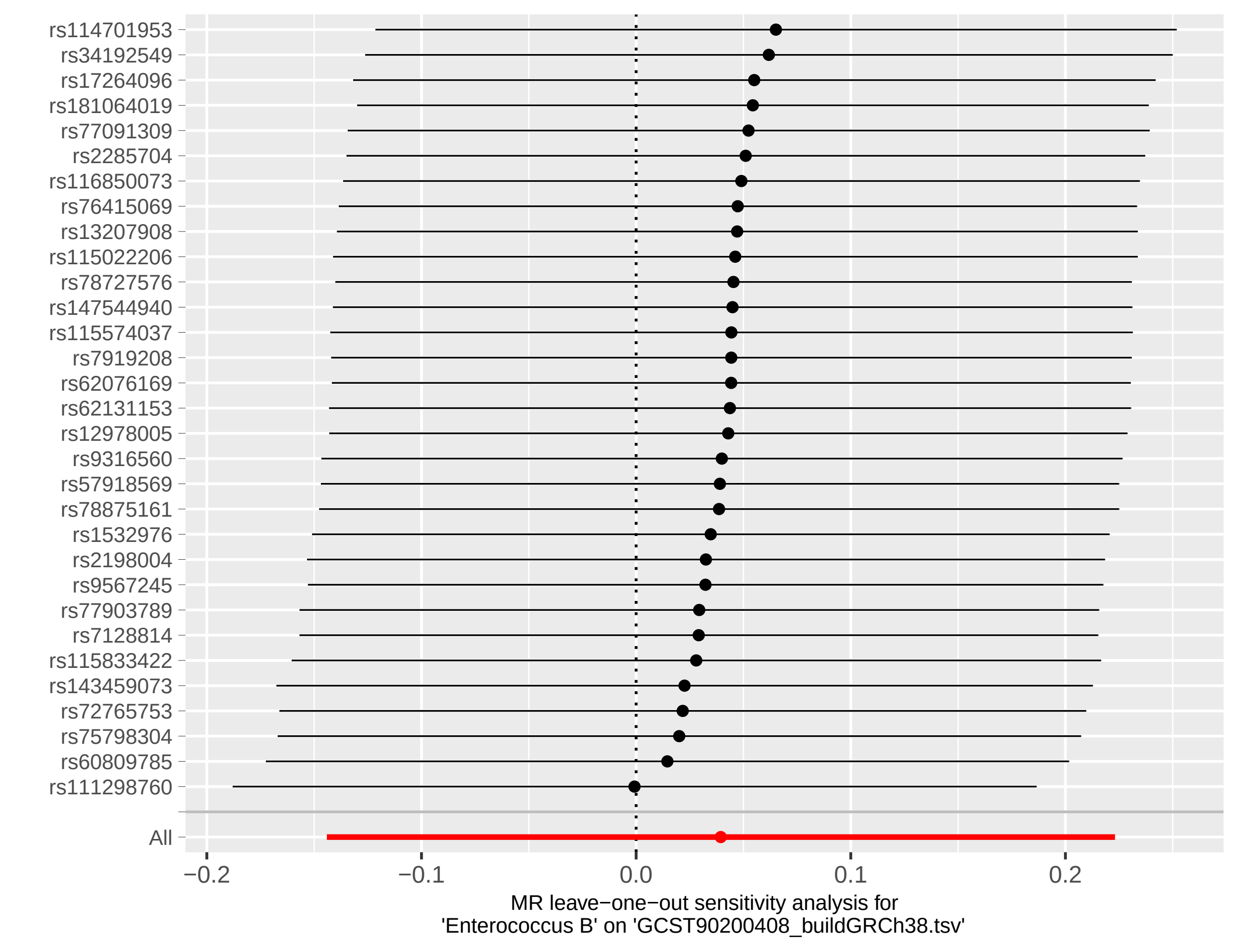

rs114701953
rs34192549
rs17264096
rs181064019
rs77091309
rs2285704
rs116850073
rs76415069
rs13207908
rs115022206
rs78727576
rs147544940
rs115574037
rs7919208
rs62076169
rs62131153
rs12978005
rs9316560
rs57918569
rs78875161
rs1532976
rs2198004
rs9567245
rs77903789
rs7128814
rs115833422
rs143459073
rs72765753
rs75798304
rs60809785
rs111298760
All
−0.2
−0.1
0.0
0.1
0.2
MR leave−one−out sensitivity analysis for
'Enterococcus B' on 'GCST90200408_buildGRCh38.tsv'

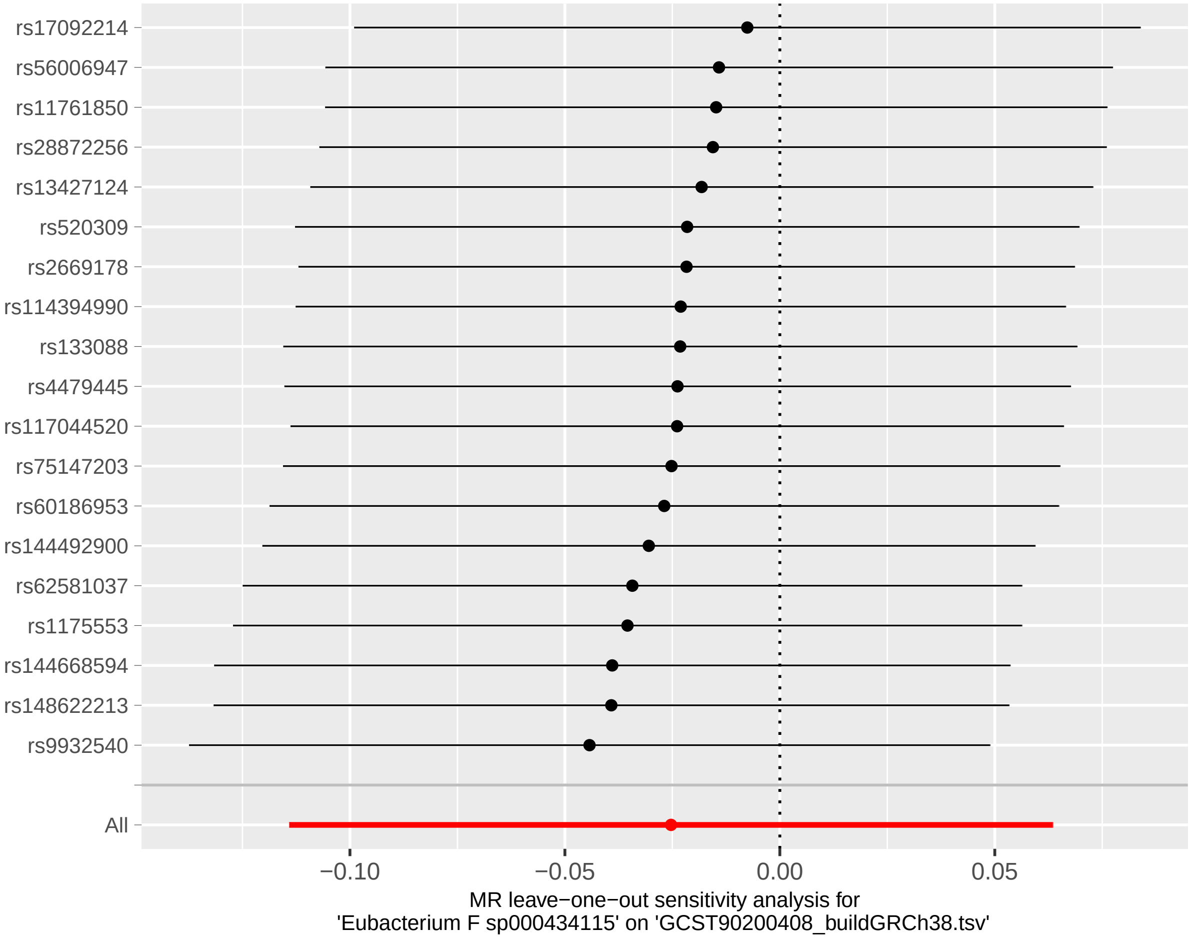

rs17092214
rs56006947
rs11761850
rs28872256
rs13427124
rs520309
rs2669178
rs114394990
rs133088
rs4479445
rs117044520
rs75147203
rs60186953
rs144492900
rs62581037
rs1175553
rs144668594
rs148622213
rs9932540
All
−0.10
−0.05
0.00
0.05
MR leave−one−out sensitivity analysis for
'Eubacterium F sp000434115' on 'GCST90200408_buildGRCh38.tsv'

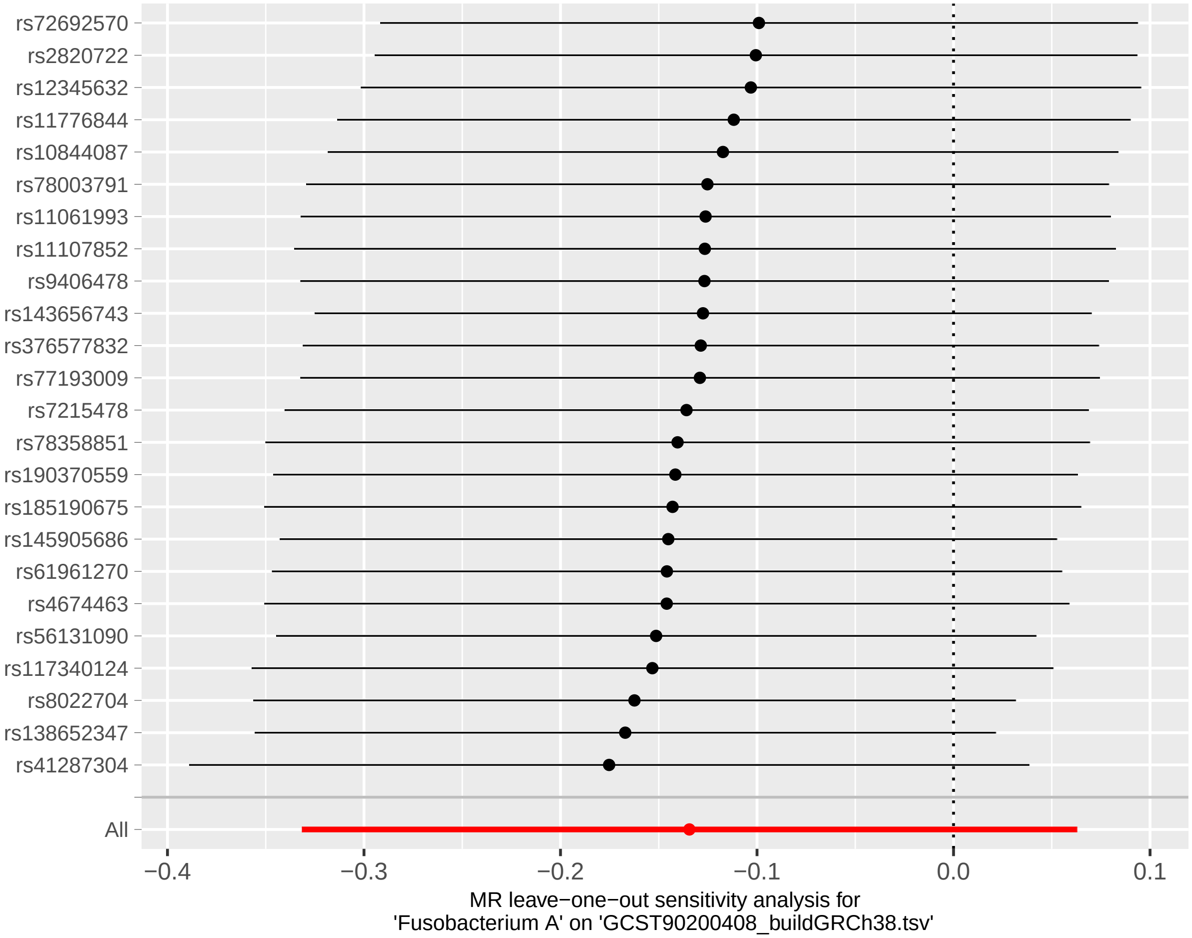

rs72692570
rs2820722
rs12345632
rs11776844
rs10844087
rs78003791
rs11061993
rs11107852
rs9406478
rs143656743
rs376577832
rs77193009
rs7215478
rs78358851
rs190370559
rs185190675
rs145905686
rs61961270
rs4674463
rs56131090
rs117340124
rs8022704
rs138652347
rs41287304
All
−0.4
−0.3
−0.2
−0.1
0.0
0.1
MR leave−one−out sensitivity analysis for
'Fusobacterium A' on 'GCST90200408_buildGRCh38.tsv'

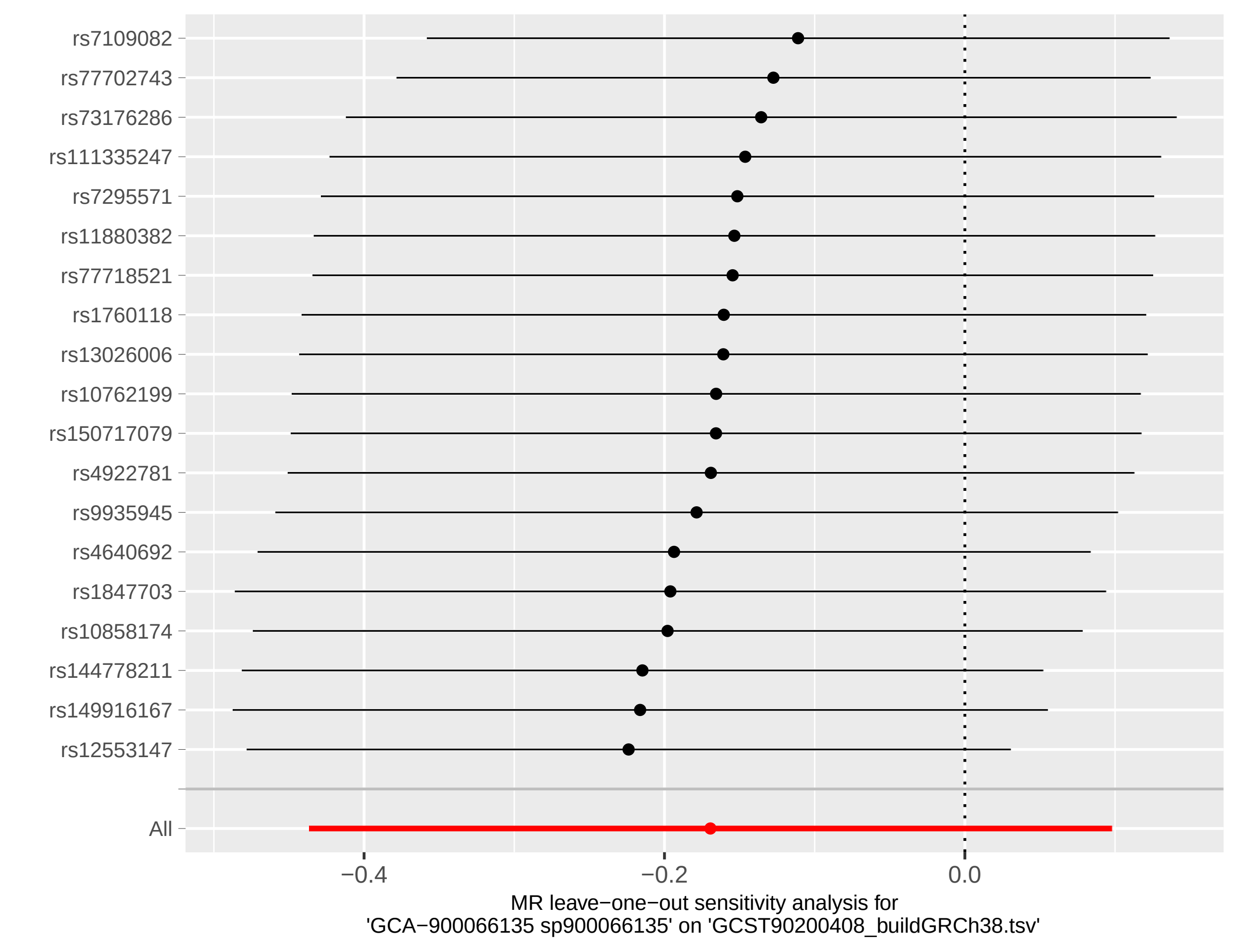

rs7109082
rs77702743
rs73176286
rs111335247
rs7295571
rs11880382
rs77718521
rs1760118
rs13026006
rs10762199
rs150717079
rs4922781
rs9935945
rs4640692
rs1847703
rs10858174
rs144778211
rs149916167
rs12553147
All
−0.4
−0.2
0.0
MR leave−one−out sensitivity analysis for
'GCA−900066135 sp900066135' on 'GCST90200408_buildGRCh38.tsv'

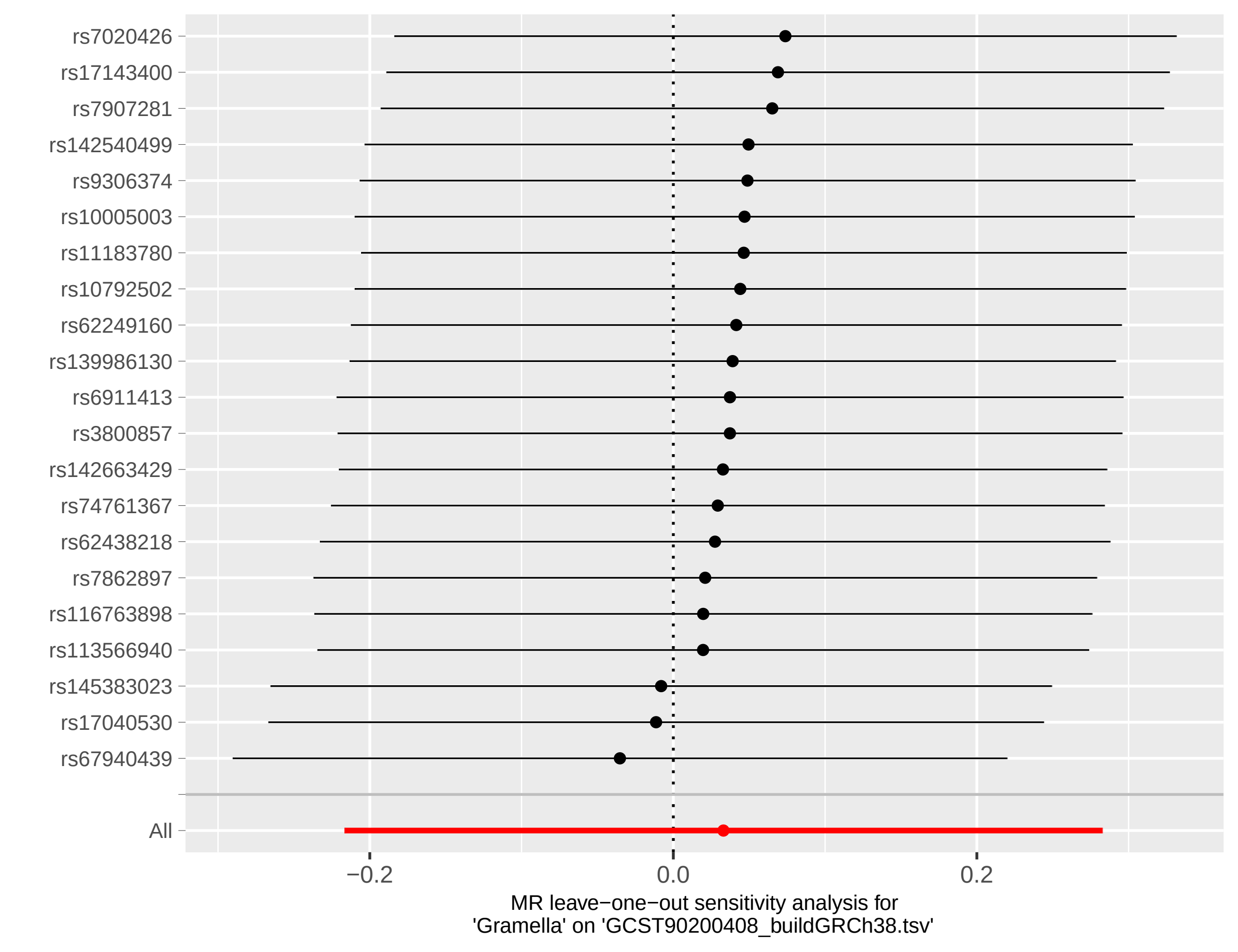
rs7020426
rs17143400
rs7907281
rs142540499
rs9306374
rs10005003
rs11183780
rs10792502
rs62249160
rs139986130
rs6911413
rs3800857
rs142663429
rs74761367
rs62438218
rs7862897
rs116763898
rs113566940
rs145383023
rs17040530
rs67940439
All
−0.2
0.0
0.2
MR leave−one−out sensitivity analysis for
'Gramella' on 'GCST90200408_buildGRCh38.tsv'

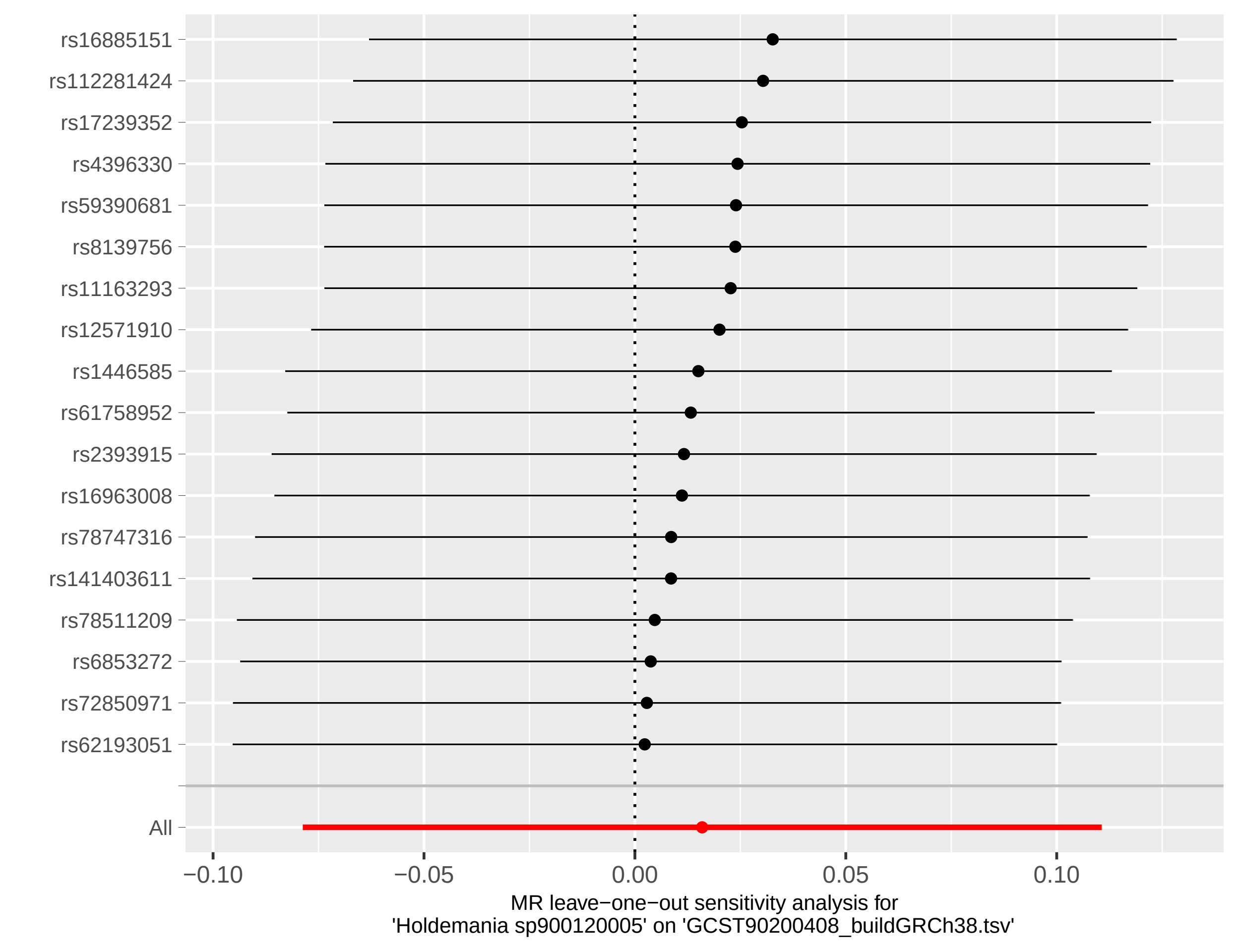

rs16885151
rs112281424
rs17239352
rs4396330
rs59390681
rs8139756
rs11163293
rs12571910
rs1446585
rs61758952
rs2393915
rs16963008
rs78747316
rs141403611
rs78511209
rs6853272
rs72850971
rs62193051
All
−0.10
−0.05
0.00
0.05
0.10
MR leave−one−out sensitivity analysis for
'Holdemania sp900120005' on 'GCST90200408_buildGRCh38.tsv'

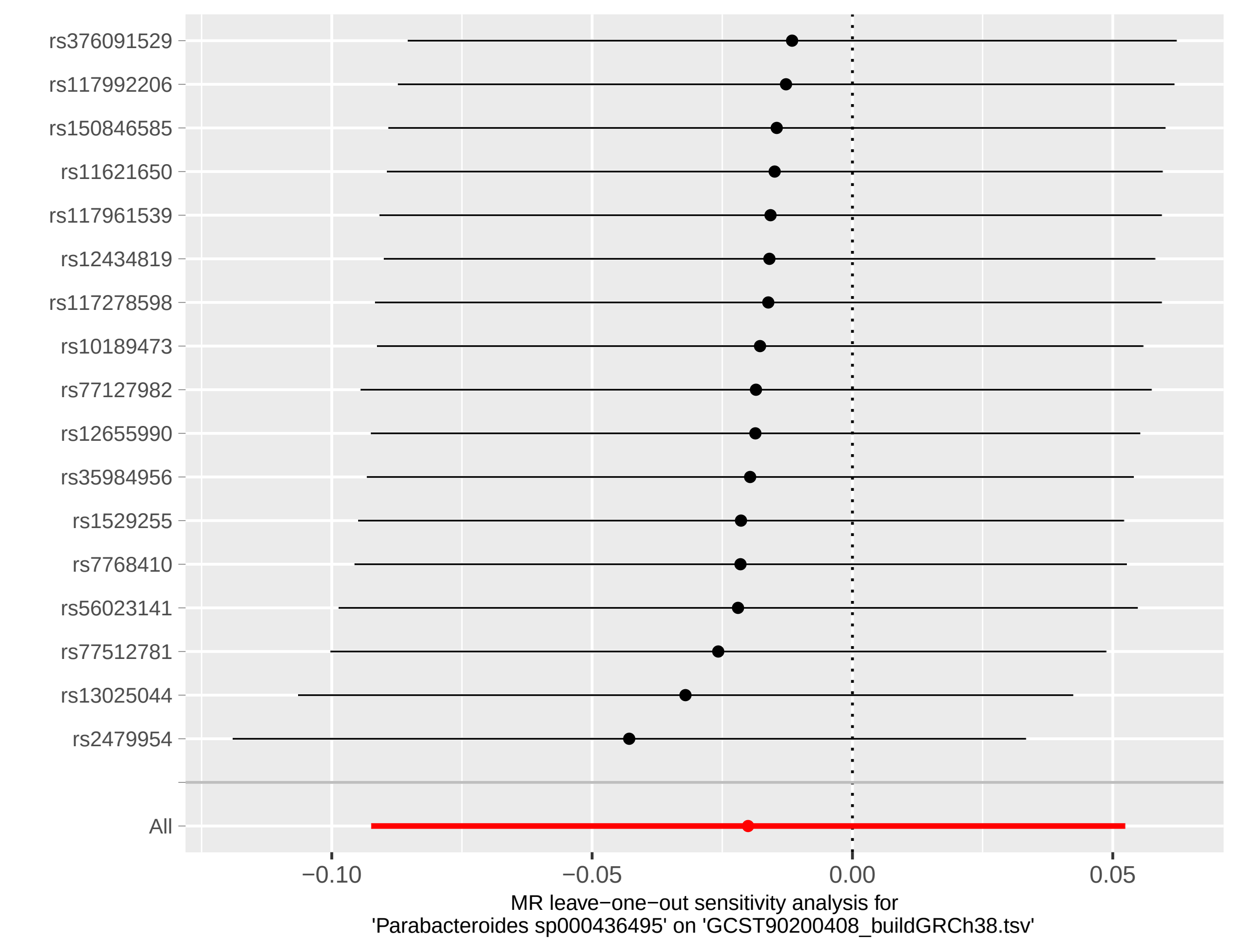
rs376091529
rs117992206
rs150846585
rs11621650
rs117961539
rs12434819
rs117278598
rs10189473
rs77127982
rs12655990
rs35984956
rs1529255
rs7768410
rs56023141
rs77512781
rs13025044
rs2479954
All
−0.10
−0.05
0.00
0.05
MR leave−one−out sensitivity analysis for
'Parabacteroides sp000436495' on 'GCST90200408_buildGRCh38.tsv'

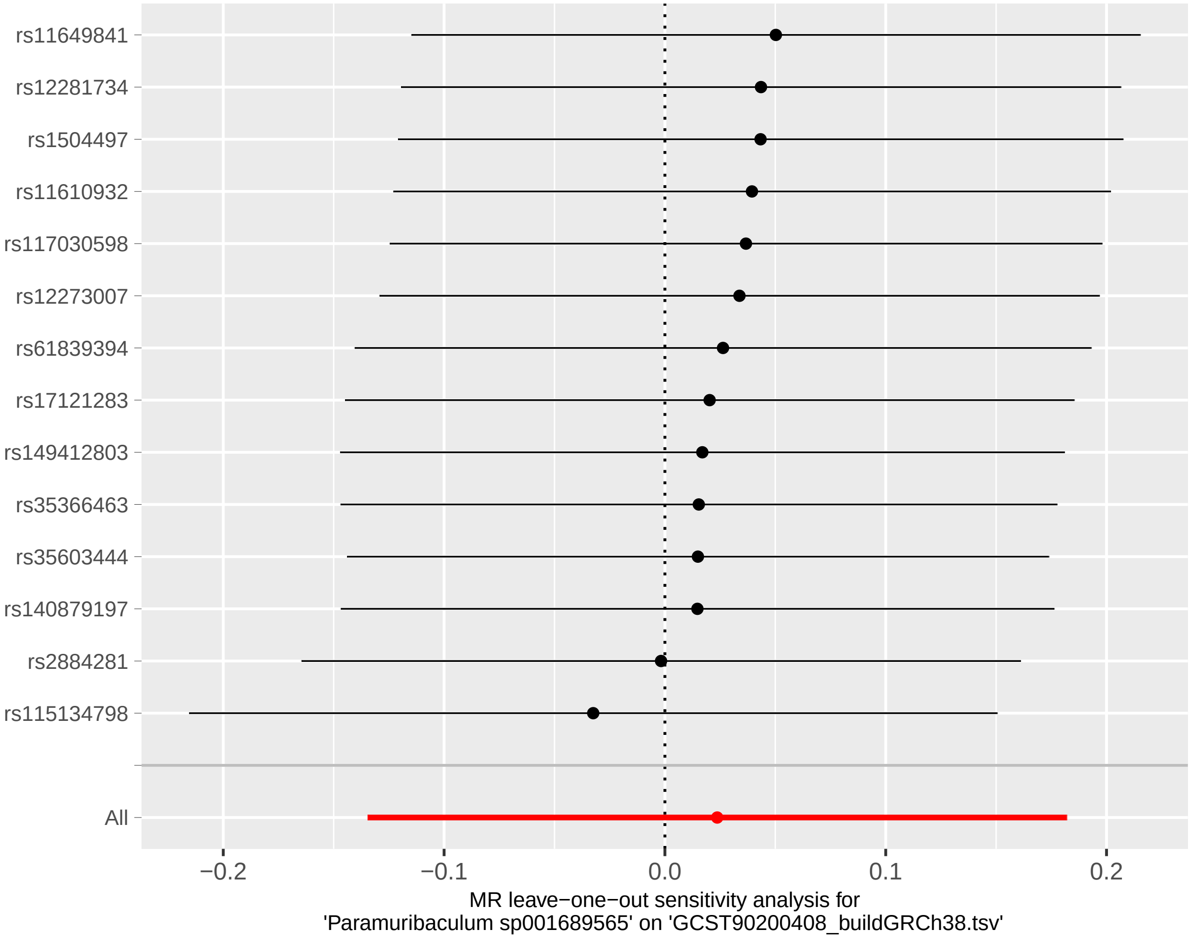
rs11649841
rs12281734
rs1504497
rs11610932
rs117030598
rs12273007
rs61839394
rs17121283
rs149412803
rs35366463
rs35603444
rs140879197
rs2884281
rs115134798
All
−0.2
−0.1
0.0
0.1
0.2
MR leave−one−out sensitivity analysis for
'Paramuribaculum sp001689565' on 'GCST90200408_buildGRCh38.tsv'

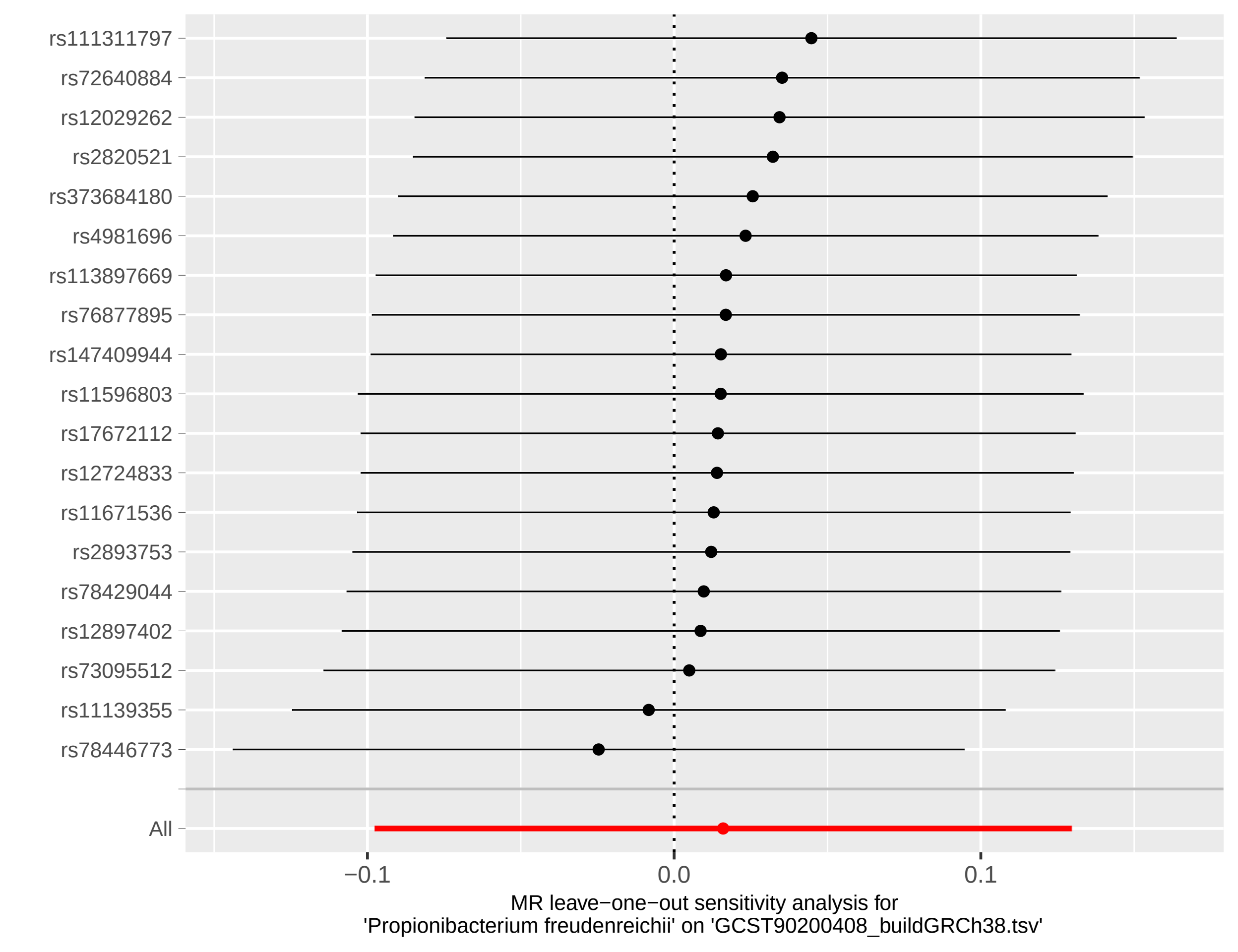

rs111311797
rs72640884
rs12029262
rs2820521
rs373684180
rs4981696
rs113897669
rs76877895
rs147409944
rs11596803
rs17672112
rs12724833
rs11671536
rs2893753
rs78429044
rs12897402
rs73095512
rs11139355
rs78446773
All
−0.1
0.0
0.1
MR leave−one−out sensitivity analysis for
'Propionibacterium freudenreichii' on 'GCST90200408_buildGRCh38.tsv'

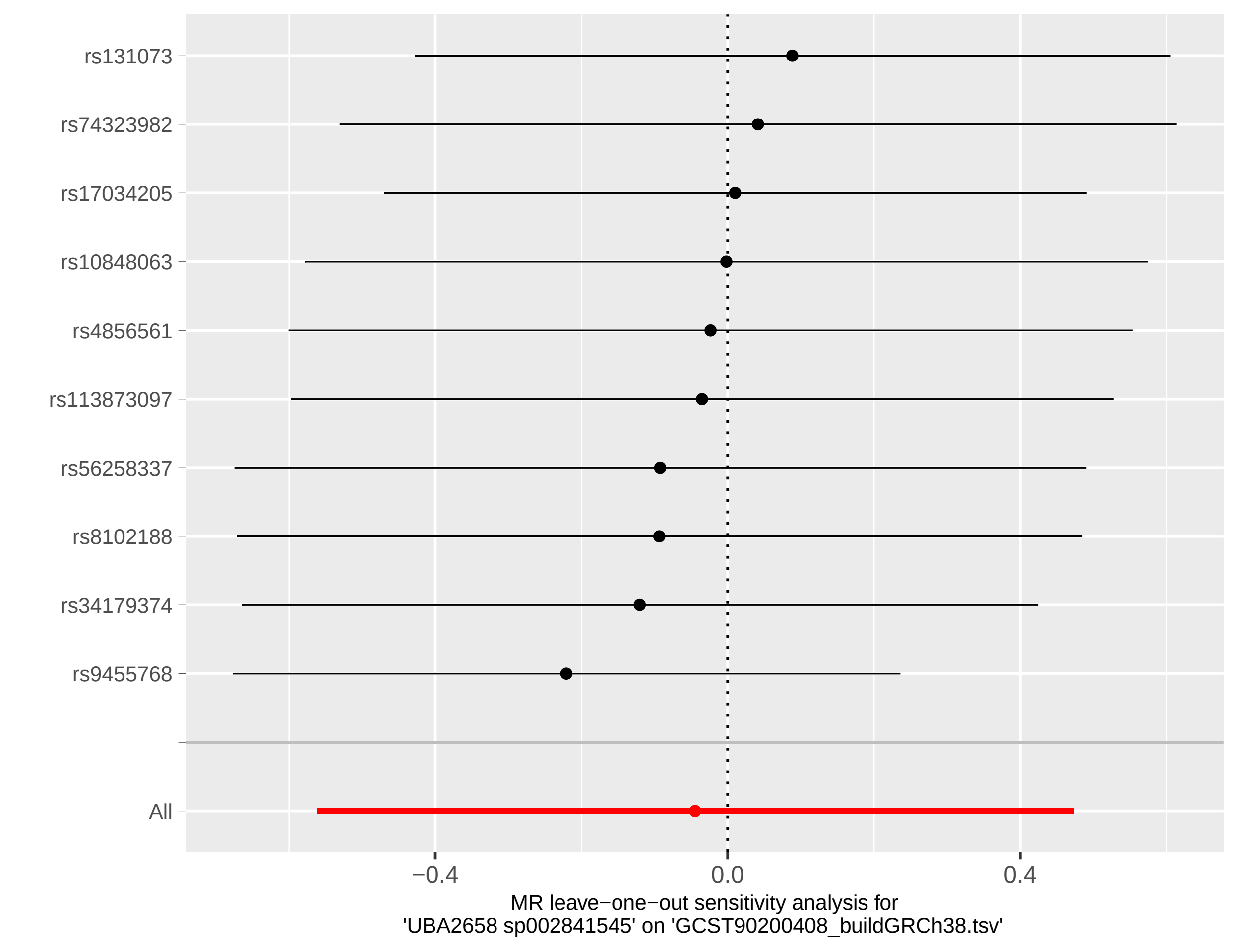

rs131073
rs74323982
rs17034205
rs10848063
rs4856561
rs113873097
rs56258337
rs8102188
rs34179374
rs9455768
All
−0.4
0.0
0.4
MR leave−one−out sensitivity analysis for
'UBA2658 sp002841545' on 'GCST90200408_buildGRCh38.tsv'

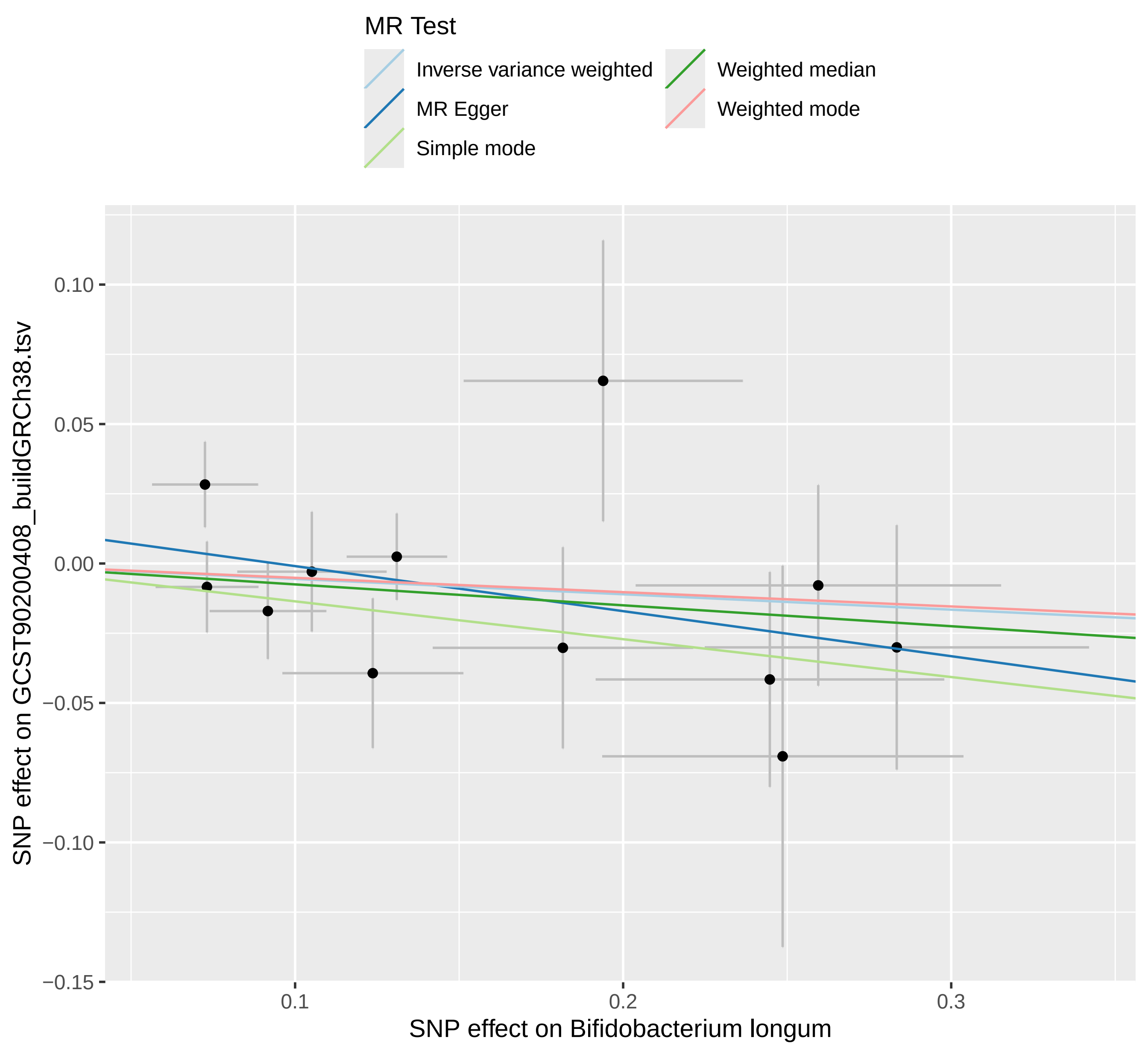
MR Test
Inverse variance weighted
MR Egger
Simple mode
Weighted median
Weighted mode
0.10
0.05
0.00
−0.05
−0.10
−0.15
SNP effect on GCST90200408_buildGRCh38.tsv
0.1
0.2
0.3
SNP effect on Bifidobacterium longum

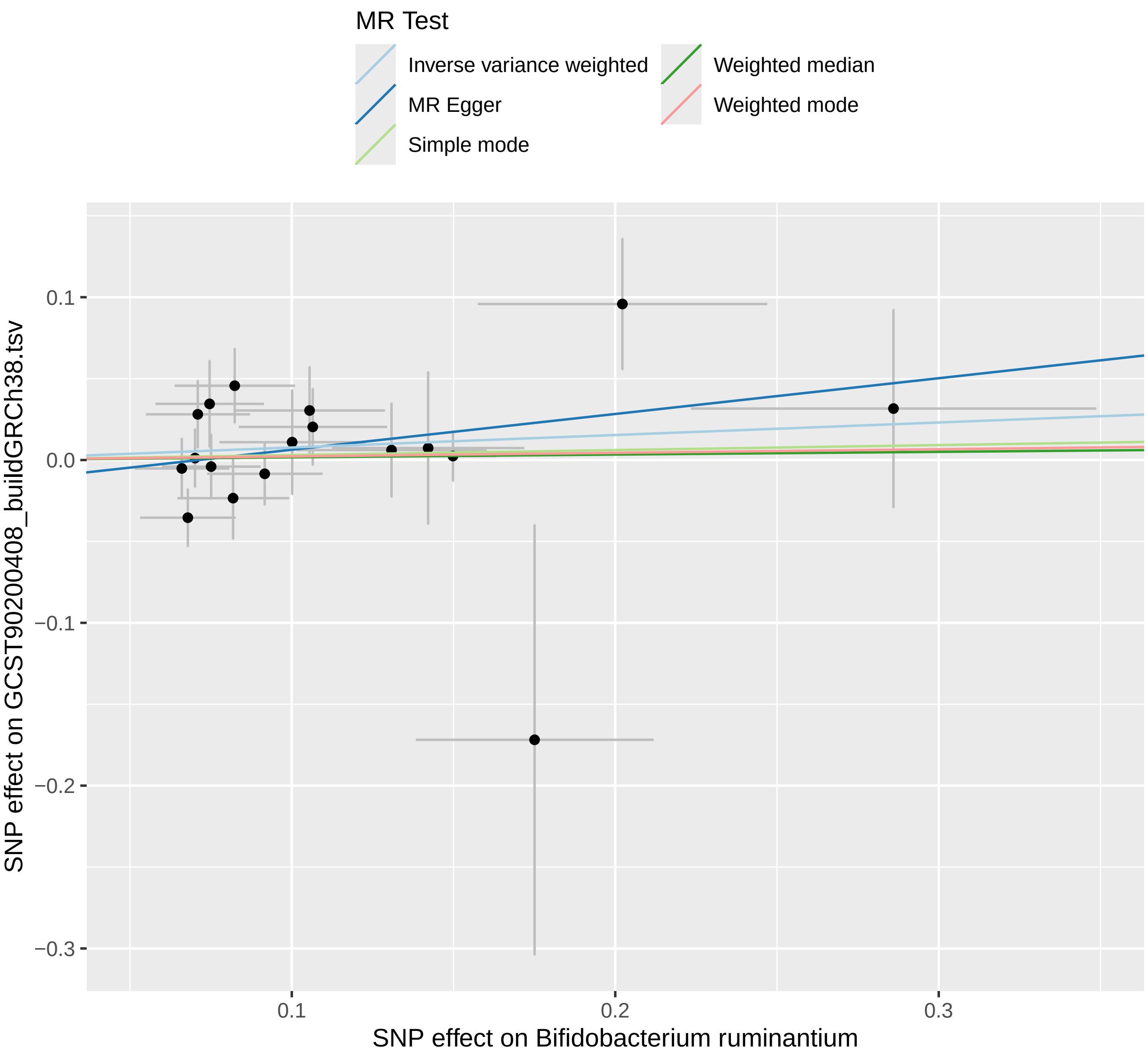

MR Test
Inverse variance weighted
MR Egger
Simple mode
Weighted median
Weighted mode
0.1
0.0
−0.1
−0.2
−0.3
0.1
0.2
0.3
SNP effect on GCST90200408_buildGRCh38.tsv
SNP effect on Bifidobacterium ruminantium

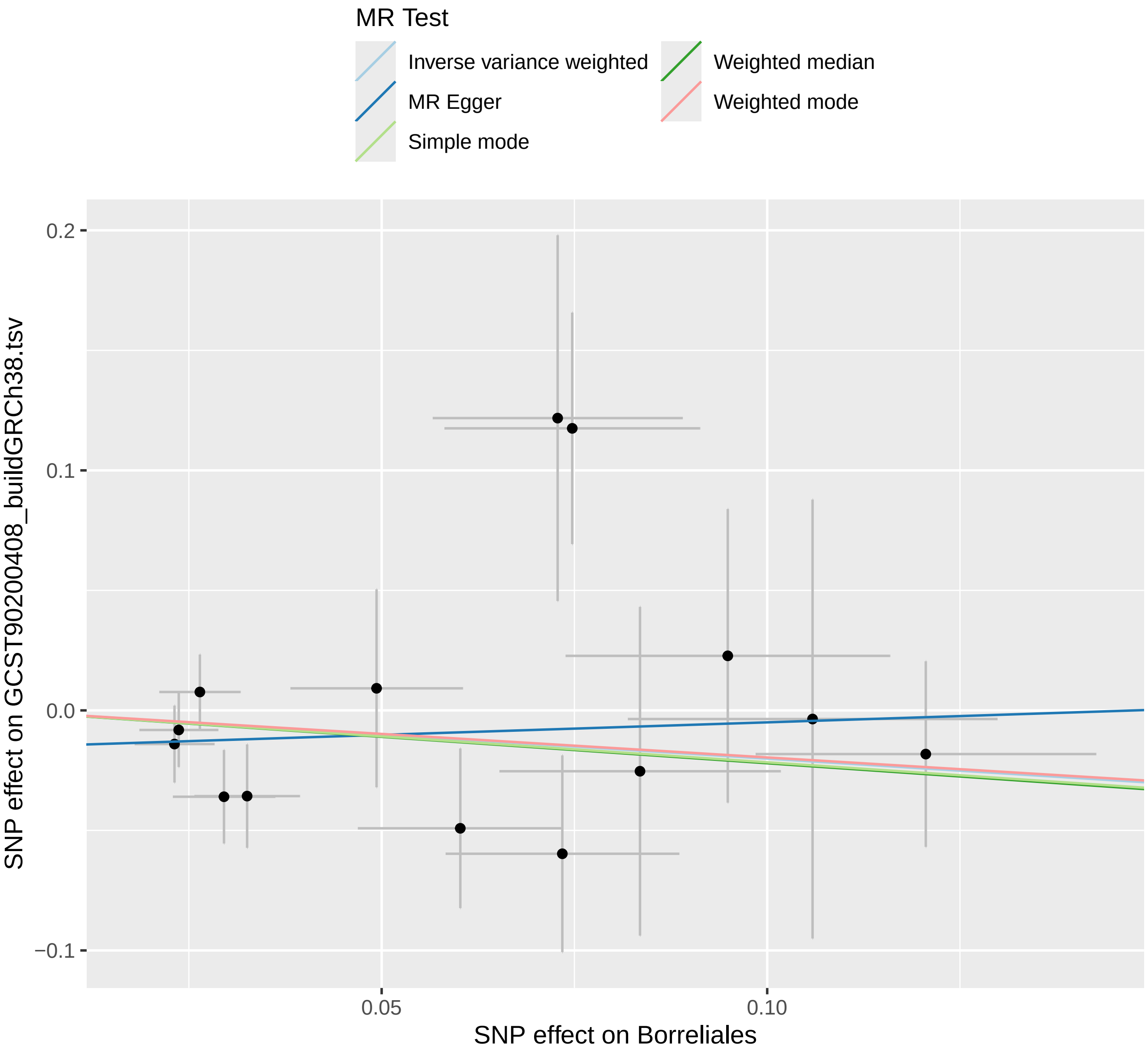
MR Test
Inverse variance weighted
MR Egger
Simple mode
Weighted median
Weighted mode
0.2
0.1
0.0
−0.1
0.05
0.10
SNP effect on GCST90200408_buildGRCh38.tsv
SNP effect on Borreliales

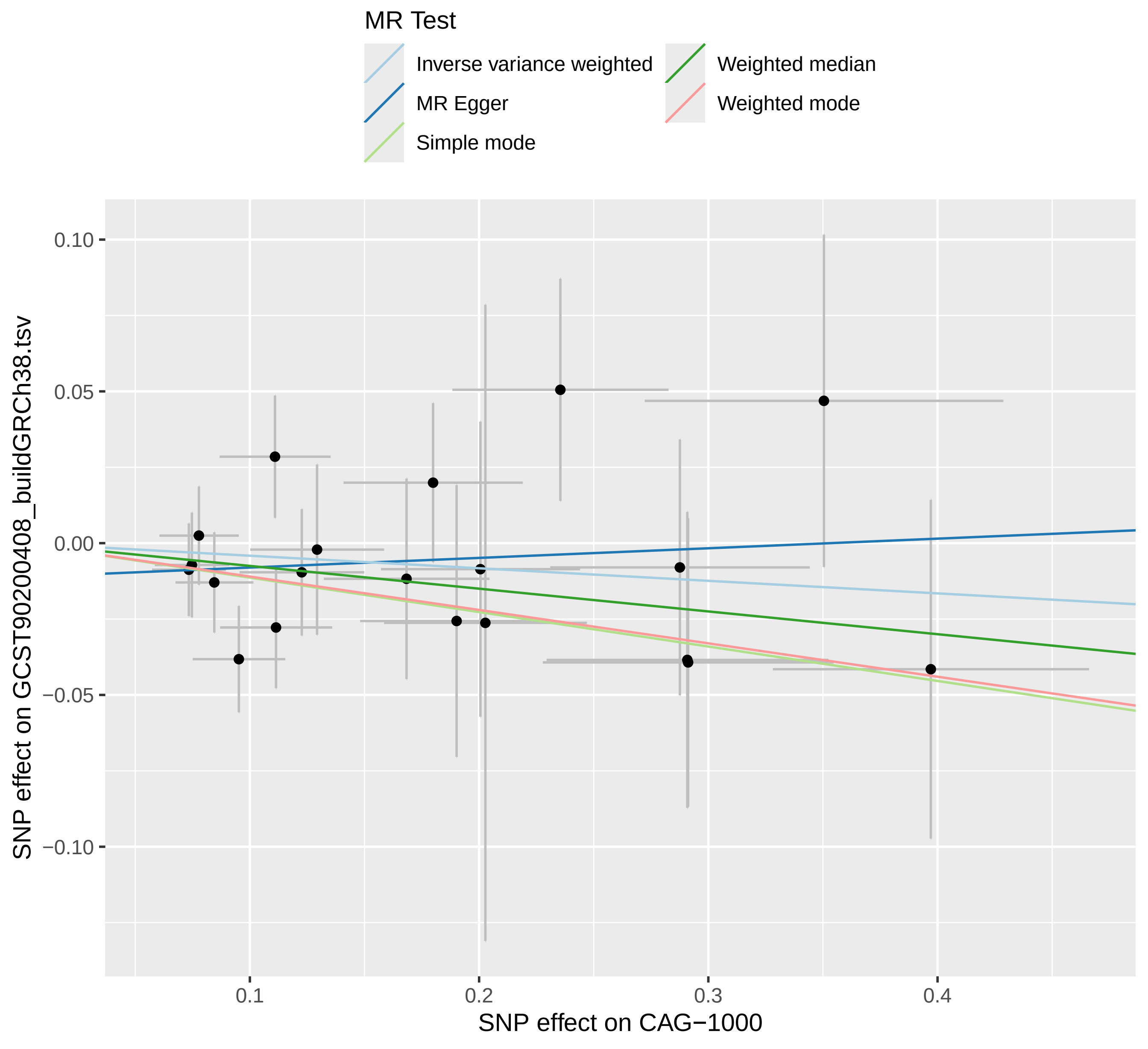

MR Test
Inverse variance weighted
MR Egger
Simple mode
Weighted median
Weighted mode
0.10
0.05
0.00
−0.05
−0.10
SNP effect on GCST90200408_buildGRCh38.tsv
0.1
0.2
0.3
0.4
SNP effect on CAG−1000

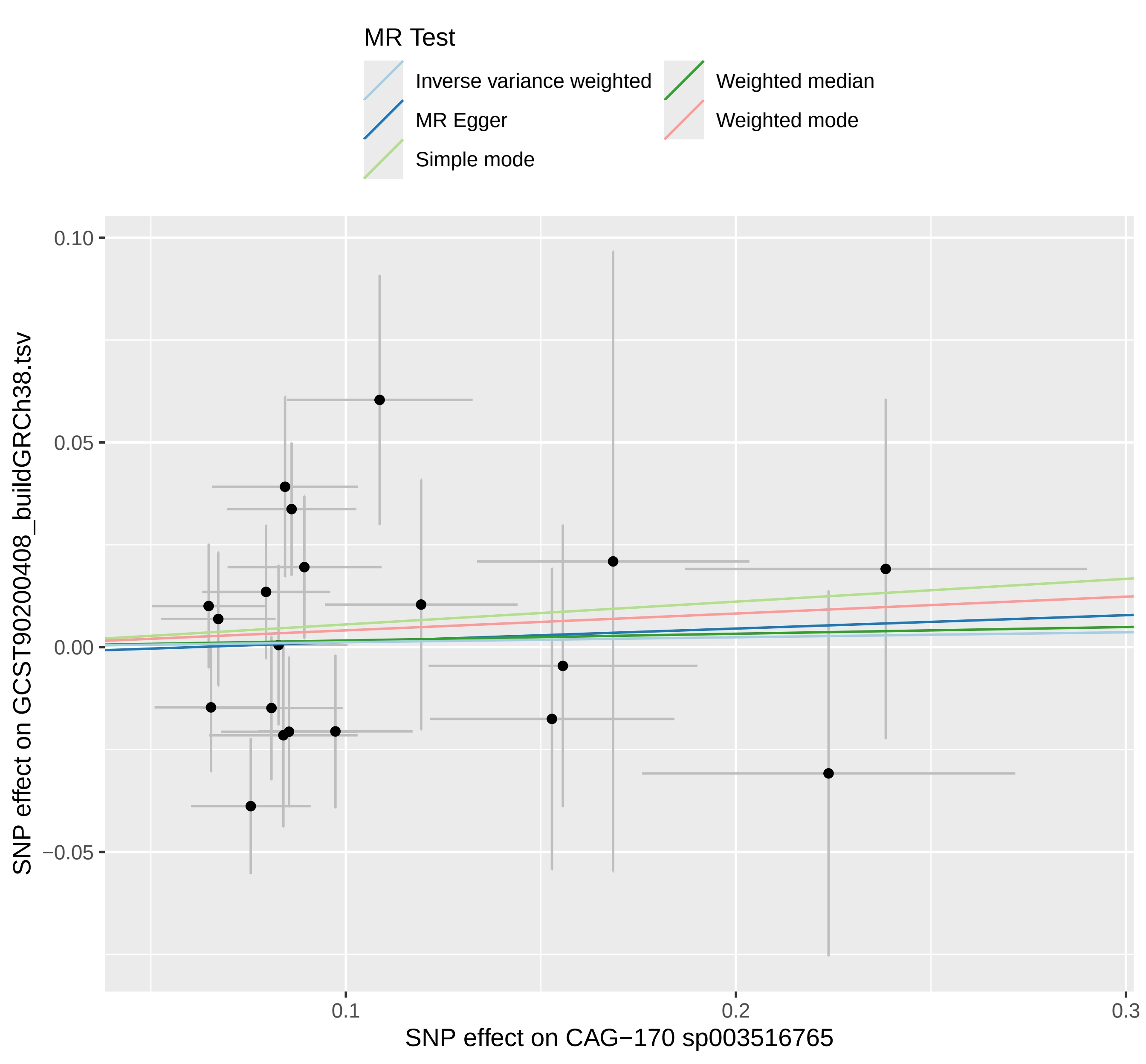
MR Test
Inverse variance weighted
MR Egger
Simple mode
Weighted median
Weighted mode
0.10
0.05
0.00
−0.05
0.1
0.2
0.3
SNP effect on GCST90200408_buildGRCh38.tsv
SNP effect on CAG−170 sp003516765

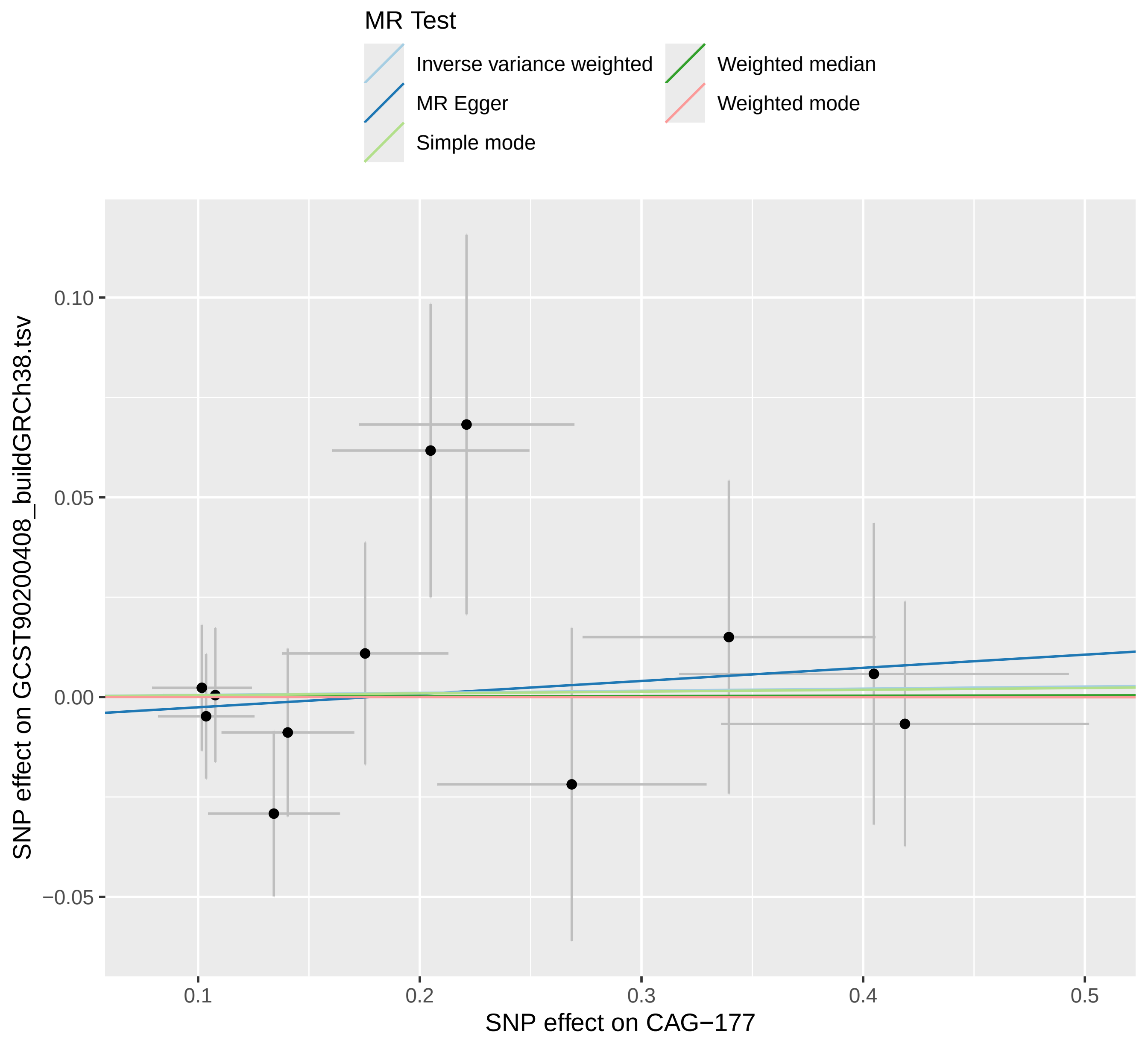
MR Test
Inverse variance weighted
MR Egger
Simple mode
Weighted median
Weighted mode
0.10
0.05
0.00
−0.05
SNP effect on GCST90200408_buildGRCh38.tsv
0.1
0.2
0.3
0.4
0.5
SNP effect on CAG−177

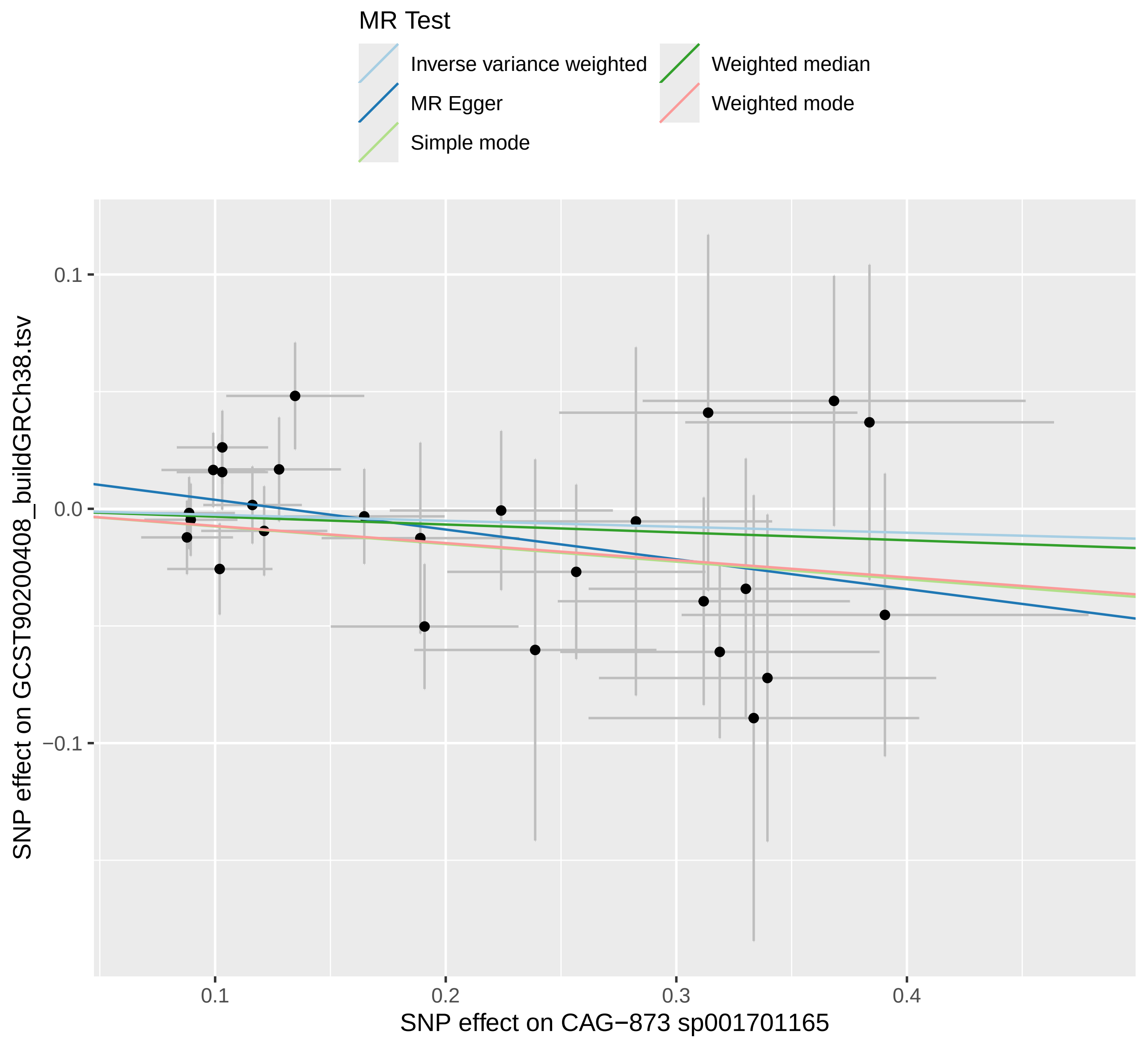
MR Test
Inverse variance weighted
MR Egger
Simple mode
Weighted median
Weighted mode
0.1
0.0
−0.1
SNP effect on GCST90200408_buildGRCh38.tsv
0.1
0.2
0.3
0.4
SNP effect on CAG−873 sp001701165

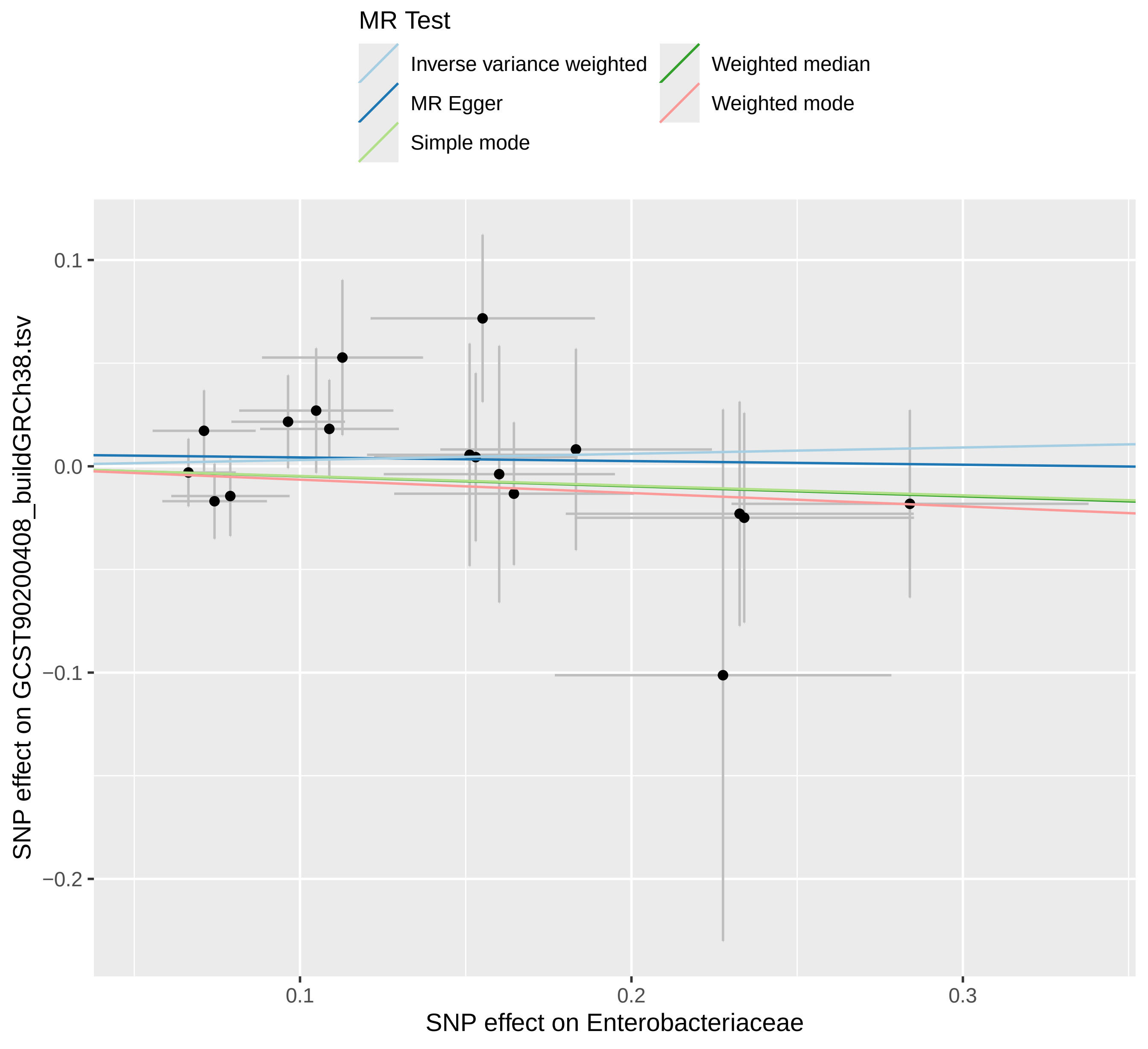

MR Test
Inverse variance weighted
MR Egger
Simple mode
Weighted median
Weighted mode
0.1
0.0
−0.1
−0.2
0.1
0.2
0.3
SNP effect on GCST90200408_buildGRCh38.tsv
SNP effect on Enterobacteriaceae

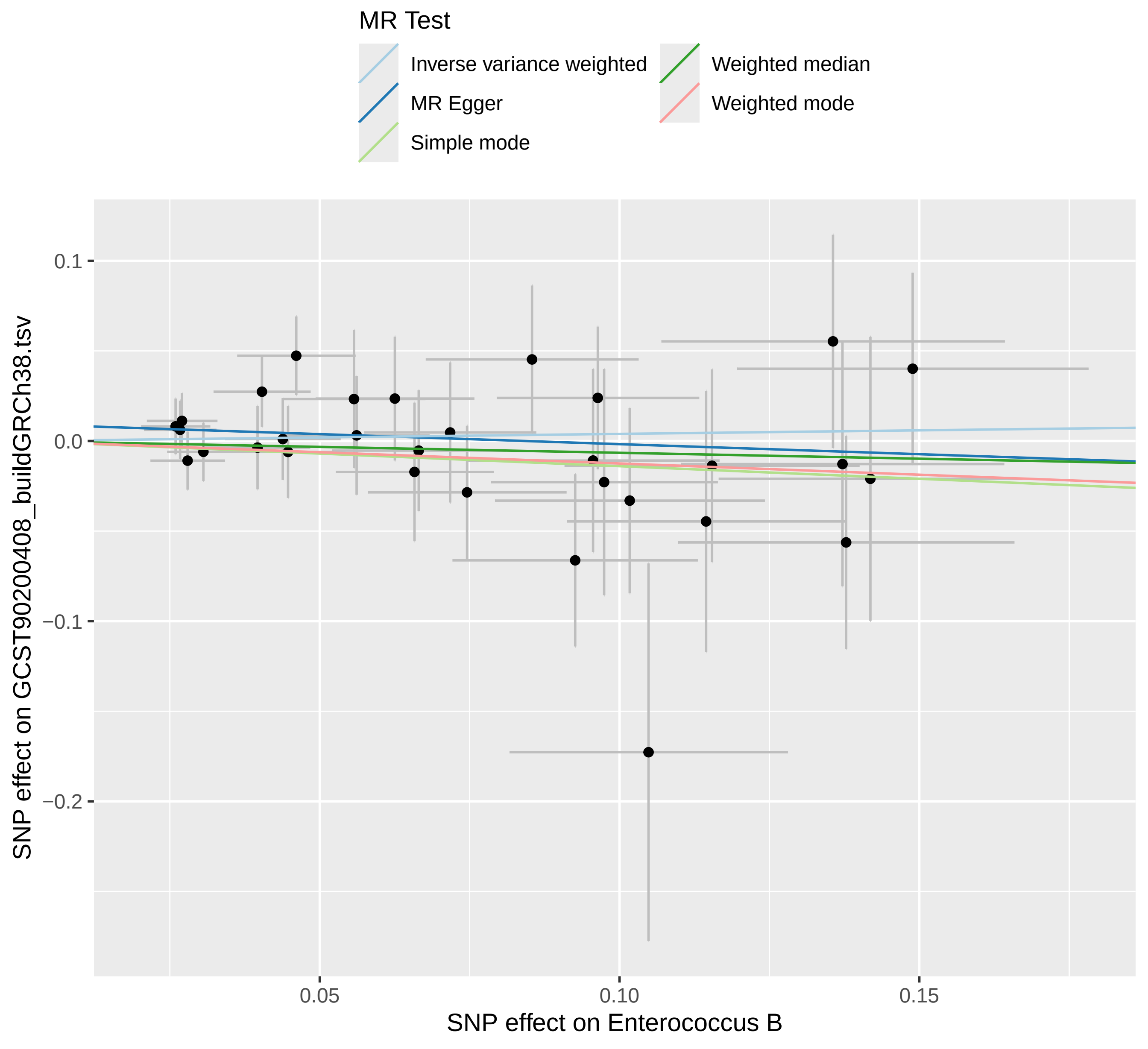
MR Test
Inverse variance weighted
MR Egger
Simple mode
Weighted median
Weighted mode
0.1
0.0
−0.1
−0.2
SNP effect on GCST90200408_buildGRCh38.tsv
0.05
0.10
0.15
SNP effect on Enterococcus B

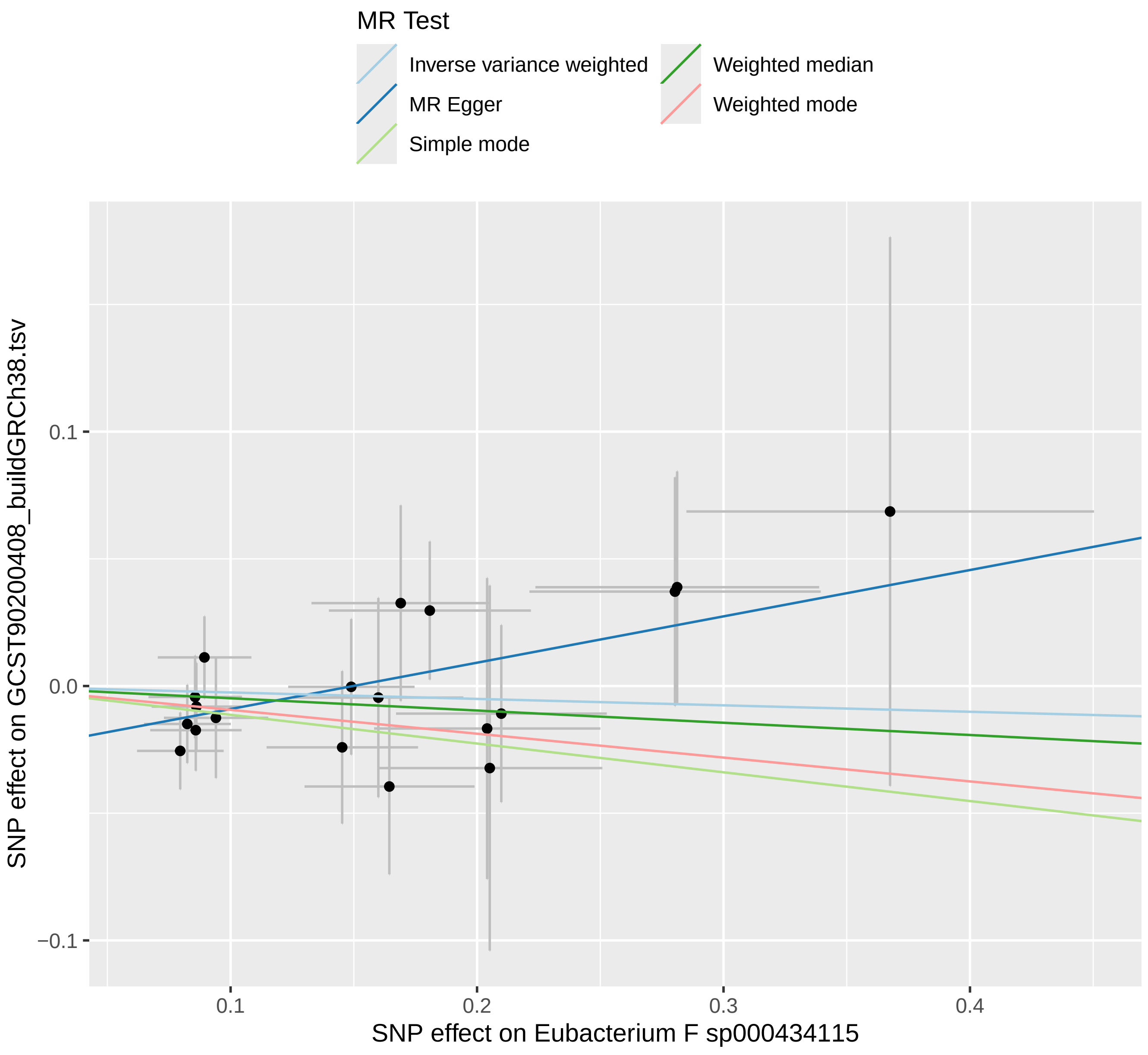

MR Test
Inverse variance weighted
MR Egger
Simple mode
Weighted median
Weighted mode
0.1
0.0
−0.1
0.1
0.2
0.3
0.4
SNP effect on GCST90200408_buildGRCh38.tsv
SNP effect on Eubacterium F sp000434115

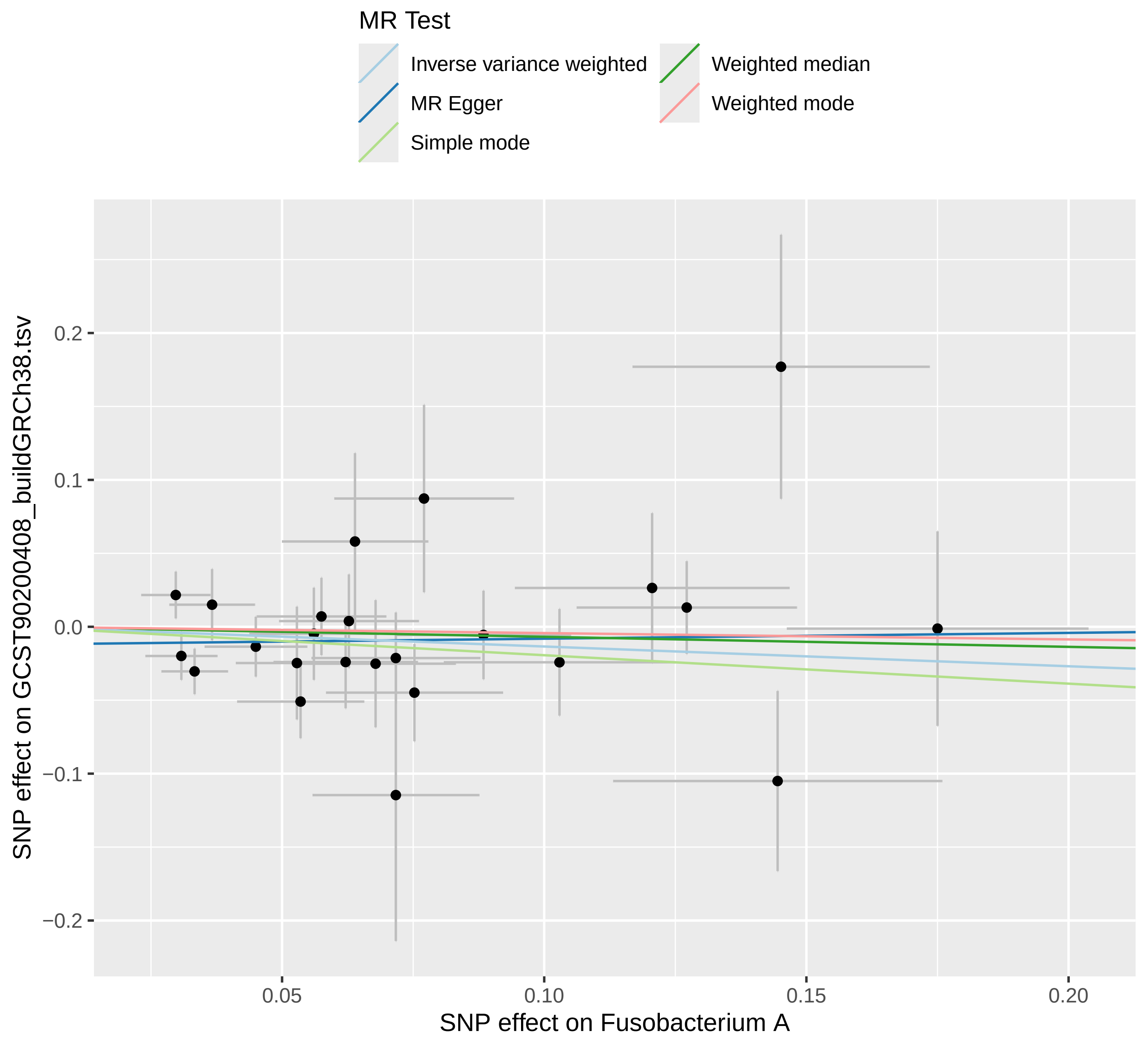

MR Test
Inverse variance weighted
MR Egger
Simple mode
Weighted median
Weighted mode
0.2
0.1
0.0
−0.1
−0.2
0.05
0.10
0.15
0.20
SNP effect on GCST90200408_buildGRCh38.tsv
SNP effect on Fusobacterium A

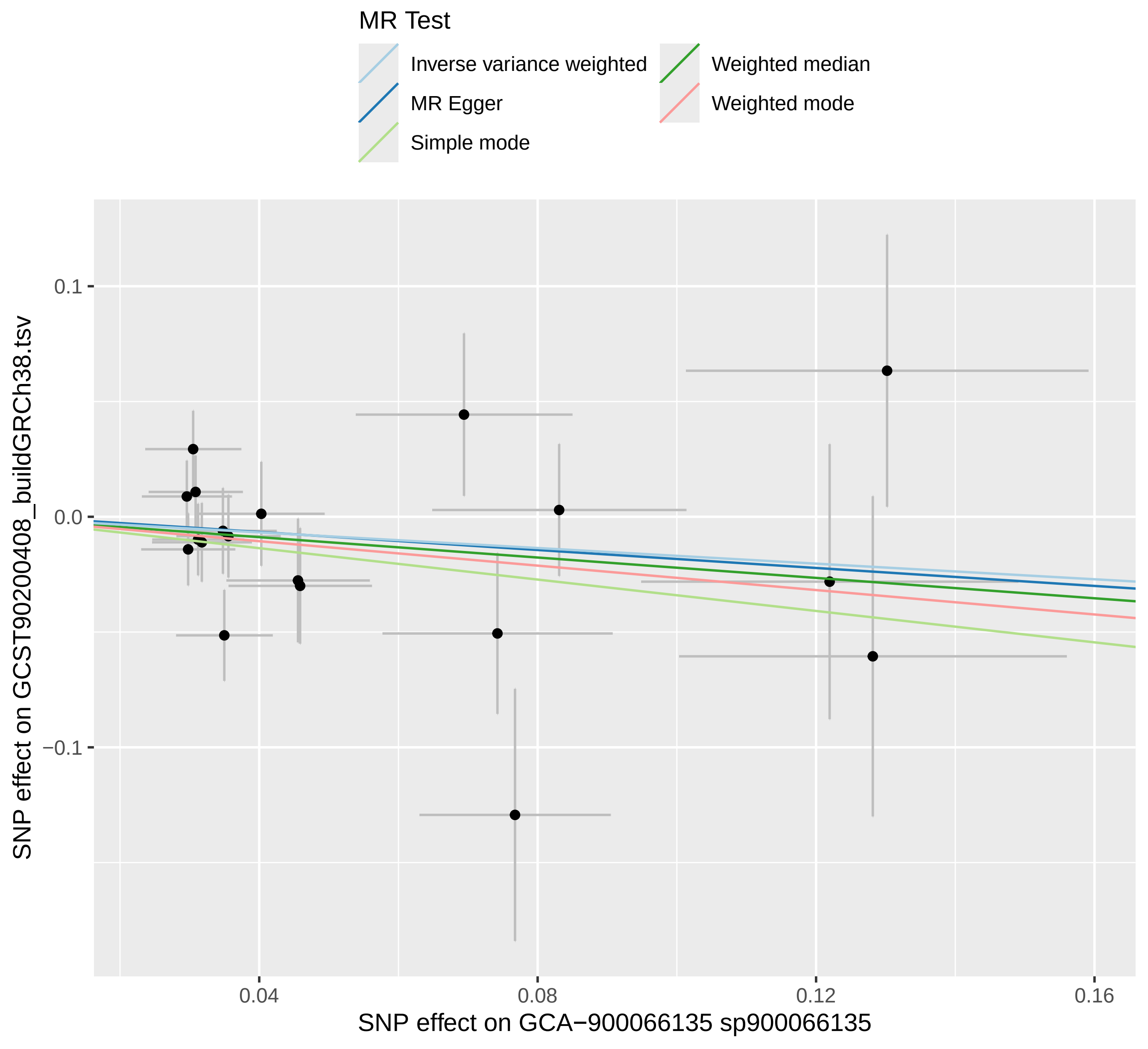

MR Test
Inverse variance weighted
MR Egger
Simple mode
Weighted median
Weighted mode
0.1
0.0
−0.1
0.04
0.08
0.12
0.16
SNP effect on GCST90200408_buildGRCh38.tsv
SNP effect on GCA−900066135 sp900066135

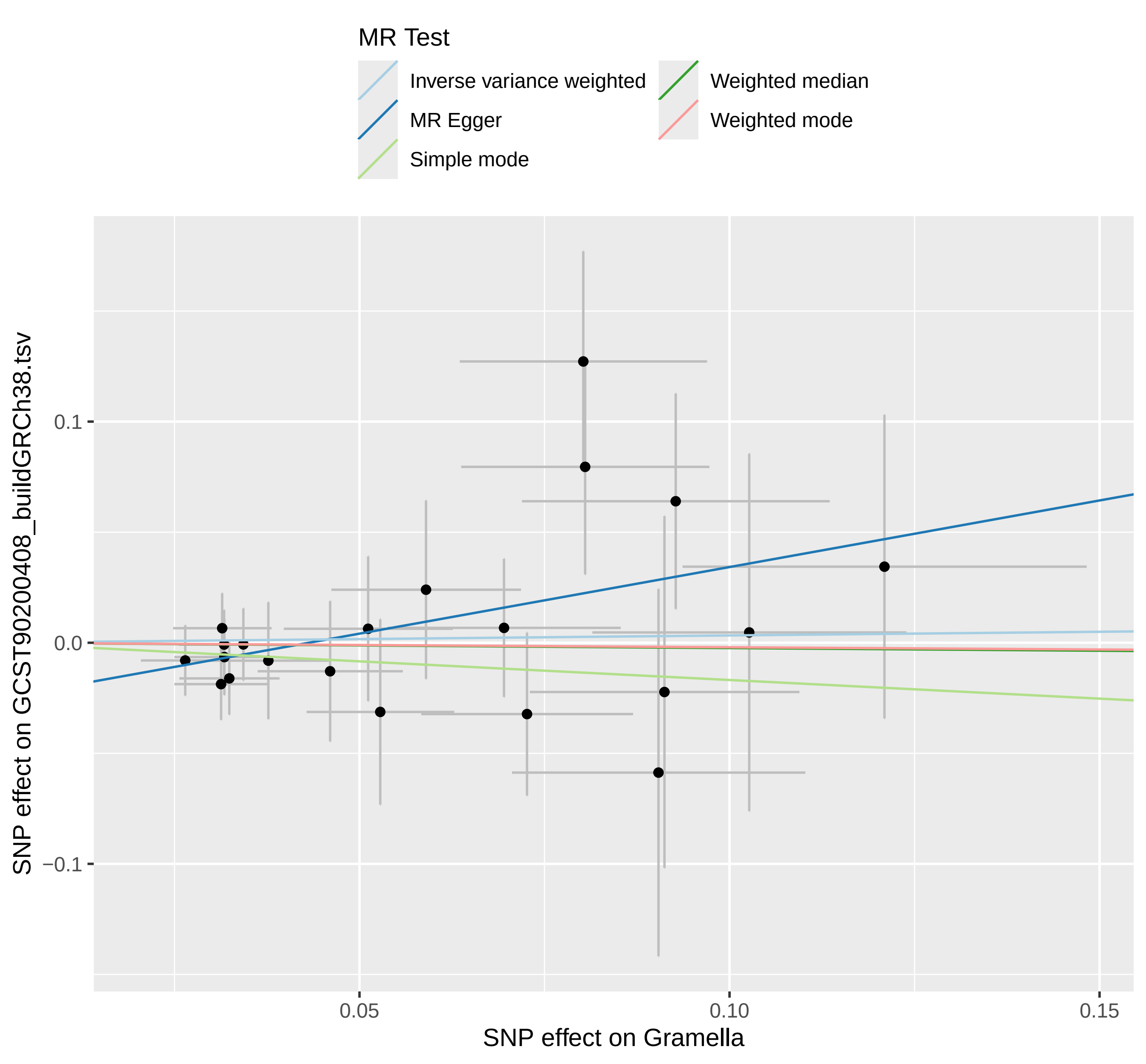
MR Test
Inverse variance weighted
MR Egger
Simple mode
Weighted median
Weighted mode
SNP effect on GCST90200408_buildGRCh38.tsv
0.1
0.0
−0.1
0.05
0.10
0.15
SNP effect on Gramella

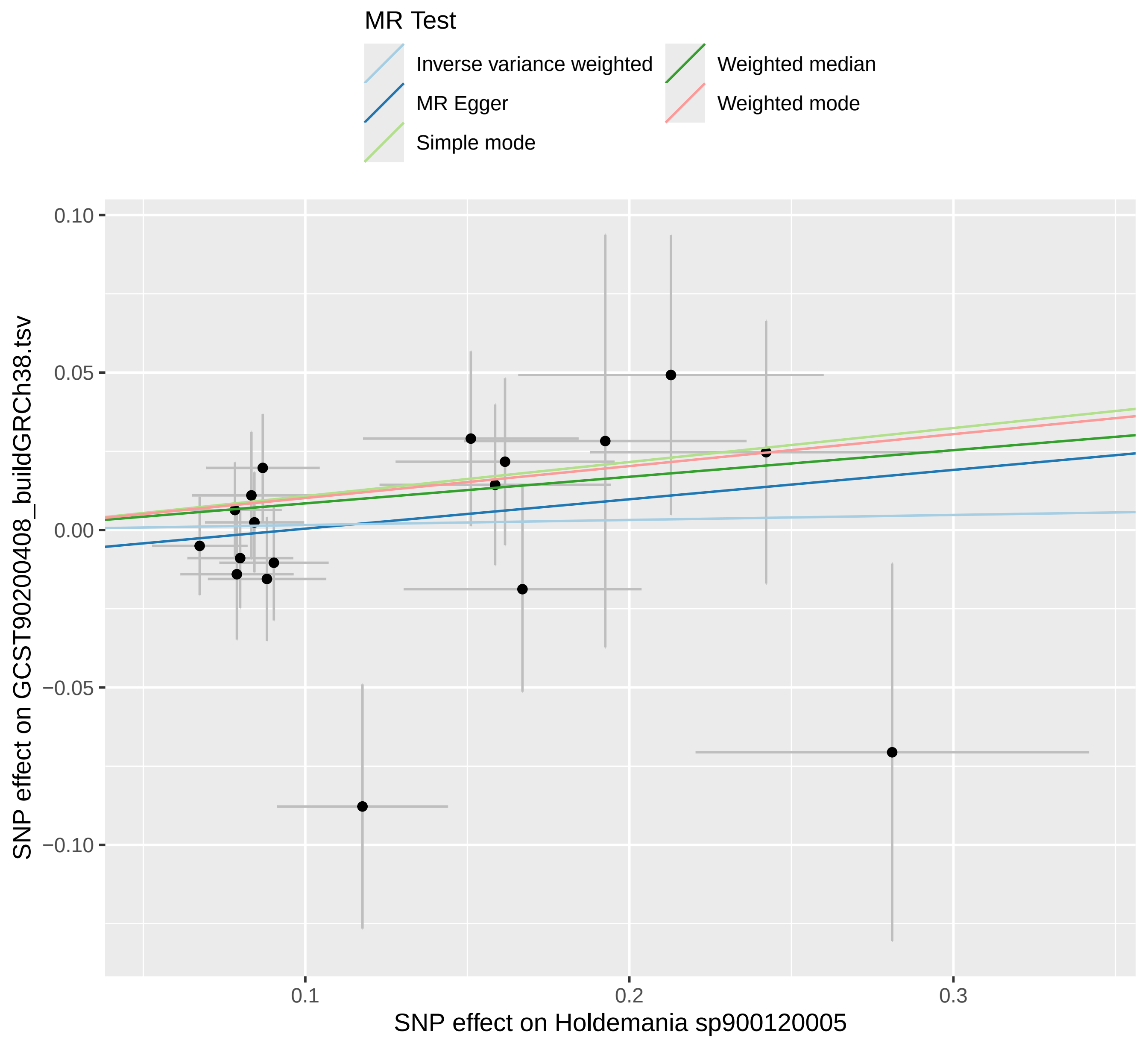
MR Test
Inverse variance weighted
MR Egger
Simple mode
Weighted median
Weighted mode
0.10
0.05
0.00
−0.05
−0.10
0.1
0.2
0.3
SNP effect on GCST90200408_buildGRCh38.tsv
SNP effect on Holdemania sp900120005

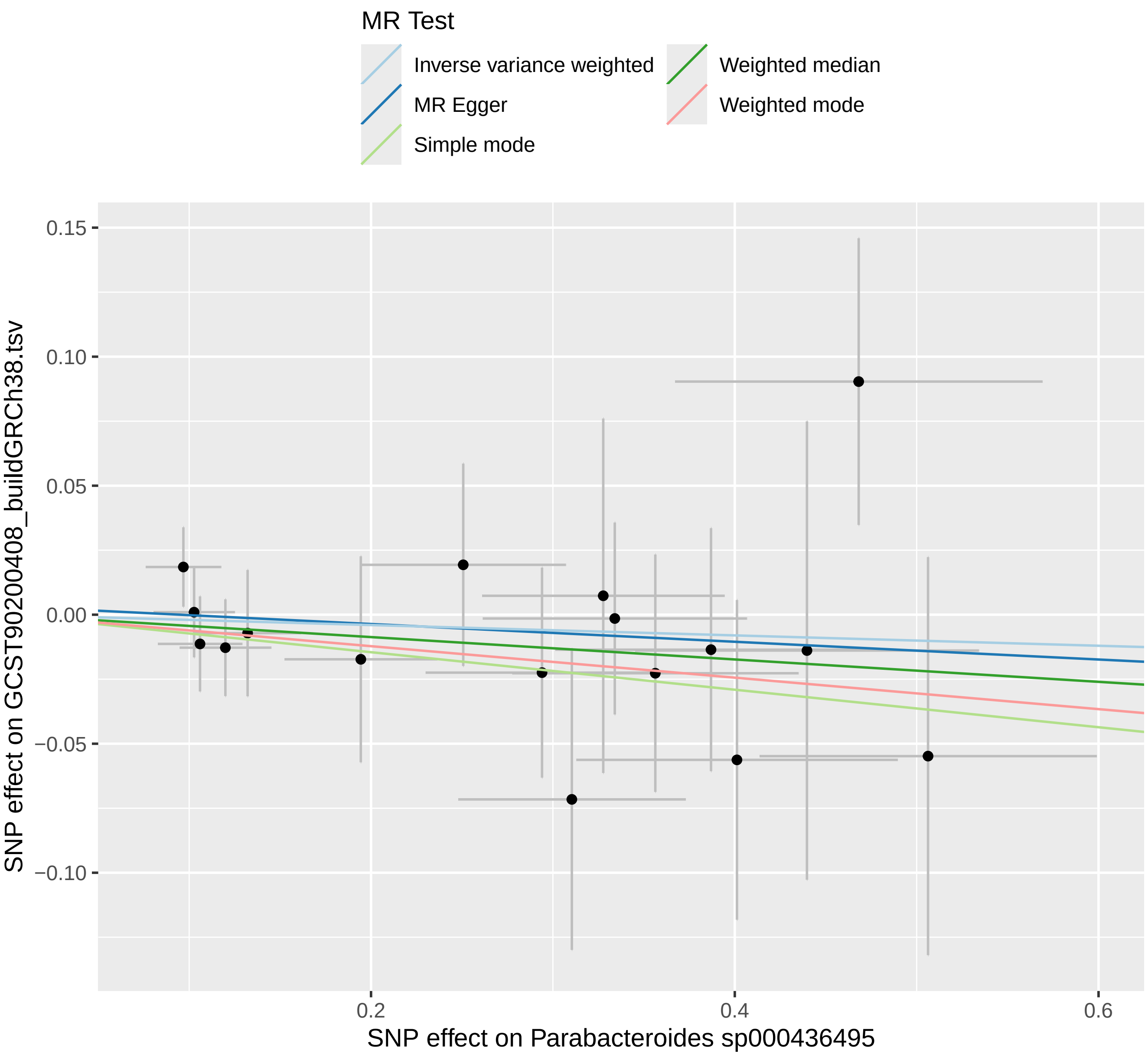
MR Test
Inverse variance weighted
MR Egger
Simple mode
Weighted median
Weighted mode
0.15
0.10
0.05
0.00
−0.05
−0.10
SNP effect on GCST90200408_buildGRCh38.tsv
0.2
0.4
0.6
SNP effect on Parabacteroides sp000436495

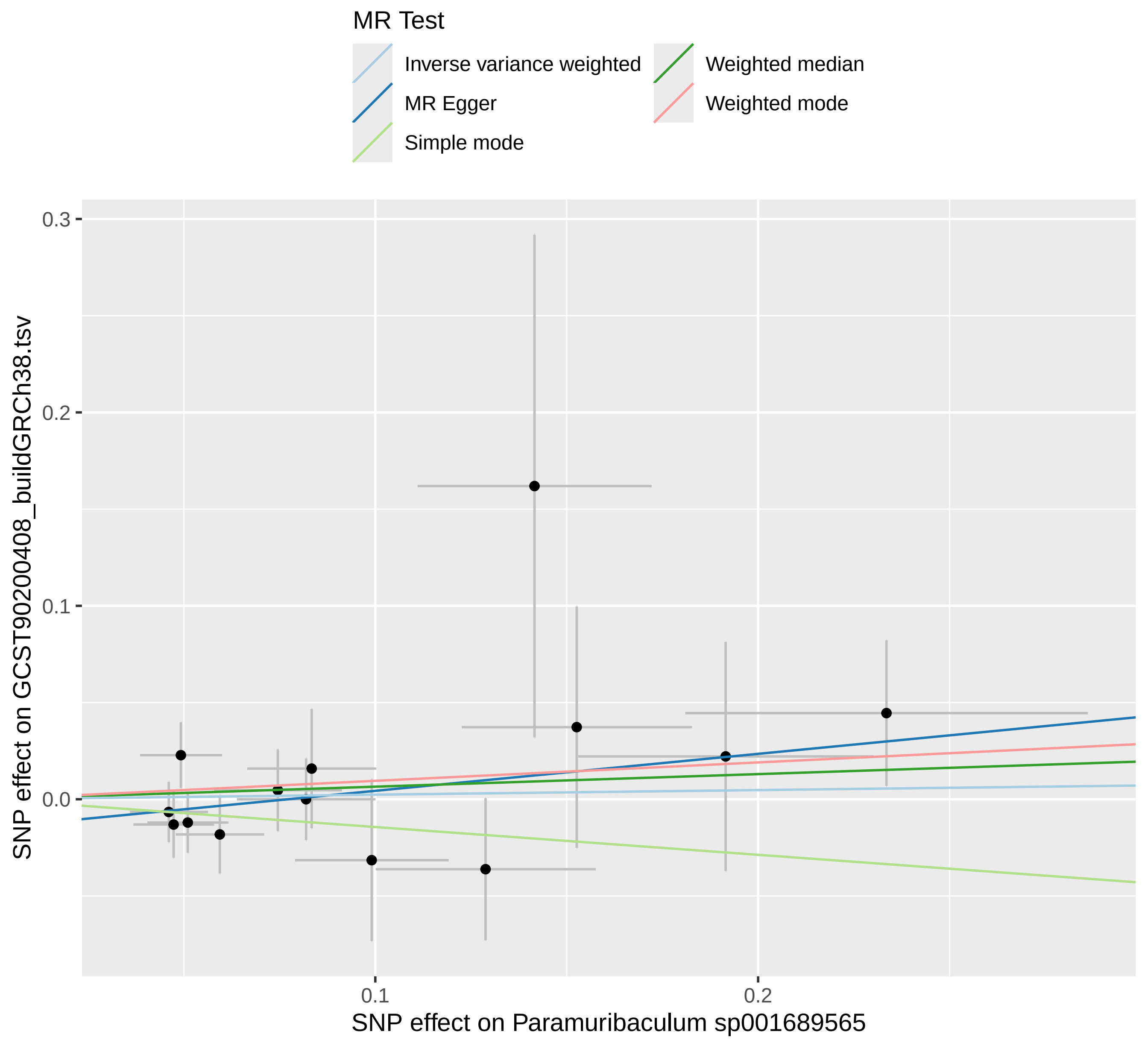

MR Test
Inverse variance weighted
MR Egger
Simple mode
Weighted median
Weighted mode
0.3
0.2
0.1
0.0
0.1
0.2
SNP effect on GCST90200408_buildGRCh38.tsv
SNP effect on Paramuribaculum sp001689565

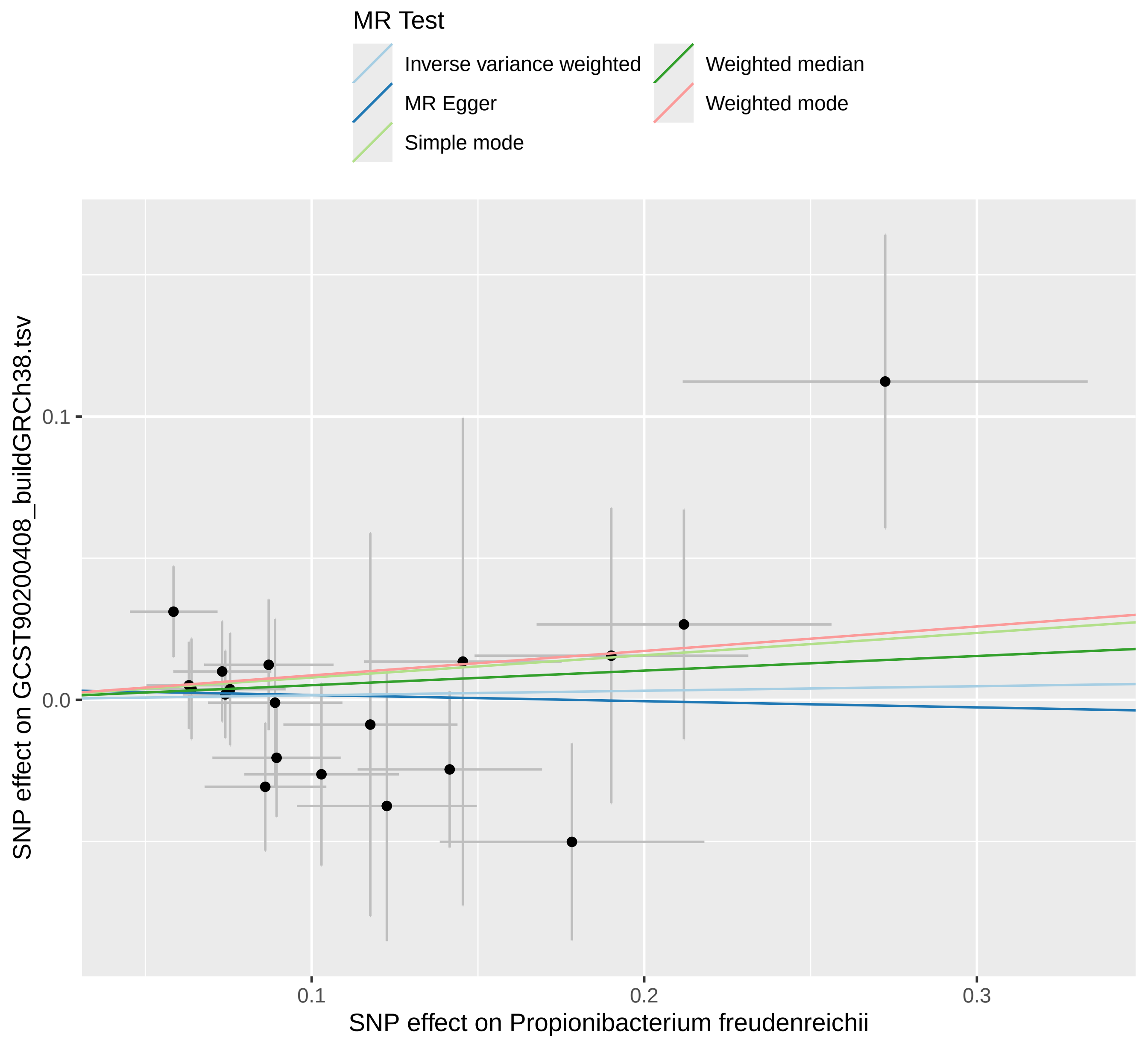

MR Test
Inverse variance weighted
MR Egger
Simple mode
Weighted median
Weighted mode
0.1
0.0
0.1
0.2
0.3
SNP effect on GCST90200408_buildGRCh38.tsv
SNP effect on Propionibacterium freudenreichii

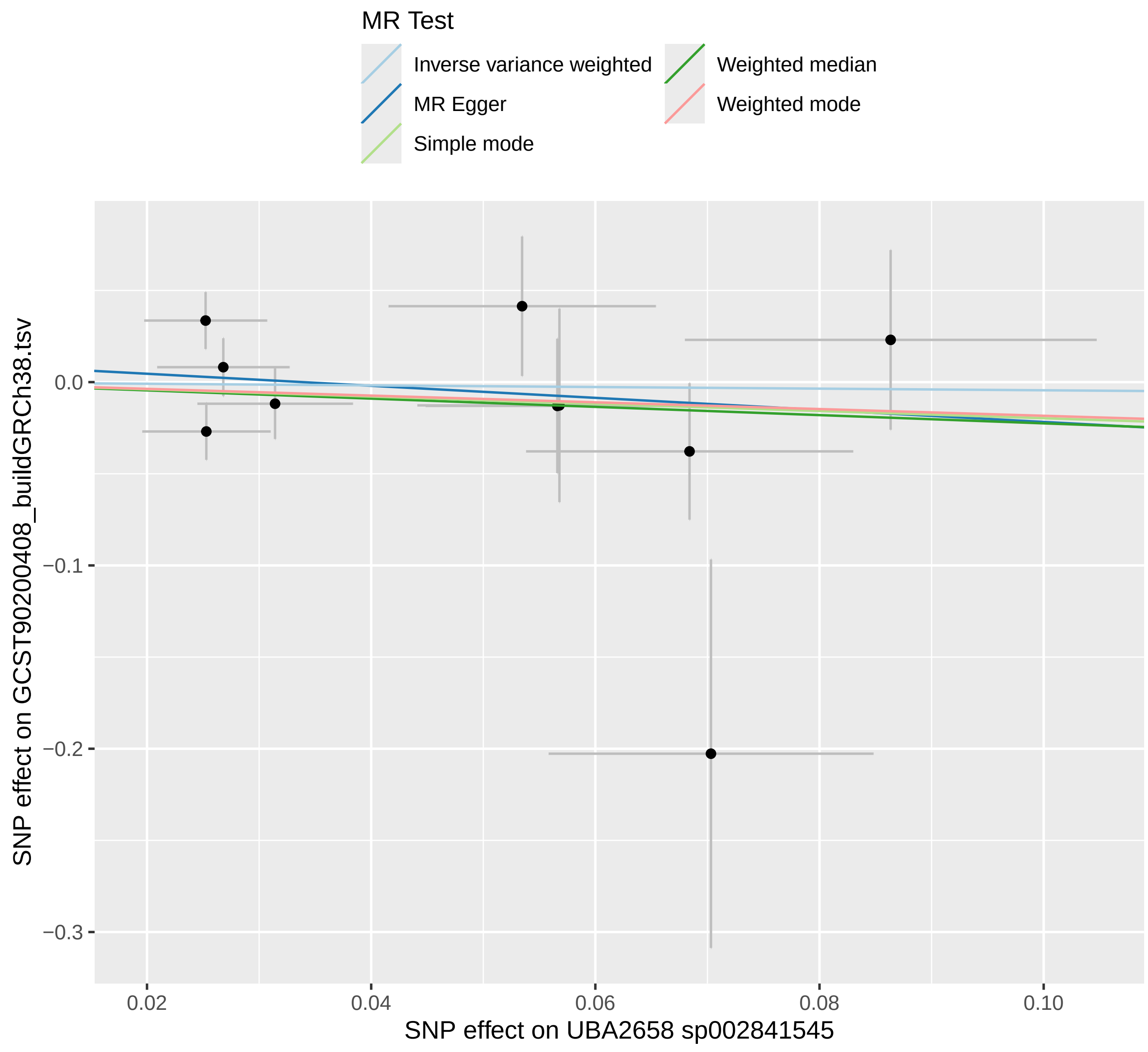

MR Test
Inverse variance weighted
MR Egger
Simple mode
Weighted median
Weighted mode
SNP effect on GCST90200408_buildGRCh38.tsv
0.0
−0.1
−0.2
−0.3
0.02
0.04
0.06
0.08
0.10
SNP effect on UBA2658 sp002841545

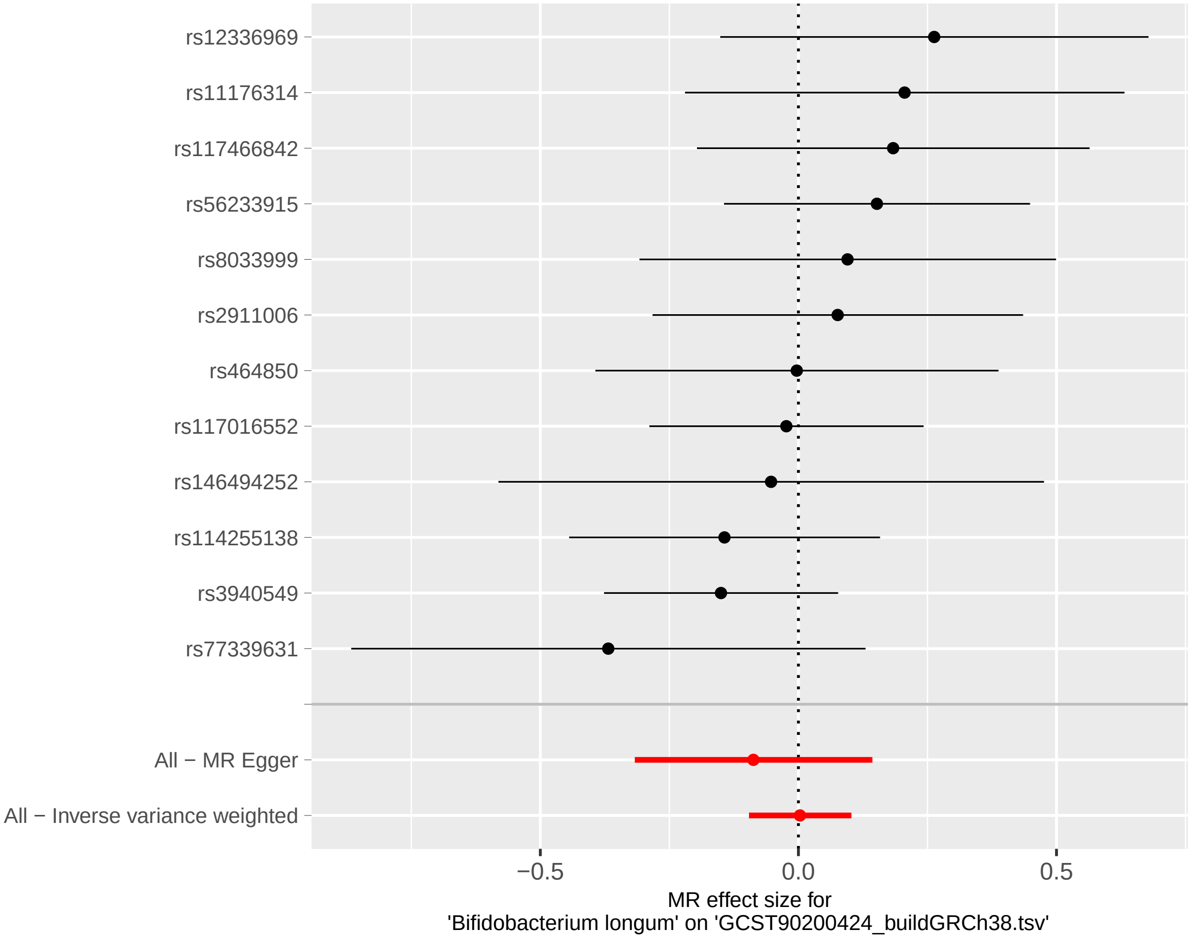
rs12336969
rs11176314
rs117466842
rs56233915
rs8033999
rs2911006
rs464850
rs117016552
rs146494252
rs114255138
rs3940549
rs77339631
All − MR Egger
All − Inverse variance weighted
−0.5
0.0
0.5
MR effect size for
'Bifidobacterium longum' on 'GCST90200424_buildGRCh38.tsv'

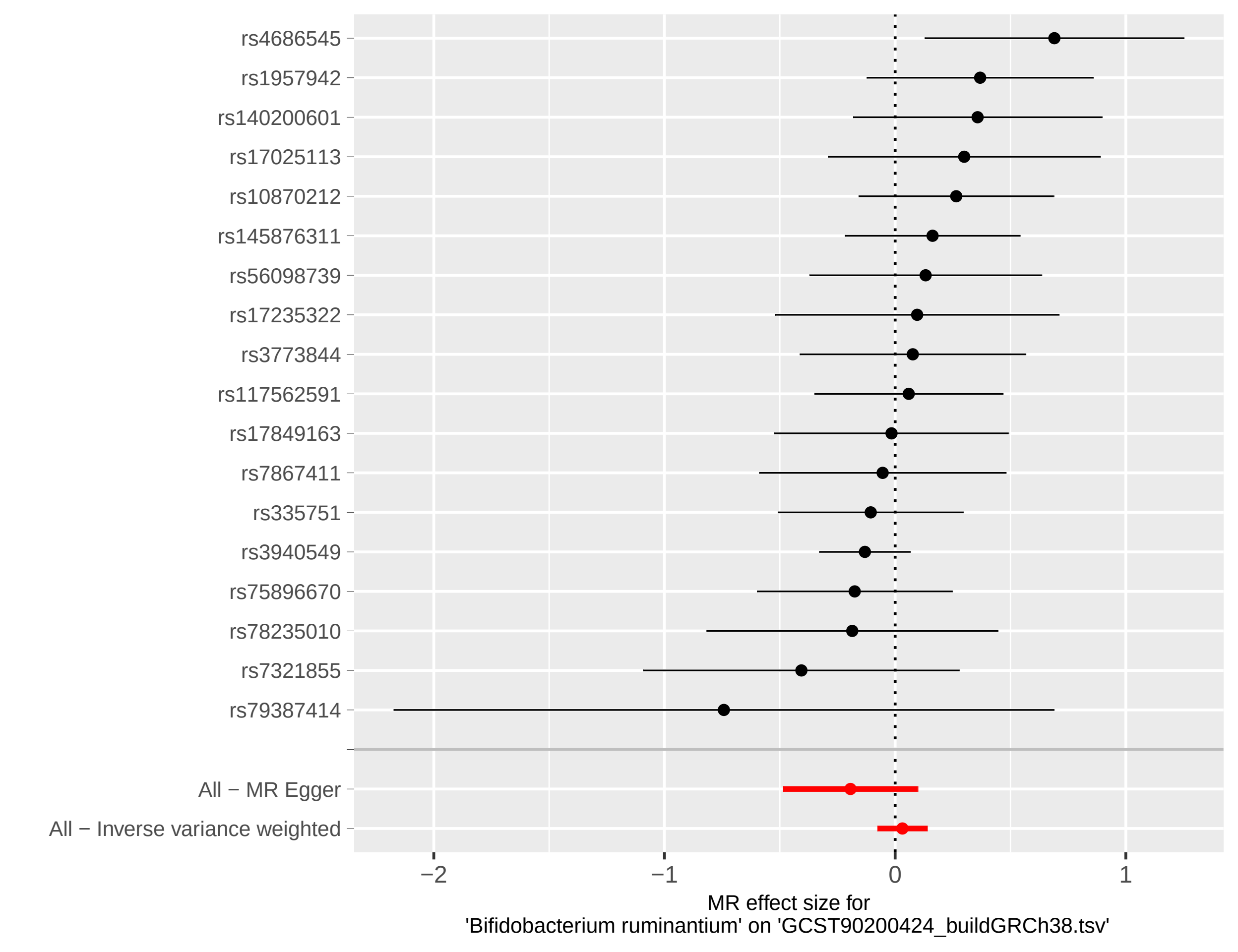
rs4686545
rs1957942
rs140200601
rs17025113
rs10870212
rs145876311
rs56098739
rs17235322
rs3773844
rs117562591
rs17849163
rs7867411
rs335751
rs3940549
rs75896670
rs78235010
rs7321855
rs79387414
All − MR Egger
All − Inverse variance weighted
−2
−1
0
1
MR effect size for
'Bifidobacterium ruminantium' on 'GCST90200424_buildGRCh38.tsv'

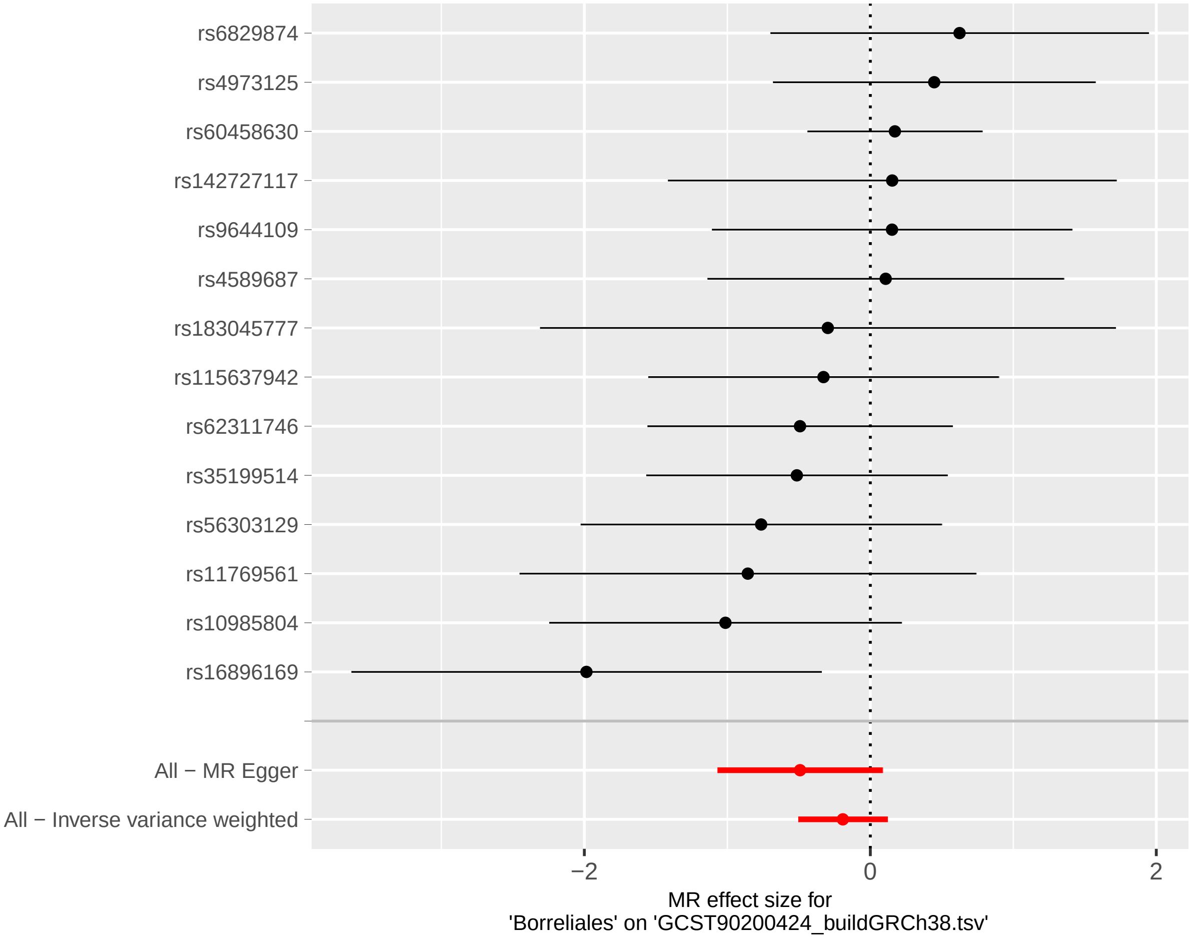
rs6829874
rs4973125
rs60458630
rs142727117
rs9644109
rs4589687
rs183045777
rs115637942
rs62311746
rs35199514
rs56303129
rs11769561
rs10985804
rs16896169
All − MR Egger
All − Inverse variance weighted
−2
0
2
MR effect size for
'Borreliales' on 'GCST90200424_buildGRCh38.tsv'

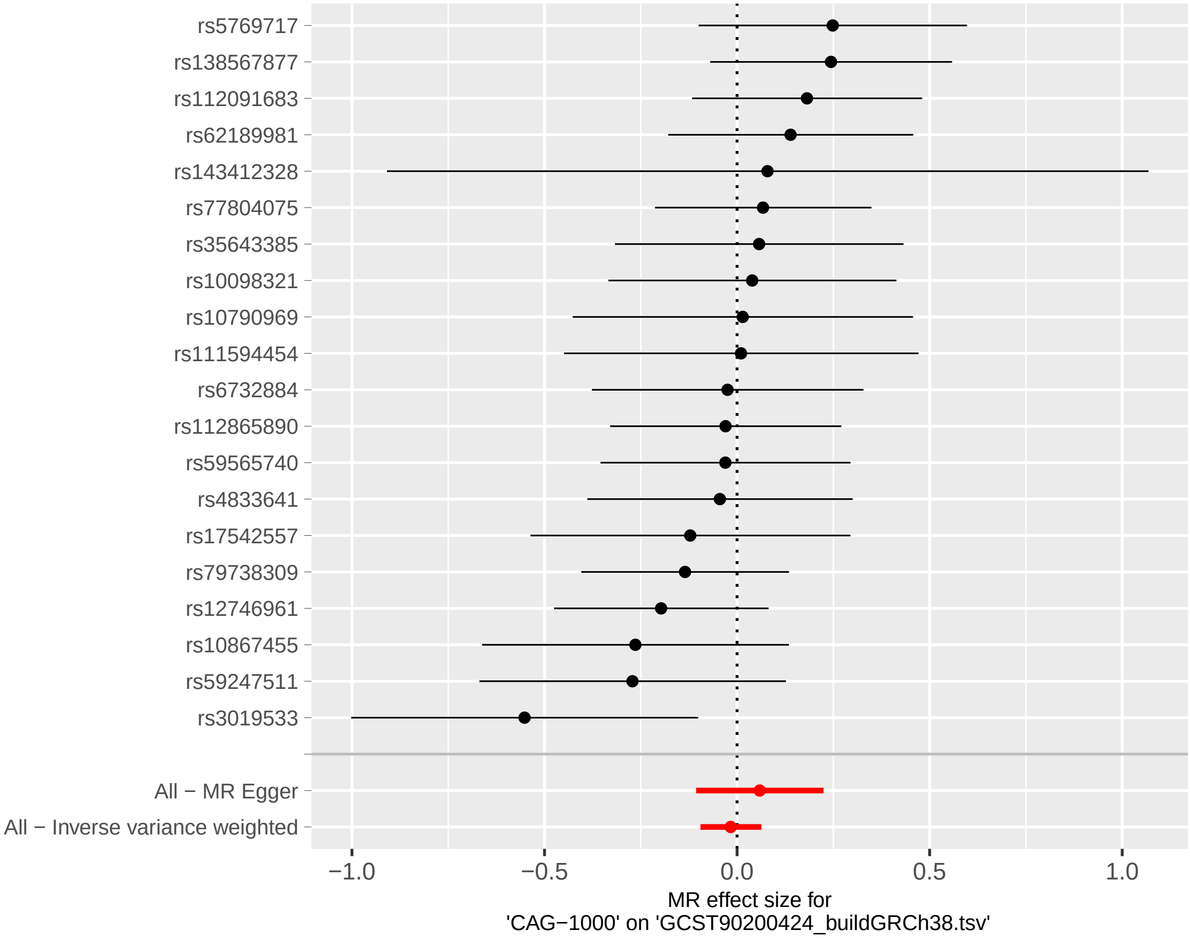

rs5769717
rs138567877
rs112091683
rs62189981
rs143412328
rs77804075
rs35643385
rs10098321
rs10790969
rs111594454
rs6732884
rs112865890
rs59565740
rs4833641
rs17542557
rs79738309
rs12746961
rs10867455
rs59247511
rs3019533
All − MR Egger
All − Inverse variance weighted
−1.0
−0.5
0.0
0.5
1.0
MR effect size for
'CAG−1000' on 'GCST90200424_buildGRCh38.tsv'

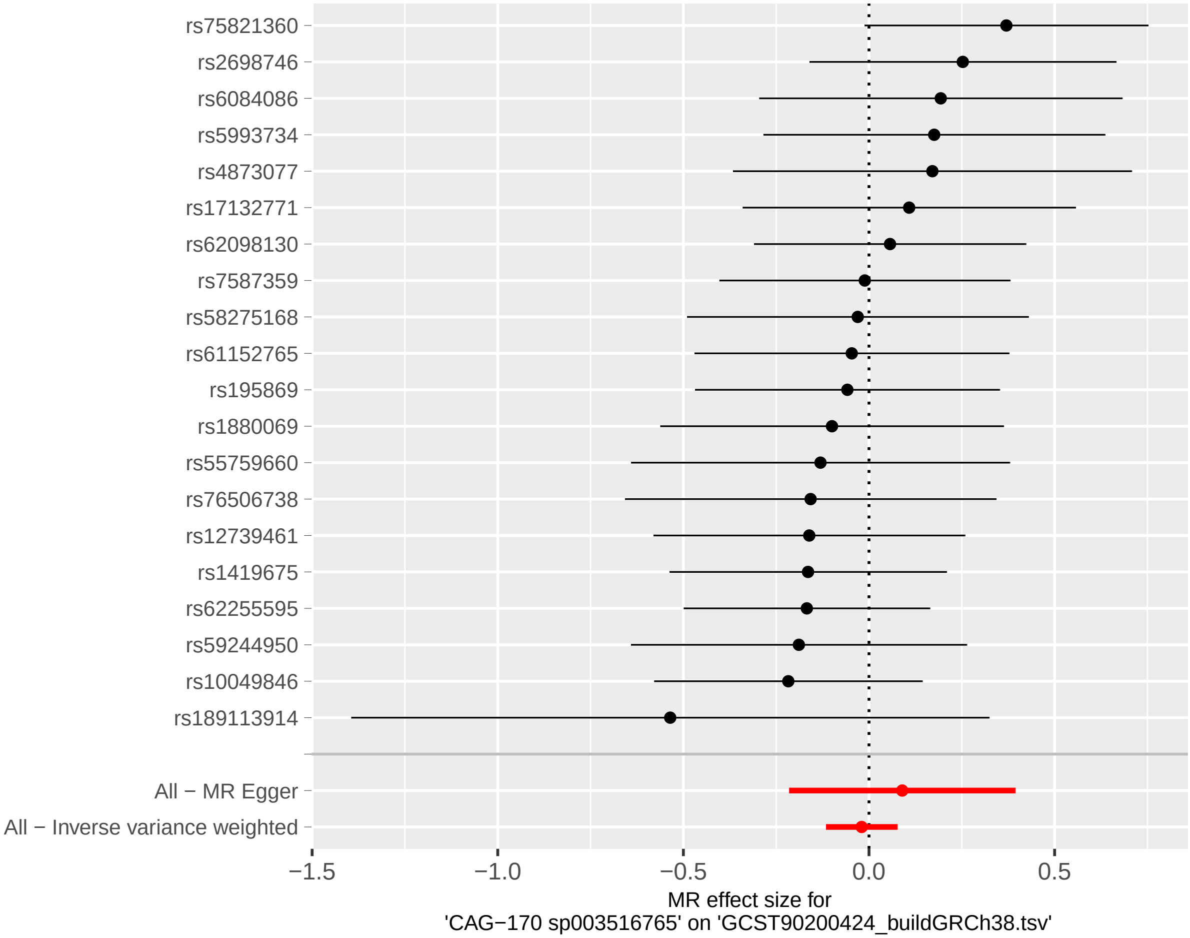
rs75821360
rs2698746
rs6084086
rs5993734
rs4873077
rs17132771
rs62098130
rs7587359
rs58275168
rs61152765
rs195869
rs1880069
rs55759660
rs76506738
rs12739461
rs1419675
rs62255595
rs59244950
rs10049846
rs189113914
All − MR Egger
All − Inverse variance weighted
−1.5
−1.0
−0.5
0.0
0.5
MR effect size for
'CAG−170 sp003516765' on 'GCST90200424_buildGRCh38.tsv'

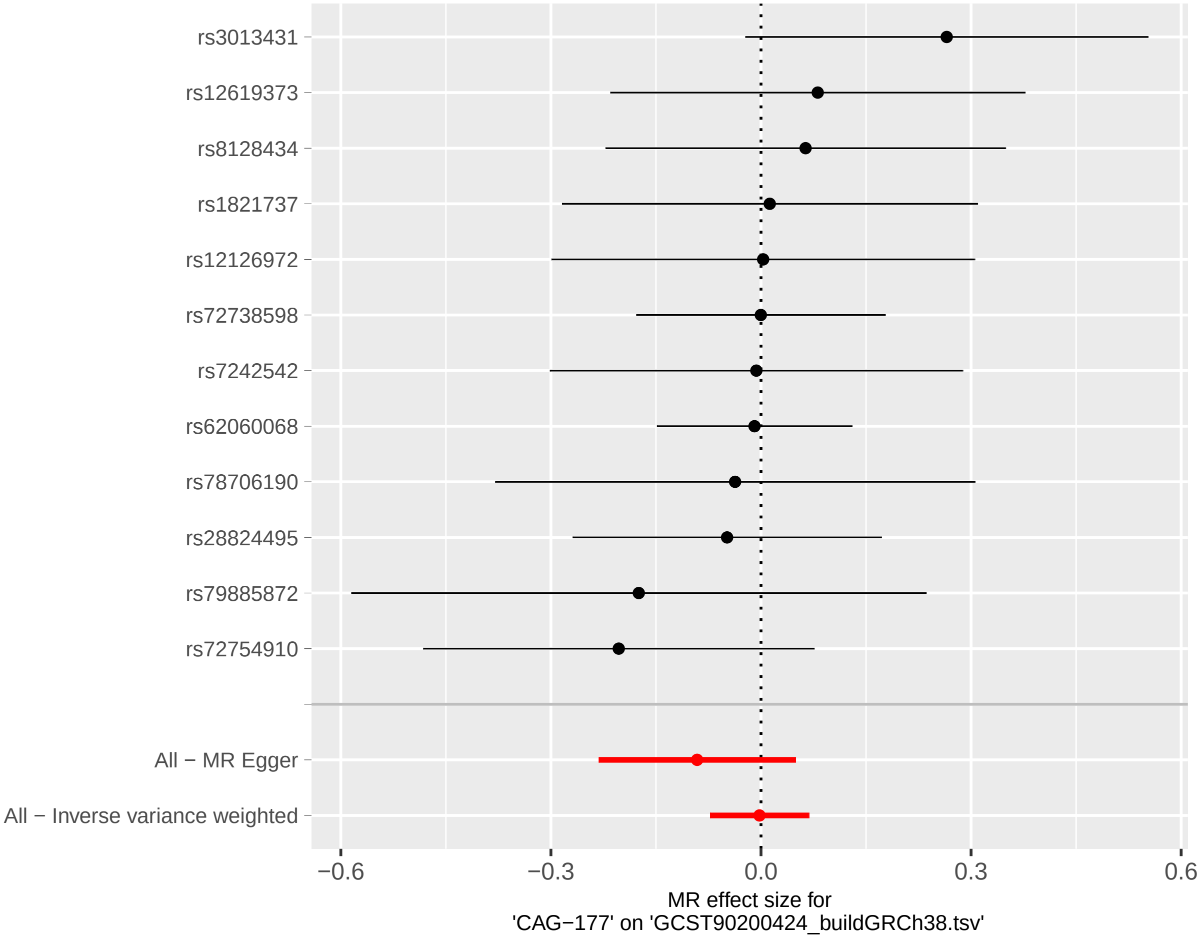

rs3013431
rs12619373
rs8128434
rs1821737
rs12126972
rs72738598
rs7242542
rs62060068
rs78706190
rs28824495
rs79885872
rs72754910
All − MR Egger
All − Inverse variance weighted
−0.6
−0.3
0.0
0.3
0.6
MR effect size for
'CAG−177' on 'GCST90200424_buildGRCh38.tsv'

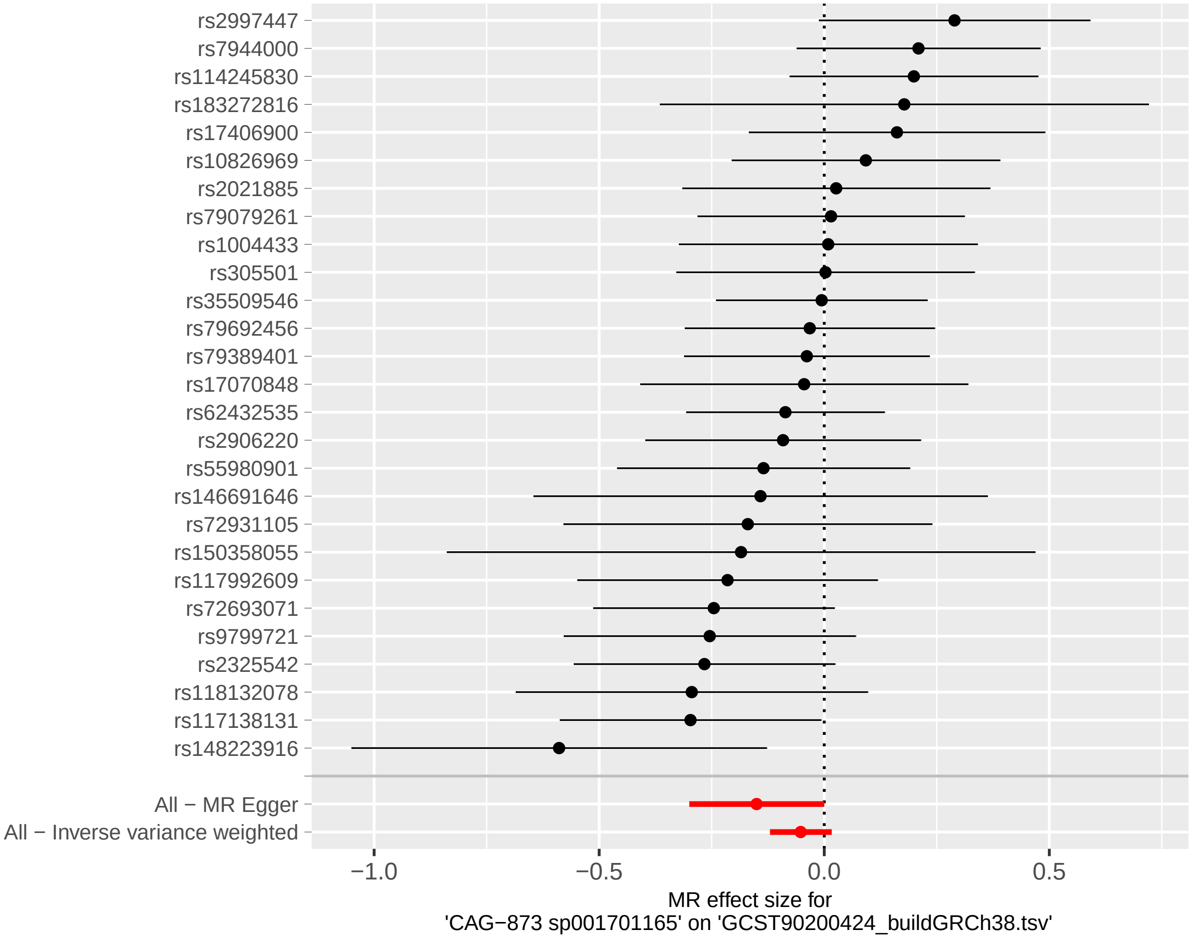

rs2997447
rs7944000
rs114245830
rs183272816
rs17406900
rs10826969
rs2021885
rs79079261
rs1004433
rs305501
rs35509546
rs79692456
rs79389401
rs17070848
rs62432535
rs2906220
rs55980901
rs146691646
rs72931105
rs150358055
rs117992609
rs72693071
rs9799721
rs2325542
rs118132078
rs117138131
rs148223916
All − MR Egger
All − Inverse variance weighted
−1.0
−0.5
0.0
0.5
MR effect size for
'CAG−873 sp001701165' on 'GCST90200424_buildGRCh38.tsv'

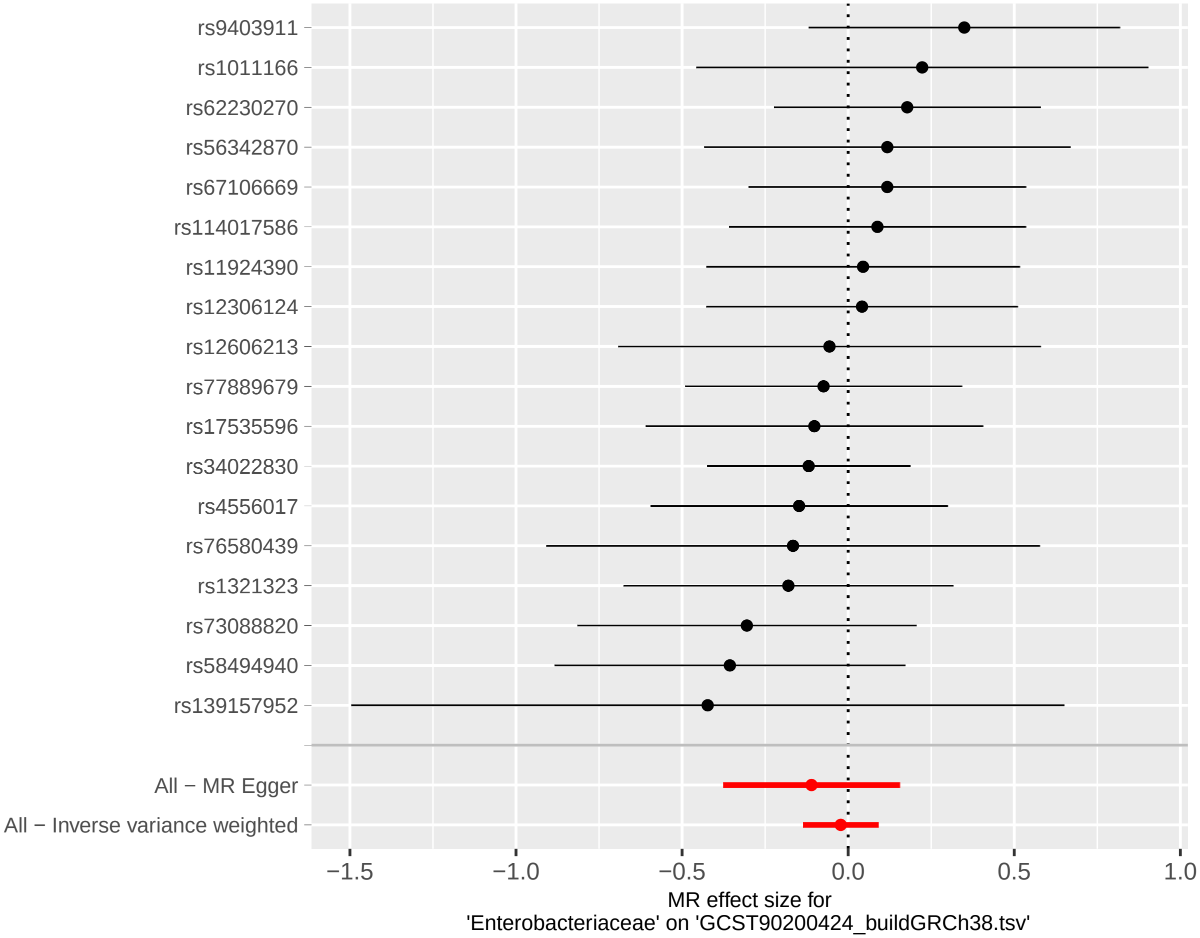

rs9403911
rs1011166
rs62230270
rs56342870
rs67106669
rs114017586
rs11924390
rs12306124
rs12606213
rs77889679
rs17535596
rs34022830
rs4556017
rs76580439
rs1321323
rs73088820
rs58494940
rs139157952
All − MR Egger
All − Inverse variance weighted
−1.5
−1.0
−0.5
0.0
0.5
1.0
MR effect size for
'Enterobacteriaceae' on 'GCST90200424_buildGRCh38.tsv'

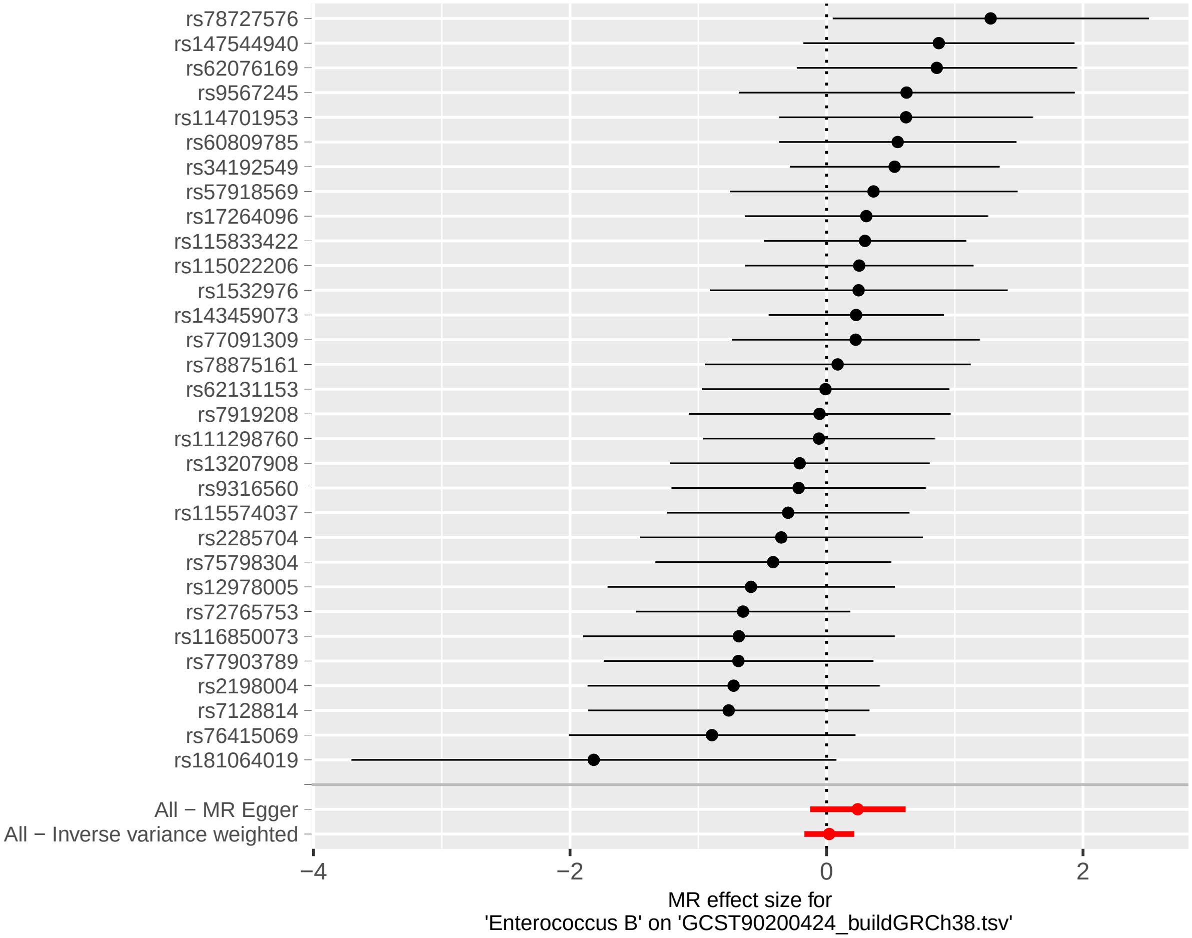

rs78727576
rs147544940
rs62076169
rs9567245
rs114701953
rs60809785
rs34192549
rs57918569
rs17264096
rs115833422
rs115022206
rs1532976
rs143459073
rs77091309
rs78875161
rs62131153
rs7919208
rs111298760
rs13207908
rs9316560
rs115574037
rs2285704
rs75798304
rs12978005
rs72765753
rs116850073
rs77903789
rs2198004
rs7128814
rs76415069
rs181064019
All − MR Egger
All − Inverse variance weighted
−4
−2
0
2
MR effect size for
'Enterococcus B' on 'GCST90200424_buildGRCh38.tsv'

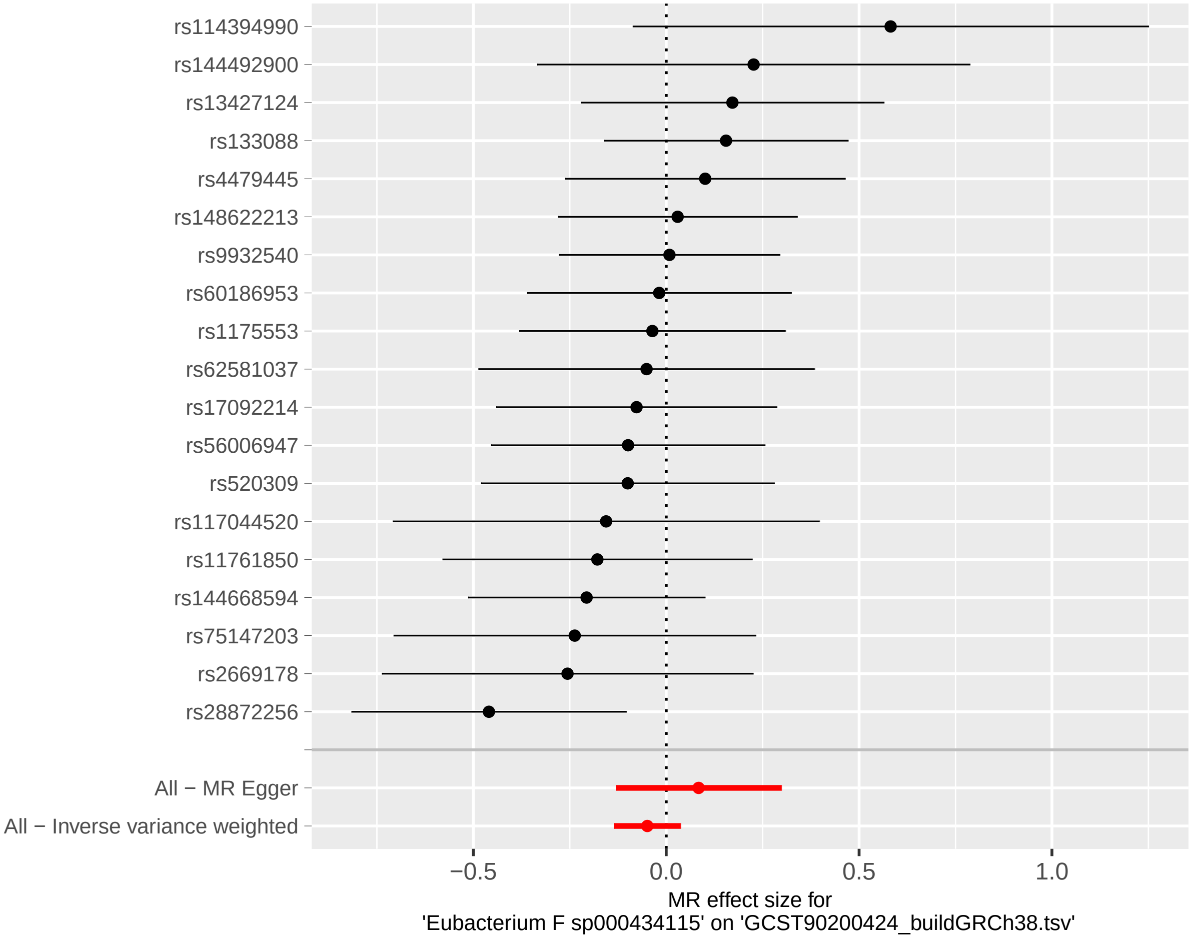

rs114394990
rs144492900
rs13427124
rs133088
rs4479445
rs148622213
rs9932540
rs60186953
rs1175553
rs62581037
rs17092214
rs56006947
rs520309
rs117044520
rs11761850
rs144668594
rs75147203
rs2669178
rs28872256
All − MR Egger
All − Inverse variance weighted
−0.5
0.0
0.5
1.0
MR effect size for
'Eubacterium F sp000434115' on 'GCST90200424_buildGRCh38.tsv'

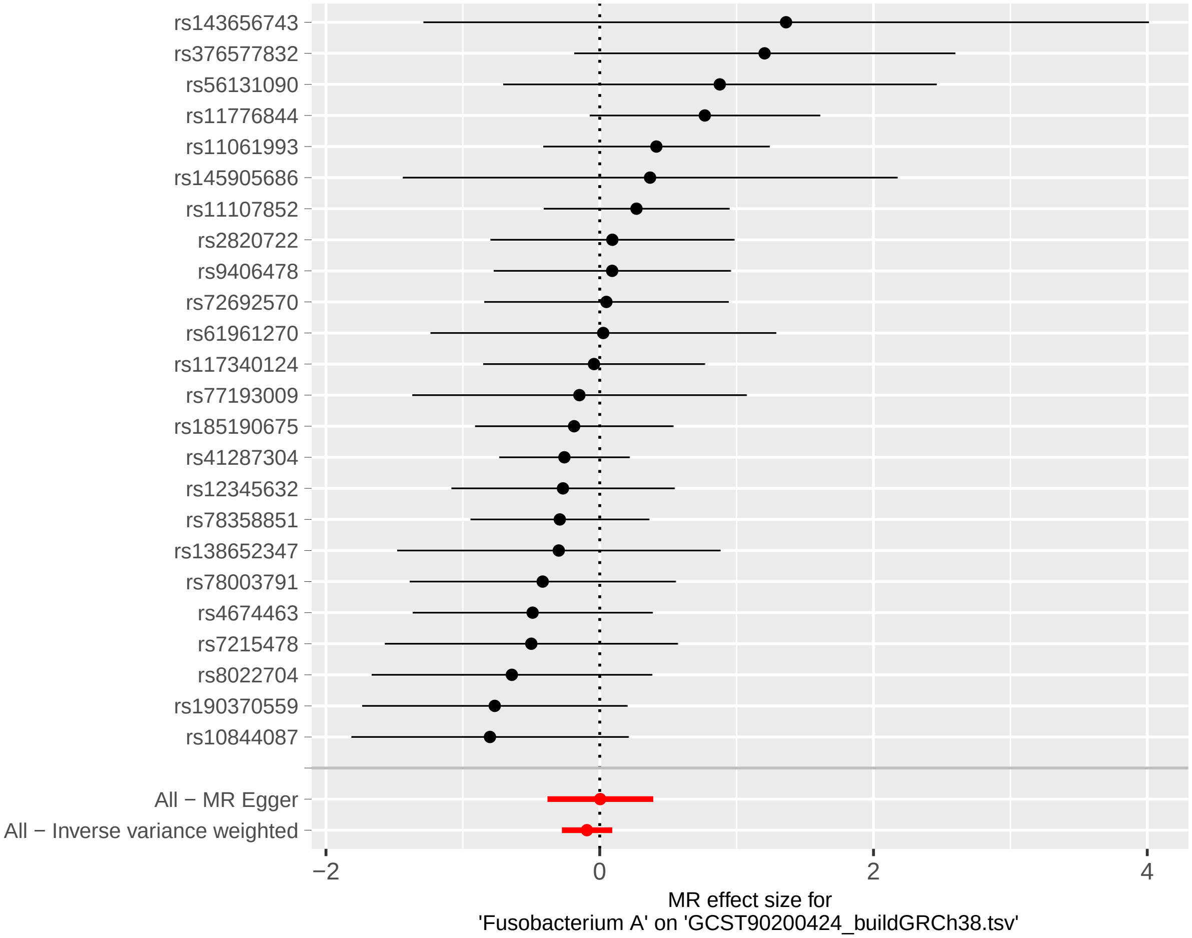

rs143656743
rs376577832
rs56131090
rs11776844
rs11061993
rs145905686
rs11107852
rs2820722
rs9406478
rs72692570
rs61961270
rs117340124
rs77193009
rs185190675
rs41287304
rs12345632
rs78358851
rs138652347
rs78003791
rs4674463
rs7215478
rs8022704
rs190370559
rs10844087
All − MR Egger
All − Inverse variance weighted
−2
0
2
4
MR effect size for
'Fusobacterium A' on 'GCST90200424_buildGRCh38.tsv'

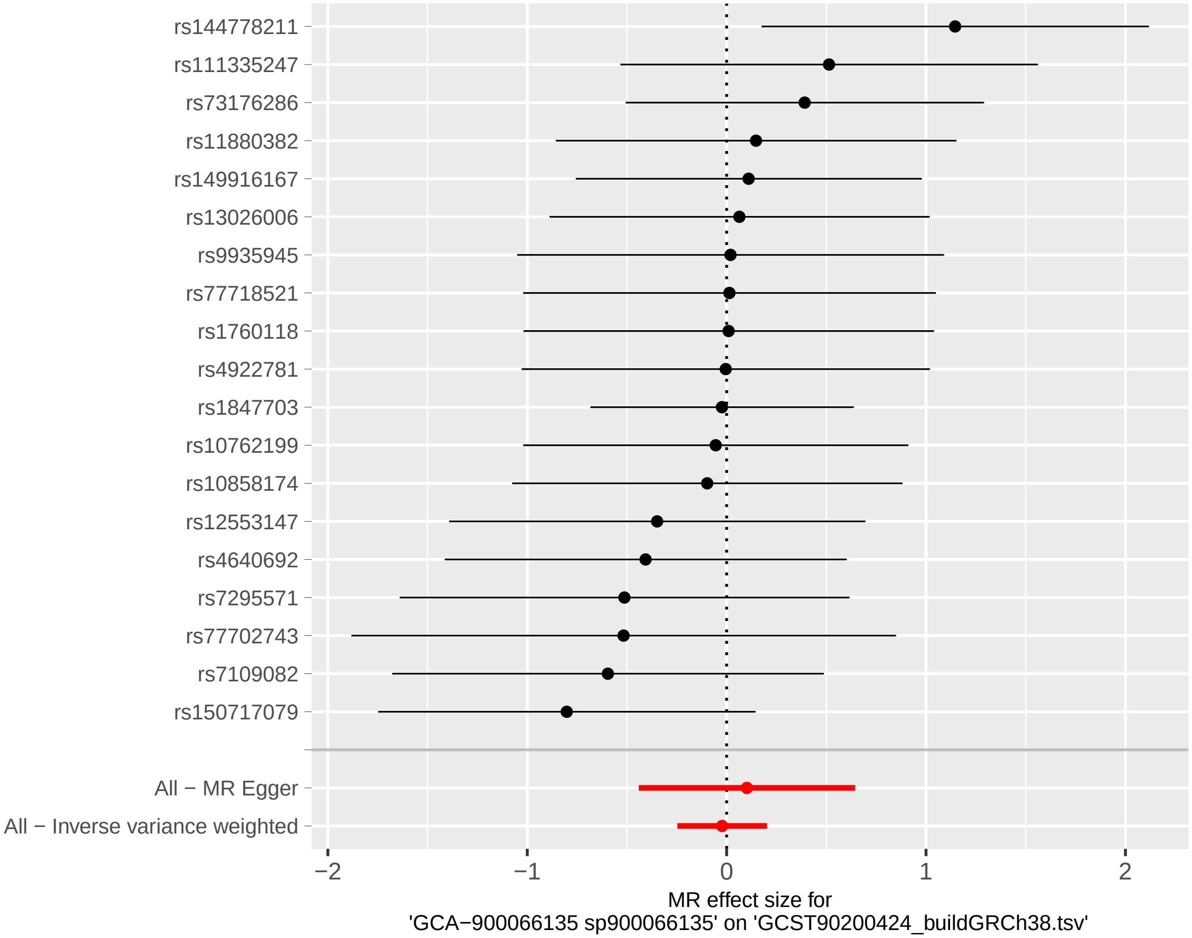

rs144778211
rs111335247
rs73176286
rs11880382
rs149916167
rs13026006
rs9935945
rs77718521
rs1760118
rs4922781
rs1847703
rs10762199
rs10858174
rs12553147
rs4640692
rs7295571
rs77702743
rs7109082
rs150717079
All − MR Egger
All − Inverse variance weighted
−2
−1
0
1
2
MR effect size for
'GCA−900066135 sp900066135' on 'GCST90200424_buildGRCh38.tsv'

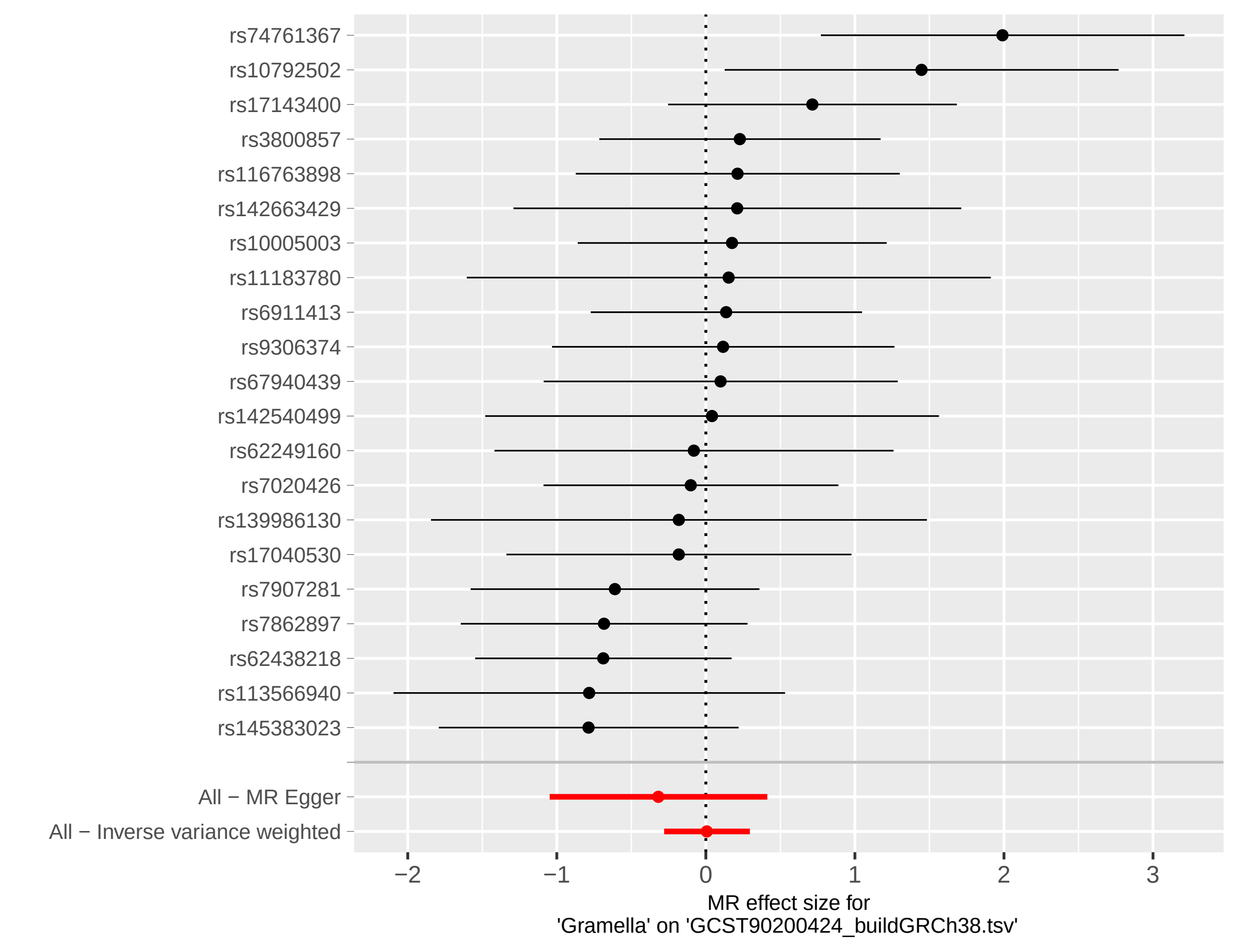

rs74761367
rs10792502
rs17143400
rs3800857
rs116763898
rs142663429
rs10005003
rs11183780
rs6911413
rs9306374
rs67940439
rs142540499
rs62249160
rs7020426
rs139986130
rs17040530
rs7907281
rs7862897
rs62438218
rs113566940
rs145383023
All − MR Egger
All − Inverse variance weighted
−2
−1
0
1
2
3
MR effect size for
'Gramella' on 'GCST90200424_buildGRCh38.tsv'

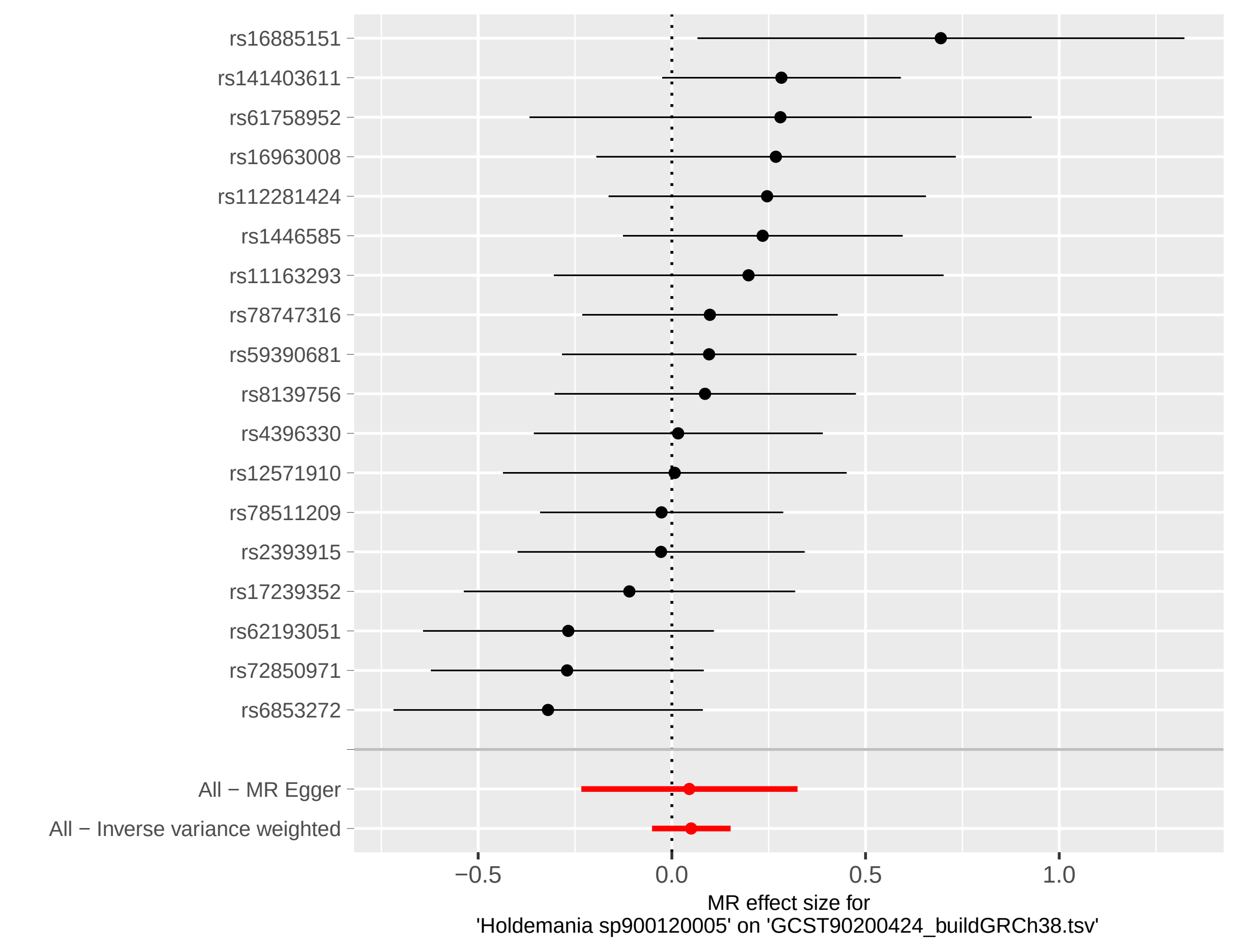

rs16885151
rs141403611
rs61758952
rs16963008
rs112281424
rs1446585
rs11163293
rs78747316
rs59390681
rs8139756
rs4396330
rs12571910
rs78511209
rs2393915
rs17239352
rs62193051
rs72850971
rs6853272
All − MR Egger
All − Inverse variance weighted
−0.5
0.0
0.5
1.0
MR effect size for
'Holdemania sp900120005' on 'GCST90200424_buildGRCh38.tsv'

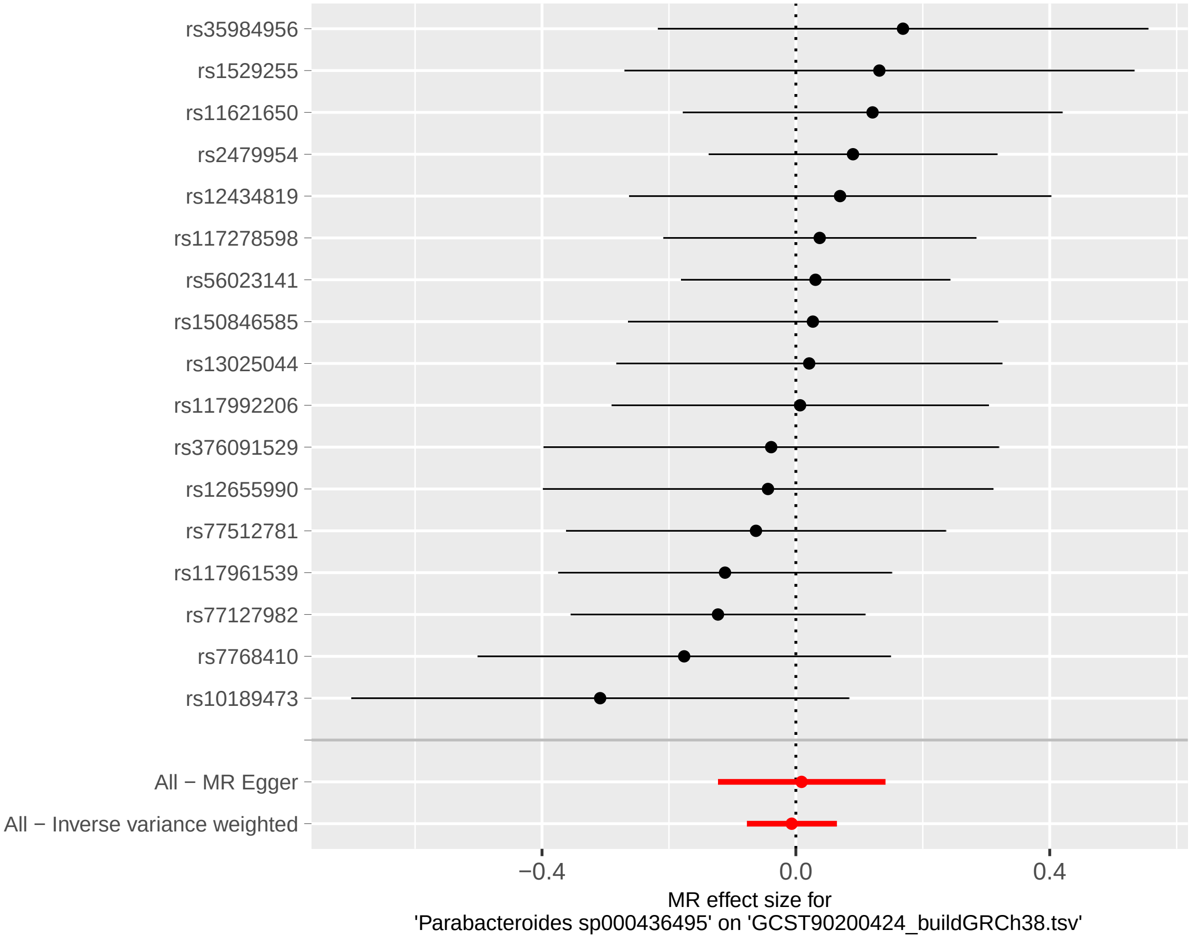

rs35984956
rs1529255
rs11621650
rs2479954
rs12434819
rs117278598
rs56023141
rs150846585
rs13025044
rs117992206
rs376091529
rs12655990
rs77512781
rs117961539
rs77127982
rs7768410
rs10189473
All − MR Egger
All − Inverse variance weighted
−0.4
0.0
0.4
MR effect size for
'Parabacteroides sp000436495' on 'GCST90200424_buildGRCh38.tsv'

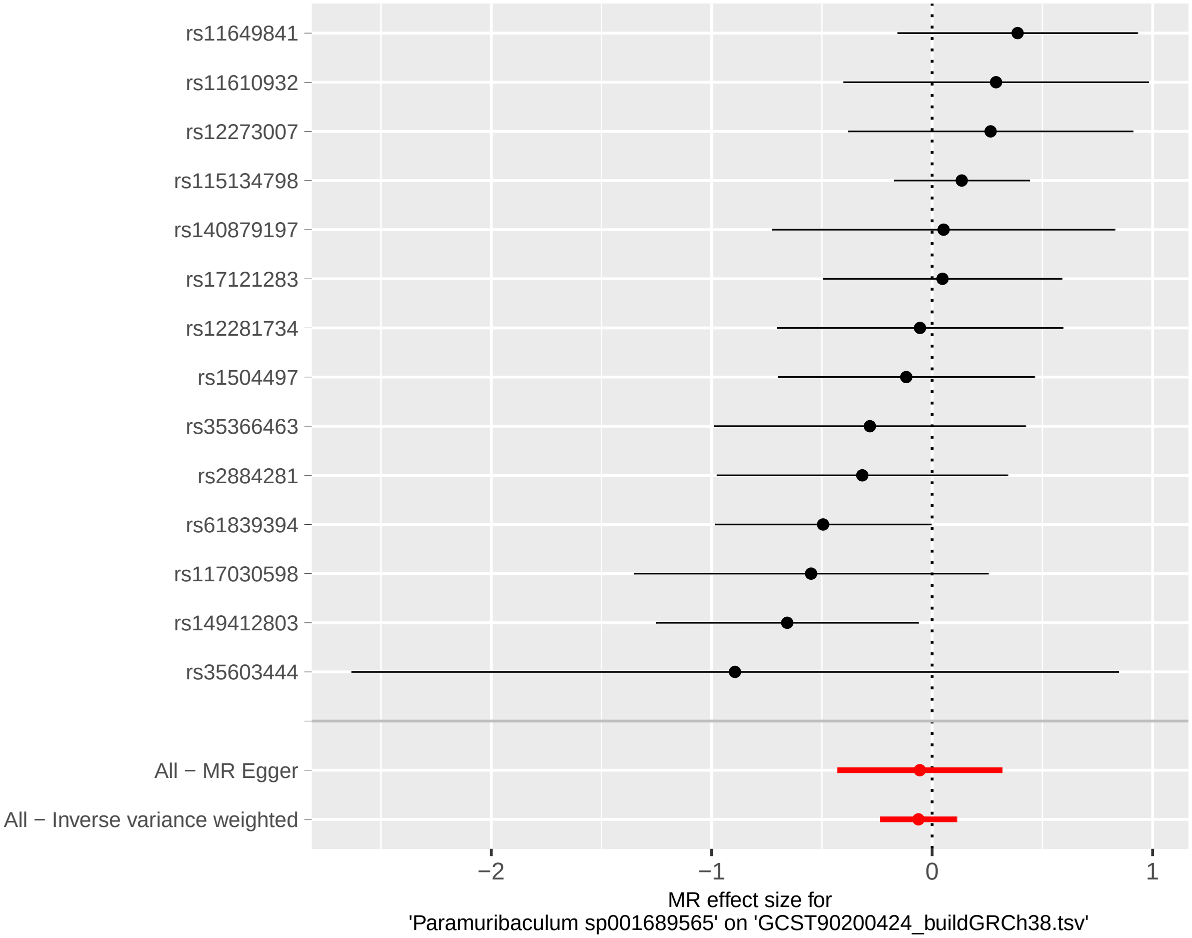

rs11649841
rs11610932
rs12273007
rs115134798
rs140879197
rs17121283
rs12281734
rs1504497
rs35366463
rs2884281
rs61839394
rs117030598
rs149412803
rs35603444
All − MR Egger
All − Inverse variance weighted
−2
−1
0
1
MR effect size for
'Paramuribaculum sp001689565' on 'GCST90200424_buildGRCh38.tsv'

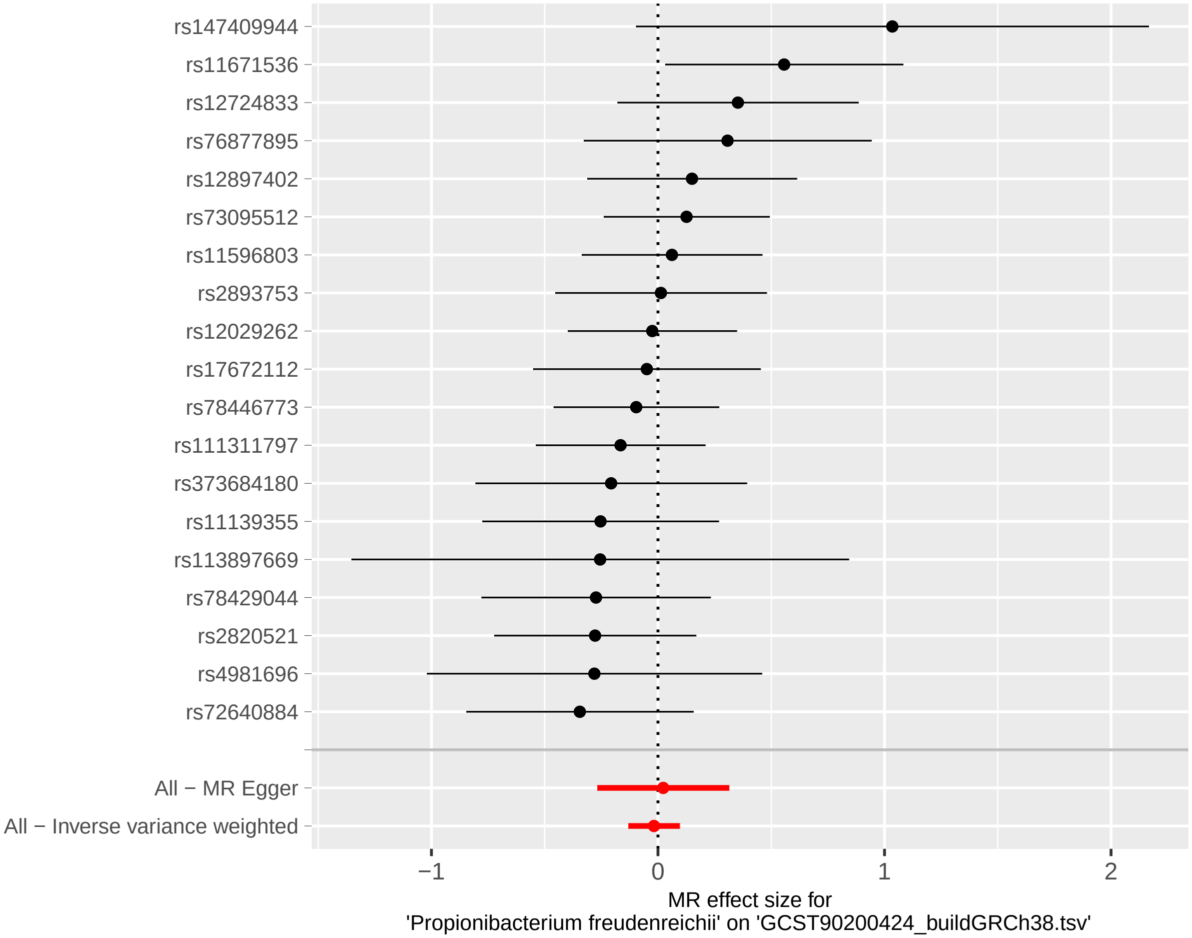

rs147409944
rs11671536
rs12724833
rs76877895
rs12897402
rs73095512
rs11596803
rs2893753
rs12029262
rs17672112
rs78446773
rs111311797
rs373684180
rs11139355
rs113897669
rs78429044
rs2820521
rs4981696
rs72640884
All − MR Egger
All − Inverse variance weighted
−1
0
1
2
MR effect size for
'Propionibacterium freudenreichii' on 'GCST90200424_buildGRCh38.tsv'

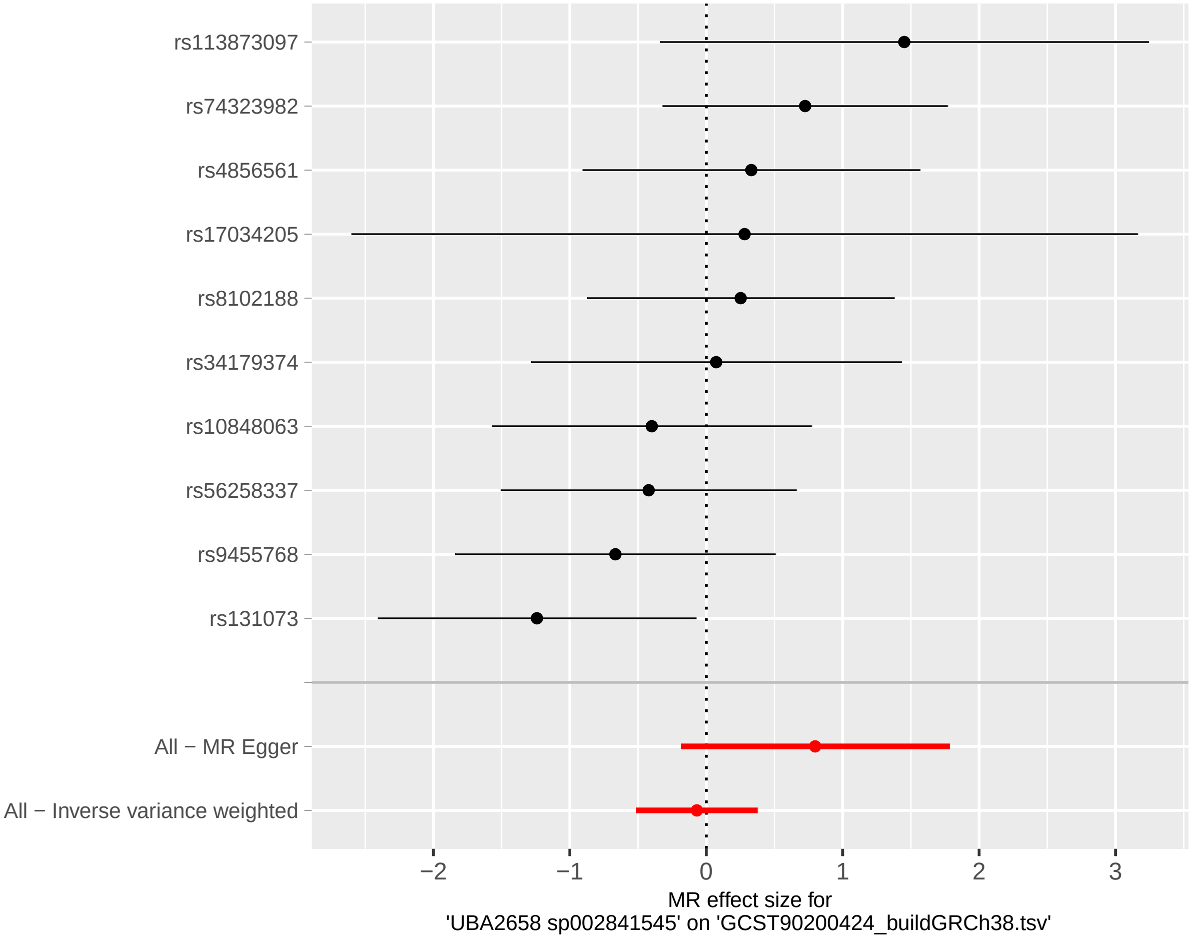

rs113873097
rs74323982
rs4856561
rs17034205
rs8102188
rs34179374
rs10848063
rs56258337
rs9455768
rs131073
All − MR Egger
All − Inverse variance weighted
−2
−1
0
1
2
3
MR effect size for
'UBA2658 sp002841545' on 'GCST90200424_buildGRCh38.tsv'

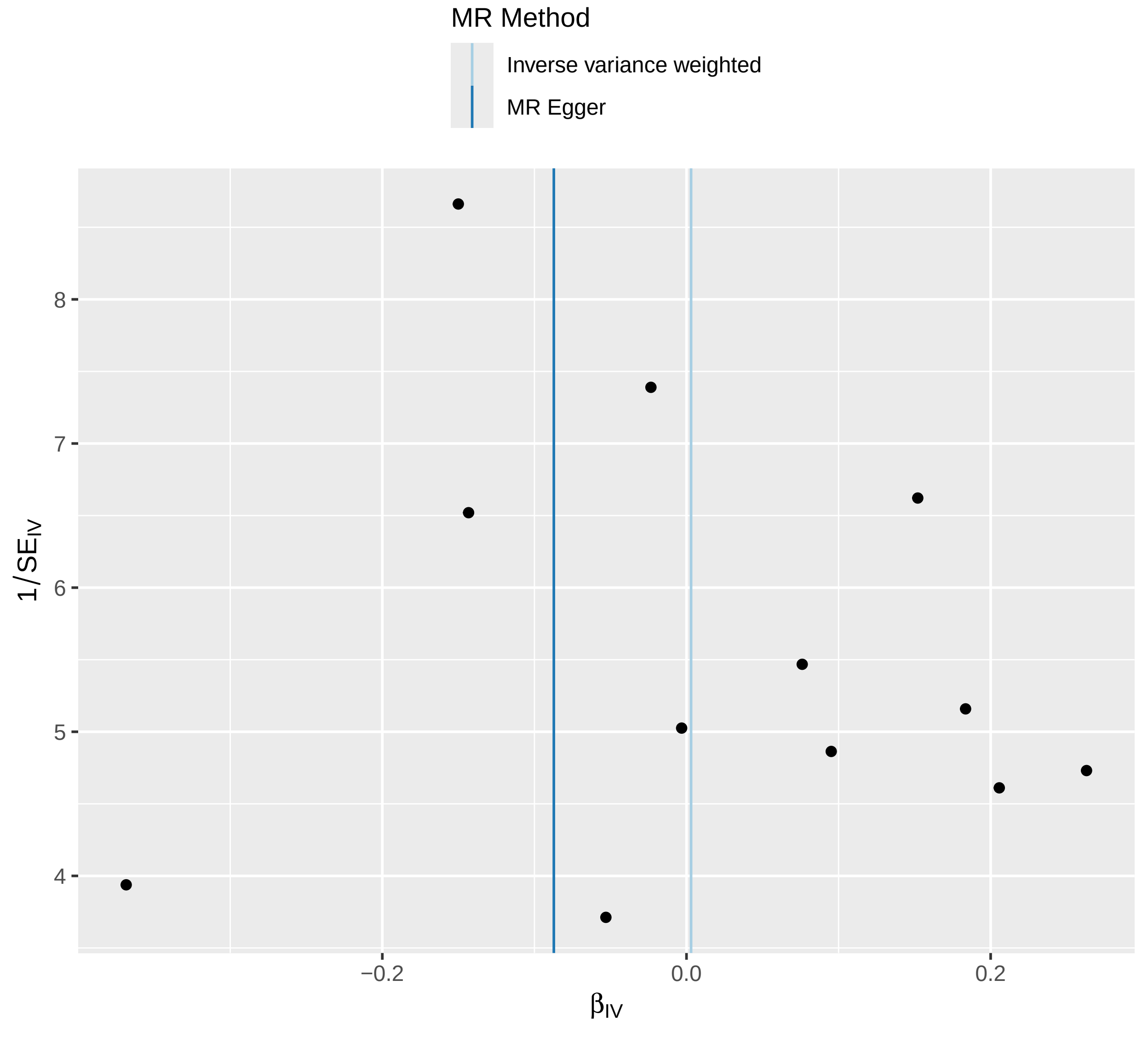

MR Method
Inverse variance weighted
MR Egger
8
7
6
5
4
1/SE$_{IV}$
−0.2
0.0
0.2
β$_{IV}$

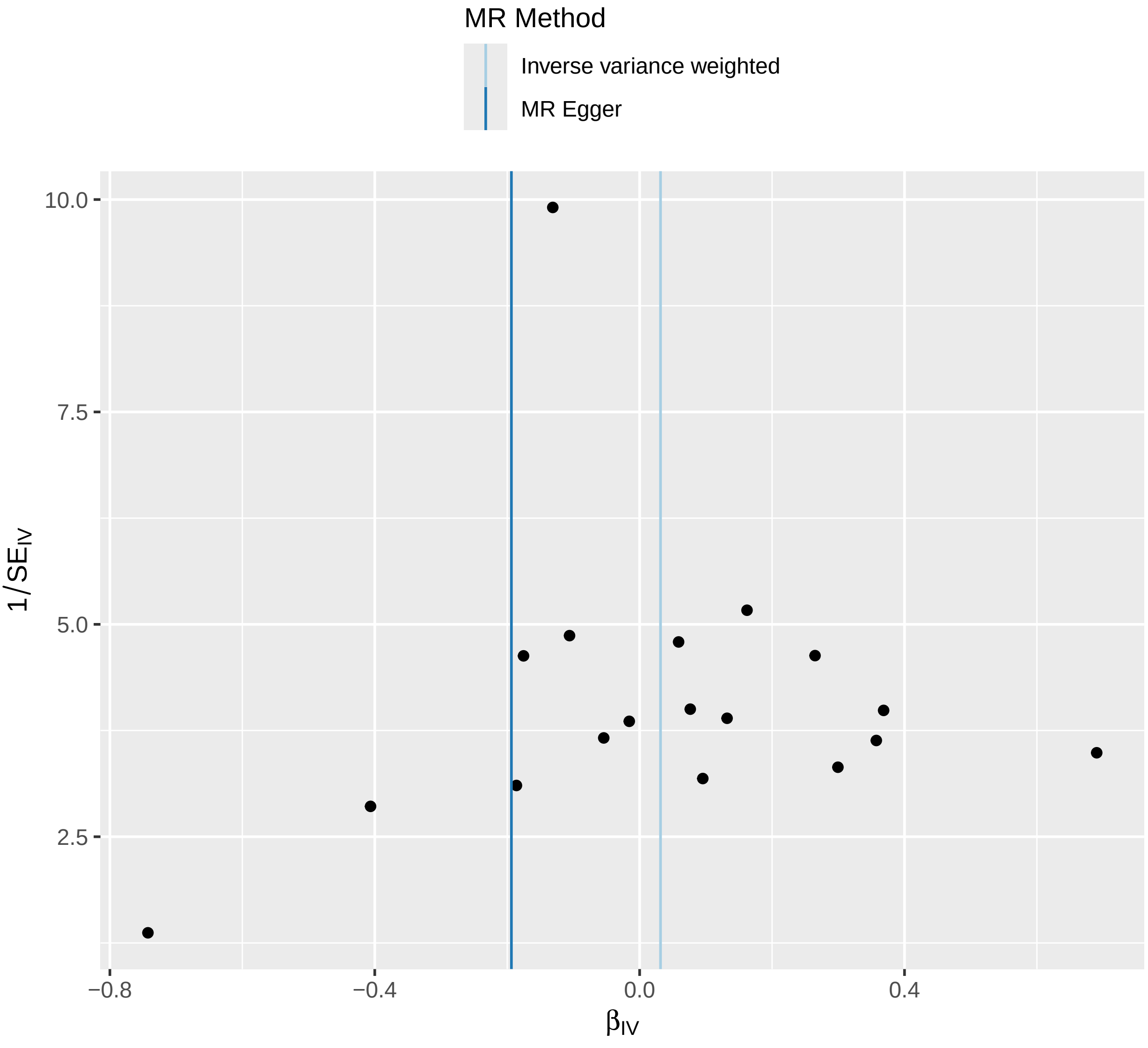

MR Method
Inverse variance weighted
MR Egger
10.0
7.5
5.0
2.5
1/SE_IV
−0.8
−0.4
0.0
0.4
β_IV

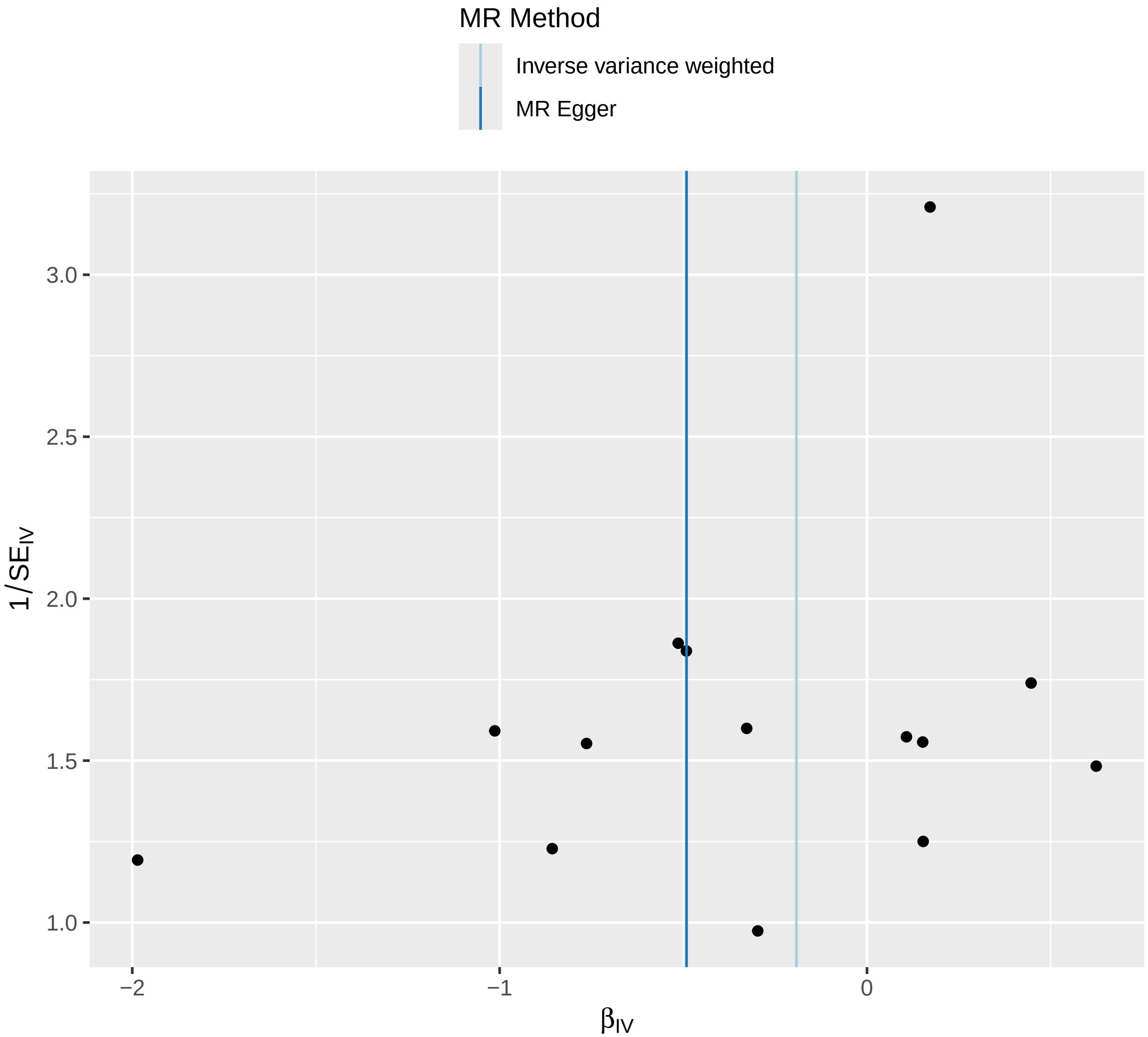

MR Method
Inverse variance weighted
MR Egger
3.0
2.5
2.0
1.5
1.0
1/SE$_{IV}$
−2
−1
0
β_{IV}

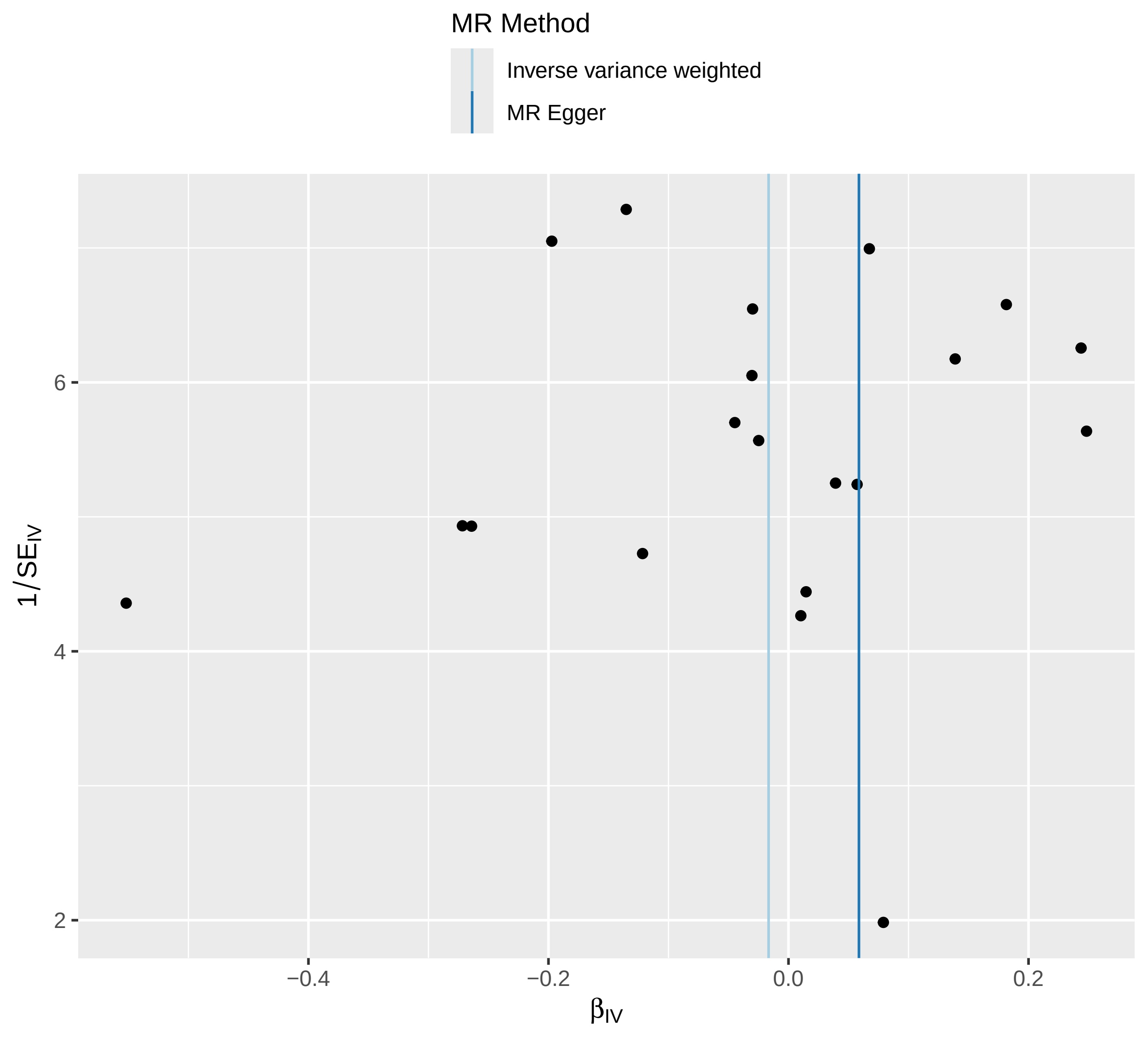
MR Method
Inverse variance weighted
MR Egger
1/SE$_{IV}$
6
4
2
−0.4
−0.2
0.0
0.2
β$_{IV}$

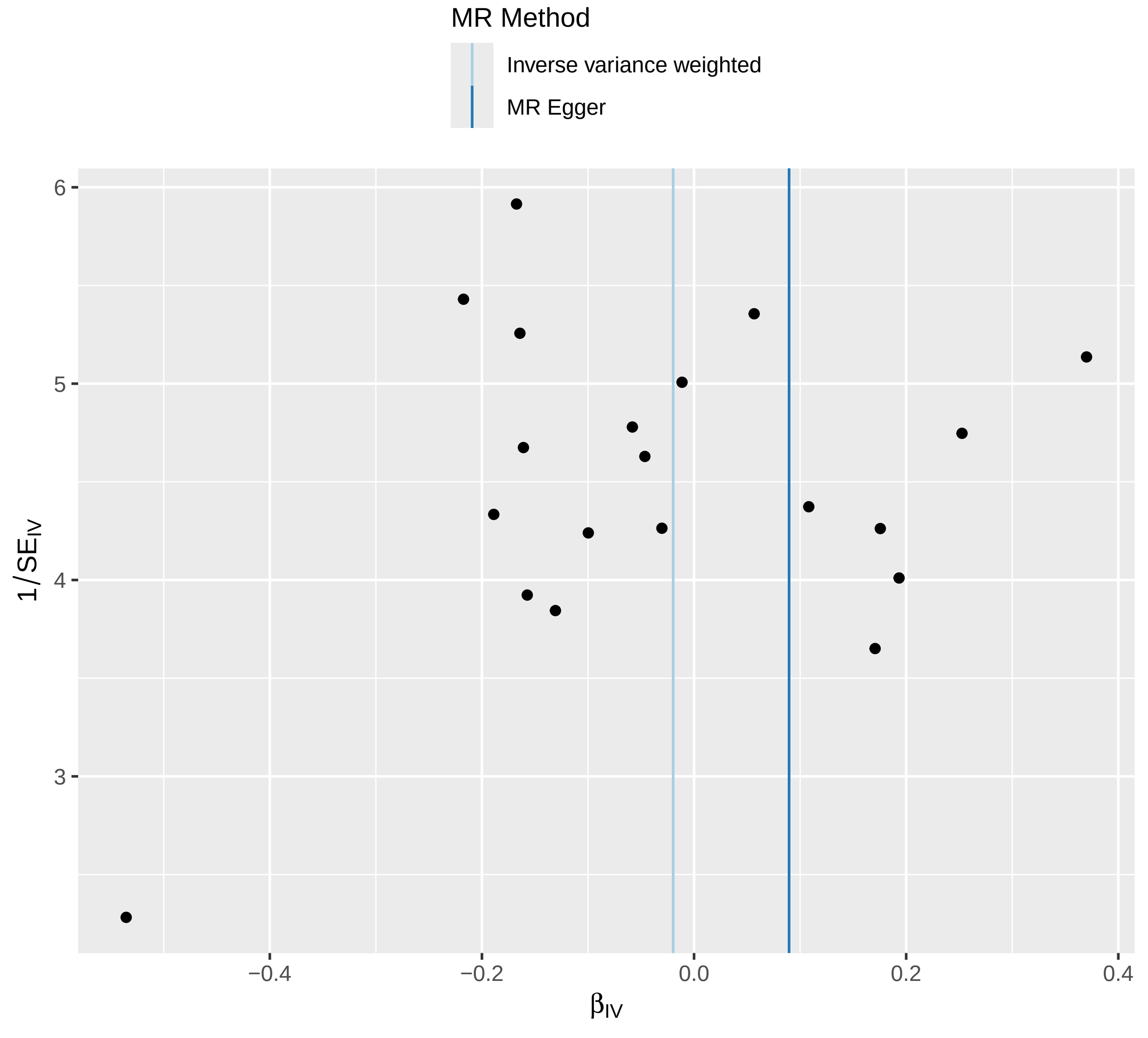
MR Method
Inverse variance weighted
MR Egger
6
5
4
3
1/SE_IV
−0.4
−0.2
0.0
0.2
0.4
β_IV

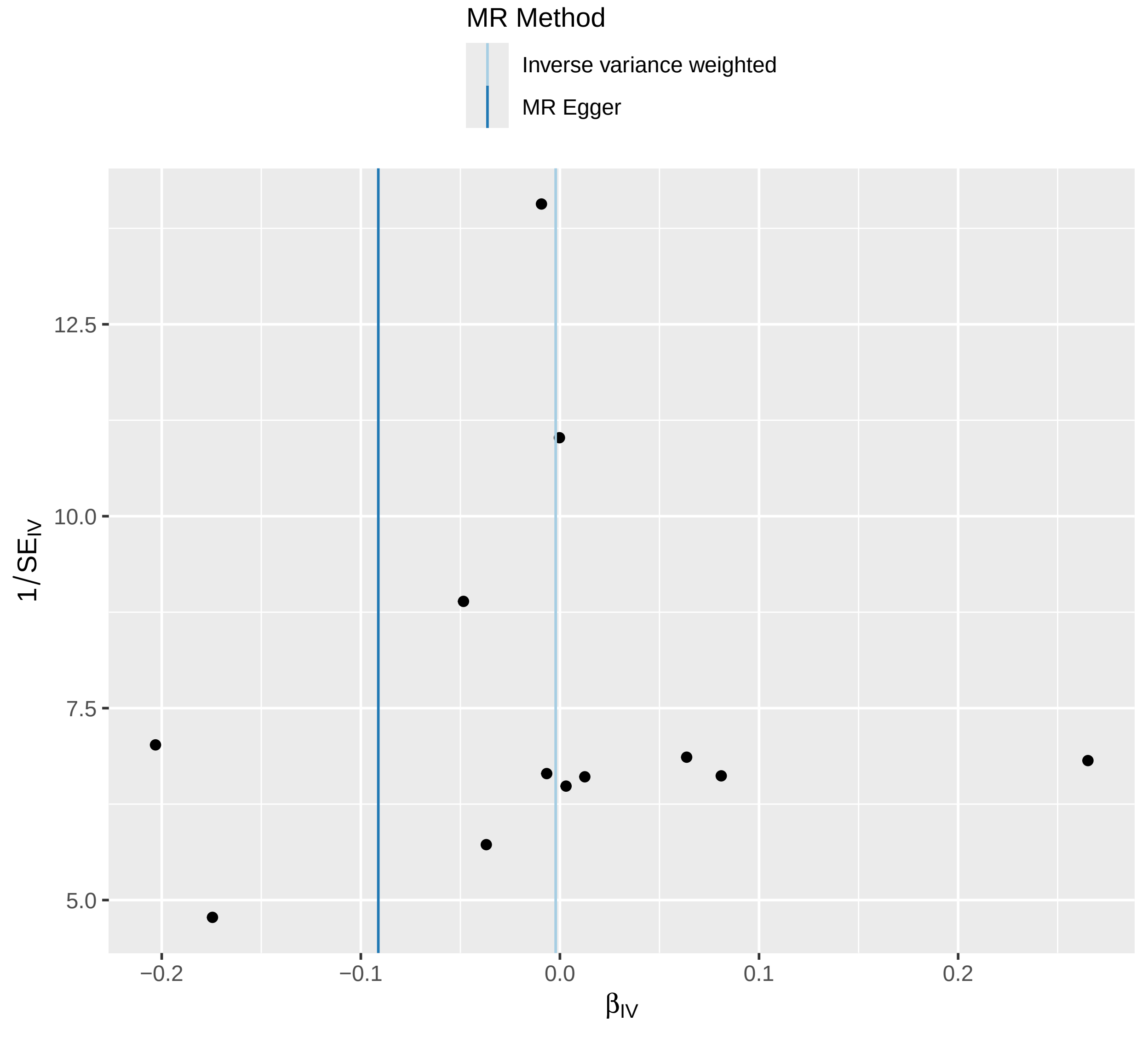
MR Method
Inverse variance weighted
MR Egger
12.5
10.0
7.5
5.0
1/SE$_{IV}$
−0.2
−0.1
0.0
0.1
0.2
β$_{IV}$

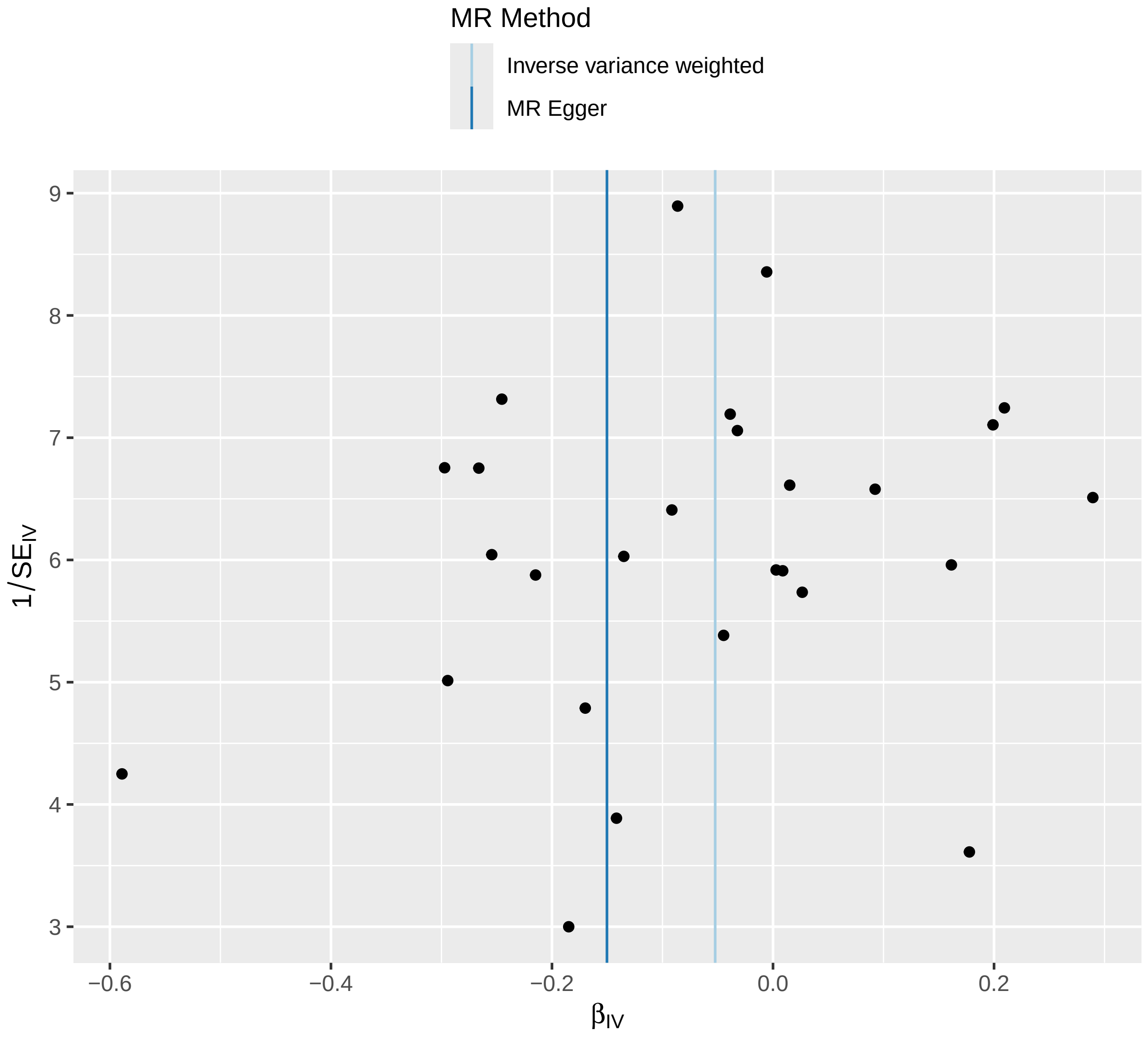
MR Method
Inverse variance weighted
MR Egger
9
8
7
6
5
4
3
1/SE_IV
−0.6
−0.4
−0.2
0.0
0.2
β_IV

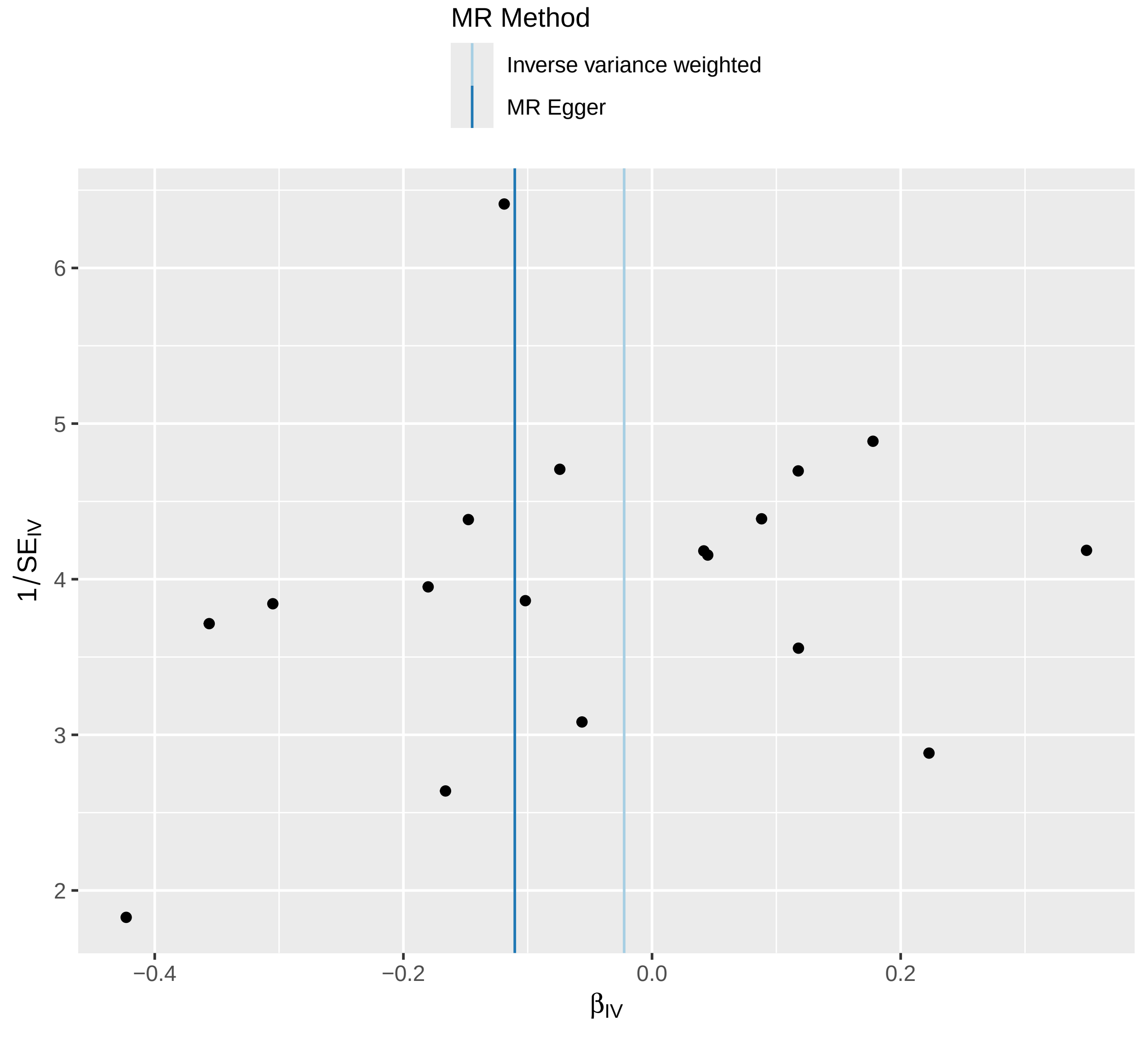
MR Method
Inverse variance weighted
MR Egger
6
5
4
3
2
1/SE_IV
−0.4
−0.2
0.0
0.2
β_IV

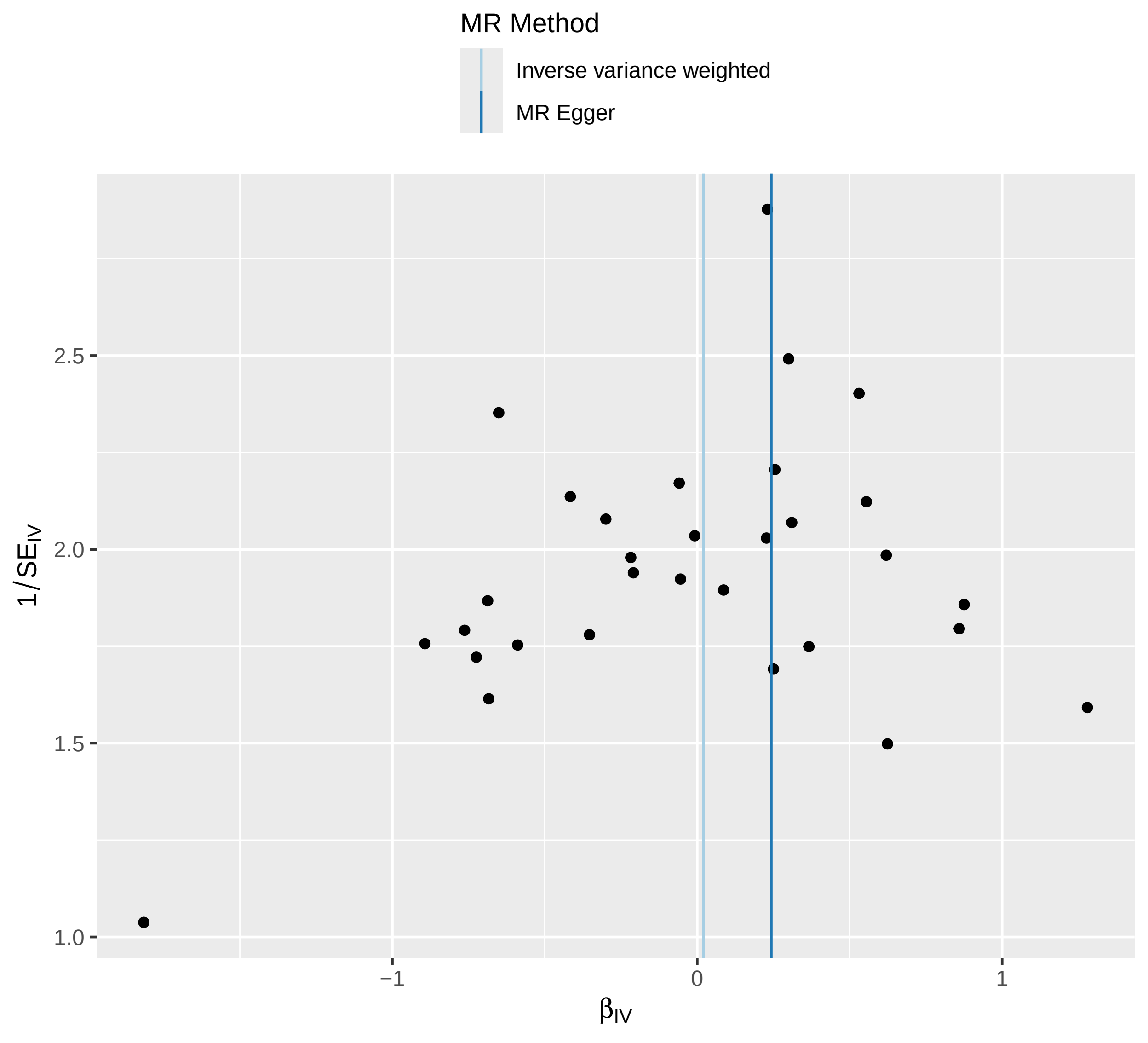
MR Method
Inverse variance weighted
MR Egger
2.5
2.0
1.5
1.0
−1
0
1
1/SE_IV
β_IV

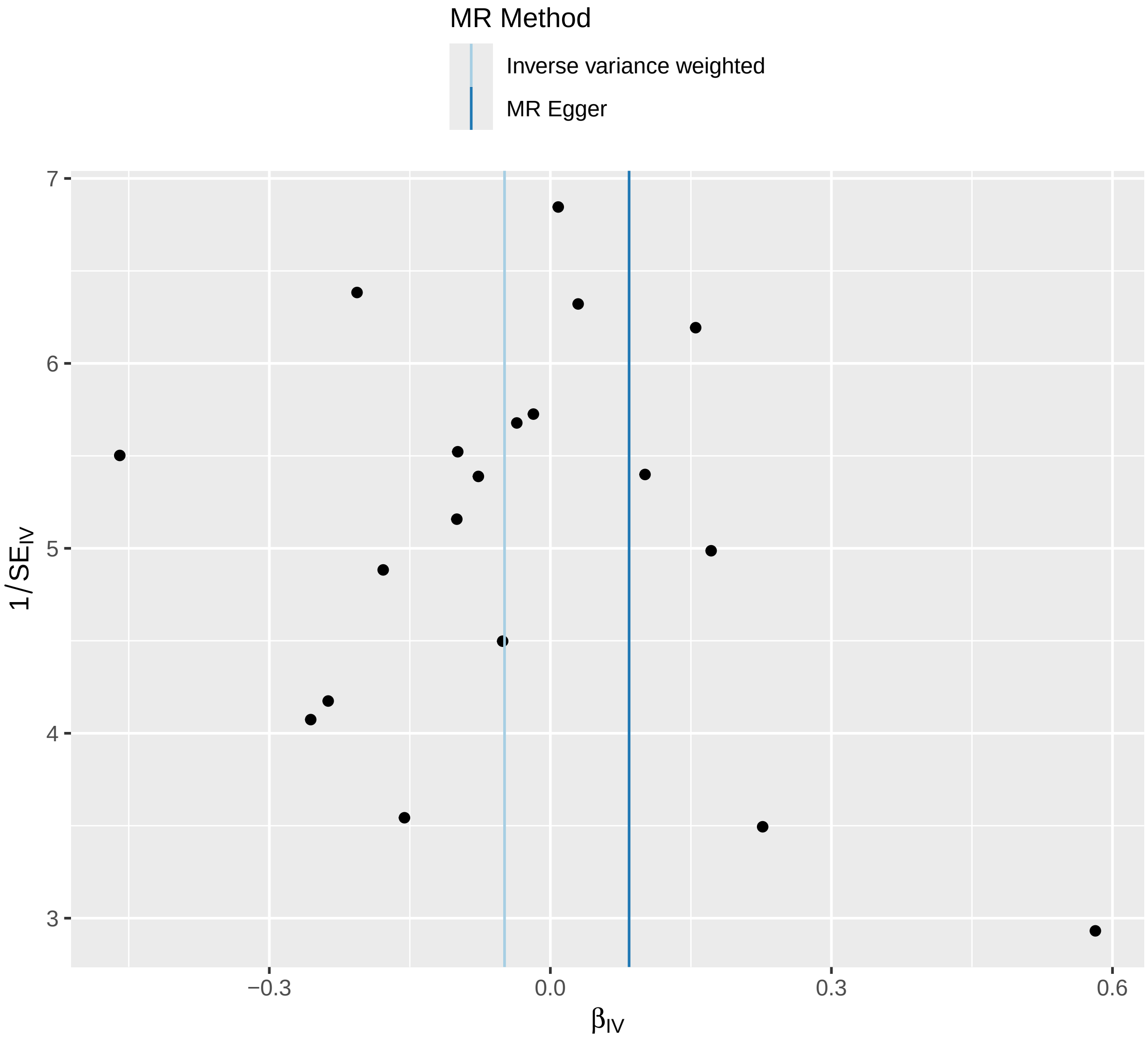
MR Method
Inverse variance weighted
MR Egger
7
6
5
4
3
1/SE_IV
−0.3
0.0
0.3
0.6
β_IV

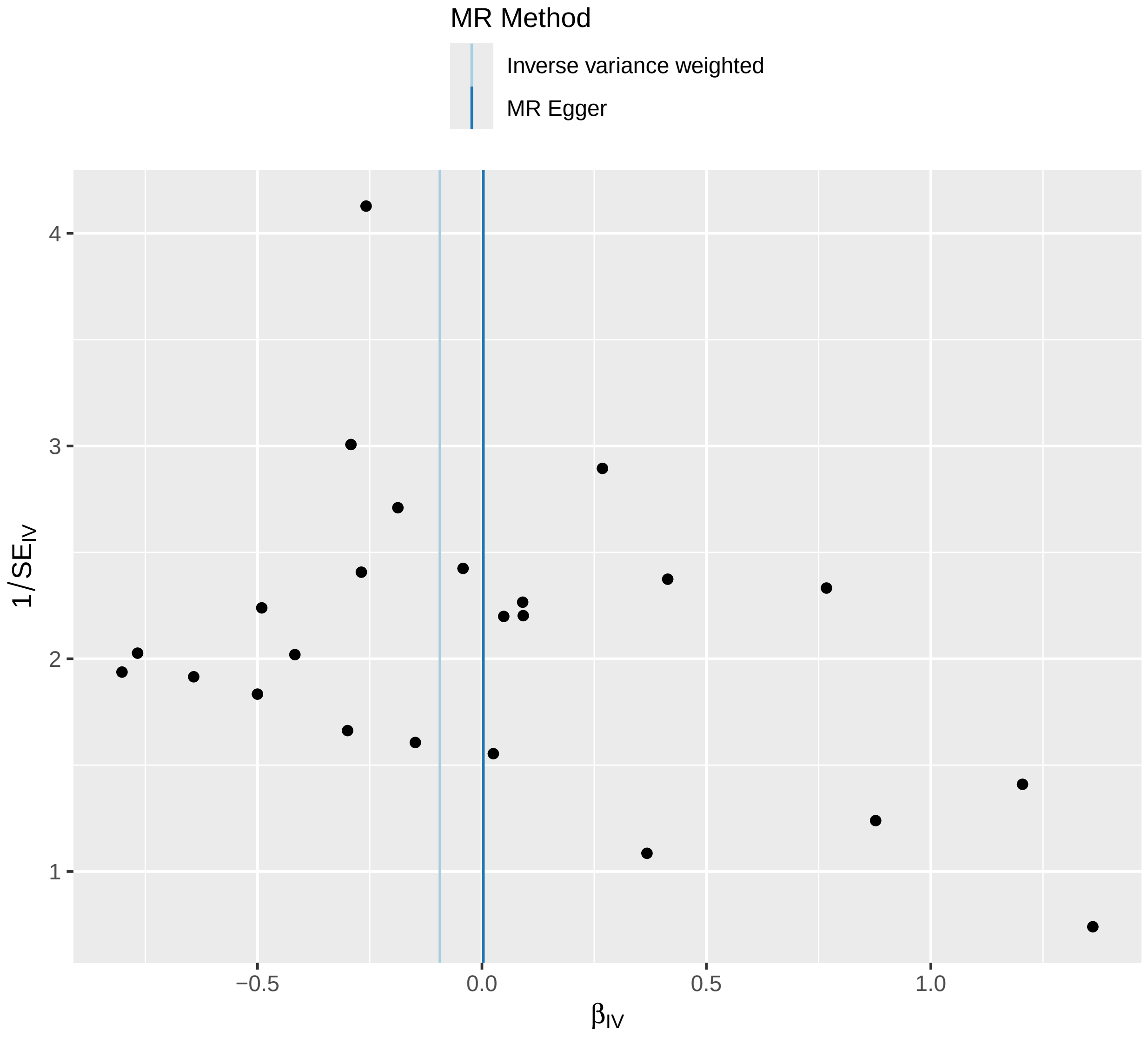
MR Method
Inverse variance weighted
MR Egger
1/SE_IV
β_IV
4
3
2
1
−0.5
0.0
0.5
1.0

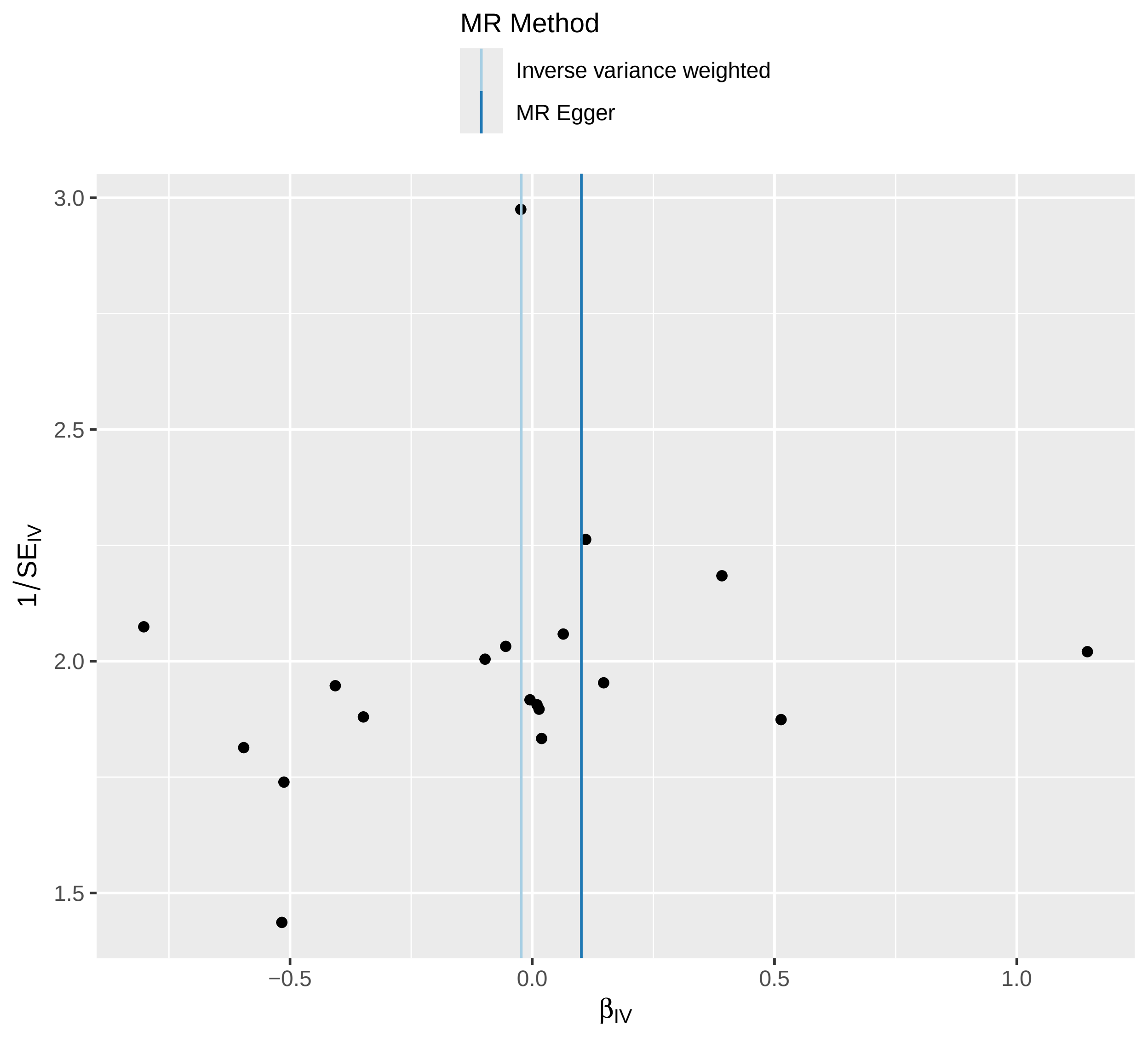
MR Method
Inverse variance weighted
MR Egger
3.0
2.5
2.0
1.5
1/SE_IV
−0.5
0.0
0.5
1.0
β_IV

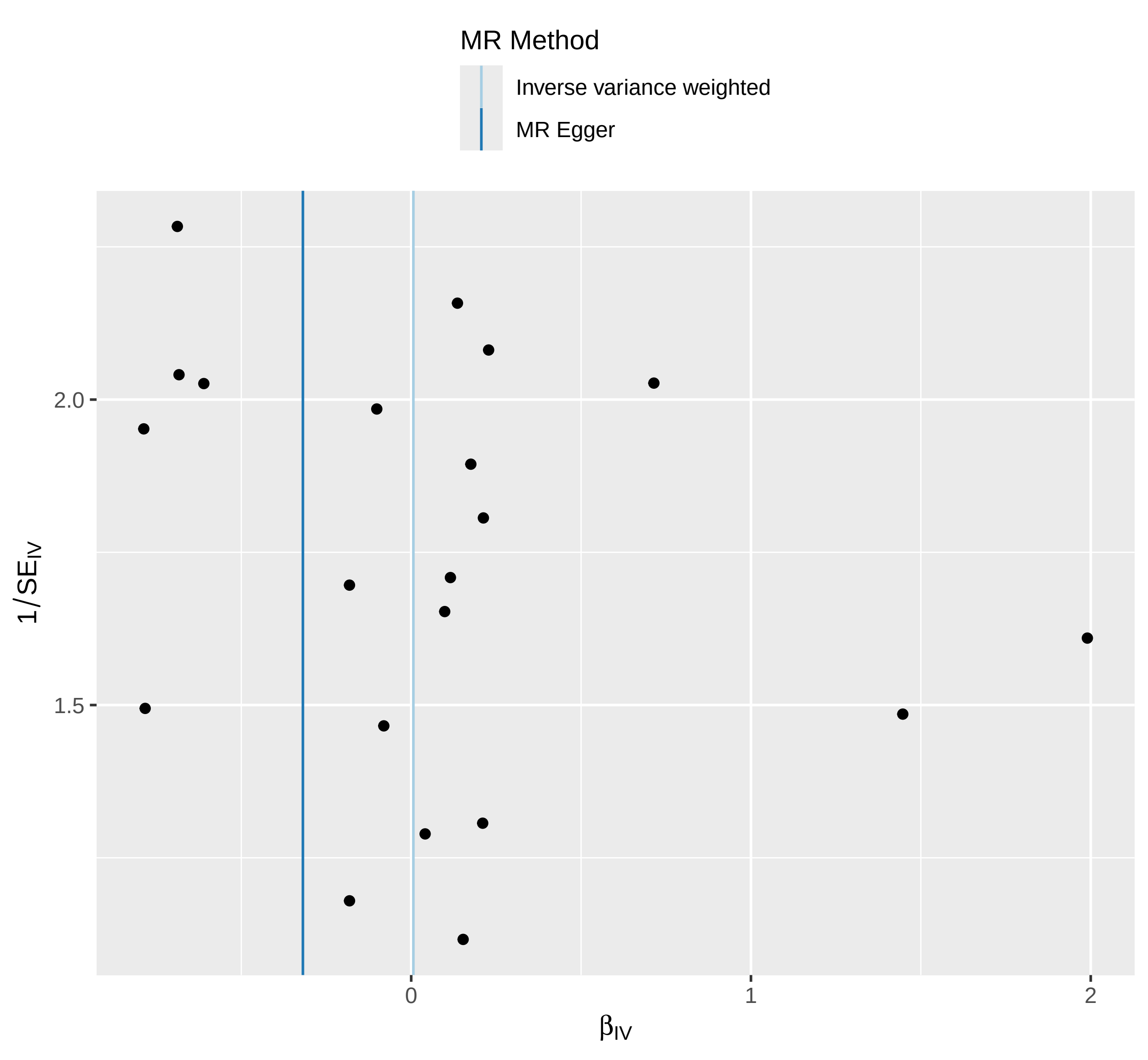

MR Method
Inverse variance weighted
MR Egger
2.0
1.5
1/SE$_{IV}$
0
1
2
β$_{IV}$

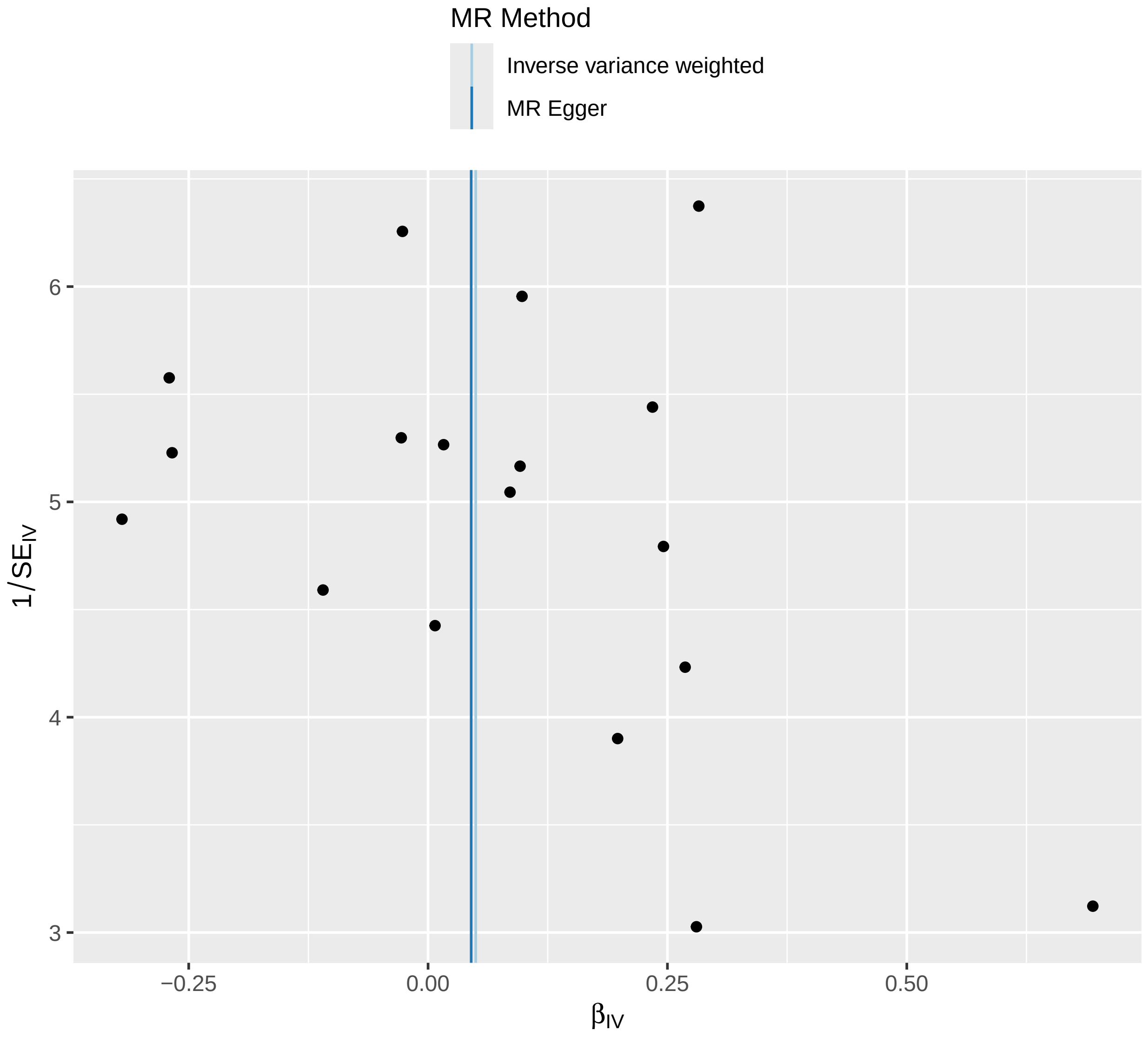
MR Method
Inverse variance weighted
MR Egger
6
5
4
3
1/SE$_{IV}$
−0.25
0.00
0.25
0.50
β$_{IV}$

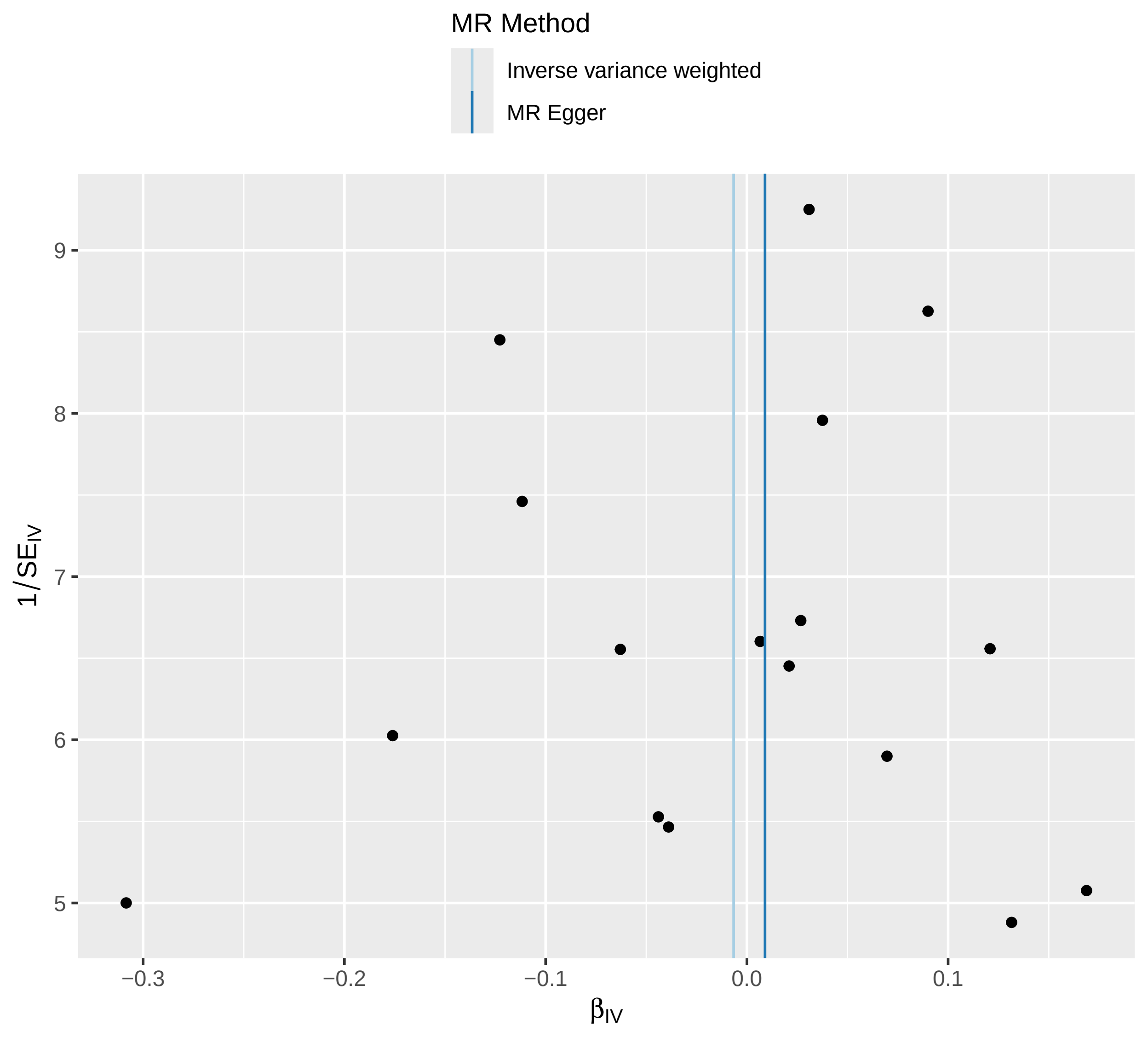

MR Method
Inverse variance weighted
MR Egger
9
8
7
6
5
$1/SE_{IV}$
−0.3
−0.2
−0.1
0.0
0.1
β_{IV}

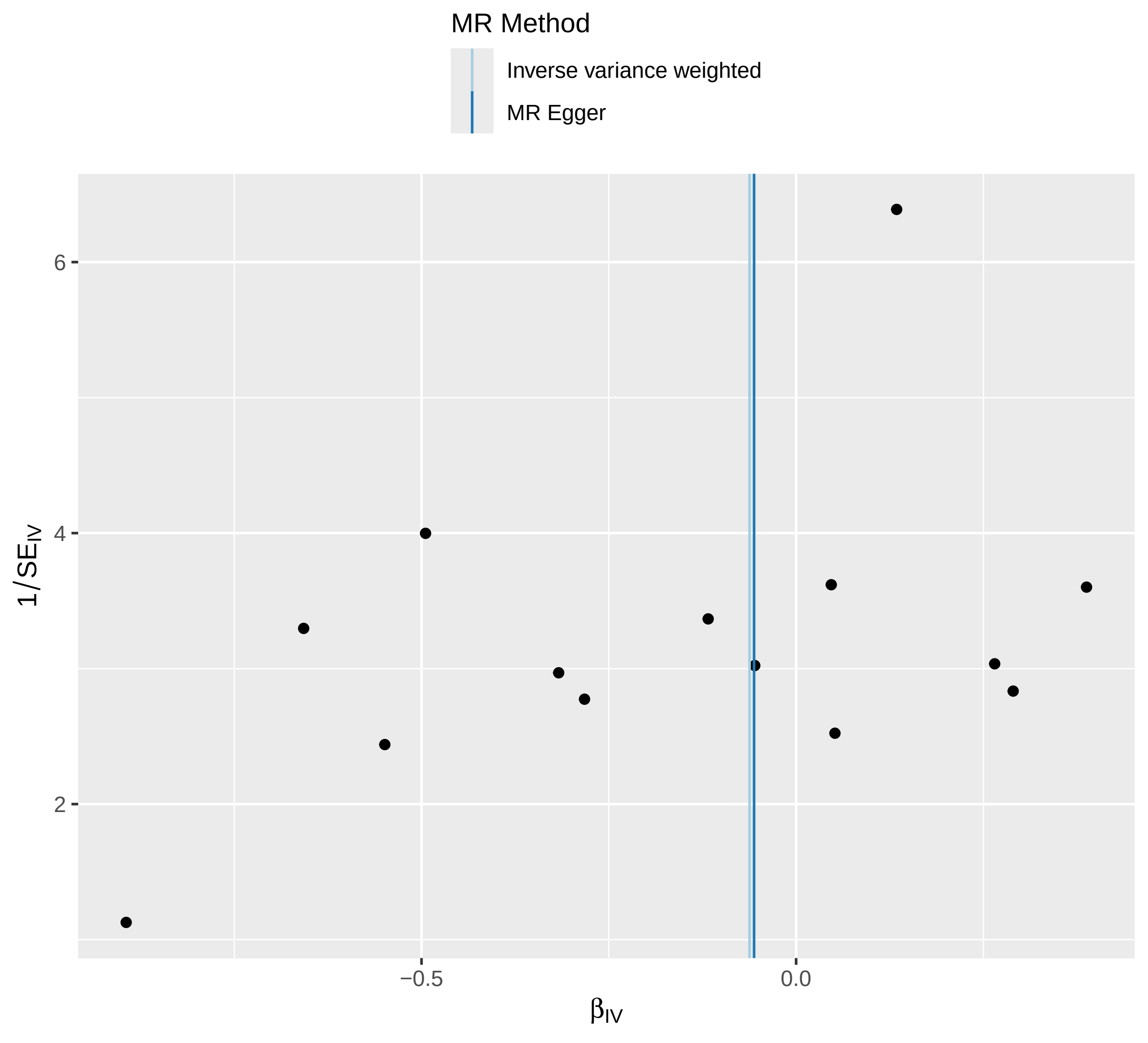

MR Method
Inverse variance weighted
MR Egger
6
4
2
$1/SE_{IV}$
−0.5
0.0
β_{IV}

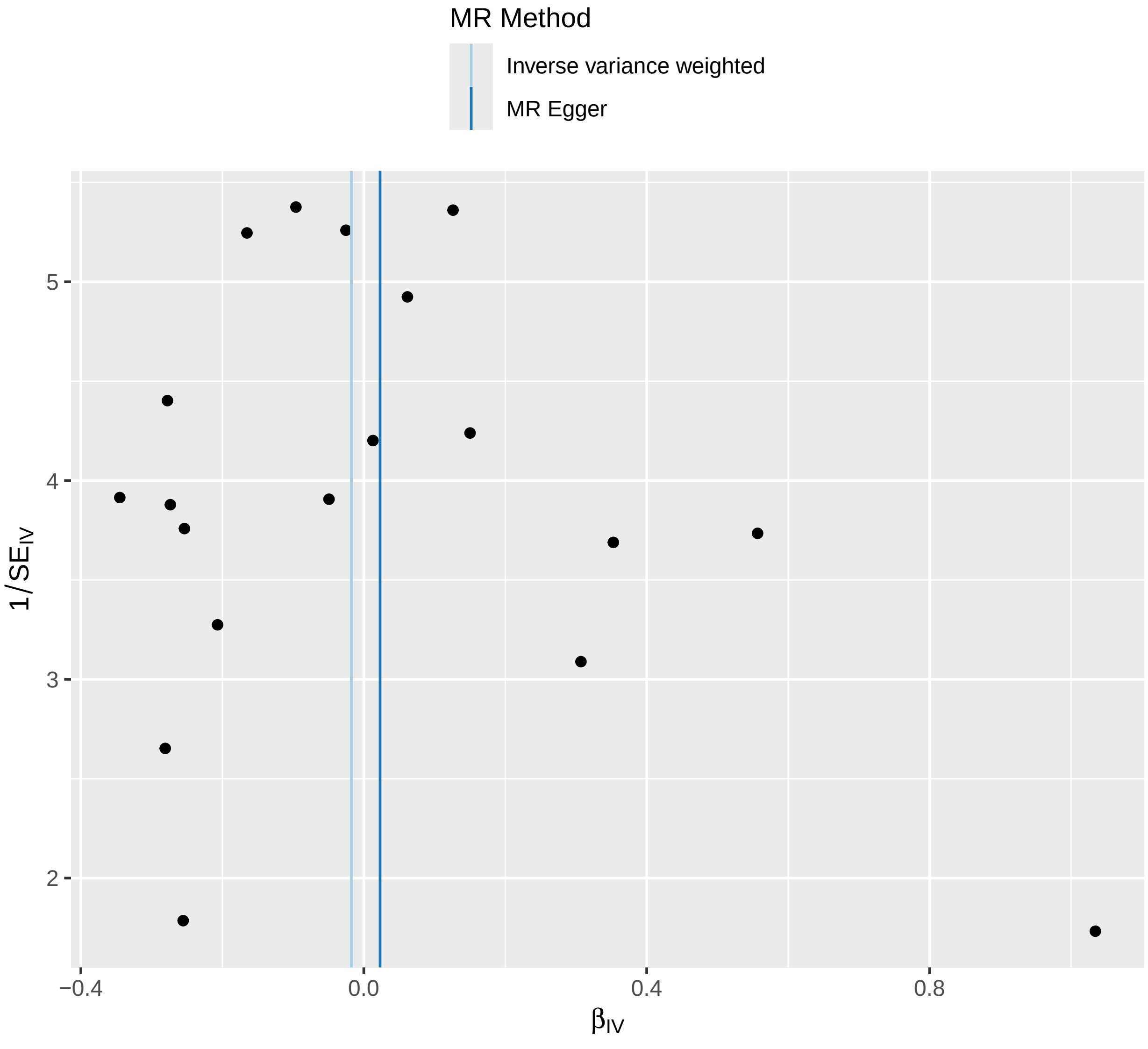
MR Method
Inverse variance weighted
MR Egger
5
4
3
2
1/SE_IV
−0.4
0.0
0.4
0.8
β_IV

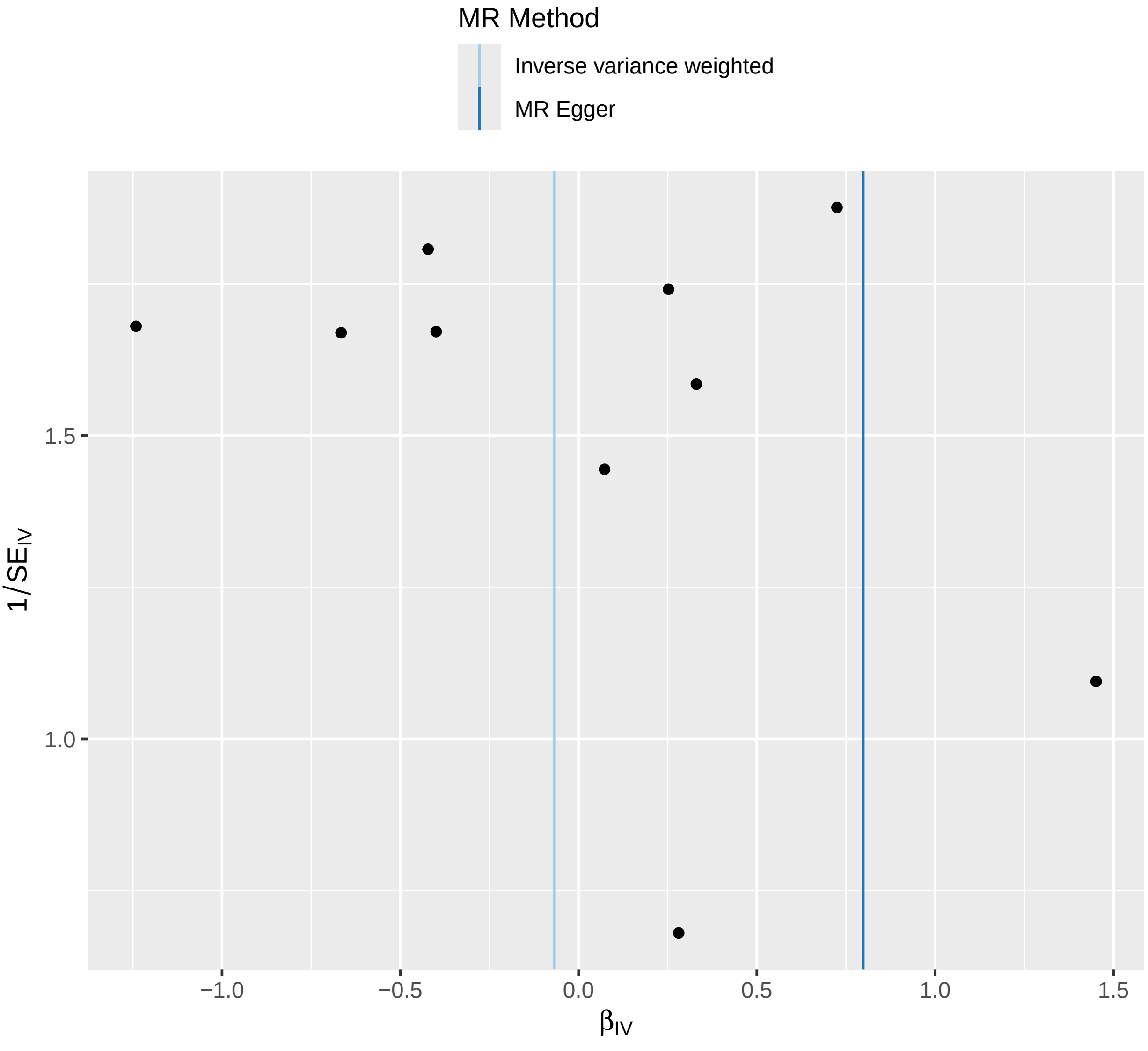
MR Method
Inverse variance weighted
MR Egger
1.5
1.0
1/SE_IV
−1.0
−0.5
0.0
0.5
1.0
1.5
β_IV

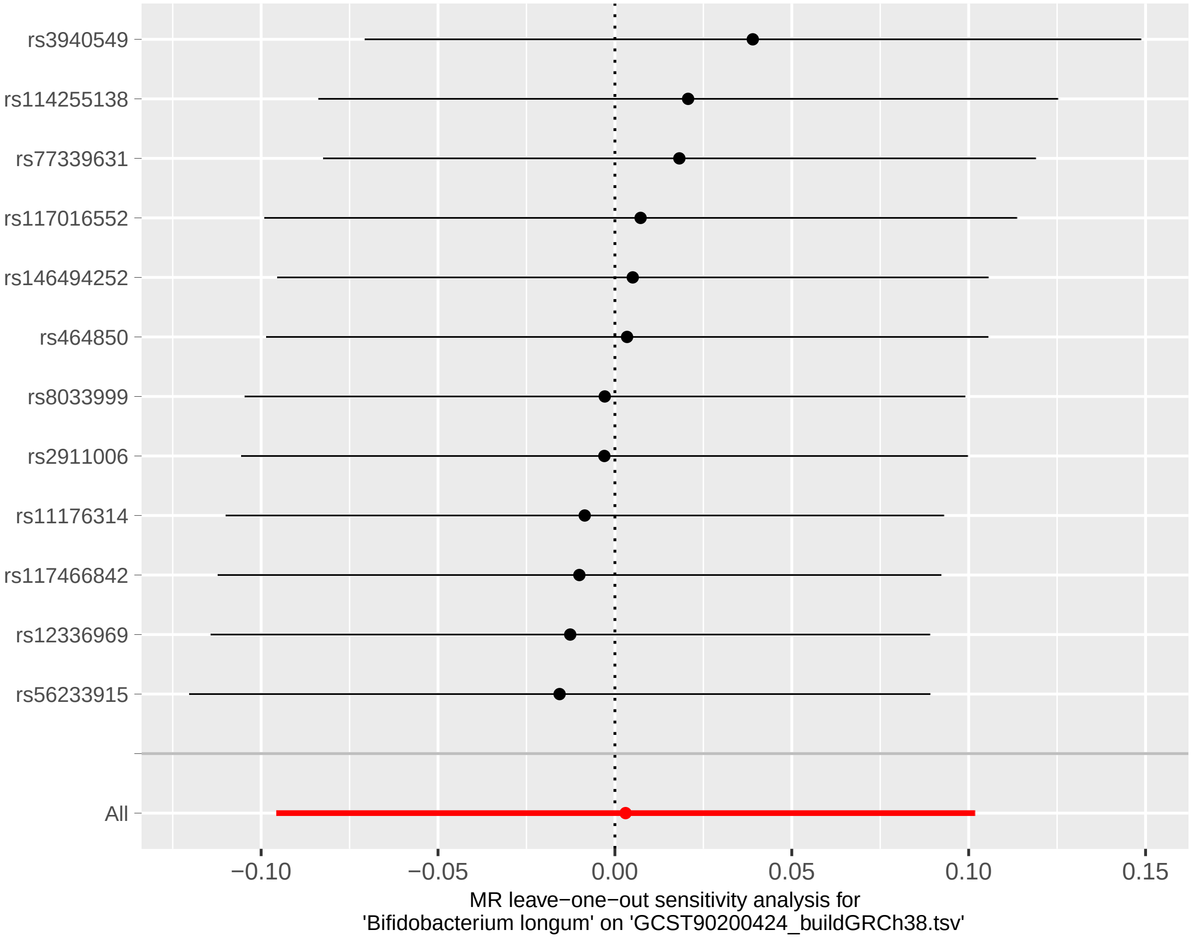

rs3940549
rs114255138
rs77339631
rs117016552
rs146494252
rs464850
rs8033999
rs2911006
rs11176314
rs117466842
rs12336969
rs56233915
All
−0.10
−0.05
0.00
0.05
0.10
0.15
MR leave−one−out sensitivity analysis for
'Bifidobacterium longum' on 'GCST90200424_buildGRCh38.tsv'

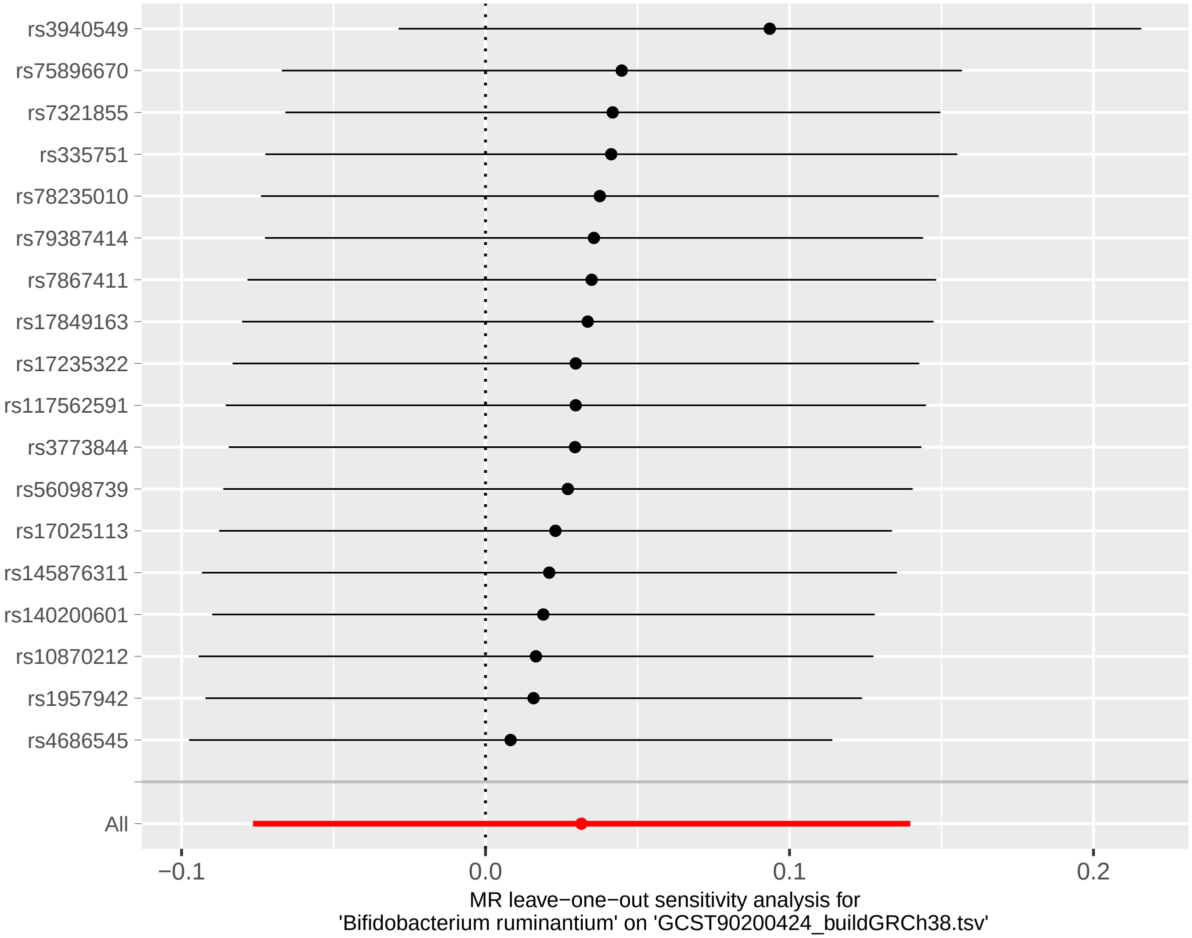

rs3940549
rs75896670
rs7321855
rs335751
rs78235010
rs79387414
rs7867411
rs17849163
rs17235322
rs117562591
rs3773844
rs56098739
rs17025113
rs145876311
rs140200601
rs10870212
rs1957942
rs4686545
All
−0.1
0.0
0.1
0.2
MR leave−one−out sensitivity analysis for
'Bifidobacterium ruminantium' on 'GCST90200424_buildGRCh38.tsv'

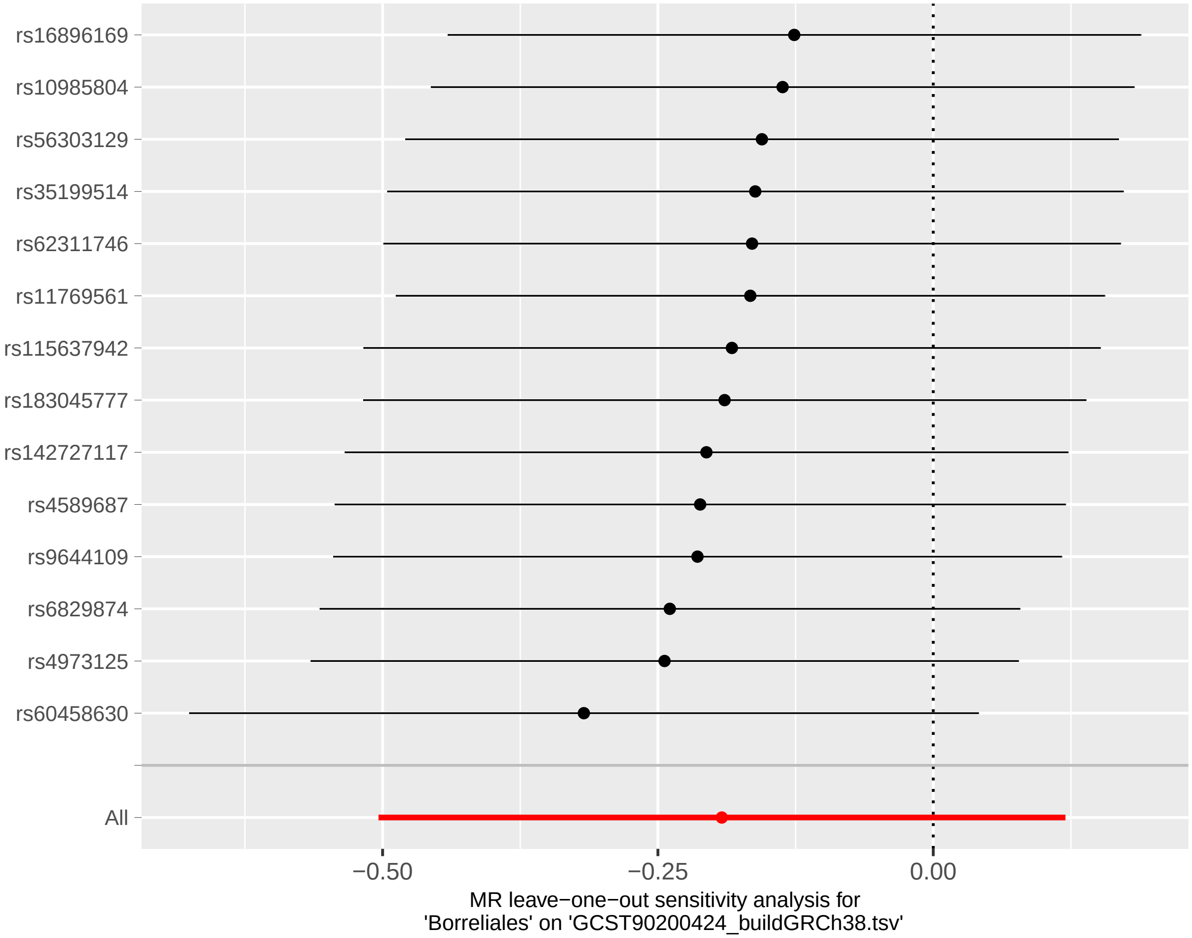

rs16896169
rs10985804
rs56303129
rs35199514
rs62311746
rs11769561
rs115637942
rs183045777
rs142727117
rs4589687
rs9644109
rs6829874
rs4973125
rs60458630
All
−0.50
−0.25
0.00
MR leave−one−out sensitivity analysis for
'Borreliales' on 'GCST90200424_buildGRCh38.tsv'

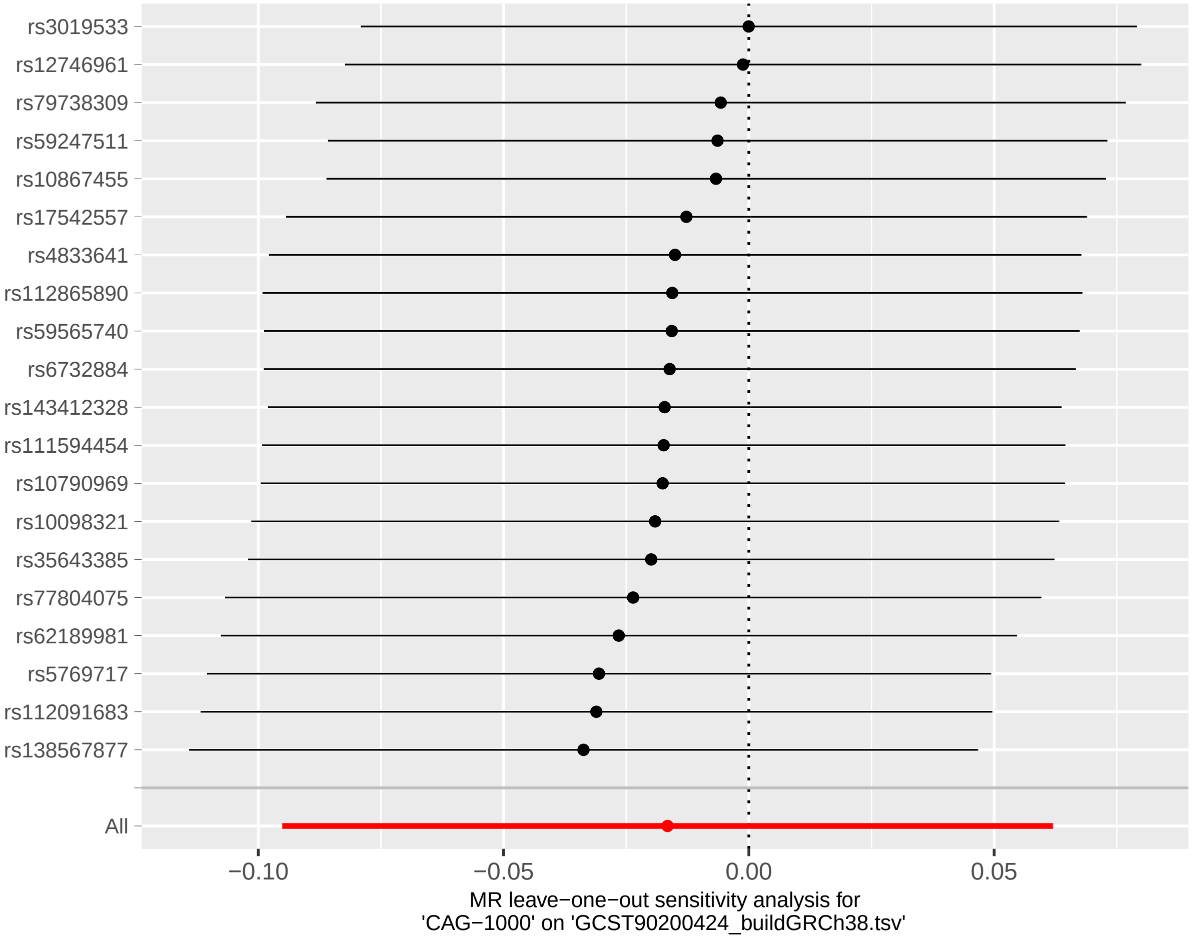
rs3019533
rs12746961
rs79738309
rs59247511
rs10867455
rs17542557
rs4833641
rs112865890
rs59565740
rs6732884
rs143412328
rs111594454
rs10790969
rs10098321
rs35643385
rs77804075
rs62189981
rs5769717
rs112091683
rs138567877
All
−0.10
−0.05
0.00
0.05
MR leave−one−out sensitivity analysis for
'CAG−1000' on 'GCST90200424_buildGRCh38.tsv'

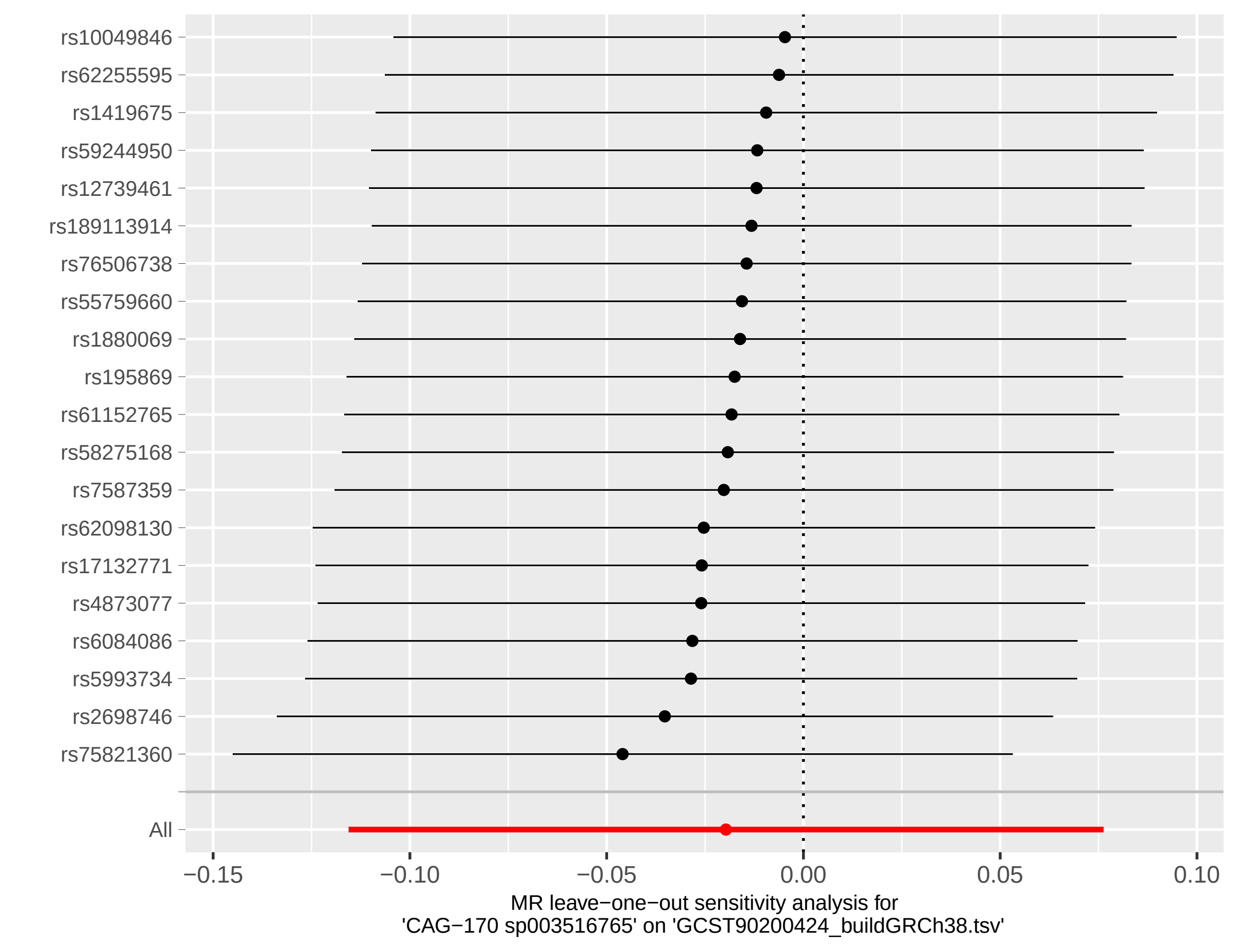

rs10049846
rs62255595
rs1419675
rs59244950
rs12739461
rs189113914
rs76506738
rs55759660
rs1880069
rs195869
rs61152765
rs58275168
rs7587359
rs62098130
rs17132771
rs4873077
rs6084086
rs5993734
rs2698746
rs75821360
All
−0.15
−0.10
−0.05
0.00
0.05
0.10
MR leave−one−out sensitivity analysis for
'CAG−170 sp003516765' on 'GCST90200424_buildGRCh38.tsv'

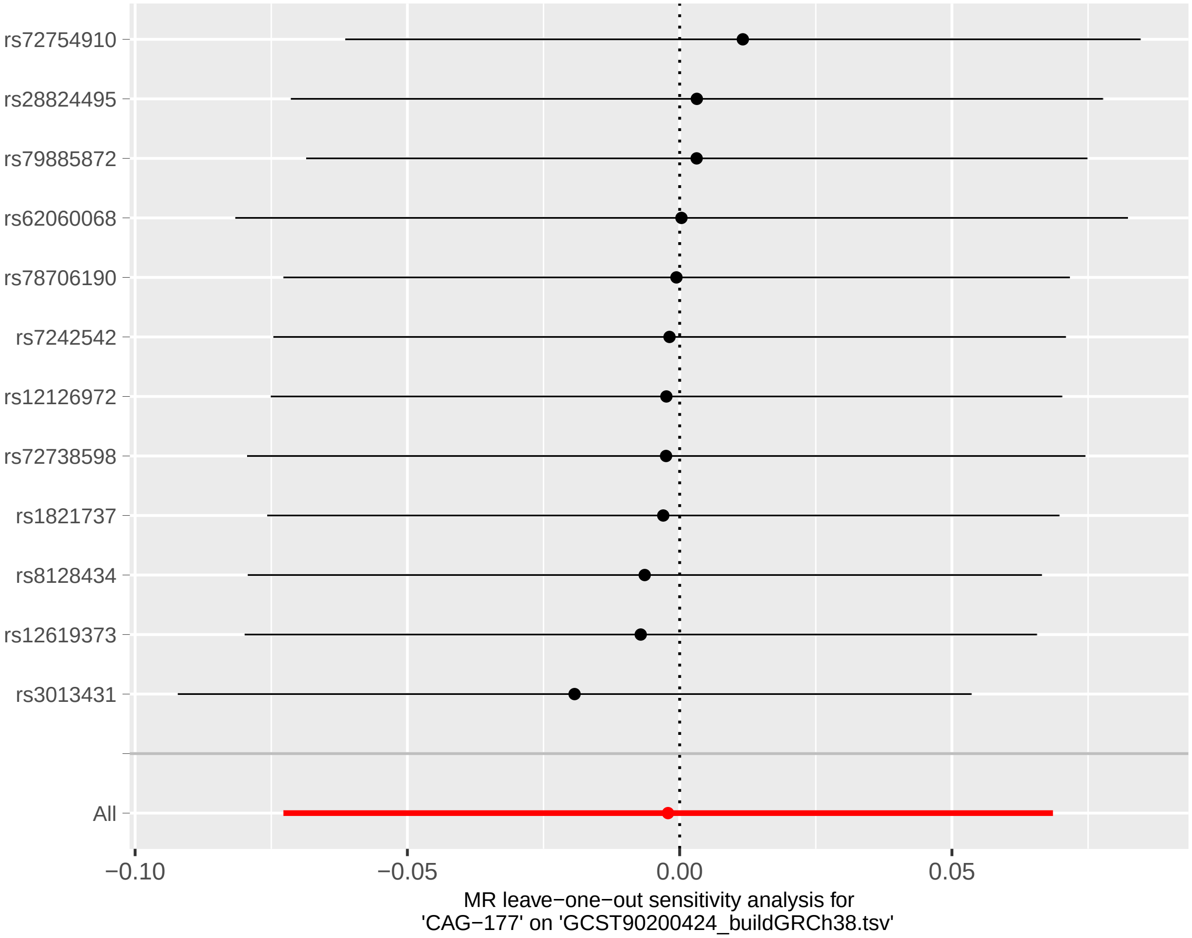
rs72754910
rs28824495
rs79885872
rs62060068
rs78706190
rs7242542
rs12126972
rs72738598
rs1821737
rs8128434
rs12619373
rs3013431
All
−0.10
−0.05
0.00
0.05
MR leave−one−out sensitivity analysis for
'CAG−177' on 'GCST90200424_buildGRCh38.tsv'

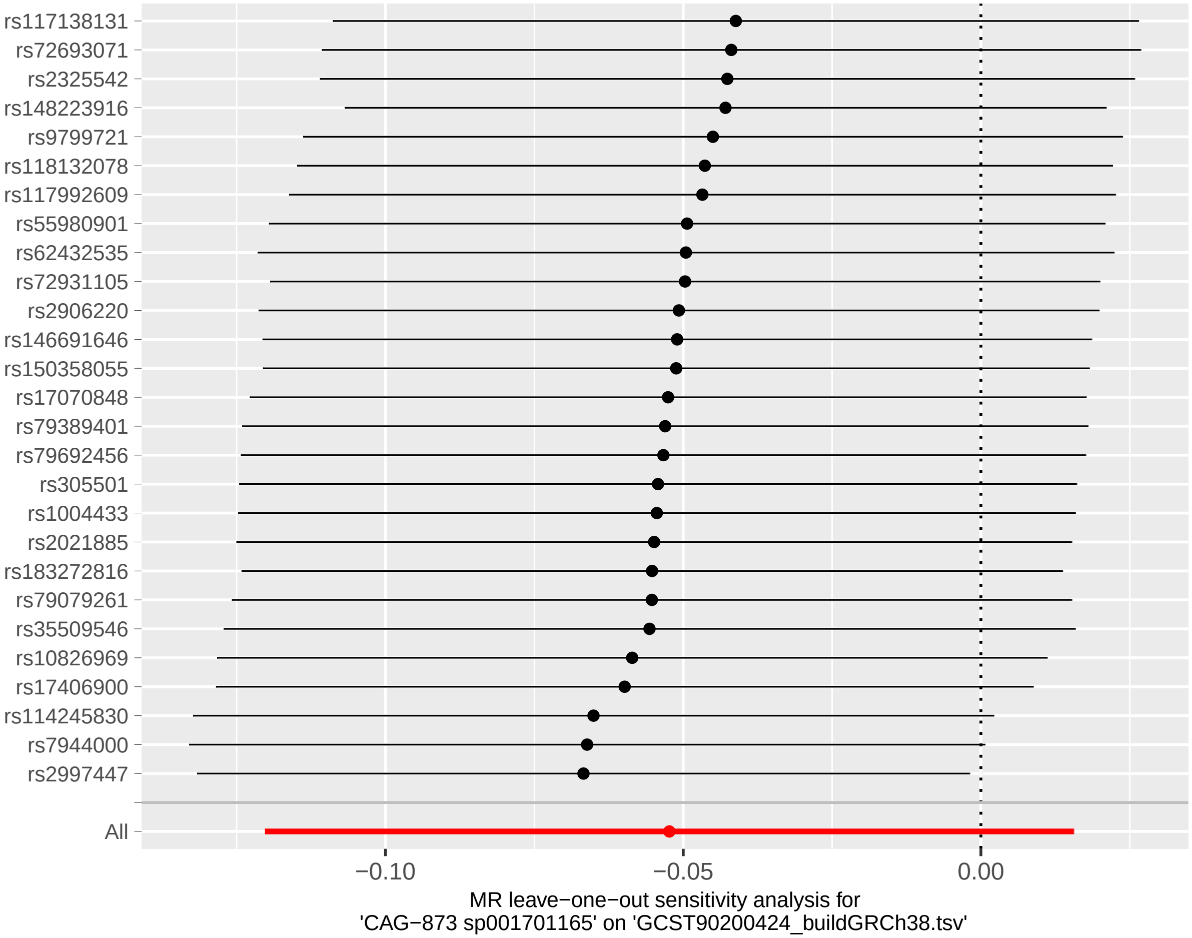

rs117138131
rs72693071
rs2325542
rs148223916
rs9799721
rs118132078
rs117992609
rs55980901
rs62432535
rs72931105
rs2906220
rs146691646
rs150358055
rs17070848
rs79389401
rs79692456
rs305501
rs1004433
rs2021885
rs183272816
rs79079261
rs35509546
rs10826969
rs17406900
rs114245830
rs7944000
rs2997447
All
−0.10
−0.05
0.00
MR leave−one−out sensitivity analysis for
'CAG−873 sp001701165' on 'GCST90200424_buildGRCh38.tsv'

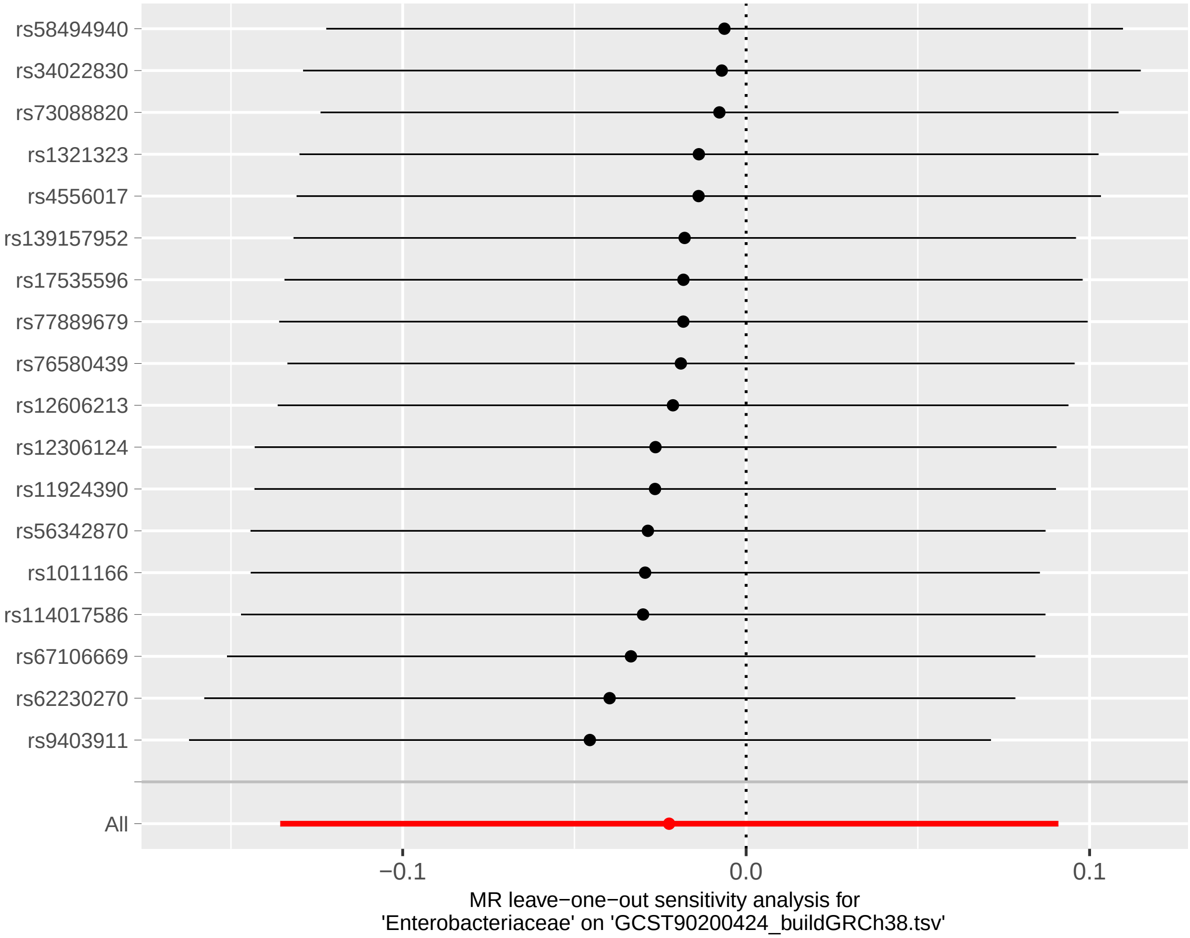

rs58494940
rs34022830
rs73088820
rs1321323
rs4556017
rs139157952
rs17535596
rs77889679
rs76580439
rs12606213
rs12306124
rs11924390
rs56342870
rs1011166
rs114017586
rs67106669
rs62230270
rs9403911
All
−0.1
0.0
0.1
MR leave−one−out sensitivity analysis for
'Enterobacteriaceae' on 'GCST90200424_buildGRCh38.tsv'

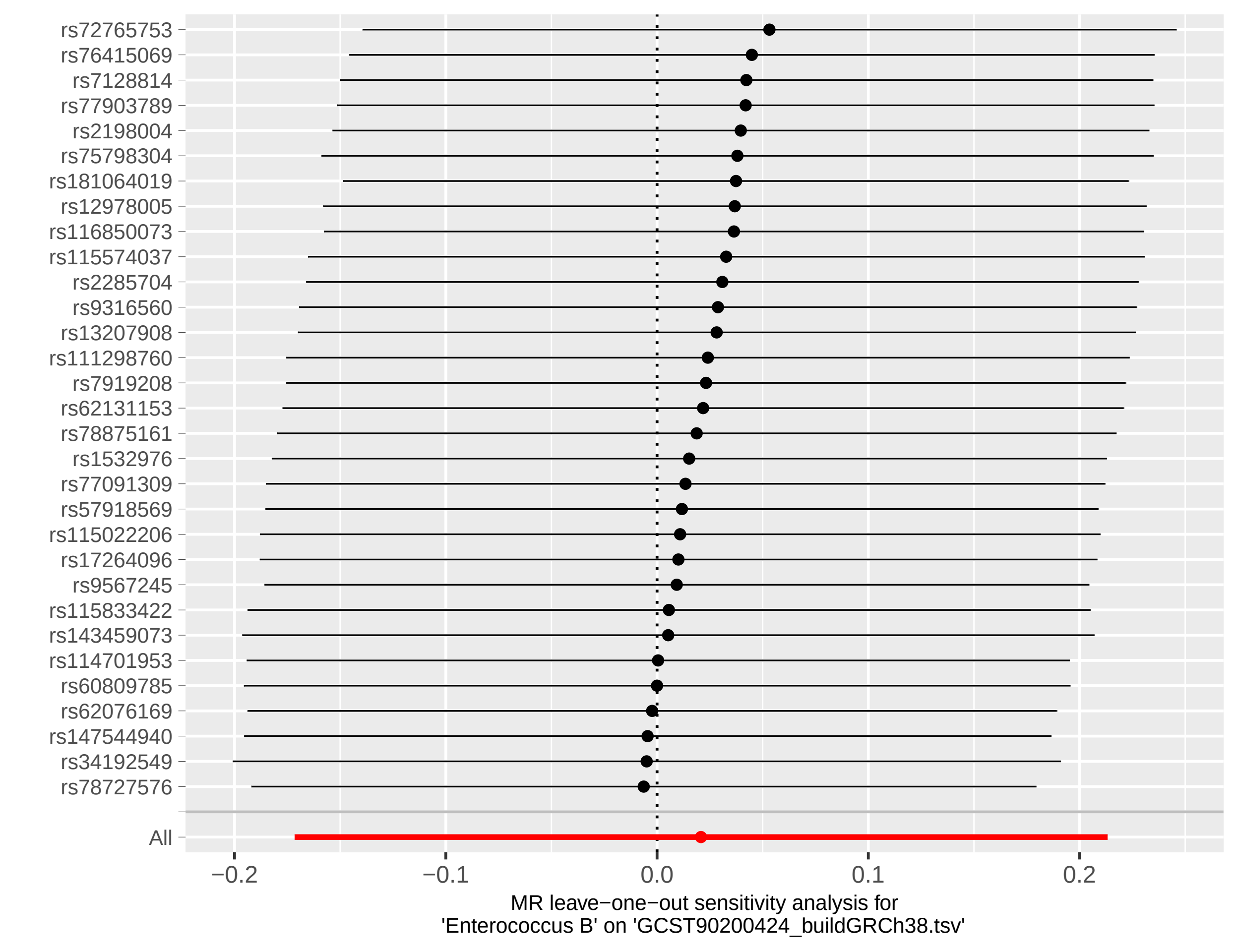
rs72765753
rs76415069
rs7128814
rs77903789
rs2198004
rs75798304
rs181064019
rs12978005
rs116850073
rs115574037
rs2285704
rs9316560
rs13207908
rs111298760
rs7919208
rs62131153
rs78875161
rs1532976
rs77091309
rs57918569
rs115022206
rs17264096
rs9567245
rs115833422
rs143459073
rs114701953
rs60809785
rs62076169
rs147544940
rs34192549
rs78727576
All
−0.2
−0.1
0.0
0.1
0.2
MR leave−one−out sensitivity analysis for
'Enterococcus B' on 'GCST90200424_buildGRCh38.tsv'

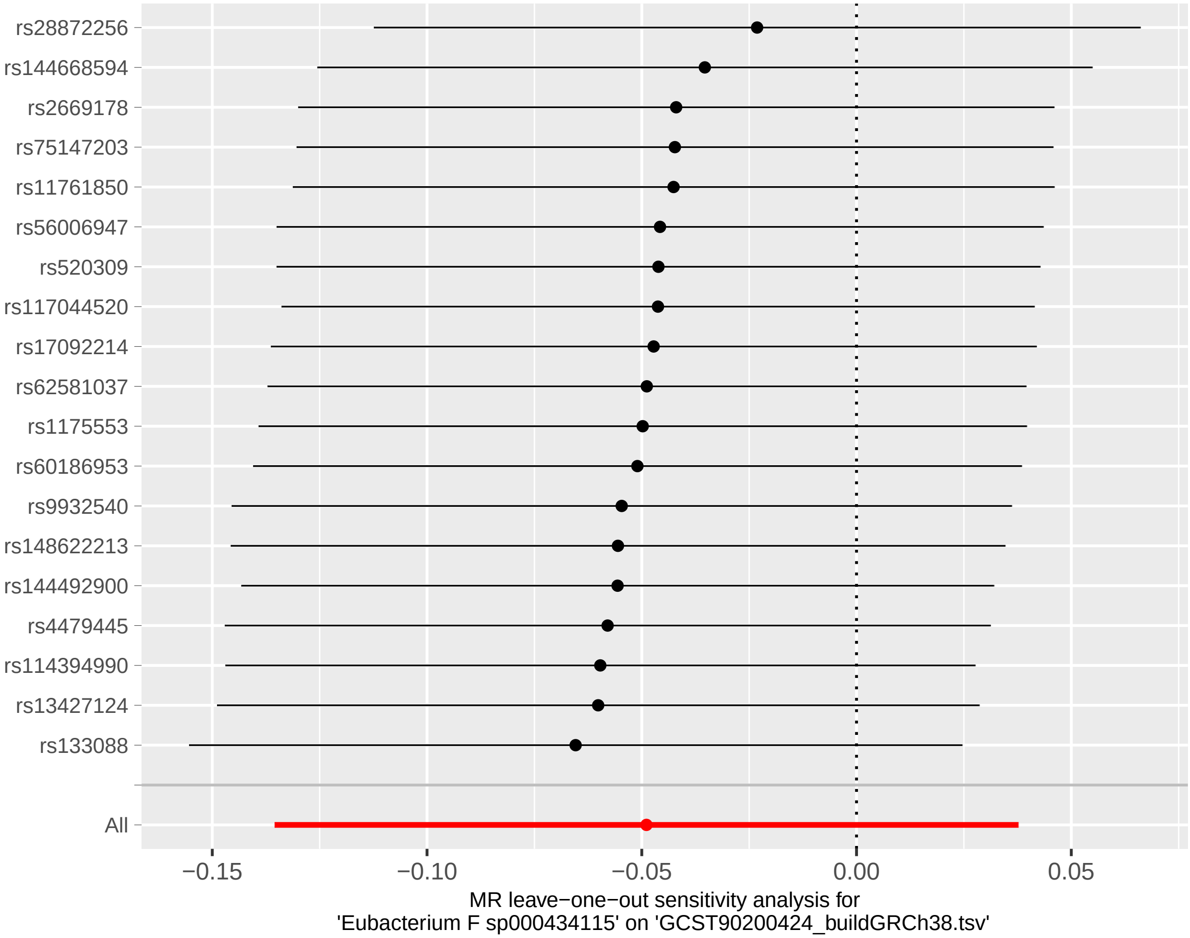
rs28872256
rs144668594
rs2669178
rs75147203
rs11761850
rs56006947
rs520309
rs117044520
rs17092214
rs62581037
rs1175553
rs60186953
rs9932540
rs148622213
rs144492900
rs4479445
rs114394990
rs13427124
rs133088
All
−0.15
−0.10
−0.05
0.00
0.05
MR leave−one−out sensitivity analysis for
'Eubacterium F sp000434115' on 'GCST90200424_buildGRCh38.tsv'

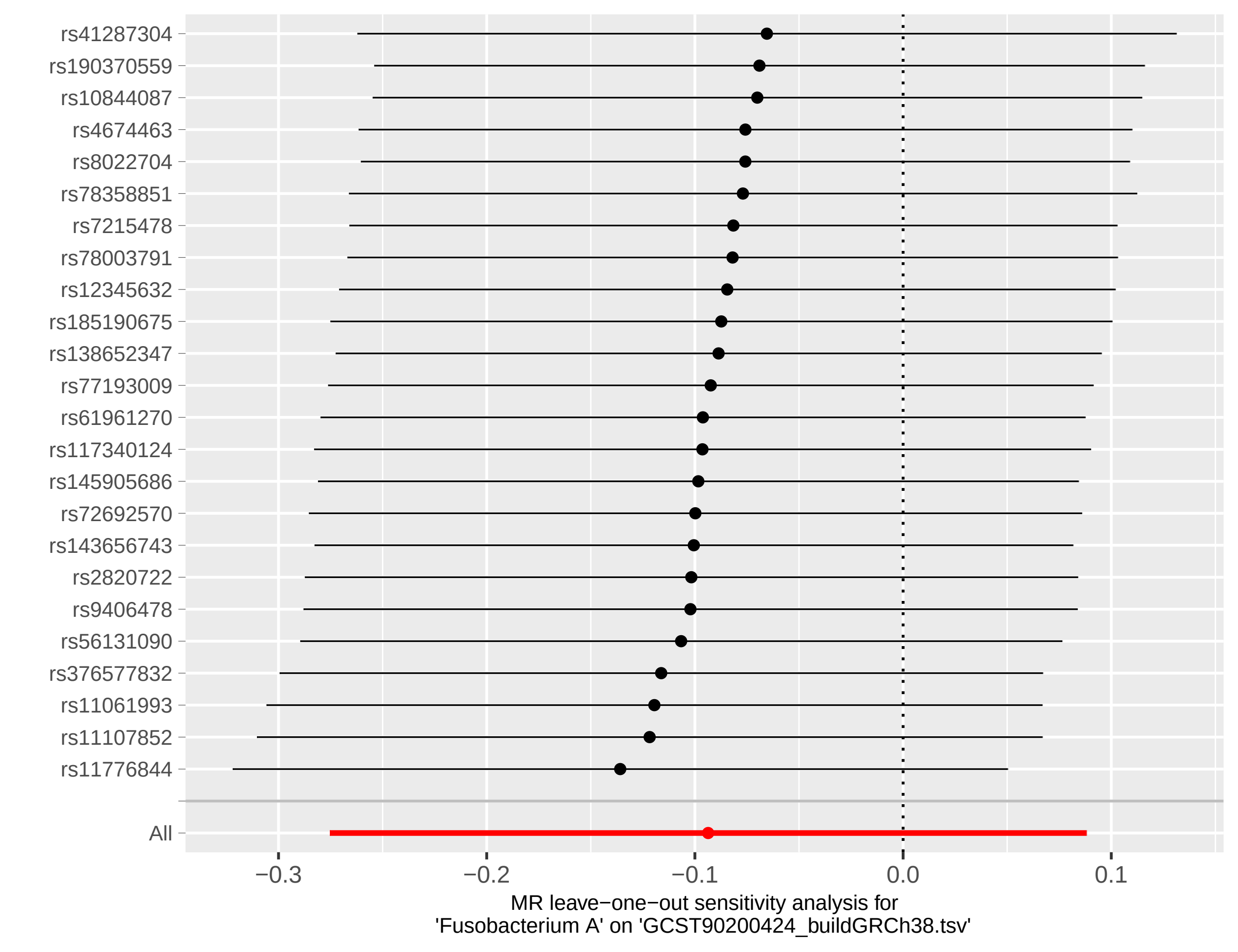

rs41287304
rs190370559
rs10844087
rs4674463
rs8022704
rs78358851
rs7215478
rs78003791
rs12345632
rs185190675
rs138652347
rs77193009
rs61961270
rs117340124
rs145905686
rs72692570
rs143656743
rs2820722
rs9406478
rs56131090
rs376577832
rs11061993
rs11107852
rs11776844
All
−0.3
−0.2
−0.1
0.0
0.1
MR leave−one−out sensitivity analysis for
'Fusobacterium A' on 'GCST90200424_buildGRCh38.tsv'

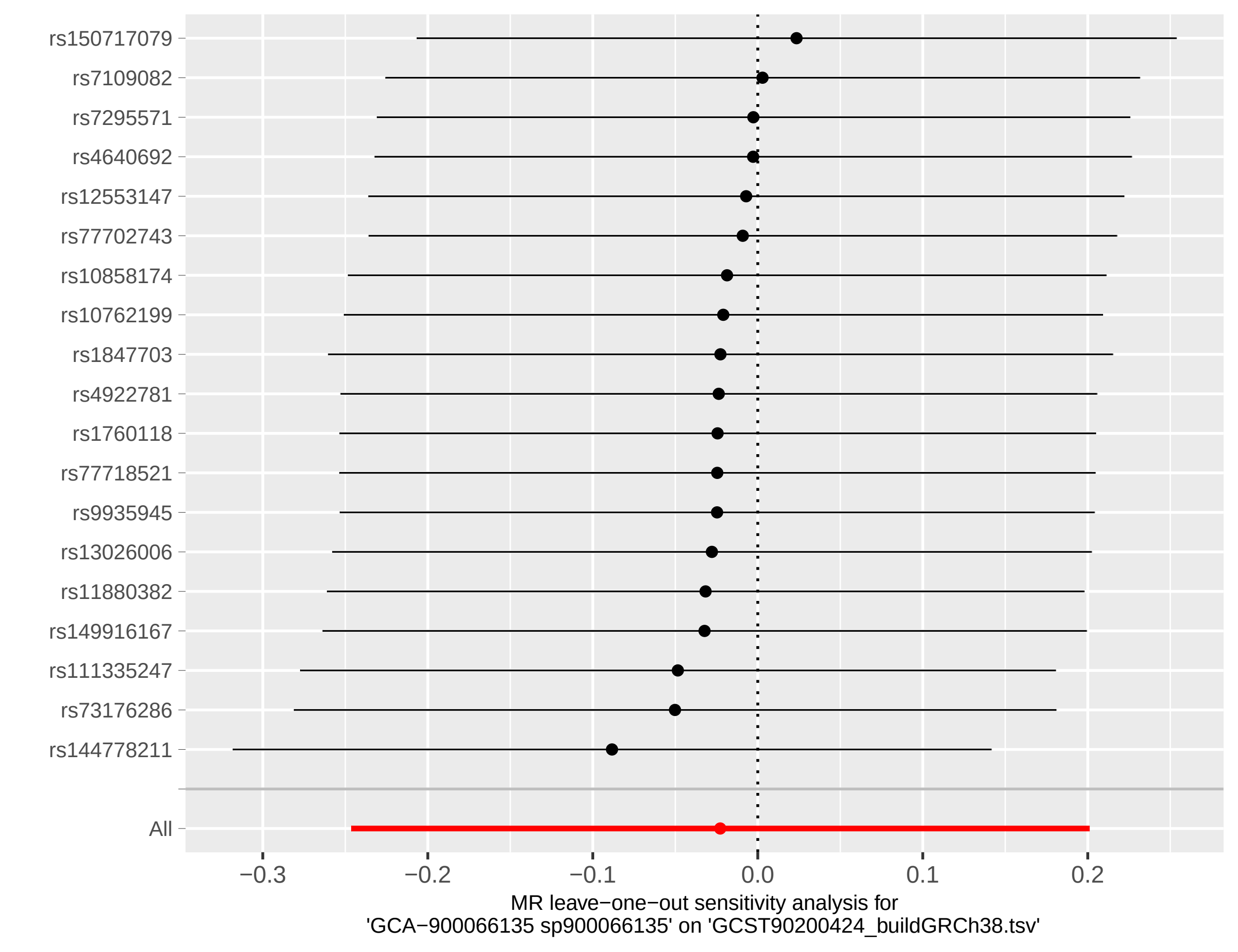

rs150717079
rs7109082
rs7295571
rs4640692
rs12553147
rs77702743
rs10858174
rs10762199
rs1847703
rs4922781
rs1760118
rs77718521
rs9935945
rs13026006
rs11880382
rs149916167
rs111335247
rs73176286
rs144778211
All
−0.3
−0.2
−0.1
0.0
0.1
0.2
MR leave−one−out sensitivity analysis for
'GCA−900066135 sp900066135' on 'GCST90200424_buildGRCh38.tsv'

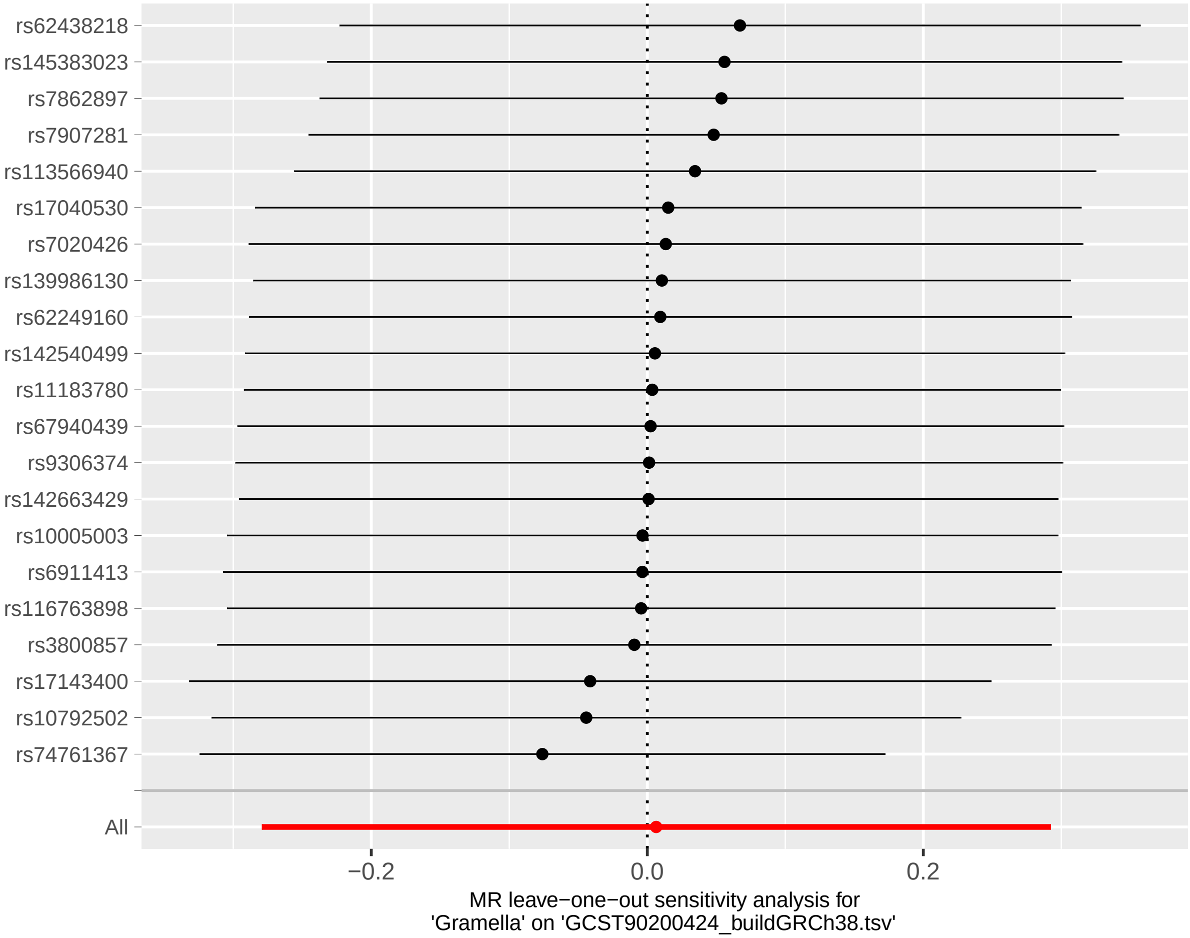

rs62438218
rs145383023
rs7862897
rs7907281
rs113566940
rs17040530
rs7020426
rs139986130
rs62249160
rs142540499
rs11183780
rs67940439
rs9306374
rs142663429
rs10005003
rs6911413
rs116763898
rs3800857
rs17143400
rs10792502
rs74761367
All
−0.2
0.0
0.2
MR leave−one−out sensitivity analysis for
'Gramella' on 'GCST90200424_buildGRCh38.tsv'

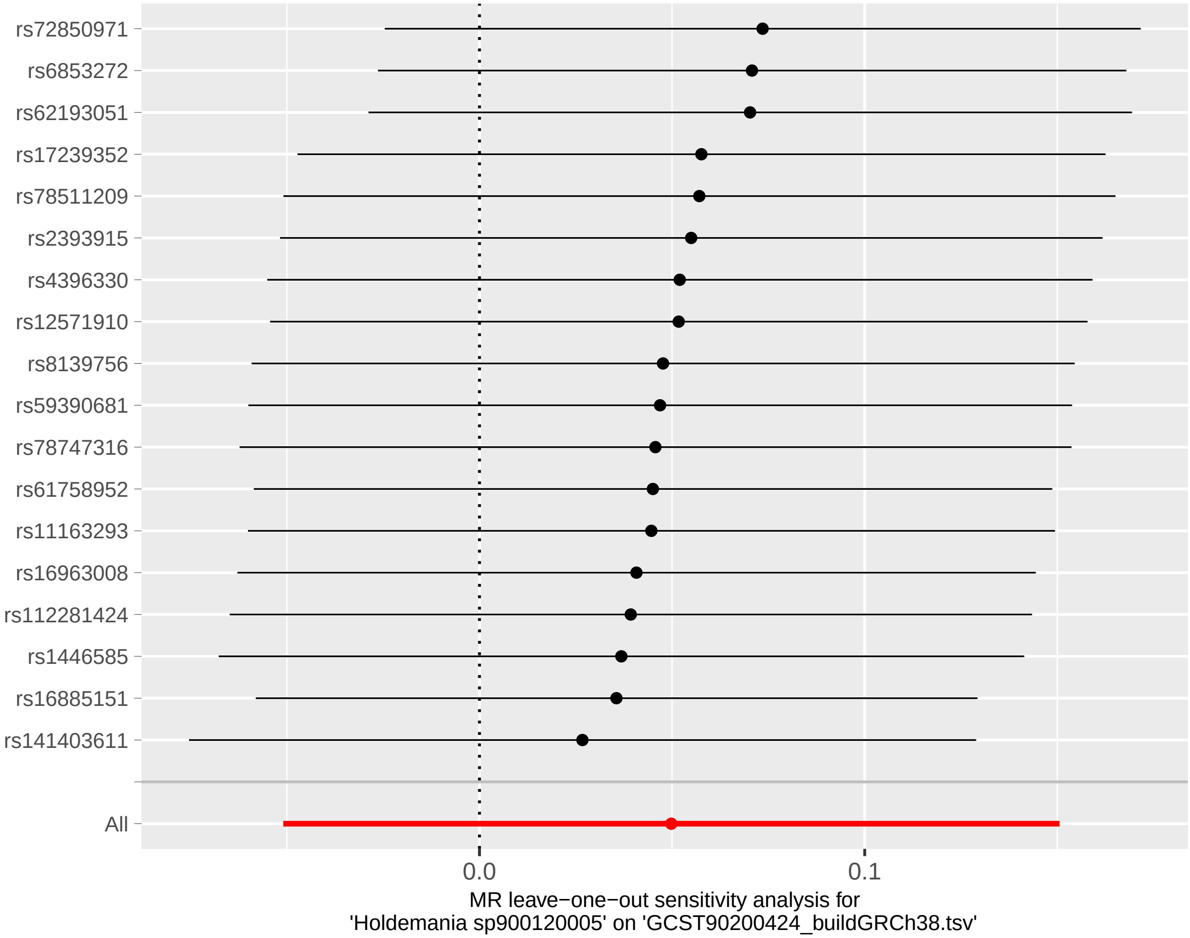

rs72850971
rs6853272
rs62193051
rs17239352
rs78511209
rs2393915
rs4396330
rs12571910
rs8139756
rs59390681
rs78747316
rs61758952
rs11163293
rs16963008
rs112281424
rs1446585
rs16885151
rs141403611
All
0.0
0.1
MR leave−one−out sensitivity analysis for
'Holdemania sp900120005' on 'GCST90200424_buildGRCh38.tsv'

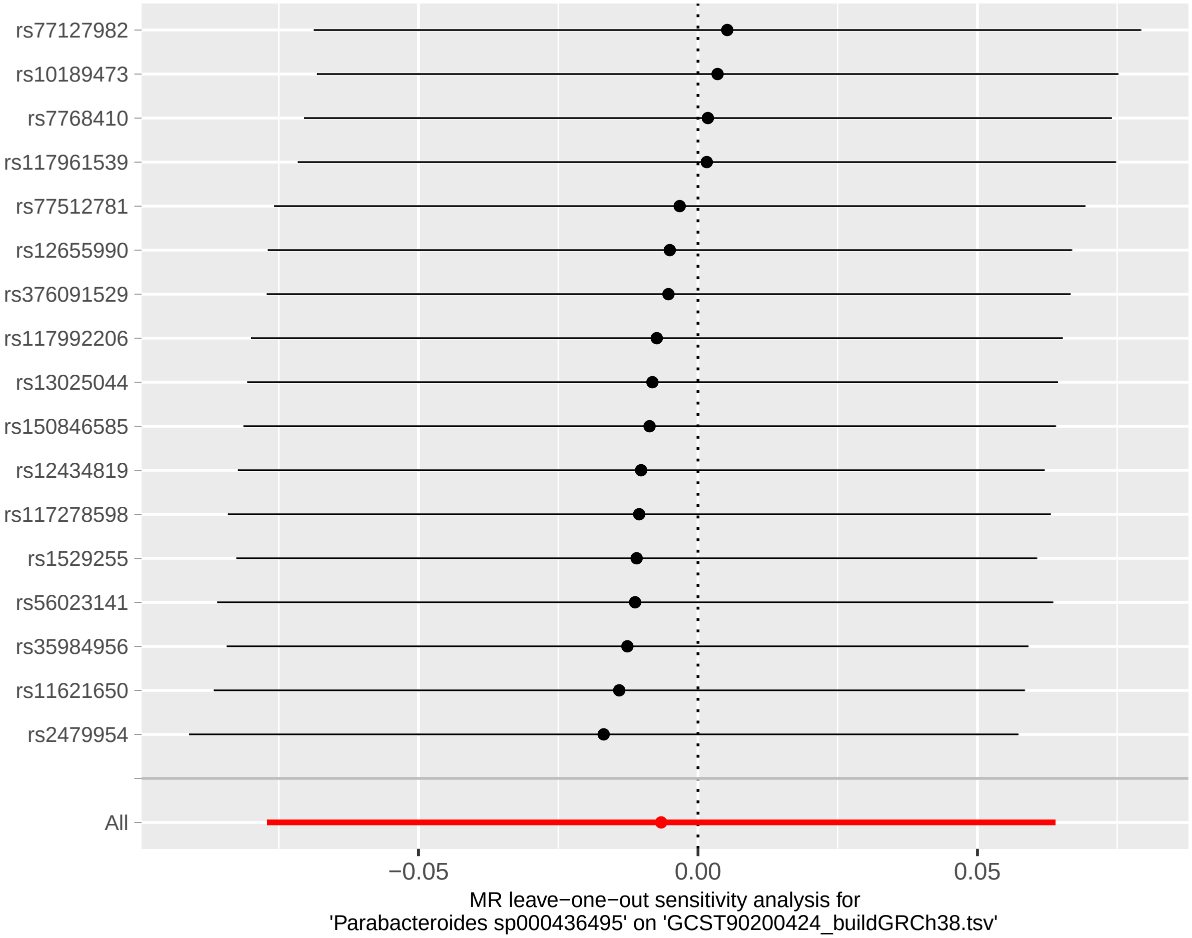
rs77127982
rs10189473
rs7768410
rs117961539
rs77512781
rs12655990
rs376091529
rs117992206
rs13025044
rs150846585
rs12434819
rs117278598
rs1529255
rs56023141
rs35984956
rs11621650
rs2479954
All
−0.05
0.00
0.05
MR leave−one−out sensitivity analysis for
'Parabacteroides sp000436495' on 'GCST90200424_buildGRCh38.tsv'

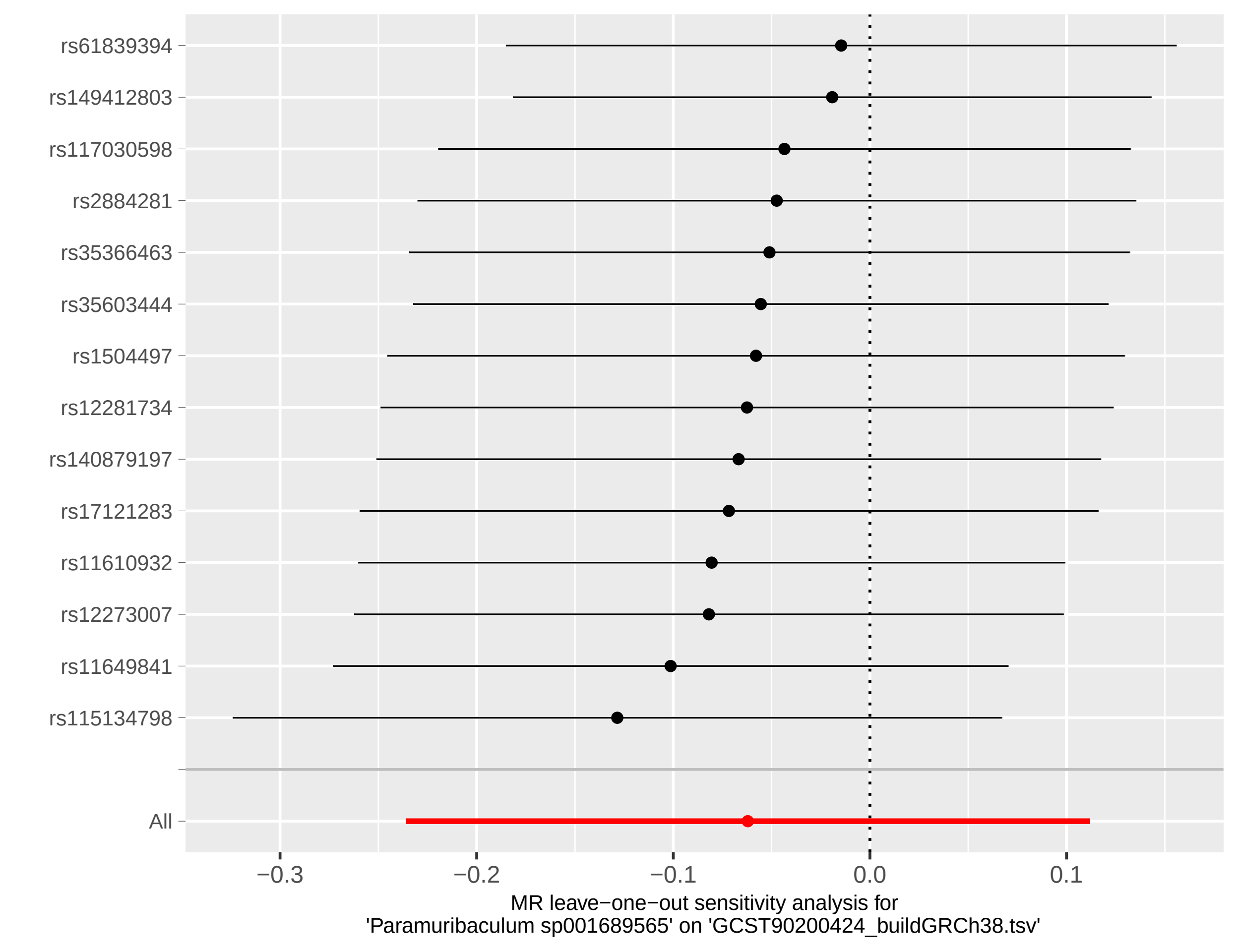

rs61839394
rs149412803
rs117030598
rs2884281
rs35366463
rs35603444
rs1504497
rs12281734
rs140879197
rs17121283
rs11610932
rs12273007
rs11649841
rs115134798
All
−0.3
−0.2
−0.1
0.0
0.1
MR leave−one−out sensitivity analysis for
'Paramuribaculum sp001689565' on 'GCST90200424_buildGRCh38.tsv'

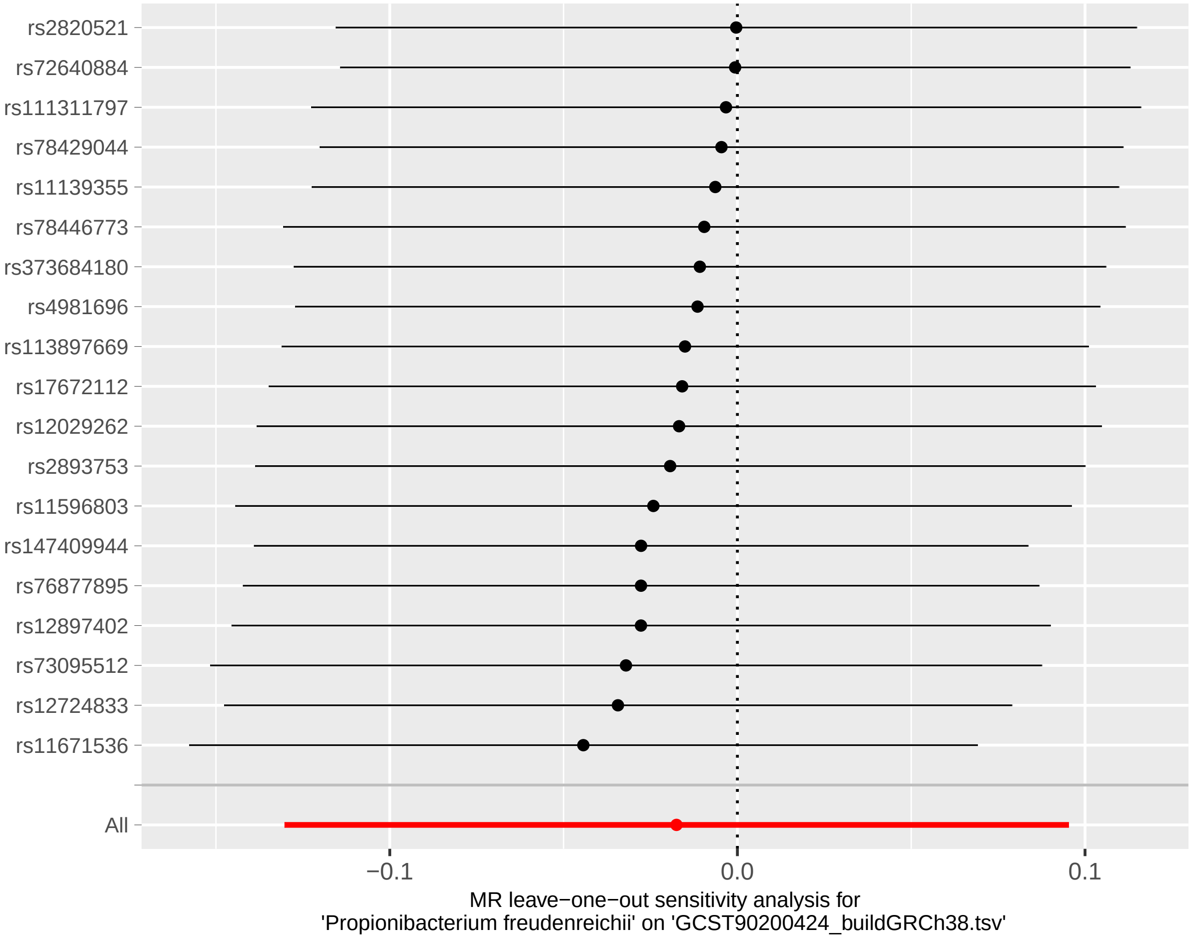
rs2820521
rs72640884
rs111311797
rs78429044
rs11139355
rs78446773
rs373684180
rs4981696
rs113897669
rs17672112
rs12029262
rs2893753
rs11596803
rs147409944
rs76877895
rs12897402
rs73095512
rs12724833
rs11671536
All
−0.1
0.0
0.1
MR leave−one−out sensitivity analysis for
'Propionibacterium freudenreichii' on 'GCST90200424_buildGRCh38.tsv'

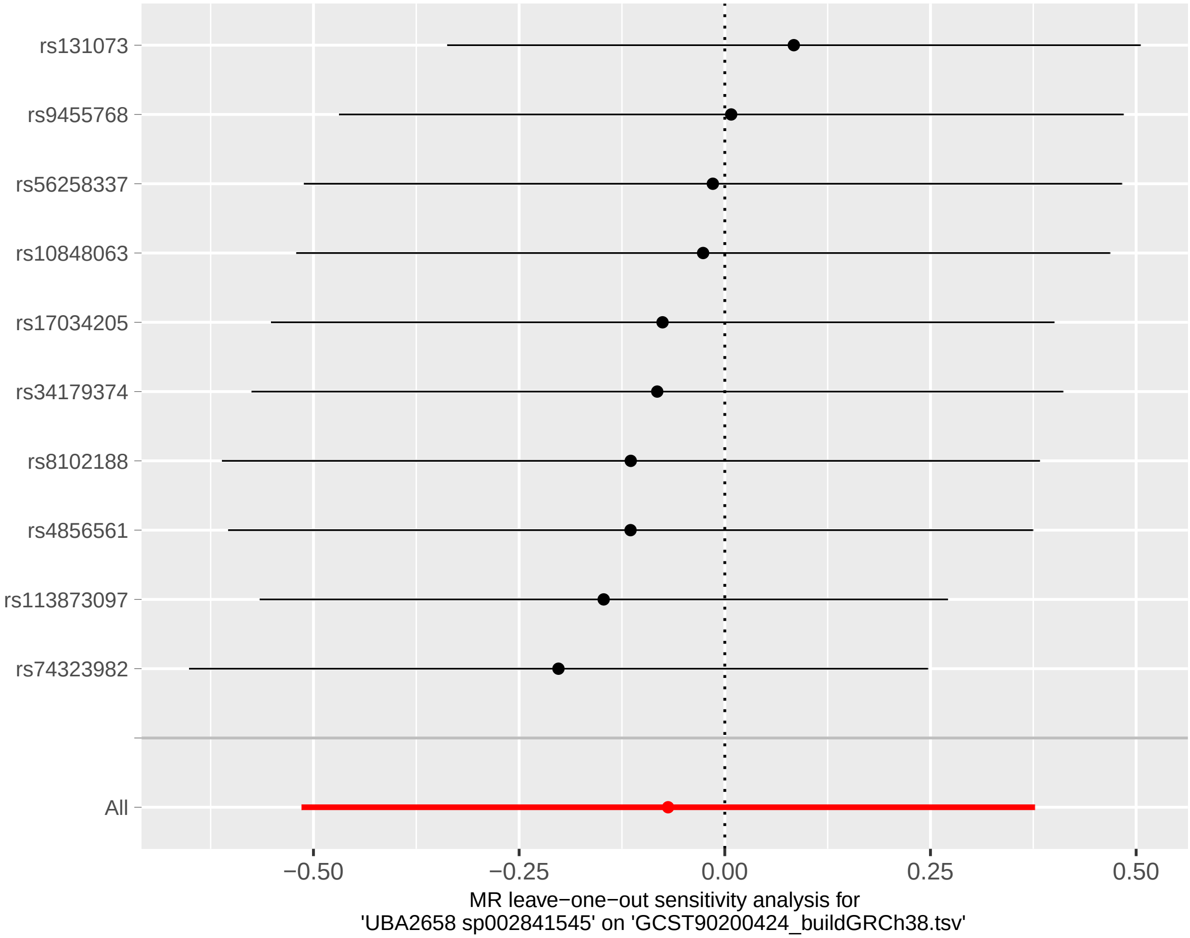
rs131073
rs9455768
rs56258337
rs10848063
rs17034205
rs34179374
rs8102188
rs4856561
rs113873097
rs74323982
All
−0.50
−0.25
0.00
0.25
0.50
MR leave−one−out sensitivity analysis for
'UBA2658 sp002841545' on 'GCST90200424_buildGRCh38.tsv'

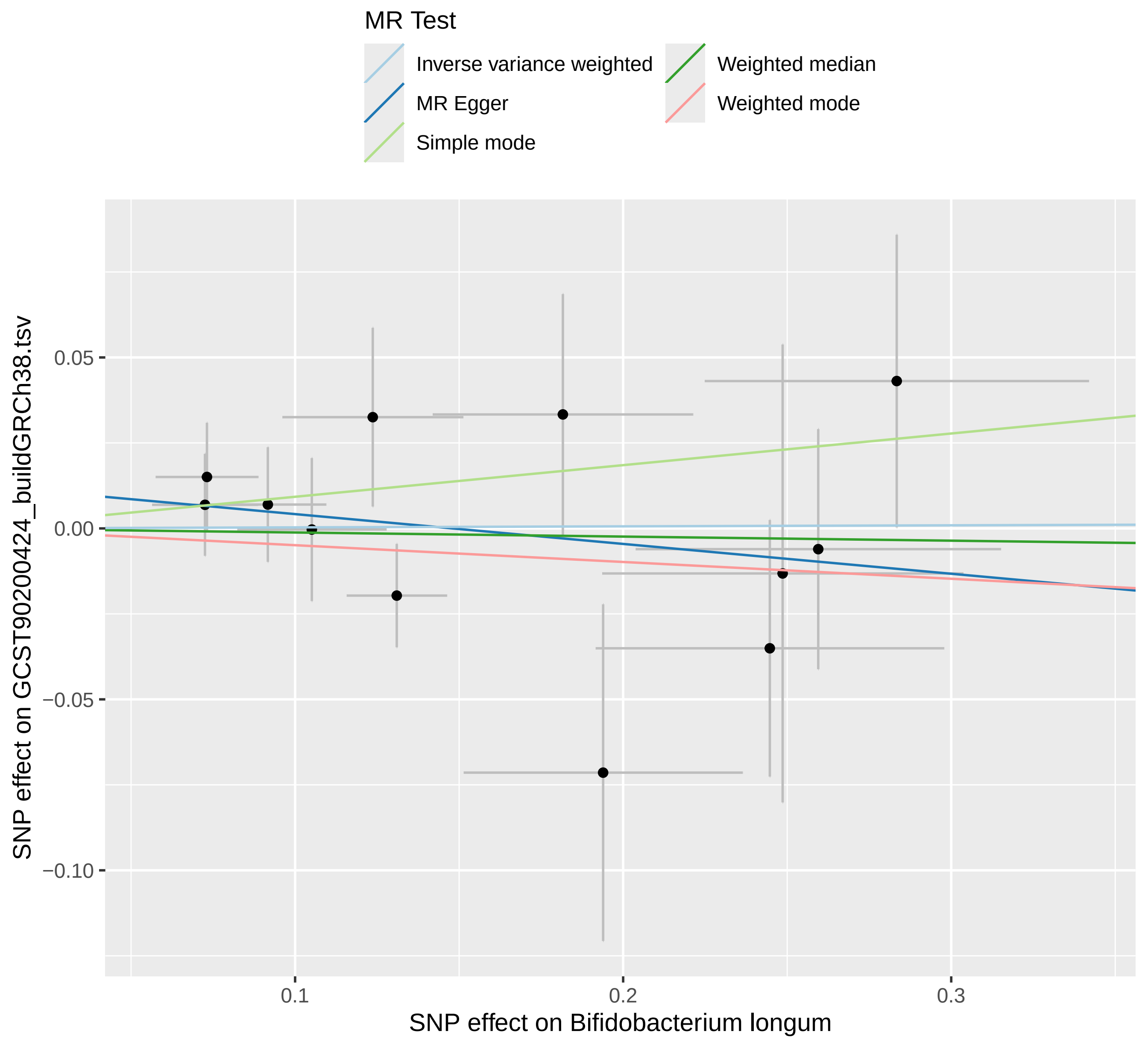

MR Test
Inverse variance weighted
MR Egger
Simple mode
Weighted median
Weighted mode
SNP effect on GCST90200424_buildGRCh38.tsv
0.05
0.00
−0.05
−0.10
0.1
0.2
0.3
SNP effect on Bifidobacterium longum

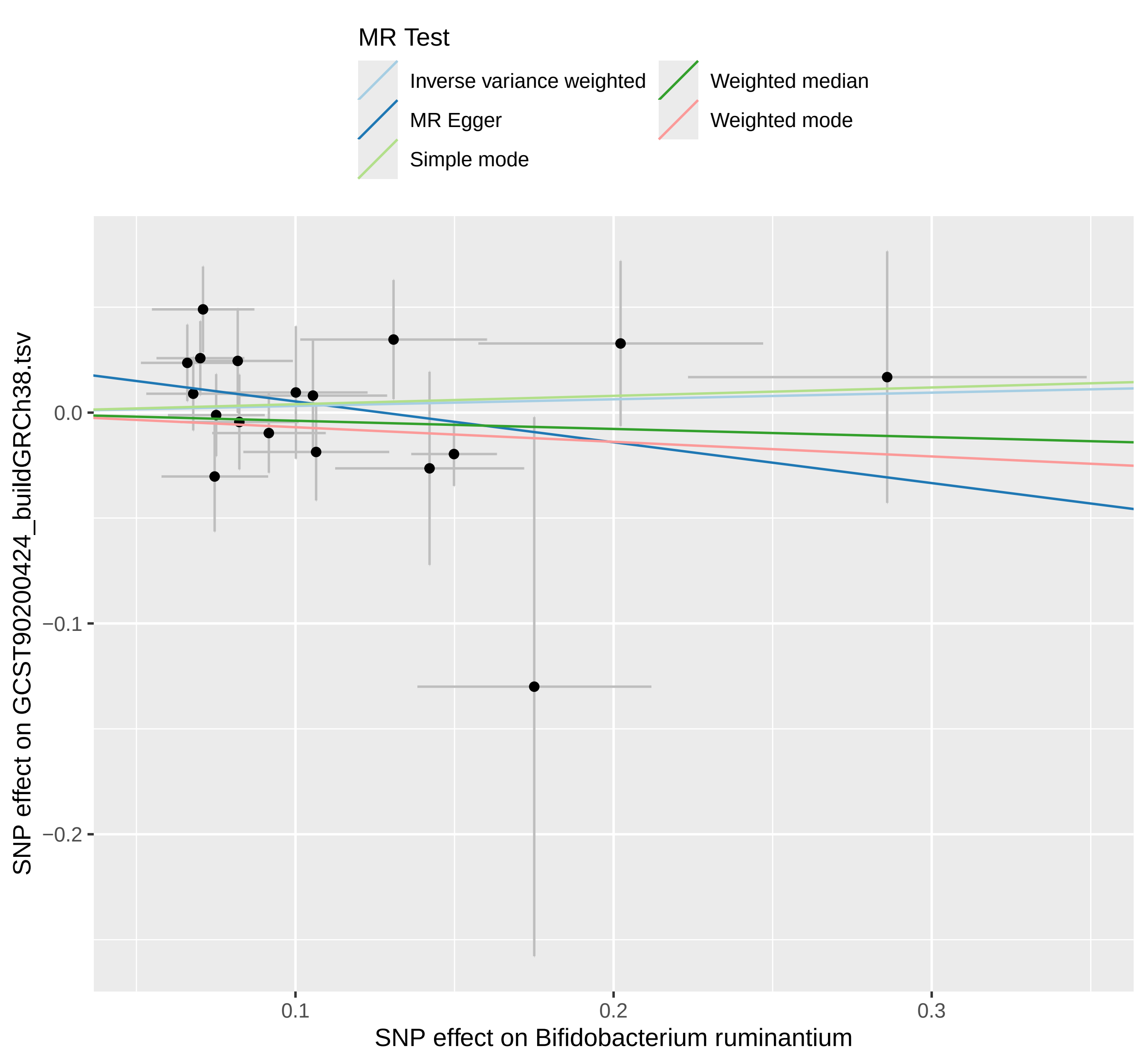

MR Test
Inverse variance weighted
MR Egger
Simple mode
Weighted median
Weighted mode
SNP effect on GCST90200424_buildGRCh38.tsv
0.0
−0.1
−0.2
0.1
0.2
0.3
SNP effect on Bifidobacterium ruminantium

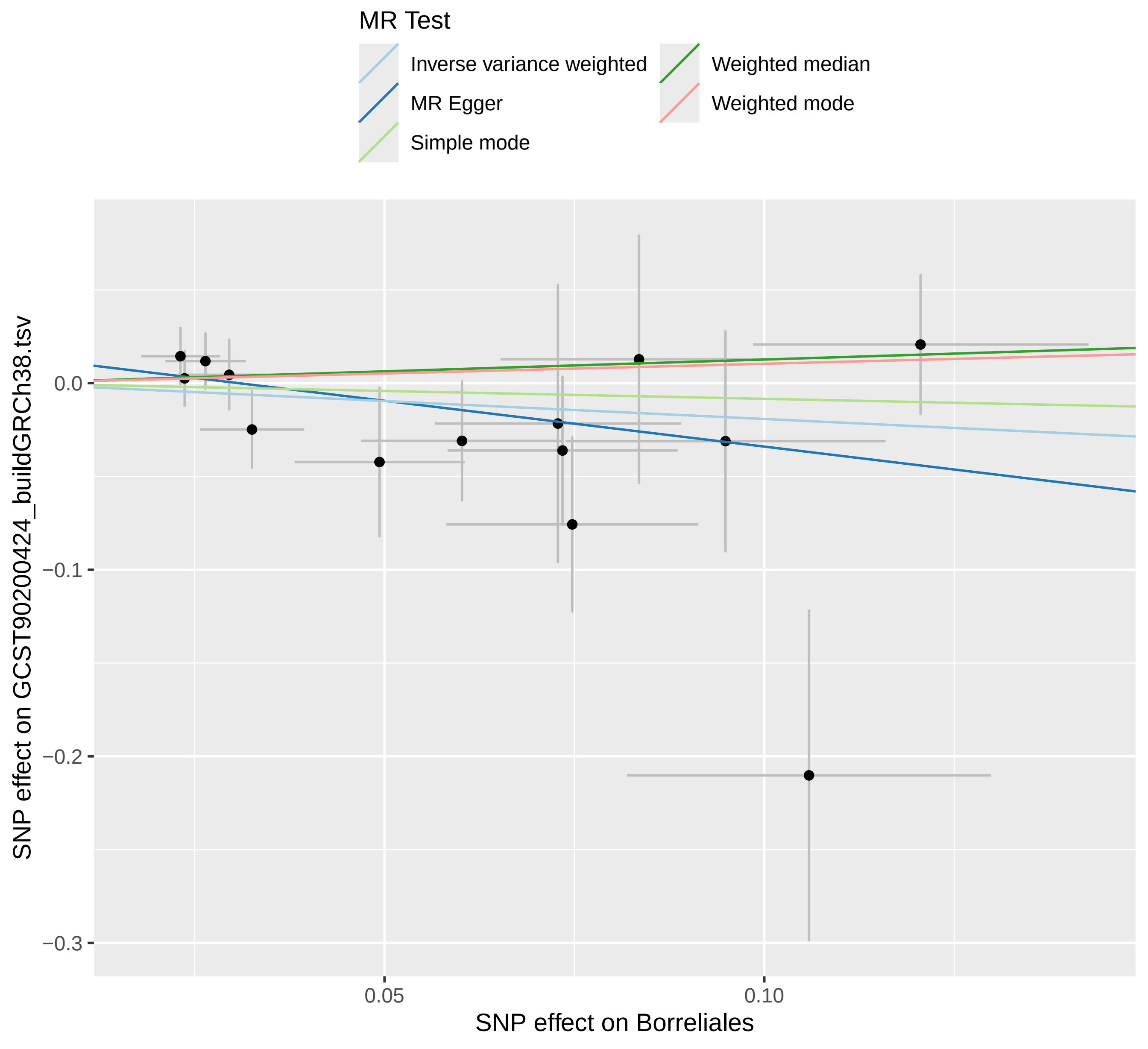
MR Test
Inverse variance weighted
MR Egger
Simple mode
Weighted median
Weighted mode
0.0
−0.1
−0.2
−0.3
0.05
0.10
SNP effect on Borreliales
SNP effect on GCST90200424_buildGRCh38.tsv

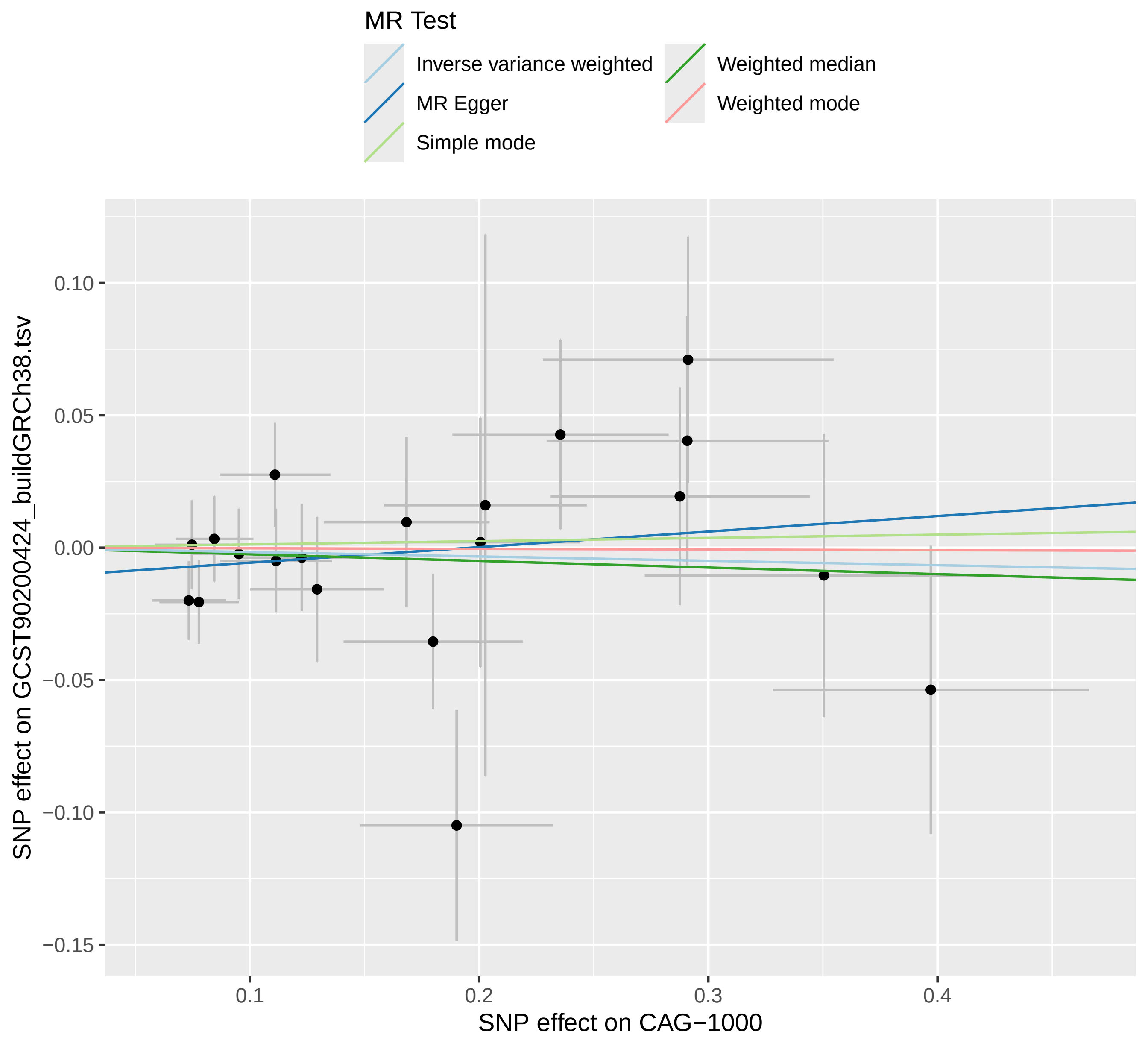

MR Test
Inverse variance weighted
MR Egger
Simple mode
Weighted median
Weighted mode
0.10
0.05
0.00
−0.05
−0.10
−0.15
0.1
0.2
0.3
0.4
SNP effect on GCST90200424_buildGRCh38.tsv
SNP effect on CAG−1000

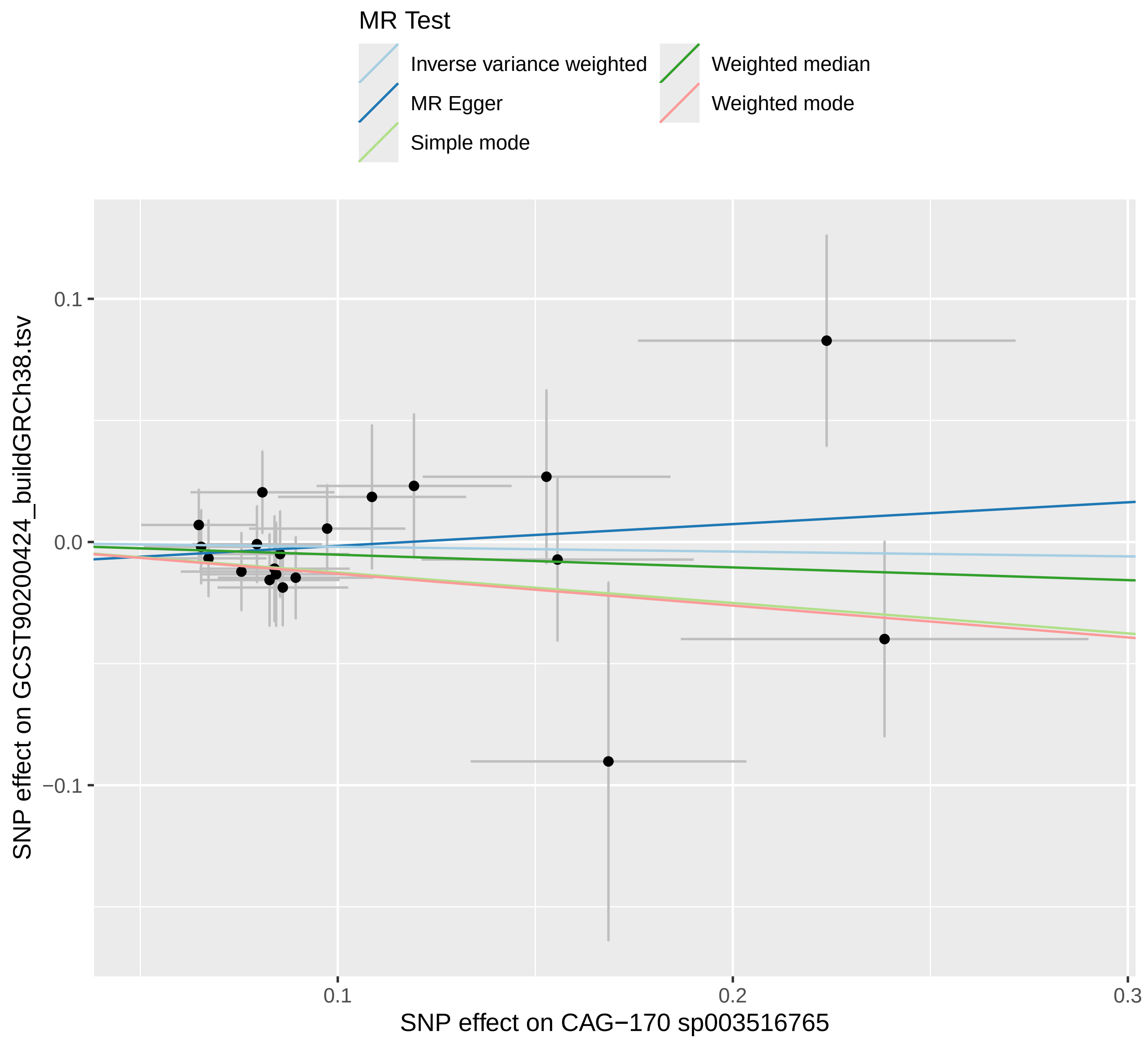
MR Test
Inverse variance weighted
MR Egger
Simple mode
Weighted median
Weighted mode
SNP effect on GCST90200424_buildGRCh38.tsv
SNP effect on CAG−170 sp003516765
0.1
0.0
−0.1
0.1
0.2
0.3

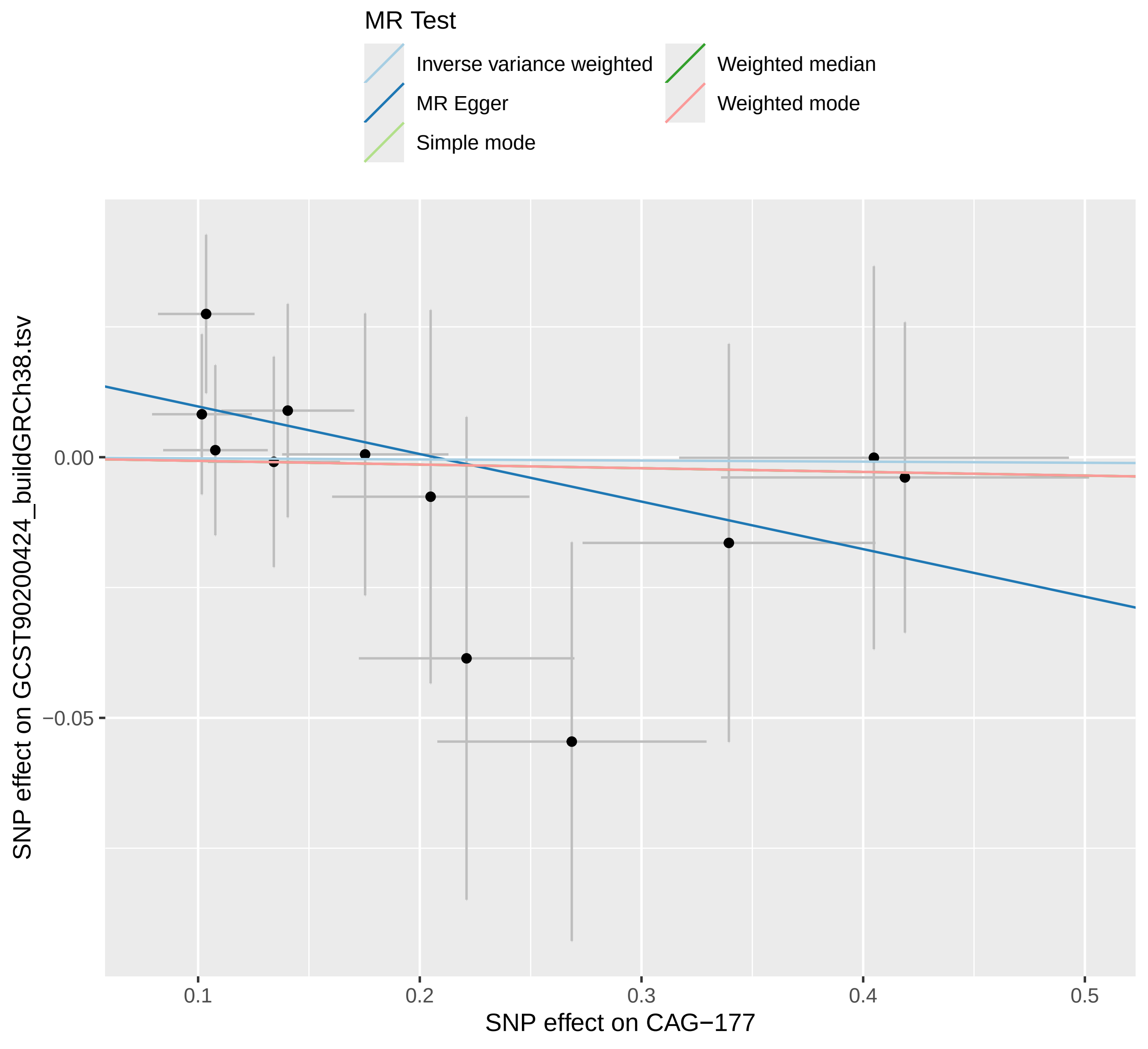
MR Test
Inverse variance weighted
MR Egger
Simple mode
Weighted median
Weighted mode
SNP effect on GCST90200424_buildGRCh38.tsv
0.00
−0.05
0.1
0.2
0.3
0.4
0.5
SNP effect on CAG−177

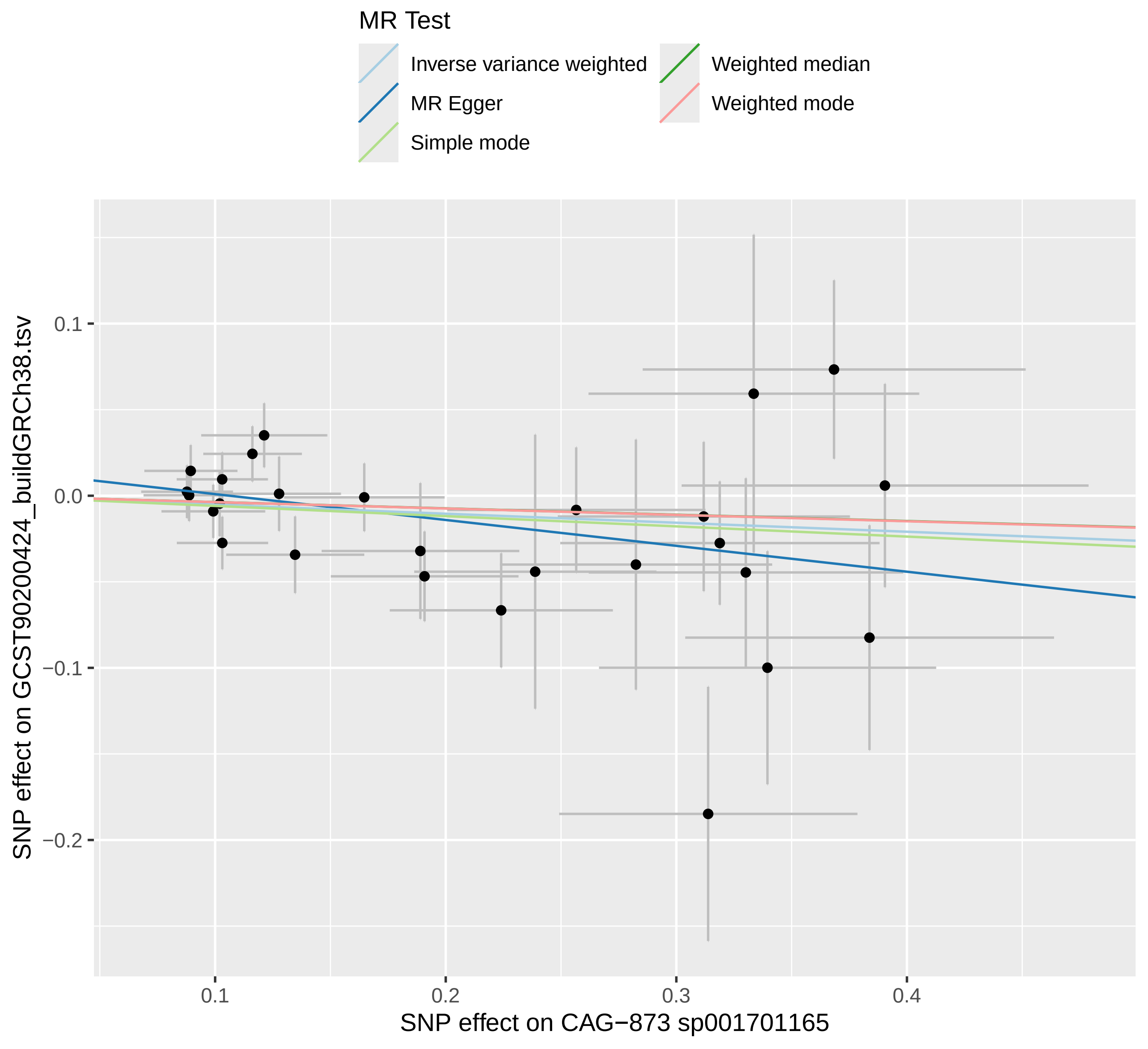
MR Test
Inverse variance weighted
MR Egger
Simple mode
Weighted median
Weighted mode
0.1
0.0
−0.1
−0.2
0.1
0.2
0.3
0.4
SNP effect on GCST90200424_buildGRCh38.tsv
SNP effect on CAG−873 sp001701165

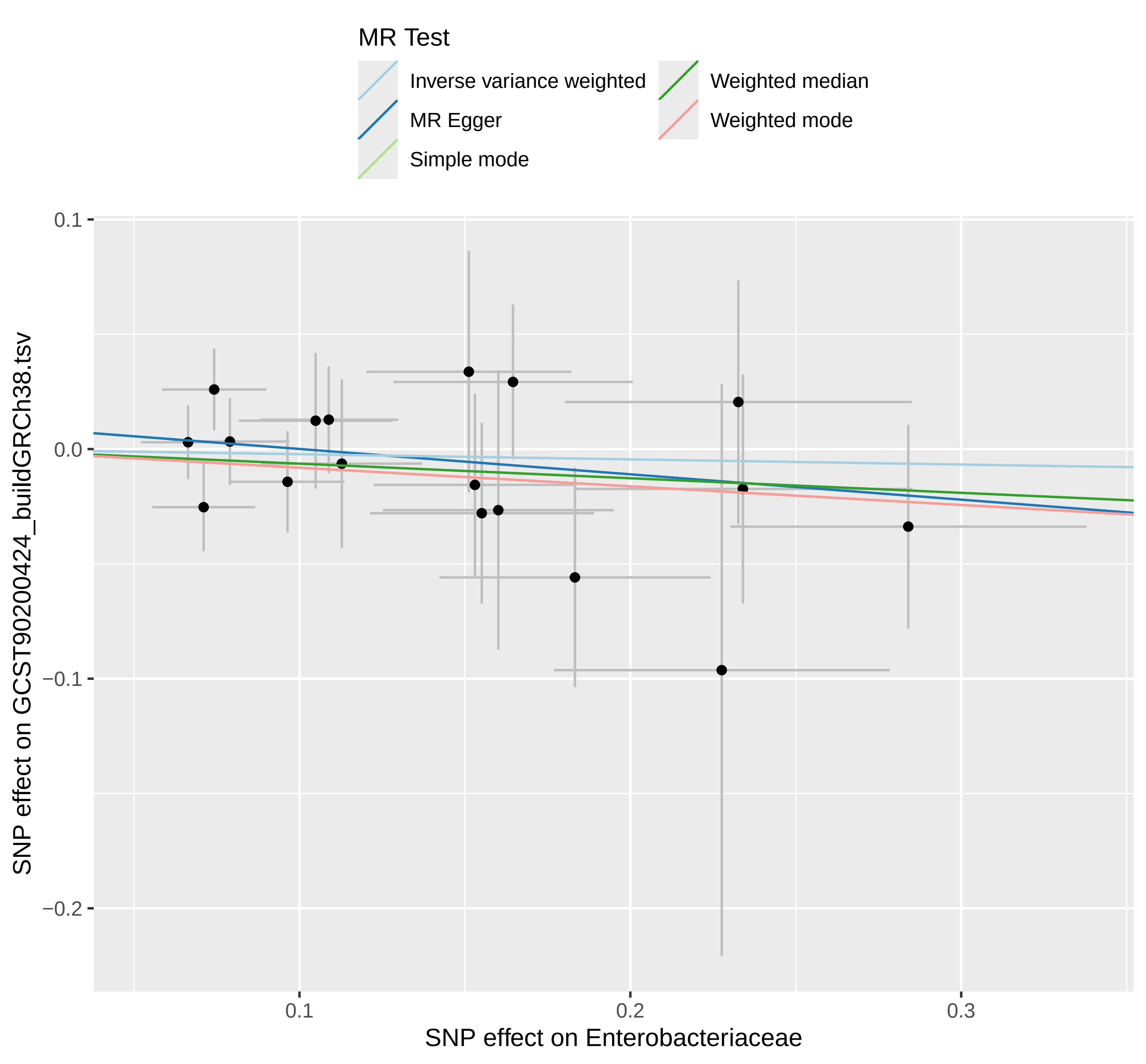

MR Test
Inverse variance weighted
MR Egger
Simple mode
Weighted median
Weighted mode
0.1
0.0
−0.1
−0.2
SNP effect on GCST90200424_buildGRCh38.tsv
0.1
0.2
0.3
SNP effect on Enterobacteriaceae

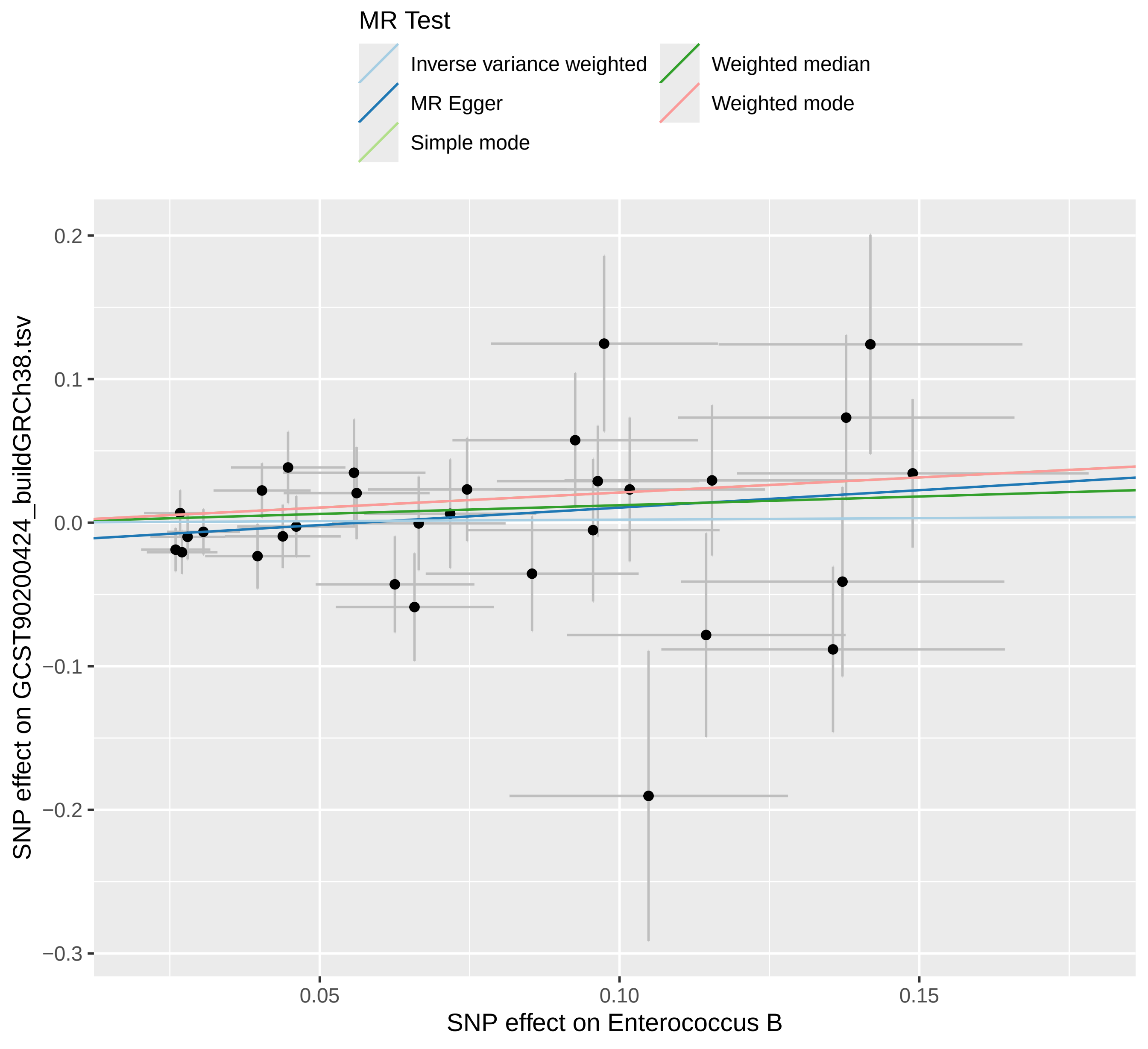
MR Test
Inverse variance weighted
MR Egger
Simple mode
Weighted median
Weighted mode
0.2
0.1
0.0
−0.1
−0.2
−0.3
SNP effect on GCST90200424_buildGRCh38.tsv
0.05
0.10
0.15
SNP effect on Enterococcus B

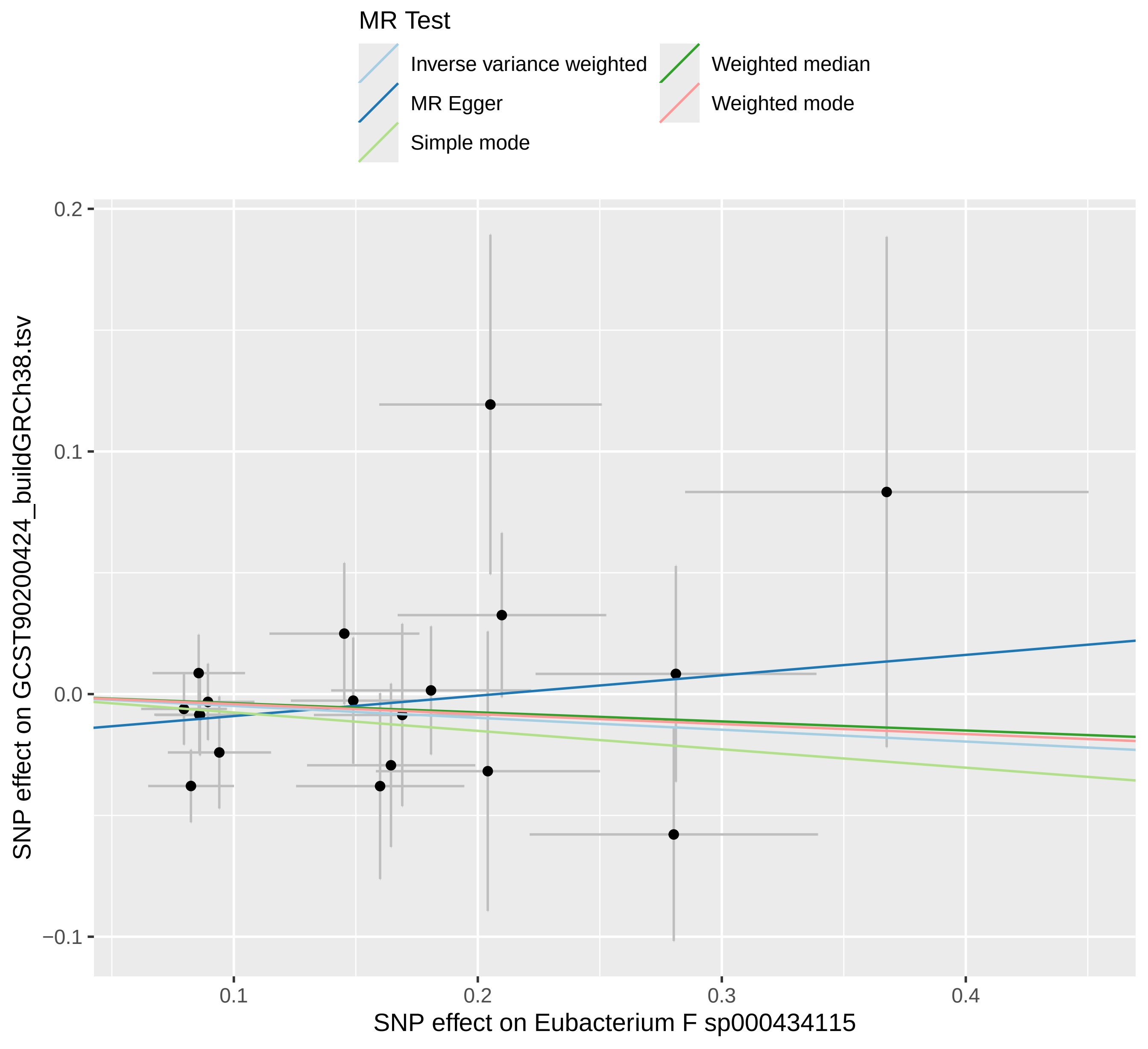
MR Test
Inverse variance weighted
MR Egger
Simple mode
Weighted median
Weighted mode
0.2
0.1
0.0
−0.1
0.1
0.2
0.3
0.4
SNP effect on GCST90200424_buildGRCh38.tsv
SNP effect on Eubacterium F sp000434115

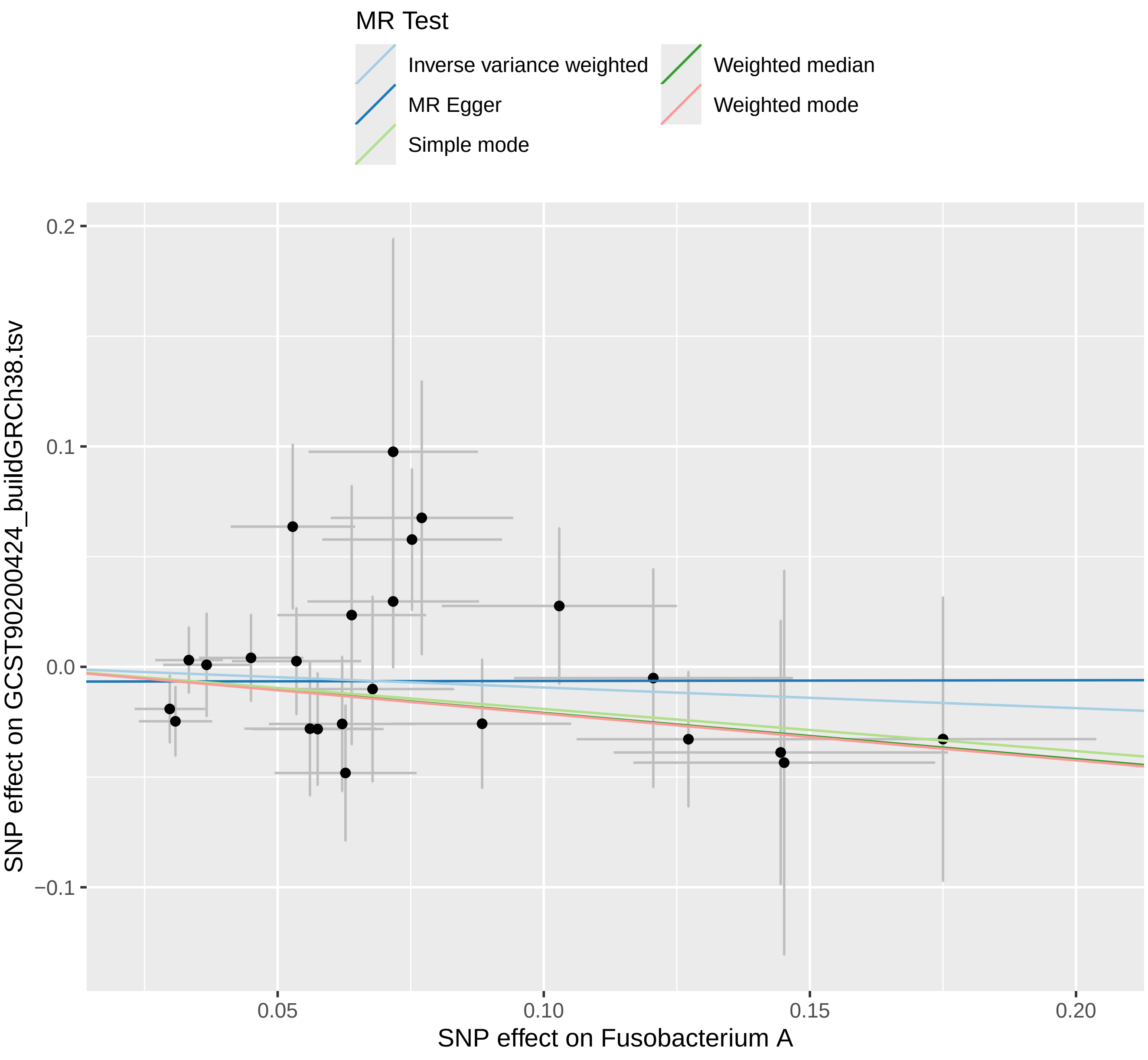
MR Test
Inverse variance weighted
MR Egger
Simple mode
Weighted median
Weighted mode
0.2
0.1
0.0
−0.1
0.05
0.10
0.15
0.20
SNP effect on GCST90200424_buildGRCh38.tsv
SNP effect on Fusobacterium A

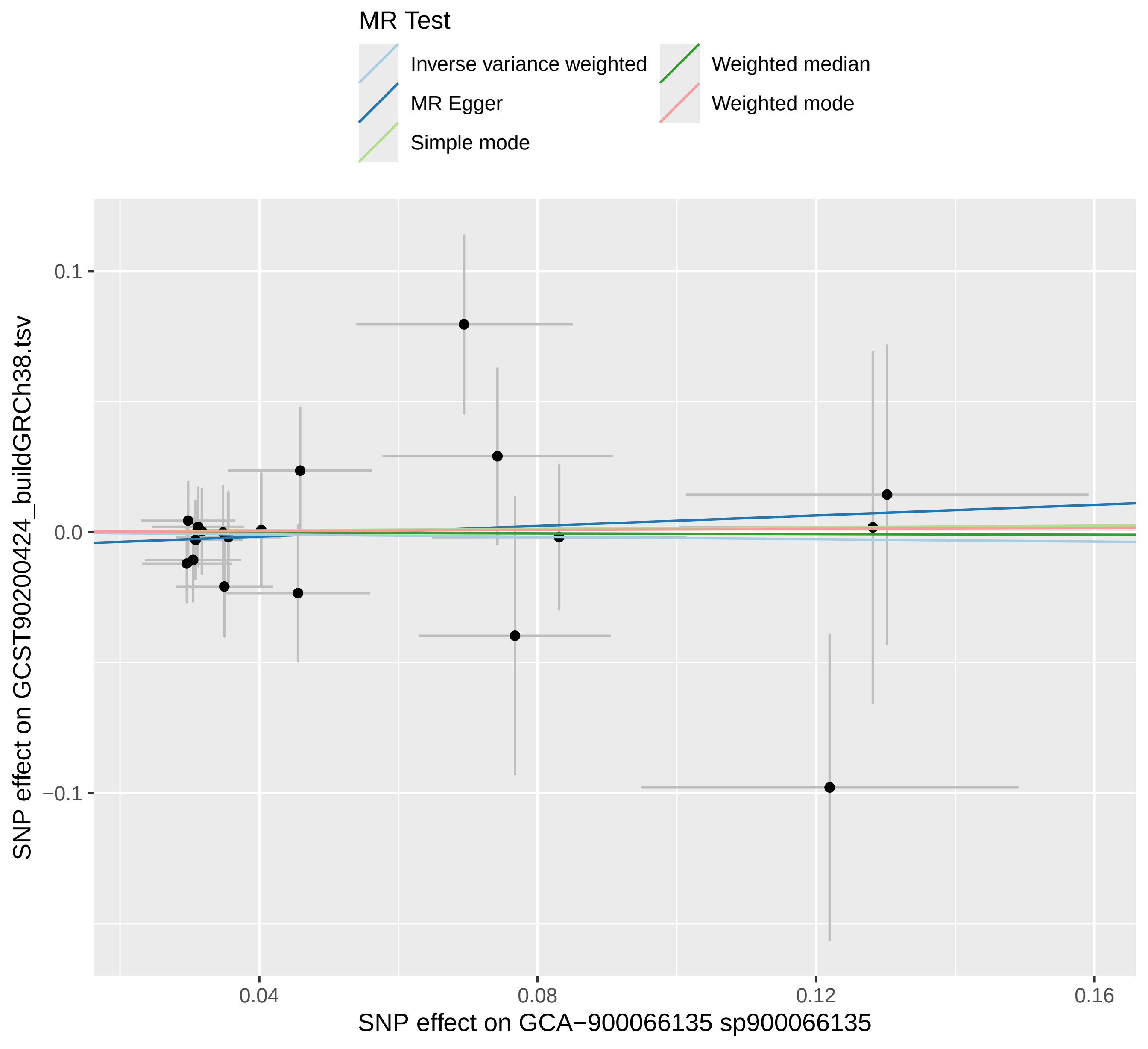

MR Test
Inverse variance weighted
MR Egger
Simple mode
Weighted median
Weighted mode
0.1
0.0
−0.1
0.04
0.08
0.12
0.16
SNP effect on GCST90200424_buildGRCh38.tsv
SNP effect on GCA−900066135 sp900066135

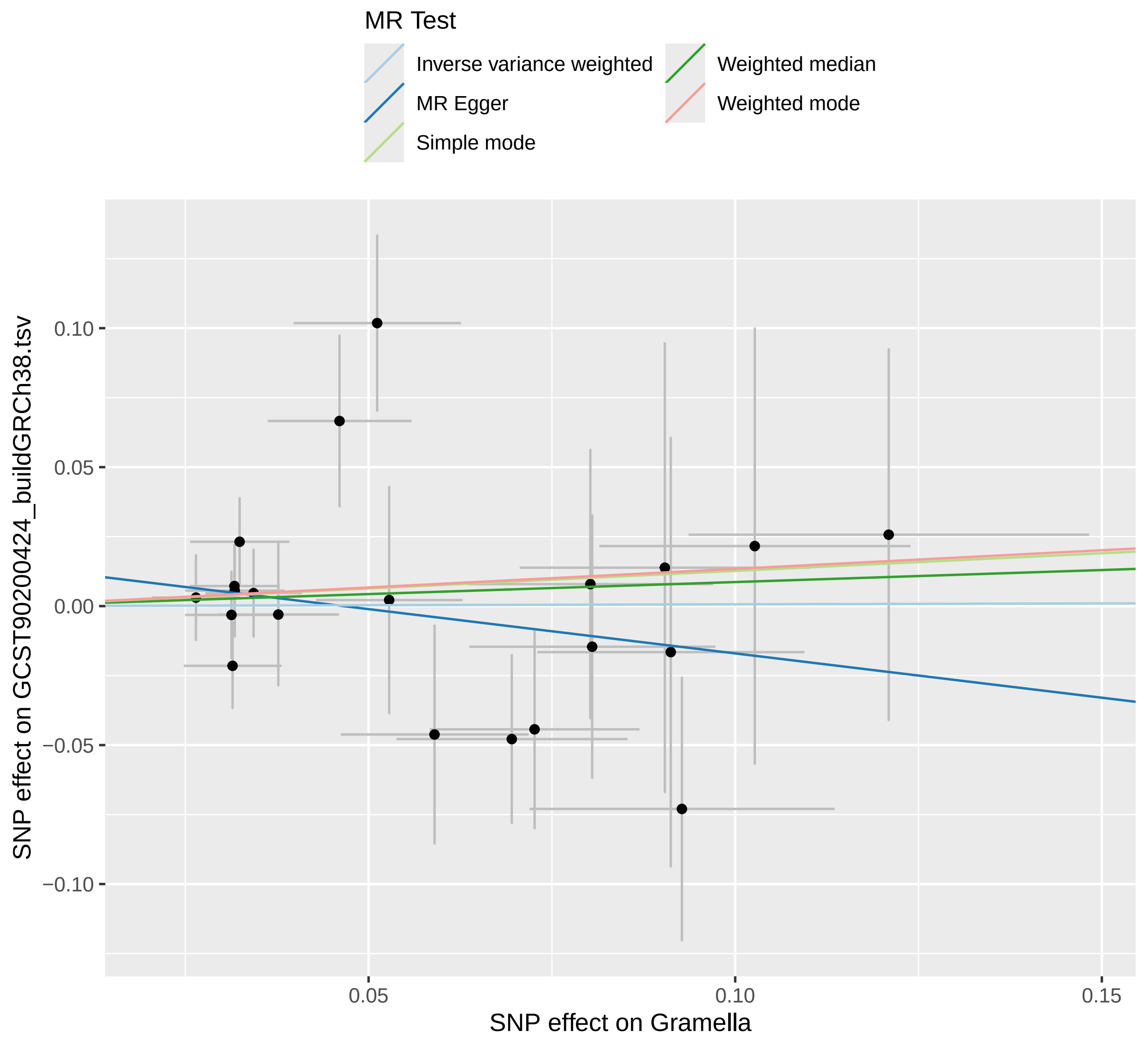

MR Test
Inverse variance weighted
MR Egger
Simple mode
Weighted median
Weighted mode
0.10
0.05
0.00
−0.05
−0.10
SNP effect on GCST90200424_buildGRCh38.tsv
0.05
0.10
0.15
SNP effect on Gramella

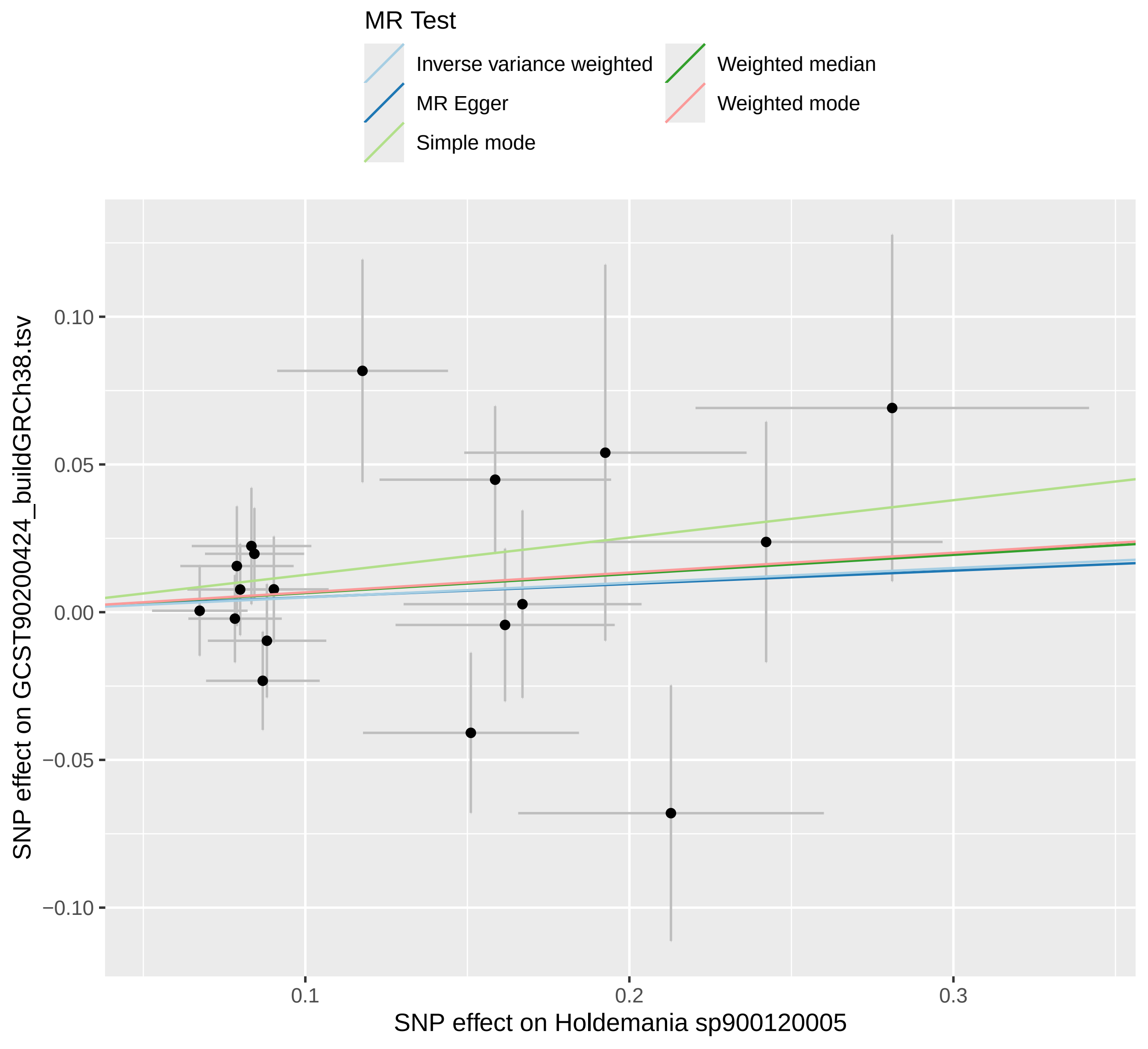

MR Test
Inverse variance weighted
MR Egger
Simple mode
Weighted median
Weighted mode
0.10
0.05
0.00
−0.05
−0.10
0.1
0.2
0.3
SNP effect on GCST90200424_buildGRCh38.tsv
SNP effect on Holdemania sp900120005

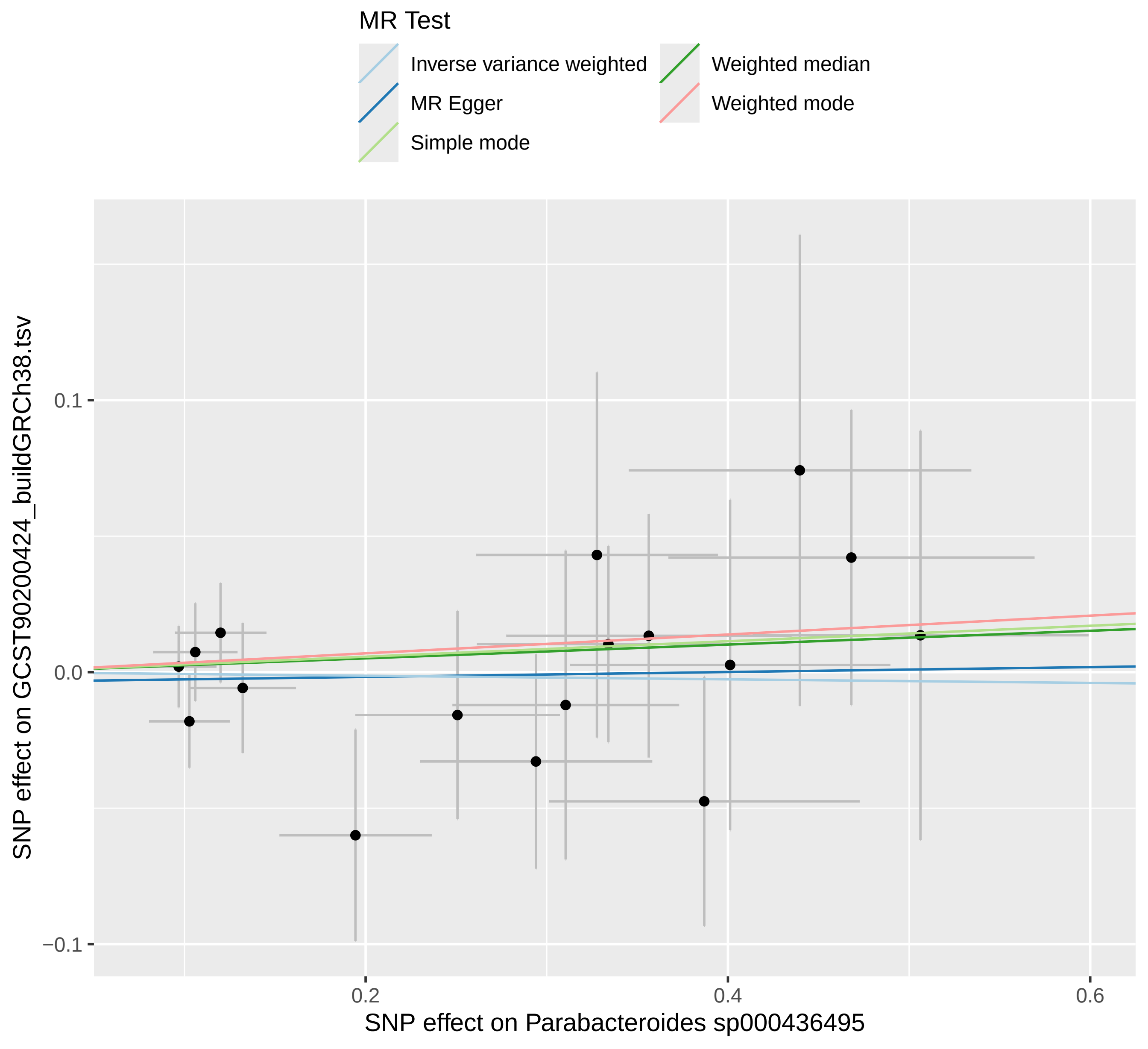

MR Test
Inverse variance weighted
MR Egger
Simple mode
Weighted median
Weighted mode
0.1
0.0
−0.1
0.2
0.4
0.6
SNP effect on GCST90200424_buildGRCh38.tsv
SNP effect on Parabacteroides sp000436495

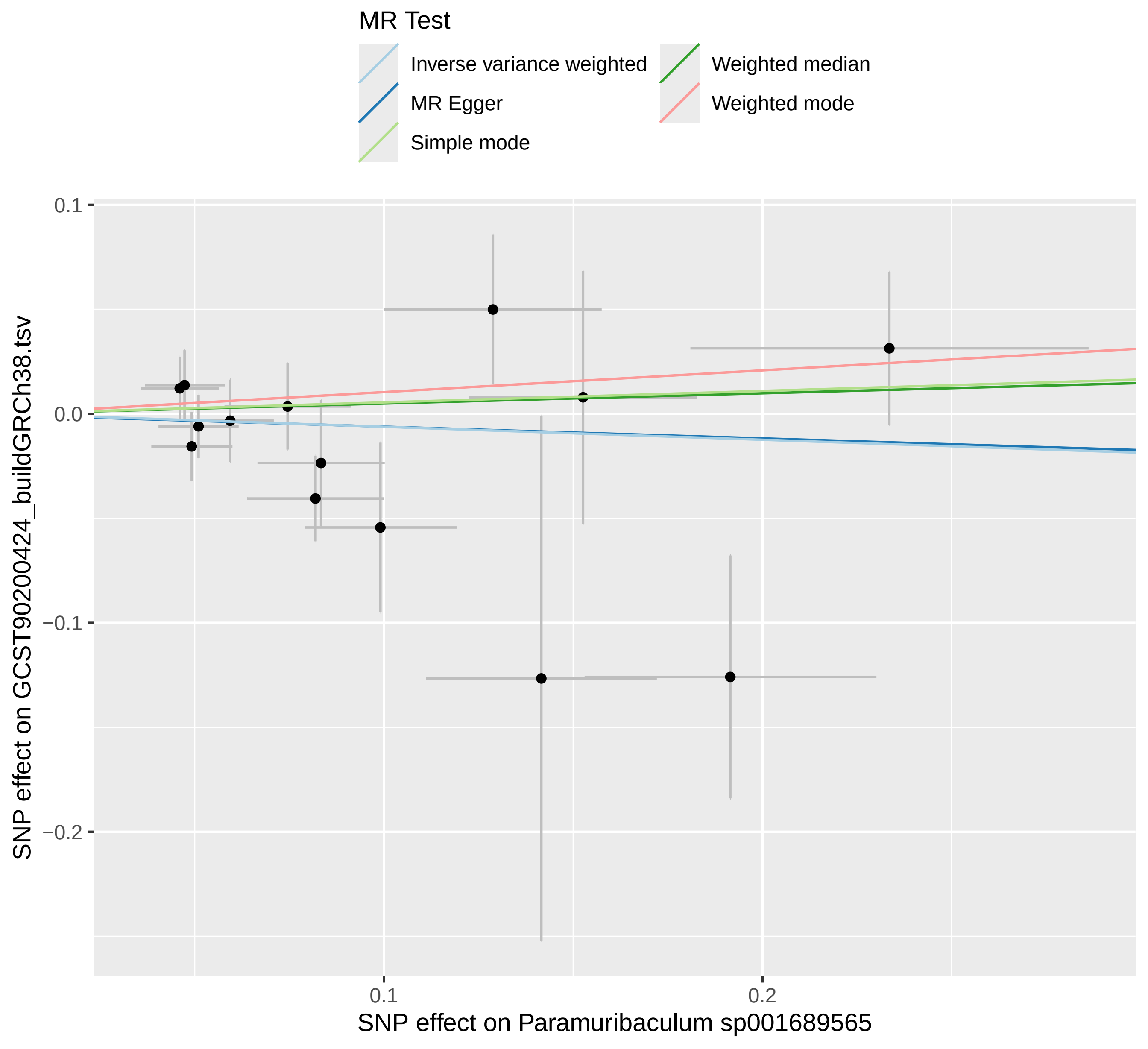
MR Test
Inverse variance weighted
MR Egger
Simple mode
Weighted median
Weighted mode
0.1
0.0
−0.1
−0.2
0.1
0.2
SNP effect on GCST90200424_buildGRCh38.tsv
SNP effect on Paramuribaculum sp001689565

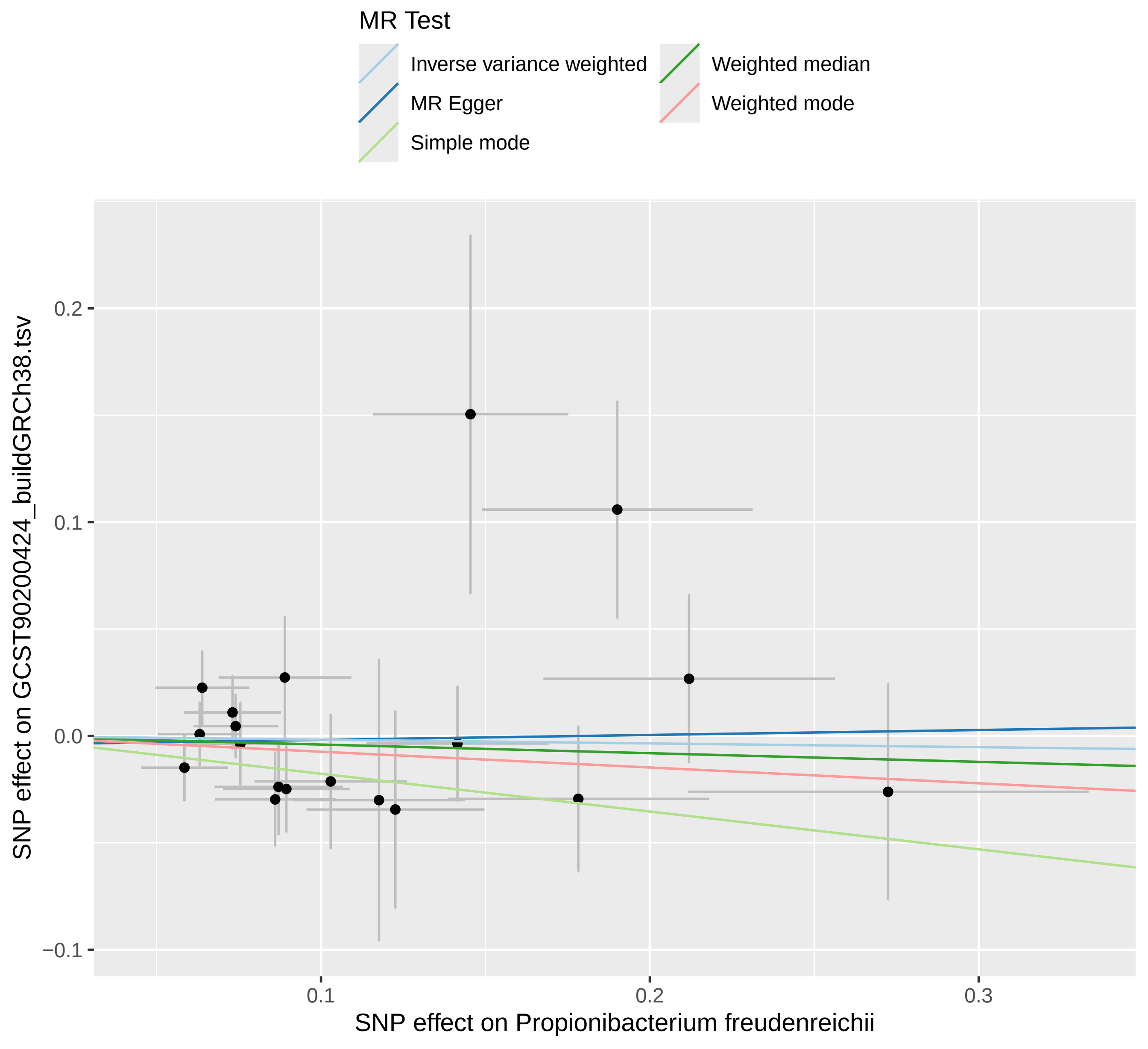
MR Test
Inverse variance weighted
MR Egger
Simple mode
Weighted median
Weighted mode
SNP effect on GCST90200424_buildGRCh38.tsv
0.2
0.1
0.0
−0.1
0.1
0.2
0.3
SNP effect on Propionibacterium freudenreichii

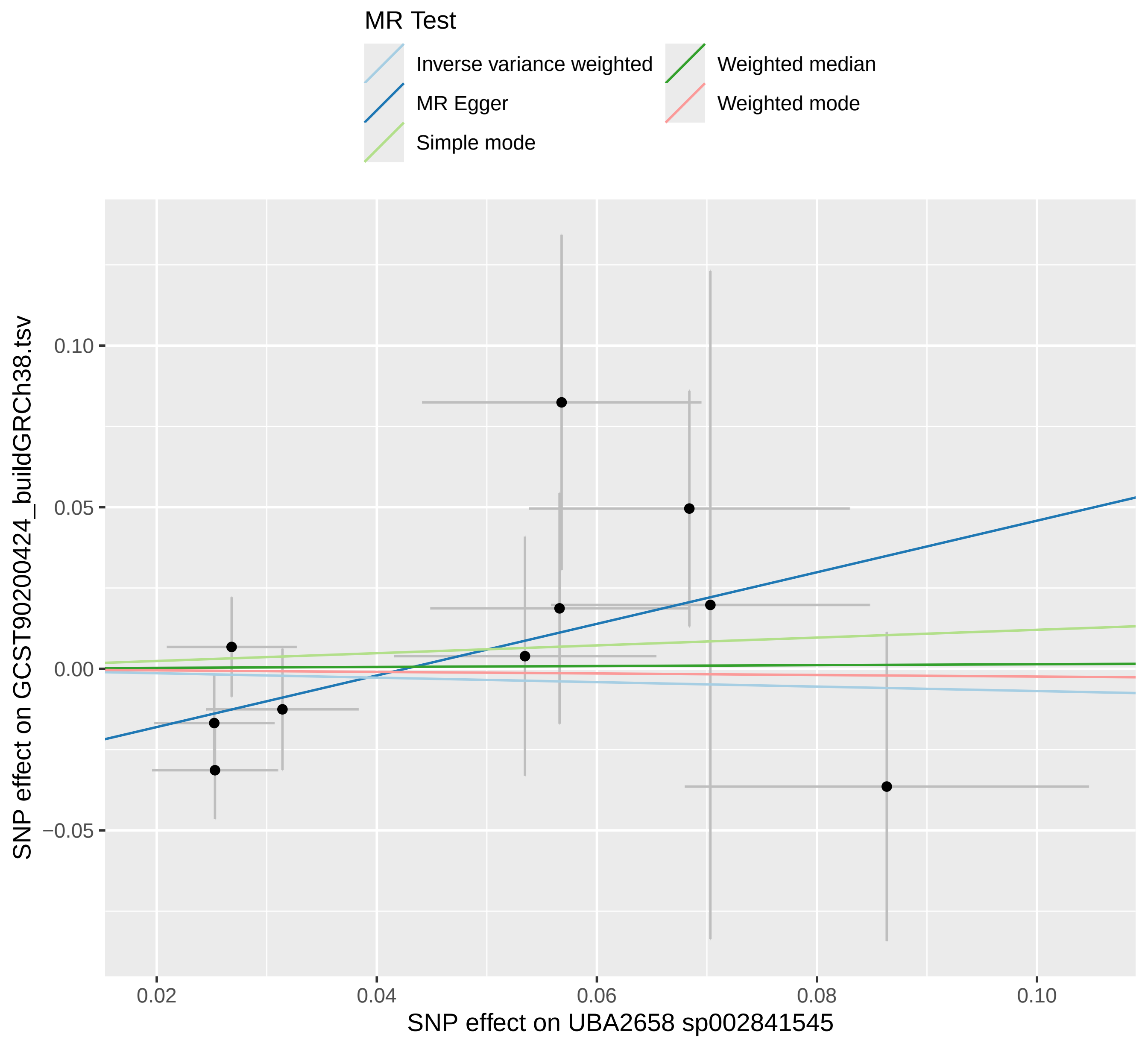

MR Test
Inverse variance weighted
MR Egger
Simple mode
Weighted median
Weighted mode
0.10
0.05
0.00
−0.05
0.02
0.04
0.06
0.08
0.10
SNP effect on GCST90200424_buildGRCh38.tsv
SNP effect on UBA2658 sp002841545

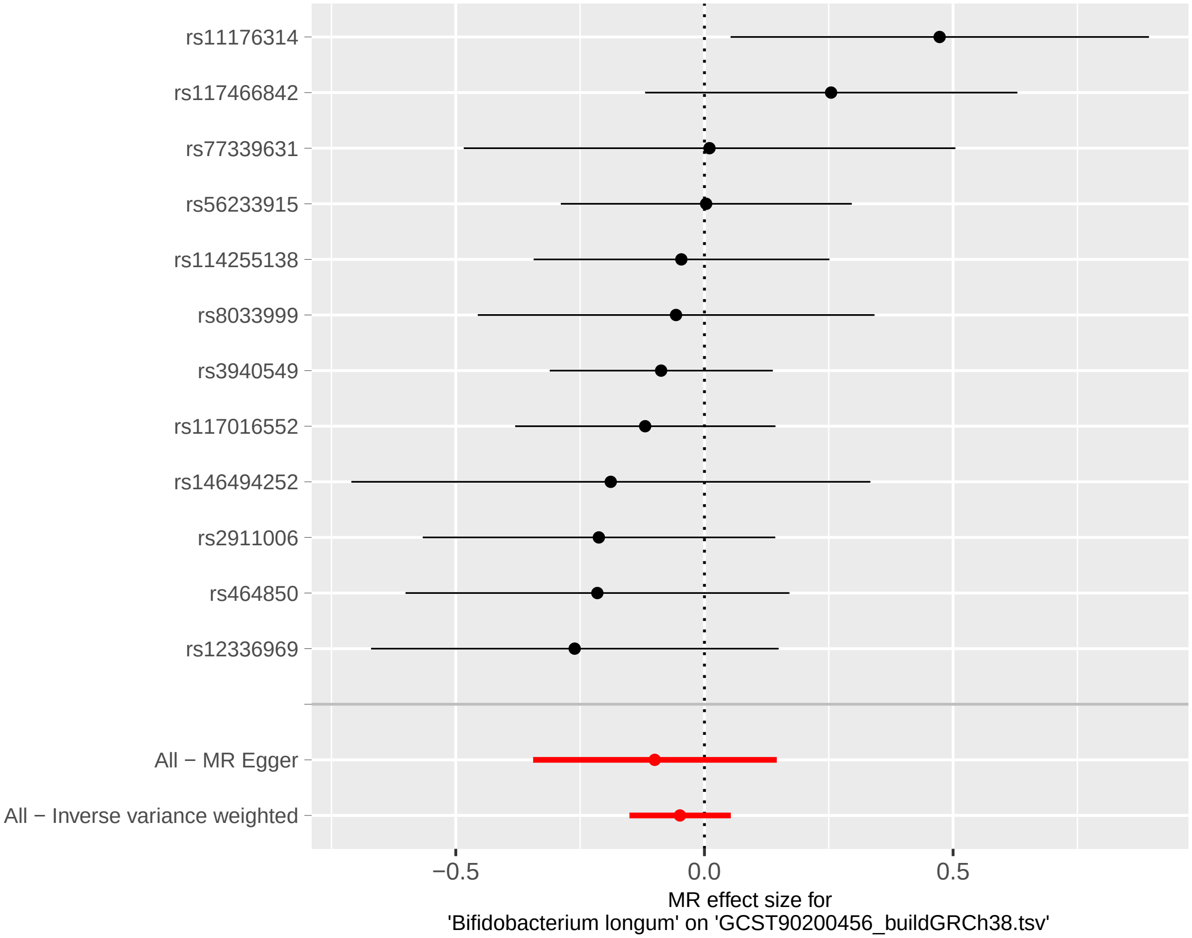

rs11176314
rs117466842
rs77339631
rs56233915
rs114255138
rs8033999
rs3940549
rs117016552
rs146494252
rs2911006
rs464850
rs12336969
All − MR Egger
All − Inverse variance weighted
−0.5
0.0
0.5
MR effect size for
'Bifidobacterium longum' on 'GCST90200456_buildGRCh38.tsv'

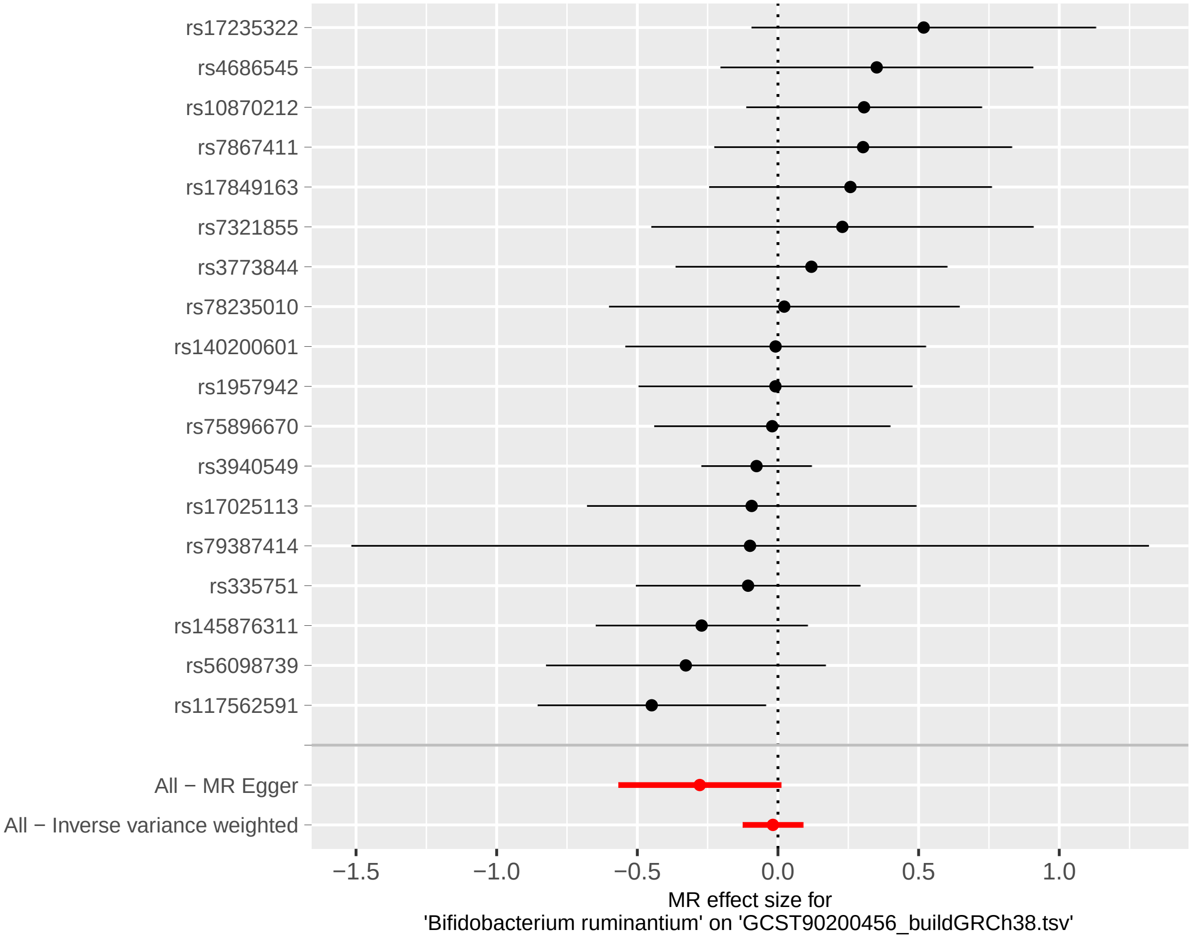
rs17235322
rs4686545
rs10870212
rs7867411
rs17849163
rs7321855
rs3773844
rs78235010
rs140200601
rs1957942
rs75896670
rs3940549
rs17025113
rs79387414
rs335751
rs145876311
rs56098739
rs117562591
All − MR Egger
All − Inverse variance weighted
−1.5
−1.0
−0.5
0.0
0.5
1.0
MR effect size for
'Bifidobacterium ruminantium' on 'GCST90200456_buildGRCh38.tsv'

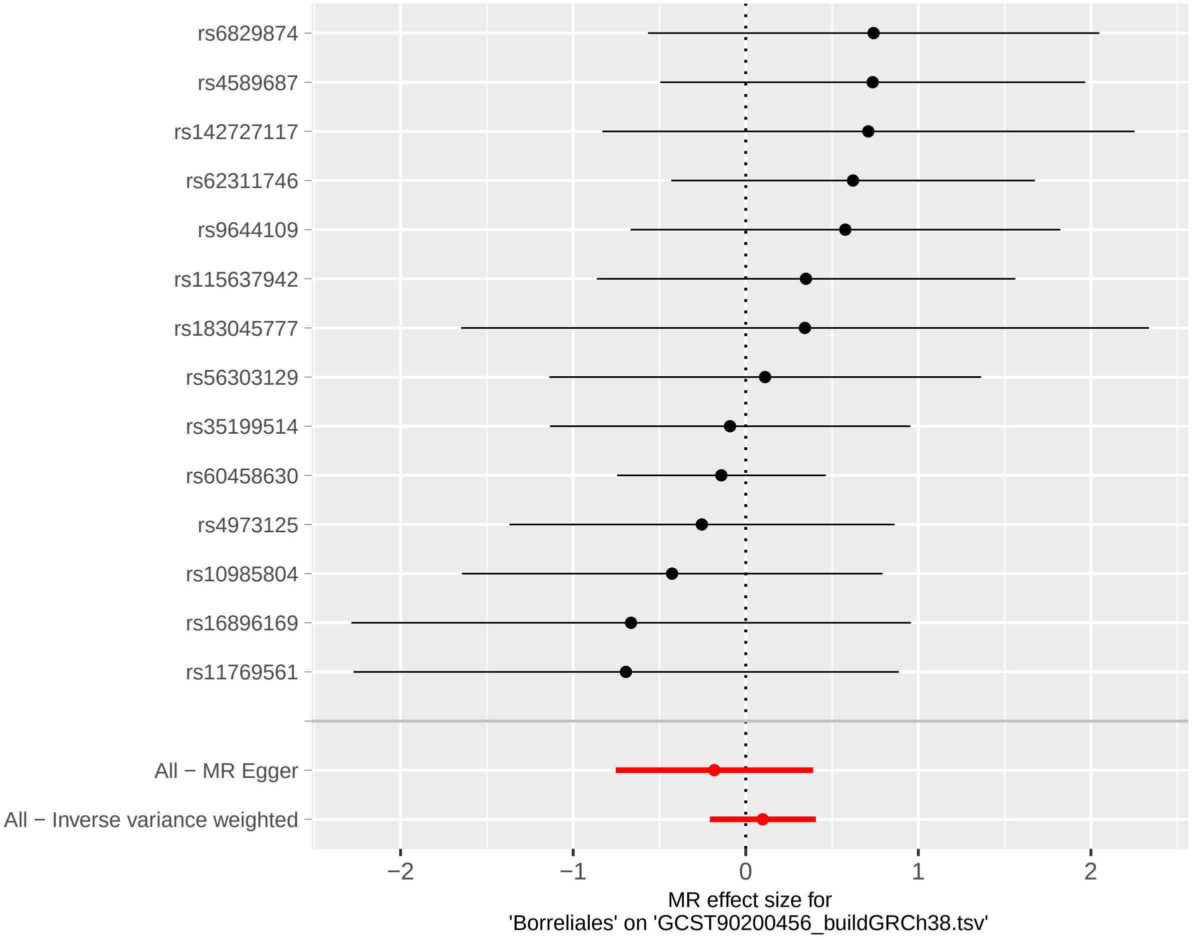
rs6829874
rs4589687
rs142727117
rs62311746
rs9644109
rs115637942
rs183045777
rs56303129
rs35199514
rs60458630
rs4973125
rs10985804
rs16896169
rs11769561
All − MR Egger
All − Inverse variance weighted
−2
−1
0
1
2
MR effect size for
'Borreliales' on 'GCST90200456_buildGRCh38.tsv'

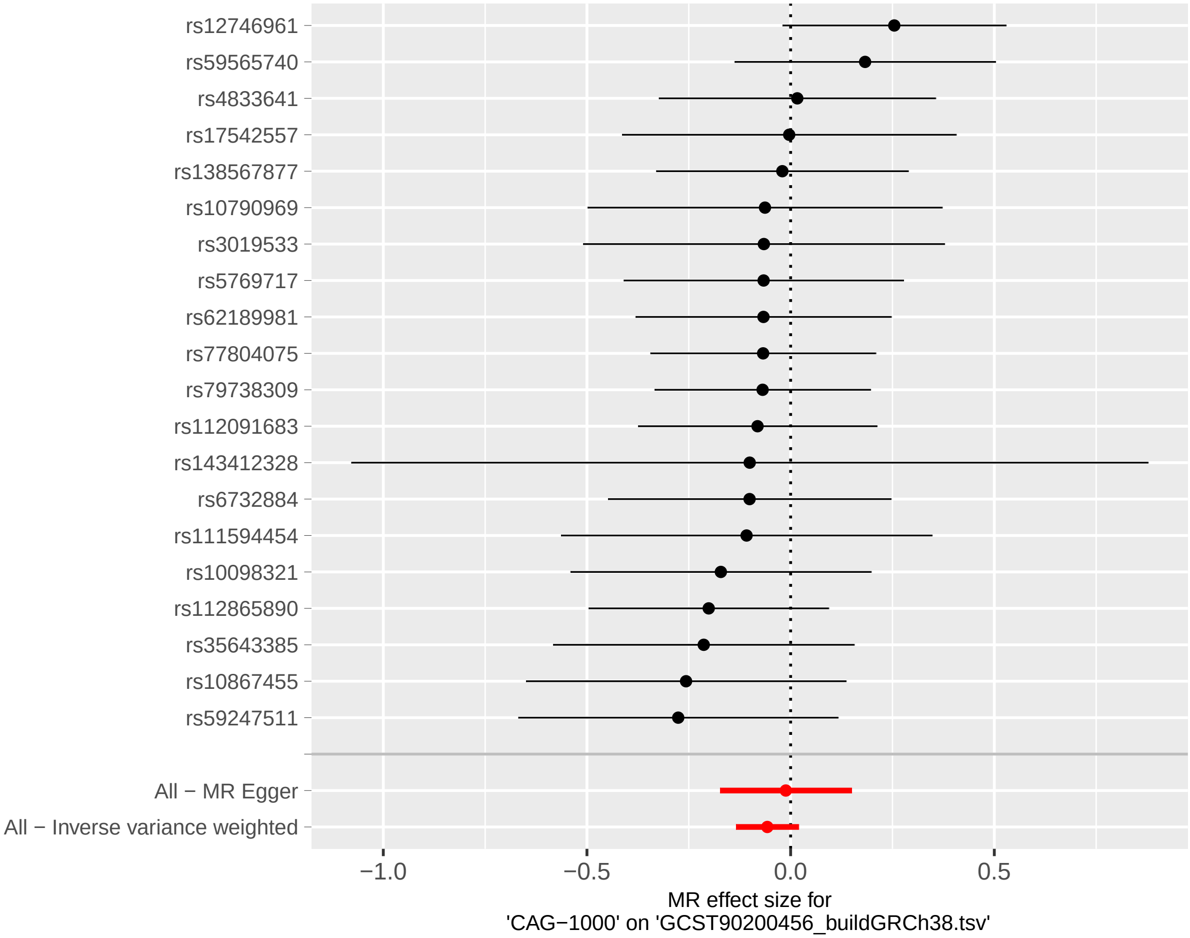
rs12746961
rs59565740
rs4833641
rs17542557
rs138567877
rs10790969
rs3019533
rs5769717
rs62189981
rs77804075
rs79738309
rs112091683
rs143412328
rs6732884
rs111594454
rs10098321
rs112865890
rs35643385
rs10867455
rs59247511
All − MR Egger
All − Inverse variance weighted
−1.0
−0.5
0.0
0.5
MR effect size for
'CAG−1000' on 'GCST90200456_buildGRCh38.tsv'

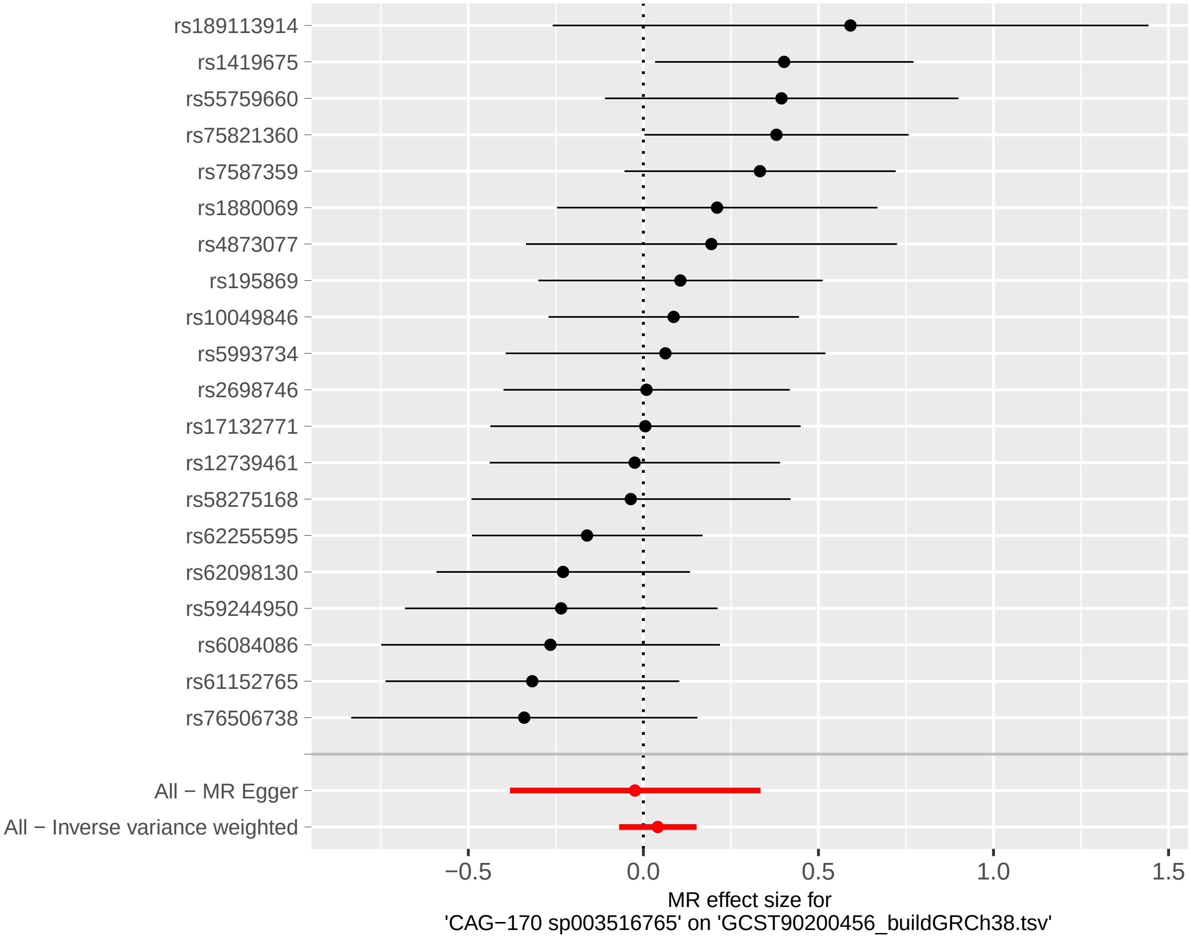

rs189113914
rs1419675
rs55759660
rs75821360
rs7587359
rs1880069
rs4873077
rs195869
rs10049846
rs5993734
rs2698746
rs17132771
rs12739461
rs58275168
rs62255595
rs62098130
rs59244950
rs6084086
rs61152765
rs76506738
All − MR Egger
All − Inverse variance weighted
−0.5
0.0
0.5
1.0
1.5
MR effect size for
'CAG−170 sp003516765' on 'GCST90200456_buildGRCh38.tsv'

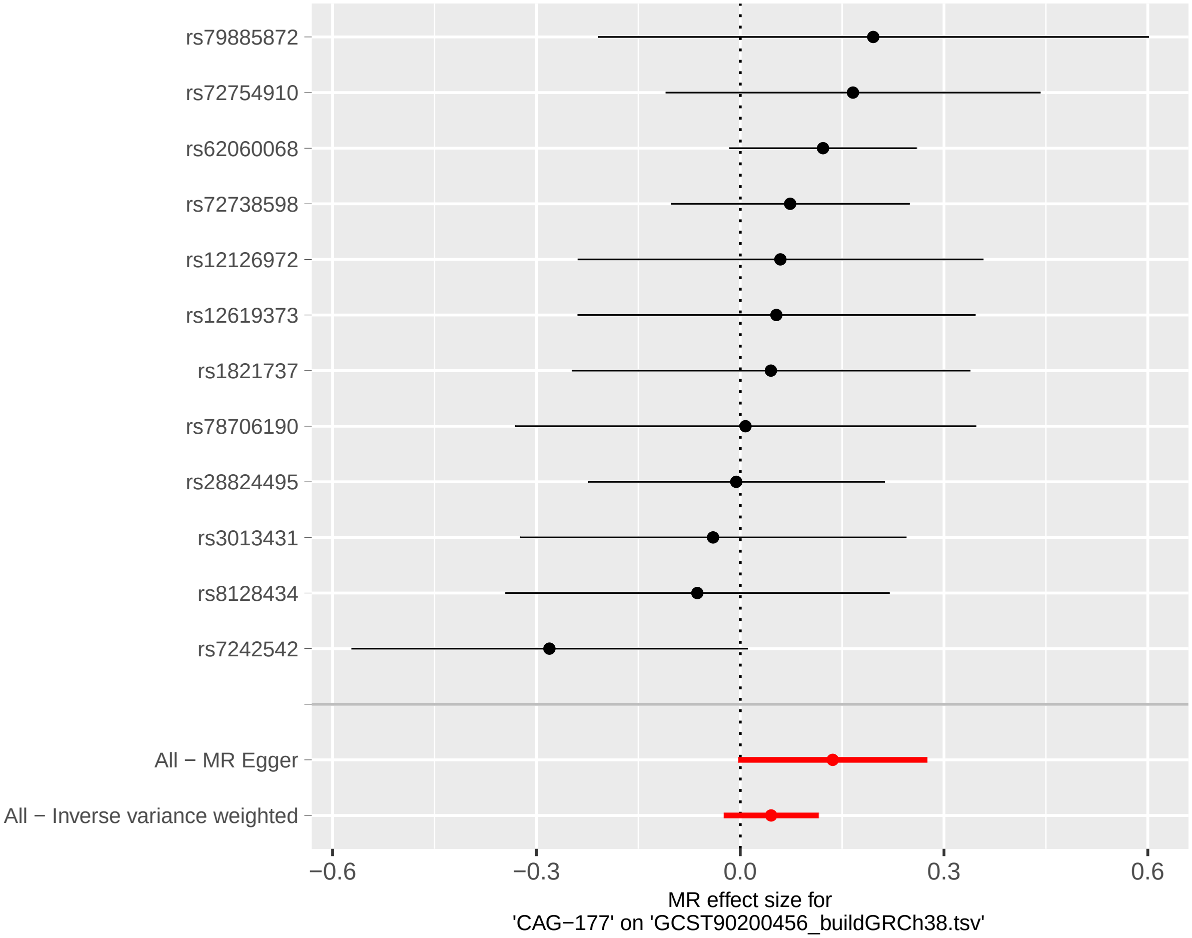

rs79885872
rs72754910
rs62060068
rs72738598
rs12126972
rs12619373
rs1821737
rs78706190
rs28824495
rs3013431
rs8128434
rs7242542
All − MR Egger
All − Inverse variance weighted
−0.6
−0.3
0.0
0.3
0.6
MR effect size for
'CAG−177' on 'GCST90200456_buildGRCh38.tsv'

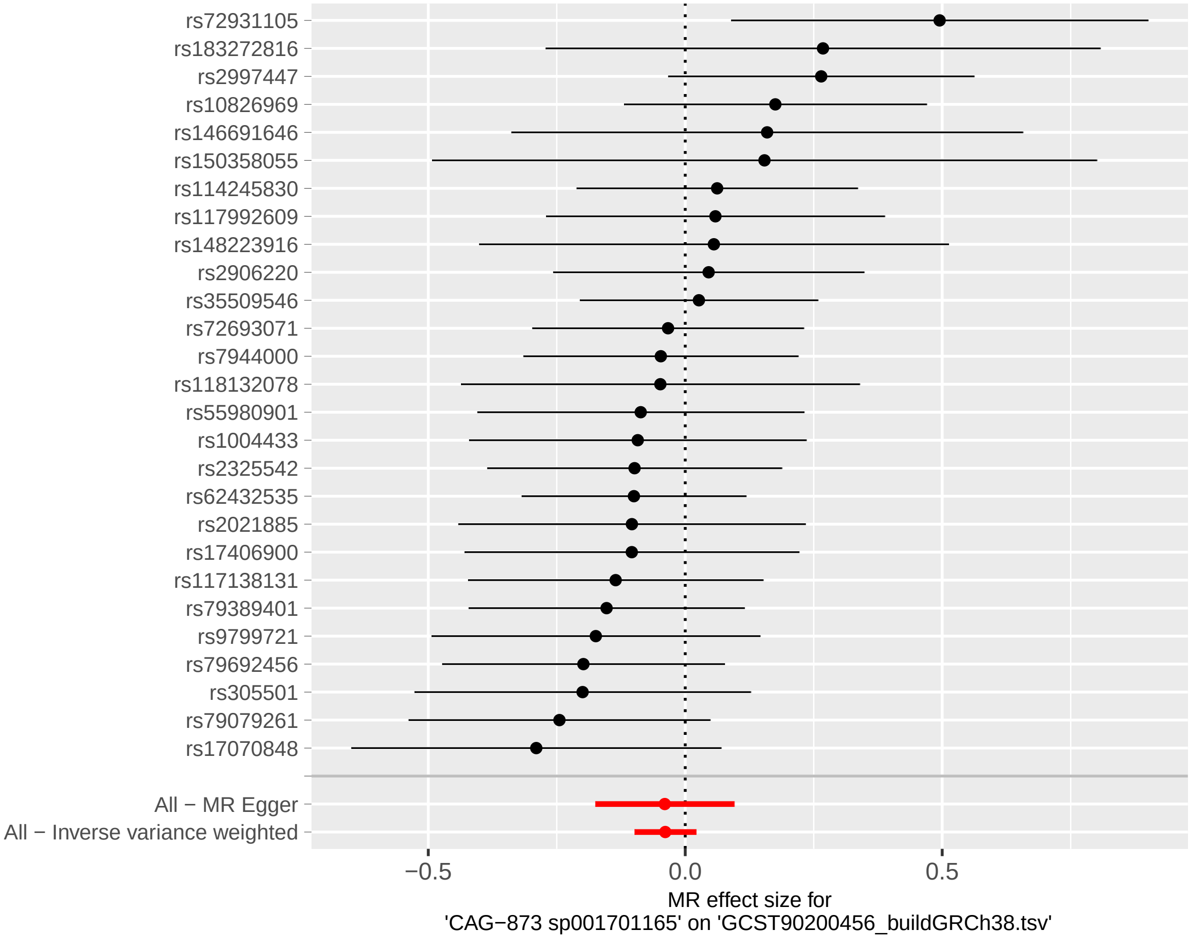
rs72931105
rs183272816
rs2997447
rs10826969
rs146691646
rs150358055
rs114245830
rs117992609
rs148223916
rs2906220
rs35509546
rs72693071
rs7944000
rs118132078
rs55980901
rs1004433
rs2325542
rs62432535
rs2021885
rs17406900
rs117138131
rs79389401
rs9799721
rs79692456
rs305501
rs79079261
rs17070848
All − MR Egger
All − Inverse variance weighted
−0.5
0.0
0.5
MR effect size for
'CAG−873 sp001701165' on 'GCST90200456_buildGRCh38.tsv'

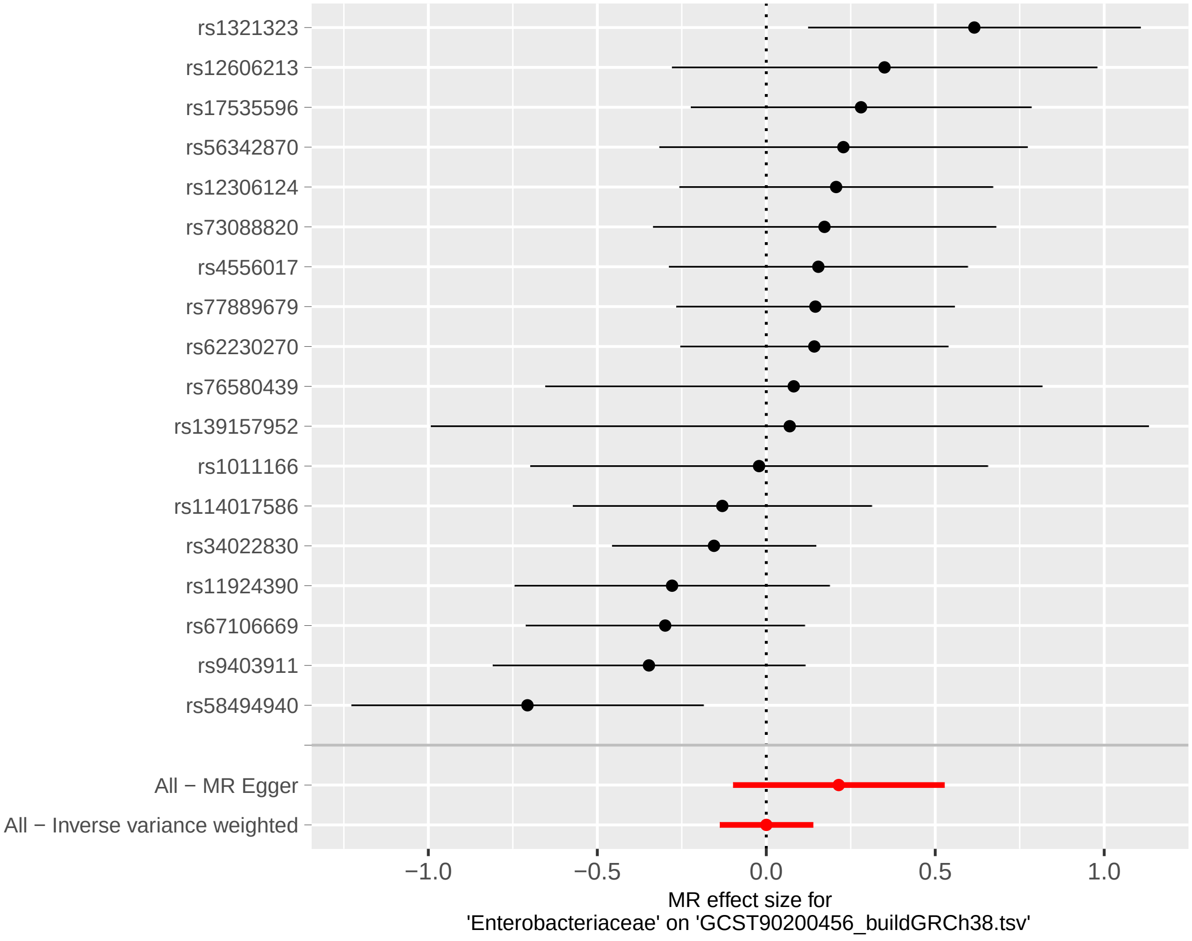
rs1321323
rs12606213
rs17535596
rs56342870
rs12306124
rs73088820
rs4556017
rs77889679
rs62230270
rs76580439
rs139157952
rs1011166
rs114017586
rs34022830
rs11924390
rs67106669
rs9403911
rs58494940
All − MR Egger
All − Inverse variance weighted
−1.0
−0.5
0.0
0.5
1.0
MR effect size for
'Enterobacteriaceae' on 'GCST90200456_buildGRCh38.tsv'

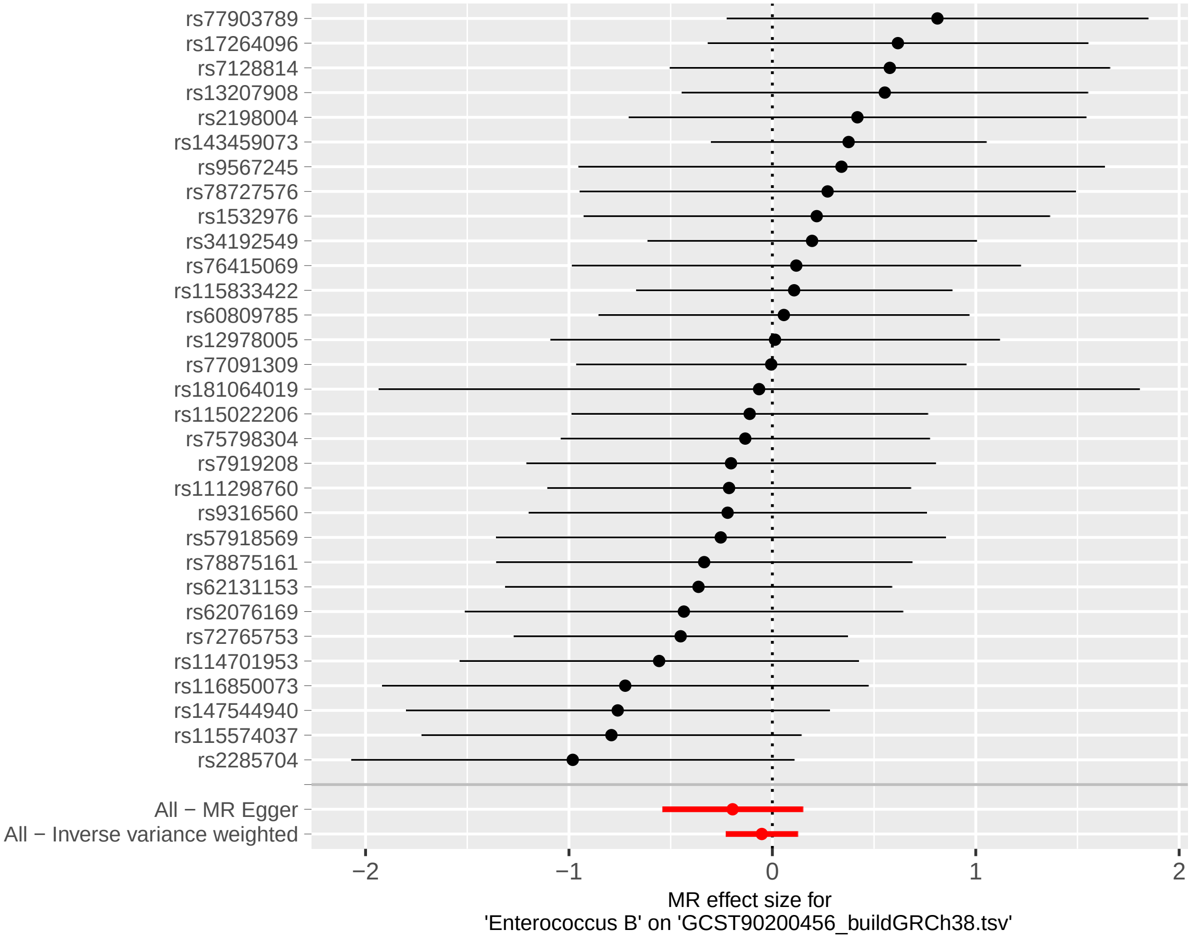
rs77903789
rs17264096
rs7128814
rs13207908
rs2198004
rs143459073
rs9567245
rs78727576
rs1532976
rs34192549
rs76415069
rs115833422
rs60809785
rs12978005
rs77091309
rs181064019
rs115022206
rs75798304
rs7919208
rs111298760
rs9316560
rs57918569
rs78875161
rs62131153
rs62076169
rs72765753
rs114701953
rs116850073
rs147544940
rs115574037
rs2285704
All − MR Egger
All − Inverse variance weighted
−2
−1
0
1
2
MR effect size for
'Enterococcus B' on 'GCST90200456_buildGRCh38.tsv'

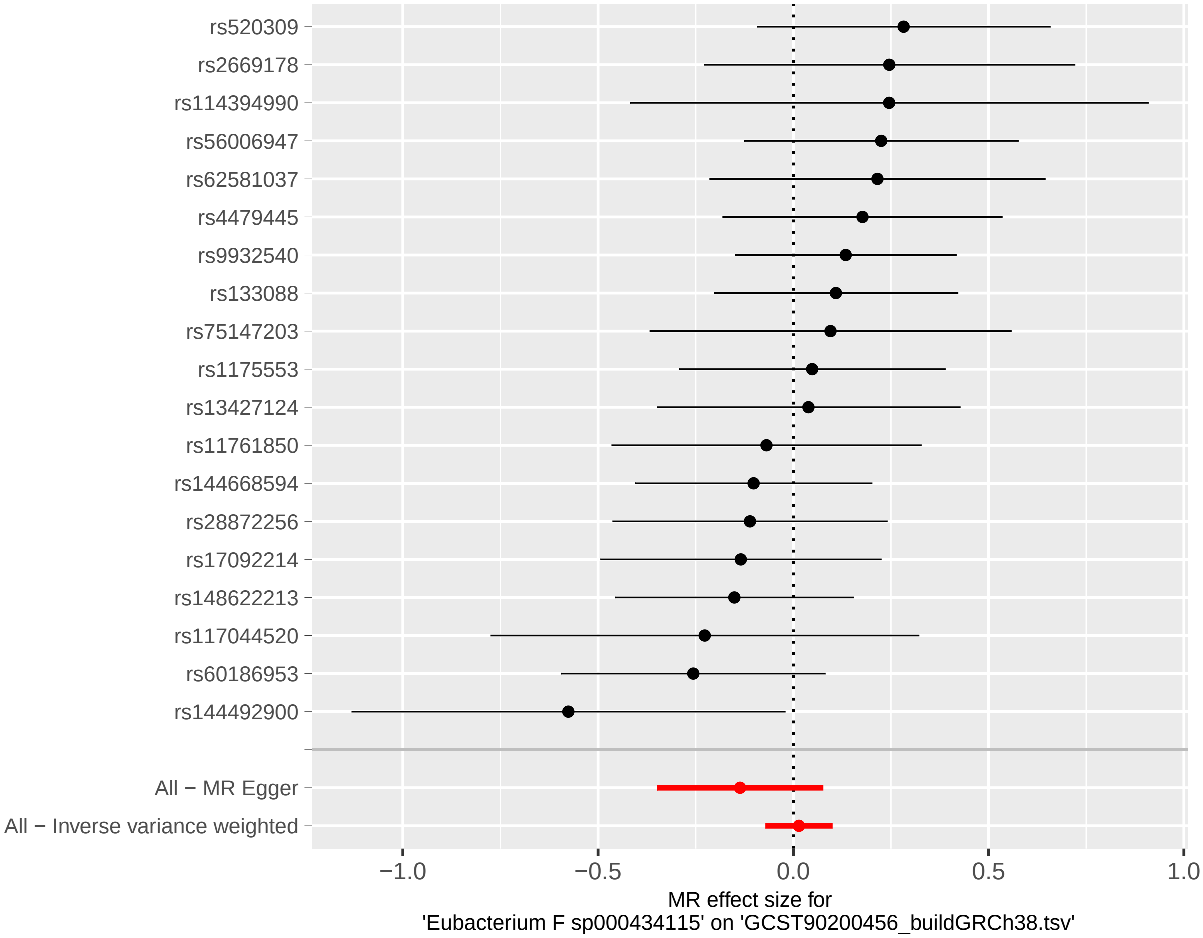
rs520309
rs2669178
rs114394990
rs56006947
rs62581037
rs4479445
rs9932540
rs133088
rs75147203
rs1175553
rs13427124
rs11761850
rs144668594
rs28872256
rs17092214
rs148622213
rs117044520
rs60186953
rs144492900
All − MR Egger
All − Inverse variance weighted
−1.0
−0.5
0.0
0.5
1.0
MR effect size for
'Eubacterium F sp000434115' on 'GCST90200456_buildGRCh38.tsv'

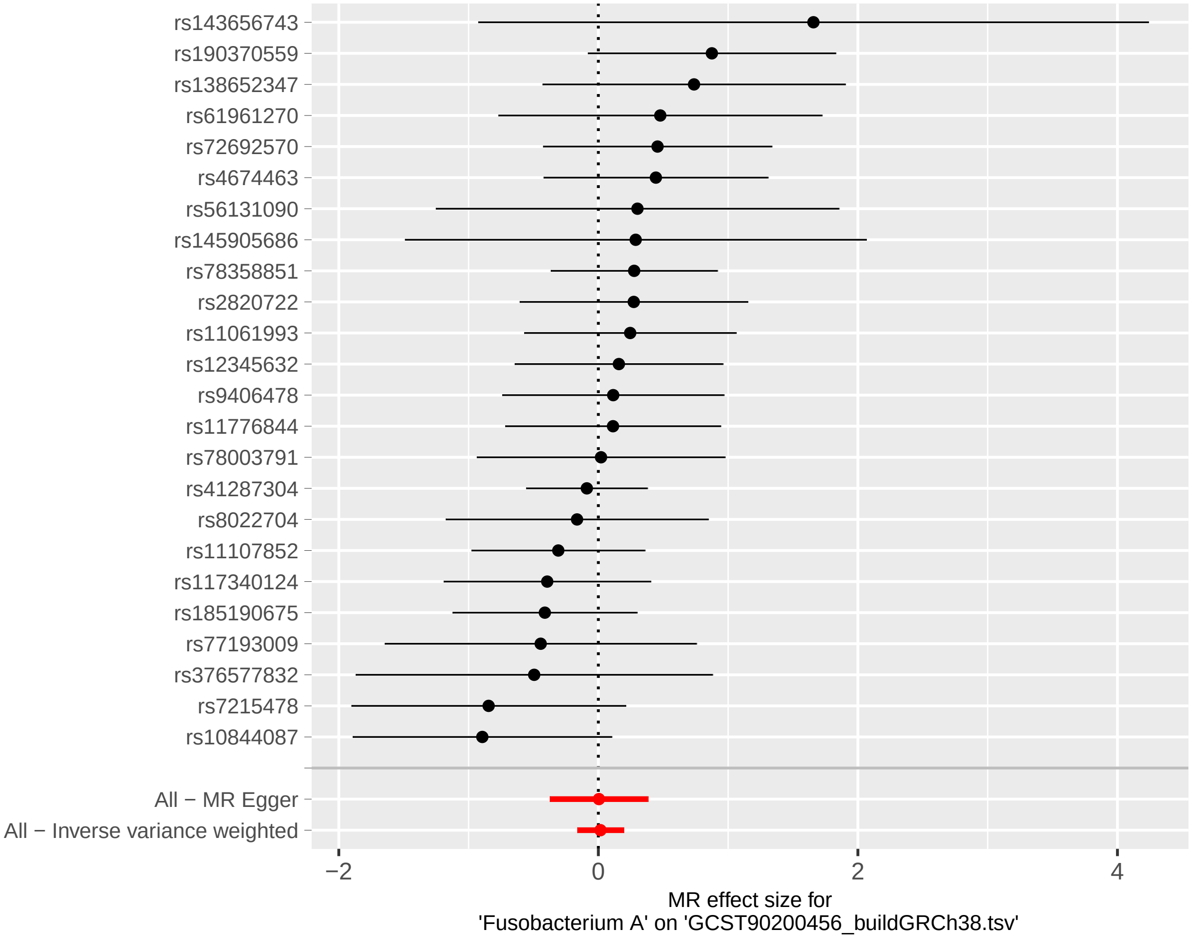

rs143656743
rs190370559
rs138652347
rs61961270
rs72692570
rs4674463
rs56131090
rs145905686
rs78358851
rs2820722
rs11061993
rs12345632
rs9406478
rs11776844
rs78003791
rs41287304
rs8022704
rs11107852
rs117340124
rs185190675
rs77193009
rs376577832
rs7215478
rs10844087
All − MR Egger
All − Inverse variance weighted
−2
0
2
4
MR effect size for
'Fusobacterium A' on 'GCST90200456_buildGRCh38.tsv'

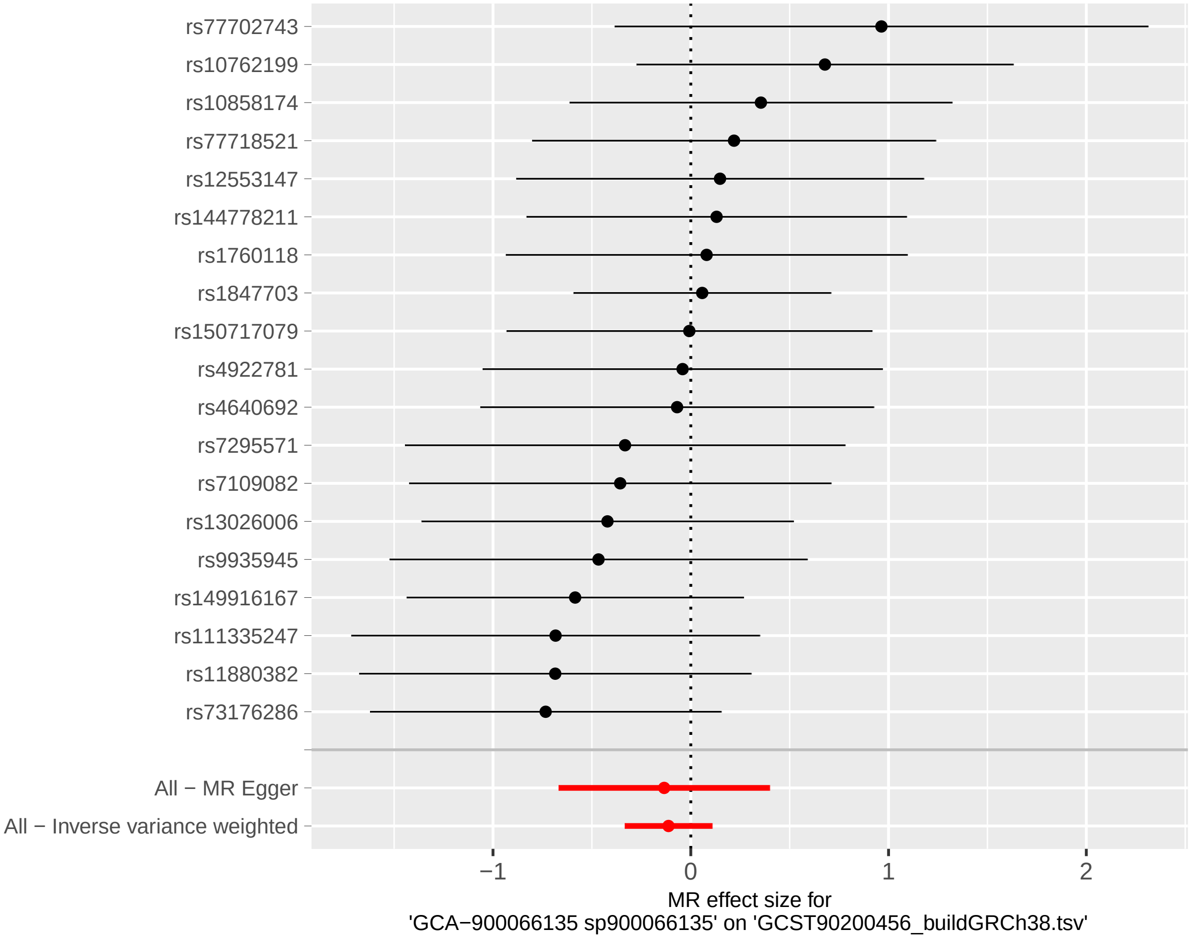
rs77702743
rs10762199
rs10858174
rs77718521
rs12553147
rs144778211
rs1760118
rs1847703
rs150717079
rs4922781
rs4640692
rs7295571
rs7109082
rs13026006
rs9935945
rs149916167
rs111335247
rs11880382
rs73176286
All − MR Egger
All − Inverse variance weighted
−1
0
1
2
MR effect size for
'GCA−900066135 sp900066135' on 'GCST90200456_buildGRCh38.tsv'

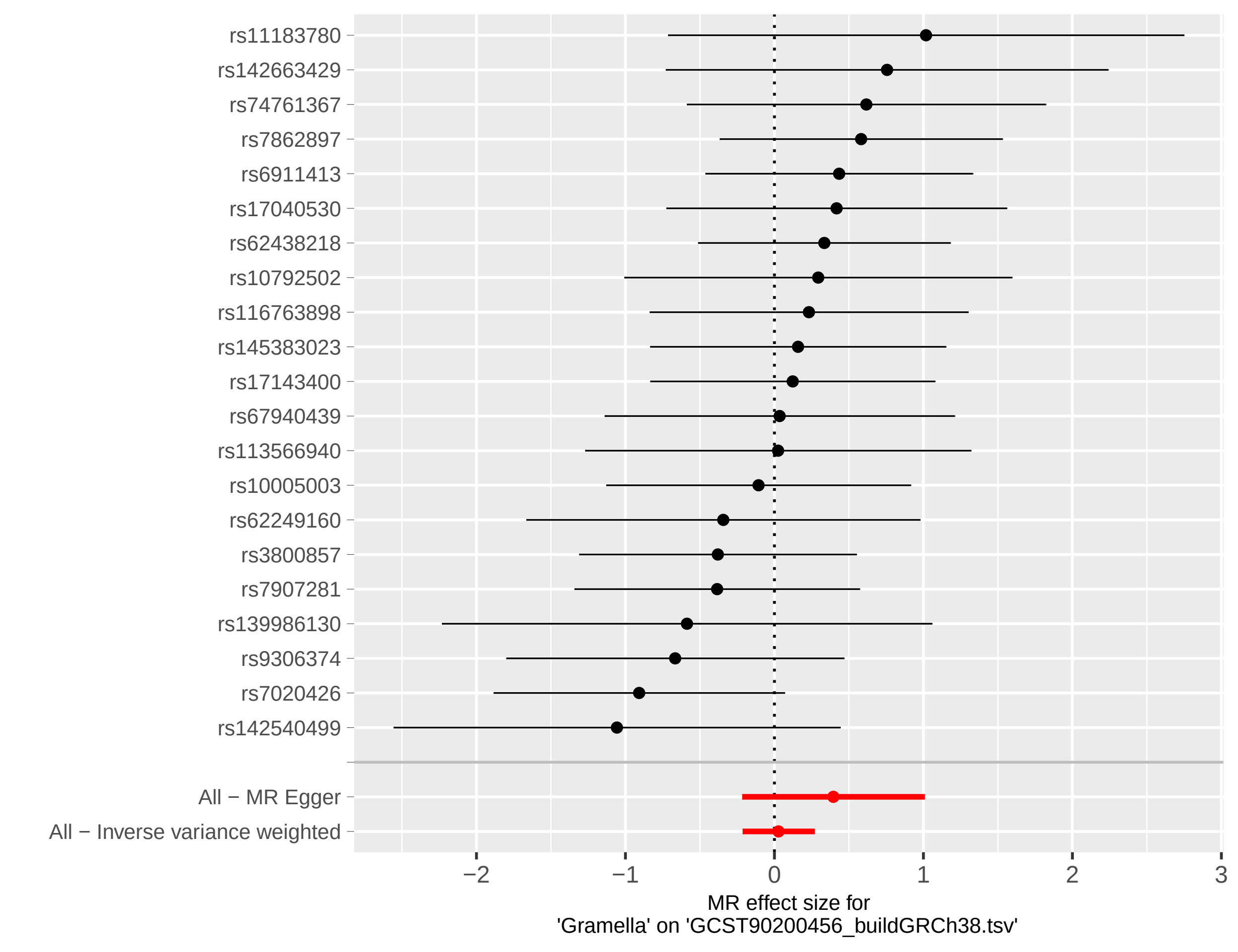
rs11183780
rs142663429
rs74761367
rs7862897
rs6911413
rs17040530
rs62438218
rs10792502
rs116763898
rs145383023
rs17143400
rs67940439
rs113566940
rs10005003
rs62249160
rs3800857
rs7907281
rs139986130
rs9306374
rs7020426
rs142540499
All − MR Egger
All − Inverse variance weighted
−2
−1
0
1
2
3
MR effect size for
'Gramella' on 'GCST90200456_buildGRCh38.tsv'

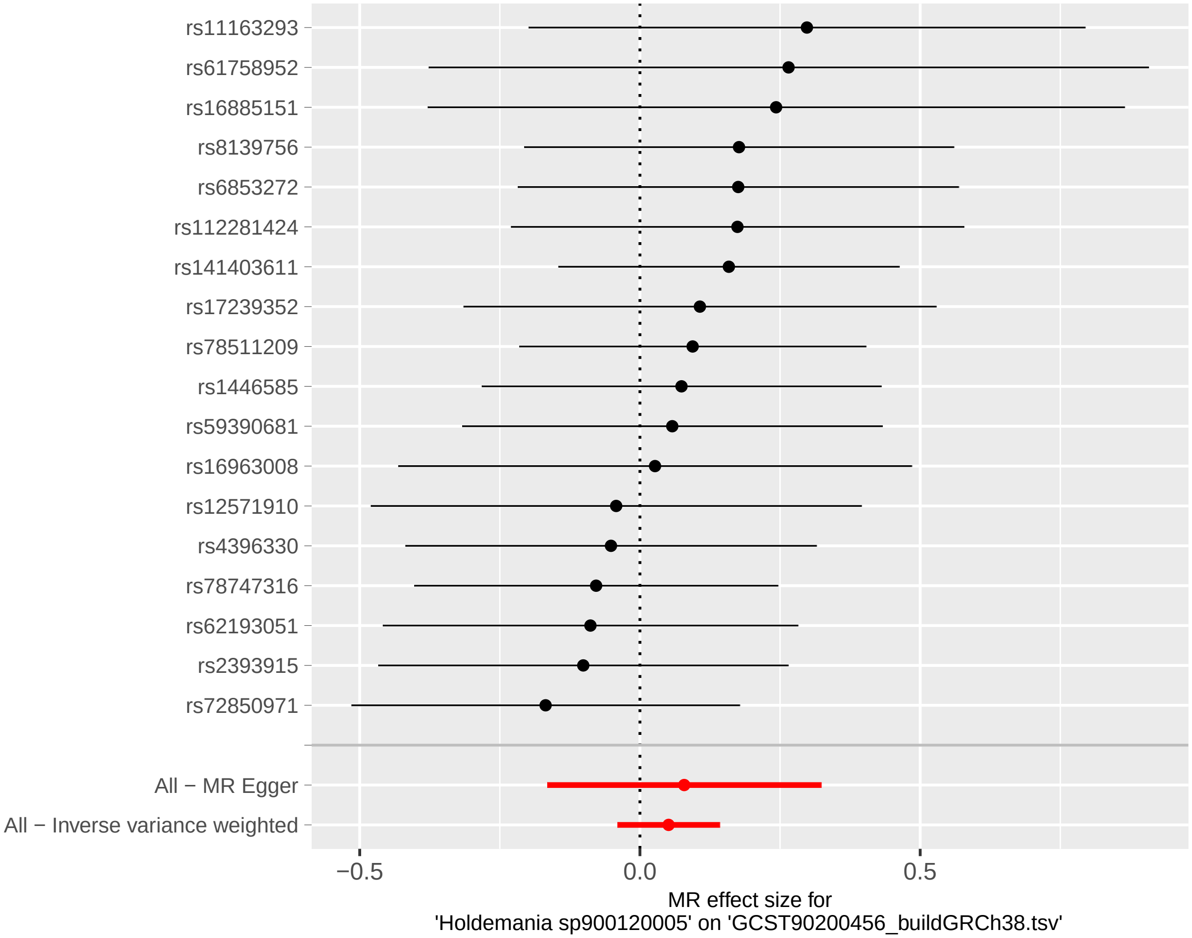
rs11163293
rs61758952
rs16885151
rs8139756
rs6853272
rs112281424
rs141403611
rs17239352
rs78511209
rs1446585
rs59390681
rs16963008
rs12571910
rs4396330
rs78747316
rs62193051
rs2393915
rs72850971
All − MR Egger
All − Inverse variance weighted
−0.5
0.0
0.5
MR effect size for
'Holdemania sp900120005' on 'GCST90200456_buildGRCh38.tsv'

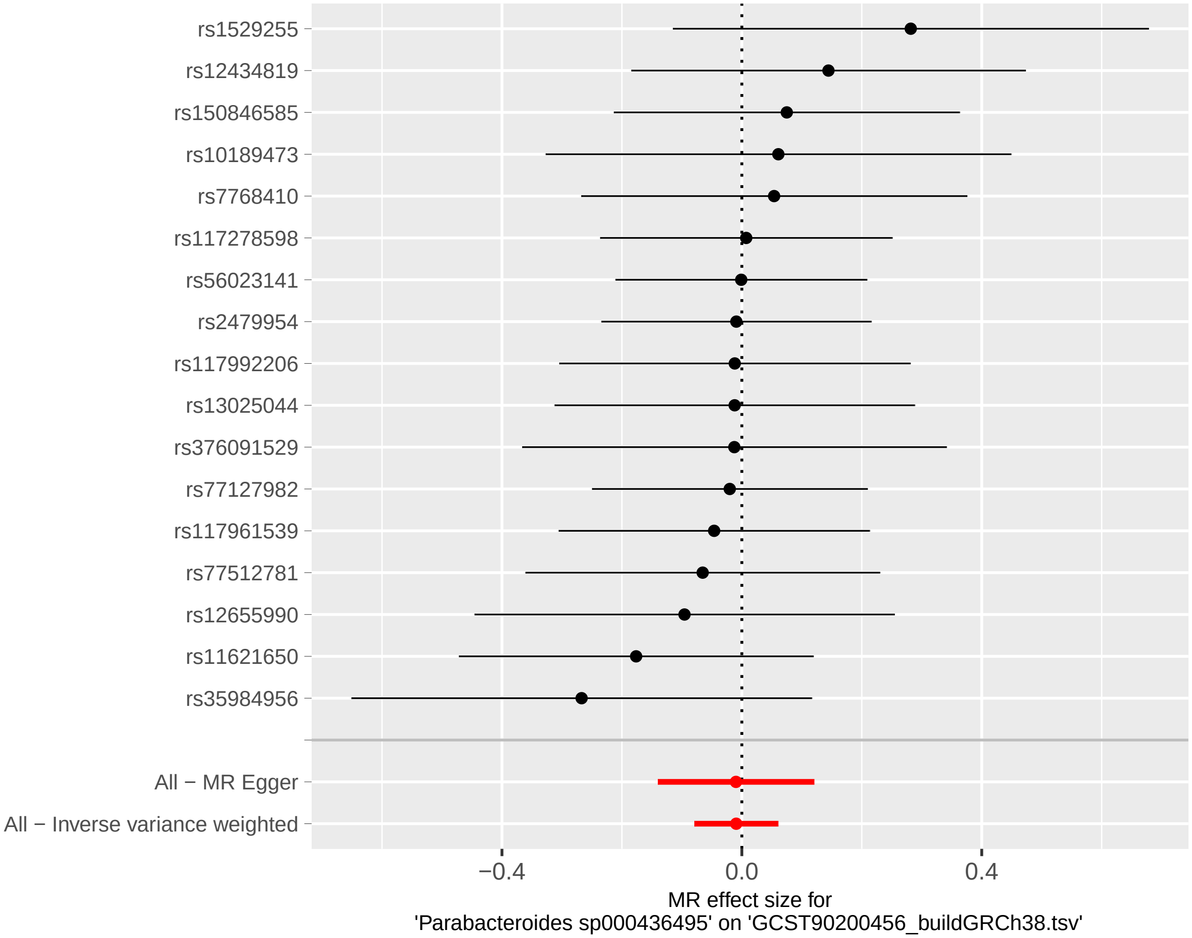
rs1529255
rs12434819
rs150846585
rs10189473
rs7768410
rs117278598
rs56023141
rs2479954
rs117992206
rs13025044
rs376091529
rs77127982
rs117961539
rs77512781
rs12655990
rs11621650
rs35984956
All − MR Egger
All − Inverse variance weighted
−0.4
0.0
0.4
MR effect size for
'Parabacteroides sp000436495' on 'GCST90200456_buildGRCh38.tsv'

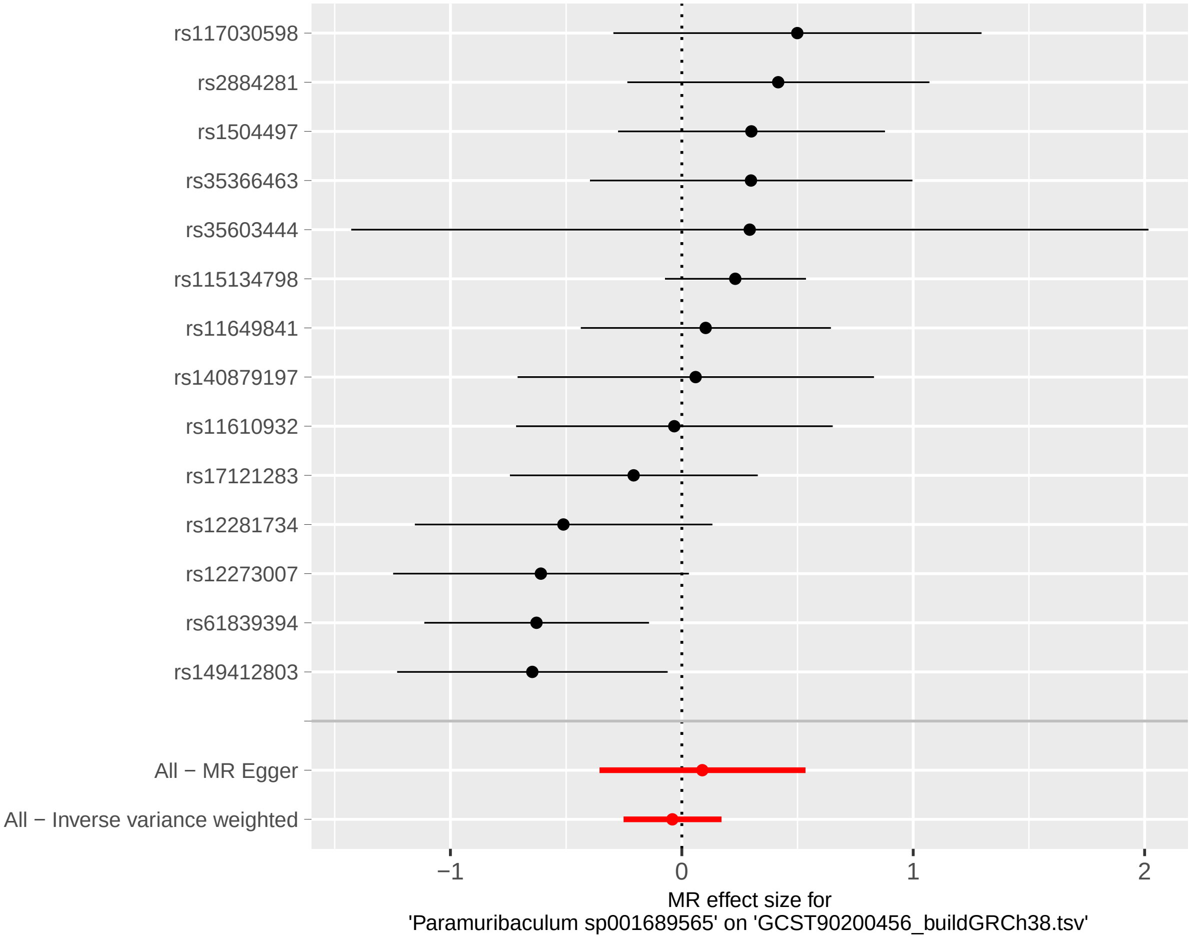

rs117030598
rs2884281
rs1504497
rs35366463
rs35603444
rs115134798
rs11649841
rs140879197
rs11610932
rs17121283
rs12281734
rs12273007
rs61839394
rs149412803
All − MR Egger
All − Inverse variance weighted
−1
0
1
2
MR effect size for
'Paramuribaculum sp001689565' on 'GCST90200456_buildGRCh38.tsv'

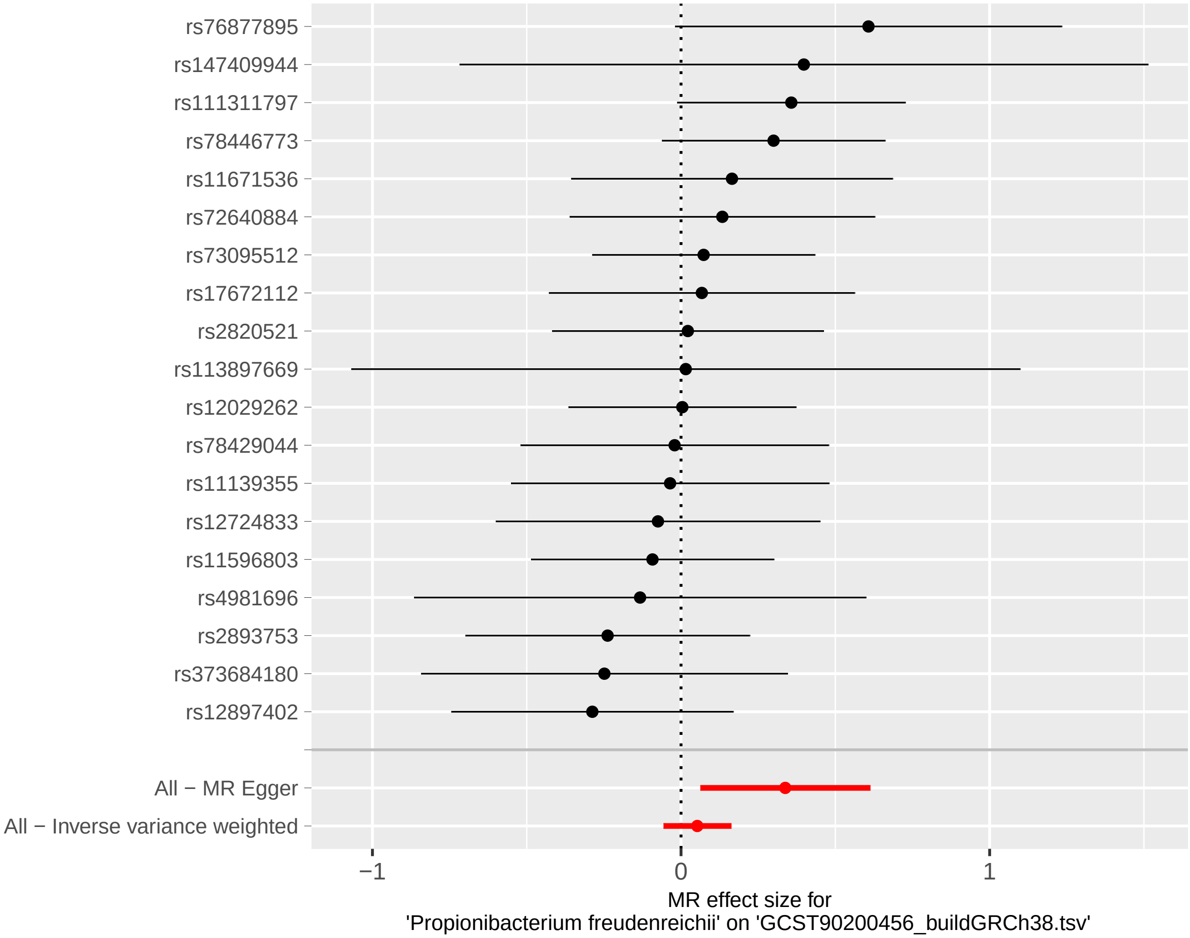

rs76877895
rs147409944
rs111311797
rs78446773
rs11671536
rs72640884
rs73095512
rs17672112
rs2820521
rs113897669
rs12029262
rs78429044
rs11139355
rs12724833
rs11596803
rs4981696
rs2893753
rs373684180
rs12897402
All − MR Egger
All − Inverse variance weighted
−1
0
1
MR effect size for
'Propionibacterium freudenreichii' on 'GCST90200456_buildGRCh38.tsv'

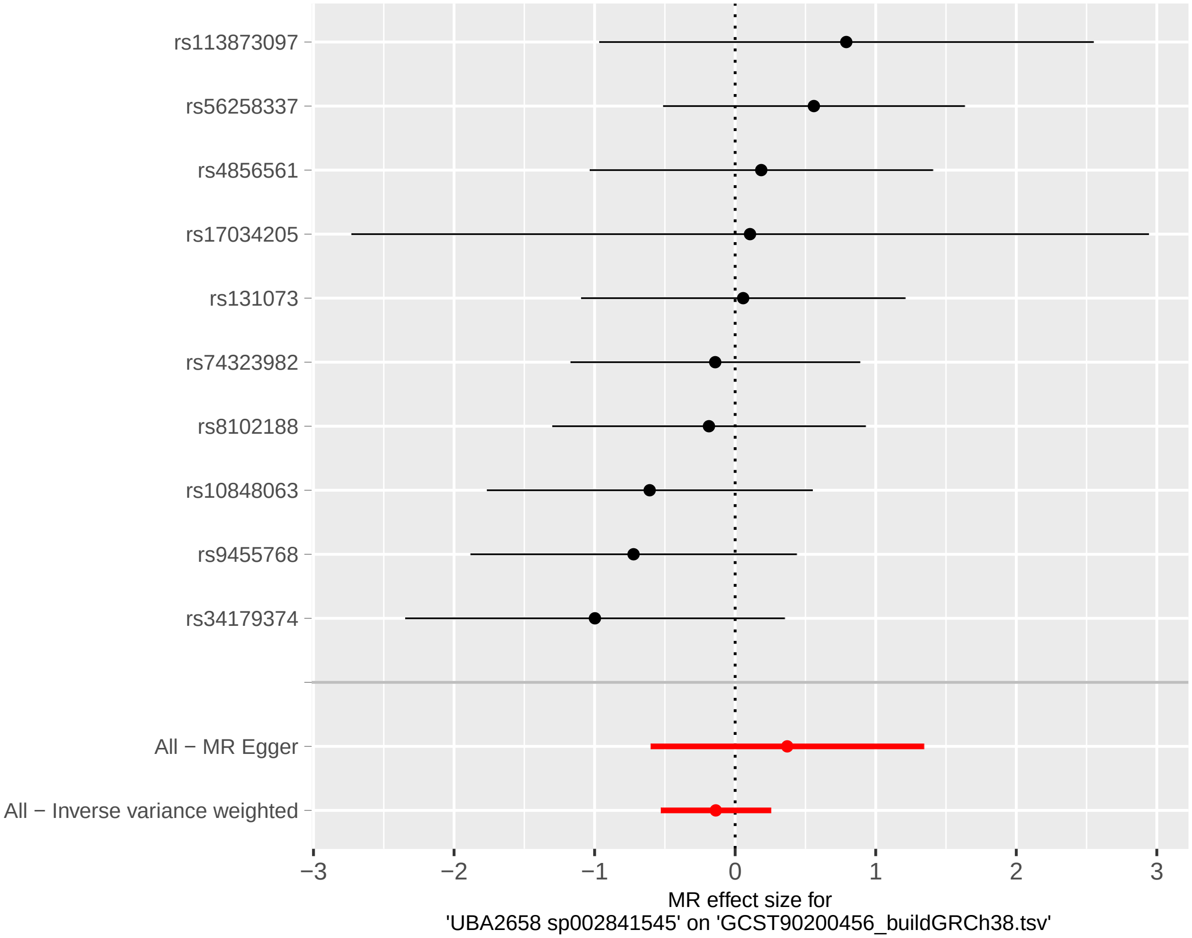
rs113873097
rs56258337
rs4856561
rs17034205
rs131073
rs74323982
rs8102188
rs10848063
rs9455768
rs34179374
All − MR Egger
All − Inverse variance weighted
−3
−2
−1
0
1
2
3
MR effect size for
'UBA2658 sp002841545' on 'GCST90200456_buildGRCh38.tsv'

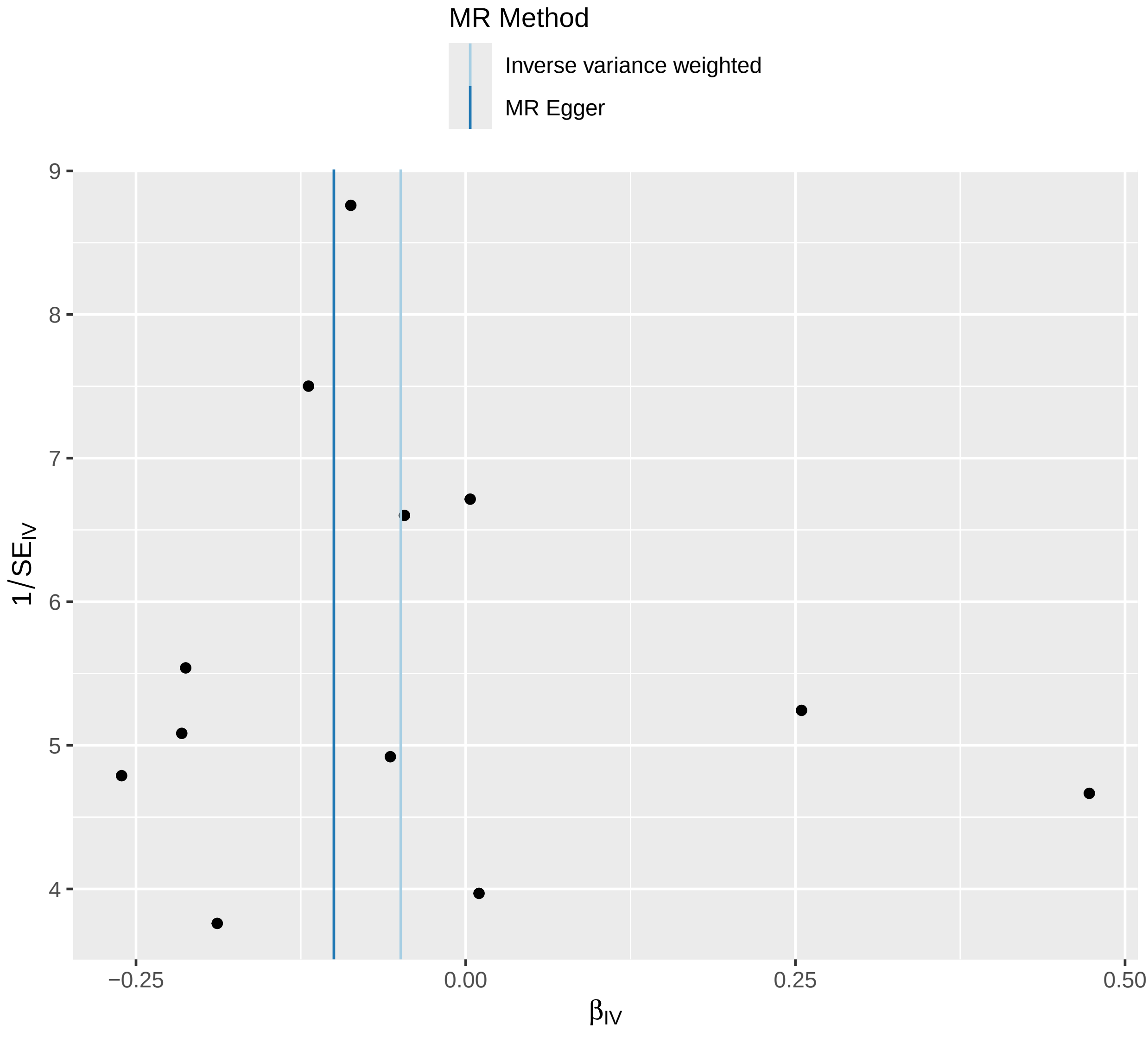

MR Method
Inverse variance weighted
MR Egger
9
8
7
6
5
4
1/SE_IV
−0.25
0.00
0.25
0.50
β_IV

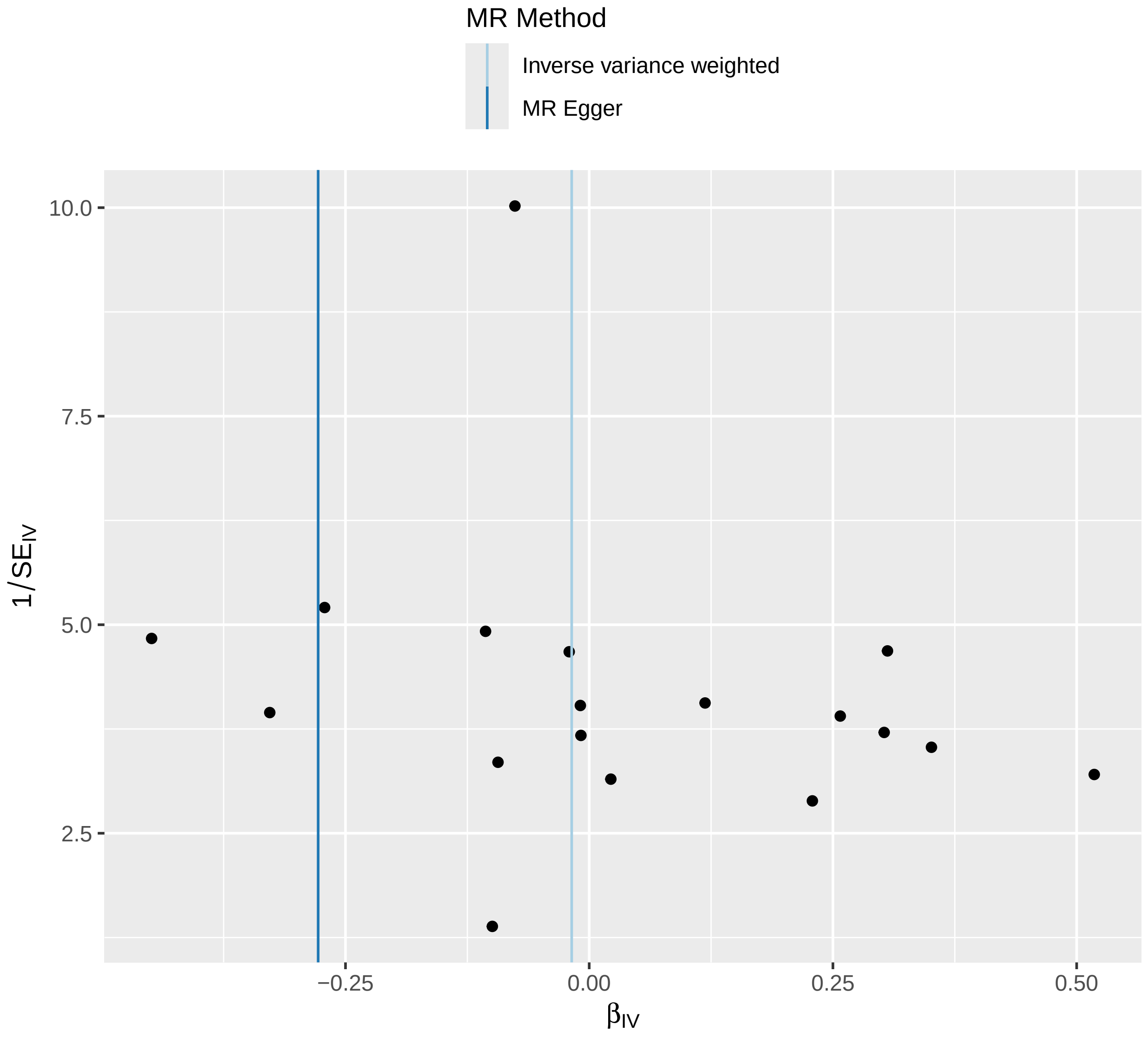
MR Method
Inverse variance weighted
MR Egger
10.0
7.5
5.0
2.5
−0.25
0.00
0.25
0.50
$1/SE_{IV}$
β_{IV}

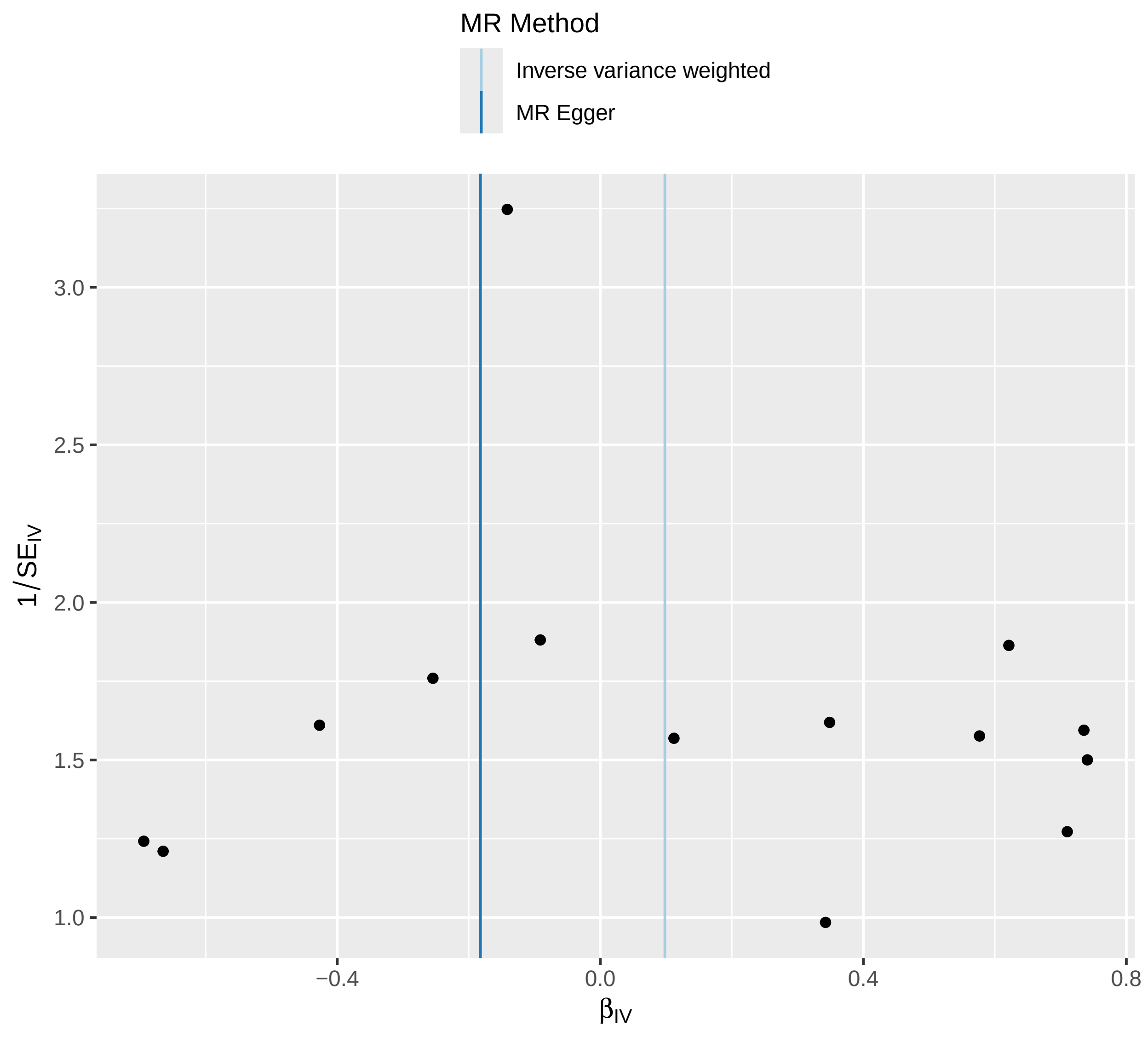
MR Method
Inverse variance weighted
MR Egger
3.0
2.5
2.0
1.5
1.0
$1/SE_{IV}$
−0.4
0.0
0.4
0.8
β_{IV}

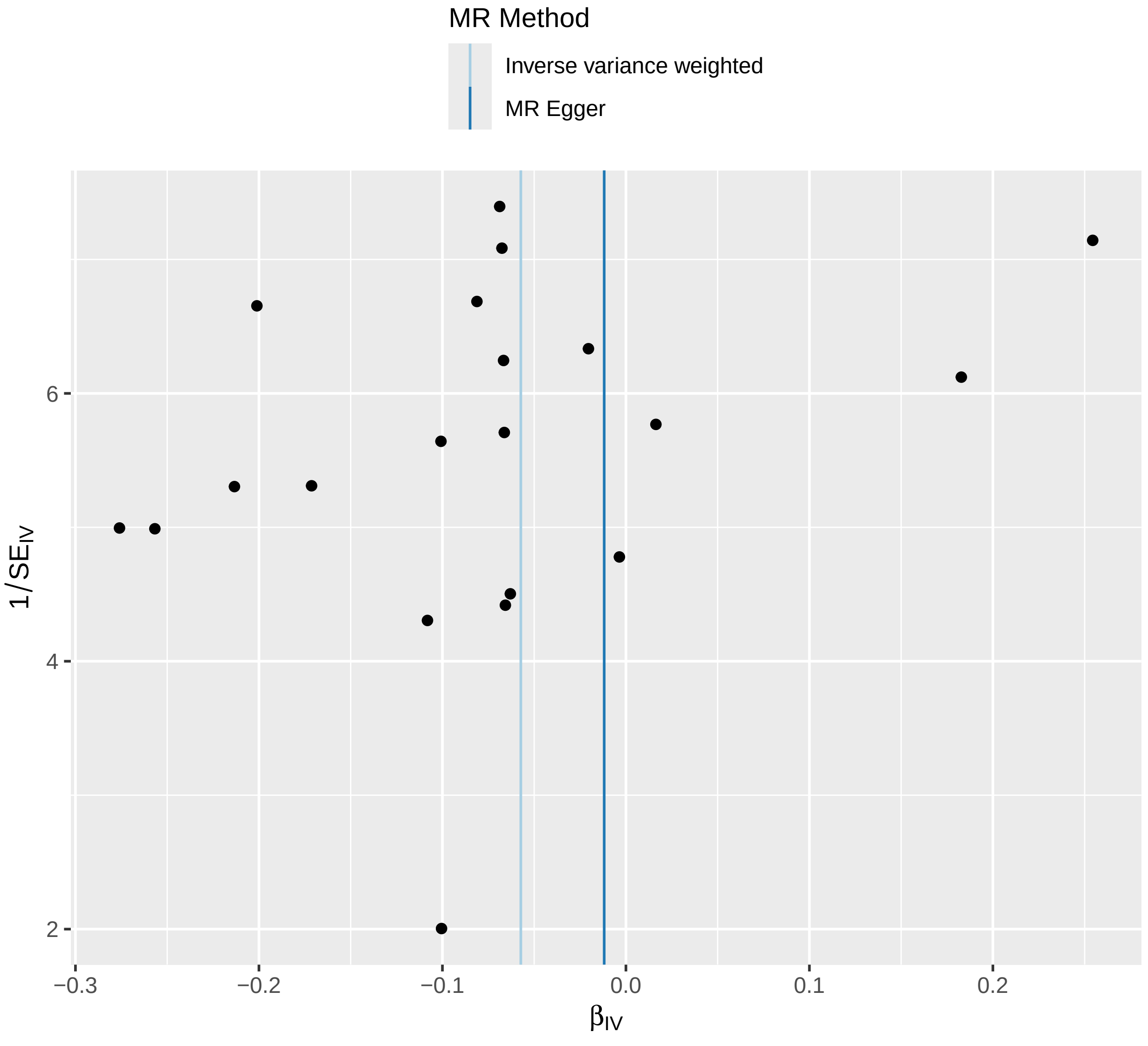

MR Method
Inverse variance weighted
MR Egger
6
4
2
−0.3
−0.2
−0.1
0.0
0.1
0.2
$1/SE_{IV}$
β_{IV}

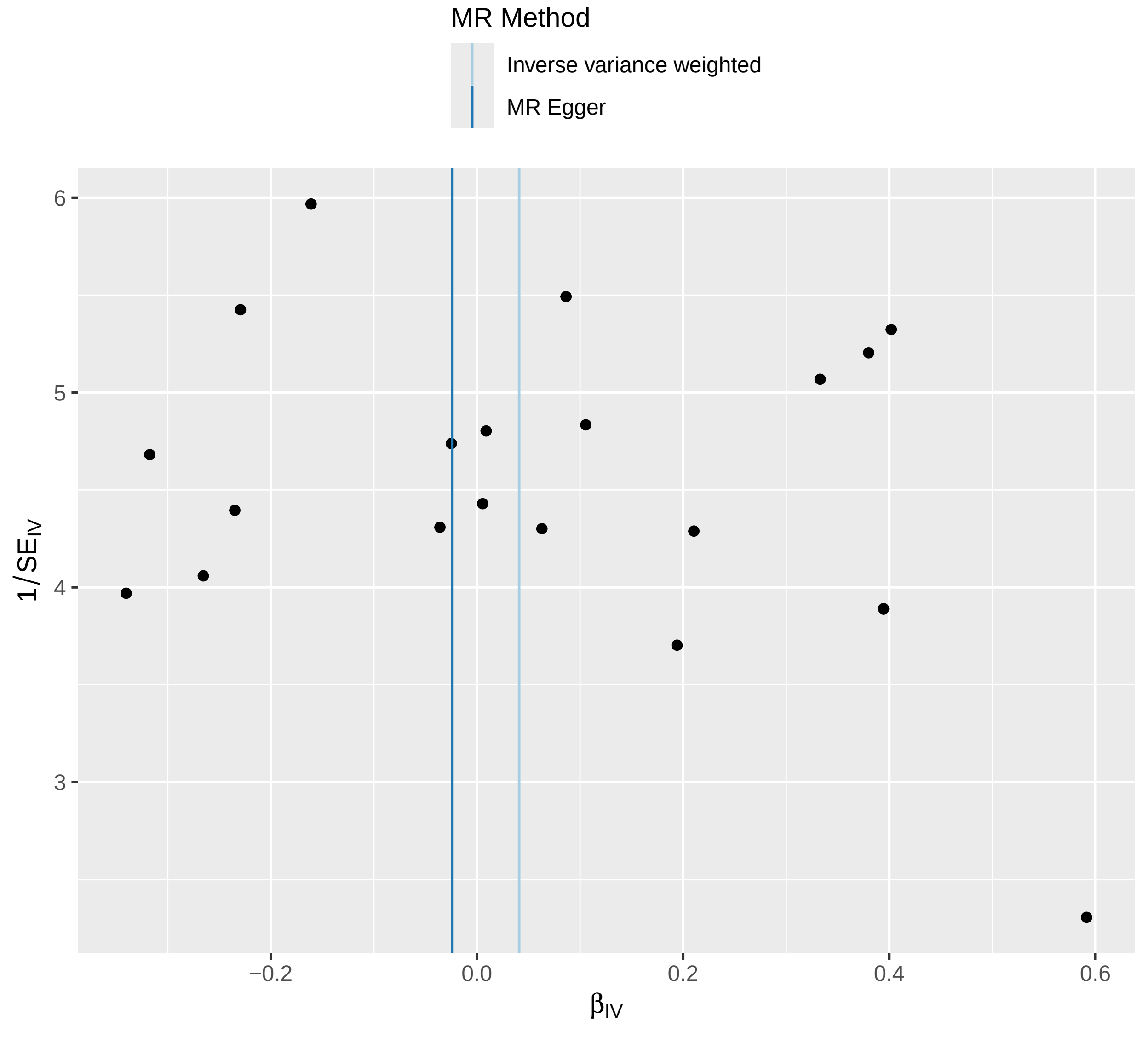
MR Method
Inverse variance weighted
MR Egger
6
5
4
3
1/SE_IV
−0.2
0.0
0.2
0.4
0.6
β_IV

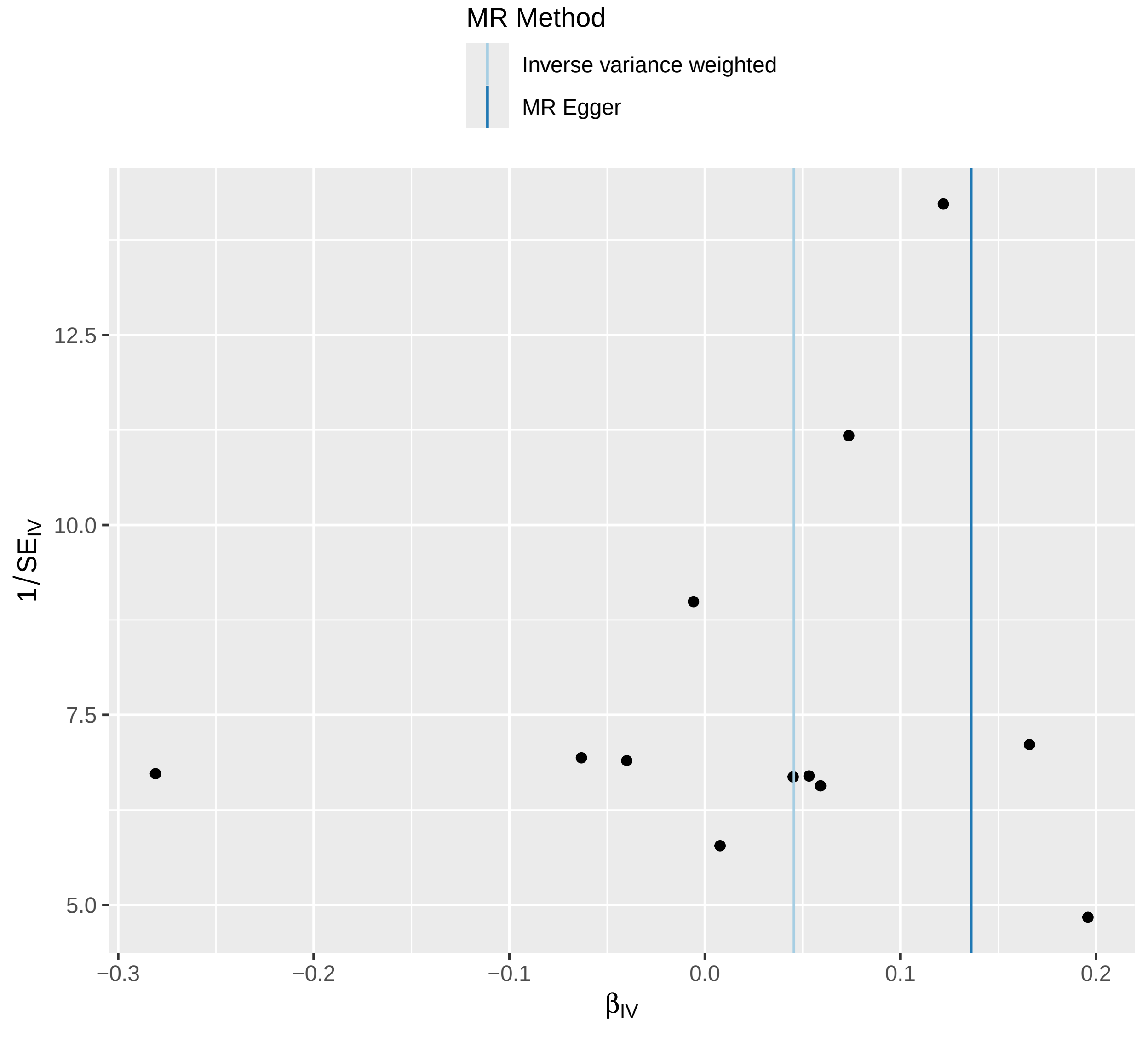
MR Method
Inverse variance weighted
MR Egger
12.5
10.0
7.5
5.0
1/SE_IV
−0.3
−0.2
−0.1
0.0
0.1
0.2
β_IV

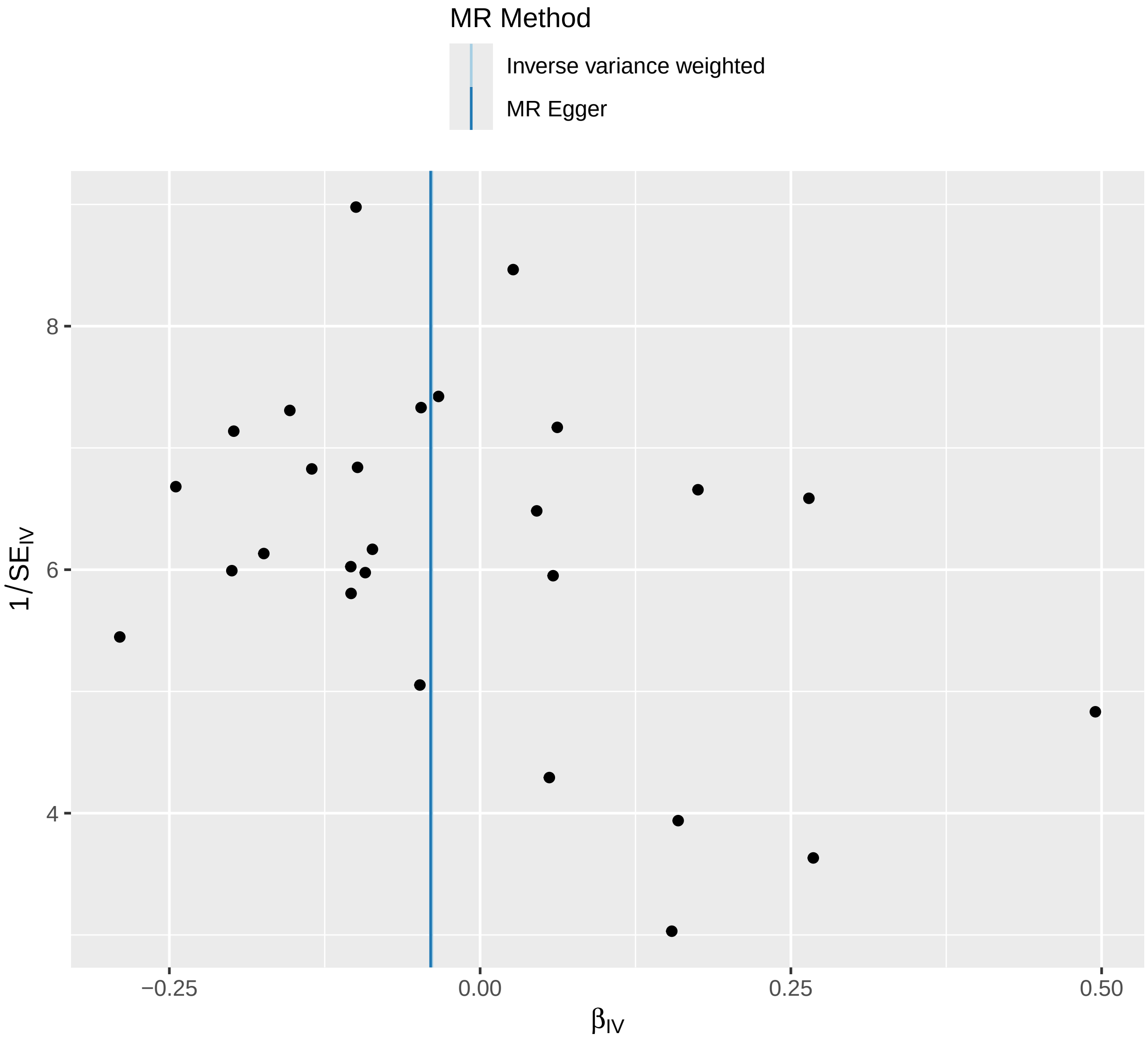
MR Method
Inverse variance weighted
MR Egger
8
6
4
1/SE_IV
−0.25
0.00
0.25
0.50
β_IV

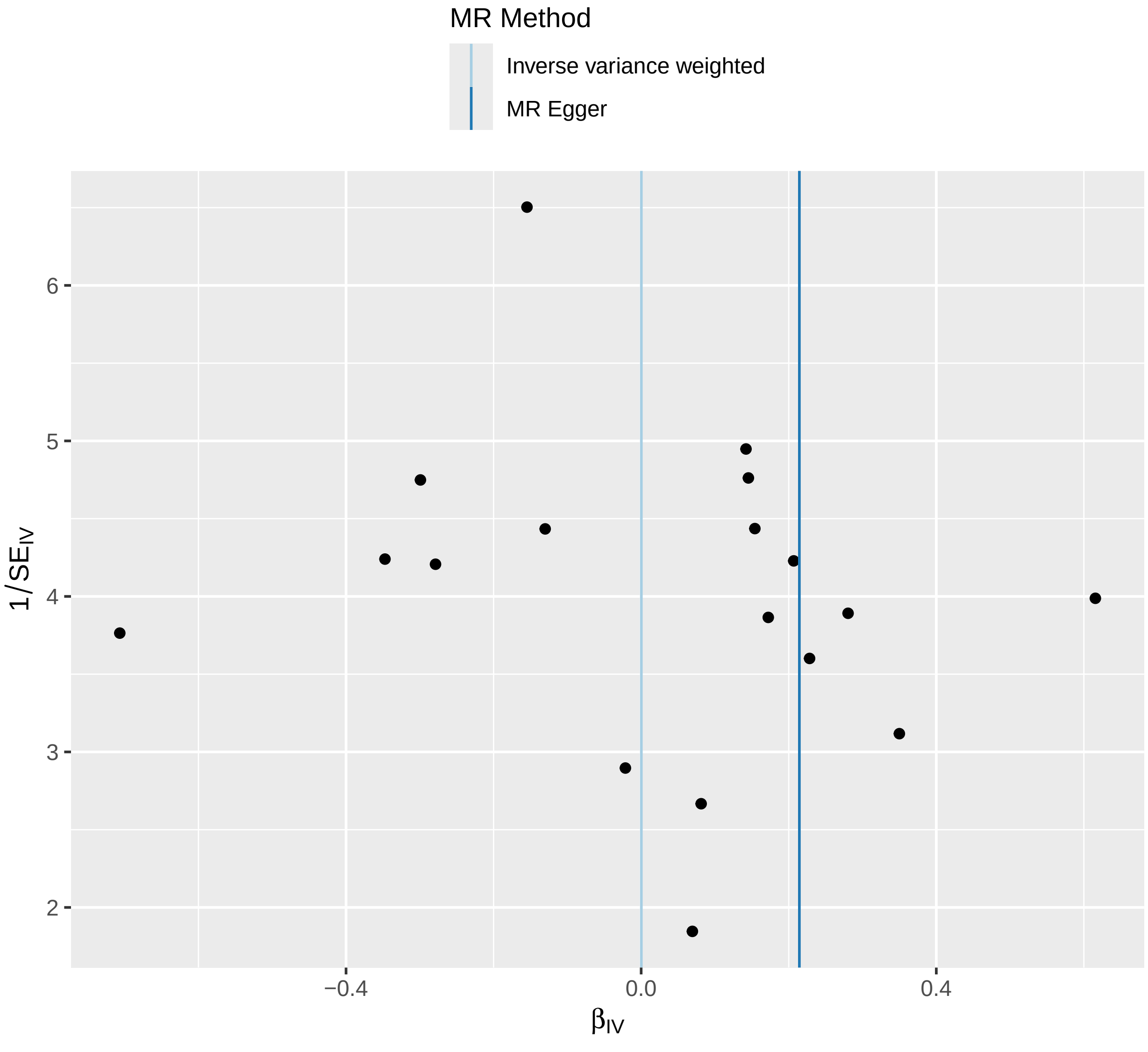

MR Method
Inverse variance weighted
MR Egger
6
5
4
3
2
1/SE_IV
−0.4
0.0
0.4
β_IV

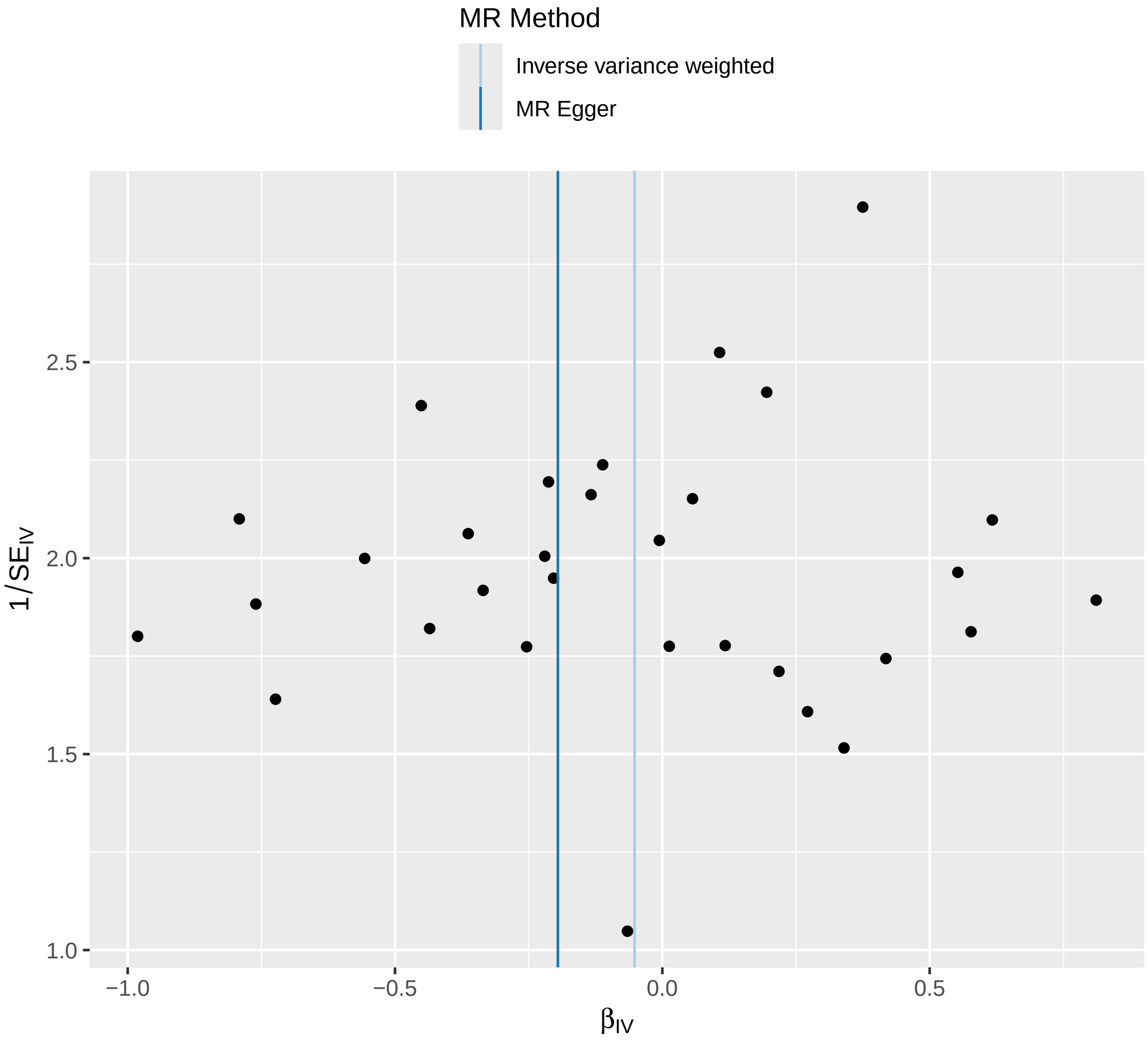
MR Method
Inverse variance weighted
MR Egger
2.5
2.0
1.5
1.0
1/SE$_{IV}$
−1.0
−0.5
0.0
0.5
β$_{IV}$

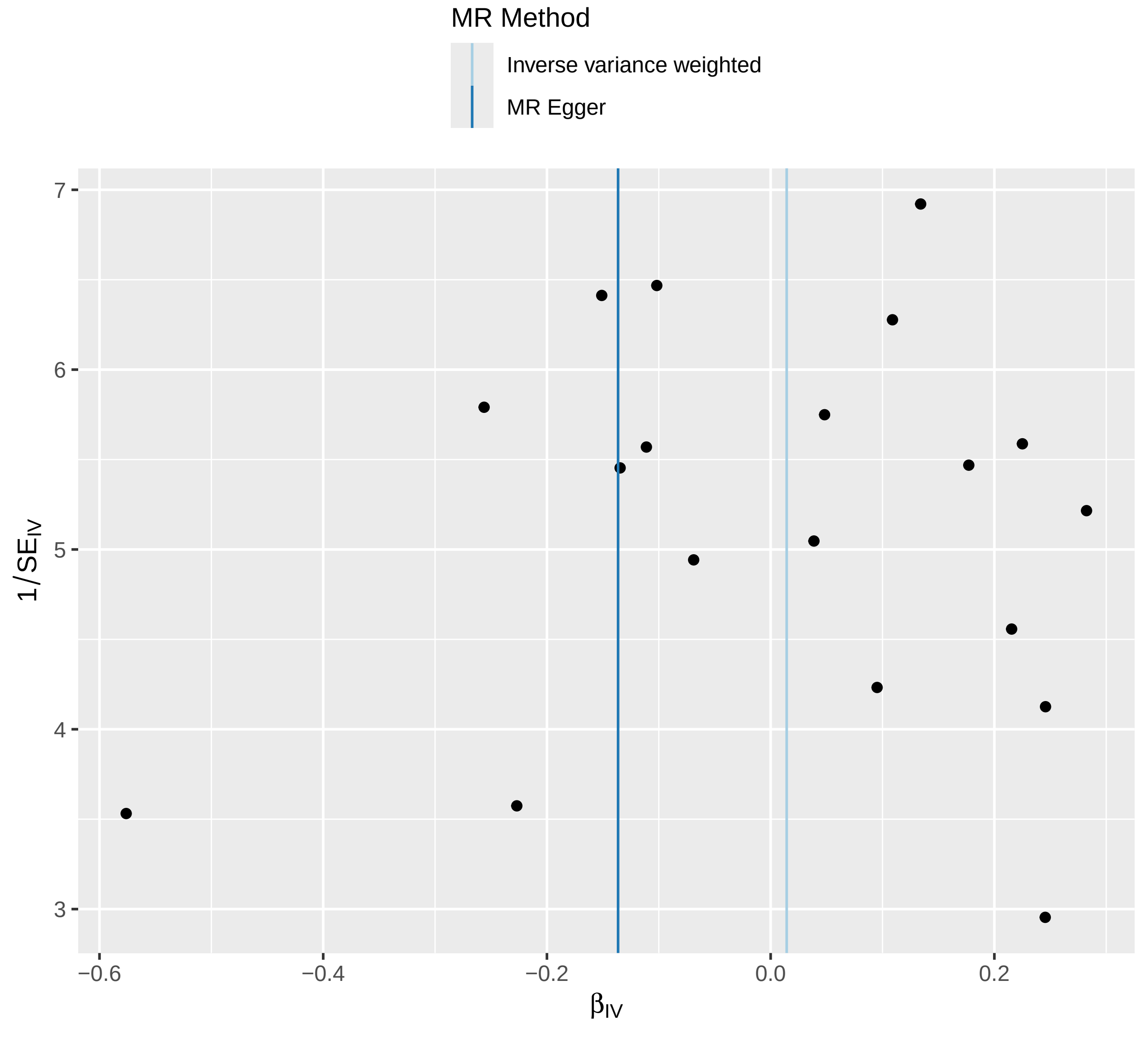

MR Method
Inverse variance weighted
MR Egger
7
6
5
4
3
$1/SE_{IV}$
−0.6
−0.4
−0.2
0.0
0.2
β_{IV}

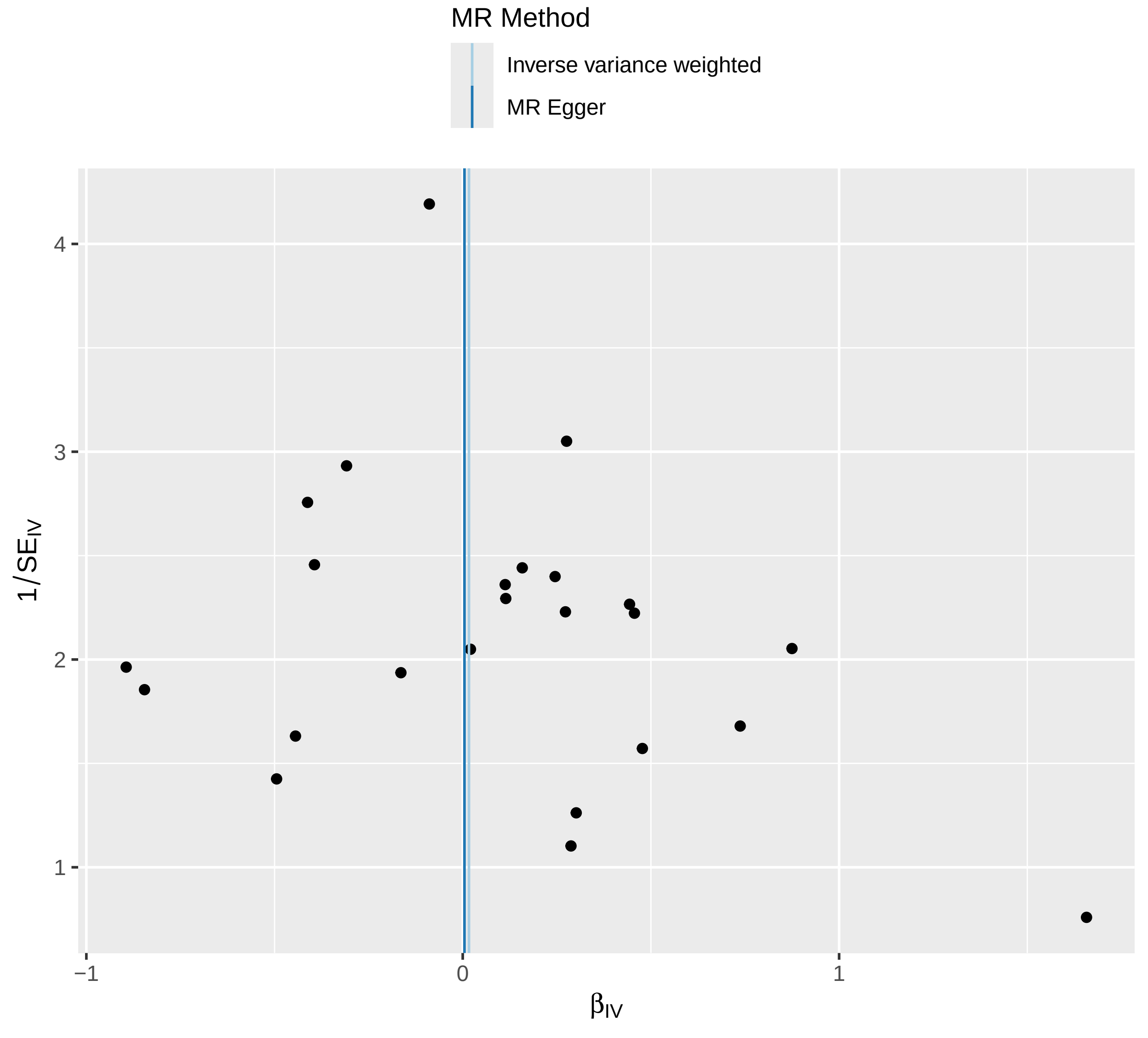
MR Method
Inverse variance weighted
MR Egger
1/SE_IV
β_IV
4
3
2
1
−1
0
1

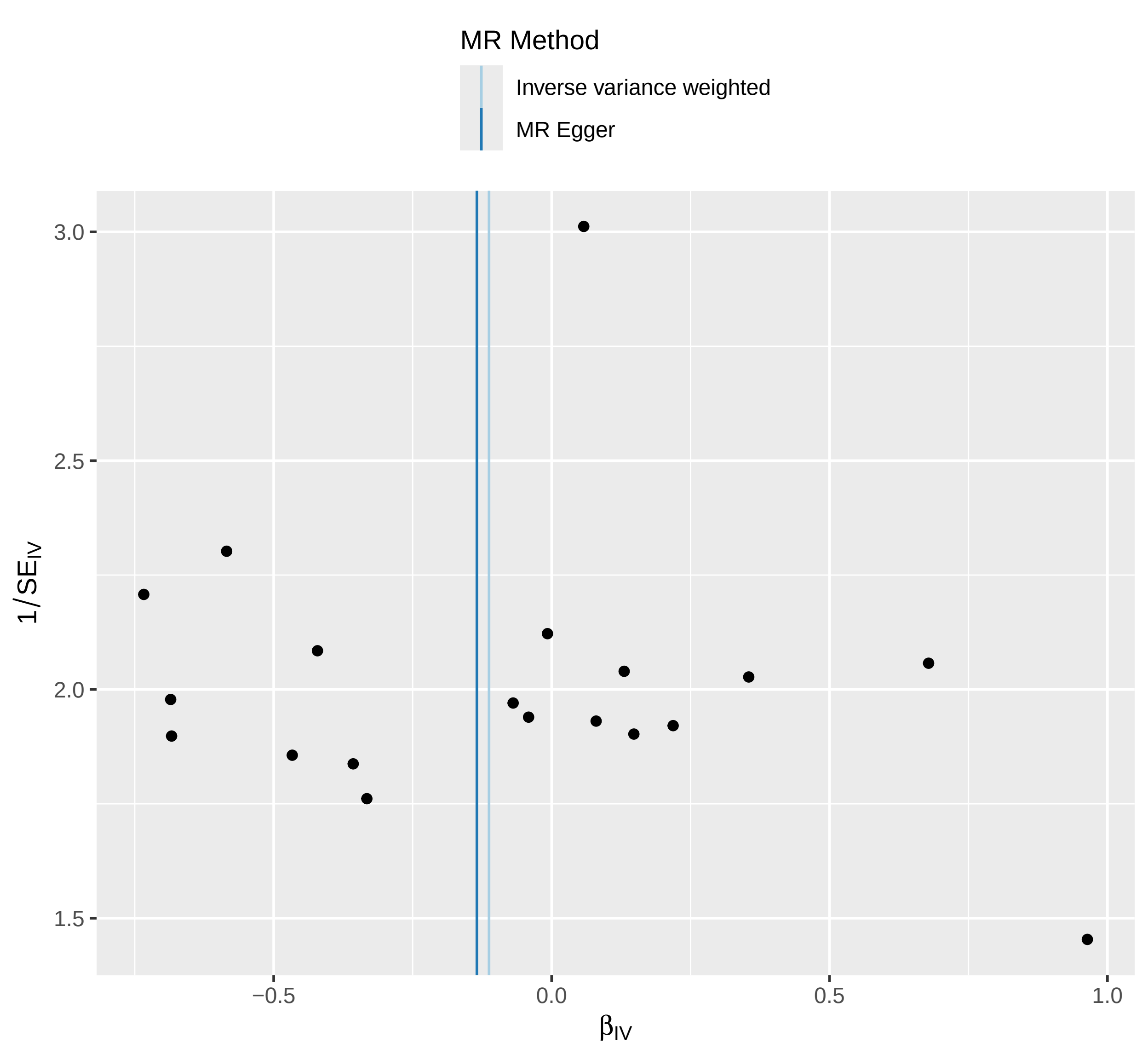
MR Method
Inverse variance weighted
MR Egger
3.0
2.5
2.0
1.5
$1/SE_{IV}$
−0.5
0.0
0.5
1.0
β_{IV}

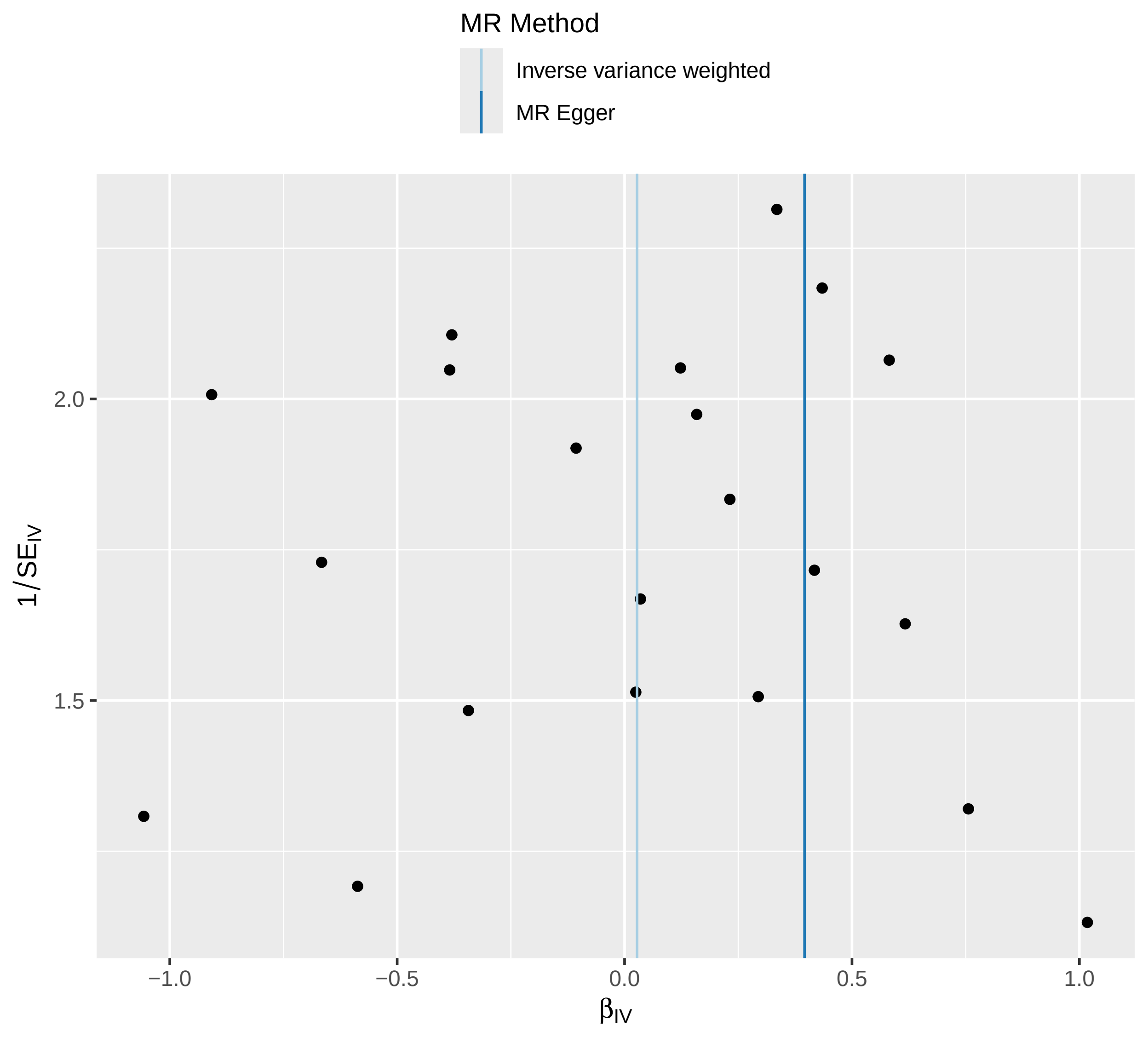
MR Method
Inverse variance weighted
MR Egger
2.0
1.5
1/SE$_{IV}$
−1.0
−0.5
0.0
0.5
1.0
β$_{IV}$

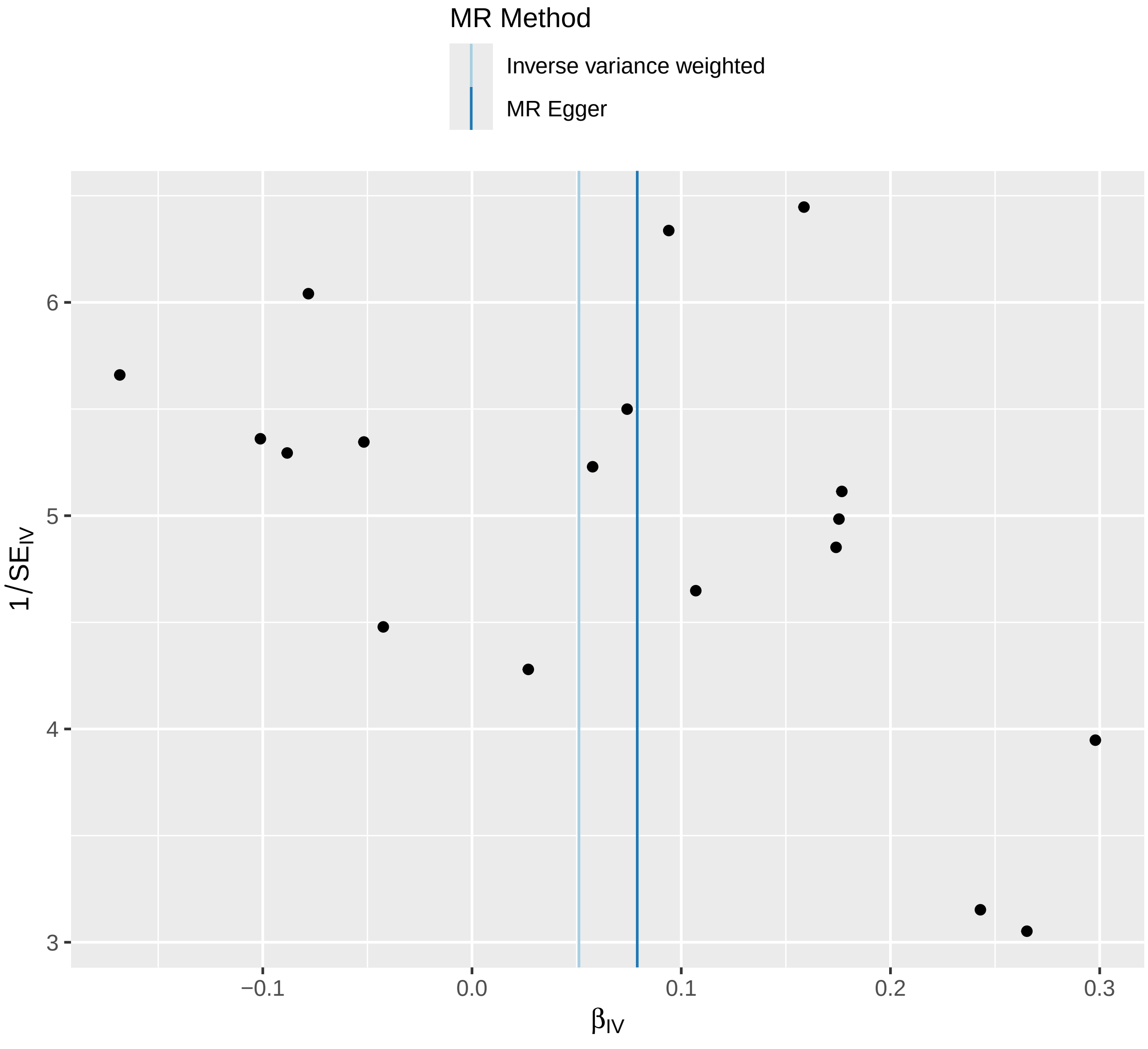
MR Method
Inverse variance weighted
MR Egger
6
5
4
3
1/SE$_{IV}$
−0.1
0.0
0.1
0.2
0.3
β$_{IV}$

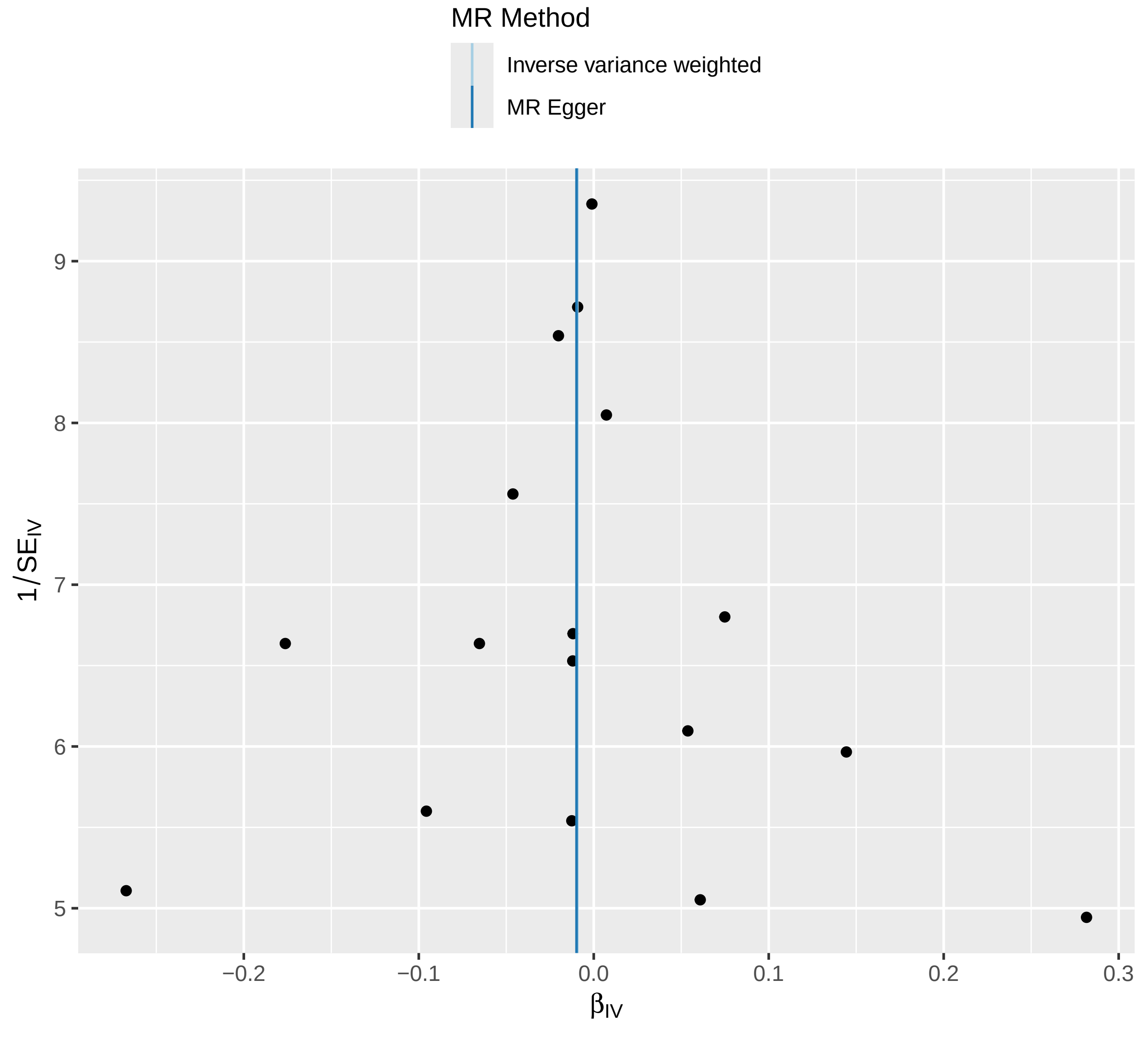

MR Method
Inverse variance weighted
MR Egger
9
8
7
6
5
1/SE_IV
−0.2
−0.1
0.0
0.1
0.2
0.3
β_IV

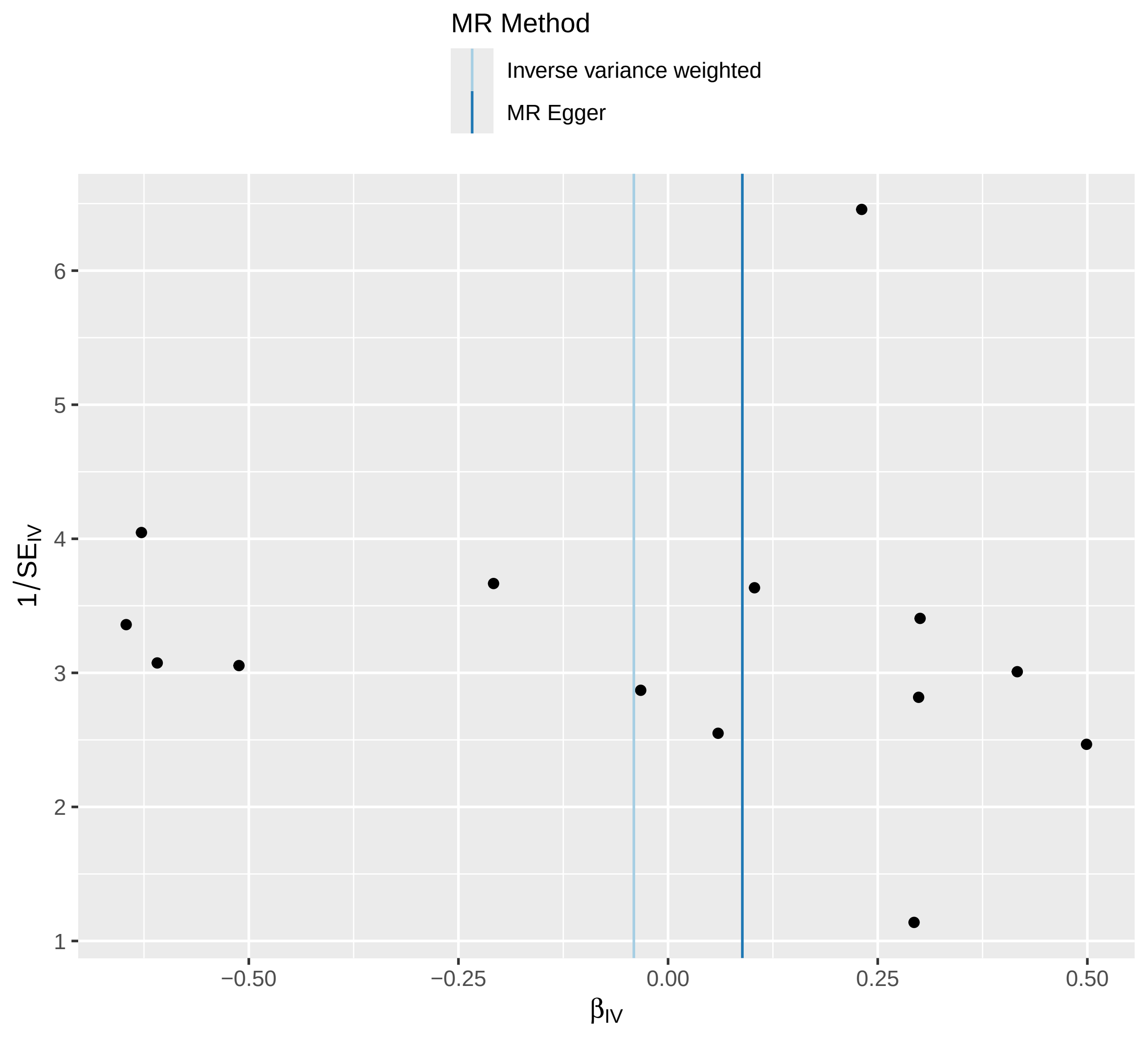

MR Method
Inverse variance weighted
MR Egger
1/SE_IV
6
5
4
3
2
1
−0.50
−0.25
0.00
0.25
0.50
β_IV

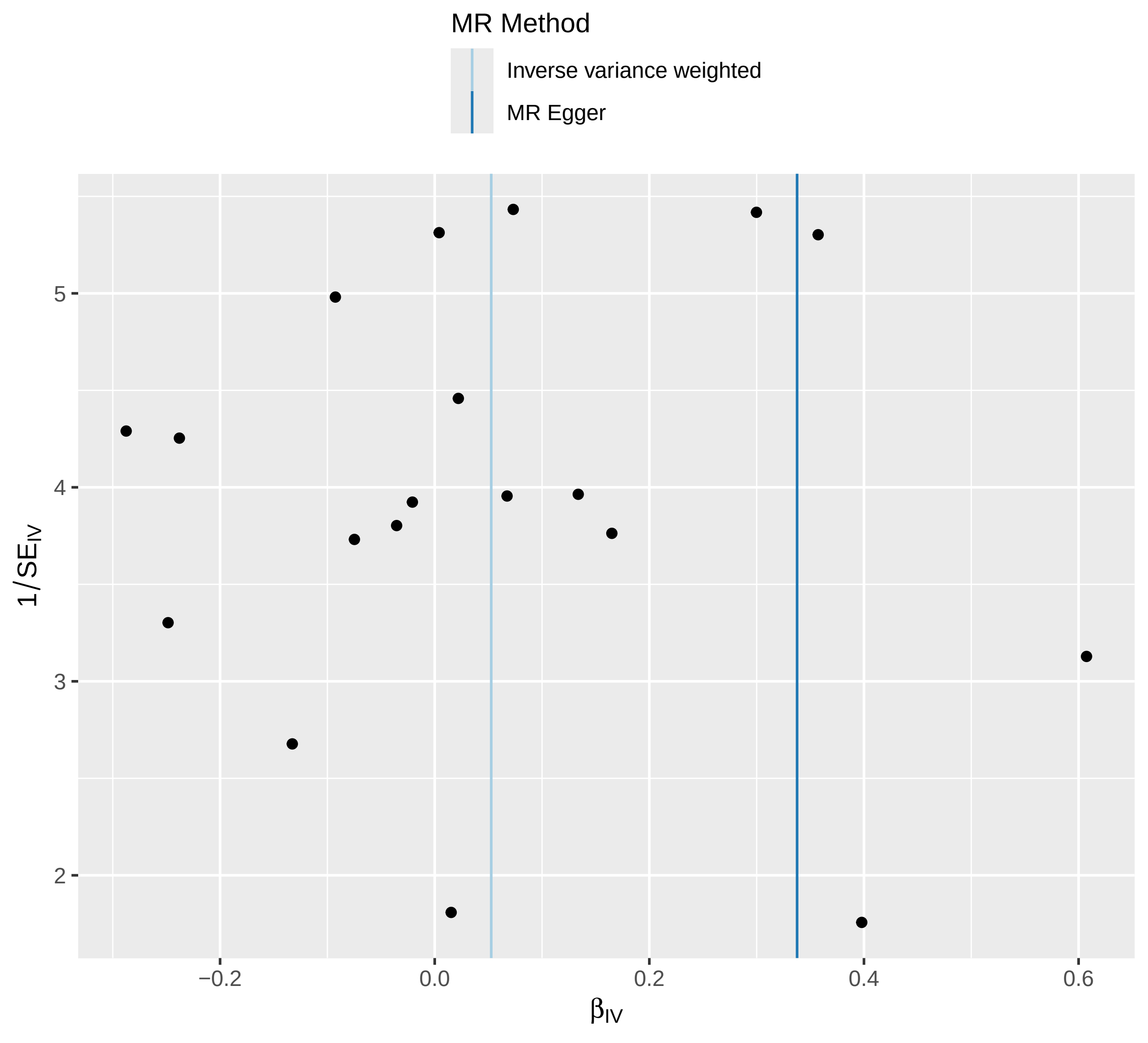

MR Method
Inverse variance weighted
MR Egger
5
4
3
2
1/SE$_{IV}$
−0.2
0.0
0.2
0.4
0.6
β$_{IV}$

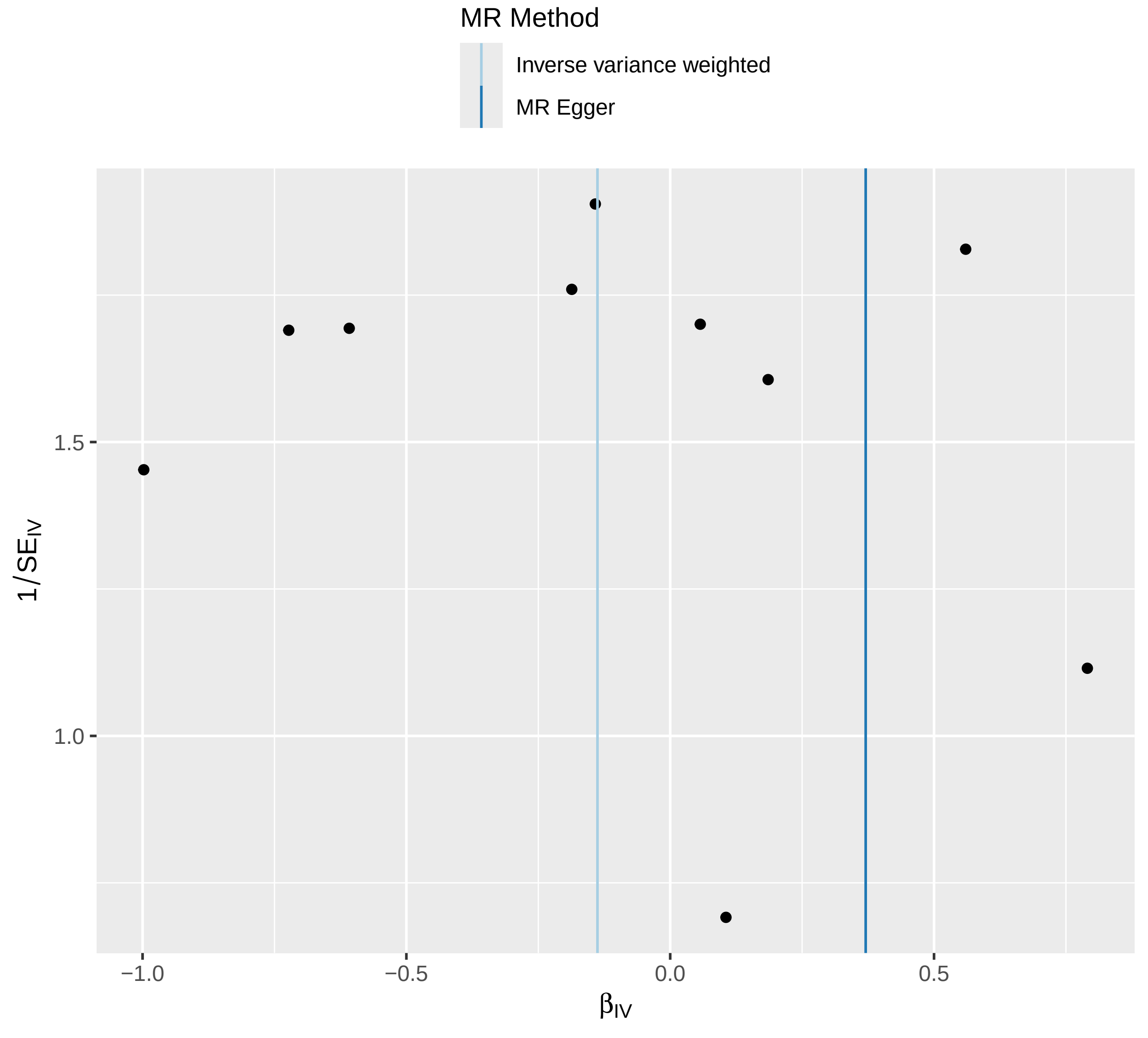
MR Method
Inverse variance weighted
MR Egger
1.5
1.0
−1.0
−0.5
0.0
0.5
$1/SE_{IV}$
β_{IV}

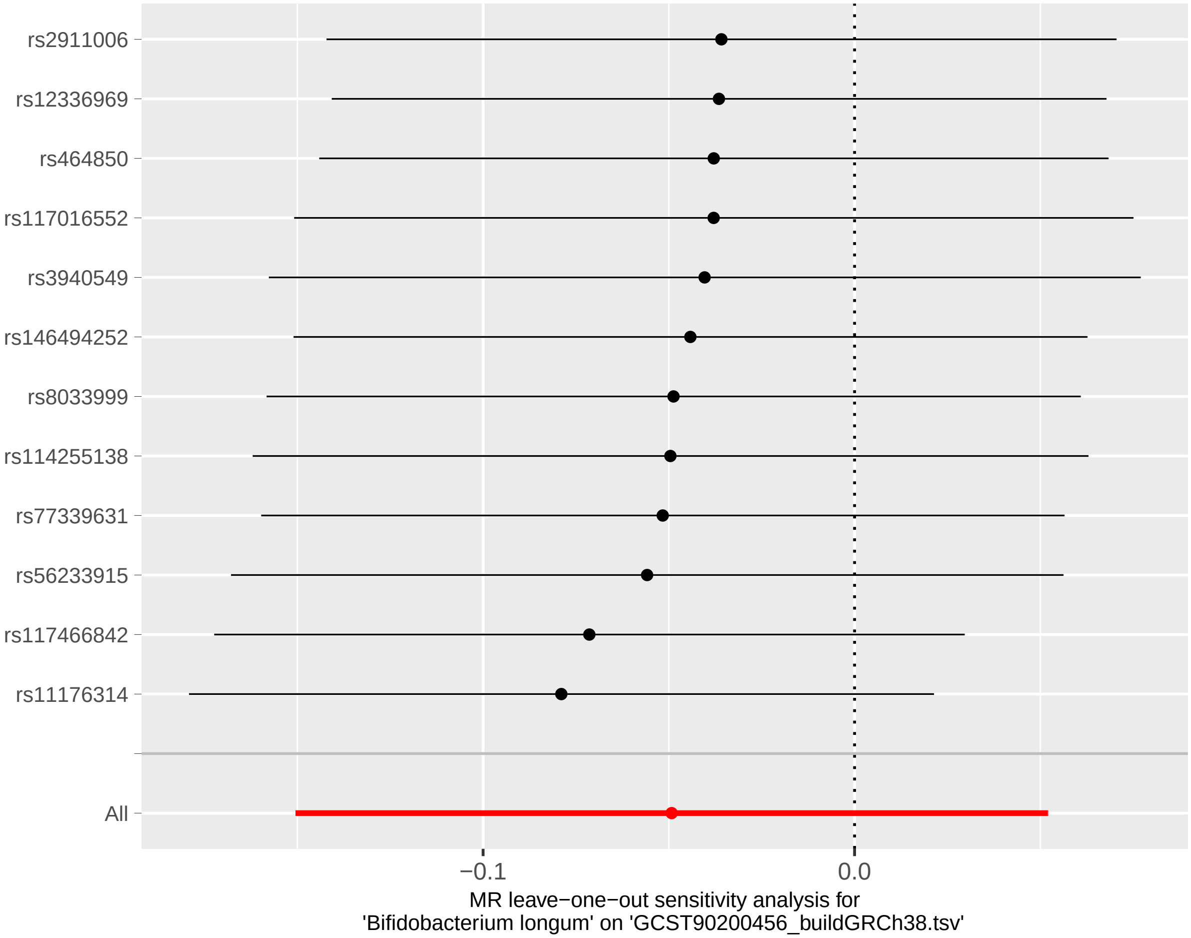

rs2911006
rs12336969
rs464850
rs117016552
rs3940549
rs146494252
rs8033999
rs114255138
rs77339631
rs56233915
rs117466842
rs11176314
All
−0.1
0.0
MR leave−one−out sensitivity analysis for
'Bifidobacterium longum' on 'GCST90200456_buildGRCh38.tsv'

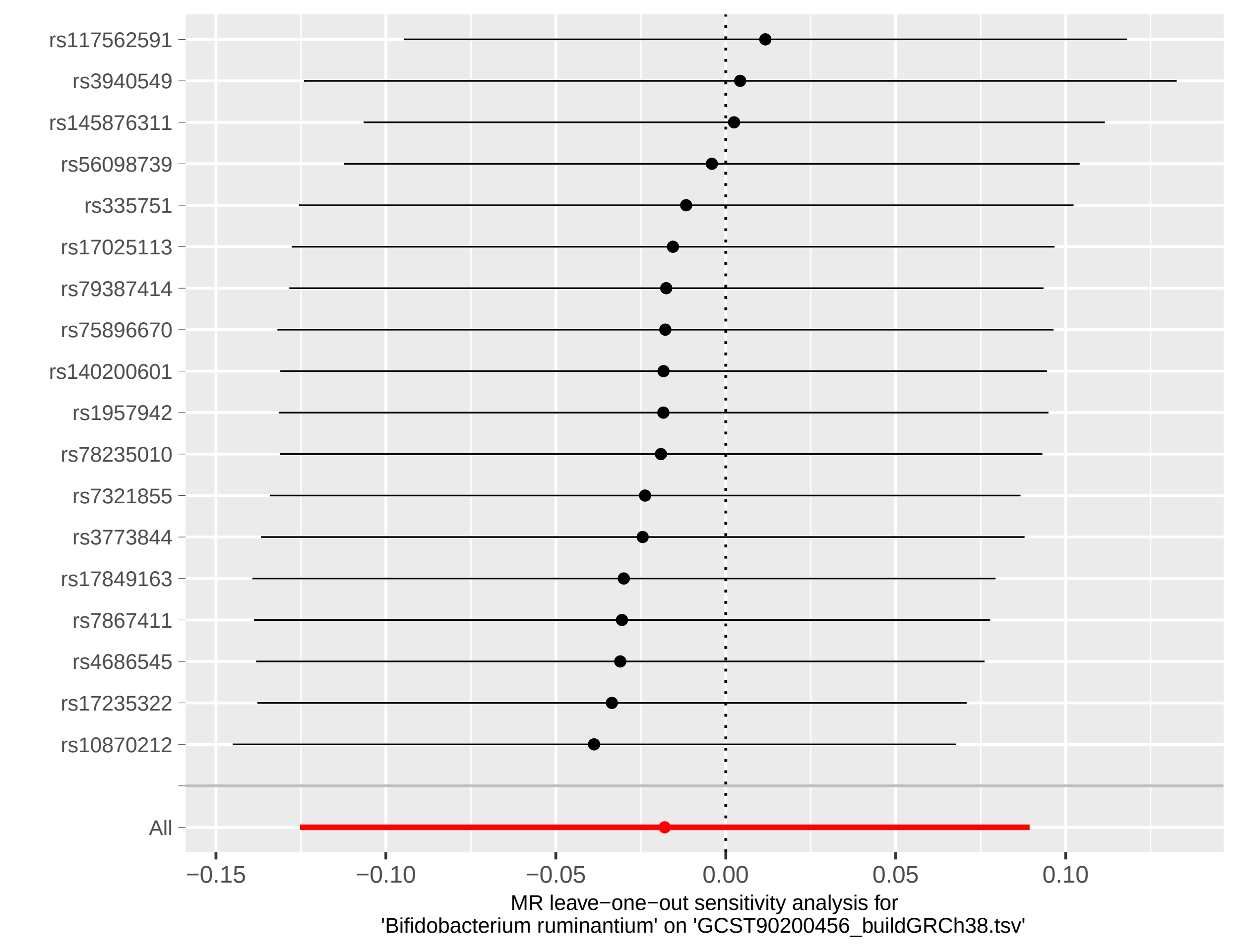
rs117562591
rs3940549
rs145876311
rs56098739
rs335751
rs17025113
rs79387414
rs75896670
rs140200601
rs1957942
rs78235010
rs7321855
rs3773844
rs17849163
rs7867411
rs4686545
rs17235322
rs10870212
All
−0.15
−0.10
−0.05
0.00
0.05
0.10
MR leave−one−out sensitivity analysis for
'Bifidobacterium ruminantium' on 'GCST90200456_buildGRCh38.tsv'

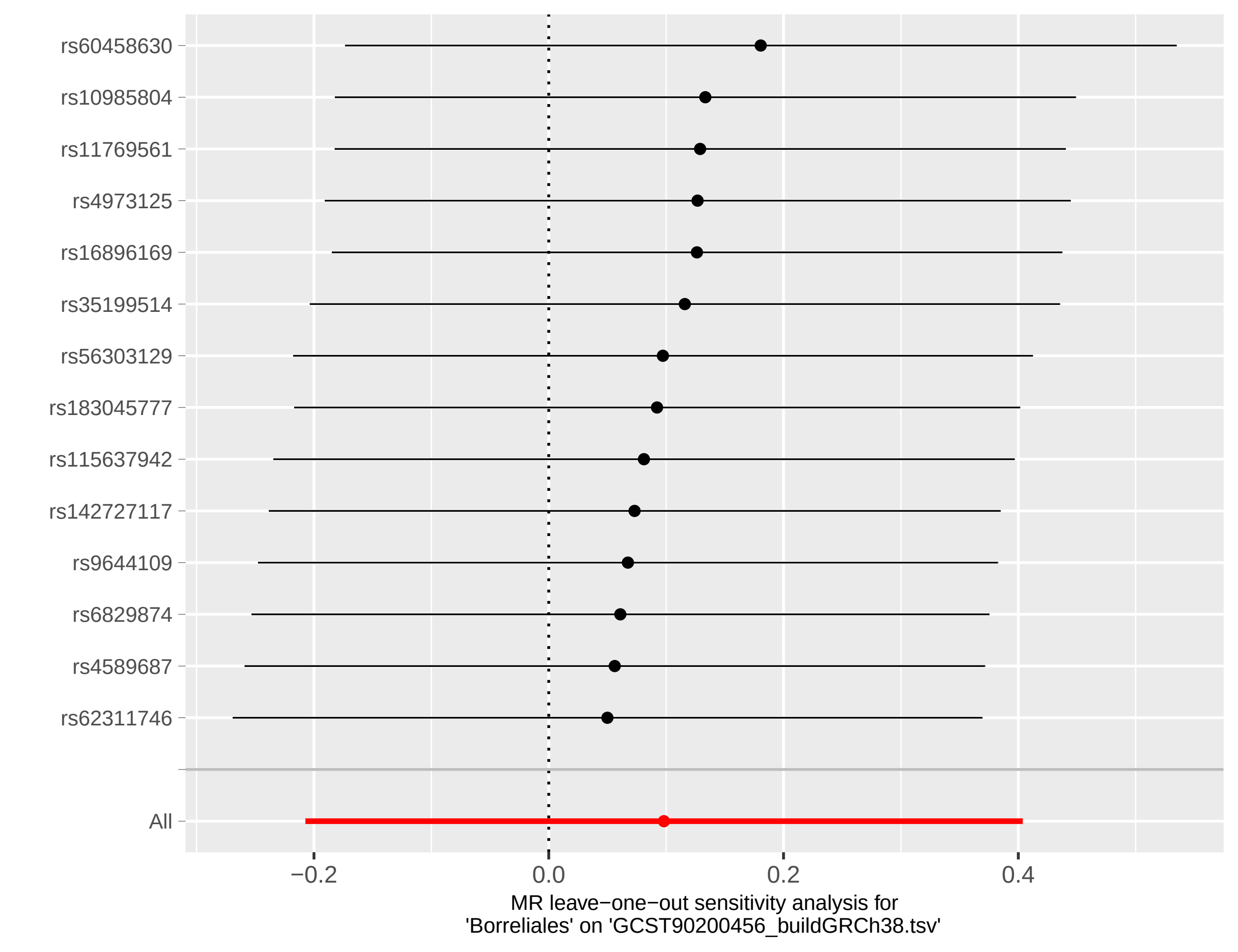

rs60458630
rs10985804
rs11769561
rs4973125
rs16896169
rs35199514
rs56303129
rs183045777
rs115637942
rs142727117
rs9644109
rs6829874
rs4589687
rs62311746
All
−0.2
0.0
0.2
0.4
MR leave−one−out sensitivity analysis for
'Borreliales' on 'GCST90200456_buildGRCh38.tsv'

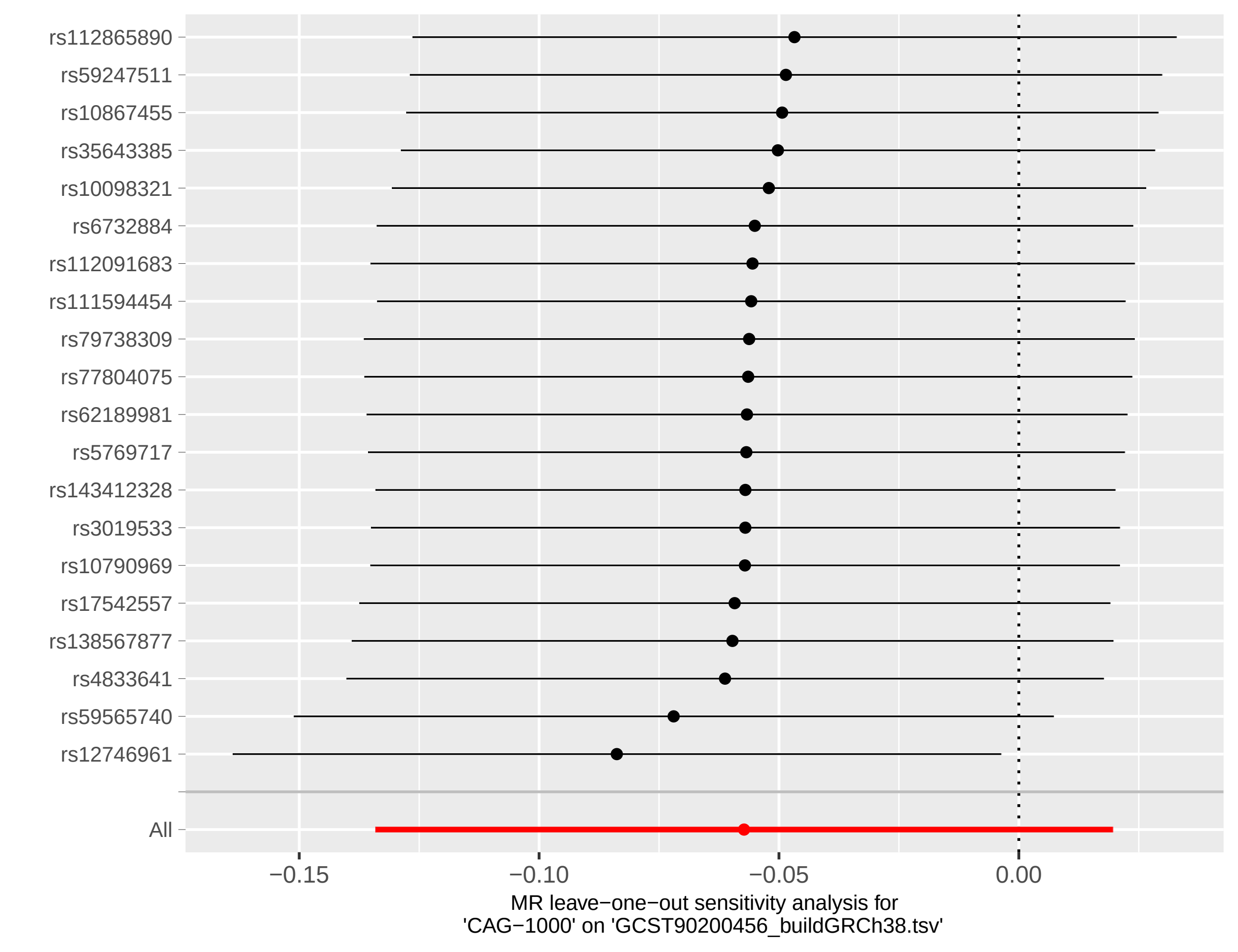

rs112865890
rs59247511
rs10867455
rs35643385
rs10098321
rs6732884
rs112091683
rs111594454
rs79738309
rs77804075
rs62189981
rs5769717
rs143412328
rs3019533
rs10790969
rs17542557
rs138567877
rs4833641
rs59565740
rs12746961
All
−0.15
−0.10
−0.05
0.00
MR leave−one−out sensitivity analysis for
'CAG−1000' on 'GCST90200456_buildGRCh38.tsv'

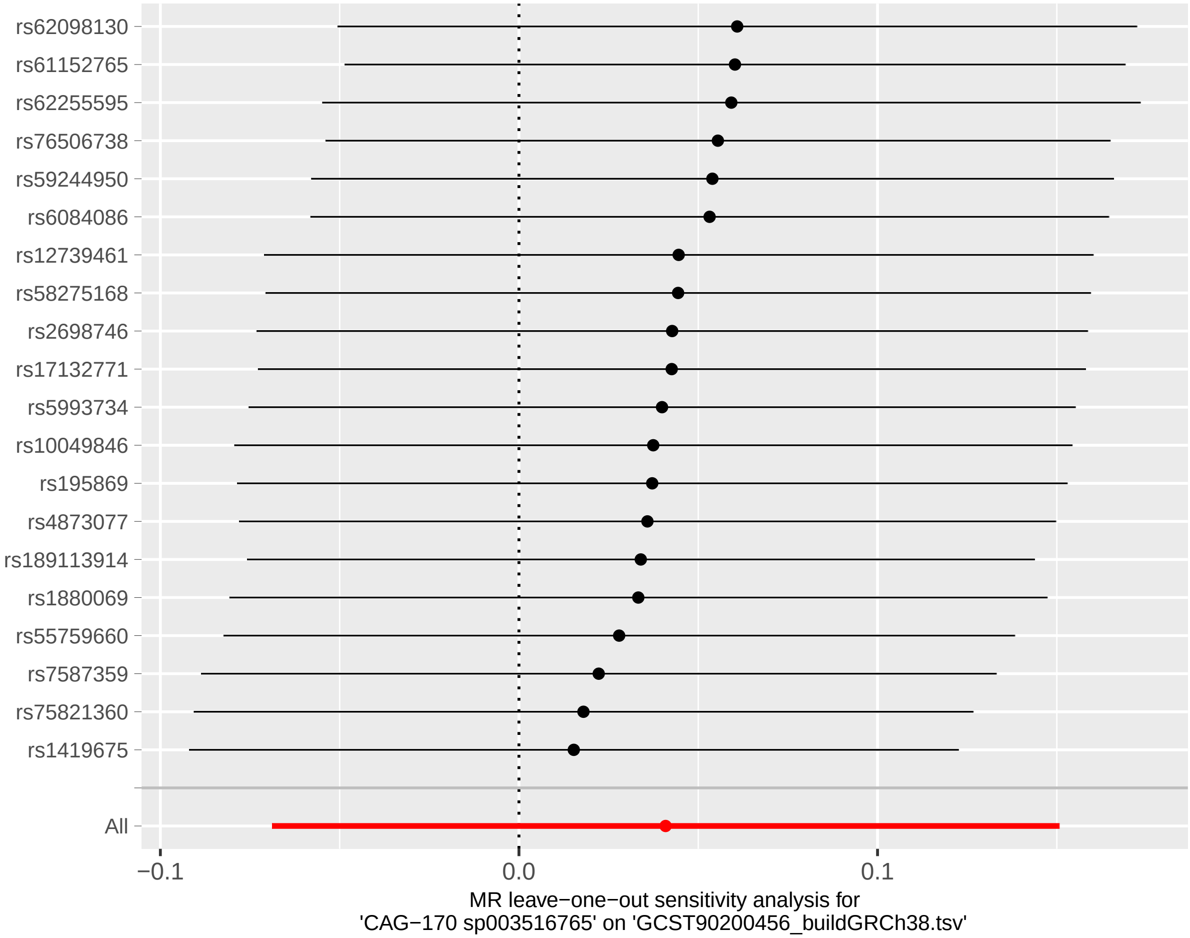

rs62098130
rs61152765
rs62255595
rs76506738
rs59244950
rs6084086
rs12739461
rs58275168
rs2698746
rs17132771
rs5993734
rs10049846
rs195869
rs4873077
rs189113914
rs1880069
rs55759660
rs7587359
rs75821360
rs1419675
All
−0.1
0.0
0.1
MR leave−one−out sensitivity analysis for
'CAG−170 sp003516765' on 'GCST90200456_buildGRCh38.tsv'

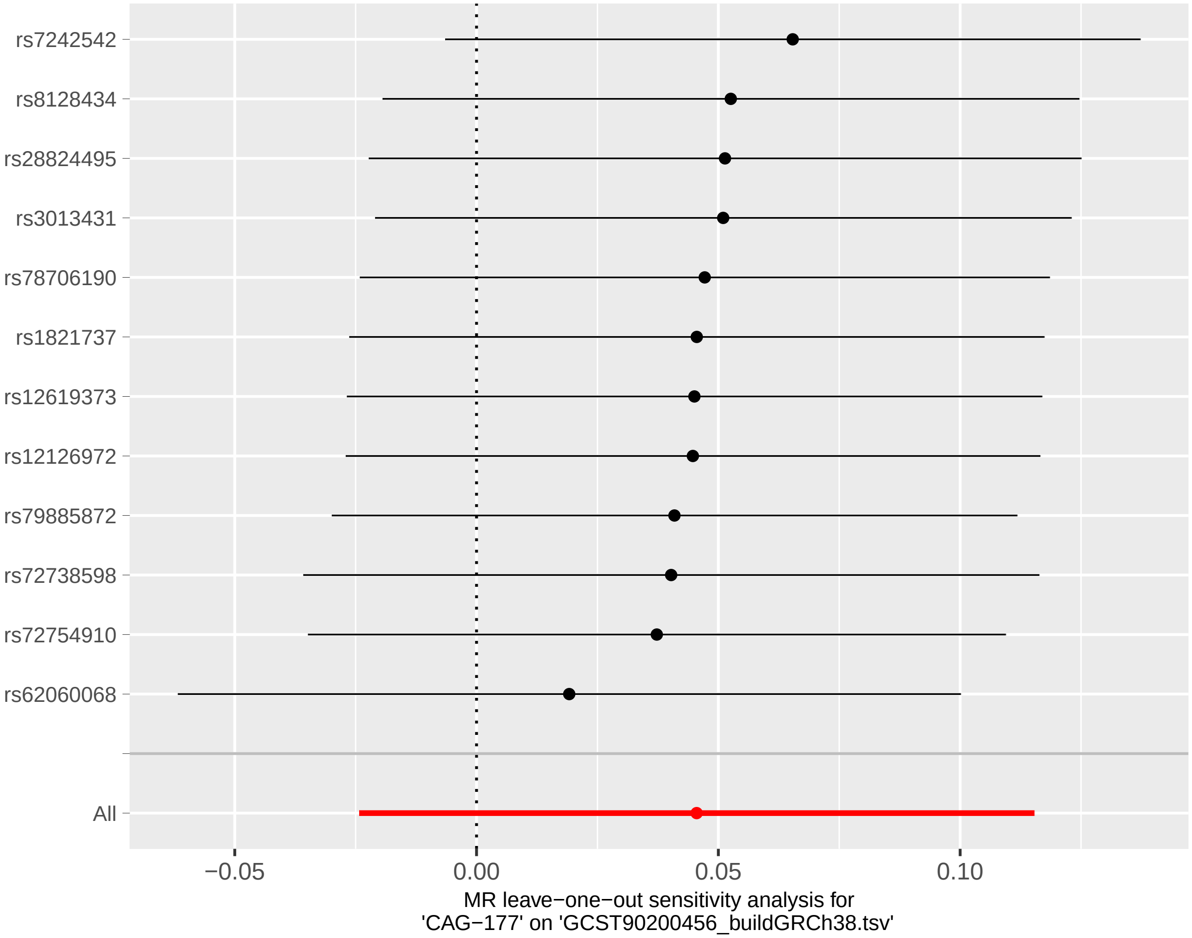
rs7242542
rs8128434
rs28824495
rs3013431
rs78706190
rs1821737
rs12619373
rs12126972
rs79885872
rs72738598
rs72754910
rs62060068
All
−0.05
0.00
0.05
0.10
MR leave−one−out sensitivity analysis for
'CAG−177' on 'GCST90200456_buildGRCh38.tsv'

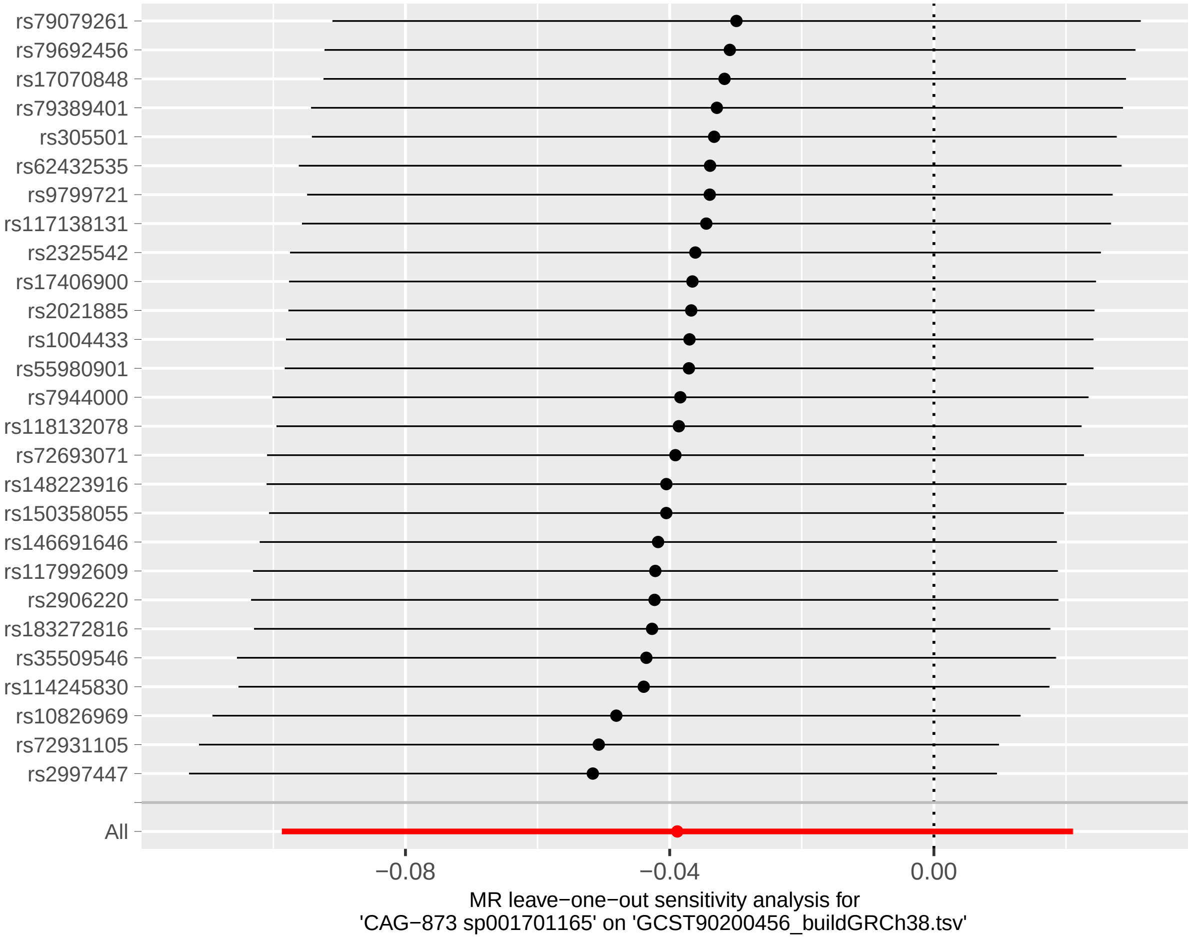
rs79079261
rs79692456
rs17070848
rs79389401
rs305501
rs62432535
rs9799721
rs117138131
rs2325542
rs17406900
rs2021885
rs1004433
rs55980901
rs7944000
rs118132078
rs72693071
rs148223916
rs150358055
rs146691646
rs117992609
rs2906220
rs183272816
rs35509546
rs114245830
rs10826969
rs72931105
rs2997447
All
−0.08
−0.04
0.00
MR leave−one−out sensitivity analysis for
'CAG−873 sp001701165' on 'GCST90200456_buildGRCh38.tsv'

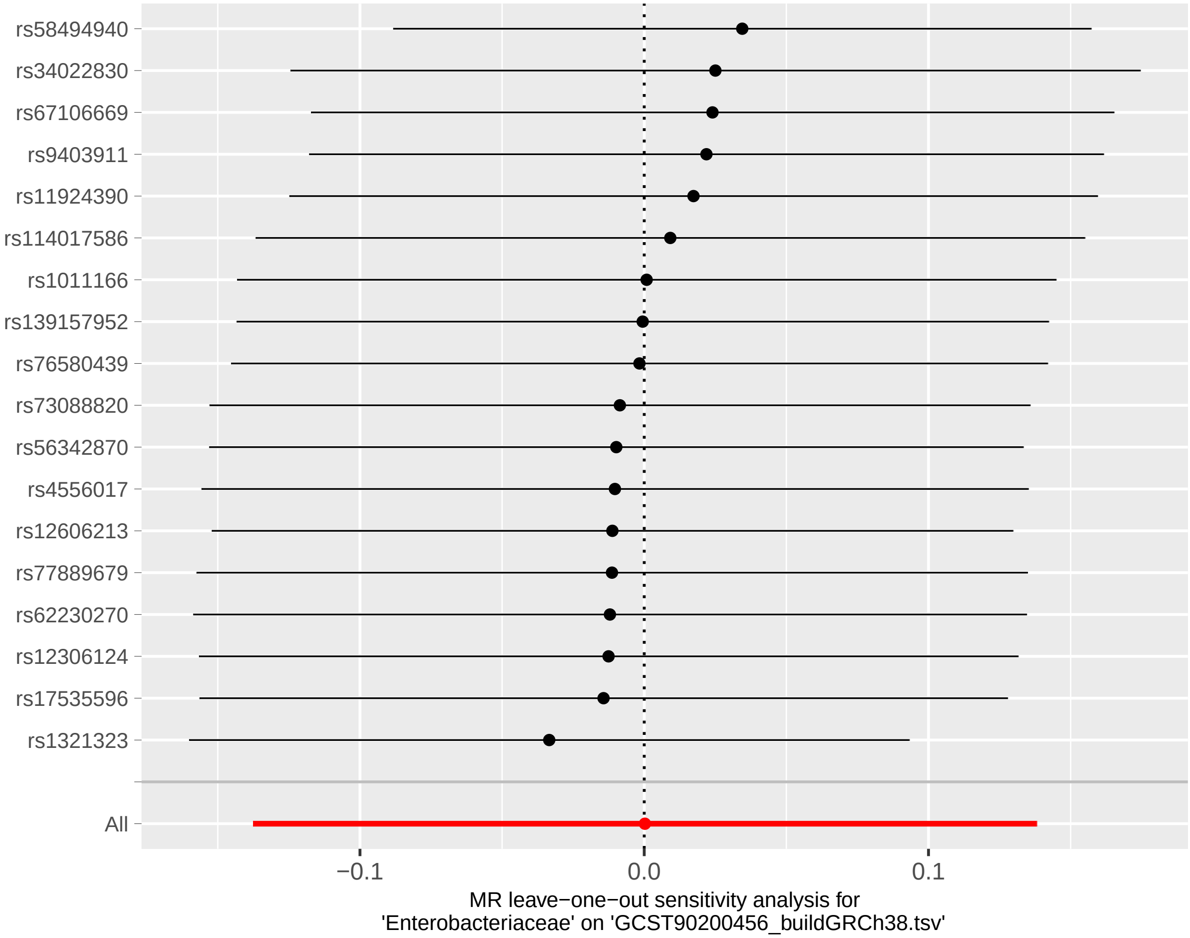
rs58494940
rs34022830
rs67106669
rs9403911
rs11924390
rs114017586
rs1011166
rs139157952
rs76580439
rs73088820
rs56342870
rs4556017
rs12606213
rs77889679
rs62230270
rs12306124
rs17535596
rs1321323
All
−0.1
0.0
0.1
MR leave−one−out sensitivity analysis for
'Enterobacteriaceae' on 'GCST90200456_buildGRCh38.tsv'

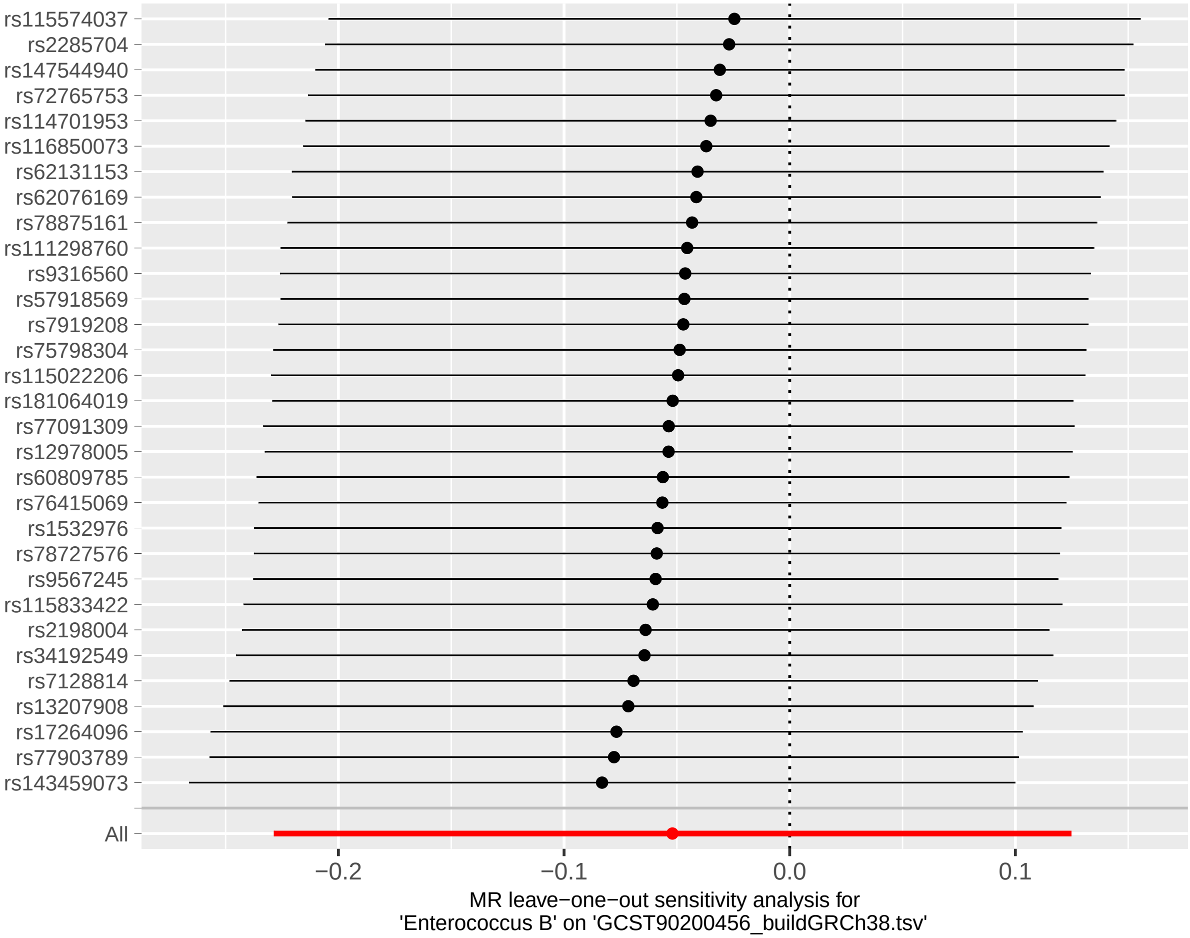
rs115574037
rs2285704
rs147544940
rs72765753
rs114701953
rs116850073
rs62131153
rs62076169
rs78875161
rs111298760
rs9316560
rs57918569
rs7919208
rs75798304
rs115022206
rs181064019
rs77091309
rs12978005
rs60809785
rs76415069
rs1532976
rs78727576
rs9567245
rs115833422
rs2198004
rs34192549
rs7128814
rs13207908
rs17264096
rs77903789
rs143459073
All
−0.2
−0.1
0.0
0.1
MR leave−one−out sensitivity analysis for
'Enterococcus B' on 'GCST90200456_buildGRCh38.tsv'

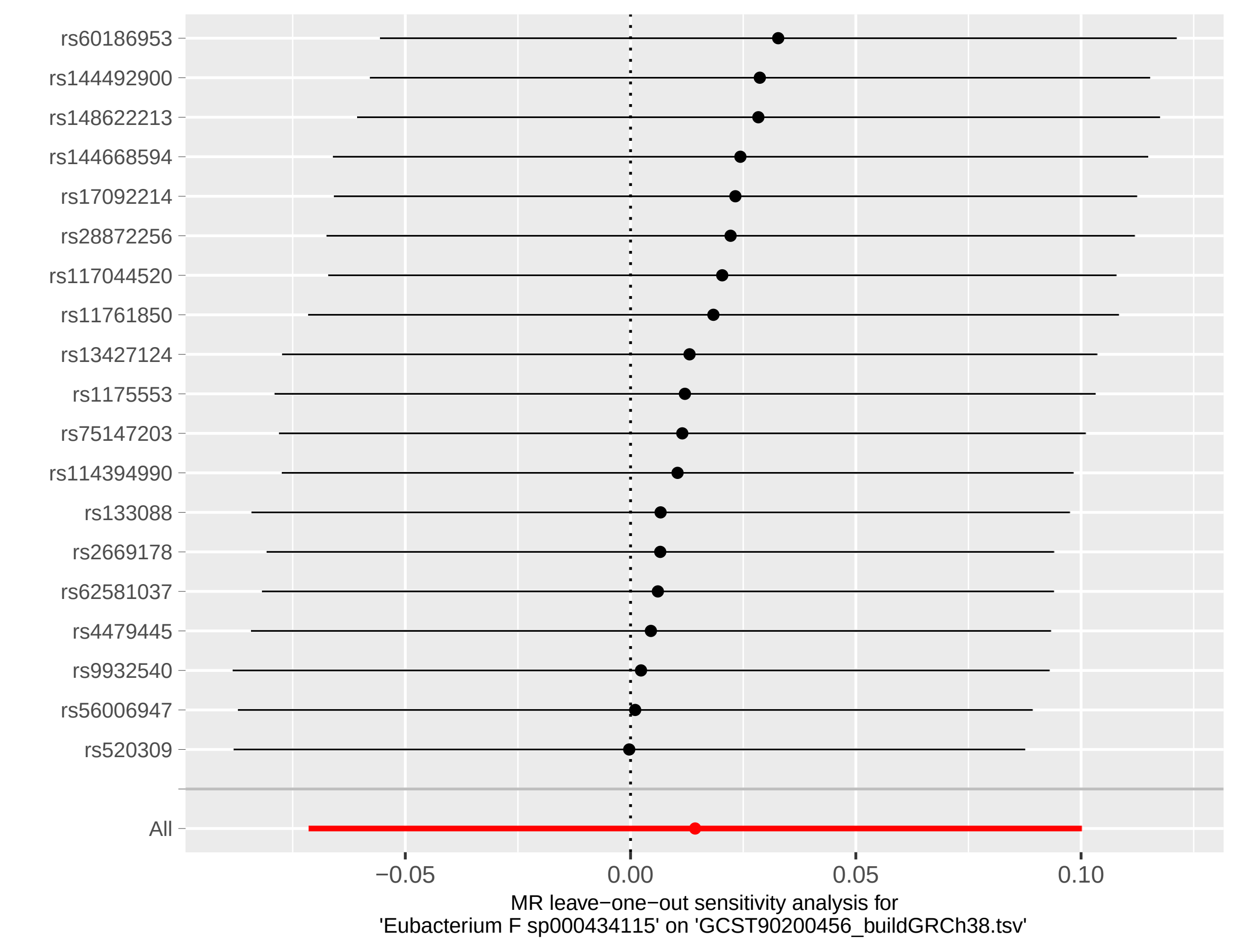

rs60186953
rs144492900
rs148622213
rs144668594
rs17092214
rs28872256
rs117044520
rs11761850
rs13427124
rs1175553
rs75147203
rs114394990
rs133088
rs2669178
rs62581037
rs4479445
rs9932540
rs56006947
rs520309
All
−0.05
0.00
0.05
0.10
MR leave−one−out sensitivity analysis for
'Eubacterium F sp000434115' on 'GCST90200456_buildGRCh38.tsv'

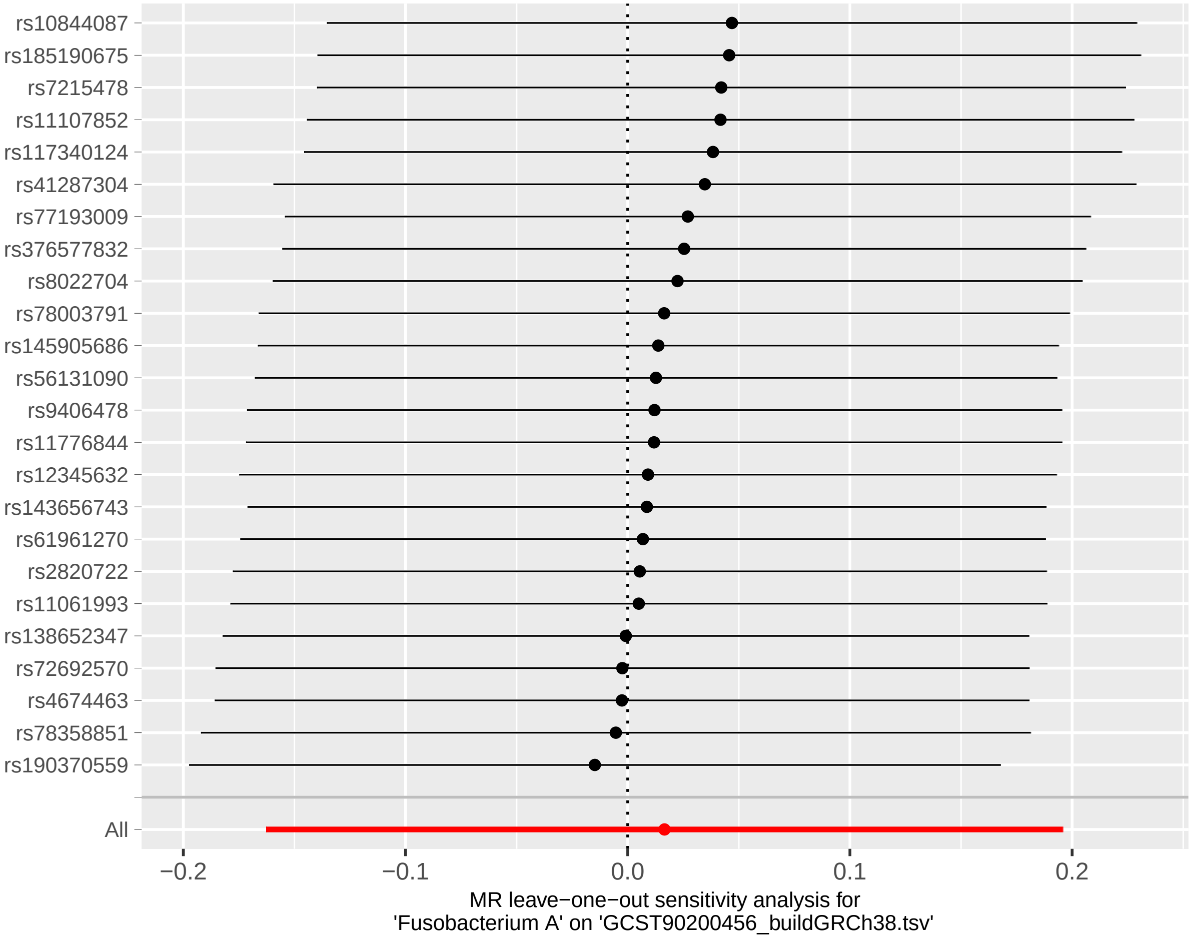
rs10844087
rs185190675
rs7215478
rs11107852
rs117340124
rs41287304
rs77193009
rs376577832
rs8022704
rs78003791
rs145905686
rs56131090
rs9406478
rs11776844
rs12345632
rs143656743
rs61961270
rs2820722
rs11061993
rs138652347
rs72692570
rs4674463
rs78358851
rs190370559
All
−0.2
−0.1
0.0
0.1
0.2
MR leave−one−out sensitivity analysis for
'Fusobacterium A' on 'GCST90200456_buildGRCh38.tsv'

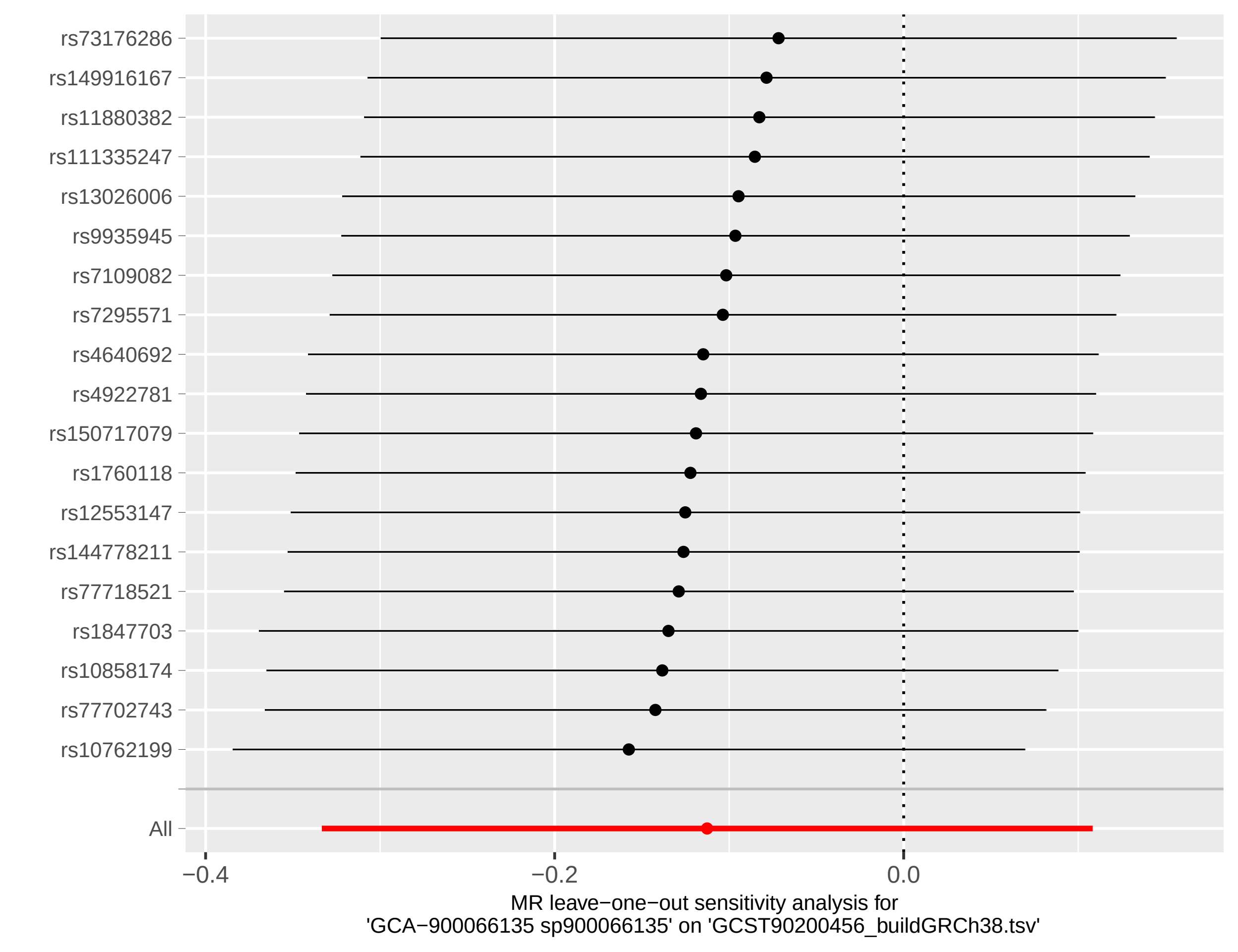
rs73176286
rs149916167
rs11880382
rs111335247
rs13026006
rs9935945
rs7109082
rs7295571
rs4640692
rs4922781
rs150717079
rs1760118
rs12553147
rs144778211
rs77718521
rs1847703
rs10858174
rs77702743
rs10762199
All
−0.4
−0.2
0.0
MR leave−one−out sensitivity analysis for
'GCA−900066135 sp900066135' on 'GCST90200456_buildGRCh38.tsv'

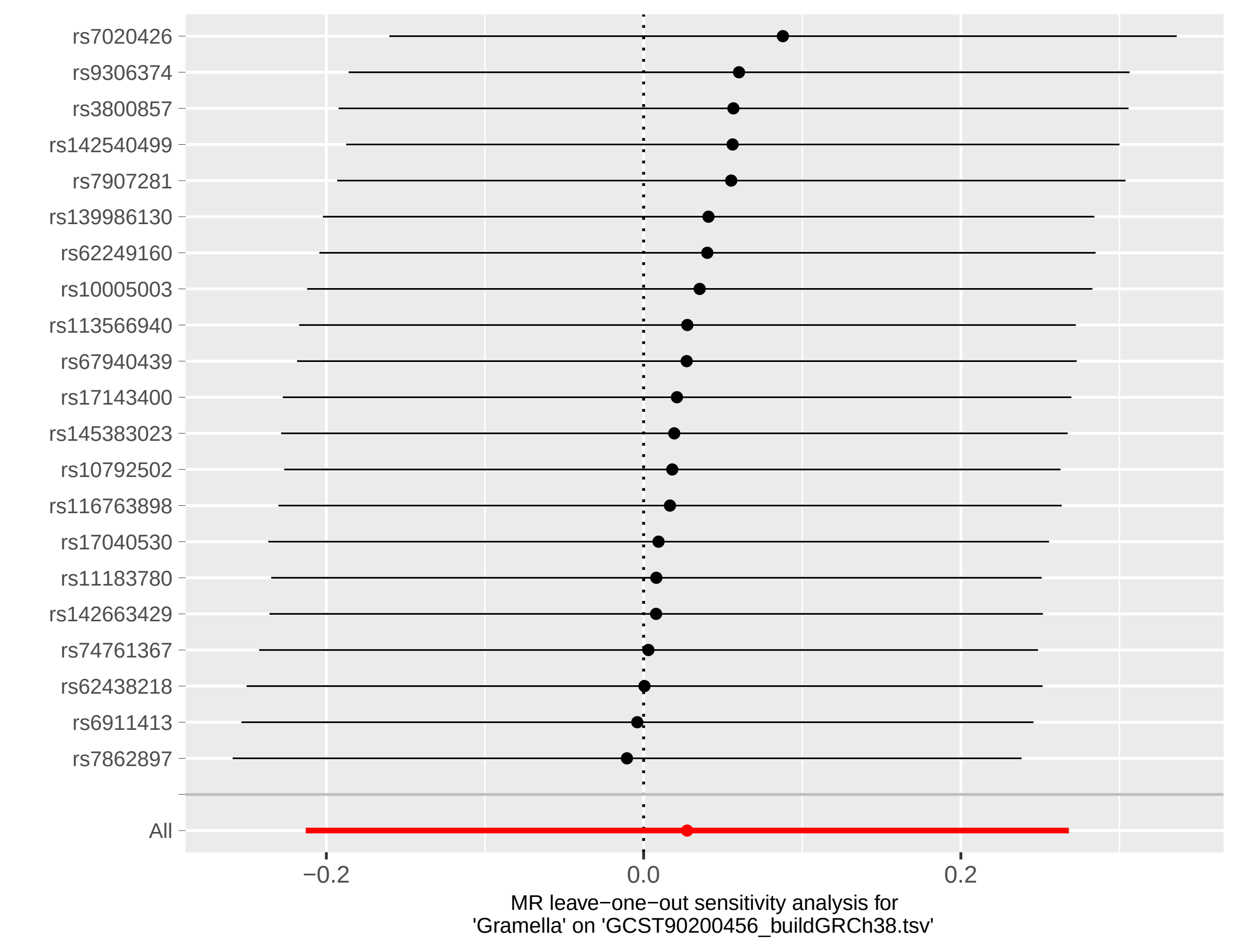

rs7020426
rs9306374
rs3800857
rs142540499
rs7907281
rs139986130
rs62249160
rs10005003
rs113566940
rs67940439
rs17143400
rs145383023
rs10792502
rs116763898
rs17040530
rs11183780
rs142663429
rs74761367
rs62438218
rs6911413
rs7862897
All
−0.2
0.0
0.2
MR leave−one−out sensitivity analysis for
'Gramella' on 'GCST90200456_buildGRCh38.tsv'

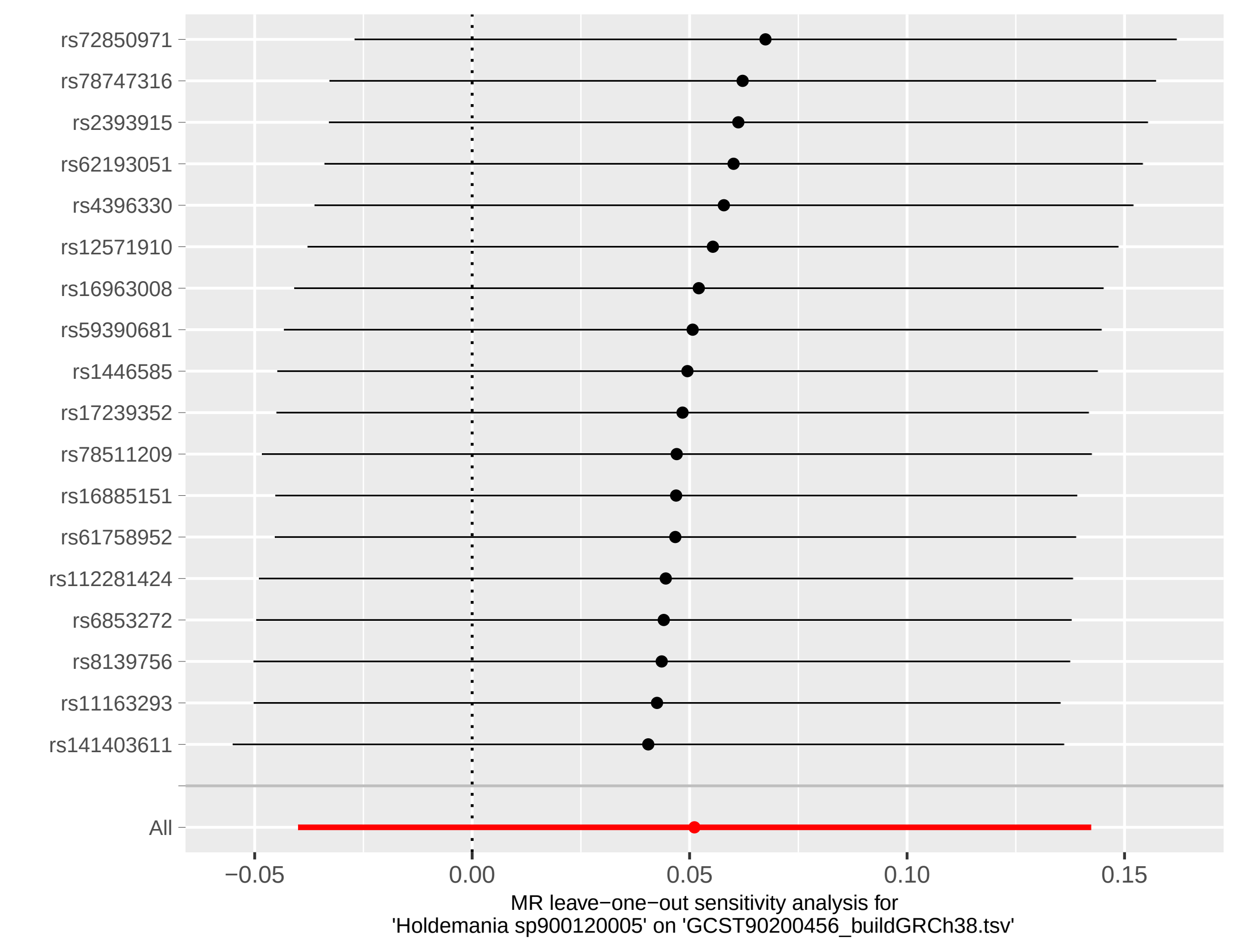
rs72850971
rs78747316
rs2393915
rs62193051
rs4396330
rs12571910
rs16963008
rs59390681
rs1446585
rs17239352
rs78511209
rs16885151
rs61758952
rs112281424
rs6853272
rs8139756
rs11163293
rs141403611
All
−0.05
0.00
0.05
0.10
0.15
MR leave−one−out sensitivity analysis for
'Holdemania sp900120005' on 'GCST90200456_buildGRCh38.tsv'

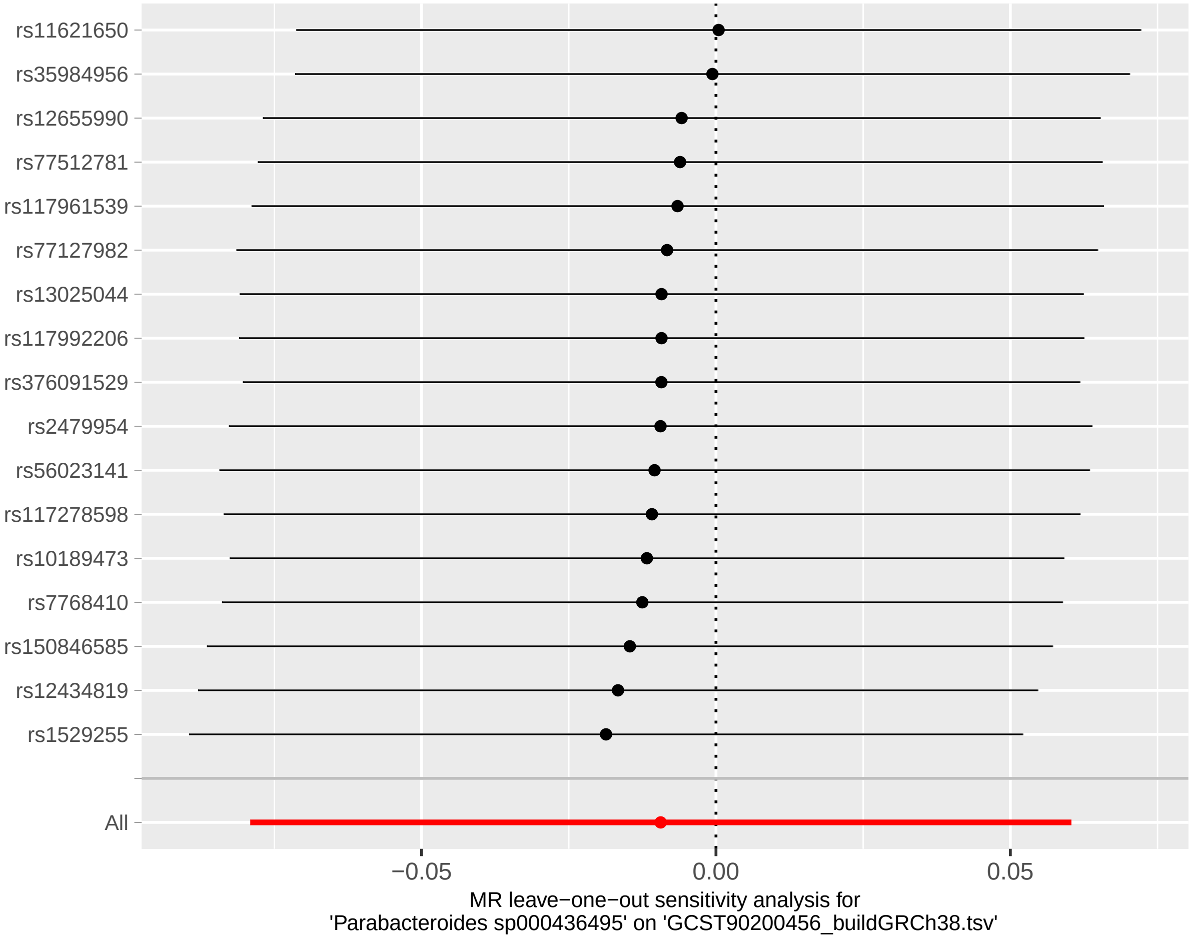

rs11621650
rs35984956
rs12655990
rs77512781
rs117961539
rs77127982
rs13025044
rs117992206
rs376091529
rs2479954
rs56023141
rs117278598
rs10189473
rs7768410
rs150846585
rs12434819
rs1529255
All
−0.05
0.00
0.05
MR leave−one−out sensitivity analysis for
'Parabacteroides sp000436495' on 'GCST90200456_buildGRCh38.tsv'

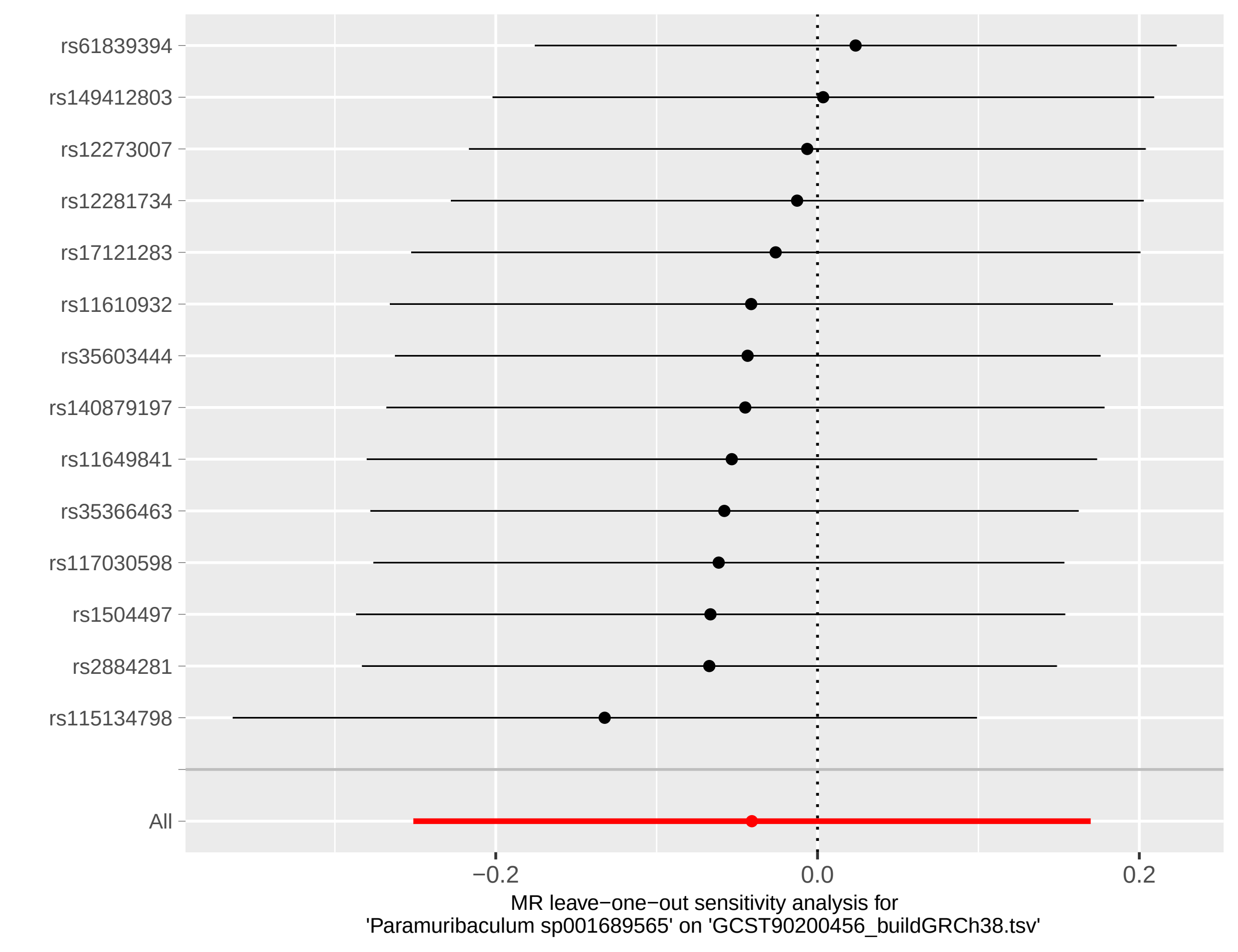
rs61839394
rs149412803
rs12273007
rs12281734
rs17121283
rs11610932
rs35603444
rs140879197
rs11649841
rs35366463
rs117030598
rs1504497
rs2884281
rs115134798
All
−0.2
0.0
0.2
MR leave−one−out sensitivity analysis for
'Paramuribaculum sp001689565' on 'GCST90200456_buildGRCh38.tsv'

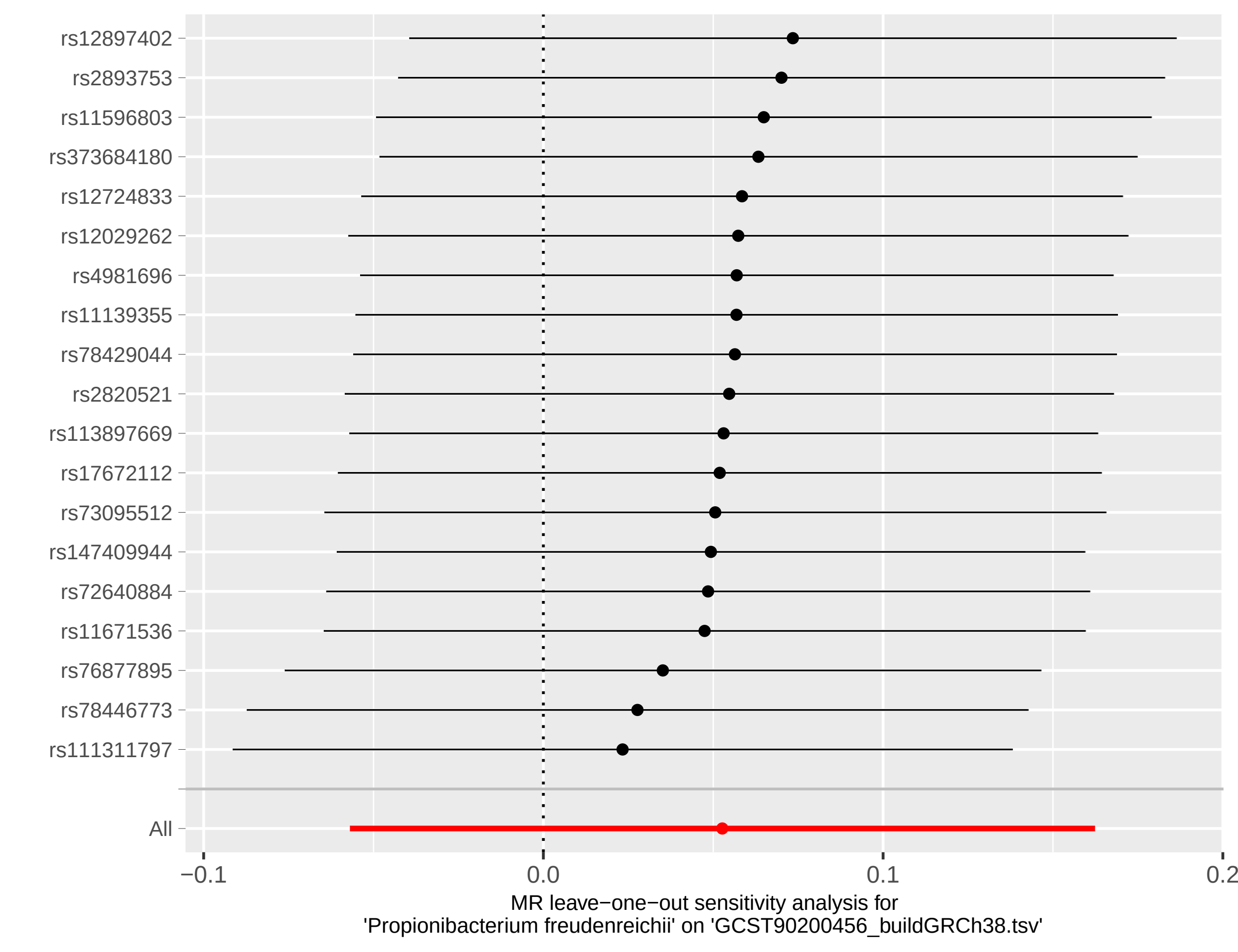

rs12897402
rs2893753
rs11596803
rs373684180
rs12724833
rs12029262
rs4981696
rs11139355
rs78429044
rs2820521
rs113897669
rs17672112
rs73095512
rs147409944
rs72640884
rs11671536
rs76877895
rs78446773
rs111311797
All
−0.1
0.0
0.1
0.2
MR leave−one−out sensitivity analysis for
'Propionibacterium freudenreichii' on 'GCST90200456_buildGRCh38.tsv'

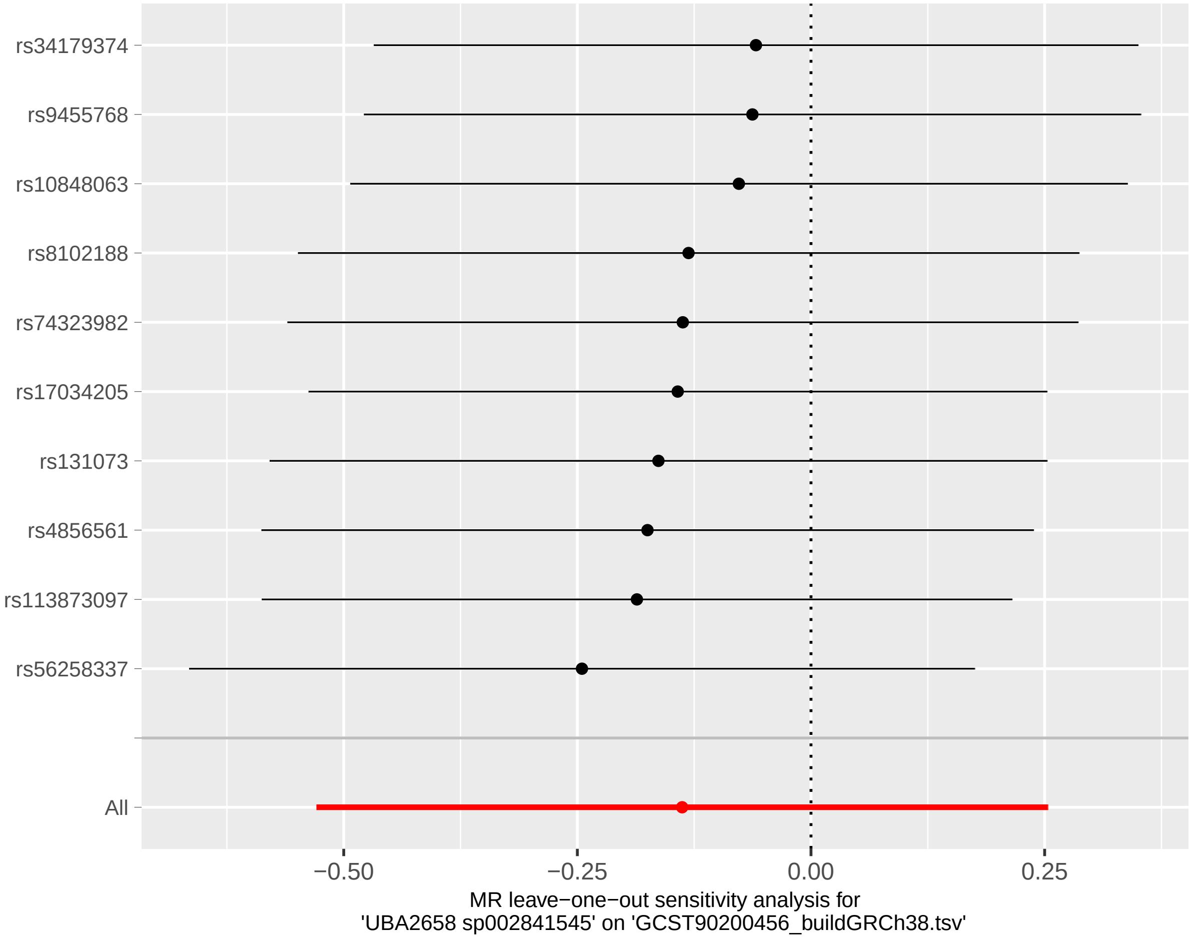

rs34179374
rs9455768
rs10848063
rs8102188
rs74323982
rs17034205
rs131073
rs4856561
rs113873097
rs56258337
All
−0.50
−0.25
0.00
0.25
MR leave−one−out sensitivity analysis for
'UBA2658 sp002841545' on 'GCST90200456_buildGRCh38.tsv'

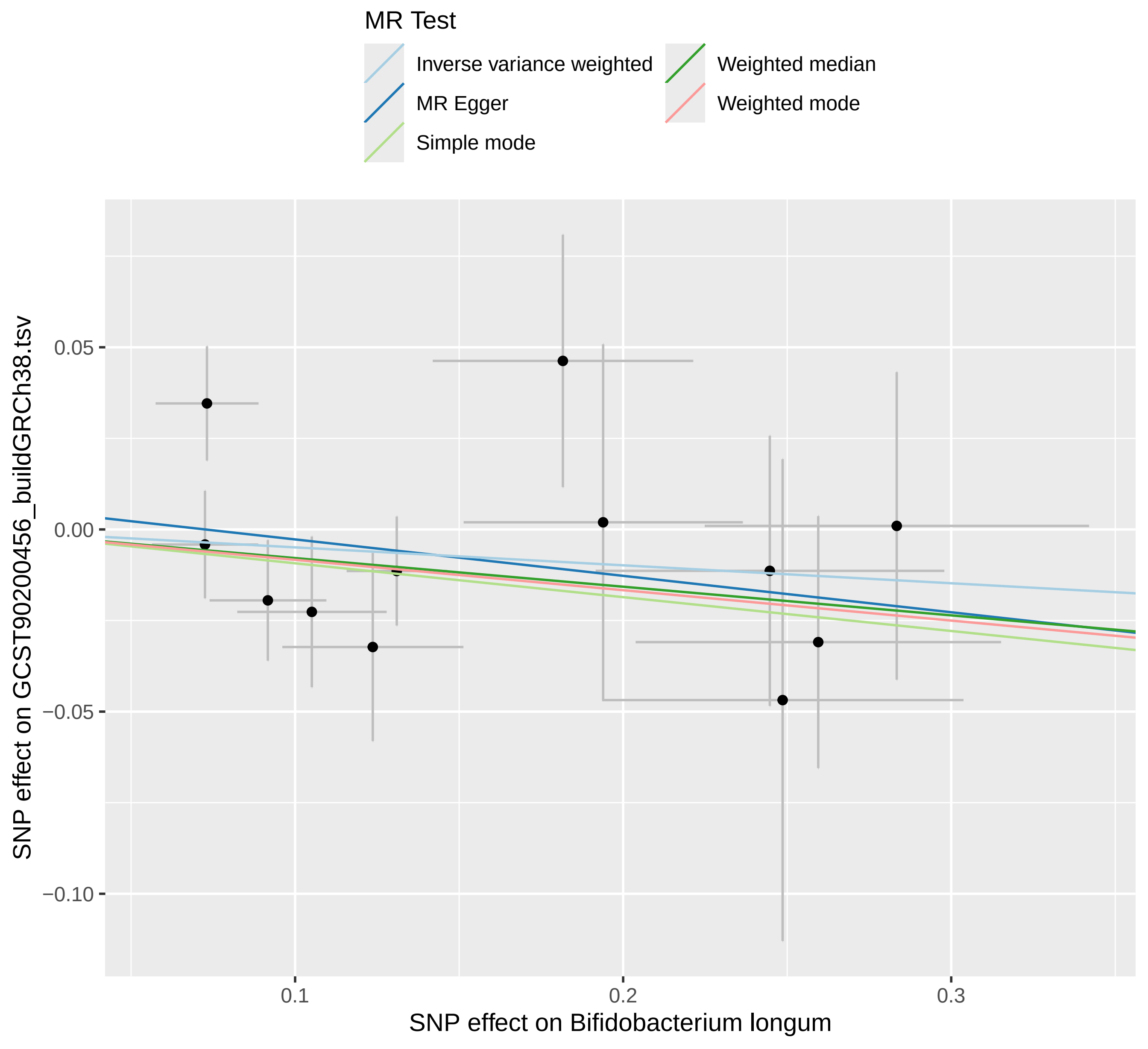

MR Test
Inverse variance weighted
MR Egger
Simple mode
Weighted median
Weighted mode
0.05
0.00
−0.05
−0.10
0.1
0.2
0.3
SNP effect on GCST90200456_buildGRCh38.tsv
SNP effect on Bifidobacterium longum

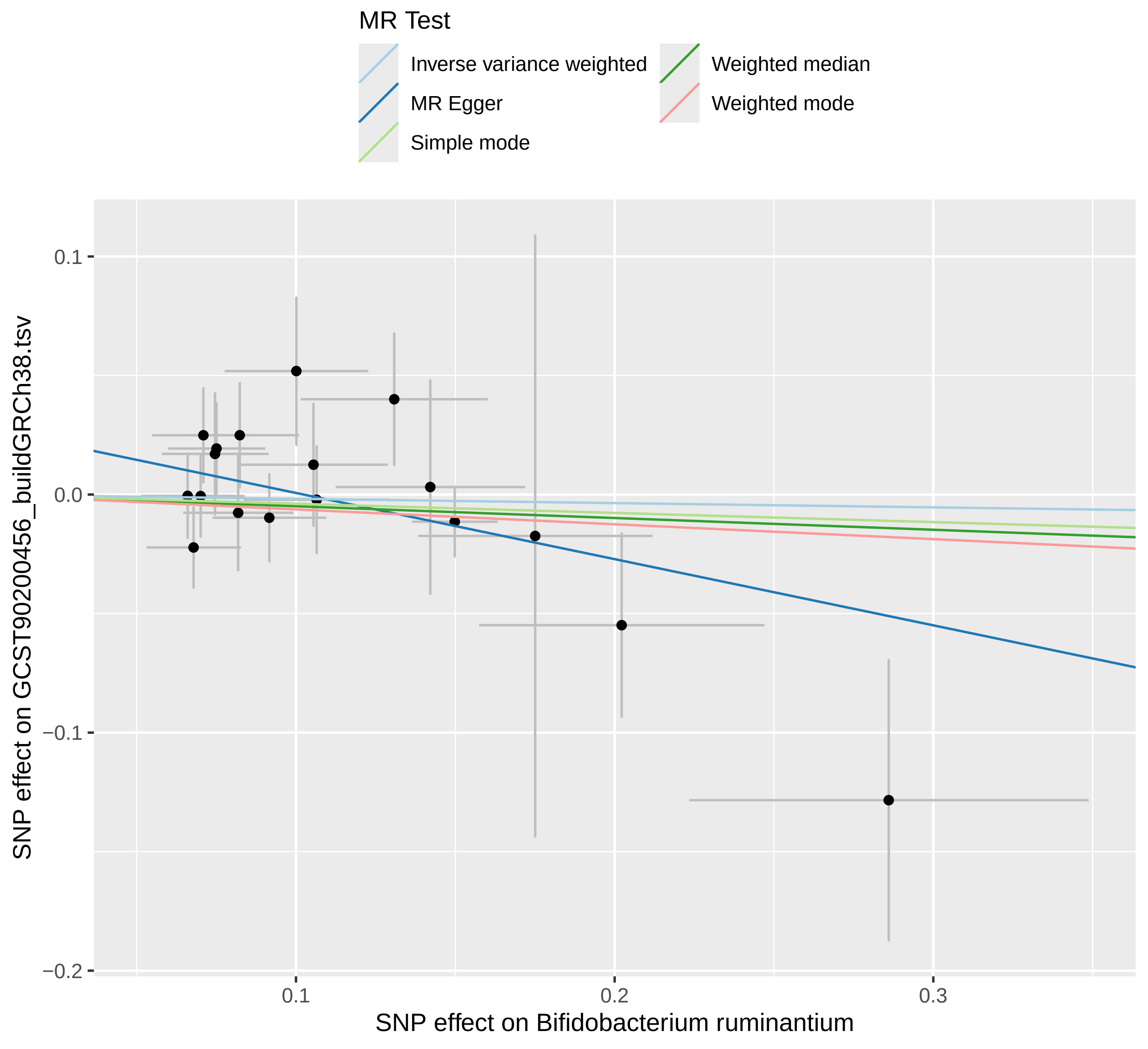
MR Test
Inverse variance weighted
MR Egger
Simple mode
Weighted median
Weighted mode
0.1
0.0
−0.1
−0.2
0.1
0.2
0.3
SNP effect on GCST90200456_buildGRCh38.tsv
SNP effect on Bifidobacterium ruminantium

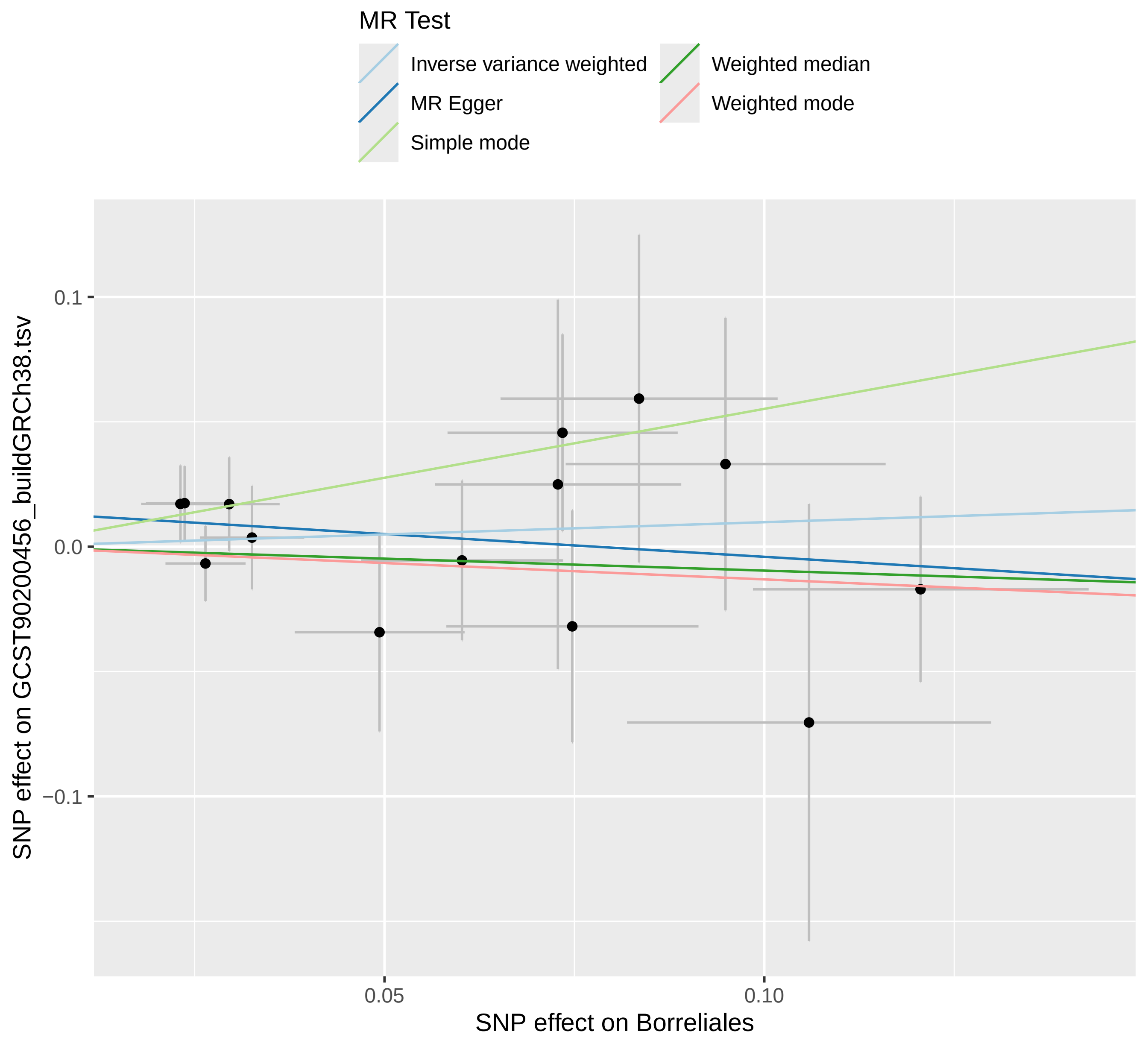

MR Test
Inverse variance weighted
MR Egger
Simple mode
Weighted median
Weighted mode
0.1
0.0
−0.1
0.05
0.10
SNP effect on GCST90200456_buildGRCh38.tsv
SNP effect on Borreliales

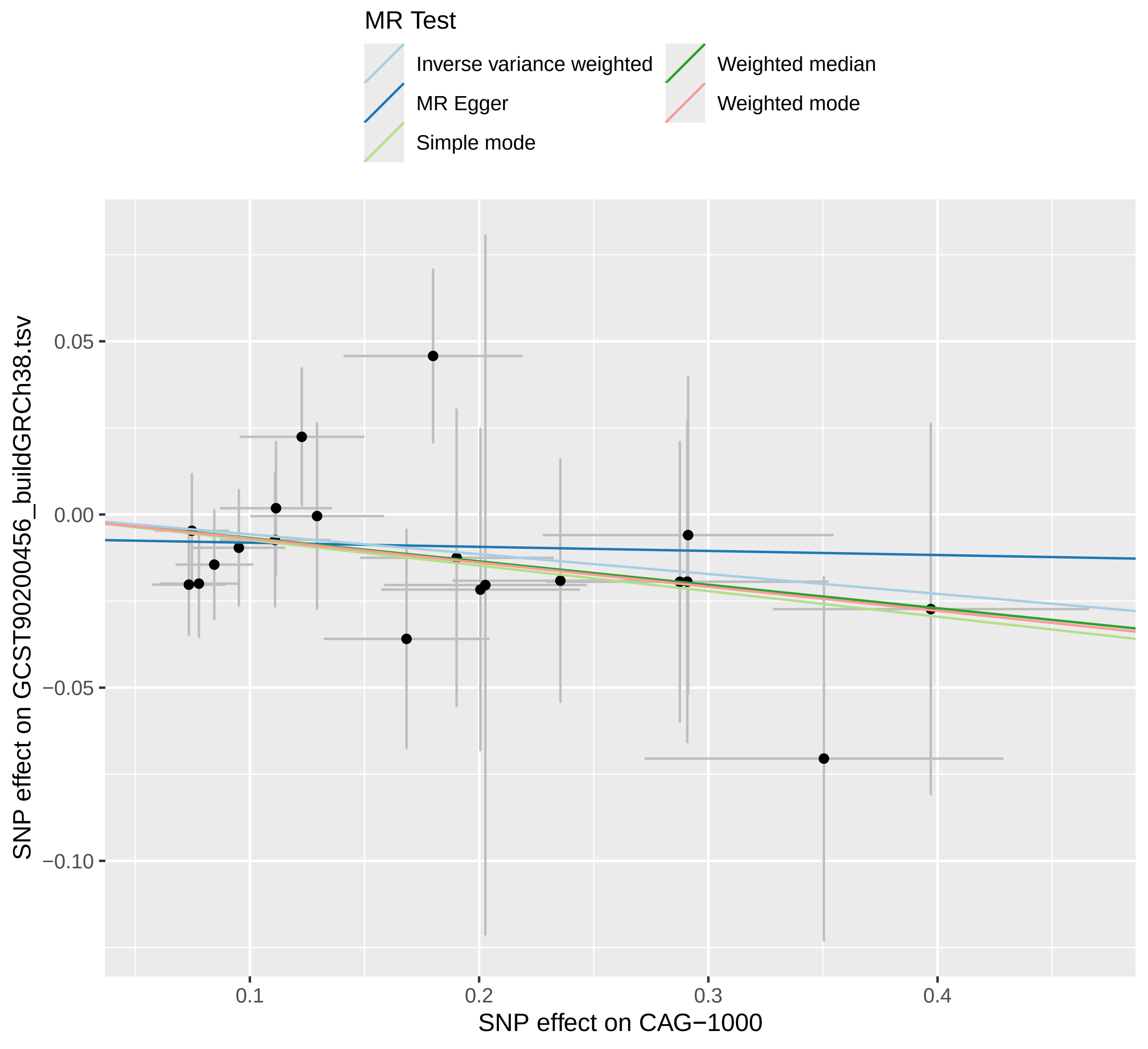
MR Test
Inverse variance weighted
MR Egger
Simple mode
Weighted median
Weighted mode
SNP effect on GCST90200456_buildGRCh38.tsv
0.05
0.00
−0.05
−0.10
0.1
0.2
0.3
0.4
SNP effect on CAG−1000

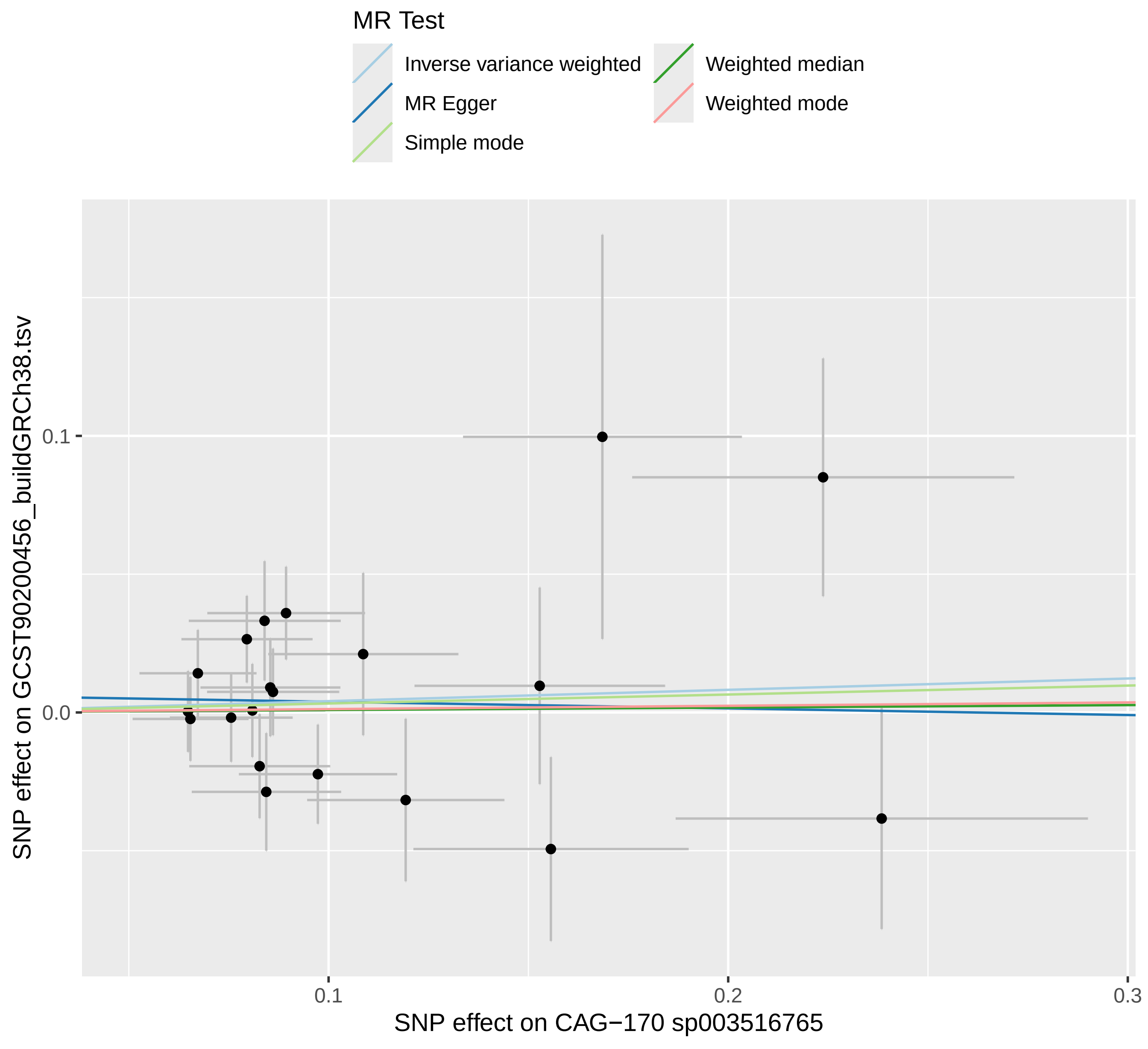

MR Test
Inverse variance weighted
MR Egger
Simple mode
Weighted median
Weighted mode
0.1
0.0
0.1
0.2
0.3
SNP effect on GCST90200456_buildGRCh38.tsv
SNP effect on CAG−170 sp003516765

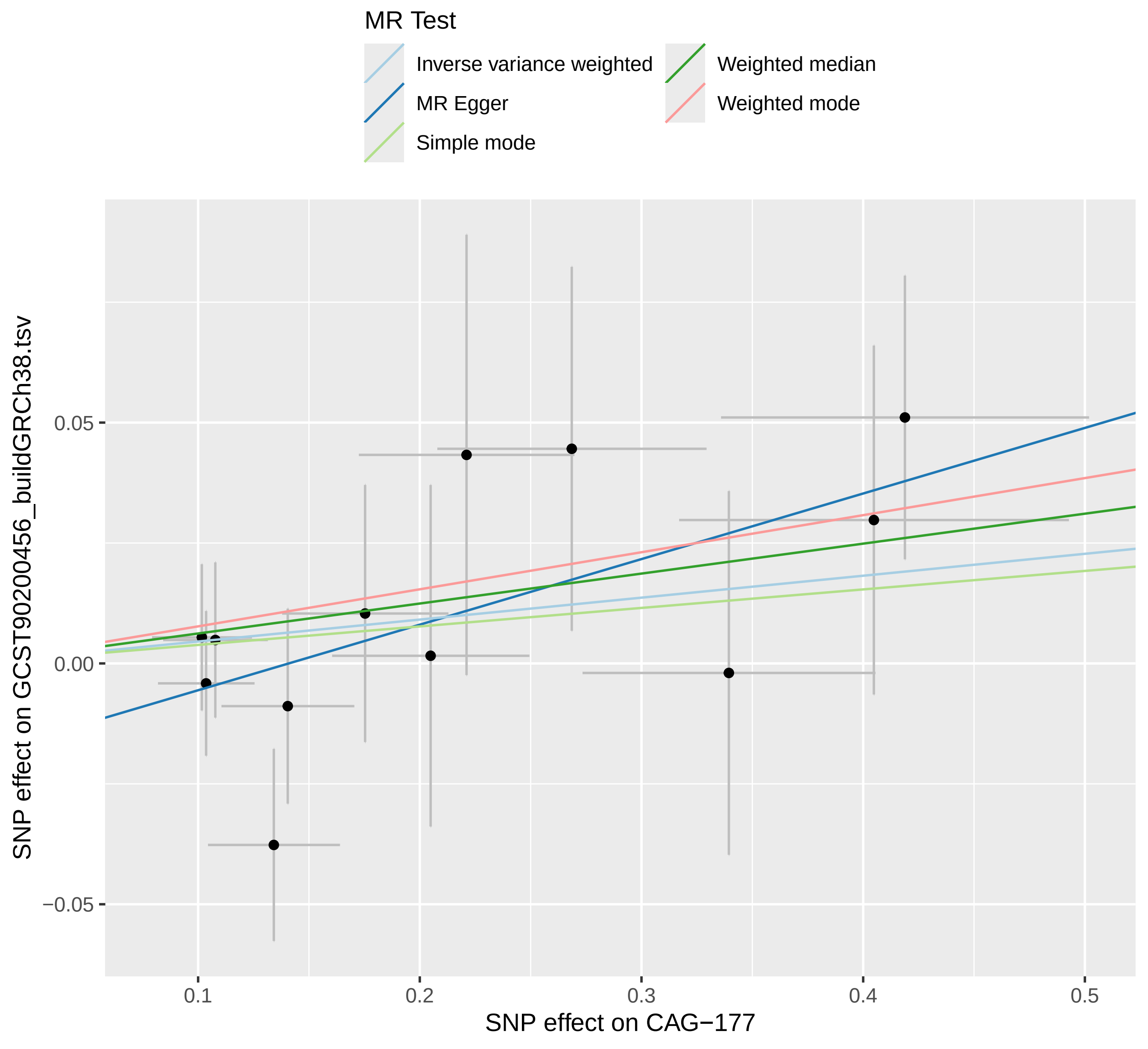
MR Test
Inverse variance weighted
MR Egger
Simple mode
Weighted median
Weighted mode
SNP effect on GCST90200456_buildGRCh38.tsv
0.05
0.00
−0.05
0.1
0.2
0.3
0.4
0.5
SNP effect on CAG−177

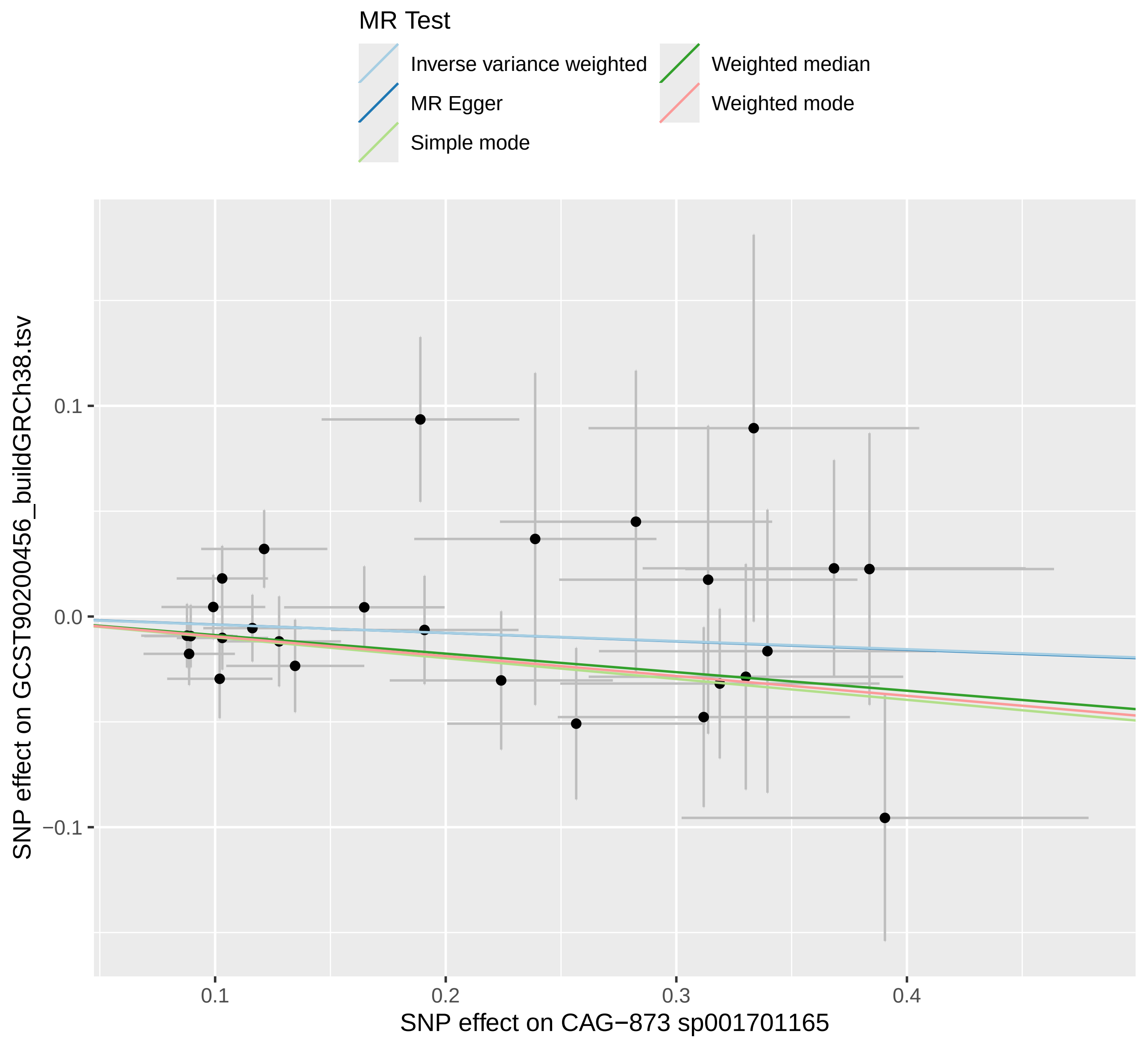

MR Test
Inverse variance weighted
MR Egger
Simple mode
Weighted median
Weighted mode
0.1
0.0
−0.1
0.1
0.2
0.3
0.4
SNP effect on GCST90200456_buildGRCh38.tsv
SNP effect on CAG−873 sp001701165

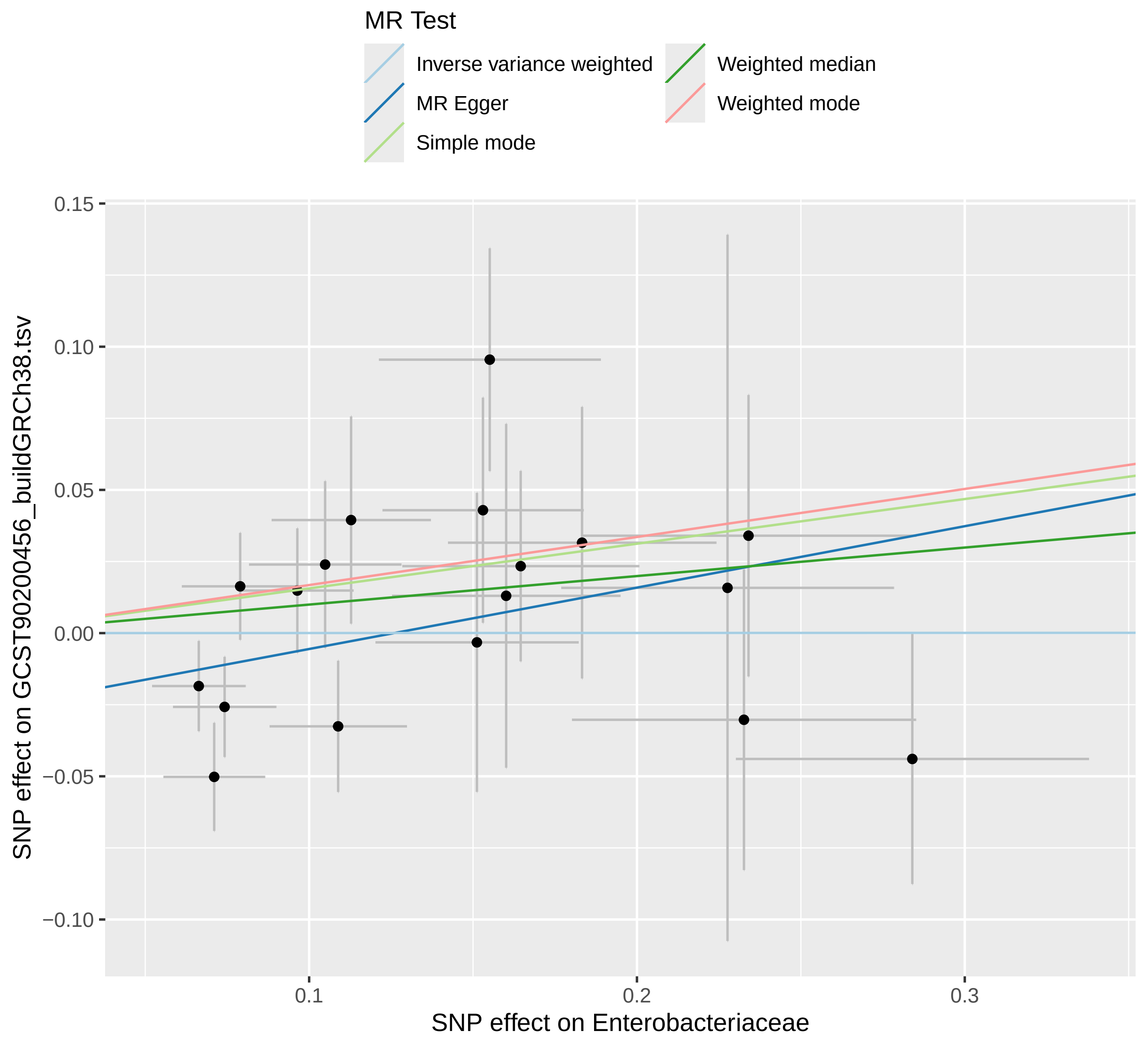
MR Test
Inverse variance weighted
MR Egger
Simple mode
Weighted median
Weighted mode
0.15
0.10
0.05
0.00
−0.05
−0.10
SNP effect on GCST90200456_buildGRCh38.tsv
0.1
0.2
0.3
SNP effect on Enterobacteriaceae

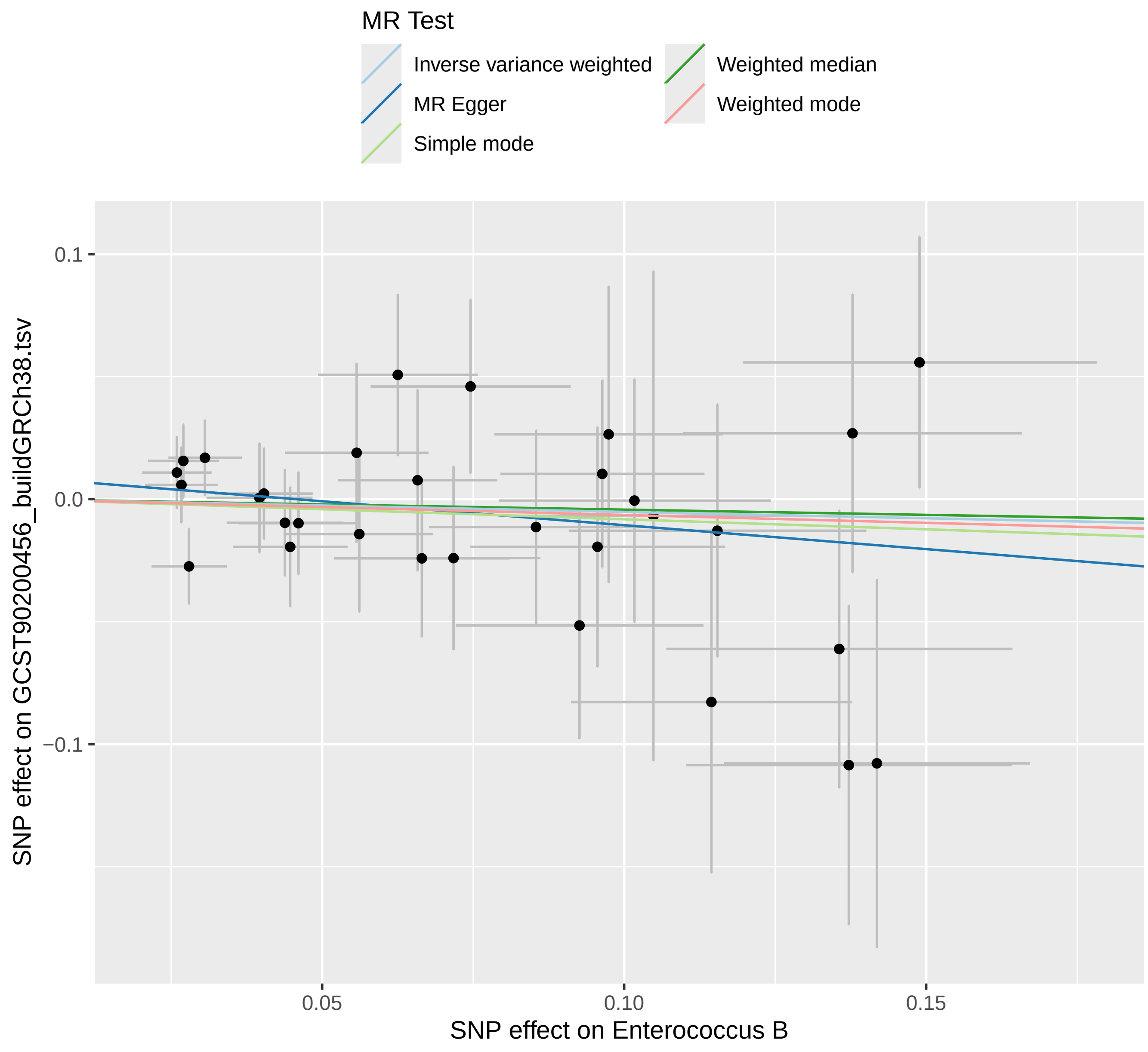

MR Test
Inverse variance weighted
MR Egger
Simple mode
Weighted median
Weighted mode
0.1
0.0
−0.1
0.05
0.10
0.15
SNP effect on GCST90200456_buildGRCh38.tsv
SNP effect on Enterococcus B

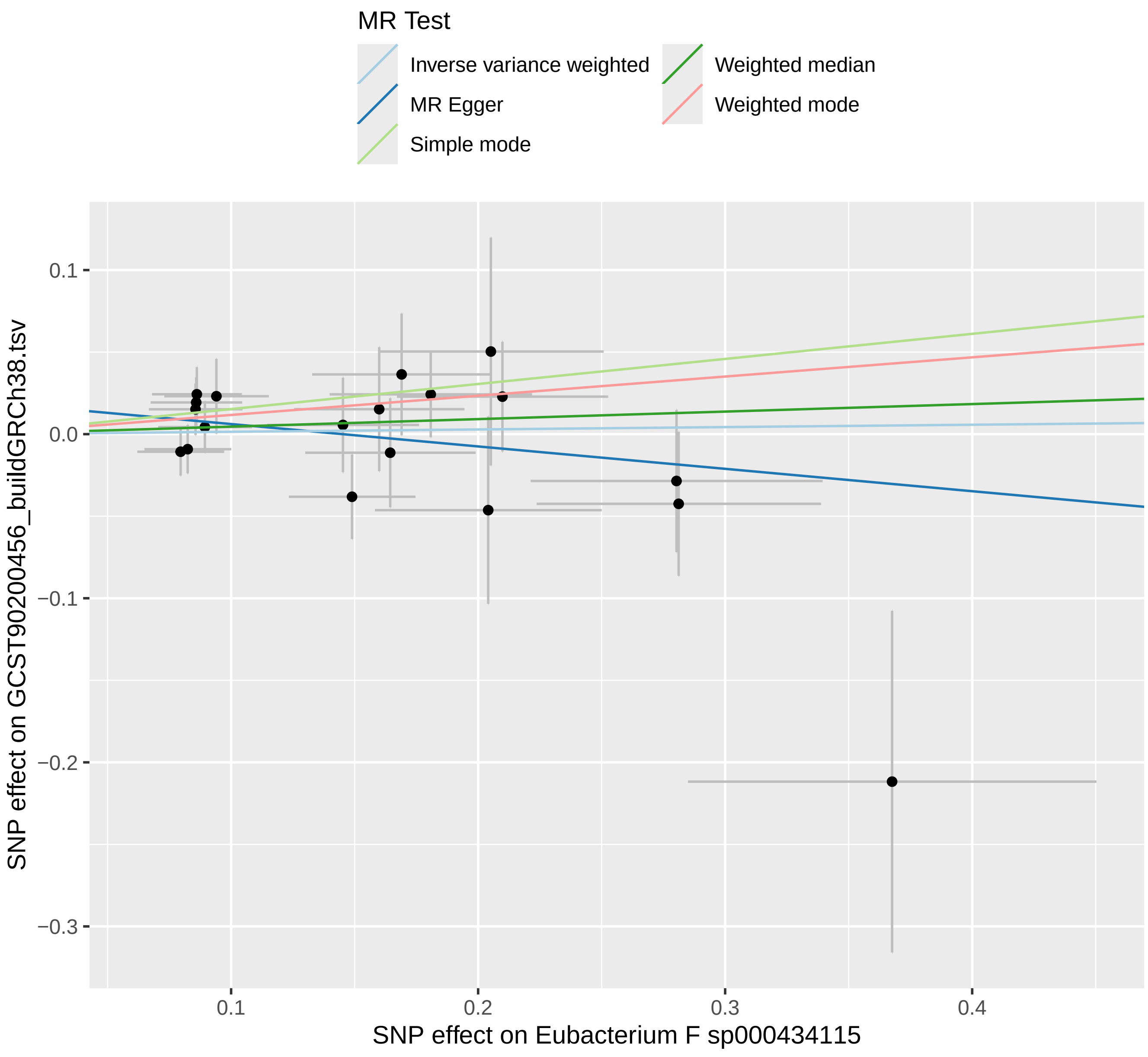
MR Test
Inverse variance weighted
MR Egger
Simple mode
Weighted median
Weighted mode
SNP effect on GCST90200456_buildGRCh38.tsv
0.1
0.0
−0.1
−0.2
−0.3
0.1
0.2
0.3
0.4
SNP effect on Eubacterium F sp000434115

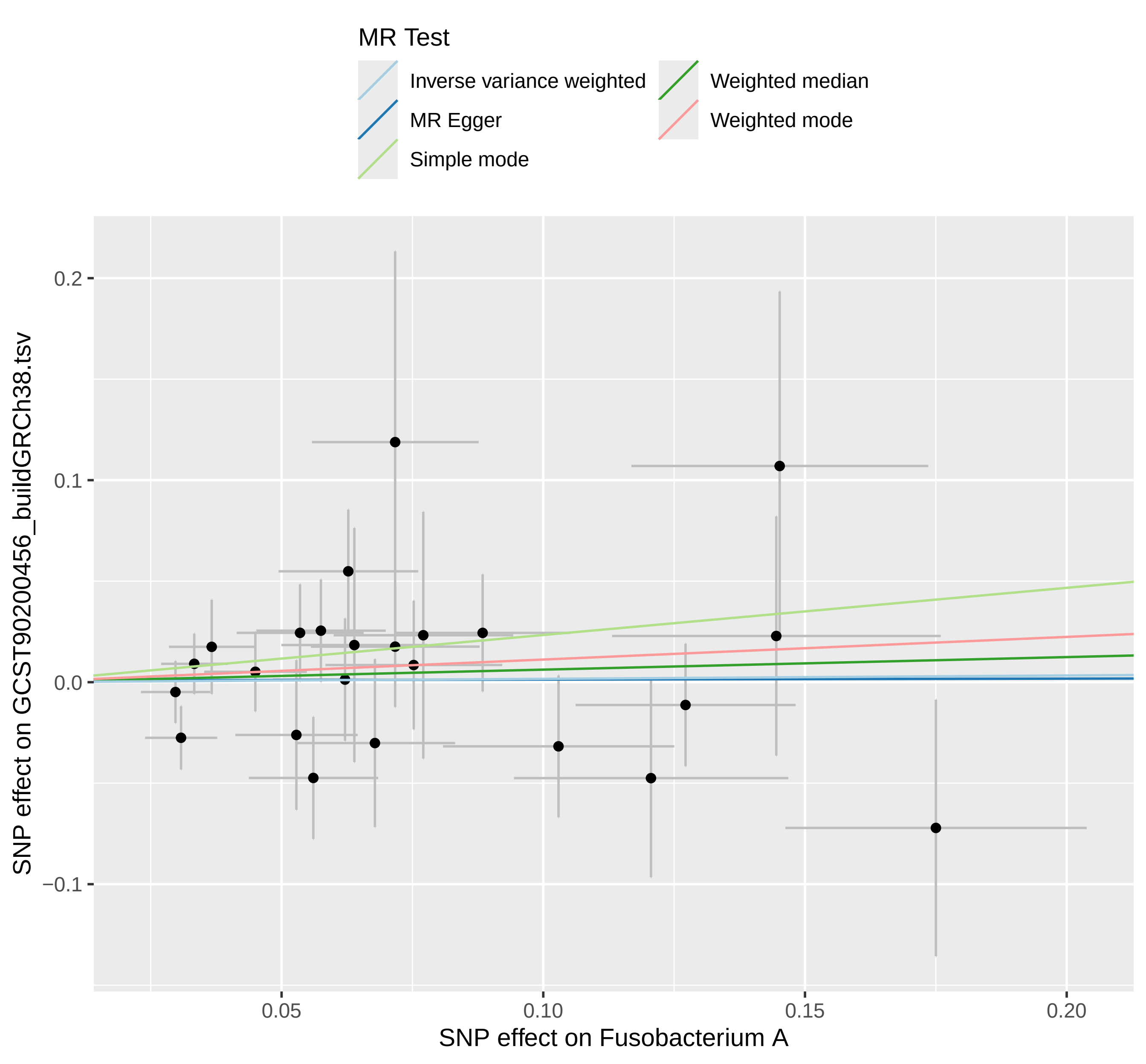

MR Test
Inverse variance weighted
MR Egger
Simple mode
Weighted median
Weighted mode
0.2
0.1
0.0
−0.1
0.05
0.10
0.15
0.20
SNP effect on GCST90200456_buildGRCh38.tsv
SNP effect on Fusobacterium A

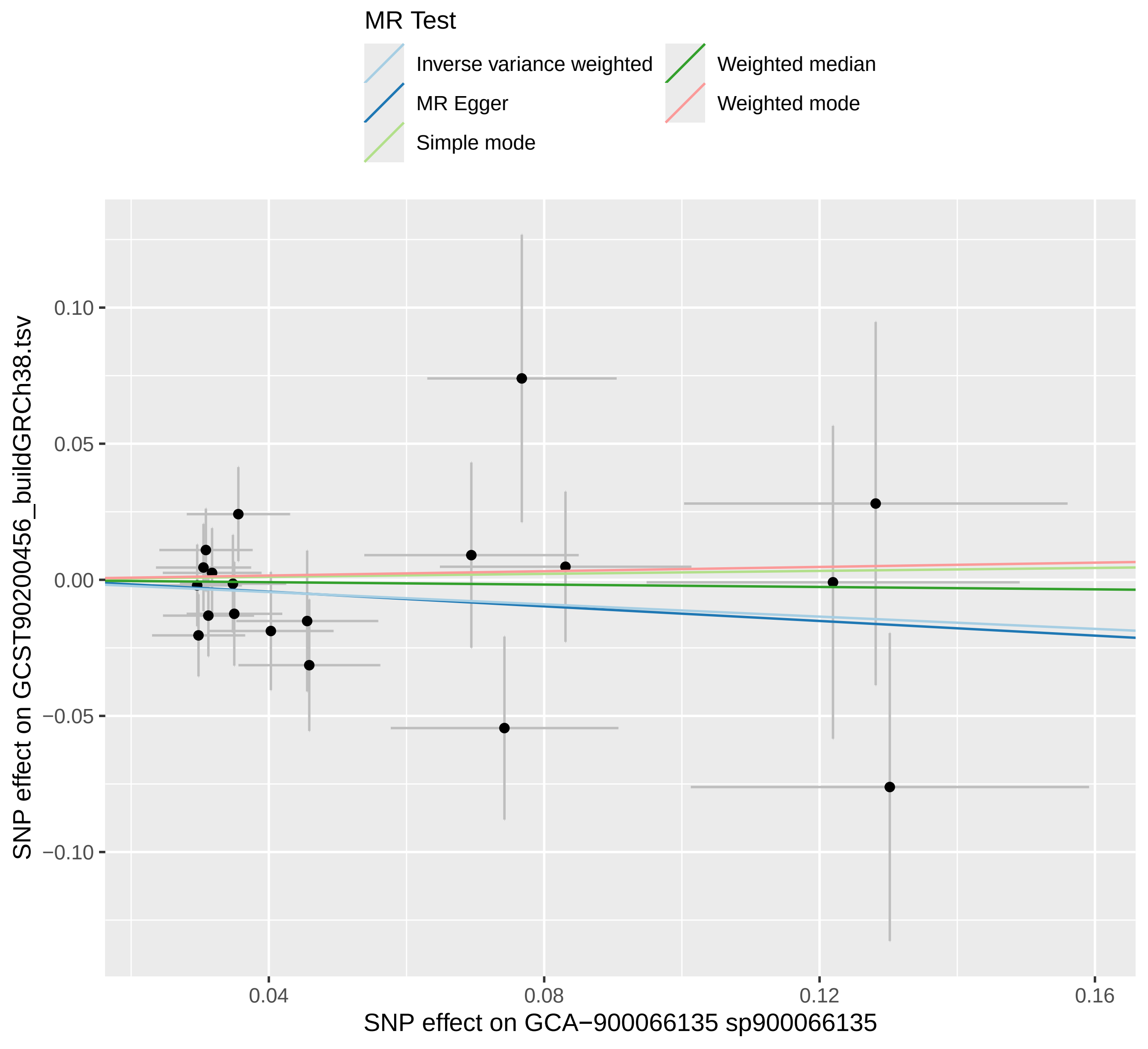

MR Test
Inverse variance weighted
MR Egger
Simple mode
Weighted median
Weighted mode
0.10
0.05
0.00
−0.05
−0.10
0.04
0.08
0.12
0.16
SNP effect on GCST90200456_buildGRCh38.tsv
SNP effect on GCA−900066135 sp900066135

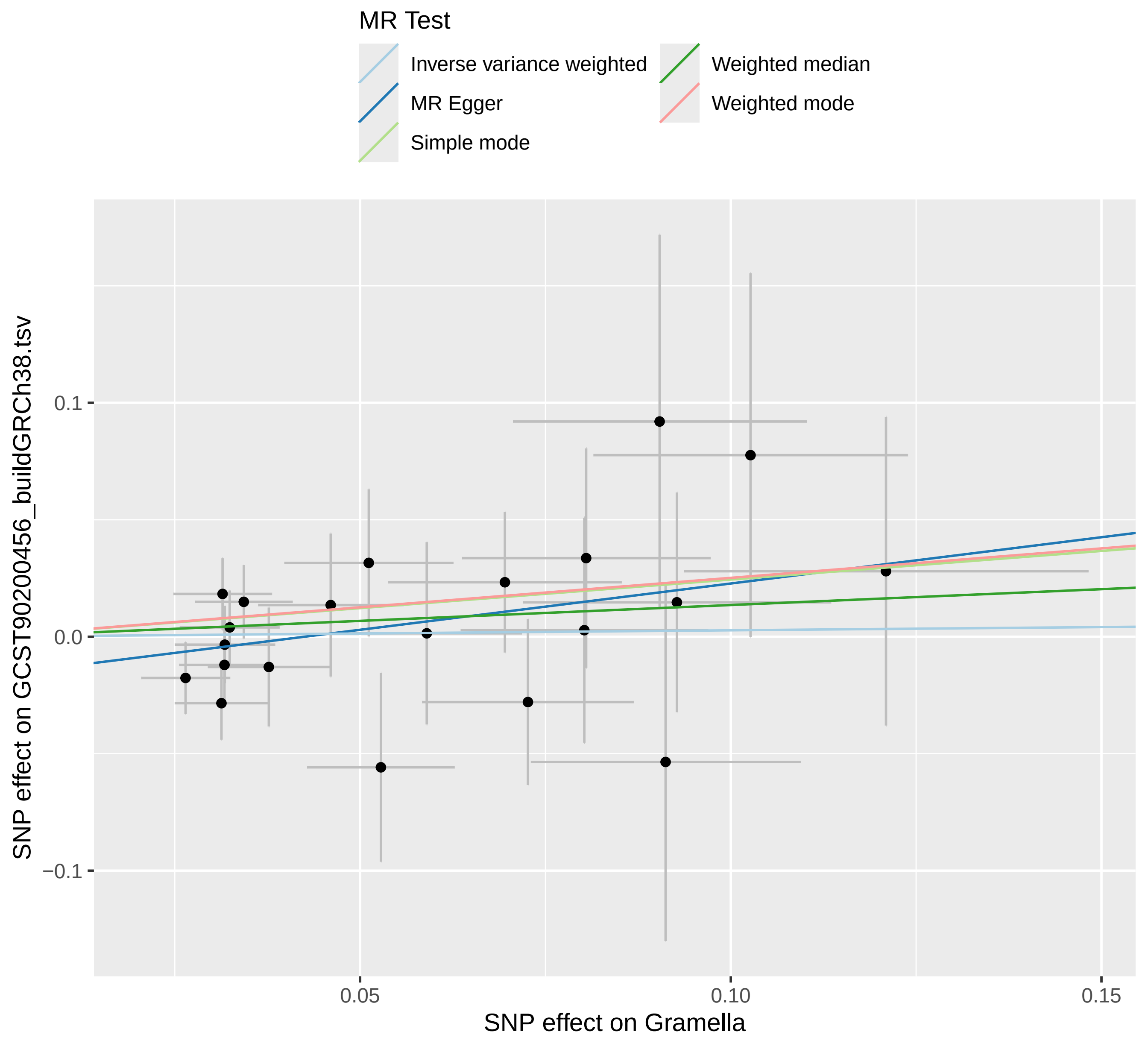

MR Test
Inverse variance weighted
MR Egger
Simple mode
Weighted median
Weighted mode
SNP effect on GCST90200456_buildGRCh38.tsv
0.1
0.0
−0.1
0.05
0.10
0.15
SNP effect on Gramella

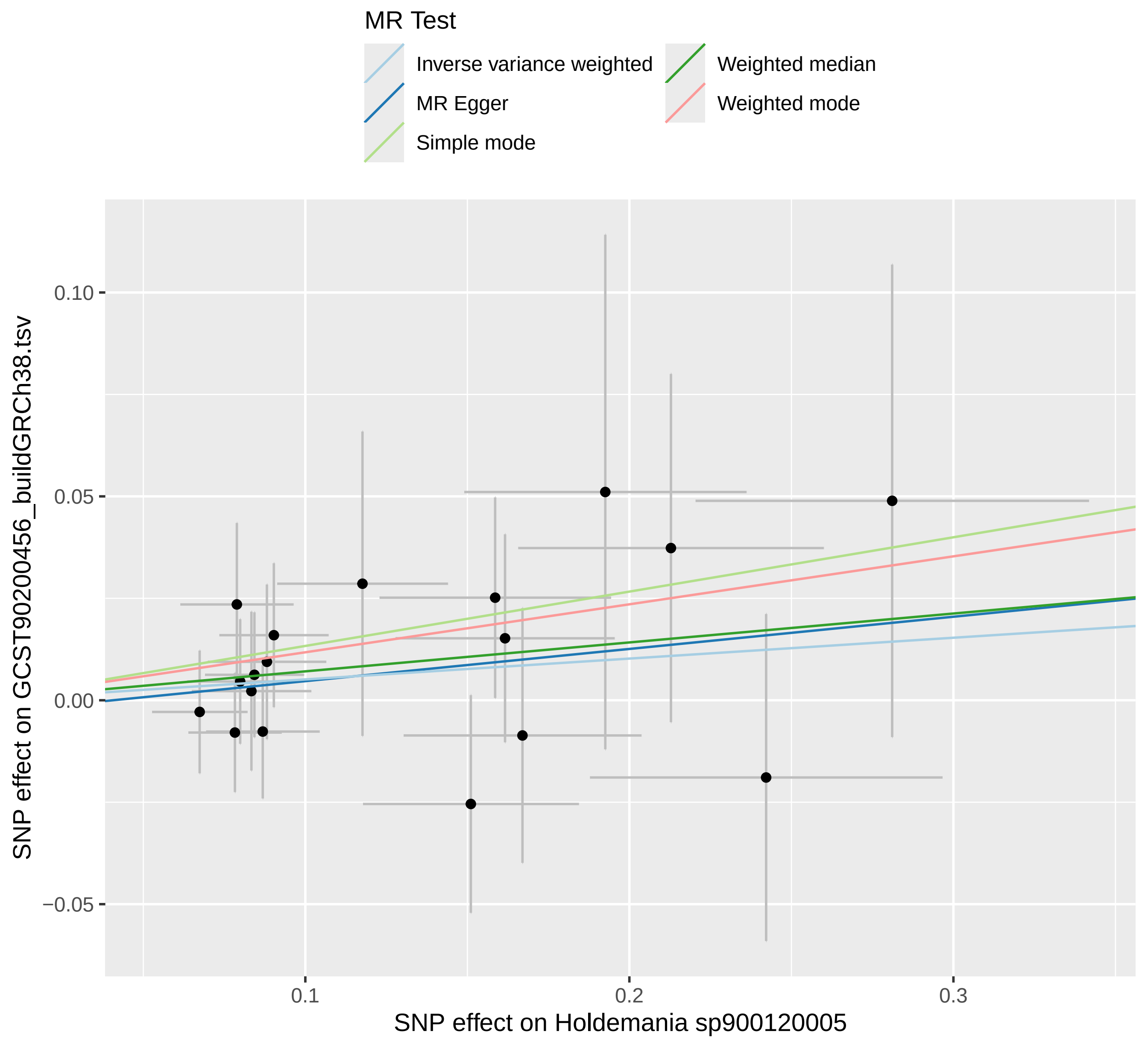
MR Test
Inverse variance weighted
MR Egger
Simple mode
Weighted median
Weighted mode
0.10
0.05
0.00
−0.05
SNP effect on GCST90200456_buildGRCh38.tsv
0.1
0.2
0.3
SNP effect on Holdemania sp900120005

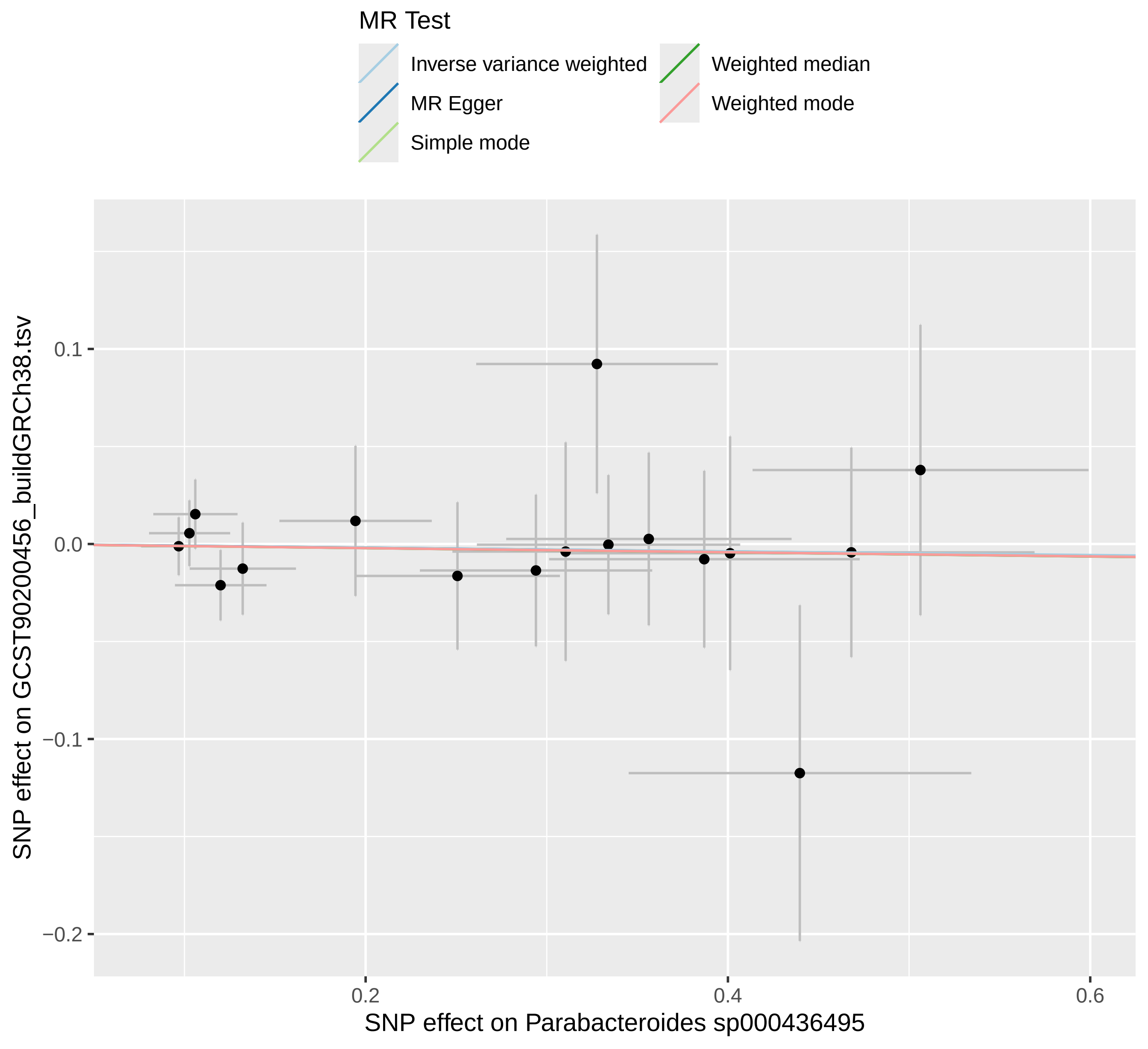
MR Test
Inverse variance weighted
MR Egger
Simple mode
Weighted median
Weighted mode
SNP effect on GCST90200456_buildGRCh38.tsv
0.1
0.0
−0.1
−0.2
0.2
0.4
0.6
SNP effect on Parabacteroides sp000436495

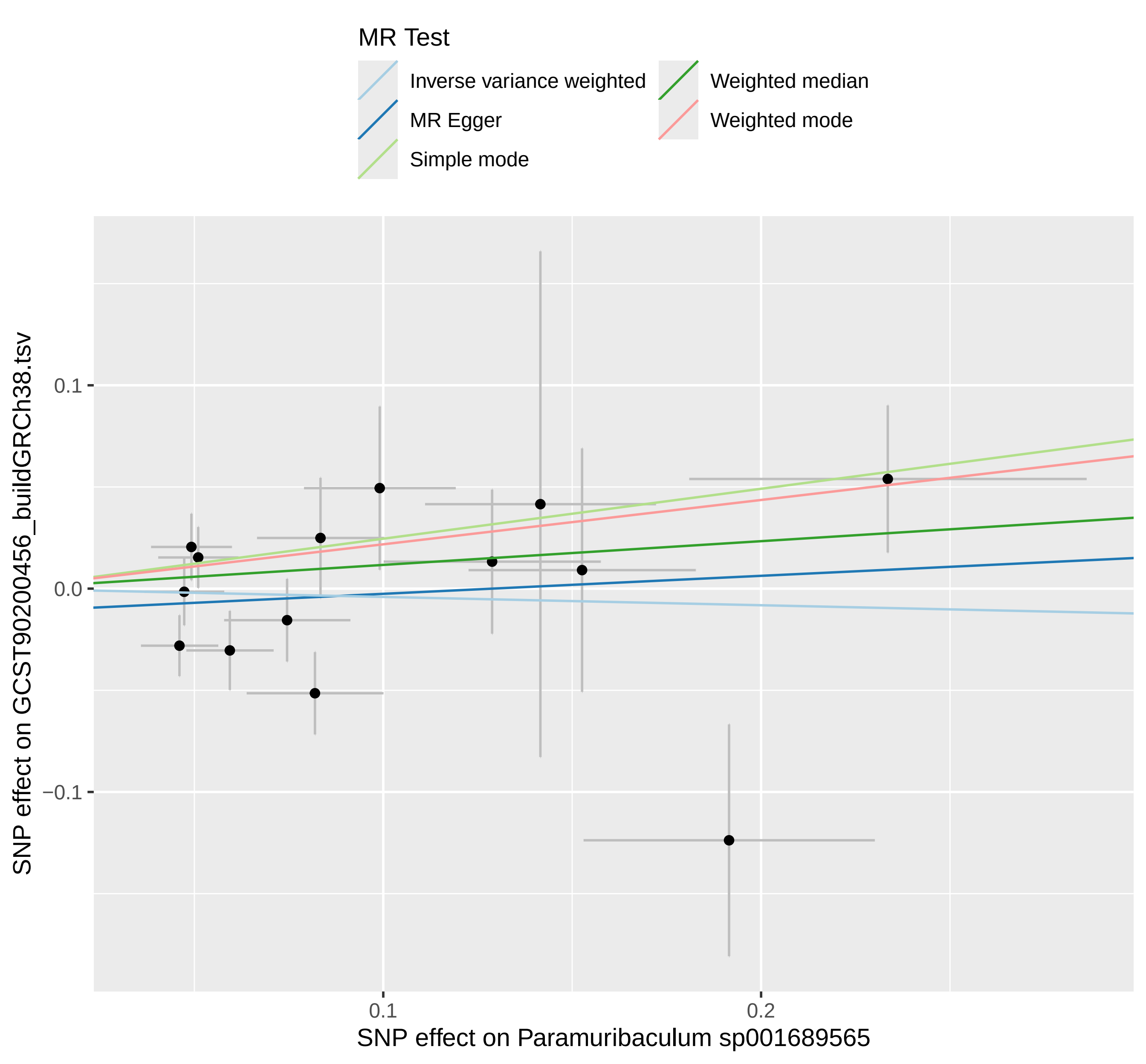
MR Test
Inverse variance weighted
MR Egger
Simple mode
Weighted median
Weighted mode
0.1
0.0
−0.1
0.1
0.2
SNP effect on GCST90200456_buildGRCh38.tsv
SNP effect on Paramuribaculum sp001689565

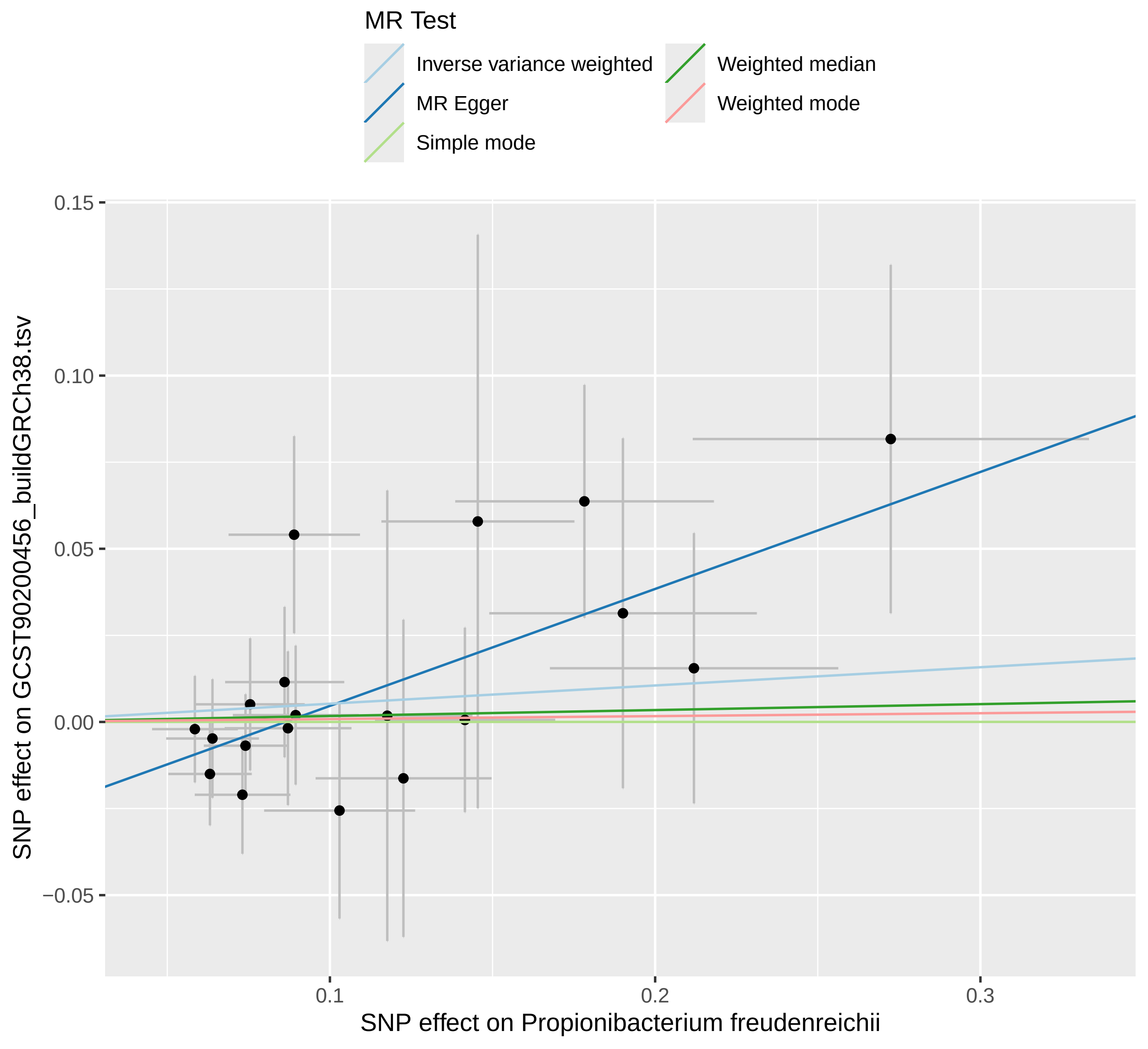
MR Test
Inverse variance weighted
MR Egger
Simple mode
Weighted median
Weighted mode
0.15
0.10
0.05
0.00
−0.05
0.1
0.2
0.3
SNP effect on GCST90200456_buildGRCh38.tsv
SNP effect on Propionibacterium freudenreichii

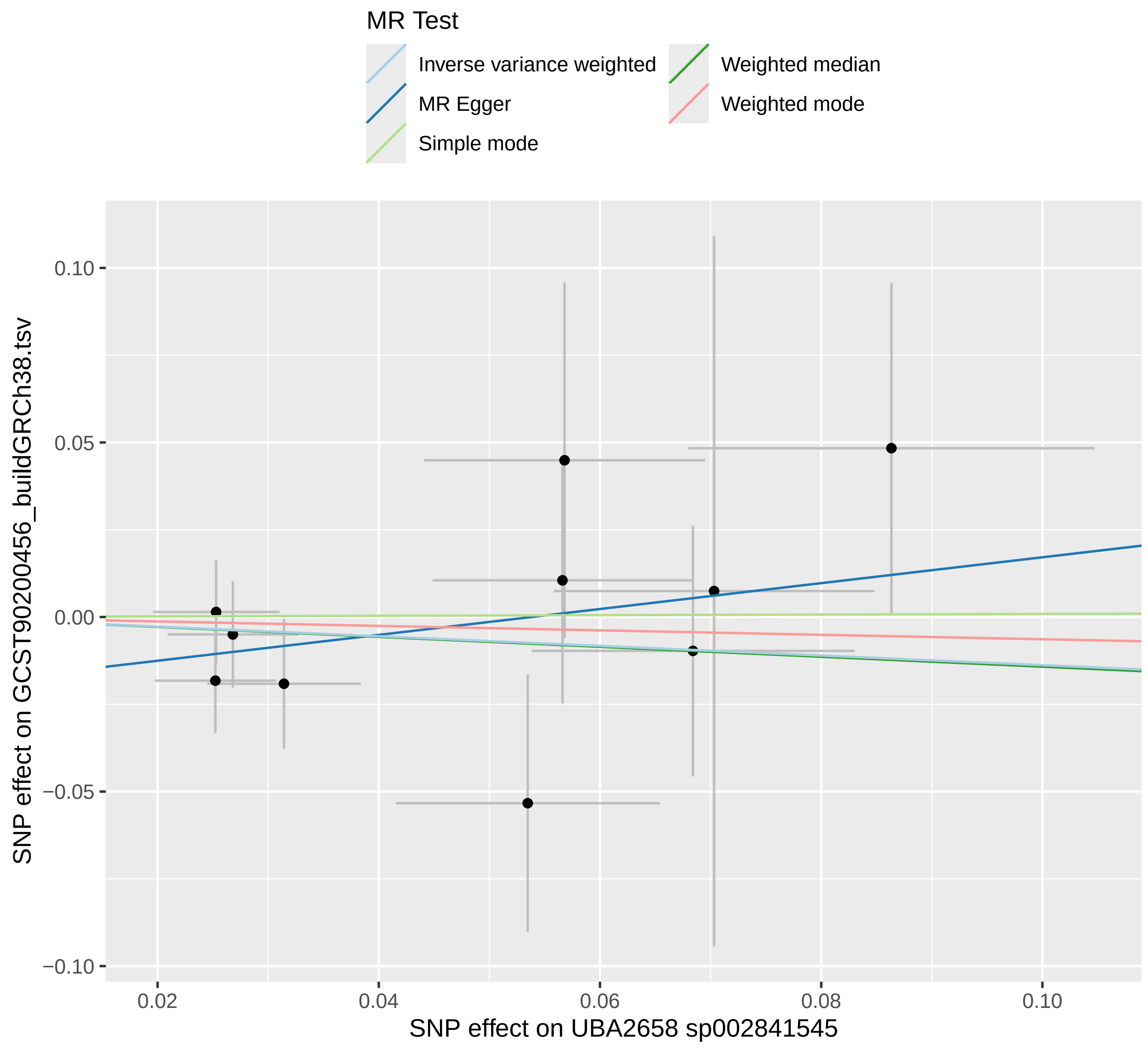

MR Test
Inverse variance weighted
MR Egger
Simple mode
Weighted median
Weighted mode
SNP effect on GCST90200456_buildGRCh38.tsv
0.10
0.05
0.00
−0.05
−0.10
0.02
0.04
0.06
0.08
0.10
SNP effect on UBA2658 sp002841545

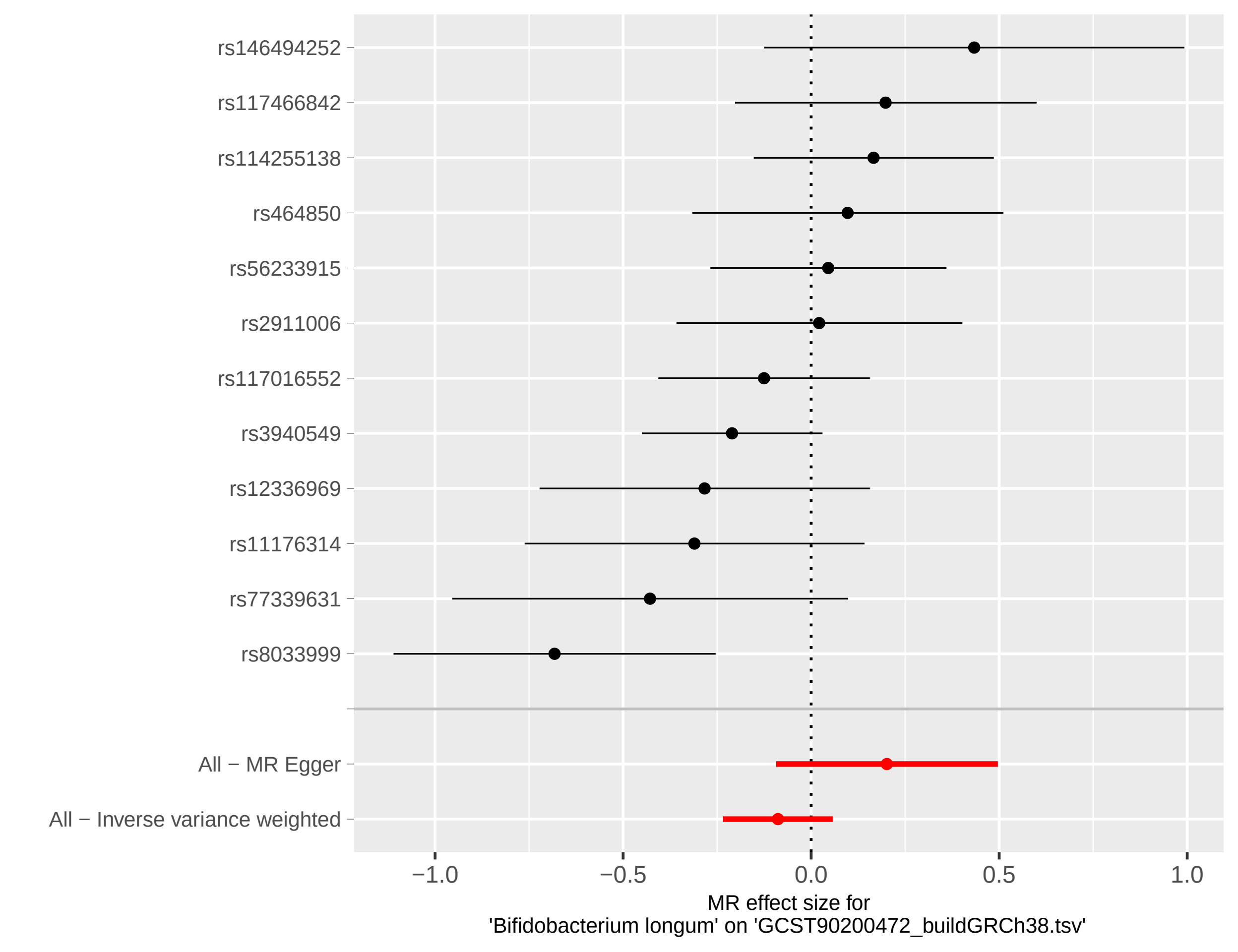
rs146494252
rs117466842
rs114255138
rs464850
rs56233915
rs2911006
rs117016552
rs3940549
rs12336969
rs11176314
rs77339631
rs8033999
All − MR Egger
All − Inverse variance weighted
−1.0
−0.5
0.0
0.5
1.0
MR effect size for
'Bifidobacterium longum' on 'GCST90200472_buildGRCh38.tsv'

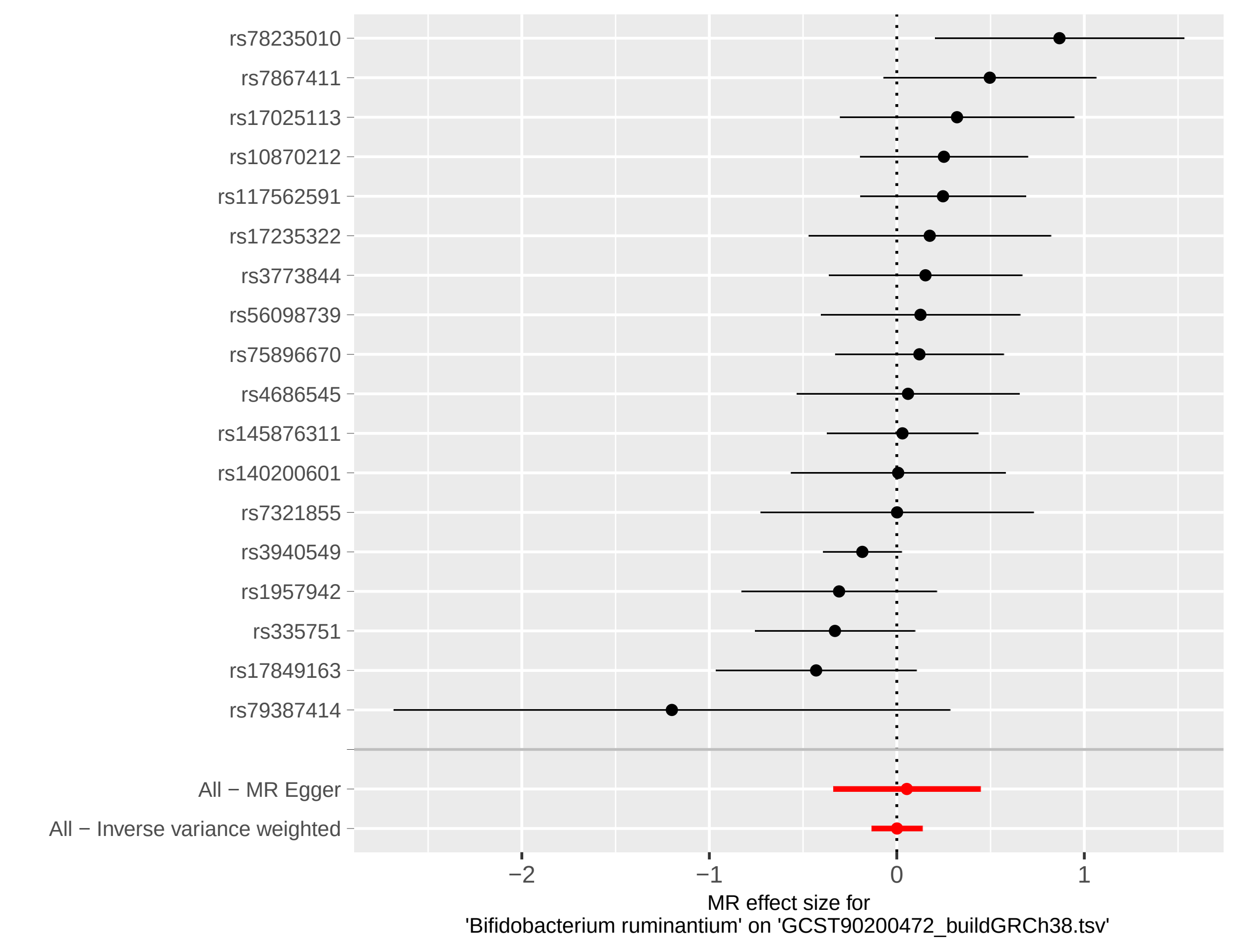
rs78235010
rs7867411
rs17025113
rs10870212
rs117562591
rs17235322
rs3773844
rs56098739
rs75896670
rs4686545
rs145876311
rs140200601
rs7321855
rs3940549
rs1957942
rs335751
rs17849163
rs79387414
All − MR Egger
All − Inverse variance weighted
−2
−1
0
1
MR effect size for
'Bifidobacterium ruminantium' on 'GCST90200472_buildGRCh38.tsv'

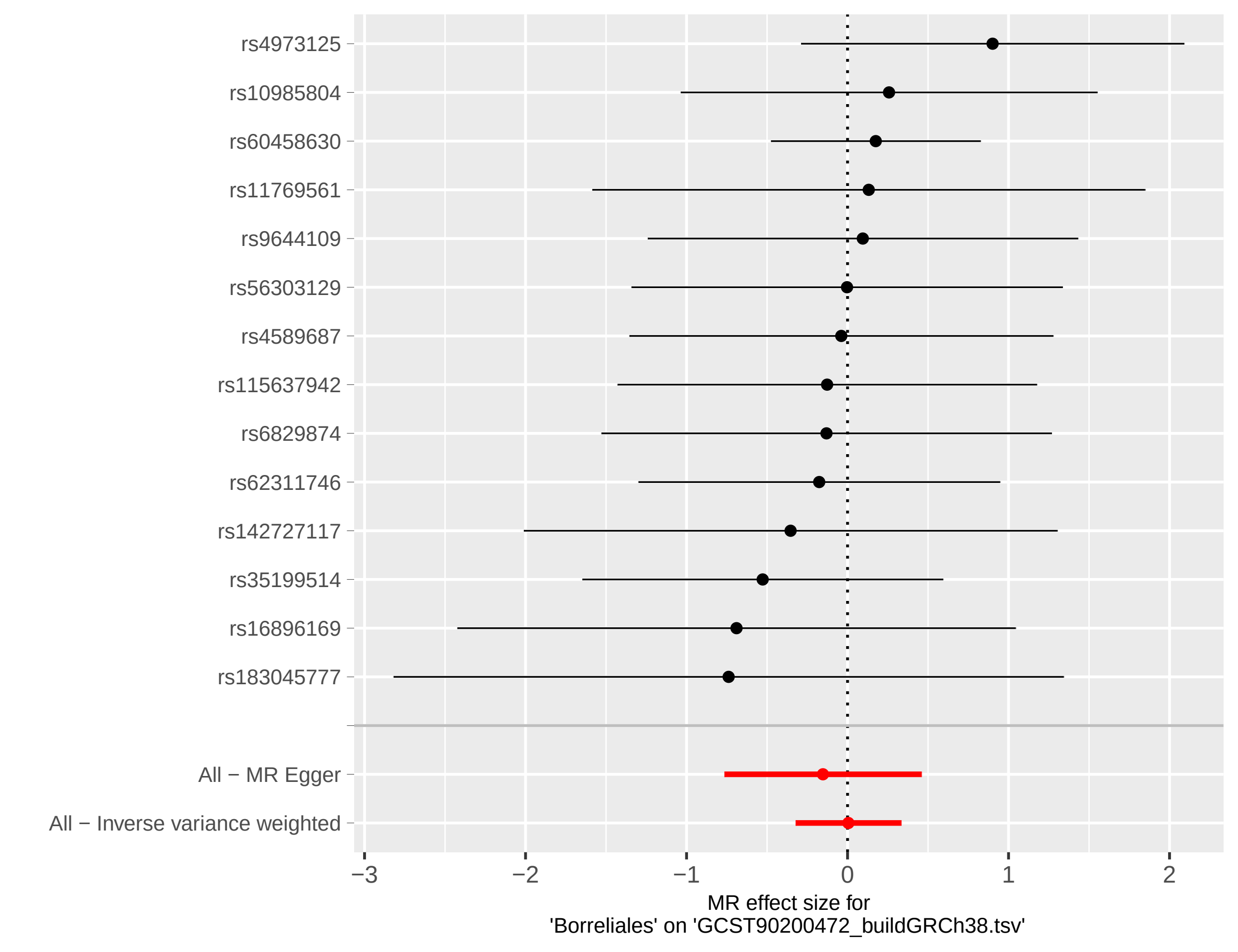
rs4973125
rs10985804
rs60458630
rs11769561
rs9644109
rs56303129
rs4589687
rs115637942
rs6829874
rs62311746
rs142727117
rs35199514
rs16896169
rs183045777
All − MR Egger
All − Inverse variance weighted
−3
−2
−1
0
1
2
MR effect size for
'Borreliales' on 'GCST90200472_buildGRCh38.tsv'

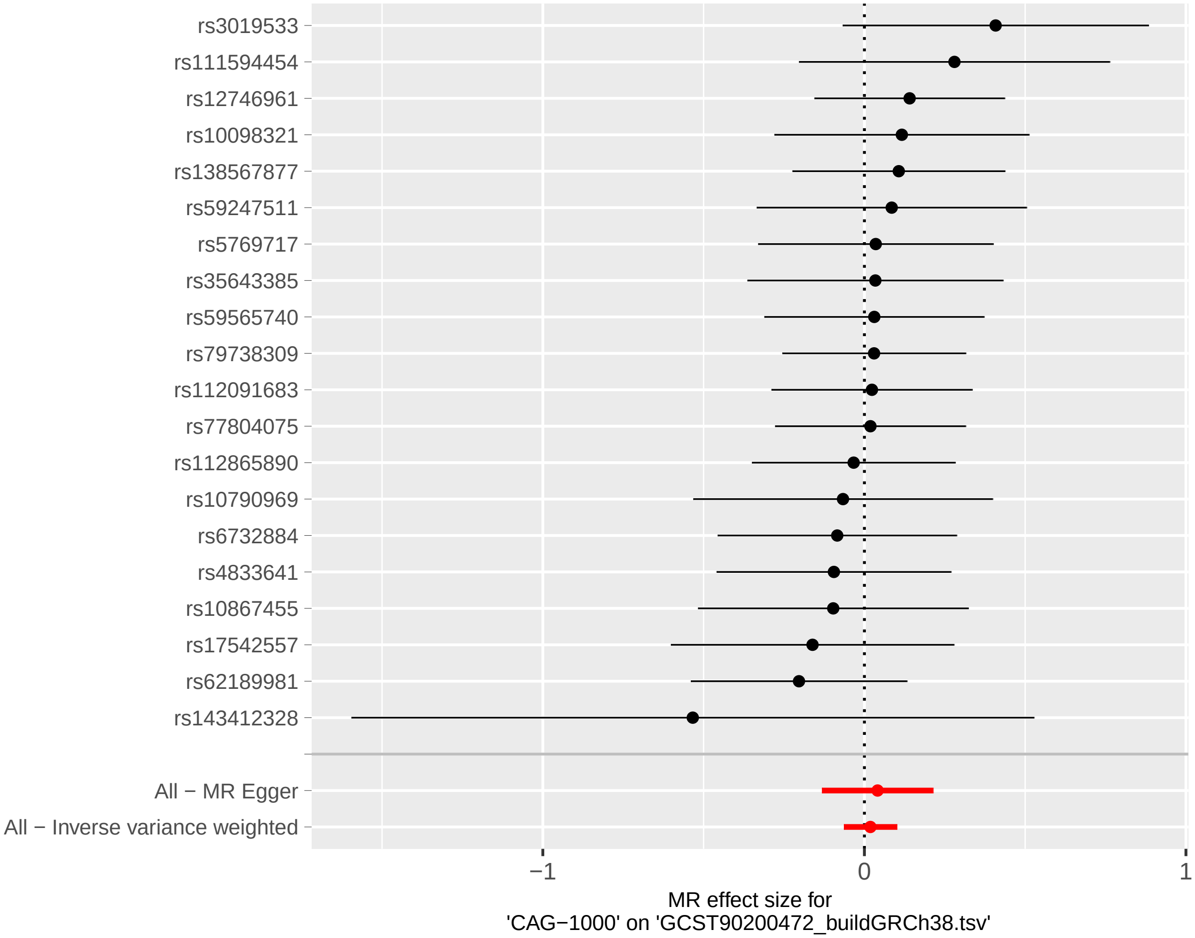

rs3019533
rs111594454
rs12746961
rs10098321
rs138567877
rs59247511
rs5769717
rs35643385
rs59565740
rs79738309
rs112091683
rs77804075
rs112865890
rs10790969
rs6732884
rs4833641
rs10867455
rs17542557
rs62189981
rs143412328
All − MR Egger
All − Inverse variance weighted
−1
0
1
MR effect size for
'CAG−1000' on 'GCST90200472_buildGRCh38.tsv'

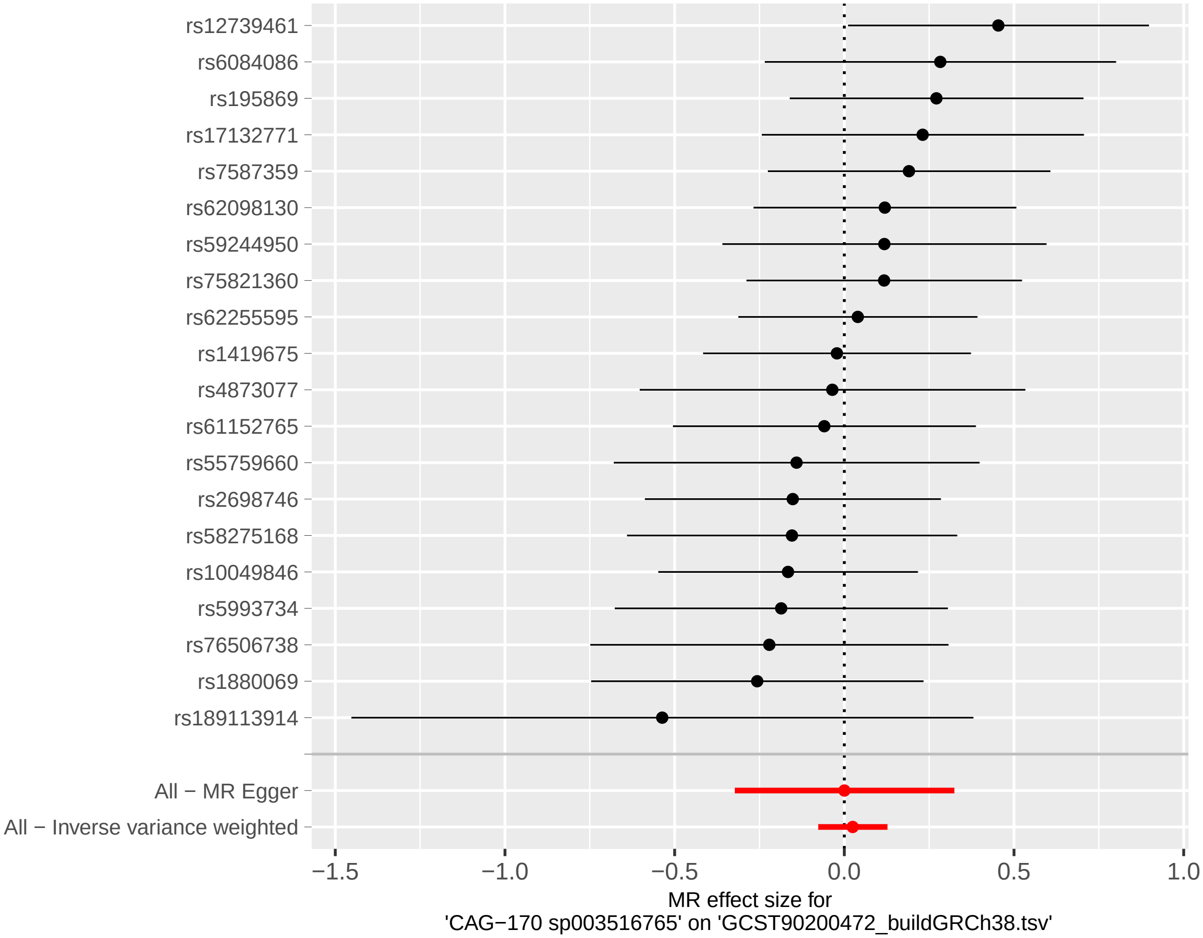

rs12739461
rs6084086
rs195869
rs17132771
rs7587359
rs62098130
rs59244950
rs75821360
rs62255595
rs1419675
rs4873077
rs61152765
rs55759660
rs2698746
rs58275168
rs10049846
rs5993734
rs76506738
rs1880069
rs189113914
All − MR Egger
All − Inverse variance weighted
−1.5
−1.0
−0.5
0.0
0.5
1.0
MR effect size for
'CAG−170 sp003516765' on 'GCST90200472_buildGRCh38.tsv'

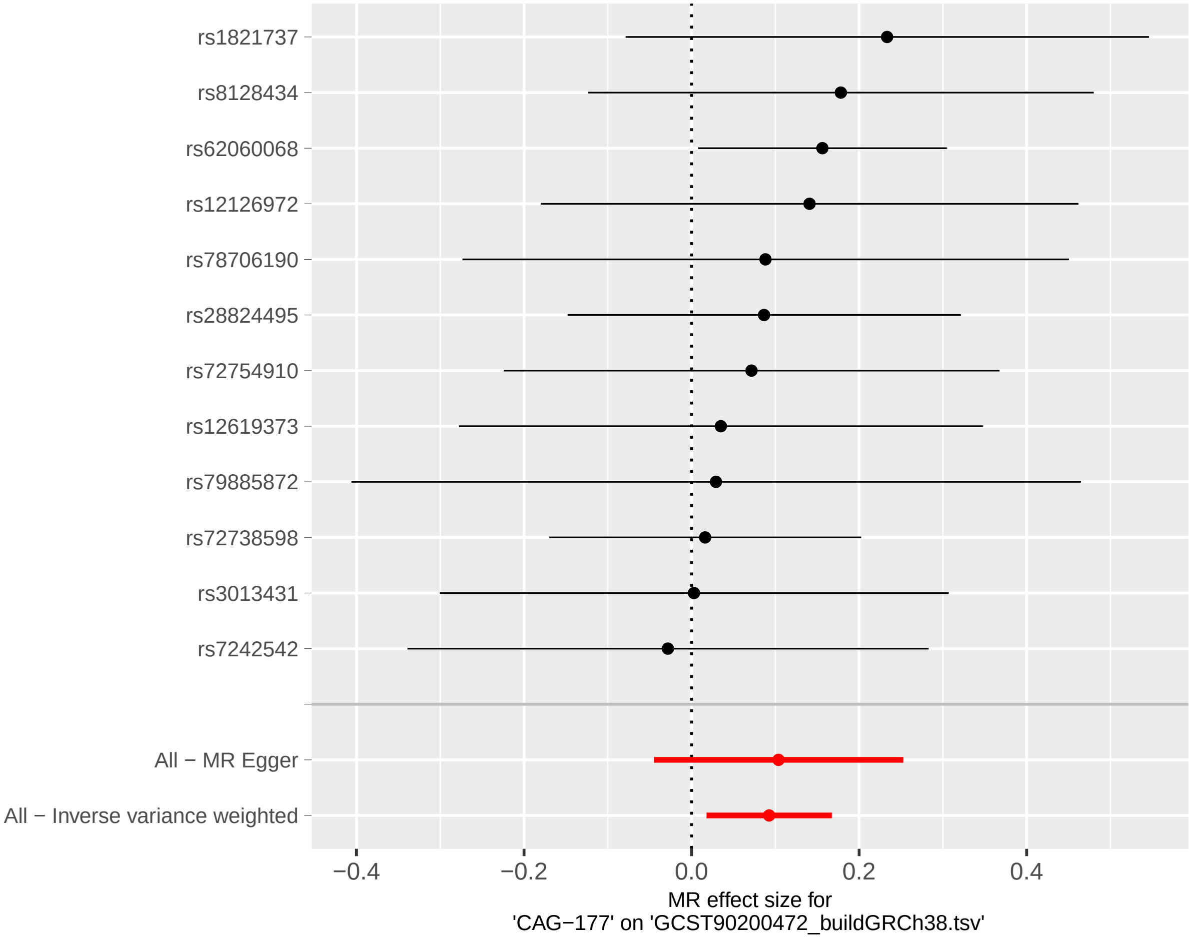

rs1821737
rs8128434
rs62060068
rs12126972
rs78706190
rs28824495
rs72754910
rs12619373
rs79885872
rs72738598
rs3013431
rs7242542
All − MR Egger
All − Inverse variance weighted
−0.4
−0.2
0.0
0.2
0.4
MR effect size for
'CAG−177' on 'GCST90200472_buildGRCh38.tsv'

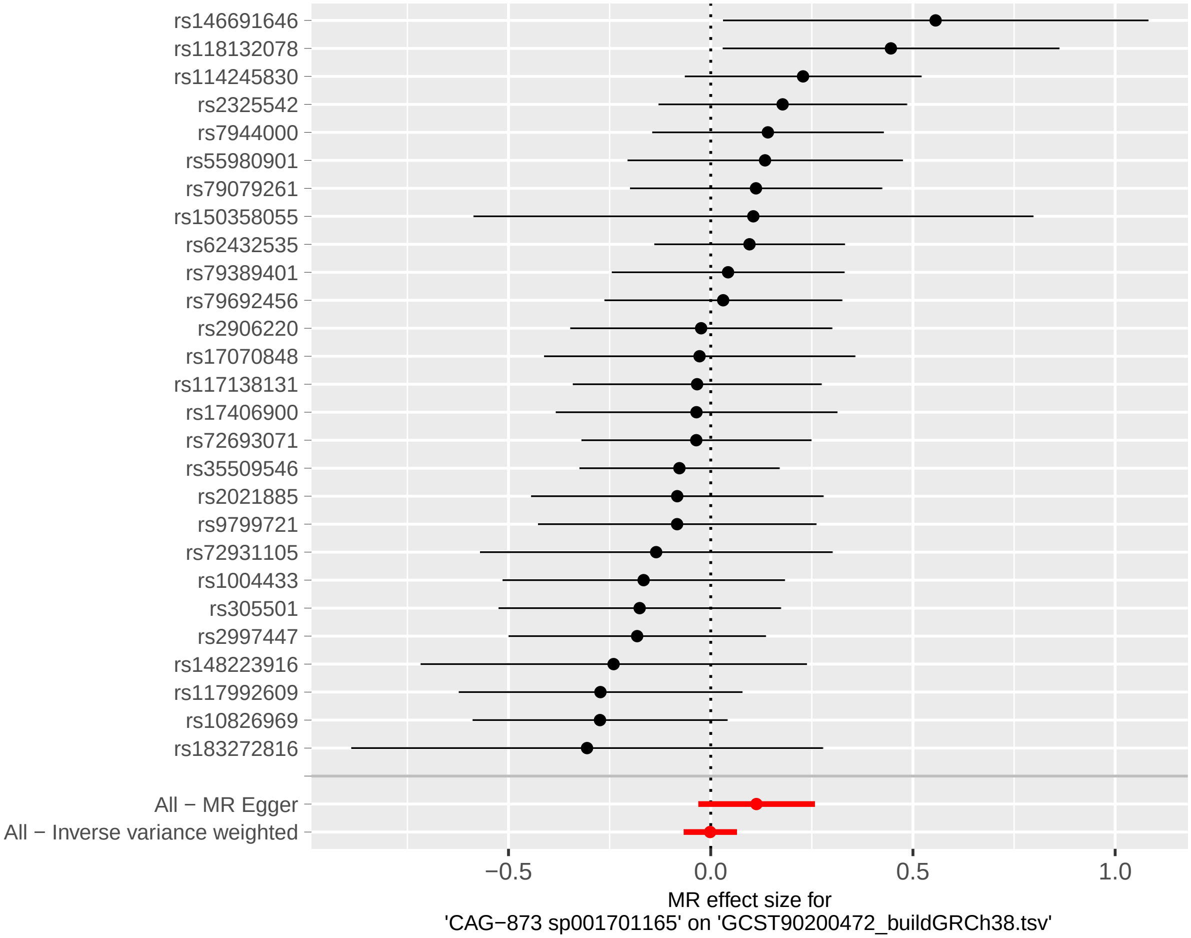
rs146691646
rs118132078
rs114245830
rs2325542
rs7944000
rs55980901
rs79079261
rs150358055
rs62432535
rs79389401
rs79692456
rs2906220
rs17070848
rs117138131
rs17406900
rs72693071
rs35509546
rs2021885
rs9799721
rs72931105
rs1004433
rs305501
rs2997447
rs148223916
rs117992609
rs10826969
rs183272816
All − MR Egger
All − Inverse variance weighted
−0.5
0.0
0.5
1.0
MR effect size for
'CAG−873 sp001701165' on 'GCST90200472_buildGRCh38.tsv'

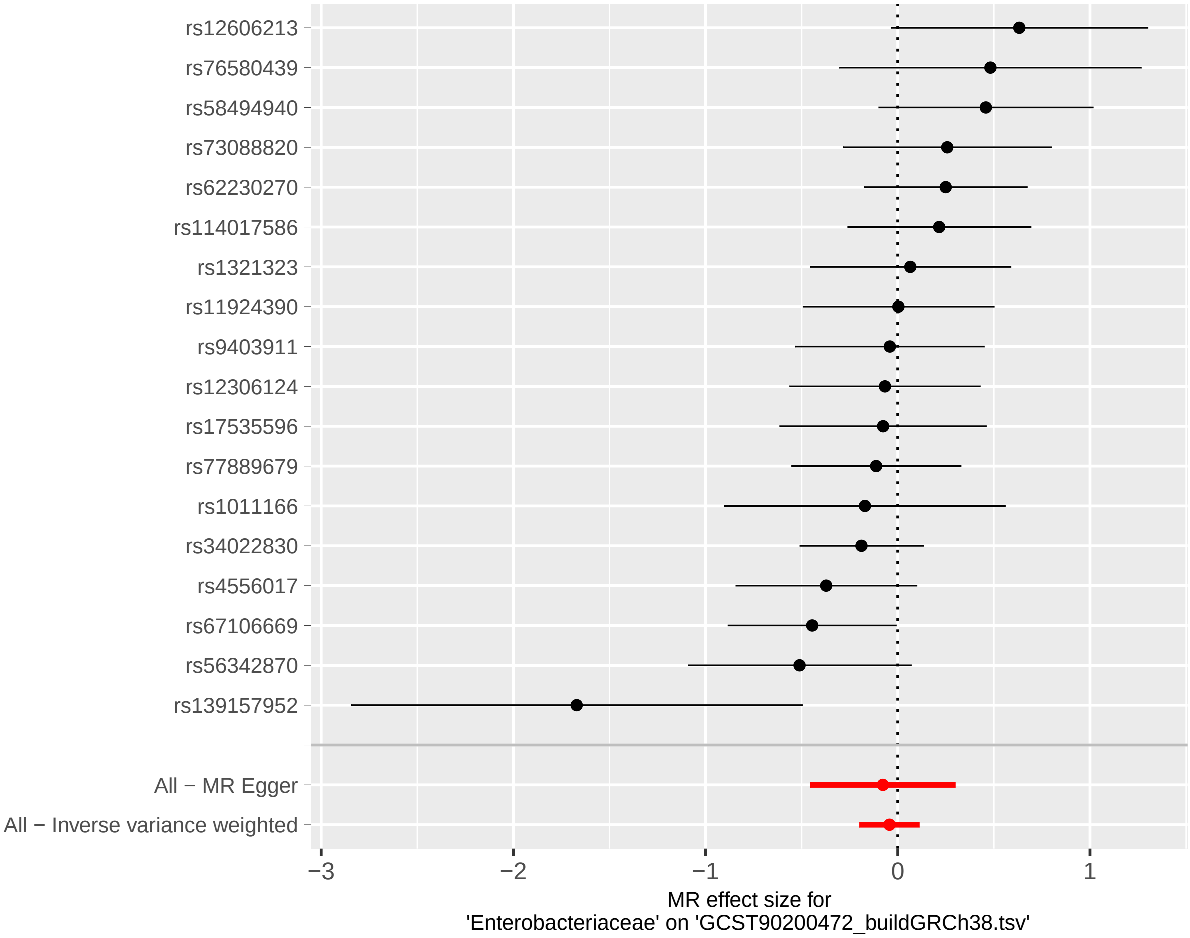

rs12606213
rs76580439
rs58494940
rs73088820
rs62230270
rs114017586
rs1321323
rs11924390
rs9403911
rs12306124
rs17535596
rs77889679
rs1011166
rs34022830
rs4556017
rs67106669
rs56342870
rs139157952
All − MR Egger
All − Inverse variance weighted
−3
−2
−1
0
1
MR effect size for
'Enterobacteriaceae' on 'GCST90200472_buildGRCh38.tsv'

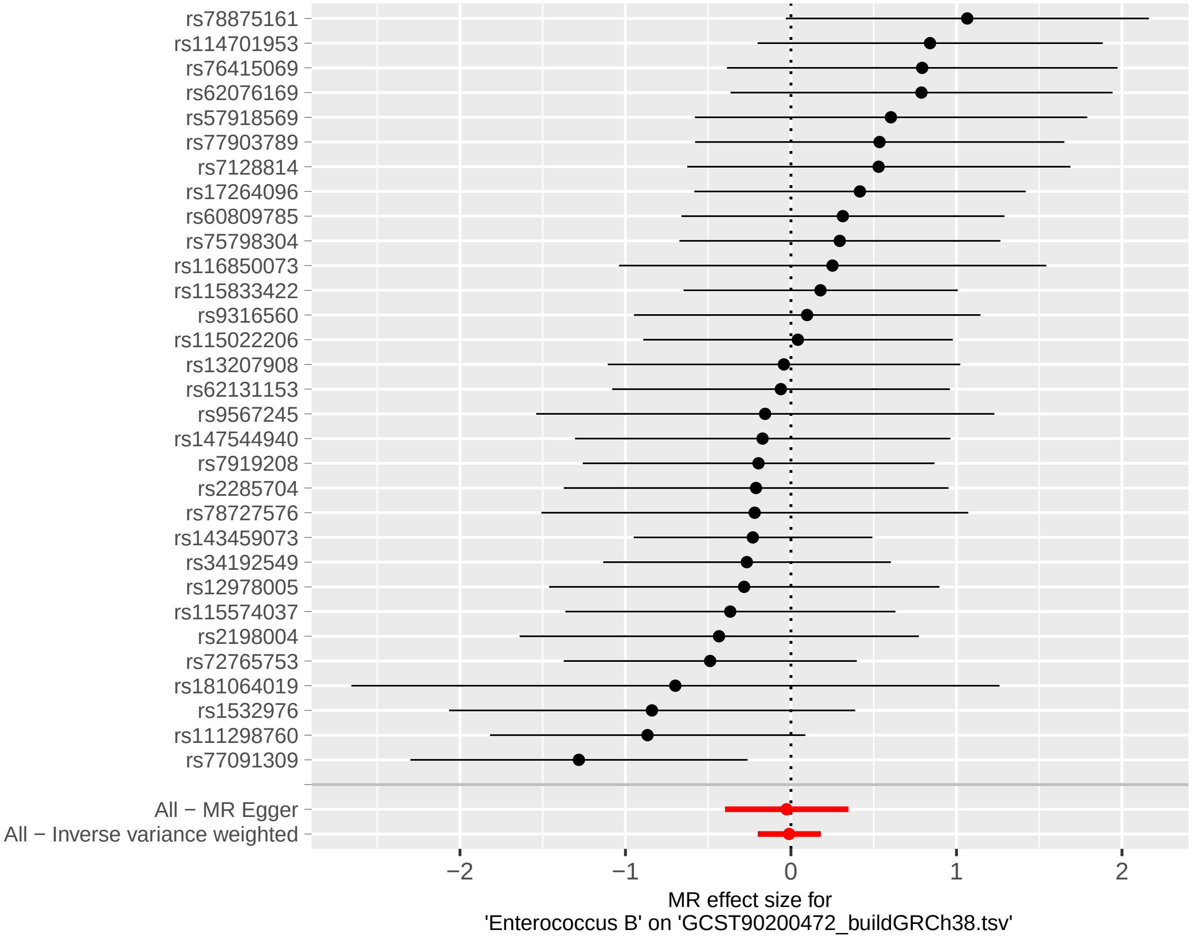
rs78875161
rs114701953
rs76415069
rs62076169
rs57918569
rs77903789
rs7128814
rs17264096
rs60809785
rs75798304
rs116850073
rs115833422
rs9316560
rs115022206
rs13207908
rs62131153
rs9567245
rs147544940
rs7919208
rs2285704
rs78727576
rs143459073
rs34192549
rs12978005
rs115574037
rs2198004
rs72765753
rs181064019
rs1532976
rs111298760
rs77091309
All − MR Egger
All − Inverse variance weighted
−2
−1
0
1
2
MR effect size for
'Enterococcus B' on 'GCST90200472_buildGRCh38.tsv'

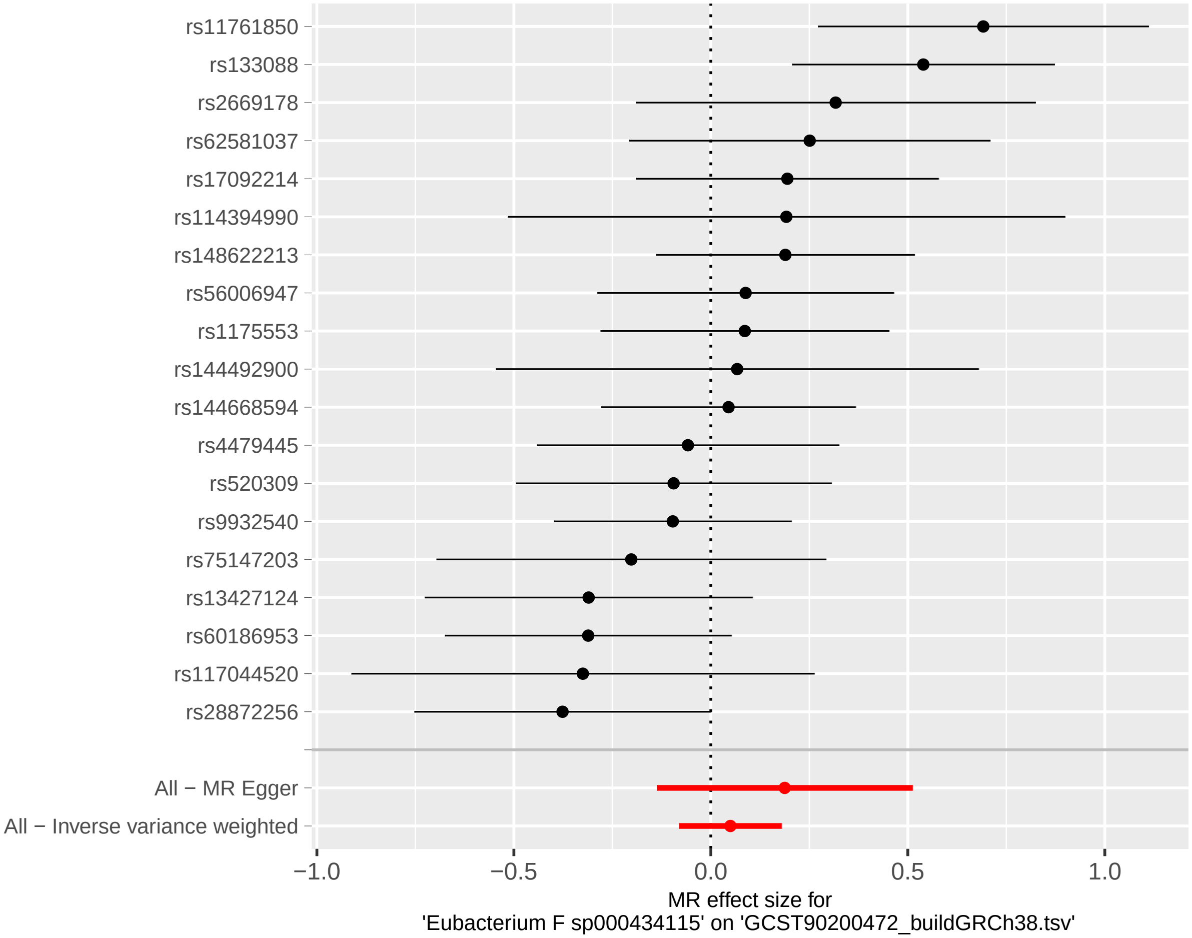
rs11761850
rs133088
rs2669178
rs62581037
rs17092214
rs114394990
rs148622213
rs56006947
rs1175553
rs144492900
rs144668594
rs4479445
rs520309
rs9932540
rs75147203
rs13427124
rs60186953
rs117044520
rs28872256
All − MR Egger
All − Inverse variance weighted
−1.0
−0.5
0.0
0.5
1.0
MR effect size for
'Eubacterium F sp000434115' on 'GCST90200472_buildGRCh38.tsv'

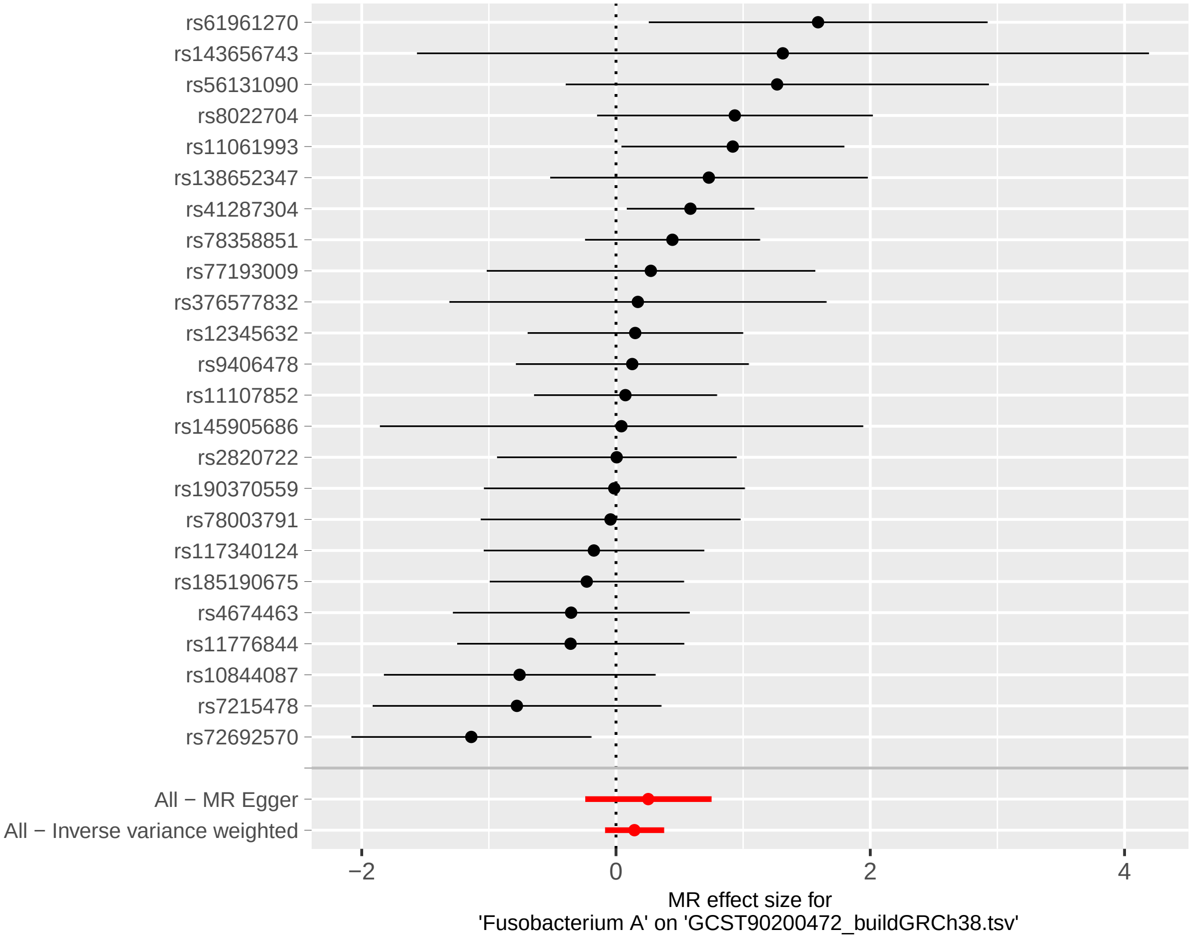

rs61961270
rs143656743
rs56131090
rs8022704
rs11061993
rs138652347
rs41287304
rs78358851
rs77193009
rs376577832
rs12345632
rs9406478
rs11107852
rs145905686
rs2820722
rs190370559
rs78003791
rs117340124
rs185190675
rs4674463
rs11776844
rs10844087
rs7215478
rs72692570
All − MR Egger
All − Inverse variance weighted
−2
0
2
4
MR effect size for
'Fusobacterium A' on 'GCST90200472_buildGRCh38.tsv'

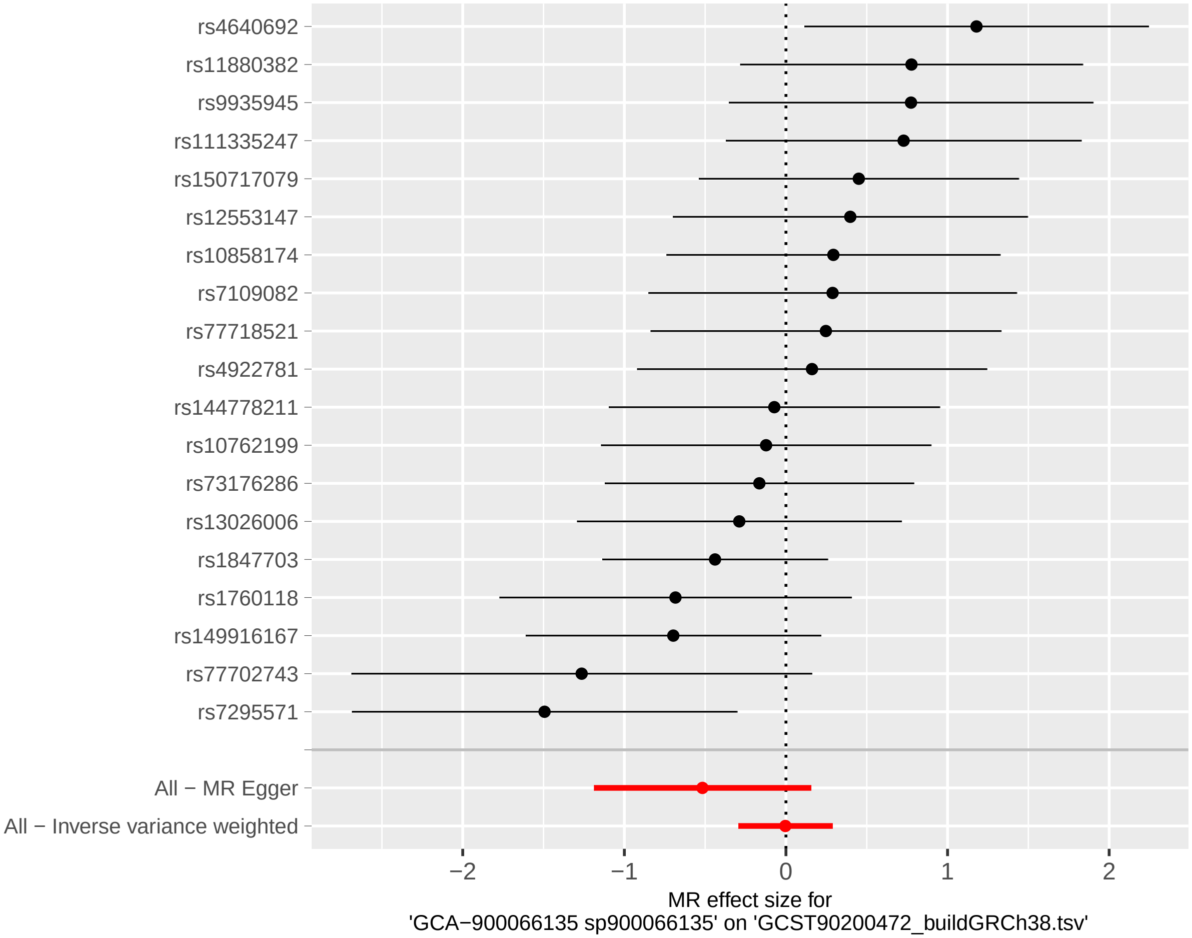
rs4640692
rs11880382
rs9935945
rs111335247
rs150717079
rs12553147
rs10858174
rs7109082
rs77718521
rs4922781
rs144778211
rs10762199
rs73176286
rs13026006
rs1847703
rs1760118
rs149916167
rs77702743
rs7295571
All − MR Egger
All − Inverse variance weighted
−2
−1
0
1
2
MR effect size for
'GCA−900066135 sp900066135' on 'GCST90200472_buildGRCh38.tsv'

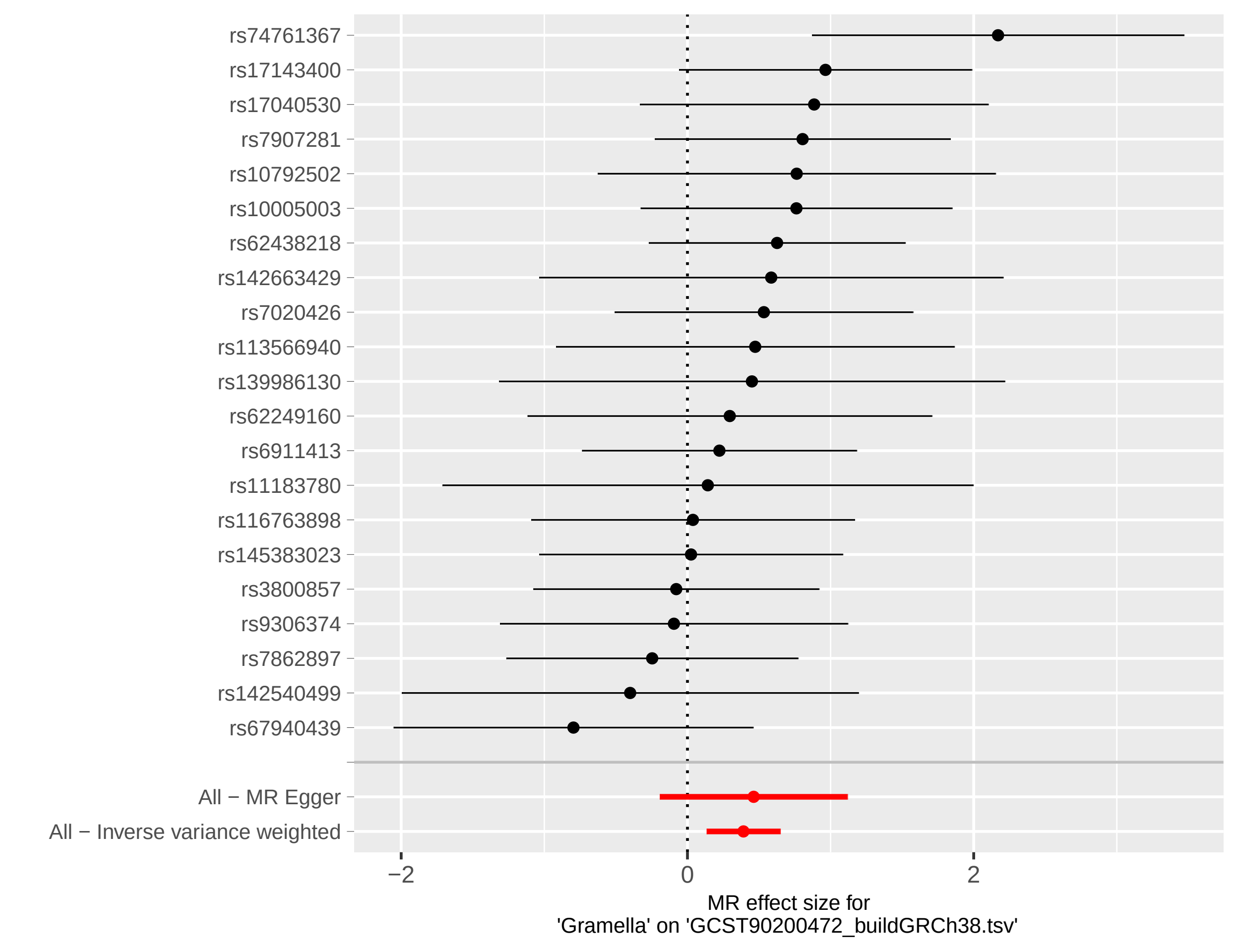

rs74761367
rs17143400
rs17040530
rs7907281
rs10792502
rs10005003
rs62438218
rs142663429
rs7020426
rs113566940
rs139986130
rs62249160
rs6911413
rs11183780
rs116763898
rs145383023
rs3800857
rs9306374
rs7862897
rs142540499
rs67940439
All − MR Egger
All − Inverse variance weighted
−2
0
2
MR effect size for
'Gramella' on 'GCST90200472_buildGRCh38.tsv'

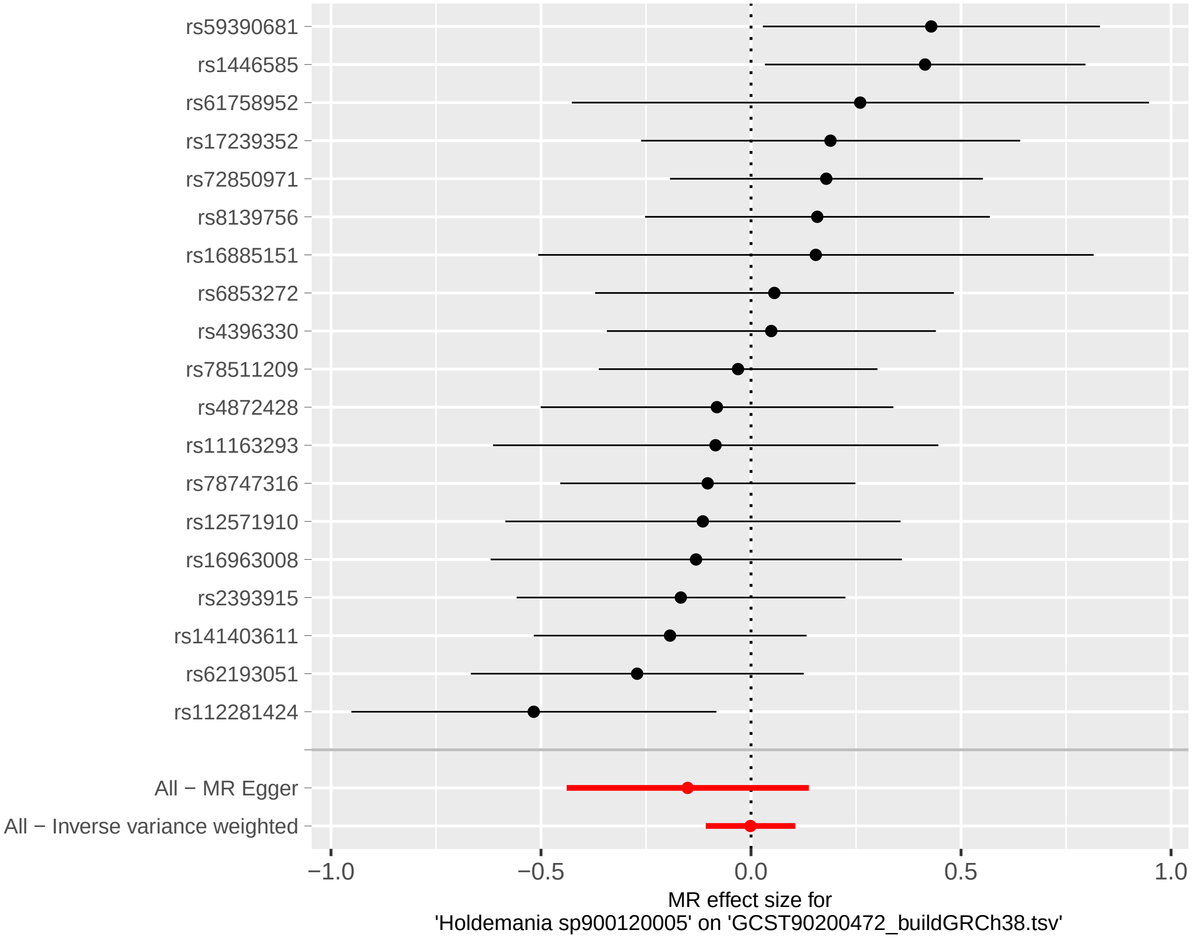
rs59390681
rs1446585
rs61758952
rs17239352
rs72850971
rs8139756
rs16885151
rs6853272
rs4396330
rs78511209
rs4872428
rs11163293
rs78747316
rs12571910
rs16963008
rs2393915
rs141403611
rs62193051
rs112281424
All − MR Egger
All − Inverse variance weighted
−1.0
−0.5
0.0
0.5
1.0
MR effect size for
'Holdemania sp900120005' on 'GCST90200472_buildGRCh38.tsv'

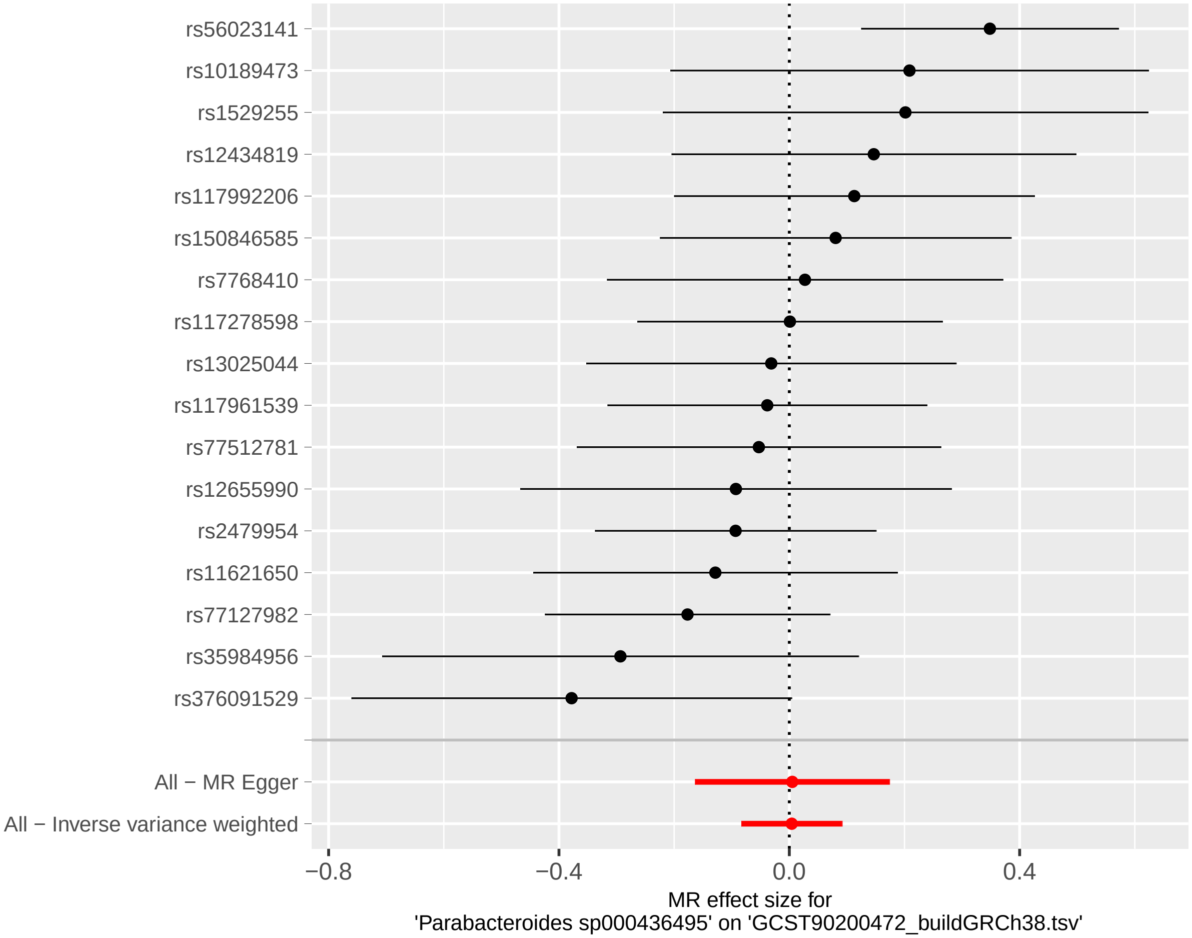

rs56023141
rs10189473
rs1529255
rs12434819
rs117992206
rs150846585
rs7768410
rs117278598
rs13025044
rs117961539
rs77512781
rs12655990
rs2479954
rs11621650
rs77127982
rs35984956
rs376091529
All − MR Egger
All − Inverse variance weighted
−0.8
−0.4
0.0
0.4
MR effect size for
'Parabacteroides sp000436495' on 'GCST90200472_buildGRCh38.tsv'

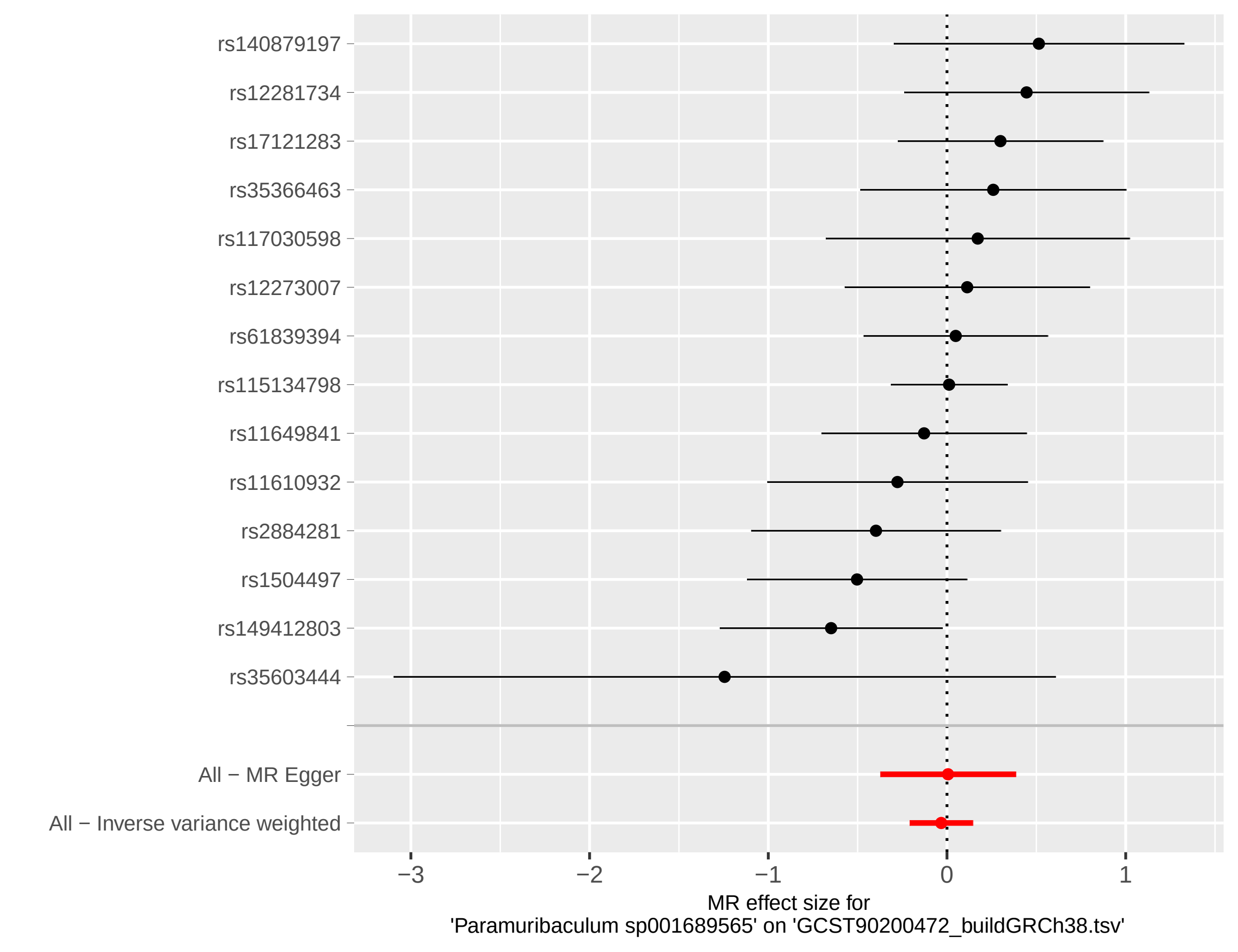

rs140879197
rs12281734
rs17121283
rs35366463
rs117030598
rs12273007
rs61839394
rs115134798
rs11649841
rs11610932
rs2884281
rs1504497
rs149412803
rs35603444
All − MR Egger
All − Inverse variance weighted
−3
−2
−1
0
1
MR effect size for
'Paramuribaculum sp001689565' on 'GCST90200472_buildGRCh38.tsv'

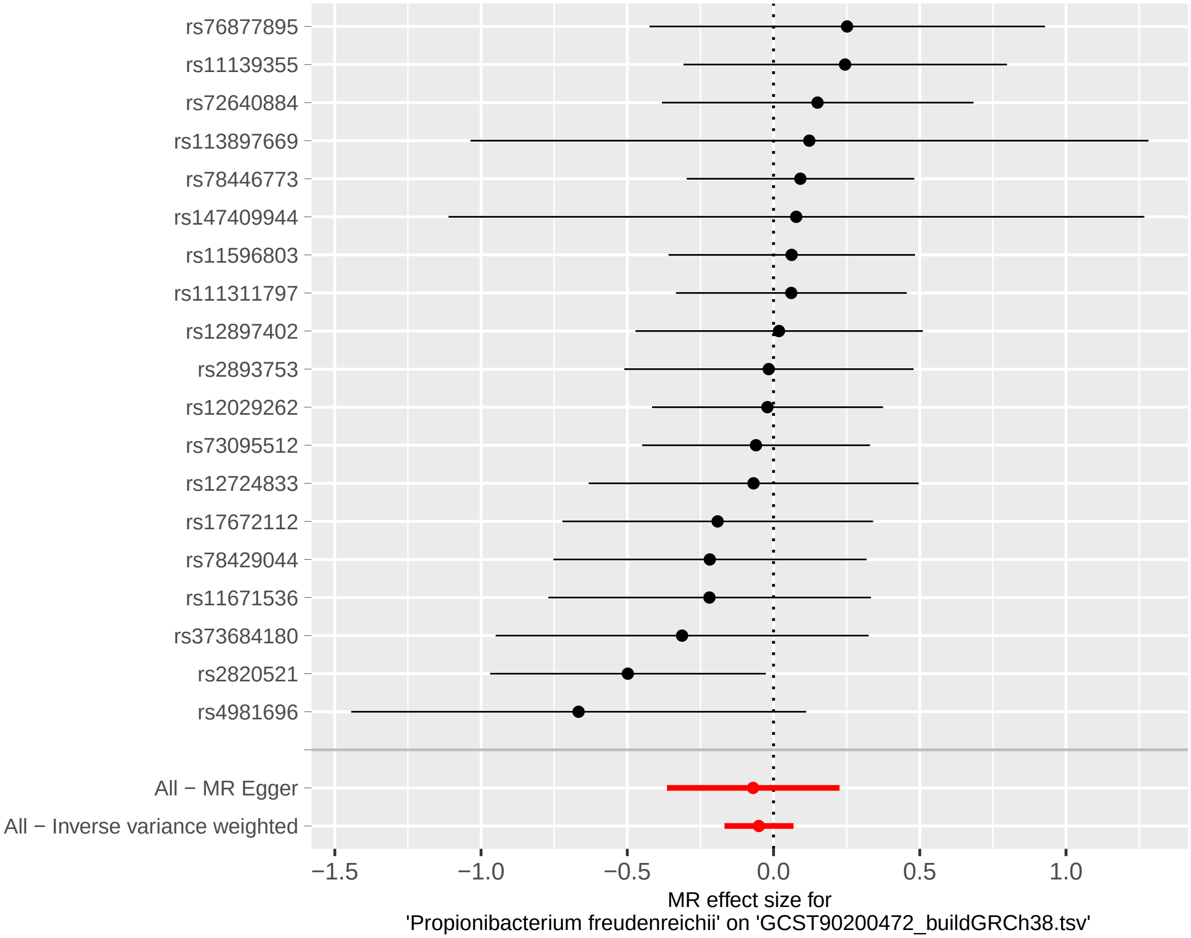

rs76877895
rs11139355
rs72640884
rs113897669
rs78446773
rs147409944
rs11596803
rs111311797
rs12897402
rs2893753
rs12029262
rs73095512
rs12724833
rs17672112
rs78429044
rs11671536
rs373684180
rs2820521
rs4981696
All − MR Egger
All − Inverse variance weighted
−1.5
−1.0
−0.5
0.0
0.5
1.0
MR effect size for
'Propionibacterium freudenreichii' on 'GCST90200472_buildGRCh38.tsv'

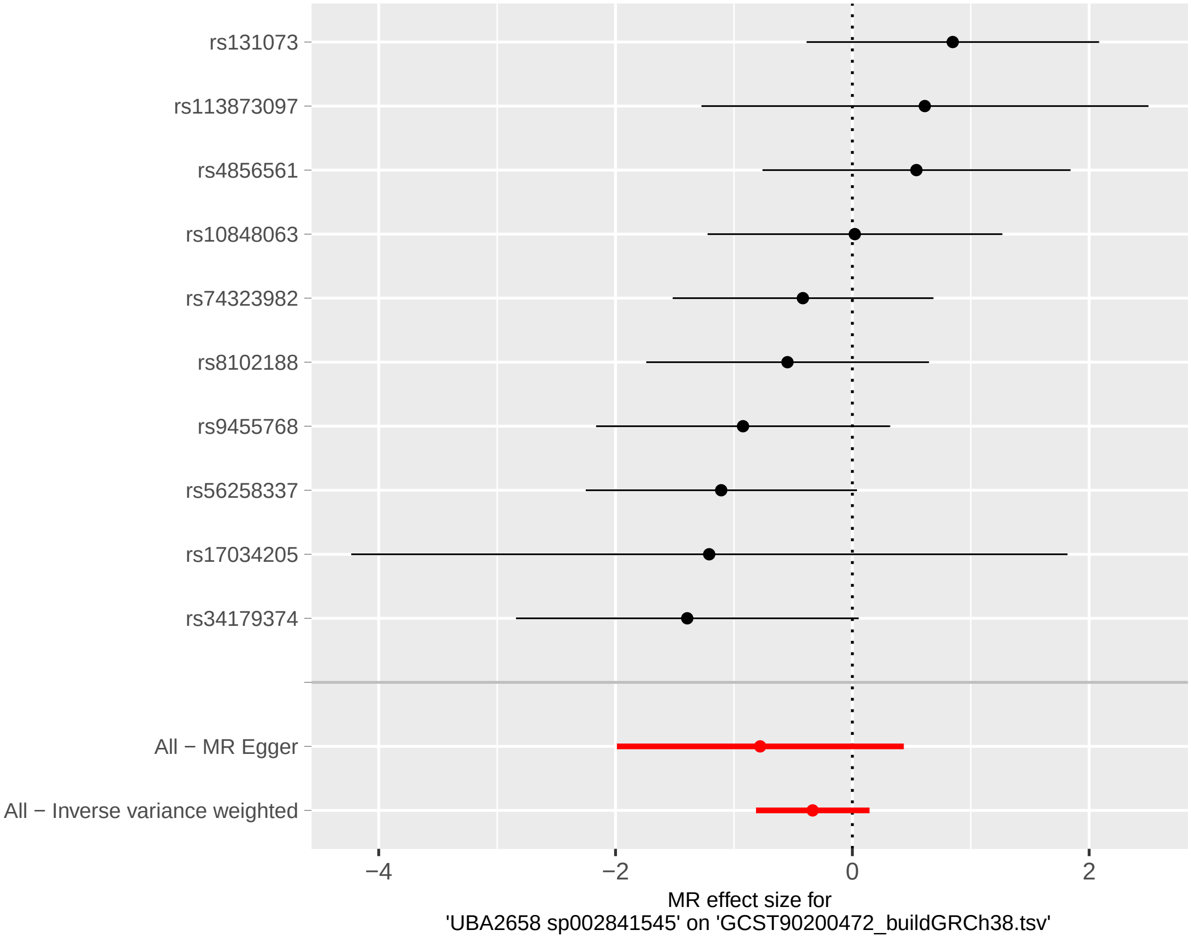

rs131073
rs113873097
rs4856561
rs10848063
rs74323982
rs8102188
rs9455768
rs56258337
rs17034205
rs34179374
All − MR Egger
All − Inverse variance weighted
−4
−2
0
2
MR effect size for
'UBA2658 sp002841545' on 'GCST90200472_buildGRCh38.tsv'

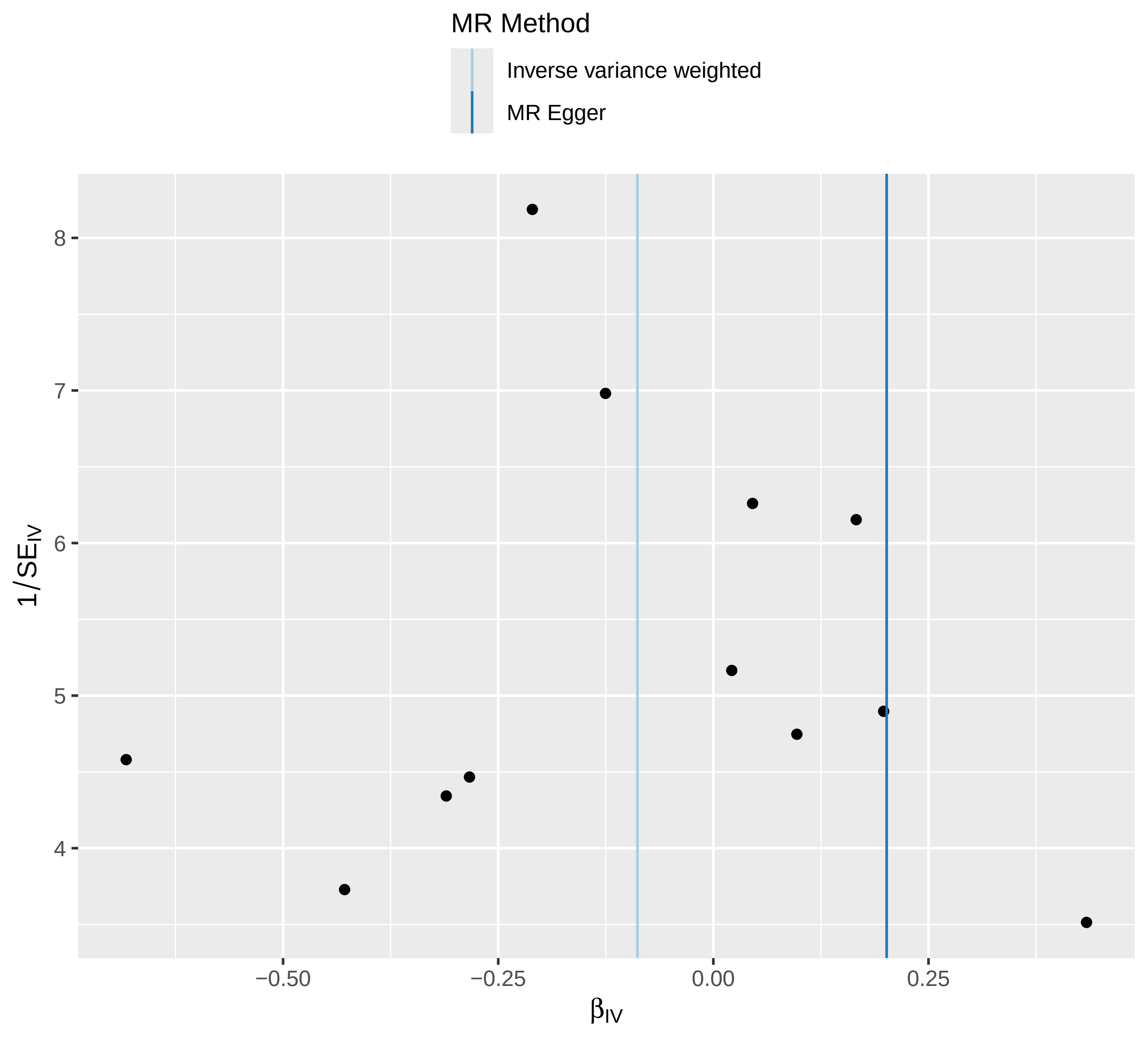
MR Method
Inverse variance weighted
MR Egger
8
7
6
5
4
1/SE$_{IV}$
−0.50
−0.25
0.00
0.25
β$_{IV}$

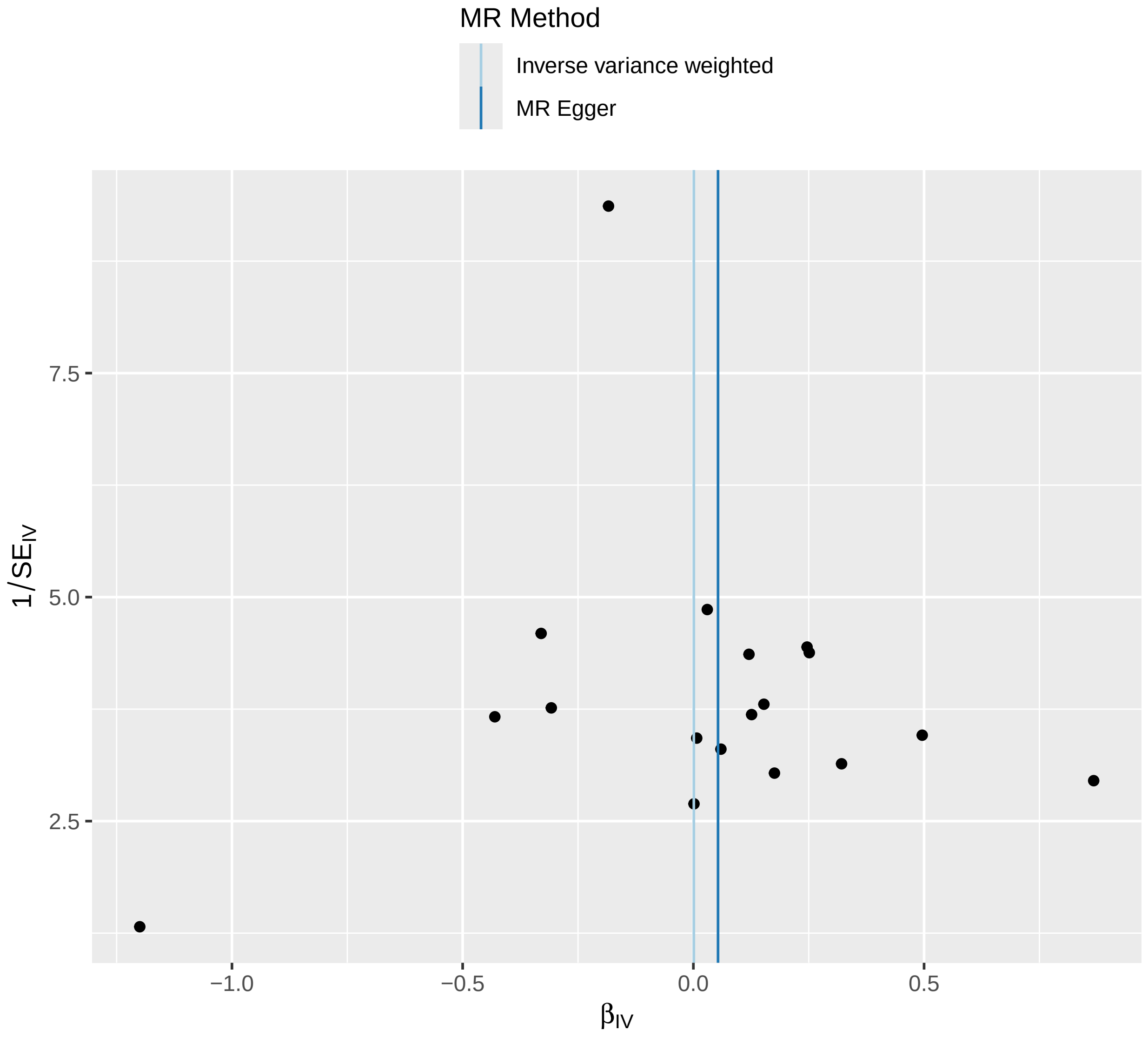

MR Method
Inverse variance weighted
MR Egger
7.5
5.0
2.5
1/SE$_{IV}$
−1.0
−0.5
0.0
0.5
β$_{IV}$

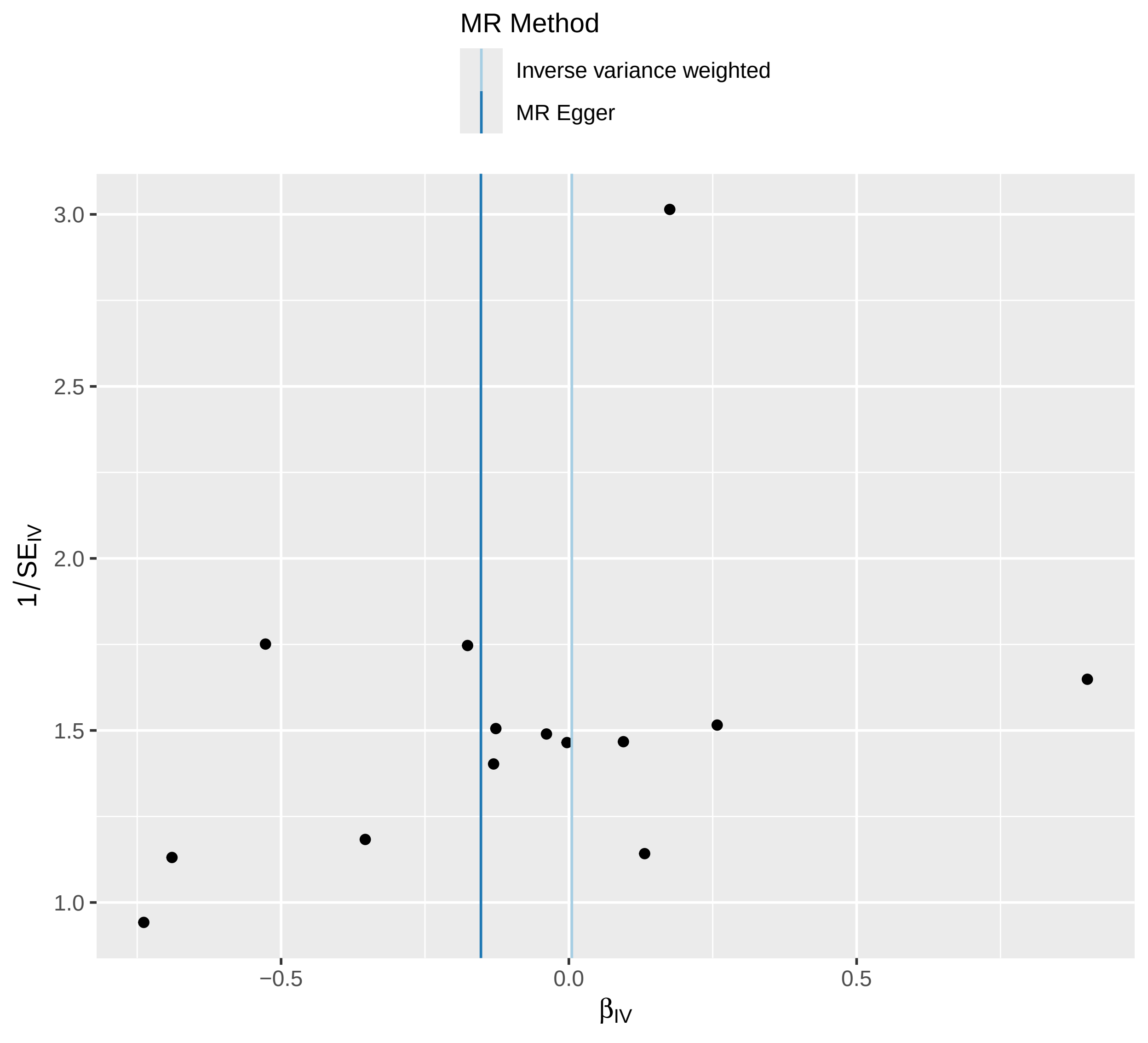
MR Method
Inverse variance weighted
MR Egger
3.0
2.5
2.0
1.5
1.0
1/SE_IV
−0.5
0.0
0.5
β_IV

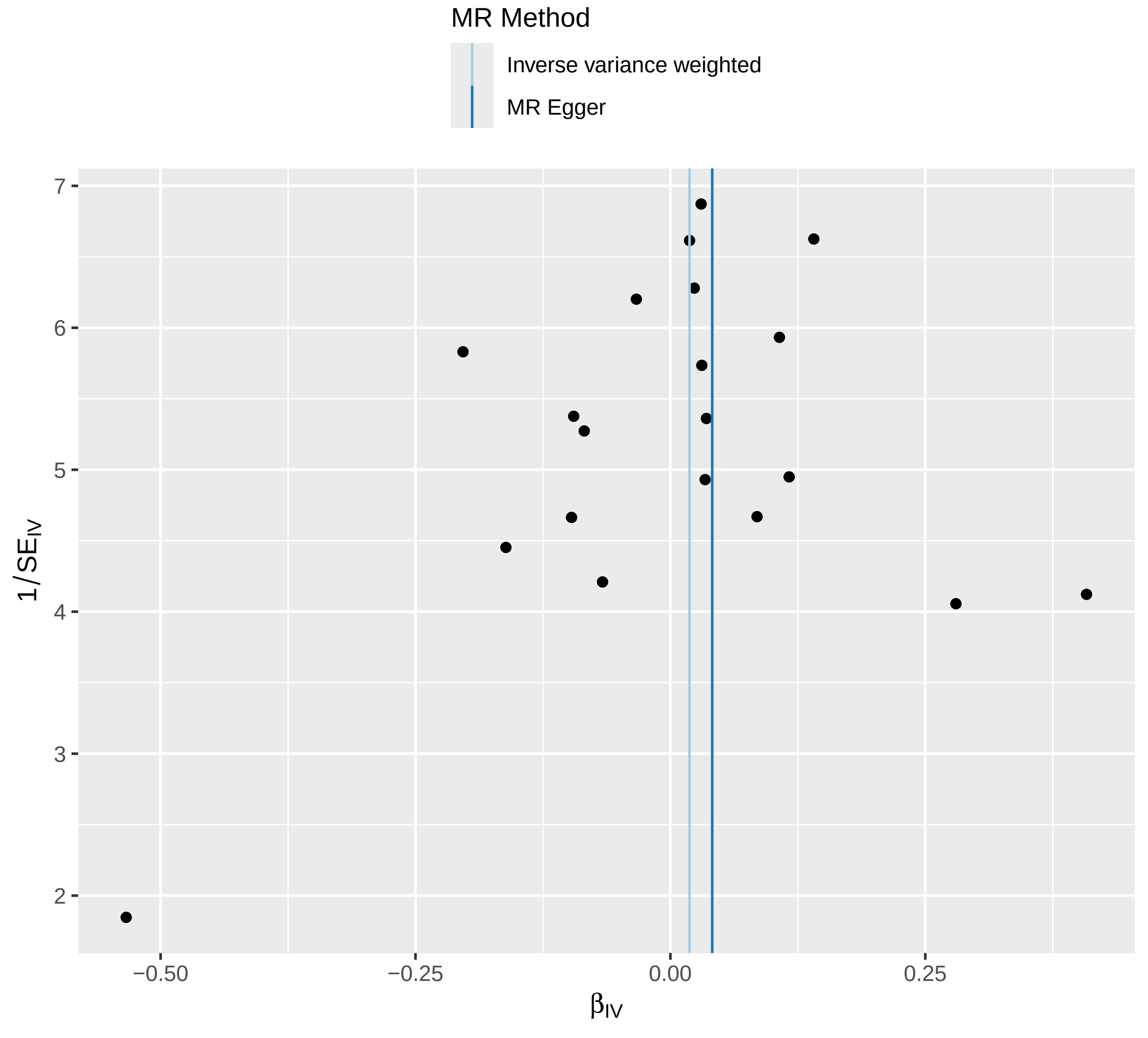

MR Method
Inverse variance weighted
MR Egger
7
6
5
4
3
2
1/SE_IV
−0.50
−0.25
0.00
0.25
β_IV

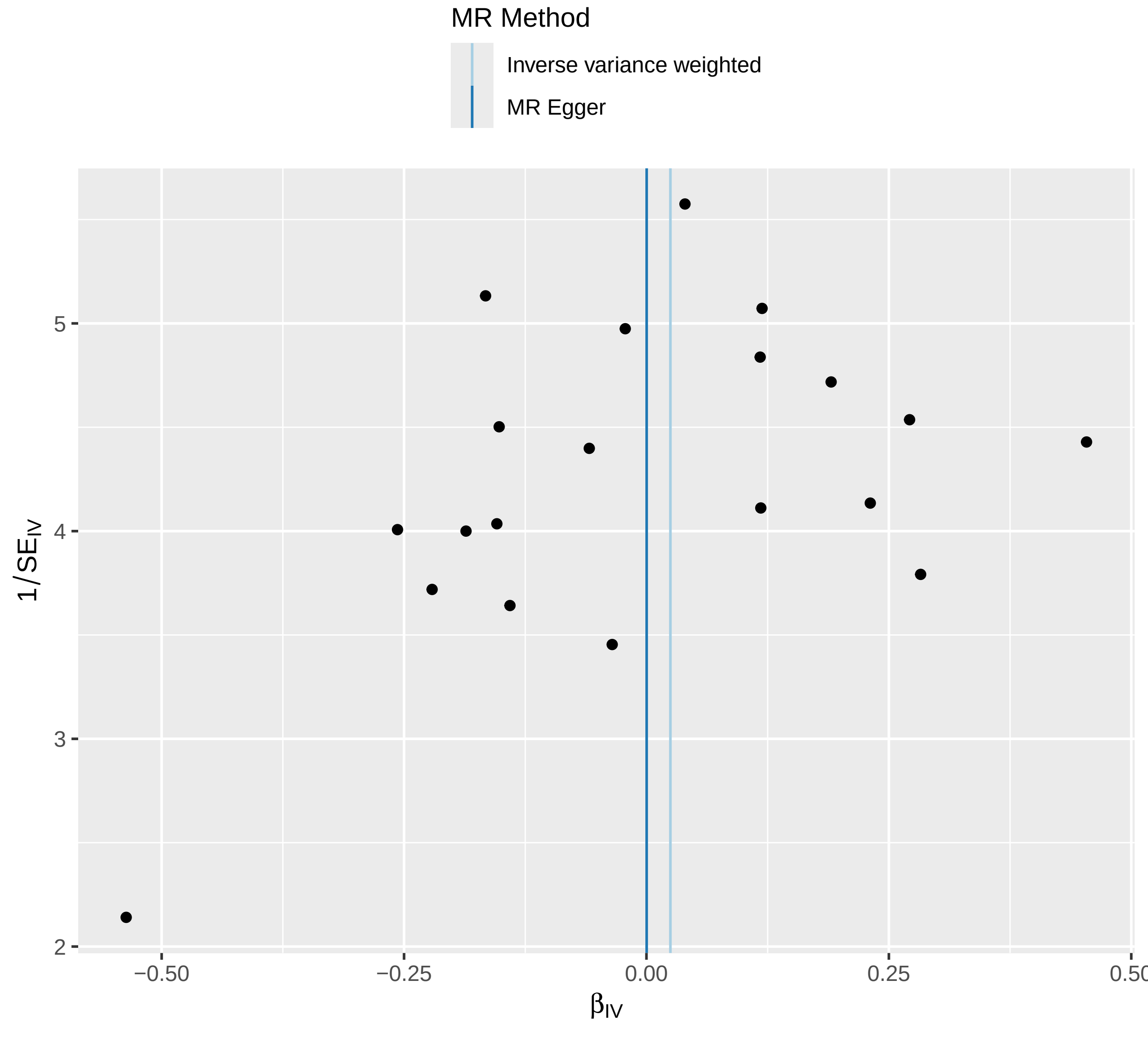

MR Method
Inverse variance weighted
MR Egger
5
4
3
2
1/SE$_{IV}$
−0.50
−0.25
0.00
0.25
0.50
β$_{IV}$

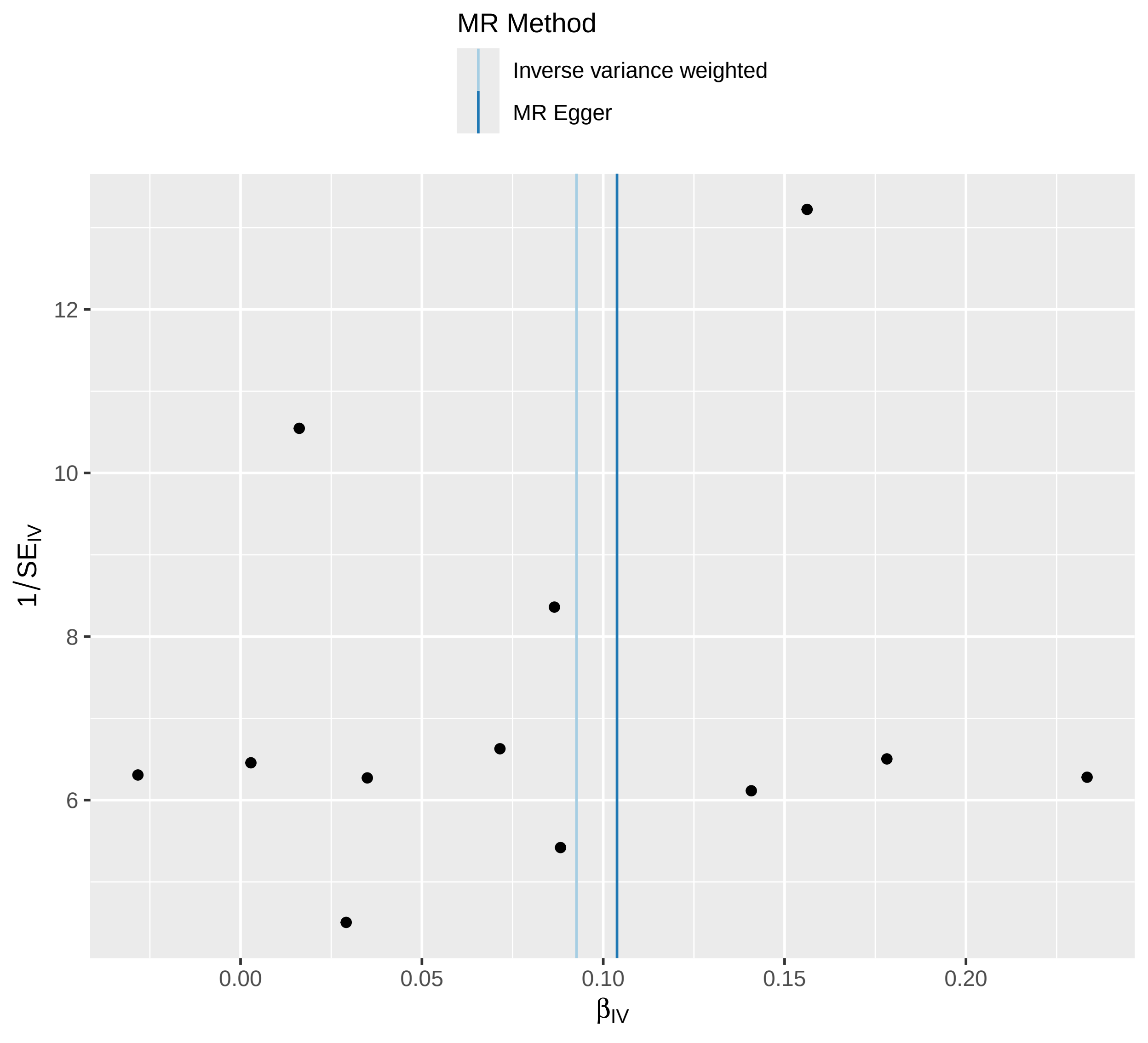
MR Method
Inverse variance weighted
MR Egger
1/SE_IV
12
10
8
6
0.00
0.05
0.10
0.15
0.20
β_IV

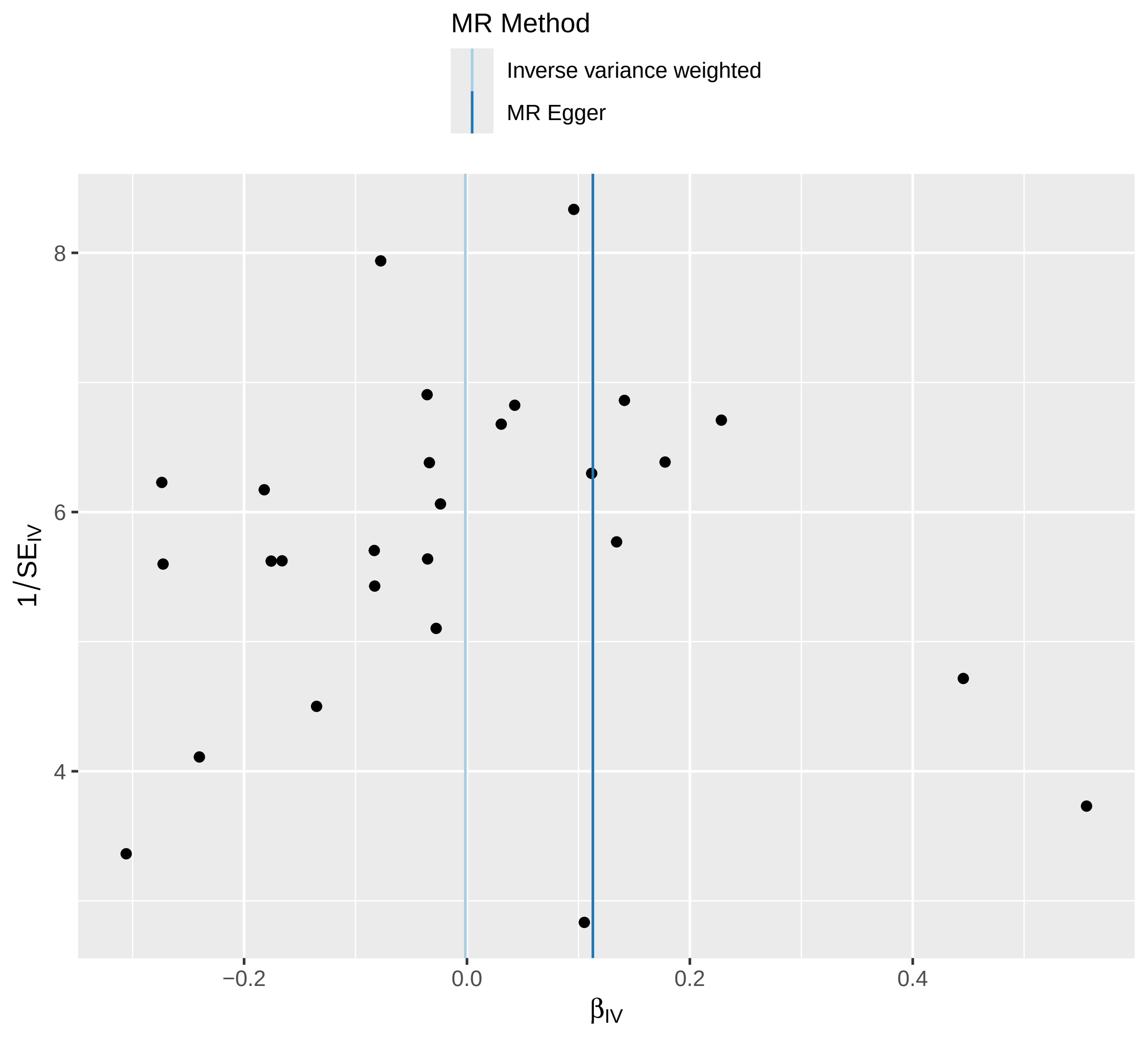
MR Method
Inverse variance weighted
MR Egger
8
6
4
$1/SE_{IV}$
−0.2
0.0
0.2
0.4
β_{IV}

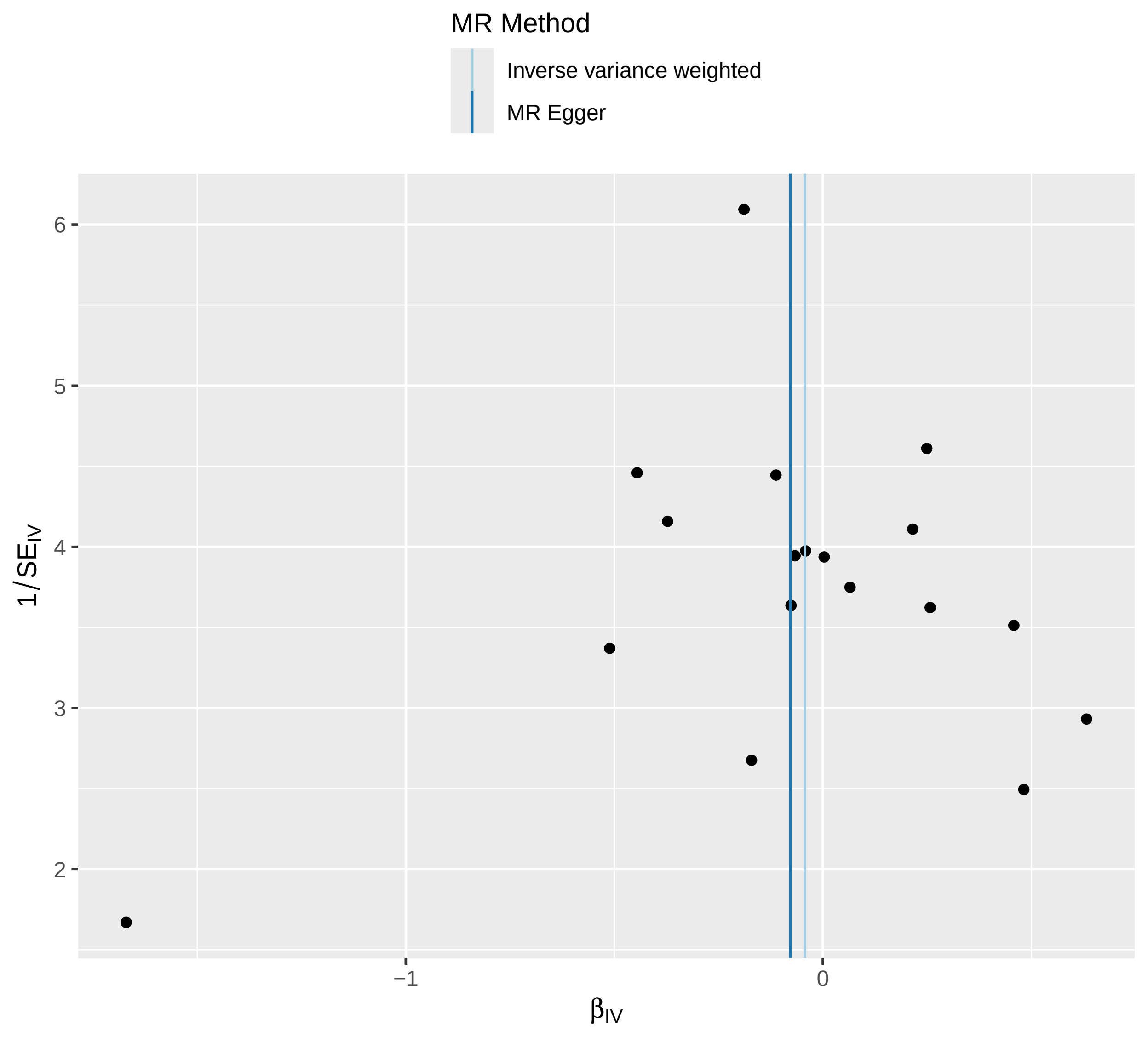

MR Method
Inverse variance weighted
MR Egger
6
5
4
3
2
1/SE_IV
−1
0
β_IV

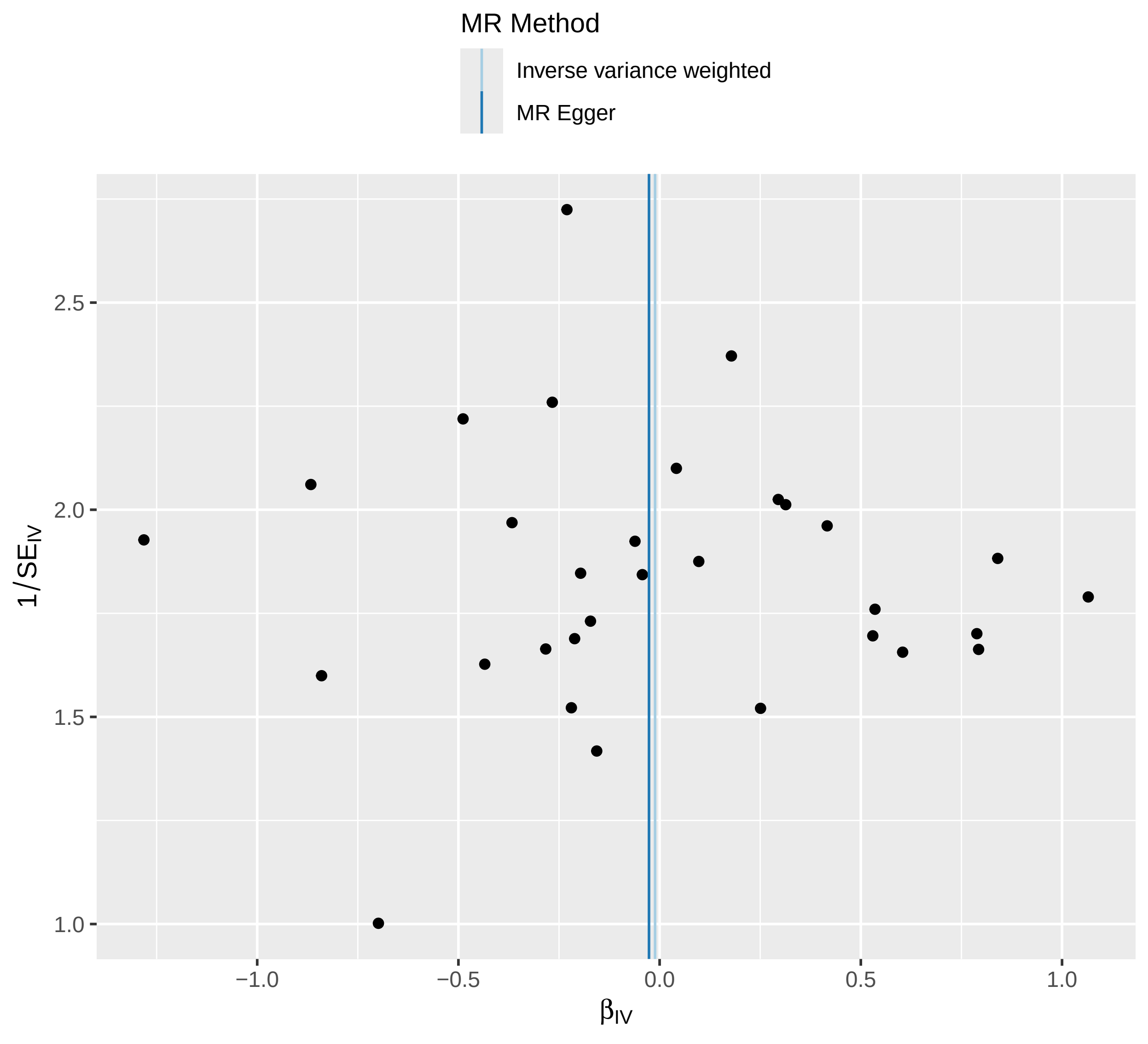
MR Method
Inverse variance weighted
MR Egger
2.5
2.0
1.5
1.0
$1/SE_{IV}$
−1.0
−0.5
0.0
0.5
1.0
β_{IV}

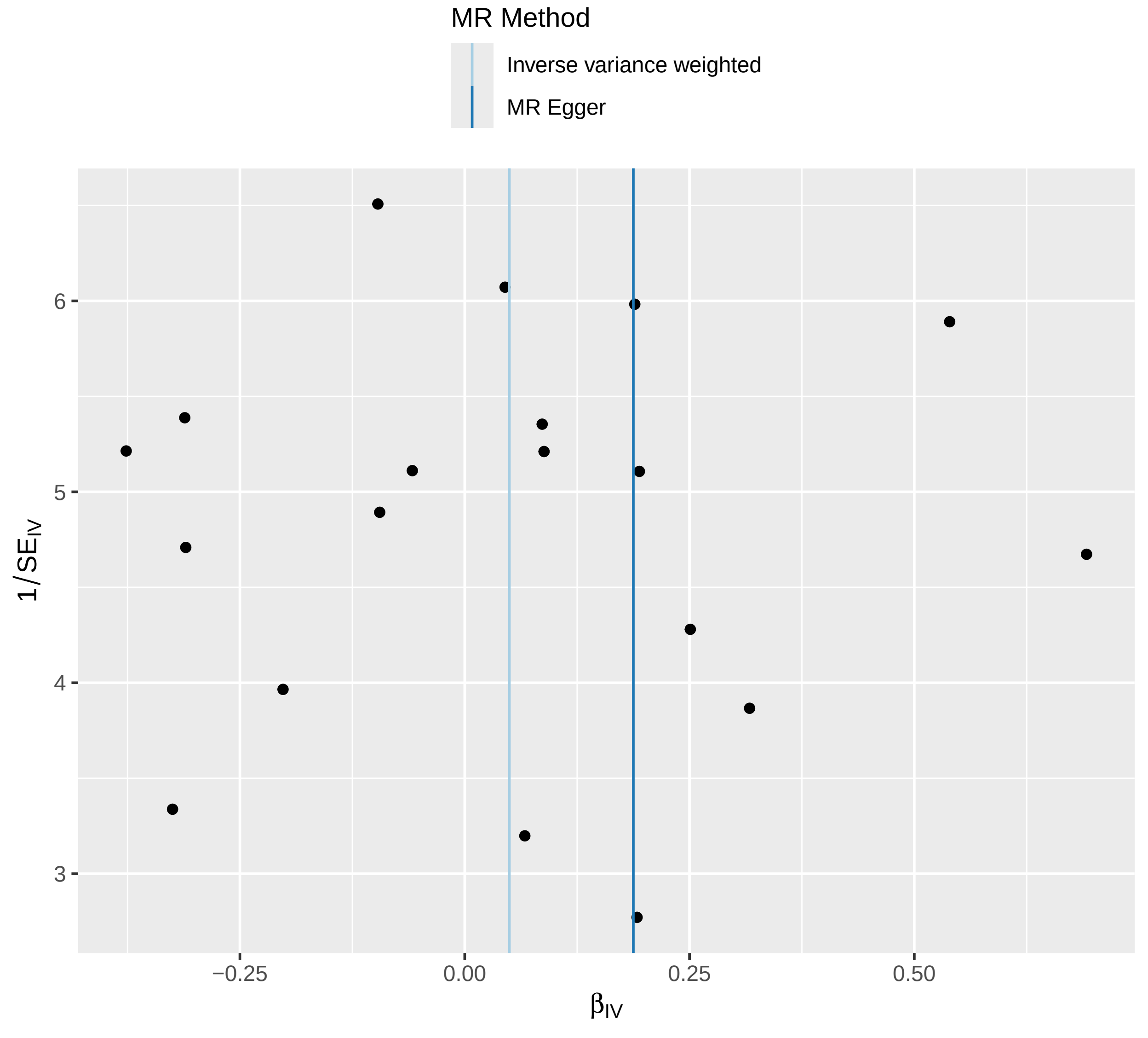

MR Method
Inverse variance weighted
MR Egger
6
5
4
3
1/SE$_{IV}$
−0.25
0.00
0.25
0.50
β$_{IV}$

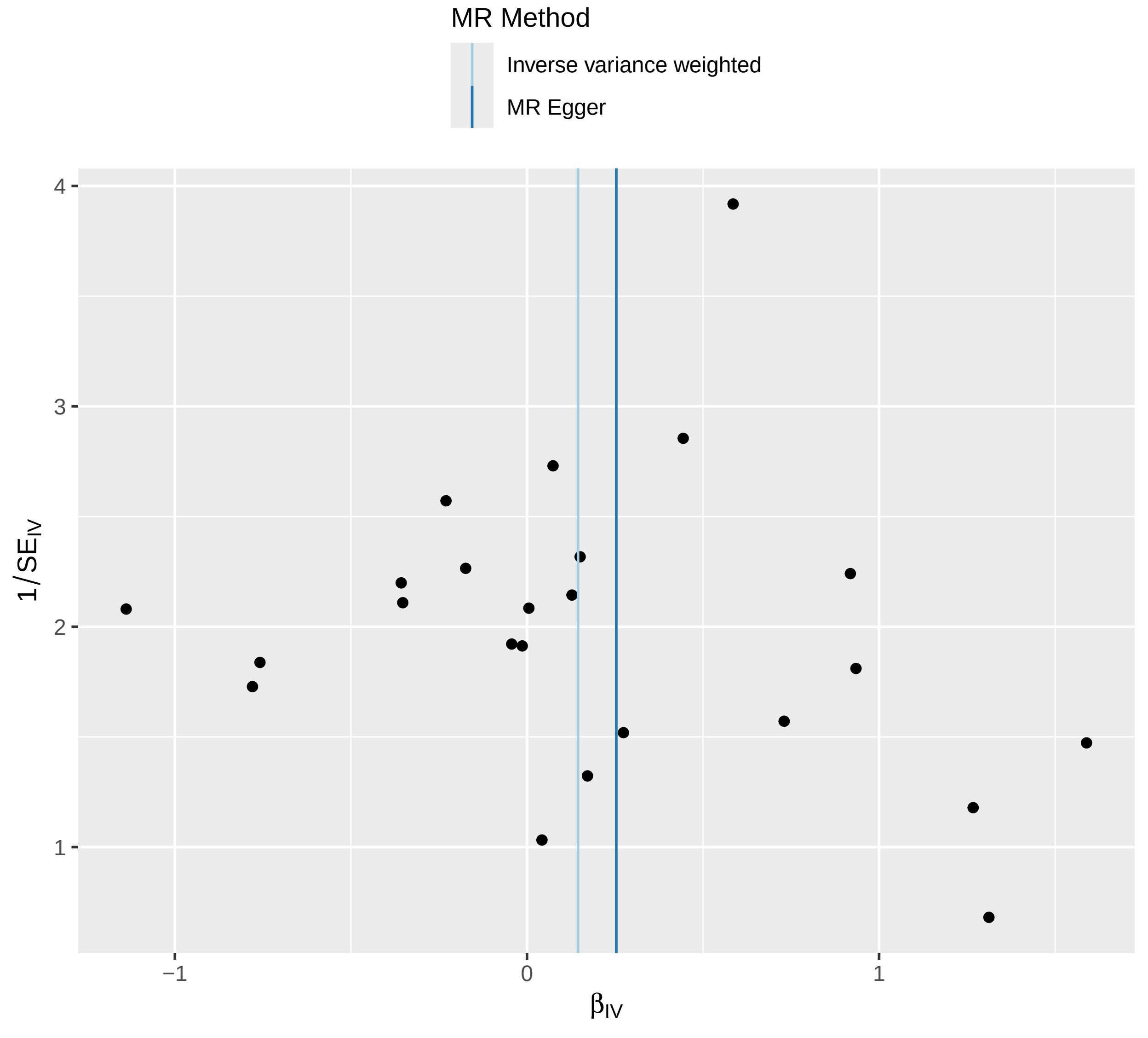
MR Method
Inverse variance weighted
MR Egger
4
3
2
1
1/SE_IV
−1
0
1
β_IV

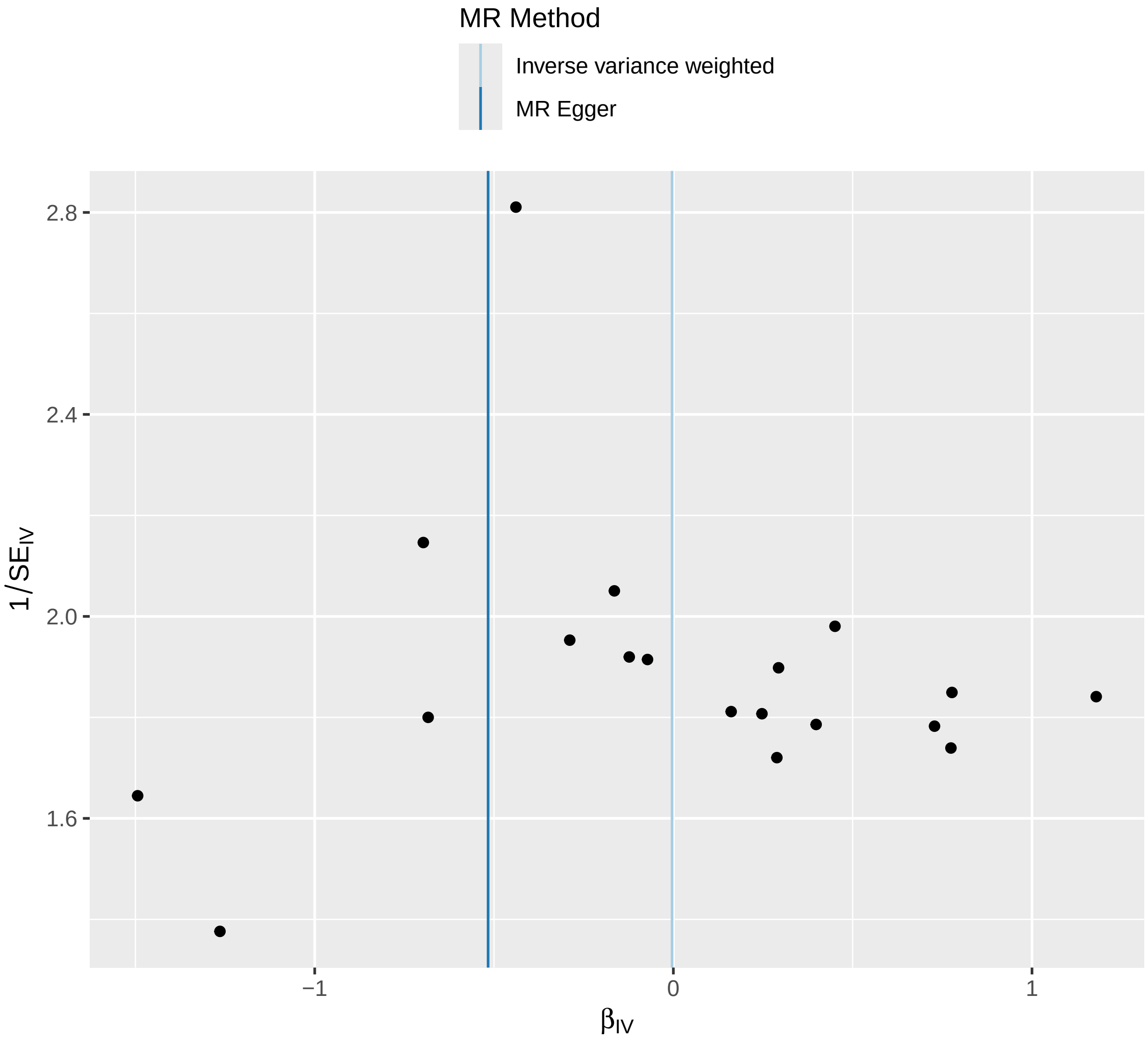

MR Method
Inverse variance weighted
MR Egger
2.8
2.4
2.0
1.6
1/SE_IV
−1
0
1
β_IV

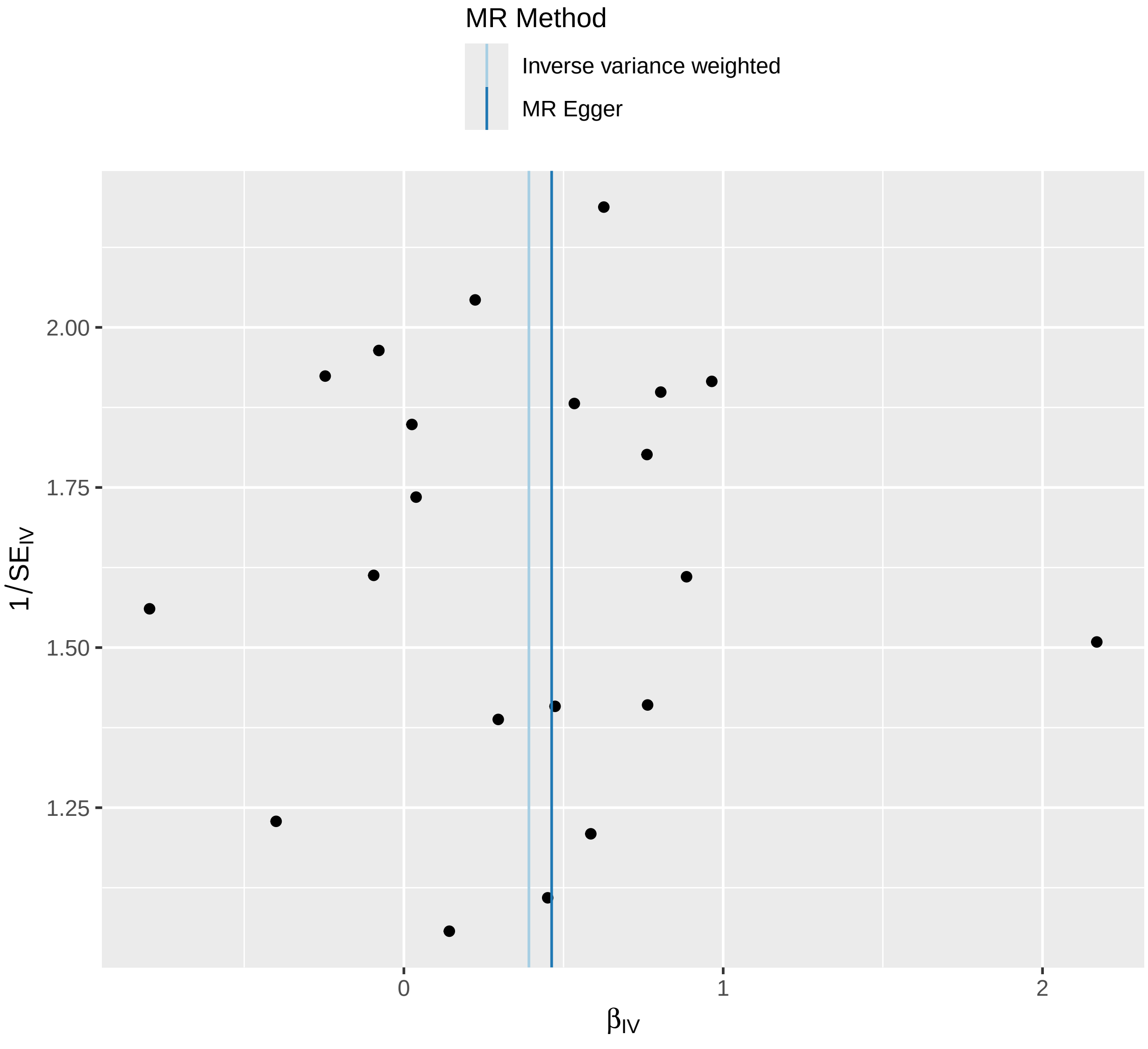
MR Method
Inverse variance weighted
MR Egger
2.00
1.75
1.50
1.25
1/SE_IV
0
1
2
β_IV

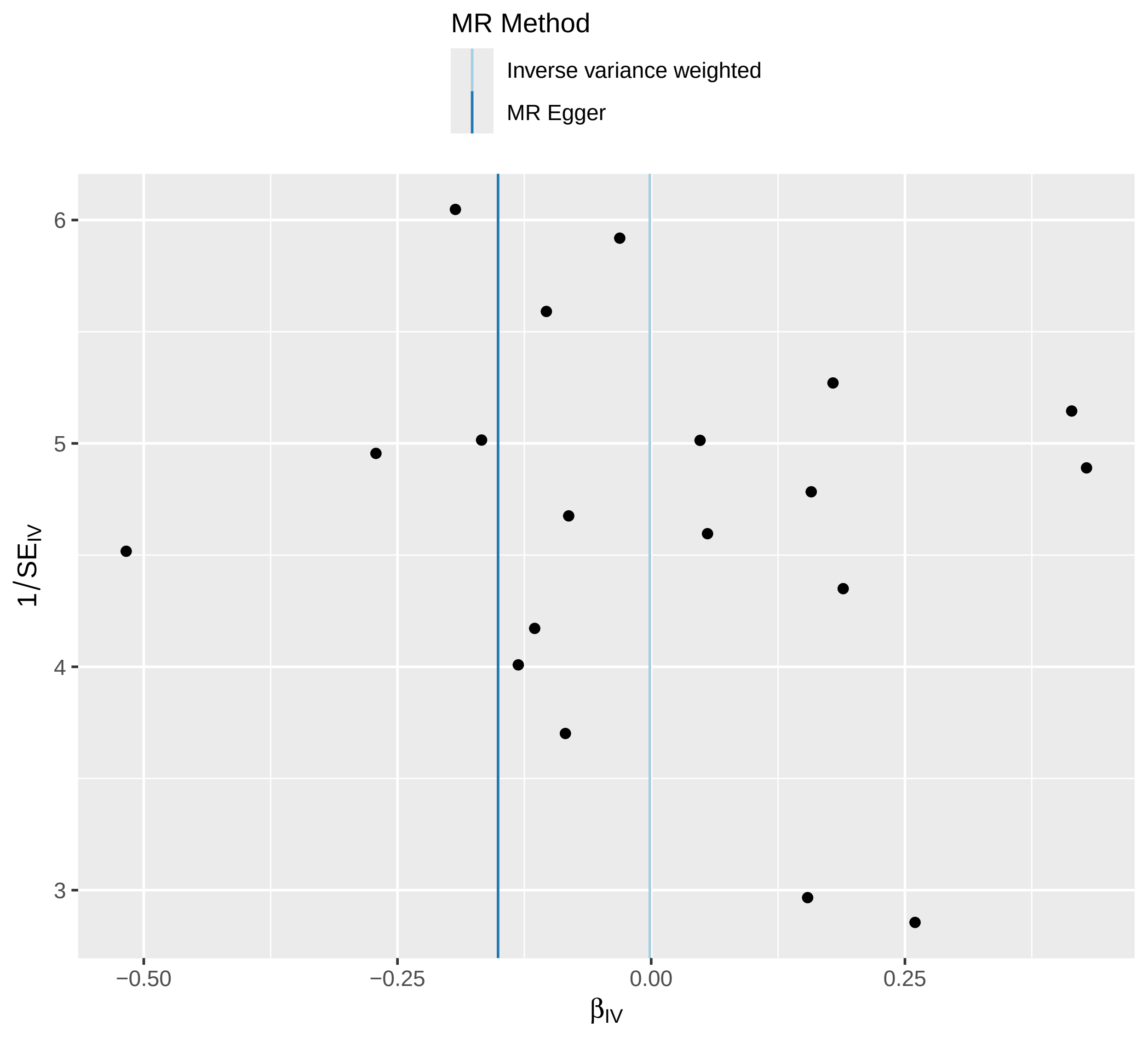
MR Method
Inverse variance weighted
MR Egger
6
5
4
3
1/SE_IV
−0.50
−0.25
0.00
0.25
β_IV

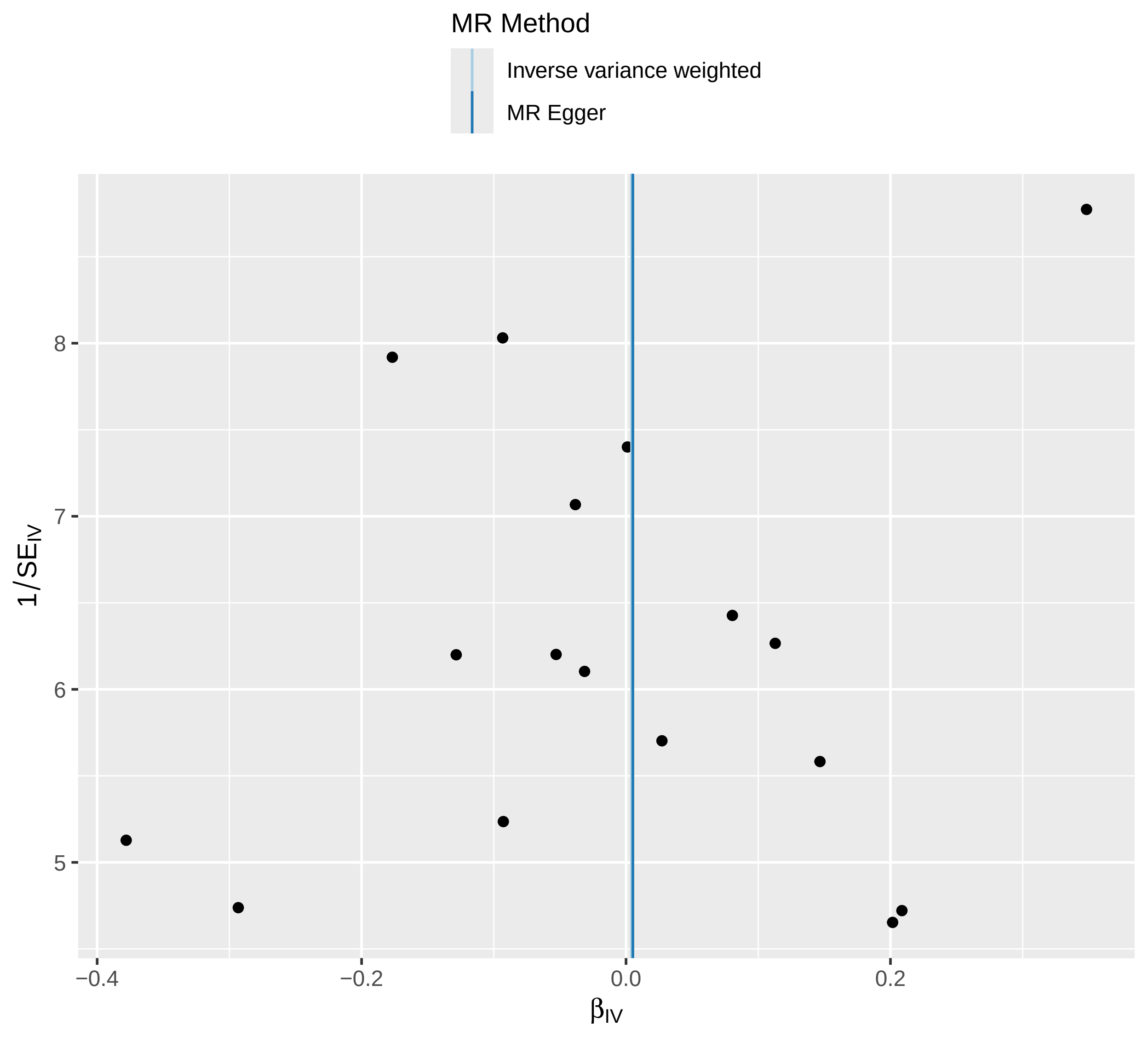
MR Method
Inverse variance weighted
MR Egger
8
7
6
5
1/SE_IV
−0.4
−0.2
0.0
0.2
β_IV

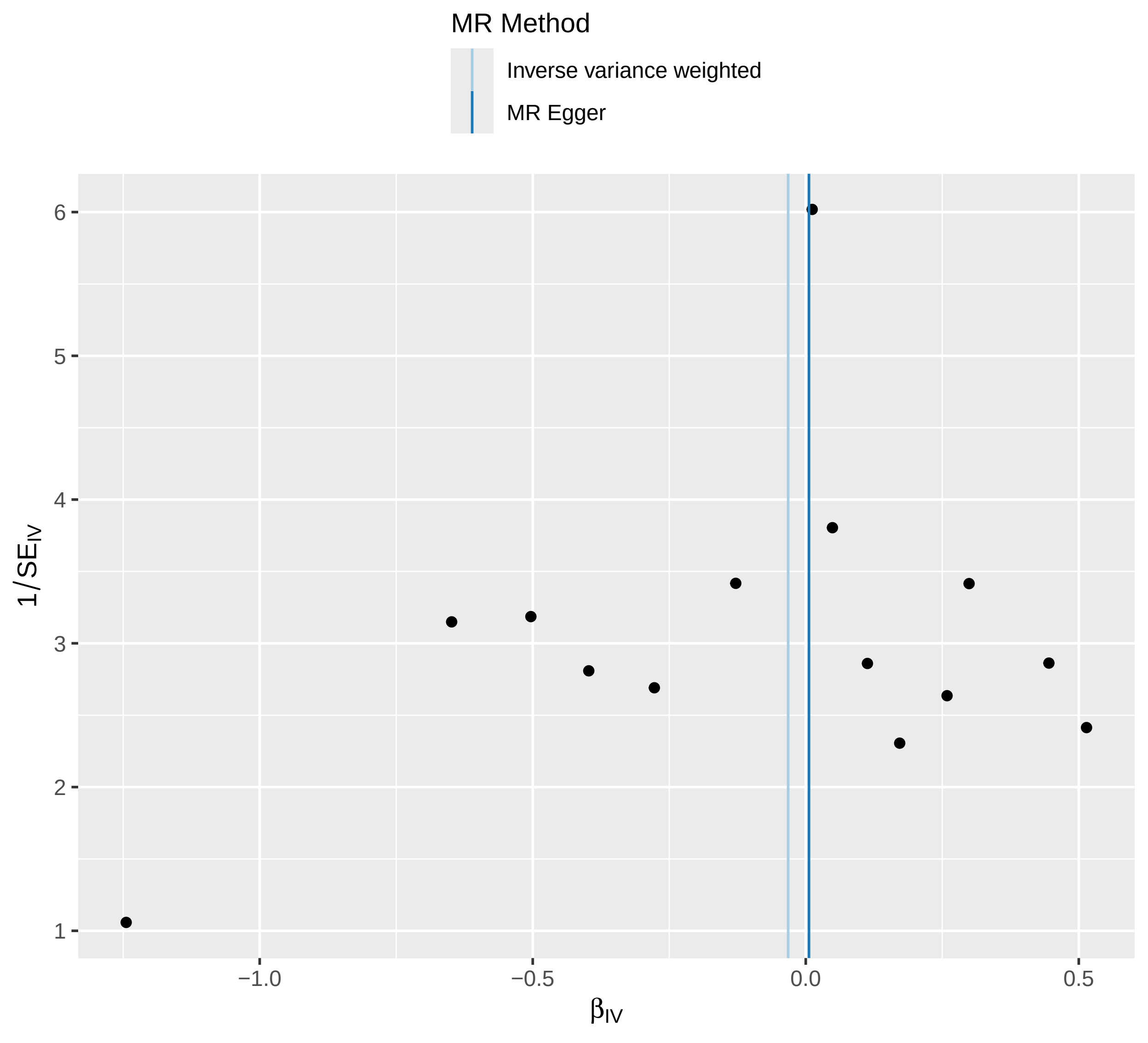

MR Method
Inverse variance weighted
MR Egger
6
5
4
3
2
1
1/SE_IV
−1.0
−0.5
0.0
0.5
β_IV

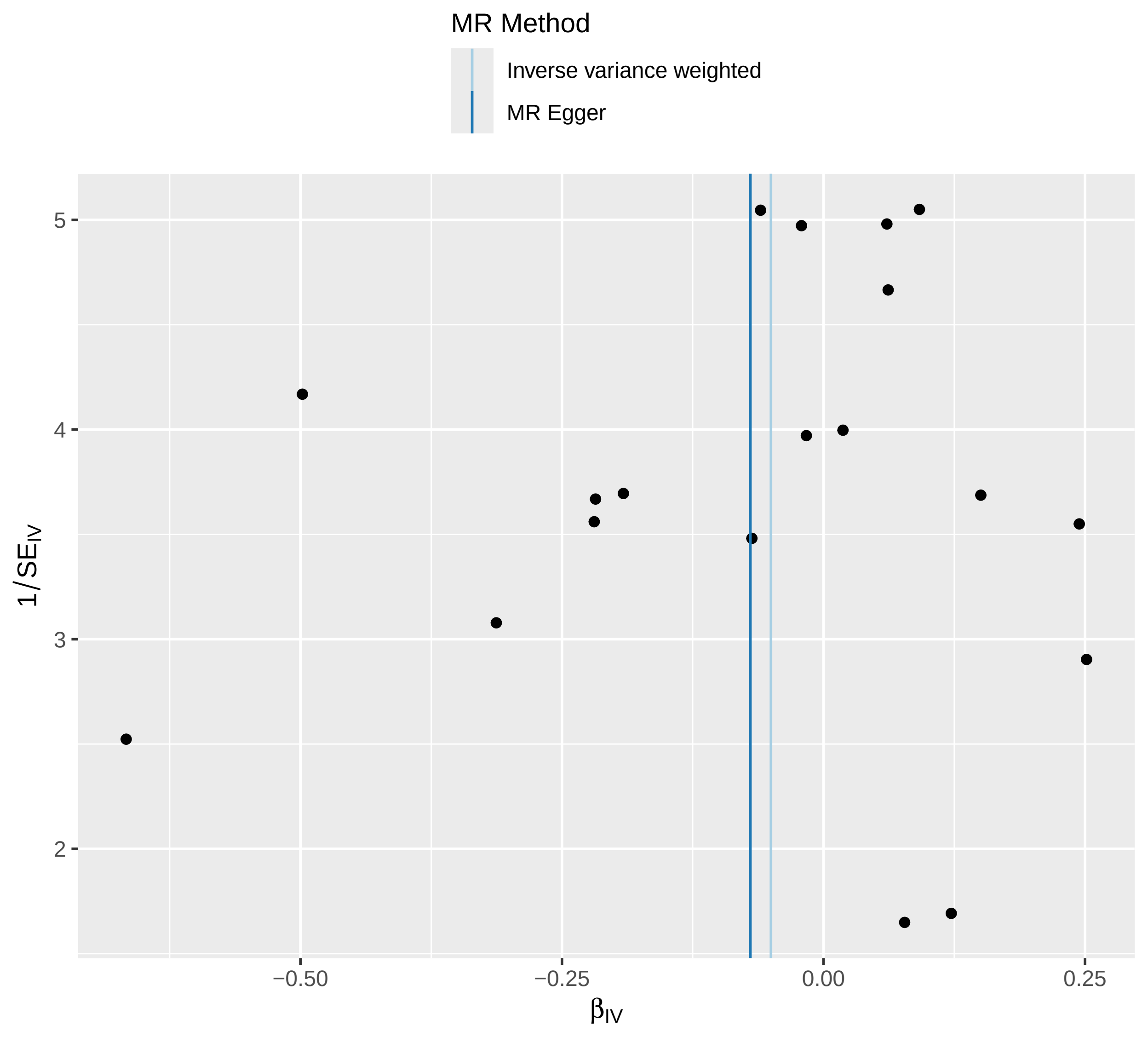

MR Method
Inverse variance weighted
MR Egger
5
4
3
2
1/SE$_{IV}$
−0.50
−0.25
0.00
0.25
β$_{IV}$

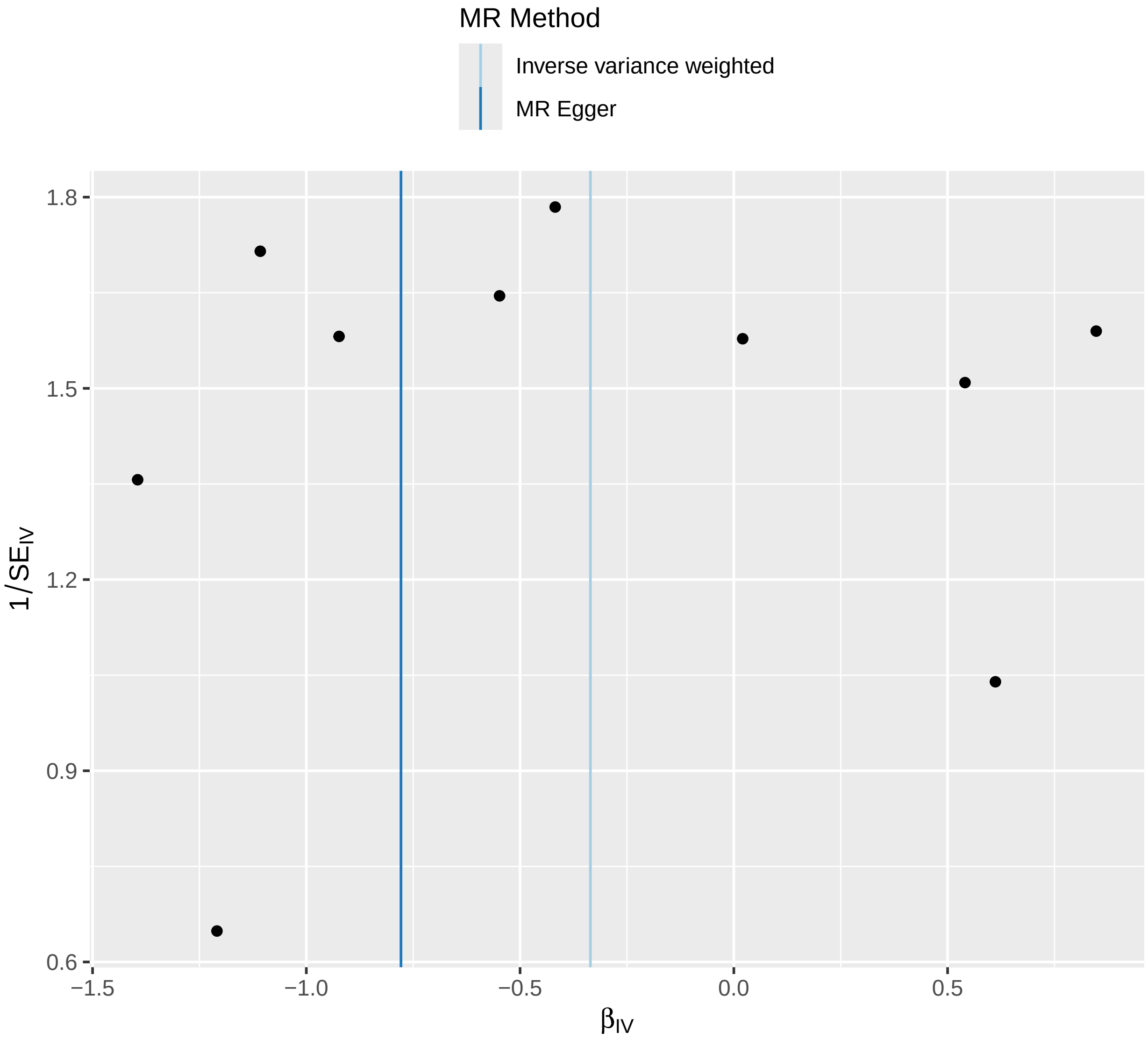

MR Method
Inverse variance weighted
MR Egger
1.8
1.5
1.2
0.9
0.6
1/SE_IV
−1.5
−1.0
−0.5
0.0
0.5
β_IV

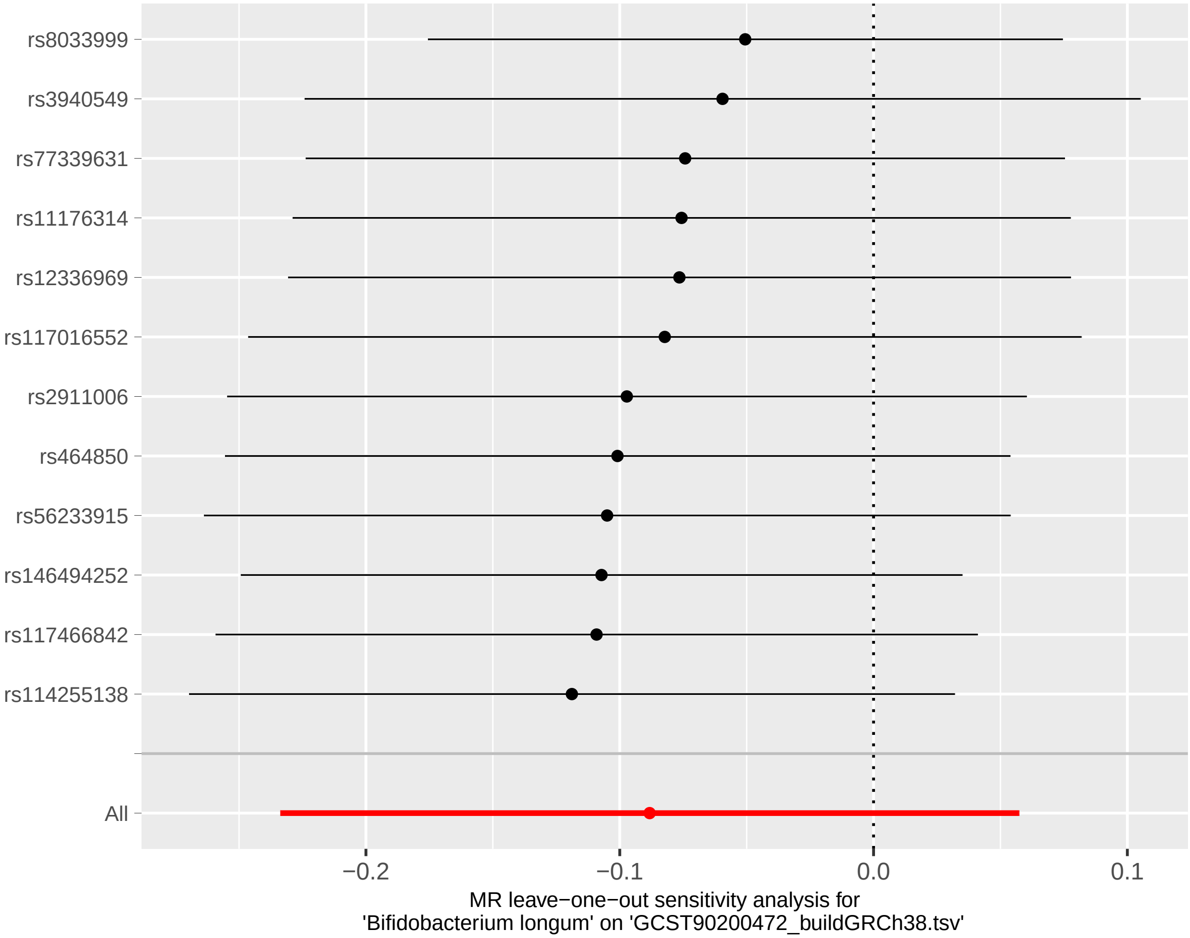

rs8033999
rs3940549
rs77339631
rs11176314
rs12336969
rs117016552
rs2911006
rs464850
rs56233915
rs146494252
rs117466842
rs114255138
All
−0.2
−0.1
0.0
0.1
MR leave−one−out sensitivity analysis for
'Bifidobacterium longum' on 'GCST90200472_buildGRCh38.tsv'

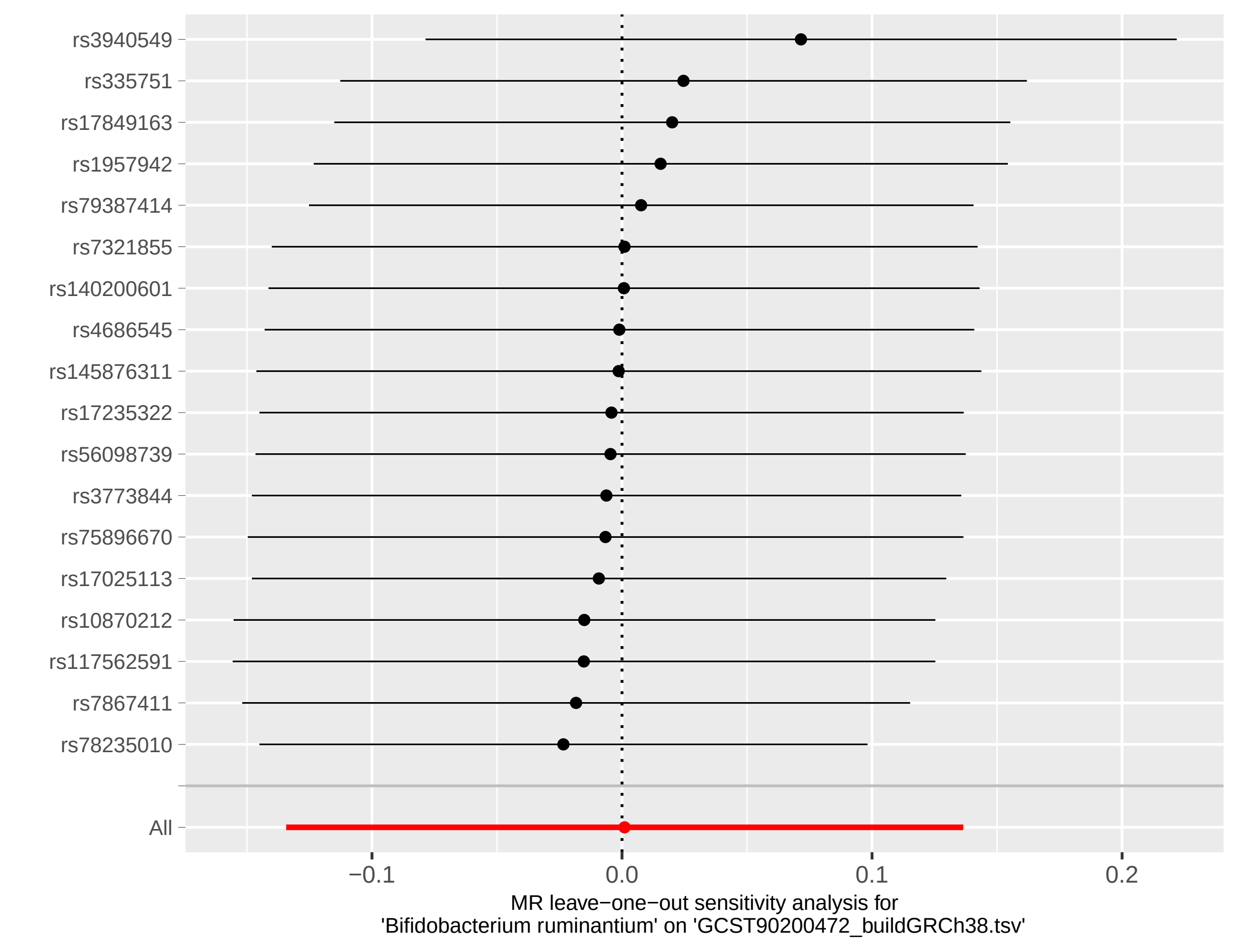
rs3940549
rs335751
rs17849163
rs1957942
rs79387414
rs7321855
rs140200601
rs4686545
rs145876311
rs17235322
rs56098739
rs3773844
rs75896670
rs17025113
rs10870212
rs117562591
rs7867411
rs78235010
All
−0.1
0.0
0.1
0.2
MR leave−one−out sensitivity analysis for
'Bifidobacterium ruminantium' on 'GCST90200472_buildGRCh38.tsv'

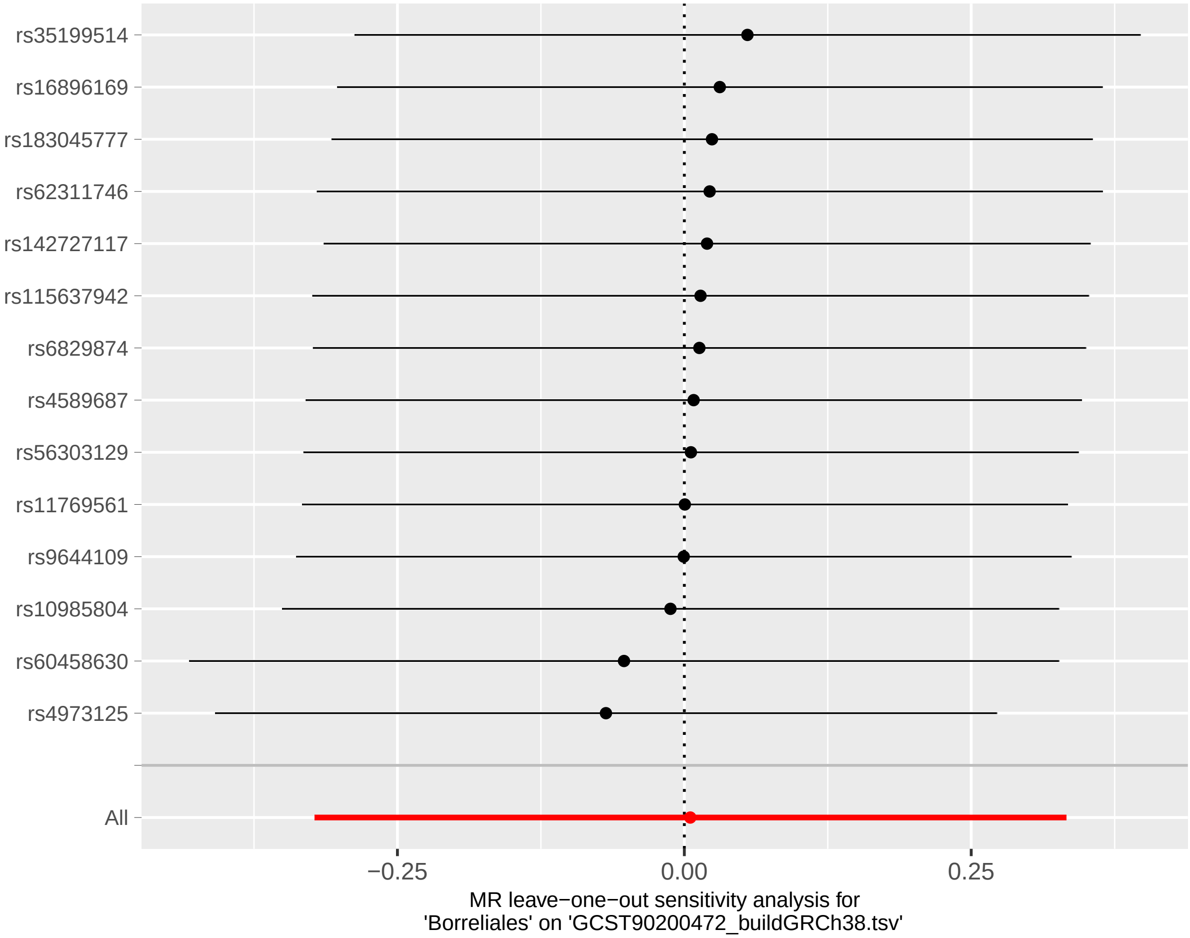
rs35199514
rs16896169
rs183045777
rs62311746
rs142727117
rs115637942
rs6829874
rs4589687
rs56303129
rs11769561
rs9644109
rs10985804
rs60458630
rs4973125
All
−0.25
0.00
0.25
MR leave−one−out sensitivity analysis for
'Borreliales' on 'GCST90200472_buildGRCh38.tsv'

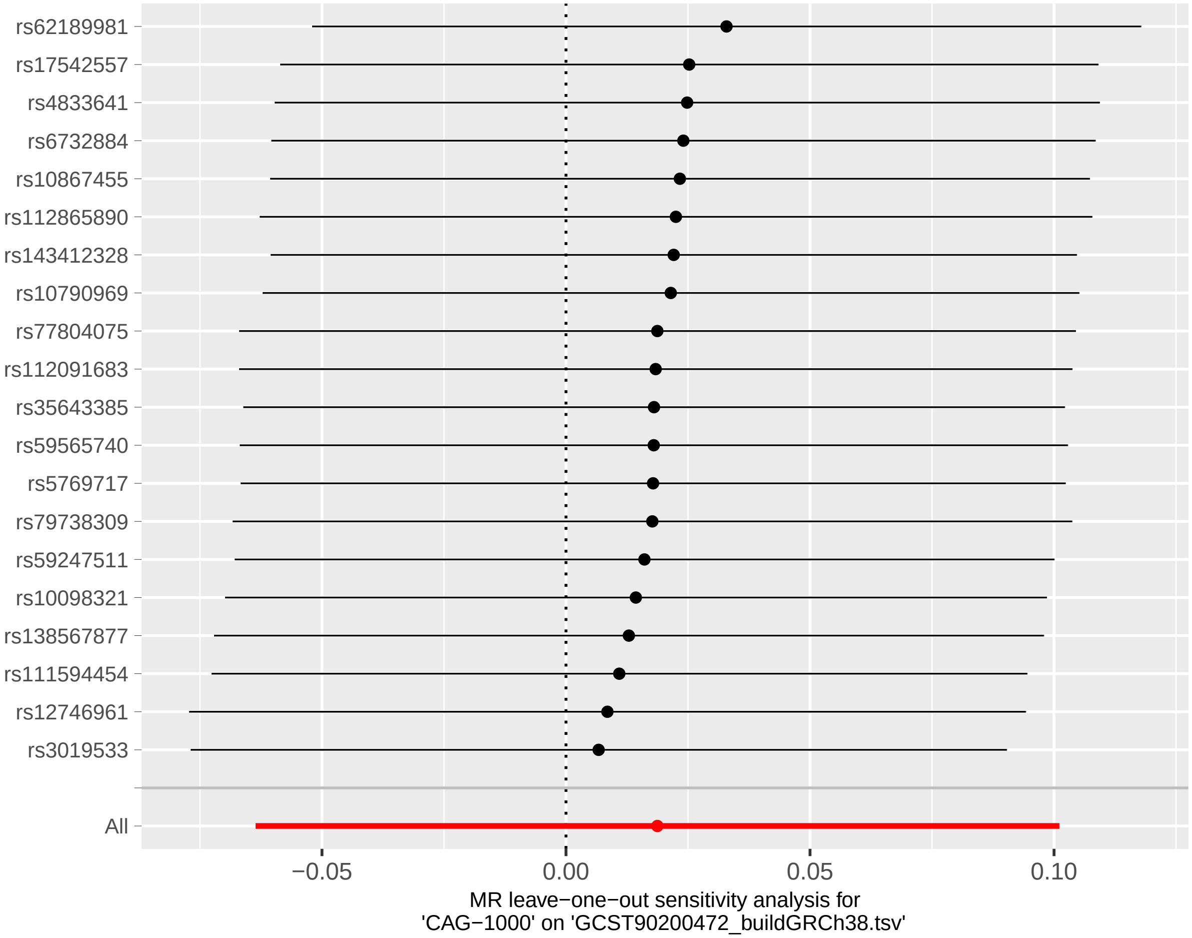
rs62189981
rs17542557
rs4833641
rs6732884
rs10867455
rs112865890
rs143412328
rs10790969
rs77804075
rs112091683
rs35643385
rs59565740
rs5769717
rs79738309
rs59247511
rs10098321
rs138567877
rs111594454
rs12746961
rs3019533
All
−0.05
0.00
0.05
0.10
MR leave−one−out sensitivity analysis for
'CAG−1000' on 'GCST90200472_buildGRCh38.tsv'

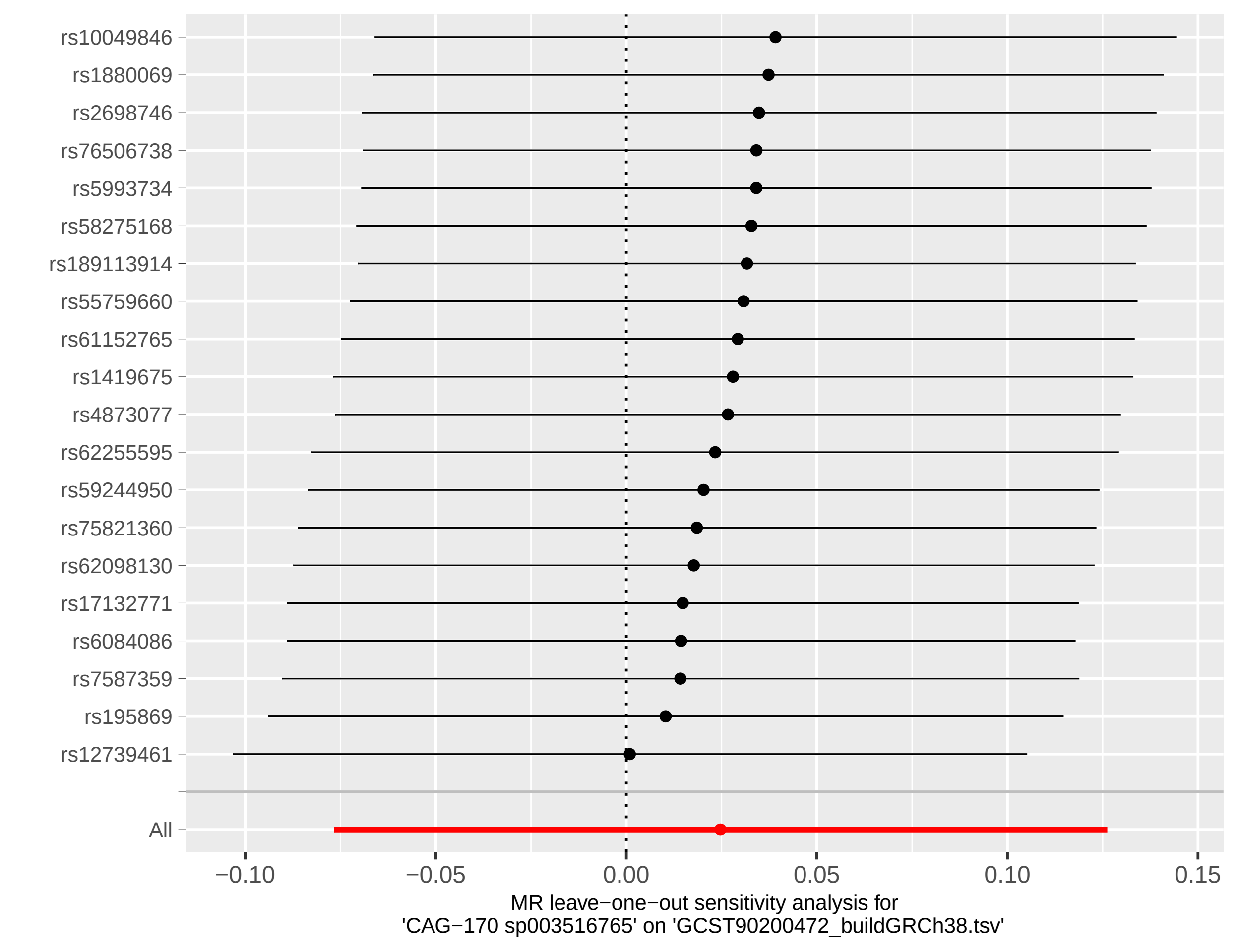

rs10049846
rs1880069
rs2698746
rs76506738
rs5993734
rs58275168
rs189113914
rs55759660
rs61152765
rs1419675
rs4873077
rs62255595
rs59244950
rs75821360
rs62098130
rs17132771
rs6084086
rs7587359
rs195869
rs12739461
All
−0.10
−0.05
0.00
0.05
0.10
0.15
MR leave−one−out sensitivity analysis for
'CAG−170 sp003516765' on 'GCST90200472_buildGRCh38.tsv'

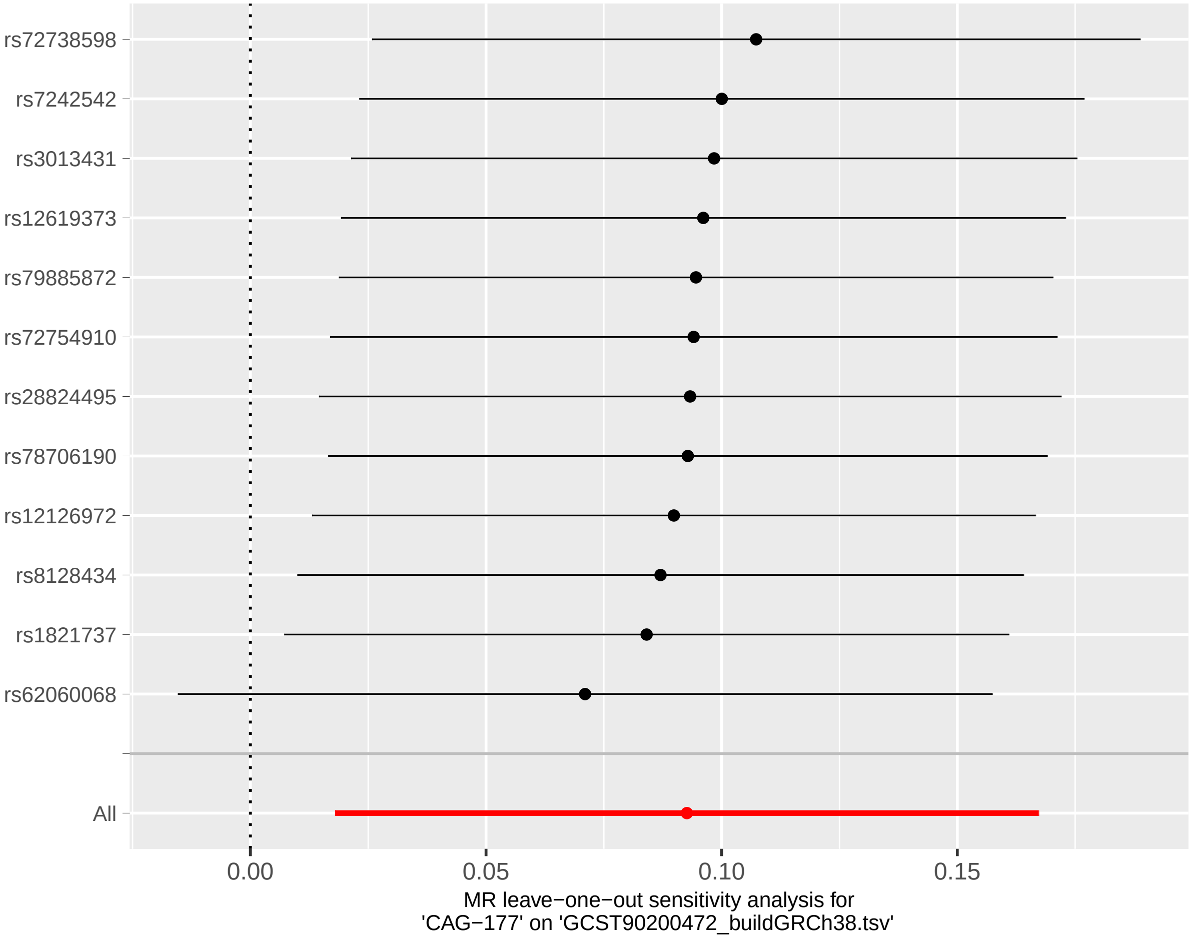

rs72738598
rs7242542
rs3013431
rs12619373
rs79885872
rs72754910
rs28824495
rs78706190
rs12126972
rs8128434
rs1821737
rs62060068
All
0.00
0.05
0.10
0.15
MR leave−one−out sensitivity analysis for
'CAG−177' on 'GCST90200472_buildGRCh38.tsv'

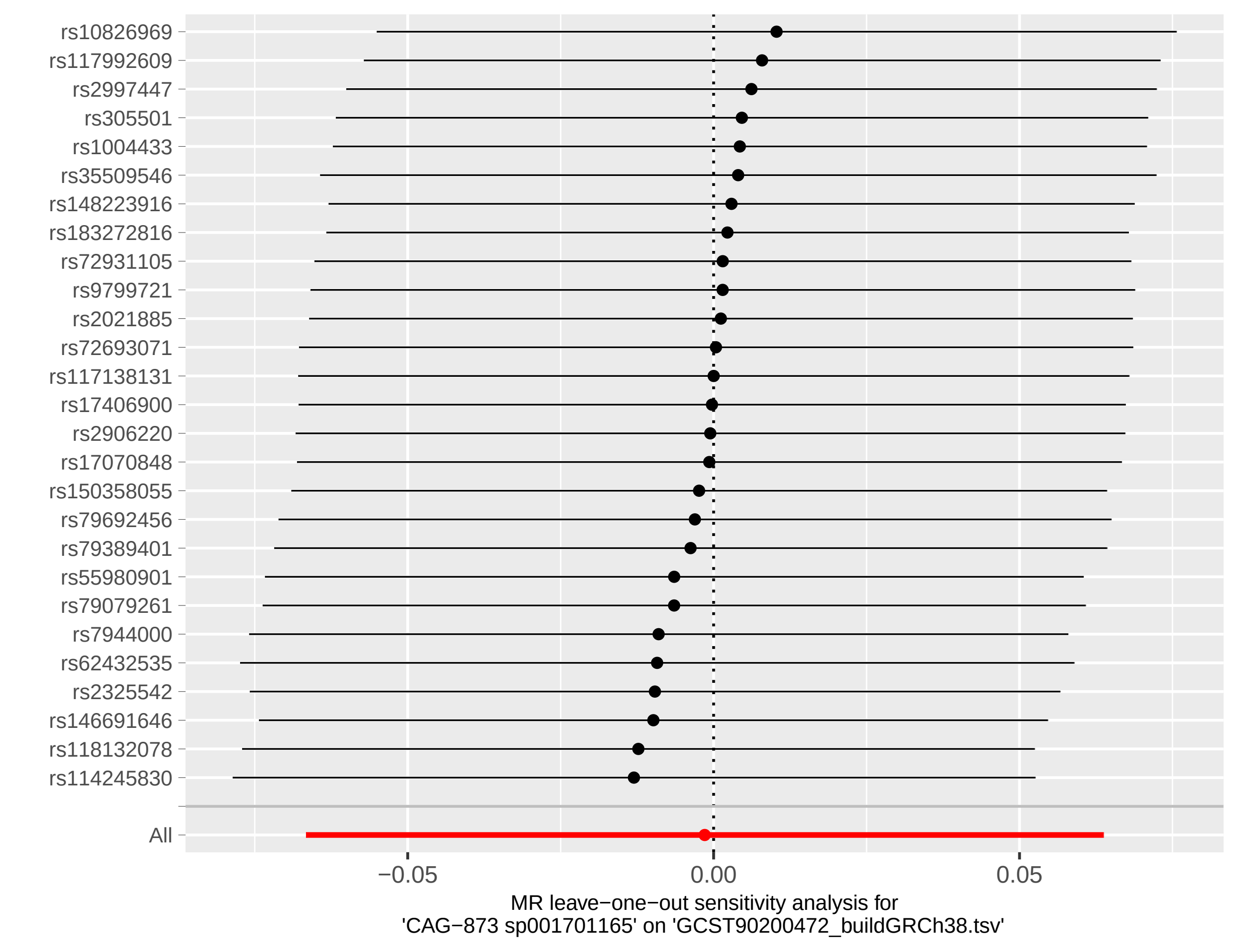
rs10826969
rs117992609
rs2997447
rs305501
rs1004433
rs35509546
rs148223916
rs183272816
rs72931105
rs9799721
rs2021885
rs72693071
rs117138131
rs17406900
rs2906220
rs17070848
rs150358055
rs79692456
rs79389401
rs55980901
rs79079261
rs7944000
rs62432535
rs2325542
rs146691646
rs118132078
rs114245830
All
−0.05
0.00
0.05
MR leave−one−out sensitivity analysis for
'CAG−873 sp001701165' on 'GCST90200472_buildGRCh38.tsv'

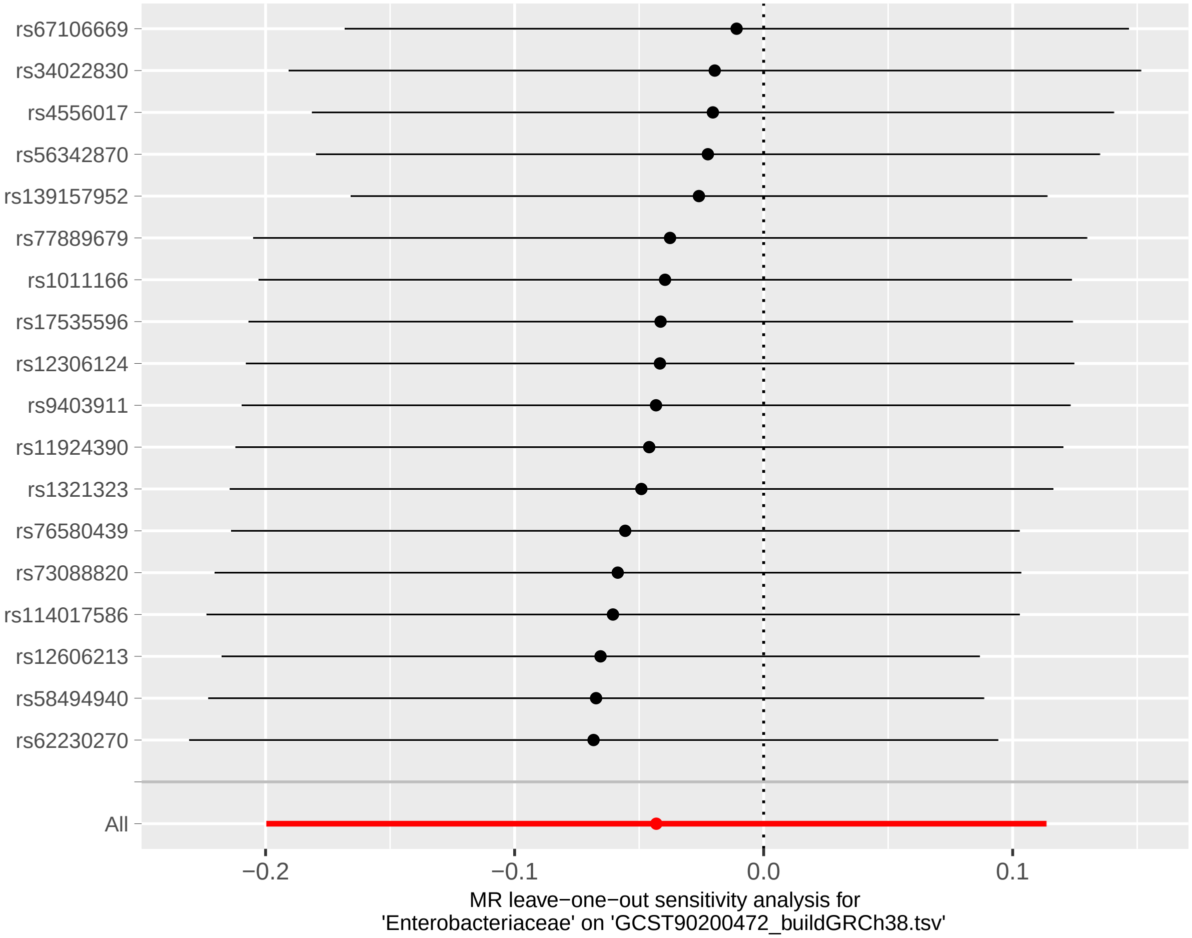
rs67106669
rs34022830
rs4556017
rs56342870
rs139157952
rs77889679
rs1011166
rs17535596
rs12306124
rs9403911
rs11924390
rs1321323
rs76580439
rs73088820
rs114017586
rs12606213
rs58494940
rs62230270
All
−0.2
−0.1
0.0
0.1
MR leave−one−out sensitivity analysis for
'Enterobacteriaceae' on 'GCST90200472_buildGRCh38.tsv'

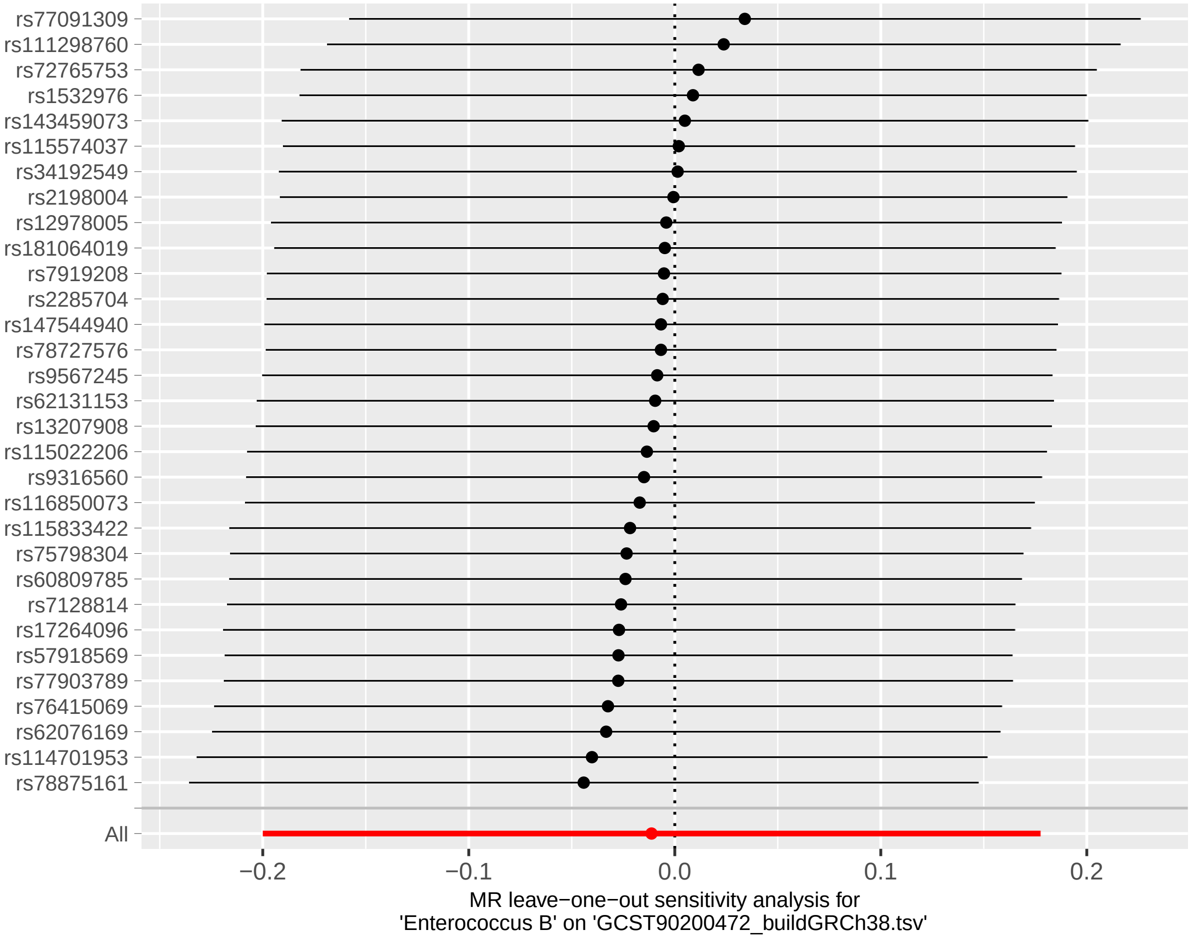

rs77091309
rs111298760
rs72765753
rs1532976
rs143459073
rs115574037
rs34192549
rs2198004
rs12978005
rs181064019
rs7919208
rs2285704
rs147544940
rs78727576
rs9567245
rs62131153
rs13207908
rs115022206
rs9316560
rs116850073
rs115833422
rs75798304
rs60809785
rs7128814
rs17264096
rs57918569
rs77903789
rs76415069
rs62076169
rs114701953
rs78875161
All
−0.2
−0.1
0.0
0.1
0.2
MR leave−one−out sensitivity analysis for
'Enterococcus B' on 'GCST90200472_buildGRCh38.tsv'

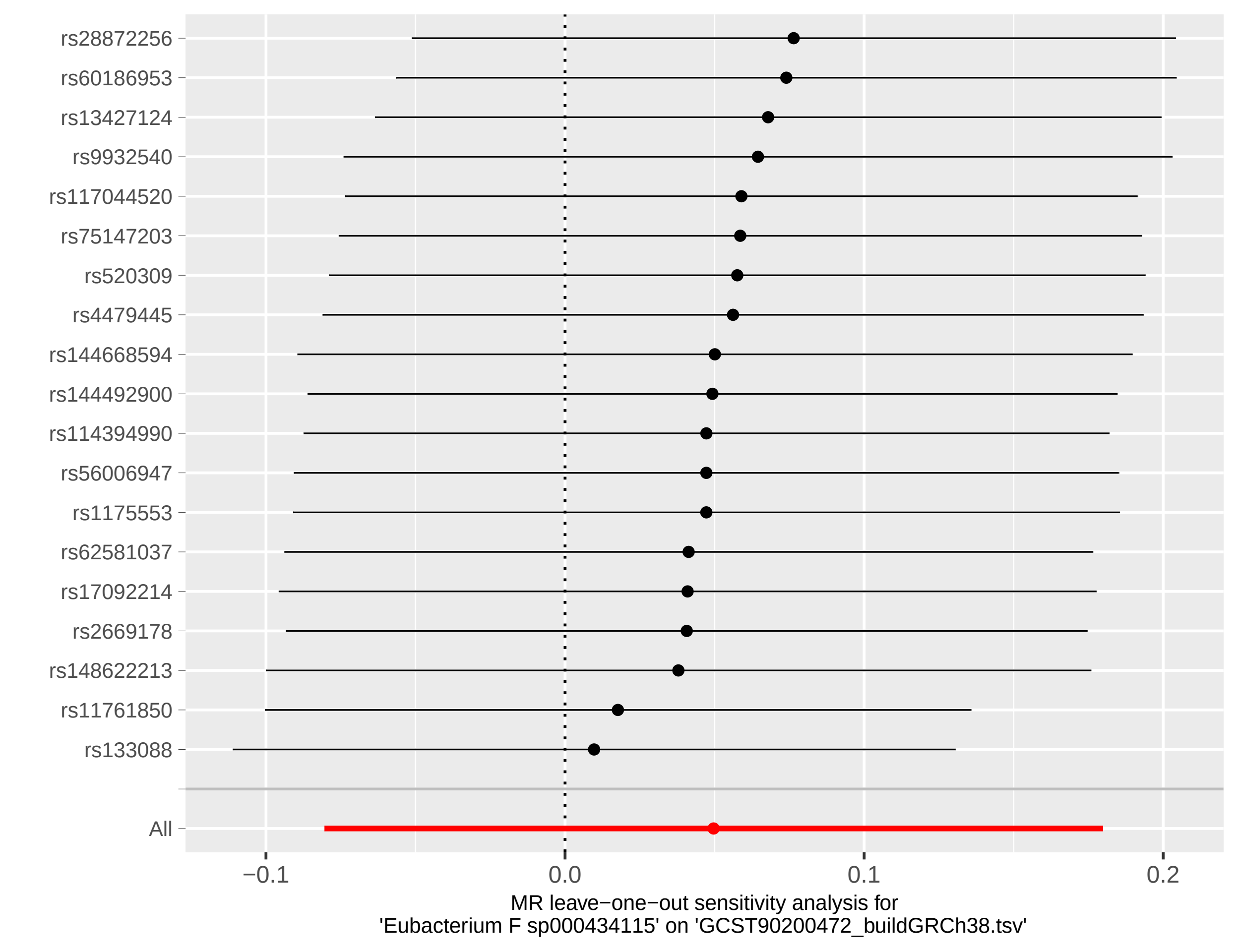

rs28872256
rs60186953
rs13427124
rs9932540
rs117044520
rs75147203
rs520309
rs4479445
rs144668594
rs144492900
rs114394990
rs56006947
rs1175553
rs62581037
rs17092214
rs2669178
rs148622213
rs11761850
rs133088
All
−0.1
0.0
0.1
0.2
MR leave−one−out sensitivity analysis for
'Eubacterium F sp000434115' on 'GCST90200472_buildGRCh38.tsv'

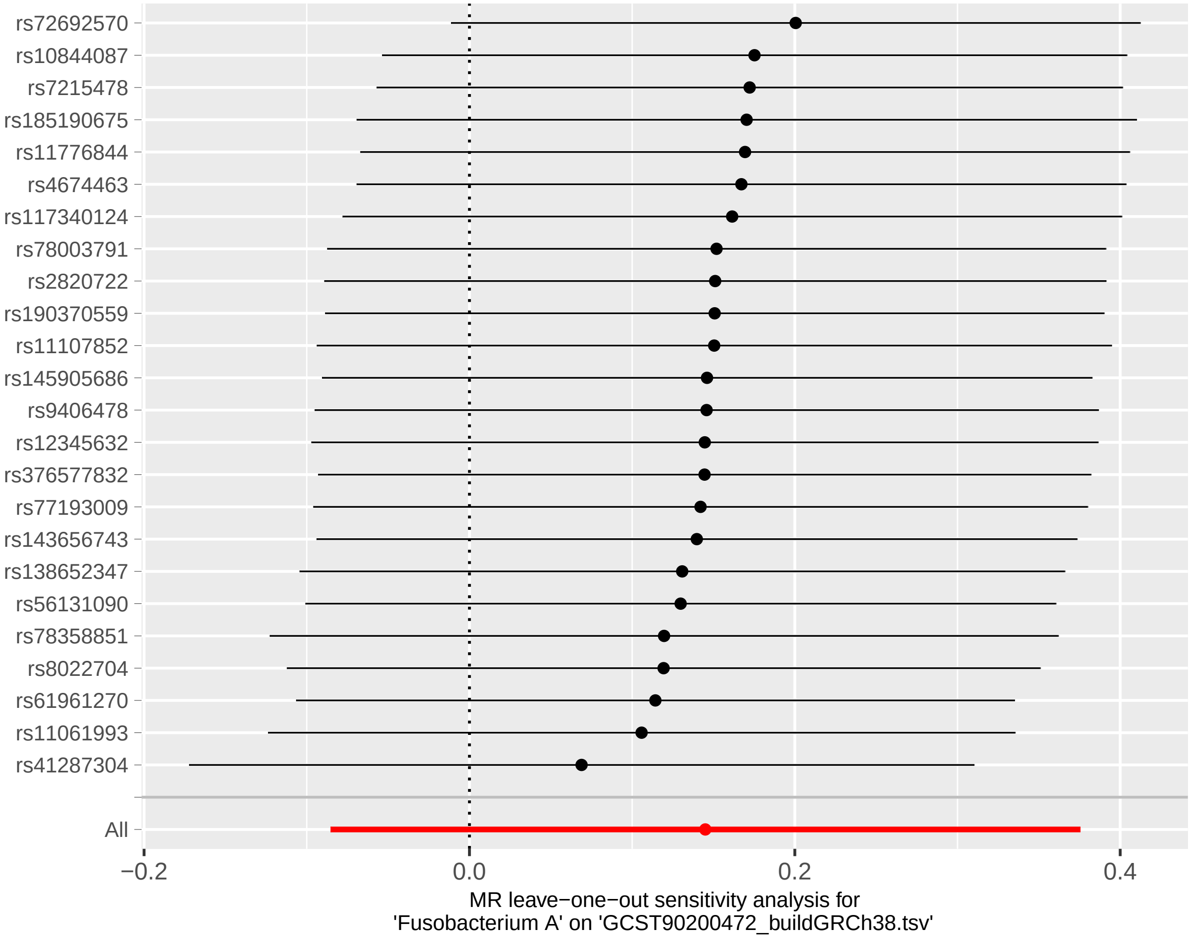

rs72692570
rs10844087
rs7215478
rs185190675
rs11776844
rs4674463
rs117340124
rs78003791
rs2820722
rs190370559
rs11107852
rs145905686
rs9406478
rs12345632
rs376577832
rs77193009
rs143656743
rs138652347
rs56131090
rs78358851
rs8022704
rs61961270
rs11061993
rs41287304
All
−0.2
0.0
0.2
0.4
MR leave−one−out sensitivity analysis for
'Fusobacterium A' on 'GCST90200472_buildGRCh38.tsv'

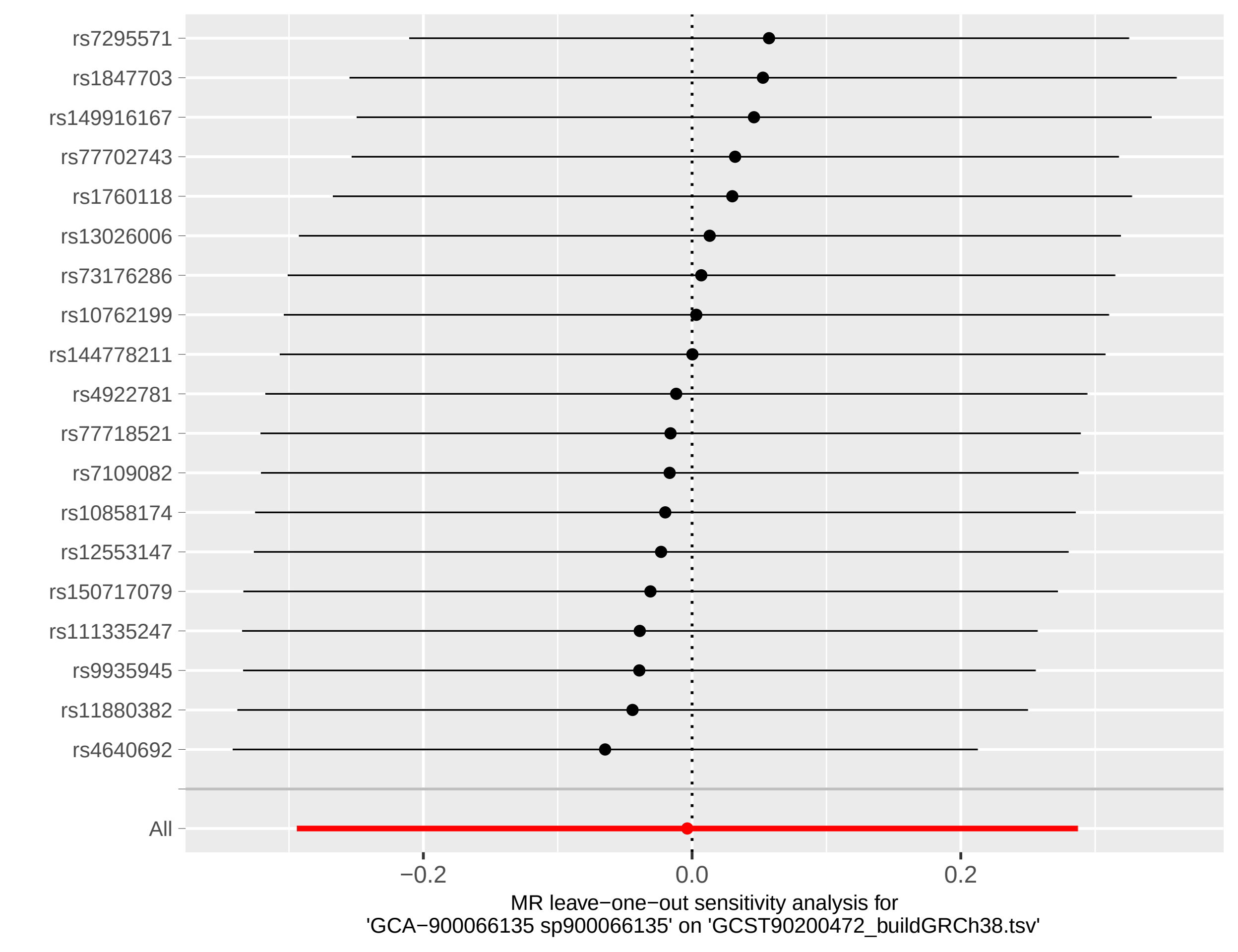
rs7295571
rs1847703
rs149916167
rs77702743
rs1760118
rs13026006
rs73176286
rs10762199
rs144778211
rs4922781
rs77718521
rs7109082
rs10858174
rs12553147
rs150717079
rs111335247
rs9935945
rs11880382
rs4640692
All
−0.2
0.0
0.2
MR leave−one−out sensitivity analysis for
'GCA−900066135 sp900066135' on 'GCST90200472_buildGRCh38.tsv'

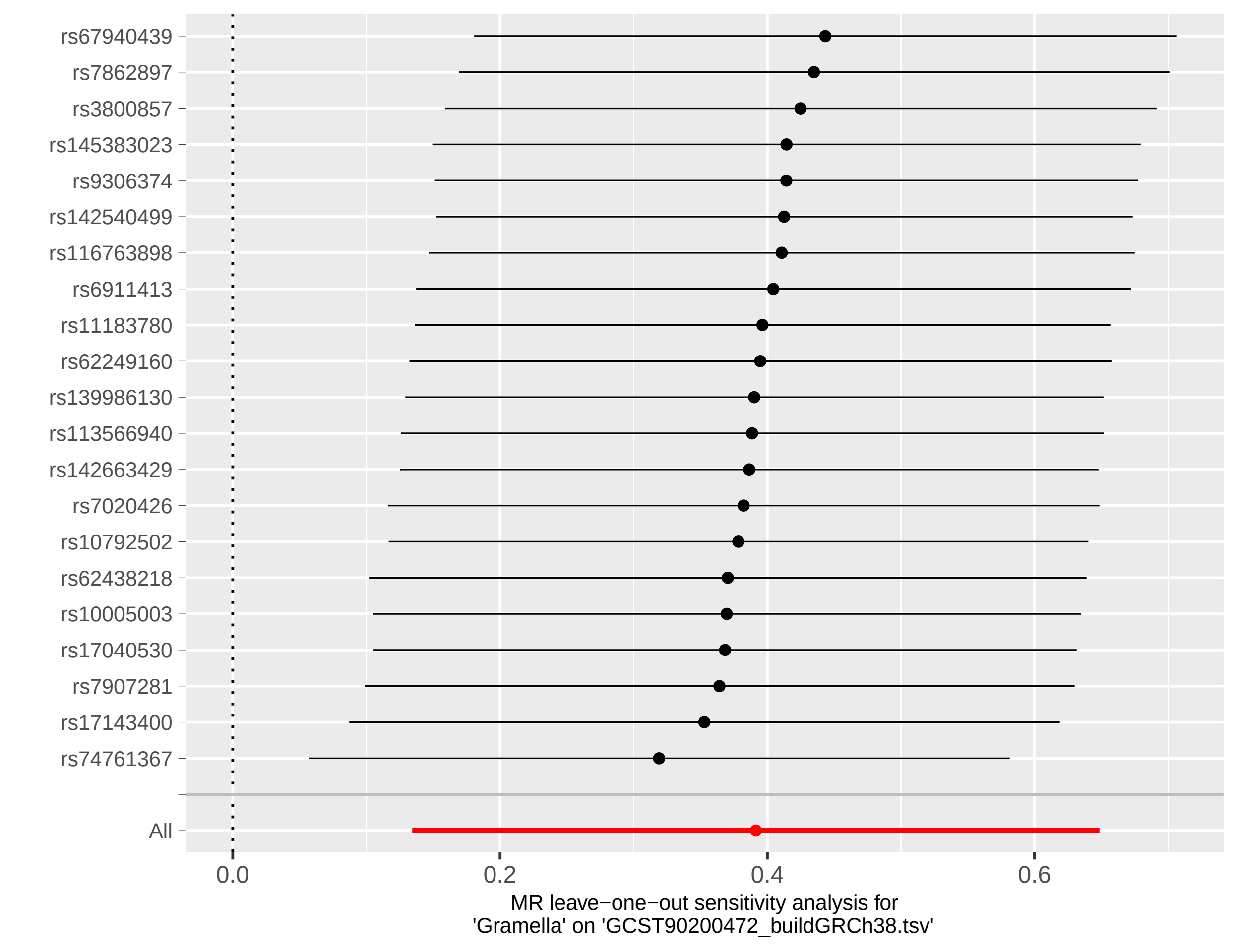
rs67940439
rs7862897
rs3800857
rs145383023
rs9306374
rs142540499
rs116763898
rs6911413
rs11183780
rs62249160
rs139986130
rs113566940
rs142663429
rs7020426
rs10792502
rs62438218
rs10005003
rs17040530
rs7907281
rs17143400
rs74761367
All
0.0
0.2
0.4
0.6
MR leave−one−out sensitivity analysis for
'Gramella' on 'GCST90200472_buildGRCh38.tsv'

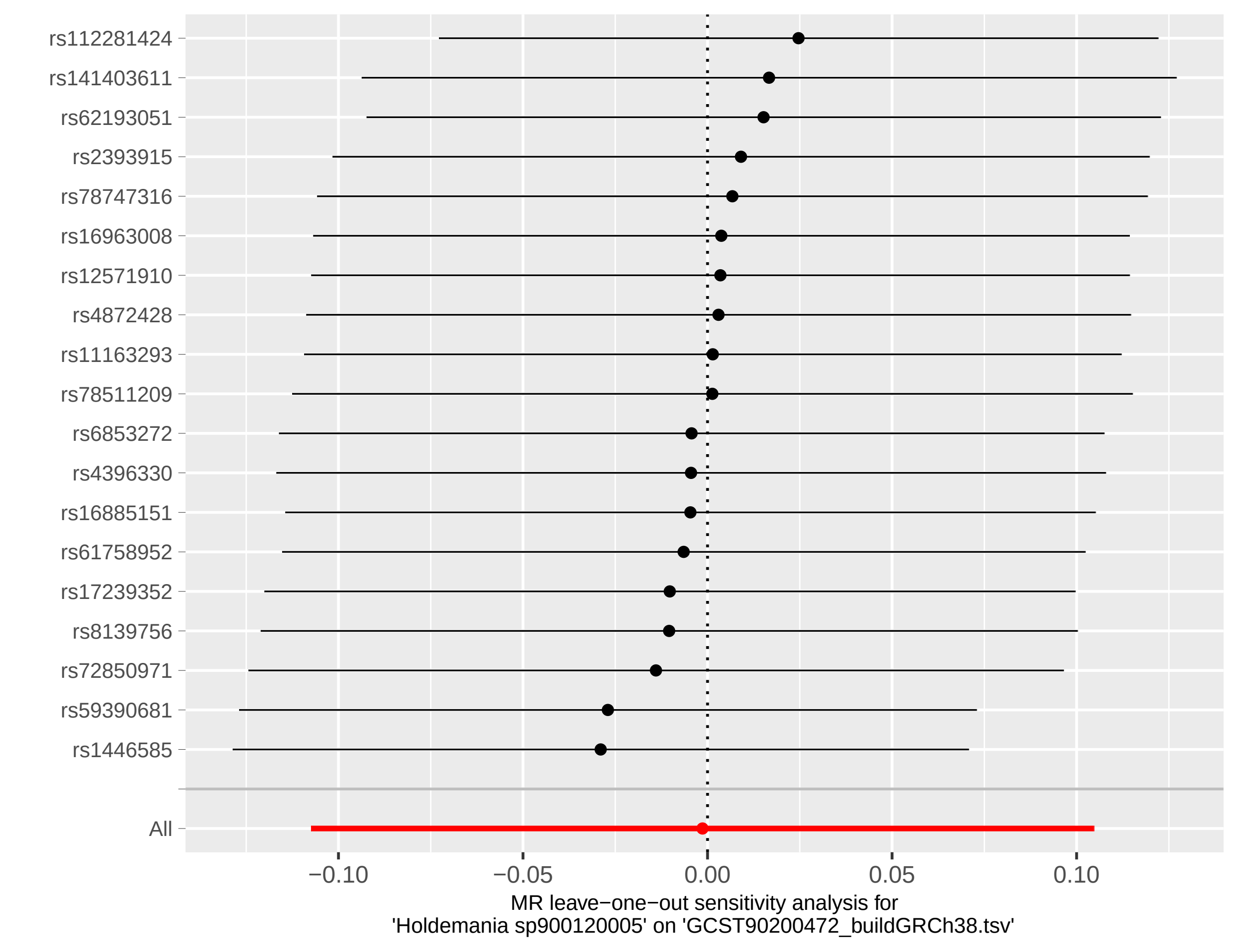
rs112281424
rs141403611
rs62193051
rs2393915
rs78747316
rs16963008
rs12571910
rs4872428
rs11163293
rs78511209
rs6853272
rs4396330
rs16885151
rs61758952
rs17239352
rs8139756
rs72850971
rs59390681
rs1446585
All
−0.10
−0.05
0.00
0.05
0.10
MR leave−one−out sensitivity analysis for
'Holdemania sp900120005' on 'GCST90200472_buildGRCh38.tsv'

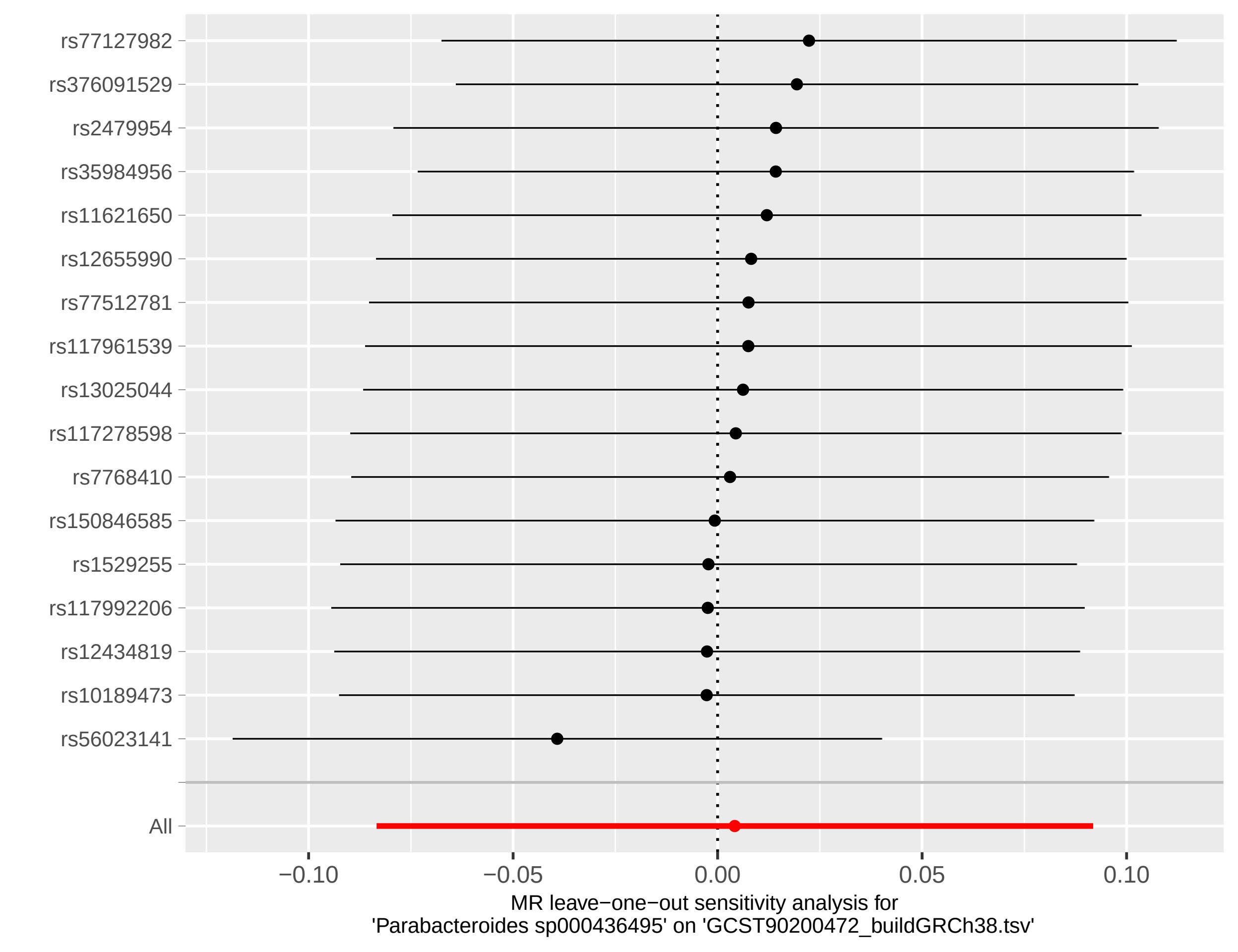
rs77127982
rs376091529
rs2479954
rs35984956
rs11621650
rs12655990
rs77512781
rs117961539
rs13025044
rs117278598
rs7768410
rs150846585
rs1529255
rs117992206
rs12434819
rs10189473
rs56023141
All
−0.10
−0.05
0.00
0.05
0.10
MR leave−one−out sensitivity analysis for
'Parabacteroides sp000436495' on 'GCST90200472_buildGRCh38.tsv'

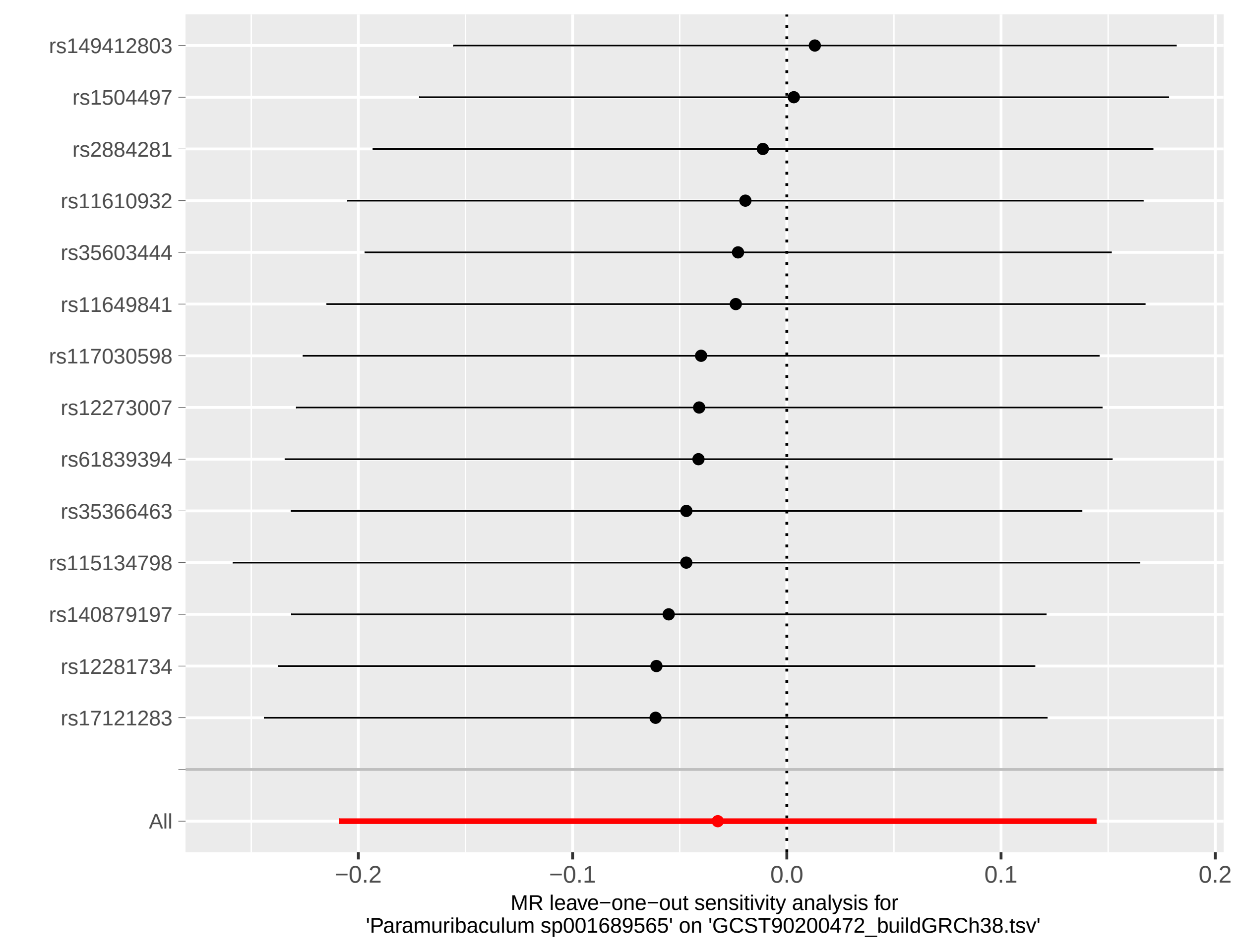
rs149412803
rs1504497
rs2884281
rs11610932
rs35603444
rs11649841
rs117030598
rs12273007
rs61839394
rs35366463
rs115134798
rs140879197
rs12281734
rs17121283
All
−0.2
−0.1
0.0
0.1
0.2
MR leave−one−out sensitivity analysis for
'Paramuribaculum sp001689565' on 'GCST90200472_buildGRCh38.tsv'

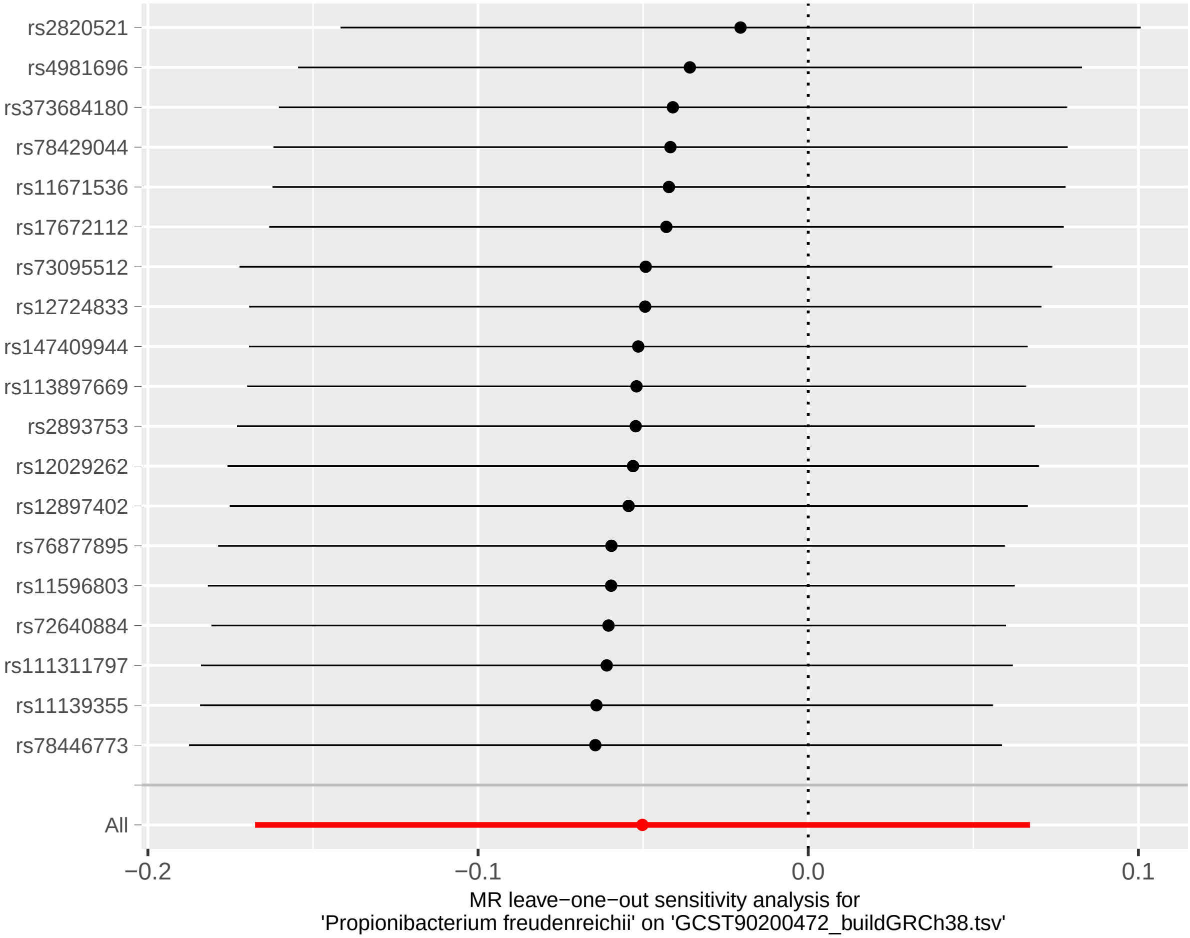

rs2820521
rs4981696
rs373684180
rs78429044
rs11671536
rs17672112
rs73095512
rs12724833
rs147409944
rs113897669
rs2893753
rs12029262
rs12897402
rs76877895
rs11596803
rs72640884
rs111311797
rs11139355
rs78446773
All
−0.2
−0.1
0.0
0.1
MR leave−one−out sensitivity analysis for
'Propionibacterium freudenreichii' on 'GCST90200472_buildGRCh38.tsv'

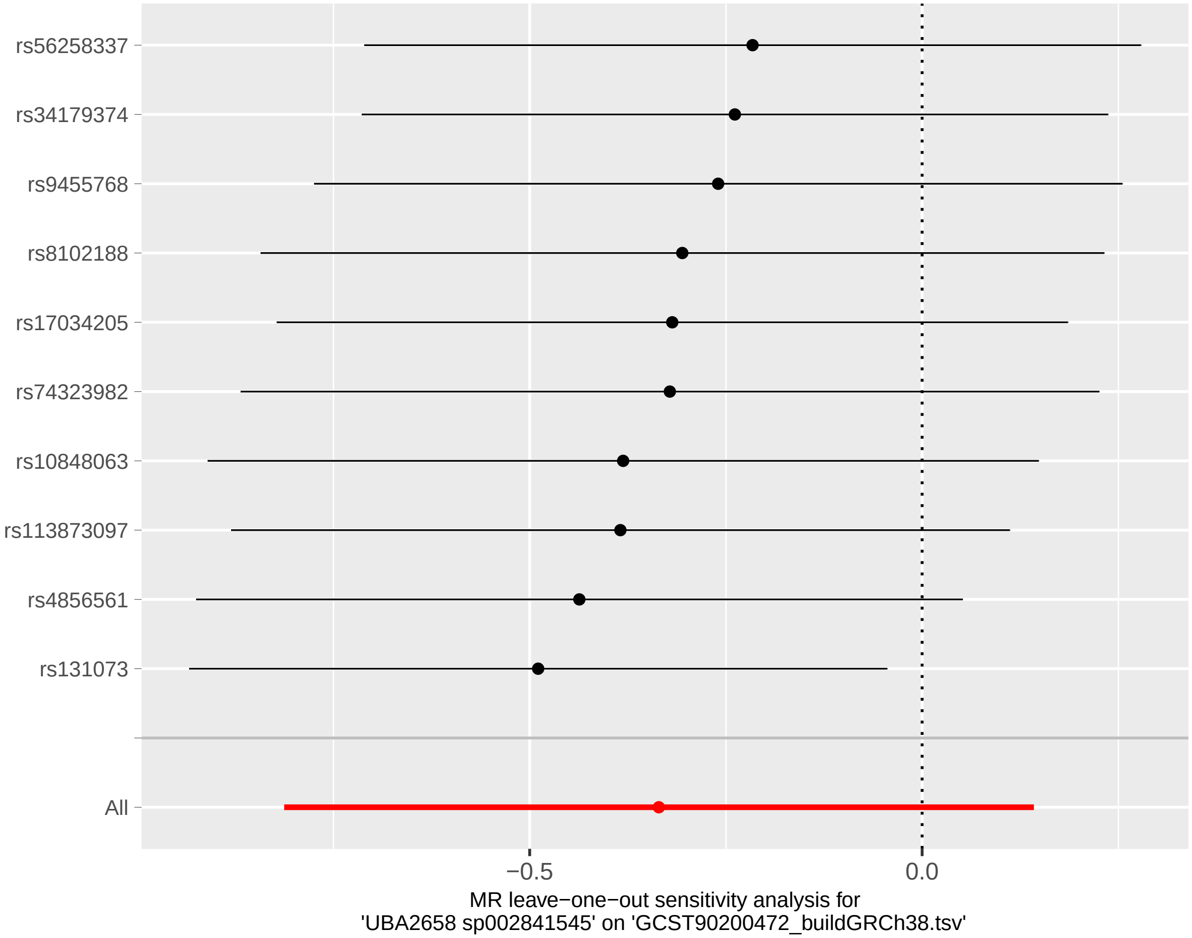
rs56258337
rs34179374
rs9455768
rs8102188
rs17034205
rs74323982
rs10848063
rs113873097
rs4856561
rs131073
All
−0.5
0.0
MR leave−one−out sensitivity analysis for
'UBA2658 sp002841545' on 'GCST90200472_buildGRCh38.tsv'

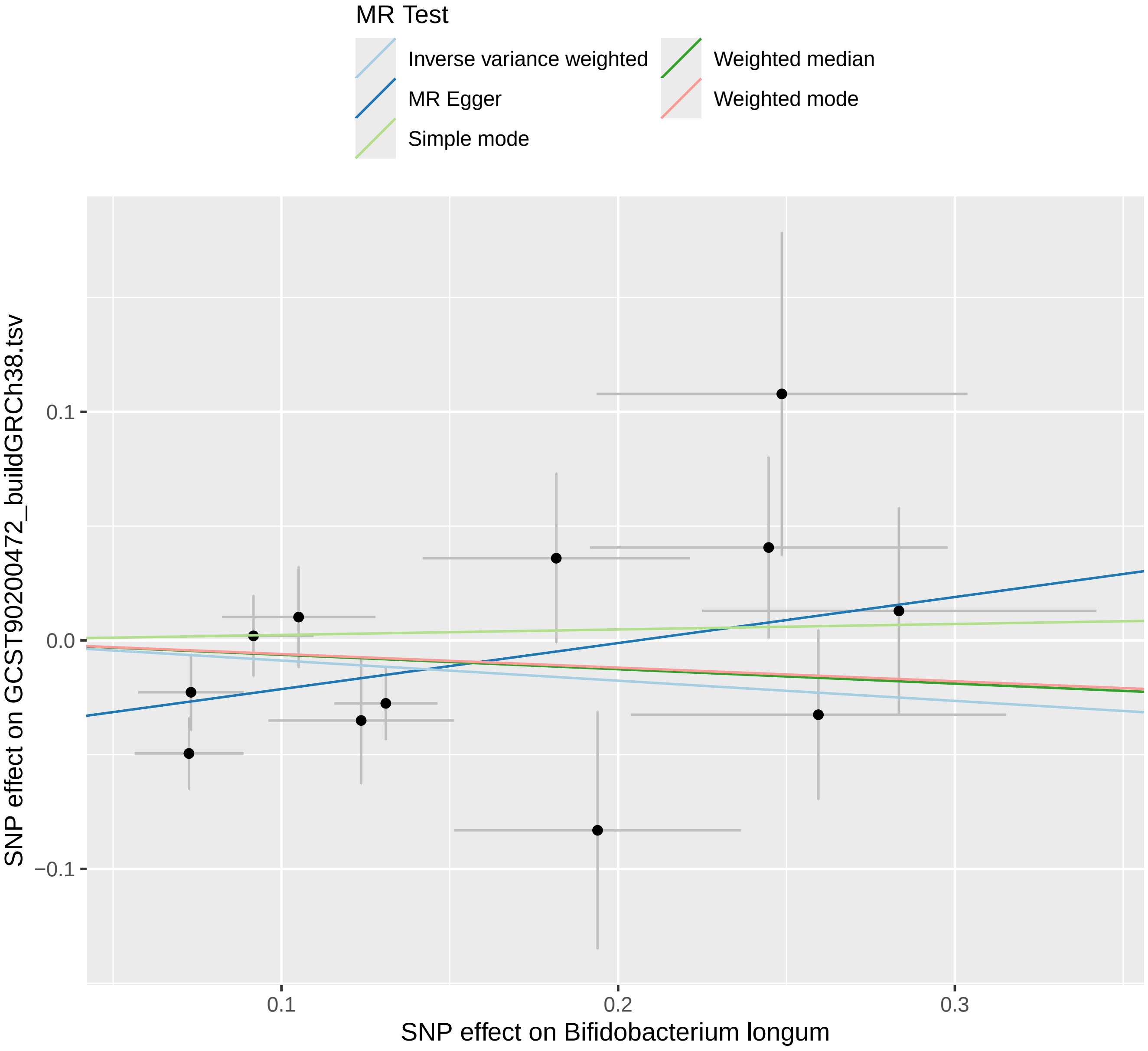
MR Test
Inverse variance weighted
MR Egger
Simple mode
Weighted median
Weighted mode
SNP effect on GCST90200472_buildGRCh38.tsv
0.1
0.0
−0.1
0.1
0.2
0.3
SNP effect on Bifidobacterium longum

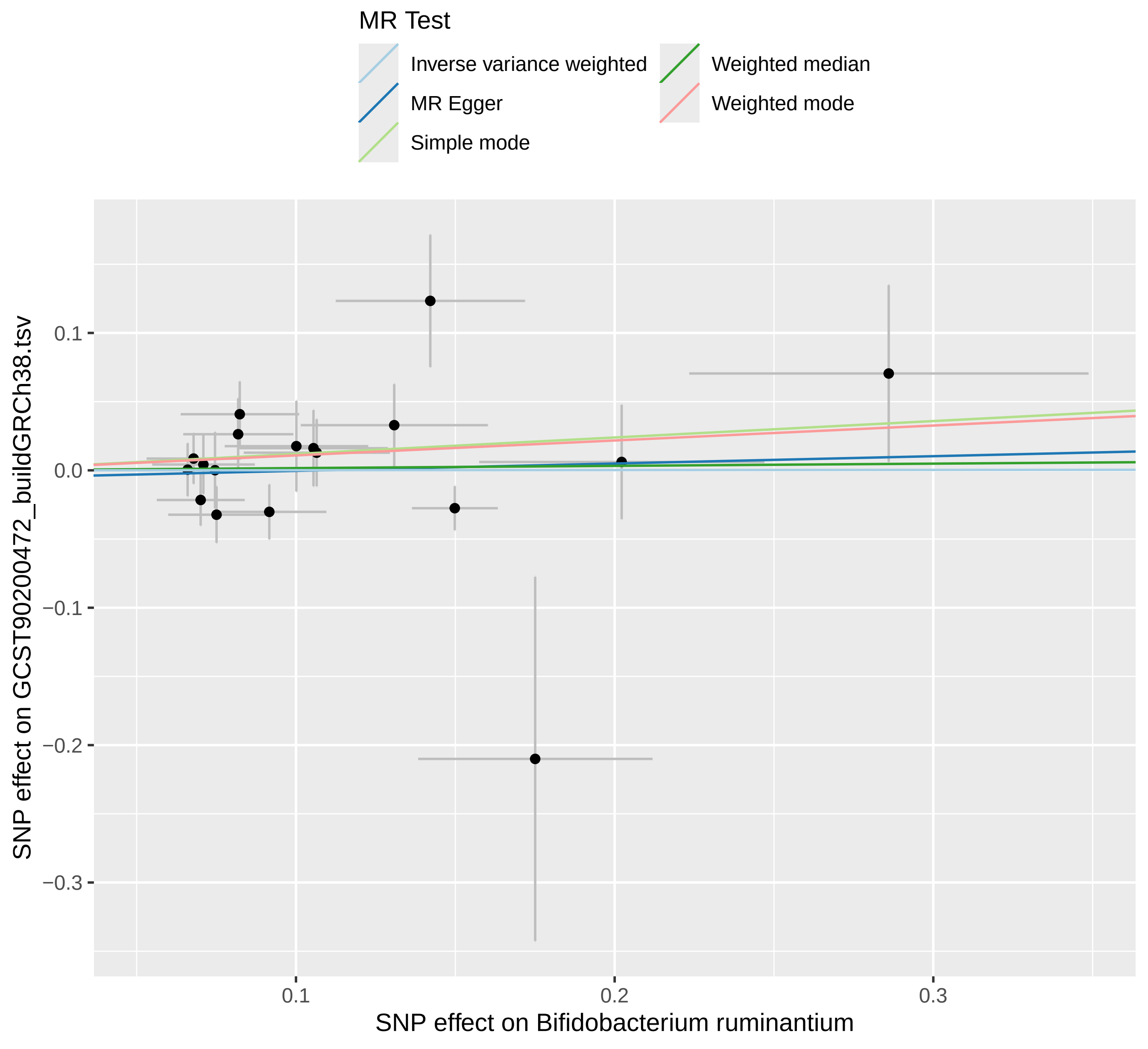
MR Test
Inverse variance weighted
MR Egger
Simple mode
Weighted median
Weighted mode
0.1
0.0
−0.1
−0.2
−0.3
SNP effect on GCST90200472_buildGRCh38.tsv
0.1
0.2
0.3
SNP effect on Bifidobacterium ruminantium

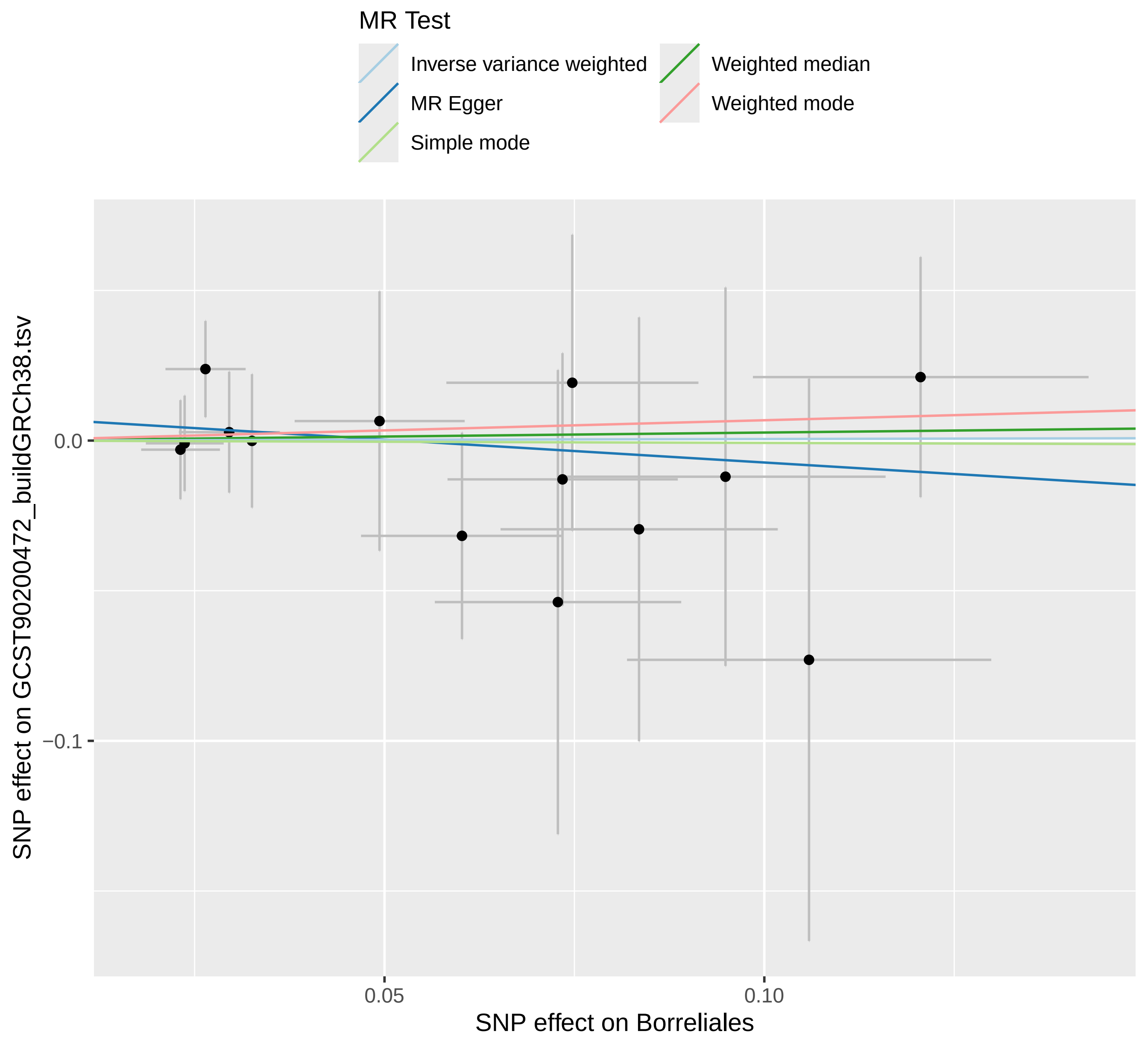

MR Test
Inverse variance weighted
MR Egger
Simple mode
Weighted median
Weighted mode
SNP effect on GCST90200472_buildGRCh38.tsv
0.0
−0.1
0.05
0.10
SNP effect on Borreliales

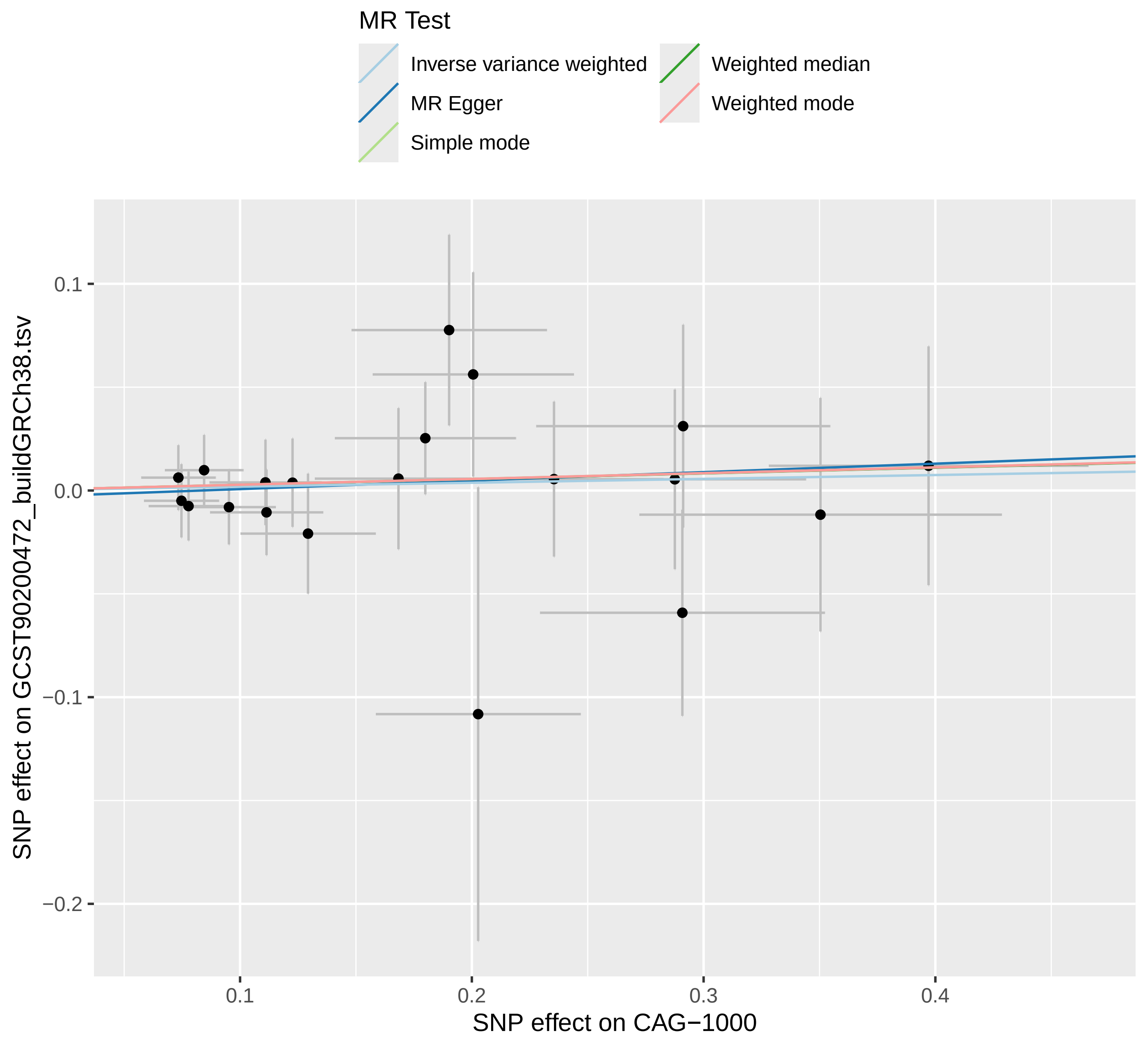
MR Test
Inverse variance weighted
MR Egger
Simple mode
Weighted median
Weighted mode
0.1
0.0
−0.1
−0.2
0.1
0.2
0.3
0.4
SNP effect on CAG−1000
SNP effect on GCST90200472_buildGRCh38.tsv

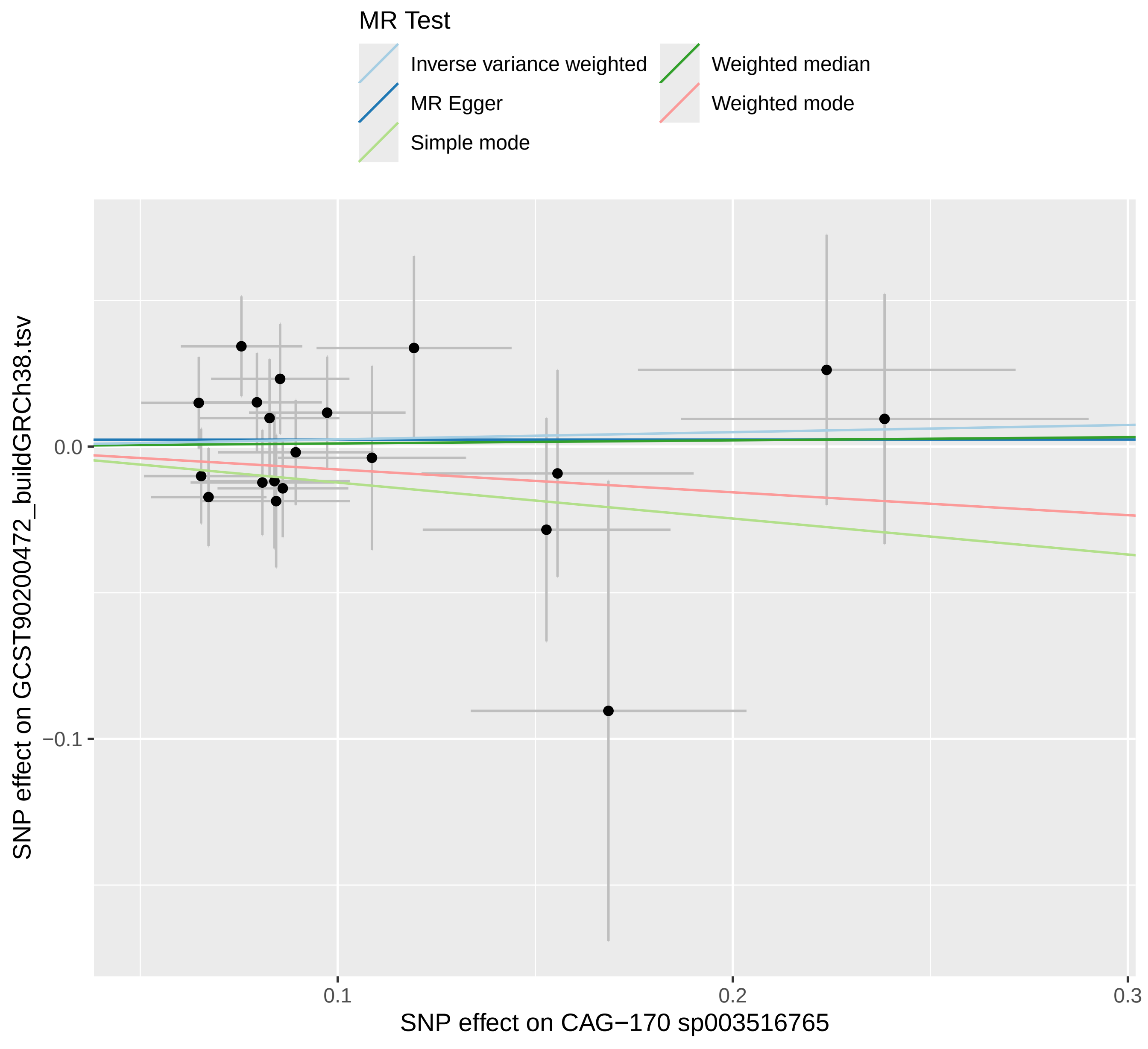
MR Test
Inverse variance weighted
MR Egger
Simple mode
Weighted median
Weighted mode
0.0
−0.1
0.1
0.2
0.3
SNP effect on GCST90200472_buildGRCh38.tsv
SNP effect on CAG−170 sp003516765

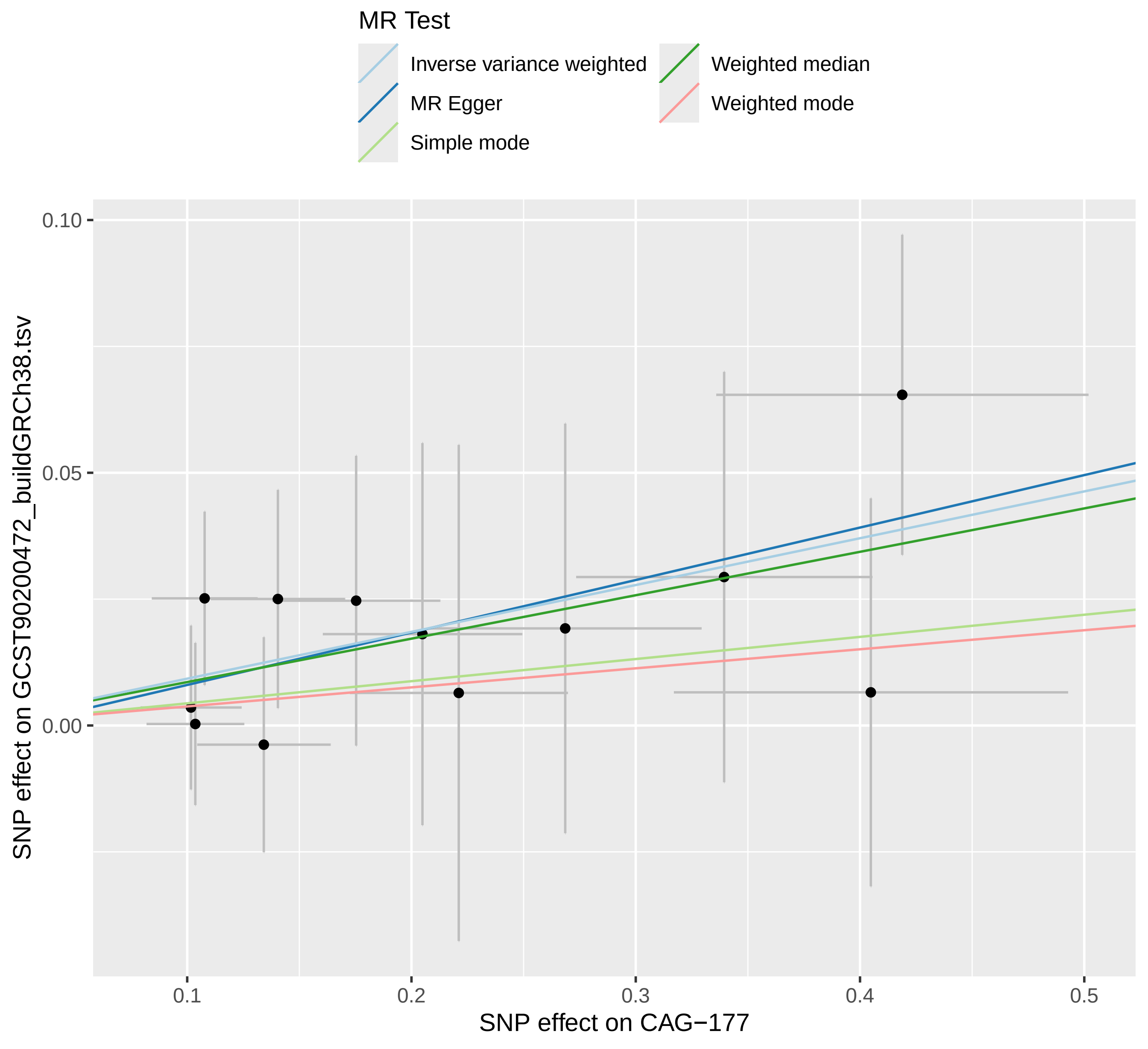

MR Test
Inverse variance weighted
MR Egger
Simple mode
Weighted median
Weighted mode
0.10
0.05
0.00
0.1
0.2
0.3
0.4
0.5
SNP effect on GCST90200472_buildGRCh38.tsv
SNP effect on CAG−177

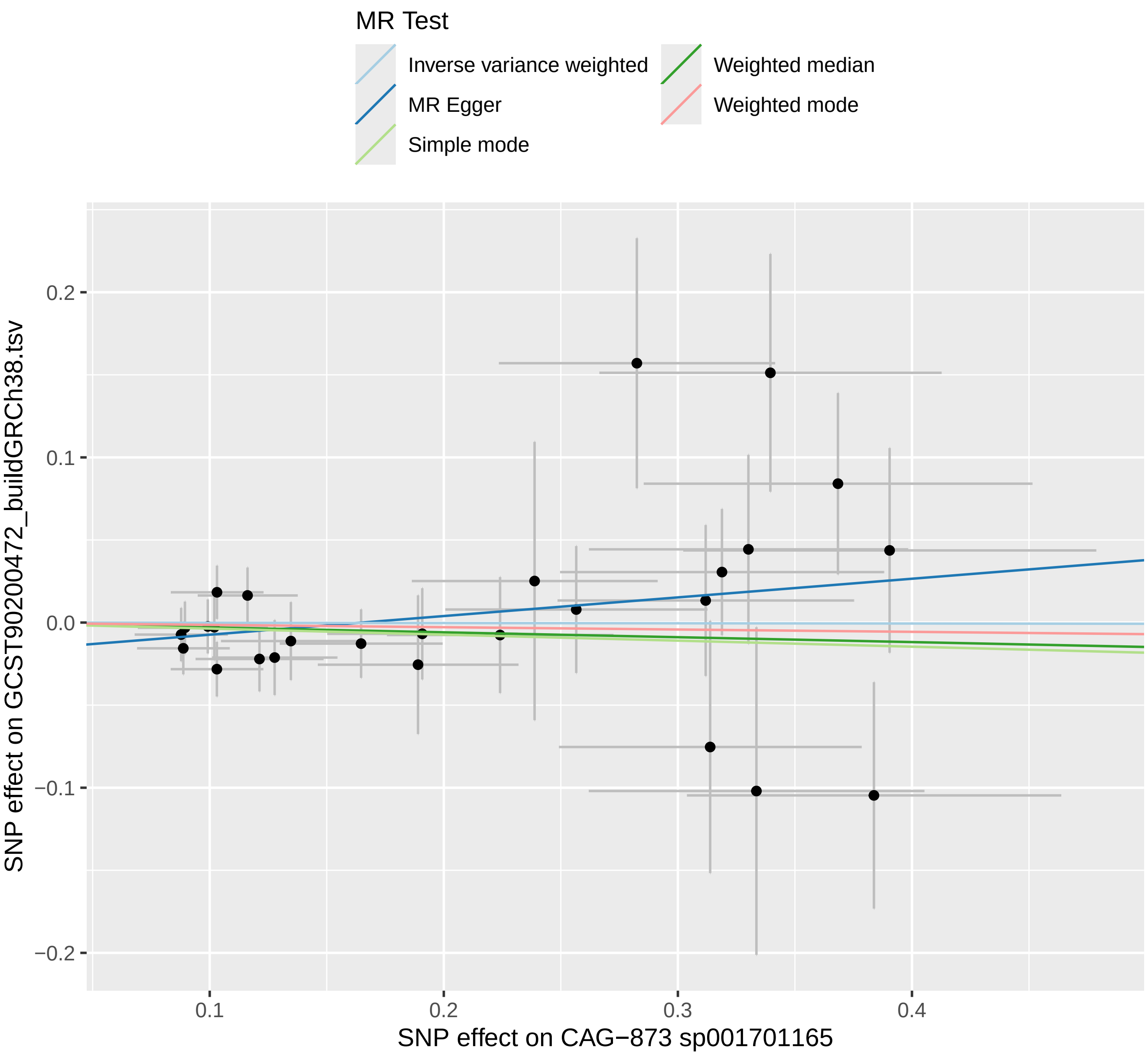
MR Test
Inverse variance weighted
MR Egger
Simple mode
Weighted median
Weighted mode
SNP effect on GCST90200472_buildGRCh38.tsv
0.2
0.1
0.0
−0.1
−0.2
0.1
0.2
0.3
0.4
SNP effect on CAG−873 sp001701165

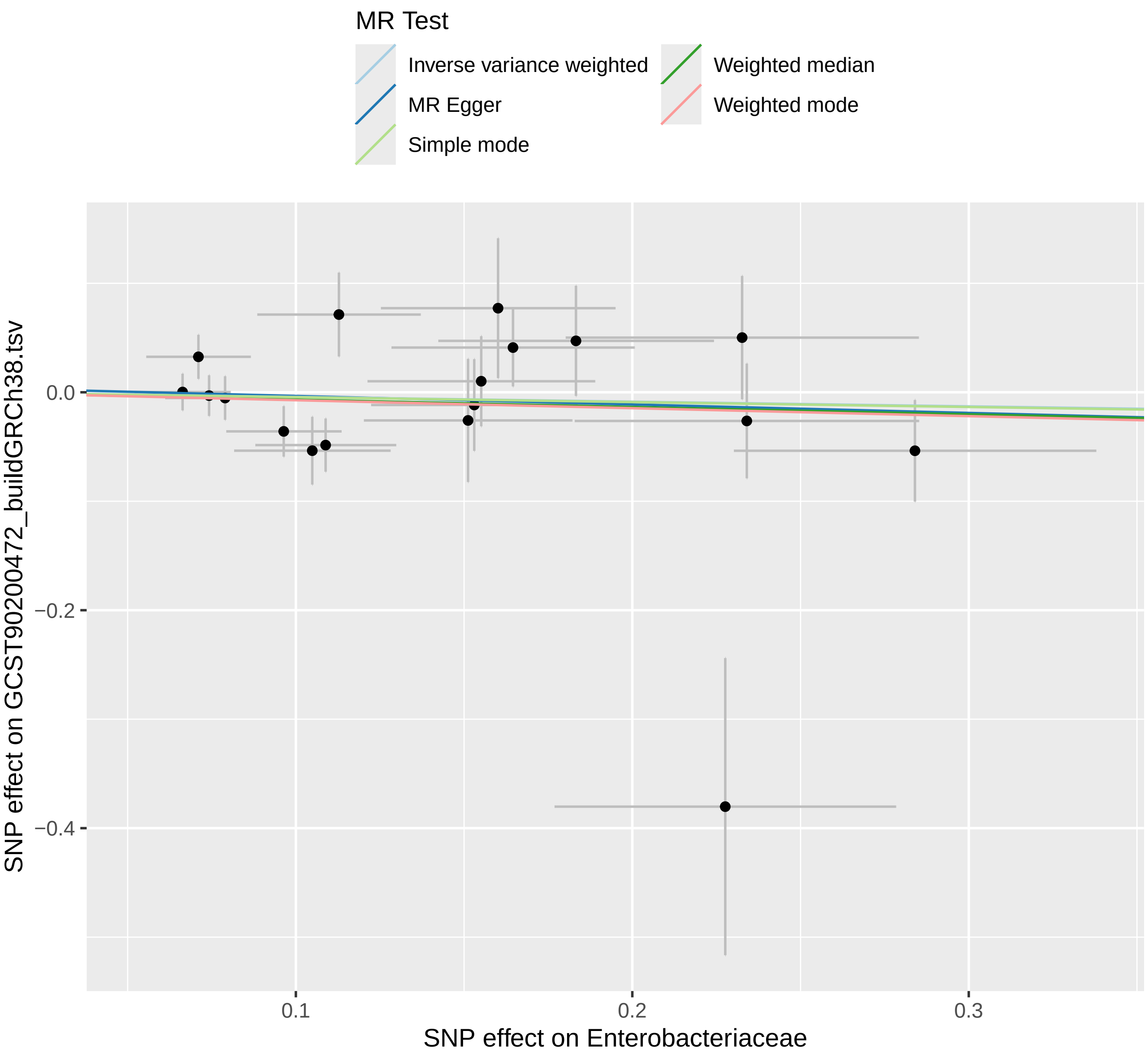
MR Test
Inverse variance weighted
MR Egger
Simple mode
Weighted median
Weighted mode
SNP effect on GCST90200472_buildGRCh38.tsv
0.0
−0.2
−0.4
0.1
0.2
0.3
SNP effect on Enterobacteriaceae

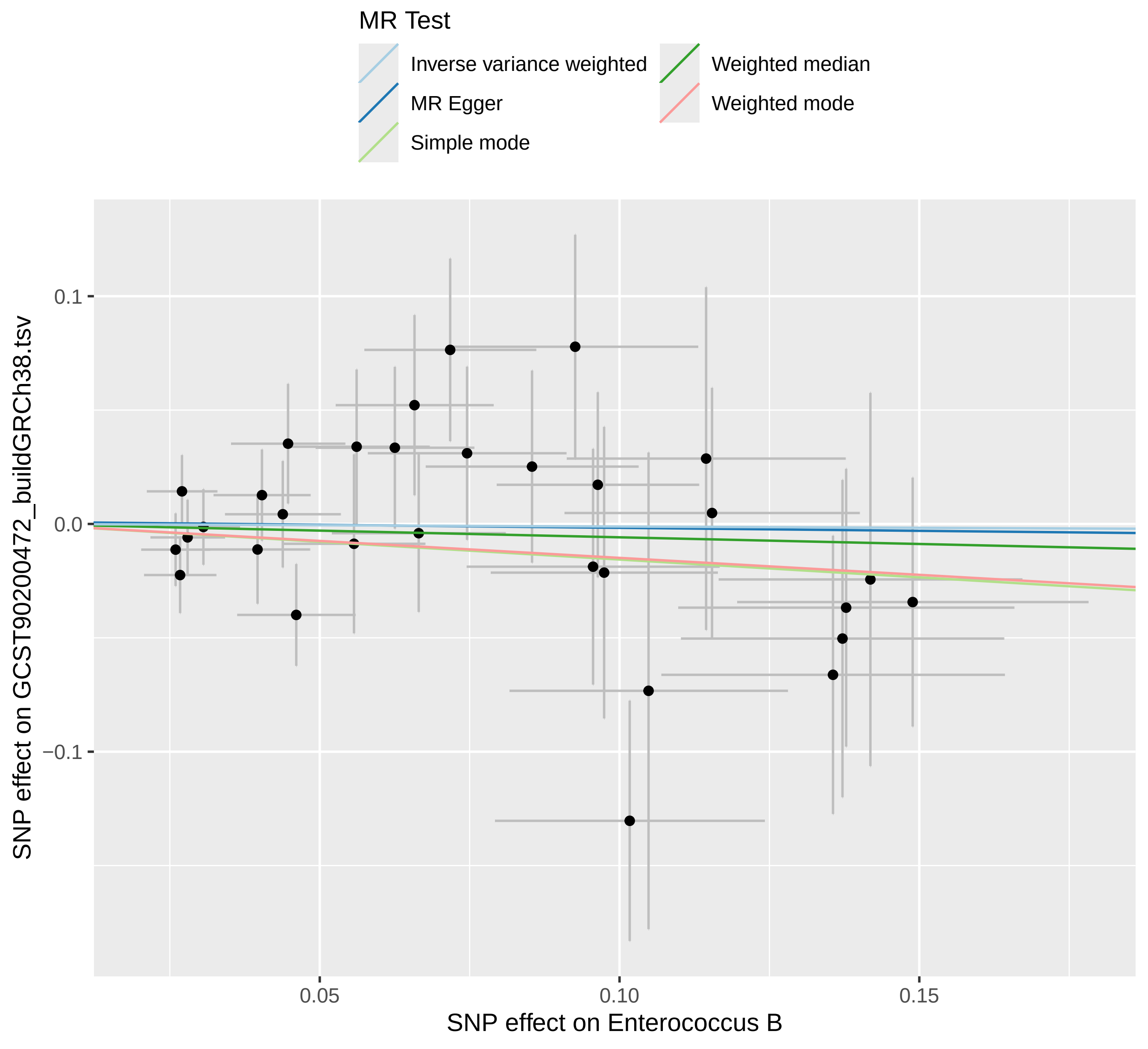
MR Test
Inverse variance weighted
MR Egger
Simple mode
Weighted median
Weighted mode
0.1
0.0
−0.1
0.05
0.10
0.15
SNP effect on GCST90200472_buildGRCh38.tsv
SNP effect on Enterococcus B

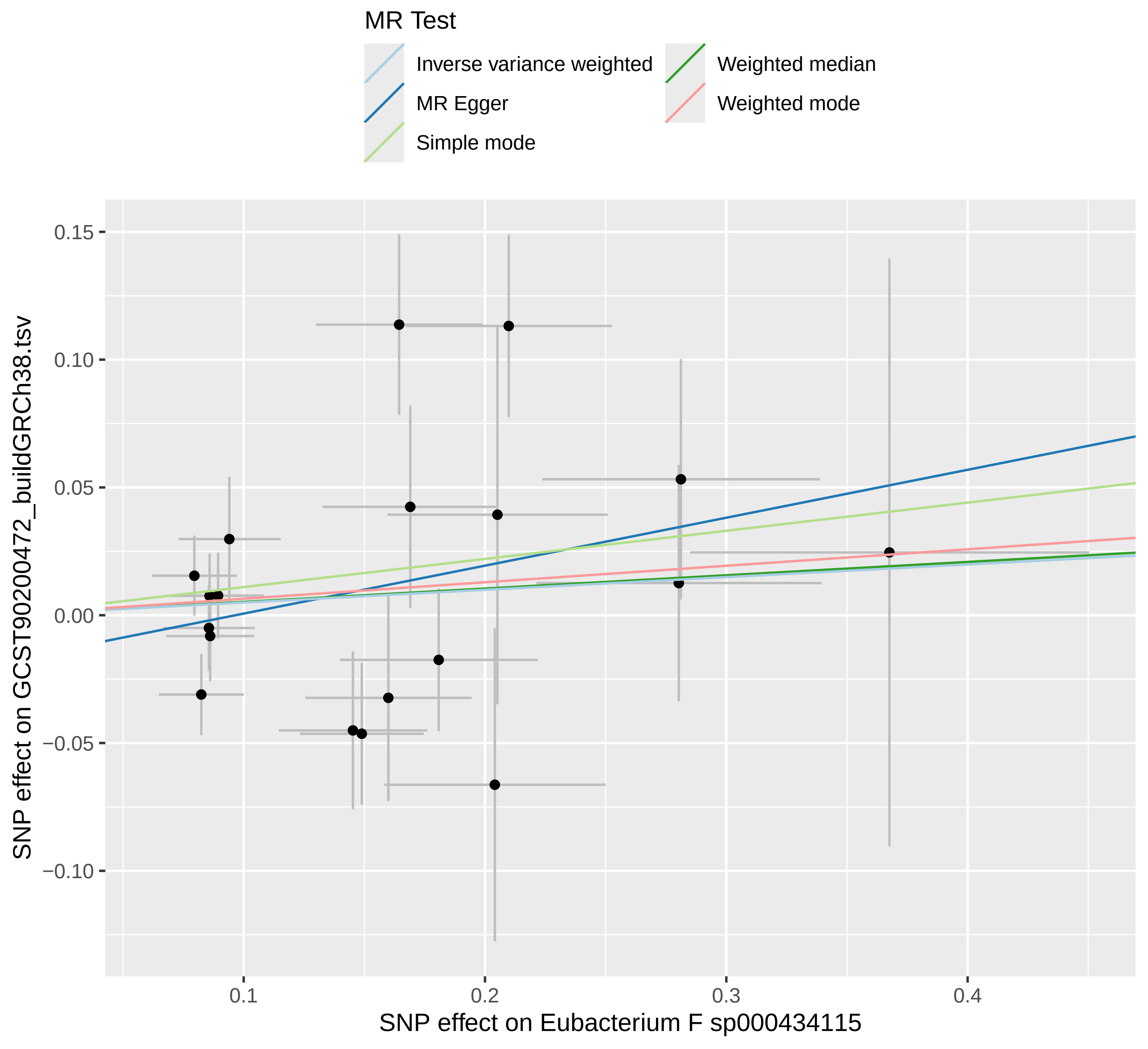
MR Test
Inverse variance weighted
MR Egger
Simple mode
Weighted median
Weighted mode
0.15
0.10
0.05
0.00
−0.05
−0.10
0.1
0.2
0.3
0.4
SNP effect on GCST90200472_buildGRCh38.tsv
SNP effect on Eubacterium F sp000434115

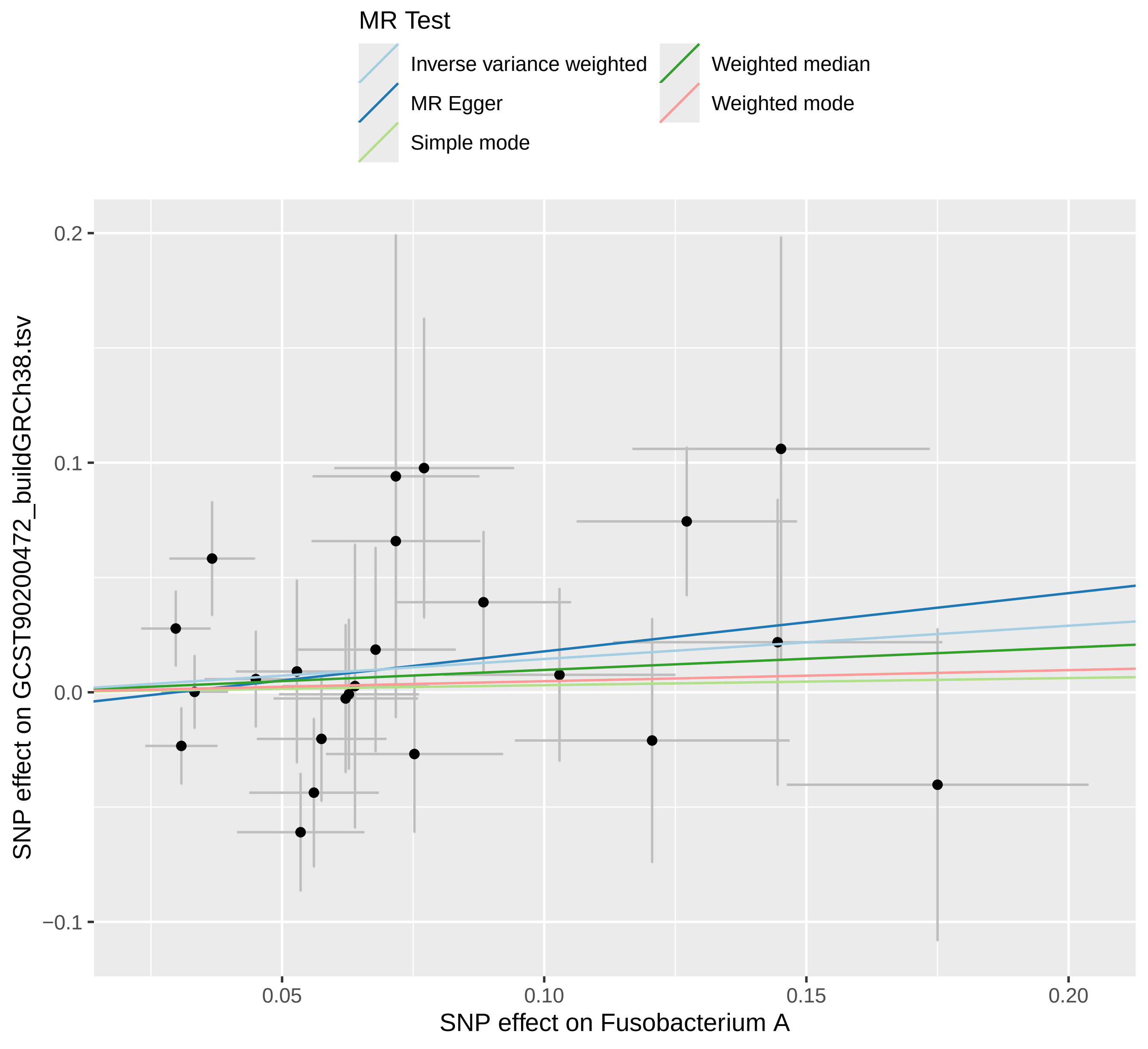

MR Test
Inverse variance weighted
MR Egger
Simple mode
Weighted median
Weighted mode
0.2
0.1
0.0
−0.1
0.05
0.10
0.15
0.20
SNP effect on GCST90200472_buildGRCh38.tsv
SNP effect on Fusobacterium A

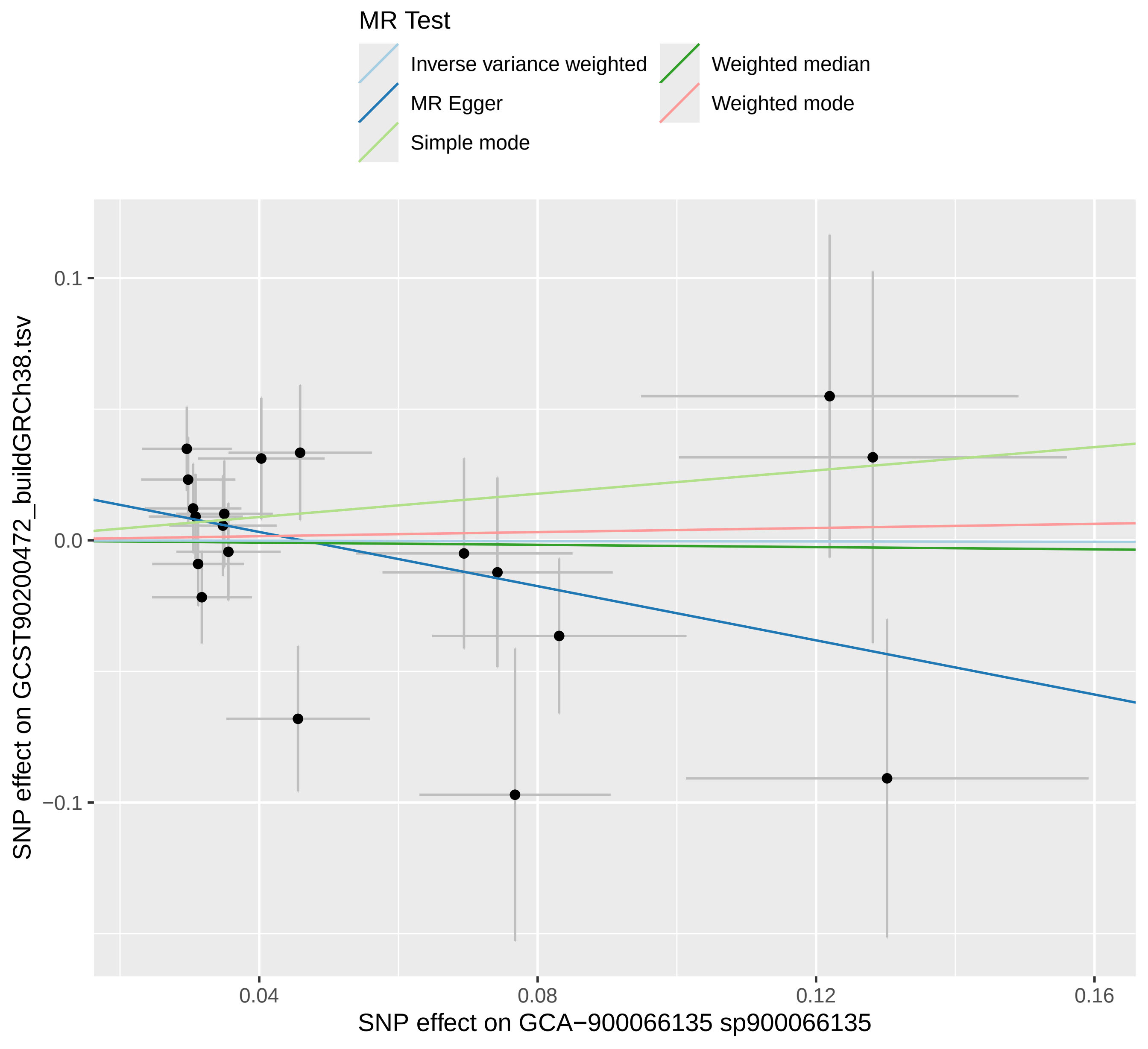
MR Test
Inverse variance weighted
MR Egger
Simple mode
Weighted median
Weighted mode
0.1
0.0
−0.1
0.04
0.08
0.12
0.16
SNP effect on GCST90200472_buildGRCh38.tsv
SNP effect on GCA−900066135 sp900066135

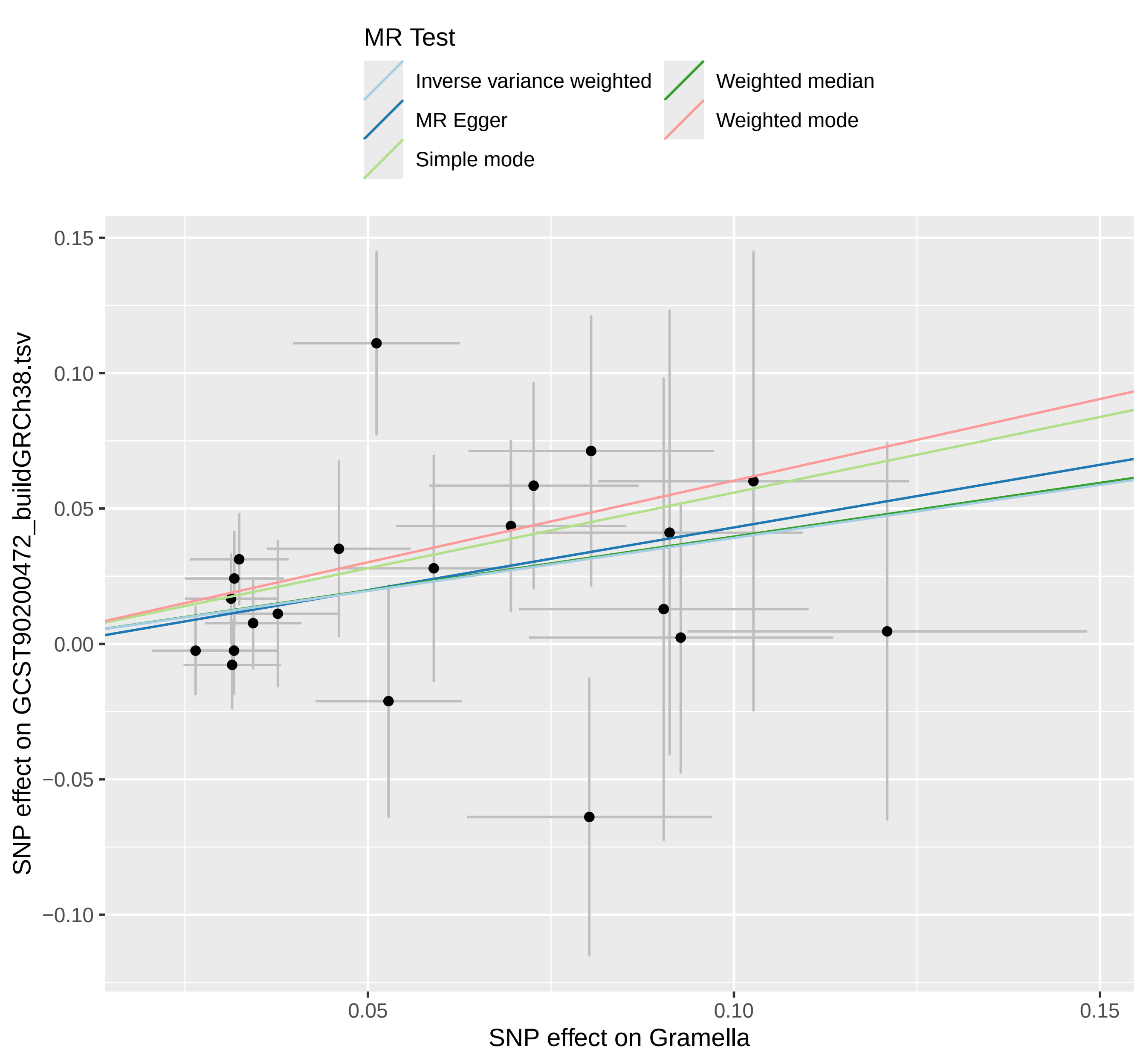
MR Test
Inverse variance weighted
MR Egger
Simple mode
Weighted median
Weighted mode
SNP effect on GCST90200472_buildGRCh38.tsv
0.15
0.10
0.05
0.00
−0.05
−0.10
0.05
0.10
0.15
SNP effect on Gramella

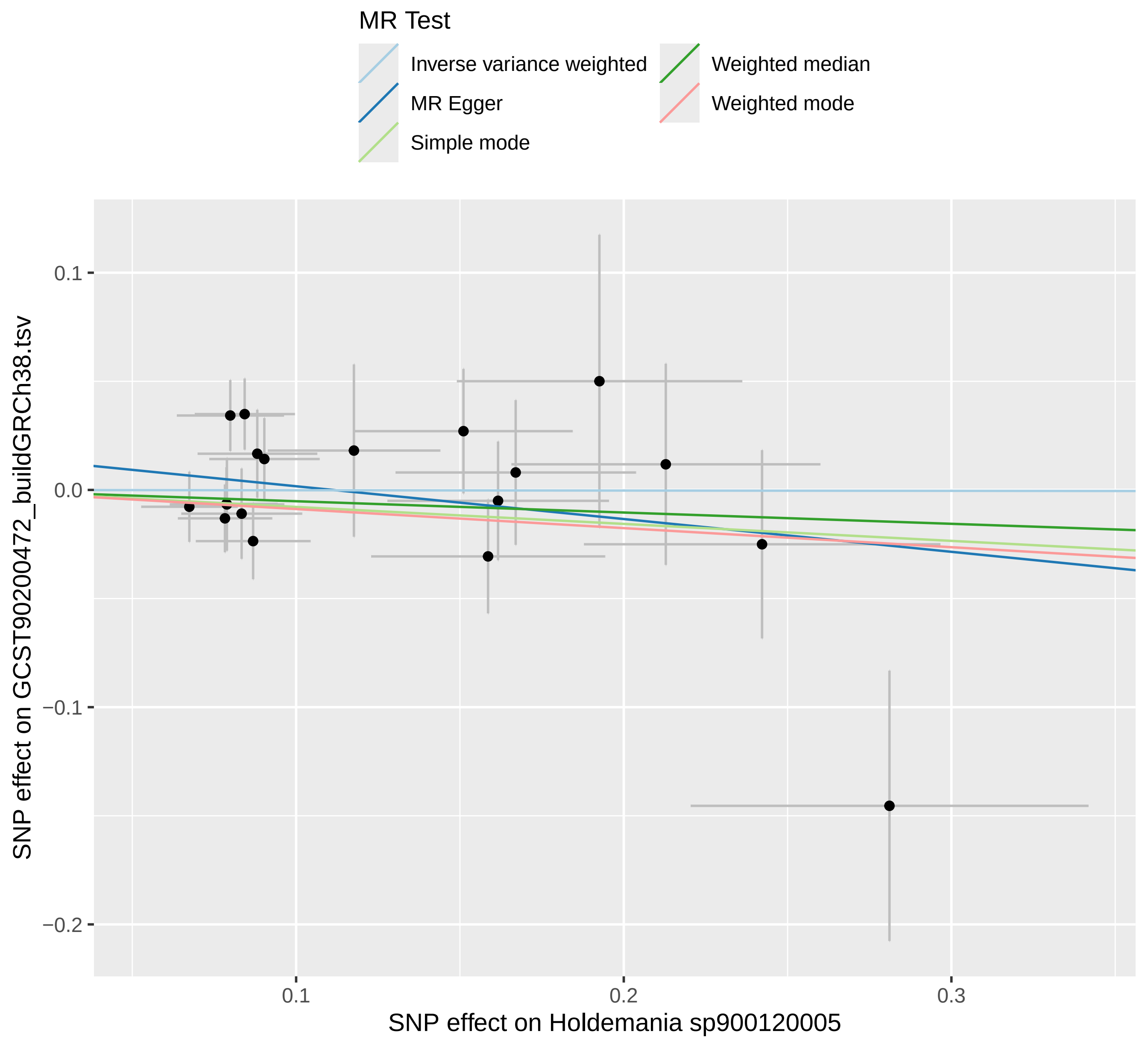

MR Test
Inverse variance weighted
MR Egger
Simple mode
Weighted median
Weighted mode
0.1
0.0
−0.1
−0.2
0.1
0.2
0.3
SNP effect on GCST90200472_buildGRCh38.tsv
SNP effect on Holdemania sp900120005

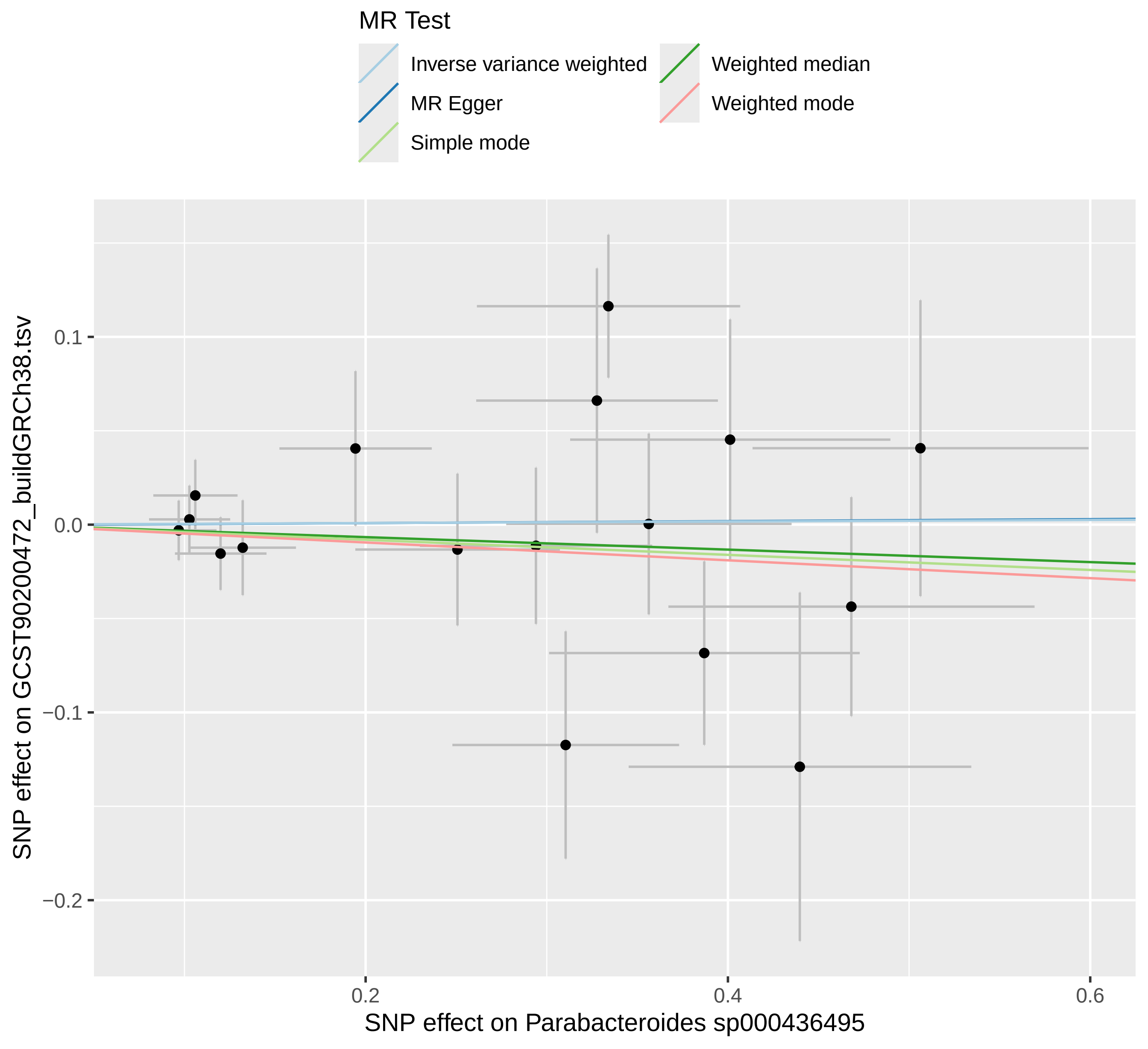
MR Test
Inverse variance weighted
MR Egger
Simple mode
Weighted median
Weighted mode
0.1
0.0
−0.1
−0.2
0.2
0.4
0.6
SNP effect on GCST90200472_buildGRCh38.tsv
SNP effect on Parabacteroides sp000436495

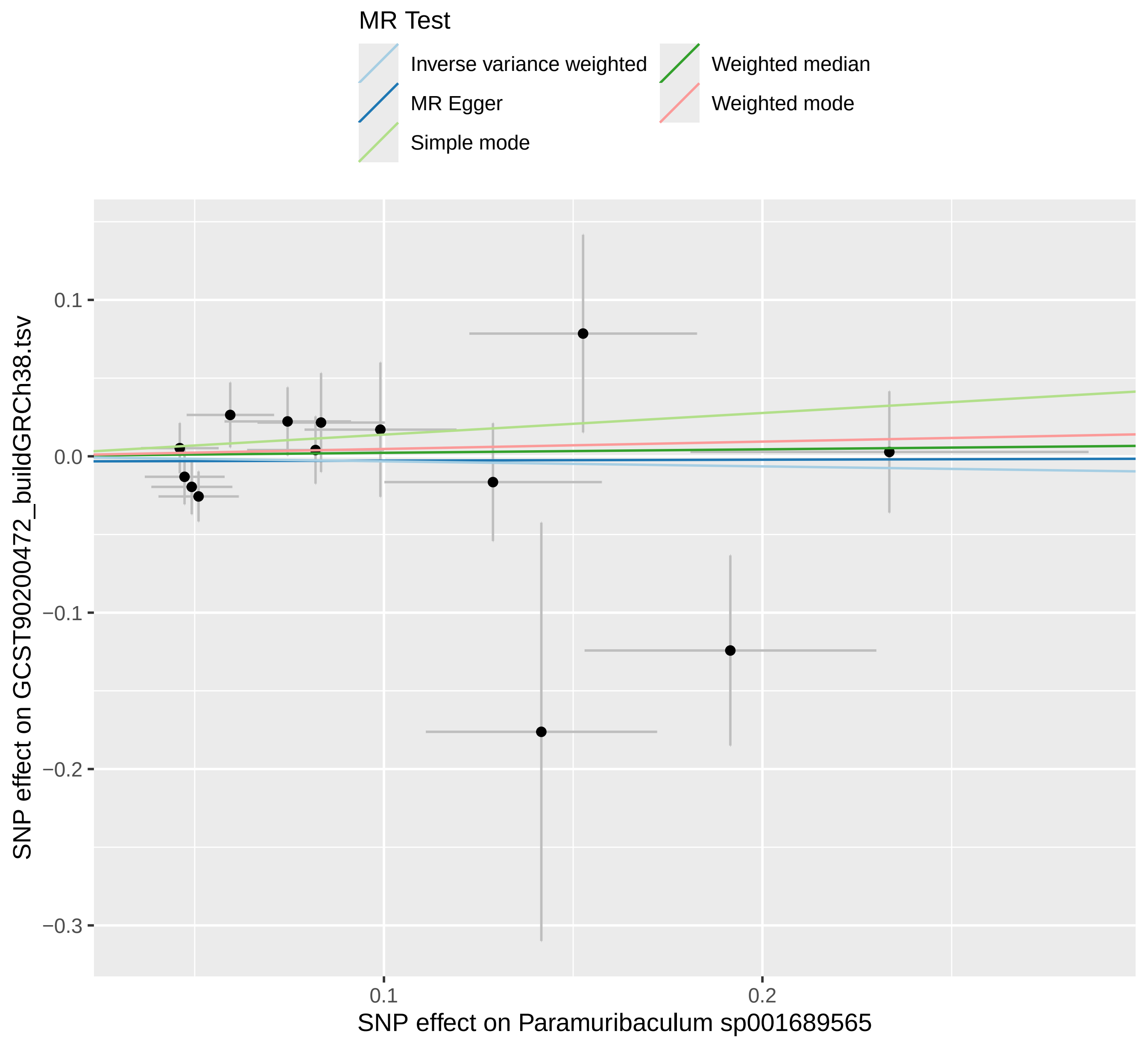

MR Test
Inverse variance weighted
MR Egger
Simple mode
Weighted median
Weighted mode
0.1
0.0
−0.1
−0.2
−0.3
0.1
0.2
SNP effect on GCST90200472_buildGRCh38.tsv
SNP effect on Paramuribaculum sp001689565

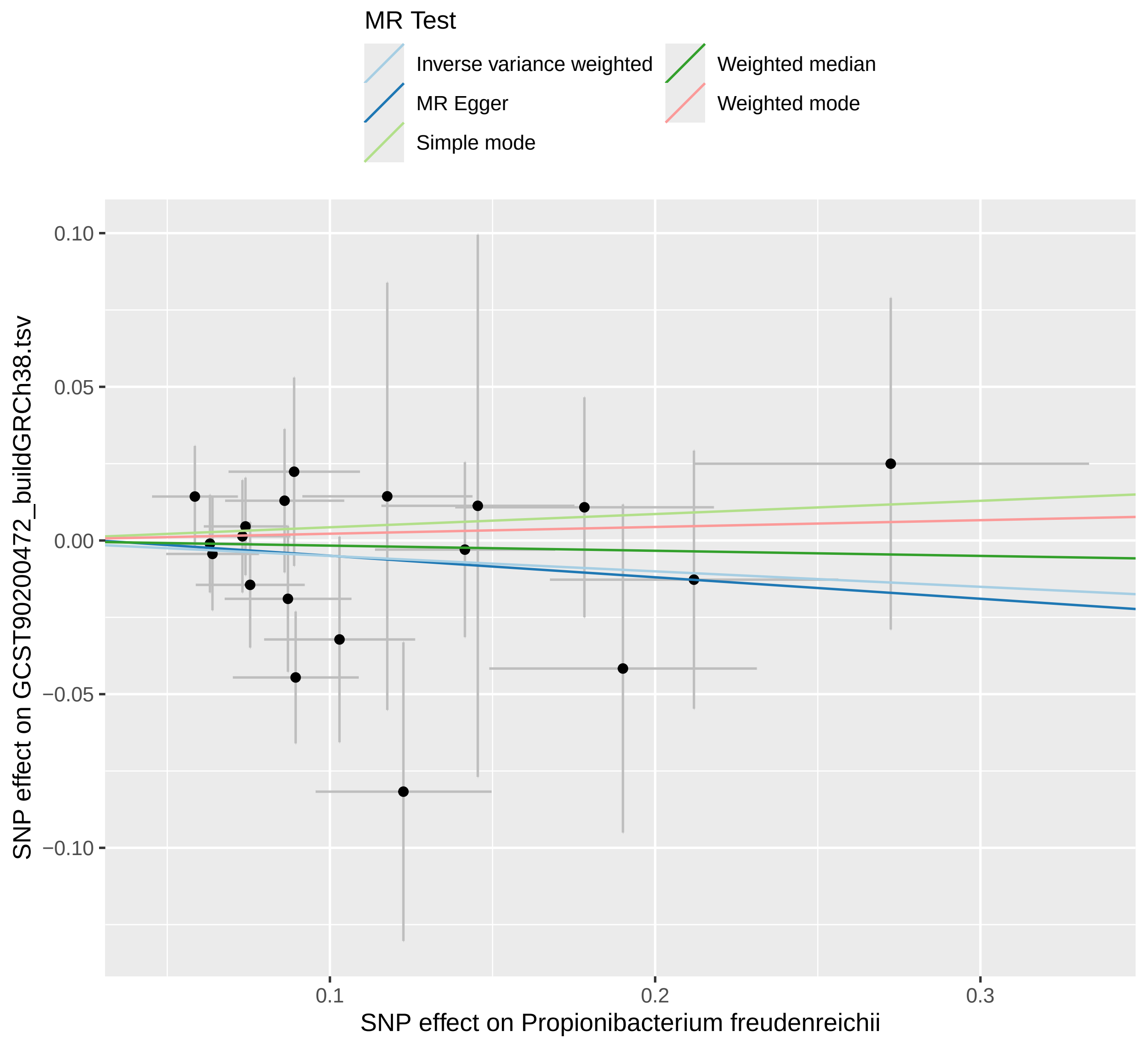
MR Test
Inverse variance weighted
MR Egger
Simple mode
Weighted median
Weighted mode
0.10
0.05
0.00
−0.05
−0.10
0.1
0.2
0.3
SNP effect on GCST90200472_buildGRCh38.tsv
SNP effect on Propionibacterium freudenreichii

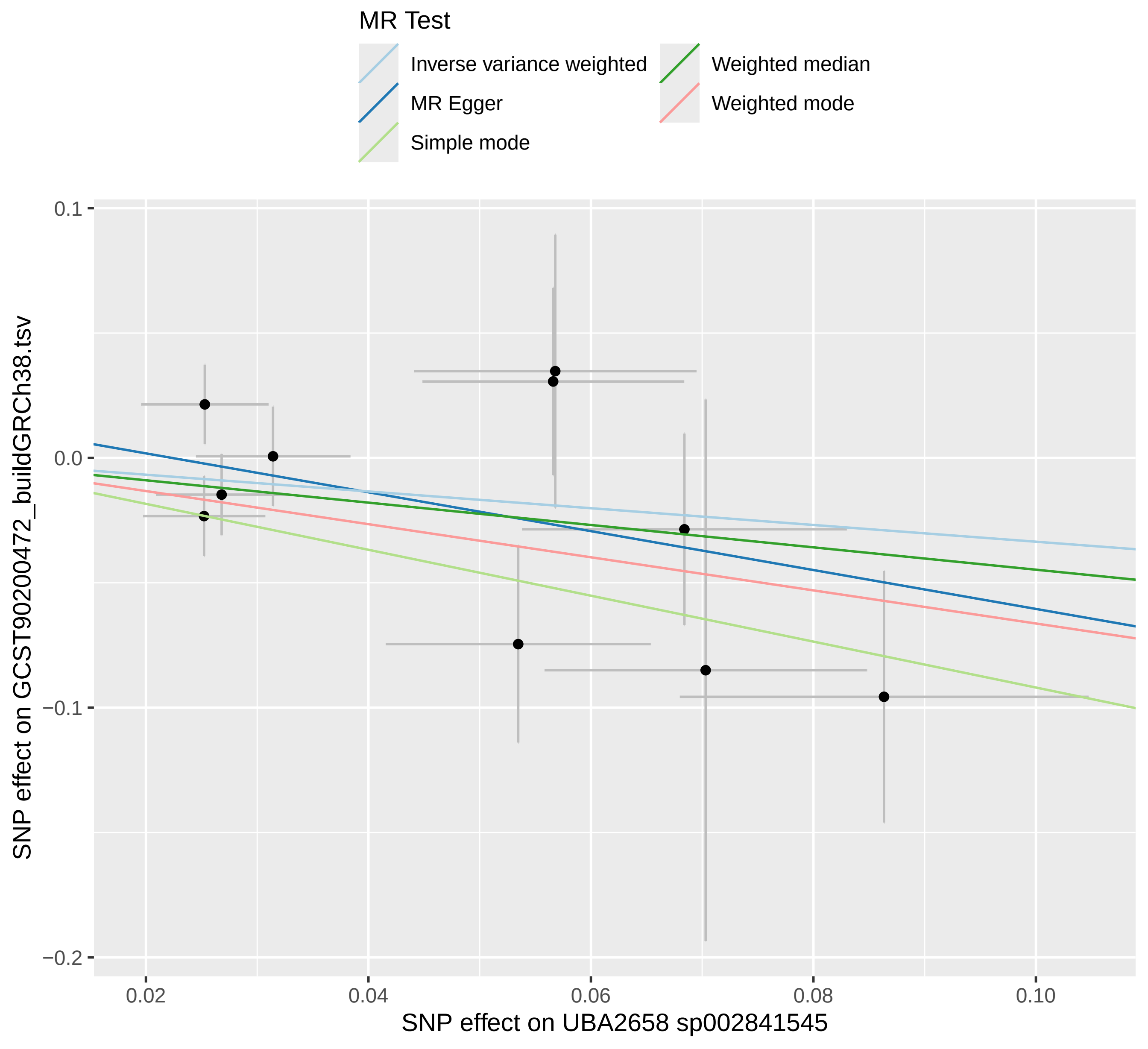

MR Test
Inverse variance weighted
MR Egger
Simple mode
Weighted median
Weighted mode
0.1
0.0
−0.1
−0.2
0.02
0.04
0.06
0.08
0.10
SNP effect on GCST90200472_buildGRCh38.tsv
SNP effect on UBA2658 sp002841545

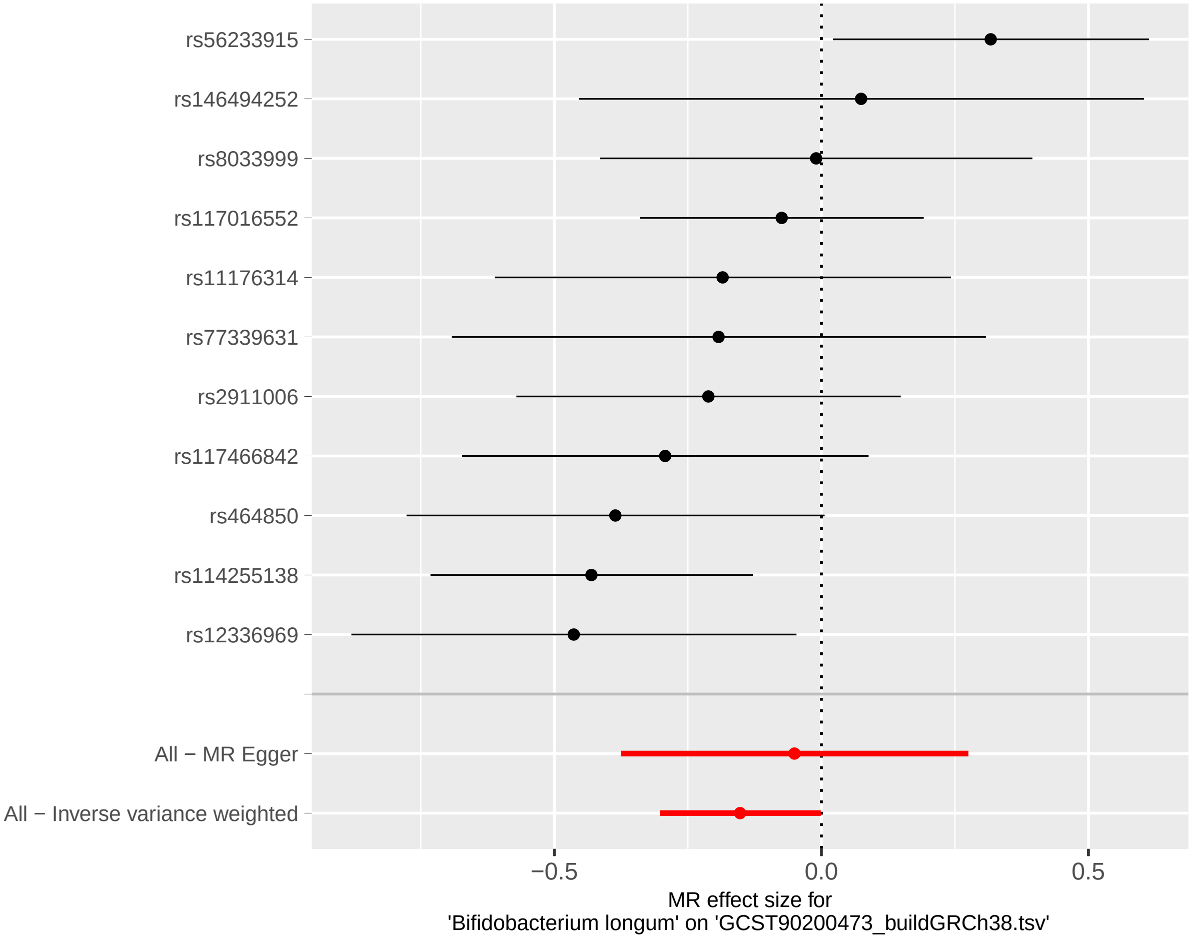

rs56233915
rs146494252
rs8033999
rs117016552
rs11176314
rs77339631
rs2911006
rs117466842
rs464850
rs114255138
rs12336969
All − MR Egger
All − Inverse variance weighted
−0.5
0.0
0.5
MR effect size for
'Bifidobacterium longum' on 'GCST90200473_buildGRCh38.tsv'

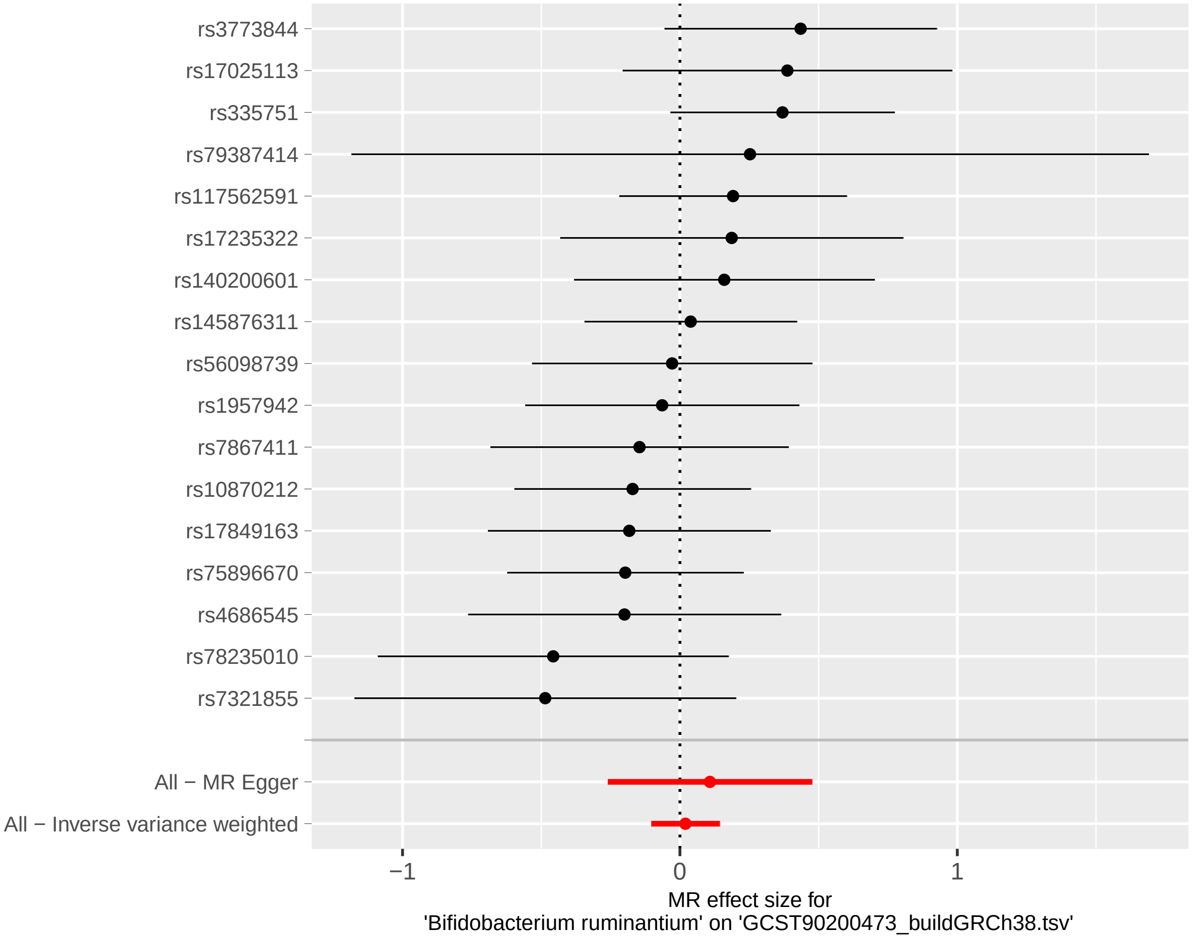
rs3773844
rs17025113
rs335751
rs79387414
rs117562591
rs17235322
rs140200601
rs145876311
rs56098739
rs1957942
rs7867411
rs10870212
rs17849163
rs75896670
rs4686545
rs78235010
rs7321855
All − MR Egger
All − Inverse variance weighted
−1
0
1
MR effect size for
'Bifidobacterium ruminantium' on 'GCST90200473_buildGRCh38.tsv'

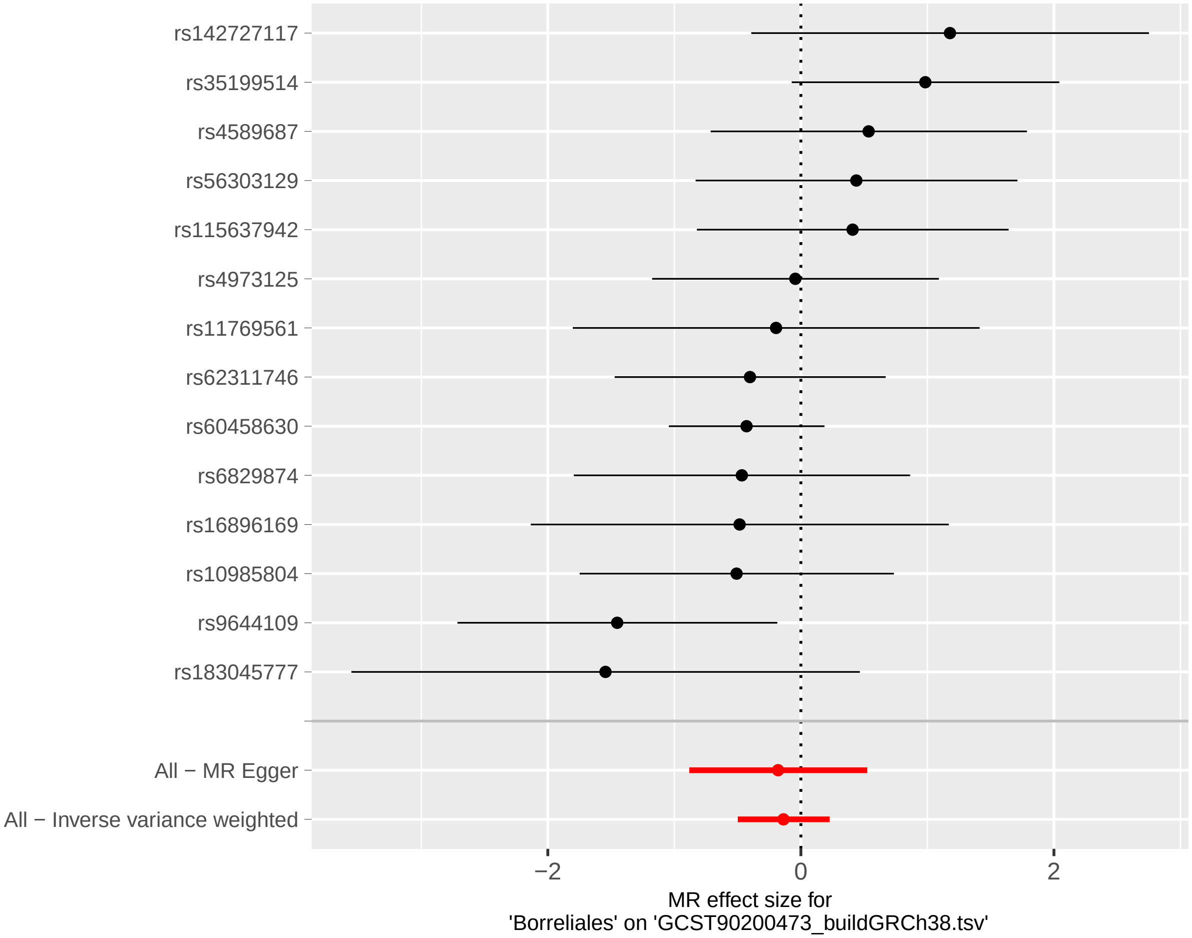

rs142727117
rs35199514
rs4589687
rs56303129
rs115637942
rs4973125
rs11769561
rs62311746
rs60458630
rs6829874
rs16896169
rs10985804
rs9644109
rs183045777
All − MR Egger
All − Inverse variance weighted
−2
0
2
MR effect size for
'Borreliales' on 'GCST90200473_buildGRCh38.tsv'

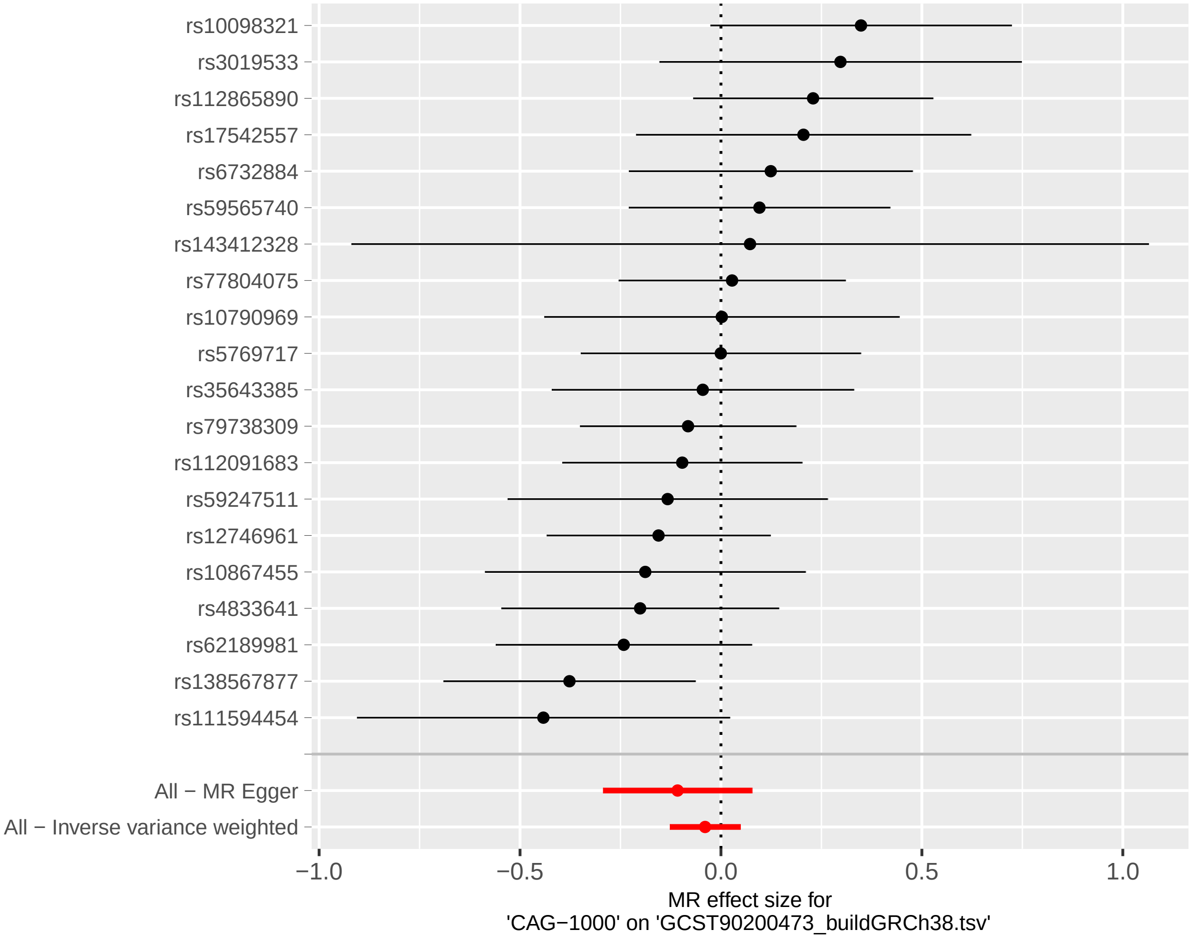

rs10098321
rs3019533
rs112865890
rs17542557
rs6732884
rs59565740
rs143412328
rs77804075
rs10790969
rs5769717
rs35643385
rs79738309
rs112091683
rs59247511
rs12746961
rs10867455
rs4833641
rs62189981
rs138567877
rs111594454
All − MR Egger
All − Inverse variance weighted
−1.0
−0.5
0.0
0.5
1.0
MR effect size for
'CAG−1000' on 'GCST90200473_buildGRCh38.tsv'

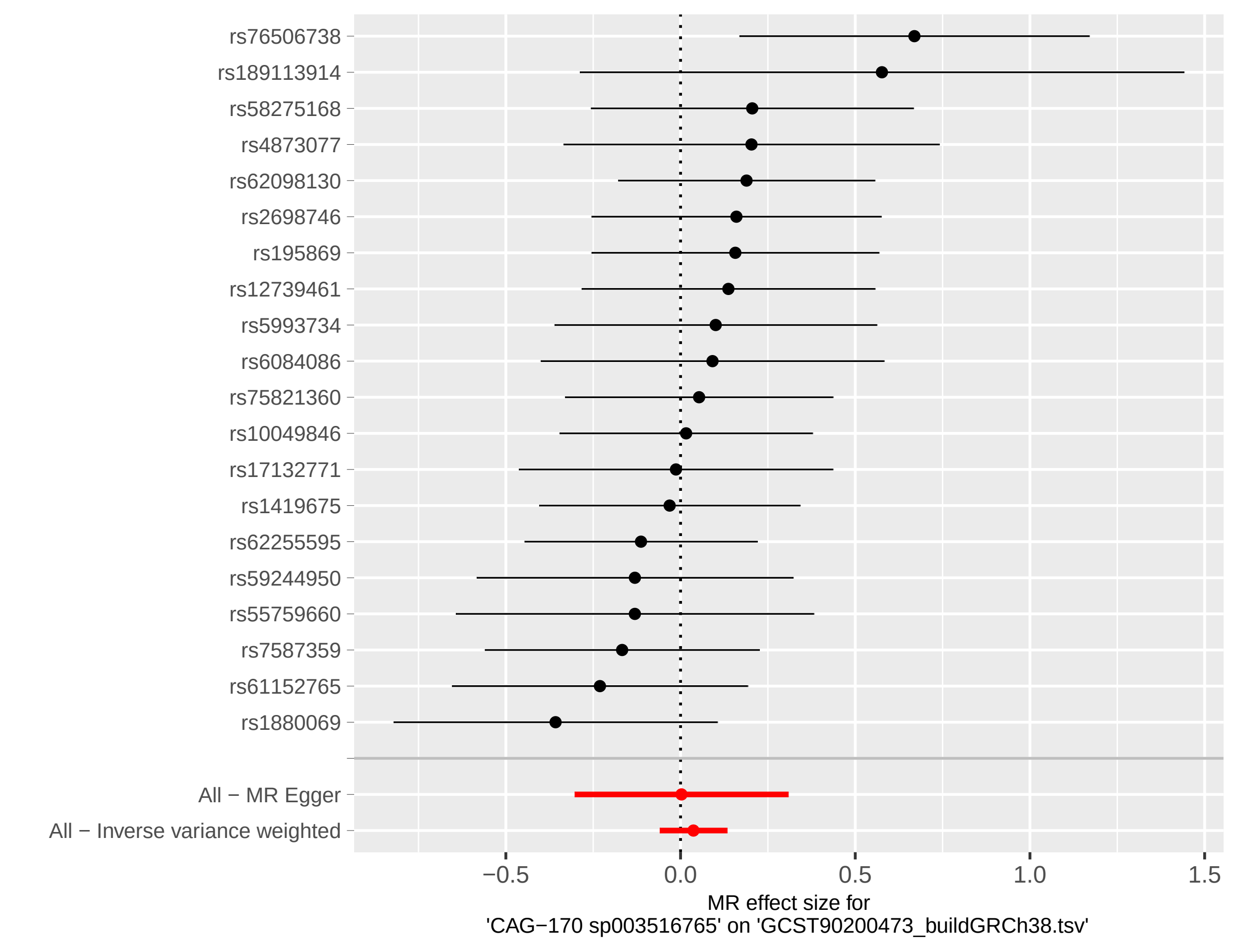

rs76506738
rs189113914
rs58275168
rs4873077
rs62098130
rs2698746
rs195869
rs12739461
rs5993734
rs6084086
rs75821360
rs10049846
rs17132771
rs1419675
rs62255595
rs59244950
rs55759660
rs7587359
rs61152765
rs1880069
All − MR Egger
All − Inverse variance weighted
−0.5
0.0
0.5
1.0
1.5
MR effect size for
'CAG−170 sp003516765' on 'GCST90200473_buildGRCh38.tsv'

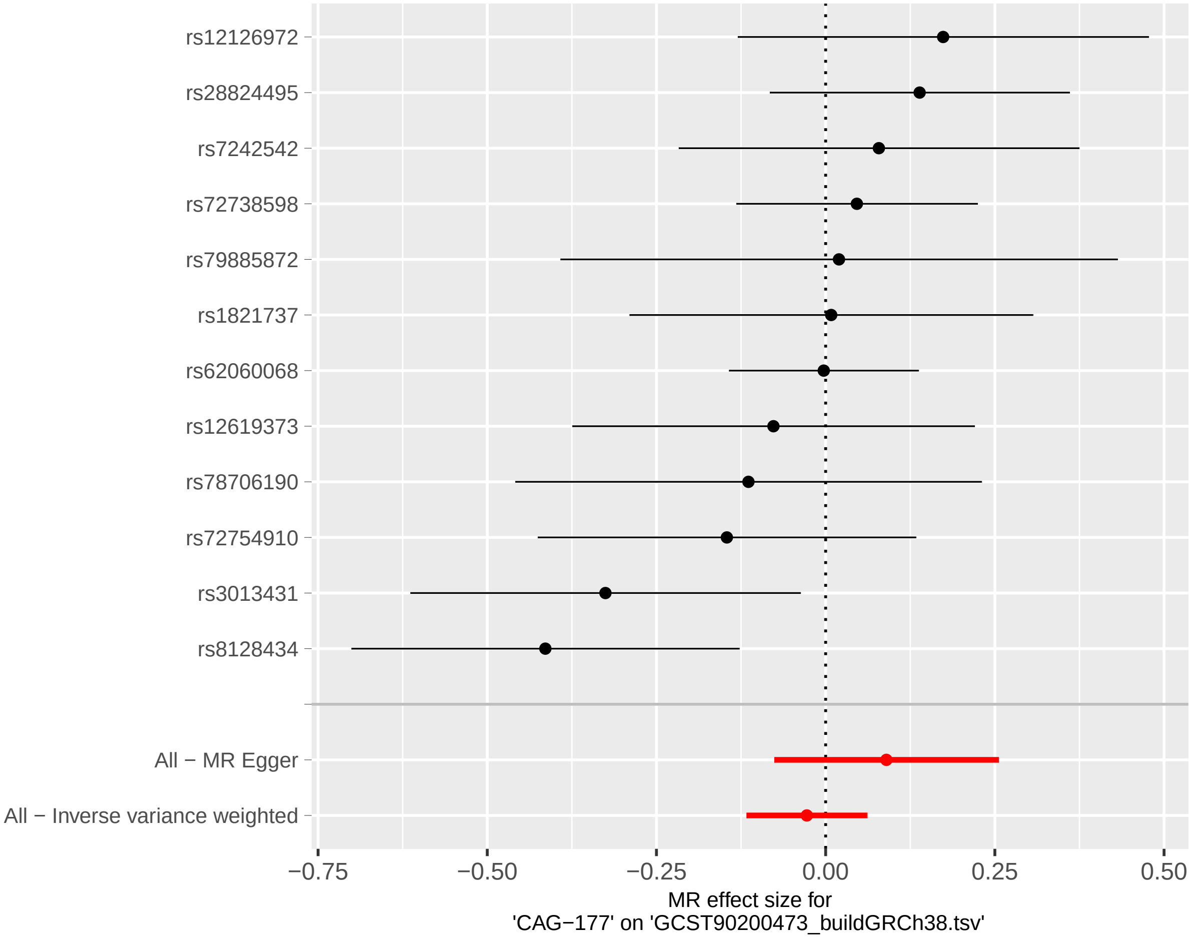

rs12126972
rs28824495
rs7242542
rs72738598
rs79885872
rs1821737
rs62060068
rs12619373
rs78706190
rs72754910
rs3013431
rs8128434
All − MR Egger
All − Inverse variance weighted
−0.75
−0.50
−0.25
0.00
0.25
0.50
MR effect size for
'CAG−177' on 'GCST90200473_buildGRCh38.tsv'

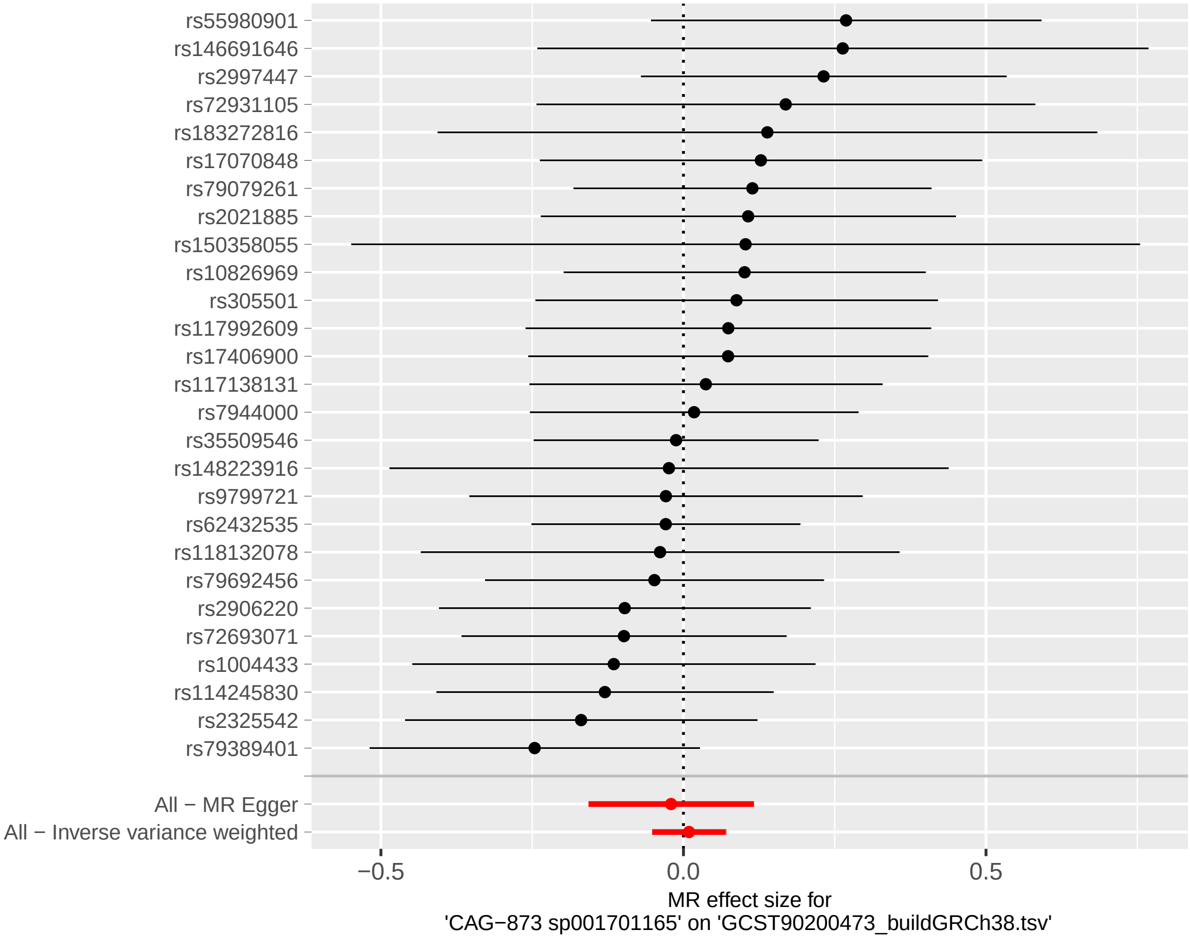
rs55980901
rs146691646
rs2997447
rs72931105
rs183272816
rs17070848
rs79079261
rs2021885
rs150358055
rs10826969
rs305501
rs117992609
rs17406900
rs117138131
rs7944000
rs35509546
rs148223916
rs9799721
rs62432535
rs118132078
rs79692456
rs2906220
rs72693071
rs1004433
rs114245830
rs2325542
rs79389401
All − MR Egger
All − Inverse variance weighted
−0.5
0.0
0.5
MR effect size for
'CAG−873 sp001701165' on 'GCST90200473_buildGRCh38.tsv'

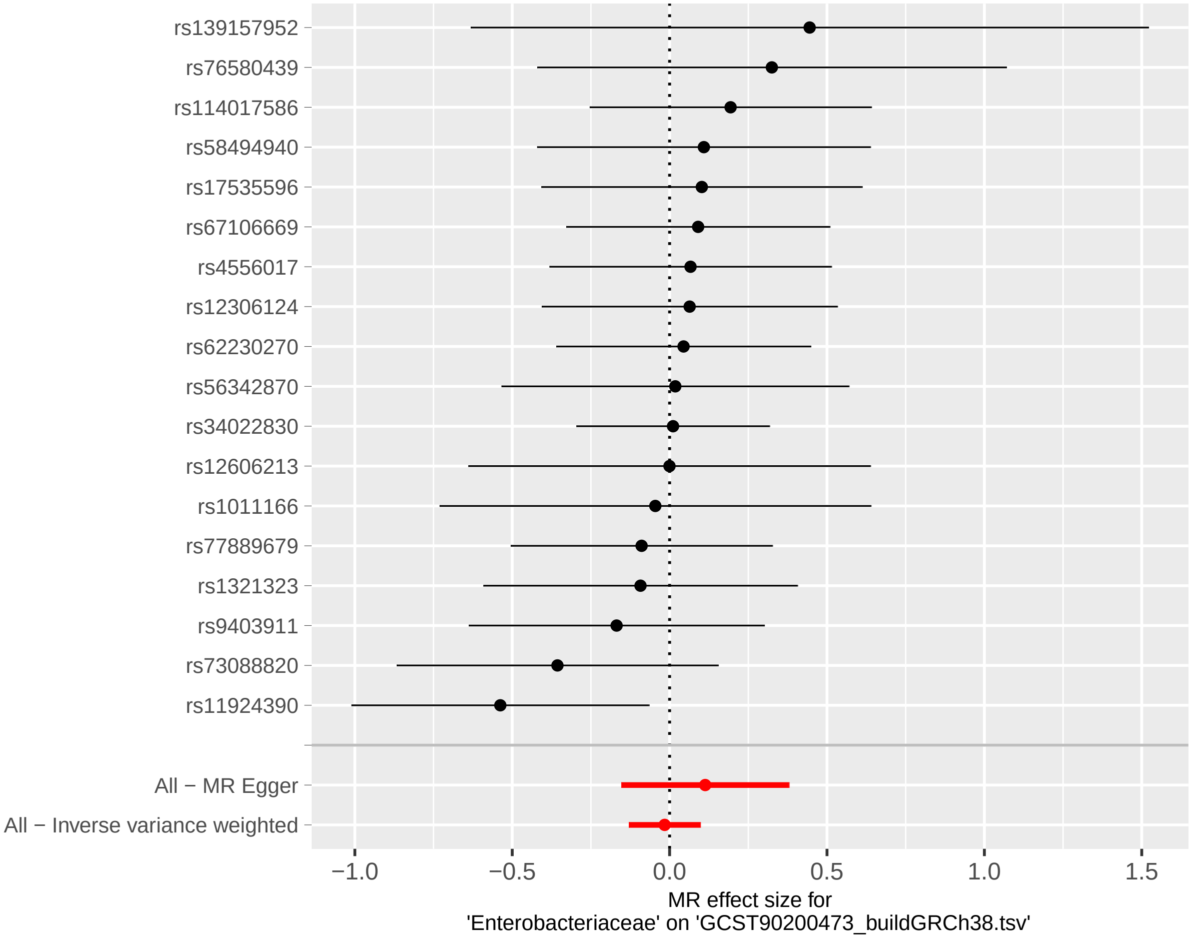
rs139157952
rs76580439
rs114017586
rs58494940
rs17535596
rs67106669
rs4556017
rs12306124
rs62230270
rs56342870
rs34022830
rs12606213
rs1011166
rs77889679
rs1321323
rs9403911
rs73088820
rs11924390
All − MR Egger
All − Inverse variance weighted
−1.0
−0.5
0.0
0.5
1.0
1.5
MR effect size for
'Enterobacteriaceae' on 'GCST90200473_buildGRCh38.tsv'

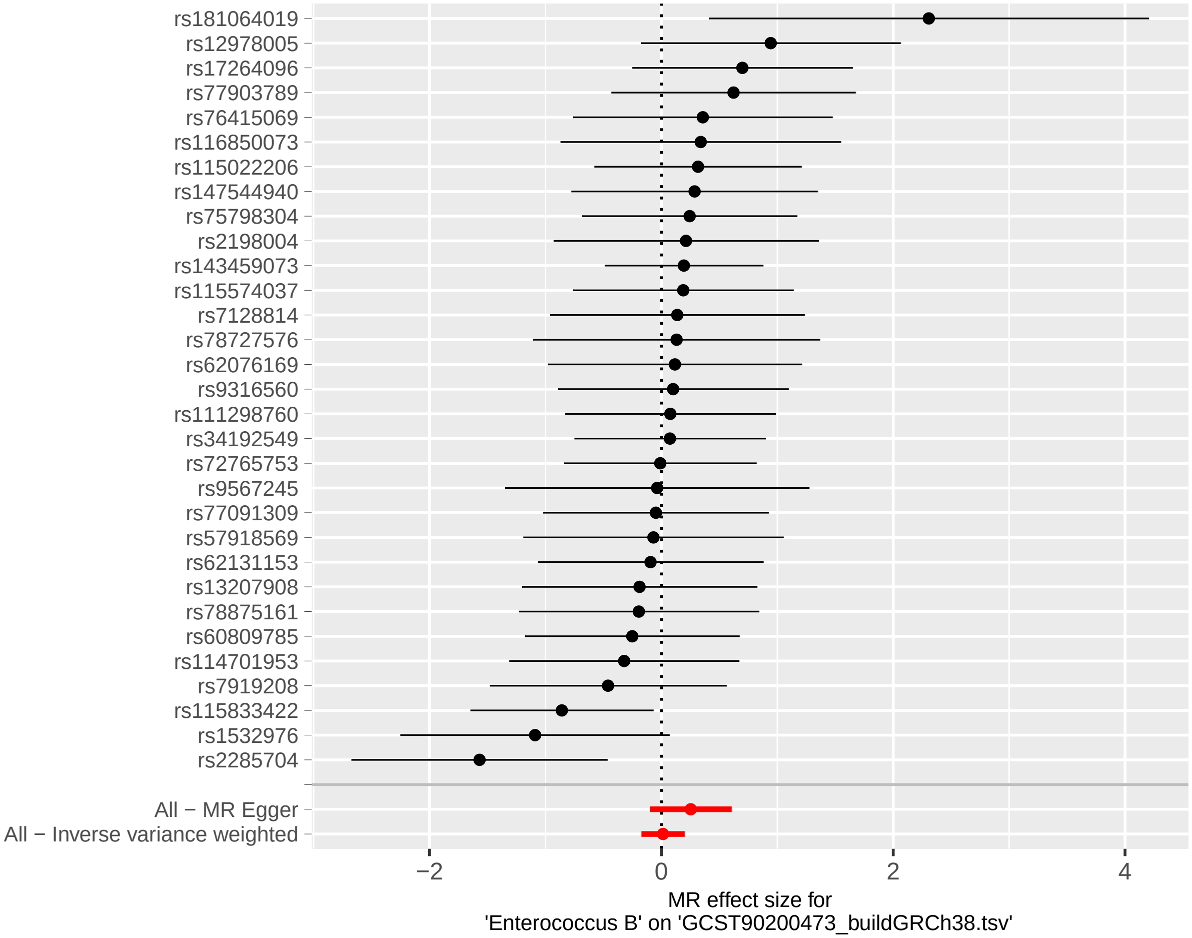
rs181064019
rs12978005
rs17264096
rs77903789
rs76415069
rs116850073
rs115022206
rs147544940
rs75798304
rs2198004
rs143459073
rs115574037
rs7128814
rs78727576
rs62076169
rs9316560
rs111298760
rs34192549
rs72765753
rs9567245
rs77091309
rs57918569
rs62131153
rs13207908
rs78875161
rs60809785
rs114701953
rs7919208
rs115833422
rs1532976
rs2285704
All − MR Egger
All − Inverse variance weighted
−2
0
2
4
MR effect size for
'Enterococcus B' on 'GCST90200473_buildGRCh38.tsv'

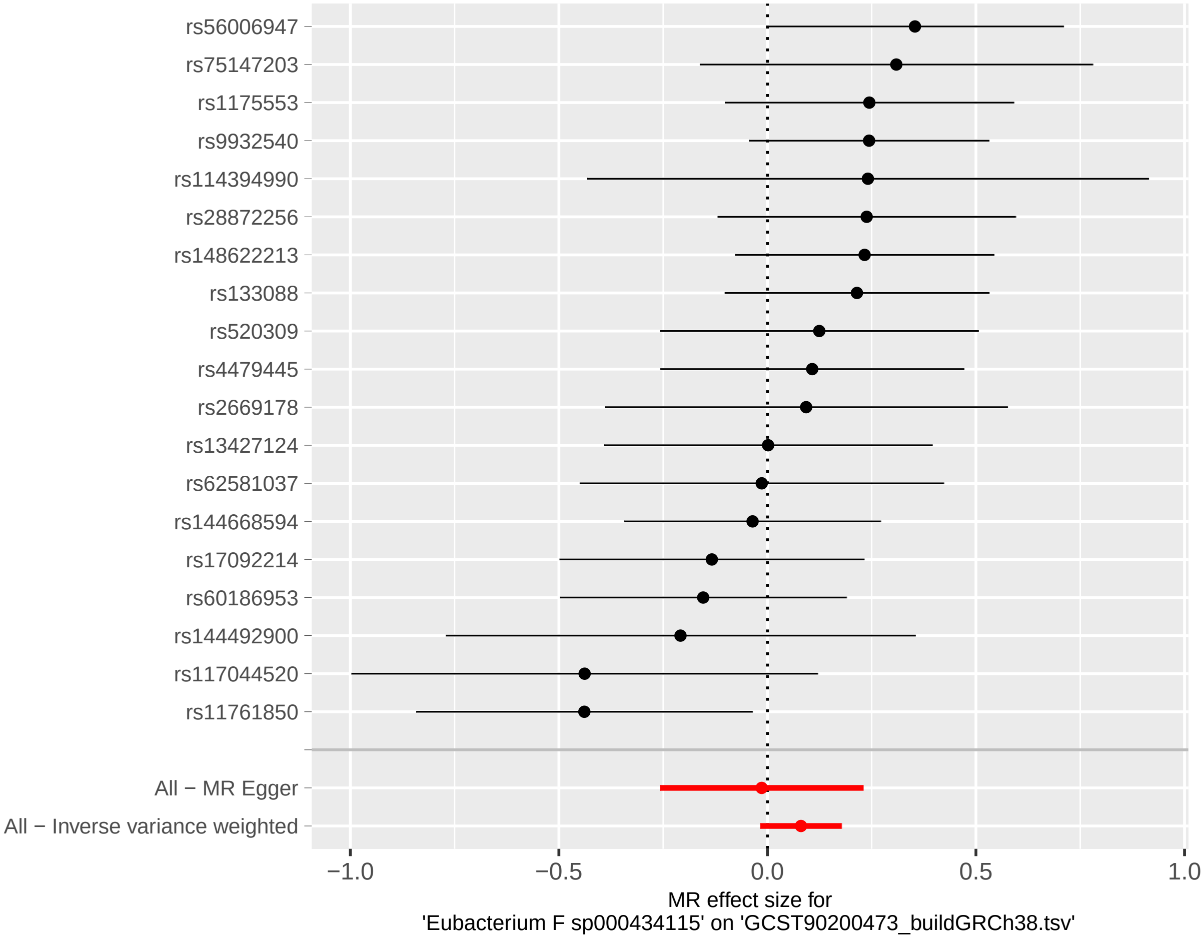
rs56006947
rs75147203
rs1175553
rs9932540
rs114394990
rs28872256
rs148622213
rs133088
rs520309
rs4479445
rs2669178
rs13427124
rs62581037
rs144668594
rs17092214
rs60186953
rs144492900
rs117044520
rs11761850
All − MR Egger
All − Inverse variance weighted
−1.0
−0.5
0.0
0.5
1.0
MR effect size for
'Eubacterium F sp000434115' on 'GCST90200473_buildGRCh38.tsv'

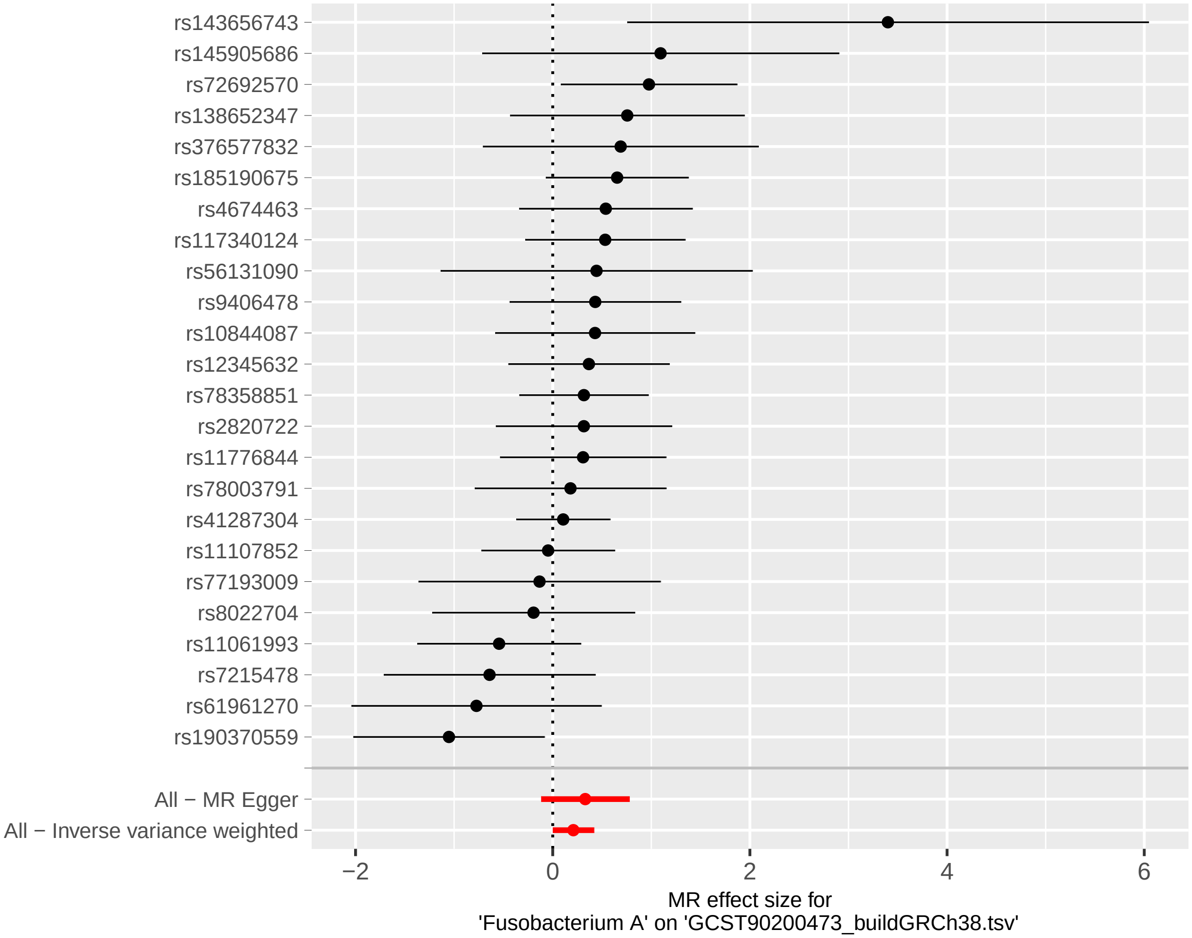

rs143656743
rs145905686
rs72692570
rs138652347
rs376577832
rs185190675
rs4674463
rs117340124
rs56131090
rs9406478
rs10844087
rs12345632
rs78358851
rs2820722
rs11776844
rs78003791
rs41287304
rs11107852
rs77193009
rs8022704
rs11061993
rs7215478
rs61961270
rs190370559
All − MR Egger
All − Inverse variance weighted
−2
0
2
4
6
MR effect size for
'Fusobacterium A' on 'GCST90200473_buildGRCh38.tsv'

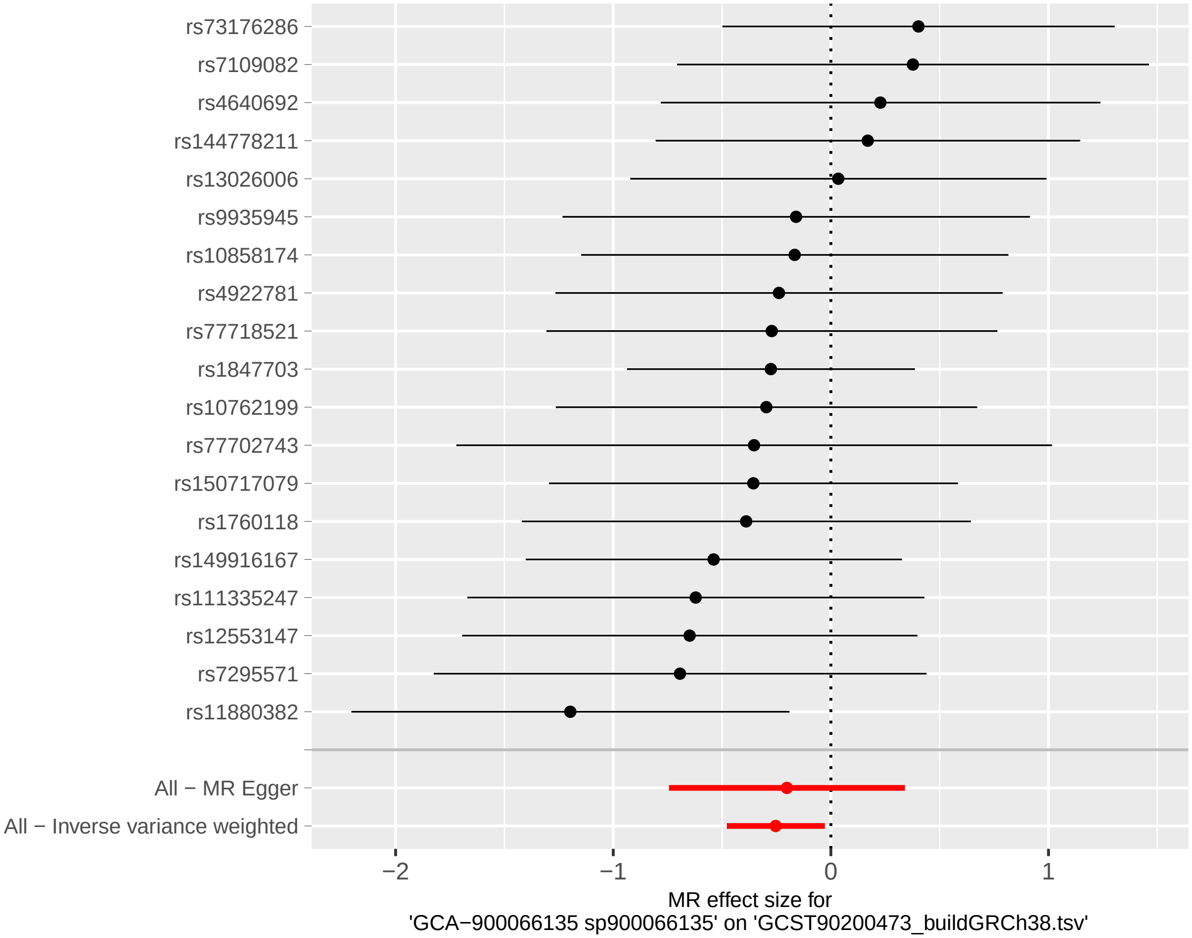
rs73176286
rs7109082
rs4640692
rs144778211
rs13026006
rs9935945
rs10858174
rs4922781
rs77718521
rs1847703
rs10762199
rs77702743
rs150717079
rs1760118
rs149916167
rs111335247
rs12553147
rs7295571
rs11880382
All − MR Egger
All − Inverse variance weighted
−2
−1
0
1
MR effect size for
'GCA−900066135 sp900066135' on 'GCST90200473_buildGRCh38.tsv'

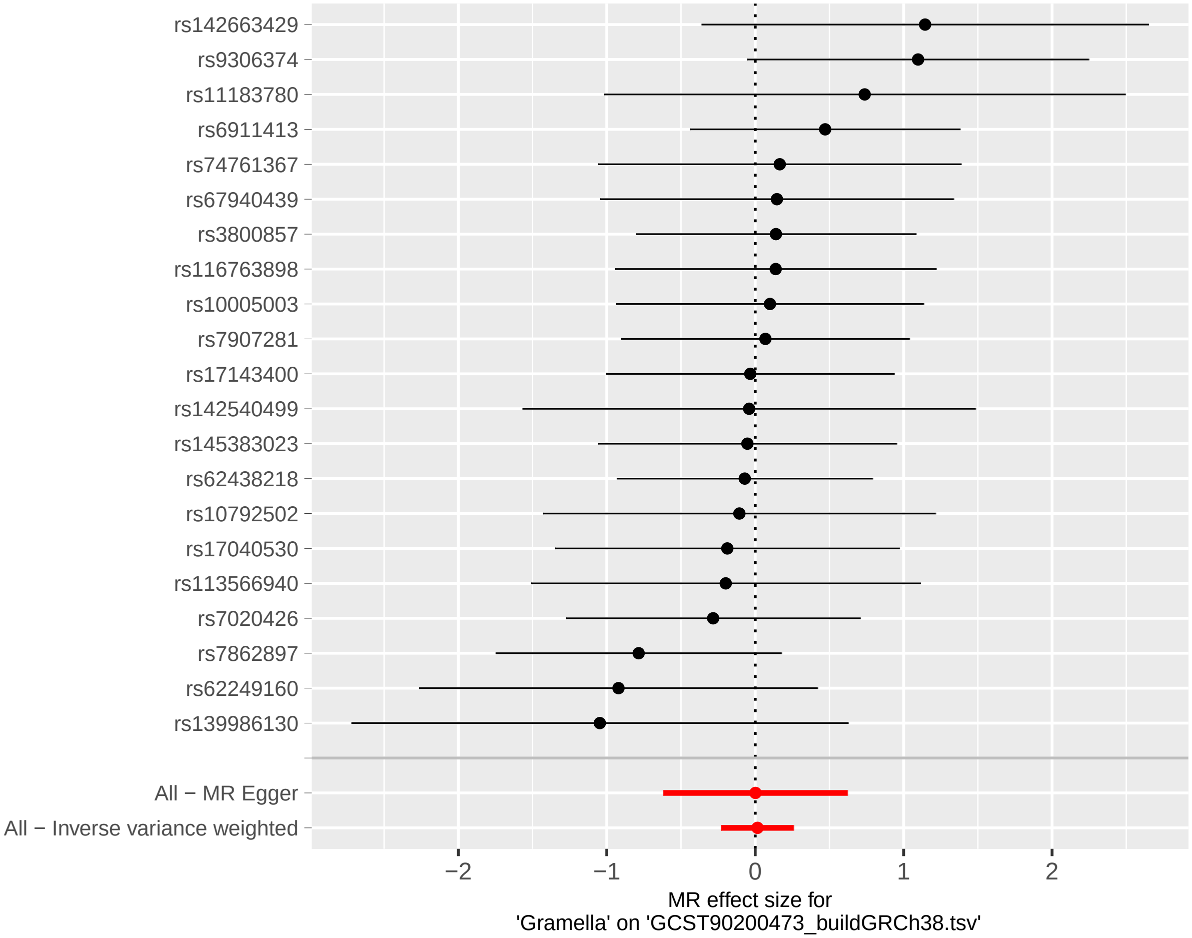
rs142663429
rs9306374
rs11183780
rs6911413
rs74761367
rs67940439
rs3800857
rs116763898
rs10005003
rs7907281
rs17143400
rs142540499
rs145383023
rs62438218
rs10792502
rs17040530
rs113566940
rs7020426
rs7862897
rs62249160
rs139986130
All − MR Egger
All − Inverse variance weighted
−2
−1
0
1
2
MR effect size for
'Gramella' on 'GCST90200473_buildGRCh38.tsv'

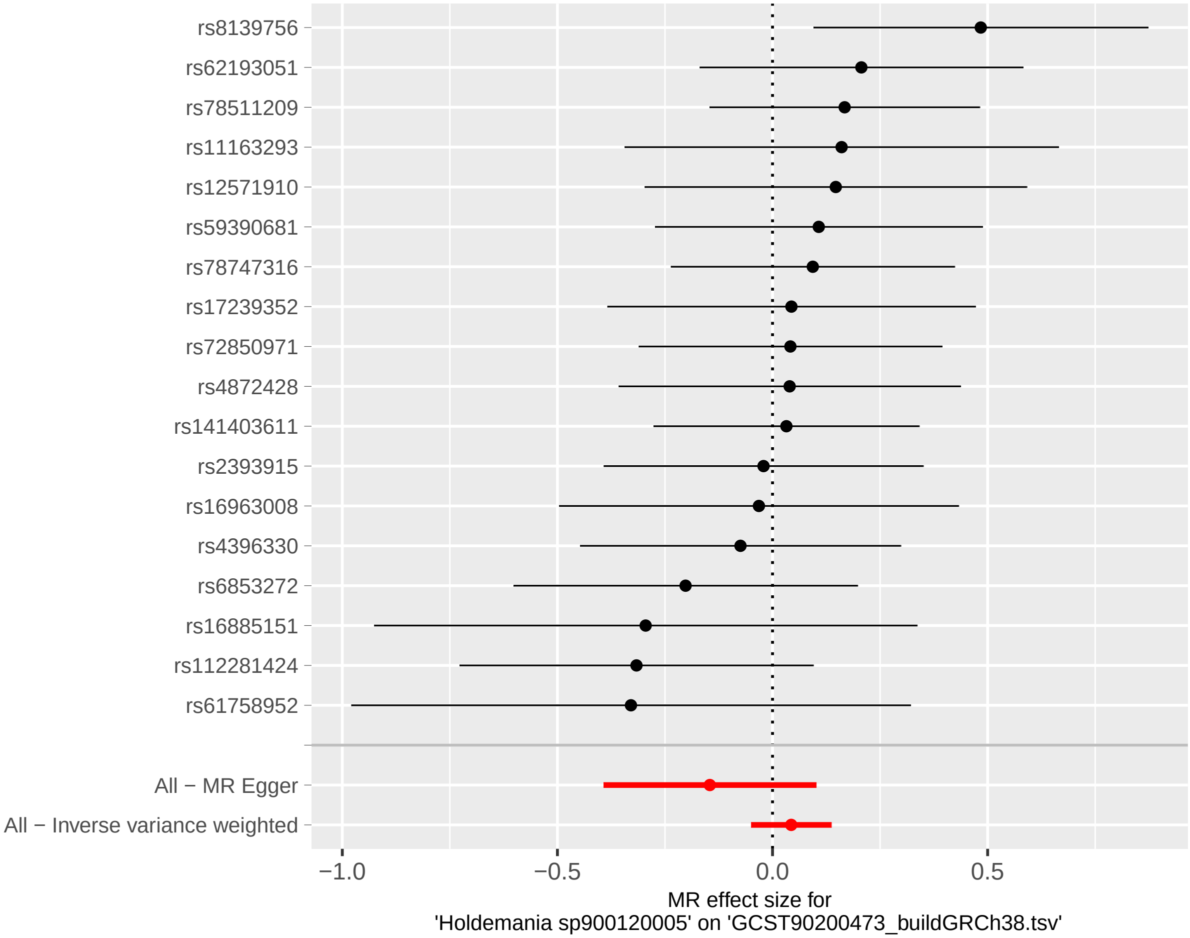

rs8139756
rs62193051
rs78511209
rs11163293
rs12571910
rs59390681
rs78747316
rs17239352
rs72850971
rs4872428
rs141403611
rs2393915
rs16963008
rs4396330
rs6853272
rs16885151
rs112281424
rs61758952
All − MR Egger
All − Inverse variance weighted
−1.0
−0.5
0.0
0.5
MR effect size for
'Holdemania sp900120005' on 'GCST90200473_buildGRCh38.tsv'

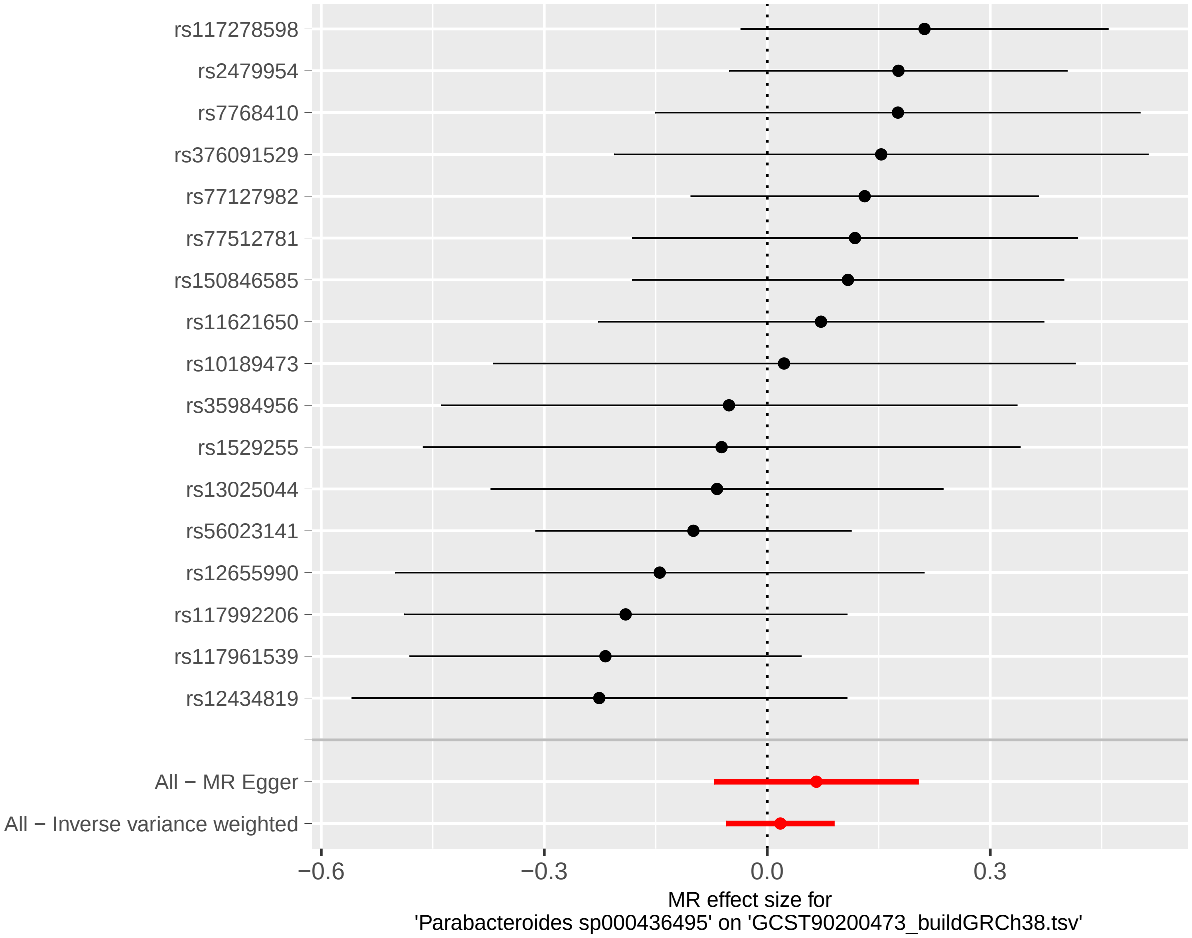
rs117278598
rs2479954
rs7768410
rs376091529
rs77127982
rs77512781
rs150846585
rs11621650
rs10189473
rs35984956
rs1529255
rs13025044
rs56023141
rs12655990
rs117992206
rs117961539
rs12434819
All − MR Egger
All − Inverse variance weighted
−0.6
−0.3
0.0
0.3
MR effect size for
'Parabacteroides sp000436495' on 'GCST90200473_buildGRCh38.tsv'

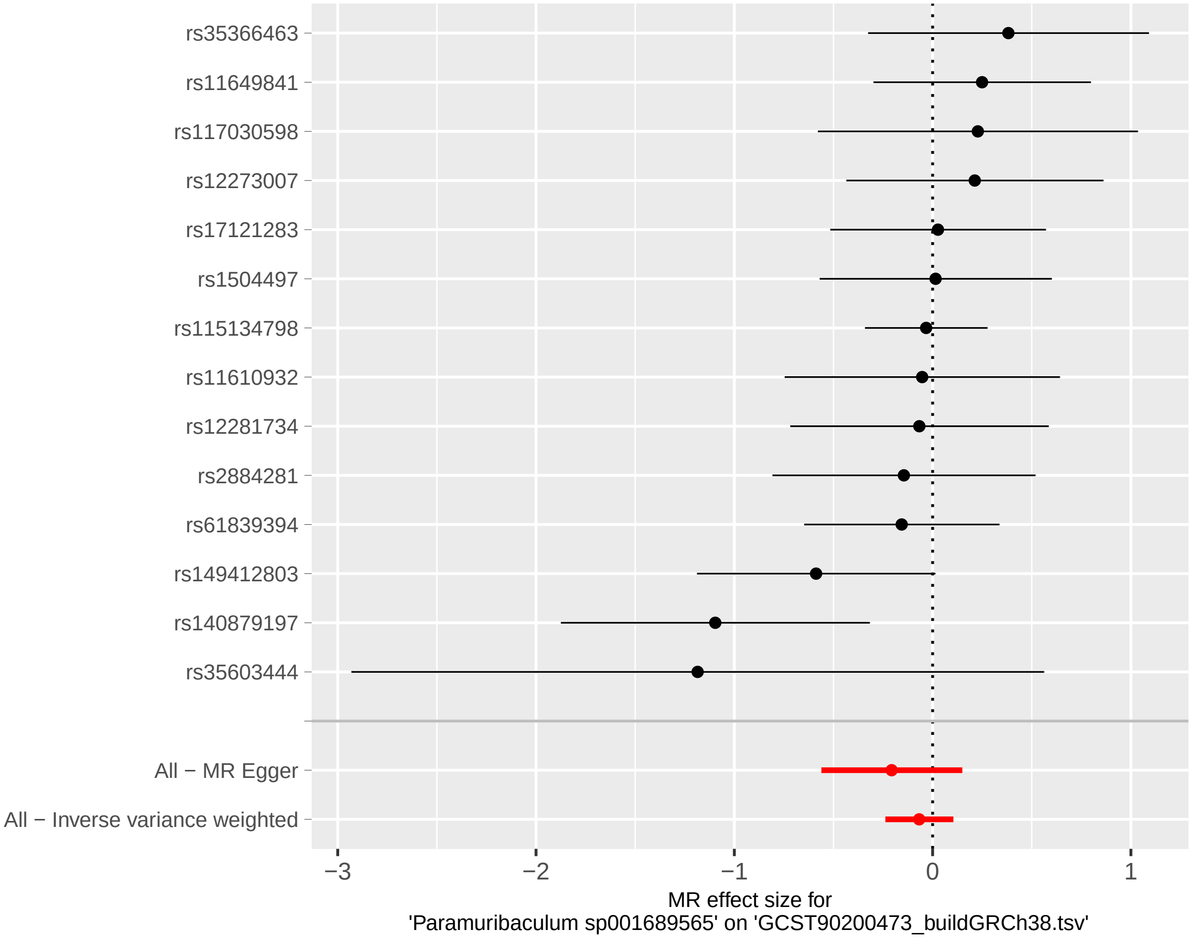
rs35366463
rs11649841
rs117030598
rs12273007
rs17121283
rs1504497
rs115134798
rs11610932
rs12281734
rs2884281
rs61839394
rs149412803
rs140879197
rs35603444
All − MR Egger
All − Inverse variance weighted
−3
−2
−1
0
1
MR effect size for
'Paramuribaculum sp001689565' on 'GCST90200473_buildGRCh38.tsv'

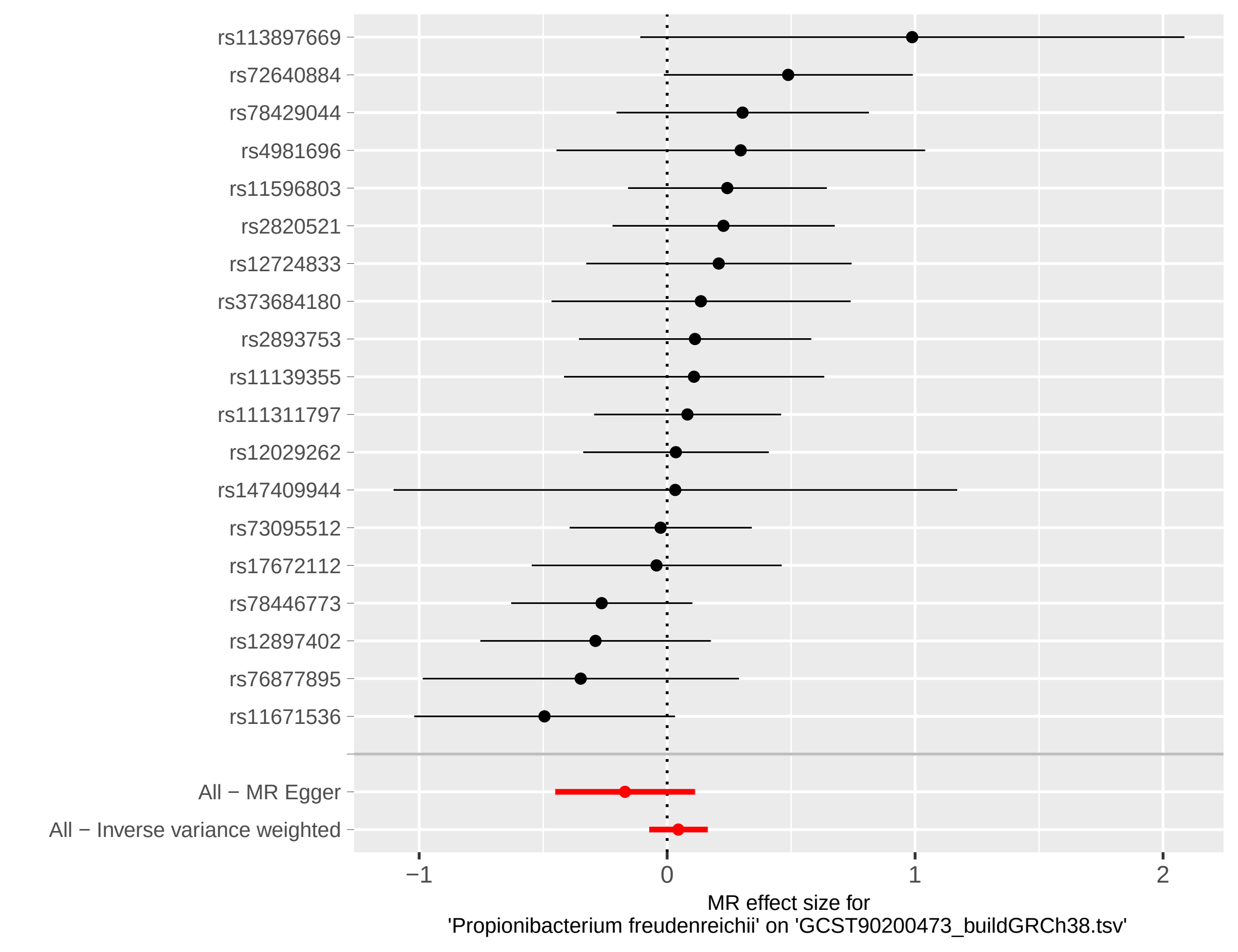

rs113897669
rs72640884
rs78429044
rs4981696
rs11596803
rs2820521
rs12724833
rs373684180
rs2893753
rs11139355
rs111311797
rs12029262
rs147409944
rs73095512
rs17672112
rs78446773
rs12897402
rs76877895
rs11671536
All − MR Egger
All − Inverse variance weighted
−1
0
1
2
MR effect size for
'Propionibacterium freudenreichii' on 'GCST90200473_buildGRCh38.tsv'

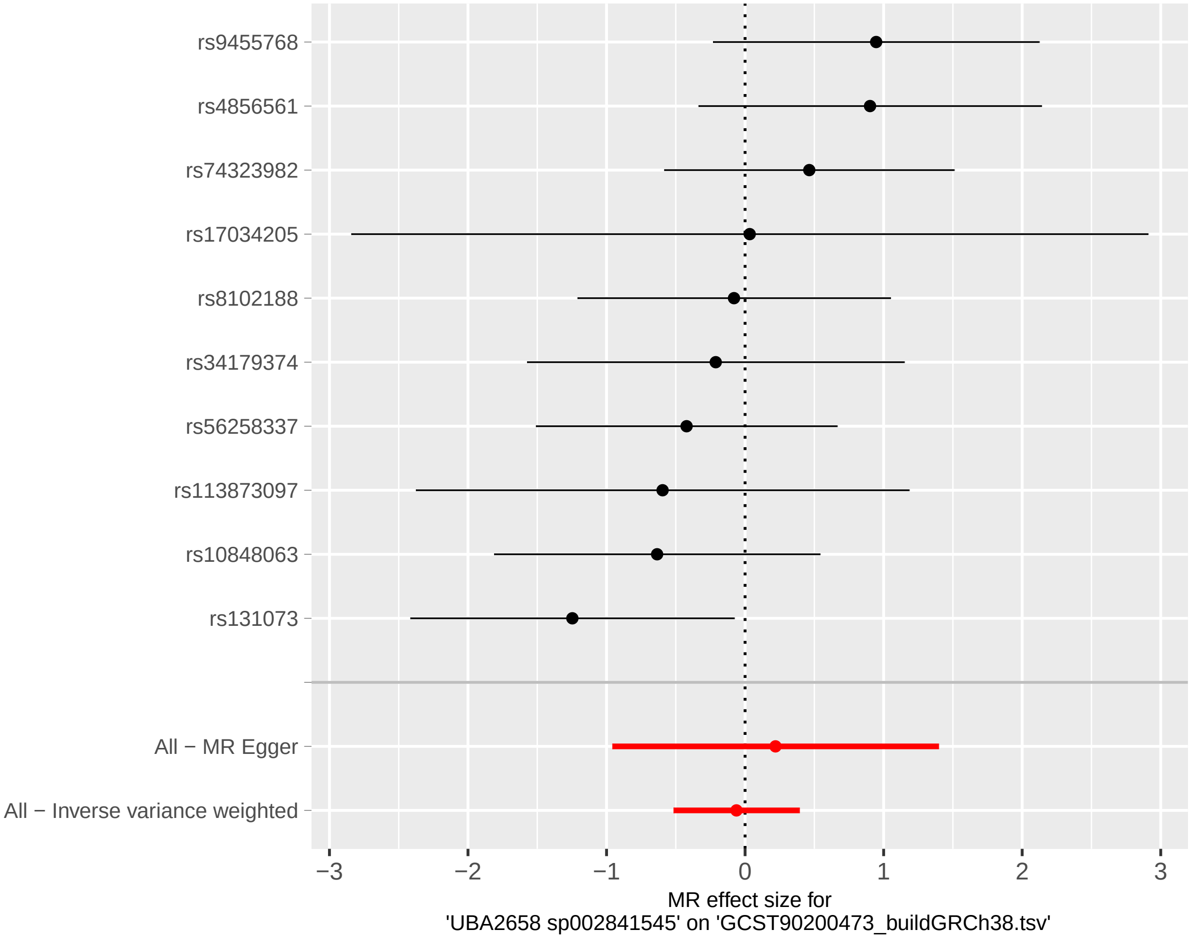

rs9455768
rs4856561
rs74323982
rs17034205
rs8102188
rs34179374
rs56258337
rs113873097
rs10848063
rs131073
All − MR Egger
All − Inverse variance weighted
−3
−2
−1
0
1
2
3
MR effect size for
'UBA2658 sp002841545' on 'GCST90200473_buildGRCh38.tsv'

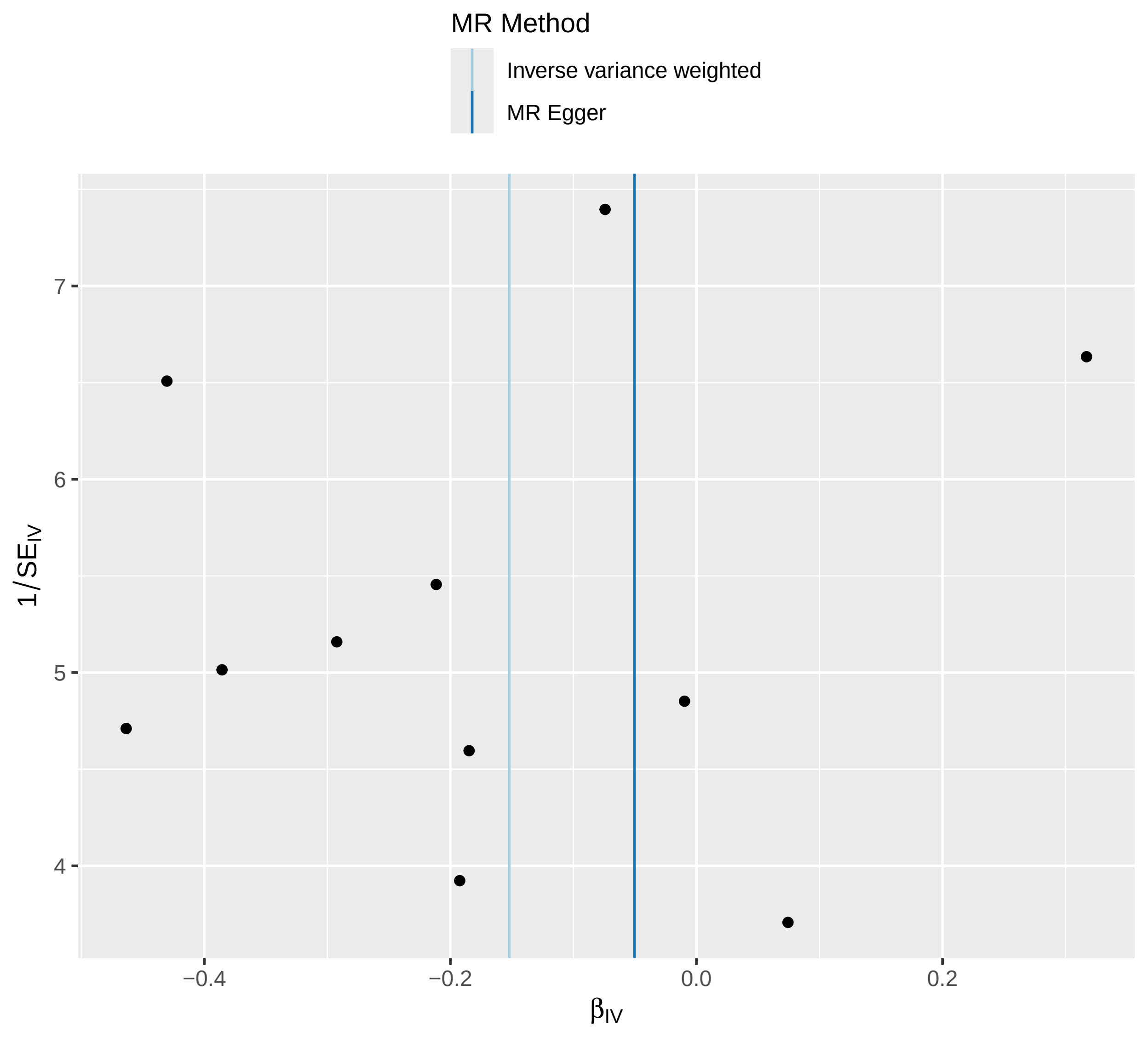
MR Method
Inverse variance weighted
MR Egger
7
6
5
4
1/SE$_{IV}$
−0.4
−0.2
0.0
0.2
β$_{IV}$

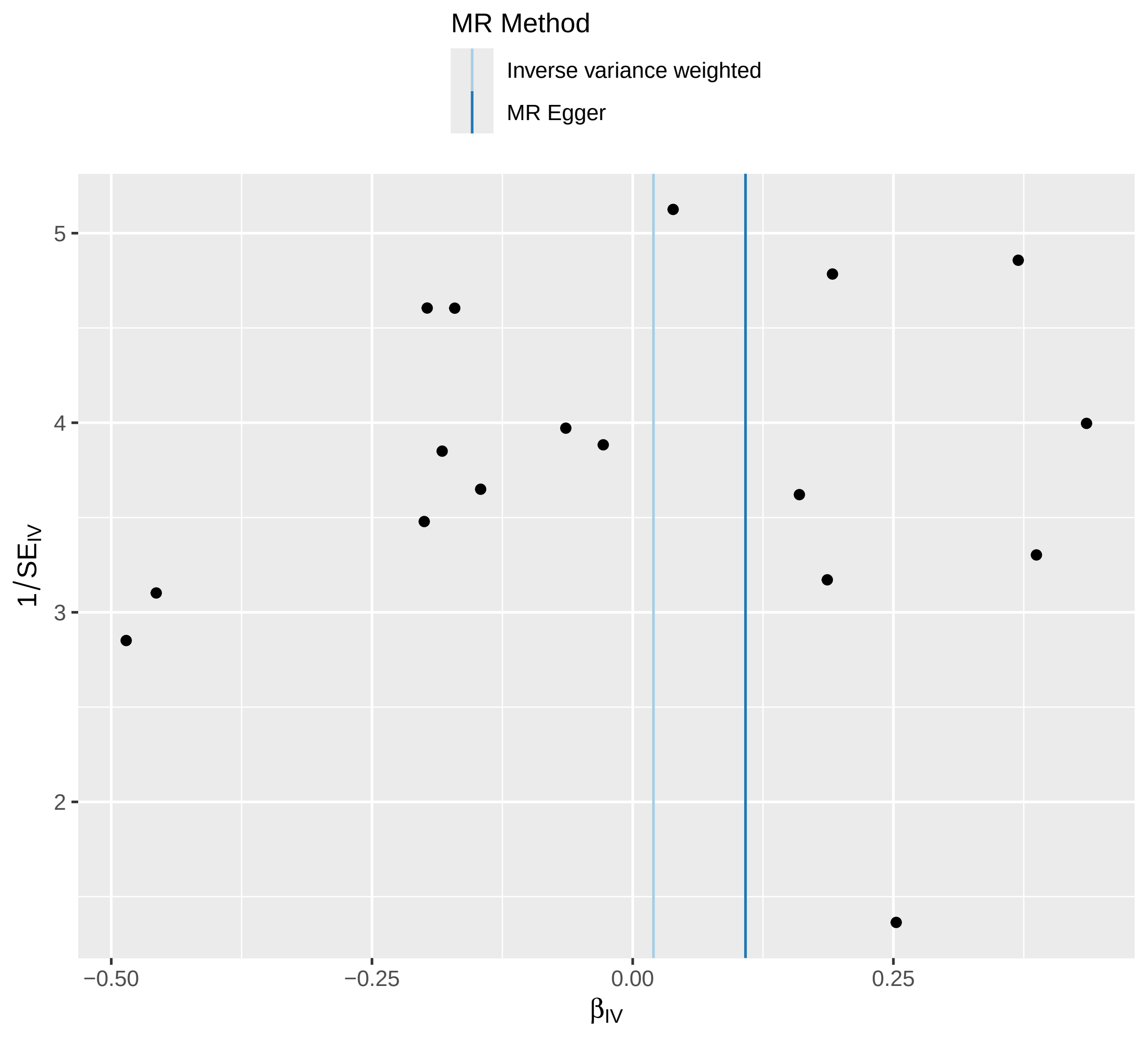
MR Method
Inverse variance weighted
MR Egger
5
4
3
2
1/SE$_{IV}$
−0.50
−0.25
0.00
0.25
β$_{IV}$

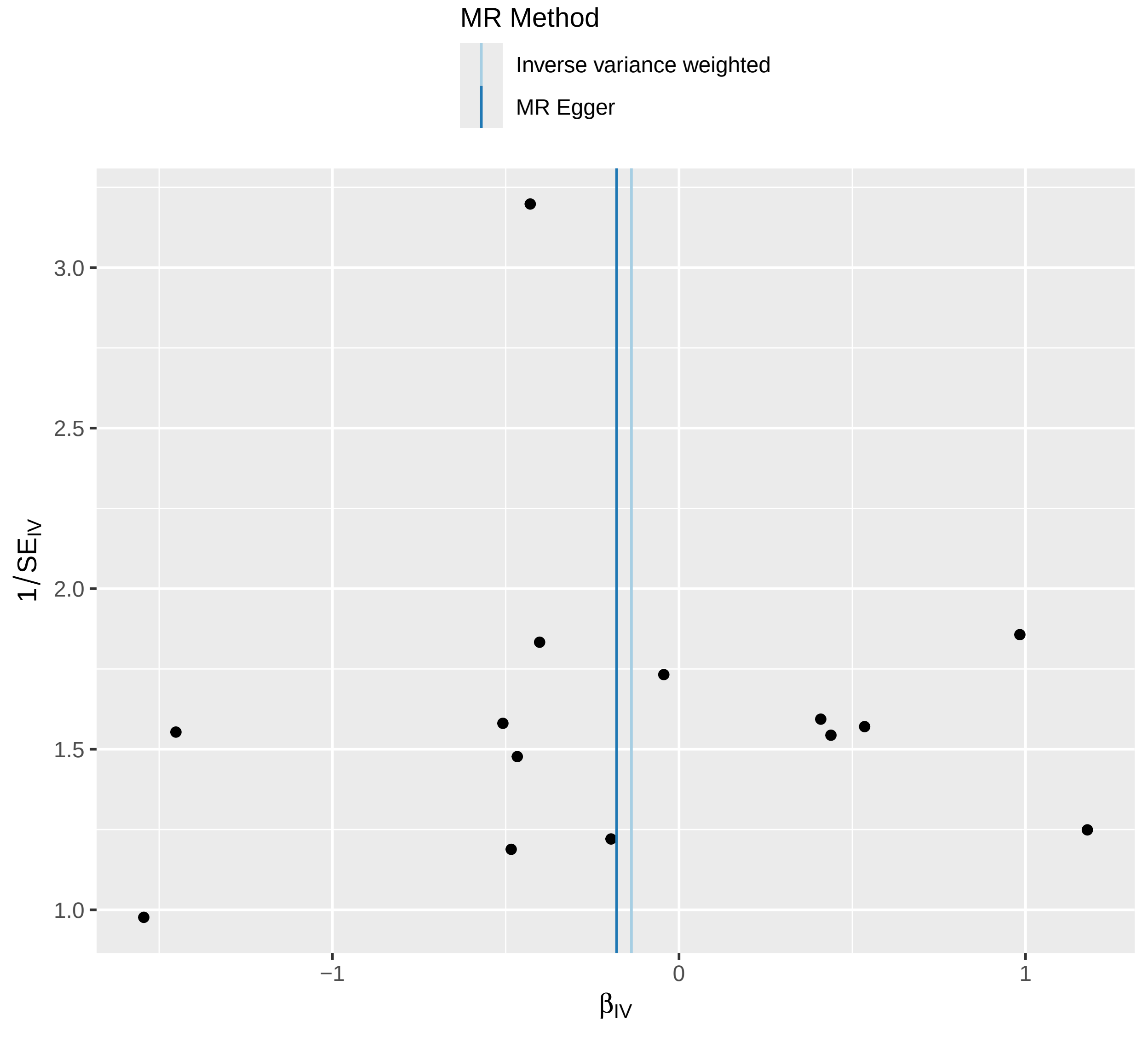

MR Method
Inverse variance weighted
MR Egger
1/SE_IV
3.0
2.5
2.0
1.5
1.0
−1
0
1
β_IV

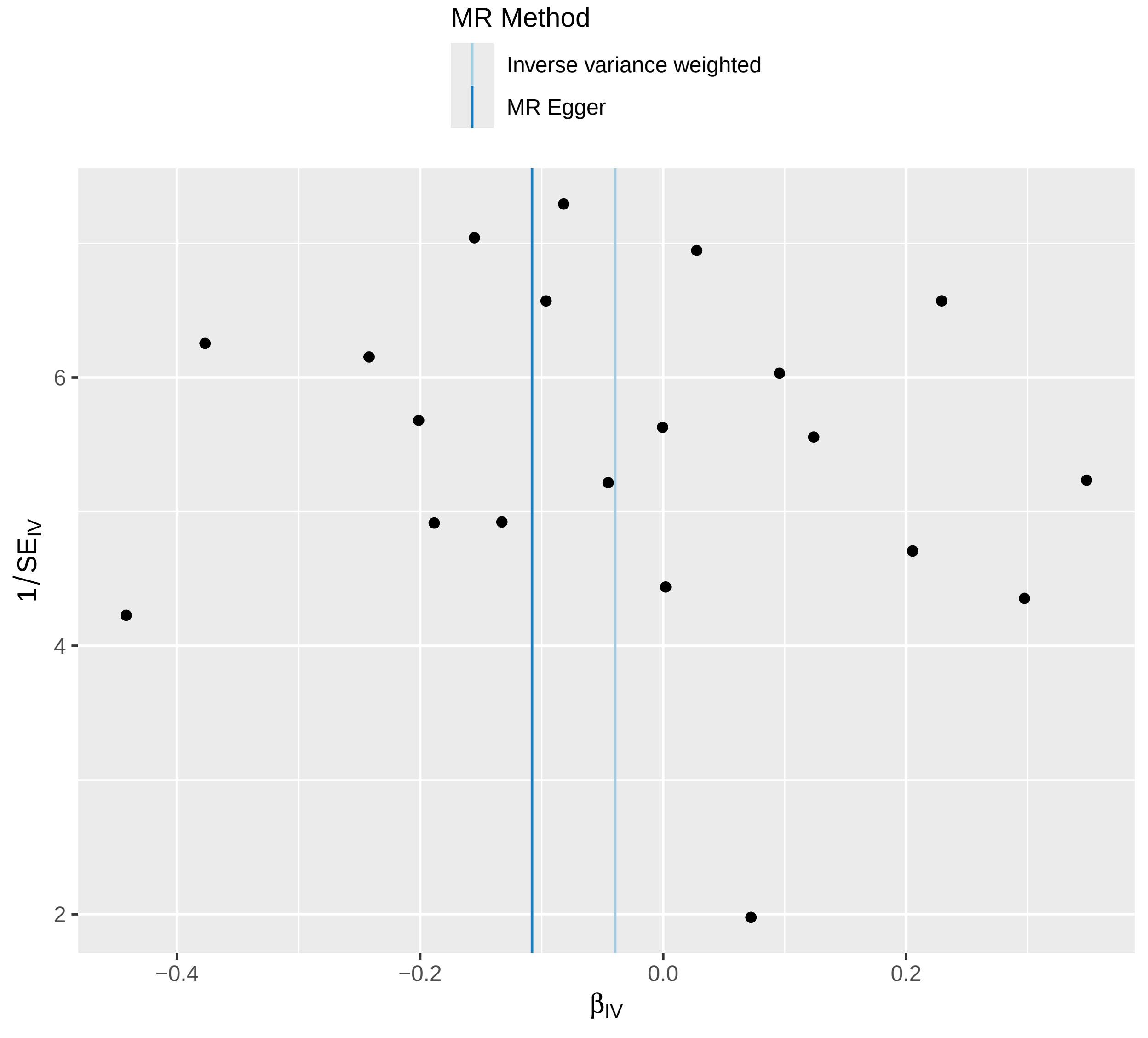
MR Method
Inverse variance weighted
MR Egger
6
4
2
1/SE_IV
−0.4
−0.2
0.0
0.2
β_IV

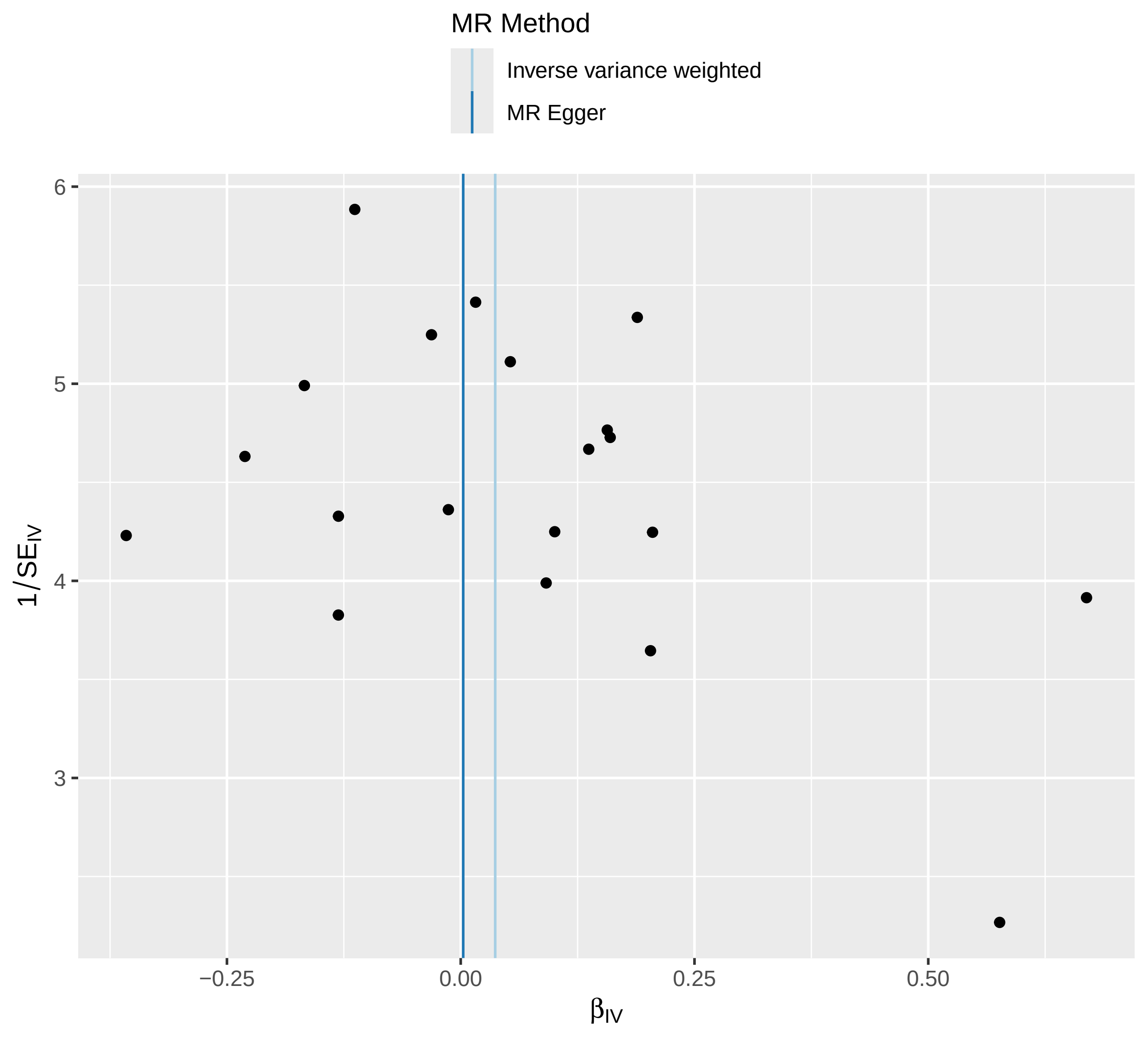
MR Method
Inverse variance weighted
MR Egger
6
5
4
3
1/SE$_{IV}$
−0.25
0.00
0.25
0.50
β$_{IV}$

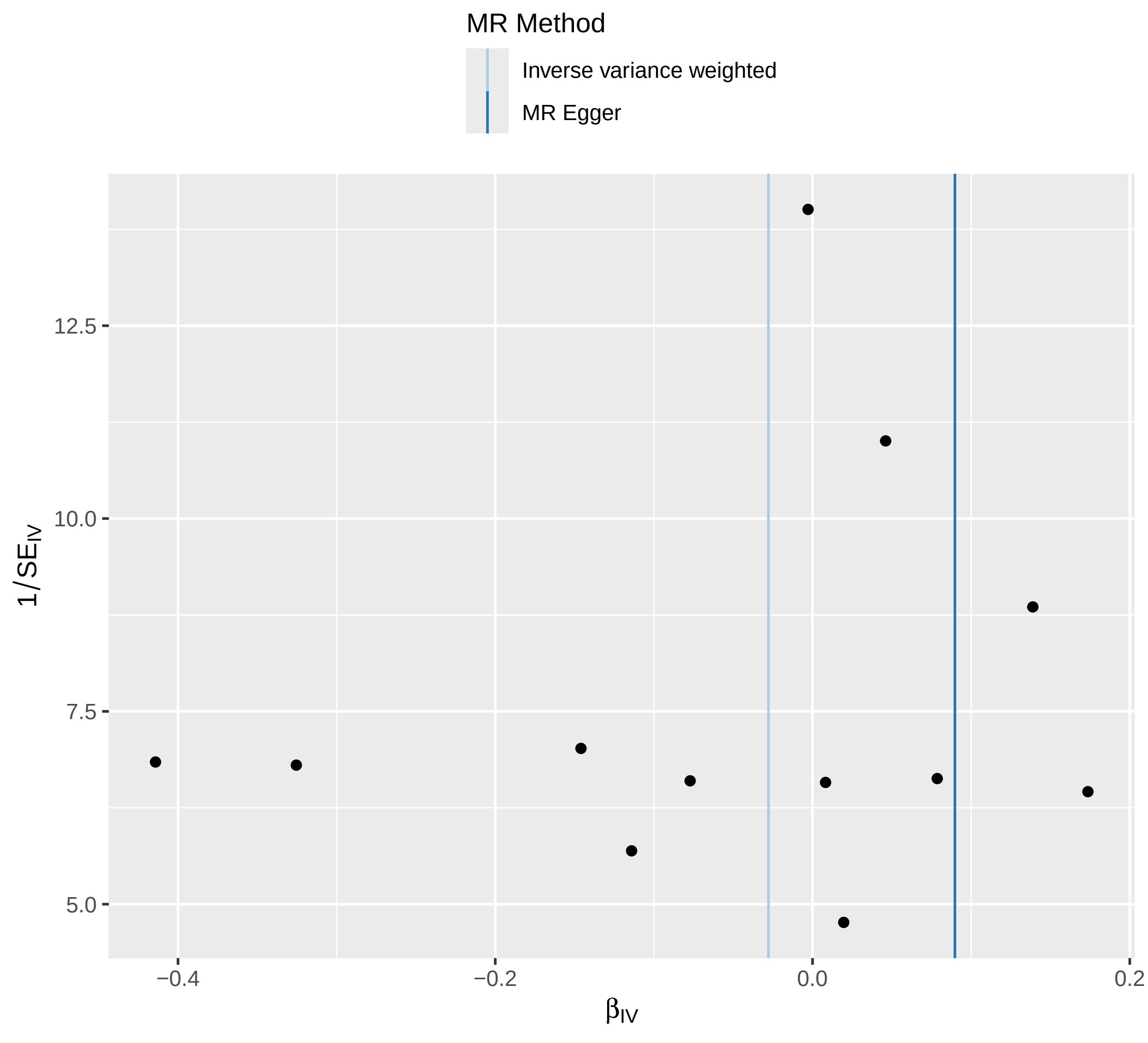

MR Method
Inverse variance weighted
MR Egger
12.5
10.0
7.5
5.0
1/SE$_{IV}$
−0.4
−0.2
0.0
0.2
β$_{IV}$

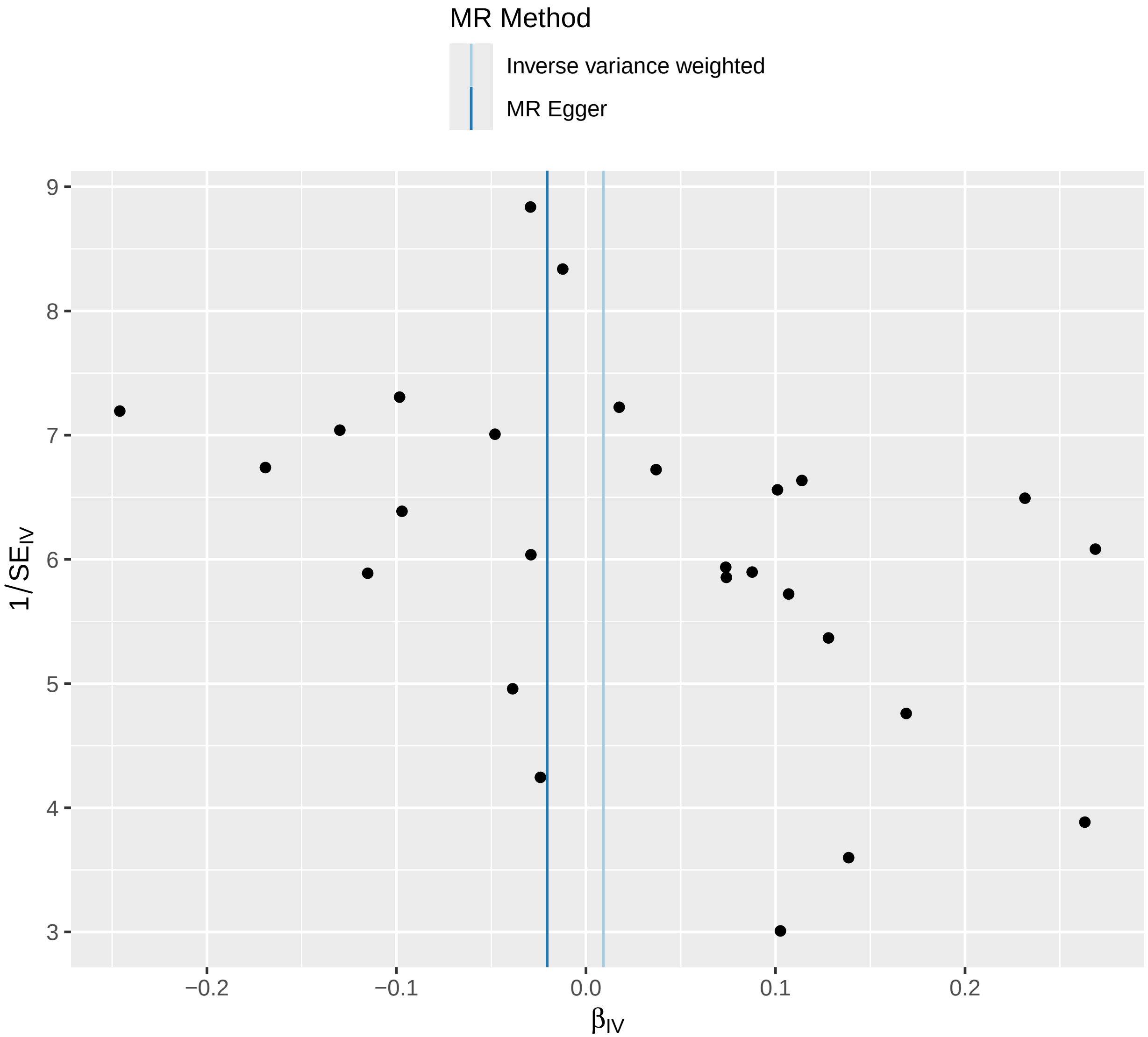
MR Method
Inverse variance weighted
MR Egger
1/SE_IV
9
8
7
6
5
4
3
−0.2
−0.1
0.0
0.1
0.2
β_IV

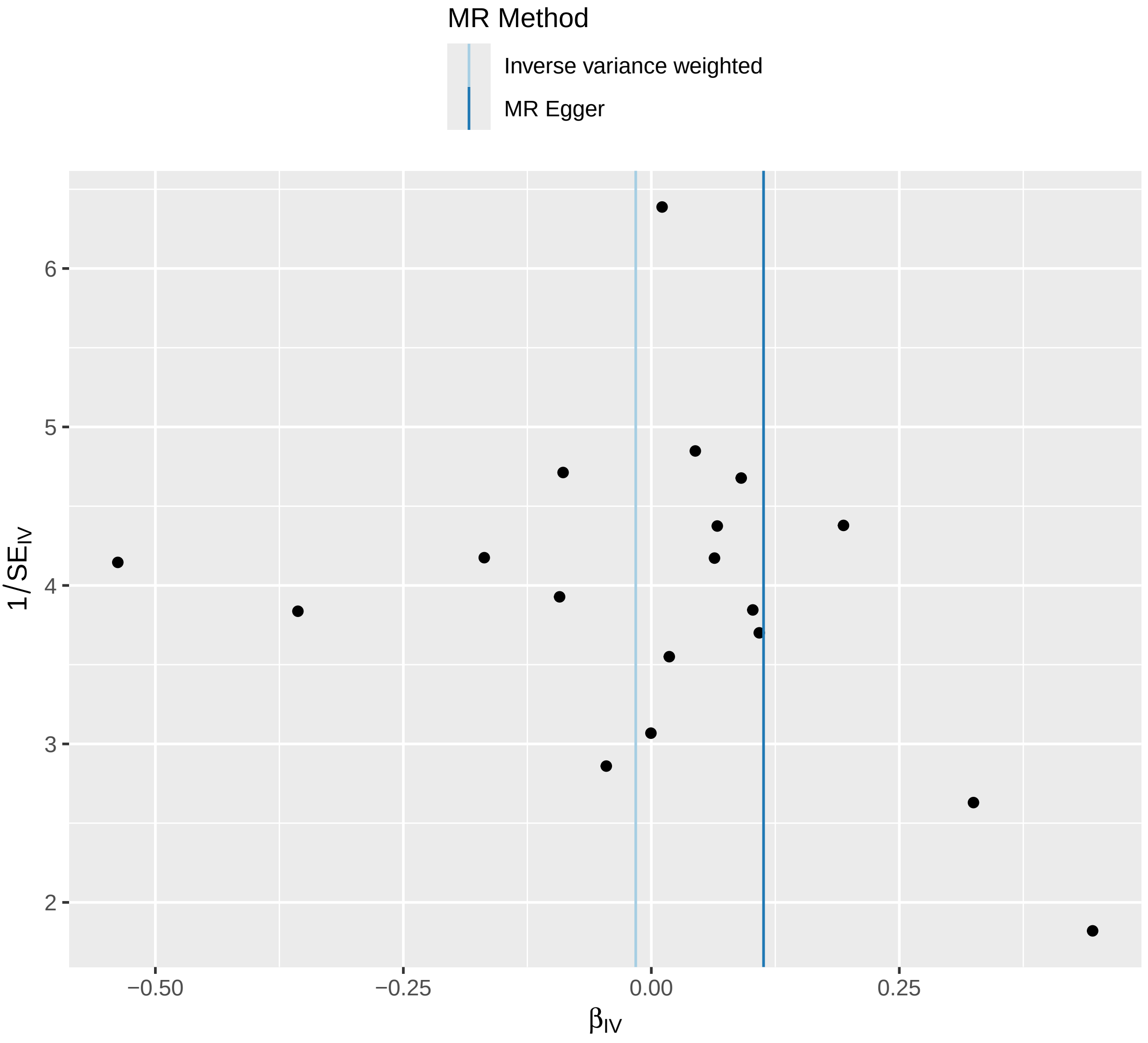
MR Method
Inverse variance weighted
MR Egger
1/SE$_{IV}$
6
5
4
3
2
−0.50
−0.25
0.00
0.25
β$_{IV}$

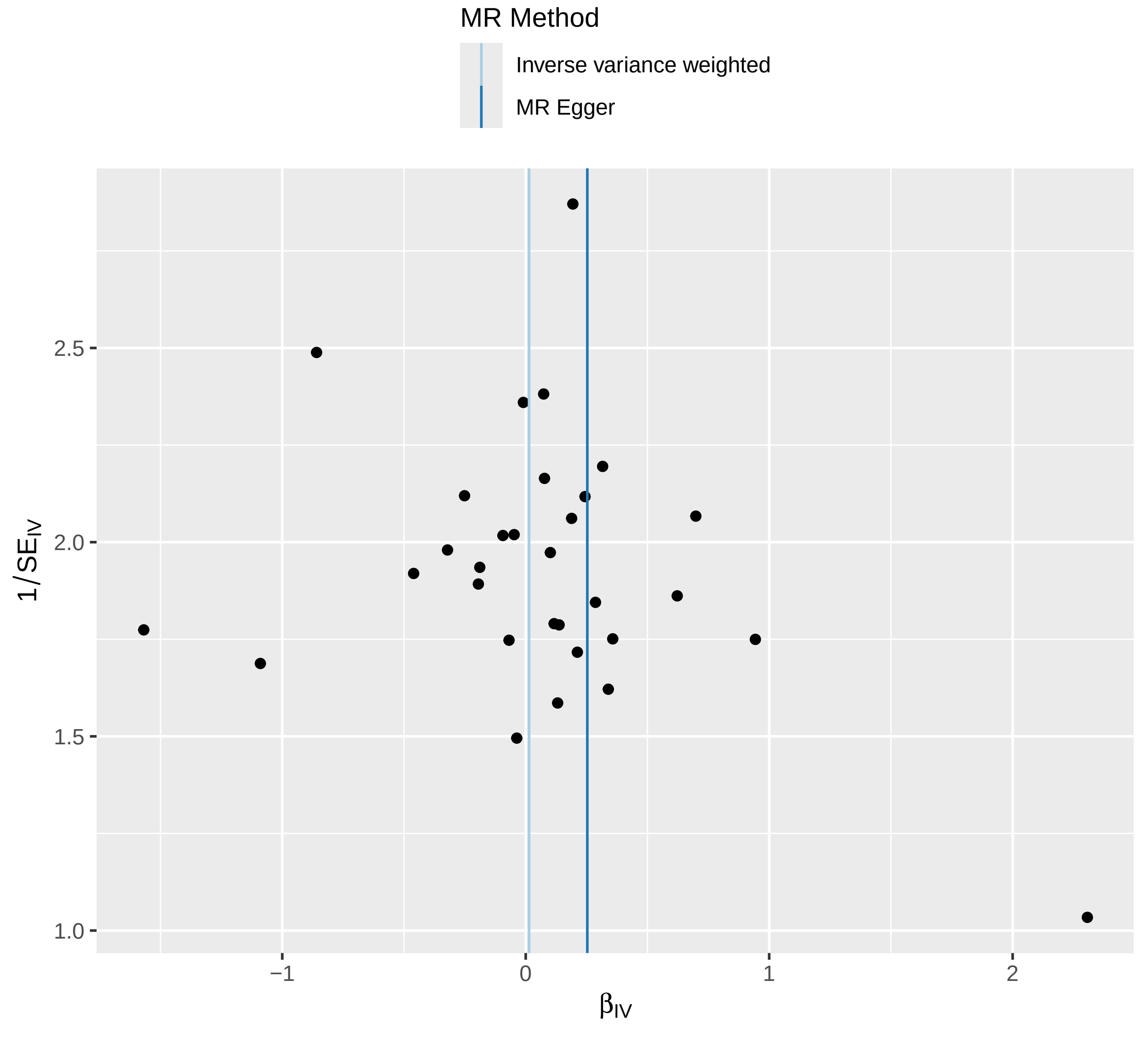
MR Method
Inverse variance weighted
MR Egger
2.5
2.0
1.5
1.0
1/SE_IV
−1
0
1
2
β_IV

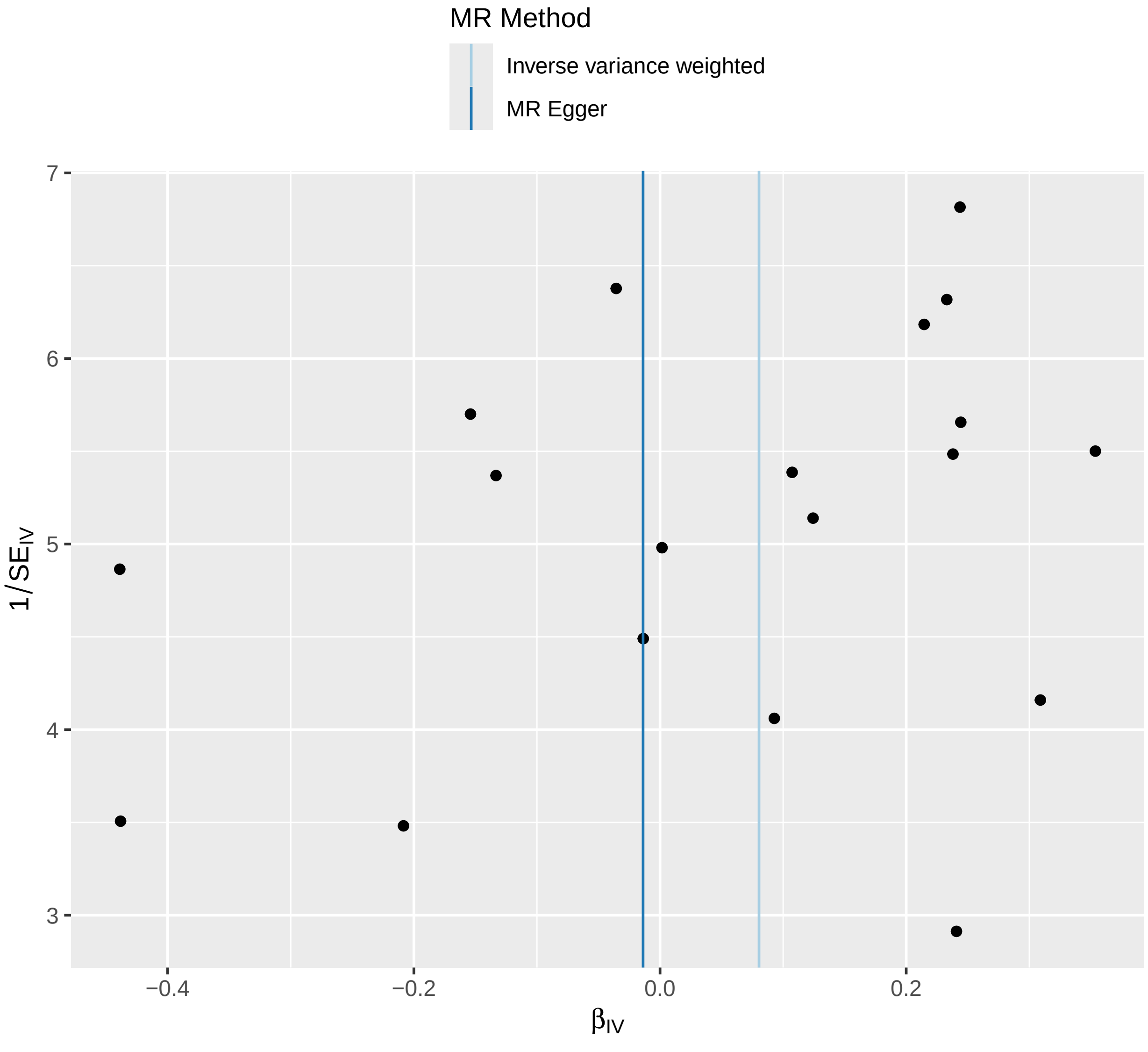
MR Method
Inverse variance weighted
MR Egger
7
6
5
4
3
1/SE_IV
−0.4
−0.2
0.0
0.2
β_IV

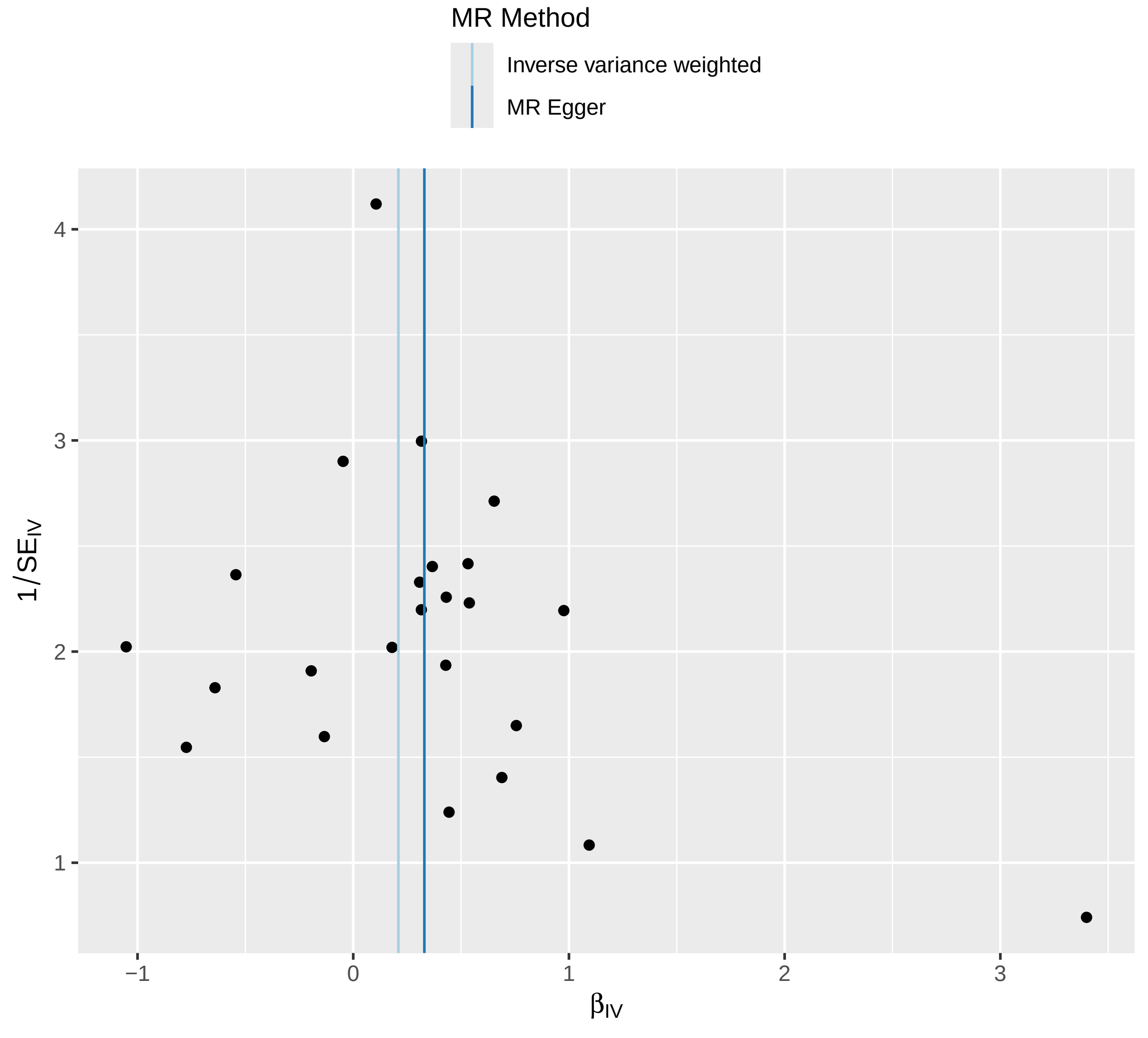
MR Method
Inverse variance weighted
MR Egger
4
3
2
1
$1/SE_{IV}$
−1
0
1
2
3
β_{IV}

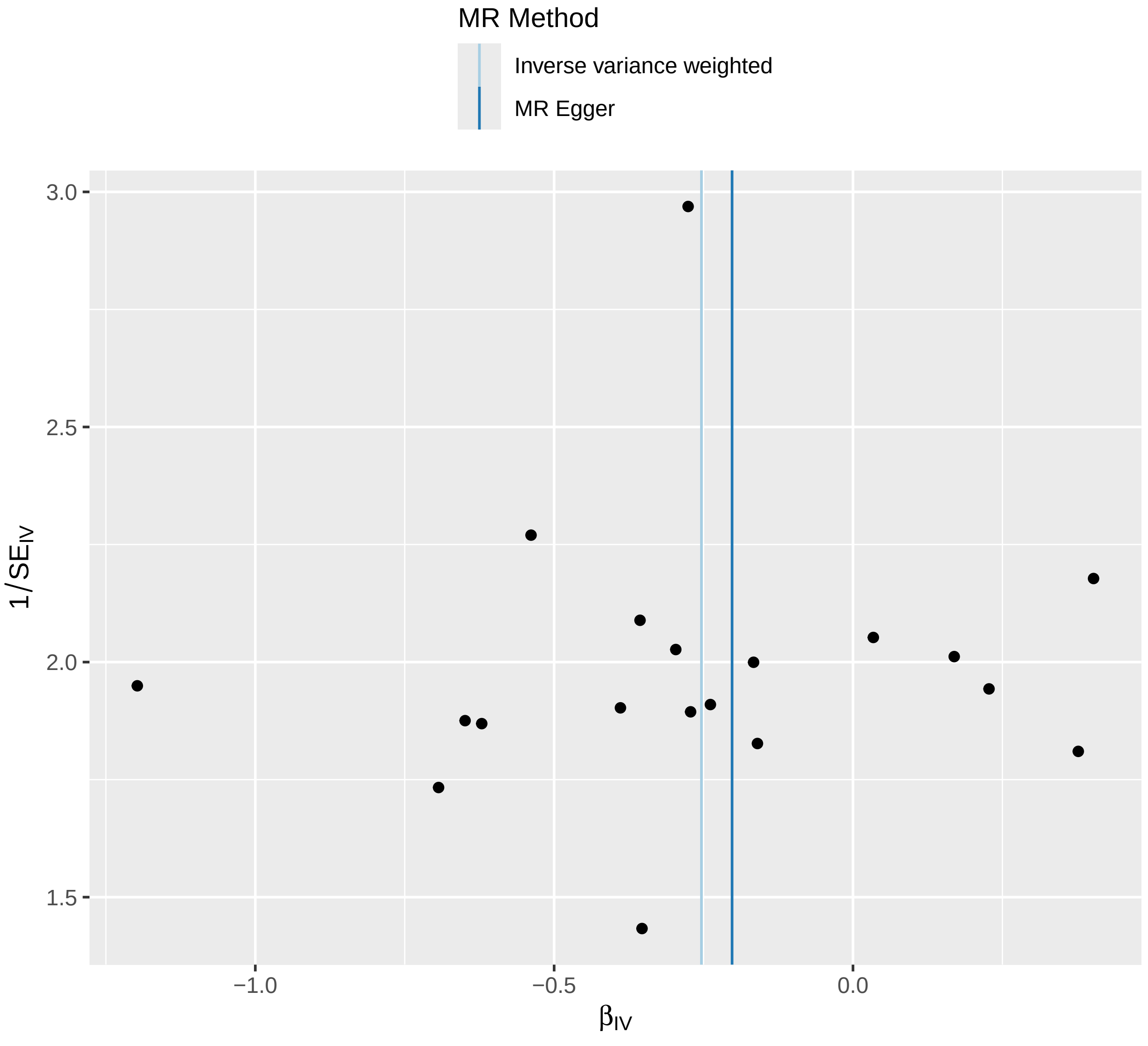

MR Method
Inverse variance weighted
MR Egger
3.0
2.5
2.0
1.5
1/SE_IV
−1.0
−0.5
0.0
β_IV

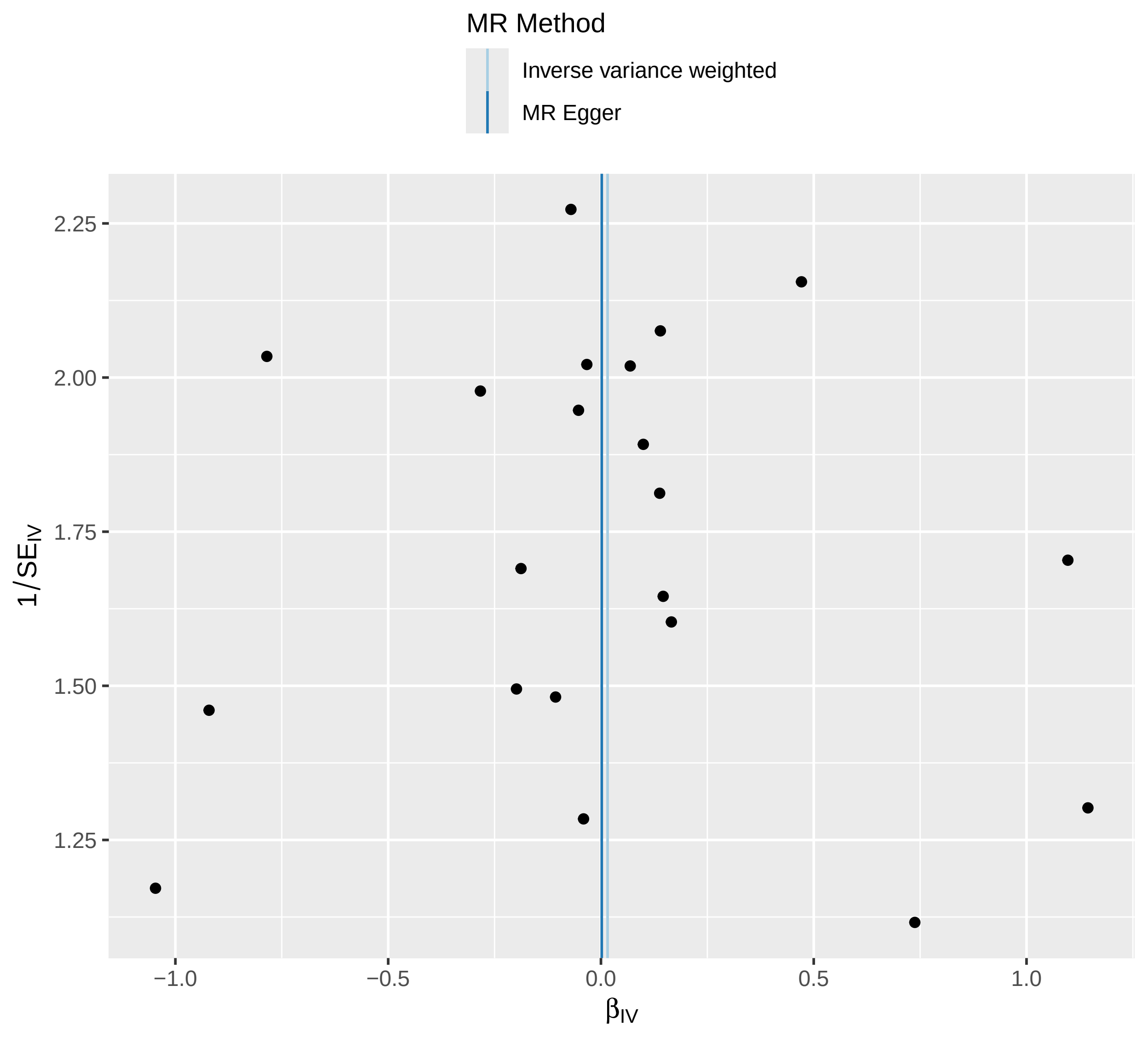

MR Method
Inverse variance weighted
MR Egger
2.25
2.00
1.75
1.50
1.25
1/SE_IV
−1.0
−0.5
0.0
0.5
1.0
β_IV

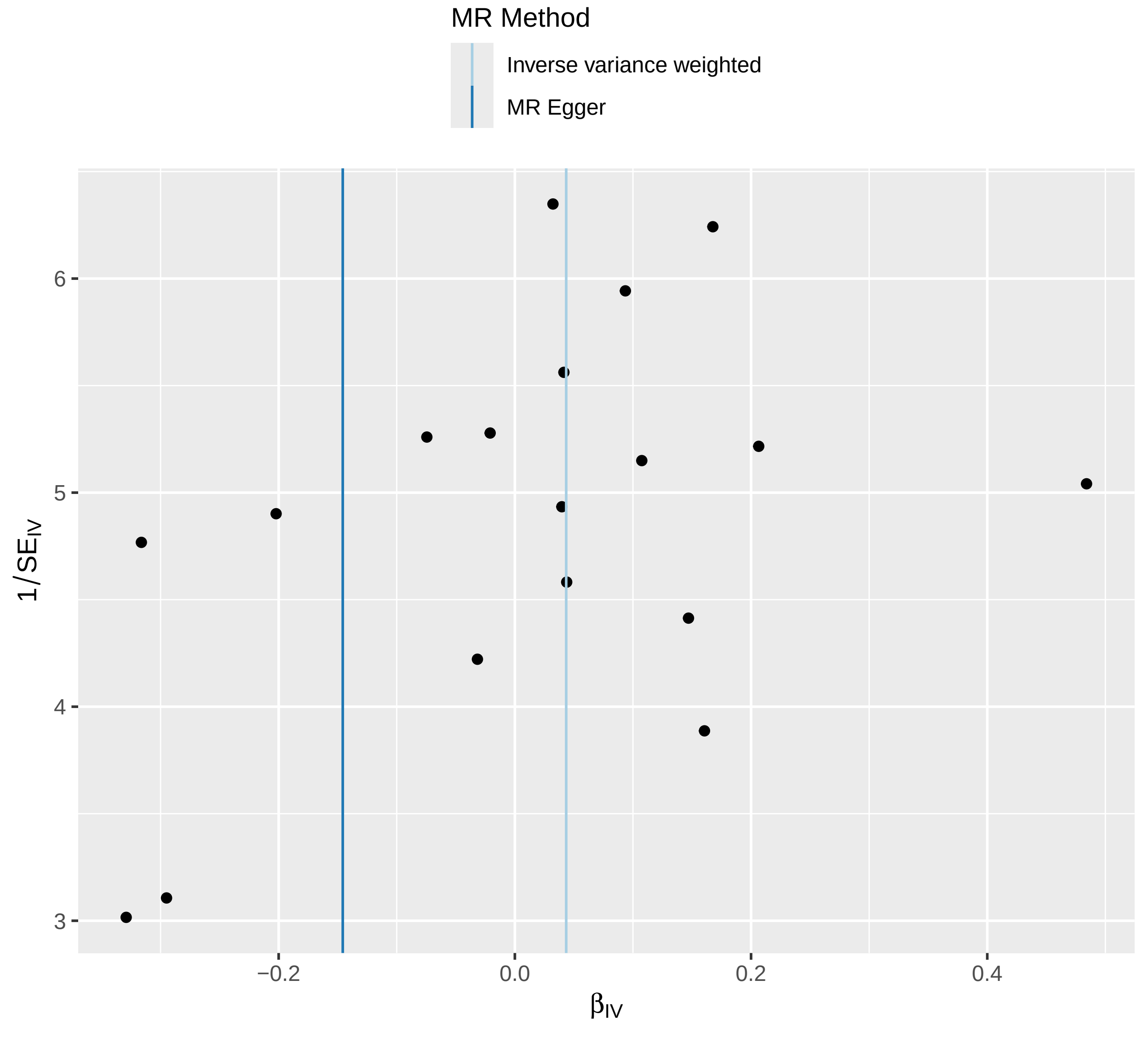
MR Method
Inverse variance weighted
MR Egger
6
5
4
3
1/SE$_{IV}$
−0.2
0.0
0.2
0.4
β$_{IV}$

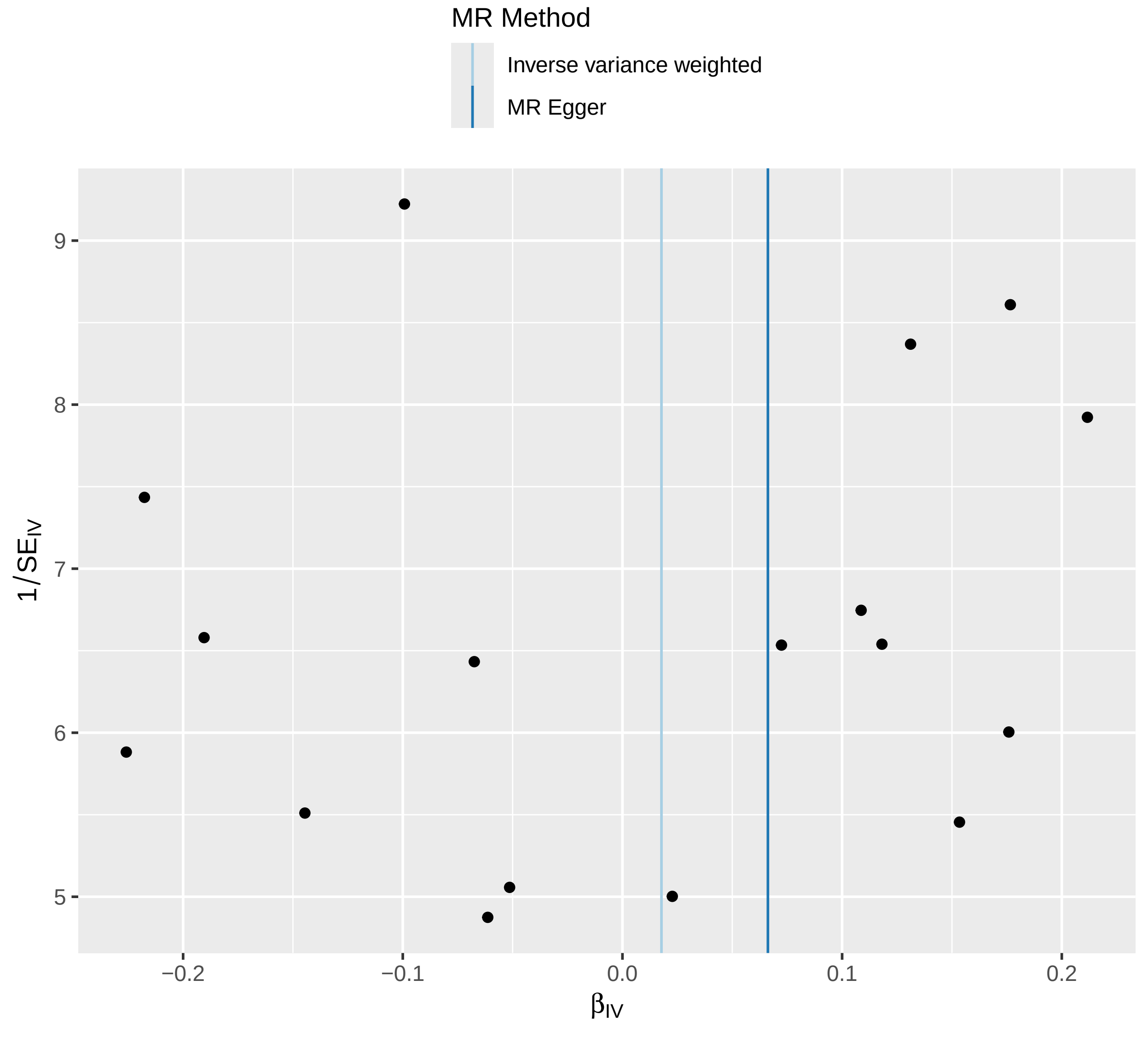

MR Method
Inverse variance weighted
MR Egger
1/SE$_{IV}$
β_{IV}
9
8
7
6
5
−0.2
−0.1
0.0
0.1
0.2

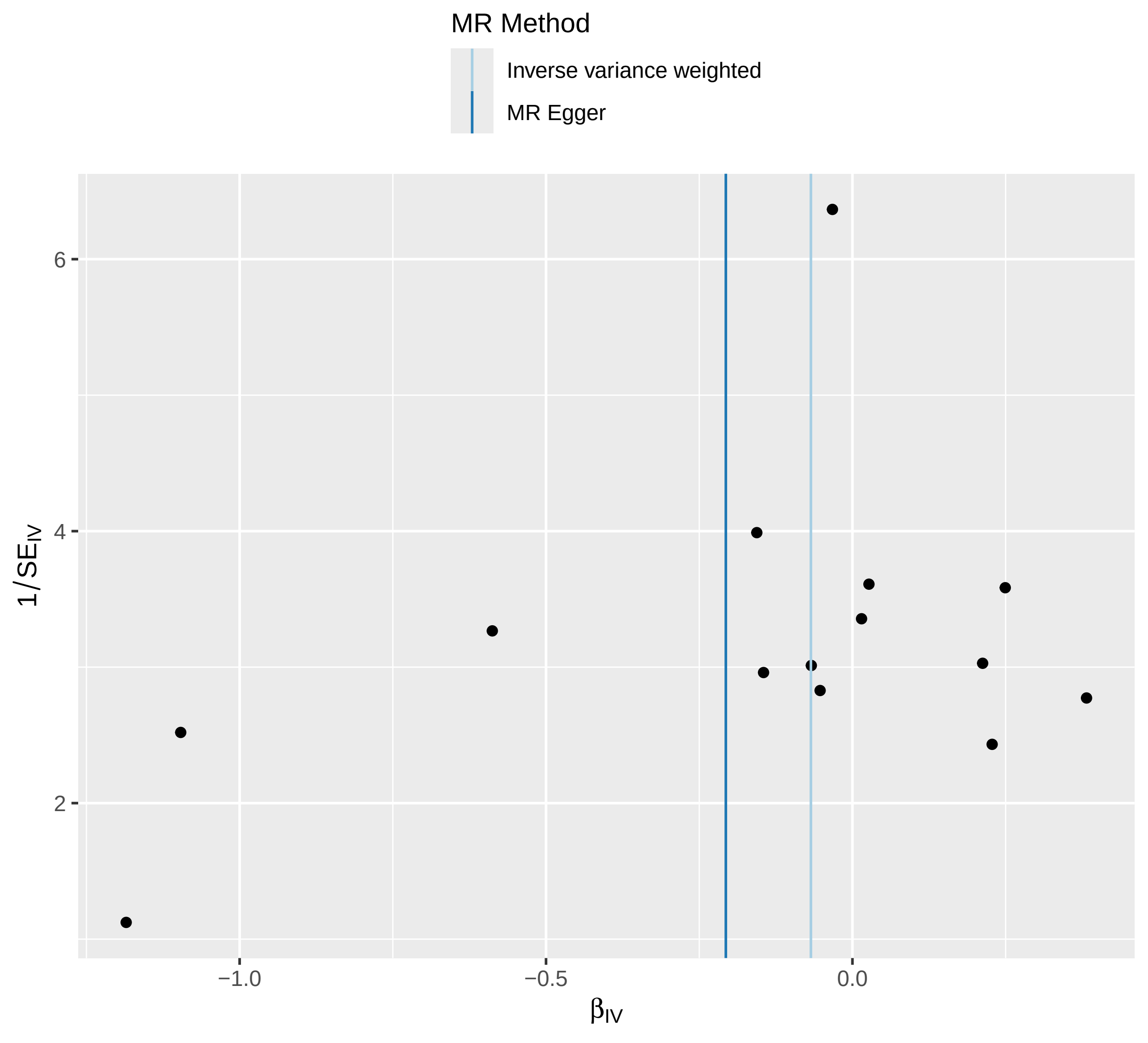
MR Method
Inverse variance weighted
MR Egger
6
4
2
1/SE_IV
−1.0
−0.5
0.0
β_IV

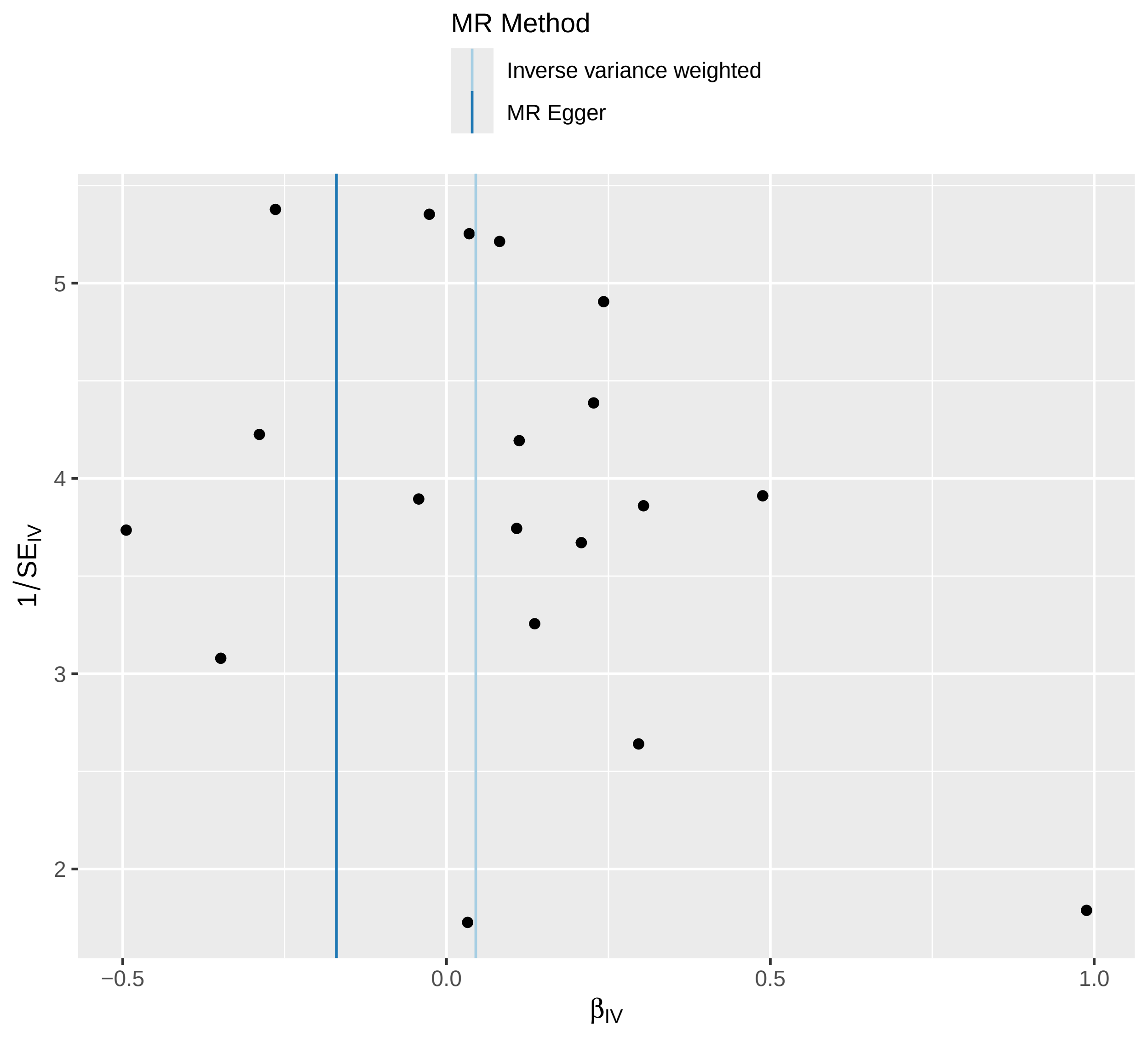
MR Method
Inverse variance weighted
MR Egger
1/SE_IV
β_IV
5
4
3
2
−0.5
0.0
0.5
1.0

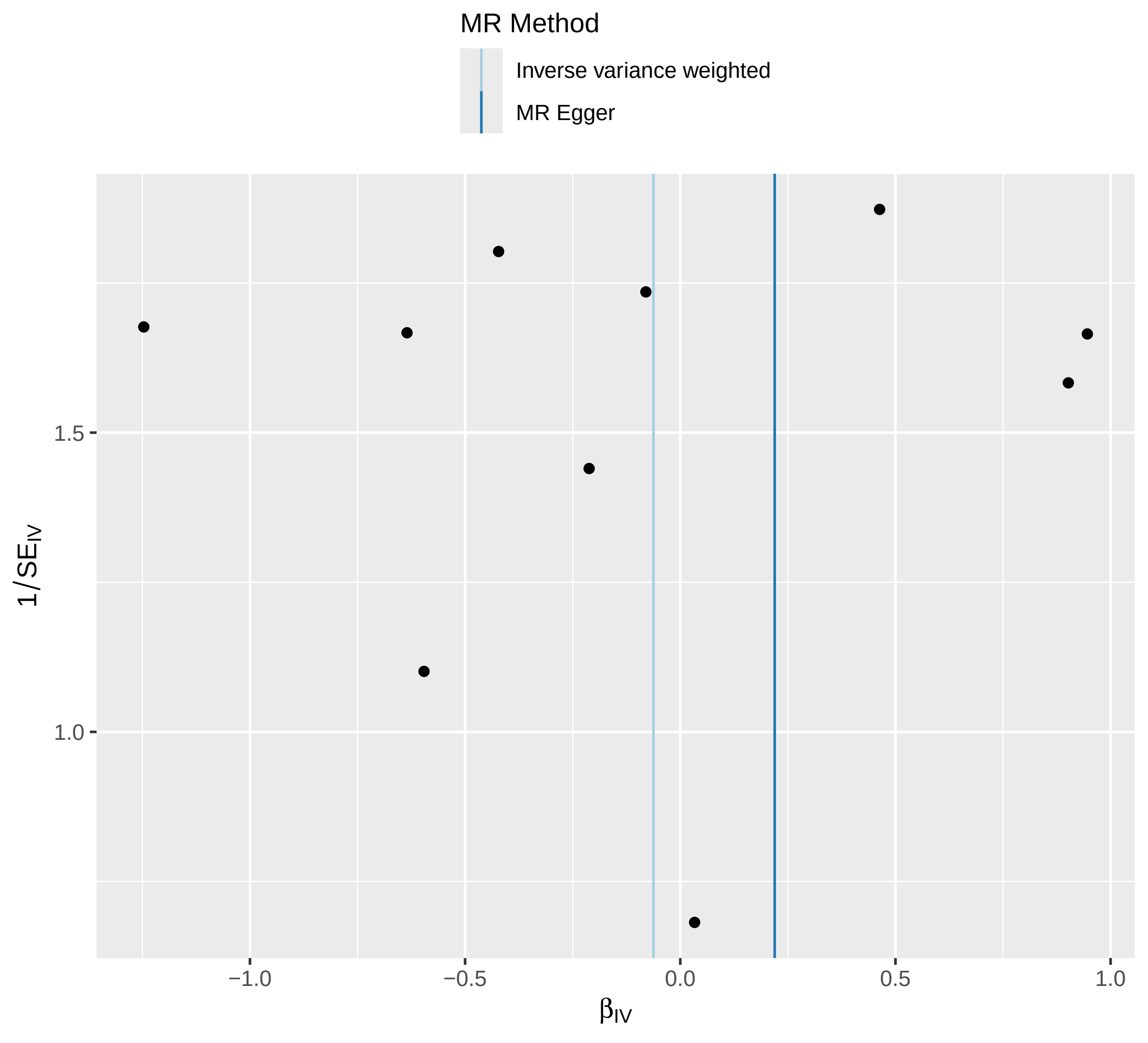

MR Method
Inverse variance weighted
MR Egger
1.5
1.0
1/SE$_{IV}$
−1.0
−0.5
0.0
0.5
1.0
β$_{IV}$

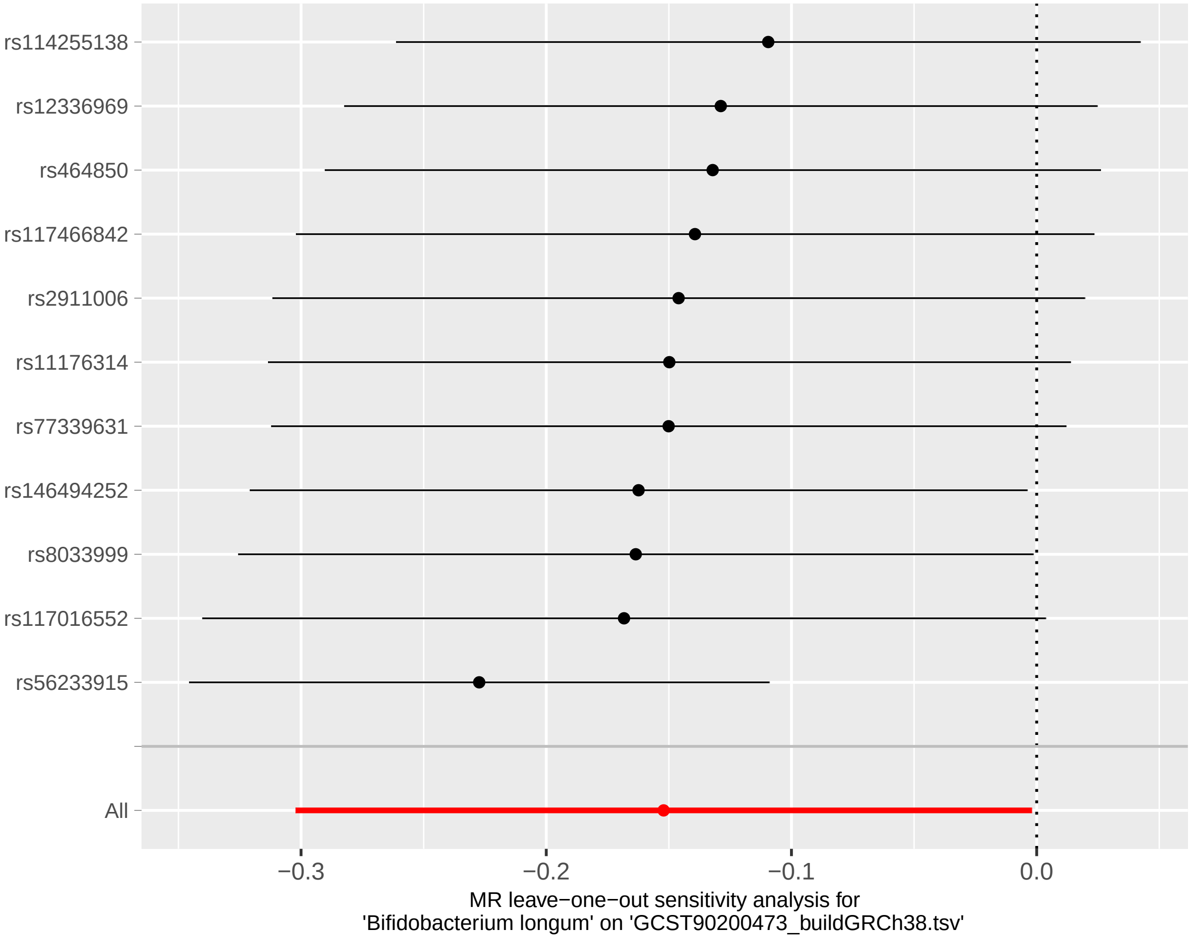
rs114255138
rs12336969
rs464850
rs117466842
rs2911006
rs11176314
rs77339631
rs146494252
rs8033999
rs117016552
rs56233915
All
−0.3
−0.2
−0.1
0.0
MR leave−one−out sensitivity analysis for
'Bifidobacterium longum' on 'GCST90200473_buildGRCh38.tsv'

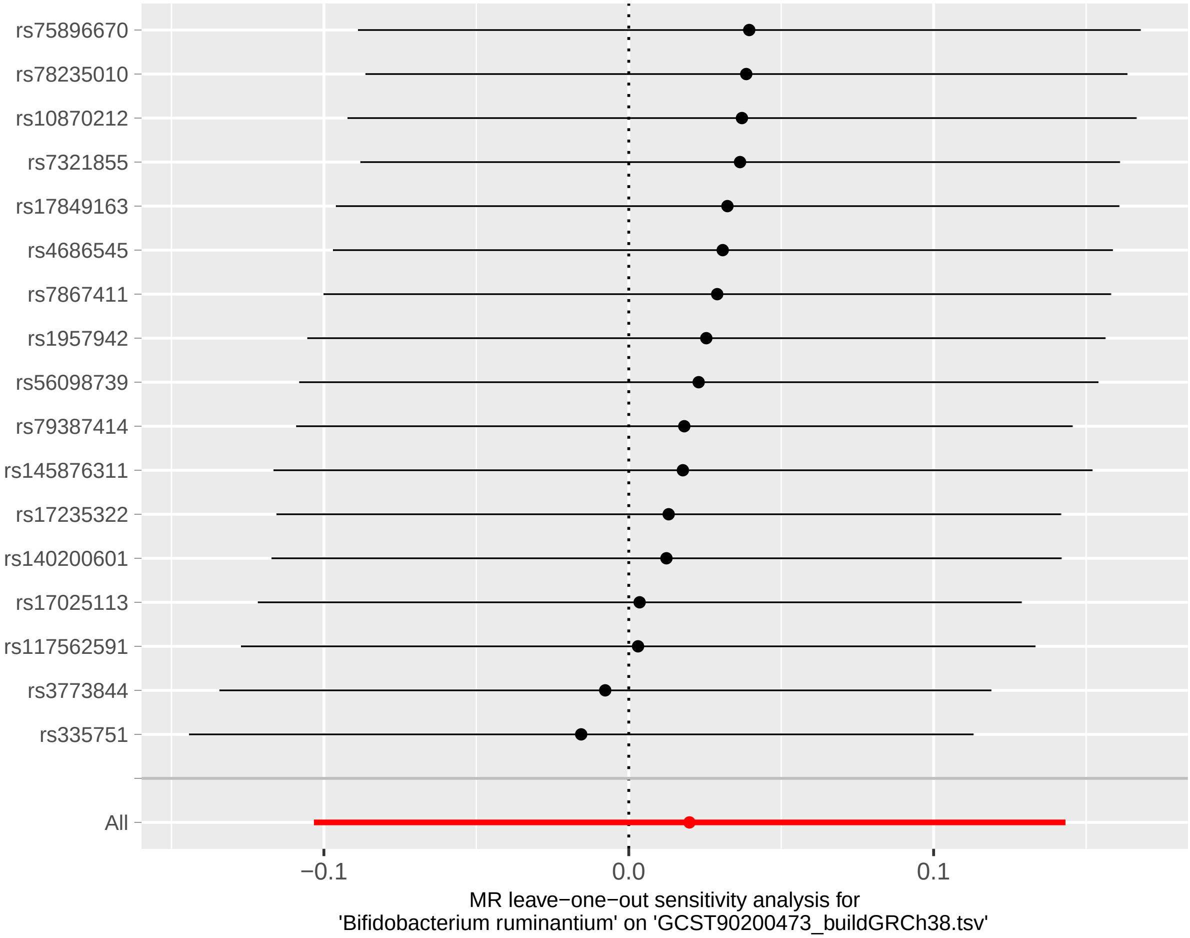
rs75896670
rs78235010
rs10870212
rs7321855
rs17849163
rs4686545
rs7867411
rs1957942
rs56098739
rs79387414
rs145876311
rs17235322
rs140200601
rs17025113
rs117562591
rs3773844
rs335751
All
−0.1
0.0
0.1
MR leave−one−out sensitivity analysis for
'Bifidobacterium ruminantium' on 'GCST90200473_buildGRCh38.tsv'

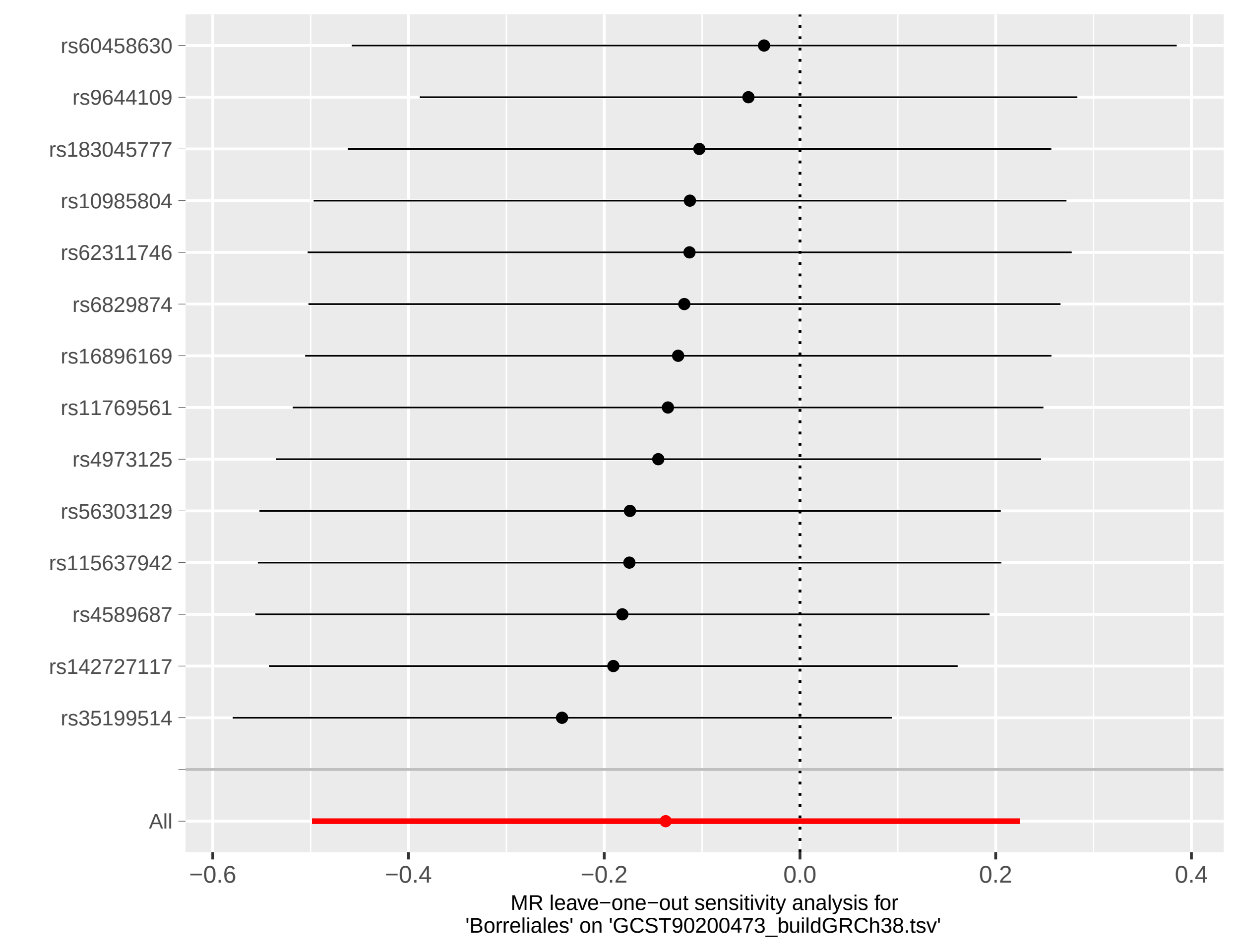

rs60458630
rs9644109
rs183045777
rs10985804
rs62311746
rs6829874
rs16896169
rs11769561
rs4973125
rs56303129
rs115637942
rs4589687
rs142727117
rs35199514
All
−0.6
−0.4
−0.2
0.0
0.2
0.4
MR leave−one−out sensitivity analysis for
'Borreliales' on 'GCST90200473_buildGRCh38.tsv'

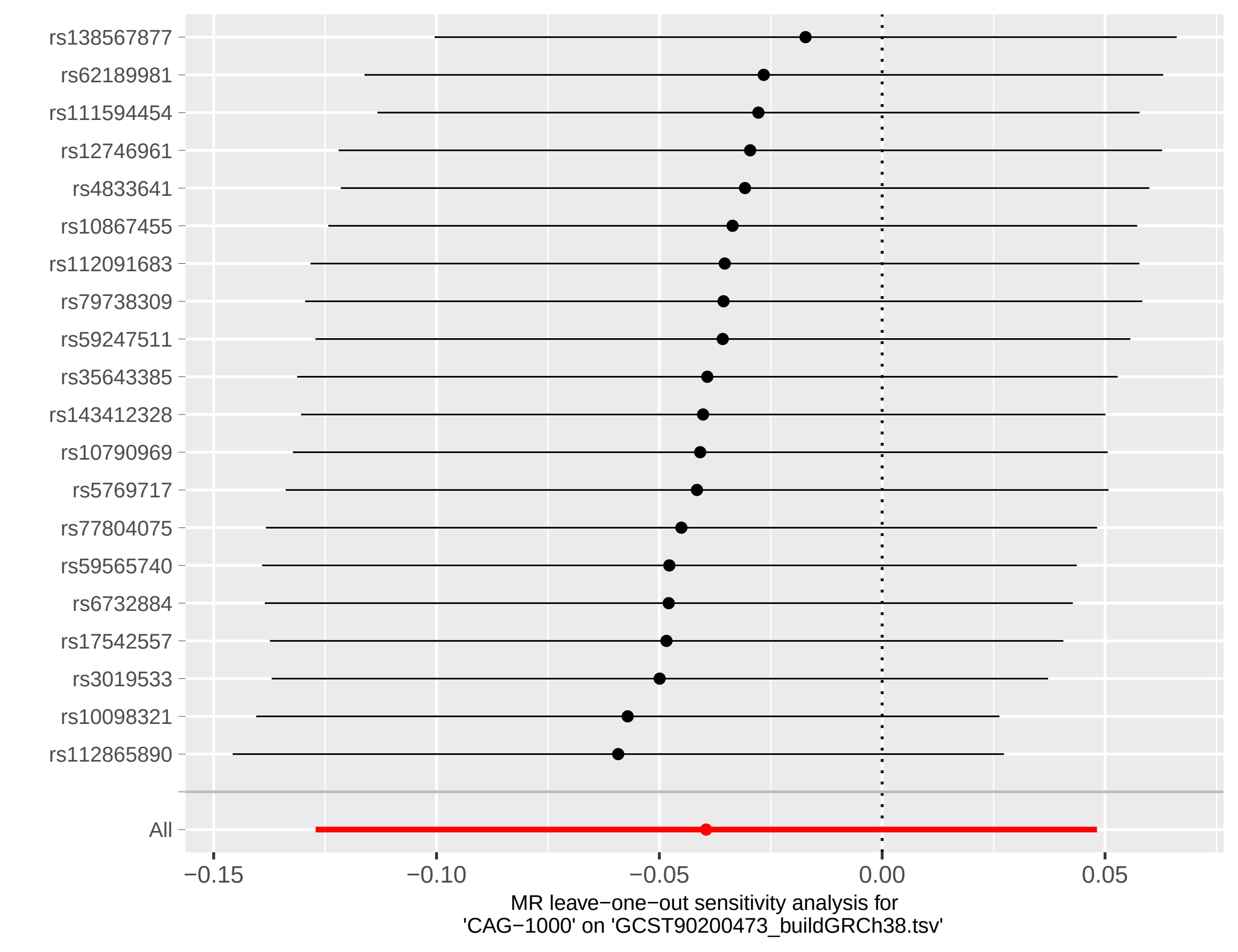
rs138567877
rs62189981
rs111594454
rs12746961
rs4833641
rs10867455
rs112091683
rs79738309
rs59247511
rs35643385
rs143412328
rs10790969
rs5769717
rs77804075
rs59565740
rs6732884
rs17542557
rs3019533
rs10098321
rs112865890
All
−0.15
−0.10
−0.05
0.00
0.05
MR leave−one−out sensitivity analysis for
'CAG−1000' on 'GCST90200473_buildGRCh38.tsv'

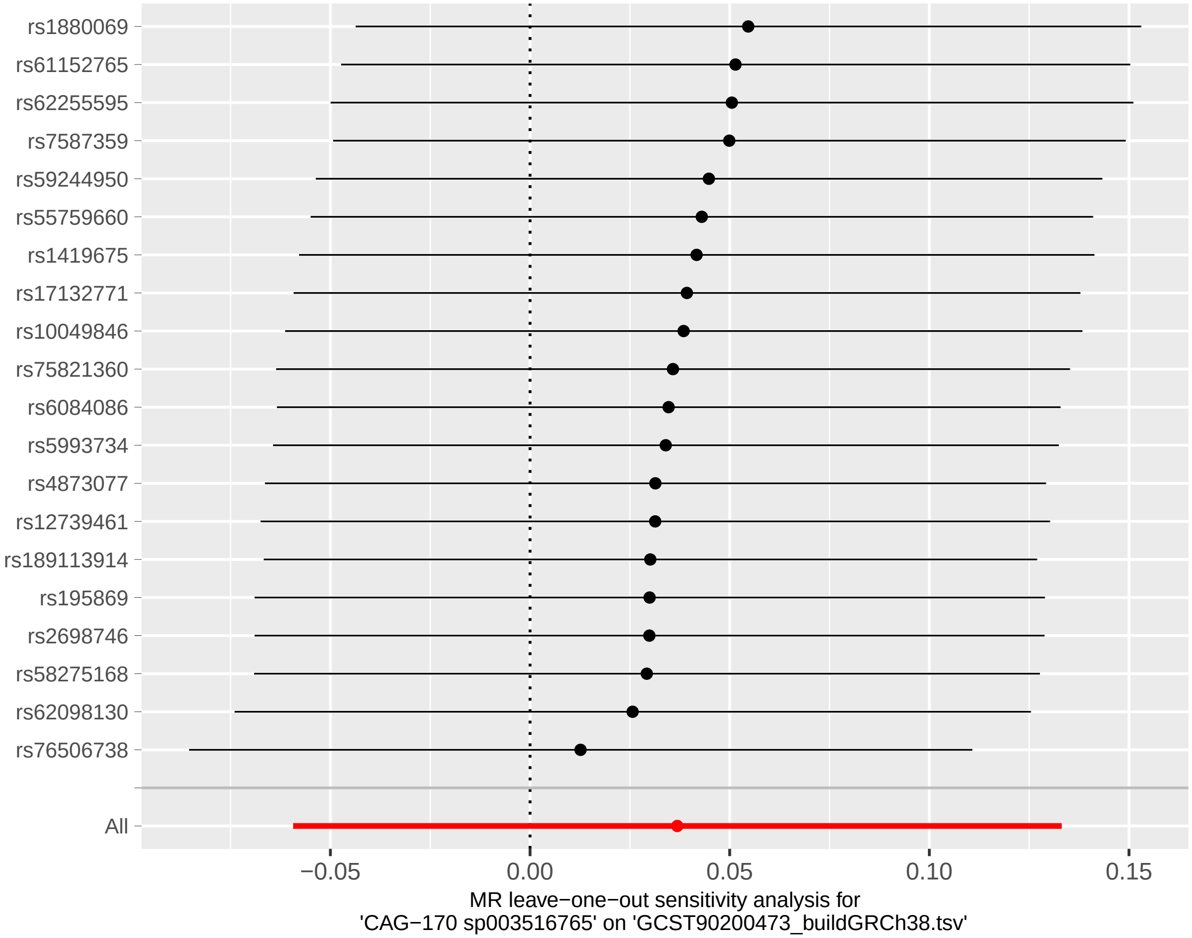

rs1880069
rs61152765
rs62255595
rs7587359
rs59244950
rs55759660
rs1419675
rs17132771
rs10049846
rs75821360
rs6084086
rs5993734
rs4873077
rs12739461
rs189113914
rs195869
rs2698746
rs58275168
rs62098130
rs76506738
All
−0.05
0.00
0.05
0.10
0.15
MR leave−one−out sensitivity analysis for
'CAG−170 sp003516765' on 'GCST90200473_buildGRCh38.tsv'

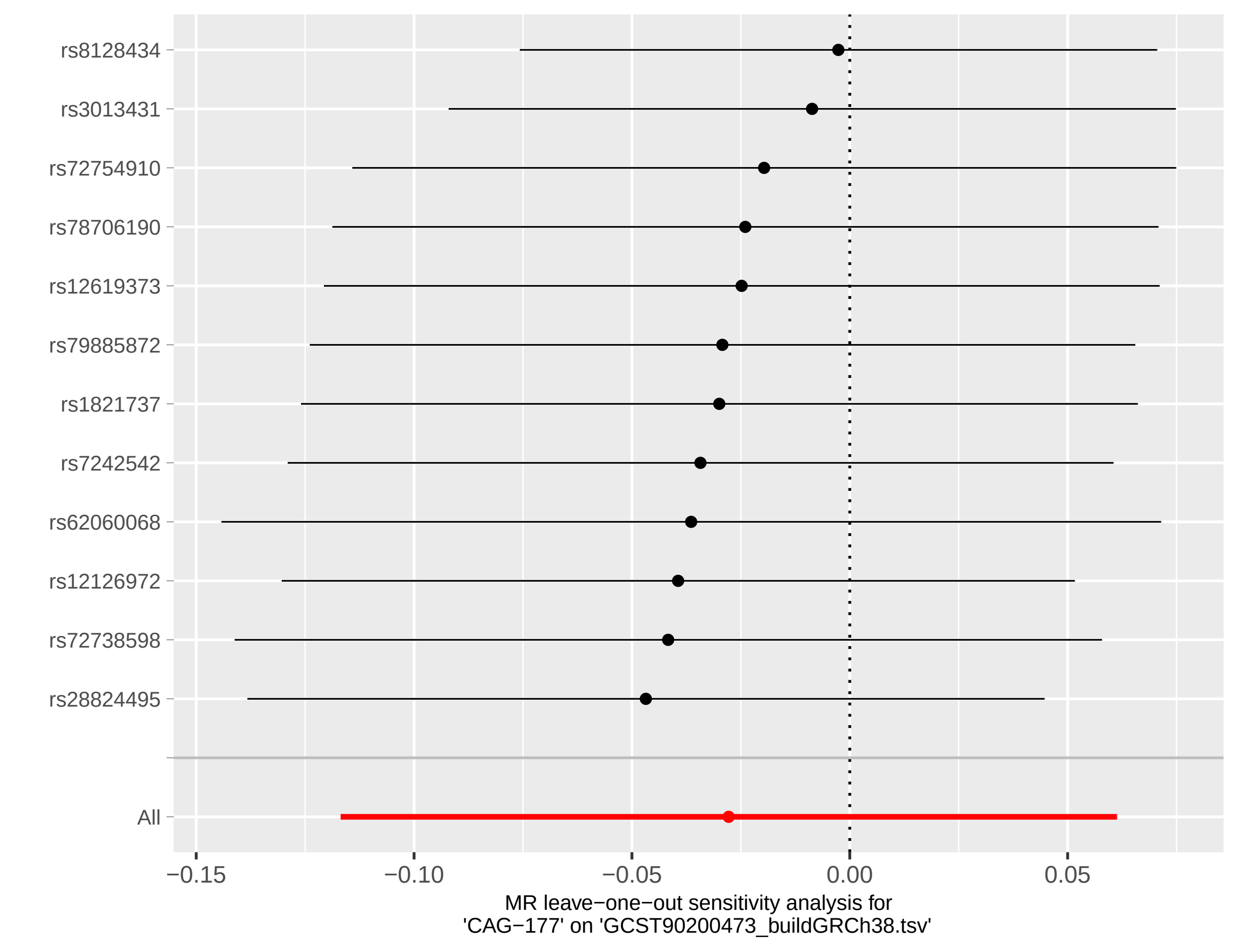

rs8128434
rs3013431
rs72754910
rs78706190
rs12619373
rs79885872
rs1821737
rs7242542
rs62060068
rs12126972
rs72738598
rs28824495
All
−0.15
−0.10
−0.05
0.00
0.05
MR leave−one−out sensitivity analysis for
'CAG−177' on 'GCST90200473_buildGRCh38.tsv'

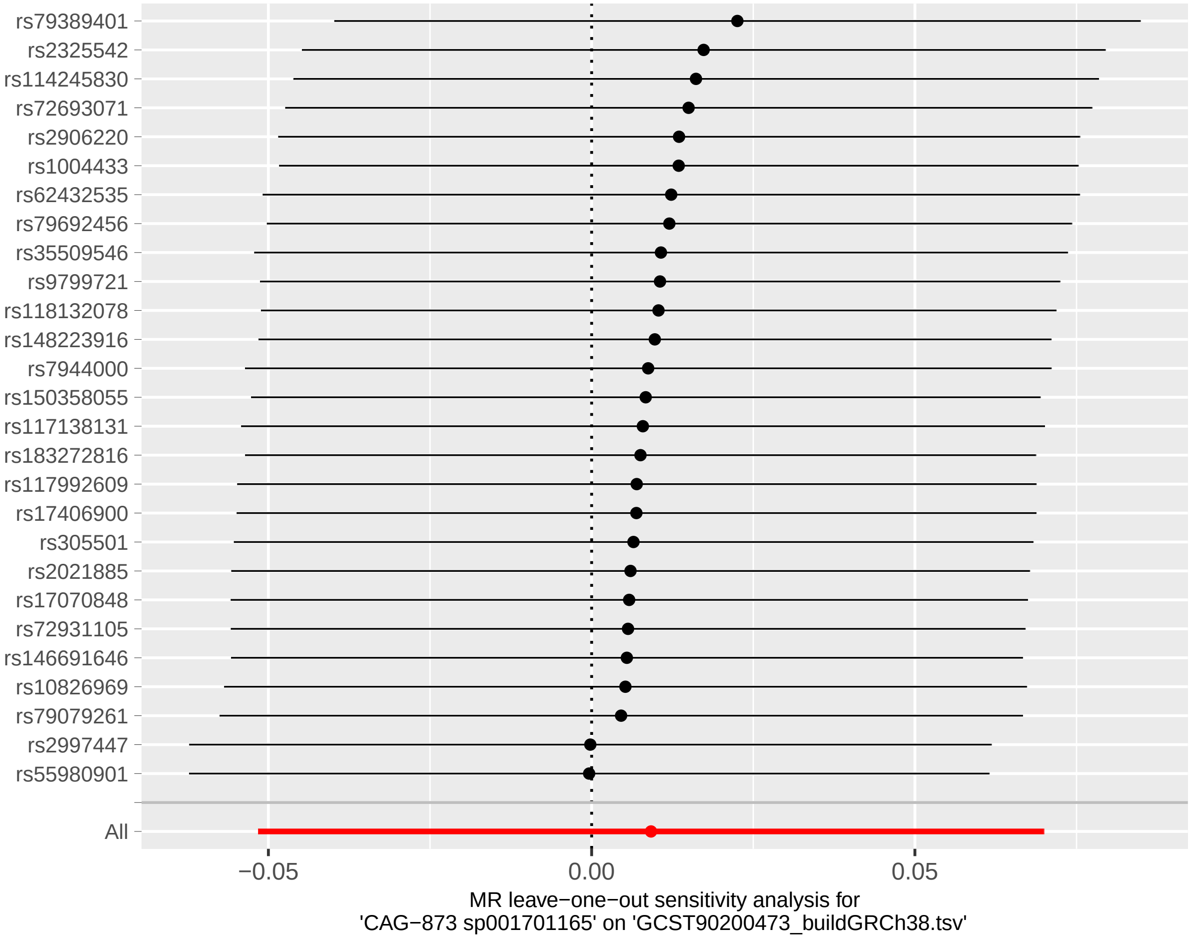

rs79389401
rs2325542
rs114245830
rs72693071
rs2906220
rs1004433
rs62432535
rs79692456
rs35509546
rs9799721
rs118132078
rs148223916
rs7944000
rs150358055
rs117138131
rs183272816
rs117992609
rs17406900
rs305501
rs2021885
rs17070848
rs72931105
rs146691646
rs10826969
rs79079261
rs2997447
rs55980901
All
−0.05
0.00
0.05
MR leave−one−out sensitivity analysis for
'CAG−873 sp001701165' on 'GCST90200473_buildGRCh38.tsv'

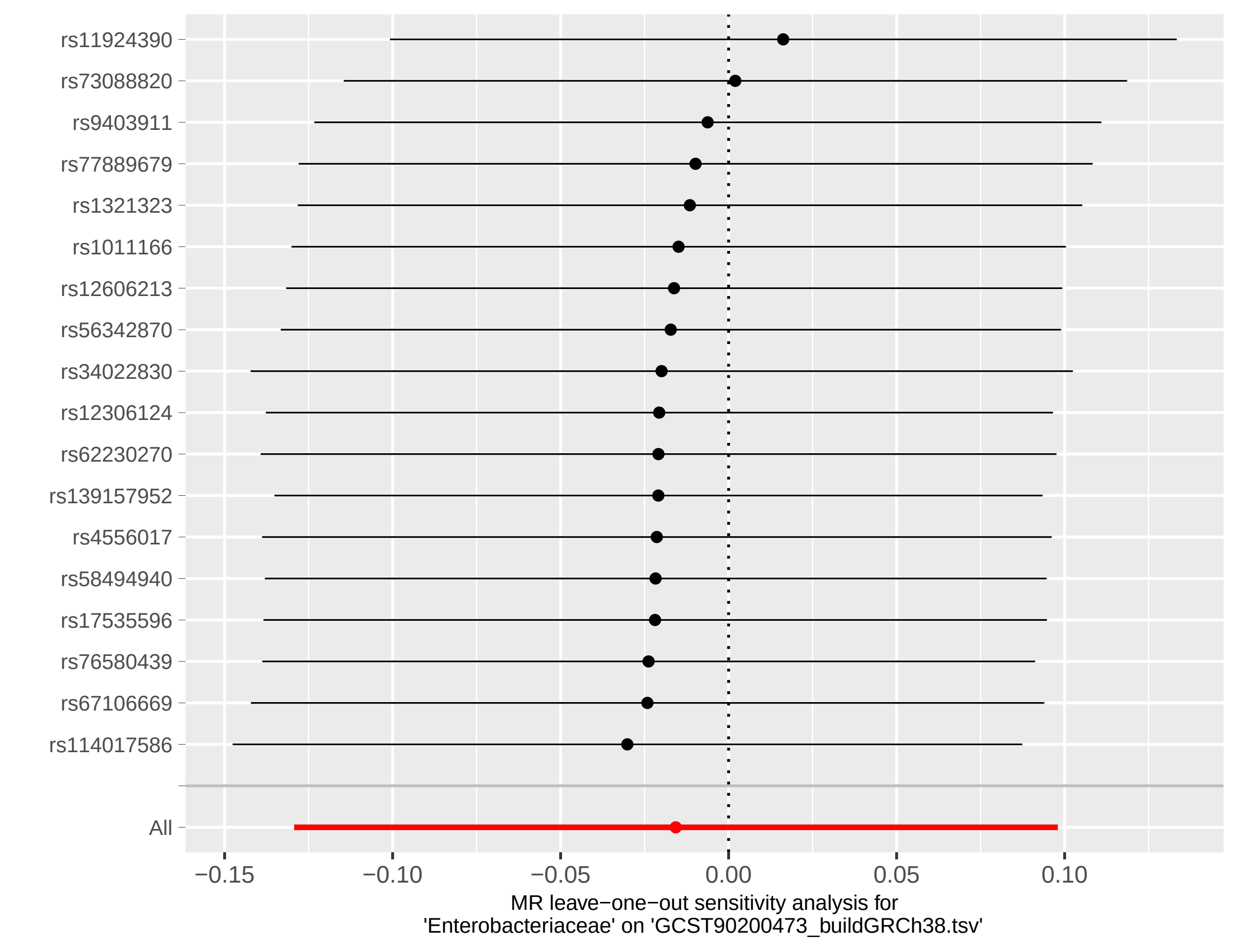

rs11924390
rs73088820
rs9403911
rs77889679
rs1321323
rs1011166
rs12606213
rs56342870
rs34022830
rs12306124
rs62230270
rs139157952
rs4556017
rs58494940
rs17535596
rs76580439
rs67106669
rs114017586
All
−0.15
−0.10
−0.05
0.00
0.05
0.10
MR leave−one−out sensitivity analysis for
'Enterobacteriaceae' on 'GCST90200473_buildGRCh38.tsv'

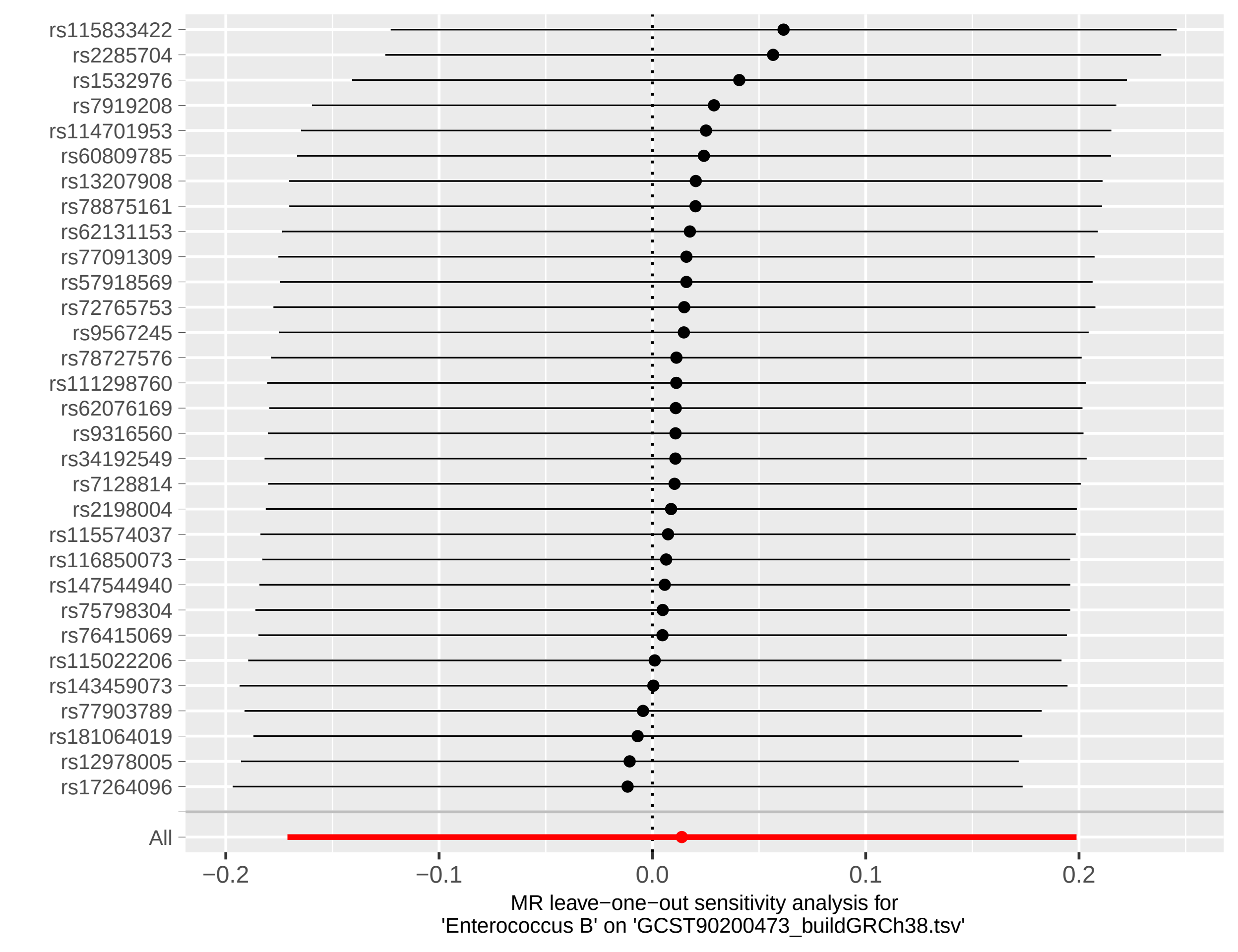
rs115833422
rs2285704
rs1532976
rs7919208
rs114701953
rs60809785
rs13207908
rs78875161
rs62131153
rs77091309
rs57918569
rs72765753
rs9567245
rs78727576
rs111298760
rs62076169
rs9316560
rs34192549
rs7128814
rs2198004
rs115574037
rs116850073
rs147544940
rs75798304
rs76415069
rs115022206
rs143459073
rs77903789
rs181064019
rs12978005
rs17264096
All
−0.2
−0.1
0.0
0.1
0.2
MR leave−one−out sensitivity analysis for
'Enterococcus B' on 'GCST90200473_buildGRCh38.tsv'

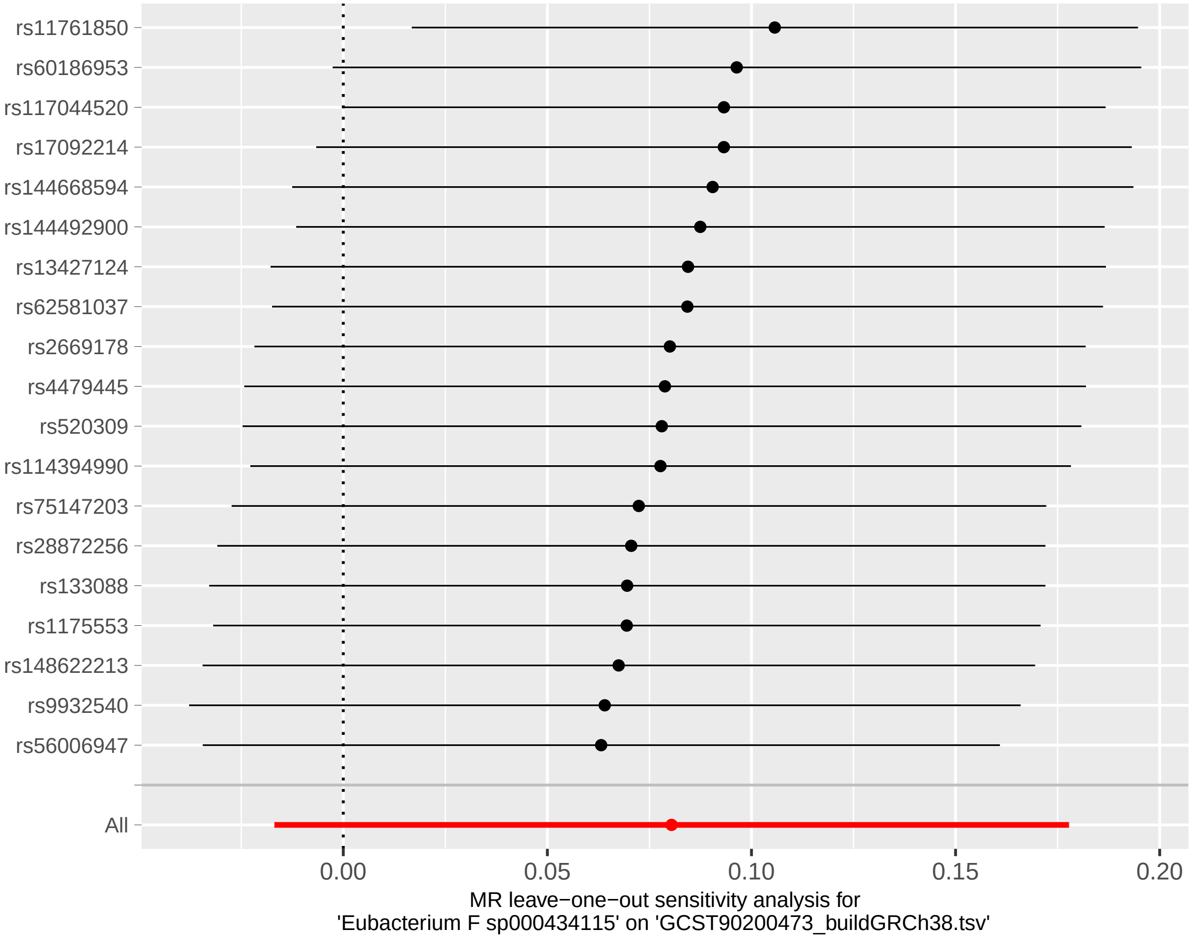
rs11761850
rs60186953
rs117044520
rs17092214
rs144668594
rs144492900
rs13427124
rs62581037
rs2669178
rs4479445
rs520309
rs114394990
rs75147203
rs28872256
rs133088
rs1175553
rs148622213
rs9932540
rs56006947
All
0.00
0.05
0.10
0.15
0.20
MR leave−one−out sensitivity analysis for
'Eubacterium F sp000434115' on 'GCST90200473_buildGRCh38.tsv'

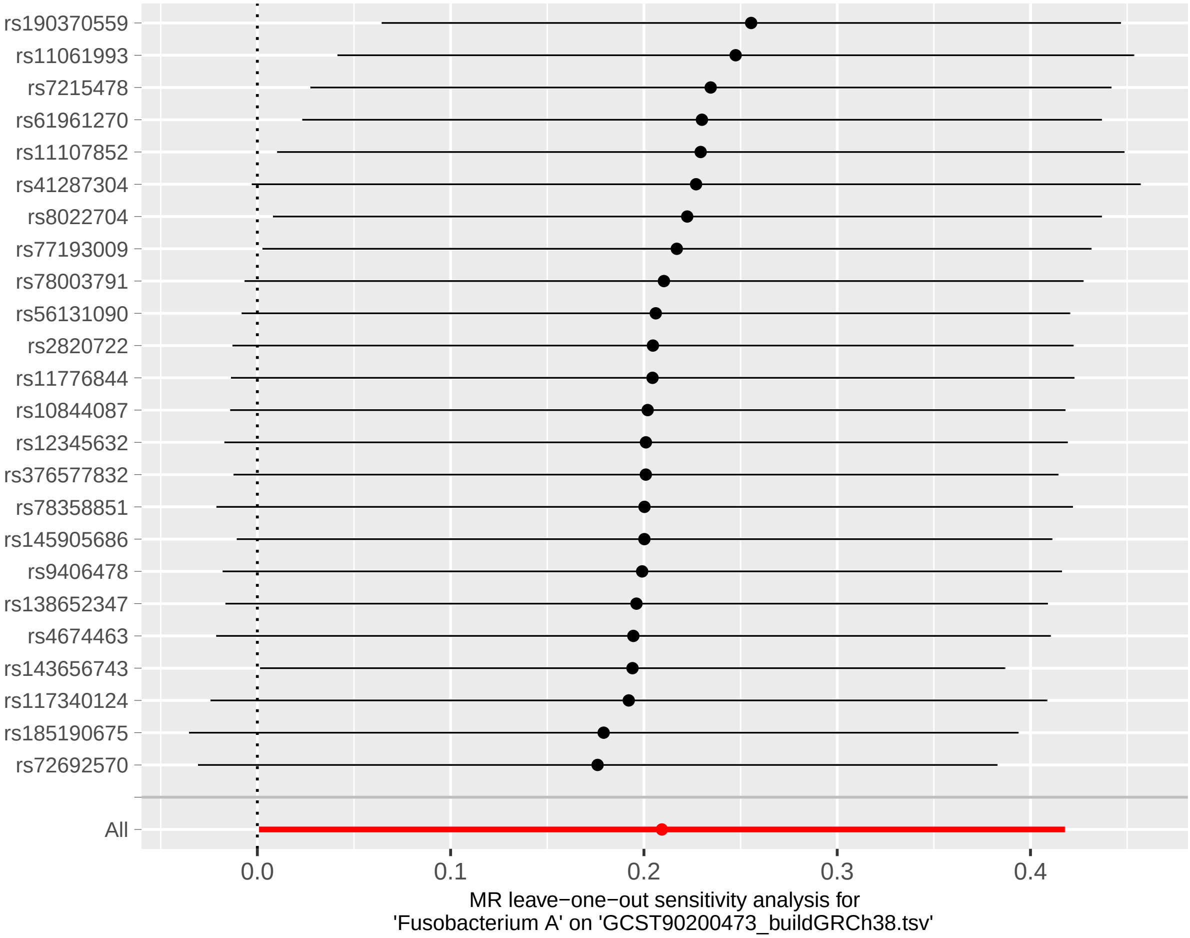
rs190370559
rs11061993
rs7215478
rs61961270
rs11107852
rs41287304
rs8022704
rs77193009
rs78003791
rs56131090
rs2820722
rs11776844
rs10844087
rs12345632
rs376577832
rs78358851
rs145905686
rs9406478
rs138652347
rs4674463
rs143656743
rs117340124
rs185190675
rs72692570
All
0.0
0.1
0.2
0.3
0.4
MR leave−one−out sensitivity analysis for
'Fusobacterium A' on 'GCST90200473_buildGRCh38.tsv'

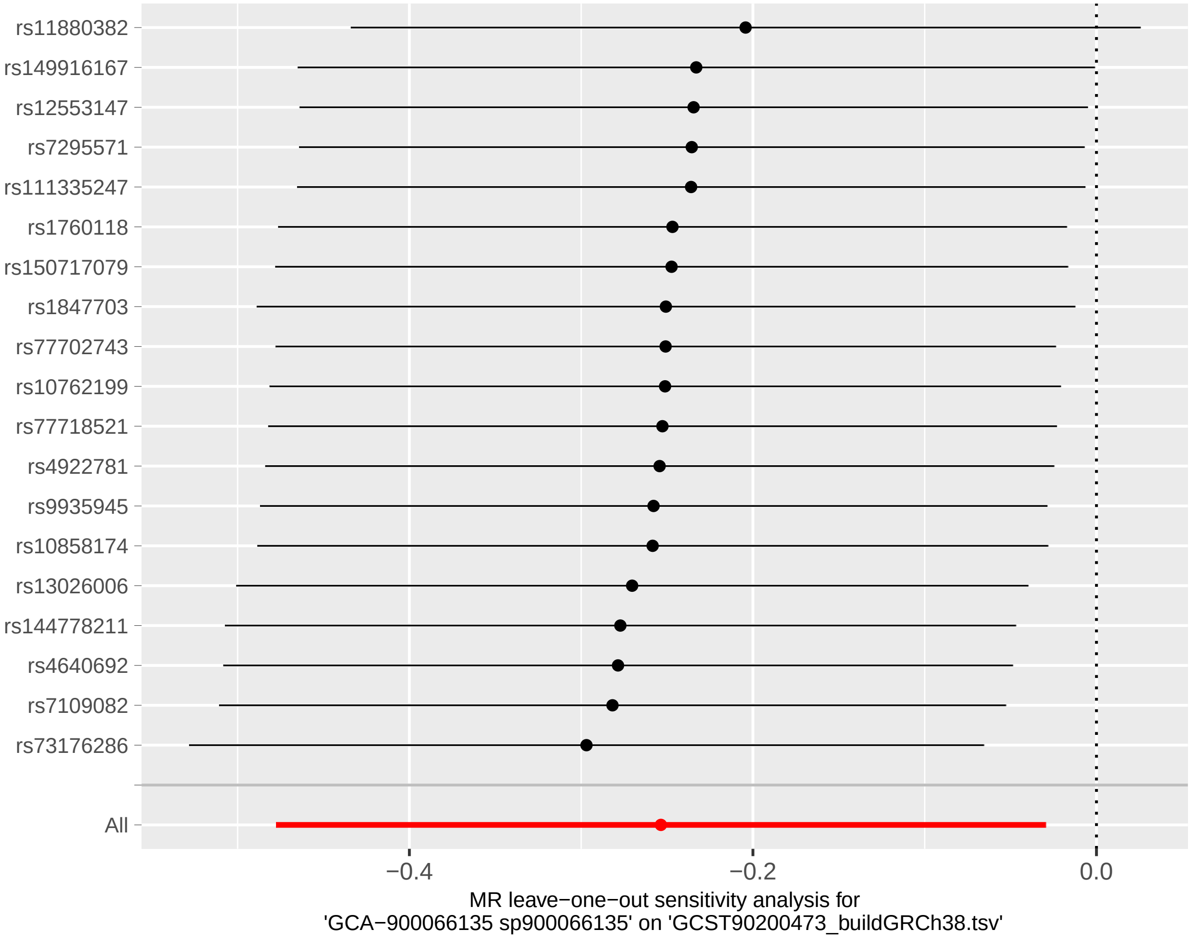
rs11880382
rs149916167
rs12553147
rs7295571
rs111335247
rs1760118
rs150717079
rs1847703
rs77702743
rs10762199
rs77718521
rs4922781
rs9935945
rs10858174
rs13026006
rs144778211
rs4640692
rs7109082
rs73176286
All
−0.4
−0.2
0.0
MR leave−one−out sensitivity analysis for
'GCA−900066135 sp900066135' on 'GCST90200473_buildGRCh38.tsv'

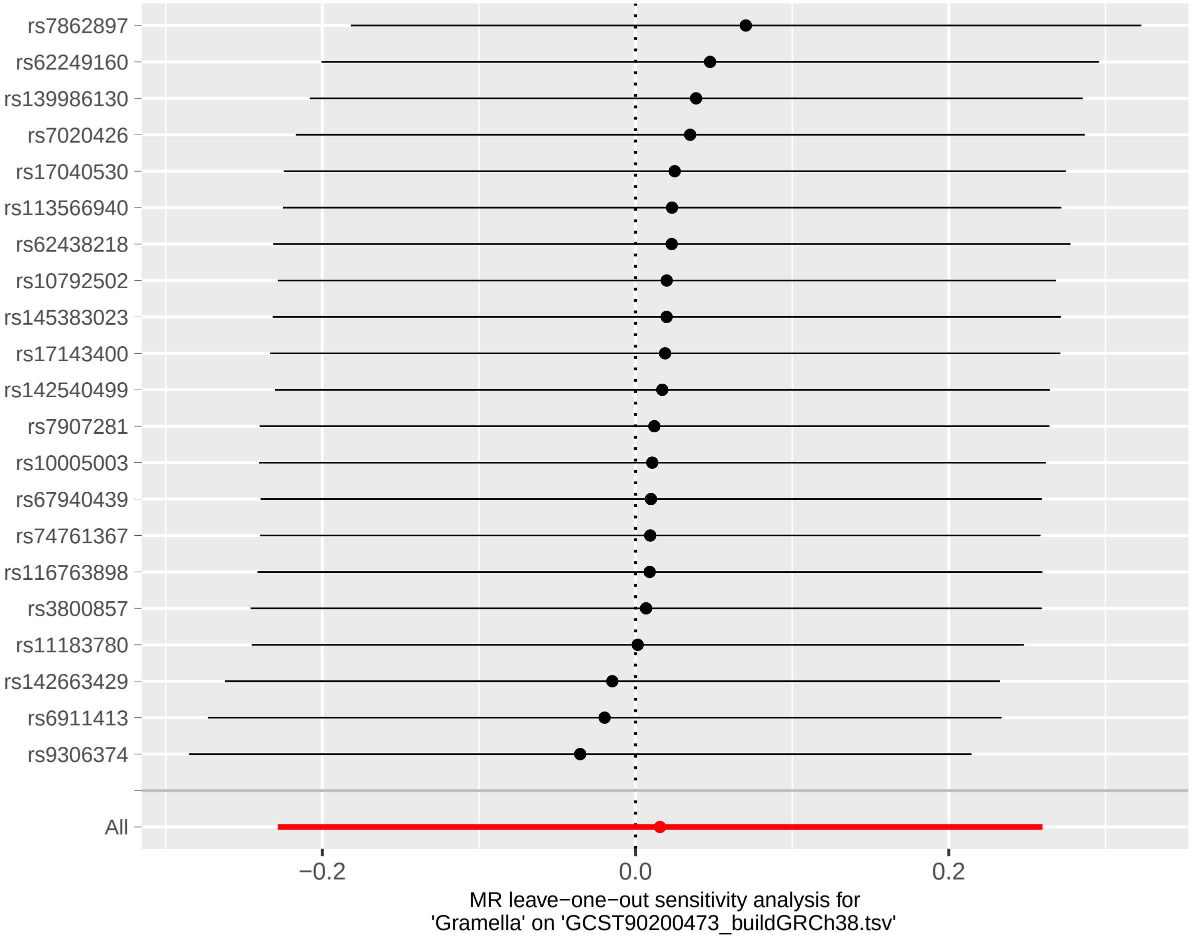
rs7862897
rs62249160
rs139986130
rs7020426
rs17040530
rs113566940
rs62438218
rs10792502
rs145383023
rs17143400
rs142540499
rs7907281
rs10005003
rs67940439
rs74761367
rs116763898
rs3800857
rs11183780
rs142663429
rs6911413
rs9306374
All
−0.2
0.0
0.2
MR leave−one−out sensitivity analysis for
'Gramella' on 'GCST90200473_buildGRCh38.tsv'

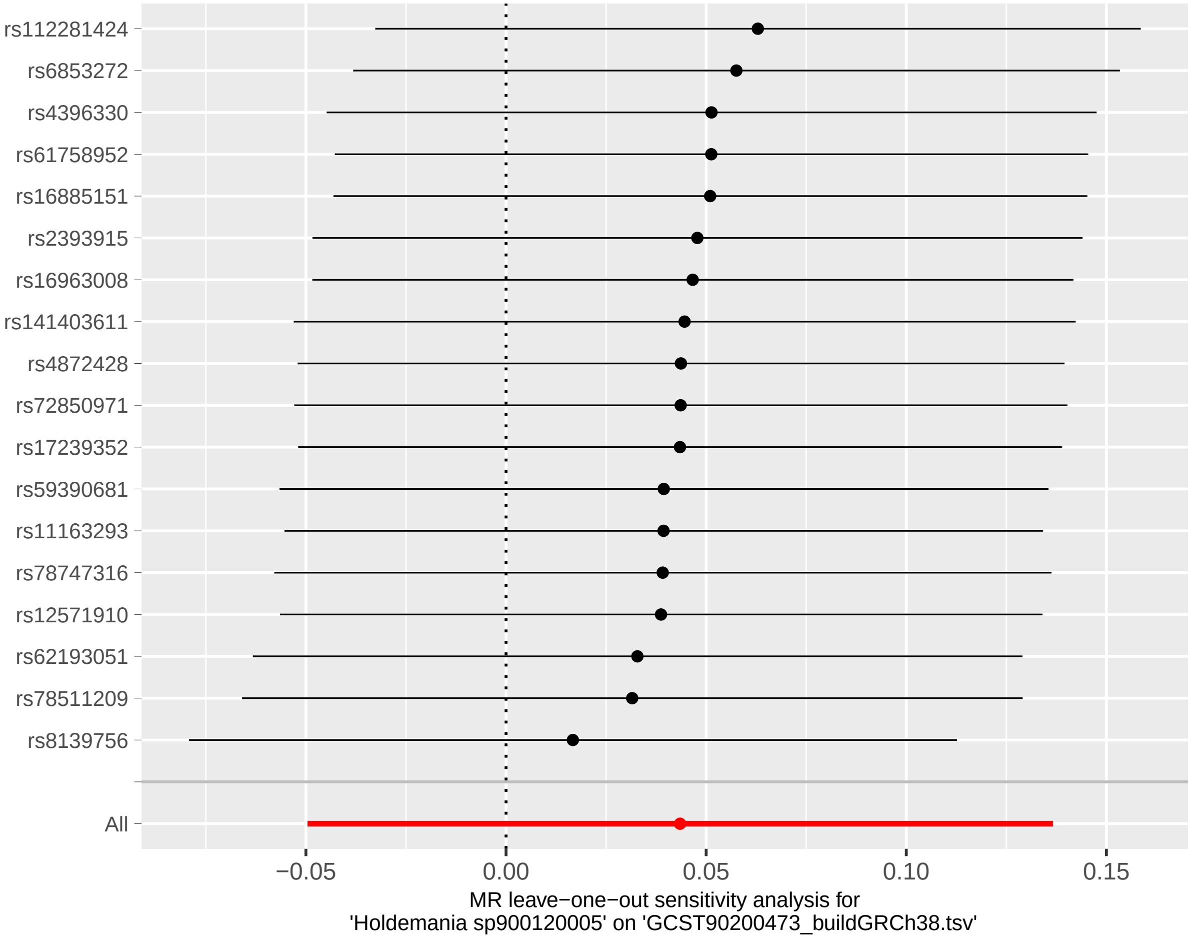

rs112281424
rs6853272
rs4396330
rs61758952
rs16885151
rs2393915
rs16963008
rs141403611
rs4872428
rs72850971
rs17239352
rs59390681
rs11163293
rs78747316
rs12571910
rs62193051
rs78511209
rs8139756
All
−0.05
0.00
0.05
0.10
0.15
MR leave−one−out sensitivity analysis for
'Holdemania sp900120005' on 'GCST90200473_buildGRCh38.tsv'

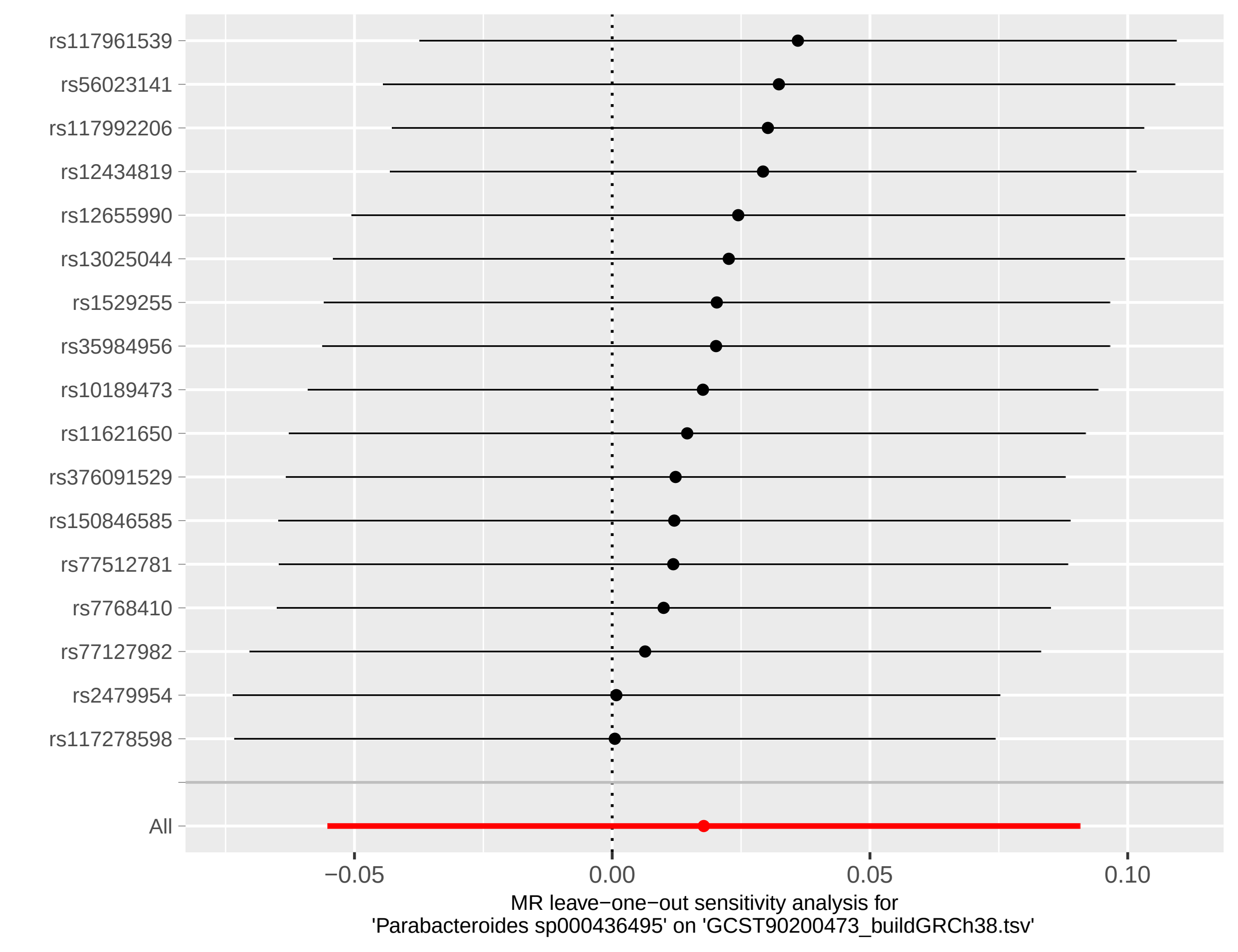
rs117961539
rs56023141
rs117992206
rs12434819
rs12655990
rs13025044
rs1529255
rs35984956
rs10189473
rs11621650
rs376091529
rs150846585
rs77512781
rs7768410
rs77127982
rs2479954
rs117278598
All
−0.05
0.00
0.05
0.10
MR leave−one−out sensitivity analysis for
'Parabacteroides sp000436495' on 'GCST90200473_buildGRCh38.tsv'

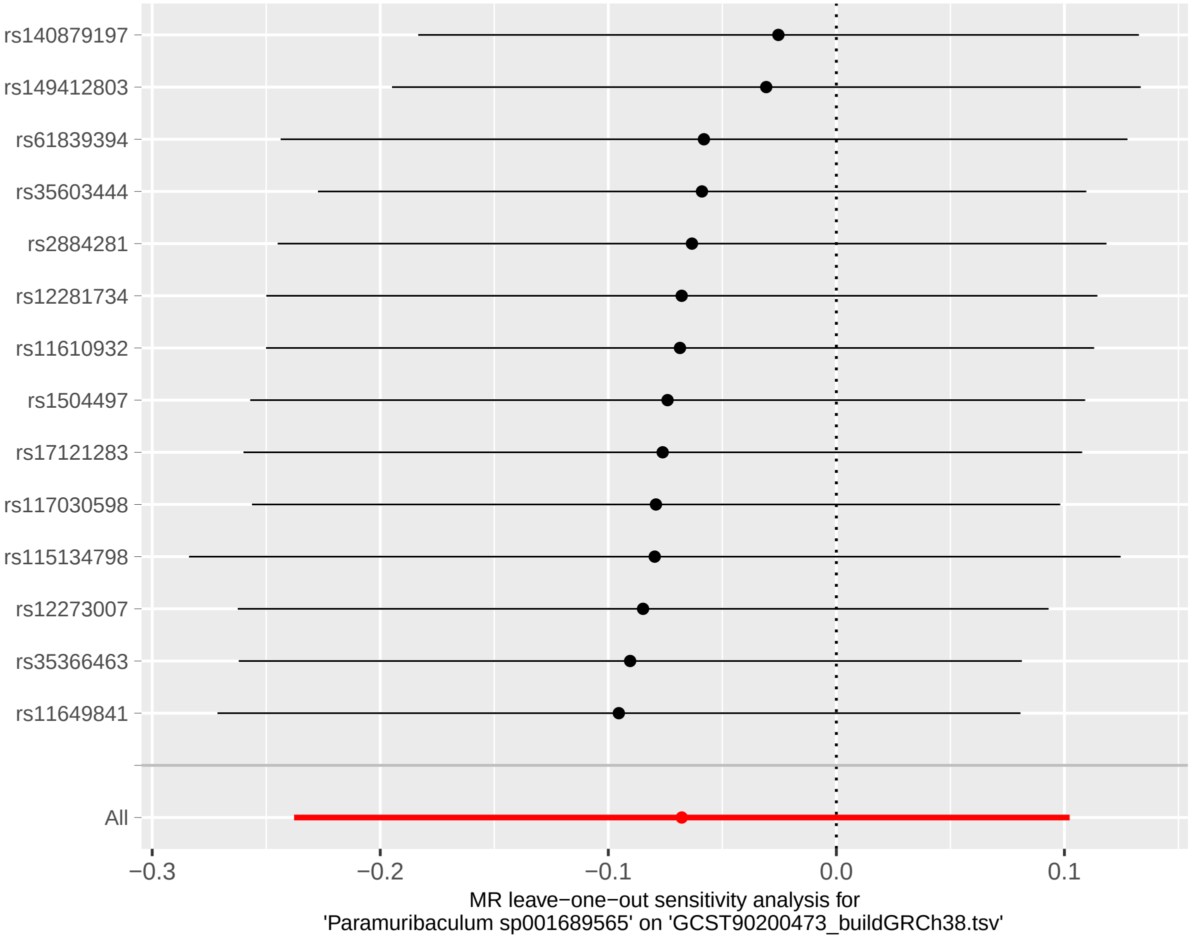
rs140879197
rs149412803
rs61839394
rs35603444
rs2884281
rs12281734
rs11610932
rs1504497
rs17121283
rs117030598
rs115134798
rs12273007
rs35366463
rs11649841
All
−0.3
−0.2
−0.1
0.0
0.1
MR leave−one−out sensitivity analysis for
'Paramuribaculum sp001689565' on 'GCST90200473_buildGRCh38.tsv'

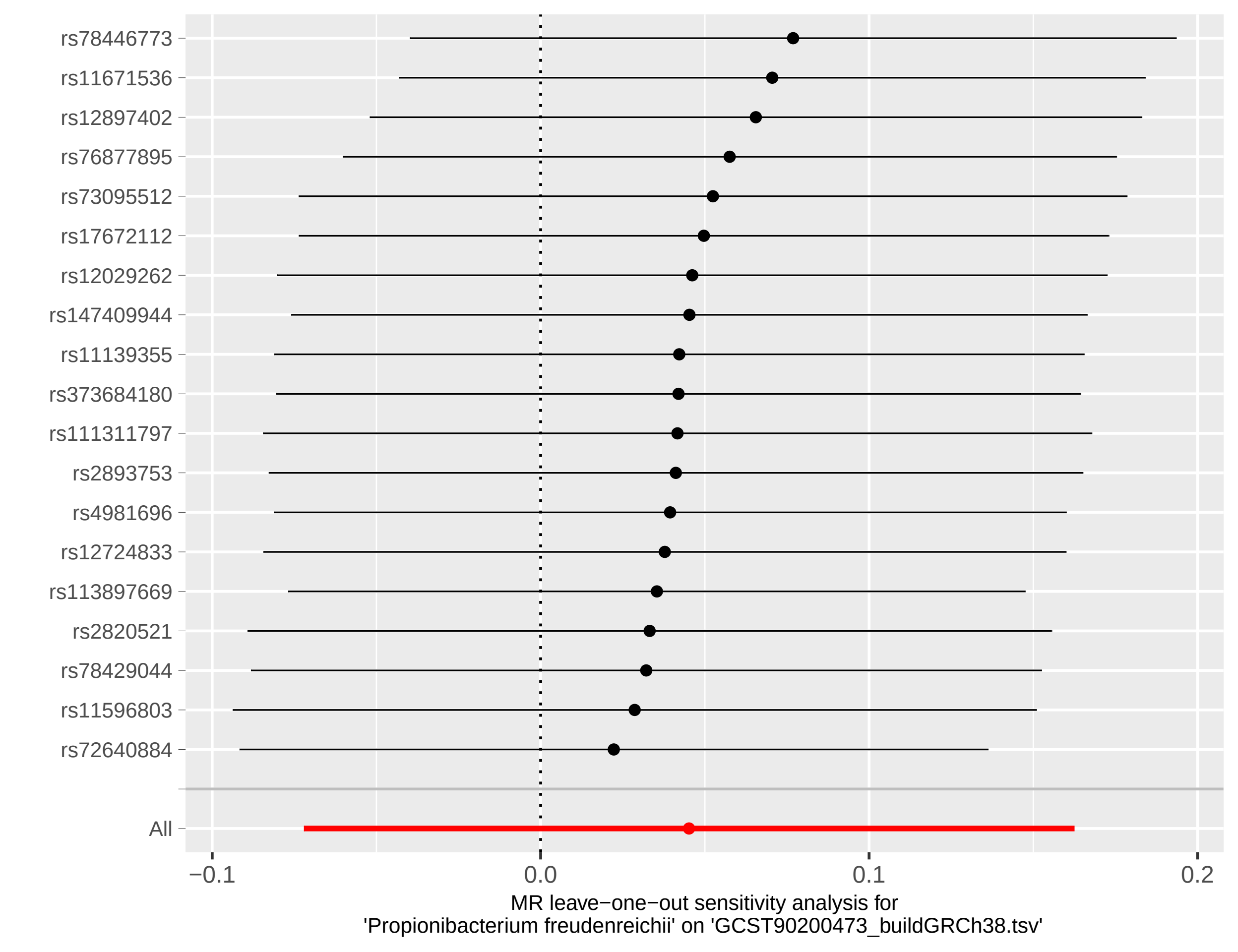
rs78446773
rs11671536
rs12897402
rs76877895
rs73095512
rs17672112
rs12029262
rs147409944
rs11139355
rs373684180
rs111311797
rs2893753
rs4981696
rs12724833
rs113897669
rs2820521
rs78429044
rs11596803
rs72640884
All
−0.1
0.0
0.1
0.2
MR leave−one−out sensitivity analysis for
'Propionibacterium freudenreichii' on 'GCST90200473_buildGRCh38.tsv'

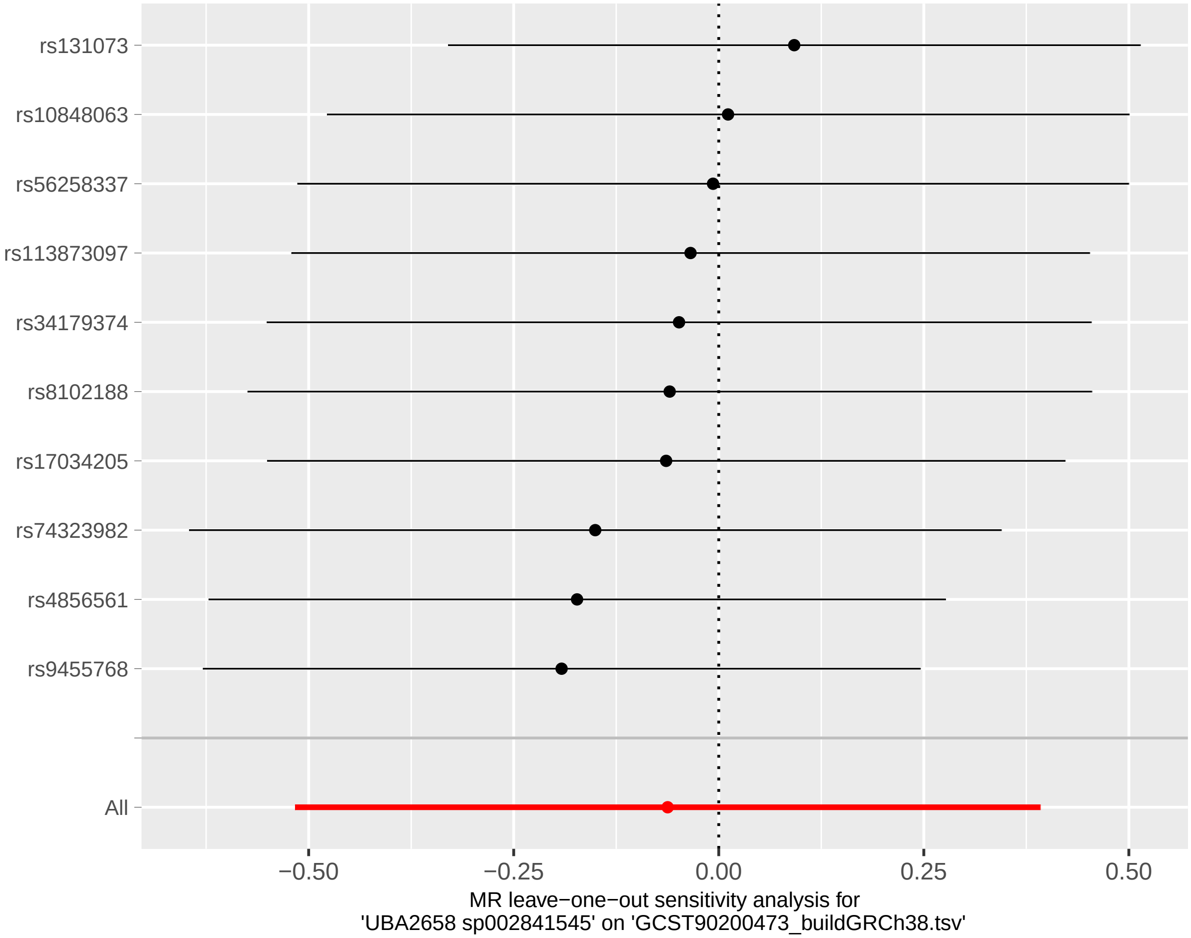
rs131073
rs10848063
rs56258337
rs113873097
rs34179374
rs8102188
rs17034205
rs74323982
rs4856561
rs9455768
All
−0.50
−0.25
0.00
0.25
0.50
MR leave−one−out sensitivity analysis for
'UBA2658 sp002841545' on 'GCST90200473_buildGRCh38.tsv'

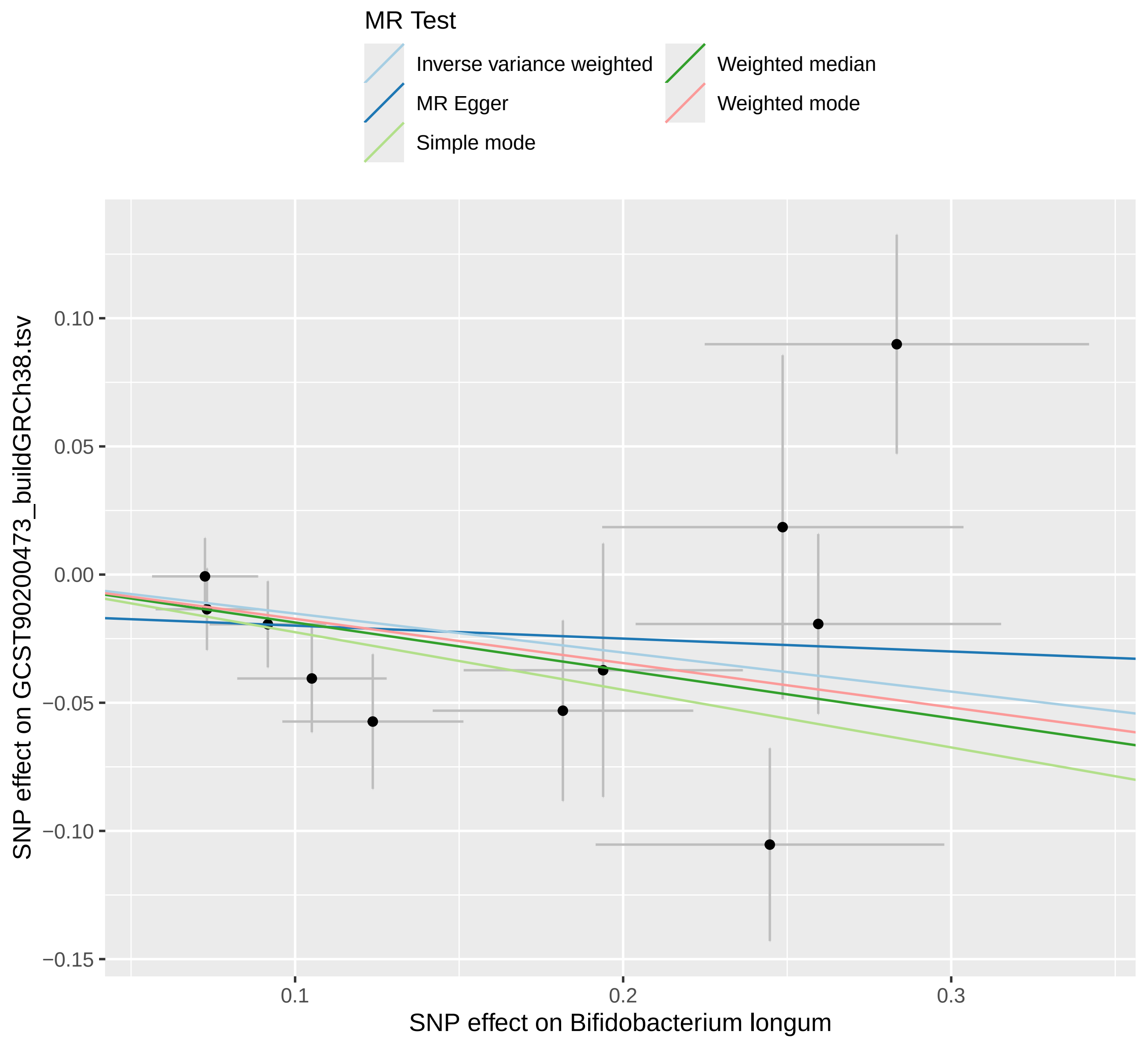

MR Test
Inverse variance weighted
MR Egger
Simple mode
Weighted median
Weighted mode
0.10
0.05
0.00
−0.05
−0.10
−0.15
SNP effect on GCST90200473_buildGRCh38.tsv
0.1
0.2
0.3
SNP effect on Bifidobacterium longum

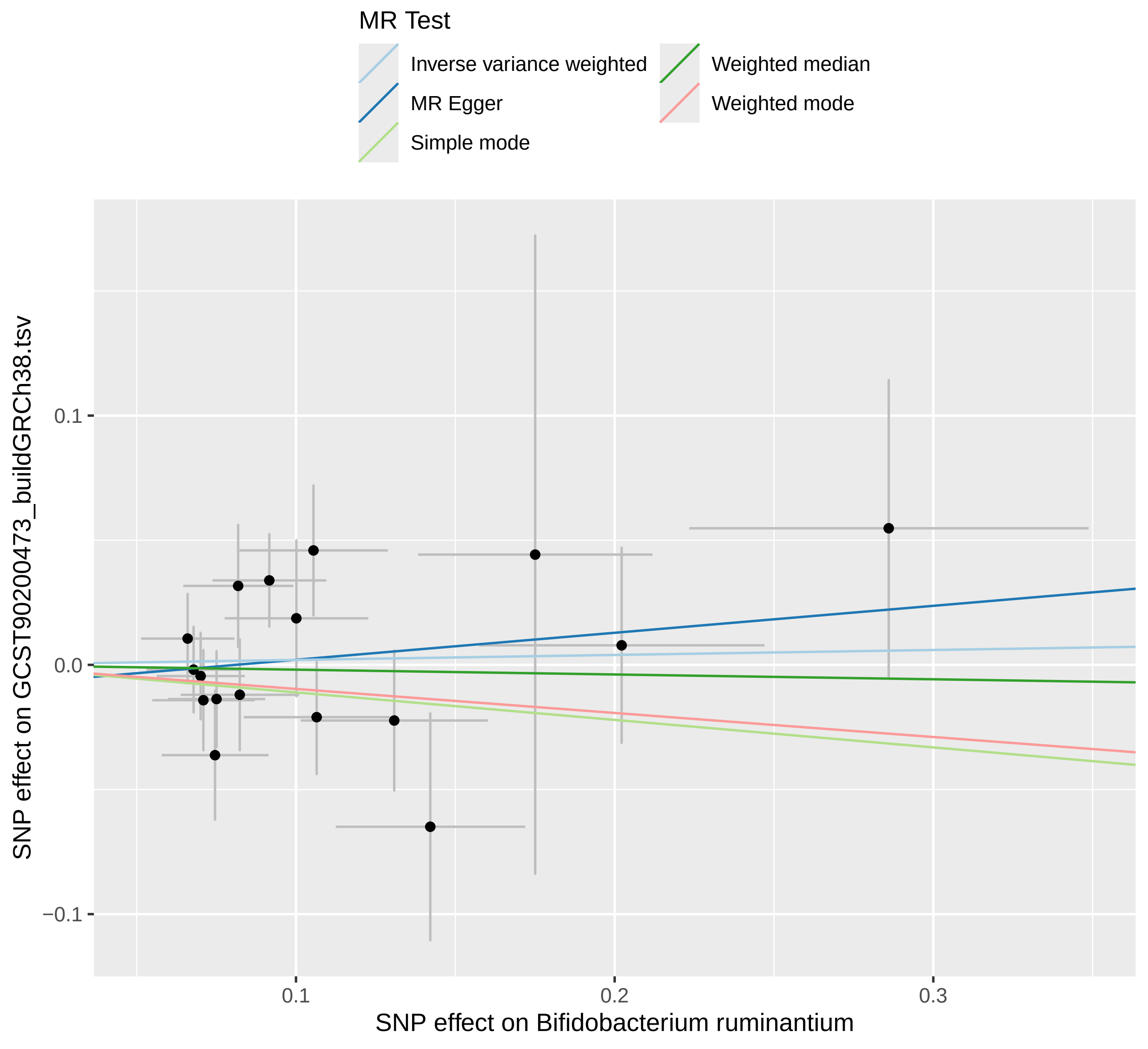
MR Test
Inverse variance weighted
MR Egger
Simple mode
Weighted median
Weighted mode
0.1
0.0
−0.1
0.1
0.2
0.3
SNP effect on GCST90200473_buildGRCh38.tsv
SNP effect on Bifidobacterium ruminantium

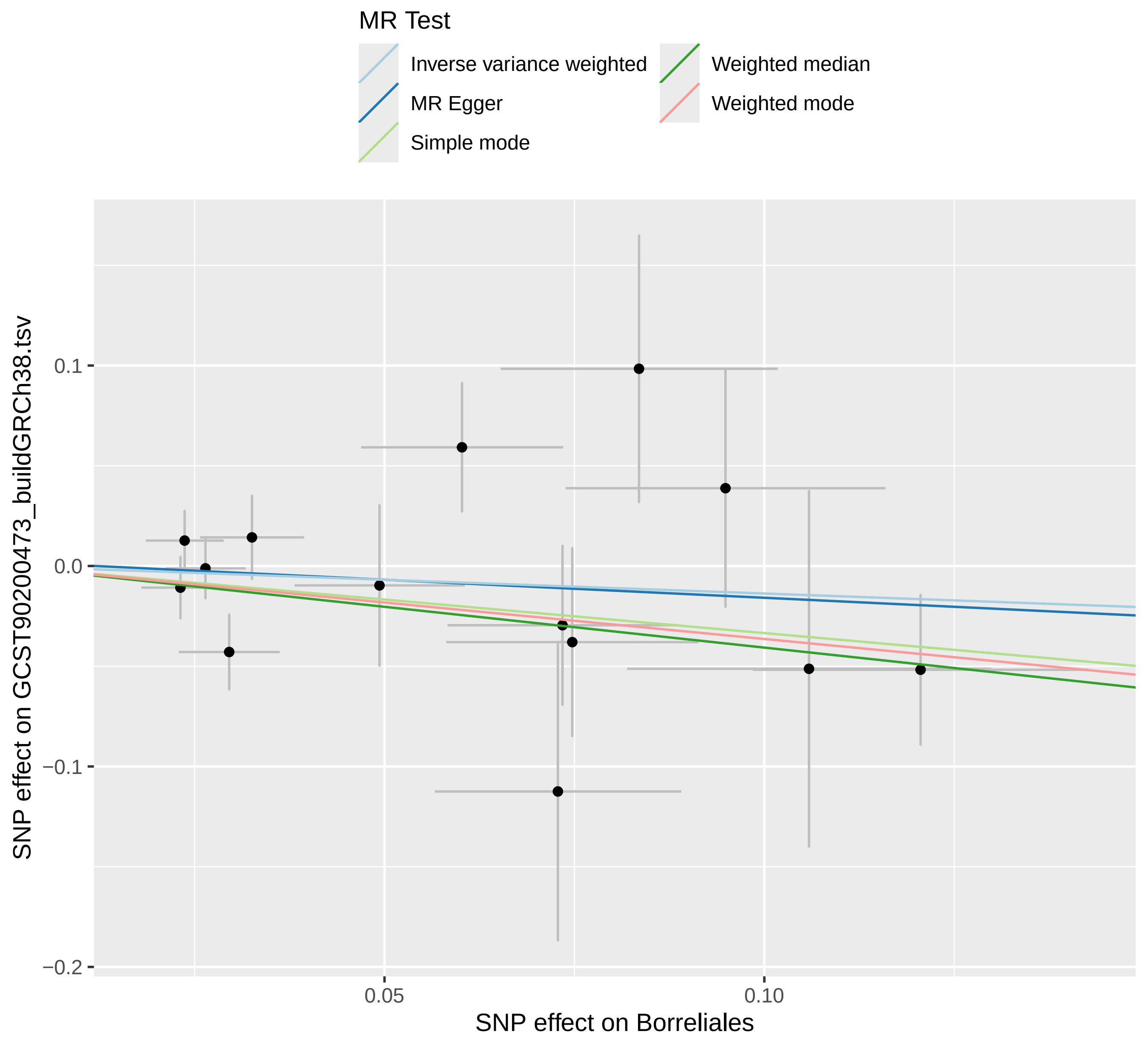

MR Test
Inverse variance weighted
MR Egger
Simple mode
Weighted median
Weighted mode
0.1
0.0
−0.1
−0.2
0.05
0.10
SNP effect on GCST90200473_buildGRCh38.tsv
SNP effect on Borreliales

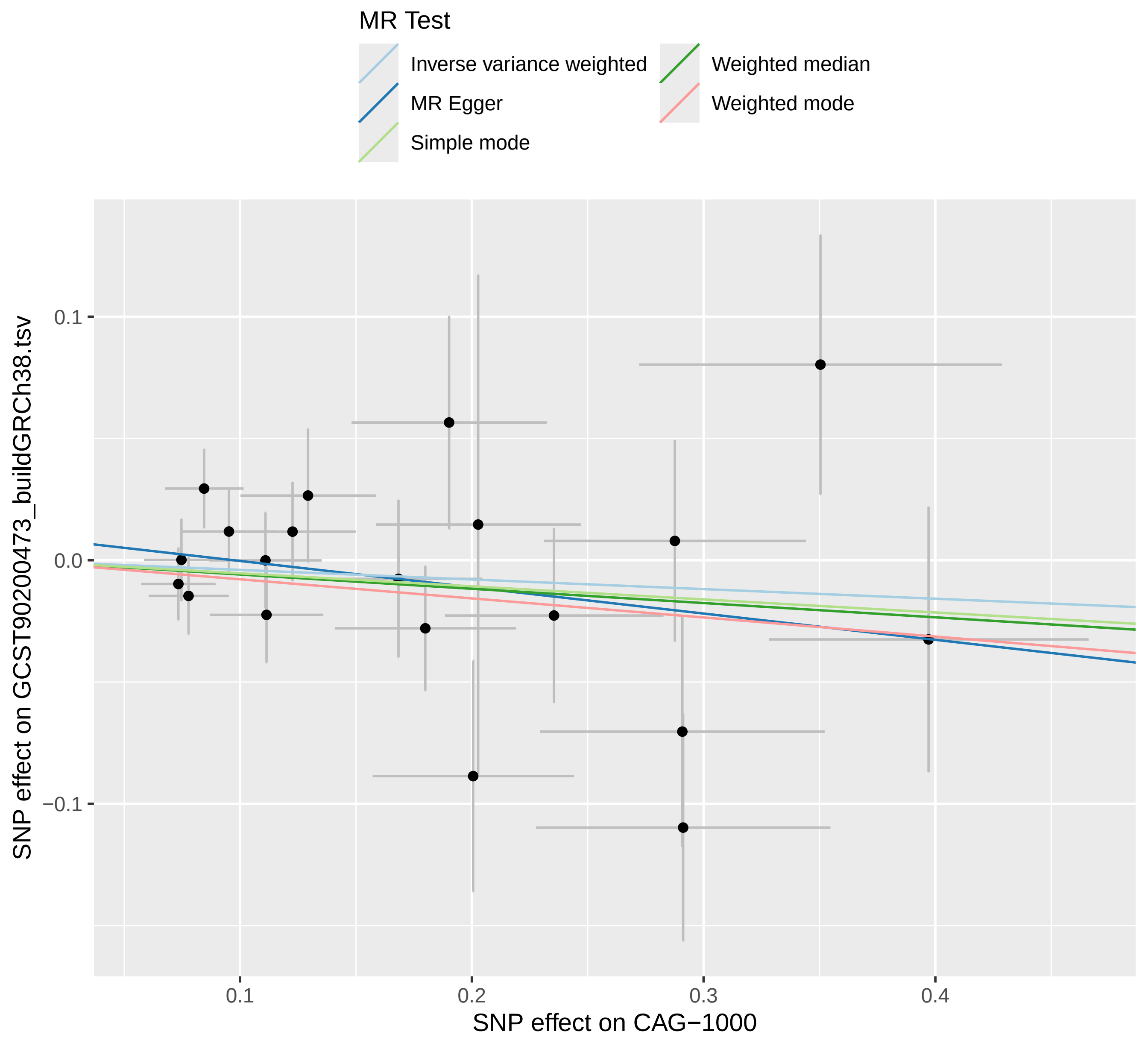
MR Test
Inverse variance weighted
MR Egger
Simple mode
Weighted median
Weighted mode
0.1
0.0
−0.1
SNP effect on GCST90200473_buildGRCh38.tsv
0.1
0.2
0.3
0.4
SNP effect on CAG−1000

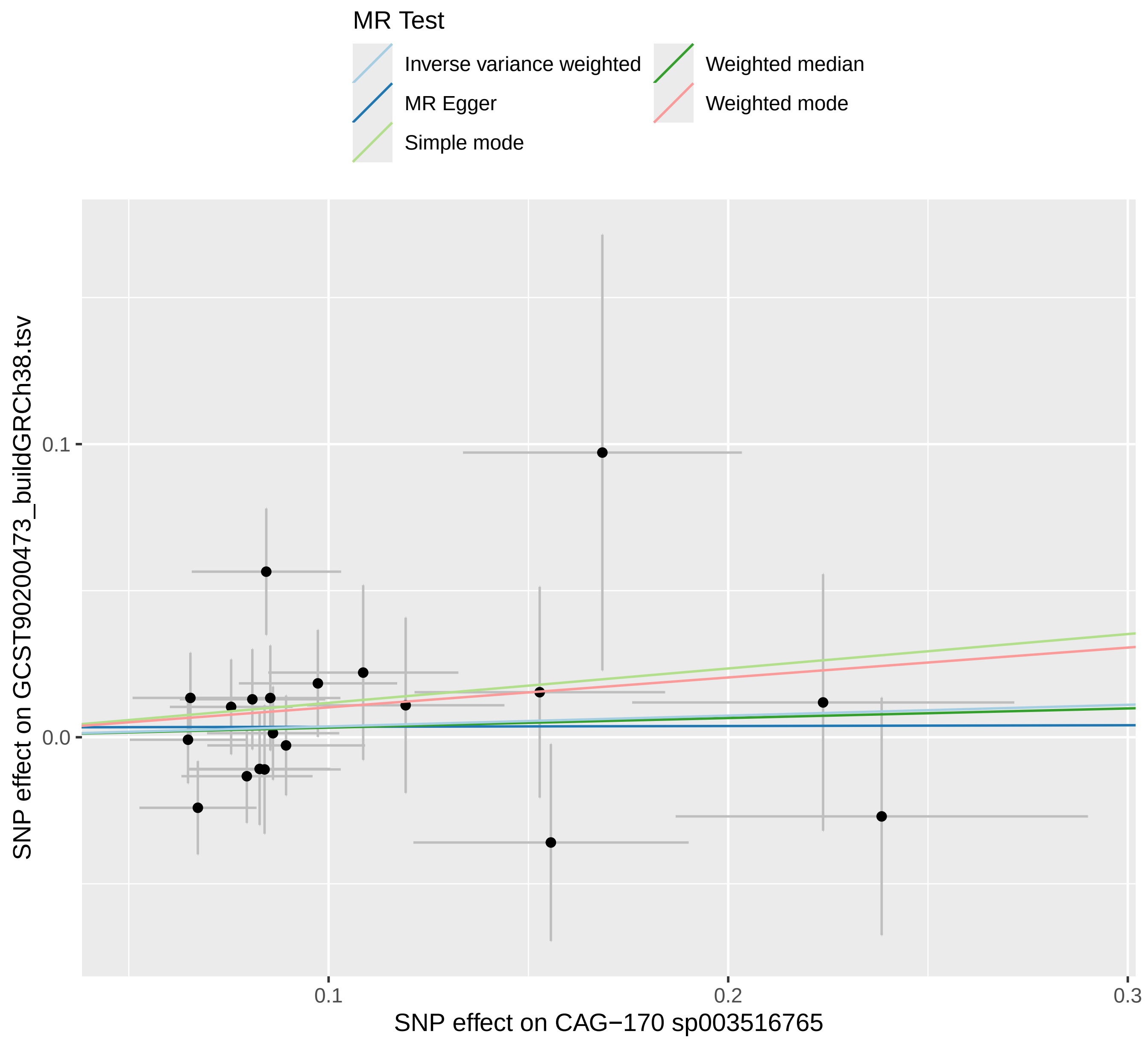
MR Test
Inverse variance weighted
MR Egger
Simple mode
Weighted median
Weighted mode
SNP effect on GCST90200473_buildGRCh38.tsv
0.1
0.0
0.1
0.2
0.3
SNP effect on CAG−170 sp003516765

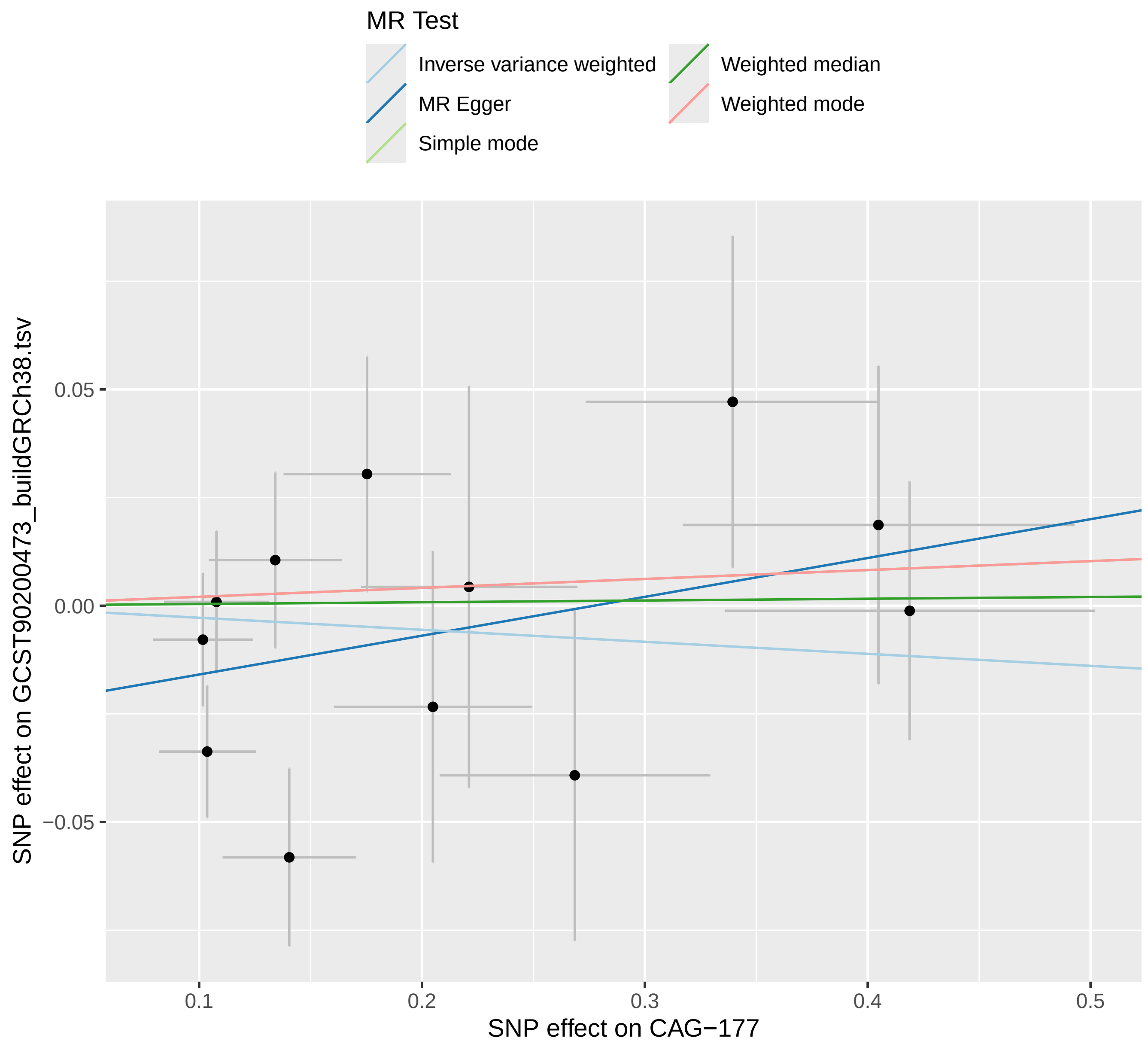

MR Test
Inverse variance weighted
MR Egger
Simple mode
Weighted median
Weighted mode
SNP effect on GCST90200473_buildGRCh38.tsv
0.05
0.00
−0.05
0.1
0.2
0.3
0.4
0.5
SNP effect on CAG−177

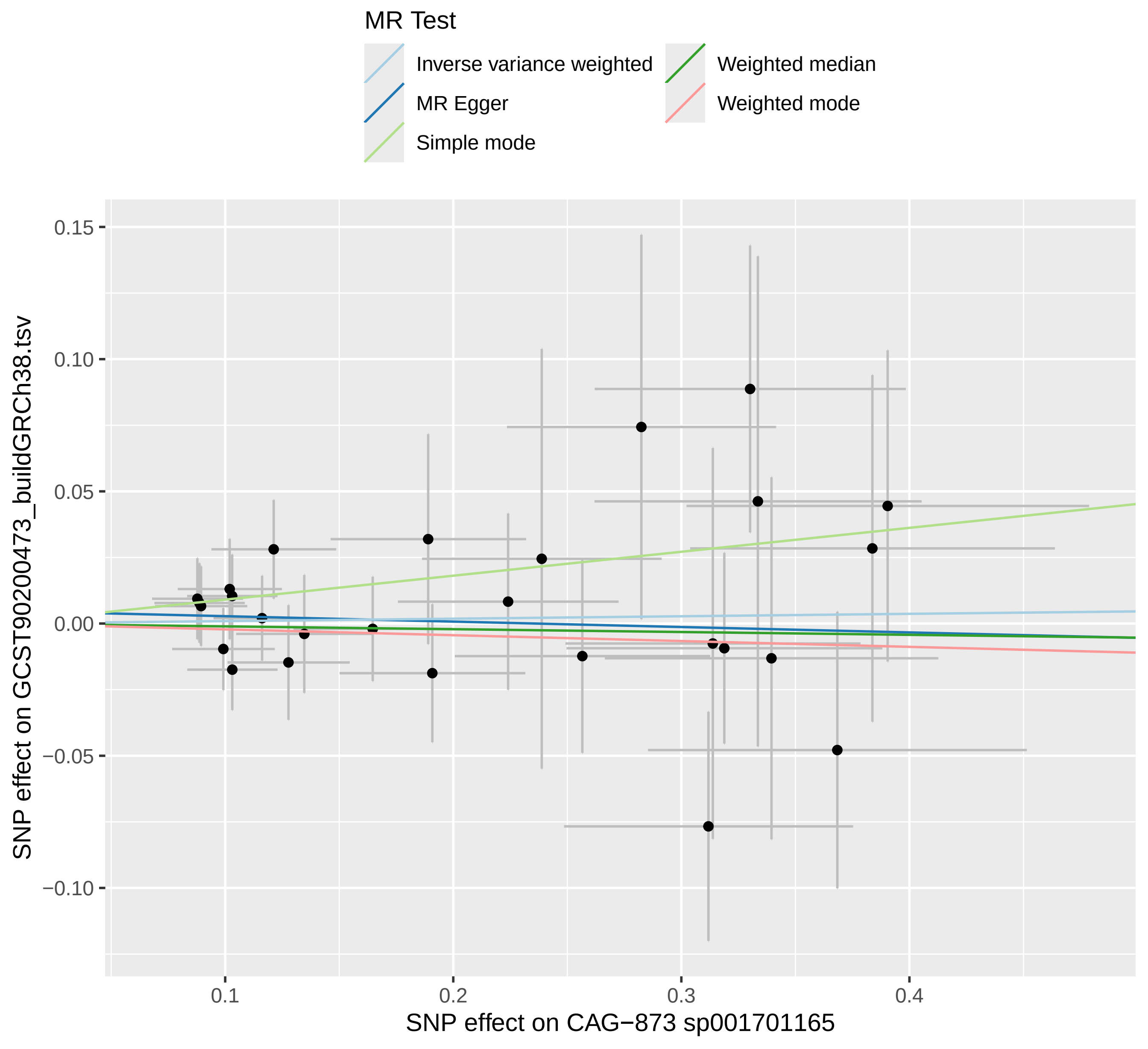

MR Test
Inverse variance weighted
MR Egger
Simple mode
Weighted median
Weighted mode
0.15
0.10
0.05
0.00
−0.05
−0.10
0.1
0.2
0.3
0.4
SNP effect on GCST90200473_buildGRCh38.tsv
SNP effect on CAG−873 sp001701165

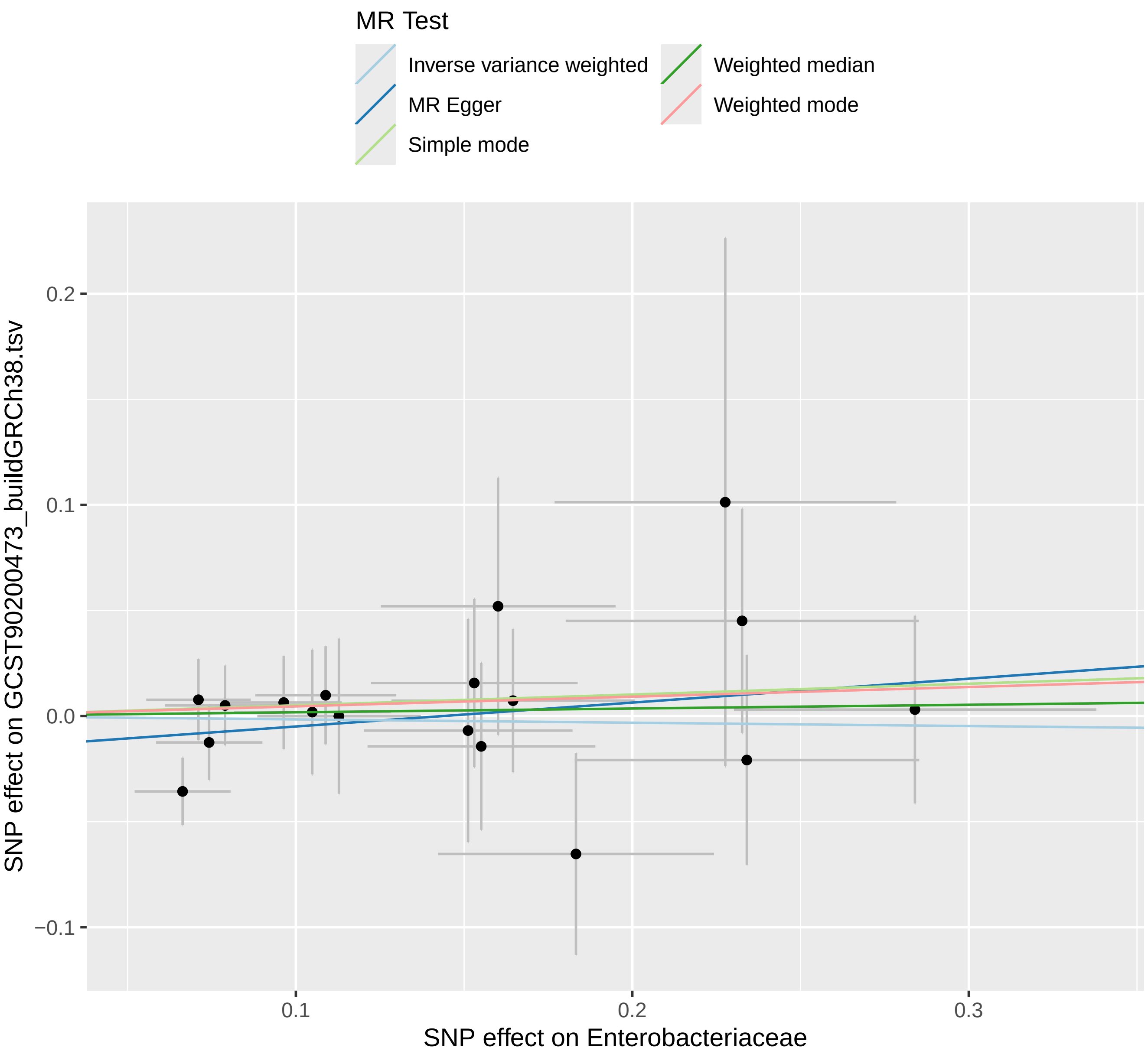

MR Test
Inverse variance weighted
MR Egger
Simple mode
Weighted median
Weighted mode
0.2
0.1
0.0
−0.1
SNP effect on GCST90200473_buildGRCh38.tsv
0.1
0.2
0.3
SNP effect on Enterobacteriaceae

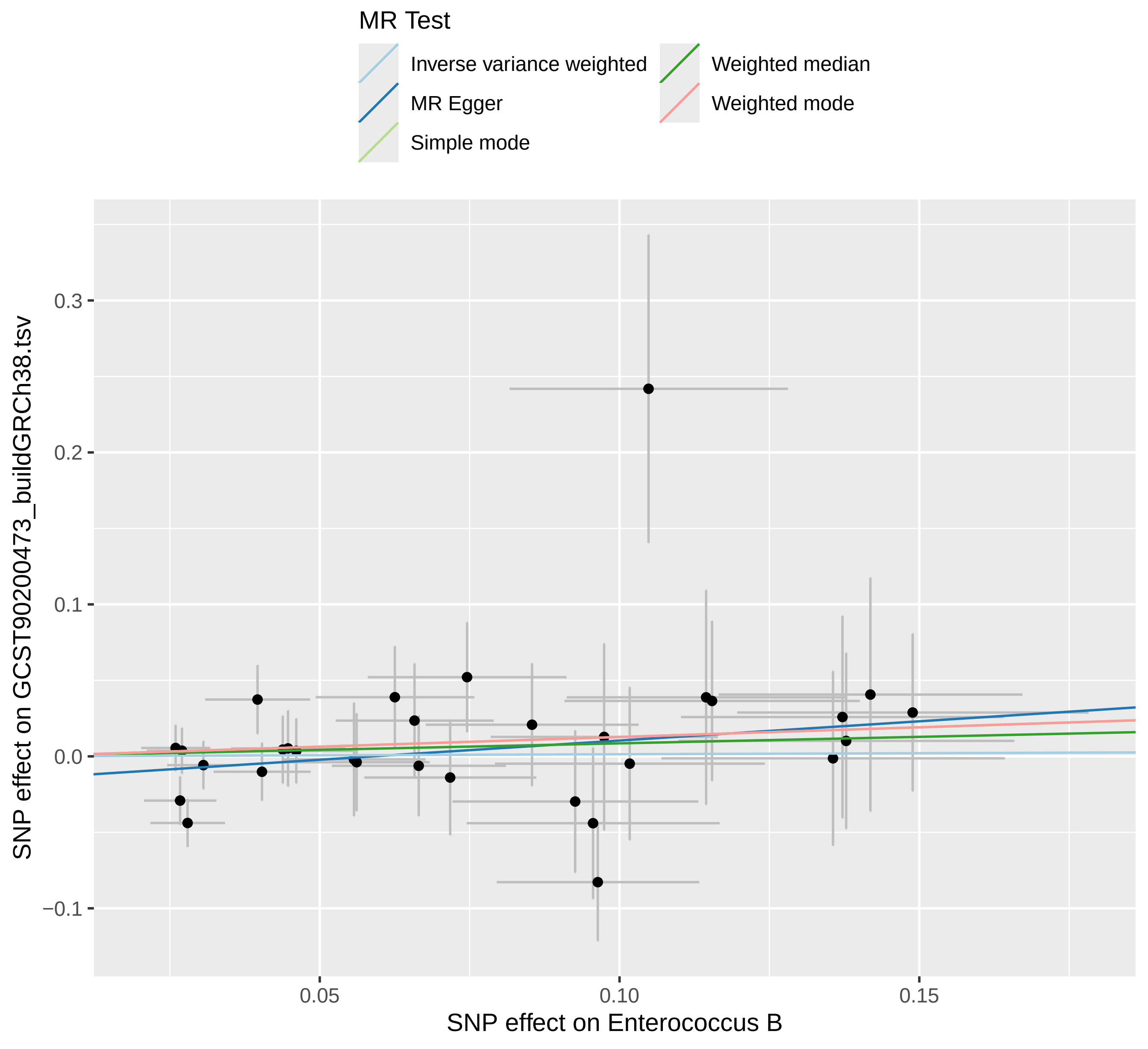
MR Test
Inverse variance weighted
MR Egger
Simple mode
Weighted median
Weighted mode
0.3
0.2
0.1
0.0
−0.1
SNP effect on GCST90200473_buildGRCh38.tsv
0.05
0.10
0.15
SNP effect on Enterococcus B

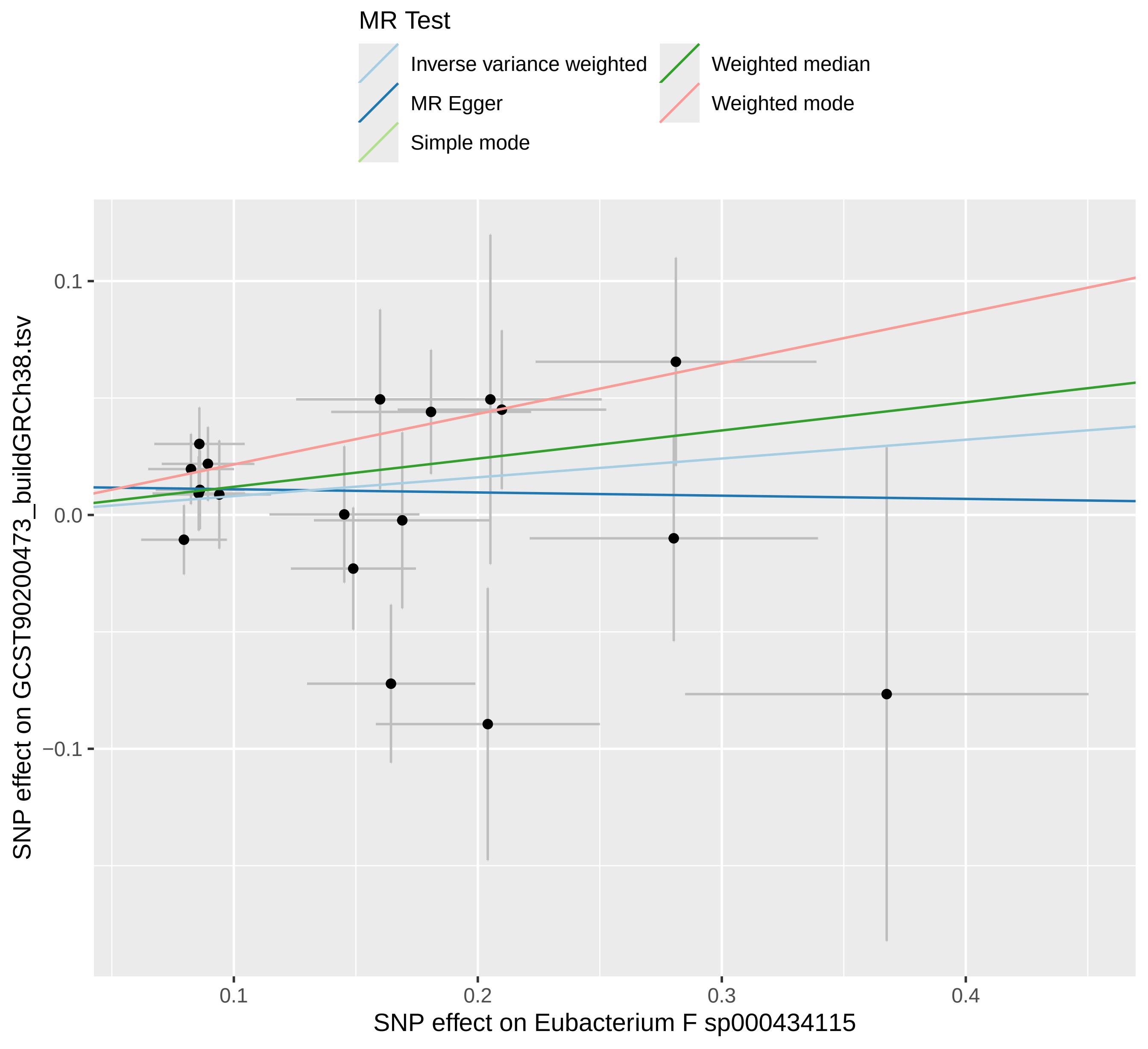
MR Test
Inverse variance weighted
MR Egger
Simple mode
Weighted median
Weighted mode
0.1
0.0
−0.1
0.1
0.2
0.3
0.4
SNP effect on GCST90200473_buildGRCh38.tsv
SNP effect on Eubacterium F sp000434115

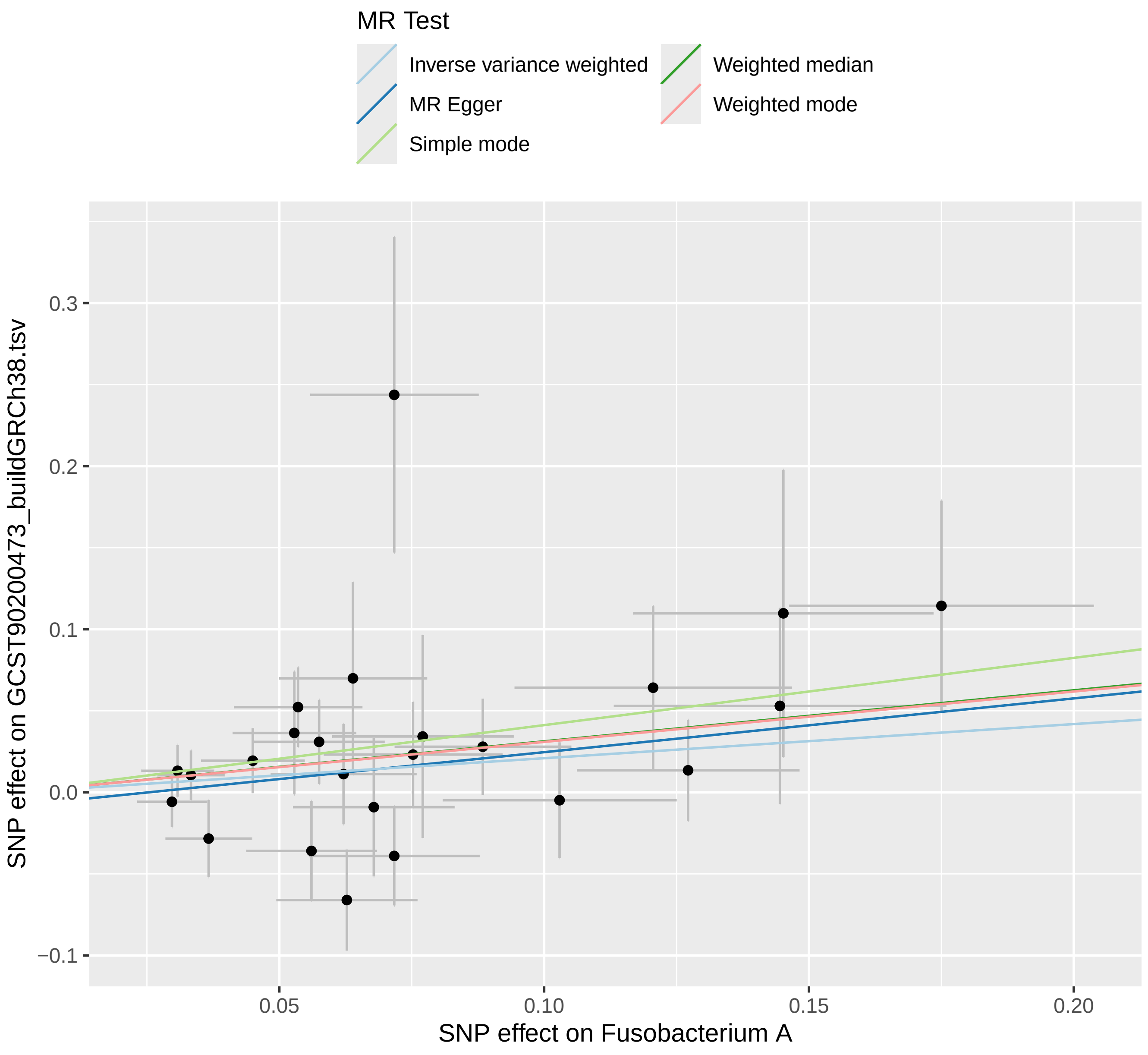

MR Test
Inverse variance weighted
MR Egger
Simple mode
Weighted median
Weighted mode
SNP effect on GCST90200473_buildGRCh38.tsv
0.3
0.2
0.1
0.0
−0.1
0.05
0.10
0.15
0.20
SNP effect on Fusobacterium A

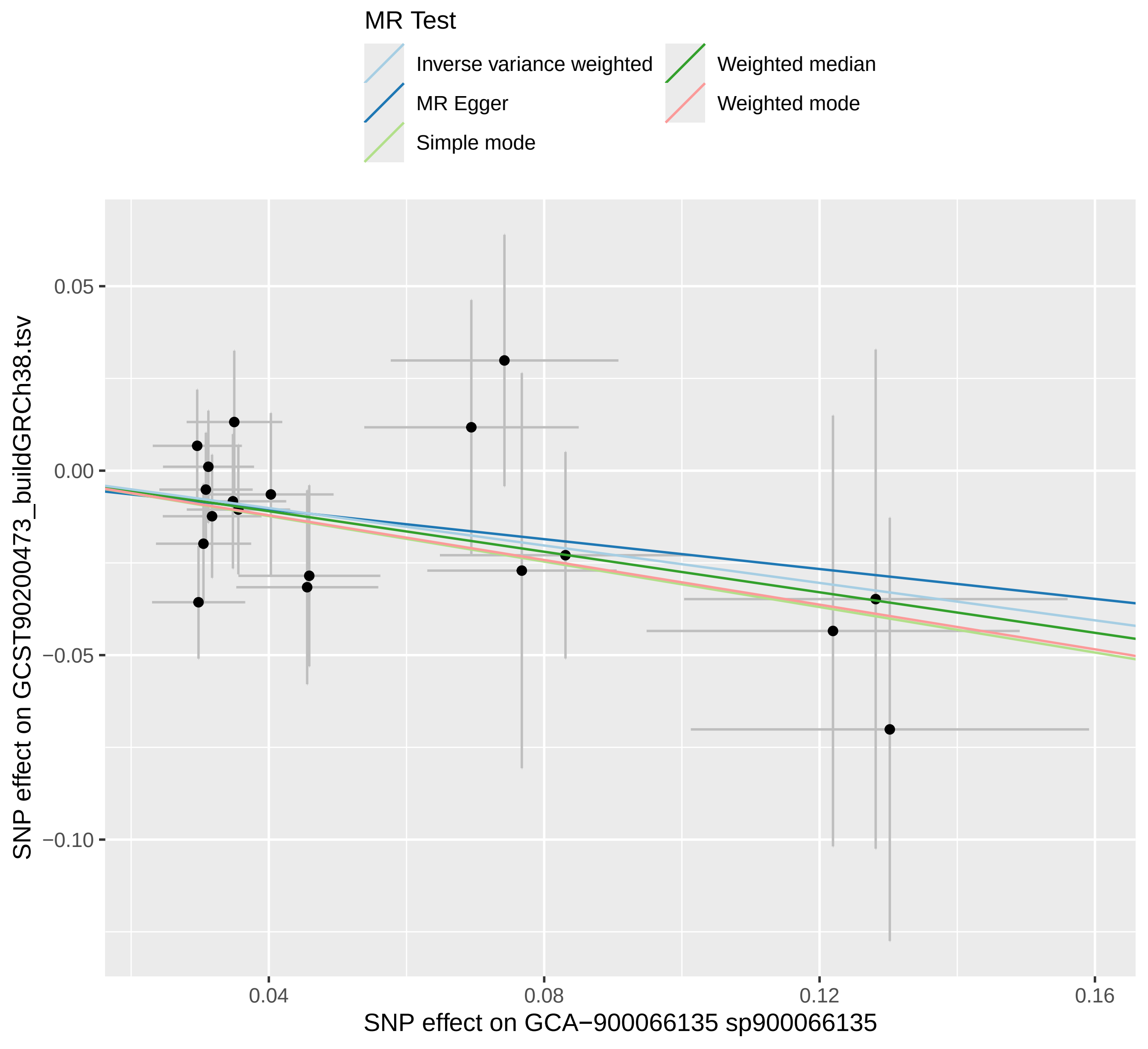
MR Test
Inverse variance weighted
MR Egger
Simple mode
Weighted median
Weighted mode
0.05
0.00
−0.05
−0.10
0.04
0.08
0.12
0.16
SNP effect on GCST90200473_buildGRCh38.tsv
SNP effect on GCA−900066135 sp900066135

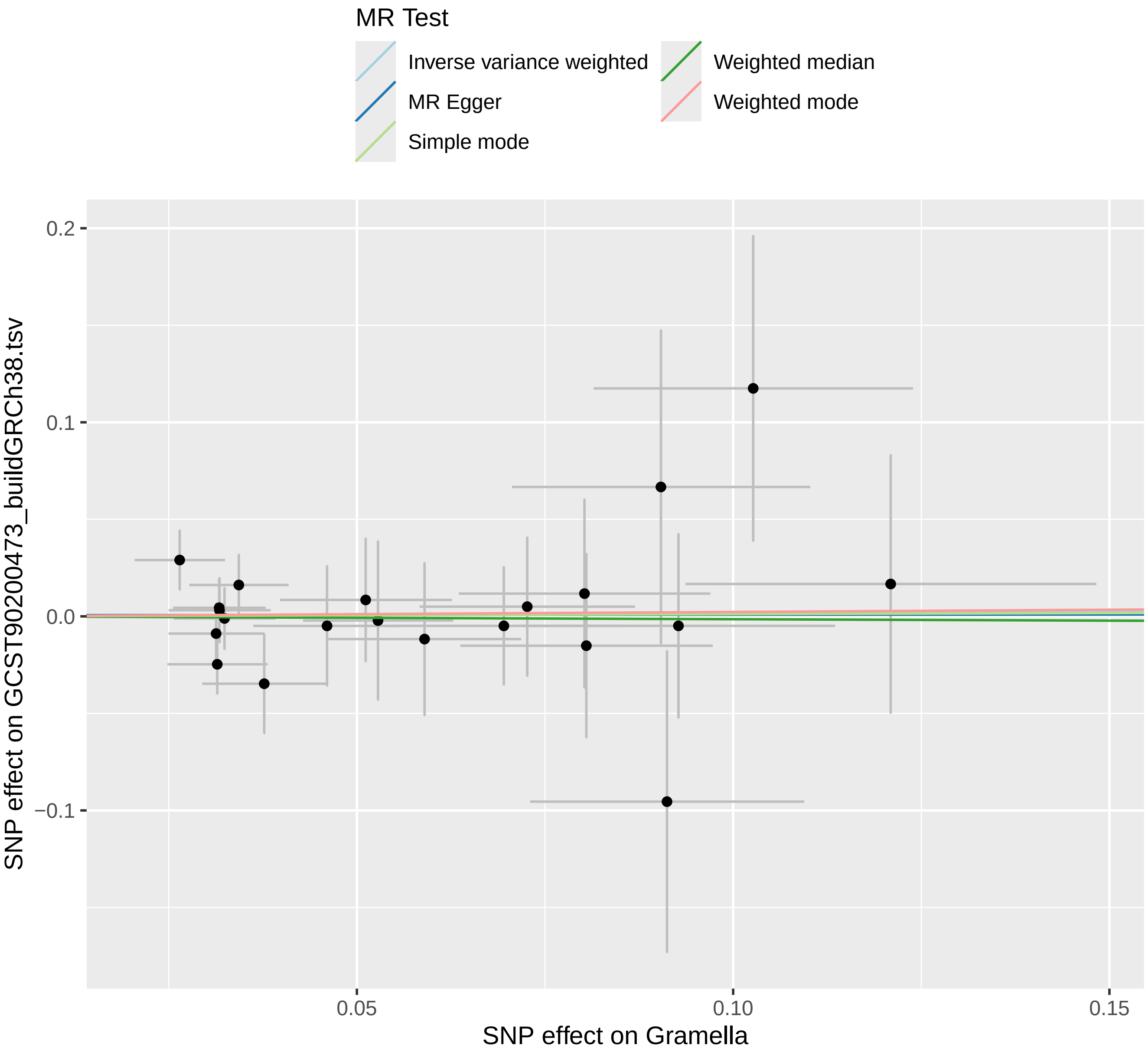
MR Test
Inverse variance weighted
MR Egger
Simple mode
Weighted median
Weighted mode
0.2
0.1
0.0
−0.1
0.05
0.10
0.15
SNP effect on GCST90200473_buildGRCh38.tsv
SNP effect on Gramella

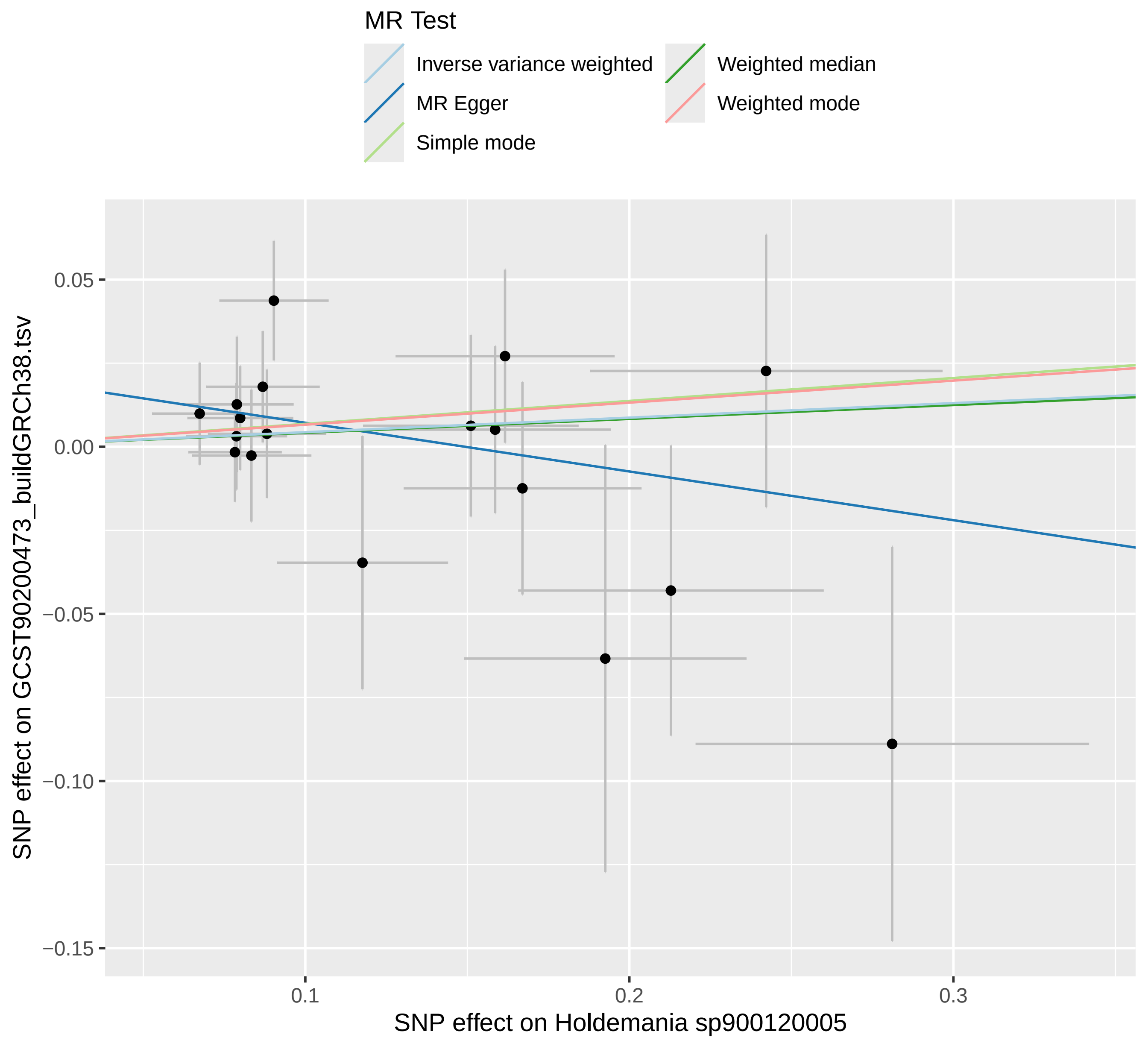
MR Test
Inverse variance weighted
MR Egger
Simple mode
Weighted median
Weighted mode
0.05
0.00
−0.05
−0.10
−0.15
0.1
0.2
0.3
SNP effect on GCST90200473_buildGRCh38.tsv
SNP effect on Holdemania sp900120005

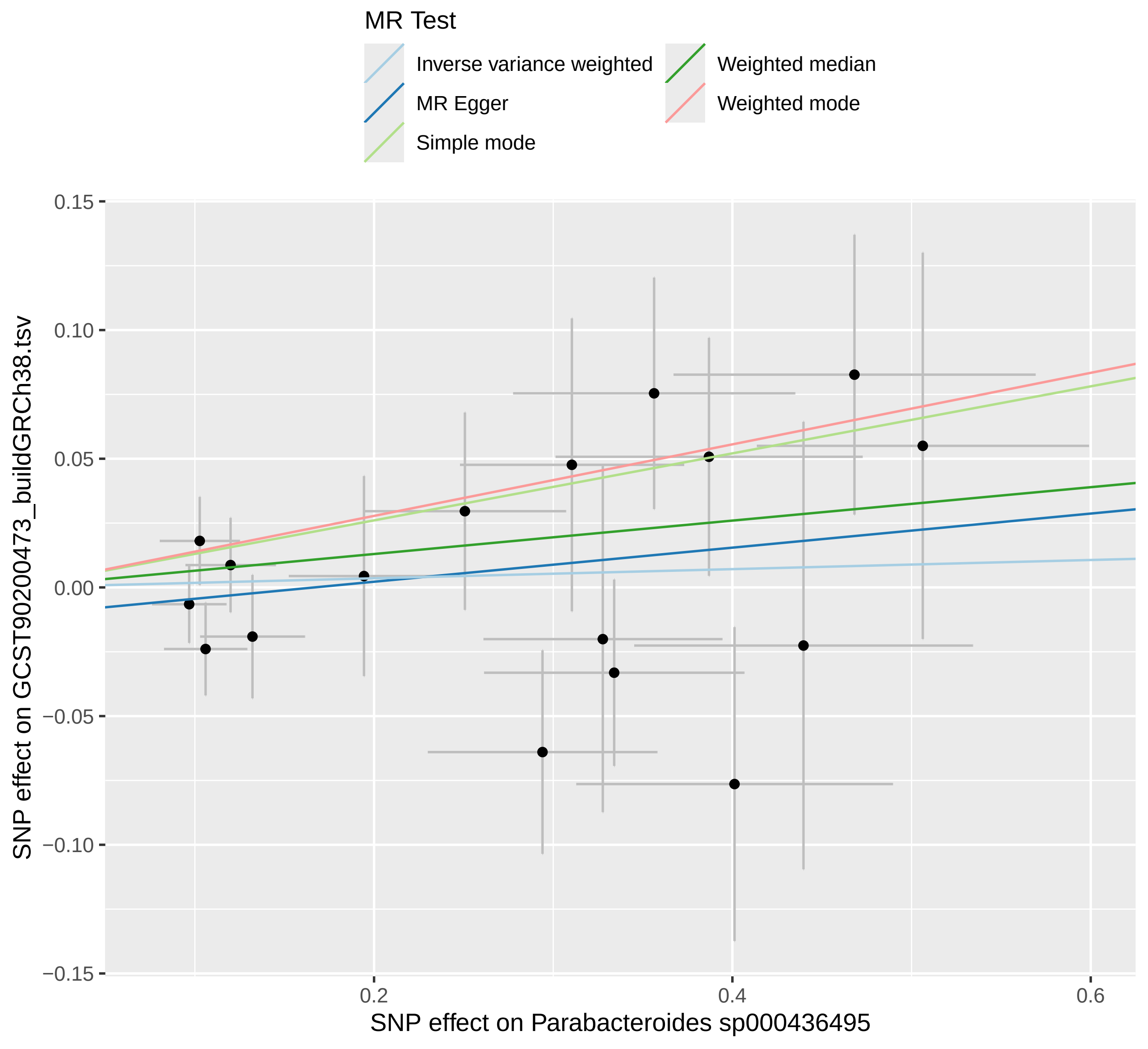
MR Test
Inverse variance weighted
MR Egger
Simple mode
Weighted median
Weighted mode
0.15
0.10
0.05
0.00
−0.05
−0.10
−0.15
0.2
0.4
0.6
SNP effect on GCST90200473_buildGRCh38.tsv
SNP effect on Parabacteroides sp000436495

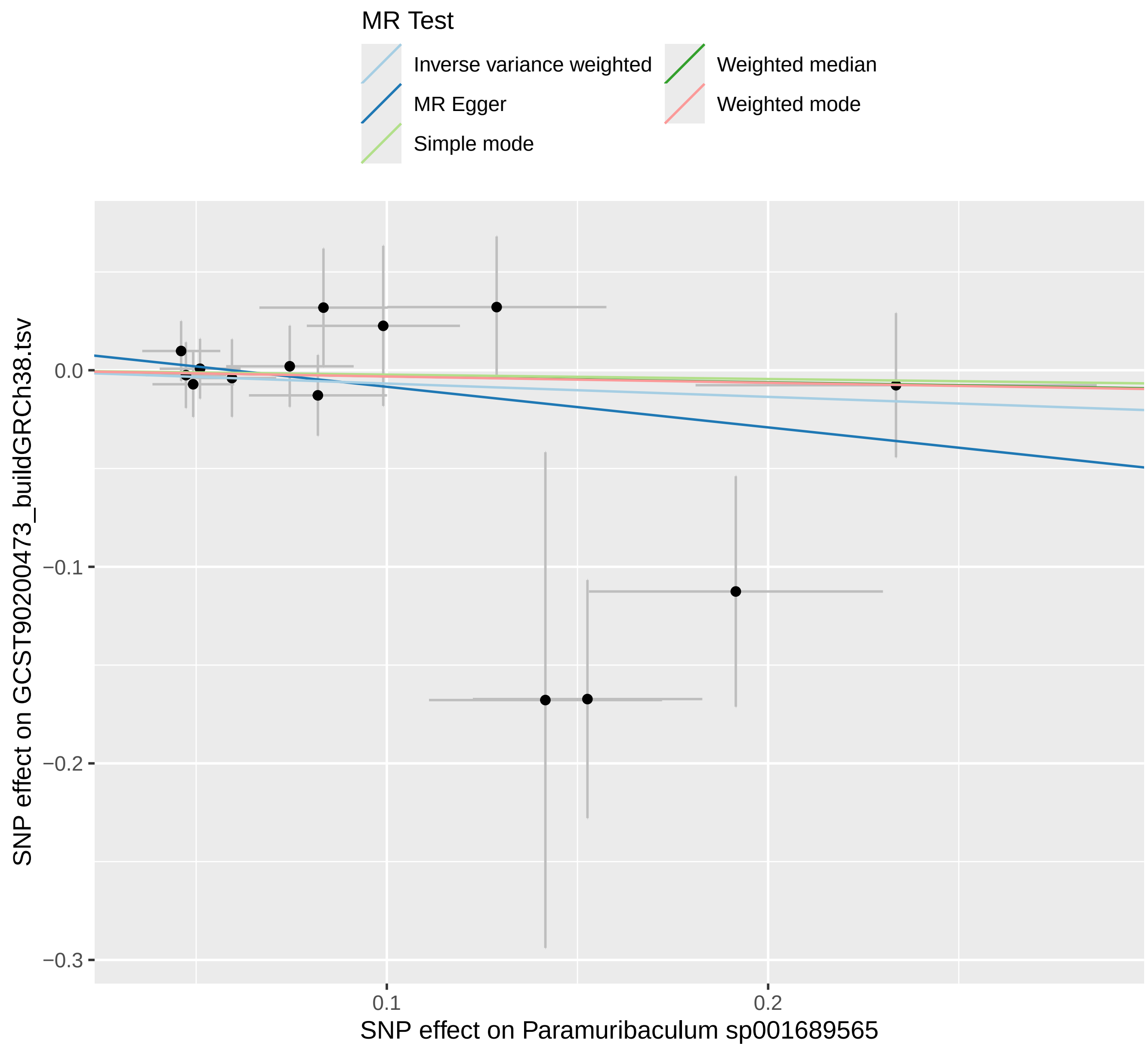
MR Test
Inverse variance weighted
MR Egger
Simple mode
Weighted median
Weighted mode
0.0
−0.1
−0.2
−0.3
0.1
0.2
SNP effect on GCST90200473_buildGRCh38.tsv
SNP effect on Paramuribaculum sp001689565

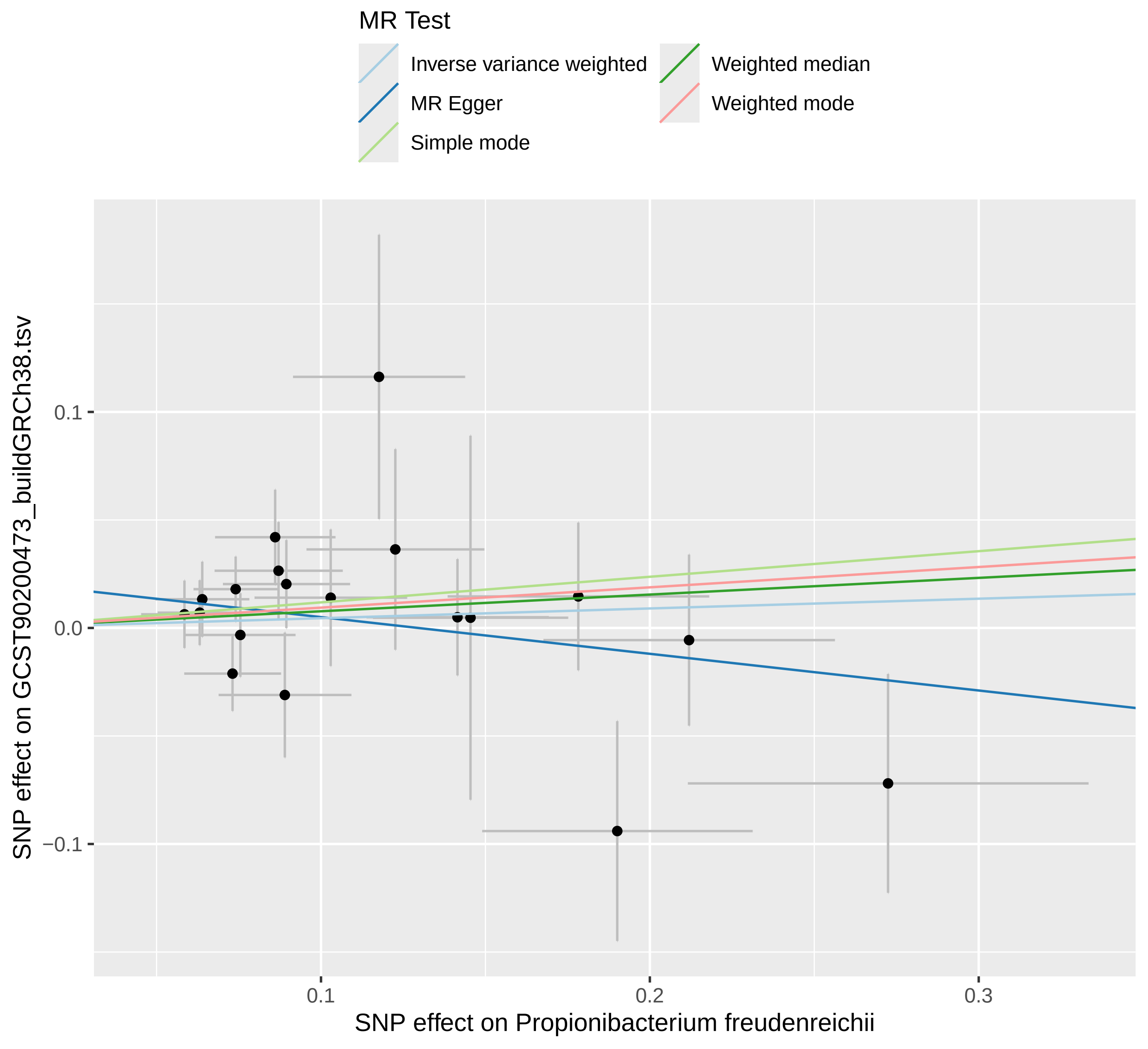

MR Test
Inverse variance weighted
MR Egger
Simple mode
Weighted median
Weighted mode
SNP effect on GCST90200473_buildGRCh38.tsv
0.1
0.0
−0.1
0.1
0.2
0.3
SNP effect on Propionibacterium freudenreichii

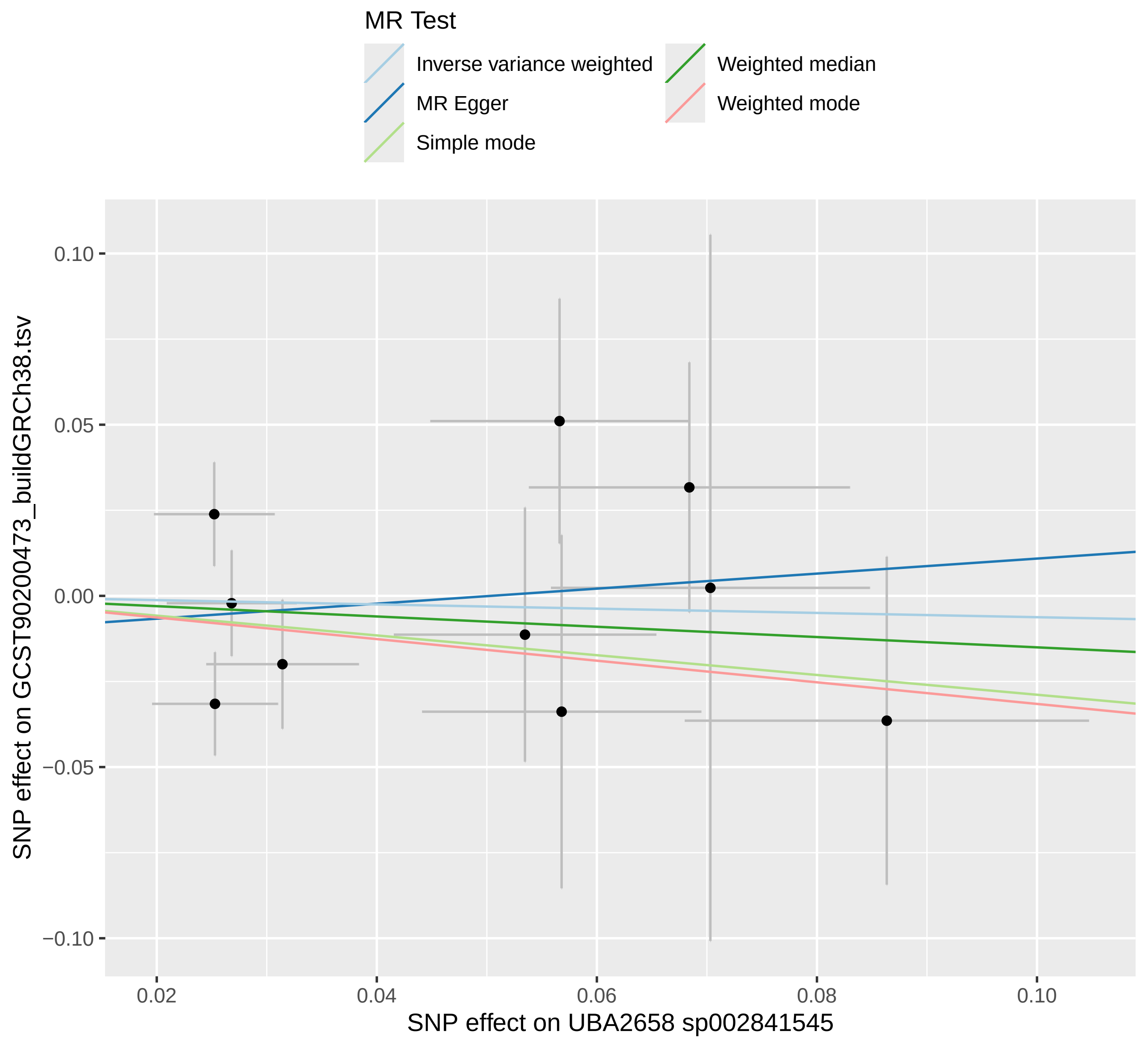

MR Test
Inverse variance weighted
MR Egger
Simple mode
Weighted median
Weighted mode
0.10
0.05
0.00
−0.05
−0.10
0.02
0.04
0.06
0.08
0.10
SNP effect on UBA2658 sp002841545
SNP effect on GCST90200473_buildGRCh38.tsv

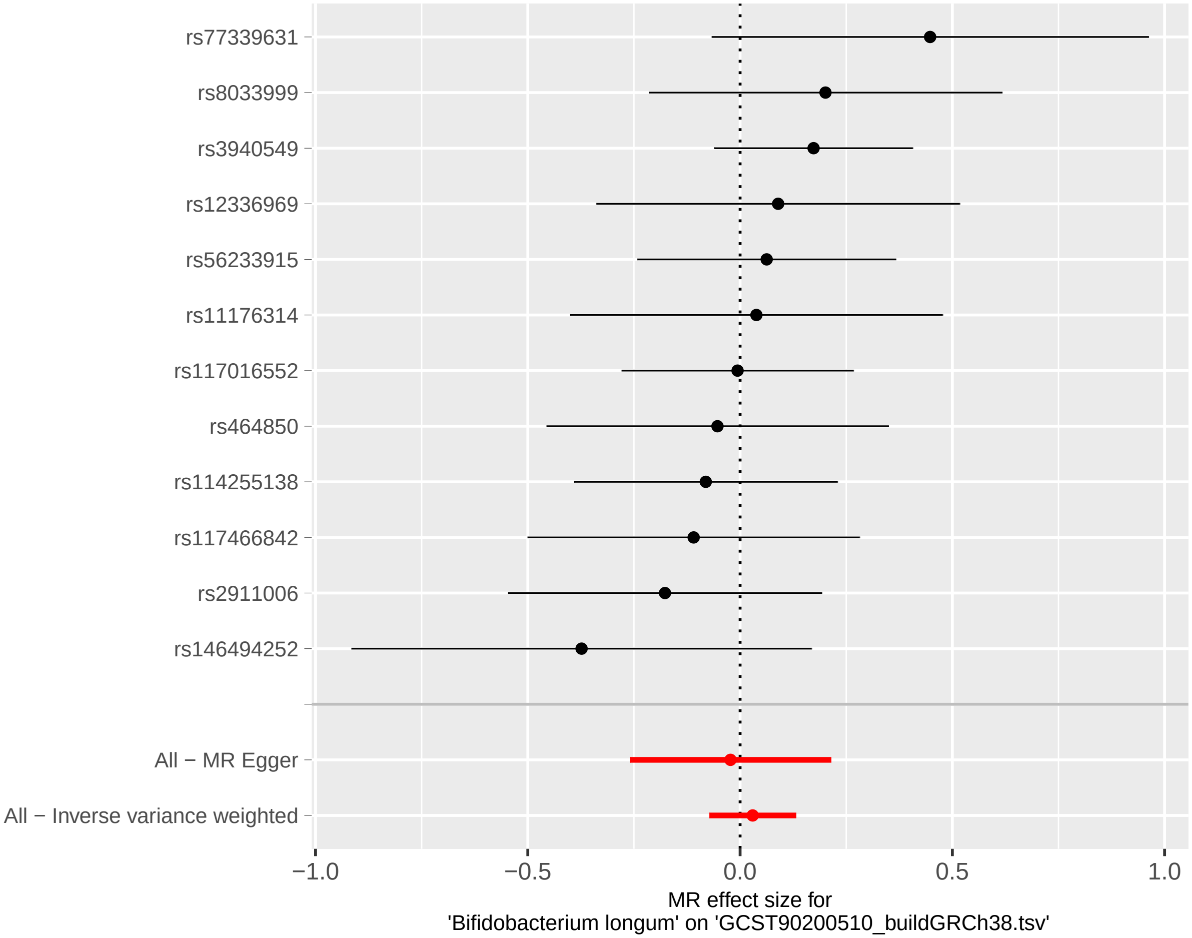

rs77339631
rs8033999
rs3940549
rs12336969
rs56233915
rs11176314
rs117016552
rs464850
rs114255138
rs117466842
rs2911006
rs146494252
All − MR Egger
All − Inverse variance weighted
−1.0
−0.5
0.0
0.5
1.0
MR effect size for
'Bifidobacterium longum' on 'GCST90200510_buildGRCh38.tsv'

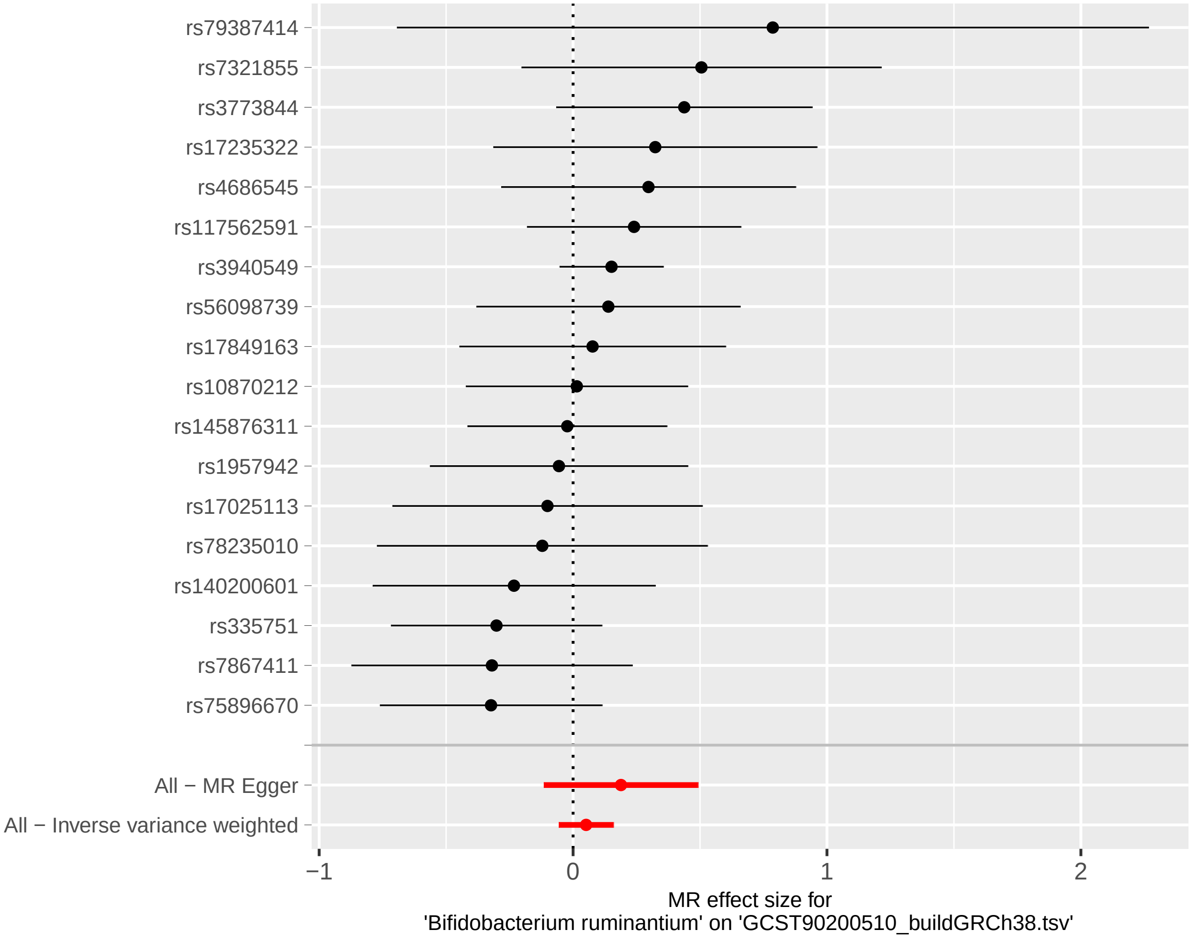
rs79387414
rs7321855
rs3773844
rs17235322
rs4686545
rs117562591
rs3940549
rs56098739
rs17849163
rs10870212
rs145876311
rs1957942
rs17025113
rs78235010
rs140200601
rs335751
rs7867411
rs75896670
All − MR Egger
All − Inverse variance weighted
−1
0
1
2
MR effect size for
'Bifidobacterium ruminantium' on 'GCST90200510_buildGRCh38.tsv'

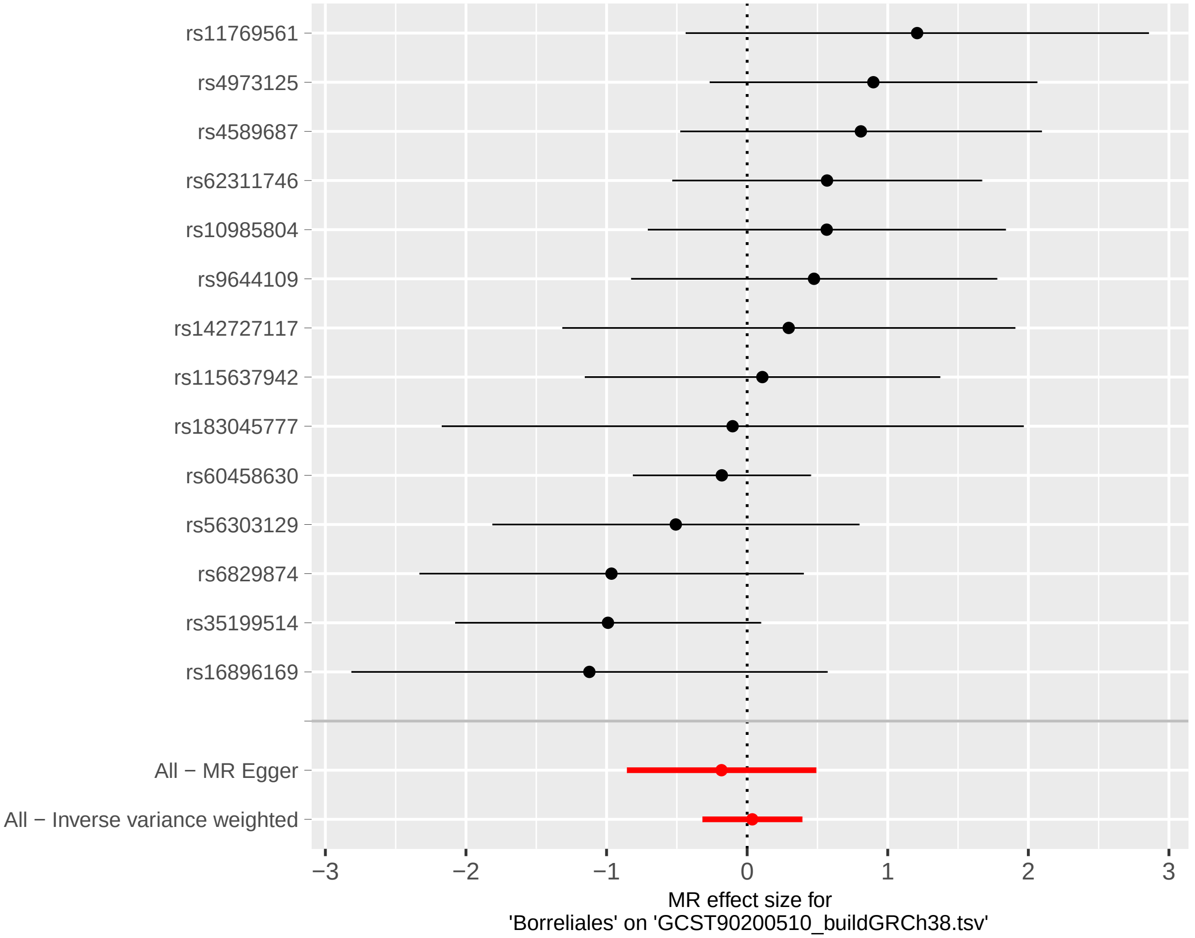

rs11769561
rs4973125
rs4589687
rs62311746
rs10985804
rs9644109
rs142727117
rs115637942
rs183045777
rs60458630
rs56303129
rs6829874
rs35199514
rs16896169
All − MR Egger
All − Inverse variance weighted
−3
−2
−1
0
1
2
3
MR effect size for
'Borreliales' on 'GCST90200510_buildGRCh38.tsv'

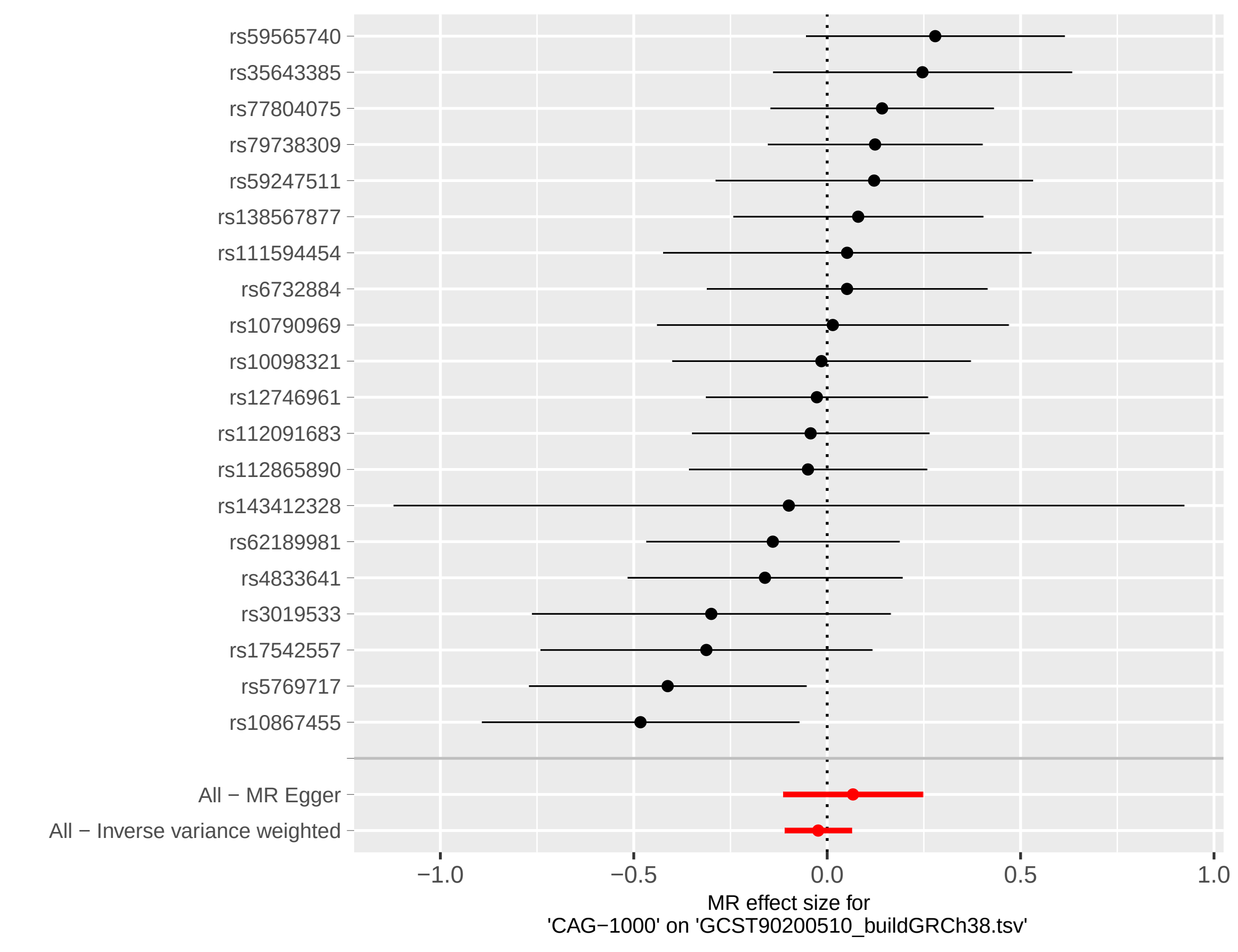
rs59565740
rs35643385
rs77804075
rs79738309
rs59247511
rs138567877
rs111594454
rs6732884
rs10790969
rs10098321
rs12746961
rs112091683
rs112865890
rs143412328
rs62189981
rs4833641
rs3019533
rs17542557
rs5769717
rs10867455
All − MR Egger
All − Inverse variance weighted
−1.0
−0.5
0.0
0.5
1.0
MR effect size for
'CAG−1000' on 'GCST90200510_buildGRCh38.tsv'

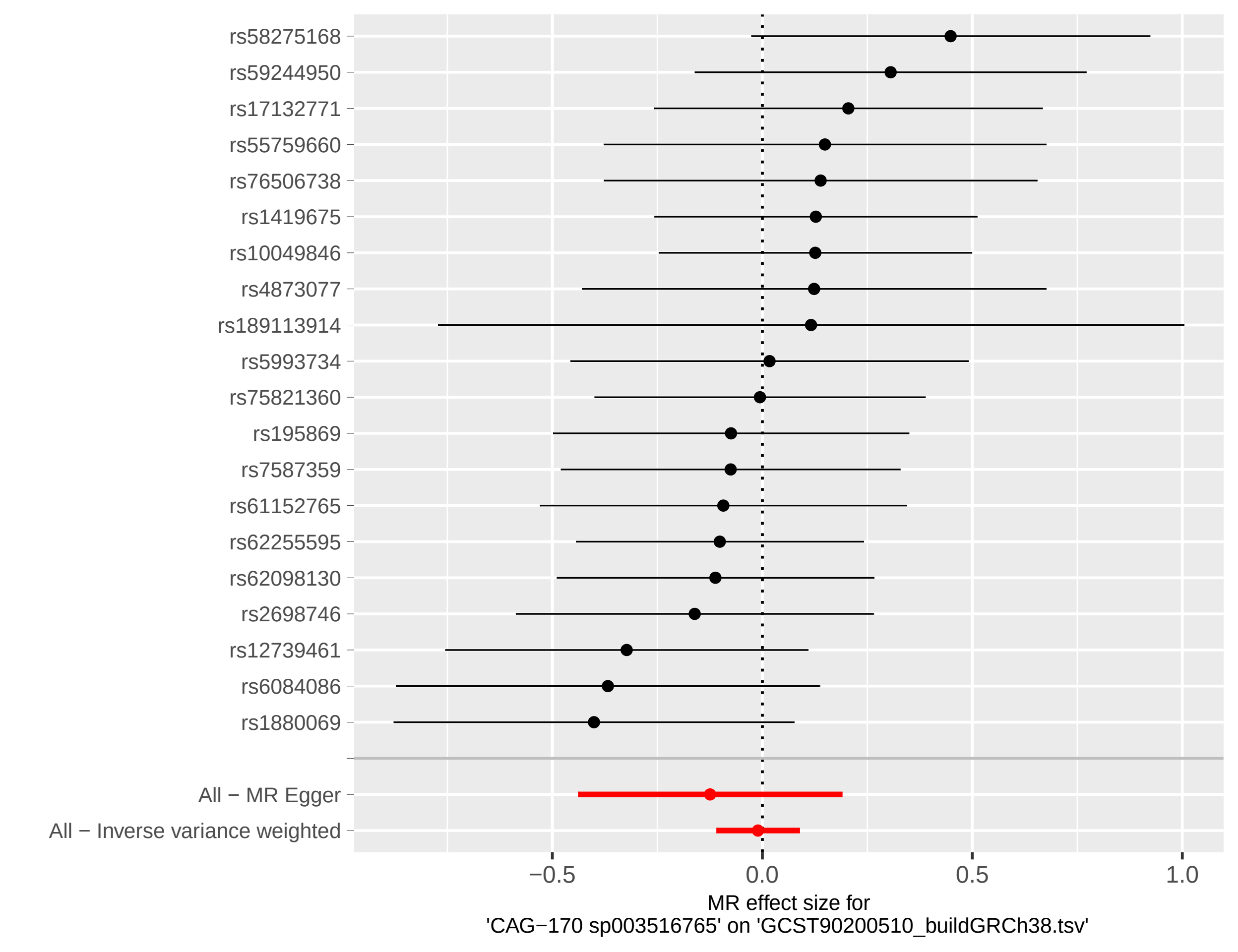
rs58275168
rs59244950
rs17132771
rs55759660
rs76506738
rs1419675
rs10049846
rs4873077
rs189113914
rs5993734
rs75821360
rs195869
rs7587359
rs61152765
rs62255595
rs62098130
rs2698746
rs12739461
rs6084086
rs1880069
All − MR Egger
All − Inverse variance weighted
−0.5
0.0
0.5
1.0
MR effect size for
'CAG−170 sp003516765' on 'GCST90200510_buildGRCh38.tsv'

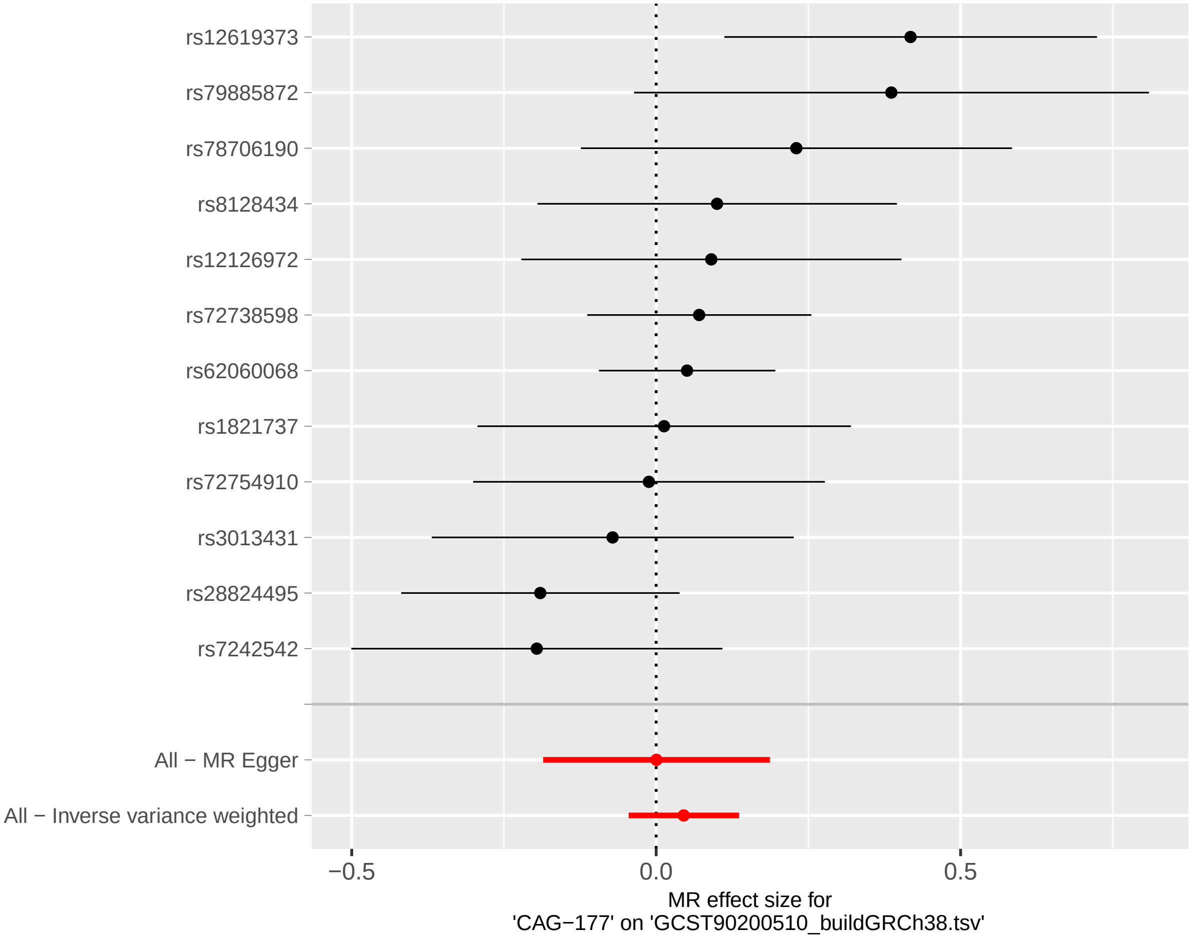

rs12619373
rs79885872
rs78706190
rs8128434
rs12126972
rs72738598
rs62060068
rs1821737
rs72754910
rs3013431
rs28824495
rs7242542
All − MR Egger
All − Inverse variance weighted
−0.5
0.0
0.5
MR effect size for
'CAG−177' on 'GCST90200510_buildGRCh38.tsv'

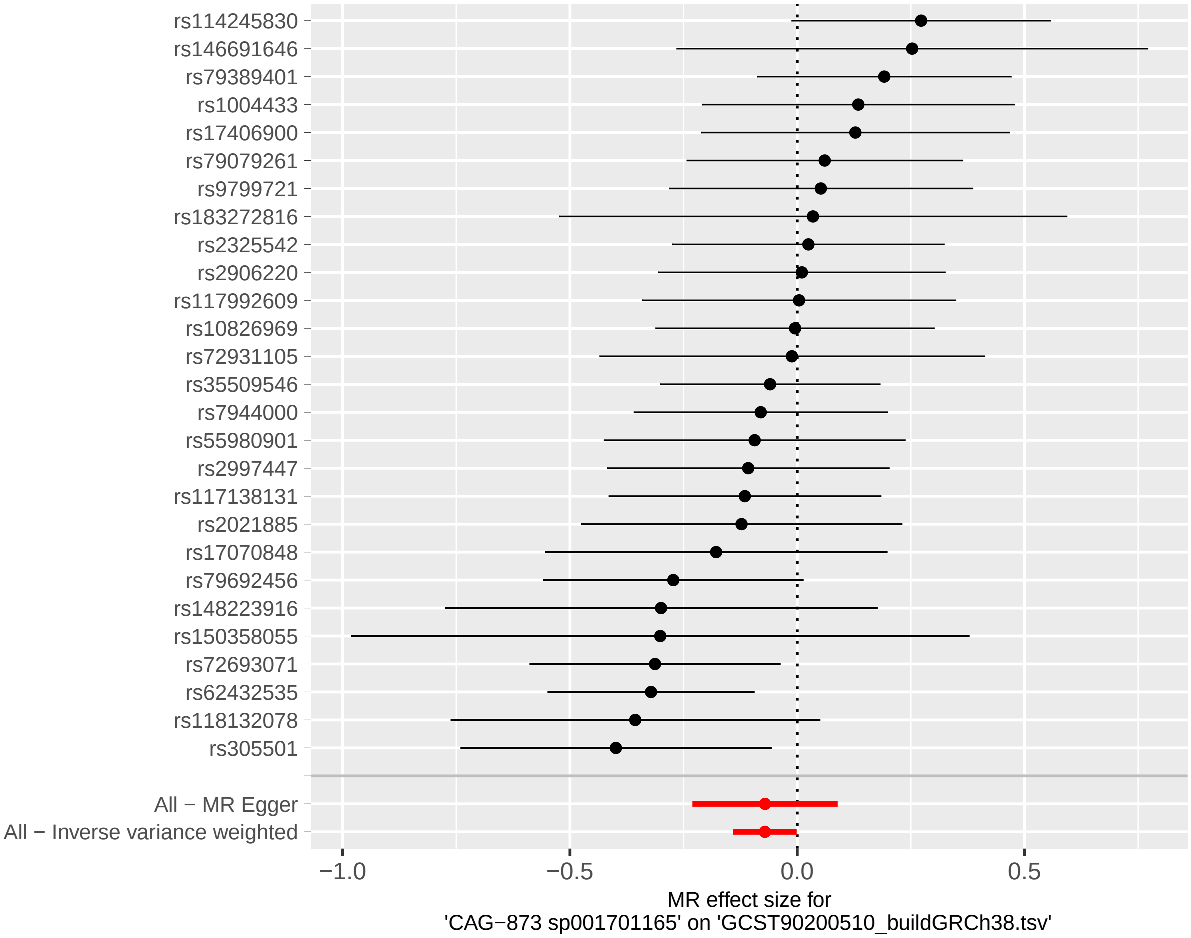

rs114245830
rs146691646
rs79389401
rs1004433
rs17406900
rs79079261
rs9799721
rs183272816
rs2325542
rs2906220
rs117992609
rs10826969
rs72931105
rs35509546
rs7944000
rs55980901
rs2997447
rs117138131
rs2021885
rs17070848
rs79692456
rs148223916
rs150358055
rs72693071
rs62432535
rs118132078
rs305501
All − MR Egger
All − Inverse variance weighted
−1.0
−0.5
0.0
0.5
MR effect size for
'CAG−873 sp001701165' on 'GCST90200510_buildGRCh38.tsv'

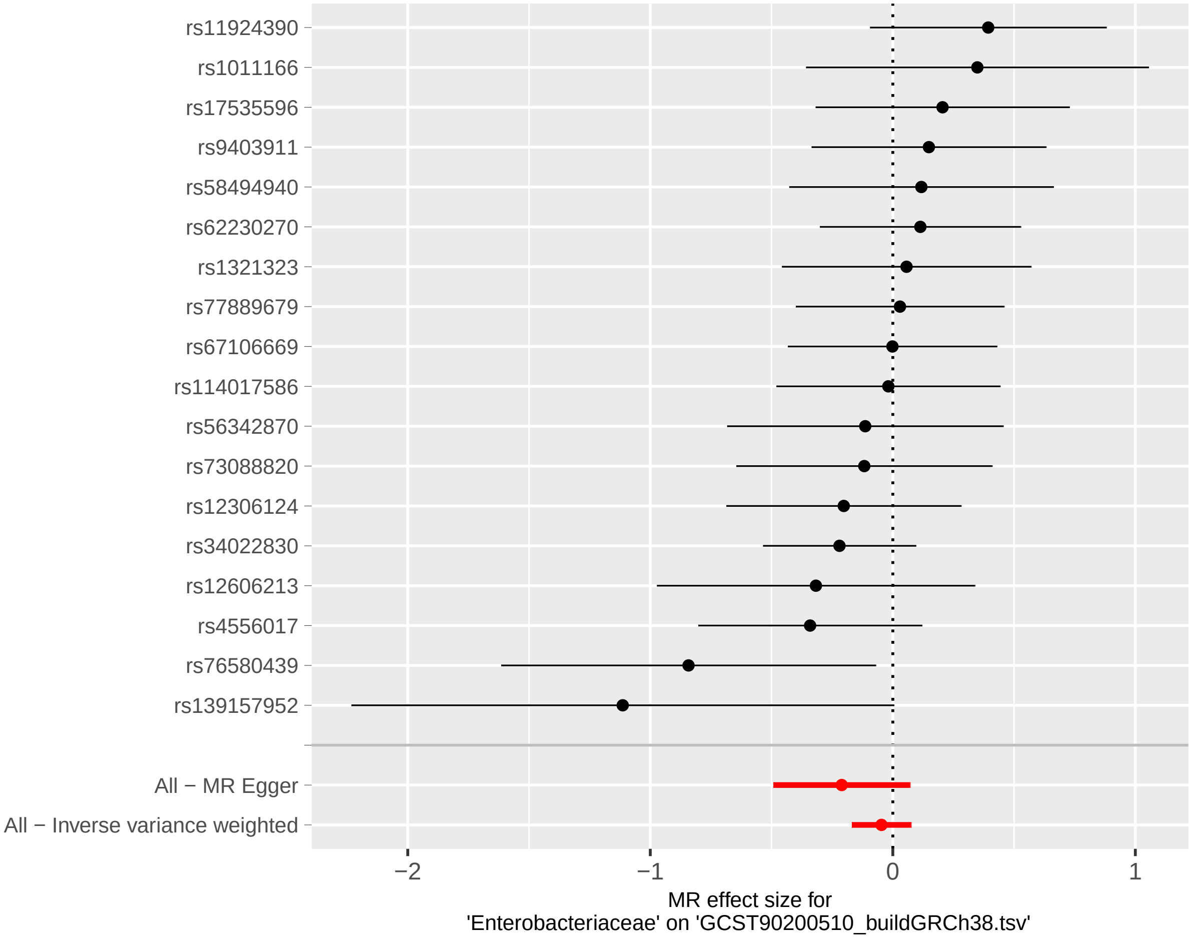
rs11924390
rs1011166
rs17535596
rs9403911
rs58494940
rs62230270
rs1321323
rs77889679
rs67106669
rs114017586
rs56342870
rs73088820
rs12306124
rs34022830
rs12606213
rs4556017
rs76580439
rs139157952
All − MR Egger
All − Inverse variance weighted
−2
−1
0
1
MR effect size for
'Enterobacteriaceae' on 'GCST90200510_buildGRCh38.tsv'

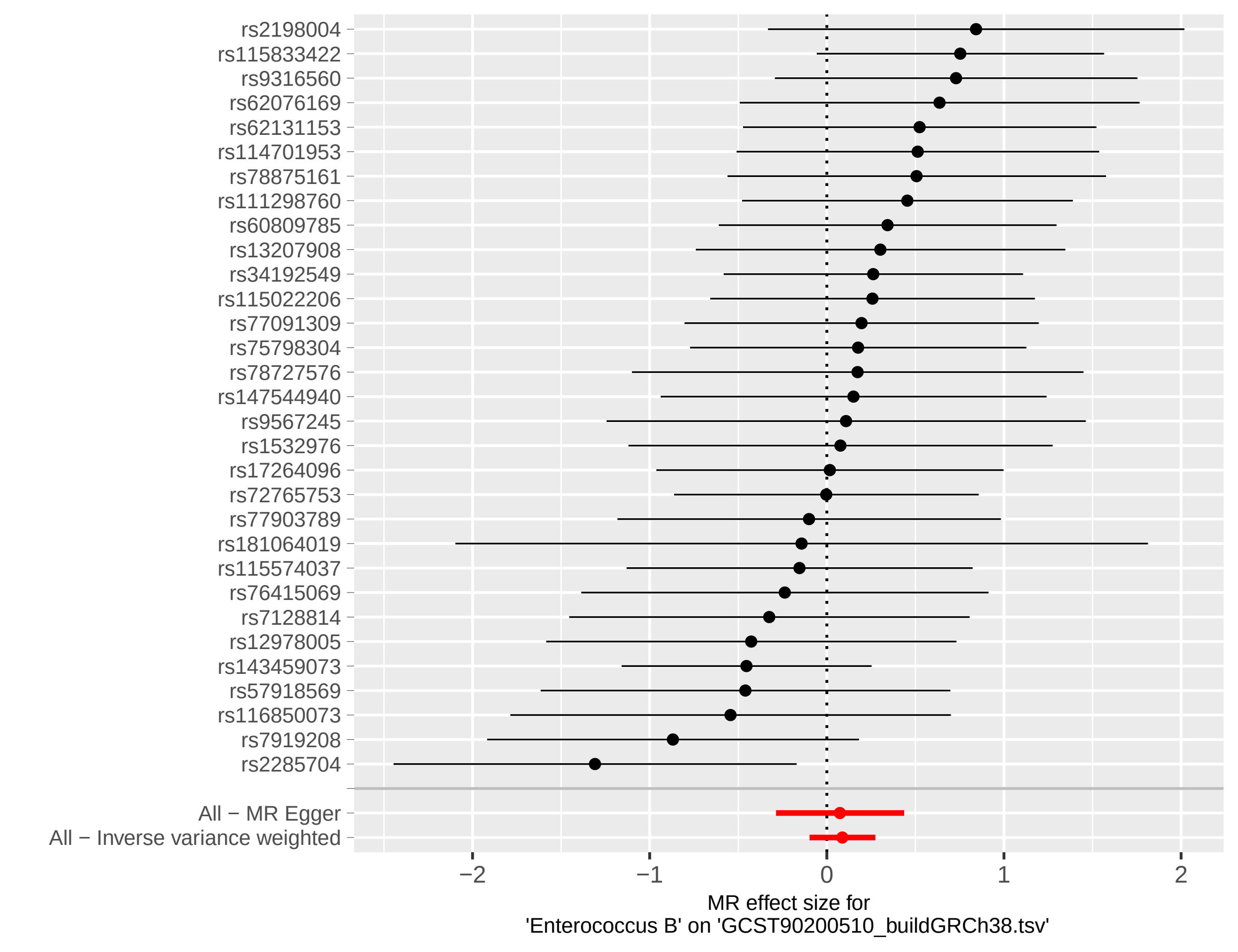
rs2198004
rs115833422
rs9316560
rs62076169
rs62131153
rs114701953
rs78875161
rs111298760
rs60809785
rs13207908
rs34192549
rs115022206
rs77091309
rs75798304
rs78727576
rs147544940
rs9567245
rs1532976
rs17264096
rs72765753
rs77903789
rs181064019
rs115574037
rs76415069
rs7128814
rs12978005
rs143459073
rs57918569
rs116850073
rs7919208
rs2285704
All − MR Egger
All − Inverse variance weighted
−2
−1
0
1
2
MR effect size for
'Enterococcus B' on 'GCST90200510_buildGRCh38.tsv'

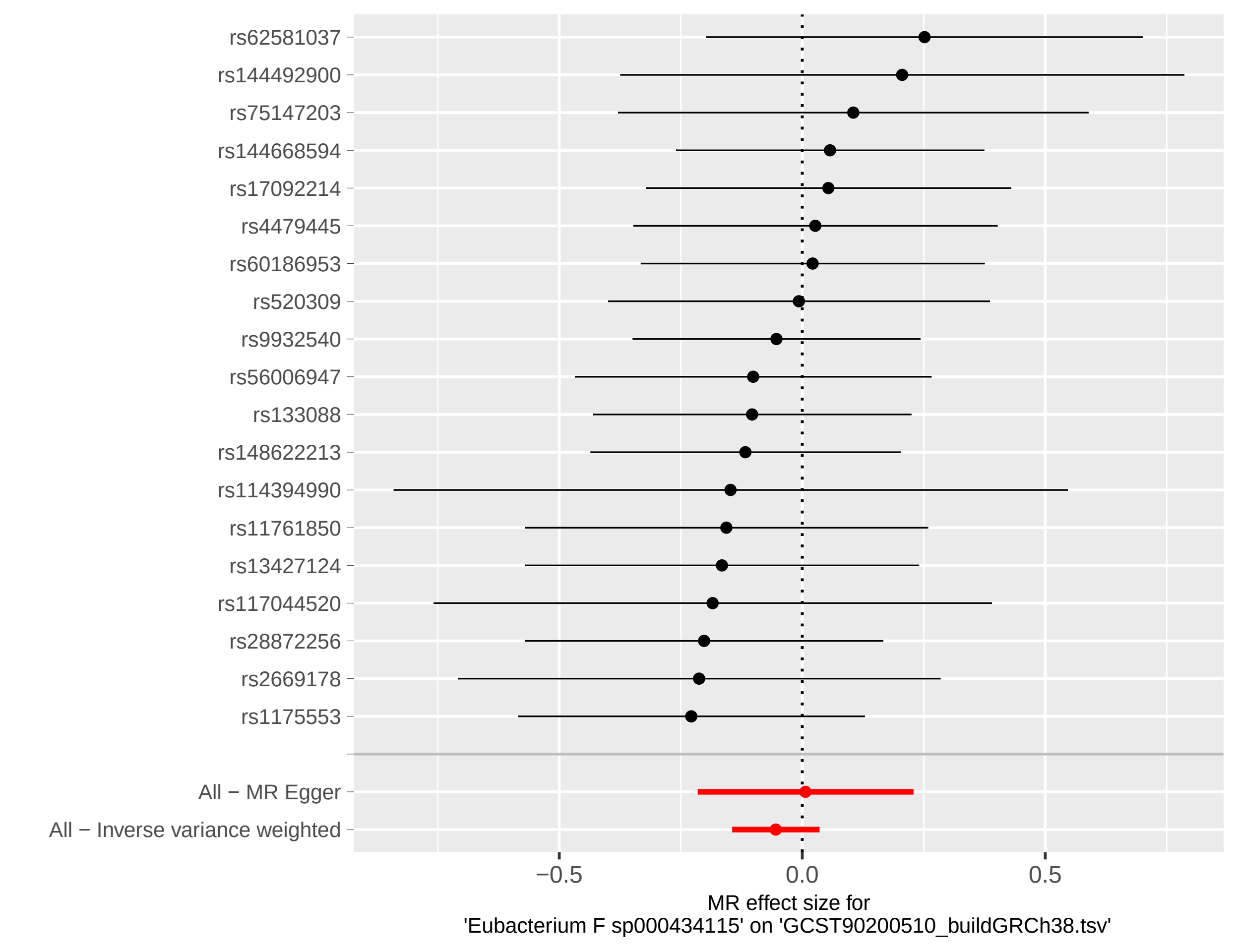
rs62581037
rs144492900
rs75147203
rs144668594
rs17092214
rs4479445
rs60186953
rs520309
rs9932540
rs56006947
rs133088
rs148622213
rs114394990
rs11761850
rs13427124
rs117044520
rs28872256
rs2669178
rs1175553
All − MR Egger
All − Inverse variance weighted
−0.5
0.0
0.5
MR effect size for
'Eubacterium F sp000434115' on 'GCST90200510_buildGRCh38.tsv'

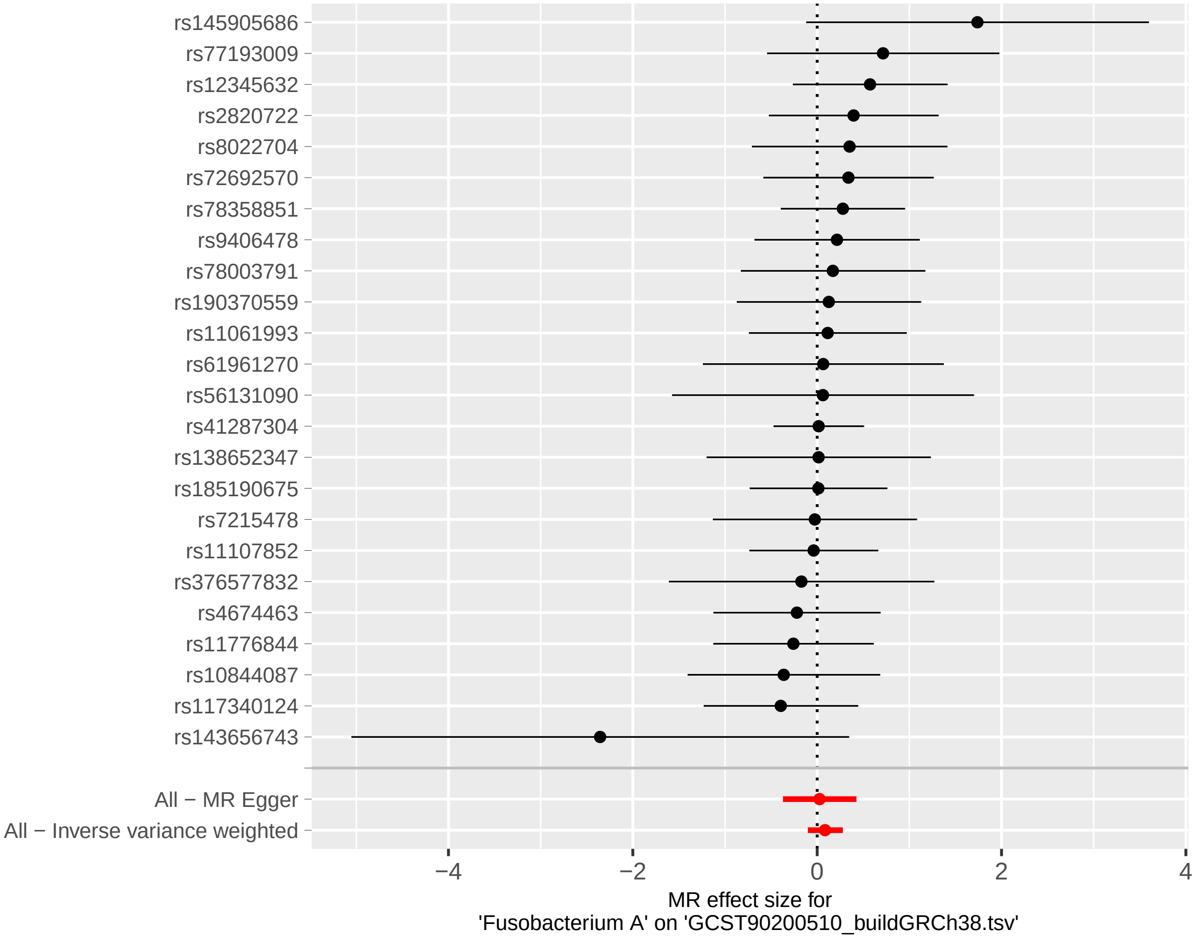

rs145905686
rs77193009
rs12345632
rs2820722
rs8022704
rs72692570
rs78358851
rs9406478
rs78003791
rs190370559
rs11061993
rs61961270
rs56131090
rs41287304
rs138652347
rs185190675
rs7215478
rs11107852
rs376577832
rs4674463
rs11776844
rs10844087
rs117340124
rs143656743
All − MR Egger
All − Inverse variance weighted
−4
−2
0
2
4
MR effect size for
'Fusobacterium A' on 'GCST90200510_buildGRCh38.tsv'

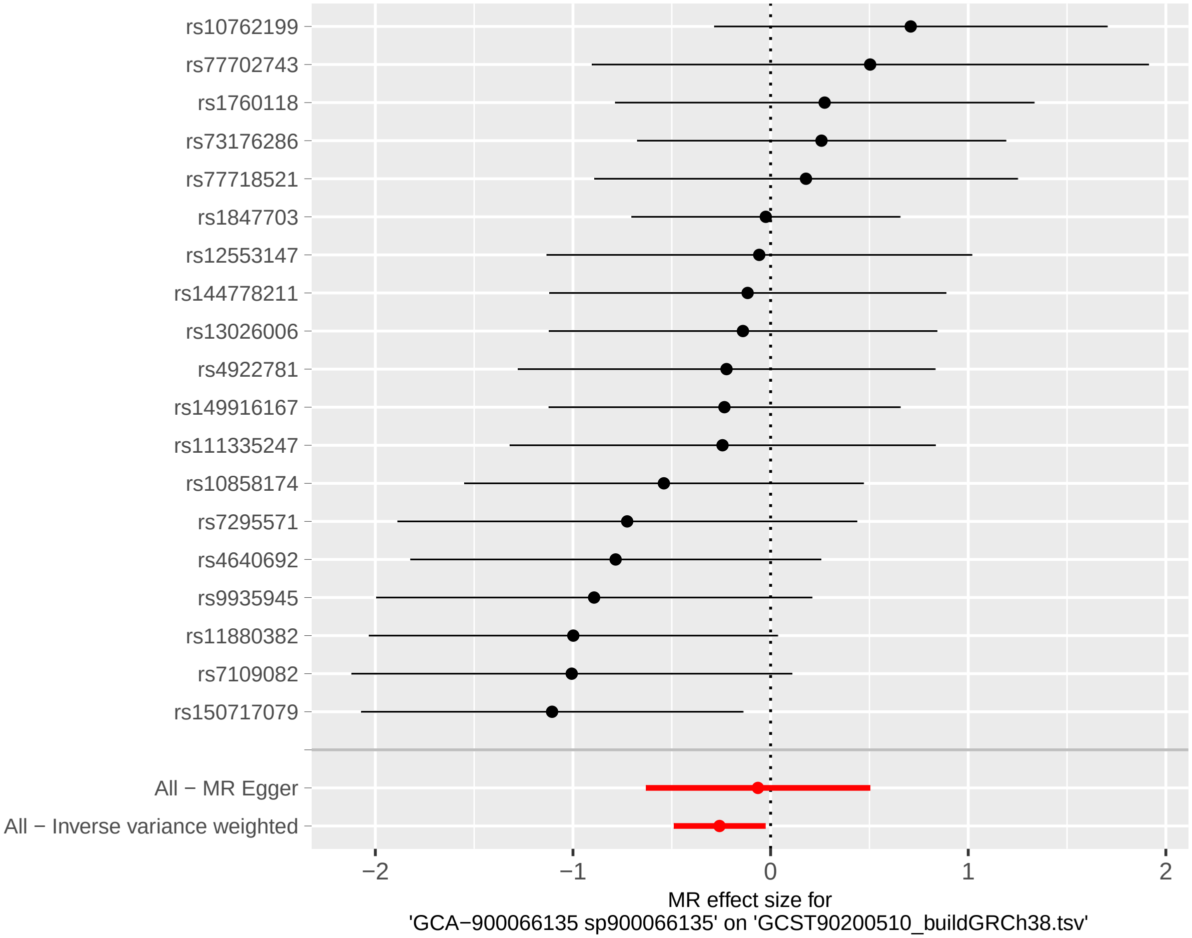

rs10762199
rs77702743
rs1760118
rs73176286
rs77718521
rs1847703
rs12553147
rs144778211
rs13026006
rs4922781
rs149916167
rs111335247
rs10858174
rs7295571
rs4640692
rs9935945
rs11880382
rs7109082
rs150717079
All − MR Egger
All − Inverse variance weighted
−2
−1
0
1
2
MR effect size for
'GCA−900066135 sp900066135' on 'GCST90200510_buildGRCh38.tsv'

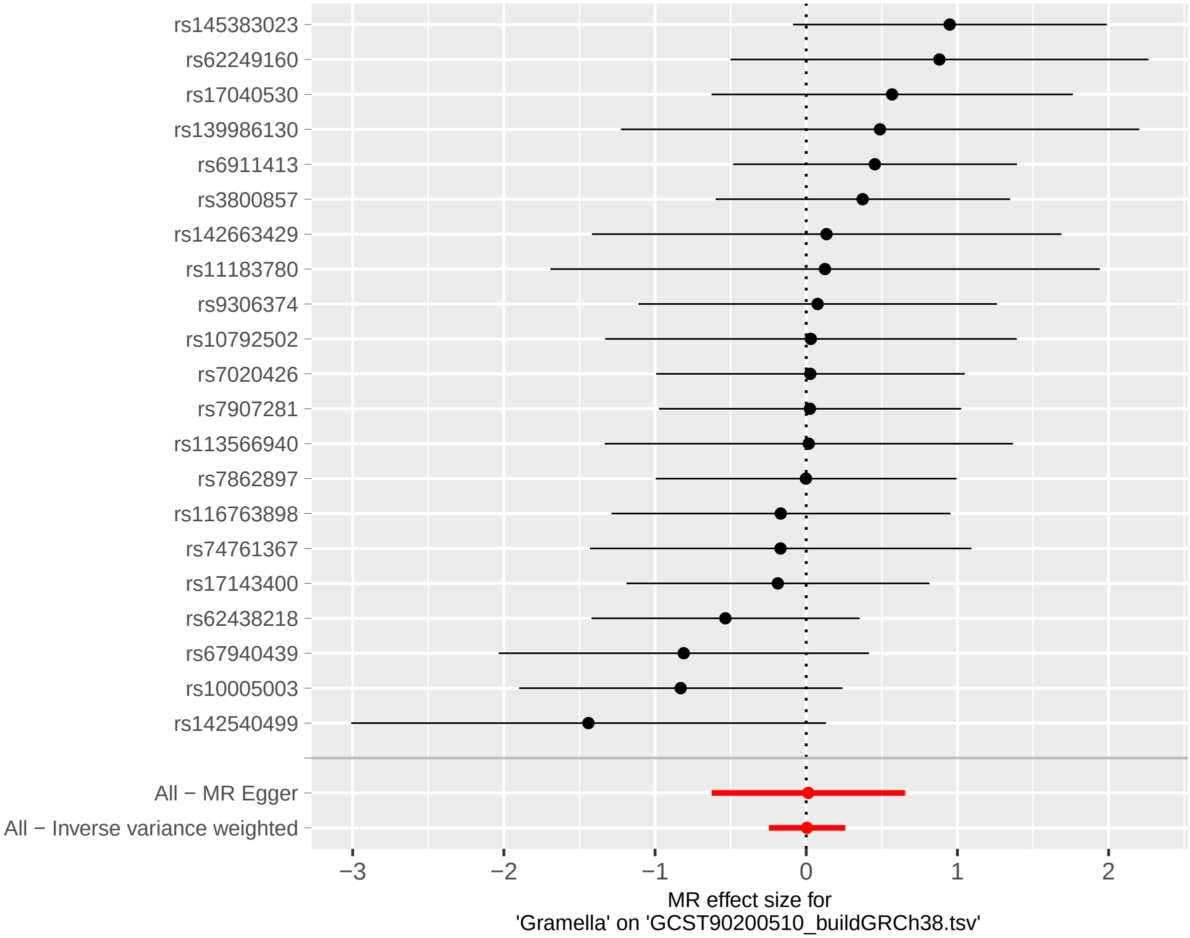
rs145383023
rs62249160
rs17040530
rs139986130
rs6911413
rs3800857
rs142663429
rs11183780
rs9306374
rs10792502
rs7020426
rs7907281
rs113566940
rs7862897
rs116763898
rs74761367
rs17143400
rs62438218
rs67940439
rs10005003
rs142540499
All − MR Egger
All − Inverse variance weighted
−3
−2
−1
0
1
2
MR effect size for
'Gramella' on 'GCST90200510_buildGRCh38.tsv'

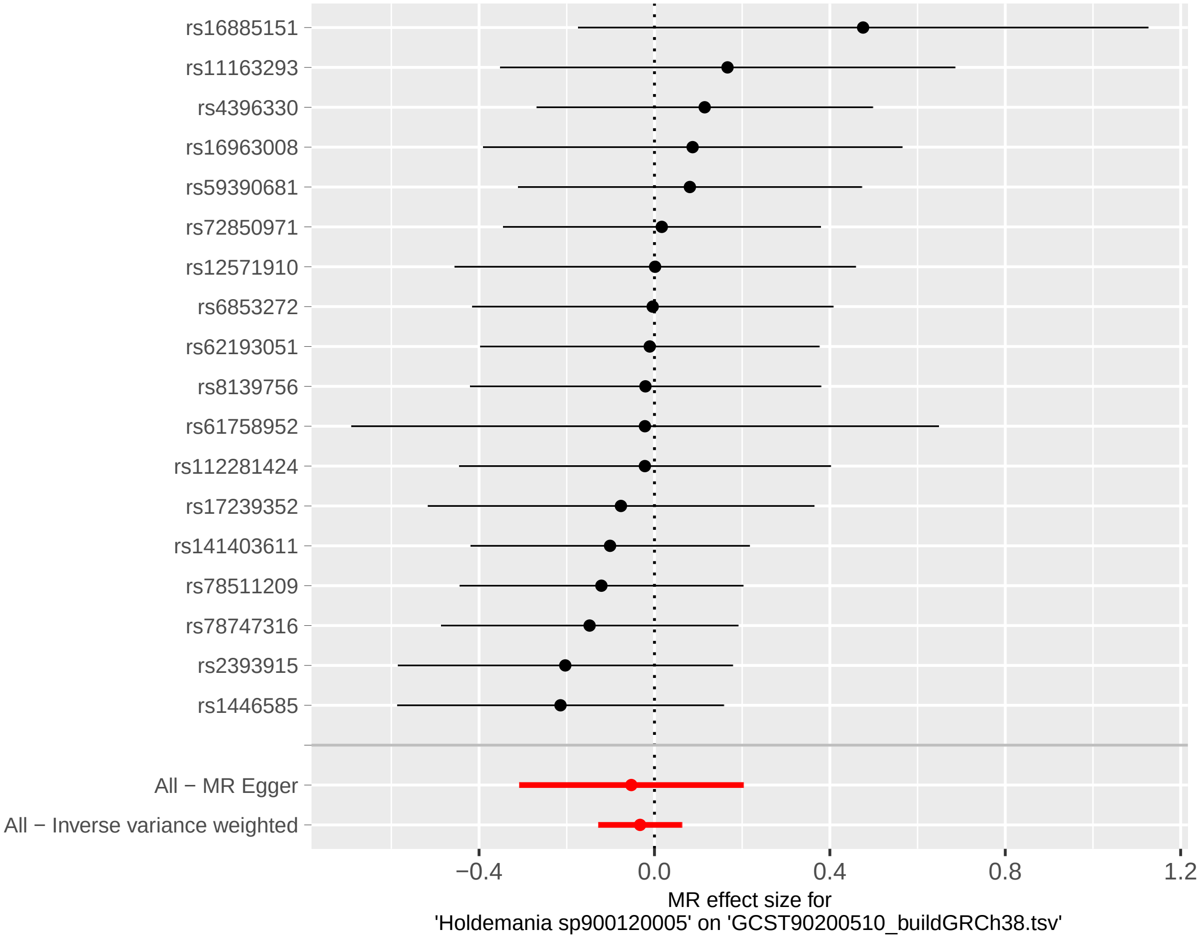
rs16885151
rs11163293
rs4396330
rs16963008
rs59390681
rs72850971
rs12571910
rs6853272
rs62193051
rs8139756
rs61758952
rs112281424
rs17239352
rs141403611
rs78511209
rs78747316
rs2393915
rs1446585
All − MR Egger
All − Inverse variance weighted
−0.4
0.0
0.4
0.8
1.2
MR effect size for
'Holdemania sp900120005' on 'GCST90200510_buildGRCh38.tsv'

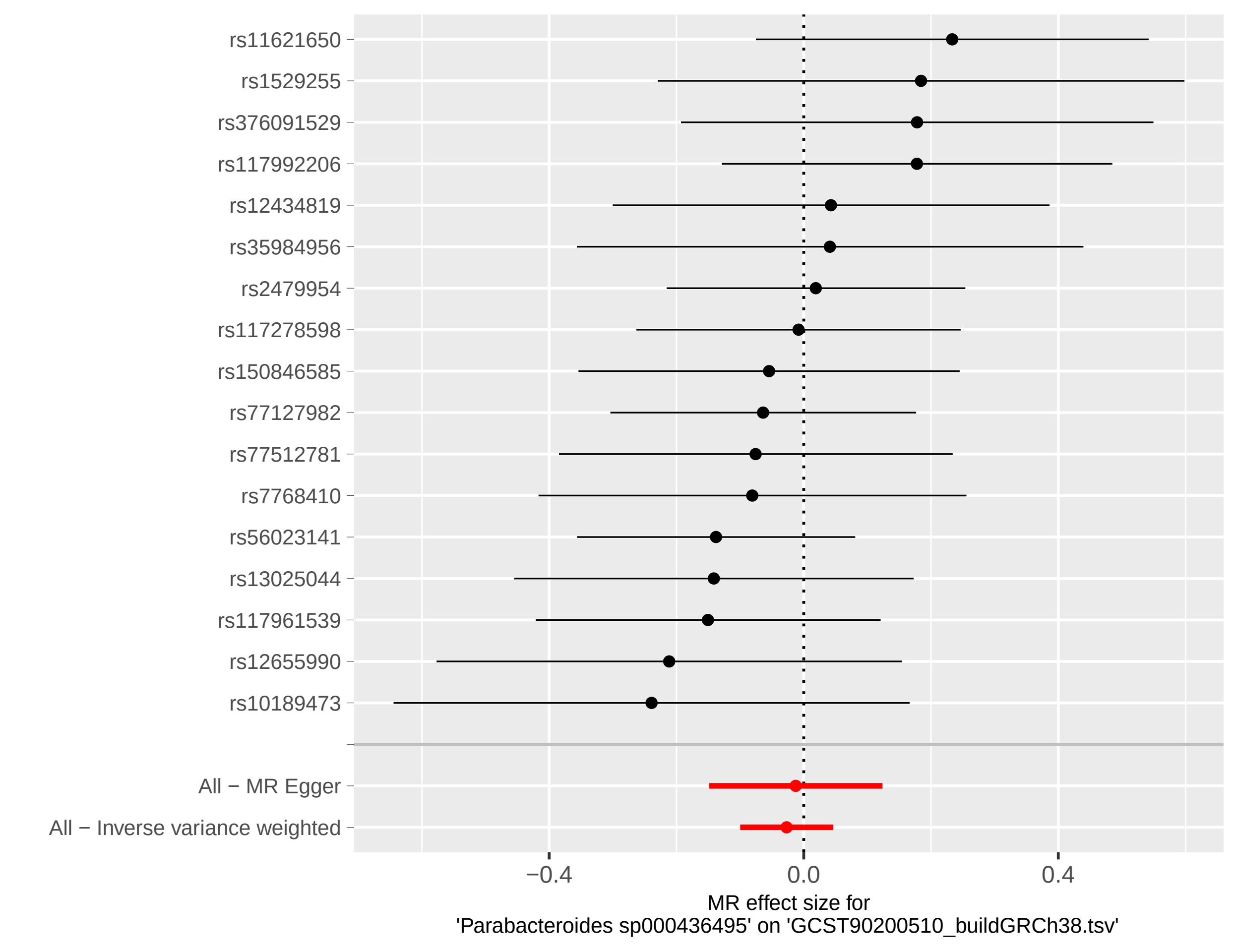

rs11621650
rs1529255
rs376091529
rs117992206
rs12434819
rs35984956
rs2479954
rs117278598
rs150846585
rs77127982
rs77512781
rs7768410
rs56023141
rs13025044
rs117961539
rs12655990
rs10189473
All − MR Egger
All − Inverse variance weighted
−0.4
0.0
0.4
MR effect size for
'Parabacteroides sp000436495' on 'GCST90200510_buildGRCh38.tsv'

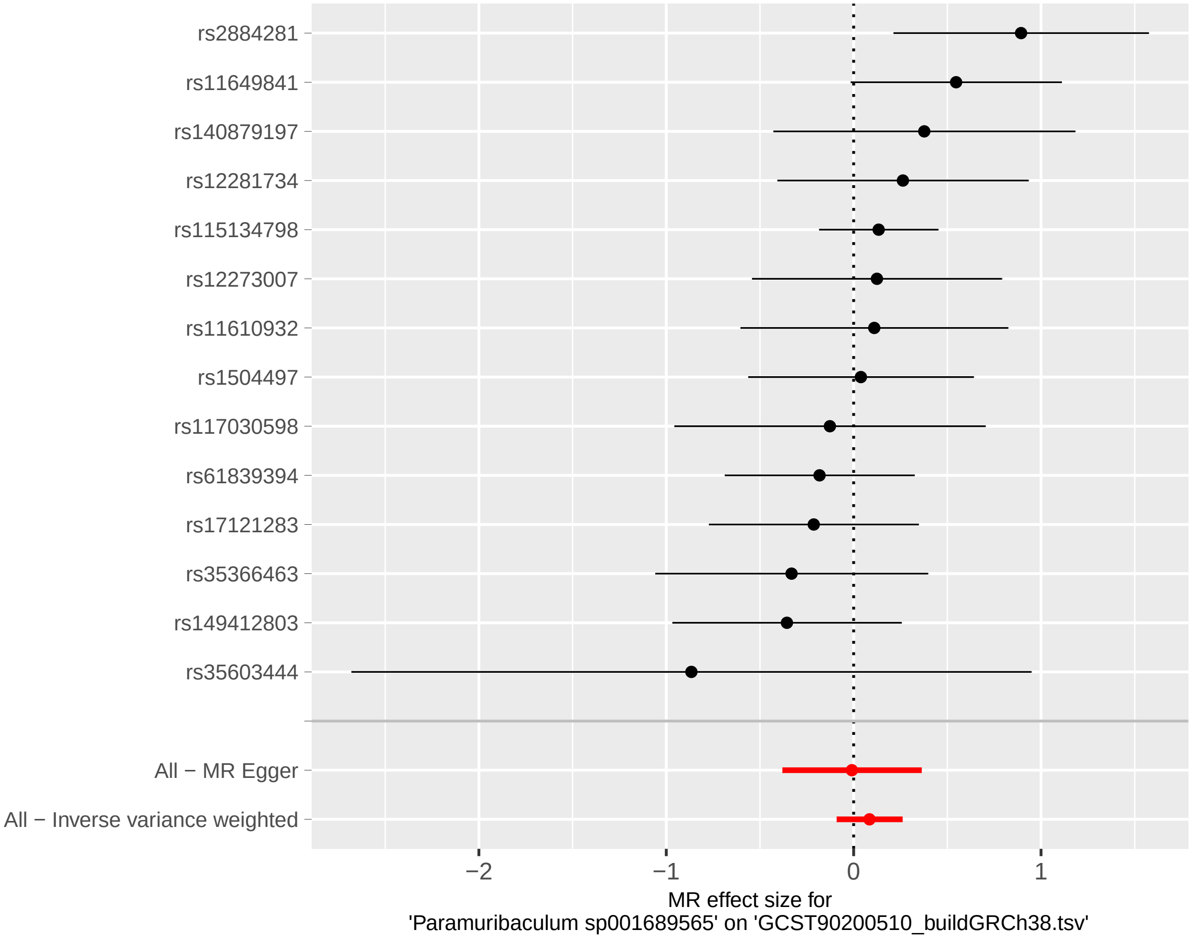

rs2884281
rs11649841
rs140879197
rs12281734
rs115134798
rs12273007
rs11610932
rs1504497
rs117030598
rs61839394
rs17121283
rs35366463
rs149412803
rs35603444
All − MR Egger
All − Inverse variance weighted
−2
−1
0
1
MR effect size for
'Paramuribaculum sp001689565' on 'GCST90200510_buildGRCh38.tsv'

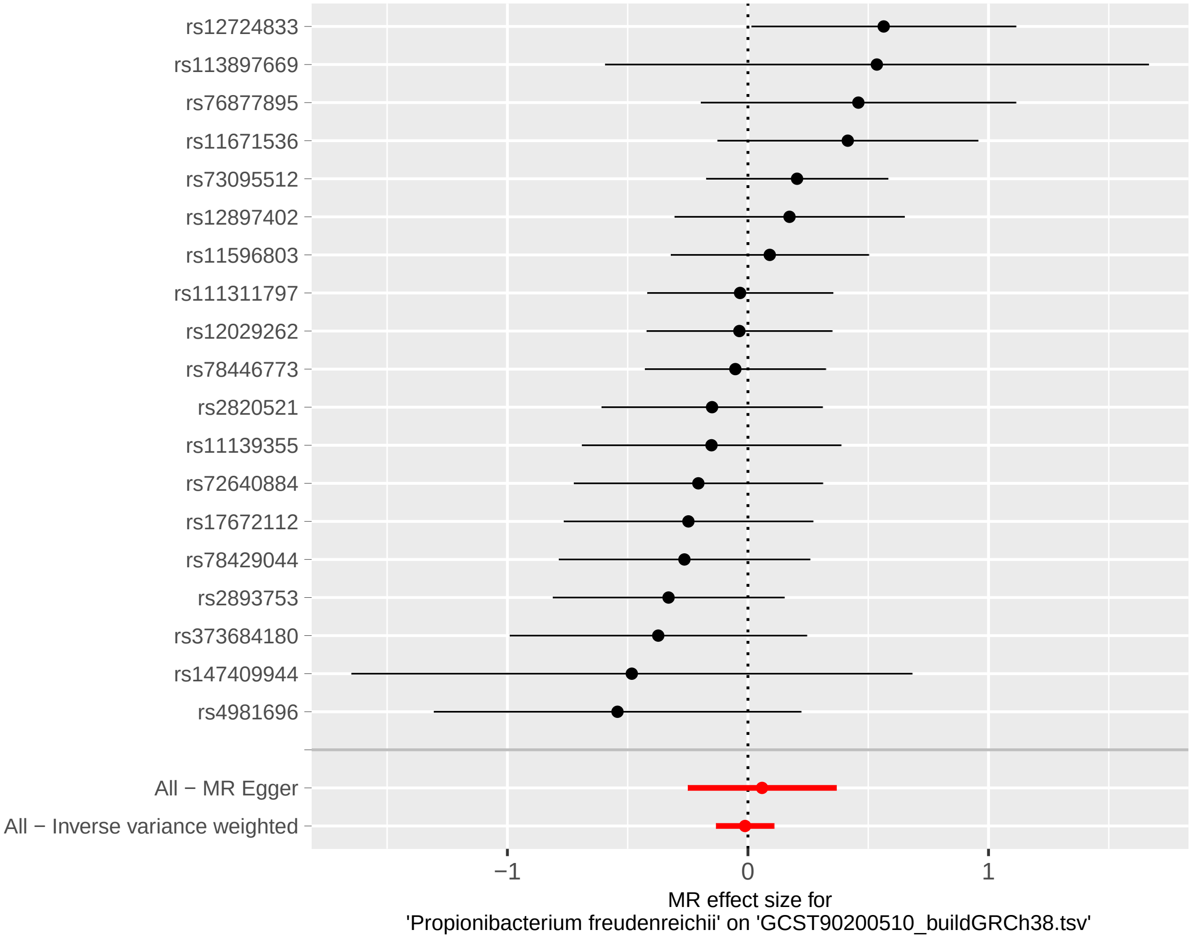
rs12724833
rs113897669
rs76877895
rs11671536
rs73095512
rs12897402
rs11596803
rs111311797
rs12029262
rs78446773
rs2820521
rs11139355
rs72640884
rs17672112
rs78429044
rs2893753
rs373684180
rs147409944
rs4981696
All − MR Egger
All − Inverse variance weighted
−1
0
1
MR effect size for
'Propionibacterium freudenreichii' on 'GCST90200510_buildGRCh38.tsv'

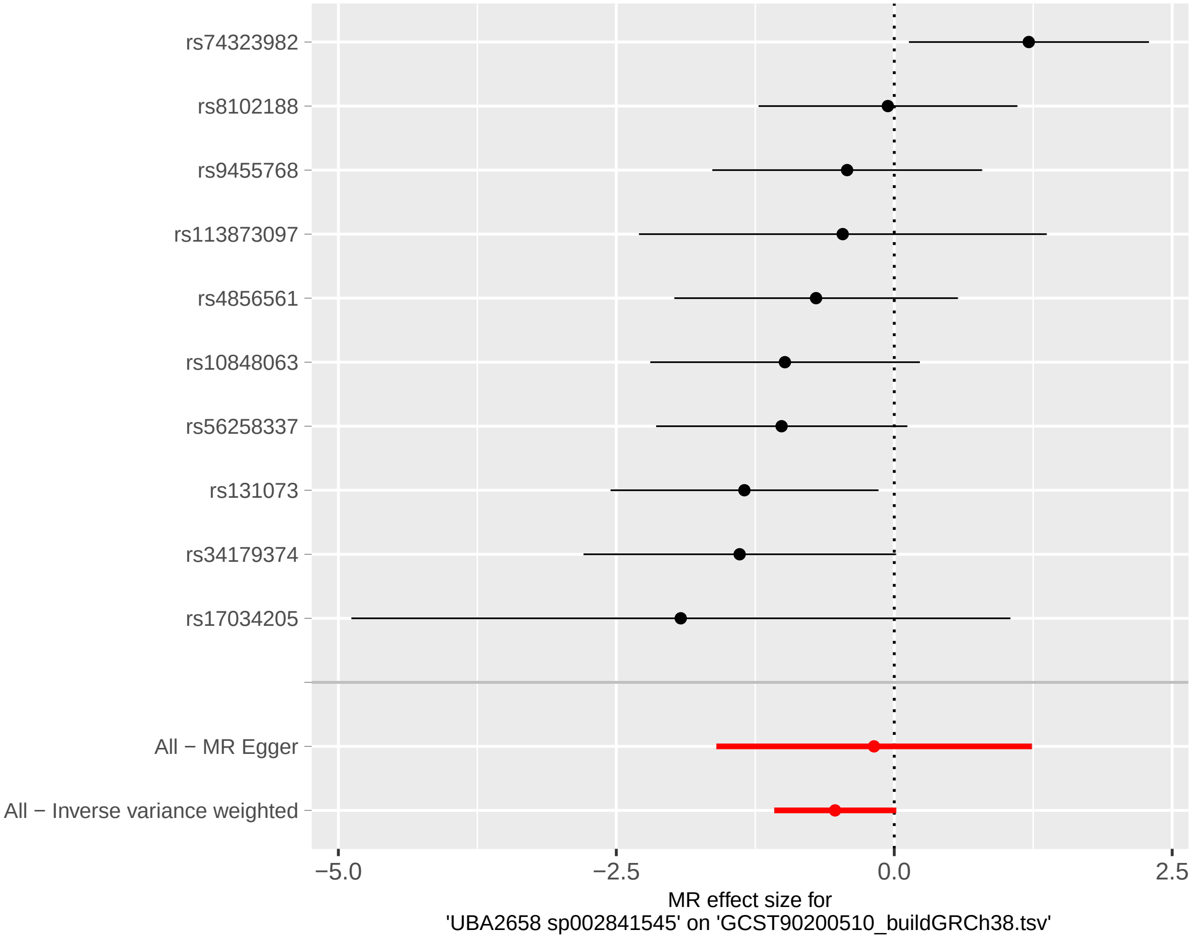

rs74323982
rs8102188
rs9455768
rs113873097
rs4856561
rs10848063
rs56258337
rs131073
rs34179374
rs17034205
All − MR Egger
All − Inverse variance weighted
−5.0
−2.5
0.0
2.5
MR effect size for
'UBA2658 sp002841545' on 'GCST90200510_buildGRCh38.tsv'

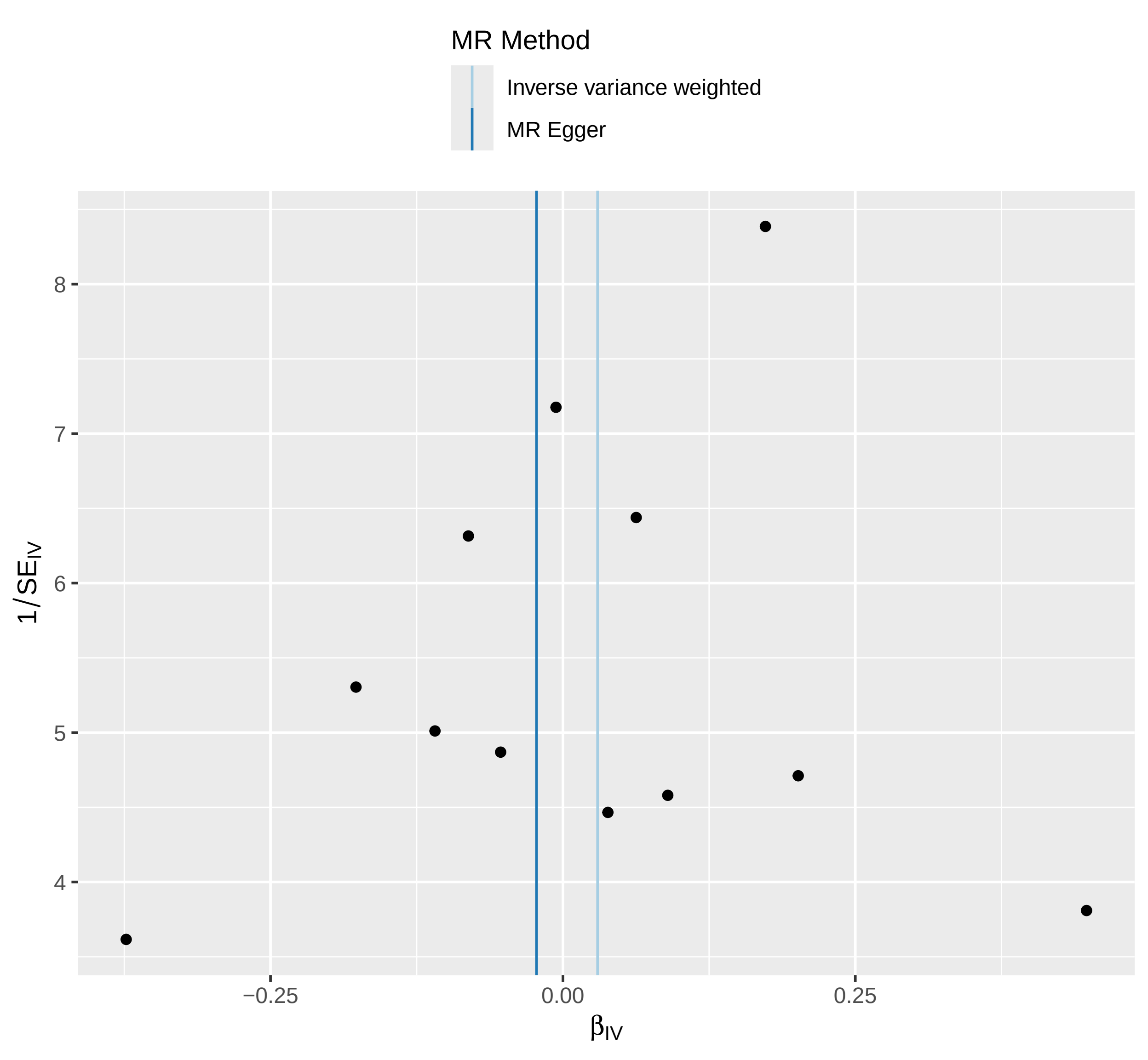
MR Method
Inverse variance weighted
MR Egger
8
7
6
5
4
1/SE_IV
−0.25
0.00
0.25
β_IV

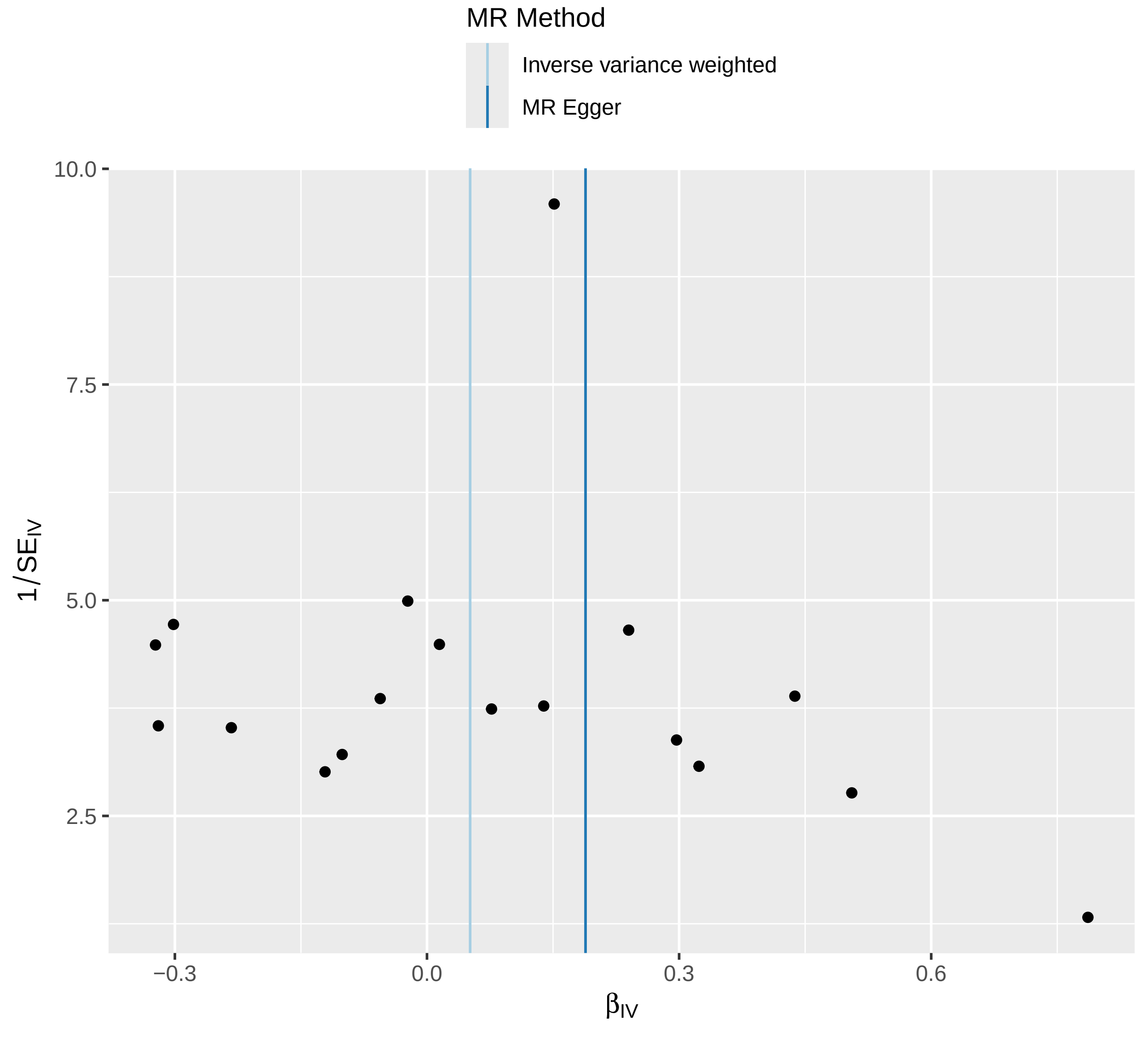

MR Method
Inverse variance weighted
MR Egger
10.0
7.5
5.0
2.5
−0.3
0.0
0.3
0.6
$1/SE_{IV}$
β_{IV}

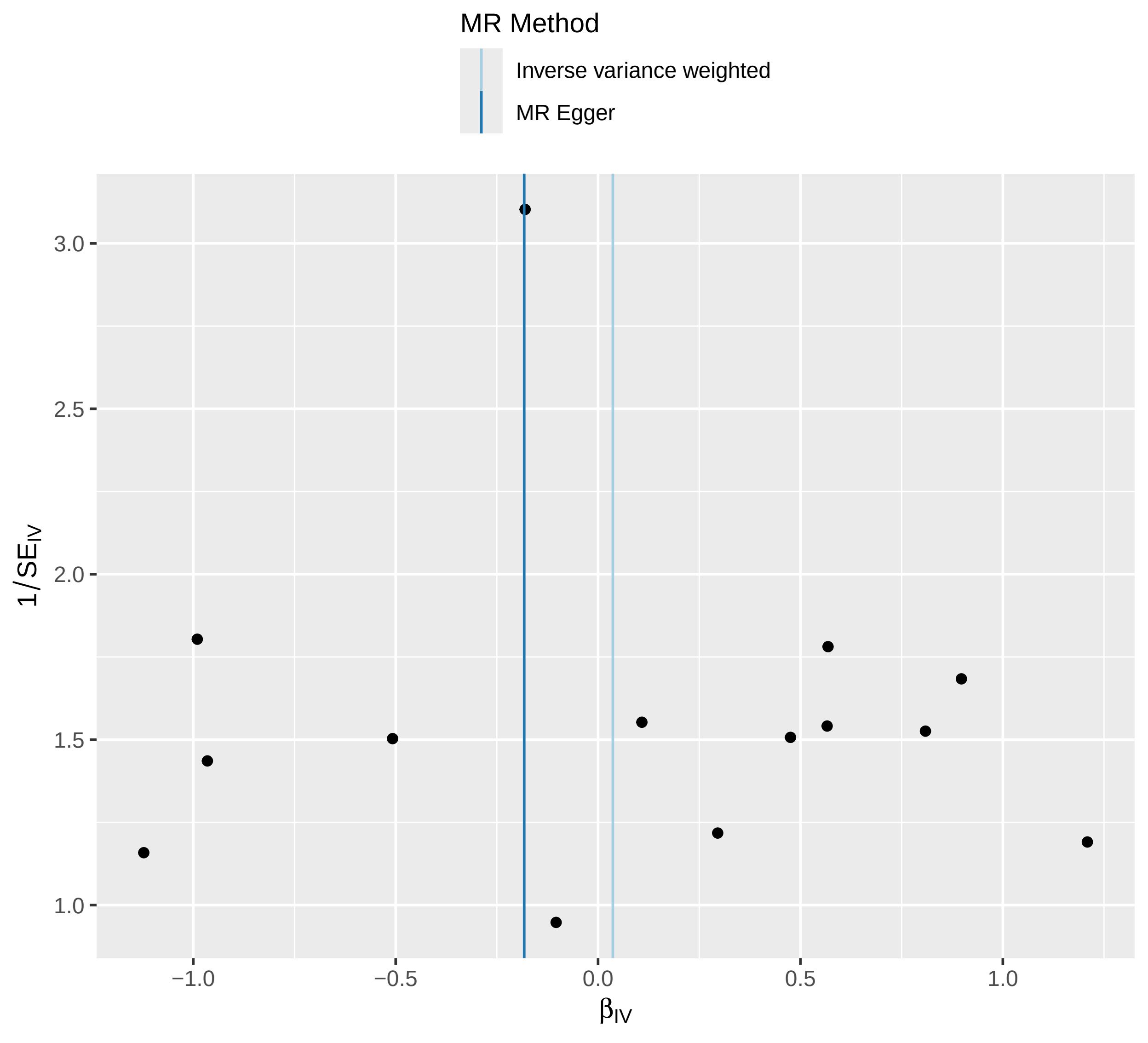
MR Method
Inverse variance weighted
MR Egger
3.0
2.5
2.0
1.5
1.0
1/SE$_{IV}$
−1.0
−0.5
0.0
0.5
1.0
β$_{IV}$

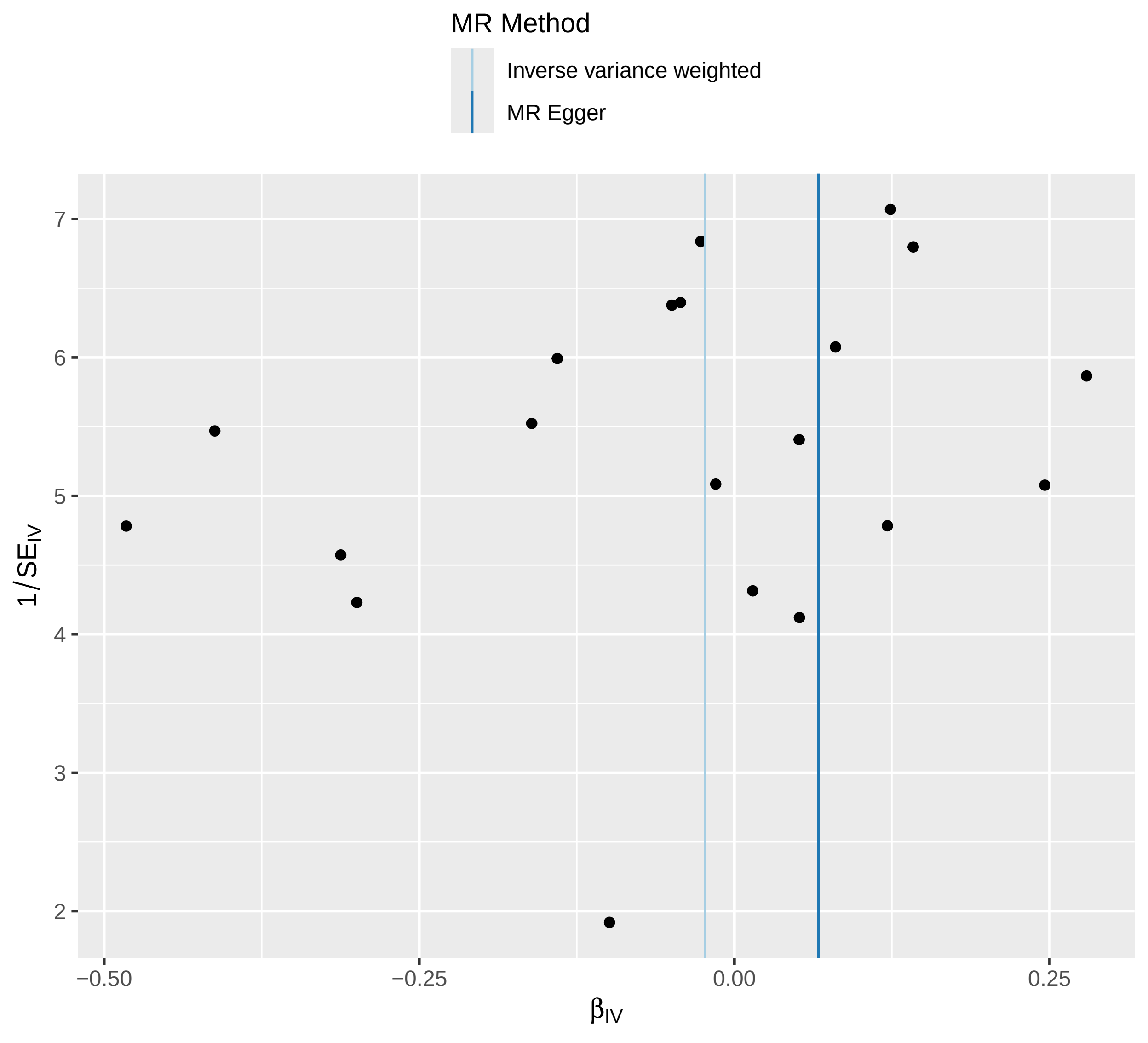
MR Method
Inverse variance weighted
MR Egger
7
6
5
4
3
2
$1/SE_{IV}$
−0.50
−0.25
0.00
0.25
β_{IV}

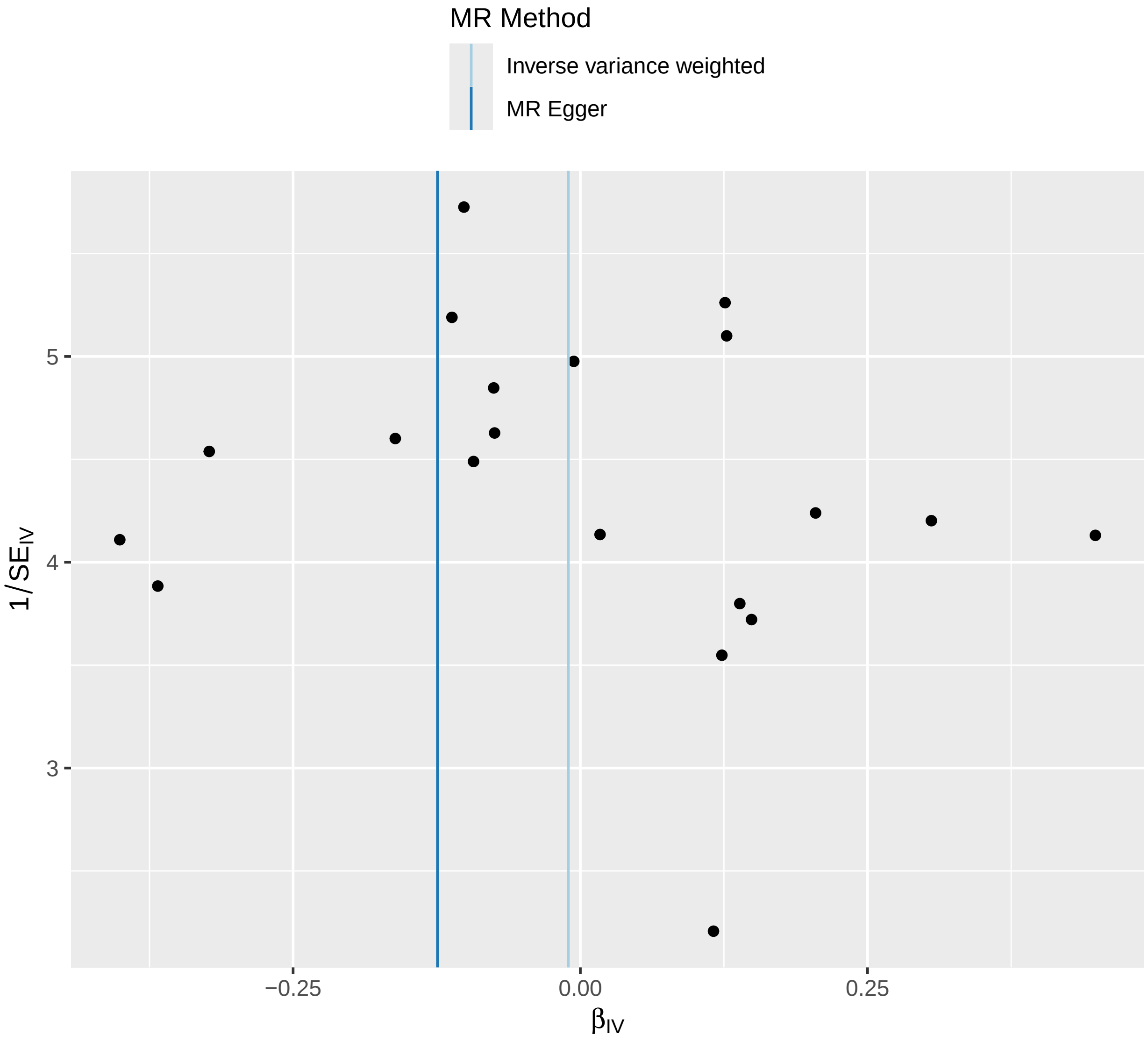
MR Method
Inverse variance weighted
MR Egger
5
4
3
1/SE_IV
−0.25
0.00
0.25
β_IV

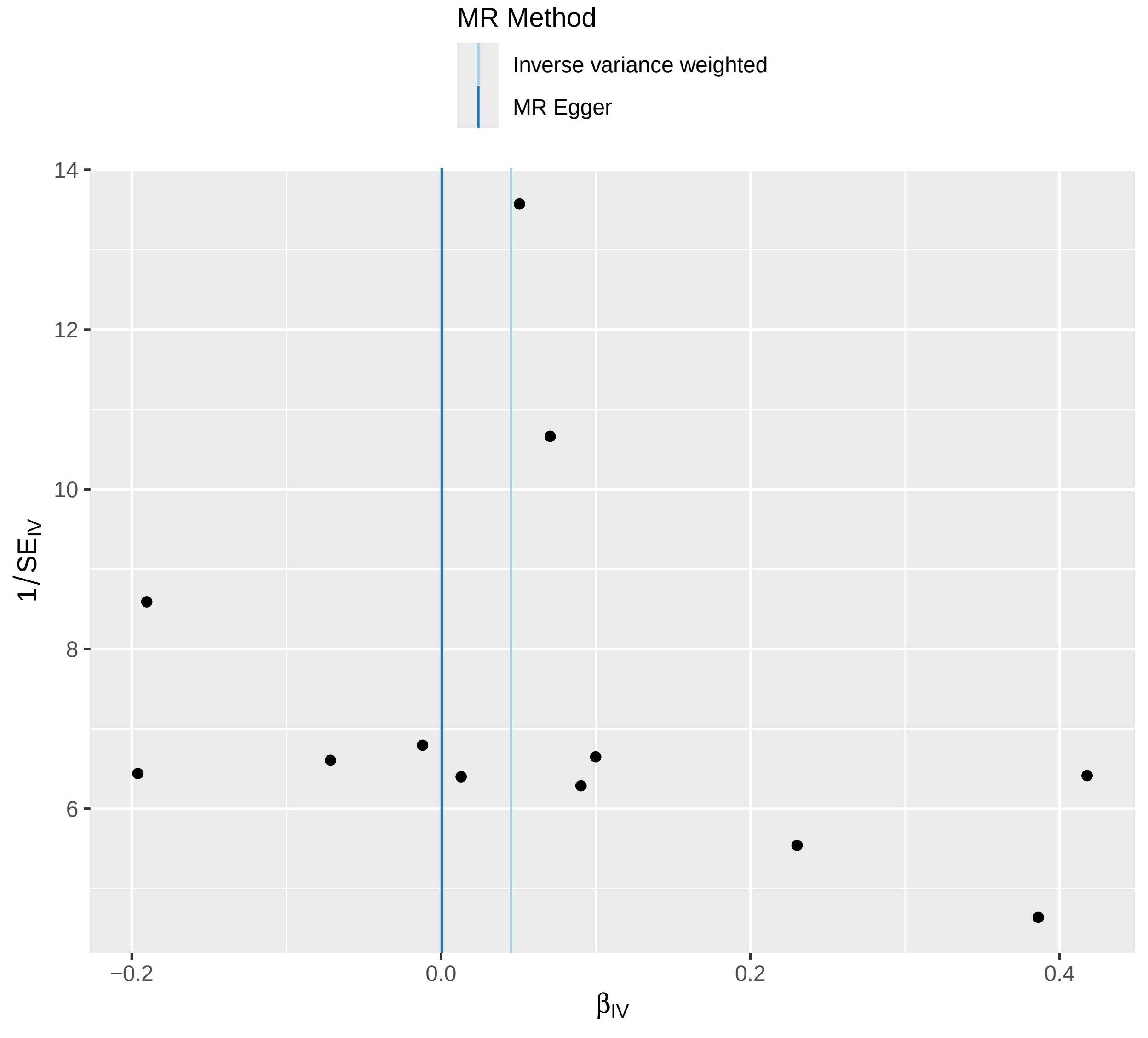
MR Method
Inverse variance weighted
MR Egger
14
12
10
8
6
1/SE$_{IV}$
−0.2
0.0
0.2
0.4
β$_{IV}$

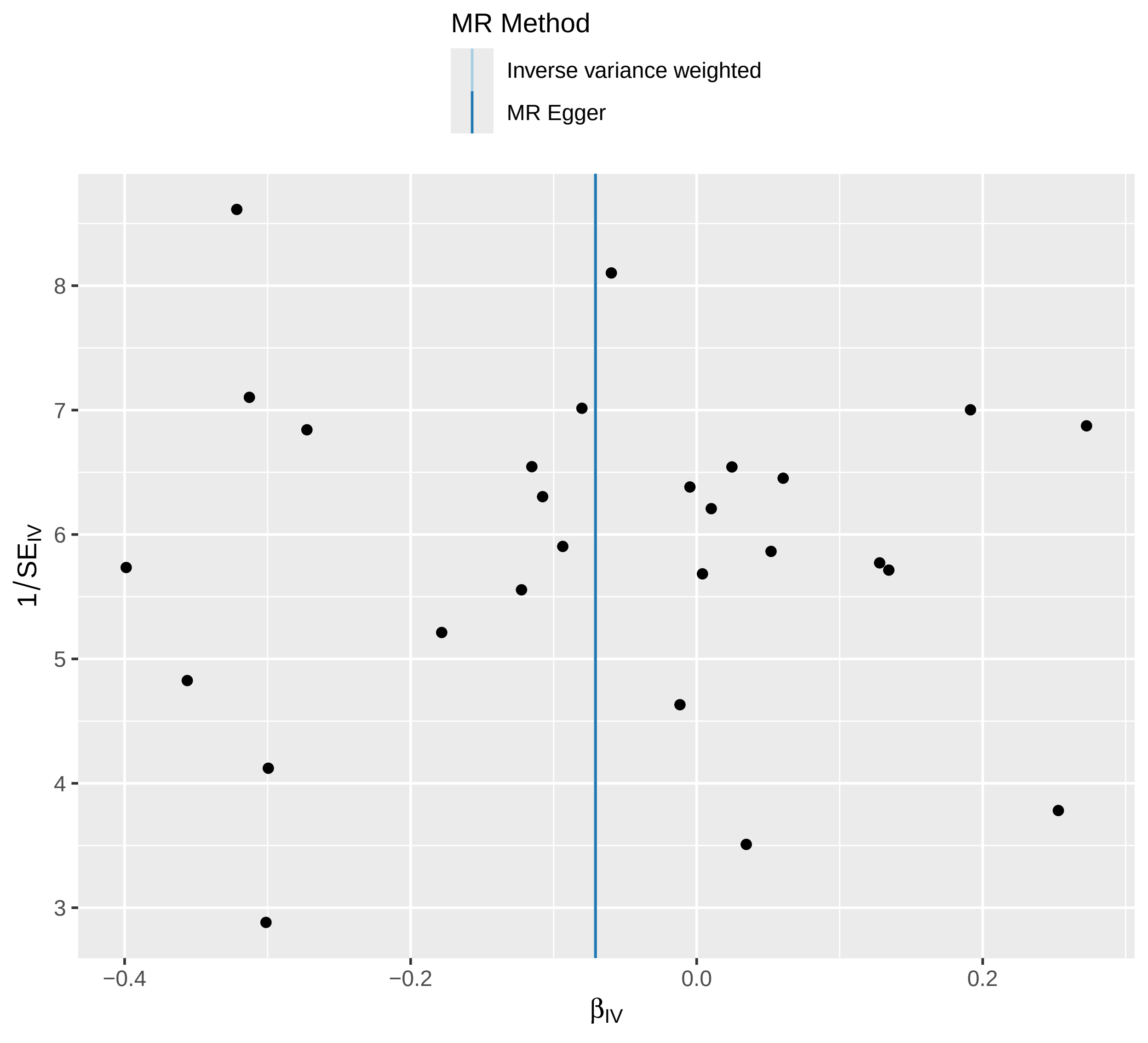

MR Method
Inverse variance weighted
MR Egger
1/SE$_{IV}$
8
7
6
5
4
3
−0.4
−0.2
0.0
0.2
β$_{IV}$

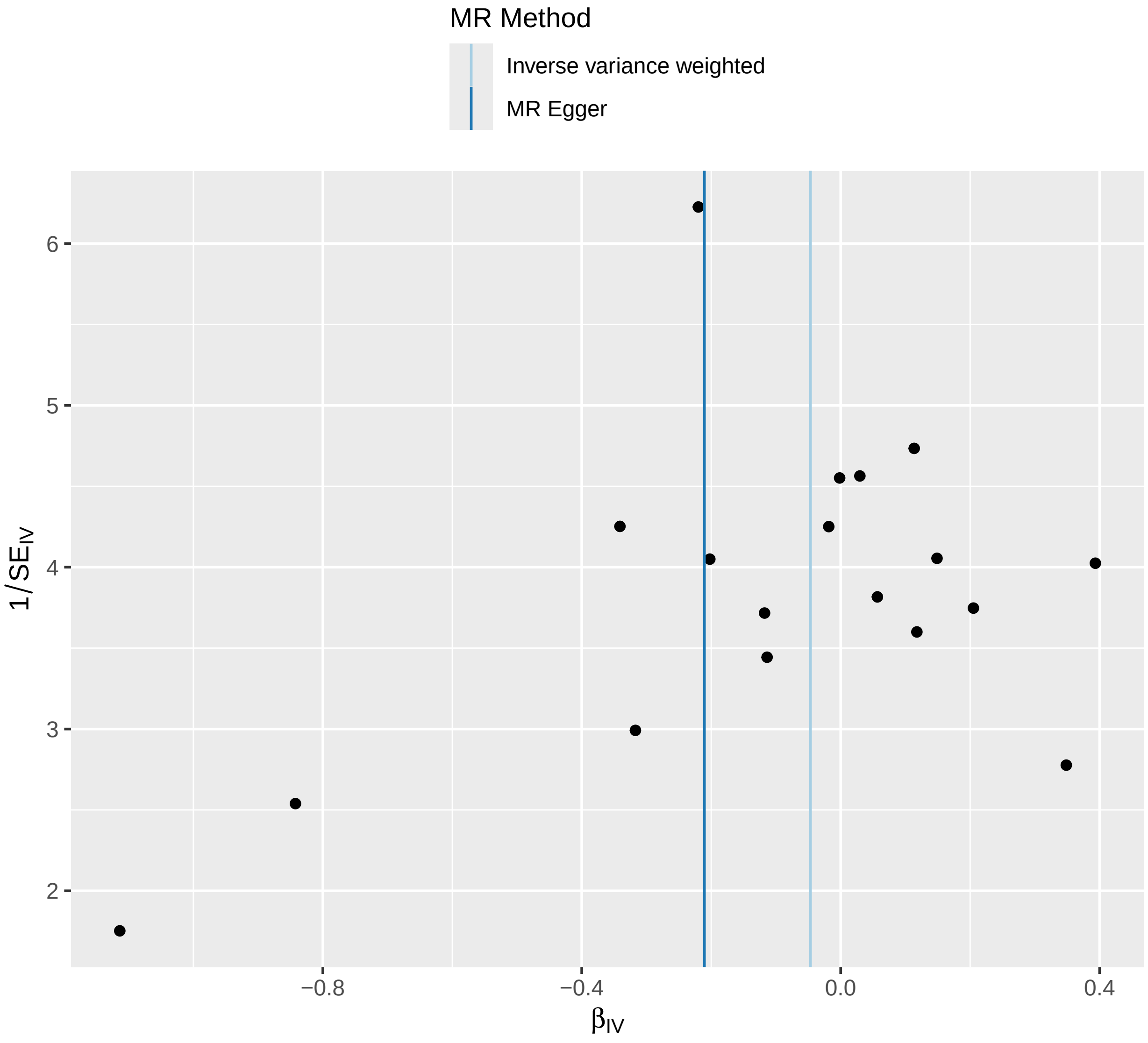

MR Method
Inverse variance weighted
MR Egger
6
5
4
3
2
1/SE$_{IV}$
−0.8
−0.4
0.0
0.4
β_{IV}

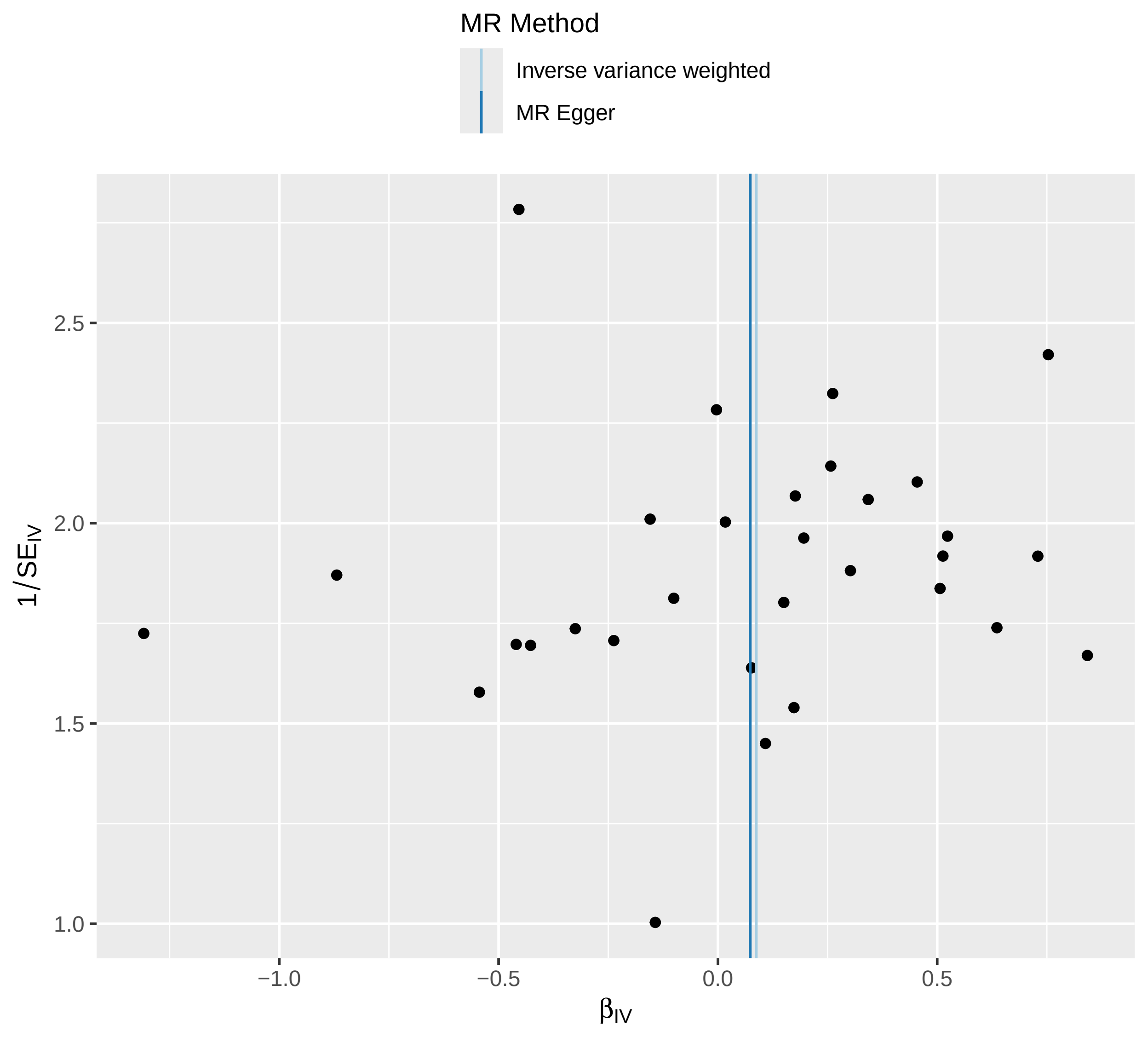

MR Method
Inverse variance weighted
MR Egger
2.5
2.0
1.5
1.0
1/SE_IV
−1.0
−0.5
0.0
0.5
β_IV

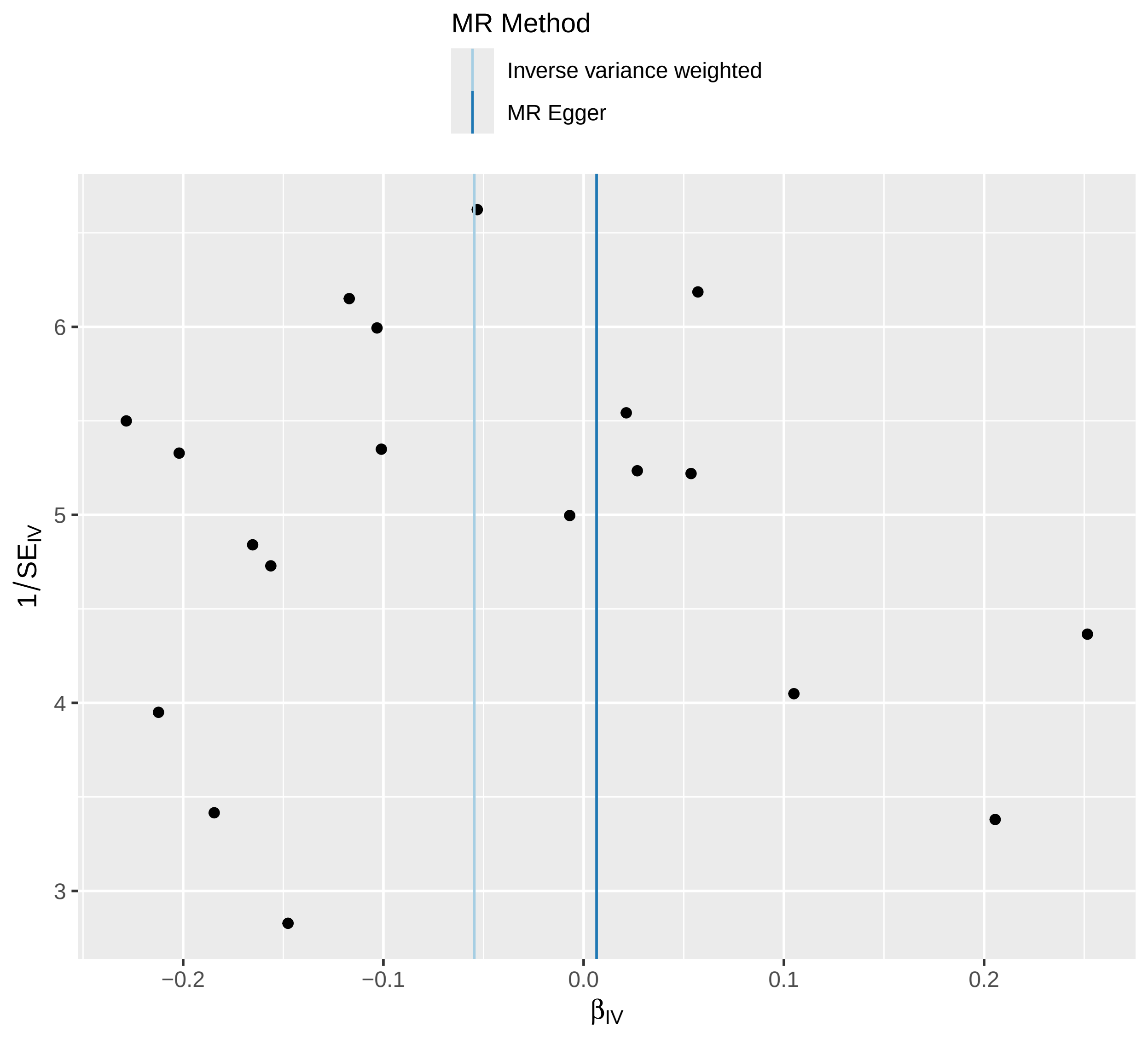
MR Method
Inverse variance weighted
MR Egger
6
5
4
3
1/SE_IV
−0.2
−0.1
0.0
0.1
0.2
β_IV

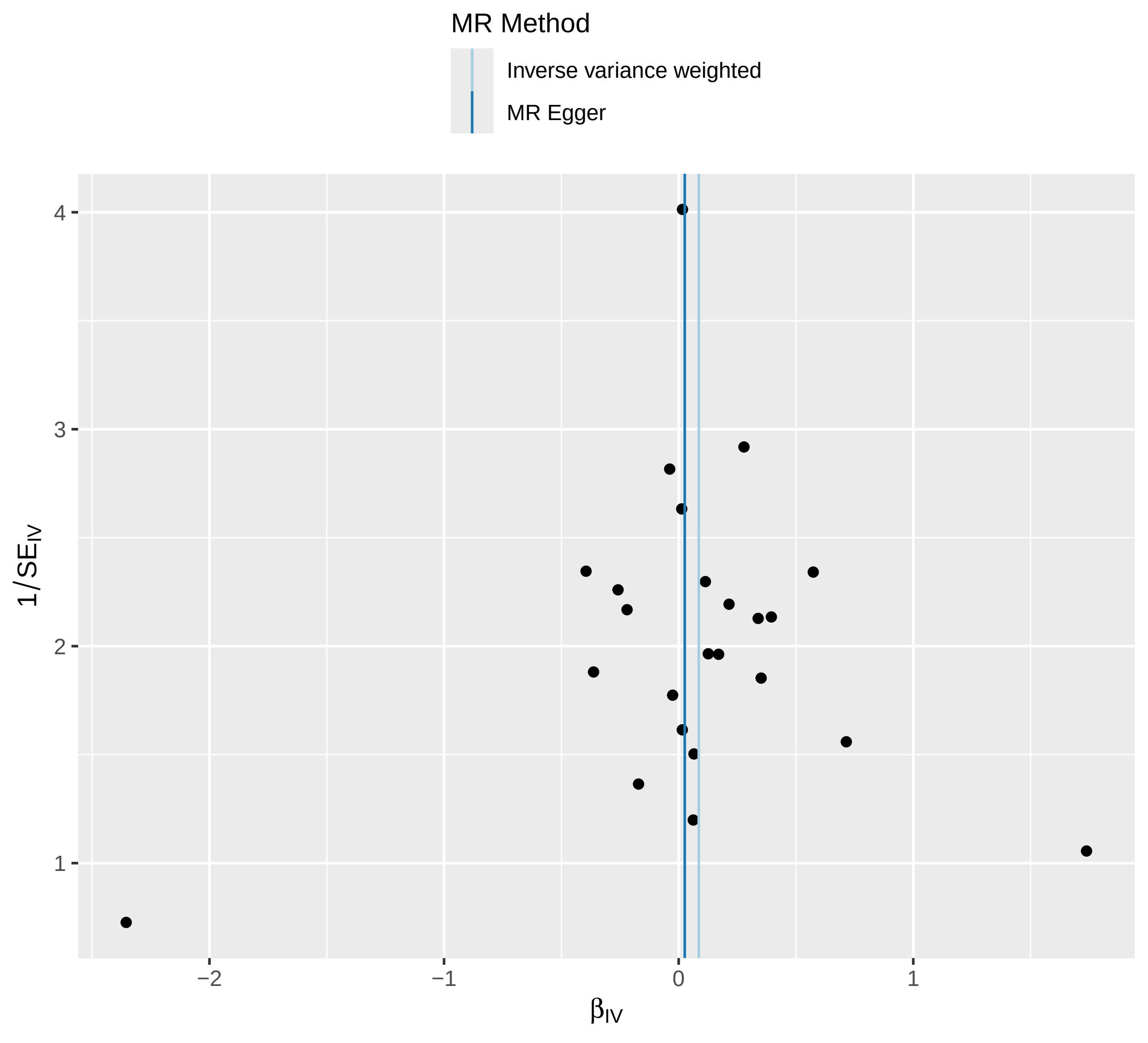
MR Method
Inverse variance weighted
MR Egger
4
3
2
1
1/SE$_{IV}$
−2
−1
0
1
β$_{IV}$

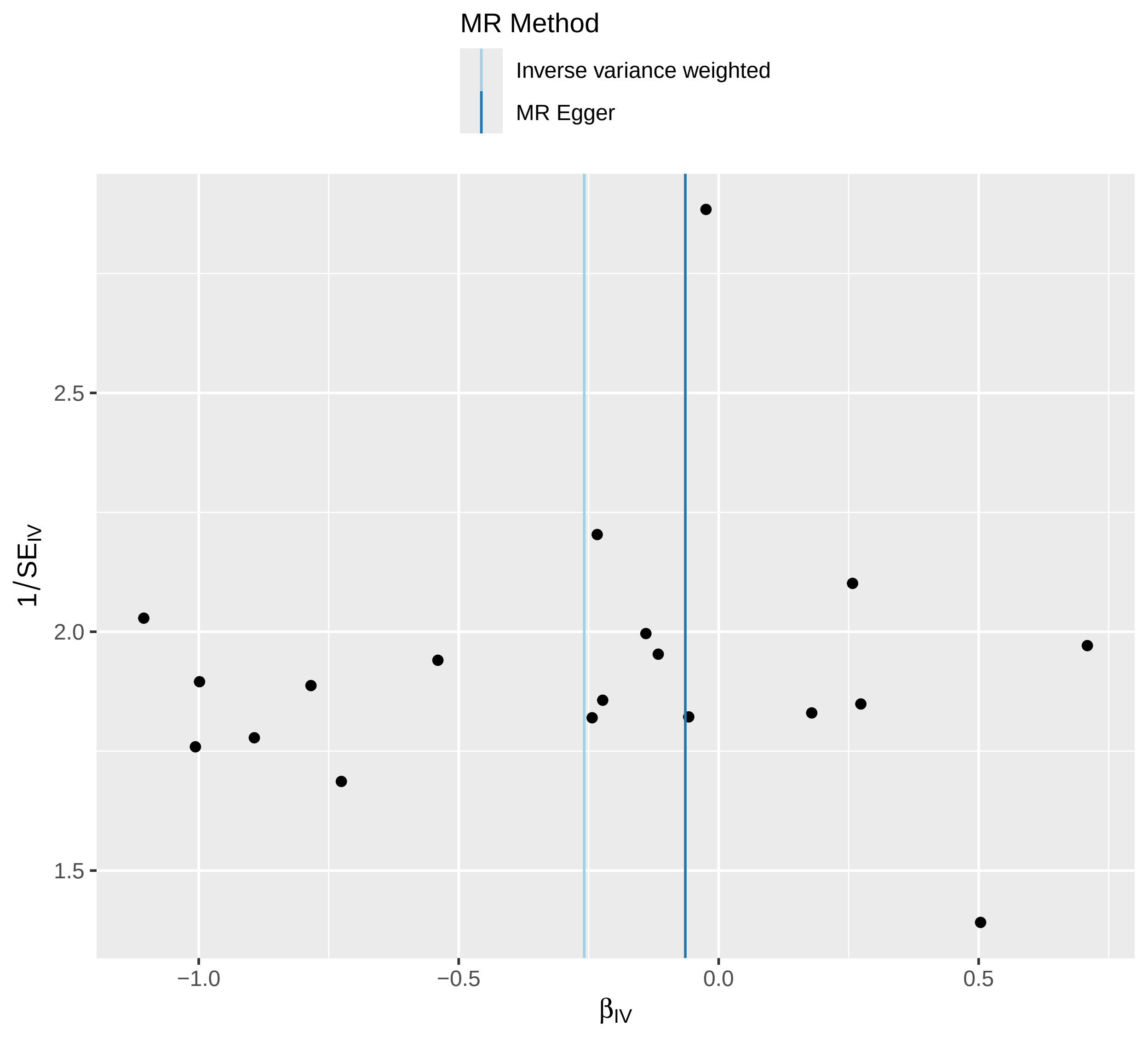
MR Method
Inverse variance weighted
MR Egger
2.5
2.0
1.5
1/SE$_{IV}$
−1.0
−0.5
0.0
0.5
β$_{IV}$

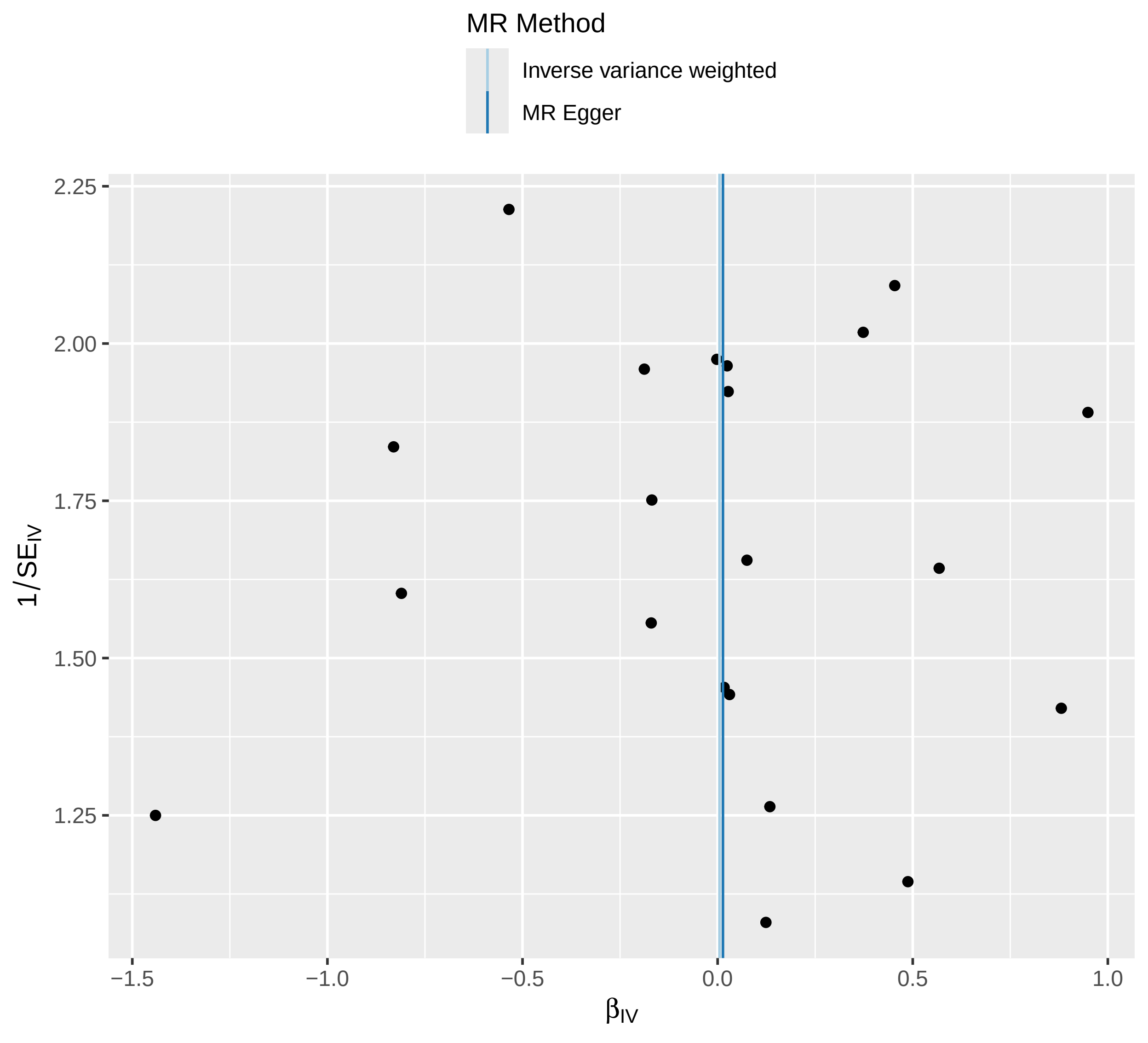

MR Method
Inverse variance weighted
MR Egger
2.25
2.00
1.75
1.50
1.25
1/SE_IV
−1.5
−1.0
−0.5
0.0
0.5
1.0
β_IV

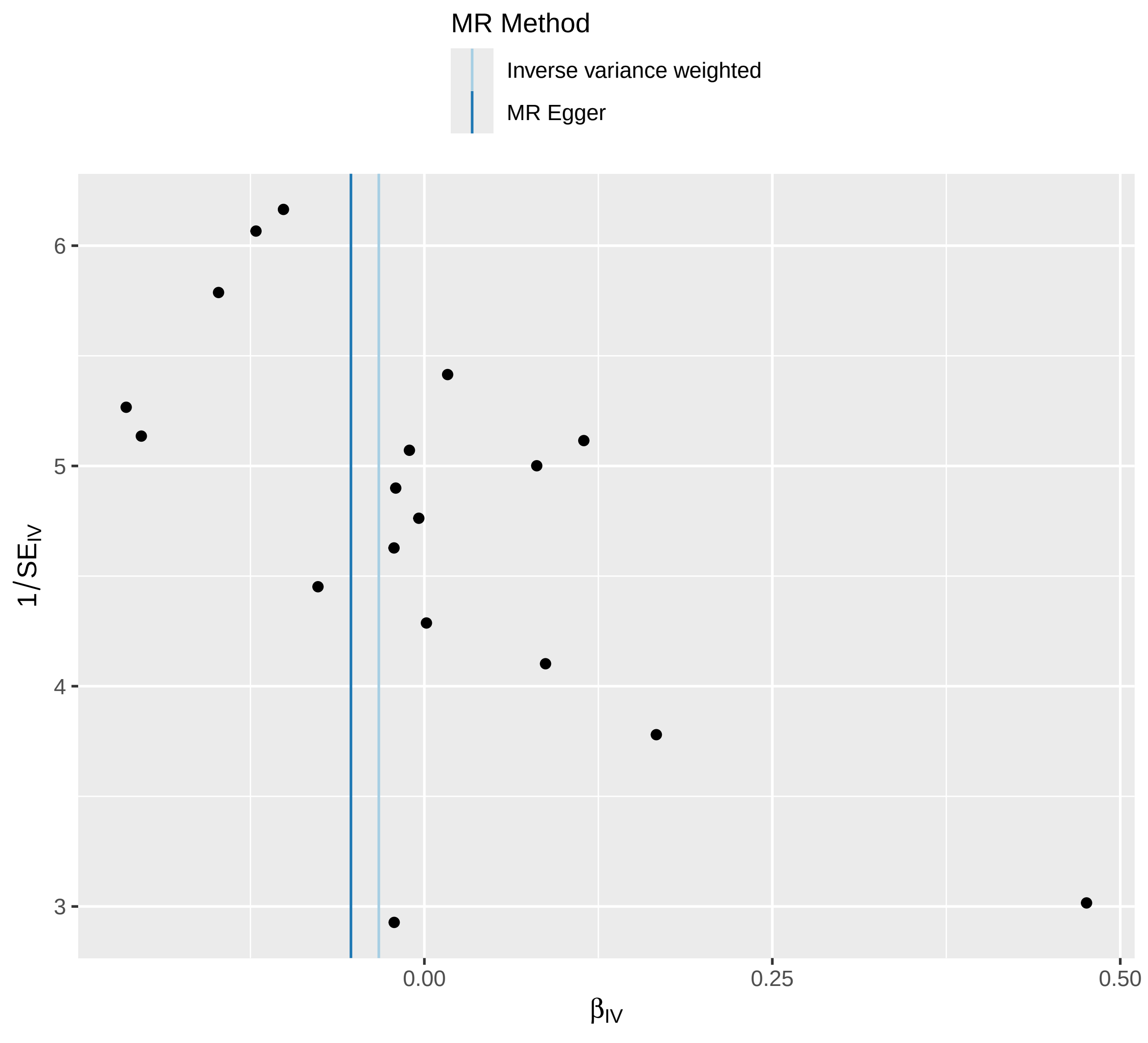

MR Method
Inverse variance weighted
MR Egger
6
5
4
3
1/SE$_{IV}$
0.00
0.25
0.50
β$_{IV}$

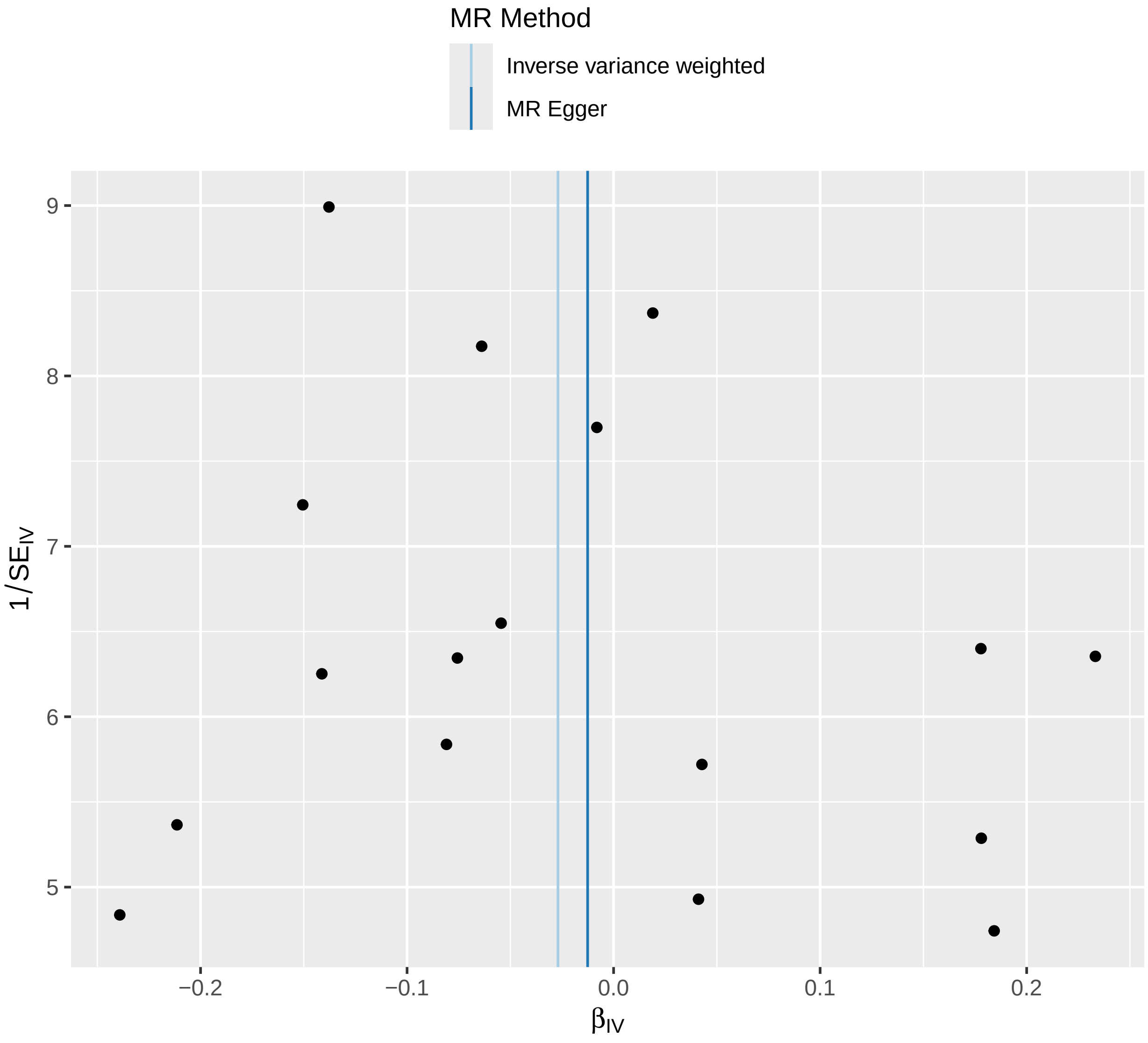
MR Method
Inverse variance weighted
MR Egger
9
8
7
6
5
1/SE$_{IV}$
−0.2
−0.1
0.0
0.1
0.2
β$_{IV}$

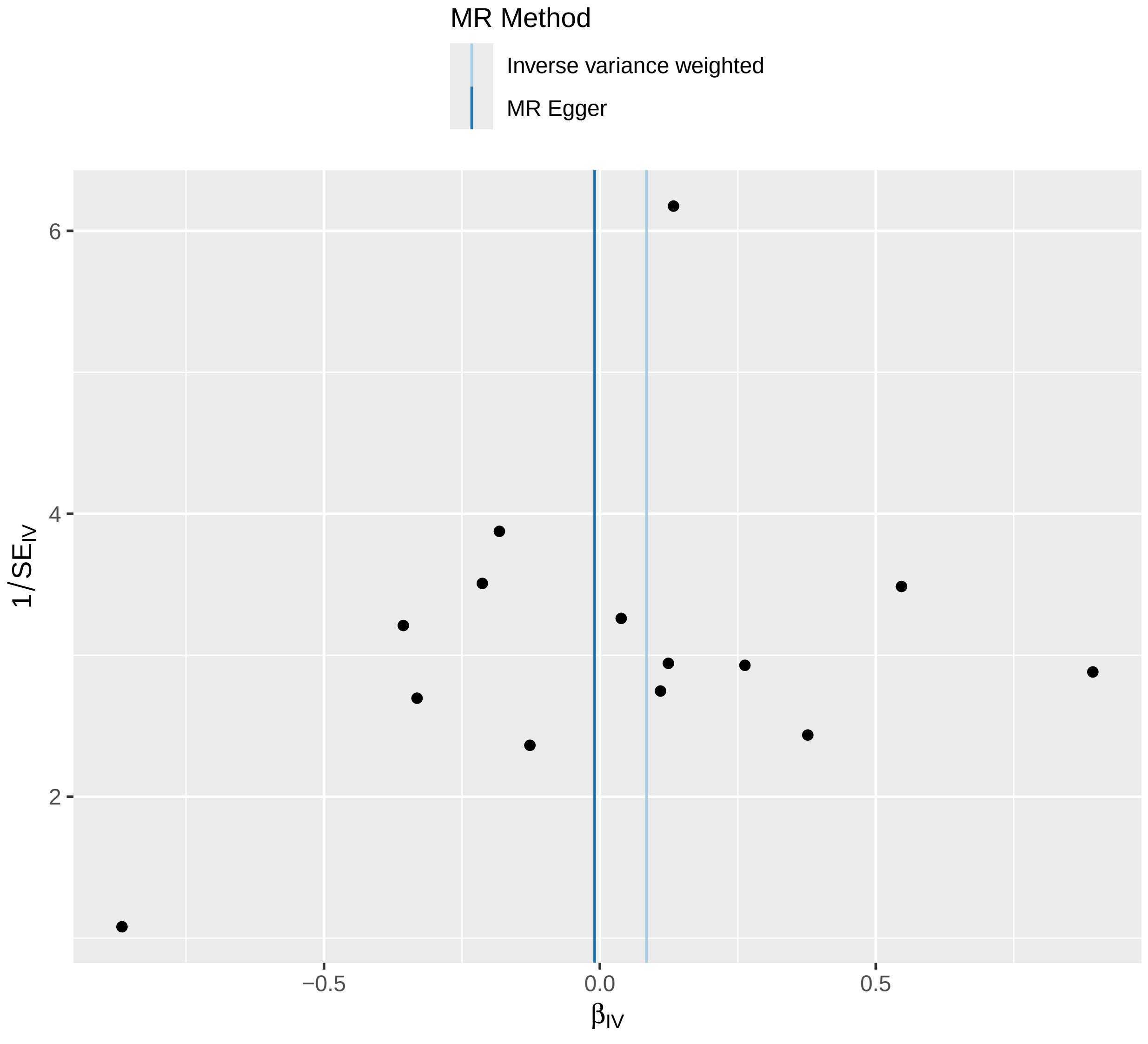
MR Method
Inverse variance weighted
MR Egger
6
4
2
1/SE$_{IV}$
−0.5
0.0
0.5
β$_{IV}$

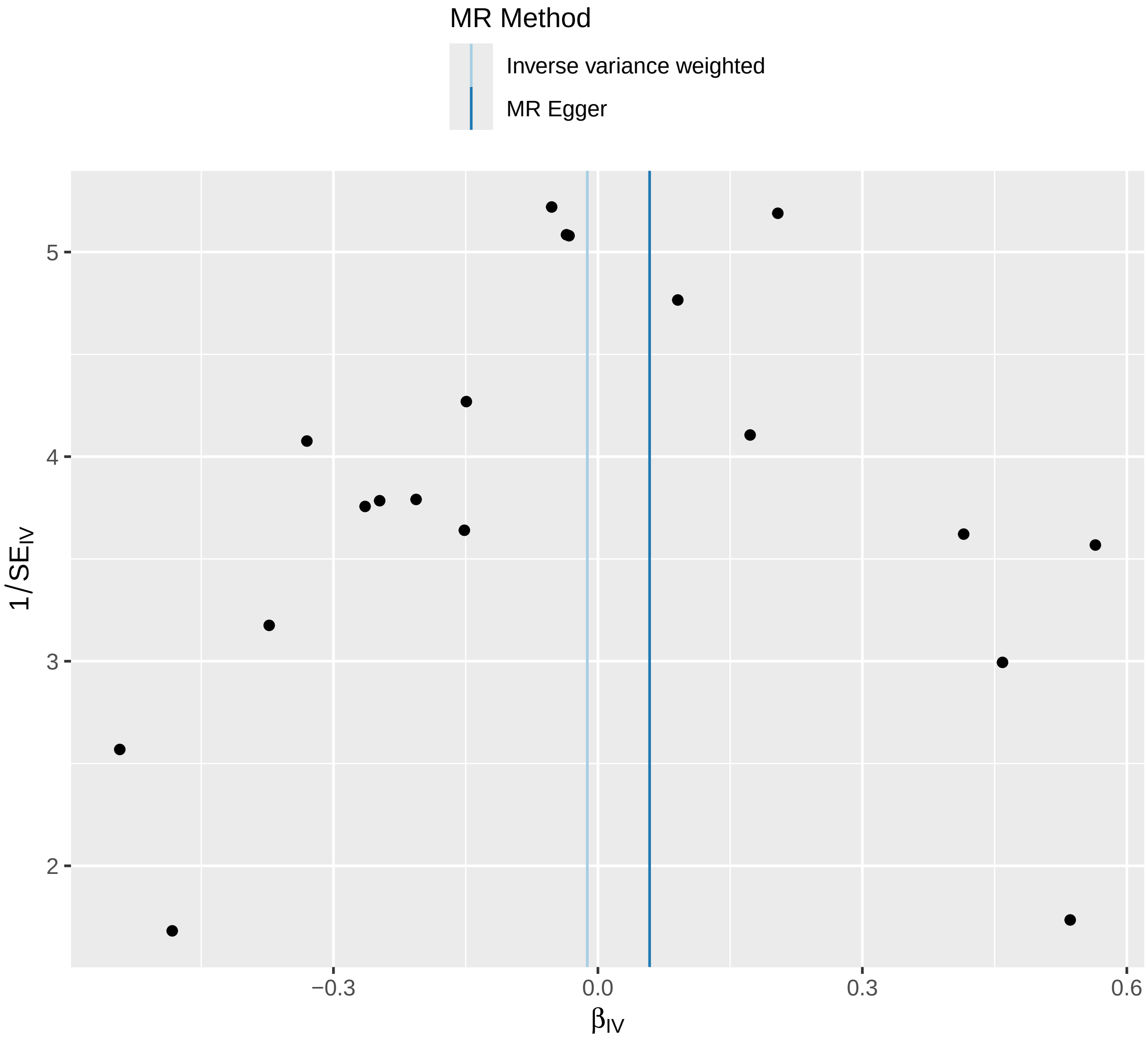

MR Method
Inverse variance weighted
MR Egger
5
4
3
2
1/SE$_{IV}$
−0.3
0.0
0.3
0.6
β$_{IV}$

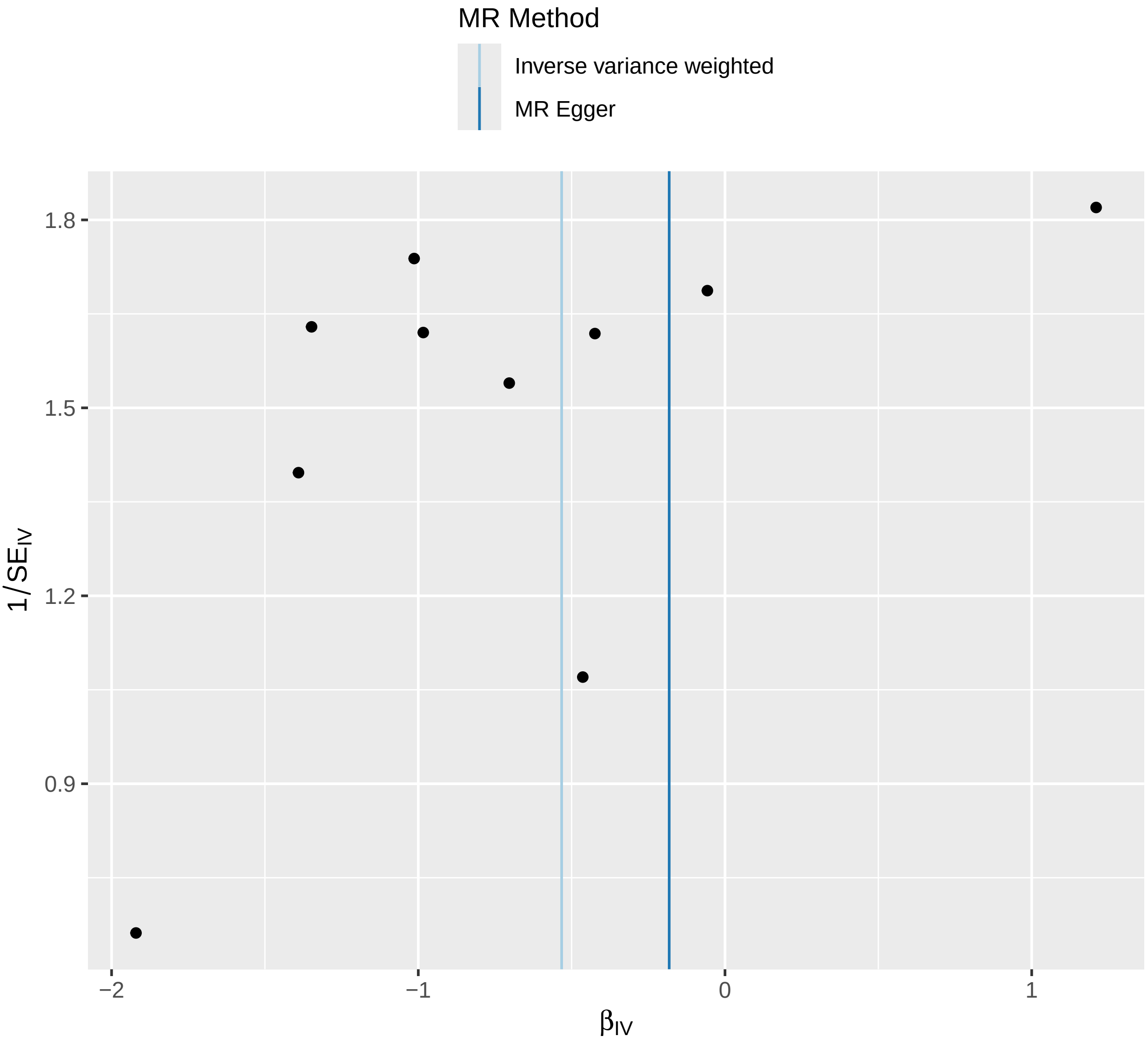

MR Method
Inverse variance weighted
MR Egger
1.8
1.5
1.2
0.9
−2
−1
0
1
1/SE_IV
β_IV

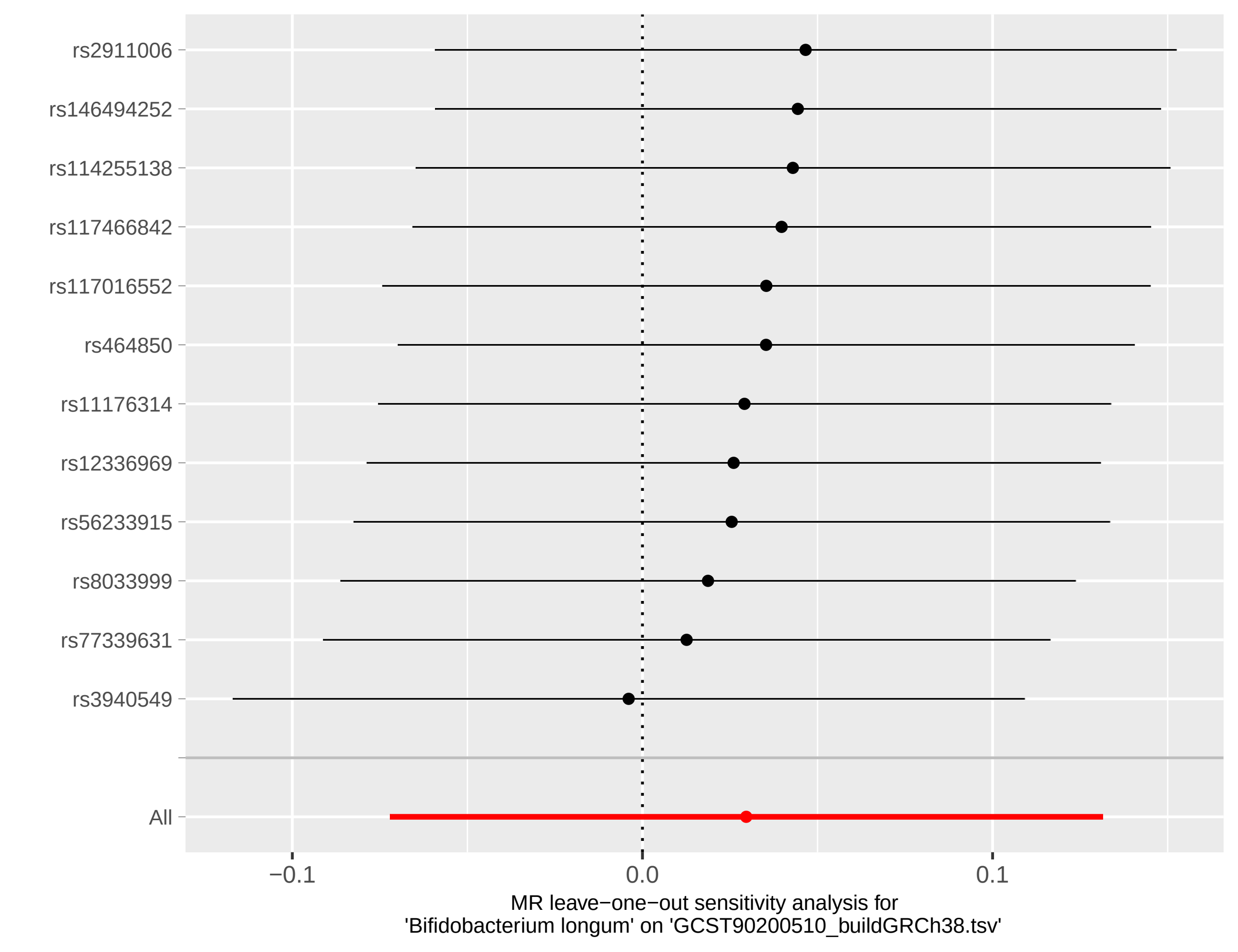
rs2911006
rs146494252
rs114255138
rs117466842
rs117016552
rs464850
rs11176314
rs12336969
rs56233915
rs8033999
rs77339631
rs3940549
All
−0.1
0.0
0.1
MR leave−one−out sensitivity analysis for
'Bifidobacterium longum' on 'GCST90200510_buildGRCh38.tsv'

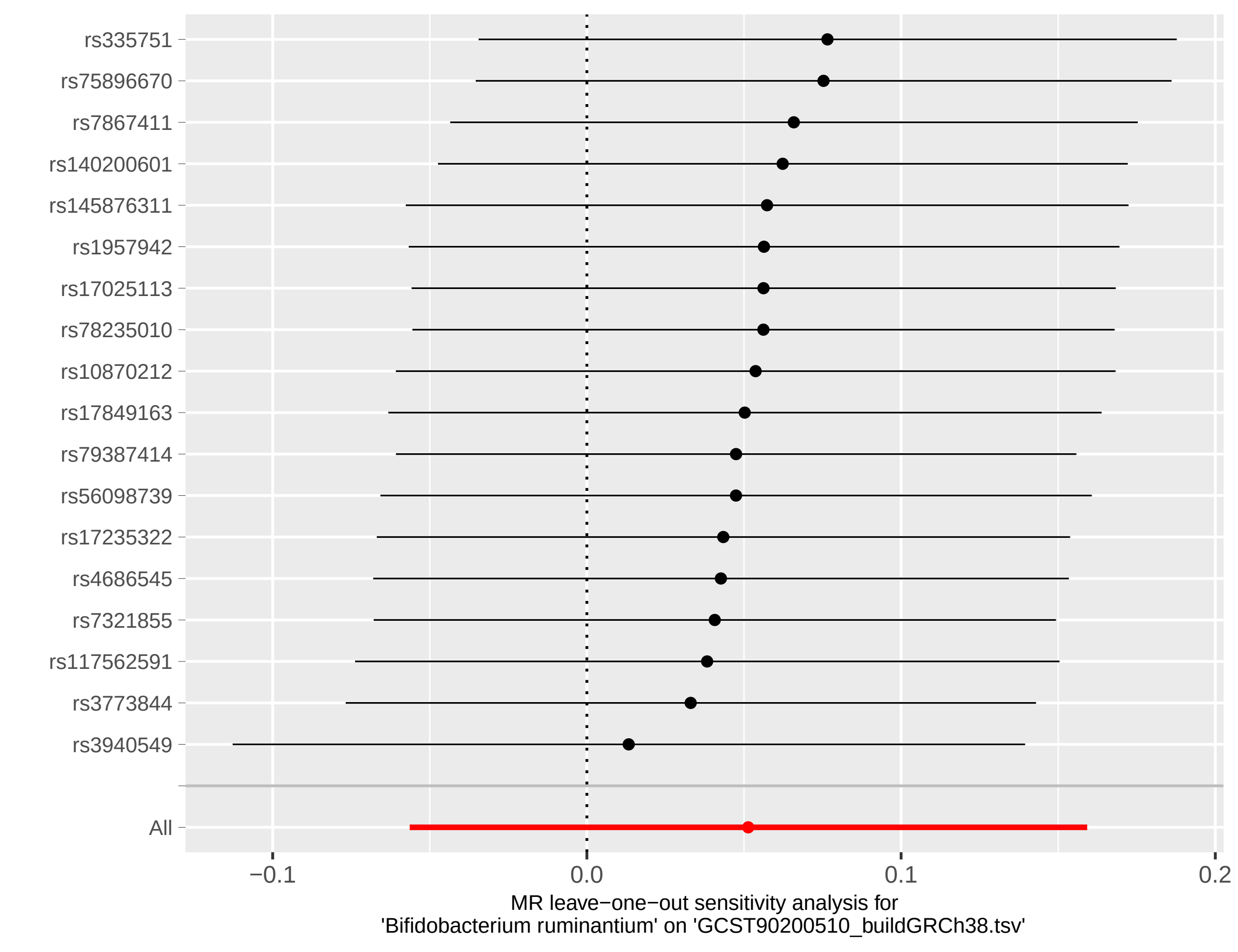
rs335751
rs75896670
rs7867411
rs140200601
rs145876311
rs1957942
rs17025113
rs78235010
rs10870212
rs17849163
rs79387414
rs56098739
rs17235322
rs4686545
rs7321855
rs117562591
rs3773844
rs3940549
All
−0.1
0.0
0.1
0.2
MR leave−one−out sensitivity analysis for
'Bifidobacterium ruminantium' on 'GCST90200510_buildGRCh38.tsv'

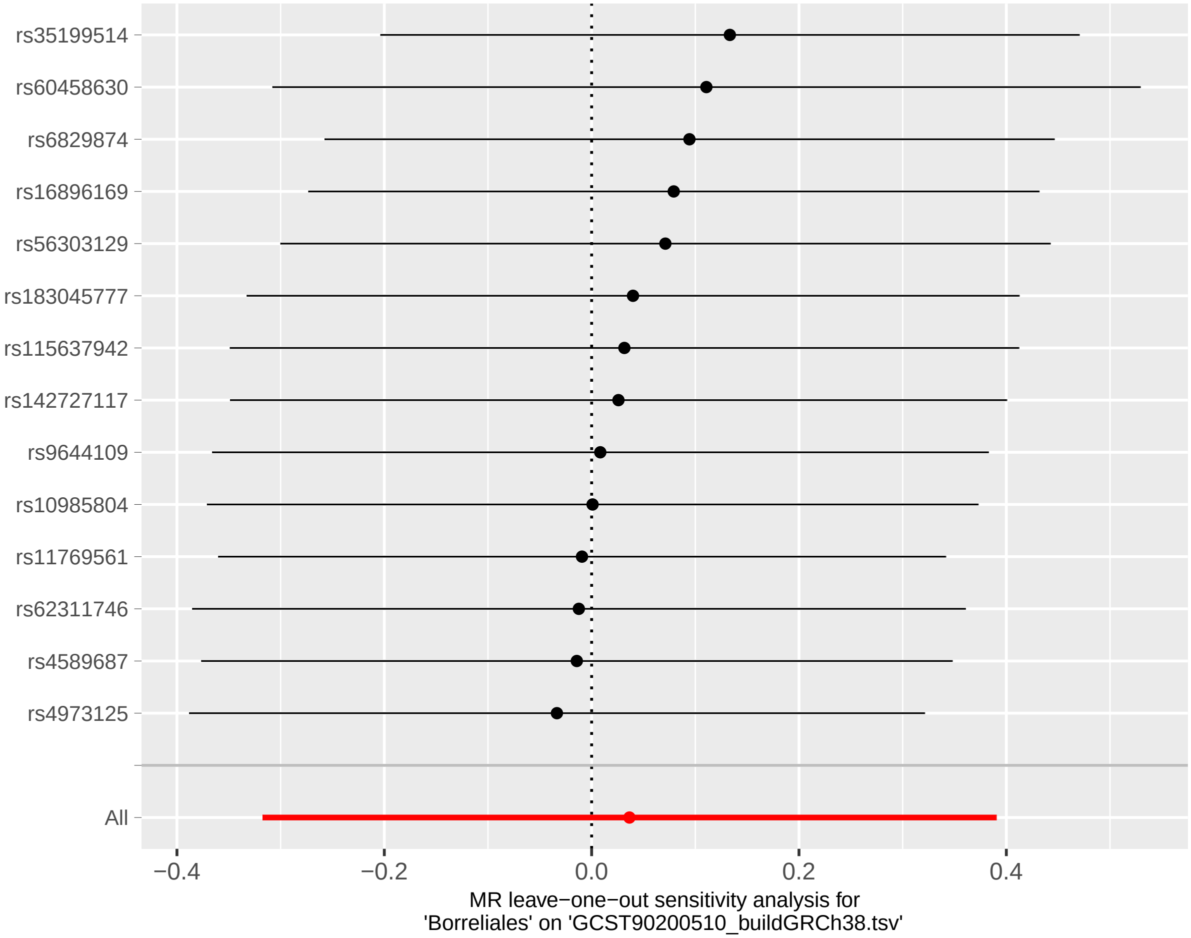
rs35199514
rs60458630
rs6829874
rs16896169
rs56303129
rs183045777
rs115637942
rs142727117
rs9644109
rs10985804
rs11769561
rs62311746
rs4589687
rs4973125
All
−0.4
−0.2
0.0
0.2
0.4
MR leave−one−out sensitivity analysis for
'Borreliales' on 'GCST90200510_buildGRCh38.tsv'

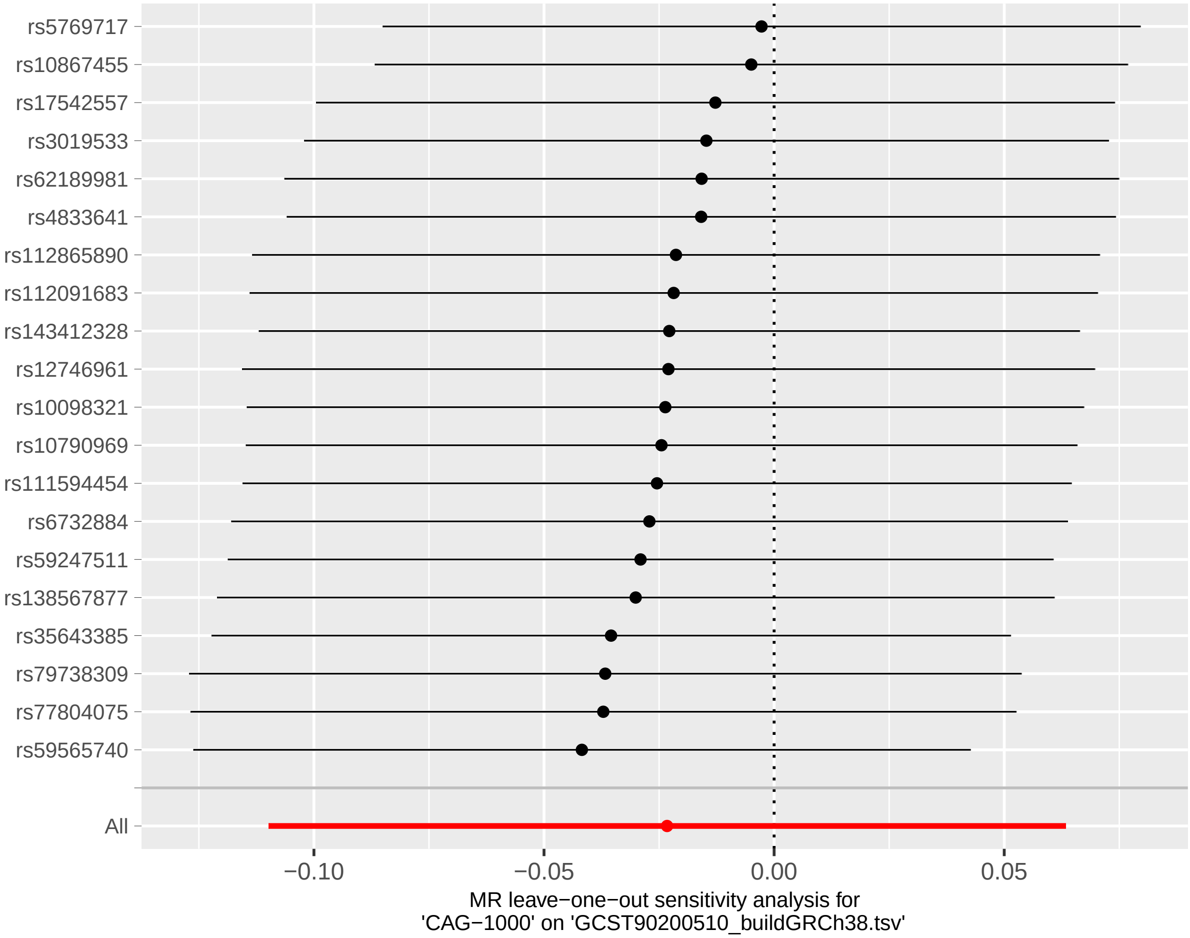
rs5769717
rs10867455
rs17542557
rs3019533
rs62189981
rs4833641
rs112865890
rs112091683
rs143412328
rs12746961
rs10098321
rs10790969
rs111594454
rs6732884
rs59247511
rs138567877
rs35643385
rs79738309
rs77804075
rs59565740
All
−0.10
−0.05
0.00
0.05
MR leave−one−out sensitivity analysis for
'CAG−1000' on 'GCST90200510_buildGRCh38.tsv'

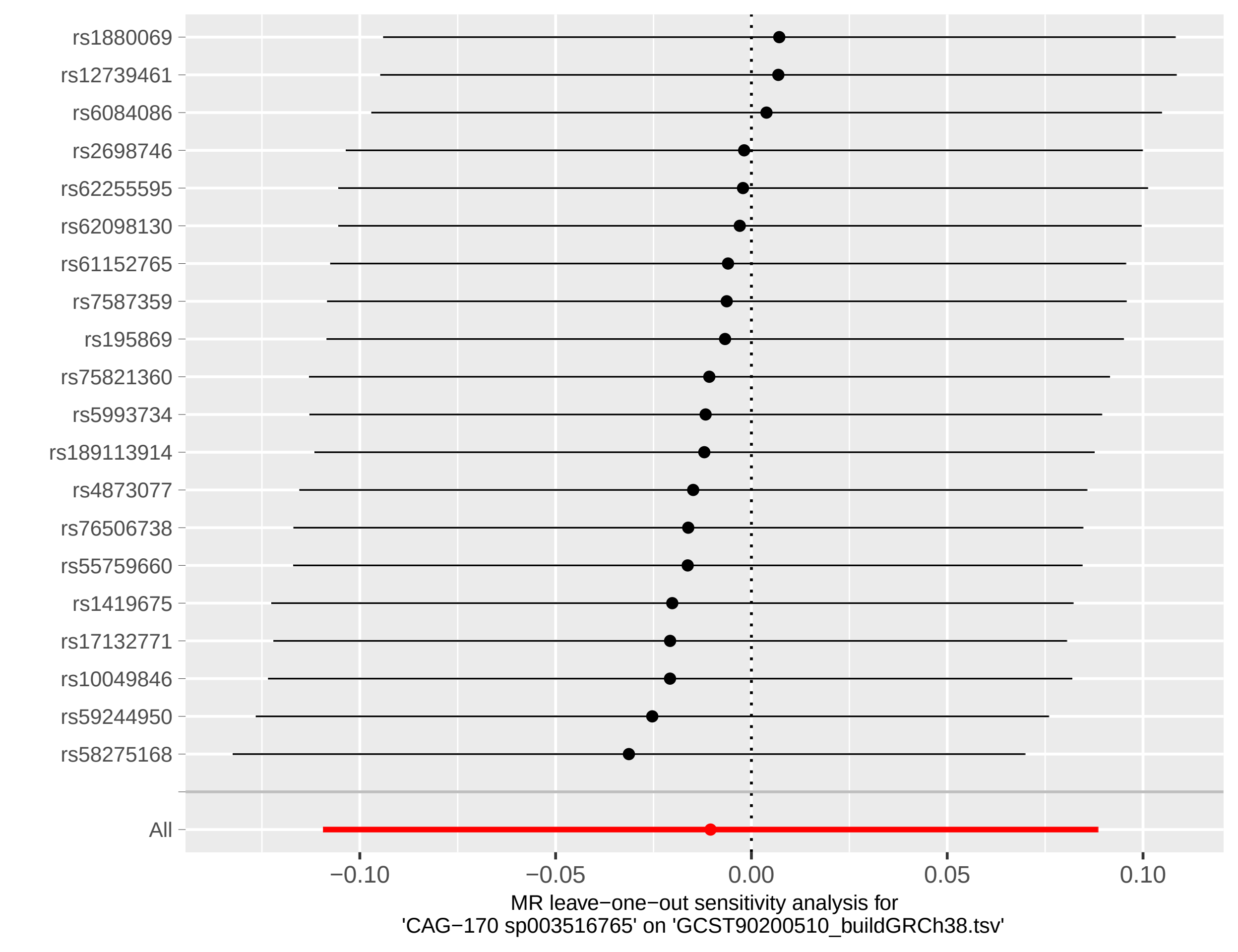

rs1880069
rs12739461
rs6084086
rs2698746
rs62255595
rs62098130
rs61152765
rs7587359
rs195869
rs75821360
rs5993734
rs189113914
rs4873077
rs76506738
rs55759660
rs1419675
rs17132771
rs10049846
rs59244950
rs58275168
All
−0.10
−0.05
0.00
0.05
0.10
MR leave−one−out sensitivity analysis for
'CAG−170 sp003516765' on 'GCST90200510_buildGRCh38.tsv'

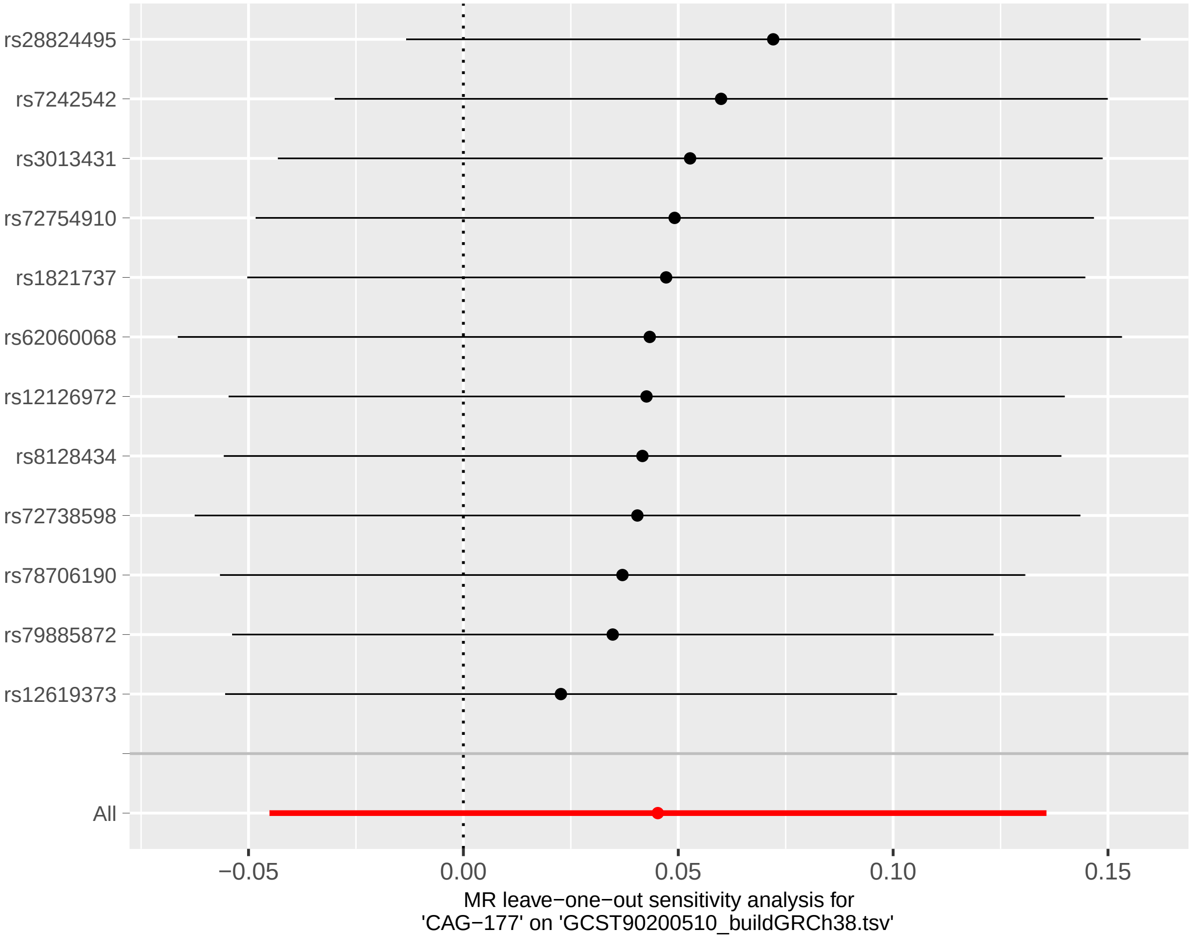

rs28824495
rs7242542
rs3013431
rs72754910
rs1821737
rs62060068
rs12126972
rs8128434
rs72738598
rs78706190
rs79885872
rs12619373
All
−0.05
0.00
0.05
0.10
0.15
MR leave−one−out sensitivity analysis for
'CAG−177' on 'GCST90200510_buildGRCh38.tsv'

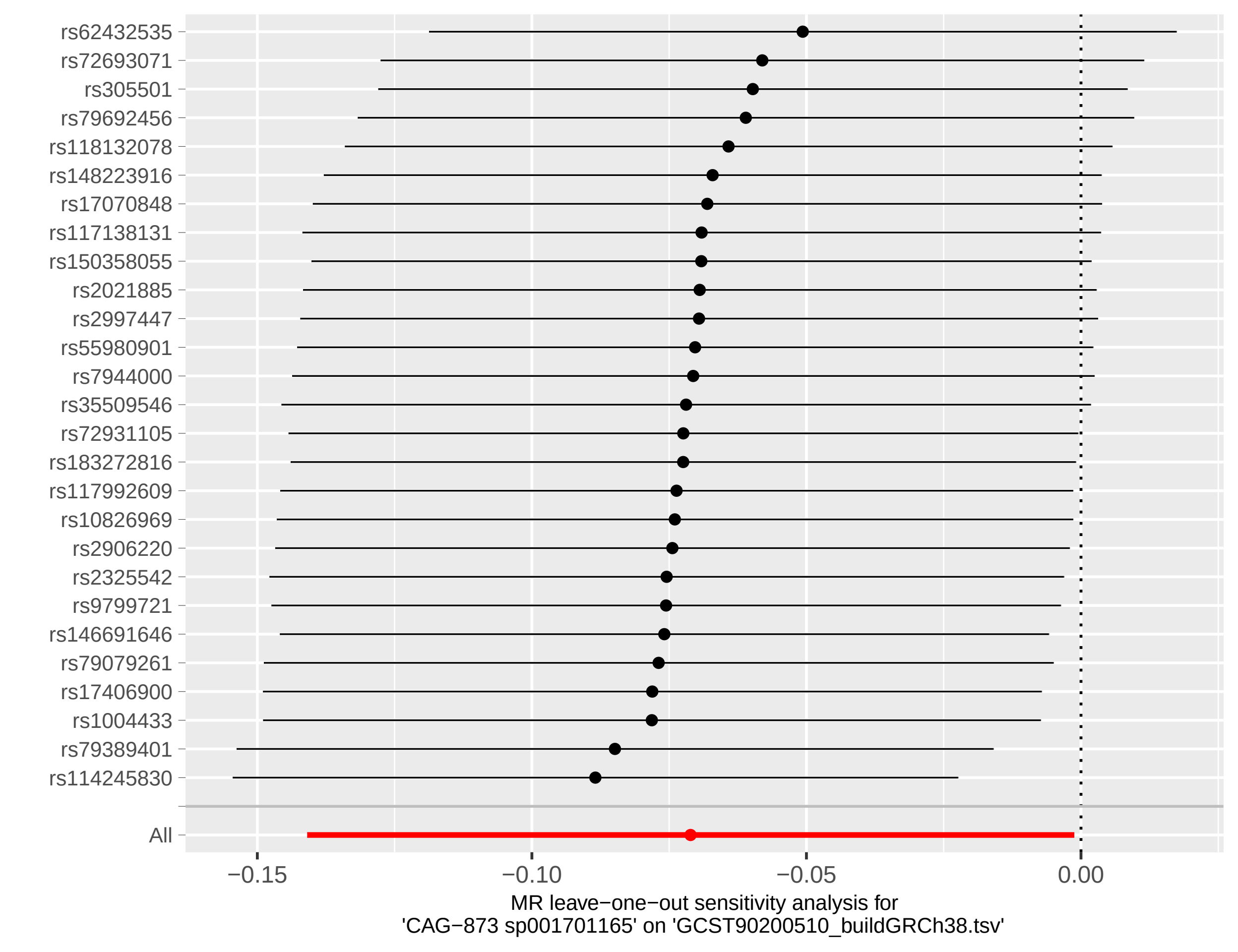
rs62432535
rs72693071
rs305501
rs79692456
rs118132078
rs148223916
rs17070848
rs117138131
rs150358055
rs2021885
rs2997447
rs55980901
rs7944000
rs35509546
rs72931105
rs183272816
rs117992609
rs10826969
rs2906220
rs2325542
rs9799721
rs146691646
rs79079261
rs17406900
rs1004433
rs79389401
rs114245830
All
−0.15
−0.10
−0.05
0.00
MR leave−one−out sensitivity analysis for
'CAG−873 sp001701165' on 'GCST90200510_buildGRCh38.tsv'

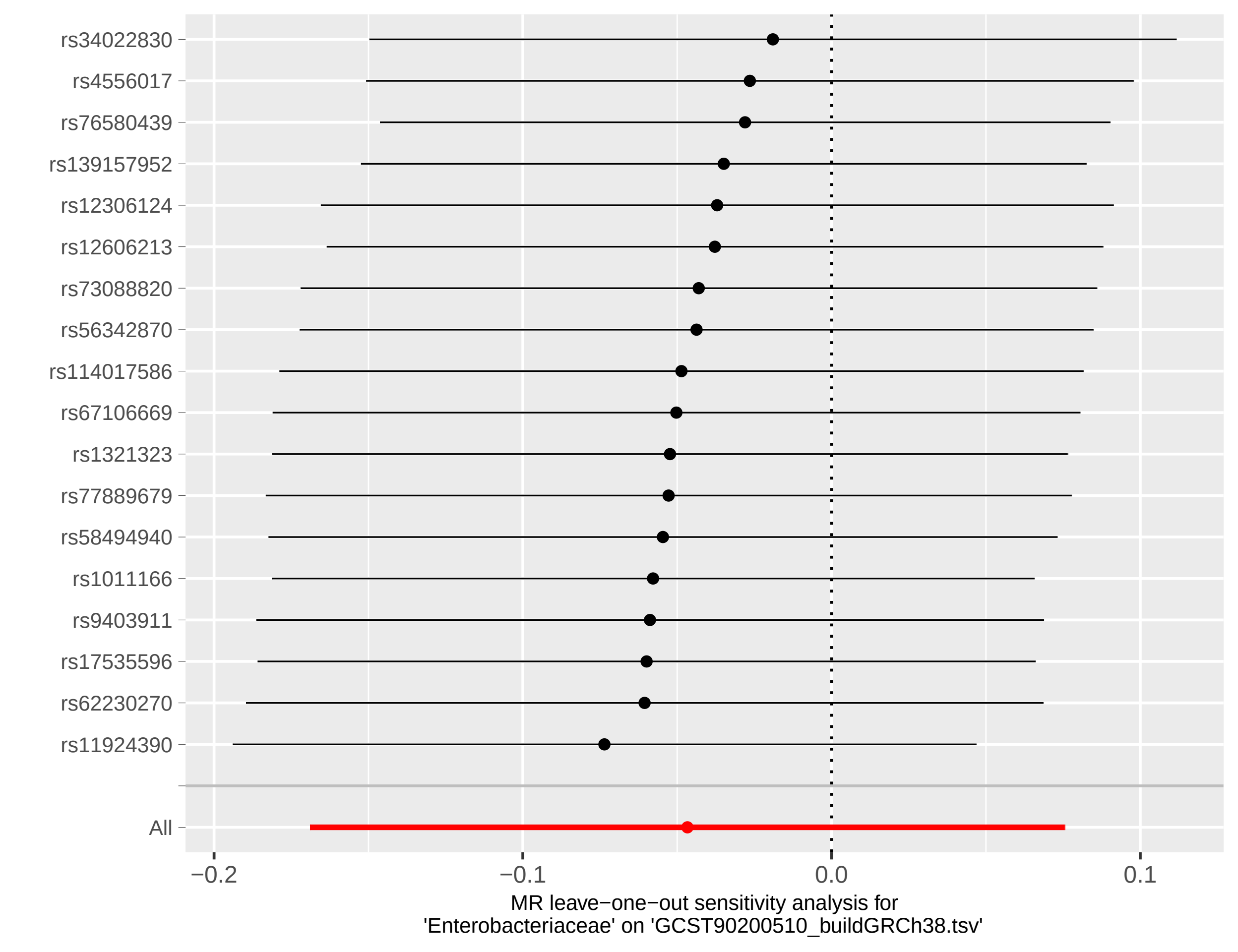
rs34022830
rs4556017
rs76580439
rs139157952
rs12306124
rs12606213
rs73088820
rs56342870
rs114017586
rs67106669
rs1321323
rs77889679
rs58494940
rs1011166
rs9403911
rs17535596
rs62230270
rs11924390
All
−0.2
−0.1
0.0
0.1
MR leave−one−out sensitivity analysis for
'Enterobacteriaceae' on 'GCST90200510_buildGRCh38.tsv'

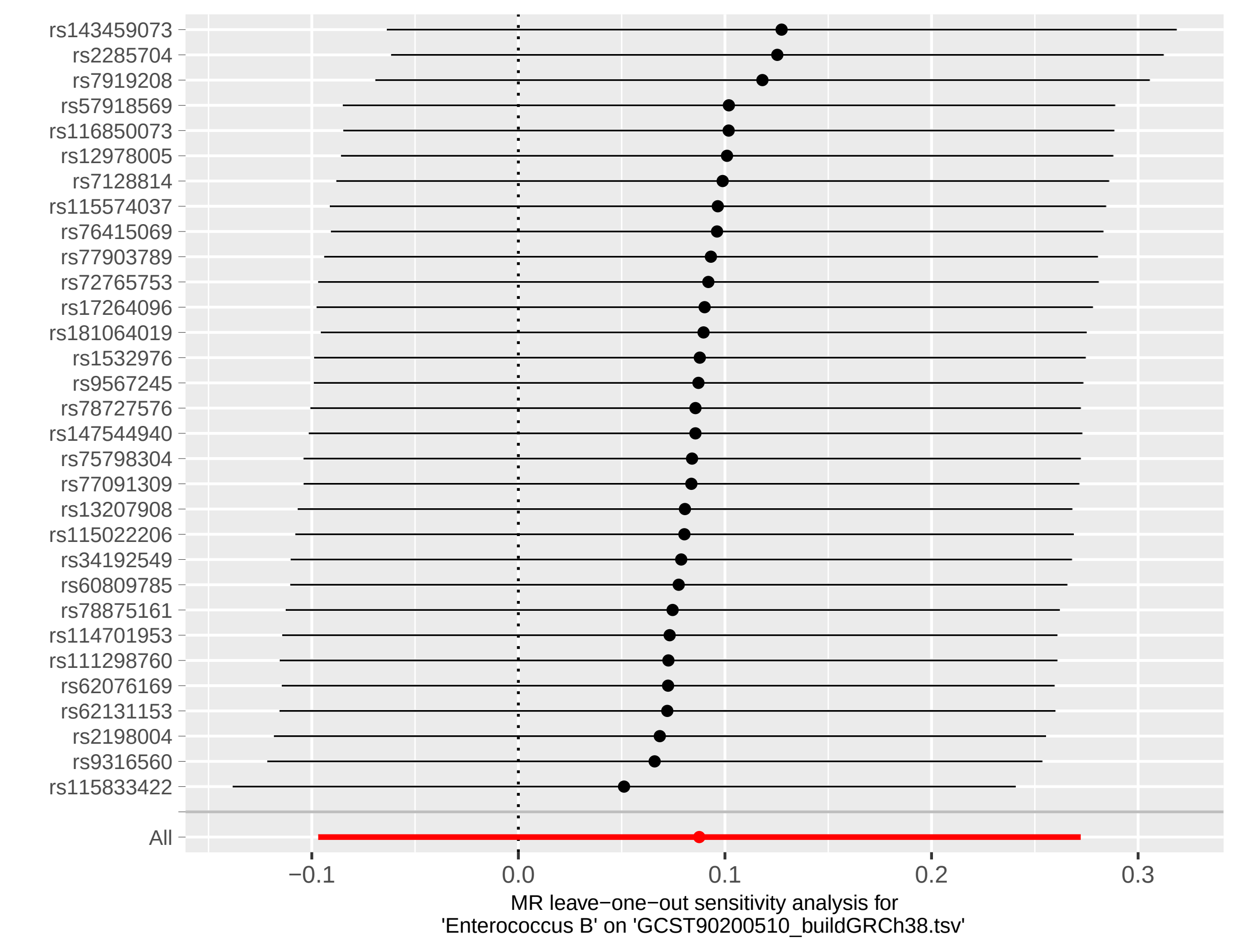
rs143459073
rs2285704
rs7919208
rs57918569
rs116850073
rs12978005
rs7128814
rs115574037
rs76415069
rs77903789
rs72765753
rs17264096
rs181064019
rs1532976
rs9567245
rs78727576
rs147544940
rs75798304
rs77091309
rs13207908
rs115022206
rs34192549
rs60809785
rs78875161
rs114701953
rs111298760
rs62076169
rs62131153
rs2198004
rs9316560
rs115833422
All
−0.1
0.0
0.1
0.2
0.3
MR leave−one−out sensitivity analysis for
'Enterococcus B' on 'GCST90200510_buildGRCh38.tsv'

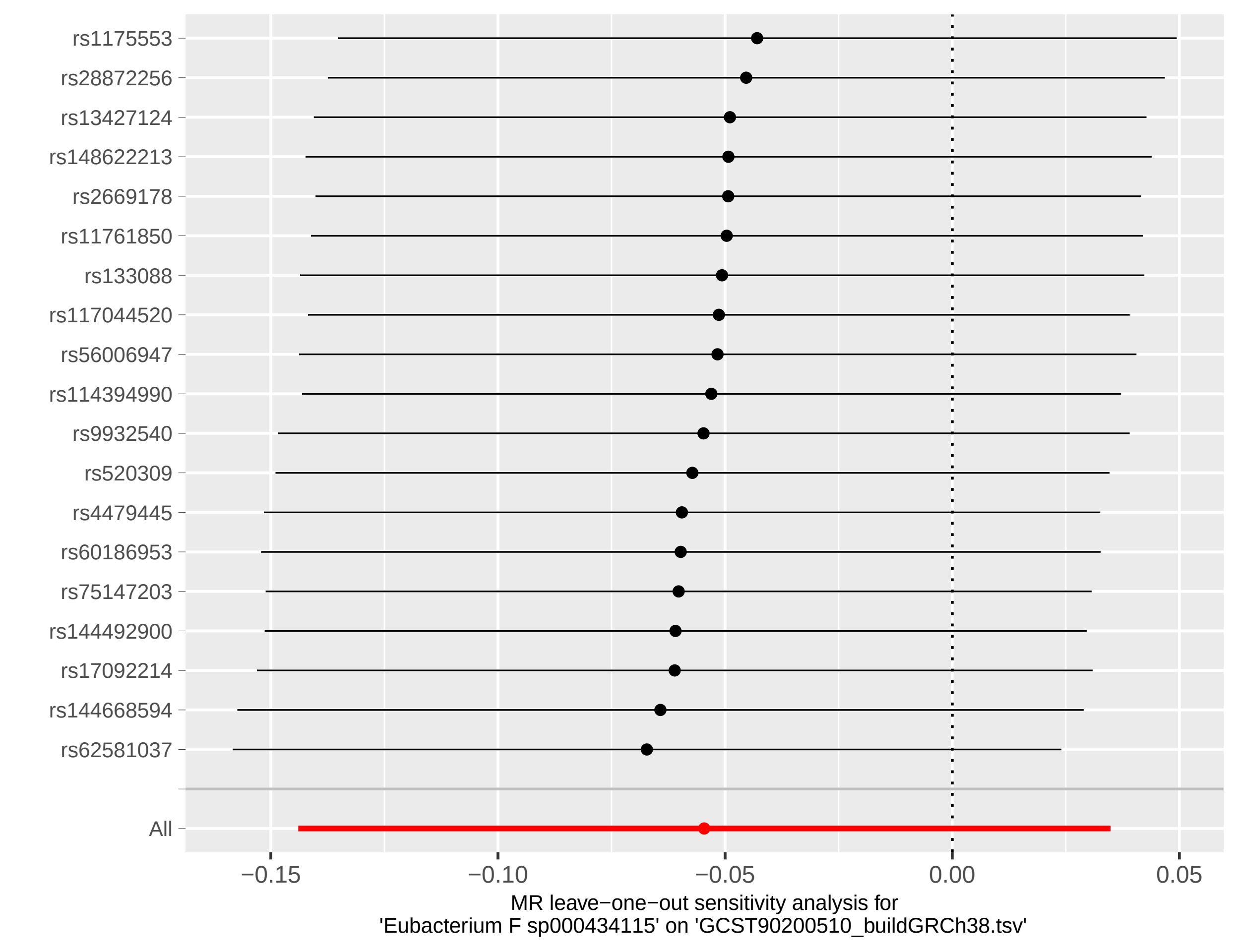

rs1175553
rs28872256
rs13427124
rs148622213
rs2669178
rs11761850
rs133088
rs117044520
rs56006947
rs114394990
rs9932540
rs520309
rs4479445
rs60186953
rs75147203
rs144492900
rs17092214
rs144668594
rs62581037
All
−0.15
−0.10
−0.05
0.00
0.05
MR leave−one−out sensitivity analysis for
'Eubacterium F sp000434115' on 'GCST90200510_buildGRCh38.tsv'

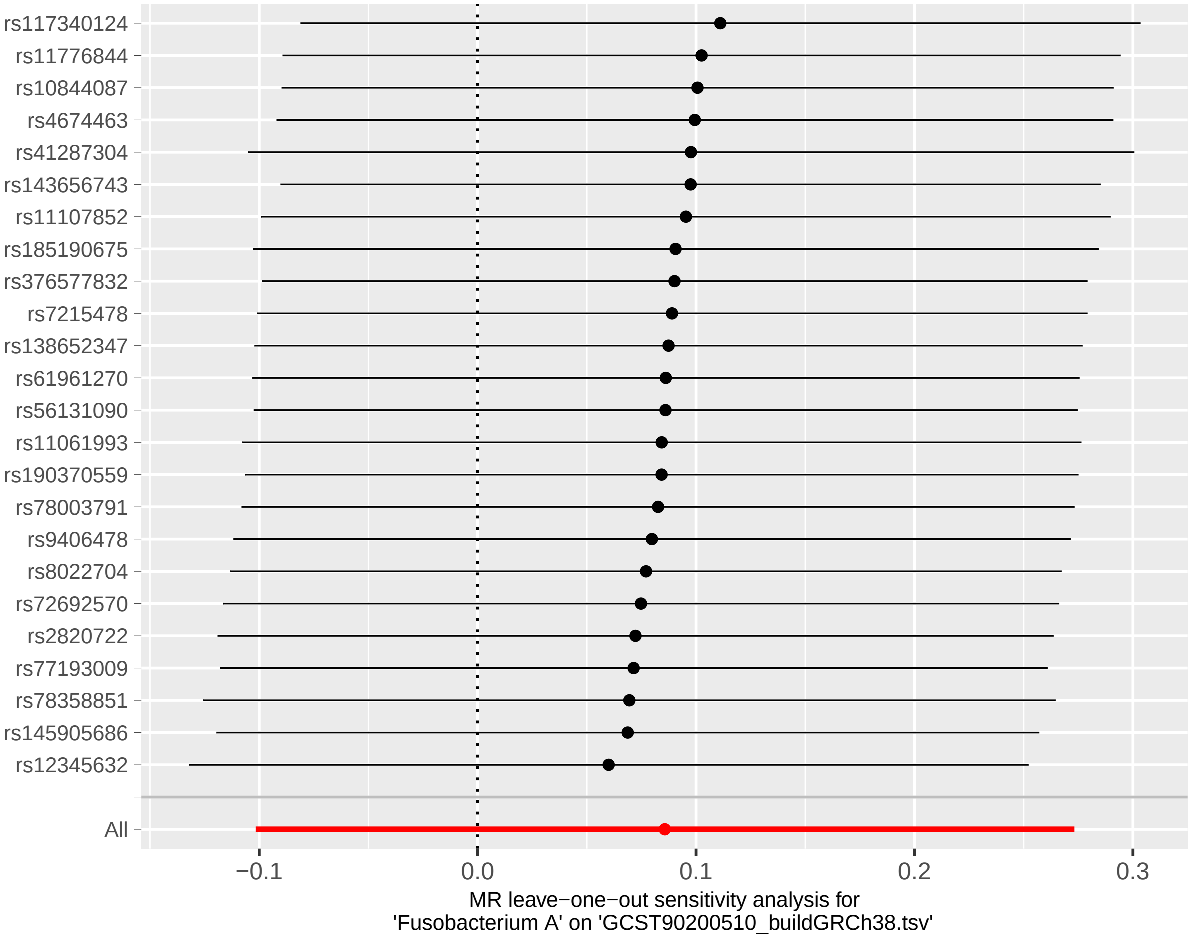

rs117340124
rs11776844
rs10844087
rs4674463
rs41287304
rs143656743
rs11107852
rs185190675
rs376577832
rs7215478
rs138652347
rs61961270
rs56131090
rs11061993
rs190370559
rs78003791
rs9406478
rs8022704
rs72692570
rs2820722
rs77193009
rs78358851
rs145905686
rs12345632
All
−0.1
0.0
0.1
0.2
0.3
MR leave−one−out sensitivity analysis for
'Fusobacterium A' on 'GCST90200510_buildGRCh38.tsv'

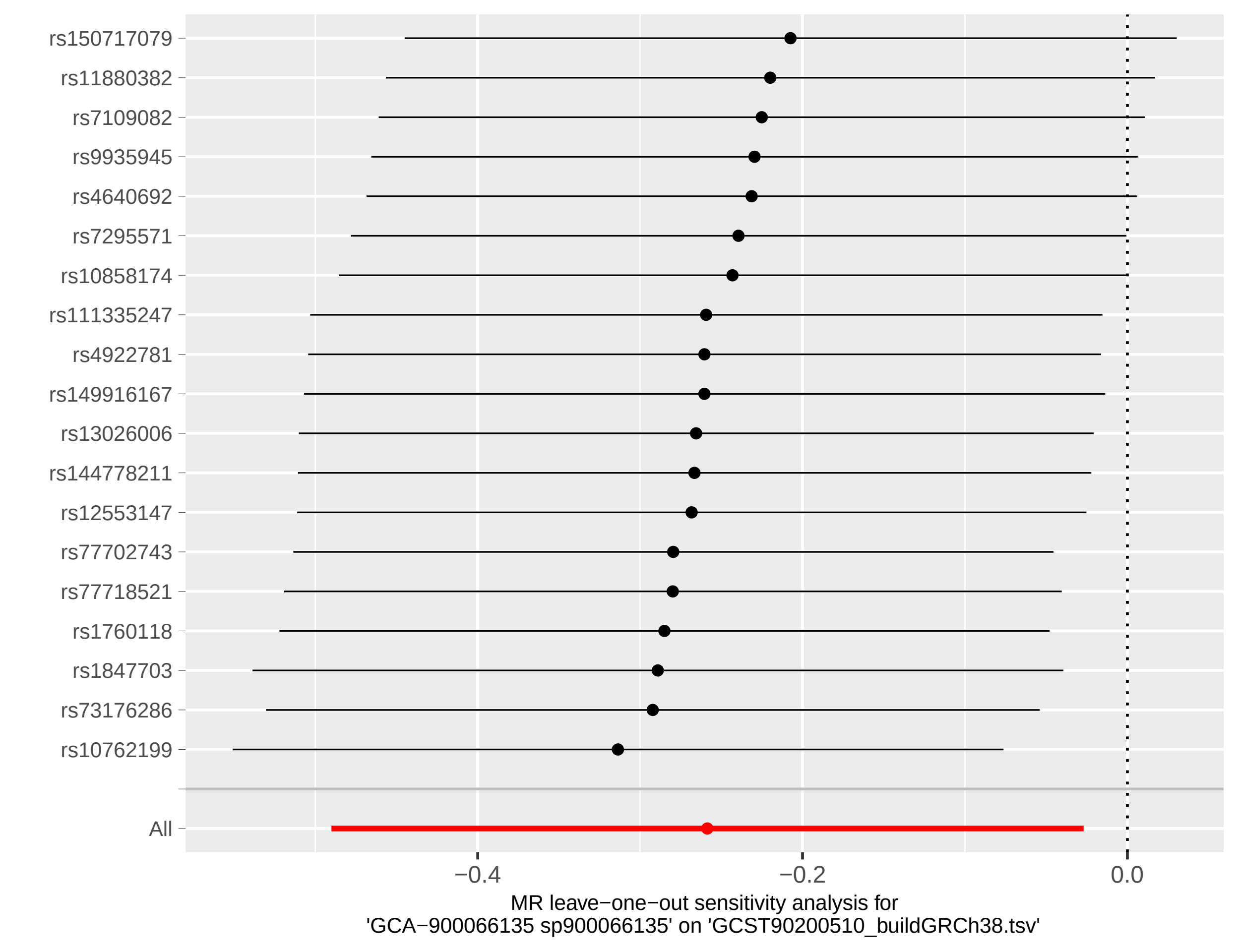
rs150717079
rs11880382
rs7109082
rs9935945
rs4640692
rs7295571
rs10858174
rs111335247
rs4922781
rs149916167
rs13026006
rs144778211
rs12553147
rs77702743
rs77718521
rs1760118
rs1847703
rs73176286
rs10762199
All
−0.4
−0.2
0.0
MR leave−one−out sensitivity analysis for
'GCA−900066135 sp900066135' on 'GCST90200510_buildGRCh38.tsv'

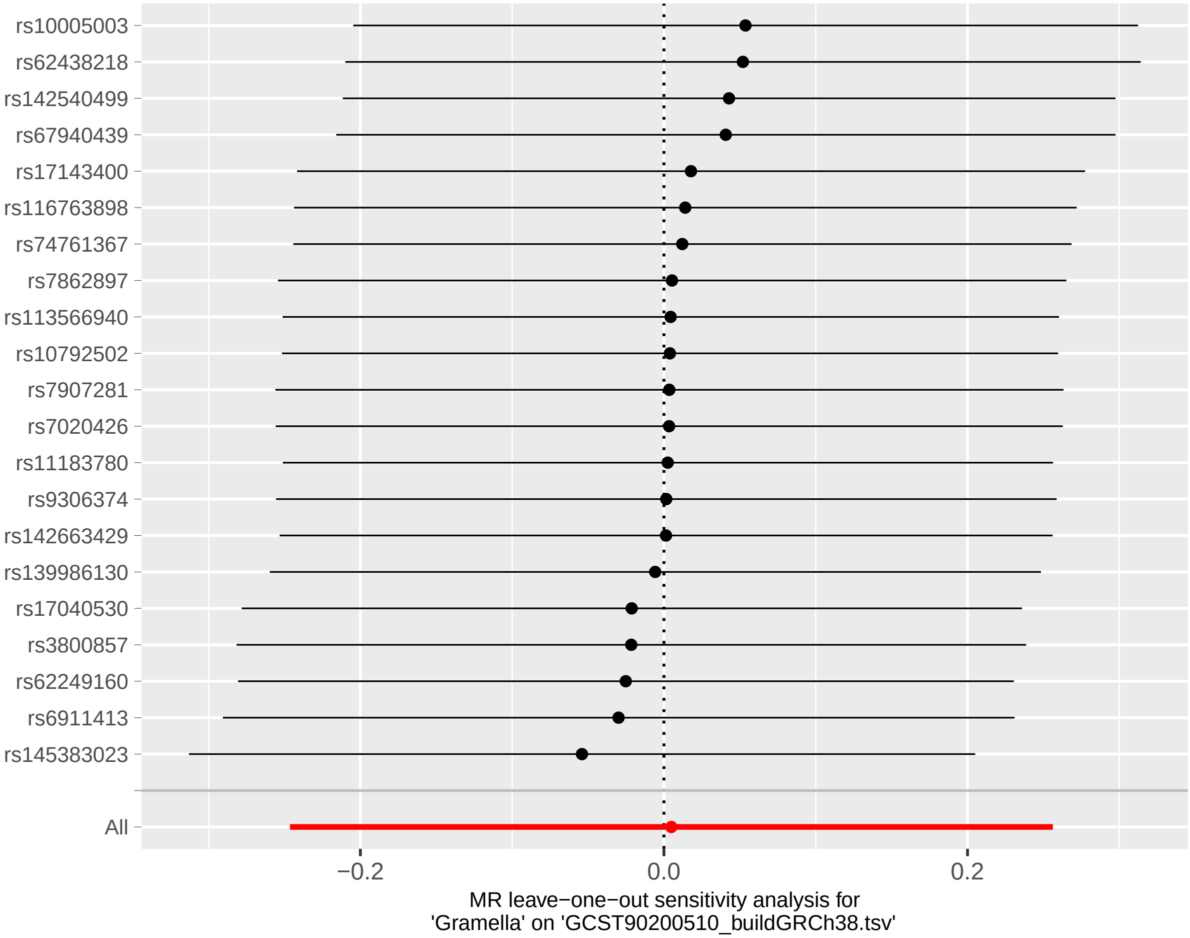
rs10005003
rs62438218
rs142540499
rs67940439
rs17143400
rs116763898
rs74761367
rs7862897
rs113566940
rs10792502
rs7907281
rs7020426
rs11183780
rs9306374
rs142663429
rs139986130
rs17040530
rs3800857
rs62249160
rs6911413
rs145383023
All
−0.2
0.0
0.2
MR leave−one−out sensitivity analysis for
'Gramella' on 'GCST90200510_buildGRCh38.tsv'

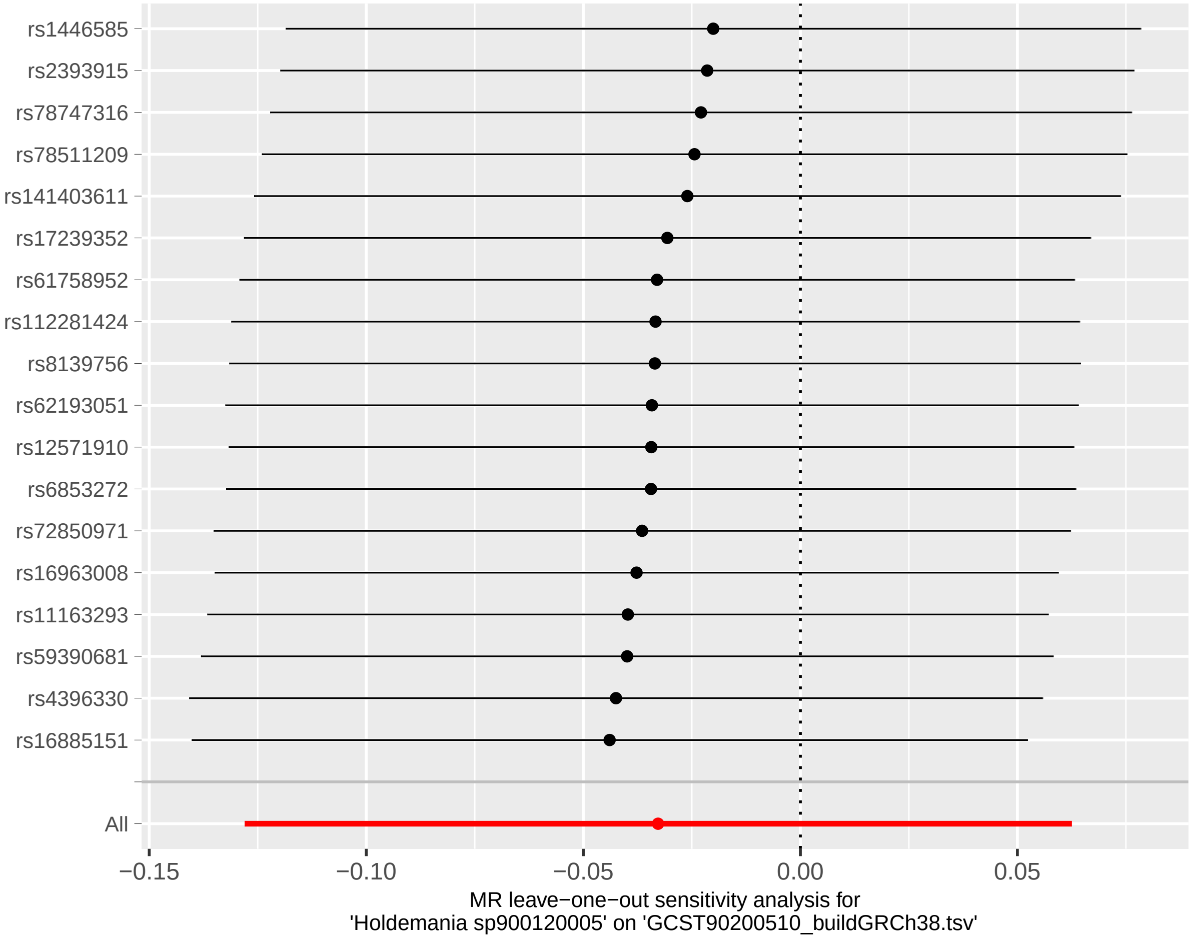

rs1446585
rs2393915
rs78747316
rs78511209
rs141403611
rs17239352
rs61758952
rs112281424
rs8139756
rs62193051
rs12571910
rs6853272
rs72850971
rs16963008
rs11163293
rs59390681
rs4396330
rs16885151
All
−0.15
−0.10
−0.05
0.00
0.05
MR leave−one−out sensitivity analysis for
'Holdemania sp900120005' on 'GCST90200510_buildGRCh38.tsv'

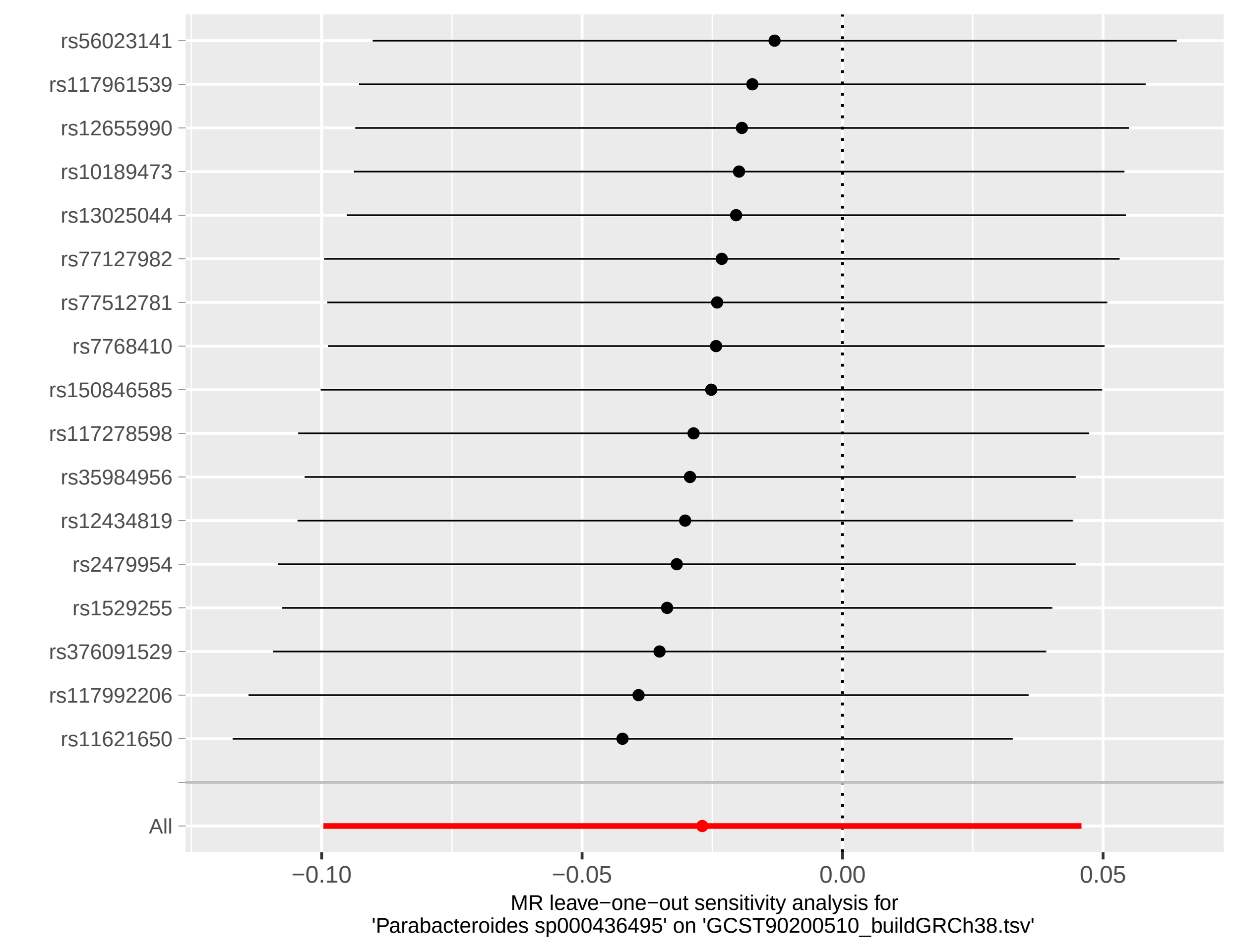
rs56023141
rs117961539
rs12655990
rs10189473
rs13025044
rs77127982
rs77512781
rs7768410
rs150846585
rs117278598
rs35984956
rs12434819
rs2479954
rs1529255
rs376091529
rs117992206
rs11621650
All
−0.10
−0.05
0.00
0.05
MR leave−one−out sensitivity analysis for
'Parabacteroides sp000436495' on 'GCST90200510_buildGRCh38.tsv'

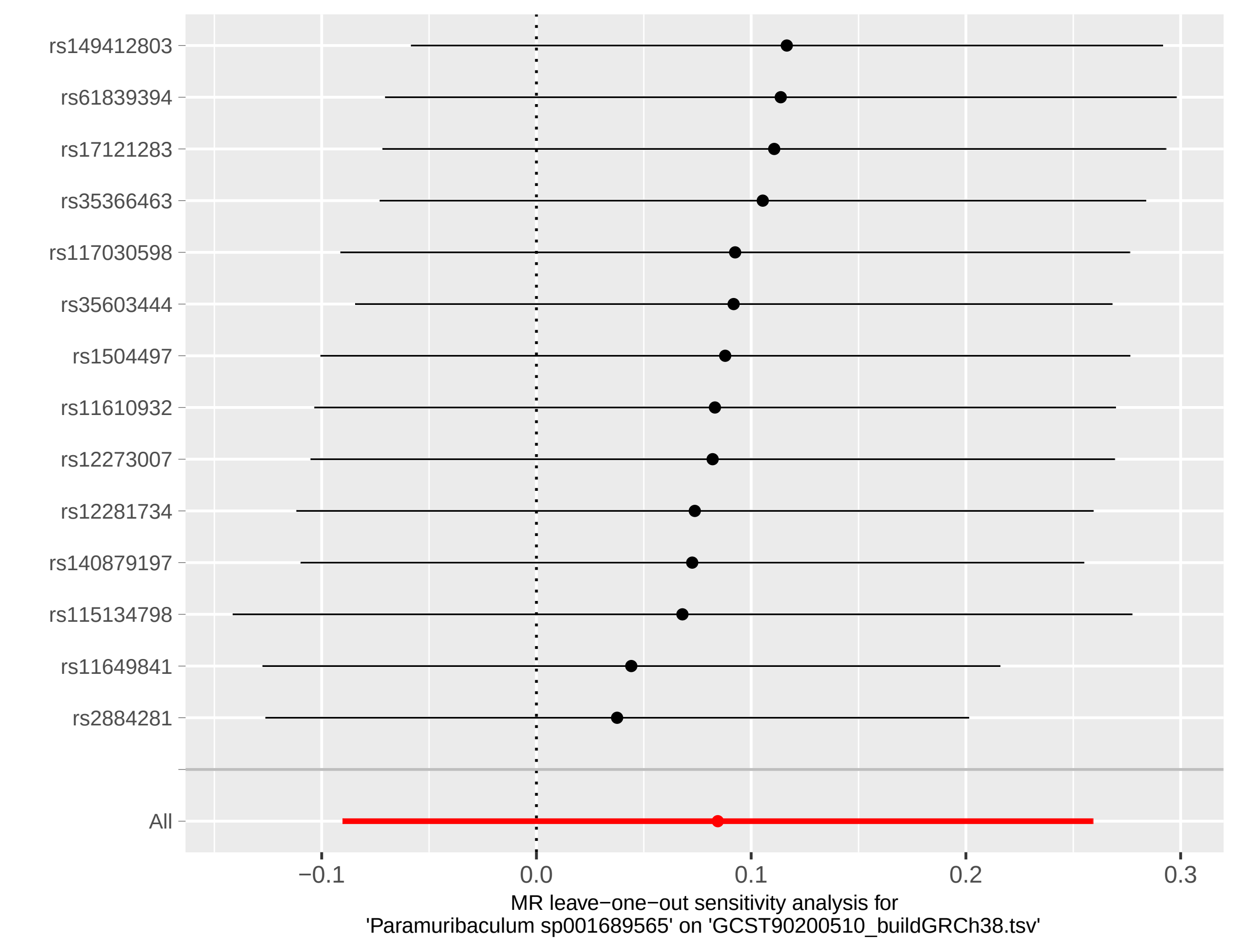
rs149412803
rs61839394
rs17121283
rs35366463
rs117030598
rs35603444
rs1504497
rs11610932
rs12273007
rs12281734
rs140879197
rs115134798
rs11649841
rs2884281
All
−0.1
0.0
0.1
0.2
0.3
MR leave−one−out sensitivity analysis for
'Paramuribaculum sp001689565' on 'GCST90200510_buildGRCh38.tsv'

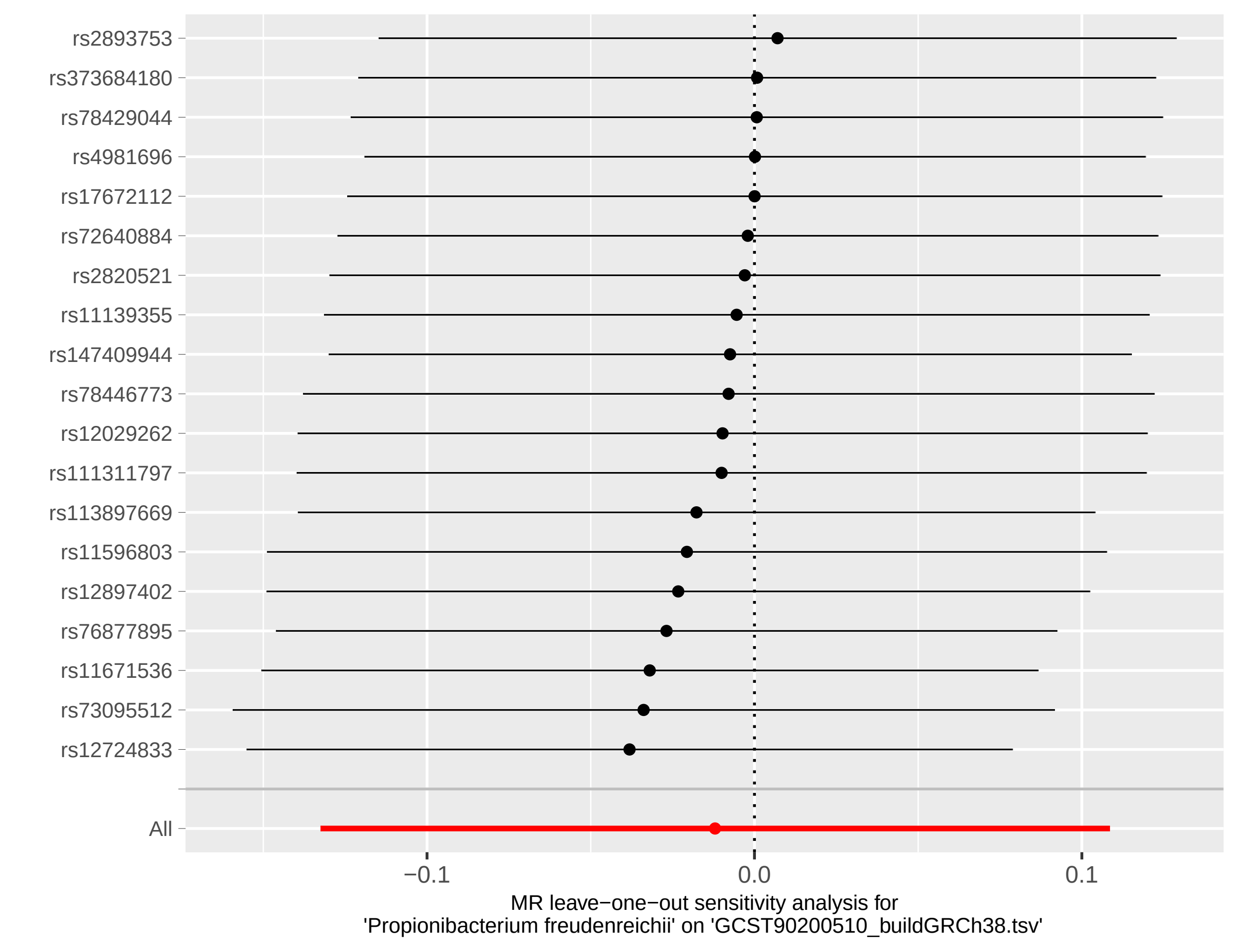
rs2893753
rs373684180
rs78429044
rs4981696
rs17672112
rs72640884
rs2820521
rs11139355
rs147409944
rs78446773
rs12029262
rs111311797
rs113897669
rs11596803
rs12897402
rs76877895
rs11671536
rs73095512
rs12724833
All
−0.1
0.0
0.1
MR leave−one−out sensitivity analysis for
'Propionibacterium freudenreichii' on 'GCST90200510_buildGRCh38.tsv'

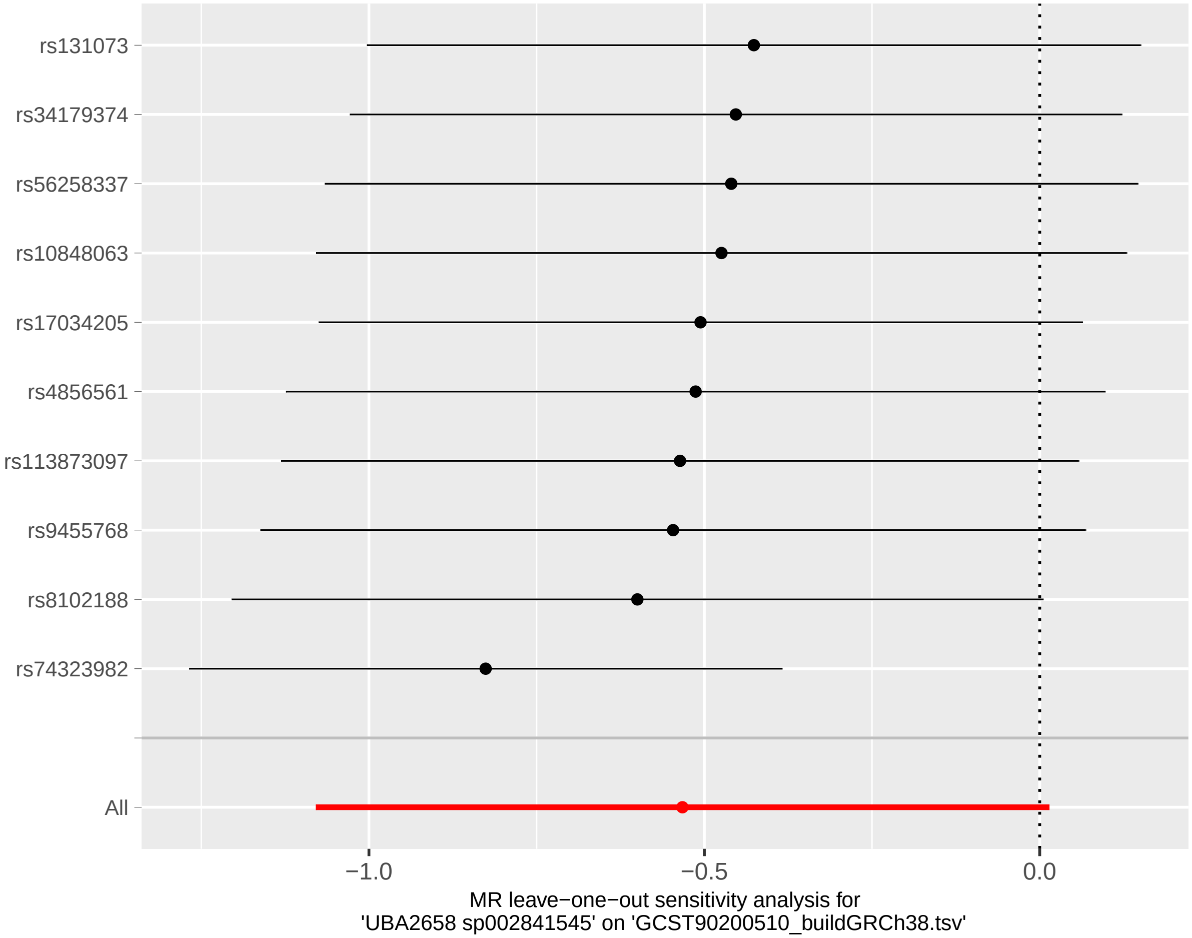
rs131073
rs34179374
rs56258337
rs10848063
rs17034205
rs4856561
rs113873097
rs9455768
rs8102188
rs74323982
All
−1.0
−0.5
0.0
MR leave−one−out sensitivity analysis for
'UBA2658 sp002841545' on 'GCST90200510_buildGRCh38.tsv'

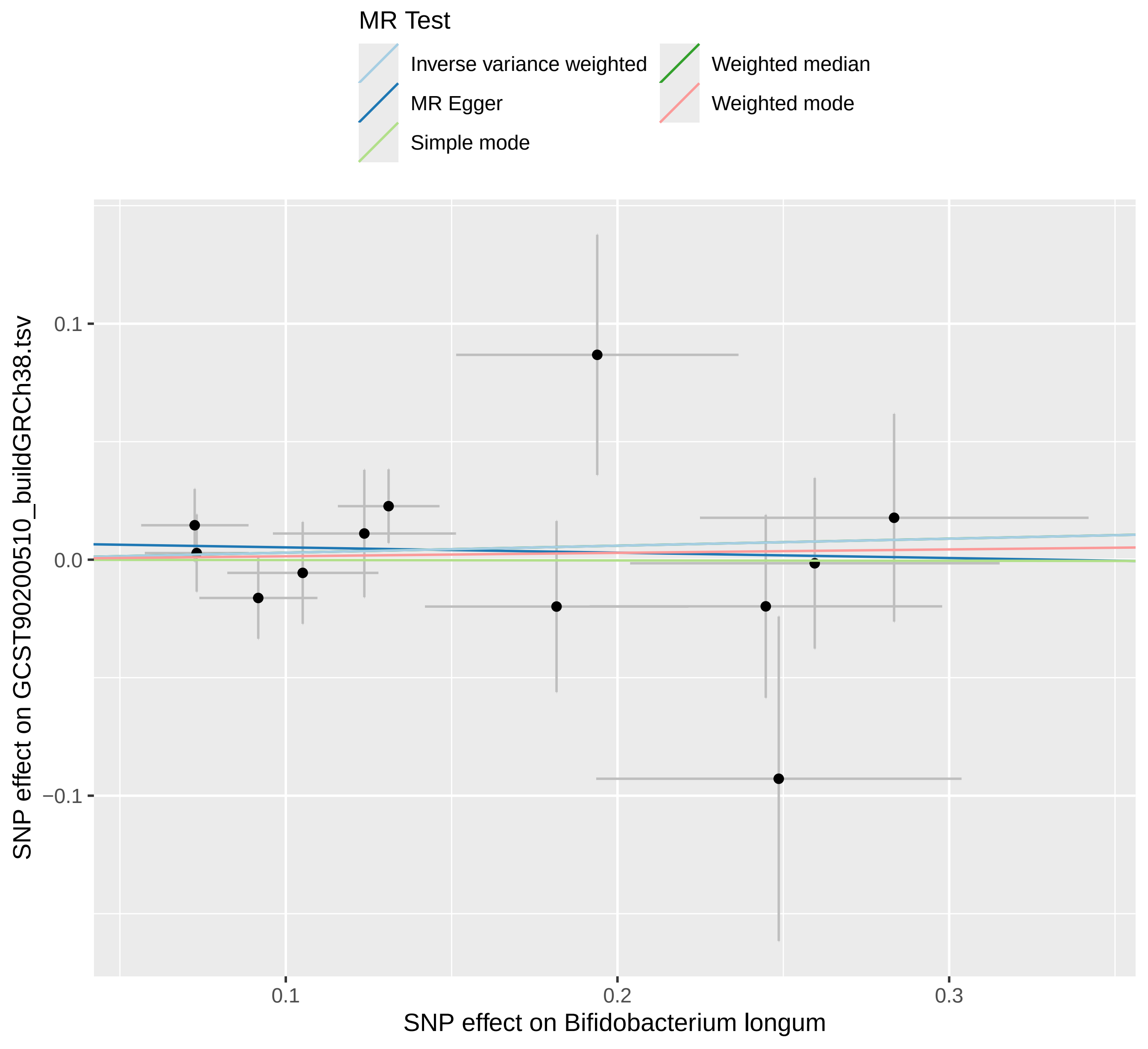
MR Test
Inverse variance weighted
MR Egger
Simple mode
Weighted median
Weighted mode
0.1
0.0
−0.1
0.1
0.2
0.3
SNP effect on GCST90200510_buildGRCh38.tsv
SNP effect on Bifidobacterium longum

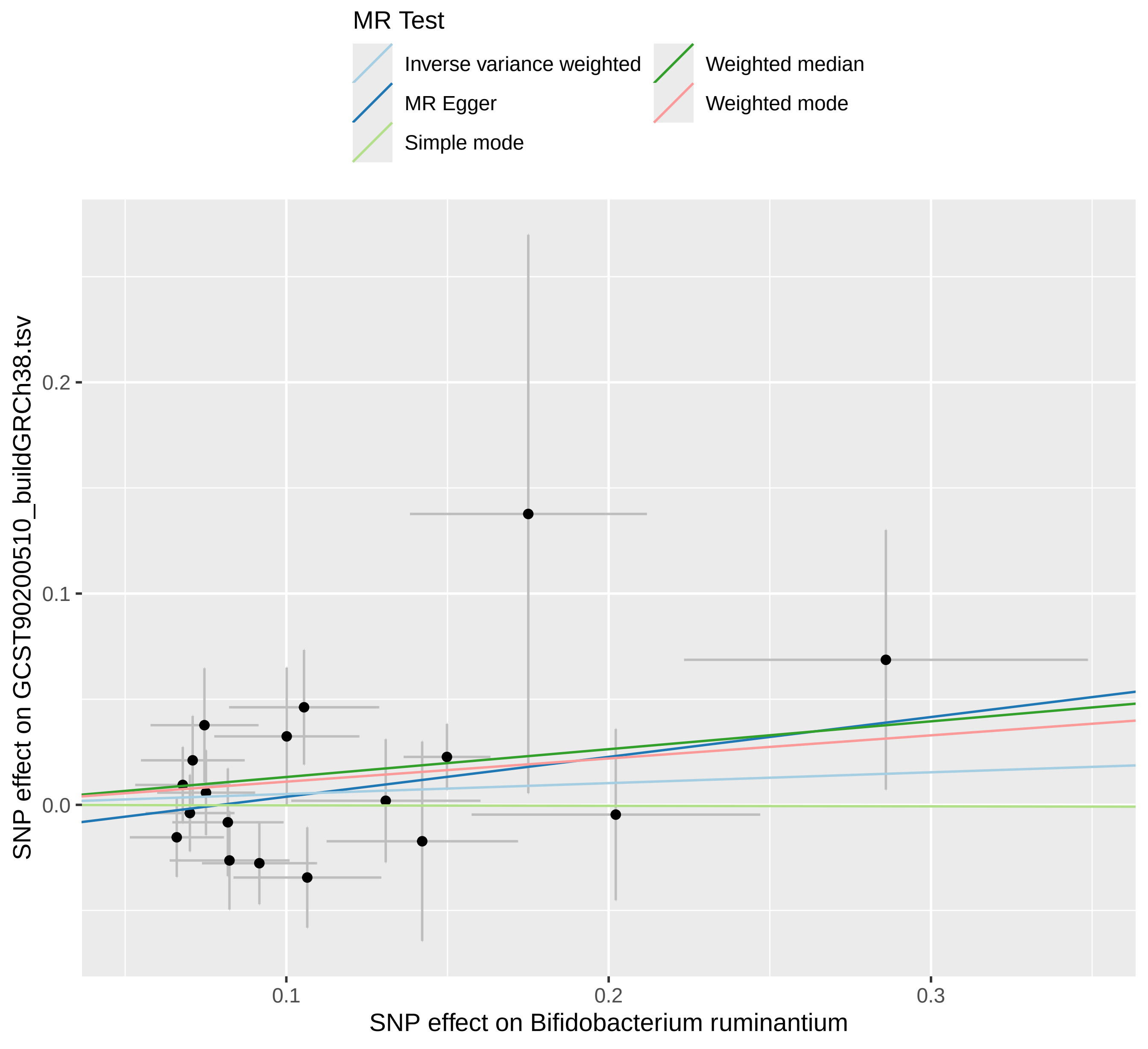
MR Test
Inverse variance weighted
MR Egger
Simple mode
Weighted median
Weighted mode
0.2
0.1
0.0
0.1
0.2
0.3
SNP effect on GCST90200510_buildGRCh38.tsv
SNP effect on Bifidobacterium ruminantium

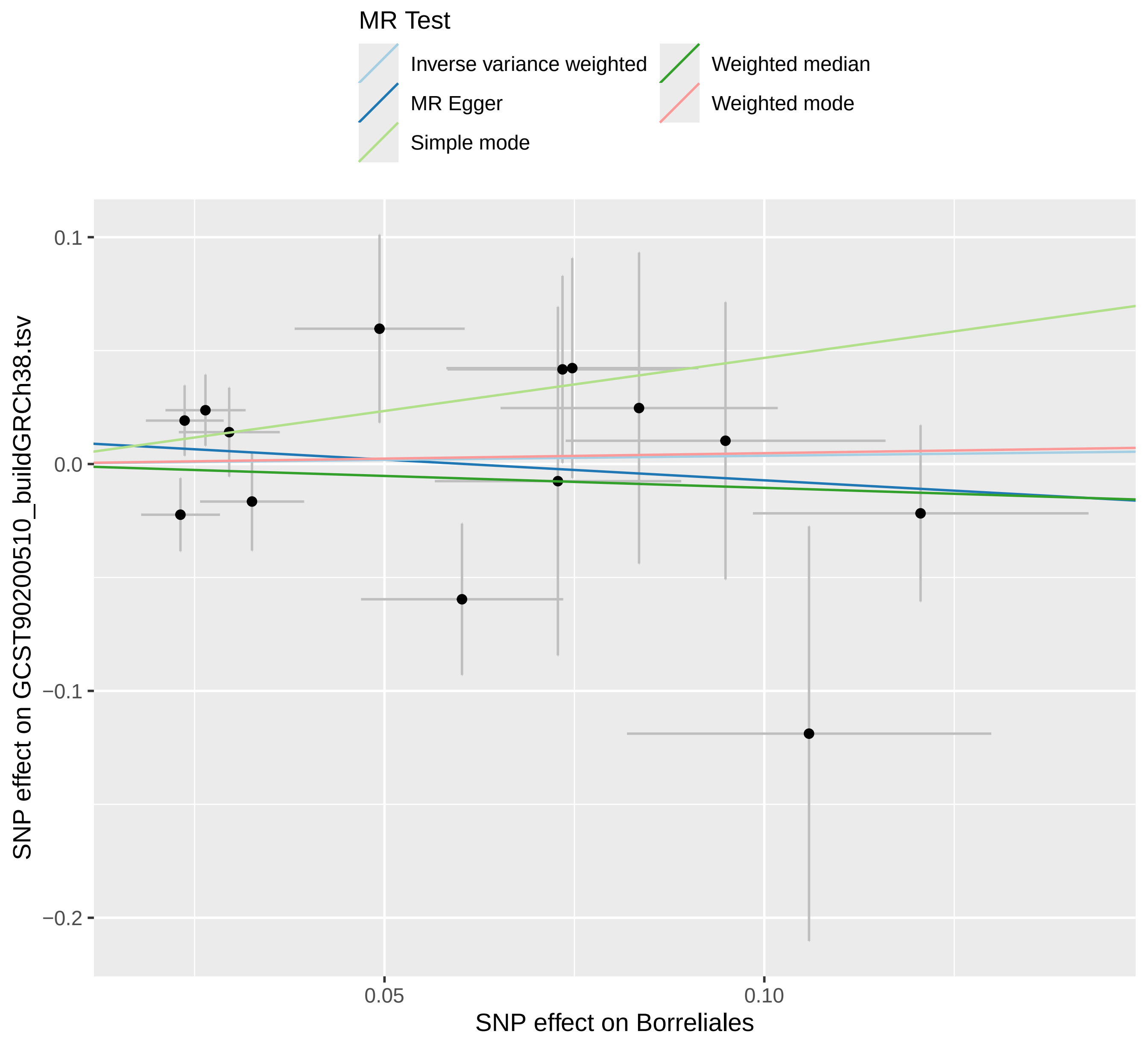

MR Test
Inverse variance weighted
MR Egger
Simple mode
Weighted median
Weighted mode
0.1
0.0
−0.1
−0.2
0.05
0.10
SNP effect on GCST90200510_buildGRCh38.tsv
SNP effect on Borreliales

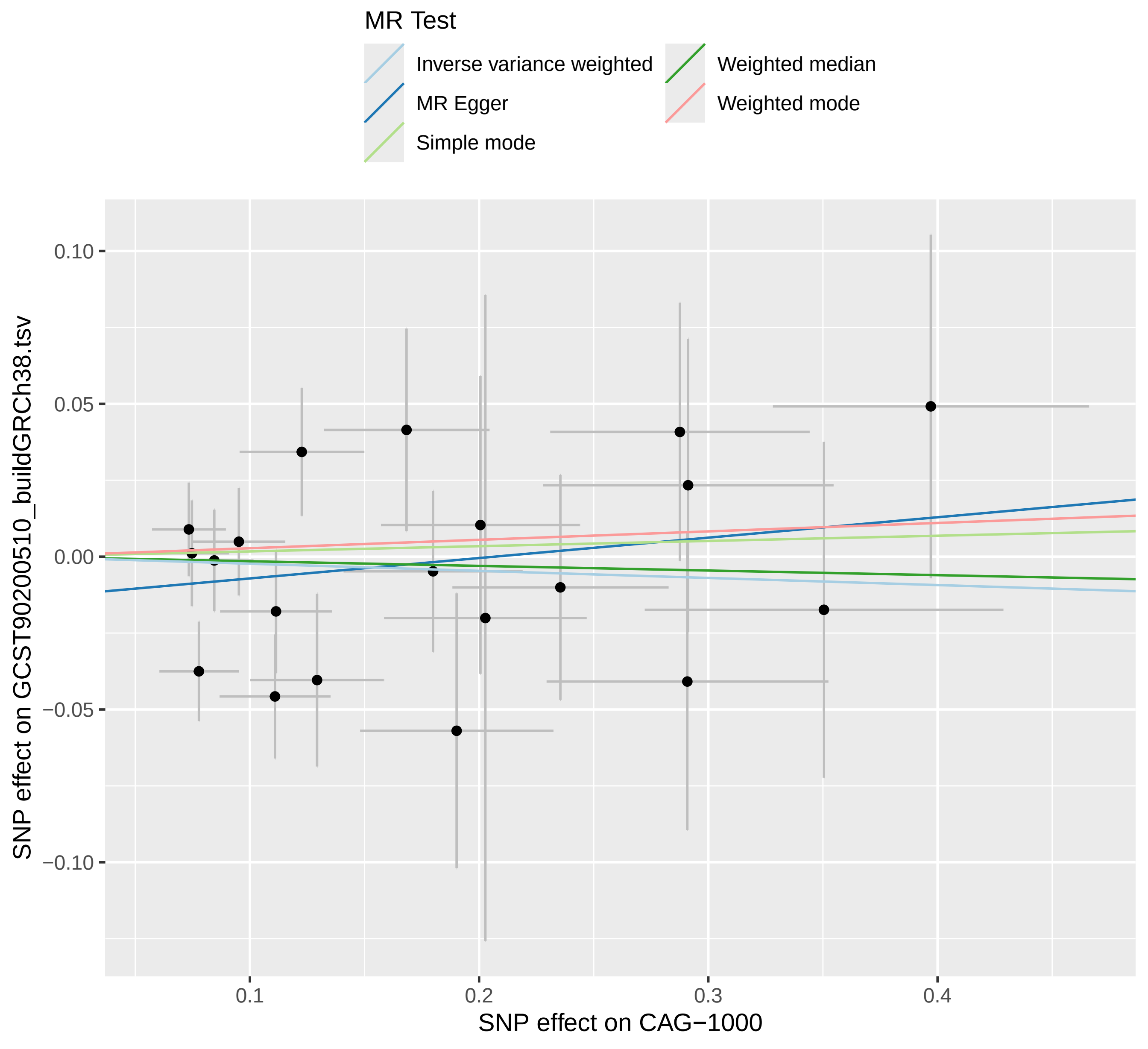
MR Test
Inverse variance weighted
MR Egger
Simple mode
Weighted median
Weighted mode
0.10
0.05
0.00
−0.05
−0.10
SNP effect on GCST90200510_buildGRCh38.tsv
0.1
0.2
0.3
0.4
SNP effect on CAG−1000

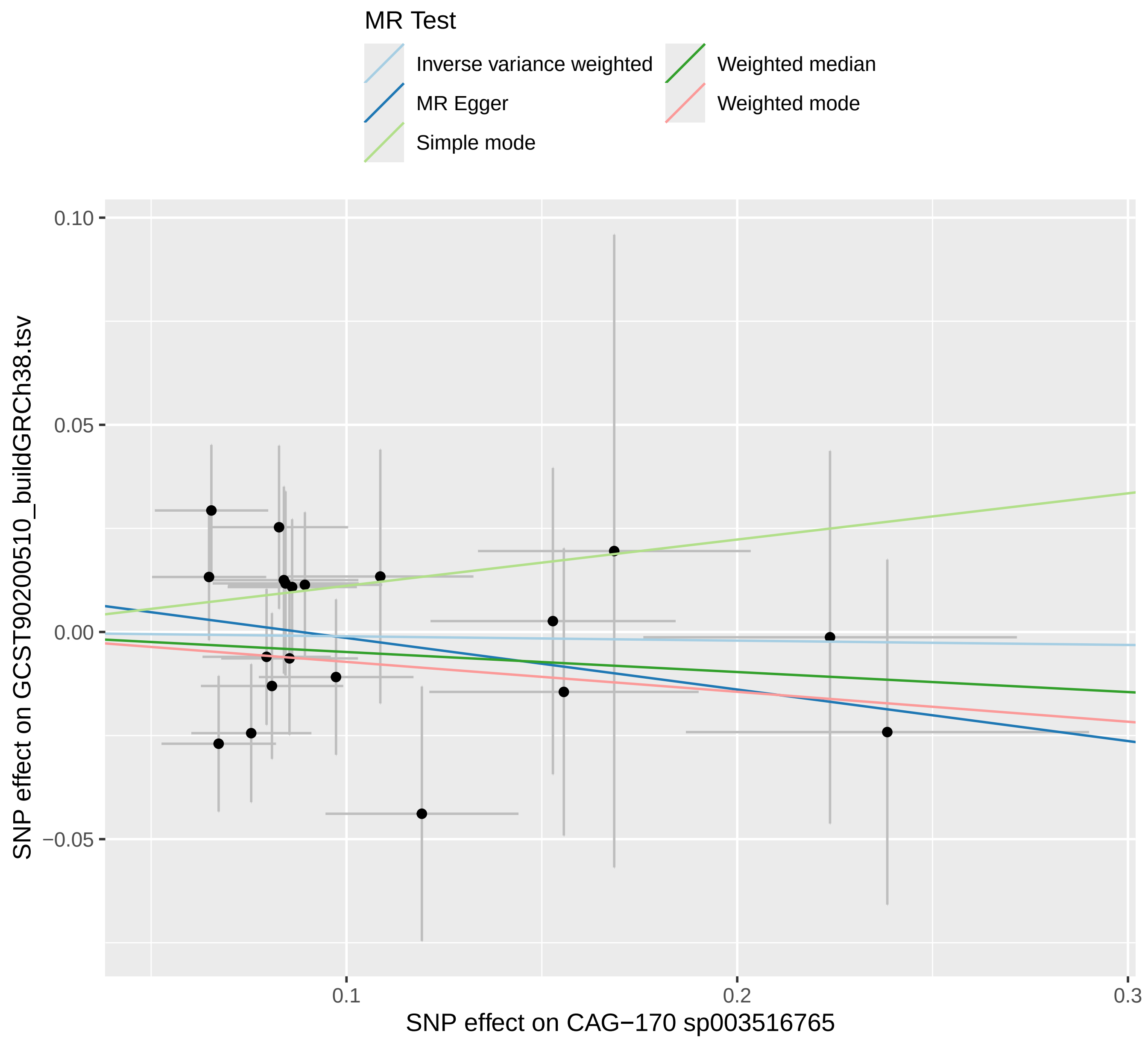
MR Test
Inverse variance weighted
MR Egger
Simple mode
Weighted median
Weighted mode
0.10
0.05
0.00
−0.05
0.1
0.2
0.3
SNP effect on GCST90200510_buildGRCh38.tsv
SNP effect on CAG−170 sp003516765

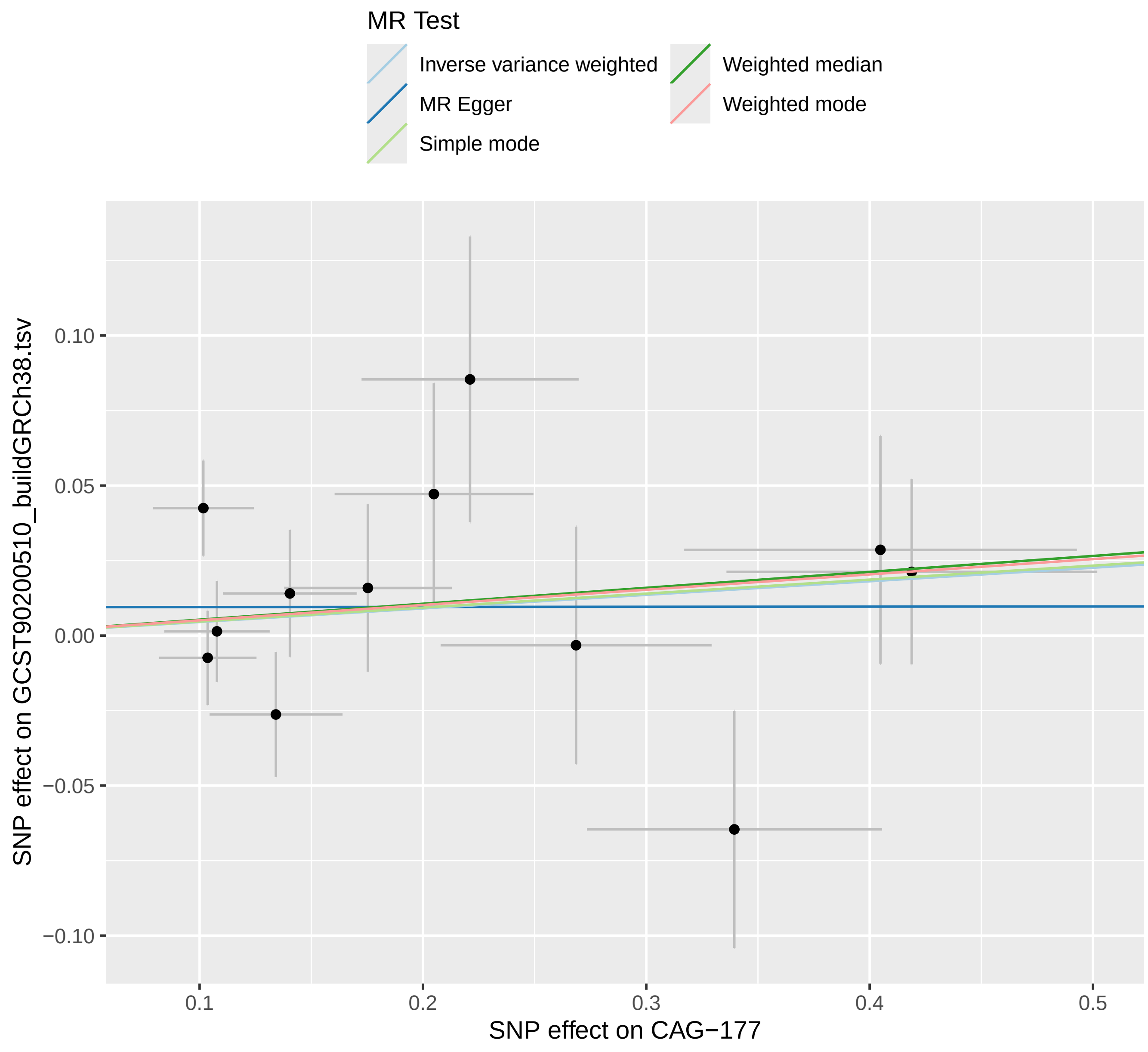

MR Test
Inverse variance weighted
MR Egger
Simple mode
Weighted median
Weighted mode
SNP effect on GCST90200510_buildGRCh38.tsv
0.10
0.05
0.00
−0.05
−0.10
0.1
0.2
0.3
0.4
0.5
SNP effect on CAG−177

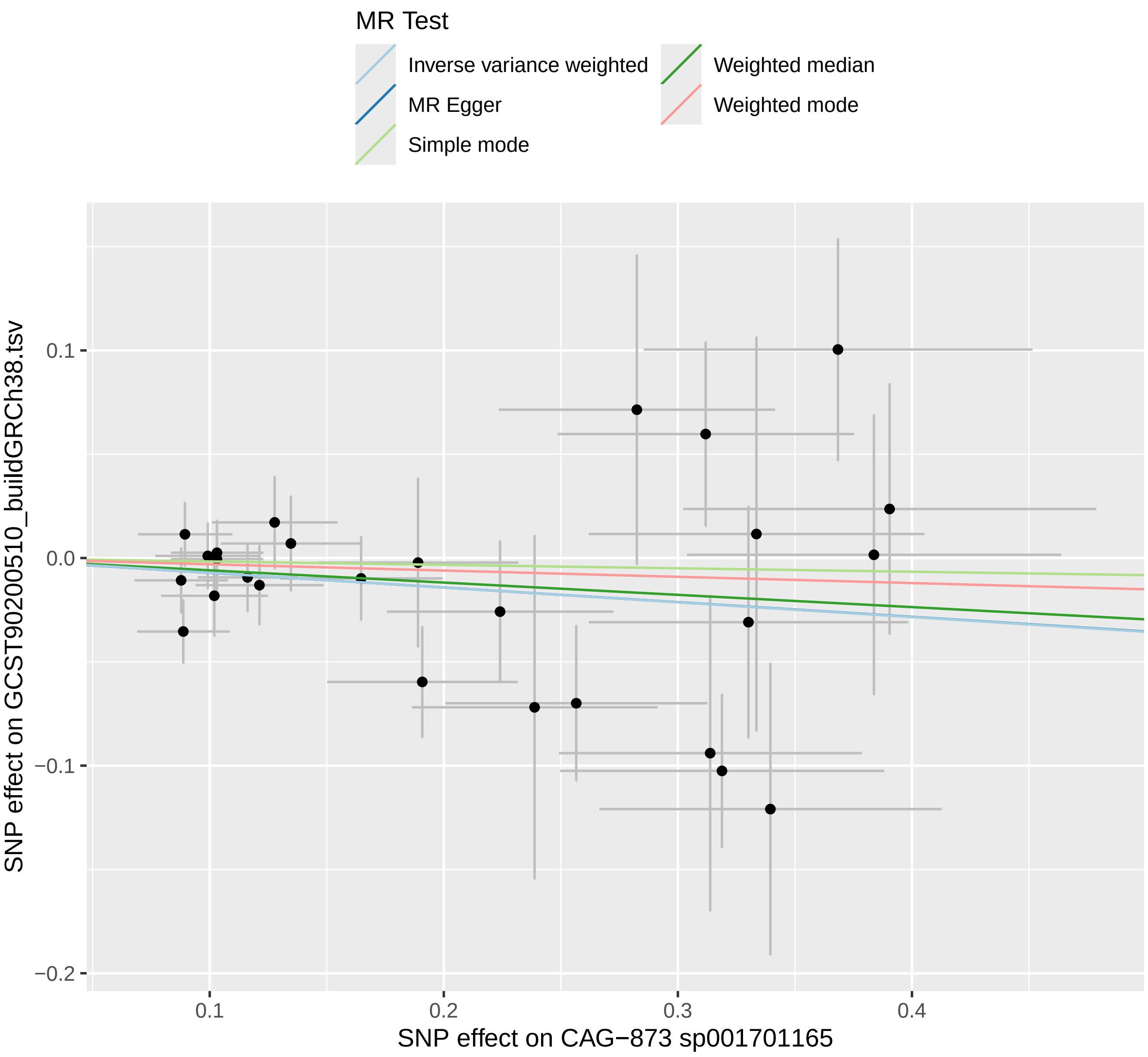
MR Test
Inverse variance weighted
MR Egger
Simple mode
Weighted median
Weighted mode
SNP effect on GCST90200510_buildGRCh38.tsv
SNP effect on CAG−873 sp001701165
0.1
0.0
−0.1
−0.2
0.1
0.2
0.3
0.4

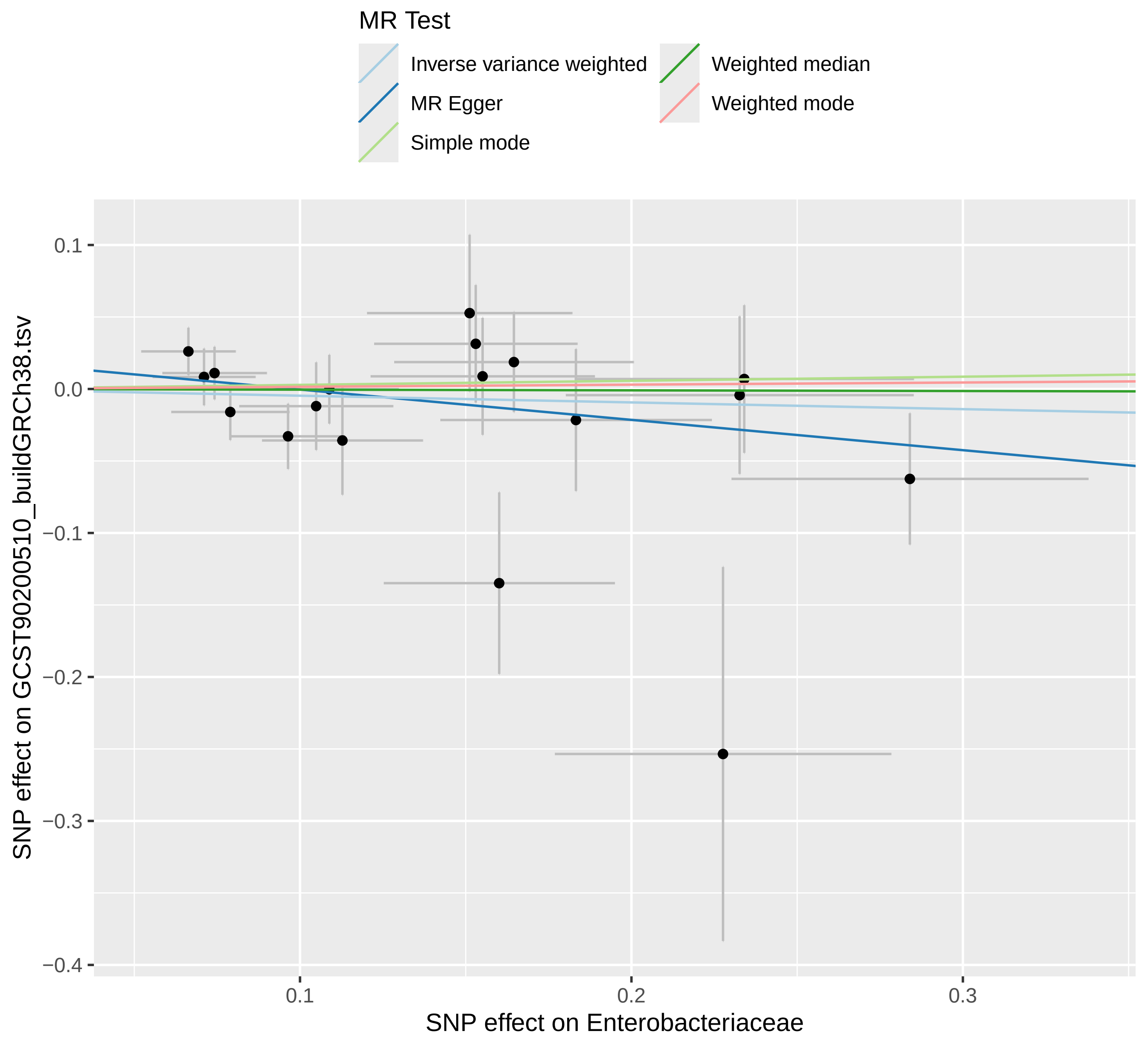

MR Test
Inverse variance weighted
MR Egger
Simple mode
Weighted median
Weighted mode
0.1
0.0
−0.1
−0.2
−0.3
−0.4
0.1
0.2
0.3
SNP effect on GCST90200510_buildGRCh38.tsv
SNP effect on Enterobacteriaceae

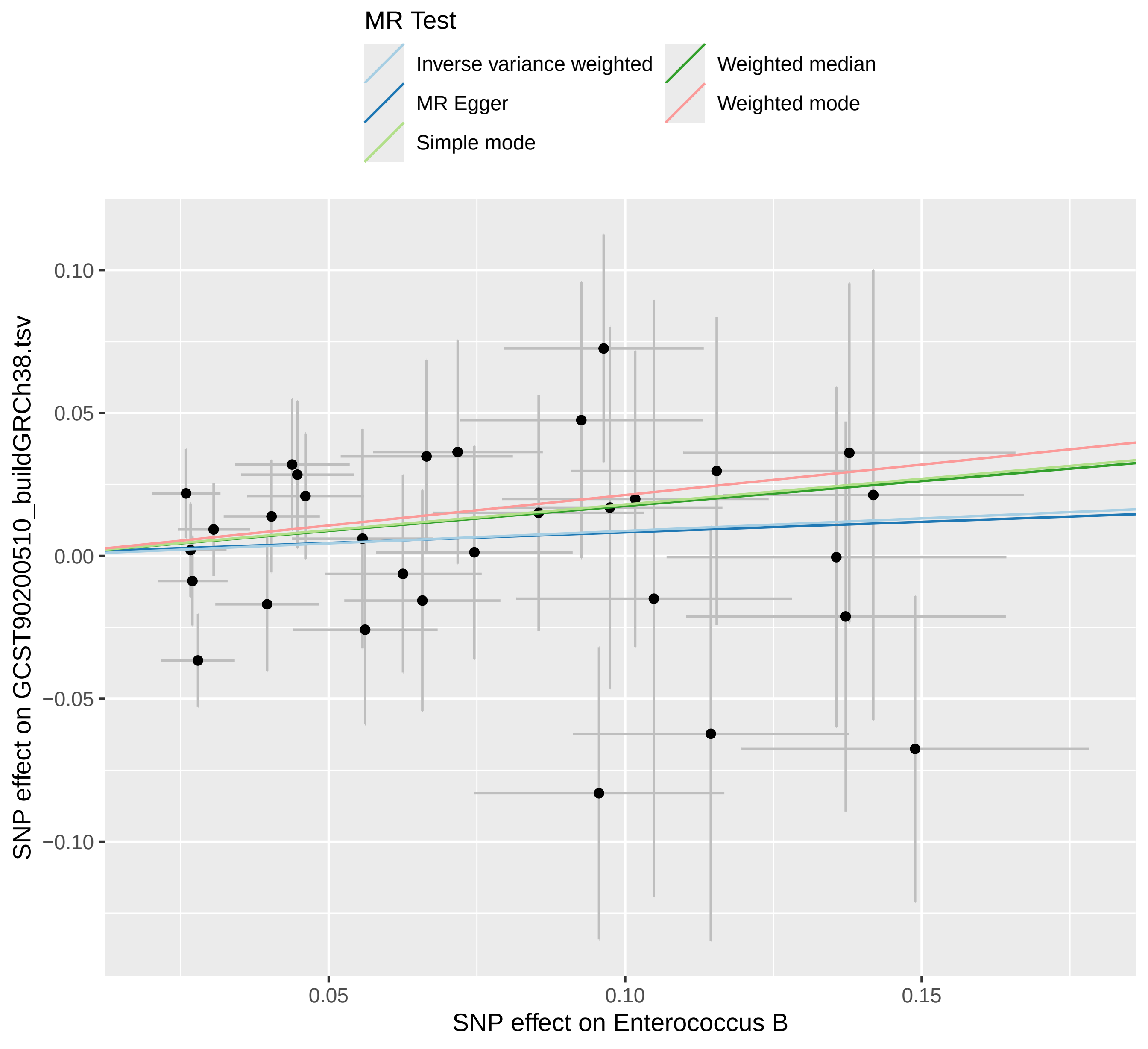

MR Test
Inverse variance weighted
MR Egger
Simple mode
Weighted median
Weighted mode
0.10
0.05
0.00
−0.05
−0.10
0.05
0.10
0.15
SNP effect on GCST90200510_buildGRCh38.tsv
SNP effect on Enterococcus B

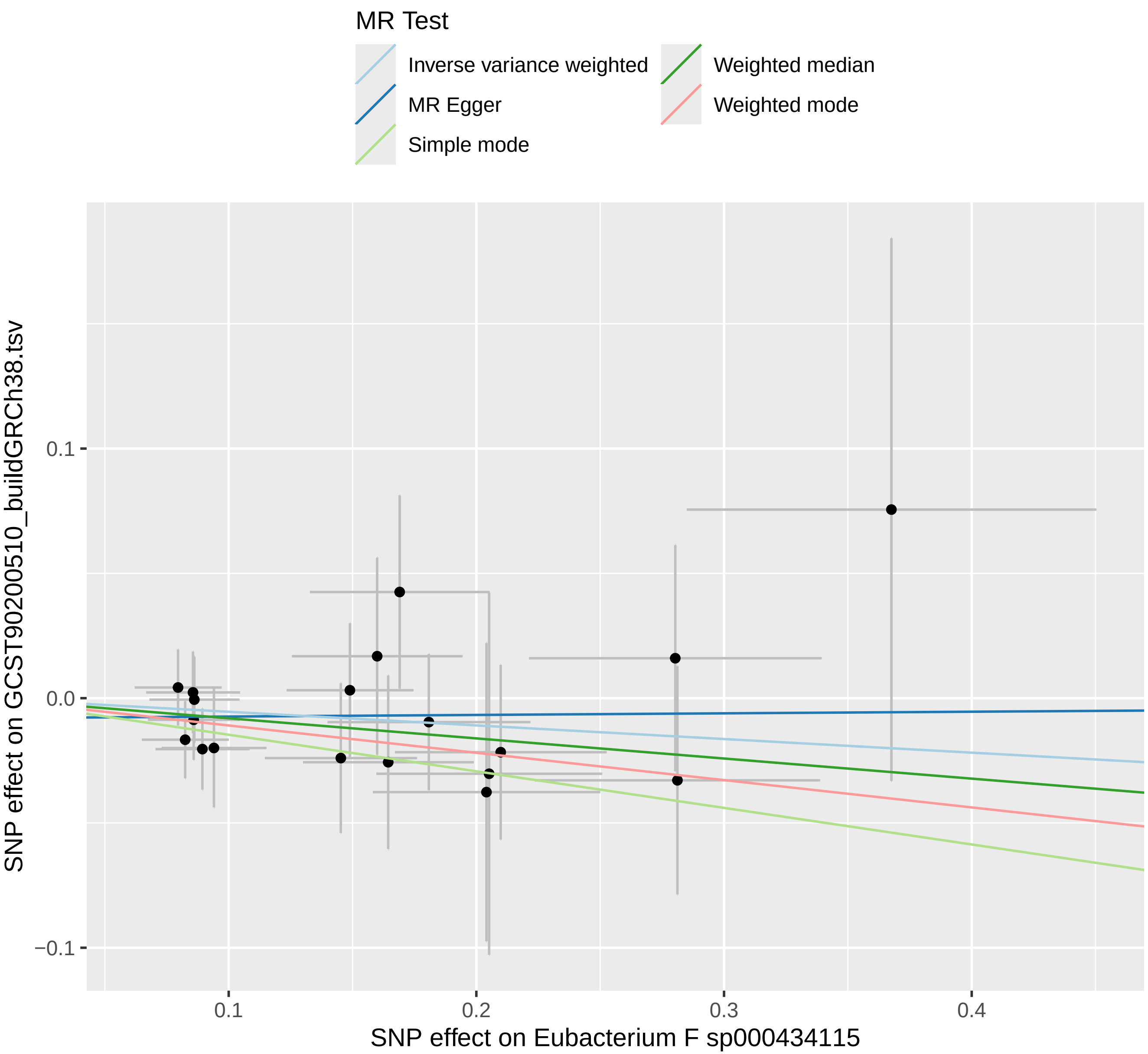

MR Test
Inverse variance weighted
MR Egger
Simple mode
Weighted median
Weighted mode
0.1
0.0
−0.1
0.1
0.2
0.3
0.4
SNP effect on GCST90200510_buildGRCh38.tsv
SNP effect on Eubacterium F sp000434115

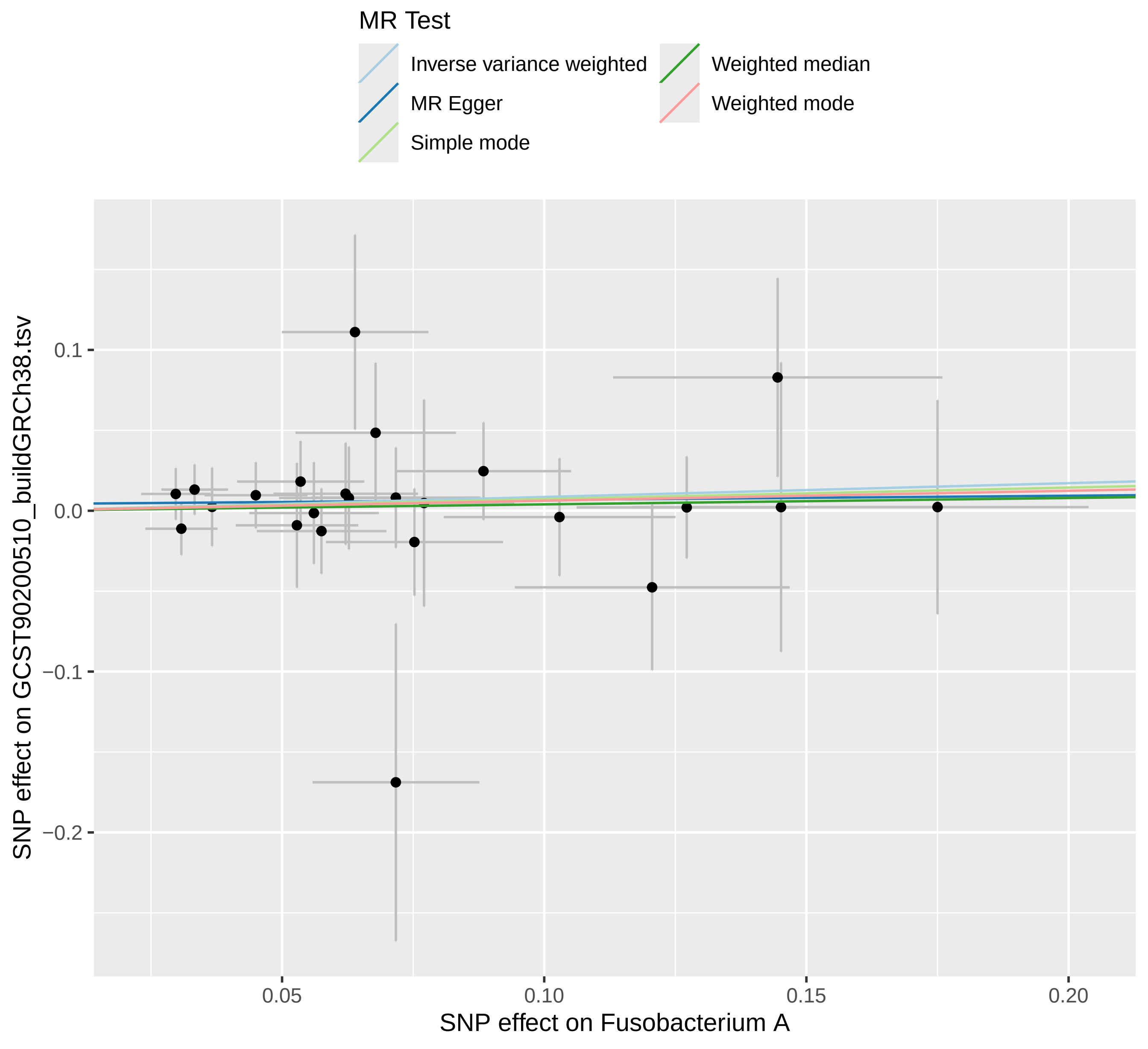

MR Test
Inverse variance weighted
MR Egger
Simple mode
Weighted median
Weighted mode
SNP effect on GCST90200510_buildGRCh38.tsv
0.1
0.0
−0.1
−0.2
0.05
0.10
0.15
0.20
SNP effect on Fusobacterium A

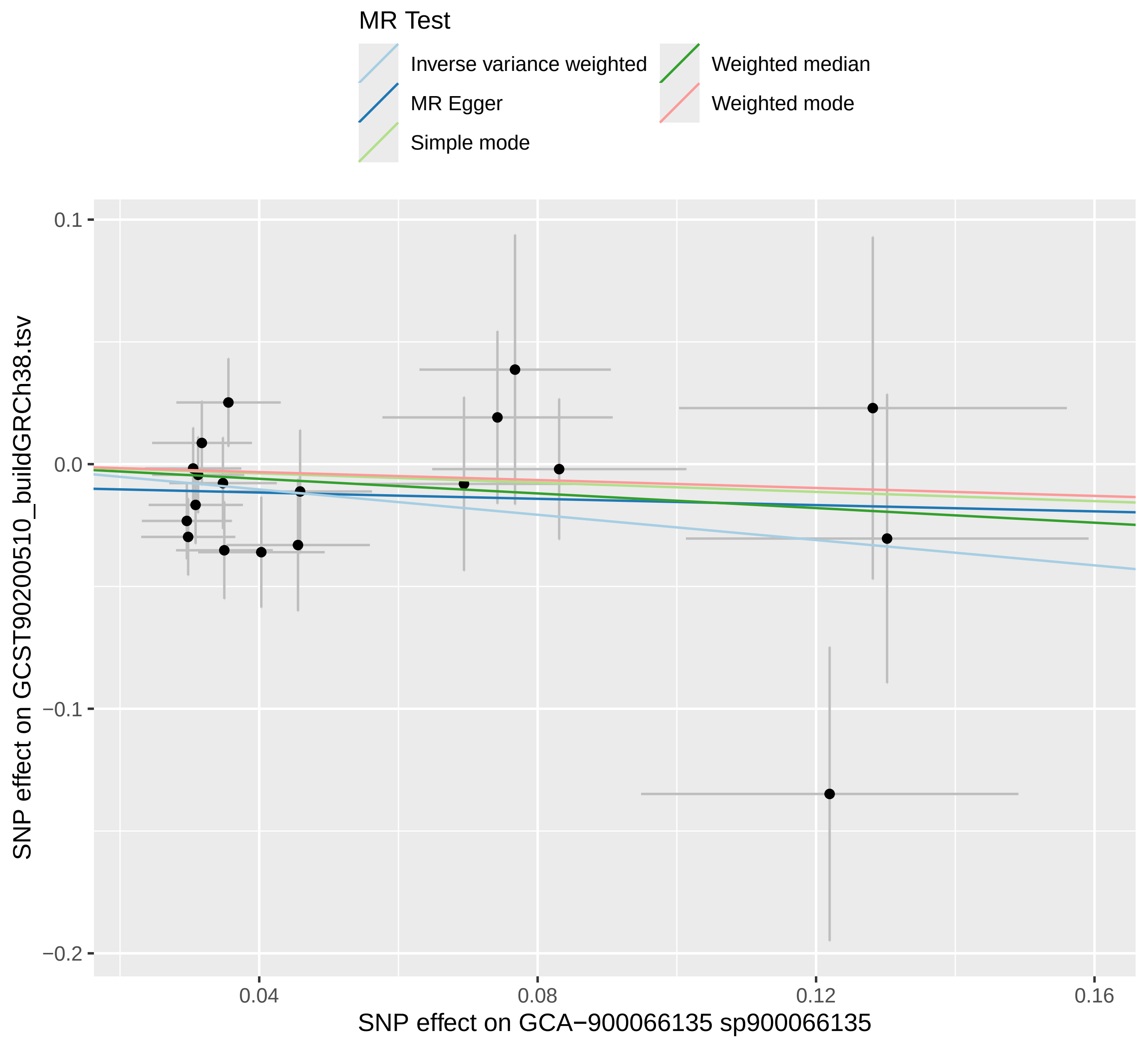

MR Test
Inverse variance weighted
MR Egger
Simple mode
Weighted median
Weighted mode
0.1
0.0
−0.1
−0.2
0.04
0.08
0.12
0.16
SNP effect on GCST90200510_buildGRCh38.tsv
SNP effect on GCA−900066135 sp900066135

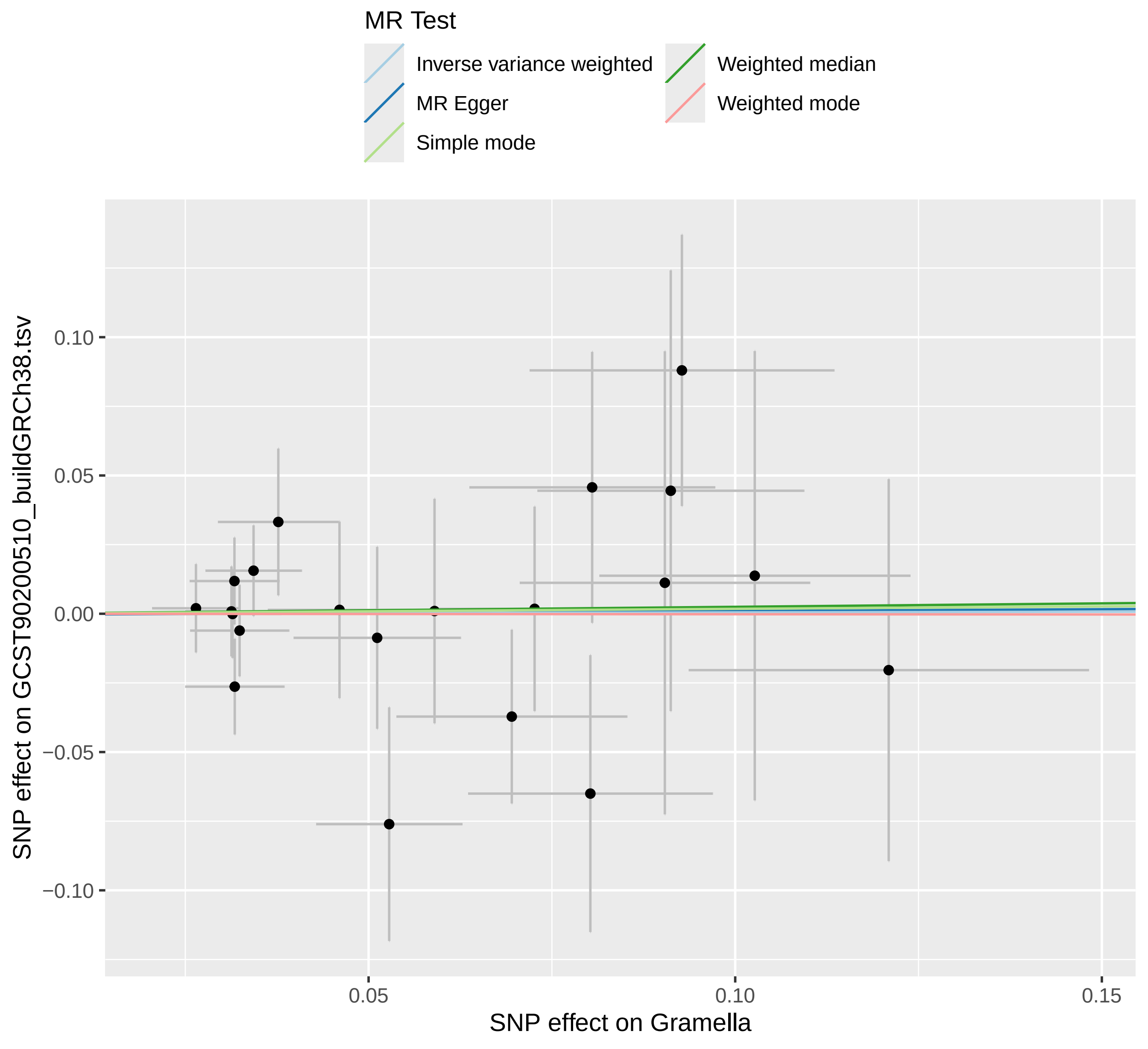
MR Test
Inverse variance weighted
MR Egger
Simple mode
Weighted median
Weighted mode
0.10
0.05
0.00
−0.05
−0.10
0.05
0.10
0.15
SNP effect on GCST90200510_buildGRCh38.tsv
SNP effect on Gramella

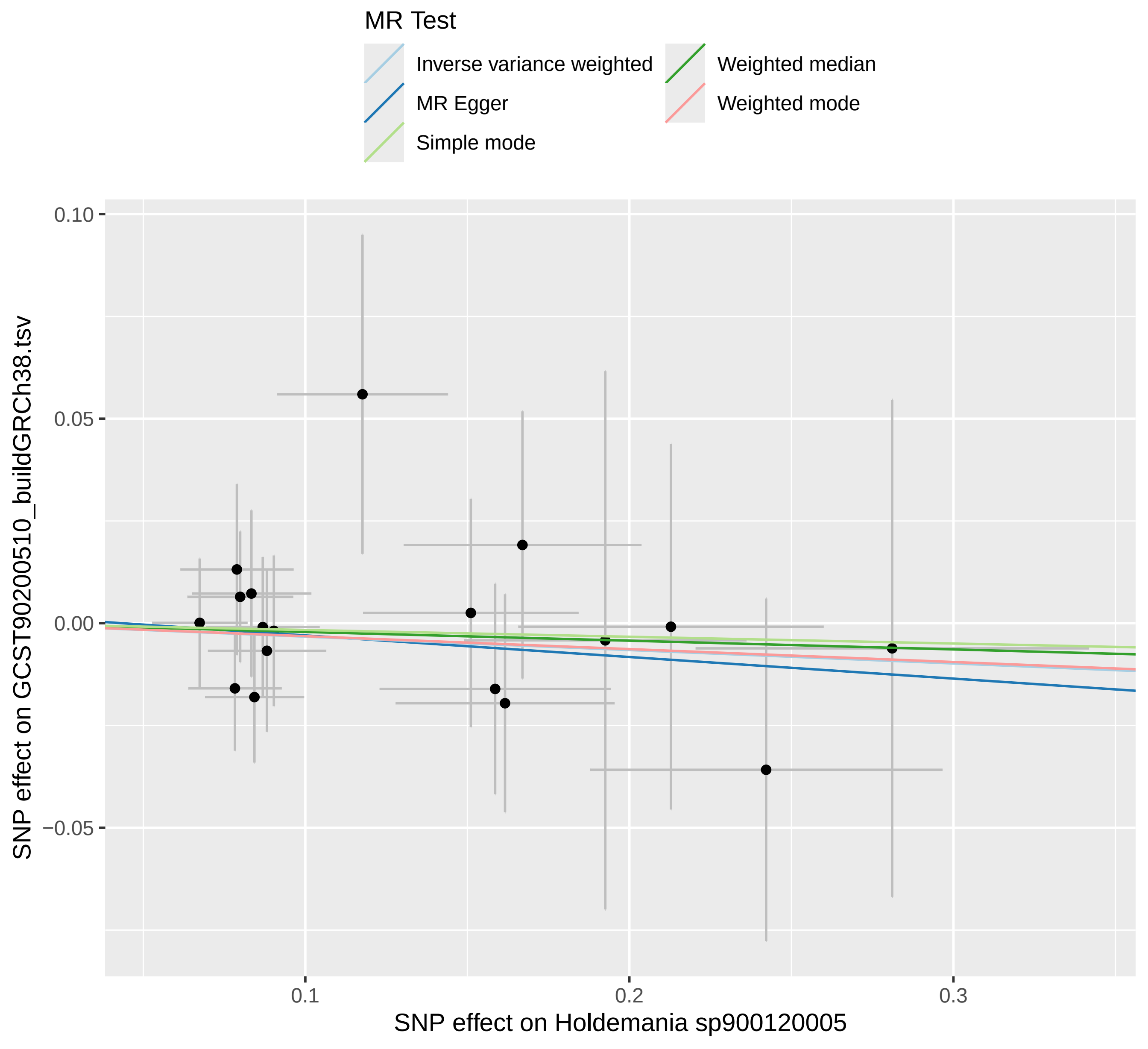

MR Test
Inverse variance weighted
MR Egger
Simple mode
Weighted median
Weighted mode
0.10
0.05
0.00
−0.05
0.1
0.2
0.3
SNP effect on GCST90200510_buildGRCh38.tsv
SNP effect on Holdemania sp900120005

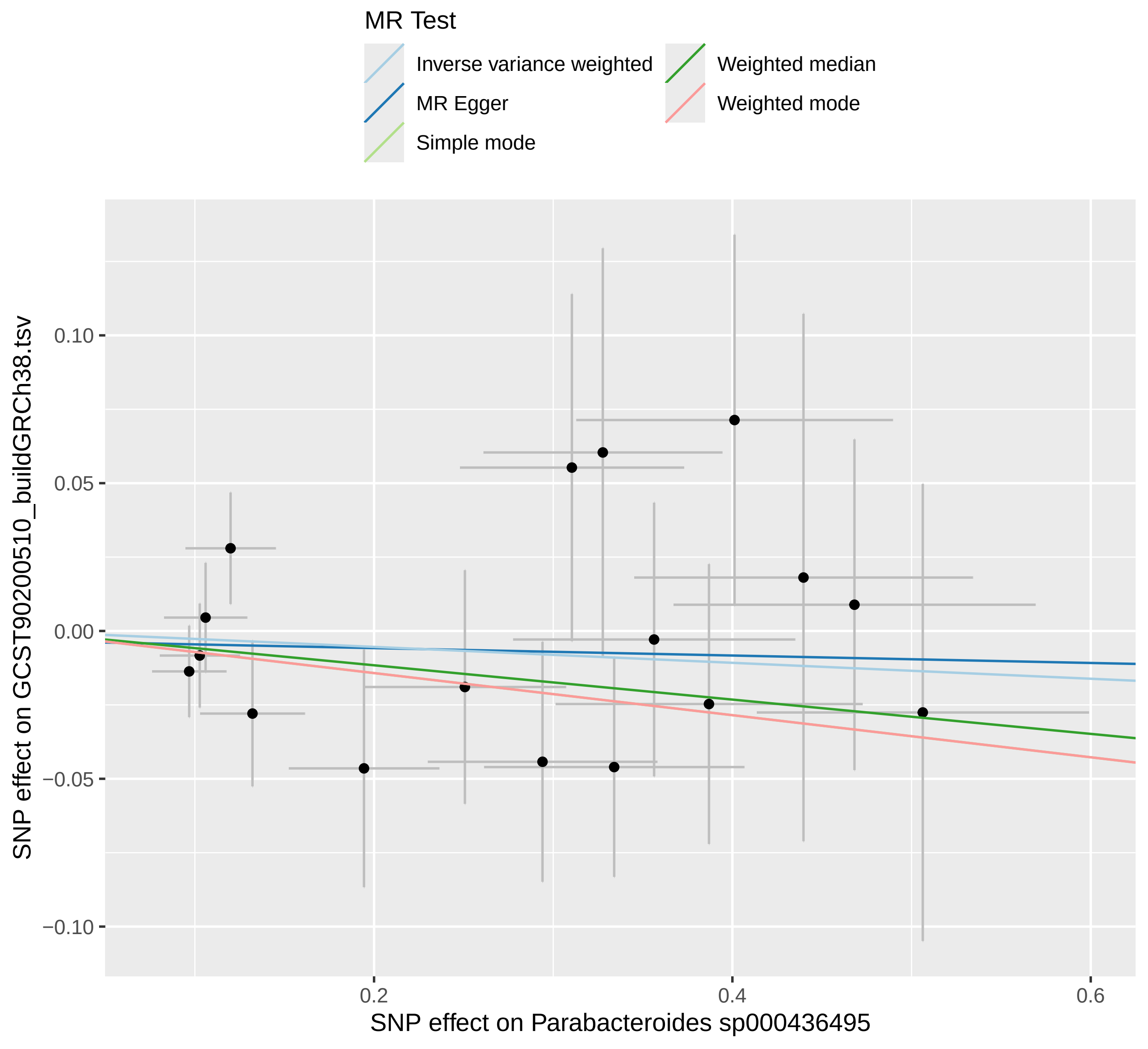
MR Test
Inverse variance weighted
MR Egger
Simple mode
Weighted median
Weighted mode
SNP effect on GCST90200510_buildGRCh38.tsv
0.10
0.05
0.00
−0.05
−0.10
0.2
0.4
0.6
SNP effect on Parabacteroides sp000436495

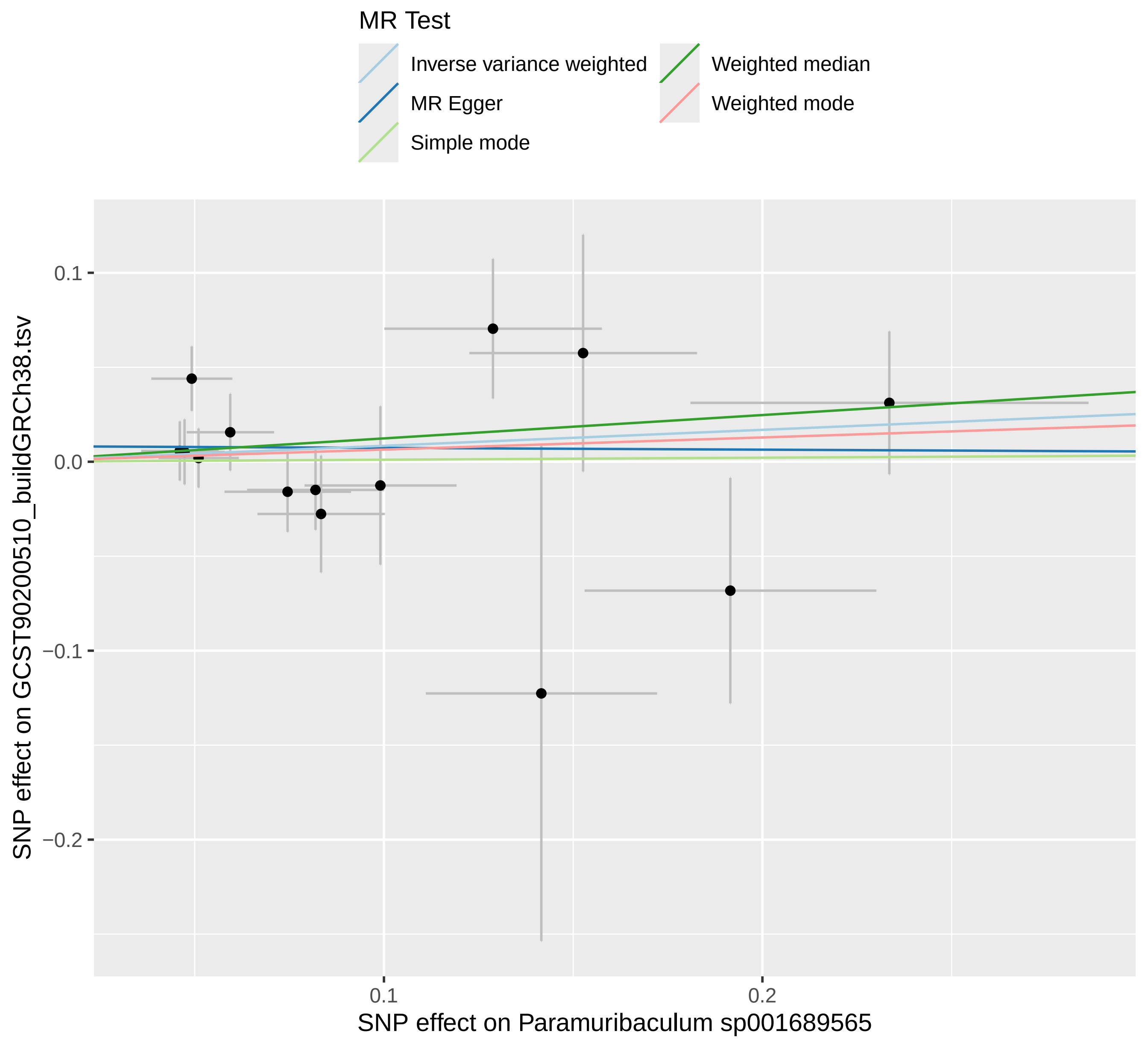

MR Test
Inverse variance weighted
MR Egger
Simple mode
Weighted median
Weighted mode
0.1
0.0
−0.1
−0.2
0.1
0.2
SNP effect on GCST90200510_buildGRCh38.tsv
SNP effect on Paramuribaculum sp001689565

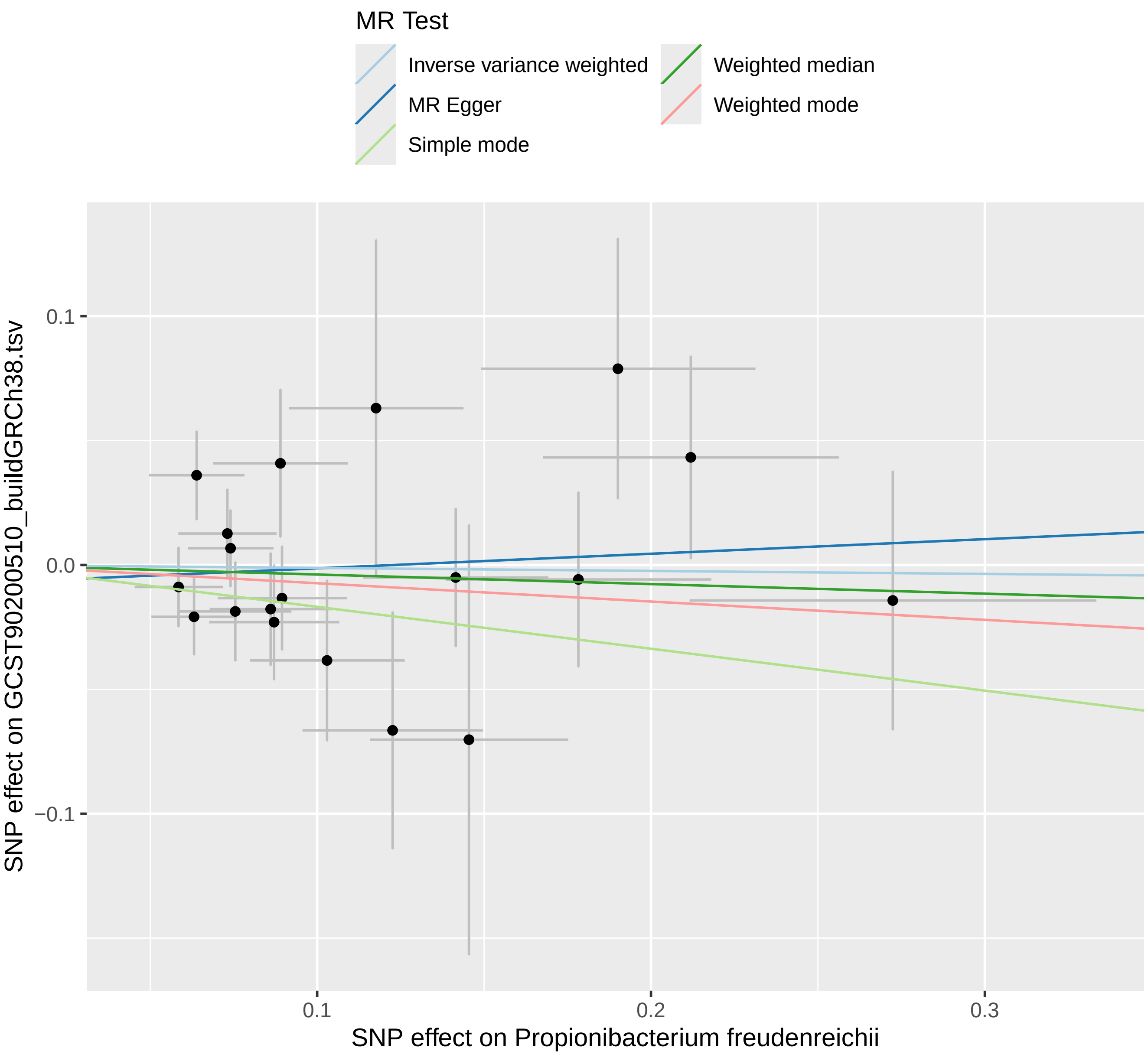
MR Test
Inverse variance weighted
MR Egger
Simple mode
Weighted median
Weighted mode
0.1
0.0
−0.1
0.1
0.2
0.3
SNP effect on GCST90200510_buildGRCh38.tsv
SNP effect on Propionibacterium freudenreichii

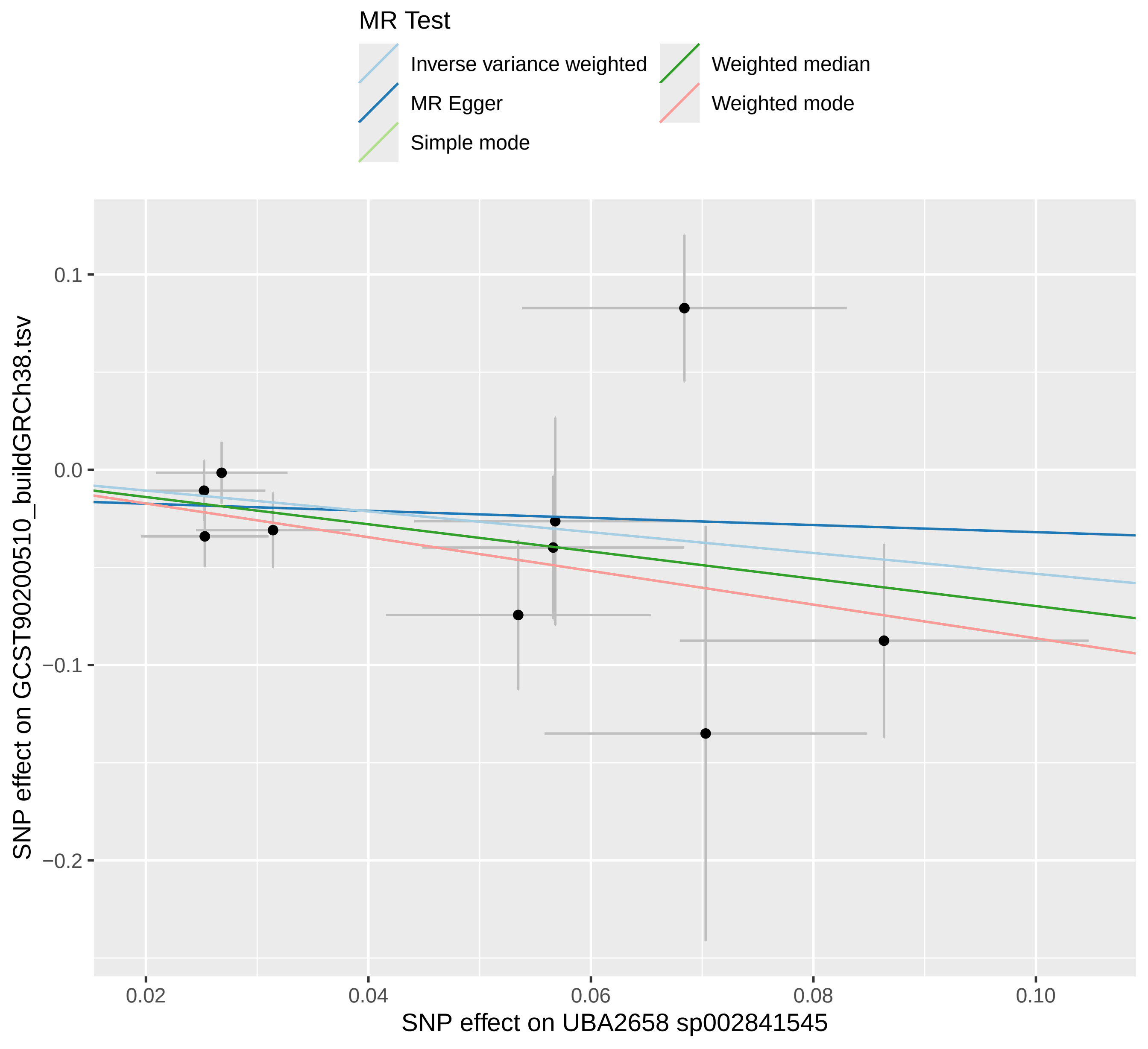

MR Test
Inverse variance weighted
MR Egger
Simple mode
Weighted median
Weighted mode
0.1
0.0
−0.1
−0.2
SNP effect on GCST90200510_buildGRCh38.tsv
0.02
0.04
0.06
0.08
0.10
SNP effect on UBA2658 sp002841545

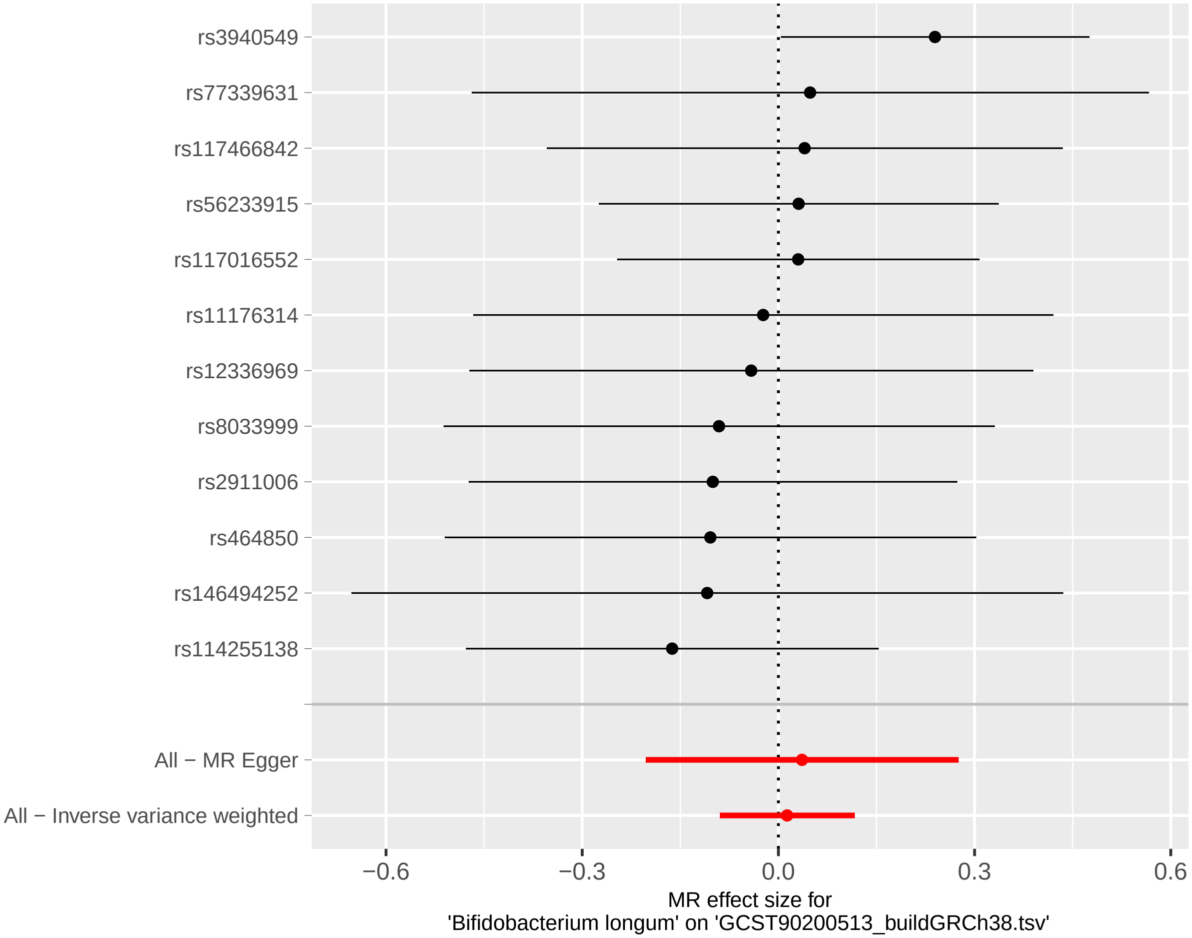
rs3940549
rs77339631
rs117466842
rs56233915
rs117016552
rs11176314
rs12336969
rs8033999
rs2911006
rs464850
rs146494252
rs114255138
All − MR Egger
All − Inverse variance weighted
−0.6
−0.3
0.0
0.3
0.6
MR effect size for
'Bifidobacterium longum' on 'GCST90200513_buildGRCh38.tsv'

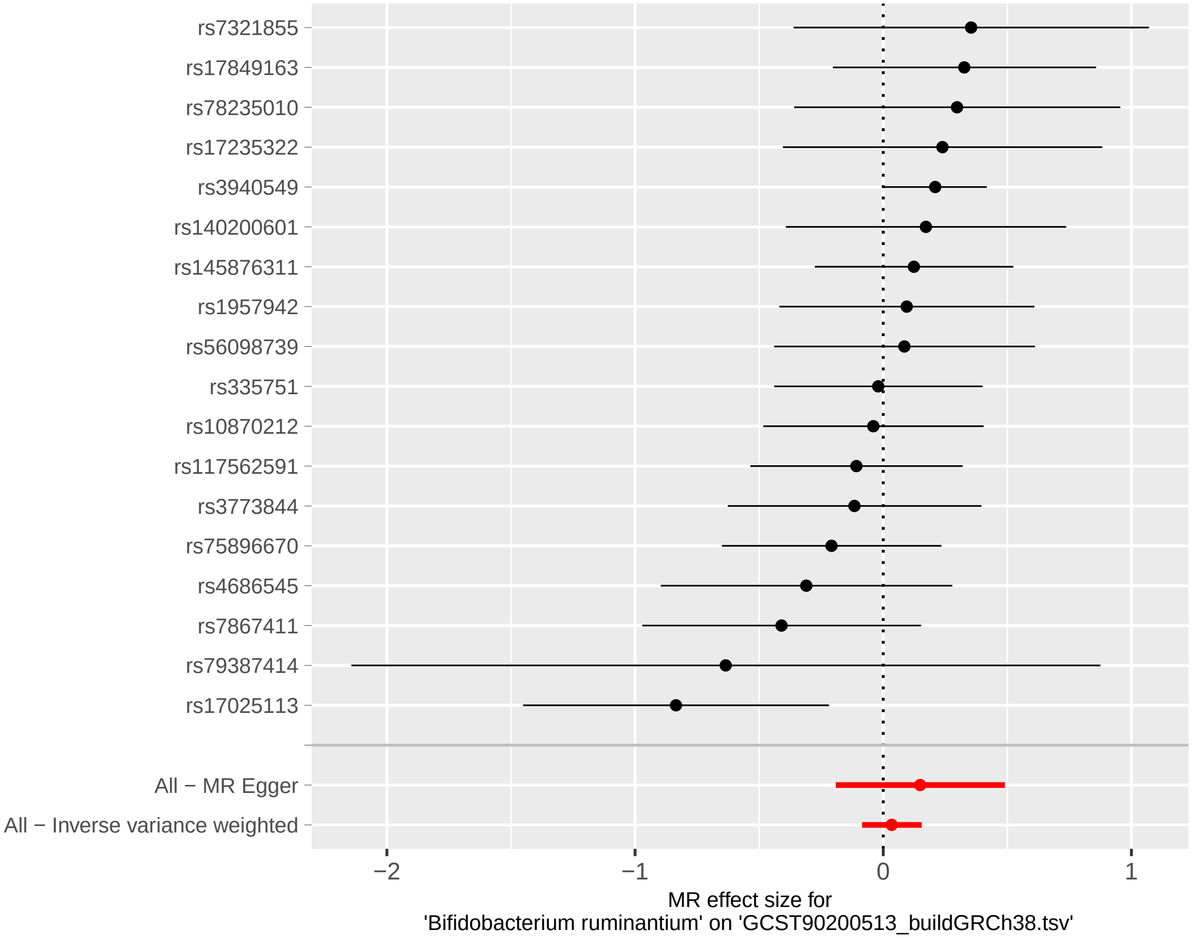
rs7321855
rs17849163
rs78235010
rs17235322
rs3940549
rs140200601
rs145876311
rs1957942
rs56098739
rs335751
rs10870212
rs117562591
rs3773844
rs75896670
rs4686545
rs7867411
rs79387414
rs17025113
All − MR Egger
All − Inverse variance weighted
−2
−1
0
1
MR effect size for
'Bifidobacterium ruminantium' on 'GCST90200513_buildGRCh38.tsv'

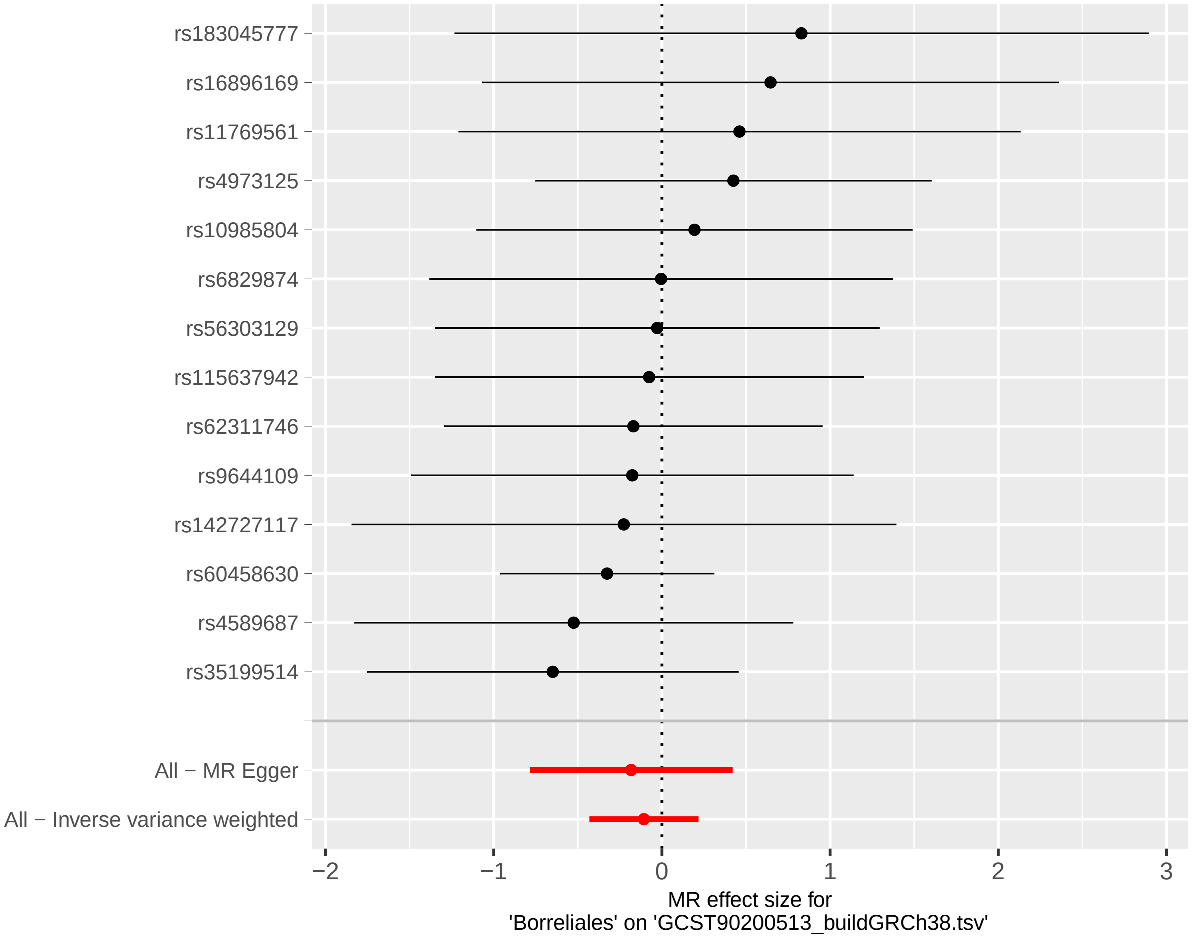

rs183045777
rs16896169
rs11769561
rs4973125
rs10985804
rs6829874
rs56303129
rs115637942
rs62311746
rs9644109
rs142727117
rs60458630
rs4589687
rs35199514
All − MR Egger
All − Inverse variance weighted
−2
−1
0
1
2
3
MR effect size for
'Borreliales' on 'GCST90200513_buildGRCh38.tsv'

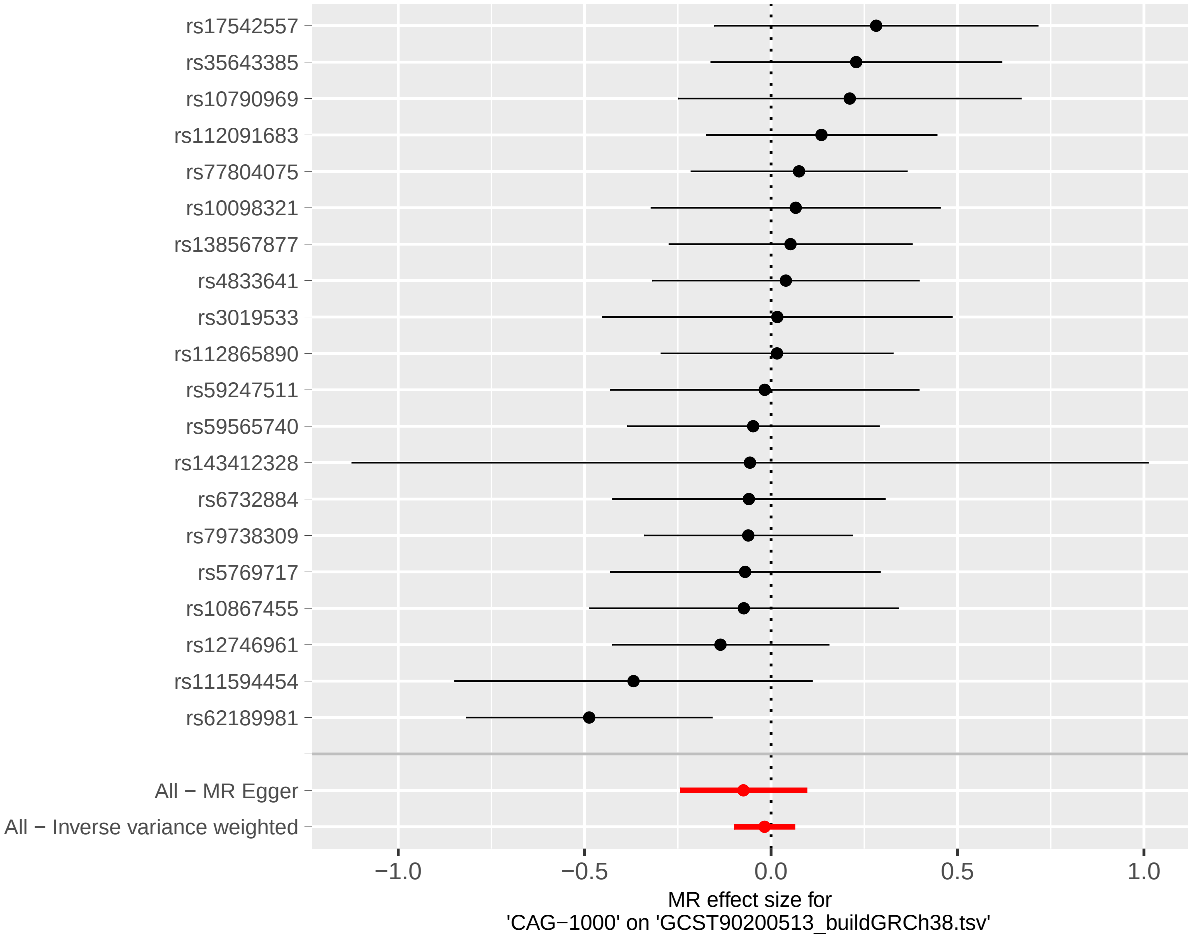
rs17542557
rs35643385
rs10790969
rs112091683
rs77804075
rs10098321
rs138567877
rs4833641
rs3019533
rs112865890
rs59247511
rs59565740
rs143412328
rs6732884
rs79738309
rs5769717
rs10867455
rs12746961
rs111594454
rs62189981
All − MR Egger
All − Inverse variance weighted
−1.0
−0.5
0.0
0.5
1.0
MR effect size for
'CAG−1000' on 'GCST90200513_buildGRCh38.tsv'

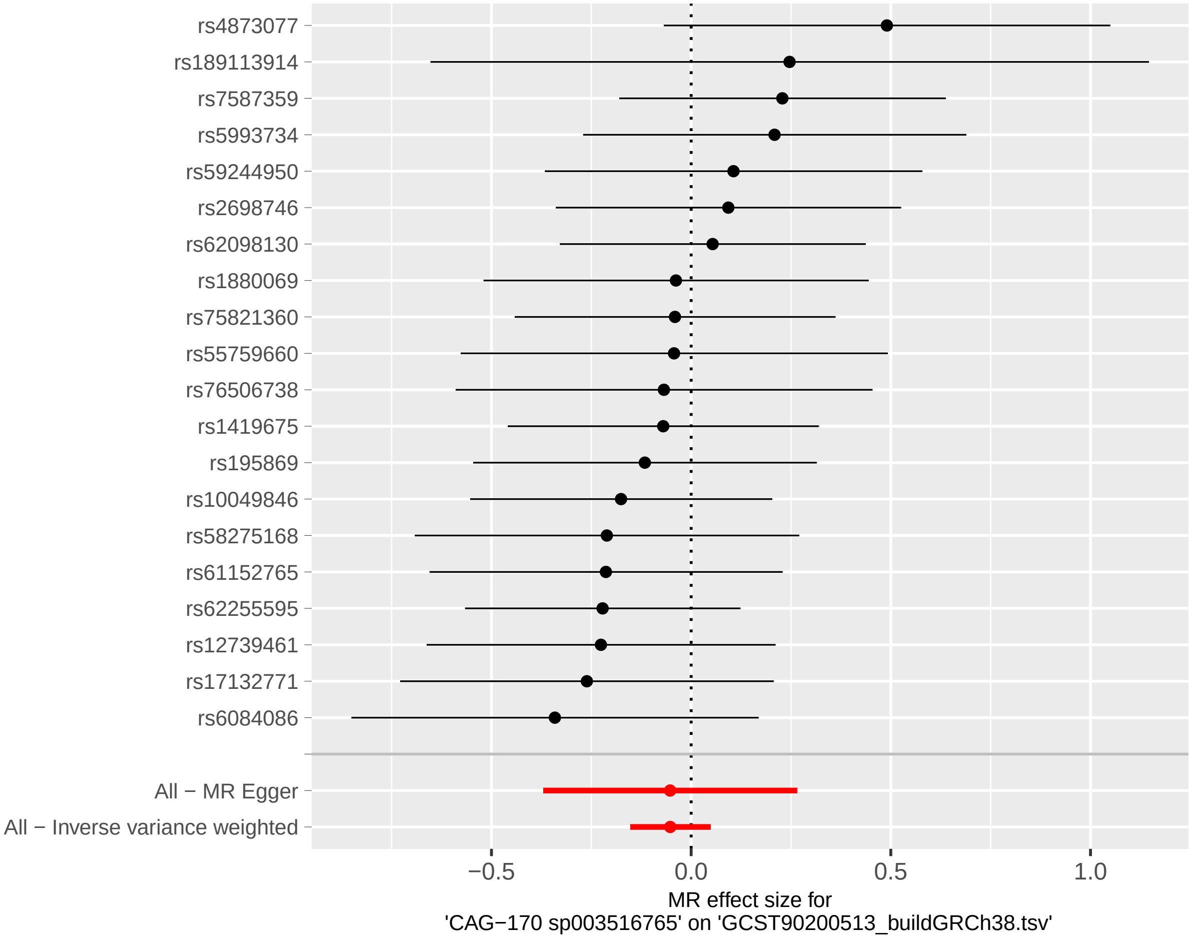
rs4873077
rs189113914
rs7587359
rs5993734
rs59244950
rs2698746
rs62098130
rs1880069
rs75821360
rs55759660
rs76506738
rs1419675
rs195869
rs10049846
rs58275168
rs61152765
rs62255595
rs12739461
rs17132771
rs6084086
All − MR Egger
All − Inverse variance weighted
−0.5
0.0
0.5
1.0
MR effect size for
'CAG−170 sp003516765' on 'GCST90200513_buildGRCh38.tsv'

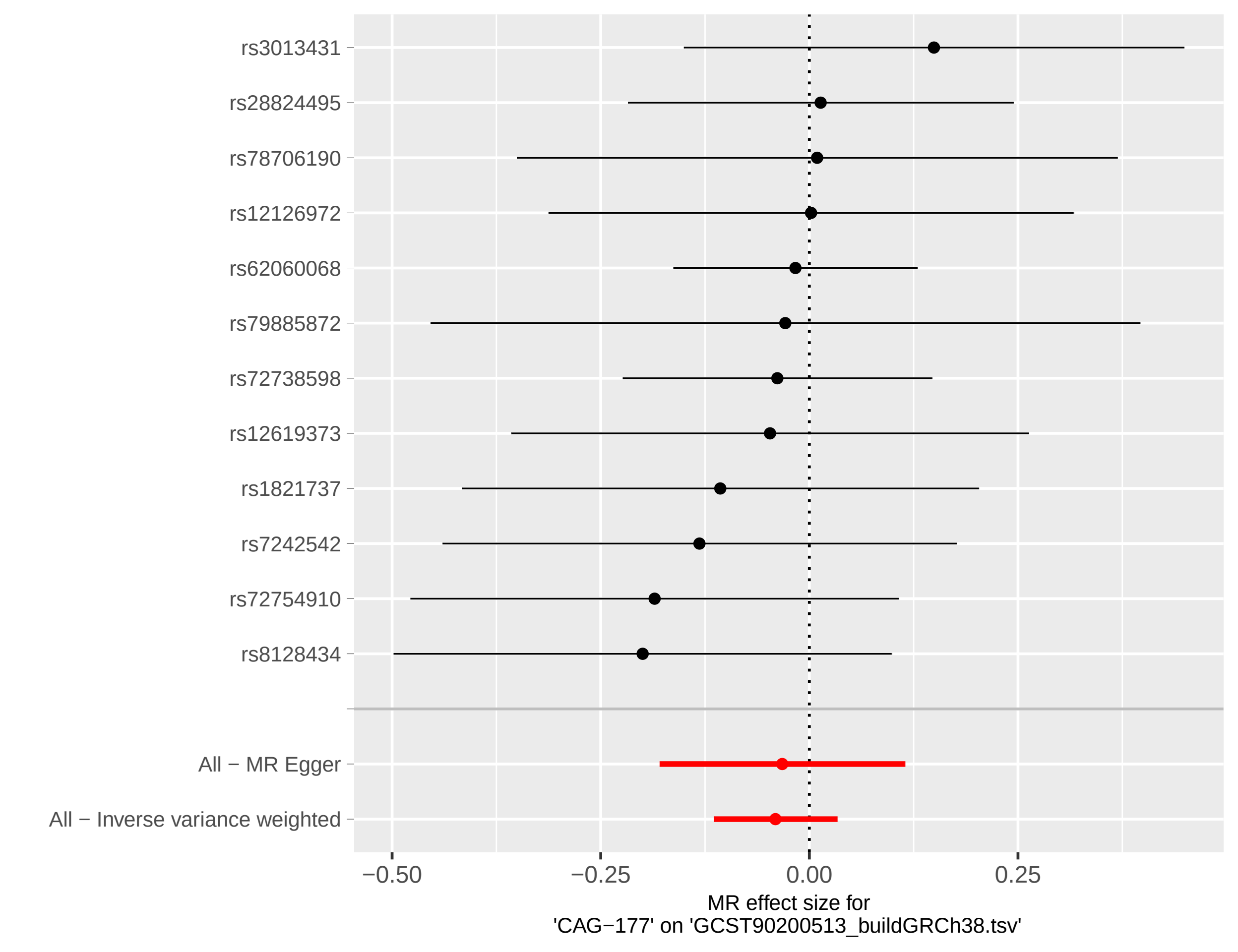

rs3013431
rs28824495
rs78706190
rs12126972
rs62060068
rs79885872
rs72738598
rs12619373
rs1821737
rs7242542
rs72754910
rs8128434
All − MR Egger
All − Inverse variance weighted
−0.50
−0.25
0.00
0.25
MR effect size for
'CAG−177' on 'GCST90200513_buildGRCh38.tsv'

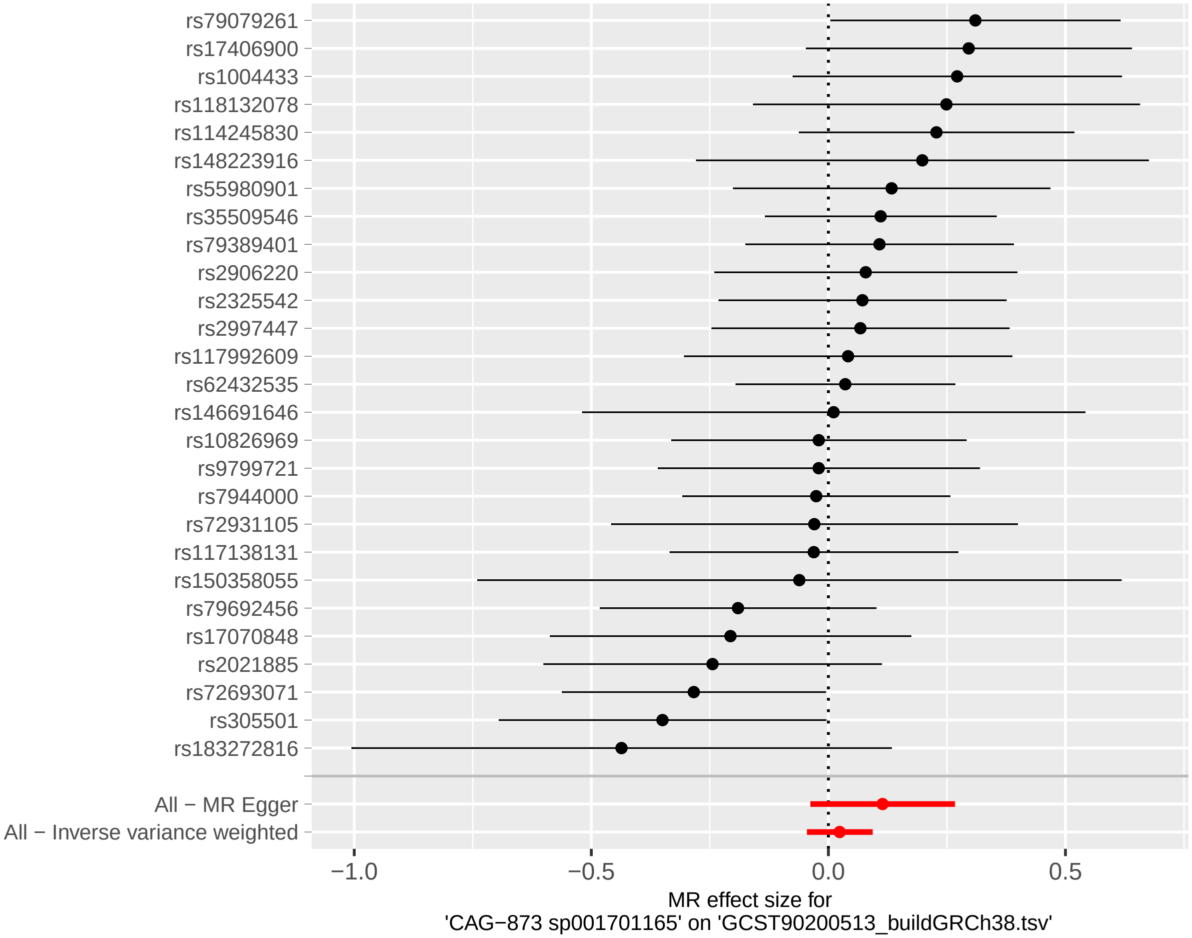
rs79079261
rs17406900
rs1004433
rs118132078
rs114245830
rs148223916
rs55980901
rs35509546
rs79389401
rs2906220
rs2325542
rs2997447
rs117992609
rs62432535
rs146691646
rs10826969
rs9799721
rs7944000
rs72931105
rs117138131
rs150358055
rs79692456
rs17070848
rs2021885
rs72693071
rs305501
rs183272816
All − MR Egger
All − Inverse variance weighted
−1.0
−0.5
0.0
0.5
MR effect size for
'CAG−873 sp001701165' on 'GCST90200513_buildGRCh38.tsv'

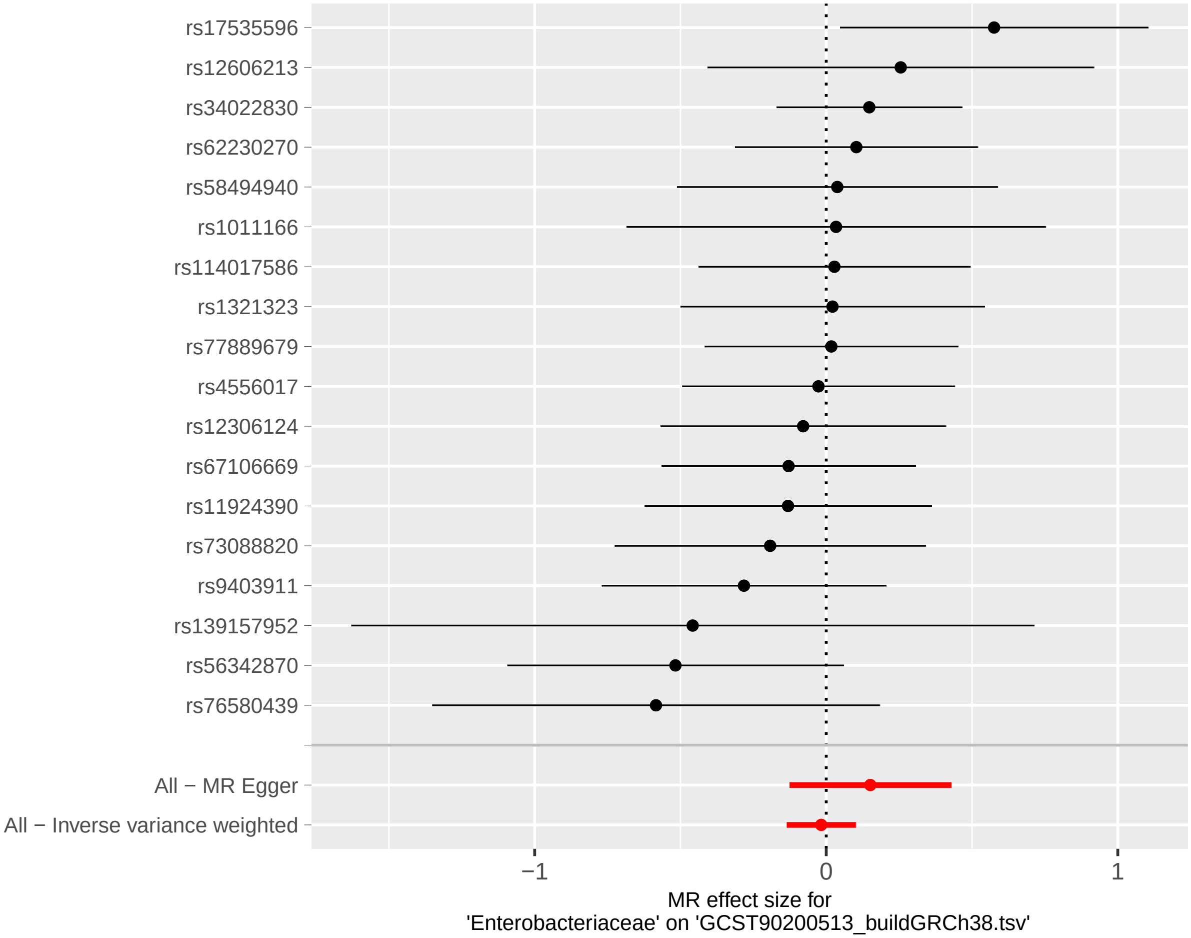

rs17535596
rs12606213
rs34022830
rs62230270
rs58494940
rs1011166
rs114017586
rs1321323
rs77889679
rs4556017
rs12306124
rs67106669
rs11924390
rs73088820
rs9403911
rs139157952
rs56342870
rs76580439
All − MR Egger
All − Inverse variance weighted
−1
0
1
MR effect size for
'Enterobacteriaceae' on 'GCST90200513_buildGRCh38.tsv'

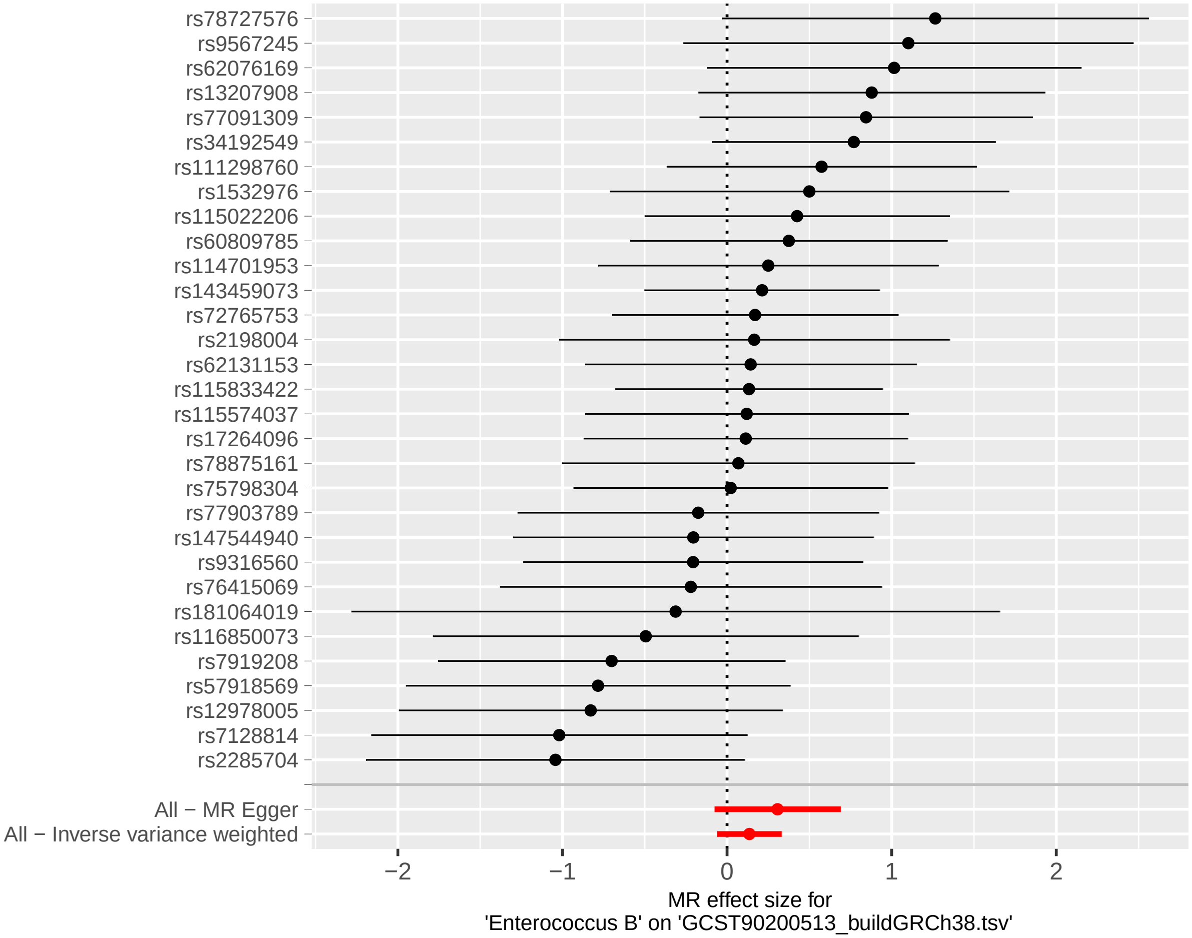
rs78727576
rs9567245
rs62076169
rs13207908
rs77091309
rs34192549
rs111298760
rs1532976
rs115022206
rs60809785
rs114701953
rs143459073
rs72765753
rs2198004
rs62131153
rs115833422
rs115574037
rs17264096
rs78875161
rs75798304
rs77903789
rs147544940
rs9316560
rs76415069
rs181064019
rs116850073
rs7919208
rs57918569
rs12978005
rs7128814
rs2285704
All − MR Egger
All − Inverse variance weighted
−2
−1
0
1
2
MR effect size for
'Enterococcus B' on 'GCST90200513_buildGRCh38.tsv'

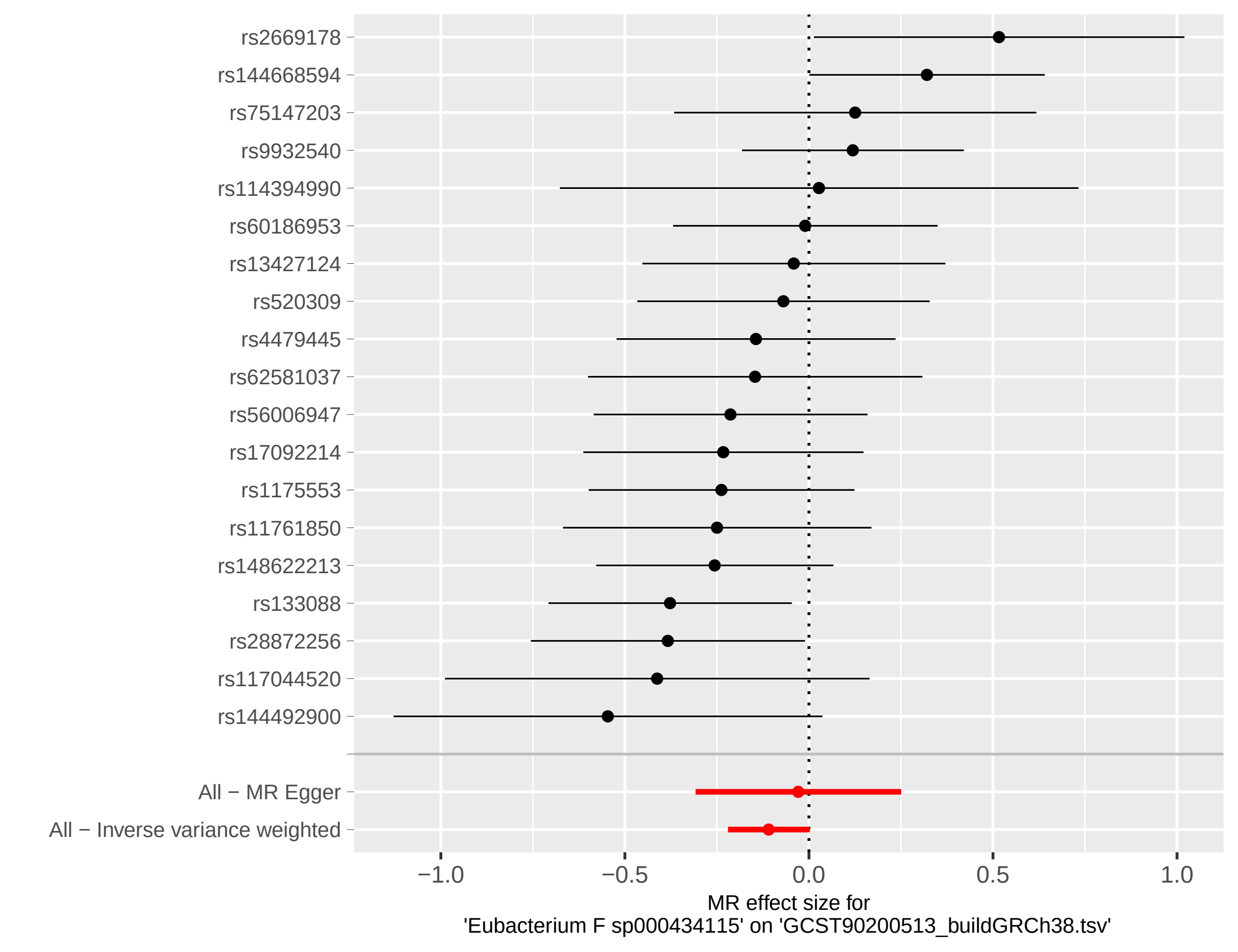
rs2669178
rs144668594
rs75147203
rs9932540
rs114394990
rs60186953
rs13427124
rs520309
rs4479445
rs62581037
rs56006947
rs17092214
rs1175553
rs11761850
rs148622213
rs133088
rs28872256
rs117044520
rs144492900
All − MR Egger
All − Inverse variance weighted
−1.0
−0.5
0.0
0.5
1.0
MR effect size for
'Eubacterium F sp000434115' on 'GCST90200513_buildGRCh38.tsv'

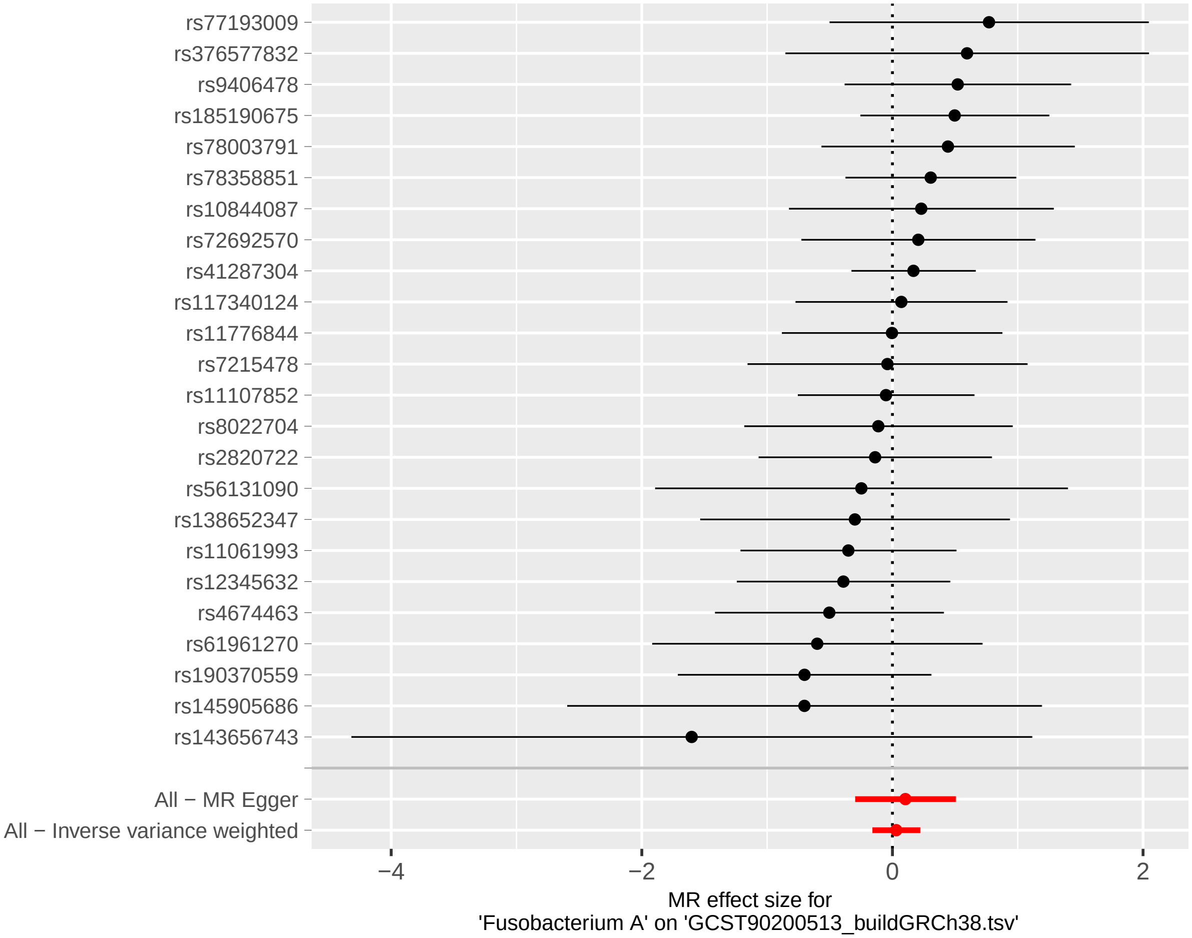

rs77193009
rs376577832
rs9406478
rs185190675
rs78003791
rs78358851
rs10844087
rs72692570
rs41287304
rs117340124
rs11776844
rs7215478
rs11107852
rs8022704
rs2820722
rs56131090
rs138652347
rs11061993
rs12345632
rs4674463
rs61961270
rs190370559
rs145905686
rs143656743
All − MR Egger
All − Inverse variance weighted
−4
−2
0
2
MR effect size for
'Fusobacterium A' on 'GCST90200513_buildGRCh38.tsv'

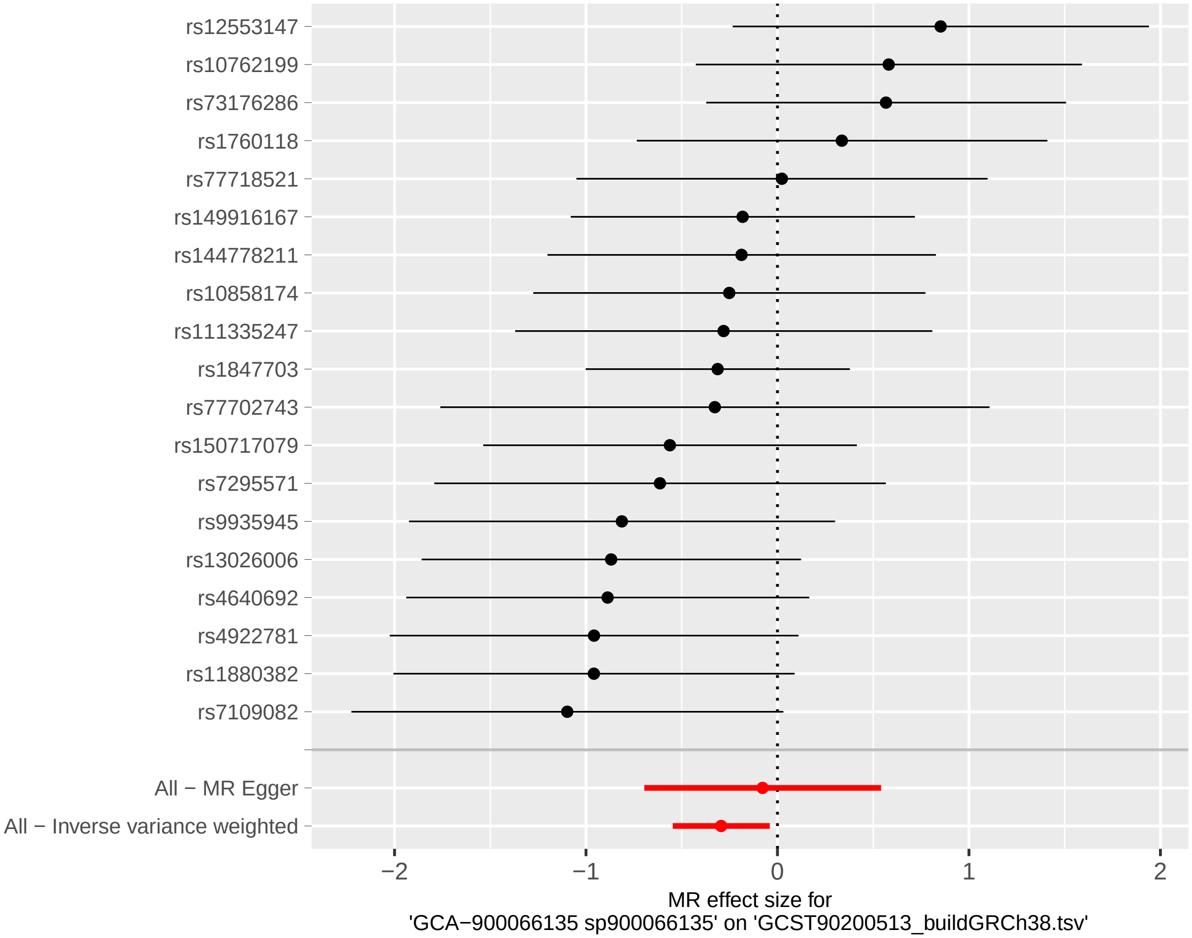

rs12553147
rs10762199
rs73176286
rs1760118
rs77718521
rs149916167
rs144778211
rs10858174
rs111335247
rs1847703
rs77702743
rs150717079
rs7295571
rs9935945
rs13026006
rs4640692
rs4922781
rs11880382
rs7109082
All − MR Egger
All − Inverse variance weighted
−2
−1
0
1
2
MR effect size for
'GCA−900066135 sp900066135' on 'GCST90200513_buildGRCh38.tsv'

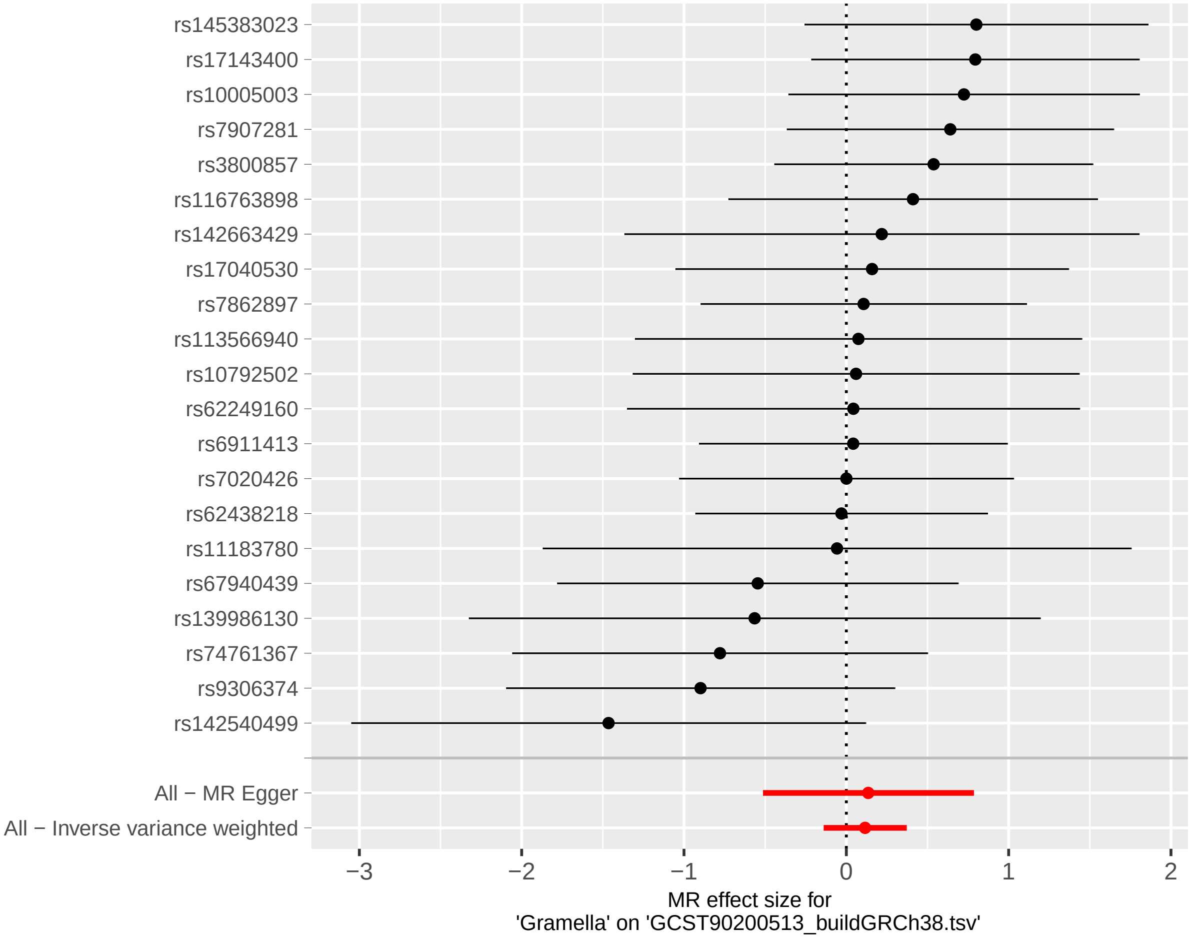

rs145383023
rs17143400
rs10005003
rs7907281
rs3800857
rs116763898
rs142663429
rs17040530
rs7862897
rs113566940
rs10792502
rs62249160
rs6911413
rs7020426
rs62438218
rs11183780
rs67940439
rs139986130
rs74761367
rs9306374
rs142540499
All − MR Egger
All − Inverse variance weighted
−3
−2
−1
0
1
2
MR effect size for
'Gramella' on 'GCST90200513_buildGRCh38.tsv'

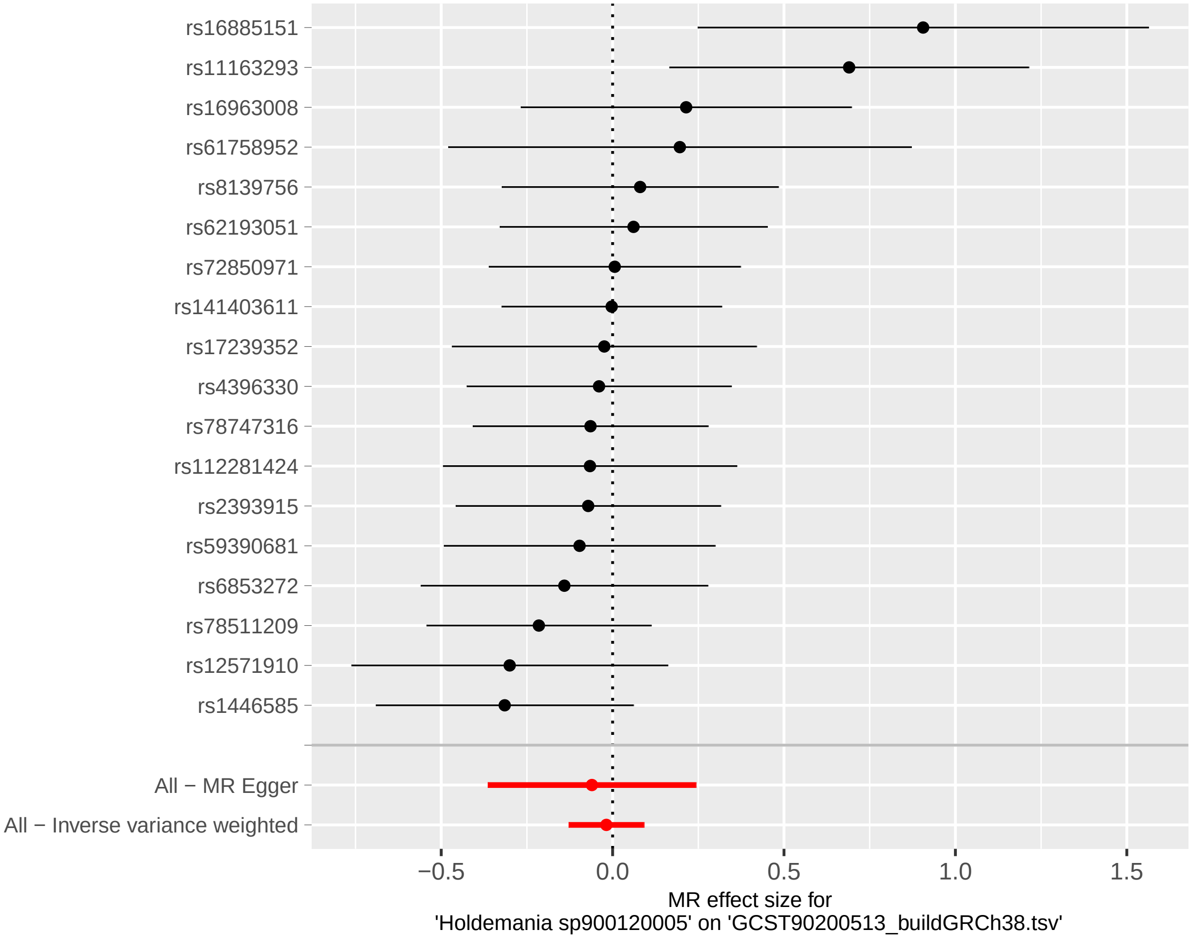
rs16885151
rs11163293
rs16963008
rs61758952
rs8139756
rs62193051
rs72850971
rs141403611
rs17239352
rs4396330
rs78747316
rs112281424
rs2393915
rs59390681
rs6853272
rs78511209
rs12571910
rs1446585
All − MR Egger
All − Inverse variance weighted
−0.5
0.0
0.5
1.0
1.5
MR effect size for
'Holdemania sp900120005' on 'GCST90200513_buildGRCh38.tsv'

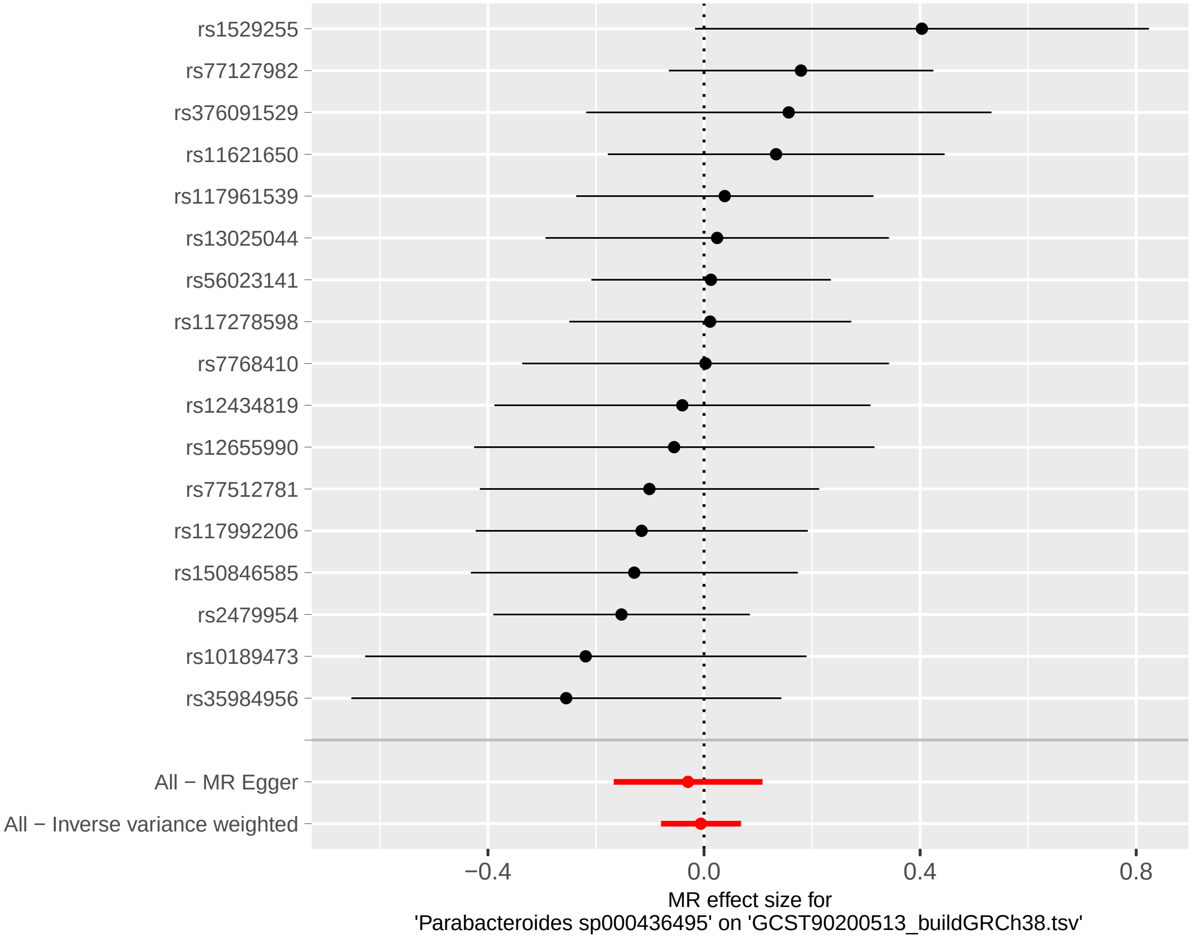
rs1529255
rs77127982
rs376091529
rs11621650
rs117961539
rs13025044
rs56023141
rs117278598
rs7768410
rs12434819
rs12655990
rs77512781
rs117992206
rs150846585
rs2479954
rs10189473
rs35984956
All − MR Egger
All − Inverse variance weighted
−0.4
0.0
0.4
0.8
MR effect size for
'Parabacteroides sp000436495' on 'GCST90200513_buildGRCh38.tsv'

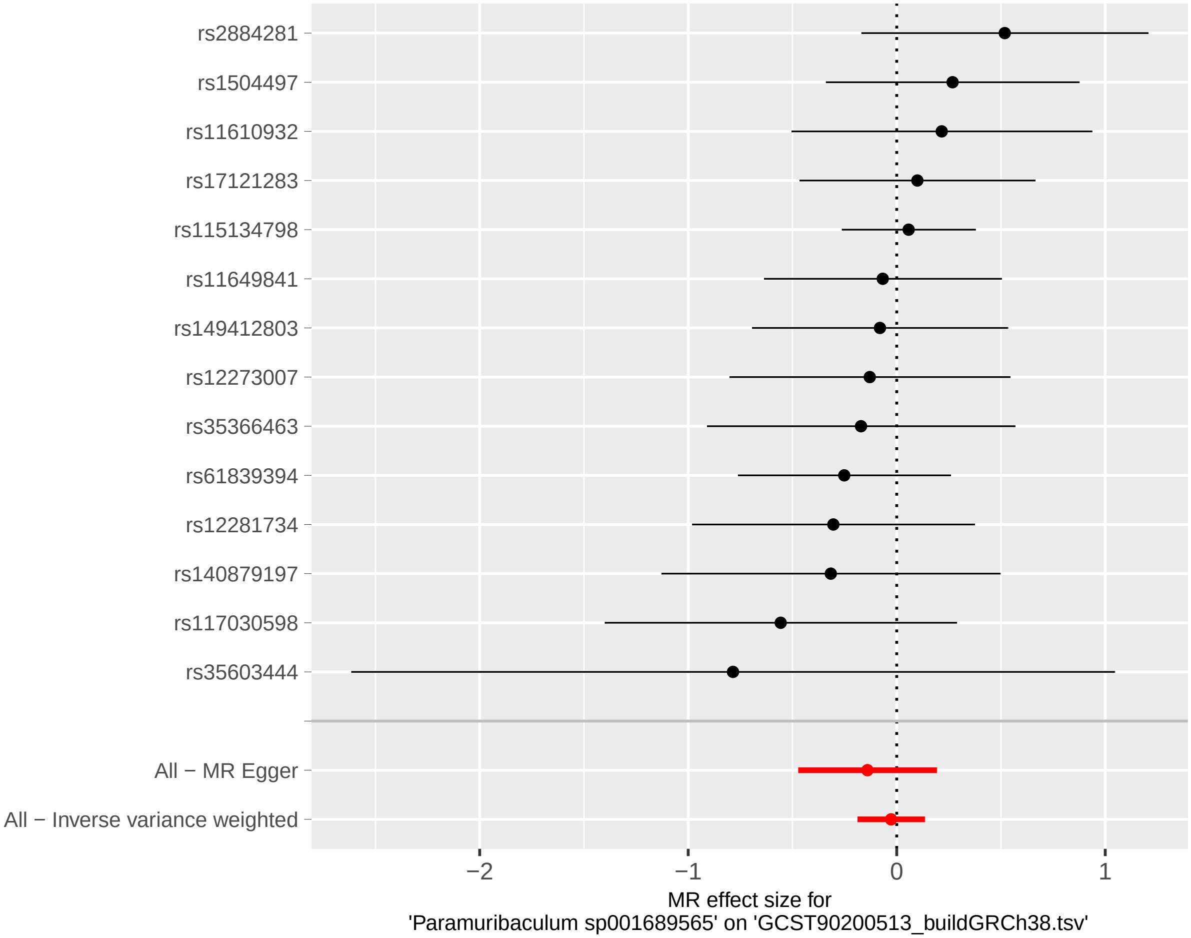
rs2884281
rs1504497
rs11610932
rs17121283
rs115134798
rs11649841
rs149412803
rs12273007
rs35366463
rs61839394
rs12281734
rs140879197
rs117030598
rs35603444
All − MR Egger
All − Inverse variance weighted
−2
−1
0
1
MR effect size for
'Paramuribaculum sp001689565' on 'GCST90200513_buildGRCh38.tsv'

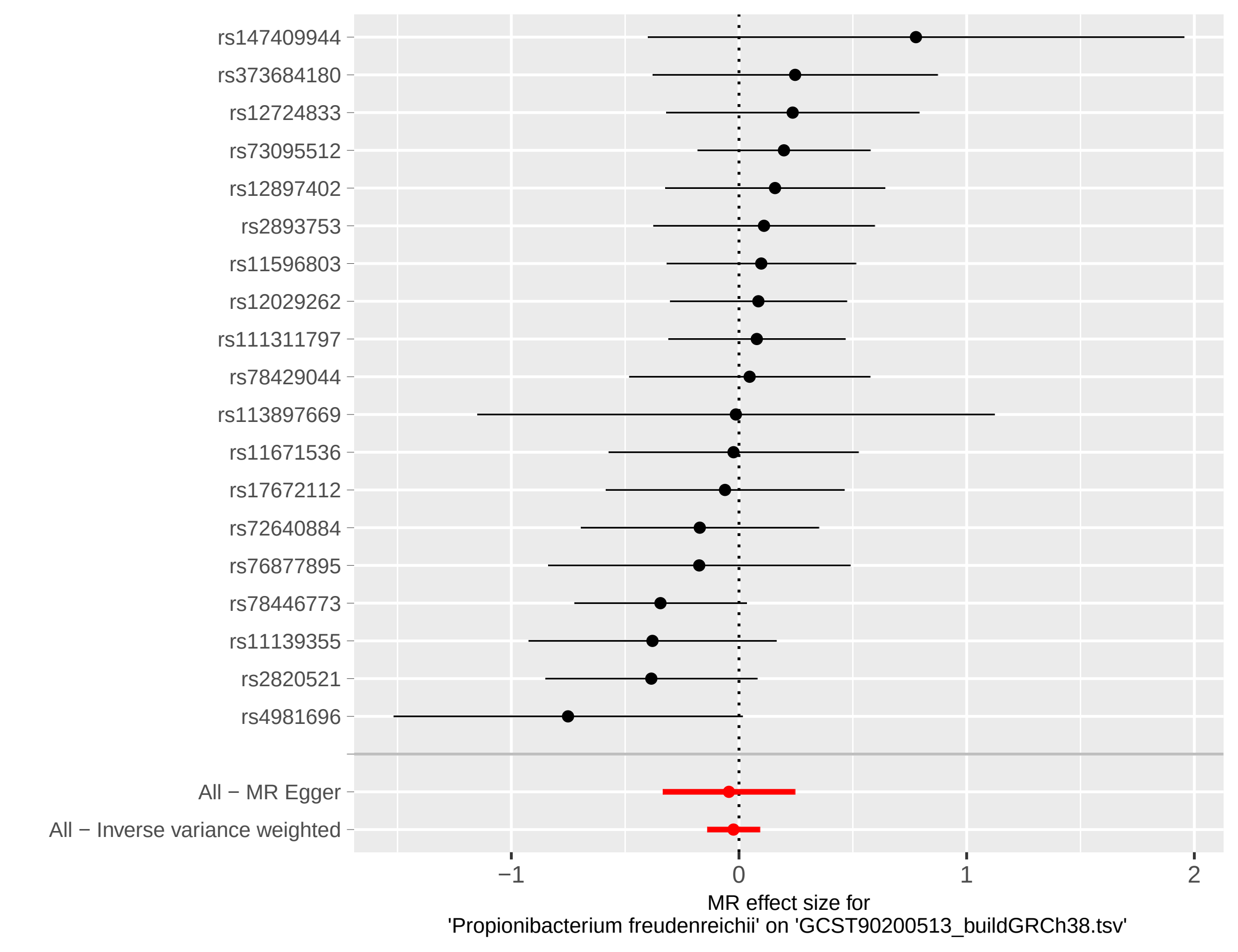
rs147409944
rs373684180
rs12724833
rs73095512
rs12897402
rs2893753
rs11596803
rs12029262
rs111311797
rs78429044
rs113897669
rs11671536
rs17672112
rs72640884
rs76877895
rs78446773
rs11139355
rs2820521
rs4981696
All − MR Egger
All − Inverse variance weighted
−1
0
1
2
MR effect size for
'Propionibacterium freudenreichii' on 'GCST90200513_buildGRCh38.tsv'

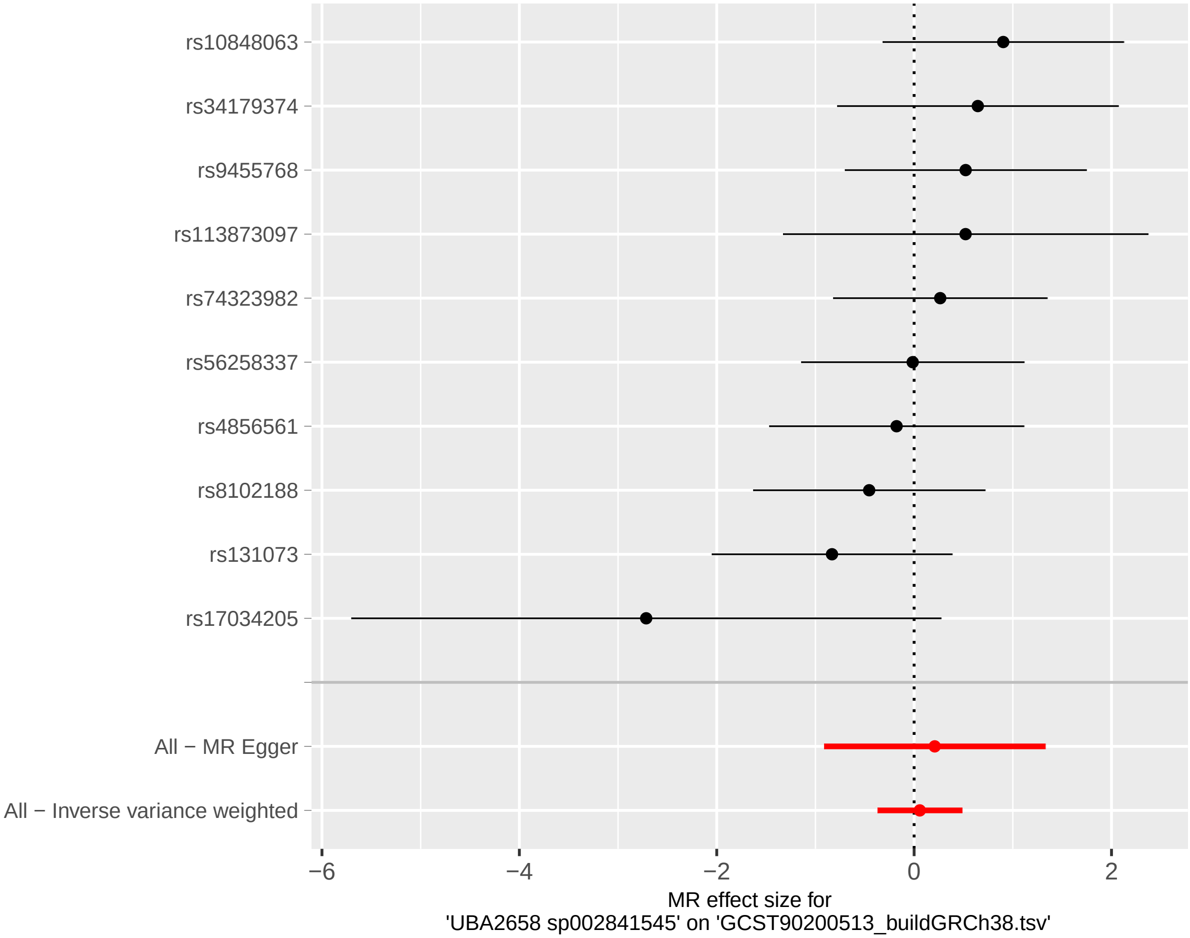
rs10848063
rs34179374
rs9455768
rs113873097
rs74323982
rs56258337
rs4856561
rs8102188
rs131073
rs17034205
All − MR Egger
All − Inverse variance weighted
−6
−4
−2
0
2
MR effect size for
'UBA2658 sp002841545' on 'GCST90200513_buildGRCh38.tsv'

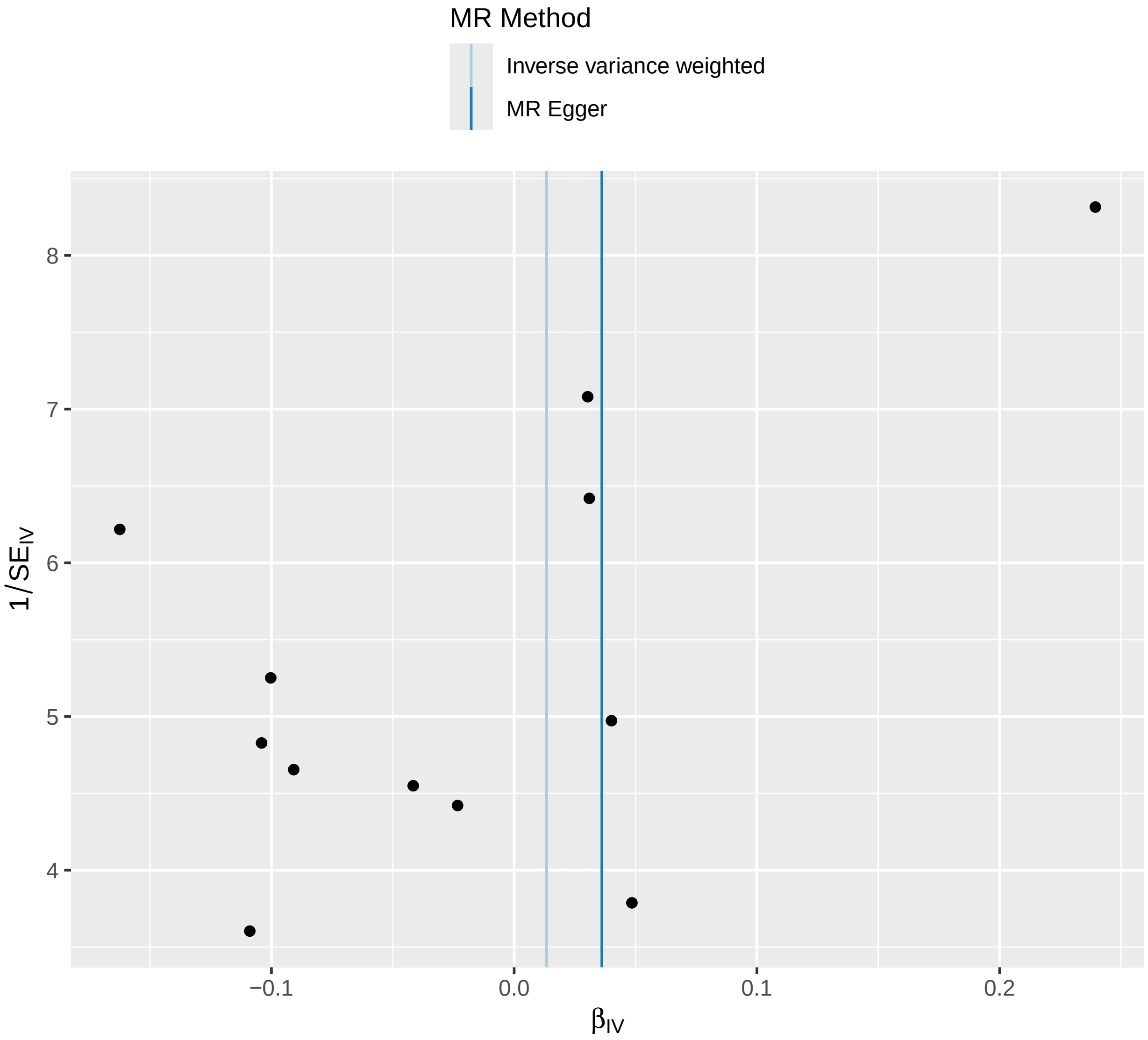

MR Method
Inverse variance weighted
MR Egger
8
7
6
5
4
1/SE_IV
−0.1
0.0
0.1
0.2
β_IV

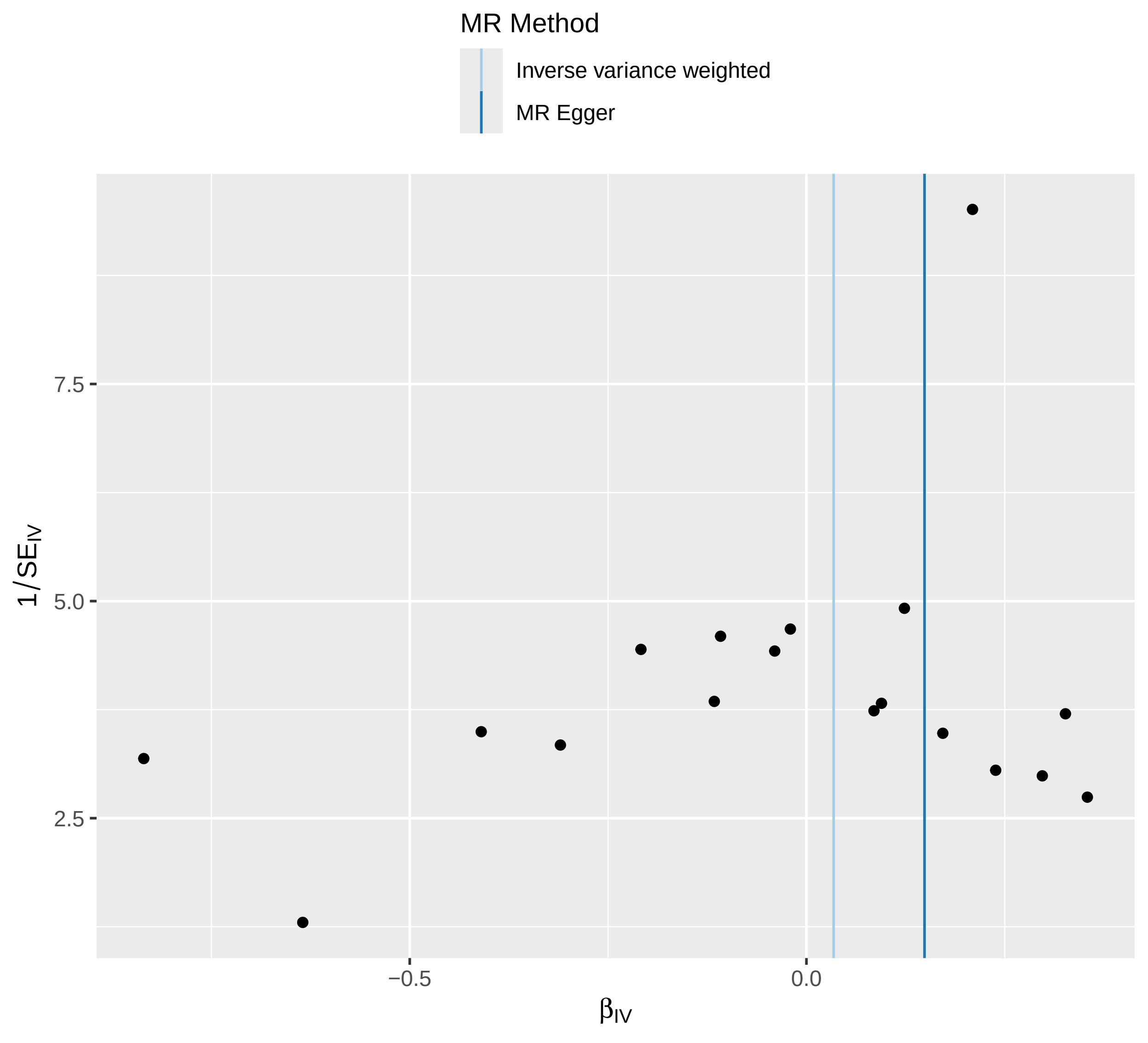
MR Method
Inverse variance weighted
MR Egger
7.5
5.0
2.5
−0.5
0.0
β_{IV}
$1/SE_{IV}$

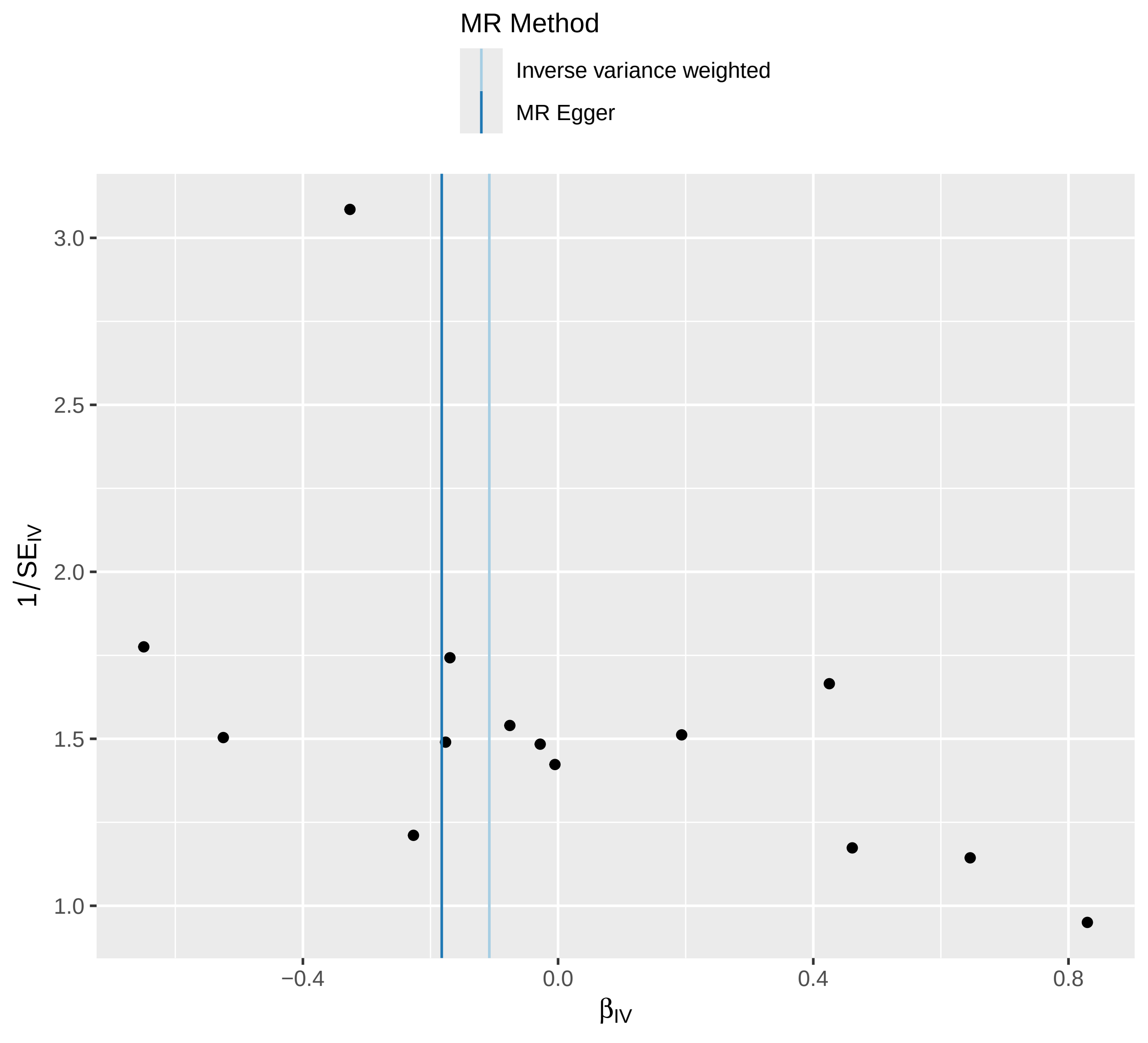

MR Method
Inverse variance weighted
MR Egger
3.0
2.5
2.0
1.5
1.0
1/SE_IV
−0.4
0.0
0.4
0.8
β_IV

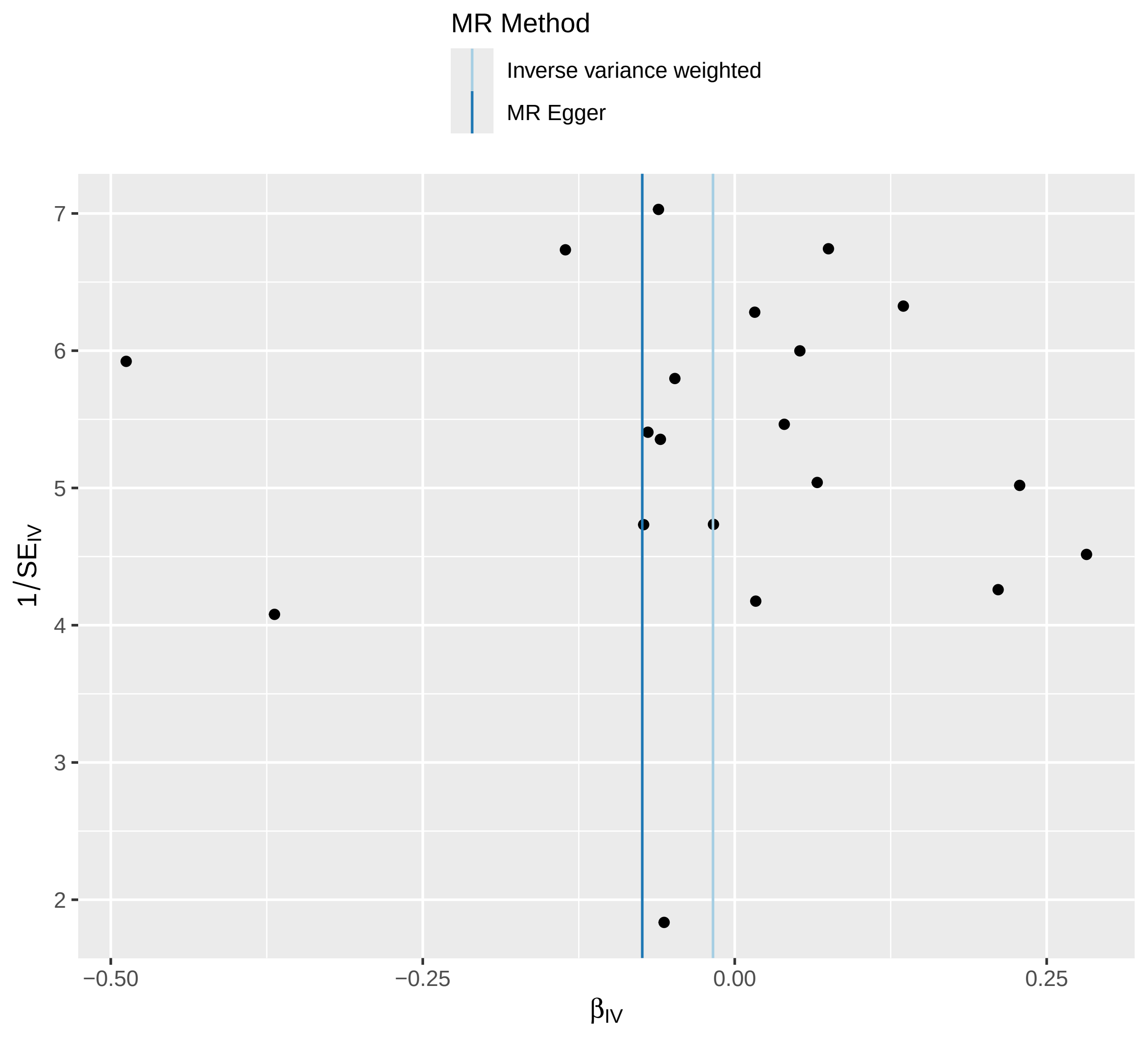

MR Method
Inverse variance weighted
MR Egger
7
6
5
4
3
2
1/SE$_{IV}$
−0.50
−0.25
0.00
0.25
β$_{IV}$

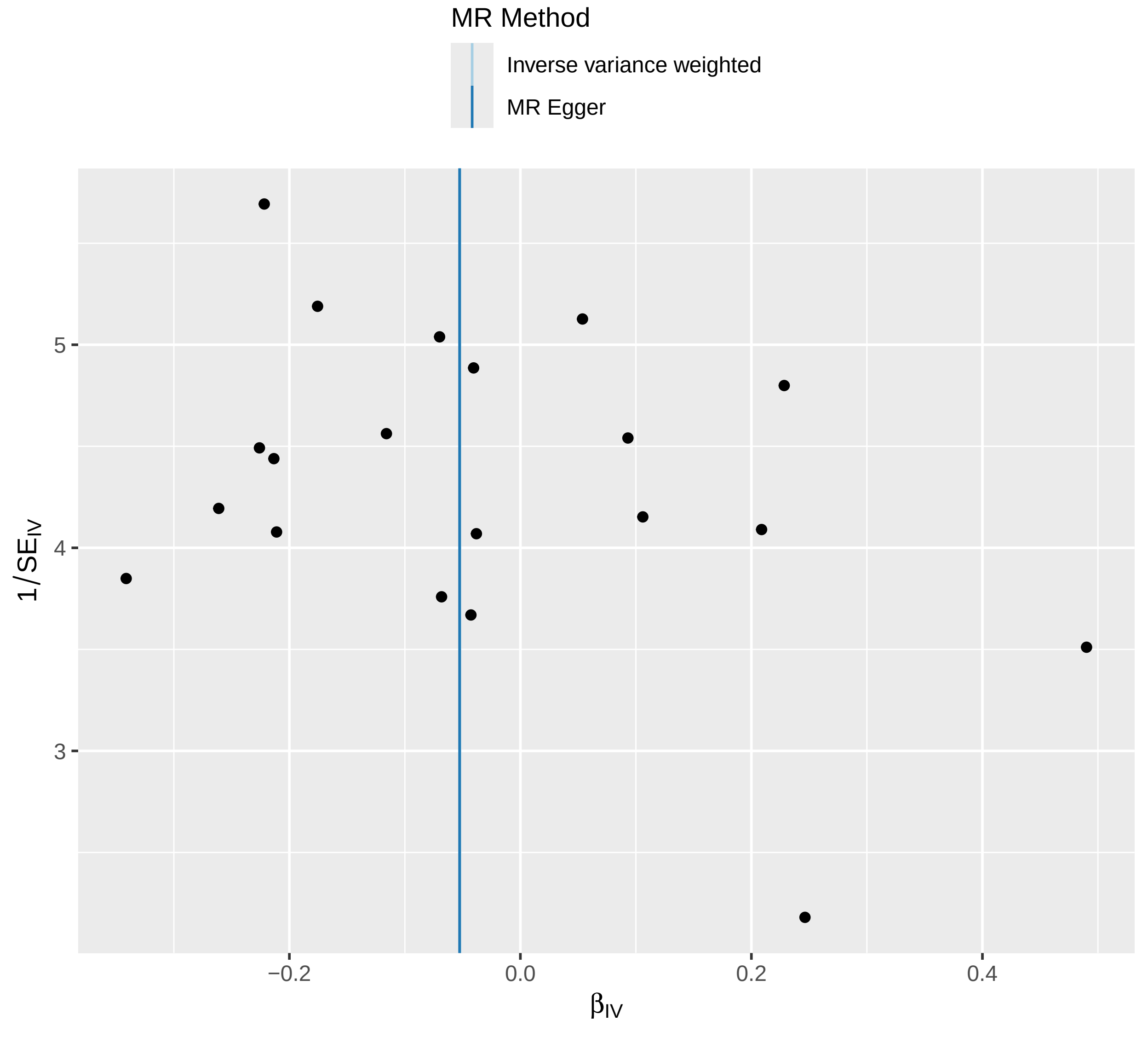

MR Method
Inverse variance weighted
MR Egger
5
4
3
1/SE_IV
−0.2
0.0
0.2
0.4
β_IV

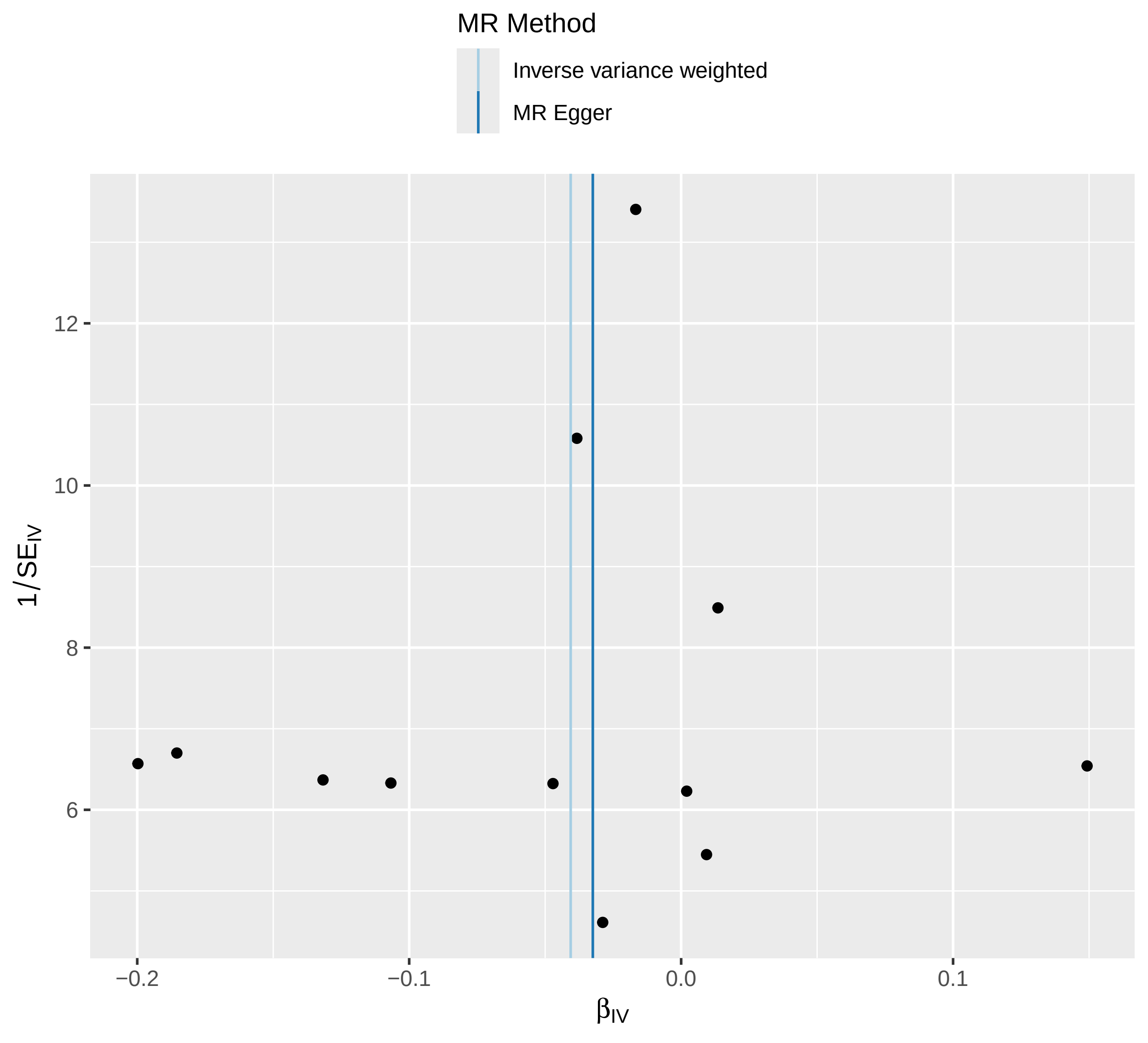
MR Method
Inverse variance weighted
MR Egger
1/SE$_{IV}$
12
10
8
6
−0.2
−0.1
0.0
0.1
β$_{IV}$

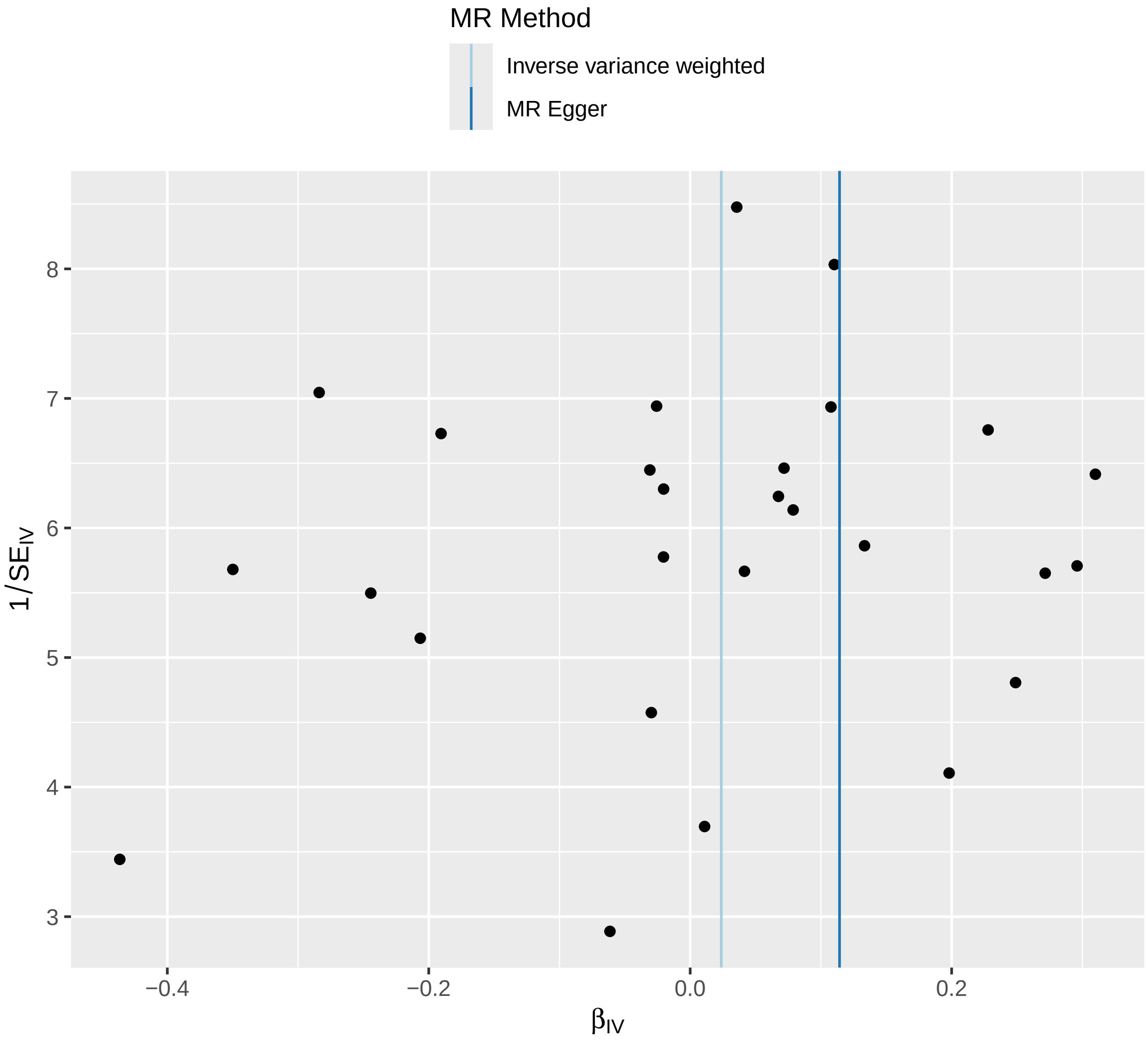

MR Method
Inverse variance weighted
MR Egger
8
7
6
5
4
3
1/SE$_{IV}$
−0.4
−0.2
0.0
0.2
β$_{IV}$

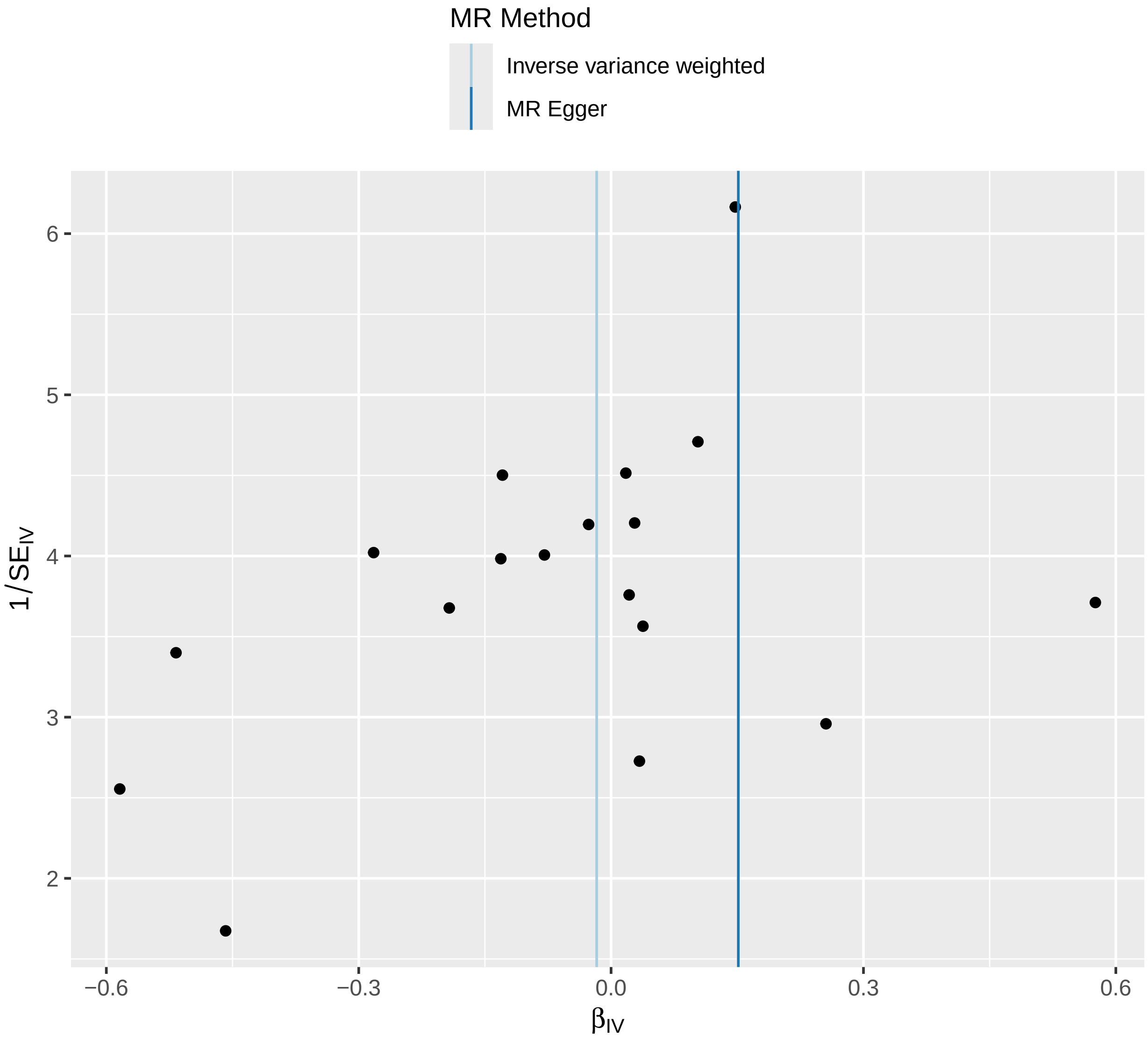

MR Method
Inverse variance weighted
MR Egger
6
5
4
3
2
1/SE_IV
−0.6
−0.3
0.0
0.3
0.6
β_IV

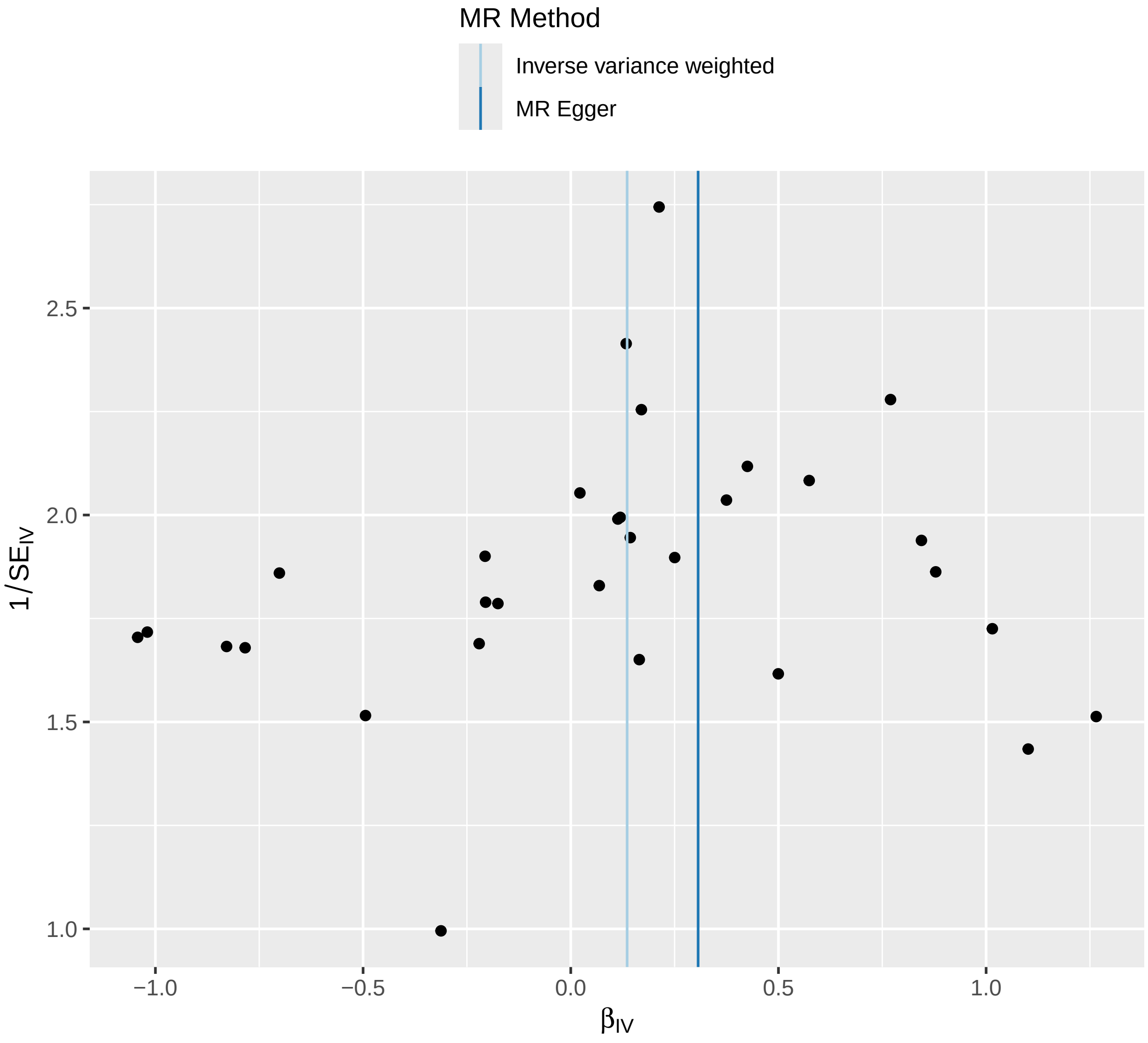
MR Method
Inverse variance weighted
MR Egger
2.5
2.0
1.5
1.0
1/SE_IV
−1.0
−0.5
0.0
0.5
1.0
β_IV

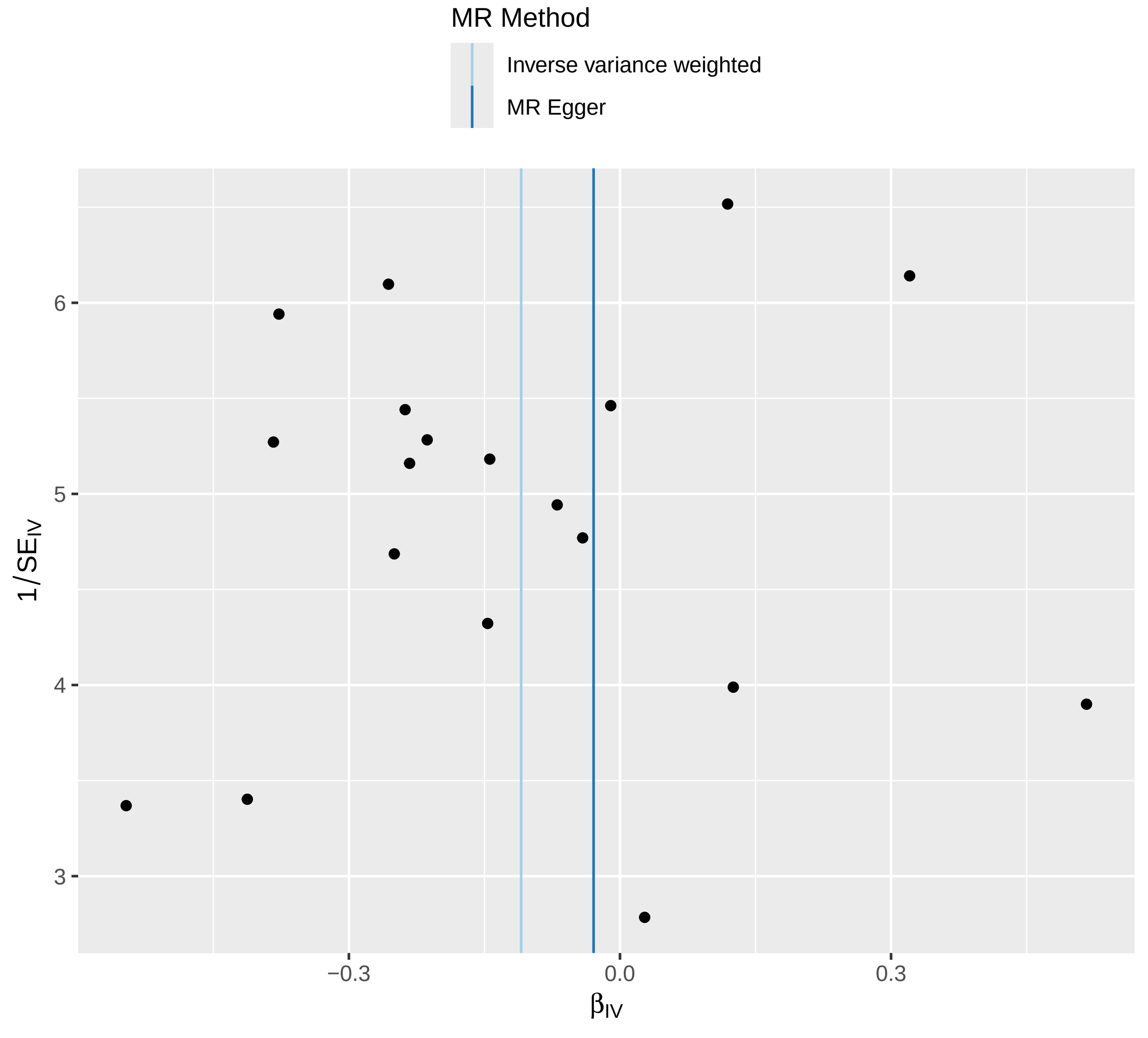
MR Method
Inverse variance weighted
MR Egger
6
5
4
3
−0.3
0.0
0.3
$1/SE_{IV}$
β_{IV}

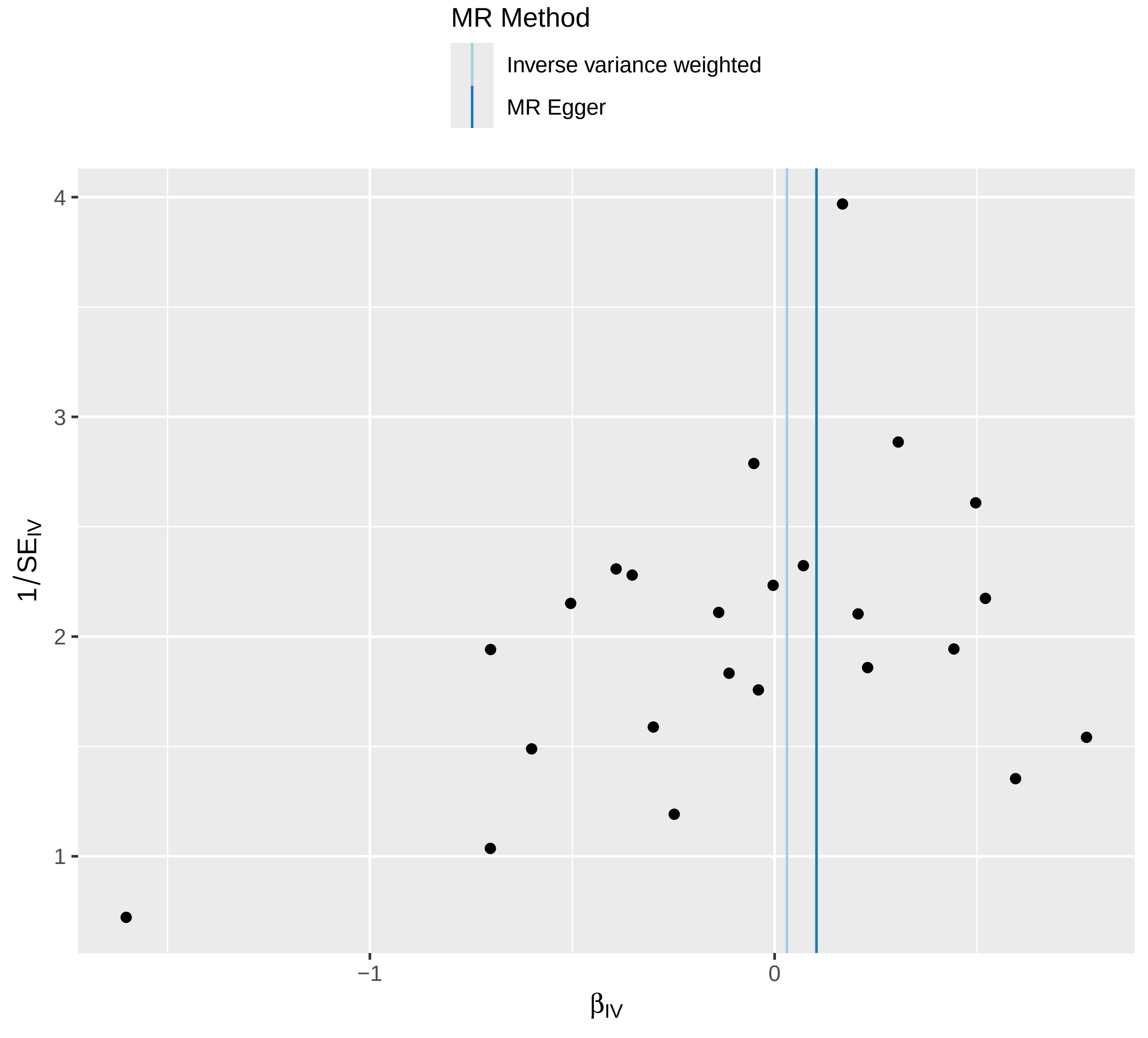

MR Method
Inverse variance weighted
MR Egger
4
3
2
1
1/SE_IV
−1
0
β_IV

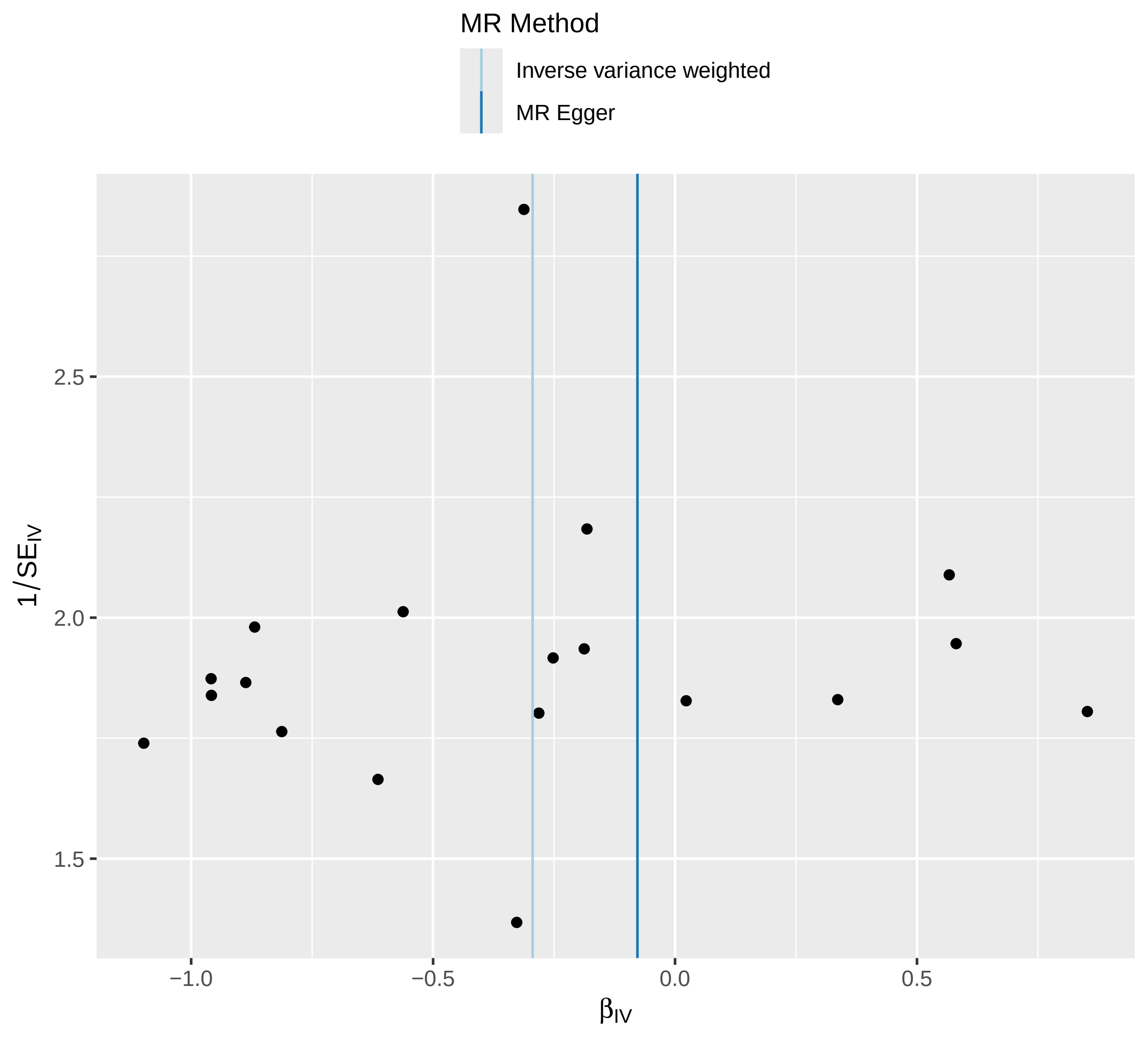
MR Method
Inverse variance weighted
MR Egger
2.5
2.0
1.5
1/SE$_{IV}$
−1.0
−0.5
0.0
0.5
β$_{IV}$

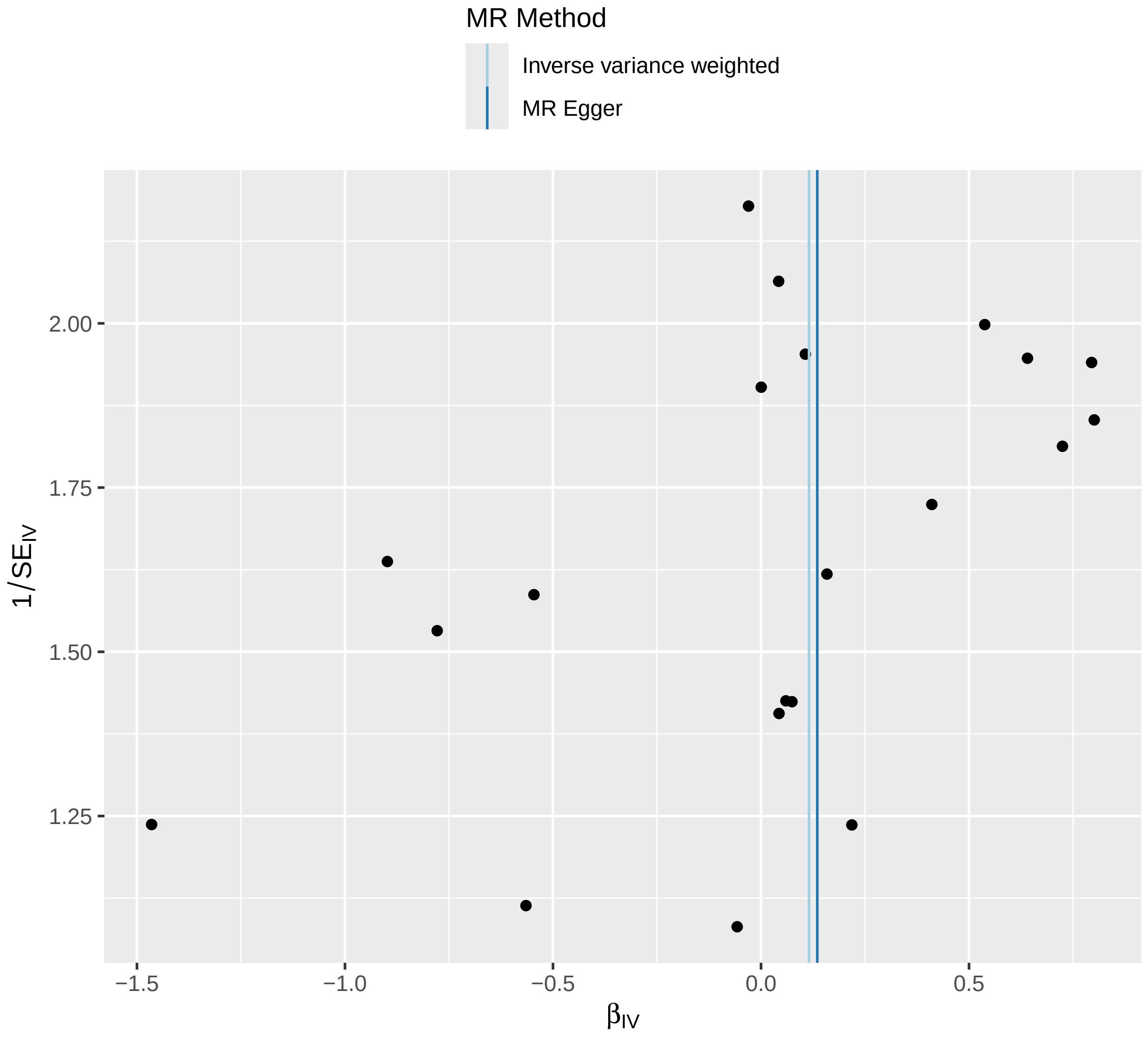
MR Method
Inverse variance weighted
MR Egger
2.00
1.75
1.50
1.25
1/SE$_{IV}$
−1.5
−1.0
−0.5
0.0
0.5
β$_{IV}$

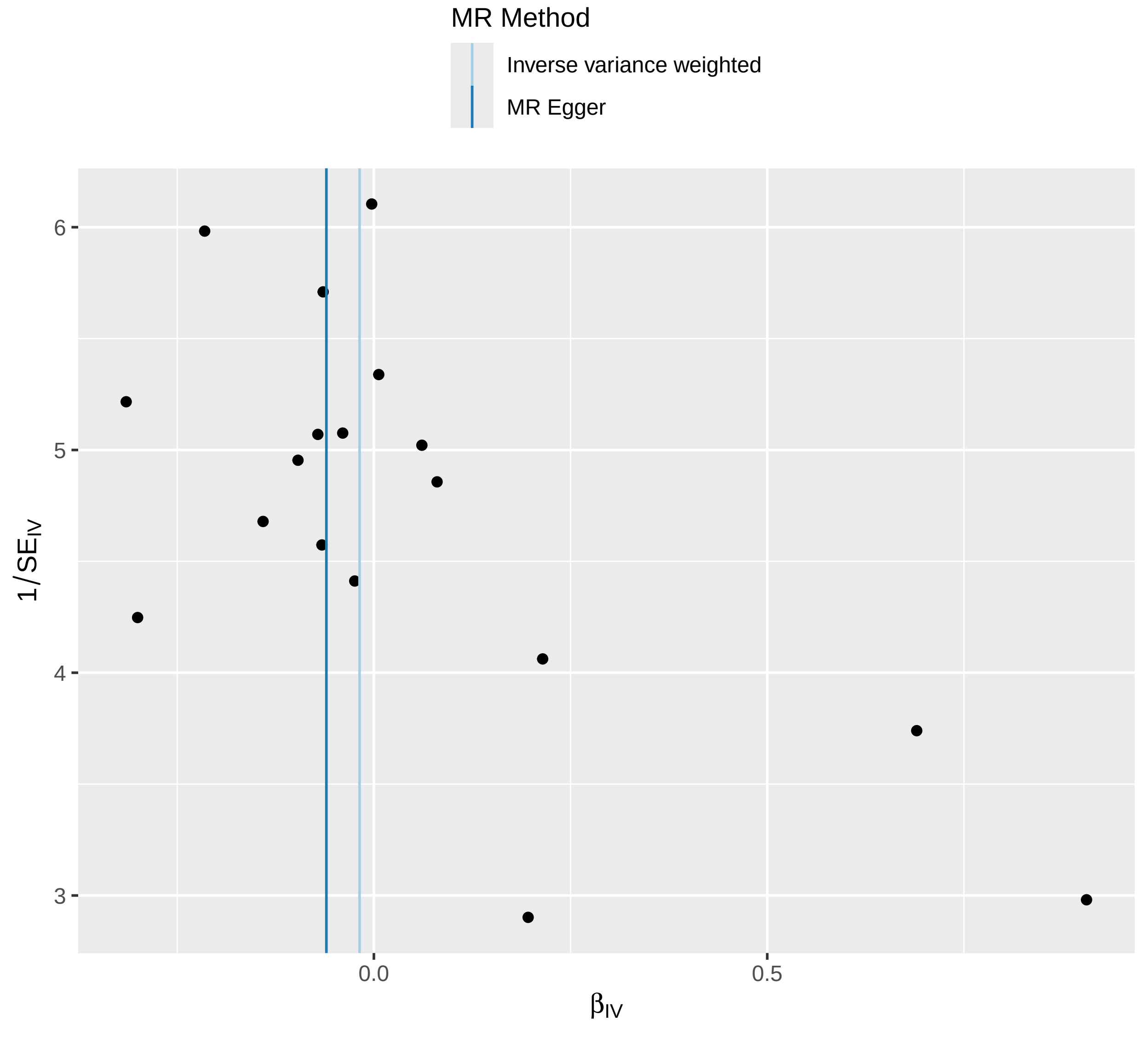
MR Method
Inverse variance weighted
MR Egger
6
5
4
3
1/SE$_{IV}$
0.0
0.5
β$_{IV}$

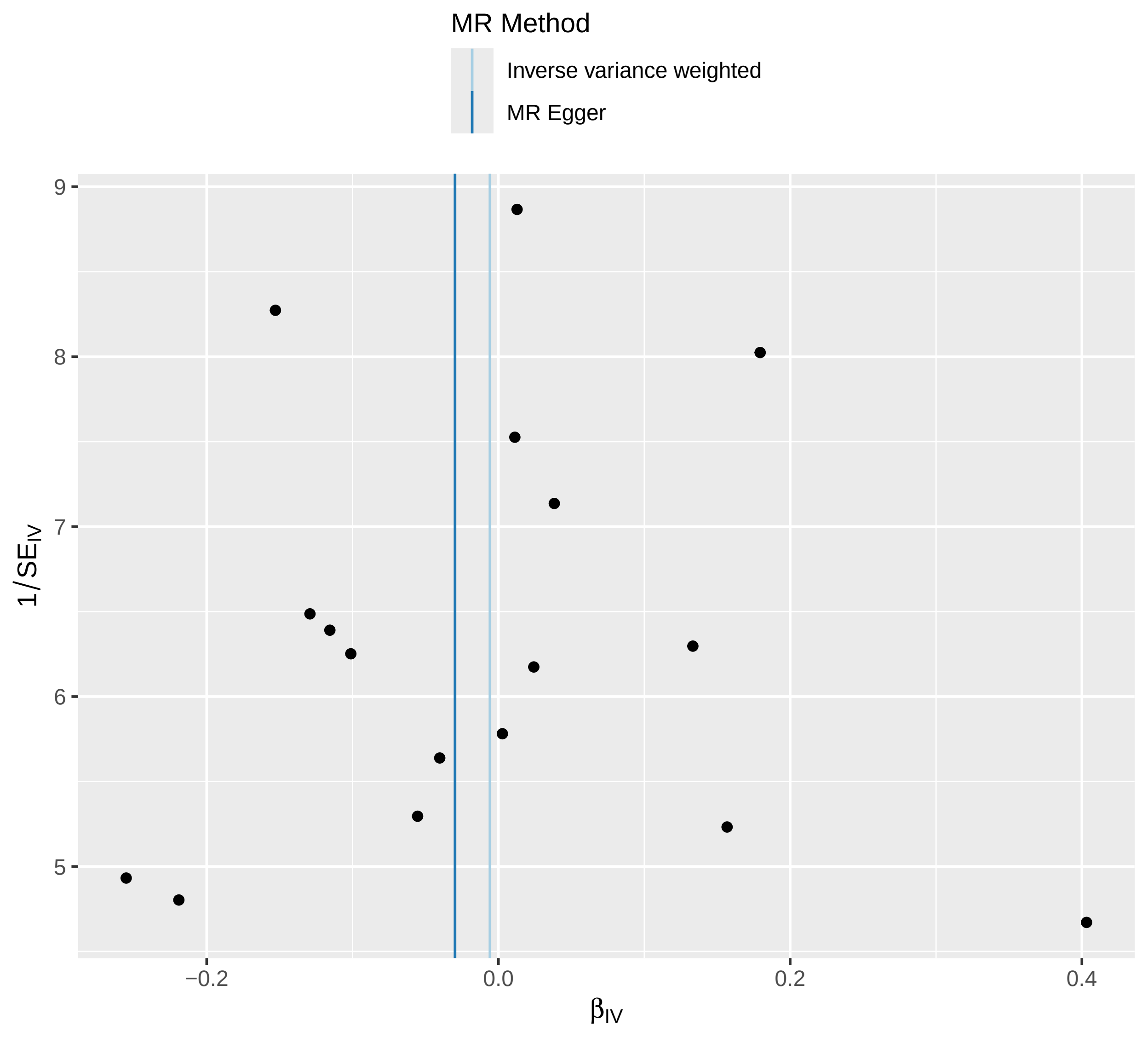
MR Method
Inverse variance weighted
MR Egger
1/SE_IV
β_IV
9
8
7
6
5
−0.2
0.0
0.2
0.4

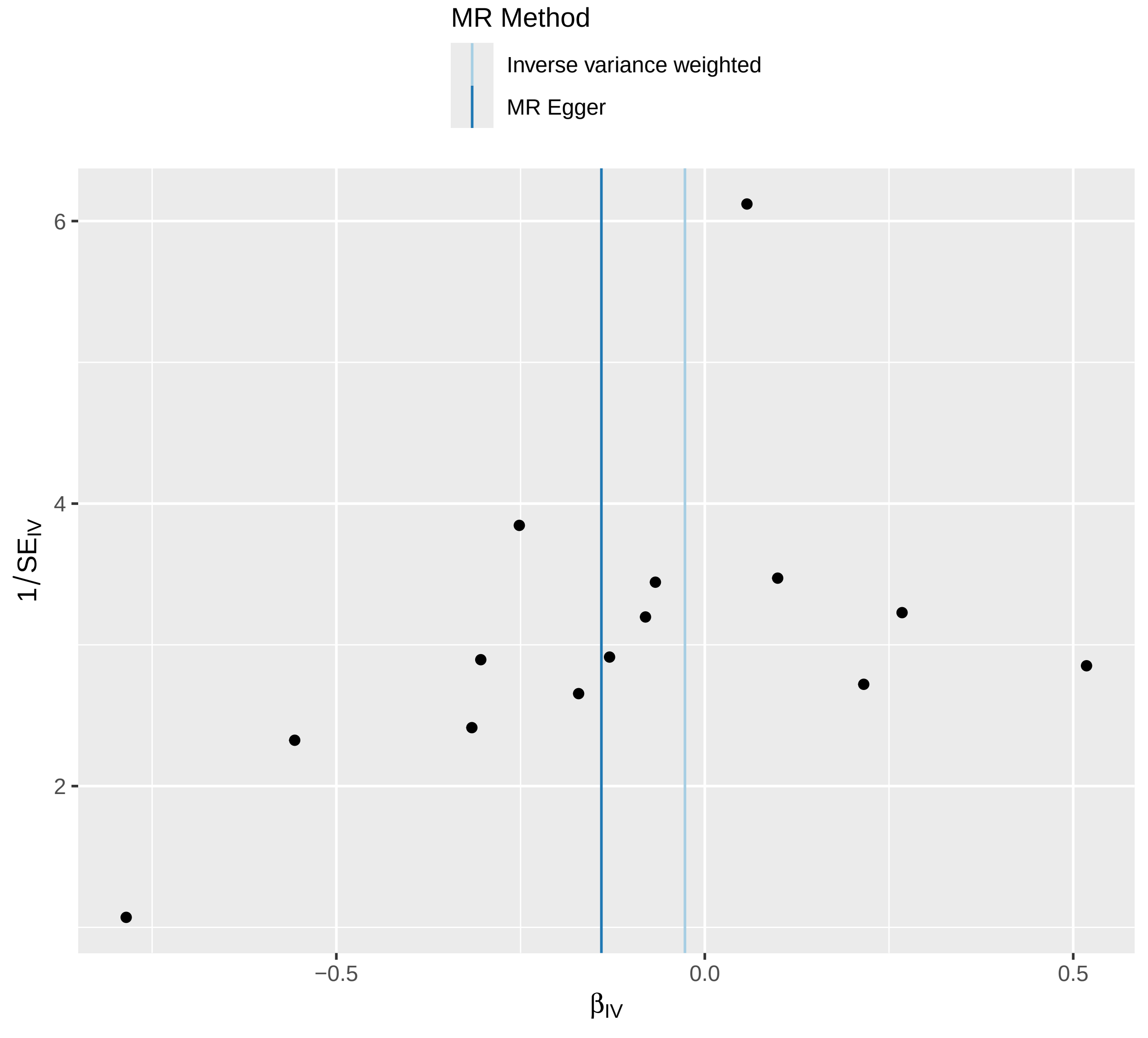

MR Method
Inverse variance weighted
MR Egger
6
4
2
1/SE_IV
−0.5
0.0
0.5
β_IV

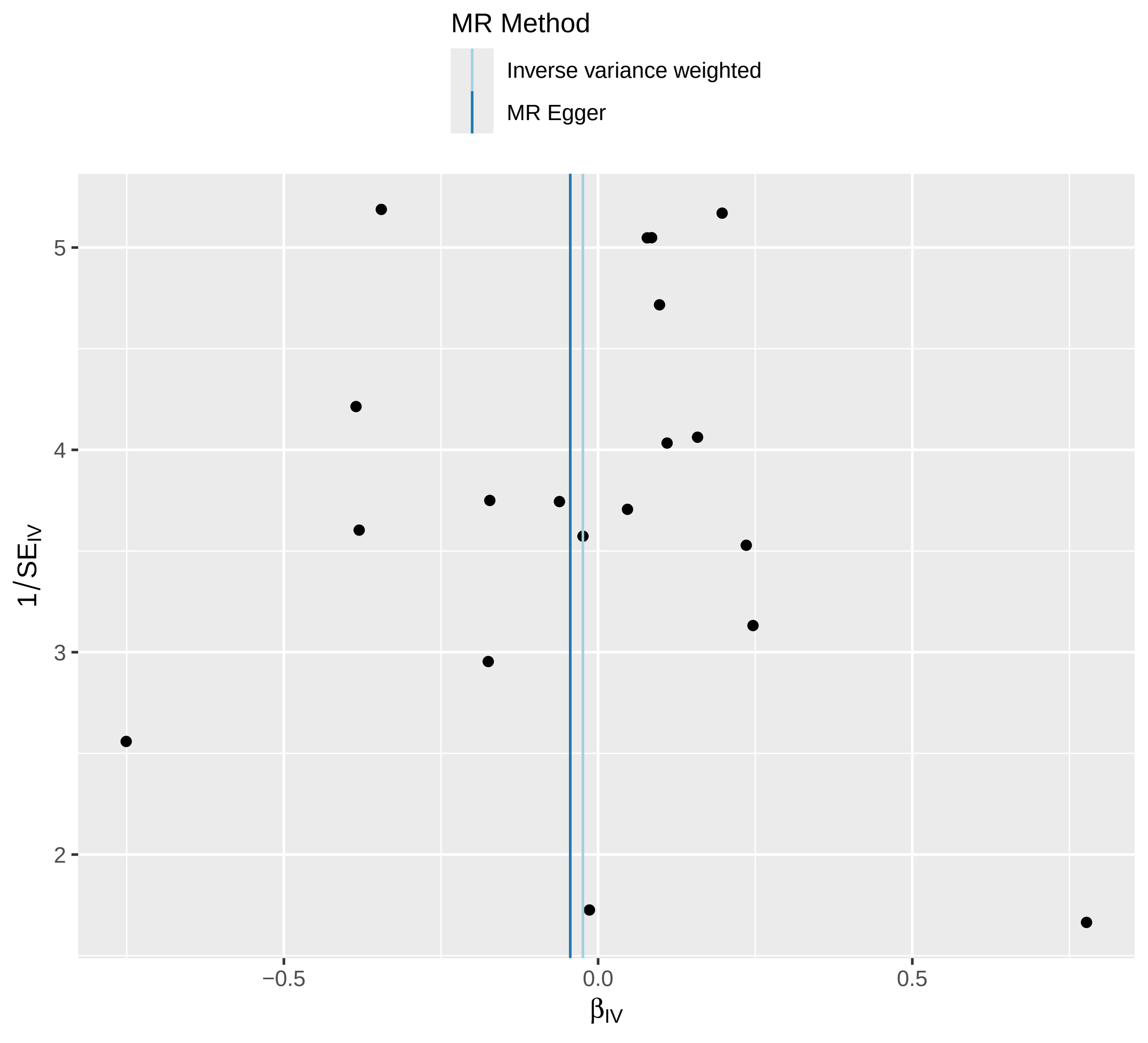

MR Method
Inverse variance weighted
MR Egger
1/SE_IV
5
4
3
2
−0.5
0.0
0.5
β_IV

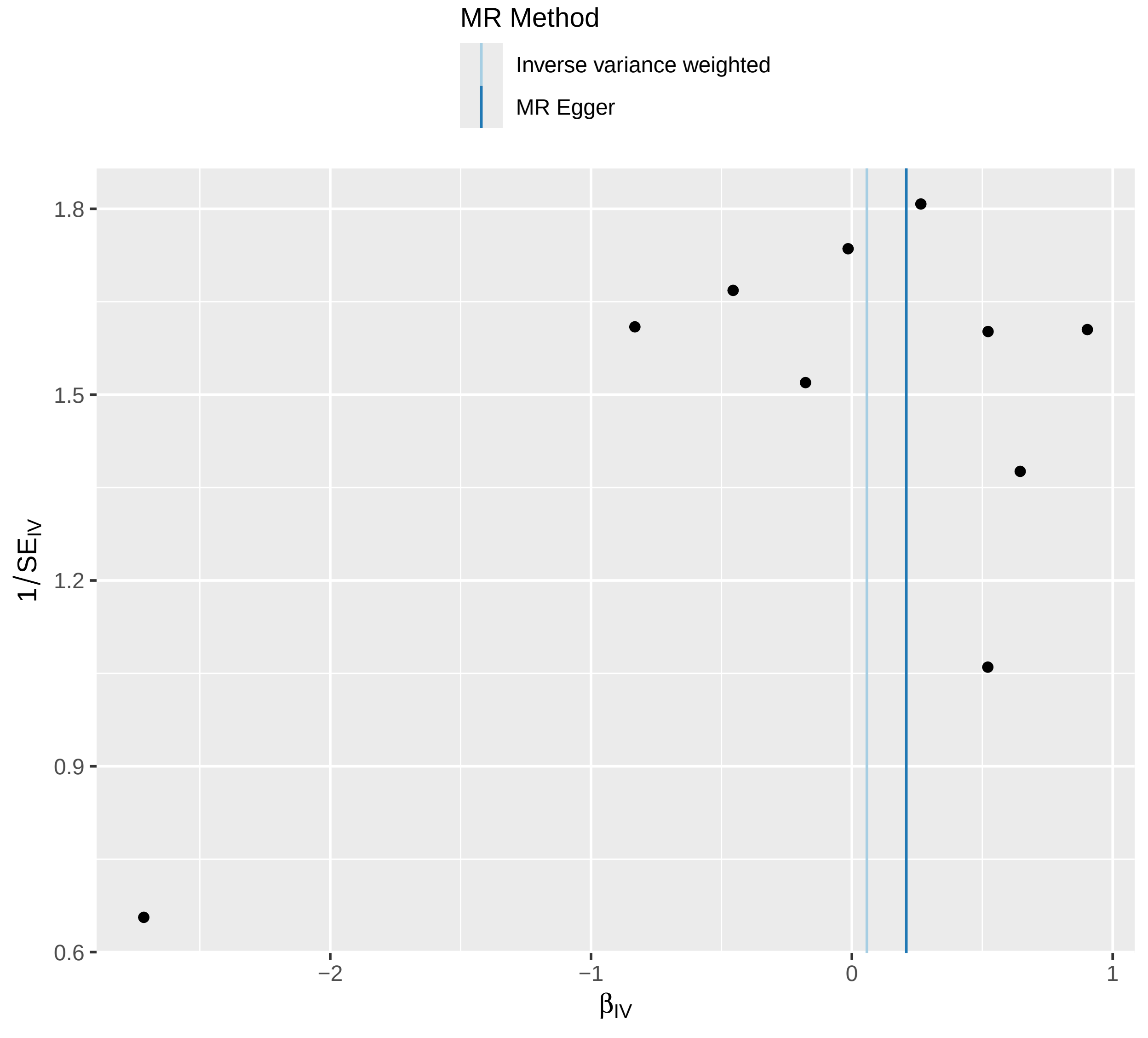

MR Method
Inverse variance weighted
MR Egger
1.8
1.5
1.2
0.9
0.6
1/SE$_{IV}$
−2
−1
0
1
β$_{IV}$

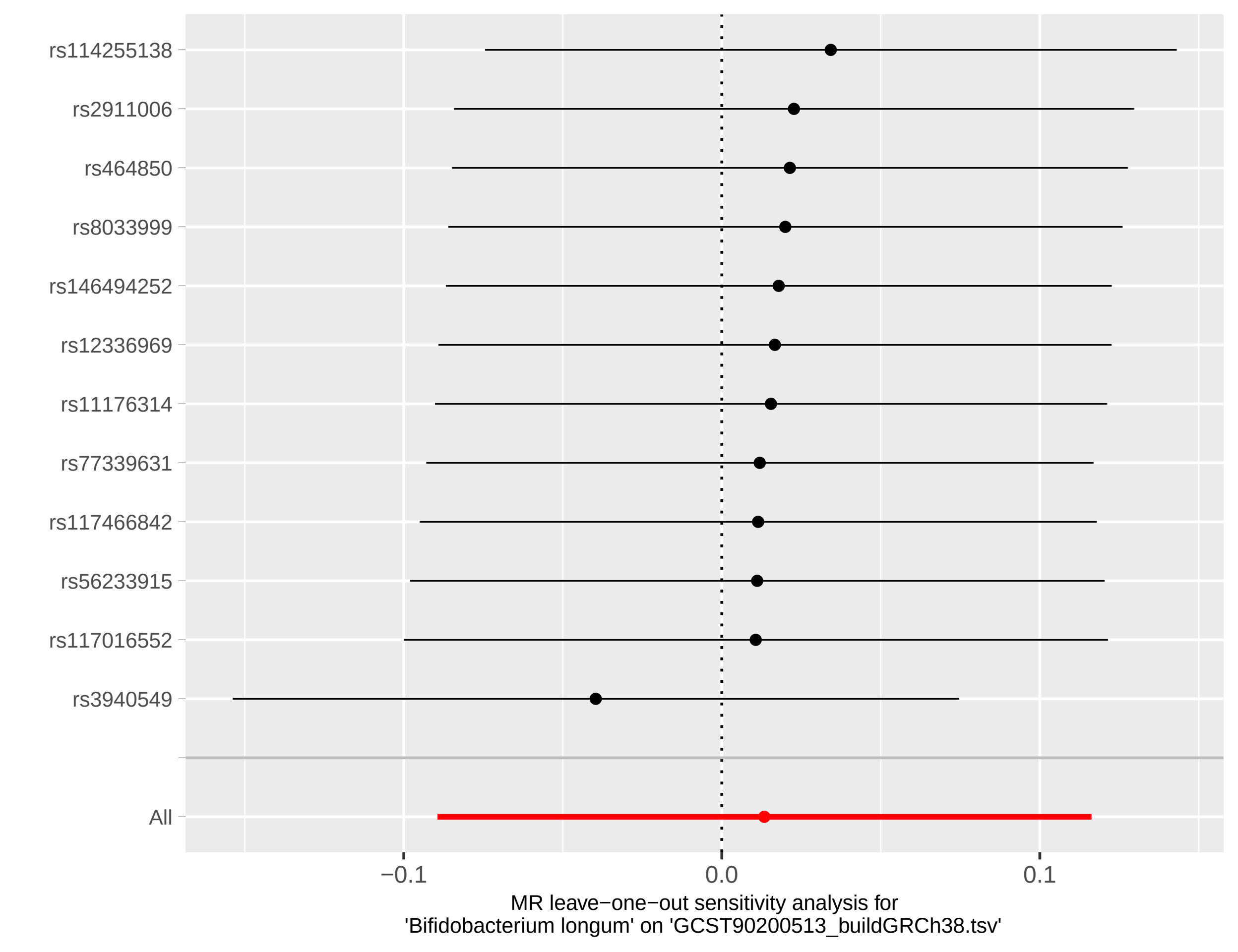
rs114255138
rs2911006
rs464850
rs8033999
rs146494252
rs12336969
rs11176314
rs77339631
rs117466842
rs56233915
rs117016552
rs3940549
All
−0.1
0.0
0.1
MR leave−one−out sensitivity analysis for
'Bifidobacterium longum' on 'GCST90200513_buildGRCh38.tsv'

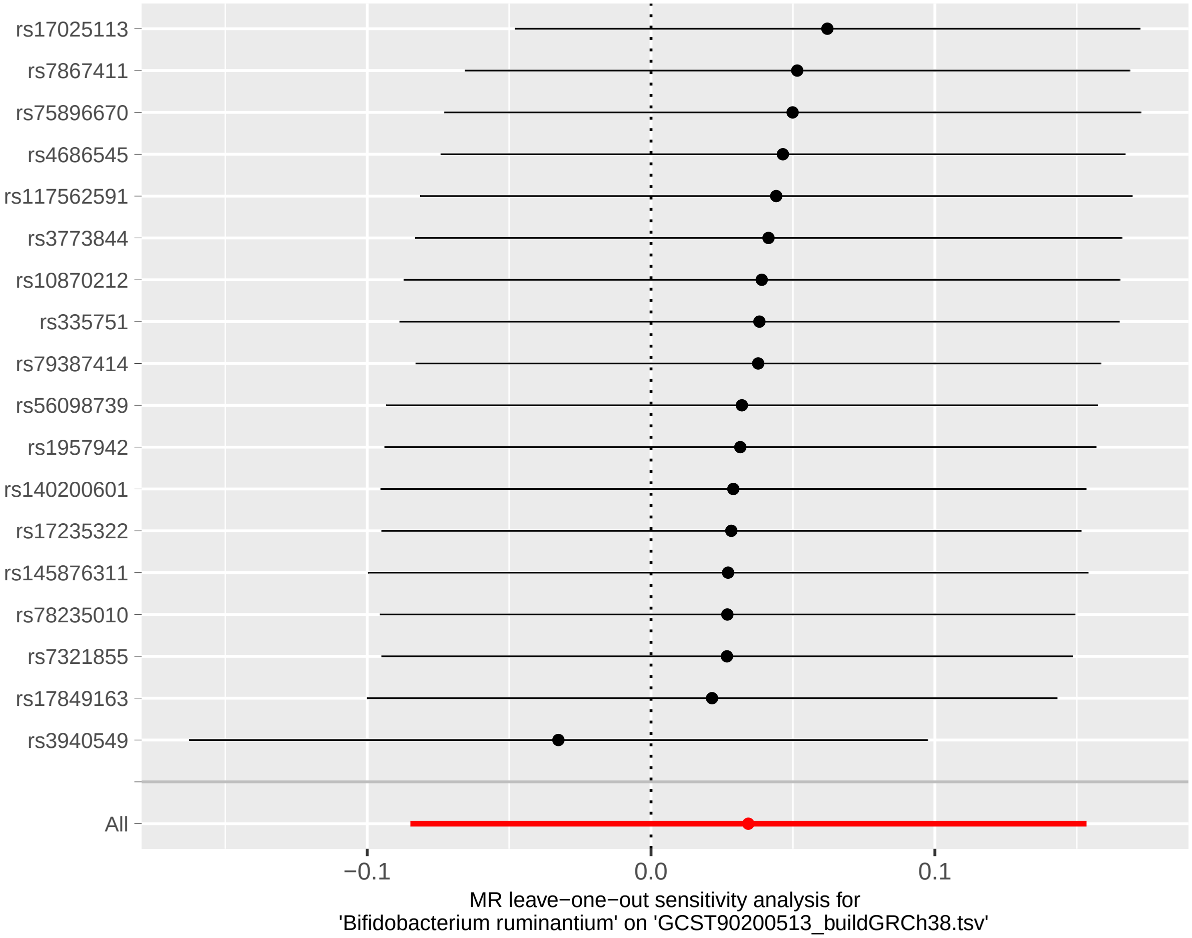

rs17025113
rs7867411
rs75896670
rs4686545
rs117562591
rs3773844
rs10870212
rs335751
rs79387414
rs56098739
rs1957942
rs140200601
rs17235322
rs145876311
rs78235010
rs7321855
rs17849163
rs3940549
All
−0.1
0.0
0.1
MR leave−one−out sensitivity analysis for
'Bifidobacterium ruminantium' on 'GCST90200513_buildGRCh38.tsv'

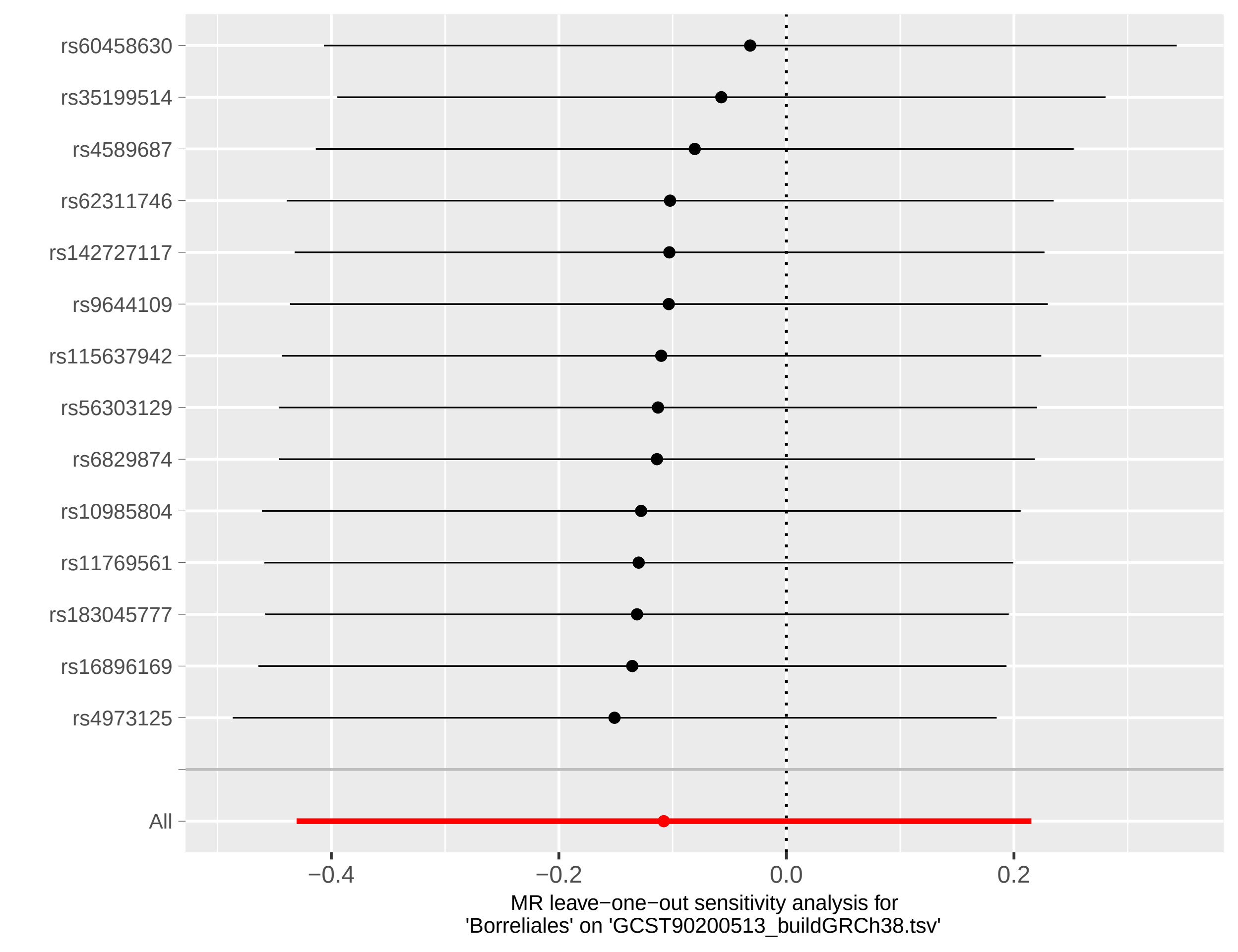

rs60458630
rs35199514
rs4589687
rs62311746
rs142727117
rs9644109
rs115637942
rs56303129
rs6829874
rs10985804
rs11769561
rs183045777
rs16896169
rs4973125
All
−0.4
−0.2
0.0
0.2
MR leave−one−out sensitivity analysis for
'Borreliales' on 'GCST90200513_buildGRCh38.tsv'

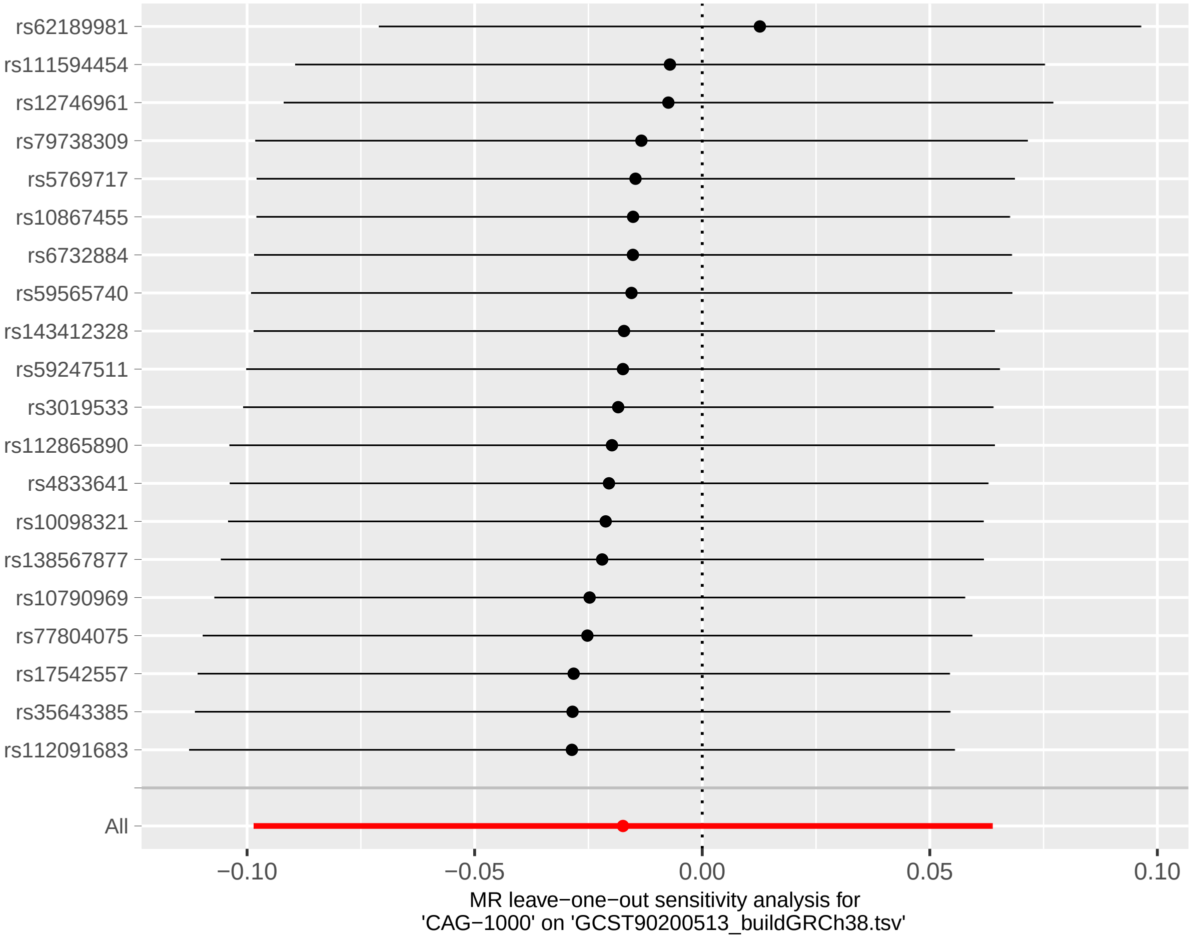

rs62189981
rs111594454
rs12746961
rs79738309
rs5769717
rs10867455
rs6732884
rs59565740
rs143412328
rs59247511
rs3019533
rs112865890
rs4833641
rs10098321
rs138567877
rs10790969
rs77804075
rs17542557
rs35643385
rs112091683
All
−0.10
−0.05
0.00
0.05
0.10
MR leave−one−out sensitivity analysis for
'CAG−1000' on 'GCST90200513_buildGRCh38.tsv'

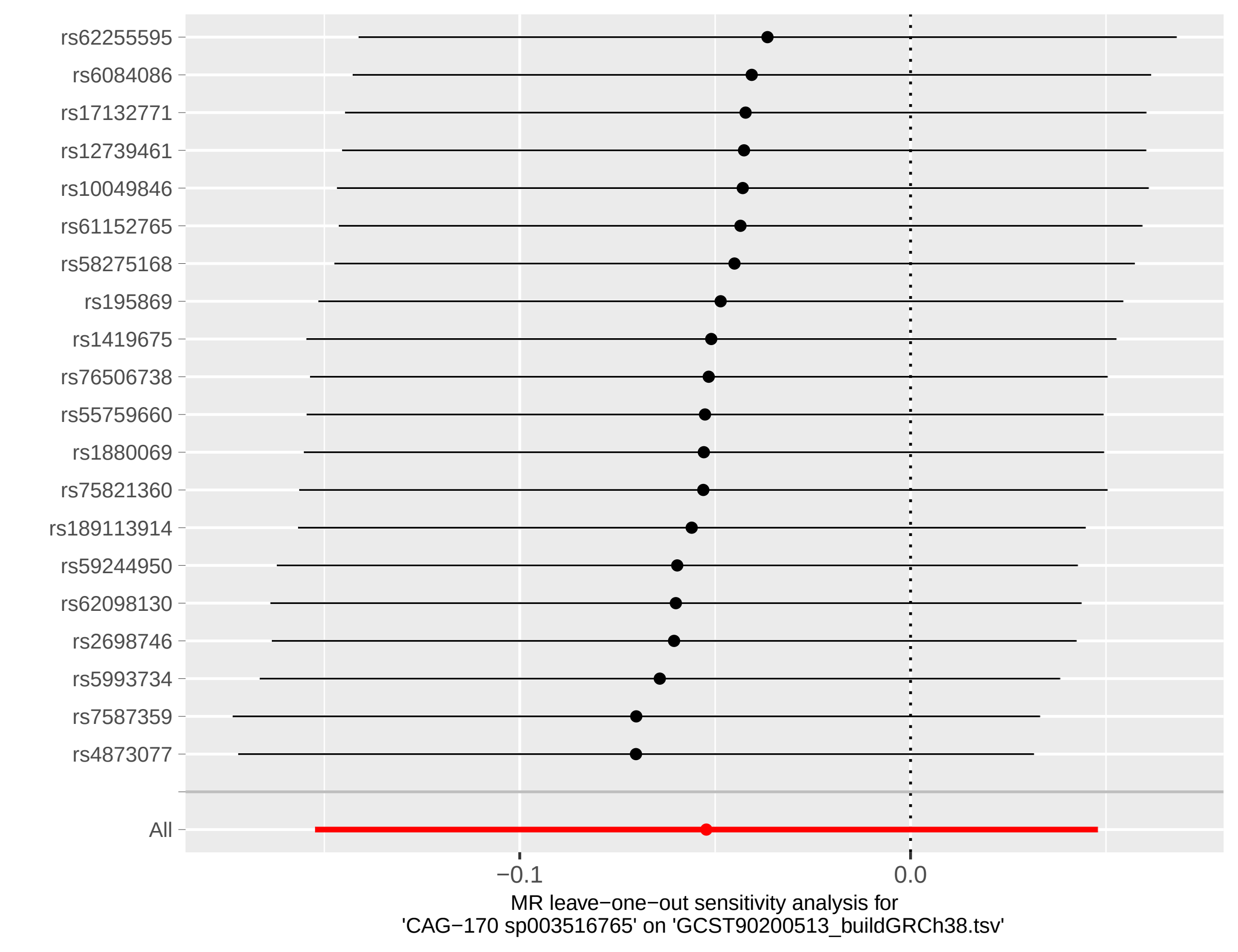
rs62255595
rs6084086
rs17132771
rs12739461
rs10049846
rs61152765
rs58275168
rs195869
rs1419675
rs76506738
rs55759660
rs1880069
rs75821360
rs189113914
rs59244950
rs62098130
rs2698746
rs5993734
rs7587359
rs4873077
All
−0.1
0.0
MR leave−one−out sensitivity analysis for
'CAG−170 sp003516765' on 'GCST90200513_buildGRCh38.tsv'

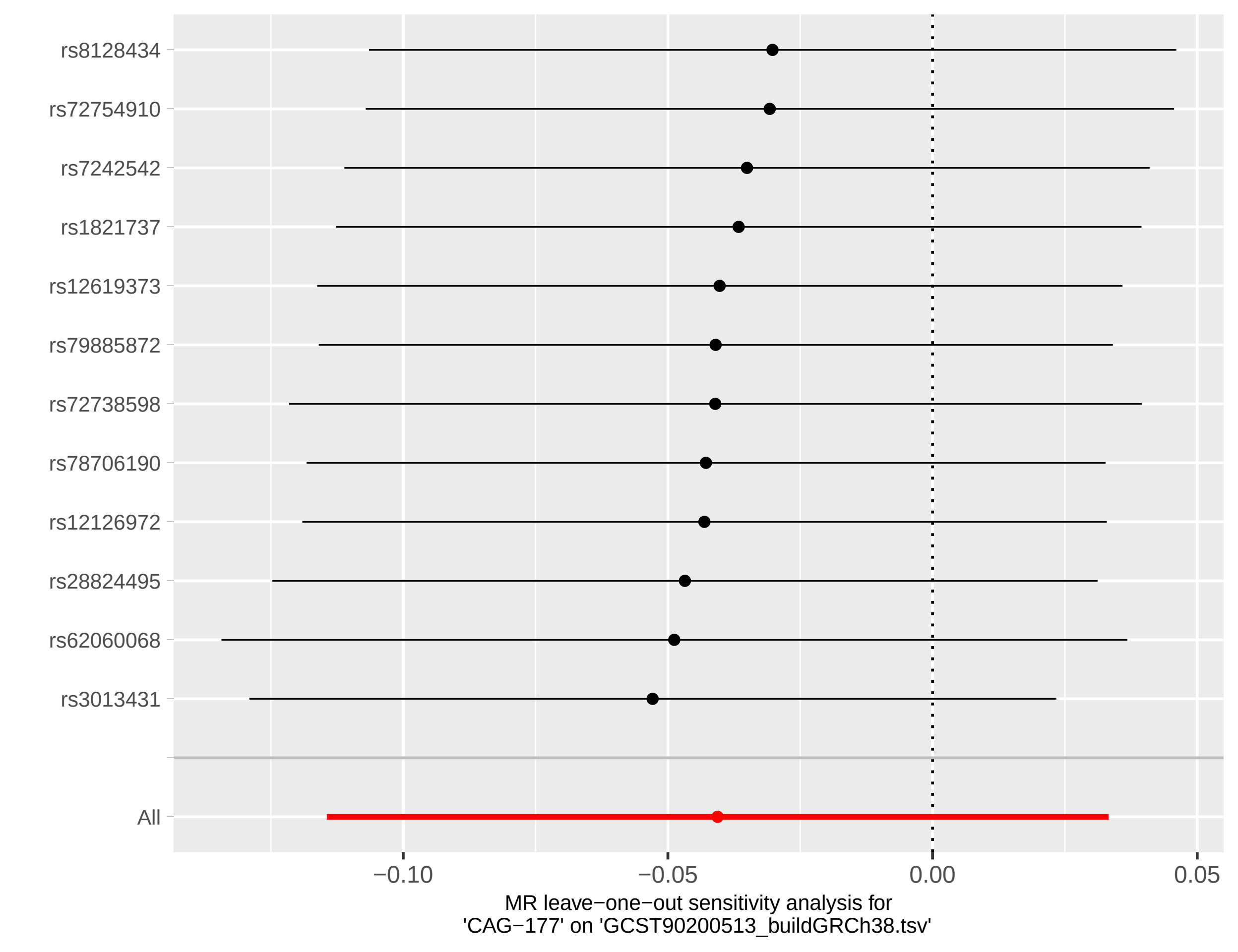
rs8128434
rs72754910
rs7242542
rs1821737
rs12619373
rs79885872
rs72738598
rs78706190
rs12126972
rs28824495
rs62060068
rs3013431
All
−0.10
−0.05
0.00
0.05
MR leave−one−out sensitivity analysis for
'CAG−177' on 'GCST90200513_buildGRCh38.tsv'

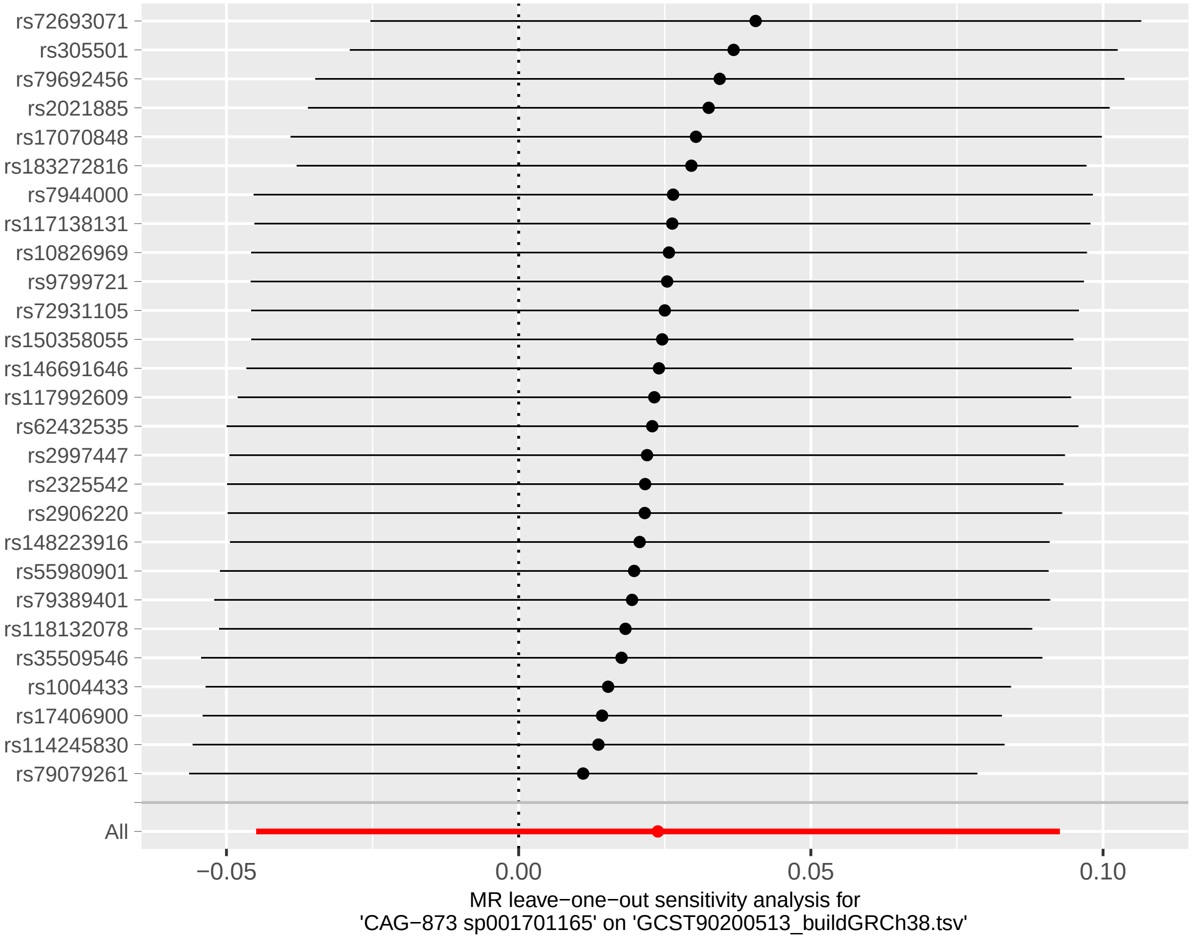
rs72693071
rs305501
rs79692456
rs2021885
rs17070848
rs183272816
rs7944000
rs117138131
rs10826969
rs9799721
rs72931105
rs150358055
rs146691646
rs117992609
rs62432535
rs2997447
rs2325542
rs2906220
rs148223916
rs55980901
rs79389401
rs118132078
rs35509546
rs1004433
rs17406900
rs114245830
rs79079261
All
−0.05
0.00
0.05
0.10
MR leave−one−out sensitivity analysis for
'CAG−873 sp001701165' on 'GCST90200513_buildGRCh38.tsv'

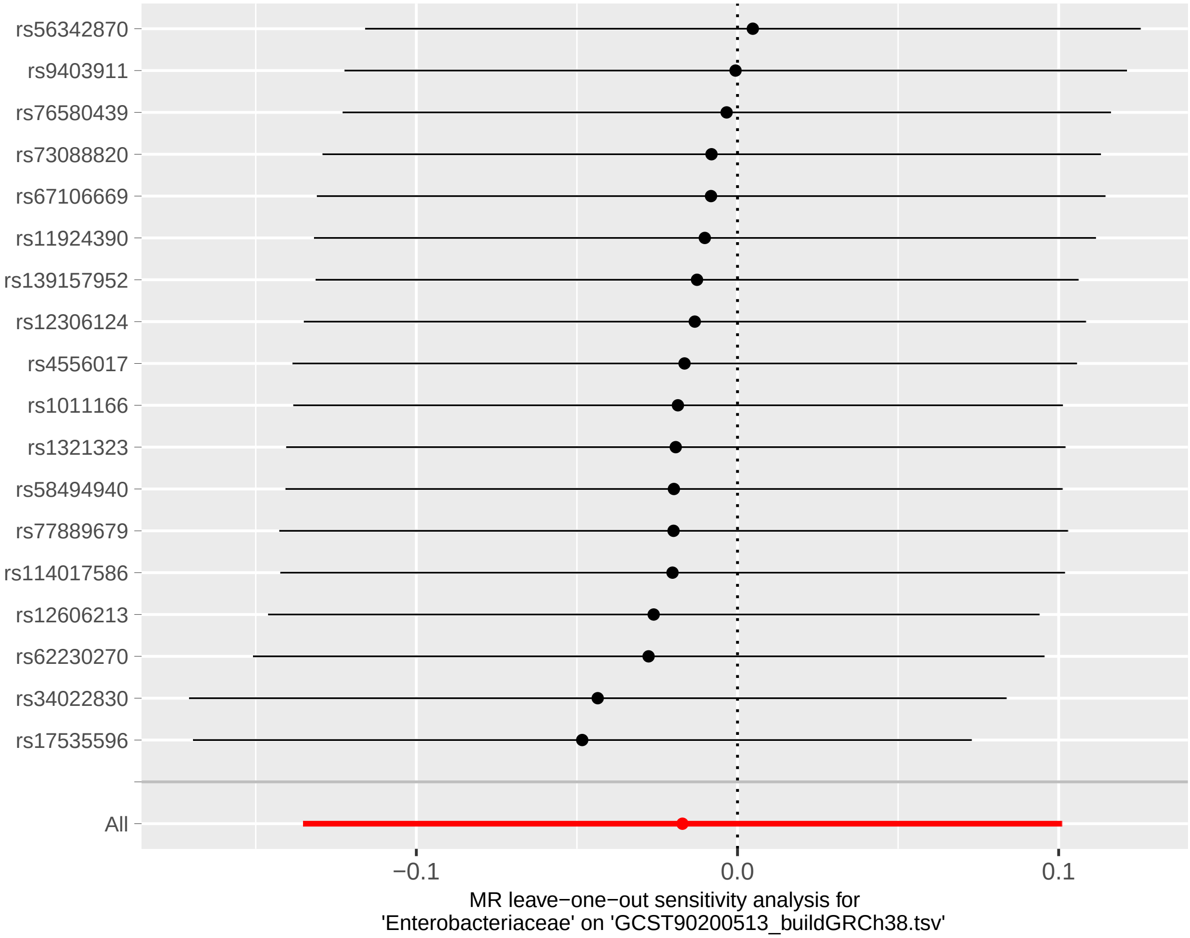
rs56342870
rs9403911
rs76580439
rs73088820
rs67106669
rs11924390
rs139157952
rs12306124
rs4556017
rs1011166
rs1321323
rs58494940
rs77889679
rs114017586
rs12606213
rs62230270
rs34022830
rs17535596
All
−0.1
0.0
0.1
MR leave−one−out sensitivity analysis for
'Enterobacteriaceae' on 'GCST90200513_buildGRCh38.tsv'

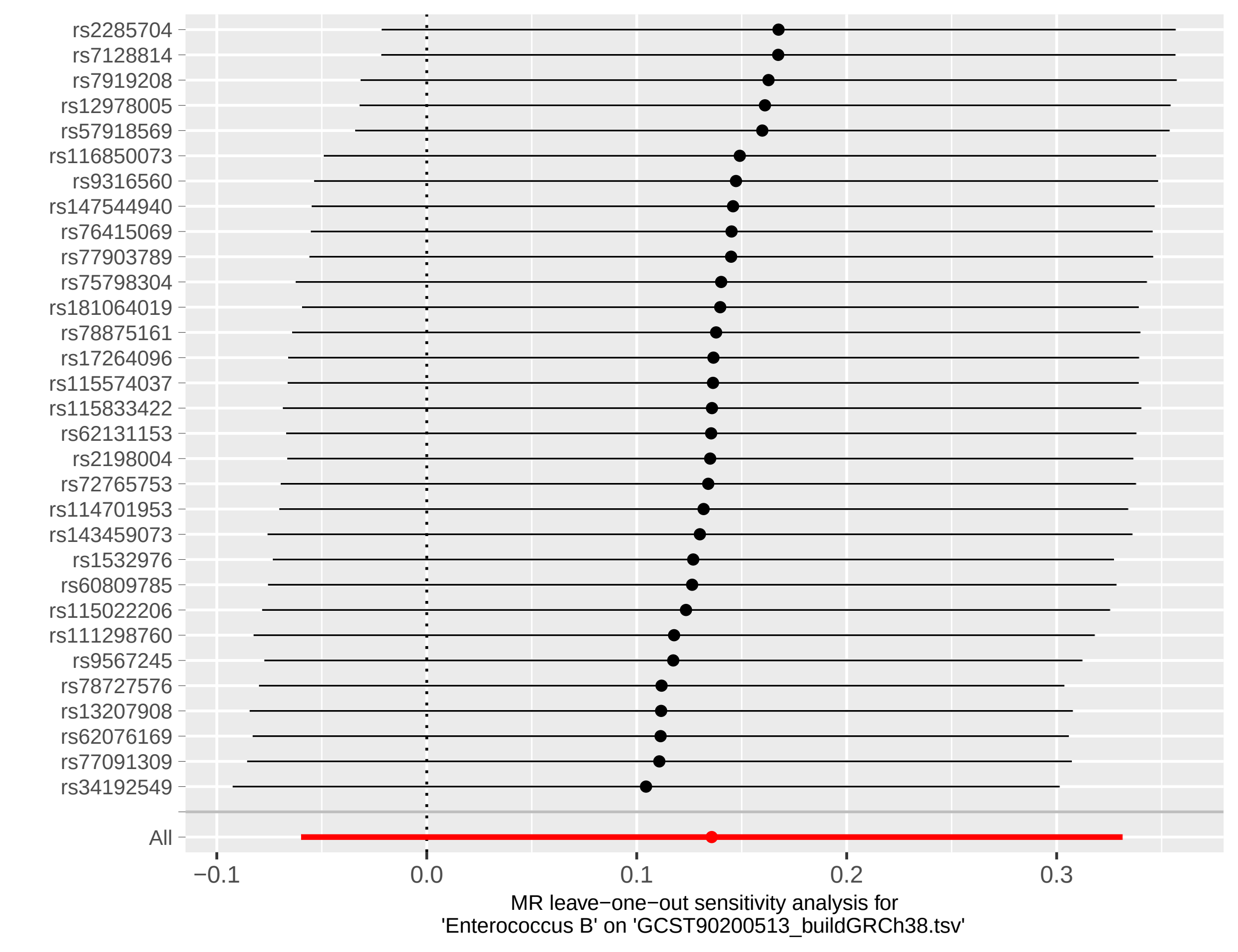
rs2285704
rs7128814
rs7919208
rs12978005
rs57918569
rs116850073
rs9316560
rs147544940
rs76415069
rs77903789
rs75798304
rs181064019
rs78875161
rs17264096
rs115574037
rs115833422
rs62131153
rs2198004
rs72765753
rs114701953
rs143459073
rs1532976
rs60809785
rs115022206
rs111298760
rs9567245
rs78727576
rs13207908
rs62076169
rs77091309
rs34192549
All
−0.1
0.0
0.1
0.2
0.3
MR leave−one−out sensitivity analysis for
'Enterococcus B' on 'GCST90200513_buildGRCh38.tsv'

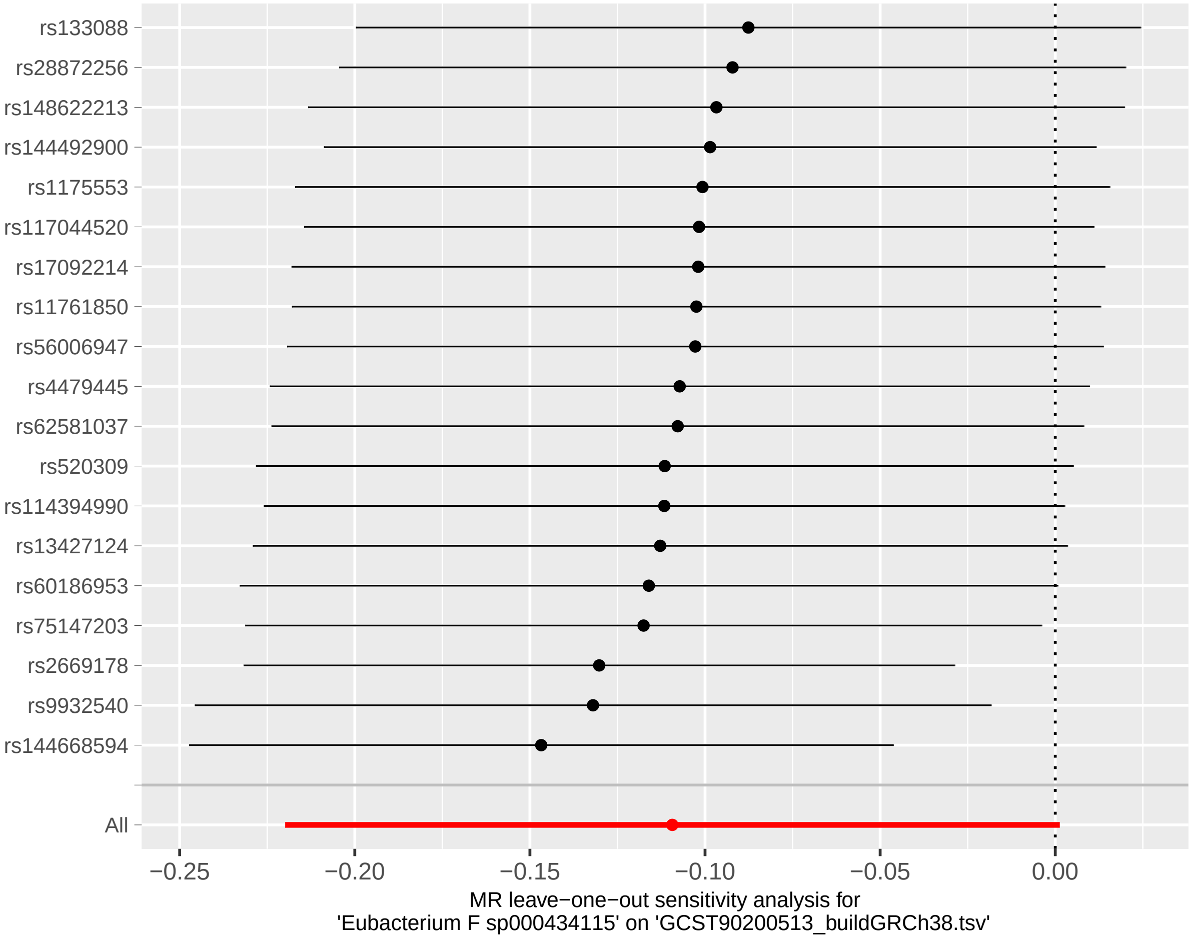

rs133088
rs28872256
rs148622213
rs144492900
rs1175553
rs117044520
rs17092214
rs11761850
rs56006947
rs4479445
rs62581037
rs520309
rs114394990
rs13427124
rs60186953
rs75147203
rs2669178
rs9932540
rs144668594
All
−0.25
−0.20
−0.15
−0.10
−0.05
0.00
MR leave−one−out sensitivity analysis for
'Eubacterium F sp000434115' on 'GCST90200513_buildGRCh38.tsv'

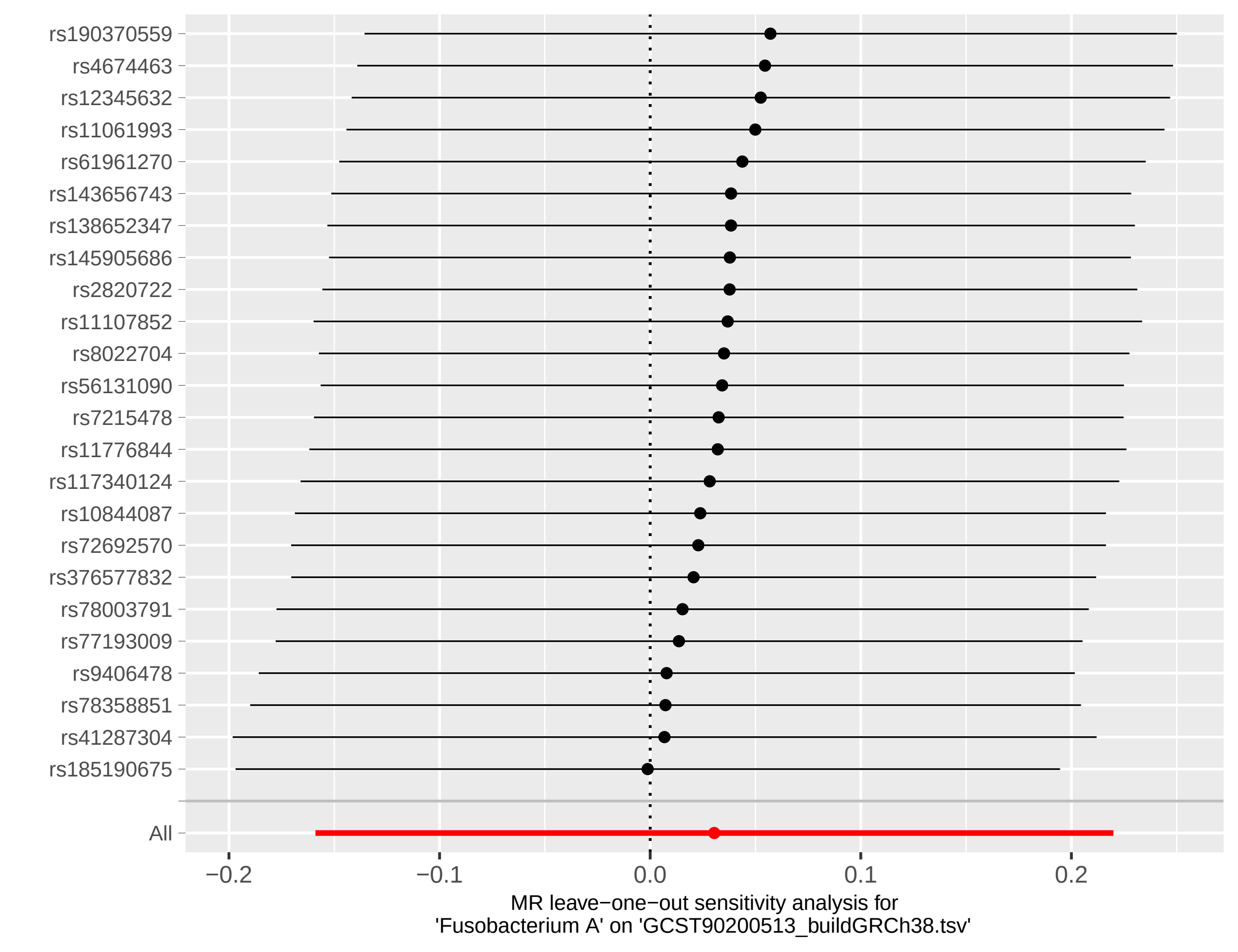
rs190370559
rs4674463
rs12345632
rs11061993
rs61961270
rs143656743
rs138652347
rs145905686
rs2820722
rs11107852
rs8022704
rs56131090
rs7215478
rs11776844
rs117340124
rs10844087
rs72692570
rs376577832
rs78003791
rs77193009
rs9406478
rs78358851
rs41287304
rs185190675
All
−0.2
−0.1
0.0
0.1
0.2
MR leave−one−out sensitivity analysis for
'Fusobacterium A' on 'GCST90200513_buildGRCh38.tsv'

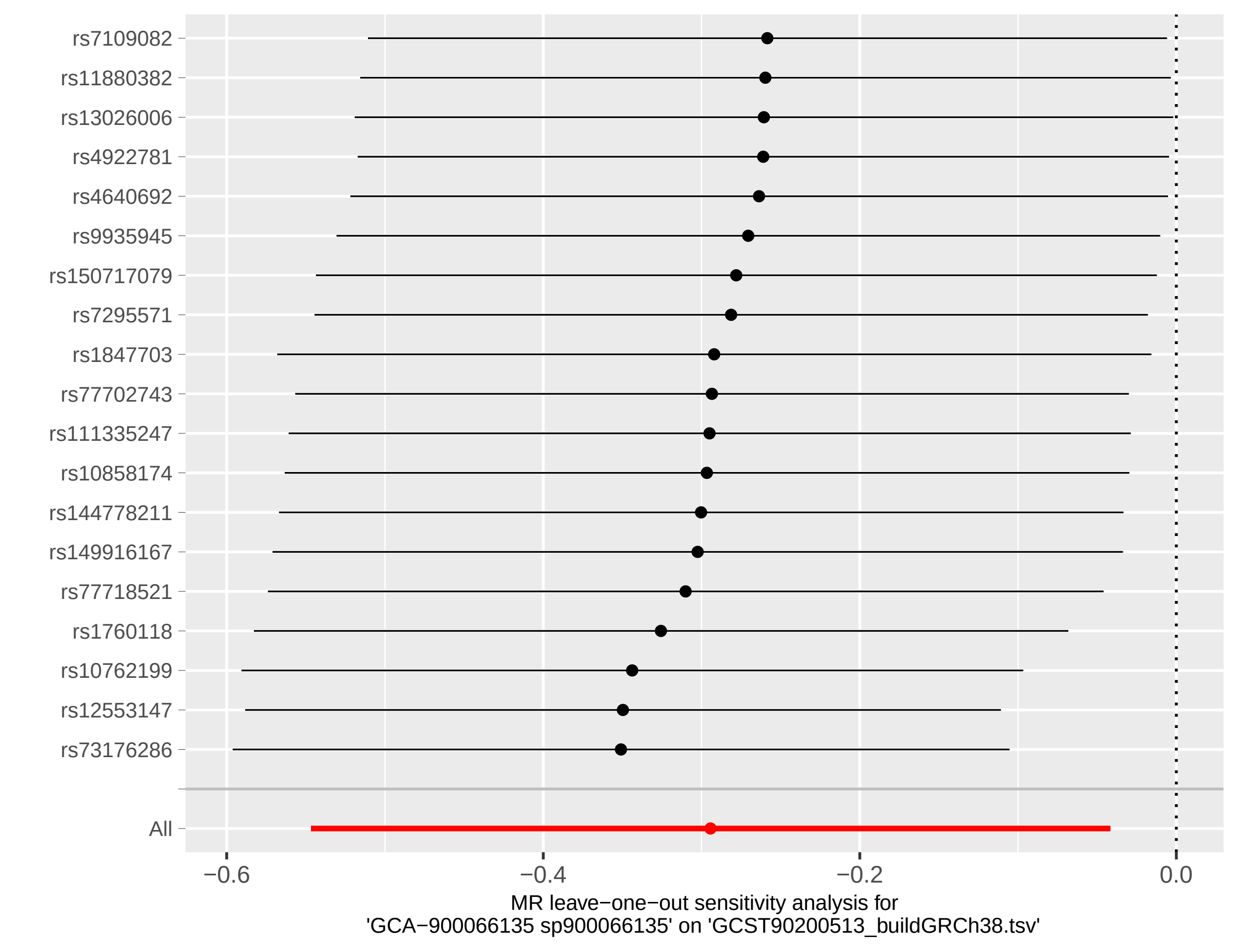
rs7109082
rs11880382
rs13026006
rs4922781
rs4640692
rs9935945
rs150717079
rs7295571
rs1847703
rs77702743
rs111335247
rs10858174
rs144778211
rs149916167
rs77718521
rs1760118
rs10762199
rs12553147
rs73176286
All
−0.6
−0.4
−0.2
0.0
MR leave−one−out sensitivity analysis for
'GCA−900066135 sp900066135' on 'GCST90200513_buildGRCh38.tsv'

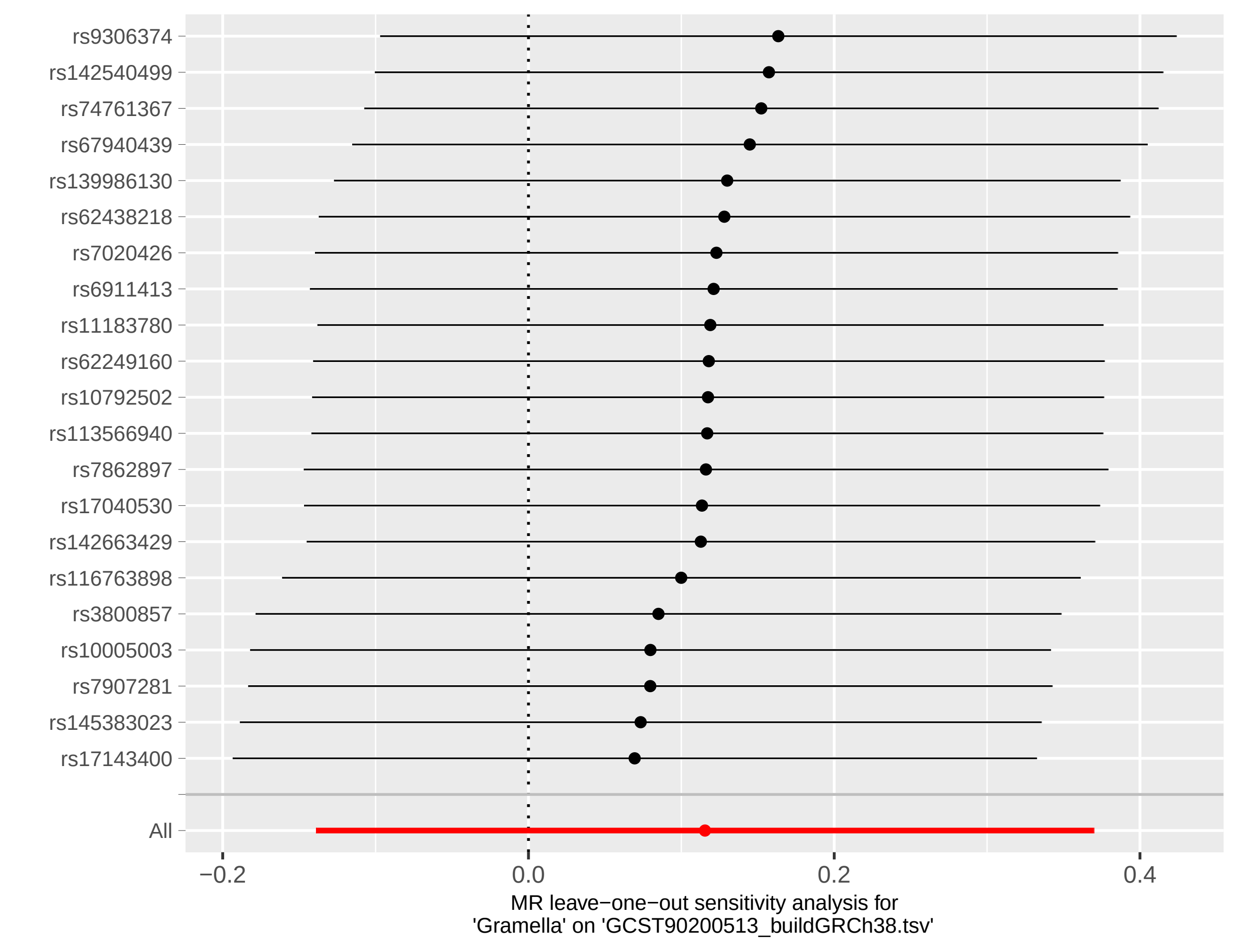
rs9306374
rs142540499
rs74761367
rs67940439
rs139986130
rs62438218
rs7020426
rs6911413
rs11183780
rs62249160
rs10792502
rs113566940
rs7862897
rs17040530
rs142663429
rs116763898
rs3800857
rs10005003
rs7907281
rs145383023
rs17143400
All
−0.2
0.0
0.2
0.4
MR leave−one−out sensitivity analysis for
'Gramella' on 'GCST90200513_buildGRCh38.tsv'

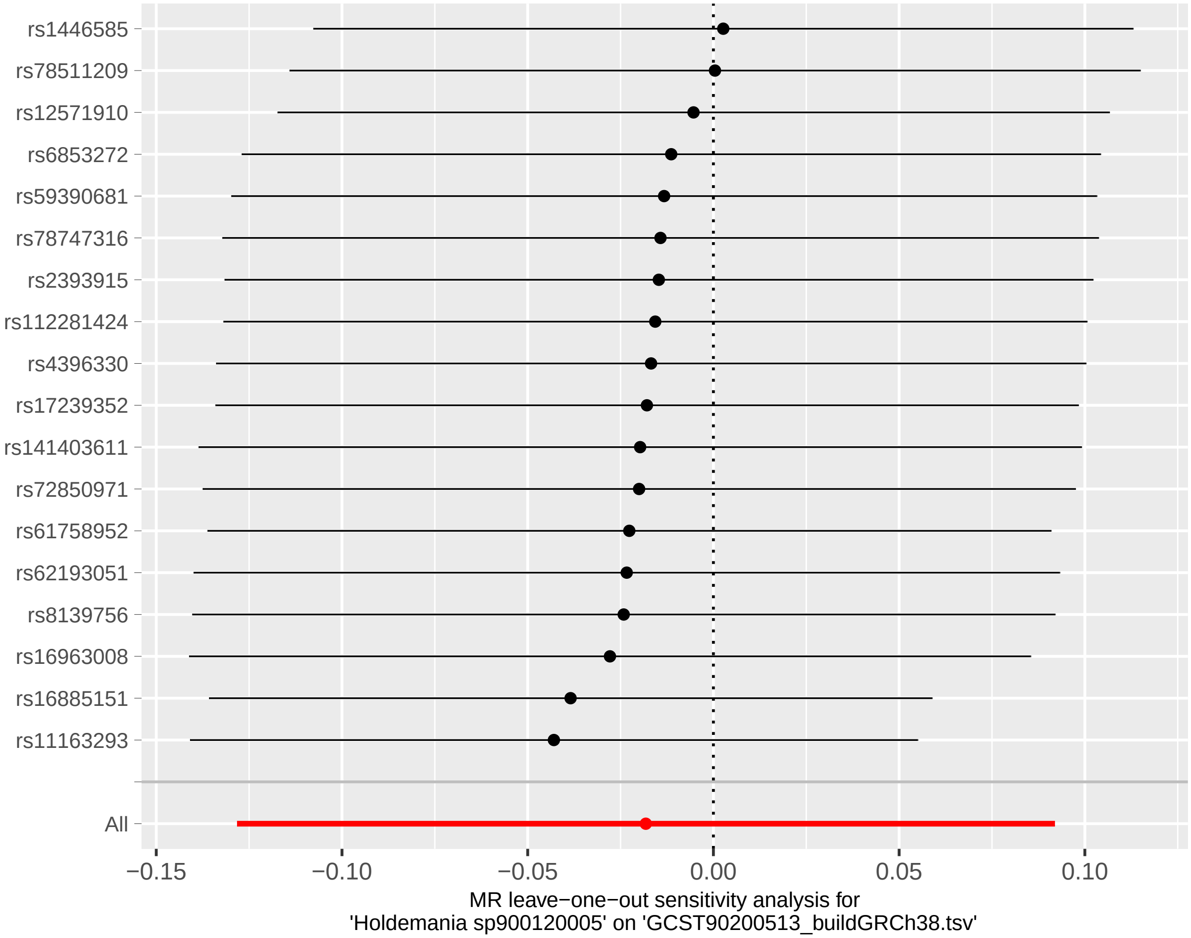
rs1446585
rs78511209
rs12571910
rs6853272
rs59390681
rs78747316
rs2393915
rs112281424
rs4396330
rs17239352
rs141403611
rs72850971
rs61758952
rs62193051
rs8139756
rs16963008
rs16885151
rs11163293
All
−0.15
−0.10
−0.05
0.00
0.05
0.10
MR leave−one−out sensitivity analysis for
'Holdemania sp900120005' on 'GCST90200513_buildGRCh38.tsv'

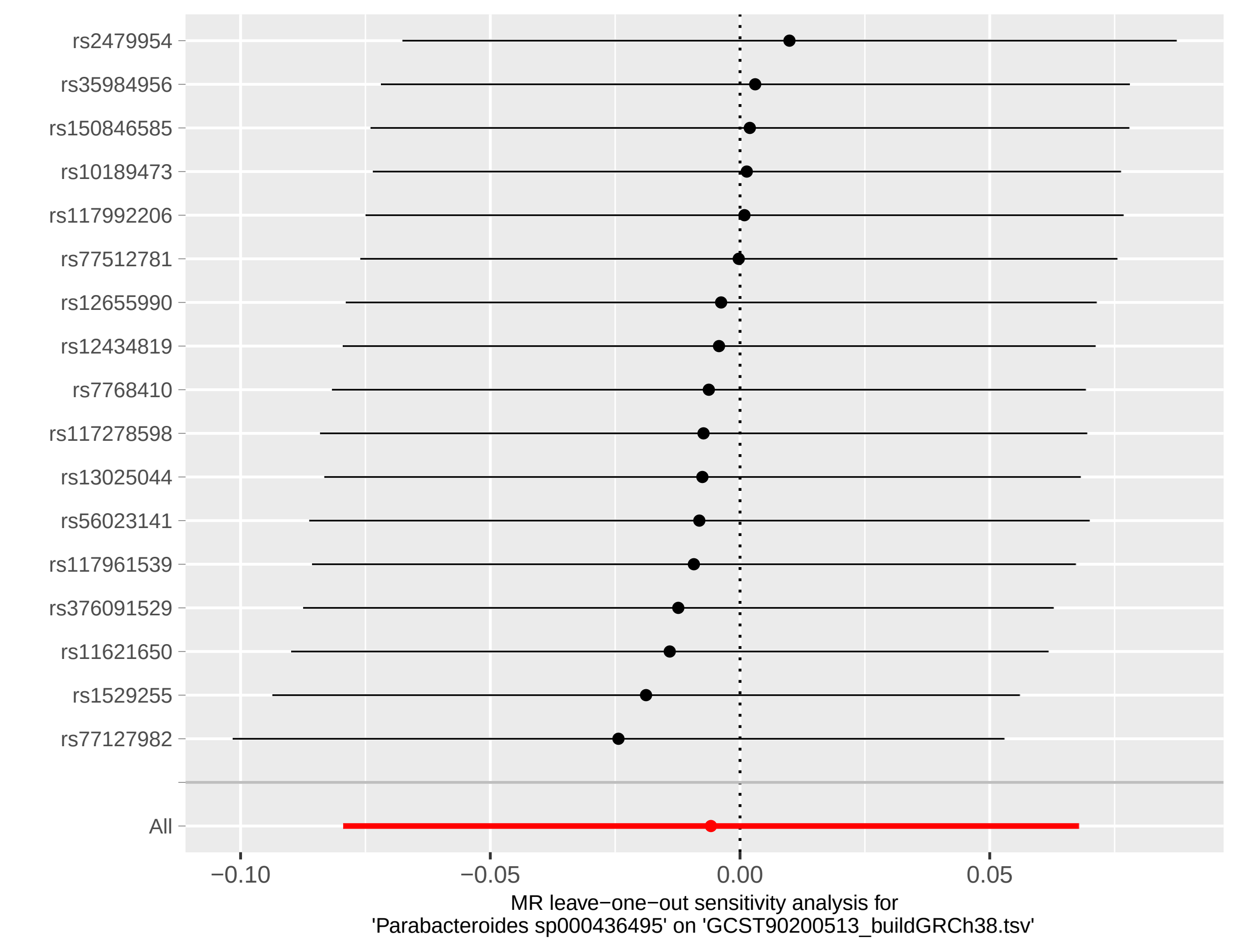
rs2479954
rs35984956
rs150846585
rs10189473
rs117992206
rs77512781
rs12655990
rs12434819
rs7768410
rs117278598
rs13025044
rs56023141
rs117961539
rs376091529
rs11621650
rs1529255
rs77127982
All
−0.10
−0.05
0.00
0.05
MR leave−one−out sensitivity analysis for
'Parabacteroides sp000436495' on 'GCST90200513_buildGRCh38.tsv'

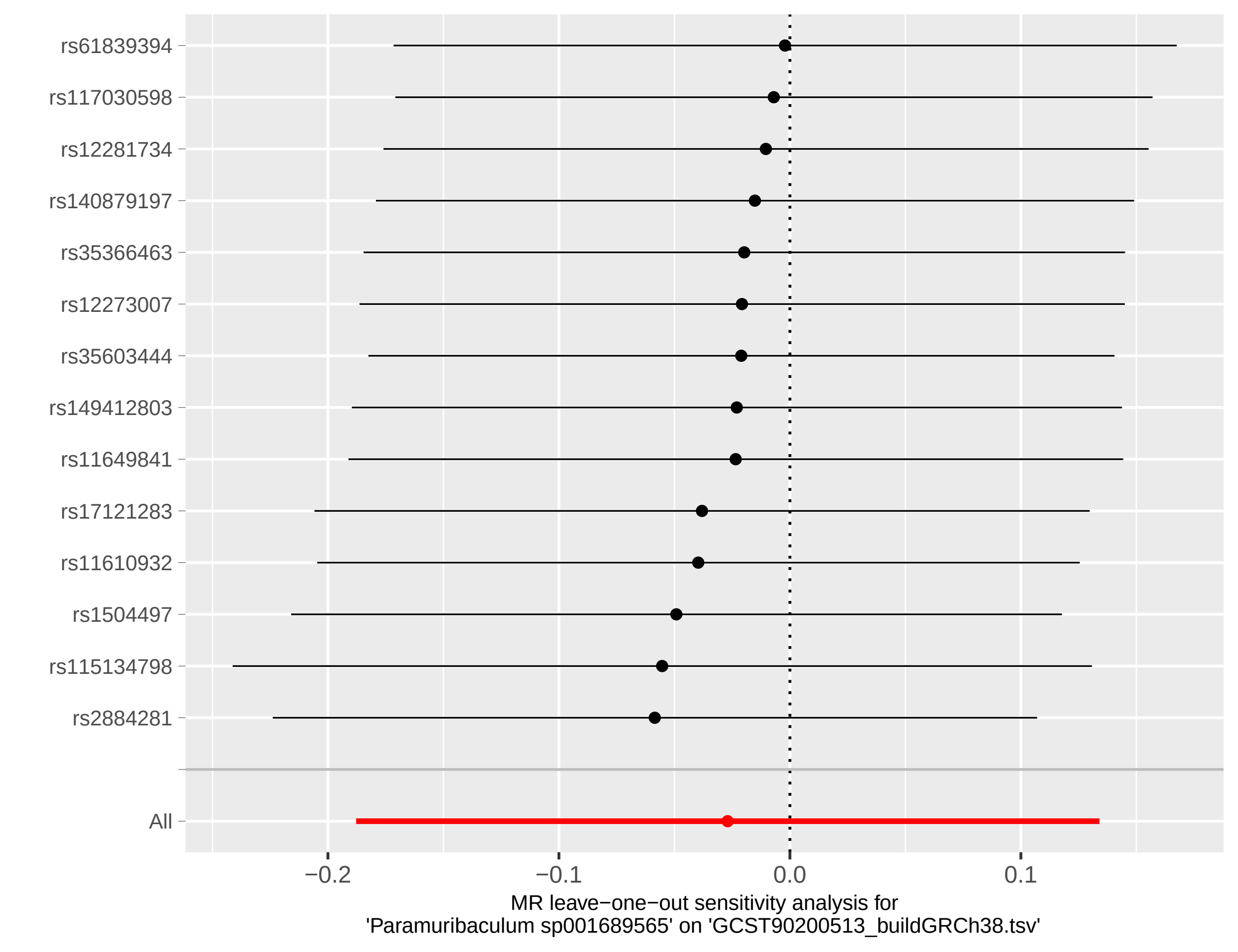
rs61839394
rs117030598
rs12281734
rs140879197
rs35366463
rs12273007
rs35603444
rs149412803
rs11649841
rs17121283
rs11610932
rs1504497
rs115134798
rs2884281
All
−0.2
−0.1
0.0
0.1
MR leave−one−out sensitivity analysis for
'Paramuribaculum sp001689565' on 'GCST90200513_buildGRCh38.tsv'

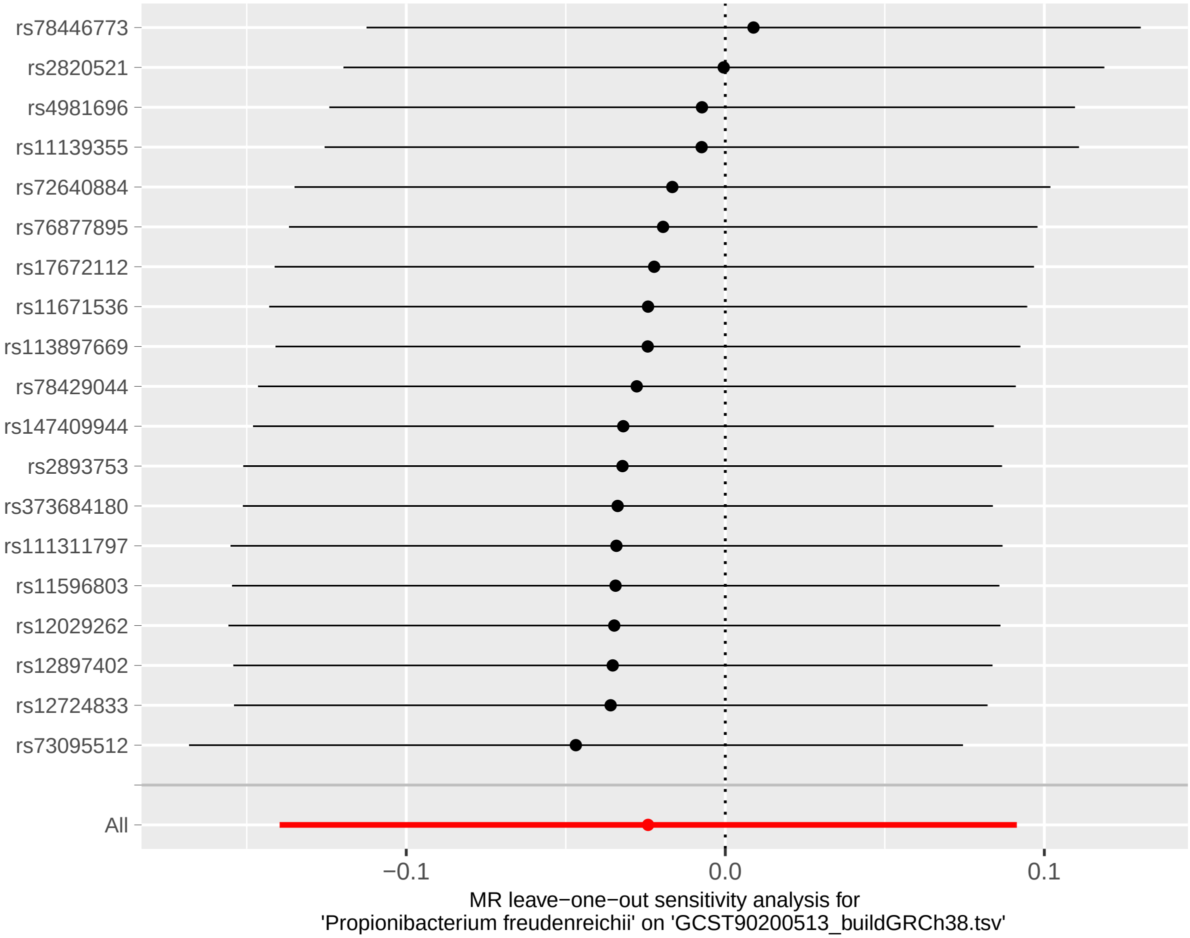
rs78446773
rs2820521
rs4981696
rs11139355
rs72640884
rs76877895
rs17672112
rs11671536
rs113897669
rs78429044
rs147409944
rs2893753
rs373684180
rs111311797
rs11596803
rs12029262
rs12897402
rs12724833
rs73095512
All
−0.1
0.0
0.1
MR leave−one−out sensitivity analysis for
'Propionibacterium freudenreichii' on 'GCST90200513_buildGRCh38.tsv'

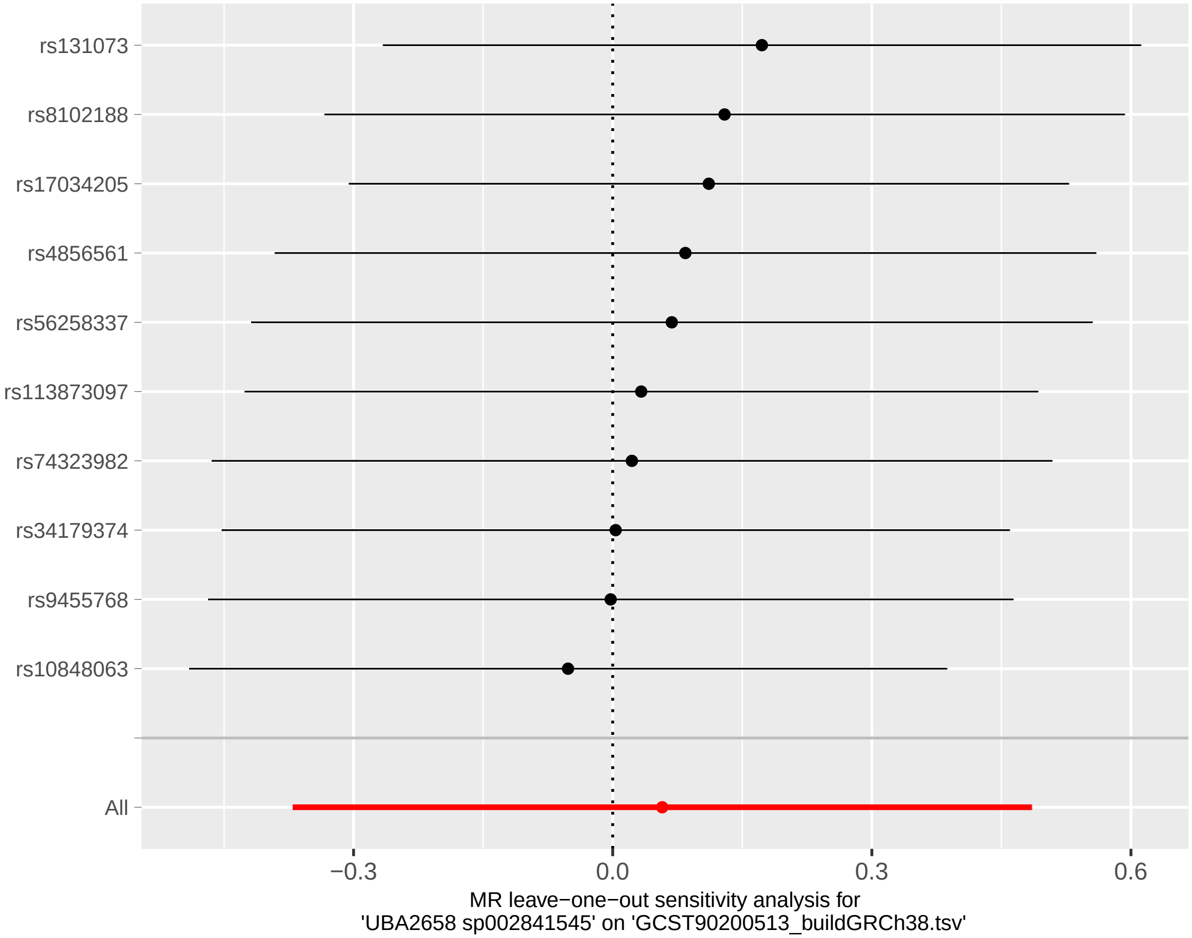
rs131073
rs8102188
rs17034205
rs4856561
rs56258337
rs113873097
rs74323982
rs34179374
rs9455768
rs10848063
All
−0.3
0.0
0.3
0.6
MR leave−one−out sensitivity analysis for
'UBA2658 sp002841545' on 'GCST90200513_buildGRCh38.tsv'

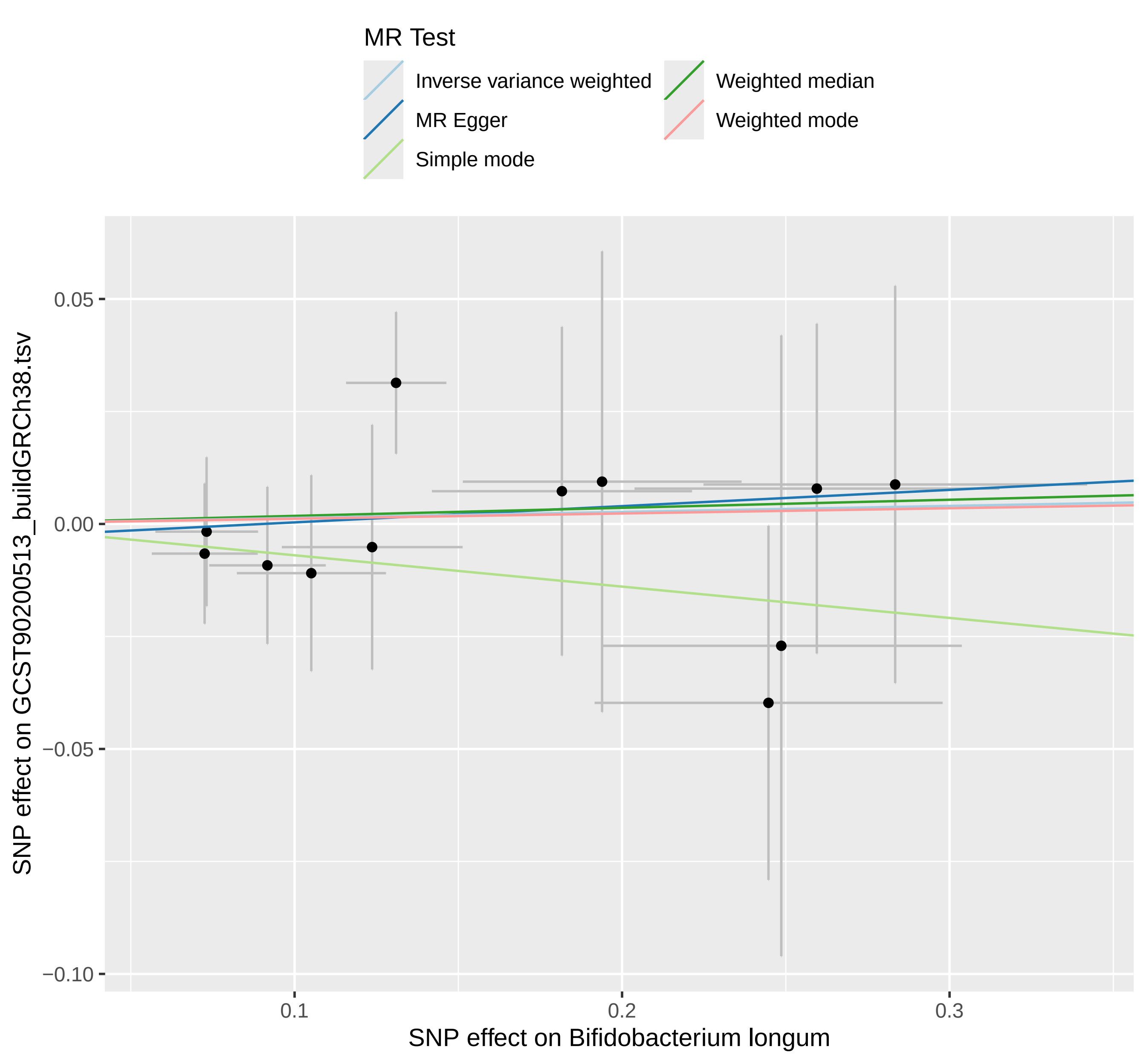
MR Test
Inverse variance weighted
MR Egger
Simple mode
Weighted median
Weighted mode
0.05
0.00
−0.05
−0.10
0.1
0.2
0.3
SNP effect on GCST90200513_buildGRCh38.tsv
SNP effect on Bifidobacterium longum

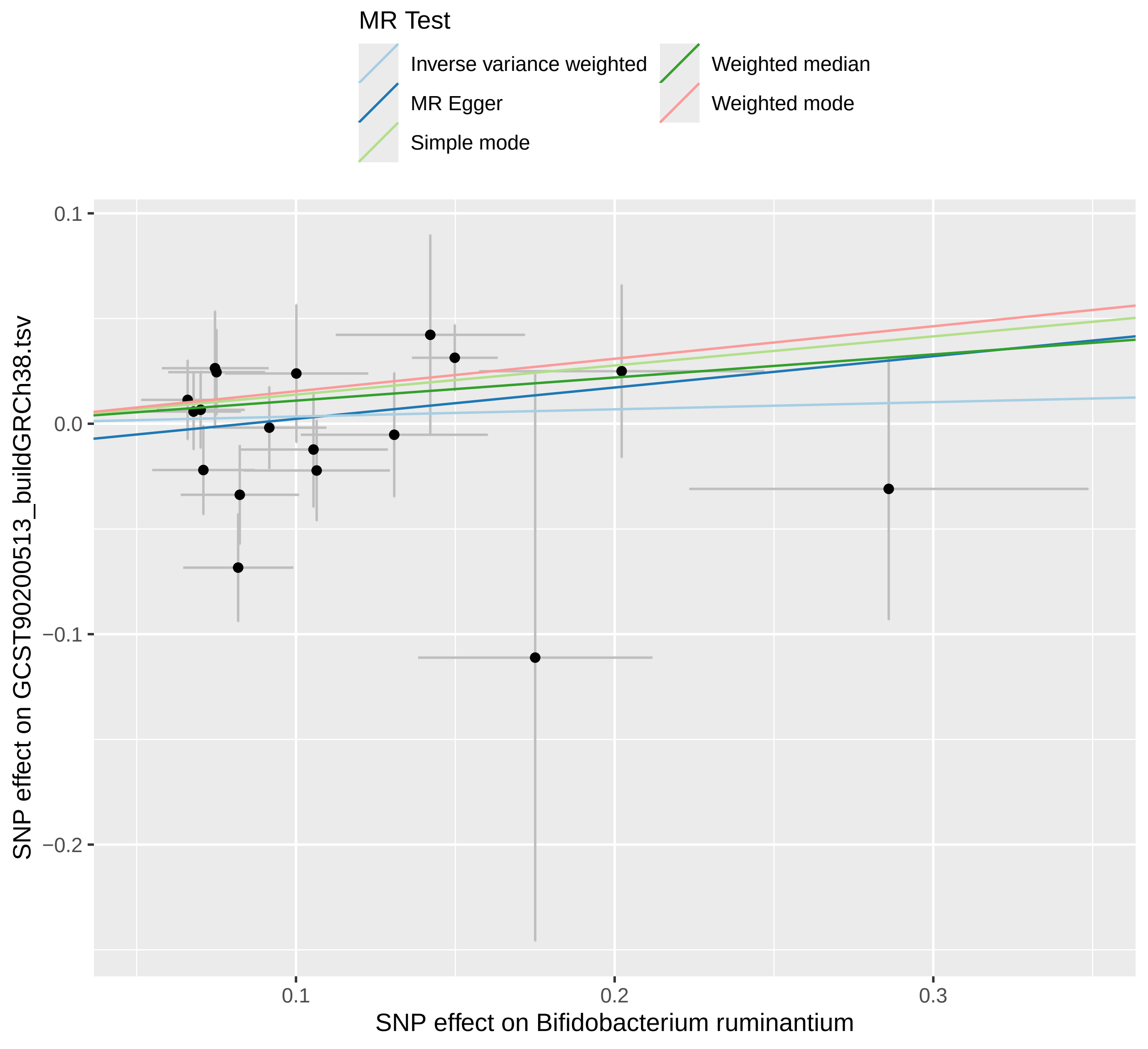

MR Test
Inverse variance weighted
MR Egger
Simple mode
Weighted median
Weighted mode
0.1
0.0
−0.1
−0.2
0.1
0.2
0.3
SNP effect on GCST90200513_buildGRCh38.tsv
SNP effect on Bifidobacterium ruminantium

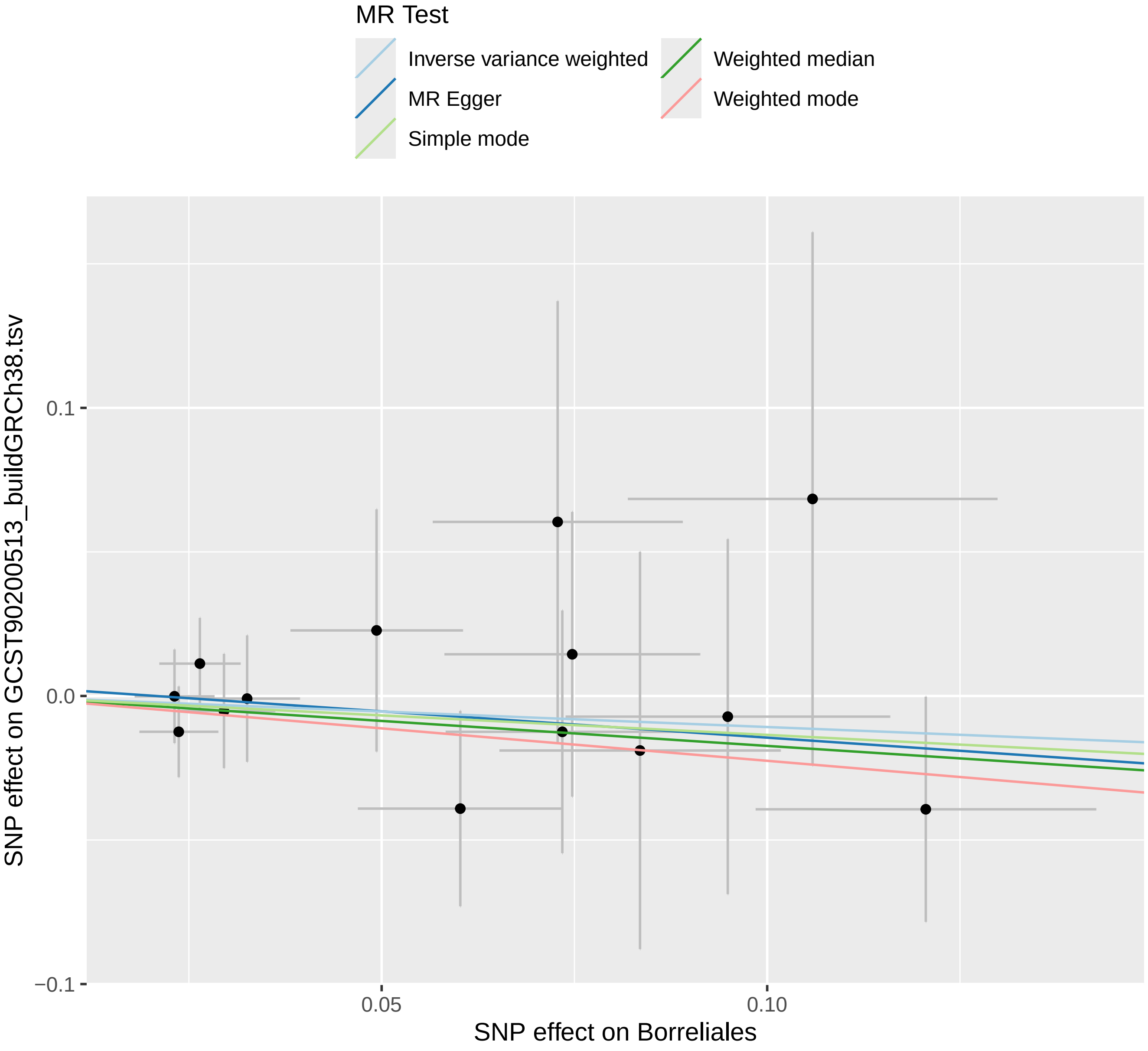

MR Test
Inverse variance weighted
MR Egger
Simple mode
Weighted median
Weighted mode
SNP effect on GCST90200513_buildGRCh38.tsv
0.1
0.0
−0.1
0.05
0.10
SNP effect on Borreliales

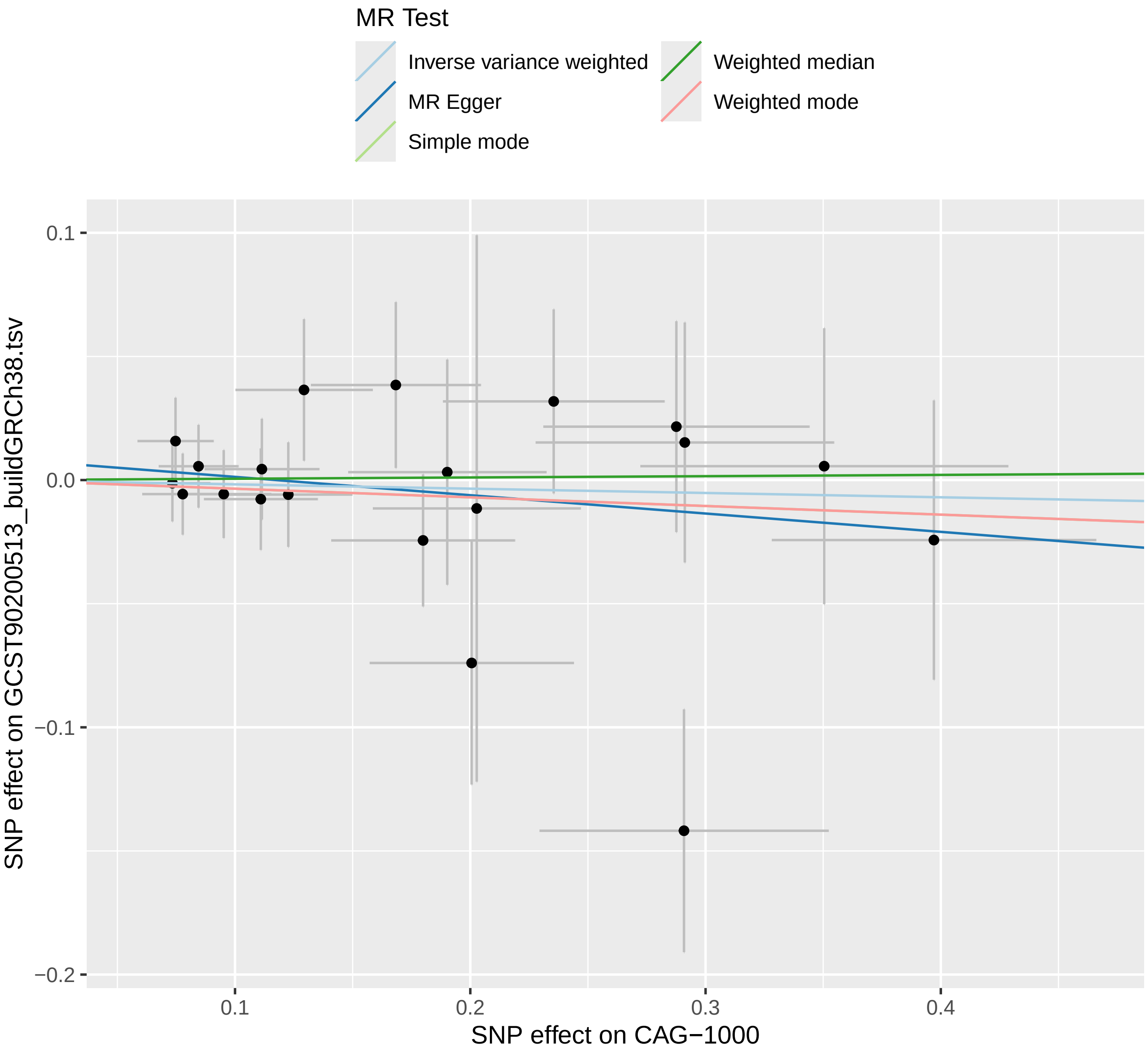

MR Test
Inverse variance weighted
MR Egger
Simple mode
Weighted median
Weighted mode
0.1
0.0
−0.1
−0.2
0.1
0.2
0.3
0.4
SNP effect on GCST90200513_buildGRCh38.tsv
SNP effect on CAG−1000

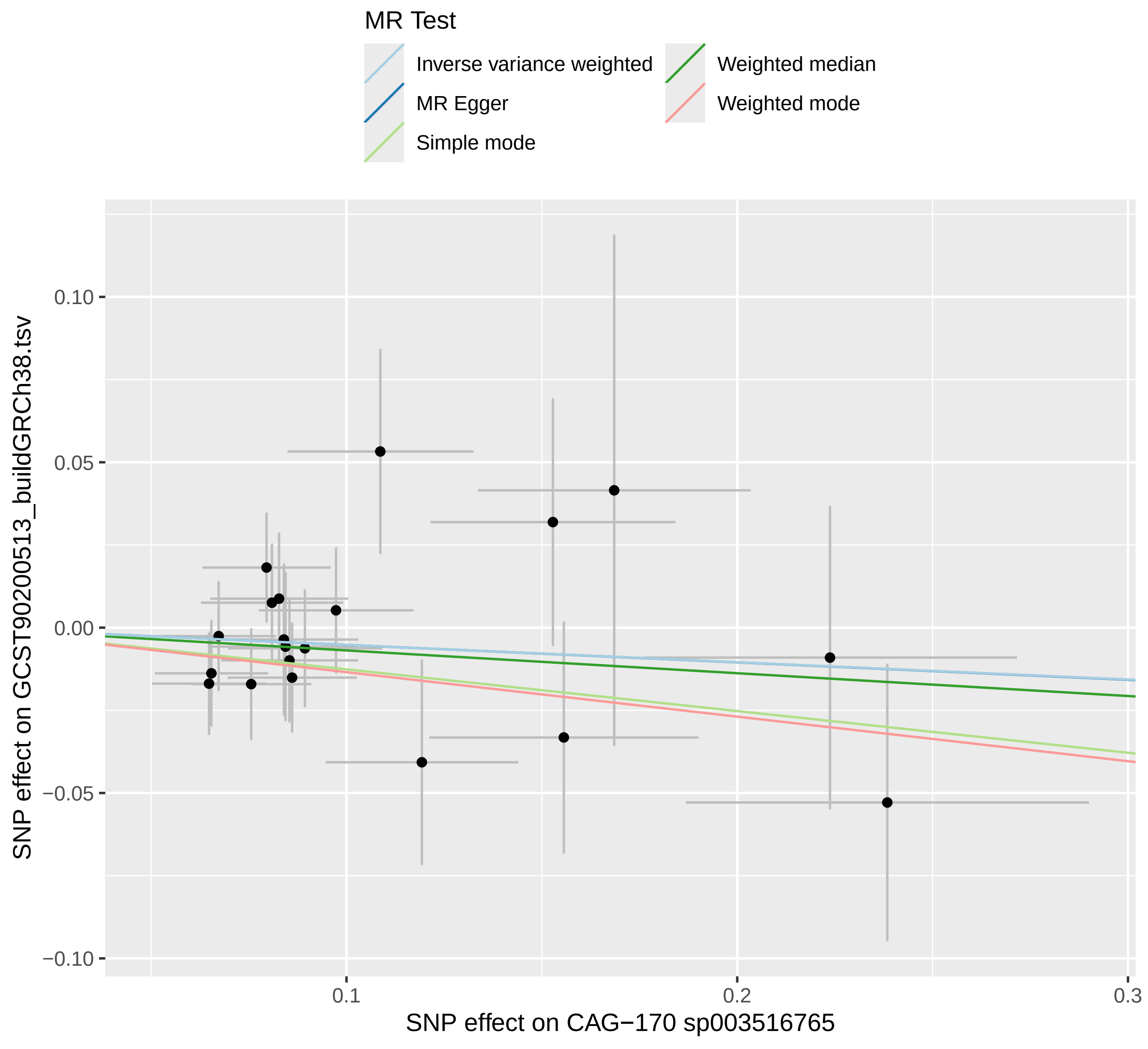

MR Test
Inverse variance weighted
MR Egger
Simple mode
Weighted median
Weighted mode
0.10
0.05
0.00
−0.05
−0.10
0.1
0.2
0.3
SNP effect on GCST90200513_buildGRCh38.tsv
SNP effect on CAG−170 sp003516765

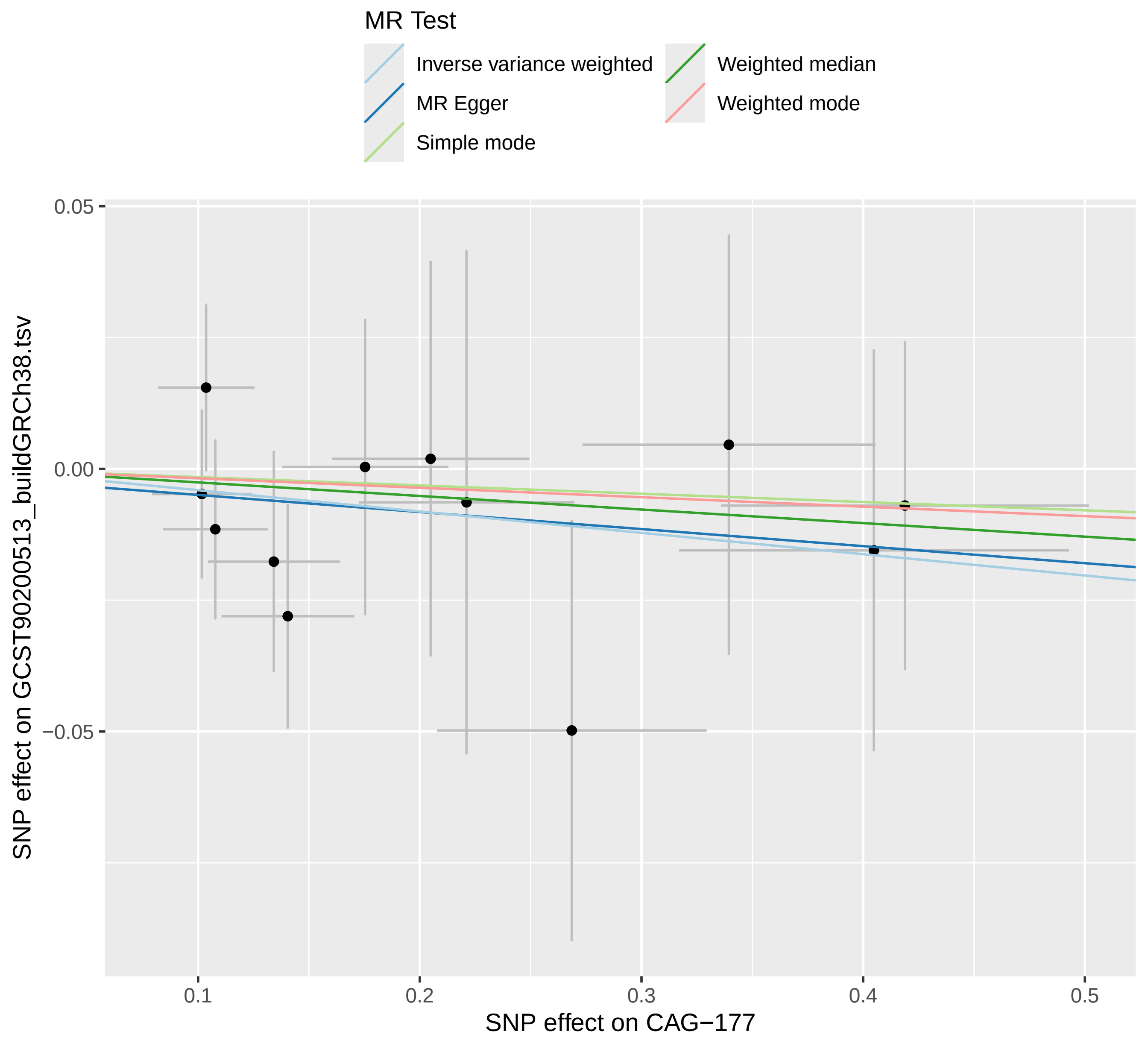

MR Test
Inverse variance weighted
MR Egger
Simple mode
Weighted median
Weighted mode
0.05
0.00
−0.05
0.1
0.2
0.3
0.4
0.5
SNP effect on GCST90200513_buildGRCh38.tsv
SNP effect on CAG−177

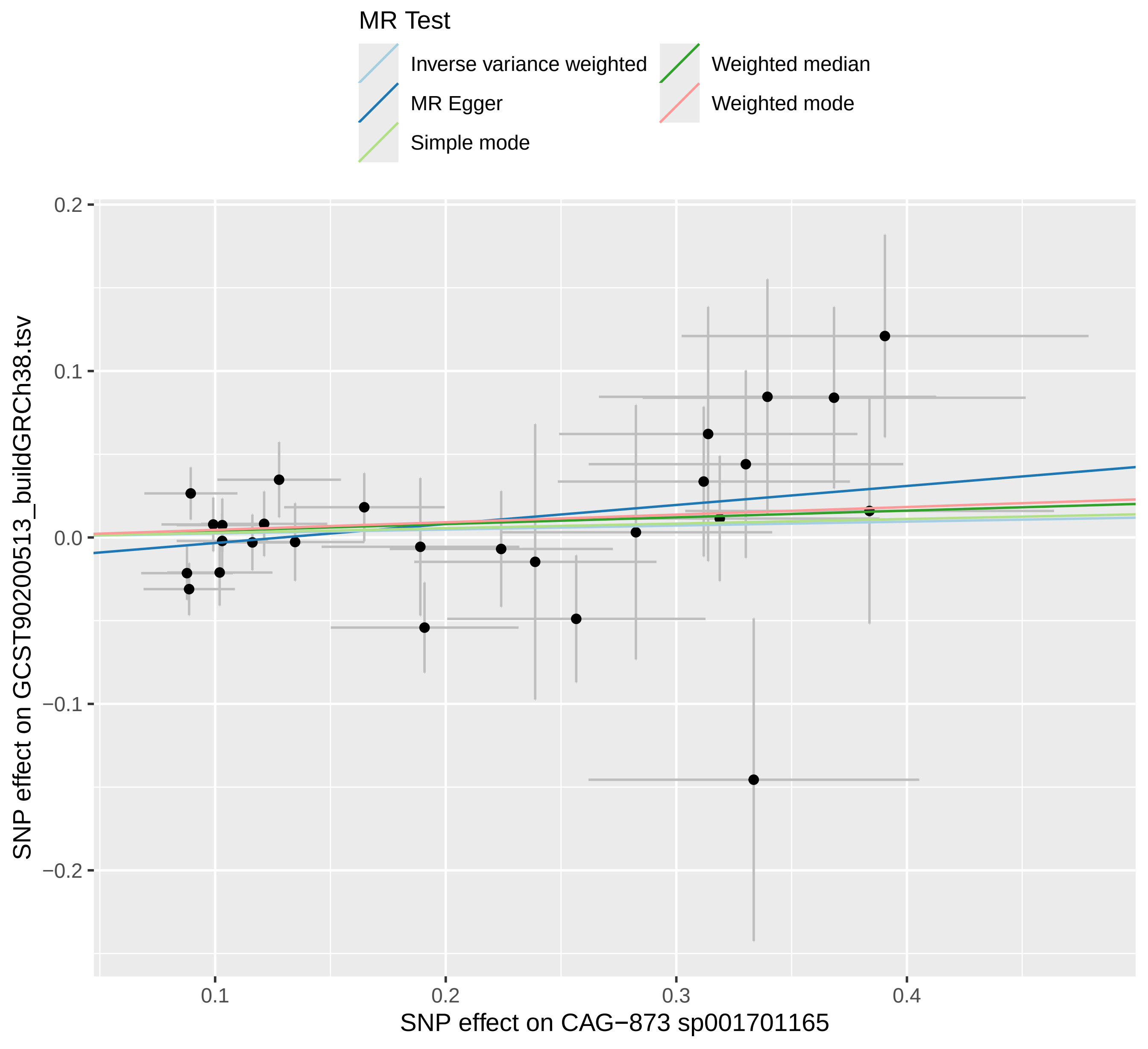
MR Test
Inverse variance weighted
MR Egger
Simple mode
Weighted median
Weighted mode
0.2
0.1
0.0
−0.1
−0.2
0.1
0.2
0.3
0.4
SNP effect on GCST90200513_buildGRCh38.tsv
SNP effect on CAG−873 sp001701165

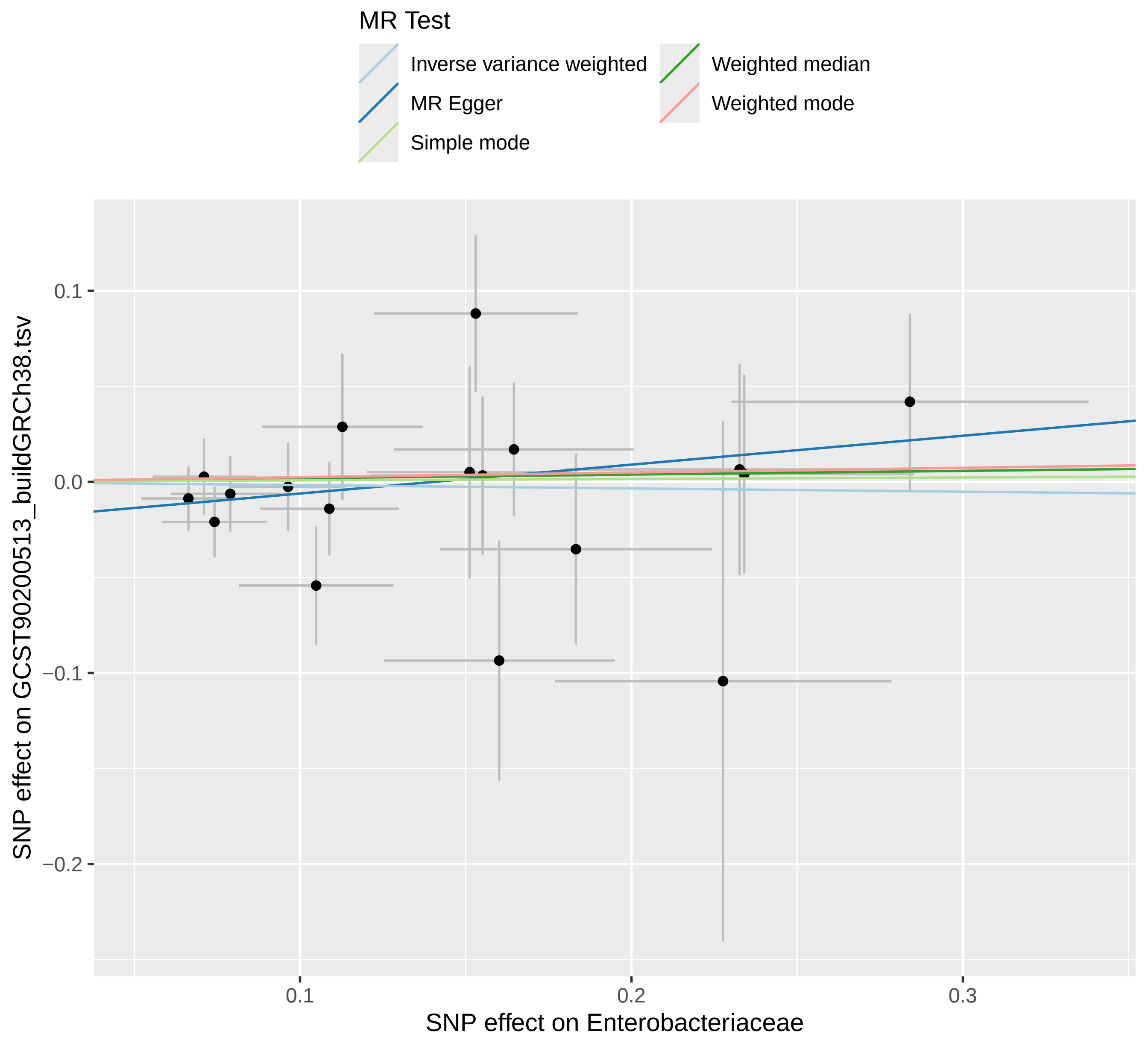
MR Test
Inverse variance weighted
MR Egger
Simple mode
Weighted median
Weighted mode
0.1
0.0
−0.1
−0.2
SNP effect on GCST90200513_buildGRCh38.tsv
0.1
0.2
0.3
SNP effect on Enterobacteriaceae

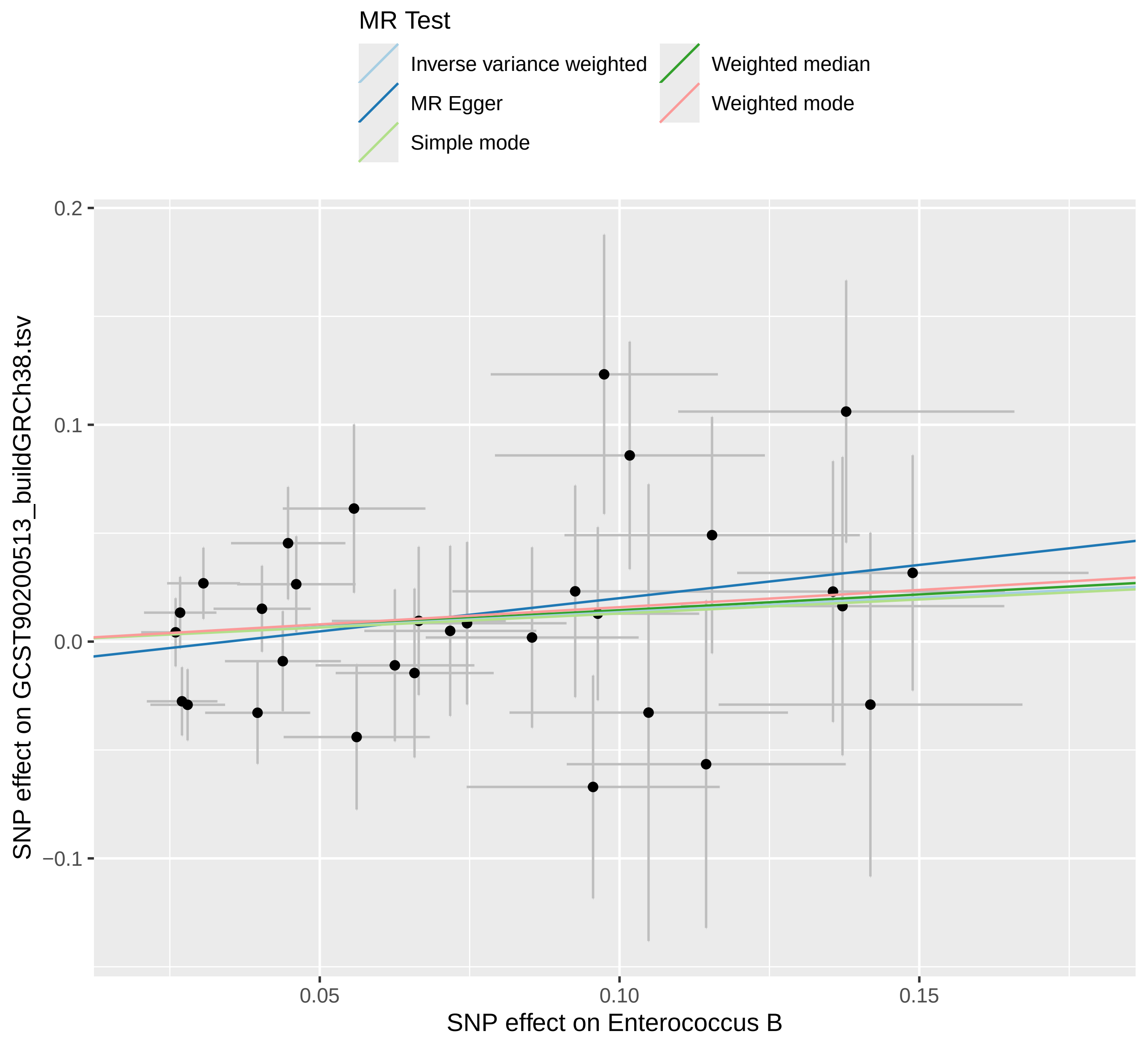
MR Test
Inverse variance weighted
MR Egger
Simple mode
Weighted median
Weighted mode
0.2
0.1
0.0
−0.1
0.05
0.10
0.15
SNP effect on GCST90200513_buildGRCh38.tsv
SNP effect on Enterococcus B

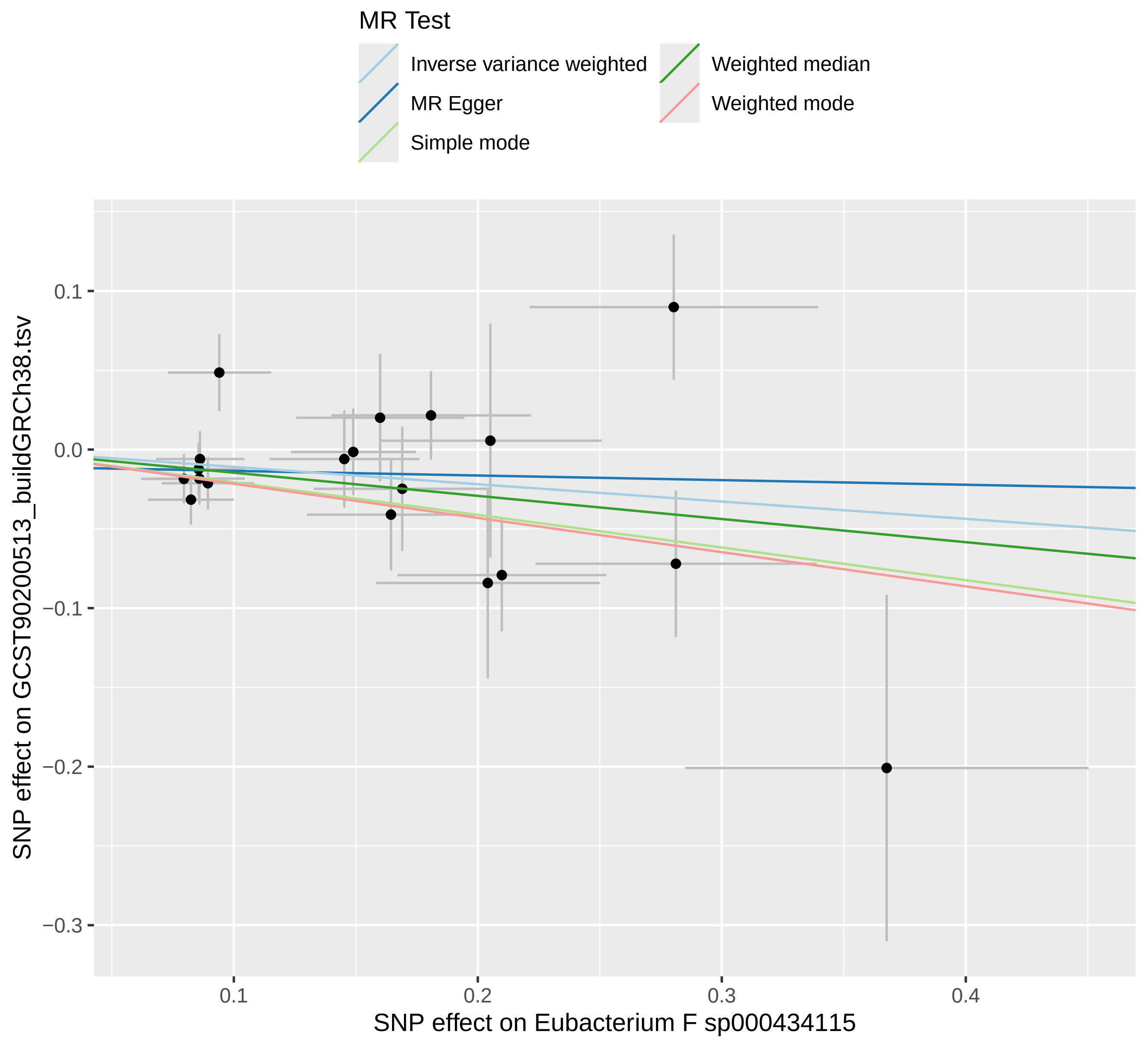
MR Test
Inverse variance weighted
MR Egger
Simple mode
Weighted median
Weighted mode
0.1
0.0
−0.1
−0.2
−0.3
SNP effect on GCST90200513_buildGRCh38.tsv
0.1
0.2
0.3
0.4
SNP effect on Eubacterium F sp000434115

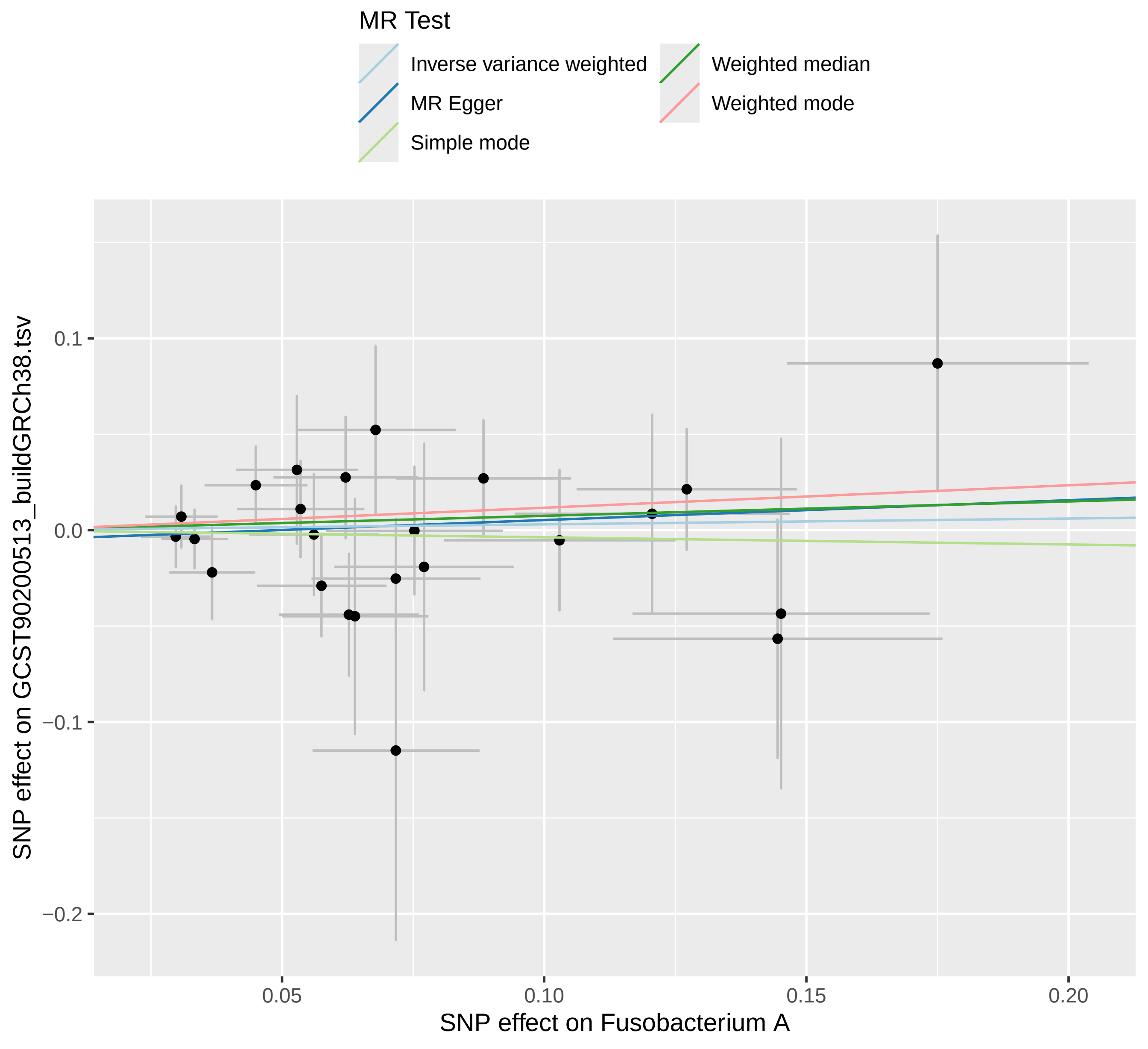

MR Test
Inverse variance weighted
MR Egger
Simple mode
Weighted median
Weighted mode
0.1
0.0
−0.1
−0.2
0.05
0.10
0.15
0.20
SNP effect on GCST90200513_buildGRCh38.tsv
SNP effect on Fusobacterium A

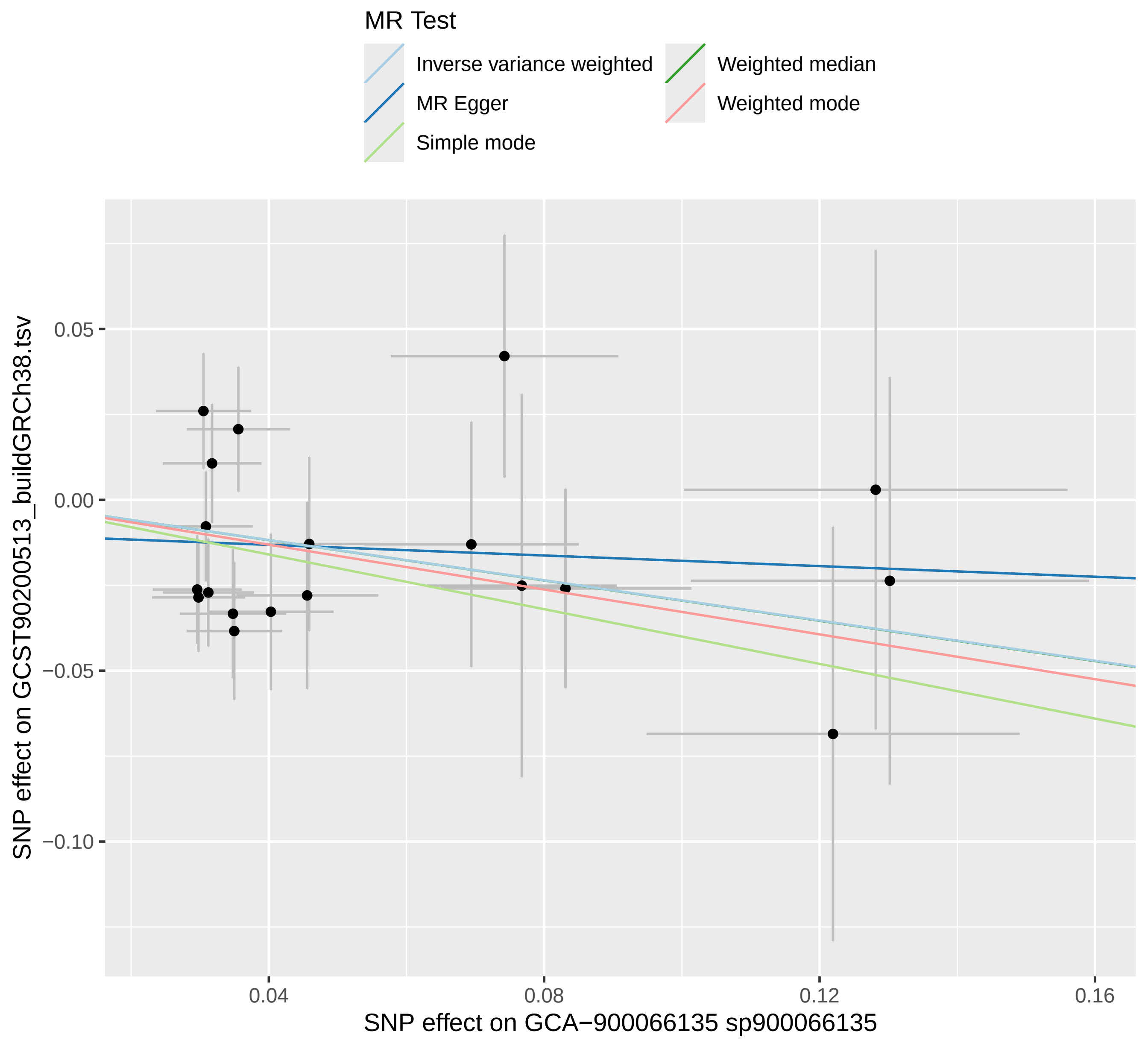
MR Test
Inverse variance weighted
MR Egger
Simple mode
Weighted median
Weighted mode
SNP effect on GCST90200513_buildGRCh38.tsv
0.05
0.00
−0.05
−0.10
0.04
0.08
0.12
0.16
SNP effect on GCA−900066135 sp900066135

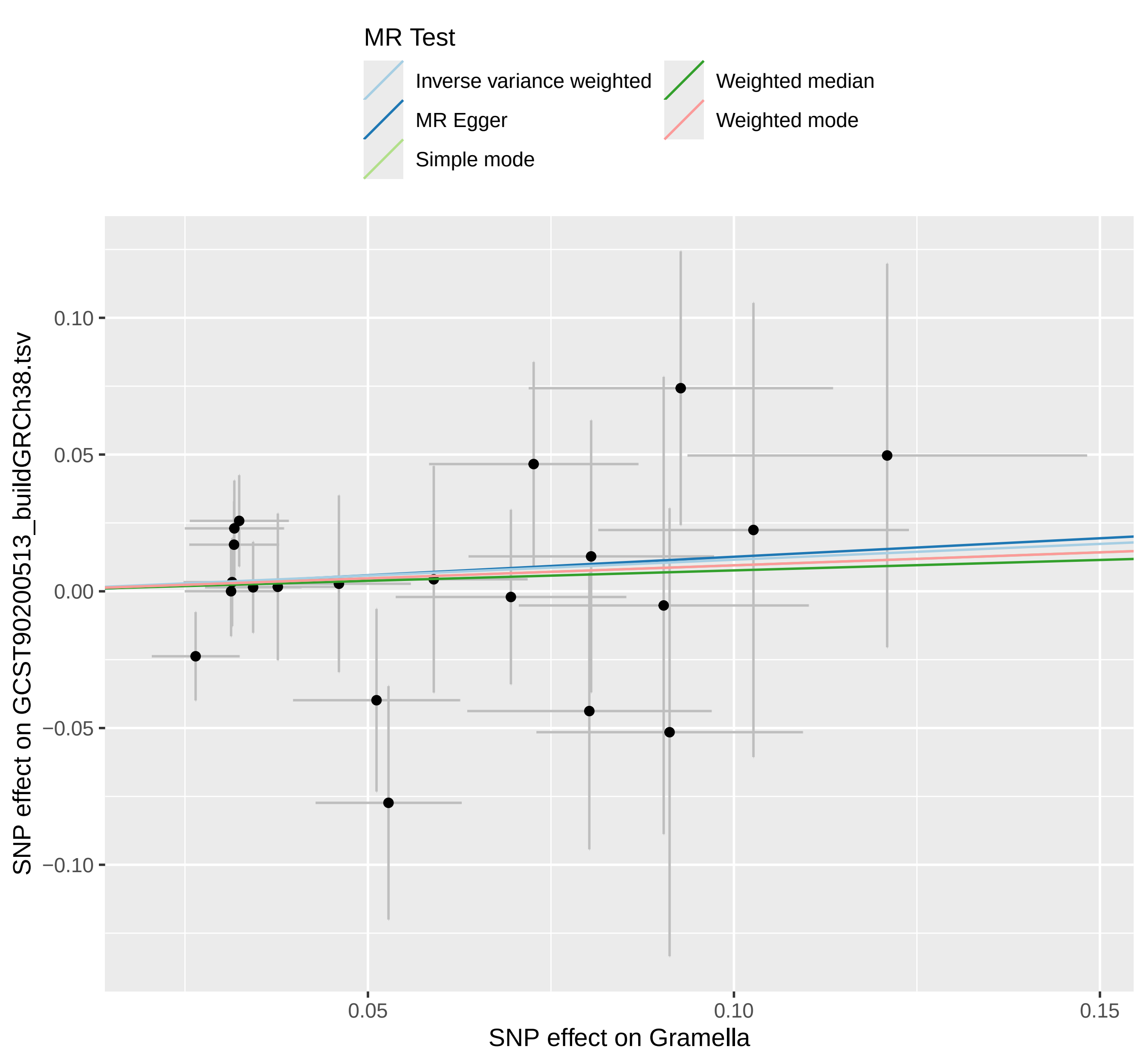
MR Test
Inverse variance weighted
MR Egger
Simple mode
Weighted median
Weighted mode
0.10
0.05
0.00
−0.05
−0.10
SNP effect on GCST90200513_buildGRCh38.tsv
0.05
0.10
0.15
SNP effect on Gramella

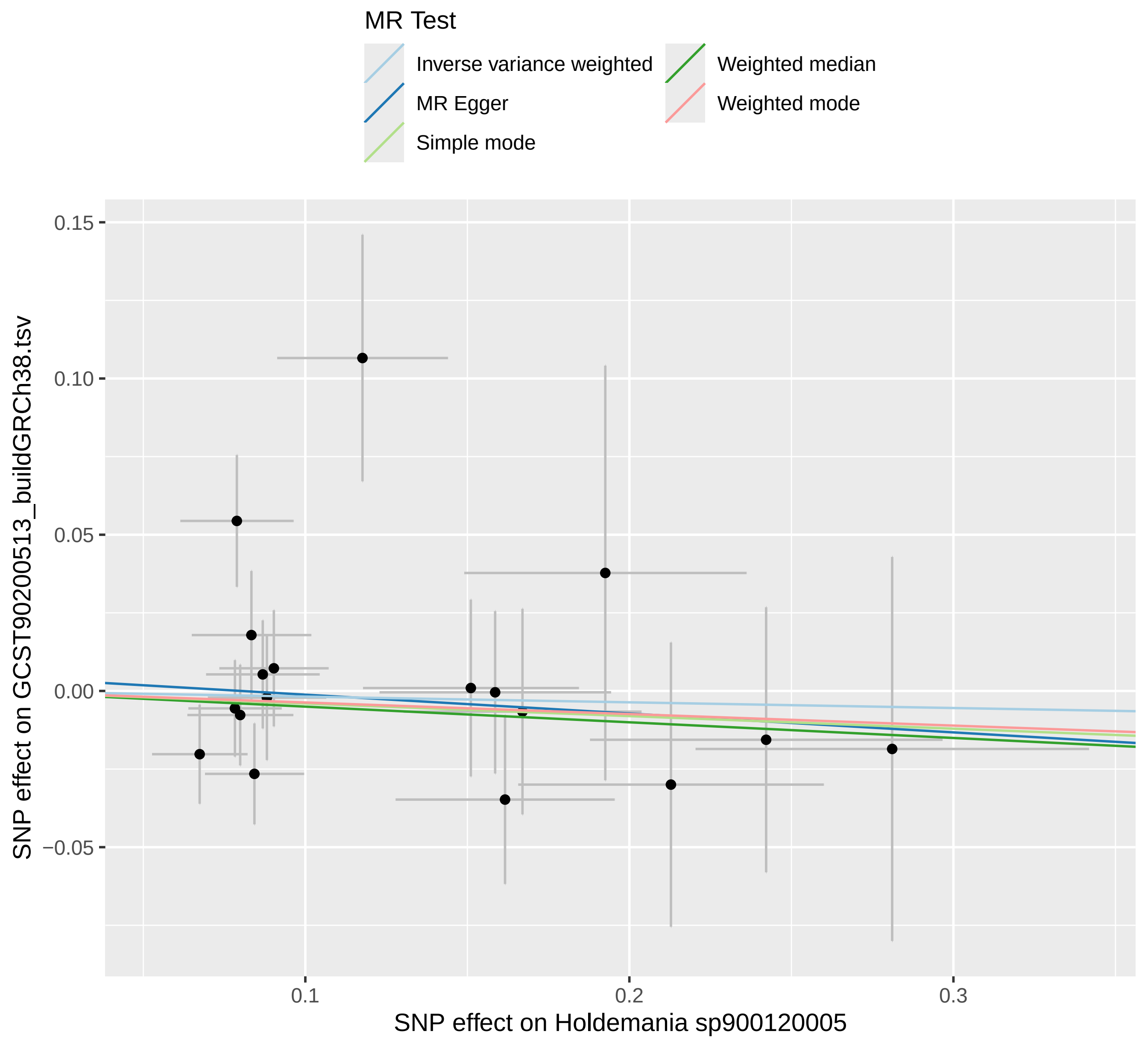
MR Test
Inverse variance weighted
MR Egger
Simple mode
Weighted median
Weighted mode
0.15
0.10
0.05
0.00
−0.05
SNP effect on GCST90200513_buildGRCh38.tsv
0.1
0.2
0.3
SNP effect on Holdemania sp900120005

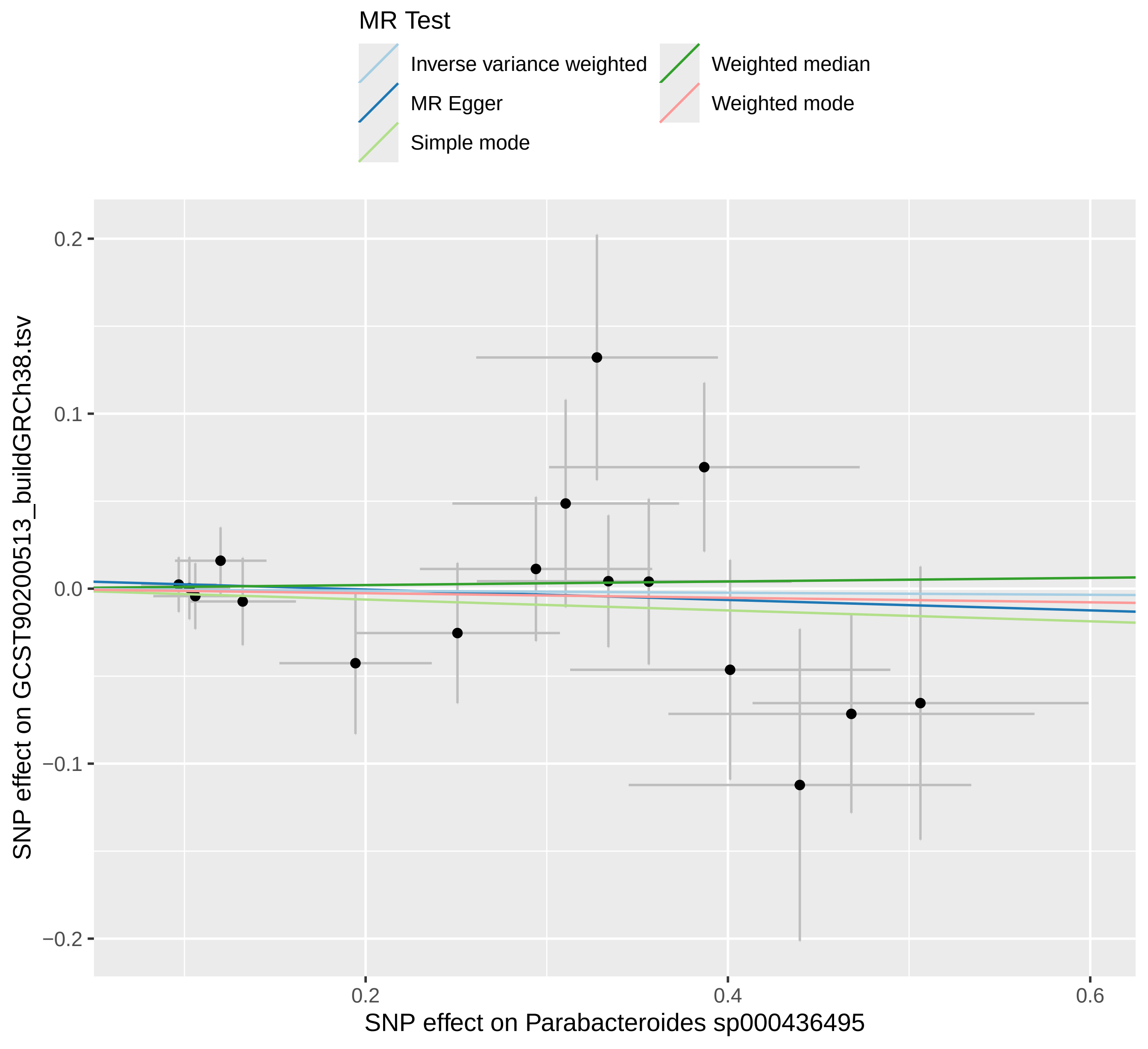

MR Test
Inverse variance weighted
MR Egger
Simple mode
Weighted median
Weighted mode
SNP effect on GCST90200513_buildGRCh38.tsv
0.2
0.1
0.0
−0.1
−0.2
0.2
0.4
0.6
SNP effect on Parabacteroides sp000436495

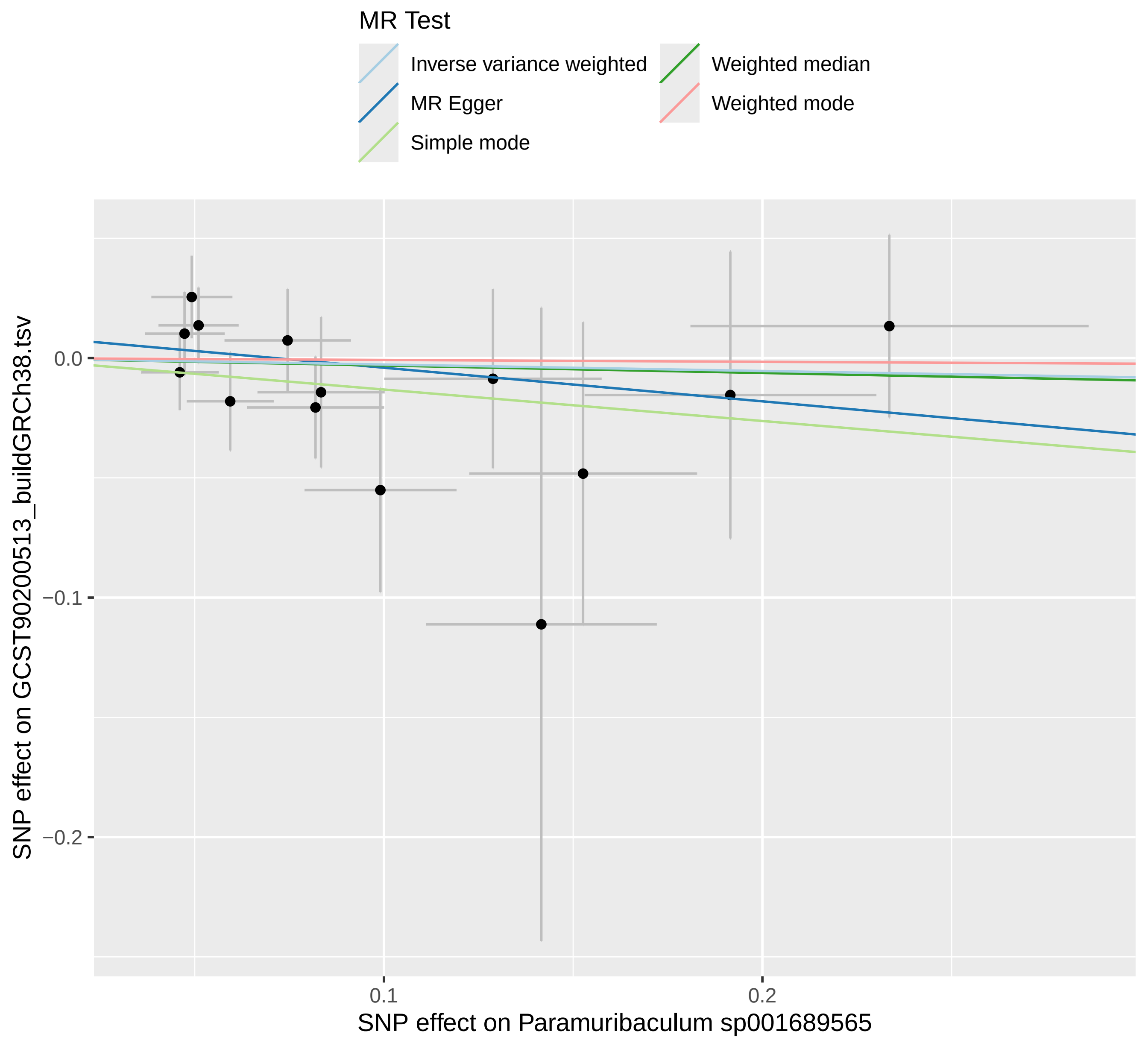
MR Test
Inverse variance weighted
MR Egger
Simple mode
Weighted median
Weighted mode
0.0
−0.1
−0.2
0.1
0.2
SNP effect on GCST90200513_buildGRCh38.tsv
SNP effect on Paramuribaculum sp001689565

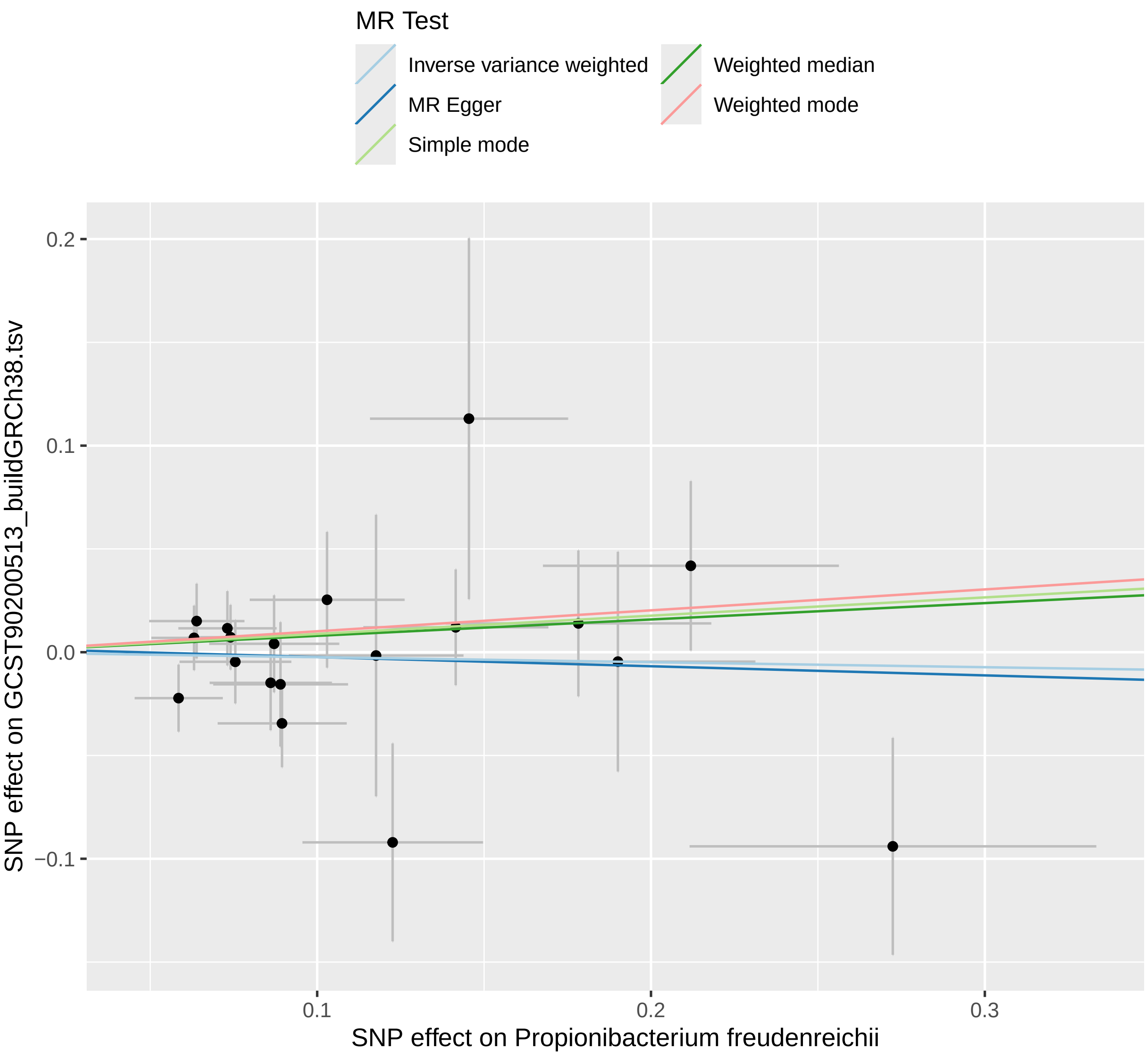

MR Test
Inverse variance weighted
MR Egger
Simple mode
Weighted median
Weighted mode
0.2
0.1
0.0
−0.1
0.1
0.2
0.3
SNP effect on GCST90200513_buildGRCh38.tsv
SNP effect on Propionibacterium freudenreichii

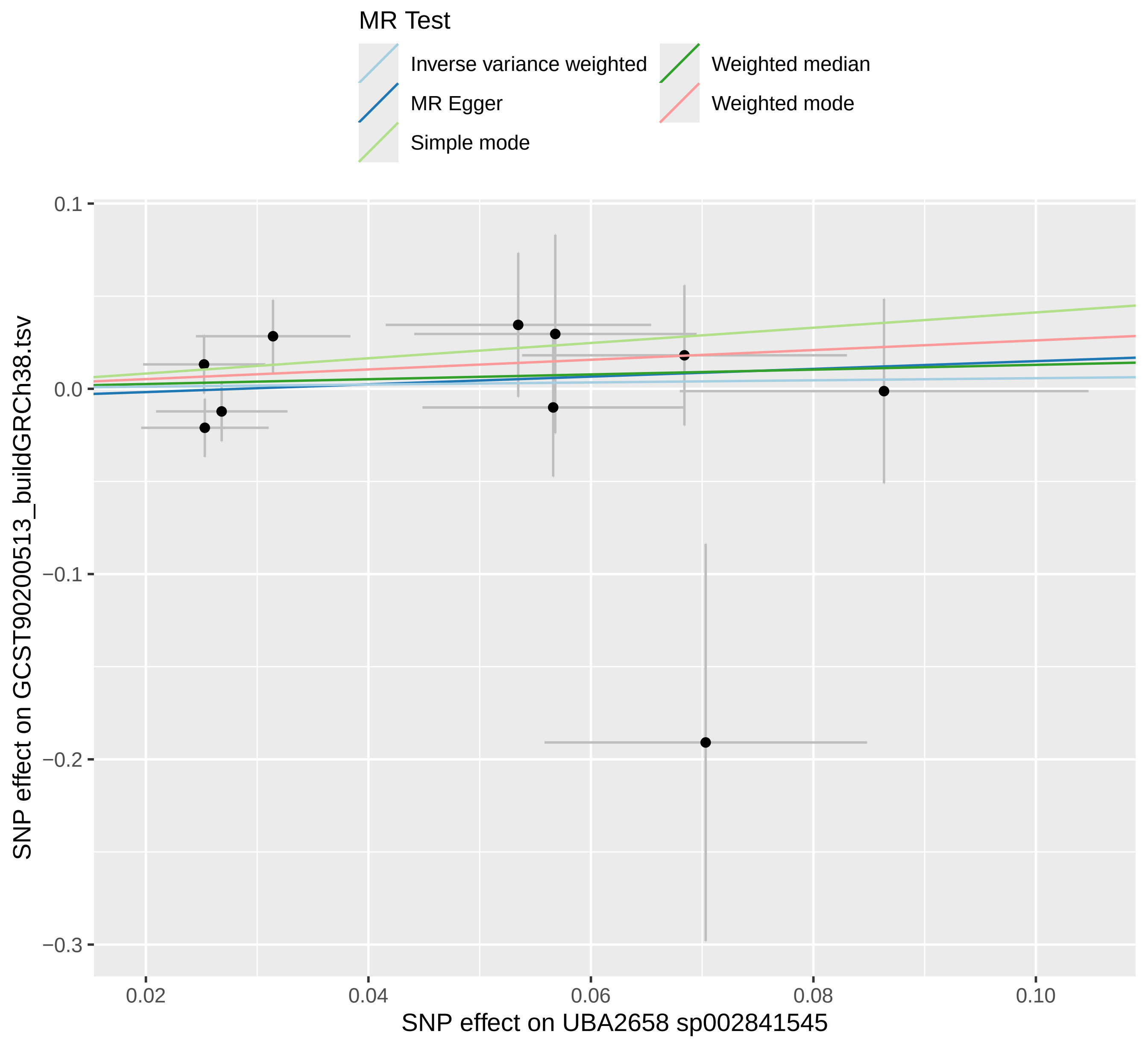
MR Test
Inverse variance weighted
MR Egger
Simple mode
Weighted median
Weighted mode
0.1
0.0
−0.1
−0.2
−0.3
0.02
0.04
0.06
0.08
0.10
SNP effect on GCST90200513_buildGRCh38.tsv
SNP effect on UBA2658 sp002841545

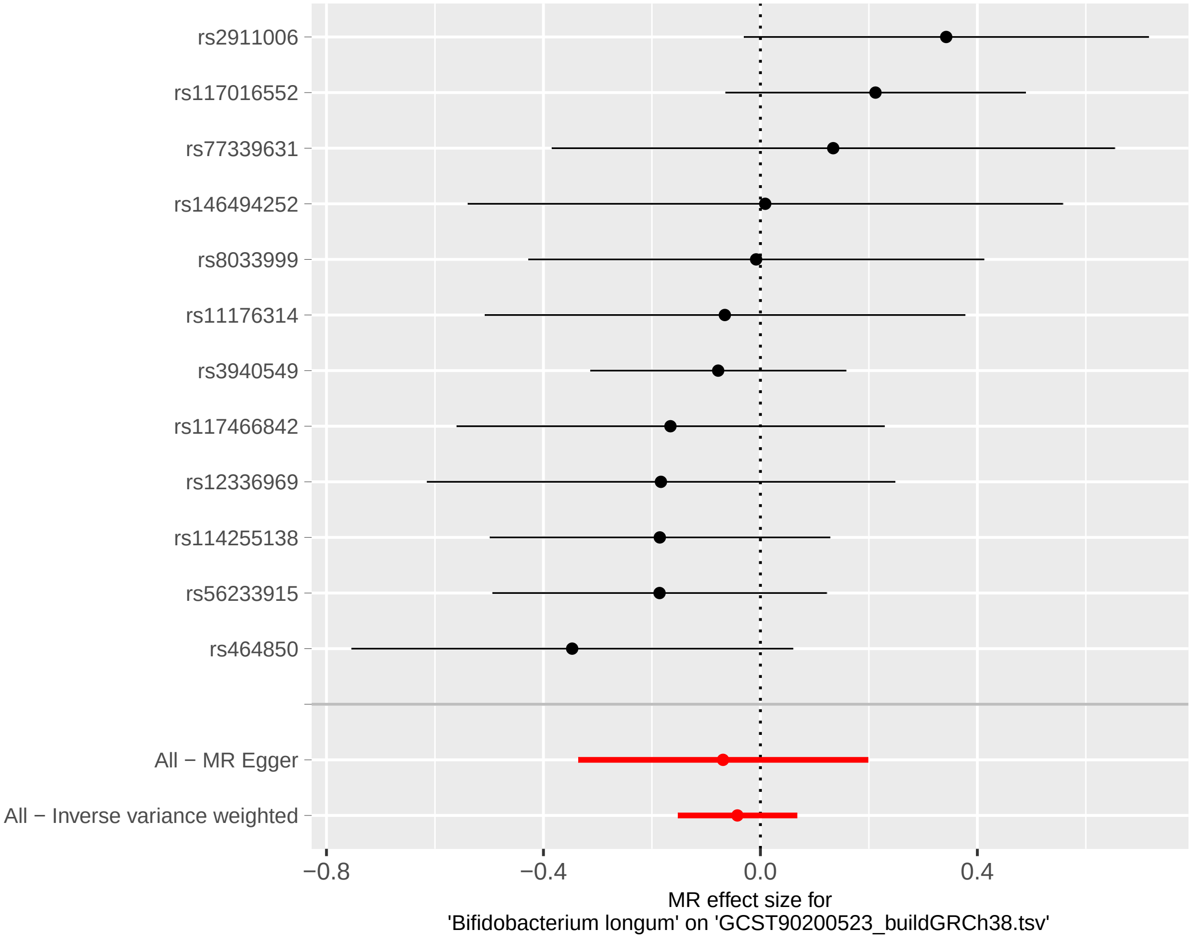

rs2911006
rs117016552
rs77339631
rs146494252
rs8033999
rs11176314
rs3940549
rs117466842
rs12336969
rs114255138
rs56233915
rs464850
All − MR Egger
All − Inverse variance weighted
−0.8
−0.4
0.0
0.4
MR effect size for
'Bifidobacterium longum' on 'GCST90200523_buildGRCh38.tsv'

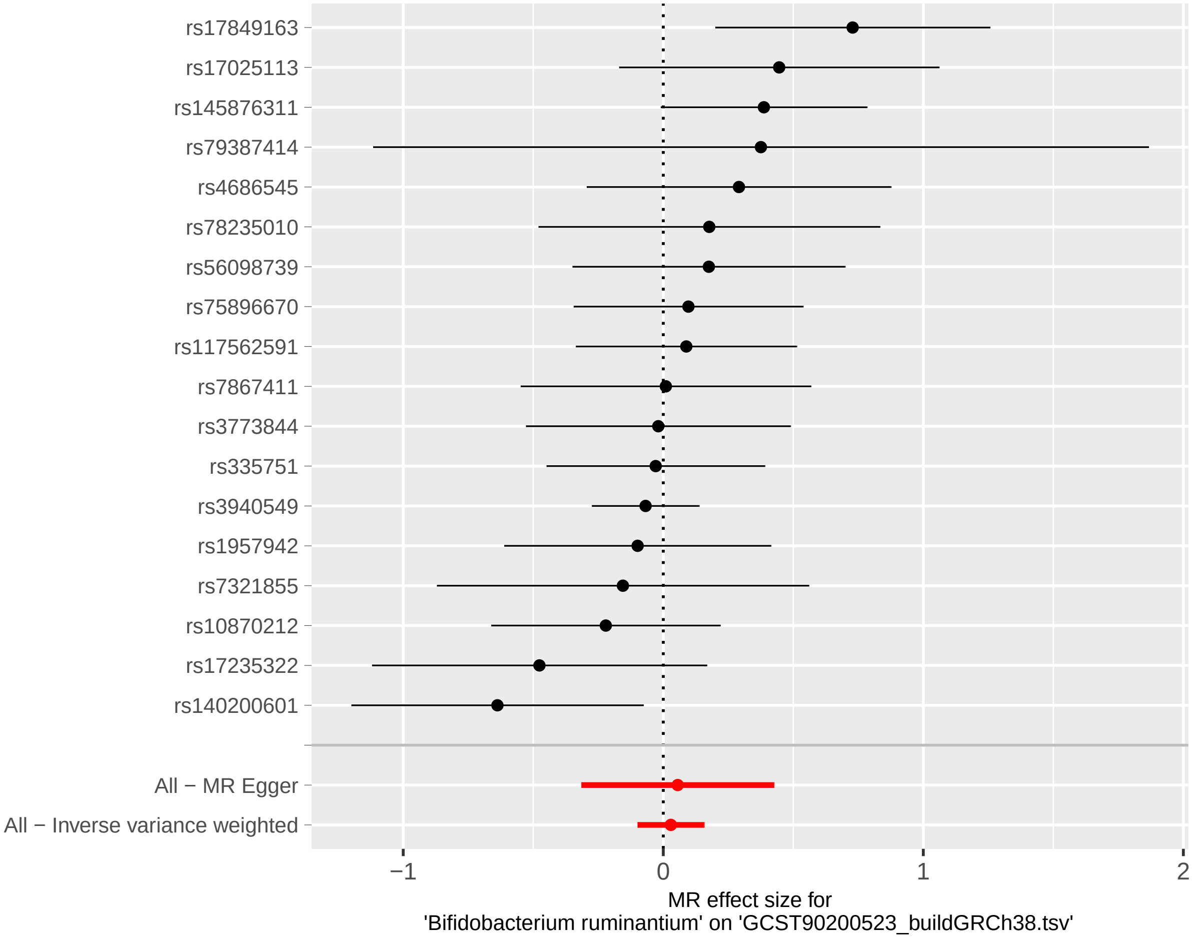
rs17849163
rs17025113
rs145876311
rs79387414
rs4686545
rs78235010
rs56098739
rs75896670
rs117562591
rs7867411
rs3773844
rs335751
rs3940549
rs1957942
rs7321855
rs10870212
rs17235322
rs140200601
All − MR Egger
All − Inverse variance weighted
−1
0
1
2
MR effect size for
'Bifidobacterium ruminantium' on 'GCST90200523_buildGRCh38.tsv'

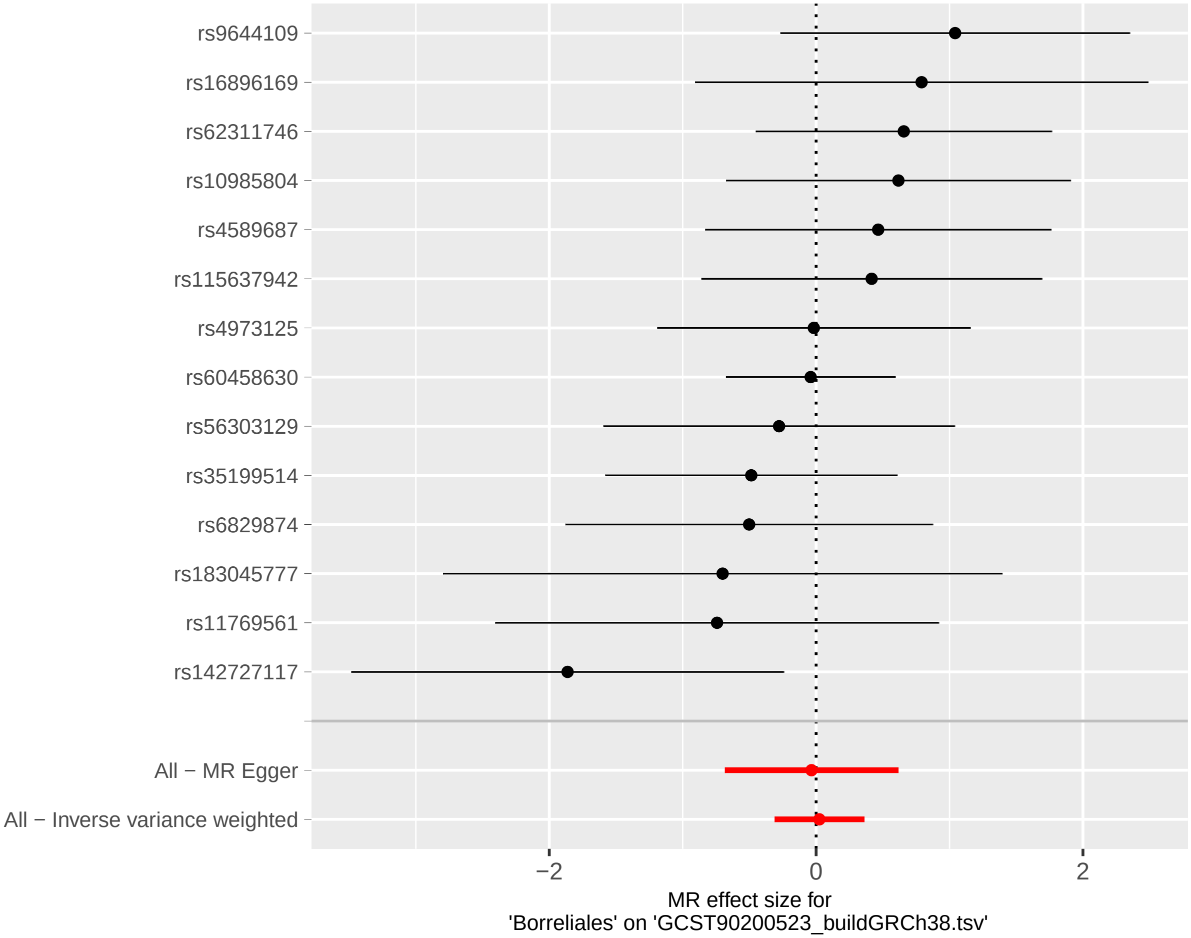

rs9644109
rs16896169
rs62311746
rs10985804
rs4589687
rs115637942
rs4973125
rs60458630
rs56303129
rs35199514
rs6829874
rs183045777
rs11769561
rs142727117
All − MR Egger
All − Inverse variance weighted
−2
0
2
MR effect size for
'Borreliales' on 'GCST90200523_buildGRCh38.tsv'

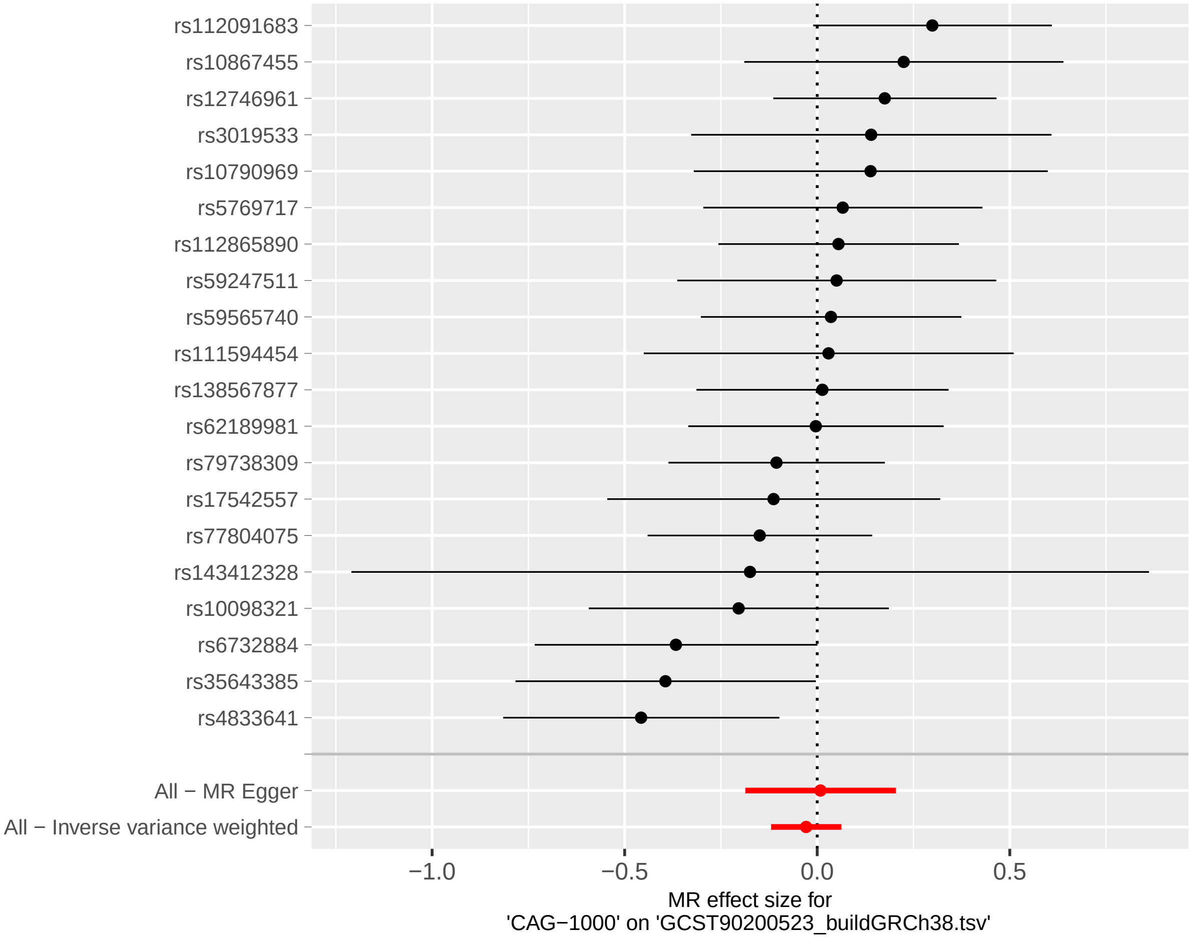

rs112091683
rs10867455
rs12746961
rs3019533
rs10790969
rs5769717
rs112865890
rs59247511
rs59565740
rs111594454
rs138567877
rs62189981
rs79738309
rs17542557
rs77804075
rs143412328
rs10098321
rs6732884
rs35643385
rs4833641
All − MR Egger
All − Inverse variance weighted
−1.0
−0.5
0.0
0.5
MR effect size for
'CAG−1000' on 'GCST90200523_buildGRCh38.tsv'

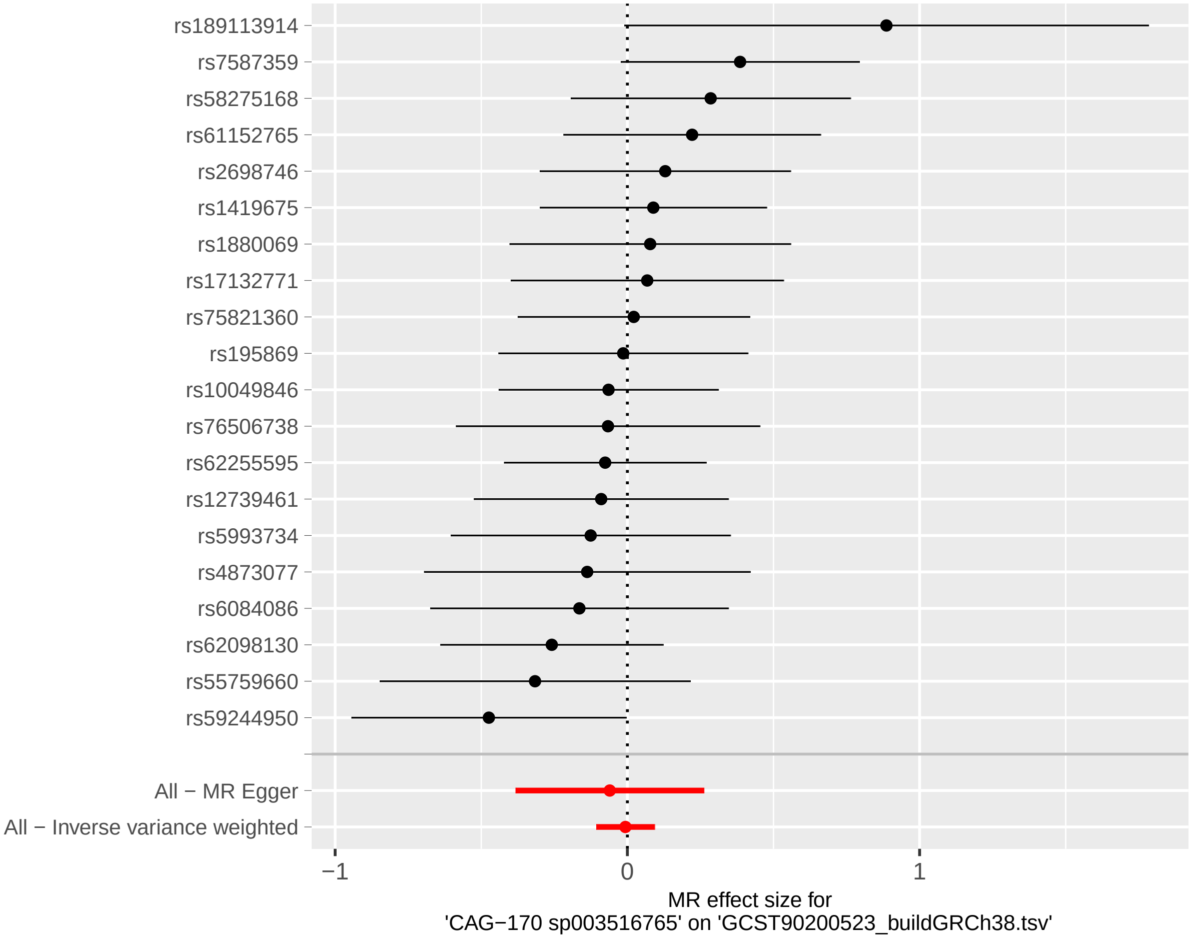

rs189113914
rs7587359
rs58275168
rs61152765
rs2698746
rs1419675
rs1880069
rs17132771
rs75821360
rs195869
rs10049846
rs76506738
rs62255595
rs12739461
rs5993734
rs4873077
rs6084086
rs62098130
rs55759660
rs59244950
All − MR Egger
All − Inverse variance weighted
−1
0
1
MR effect size for
'CAG−170 sp003516765' on 'GCST90200523_buildGRCh38.tsv'

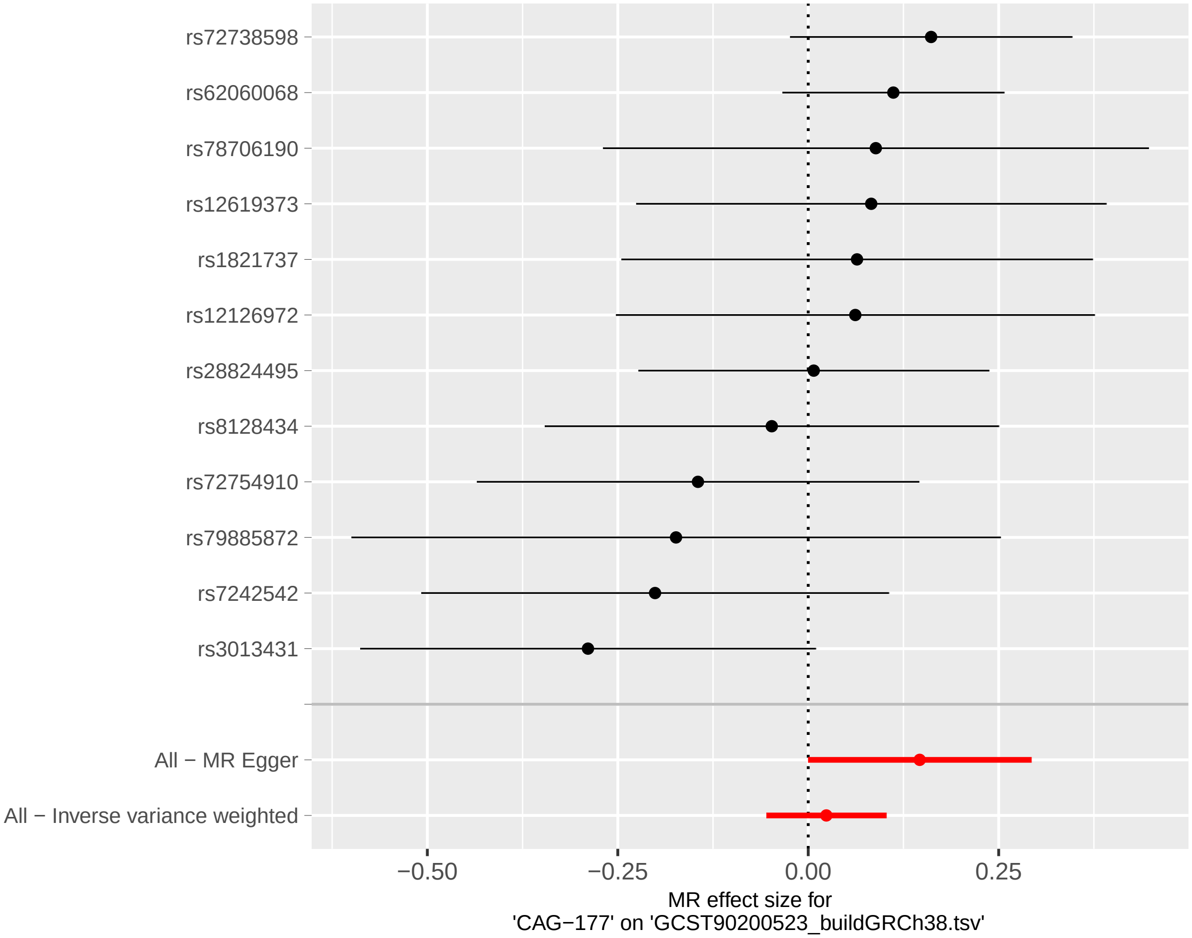
rs72738598
rs62060068
rs78706190
rs12619373
rs1821737
rs12126972
rs28824495
rs8128434
rs72754910
rs79885872
rs7242542
rs3013431
All − MR Egger
All − Inverse variance weighted
−0.50
−0.25
0.00
0.25
MR effect size for
'CAG−177' on 'GCST90200523_buildGRCh38.tsv'

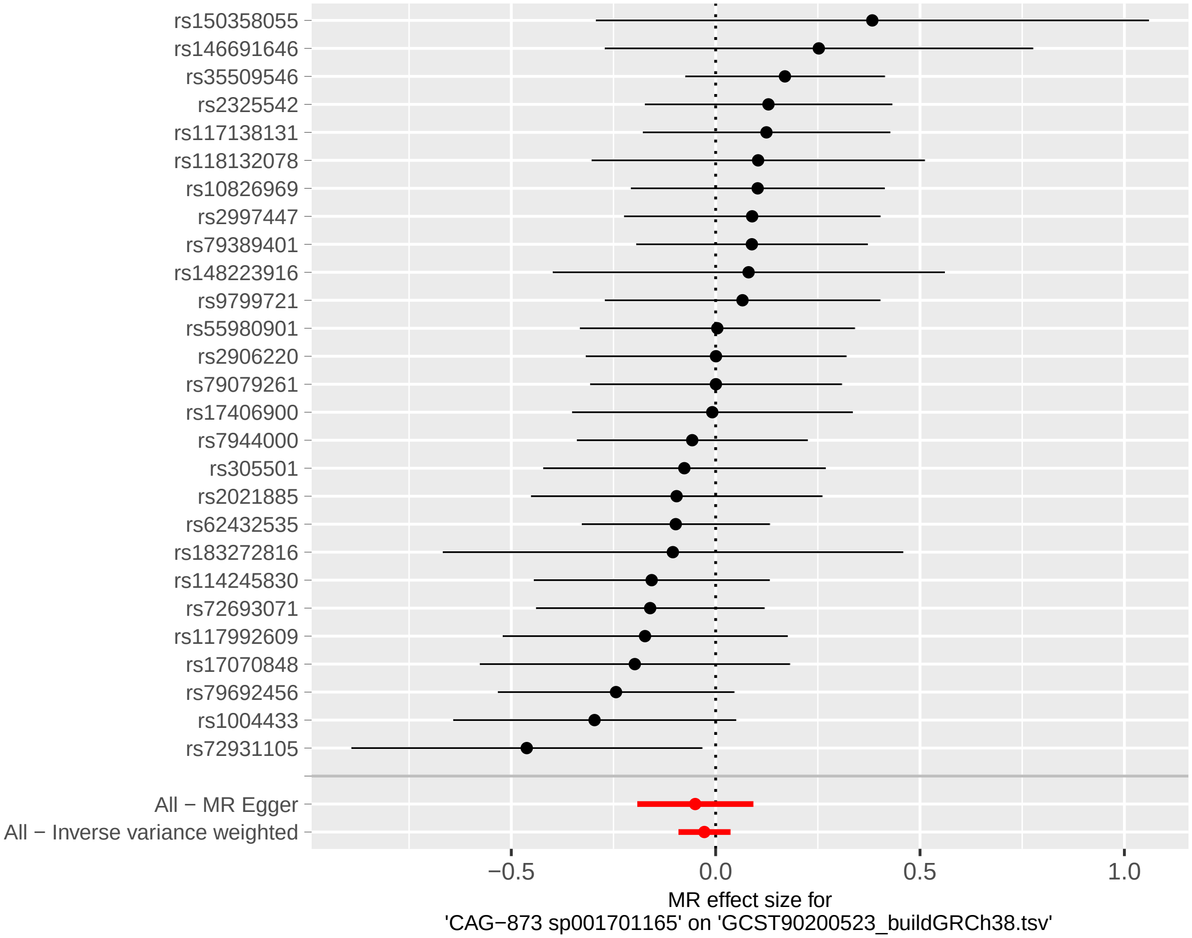
rs150358055
rs146691646
rs35509546
rs2325542
rs117138131
rs118132078
rs10826969
rs2997447
rs79389401
rs148223916
rs9799721
rs55980901
rs2906220
rs79079261
rs17406900
rs7944000
rs305501
rs2021885
rs62432535
rs183272816
rs114245830
rs72693071
rs117992609
rs17070848
rs79692456
rs1004433
rs72931105
All − MR Egger
All − Inverse variance weighted
−0.5
0.0
0.5
1.0
MR effect size for
'CAG−873 sp001701165' on 'GCST90200523_buildGRCh38.tsv'

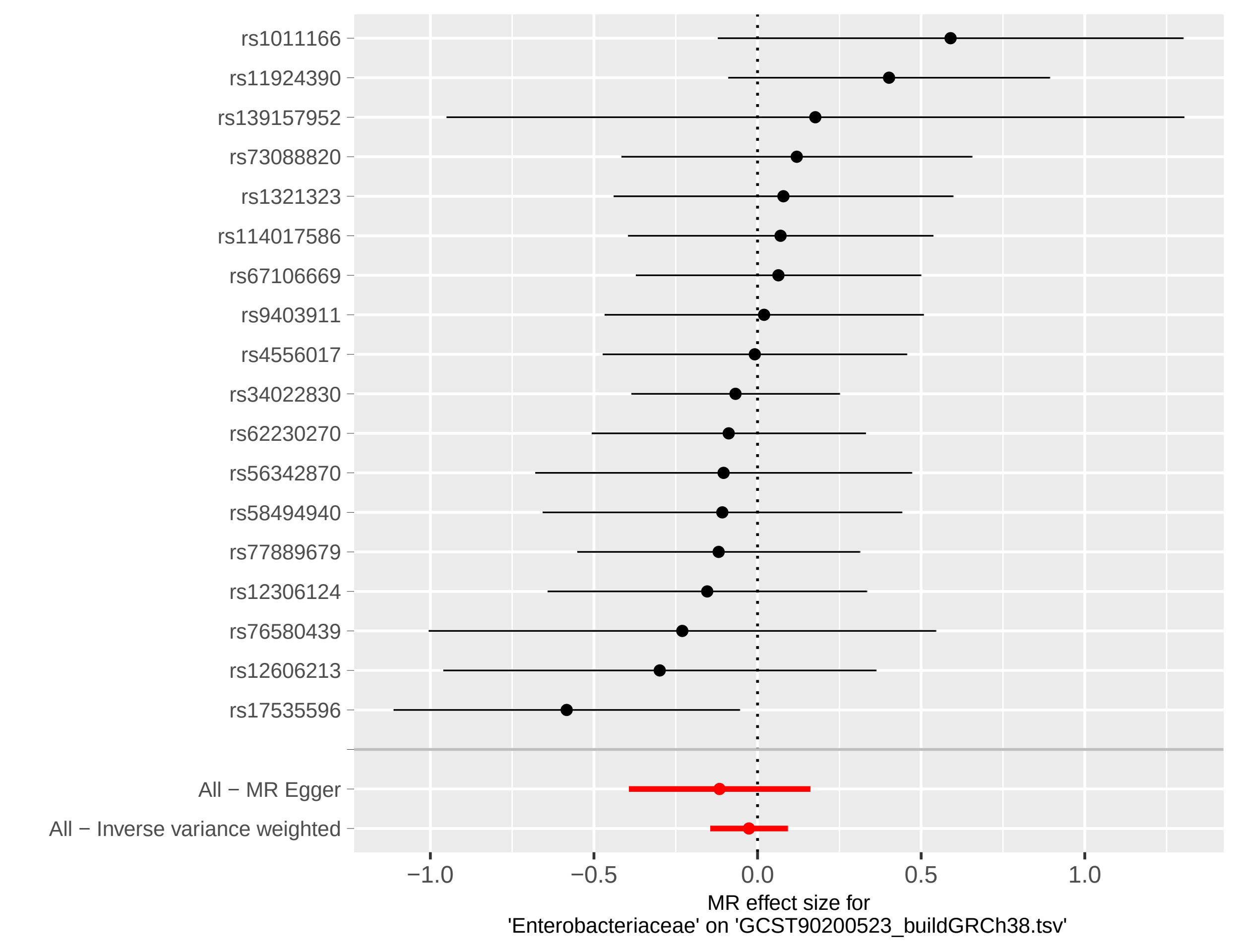
rs1011166
rs11924390
rs139157952
rs73088820
rs1321323
rs114017586
rs67106669
rs9403911
rs4556017
rs34022830
rs62230270
rs56342870
rs58494940
rs77889679
rs12306124
rs76580439
rs12606213
rs17535596
All − MR Egger
All − Inverse variance weighted
−1.0
−0.5
0.0
0.5
1.0
MR effect size for
'Enterobacteriaceae' on 'GCST90200523_buildGRCh38.tsv'

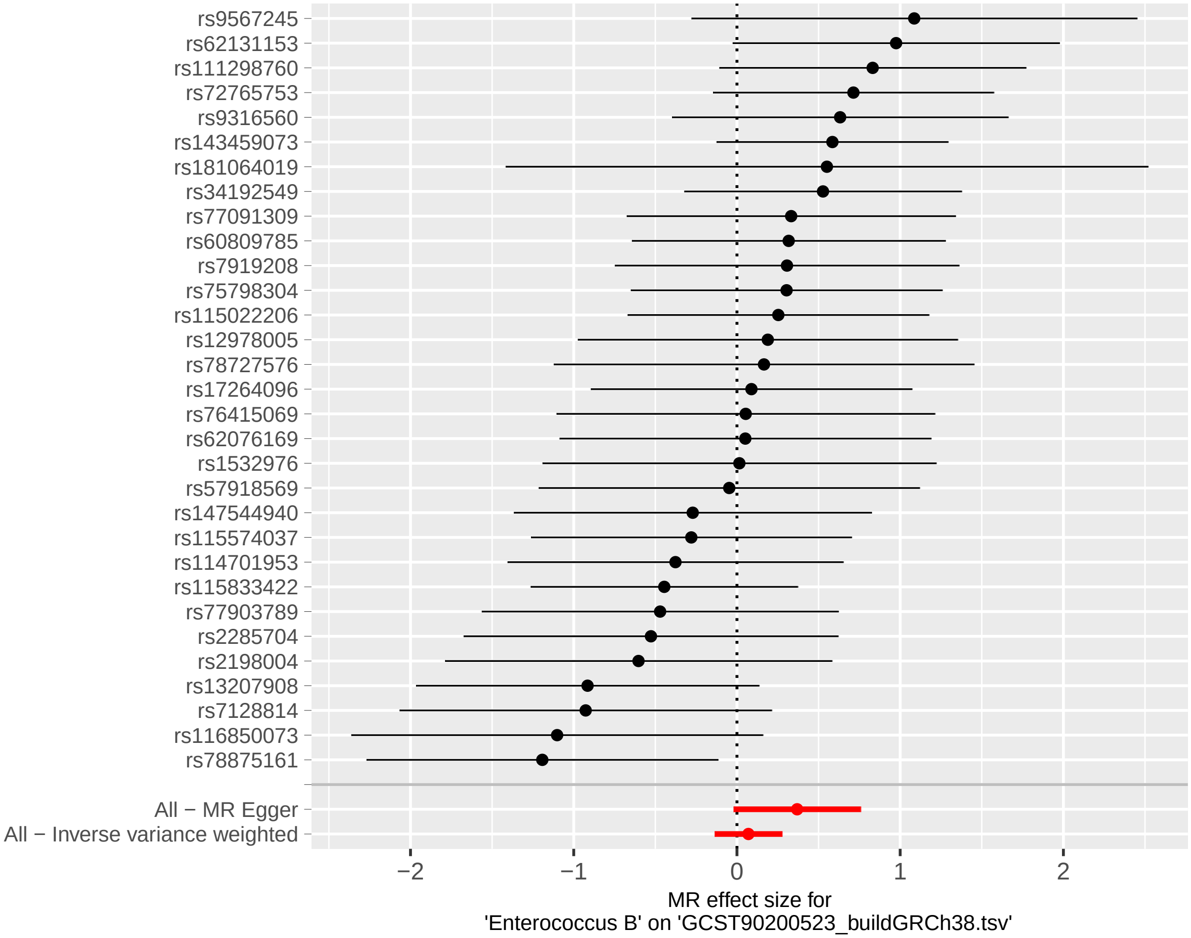
rs9567245
rs62131153
rs111298760
rs72765753
rs9316560
rs143459073
rs181064019
rs34192549
rs77091309
rs60809785
rs7919208
rs75798304
rs115022206
rs12978005
rs78727576
rs17264096
rs76415069
rs62076169
rs1532976
rs57918569
rs147544940
rs115574037
rs114701953
rs115833422
rs77903789
rs2285704
rs2198004
rs13207908
rs7128814
rs116850073
rs78875161
All − MR Egger
All − Inverse variance weighted
−2
−1
0
1
2
MR effect size for
'Enterococcus B' on 'GCST90200523_buildGRCh38.tsv'

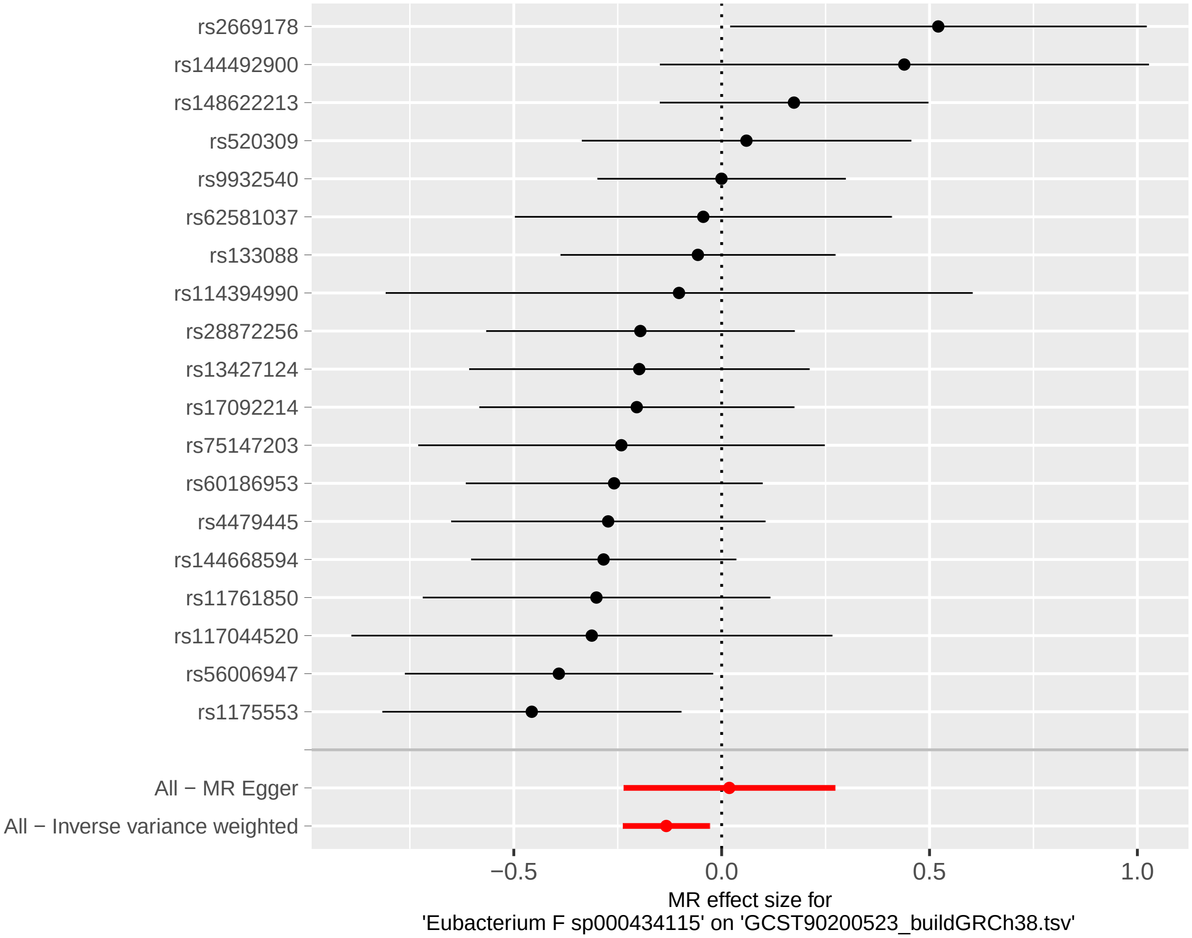
rs2669178
rs144492900
rs148622213
rs520309
rs9932540
rs62581037
rs133088
rs114394990
rs28872256
rs13427124
rs17092214
rs75147203
rs60186953
rs4479445
rs144668594
rs11761850
rs117044520
rs56006947
rs1175553
All − MR Egger
All − Inverse variance weighted
−0.5
0.0
0.5
1.0
MR effect size for
'Eubacterium F sp000434115' on 'GCST90200523_buildGRCh38.tsv'

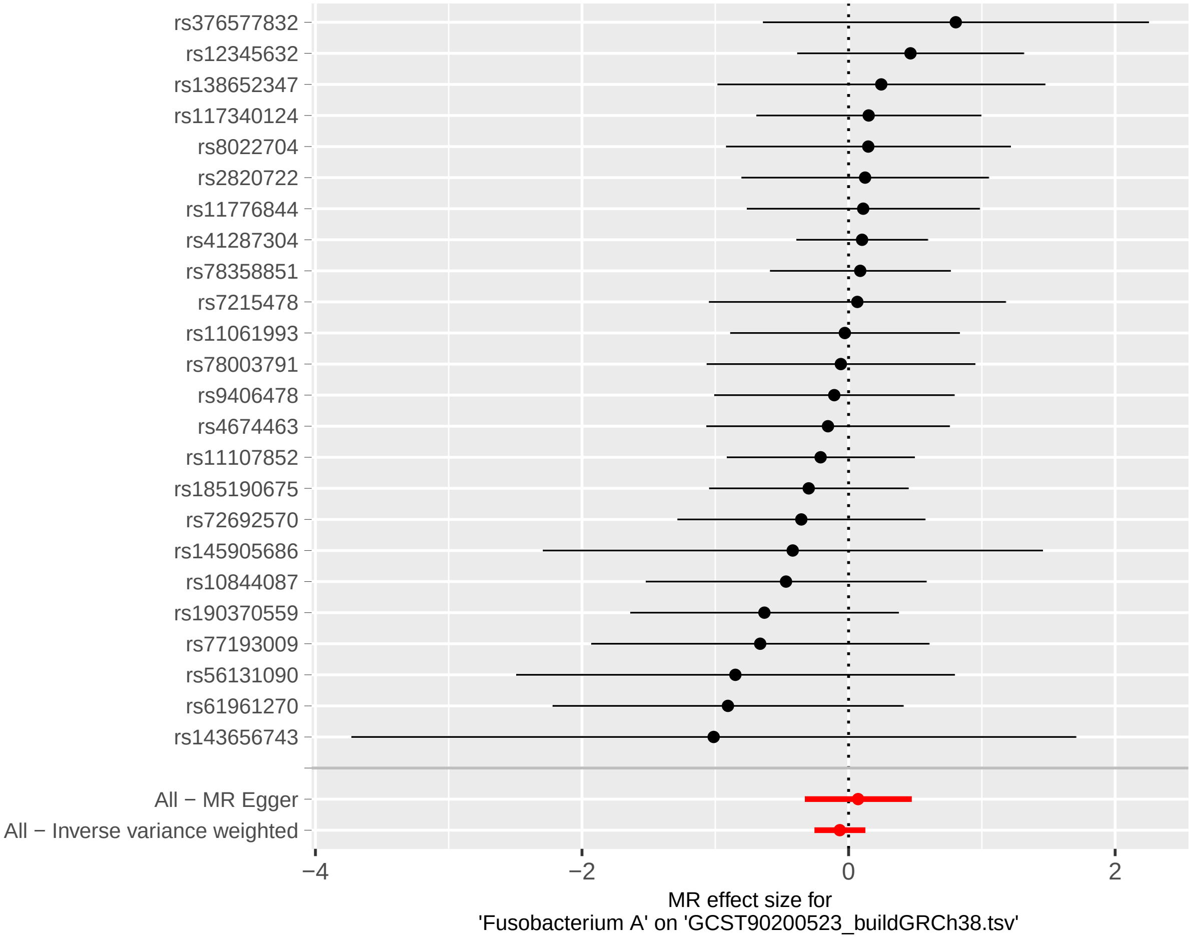

rs376577832
rs12345632
rs138652347
rs117340124
rs8022704
rs2820722
rs11776844
rs41287304
rs78358851
rs7215478
rs11061993
rs78003791
rs9406478
rs4674463
rs11107852
rs185190675
rs72692570
rs145905686
rs10844087
rs190370559
rs77193009
rs56131090
rs61961270
rs143656743
All − MR Egger
All − Inverse variance weighted
−4
−2
0
2
MR effect size for
'Fusobacterium A' on 'GCST90200523_buildGRCh38.tsv'

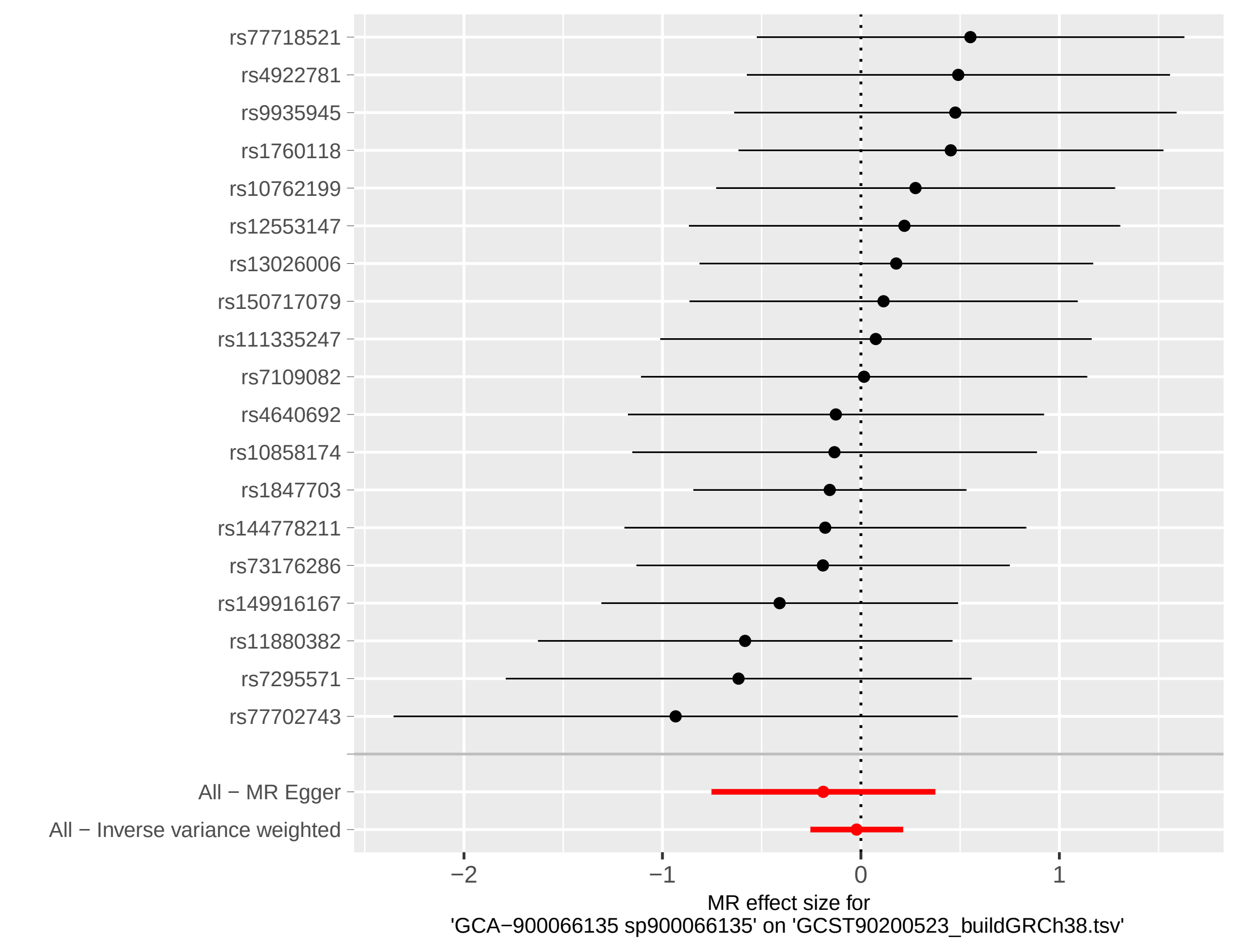
rs77718521
rs4922781
rs9935945
rs1760118
rs10762199
rs12553147
rs13026006
rs150717079
rs111335247
rs7109082
rs4640692
rs10858174
rs1847703
rs144778211
rs73176286
rs149916167
rs11880382
rs7295571
rs77702743
All − MR Egger
All − Inverse variance weighted
−2
−1
0
1
MR effect size for
'GCA−900066135 sp900066135' on 'GCST90200523_buildGRCh38.tsv'

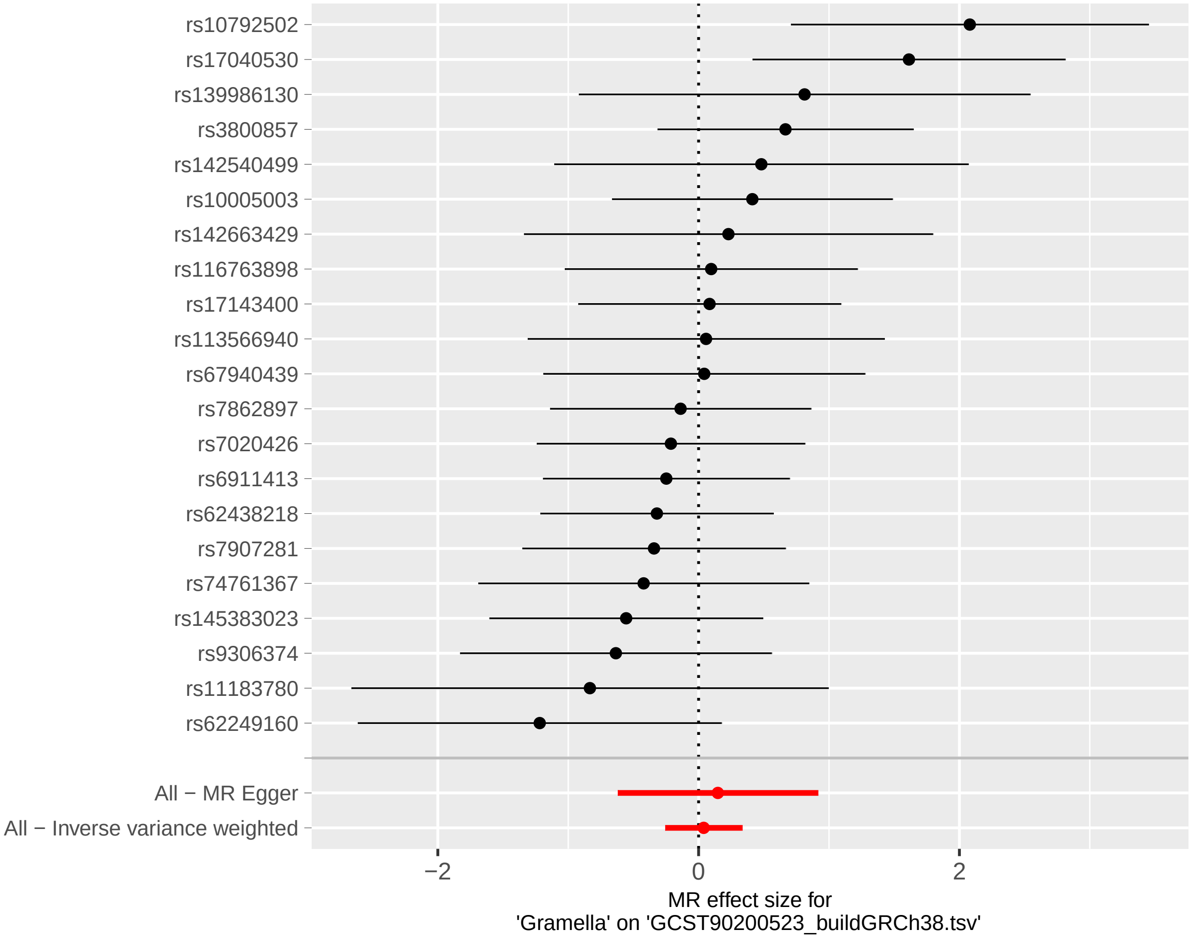

rs10792502
rs17040530
rs139986130
rs3800857
rs142540499
rs10005003
rs142663429
rs116763898
rs17143400
rs113566940
rs67940439
rs7862897
rs7020426
rs6911413
rs62438218
rs7907281
rs74761367
rs145383023
rs9306374
rs11183780
rs62249160
All − MR Egger
All − Inverse variance weighted
−2
0
2
MR effect size for
'Gramella' on 'GCST90200523_buildGRCh38.tsv'

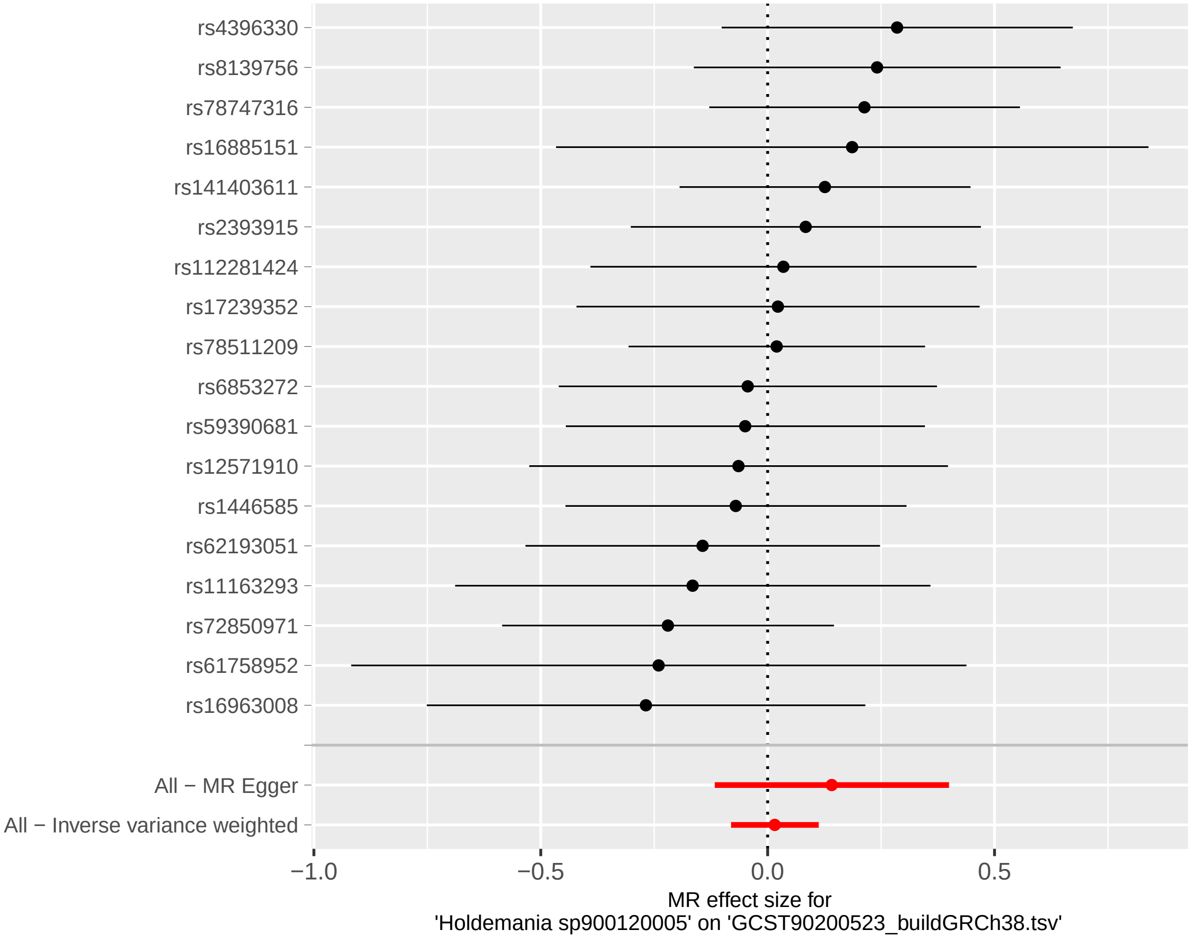
rs4396330
rs8139756
rs78747316
rs16885151
rs141403611
rs2393915
rs112281424
rs17239352
rs78511209
rs6853272
rs59390681
rs12571910
rs1446585
rs62193051
rs11163293
rs72850971
rs61758952
rs16963008
All − MR Egger
All − Inverse variance weighted
−1.0
−0.5
0.0
0.5
MR effect size for
'Holdemania sp900120005' on 'GCST90200523_buildGRCh38.tsv'

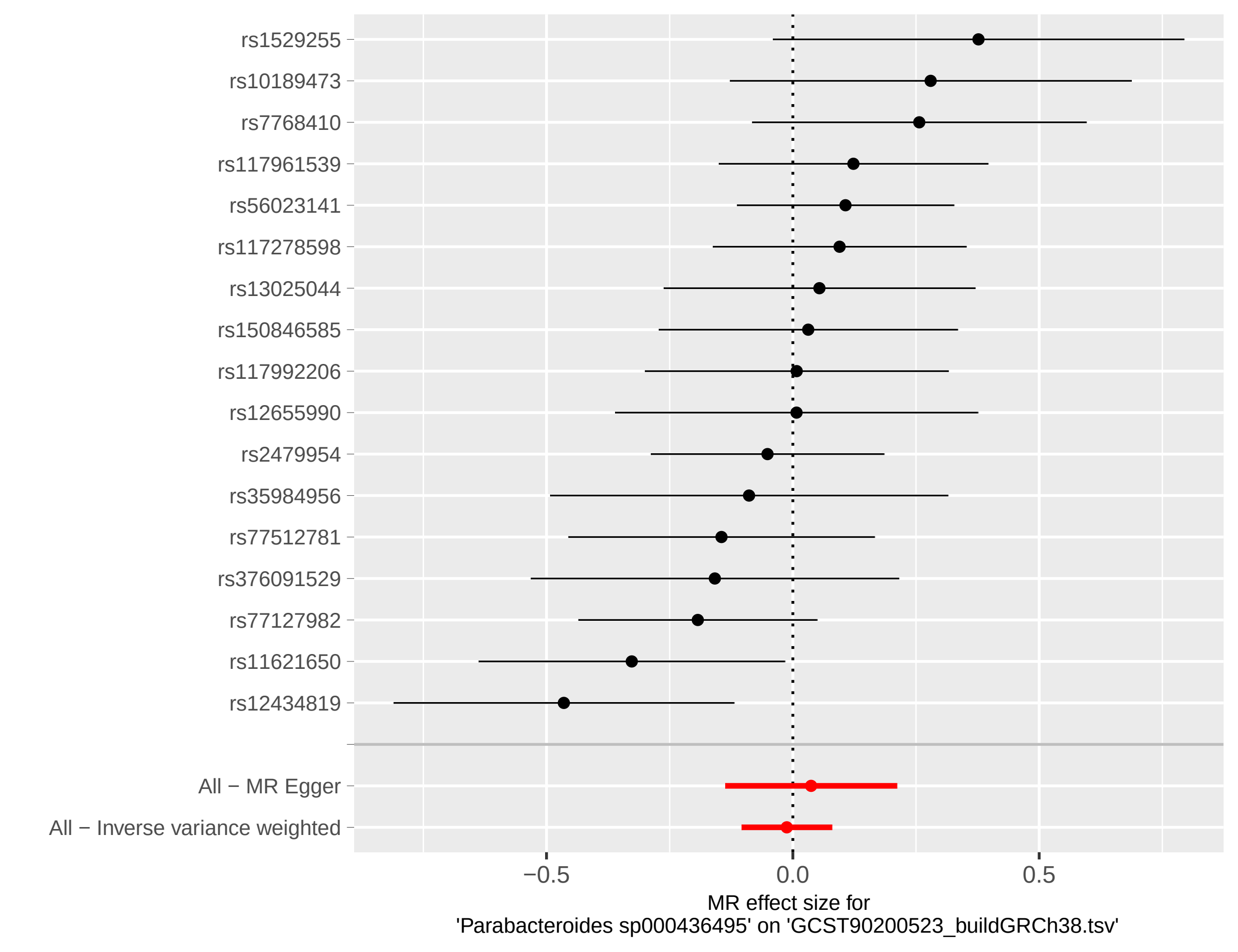
rs1529255
rs10189473
rs7768410
rs117961539
rs56023141
rs117278598
rs13025044
rs150846585
rs117992206
rs12655990
rs2479954
rs35984956
rs77512781
rs376091529
rs77127982
rs11621650
rs12434819
All − MR Egger
All − Inverse variance weighted
−0.5
0.0
0.5
MR effect size for
'Parabacteroides sp000436495' on 'GCST90200523_buildGRCh38.tsv'

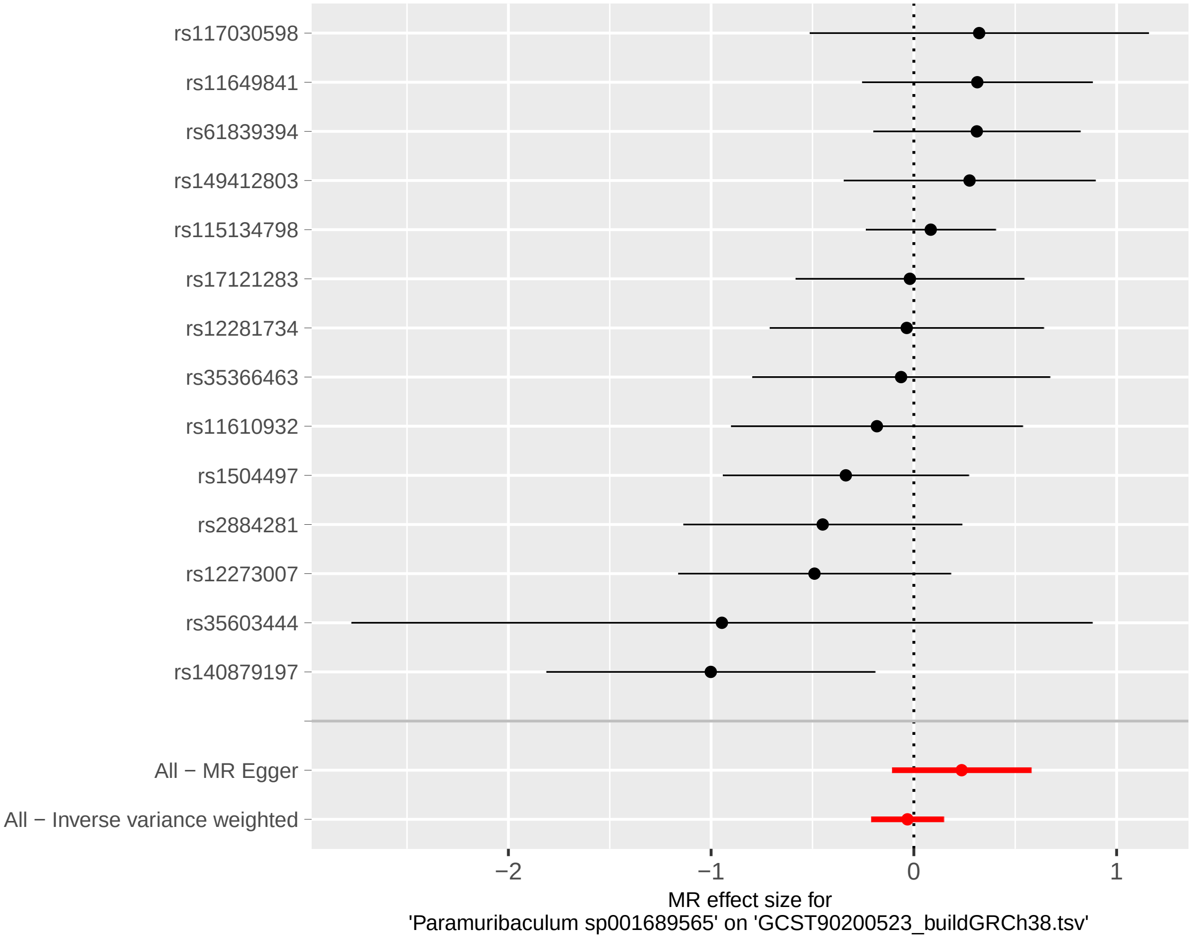
rs117030598
rs11649841
rs61839394
rs149412803
rs115134798
rs17121283
rs12281734
rs35366463
rs11610932
rs1504497
rs2884281
rs12273007
rs35603444
rs140879197
All − MR Egger
All − Inverse variance weighted
−2
−1
0
1
MR effect size for
'Paramuribaculum sp001689565' on 'GCST90200523_buildGRCh38.tsv'

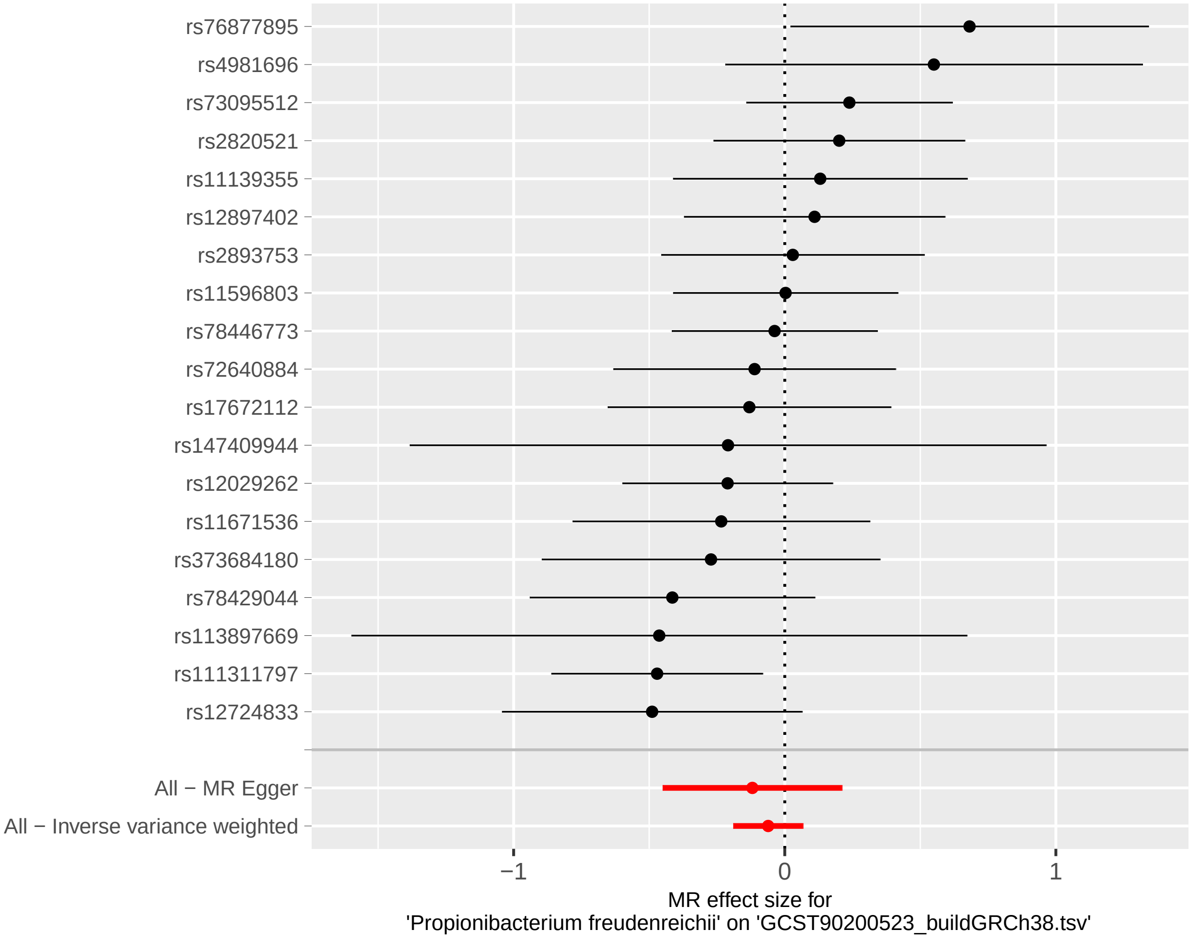

rs76877895
rs4981696
rs73095512
rs2820521
rs11139355
rs12897402
rs2893753
rs11596803
rs78446773
rs72640884
rs17672112
rs147409944
rs12029262
rs11671536
rs373684180
rs78429044
rs113897669
rs111311797
rs12724833
All − MR Egger
All − Inverse variance weighted
−1
0
1
MR effect size for
'Propionibacterium freudenreichii' on 'GCST90200523_buildGRCh38.tsv'

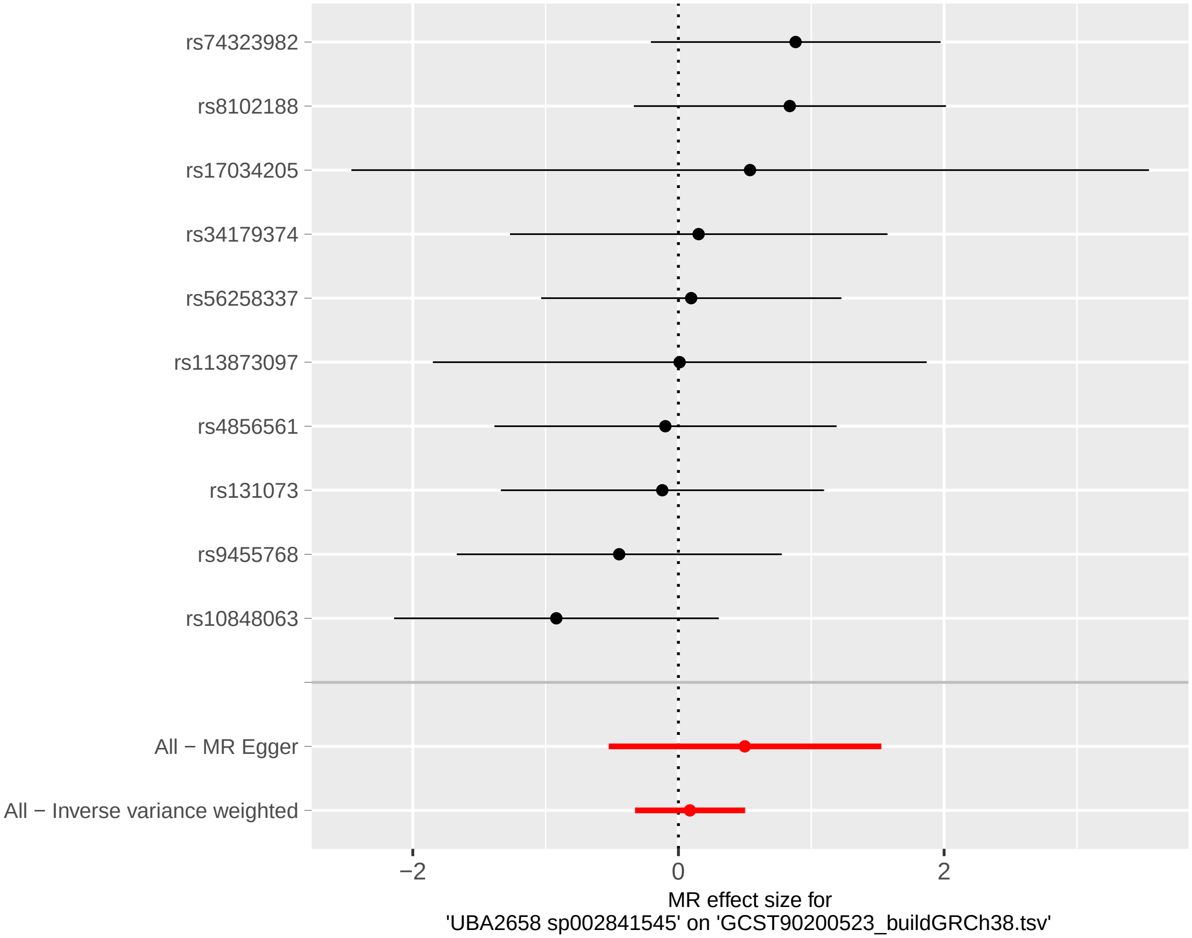
rs74323982
rs8102188
rs17034205
rs34179374
rs56258337
rs113873097
rs4856561
rs131073
rs9455768
rs10848063
All − MR Egger
All − Inverse variance weighted
−2
0
2
MR effect size for
'UBA2658 sp002841545' on 'GCST90200523_buildGRCh38.tsv'

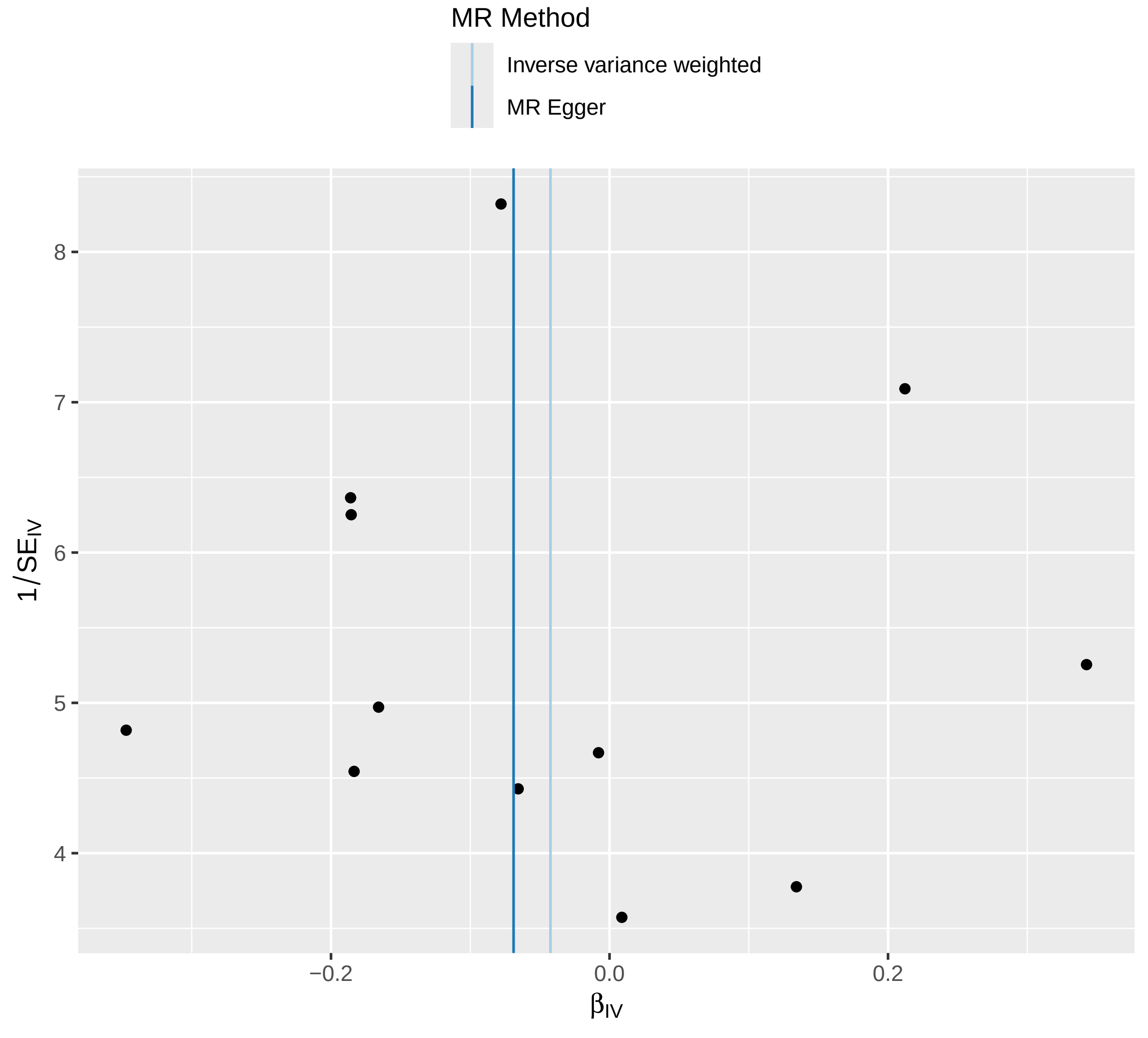

MR Method
Inverse variance weighted
MR Egger
8
7
6
5
4
1/SE_IV
−0.2
0.0
0.2
β_IV

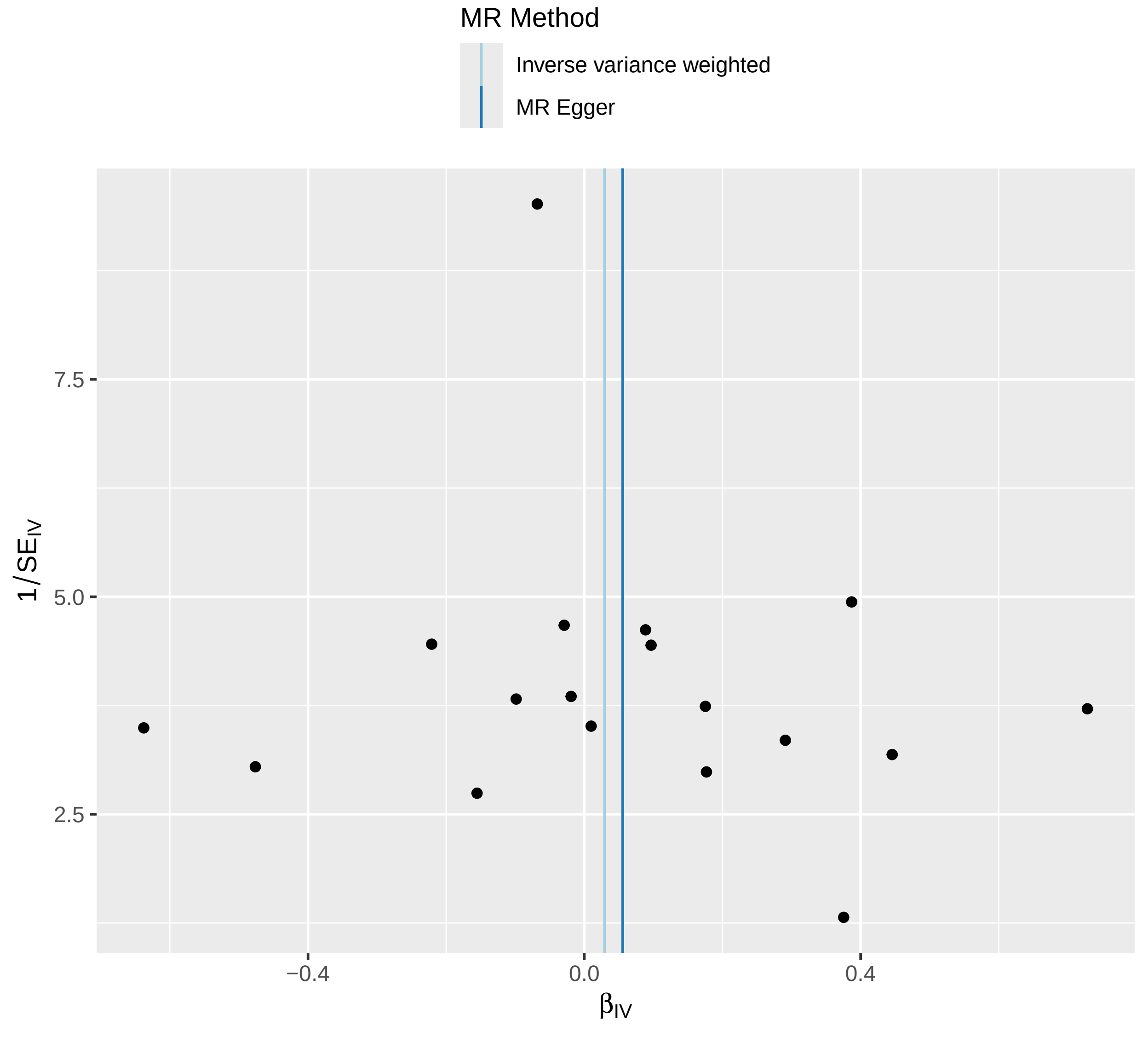

MR Method
Inverse variance weighted
MR Egger
7.5
5.0
2.5
1/SE_IV
−0.4
0.0
0.4
β_IV

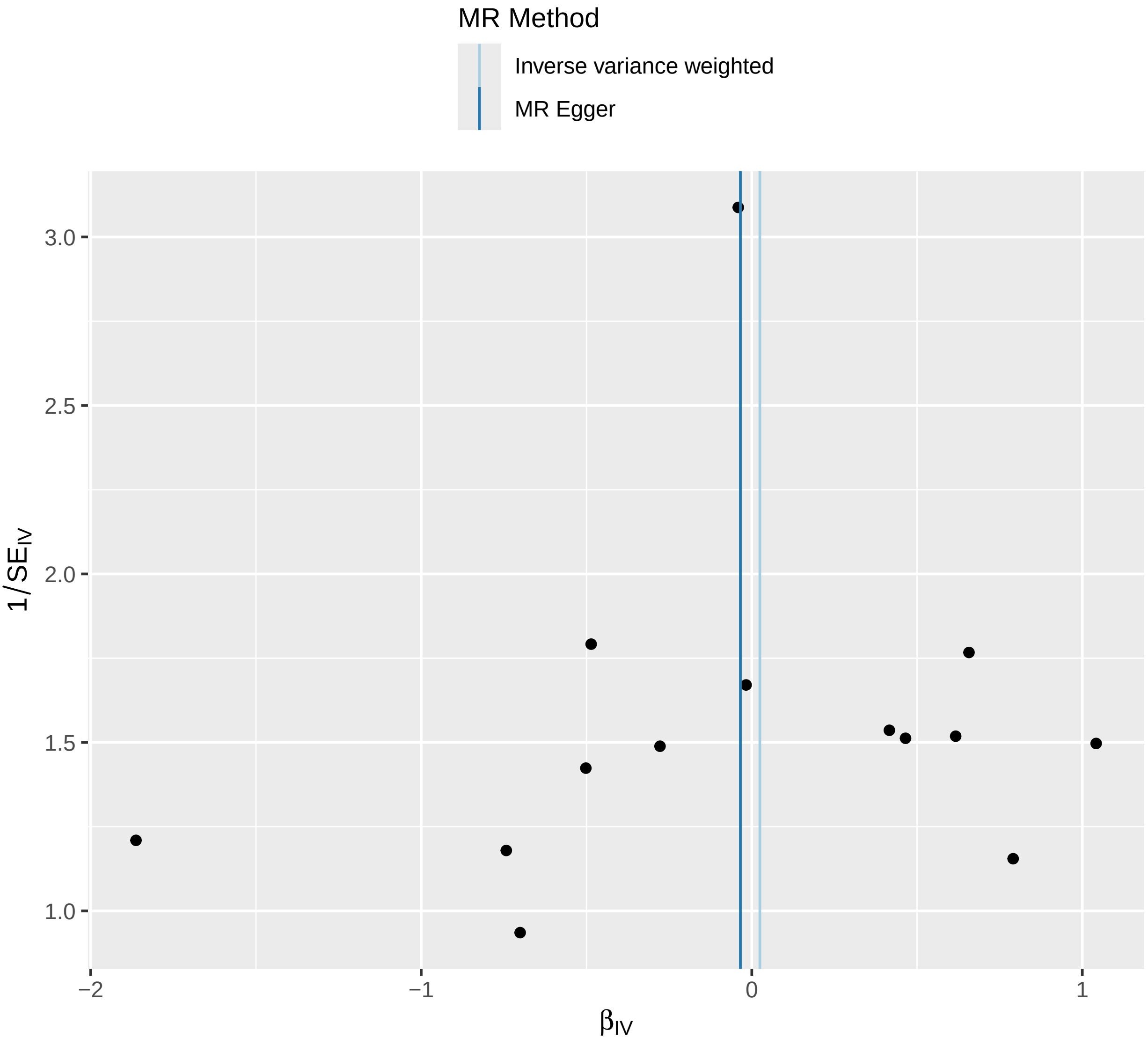
MR Method
Inverse variance weighted
MR Egger
3.0
2.5
2.0
1.5
1.0
$1/SE_{IV}$
−2
−1
0
1
β_{IV}

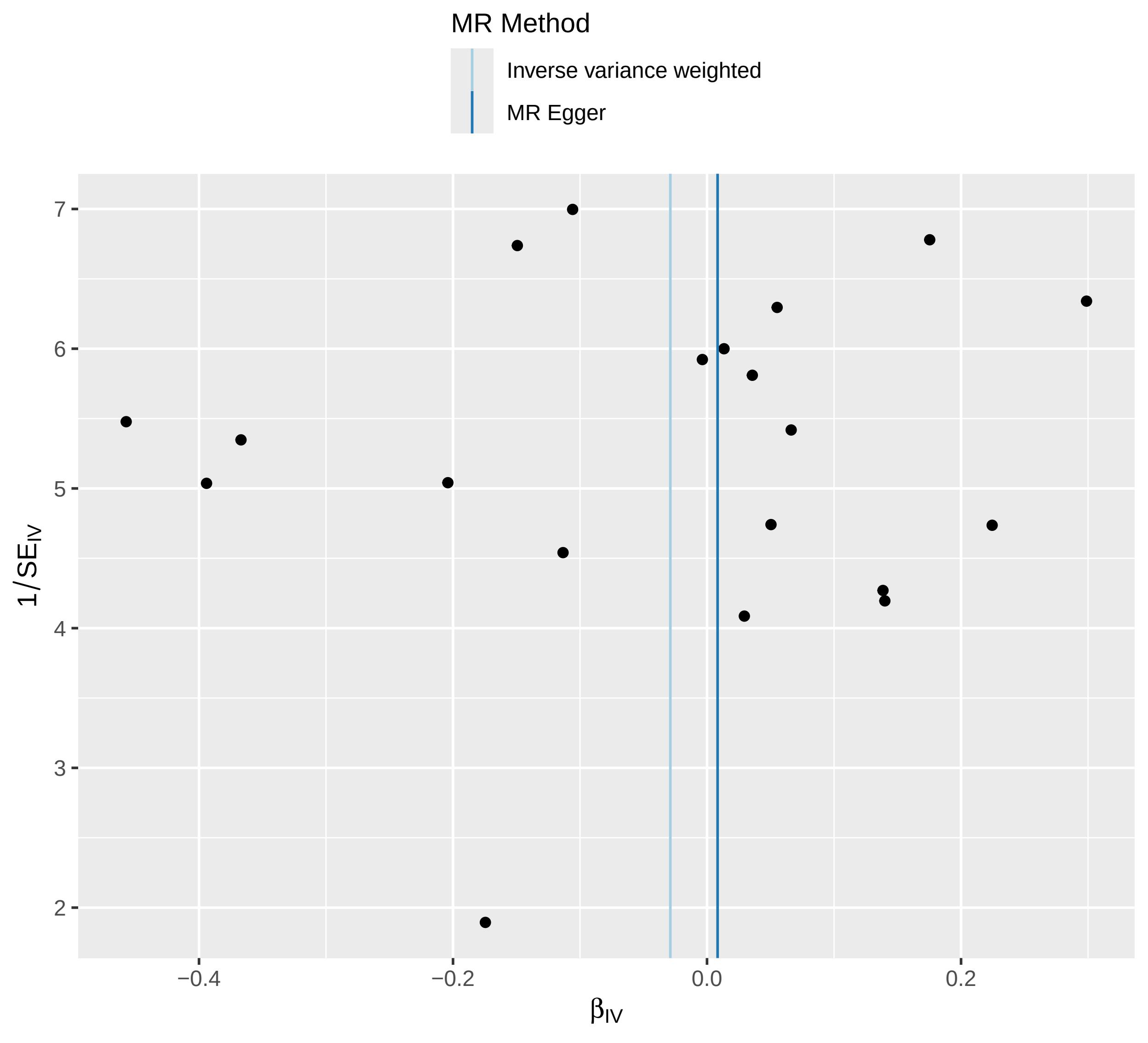
MR Method
Inverse variance weighted
MR Egger
7
6
5
4
3
2
$1/SE_{IV}$
−0.4
−0.2
0.0
0.2
β_{IV}

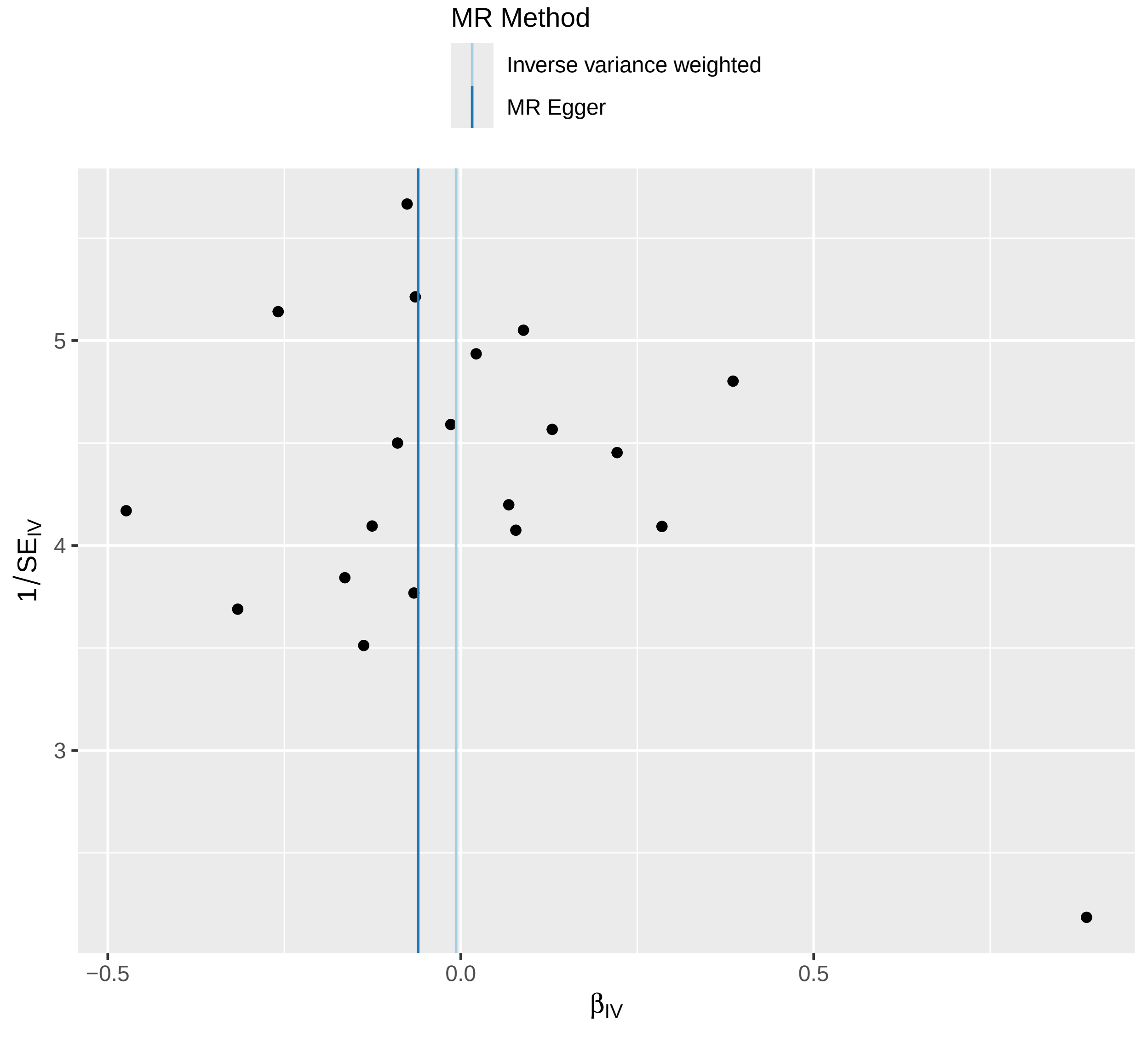
MR Method
Inverse variance weighted
MR Egger
5
4
3
1/SE$_{IV}$
−0.5
0.0
0.5
β$_{IV}$

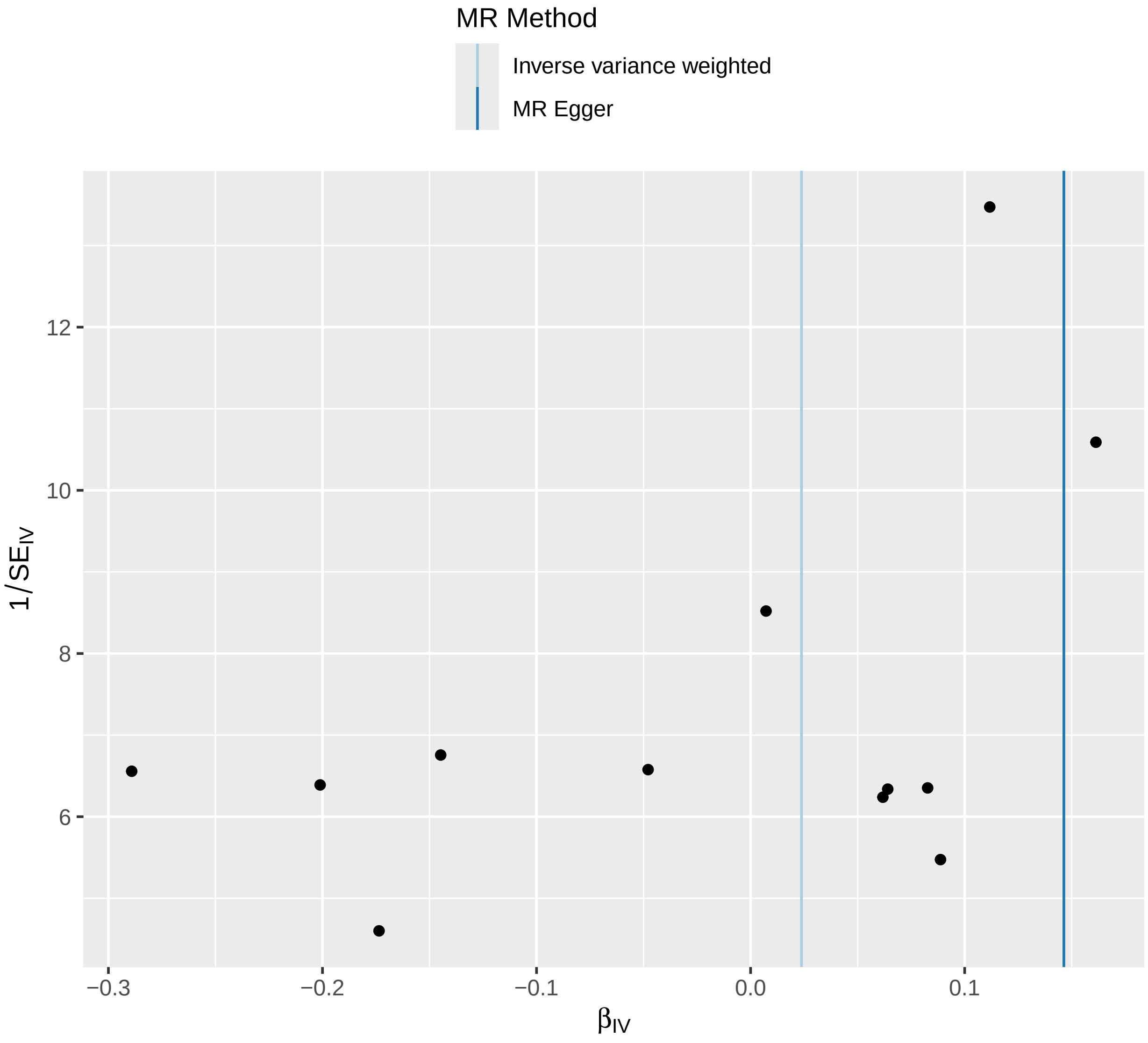
MR Method
Inverse variance weighted
MR Egger
1/SE_IV
12
10
8
6
−0.3
−0.2
−0.1
0.0
0.1
β_IV

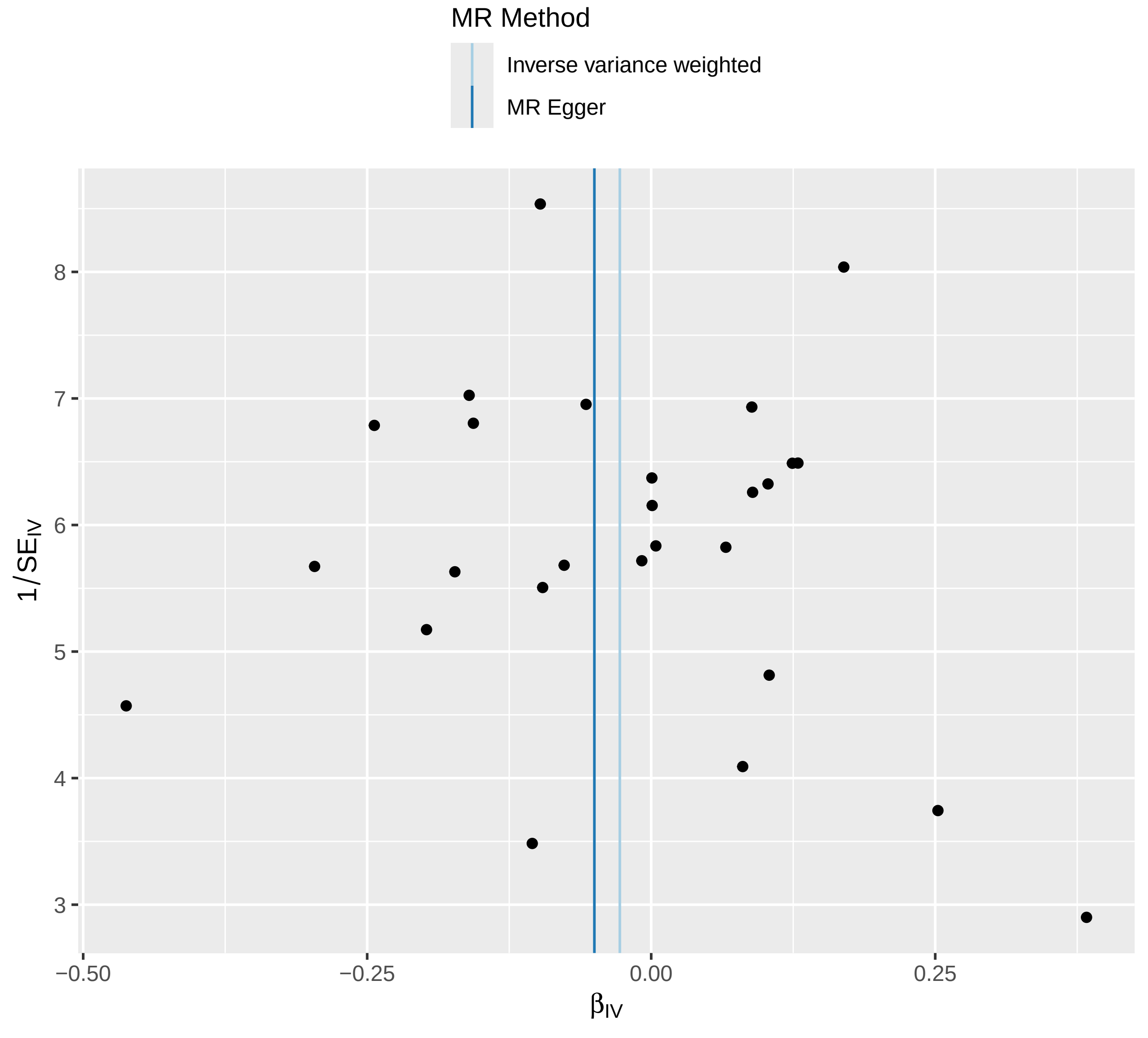

MR Method
Inverse variance weighted
MR Egger
8
7
6
5
4
3
1/SE_IV
−0.50
−0.25
0.00
0.25
β_IV

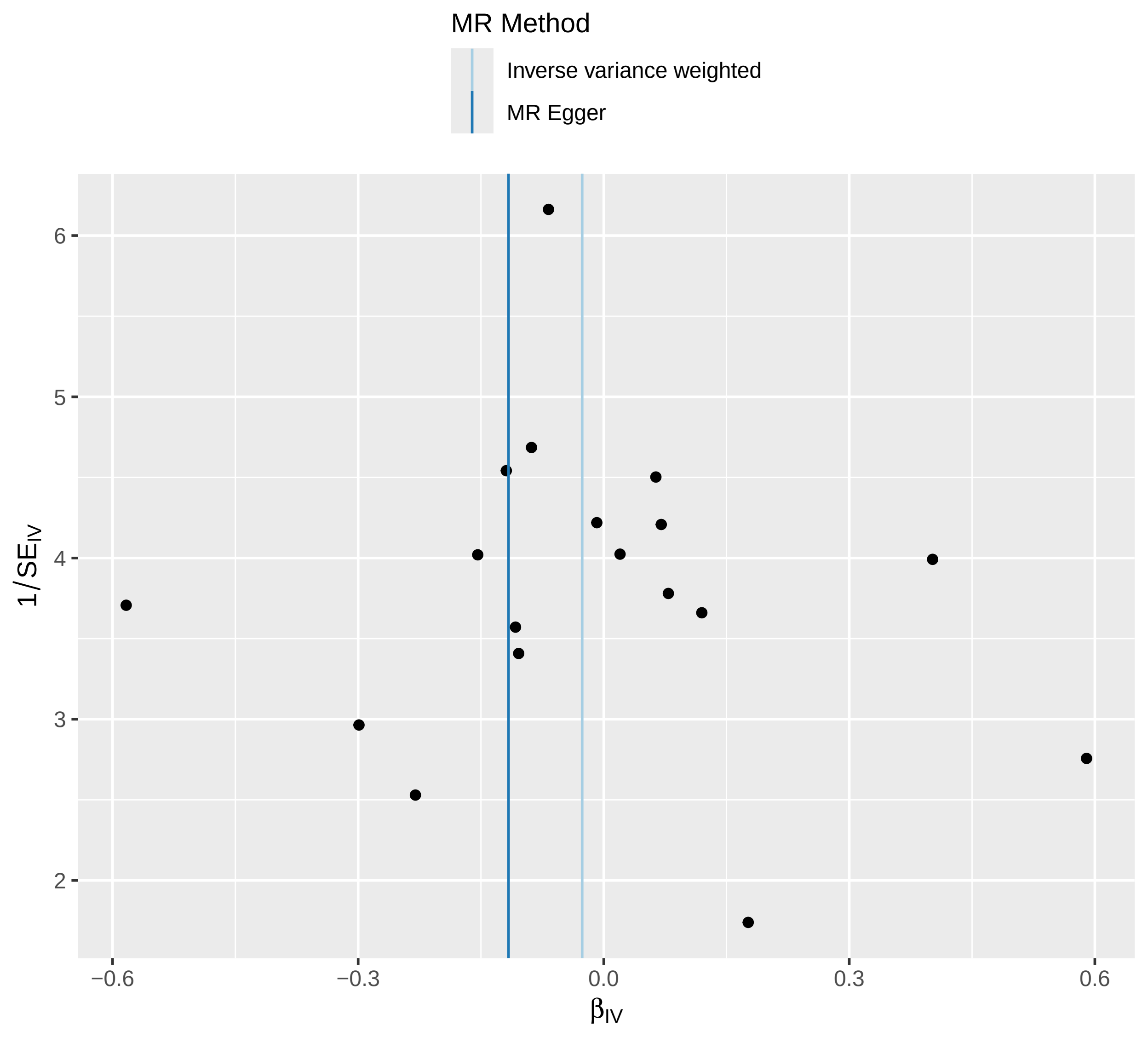
MR Method
Inverse variance weighted
MR Egger
6
5
4
3
2
1/SE_IV
−0.6
−0.3
0.0
0.3
0.6
β_IV

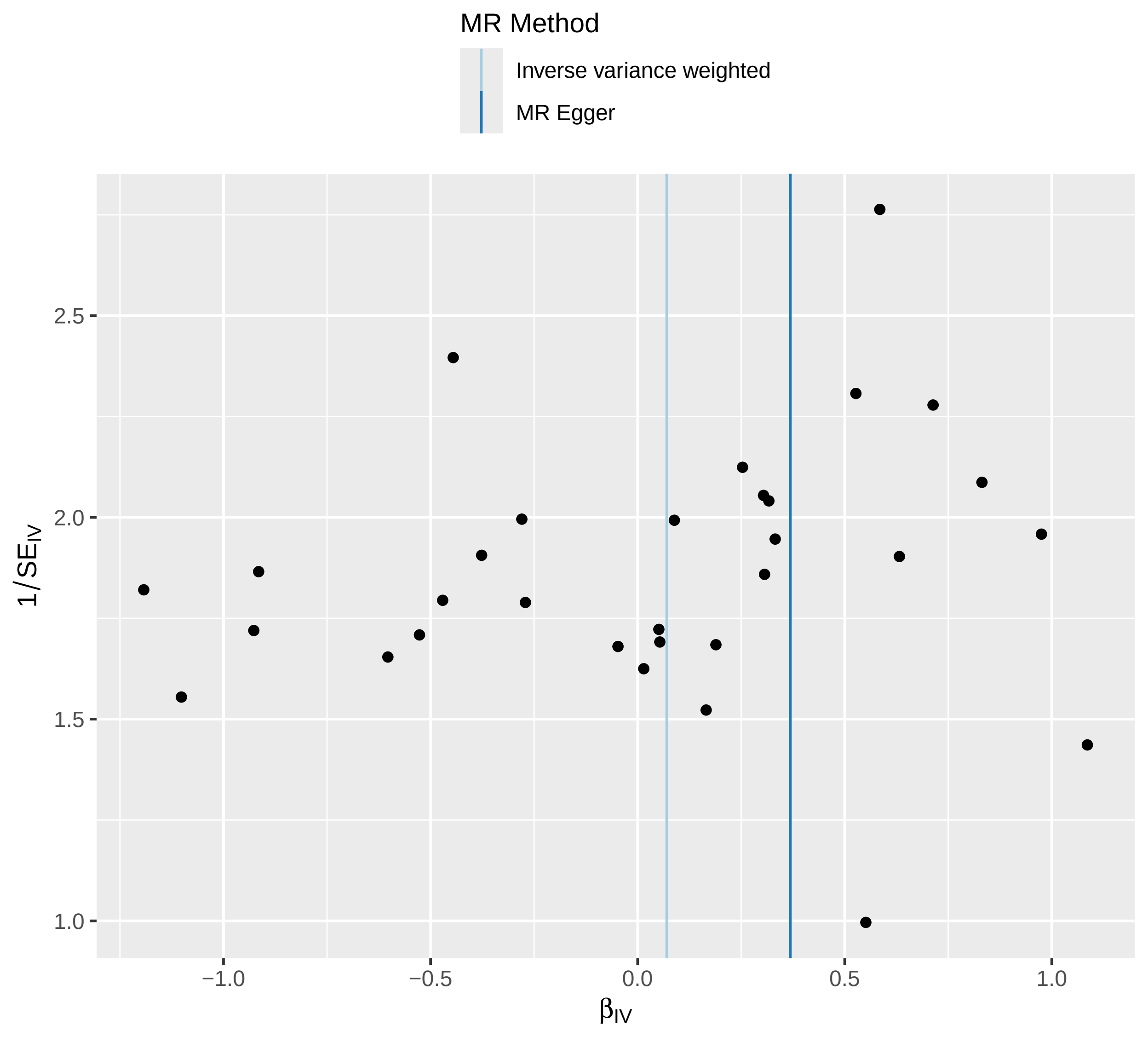
MR Method
Inverse variance weighted
MR Egger
2.5
2.0
1.5
1.0
1/SE_IV
−1.0
−0.5
0.0
0.5
1.0
β_IV

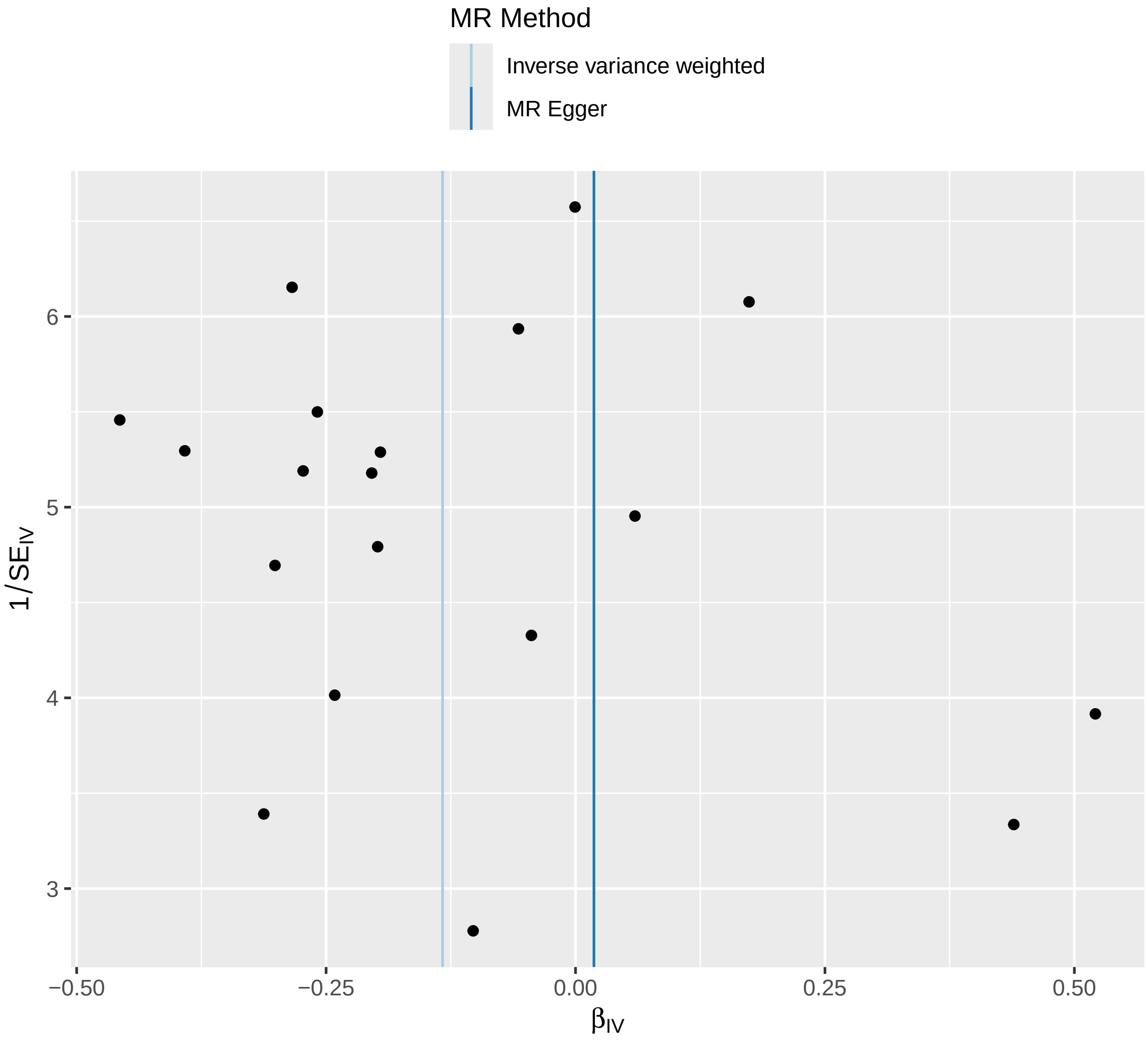
MR Method
Inverse variance weighted
MR Egger
6
5
4
3
1/SE_IV
−0.50
−0.25
0.00
0.25
0.50
β_IV

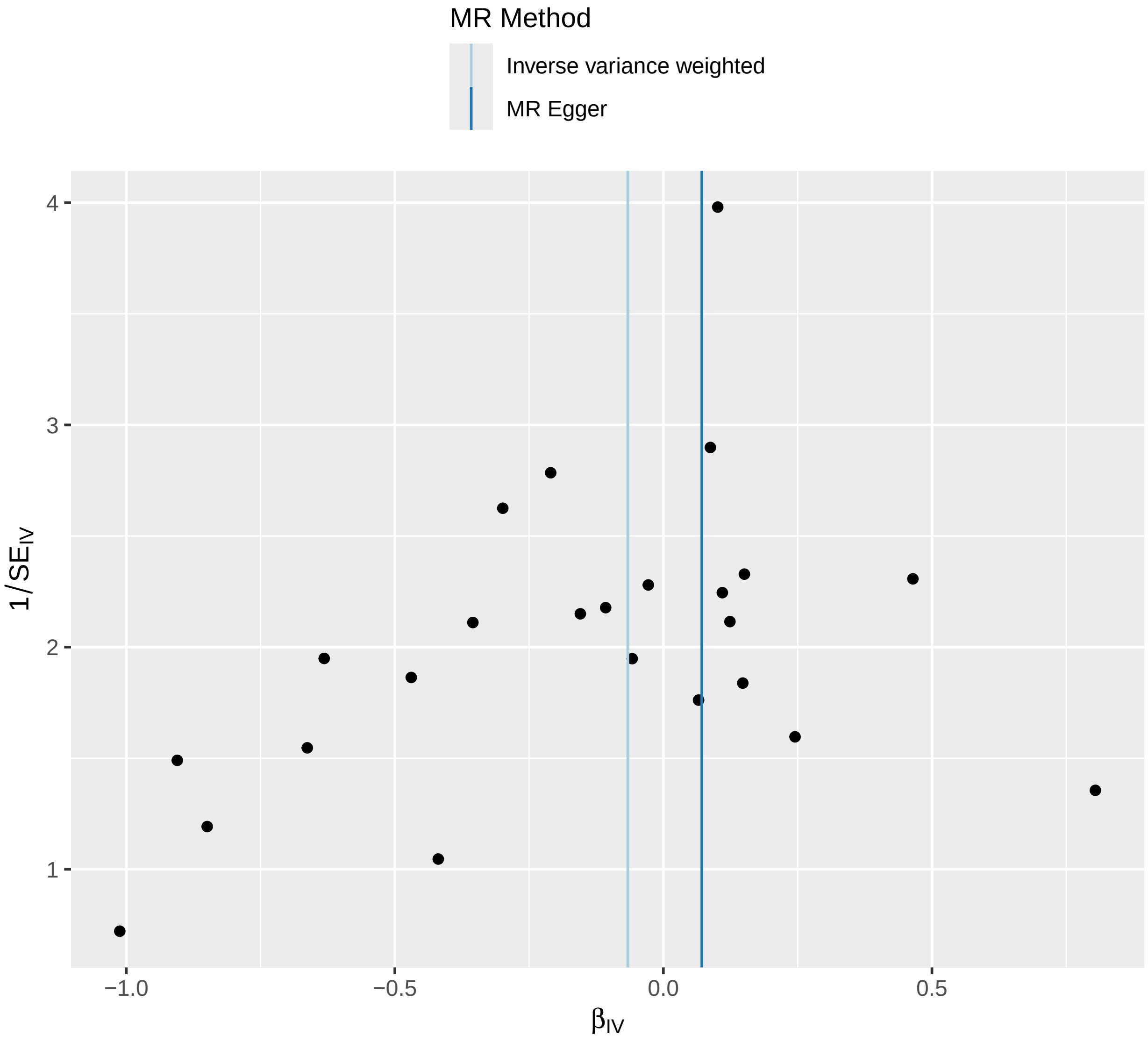

MR Method
Inverse variance weighted
MR Egger
4
3
2
1
1/SE$_{IV}$
−1.0
−0.5
0.0
0.5
β_{IV}

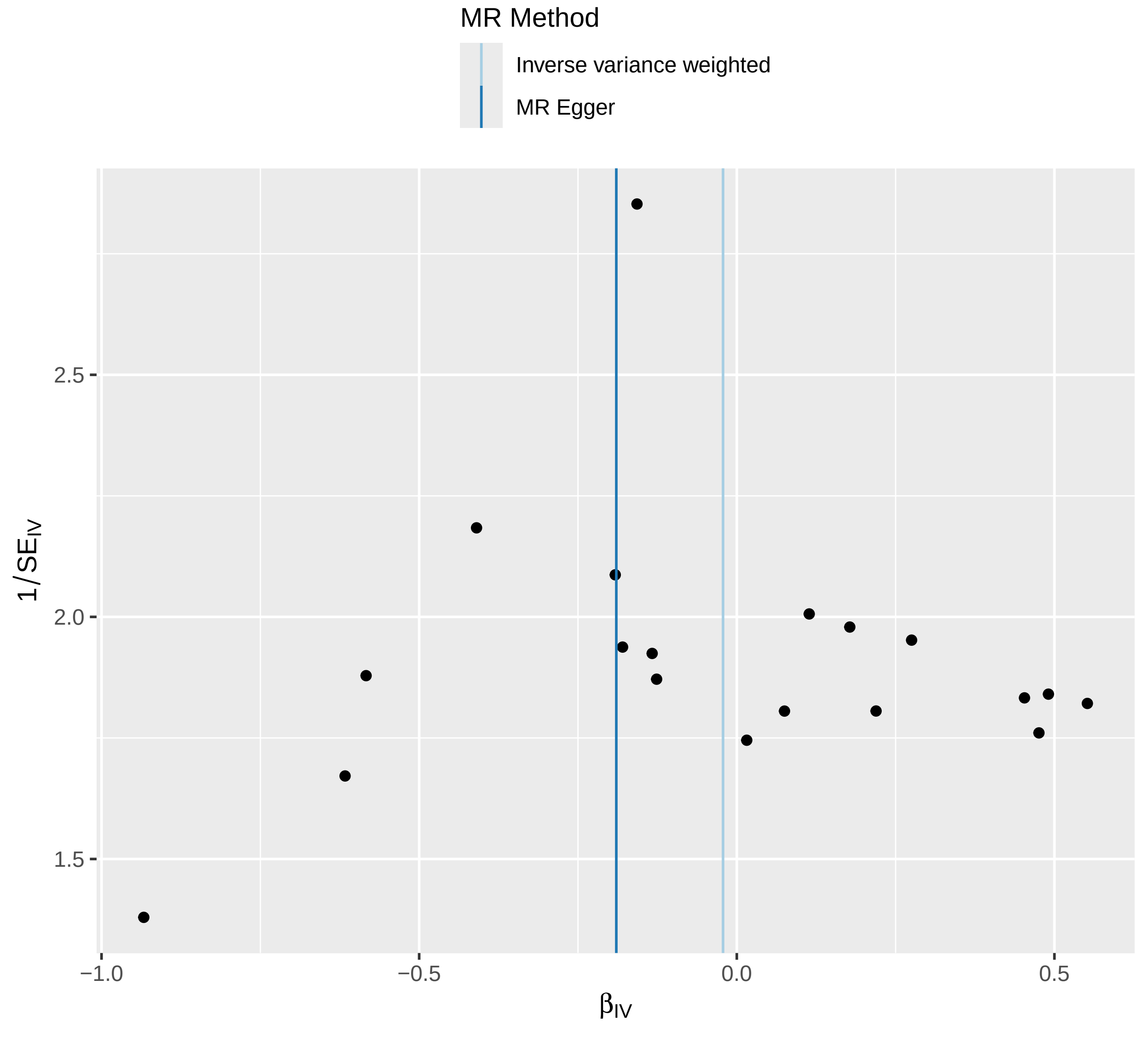

MR Method
Inverse variance weighted
MR Egger
2.5
2.0
1.5
1/SE$_{IV}$
−1.0
−0.5
0.0
0.5
β$_{IV}$

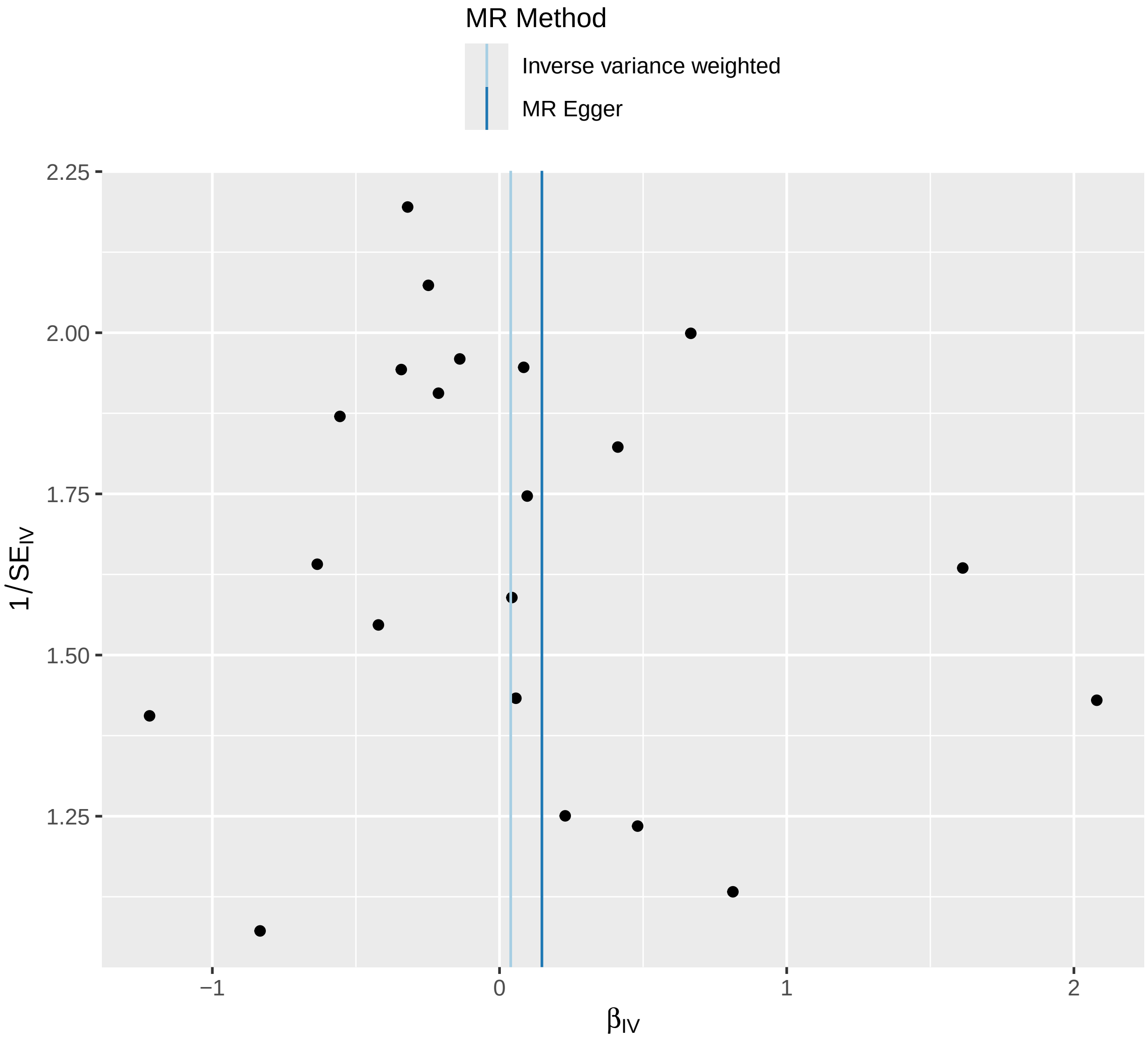

MR Method
Inverse variance weighted
MR Egger
2.25
2.00
1.75
1.50
1.25
1/SE_IV
−1
0
1
2
β_IV

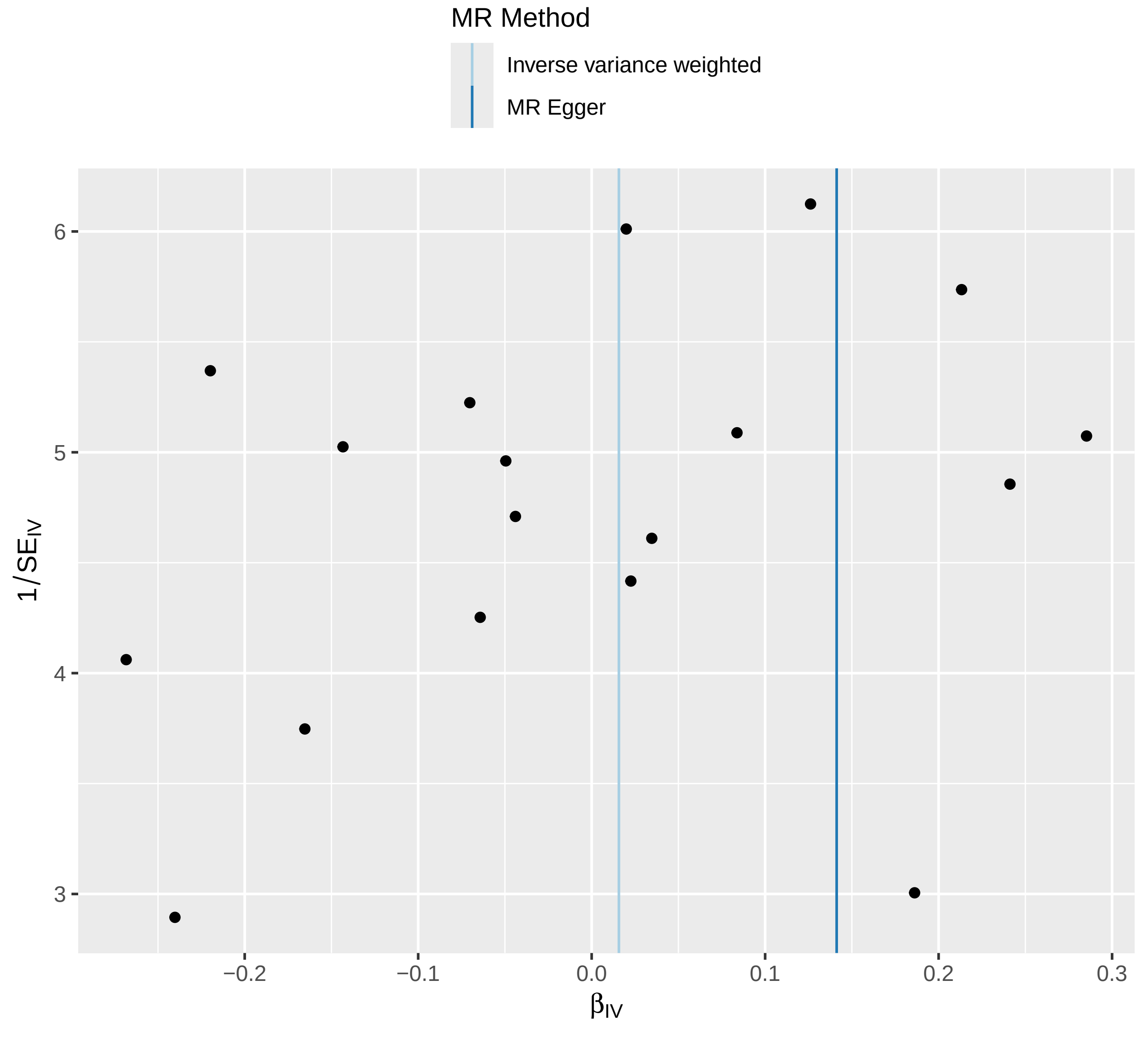
MR Method
Inverse variance weighted
MR Egger
6
5
4
3
1/SE$_{IV}$
−0.2
−0.1
0.0
0.1
0.2
0.3
β$_{IV}$

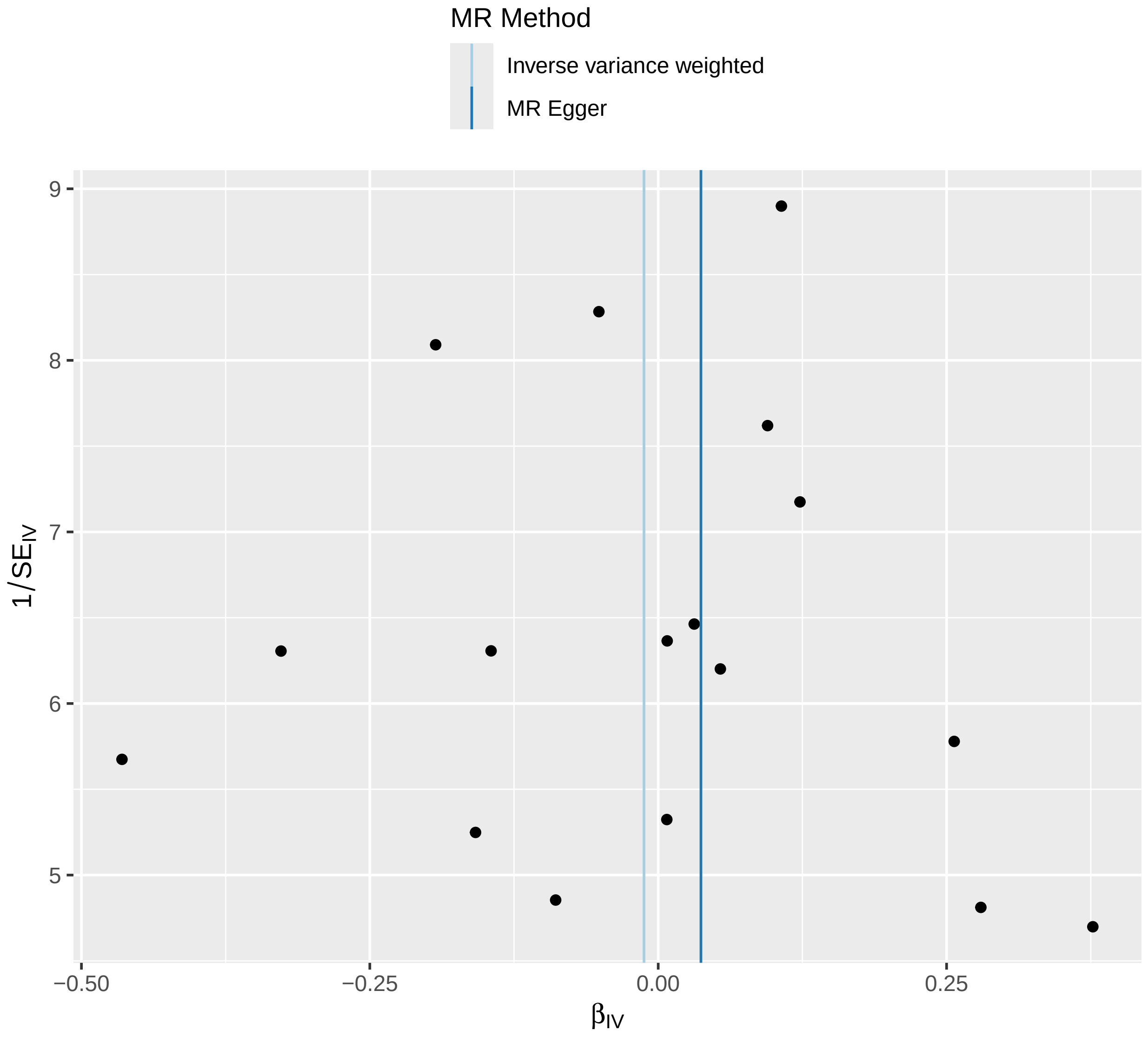

MR Method
Inverse variance weighted
MR Egger
9
8
7
6
5
1/SE_IV
−0.50
−0.25
0.00
0.25
β_IV

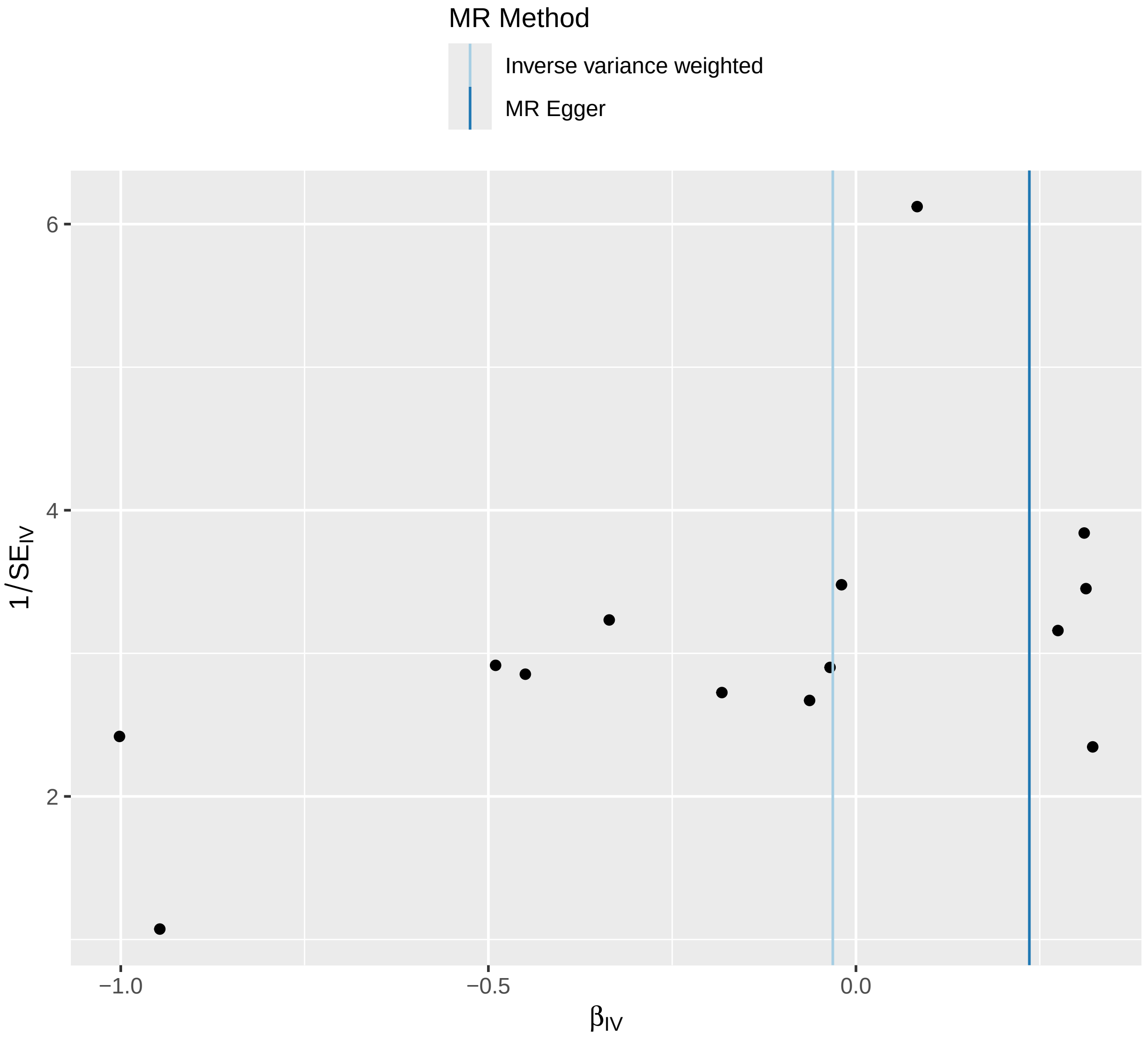
MR Method
Inverse variance weighted
MR Egger
6
4
2
1/SE$_{IV}$
−1.0
−0.5
0.0
β$_{IV}$

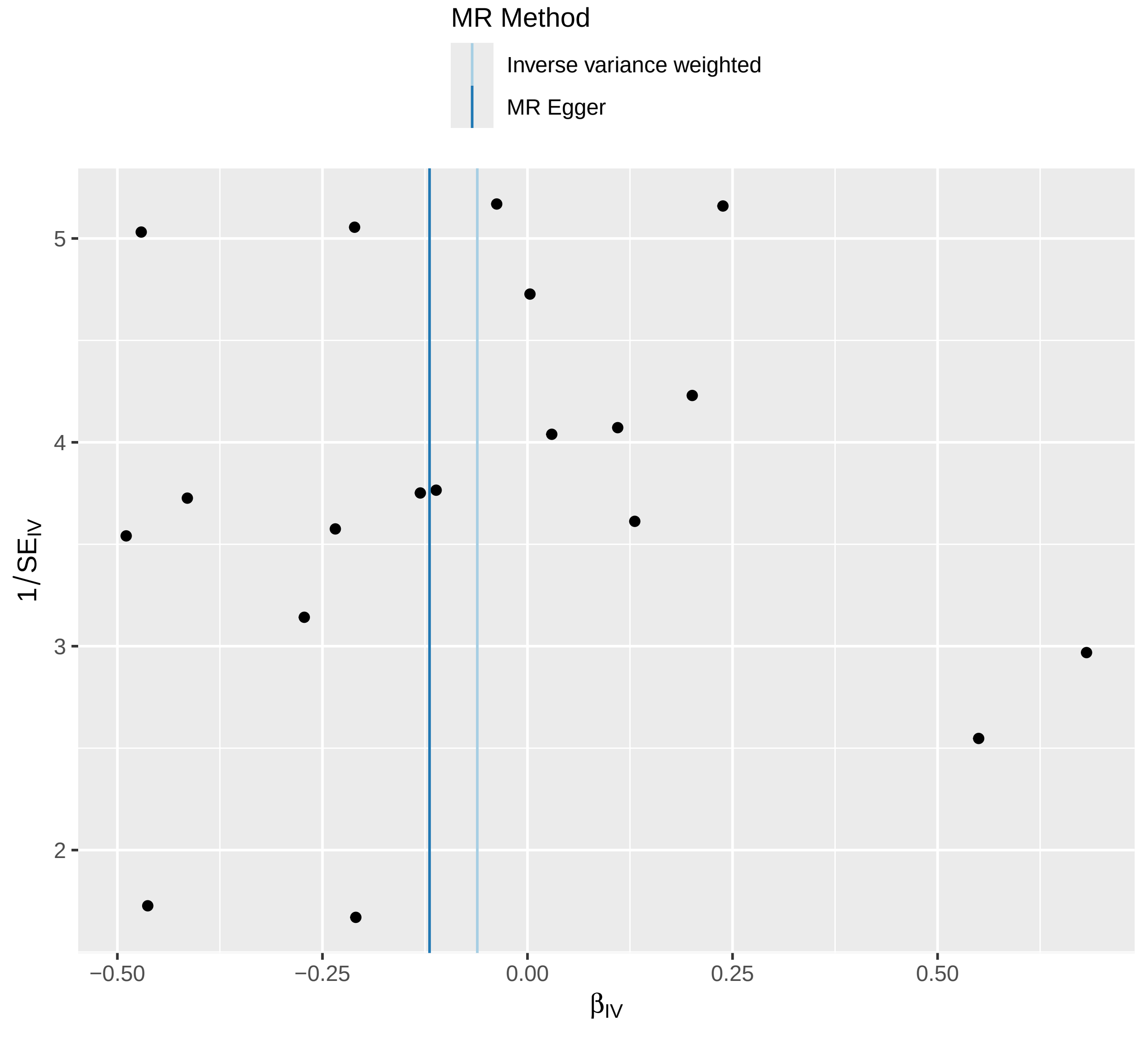

MR Method
Inverse variance weighted
MR Egger
1/SE$_{IV}$
5
4
3
2
−0.50
−0.25
0.00
0.25
0.50
β_{IV}

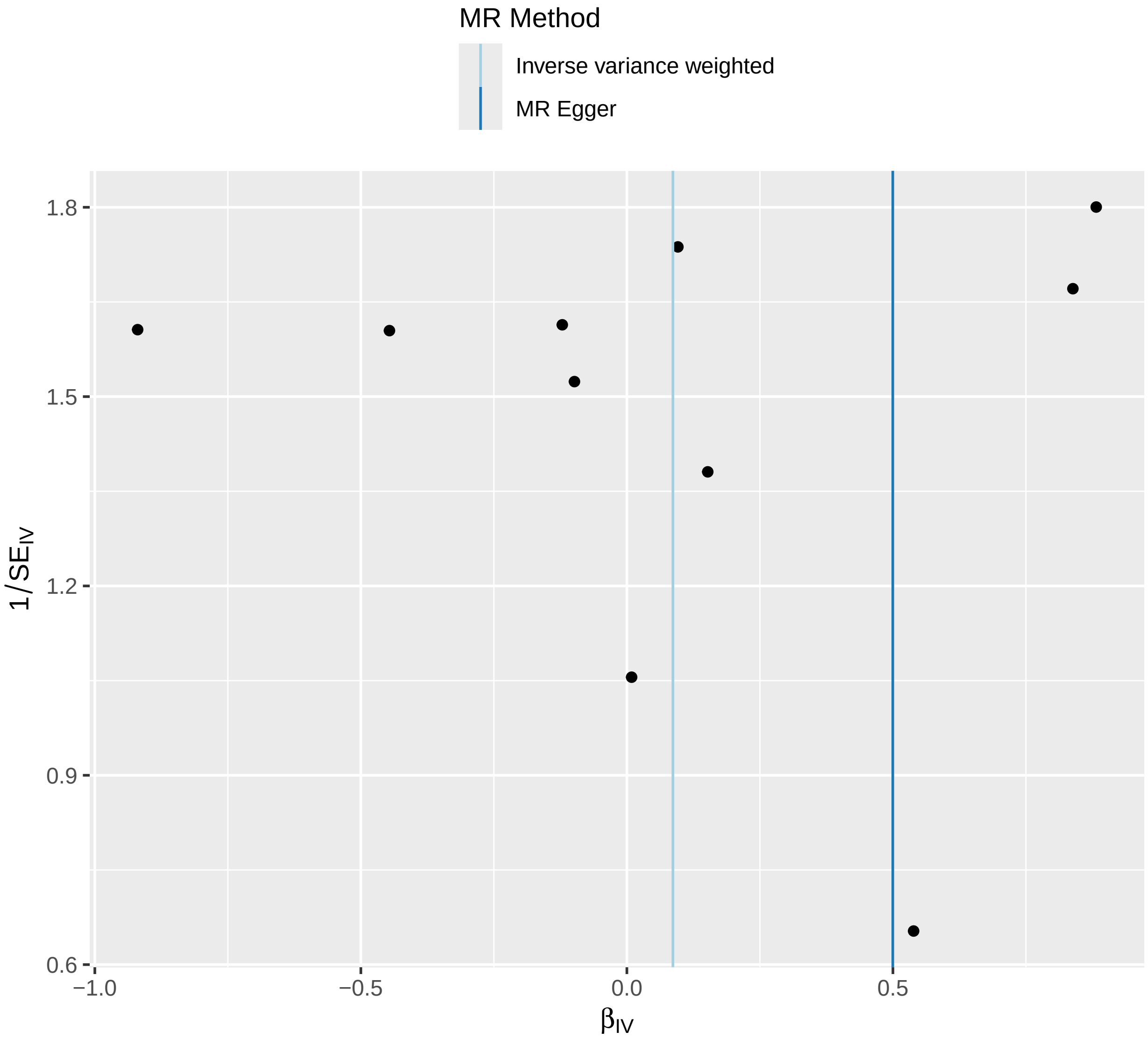
MR Method
Inverse variance weighted
MR Egger
1.8
1.5
1.2
0.9
0.6
−1.0
−0.5
0.0
0.5
$1/SE_{IV}$
β_{IV}

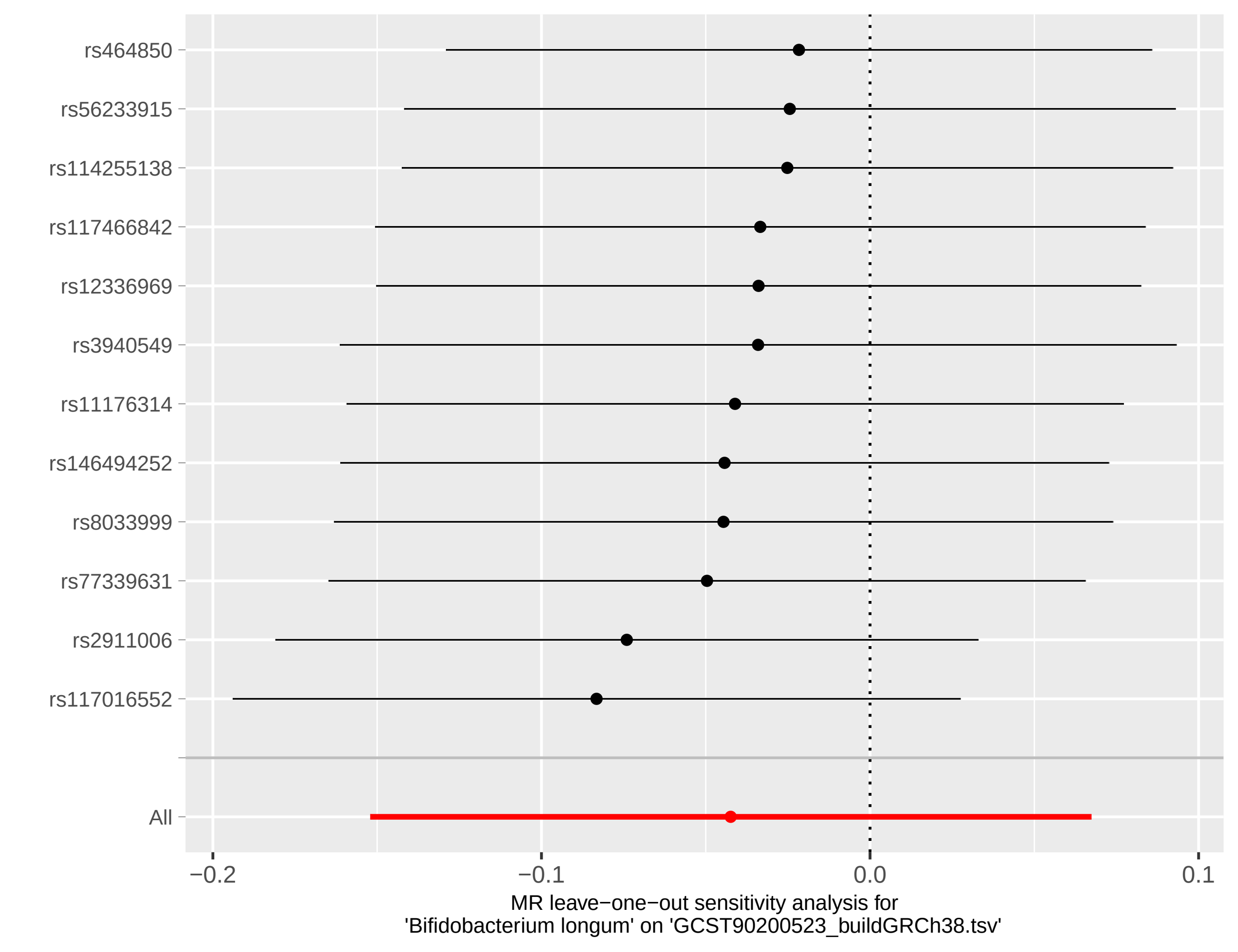
rs464850
rs56233915
rs114255138
rs117466842
rs12336969
rs3940549
rs11176314
rs146494252
rs8033999
rs77339631
rs2911006
rs117016552
All
−0.2
−0.1
0.0
0.1
MR leave−one−out sensitivity analysis for
'Bifidobacterium longum' on 'GCST90200523_buildGRCh38.tsv'

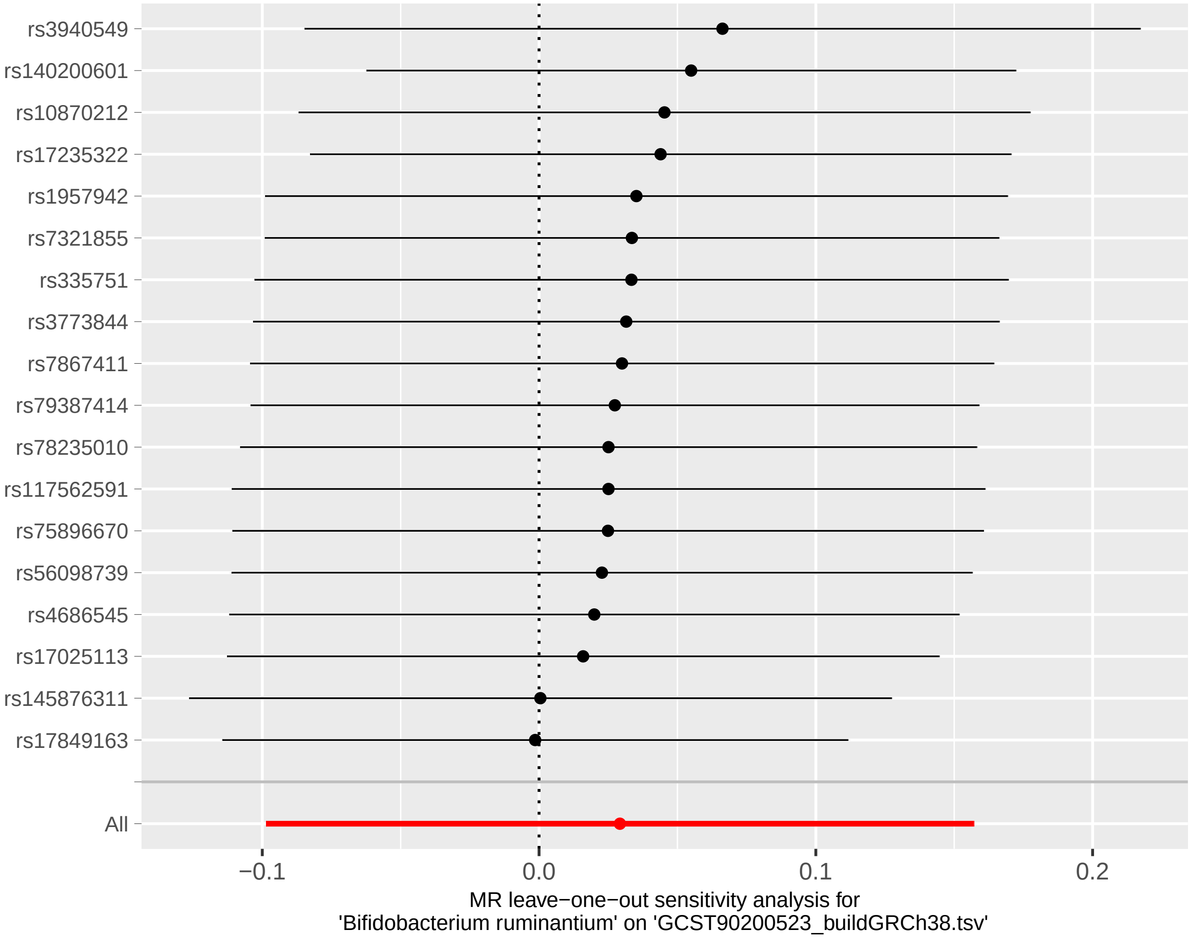

rs3940549
rs140200601
rs10870212
rs17235322
rs1957942
rs7321855
rs335751
rs3773844
rs7867411
rs79387414
rs78235010
rs117562591
rs75896670
rs56098739
rs4686545
rs17025113
rs145876311
rs17849163
All
−0.1
0.0
0.1
0.2
MR leave−one−out sensitivity analysis for
'Bifidobacterium ruminantium' on 'GCST90200523_buildGRCh38.tsv'

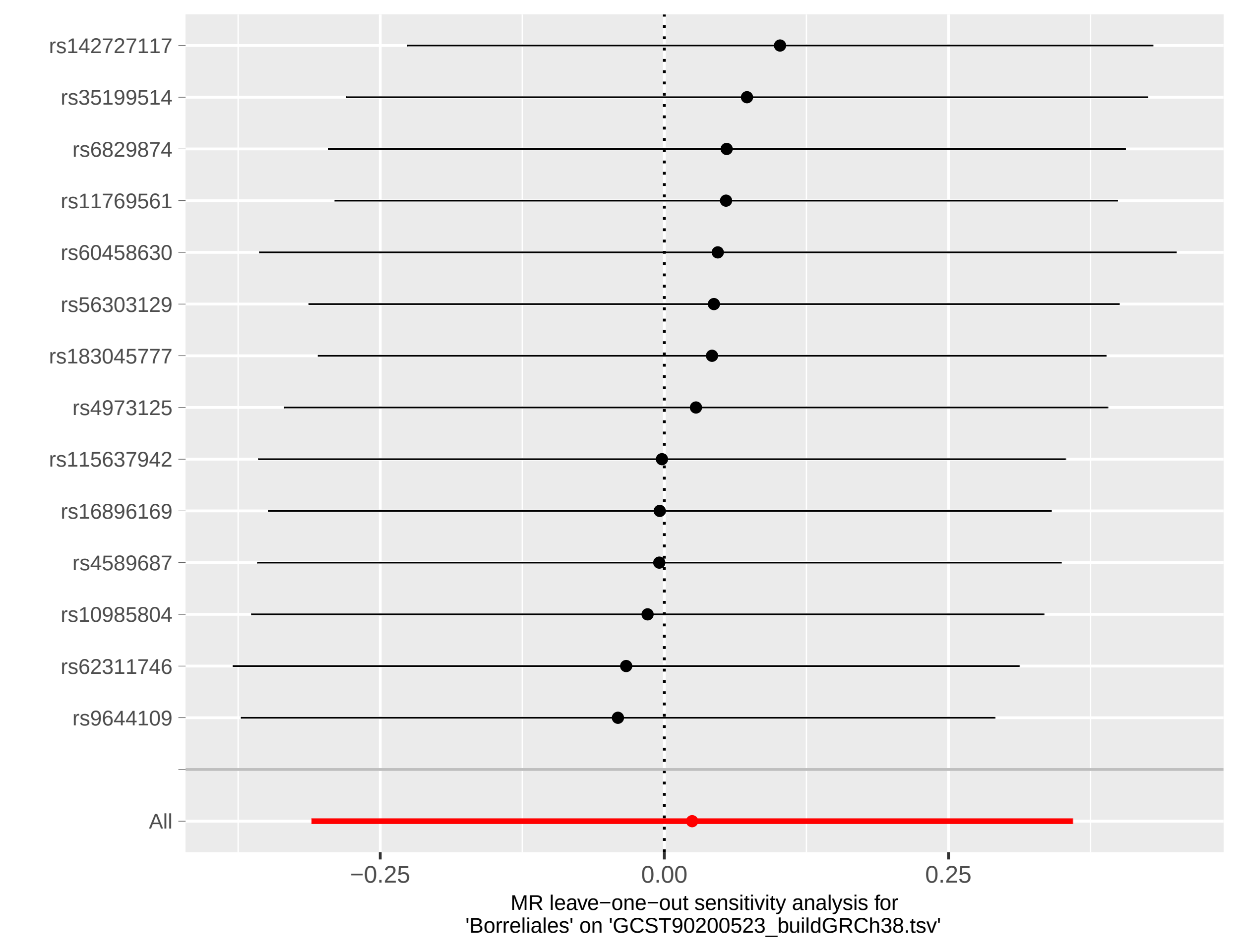
rs142727117
rs35199514
rs6829874
rs11769561
rs60458630
rs56303129
rs183045777
rs4973125
rs115637942
rs16896169
rs4589687
rs10985804
rs62311746
rs9644109
All
−0.25
0.00
0.25
MR leave−one−out sensitivity analysis for
'Borreliales' on 'GCST90200523_buildGRCh38.tsv'

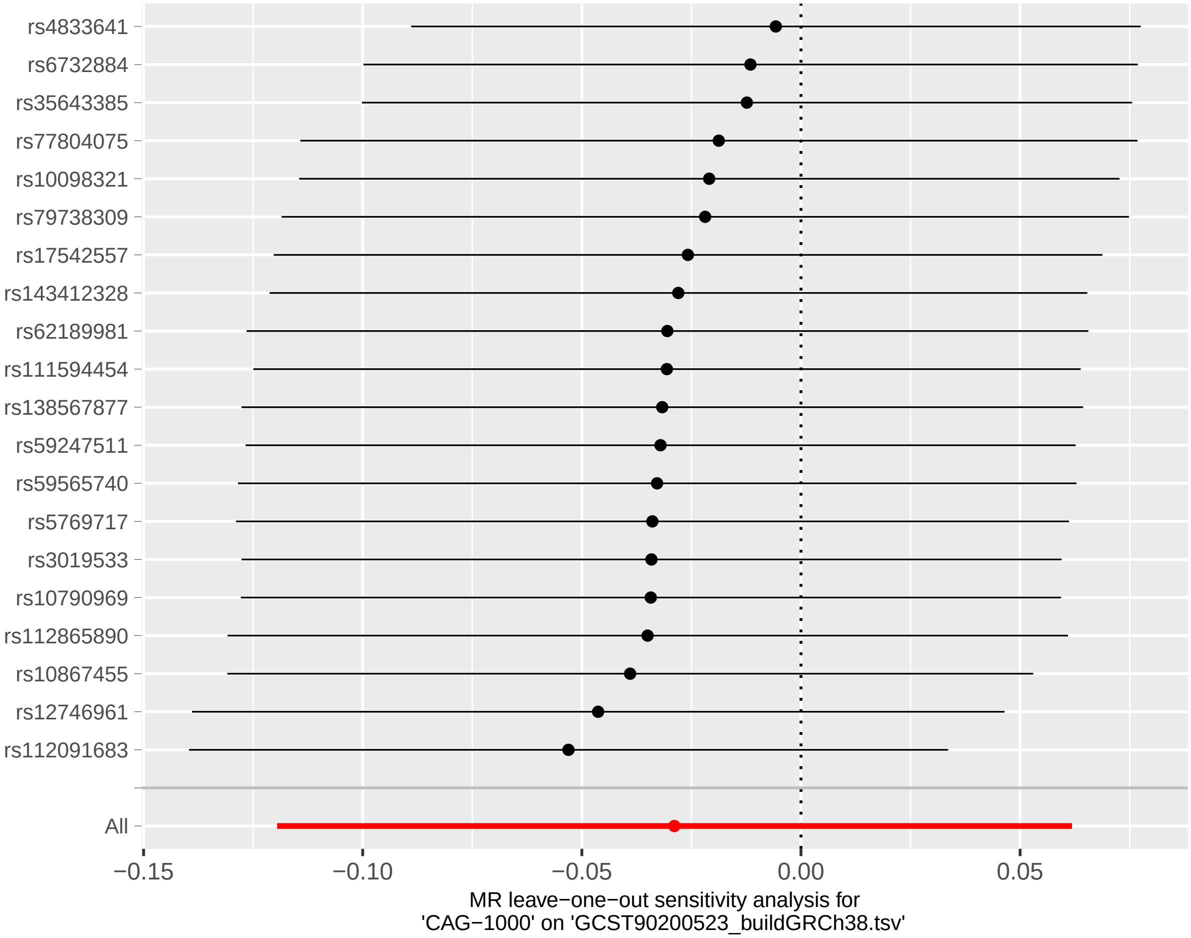

rs4833641
rs6732884
rs35643385
rs77804075
rs10098321
rs79738309
rs17542557
rs143412328
rs62189981
rs111594454
rs138567877
rs59247511
rs59565740
rs5769717
rs3019533
rs10790969
rs112865890
rs10867455
rs12746961
rs112091683
All
−0.15
−0.10
−0.05
0.00
0.05
MR leave−one−out sensitivity analysis for
'CAG−1000' on 'GCST90200523_buildGRCh38.tsv'

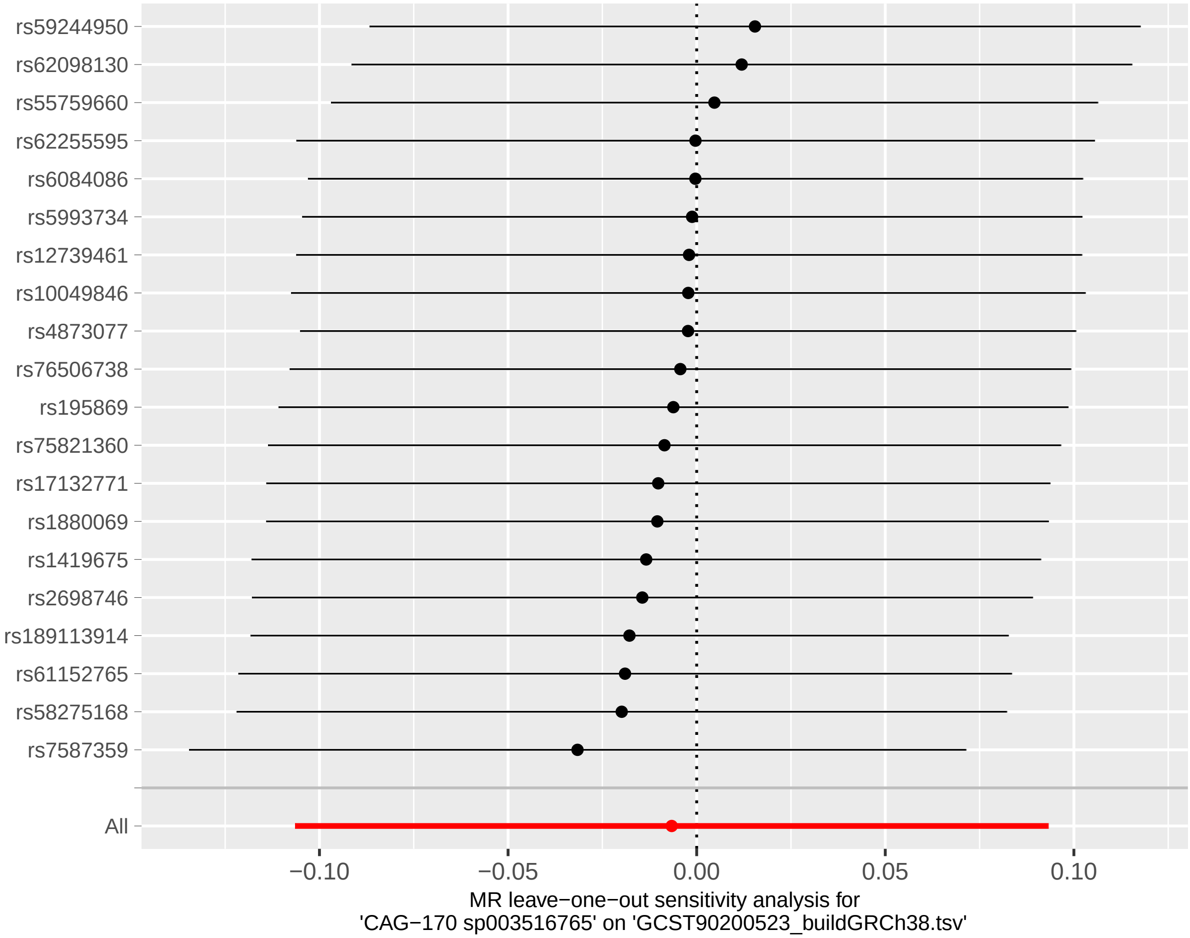
rs59244950
rs62098130
rs55759660
rs62255595
rs6084086
rs5993734
rs12739461
rs10049846
rs4873077
rs76506738
rs195869
rs75821360
rs17132771
rs1880069
rs1419675
rs2698746
rs189113914
rs61152765
rs58275168
rs7587359
All
−0.10
−0.05
0.00
0.05
0.10
MR leave−one−out sensitivity analysis for
'CAG−170 sp003516765' on 'GCST90200523_buildGRCh38.tsv'

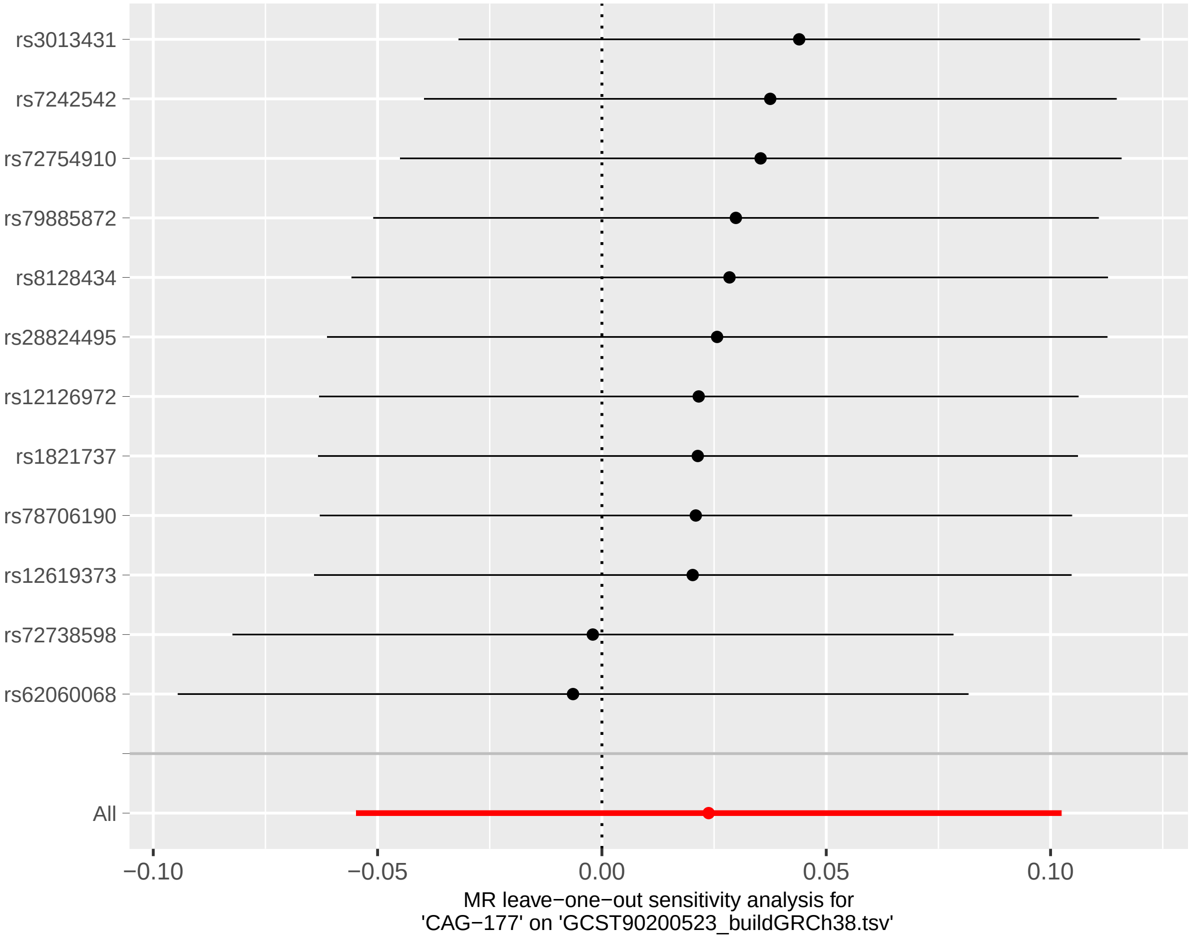
rs3013431
rs7242542
rs72754910
rs79885872
rs8128434
rs28824495
rs12126972
rs1821737
rs78706190
rs12619373
rs72738598
rs62060068
All
−0.10
−0.05
0.00
0.05
0.10
MR leave−one−out sensitivity analysis for
'CAG−177' on 'GCST90200523_buildGRCh38.tsv'

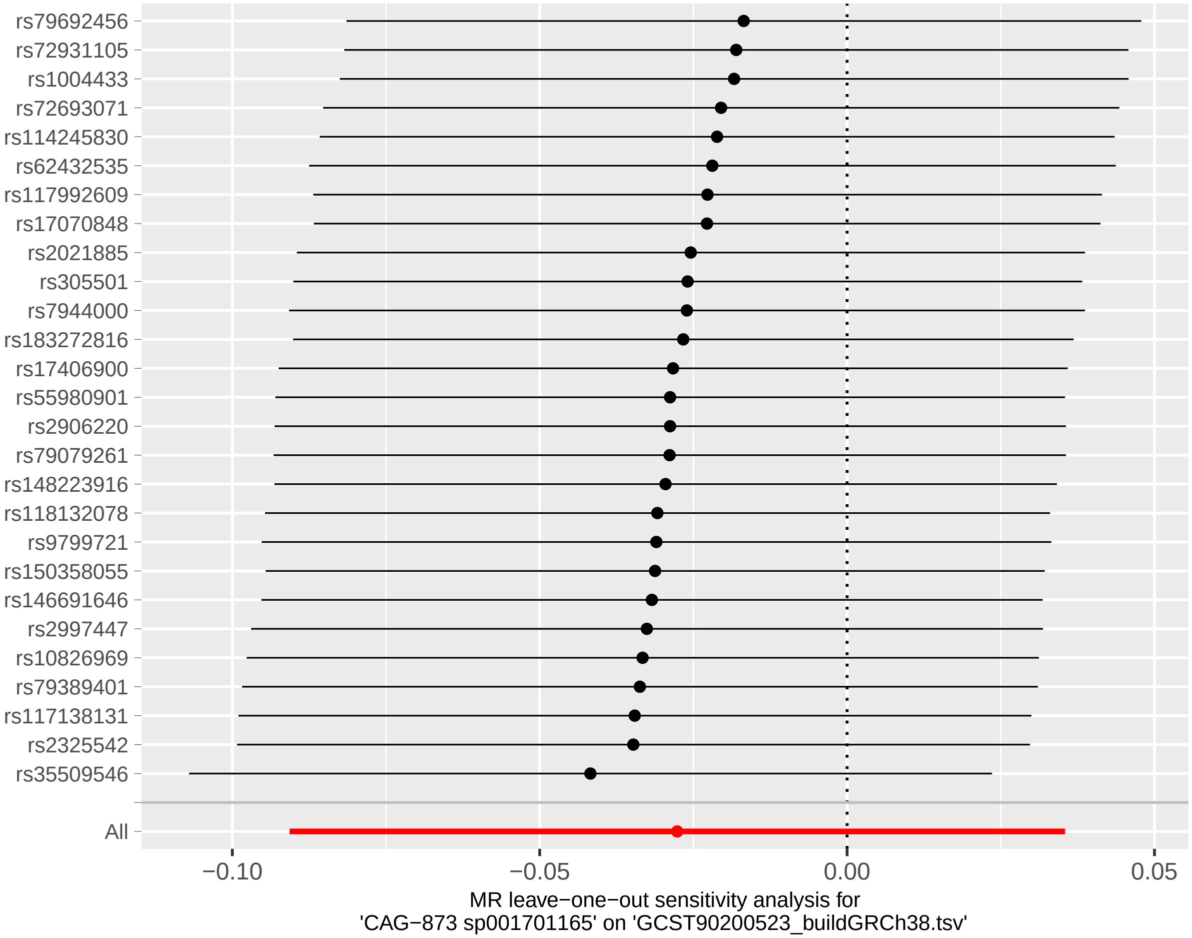
rs79692456
rs72931105
rs1004433
rs72693071
rs114245830
rs62432535
rs117992609
rs17070848
rs2021885
rs305501
rs7944000
rs183272816
rs17406900
rs55980901
rs2906220
rs79079261
rs148223916
rs118132078
rs9799721
rs150358055
rs146691646
rs2997447
rs10826969
rs79389401
rs117138131
rs2325542
rs35509546
All
−0.10
−0.05
0.00
0.05
MR leave−one−out sensitivity analysis for
'CAG−873 sp001701165' on 'GCST90200523_buildGRCh38.tsv'

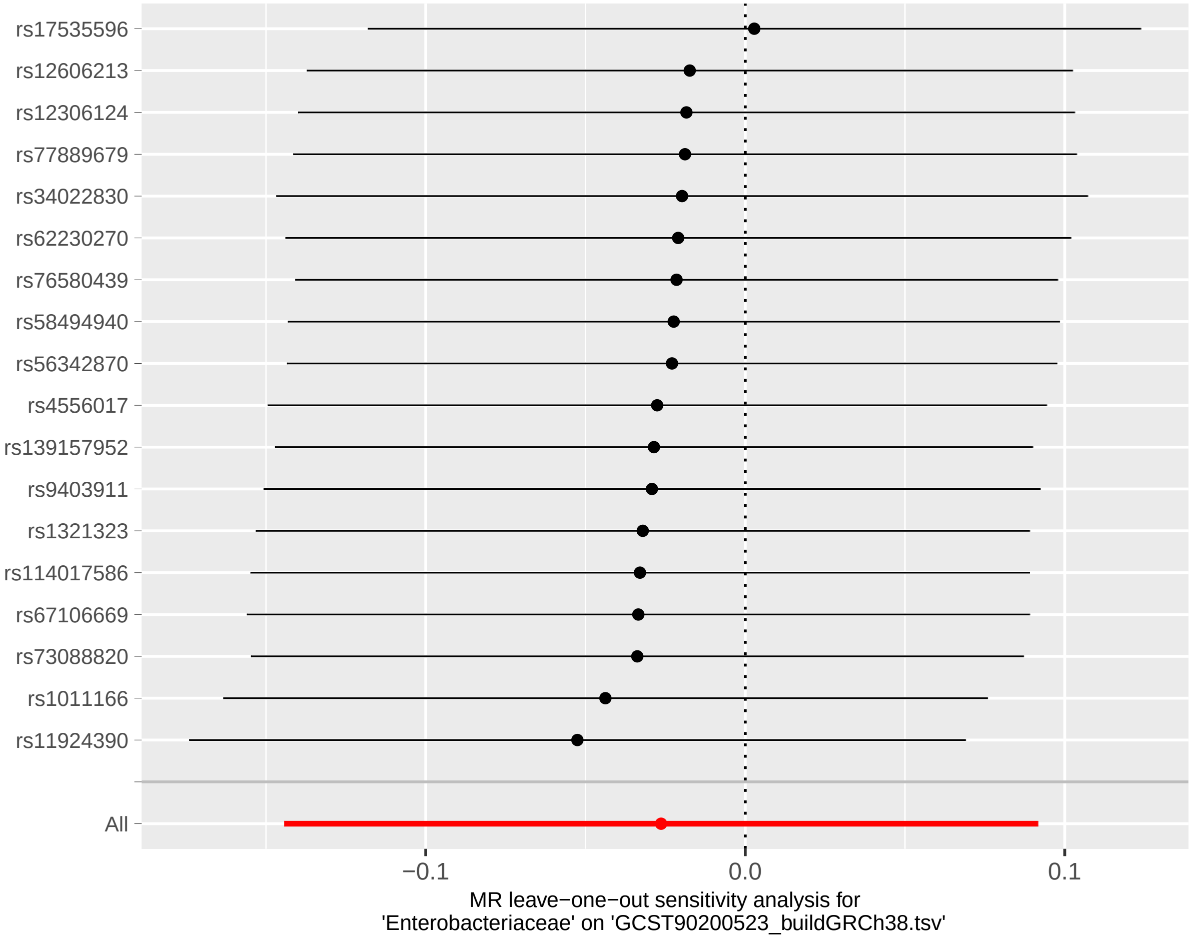
rs17535596
rs12606213
rs12306124
rs77889679
rs34022830
rs62230270
rs76580439
rs58494940
rs56342870
rs4556017
rs139157952
rs9403911
rs1321323
rs114017586
rs67106669
rs73088820
rs1011166
rs11924390
All
−0.1
0.0
0.1
MR leave−one−out sensitivity analysis for
'Enterobacteriaceae' on 'GCST90200523_buildGRCh38.tsv'

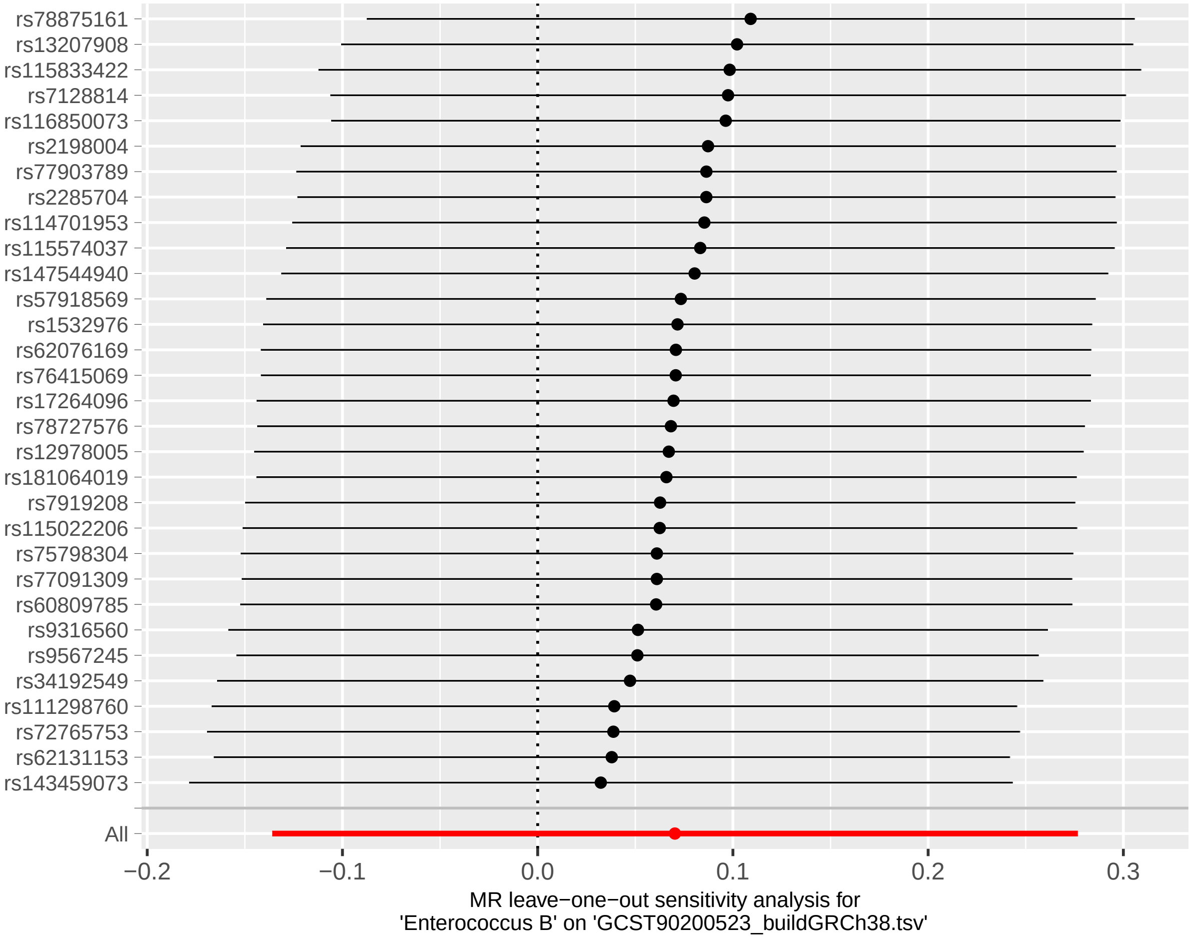
rs78875161
rs13207908
rs115833422
rs7128814
rs116850073
rs2198004
rs77903789
rs2285704
rs114701953
rs115574037
rs147544940
rs57918569
rs1532976
rs62076169
rs76415069
rs17264096
rs78727576
rs12978005
rs181064019
rs7919208
rs115022206
rs75798304
rs77091309
rs60809785
rs9316560
rs9567245
rs34192549
rs111298760
rs72765753
rs62131153
rs143459073
All
−0.2
−0.1
0.0
0.1
0.2
0.3
MR leave−one−out sensitivity analysis for
'Enterococcus B' on 'GCST90200523_buildGRCh38.tsv'

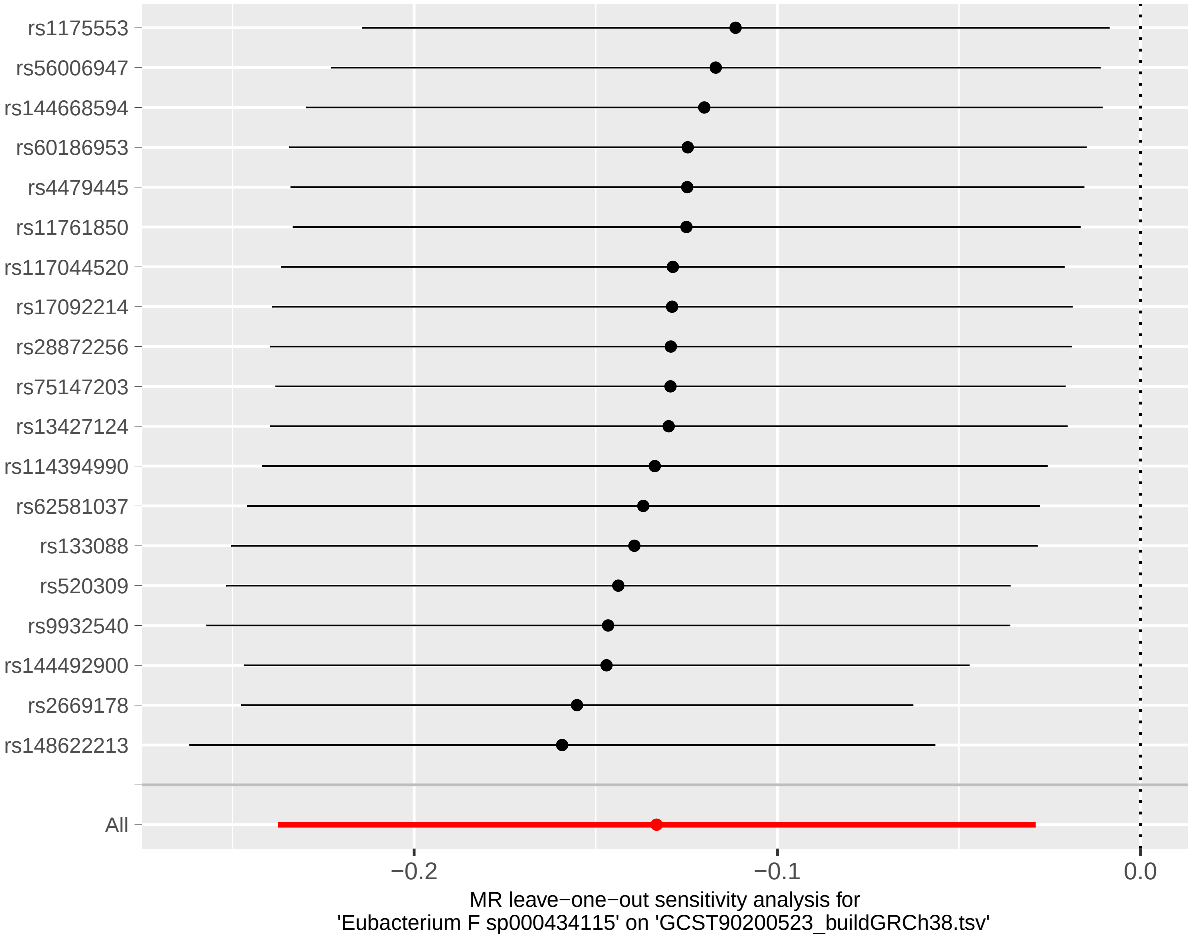
rs1175553
rs56006947
rs144668594
rs60186953
rs4479445
rs11761850
rs117044520
rs17092214
rs28872256
rs75147203
rs13427124
rs114394990
rs62581037
rs133088
rs520309
rs9932540
rs144492900
rs2669178
rs148622213
All
−0.2
−0.1
0.0
MR leave−one−out sensitivity analysis for
'Eubacterium F sp000434115' on 'GCST90200523_buildGRCh38.tsv'

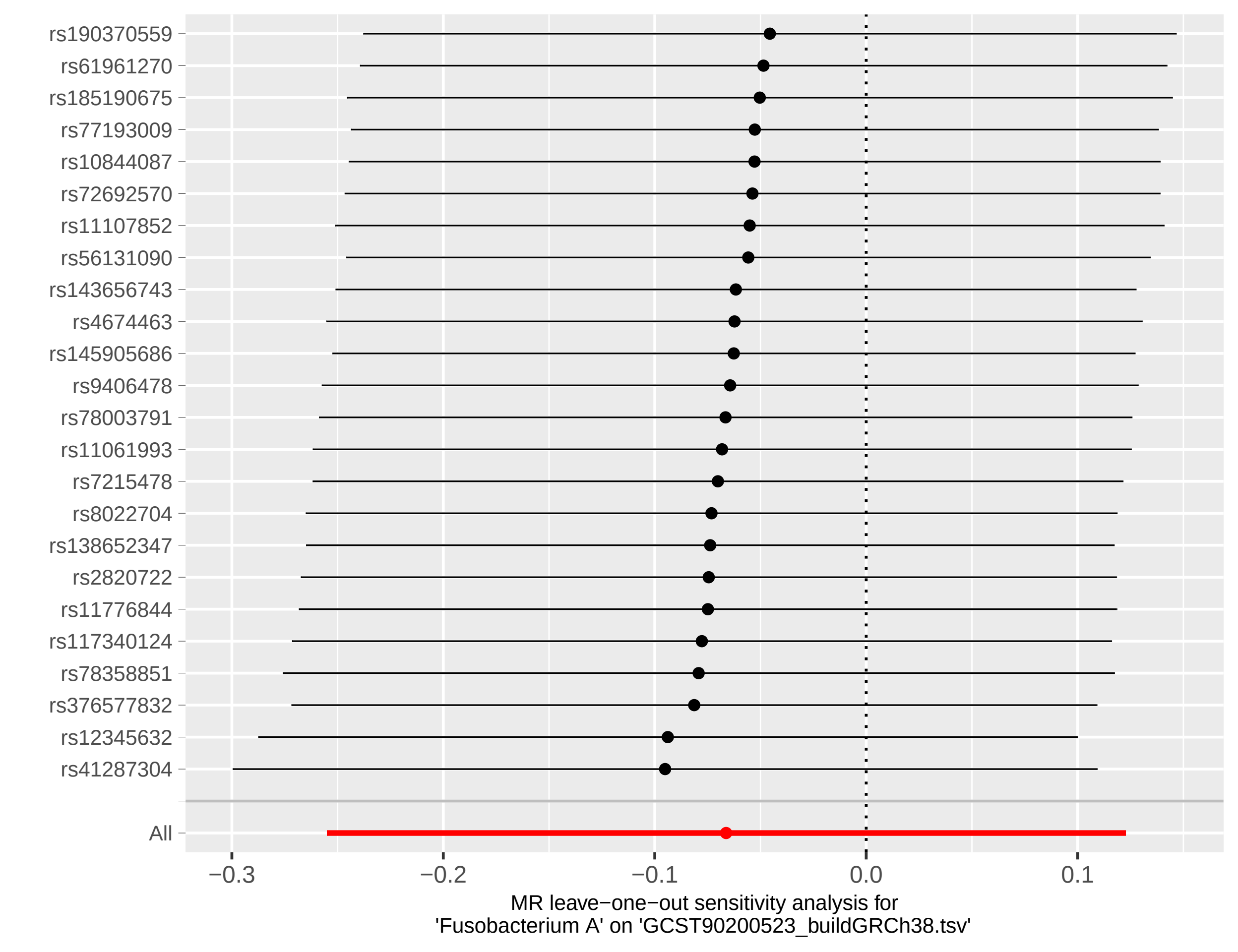
rs190370559
rs61961270
rs185190675
rs77193009
rs10844087
rs72692570
rs11107852
rs56131090
rs143656743
rs4674463
rs145905686
rs9406478
rs78003791
rs11061993
rs7215478
rs8022704
rs138652347
rs2820722
rs11776844
rs117340124
rs78358851
rs376577832
rs12345632
rs41287304
All
−0.3
−0.2
−0.1
0.0
0.1
MR leave−one−out sensitivity analysis for
'Fusobacterium A' on 'GCST90200523_buildGRCh38.tsv'

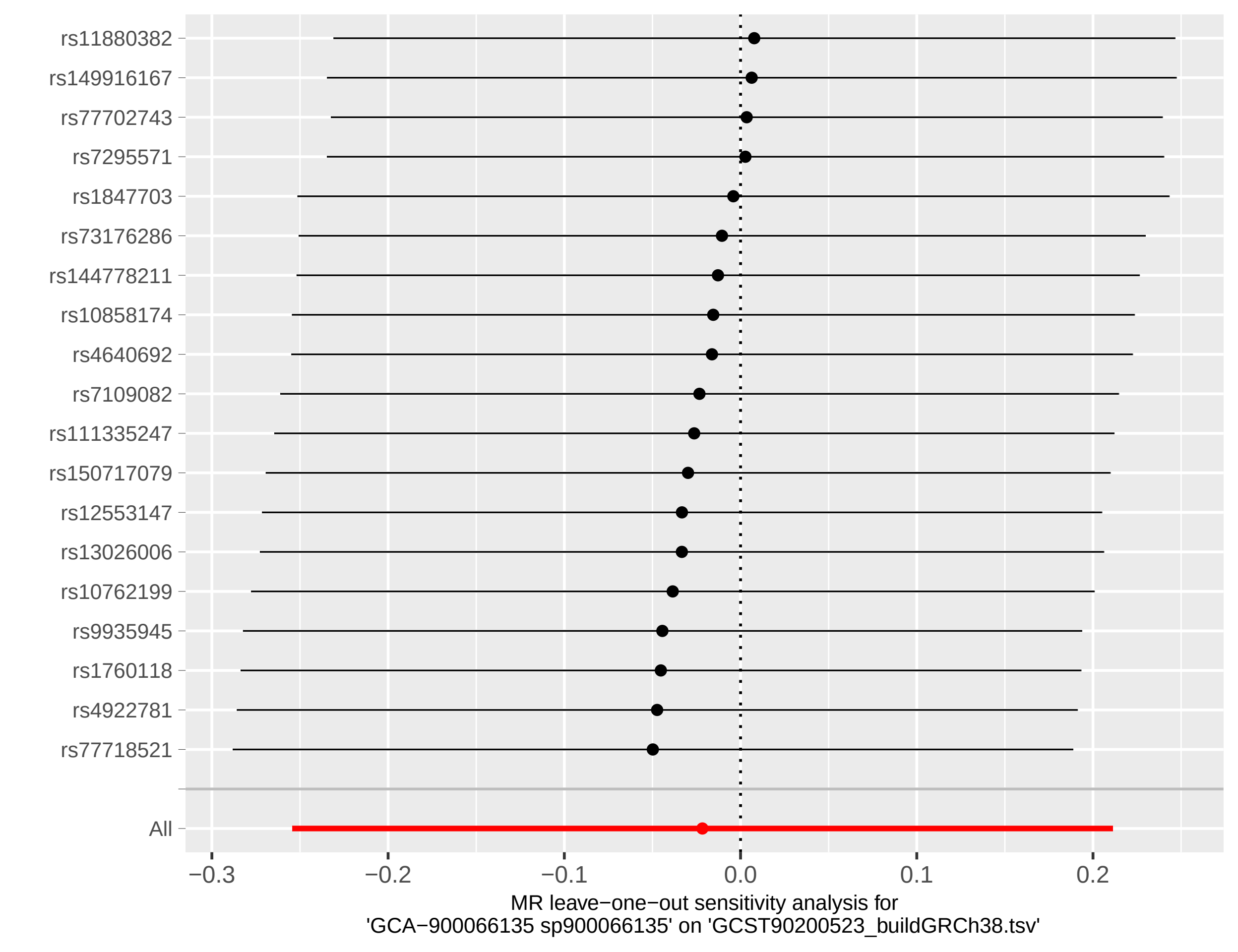
rs11880382
rs149916167
rs77702743
rs7295571
rs1847703
rs73176286
rs144778211
rs10858174
rs4640692
rs7109082
rs111335247
rs150717079
rs12553147
rs13026006
rs10762199
rs9935945
rs1760118
rs4922781
rs77718521
All
−0.3
−0.2
−0.1
0.0
0.1
0.2
MR leave−one−out sensitivity analysis for
'GCA−900066135 sp900066135' on 'GCST90200523_buildGRCh38.tsv'

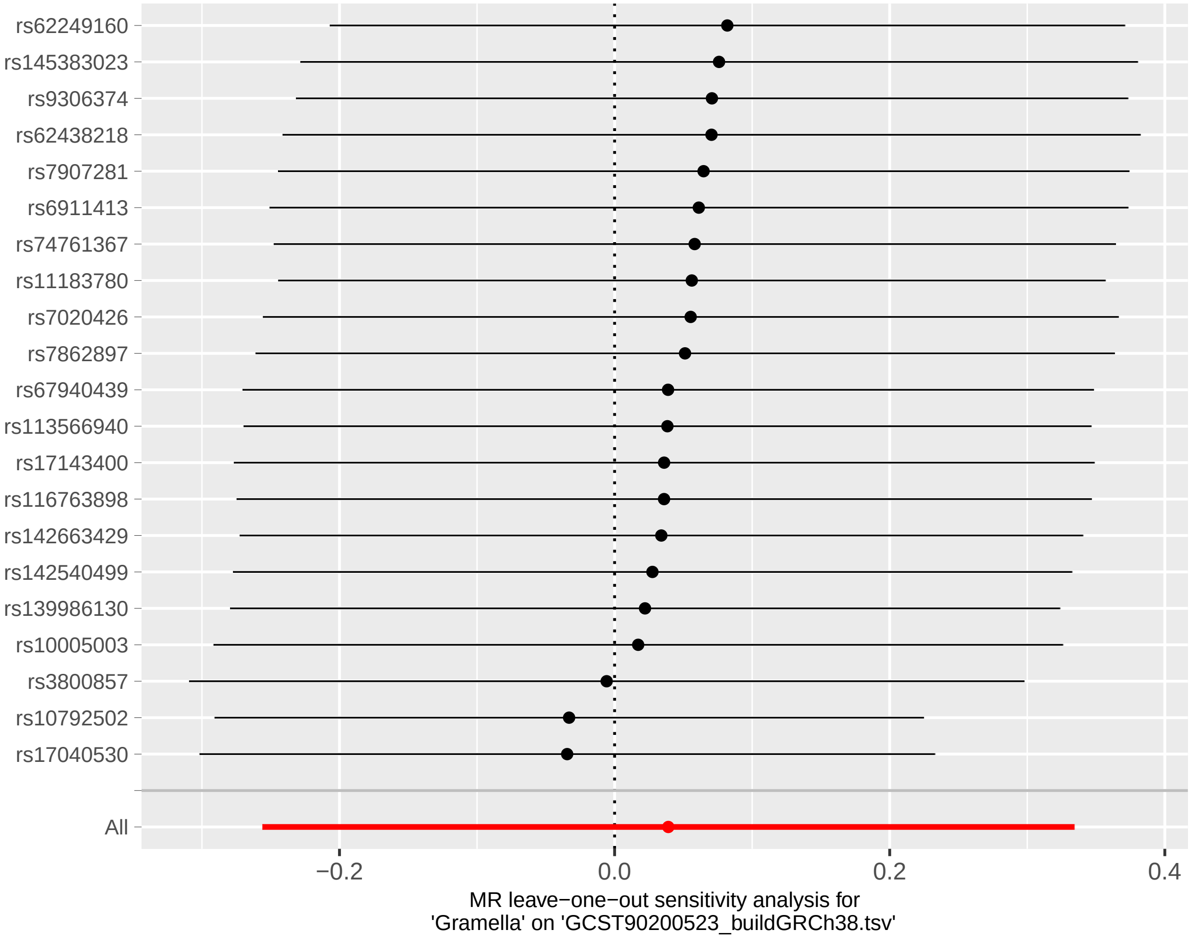
rs62249160
rs145383023
rs9306374
rs62438218
rs7907281
rs6911413
rs74761367
rs11183780
rs7020426
rs7862897
rs67940439
rs113566940
rs17143400
rs116763898
rs142663429
rs142540499
rs139986130
rs10005003
rs3800857
rs10792502
rs17040530
All
−0.2
0.0
0.2
0.4
MR leave−one−out sensitivity analysis for
'Gramella' on 'GCST90200523_buildGRCh38.tsv'

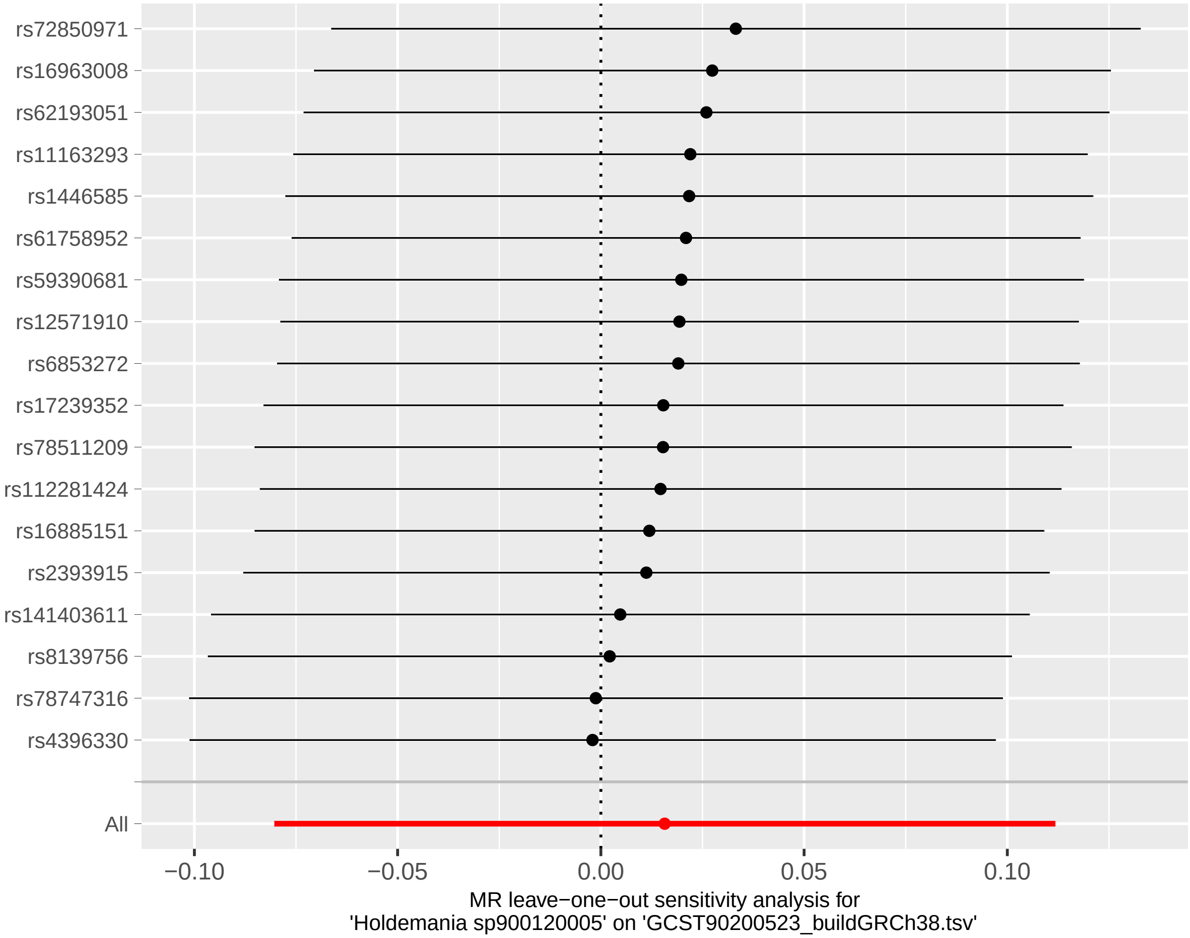
rs72850971
rs16963008
rs62193051
rs11163293
rs1446585
rs61758952
rs59390681
rs12571910
rs6853272
rs17239352
rs78511209
rs112281424
rs16885151
rs2393915
rs141403611
rs8139756
rs78747316
rs4396330
All
−0.10
−0.05
0.00
0.05
0.10
MR leave−one−out sensitivity analysis for
'Holdemania sp900120005' on 'GCST90200523_buildGRCh38.tsv'

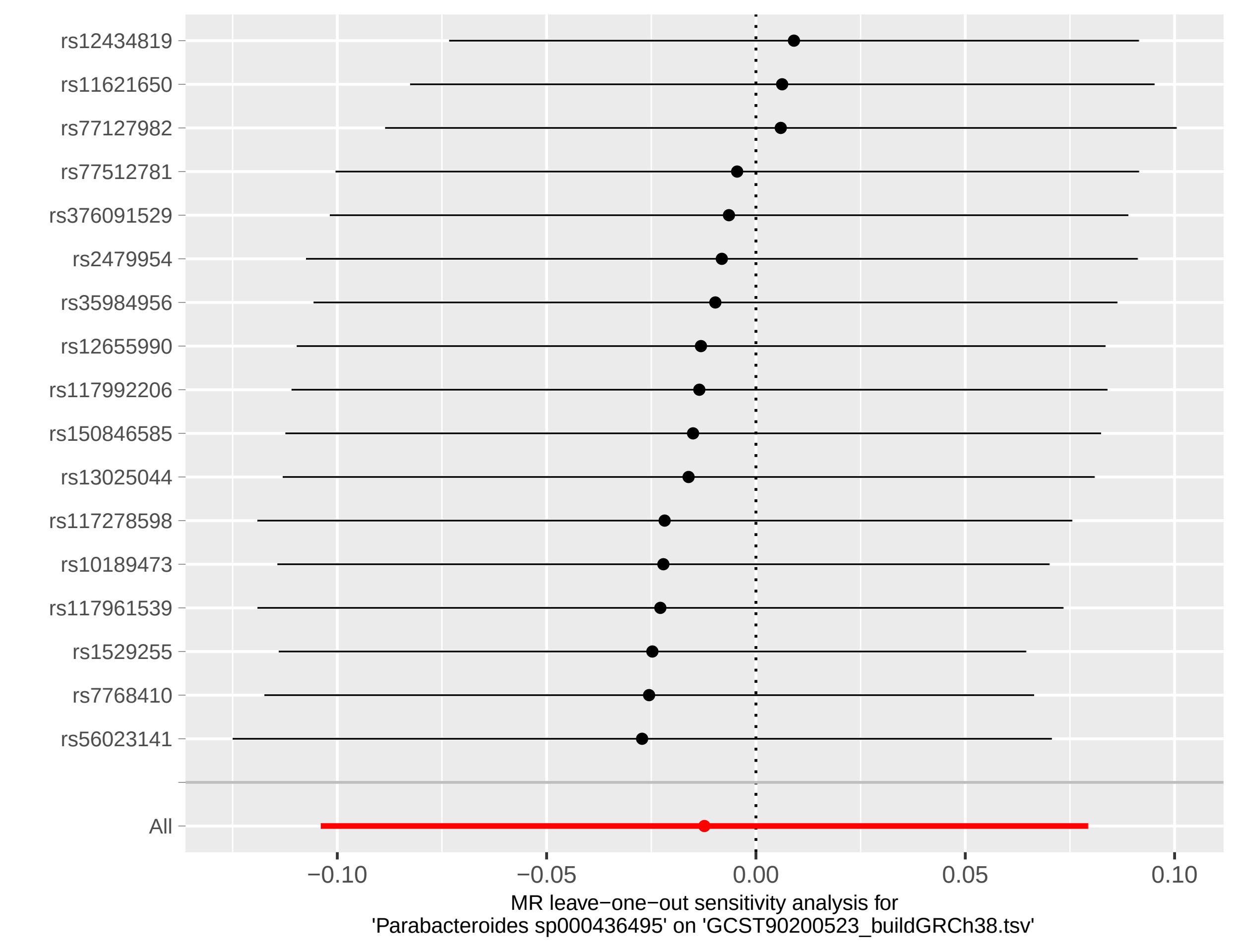
rs12434819
rs11621650
rs77127982
rs77512781
rs376091529
rs2479954
rs35984956
rs12655990
rs117992206
rs150846585
rs13025044
rs117278598
rs10189473
rs117961539
rs1529255
rs7768410
rs56023141
All
−0.10
−0.05
0.00
0.05
0.10
MR leave−one−out sensitivity analysis for
'Parabacteroides sp000436495' on 'GCST90200523_buildGRCh38.tsv'

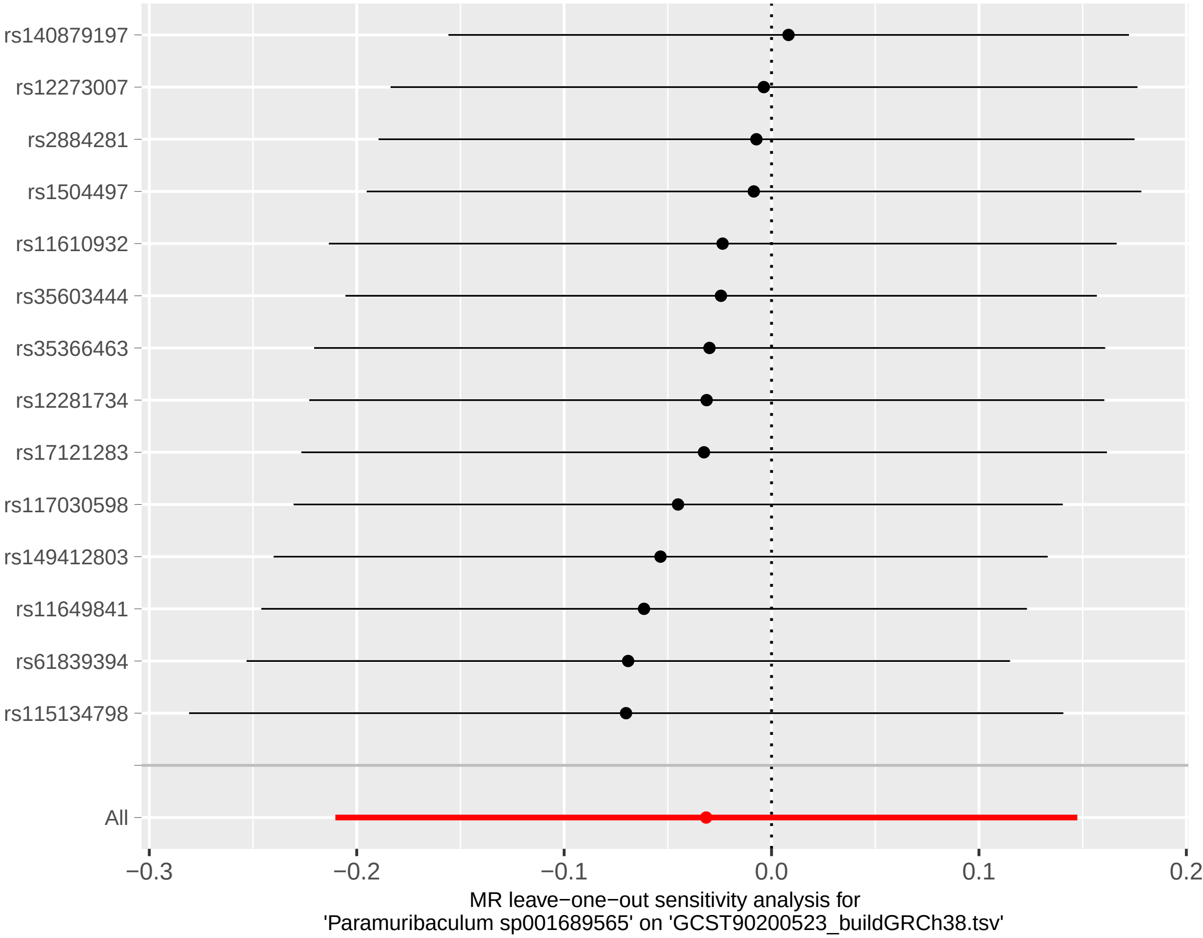

rs140879197
rs12273007
rs2884281
rs1504497
rs11610932
rs35603444
rs35366463
rs12281734
rs17121283
rs117030598
rs149412803
rs11649841
rs61839394
rs115134798
All
−0.3
−0.2
−0.1
0.0
0.1
0.2
MR leave−one−out sensitivity analysis for
'Paramuribaculum sp001689565' on 'GCST90200523_buildGRCh38.tsv'

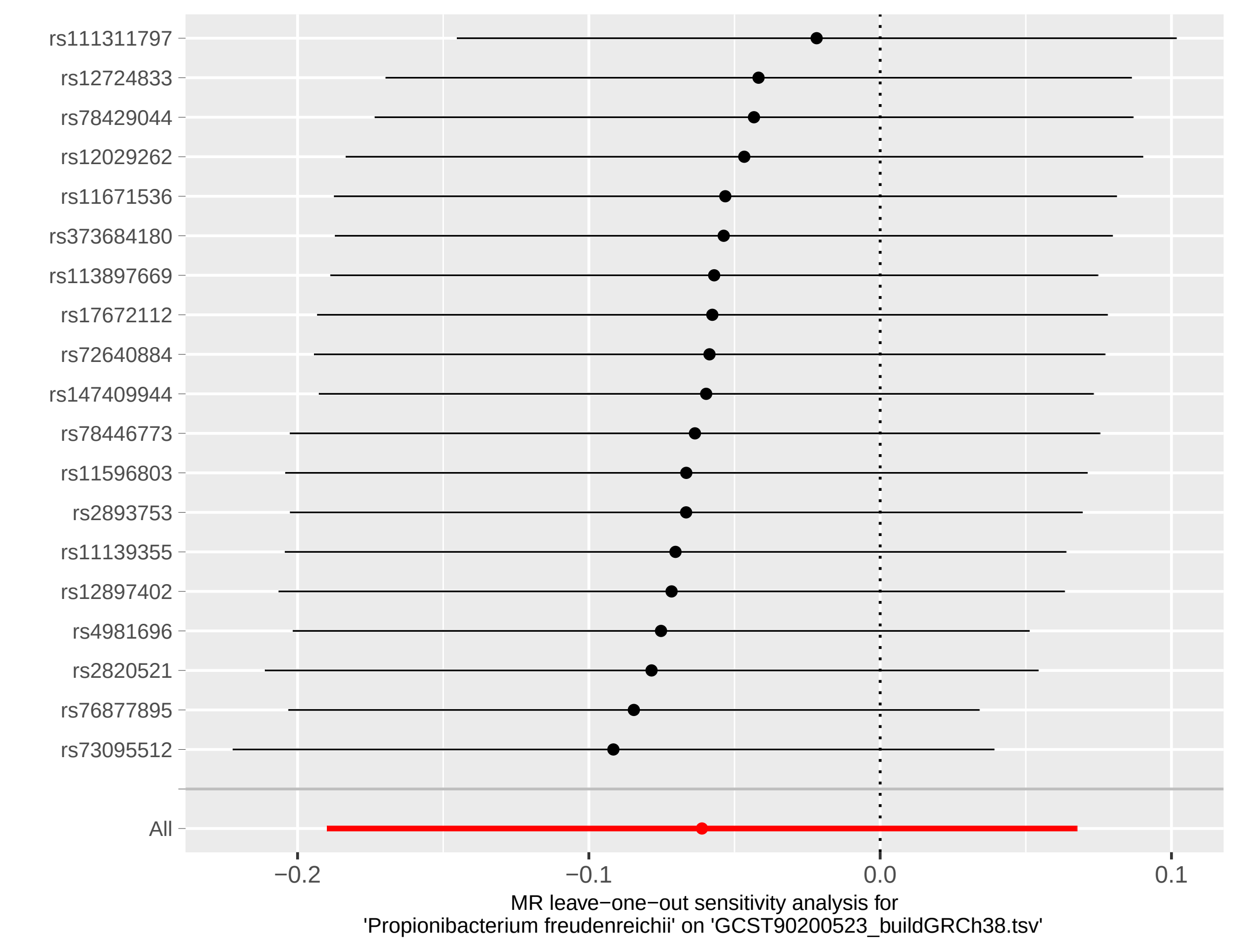
rs111311797
rs12724833
rs78429044
rs12029262
rs11671536
rs373684180
rs113897669
rs17672112
rs72640884
rs147409944
rs78446773
rs11596803
rs2893753
rs11139355
rs12897402
rs4981696
rs2820521
rs76877895
rs73095512
All
−0.2
−0.1
0.0
0.1
MR leave−one−out sensitivity analysis for
'Propionibacterium freudenreichii' on 'GCST90200523_buildGRCh38.tsv'

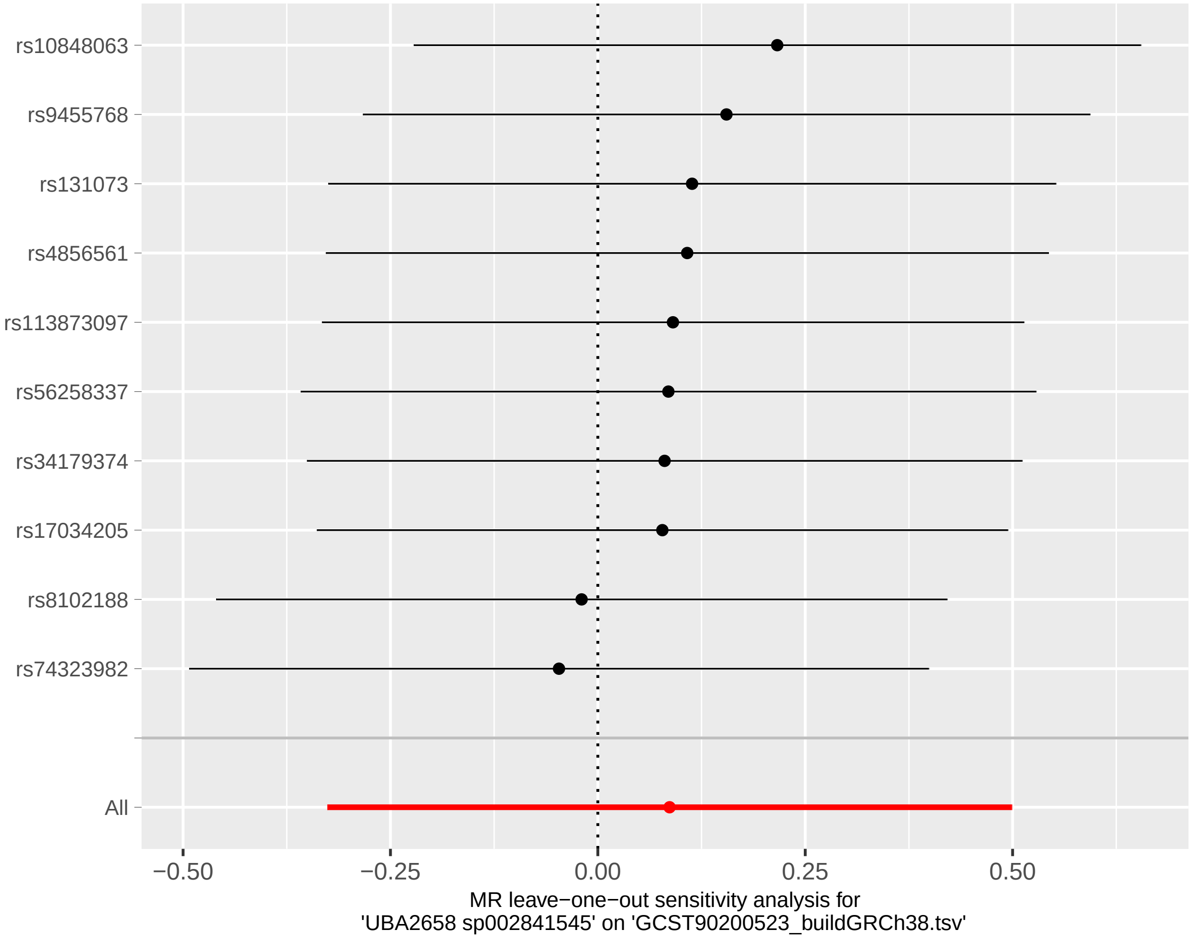
rs10848063
rs9455768
rs131073
rs4856561
rs113873097
rs56258337
rs34179374
rs17034205
rs8102188
rs74323982
All
−0.50
−0.25
0.00
0.25
0.50
MR leave−one−out sensitivity analysis for
'UBA2658 sp002841545' on 'GCST90200523_buildGRCh38.tsv'

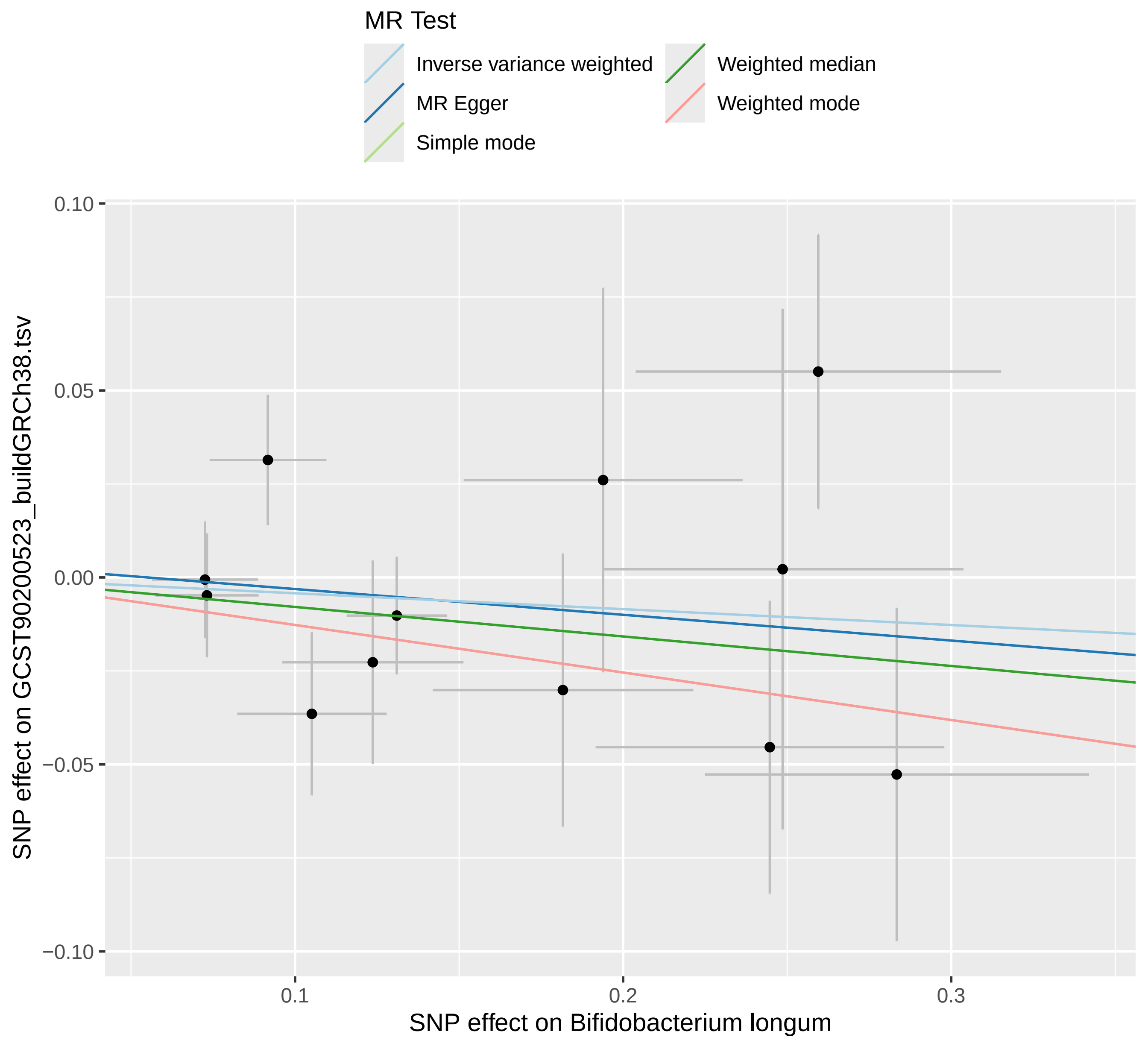
MR Test
Inverse variance weighted
MR Egger
Simple mode
Weighted median
Weighted mode
0.10
0.05
0.00
−0.05
−0.10
0.1
0.2
0.3
SNP effect on GCST90200523_buildGRCh38.tsv
SNP effect on Bifidobacterium longum

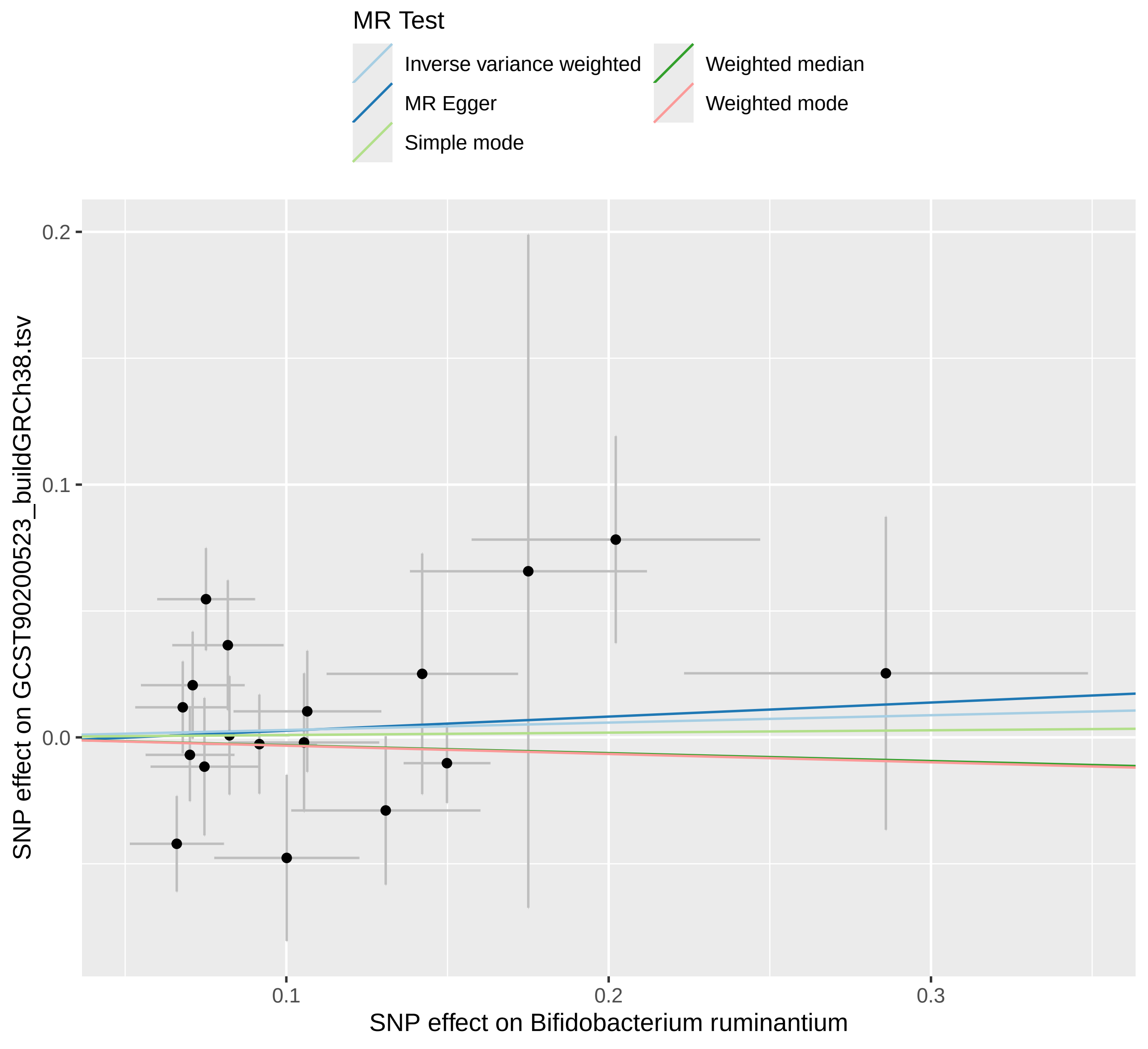
MR Test
Inverse variance weighted
MR Egger
Simple mode
Weighted median
Weighted mode
0.2
0.1
0.0
SNP effect on GCST90200523_buildGRCh38.tsv
0.1
0.2
0.3
SNP effect on Bifidobacterium ruminantium

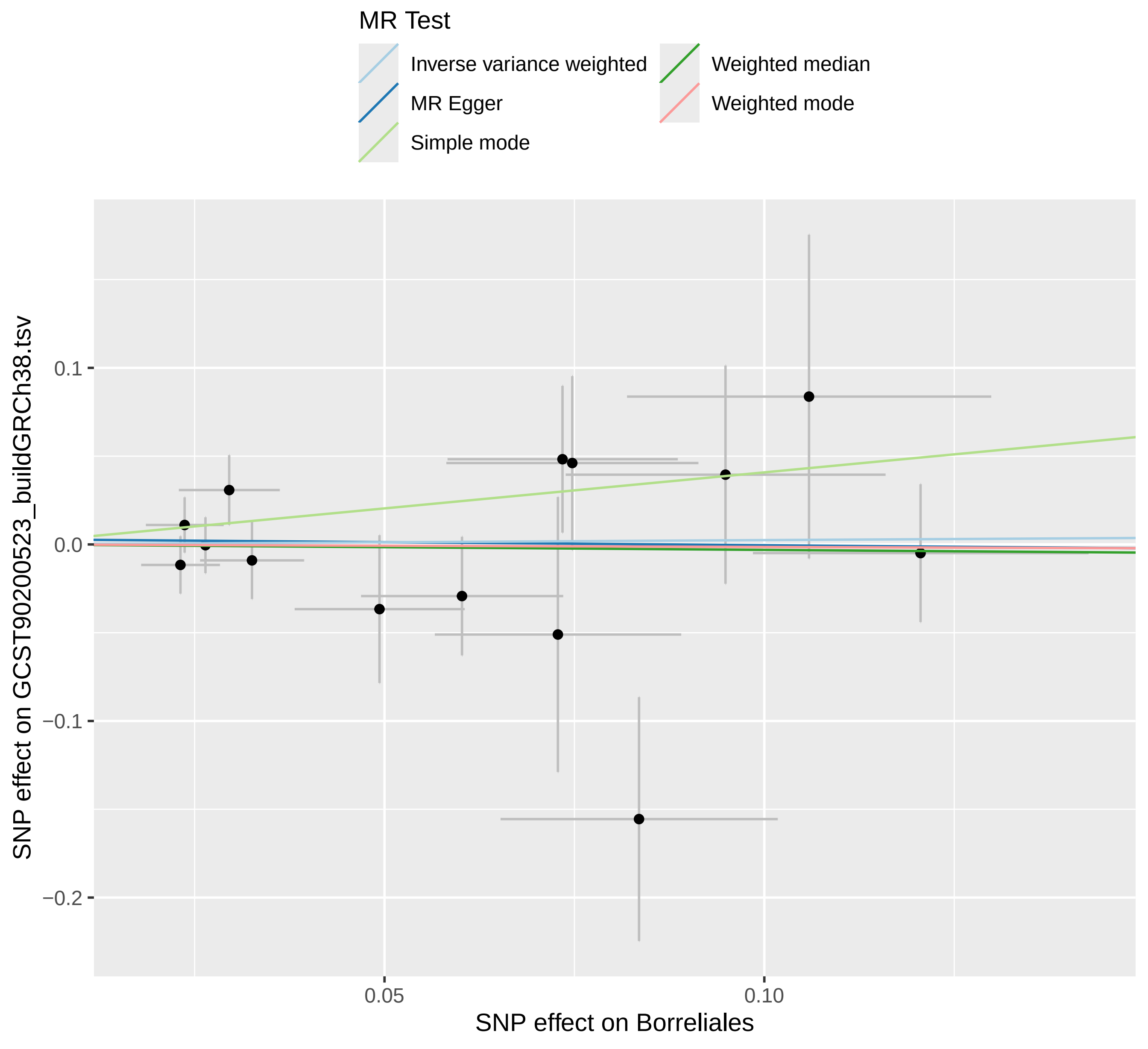
MR Test
Inverse variance weighted
MR Egger
Simple mode
Weighted median
Weighted mode
SNP effect on GCST90200523_buildGRCh38.tsv
0.1
0.0
−0.1
−0.2
0.05
0.10
SNP effect on Borreliales

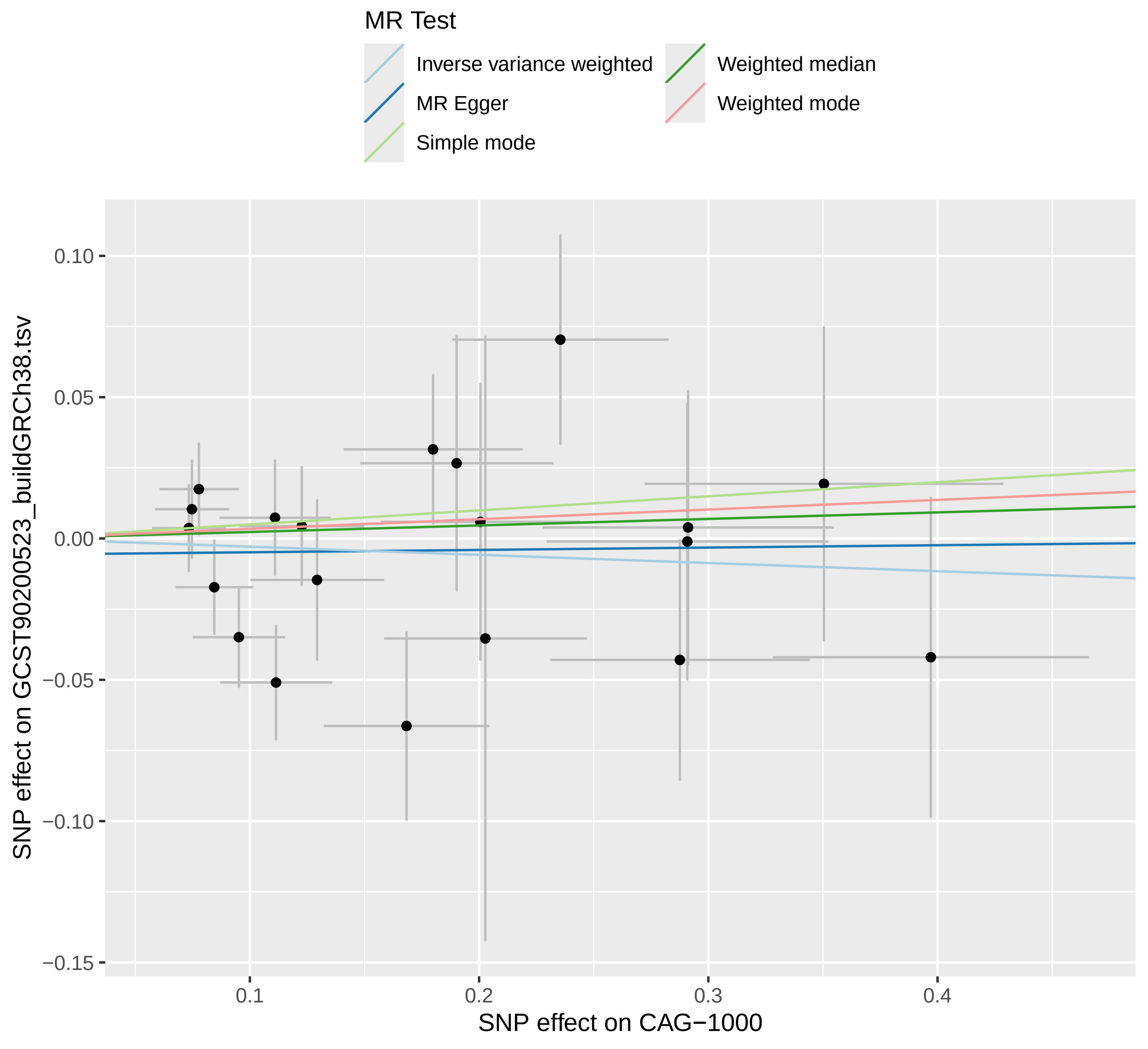
MR Test
Inverse variance weighted
MR Egger
Simple mode
Weighted median
Weighted mode
0.10
0.05
0.00
−0.05
−0.10
−0.15
0.1
0.2
0.3
0.4
SNP effect on GCST90200523_buildGRCh38.tsv
SNP effect on CAG−1000

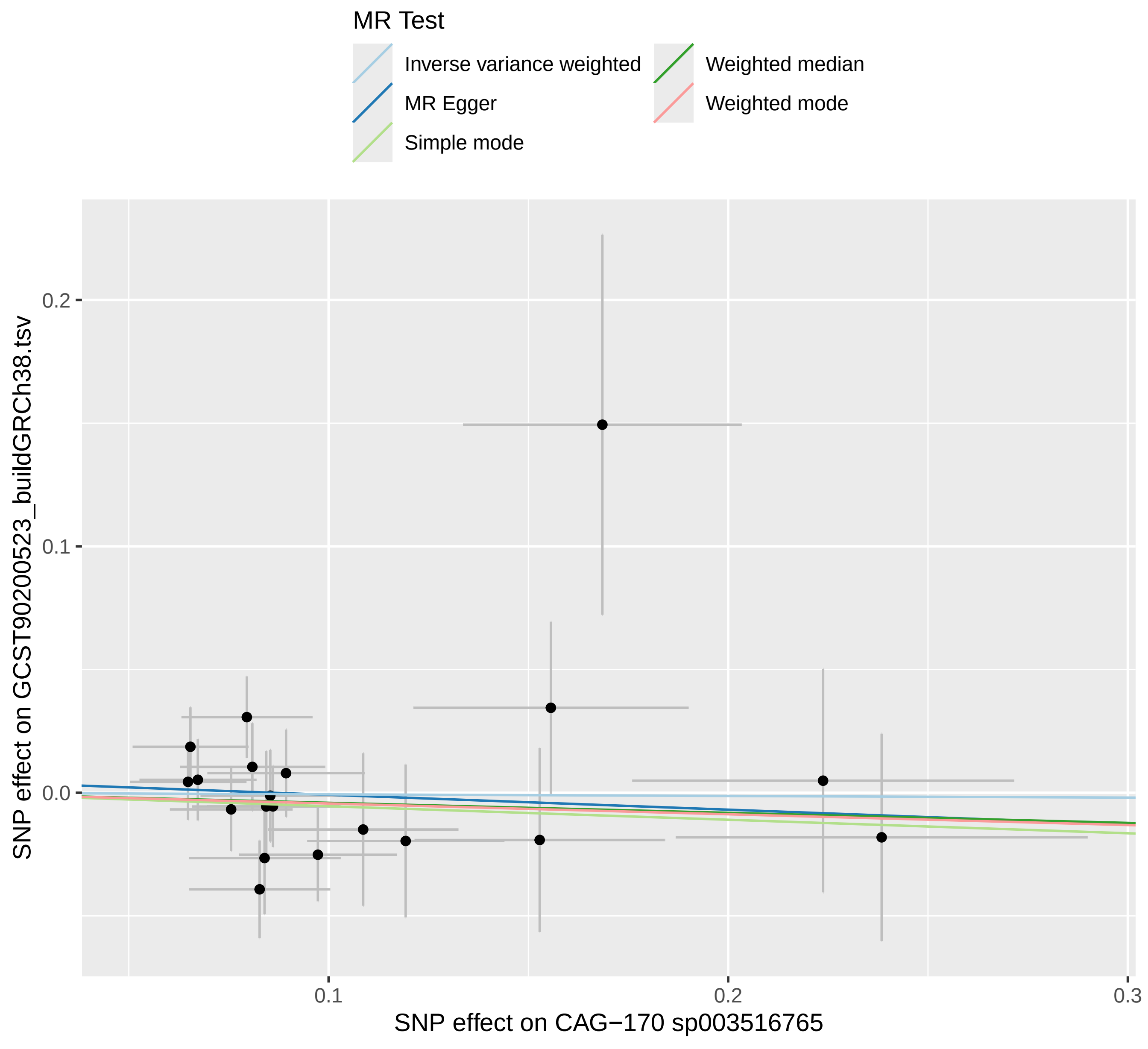

MR Test
Inverse variance weighted
MR Egger
Simple mode
Weighted median
Weighted mode
SNP effect on GCST90200523_buildGRCh38.tsv
0.2
0.1
0.0
0.1
0.2
0.3
SNP effect on CAG−170 sp003516765

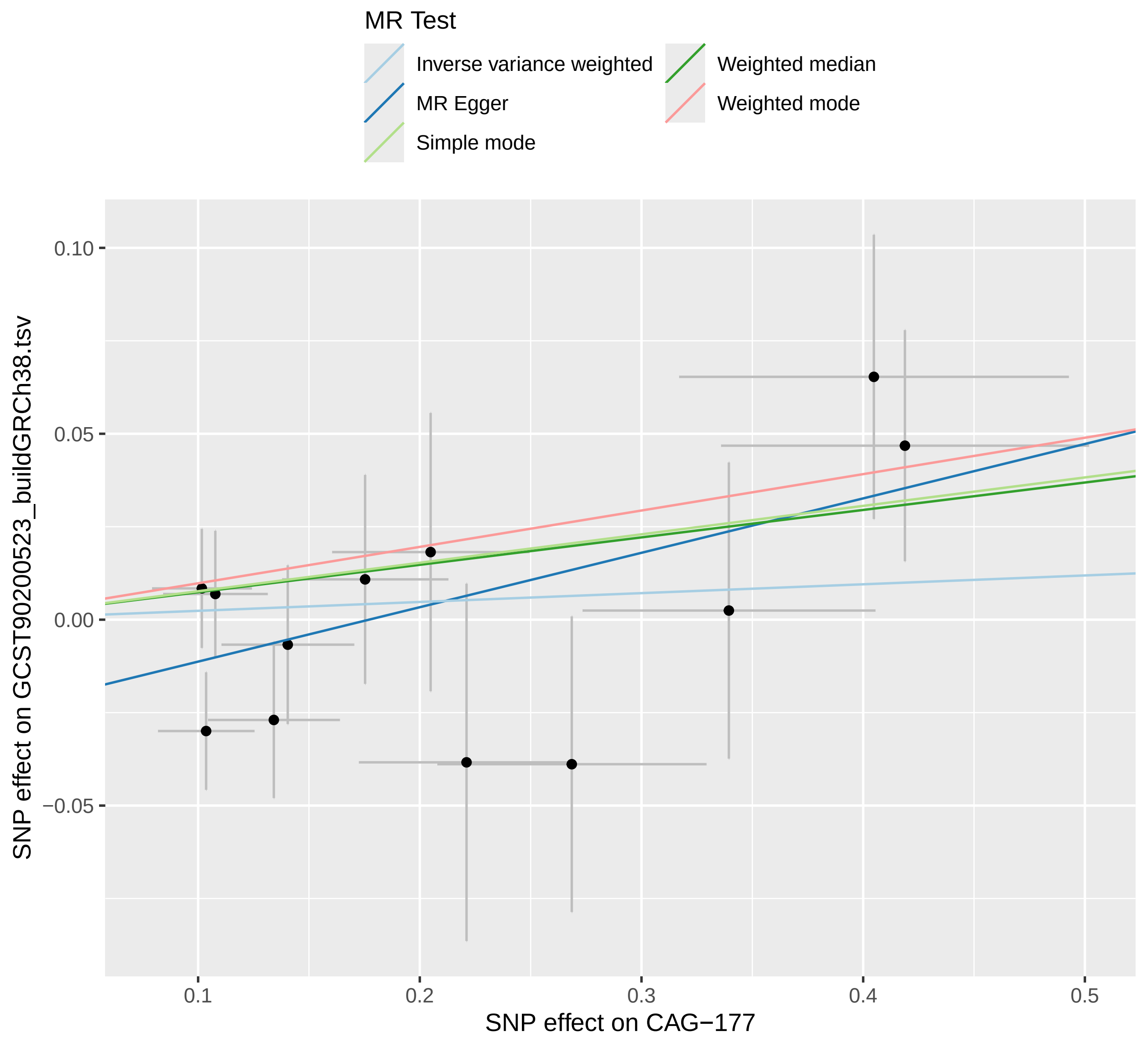

MR Test
Inverse variance weighted
MR Egger
Simple mode
Weighted median
Weighted mode
0.10
0.05
0.00
−0.05
0.1
0.2
0.3
0.4
0.5
SNP effect on GCST90200523_buildGRCh38.tsv
SNP effect on CAG−177

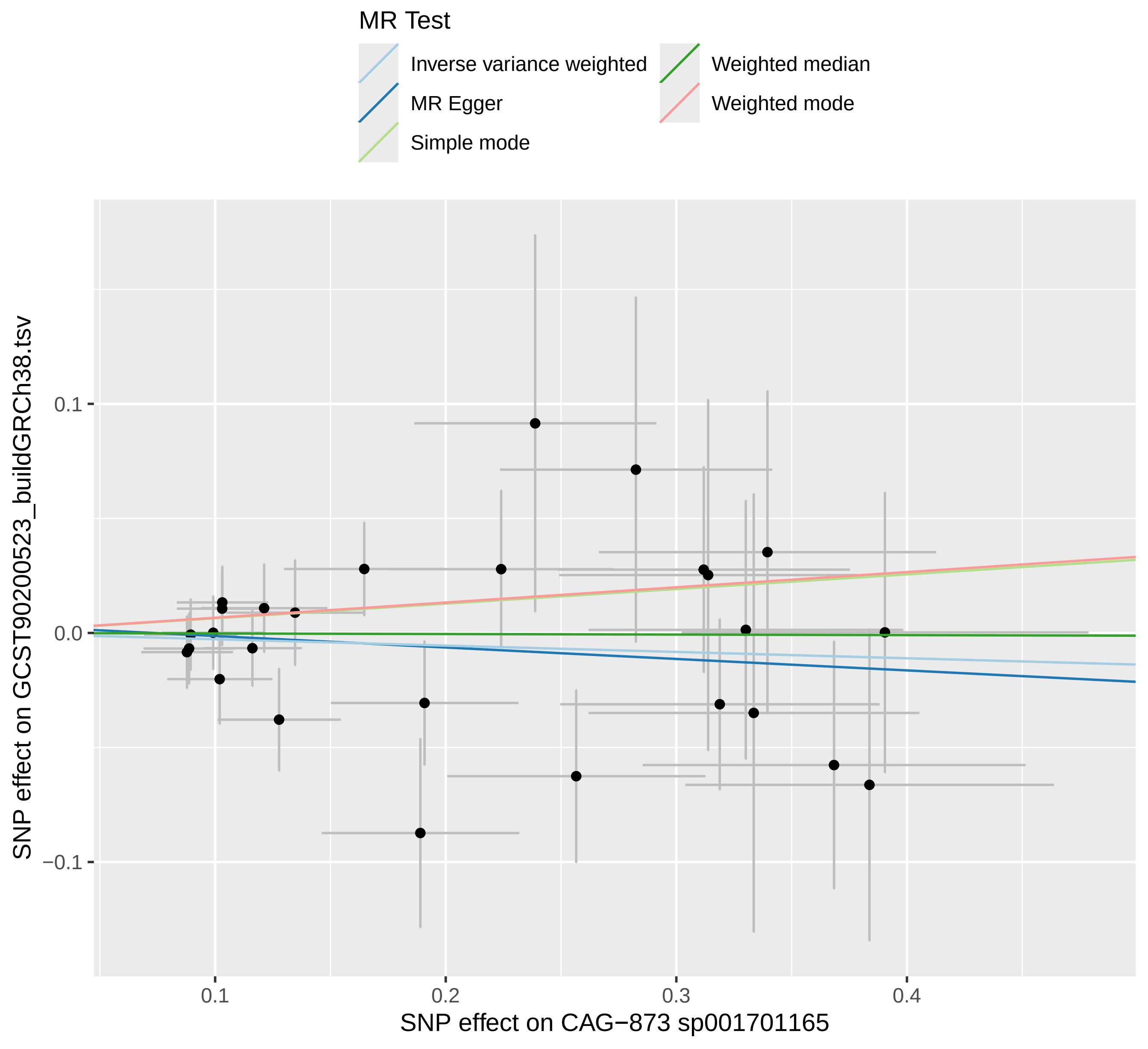

MR Test
Inverse variance weighted
MR Egger
Simple mode
Weighted median
Weighted mode
SNP effect on GCST90200523_buildGRCh38.tsv
0.1
0.0
−0.1
0.1
0.2
0.3
0.4
SNP effect on CAG−873 sp001701165

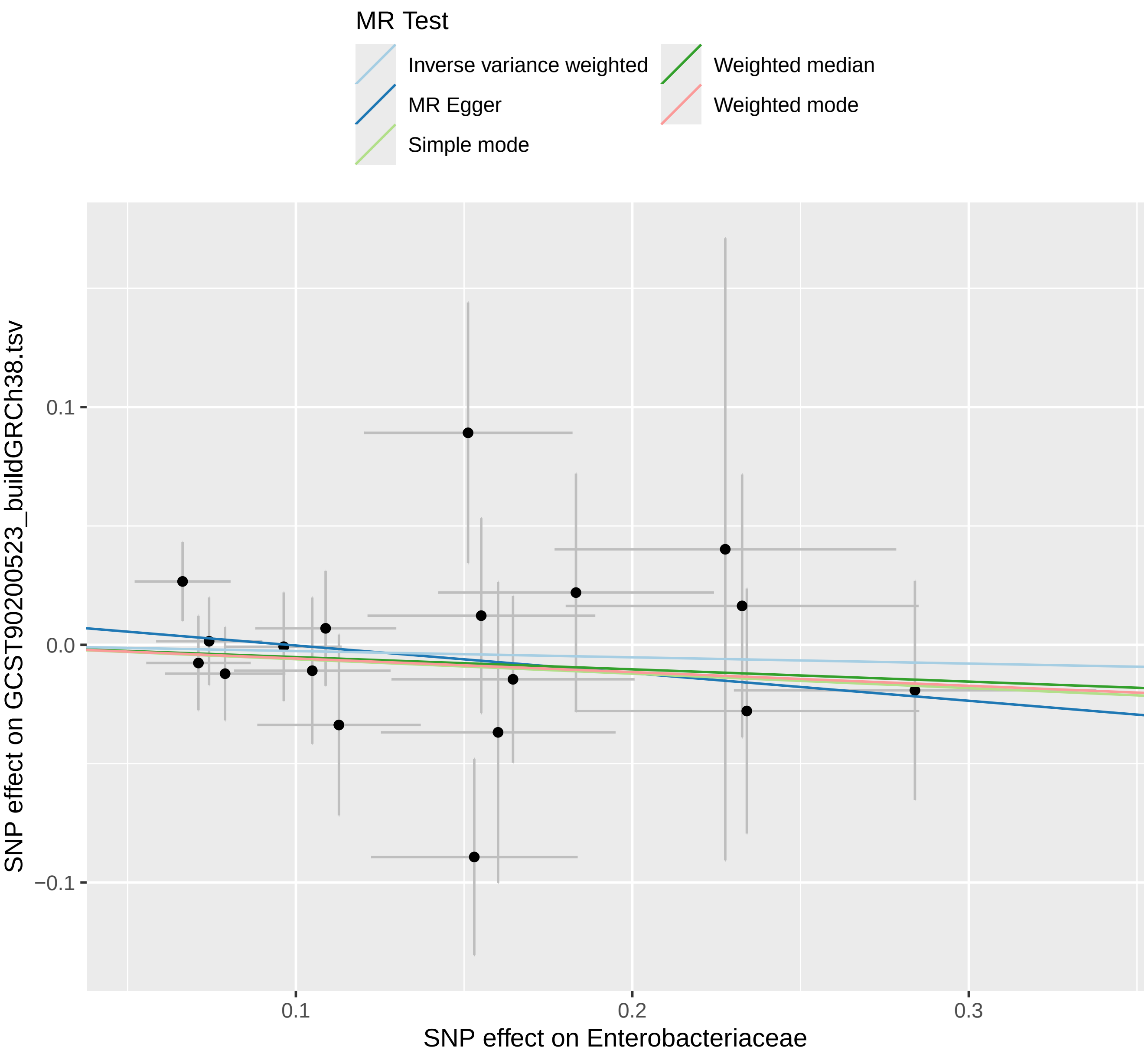
MR Test
Inverse variance weighted
MR Egger
Simple mode
Weighted median
Weighted mode
0.1
0.0
−0.1
0.1
0.2
0.3
SNP effect on GCST90200523_buildGRCh38.tsv
SNP effect on Enterobacteriaceae

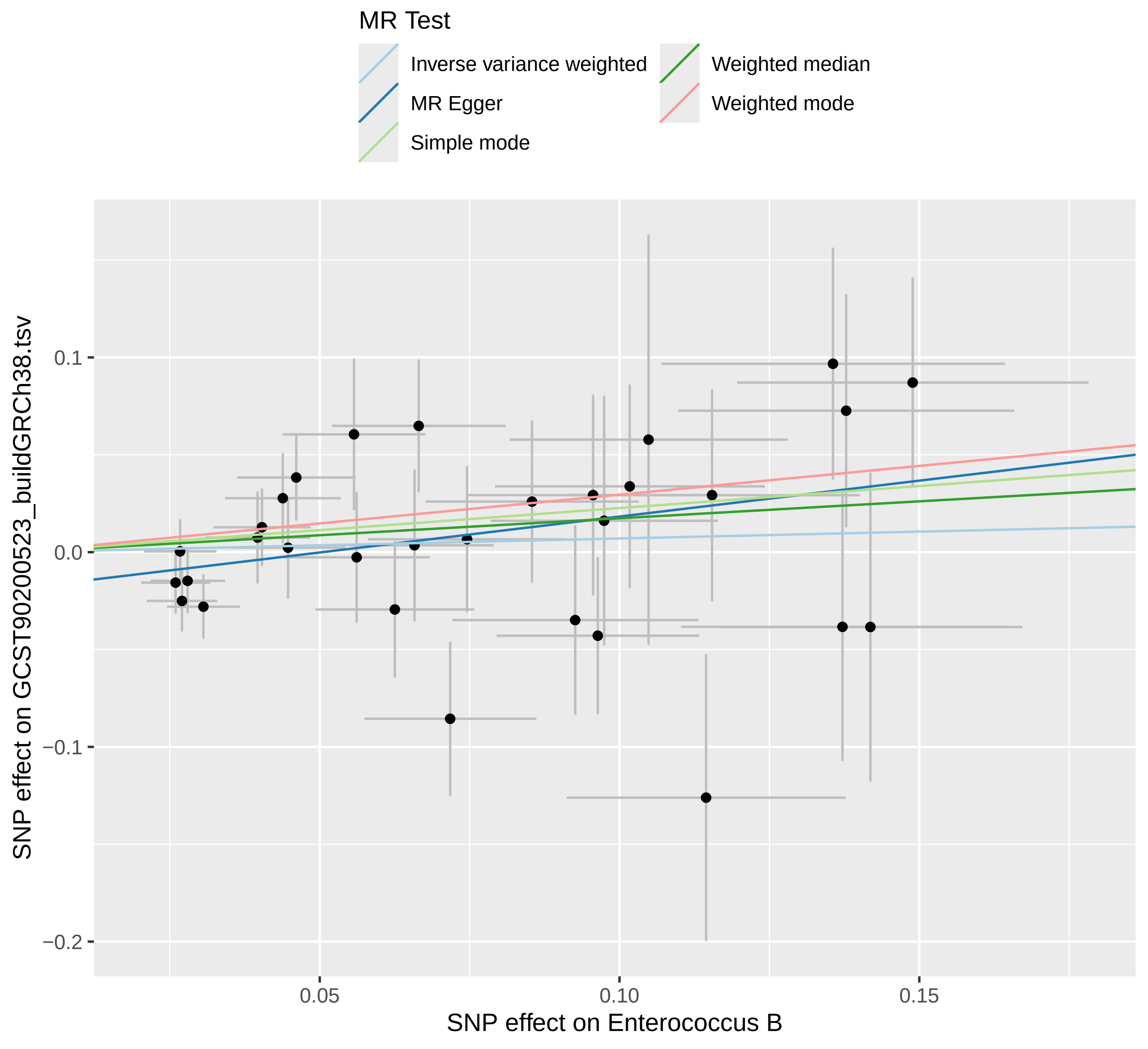

MR Test
Inverse variance weighted
MR Egger
Simple mode
Weighted median
Weighted mode
SNP effect on GCST90200523_buildGRCh38.tsv
0.1
0.0
−0.1
−0.2
0.05
0.10
0.15
SNP effect on Enterococcus B

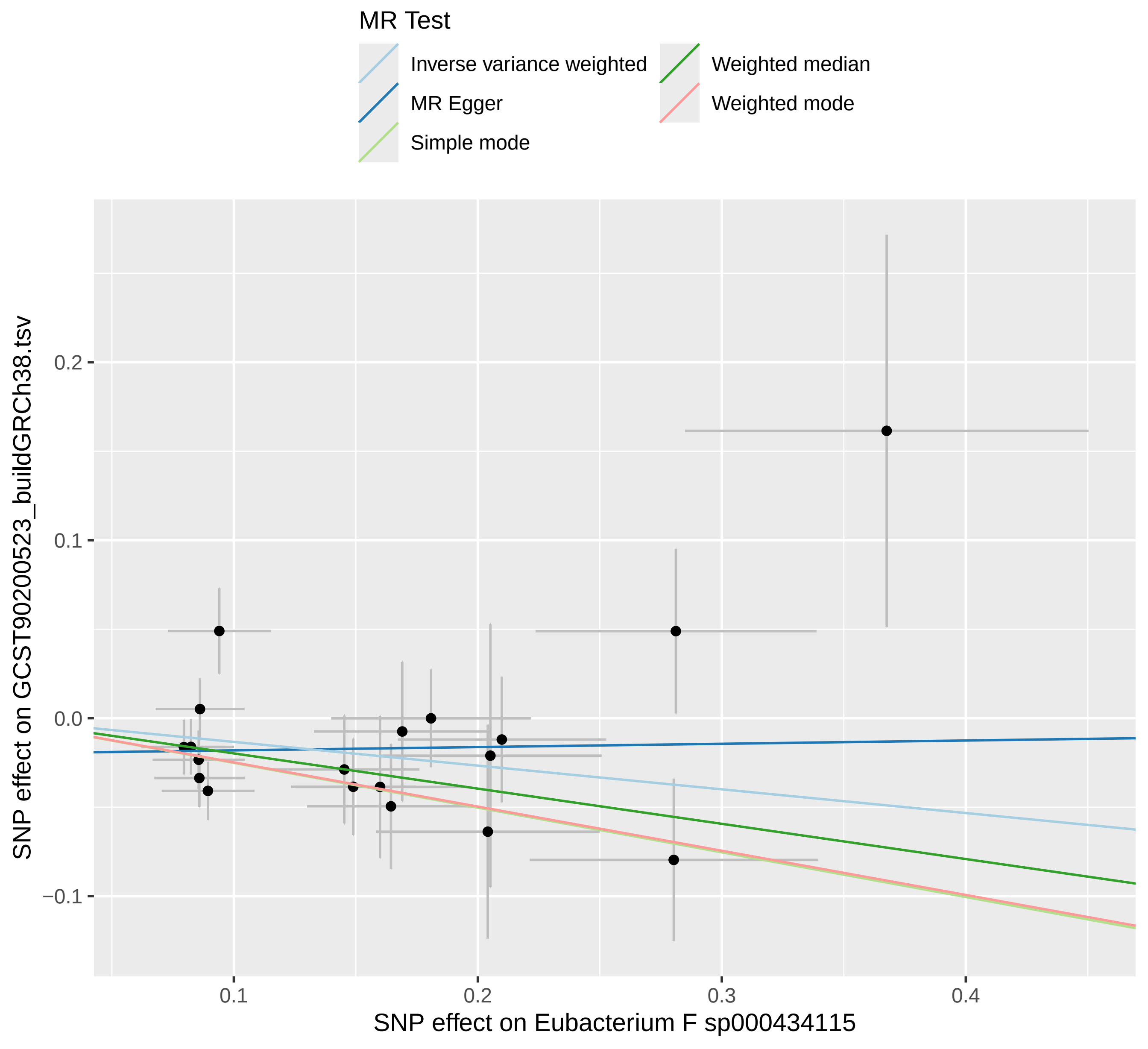
MR Test
Inverse variance weighted
MR Egger
Simple mode
Weighted median
Weighted mode
SNP effect on GCST90200523_buildGRCh38.tsv
0.2
0.1
0.0
−0.1
0.1
0.2
0.3
0.4
SNP effect on Eubacterium F sp000434115

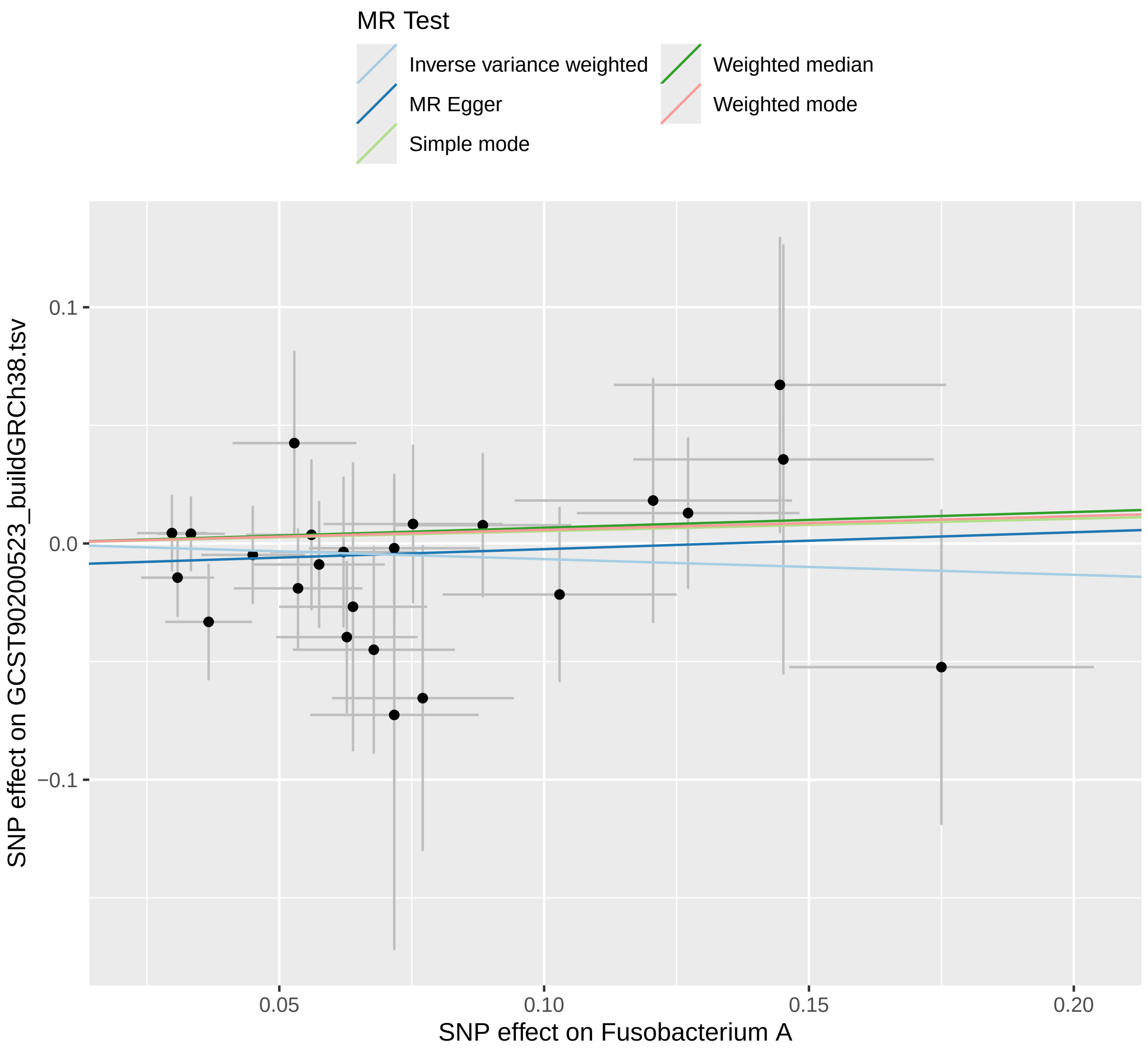
MR Test
Inverse variance weighted
MR Egger
Simple mode
Weighted median
Weighted mode
0.1
0.0
−0.1
0.05
0.10
0.15
0.20
SNP effect on GCST90200523_buildGRCh38.tsv
SNP effect on Fusobacterium A

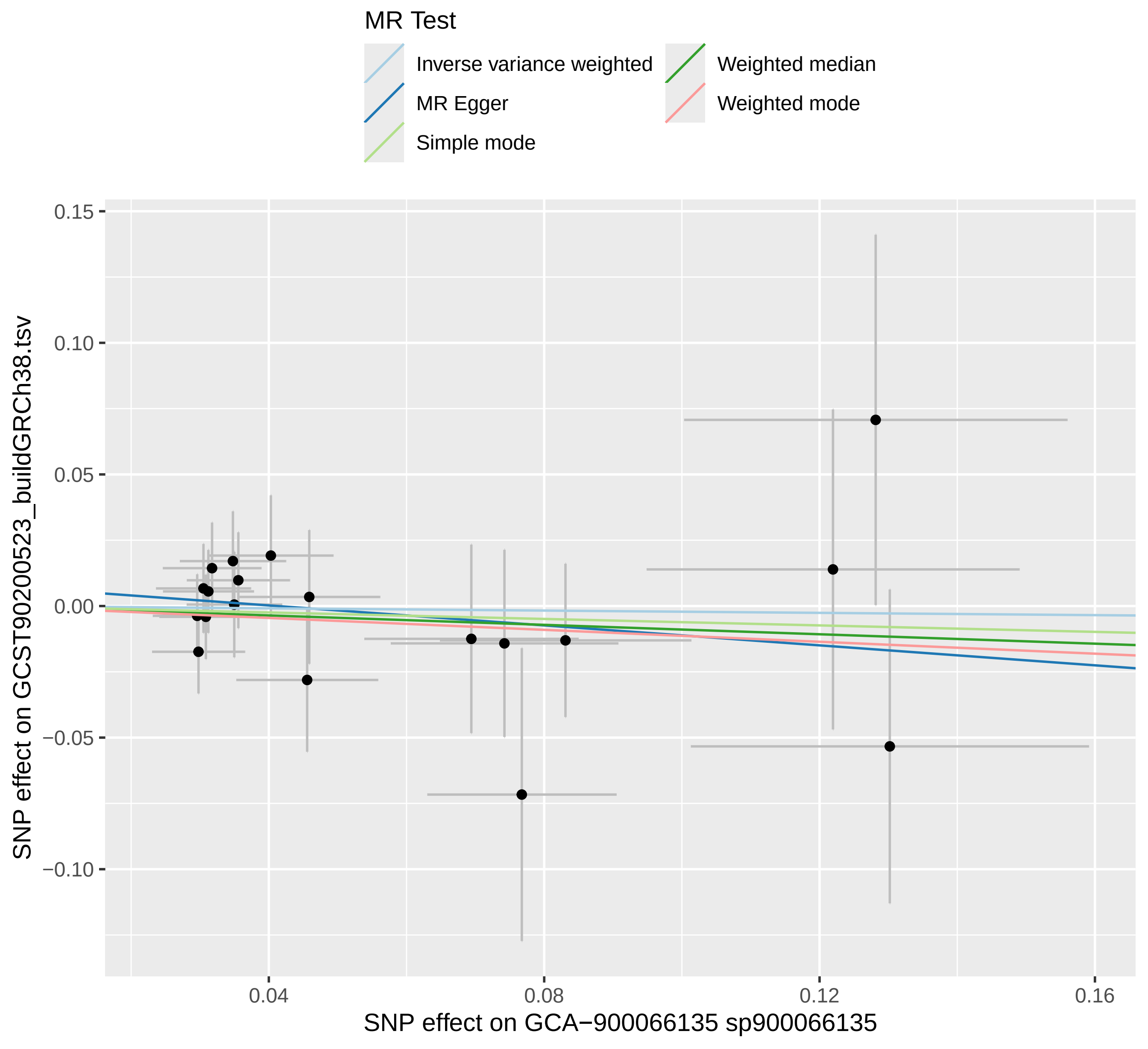

MR Test
Inverse variance weighted
MR Egger
Simple mode
Weighted median
Weighted mode
0.15
0.10
0.05
0.00
−0.05
−0.10
SNP effect on GCST90200523_buildGRCh38.tsv
0.04
0.08
0.12
0.16
SNP effect on GCA−900066135 sp900066135

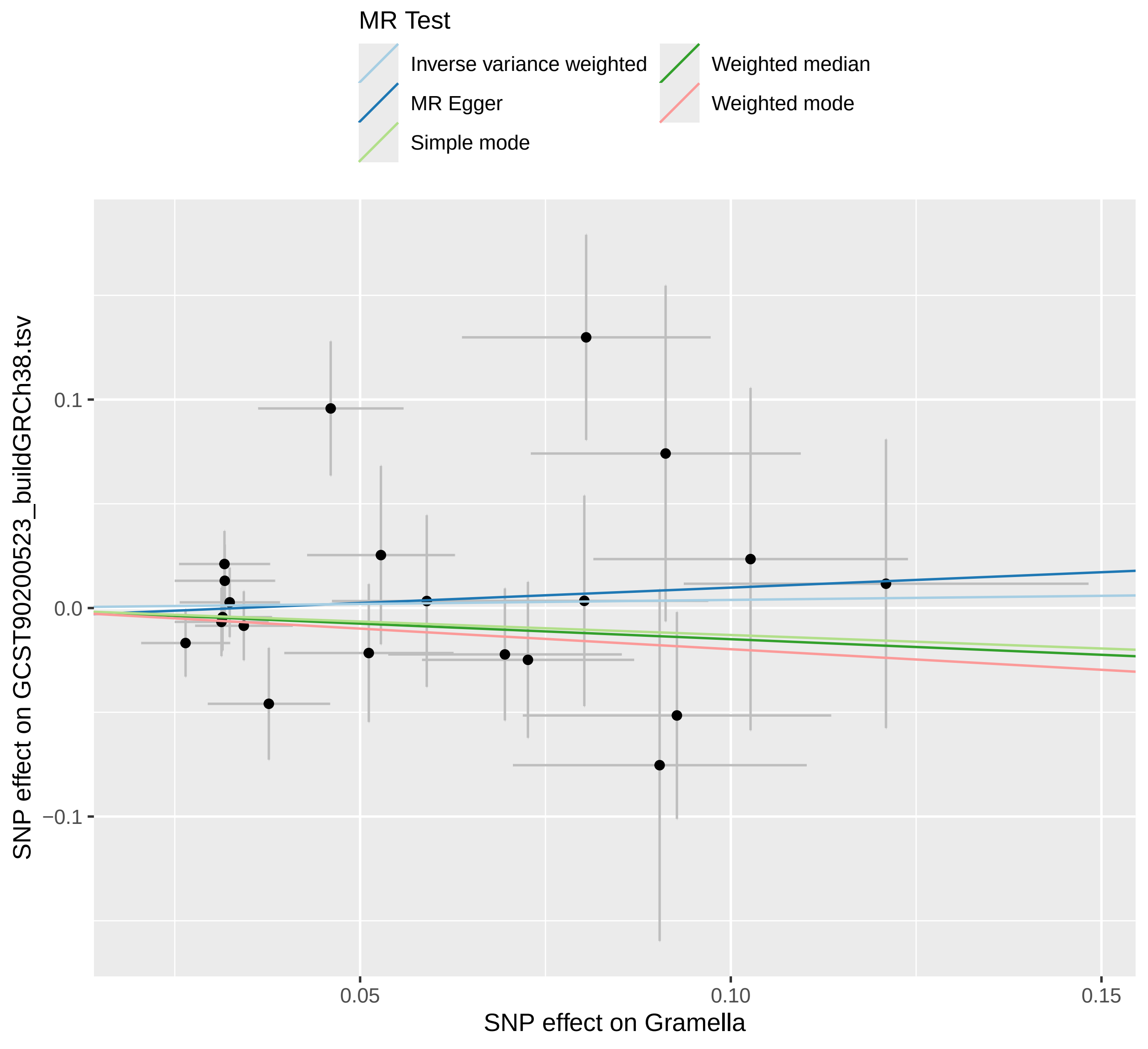

MR Test
Inverse variance weighted
MR Egger
Simple mode
Weighted median
Weighted mode
SNP effect on GCST90200523_buildGRCh38.tsv
0.1
0.0
−0.1
0.05
0.10
0.15
SNP effect on Gramella

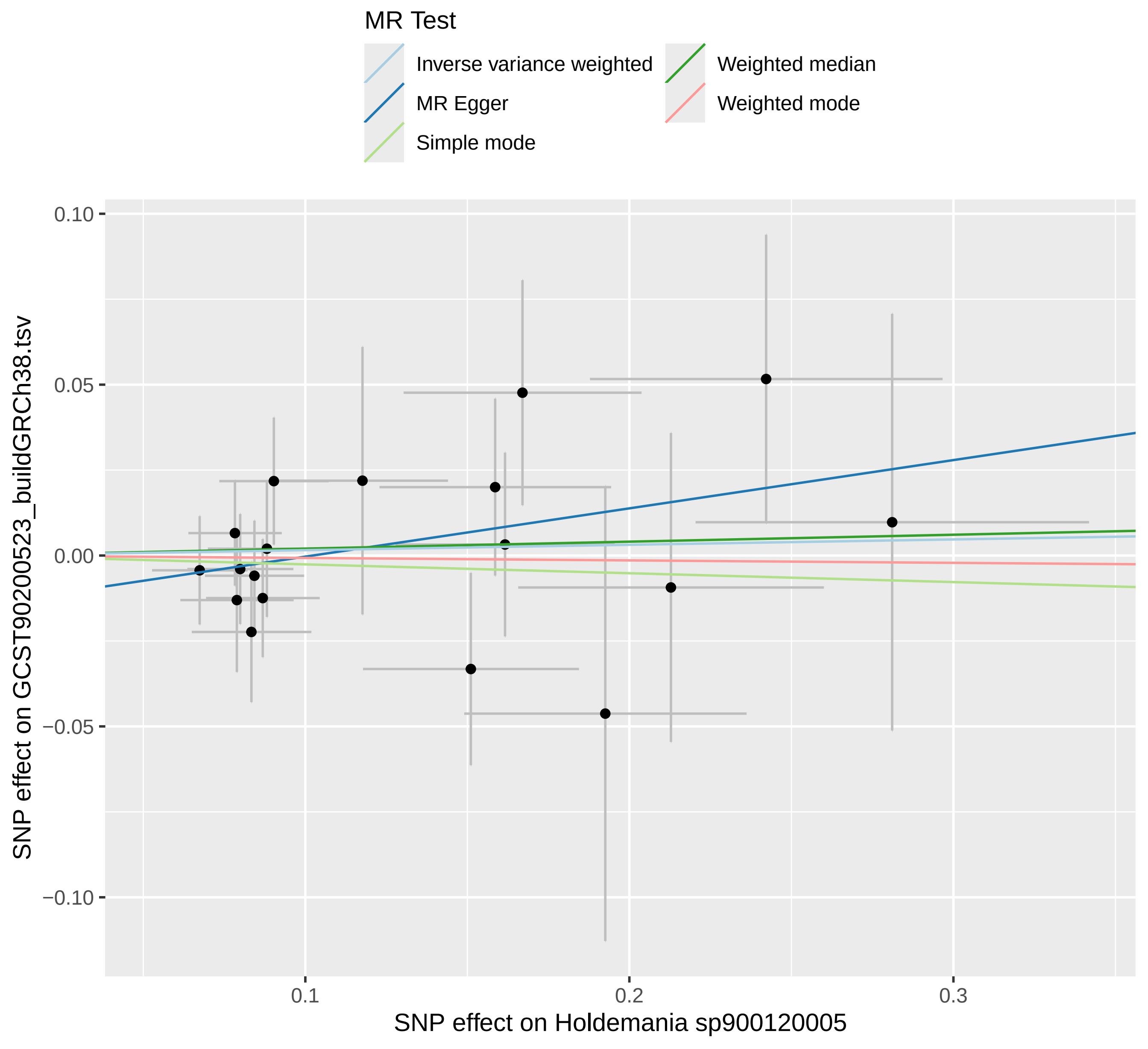
MR Test
Inverse variance weighted
MR Egger
Simple mode
Weighted median
Weighted mode
0.10
0.05
0.00
−0.05
−0.10
0.1
0.2
0.3
SNP effect on GCST90200523_buildGRCh38.tsv
SNP effect on Holdemania sp900120005

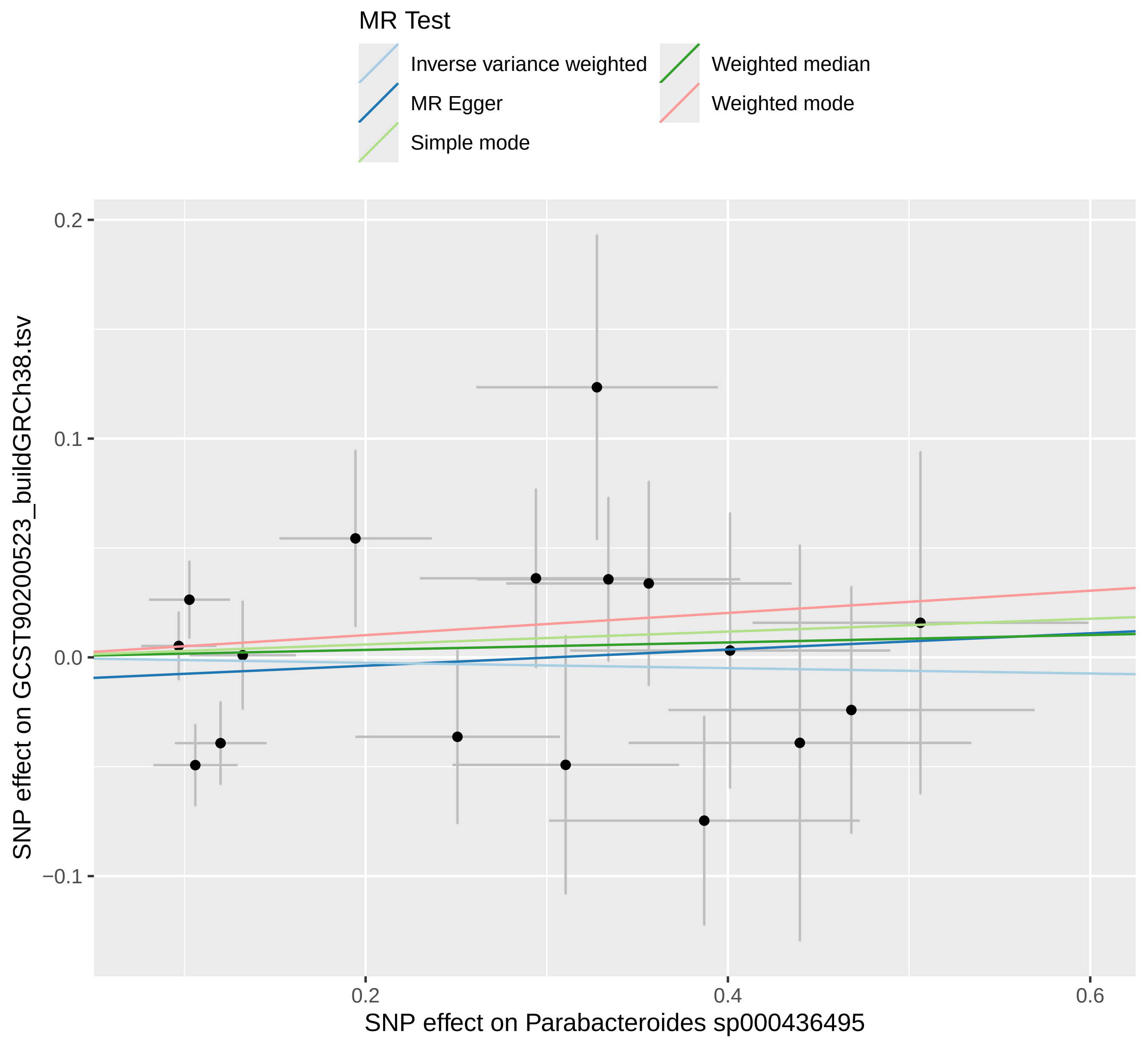
MR Test
Inverse variance weighted
MR Egger
Simple mode
Weighted median
Weighted mode
0.2
0.1
0.0
−0.1
0.2
0.4
0.6
SNP effect on GCST90200523_buildGRCh38.tsv
SNP effect on Parabacteroides sp000436495

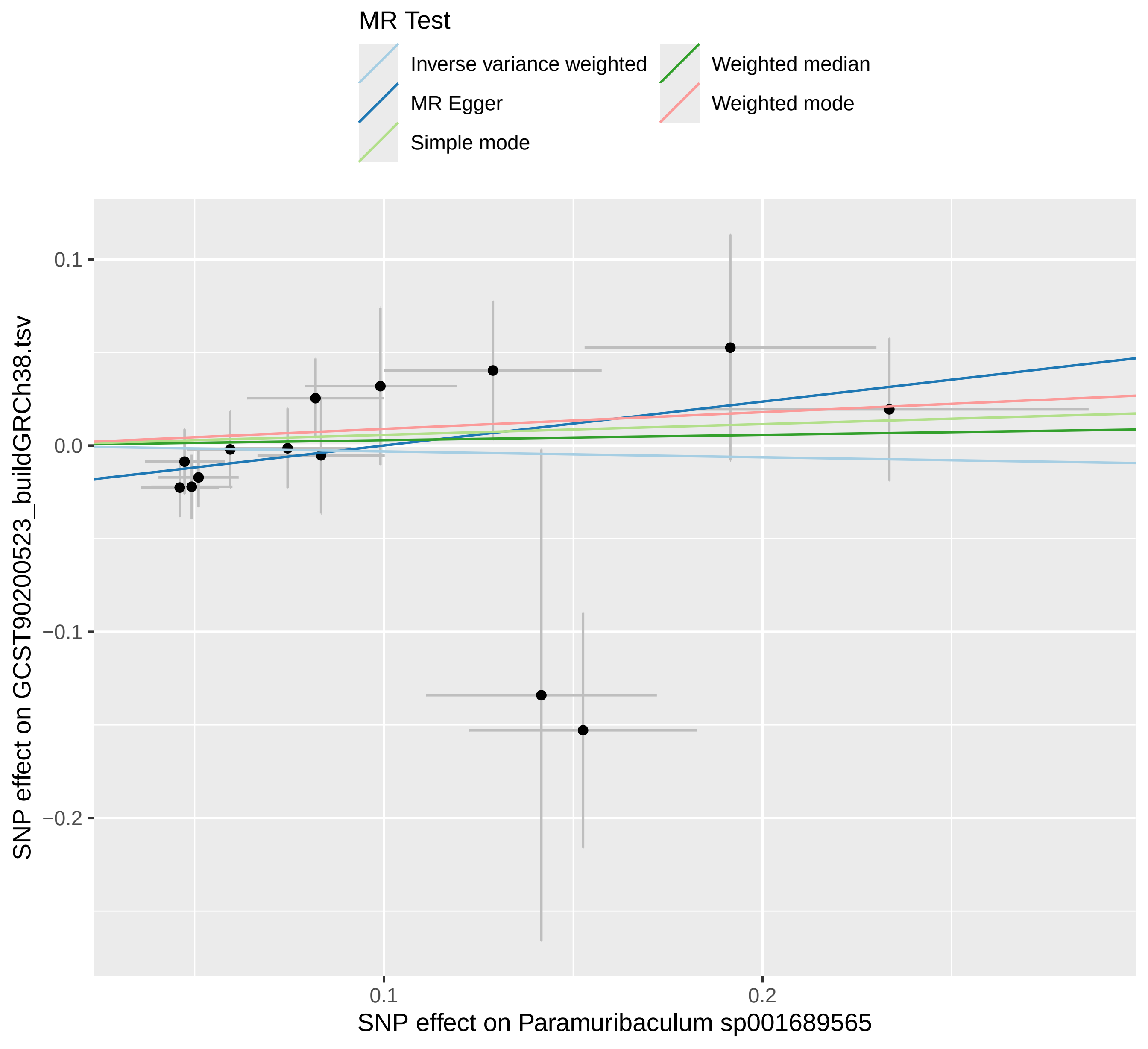
MR Test
Inverse variance weighted
MR Egger
Simple mode
Weighted median
Weighted mode
0.1
0.0
−0.1
−0.2
0.1
0.2
SNP effect on GCST90200523_buildGRCh38.tsv
SNP effect on Paramuribaculum sp001689565

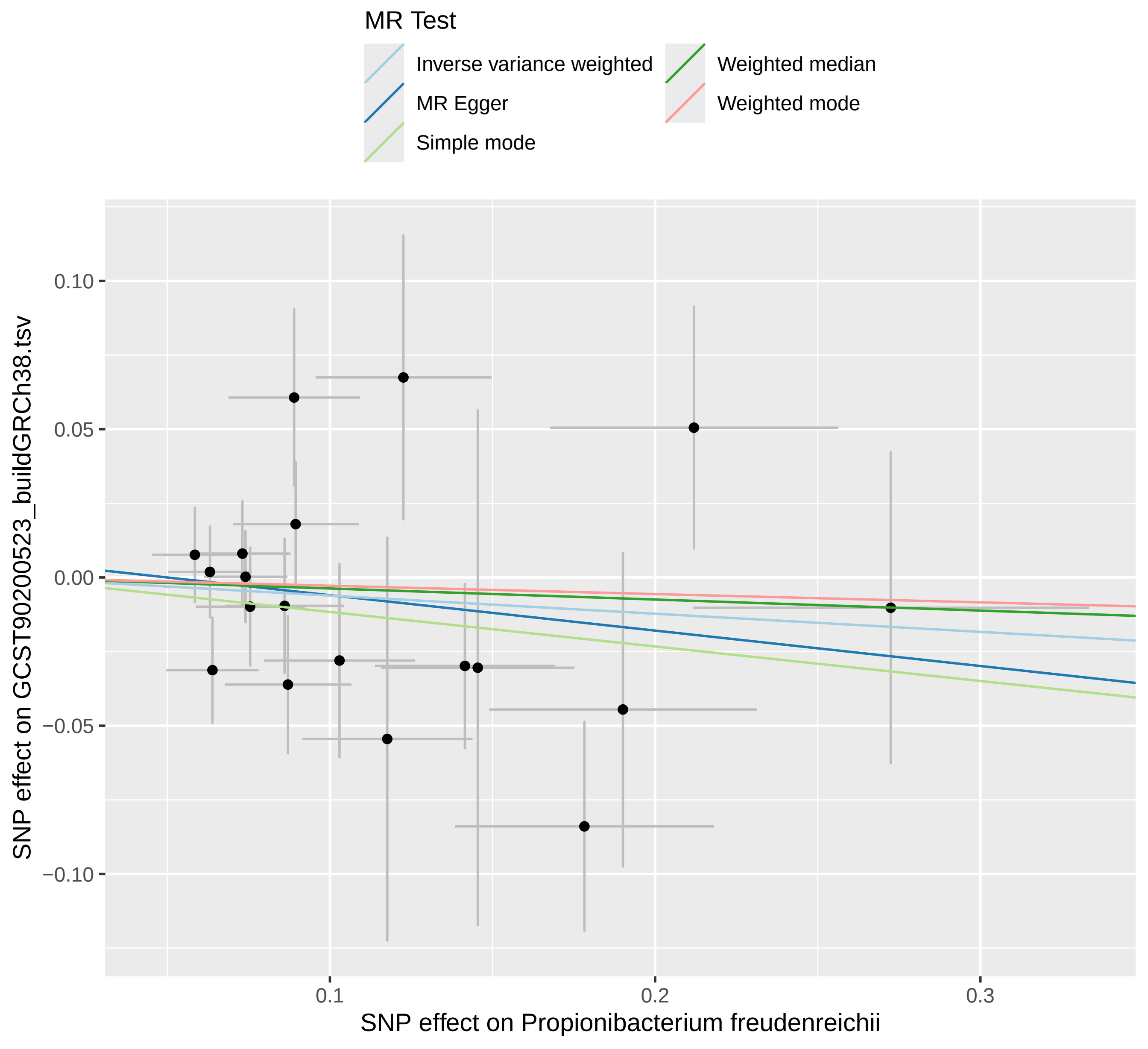
MR Test
Inverse variance weighted
MR Egger
Simple mode
Weighted median
Weighted mode
0.10
0.05
0.00
−0.05
−0.10
SNP effect on GCST90200523_buildGRCh38.tsv
0.1
0.2
0.3
SNP effect on Propionibacterium freudenreichii

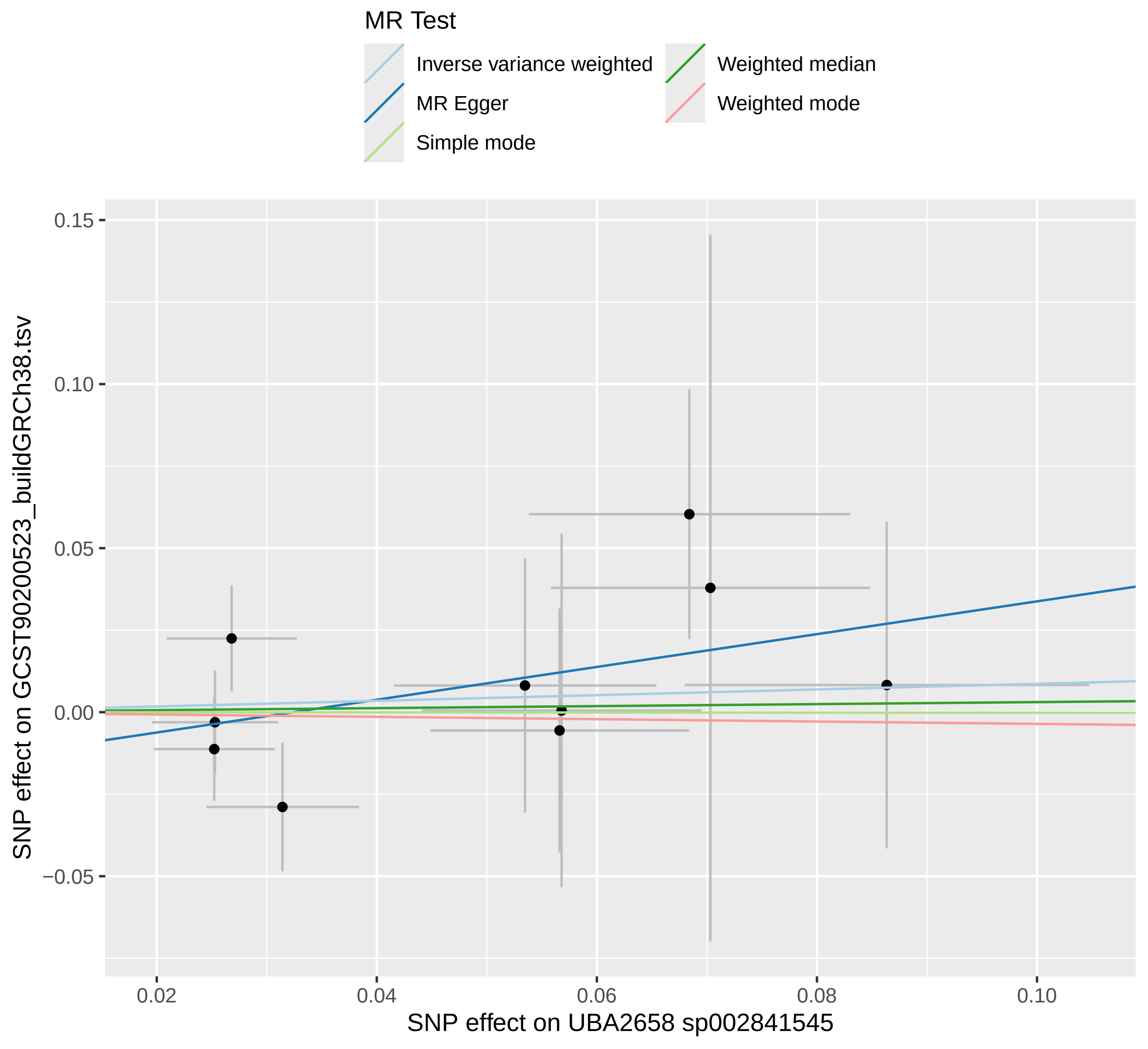
MR Test
Inverse variance weighted
MR Egger
Simple mode
Weighted median
Weighted mode
0.15
0.10
0.05
0.00
−0.05
0.02
0.04
0.06
0.08
0.10
SNP effect on GCST90200523_buildGRCh38.tsv
SNP effect on UBA2658 sp002841545

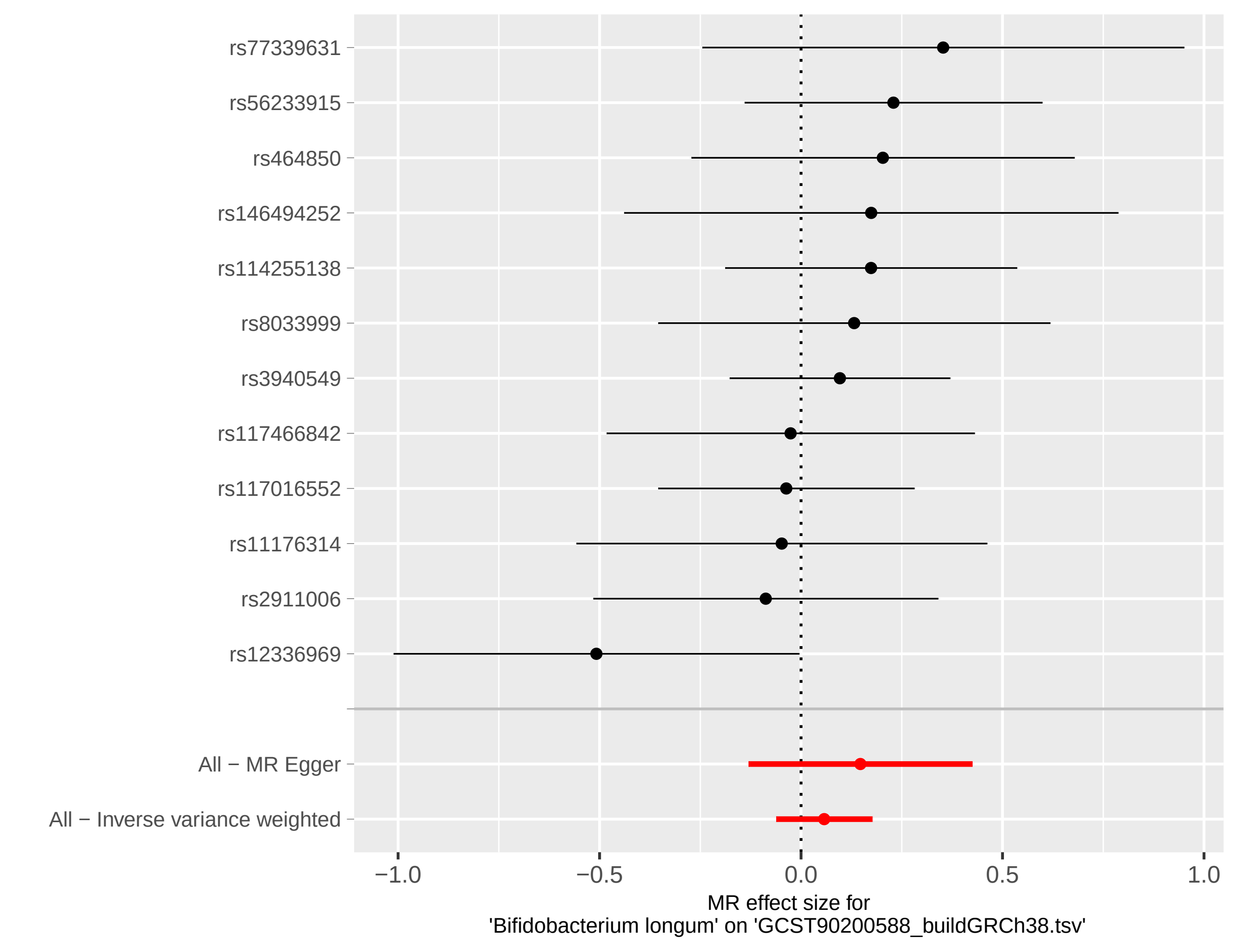
rs77339631
rs56233915
rs464850
rs146494252
rs114255138
rs8033999
rs3940549
rs117466842
rs117016552
rs11176314
rs2911006
rs12336969
All − MR Egger
All − Inverse variance weighted
−1.0
−0.5
0.0
0.5
1.0
MR effect size for
'Bifidobacterium longum' on 'GCST90200588_buildGRCh38.tsv'

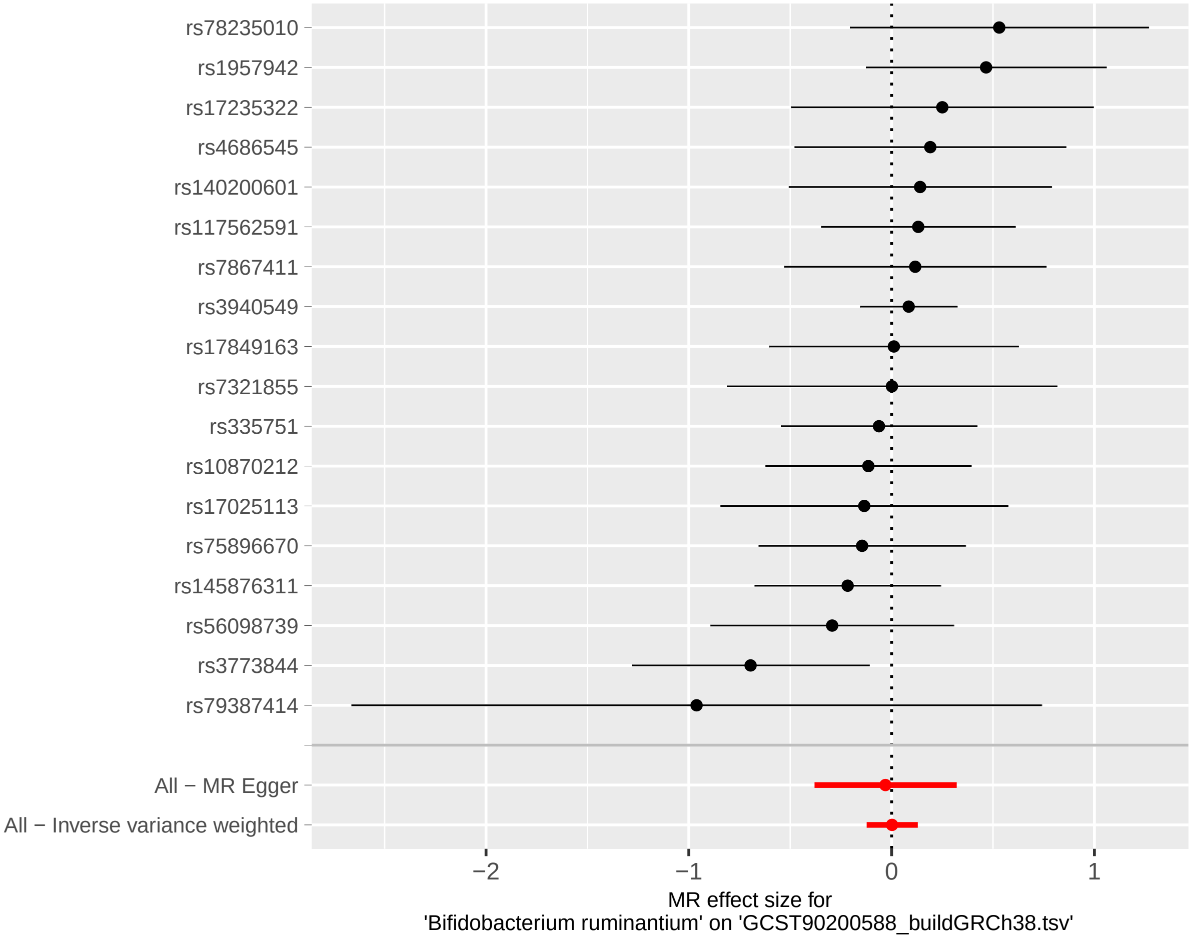
rs78235010
rs1957942
rs17235322
rs4686545
rs140200601
rs117562591
rs7867411
rs3940549
rs17849163
rs7321855
rs335751
rs10870212
rs17025113
rs75896670
rs145876311
rs56098739
rs3773844
rs79387414
All − MR Egger
All − Inverse variance weighted
−2
−1
0
1
MR effect size for
'Bifidobacterium ruminantium' on 'GCST90200588_buildGRCh38.tsv'

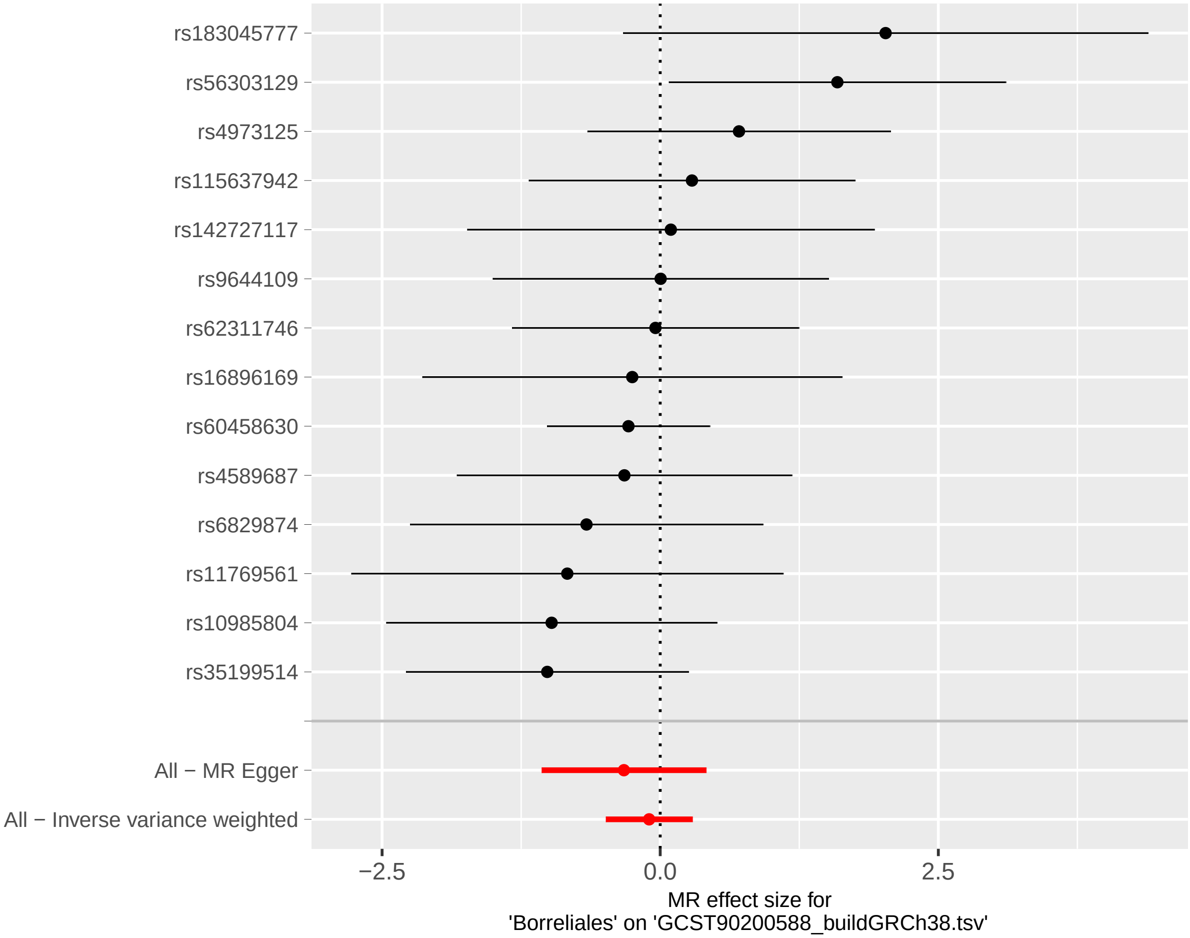
rs183045777
rs56303129
rs4973125
rs115637942
rs142727117
rs9644109
rs62311746
rs16896169
rs60458630
rs4589687
rs6829874
rs11769561
rs10985804
rs35199514
All − MR Egger
All − Inverse variance weighted
−2.5
0.0
2.5
MR effect size for
'Borreliales' on 'GCST90200588_buildGRCh38.tsv'

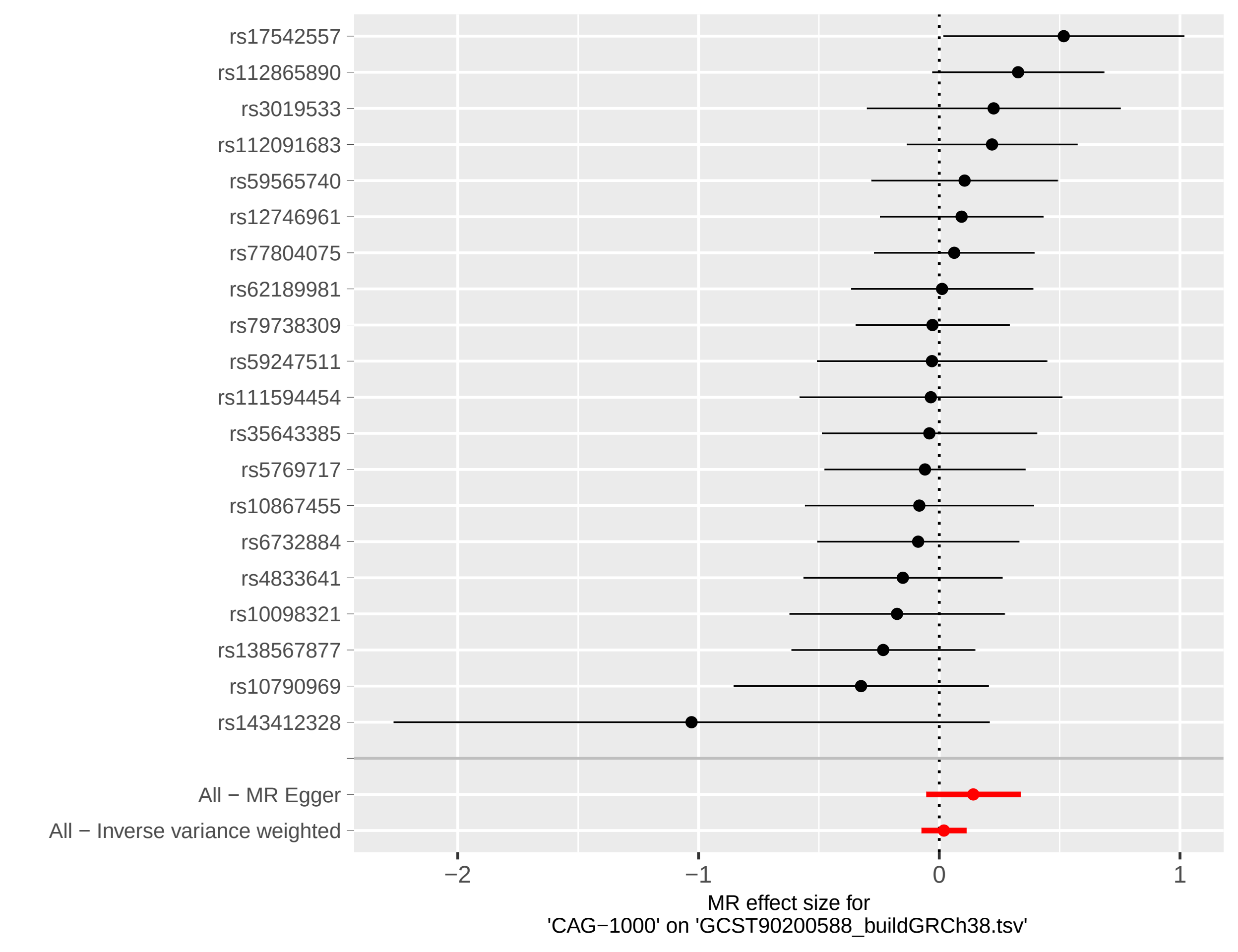

rs17542557
rs112865890
rs3019533
rs112091683
rs59565740
rs12746961
rs77804075
rs62189981
rs79738309
rs59247511
rs111594454
rs35643385
rs5769717
rs10867455
rs6732884
rs4833641
rs10098321
rs138567877
rs10790969
rs143412328
All − MR Egger
All − Inverse variance weighted
−2
−1
0
1
MR effect size for
'CAG−1000' on 'GCST90200588_buildGRCh38.tsv'

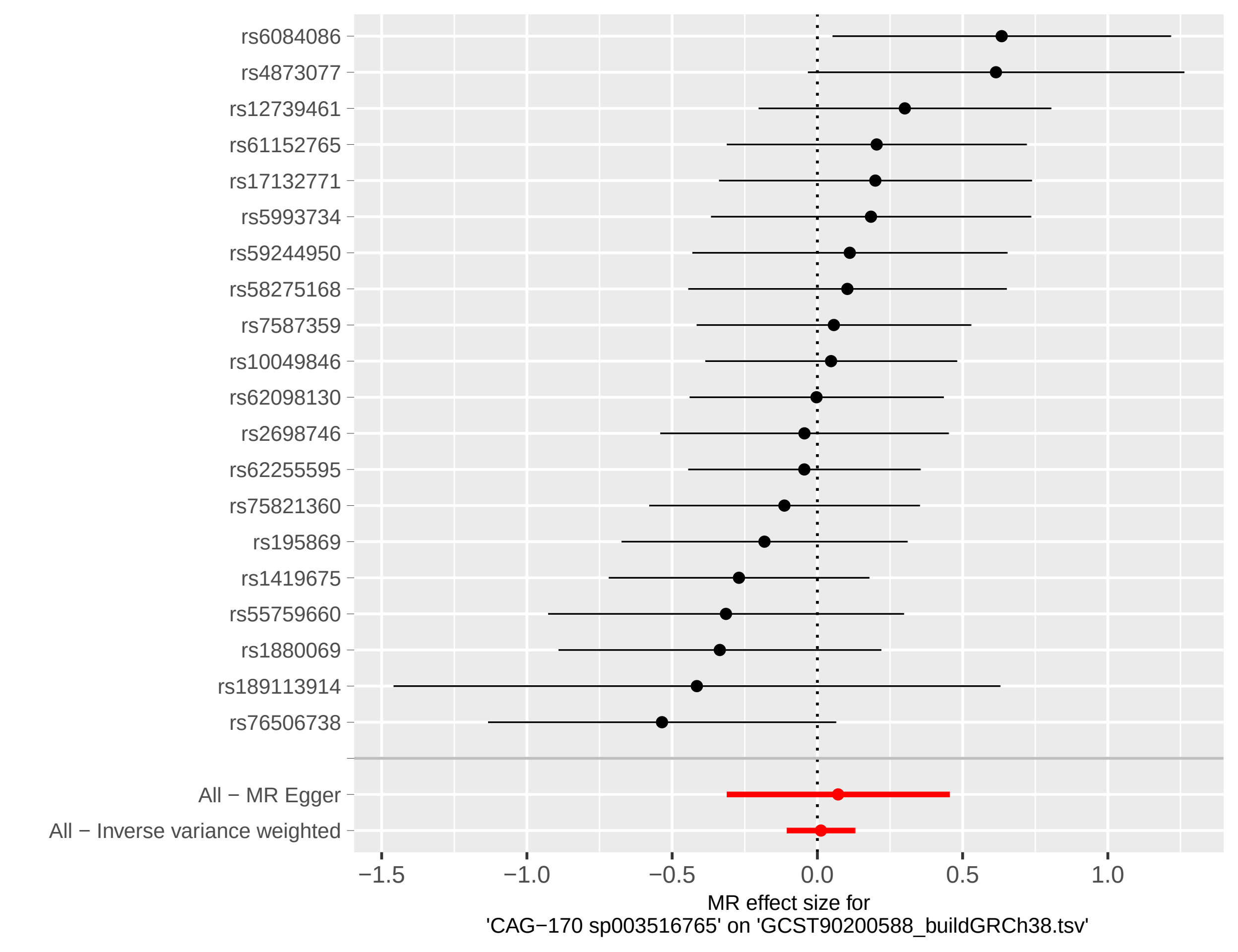
rs6084086
rs4873077
rs12739461
rs61152765
rs17132771
rs5993734
rs59244950
rs58275168
rs7587359
rs10049846
rs62098130
rs2698746
rs62255595
rs75821360
rs195869
rs1419675
rs55759660
rs1880069
rs189113914
rs76506738
All − MR Egger
All − Inverse variance weighted
−1.5
−1.0
−0.5
0.0
0.5
1.0
MR effect size for
'CAG−170 sp003516765' on 'GCST90200588_buildGRCh38.tsv'

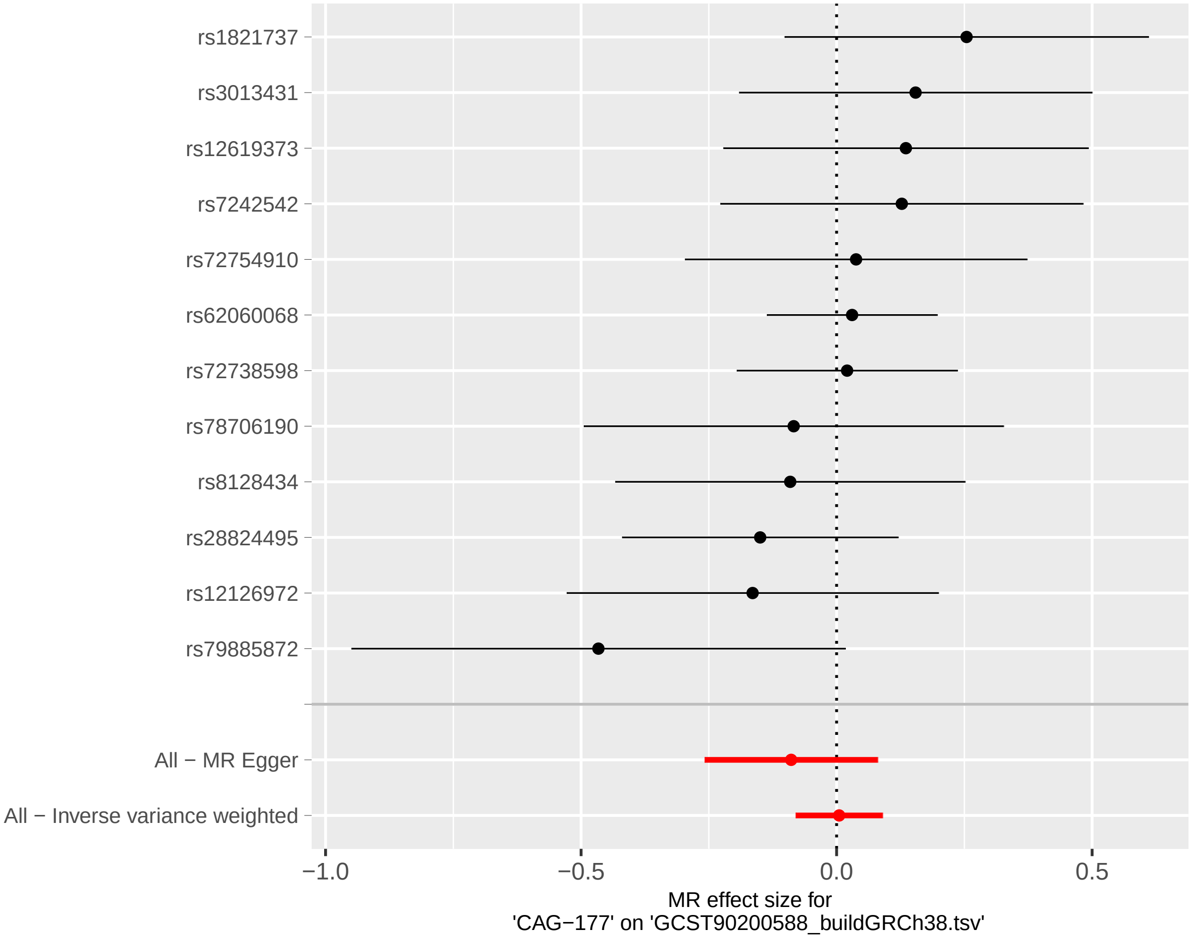

rs1821737
rs3013431
rs12619373
rs7242542
rs72754910
rs62060068
rs72738598
rs78706190
rs8128434
rs28824495
rs12126972
rs79885872
All − MR Egger
All − Inverse variance weighted
−1.0
−0.5
0.0
0.5
MR effect size for
'CAG−177' on 'GCST90200588_buildGRCh38.tsv'

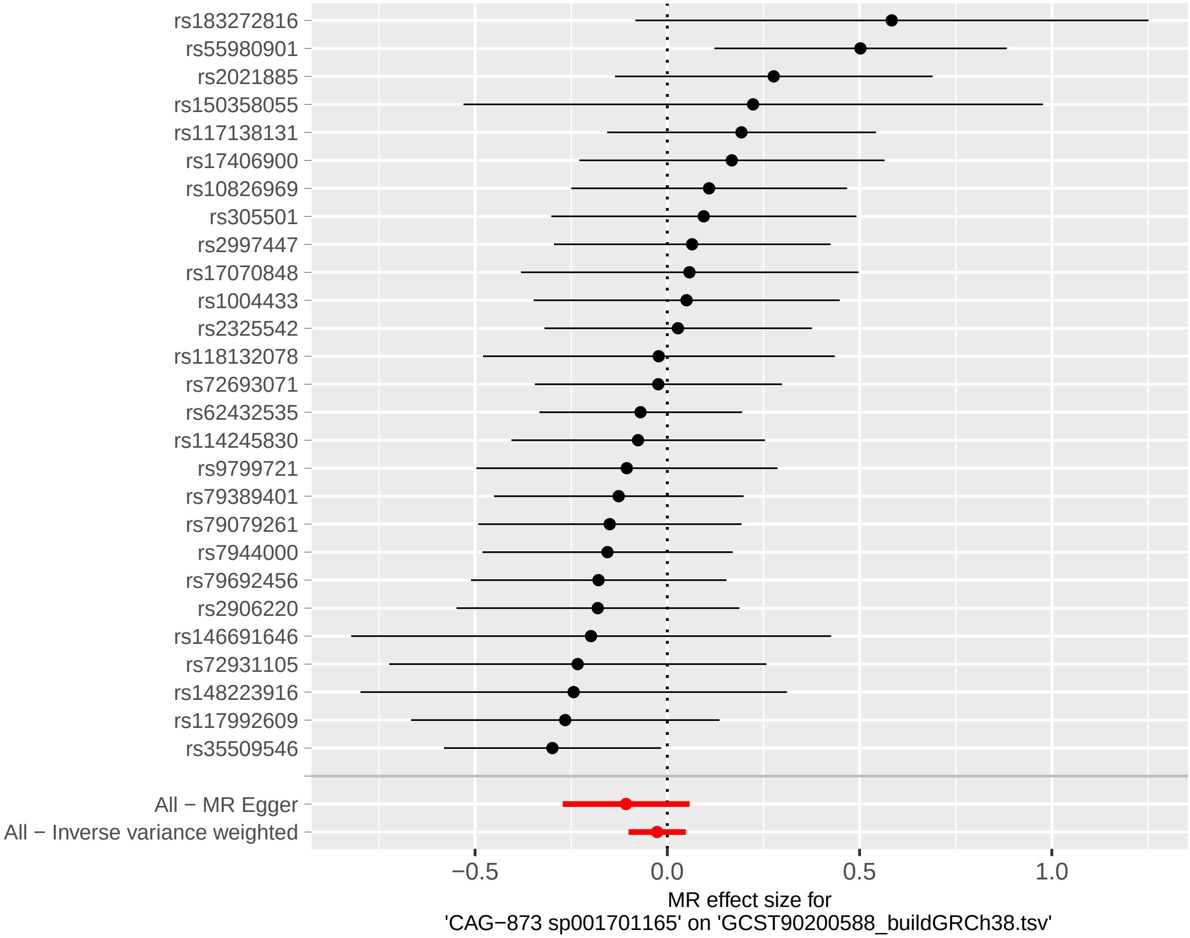

rs183272816
rs55980901
rs2021885
rs150358055
rs117138131
rs17406900
rs10826969
rs305501
rs2997447
rs17070848
rs1004433
rs2325542
rs118132078
rs72693071
rs62432535
rs114245830
rs9799721
rs79389401
rs79079261
rs7944000
rs79692456
rs2906220
rs146691646
rs72931105
rs148223916
rs117992609
rs35509546
All − MR Egger
All − Inverse variance weighted
−0.5
0.0
0.5
1.0
MR effect size for
'CAG−873 sp001701165' on 'GCST90200588_buildGRCh38.tsv'

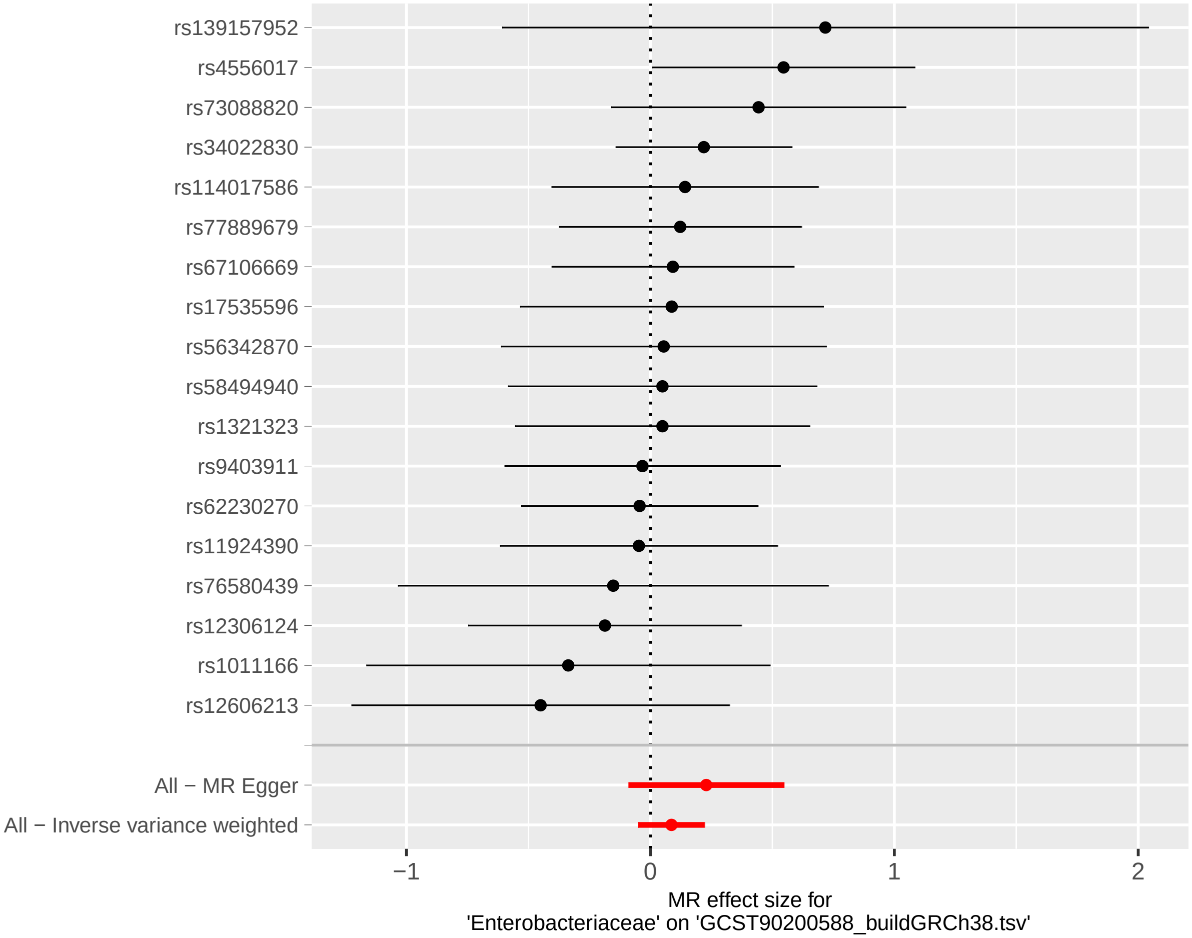

rs139157952
rs4556017
rs73088820
rs34022830
rs114017586
rs77889679
rs67106669
rs17535596
rs56342870
rs58494940
rs1321323
rs9403911
rs62230270
rs11924390
rs76580439
rs12306124
rs1011166
rs12606213
All − MR Egger
All − Inverse variance weighted
−1
0
1
2
MR effect size for
'Enterobacteriaceae' on 'GCST90200588_buildGRCh38.tsv'

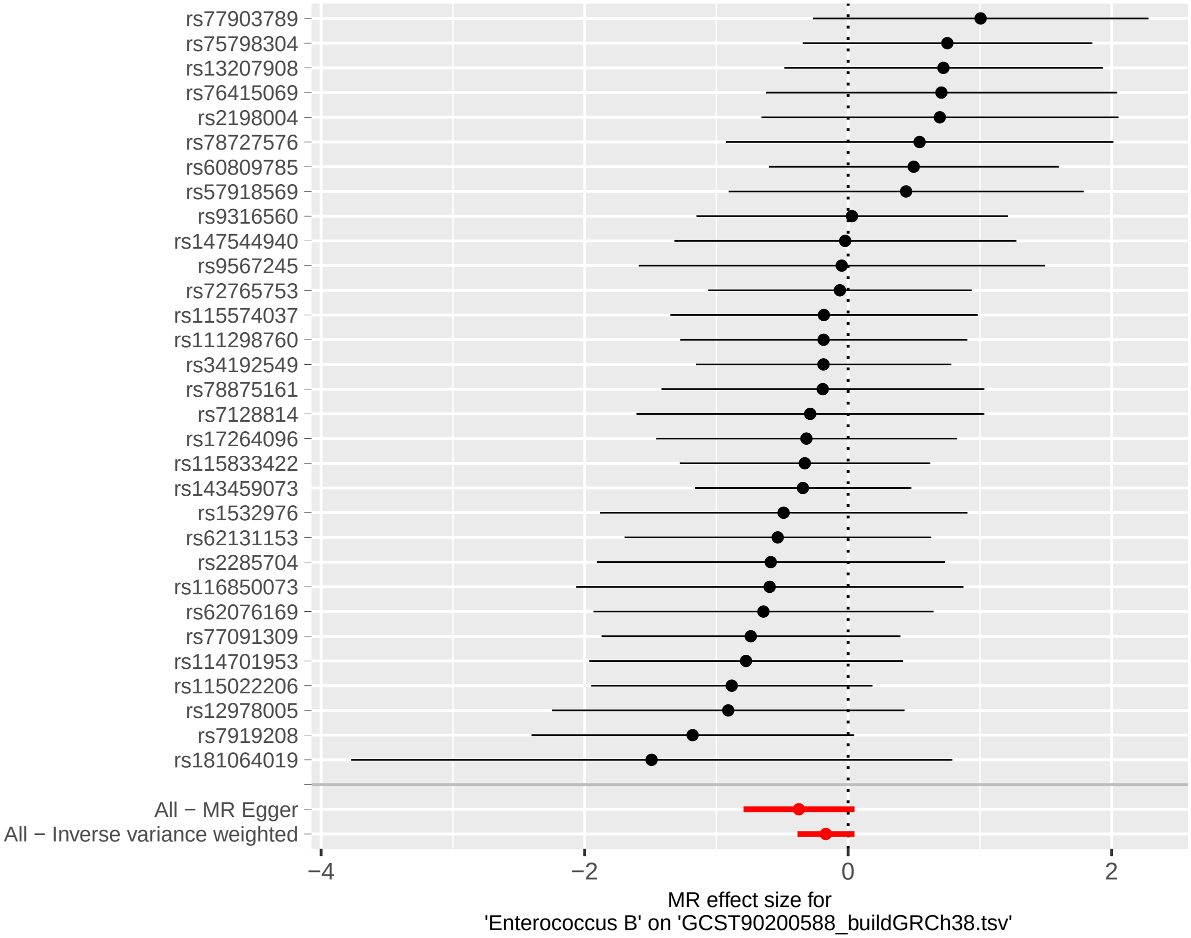
rs77903789
rs75798304
rs13207908
rs76415069
rs2198004
rs78727576
rs60809785
rs57918569
rs9316560
rs147544940
rs9567245
rs72765753
rs115574037
rs111298760
rs34192549
rs78875161
rs7128814
rs17264096
rs115833422
rs143459073
rs1532976
rs62131153
rs2285704
rs116850073
rs62076169
rs77091309
rs114701953
rs115022206
rs12978005
rs7919208
rs181064019
All − MR Egger
All − Inverse variance weighted
−4
−2
0
2
MR effect size for
'Enterococcus B' on 'GCST90200588_buildGRCh38.tsv'

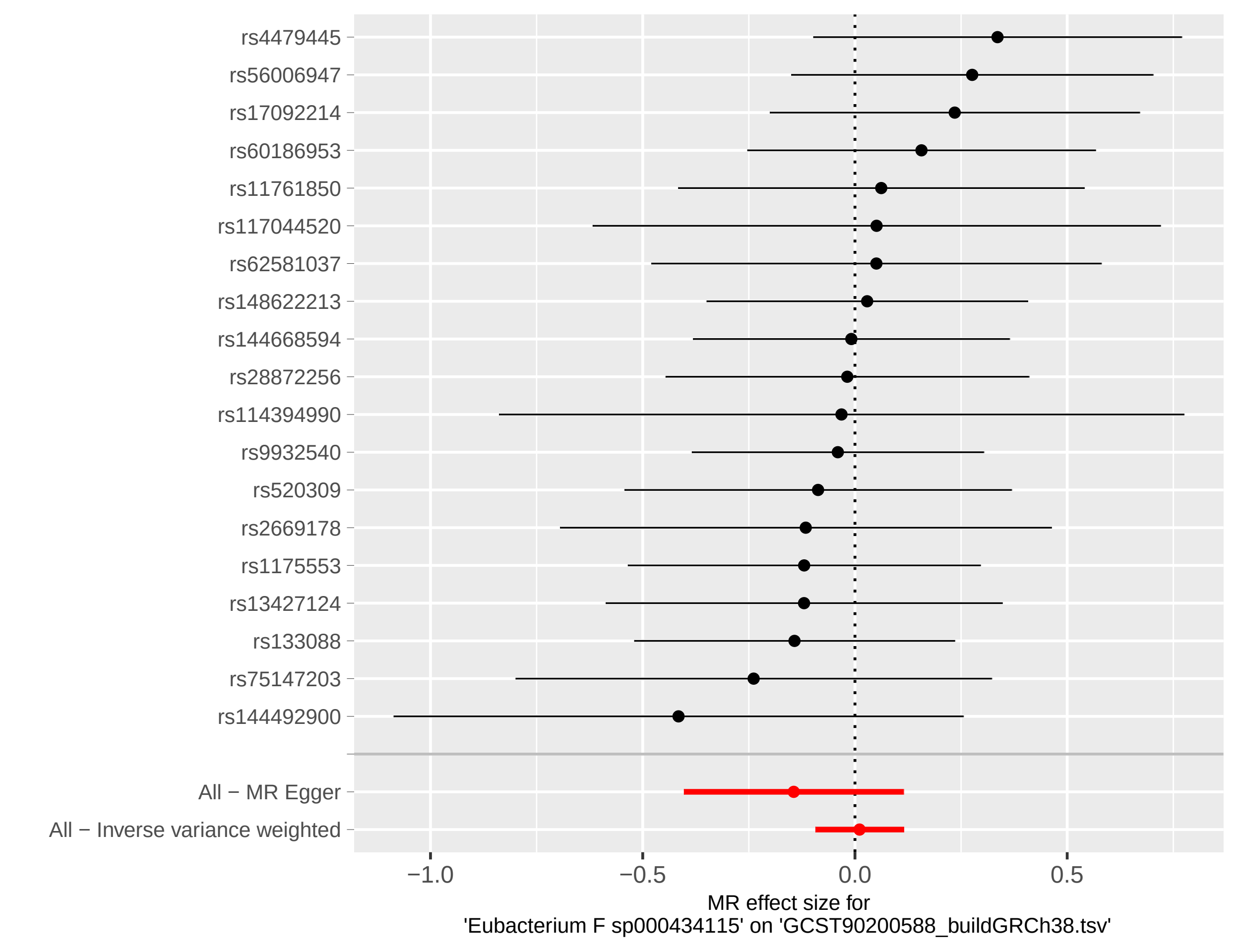

rs4479445
rs56006947
rs17092214
rs60186953
rs11761850
rs117044520
rs62581037
rs148622213
rs144668594
rs28872256
rs114394990
rs9932540
rs520309
rs2669178
rs1175553
rs13427124
rs133088
rs75147203
rs144492900
All − MR Egger
All − Inverse variance weighted
−1.0
−0.5
0.0
0.5
MR effect size for
'Eubacterium F sp000434115' on 'GCST90200588_buildGRCh38.tsv'

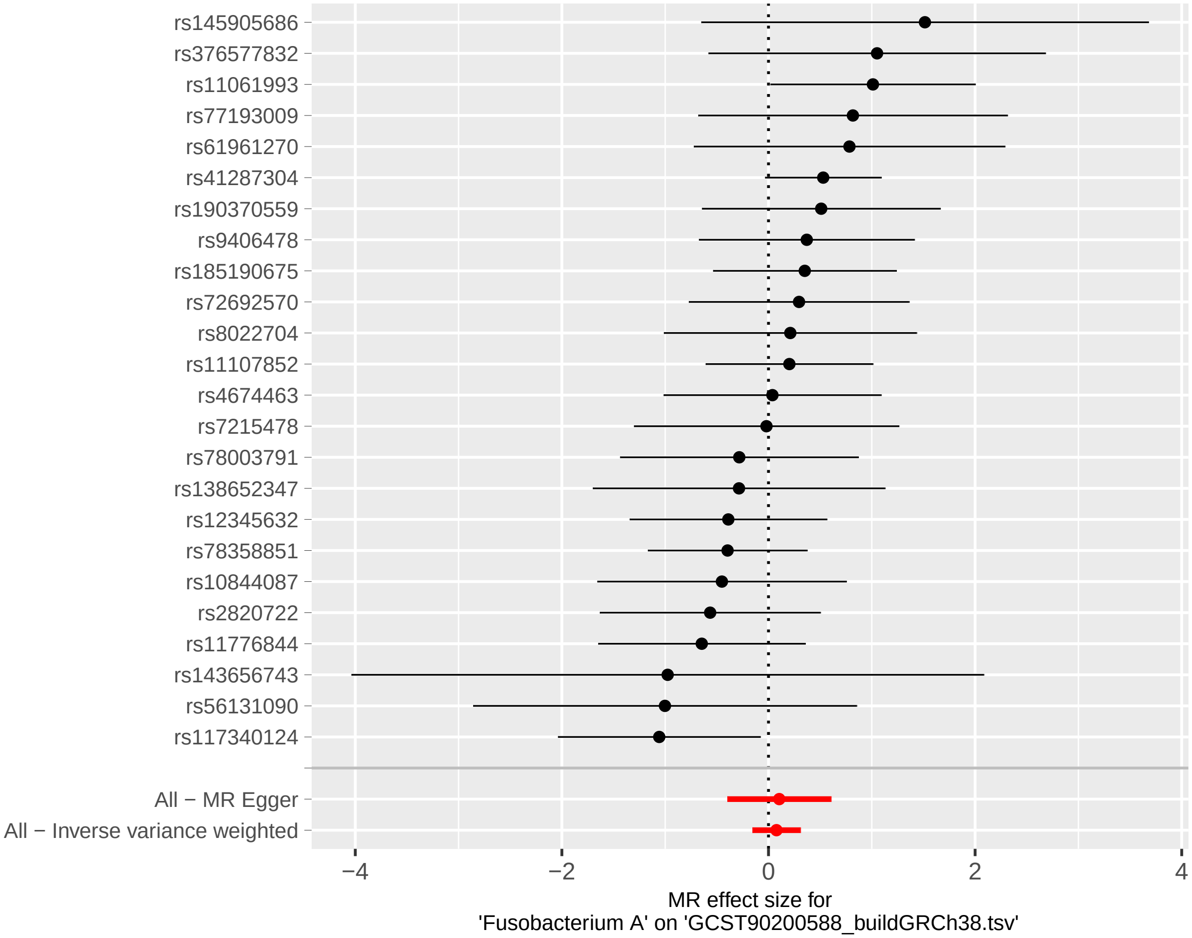
rs145905686
rs376577832
rs11061993
rs77193009
rs61961270
rs41287304
rs190370559
rs9406478
rs185190675
rs72692570
rs8022704
rs11107852
rs4674463
rs7215478
rs78003791
rs138652347
rs12345632
rs78358851
rs10844087
rs2820722
rs11776844
rs143656743
rs56131090
rs117340124
All − MR Egger
All − Inverse variance weighted
−4
−2
0
2
4
MR effect size for
'Fusobacterium A' on 'GCST90200588_buildGRCh38.tsv'

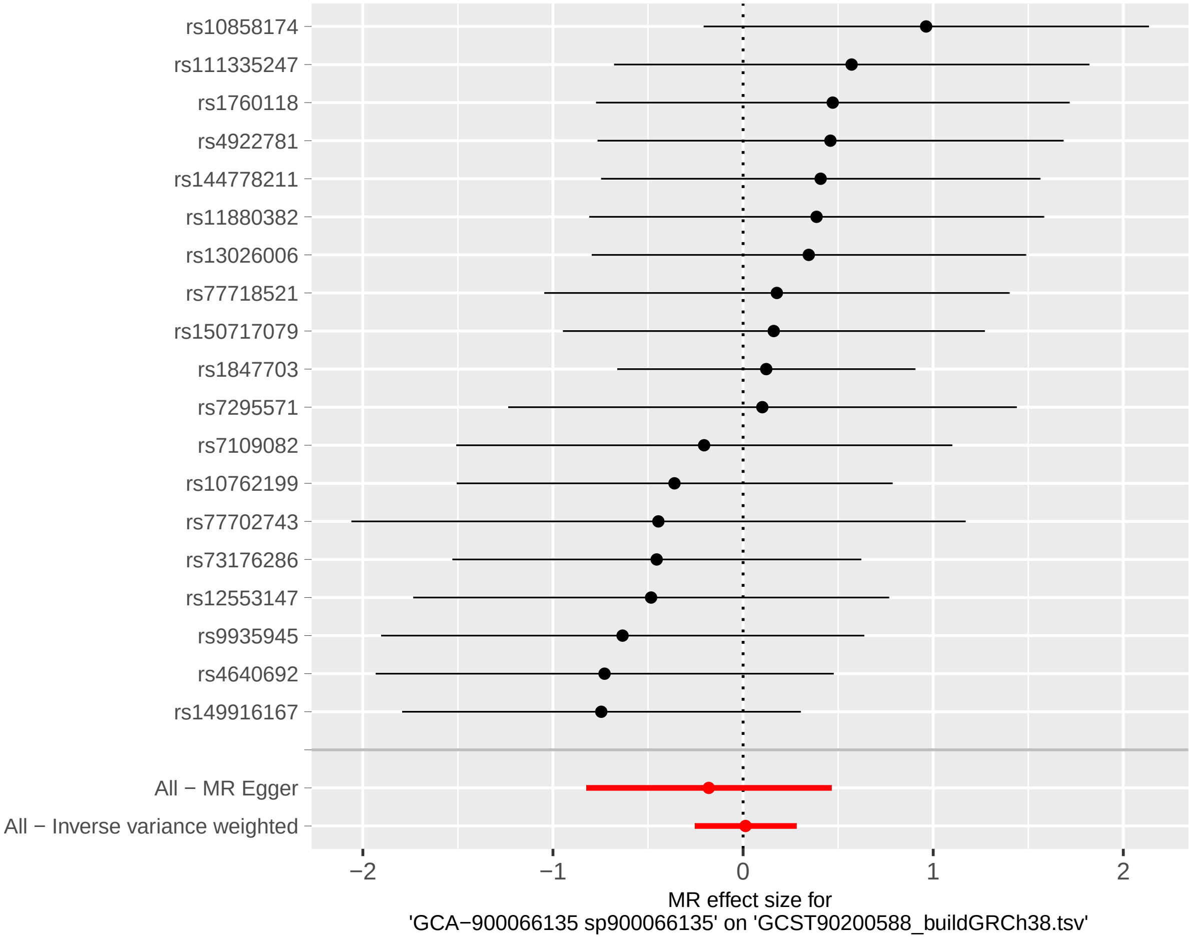

rs10858174
rs111335247
rs1760118
rs4922781
rs144778211
rs11880382
rs13026006
rs77718521
rs150717079
rs1847703
rs7295571
rs7109082
rs10762199
rs77702743
rs73176286
rs12553147
rs9935945
rs4640692
rs149916167
All − MR Egger
All − Inverse variance weighted
−2
−1
0
1
2
MR effect size for
'GCA−900066135 sp900066135' on 'GCST90200588_buildGRCh38.tsv'

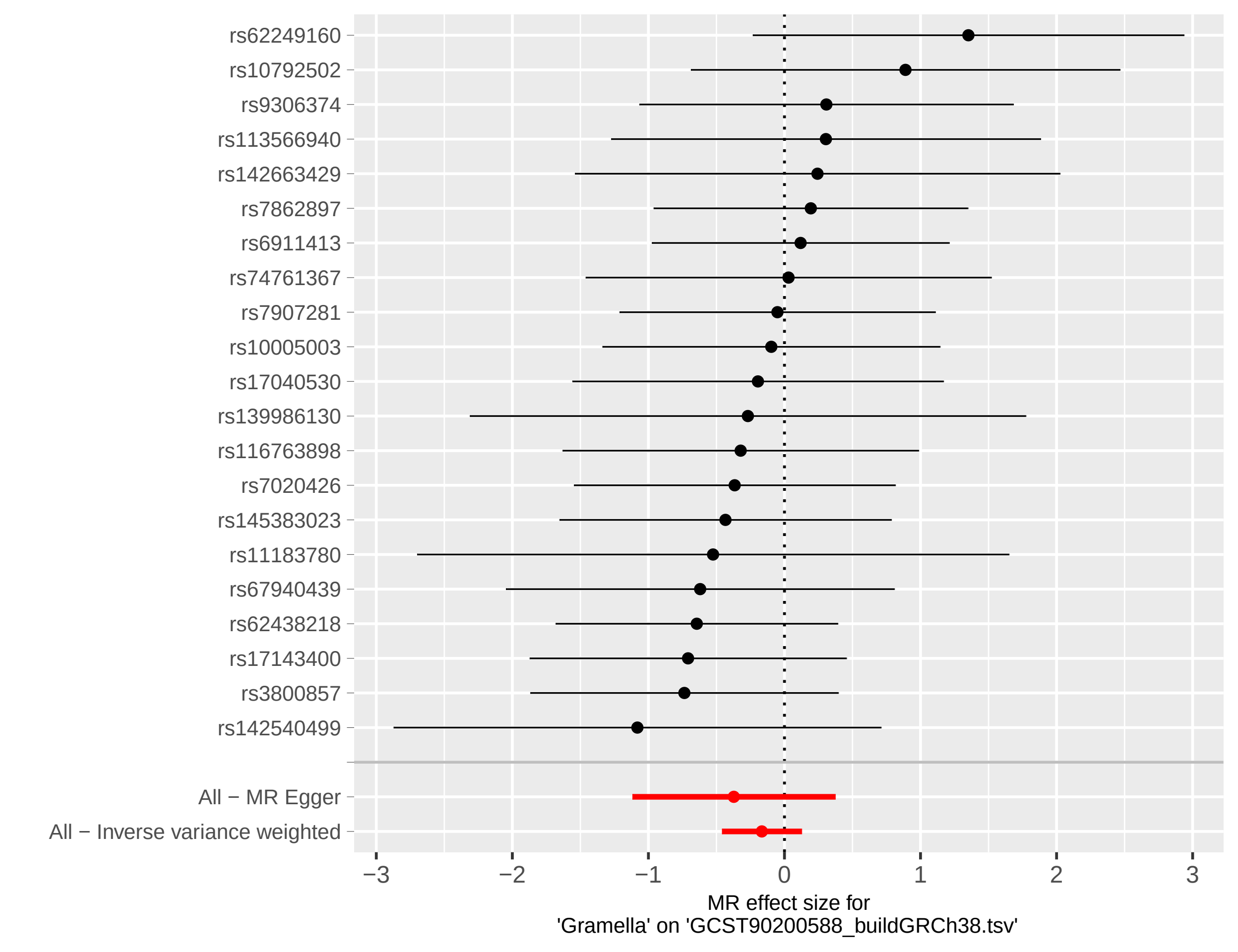

rs62249160
rs10792502
rs9306374
rs113566940
rs142663429
rs7862897
rs6911413
rs74761367
rs7907281
rs10005003
rs17040530
rs139986130
rs116763898
rs7020426
rs145383023
rs11183780
rs67940439
rs62438218
rs17143400
rs3800857
rs142540499
All − MR Egger
All − Inverse variance weighted
−3
−2
−1
0
1
2
3
MR effect size for
'Gramella' on 'GCST90200588_buildGRCh38.tsv'

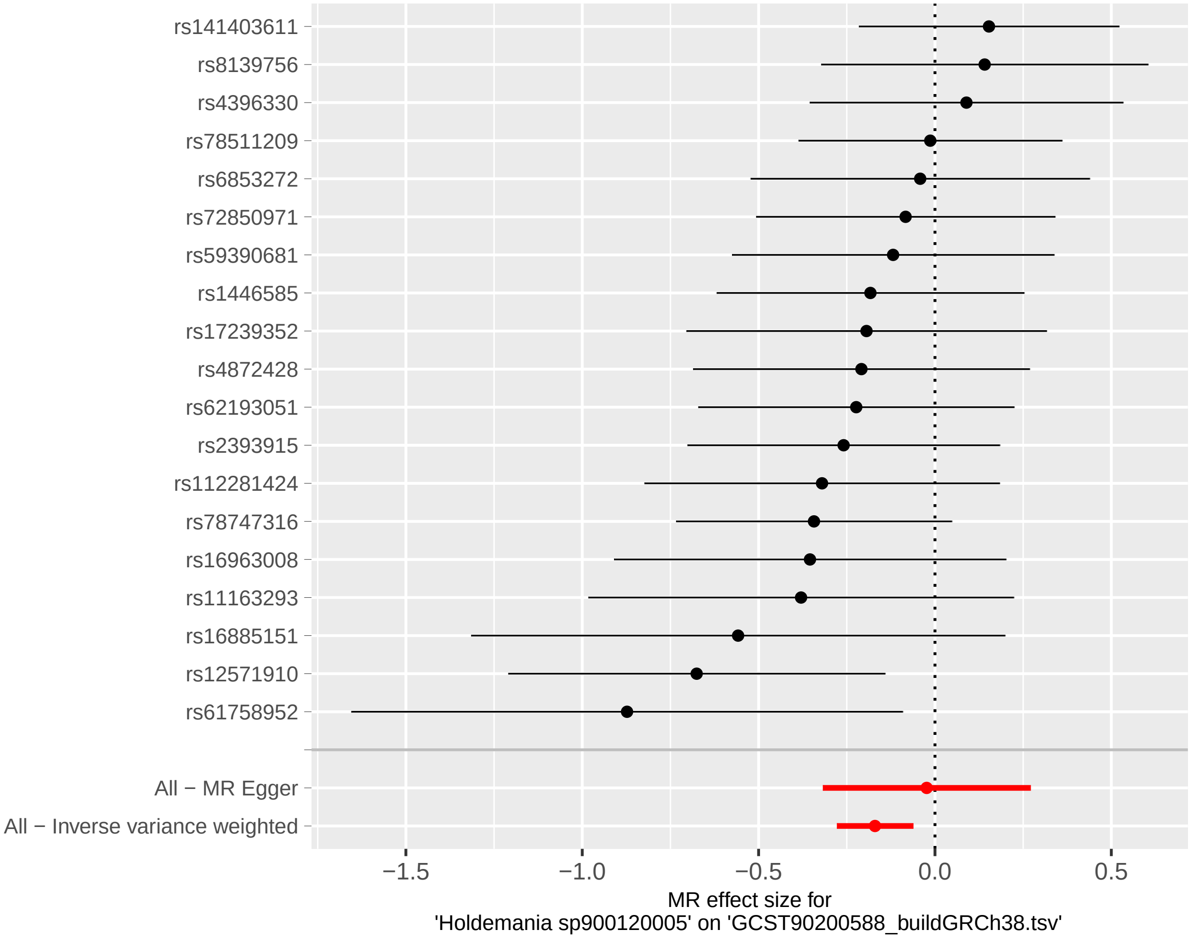
rs141403611
rs8139756
rs4396330
rs78511209
rs6853272
rs72850971
rs59390681
rs1446585
rs17239352
rs4872428
rs62193051
rs2393915
rs112281424
rs78747316
rs16963008
rs11163293
rs16885151
rs12571910
rs61758952
All − MR Egger
All − Inverse variance weighted
−1.5
−1.0
−0.5
0.0
0.5
MR effect size for
'Holdemania sp900120005' on 'GCST90200588_buildGRCh38.tsv'

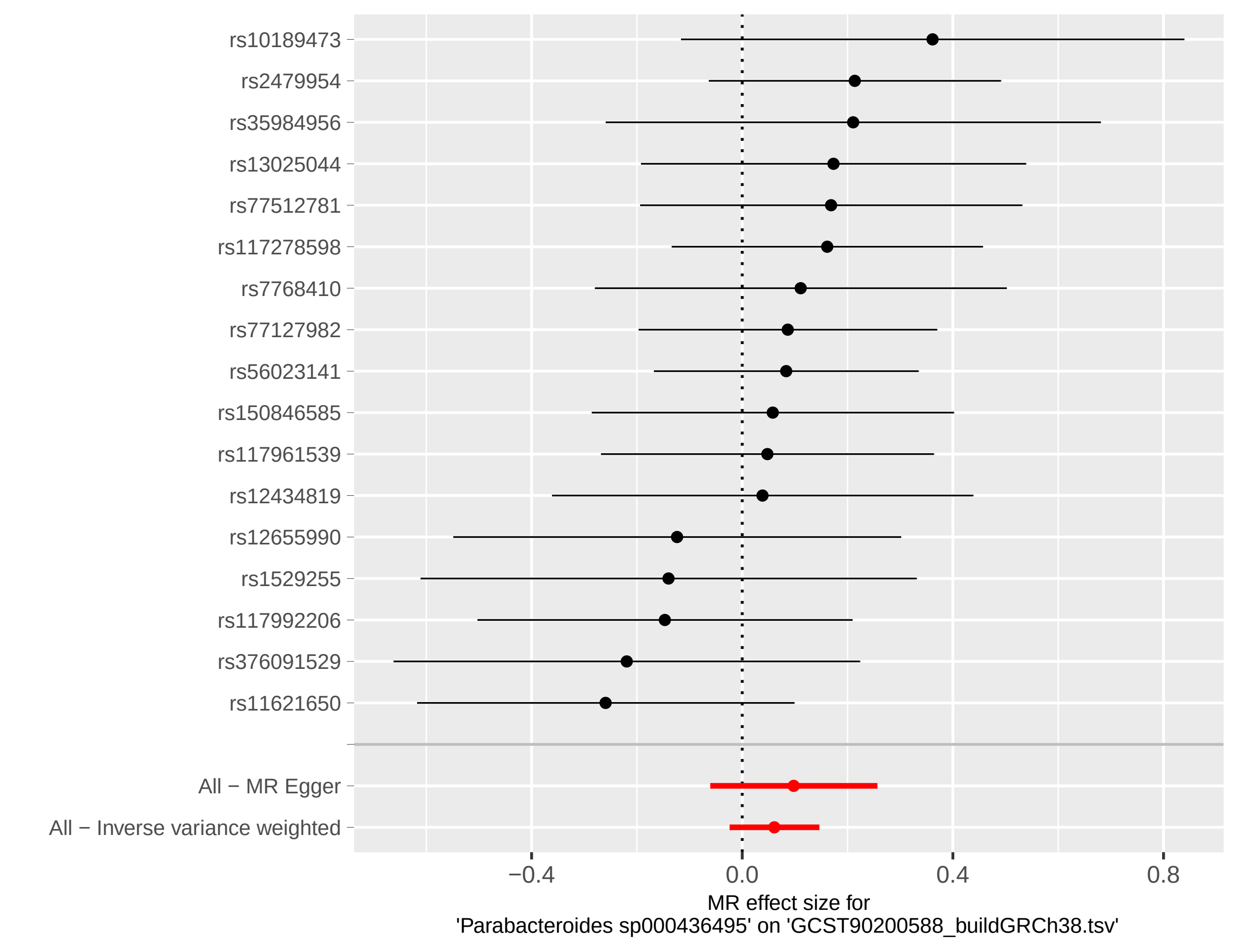

rs10189473
rs2479954
rs35984956
rs13025044
rs77512781
rs117278598
rs7768410
rs77127982
rs56023141
rs150846585
rs117961539
rs12434819
rs12655990
rs1529255
rs117992206
rs376091529
rs11621650
All − MR Egger
All − Inverse variance weighted
−0.4
0.0
0.4
0.8
MR effect size for
'Parabacteroides sp000436495' on 'GCST90200588_buildGRCh38.tsv'

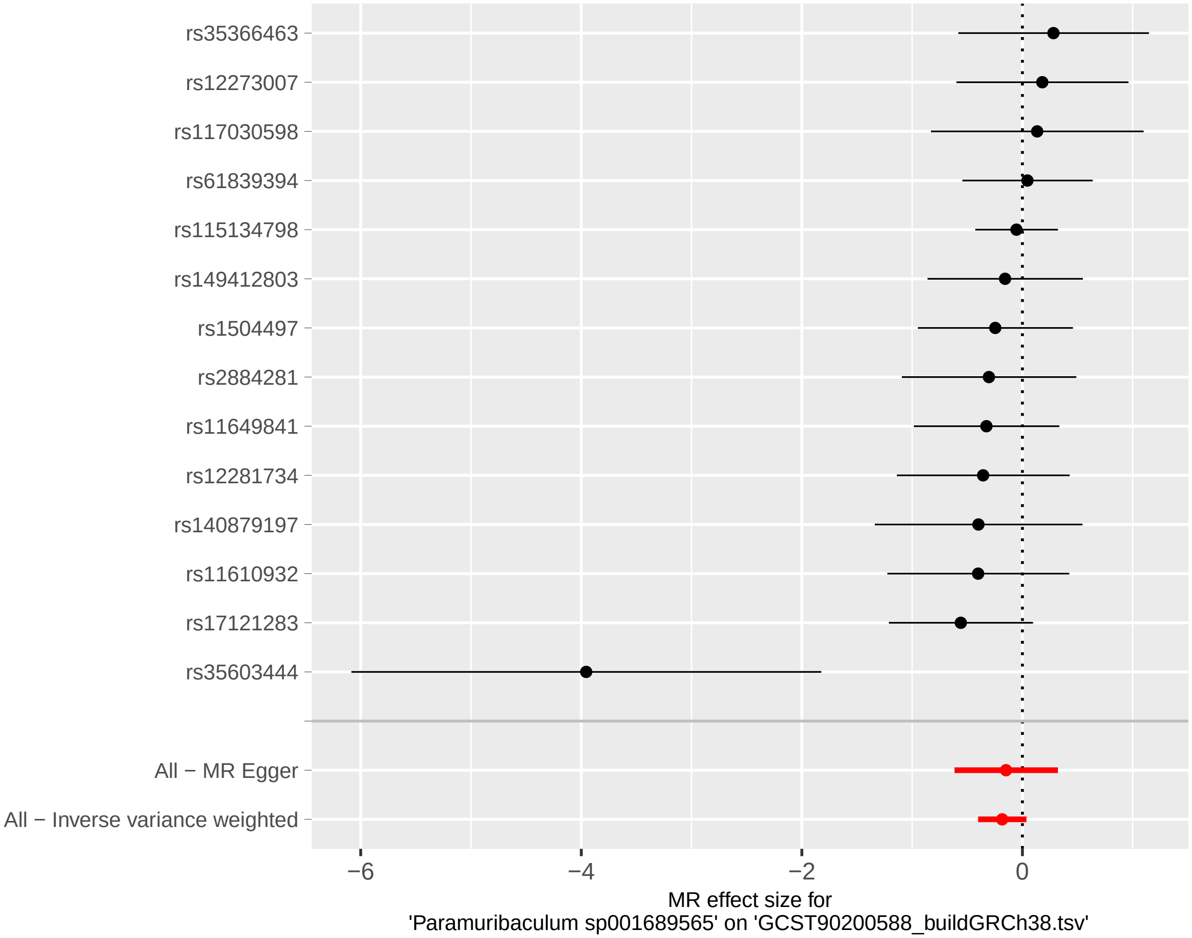
rs35366463
rs12273007
rs117030598
rs61839394
rs115134798
rs149412803
rs1504497
rs2884281
rs11649841
rs12281734
rs140879197
rs11610932
rs17121283
rs35603444
All − MR Egger
All − Inverse variance weighted
−6
−4
−2
0
MR effect size for
'Paramuribaculum sp001689565' on 'GCST90200588_buildGRCh38.tsv'

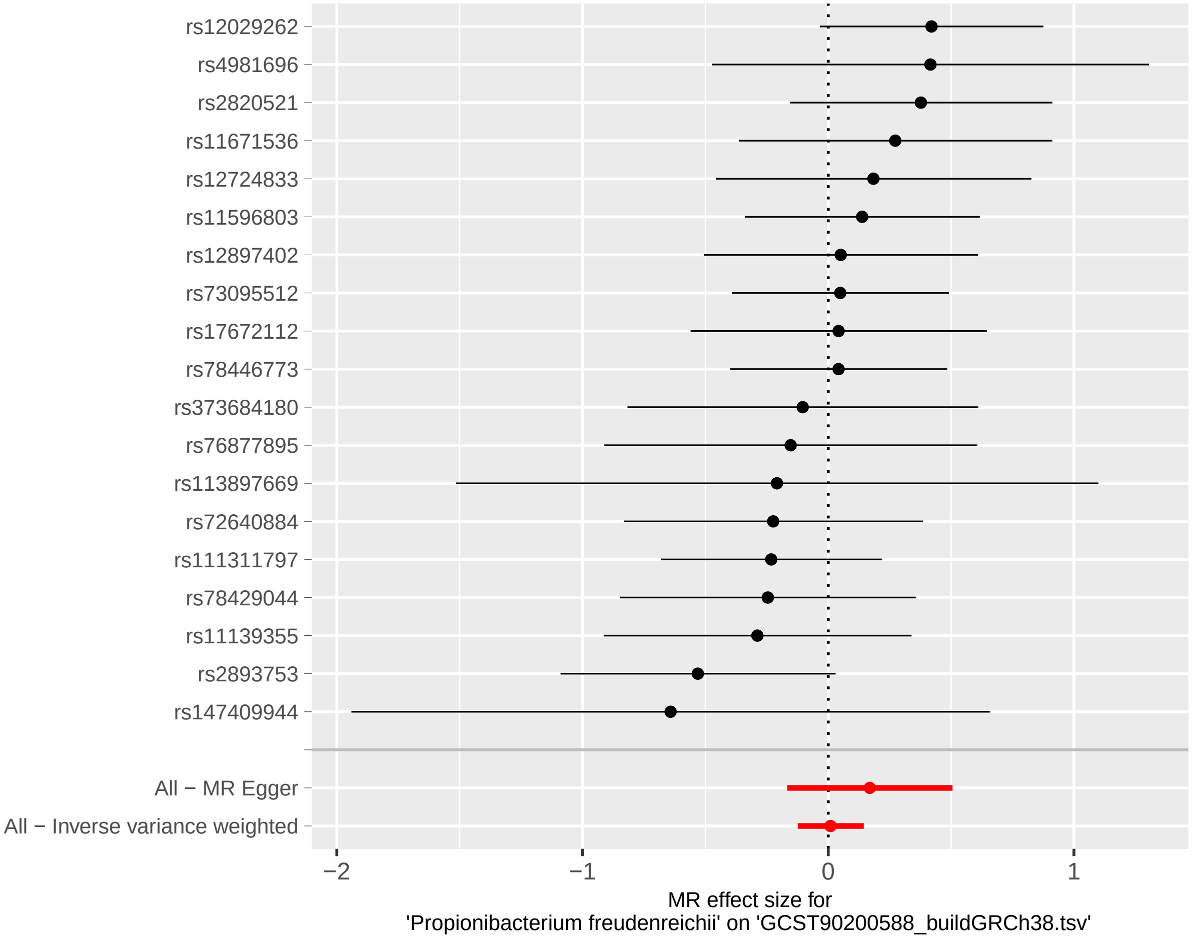
rs12029262
rs4981696
rs2820521
rs11671536
rs12724833
rs11596803
rs12897402
rs73095512
rs17672112
rs78446773
rs373684180
rs76877895
rs113897669
rs72640884
rs111311797
rs78429044
rs11139355
rs2893753
rs147409944
All − MR Egger
All − Inverse variance weighted
−2
−1
0
1
MR effect size for
'Propionibacterium freudenreichii' on 'GCST90200588_buildGRCh38.tsv'

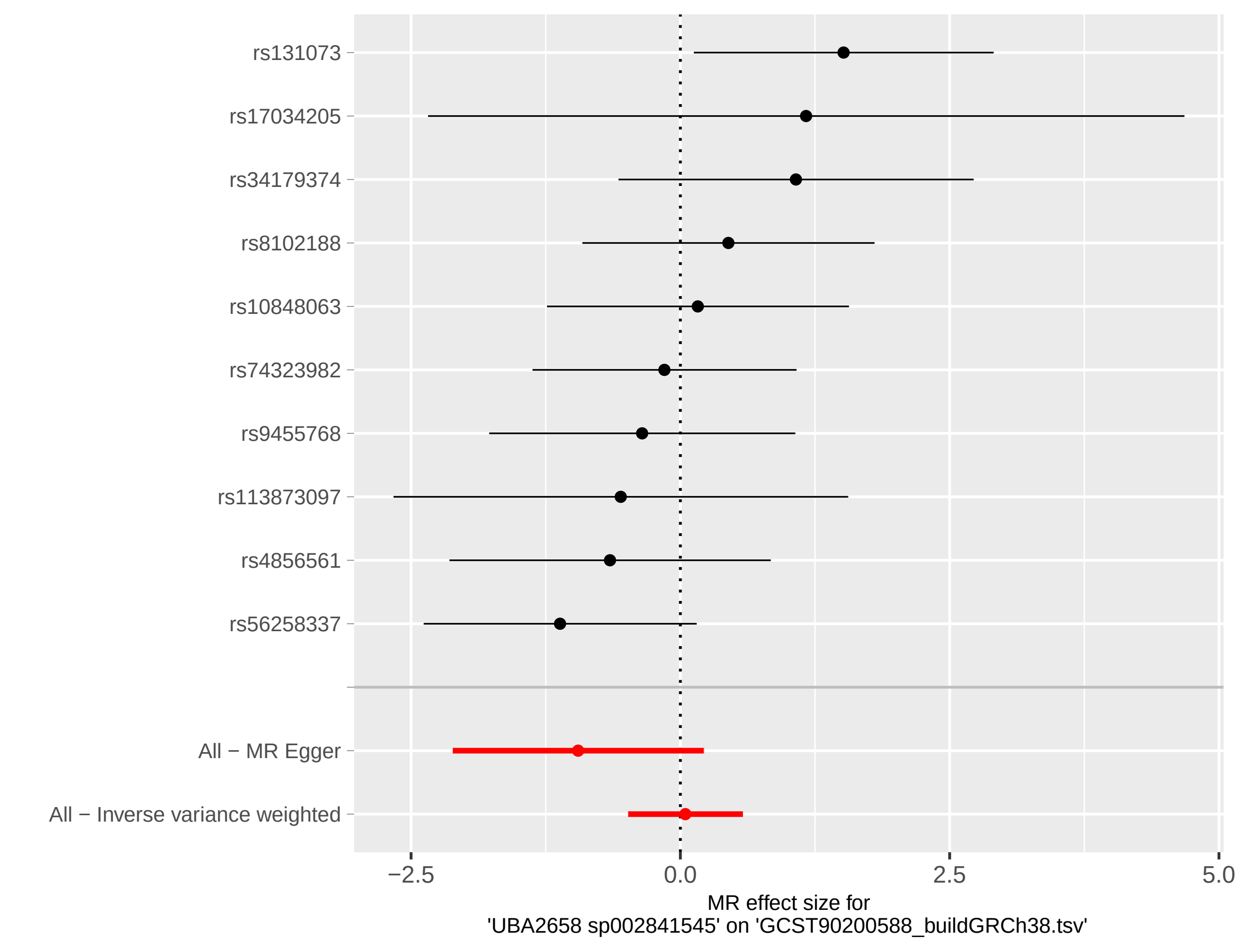

rs131073
rs17034205
rs34179374
rs8102188
rs10848063
rs74323982
rs9455768
rs113873097
rs4856561
rs56258337
All − MR Egger
All − Inverse variance weighted
−2.5
0.0
2.5
5.0
MR effect size for
'UBA2658 sp002841545' on 'GCST90200588_buildGRCh38.tsv'

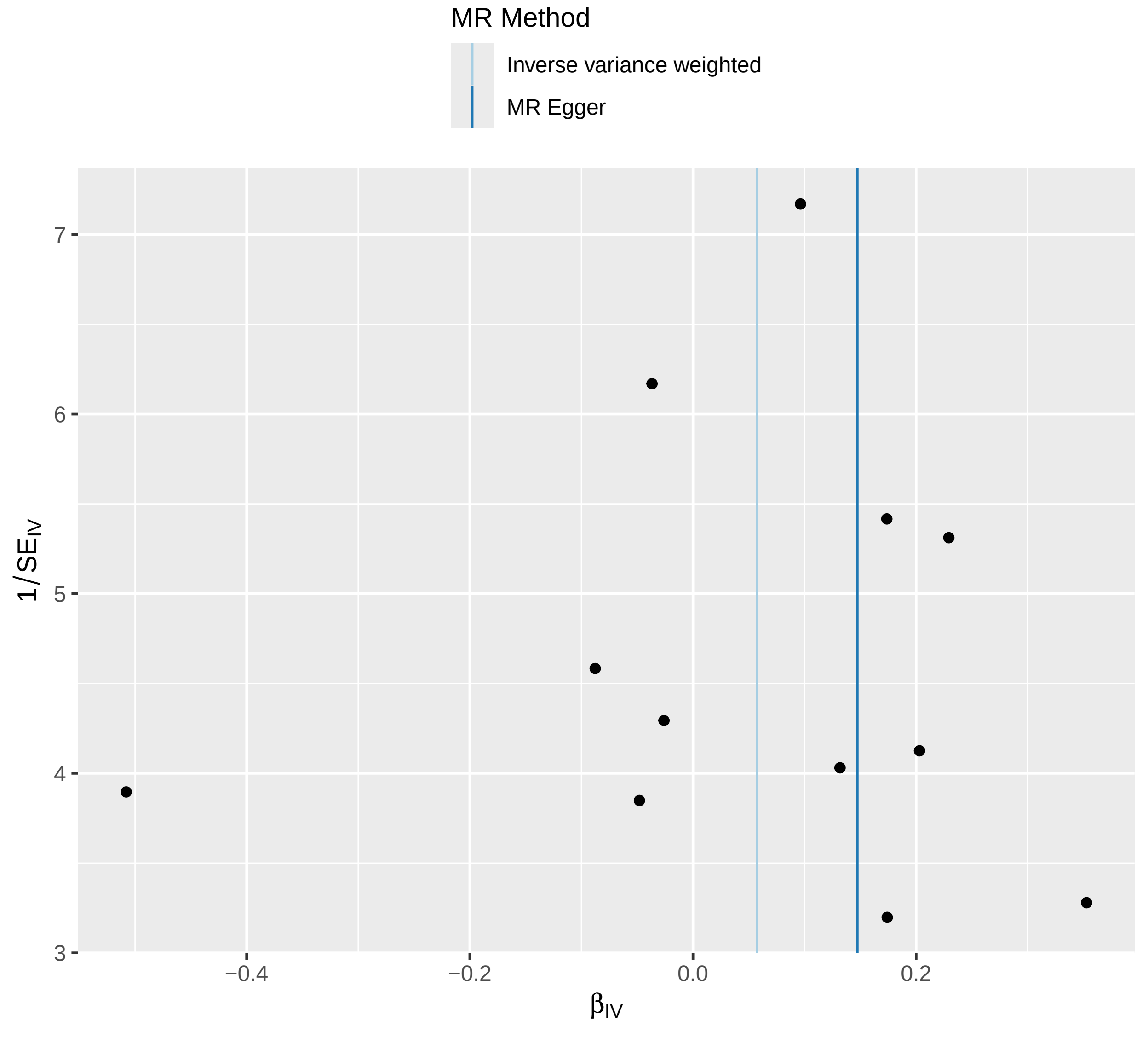
MR Method
Inverse variance weighted
MR Egger
1/SE_IV
β_IV
7
6
5
4
3
−0.4
−0.2
0.0
0.2

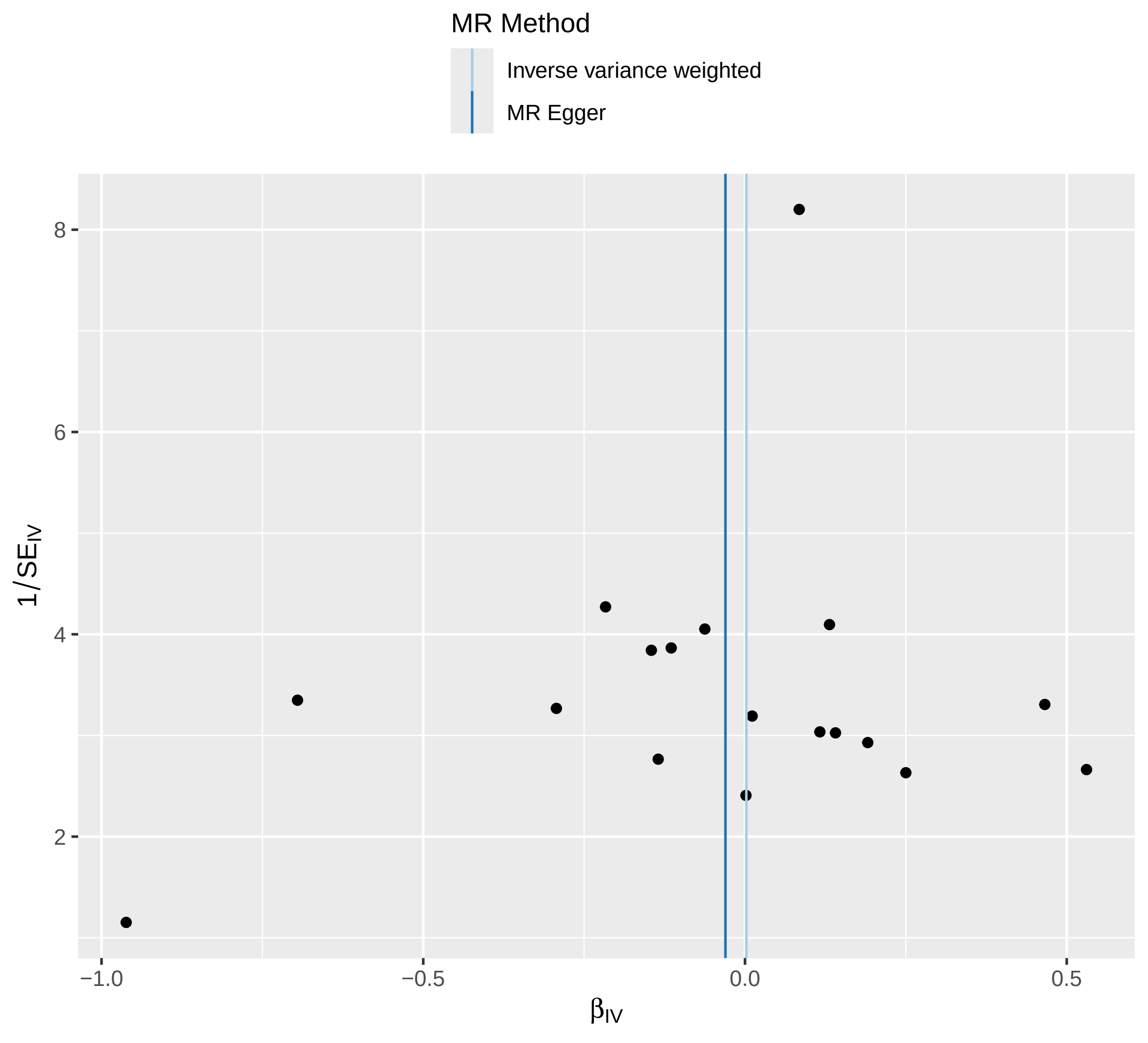
MR Method
Inverse variance weighted
MR Egger
8
6
4
2
1/SE_IV
−1.0
−0.5
0.0
0.5
β_IV

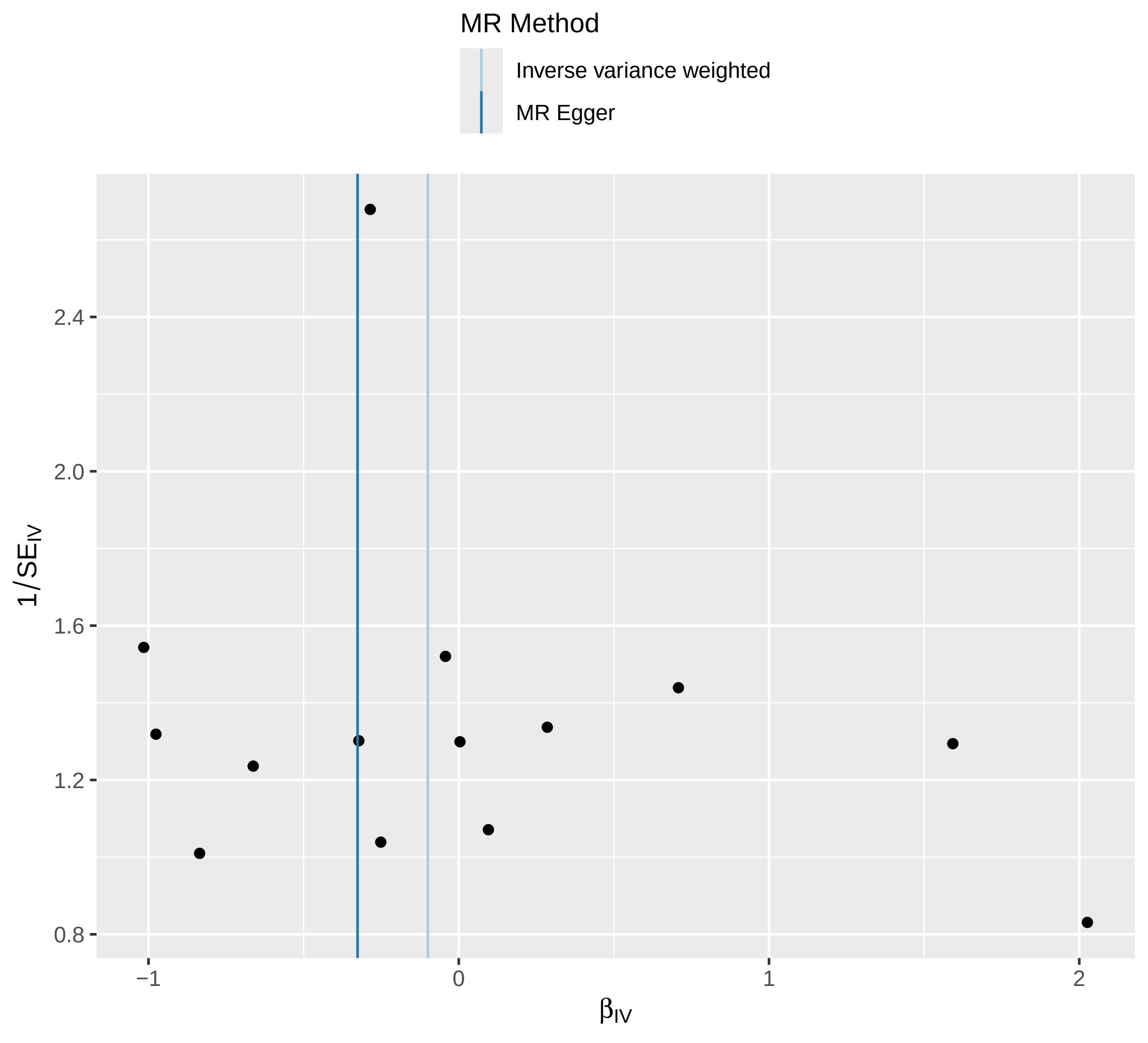
MR Method
Inverse variance weighted
MR Egger
2.4
2.0
1.6
1.2
0.8
1/SE_IV
−1
0
1
2
β_IV

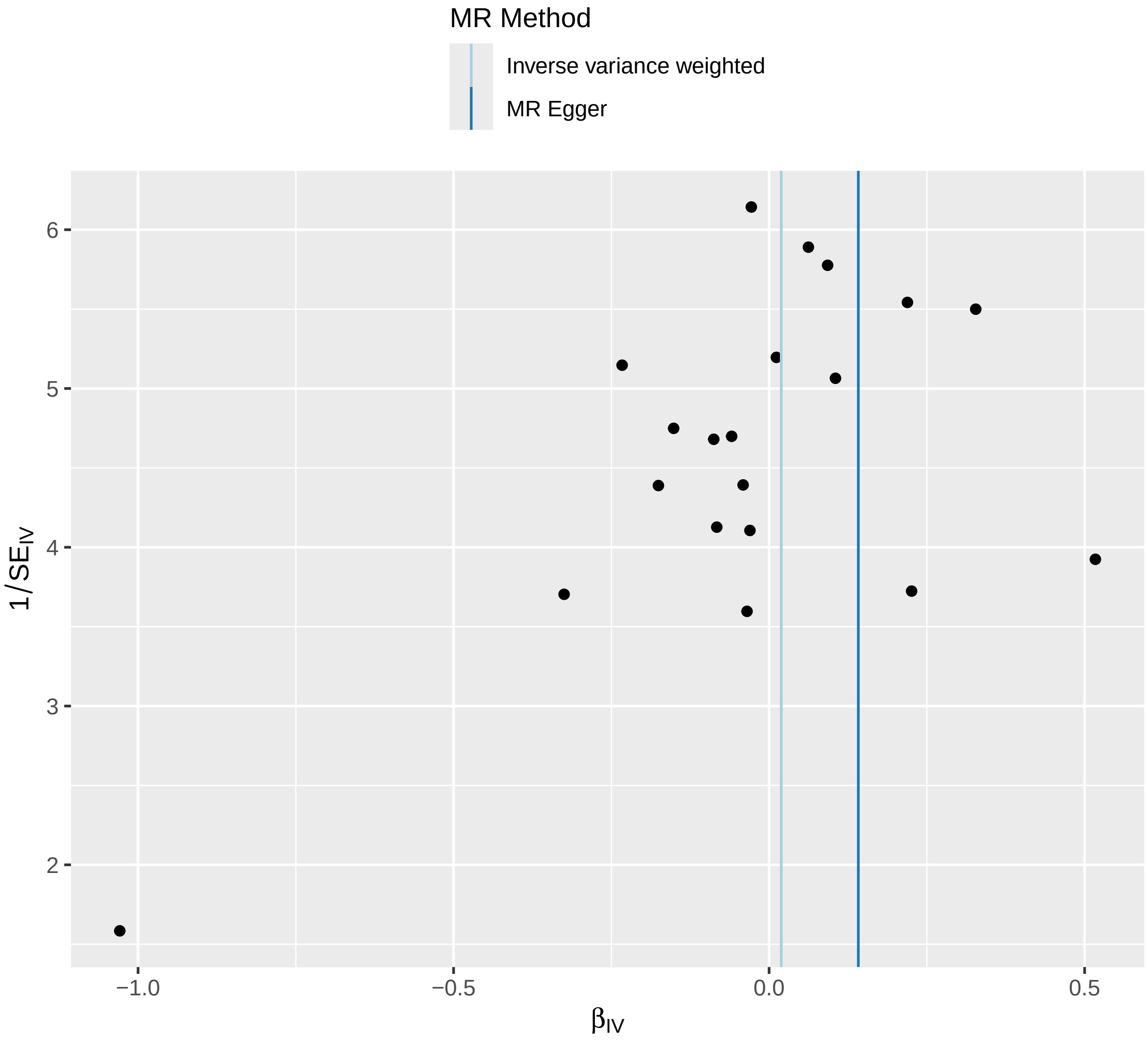
MR Method
Inverse variance weighted
MR Egger
6
5
4
3
2
1/SE_IV
−1.0
−0.5
0.0
0.5
β_IV

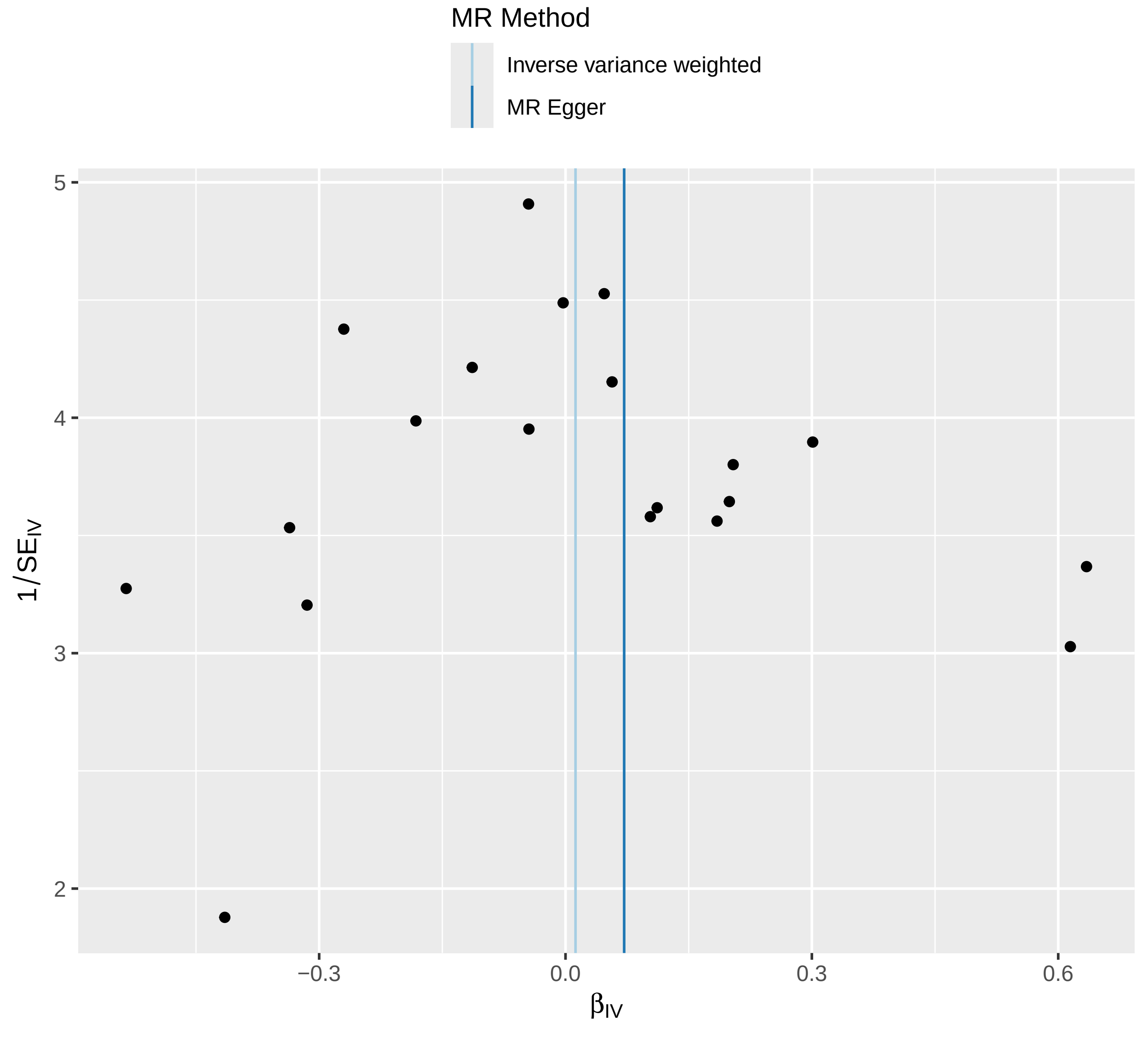
MR Method
Inverse variance weighted
MR Egger
5
4
3
2
1/SE_IV
−0.3
0.0
0.3
0.6
β_IV

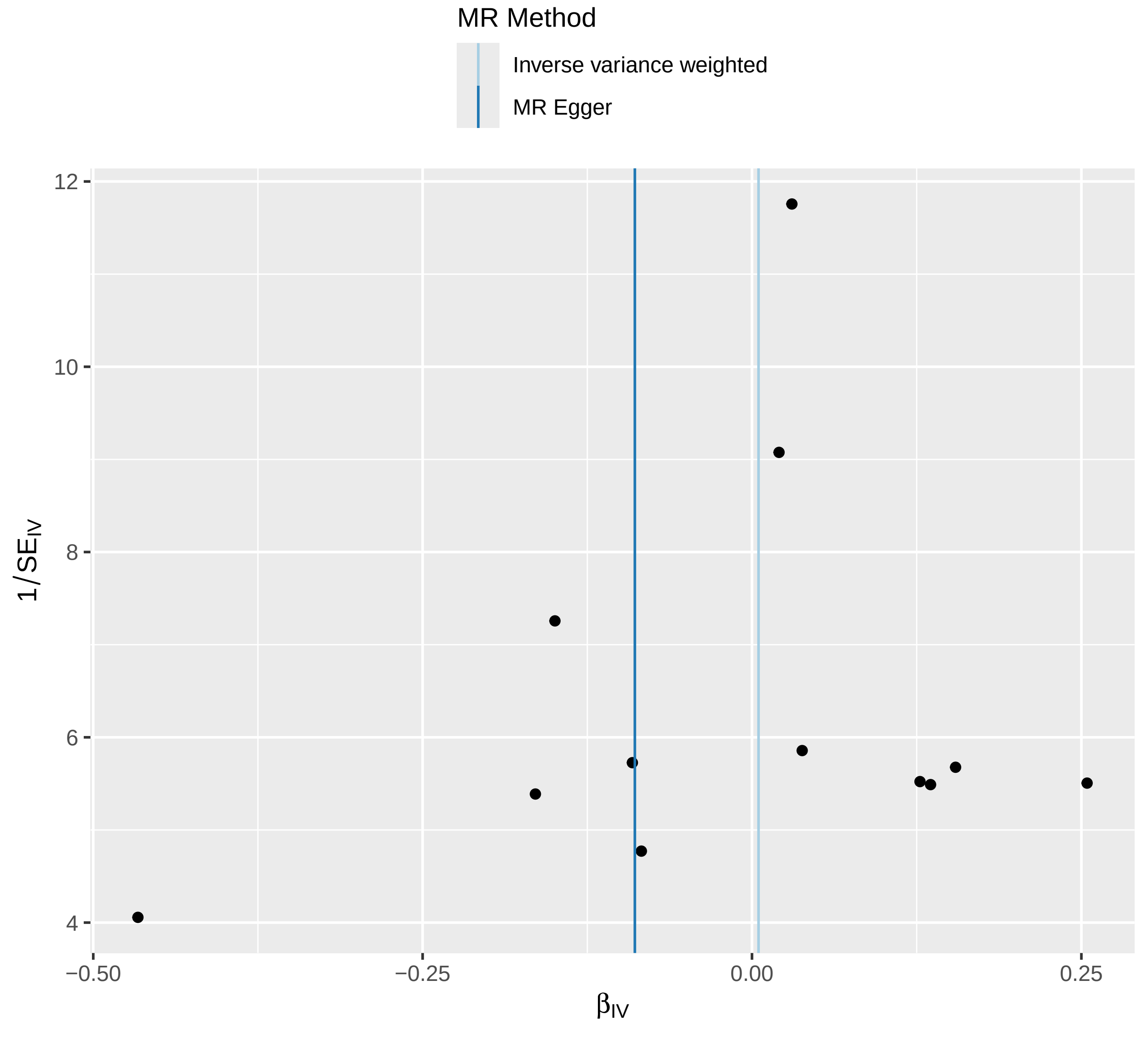

MR Method
Inverse variance weighted
MR Egger
12
10
8
6
4
1/SE$_{IV}$
−0.50
−0.25
0.00
0.25
β$_{IV}$

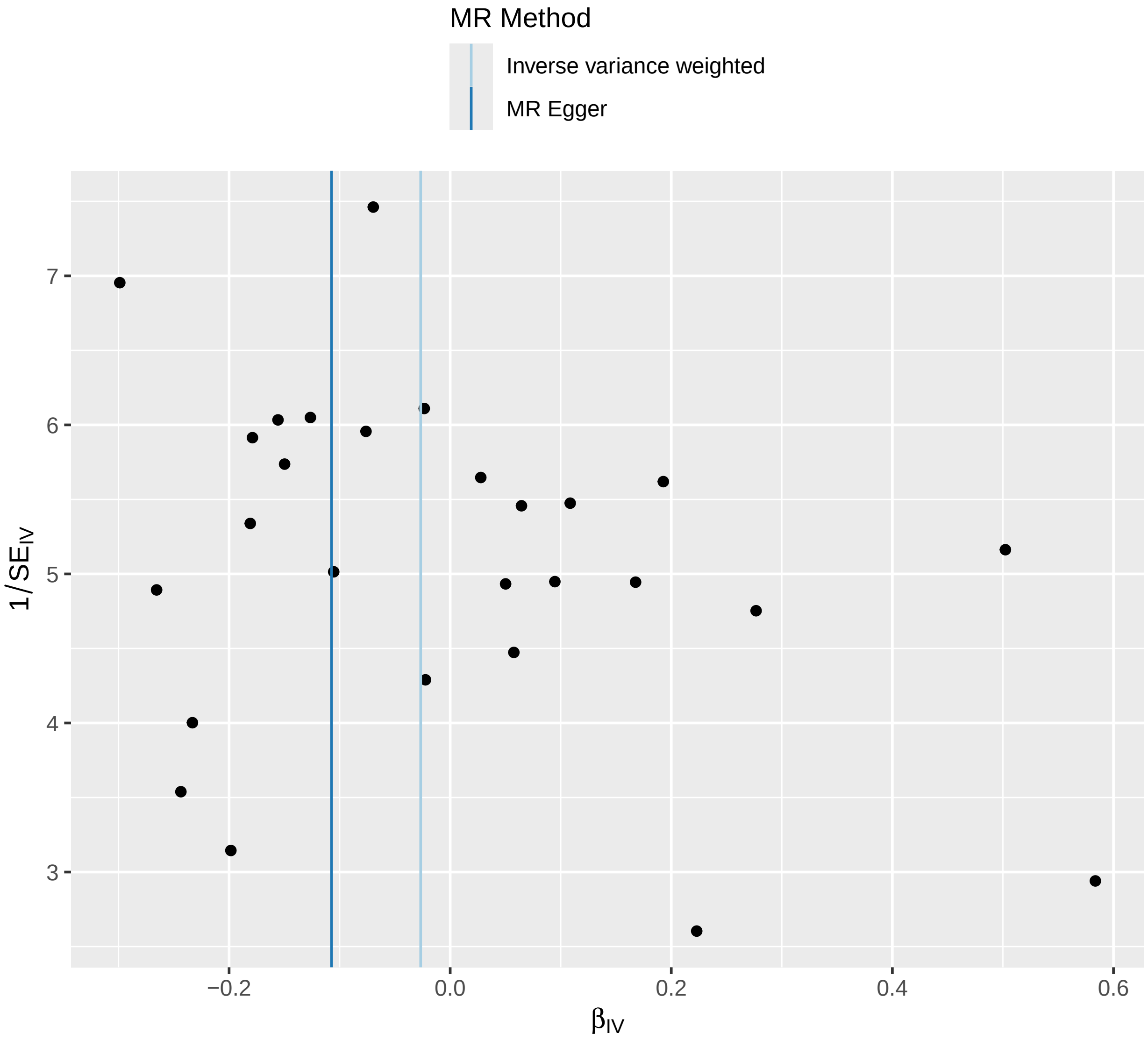

MR Method
Inverse variance weighted
MR Egger
7
6
5
4
3
1/SE_IV
−0.2
0.0
0.2
0.4
0.6
β_IV

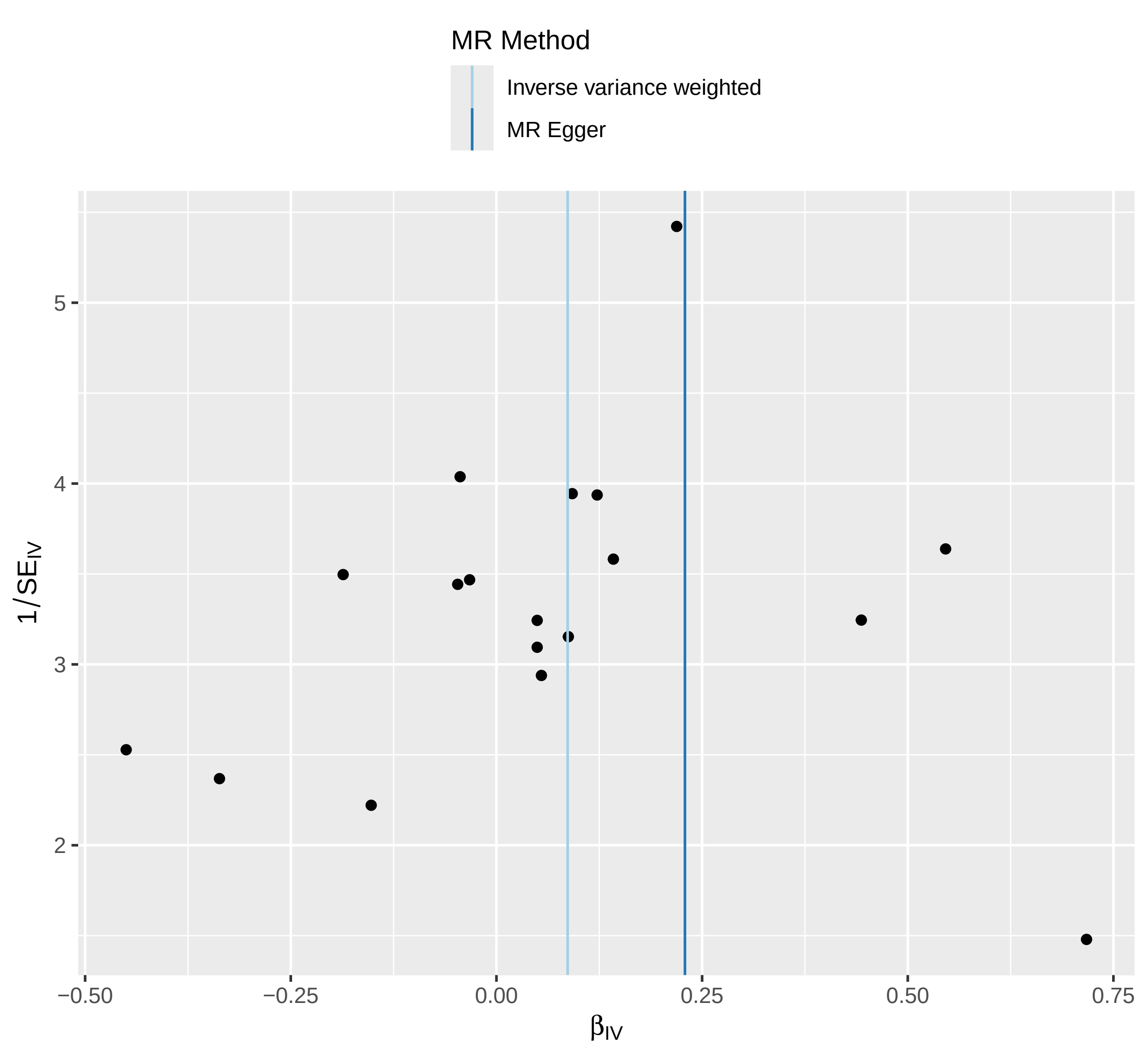
MR Method
Inverse variance weighted
MR Egger
5
4
3
2
1/SE_IV
−0.50
−0.25
0.00
0.25
0.50
0.75
β_IV

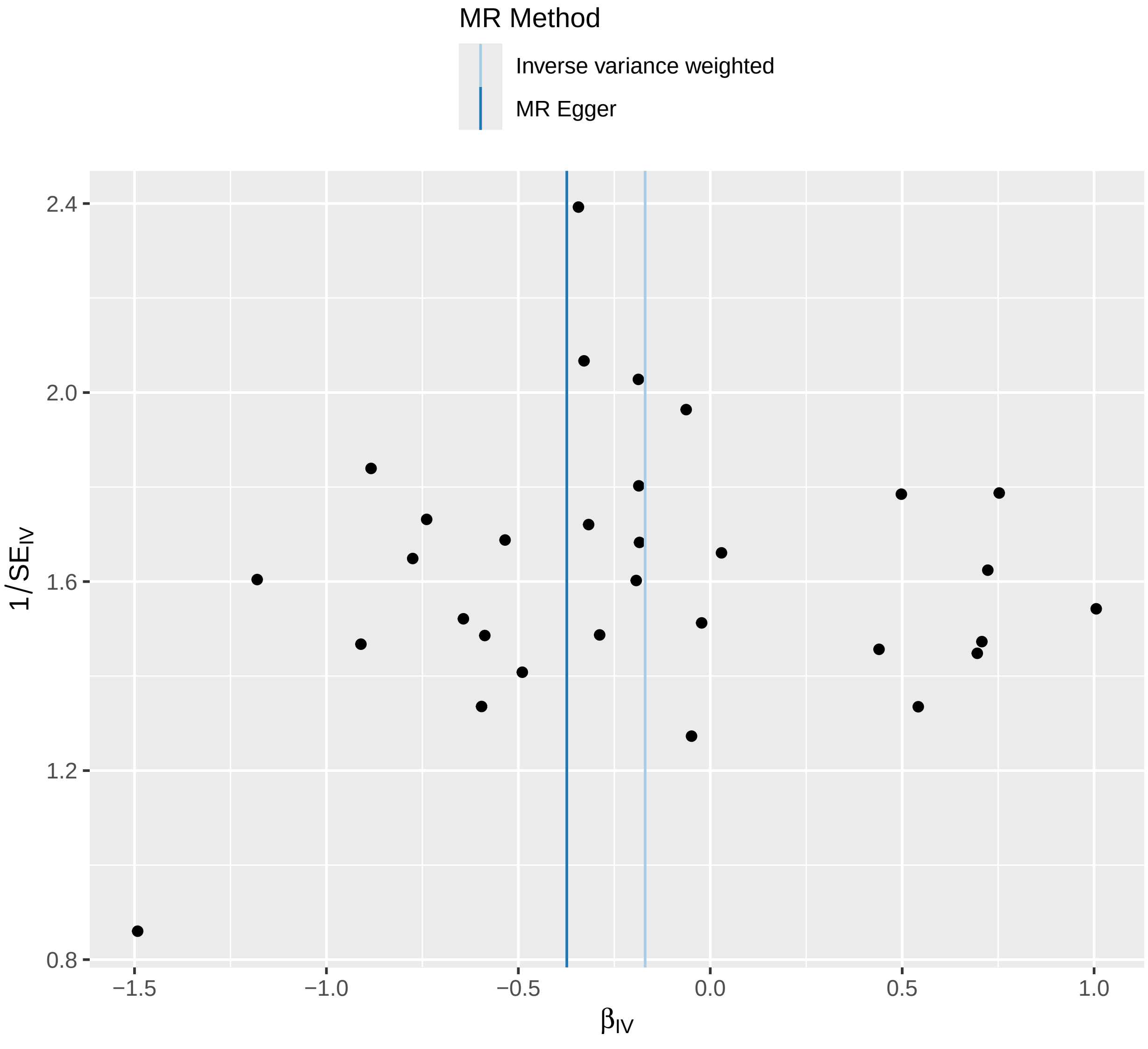
MR Method
Inverse variance weighted
MR Egger
2.4
2.0
1.6
1.2
0.8
$1/SE_{IV}$
−1.5
−1.0
−0.5
0.0
0.5
1.0
β_{IV}

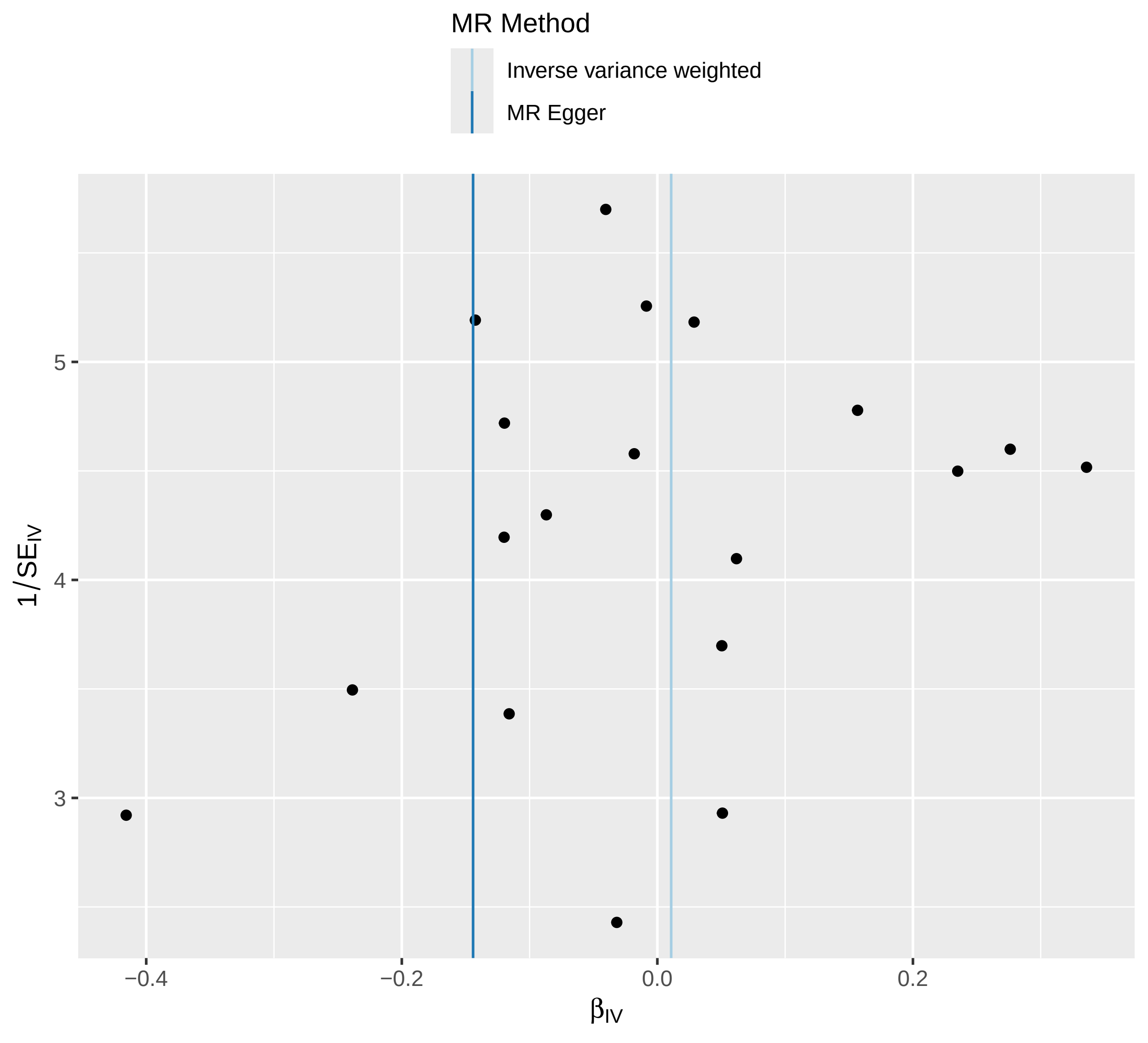
MR Method
Inverse variance weighted
MR Egger
5
4
3
1/SE$_{IV}$
−0.4
−0.2
0.0
0.2
β$_{IV}$

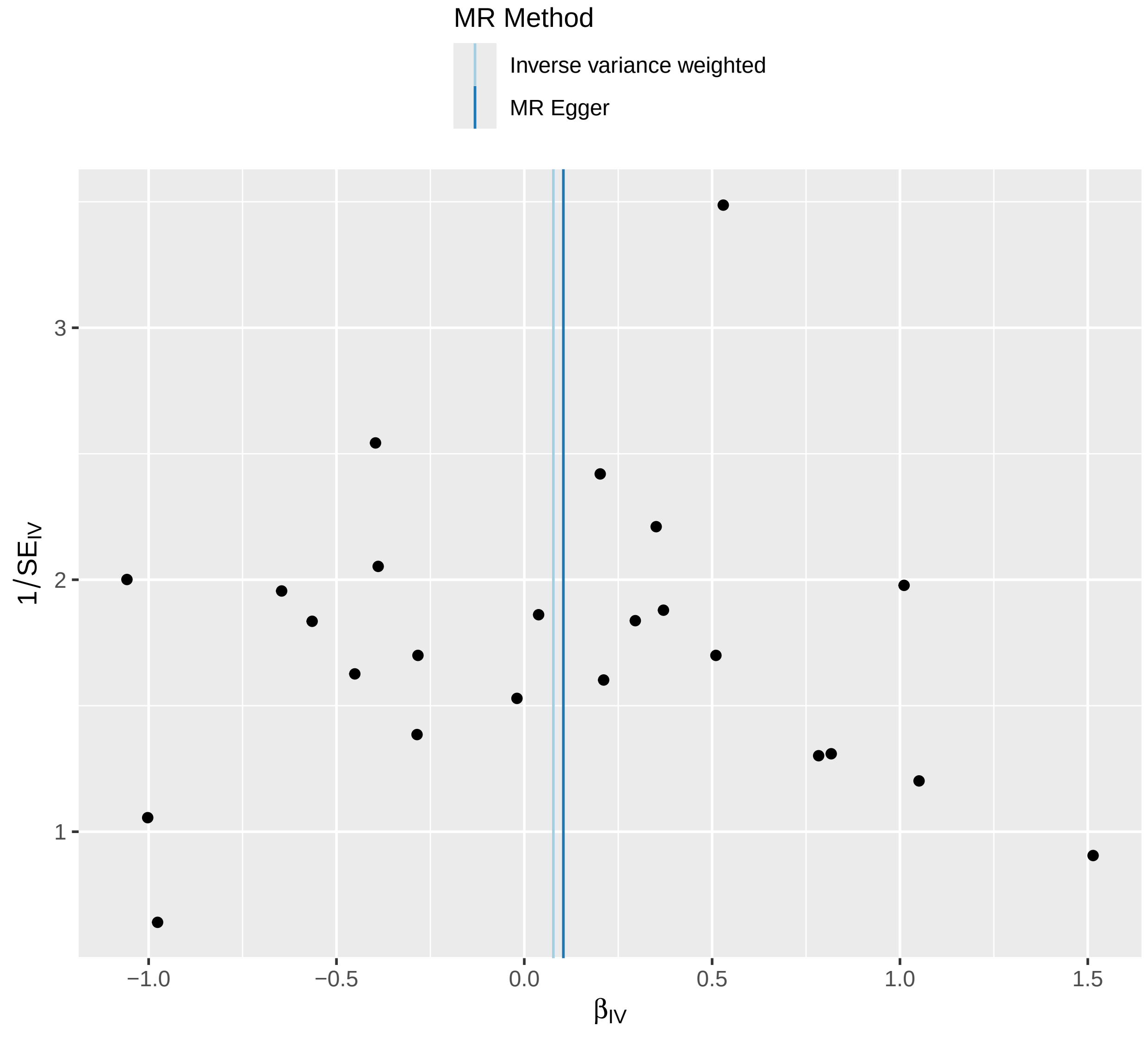
MR Method
Inverse variance weighted
MR Egger
1/SE$_{IV}$
3
2
1
−1.0
−0.5
0.0
0.5
1.0
1.5
β$_{IV}$

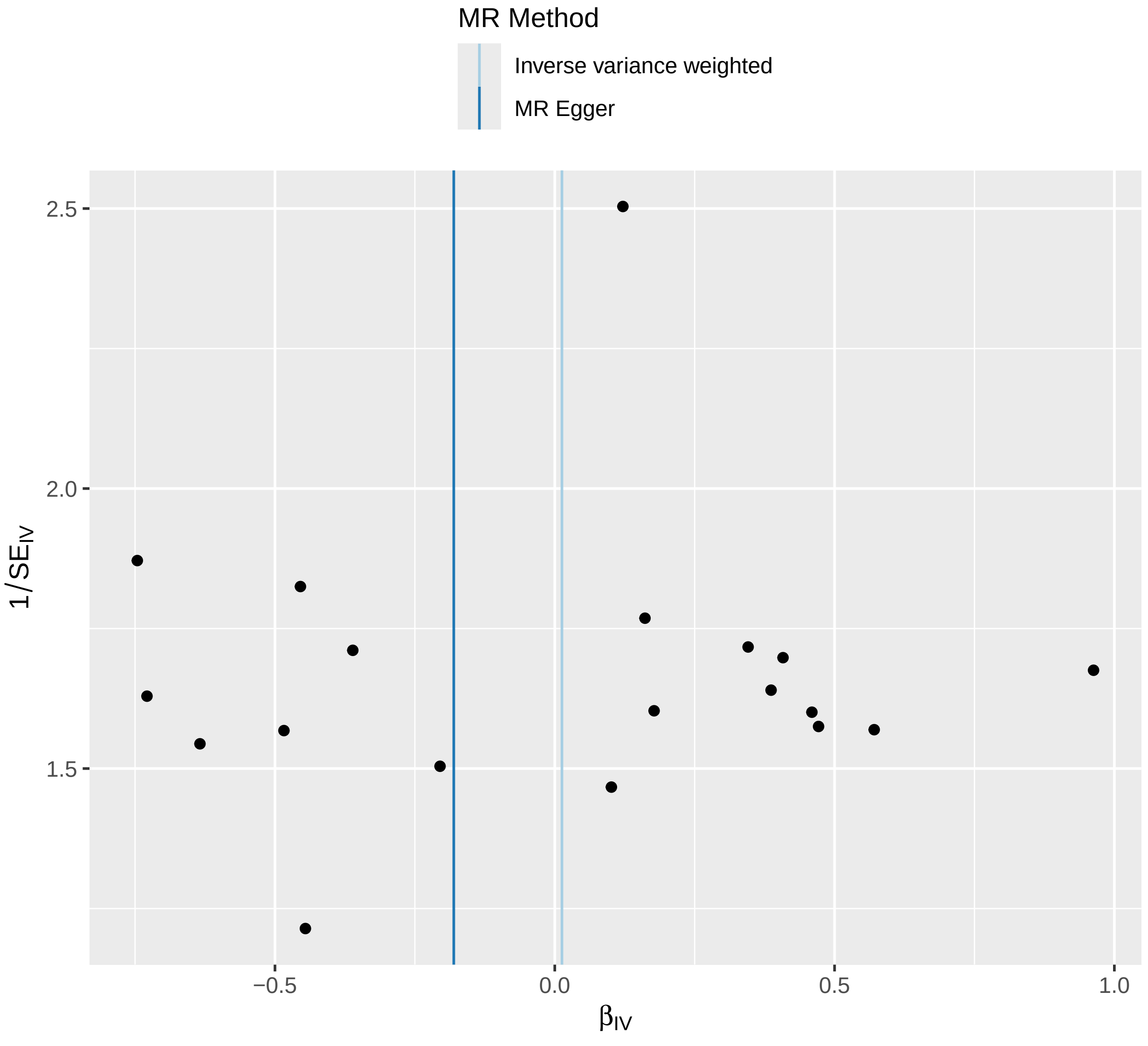
MR Method
Inverse variance weighted
MR Egger
2.5
2.0
1.5
1/SE$_{IV}$
−0.5
0.0
0.5
1.0
β$_{IV}$

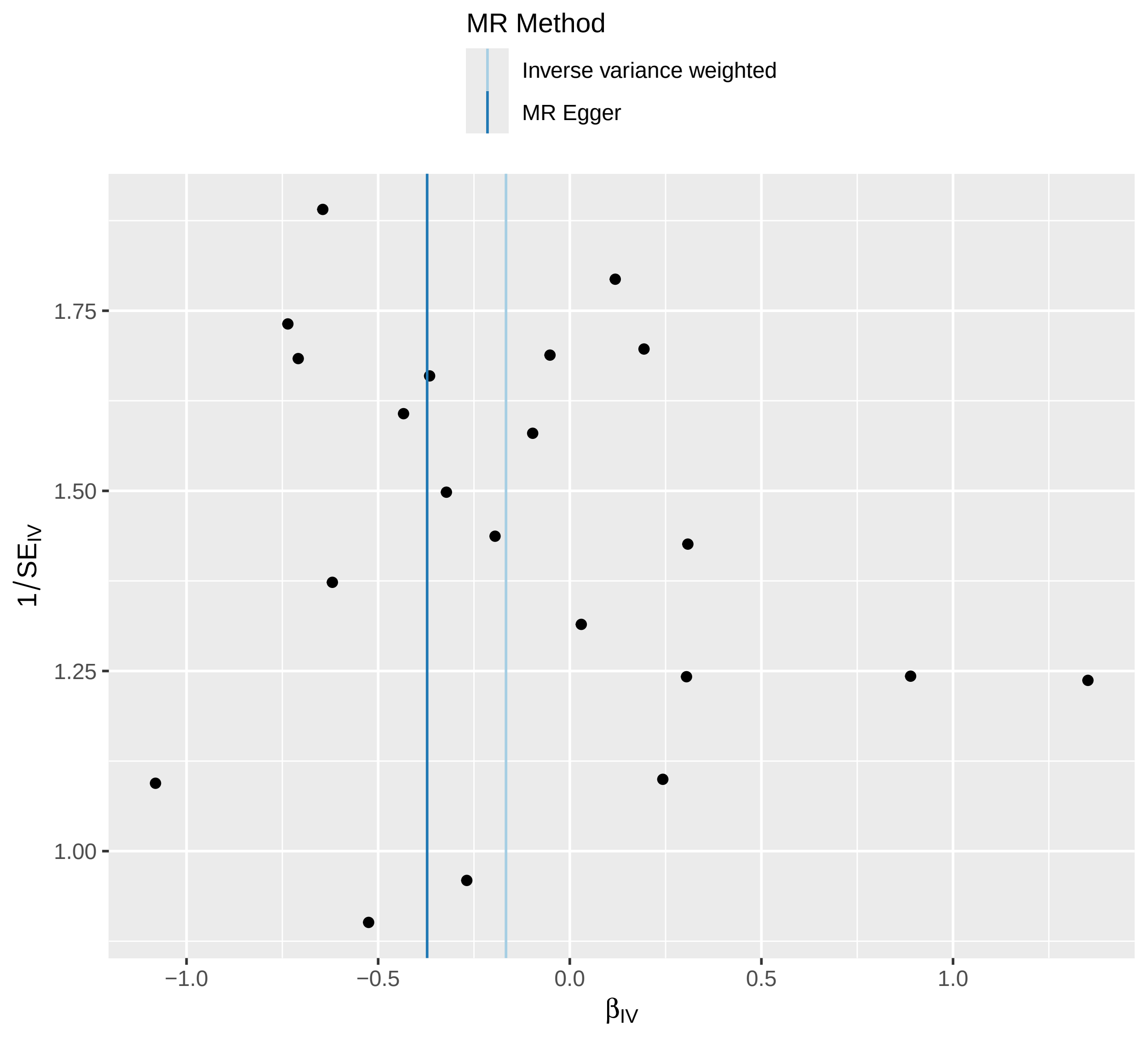
MR Method
Inverse variance weighted
MR Egger
1.75
1.50
1.25
1.00
$1/SE_{IV}$
−1.0
−0.5
0.0
0.5
1.0
β_{IV}

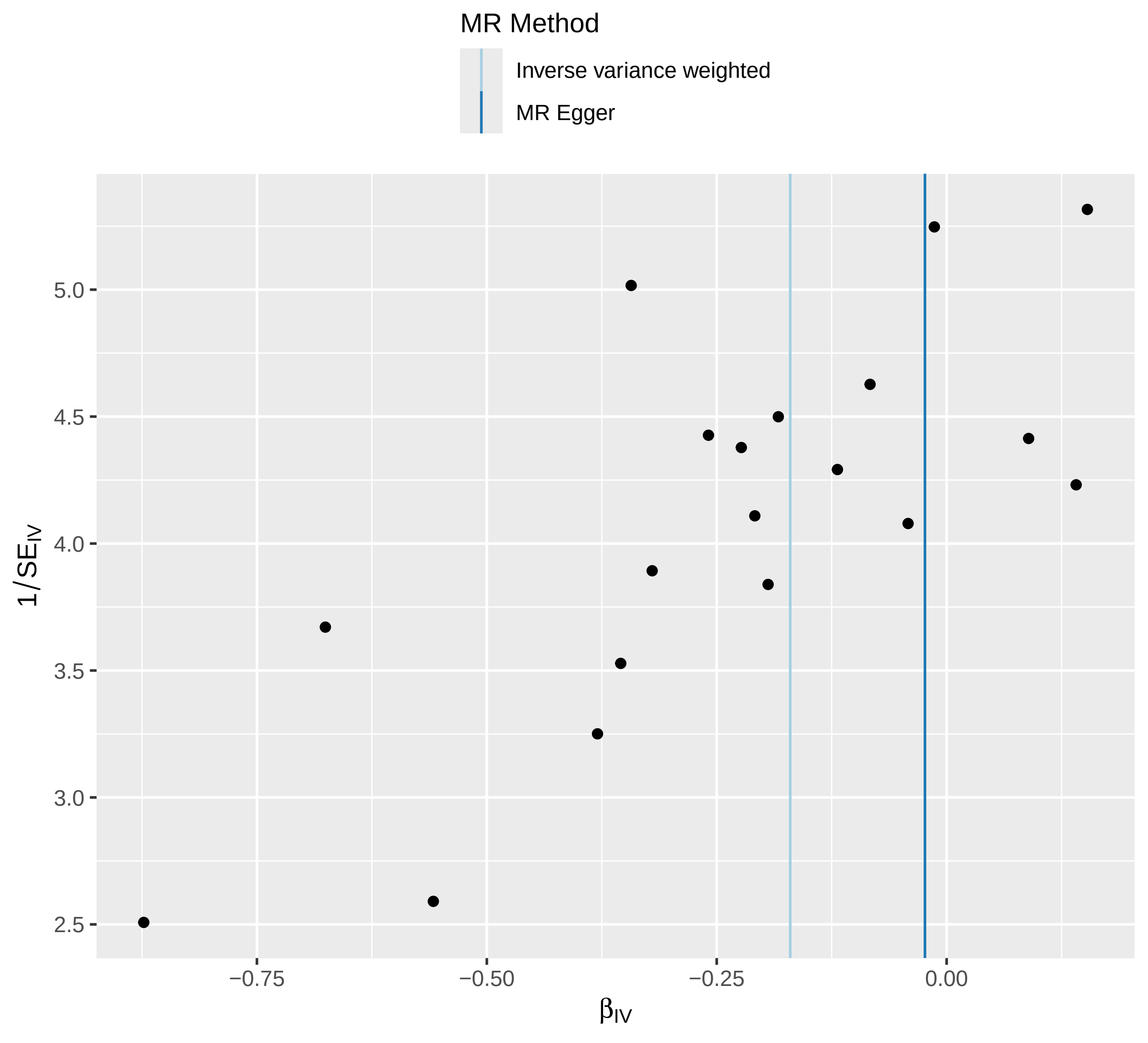
MR Method
Inverse variance weighted
MR Egger
5.0
4.5
4.0
3.5
3.0
2.5
$1/SE_{IV}$
−0.75
−0.50
−0.25
0.00
β_{IV}

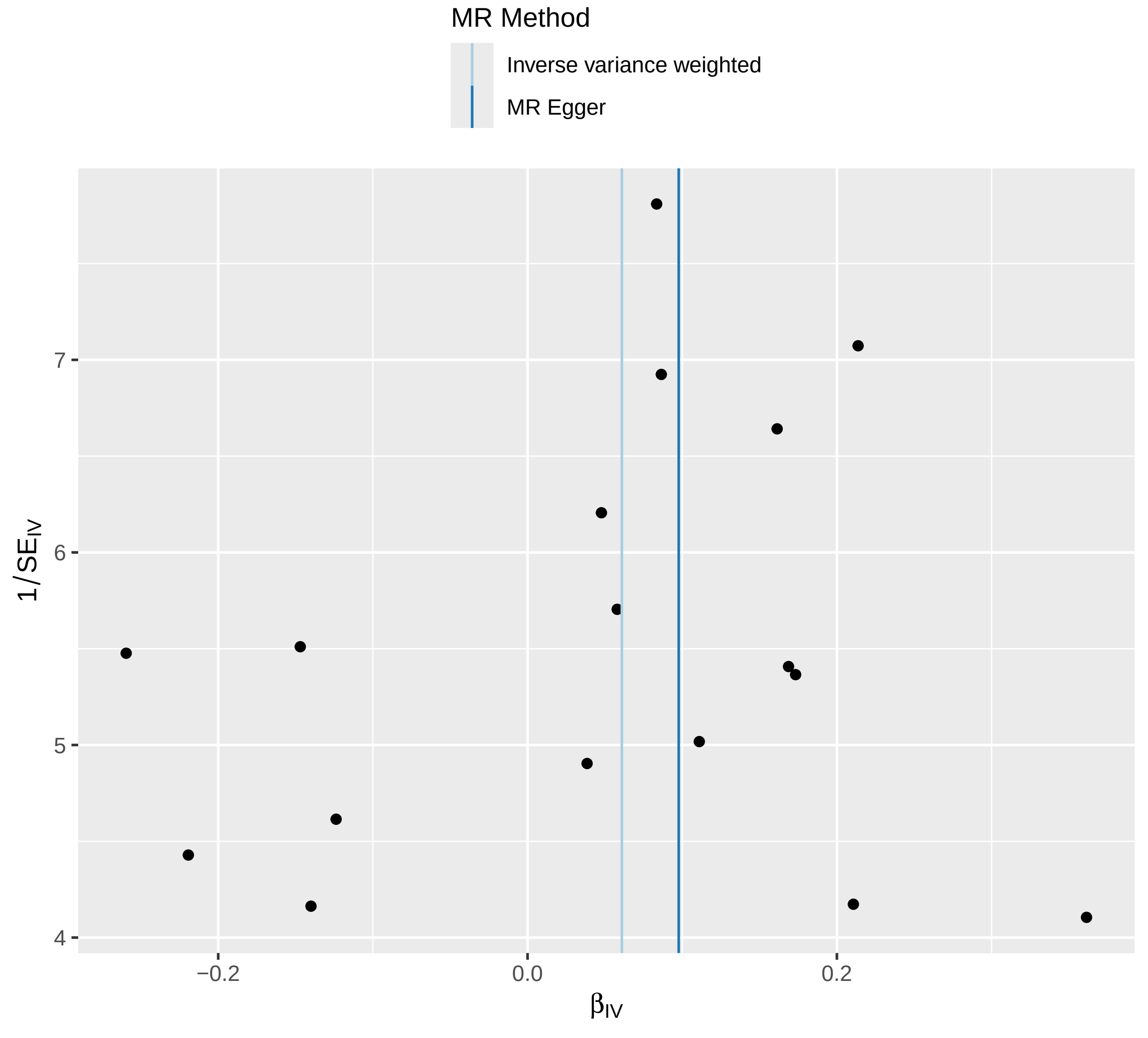
MR Method
Inverse variance weighted
MR Egger
7
6
5
4
−0.2
0.0
0.2
1/SE_IV
β_IV

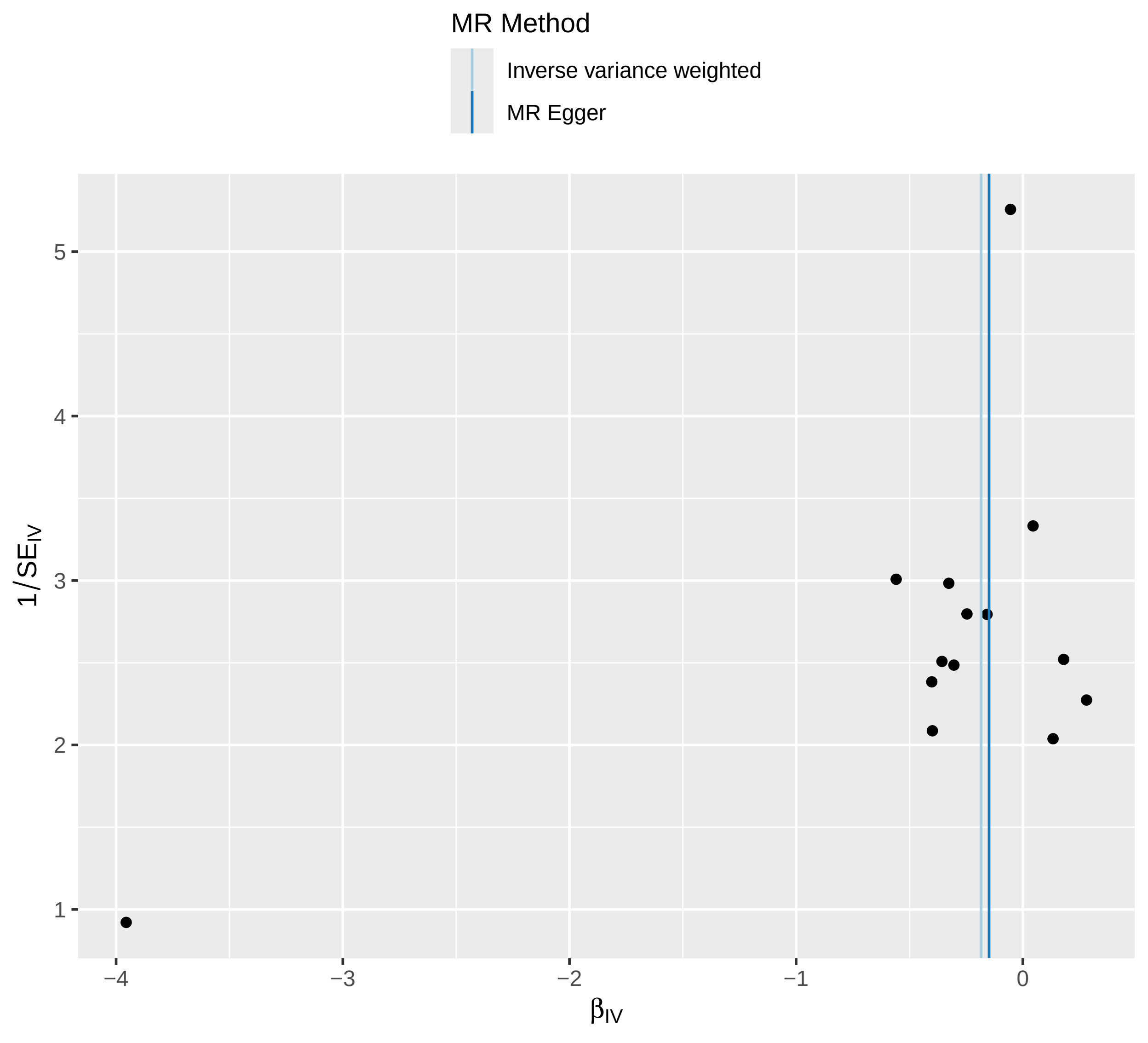

MR Method
Inverse variance weighted
MR Egger
1/SE_IV
5
4
3
2
1
−4
−3
−2
−1
0
β_IV

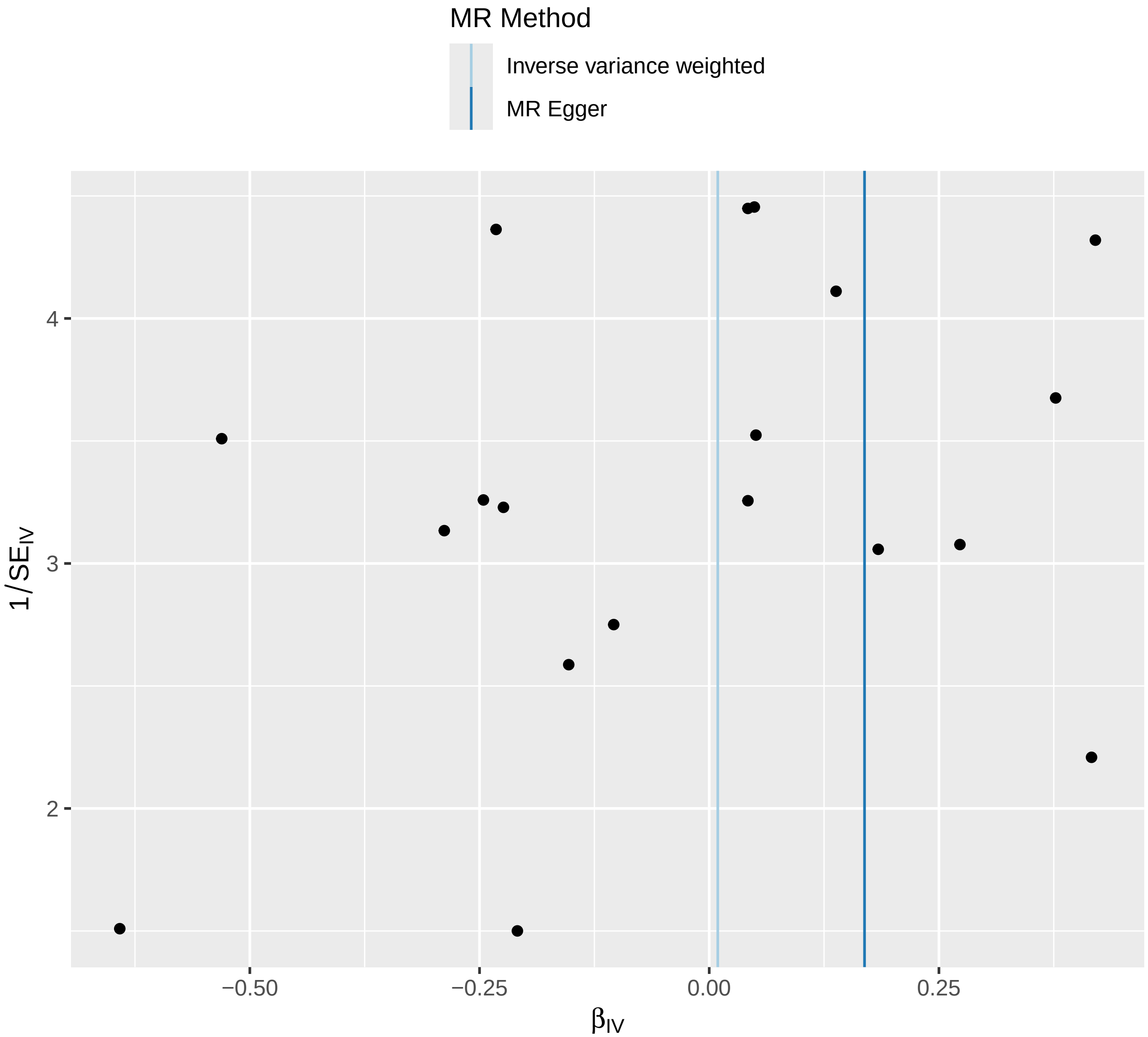

MR Method
Inverse variance weighted
MR Egger
4
3
2
1/SE_IV
−0.50
−0.25
0.00
0.25
β_IV

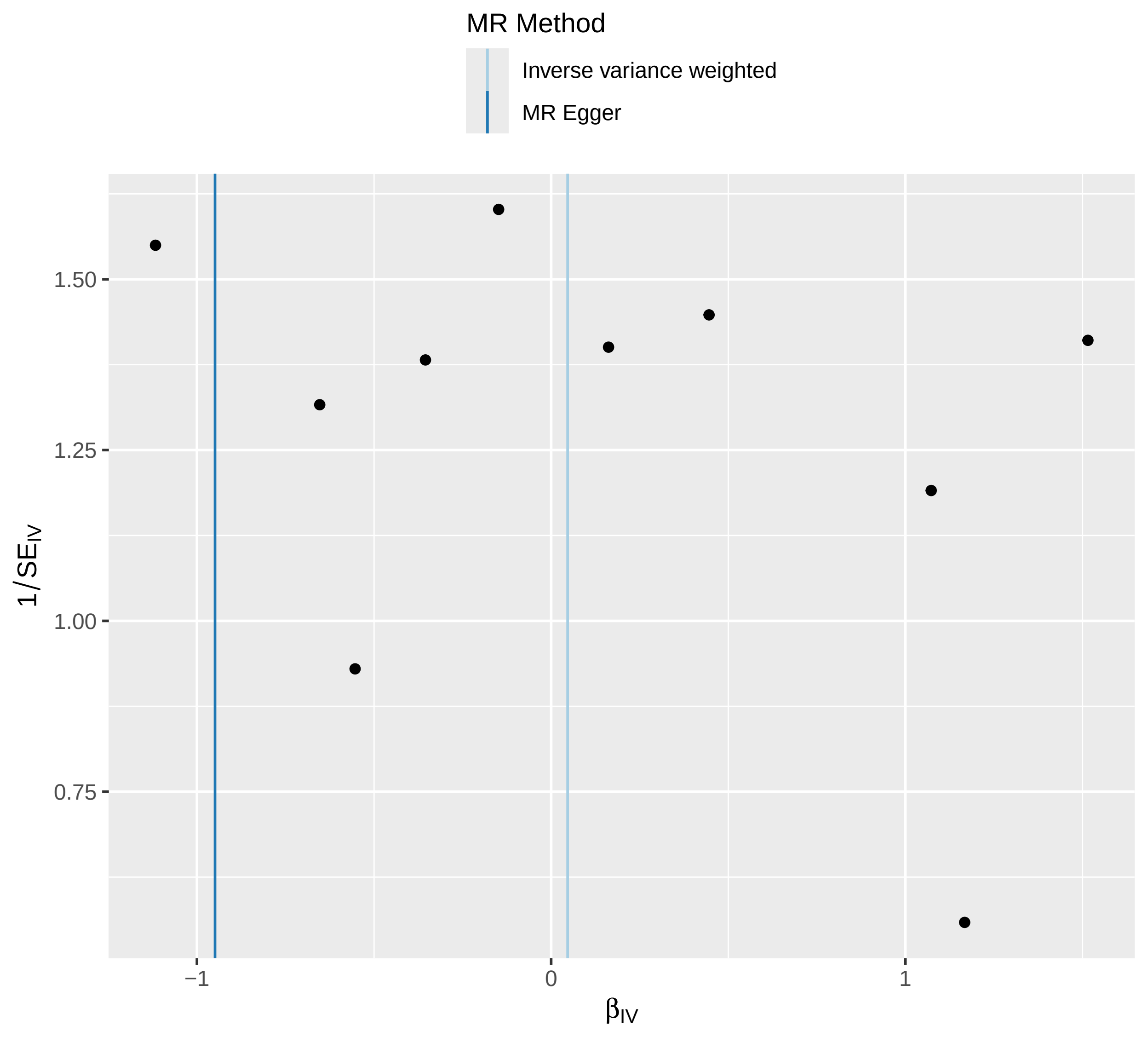

MR Method
Inverse variance weighted
MR Egger
1.50
1.25
1.00
0.75
1/SE_IV
−1
0
1
β_IV

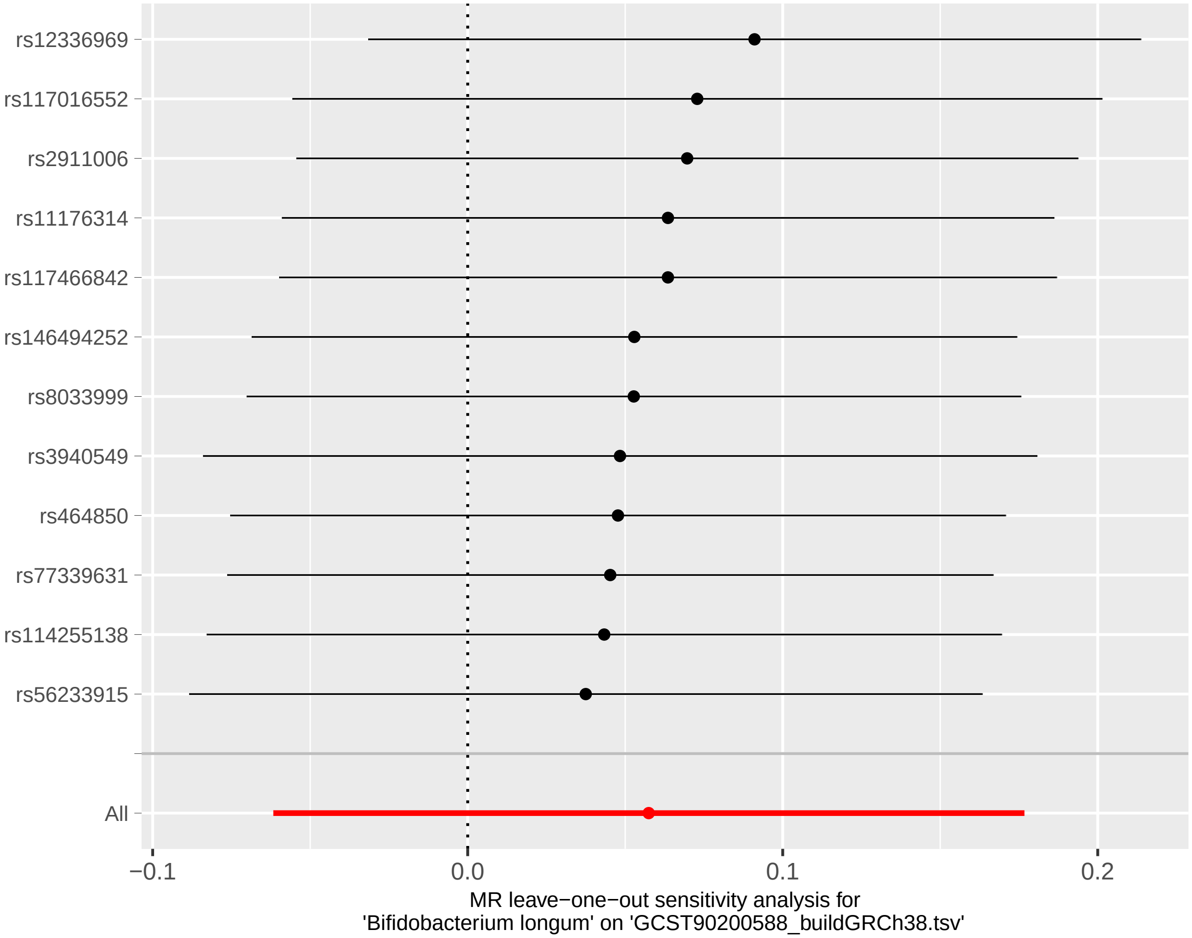
rs12336969
rs117016552
rs2911006
rs11176314
rs117466842
rs146494252
rs8033999
rs3940549
rs464850
rs77339631
rs114255138
rs56233915
All
−0.1
0.0
0.1
0.2
MR leave−one−out sensitivity analysis for
'Bifidobacterium longum' on 'GCST90200588_buildGRCh38.tsv'

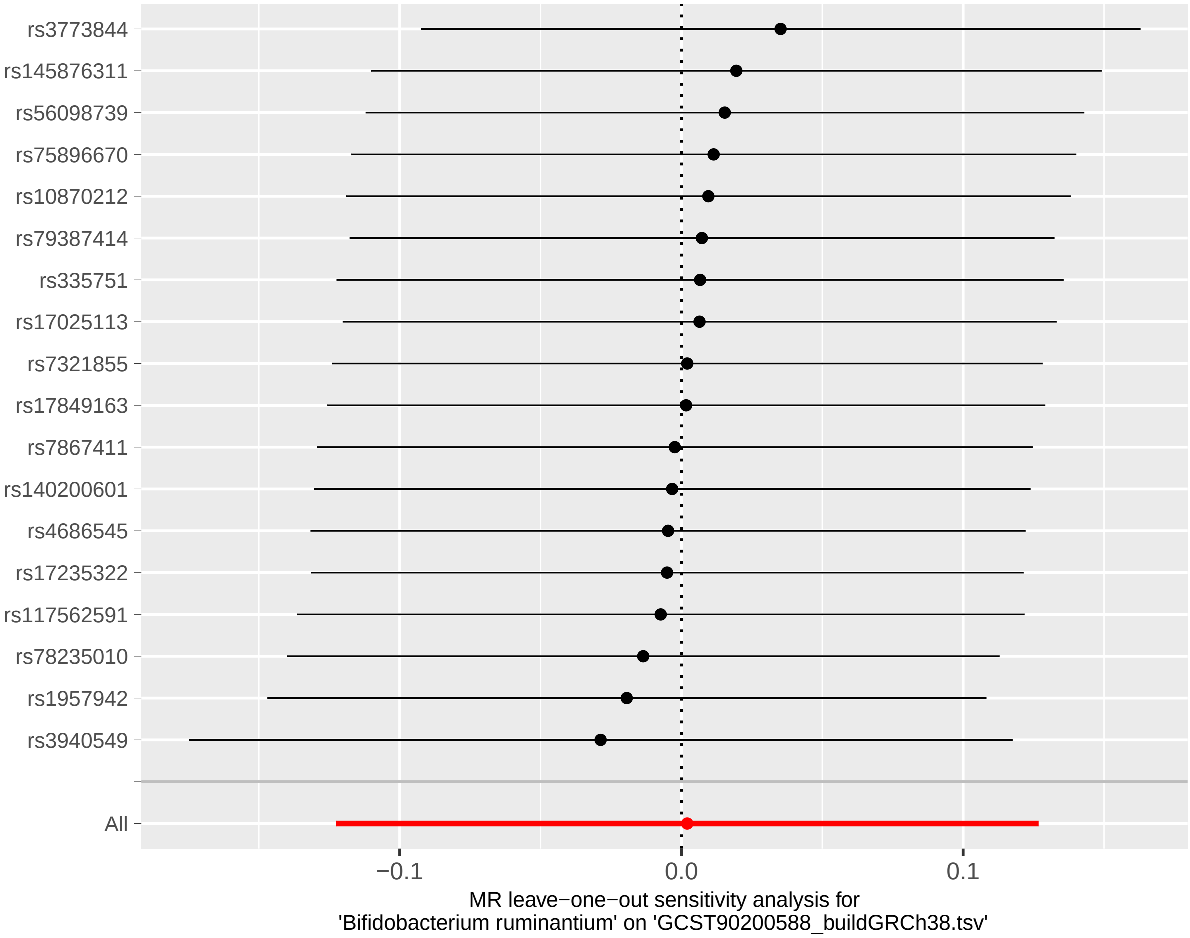
rs3773844
rs145876311
rs56098739
rs75896670
rs10870212
rs79387414
rs335751
rs17025113
rs7321855
rs17849163
rs7867411
rs140200601
rs4686545
rs17235322
rs117562591
rs78235010
rs1957942
rs3940549
All
−0.1
0.0
0.1
MR leave−one−out sensitivity analysis for
'Bifidobacterium ruminantium' on 'GCST90200588_buildGRCh38.tsv'

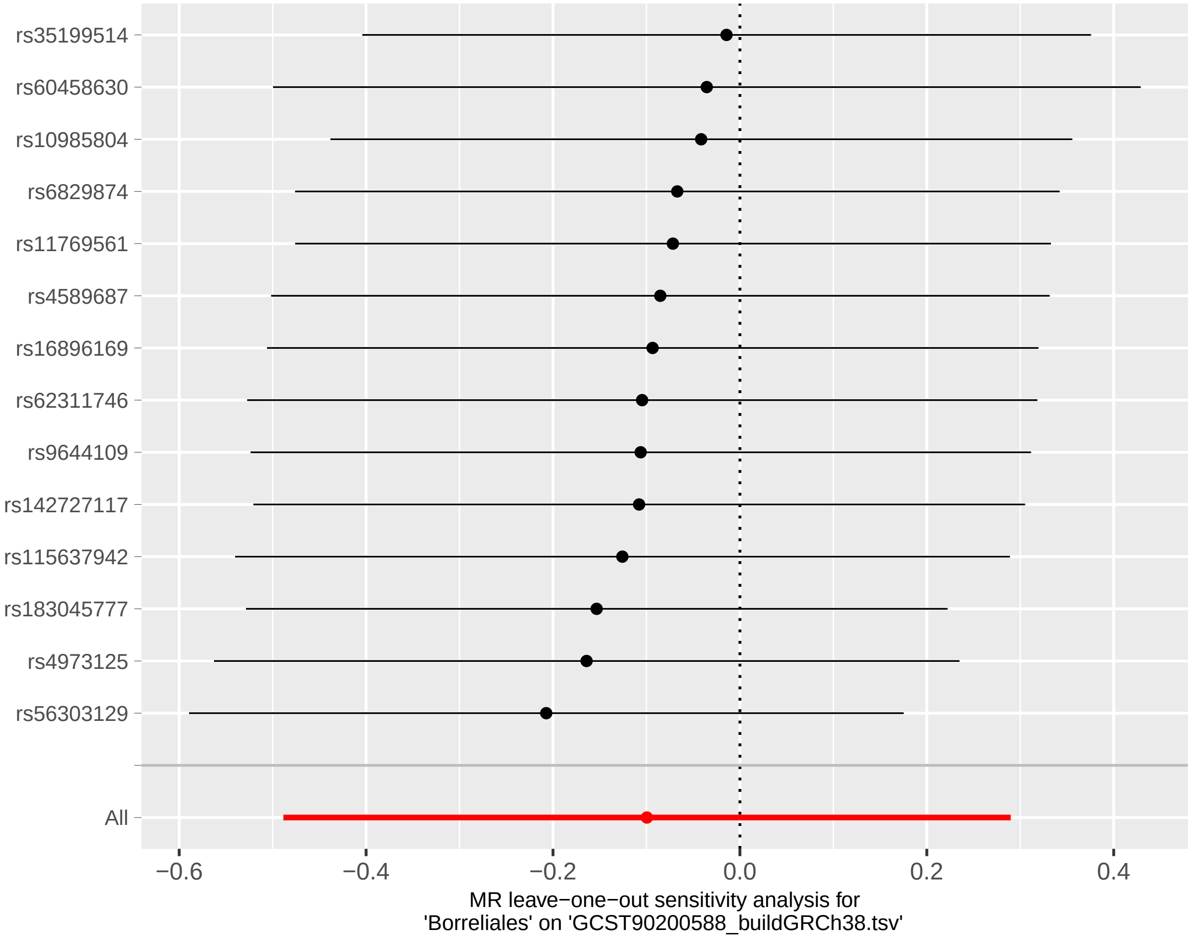

rs35199514
rs60458630
rs10985804
rs6829874
rs11769561
rs4589687
rs16896169
rs62311746
rs9644109
rs142727117
rs115637942
rs183045777
rs4973125
rs56303129
All
−0.6
−0.4
−0.2
0.0
0.2
0.4
MR leave−one−out sensitivity analysis for
'Borreliales' on 'GCST90200588_buildGRCh38.tsv'

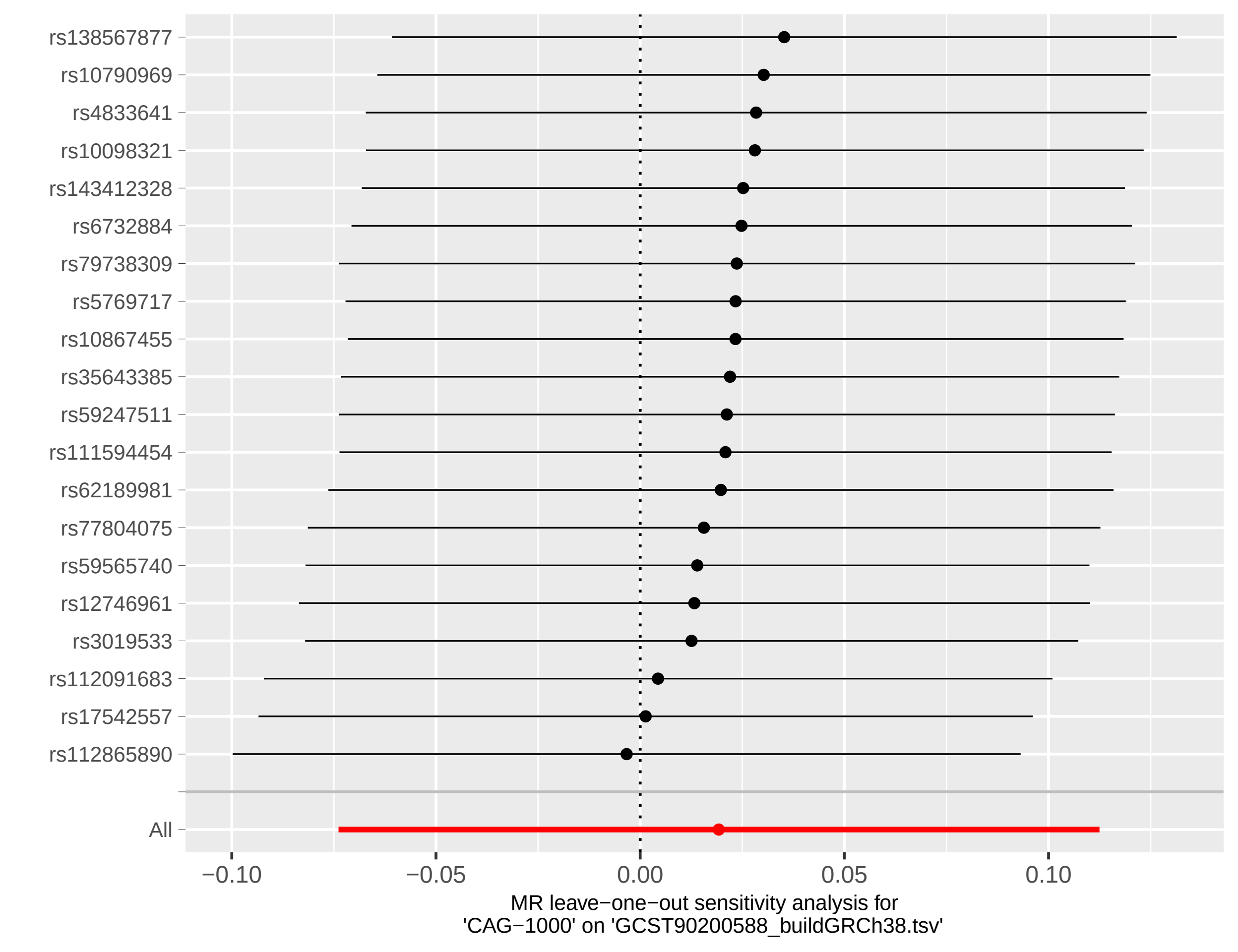

rs138567877
rs10790969
rs4833641
rs10098321
rs143412328
rs6732884
rs79738309
rs5769717
rs10867455
rs35643385
rs59247511
rs111594454
rs62189981
rs77804075
rs59565740
rs12746961
rs3019533
rs112091683
rs17542557
rs112865890
All
−0.10
−0.05
0.00
0.05
0.10
MR leave−one−out sensitivity analysis for
'CAG−1000' on 'GCST90200588_buildGRCh38.tsv'

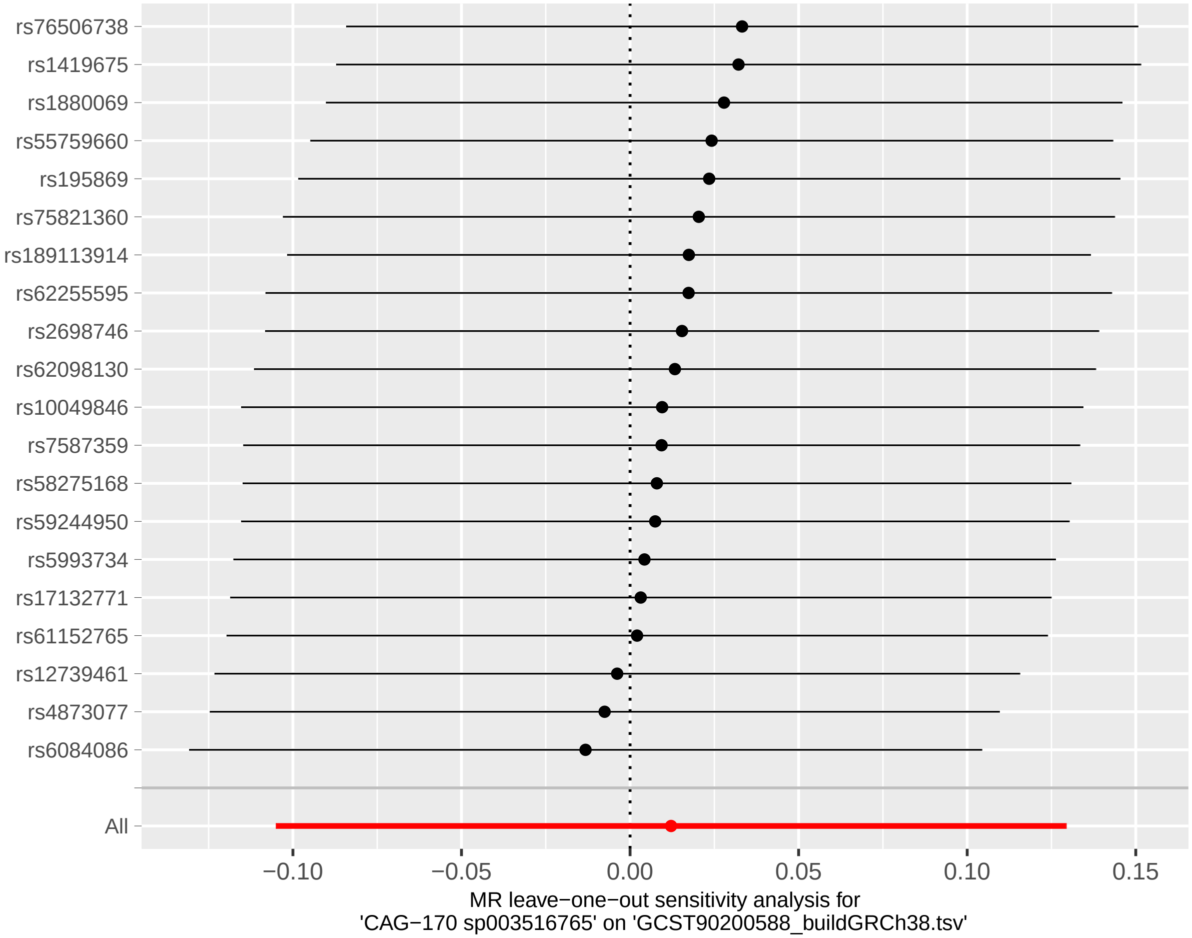

rs76506738
rs1419675
rs1880069
rs55759660
rs195869
rs75821360
rs189113914
rs62255595
rs2698746
rs62098130
rs10049846
rs7587359
rs58275168
rs59244950
rs5993734
rs17132771
rs61152765
rs12739461
rs4873077
rs6084086
All
−0.10
−0.05
0.00
0.05
0.10
0.15
MR leave−one−out sensitivity analysis for
'CAG−170 sp003516765' on 'GCST90200588_buildGRCh38.tsv'

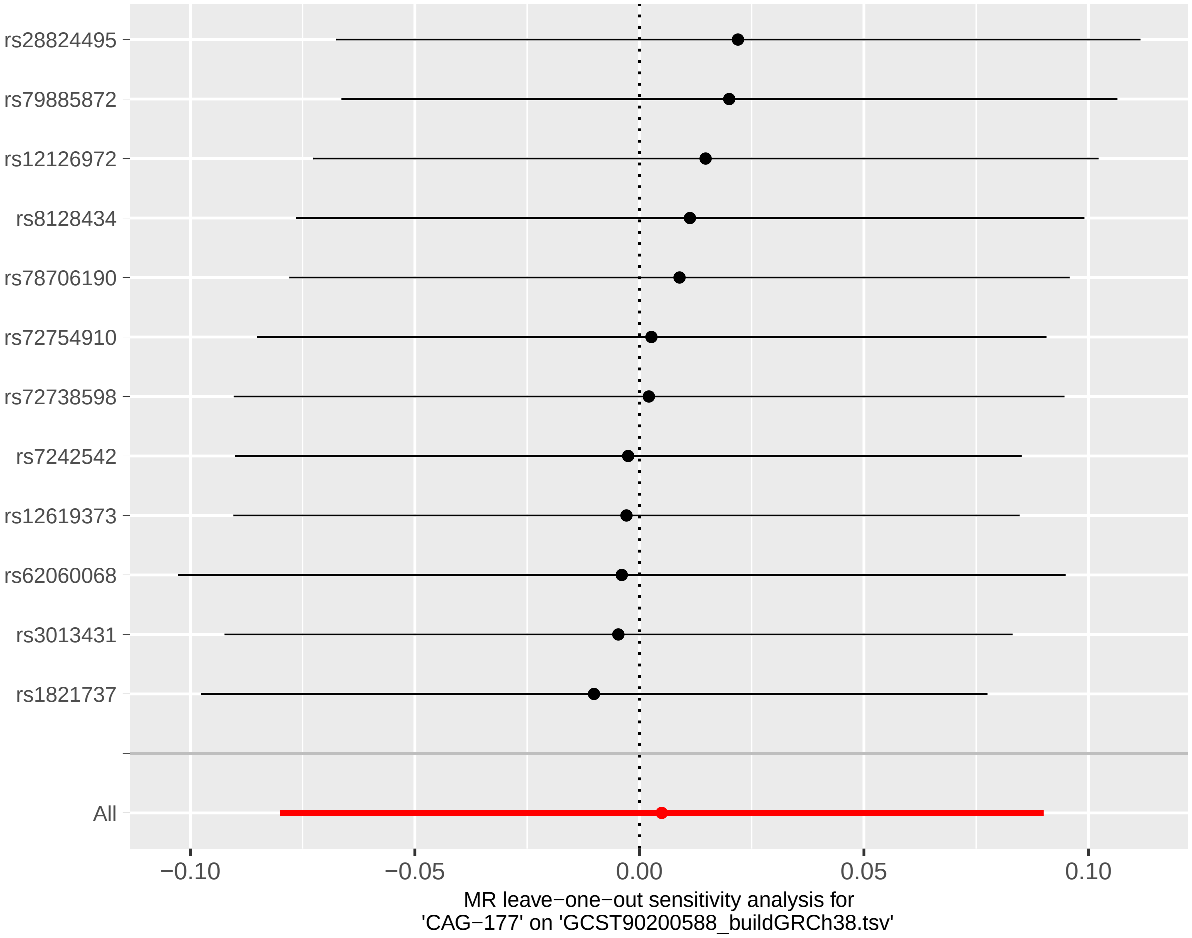

rs28824495
rs79885872
rs12126972
rs8128434
rs78706190
rs72754910
rs72738598
rs7242542
rs12619373
rs62060068
rs3013431
rs1821737
All
−0.10
−0.05
0.00
0.05
0.10
MR leave−one−out sensitivity analysis for
'CAG−177' on 'GCST90200588_buildGRCh38.tsv'

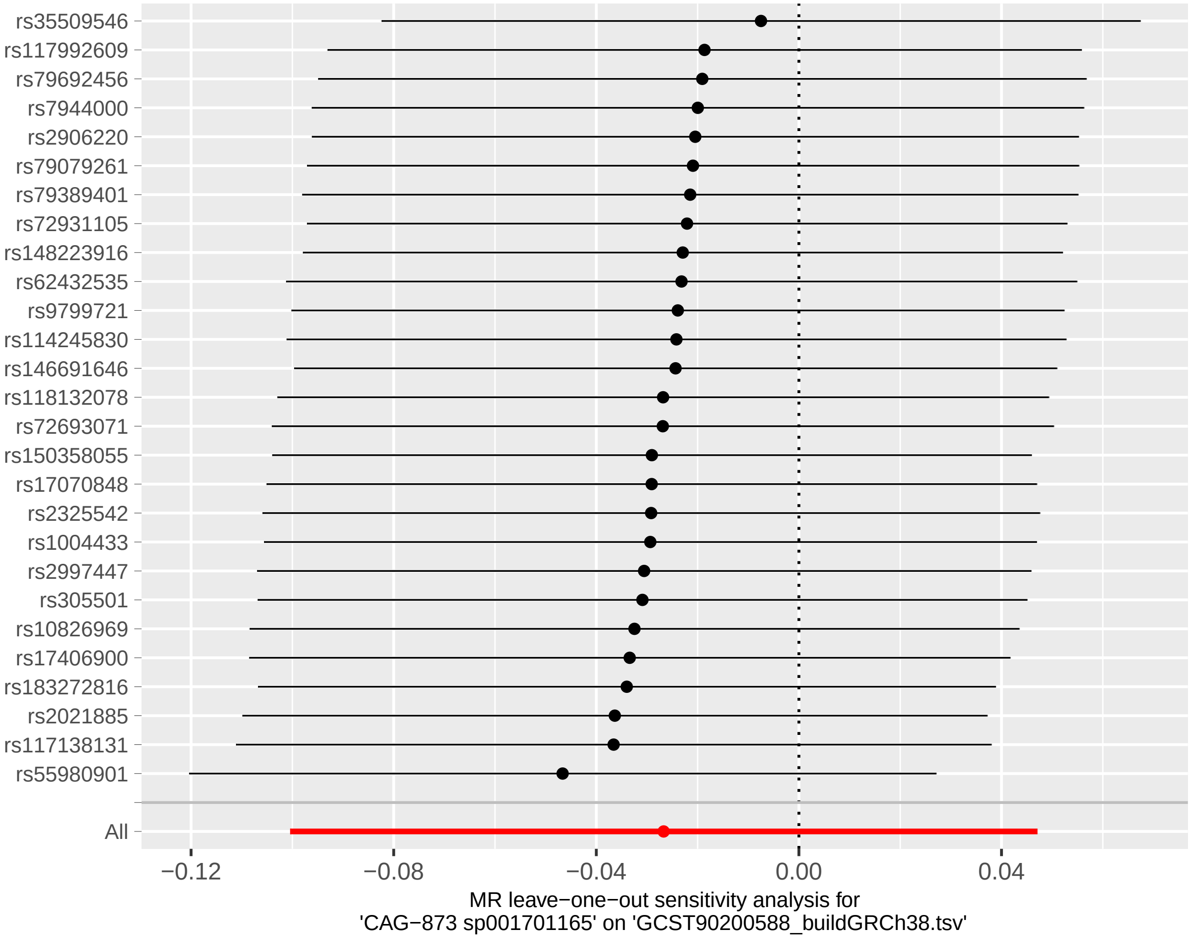
rs35509546
rs117992609
rs79692456
rs7944000
rs2906220
rs79079261
rs79389401
rs72931105
rs148223916
rs62432535
rs9799721
rs114245830
rs146691646
rs118132078
rs72693071
rs150358055
rs17070848
rs2325542
rs1004433
rs2997447
rs305501
rs10826969
rs17406900
rs183272816
rs2021885
rs117138131
rs55980901
All
−0.12
−0.08
−0.04
0.00
0.04
MR leave−one−out sensitivity analysis for
'CAG−873 sp001701165' on 'GCST90200588_buildGRCh38.tsv'

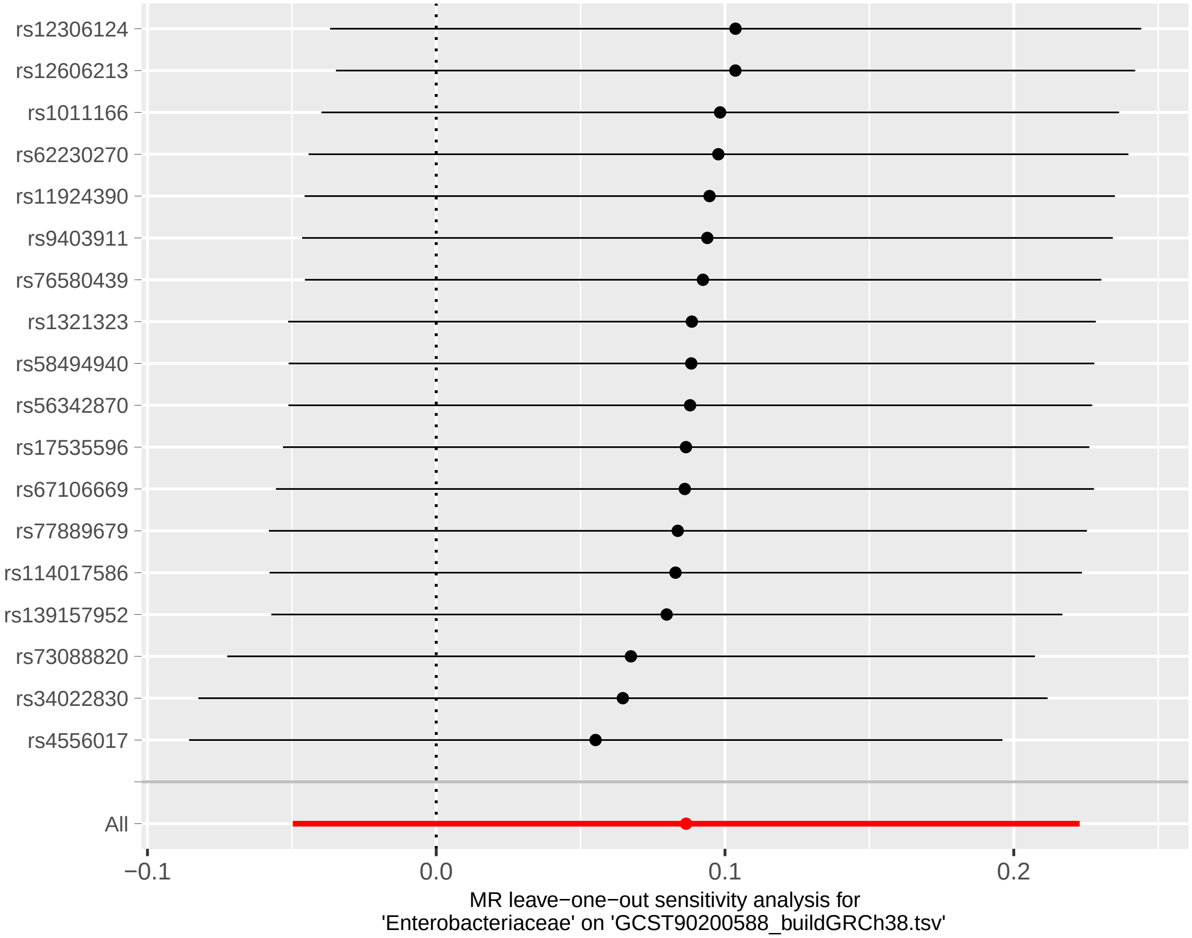
rs12306124
rs12606213
rs1011166
rs62230270
rs11924390
rs9403911
rs76580439
rs1321323
rs58494940
rs56342870
rs17535596
rs67106669
rs77889679
rs114017586
rs139157952
rs73088820
rs34022830
rs4556017
All
−0.1
0.0
0.1
0.2
MR leave−one−out sensitivity analysis for
'Enterobacteriaceae' on 'GCST90200588_buildGRCh38.tsv'

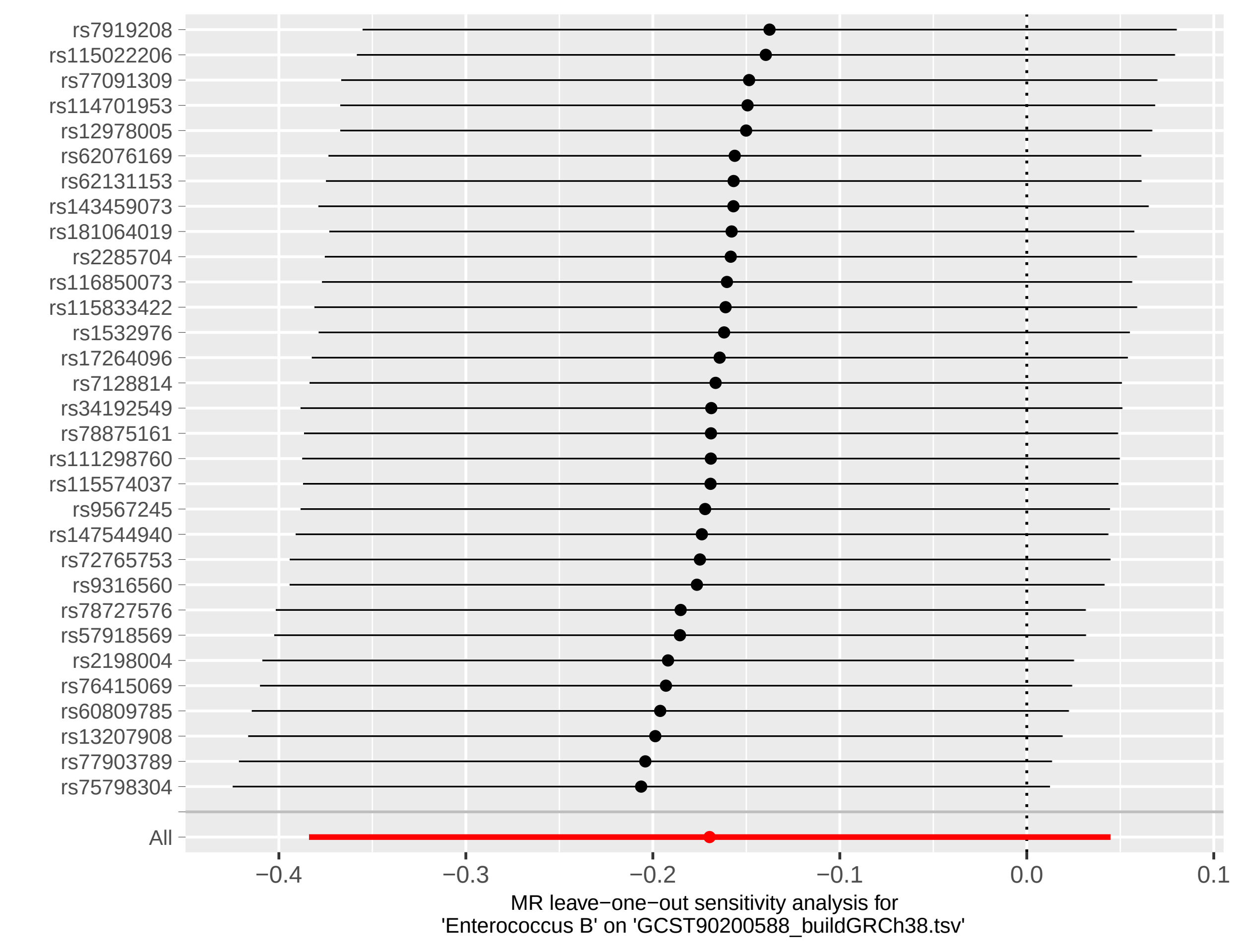
rs7919208
rs115022206
rs77091309
rs114701953
rs12978005
rs62076169
rs62131153
rs143459073
rs181064019
rs2285704
rs116850073
rs115833422
rs1532976
rs17264096
rs7128814
rs34192549
rs78875161
rs111298760
rs115574037
rs9567245
rs147544940
rs72765753
rs9316560
rs78727576
rs57918569
rs2198004
rs76415069
rs60809785
rs13207908
rs77903789
rs75798304
All
−0.4
−0.3
−0.2
−0.1
0.0
0.1
MR leave−one−out sensitivity analysis for
'Enterococcus B' on 'GCST90200588_buildGRCh38.tsv'

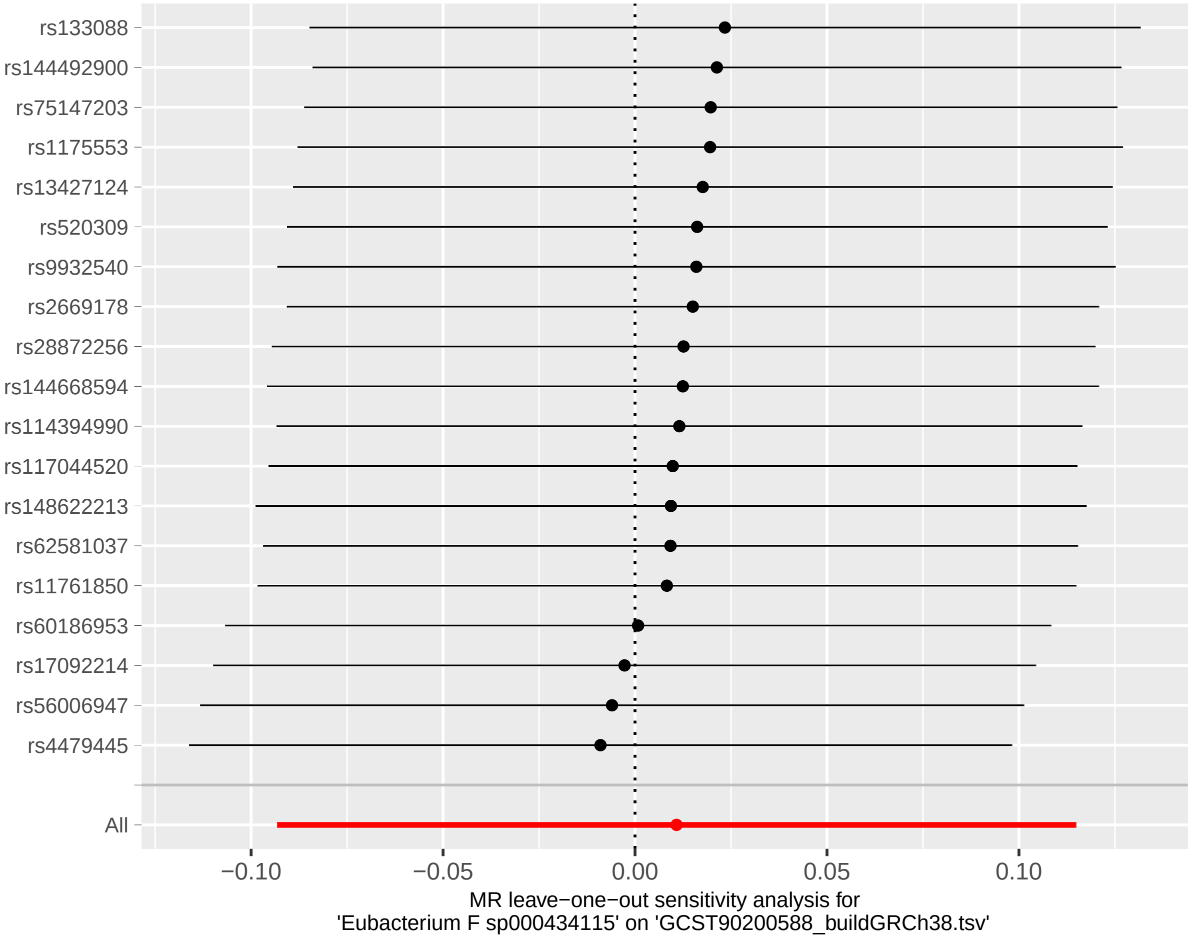
rs133088
rs144492900
rs75147203
rs1175553
rs13427124
rs520309
rs9932540
rs2669178
rs28872256
rs144668594
rs114394990
rs117044520
rs148622213
rs62581037
rs11761850
rs60186953
rs17092214
rs56006947
rs4479445
All
−0.10
−0.05
0.00
0.05
0.10
MR leave−one−out sensitivity analysis for
'Eubacterium F sp000434115' on 'GCST90200588_buildGRCh38.tsv'

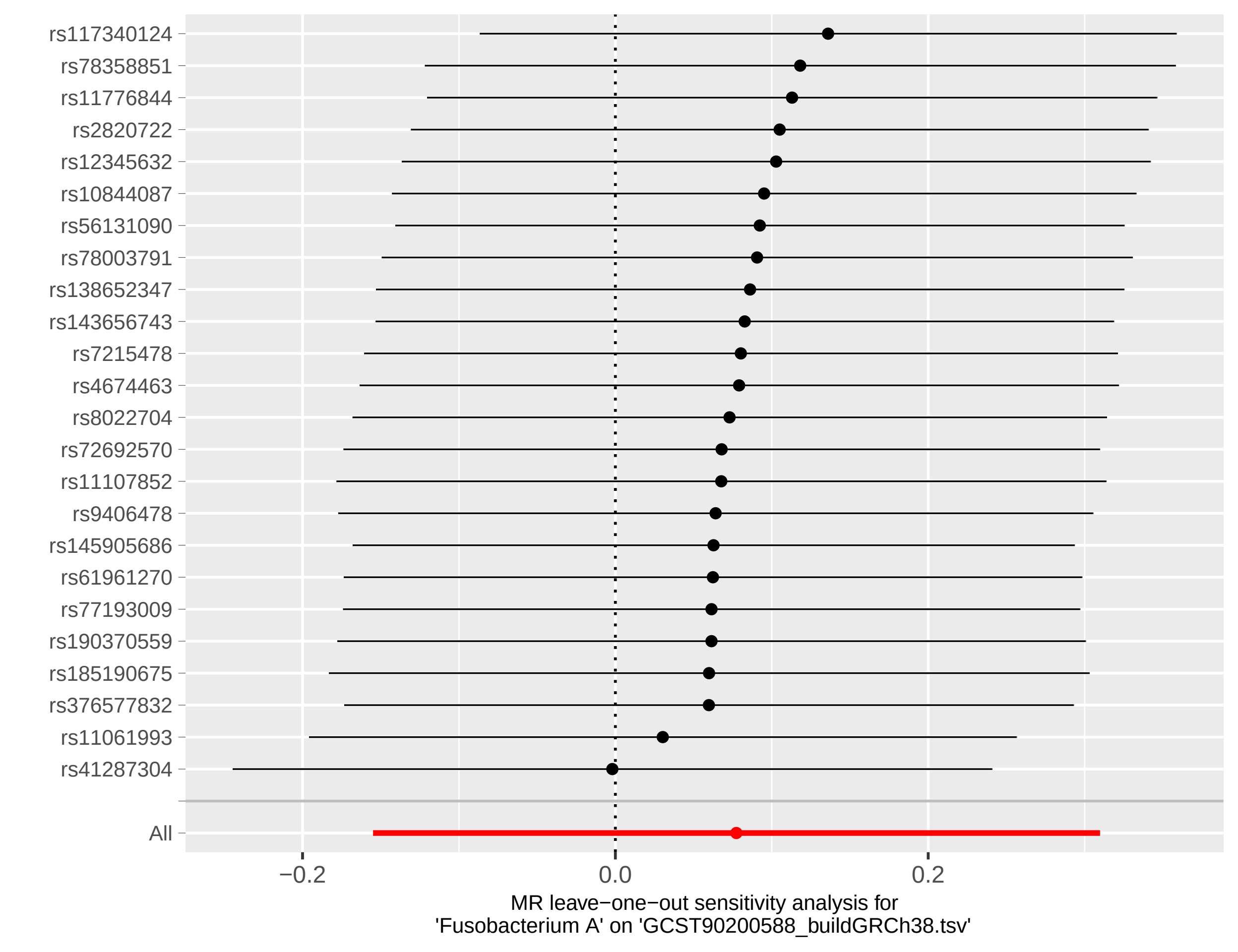

rs117340124
rs78358851
rs11776844
rs2820722
rs12345632
rs10844087
rs56131090
rs78003791
rs138652347
rs143656743
rs7215478
rs4674463
rs8022704
rs72692570
rs11107852
rs9406478
rs145905686
rs61961270
rs77193009
rs190370559
rs185190675
rs376577832
rs11061993
rs41287304
All
−0.2
0.0
0.2
MR leave−one−out sensitivity analysis for
'Fusobacterium A' on 'GCST90200588_buildGRCh38.tsv'

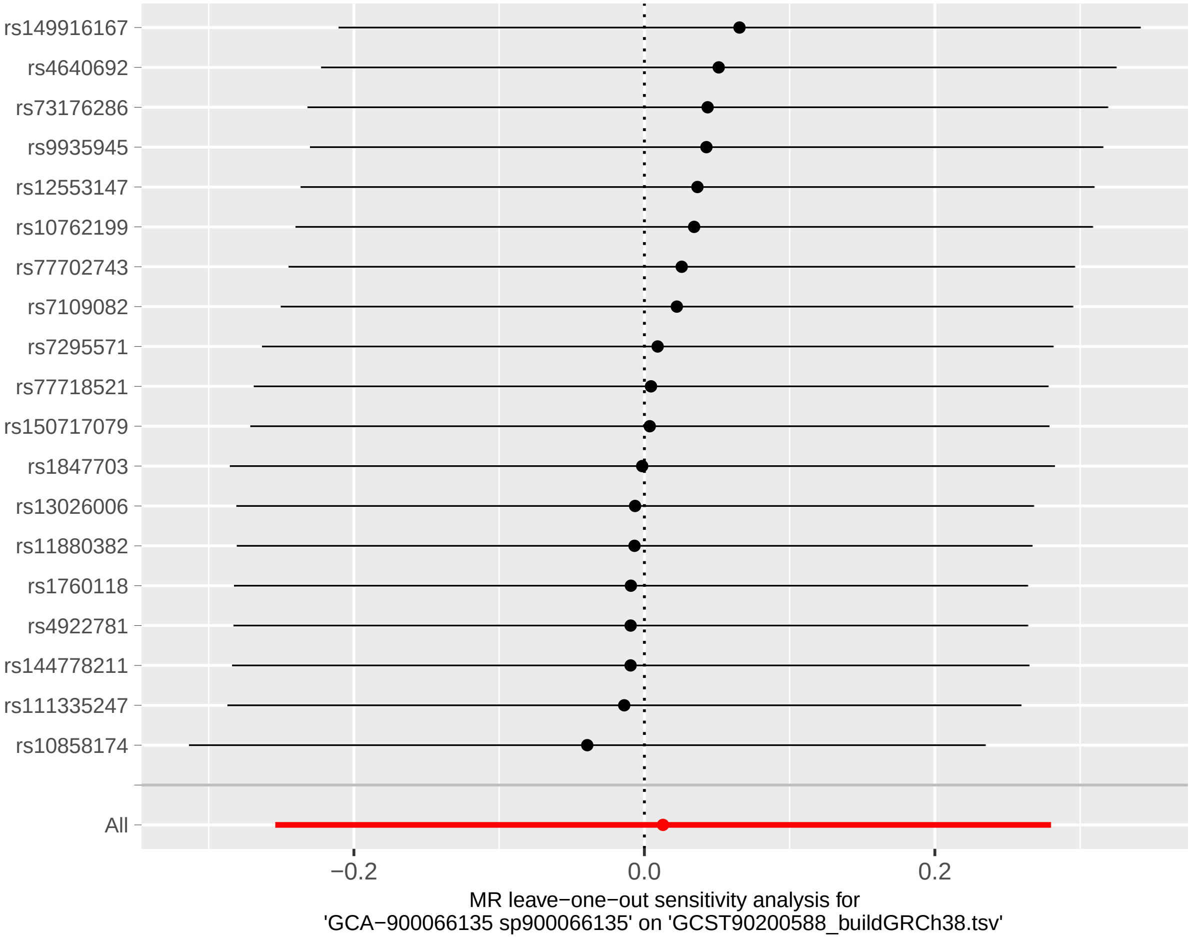

rs149916167
rs4640692
rs73176286
rs9935945
rs12553147
rs10762199
rs77702743
rs7109082
rs7295571
rs77718521
rs150717079
rs1847703
rs13026006
rs11880382
rs1760118
rs4922781
rs144778211
rs111335247
rs10858174
All
−0.2
0.0
0.2
MR leave−one−out sensitivity analysis for
'GCA−900066135 sp900066135' on 'GCST90200588_buildGRCh38.tsv'

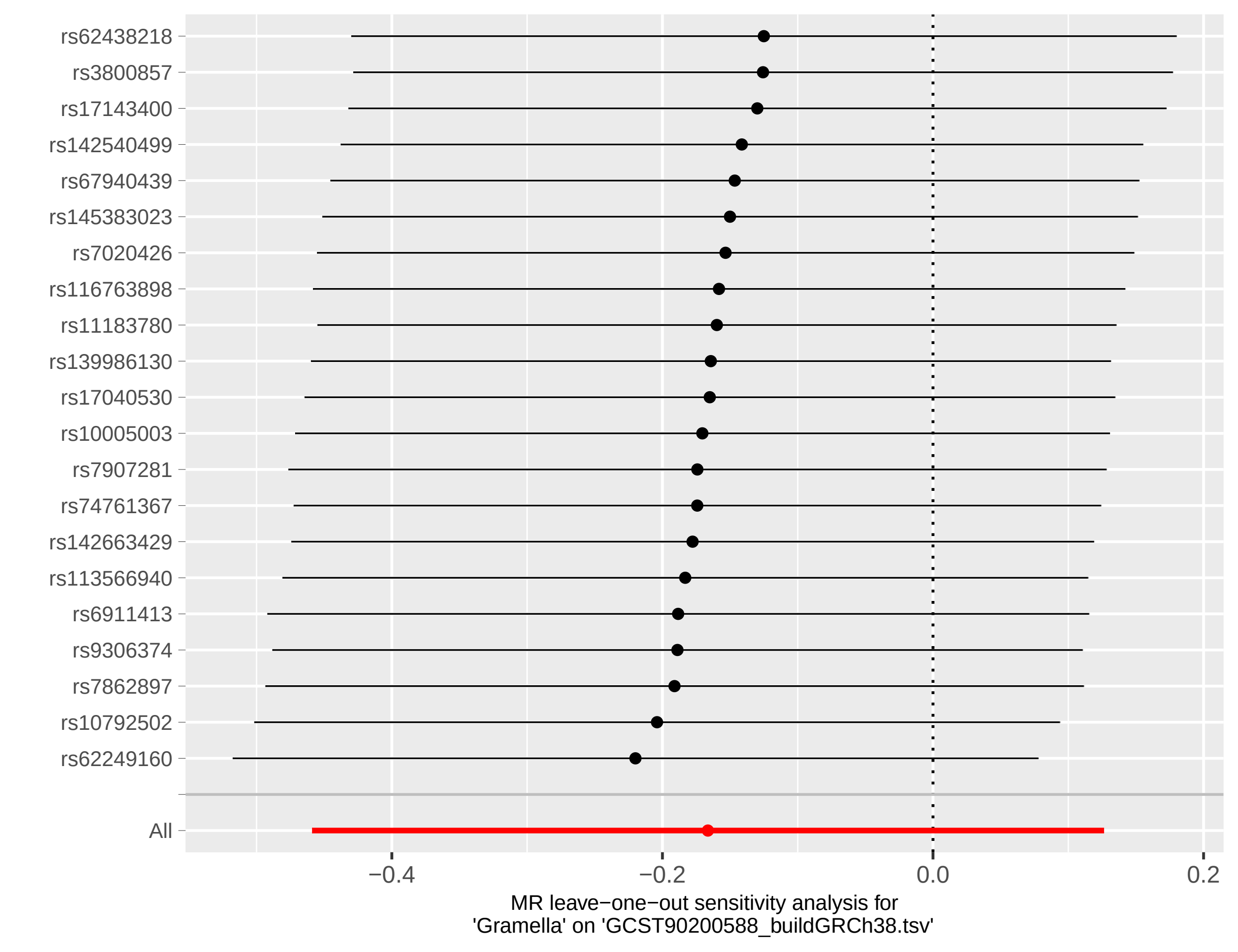
rs62438218
rs3800857
rs17143400
rs142540499
rs67940439
rs145383023
rs7020426
rs116763898
rs11183780
rs139986130
rs17040530
rs10005003
rs7907281
rs74761367
rs142663429
rs113566940
rs6911413
rs9306374
rs7862897
rs10792502
rs62249160
All
−0.4
−0.2
0.0
0.2
MR leave−one−out sensitivity analysis for
'Gramella' on 'GCST90200588_buildGRCh38.tsv'

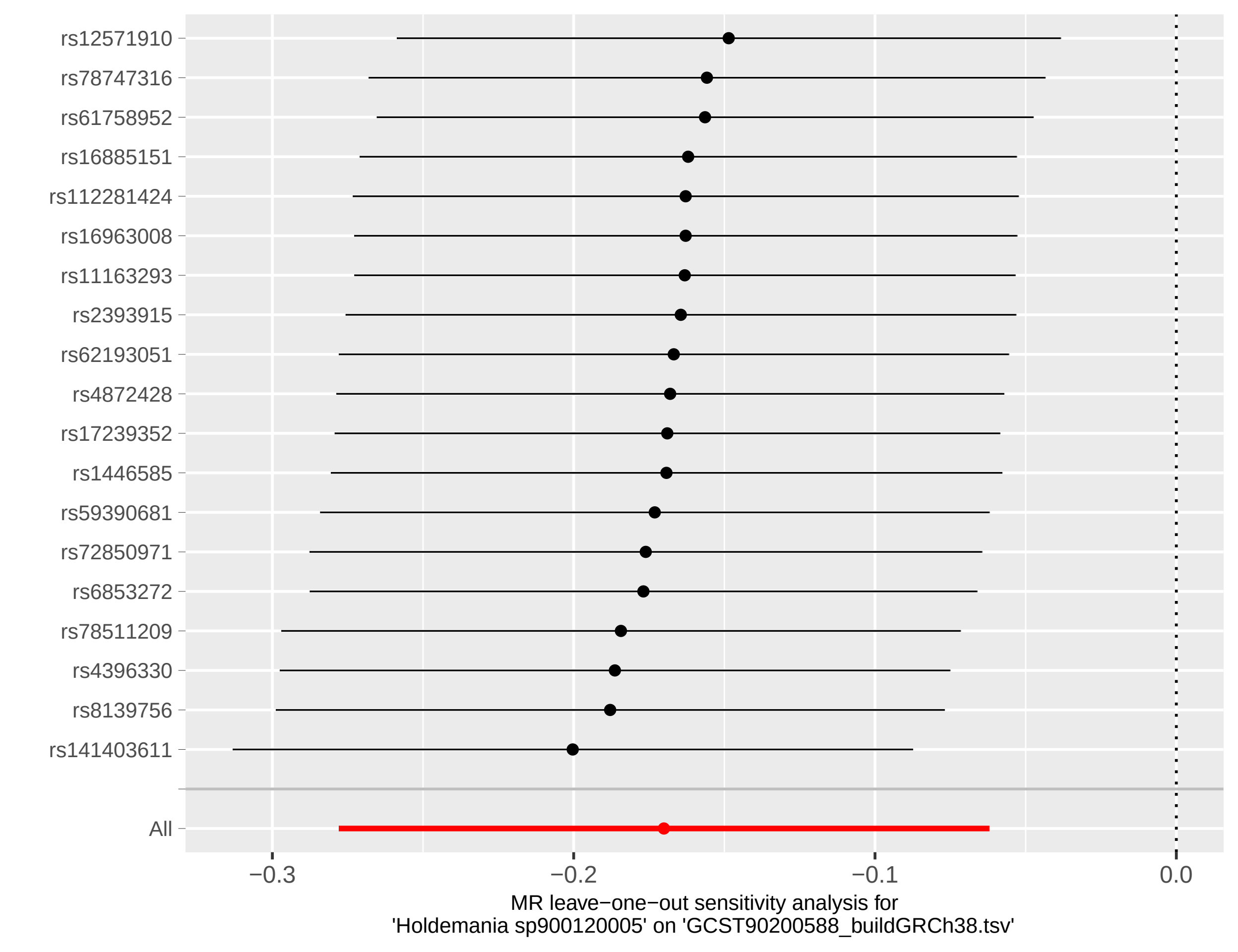
rs12571910
rs78747316
rs61758952
rs16885151
rs112281424
rs16963008
rs11163293
rs2393915
rs62193051
rs4872428
rs17239352
rs1446585
rs59390681
rs72850971
rs6853272
rs78511209
rs4396330
rs8139756
rs141403611
All
−0.3
−0.2
−0.1
0.0
MR leave−one−out sensitivity analysis for
'Holdemania sp900120005' on 'GCST90200588_buildGRCh38.tsv'

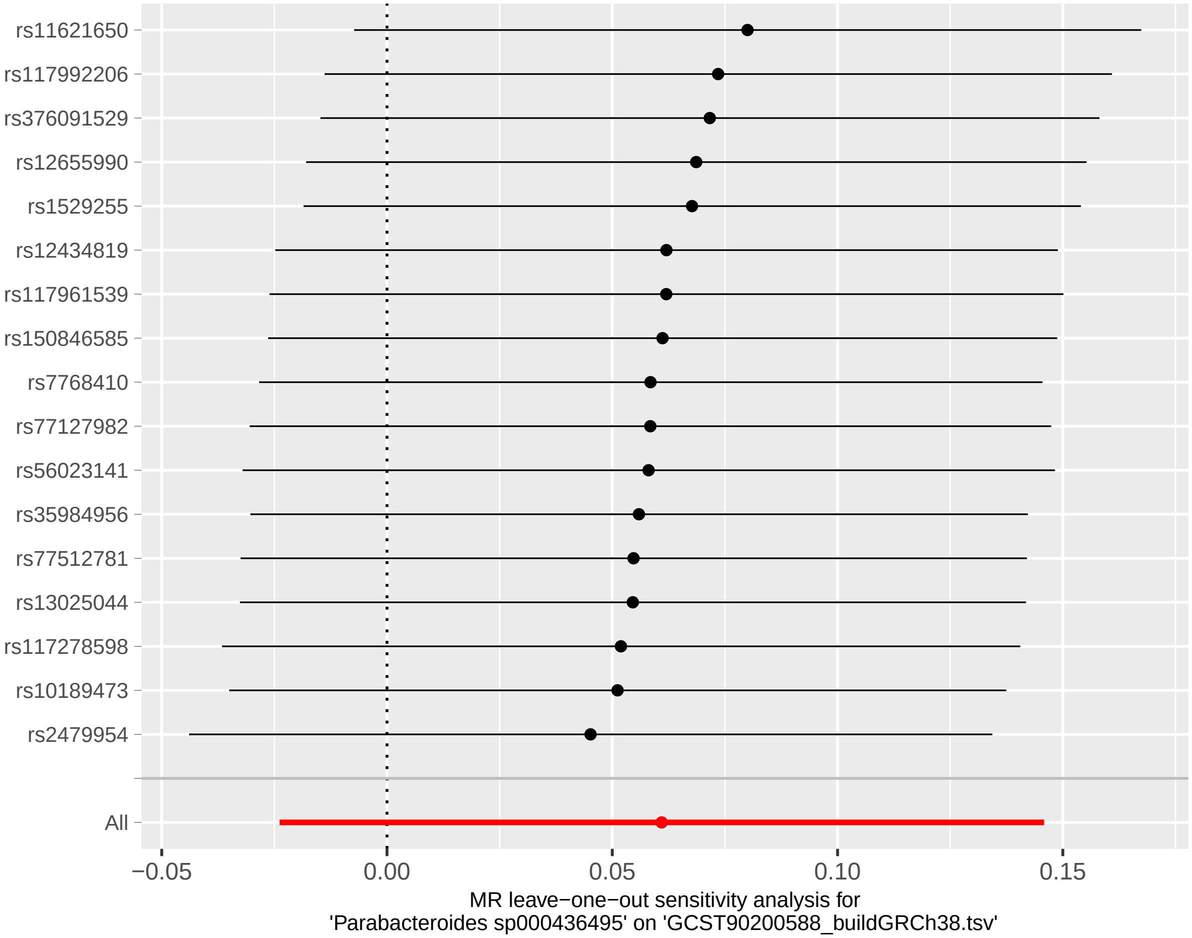

rs11621650
rs117992206
rs376091529
rs12655990
rs1529255
rs12434819
rs117961539
rs150846585
rs7768410
rs77127982
rs56023141
rs35984956
rs77512781
rs13025044
rs117278598
rs10189473
rs2479954
All
−0.05
0.00
0.05
0.10
0.15
MR leave−one−out sensitivity analysis for
'Parabacteroides sp000436495' on 'GCST90200588_buildGRCh38.tsv'

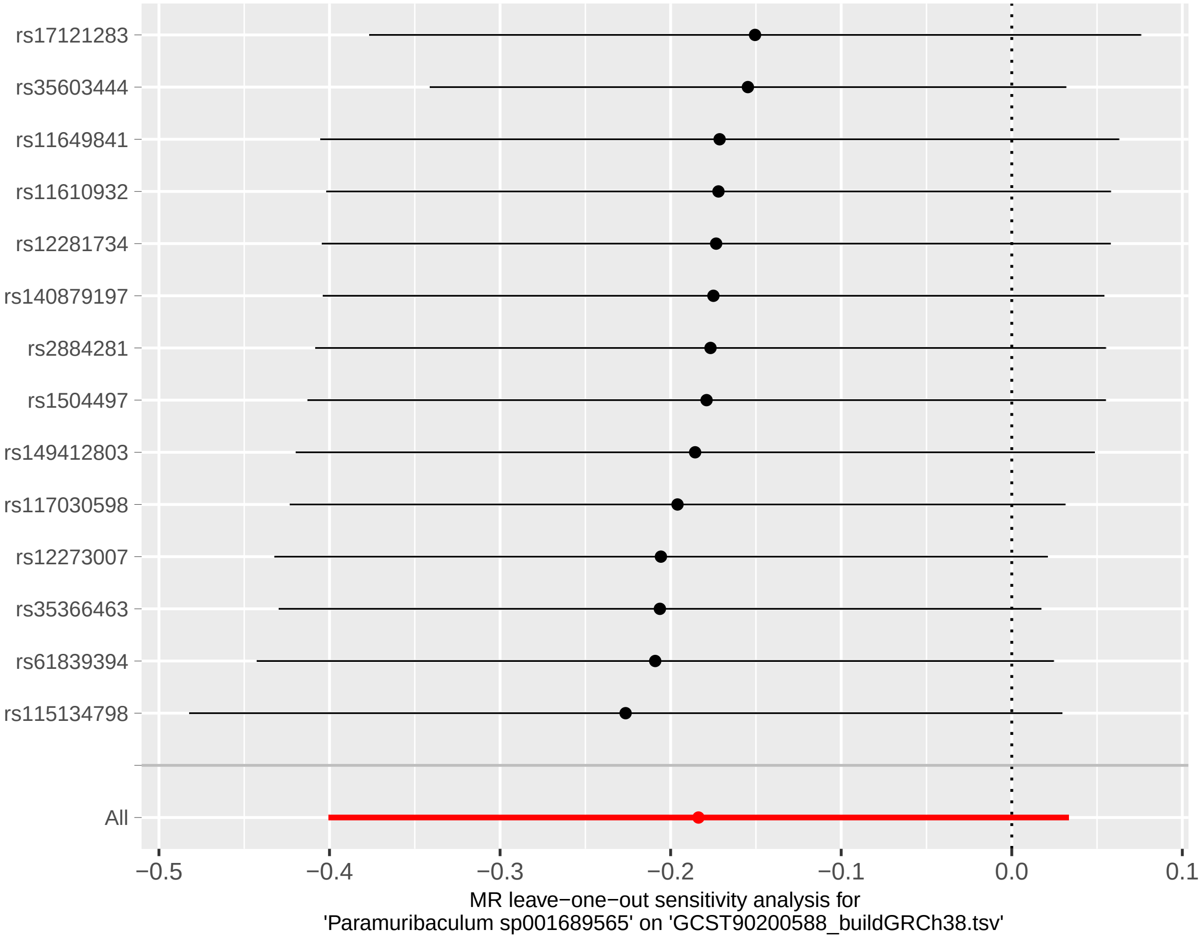
rs17121283
rs35603444
rs11649841
rs11610932
rs12281734
rs140879197
rs2884281
rs1504497
rs149412803
rs117030598
rs12273007
rs35366463
rs61839394
rs115134798
All
−0.5
−0.4
−0.3
−0.2
−0.1
0.0
0.1
MR leave−one−out sensitivity analysis for
'Paramuribaculum sp001689565' on 'GCST90200588_buildGRCh38.tsv'

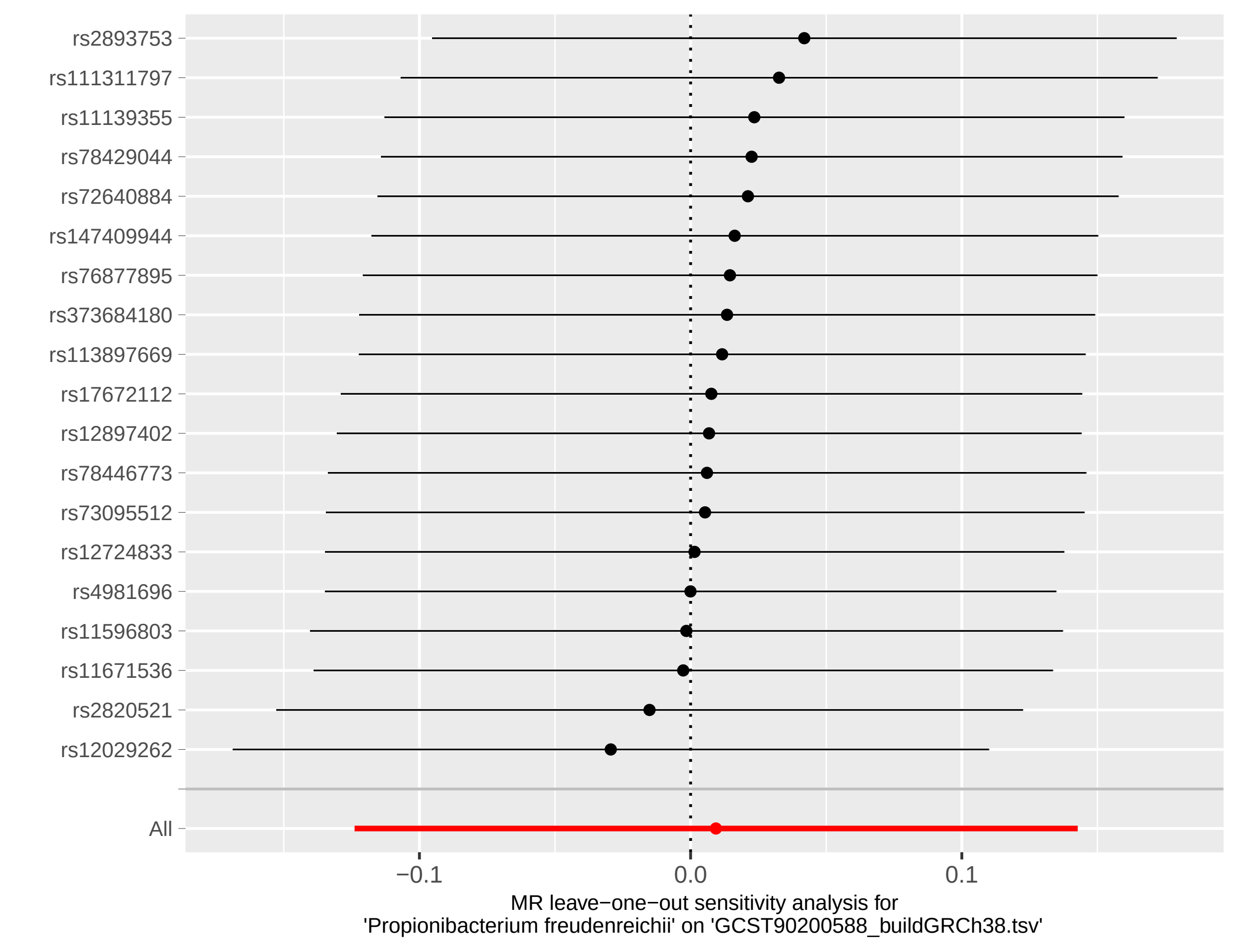

rs2893753
rs111311797
rs11139355
rs78429044
rs72640884
rs147409944
rs76877895
rs373684180
rs113897669
rs17672112
rs12897402
rs78446773
rs73095512
rs12724833
rs4981696
rs11596803
rs11671536
rs2820521
rs12029262
All
−0.1
0.0
0.1
MR leave−one−out sensitivity analysis for
'Propionibacterium freudenreichii' on 'GCST90200588_buildGRCh38.tsv'

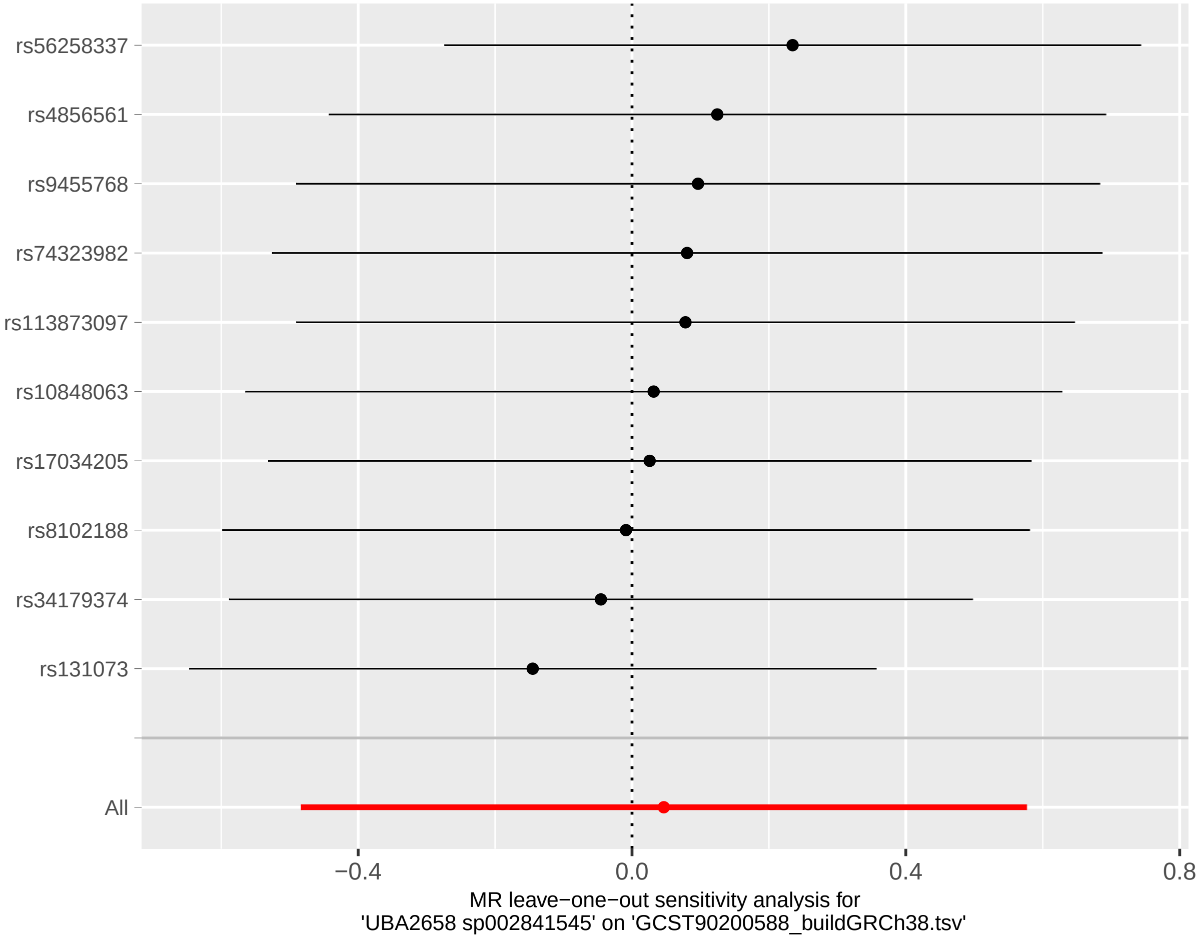
rs56258337
rs4856561
rs9455768
rs74323982
rs113873097
rs10848063
rs17034205
rs8102188
rs34179374
rs131073
All
−0.4
0.0
0.4
0.8
MR leave−one−out sensitivity analysis for
'UBA2658 sp002841545' on 'GCST90200588_buildGRCh38.tsv'

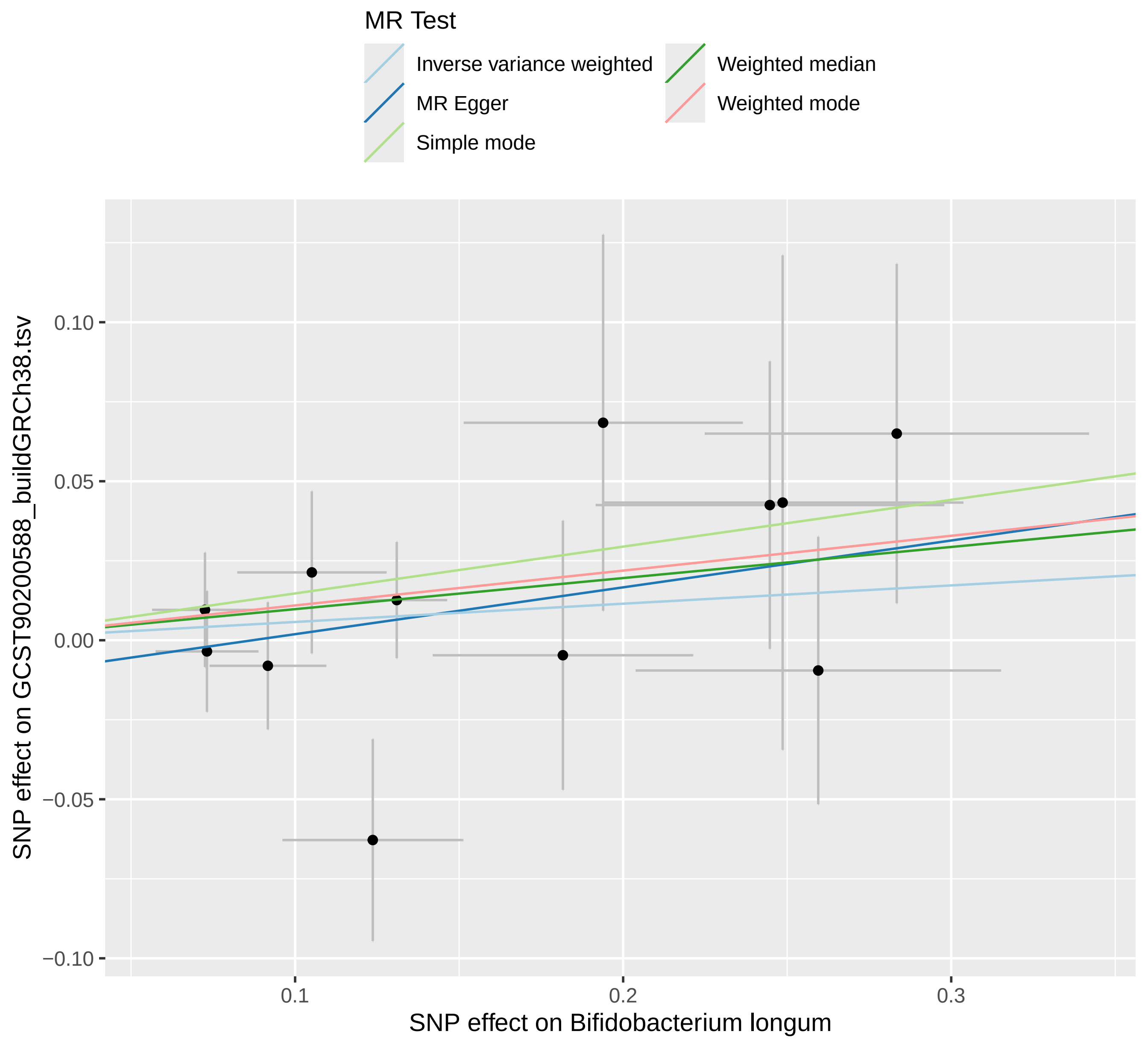

MR Test
Inverse variance weighted
MR Egger
Simple mode
Weighted median
Weighted mode
0.10
0.05
0.00
−0.05
−0.10
0.1
0.2
0.3
SNP effect on GCST90200588_buildGRCh38.tsv
SNP effect on Bifidobacterium longum

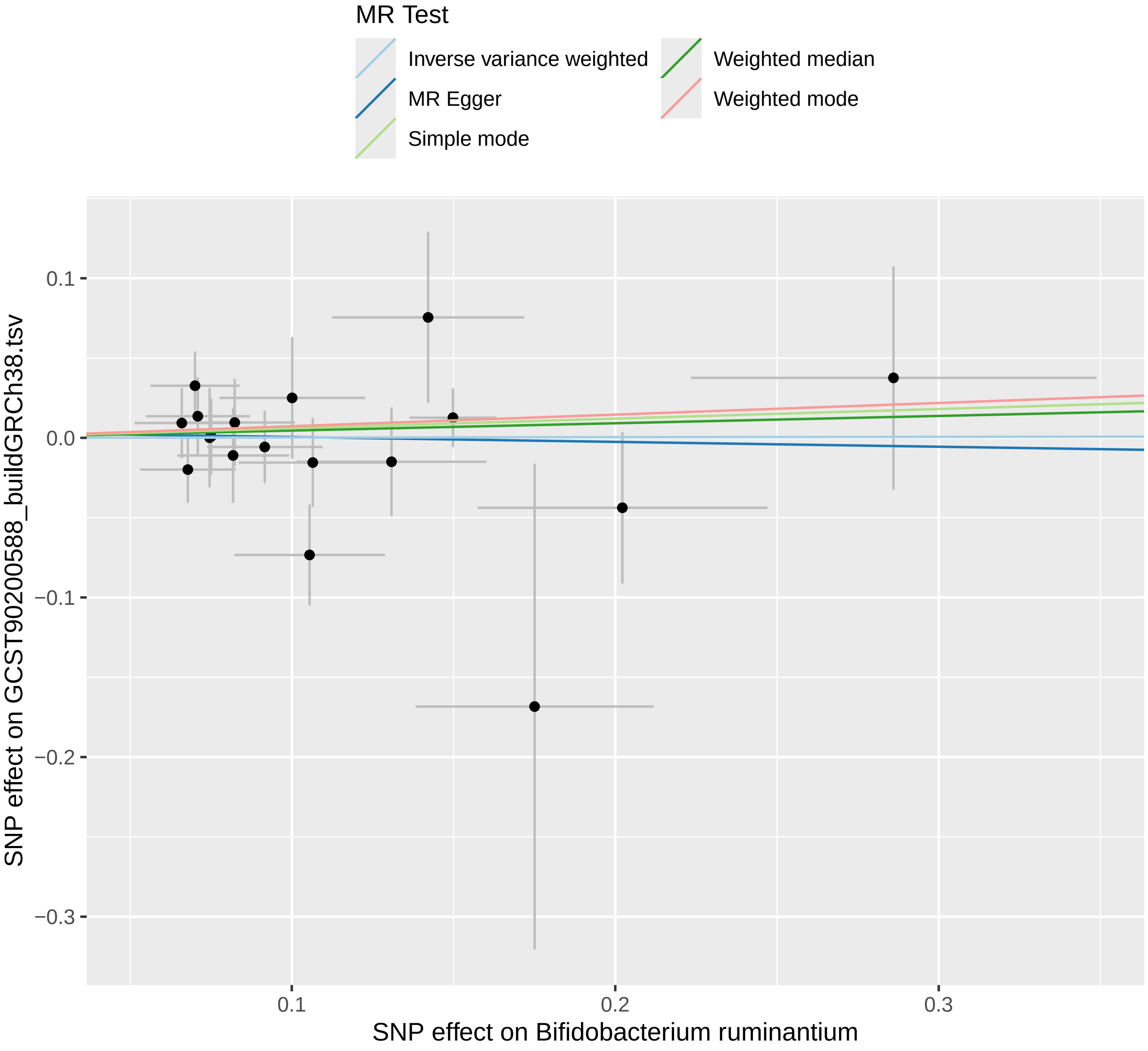

MR Test
Inverse variance weighted
MR Egger
Simple mode
Weighted median
Weighted mode
0.1
0.0
−0.1
−0.2
−0.3
0.1
0.2
0.3
SNP effect on GCST90200588_buildGRCh38.tsv
SNP effect on Bifidobacterium ruminantium

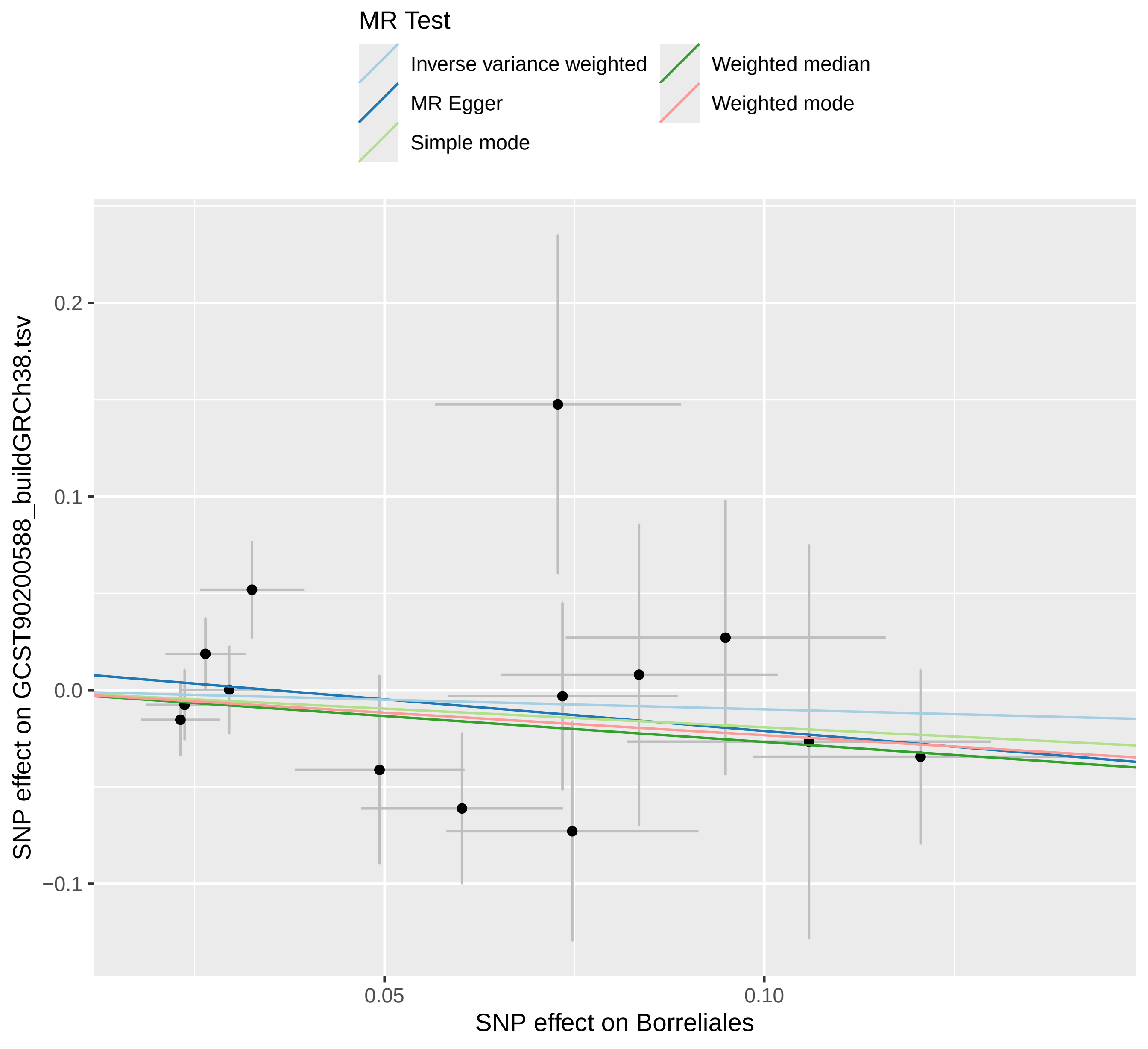

MR Test
Inverse variance weighted
MR Egger
Simple mode
Weighted median
Weighted mode
0.2
0.1
0.0
−0.1
0.05
0.10
SNP effect on GCST90200588_buildGRCh38.tsv
SNP effect on Borreliales

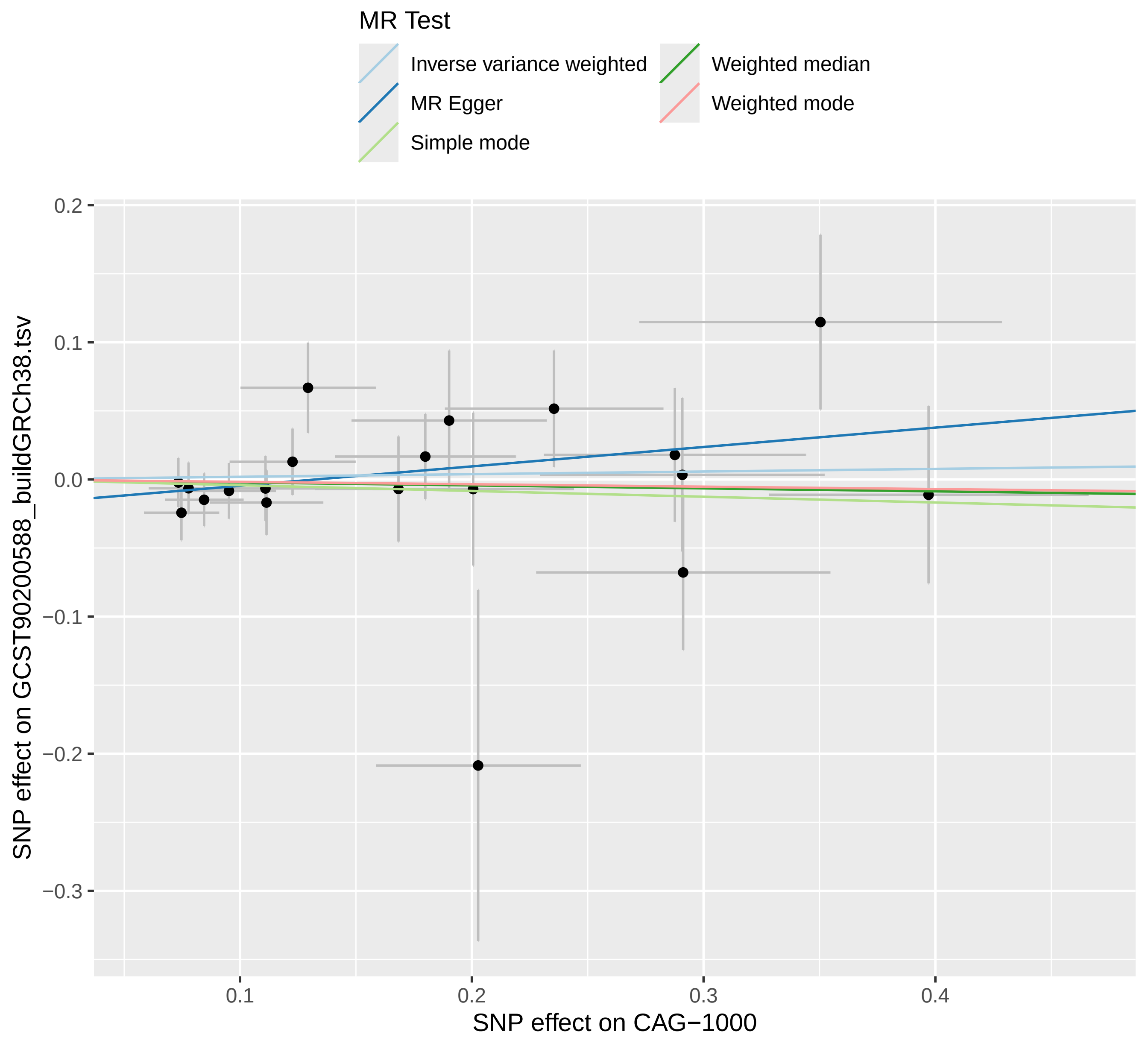

MR Test
Inverse variance weighted
MR Egger
Simple mode
Weighted median
Weighted mode
0.2
0.1
0.0
−0.1
−0.2
−0.3
SNP effect on GCST90200588_buildGRCh38.tsv
0.1
0.2
0.3
0.4
SNP effect on CAG−1000

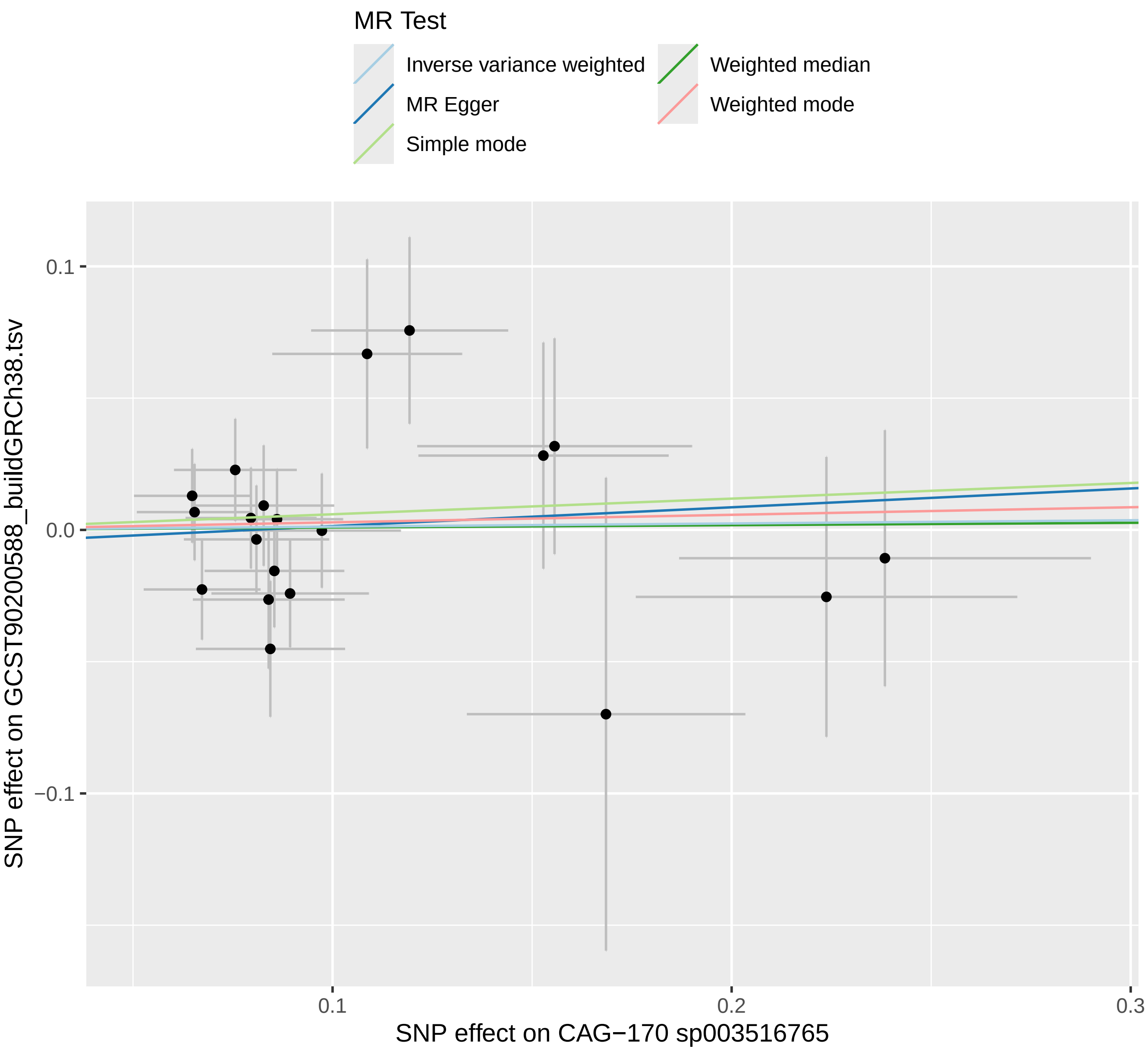

MR Test
Inverse variance weighted
MR Egger
Simple mode
Weighted median
Weighted mode
0.1
0.0
−0.1
0.1
0.2
0.3
SNP effect on GCST90200588_buildGRCh38.tsv
SNP effect on CAG−170 sp003516765

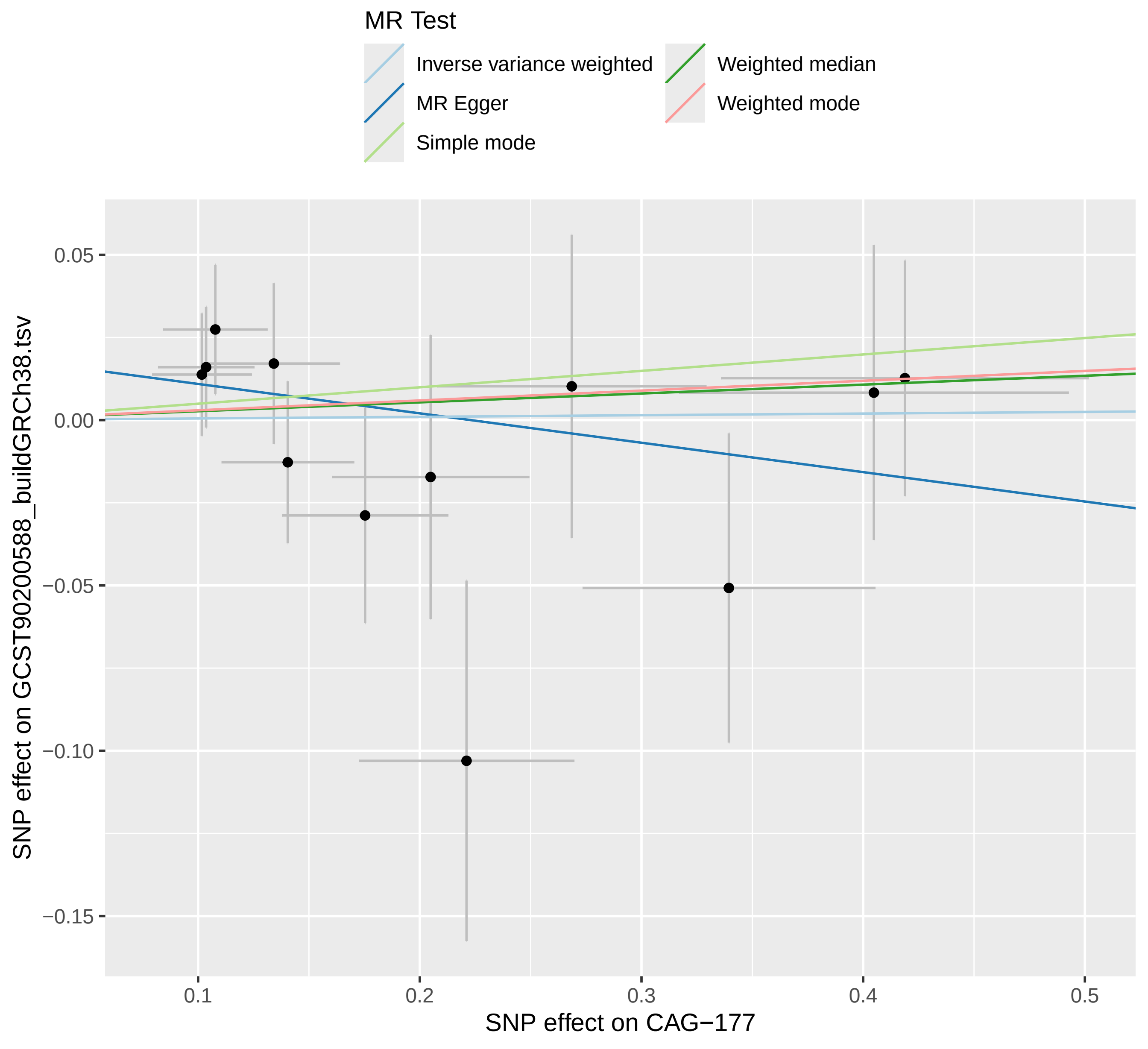
MR Test
Inverse variance weighted
MR Egger
Simple mode
Weighted median
Weighted mode
0.05
0.00
−0.05
−0.10
−0.15
0.1
0.2
0.3
0.4
0.5
SNP effect on GCST90200588_buildGRCh38.tsv
SNP effect on CAG−177

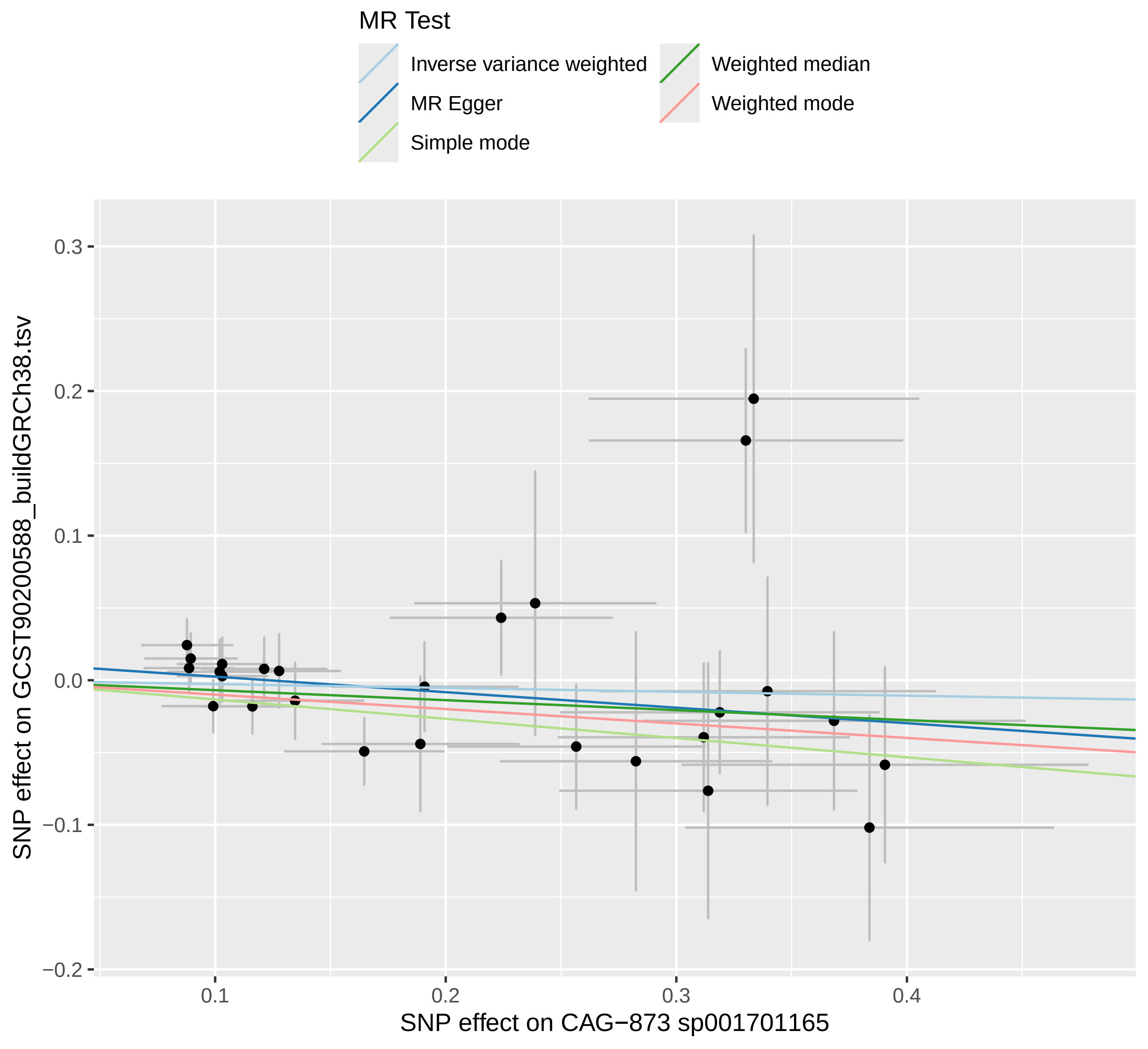

MR Test
Inverse variance weighted
MR Egger
Simple mode
Weighted median
Weighted mode
SNP effect on GCST90200588_buildGRCh38.tsv
SNP effect on CAG−873 sp001701165
0.3
0.2
0.1
0.0
−0.1
−0.2
0.1
0.2
0.3
0.4

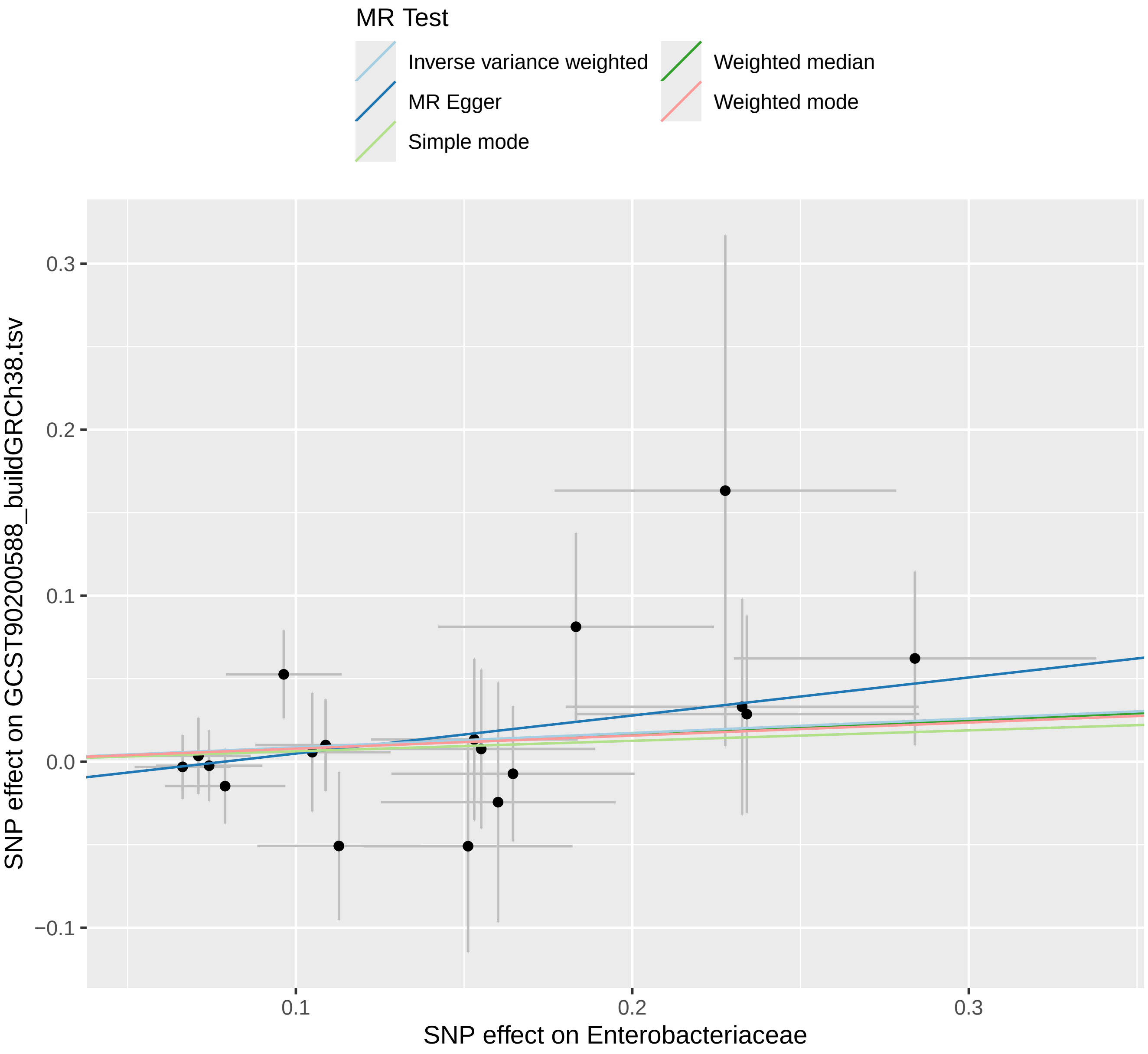
MR Test
Inverse variance weighted
MR Egger
Simple mode
Weighted median
Weighted mode
0.3
0.2
0.1
0.0
−0.1
SNP effect on GCST90200588_buildGRCh38.tsv
0.1
0.2
0.3
SNP effect on Enterobacteriaceae

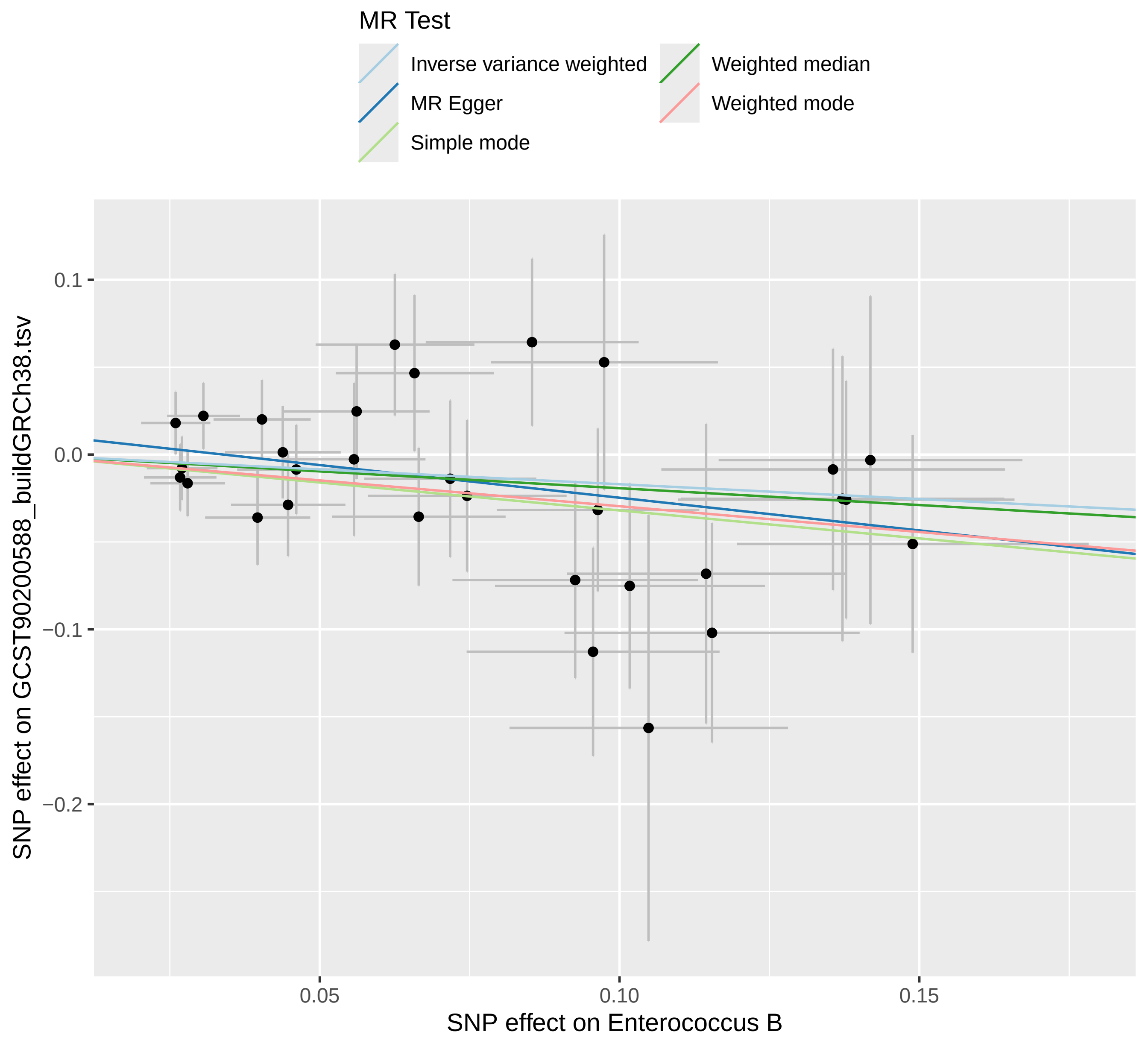
MR Test
Inverse variance weighted
MR Egger
Simple mode
Weighted median
Weighted mode
0.1
0.0
−0.1
−0.2
0.05
0.10
0.15
SNP effect on GCST90200588_buildGRCh38.tsv
SNP effect on Enterococcus B

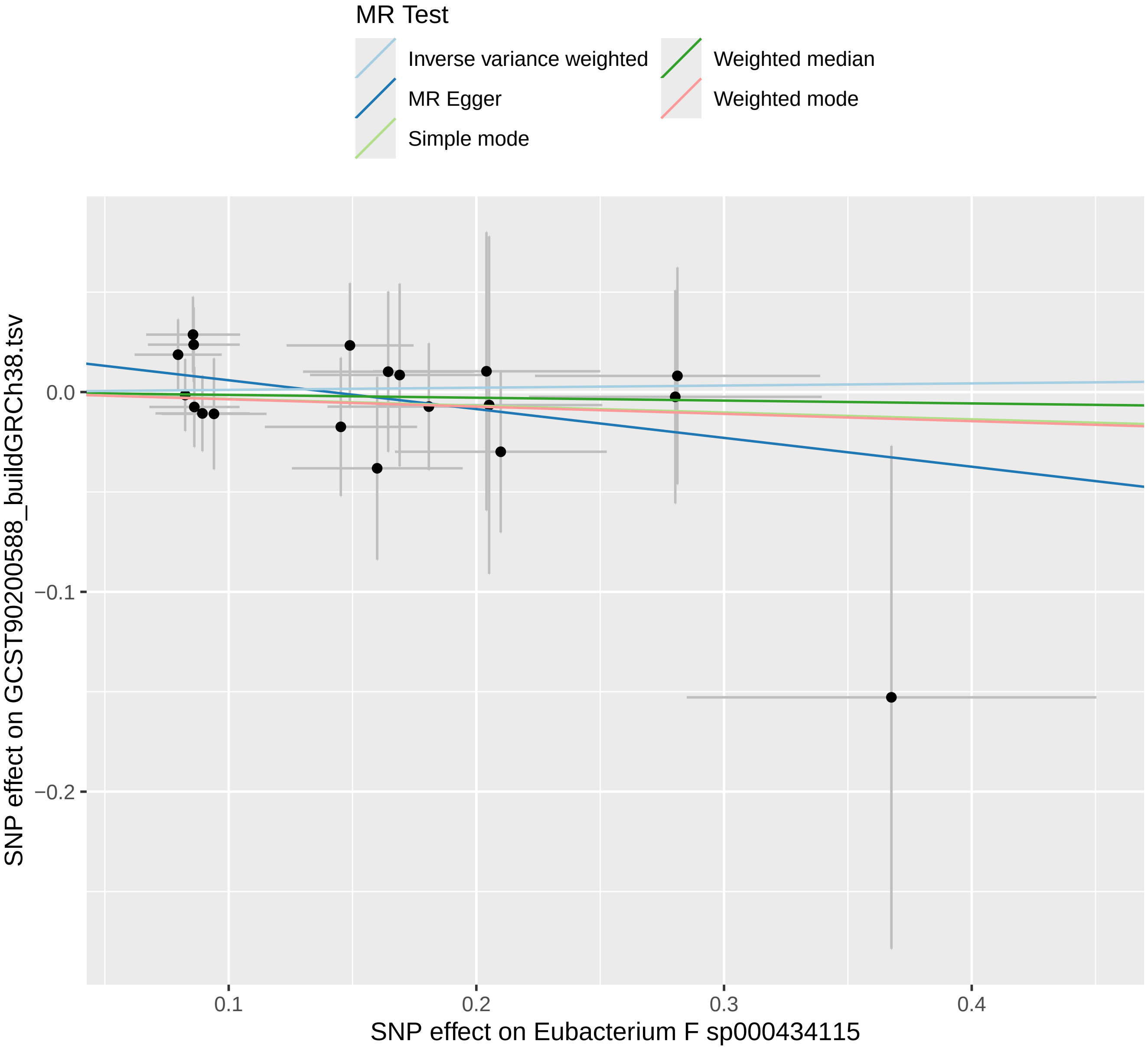

MR Test
Inverse variance weighted
MR Egger
Simple mode
Weighted median
Weighted mode
SNP effect on GCST90200588_buildGRCh38.tsv
0.0
−0.1
−0.2
0.1
0.2
0.3
0.4
SNP effect on Eubacterium F sp000434115

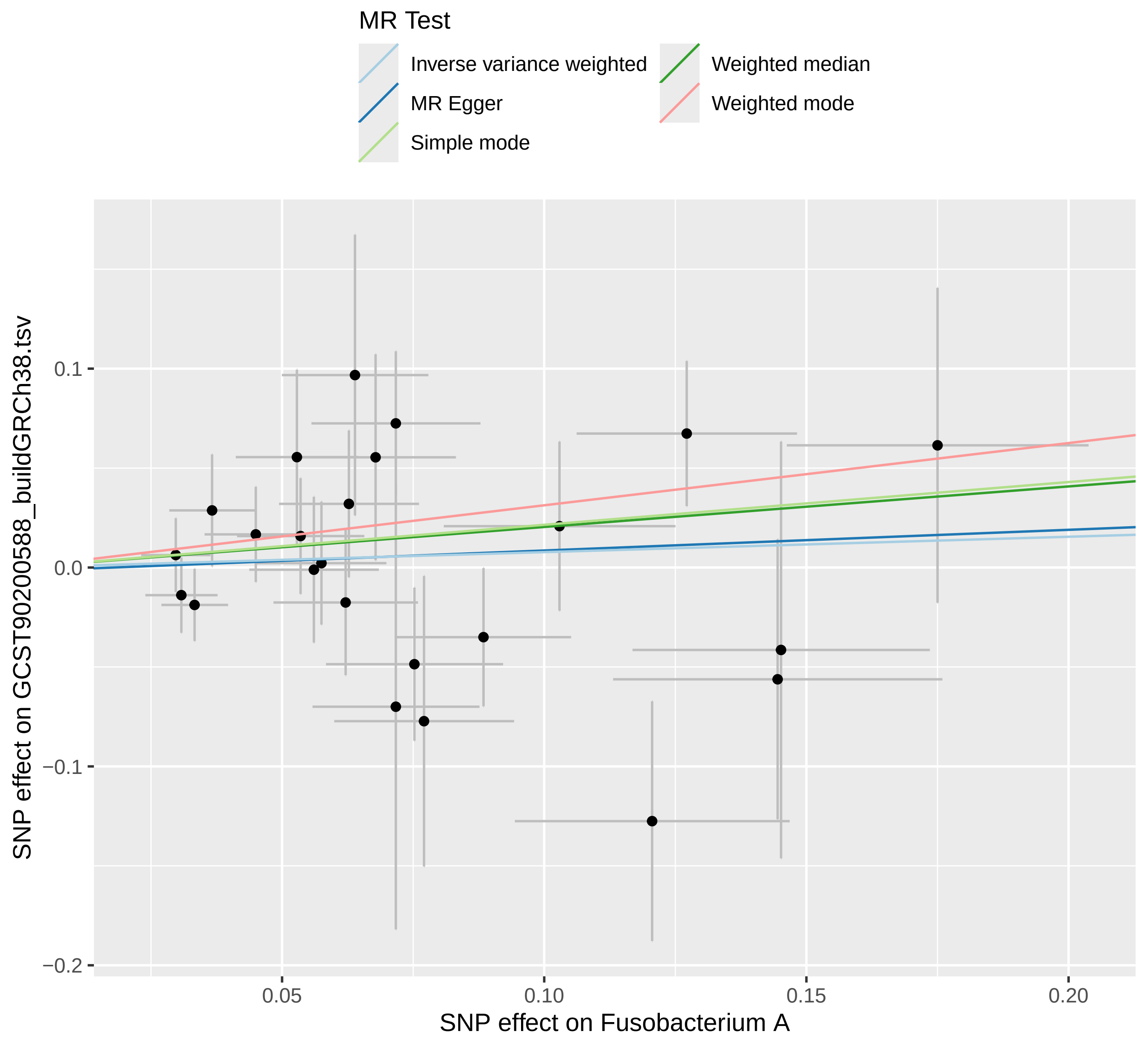

MR Test
Inverse variance weighted
MR Egger
Simple mode
Weighted median
Weighted mode
SNP effect on GCST90200588_buildGRCh38.tsv
0.1
0.0
−0.1
−0.2
0.05
0.10
0.15
0.20
SNP effect on Fusobacterium A

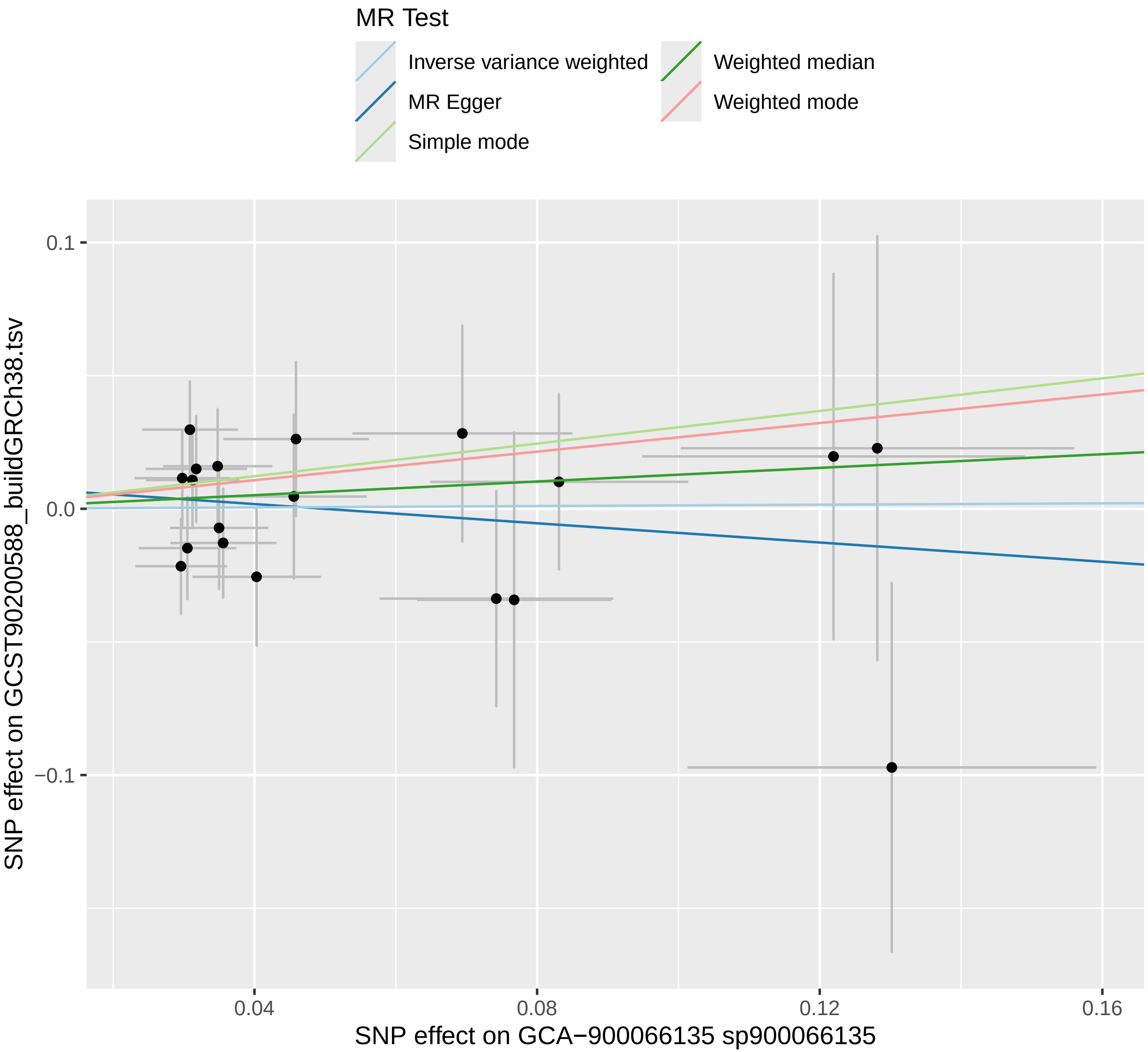
MR Test
Inverse variance weighted
MR Egger
Simple mode
Weighted median
Weighted mode
0.1
0.0
−0.1
0.04
0.08
0.12
0.16
SNP effect on GCST90200588_buildGRCh38.tsv
SNP effect on GCA−900066135 sp900066135

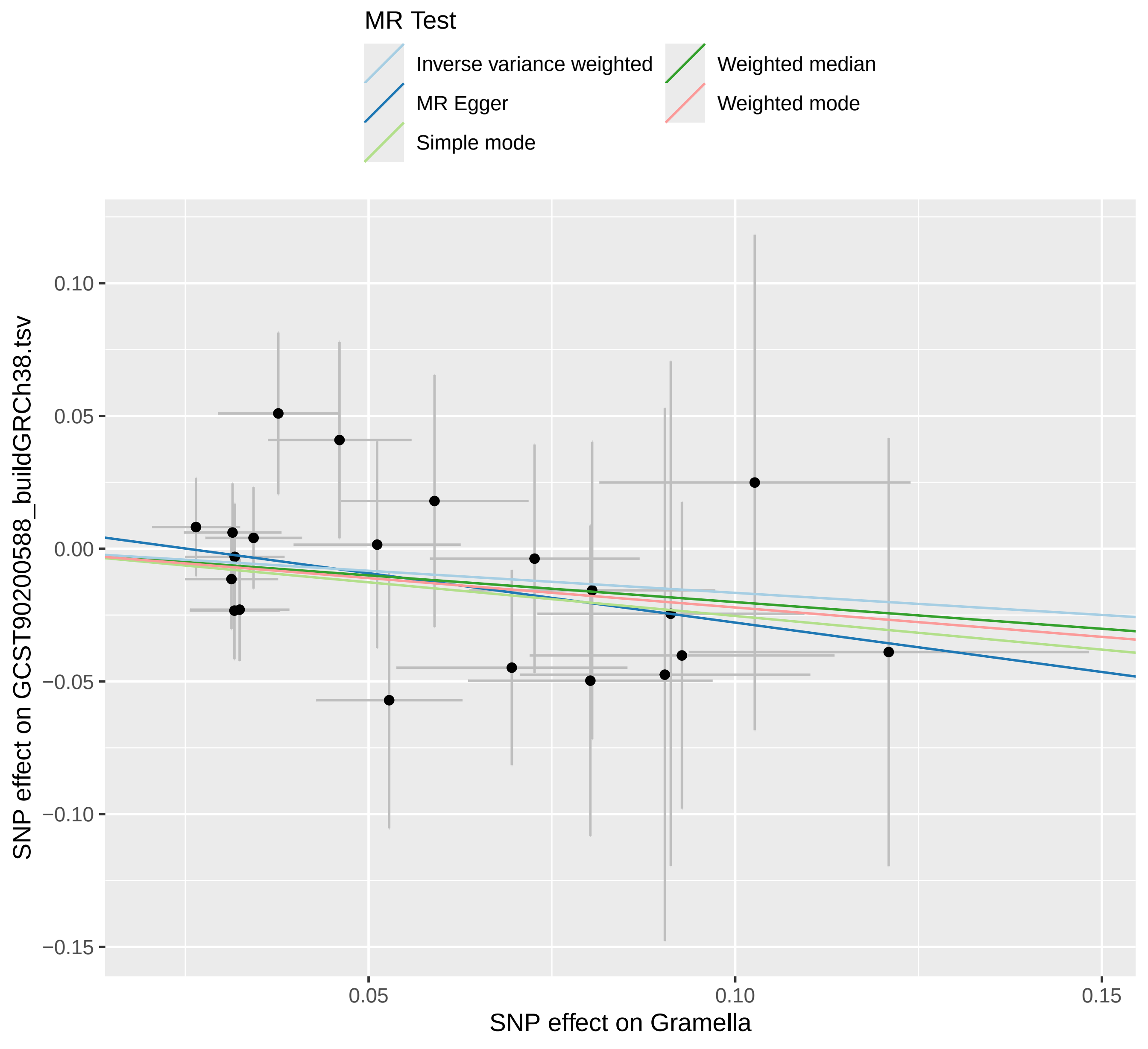

MR Test
Inverse variance weighted
MR Egger
Simple mode
Weighted median
Weighted mode
0.10
0.05
0.00
−0.05
−0.10
−0.15
SNP effect on GCST90200588_buildGRCh38.tsv
0.05
0.10
0.15
SNP effect on Gramella

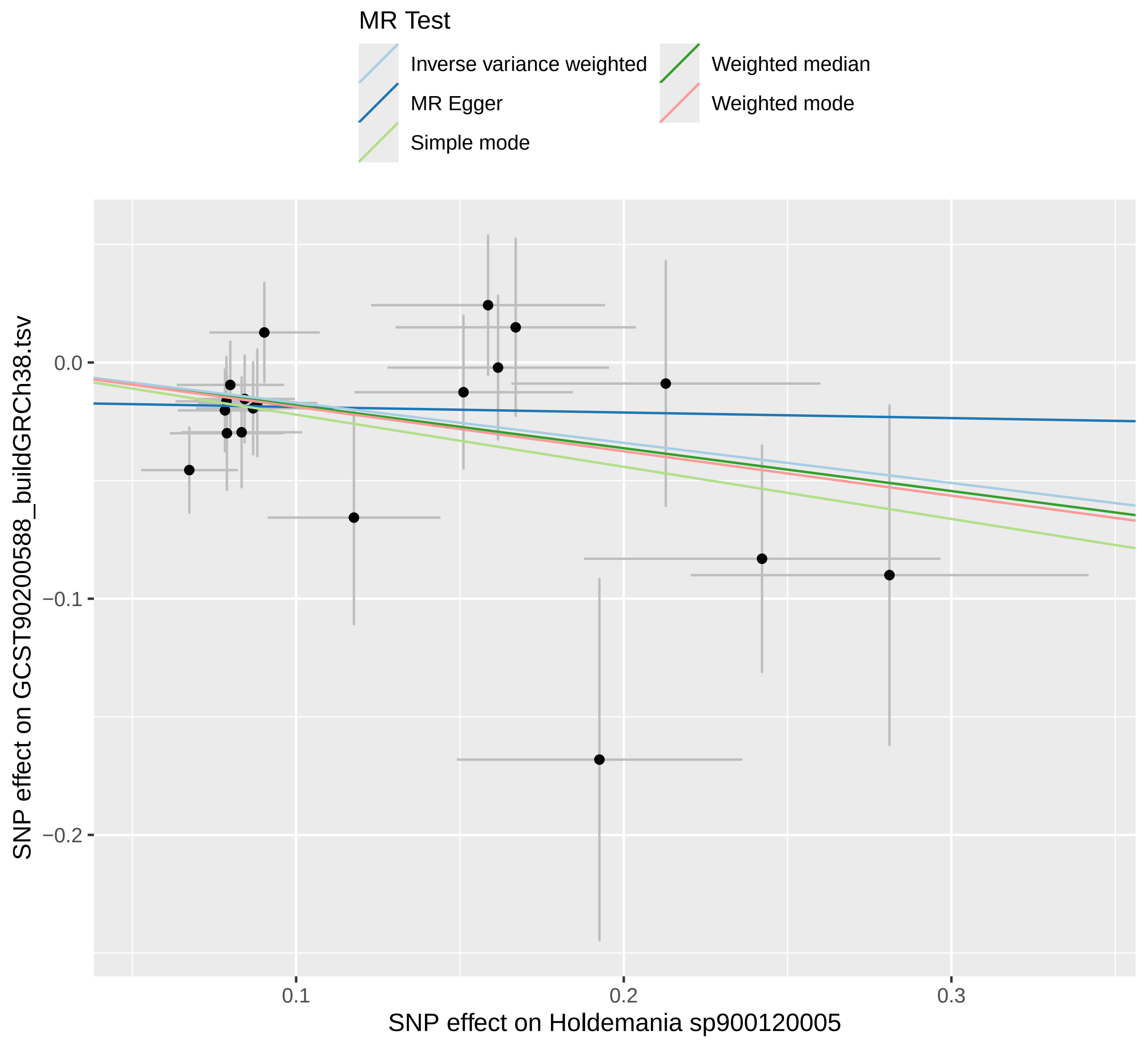
MR Test
Inverse variance weighted
MR Egger
Simple mode
Weighted median
Weighted mode
0.0
−0.1
−0.2
SNP effect on GCST90200588_buildGRCh38.tsv
0.1
0.2
0.3
SNP effect on Holdemania sp900120005

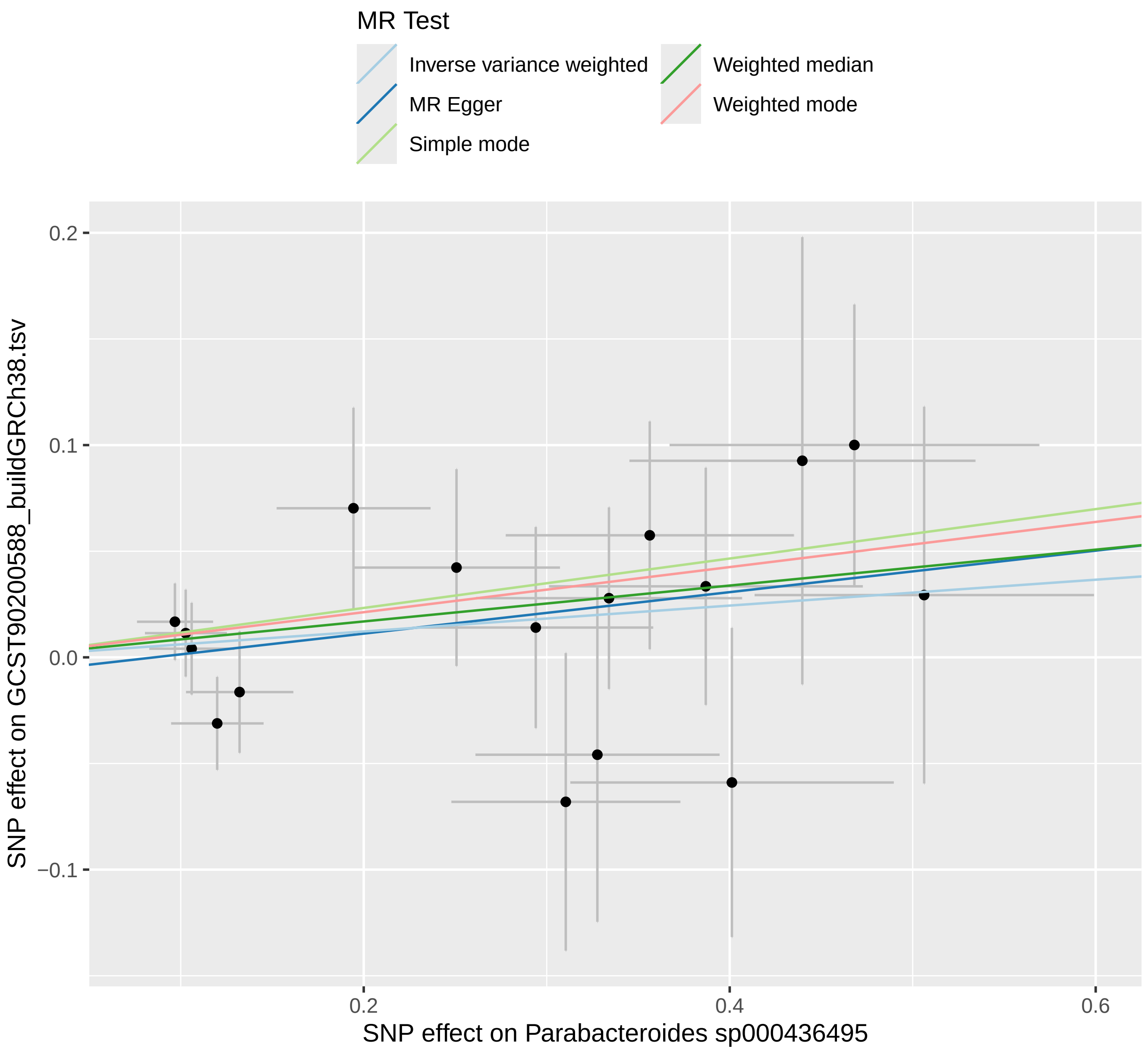
MR Test
Inverse variance weighted
MR Egger
Simple mode
Weighted median
Weighted mode
0.2
0.1
0.0
−0.1
0.2
0.4
0.6
SNP effect on GCST90200588_buildGRCh38.tsv
SNP effect on Parabacteroides sp000436495

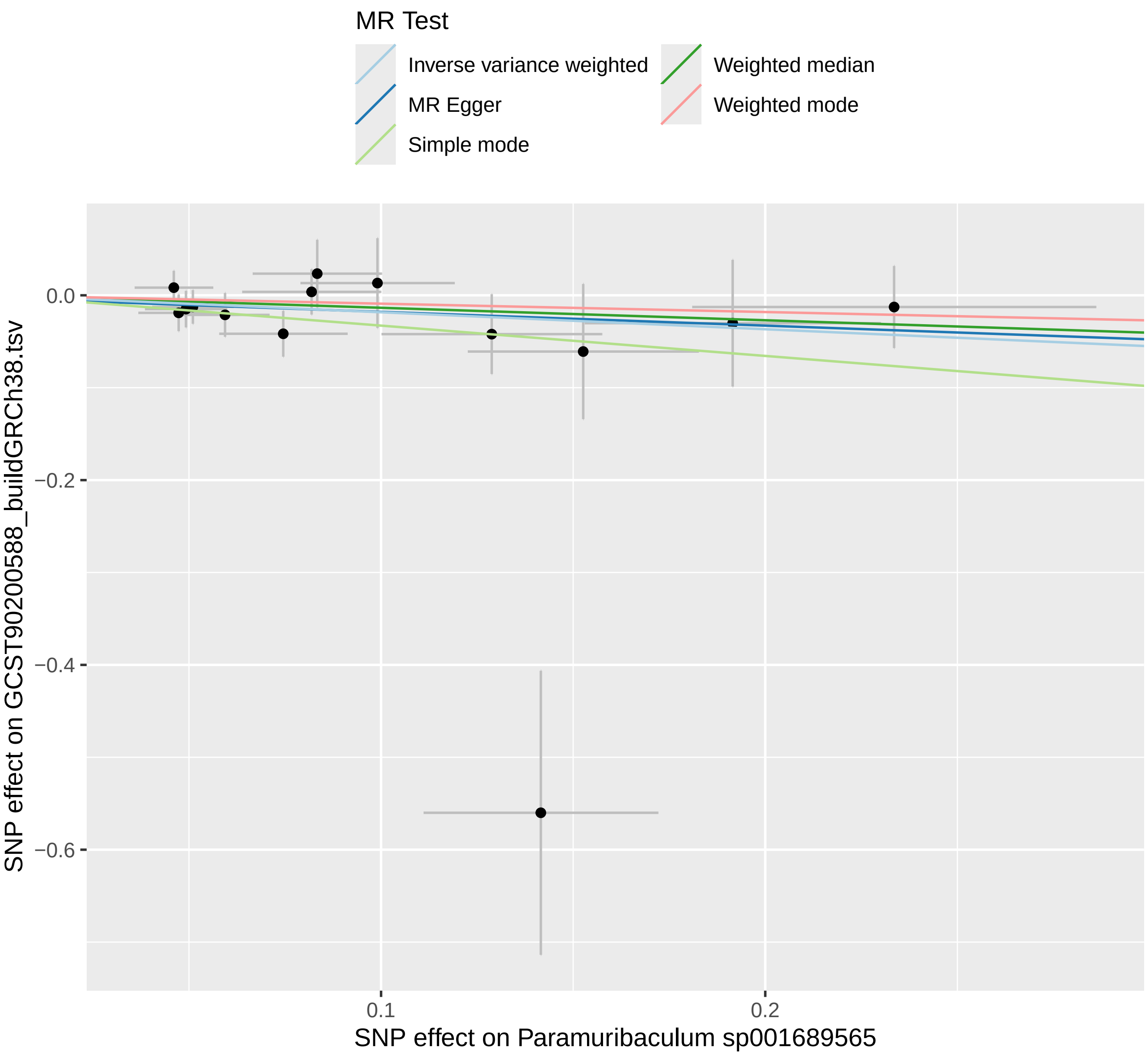
MR Test
Inverse variance weighted
MR Egger
Simple mode
Weighted median
Weighted mode
0.0
−0.2
−0.4
−0.6
SNP effect on GCST90200588_buildGRCh38.tsv
0.1
0.2
SNP effect on Paramuribaculum sp001689565

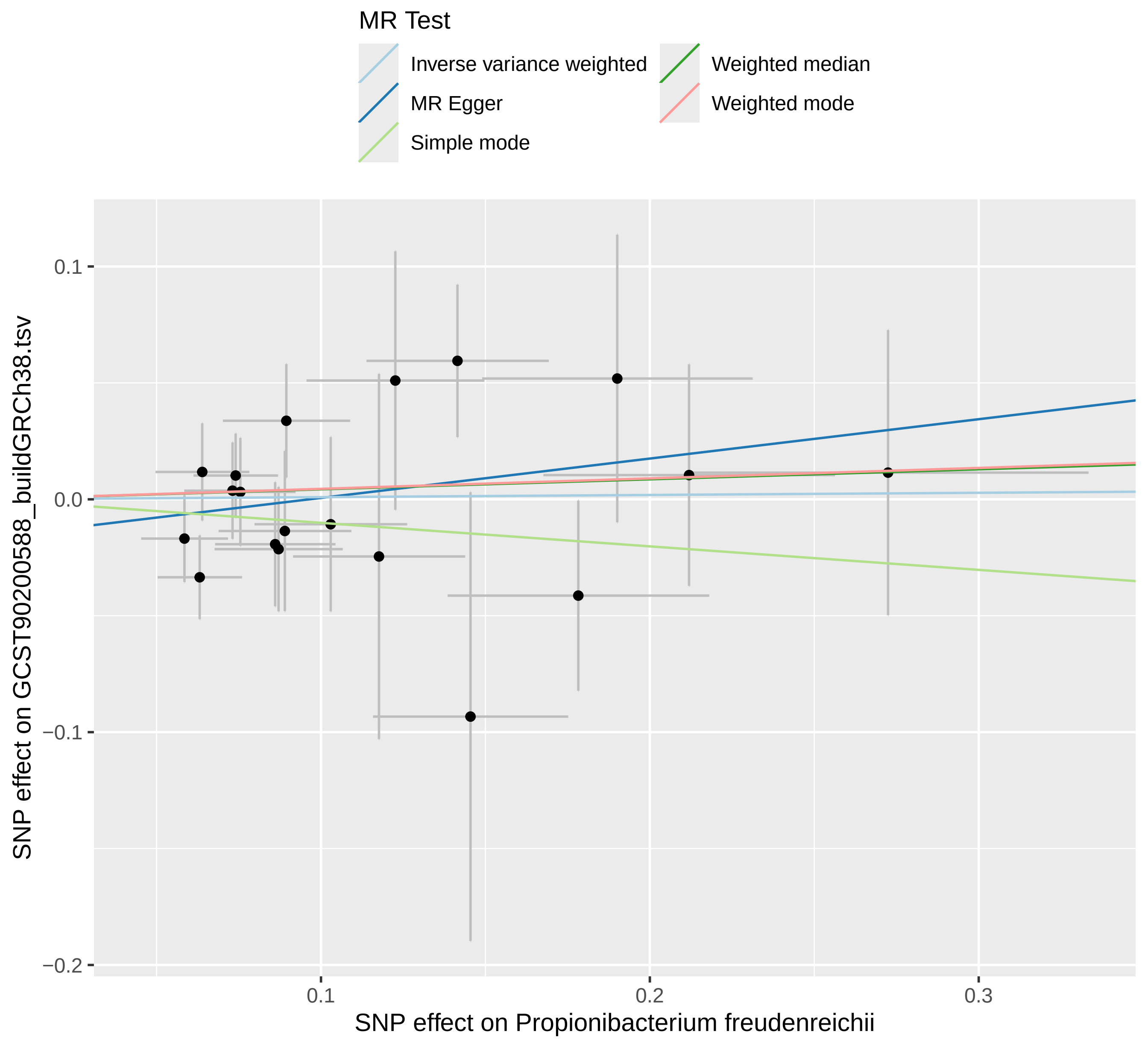
MR Test
Inverse variance weighted
MR Egger
Simple mode
Weighted median
Weighted mode
0.1
0.0
−0.1
−0.2
0.1
0.2
0.3
SNP effect on GCST90200588_buildGRCh38.tsv
SNP effect on Propionibacterium freudenreichii

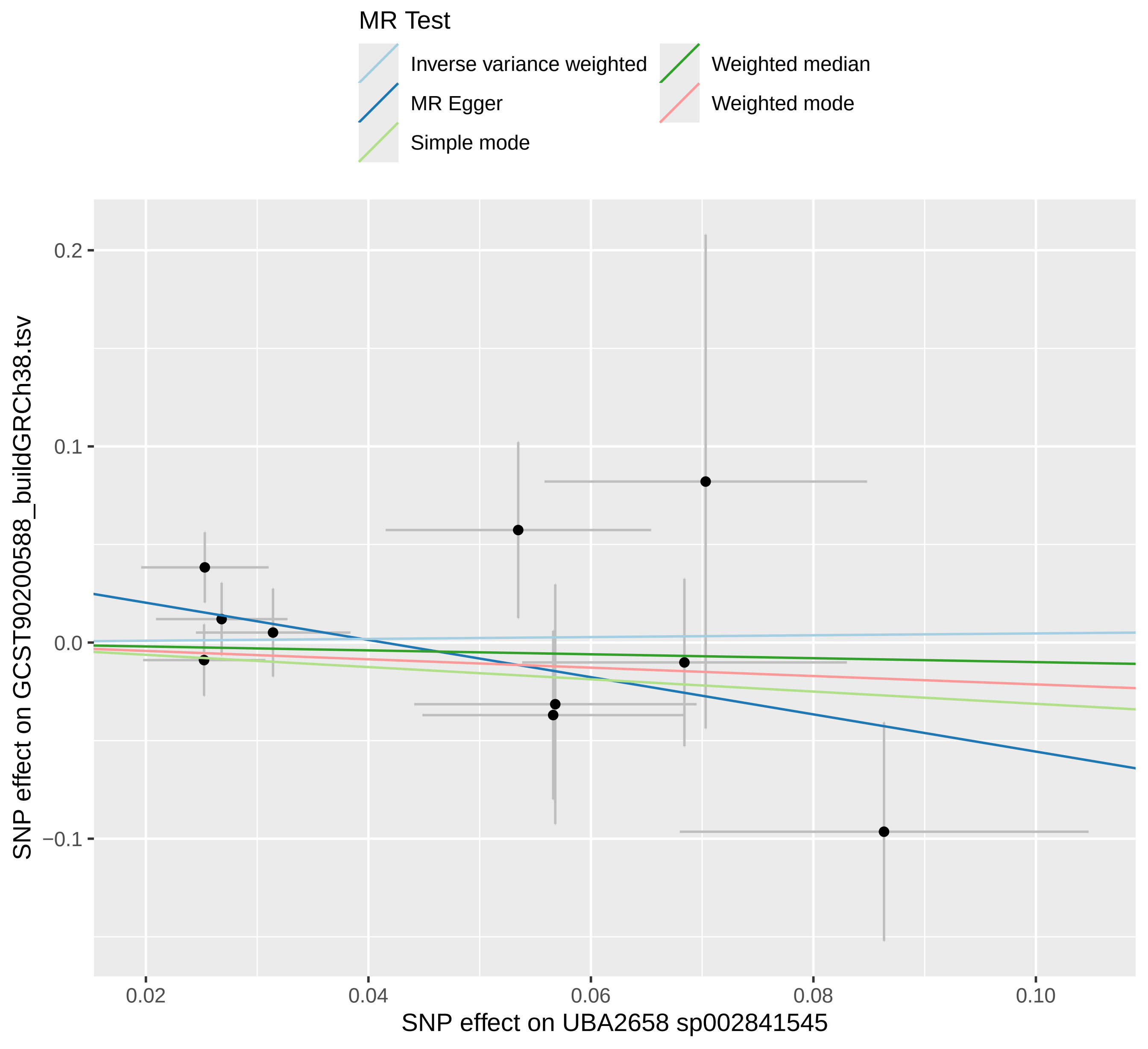
MR Test
Inverse variance weighted
MR Egger
Simple mode
Weighted median
Weighted mode
0.2
0.1
0.0
−0.1
0.02
0.04
0.06
0.08
0.10
SNP effect on GCST90200588_buildGRCh38.tsv
SNP effect on UBA2658 sp002841545

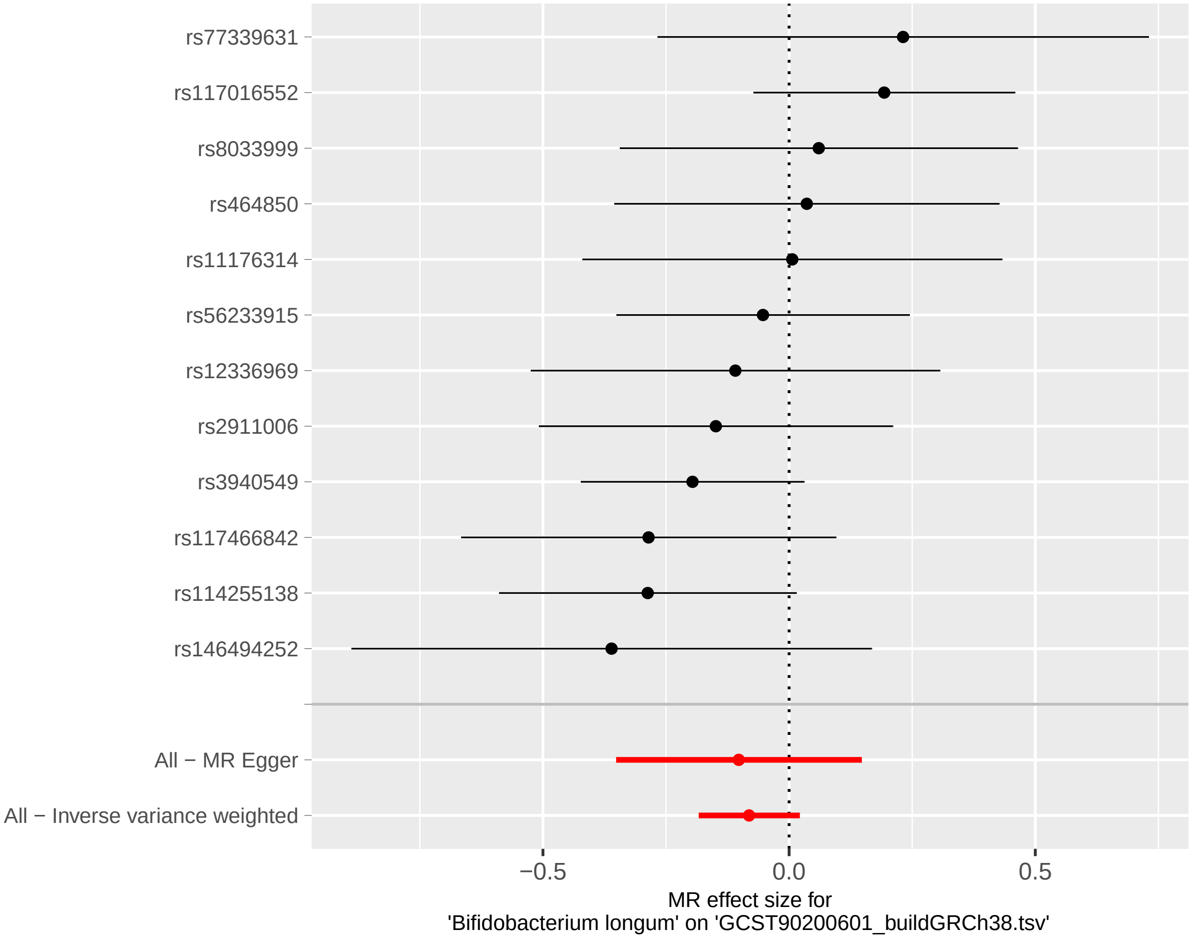

rs77339631
rs117016552
rs8033999
rs464850
rs11176314
rs56233915
rs12336969
rs2911006
rs3940549
rs117466842
rs114255138
rs146494252
All − MR Egger
All − Inverse variance weighted
−0.5
0.0
0.5
MR effect size for
'Bifidobacterium longum' on 'GCST90200601_buildGRCh38.tsv'

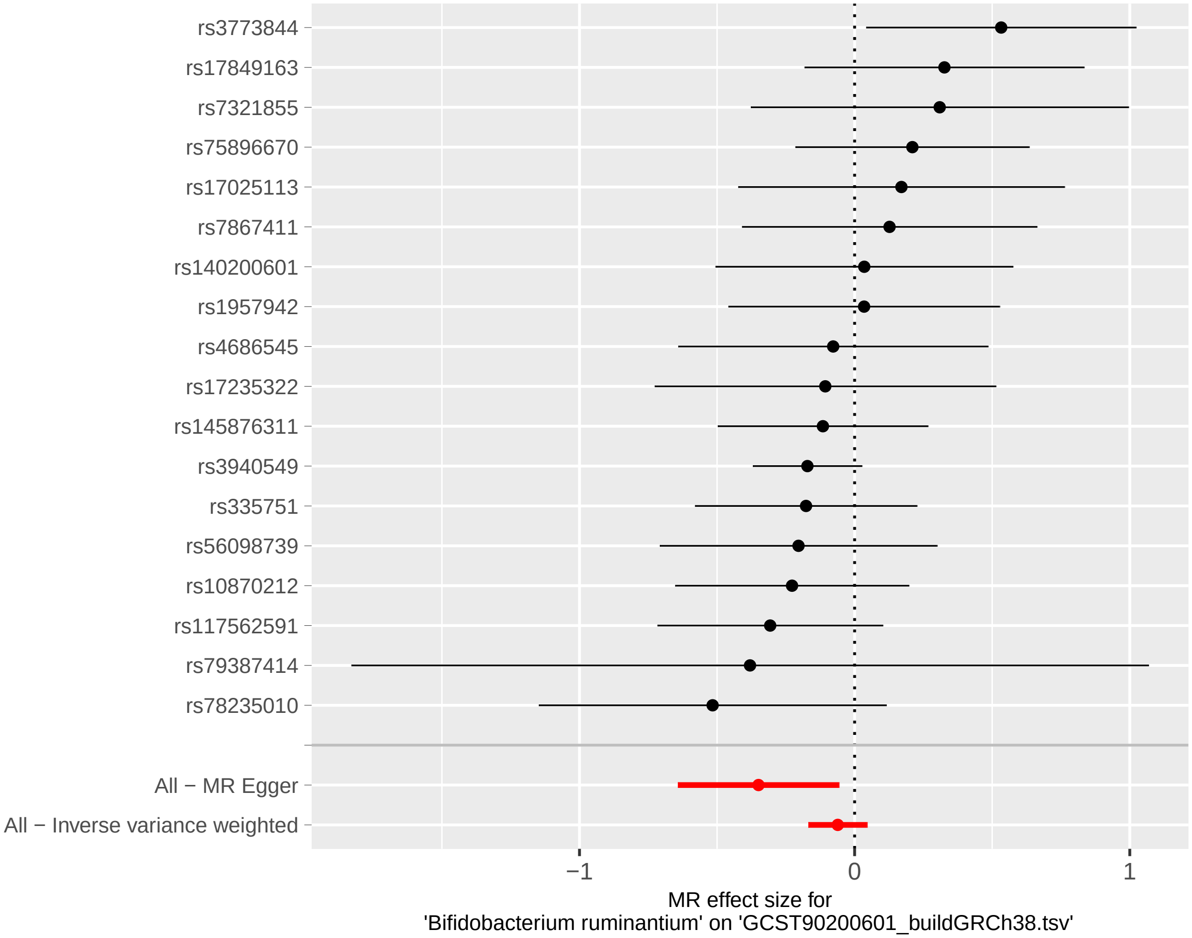
rs3773844
rs17849163
rs7321855
rs75896670
rs17025113
rs7867411
rs140200601
rs1957942
rs4686545
rs17235322
rs145876311
rs3940549
rs335751
rs56098739
rs10870212
rs117562591
rs79387414
rs78235010
All − MR Egger
All − Inverse variance weighted
−1
0
1
MR effect size for
'Bifidobacterium ruminantium' on 'GCST90200601_buildGRCh38.tsv'

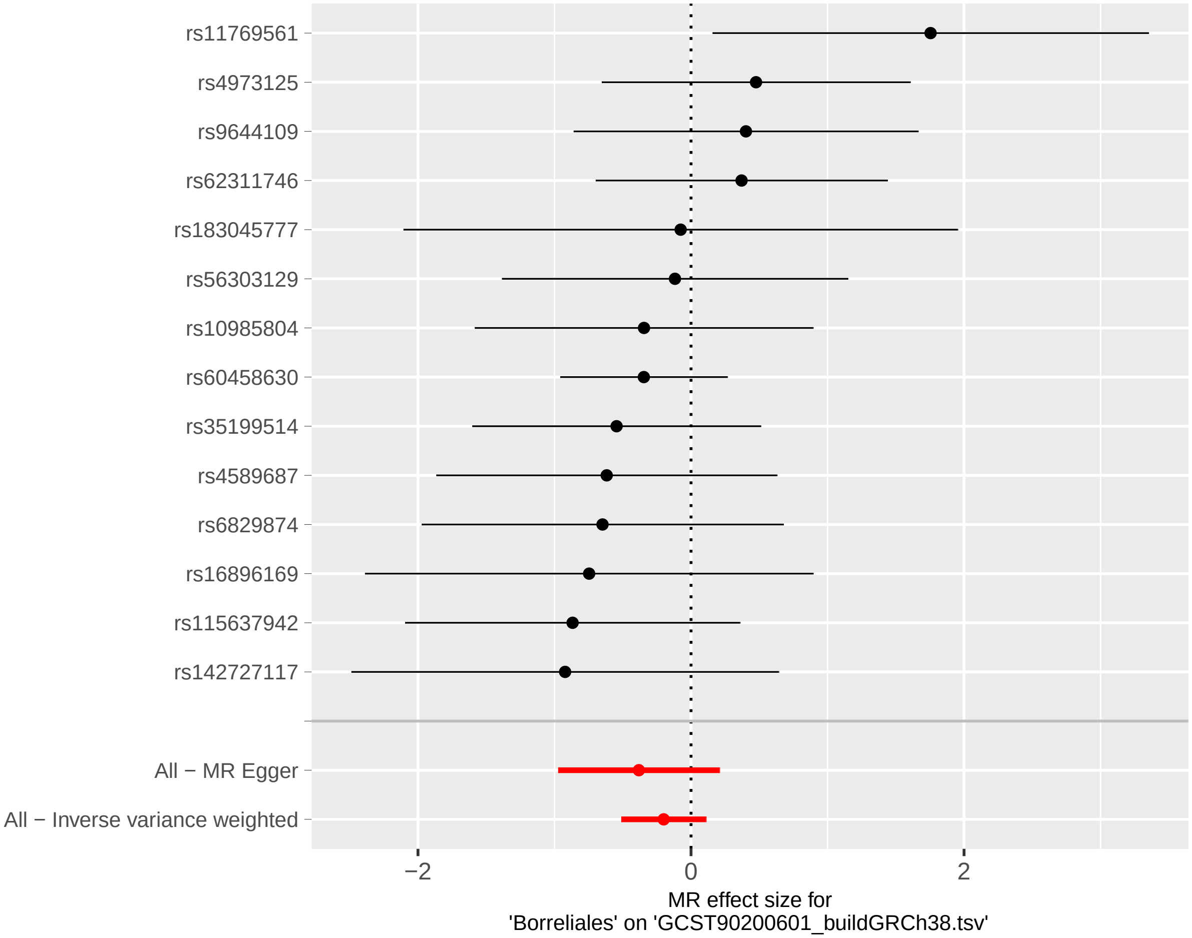
rs11769561
rs4973125
rs9644109
rs62311746
rs183045777
rs56303129
rs10985804
rs60458630
rs35199514
rs4589687
rs6829874
rs16896169
rs115637942
rs142727117
All − MR Egger
All − Inverse variance weighted
−2
0
2
MR effect size for
'Borreliales' on 'GCST90200601_buildGRCh38.tsv'

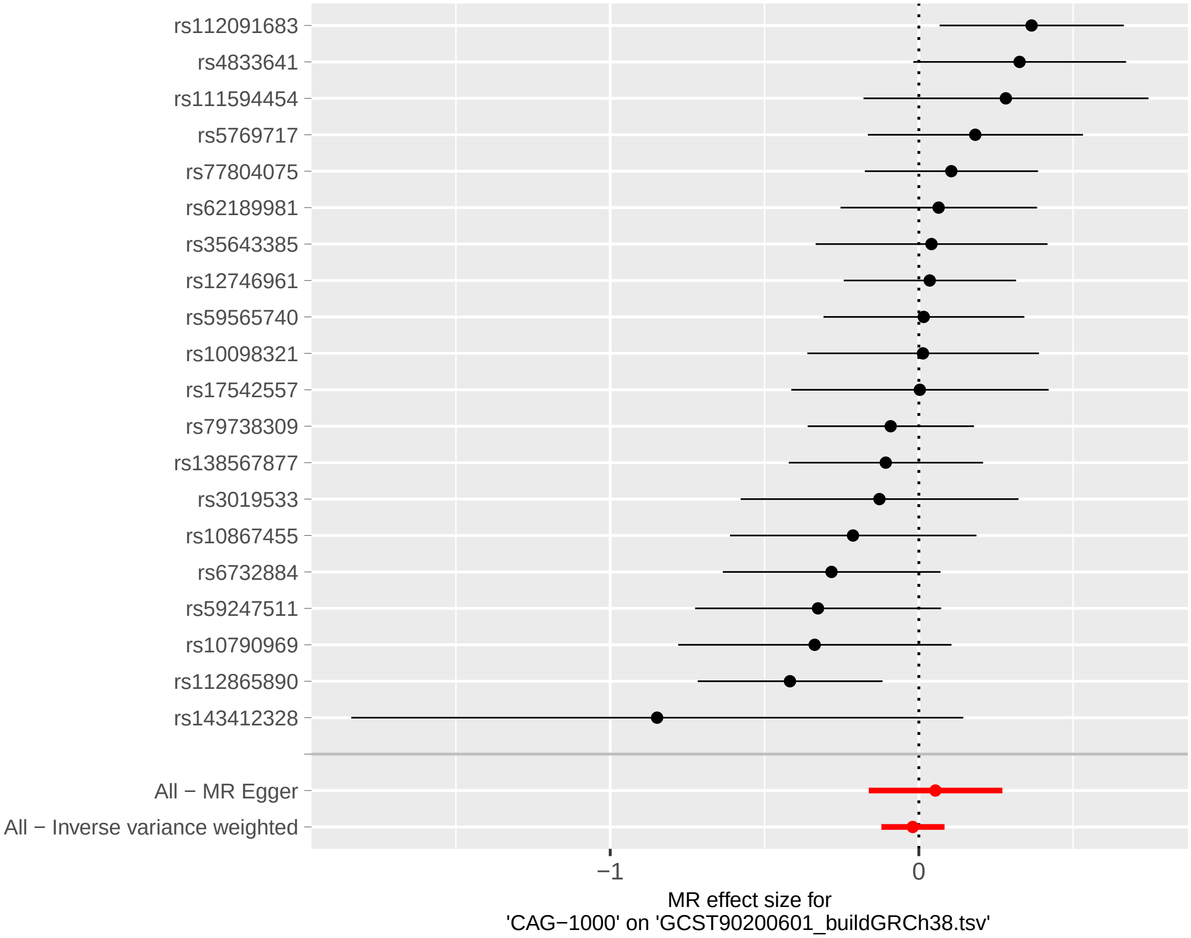
rs112091683
rs4833641
rs111594454
rs5769717
rs77804075
rs62189981
rs35643385
rs12746961
rs59565740
rs10098321
rs17542557
rs79738309
rs138567877
rs3019533
rs10867455
rs6732884
rs59247511
rs10790969
rs112865890
rs143412328
All − MR Egger
All − Inverse variance weighted
−1
0
MR effect size for
'CAG−1000' on 'GCST90200601_buildGRCh38.tsv'

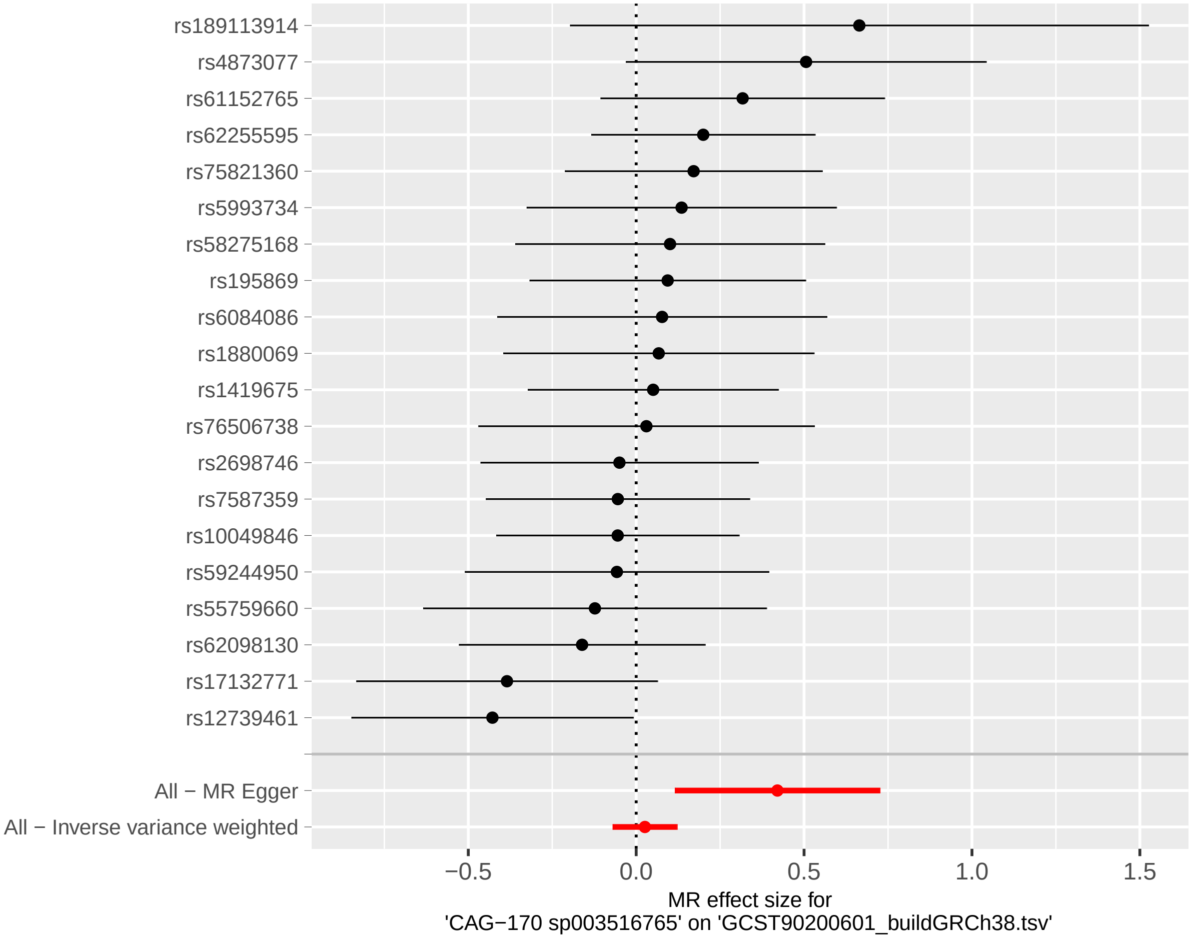

rs189113914
rs4873077
rs61152765
rs62255595
rs75821360
rs5993734
rs58275168
rs195869
rs6084086
rs1880069
rs1419675
rs76506738
rs2698746
rs7587359
rs10049846
rs59244950
rs55759660
rs62098130
rs17132771
rs12739461
All − MR Egger
All − Inverse variance weighted
−0.5
0.0
0.5
1.0
1.5
MR effect size for
'CAG−170 sp003516765' on 'GCST90200601_buildGRCh38.tsv'

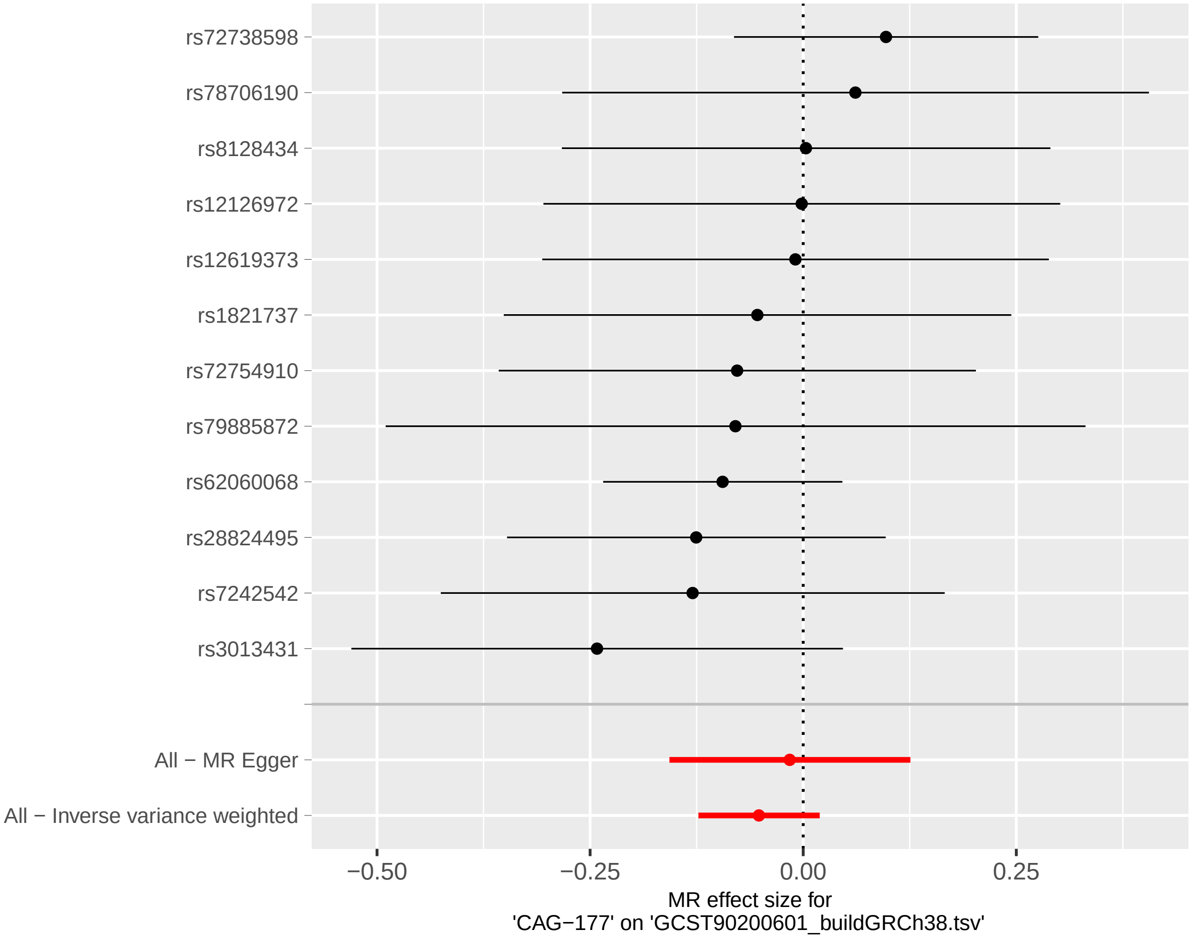

rs72738598
rs78706190
rs8128434
rs12126972
rs12619373
rs1821737
rs72754910
rs79885872
rs62060068
rs28824495
rs7242542
rs3013431
All − MR Egger
All − Inverse variance weighted
−0.50
−0.25
0.00
0.25
MR effect size for
'CAG−177' on 'GCST90200601_buildGRCh38.tsv'

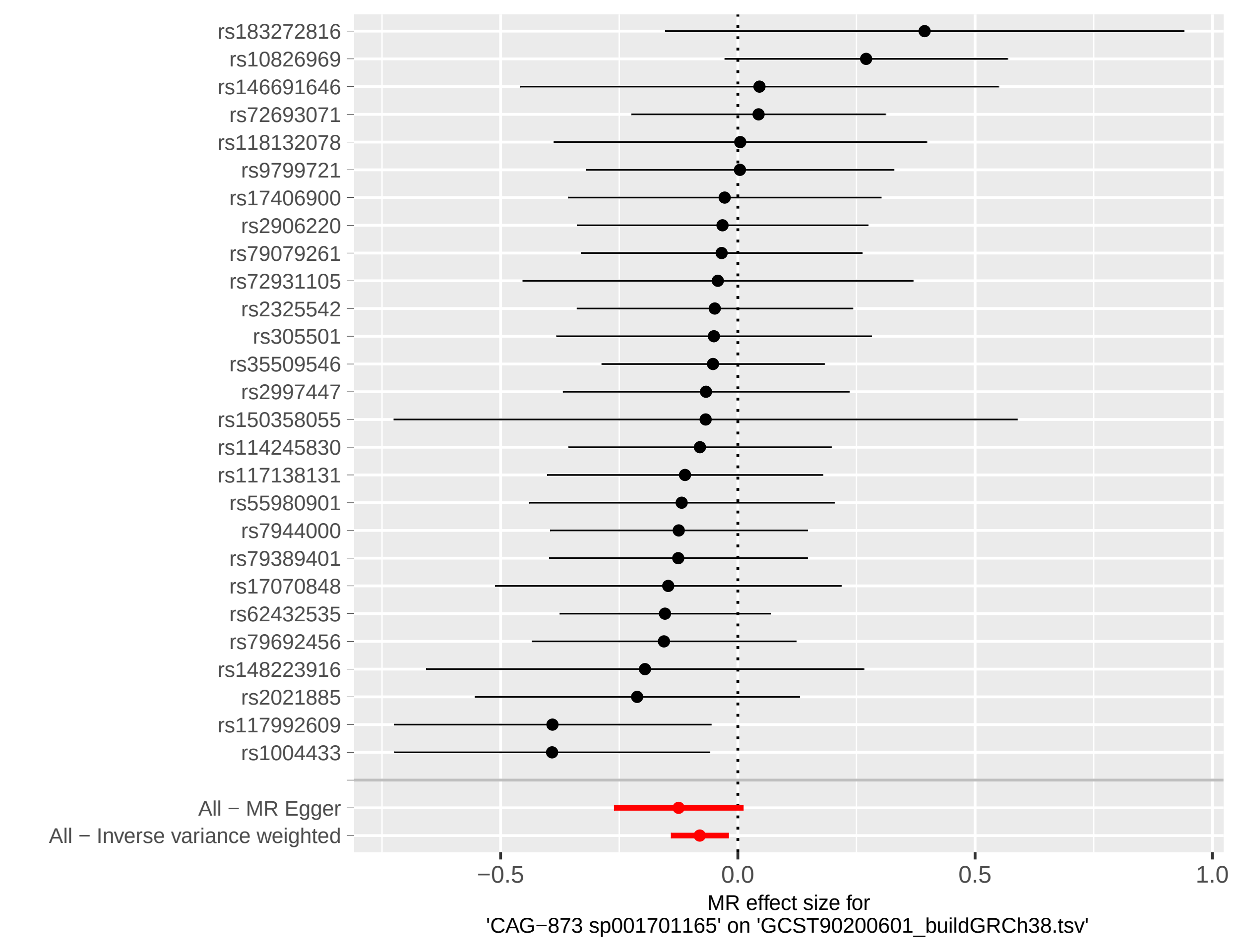
rs183272816
rs10826969
rs146691646
rs72693071
rs118132078
rs9799721
rs17406900
rs2906220
rs79079261
rs72931105
rs2325542
rs305501
rs35509546
rs2997447
rs150358055
rs114245830
rs117138131
rs55980901
rs7944000
rs79389401
rs17070848
rs62432535
rs79692456
rs148223916
rs2021885
rs117992609
rs1004433
All − MR Egger
All − Inverse variance weighted
−0.5
0.0
0.5
1.0
MR effect size for
'CAG−873 sp001701165' on 'GCST90200601_buildGRCh38.tsv'

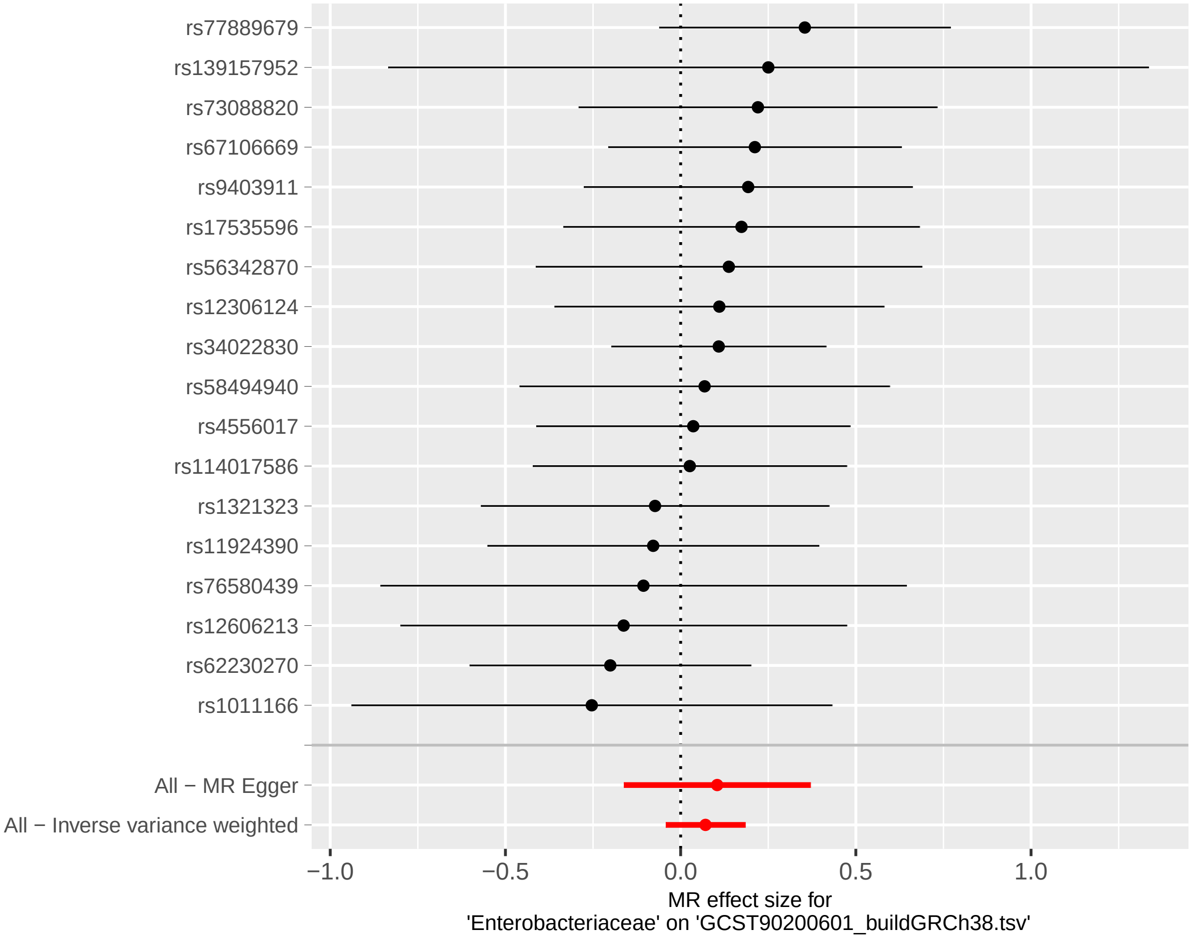
rs77889679
rs139157952
rs73088820
rs67106669
rs9403911
rs17535596
rs56342870
rs12306124
rs34022830
rs58494940
rs4556017
rs114017586
rs1321323
rs11924390
rs76580439
rs12606213
rs62230270
rs1011166
All − MR Egger
All − Inverse variance weighted
−1.0
−0.5
0.0
0.5
1.0
MR effect size for
'Enterobacteriaceae' on 'GCST90200601_buildGRCh38.tsv'

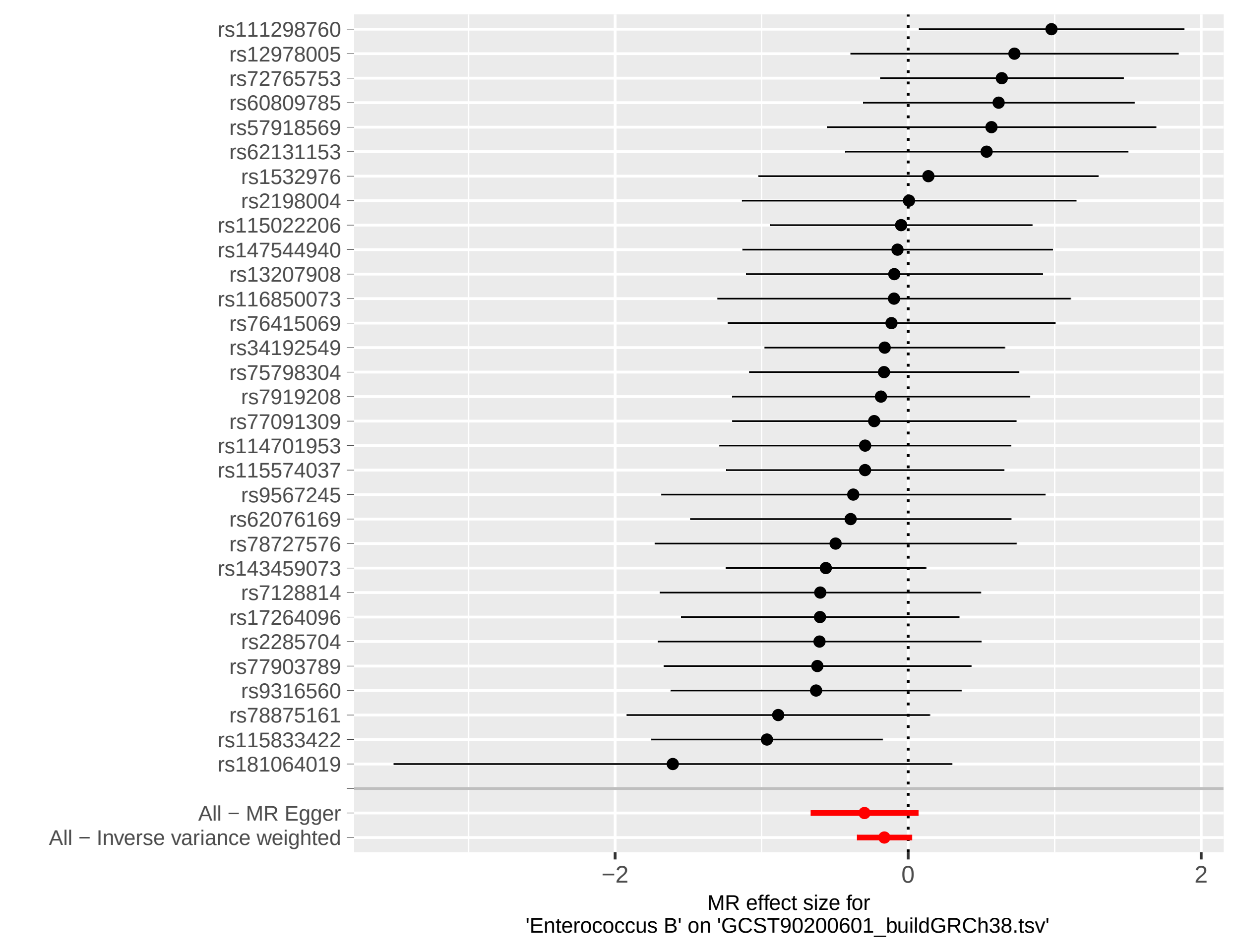
rs111298760
rs12978005
rs72765753
rs60809785
rs57918569
rs62131153
rs1532976
rs2198004
rs115022206
rs147544940
rs13207908
rs116850073
rs76415069
rs34192549
rs75798304
rs7919208
rs77091309
rs114701953
rs115574037
rs9567245
rs62076169
rs78727576
rs143459073
rs7128814
rs17264096
rs2285704
rs77903789
rs9316560
rs78875161
rs115833422
rs181064019
All − MR Egger
All − Inverse variance weighted
−2
0
2
MR effect size for
'Enterococcus B' on 'GCST90200601_buildGRCh38.tsv'

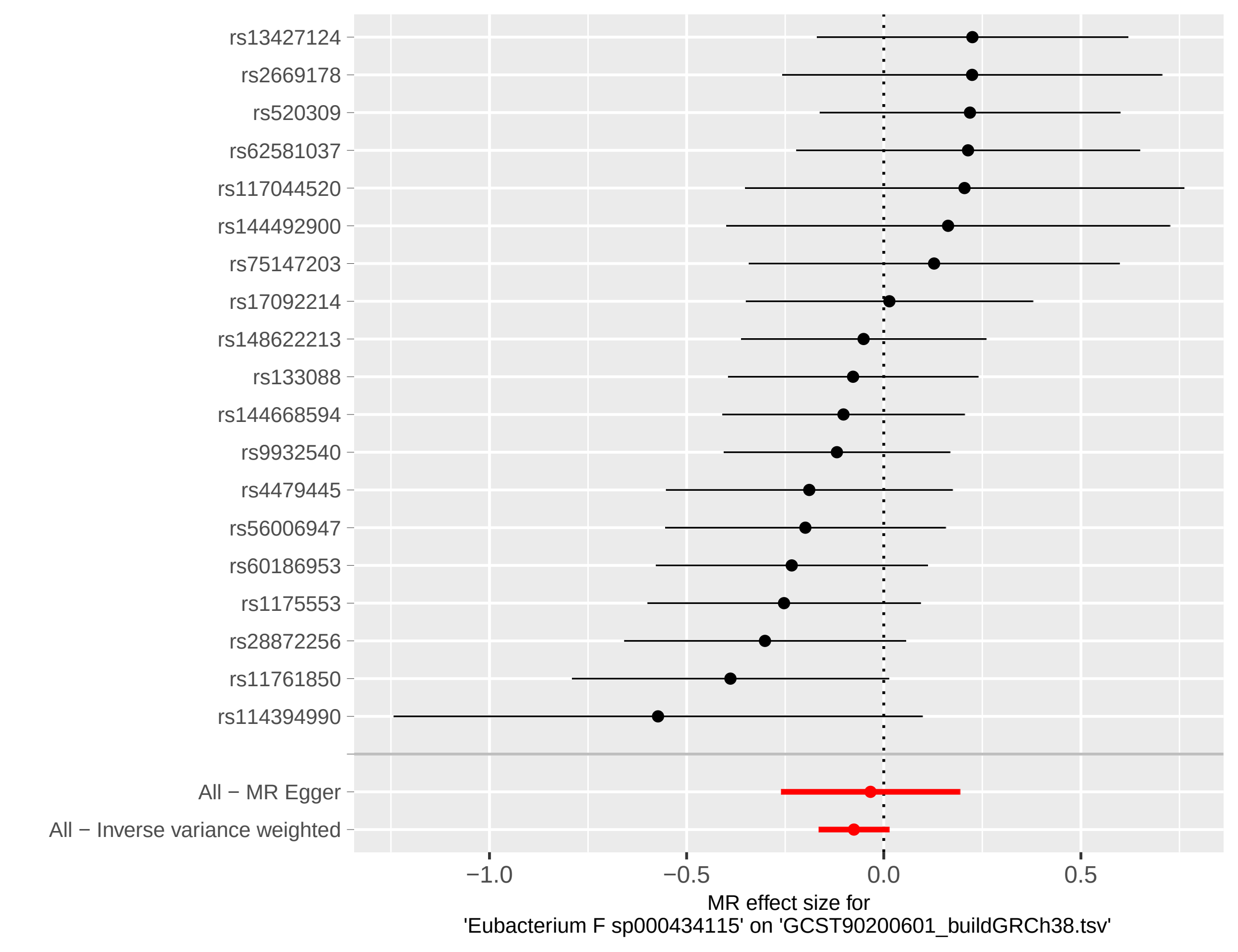
rs13427124
rs2669178
rs520309
rs62581037
rs117044520
rs144492900
rs75147203
rs17092214
rs148622213
rs133088
rs144668594
rs9932540
rs4479445
rs56006947
rs60186953
rs1175553
rs28872256
rs11761850
rs114394990
All − MR Egger
All − Inverse variance weighted
−1.0
−0.5
0.0
0.5
MR effect size for
'Eubacterium F sp000434115' on 'GCST90200601_buildGRCh38.tsv'

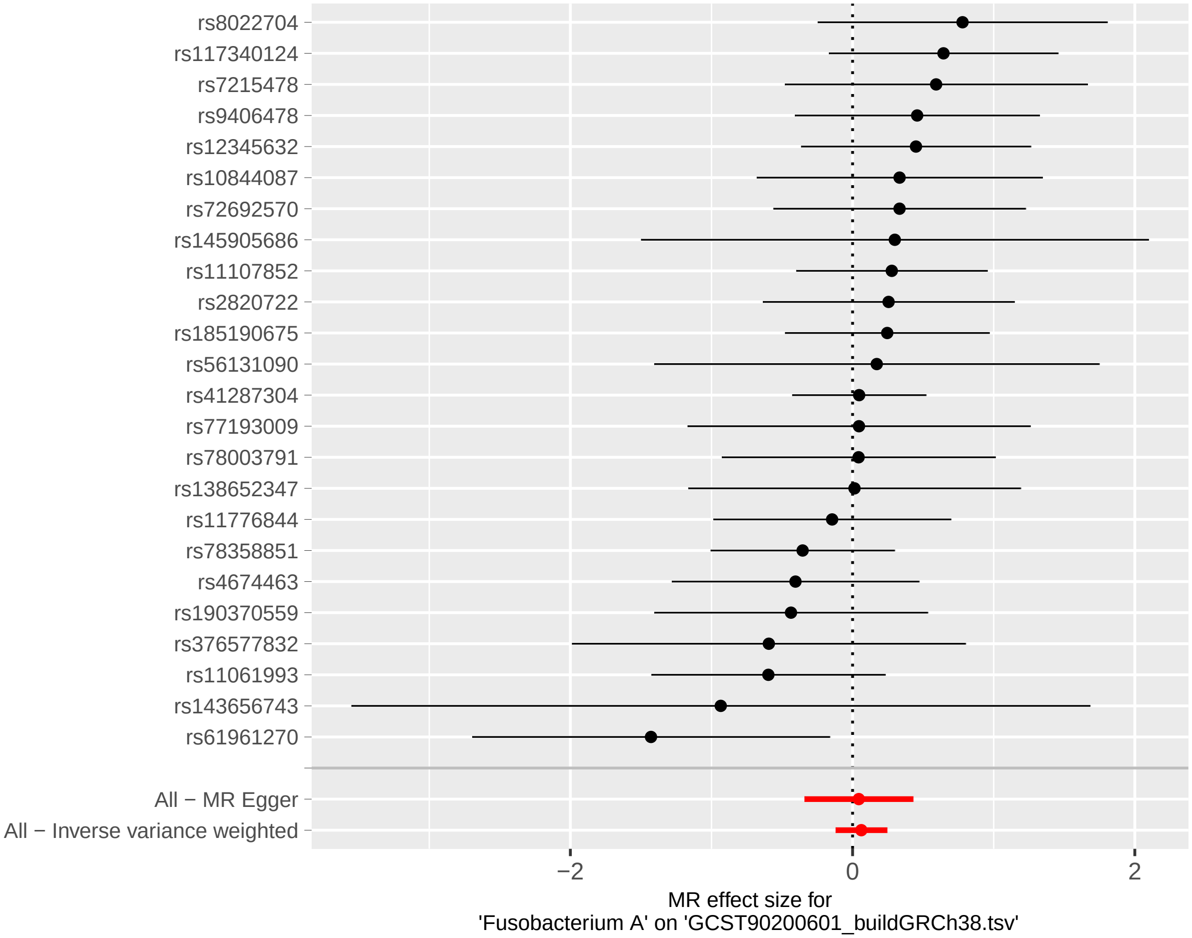

rs8022704
rs117340124
rs7215478
rs9406478
rs12345632
rs10844087
rs72692570
rs145905686
rs11107852
rs2820722
rs185190675
rs56131090
rs41287304
rs77193009
rs78003791
rs138652347
rs11776844
rs78358851
rs4674463
rs190370559
rs376577832
rs11061993
rs143656743
rs61961270
All − MR Egger
All − Inverse variance weighted
−2
0
2
MR effect size for
'Fusobacterium A' on 'GCST90200601_buildGRCh38.tsv'

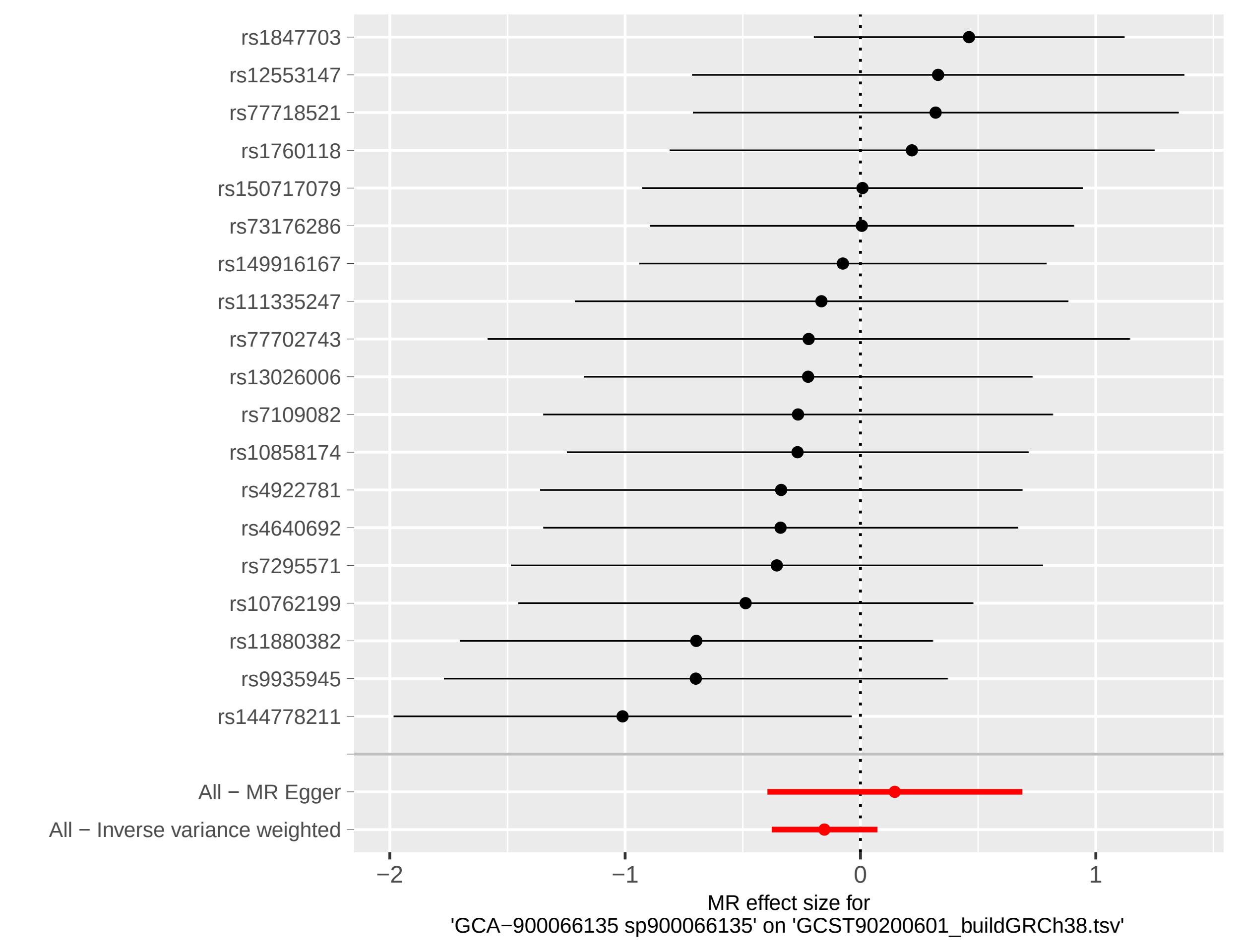
rs1847703
rs12553147
rs77718521
rs1760118
rs150717079
rs73176286
rs149916167
rs111335247
rs77702743
rs13026006
rs7109082
rs10858174
rs4922781
rs4640692
rs7295571
rs10762199
rs11880382
rs9935945
rs144778211
All − MR Egger
All − Inverse variance weighted
−2
−1
0
1
MR effect size for
'GCA−900066135 sp900066135' on 'GCST90200601_buildGRCh38.tsv'

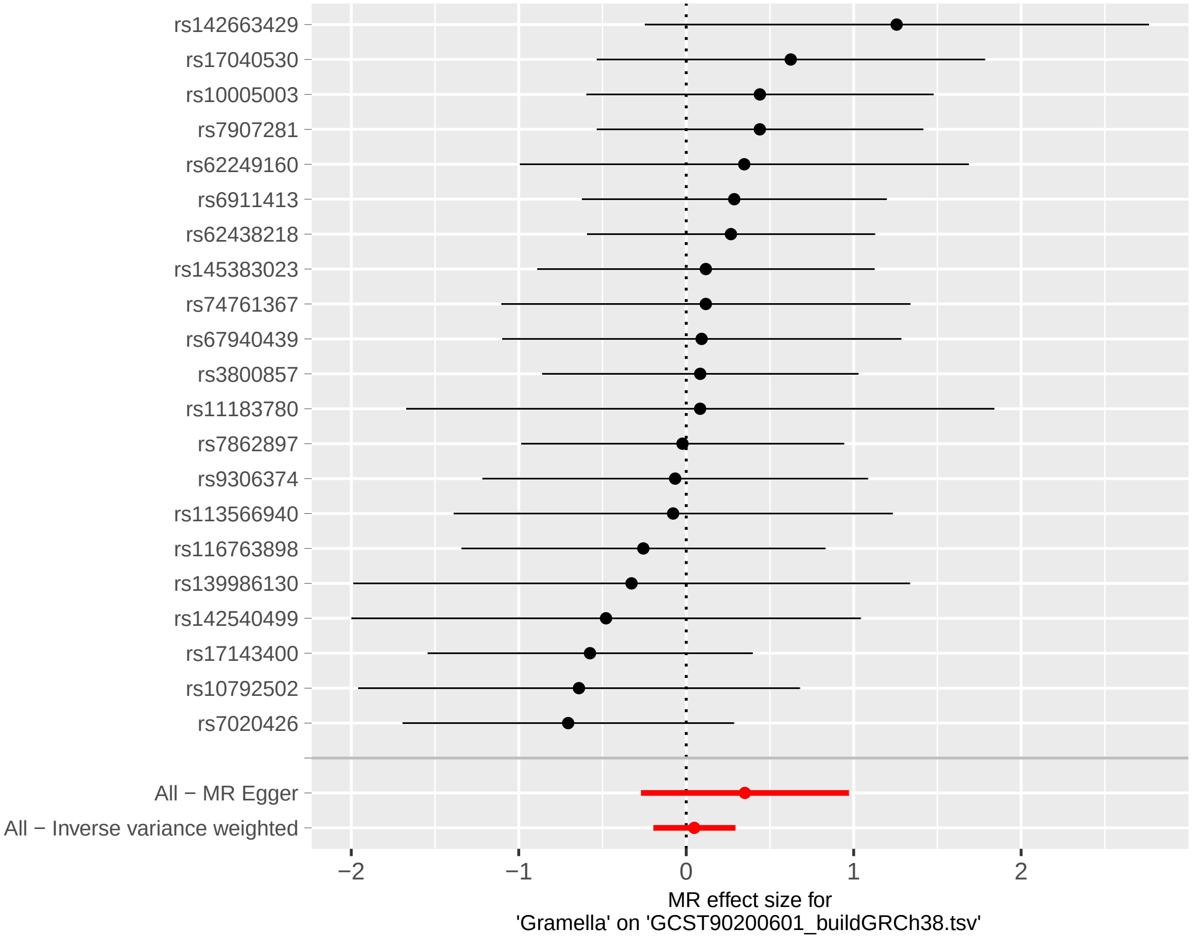
rs142663429
rs17040530
rs10005003
rs7907281
rs62249160
rs6911413
rs62438218
rs145383023
rs74761367
rs67940439
rs3800857
rs11183780
rs7862897
rs9306374
rs113566940
rs116763898
rs139986130
rs142540499
rs17143400
rs10792502
rs7020426
All − MR Egger
All − Inverse variance weighted
−2
−1
0
1
2
MR effect size for
'Gramella' on 'GCST90200601_buildGRCh38.tsv'

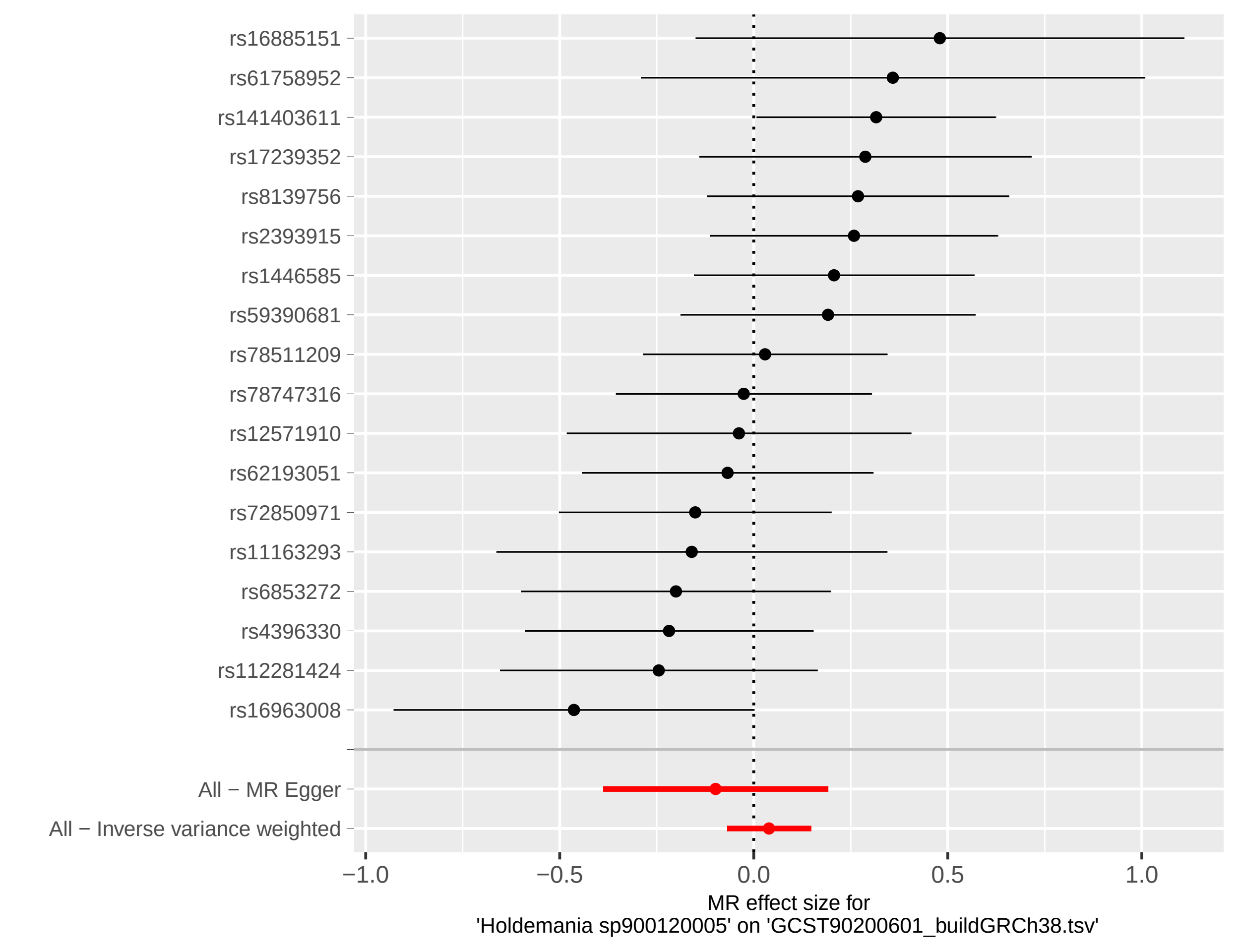

rs16885151
rs61758952
rs141403611
rs17239352
rs8139756
rs2393915
rs1446585
rs59390681
rs78511209
rs78747316
rs12571910
rs62193051
rs72850971
rs11163293
rs6853272
rs4396330
rs112281424
rs16963008
All − MR Egger
All − Inverse variance weighted
−1.0
−0.5
0.0
0.5
1.0
MR effect size for
'Holdemania sp900120005' on 'GCST90200601_buildGRCh38.tsv'

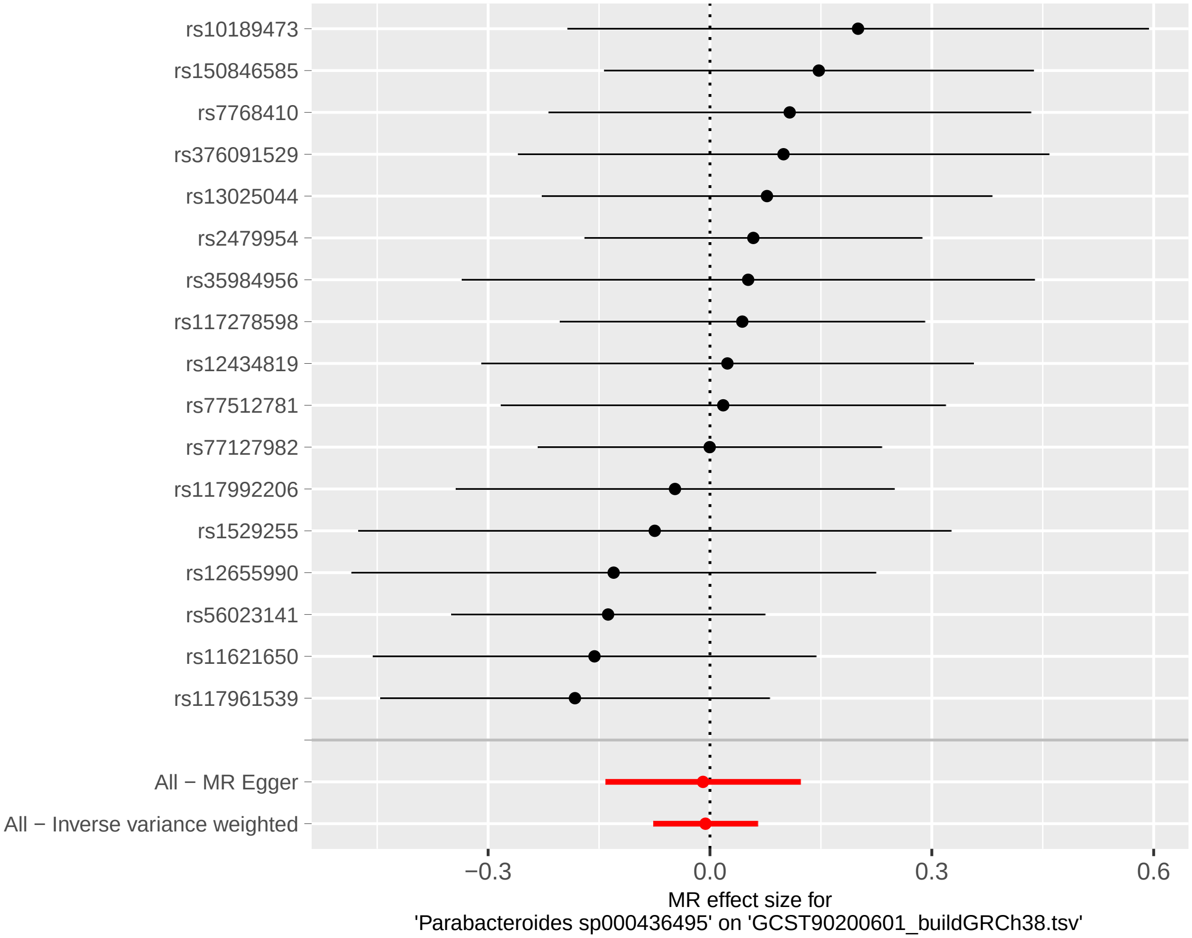
rs10189473
rs150846585
rs7768410
rs376091529
rs13025044
rs2479954
rs35984956
rs117278598
rs12434819
rs77512781
rs77127982
rs117992206
rs1529255
rs12655990
rs56023141
rs11621650
rs117961539
All − MR Egger
All − Inverse variance weighted
−0.3
0.0
0.3
0.6
MR effect size for
'Parabacteroides sp000436495' on 'GCST90200601_buildGRCh38.tsv'

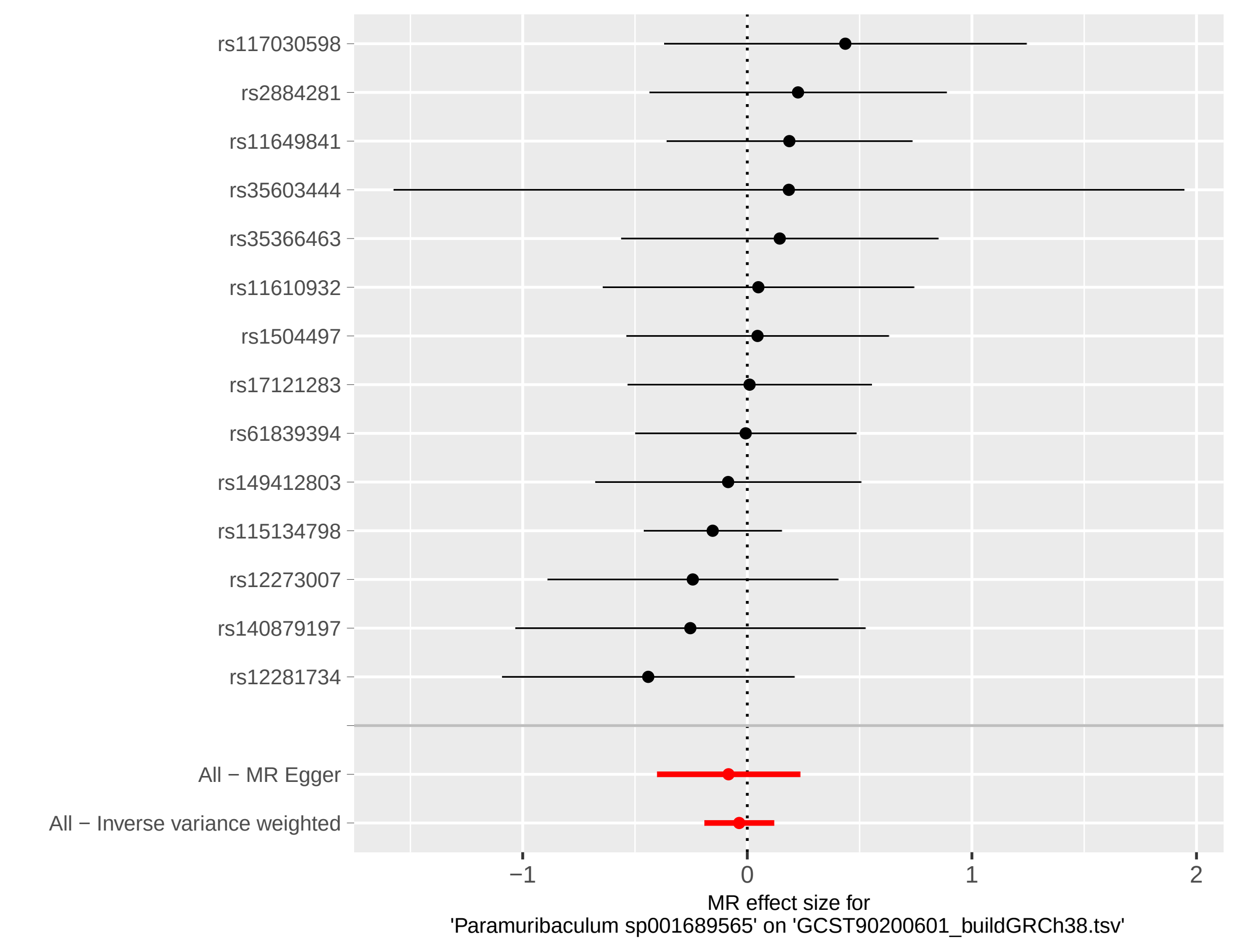
rs117030598
rs2884281
rs11649841
rs35603444
rs35366463
rs11610932
rs1504497
rs17121283
rs61839394
rs149412803
rs115134798
rs12273007
rs140879197
rs12281734
All − MR Egger
All − Inverse variance weighted
−1
0
1
2
MR effect size for
'Paramuribaculum sp001689565' on 'GCST90200601_buildGRCh38.tsv'

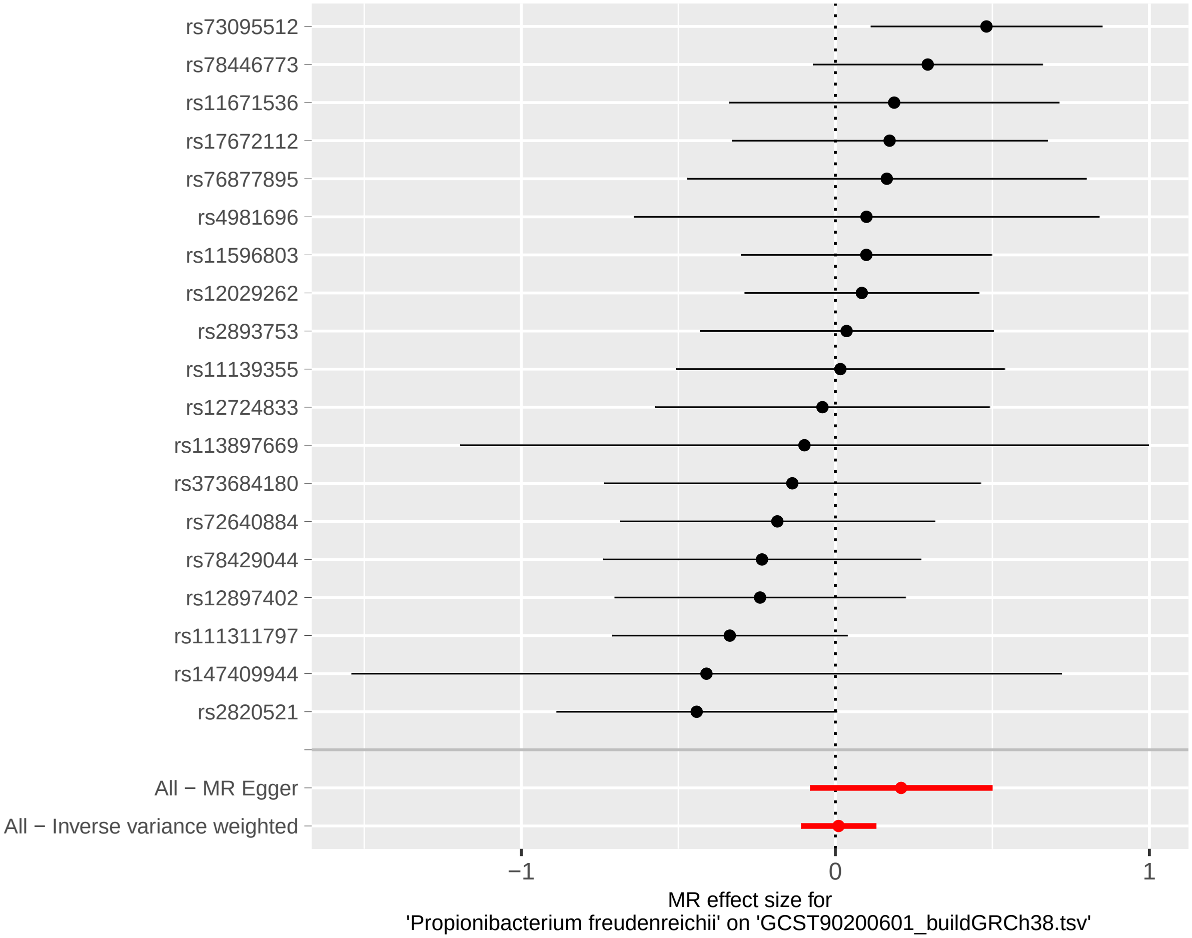
rs73095512
rs78446773
rs11671536
rs17672112
rs76877895
rs4981696
rs11596803
rs12029262
rs2893753
rs11139355
rs12724833
rs113897669
rs373684180
rs72640884
rs78429044
rs12897402
rs111311797
rs147409944
rs2820521
All − MR Egger
All − Inverse variance weighted
−1
0
1
MR effect size for
'Propionibacterium freudenreichii' on 'GCST90200601_buildGRCh38.tsv'

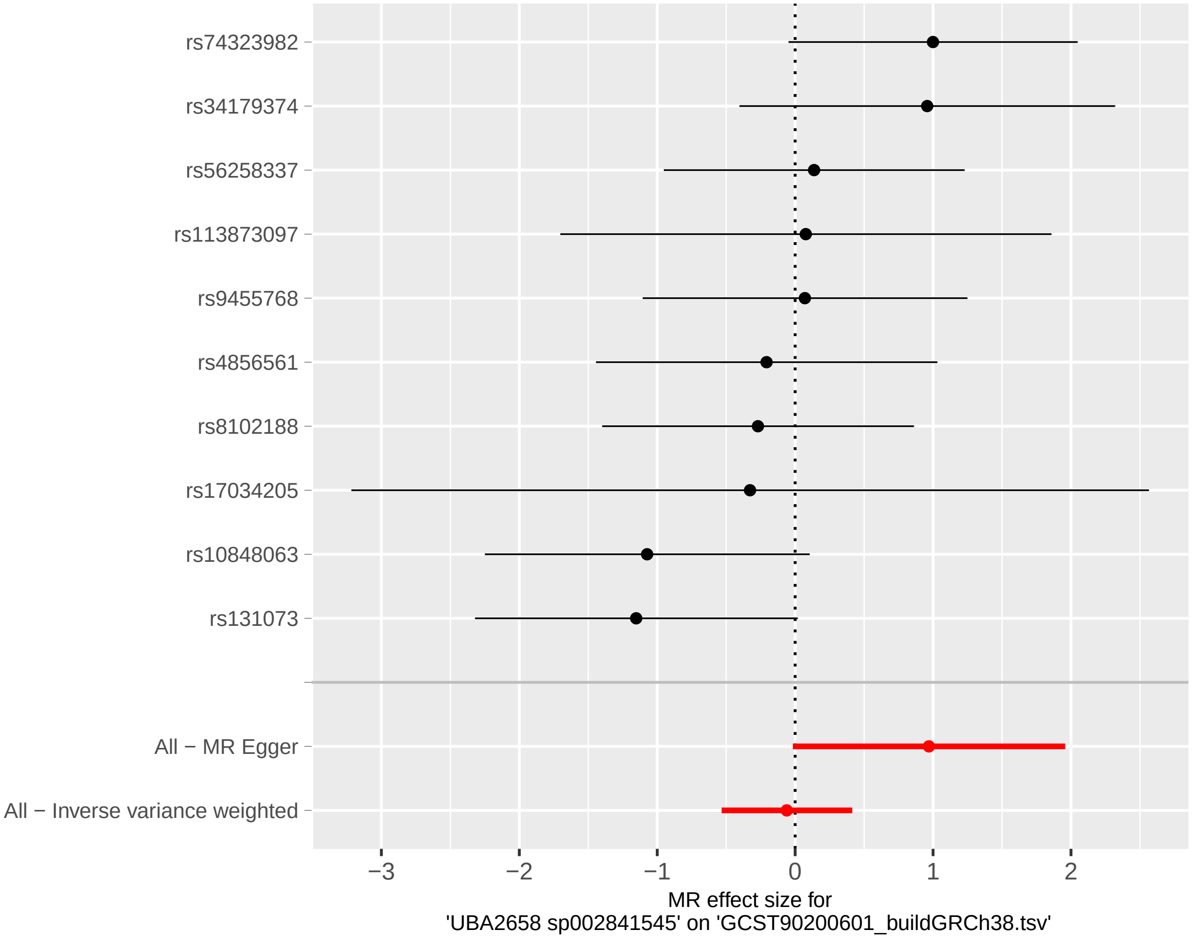
rs74323982
rs34179374
rs56258337
rs113873097
rs9455768
rs4856561
rs8102188
rs17034205
rs10848063
rs131073
All − MR Egger
All − Inverse variance weighted
−3
−2
−1
0
1
2
MR effect size for
'UBA2658 sp002841545' on 'GCST90200601_buildGRCh38.tsv'

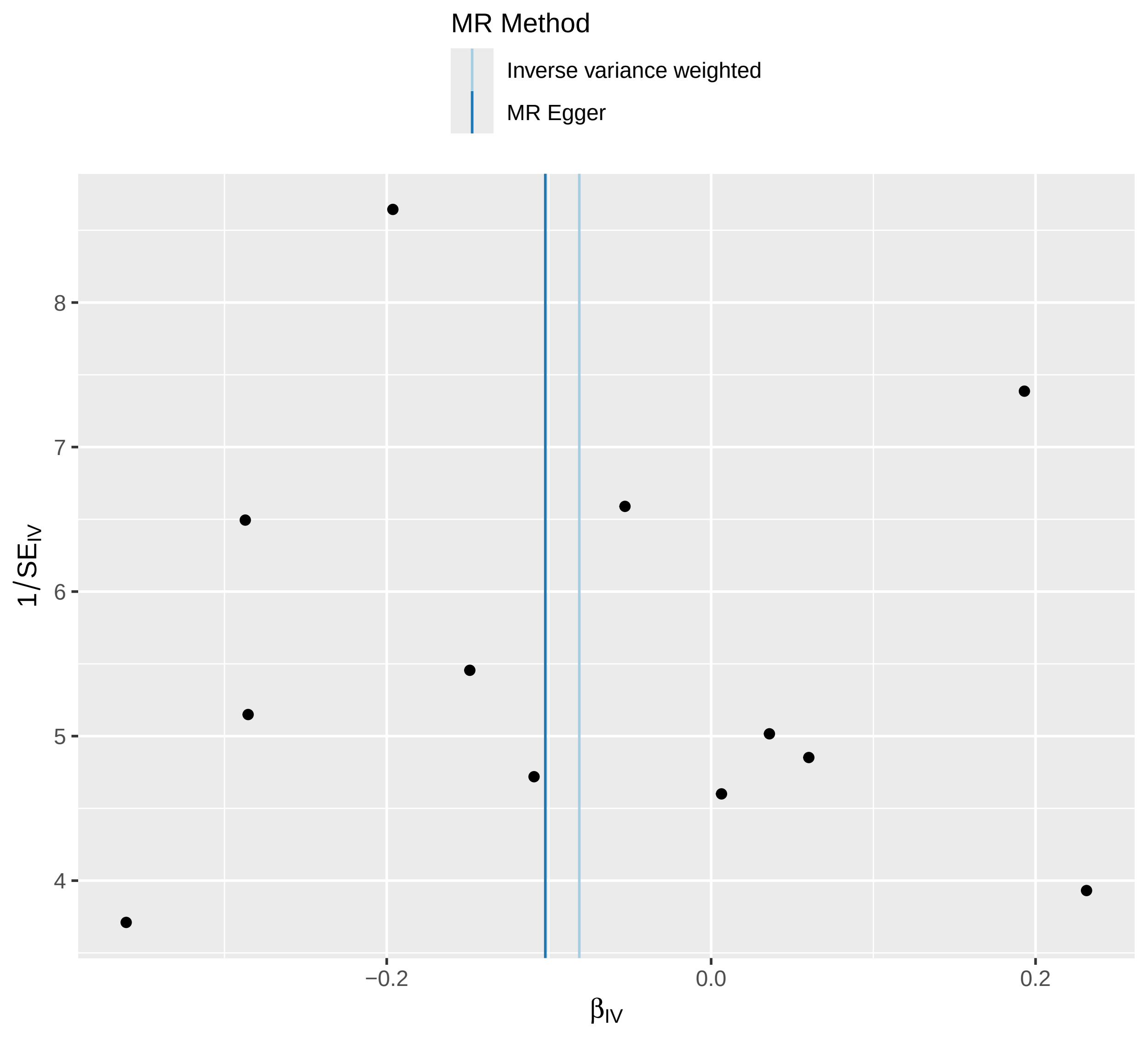

MR Method
Inverse variance weighted
MR Egger
8
7
6
5
4
−0.2
0.0
0.2
1/SE_IV
β_IV

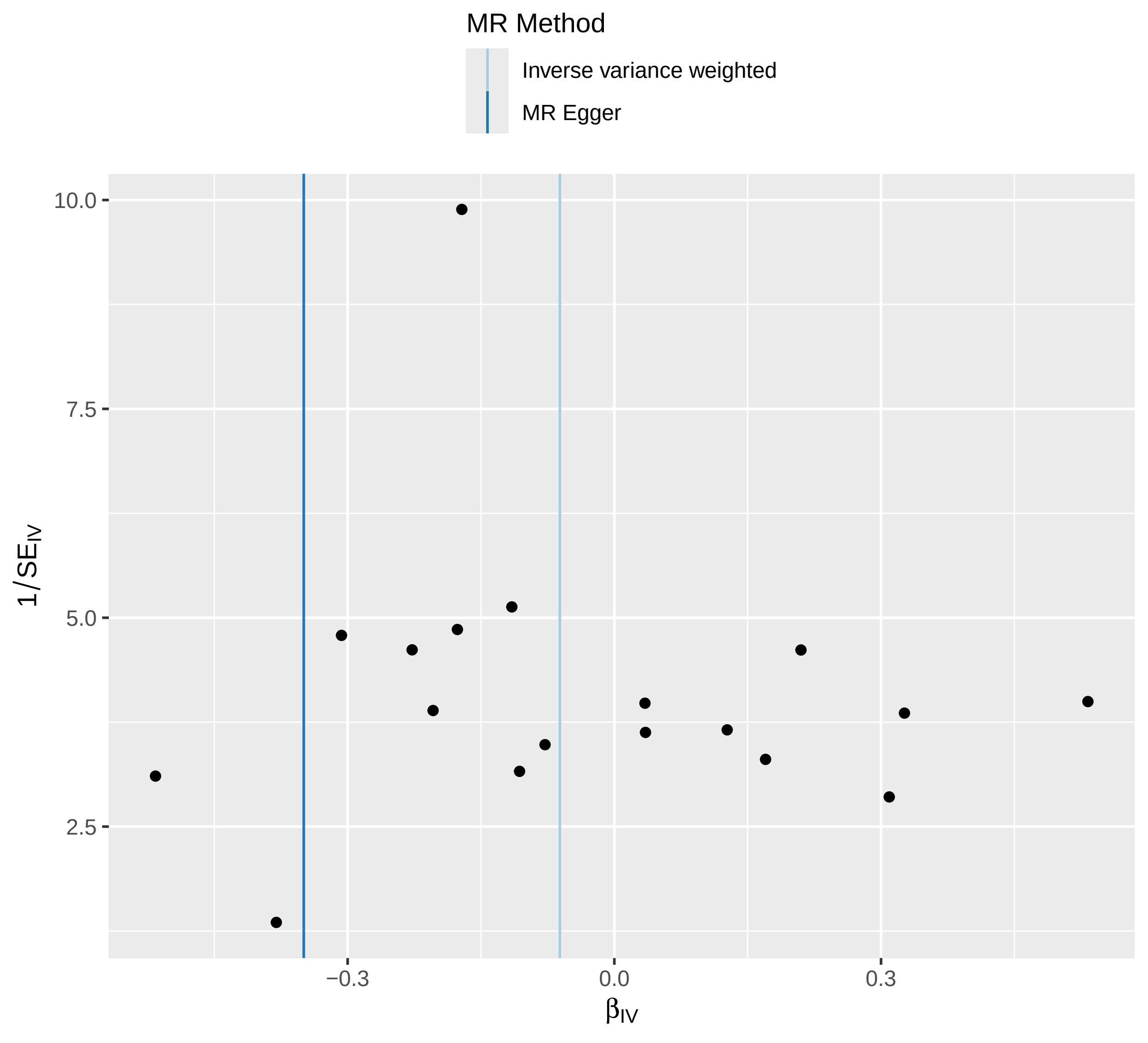

MR Method
Inverse variance weighted
MR Egger
10.0
7.5
5.0
2.5
1/SE_IV
−0.3
0.0
0.3
β_IV

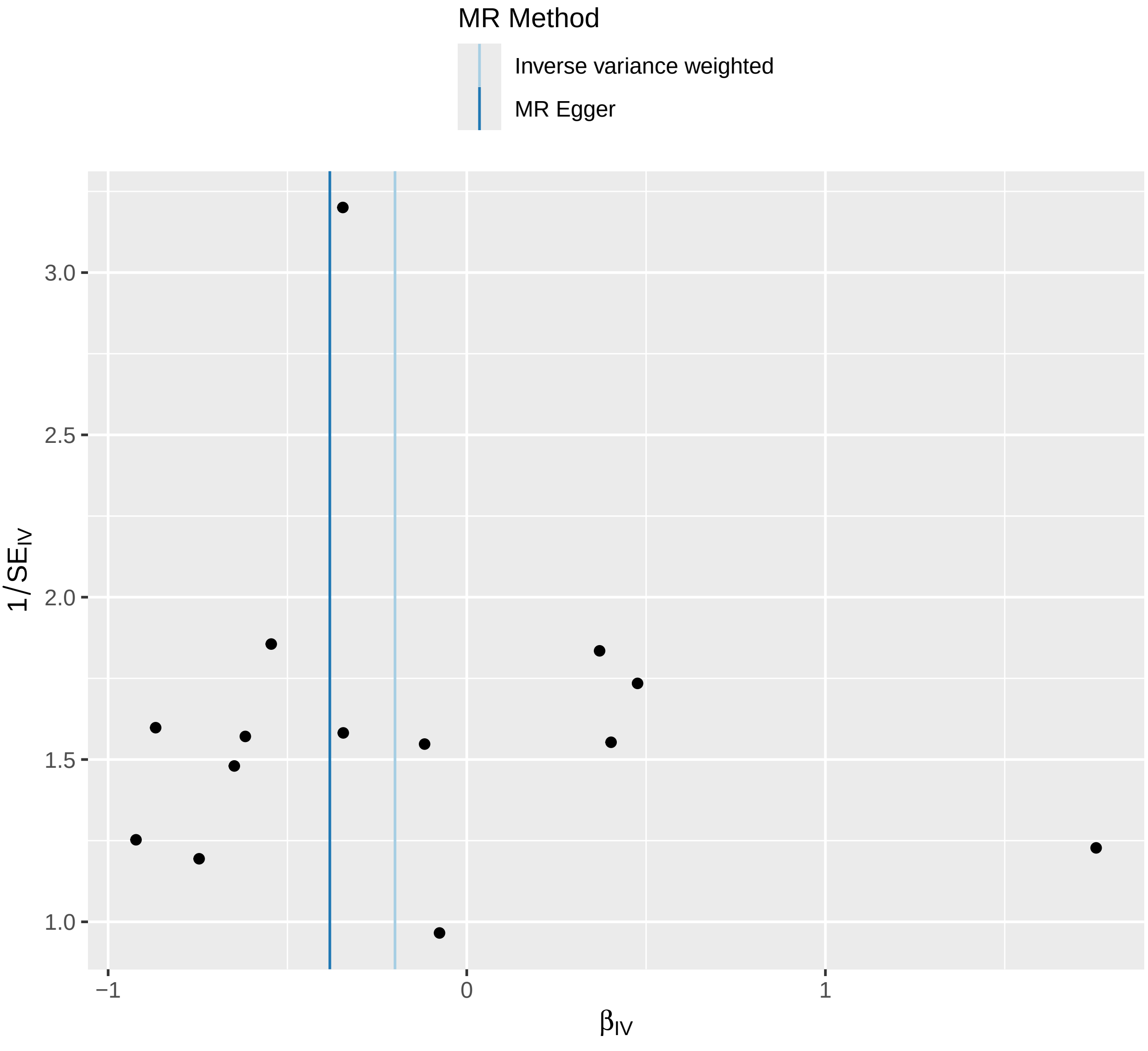
MR Method
Inverse variance weighted
MR Egger
3.0
2.5
2.0
1.5
1.0
$1/SE_{IV}$
−1
0
1
β_{IV}

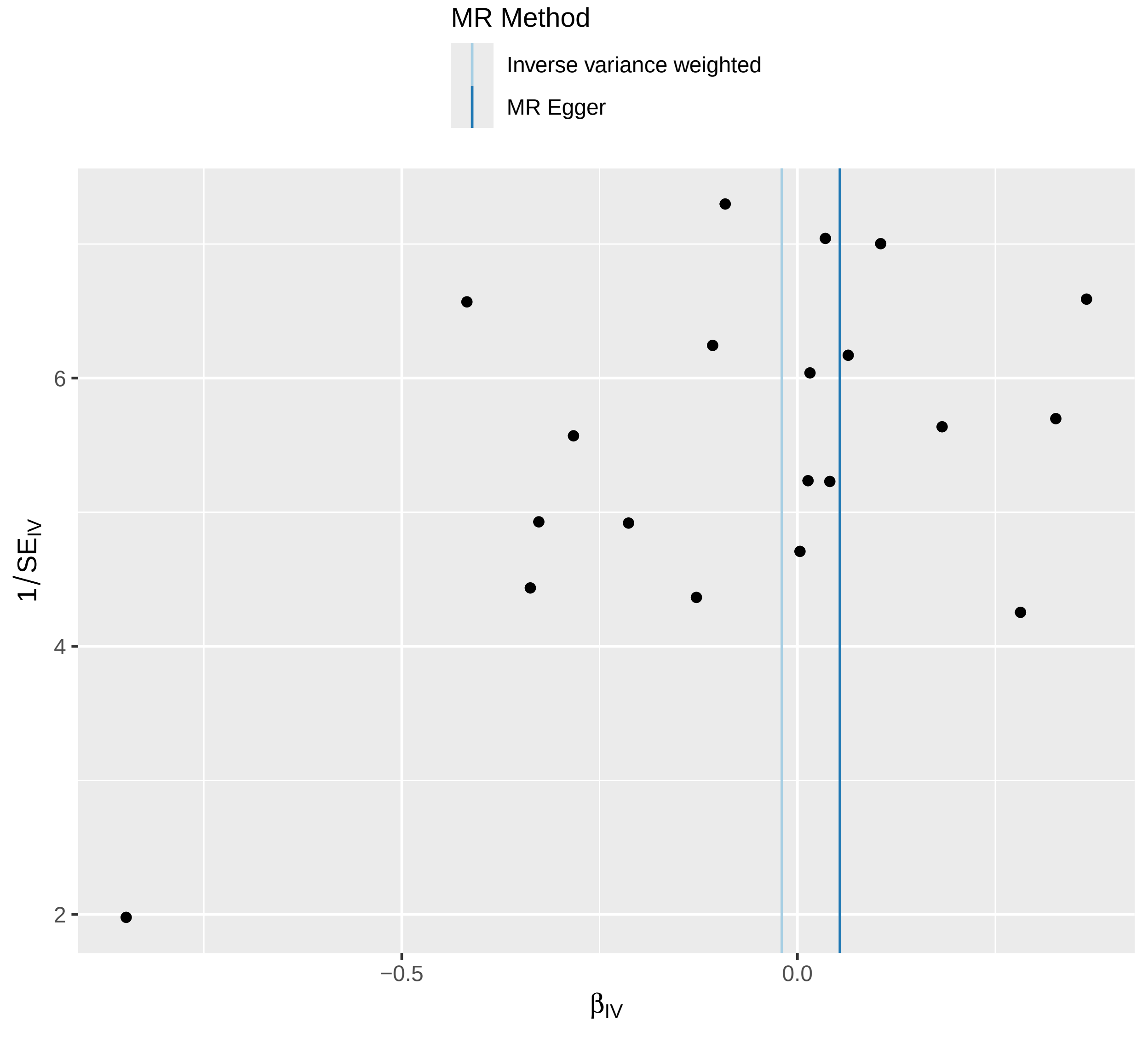

MR Method
Inverse variance weighted
MR Egger
6
4
2
1/SE$_{IV}$
−0.5
0.0
β$_{IV}$

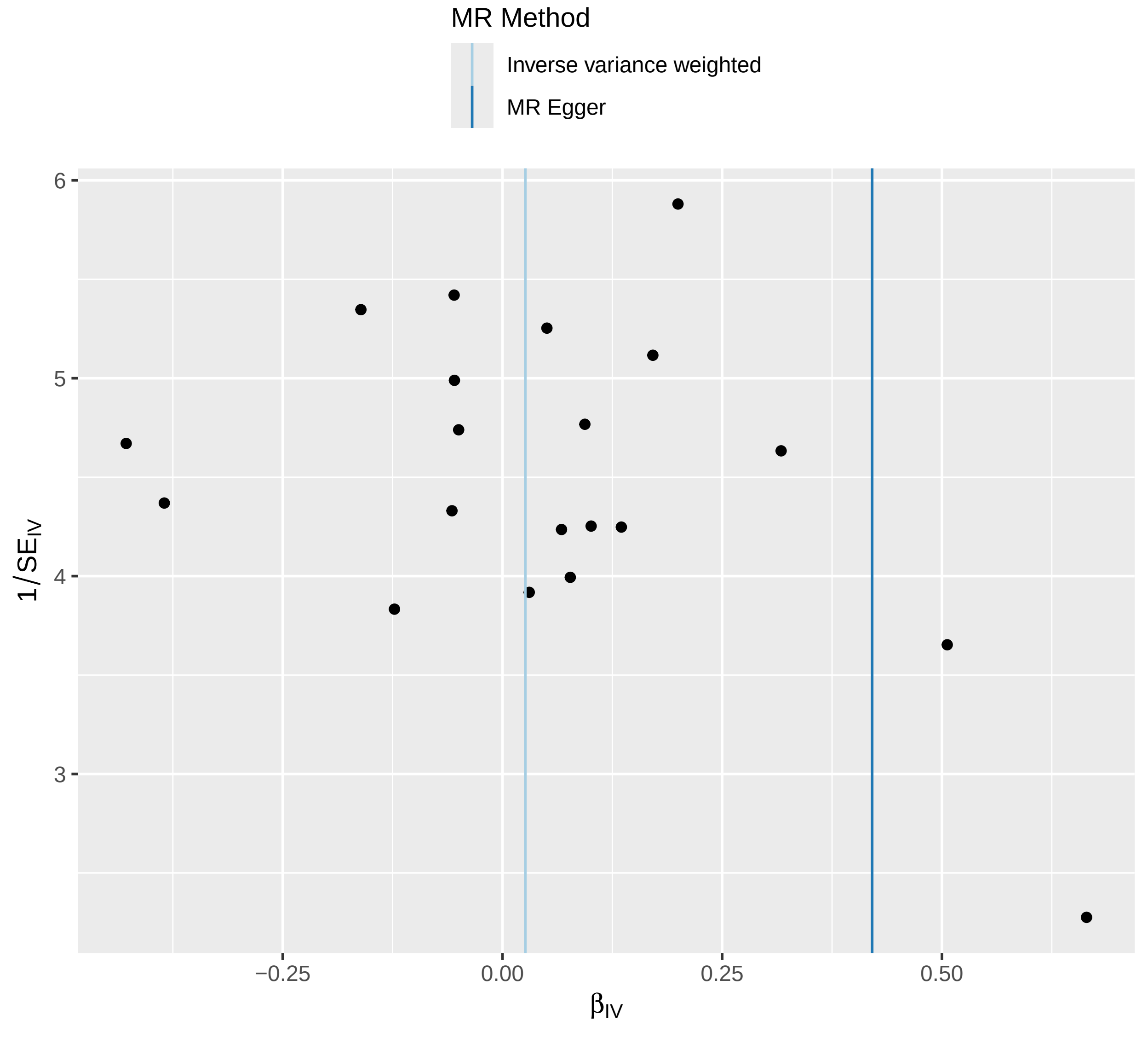
MR Method
Inverse variance weighted
MR Egger
6
5
4
3
1/SE$_{IV}$
−0.25
0.00
0.25
0.50
β$_{IV}$

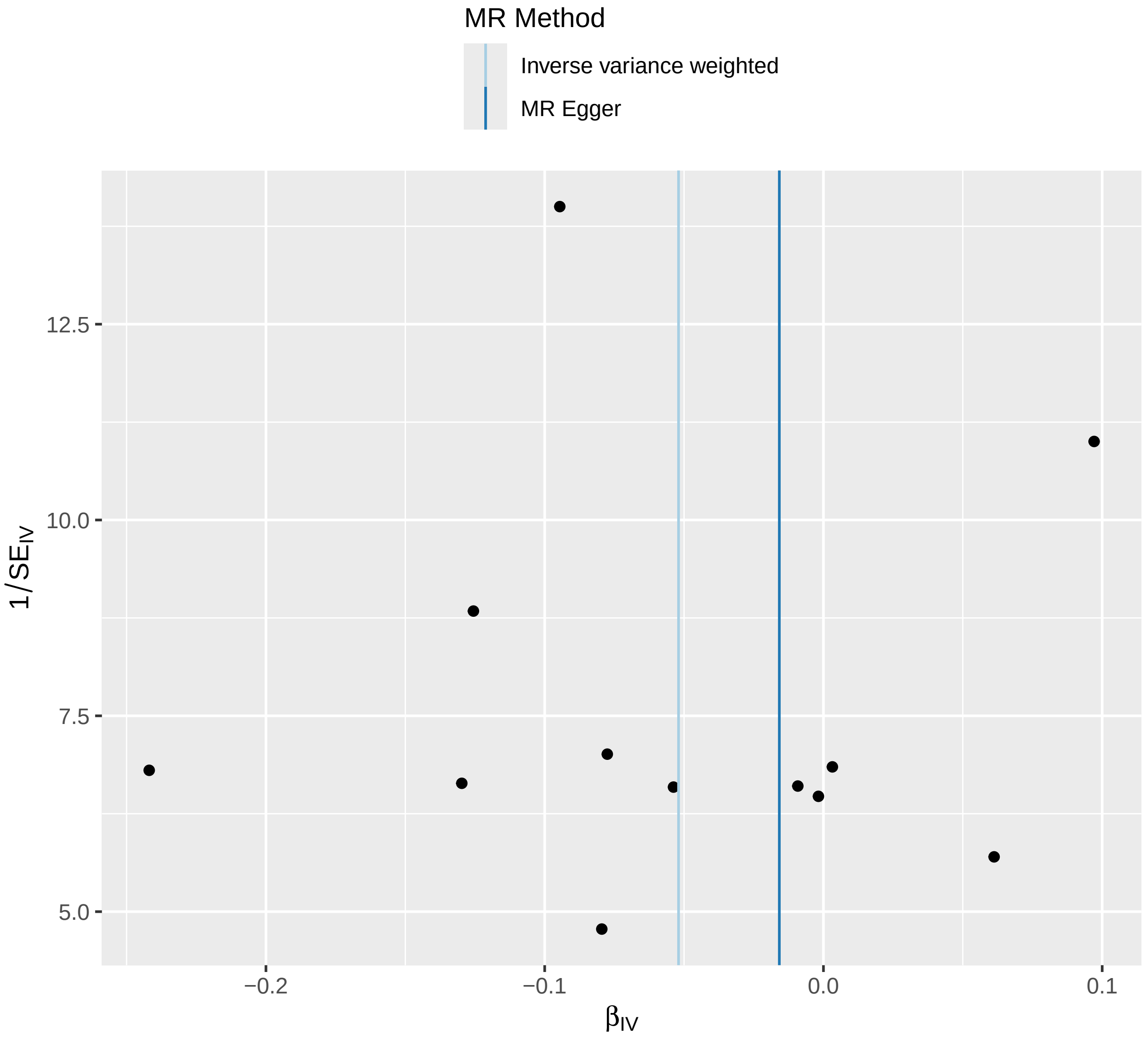
MR Method
Inverse variance weighted
MR Egger
12.5
10.0
7.5
5.0
1/SE$_{IV}$
−0.2
−0.1
0.0
0.1
β$_{IV}$

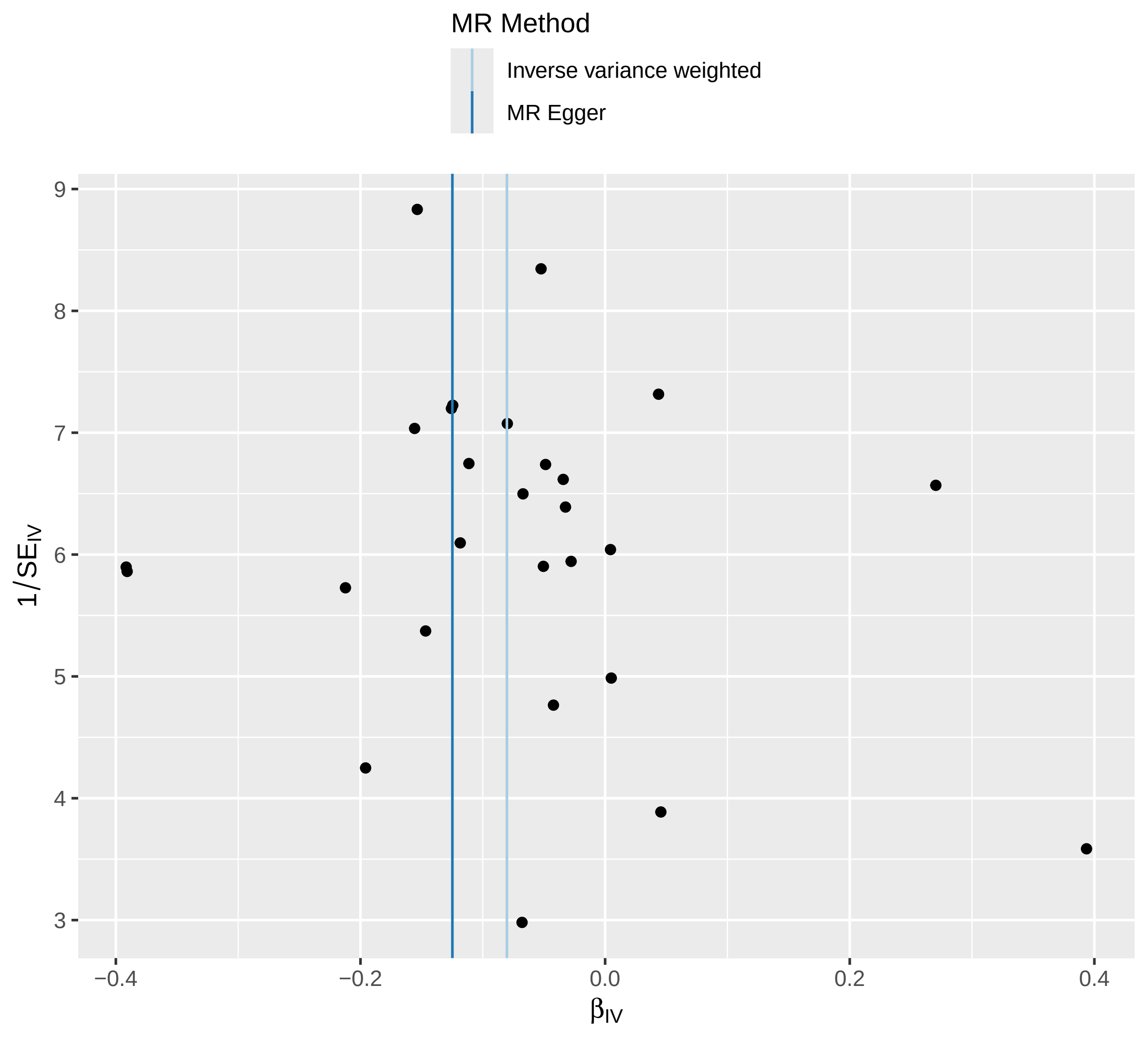
MR Method
Inverse variance weighted
MR Egger
9
8
7
6
5
4
3
1/SE$_{IV}$
−0.4
−0.2
0.0
0.2
0.4
β$_{IV}$

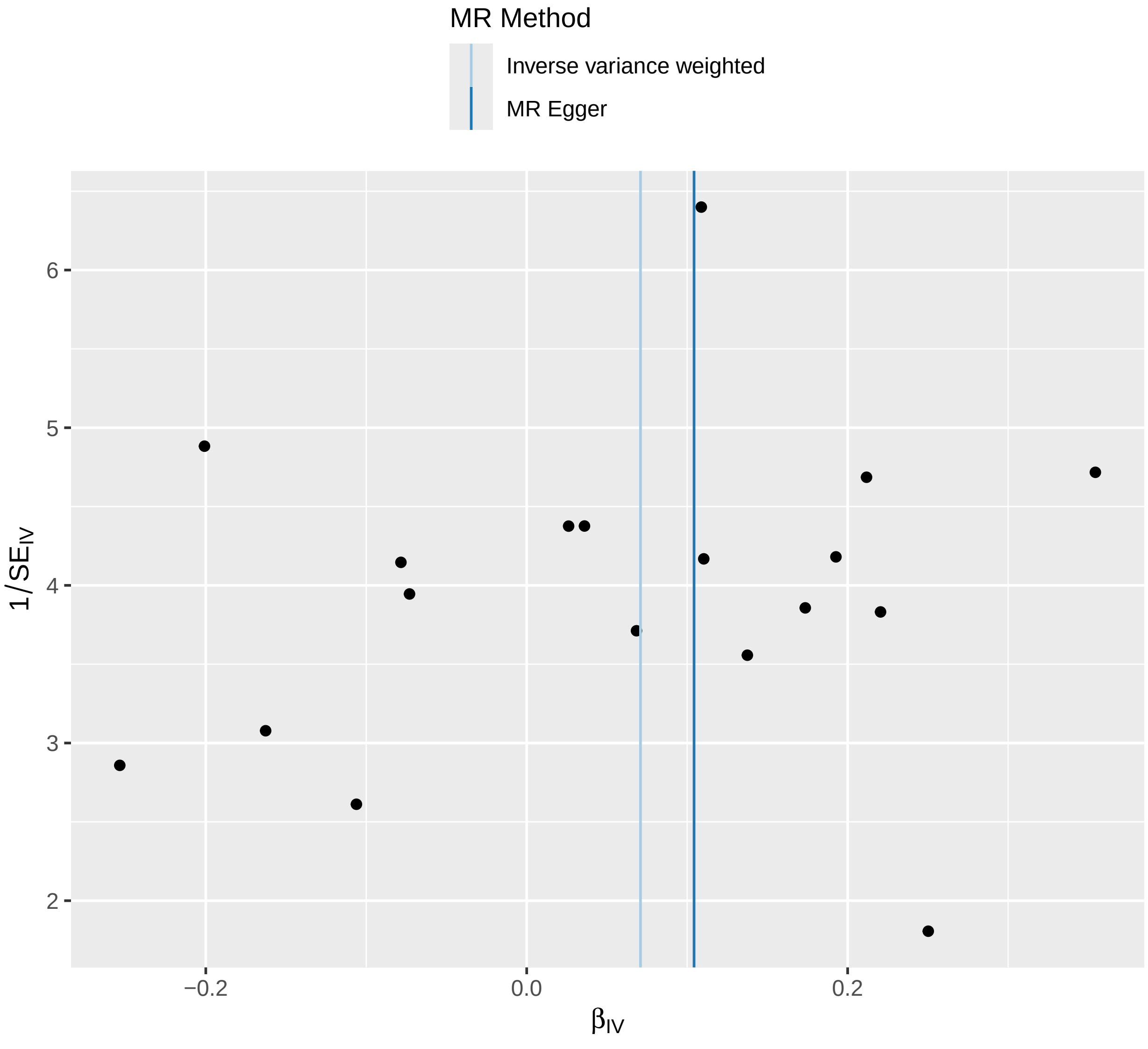
MR Method
Inverse variance weighted
MR Egger
6
5
4
3
2
1/SE_IV
−0.2
0.0
0.2
β_IV

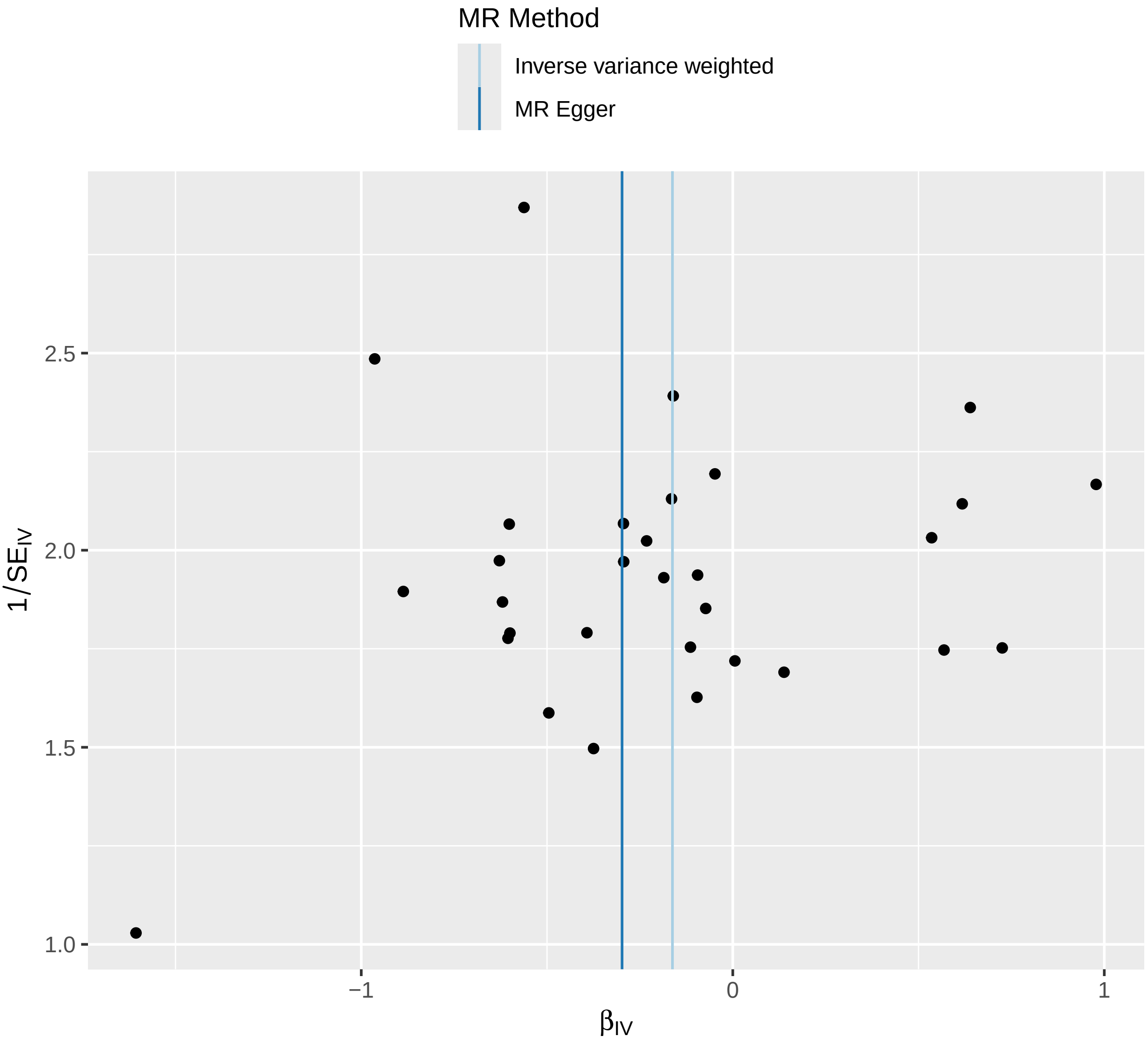

MR Method
Inverse variance weighted
MR Egger
2.5
2.0
1.5
1.0
$1/SE_{IV}$
−1
0
1
β_{IV}

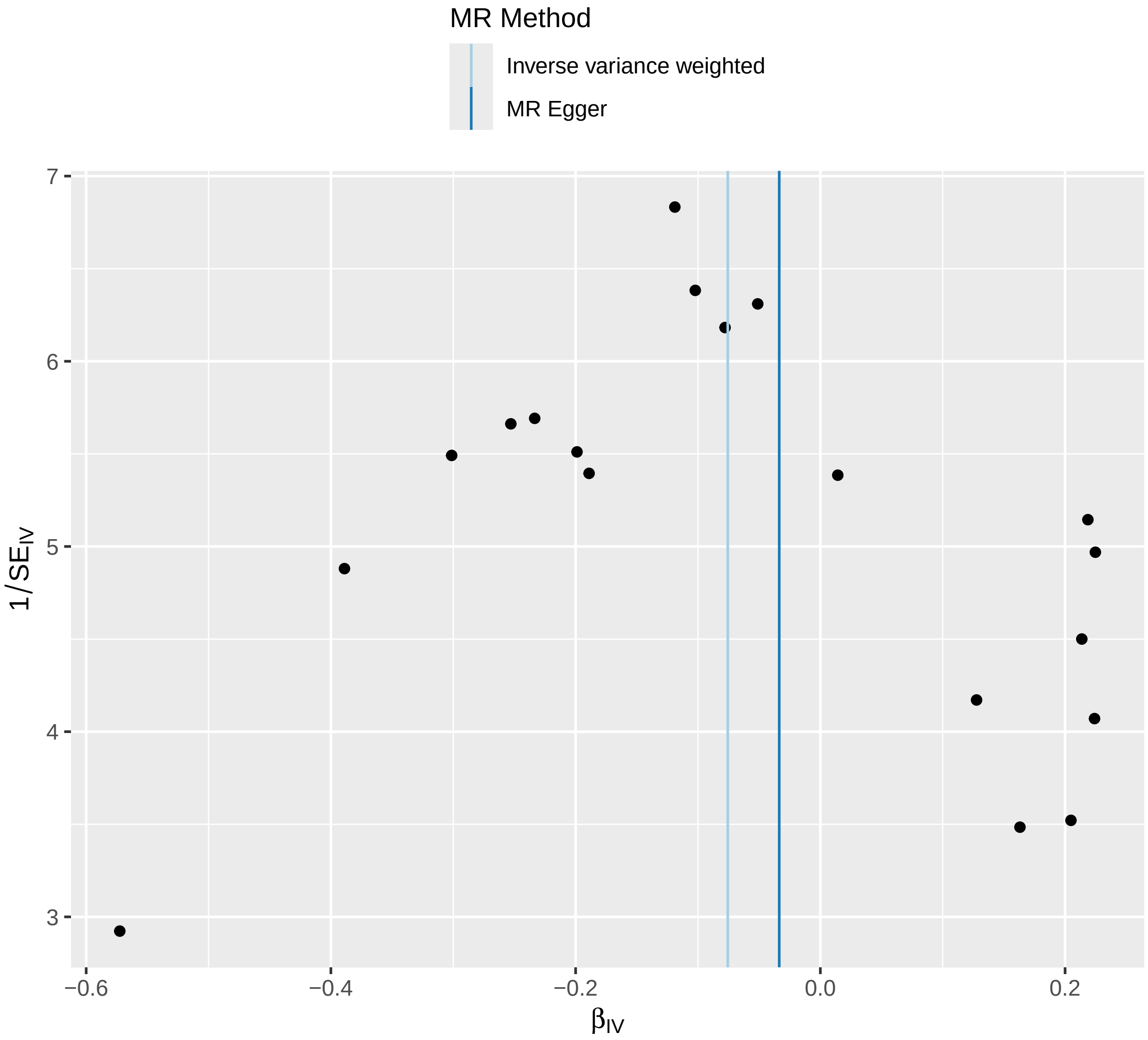
MR Method
Inverse variance weighted
MR Egger
1/SE_IV
β_IV
7
6
5
4
3
−0.6
−0.4
−0.2
0.0
0.2

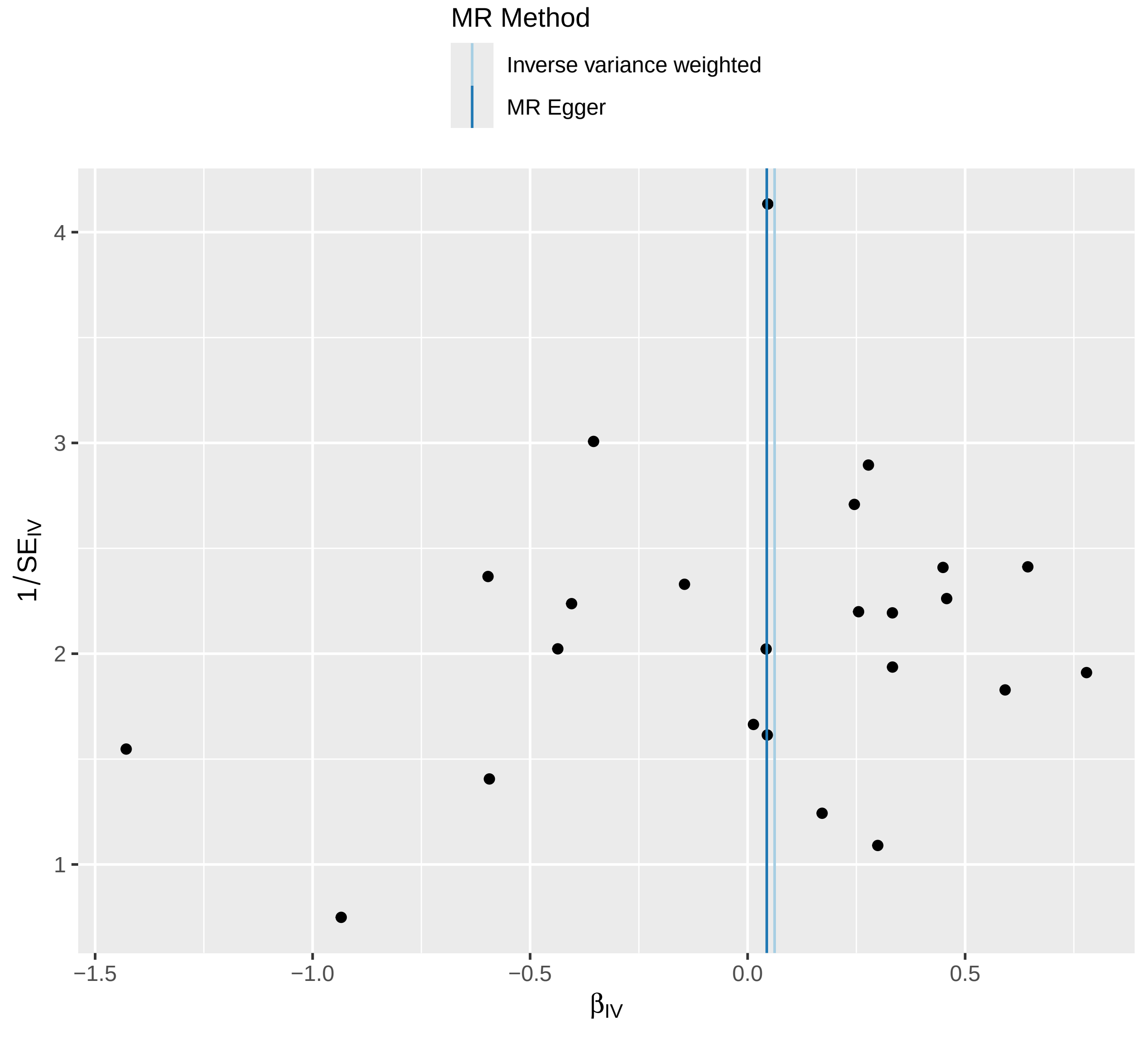
MR Method
Inverse variance weighted
MR Egger
4
3
2
1
1/SE_IV
−1.5
−1.0
−0.5
0.0
0.5
β_IV

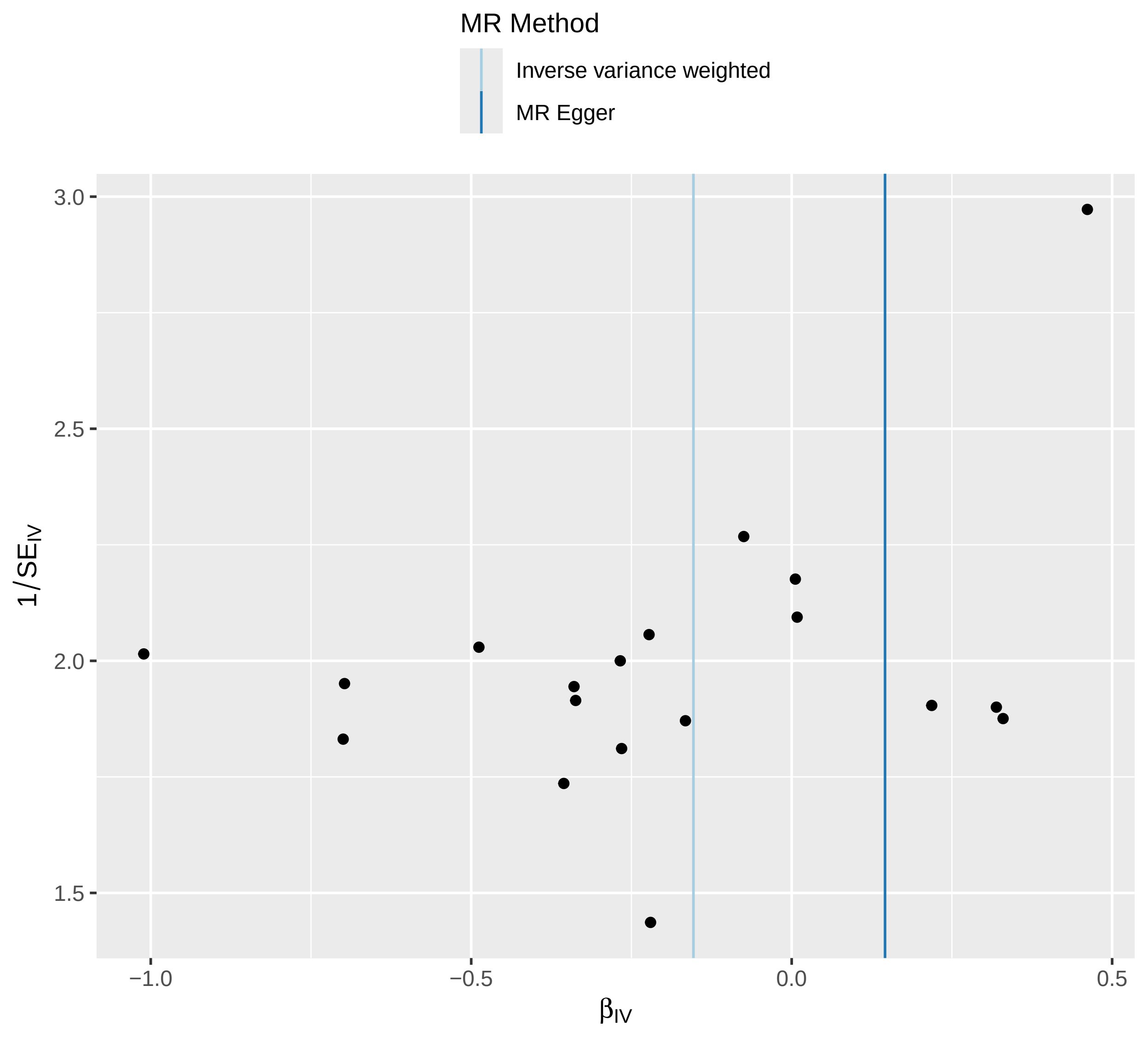
MR Method
Inverse variance weighted
MR Egger
3.0
2.5
2.0
1.5
$1/SE_{IV}$
−1.0
−0.5
0.0
0.5
β_{IV}

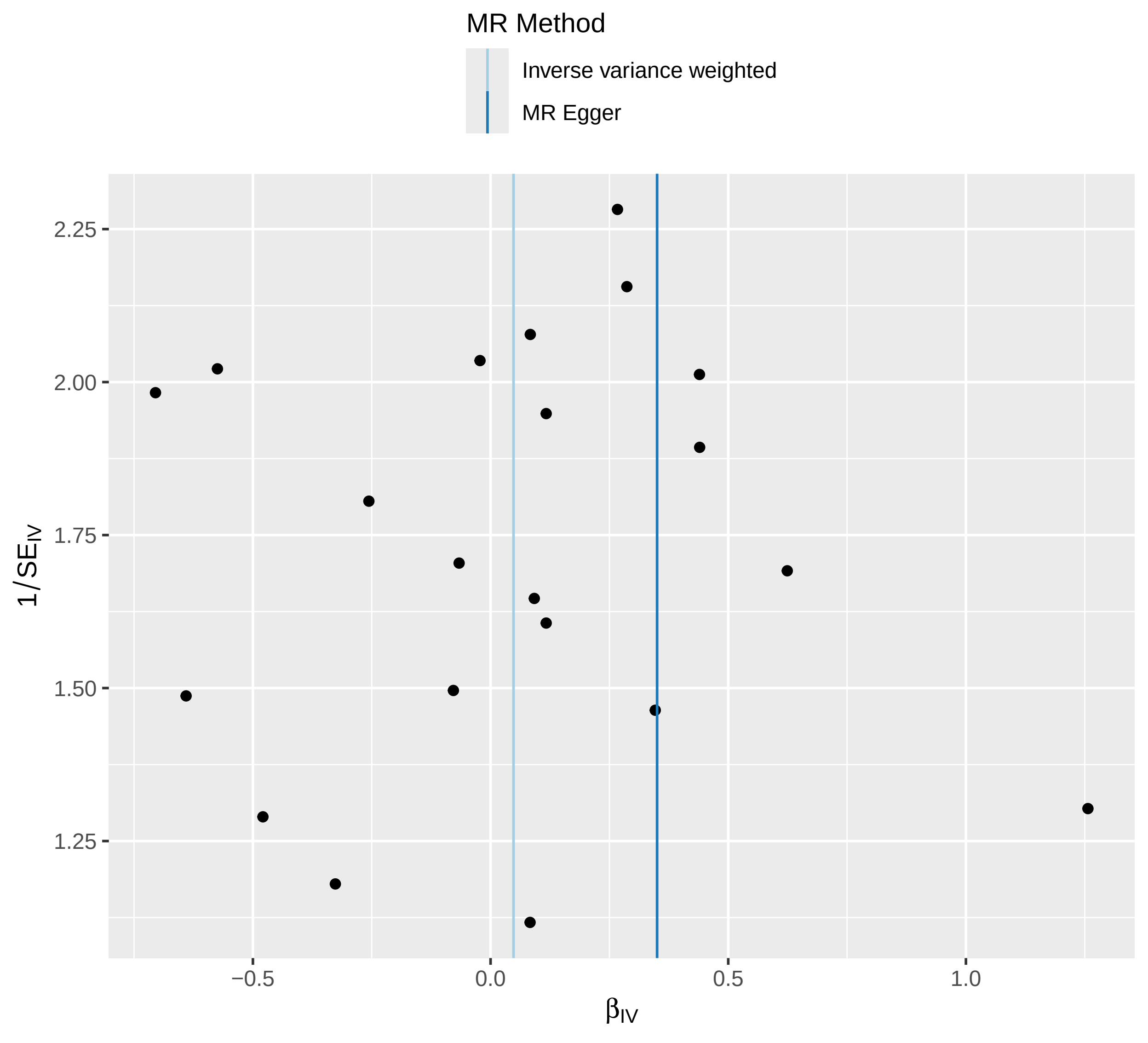

MR Method
Inverse variance weighted
MR Egger
2.25
2.00
1.75
1.50
1.25
1/SE$_{IV}$
−0.5
0.0
0.5
1.0
β$_{IV}$

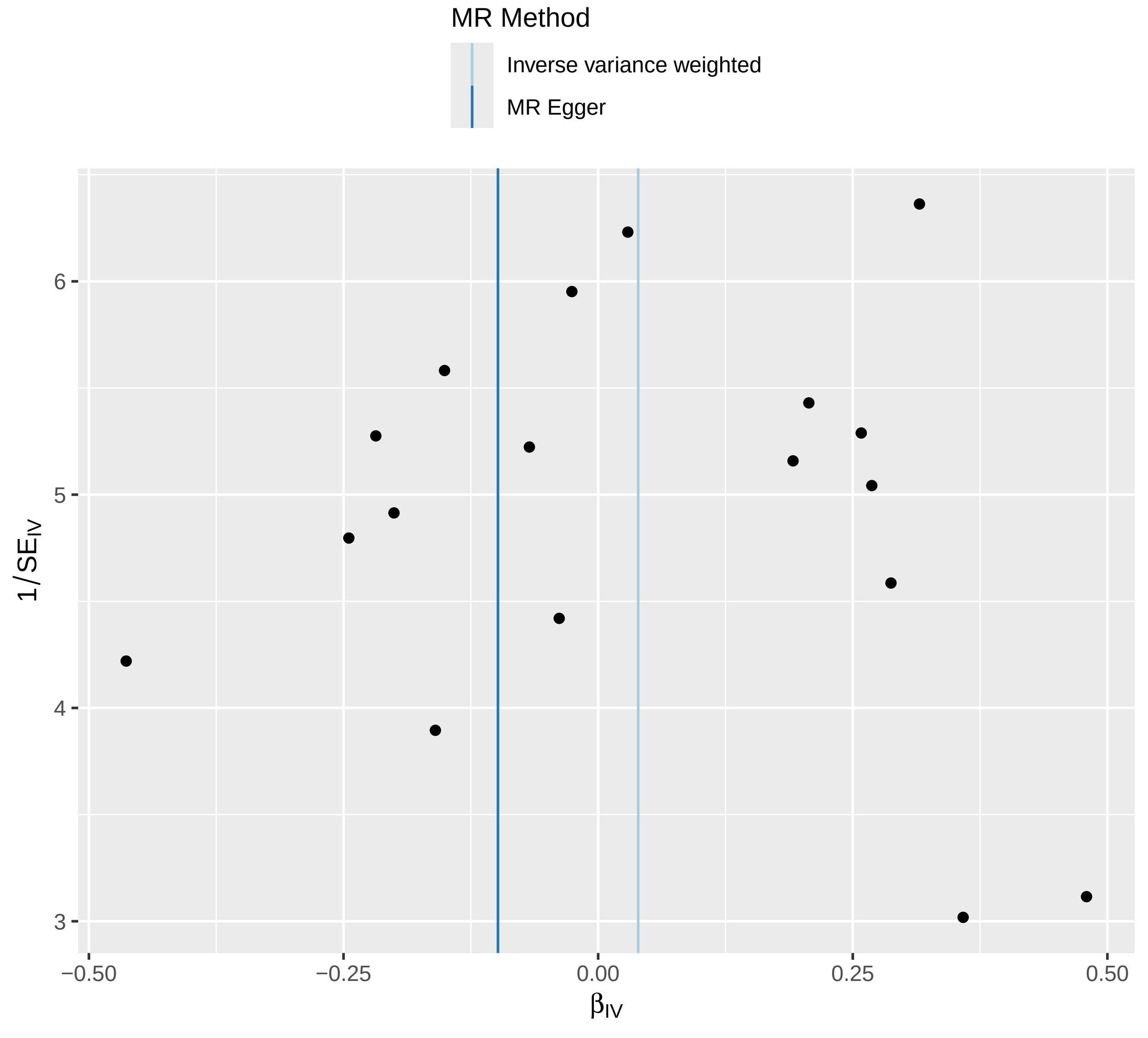
MR Method
Inverse variance weighted
MR Egger
6
5
4
3
1/SE_IV
−0.50
−0.25
0.00
0.25
0.50
β_IV

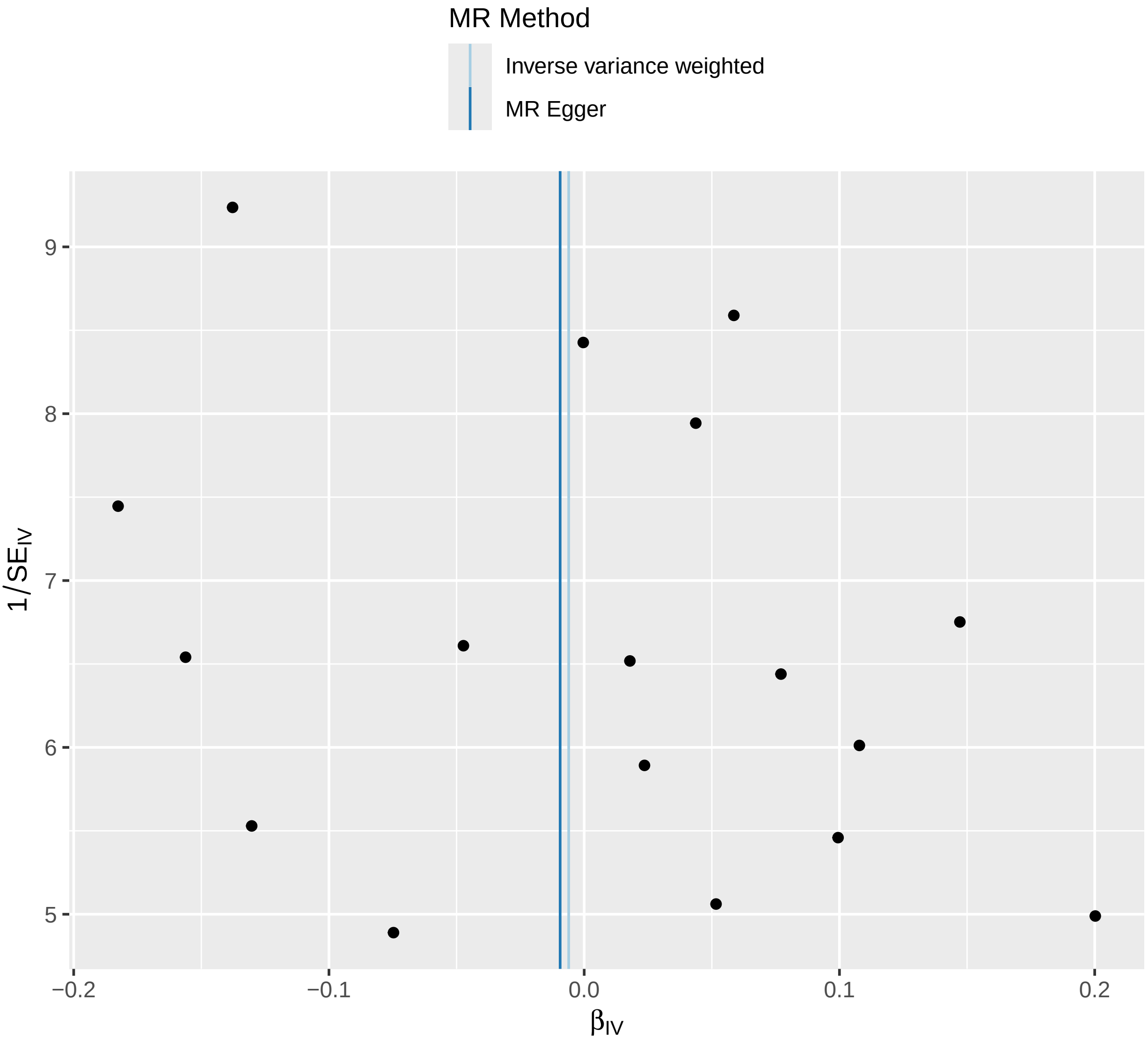

MR Method
Inverse variance weighted
MR Egger
9
8
7
6
5
1/SE_IV
−0.2
−0.1
0.0
0.1
0.2
β_IV

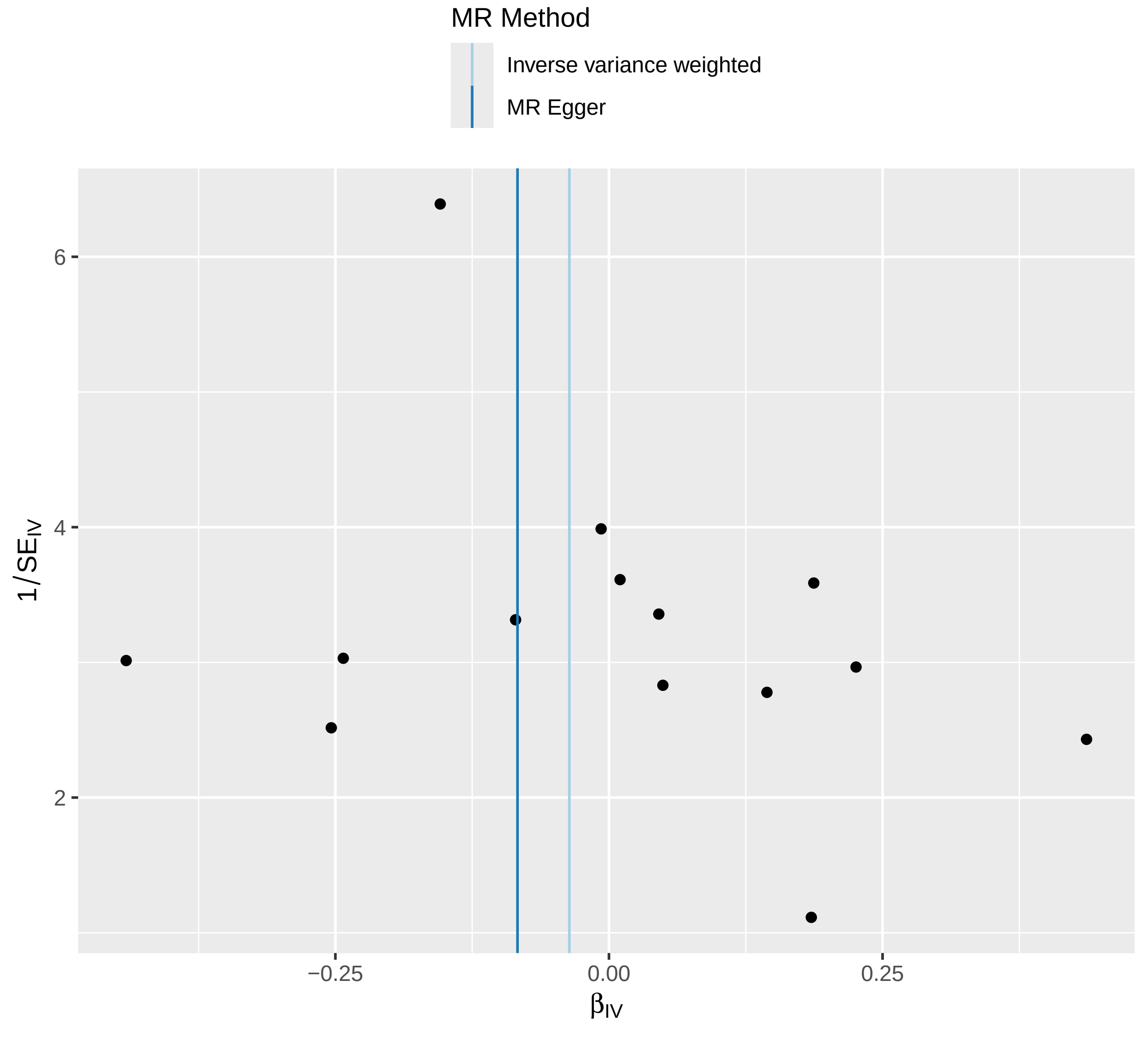

MR Method
Inverse variance weighted
MR Egger
6
4
2
1/SE$_{IV}$
−0.25
0.00
0.25
β$_{IV}$

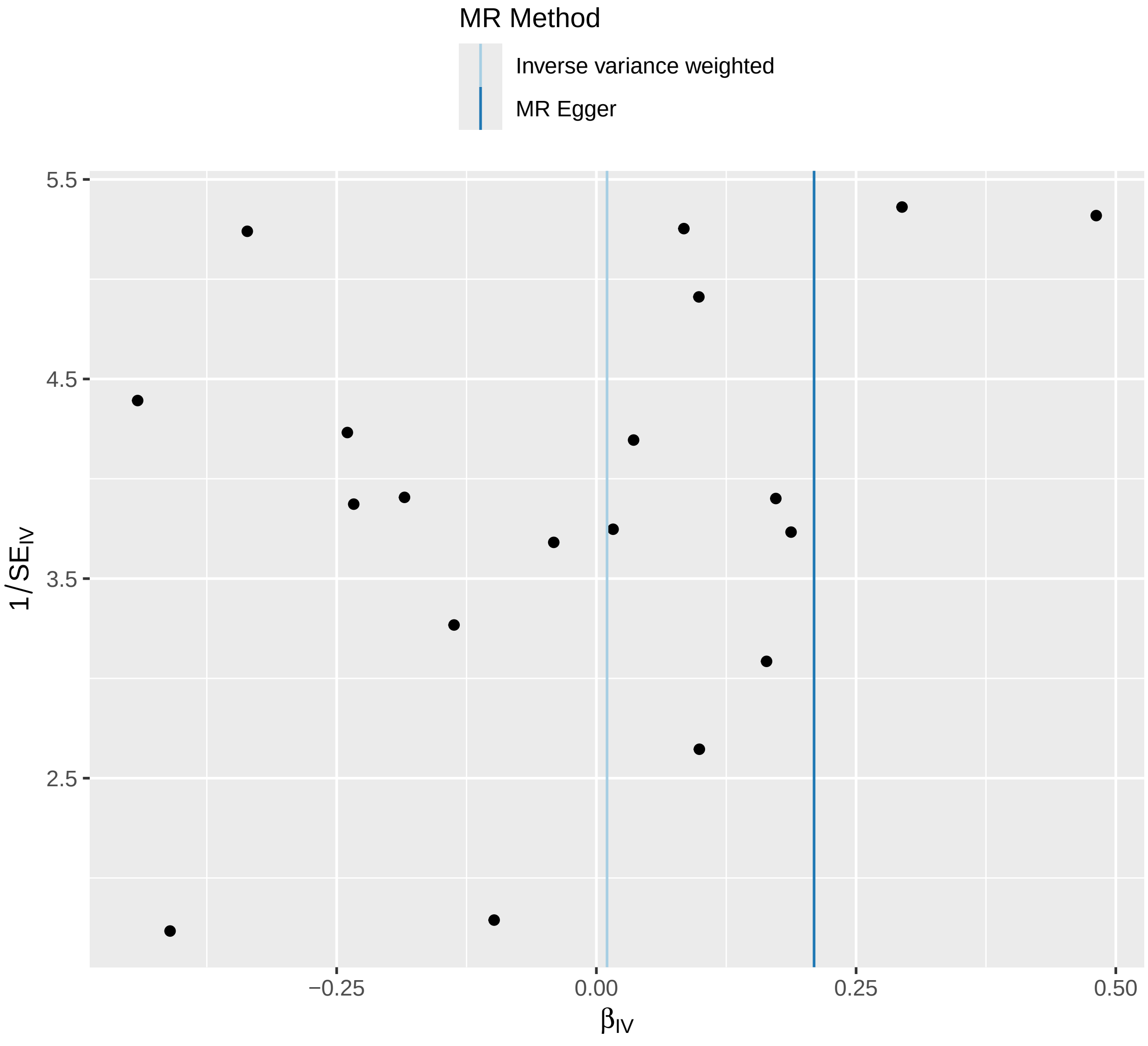

MR Method
Inverse variance weighted
MR Egger
5.5
4.5
3.5
2.5
1/SE_IV
−0.25
0.00
0.25
0.50
β_IV

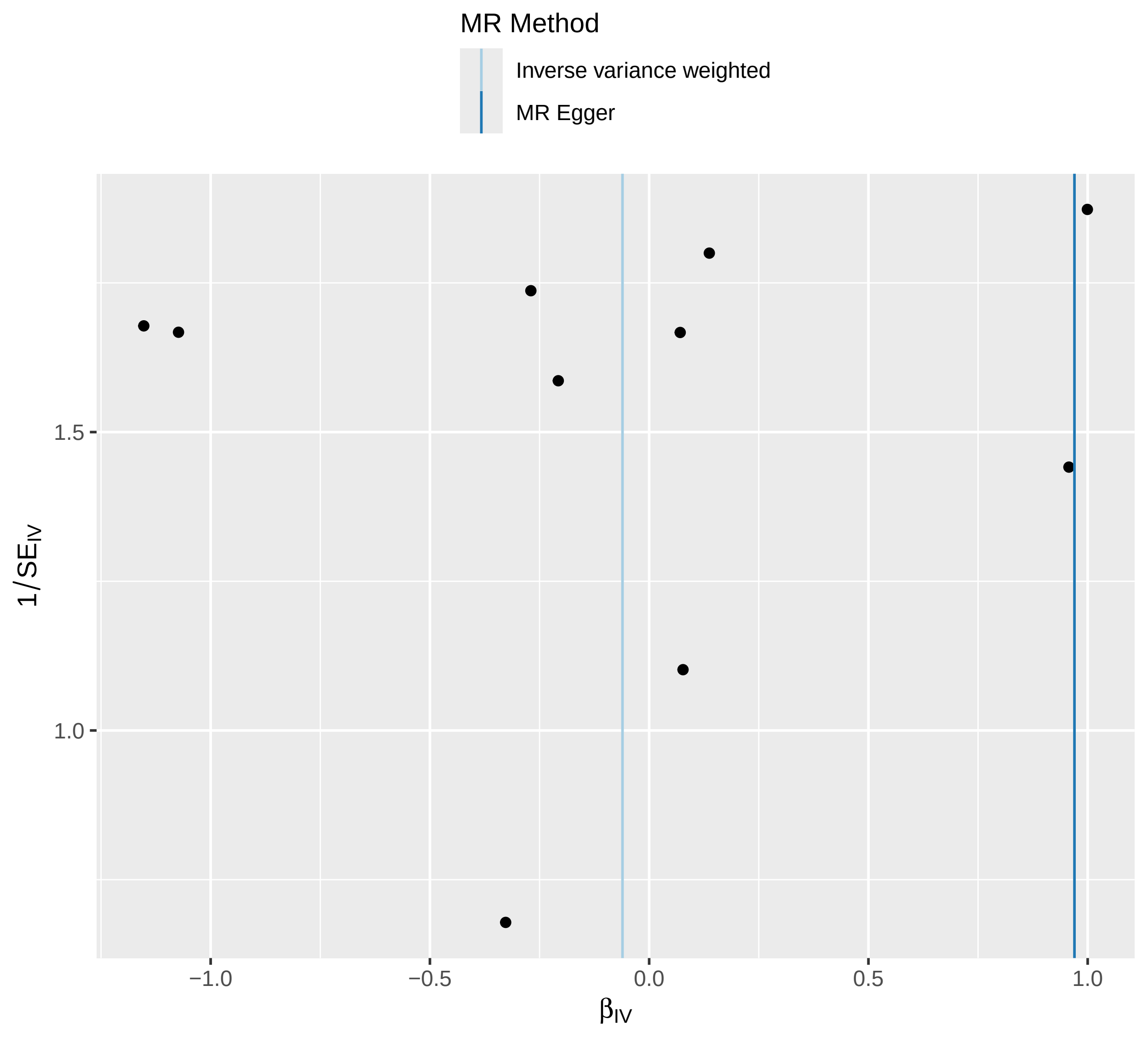

MR Method
Inverse variance weighted
MR Egger
1.5
1.0
−1.0
−0.5
0.0
0.5
1.0
$1/SE_{IV}$
β_{IV}

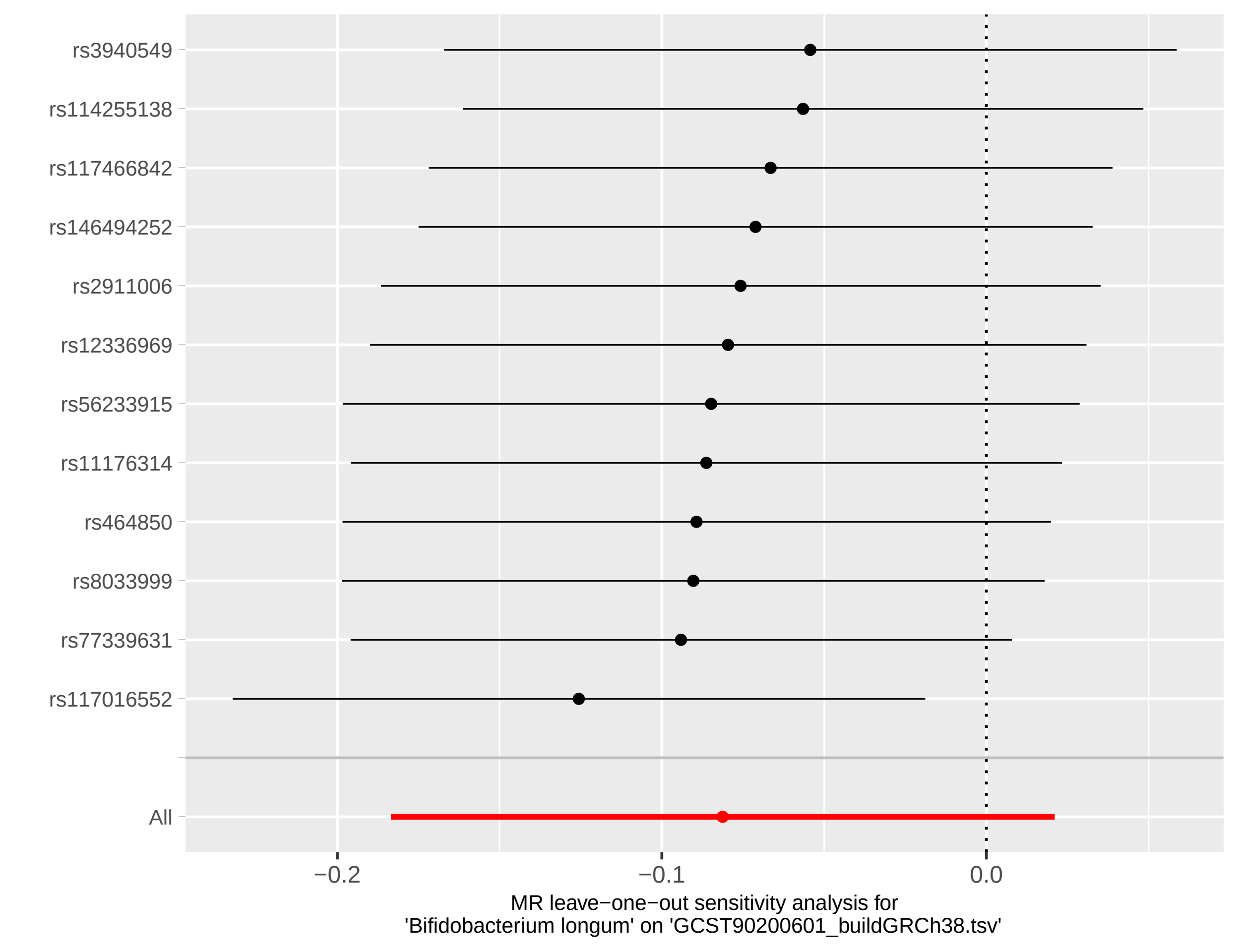

rs3940549
rs114255138
rs117466842
rs146494252
rs2911006
rs12336969
rs56233915
rs11176314
rs464850
rs8033999
rs77339631
rs117016552
All
−0.2
−0.1
0.0
MR leave−one−out sensitivity analysis for
'Bifidobacterium longum' on 'GCST90200601_buildGRCh38.tsv'

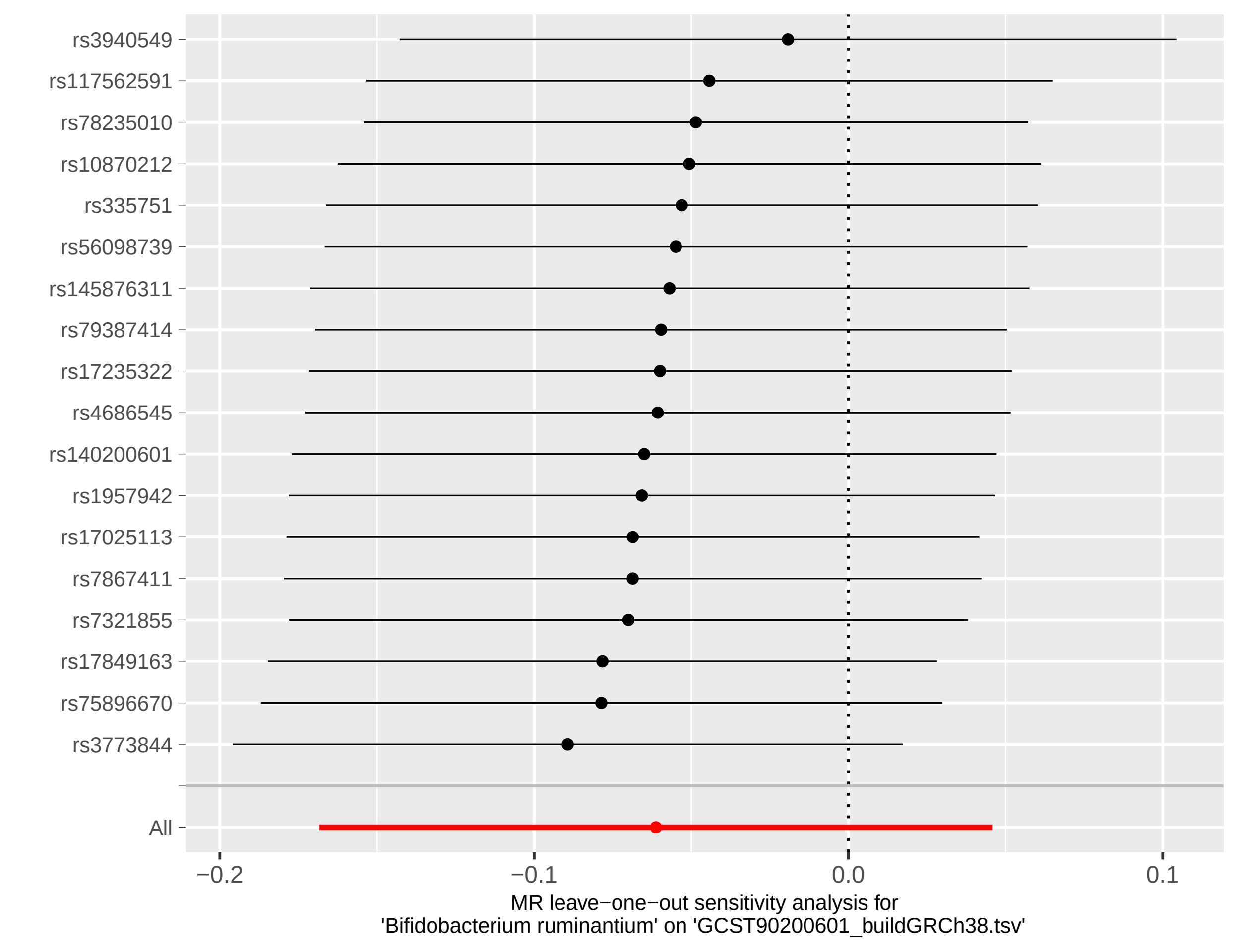
rs3940549
rs117562591
rs78235010
rs10870212
rs335751
rs56098739
rs145876311
rs79387414
rs17235322
rs4686545
rs140200601
rs1957942
rs17025113
rs7867411
rs7321855
rs17849163
rs75896670
rs3773844
All
−0.2
−0.1
0.0
0.1
MR leave−one−out sensitivity analysis for
'Bifidobacterium ruminantium' on 'GCST90200601_buildGRCh38.tsv'

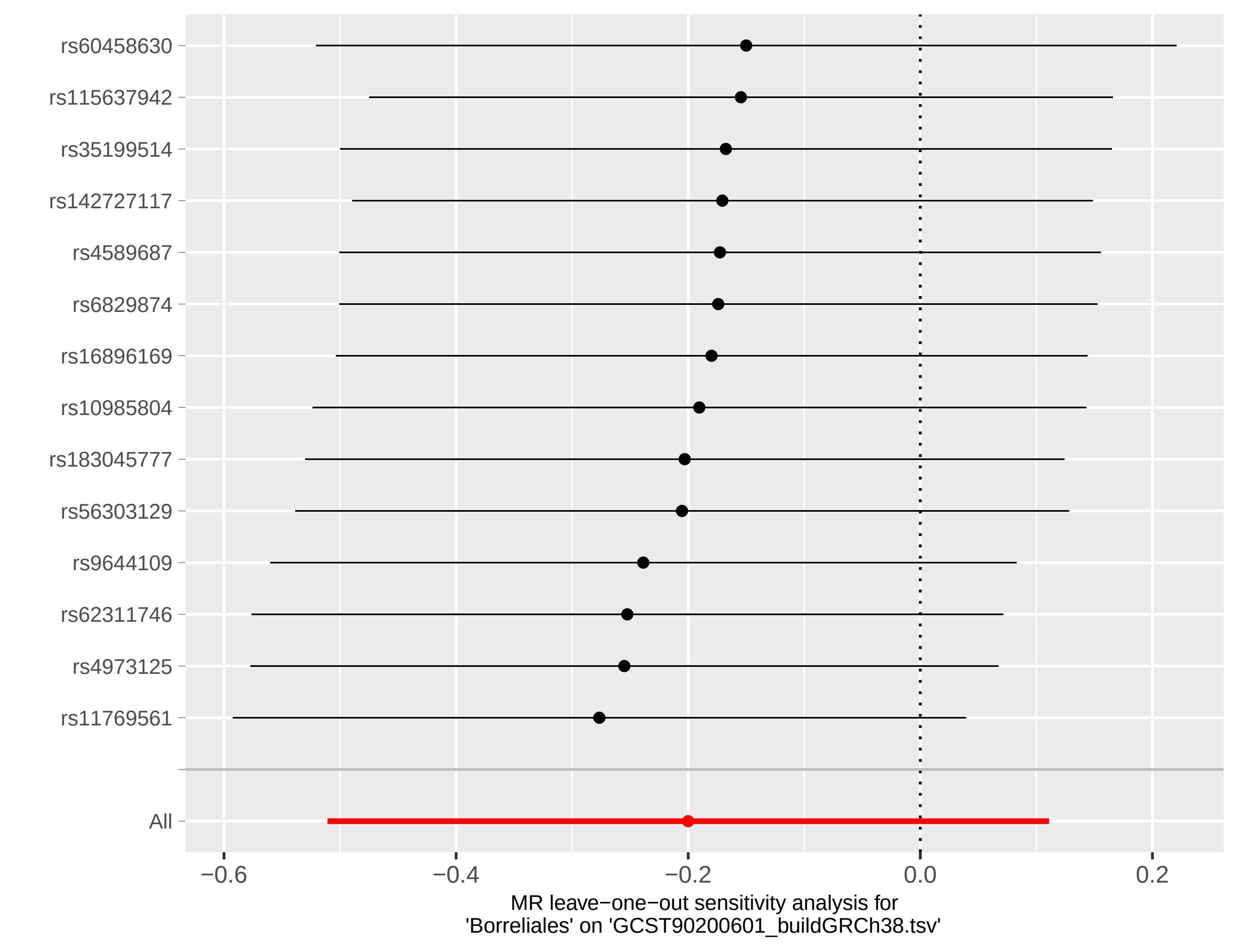
rs60458630
rs115637942
rs35199514
rs142727117
rs4589687
rs6829874
rs16896169
rs10985804
rs183045777
rs56303129
rs9644109
rs62311746
rs4973125
rs11769561
All
−0.6
−0.4
−0.2
0.0
0.2
MR leave−one−out sensitivity analysis for
'Borreliales' on 'GCST90200601_buildGRCh38.tsv'

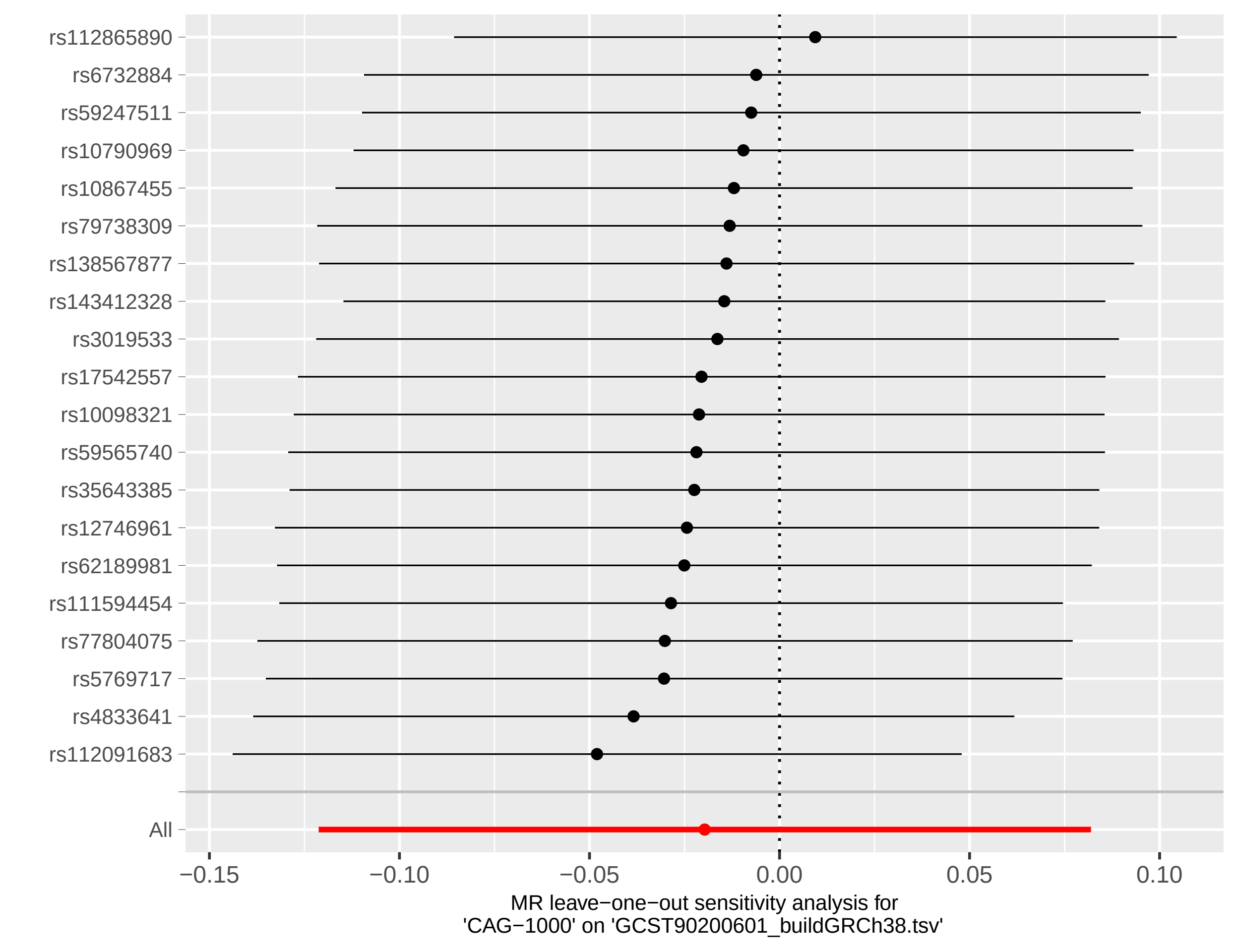

rs112865890
rs6732884
rs59247511
rs10790969
rs10867455
rs79738309
rs138567877
rs143412328
rs3019533
rs17542557
rs10098321
rs59565740
rs35643385
rs12746961
rs62189981
rs111594454
rs77804075
rs5769717
rs4833641
rs112091683
All
−0.15
−0.10
−0.05
0.00
0.05
0.10
MR leave−one−out sensitivity analysis for
'CAG−1000' on 'GCST90200601_buildGRCh38.tsv'

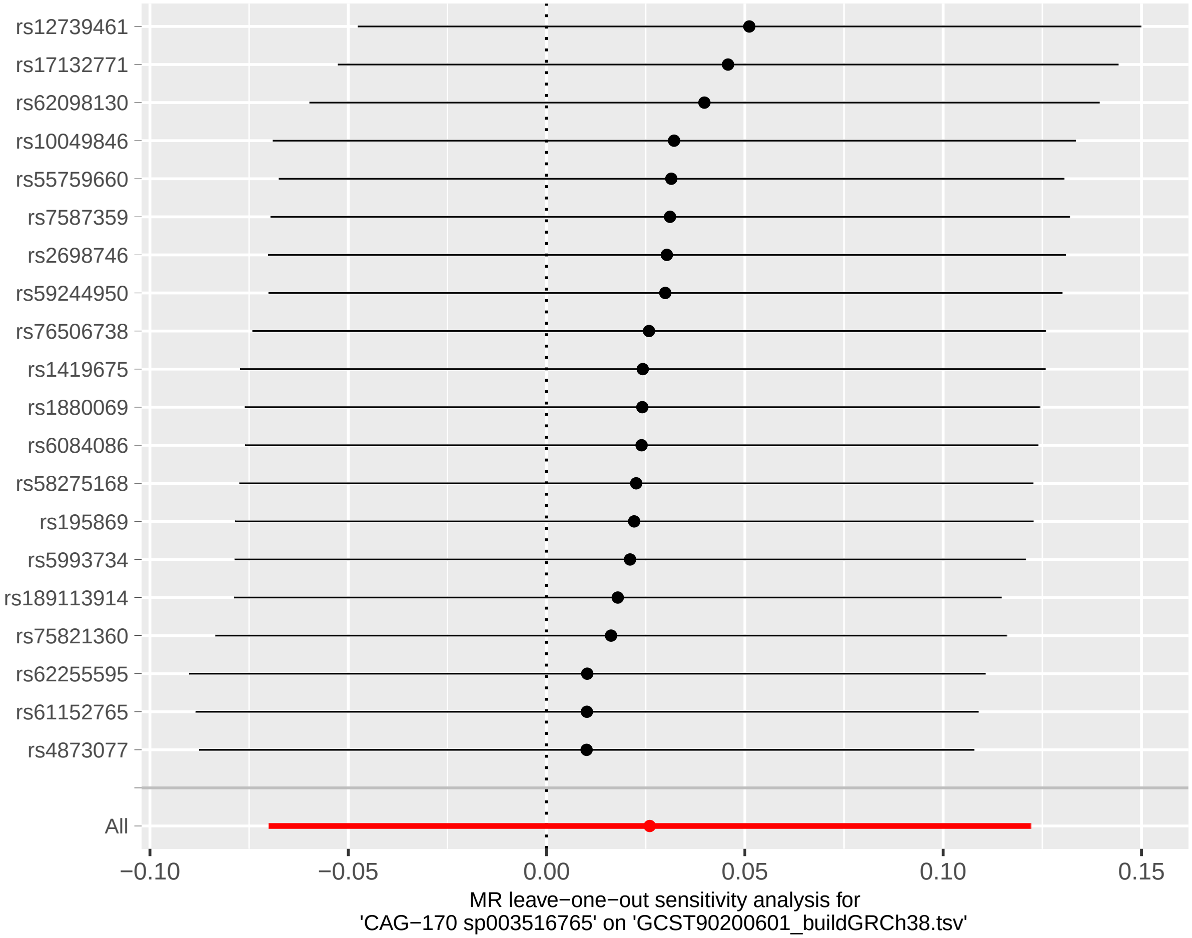
rs12739461
rs17132771
rs62098130
rs10049846
rs55759660
rs7587359
rs2698746
rs59244950
rs76506738
rs1419675
rs1880069
rs6084086
rs58275168
rs195869
rs5993734
rs189113914
rs75821360
rs62255595
rs61152765
rs4873077
All
−0.10
−0.05
0.00
0.05
0.10
0.15
MR leave−one−out sensitivity analysis for
'CAG−170 sp003516765' on 'GCST90200601_buildGRCh38.tsv'

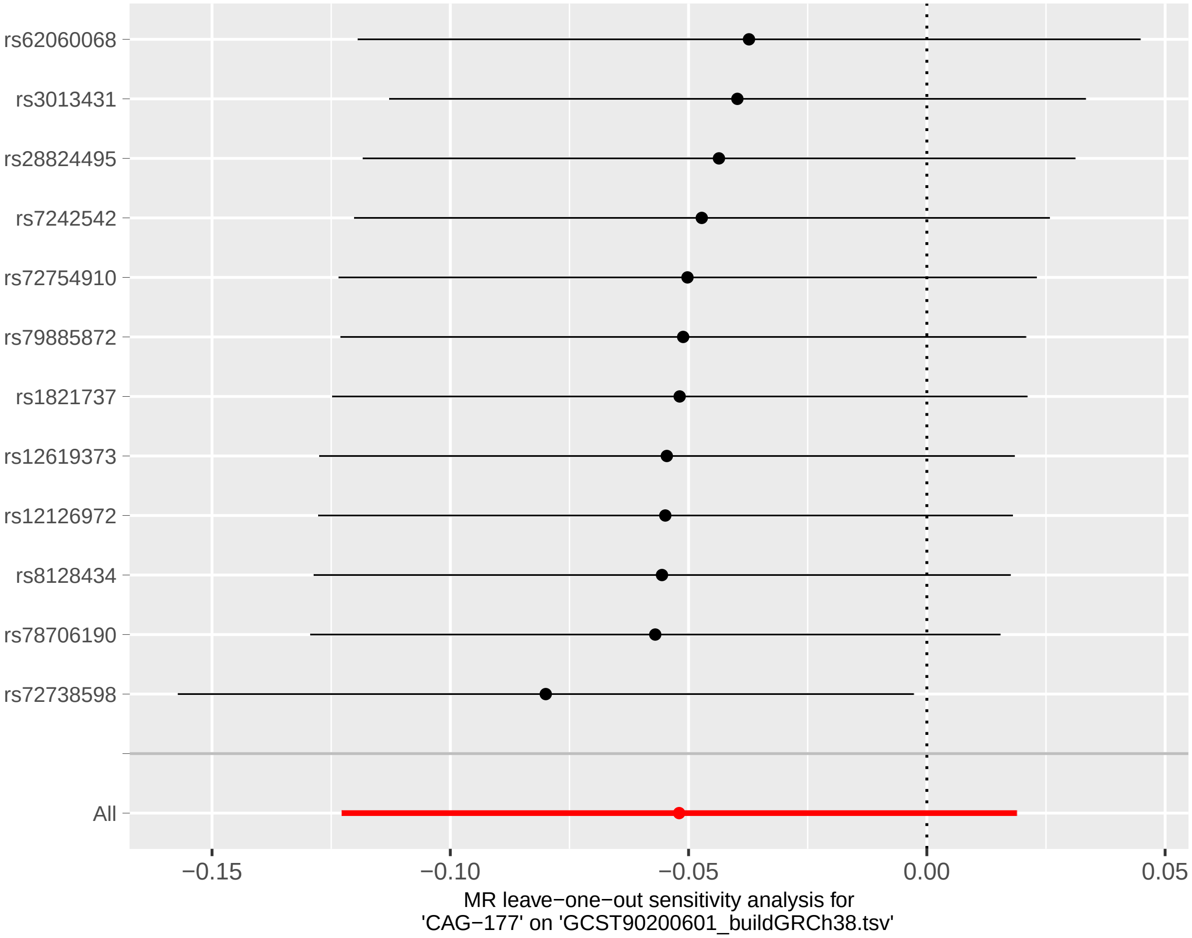
rs62060068
rs3013431
rs28824495
rs7242542
rs72754910
rs79885872
rs1821737
rs12619373
rs12126972
rs8128434
rs78706190
rs72738598
All
−0.15
−0.10
−0.05
0.00
0.05
MR leave−one−out sensitivity analysis for
'CAG−177' on 'GCST90200601_buildGRCh38.tsv'

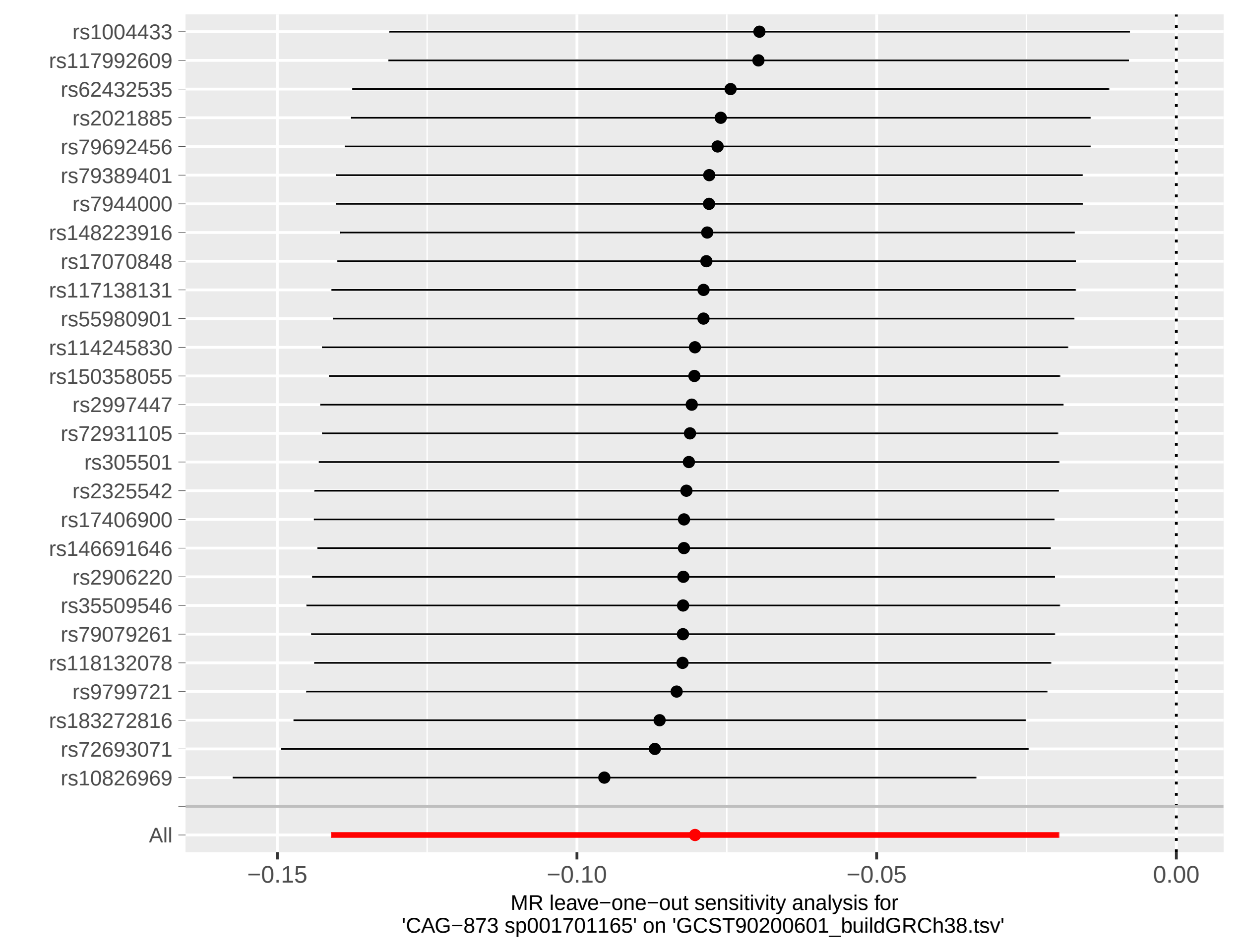

rs1004433
rs117992609
rs62432535
rs2021885
rs79692456
rs79389401
rs7944000
rs148223916
rs17070848
rs117138131
rs55980901
rs114245830
rs150358055
rs2997447
rs72931105
rs305501
rs2325542
rs17406900
rs146691646
rs2906220
rs35509546
rs79079261
rs118132078
rs9799721
rs183272816
rs72693071
rs10826969
All
−0.15
−0.10
−0.05
0.00
MR leave−one−out sensitivity analysis for
'CAG−873 sp001701165' on 'GCST90200601_buildGRCh38.tsv'

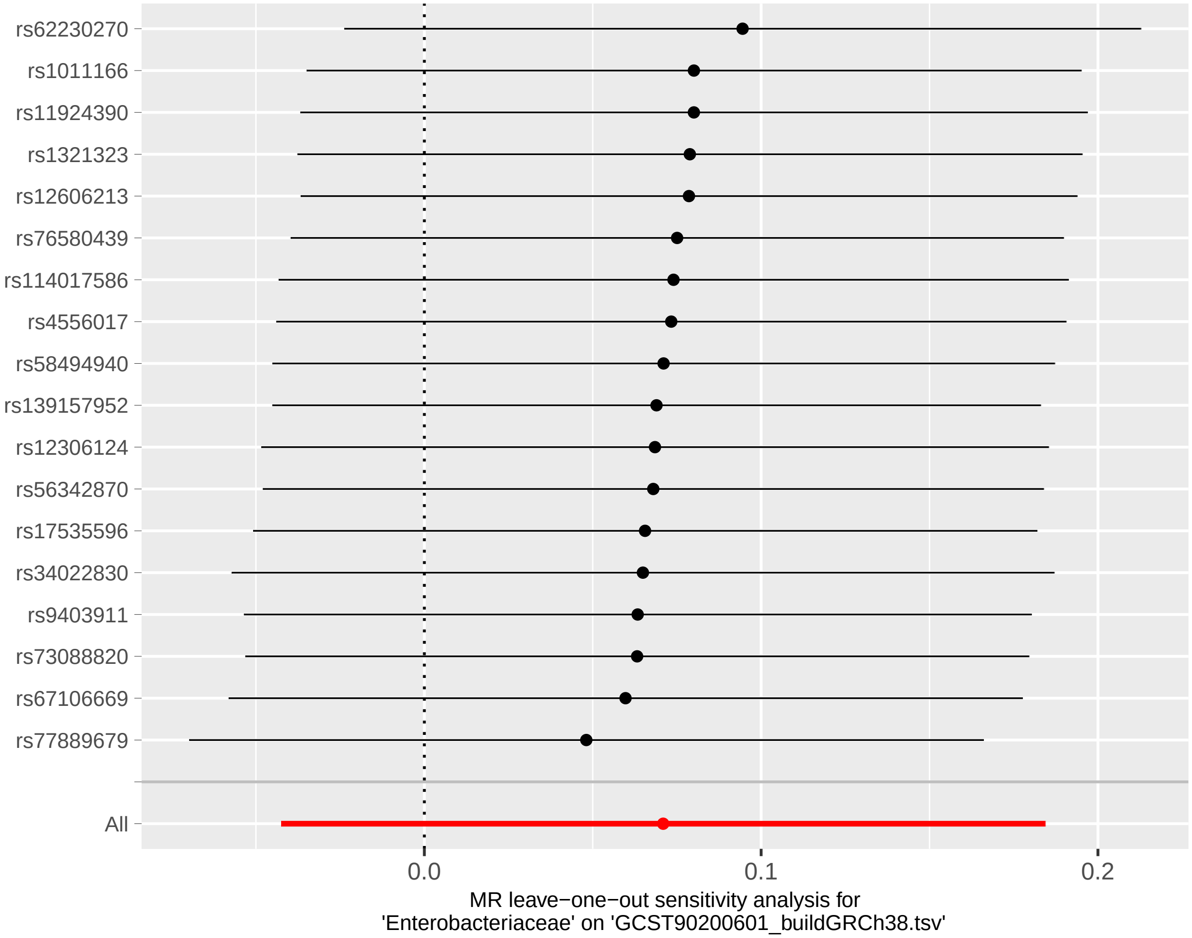

rs62230270
rs1011166
rs11924390
rs1321323
rs12606213
rs76580439
rs114017586
rs4556017
rs58494940
rs139157952
rs12306124
rs56342870
rs17535596
rs34022830
rs9403911
rs73088820
rs67106669
rs77889679
All
0.0
0.1
0.2
MR leave−one−out sensitivity analysis for
'Enterobacteriaceae' on 'GCST90200601_buildGRCh38.tsv'

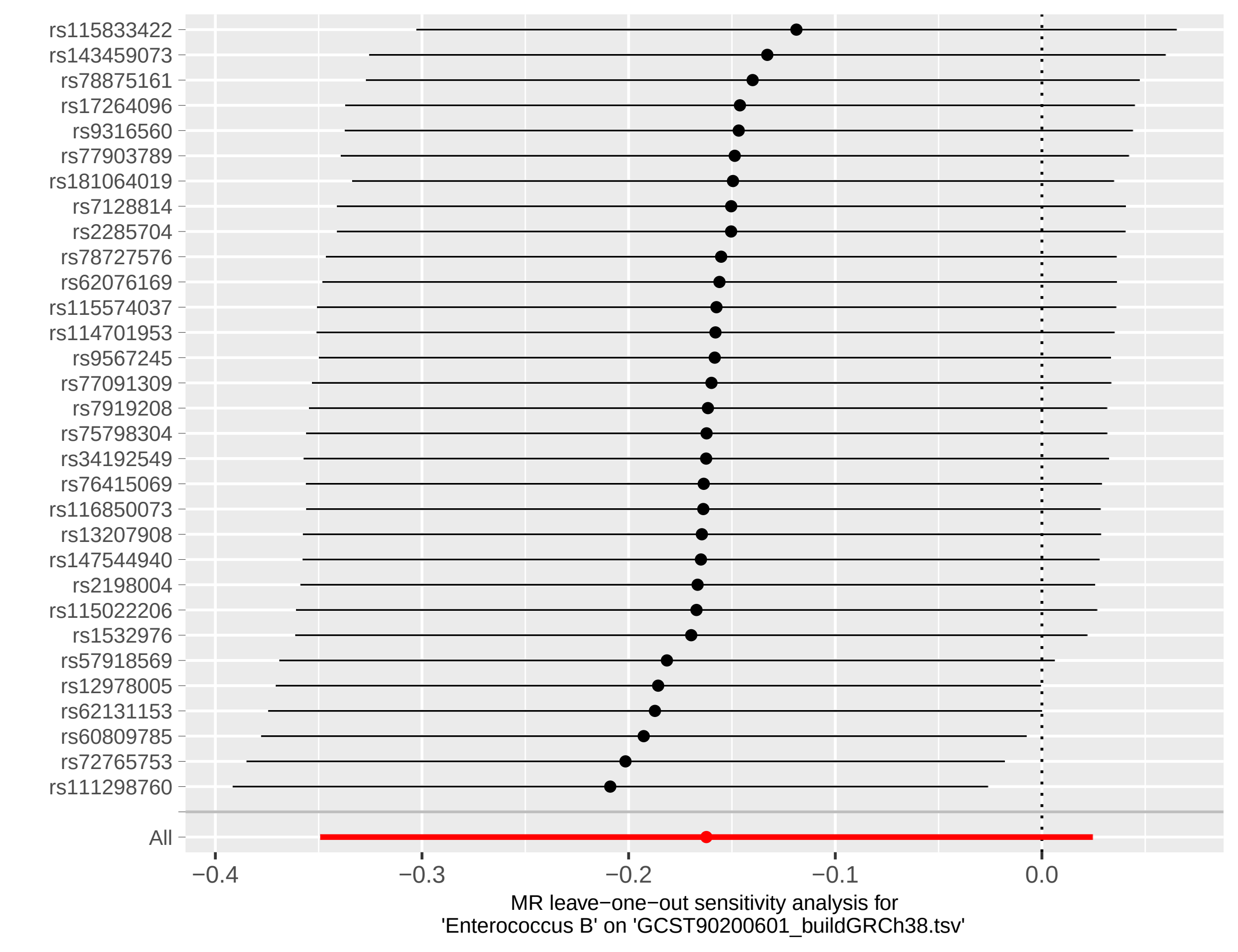

rs115833422
rs143459073
rs78875161
rs17264096
rs9316560
rs77903789
rs181064019
rs7128814
rs2285704
rs78727576
rs62076169
rs115574037
rs114701953
rs9567245
rs77091309
rs7919208
rs75798304
rs34192549
rs76415069
rs116850073
rs13207908
rs147544940
rs2198004
rs115022206
rs1532976
rs57918569
rs12978005
rs62131153
rs60809785
rs72765753
rs111298760
All
−0.4
−0.3
−0.2
−0.1
0.0
MR leave−one−out sensitivity analysis for
'Enterococcus B' on 'GCST90200601_buildGRCh38.tsv'

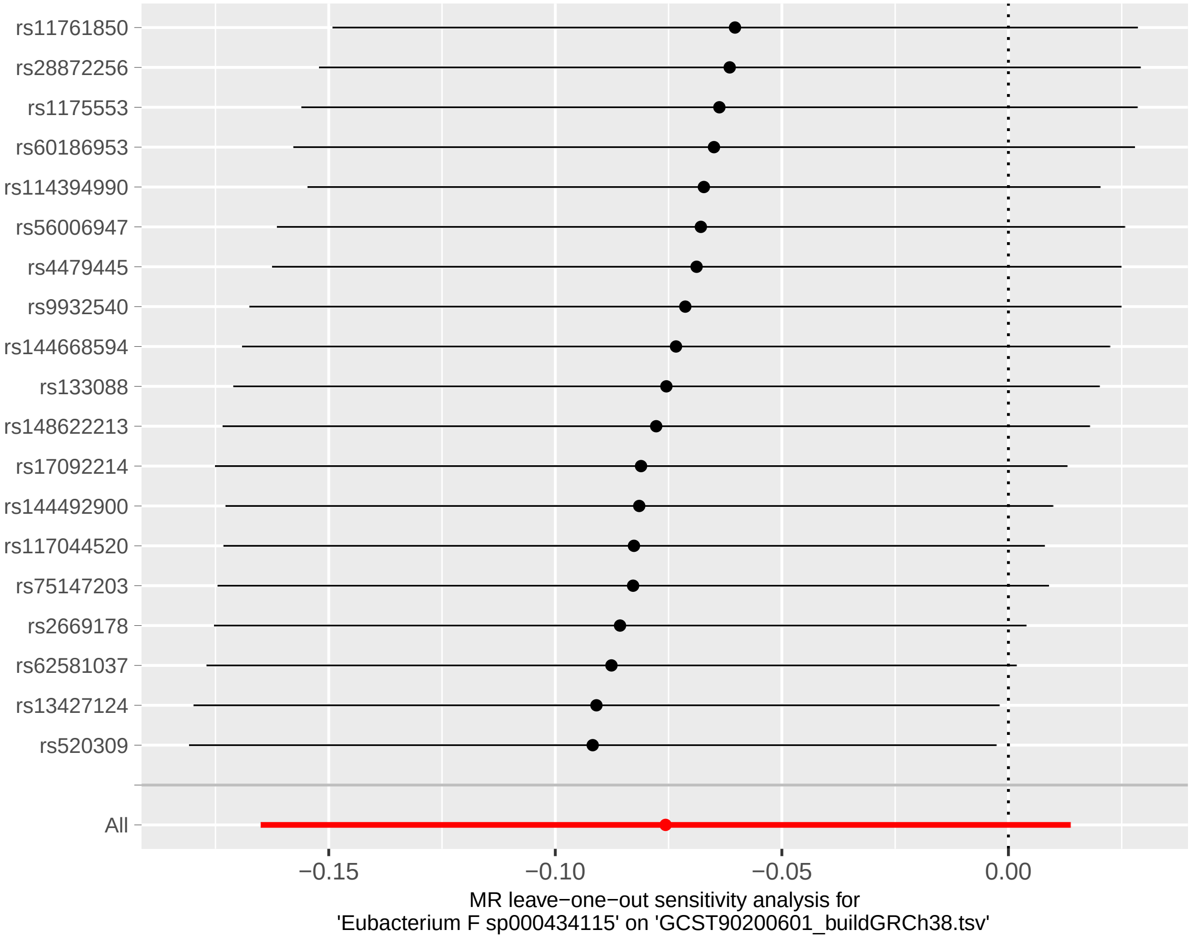
rs11761850
rs28872256
rs1175553
rs60186953
rs114394990
rs56006947
rs4479445
rs9932540
rs144668594
rs133088
rs148622213
rs17092214
rs144492900
rs117044520
rs75147203
rs2669178
rs62581037
rs13427124
rs520309
All
−0.15
−0.10
−0.05
0.00
MR leave−one−out sensitivity analysis for
'Eubacterium F sp000434115' on 'GCST90200601_buildGRCh38.tsv'

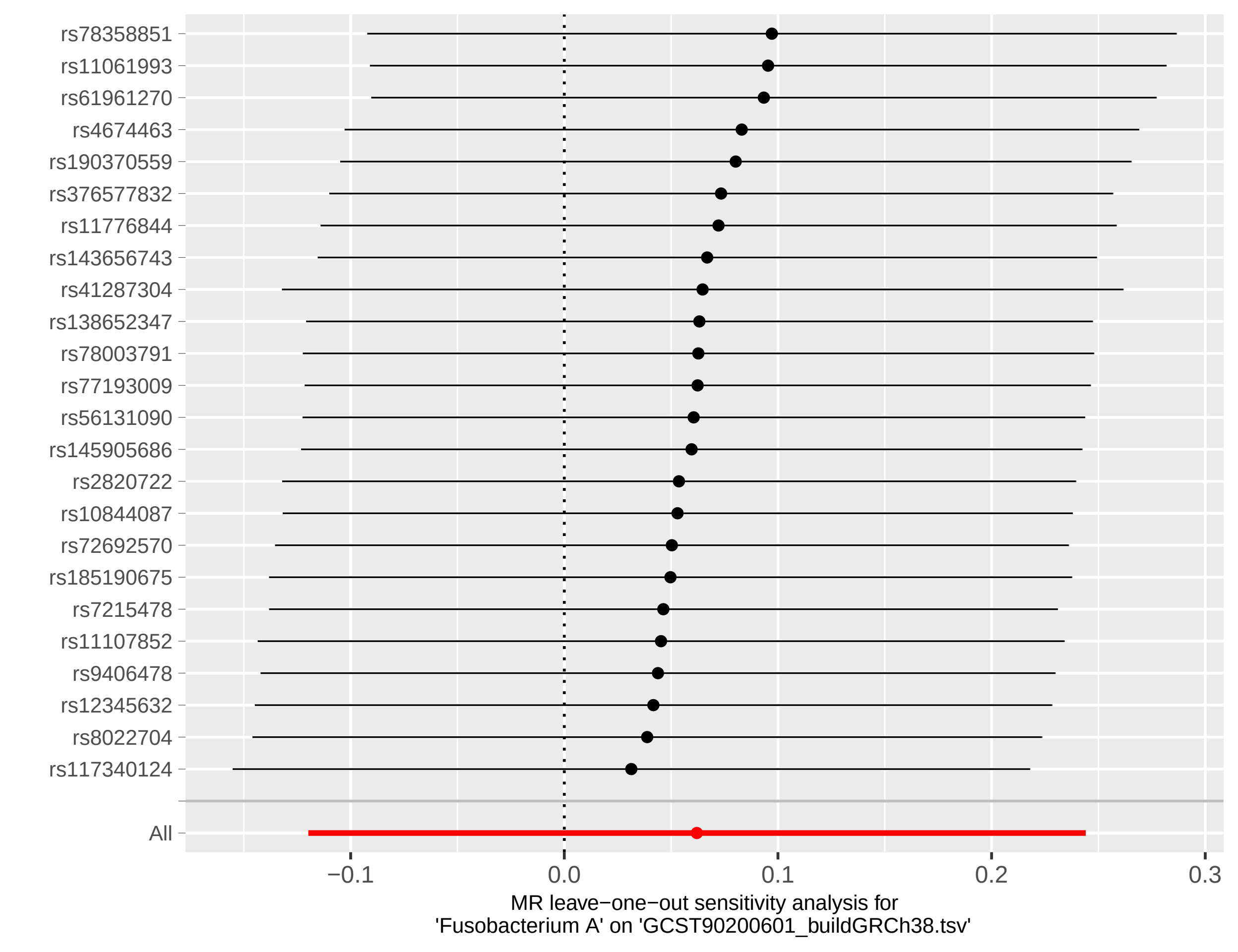
rs78358851
rs11061993
rs61961270
rs4674463
rs190370559
rs376577832
rs11776844
rs143656743
rs41287304
rs138652347
rs78003791
rs77193009
rs56131090
rs145905686
rs2820722
rs10844087
rs72692570
rs185190675
rs7215478
rs11107852
rs9406478
rs12345632
rs8022704
rs117340124
All
−0.1
0.0
0.1
0.2
0.3
MR leave−one−out sensitivity analysis for
'Fusobacterium A' on 'GCST90200601_buildGRCh38.tsv'

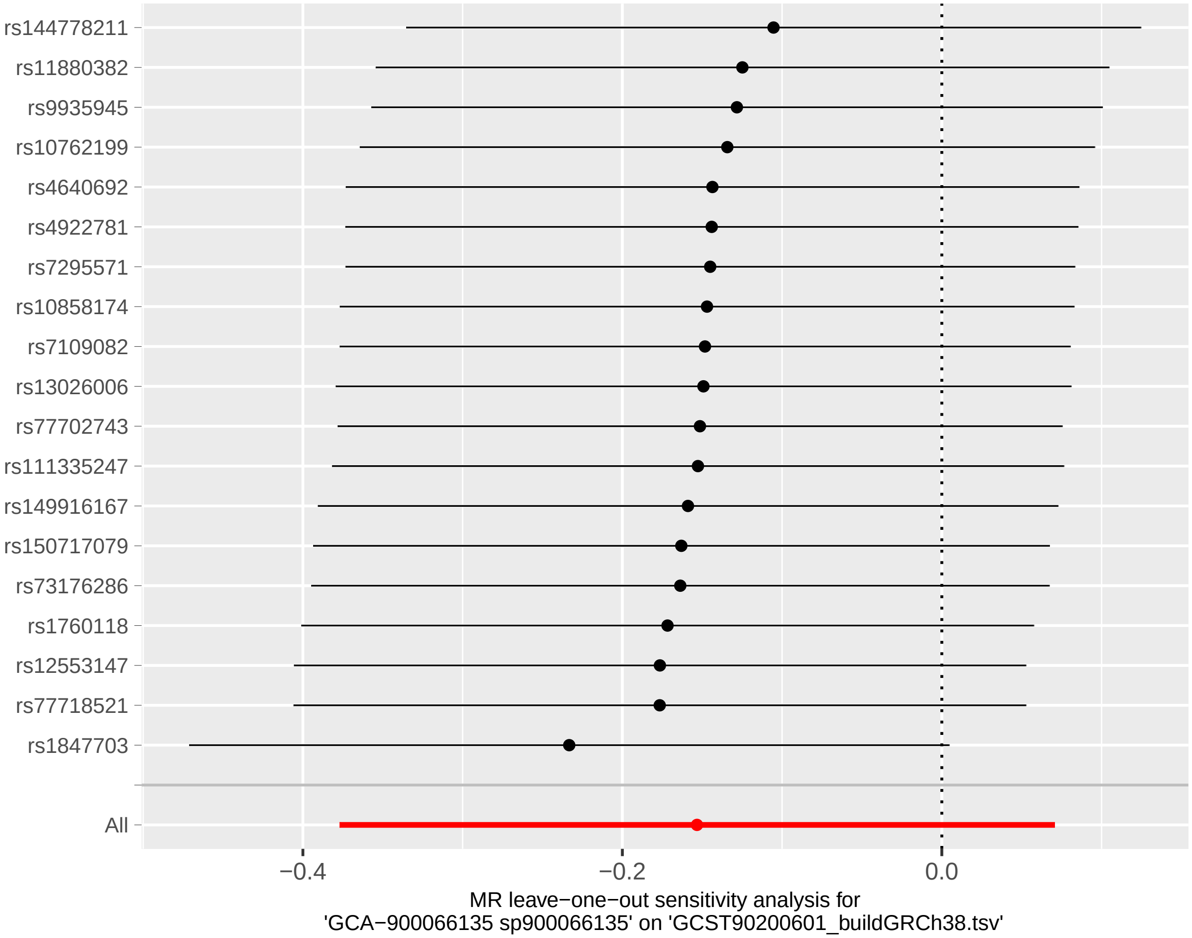
rs144778211
rs11880382
rs9935945
rs10762199
rs4640692
rs4922781
rs7295571
rs10858174
rs7109082
rs13026006
rs77702743
rs111335247
rs149916167
rs150717079
rs73176286
rs1760118
rs12553147
rs77718521
rs1847703
All
−0.4
−0.2
0.0
MR leave−one−out sensitivity analysis for
'GCA−900066135 sp900066135' on 'GCST90200601_buildGRCh38.tsv'

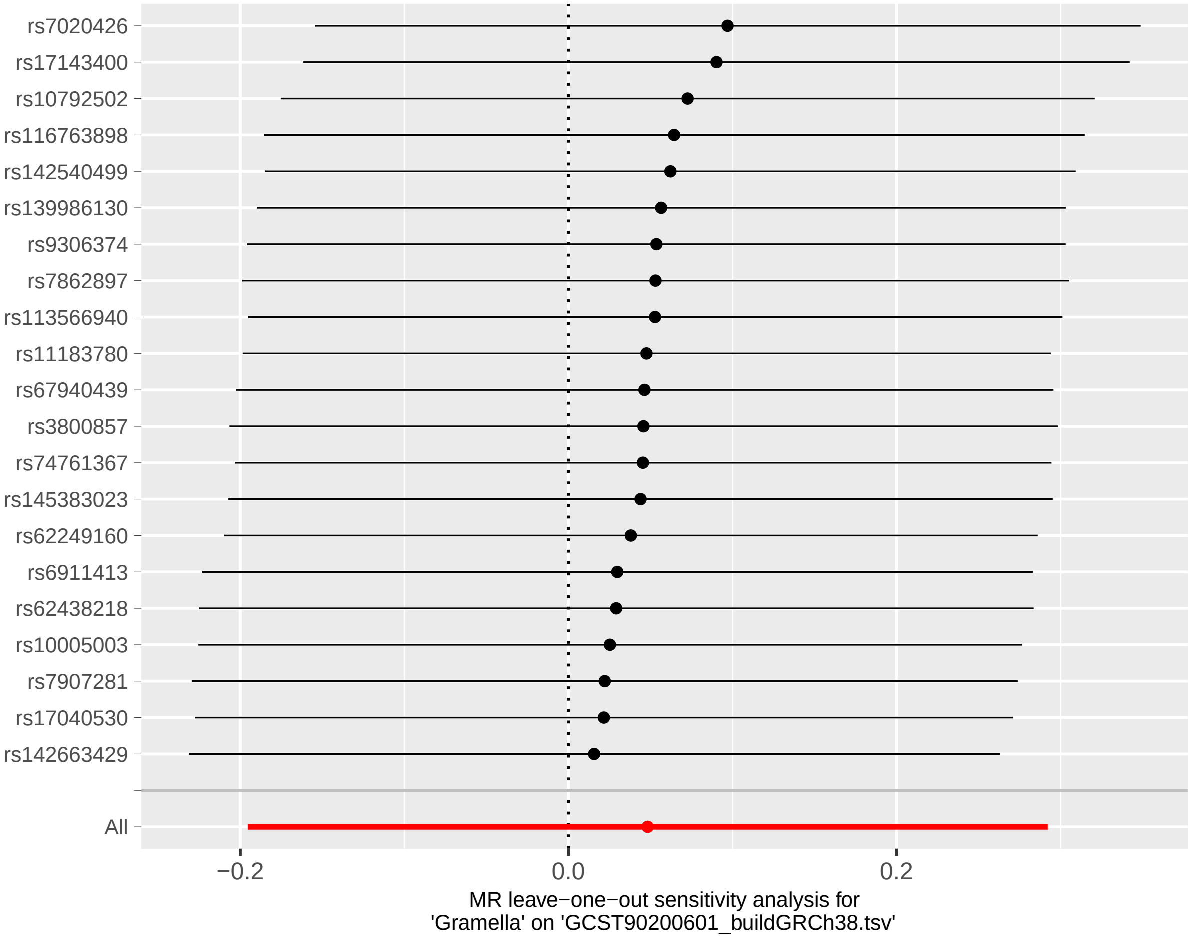
rs7020426
rs17143400
rs10792502
rs116763898
rs142540499
rs139986130
rs9306374
rs7862897
rs113566940
rs11183780
rs67940439
rs3800857
rs74761367
rs145383023
rs62249160
rs6911413
rs62438218
rs10005003
rs7907281
rs17040530
rs142663429
All
−0.2
0.0
0.2
MR leave−one−out sensitivity analysis for
'Gramella' on 'GCST90200601_buildGRCh38.tsv'

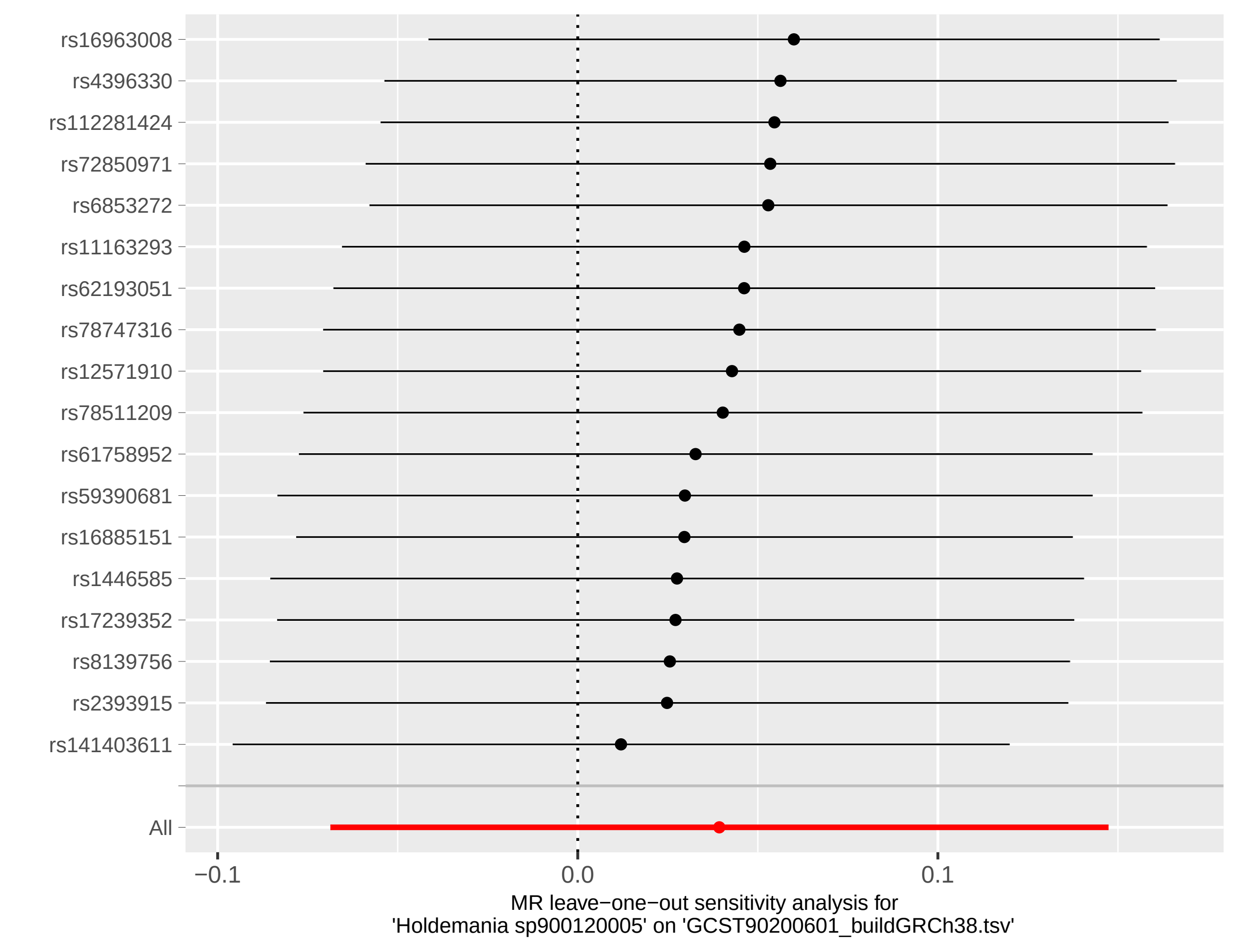
rs16963008
rs4396330
rs112281424
rs72850971
rs6853272
rs11163293
rs62193051
rs78747316
rs12571910
rs78511209
rs61758952
rs59390681
rs16885151
rs1446585
rs17239352
rs8139756
rs2393915
rs141403611
All
−0.1
0.0
0.1
MR leave−one−out sensitivity analysis for
'Holdemania sp900120005' on 'GCST90200601_buildGRCh38.tsv'

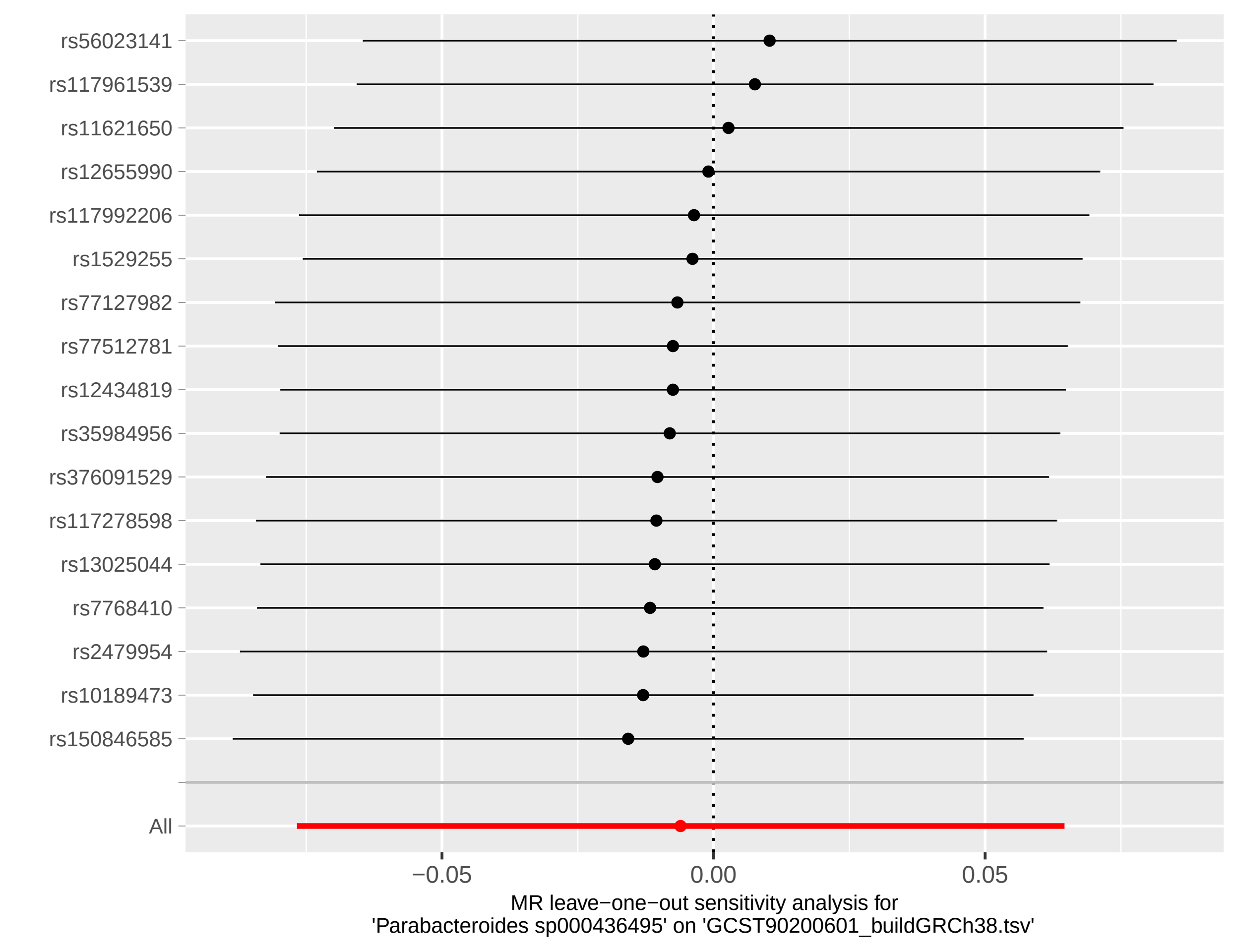

rs56023141
rs117961539
rs11621650
rs12655990
rs117992206
rs1529255
rs77127982
rs77512781
rs12434819
rs35984956
rs376091529
rs117278598
rs13025044
rs7768410
rs2479954
rs10189473
rs150846585
All
−0.05
0.00
0.05
MR leave−one−out sensitivity analysis for
'Parabacteroides sp000436495' on 'GCST90200601_buildGRCh38.tsv'

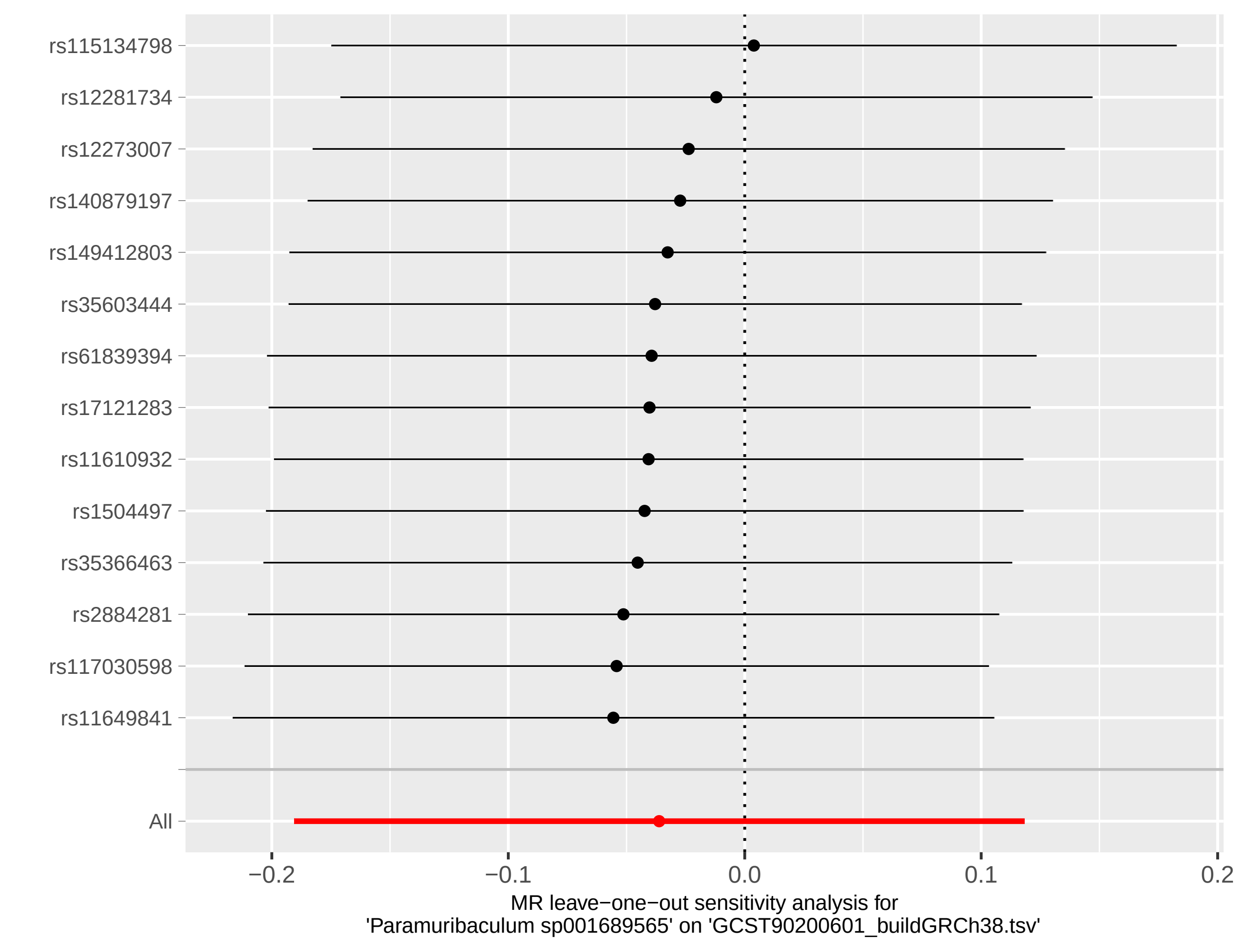

rs115134798
rs12281734
rs12273007
rs140879197
rs149412803
rs35603444
rs61839394
rs17121283
rs11610932
rs1504497
rs35366463
rs2884281
rs117030598
rs11649841
All
−0.2
−0.1
0.0
0.1
0.2
MR leave−one−out sensitivity analysis for
'Paramuribaculum sp001689565' on 'GCST90200601_buildGRCh38.tsv'

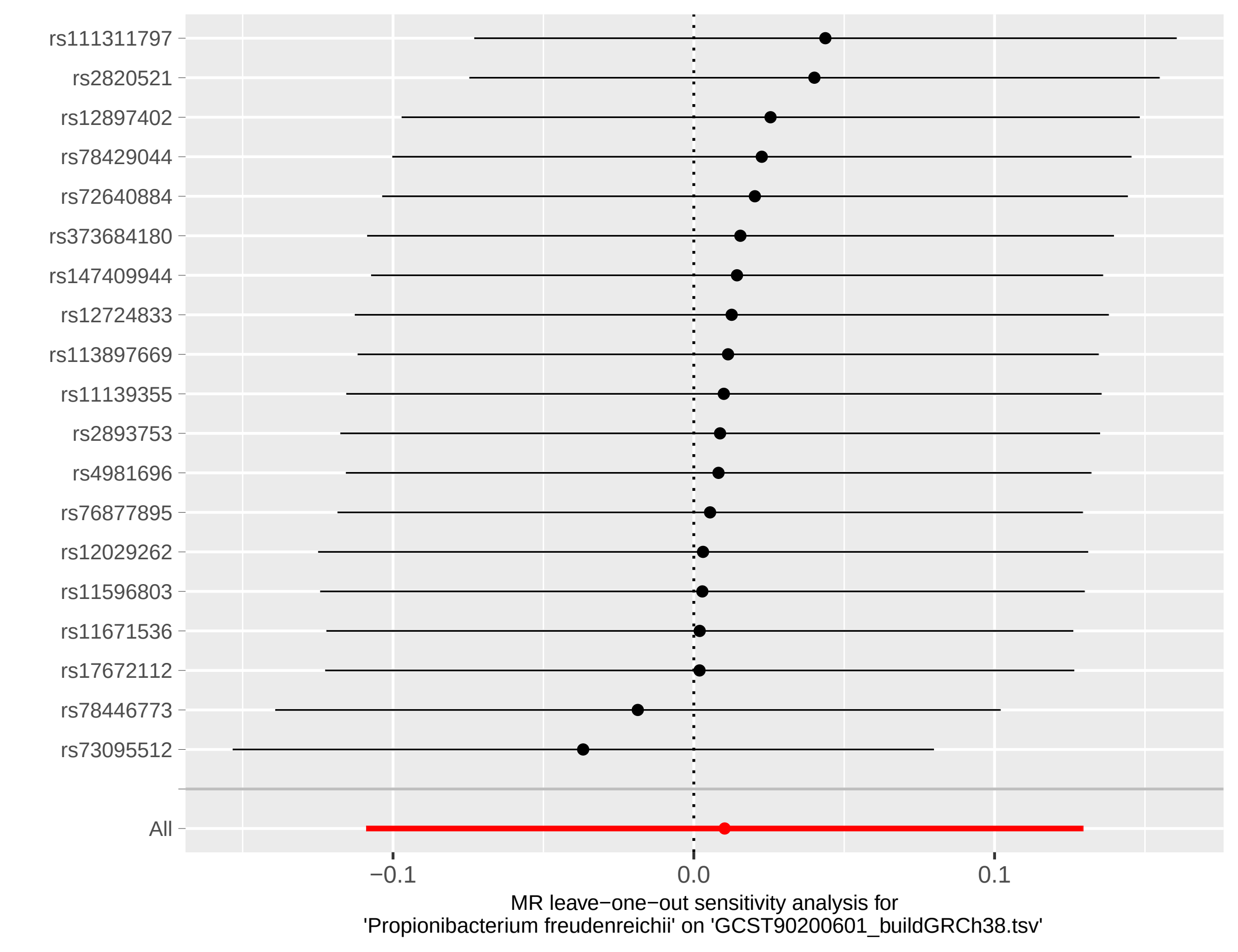
rs111311797
rs2820521
rs12897402
rs78429044
rs72640884
rs373684180
rs147409944
rs12724833
rs113897669
rs11139355
rs2893753
rs4981696
rs76877895
rs12029262
rs11596803
rs11671536
rs17672112
rs78446773
rs73095512
All
−0.1
0.0
0.1
MR leave−one−out sensitivity analysis for
'Propionibacterium freudenreichii' on 'GCST90200601_buildGRCh38.tsv'

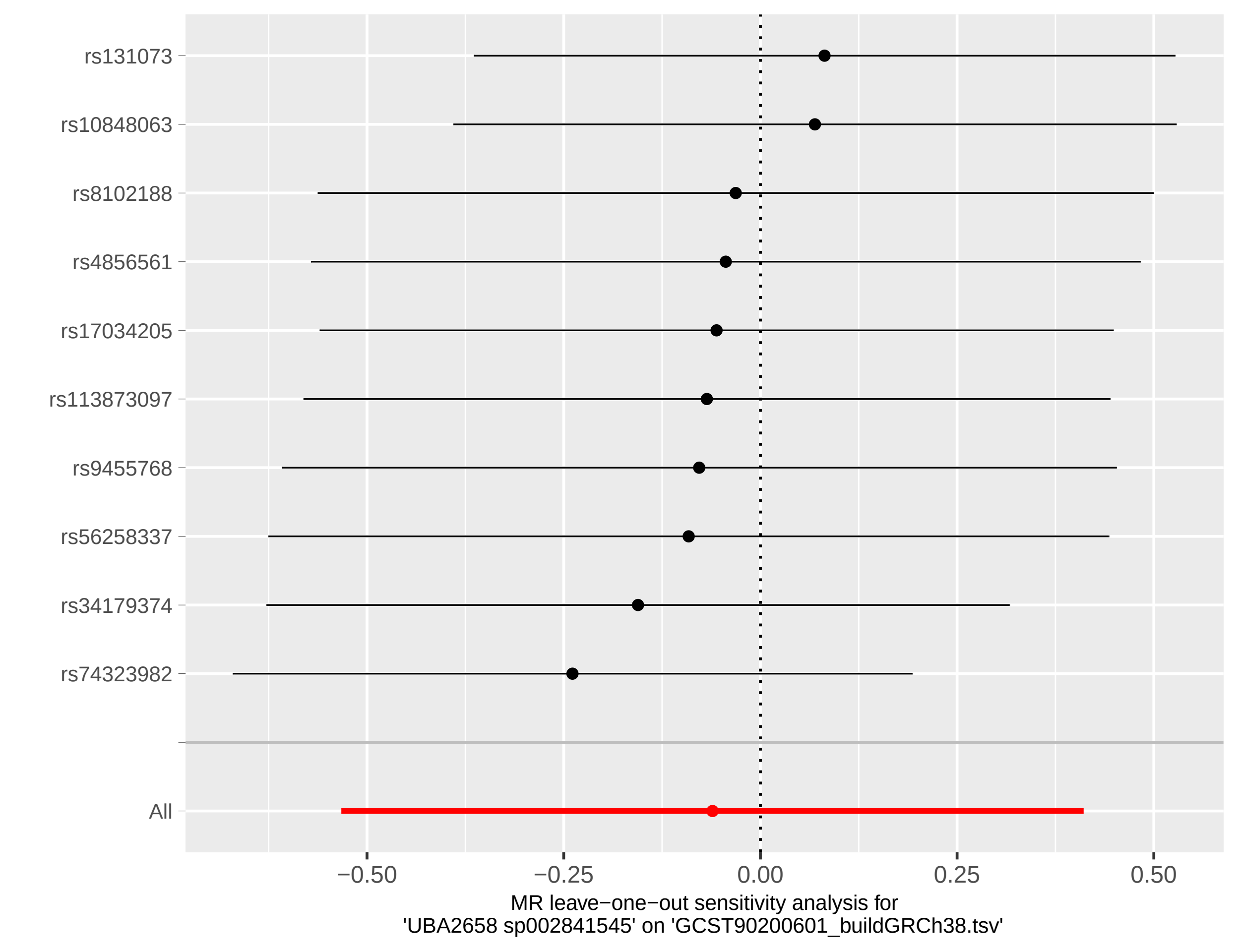
rs131073
rs10848063
rs8102188
rs4856561
rs17034205
rs113873097
rs9455768
rs56258337
rs34179374
rs74323982
All
−0.50
−0.25
0.00
0.25
0.50
MR leave−one−out sensitivity analysis for
'UBA2658 sp002841545' on 'GCST90200601_buildGRCh38.tsv'

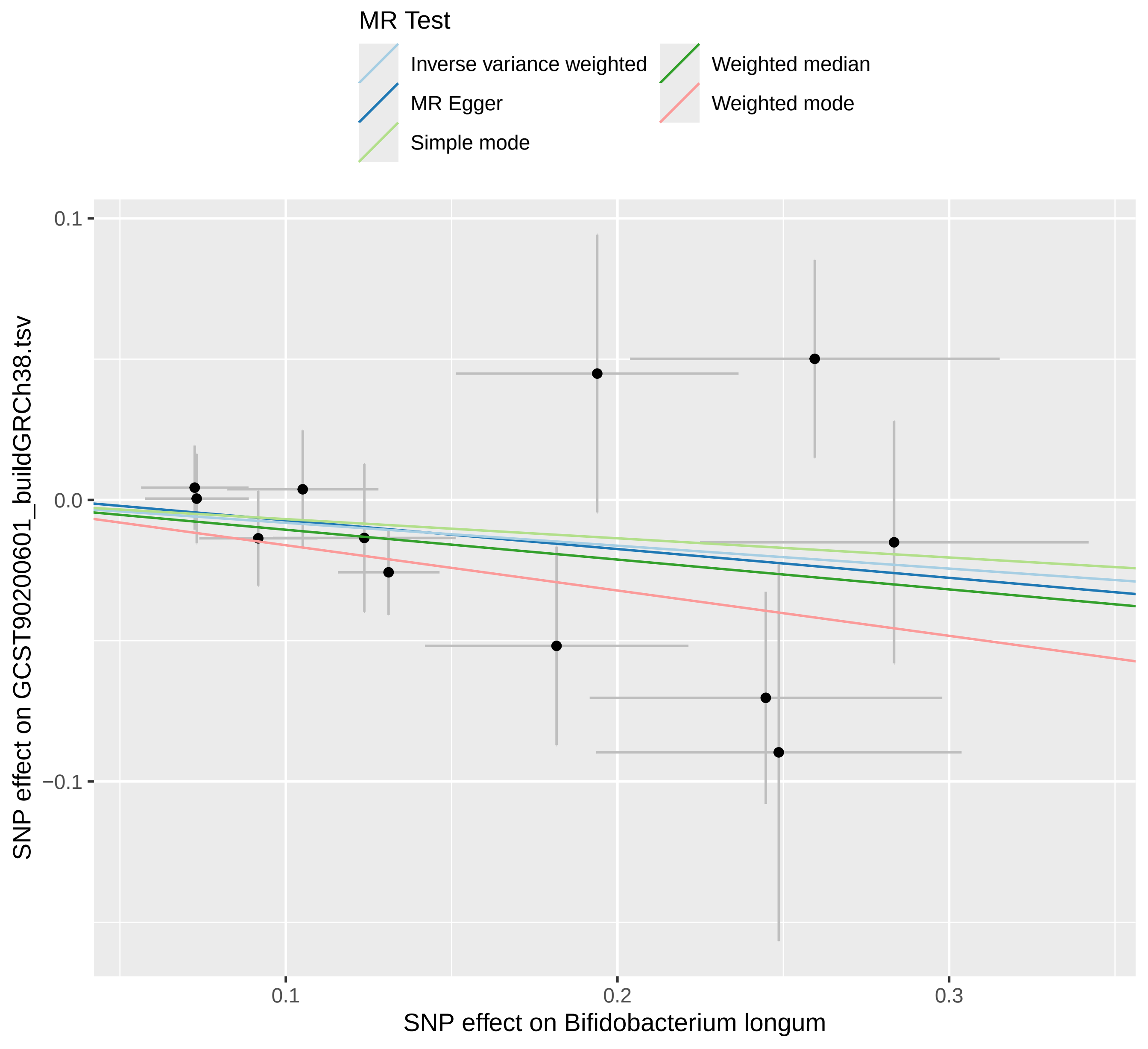
MR Test
Inverse variance weighted
MR Egger
Simple mode
Weighted median
Weighted mode
0.1
0.0
−0.1
0.1
0.2
0.3
SNP effect on GCST90200601_buildGRCh38.tsv
SNP effect on Bifidobacterium longum

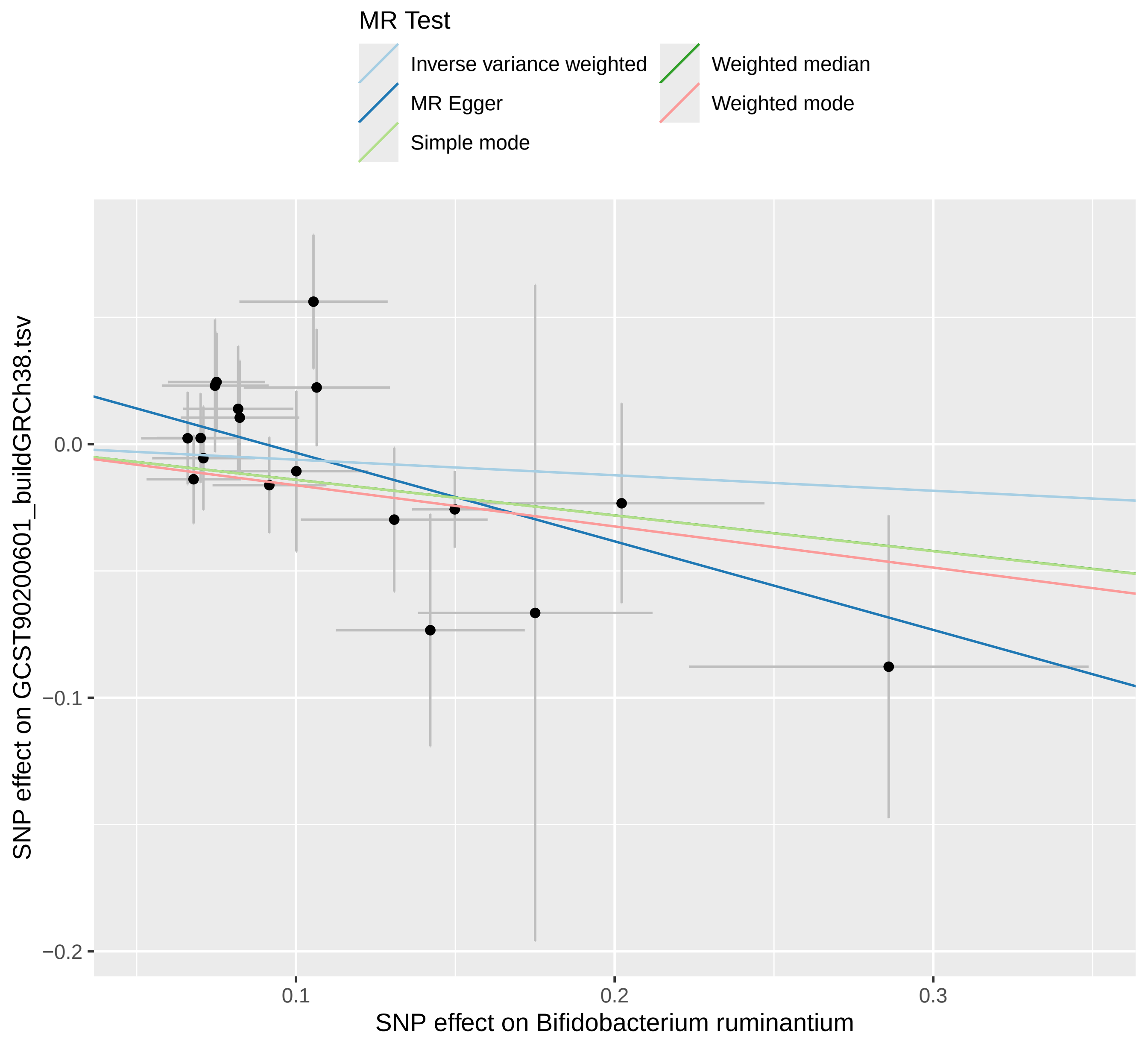

MR Test
Inverse variance weighted
MR Egger
Simple mode
Weighted median
Weighted mode
0.0
−0.1
−0.2
0.1
0.2
0.3
SNP effect on GCST90200601_buildGRCh38.tsv
SNP effect on Bifidobacterium ruminantium

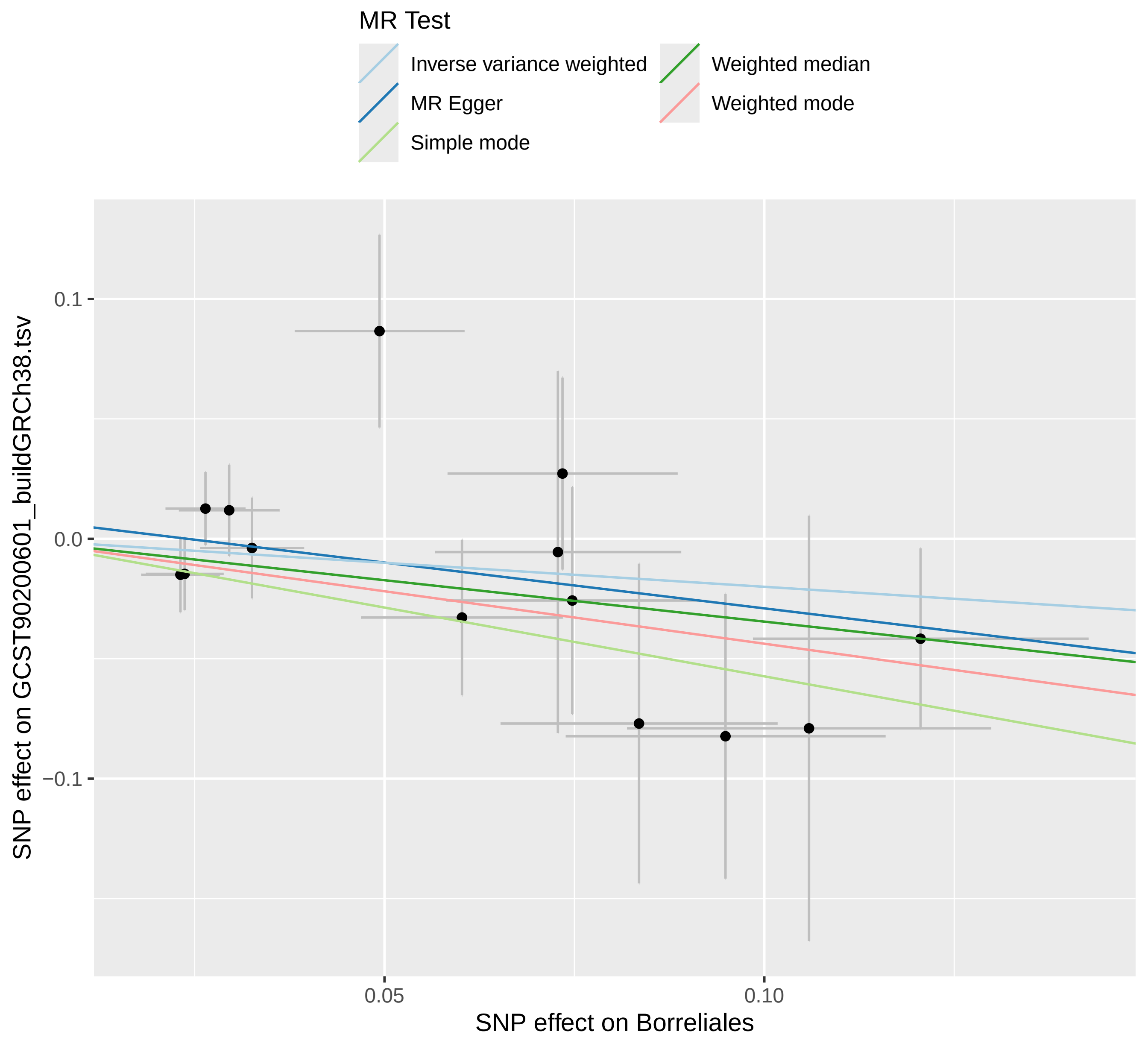

MR Test
Inverse variance weighted
MR Egger
Simple mode
Weighted median
Weighted mode
0.1
0.0
−0.1
0.05
0.10
SNP effect on GCST90200601_buildGRCh38.tsv
SNP effect on Borreliales

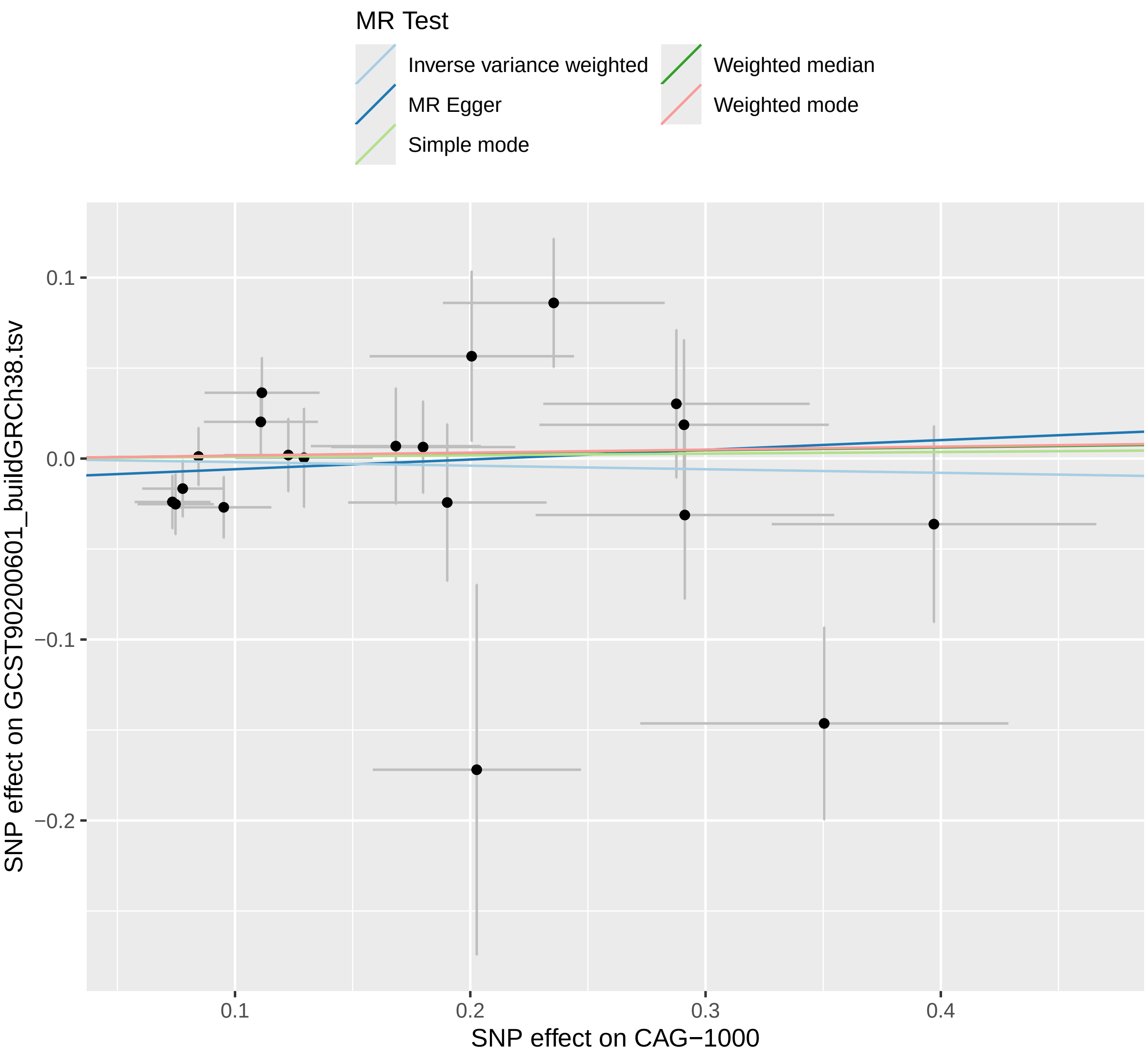

MR Test
Inverse variance weighted
MR Egger
Simple mode
Weighted median
Weighted mode
0.1
0.0
−0.1
−0.2
0.1
0.2
0.3
0.4
SNP effect on GCST90200601_buildGRCh38.tsv
SNP effect on CAG−1000

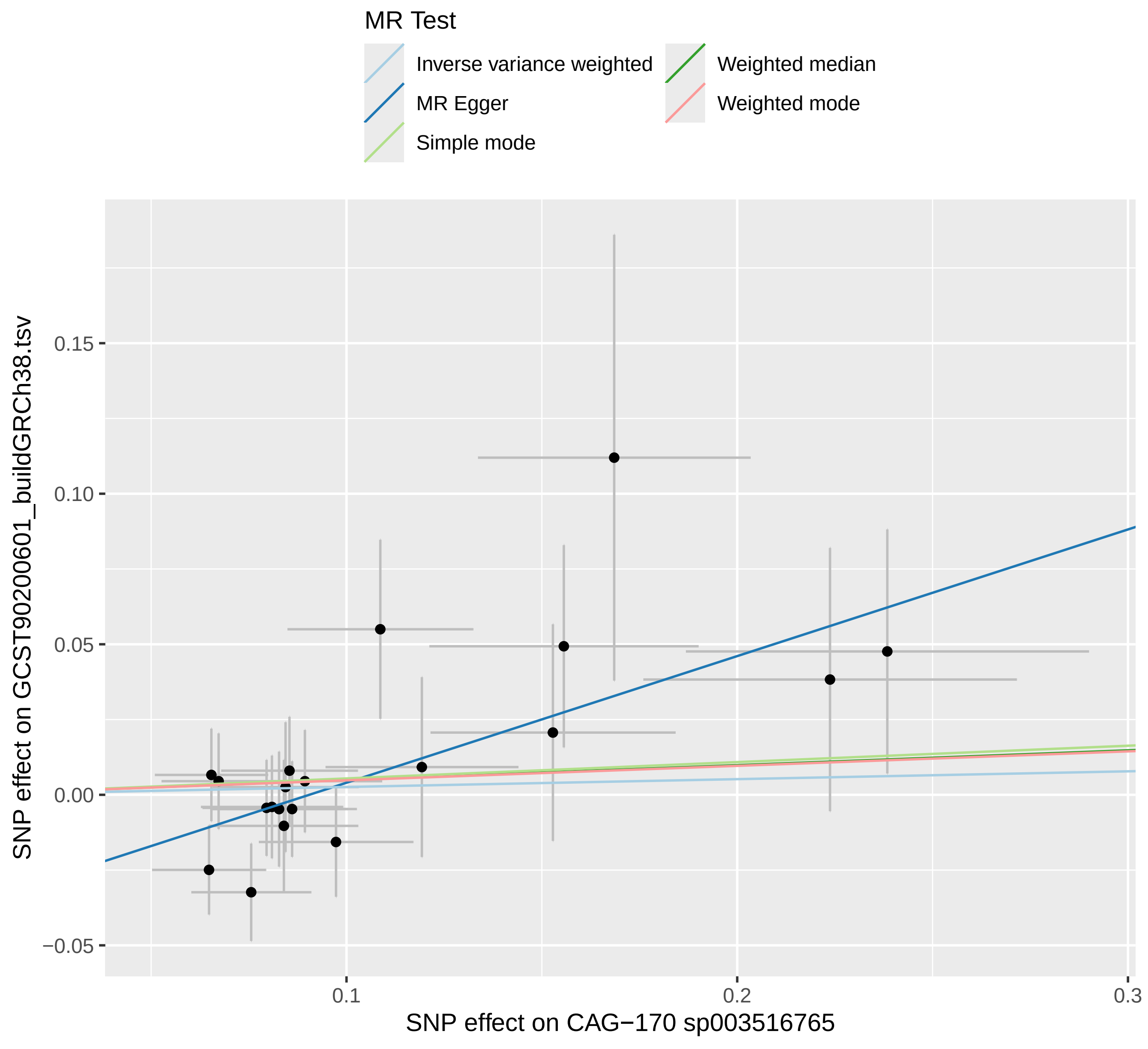
MR Test
Inverse variance weighted
MR Egger
Simple mode
Weighted median
Weighted mode
SNP effect on GCST90200601_buildGRCh38.tsv
0.15
0.10
0.05
0.00
−0.05
0.1
0.2
0.3
SNP effect on CAG−170 sp003516765

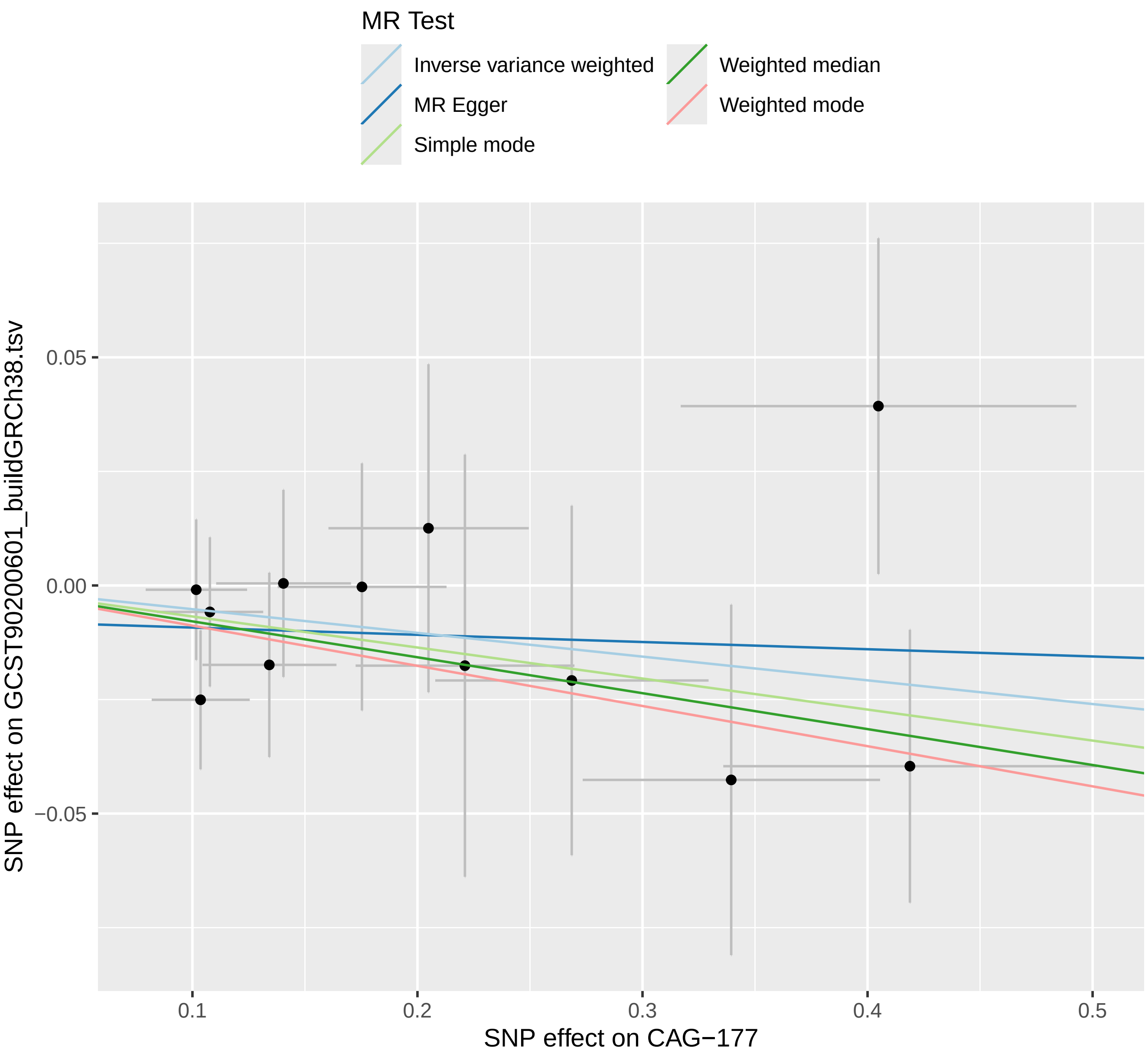

MR Test
Inverse variance weighted
MR Egger
Simple mode
Weighted median
Weighted mode
0.05
0.00
−0.05
0.1
0.2
0.3
0.4
0.5
SNP effect on GCST90200601_buildGRCh38.tsv
SNP effect on CAG−177

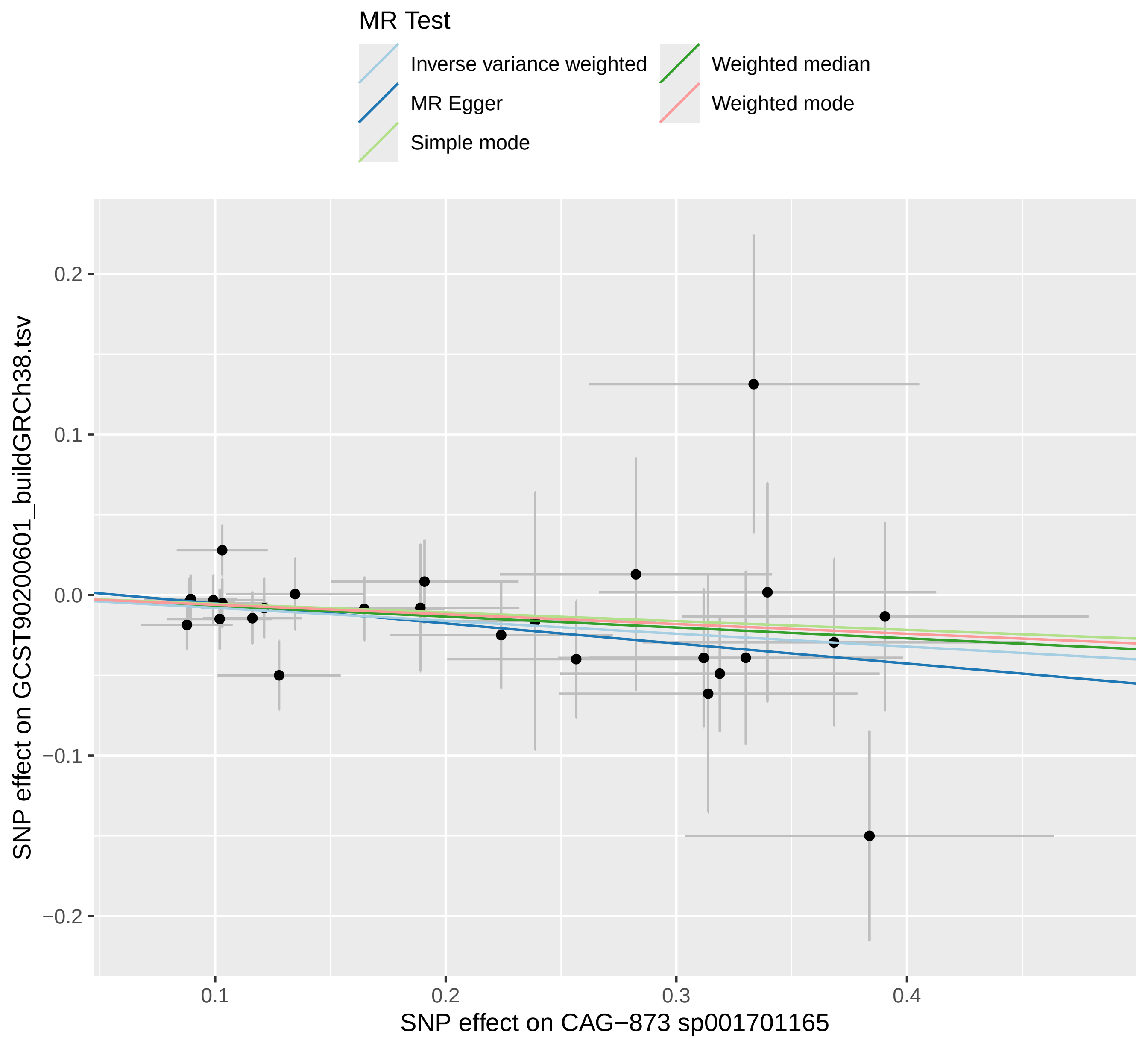
MR Test
Inverse variance weighted
MR Egger
Simple mode
Weighted median
Weighted mode
0.2
0.1
0.0
−0.1
−0.2
0.1
0.2
0.3
0.4
SNP effect on GCST90200601_buildGRCh38.tsv
SNP effect on CAG−873 sp001701165

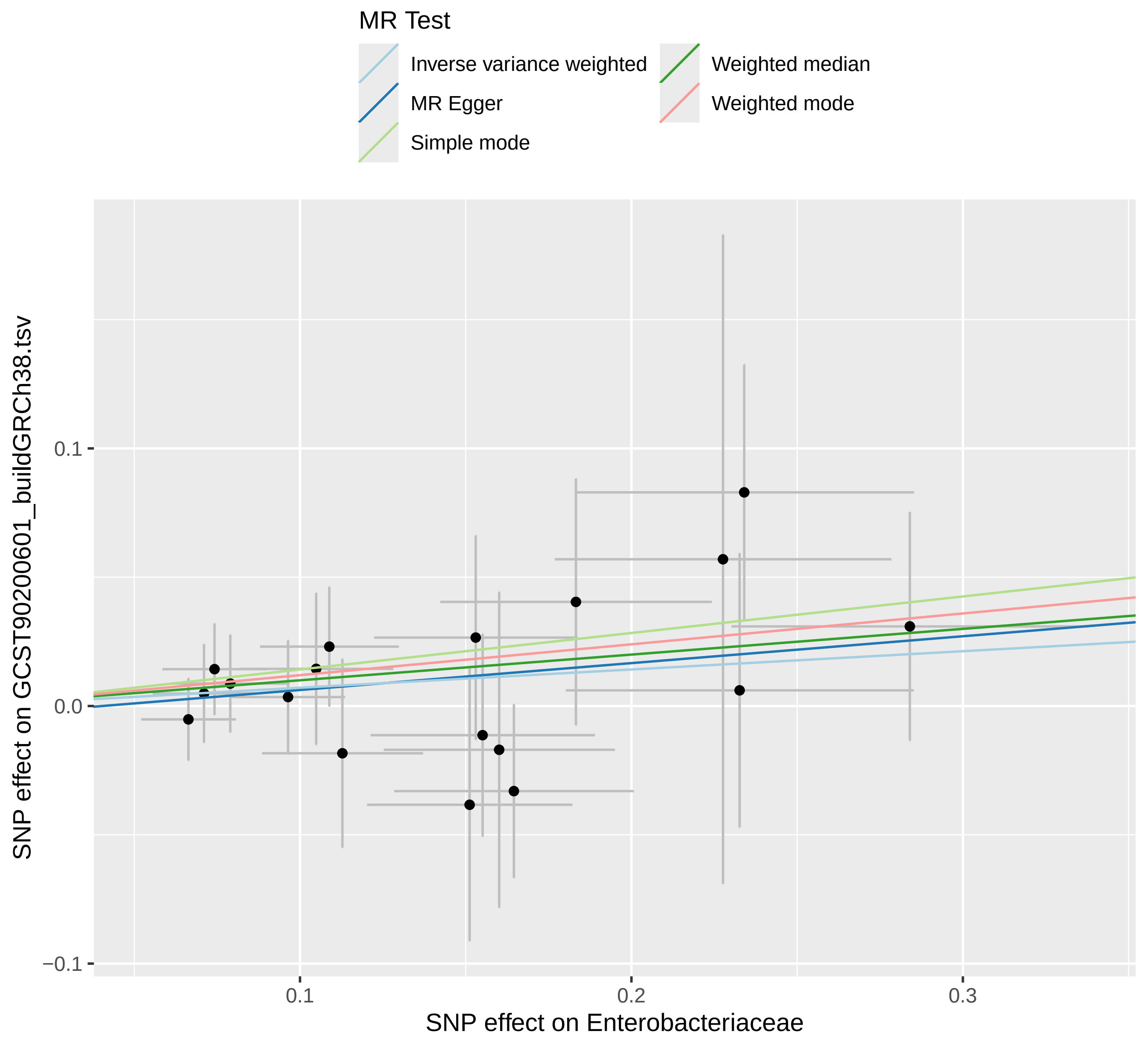
MR Test
Inverse variance weighted
MR Egger
Simple mode
Weighted median
Weighted mode
SNP effect on GCST90200601_buildGRCh38.tsv
0.1
0.0
−0.1
0.1
0.2
0.3
SNP effect on Enterobacteriaceae

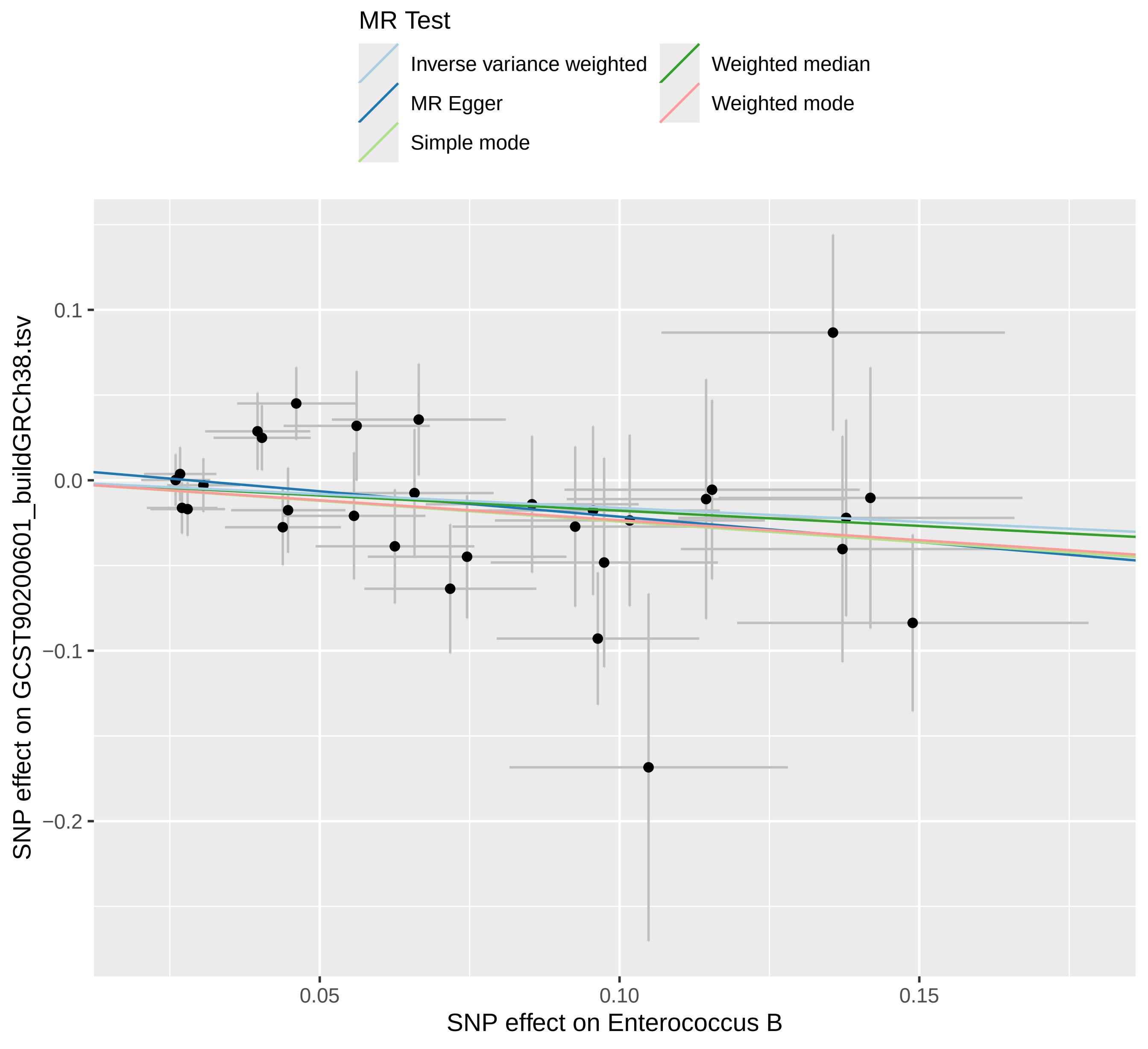
MR Test
Inverse variance weighted
MR Egger
Simple mode
Weighted median
Weighted mode
0.1
0.0
−0.1
−0.2
0.05
0.10
0.15
SNP effect on GCST90200601_buildGRCh38.tsv
SNP effect on Enterococcus B

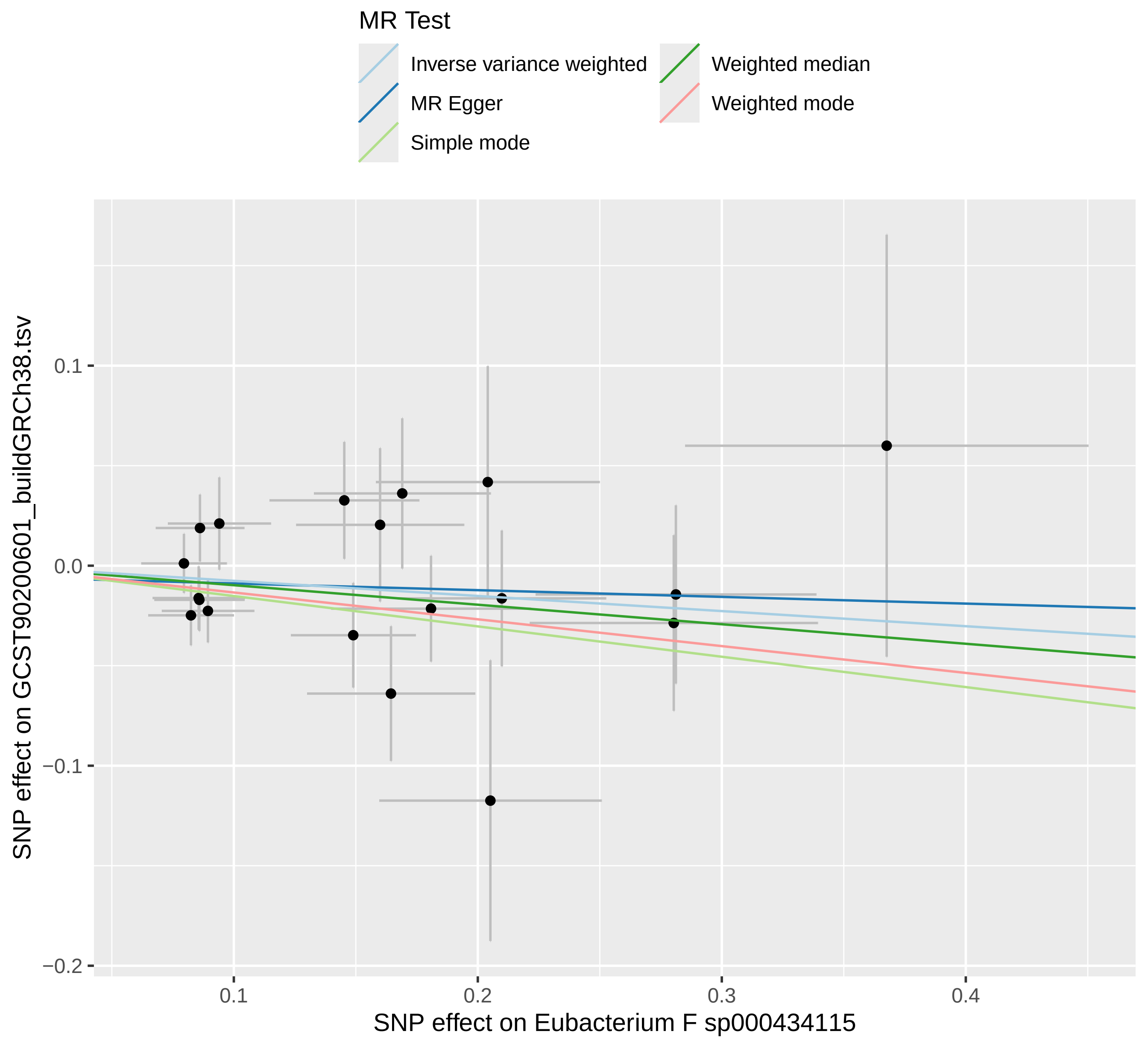
MR Test
Inverse variance weighted
MR Egger
Simple mode
Weighted median
Weighted mode
0.1
0.0
−0.1
−0.2
0.1
0.2
0.3
0.4
SNP effect on GCST90200601_buildGRCh38.tsv
SNP effect on Eubacterium F sp000434115

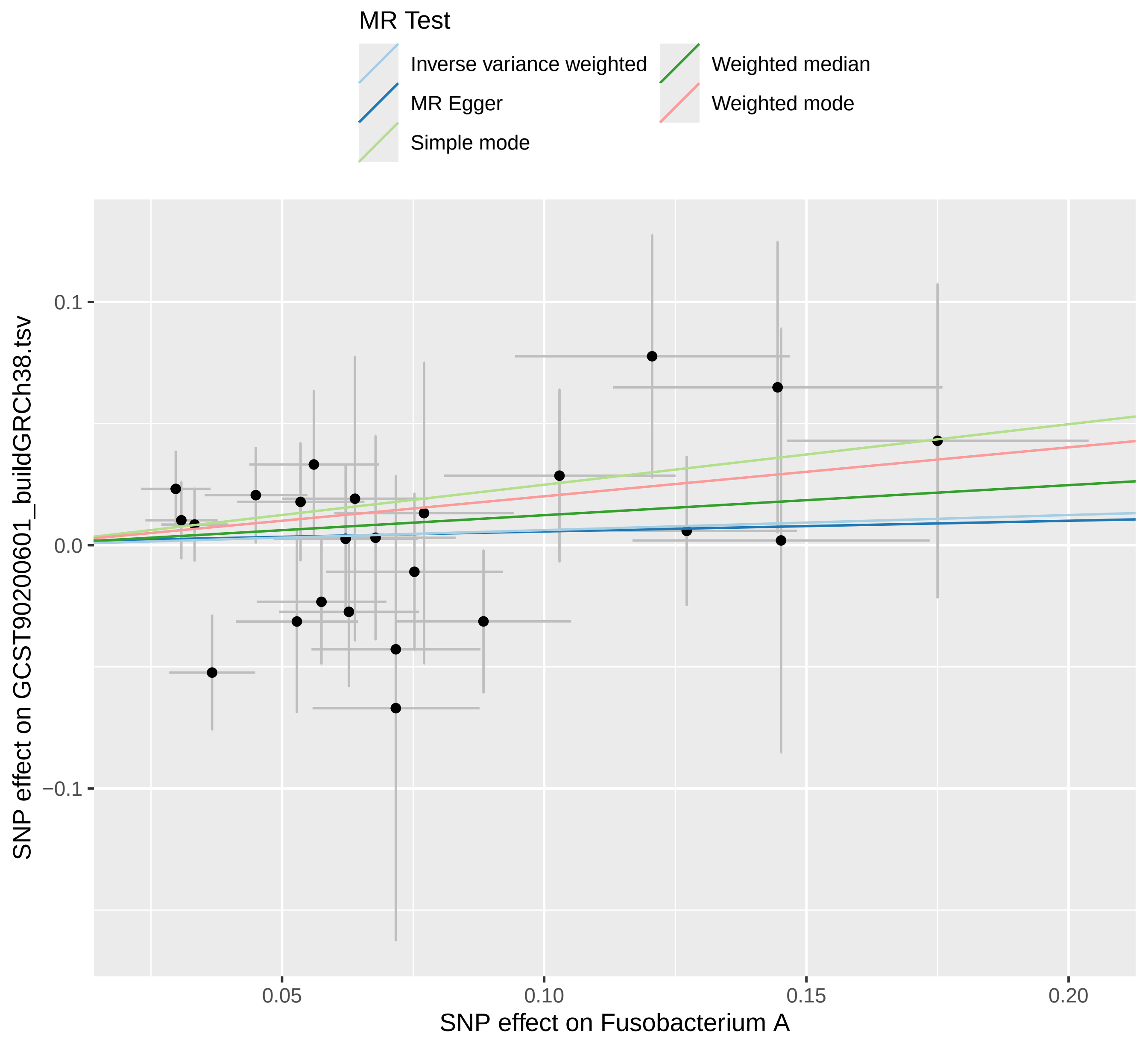
MR Test
Inverse variance weighted
MR Egger
Simple mode
Weighted median
Weighted mode
0.1
0.0
−0.1
0.05
0.10
0.15
0.20
SNP effect on GCST90200601_buildGRCh38.tsv
SNP effect on Fusobacterium A

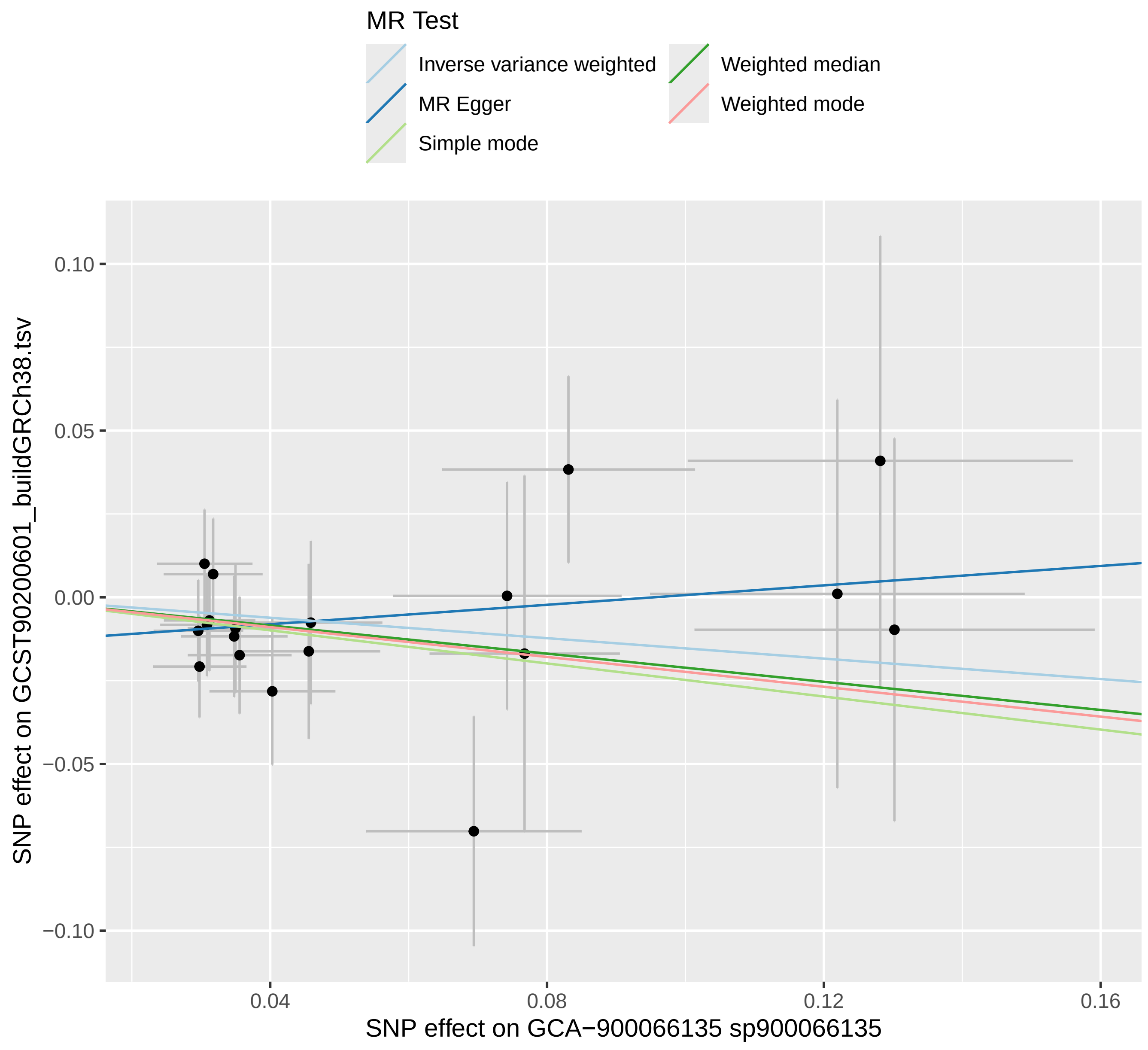
MR Test
Inverse variance weighted
MR Egger
Simple mode
Weighted median
Weighted mode
0.10
0.05
0.00
−0.05
−0.10
SNP effect on GCST90200601_buildGRCh38.tsv
0.04
0.08
0.12
0.16
SNP effect on GCA−900066135 sp900066135

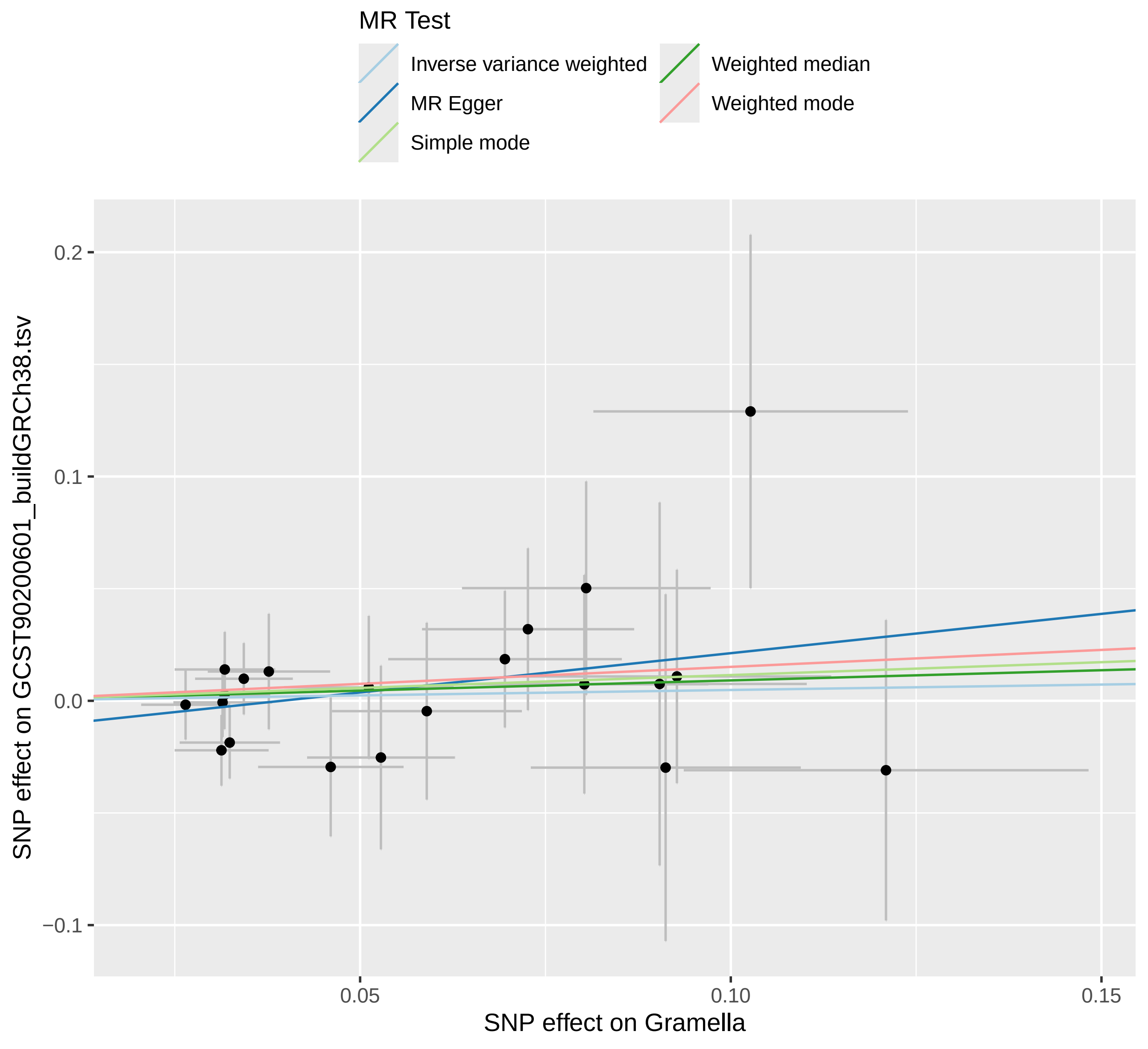
MR Test
Inverse variance weighted
MR Egger
Simple mode
Weighted median
Weighted mode
0.2
0.1
0.0
−0.1
0.05
0.10
0.15
SNP effect on GCST90200601_buildGRCh38.tsv
SNP effect on Gramella

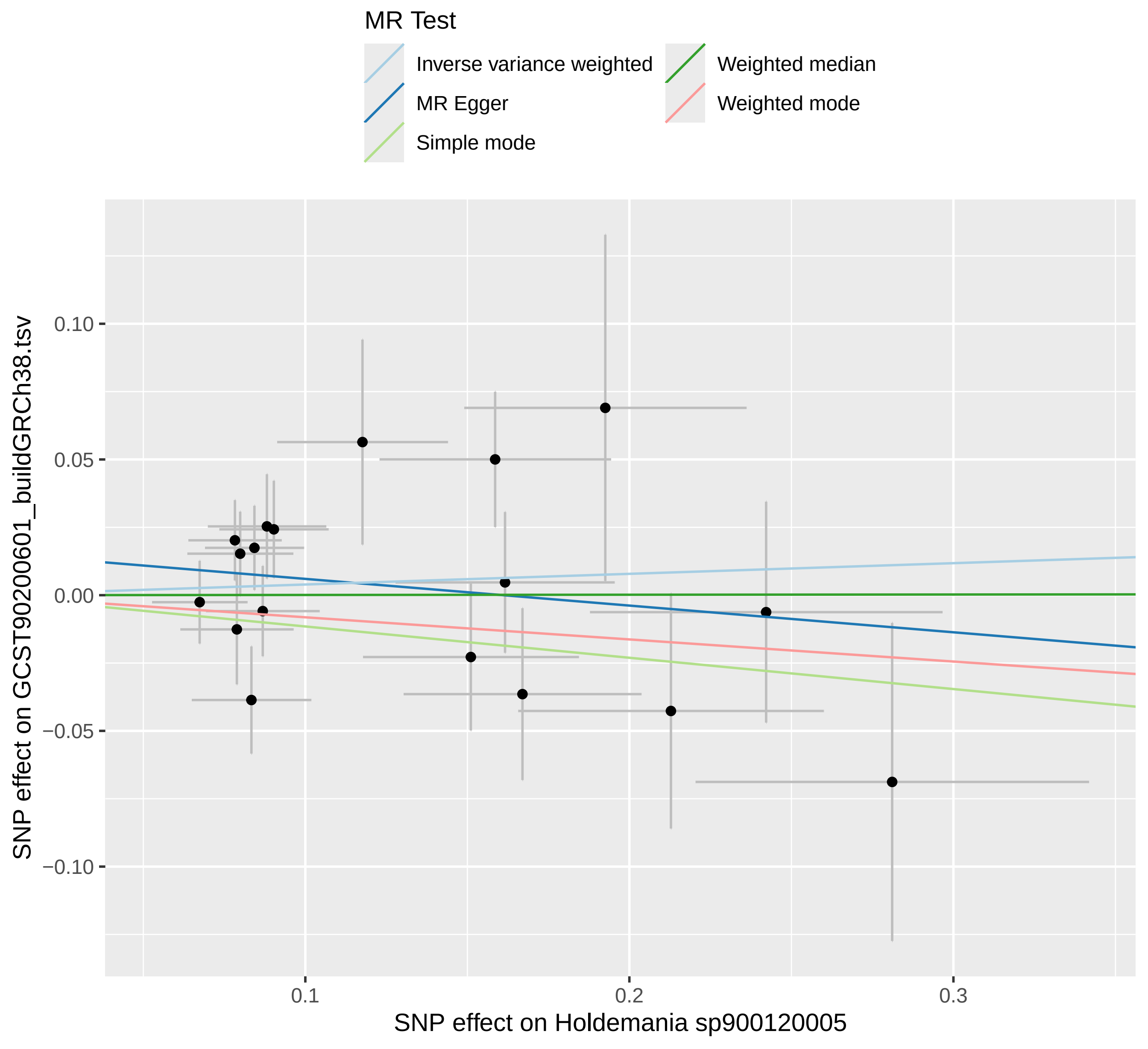

MR Test
Inverse variance weighted
MR Egger
Simple mode
Weighted median
Weighted mode
0.10
0.05
0.00
−0.05
−0.10
0.1
0.2
0.3
SNP effect on GCST90200601_buildGRCh38.tsv
SNP effect on Holdemania sp900120005

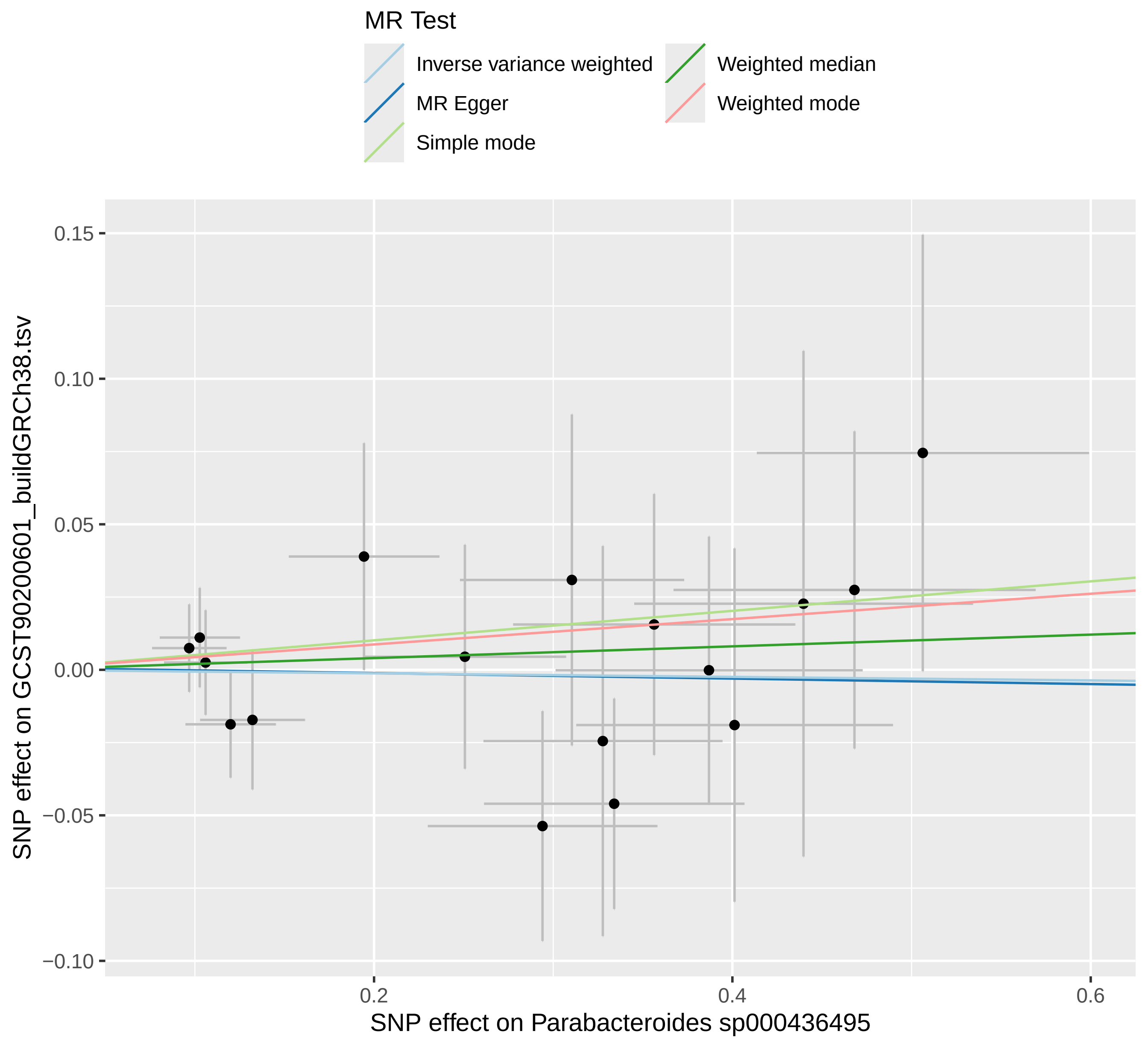
MR Test
Inverse variance weighted
MR Egger
Simple mode
Weighted median
Weighted mode
0.15
0.10
0.05
0.00
−0.05
−0.10
0.2
0.4
0.6
SNP effect on GCST90200601_buildGRCh38.tsv
SNP effect on Parabacteroides sp000436495

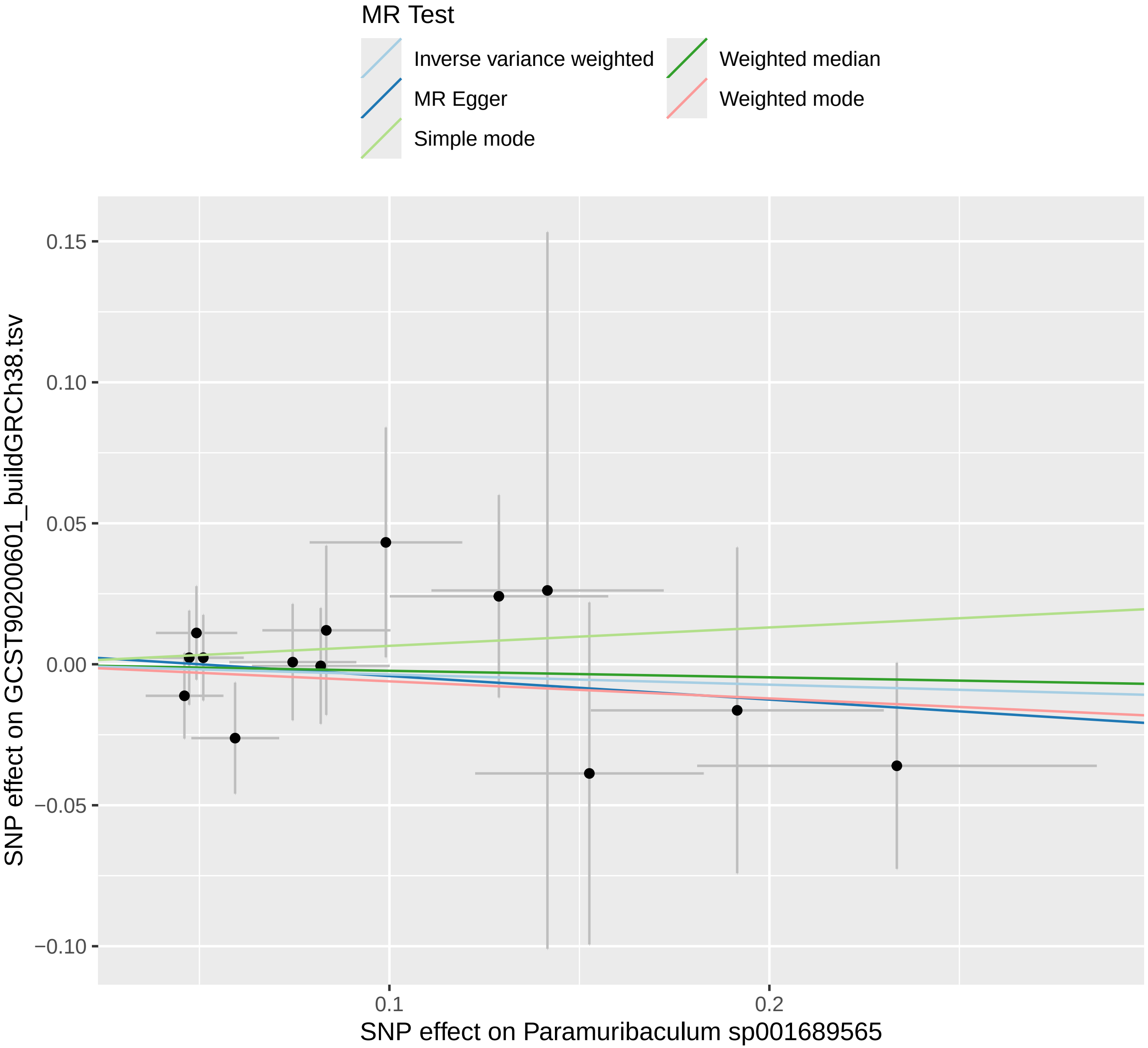

MR Test
Inverse variance weighted
MR Egger
Simple mode
Weighted median
Weighted mode
0.15
0.10
0.05
0.00
−0.05
−0.10
0.1
0.2
SNP effect on GCST90200601_buildGRCh38.tsv
SNP effect on Paramuribaculum sp001689565

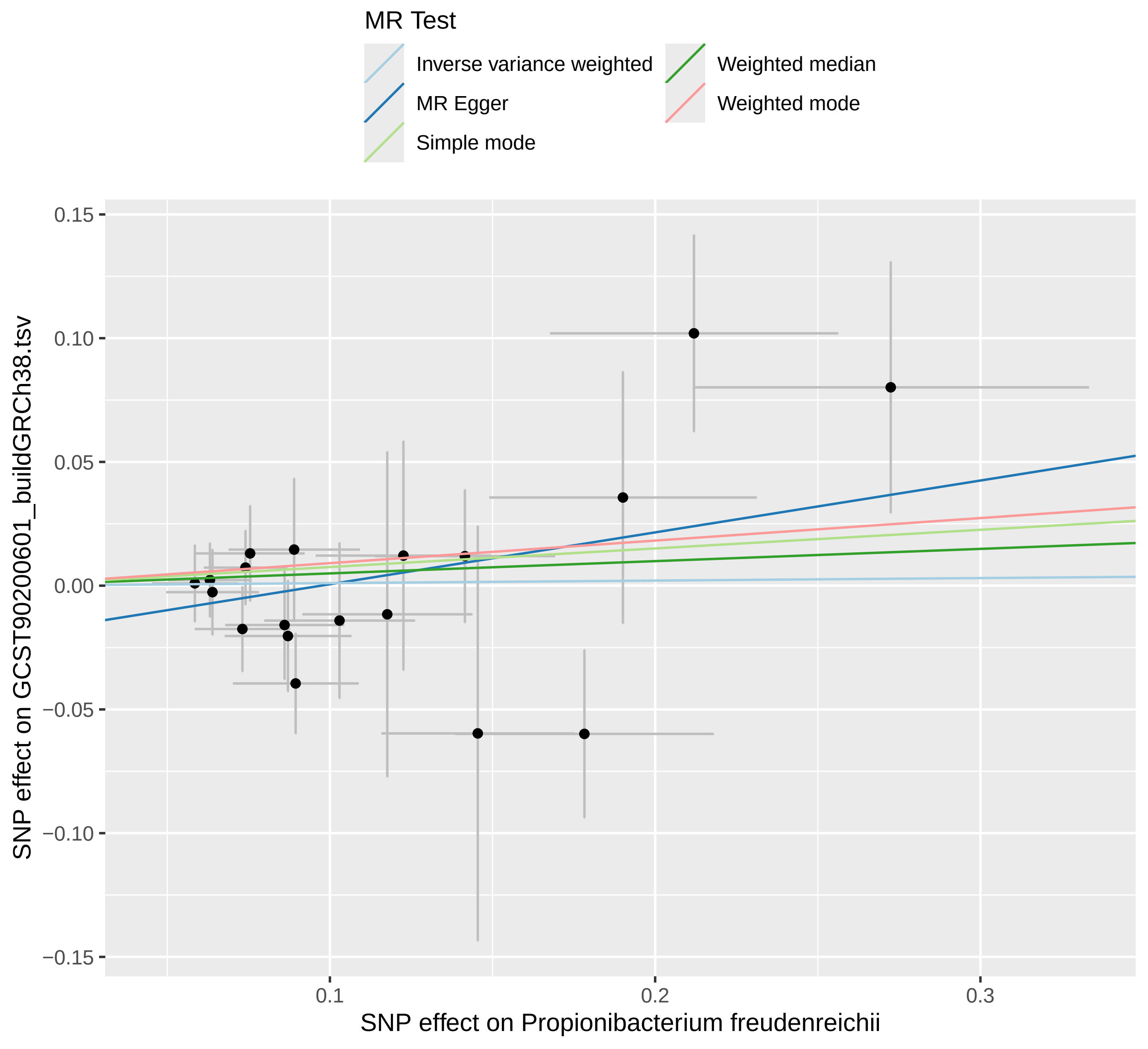

MR Test
Inverse variance weighted
MR Egger
Simple mode
Weighted median
Weighted mode
0.15
0.10
0.05
0.00
−0.05
−0.10
−0.15
0.1
0.2
0.3
SNP effect on GCST90200601_buildGRCh38.tsv
SNP effect on Propionibacterium freudenreichii

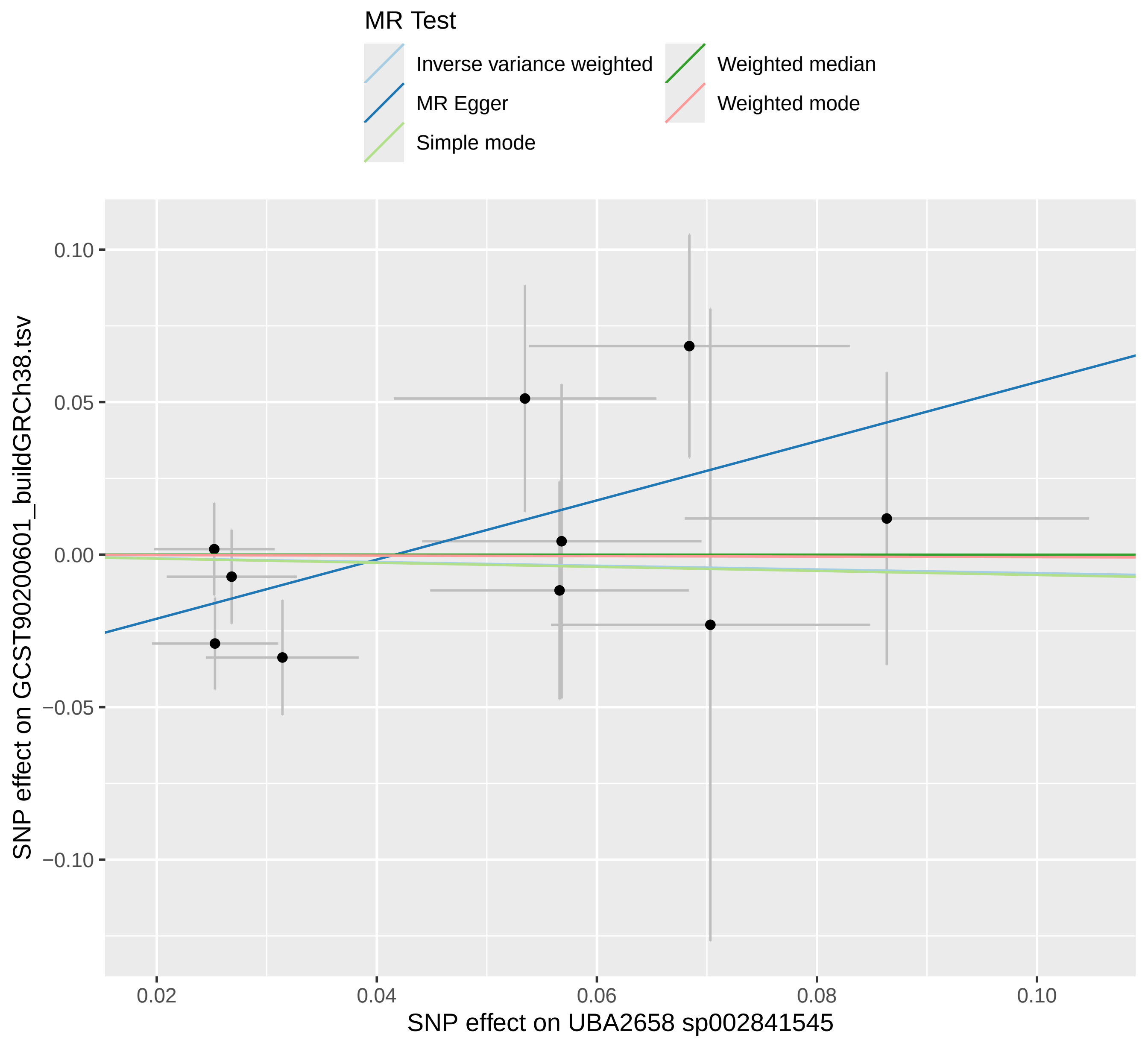

MR Test
Inverse variance weighted
MR Egger
Simple mode
Weighted median
Weighted mode
0.10
0.05
0.00
−0.05
−0.10
0.02
0.04
0.06
0.08
0.10
SNP effect on GCST90200601_buildGRCh38.tsv
SNP effect on UBA2658 sp002841545

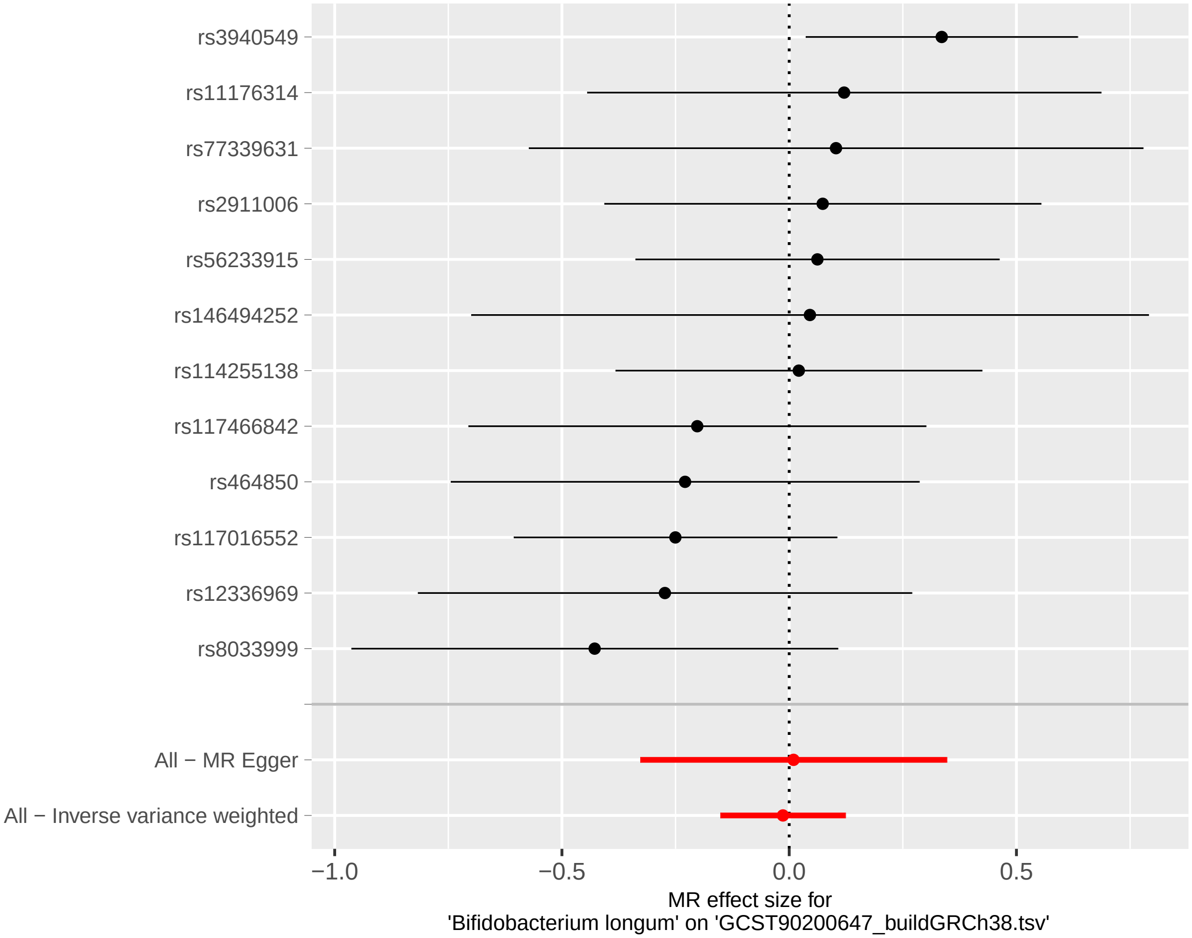
rs3940549
rs11176314
rs77339631
rs2911006
rs56233915
rs146494252
rs114255138
rs117466842
rs464850
rs117016552
rs12336969
rs8033999
All − MR Egger
All − Inverse variance weighted
−1.0
−0.5
0.0
0.5
MR effect size for
'Bifidobacterium longum' on 'GCST90200647_buildGRCh38.tsv'

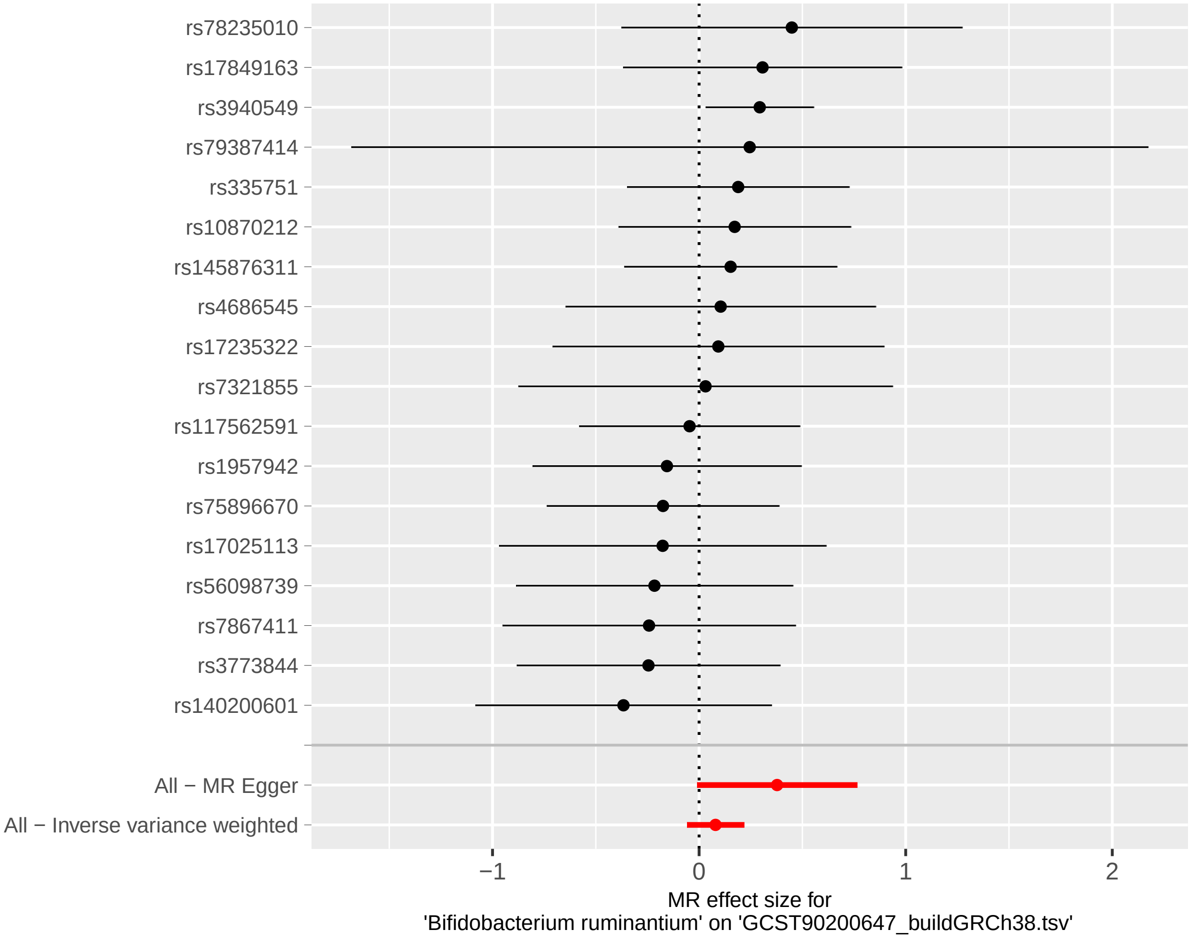
rs78235010
rs17849163
rs3940549
rs79387414
rs335751
rs10870212
rs145876311
rs4686545
rs17235322
rs7321855
rs117562591
rs1957942
rs75896670
rs17025113
rs56098739
rs7867411
rs3773844
rs140200601
All − MR Egger
All − Inverse variance weighted
−1
0
1
2
MR effect size for
'Bifidobacterium ruminantium' on 'GCST90200647_buildGRCh38.tsv'

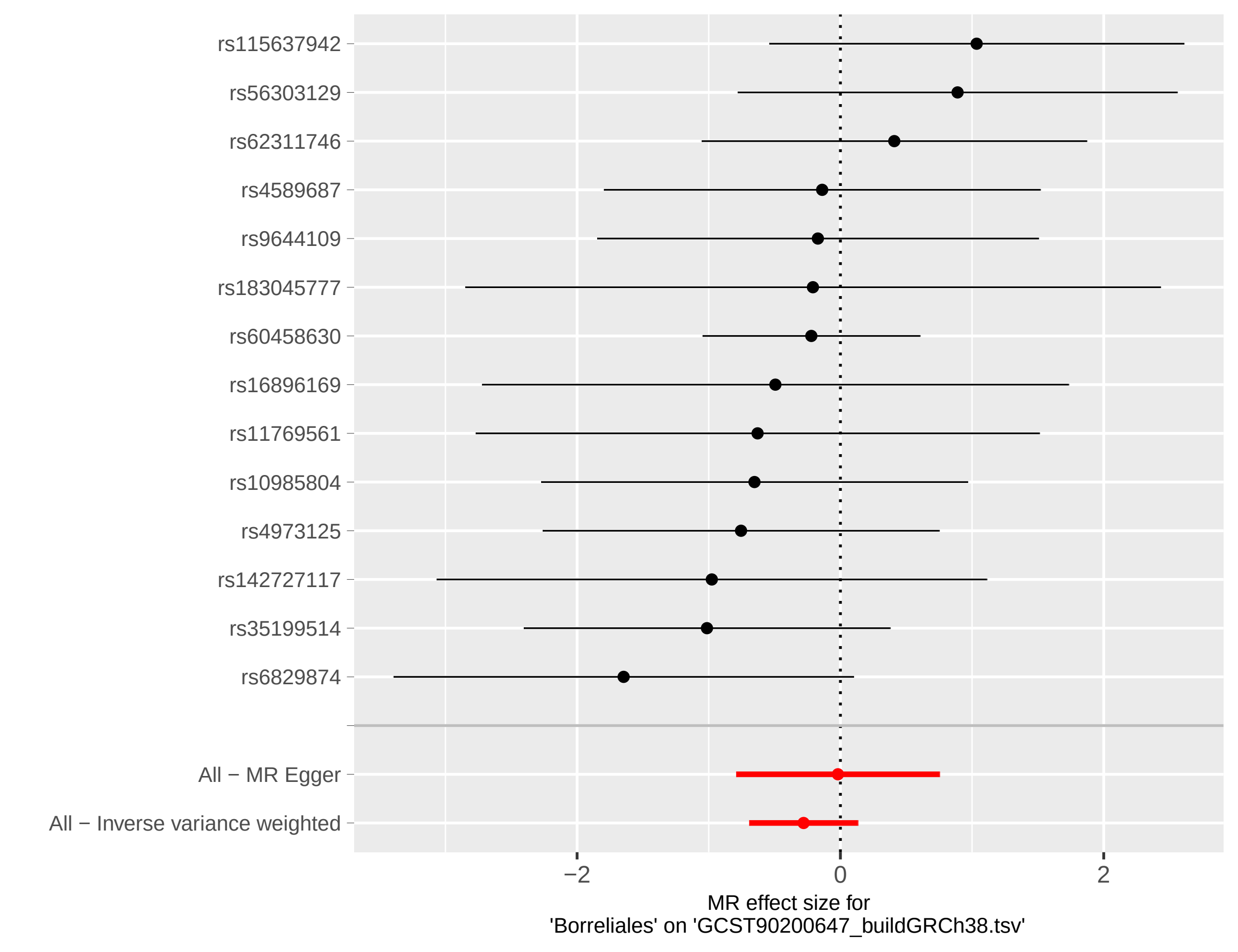
rs115637942
rs56303129
rs62311746
rs4589687
rs9644109
rs183045777
rs60458630
rs16896169
rs11769561
rs10985804
rs4973125
rs142727117
rs35199514
rs6829874
All − MR Egger
All − Inverse variance weighted
−2
0
2
MR effect size for
'Borreliales' on 'GCST90200647_buildGRCh38.tsv'

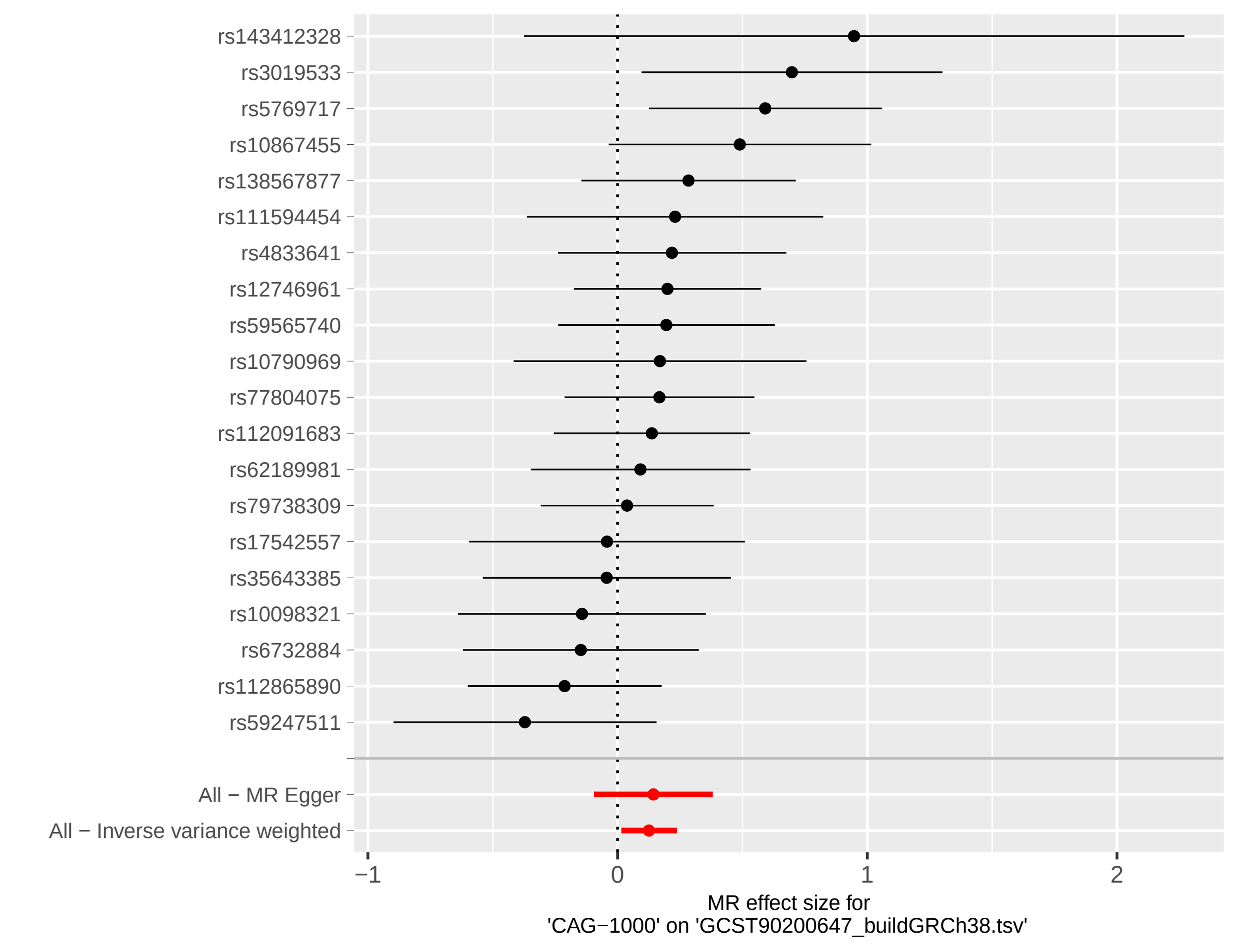
rs143412328
rs3019533
rs5769717
rs10867455
rs138567877
rs111594454
rs4833641
rs12746961
rs59565740
rs10790969
rs77804075
rs112091683
rs62189981
rs79738309
rs17542557
rs35643385
rs10098321
rs6732884
rs112865890
rs59247511
All − MR Egger
All − Inverse variance weighted
−1
0
1
2
MR effect size for
'CAG−1000' on 'GCST90200647_buildGRCh38.tsv'

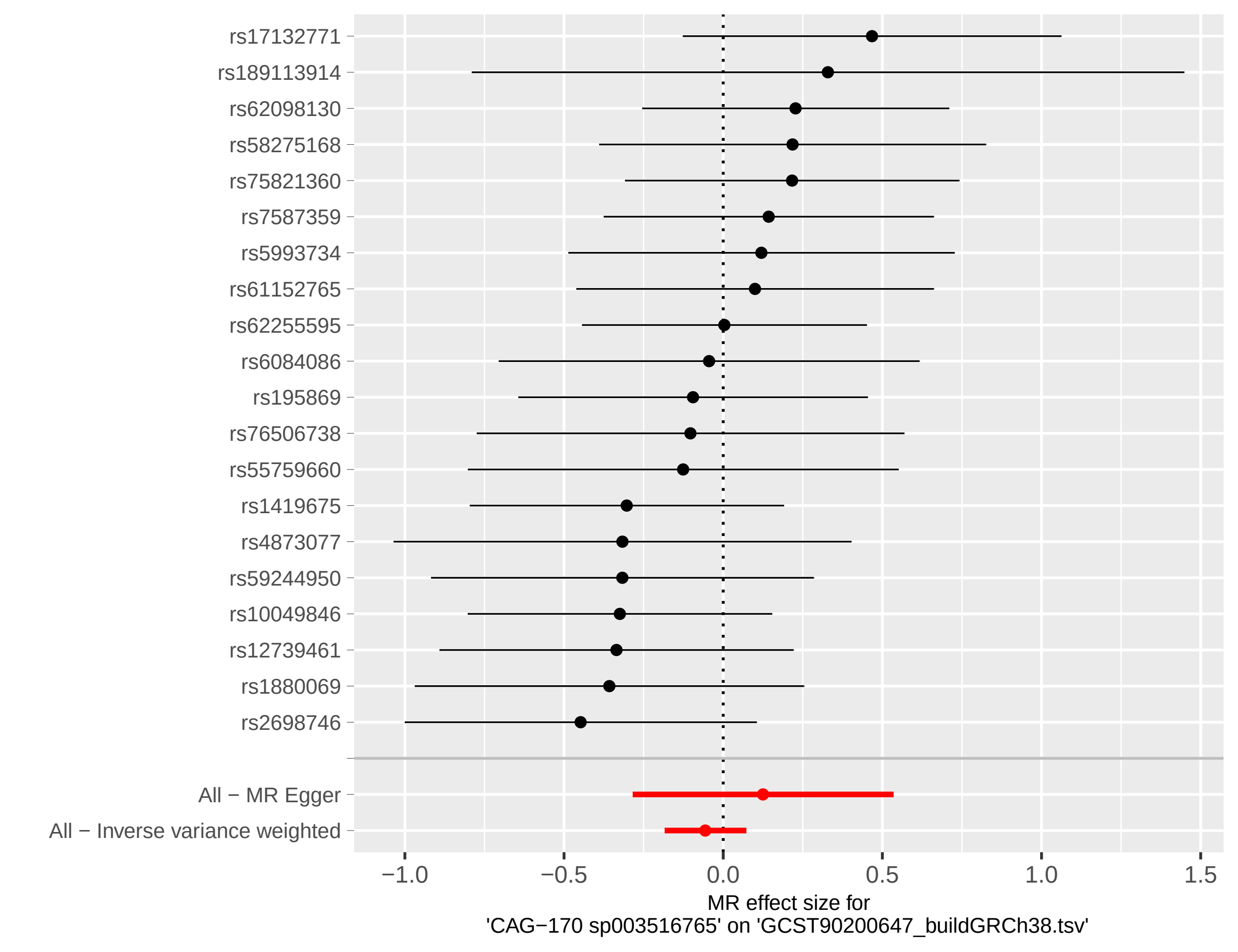
rs17132771
rs189113914
rs62098130
rs58275168
rs75821360
rs7587359
rs5993734
rs61152765
rs62255595
rs6084086
rs195869
rs76506738
rs55759660
rs1419675
rs4873077
rs59244950
rs10049846
rs12739461
rs1880069
rs2698746
All − MR Egger
All − Inverse variance weighted
−1.0
−0.5
0.0
0.5
1.0
1.5
MR effect size for
'CAG−170 sp003516765' on 'GCST90200647_buildGRCh38.tsv'

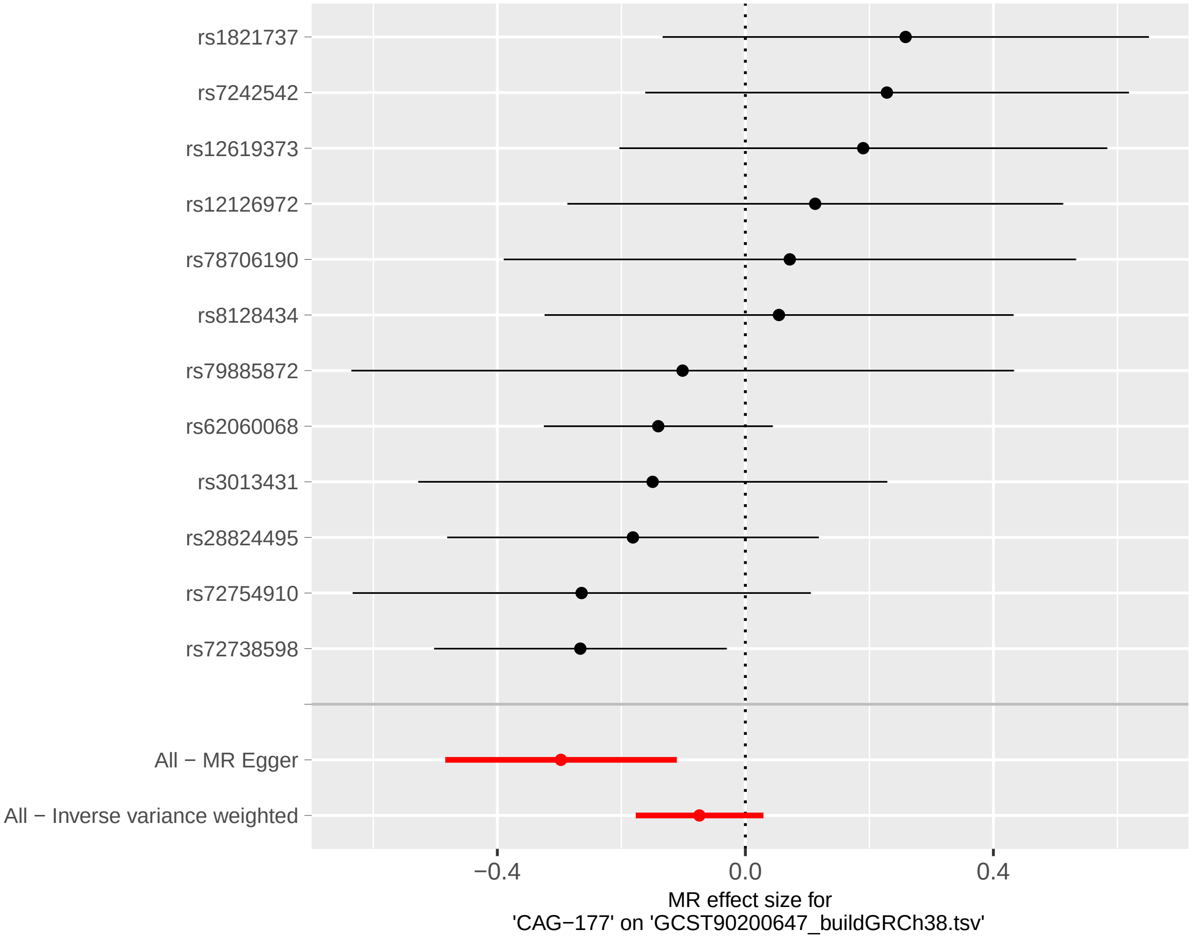

rs1821737
rs7242542
rs12619373
rs12126972
rs78706190
rs8128434
rs79885872
rs62060068
rs3013431
rs28824495
rs72754910
rs72738598
All − MR Egger
All − Inverse variance weighted
−0.4
0.0
0.4
MR effect size for
'CAG−177' on 'GCST90200647_buildGRCh38.tsv'

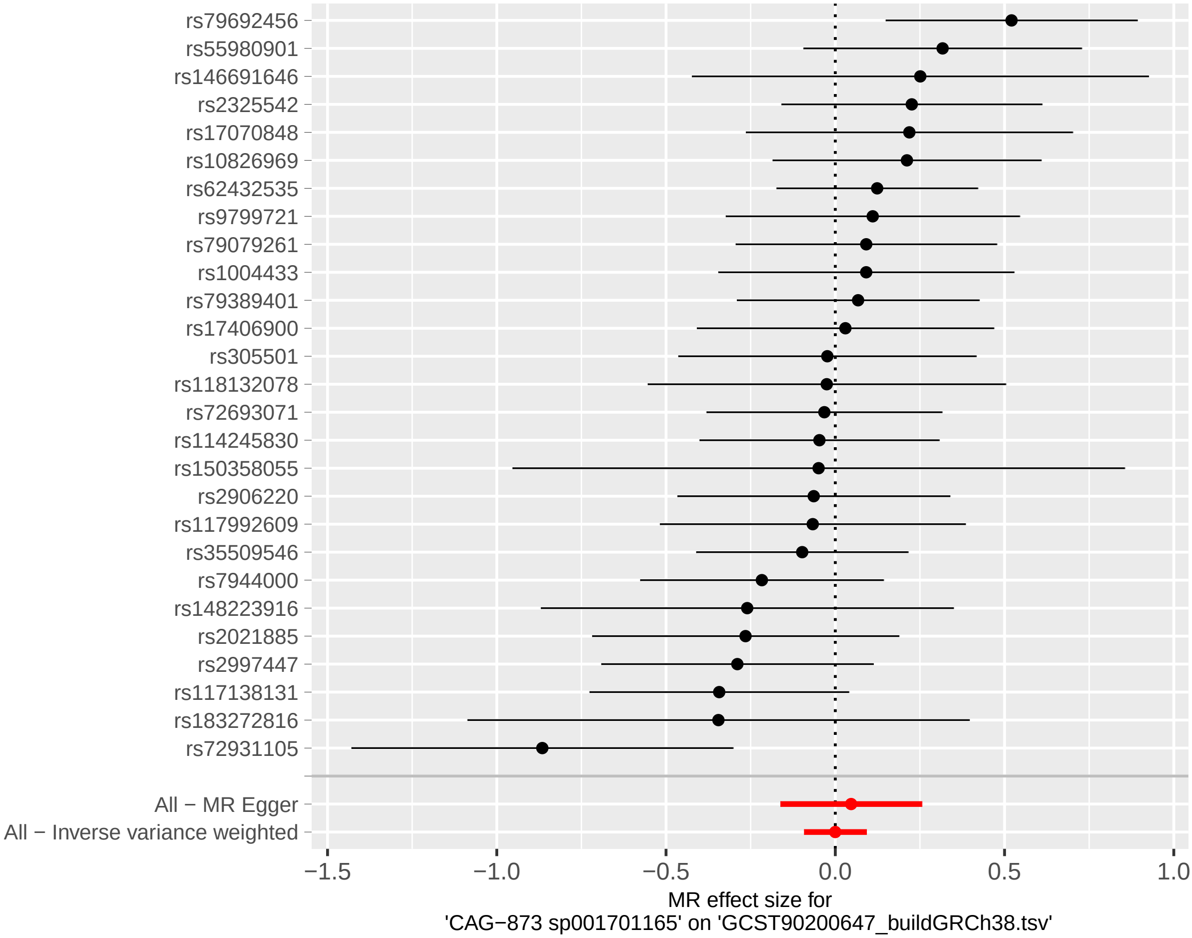
rs79692456
rs55980901
rs146691646
rs2325542
rs17070848
rs10826969
rs62432535
rs9799721
rs79079261
rs1004433
rs79389401
rs17406900
rs305501
rs118132078
rs72693071
rs114245830
rs150358055
rs2906220
rs117992609
rs35509546
rs7944000
rs148223916
rs2021885
rs2997447
rs117138131
rs183272816
rs72931105
All − MR Egger
All − Inverse variance weighted
−1.5
−1.0
−0.5
0.0
0.5
1.0
MR effect size for
'CAG−873 sp001701165' on 'GCST90200647_buildGRCh38.tsv'

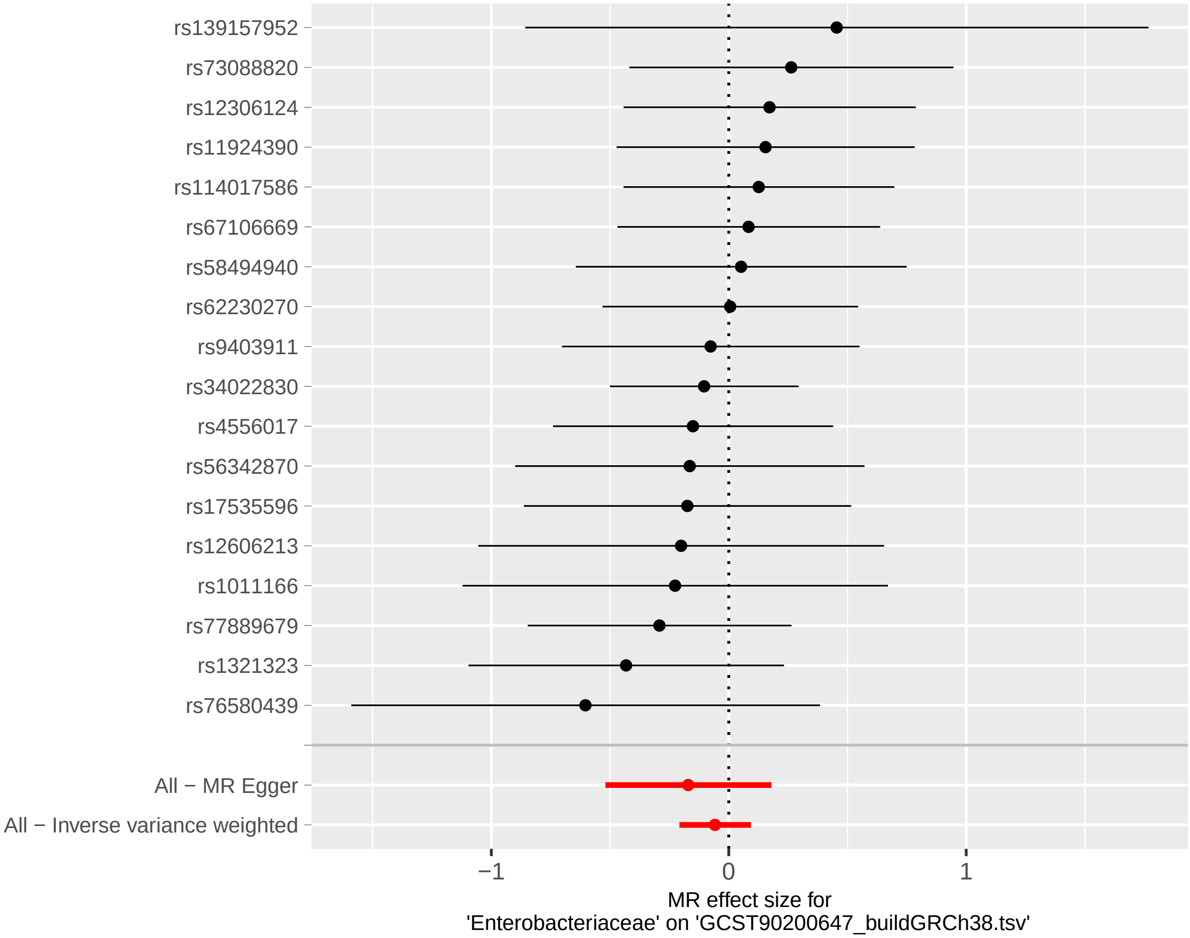

rs139157952
rs73088820
rs12306124
rs11924390
rs114017586
rs67106669
rs58494940
rs62230270
rs9403911
rs34022830
rs4556017
rs56342870
rs17535596
rs12606213
rs1011166
rs77889679
rs1321323
rs76580439
All − MR Egger
All − Inverse variance weighted
−1
0
1
MR effect size for
'Enterobacteriaceae' on 'GCST90200647_buildGRCh38.tsv'

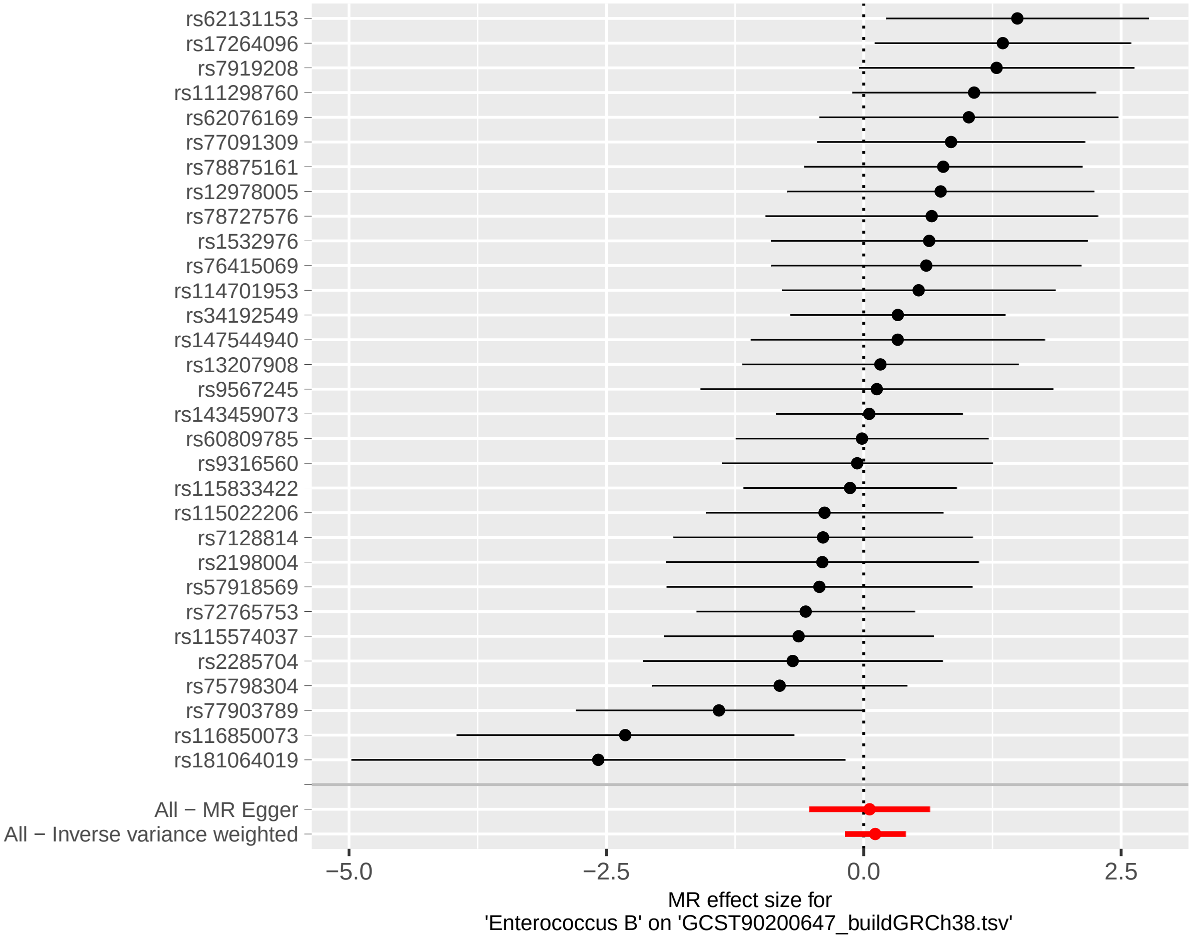
rs62131153
rs17264096
rs7919208
rs111298760
rs62076169
rs77091309
rs78875161
rs12978005
rs78727576
rs1532976
rs76415069
rs114701953
rs34192549
rs147544940
rs13207908
rs9567245
rs143459073
rs60809785
rs9316560
rs115833422
rs115022206
rs7128814
rs2198004
rs57918569
rs72765753
rs115574037
rs2285704
rs75798304
rs77903789
rs116850073
rs181064019
All − MR Egger
All − Inverse variance weighted
−5.0
−2.5
0.0
2.5
MR effect size for
'Enterococcus B' on 'GCST90200647_buildGRCh38.tsv'

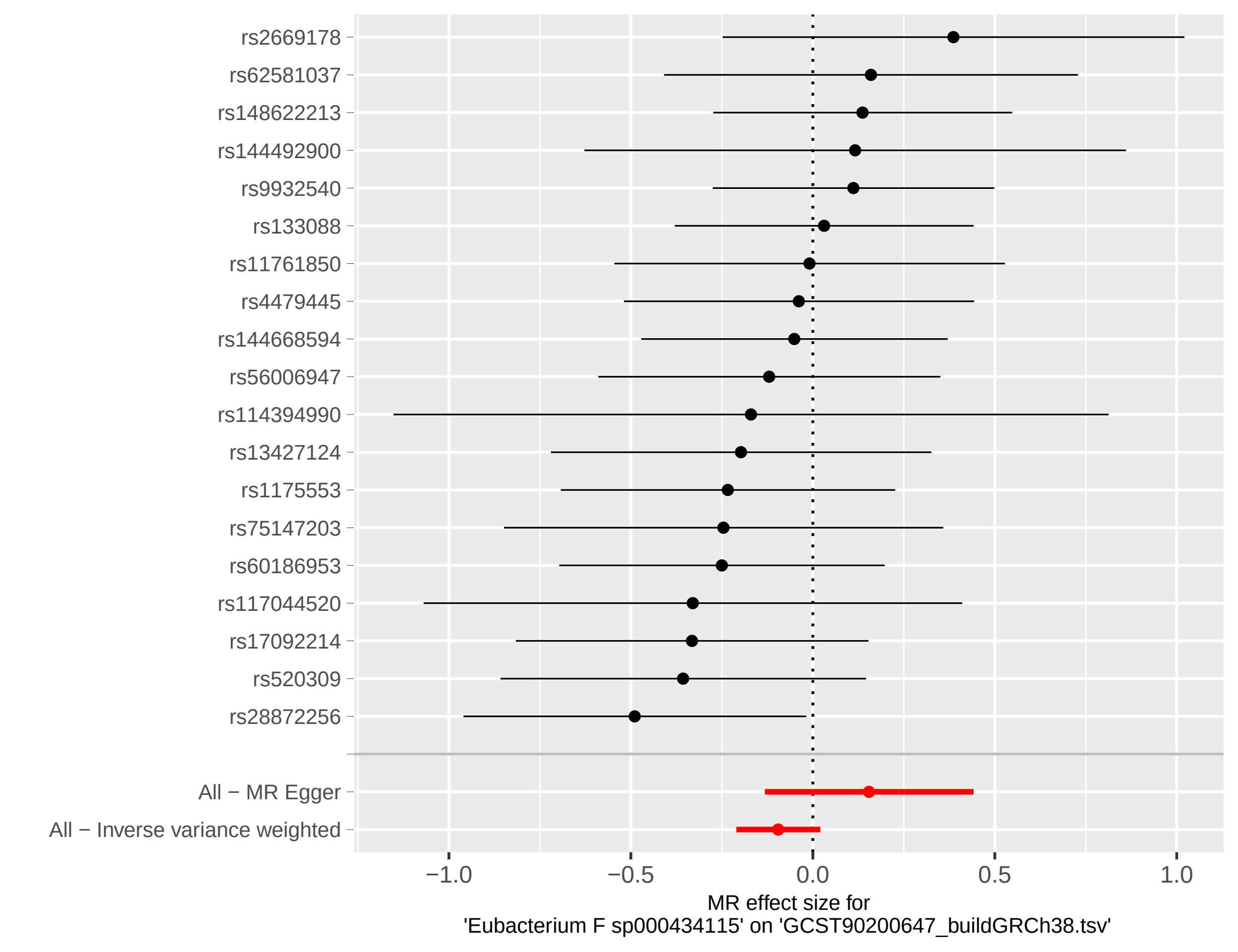
rs2669178
rs62581037
rs148622213
rs144492900
rs9932540
rs133088
rs11761850
rs4479445
rs144668594
rs56006947
rs114394990
rs13427124
rs1175553
rs75147203
rs60186953
rs117044520
rs17092214
rs520309
rs28872256
All − MR Egger
All − Inverse variance weighted
−1.0
−0.5
0.0
0.5
1.0
MR effect size for
'Eubacterium F sp000434115' on 'GCST90200647_buildGRCh38.tsv'

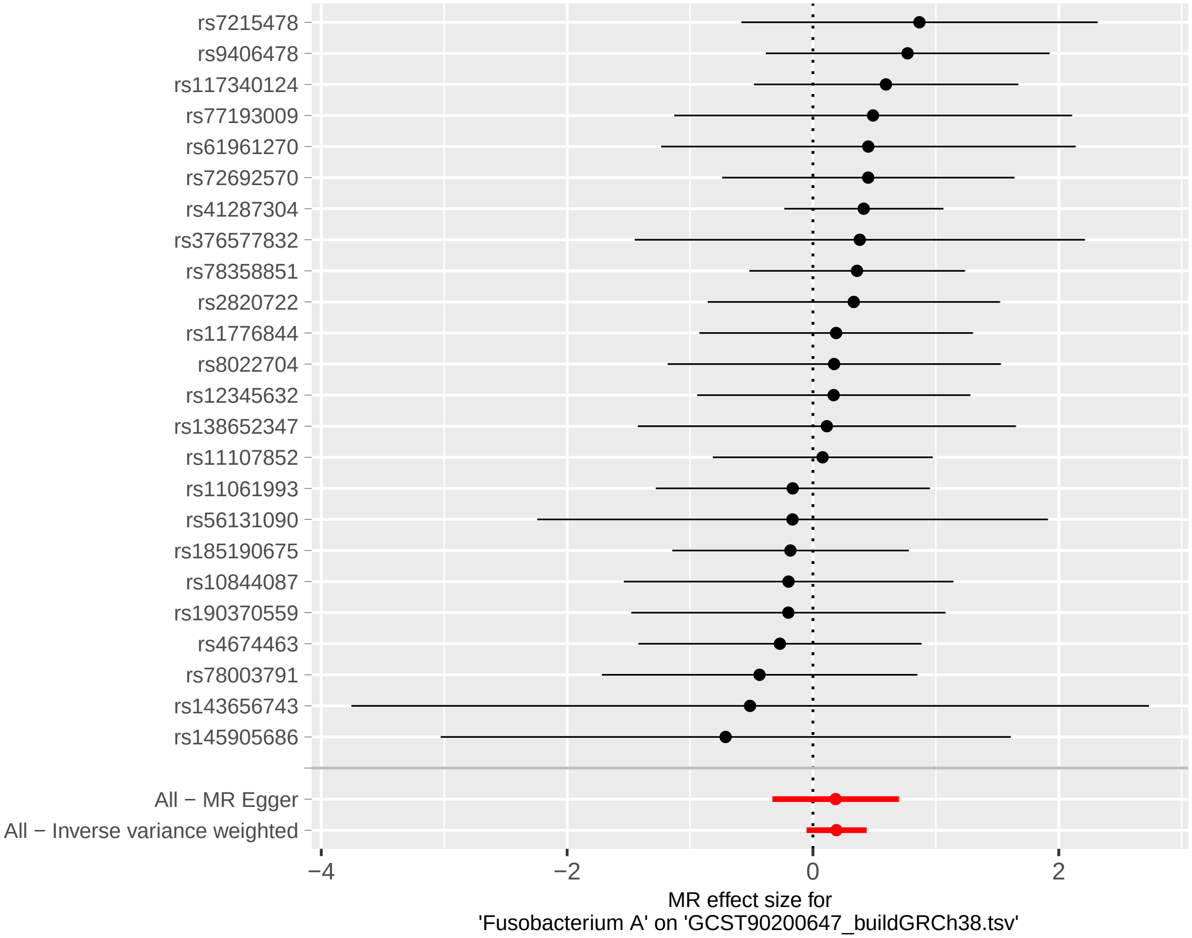
rs7215478
rs9406478
rs117340124
rs77193009
rs61961270
rs72692570
rs41287304
rs376577832
rs78358851
rs2820722
rs11776844
rs8022704
rs12345632
rs138652347
rs11107852
rs11061993
rs56131090
rs185190675
rs10844087
rs190370559
rs4674463
rs78003791
rs143656743
rs145905686
All − MR Egger
All − Inverse variance weighted
−4
−2
0
2
MR effect size for
'Fusobacterium A' on 'GCST90200647_buildGRCh38.tsv'

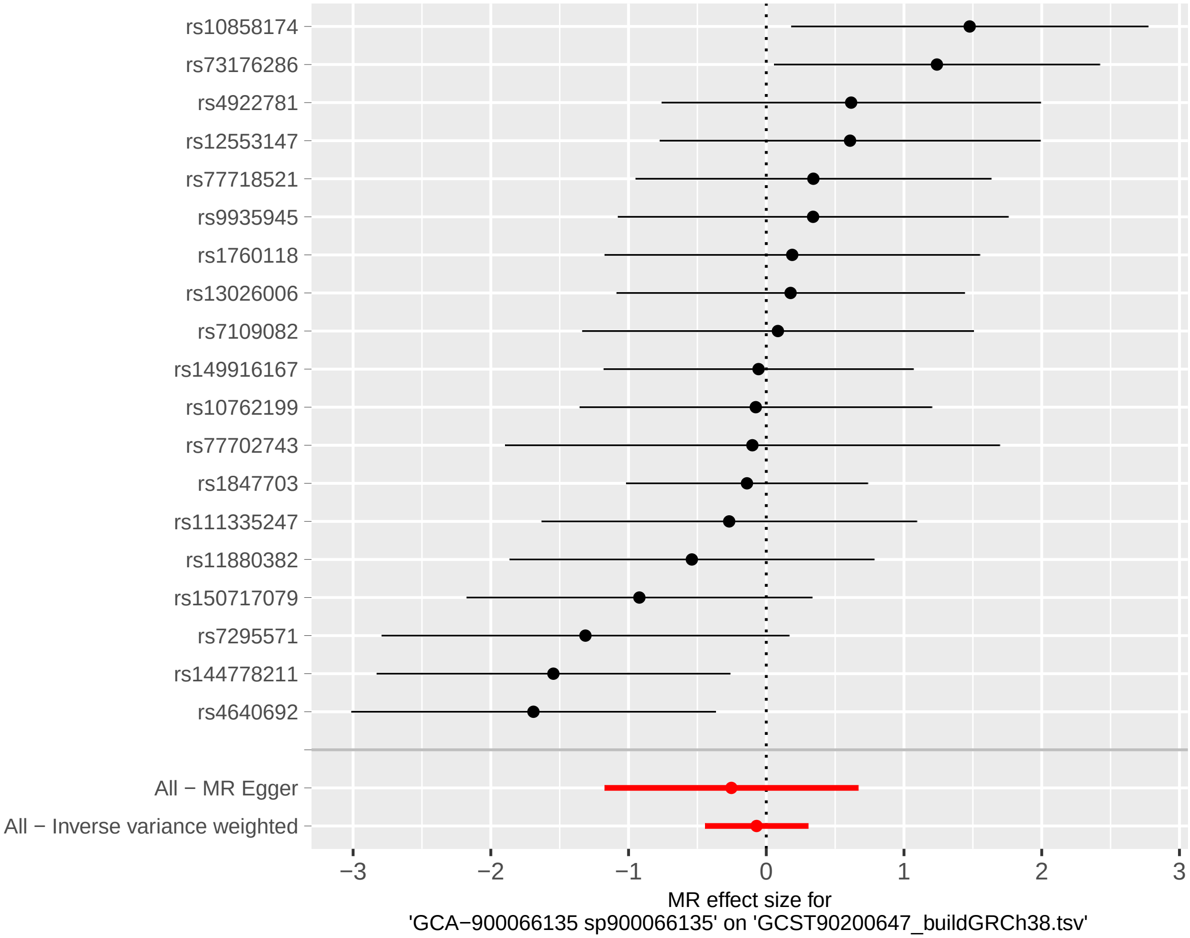
rs10858174
rs73176286
rs4922781
rs12553147
rs77718521
rs9935945
rs1760118
rs13026006
rs7109082
rs149916167
rs10762199
rs77702743
rs1847703
rs111335247
rs11880382
rs150717079
rs7295571
rs144778211
rs4640692
All − MR Egger
All − Inverse variance weighted
−3
−2
−1
0
1
2
3
MR effect size for
'GCA−900066135 sp900066135' on 'GCST90200647_buildGRCh38.tsv'

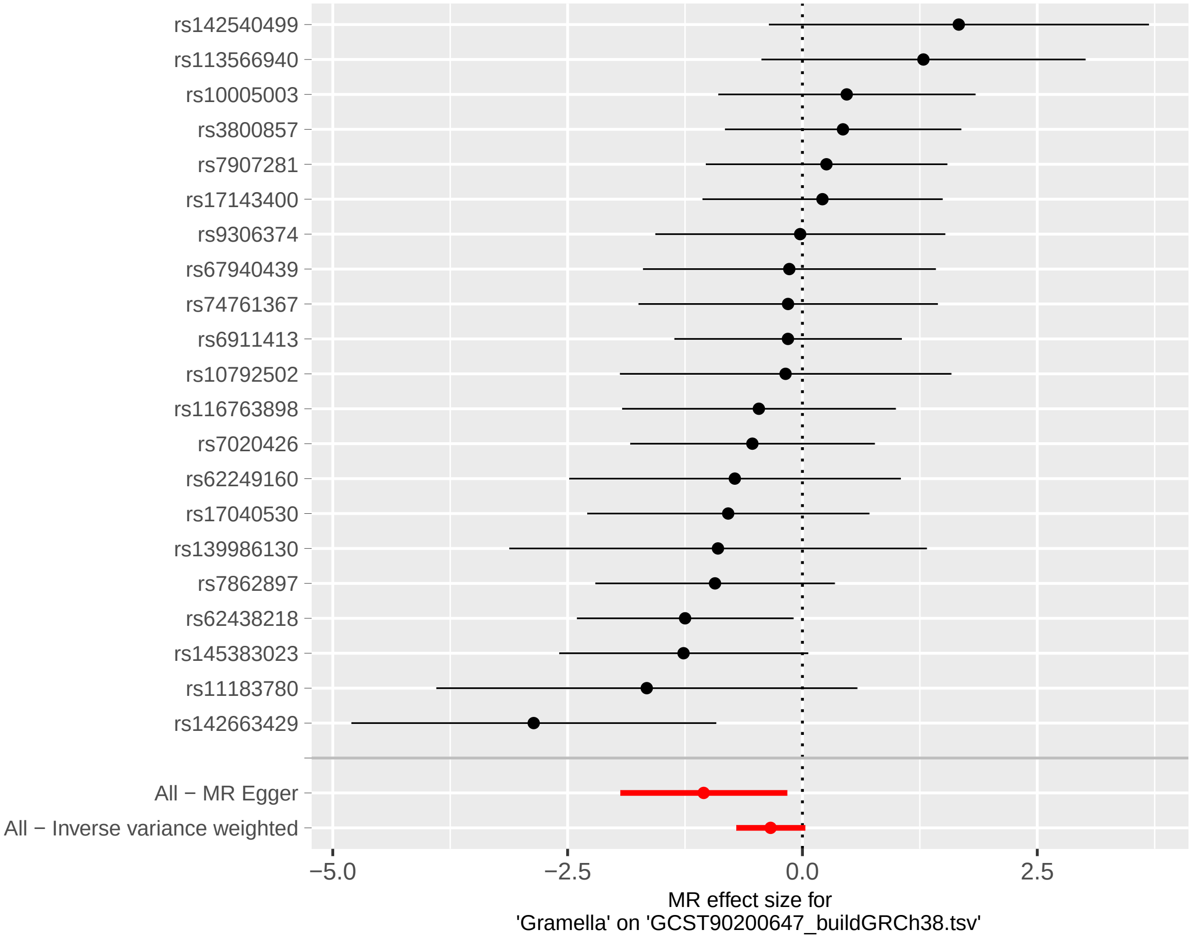

rs142540499
rs113566940
rs10005003
rs3800857
rs7907281
rs17143400
rs9306374
rs67940439
rs74761367
rs6911413
rs10792502
rs116763898
rs7020426
rs62249160
rs17040530
rs139986130
rs7862897
rs62438218
rs145383023
rs11183780
rs142663429
All − MR Egger
All − Inverse variance weighted
−5.0
−2.5
0.0
2.5
MR effect size for
'Gramella' on 'GCST90200647_buildGRCh38.tsv'

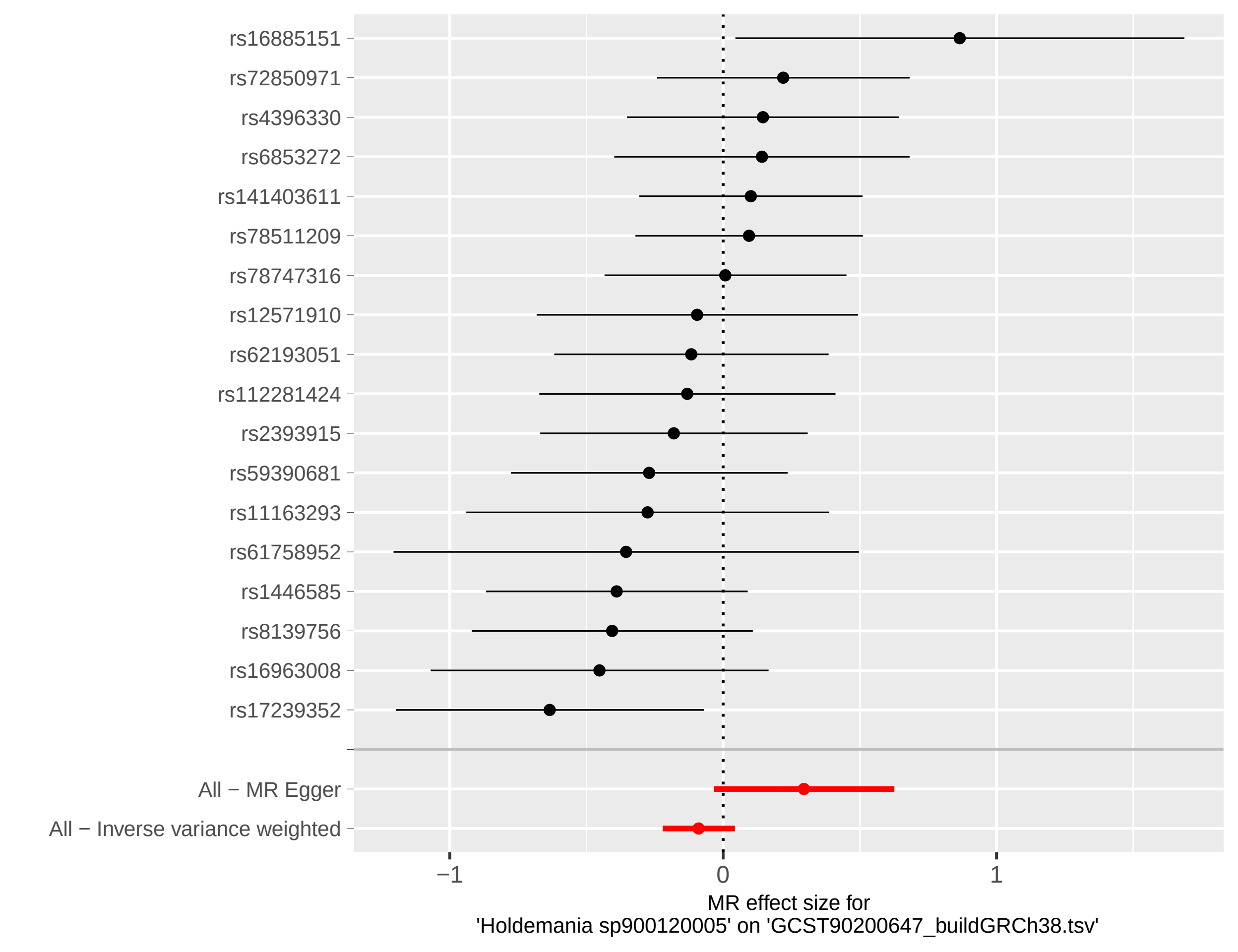

rs16885151
rs72850971
rs4396330
rs6853272
rs141403611
rs78511209
rs78747316
rs12571910
rs62193051
rs112281424
rs2393915
rs59390681
rs11163293
rs61758952
rs1446585
rs8139756
rs16963008
rs17239352
All − MR Egger
All − Inverse variance weighted
−1
0
1
MR effect size for
'Holdemania sp900120005' on 'GCST90200647_buildGRCh38.tsv'

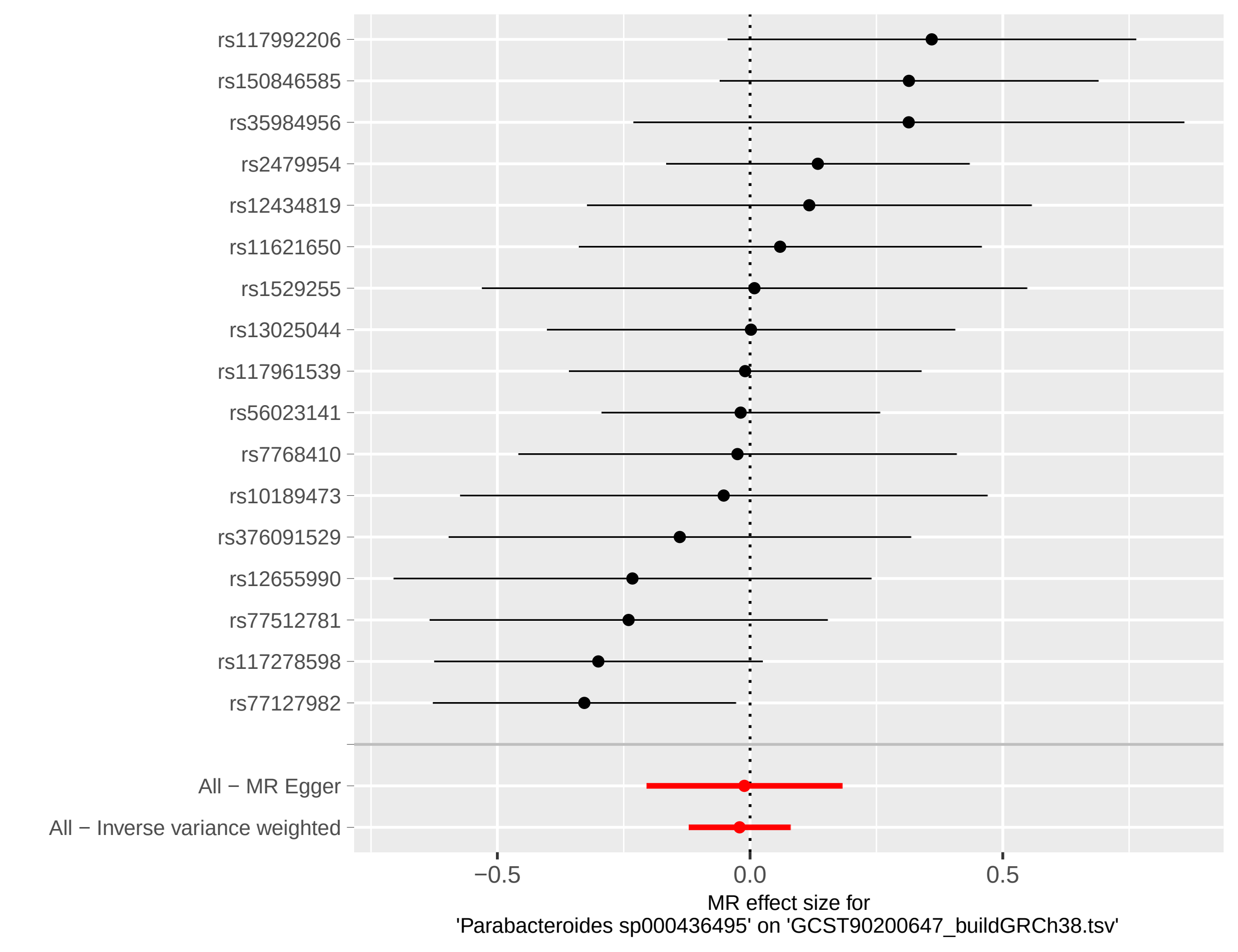
rs117992206
rs150846585
rs35984956
rs2479954
rs12434819
rs11621650
rs1529255
rs13025044
rs117961539
rs56023141
rs7768410
rs10189473
rs376091529
rs12655990
rs77512781
rs117278598
rs77127982
All − MR Egger
All − Inverse variance weighted
−0.5
0.0
0.5
MR effect size for
'Parabacteroides sp000436495' on 'GCST90200647_buildGRCh38.tsv'

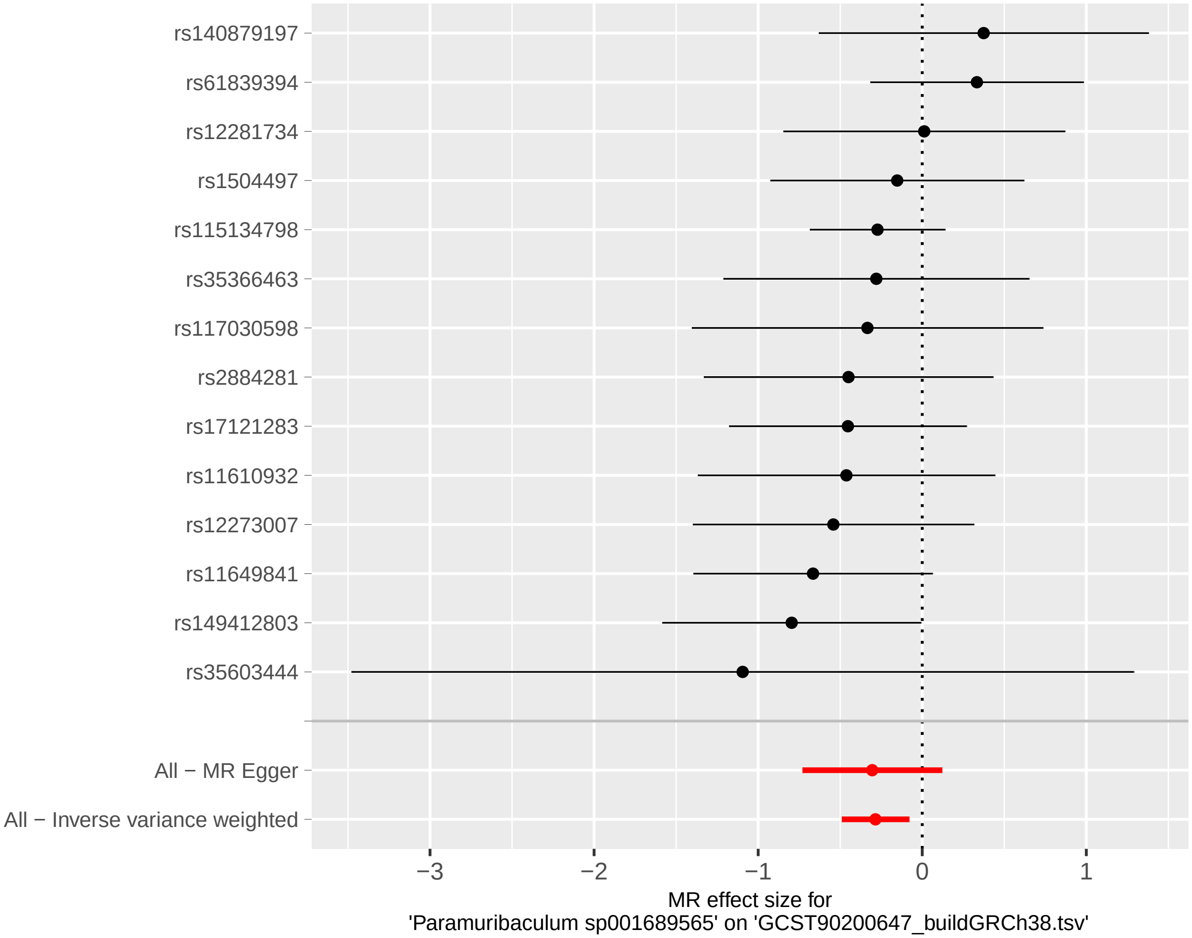
rs140879197
rs61839394
rs12281734
rs1504497
rs115134798
rs35366463
rs117030598
rs2884281
rs17121283
rs11610932
rs12273007
rs11649841
rs149412803
rs35603444
All − MR Egger
All − Inverse variance weighted
−3
−2
−1
0
1
MR effect size for
'Paramuribaculum sp001689565' on 'GCST90200647_buildGRCh38.tsv'

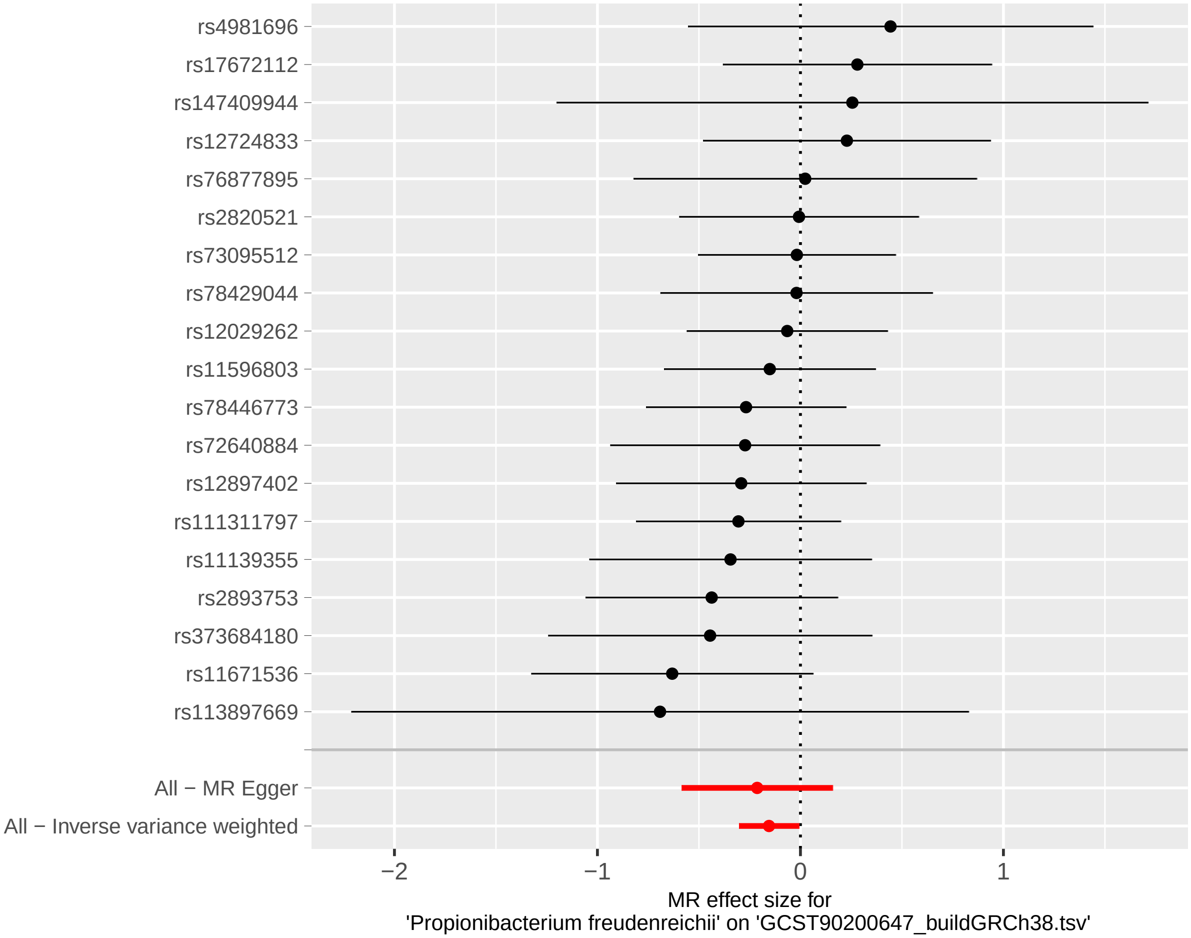
rs4981696
rs17672112
rs147409944
rs12724833
rs76877895
rs2820521
rs73095512
rs78429044
rs12029262
rs11596803
rs78446773
rs72640884
rs12897402
rs111311797
rs11139355
rs2893753
rs373684180
rs11671536
rs113897669
All − MR Egger
All − Inverse variance weighted
−2
−1
0
1
MR effect size for
'Propionibacterium freudenreichii' on 'GCST90200647_buildGRCh38.tsv'

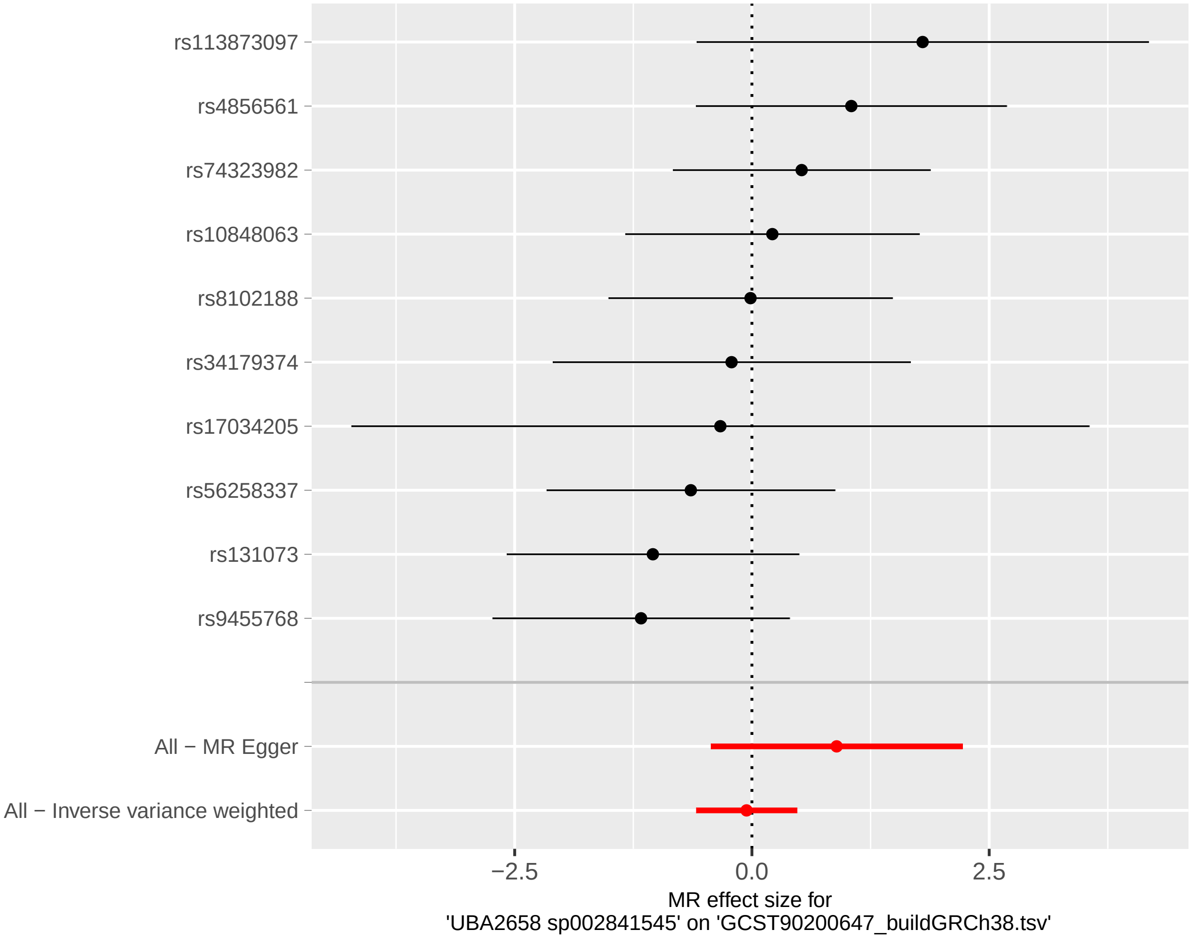

rs113873097
rs4856561
rs74323982
rs10848063
rs8102188
rs34179374
rs17034205
rs56258337
rs131073
rs9455768
All − MR Egger
All − Inverse variance weighted
−2.5
0.0
2.5
MR effect size for
'UBA2658 sp002841545' on 'GCST90200647_buildGRCh38.tsv'

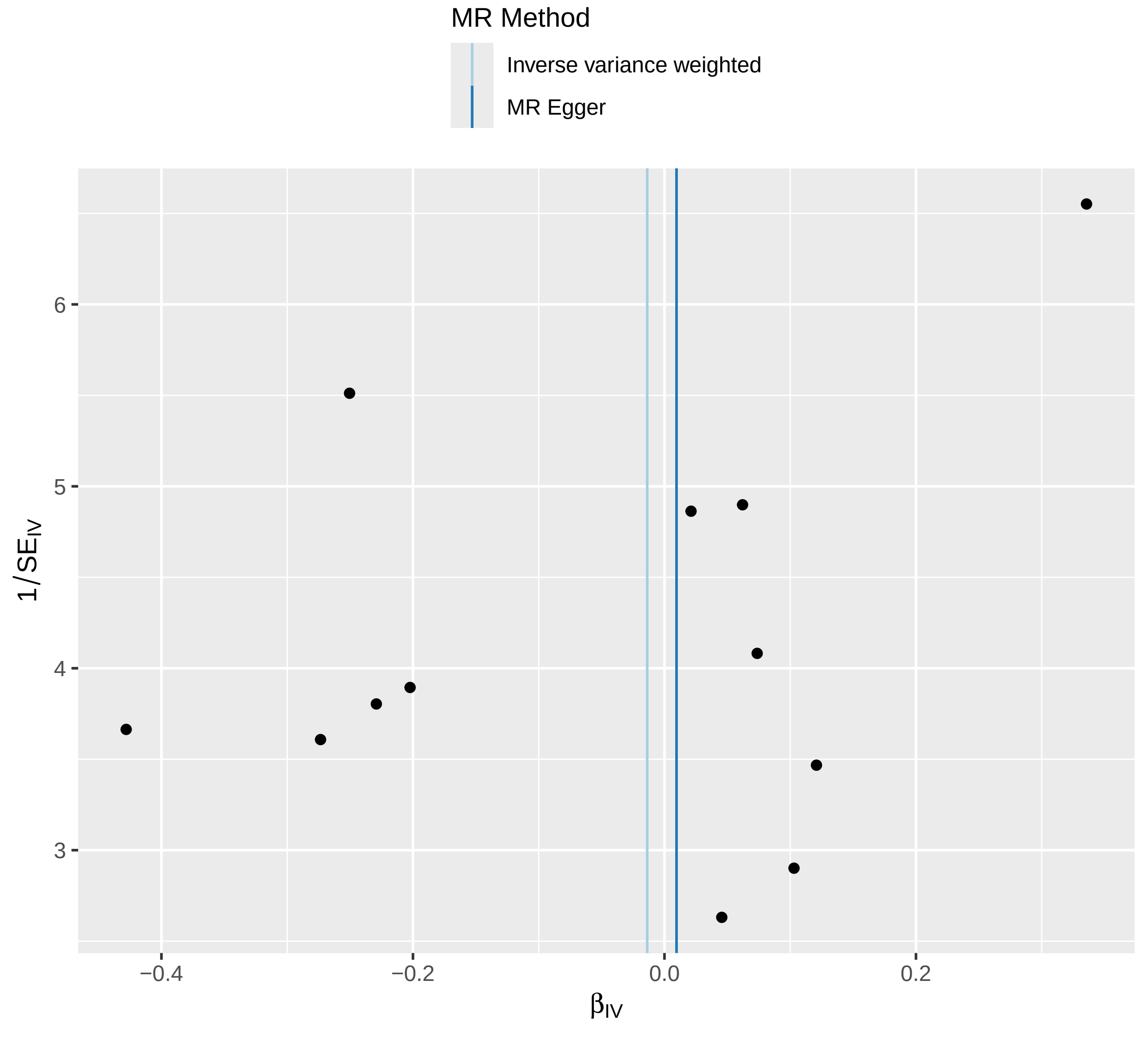
MR Method
Inverse variance weighted
MR Egger
6
5
4
3
1/SE$_{IV}$
−0.4
−0.2
0.0
0.2
β$_{IV}$

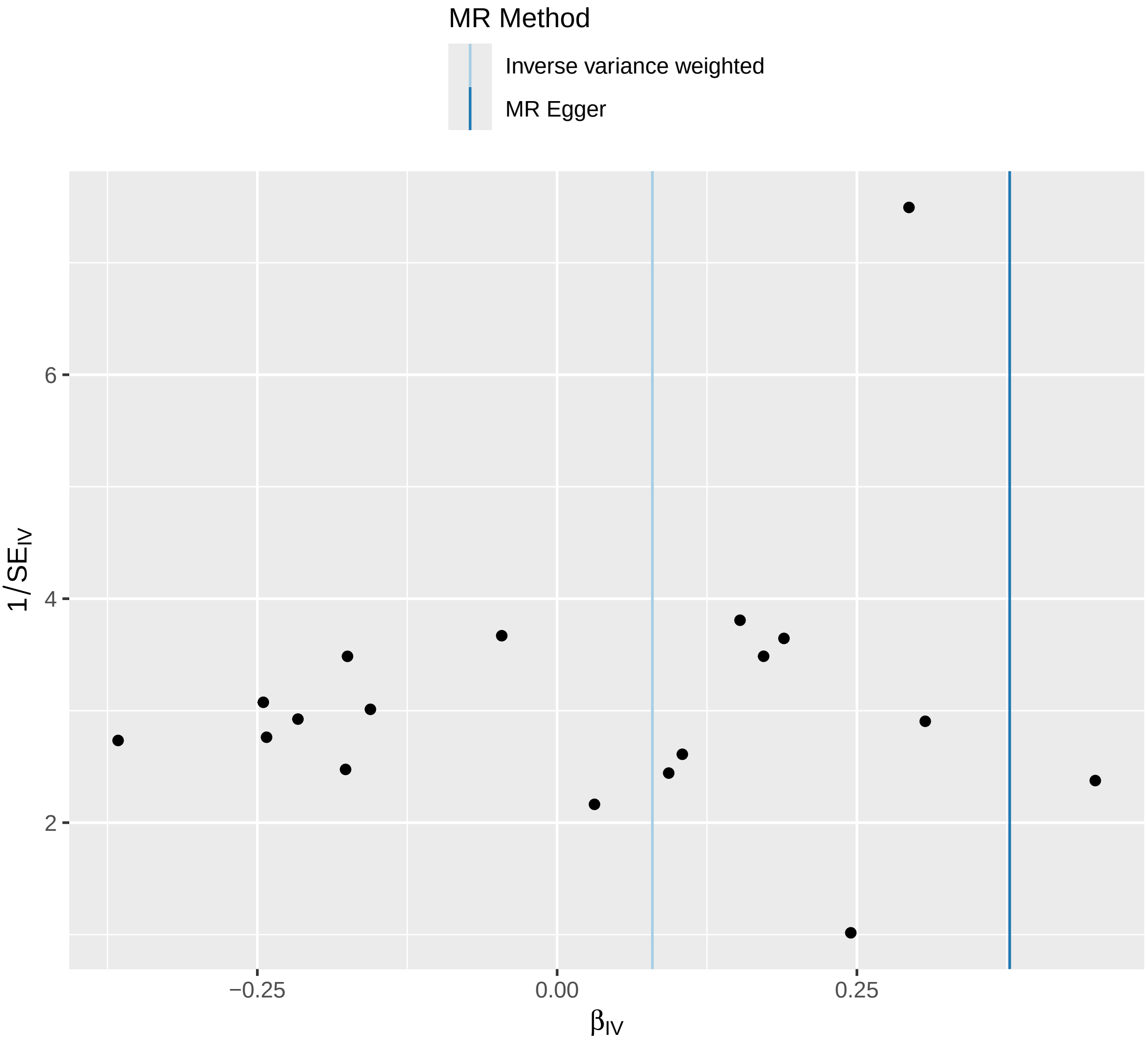

MR Method
Inverse variance weighted
MR Egger
6
4
2
1/SE_IV
−0.25
0.00
0.25
β_IV

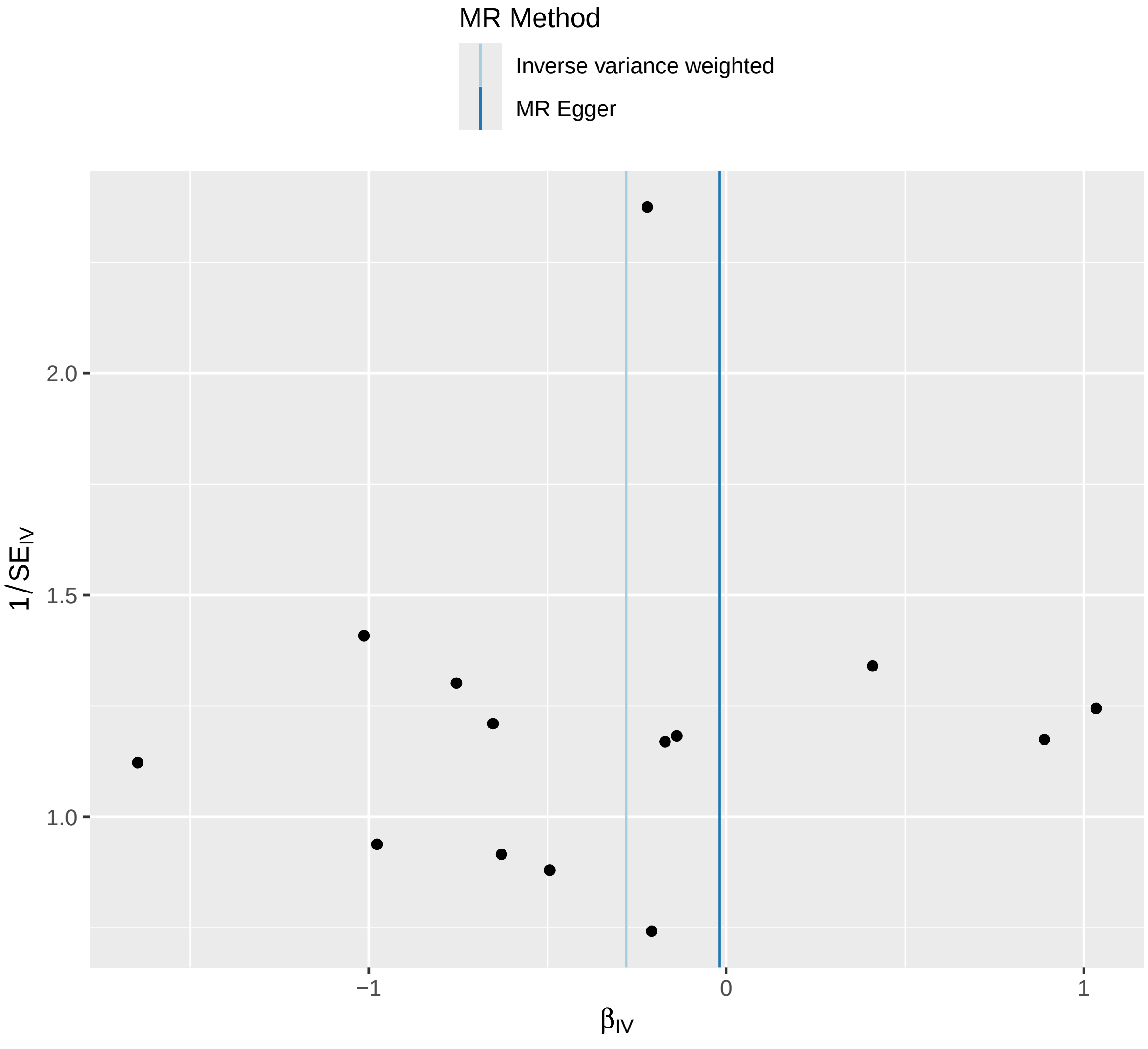
MR Method
Inverse variance weighted
MR Egger
2.0
1.5
1.0
1/SE_IV
−1
0
1
β_IV

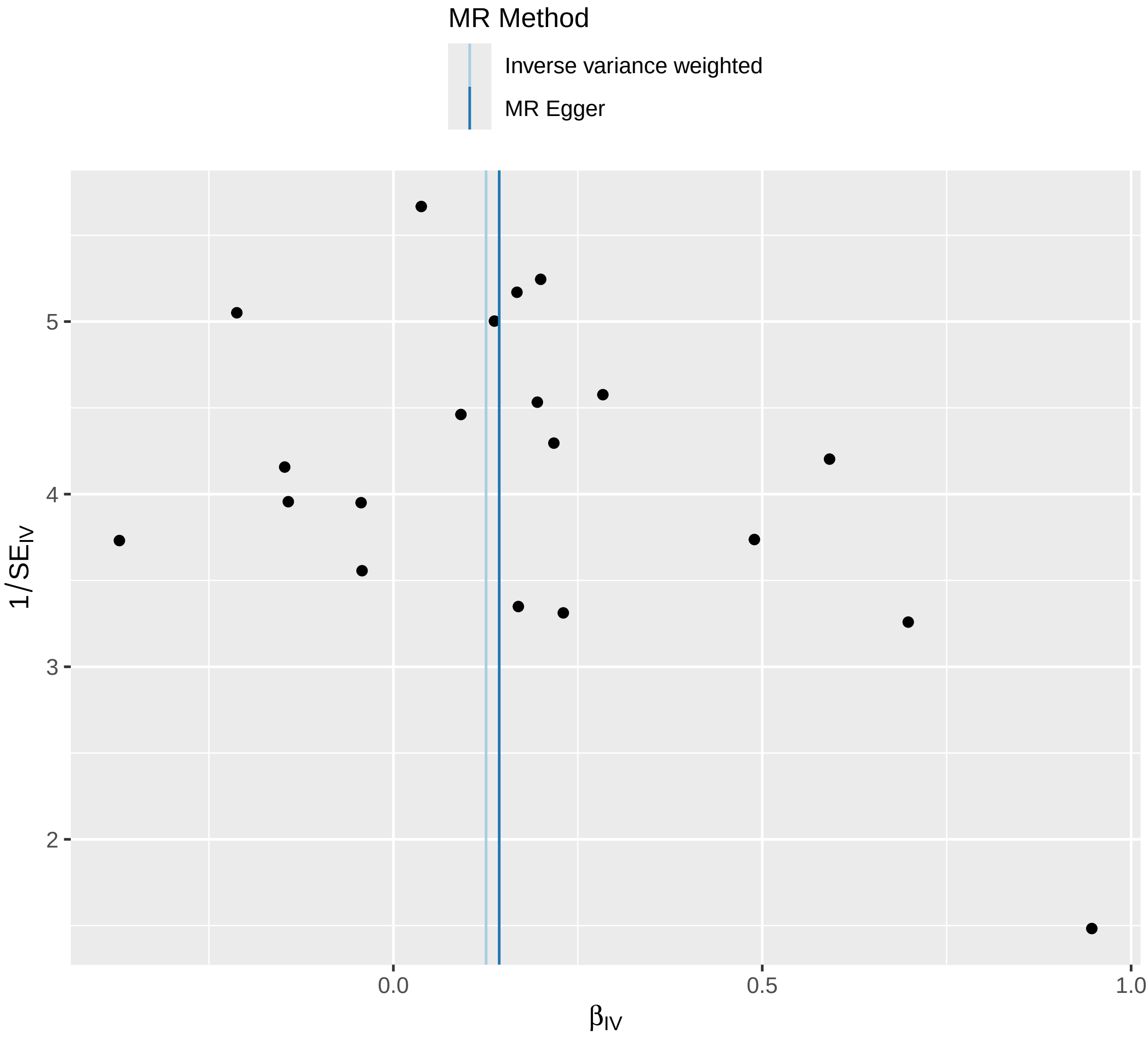
MR Method
Inverse variance weighted
MR Egger
1/SE_IV
5
4
3
2
0.0
0.5
1.0
β_IV

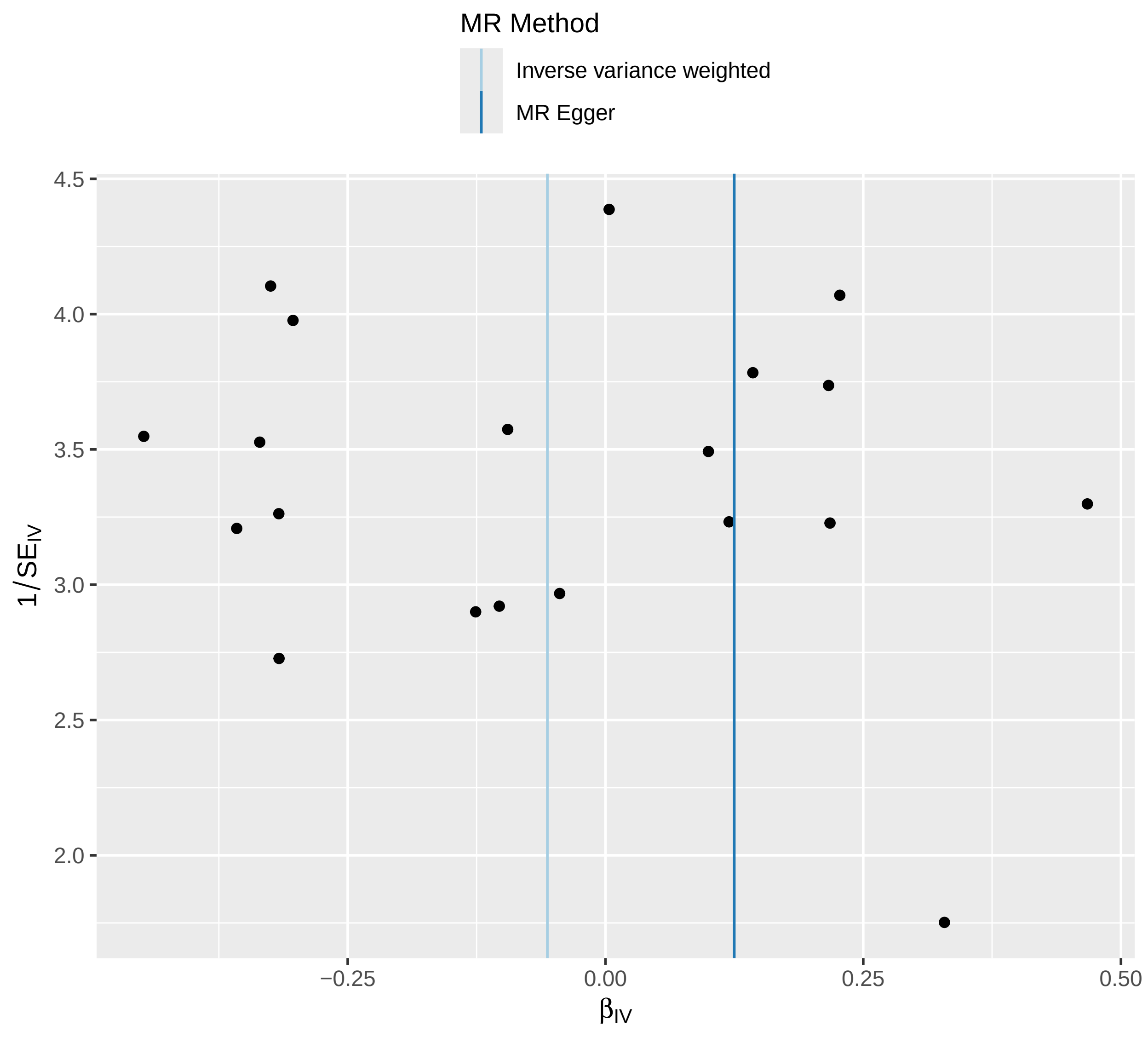

MR Method
Inverse variance weighted
MR Egger
4.5
4.0
3.5
3.0
2.5
2.0
1/SE_IV
−0.25
0.00
0.25
0.50
β_IV

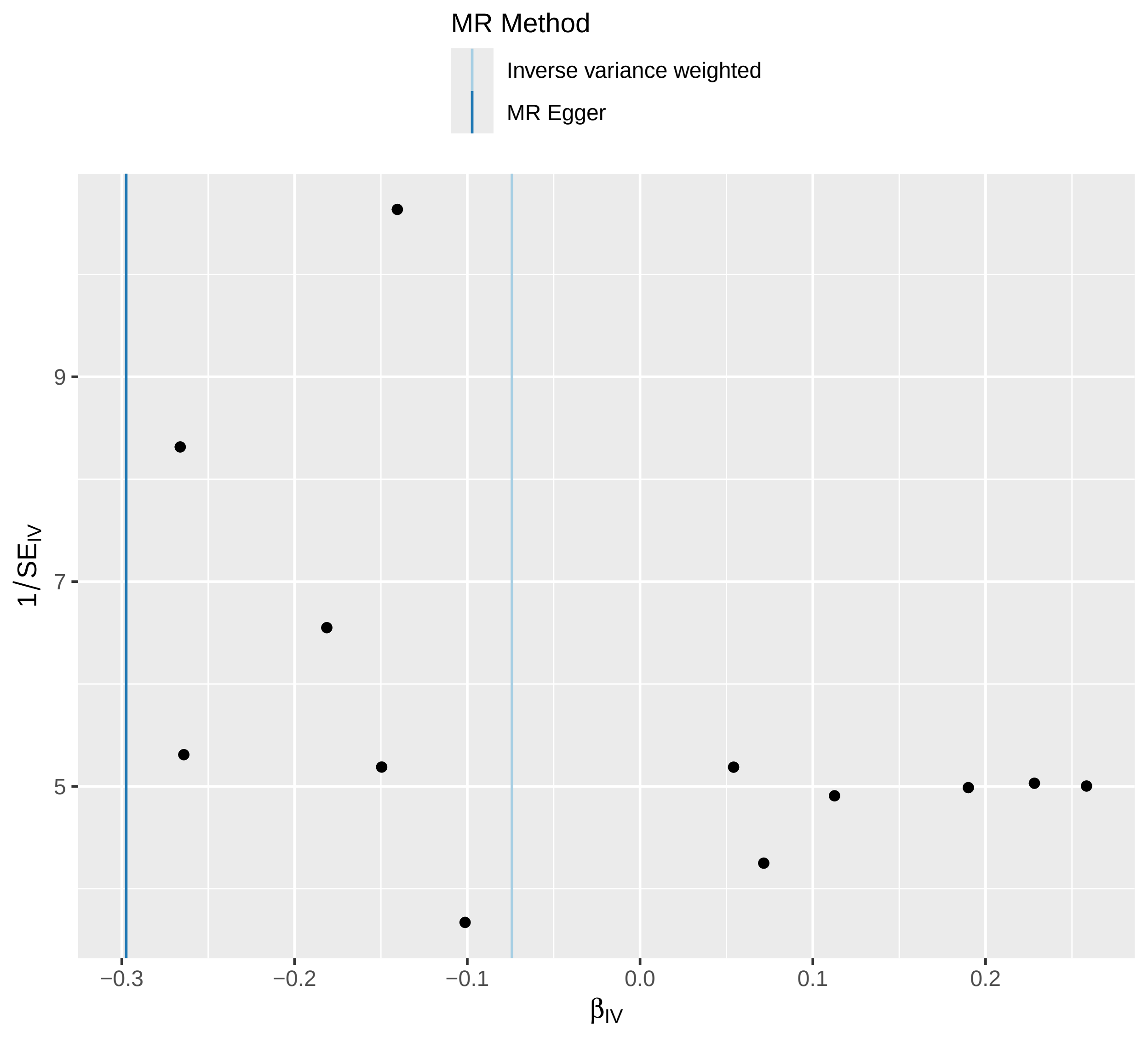
MR Method
Inverse variance weighted
MR Egger
9
7
5
1/SE_IV
−0.3
−0.2
−0.1
0.0
0.1
0.2
β_IV

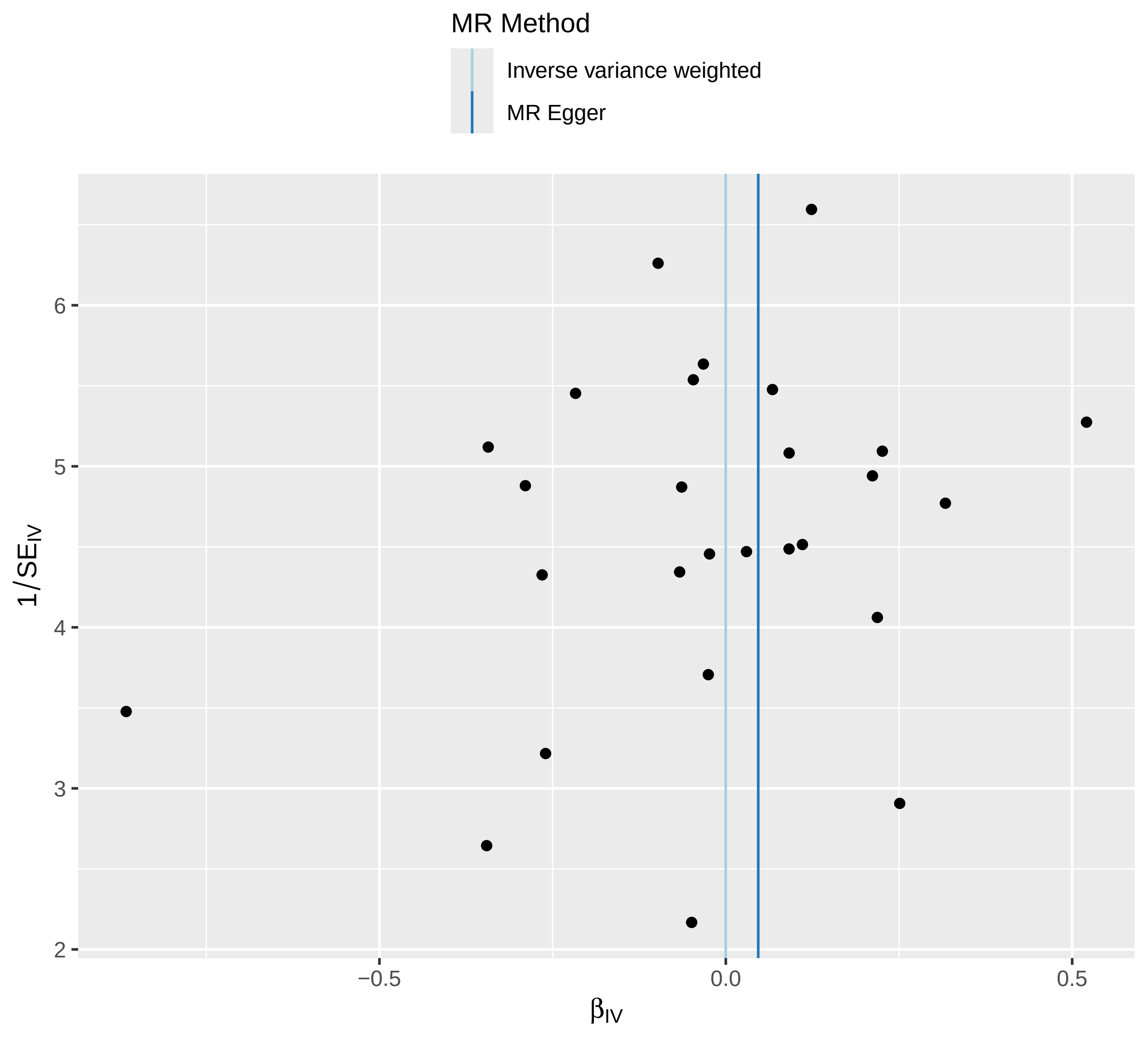
MR Method
Inverse variance weighted
MR Egger
6
5
4
3
2
1/SE$_{IV}$
−0.5
0.0
0.5
β$_{IV}$

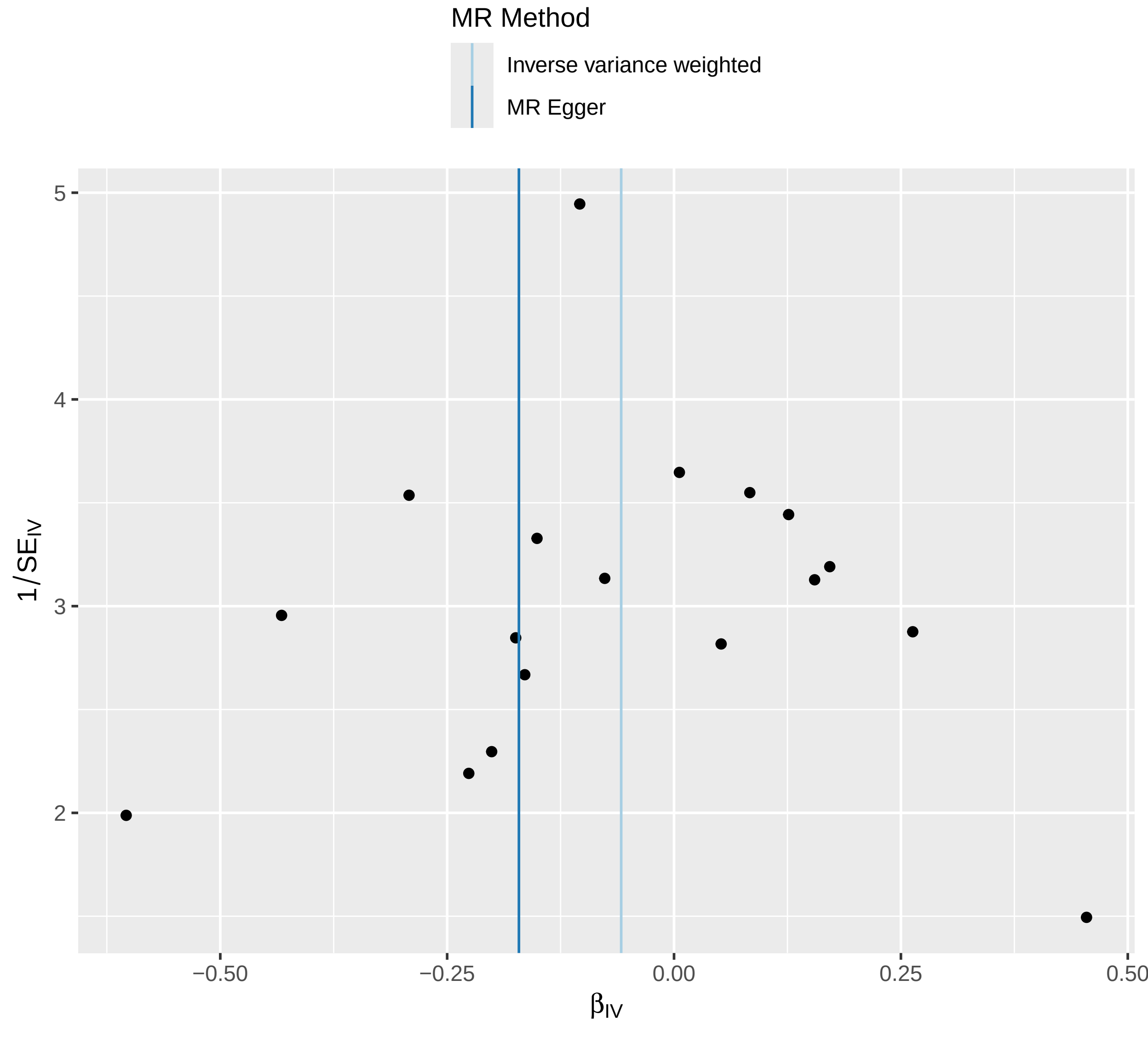

MR Method
Inverse variance weighted
MR Egger
1/SE_IV
β_IV
5
4
3
2
−0.50
−0.25
0.00
0.25
0.50

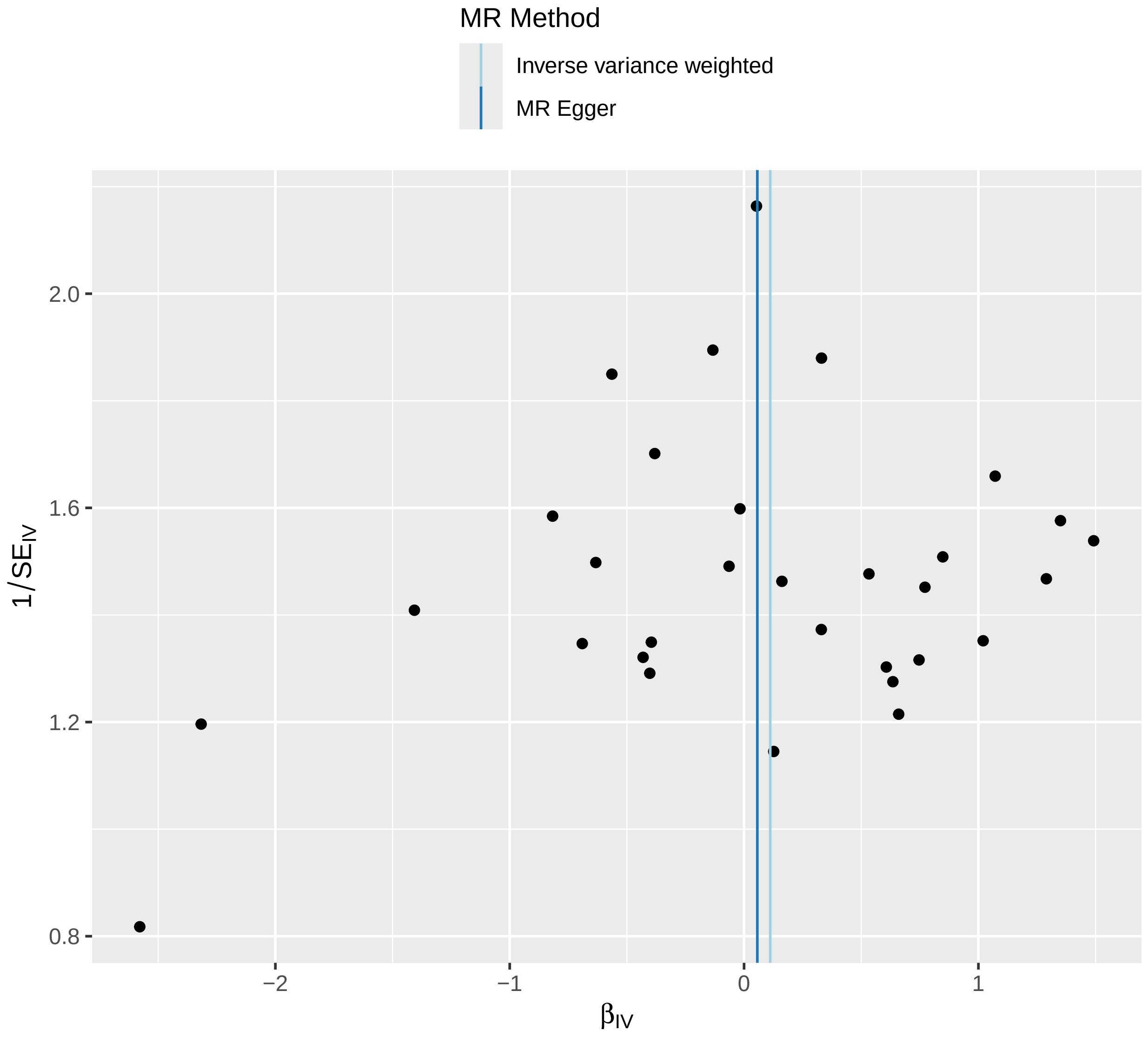

MR Method
Inverse variance weighted
MR Egger
2.0
1.6
1.2
0.8
1/SE_IV
−2
−1
0
1
β_IV

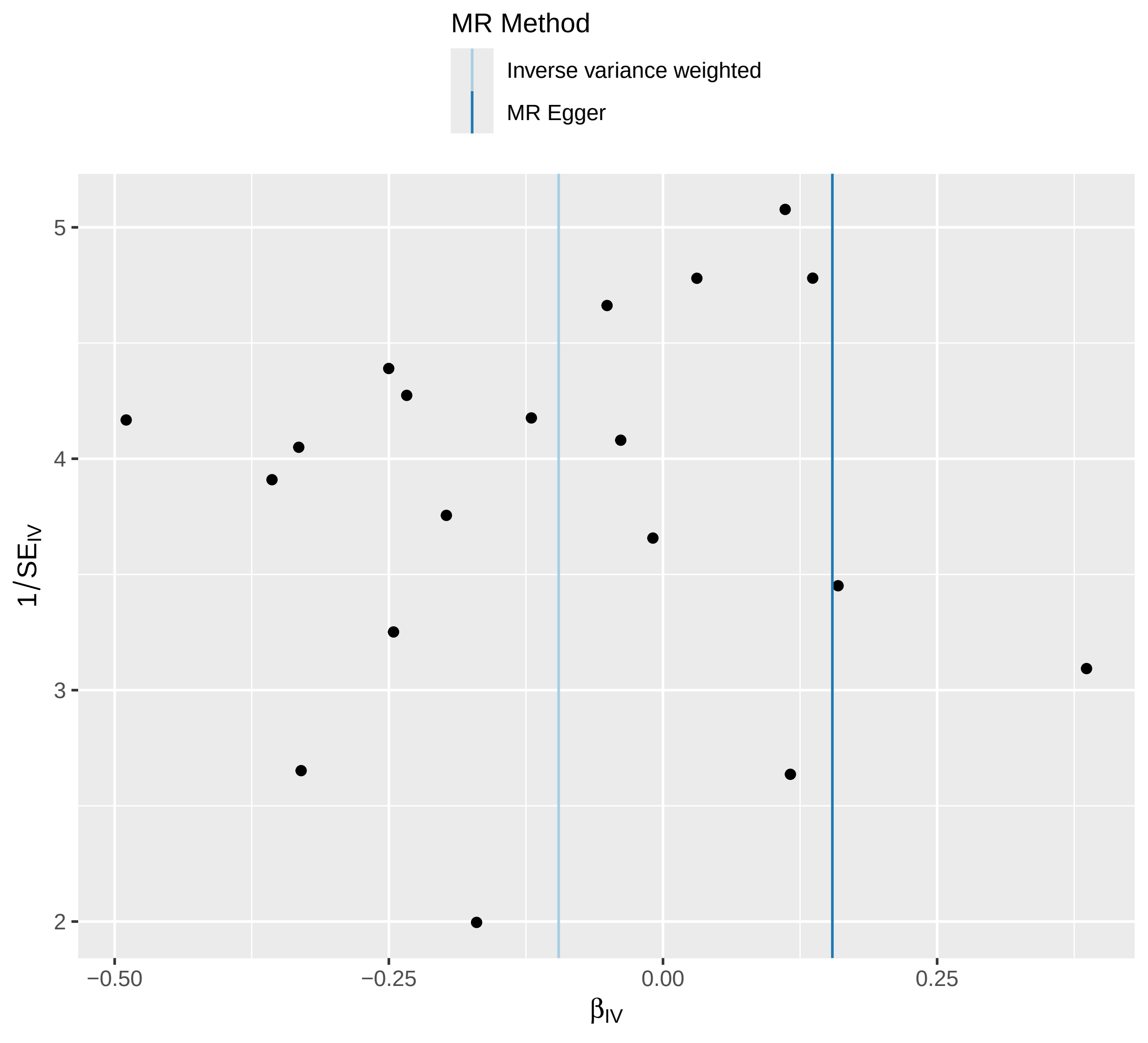
MR Method
Inverse variance weighted
MR Egger
5
4
3
2
1/SE_IV
−0.50
−0.25
0.00
0.25
β_IV

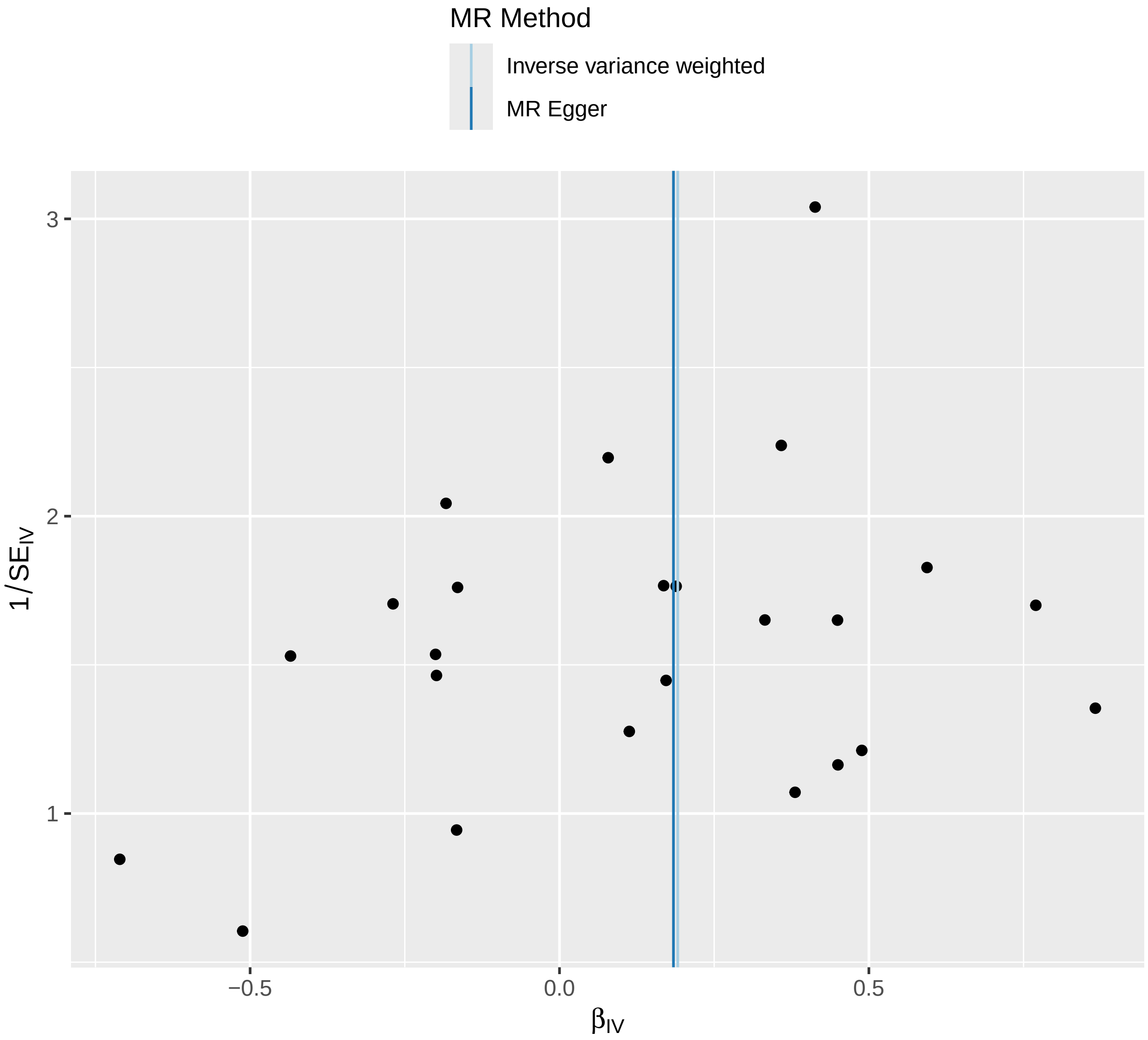
MR Method
Inverse variance weighted
MR Egger
3
2
1
$1/SE_{IV}$
−0.5
0.0
0.5
β_{IV}

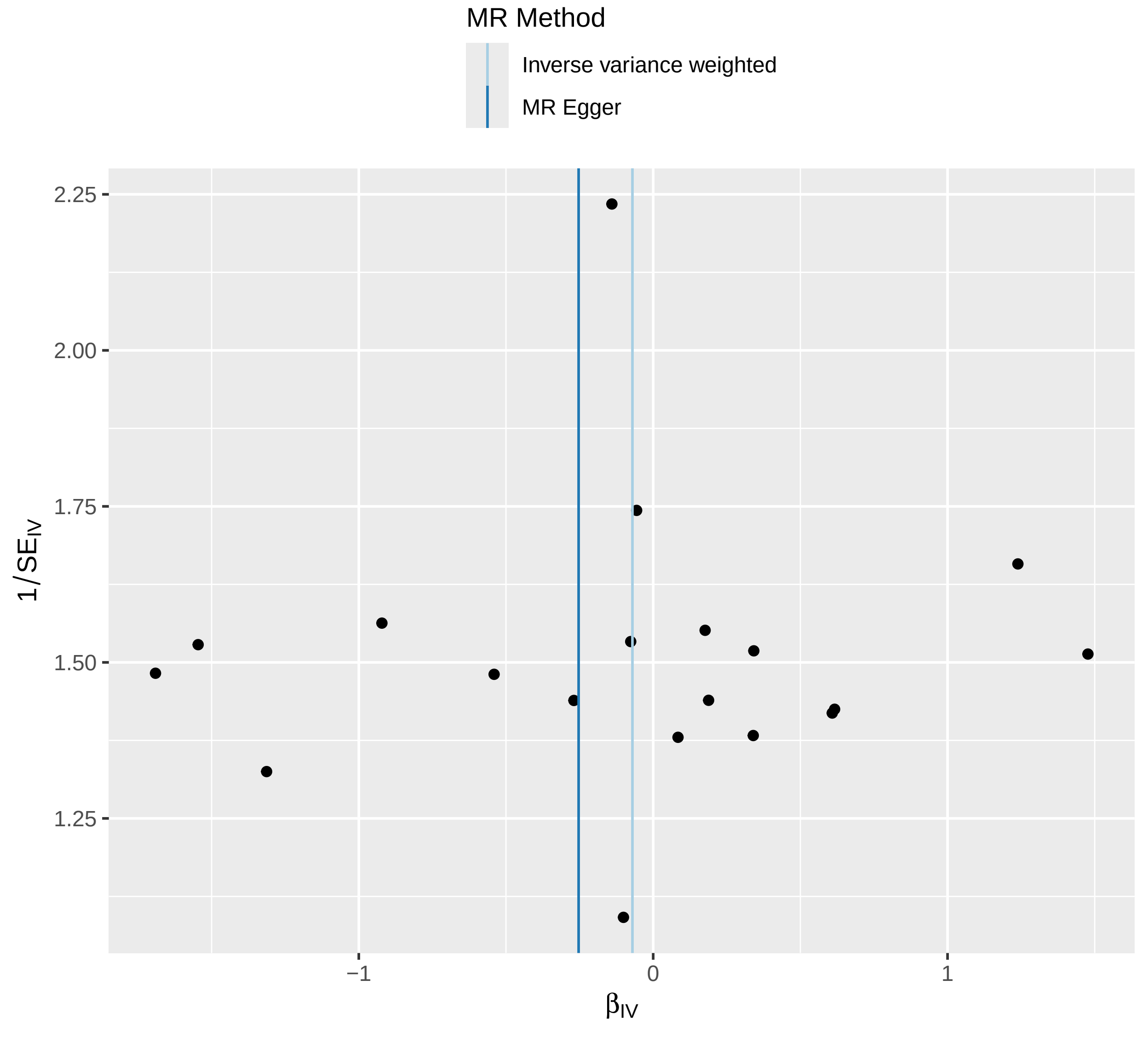
MR Method
Inverse variance weighted
MR Egger
2.25
2.00
1.75
1.50
1.25
$1/SE_{IV}$
−1
0
1
β_{IV}

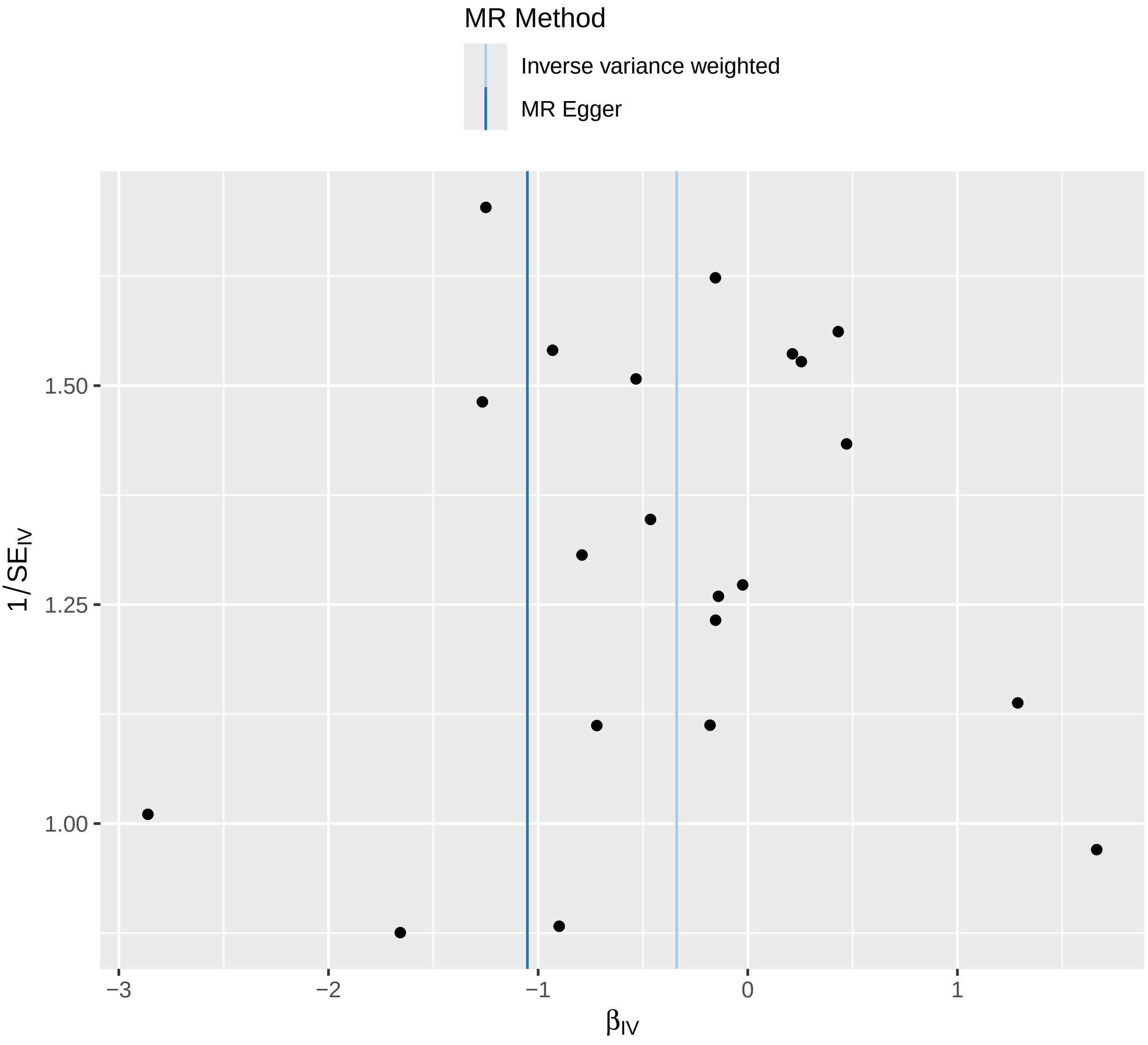
MR Method
Inverse variance weighted
MR Egger
1.50
1.25
1.00
$1/SE_{IV}$
−3
−2
−1
0
1
β_{IV}

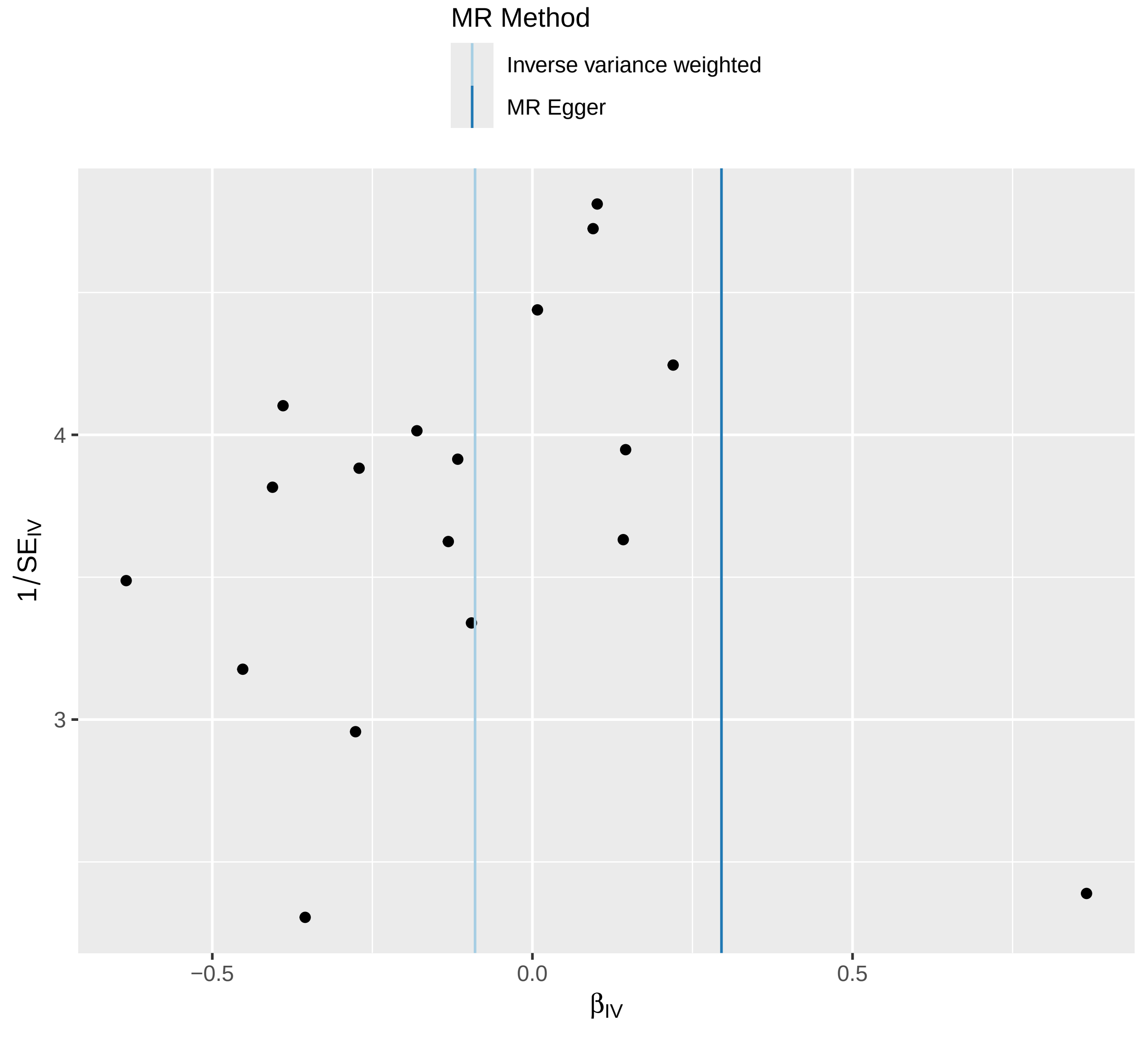
MR Method
Inverse variance weighted
MR Egger
1/SE$_{IV}$
4
3
−0.5
0.0
0.5
β$_{IV}$

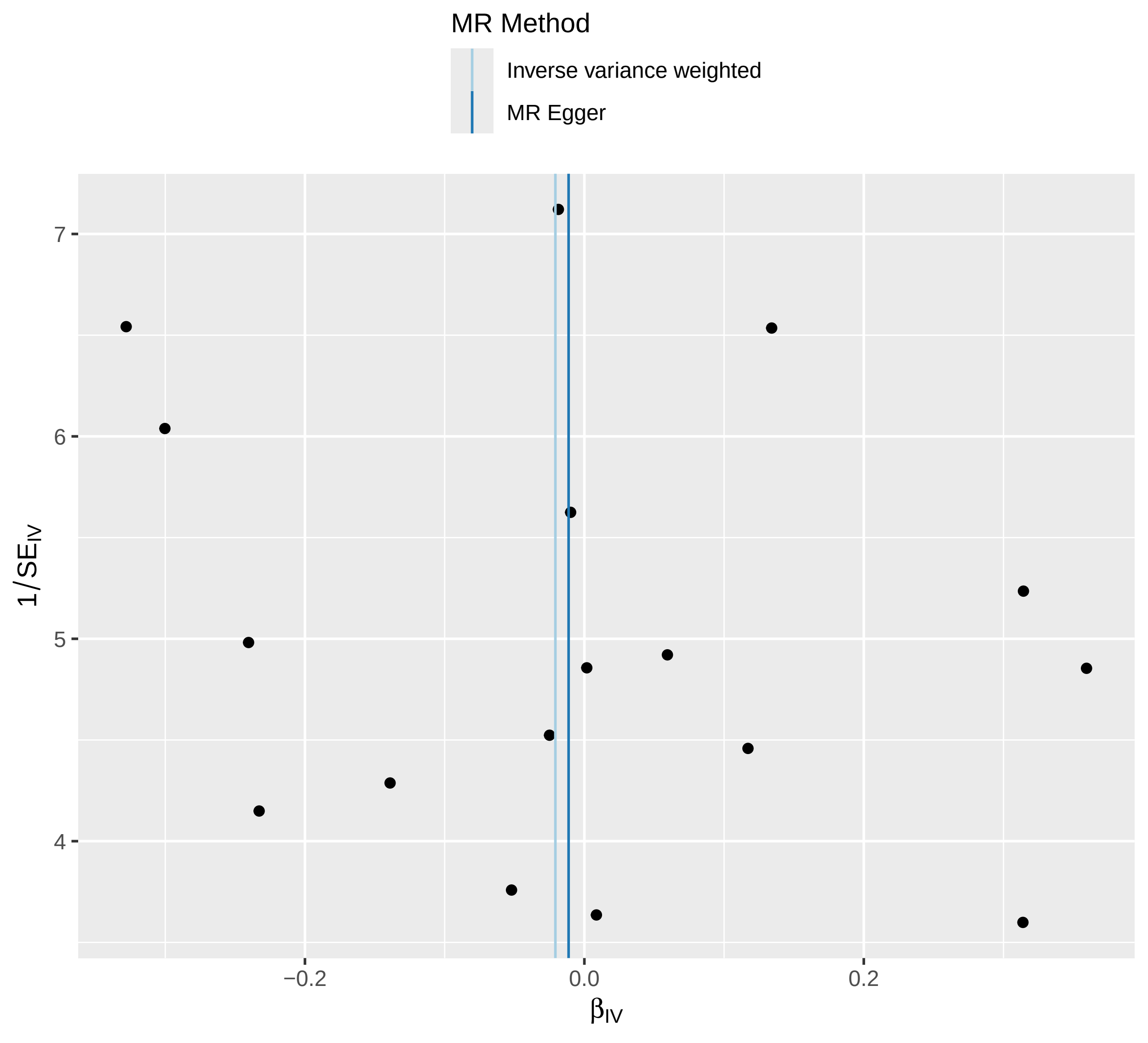
MR Method
Inverse variance weighted
MR Egger
7
6
5
4
$1/SE_{IV}$
−0.2
0.0
0.2
β_{IV}

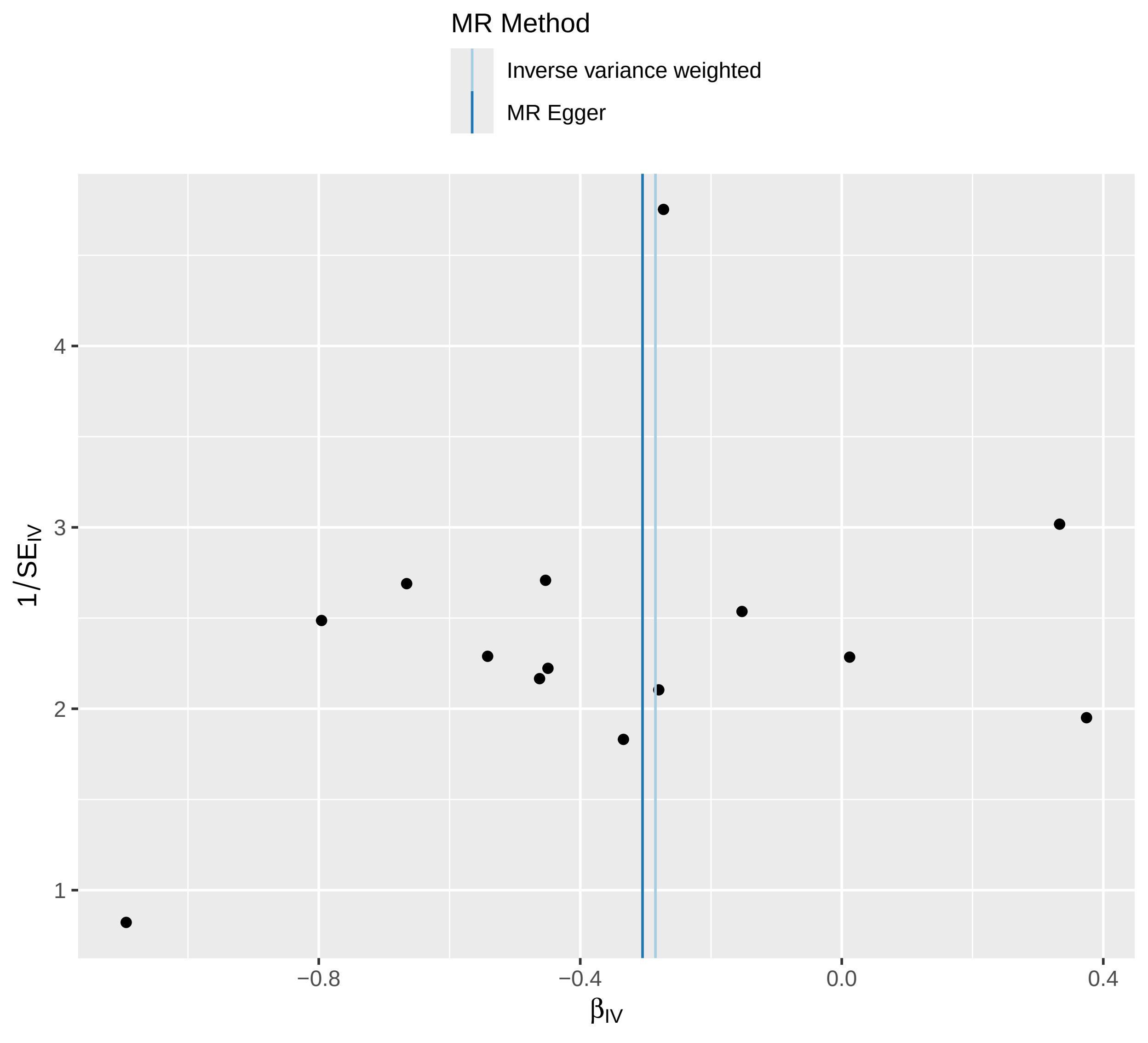
MR Method
Inverse variance weighted
MR Egger
$1/SE_{IV}$
4
3
2
1
−0.8
−0.4
0.0
0.4
β_{IV}

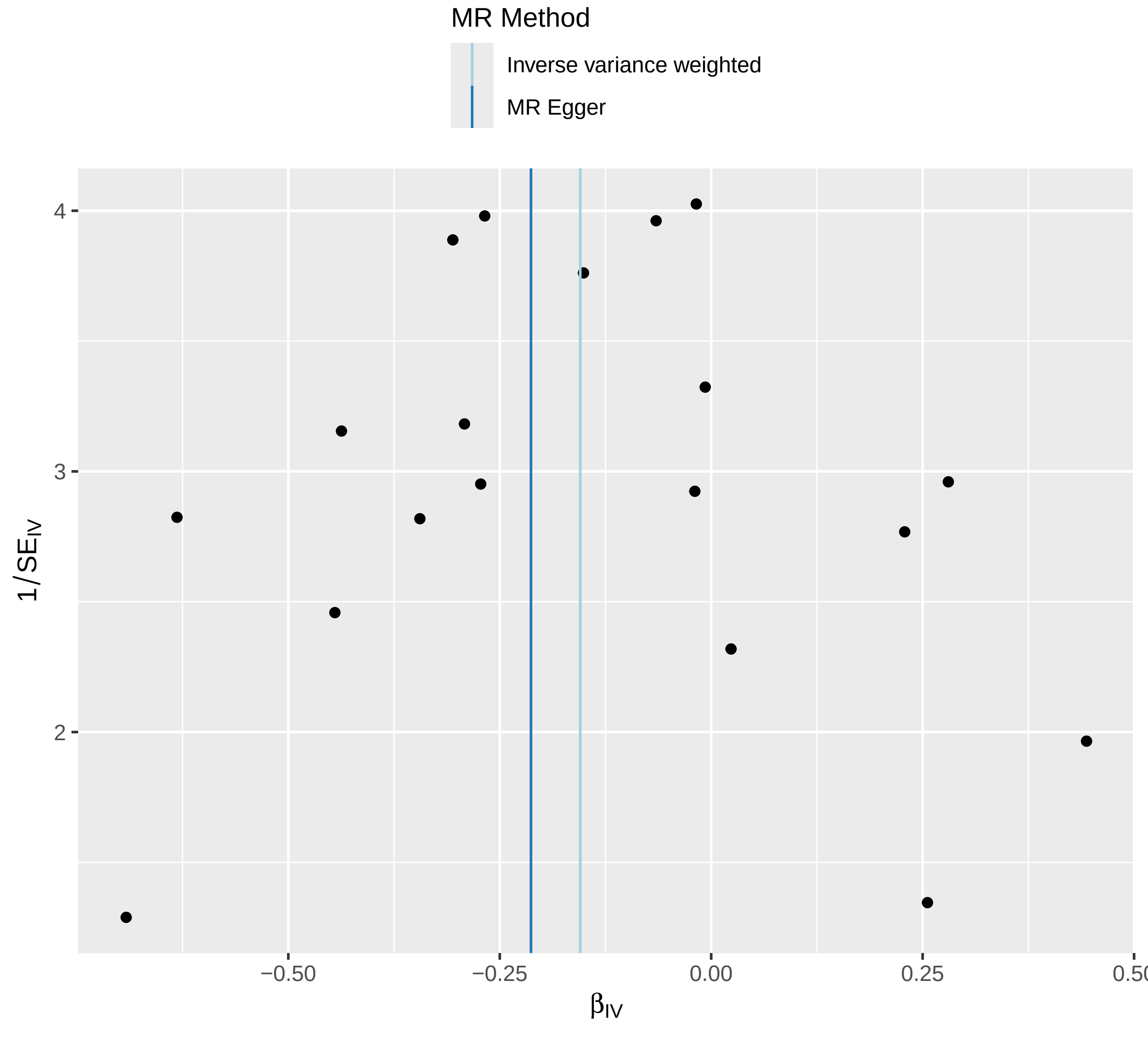

MR Method
Inverse variance weighted
MR Egger
4
3
2
1/SE$_{IV}$
−0.50
−0.25
0.00
0.25
0.50
β$_{IV}$

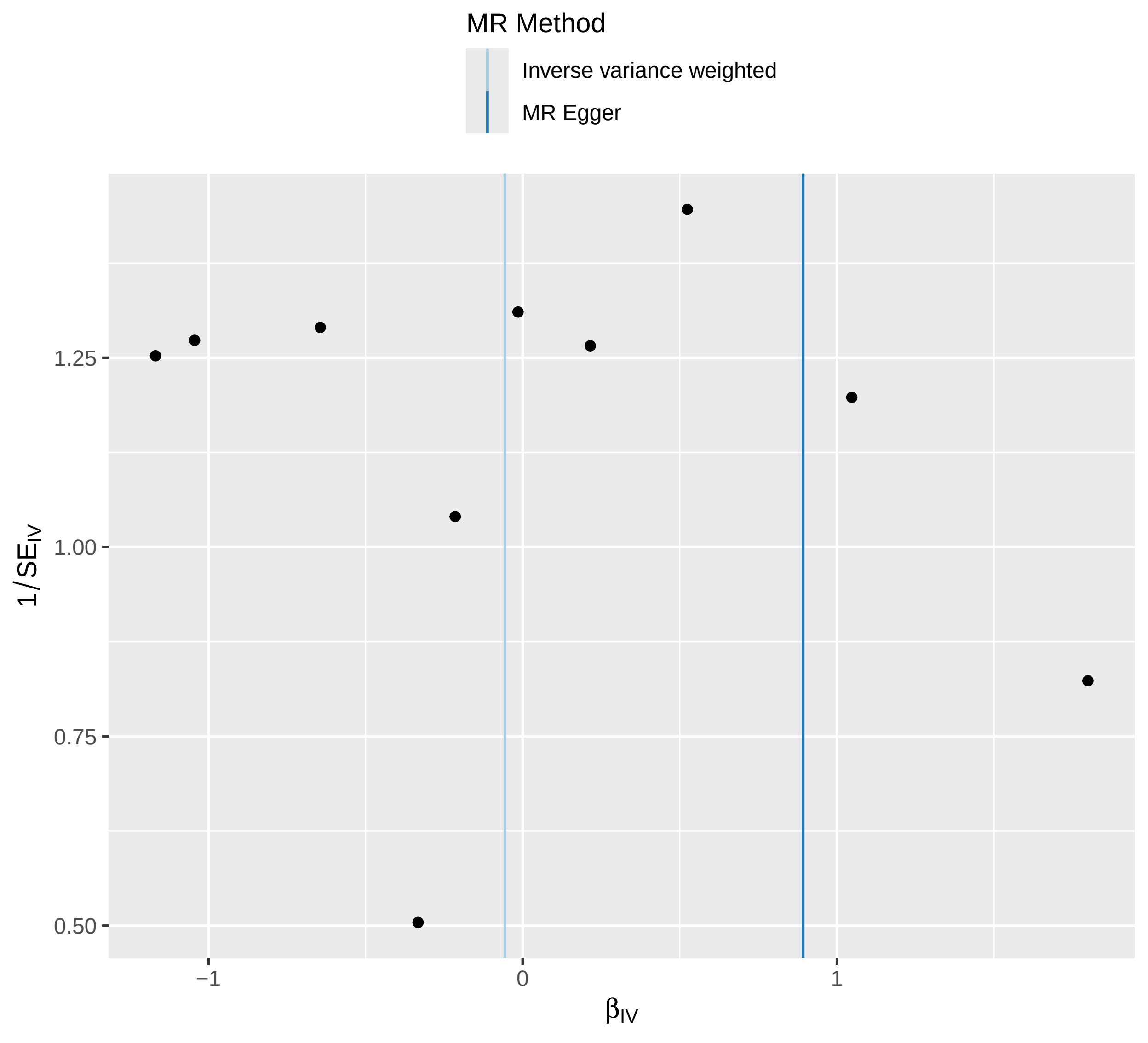

MR Method
Inverse variance weighted
MR Egger
1.25
1.00
0.75
0.50
1/SE_IV
−1
0
1
β_IV

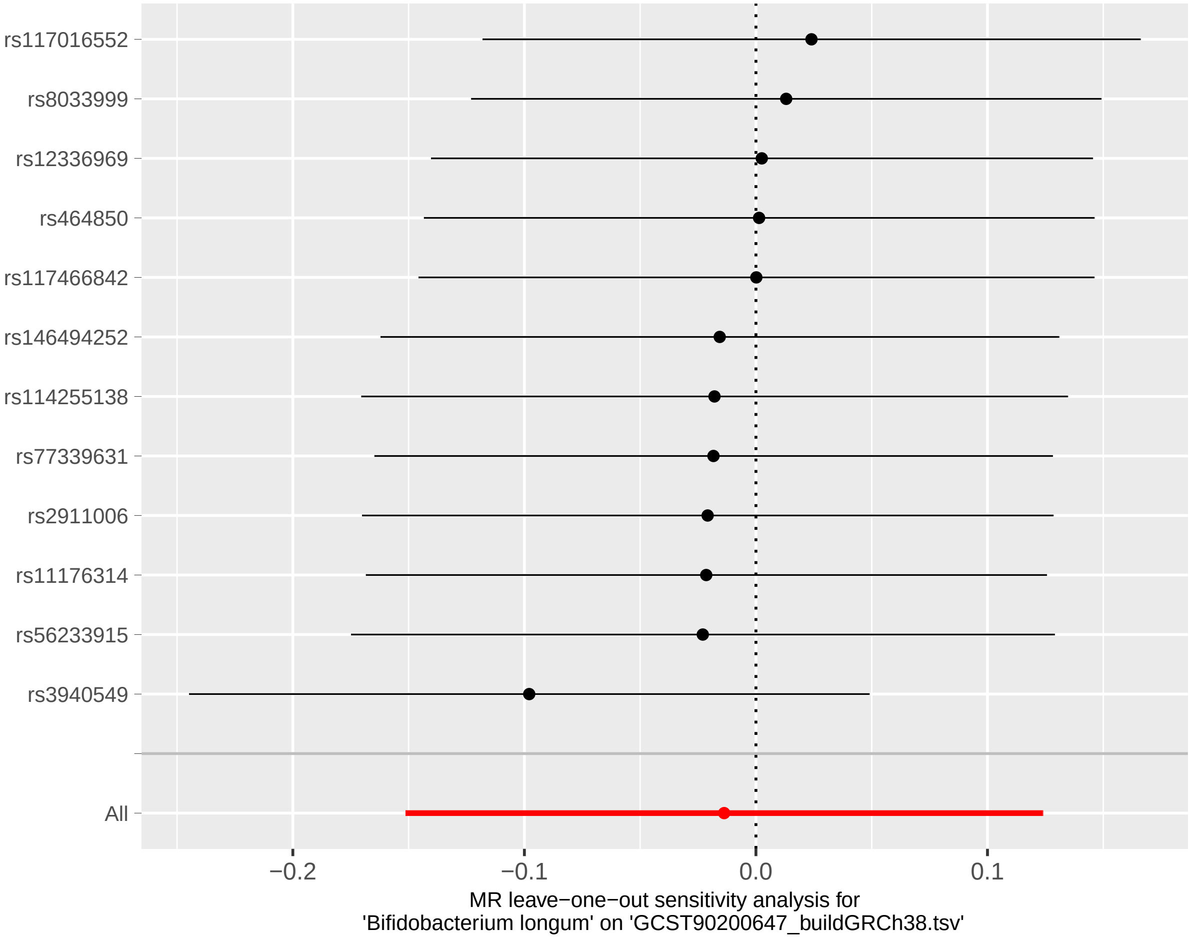

rs117016552
rs8033999
rs12336969
rs464850
rs117466842
rs146494252
rs114255138
rs77339631
rs2911006
rs11176314
rs56233915
rs3940549
All
−0.2
−0.1
0.0
0.1
MR leave−one−out sensitivity analysis for
'Bifidobacterium longum' on 'GCST90200647_buildGRCh38.tsv'

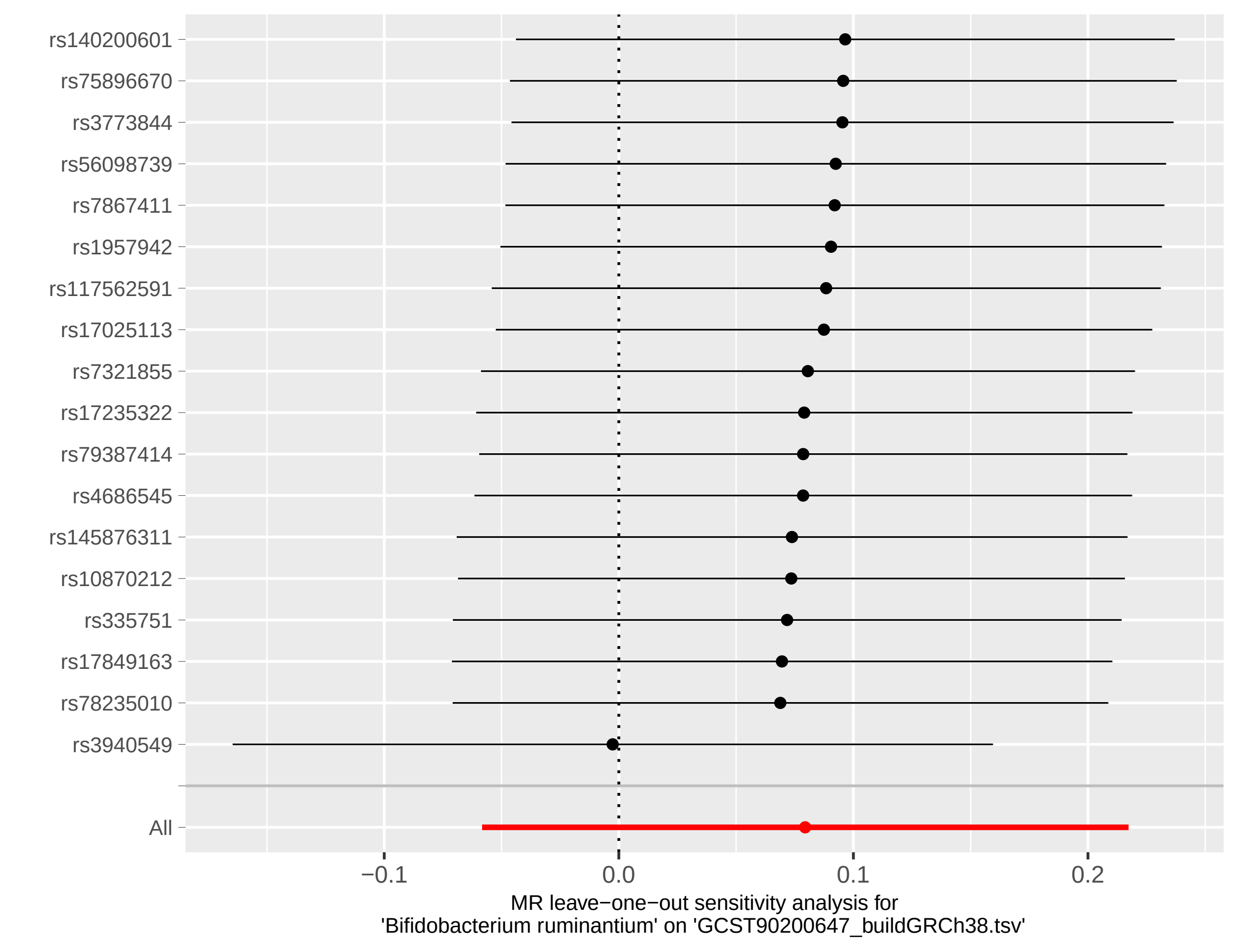

rs140200601
rs75896670
rs3773844
rs56098739
rs7867411
rs1957942
rs117562591
rs17025113
rs7321855
rs17235322
rs79387414
rs4686545
rs145876311
rs10870212
rs335751
rs17849163
rs78235010
rs3940549
All
−0.1
0.0
0.1
0.2
MR leave−one−out sensitivity analysis for
'Bifidobacterium ruminantium' on 'GCST90200647_buildGRCh38.tsv'

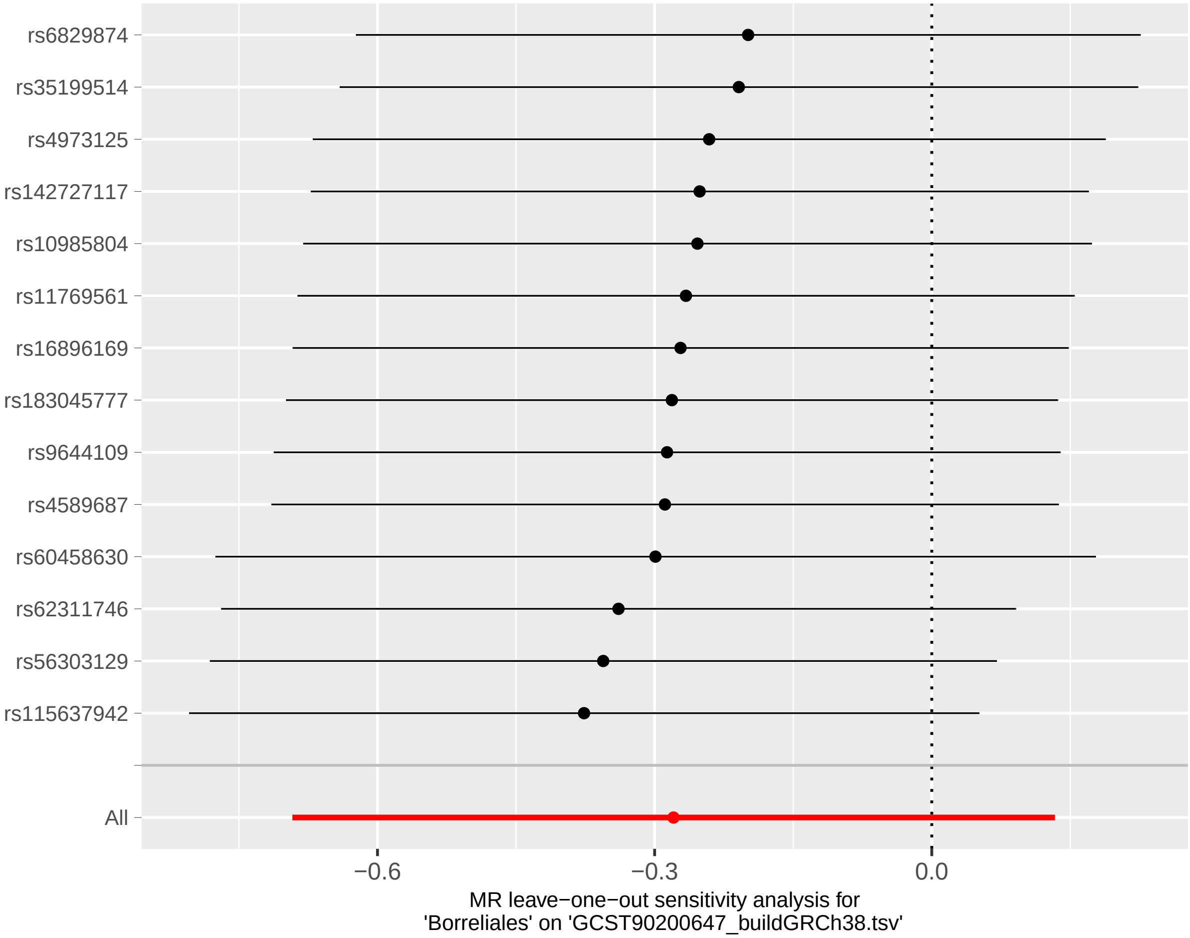
rs6829874
rs35199514
rs4973125
rs142727117
rs10985804
rs11769561
rs16896169
rs183045777
rs9644109
rs4589687
rs60458630
rs62311746
rs56303129
rs115637942
All
−0.6
−0.3
0.0
MR leave−one−out sensitivity analysis for
'Borreliales' on 'GCST90200647_buildGRCh38.tsv'

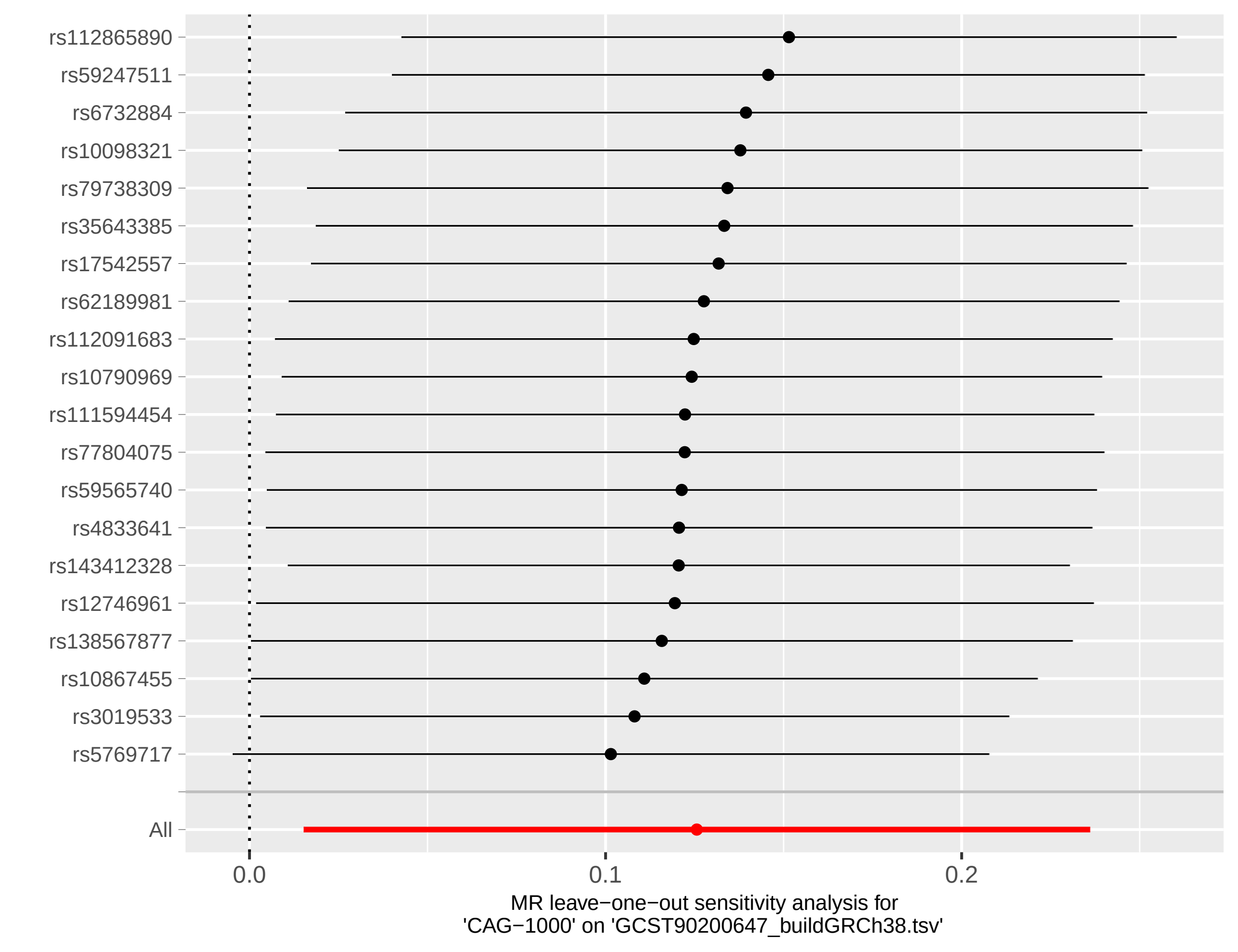
rs112865890
rs59247511
rs6732884
rs10098321
rs79738309
rs35643385
rs17542557
rs62189981
rs112091683
rs10790969
rs111594454
rs77804075
rs59565740
rs4833641
rs143412328
rs12746961
rs138567877
rs10867455
rs3019533
rs5769717
All
0.0
0.1
0.2
MR leave−one−out sensitivity analysis for
'CAG−1000' on 'GCST90200647_buildGRCh38.tsv'

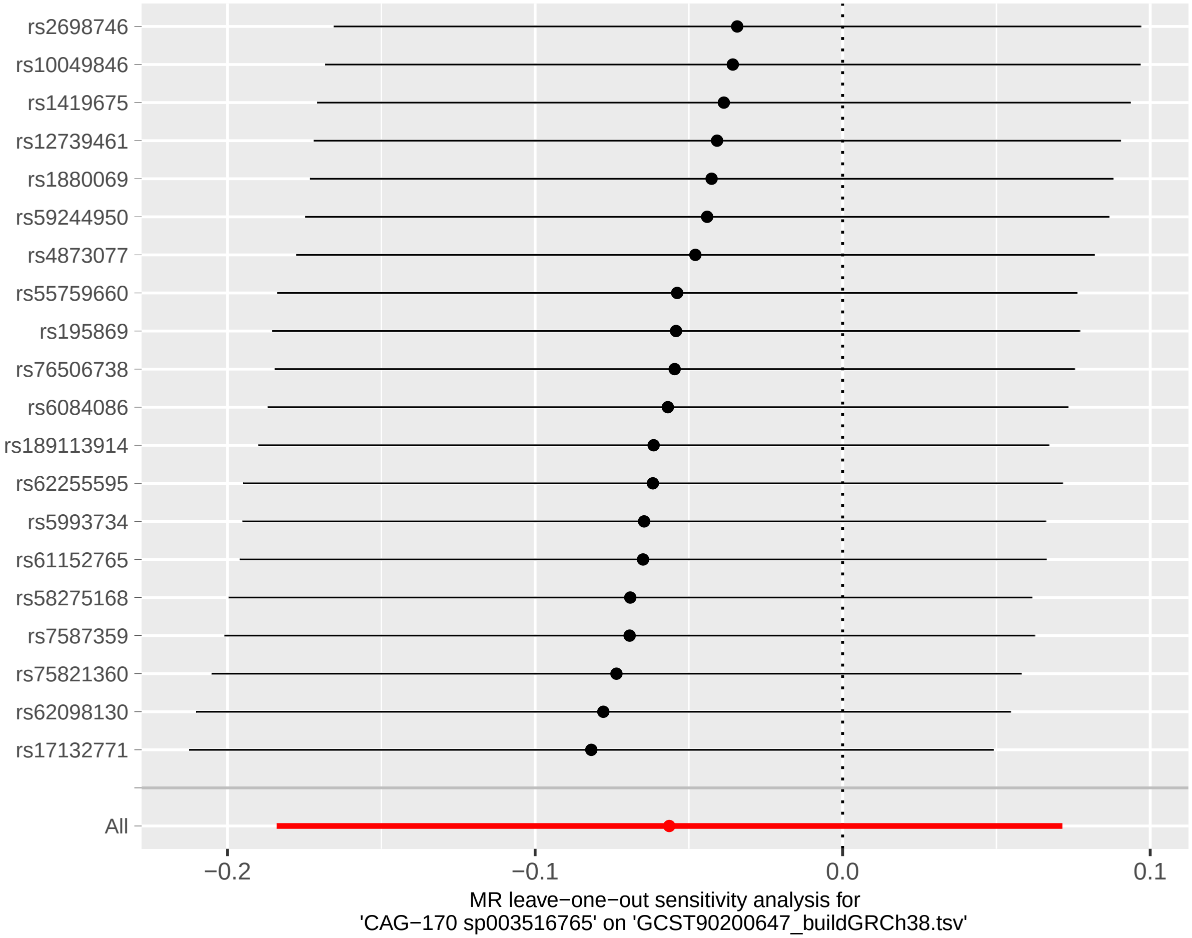

rs2698746
rs10049846
rs1419675
rs12739461
rs1880069
rs59244950
rs4873077
rs55759660
rs195869
rs76506738
rs6084086
rs189113914
rs62255595
rs5993734
rs61152765
rs58275168
rs7587359
rs75821360
rs62098130
rs17132771
All
−0.2
−0.1
0.0
0.1
MR leave−one−out sensitivity analysis for
'CAG−170 sp003516765' on 'GCST90200647_buildGRCh38.tsv'

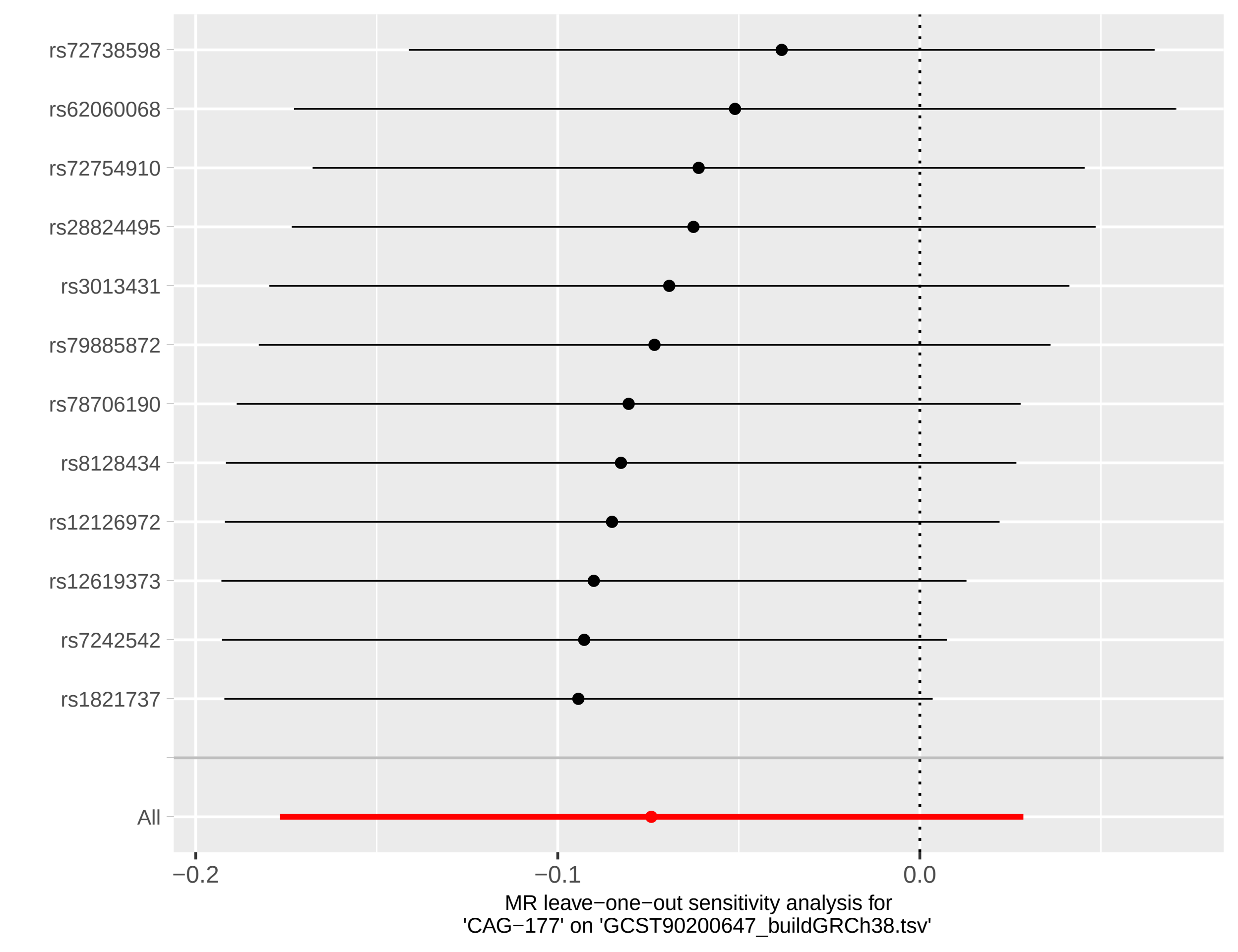
rs72738598
rs62060068
rs72754910
rs28824495
rs3013431
rs79885872
rs78706190
rs8128434
rs12126972
rs12619373
rs7242542
rs1821737
All
−0.2
−0.1
0.0
MR leave−one−out sensitivity analysis for
'CAG−177' on 'GCST90200647_buildGRCh38.tsv'

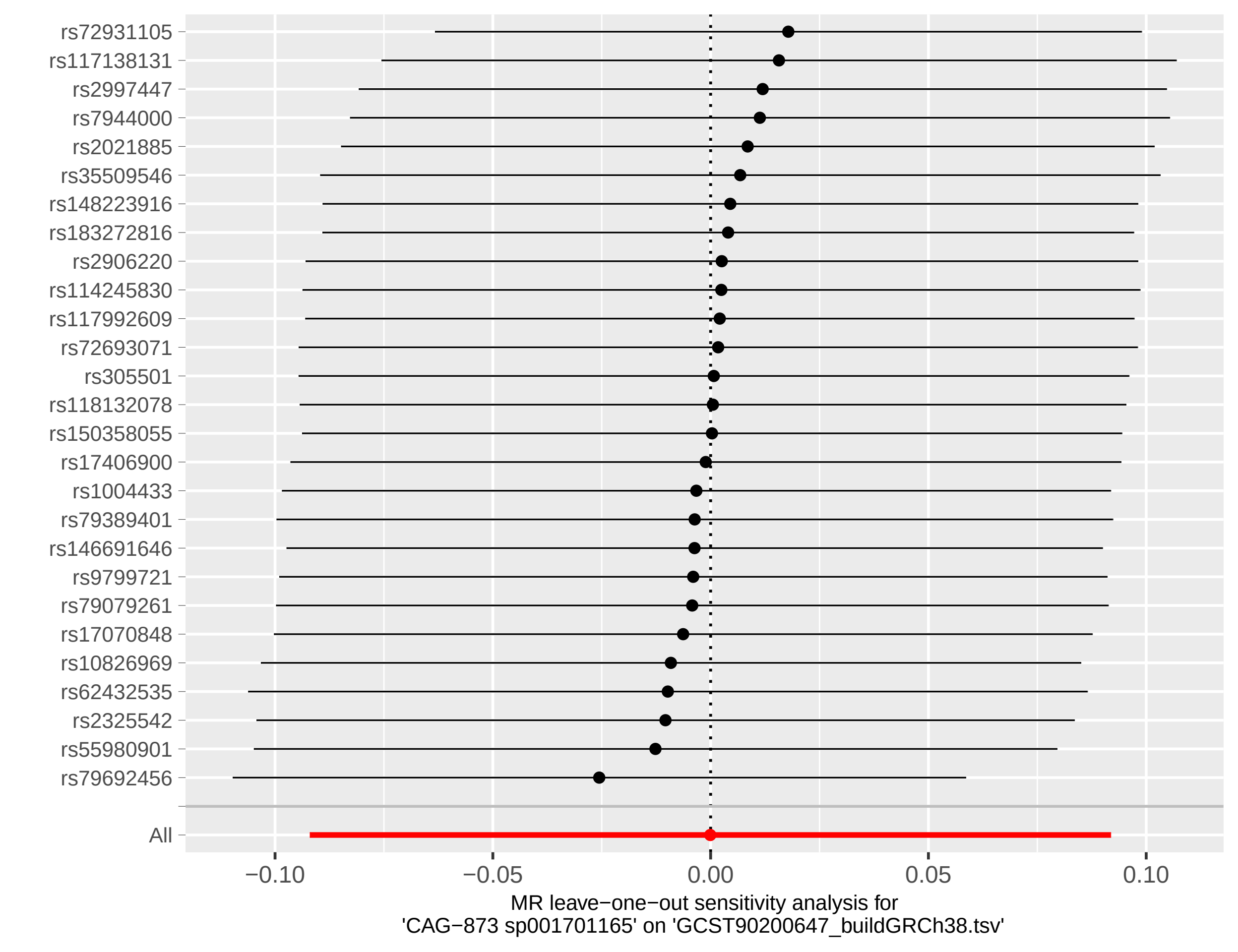

rs72931105
rs117138131
rs2997447
rs7944000
rs2021885
rs35509546
rs148223916
rs183272816
rs2906220
rs114245830
rs117992609
rs72693071
rs305501
rs118132078
rs150358055
rs17406900
rs1004433
rs79389401
rs146691646
rs9799721
rs79079261
rs17070848
rs10826969
rs62432535
rs2325542
rs55980901
rs79692456
All
−0.10
−0.05
0.00
0.05
0.10
MR leave−one−out sensitivity analysis for
'CAG−873 sp001701165' on 'GCST90200647_buildGRCh38.tsv'

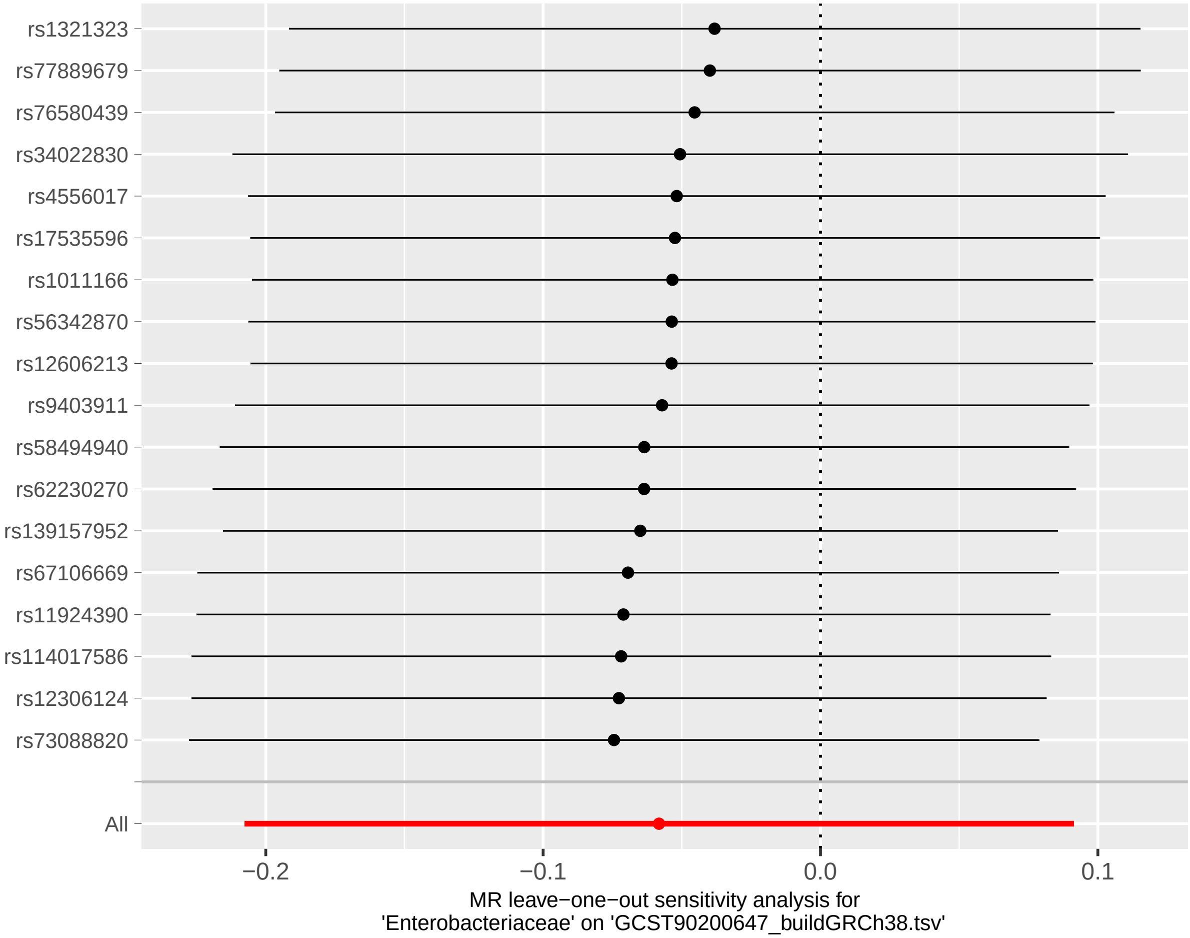
rs1321323
rs77889679
rs76580439
rs34022830
rs4556017
rs17535596
rs1011166
rs56342870
rs12606213
rs9403911
rs58494940
rs62230270
rs139157952
rs67106669
rs11924390
rs114017586
rs12306124
rs73088820
All
−0.2
−0.1
0.0
0.1
MR leave−one−out sensitivity analysis for
'Enterobacteriaceae' on 'GCST90200647_buildGRCh38.tsv'

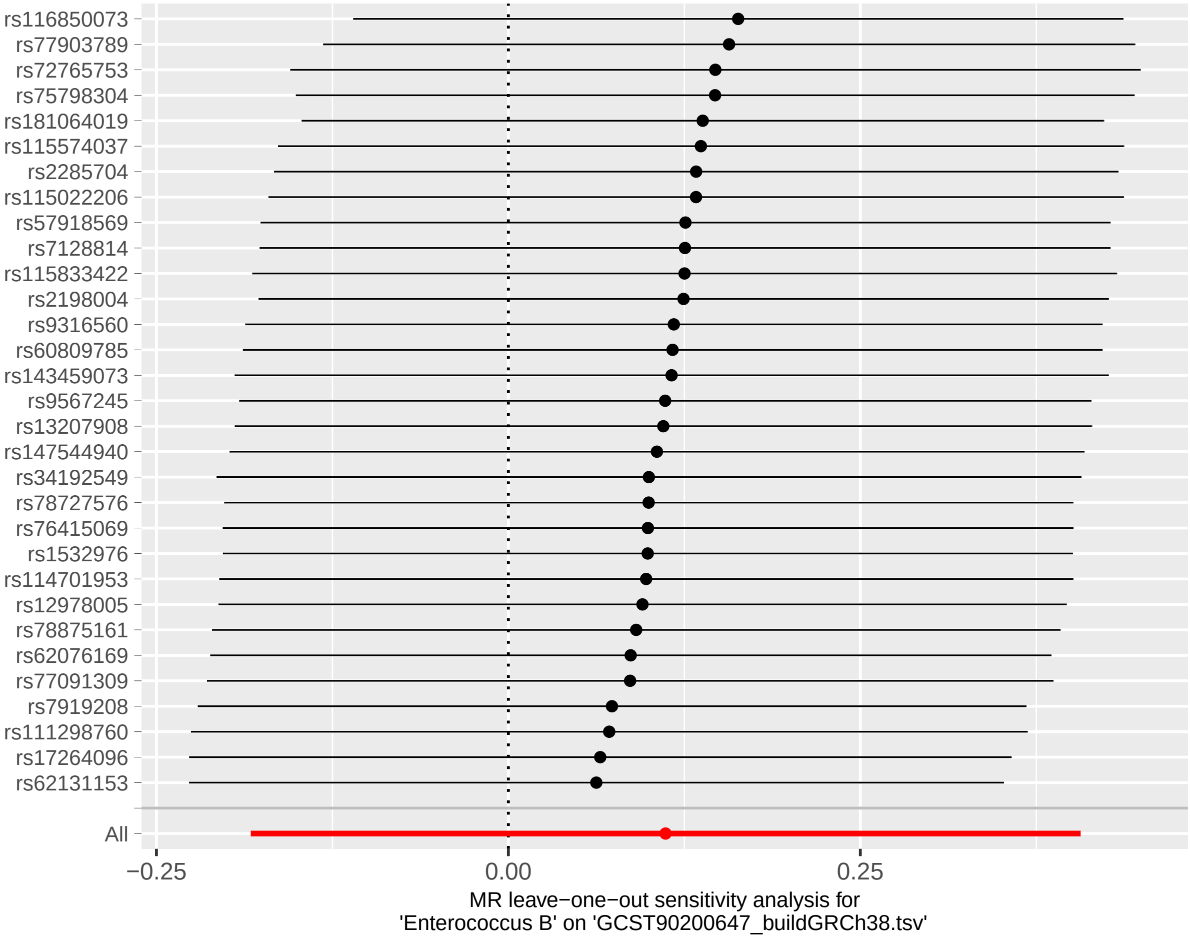

rs116850073
rs77903789
rs72765753
rs75798304
rs181064019
rs115574037
rs2285704
rs115022206
rs57918569
rs7128814
rs115833422
rs2198004
rs9316560
rs60809785
rs143459073
rs9567245
rs13207908
rs147544940
rs34192549
rs78727576
rs76415069
rs1532976
rs114701953
rs12978005
rs78875161
rs62076169
rs77091309
rs7919208
rs111298760
rs17264096
rs62131153
All
−0.25
0.00
0.25
MR leave−one−out sensitivity analysis for
'Enterococcus B' on 'GCST90200647_buildGRCh38.tsv'

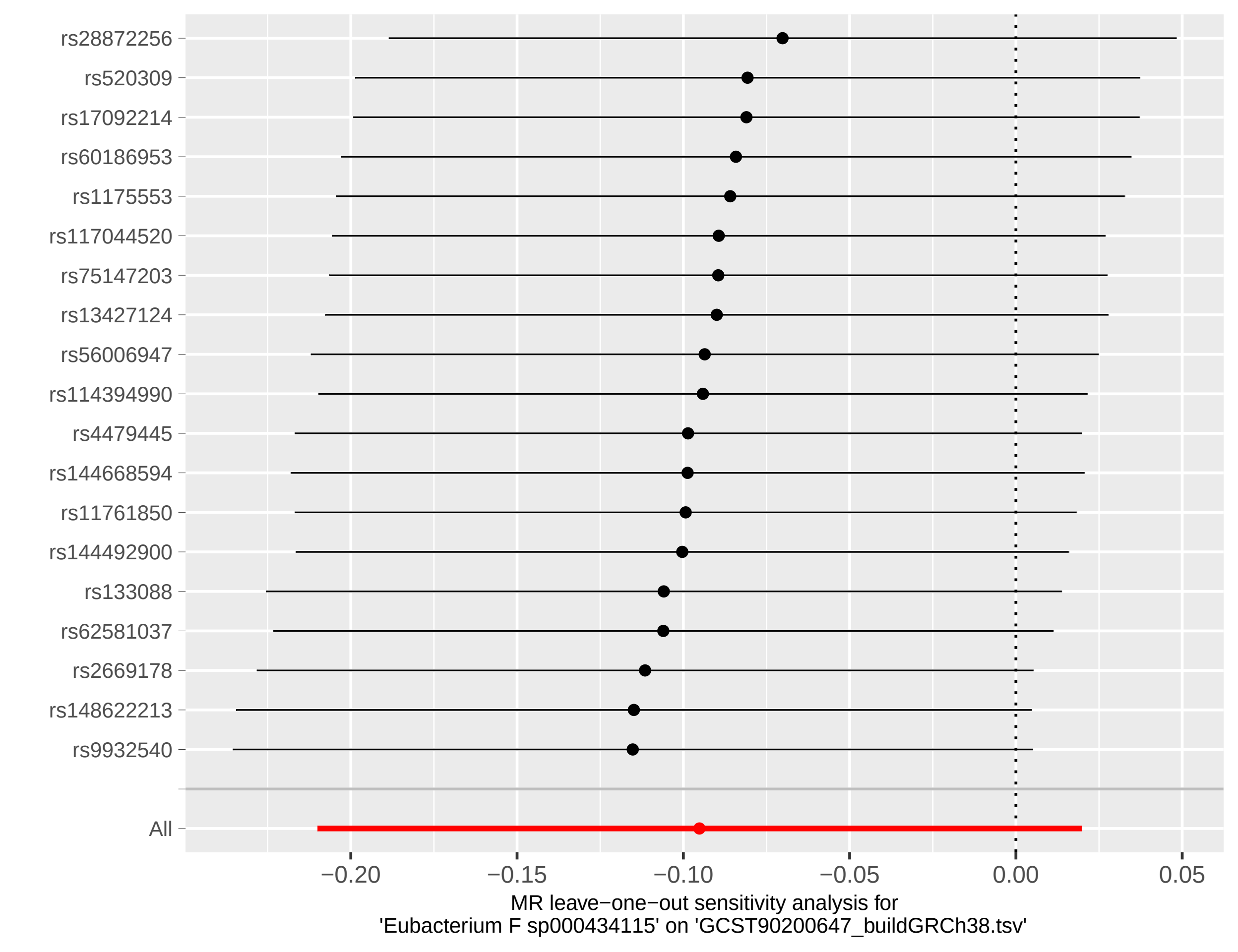
rs28872256
rs520309
rs17092214
rs60186953
rs1175553
rs117044520
rs75147203
rs13427124
rs56006947
rs114394990
rs4479445
rs144668594
rs11761850
rs144492900
rs133088
rs62581037
rs2669178
rs148622213
rs9932540
All
−0.20
−0.15
−0.10
−0.05
0.00
0.05
MR leave−one−out sensitivity analysis for
'Eubacterium F sp000434115' on 'GCST90200647_buildGRCh38.tsv'

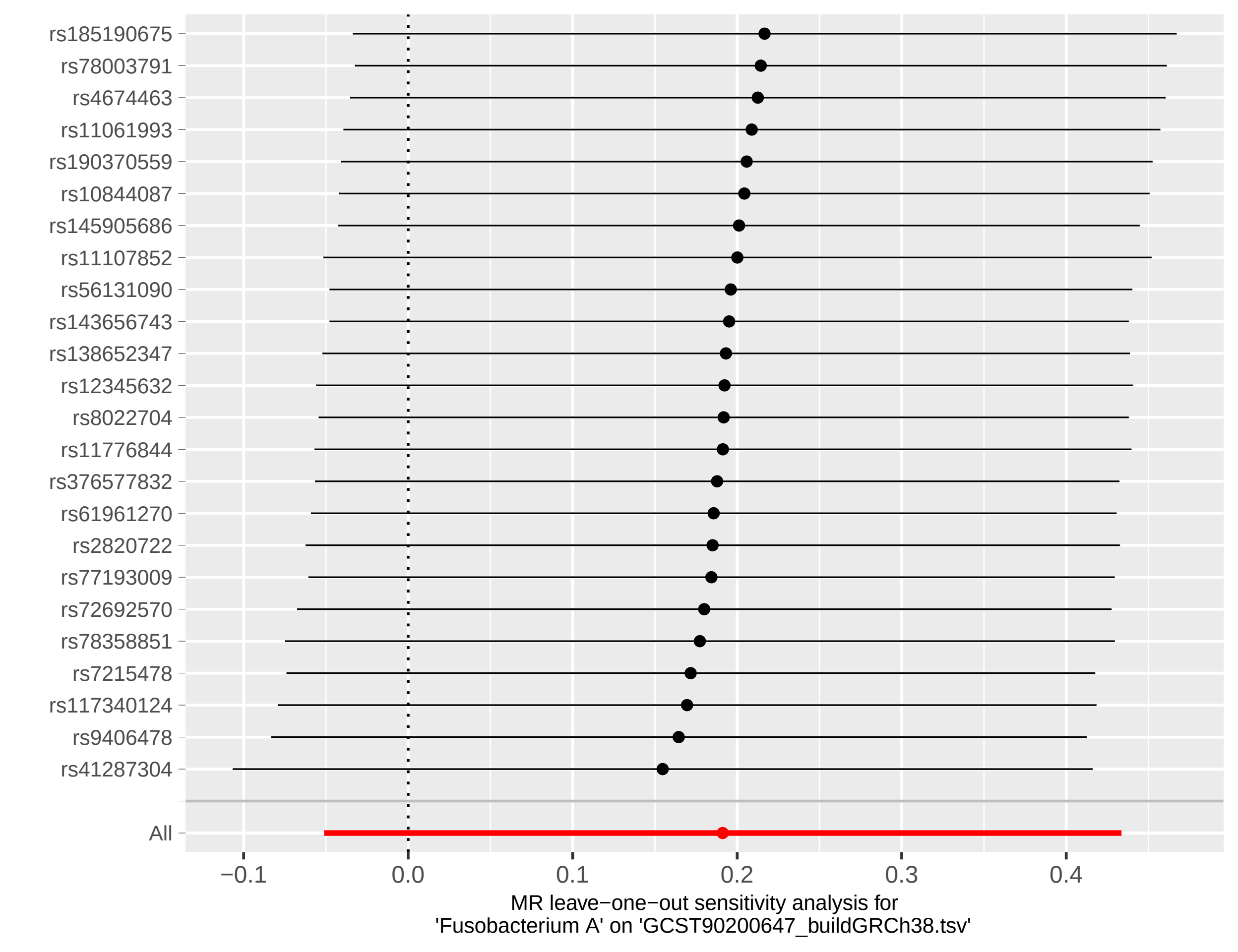

rs185190675
rs78003791
rs4674463
rs11061993
rs190370559
rs10844087
rs145905686
rs11107852
rs56131090
rs143656743
rs138652347
rs12345632
rs8022704
rs11776844
rs376577832
rs61961270
rs2820722
rs77193009
rs72692570
rs78358851
rs7215478
rs117340124
rs9406478
rs41287304
All
−0.1
0.0
0.1
0.2
0.3
0.4
MR leave−one−out sensitivity analysis for
'Fusobacterium A' on 'GCST90200647_buildGRCh38.tsv'

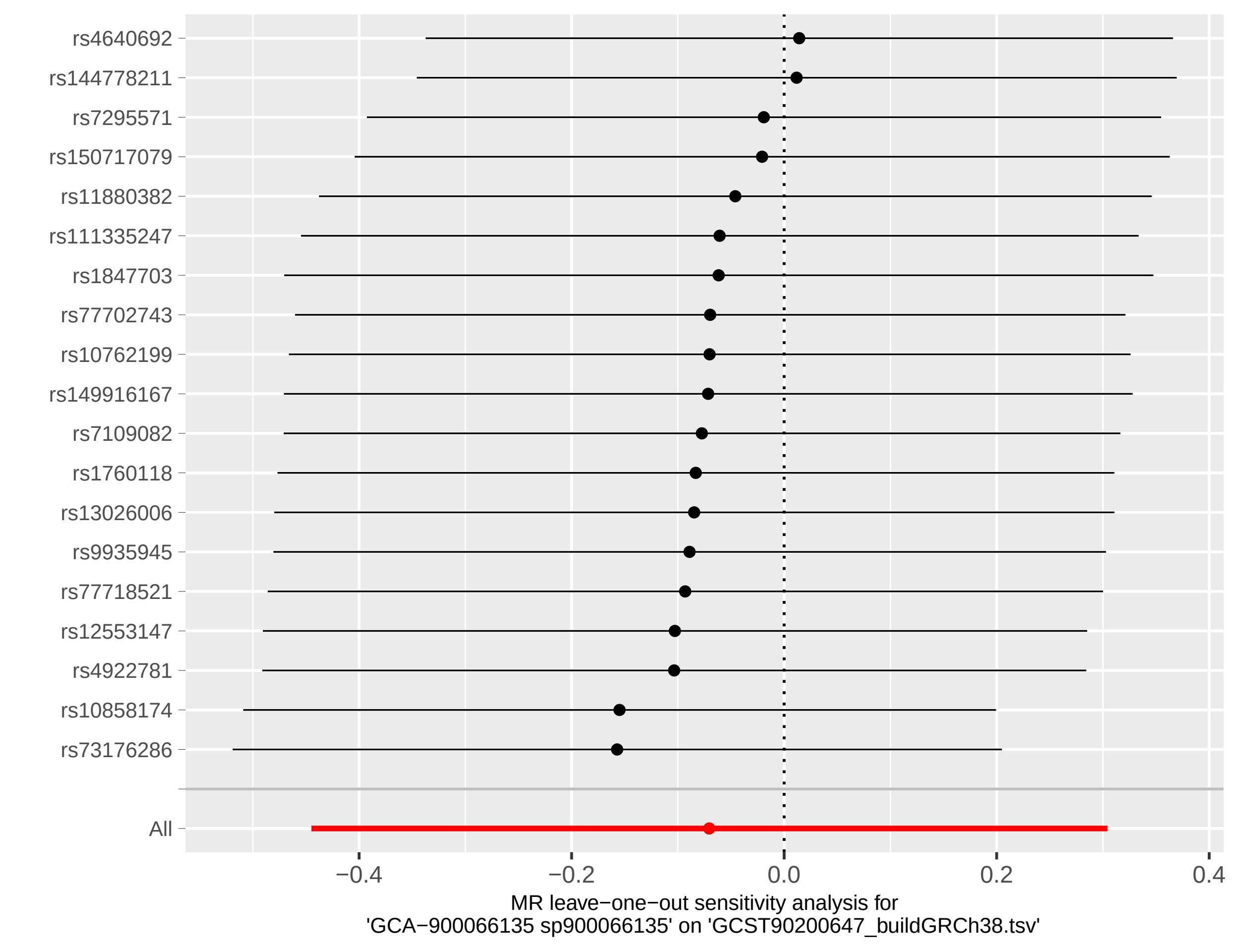

rs4640692
rs144778211
rs7295571
rs150717079
rs11880382
rs111335247
rs1847703
rs77702743
rs10762199
rs149916167
rs7109082
rs1760118
rs13026006
rs9935945
rs77718521
rs12553147
rs4922781
rs10858174
rs73176286
All
−0.4
−0.2
0.0
0.2
0.4
MR leave−one−out sensitivity analysis for
'GCA−900066135 sp900066135' on 'GCST90200647_buildGRCh38.tsv'

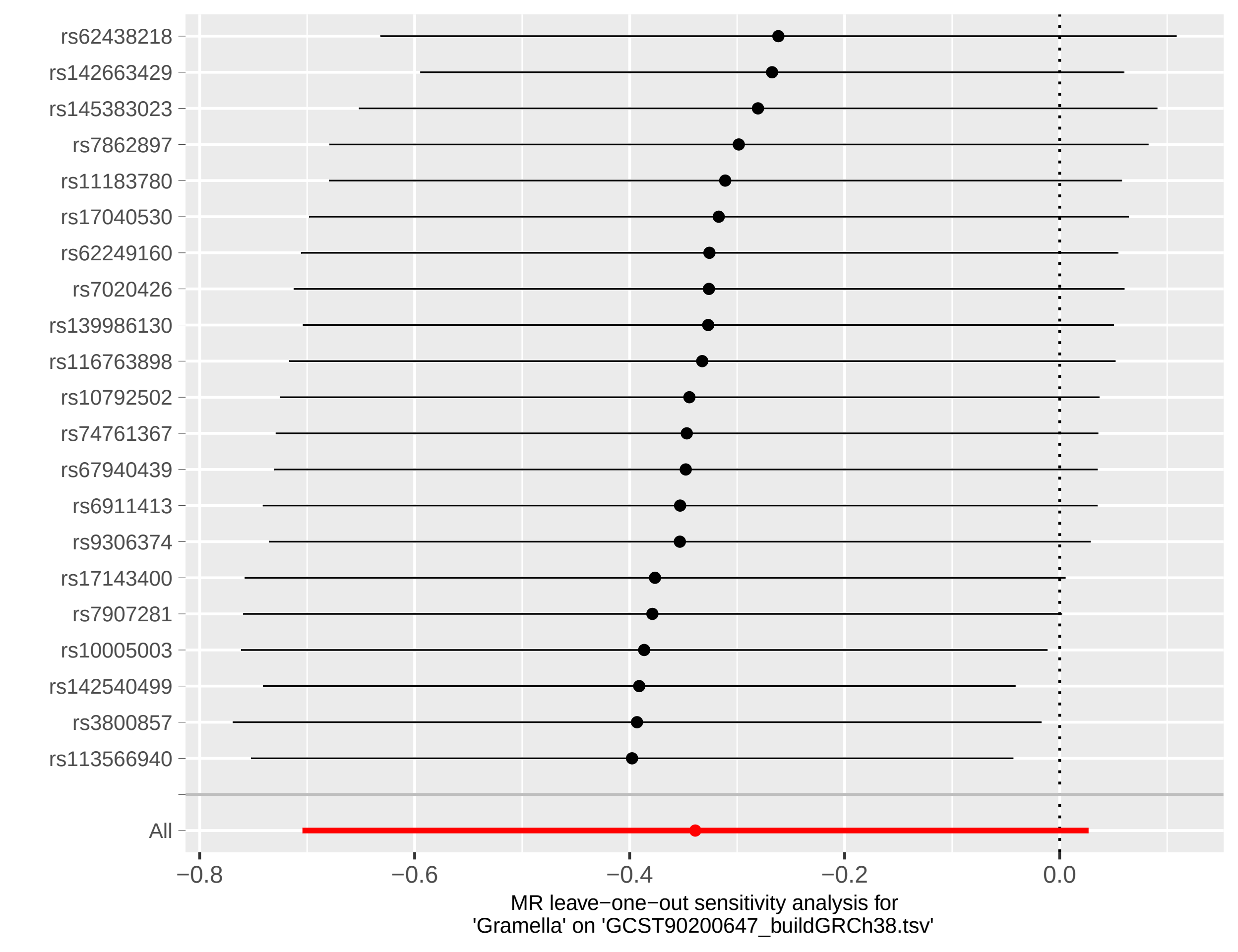

rs62438218
rs142663429
rs145383023
rs7862897
rs11183780
rs17040530
rs62249160
rs7020426
rs139986130
rs116763898
rs10792502
rs74761367
rs67940439
rs6911413
rs9306374
rs17143400
rs7907281
rs10005003
rs142540499
rs3800857
rs113566940
All
−0.8
−0.6
−0.4
−0.2
0.0
MR leave−one−out sensitivity analysis for
'Gramella' on 'GCST90200647_buildGRCh38.tsv'

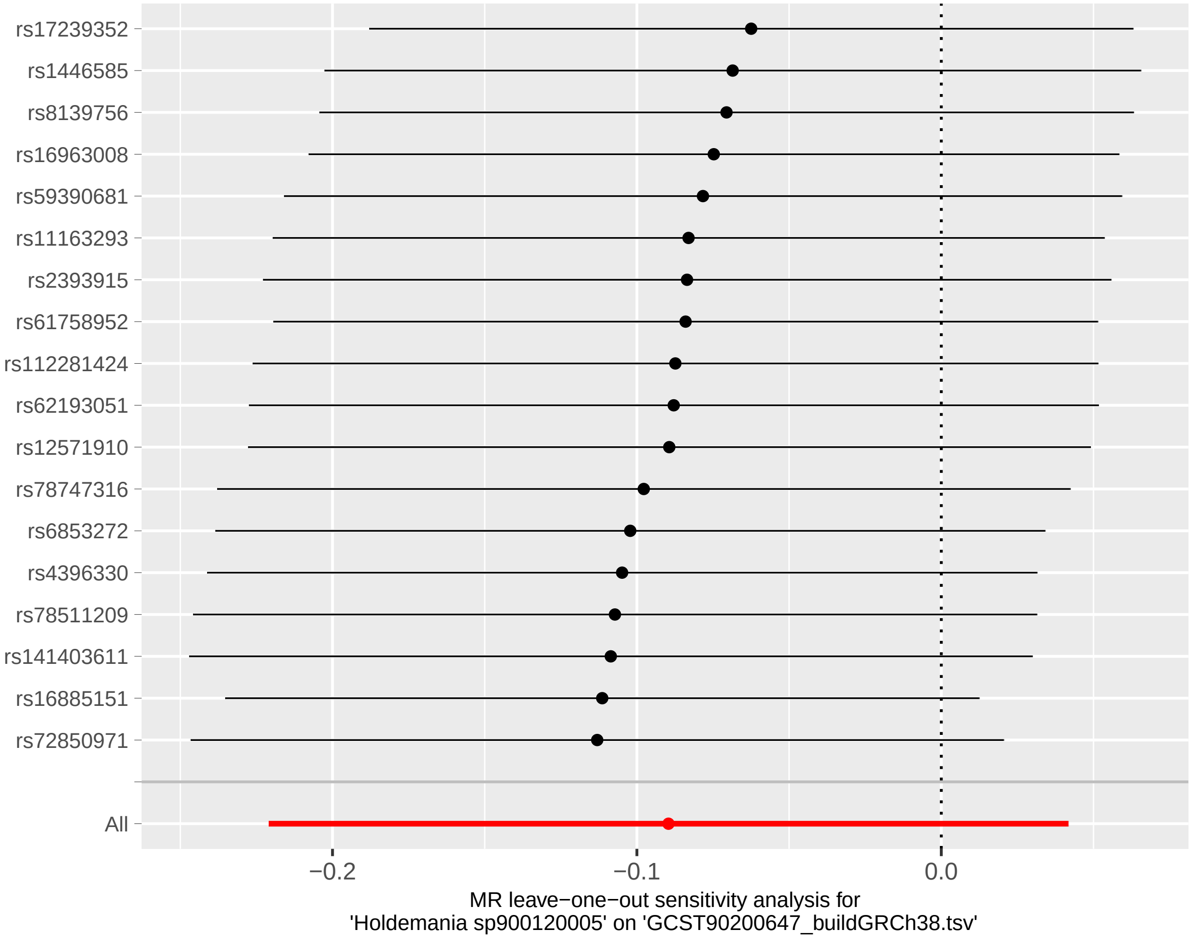
rs17239352
rs1446585
rs8139756
rs16963008
rs59390681
rs11163293
rs2393915
rs61758952
rs112281424
rs62193051
rs12571910
rs78747316
rs6853272
rs4396330
rs78511209
rs141403611
rs16885151
rs72850971
All
−0.2
−0.1
0.0
MR leave−one−out sensitivity analysis for
'Holdemania sp900120005' on 'GCST90200647_buildGRCh38.tsv'

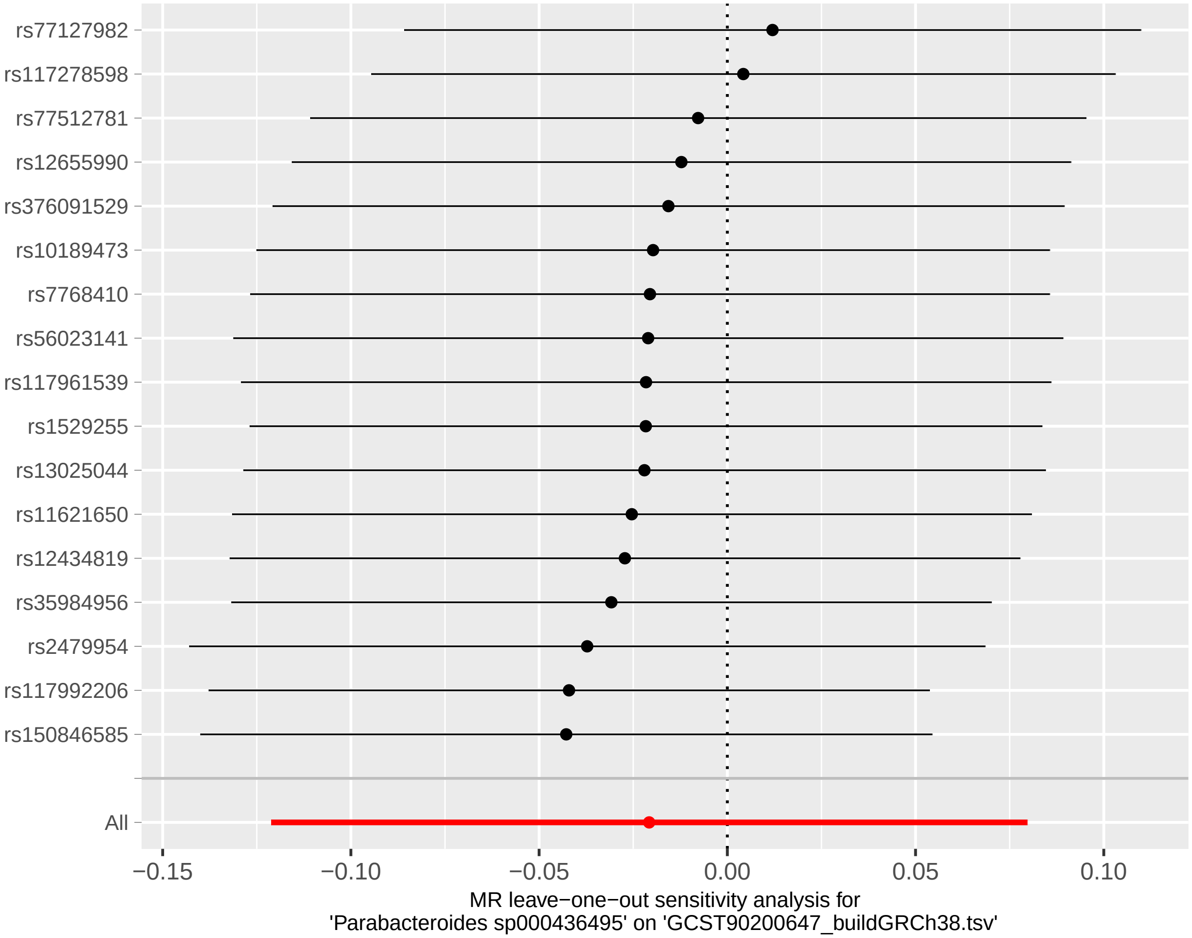
rs77127982
rs117278598
rs77512781
rs12655990
rs376091529
rs10189473
rs7768410
rs56023141
rs117961539
rs1529255
rs13025044
rs11621650
rs12434819
rs35984956
rs2479954
rs117992206
rs150846585
All
−0.15
−0.10
−0.05
0.00
0.05
0.10
MR leave−one−out sensitivity analysis for
'Parabacteroides sp000436495' on 'GCST90200647_buildGRCh38.tsv'

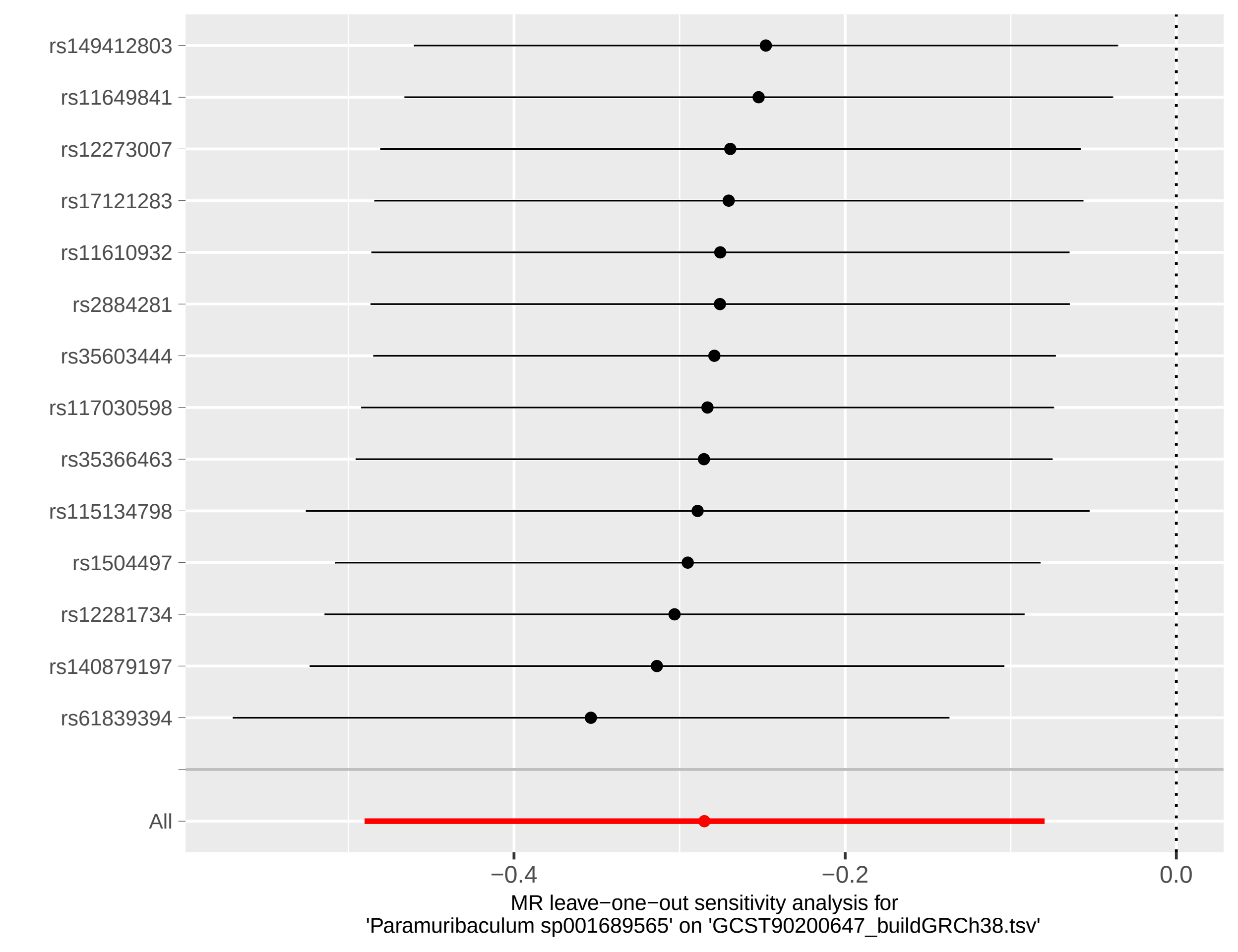
rs149412803
rs11649841
rs12273007
rs17121283
rs11610932
rs2884281
rs35603444
rs117030598
rs35366463
rs115134798
rs1504497
rs12281734
rs140879197
rs61839394
All
−0.4
−0.2
0.0
MR leave−one−out sensitivity analysis for
'Paramuribaculum sp001689565' on 'GCST90200647_buildGRCh38.tsv'

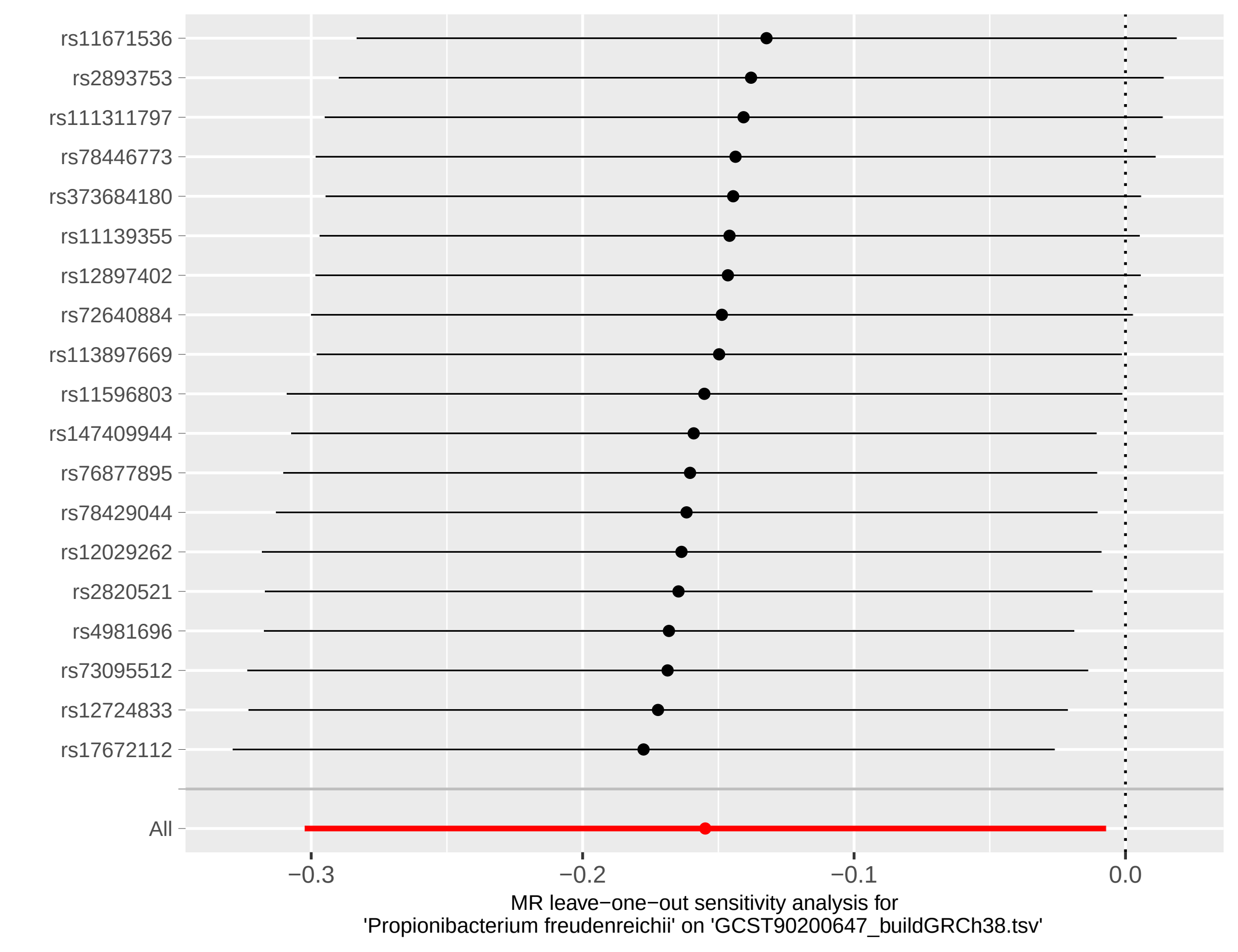

rs11671536
rs2893753
rs111311797
rs78446773
rs373684180
rs11139355
rs12897402
rs72640884
rs113897669
rs11596803
rs147409944
rs76877895
rs78429044
rs12029262
rs2820521
rs4981696
rs73095512
rs12724833
rs17672112
All
−0.3
−0.2
−0.1
0.0
MR leave−one−out sensitivity analysis for
'Propionibacterium freudenreichii' on 'GCST90200647_buildGRCh38.tsv'

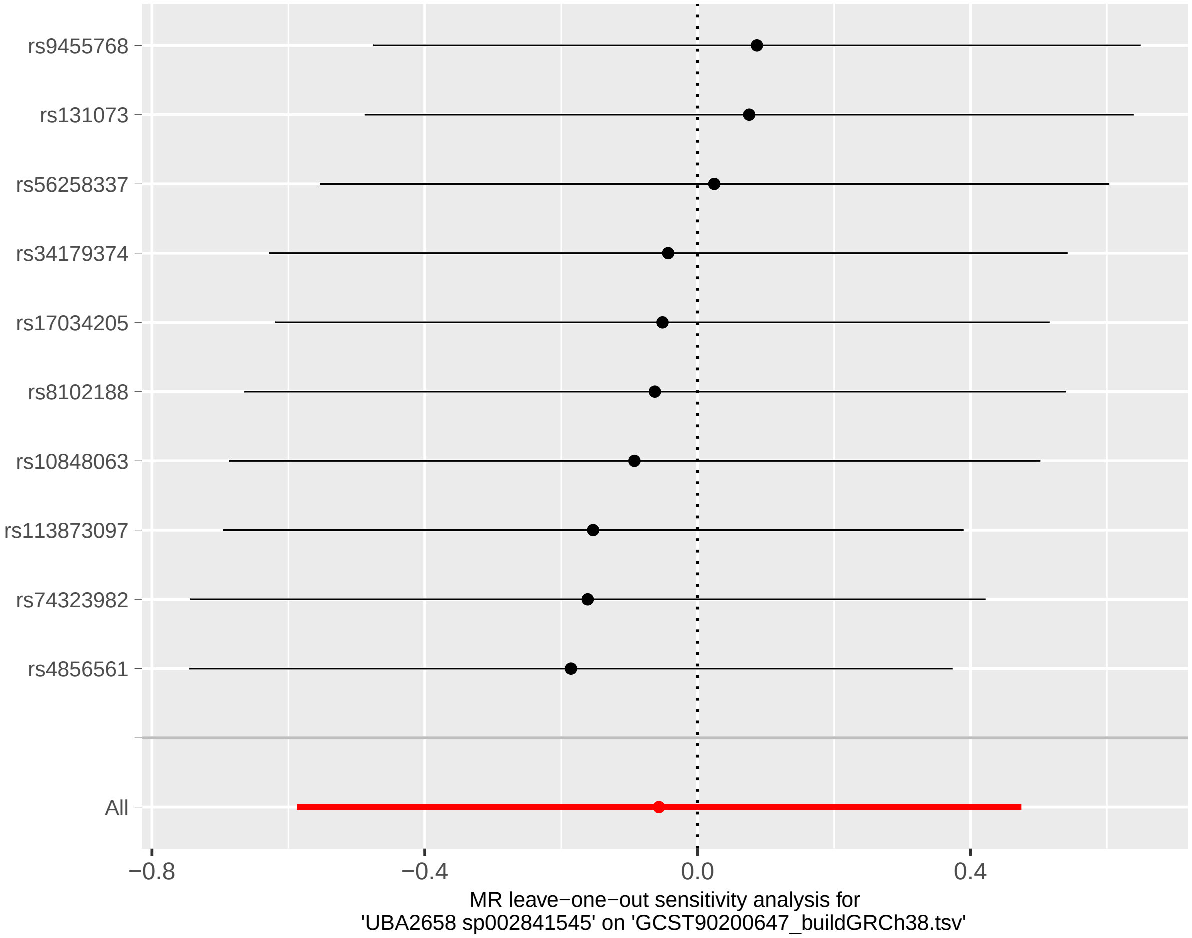

rs9455768
rs131073
rs56258337
rs34179374
rs17034205
rs8102188
rs10848063
rs113873097
rs74323982
rs4856561
All
−0.8
−0.4
0.0
0.4
MR leave−one−out sensitivity analysis for
'UBA2658 sp002841545' on 'GCST90200647_buildGRCh38.tsv'

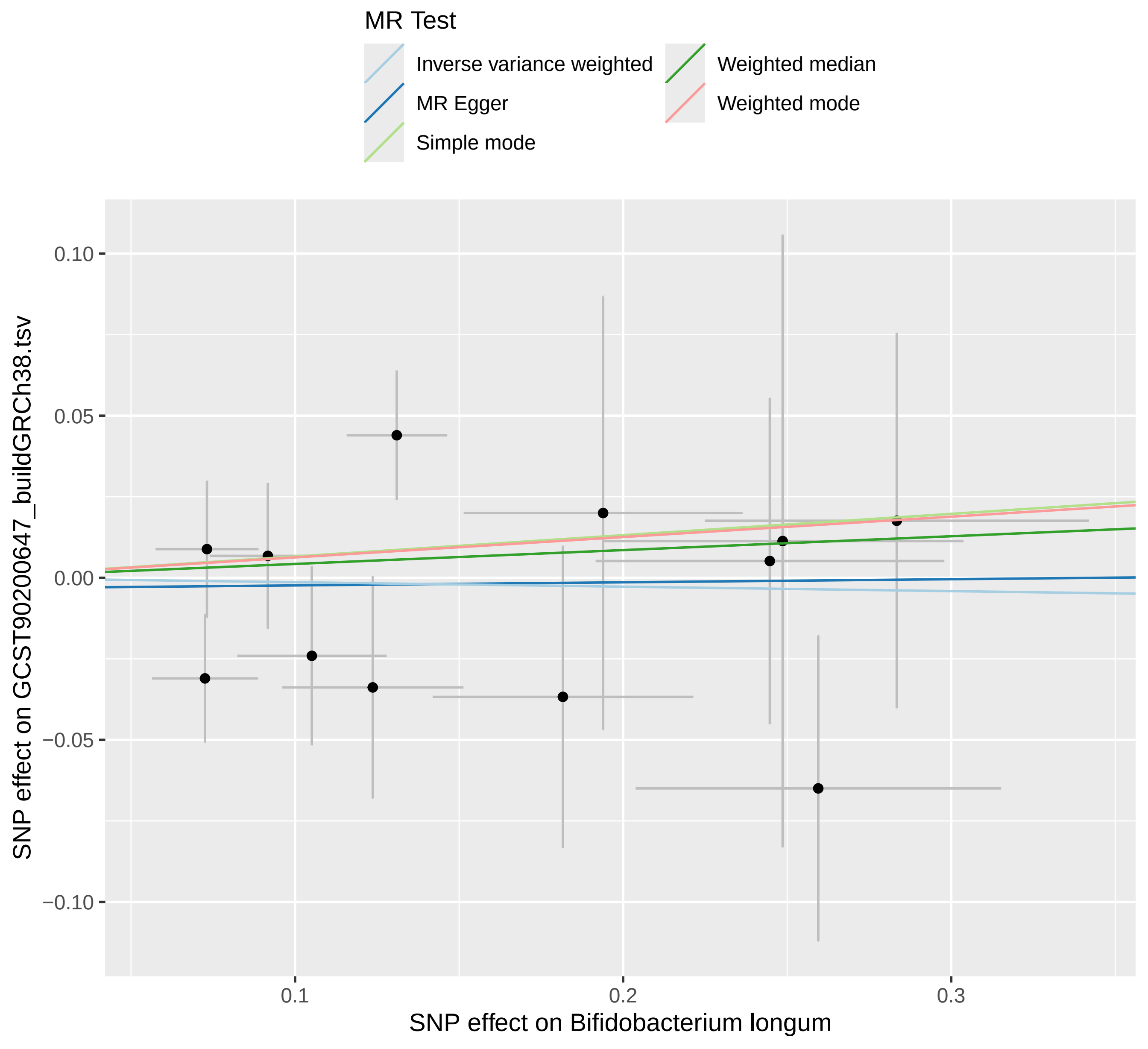

MR Test
Inverse variance weighted
MR Egger
Simple mode
Weighted median
Weighted mode
0.10
0.05
0.00
−0.05
−0.10
SNP effect on GCST90200647_buildGRCh38.tsv
0.1
0.2
0.3
SNP effect on Bifidobacterium longum

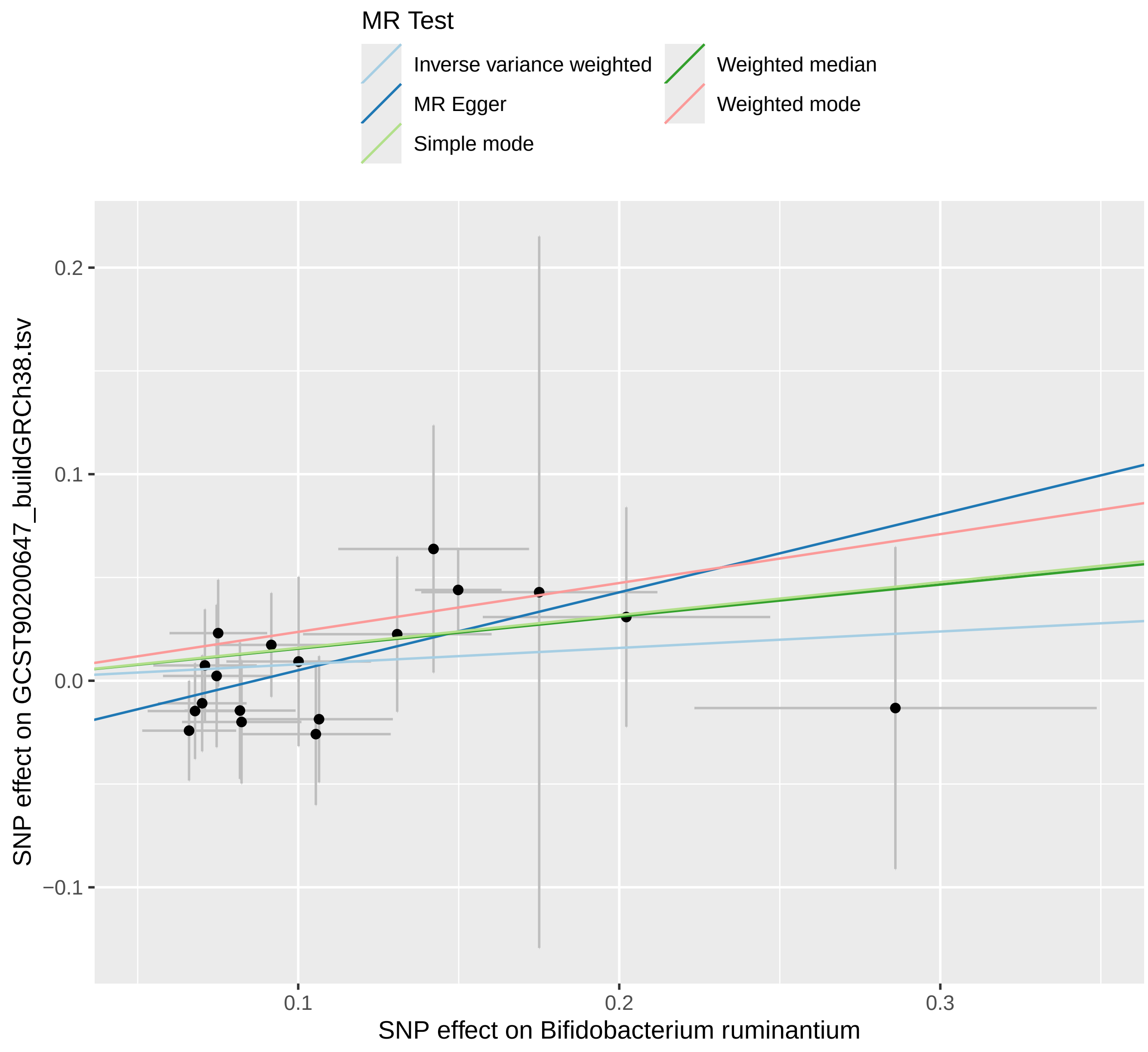
MR Test
Inverse variance weighted
MR Egger
Simple mode
Weighted median
Weighted mode
SNP effect on GCST90200647_buildGRCh38.tsv
0.2
0.1
0.0
−0.1
0.1
0.2
0.3
SNP effect on Bifidobacterium ruminantium

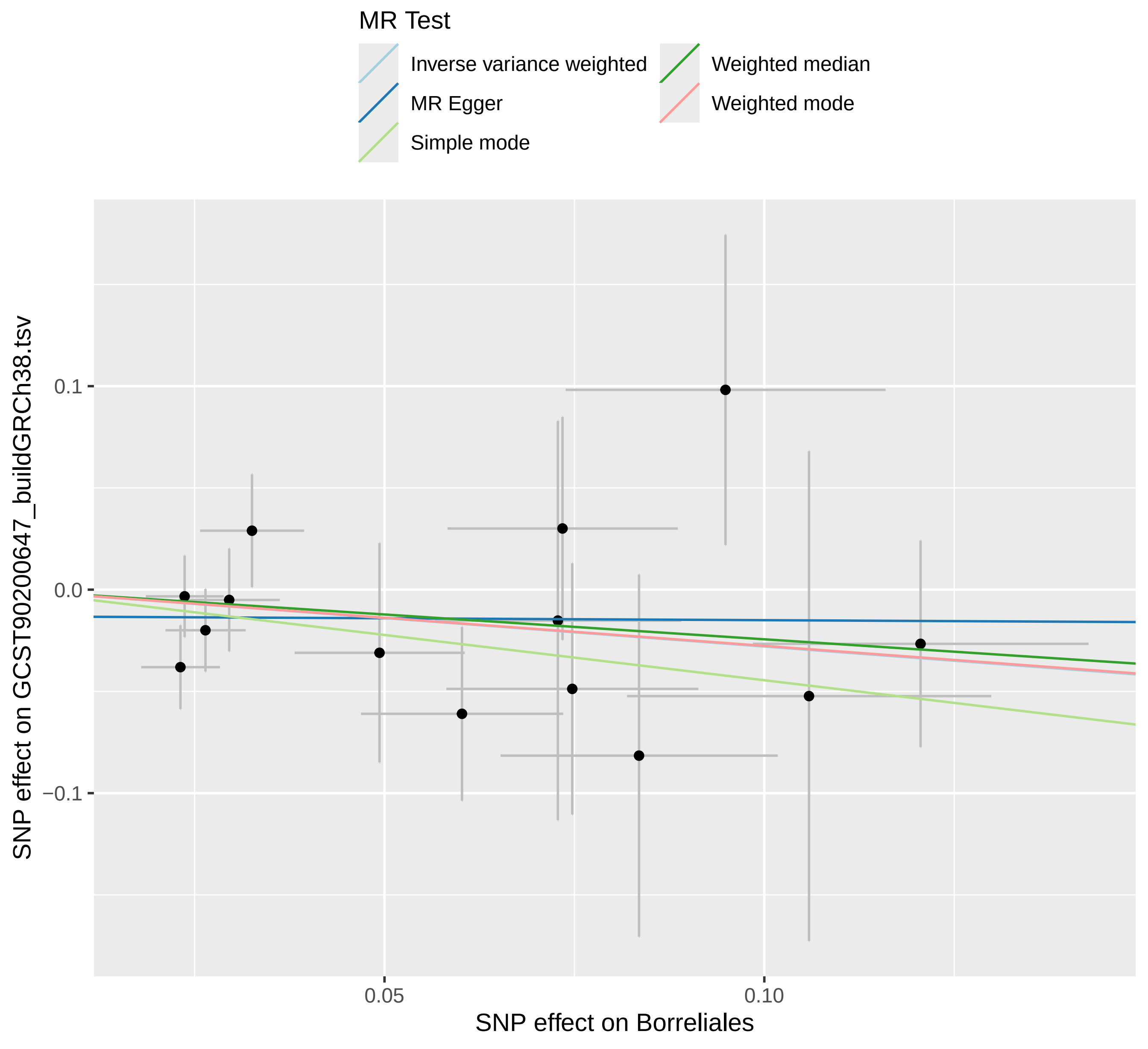

MR Test
Inverse variance weighted
MR Egger
Simple mode
Weighted median
Weighted mode
0.1
0.0
−0.1
0.05
0.10
SNP effect on GCST90200647_buildGRCh38.tsv
SNP effect on Borreliales

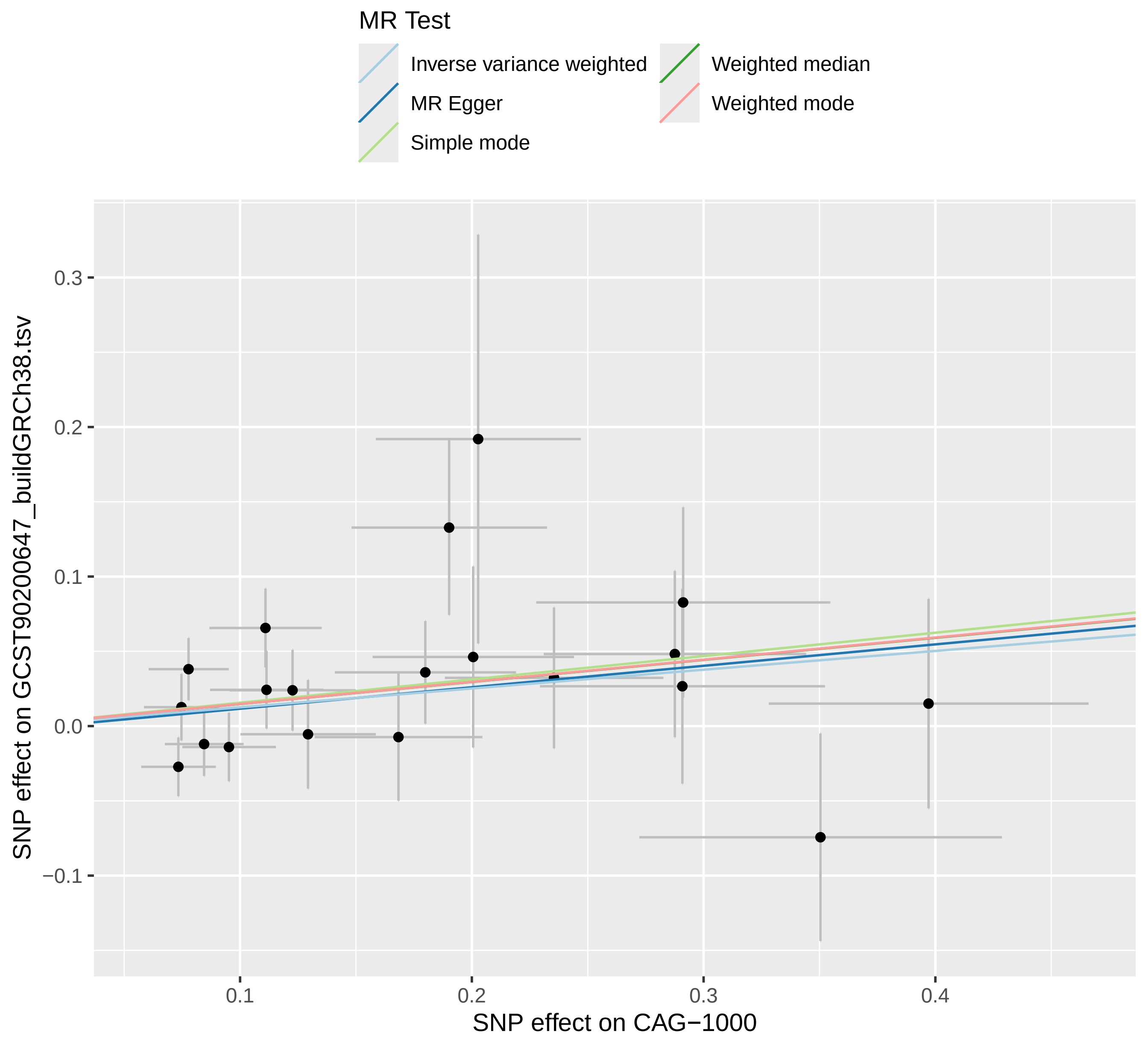
MR Test
Inverse variance weighted
MR Egger
Simple mode
Weighted median
Weighted mode
0.3
0.2
0.1
0.0
−0.1
0.1
0.2
0.3
0.4
SNP effect on GCST90200647_buildGRCh38.tsv
SNP effect on CAG−1000

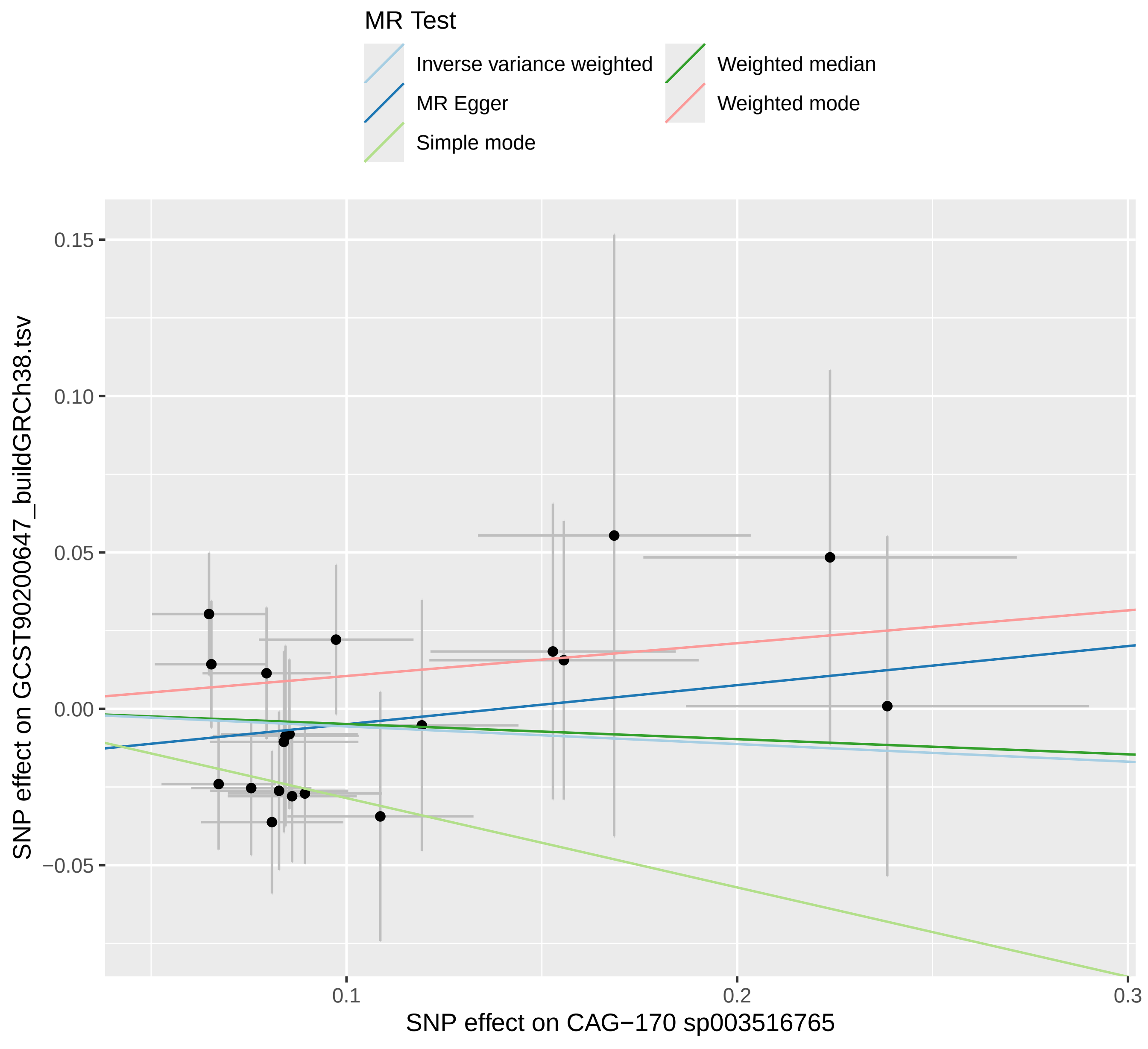

MR Test
Inverse variance weighted
MR Egger
Simple mode
Weighted median
Weighted mode
0.15
0.10
0.05
0.00
−0.05
0.1
0.2
0.3
SNP effect on GCST90200647_buildGRCh38.tsv
SNP effect on CAG−170 sp003516765

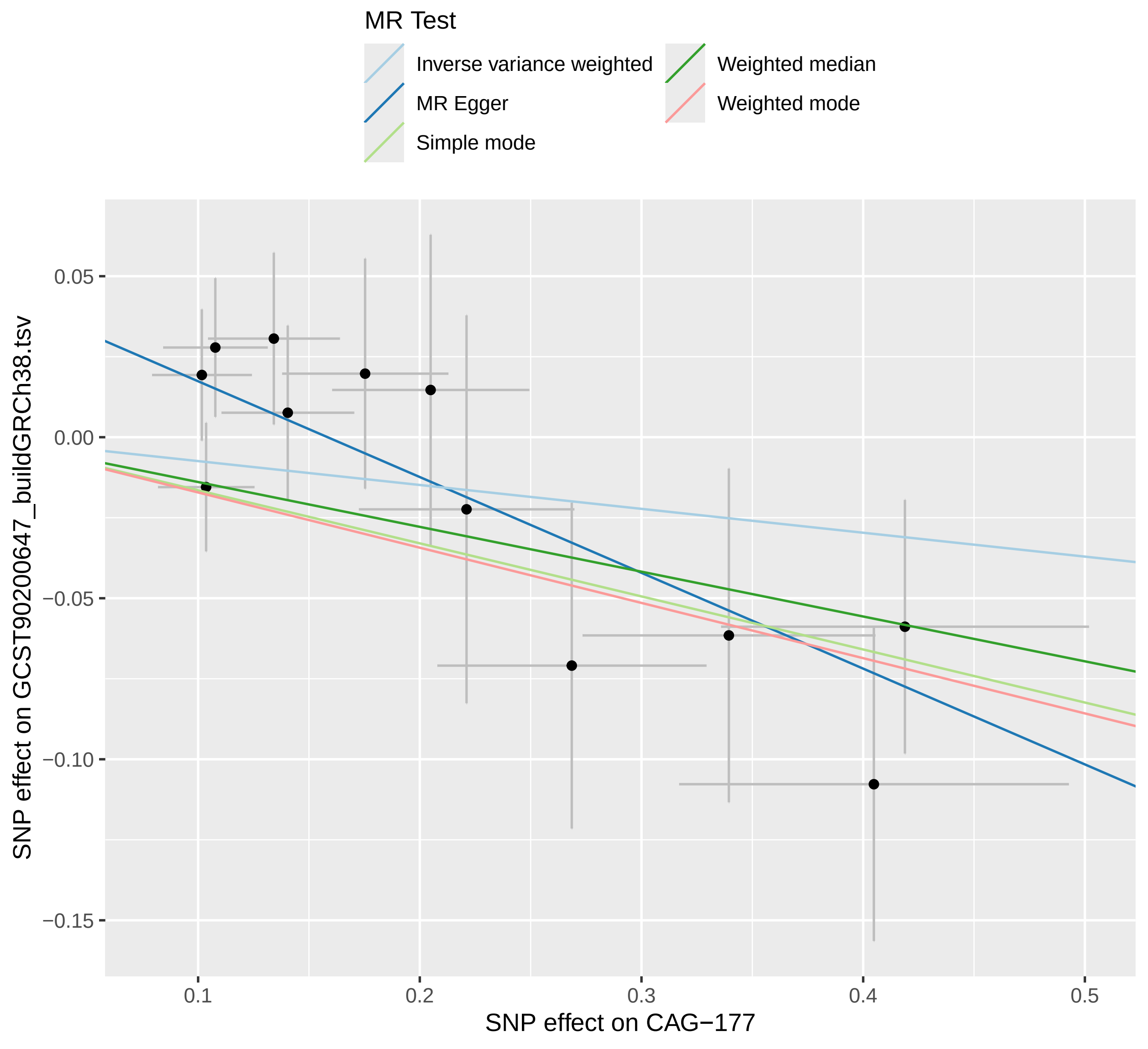

MR Test
Inverse variance weighted
MR Egger
Simple mode
Weighted median
Weighted mode
0.05
0.00
−0.05
−0.10
−0.15
SNP effect on GCST90200647_buildGRCh38.tsv
0.1
0.2
0.3
0.4
0.5
SNP effect on CAG−177

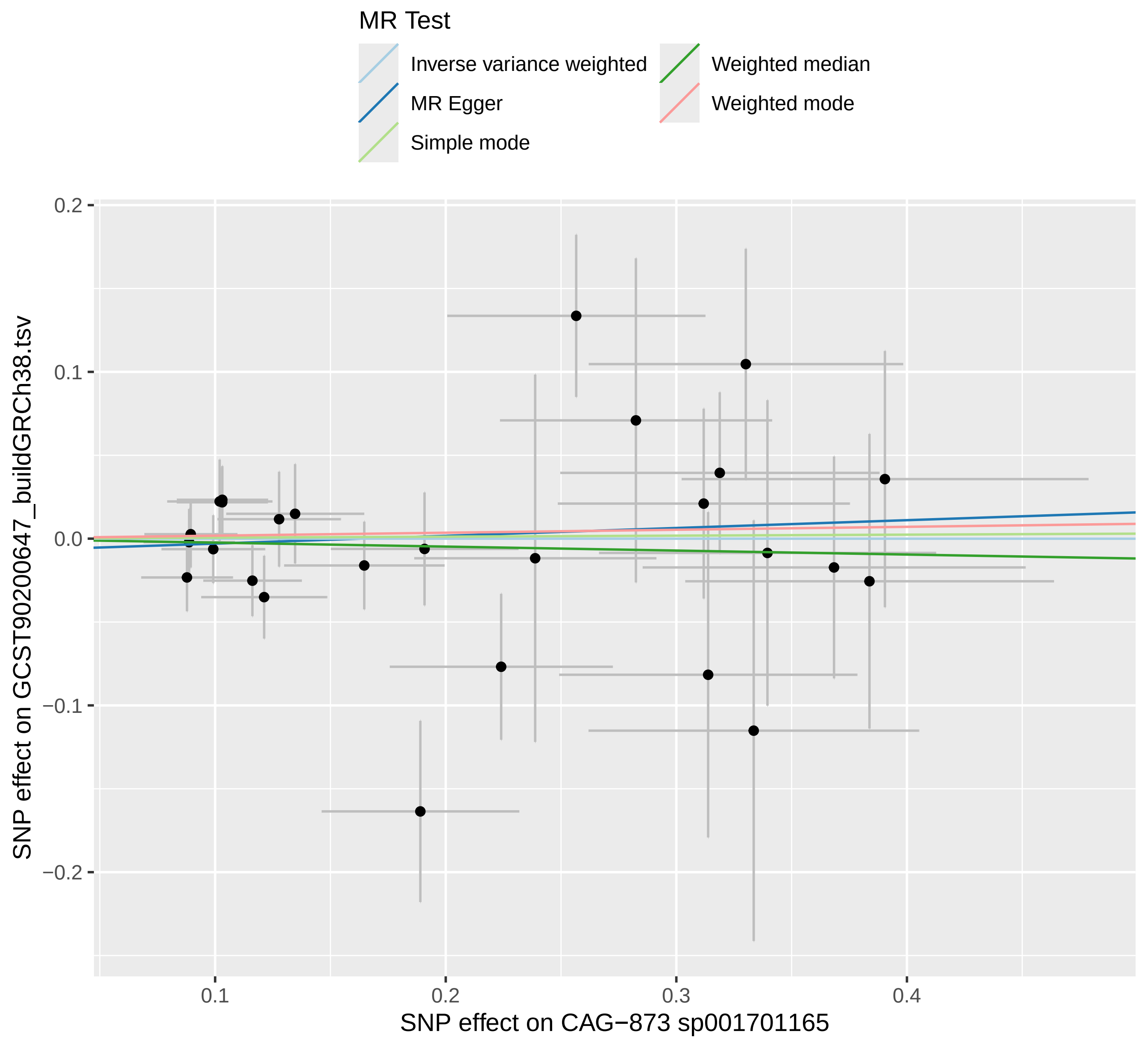

MR Test
Inverse variance weighted
MR Egger
Simple mode
Weighted median
Weighted mode
0.2
0.1
0.0
−0.1
−0.2
SNP effect on GCST90200647_buildGRCh38.tsv
0.1
0.2
0.3
0.4
SNP effect on CAG−873 sp001701165

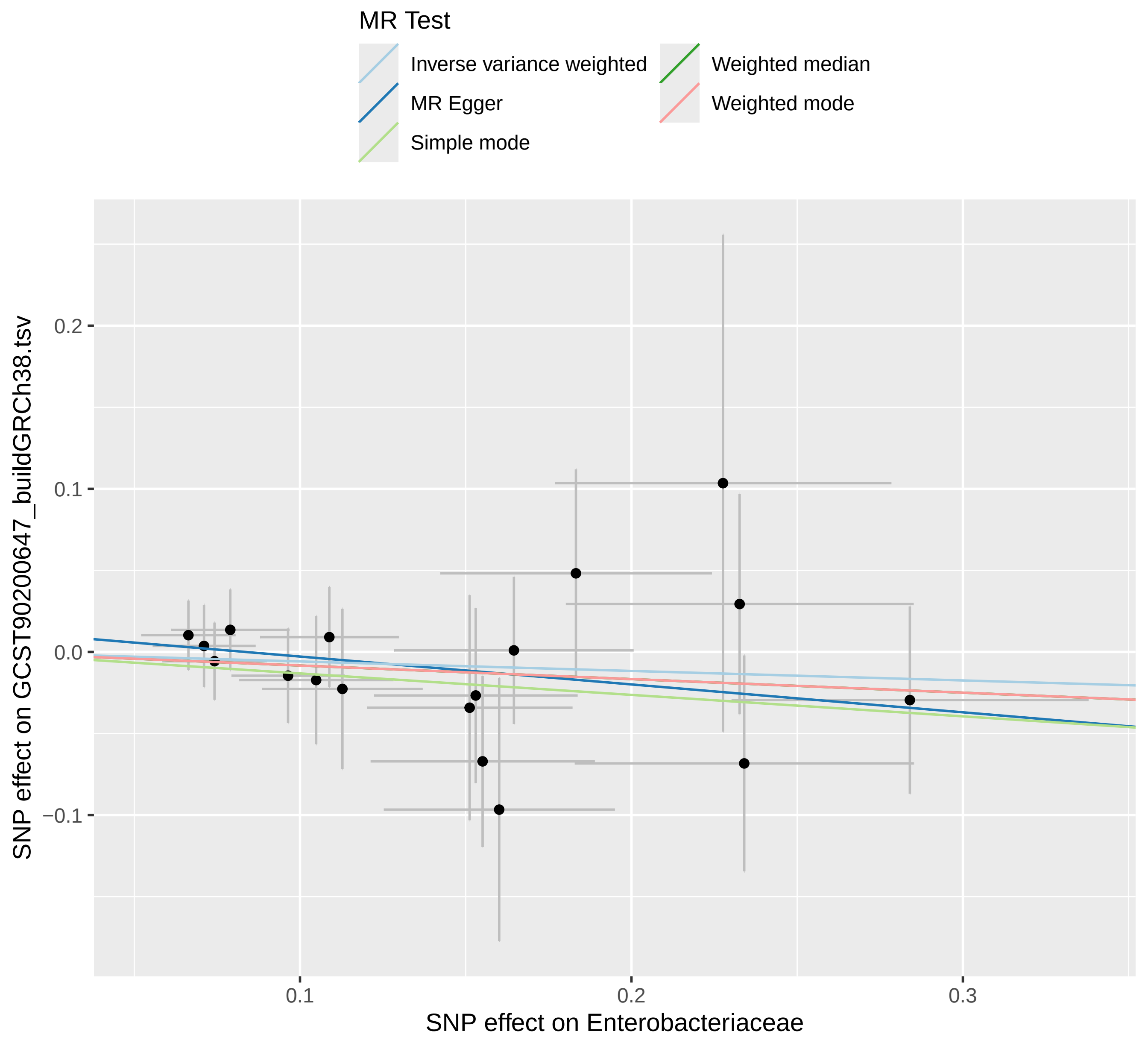

MR Test
Inverse variance weighted
MR Egger
Simple mode
Weighted median
Weighted mode
0.2
0.1
0.0
−0.1
SNP effect on GCST90200647_buildGRCh38.tsv
0.1
0.2
0.3
SNP effect on Enterobacteriaceae

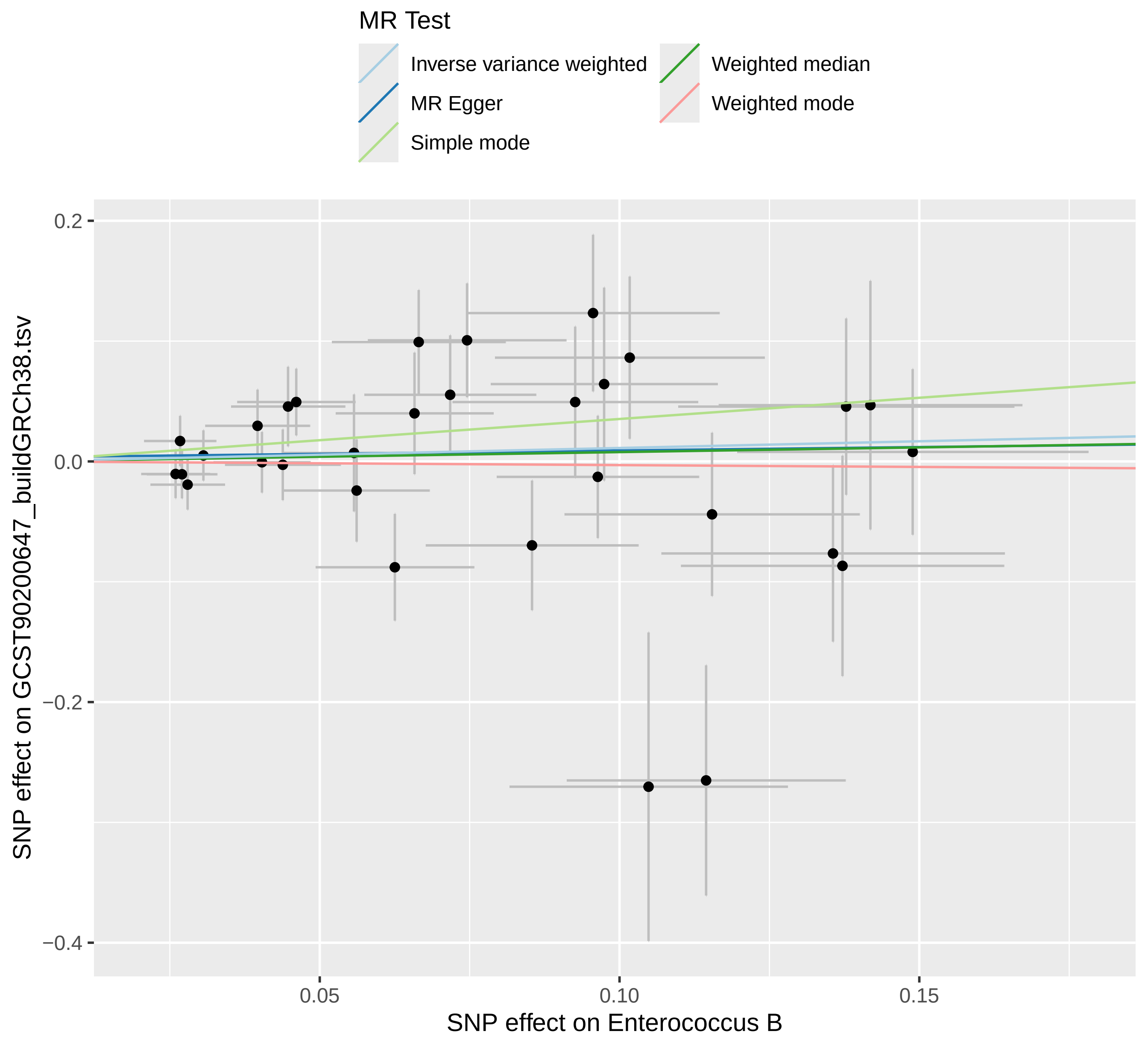

MR Test
Inverse variance weighted
MR Egger
Simple mode
Weighted median
Weighted mode
0.2
0.0
−0.2
−0.4
0.05
0.10
0.15
SNP effect on GCST90200647_buildGRCh38.tsv
SNP effect on Enterococcus B

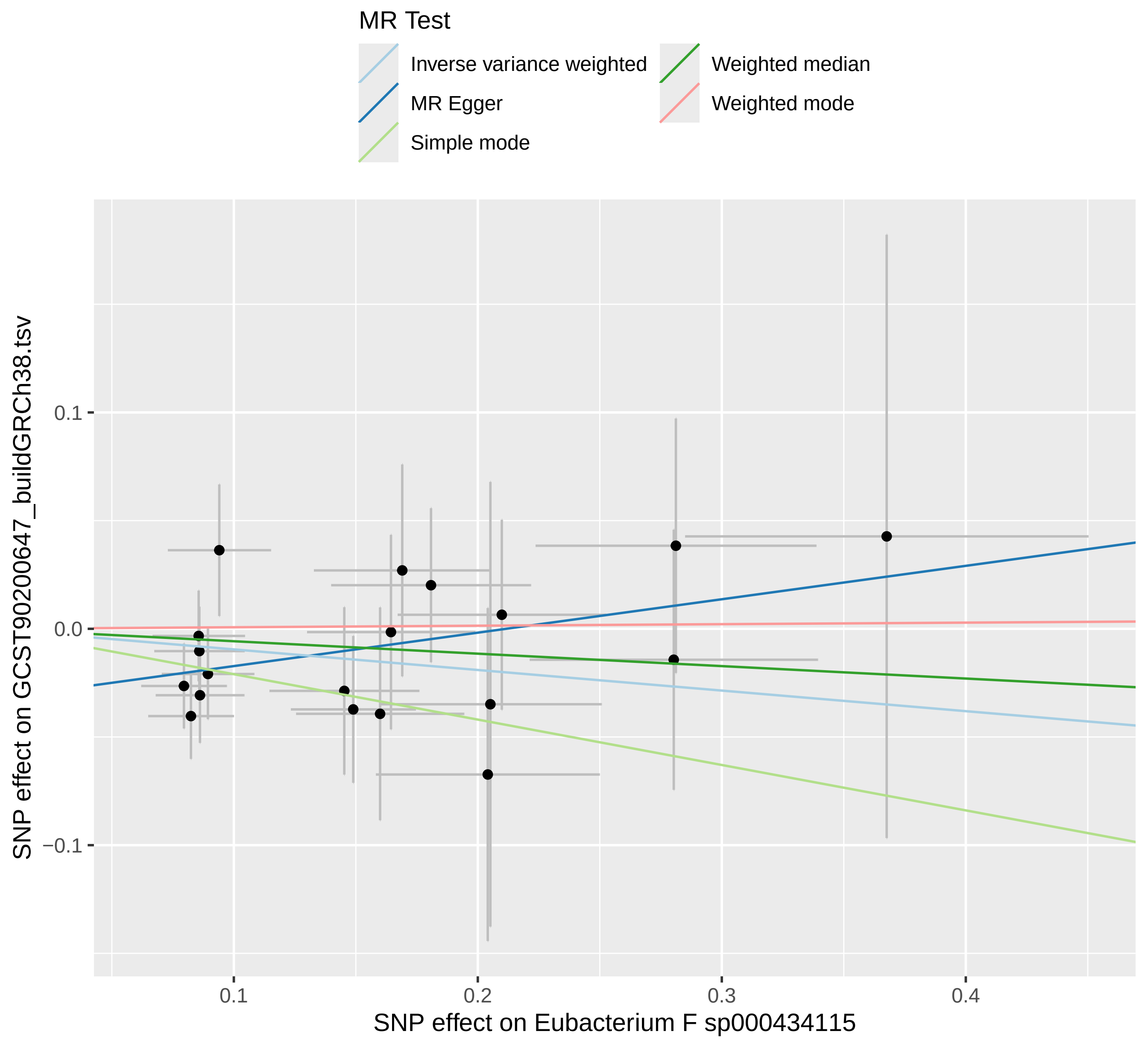
MR Test
Inverse variance weighted
MR Egger
Simple mode
Weighted median
Weighted mode
SNP effect on GCST90200647_buildGRCh38.tsv
0.1
0.0
−0.1
0.1
0.2
0.3
0.4
SNP effect on Eubacterium F sp000434115

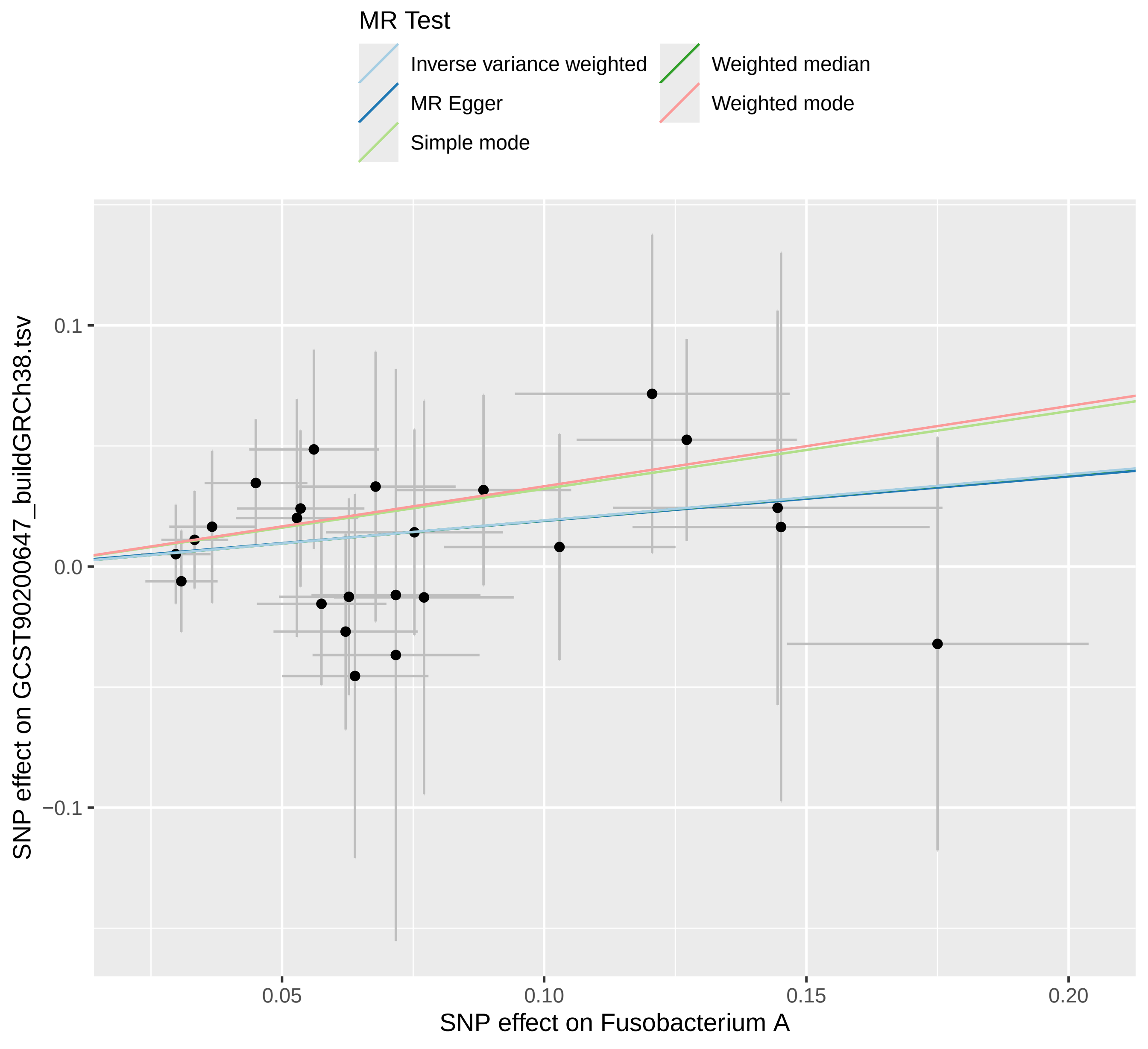

MR Test
Inverse variance weighted
MR Egger
Simple mode
Weighted median
Weighted mode
0.1
0.0
−0.1
0.05
0.10
0.15
0.20
SNP effect on GCST90200647_buildGRCh38.tsv
SNP effect on Fusobacterium A

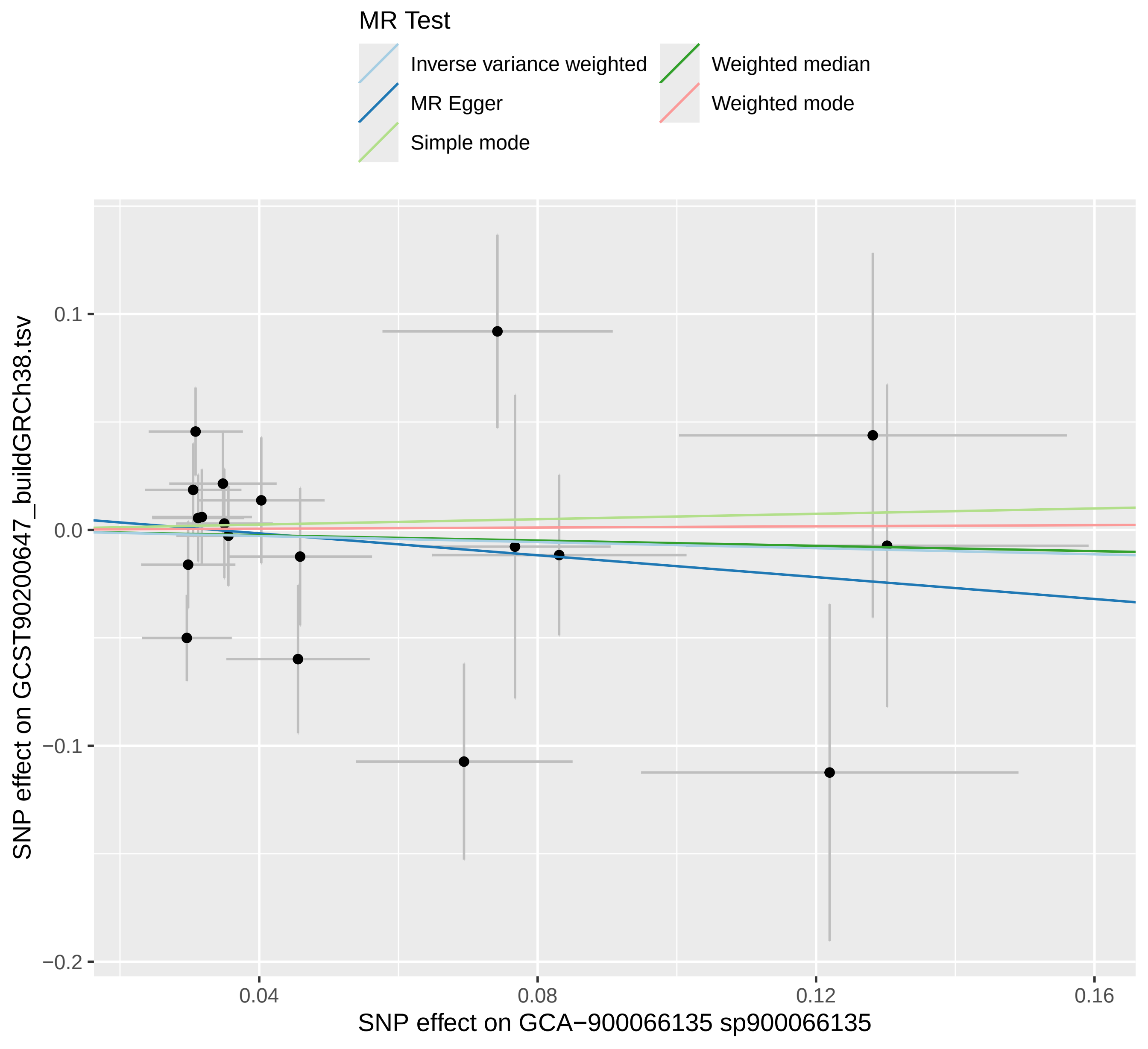
MR Test
Inverse variance weighted
MR Egger
Simple mode
Weighted median
Weighted mode
0.1
0.0
−0.1
−0.2
0.04
0.08
0.12
0.16
SNP effect on GCST90200647_buildGRCh38.tsv
SNP effect on GCA−900066135 sp900066135

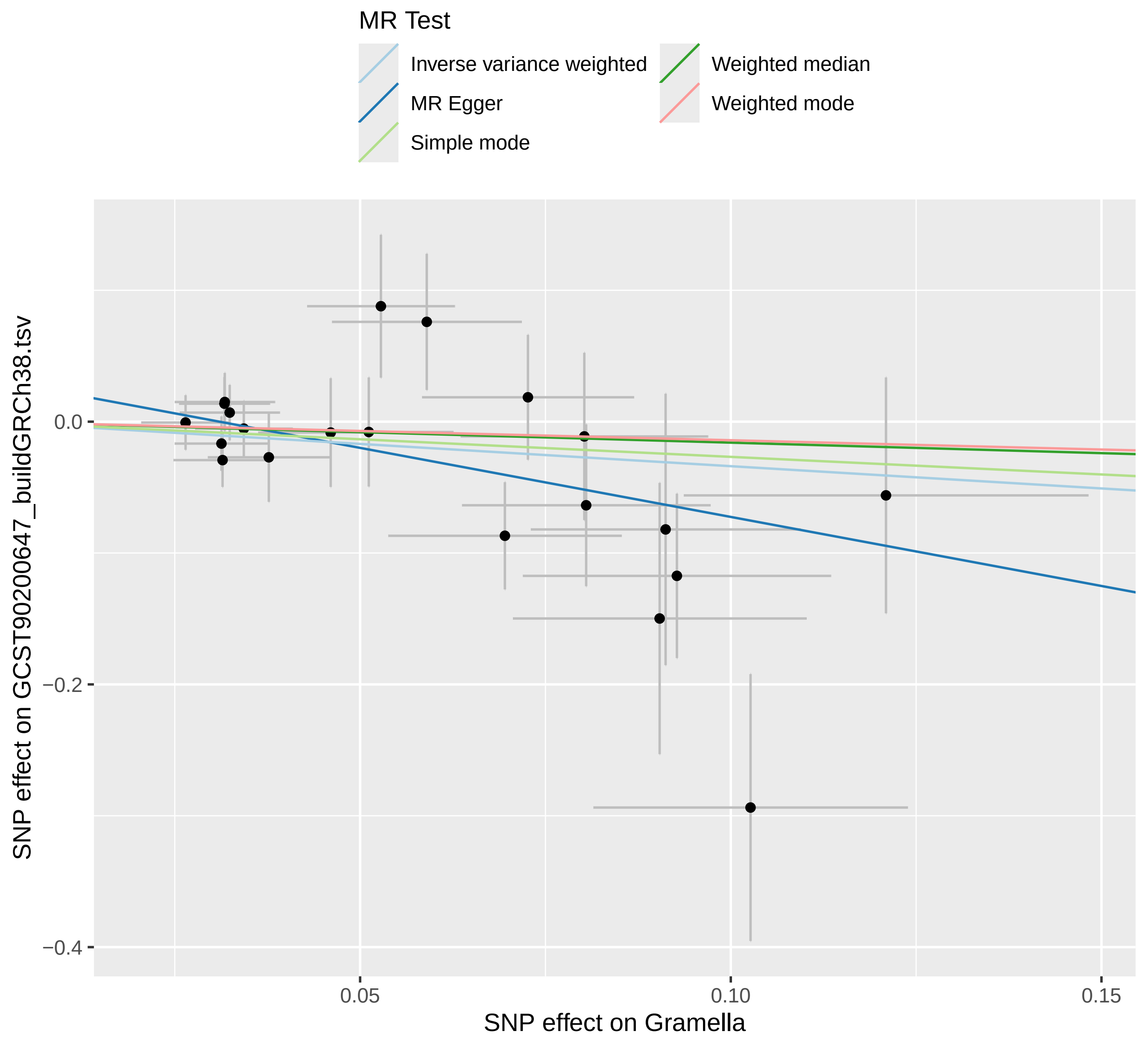
MR Test
Inverse variance weighted
MR Egger
Simple mode
Weighted median
Weighted mode
SNP effect on GCST90200647_buildGRCh38.tsv
0.0
−0.2
−0.4
0.05
0.10
0.15
SNP effect on Gramella

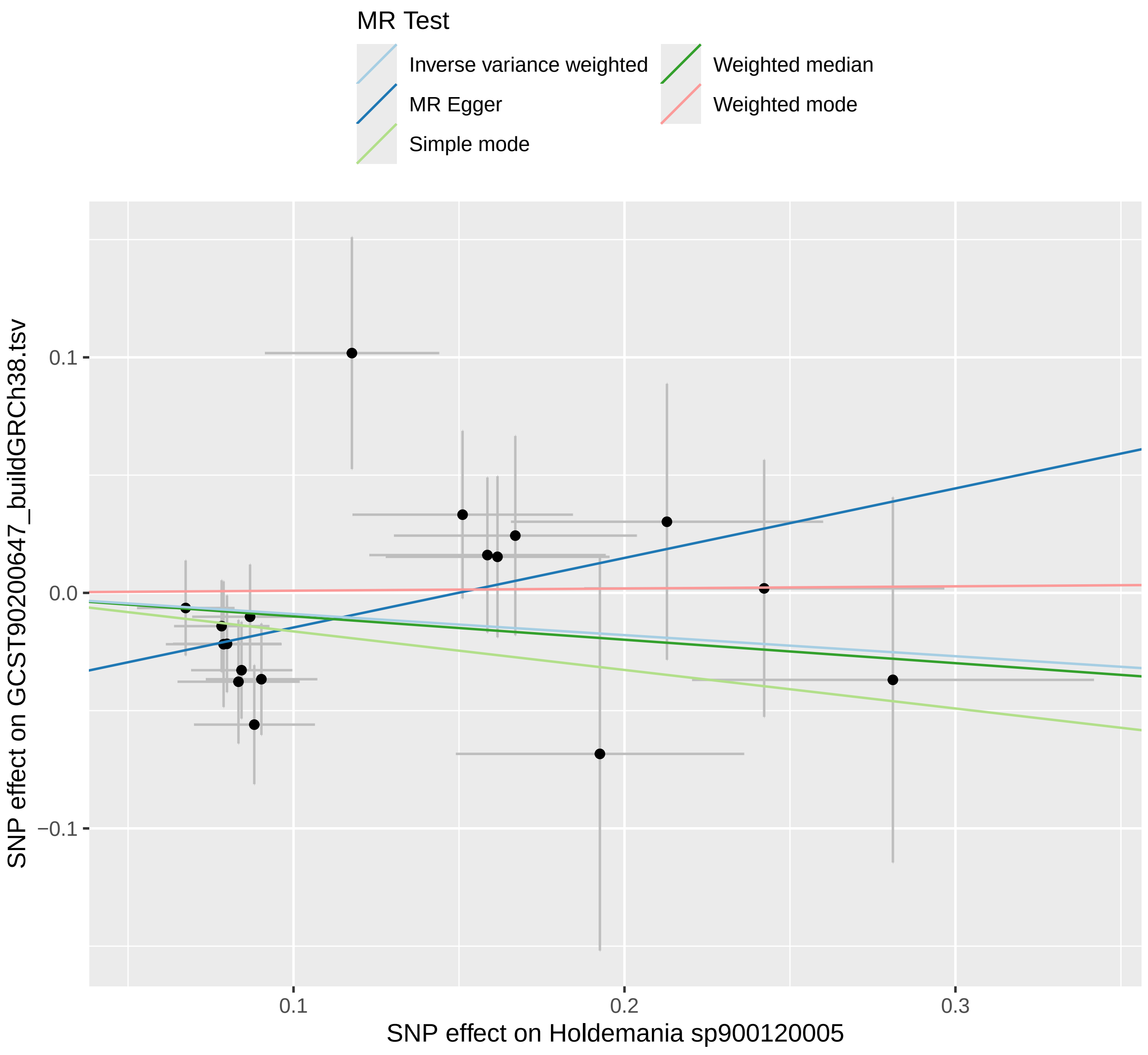

MR Test
Inverse variance weighted
MR Egger
Simple mode
Weighted median
Weighted mode
0.1
0.0
−0.1
0.1
0.2
0.3
SNP effect on GCST90200647_buildGRCh38.tsv
SNP effect on Holdemania sp900120005

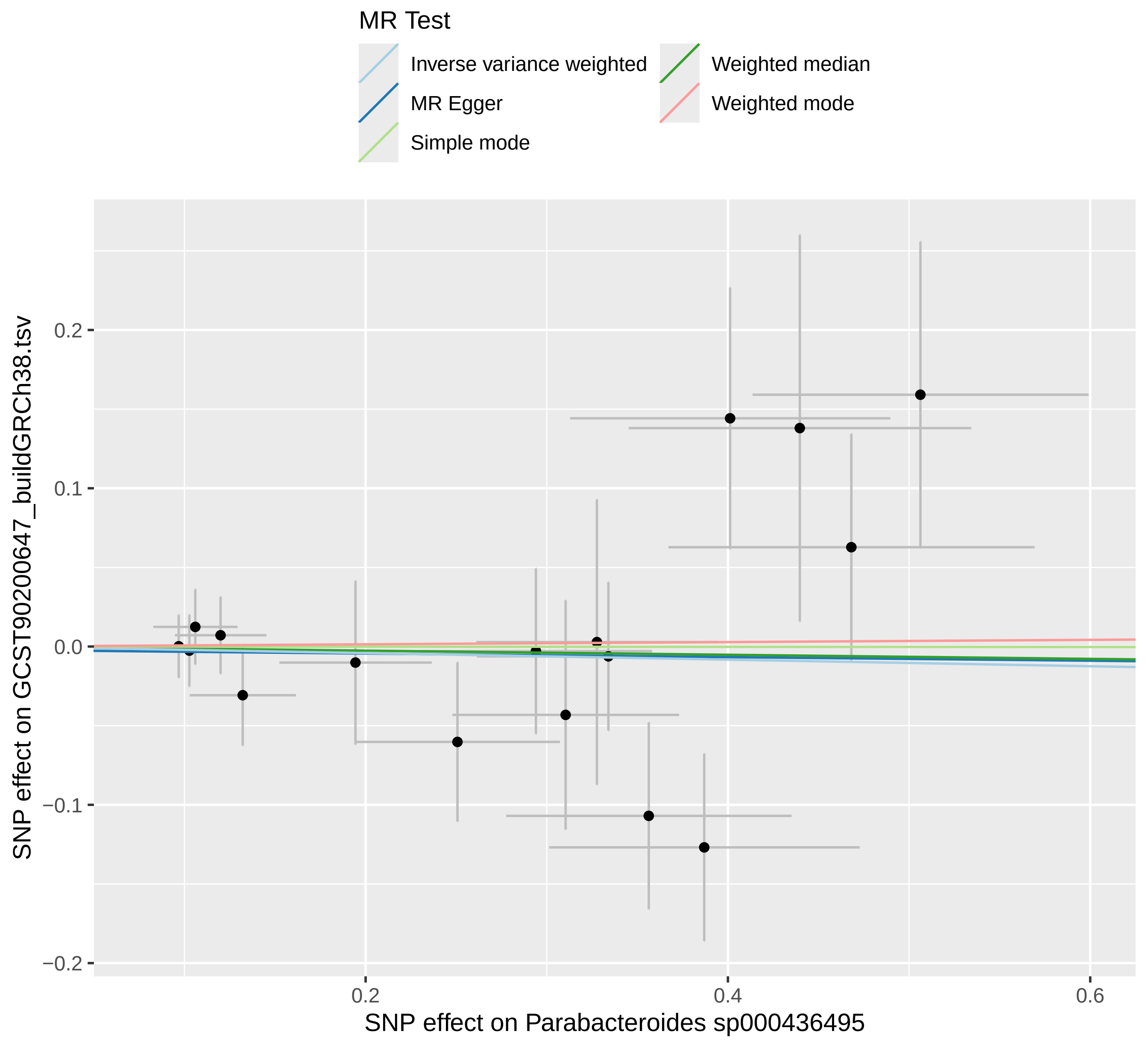

MR Test
Inverse variance weighted
MR Egger
Simple mode
Weighted median
Weighted mode
SNP effect on GCST90200647_buildGRCh38.tsv
0.2
0.1
0.0
−0.1
−0.2
0.2
0.4
0.6
SNP effect on Parabacteroides sp000436495

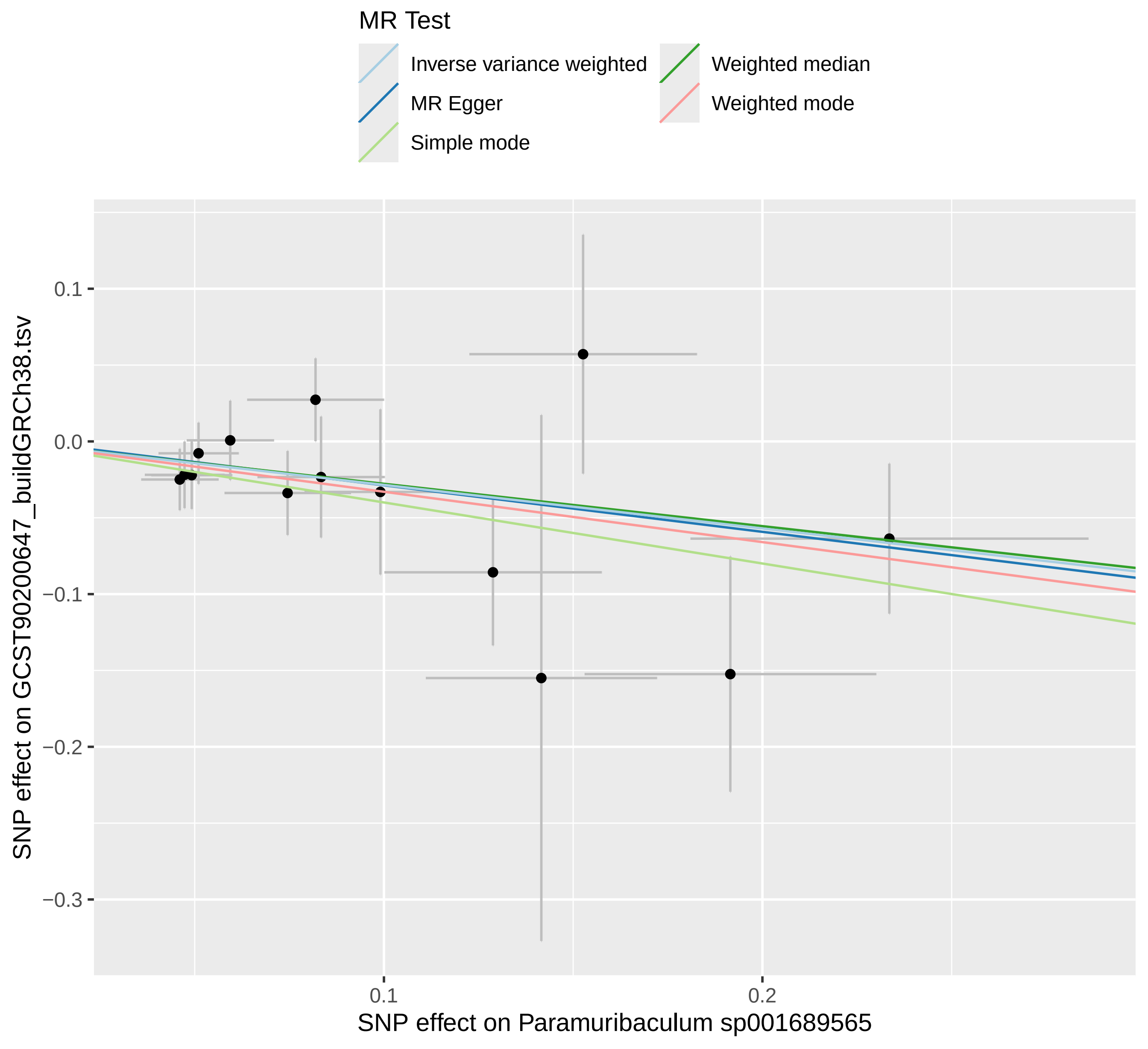
MR Test
Inverse variance weighted
MR Egger
Simple mode
Weighted median
Weighted mode
0.1
0.0
−0.1
−0.2
−0.3
SNP effect on GCST90200647_buildGRCh38.tsv
0.1
0.2
SNP effect on Paramuribaculum sp001689565

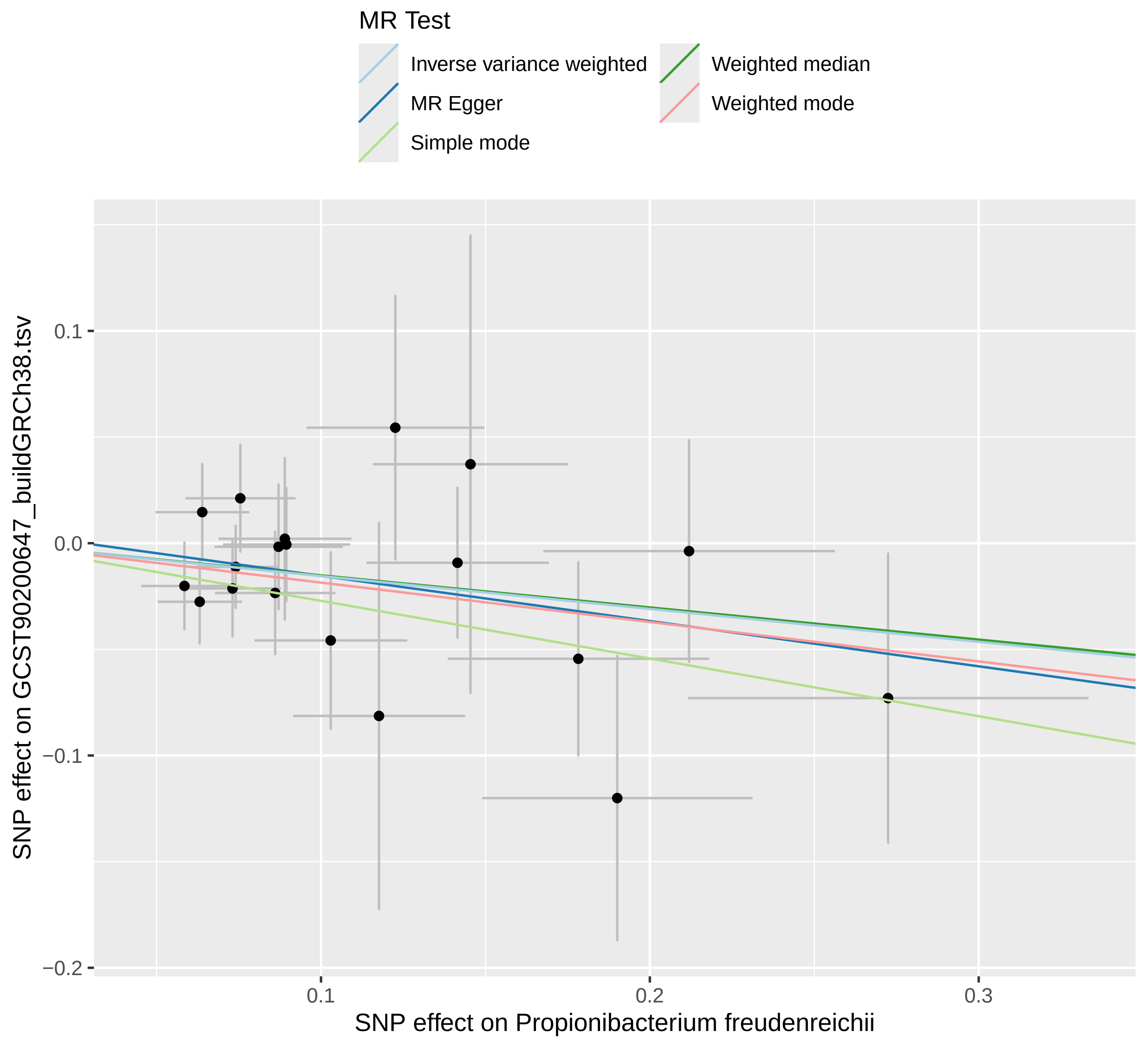
MR Test
Inverse variance weighted
MR Egger
Simple mode
Weighted median
Weighted mode
SNP effect on GCST90200647_buildGRCh38.tsv
0.1
0.0
−0.1
−0.2
0.1
0.2
0.3
SNP effect on Propionibacterium freudenreichii

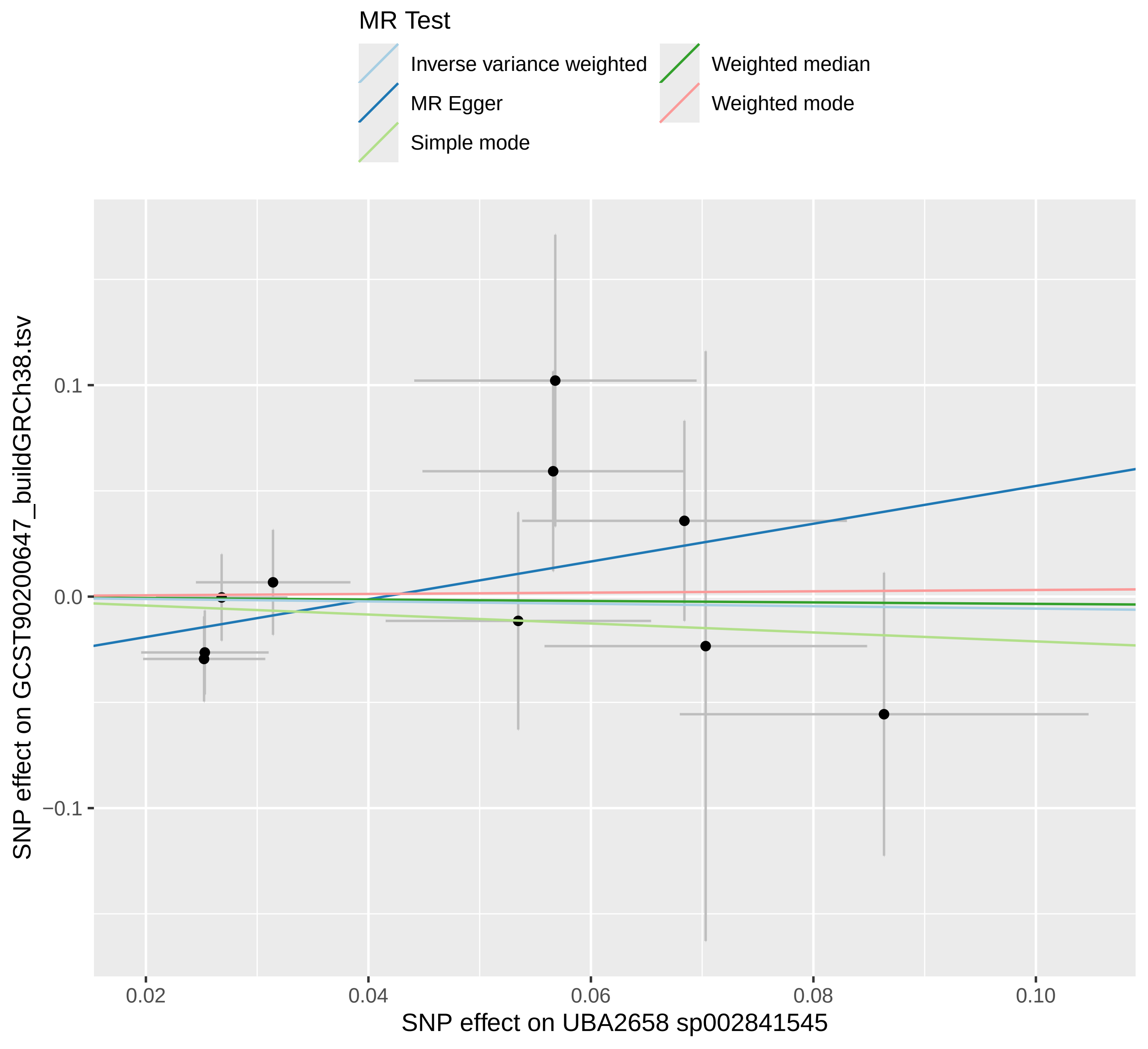
MR Test
Inverse variance weighted
MR Egger
Simple mode
Weighted median
Weighted mode
SNP effect on GCST90200647_buildGRCh38.tsv
0.1
0.0
−0.1
0.02
0.04
0.06
0.08
0.10
SNP effect on UBA2658 sp002841545

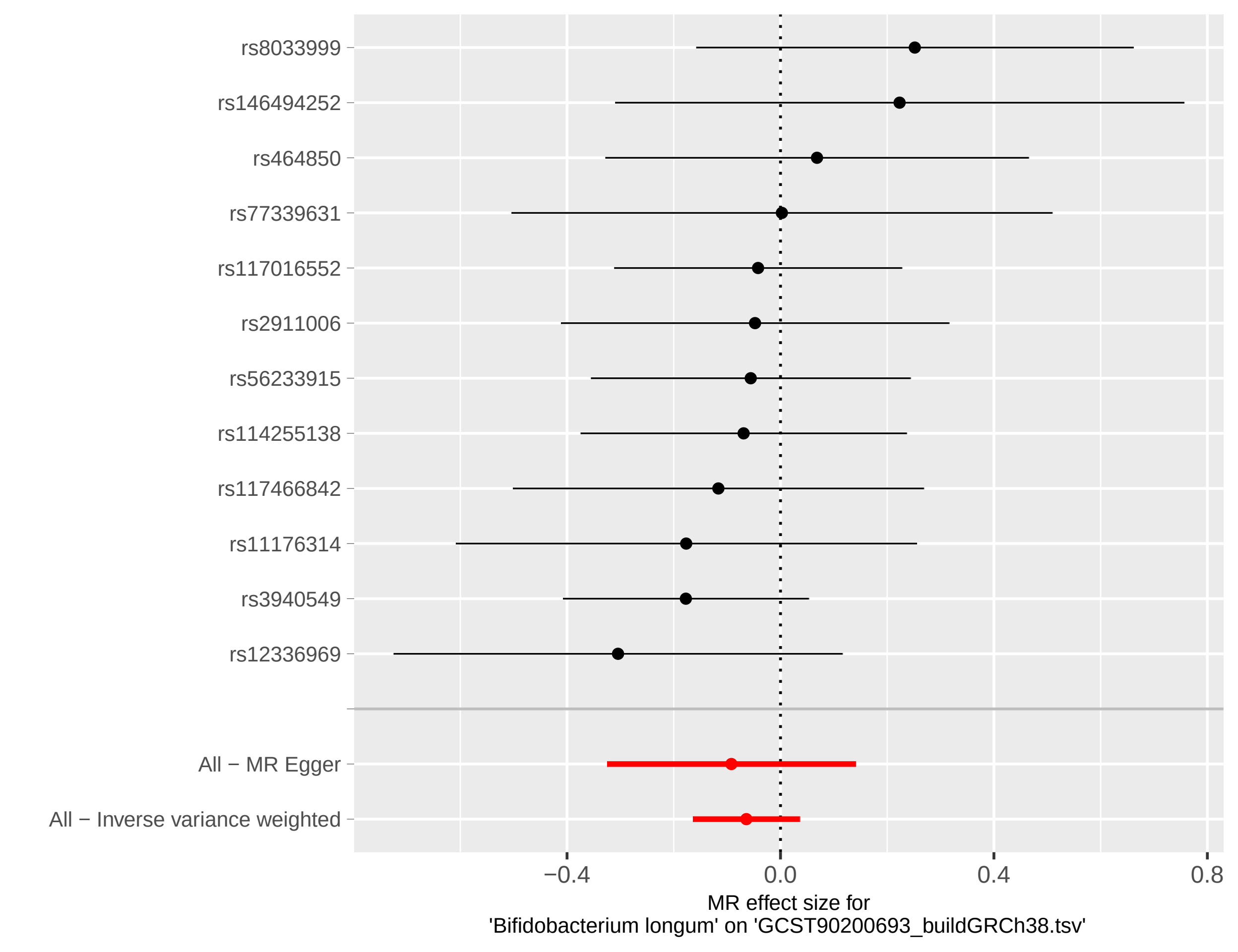

rs8033999
rs146494252
rs464850
rs77339631
rs117016552
rs2911006
rs56233915
rs114255138
rs117466842
rs11176314
rs3940549
rs12336969
All − MR Egger
All − Inverse variance weighted
−0.4
0.0
0.4
0.8
MR effect size for
'Bifidobacterium longum' on 'GCST90200693_buildGRCh38.tsv'

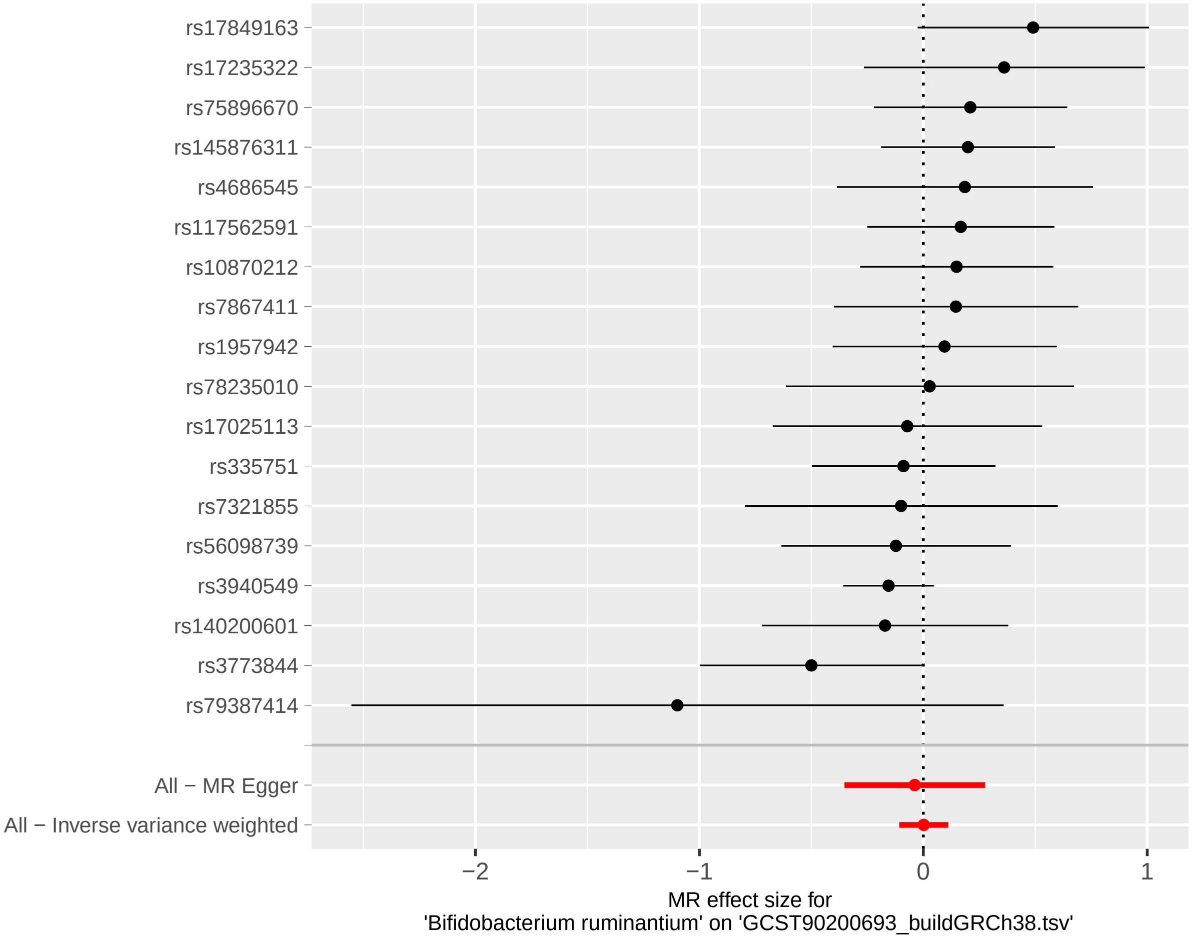
rs17849163
rs17235322
rs75896670
rs145876311
rs4686545
rs117562591
rs10870212
rs7867411
rs1957942
rs78235010
rs17025113
rs335751
rs7321855
rs56098739
rs3940549
rs140200601
rs3773844
rs79387414
All − MR Egger
All − Inverse variance weighted
−2
−1
0
1
MR effect size for
'Bifidobacterium ruminantium' on 'GCST90200693_buildGRCh38.tsv'

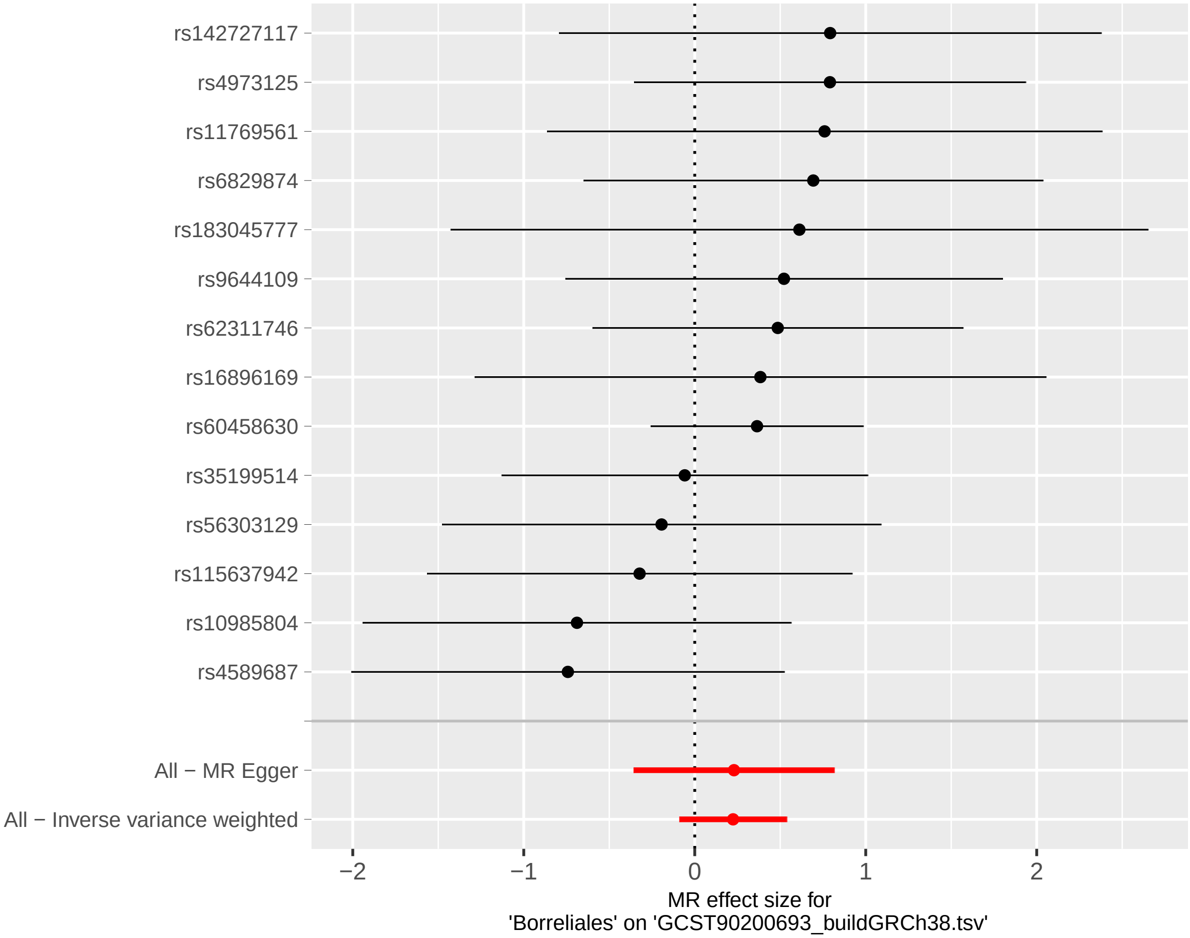
rs142727117
rs4973125
rs11769561
rs6829874
rs183045777
rs9644109
rs62311746
rs16896169
rs60458630
rs35199514
rs56303129
rs115637942
rs10985804
rs4589687
All − MR Egger
All − Inverse variance weighted
−2
−1
0
1
2
MR effect size for
'Borreliales' on 'GCST90200693_buildGRCh38.tsv'

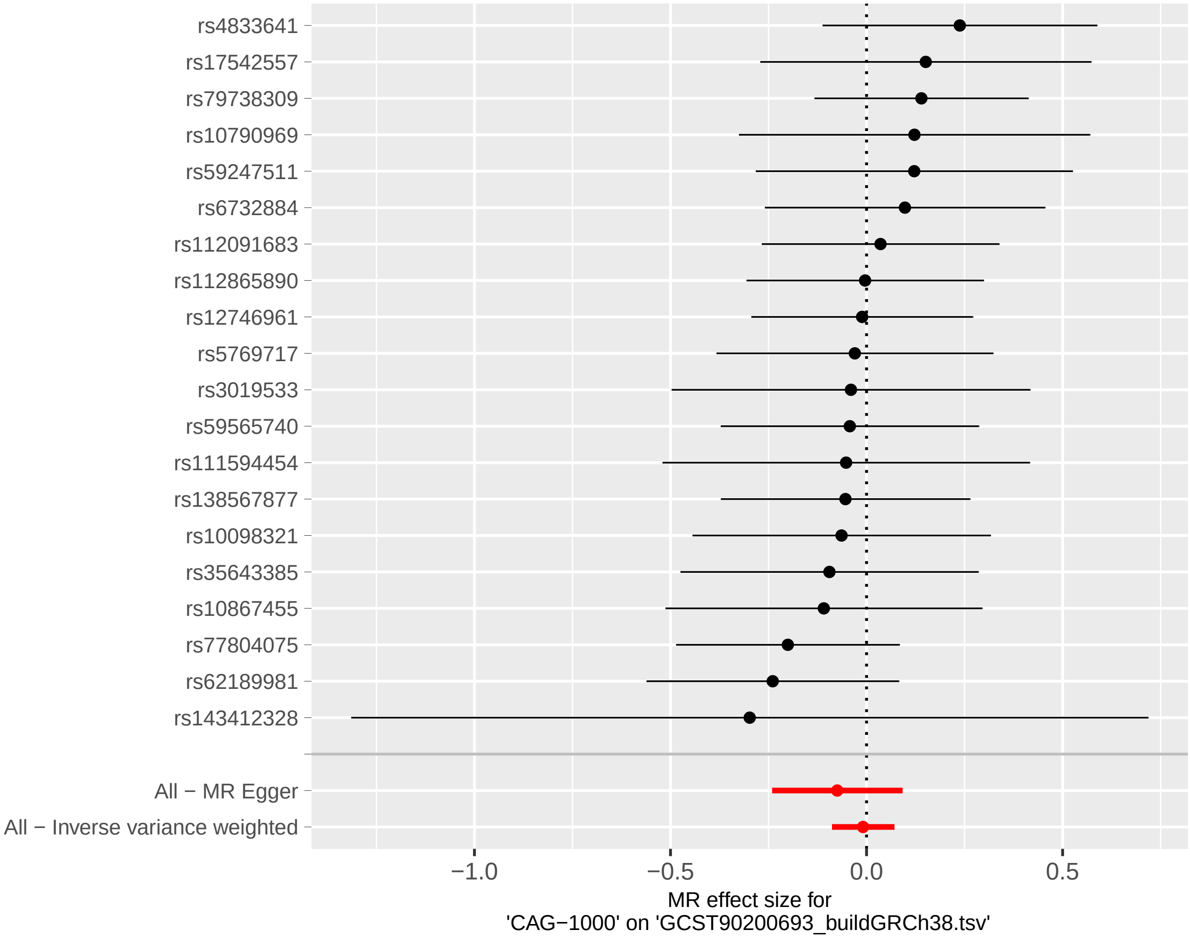
rs4833641
rs17542557
rs79738309
rs10790969
rs59247511
rs6732884
rs112091683
rs112865890
rs12746961
rs5769717
rs3019533
rs59565740
rs111594454
rs138567877
rs10098321
rs35643385
rs10867455
rs77804075
rs62189981
rs143412328
All − MR Egger
All − Inverse variance weighted
−1.0
−0.5
0.0
0.5
MR effect size for
'CAG−1000' on 'GCST90200693_buildGRCh38.tsv'

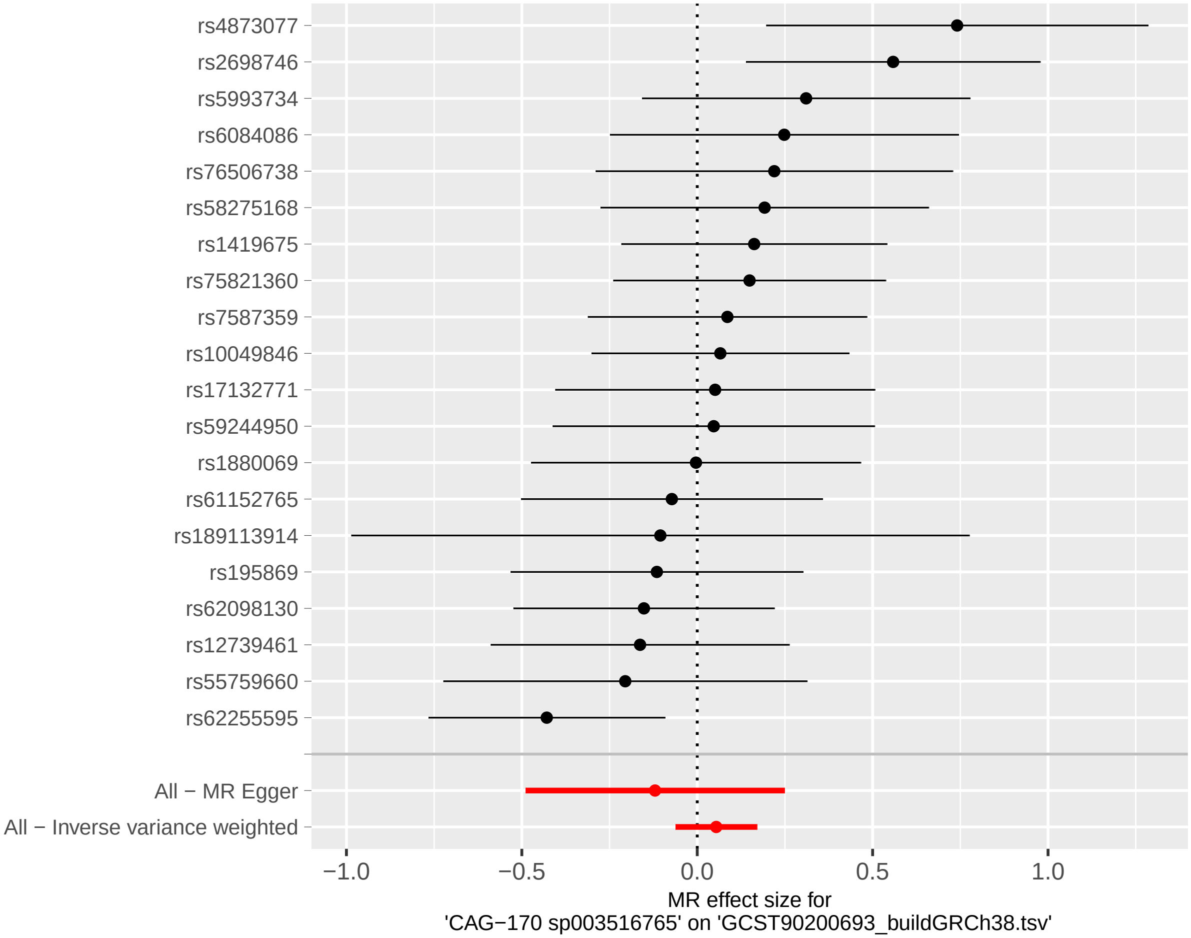
rs4873077
rs2698746
rs5993734
rs6084086
rs76506738
rs58275168
rs1419675
rs75821360
rs7587359
rs10049846
rs17132771
rs59244950
rs1880069
rs61152765
rs189113914
rs195869
rs62098130
rs12739461
rs55759660
rs62255595
All − MR Egger
All − Inverse variance weighted
−1.0
−0.5
0.0
0.5
1.0
MR effect size for
'CAG−170 sp003516765' on 'GCST90200693_buildGRCh38.tsv'

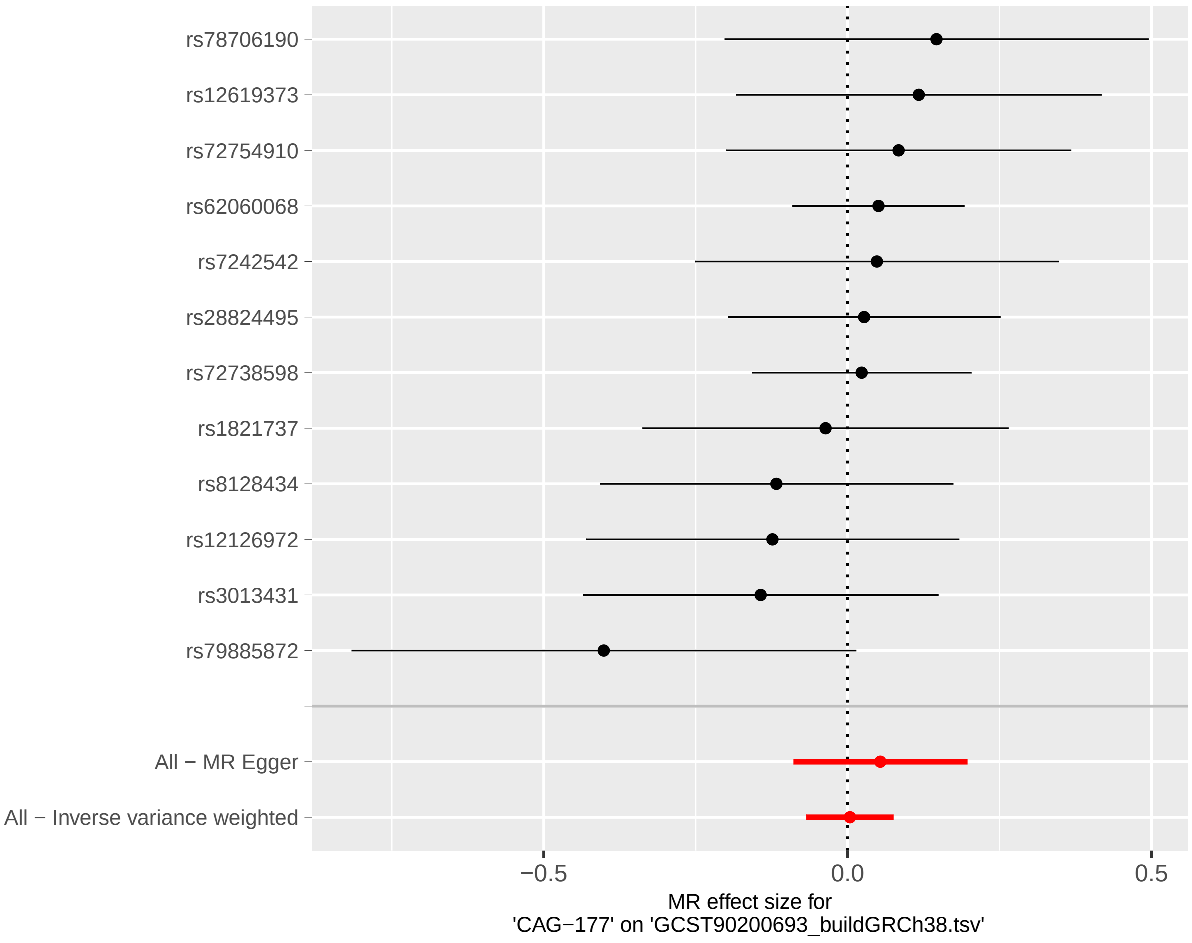
rs78706190
rs12619373
rs72754910
rs62060068
rs7242542
rs28824495
rs72738598
rs1821737
rs8128434
rs12126972
rs3013431
rs79885872
All − MR Egger
All − Inverse variance weighted
−0.5
0.0
0.5
MR effect size for
'CAG−177' on 'GCST90200693_buildGRCh38.tsv'

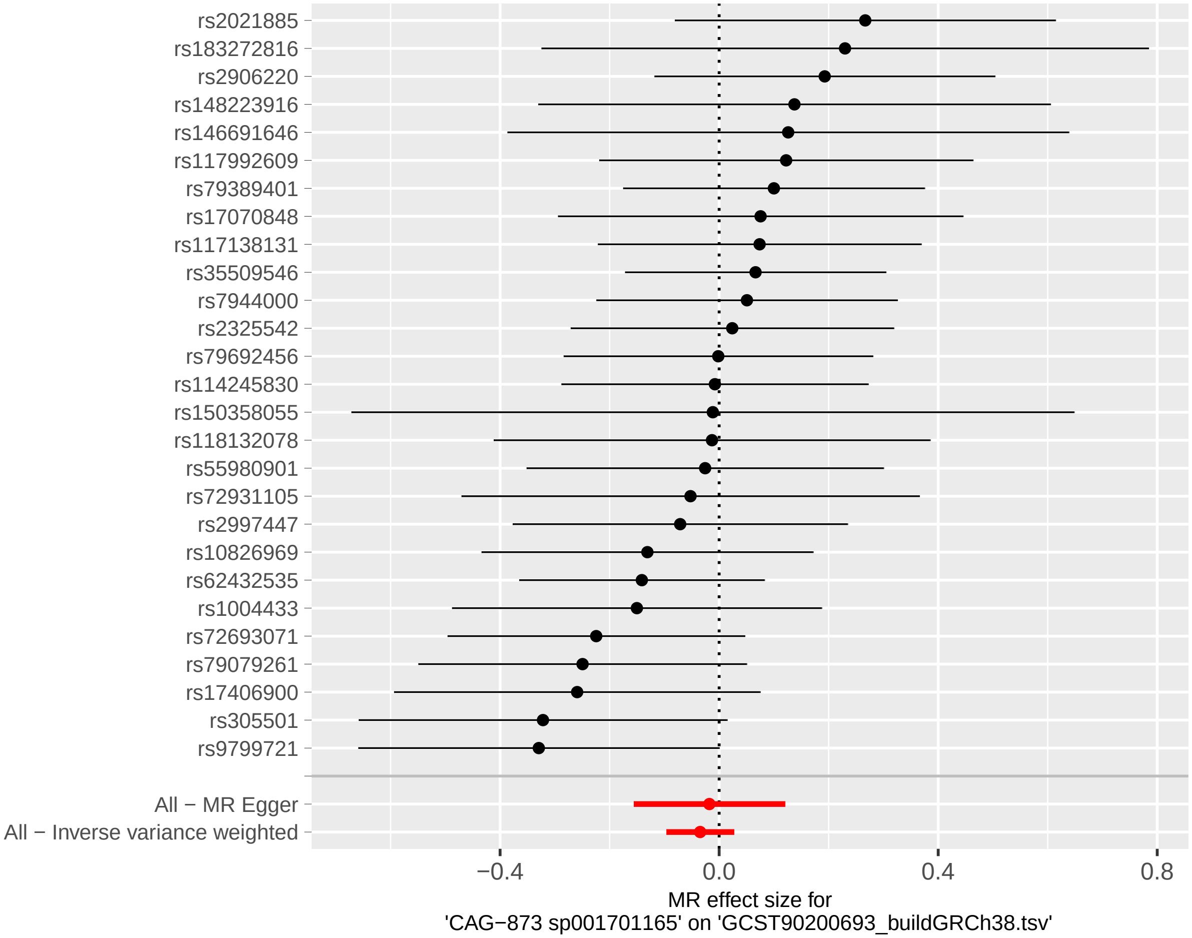
rs2021885
rs183272816
rs2906220
rs148223916
rs146691646
rs117992609
rs79389401
rs17070848
rs117138131
rs35509546
rs7944000
rs2325542
rs79692456
rs114245830
rs150358055
rs118132078
rs55980901
rs72931105
rs2997447
rs10826969
rs62432535
rs1004433
rs72693071
rs79079261
rs17406900
rs305501
rs9799721
All − MR Egger
All − Inverse variance weighted
−0.4
0.0
0.4
0.8
MR effect size for
'CAG−873 sp001701165' on 'GCST90200693_buildGRCh38.tsv'

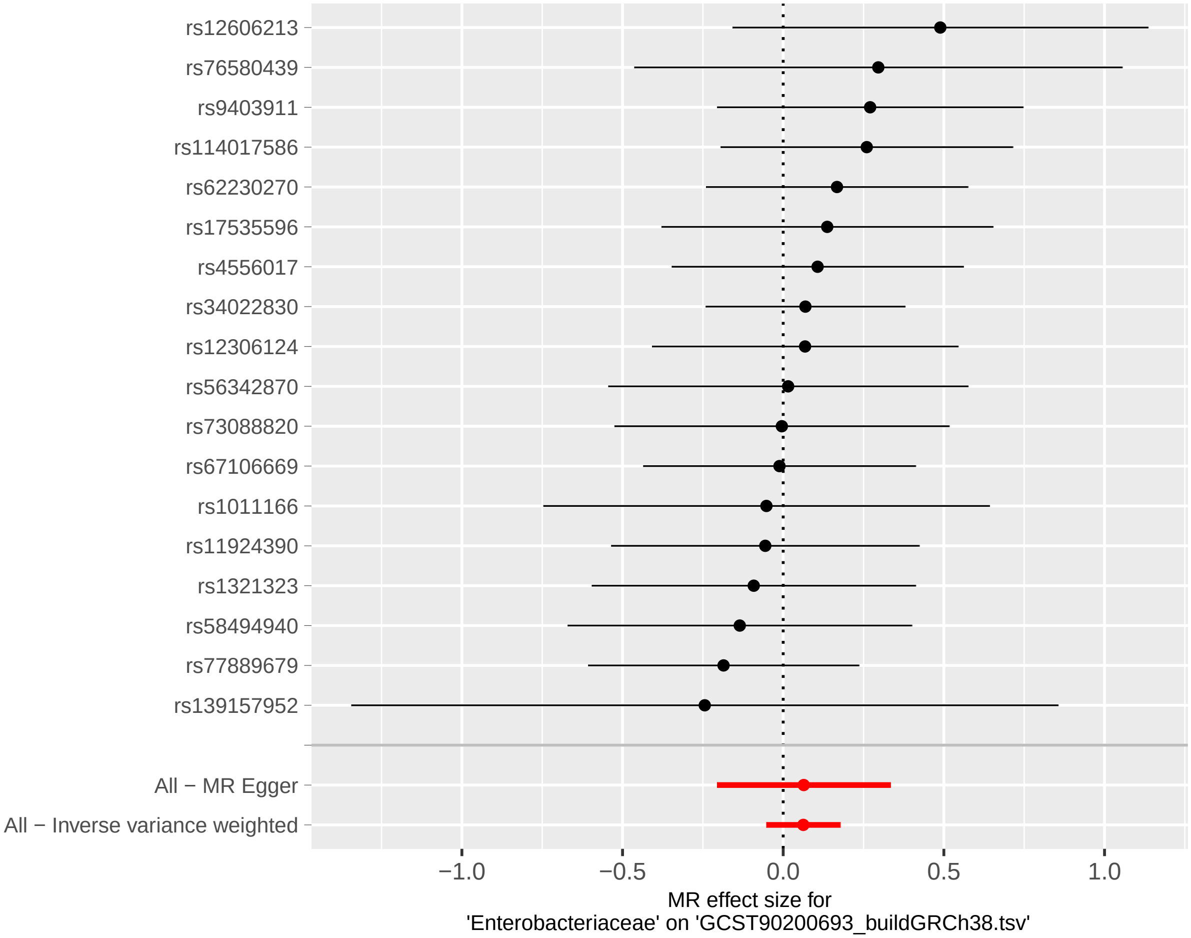
rs12606213
rs76580439
rs9403911
rs114017586
rs62230270
rs17535596
rs4556017
rs34022830
rs12306124
rs56342870
rs73088820
rs67106669
rs1011166
rs11924390
rs1321323
rs58494940
rs77889679
rs139157952
All − MR Egger
All − Inverse variance weighted
−1.0
−0.5
0.0
0.5
1.0
MR effect size for
'Enterobacteriaceae' on 'GCST90200693_buildGRCh38.tsv'

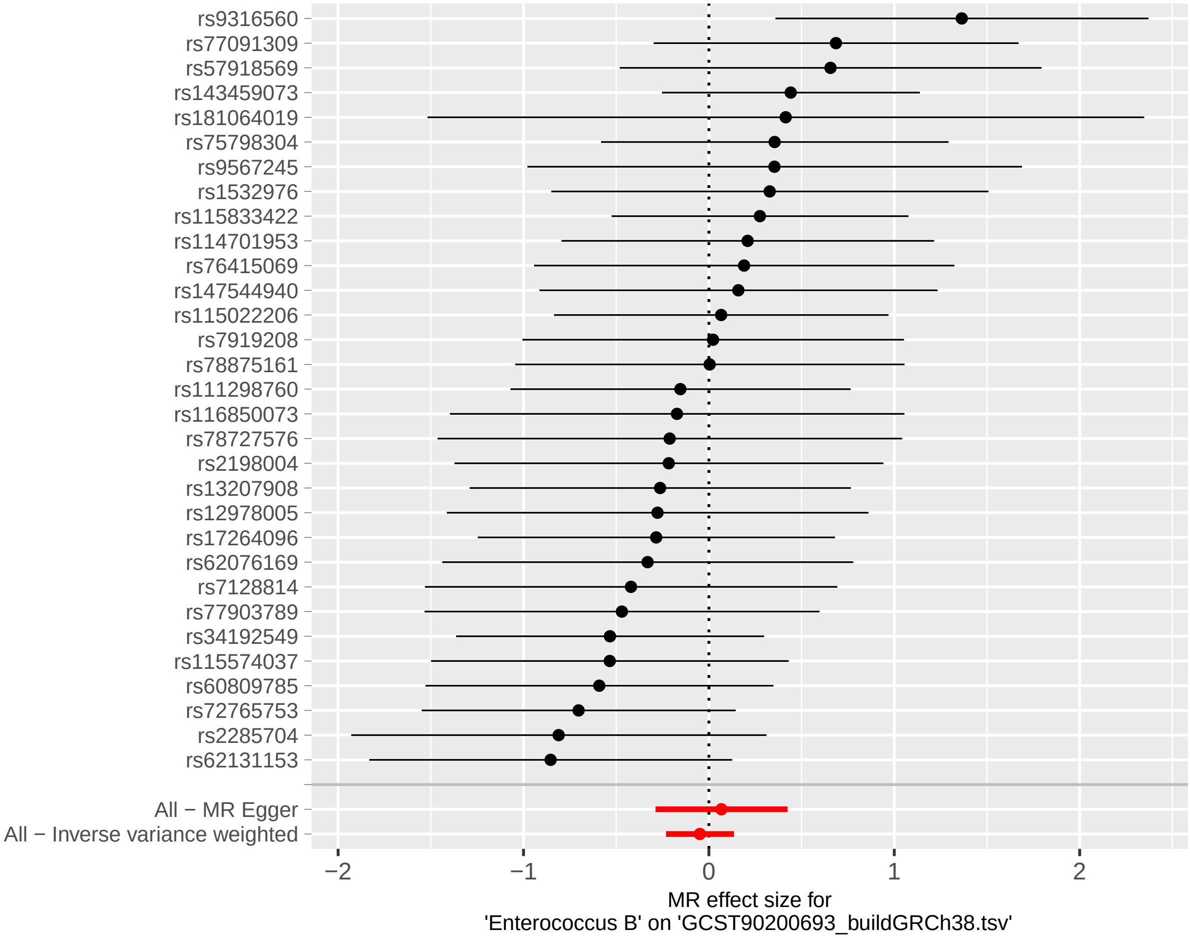
rs9316560
rs77091309
rs57918569
rs143459073
rs181064019
rs75798304
rs9567245
rs1532976
rs115833422
rs114701953
rs76415069
rs147544940
rs115022206
rs7919208
rs78875161
rs111298760
rs116850073
rs78727576
rs2198004
rs13207908
rs12978005
rs17264096
rs62076169
rs7128814
rs77903789
rs34192549
rs115574037
rs60809785
rs72765753
rs2285704
rs62131153
All − MR Egger
All − Inverse variance weighted
−2
−1
0
1
2
MR effect size for
'Enterococcus B' on 'GCST90200693_buildGRCh38.tsv'

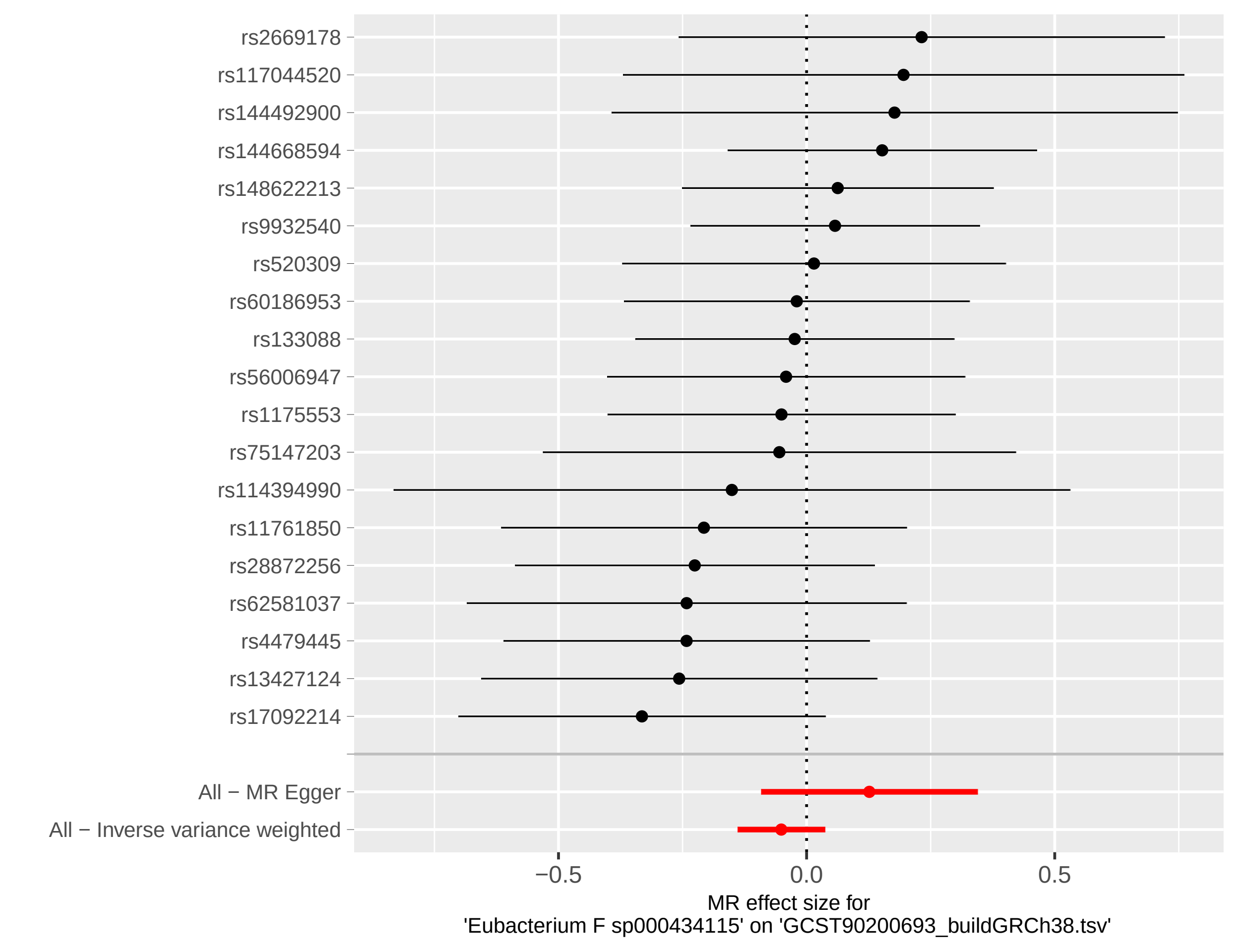
rs2669178
rs117044520
rs144492900
rs144668594
rs148622213
rs9932540
rs520309
rs60186953
rs133088
rs56006947
rs1175553
rs75147203
rs114394990
rs11761850
rs28872256
rs62581037
rs4479445
rs13427124
rs17092214
All − MR Egger
All − Inverse variance weighted
−0.5
0.0
0.5
MR effect size for
'Eubacterium F sp000434115' on 'GCST90200693_buildGRCh38.tsv'

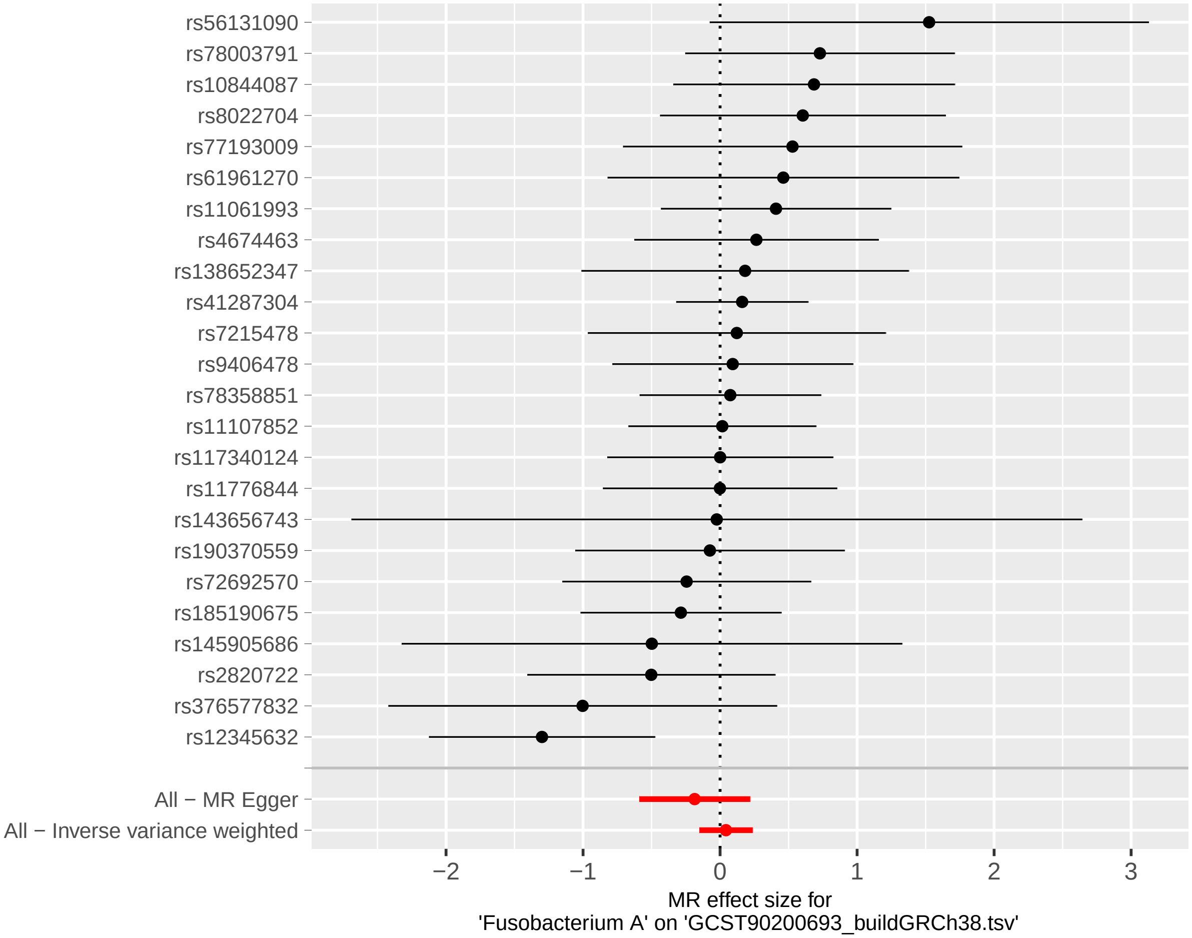

rs56131090
rs78003791
rs10844087
rs8022704
rs77193009
rs61961270
rs11061993
rs4674463
rs138652347
rs41287304
rs7215478
rs9406478
rs78358851
rs11107852
rs117340124
rs11776844
rs143656743
rs190370559
rs72692570
rs185190675
rs145905686
rs2820722
rs376577832
rs12345632
All − MR Egger
All − Inverse variance weighted
−2
−1
0
1
2
3
MR effect size for
'Fusobacterium A' on 'GCST90200693_buildGRCh38.tsv'

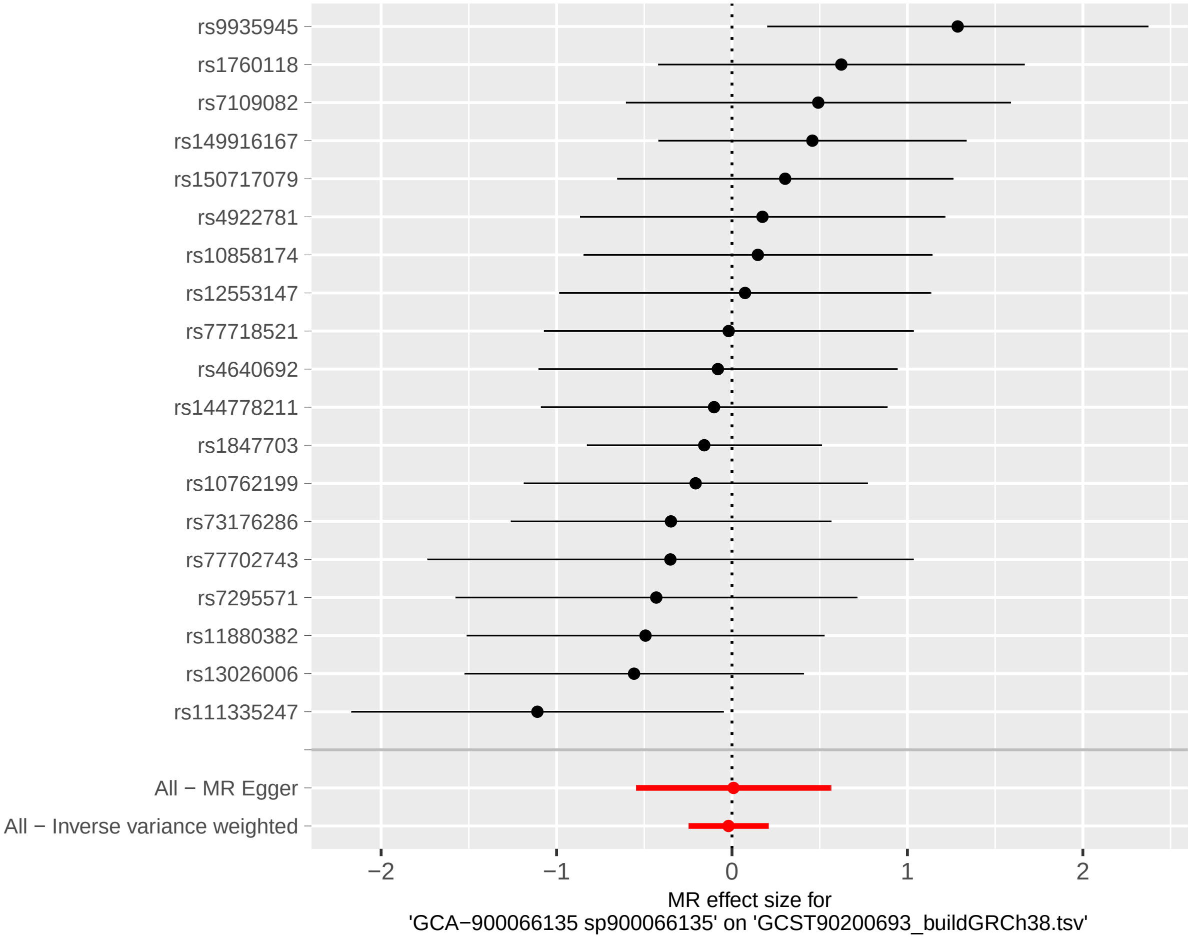
rs9935945
rs1760118
rs7109082
rs149916167
rs150717079
rs4922781
rs10858174
rs12553147
rs77718521
rs4640692
rs144778211
rs1847703
rs10762199
rs73176286
rs77702743
rs7295571
rs11880382
rs13026006
rs111335247
All − MR Egger
All − Inverse variance weighted
−2
−1
0
1
2
MR effect size for
'GCA−900066135 sp900066135' on 'GCST90200693_buildGRCh38.tsv'

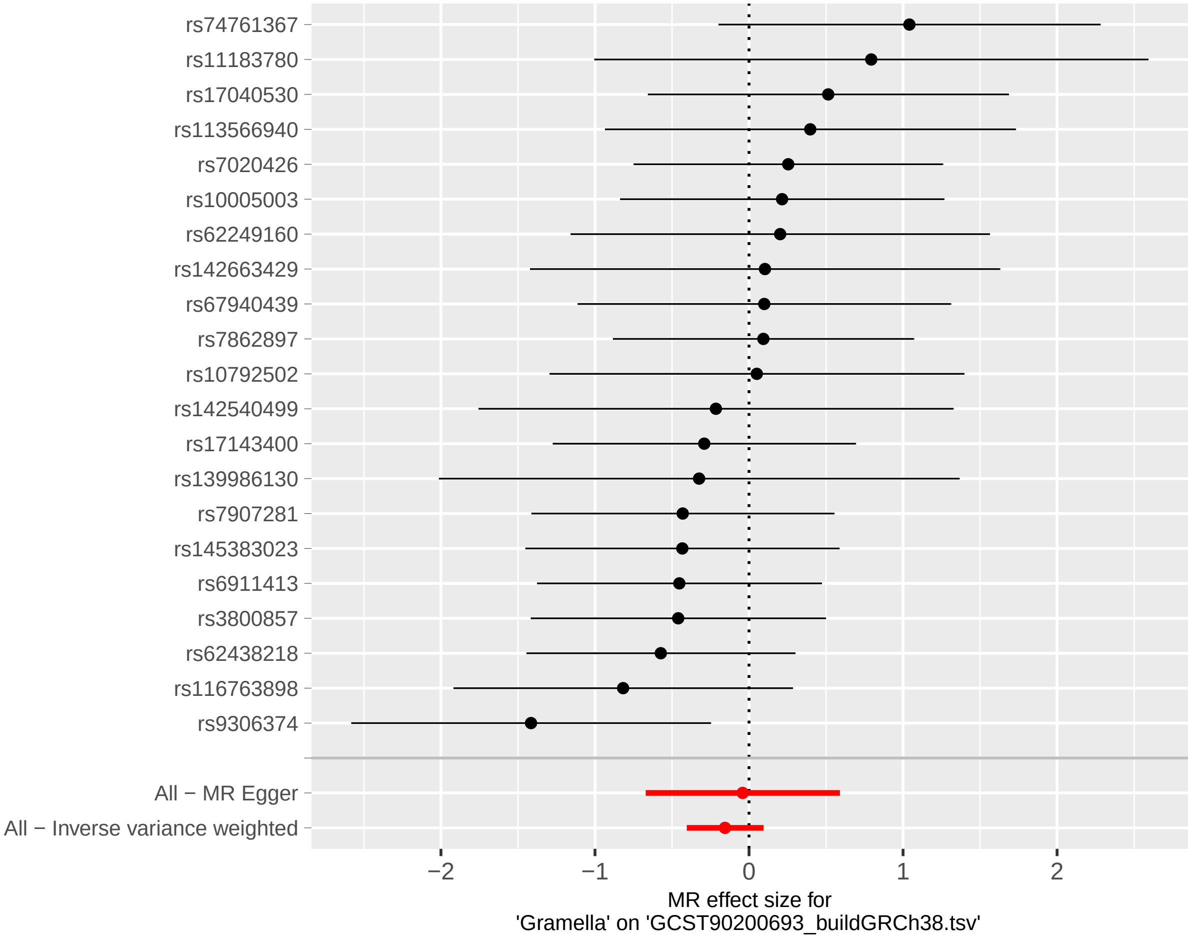
rs74761367
rs11183780
rs17040530
rs113566940
rs7020426
rs10005003
rs62249160
rs142663429
rs67940439
rs7862897
rs10792502
rs142540499
rs17143400
rs139986130
rs7907281
rs145383023
rs6911413
rs3800857
rs62438218
rs116763898
rs9306374
All − MR Egger
All − Inverse variance weighted
−2
−1
0
1
2
MR effect size for
'Gramella' on 'GCST90200693_buildGRCh38.tsv'

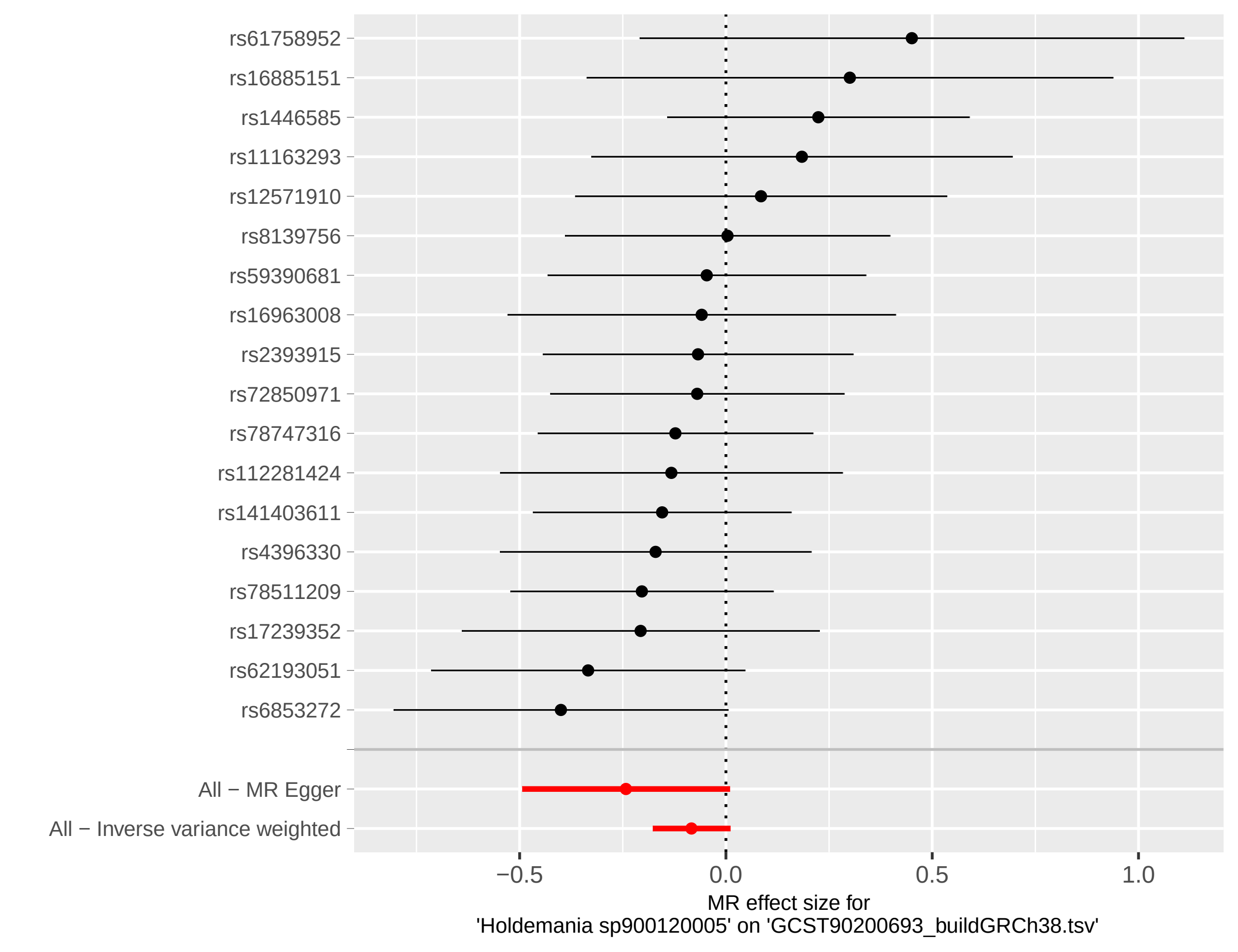

rs61758952
rs16885151
rs1446585
rs11163293
rs12571910
rs8139756
rs59390681
rs16963008
rs2393915
rs72850971
rs78747316
rs112281424
rs141403611
rs4396330
rs78511209
rs17239352
rs62193051
rs6853272
All − MR Egger
All − Inverse variance weighted
−0.5
0.0
0.5
1.0
MR effect size for
'Holdemania sp900120005' on 'GCST90200693_buildGRCh38.tsv'

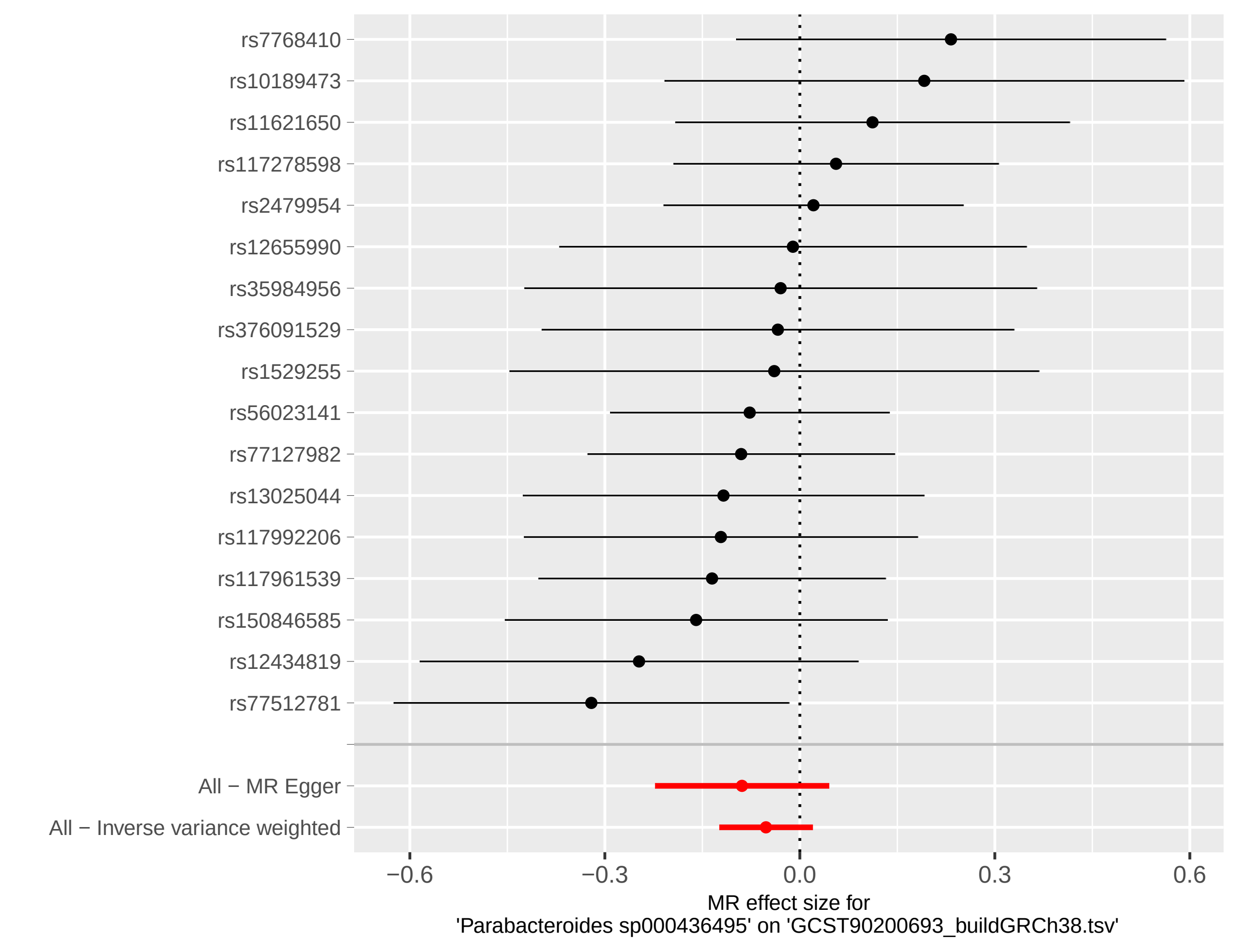

rs7768410
rs10189473
rs11621650
rs117278598
rs2479954
rs12655990
rs35984956
rs376091529
rs1529255
rs56023141
rs77127982
rs13025044
rs117992206
rs117961539
rs150846585
rs12434819
rs77512781
All − MR Egger
All − Inverse variance weighted
−0.6
−0.3
0.0
0.3
0.6
MR effect size for
'Parabacteroides sp000436495' on 'GCST90200693_buildGRCh38.tsv'

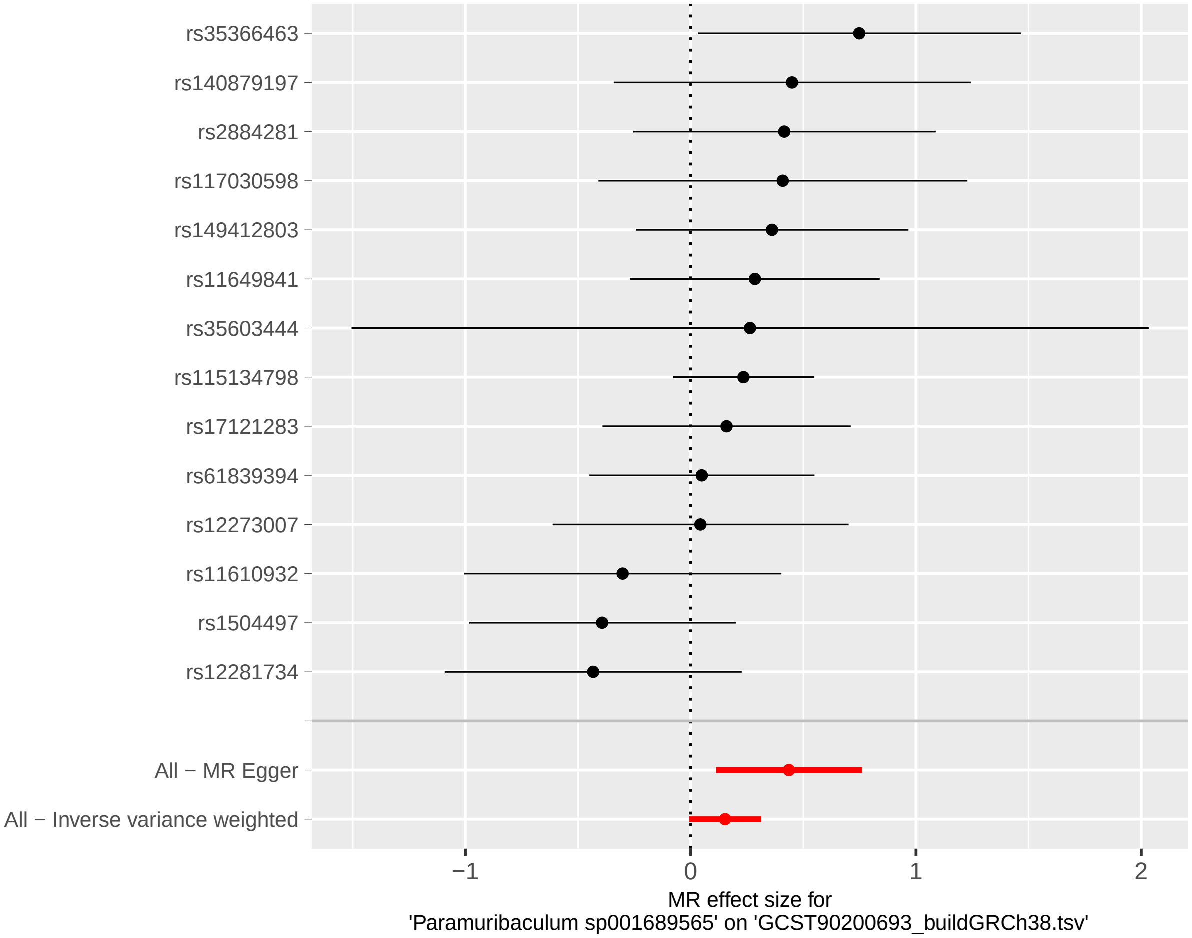
rs35366463
rs140879197
rs2884281
rs117030598
rs149412803
rs11649841
rs35603444
rs115134798
rs17121283
rs61839394
rs12273007
rs11610932
rs1504497
rs12281734
All − MR Egger
All − Inverse variance weighted
−1
0
1
2
MR effect size for
'Paramuribaculum sp001689565' on 'GCST90200693_buildGRCh38.tsv'

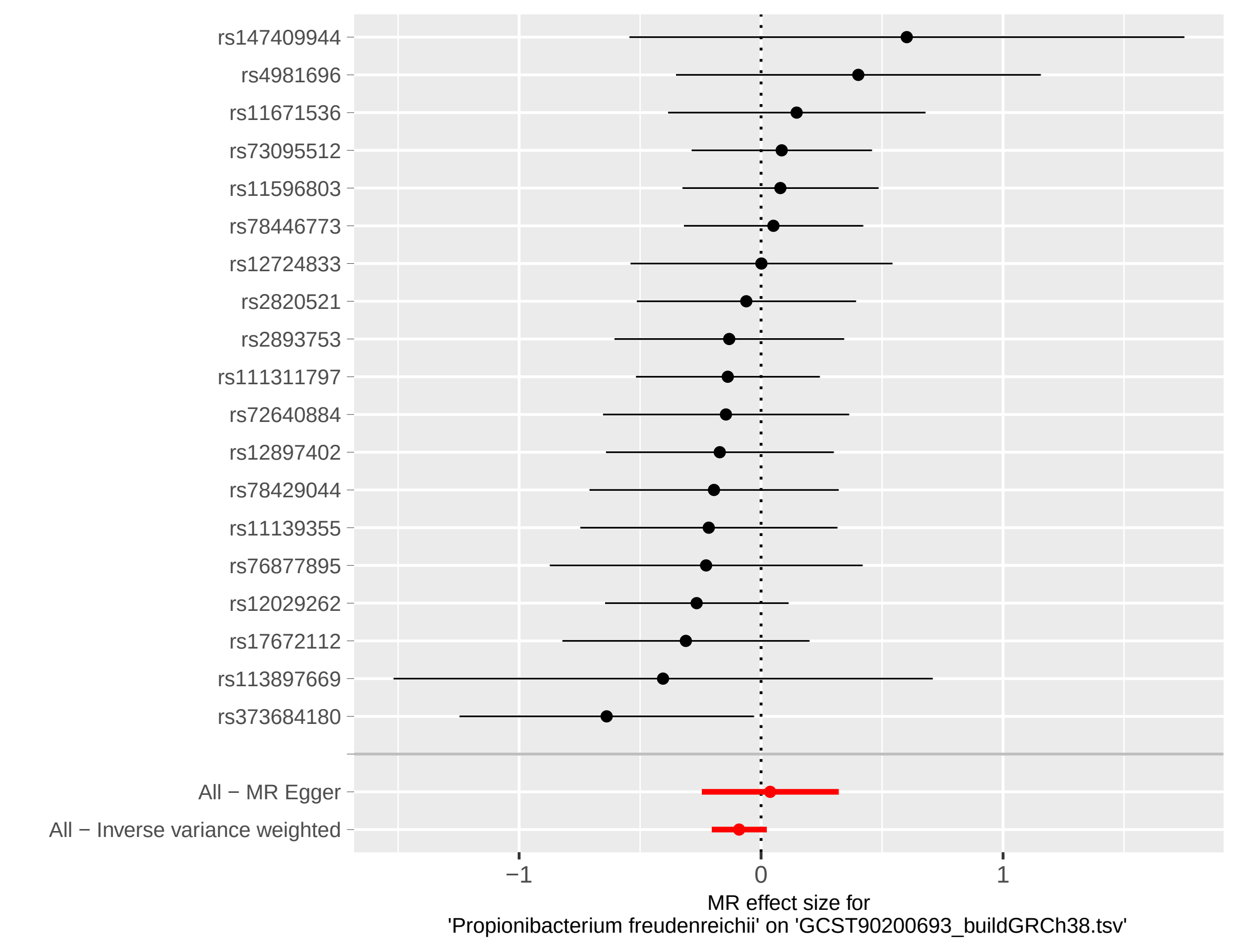
rs147409944
rs4981696
rs11671536
rs73095512
rs11596803
rs78446773
rs12724833
rs2820521
rs2893753
rs111311797
rs72640884
rs12897402
rs78429044
rs11139355
rs76877895
rs12029262
rs17672112
rs113897669
rs373684180
All − MR Egger
All − Inverse variance weighted
−1
0
1
MR effect size for
'Propionibacterium freudenreichii' on 'GCST90200693_buildGRCh38.tsv'

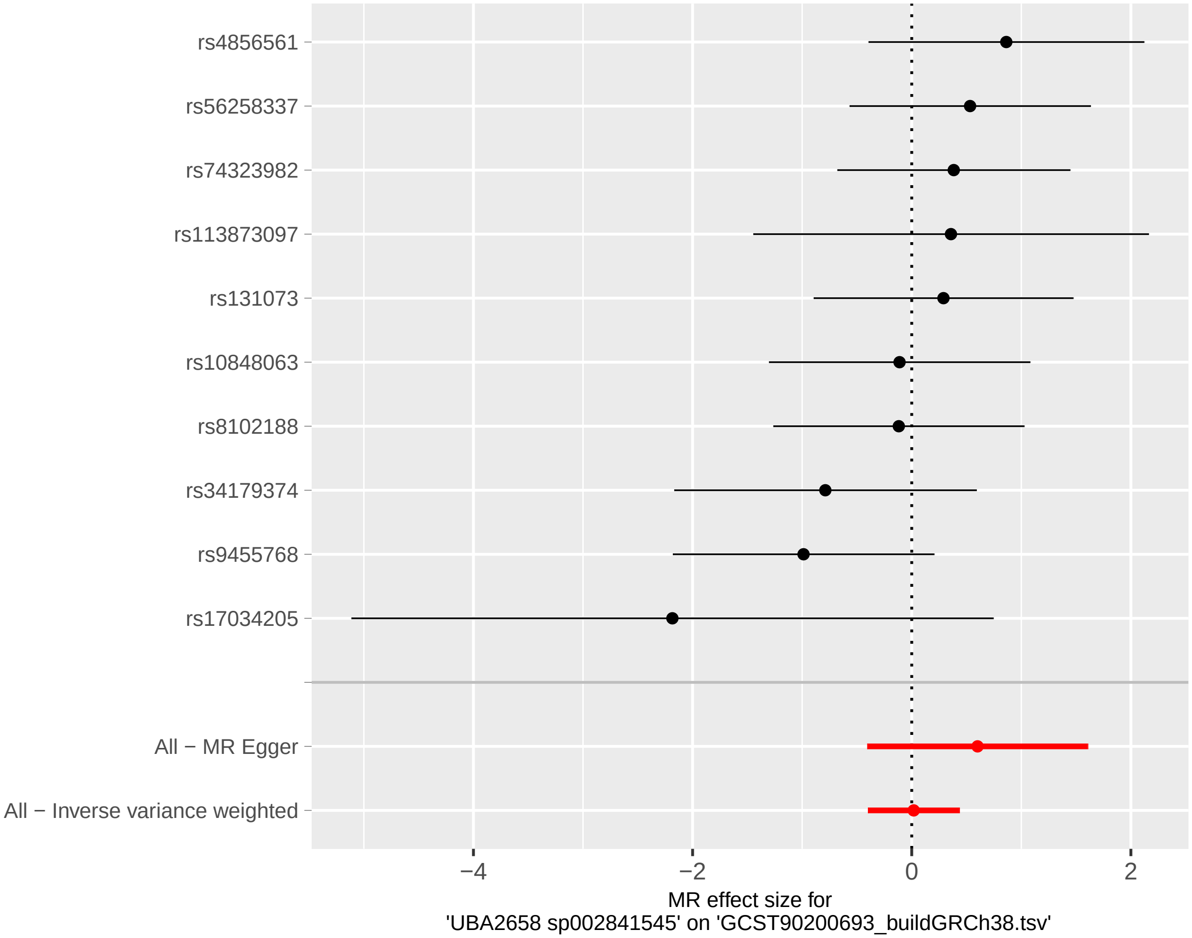
rs4856561
rs56258337
rs74323982
rs113873097
rs131073
rs10848063
rs8102188
rs34179374
rs9455768
rs17034205
All − MR Egger
All − Inverse variance weighted
−4
−2
0
2
MR effect size for
'UBA2658 sp002841545' on 'GCST90200693_buildGRCh38.tsv'

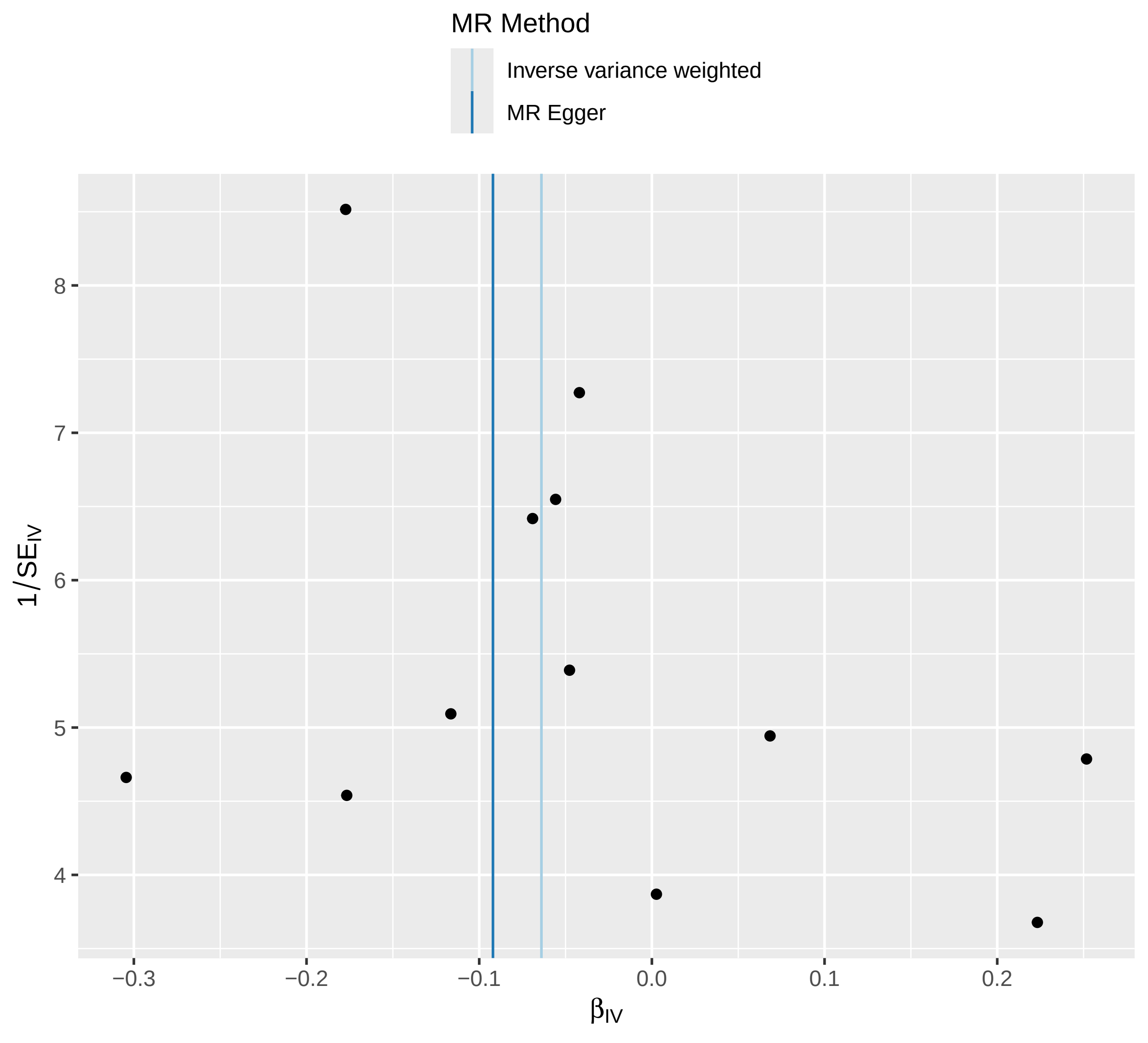
MR Method
Inverse variance weighted
MR Egger
8
7
6
5
4
1/SE_IV
−0.3
−0.2
−0.1
0.0
0.1
0.2
β_IV

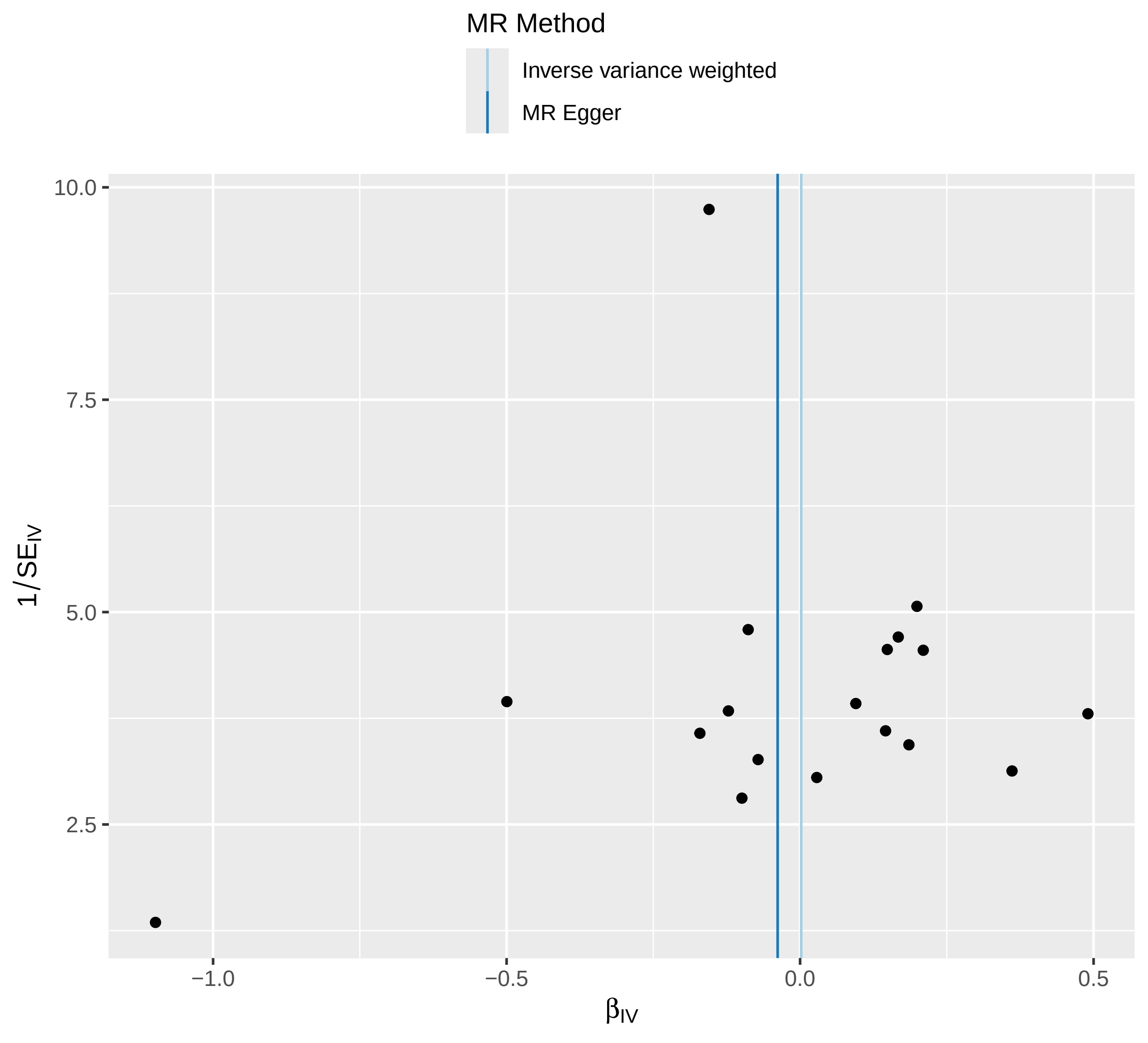
MR Method
Inverse variance weighted
MR Egger
10.0
7.5
5.0
2.5
$1/SE_{IV}$
−1.0
−0.5
0.0
0.5
β_{IV}

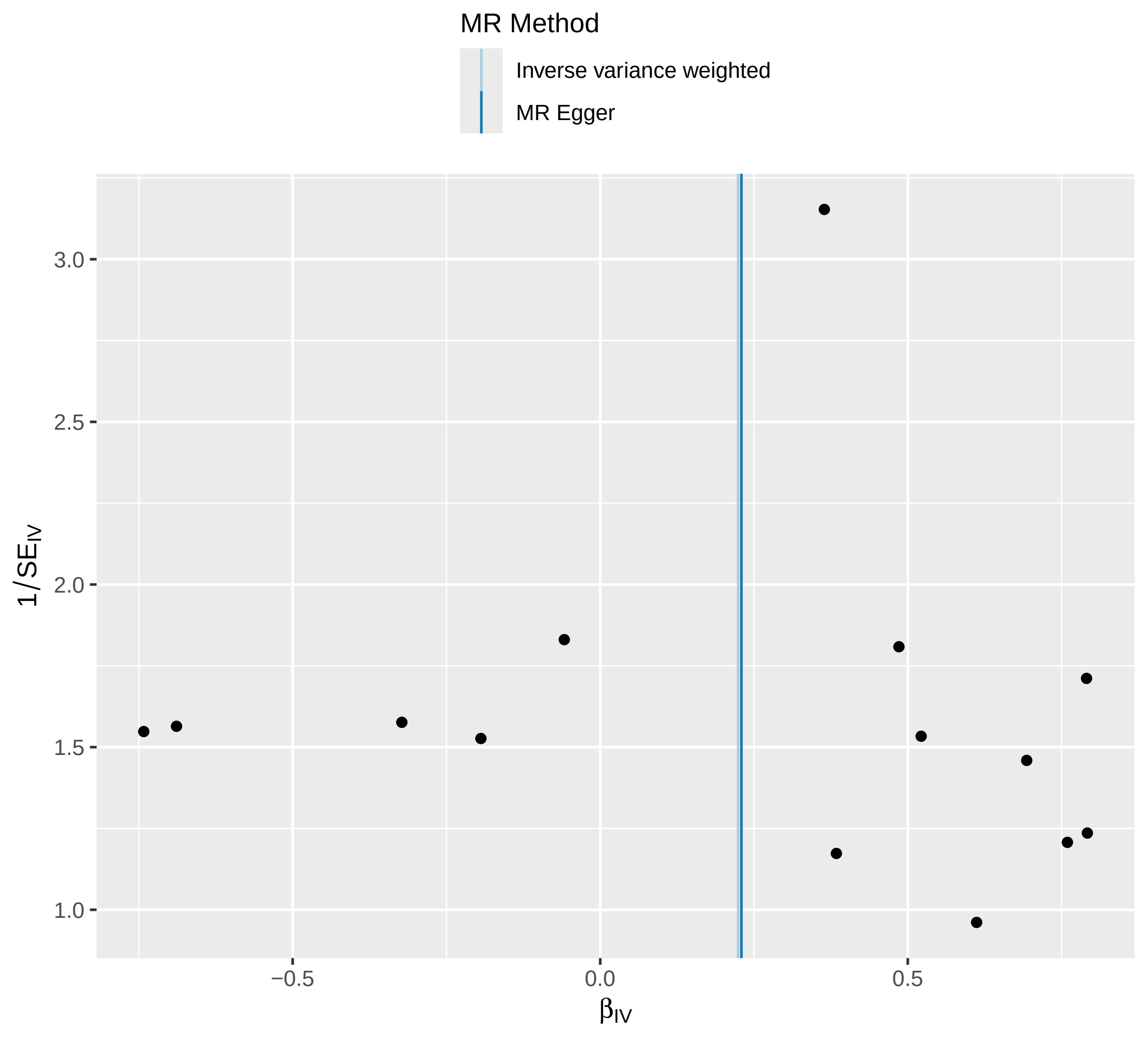

MR Method
Inverse variance weighted
MR Egger
3.0
2.5
2.0
1.5
1.0
1/SE$_{IV}$
−0.5
0.0
0.5
β$_{IV}$

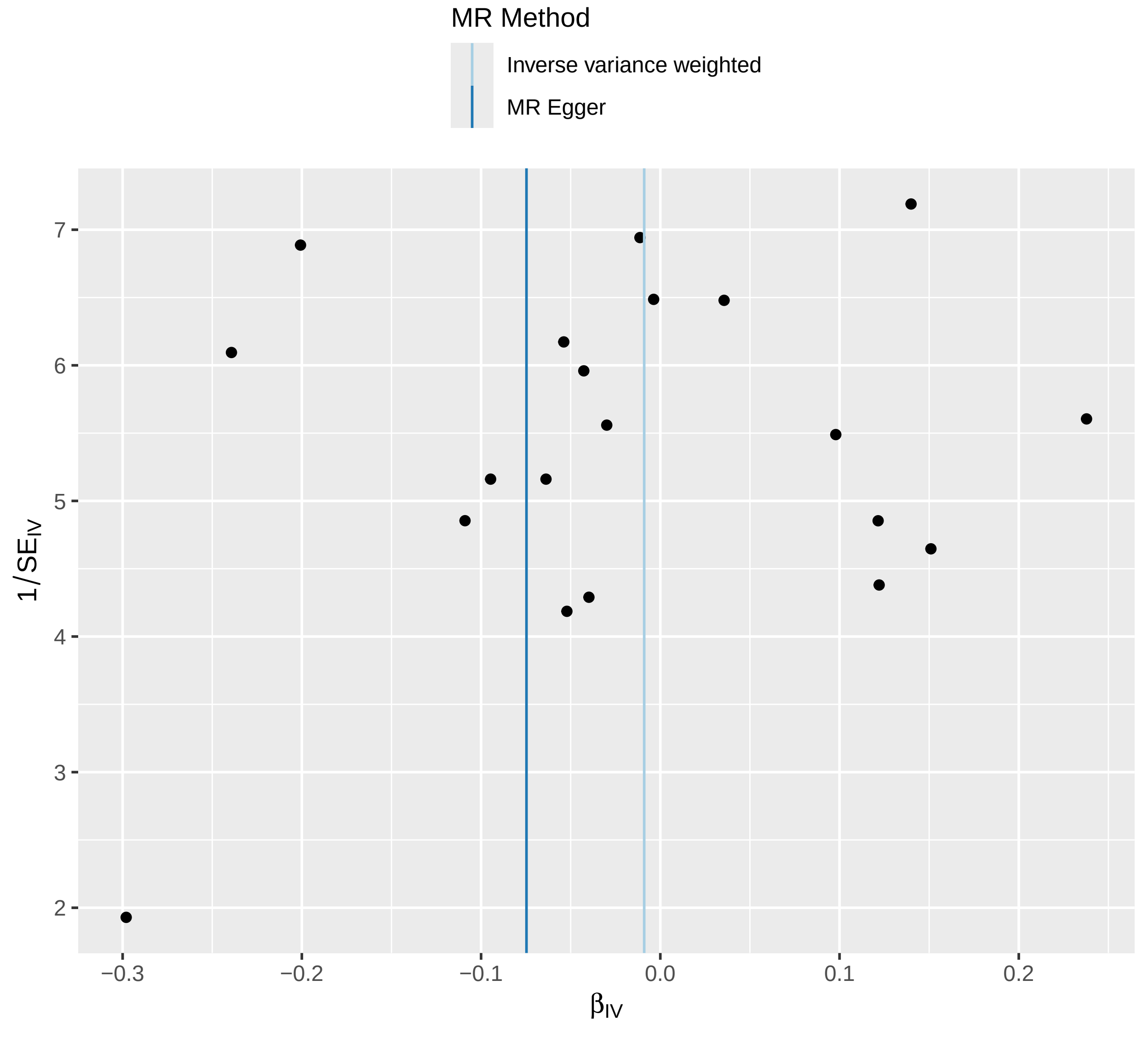

MR Method
Inverse variance weighted
MR Egger
7
6
5
4
3
2
1/SE_IV
−0.3
−0.2
−0.1
0.0
0.1
0.2
β_IV

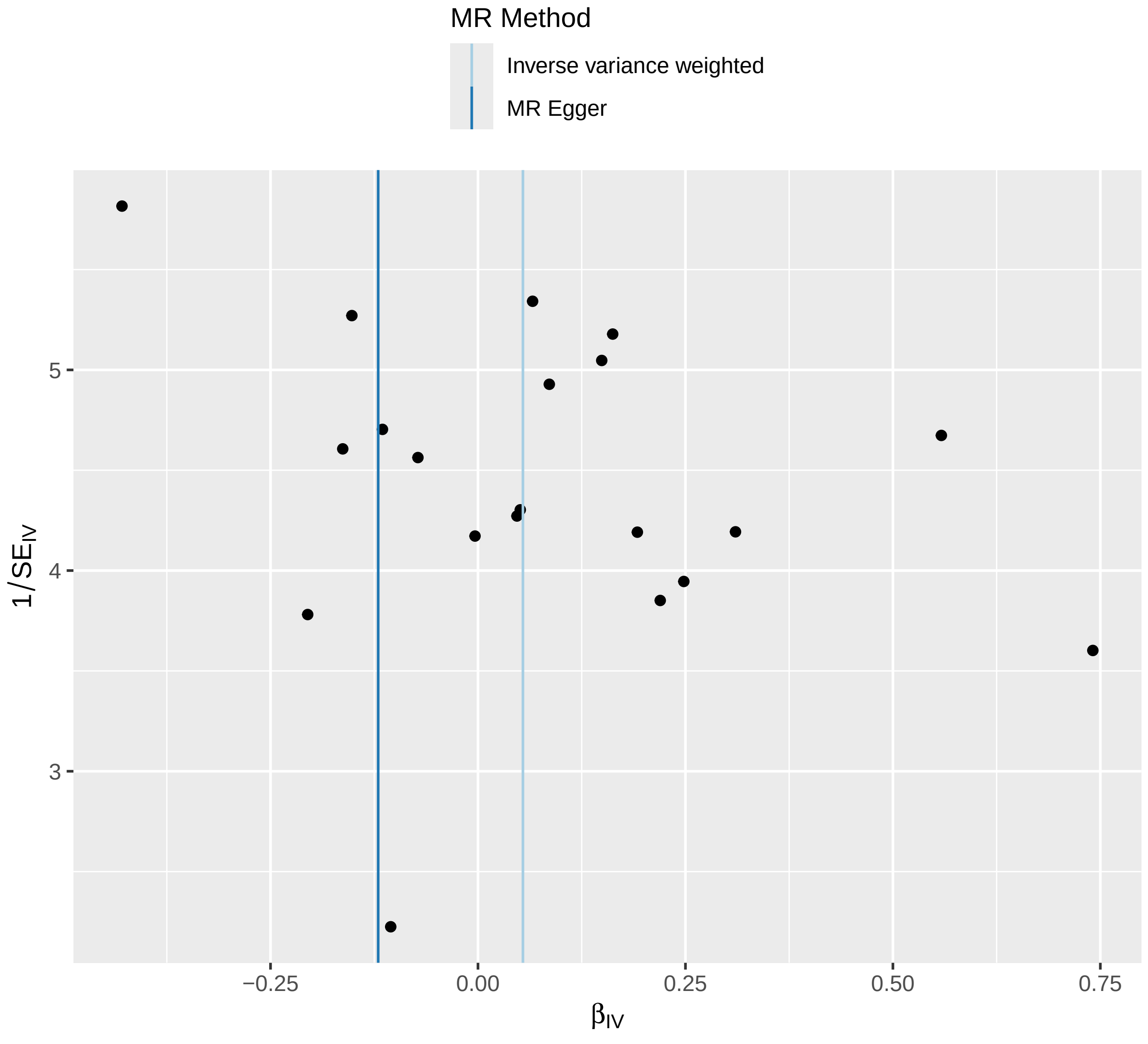

MR Method
Inverse variance weighted
MR Egger
5
4
3
1/SE$_{IV}$
−0.25
0.00
0.25
0.50
0.75
β$_{IV}$

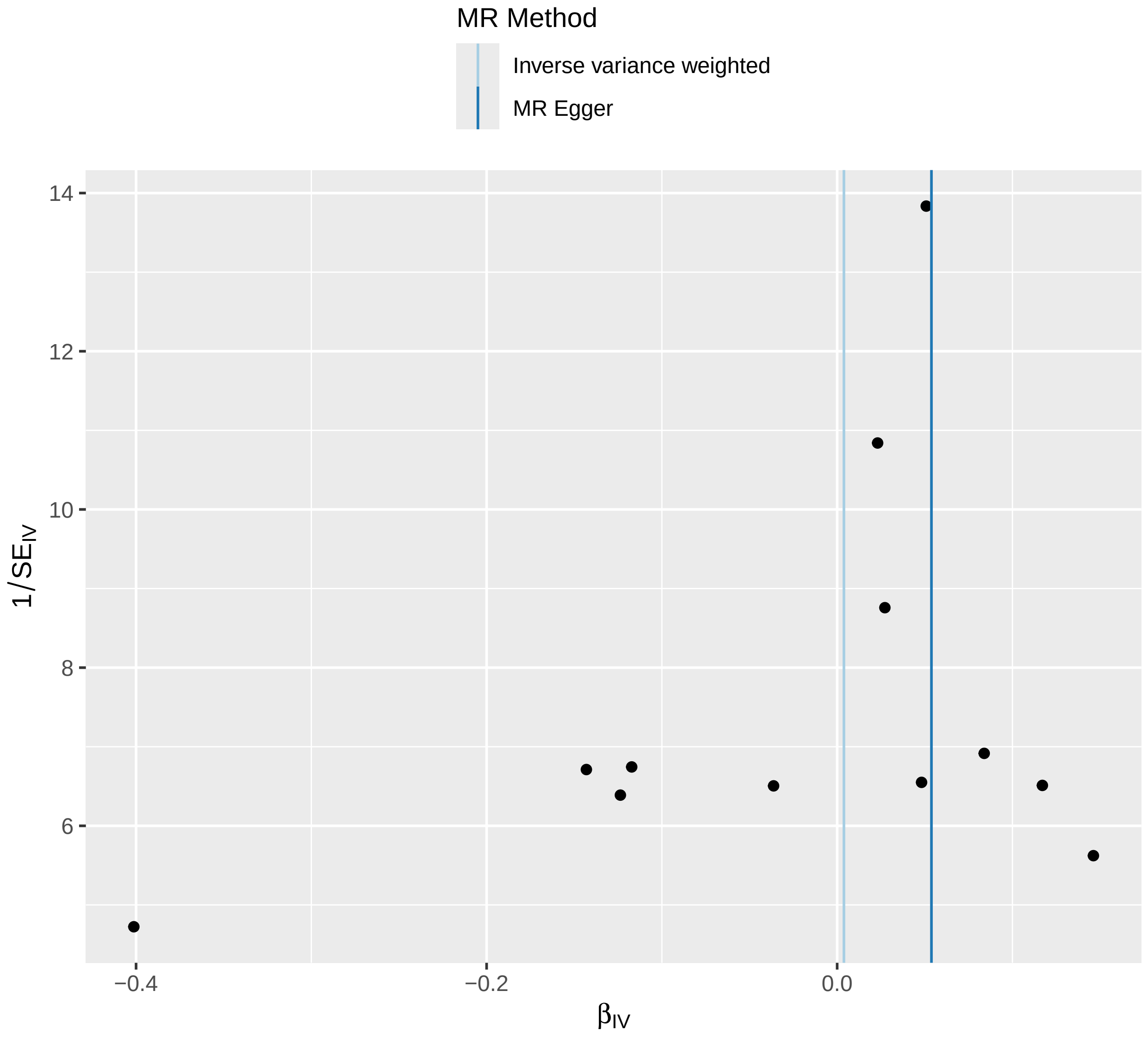

MR Method
Inverse variance weighted
MR Egger
14
12
10
8
6
1/SE$_{IV}$
−0.4
−0.2
0.0
β$_{IV}$

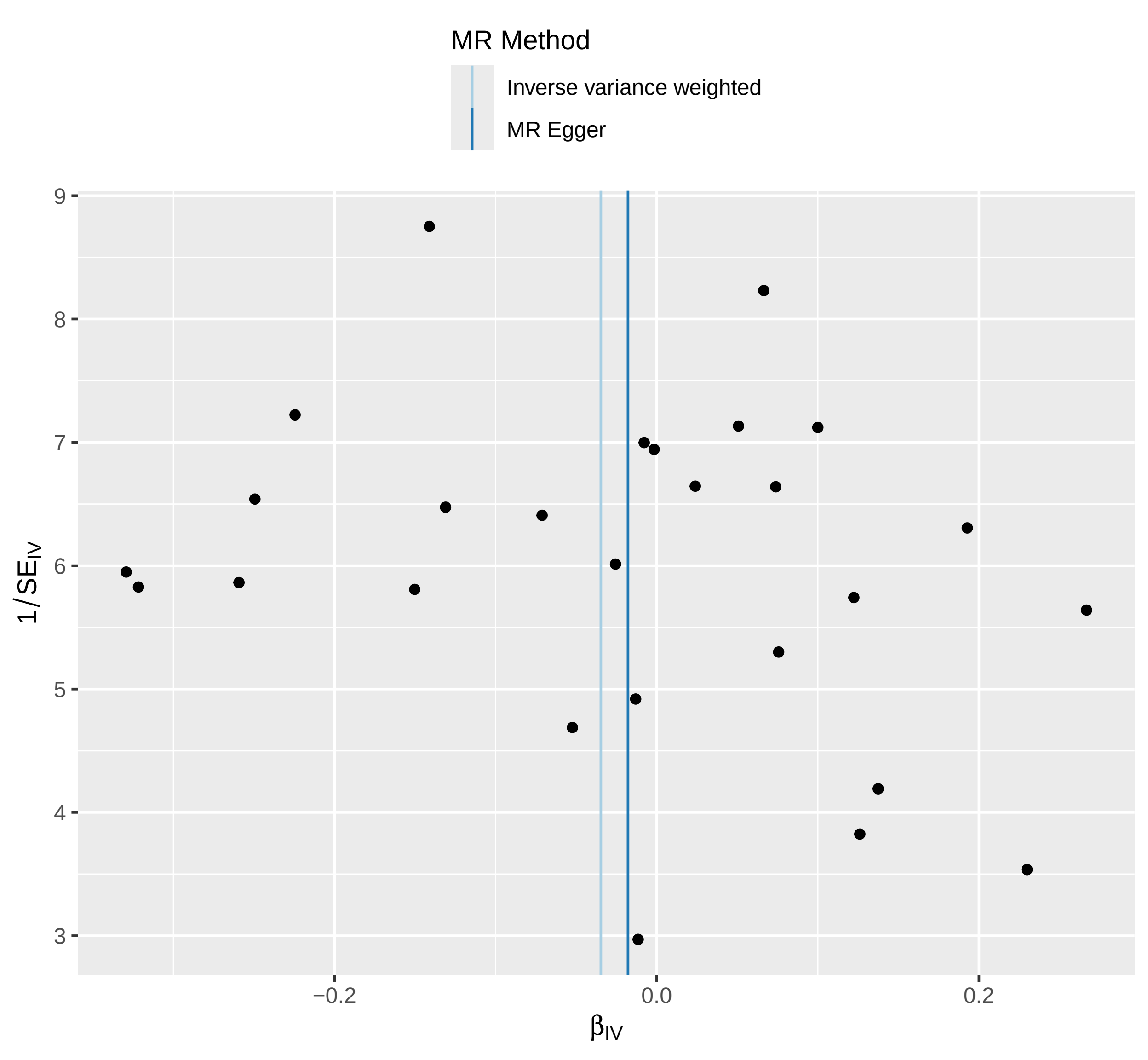
MR Method
Inverse variance weighted
MR Egger
9
8
7
6
5
4
3
1/SE_IV
−0.2
0.0
0.2
β_IV

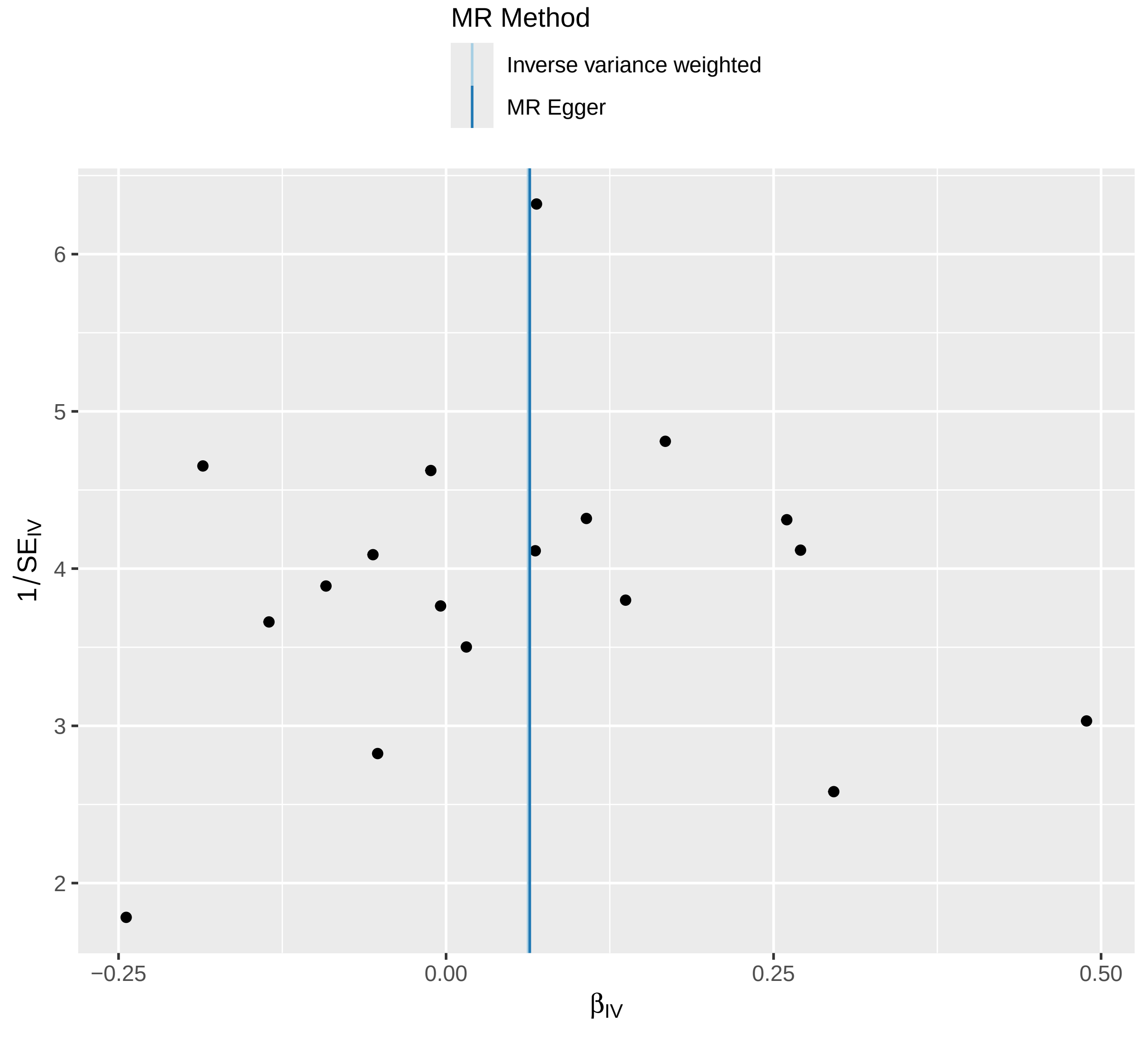

MR Method
Inverse variance weighted
MR Egger
6
5
4
3
2
1/SE_IV
−0.25
0.00
0.25
0.50
β_IV

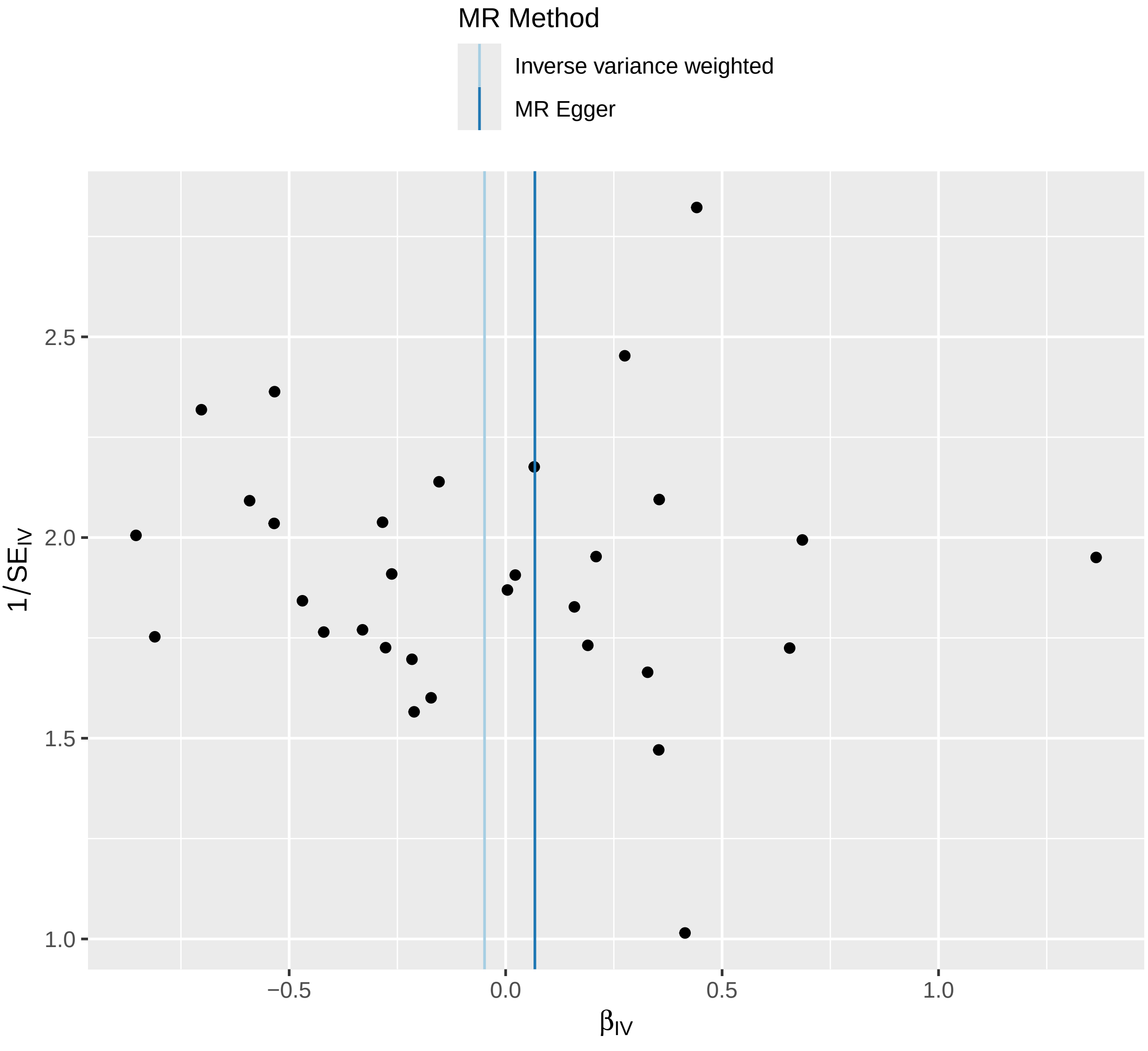
MR Method
Inverse variance weighted
MR Egger
2.5
2.0
1.5
1.0
−0.5
0.0
0.5
1.0
$1/SE_{IV}$
β_{IV}

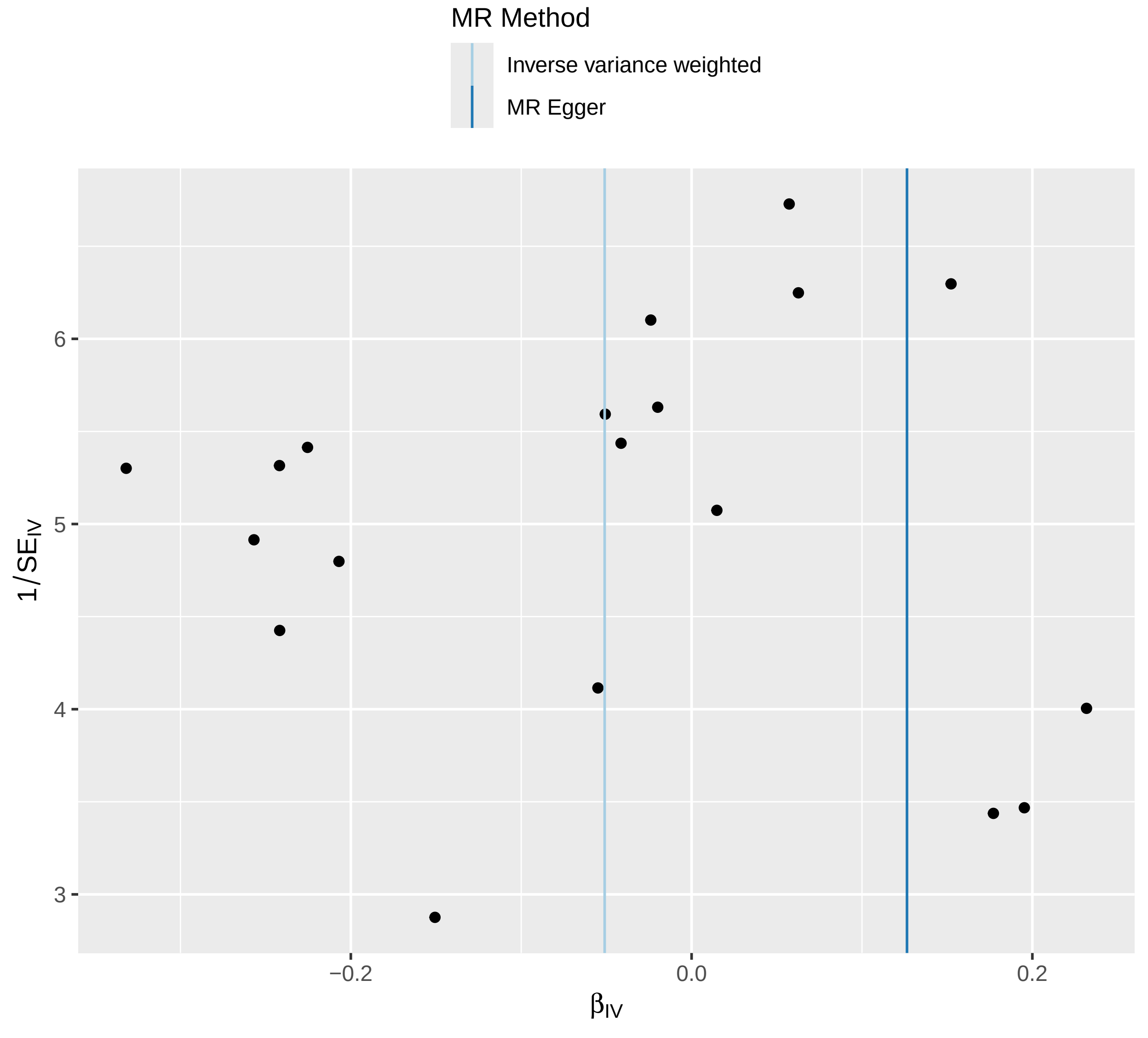
MR Method
Inverse variance weighted
MR Egger
6
5
4
3
−0.2
0.0
0.2
$1/SE_{IV}$
β_{IV}

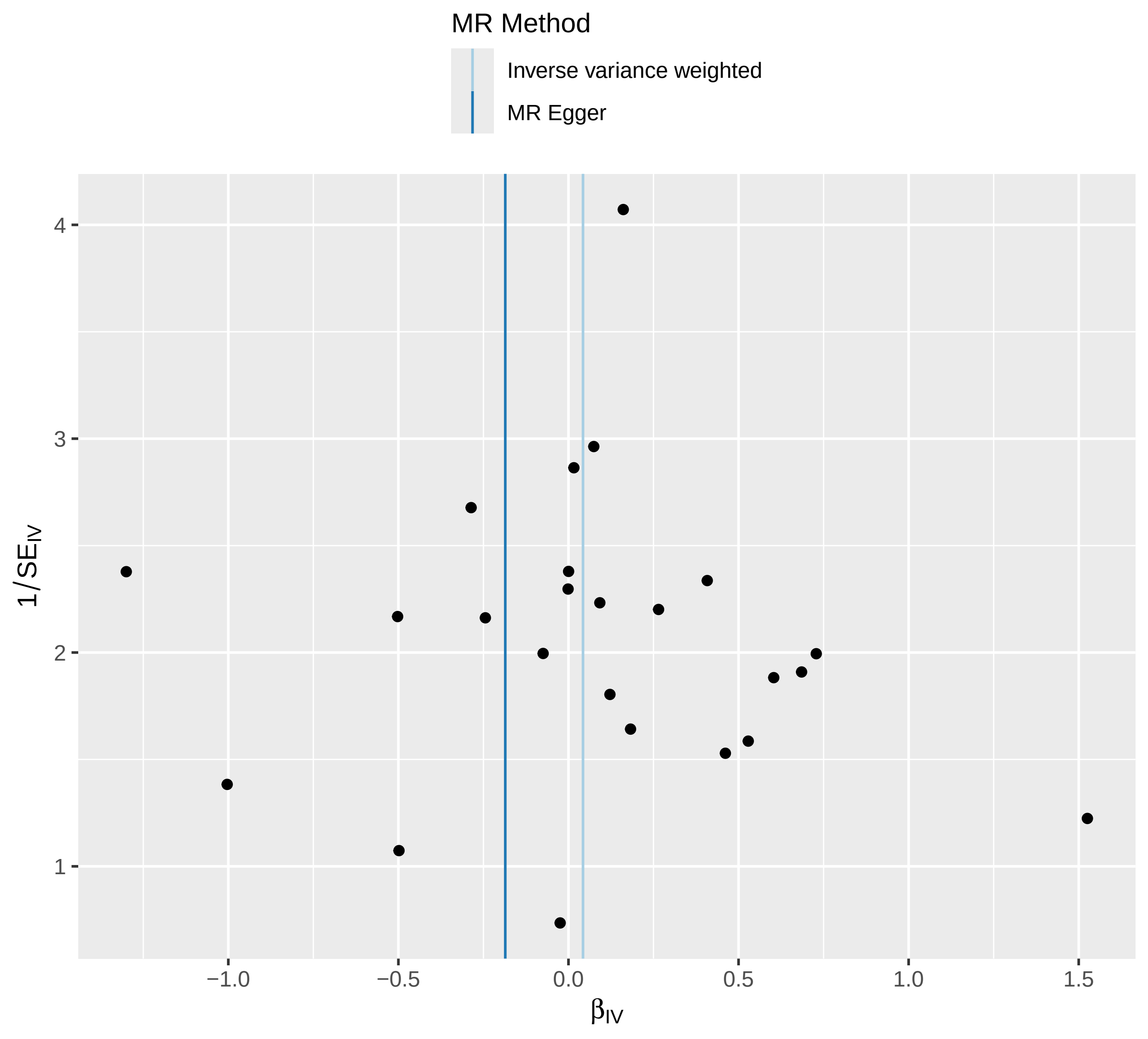
MR Method
Inverse variance weighted
MR Egger
1/SE_{IV}
4
3
2
1
−1.0
−0.5
0.0
0.5
1.0
1.5
β_{IV}

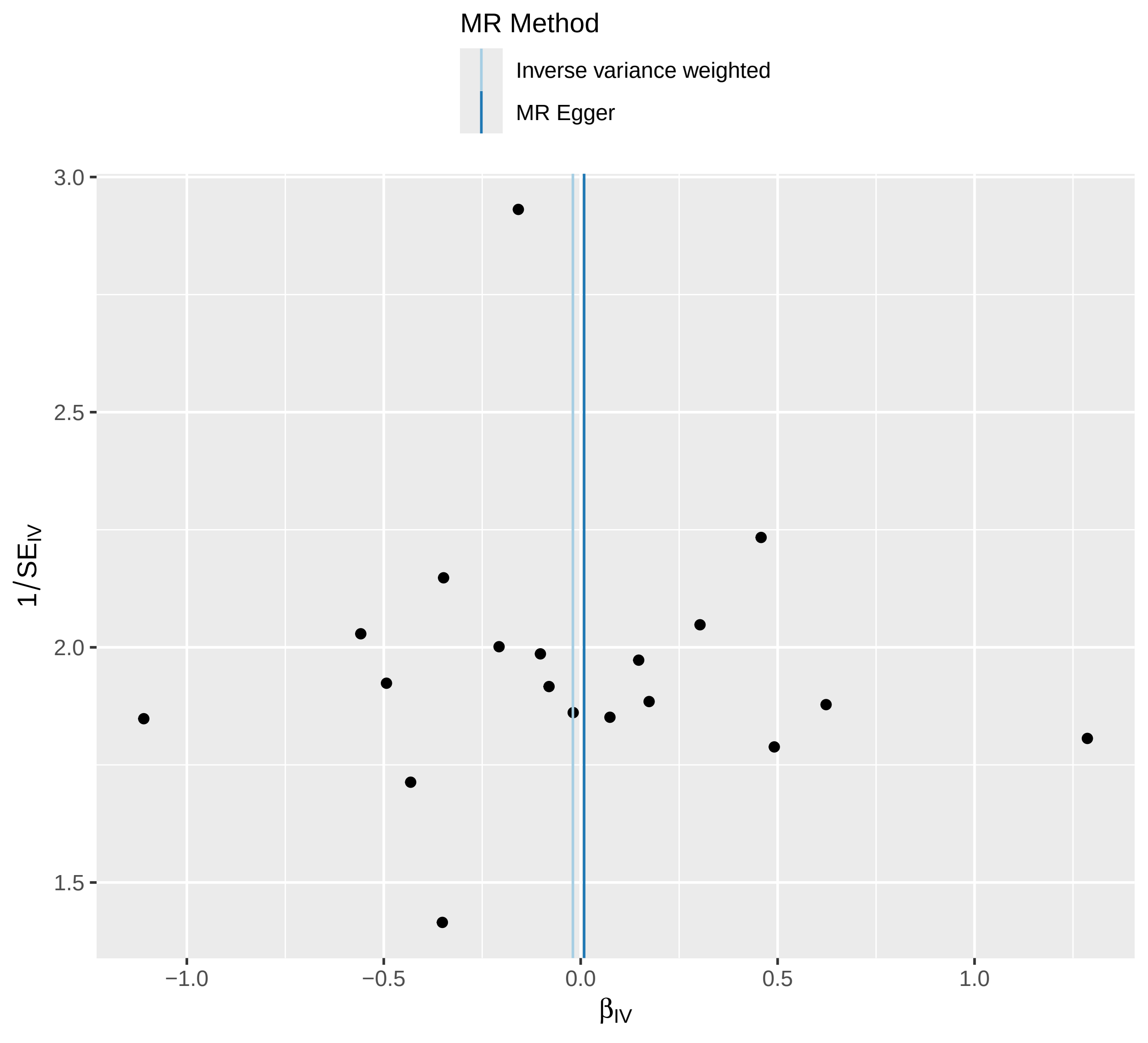

MR Method
Inverse variance weighted
MR Egger
3.0
2.5
2.0
1.5
1/SE_IV
−1.0
−0.5
0.0
0.5
1.0
β_IV

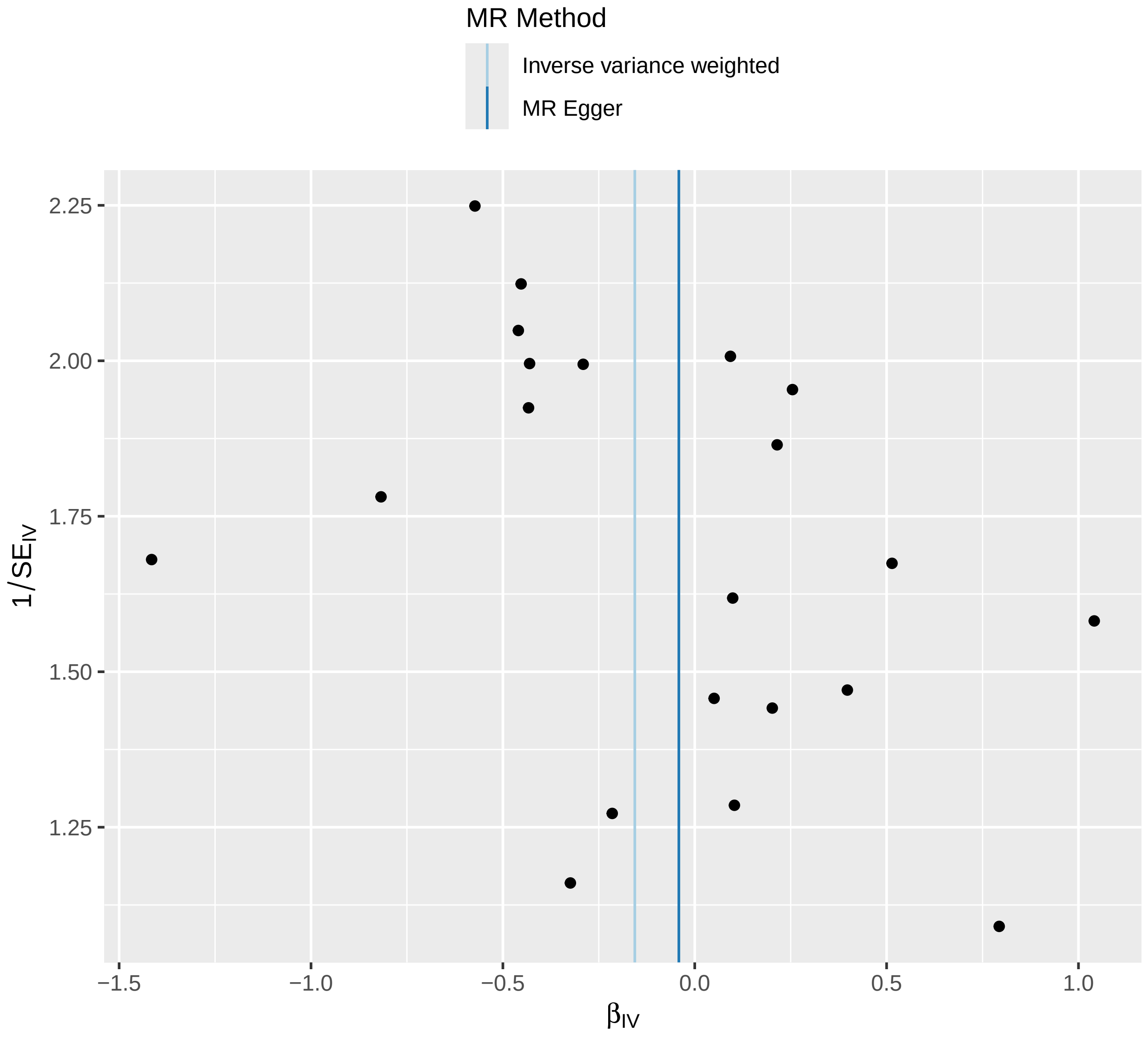

MR Method
Inverse variance weighted
MR Egger
2.25
2.00
1.75
1.50
1.25
1/SE$_{IV}$
−1.5
−1.0
−0.5
0.0
0.5
1.0
β$_{IV}$

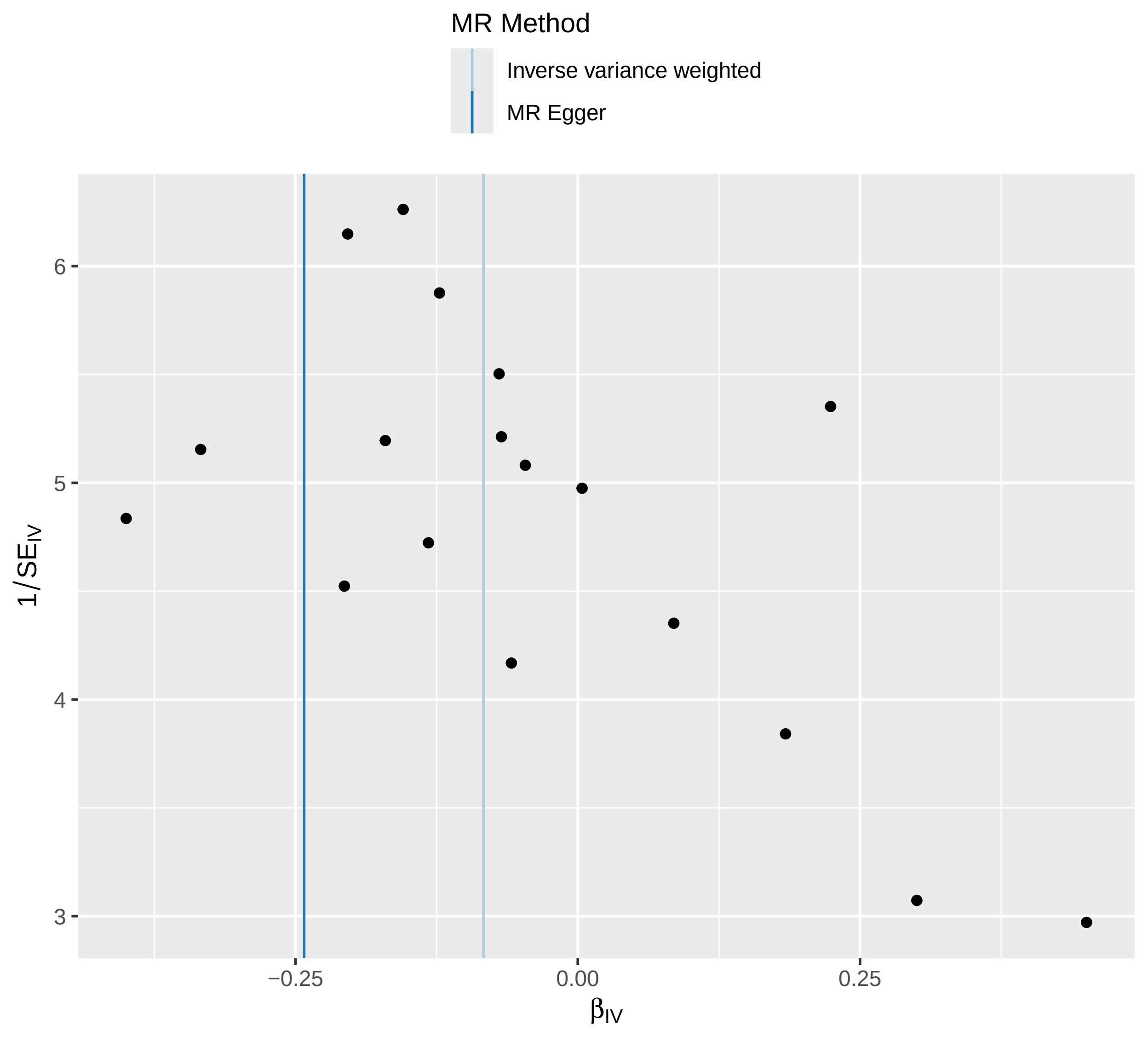
MR Method
Inverse variance weighted
MR Egger
6
5
4
3
1/SE$_{IV}$
−0.25
0.00
0.25
β$_{IV}$

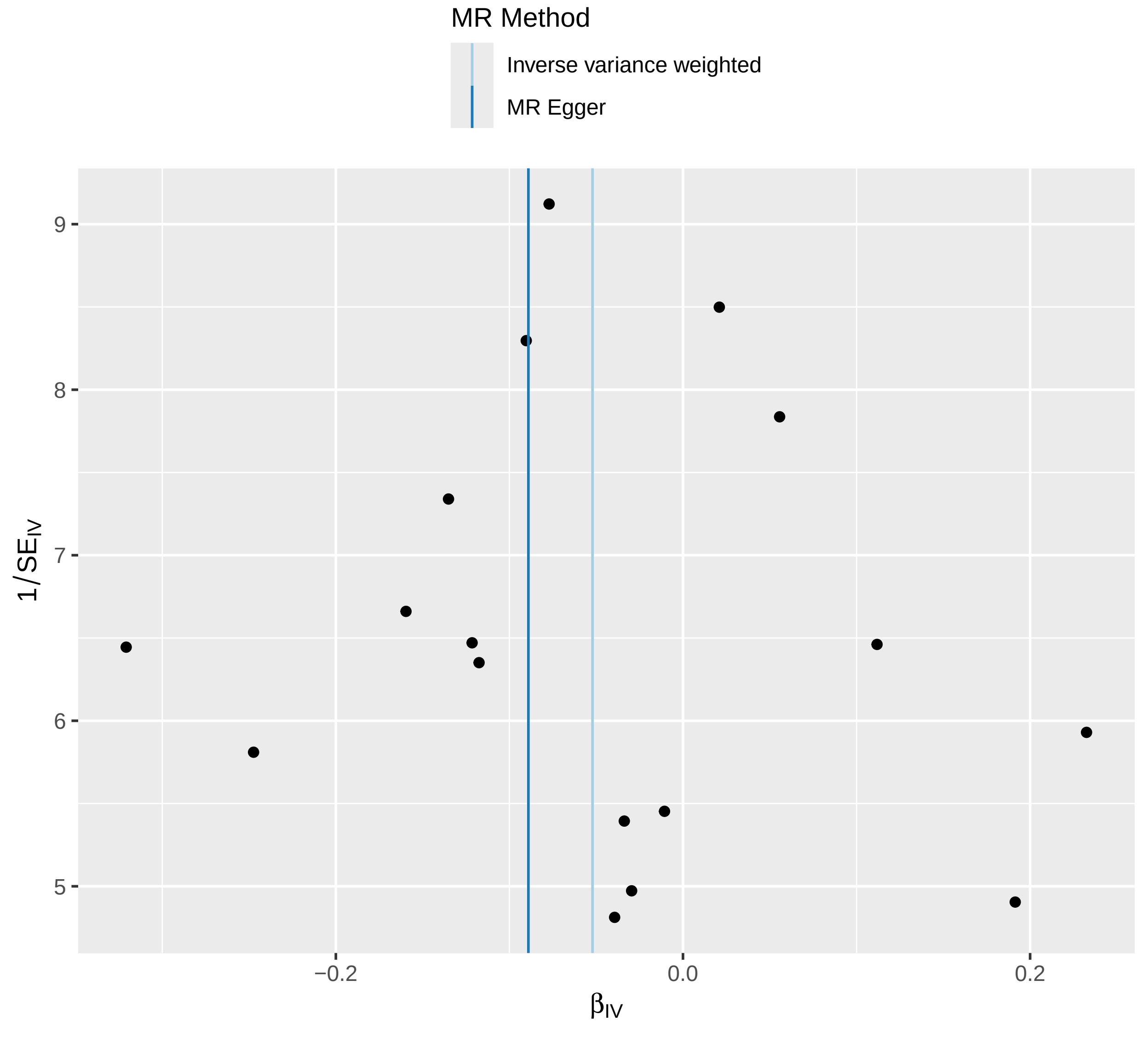
MR Method
Inverse variance weighted
MR Egger
9
8
7
6
5
1/SE$_{IV}$
−0.2
0.0
0.2
β$_{IV}$

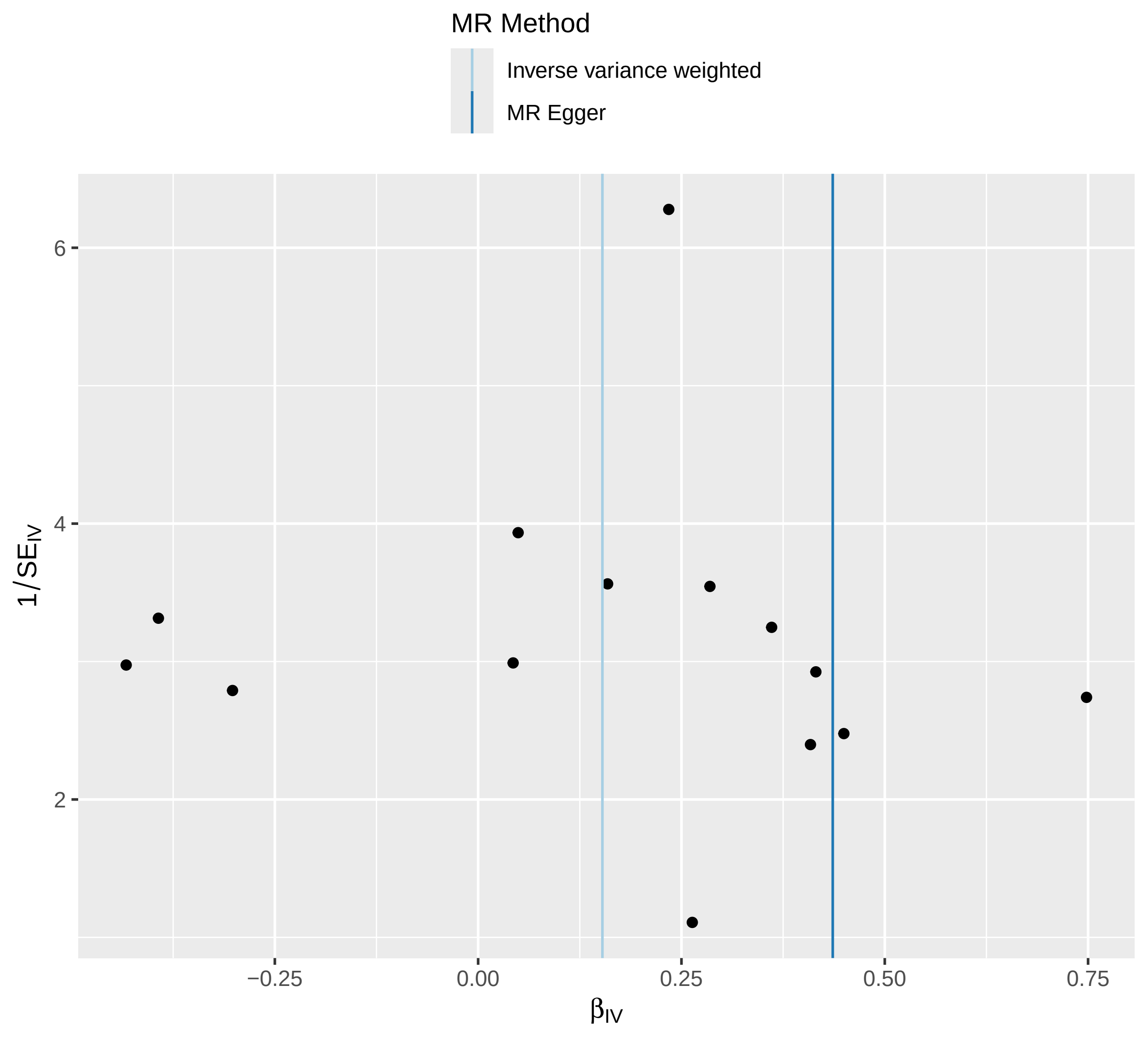
MR Method
Inverse variance weighted
MR Egger
6
4
2
1/SE_IV
−0.25
0.00
0.25
0.50
0.75
β_IV

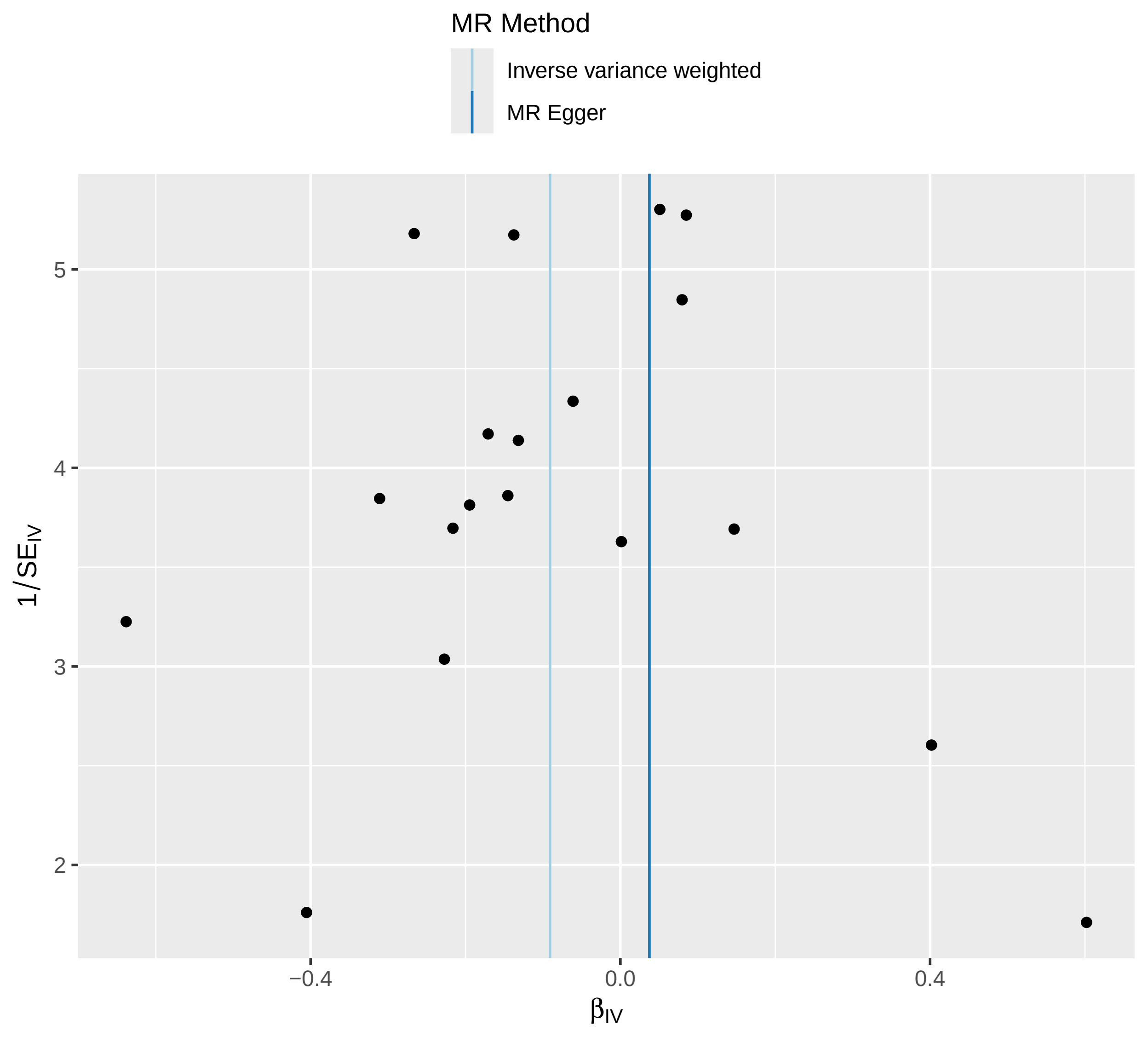
MR Method
Inverse variance weighted
MR Egger
5
4
3
2
1/SE$_{IV}$
−0.4
0.0
0.4
β$_{IV}$

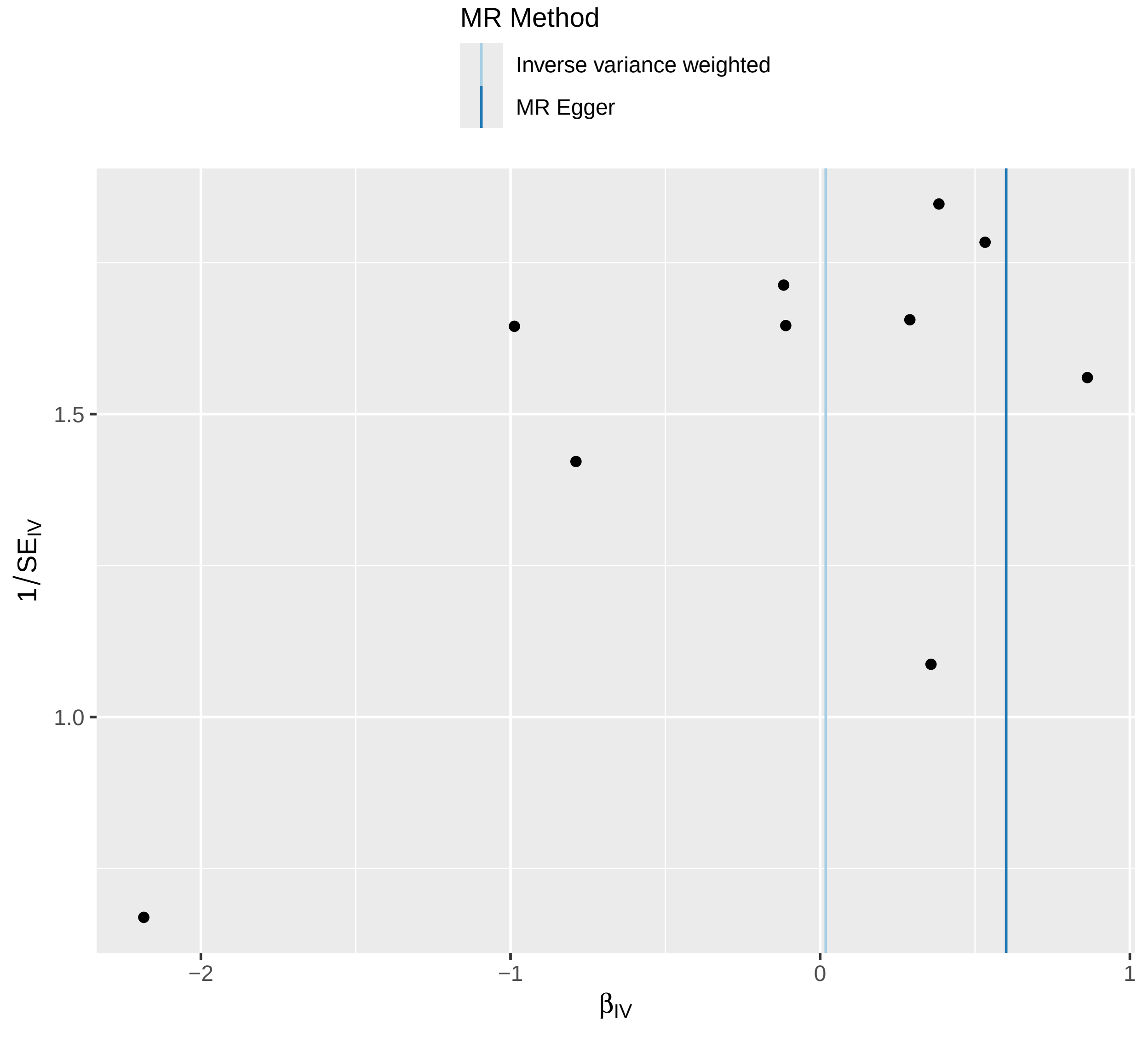
MR Method
Inverse variance weighted
MR Egger
1.5
1.0
1/SE_{IV}
−2
−1
0
1
β_{IV}

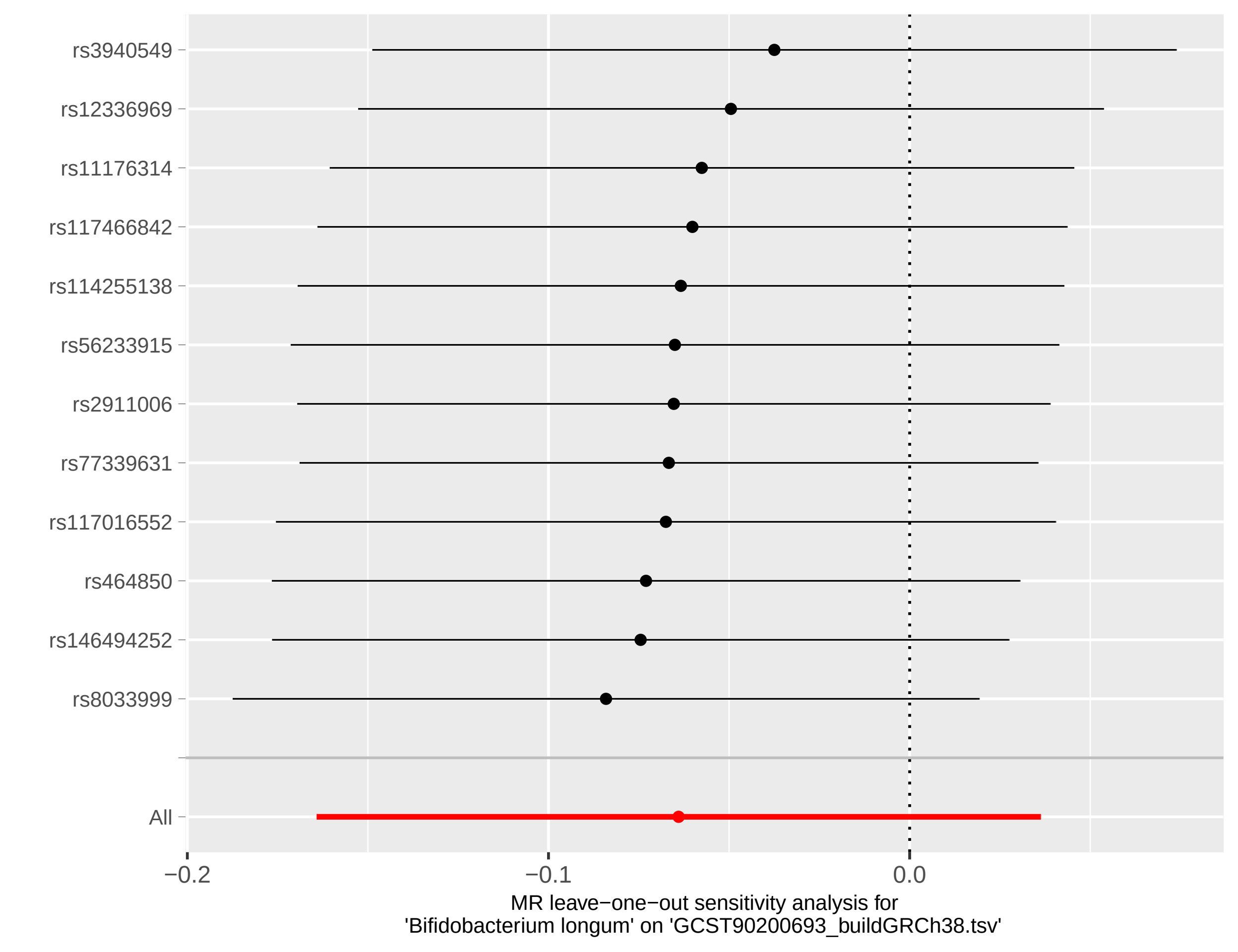
rs3940549
rs12336969
rs11176314
rs117466842
rs114255138
rs56233915
rs2911006
rs77339631
rs117016552
rs464850
rs146494252
rs8033999
All
−0.2
−0.1
0.0
MR leave−one−out sensitivity analysis for
'Bifidobacterium longum' on 'GCST90200693_buildGRCh38.tsv'

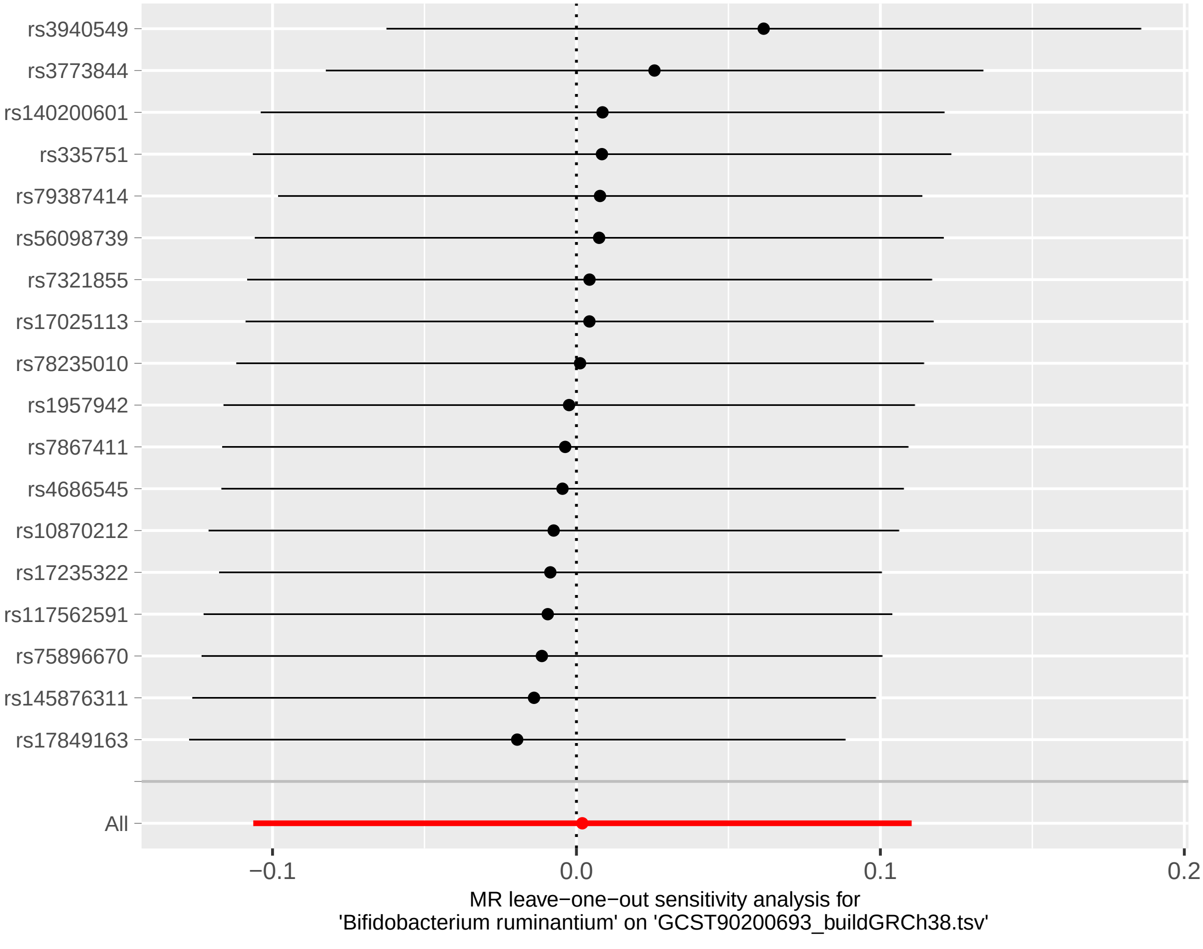
rs3940549
rs3773844
rs140200601
rs335751
rs79387414
rs56098739
rs7321855
rs17025113
rs78235010
rs1957942
rs7867411
rs4686545
rs10870212
rs17235322
rs117562591
rs75896670
rs145876311
rs17849163
All
−0.1
0.0
0.1
0.2
MR leave−one−out sensitivity analysis for
'Bifidobacterium ruminantium' on 'GCST90200693_buildGRCh38.tsv'

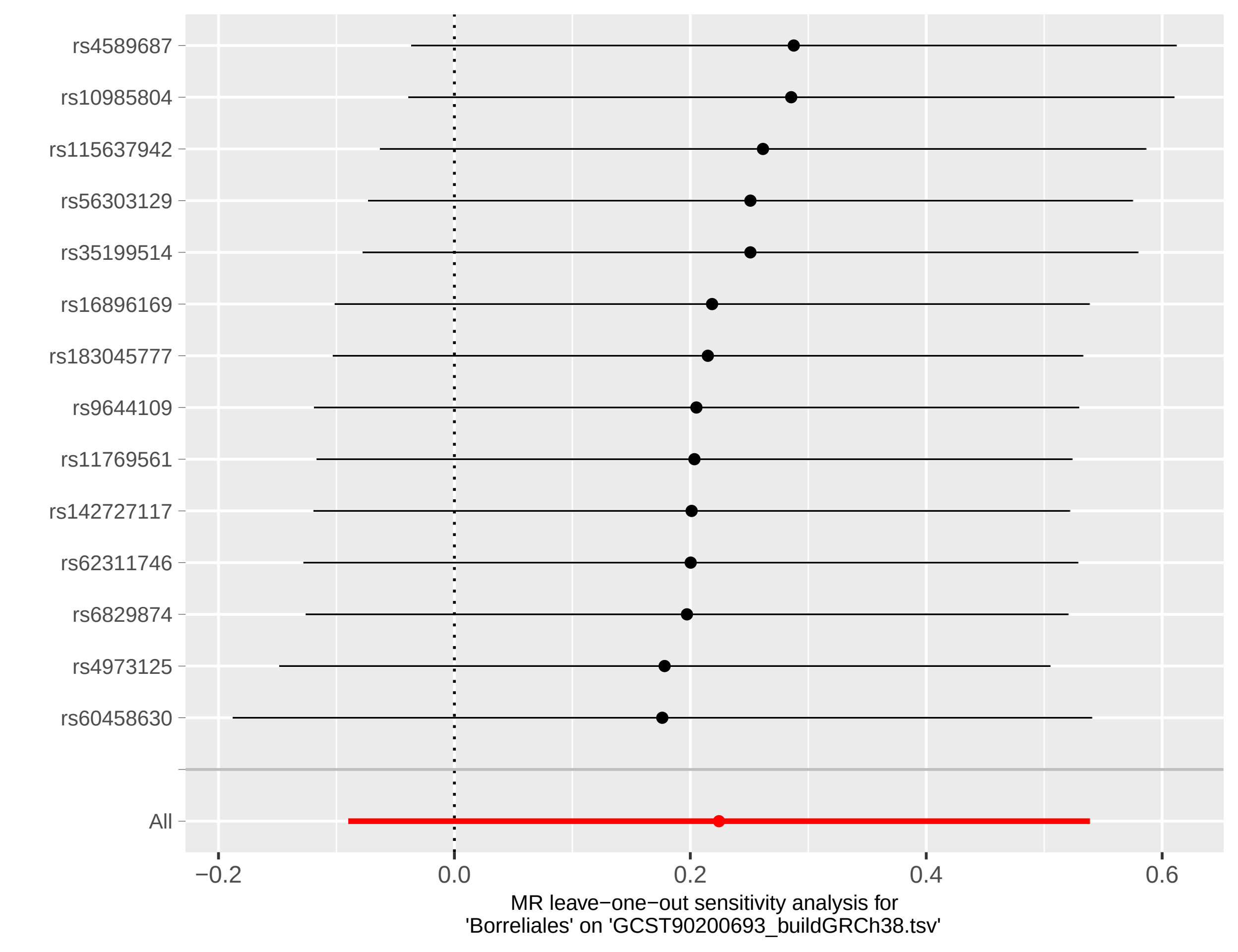

rs4589687
rs10985804
rs115637942
rs56303129
rs35199514
rs16896169
rs183045777
rs9644109
rs11769561
rs142727117
rs62311746
rs6829874
rs4973125
rs60458630
All
−0.2
0.0
0.2
0.4
0.6
MR leave−one−out sensitivity analysis for
'Borreliales' on 'GCST90200693_buildGRCh38.tsv'

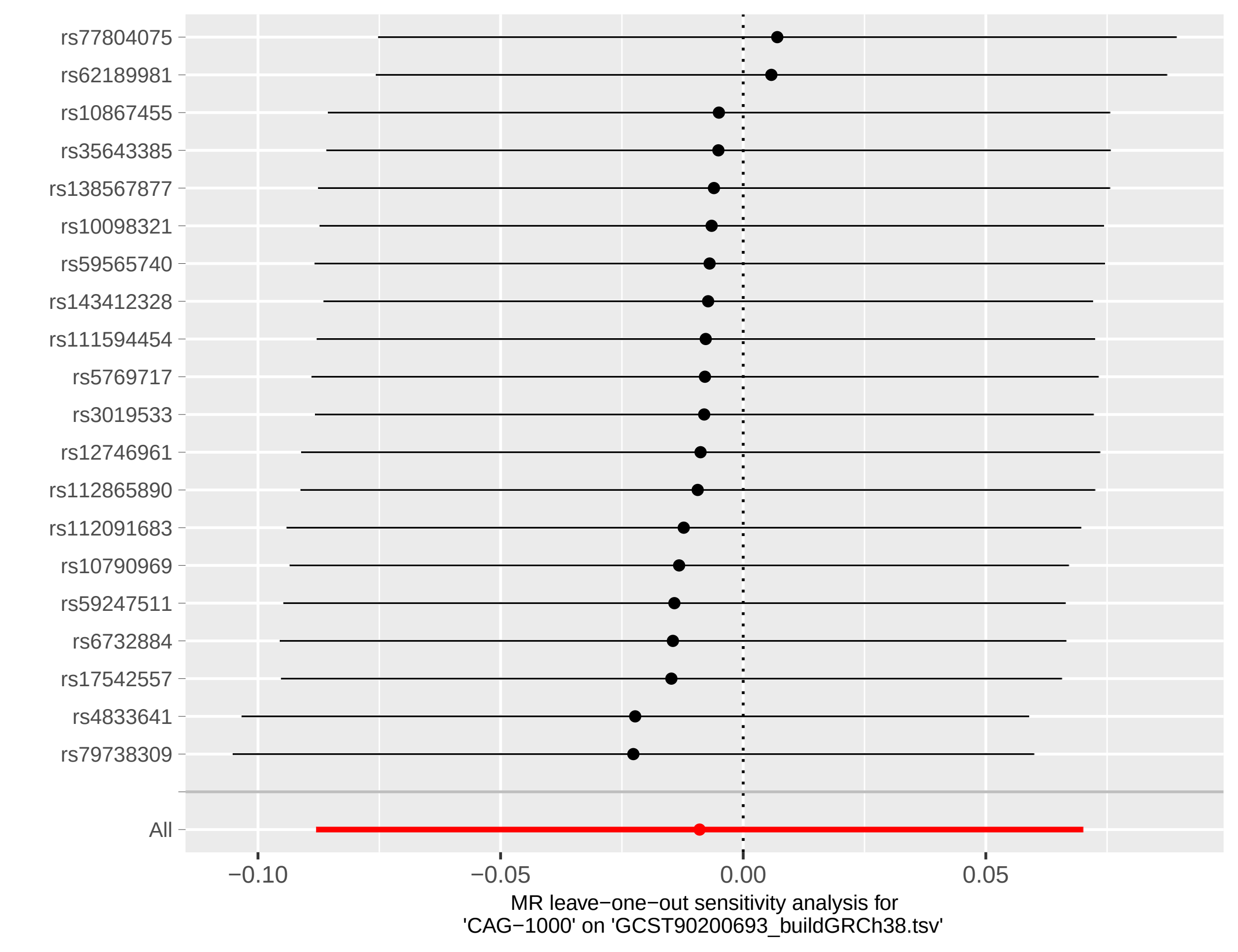
rs77804075
rs62189981
rs10867455
rs35643385
rs138567877
rs10098321
rs59565740
rs143412328
rs111594454
rs5769717
rs3019533
rs12746961
rs112865890
rs112091683
rs10790969
rs59247511
rs6732884
rs17542557
rs4833641
rs79738309
All
−0.10
−0.05
0.00
0.05
MR leave−one−out sensitivity analysis for
'CAG−1000' on 'GCST90200693_buildGRCh38.tsv'

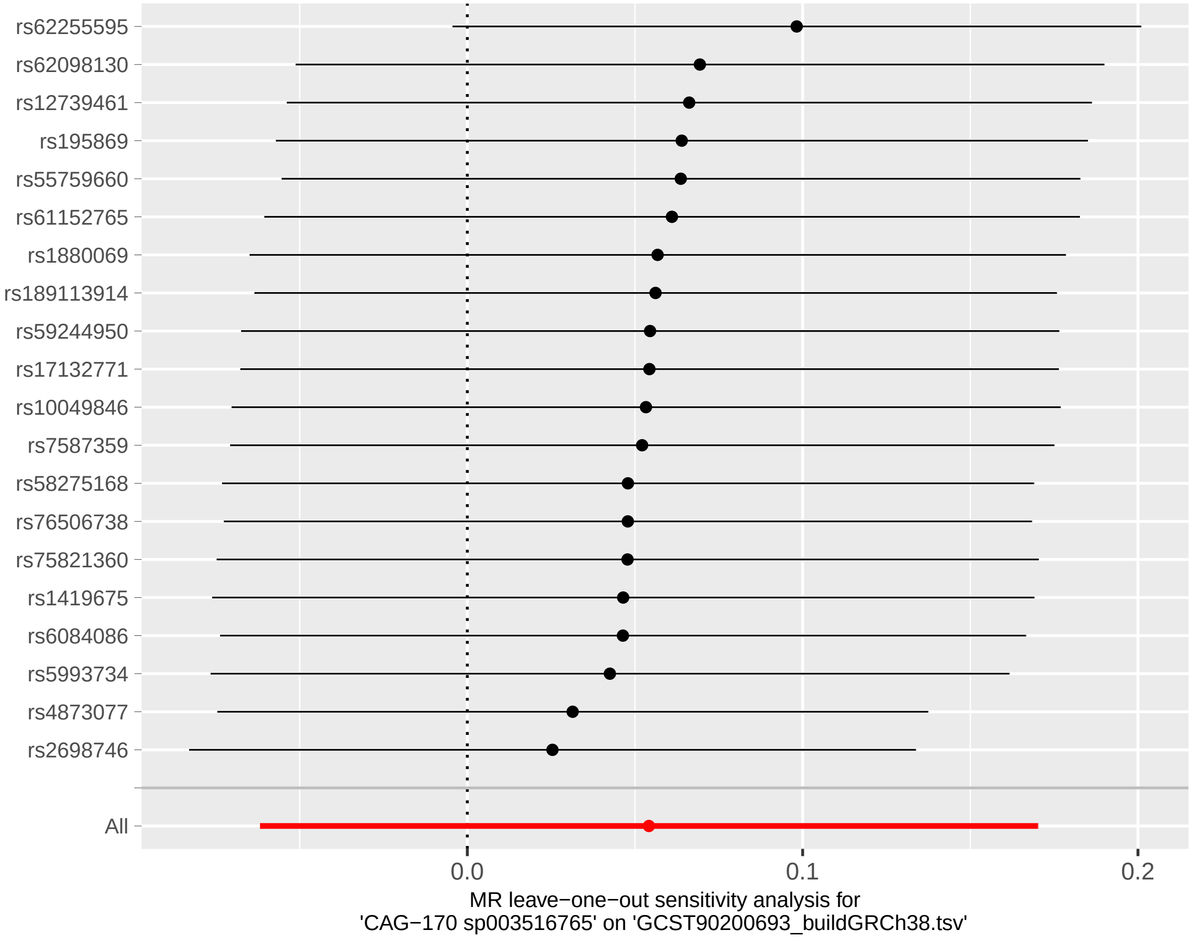
rs62255595
rs62098130
rs12739461
rs195869
rs55759660
rs61152765
rs1880069
rs189113914
rs59244950
rs17132771
rs10049846
rs7587359
rs58275168
rs76506738
rs75821360
rs1419675
rs6084086
rs5993734
rs4873077
rs2698746
All
0.0
0.1
0.2
MR leave−one−out sensitivity analysis for
'CAG−170 sp003516765' on 'GCST90200693_buildGRCh38.tsv'

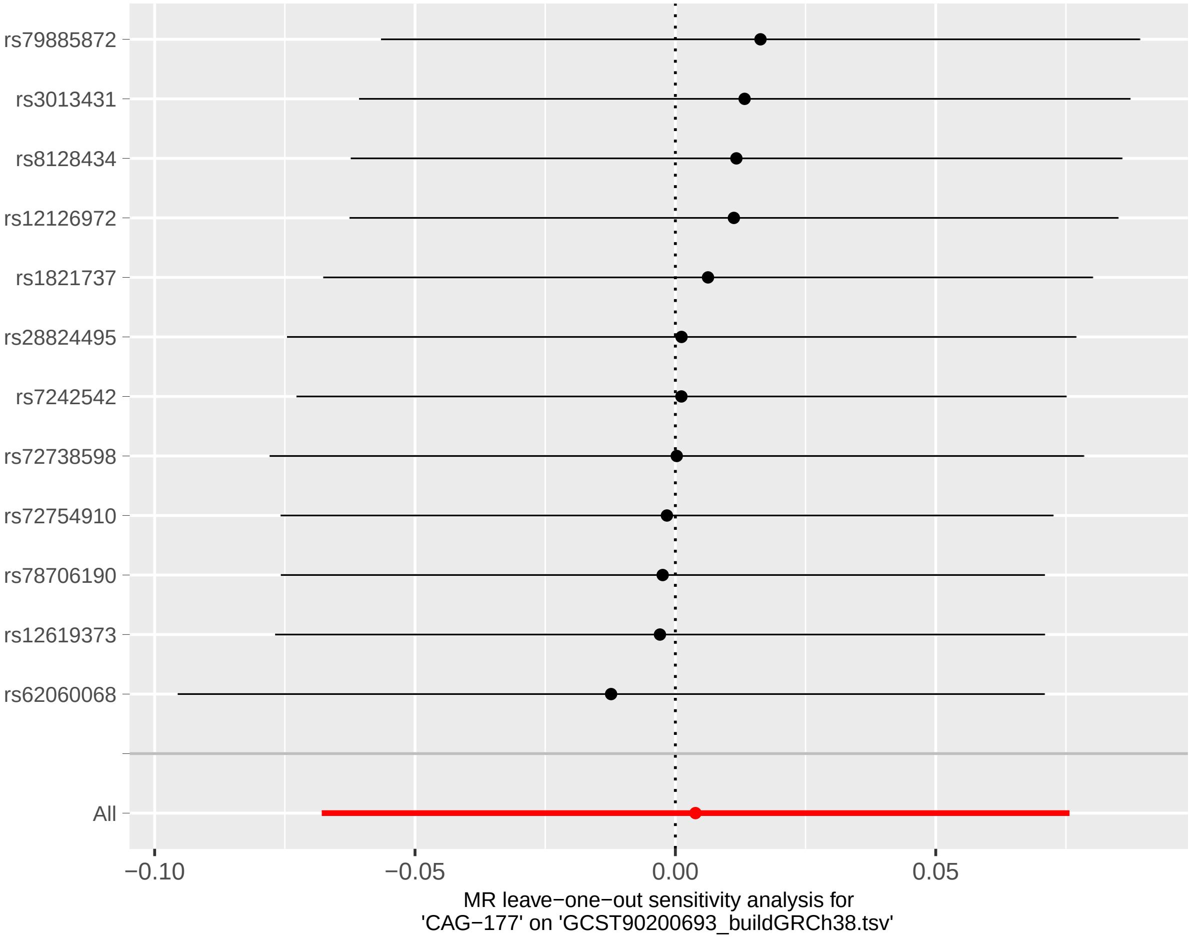
rs79885872
rs3013431
rs8128434
rs12126972
rs1821737
rs28824495
rs7242542
rs72738598
rs72754910
rs78706190
rs12619373
rs62060068
All
−0.10
−0.05
0.00
0.05
MR leave−one−out sensitivity analysis for
'CAG−177' on 'GCST90200693_buildGRCh38.tsv'

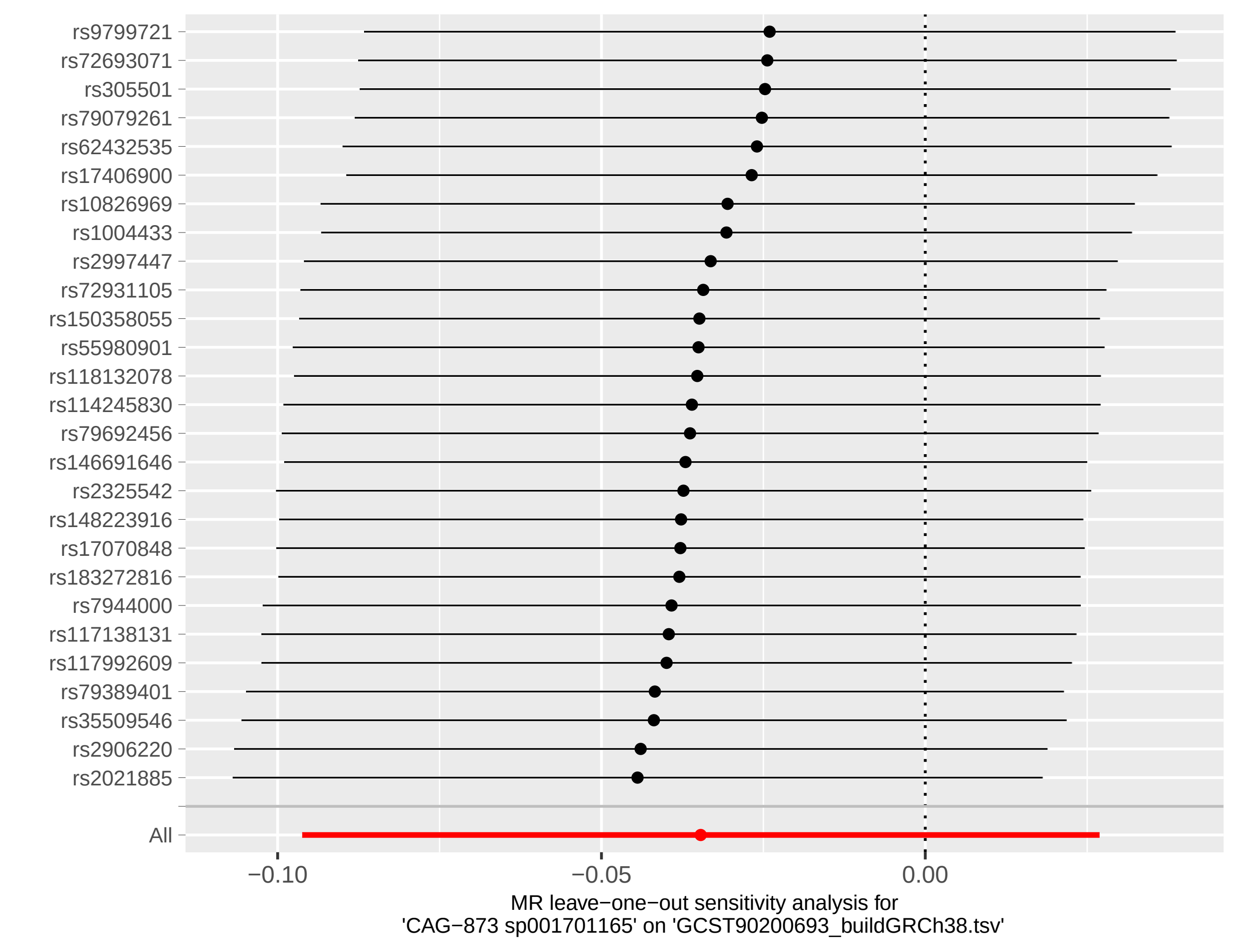
rs9799721
rs72693071
rs305501
rs79079261
rs62432535
rs17406900
rs10826969
rs1004433
rs2997447
rs72931105
rs150358055
rs55980901
rs118132078
rs114245830
rs79692456
rs146691646
rs2325542
rs148223916
rs17070848
rs183272816
rs7944000
rs117138131
rs117992609
rs79389401
rs35509546
rs2906220
rs2021885
All
−0.10
−0.05
0.00
MR leave−one−out sensitivity analysis for
'CAG−873 sp001701165' on 'GCST90200693_buildGRCh38.tsv'

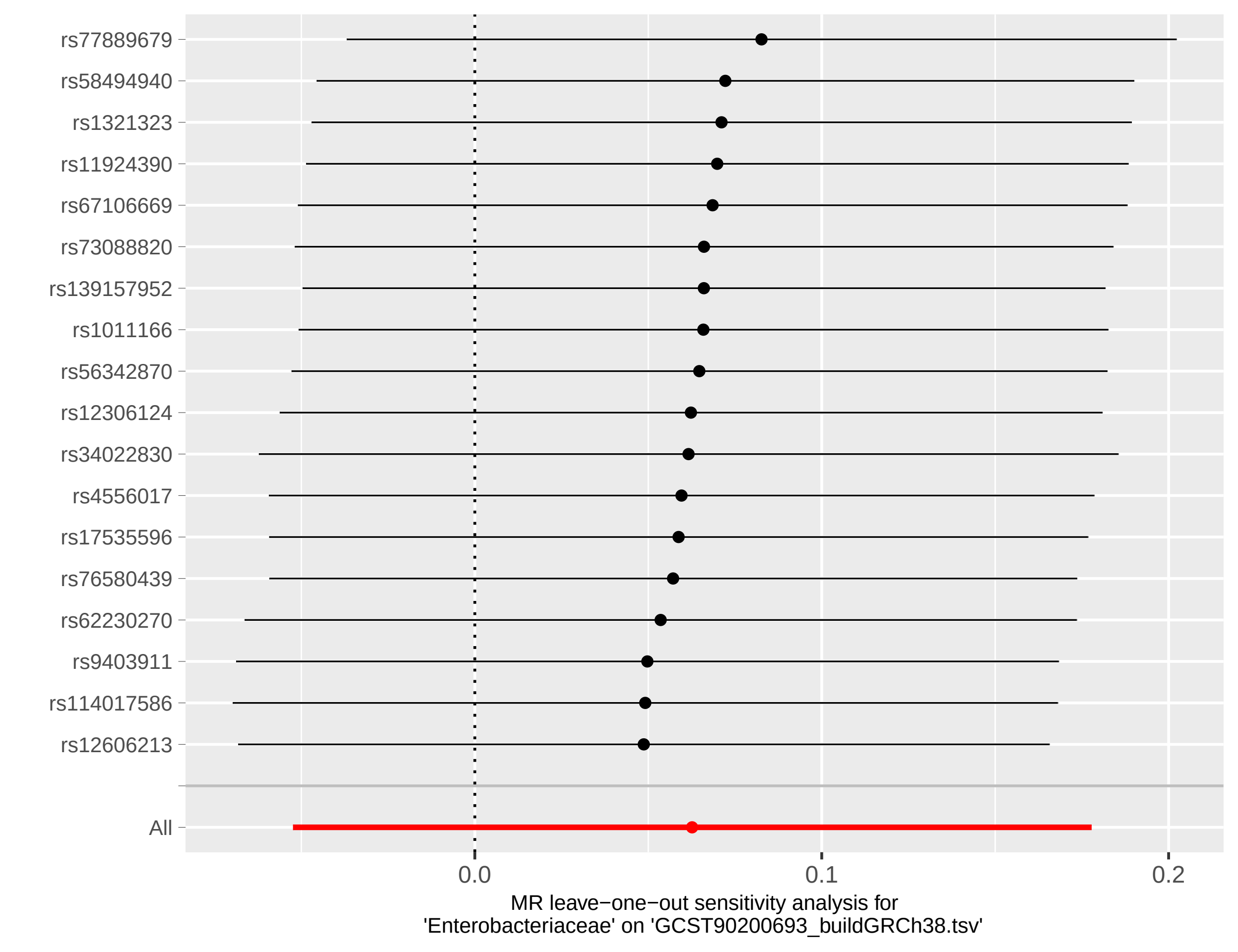
rs77889679
rs58494940
rs1321323
rs11924390
rs67106669
rs73088820
rs139157952
rs1011166
rs56342870
rs12306124
rs34022830
rs4556017
rs17535596
rs76580439
rs62230270
rs9403911
rs114017586
rs12606213
All
0.0
0.1
0.2
MR leave−one−out sensitivity analysis for
'Enterobacteriaceae' on 'GCST90200693_buildGRCh38.tsv'

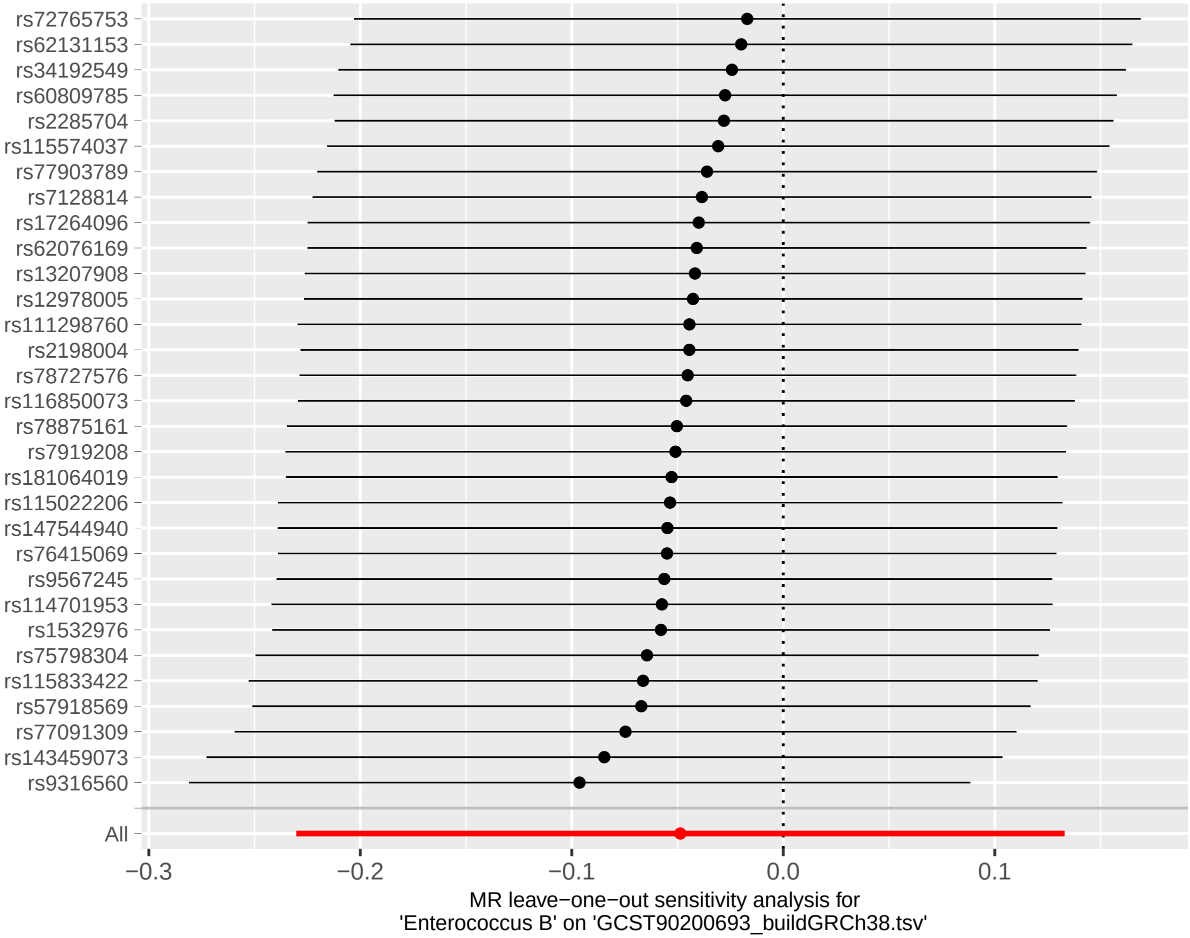

rs72765753
rs62131153
rs34192549
rs60809785
rs2285704
rs115574037
rs77903789
rs7128814
rs17264096
rs62076169
rs13207908
rs12978005
rs111298760
rs2198004
rs78727576
rs116850073
rs78875161
rs7919208
rs181064019
rs115022206
rs147544940
rs76415069
rs9567245
rs114701953
rs1532976
rs75798304
rs115833422
rs57918569
rs77091309
rs143459073
rs9316560
All
−0.3
−0.2
−0.1
0.0
0.1
MR leave−one−out sensitivity analysis for
'Enterococcus B' on 'GCST90200693_buildGRCh38.tsv'

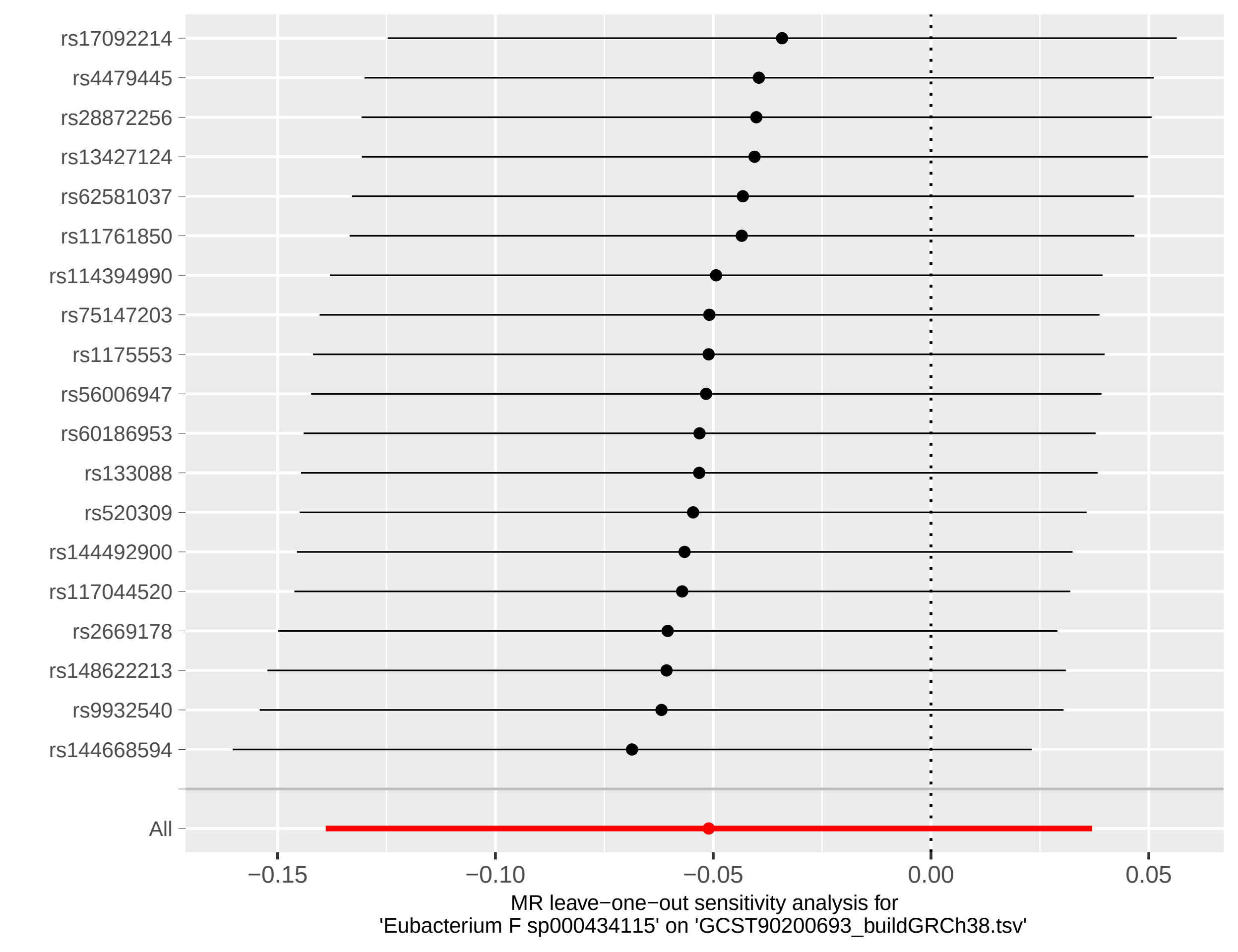
rs17092214
rs4479445
rs28872256
rs13427124
rs62581037
rs11761850
rs114394990
rs75147203
rs1175553
rs56006947
rs60186953
rs133088
rs520309
rs144492900
rs117044520
rs2669178
rs148622213
rs9932540
rs144668594
All
−0.15
−0.10
−0.05
0.00
0.05
MR leave−one−out sensitivity analysis for
'Eubacterium F sp000434115' on 'GCST90200693_buildGRCh38.tsv'

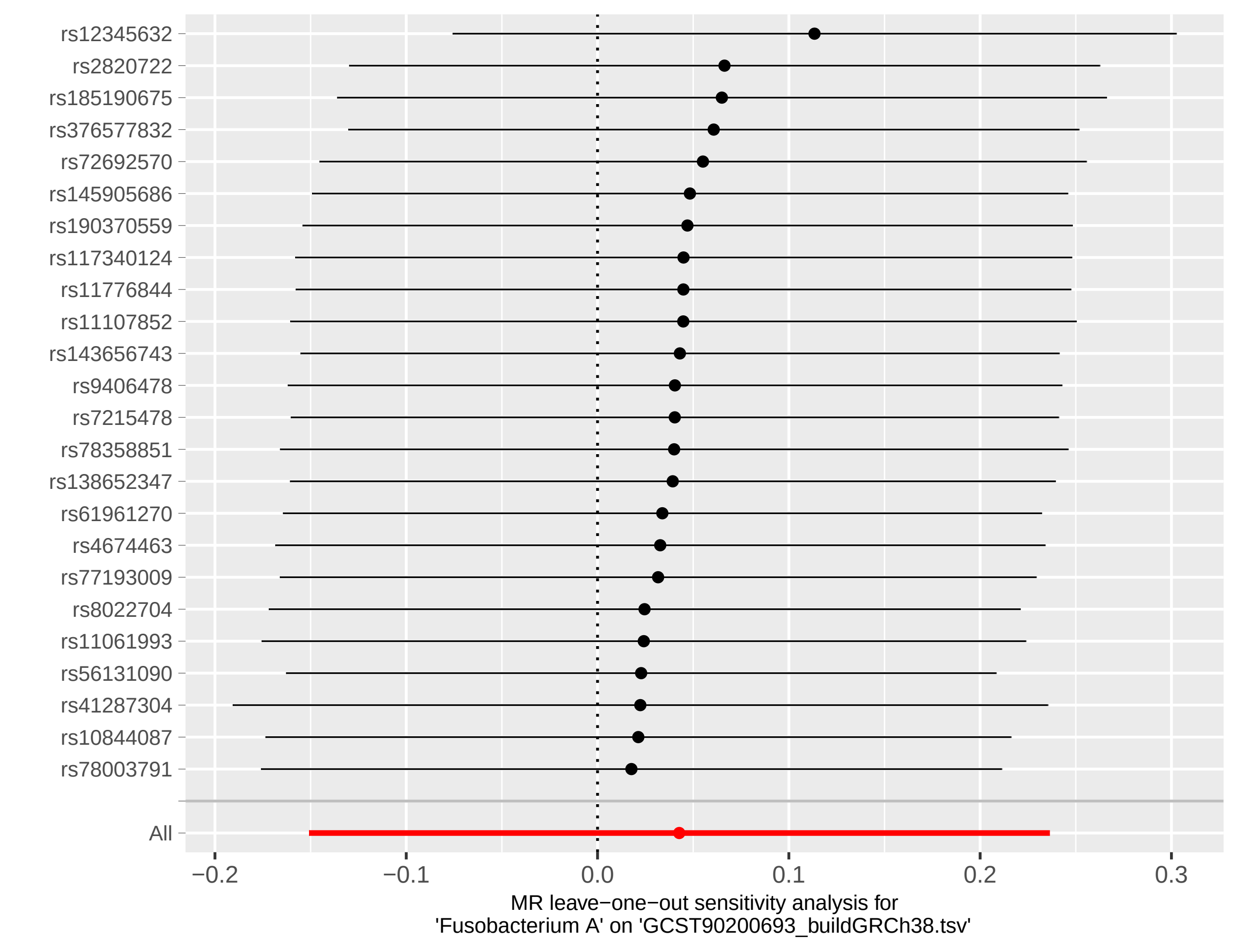

rs12345632
rs2820722
rs185190675
rs376577832
rs72692570
rs145905686
rs190370559
rs117340124
rs11776844
rs11107852
rs143656743
rs9406478
rs7215478
rs78358851
rs138652347
rs61961270
rs4674463
rs77193009
rs8022704
rs11061993
rs56131090
rs41287304
rs10844087
rs78003791
All
−0.2
−0.1
0.0
0.1
0.2
0.3
MR leave−one−out sensitivity analysis for
'Fusobacterium A' on 'GCST90200693_buildGRCh38.tsv'

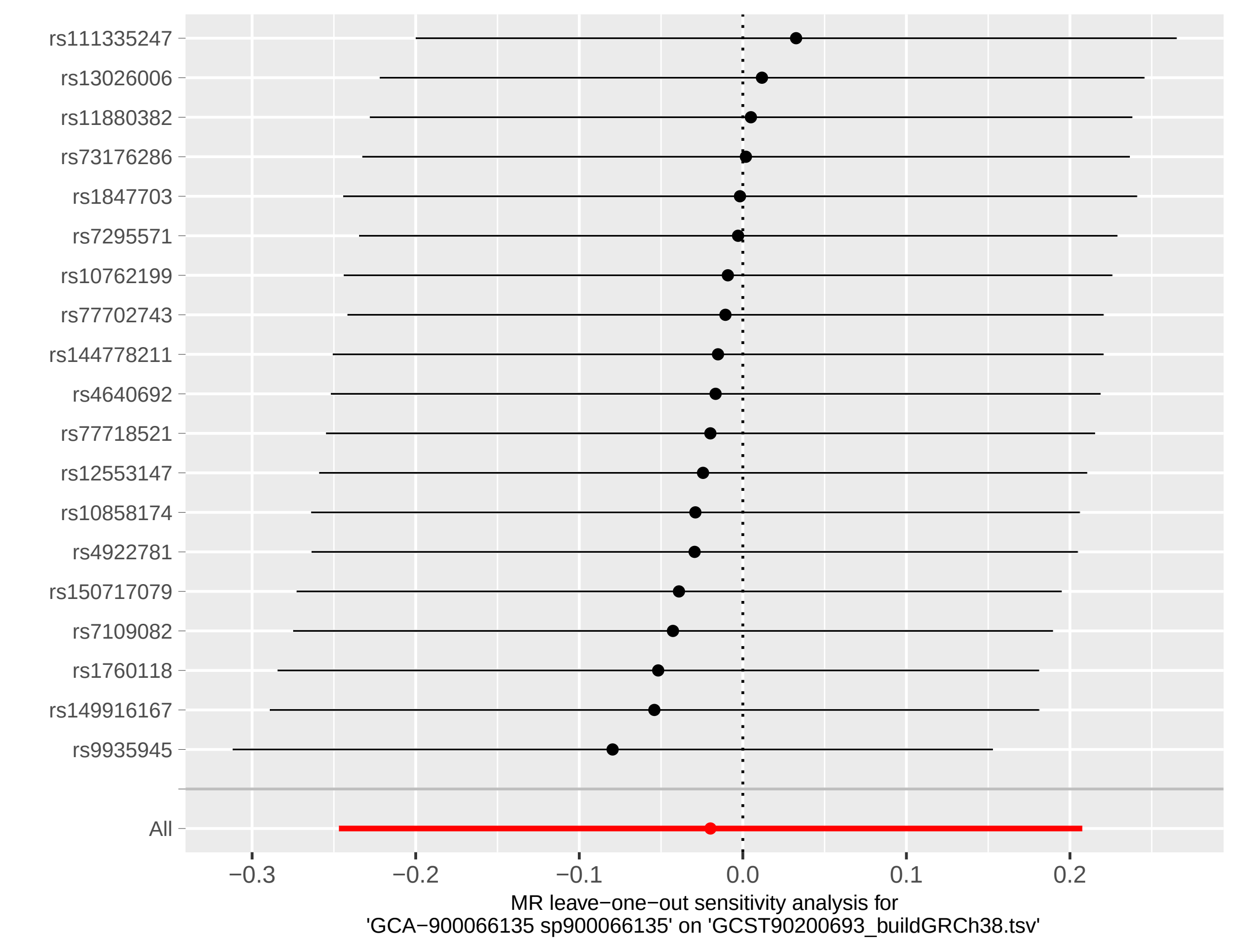
rs111335247
rs13026006
rs11880382
rs73176286
rs1847703
rs7295571
rs10762199
rs77702743
rs144778211
rs4640692
rs77718521
rs12553147
rs10858174
rs4922781
rs150717079
rs7109082
rs1760118
rs149916167
rs9935945
All
−0.3
−0.2
−0.1
0.0
0.1
0.2
MR leave−one−out sensitivity analysis for
'GCA−900066135 sp900066135' on 'GCST90200693_buildGRCh38.tsv'

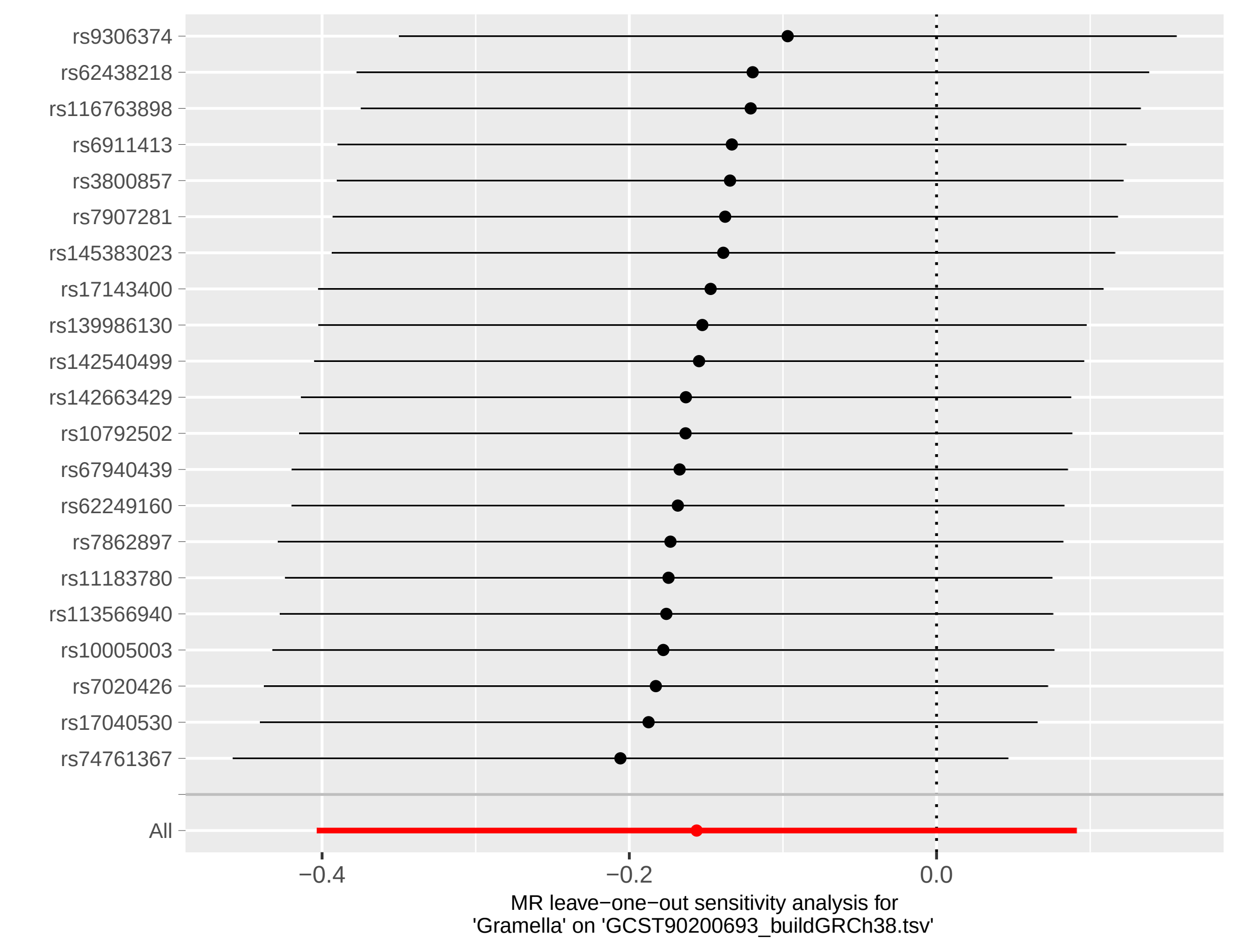
rs9306374
rs62438218
rs116763898
rs6911413
rs3800857
rs7907281
rs145383023
rs17143400
rs139986130
rs142540499
rs142663429
rs10792502
rs67940439
rs62249160
rs7862897
rs11183780
rs113566940
rs10005003
rs7020426
rs17040530
rs74761367
All
−0.4
−0.2
0.0
MR leave−one−out sensitivity analysis for
'Gramella' on 'GCST90200693_buildGRCh38.tsv'

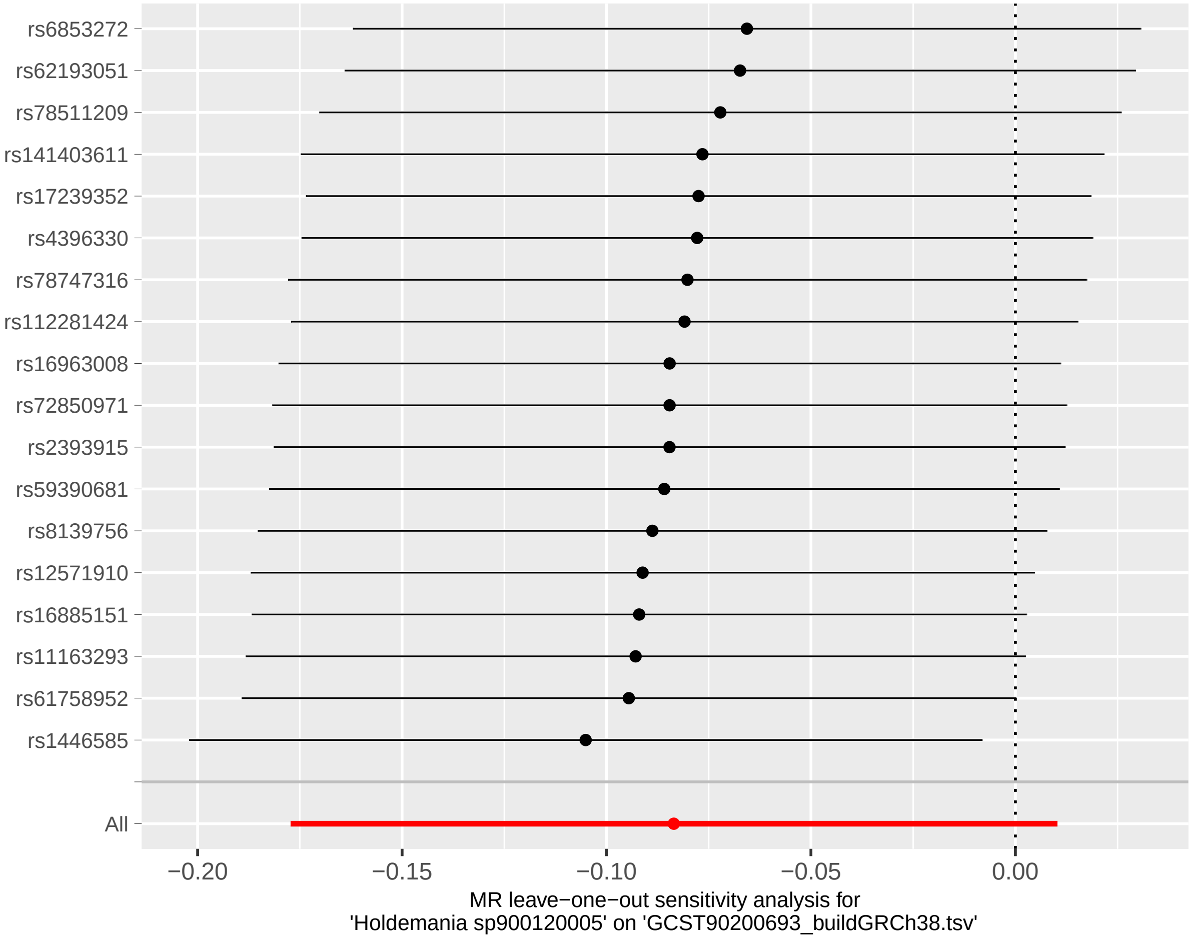
rs6853272
rs62193051
rs78511209
rs141403611
rs17239352
rs4396330
rs78747316
rs112281424
rs16963008
rs72850971
rs2393915
rs59390681
rs8139756
rs12571910
rs16885151
rs11163293
rs61758952
rs1446585
All
−0.20
−0.15
−0.10
−0.05
0.00
MR leave−one−out sensitivity analysis for
'Holdemania sp900120005' on 'GCST90200693_buildGRCh38.tsv'

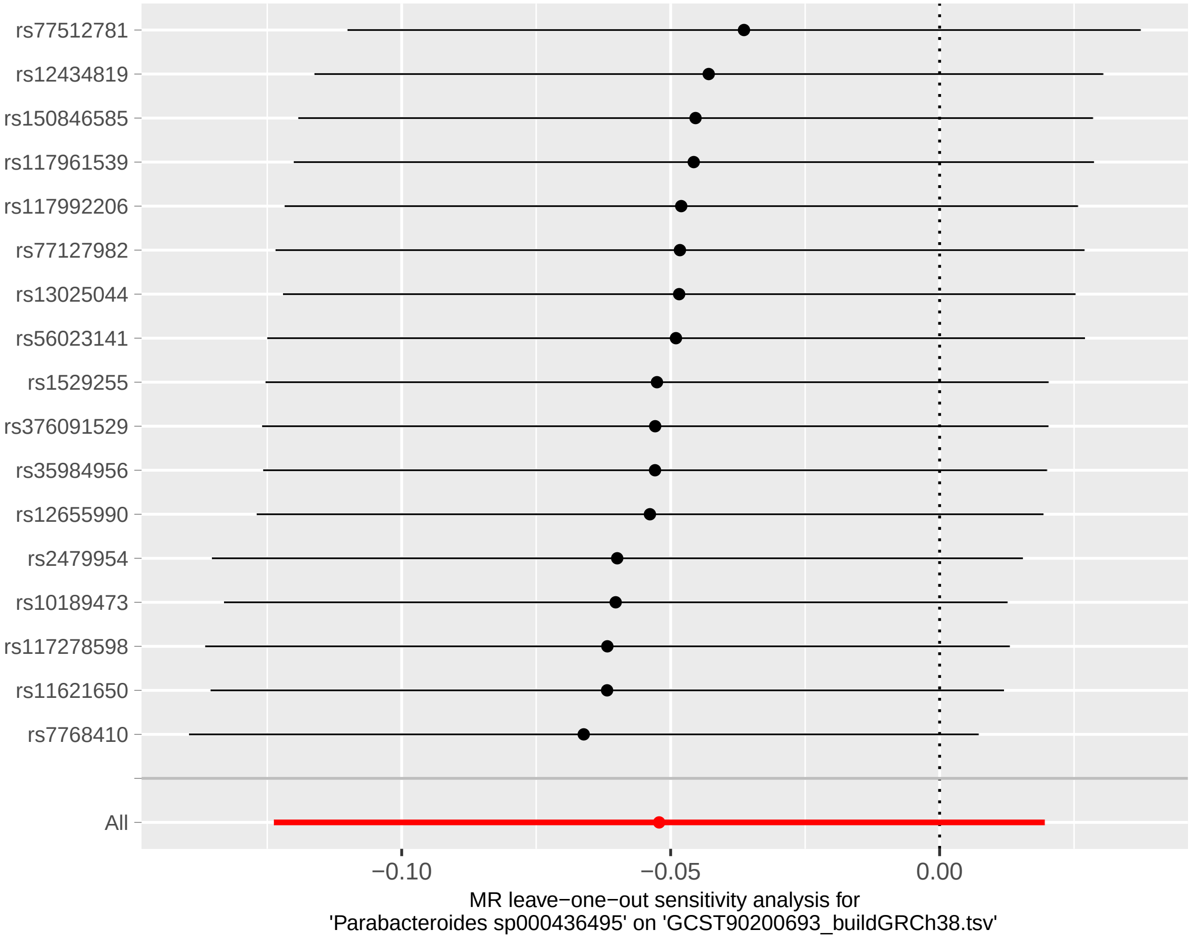
rs77512781
rs12434819
rs150846585
rs117961539
rs117992206
rs77127982
rs13025044
rs56023141
rs1529255
rs376091529
rs35984956
rs12655990
rs2479954
rs10189473
rs117278598
rs11621650
rs7768410
All
−0.10
−0.05
0.00
MR leave−one−out sensitivity analysis for
'Parabacteroides sp000436495' on 'GCST90200693_buildGRCh38.tsv'

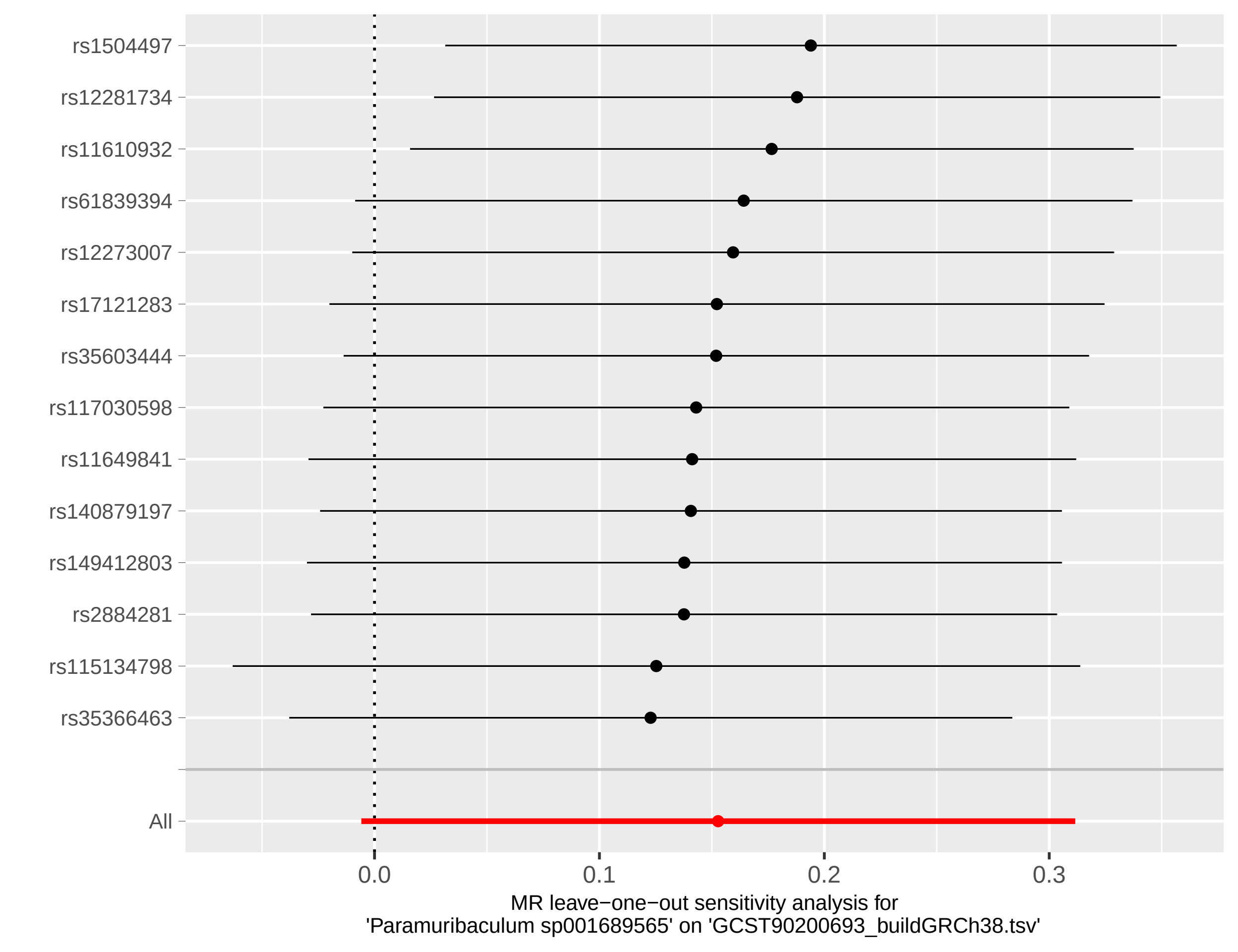
rs1504497
rs12281734
rs11610932
rs61839394
rs12273007
rs17121283
rs35603444
rs117030598
rs11649841
rs140879197
rs149412803
rs2884281
rs115134798
rs35366463
All
0.0
0.1
0.2
0.3
MR leave−one−out sensitivity analysis for
'Paramuribaculum sp001689565' on 'GCST90200693_buildGRCh38.tsv'

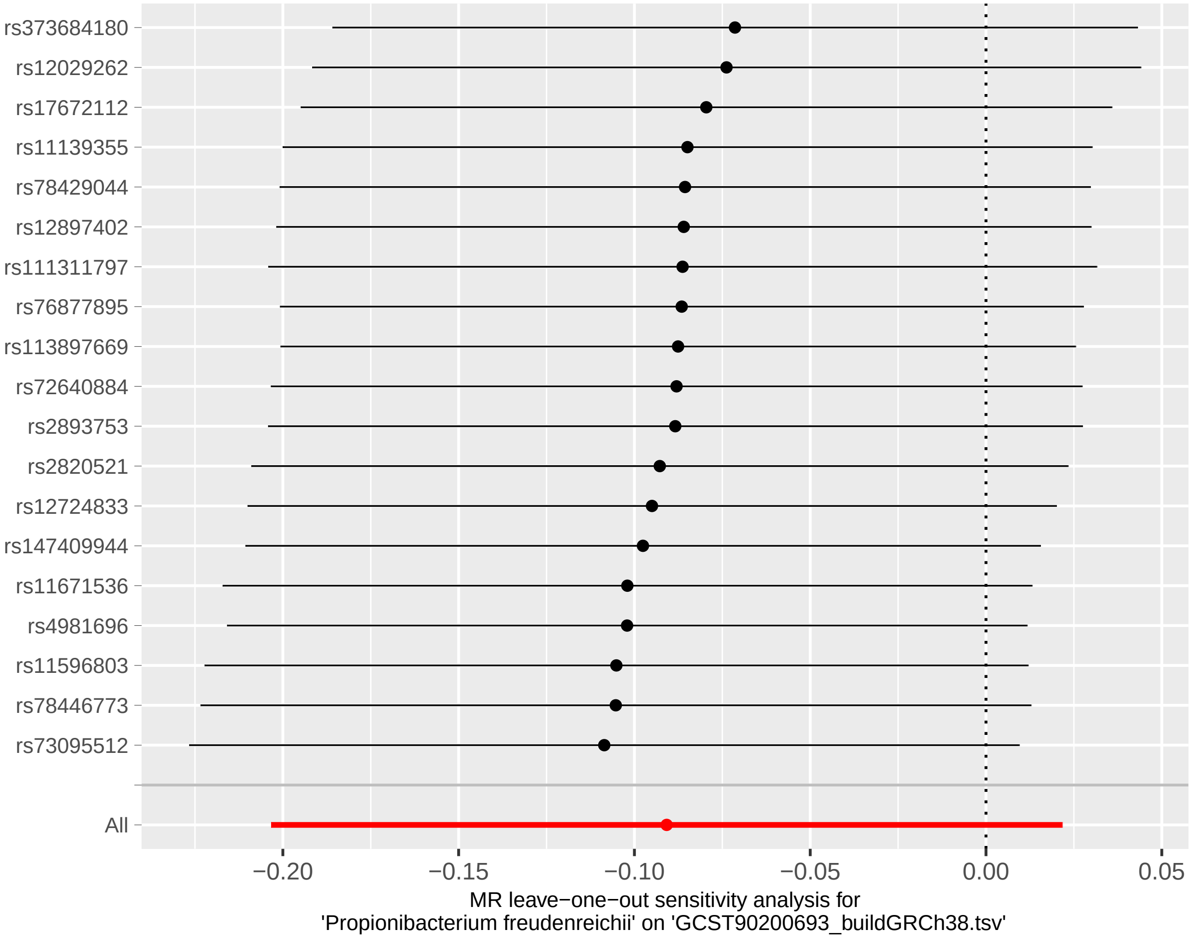
rs373684180
rs12029262
rs17672112
rs11139355
rs78429044
rs12897402
rs111311797
rs76877895
rs113897669
rs72640884
rs2893753
rs2820521
rs12724833
rs147409944
rs11671536
rs4981696
rs11596803
rs78446773
rs73095512
All
−0.20
−0.15
−0.10
−0.05
0.00
0.05
MR leave−one−out sensitivity analysis for
'Propionibacterium freudenreichii' on 'GCST90200693_buildGRCh38.tsv'

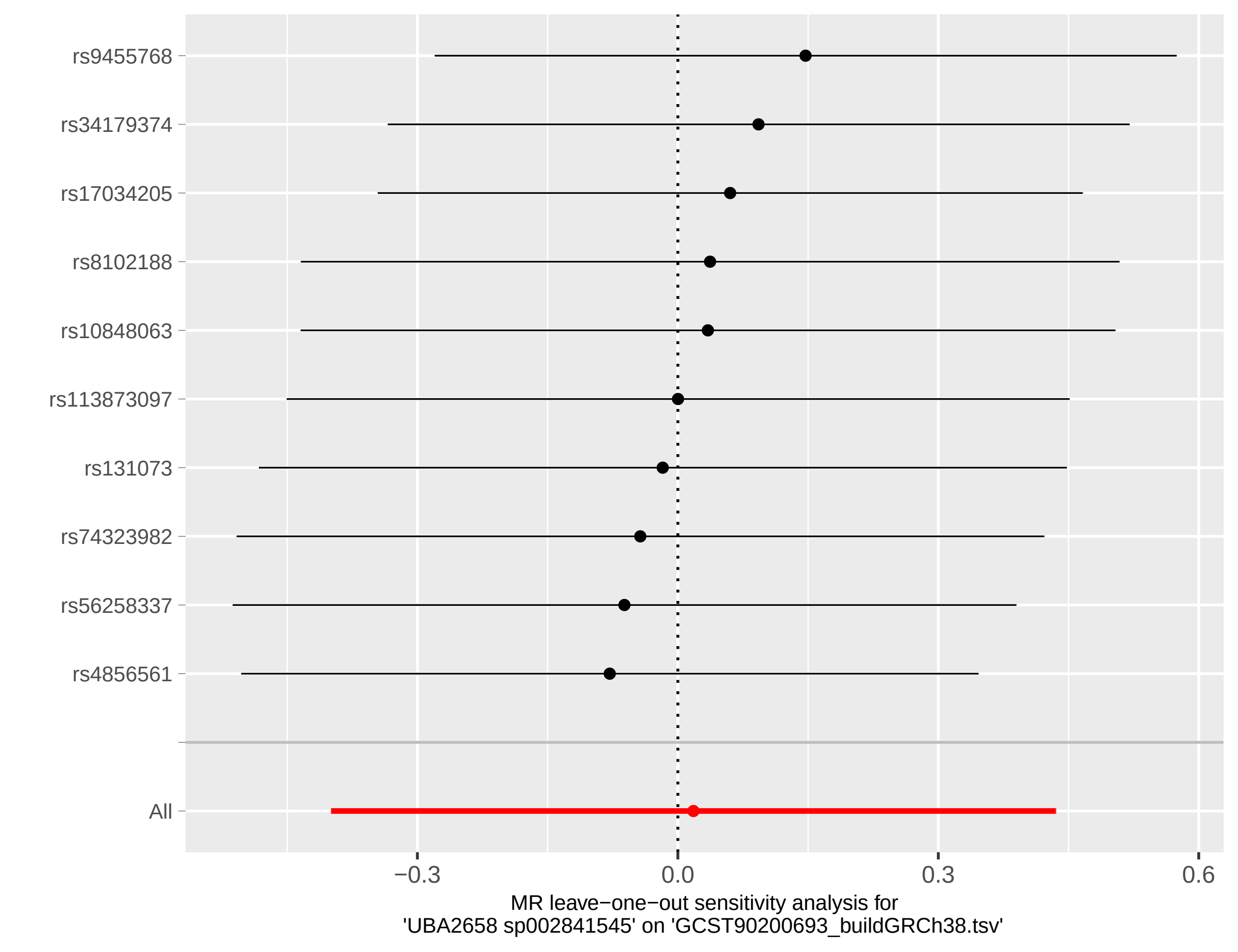
rs9455768
rs34179374
rs17034205
rs8102188
rs10848063
rs113873097
rs131073
rs74323982
rs56258337
rs4856561
All
−0.3
0.0
0.3
0.6
MR leave−one−out sensitivity analysis for
'UBA2658 sp002841545' on 'GCST90200693_buildGRCh38.tsv'

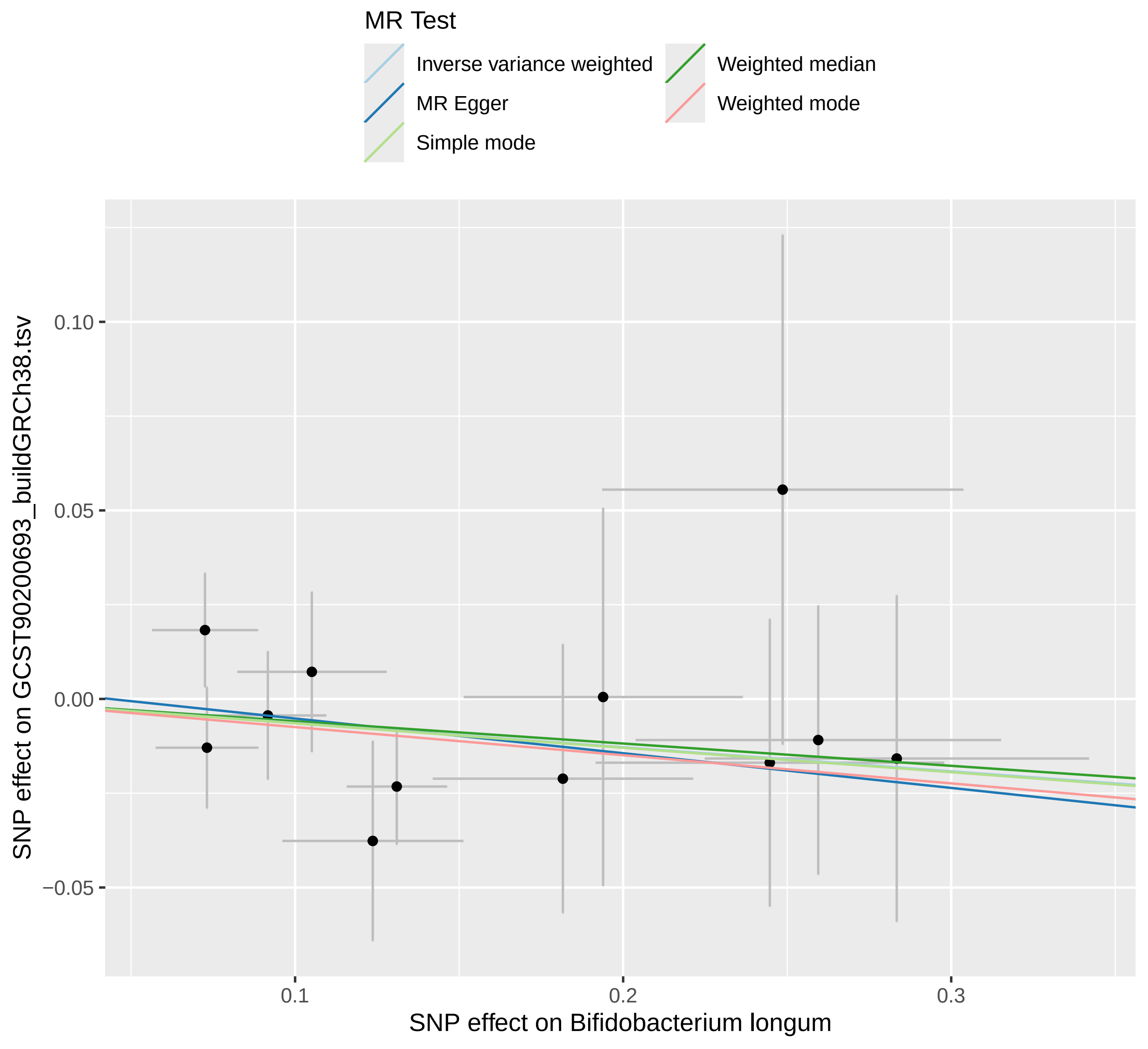
MR Test
Inverse variance weighted
MR Egger
Simple mode
Weighted median
Weighted mode
0.10
0.05
0.00
−0.05
0.1
0.2
0.3
SNP effect on Bifidobacterium longum
SNP effect on GCST90200693_buildGRCh38.tsv

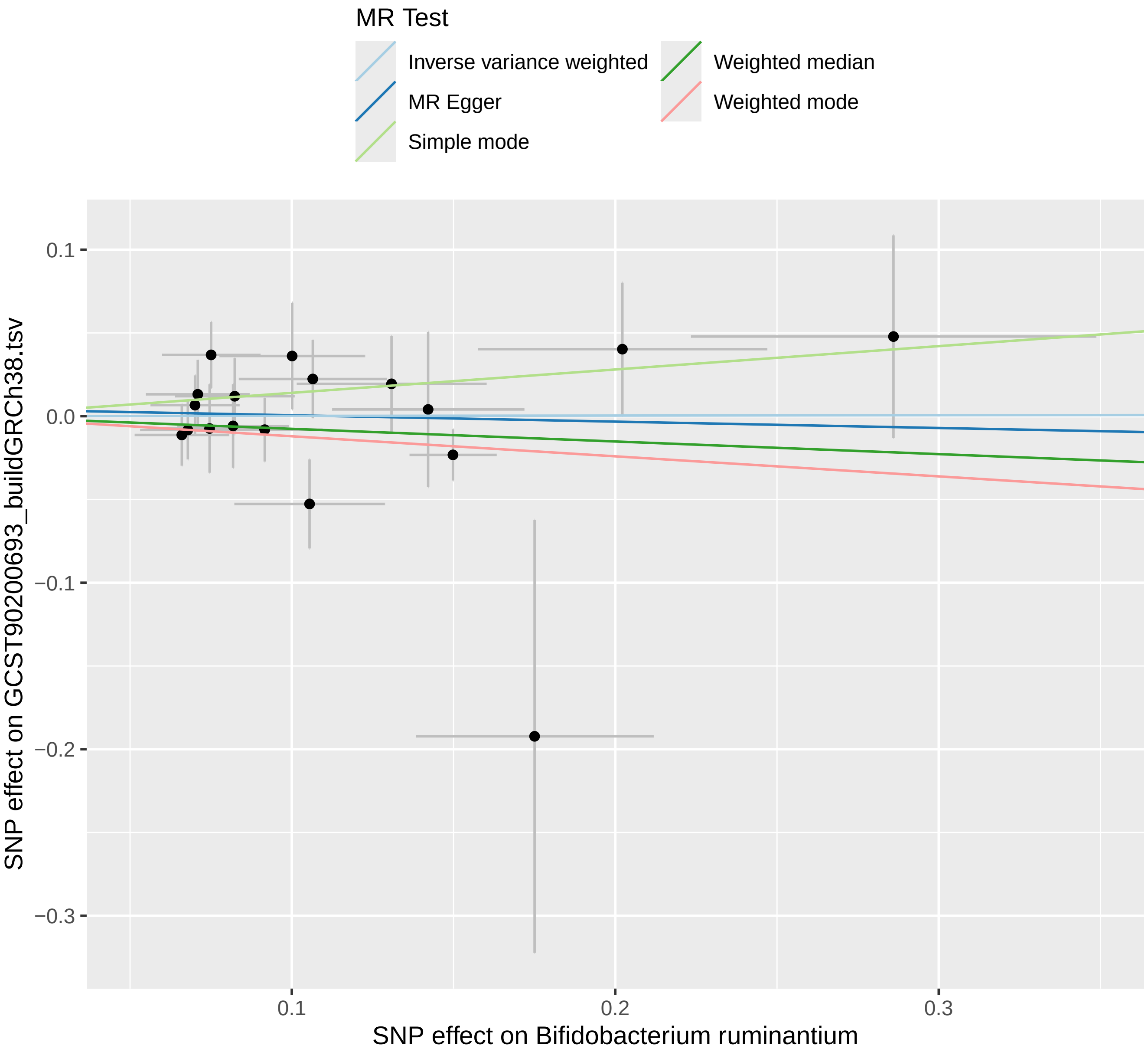
MR Test
Inverse variance weighted
MR Egger
Simple mode
Weighted median
Weighted mode
SNP effect on GCST90200693_buildGRCh38.tsv
0.1
0.0
−0.1
−0.2
−0.3
0.1
0.2
0.3
SNP effect on Bifidobacterium ruminantium

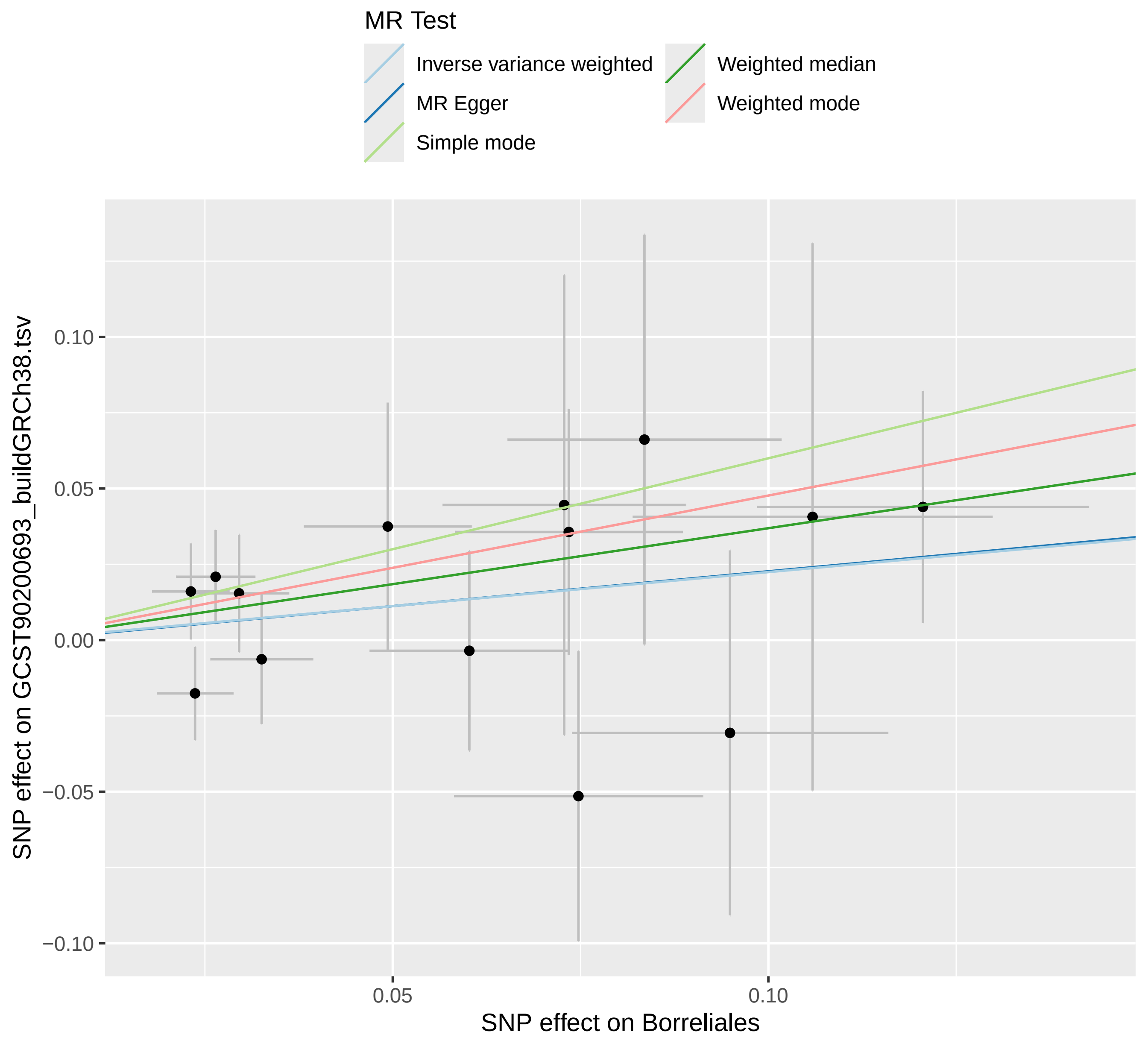
MR Test
Inverse variance weighted
MR Egger
Simple mode
Weighted median
Weighted mode
0.10
0.05
0.00
−0.05
−0.10
0.05
0.10
SNP effect on GCST90200693_buildGRCh38.tsv
SNP effect on Borreliales

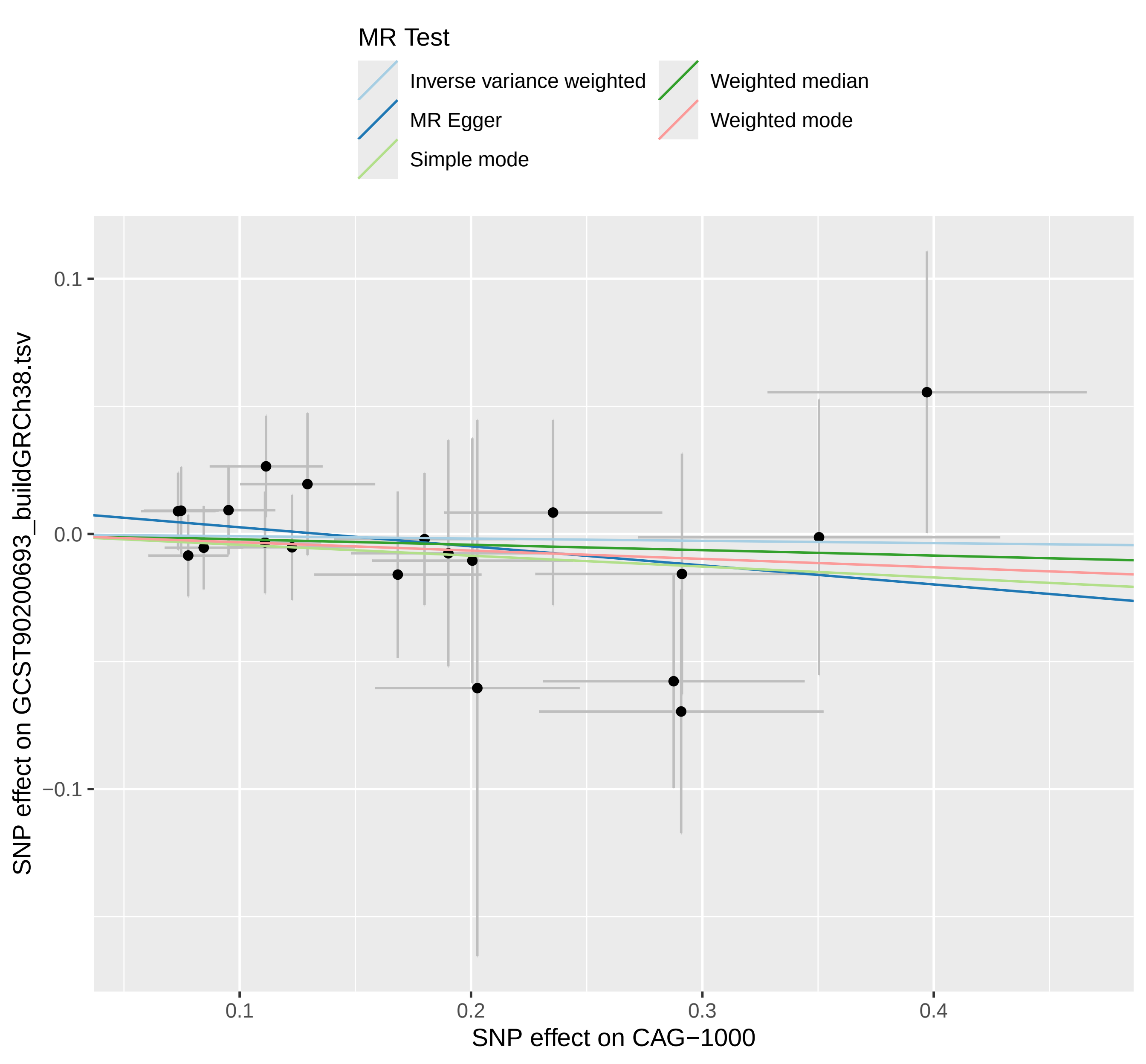
MR Test
Inverse variance weighted
MR Egger
Simple mode
Weighted median
Weighted mode
0.1
0.0
−0.1
0.1
0.2
0.3
0.4
SNP effect on GCST90200693_buildGRCh38.tsv
SNP effect on CAG−1000

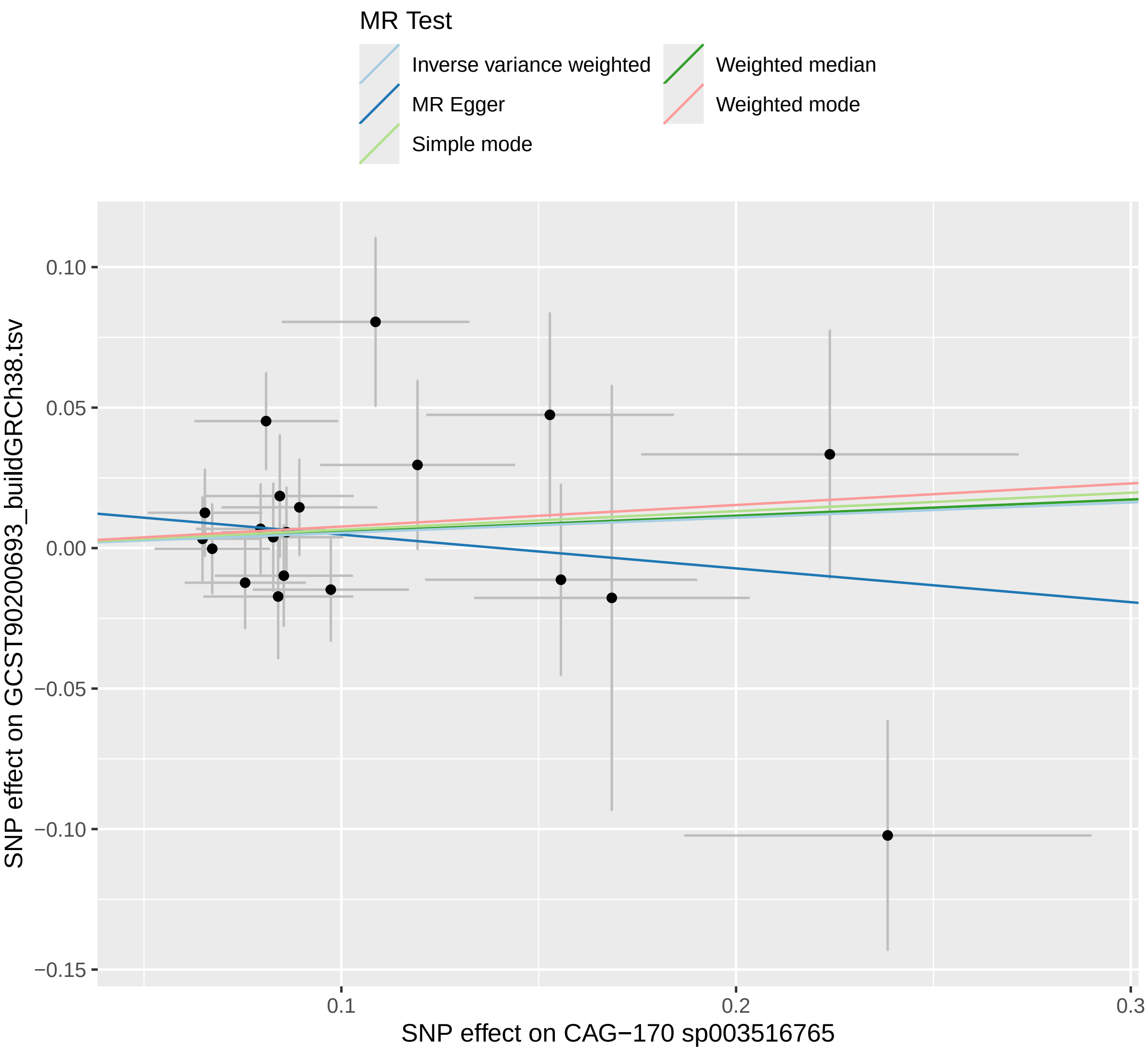
MR Test
Inverse variance weighted
MR Egger
Simple mode
Weighted median
Weighted mode
0.10
0.05
0.00
−0.05
−0.10
−0.15
0.1
0.2
0.3
SNP effect on GCST90200693_buildGRCh38.tsv
SNP effect on CAG−170 sp003516765

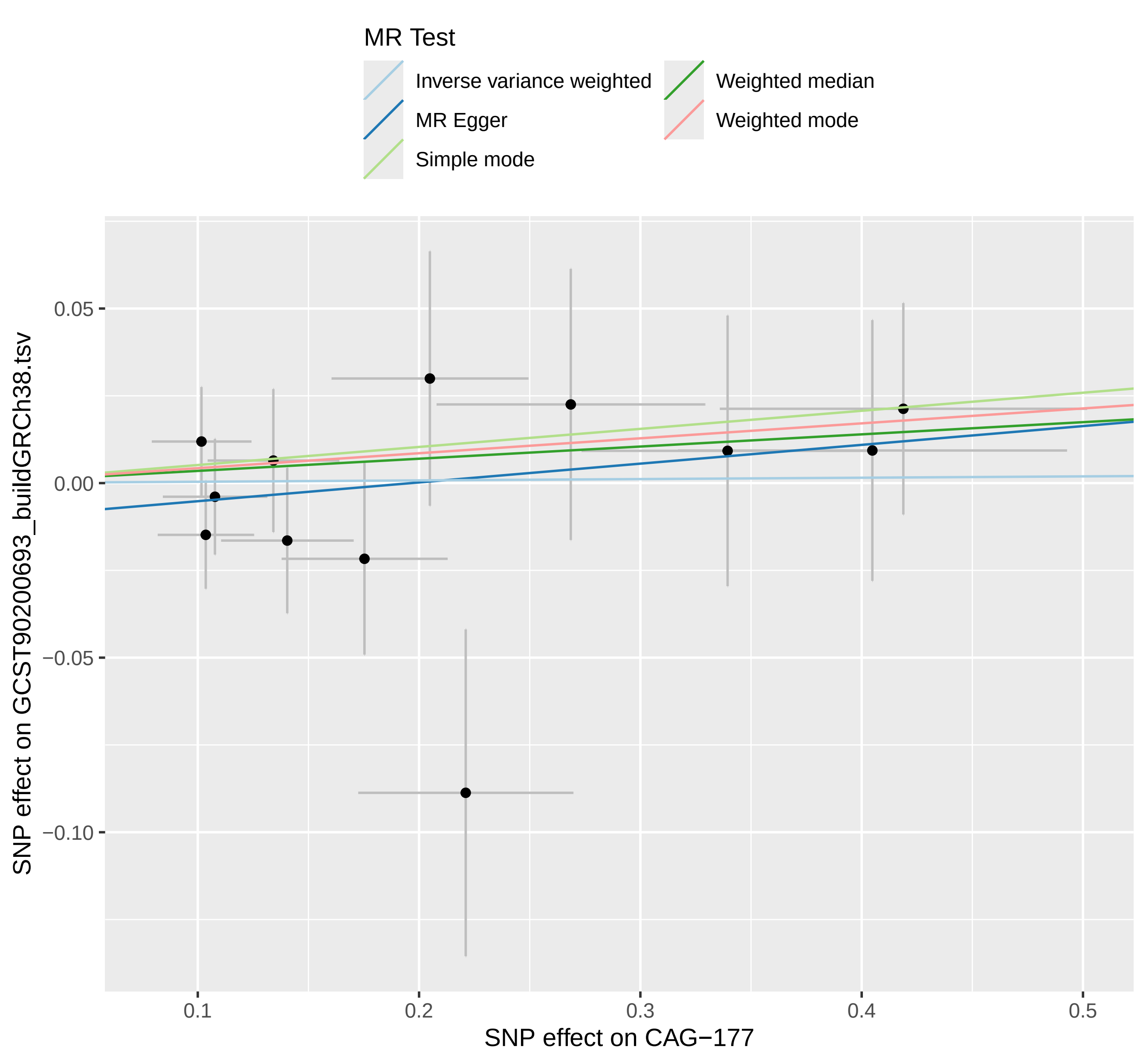
MR Test
Inverse variance weighted
MR Egger
Simple mode
Weighted median
Weighted mode
0.05
0.00
−0.05
−0.10
SNP effect on GCST90200693_buildGRCh38.tsv
0.1
0.2
0.3
0.4
0.5
SNP effect on CAG−177

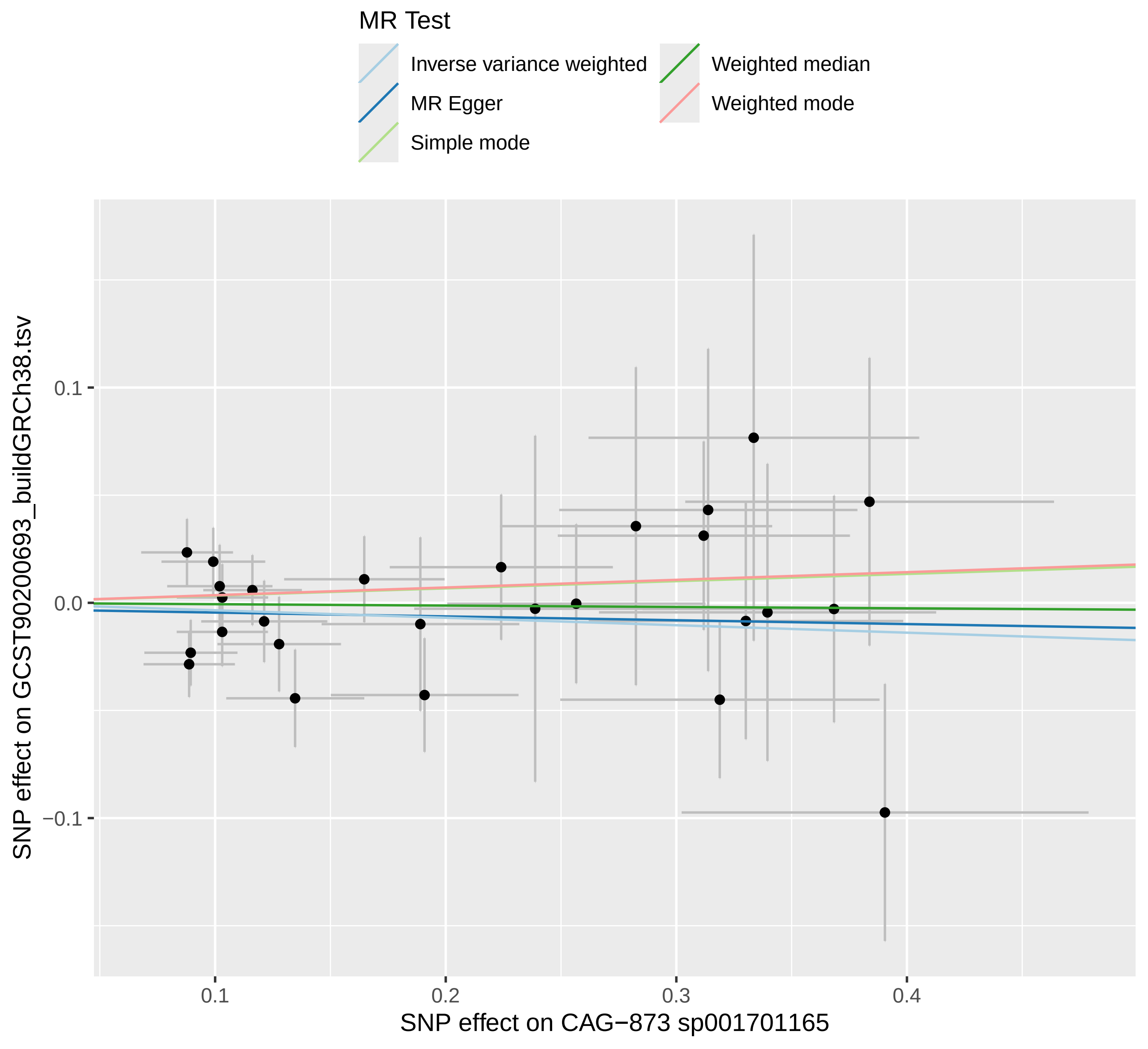
MR Test
Inverse variance weighted
MR Egger
Simple mode
Weighted median
Weighted mode
0.1
0.0
−0.1
0.1
0.2
0.3
0.4
SNP effect on GCST90200693_buildGRCh38.tsv
SNP effect on CAG−873 sp001701165

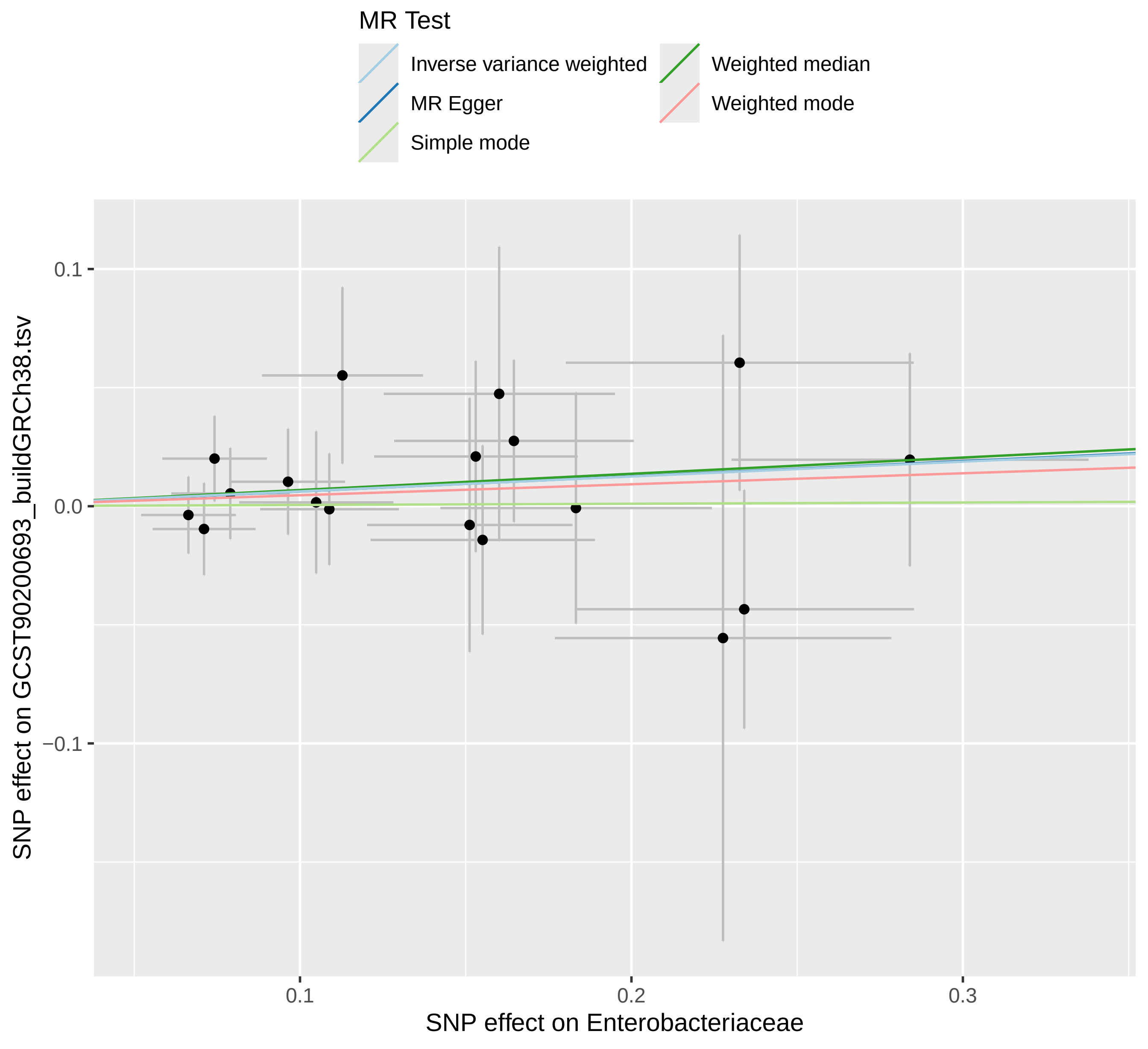
MR Test
Inverse variance weighted
MR Egger
Simple mode
Weighted median
Weighted mode
0.1
0.0
−0.1
0.1
0.2
0.3
SNP effect on GCST90200693_buildGRCh38.tsv
SNP effect on Enterobacteriaceae

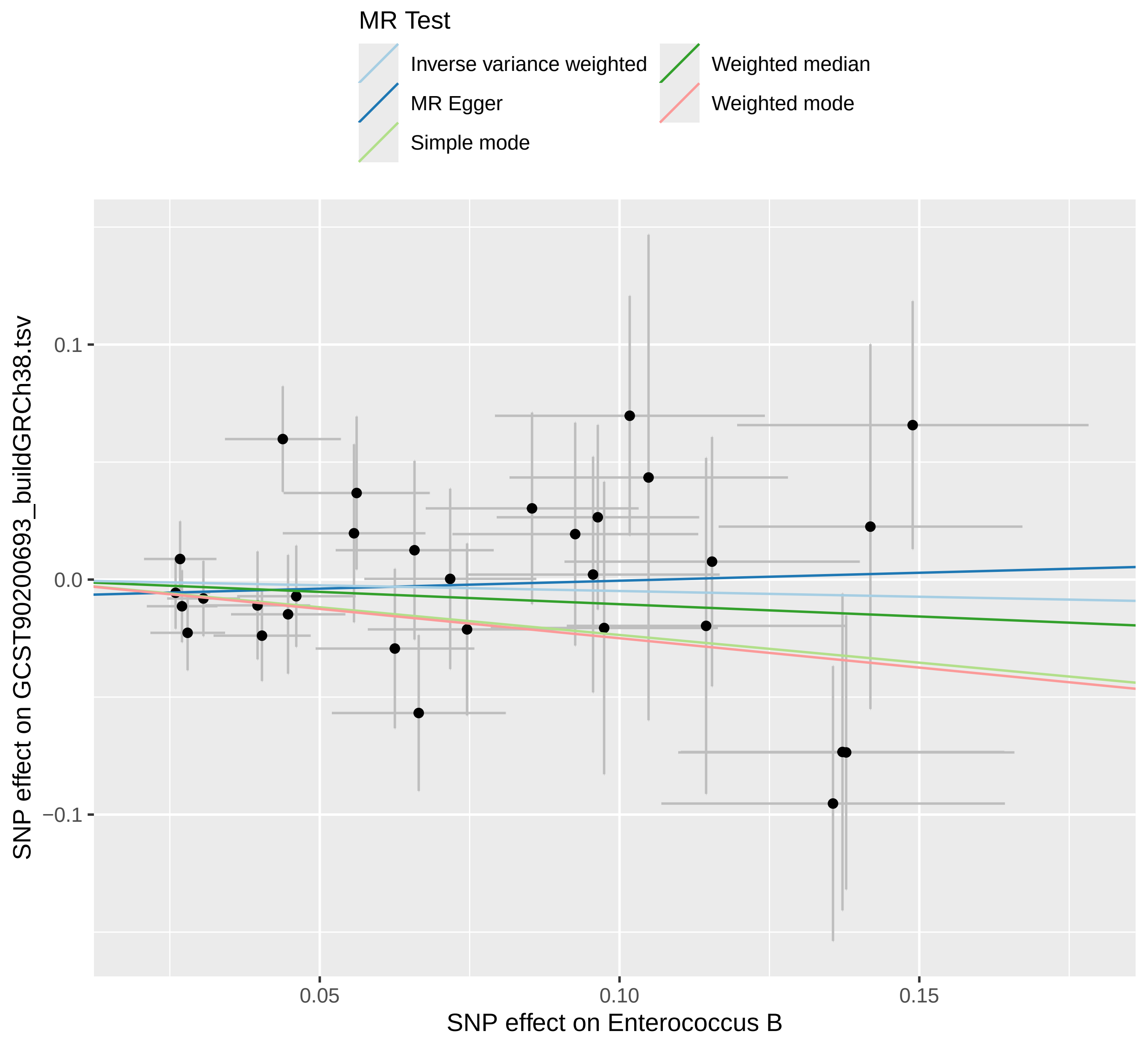
MR Test
Inverse variance weighted
MR Egger
Simple mode
Weighted median
Weighted mode
0.1
0.0
−0.1
0.05
0.10
0.15
SNP effect on GCST90200693_buildGRCh38.tsv
SNP effect on Enterococcus B

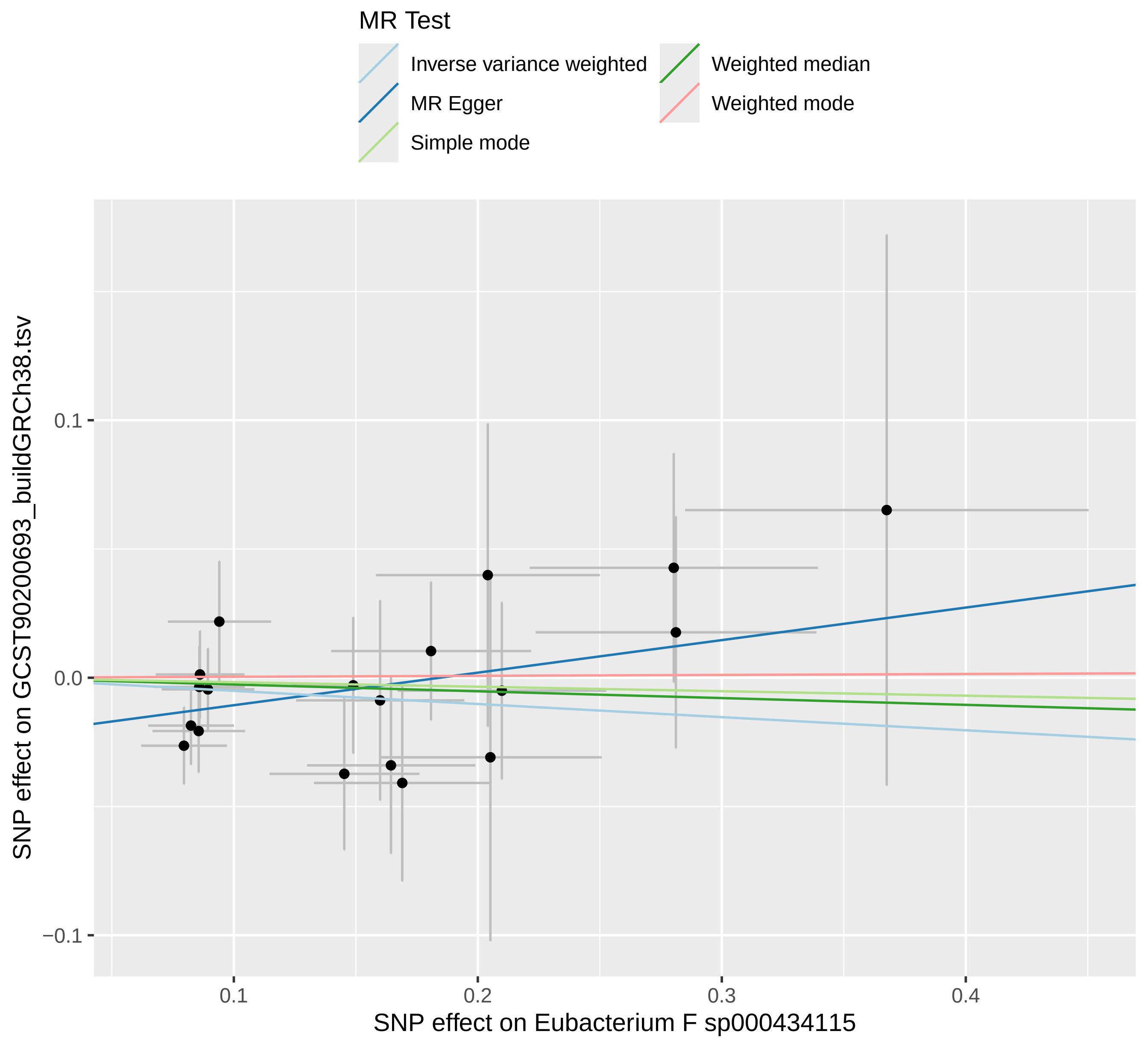
MR Test
Inverse variance weighted
MR Egger
Simple mode
Weighted median
Weighted mode
0.1
0.0
−0.1
0.1
0.2
0.3
0.4
SNP effect on GCST90200693_buildGRCh38.tsv
SNP effect on Eubacterium F sp000434115

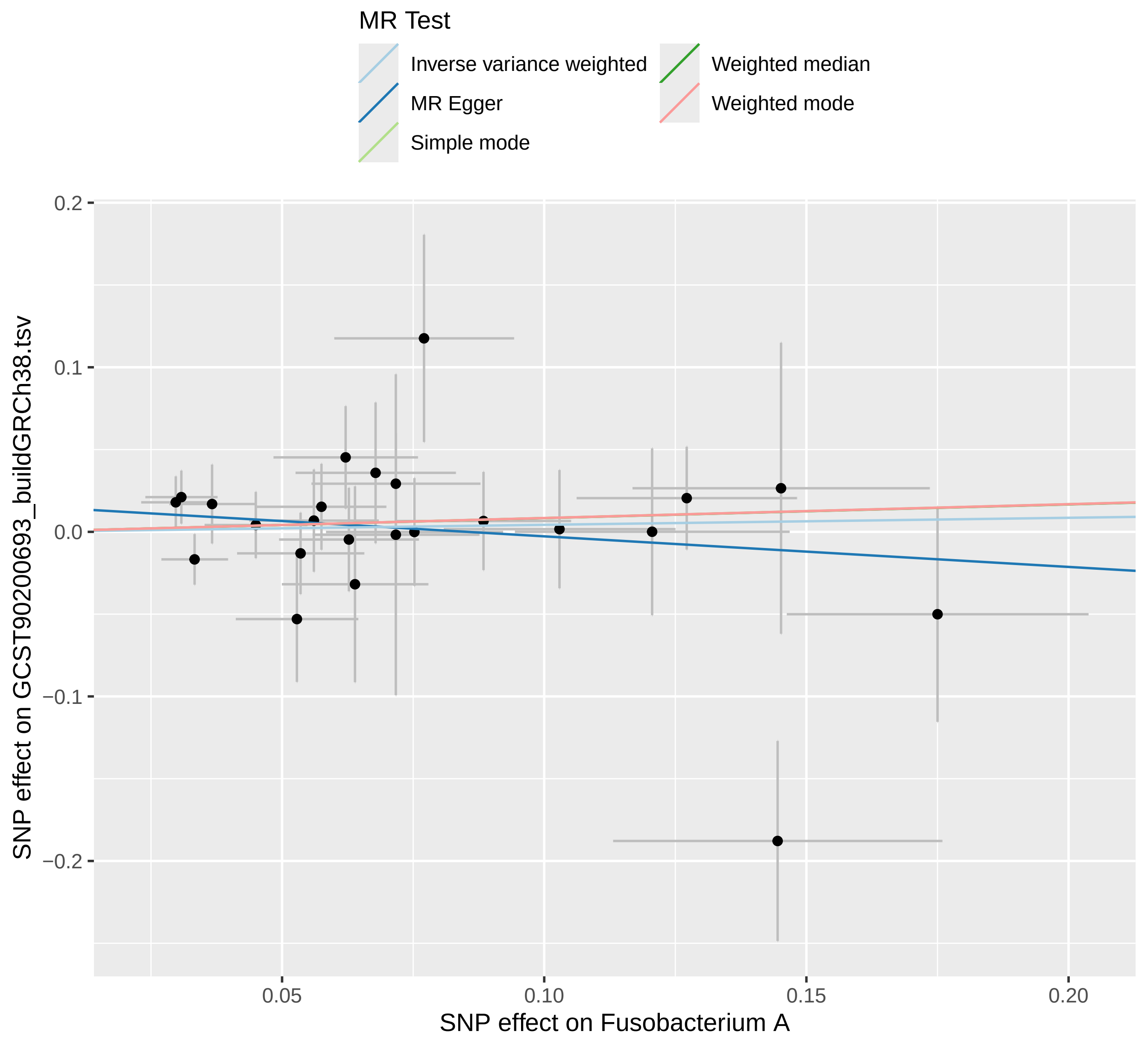

MR Test
Inverse variance weighted
MR Egger
Simple mode
Weighted median
Weighted mode
SNP effect on GCST90200693_buildGRCh38.tsv
SNP effect on Fusobacterium A
0.2
0.1
0.0
−0.1
−0.2
0.05
0.10
0.15
0.20

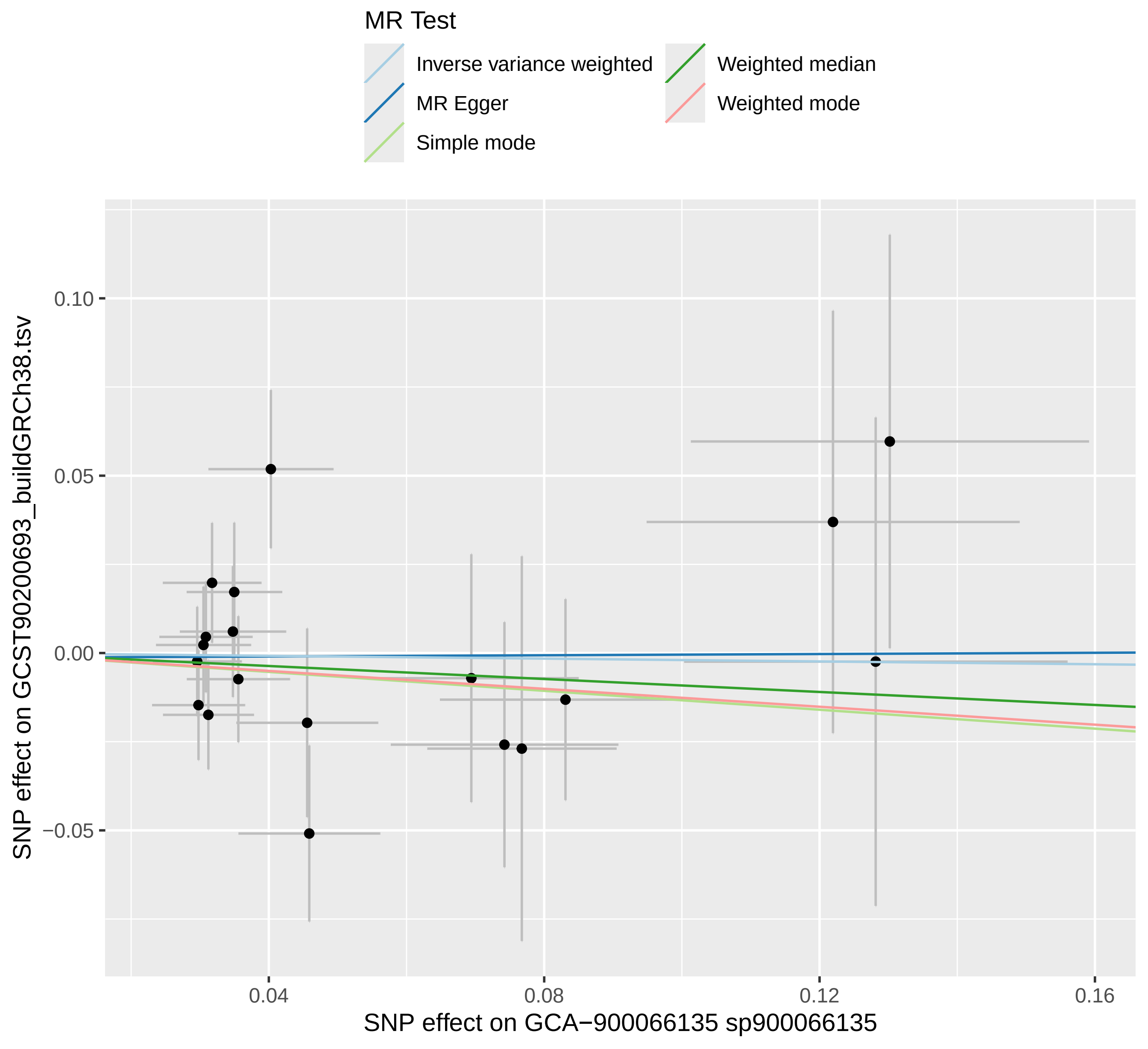

MR Test
Inverse variance weighted
MR Egger
Simple mode
Weighted median
Weighted mode
SNP effect on GCST90200693_buildGRCh38.tsv
0.10
0.05
0.00
−0.05
0.04
0.08
0.12
0.16
SNP effect on GCA−900066135 sp900066135

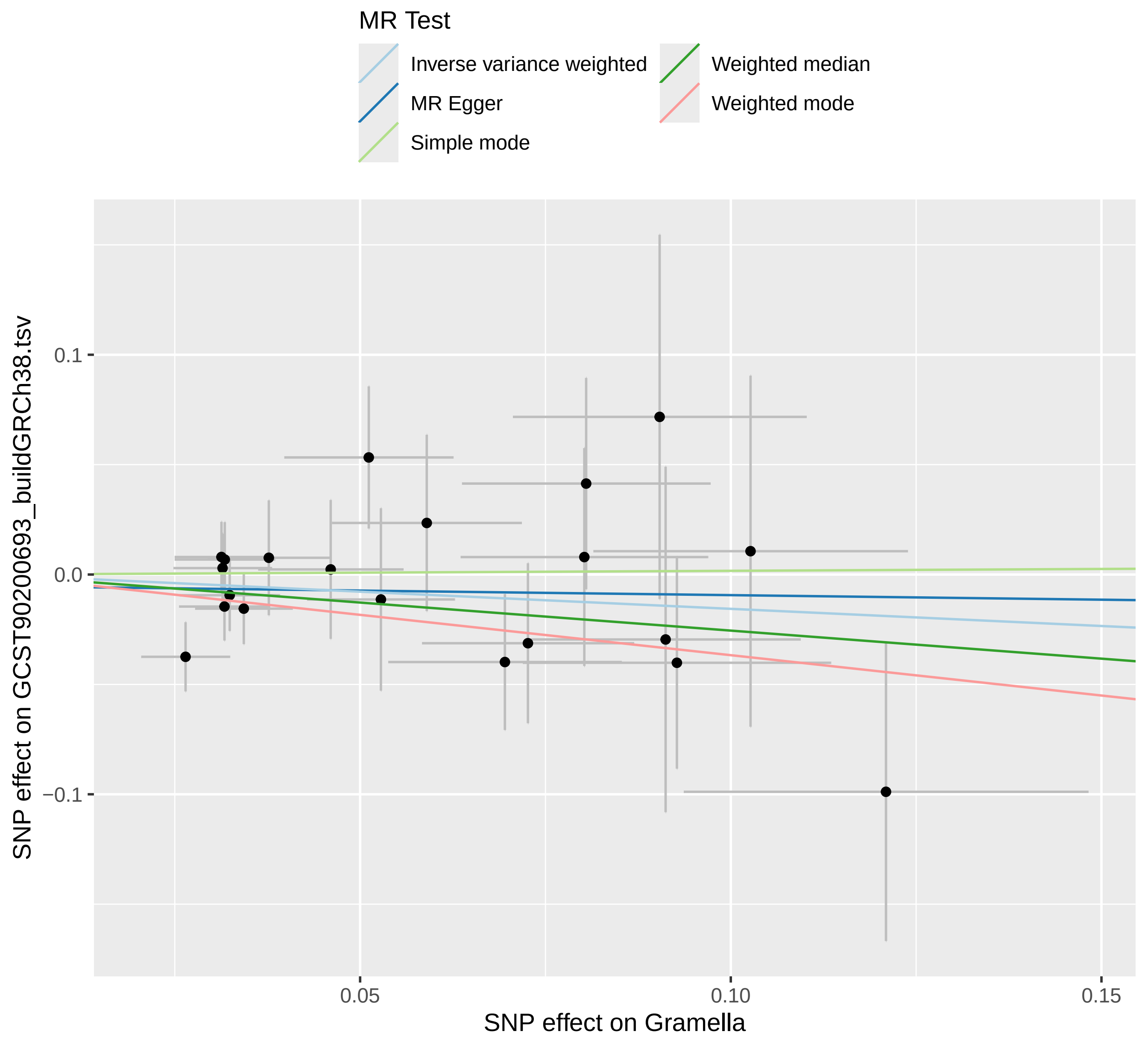
MR Test
Inverse variance weighted
MR Egger
Simple mode
Weighted median
Weighted mode
0.1
0.0
−0.1
0.05
0.10
0.15
SNP effect on Gramella
SNP effect on GCST90200693_buildGRCh38.tsv

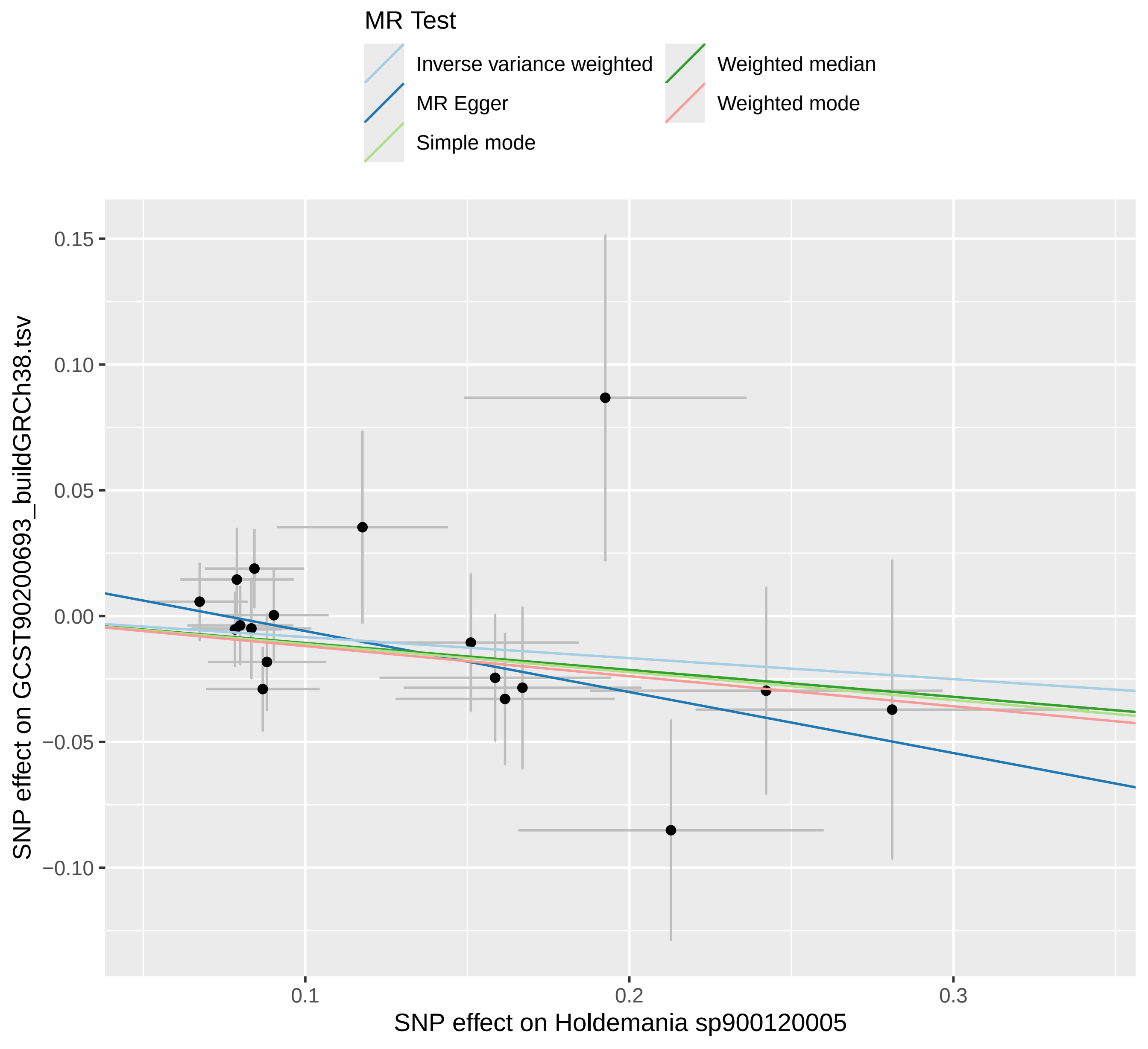

MR Test
Inverse variance weighted
MR Egger
Simple mode
Weighted median
Weighted mode
0.15
0.10
0.05
0.00
−0.05
−0.10
0.1
0.2
0.3
SNP effect on GCST90200693_buildGRCh38.tsv
SNP effect on Holdemania sp900120005

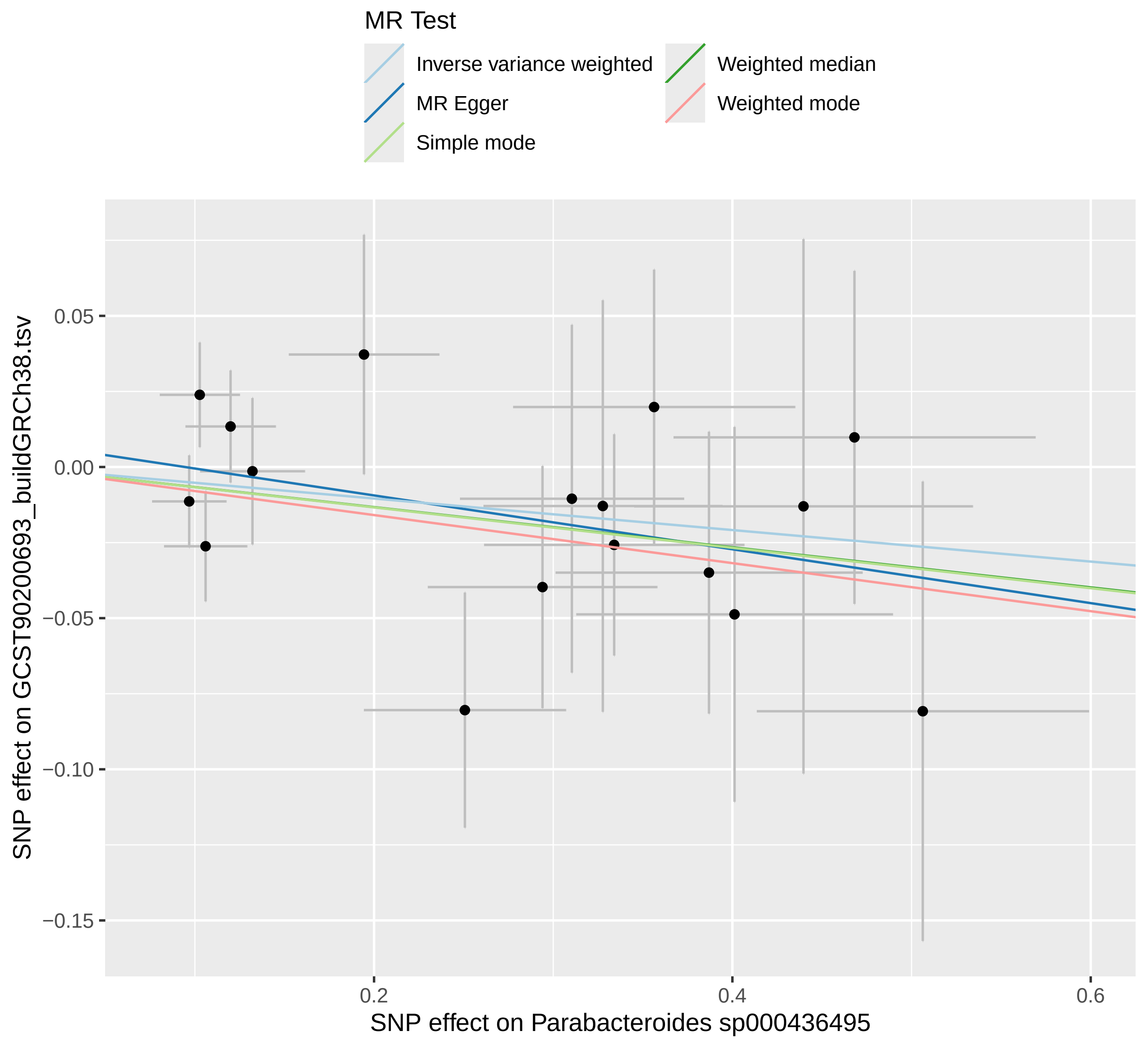
MR Test
Inverse variance weighted
MR Egger
Simple mode
Weighted median
Weighted mode
0.05
0.00
−0.05
−0.10
−0.15
0.2
0.4
0.6
SNP effect on GCST90200693_buildGRCh38.tsv
SNP effect on Parabacteroides sp000436495

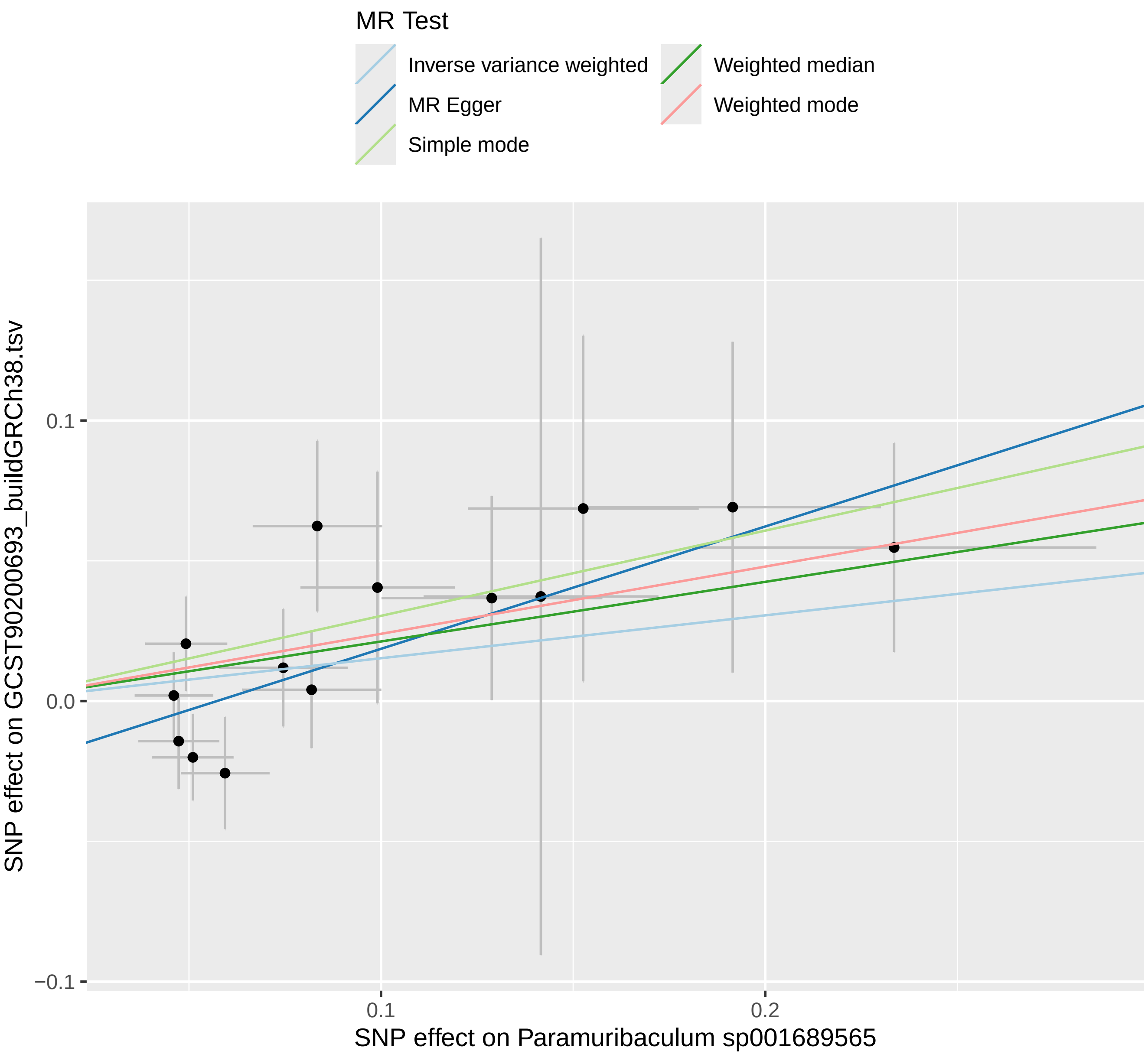
MR Test
Inverse variance weighted
MR Egger
Simple mode
Weighted median
Weighted mode
SNP effect on GCST90200693_buildGRCh38.tsv
0.1
0.0
−0.1
0.1
0.2
SNP effect on Paramuribaculum sp001689565

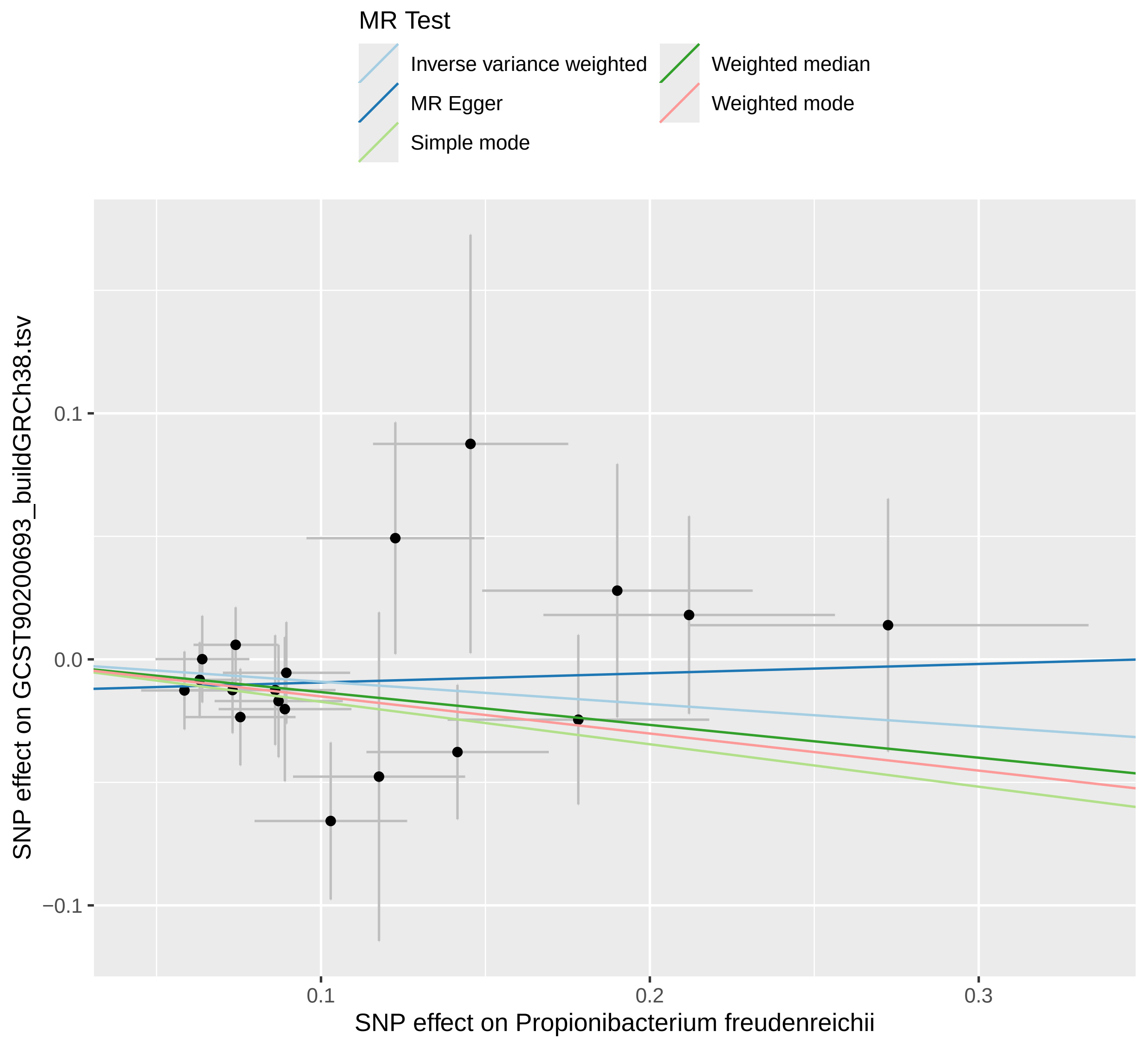
MR Test
Inverse variance weighted
MR Egger
Simple mode
Weighted median
Weighted mode
0.1
0.0
−0.1
0.1
0.2
0.3
SNP effect on Propionibacterium freudenreichii
SNP effect on GCST90200693_buildGRCh38.tsv

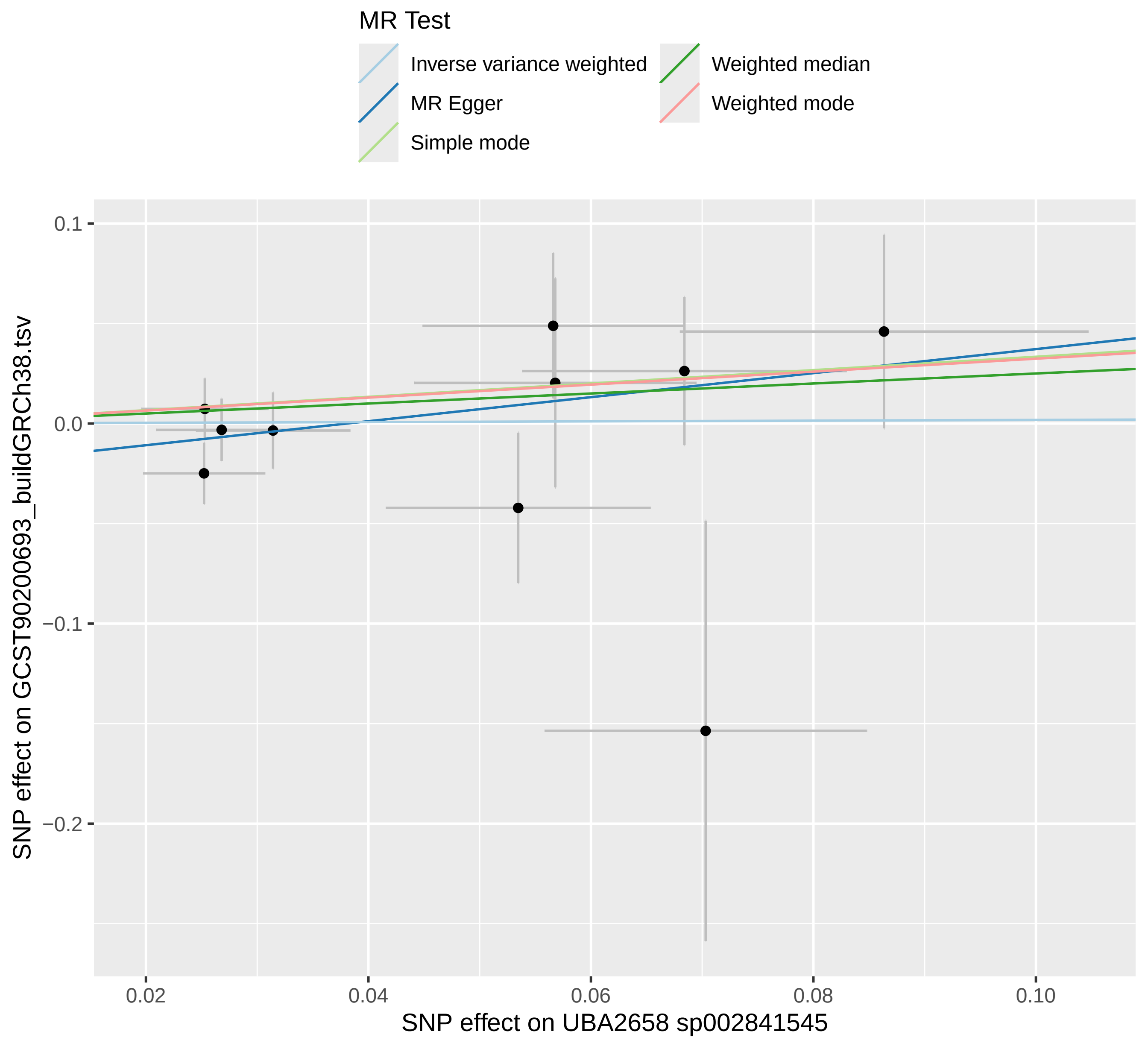

MR Test
Inverse variance weighted
MR Egger
Simple mode
Weighted median
Weighted mode
0.1
0.0
−0.1
−0.2
0.02
0.04
0.06
0.08
0.10
SNP effect on GCST90200693_buildGRCh38.tsv
SNP effect on UBA2658 sp002841545

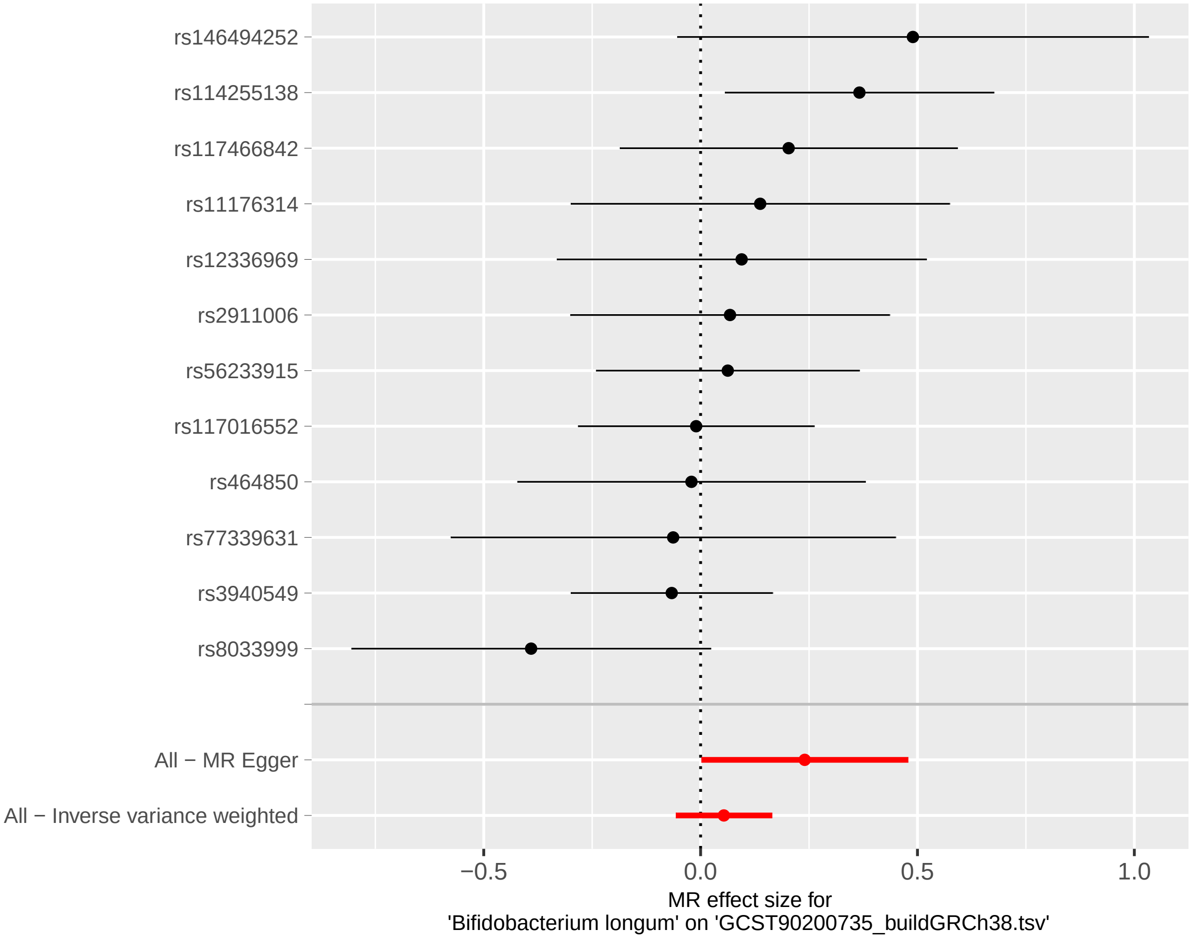
rs146494252
rs114255138
rs117466842
rs11176314
rs12336969
rs2911006
rs56233915
rs117016552
rs464850
rs77339631
rs3940549
rs8033999
All − MR Egger
All − Inverse variance weighted
−0.5
0.0
0.5
1.0
MR effect size for
'Bifidobacterium longum' on 'GCST90200735_buildGRCh38.tsv'

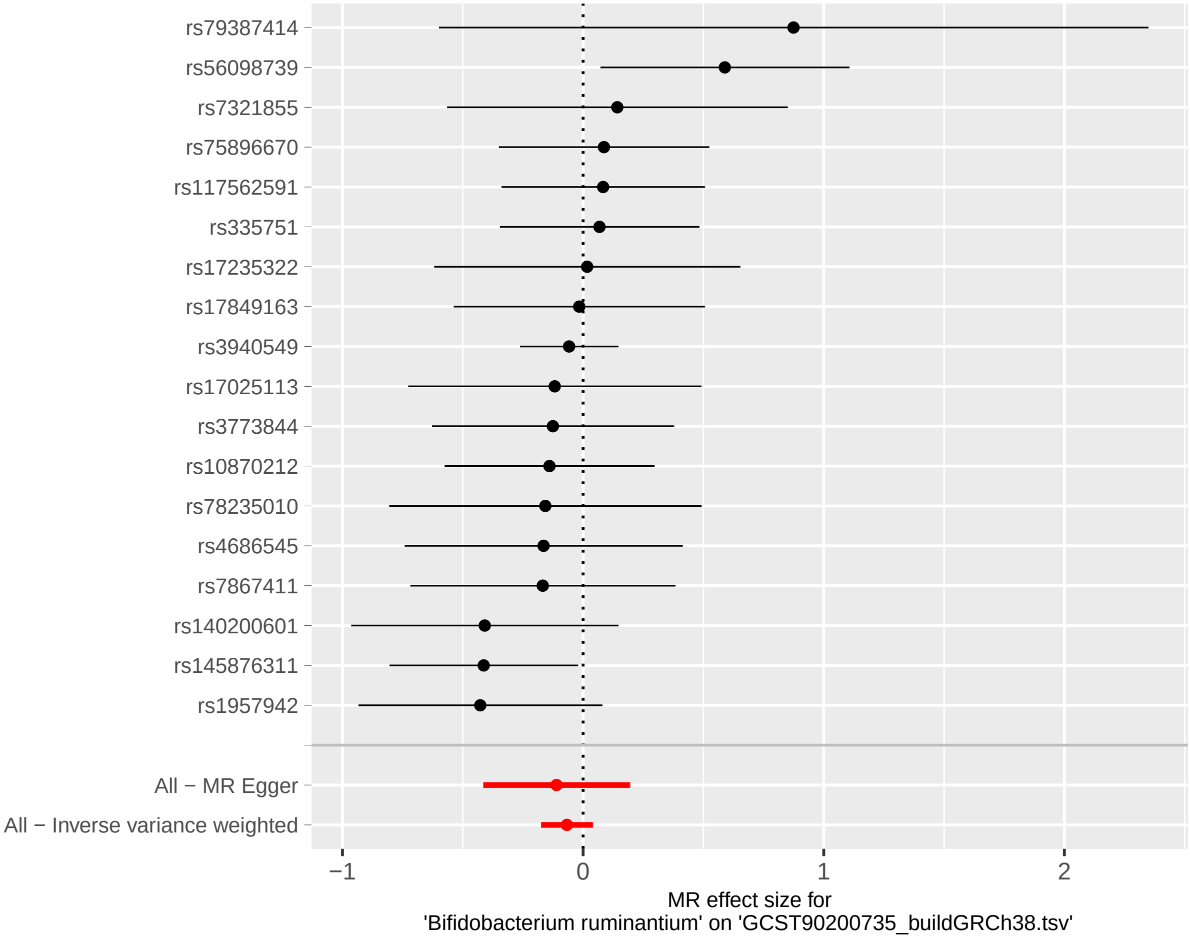

rs79387414
rs56098739
rs7321855
rs75896670
rs117562591
rs335751
rs17235322
rs17849163
rs3940549
rs17025113
rs3773844
rs10870212
rs78235010
rs4686545
rs7867411
rs140200601
rs145876311
rs1957942
All − MR Egger
All − Inverse variance weighted
−1
0
1
2
MR effect size for
'Bifidobacterium ruminantium' on 'GCST90200735_buildGRCh38.tsv'

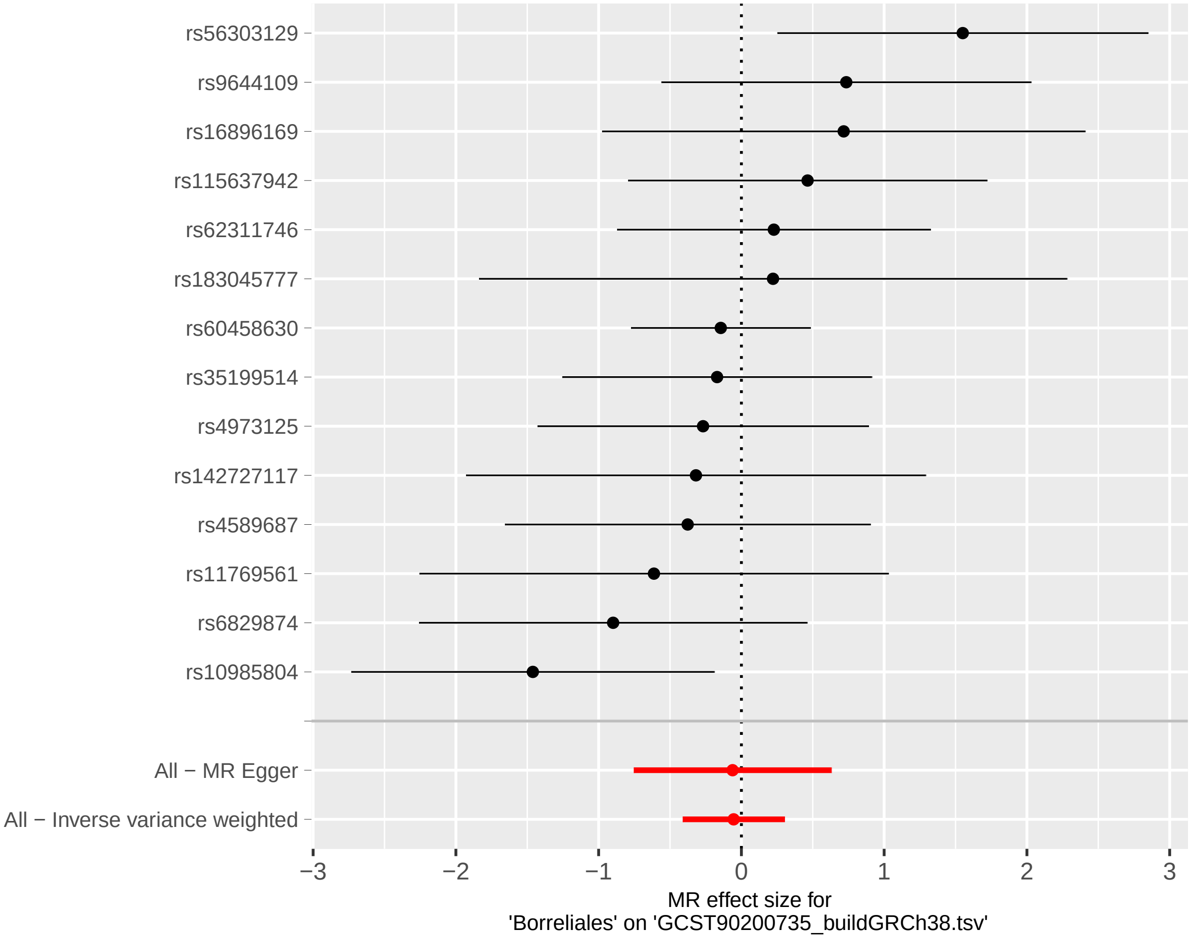
rs56303129
rs9644109
rs16896169
rs115637942
rs62311746
rs183045777
rs60458630
rs35199514
rs4973125
rs142727117
rs4589687
rs11769561
rs6829874
rs10985804
All − MR Egger
All − Inverse variance weighted
−3
−2
−1
0
1
2
3
MR effect size for
'Borreliales' on 'GCST90200735_buildGRCh38.tsv'

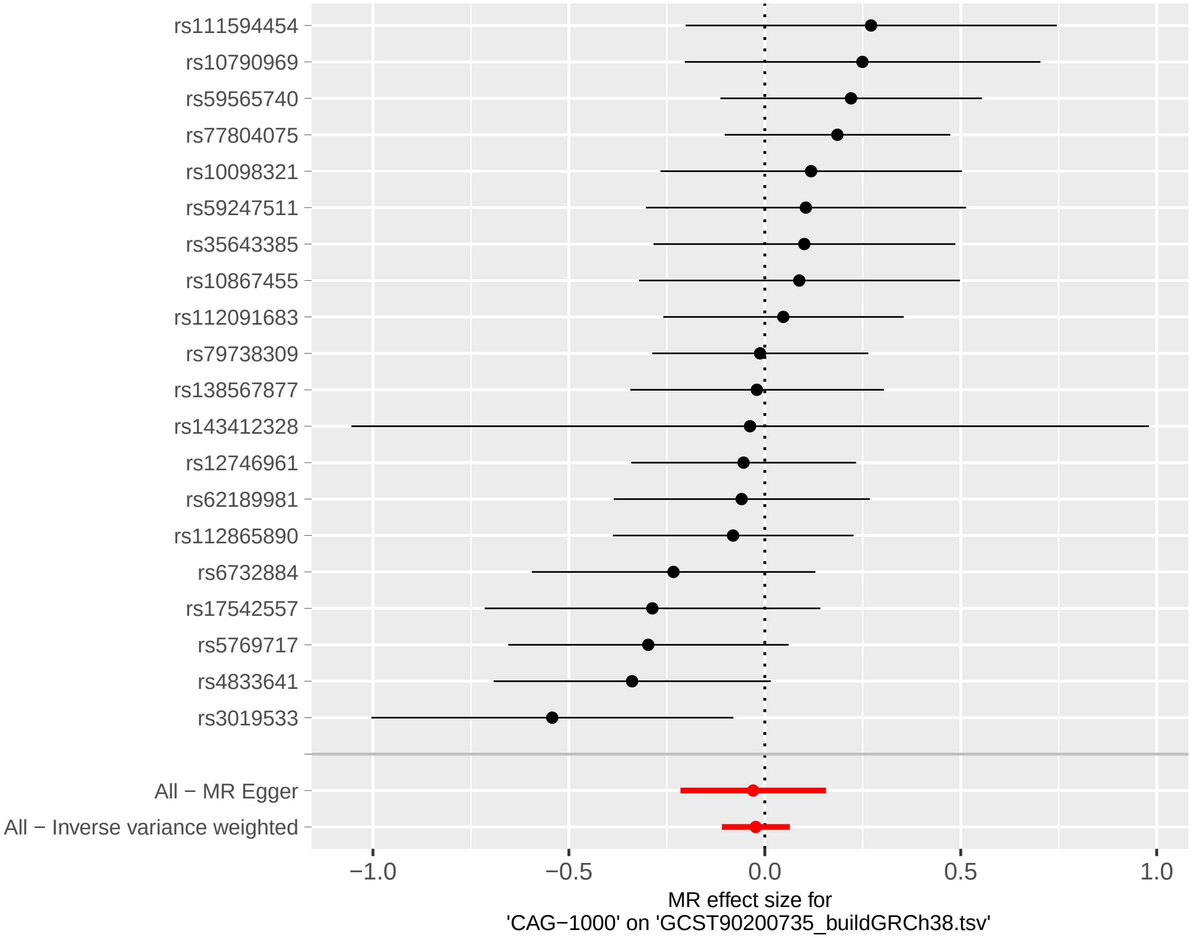
rs111594454
rs10790969
rs59565740
rs77804075
rs10098321
rs59247511
rs35643385
rs10867455
rs112091683
rs79738309
rs138567877
rs143412328
rs12746961
rs62189981
rs112865890
rs6732884
rs17542557
rs5769717
rs4833641
rs3019533
All − MR Egger
All − Inverse variance weighted
−1.0
−0.5
0.0
0.5
1.0
MR effect size for
'CAG−1000' on 'GCST90200735_buildGRCh38.tsv'

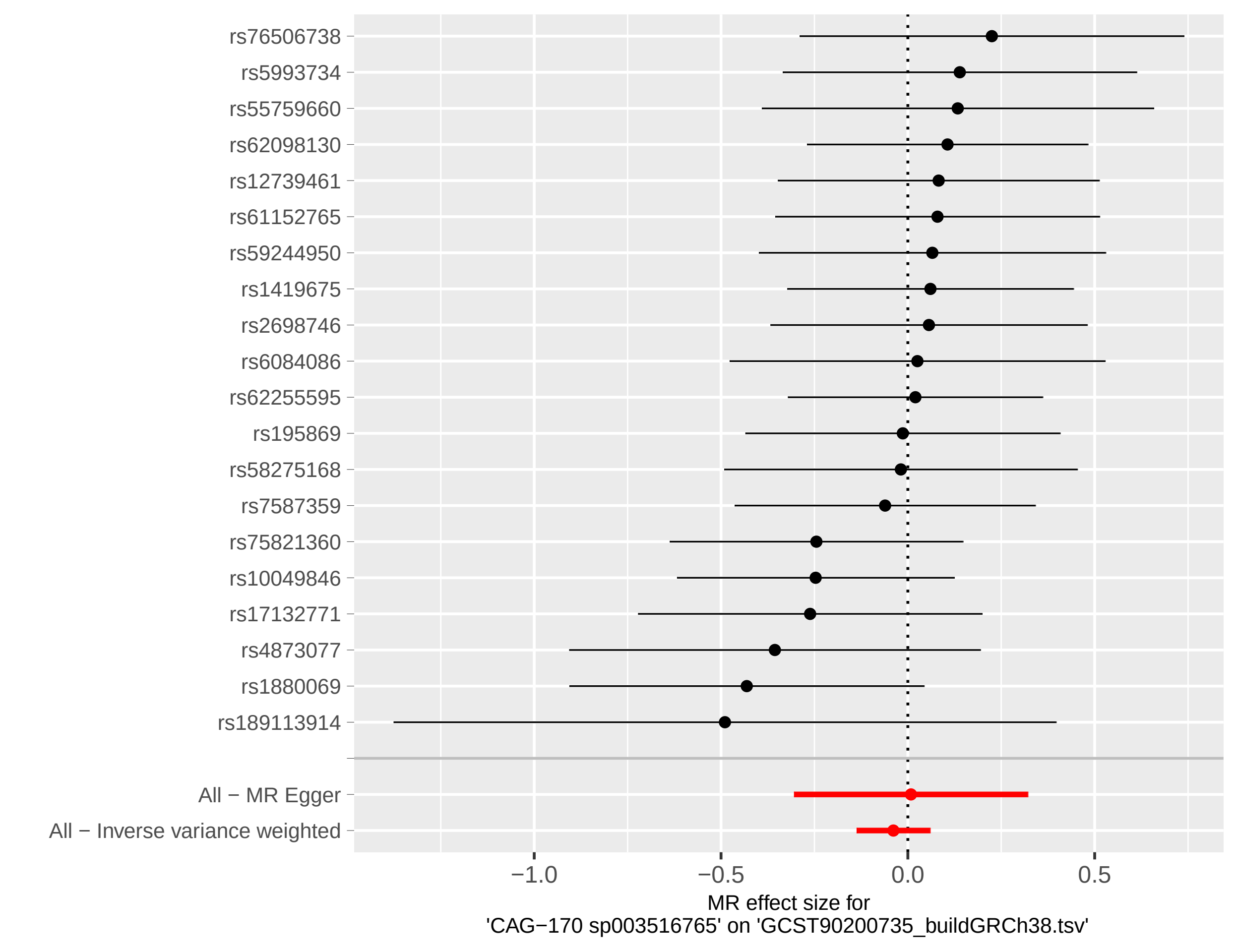
rs76506738
rs5993734
rs55759660
rs62098130
rs12739461
rs61152765
rs59244950
rs1419675
rs2698746
rs6084086
rs62255595
rs195869
rs58275168
rs7587359
rs75821360
rs10049846
rs17132771
rs4873077
rs1880069
rs189113914
All − MR Egger
All − Inverse variance weighted
−1.0
−0.5
0.0
0.5
MR effect size for
'CAG−170 sp003516765' on 'GCST90200735_buildGRCh38.tsv'

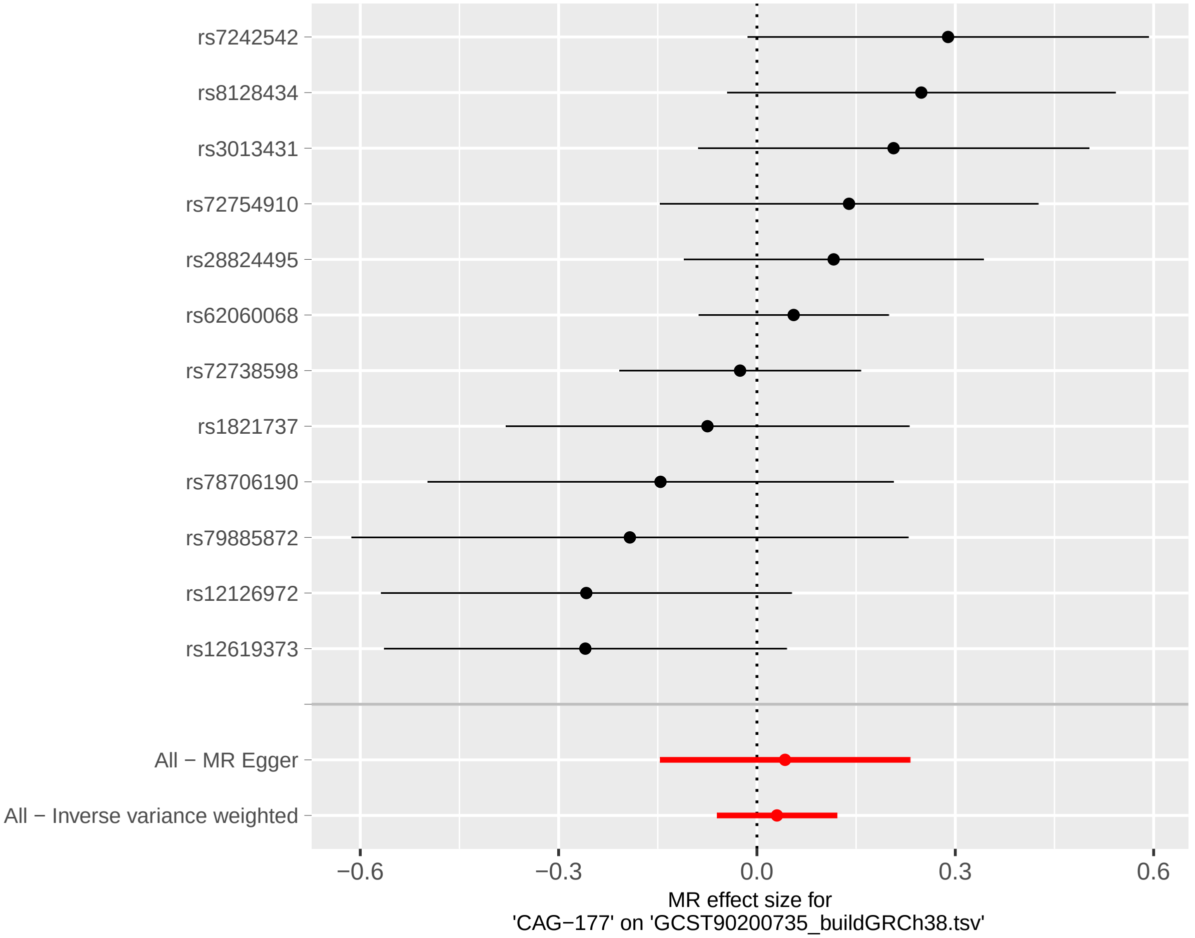

rs7242542
rs8128434
rs3013431
rs72754910
rs28824495
rs62060068
rs72738598
rs1821737
rs78706190
rs79885872
rs12126972
rs12619373
All − MR Egger
All − Inverse variance weighted
−0.6
−0.3
0.0
0.3
0.6
MR effect size for
'CAG−177' on 'GCST90200735_buildGRCh38.tsv'

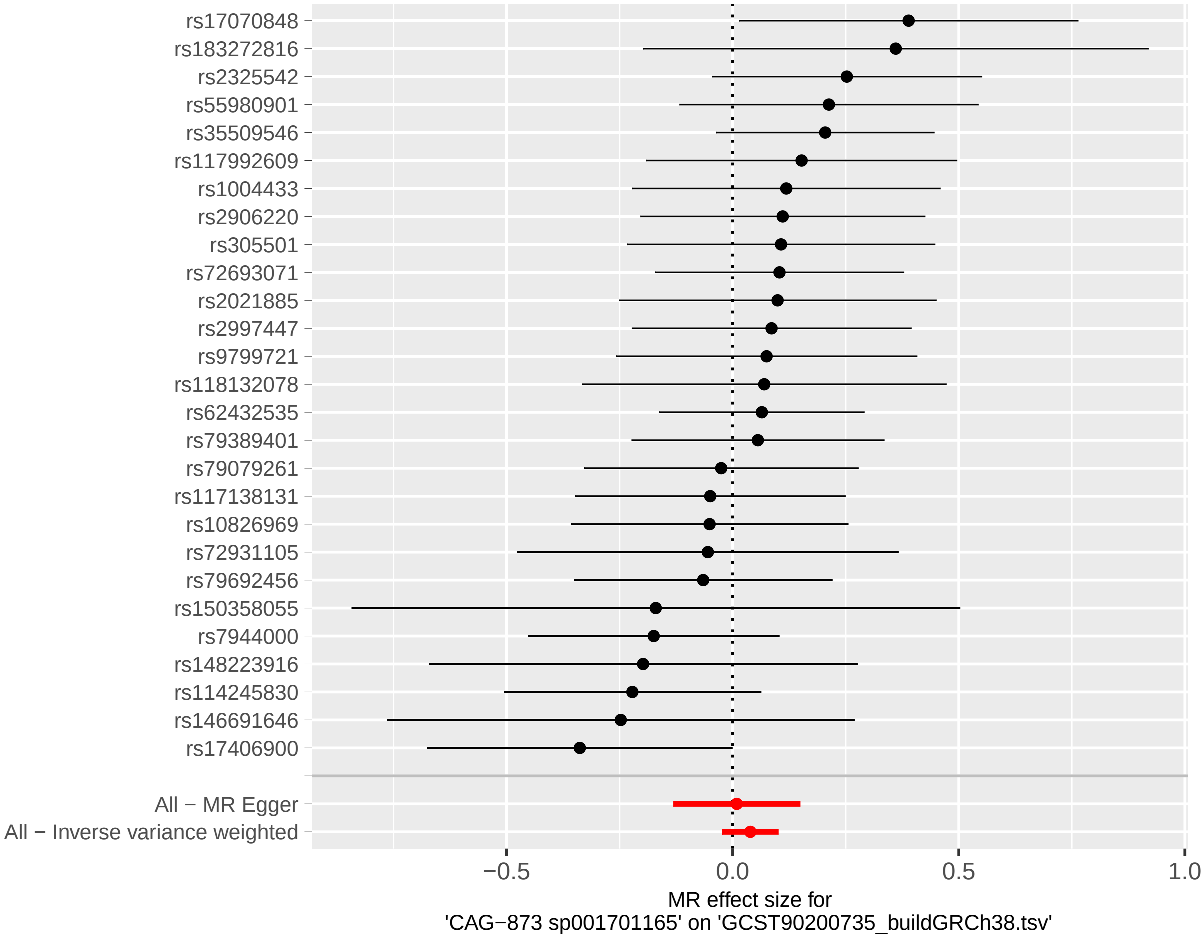

rs17070848
rs183272816
rs2325542
rs55980901
rs35509546
rs117992609
rs1004433
rs2906220
rs305501
rs72693071
rs2021885
rs2997447
rs9799721
rs118132078
rs62432535
rs79389401
rs79079261
rs117138131
rs10826969
rs72931105
rs79692456
rs150358055
rs7944000
rs148223916
rs114245830
rs146691646
rs17406900
All − MR Egger
All − Inverse variance weighted
−0.5
0.0
0.5
1.0
MR effect size for
'CAG−873 sp001701165' on 'GCST90200735_buildGRCh38.tsv'

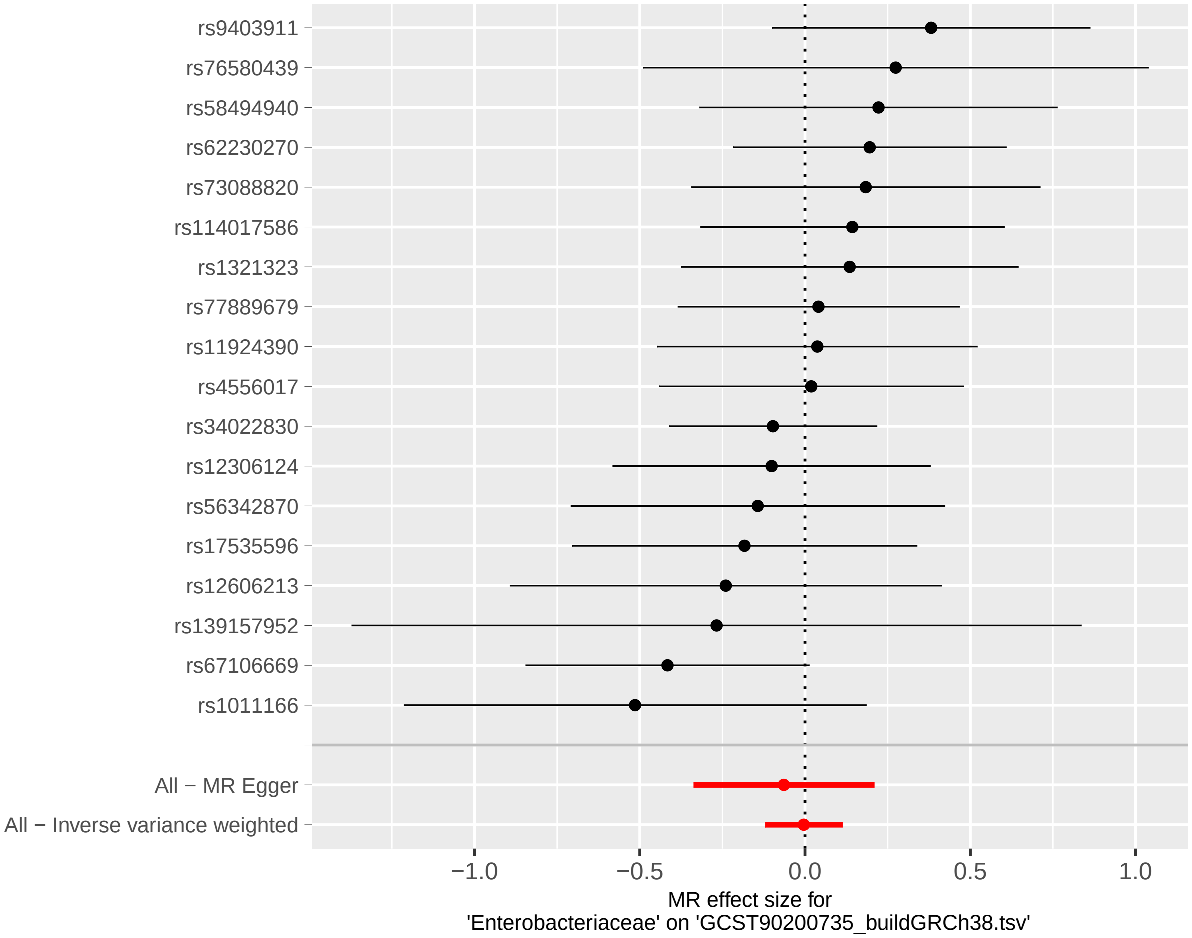

rs9403911
rs76580439
rs58494940
rs62230270
rs73088820
rs114017586
rs1321323
rs77889679
rs11924390
rs4556017
rs34022830
rs12306124
rs56342870
rs17535596
rs12606213
rs139157952
rs67106669
rs1011166
All − MR Egger
All − Inverse variance weighted
−1.0
−0.5
0.0
0.5
1.0
MR effect size for
'Enterobacteriaceae' on 'GCST90200735_buildGRCh38.tsv'

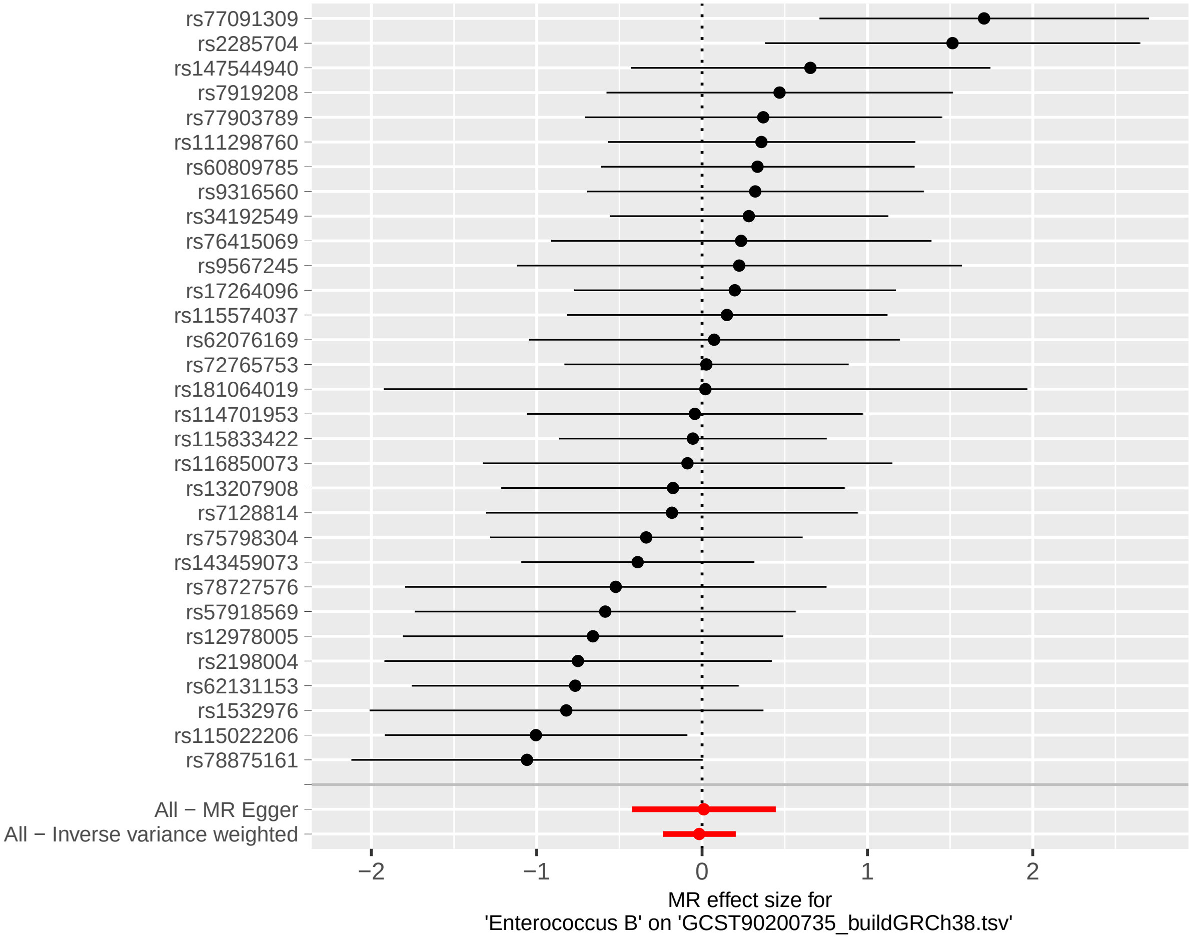
rs77091309
rs2285704
rs147544940
rs7919208
rs77903789
rs111298760
rs60809785
rs9316560
rs34192549
rs76415069
rs9567245
rs17264096
rs115574037
rs62076169
rs72765753
rs181064019
rs114701953
rs115833422
rs116850073
rs13207908
rs7128814
rs75798304
rs143459073
rs78727576
rs57918569
rs12978005
rs2198004
rs62131153
rs1532976
rs115022206
rs78875161
All − MR Egger
All − Inverse variance weighted
−2
−1
0
1
2
MR effect size for
'Enterococcus B' on 'GCST90200735_buildGRCh38.tsv'

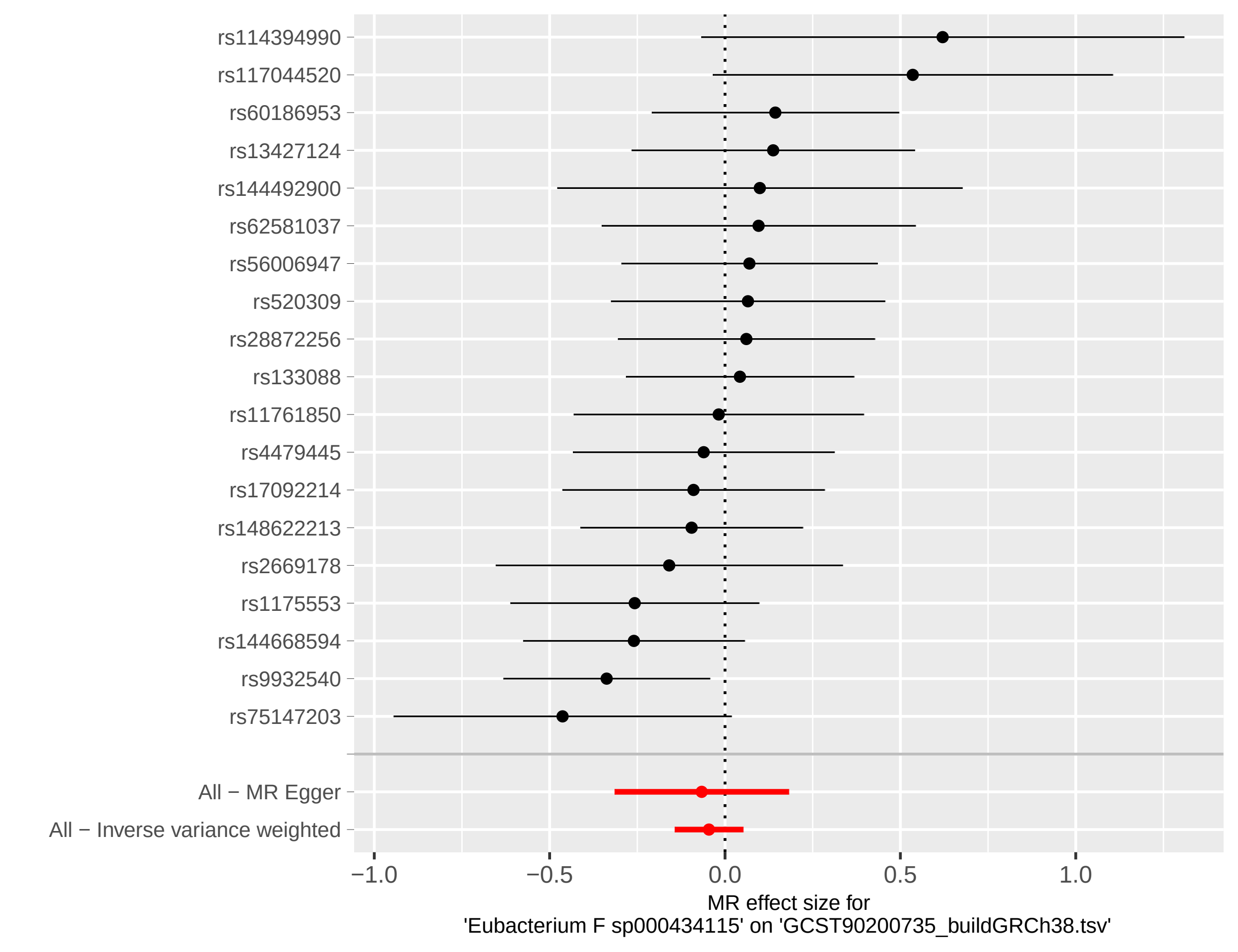
rs114394990
rs117044520
rs60186953
rs13427124
rs144492900
rs62581037
rs56006947
rs520309
rs28872256
rs133088
rs11761850
rs4479445
rs17092214
rs148622213
rs2669178
rs1175553
rs144668594
rs9932540
rs75147203
All − MR Egger
All − Inverse variance weighted
−1.0
−0.5
0.0
0.5
1.0
MR effect size for
'Eubacterium F sp000434115' on 'GCST90200735_buildGRCh38.tsv'

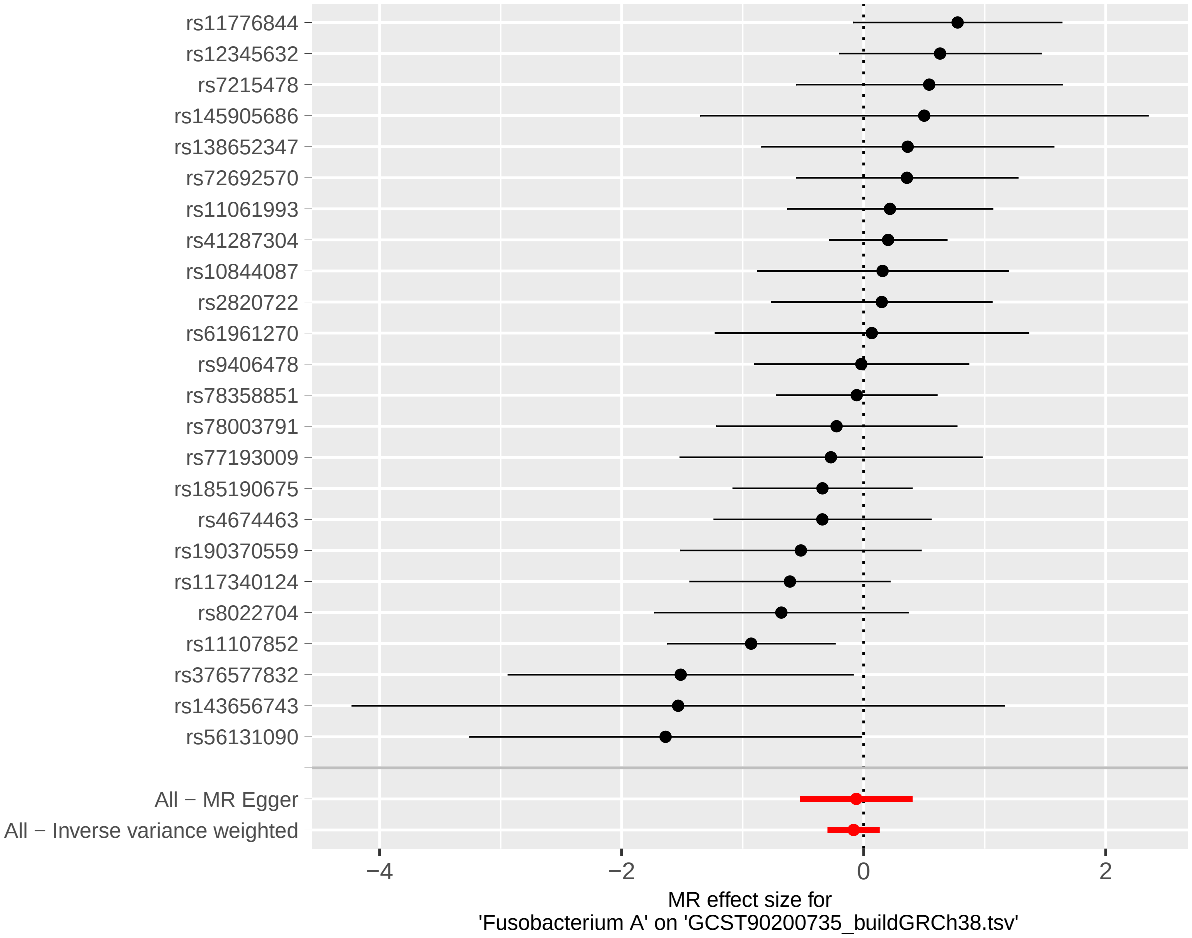

rs11776844
rs12345632
rs7215478
rs145905686
rs138652347
rs72692570
rs11061993
rs41287304
rs10844087
rs2820722
rs61961270
rs9406478
rs78358851
rs78003791
rs77193009
rs185190675
rs4674463
rs190370559
rs117340124
rs8022704
rs11107852
rs376577832
rs143656743
rs56131090
All − MR Egger
All − Inverse variance weighted
−4
−2
0
2
MR effect size for
'Fusobacterium A' on 'GCST90200735_buildGRCh38.tsv'

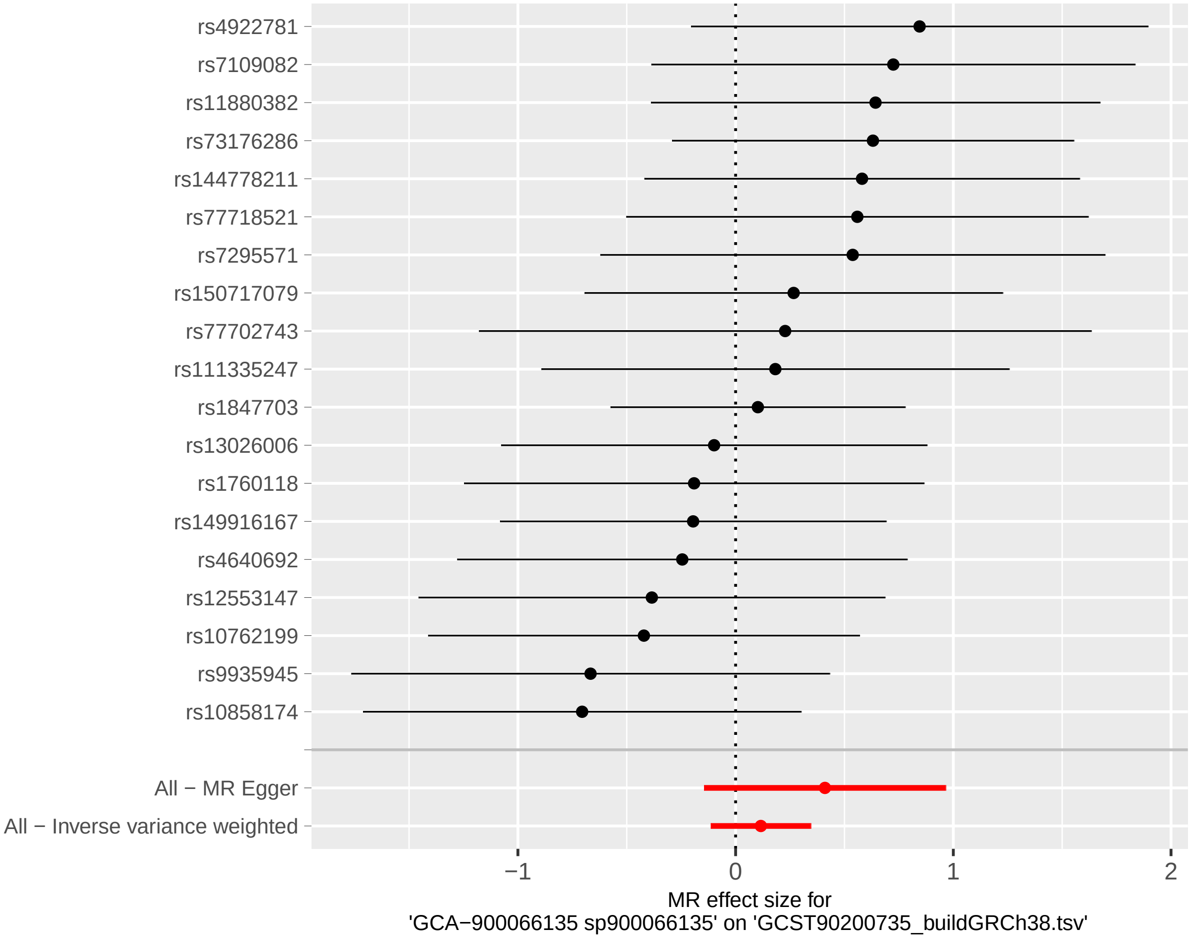
rs4922781
rs7109082
rs11880382
rs73176286
rs144778211
rs77718521
rs7295571
rs150717079
rs77702743
rs111335247
rs1847703
rs13026006
rs1760118
rs149916167
rs4640692
rs12553147
rs10762199
rs9935945
rs10858174
All − MR Egger
All − Inverse variance weighted
−1
0
1
2
MR effect size for
'GCA−900066135 sp900066135' on 'GCST90200735_buildGRCh38.tsv'

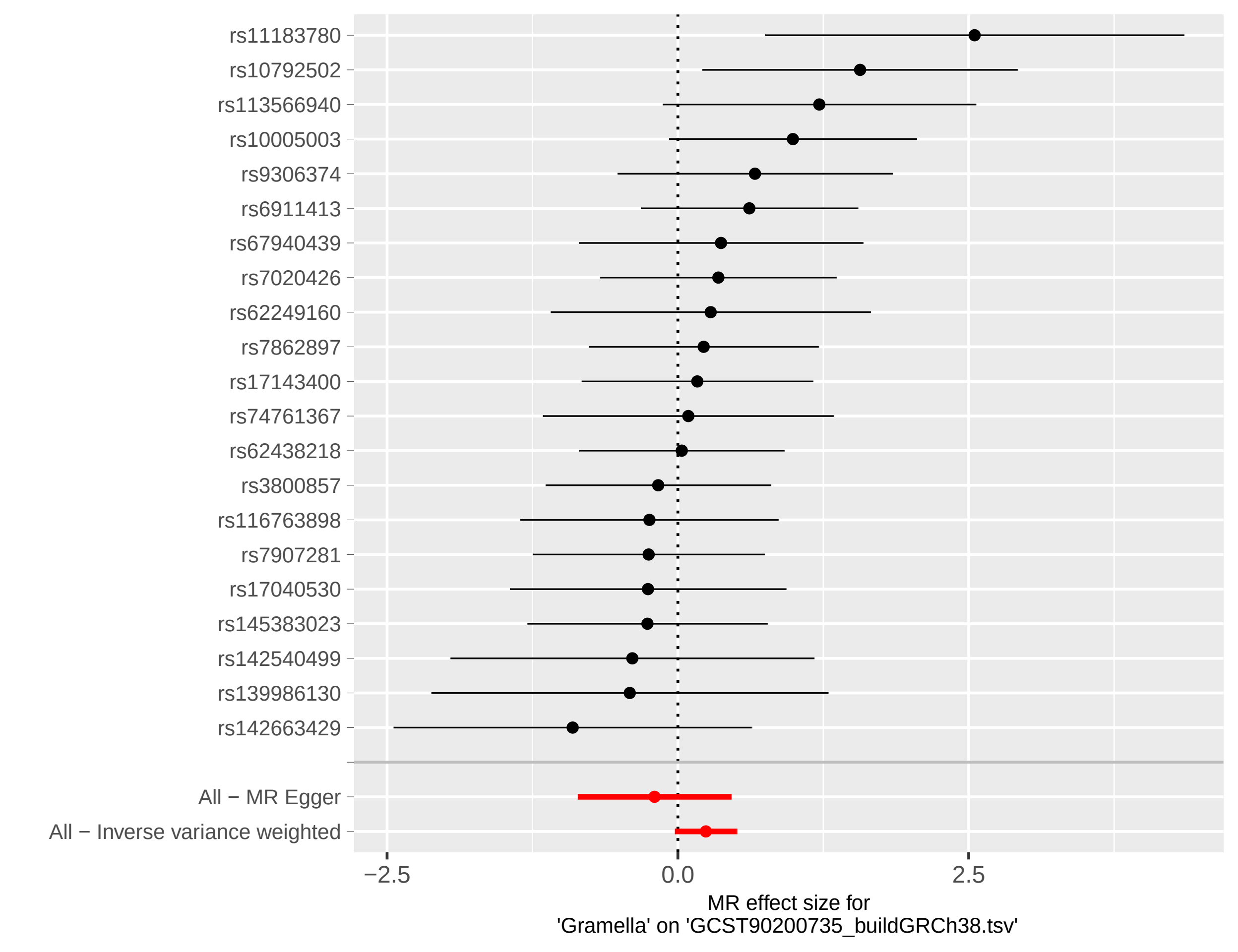

rs11183780
rs10792502
rs113566940
rs10005003
rs9306374
rs6911413
rs67940439
rs7020426
rs62249160
rs7862897
rs17143400
rs74761367
rs62438218
rs3800857
rs116763898
rs7907281
rs17040530
rs145383023
rs142540499
rs139986130
rs142663429
All − MR Egger
All − Inverse variance weighted
−2.5
0.0
2.5
MR effect size for
'Gramella' on 'GCST90200735_buildGRCh38.tsv'

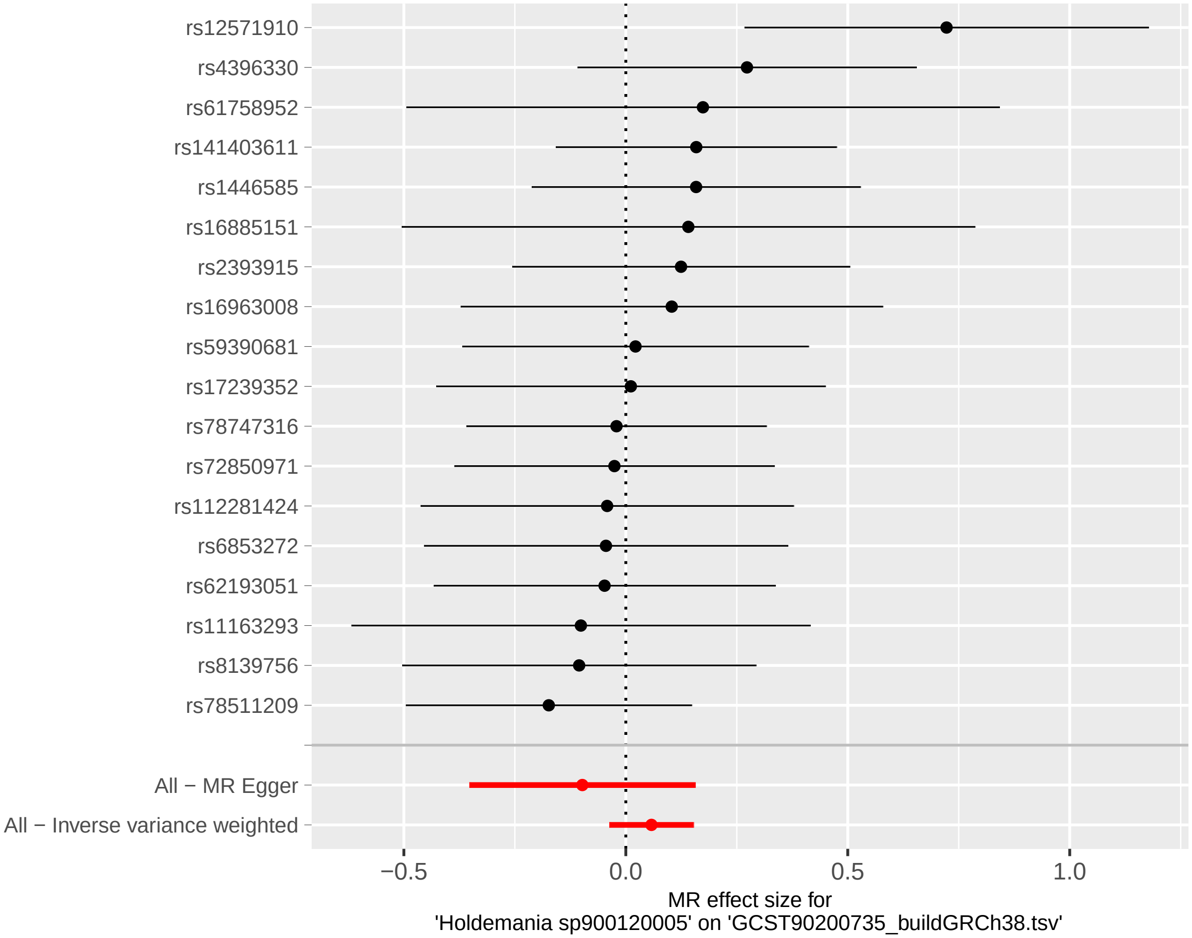
rs12571910
rs4396330
rs61758952
rs141403611
rs1446585
rs16885151
rs2393915
rs16963008
rs59390681
rs17239352
rs78747316
rs72850971
rs112281424
rs6853272
rs62193051
rs11163293
rs8139756
rs78511209
All − MR Egger
All − Inverse variance weighted
−0.5
0.0
0.5
1.0
MR effect size for
'Holdemania sp900120005' on 'GCST90200735_buildGRCh38.tsv'

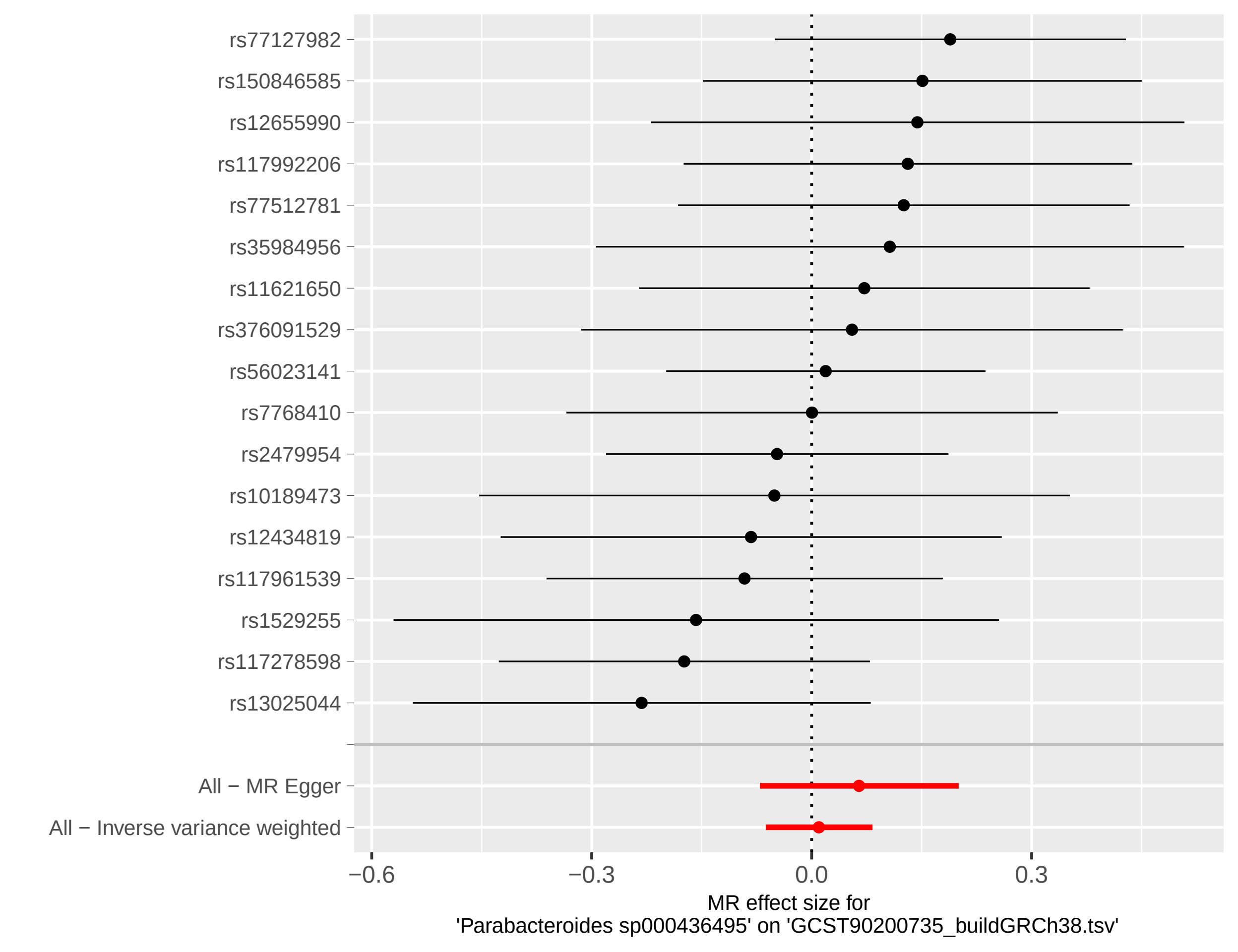

rs77127982
rs150846585
rs12655990
rs117992206
rs77512781
rs35984956
rs11621650
rs376091529
rs56023141
rs7768410
rs2479954
rs10189473
rs12434819
rs117961539
rs1529255
rs117278598
rs13025044
All − MR Egger
All − Inverse variance weighted
−0.6
−0.3
0.0
0.3
MR effect size for
'Parabacteroides sp000436495' on 'GCST90200735_buildGRCh38.tsv'

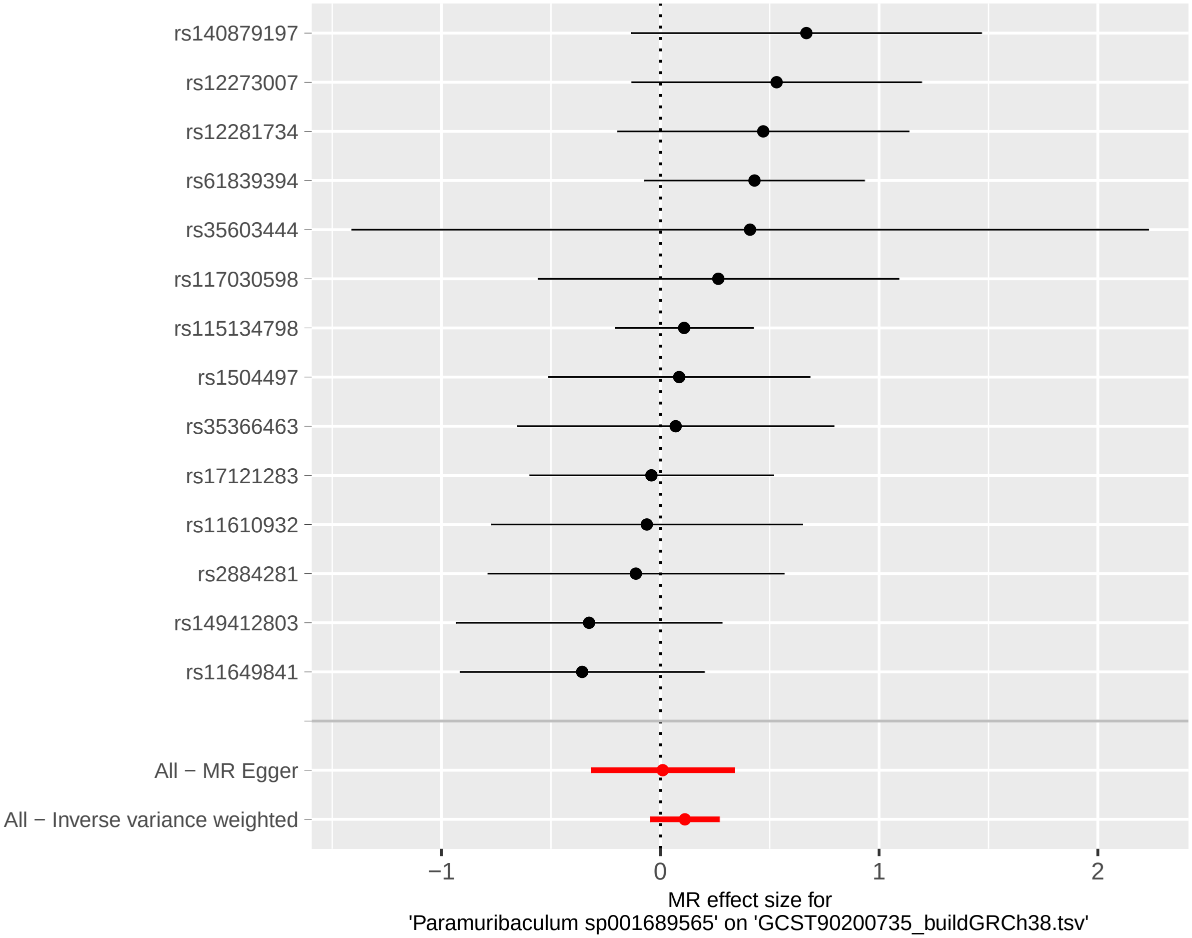
rs140879197
rs12273007
rs12281734
rs61839394
rs35603444
rs117030598
rs115134798
rs1504497
rs35366463
rs17121283
rs11610932
rs2884281
rs149412803
rs11649841
All − MR Egger
All − Inverse variance weighted
−1
0
1
2
MR effect size for
'Paramuribaculum sp001689565' on 'GCST90200735_buildGRCh38.tsv'

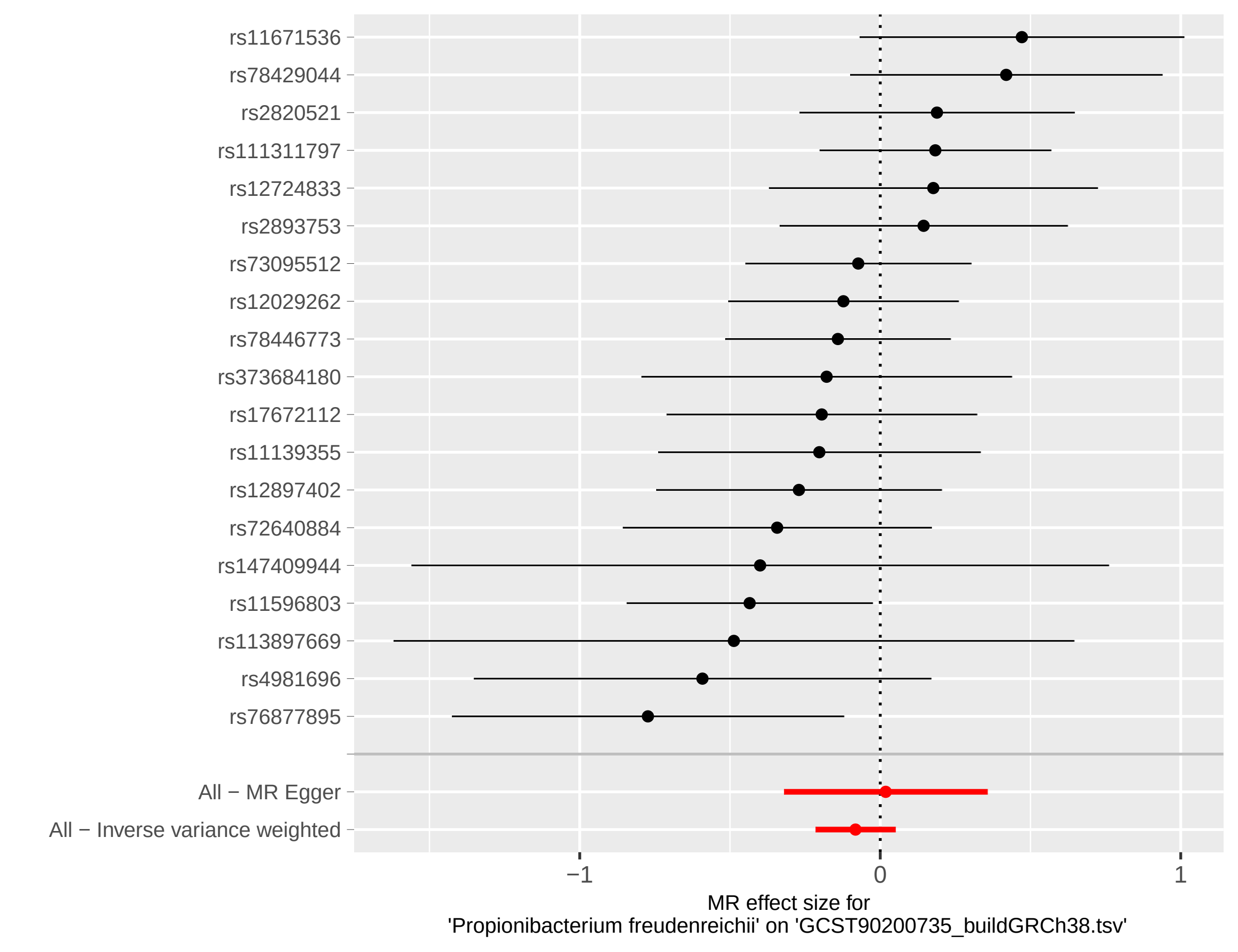
rs11671536
rs78429044
rs2820521
rs111311797
rs12724833
rs2893753
rs73095512
rs12029262
rs78446773
rs373684180
rs17672112
rs11139355
rs12897402
rs72640884
rs147409944
rs11596803
rs113897669
rs4981696
rs76877895
All − MR Egger
All − Inverse variance weighted
−1
0
1
MR effect size for
'Propionibacterium freudenreichii' on 'GCST90200735_buildGRCh38.tsv'

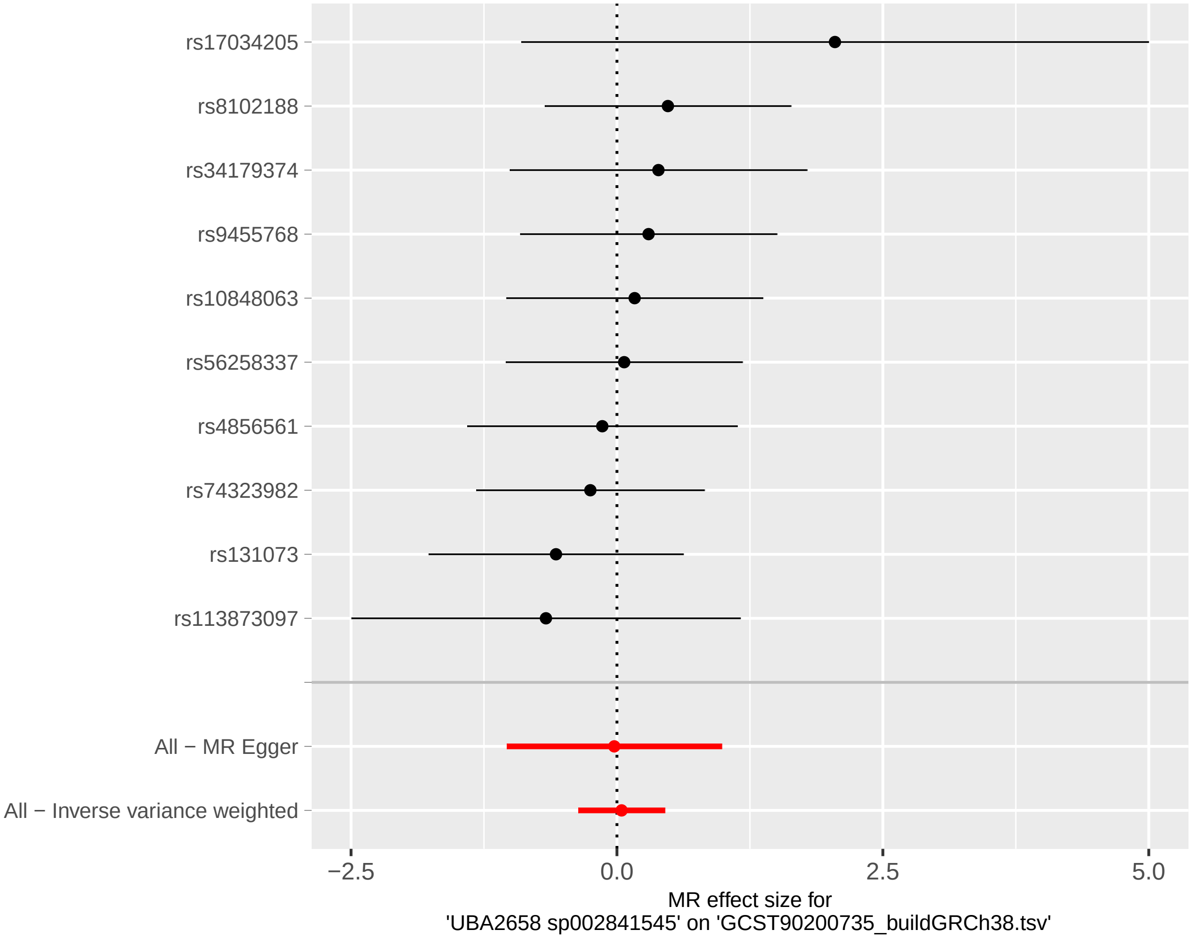
rs17034205
rs8102188
rs34179374
rs9455768
rs10848063
rs56258337
rs4856561
rs74323982
rs131073
rs113873097
All − MR Egger
All − Inverse variance weighted
−2.5
0.0
2.5
5.0
MR effect size for
'UBA2658 sp002841545' on 'GCST90200735_buildGRCh38.tsv'

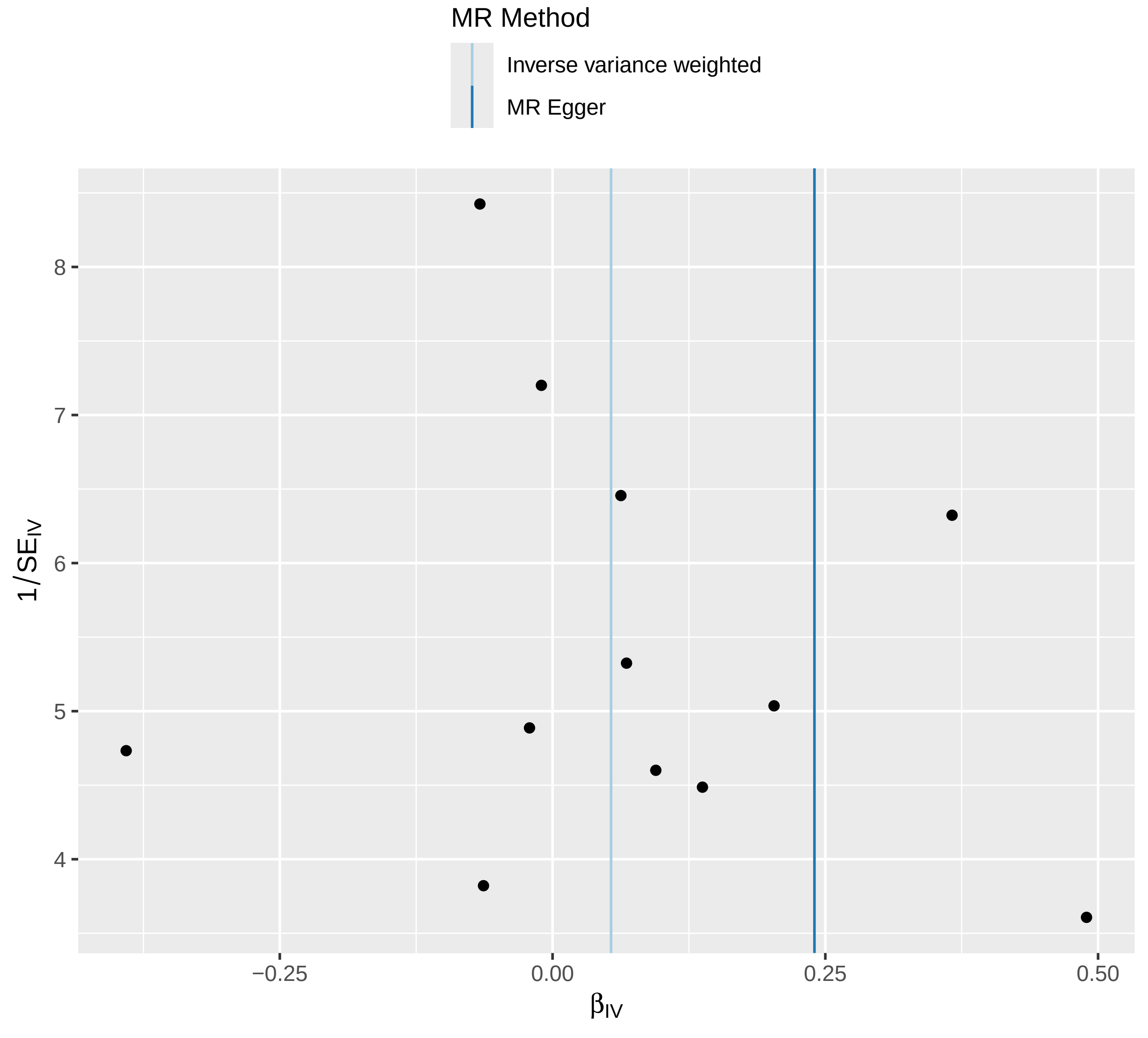

MR Method
Inverse variance weighted
MR Egger
8
7
6
5
4
1/SE_IV
−0.25
0.00
0.25
0.50
β_IV

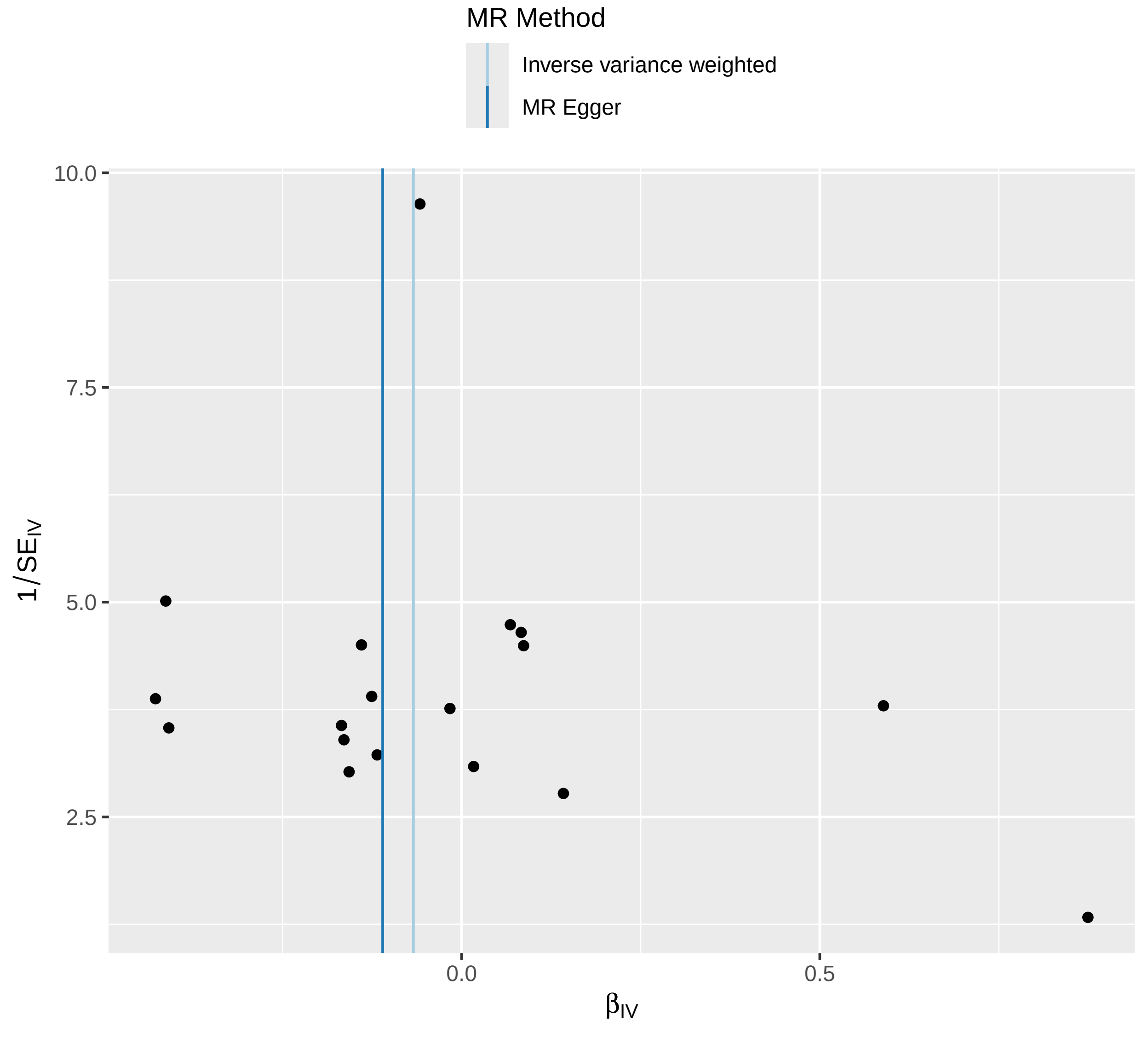

MR Method
Inverse variance weighted
MR Egger
10.0
7.5
5.0
2.5
1/SE$_{IV}$
0.0
0.5
β$_{IV}$

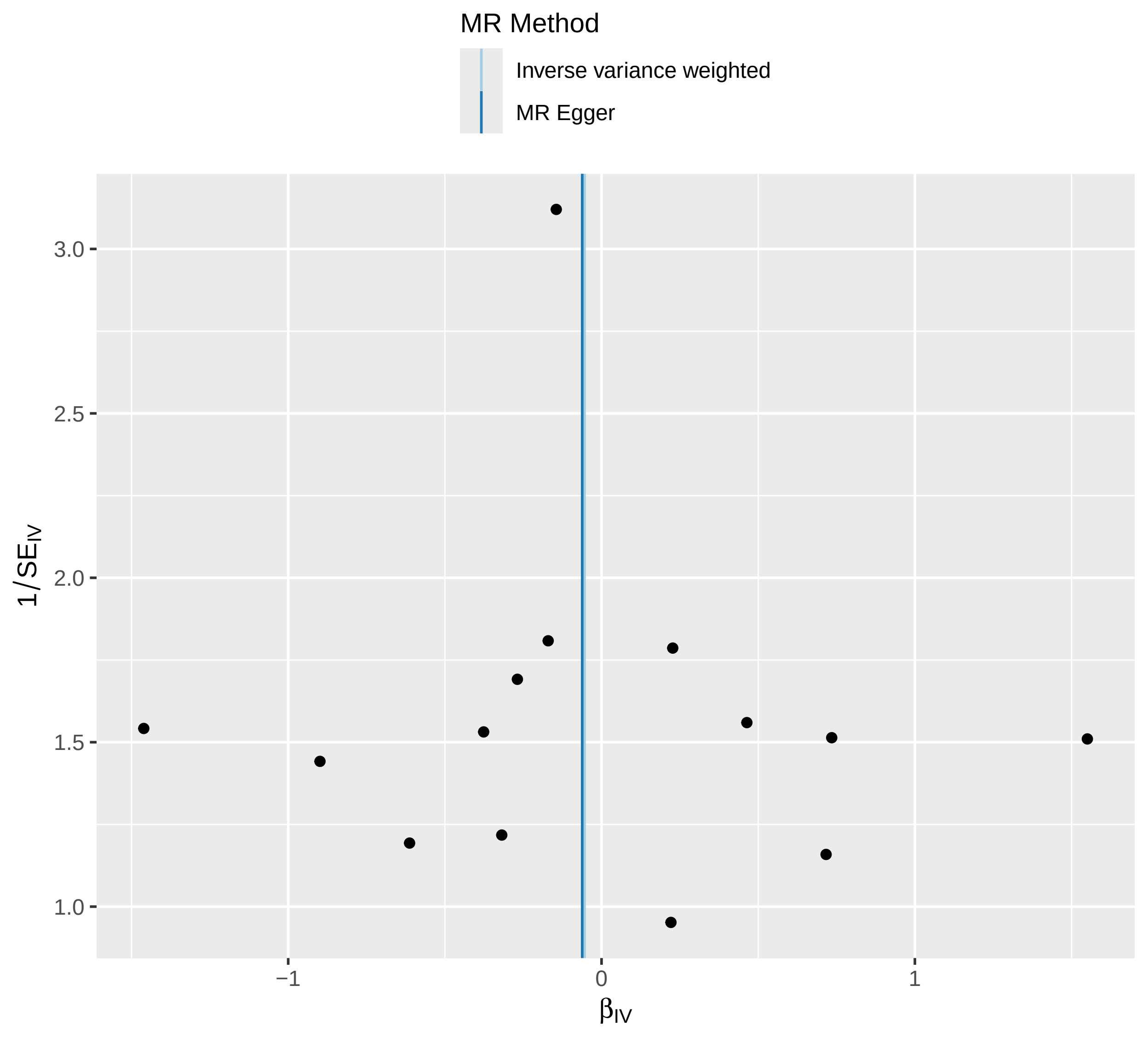

MR Method
Inverse variance weighted
MR Egger
3.0
2.5
2.0
1.5
1.0
1/SE_IV
−1
0
1
β_IV

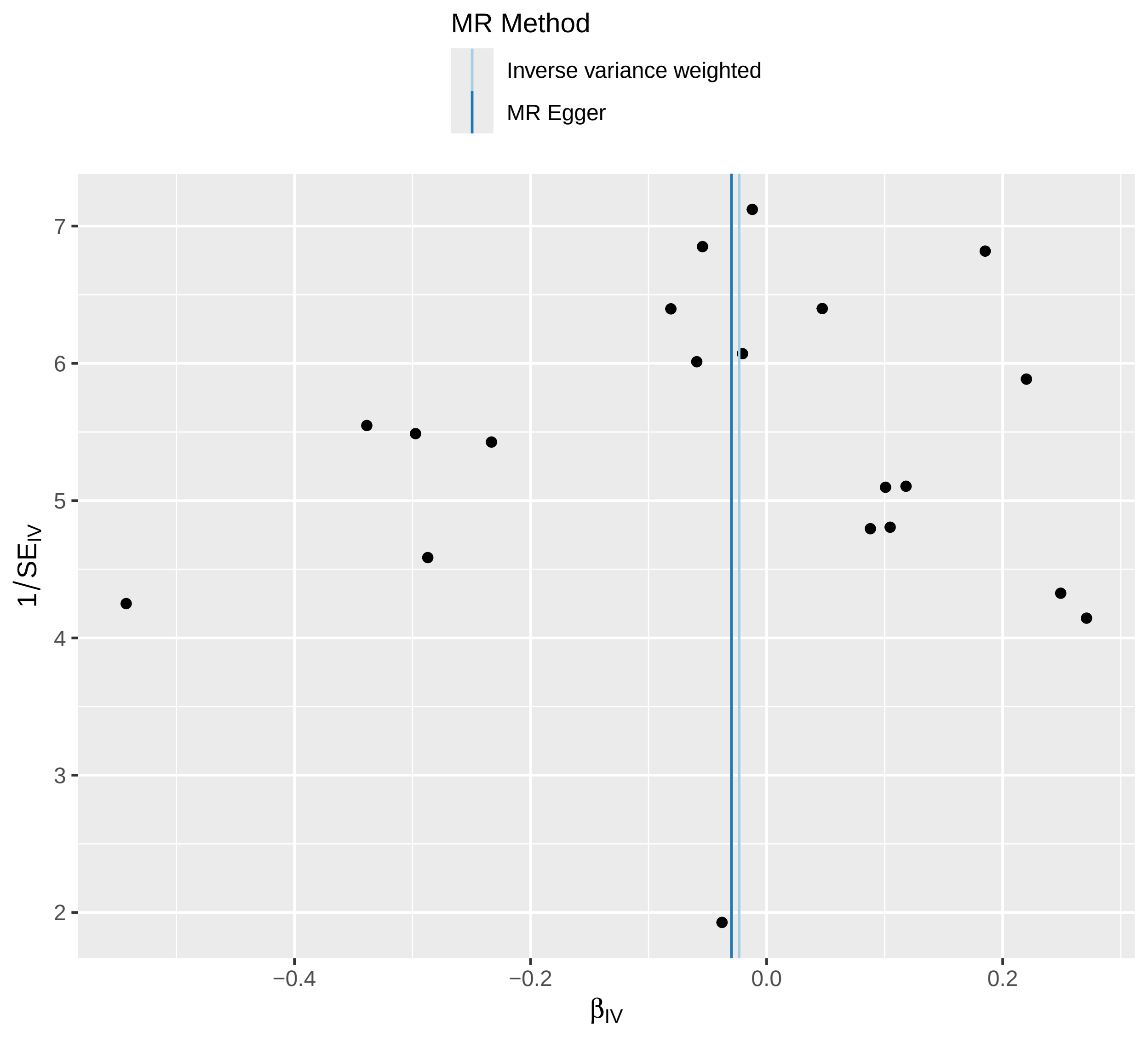

MR Method
Inverse variance weighted
MR Egger
1/SE$_{IV}$
β_{IV}
7
6
5
4
3
2
−0.4
−0.2
0.0
0.2

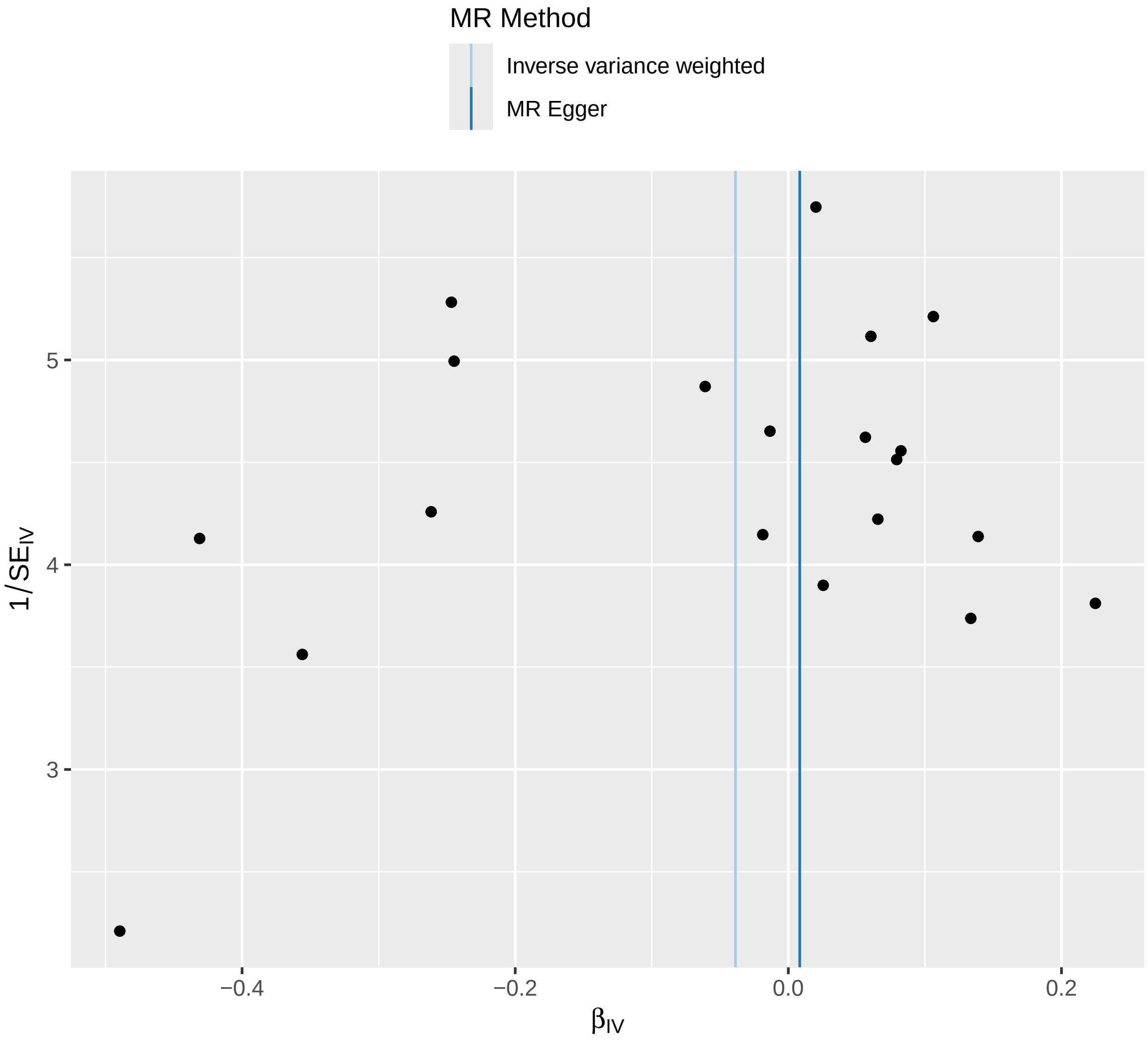

MR Method
Inverse variance weighted
MR Egger
5
4
3
1/SE_IV
−0.4
−0.2
0.0
0.2
β_IV

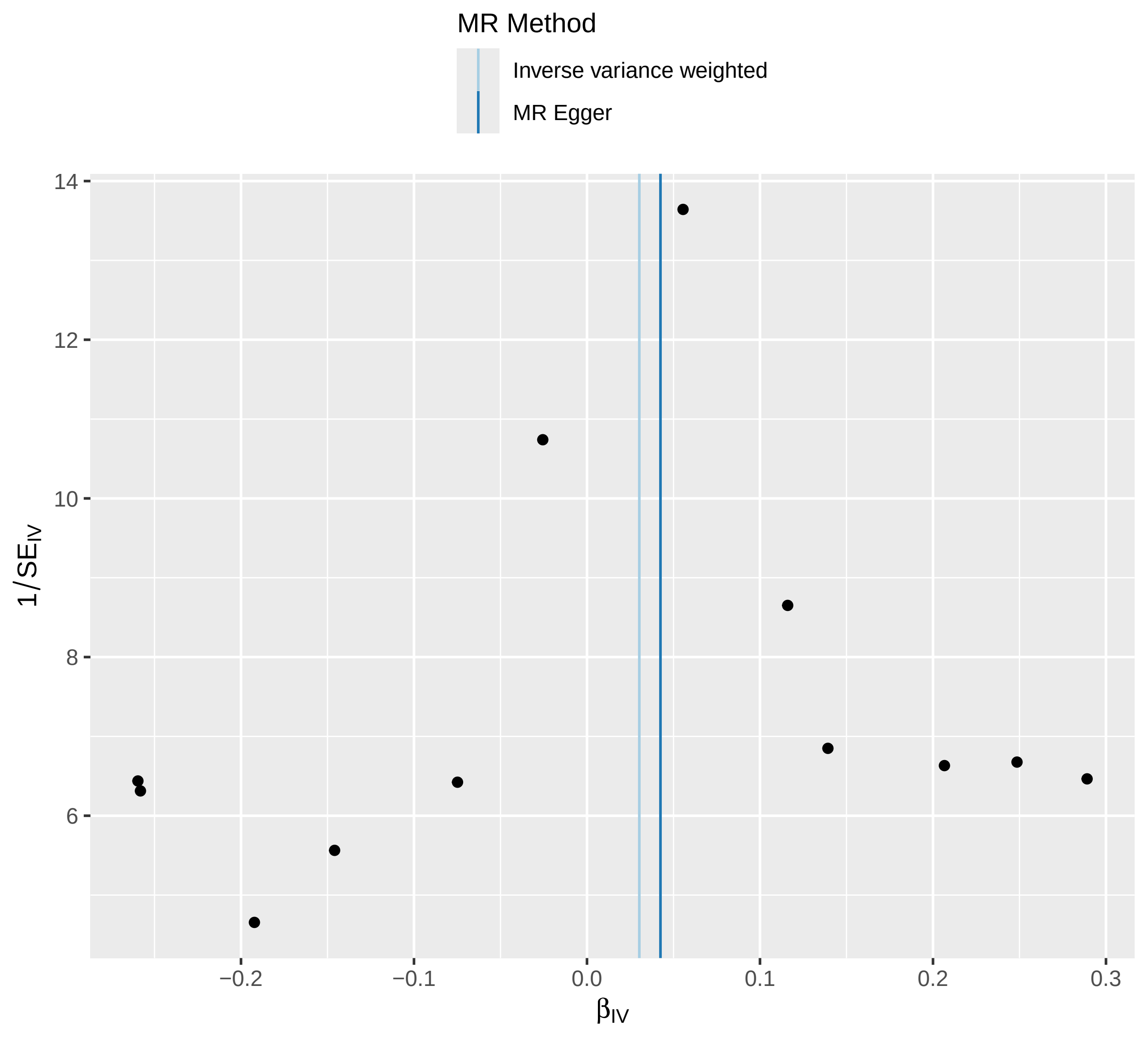
MR Method
Inverse variance weighted
MR Egger
14
12
10
8
6
1/SE_IV
−0.2
−0.1
0.0
0.1
0.2
0.3
β_IV

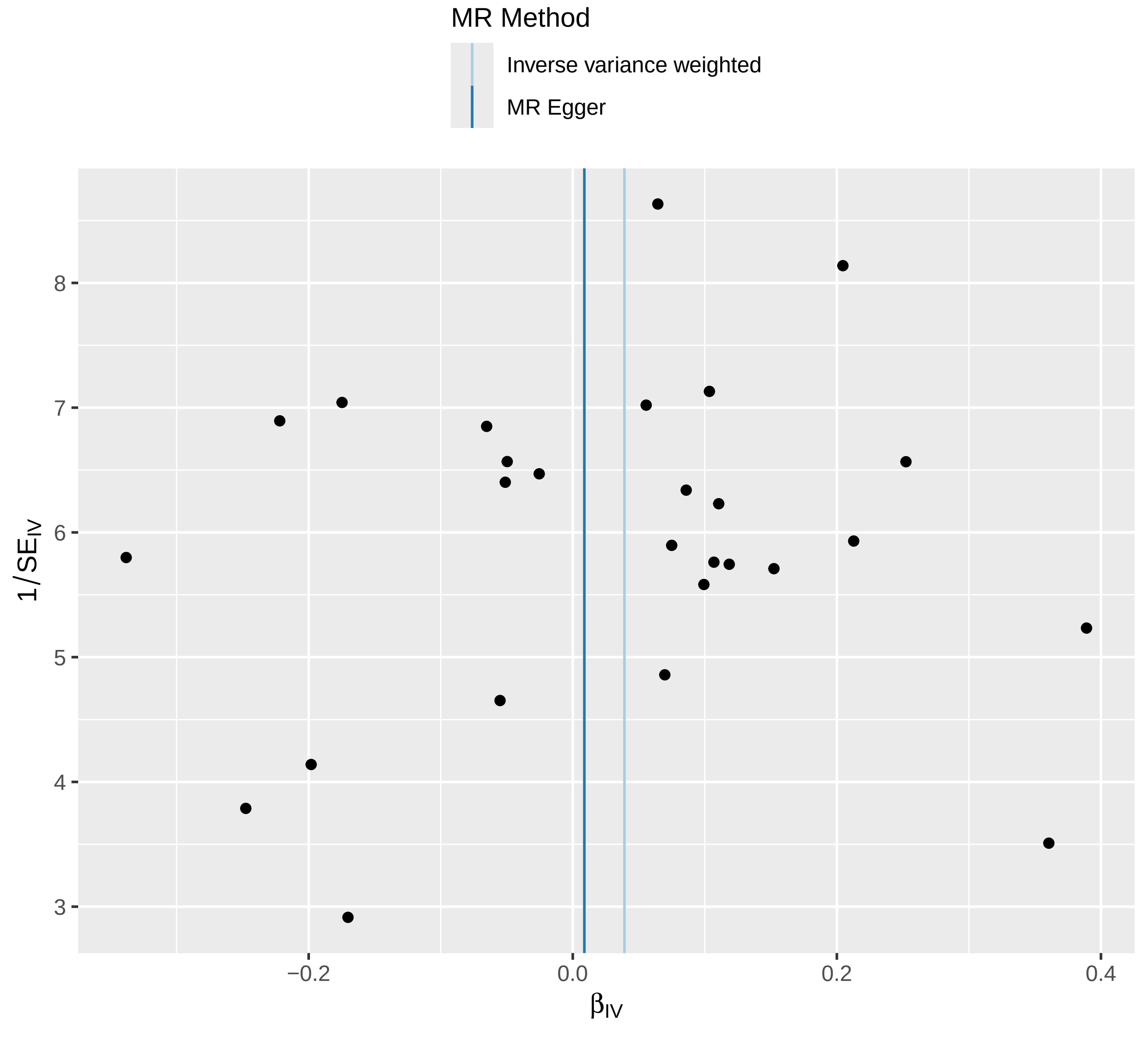

MR Method
Inverse variance weighted
MR Egger
1/SE_IV
β_IV
−0.2
0.0
0.2
0.4
3
4
5
6
7
8

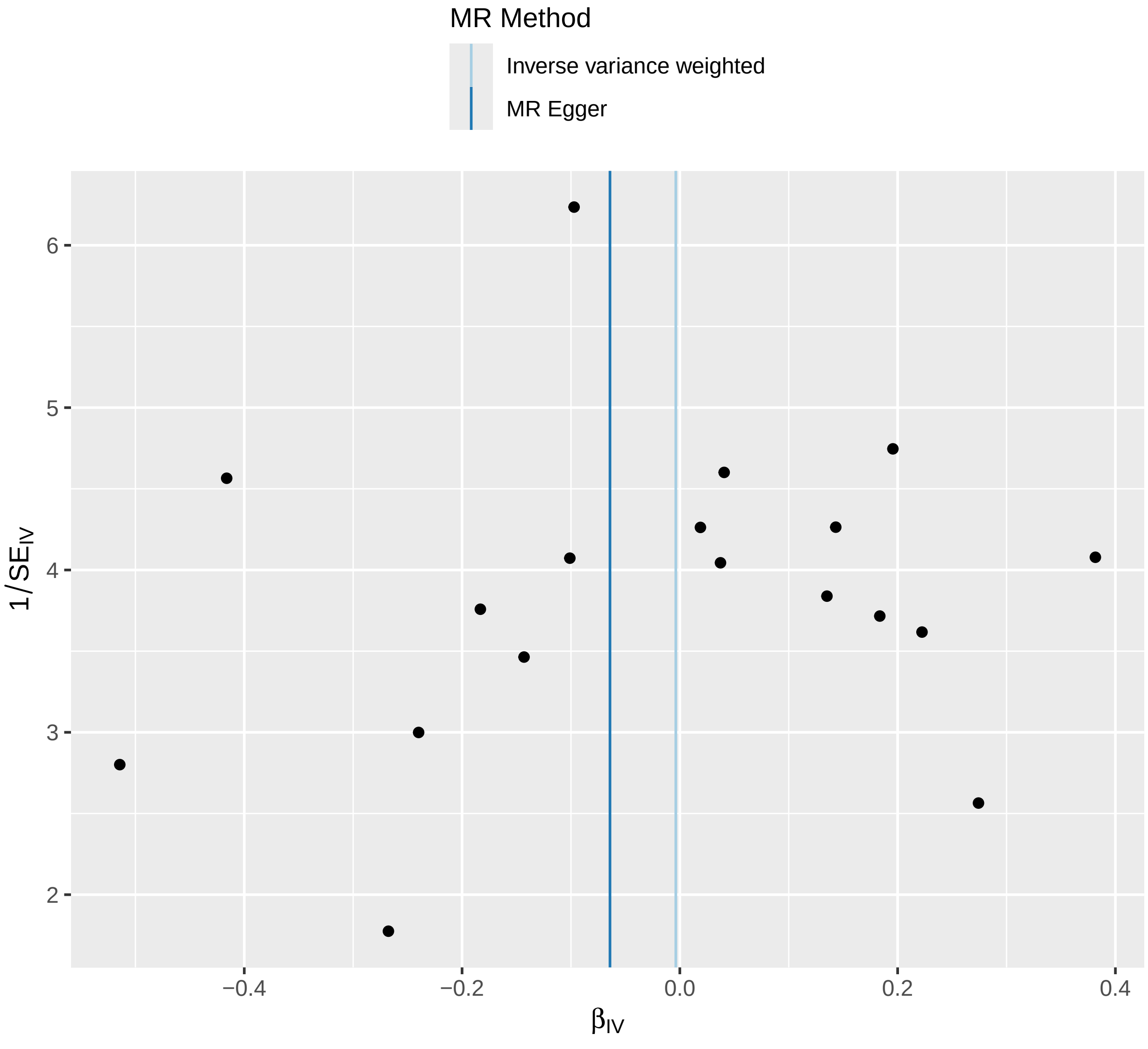
MR Method
Inverse variance weighted
MR Egger
6
5
4
3
2
1/SE_IV
−0.4
−0.2
0.0
0.2
0.4
β_IV

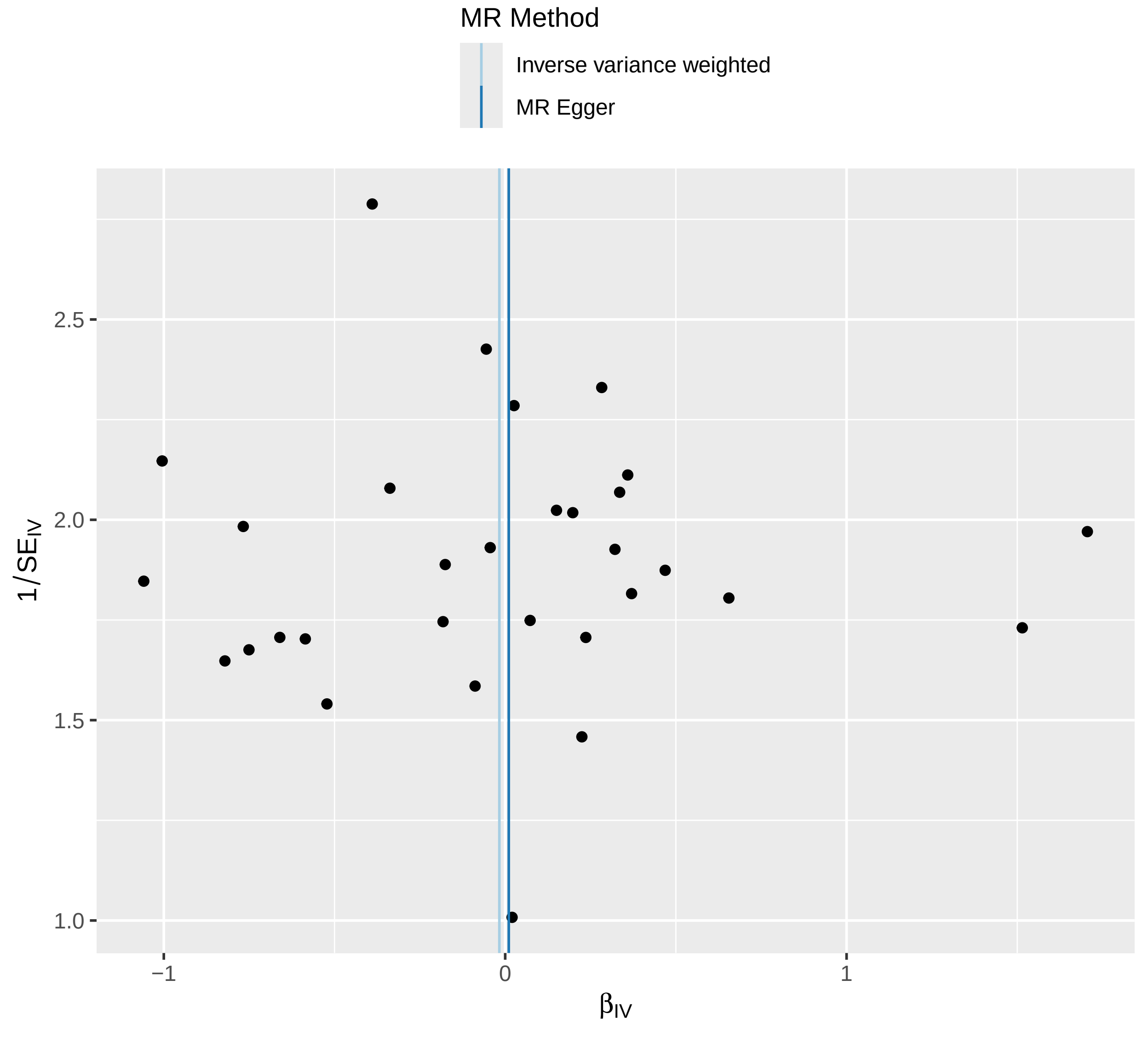
MR Method
Inverse variance weighted
MR Egger
2.5
2.0
1.5
1.0
1/SE_IV
−1
0
1
β_IV

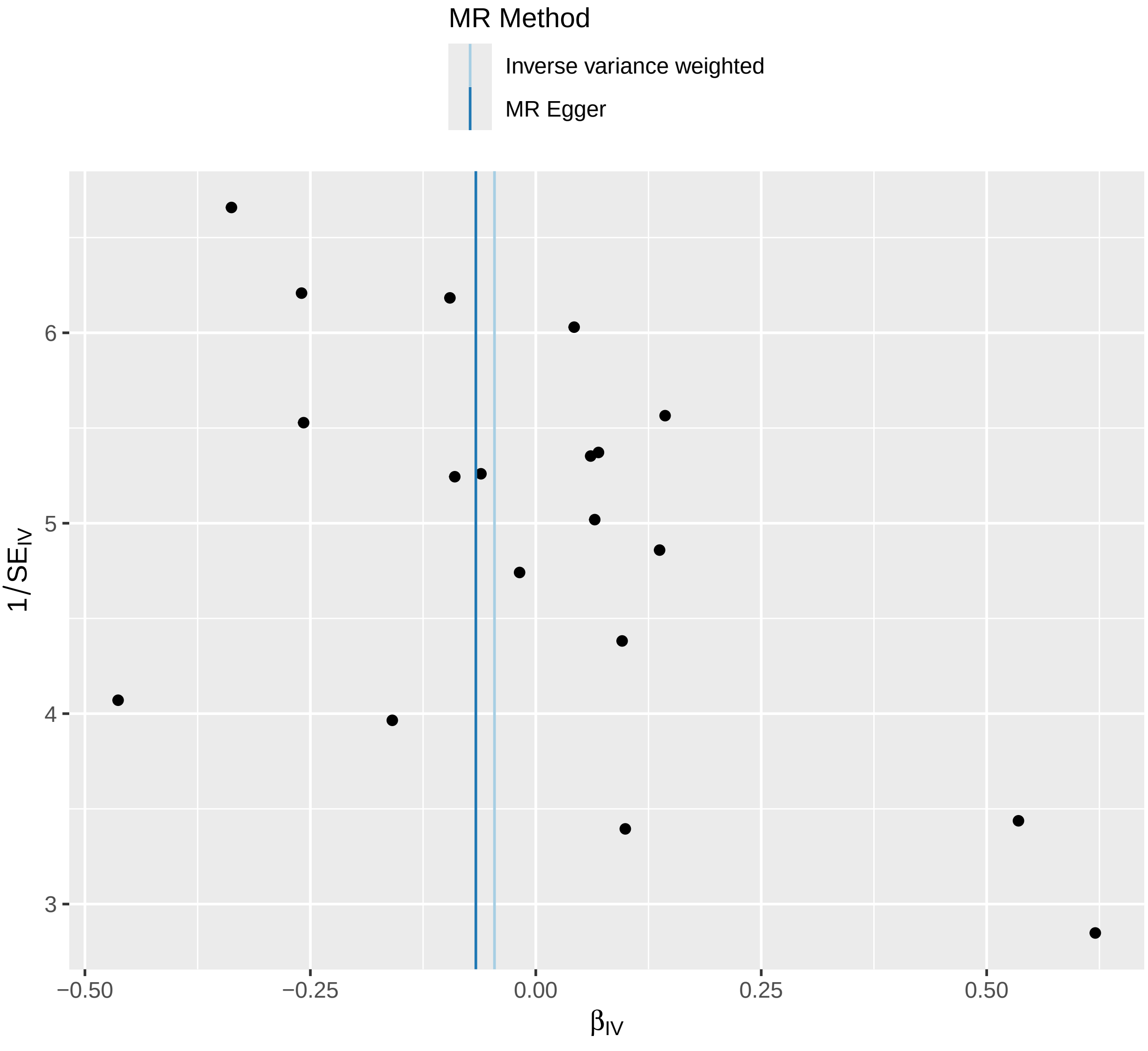

MR Method
Inverse variance weighted
MR Egger
6
5
4
3
1/SE_IV
−0.50
−0.25
0.00
0.25
0.50
β_IV

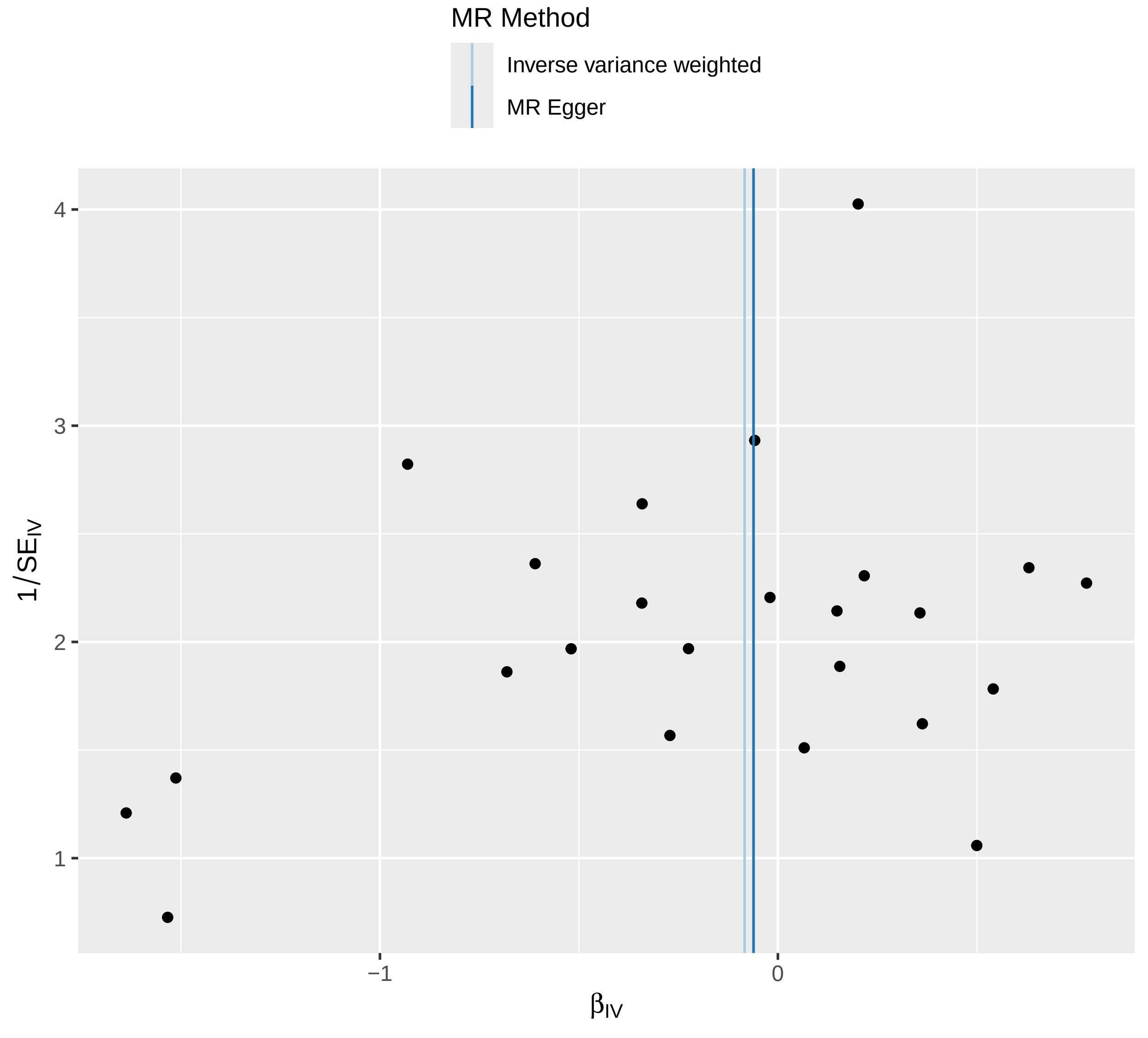
MR Method
Inverse variance weighted
MR Egger
4
3
2
1
−1
0
β_{IV}
$1/SE_{IV}$

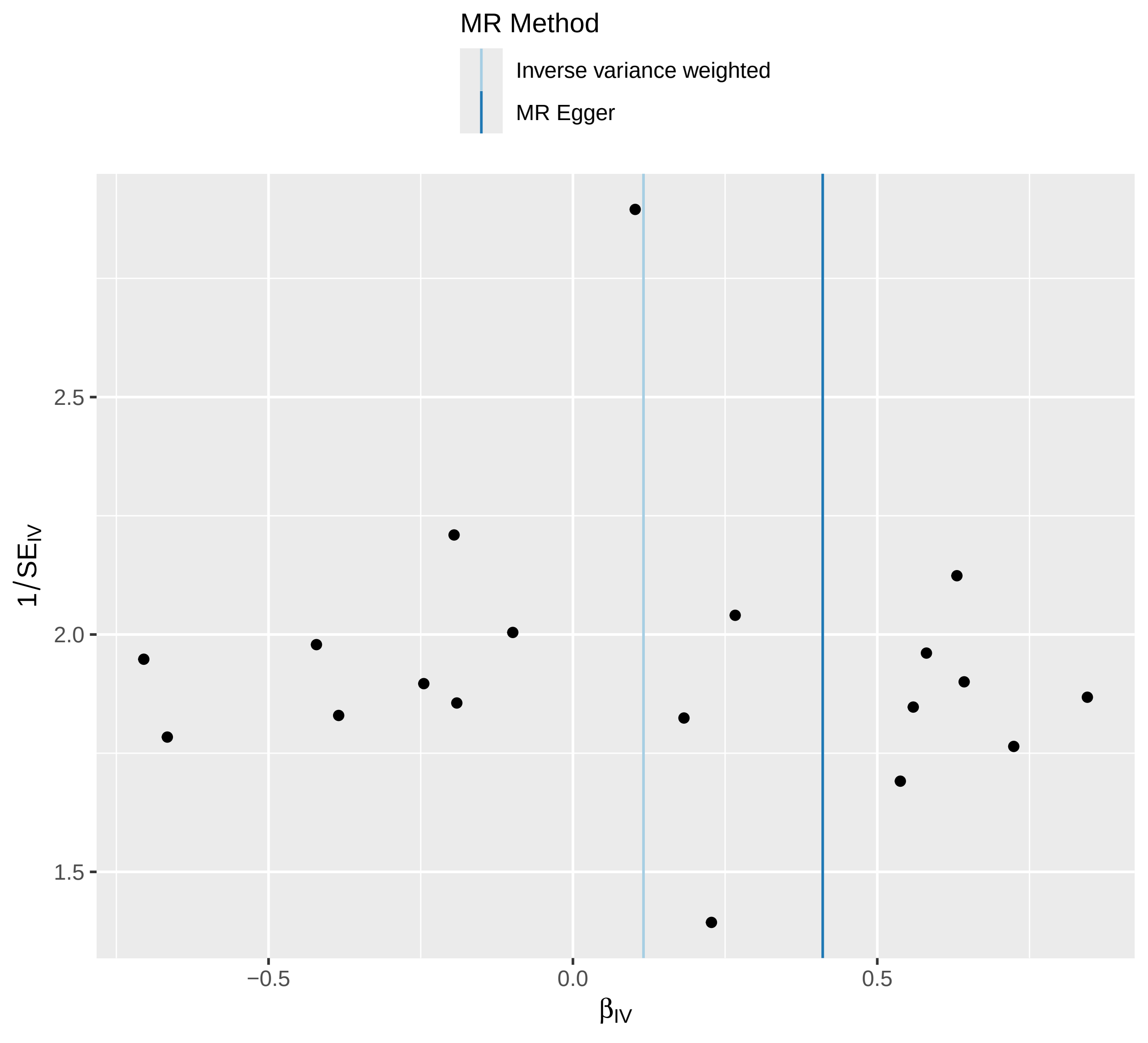

MR Method
Inverse variance weighted
MR Egger
2.5
2.0
1.5
1/SE_IV
−0.5
0.0
0.5
β_IV

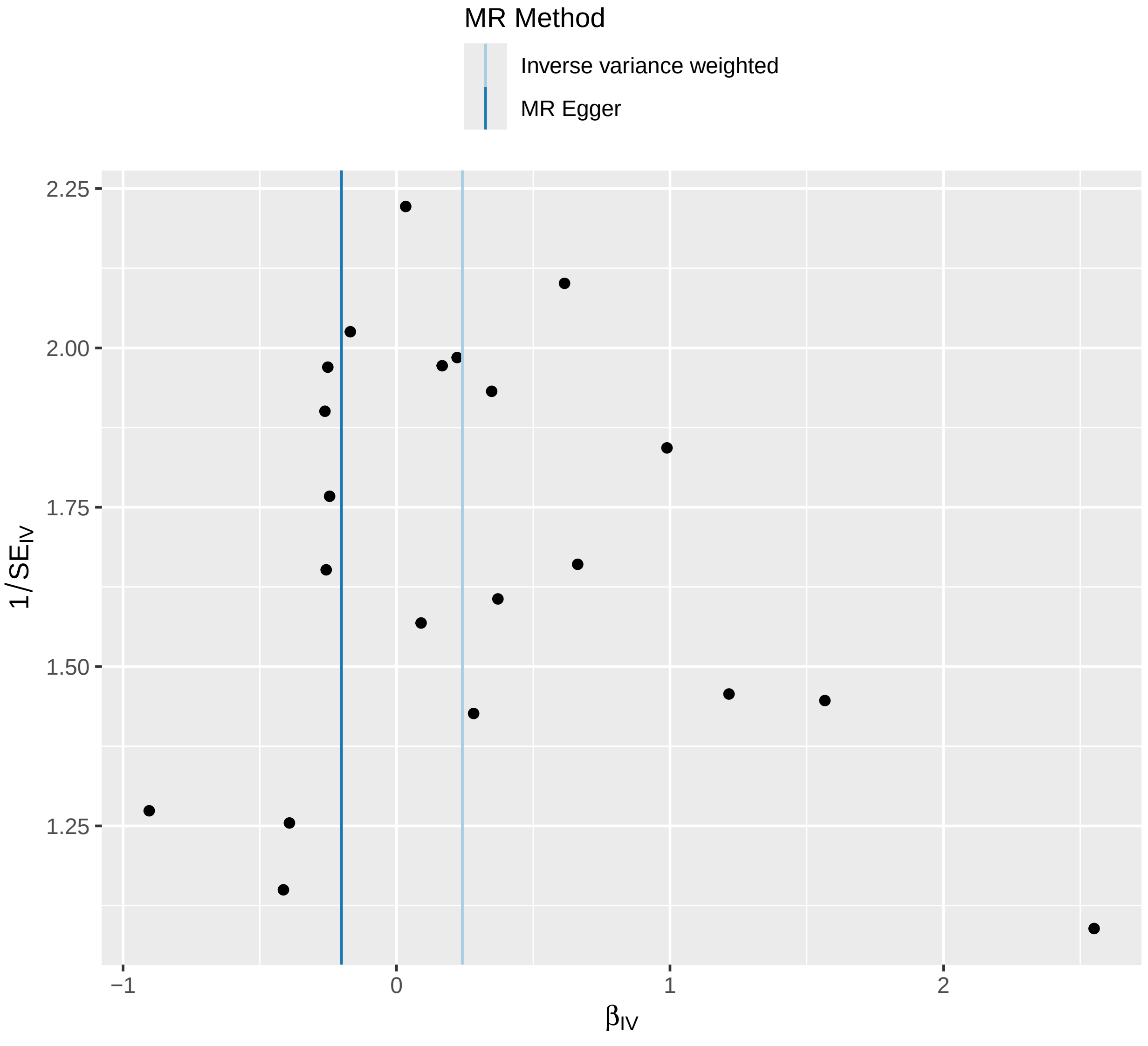
MR Method
Inverse variance weighted
MR Egger
2.25
2.00
1.75
1.50
1.25
$1/SE_{IV}$
−1
0
1
2
β_{IV}

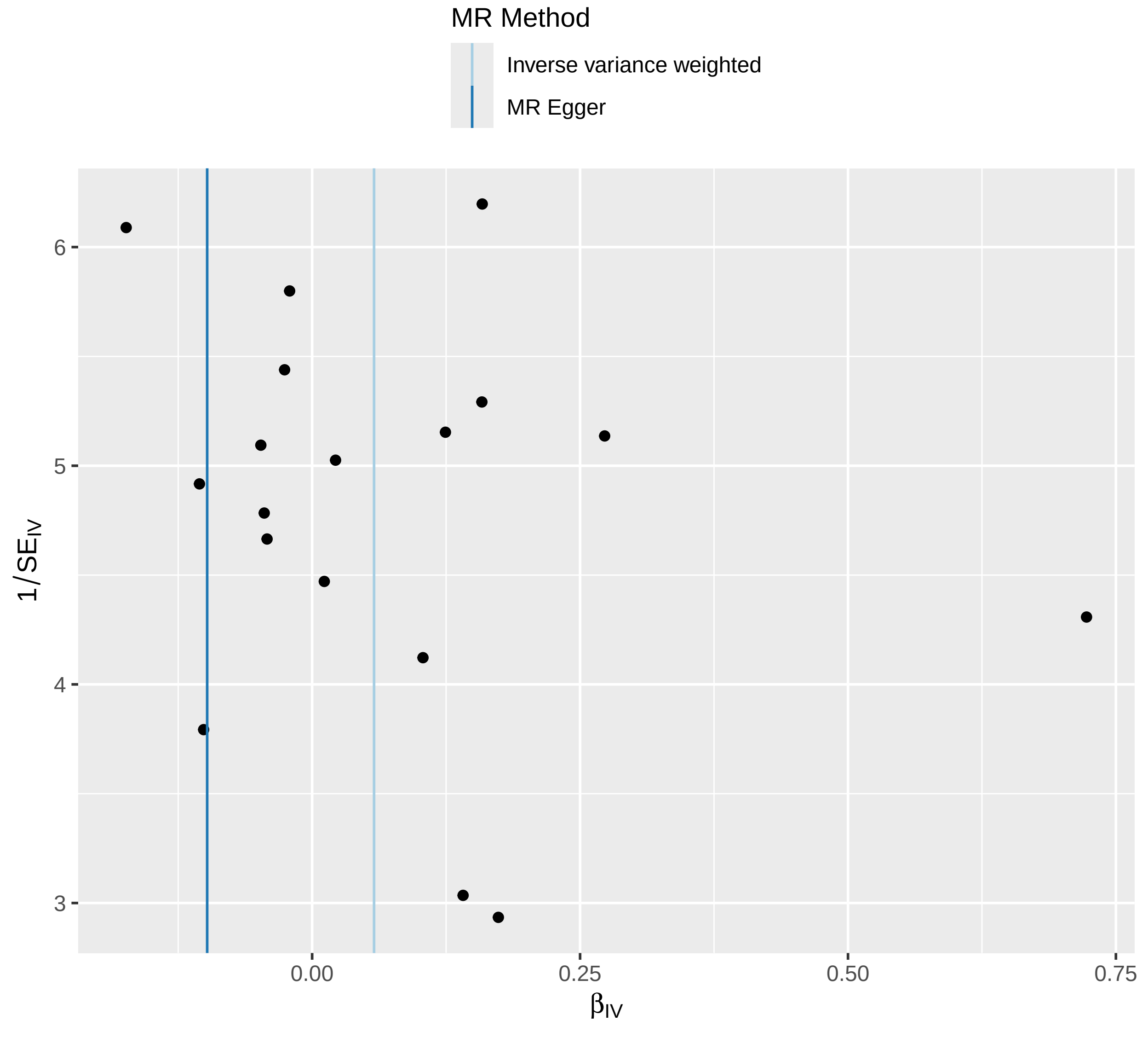
MR Method
Inverse variance weighted
MR Egger
6
5
4
3
1/SE$_{IV}$
0.00
0.25
0.50
0.75
β$_{IV}$

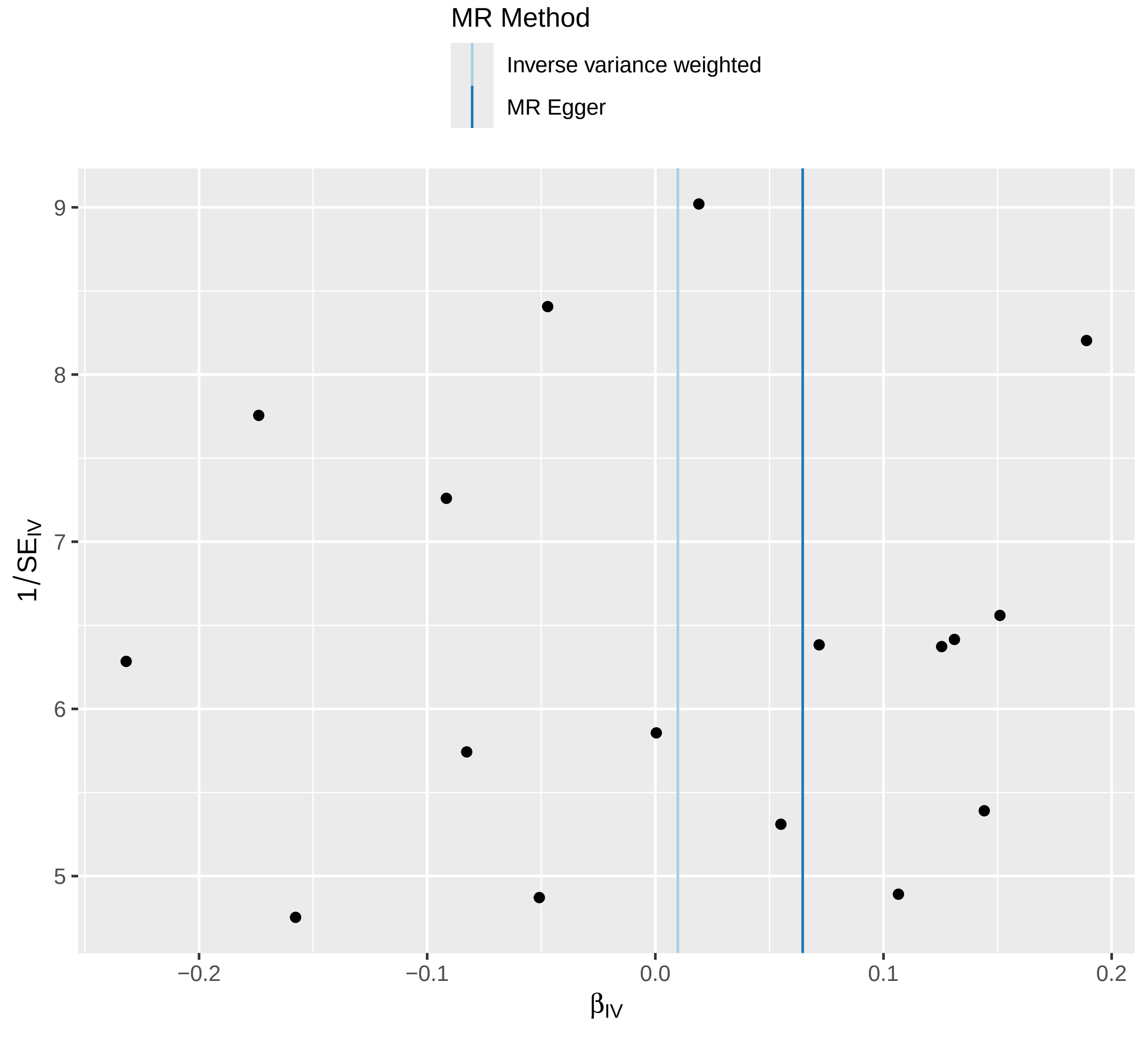
MR Method
Inverse variance weighted
MR Egger
9
8
7
6
5
1/SE$_{IV}$
−0.2
−0.1
0.0
0.1
0.2
β$_{IV}$

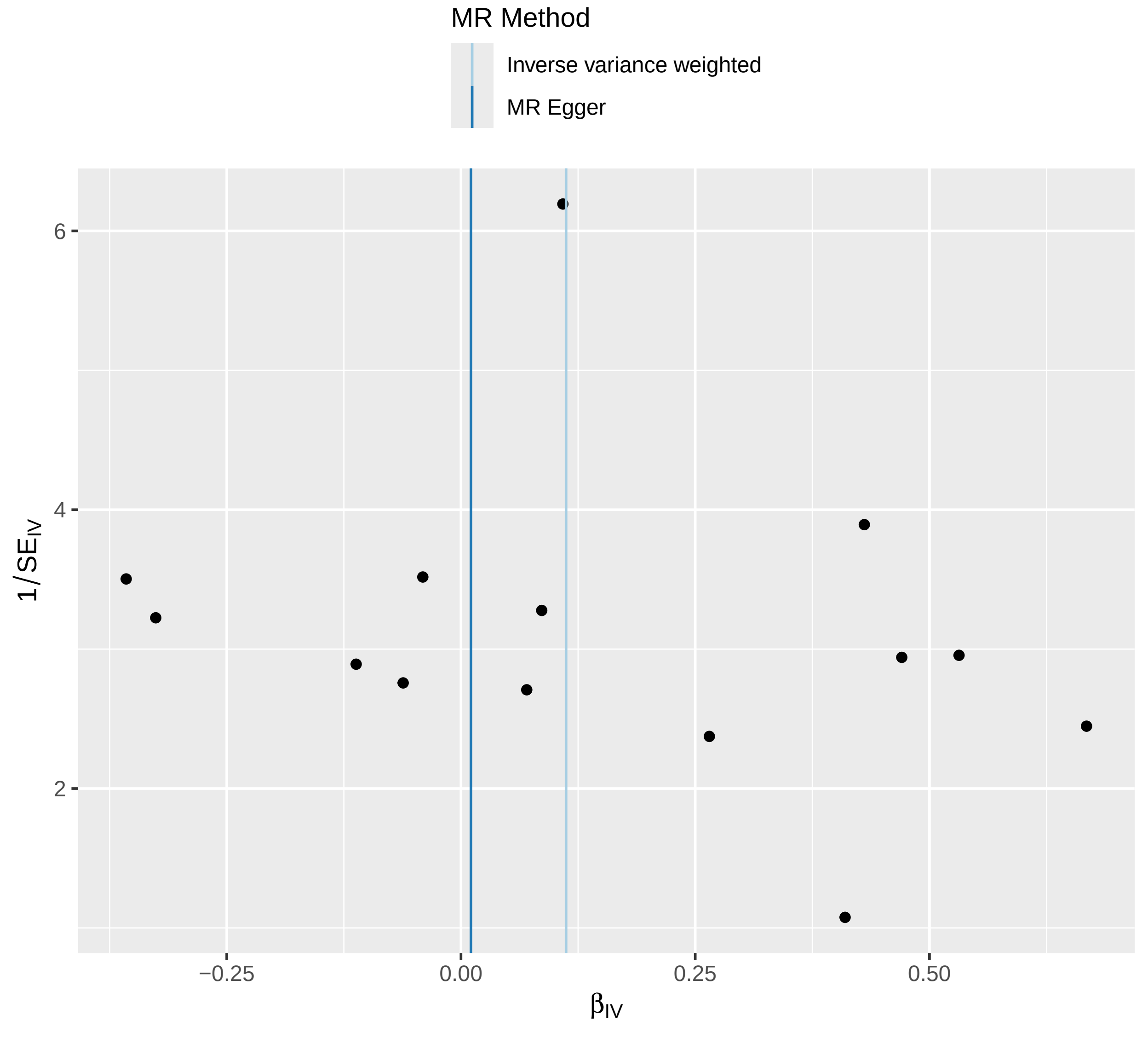

MR Method
Inverse variance weighted
MR Egger
6
4
2
$1/SE_{IV}$
−0.25
0.00
0.25
0.50
β_{IV}

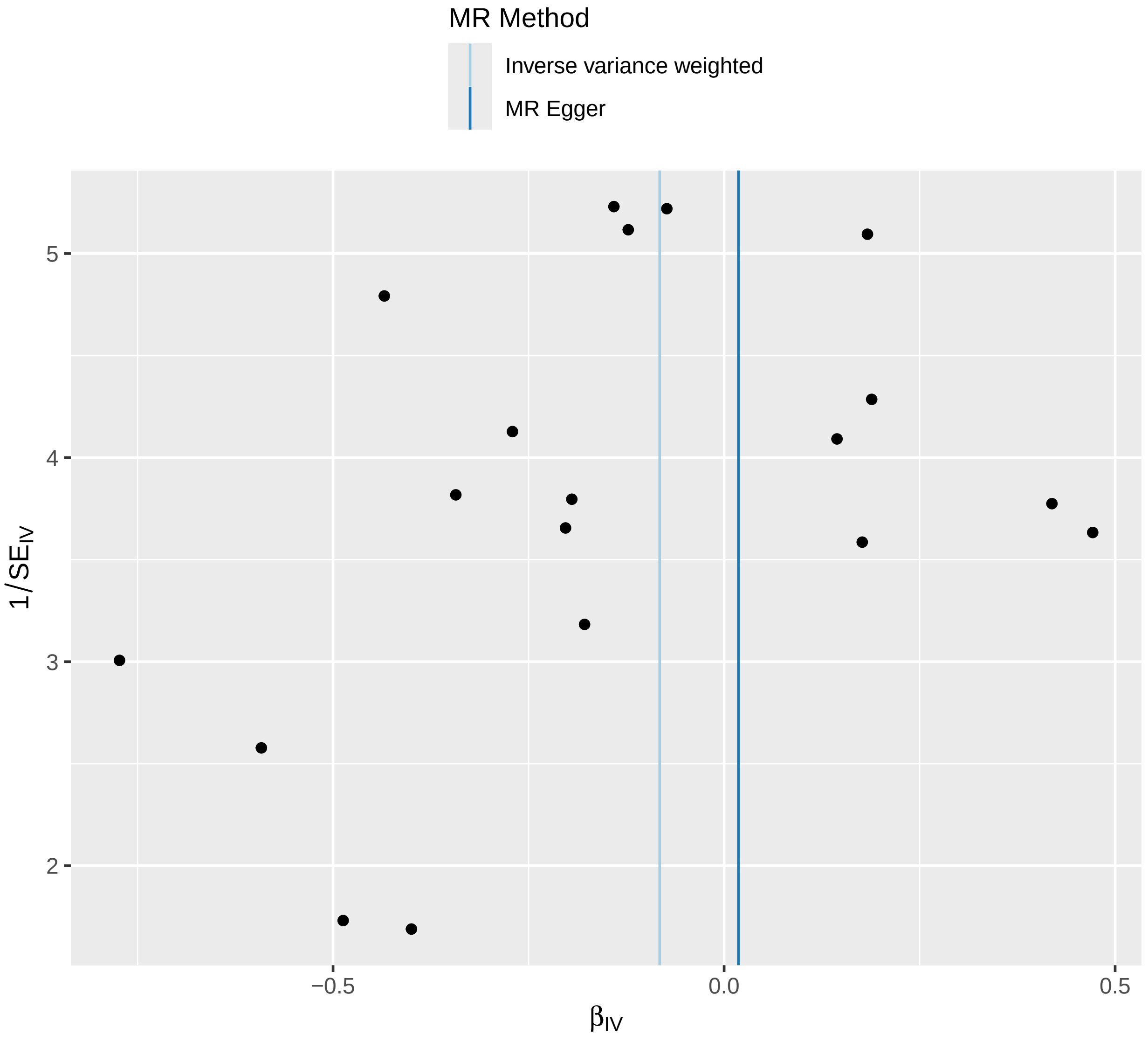

MR Method
Inverse variance weighted
MR Egger
5
4
3
2
−0.5
0.0
0.5
$1/SE_{IV}$
β_{IV}

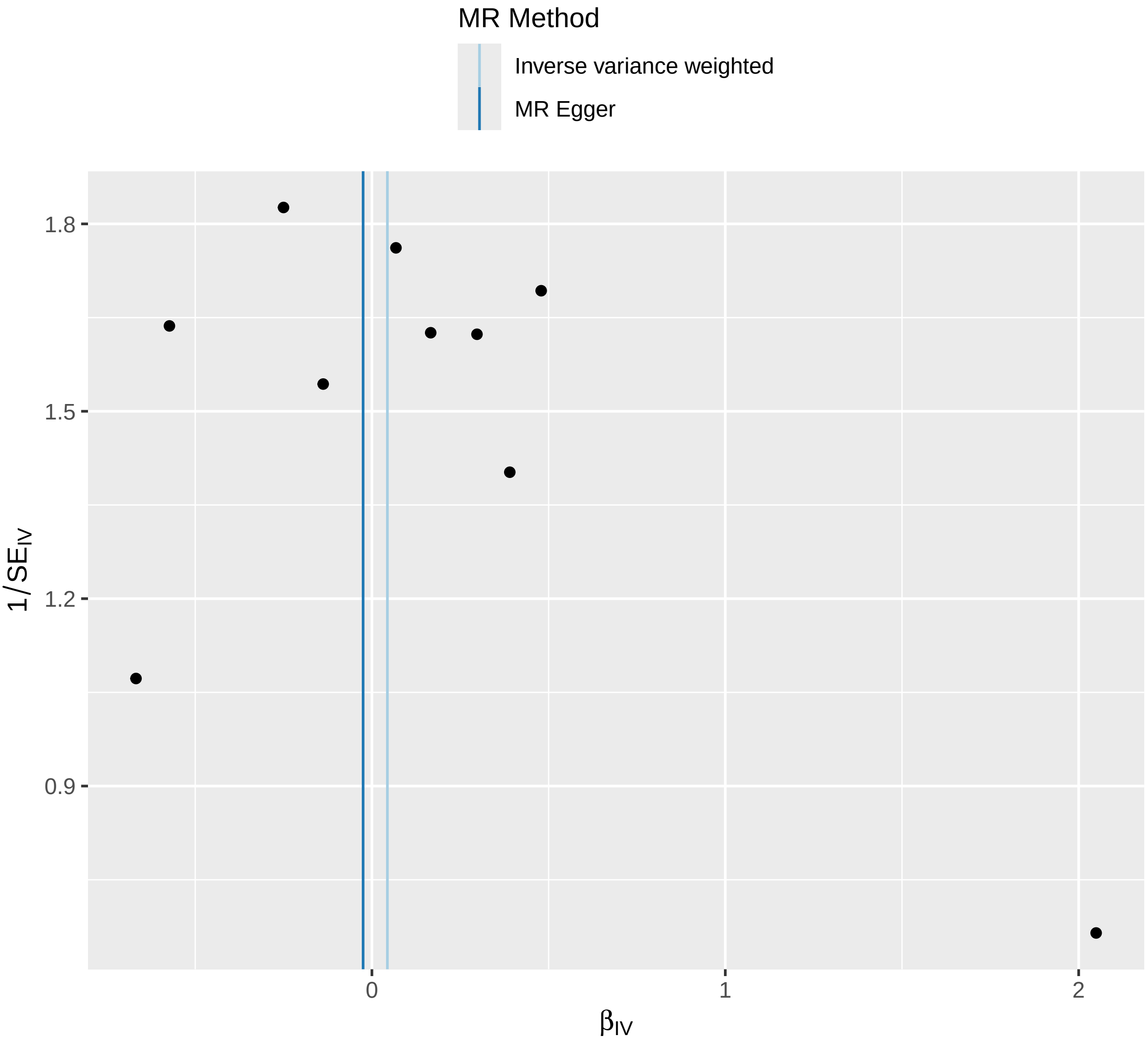

MR Method
Inverse variance weighted
MR Egger
1.8
1.5
1.2
0.9
$1/SE_{IV}$
0
1
2
β_{IV}

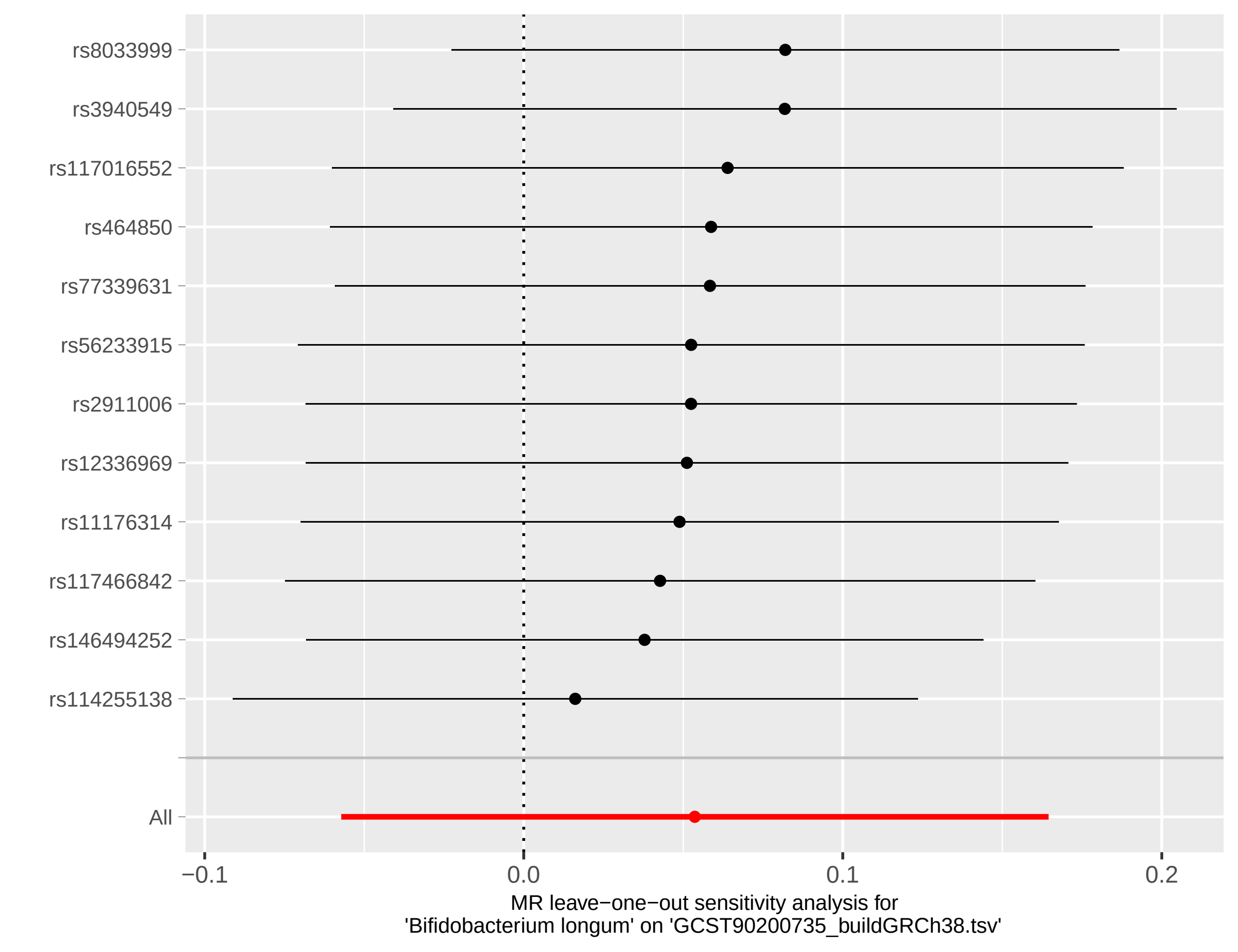
rs8033999
rs3940549
rs117016552
rs464850
rs77339631
rs56233915
rs2911006
rs12336969
rs11176314
rs117466842
rs146494252
rs114255138
All
−0.1
0.0
0.1
0.2
MR leave−one−out sensitivity analysis for
'Bifidobacterium longum' on 'GCST90200735_buildGRCh38.tsv'

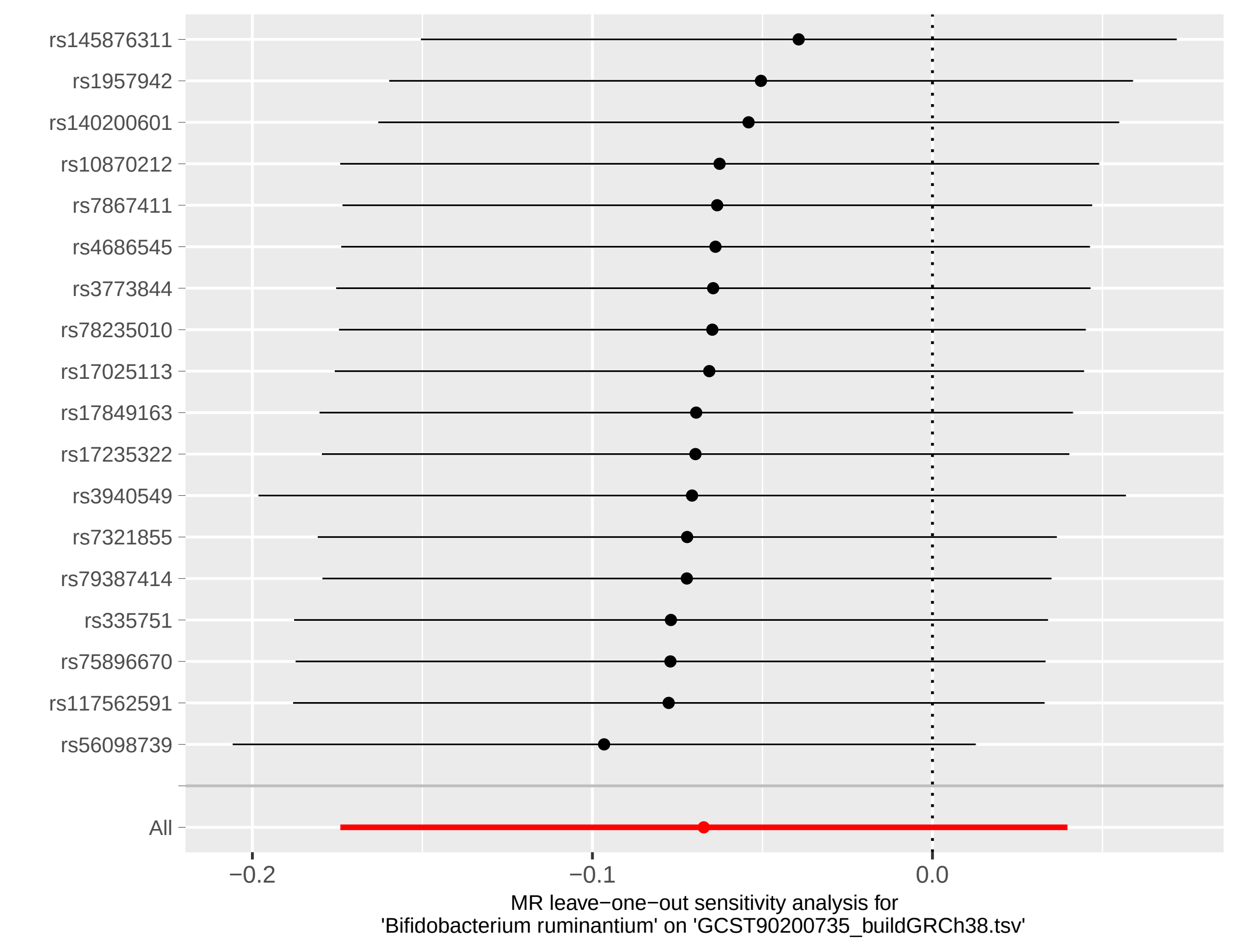
rs145876311
rs1957942
rs140200601
rs10870212
rs7867411
rs4686545
rs3773844
rs78235010
rs17025113
rs17849163
rs17235322
rs3940549
rs7321855
rs79387414
rs335751
rs75896670
rs117562591
rs56098739
All
−0.2
−0.1
0.0
MR leave−one−out sensitivity analysis for
'Bifidobacterium ruminantium' on 'GCST90200735_buildGRCh38.tsv'

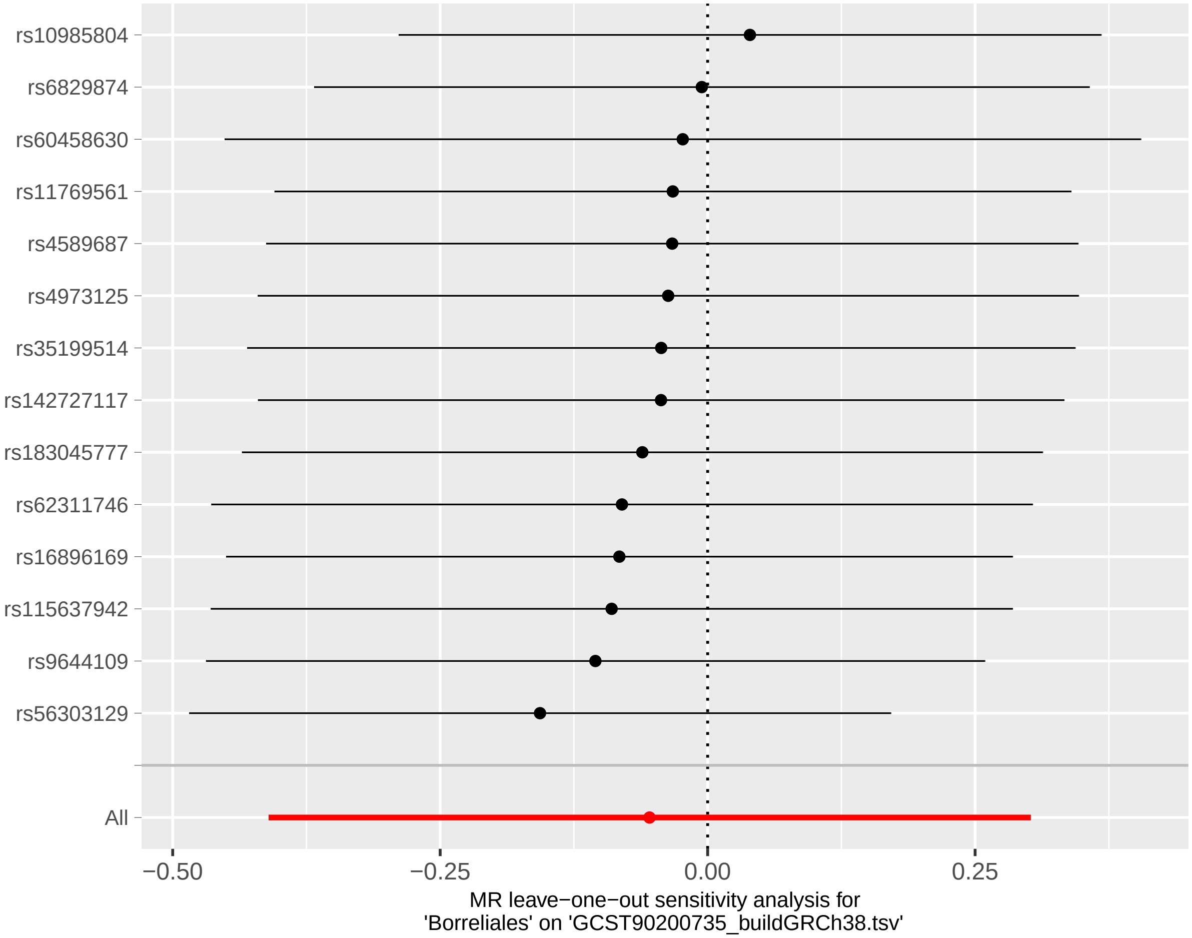
rs10985804
rs6829874
rs60458630
rs11769561
rs4589687
rs4973125
rs35199514
rs142727117
rs183045777
rs62311746
rs16896169
rs115637942
rs9644109
rs56303129
All
−0.50
−0.25
0.00
0.25
MR leave−one−out sensitivity analysis for
'Borreliales' on 'GCST90200735_buildGRCh38.tsv'

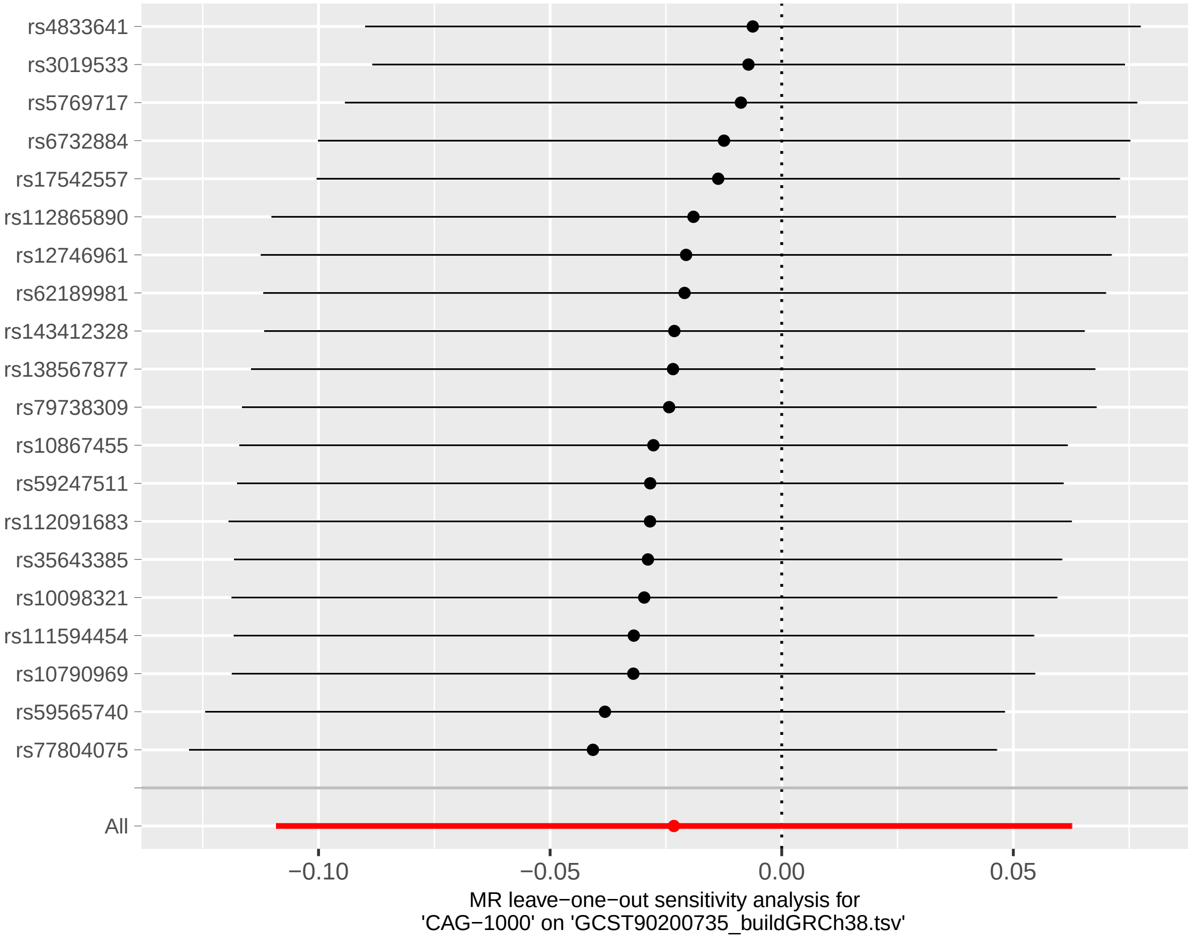
rs4833641
rs3019533
rs5769717
rs6732884
rs17542557
rs112865890
rs12746961
rs62189981
rs143412328
rs138567877
rs79738309
rs10867455
rs59247511
rs112091683
rs35643385
rs10098321
rs111594454
rs10790969
rs59565740
rs77804075
All
−0.10
−0.05
0.00
0.05
MR leave−one−out sensitivity analysis for
'CAG−1000' on 'GCST90200735_buildGRCh38.tsv'

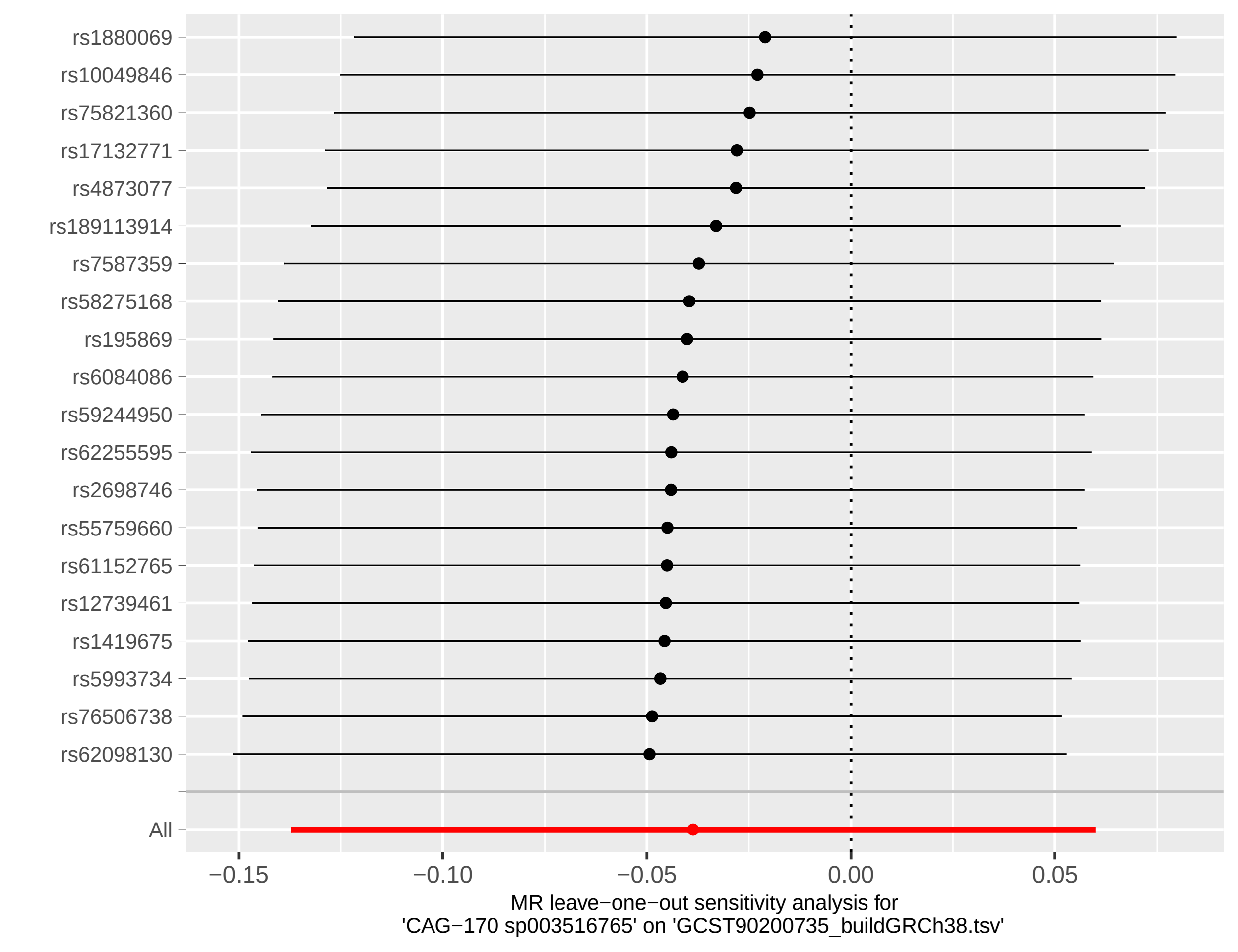
rs1880069
rs10049846
rs75821360
rs17132771
rs4873077
rs189113914
rs7587359
rs58275168
rs195869
rs6084086
rs59244950
rs62255595
rs2698746
rs55759660
rs61152765
rs12739461
rs1419675
rs5993734
rs76506738
rs62098130
All
−0.15
−0.10
−0.05
0.00
0.05
MR leave−one−out sensitivity analysis for
'CAG−170 sp003516765' on 'GCST90200735_buildGRCh38.tsv'

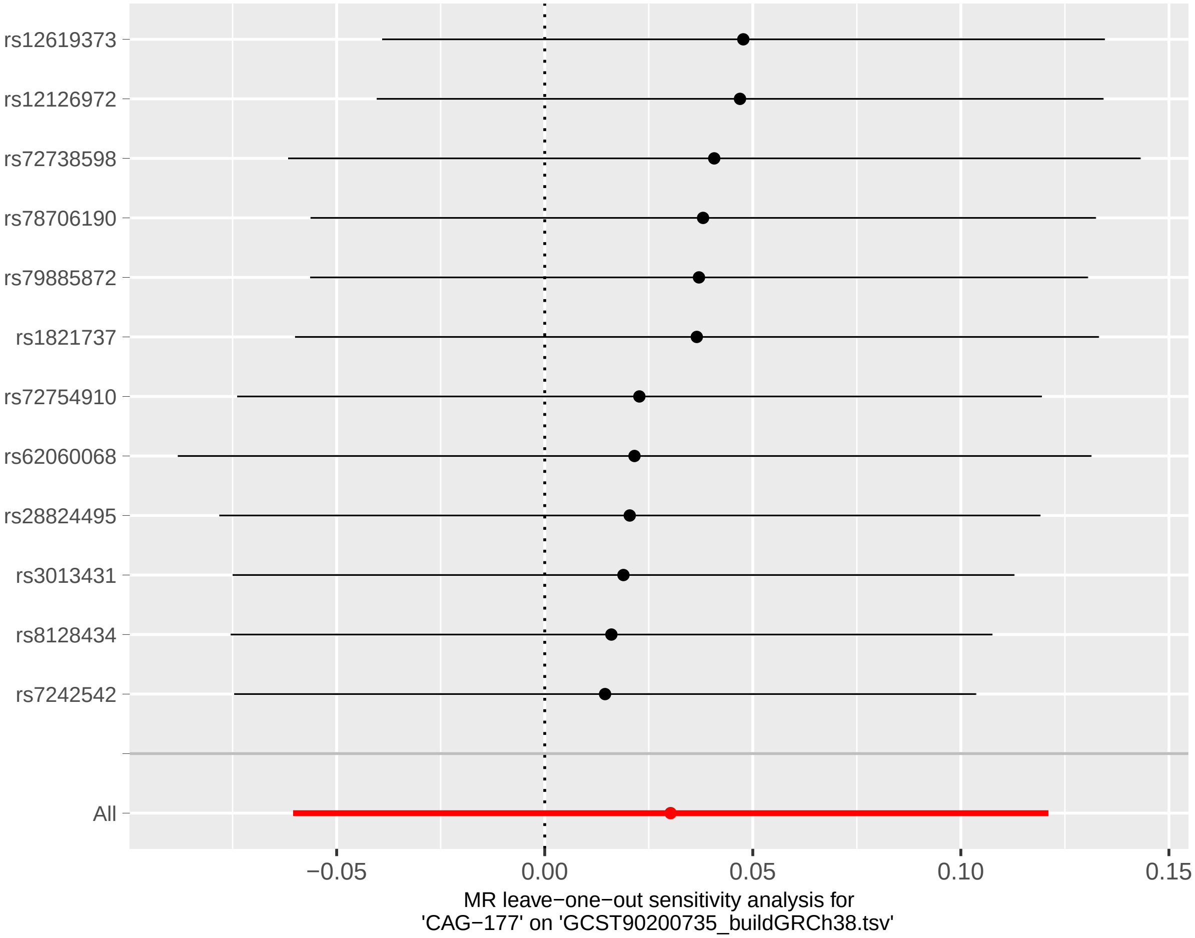
rs12619373
rs12126972
rs72738598
rs78706190
rs79885872
rs1821737
rs72754910
rs62060068
rs28824495
rs3013431
rs8128434
rs7242542
All
−0.05
0.00
0.05
0.10
0.15
MR leave−one−out sensitivity analysis for
'CAG−177' on 'GCST90200735_buildGRCh38.tsv'

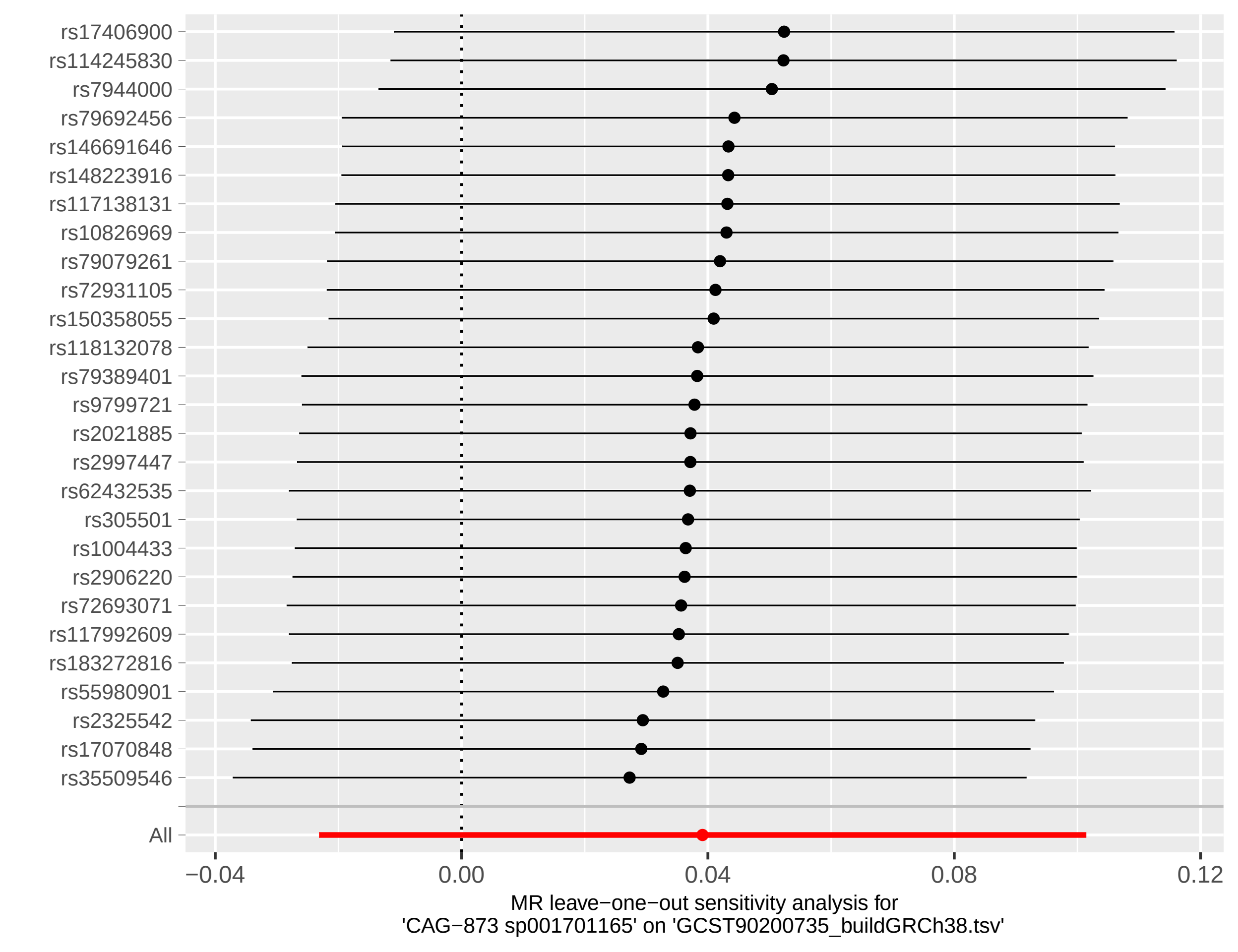
rs17406900
rs114245830
rs7944000
rs79692456
rs146691646
rs148223916
rs117138131
rs10826969
rs79079261
rs72931105
rs150358055
rs118132078
rs79389401
rs9799721
rs2021885
rs2997447
rs62432535
rs305501
rs1004433
rs2906220
rs72693071
rs117992609
rs183272816
rs55980901
rs2325542
rs17070848
rs35509546
All
−0.04
0.00
0.04
0.08
0.12
MR leave−one−out sensitivity analysis for
'CAG−873 sp001701165' on 'GCST90200735_buildGRCh38.tsv'

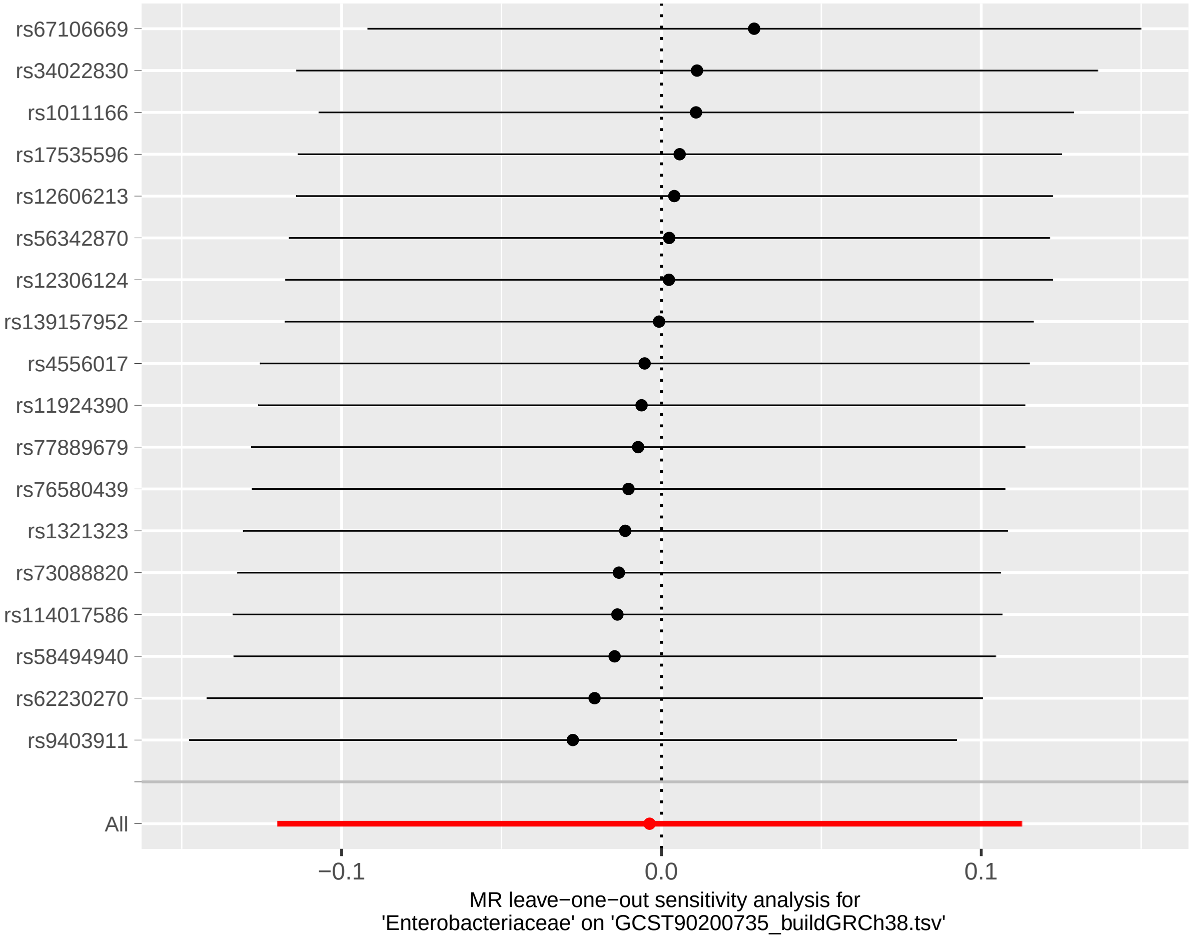
rs67106669
rs34022830
rs1011166
rs17535596
rs12606213
rs56342870
rs12306124
rs139157952
rs4556017
rs11924390
rs77889679
rs76580439
rs1321323
rs73088820
rs114017586
rs58494940
rs62230270
rs9403911
All
−0.1
0.0
0.1
MR leave−one−out sensitivity analysis for
'Enterobacteriaceae' on 'GCST90200735_buildGRCh38.tsv'

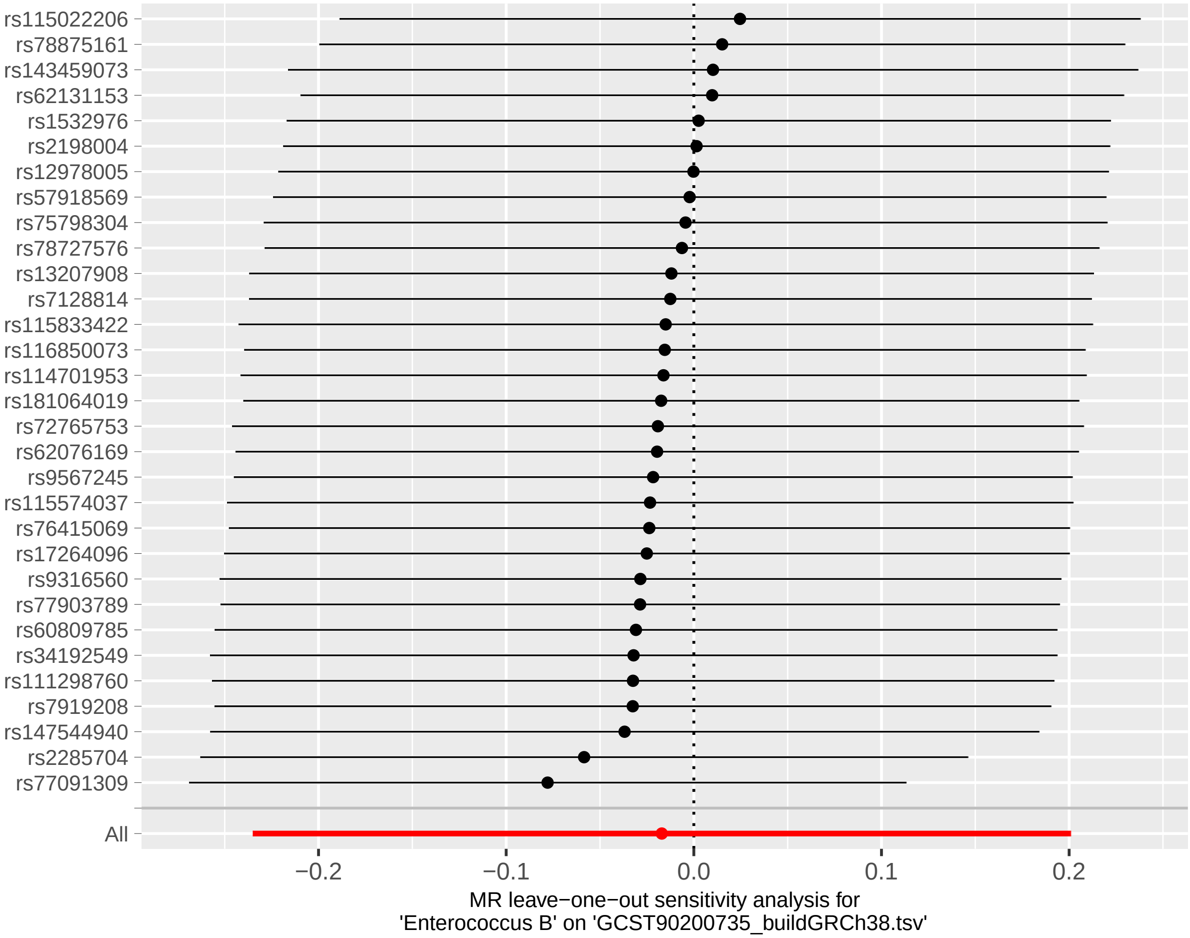
rs115022206
rs78875161
rs143459073
rs62131153
rs1532976
rs2198004
rs12978005
rs57918569
rs75798304
rs78727576
rs13207908
rs7128814
rs115833422
rs116850073
rs114701953
rs181064019
rs72765753
rs62076169
rs9567245
rs115574037
rs76415069
rs17264096
rs9316560
rs77903789
rs60809785
rs34192549
rs111298760
rs7919208
rs147544940
rs2285704
rs77091309
All
−0.2
−0.1
0.0
0.1
0.2
MR leave−one−out sensitivity analysis for
'Enterococcus B' on 'GCST90200735_buildGRCh38.tsv'

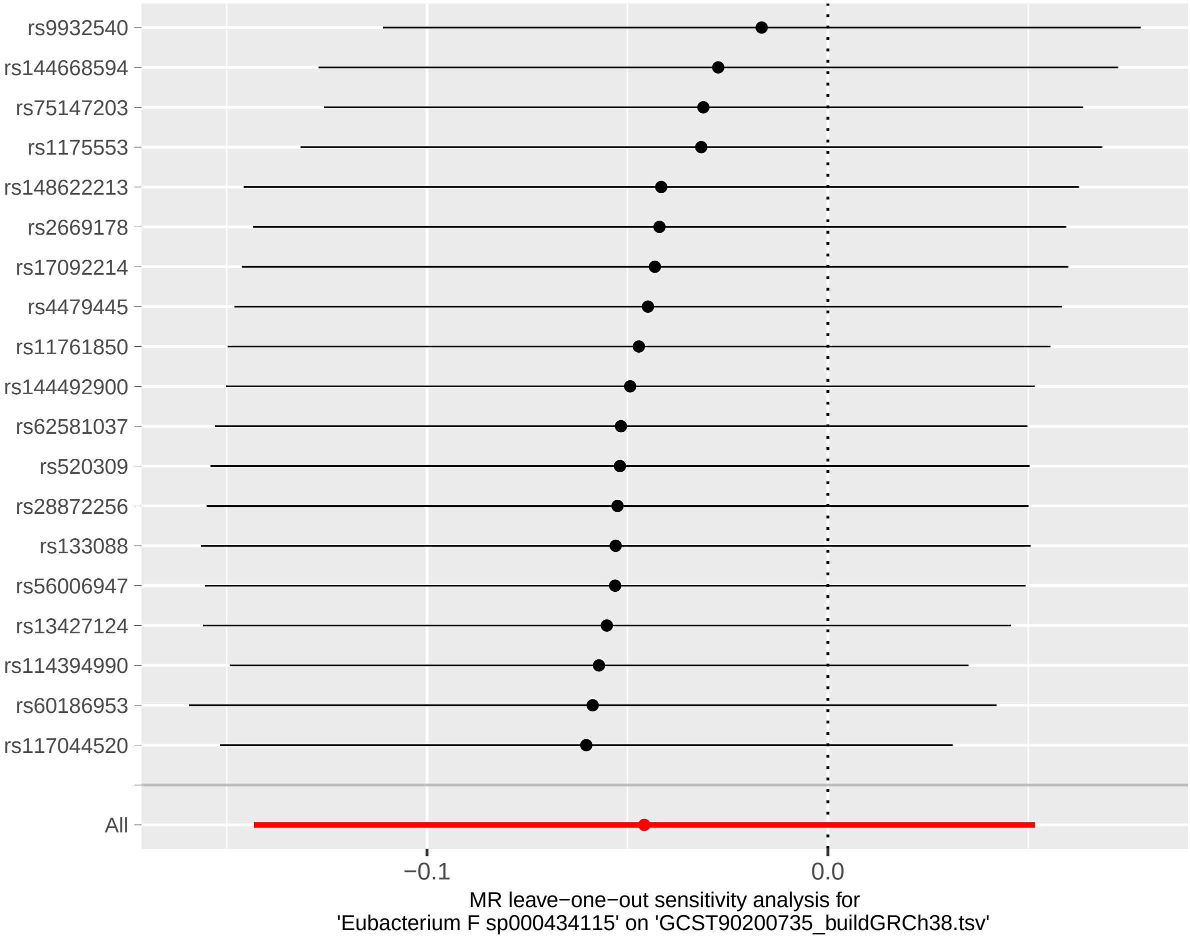
rs9932540
rs144668594
rs75147203
rs1175553
rs148622213
rs2669178
rs17092214
rs4479445
rs11761850
rs144492900
rs62581037
rs520309
rs28872256
rs133088
rs56006947
rs13427124
rs114394990
rs60186953
rs117044520
All
−0.1
0.0
MR leave−one−out sensitivity analysis for
'Eubacterium F sp000434115' on 'GCST90200735_buildGRCh38.tsv'

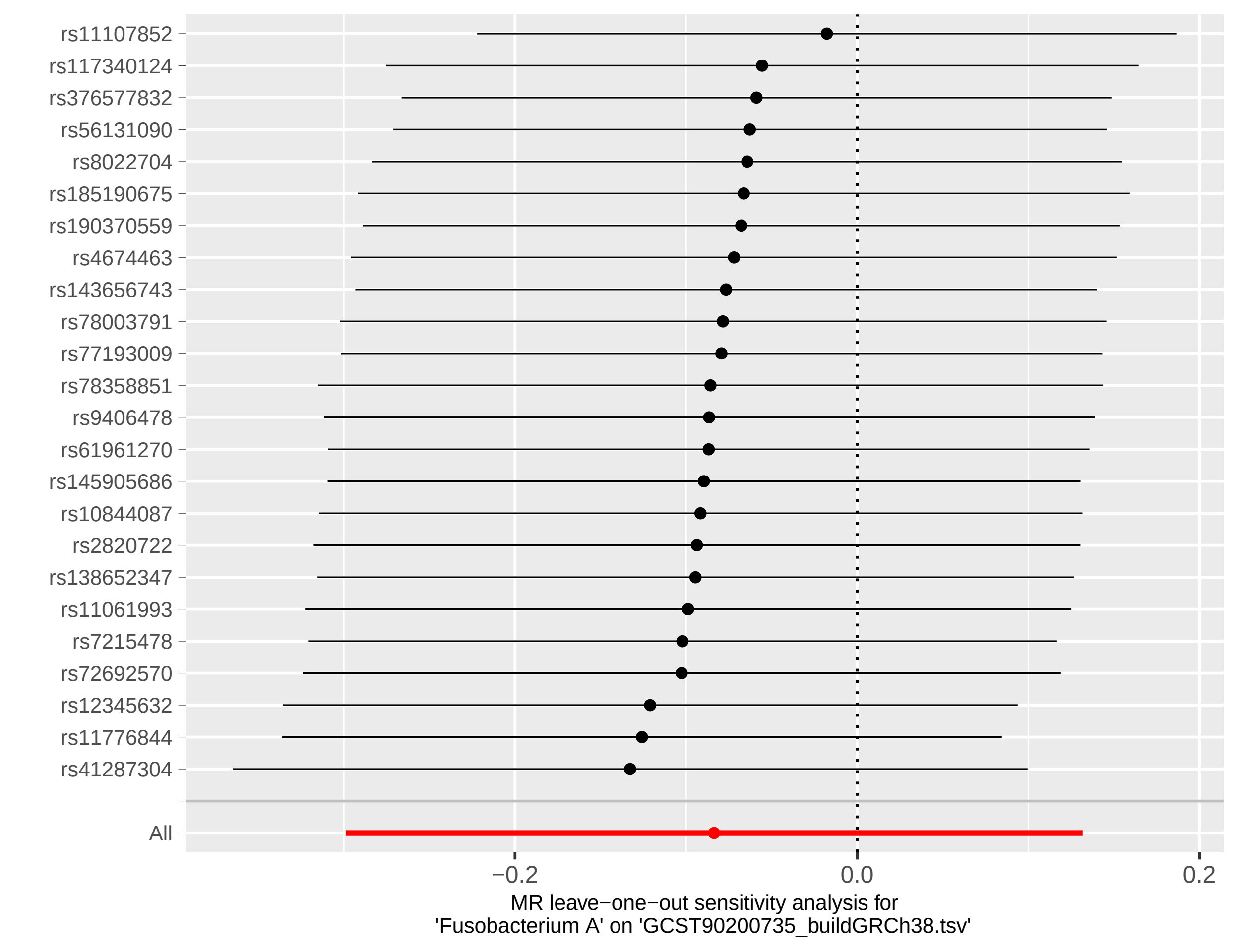
rs11107852
rs117340124
rs376577832
rs56131090
rs8022704
rs185190675
rs190370559
rs4674463
rs143656743
rs78003791
rs77193009
rs78358851
rs9406478
rs61961270
rs145905686
rs10844087
rs2820722
rs138652347
rs11061993
rs7215478
rs72692570
rs12345632
rs11776844
rs41287304
All
−0.2
0.0
0.2
MR leave−one−out sensitivity analysis for
'Fusobacterium A' on 'GCST90200735_buildGRCh38.tsv'

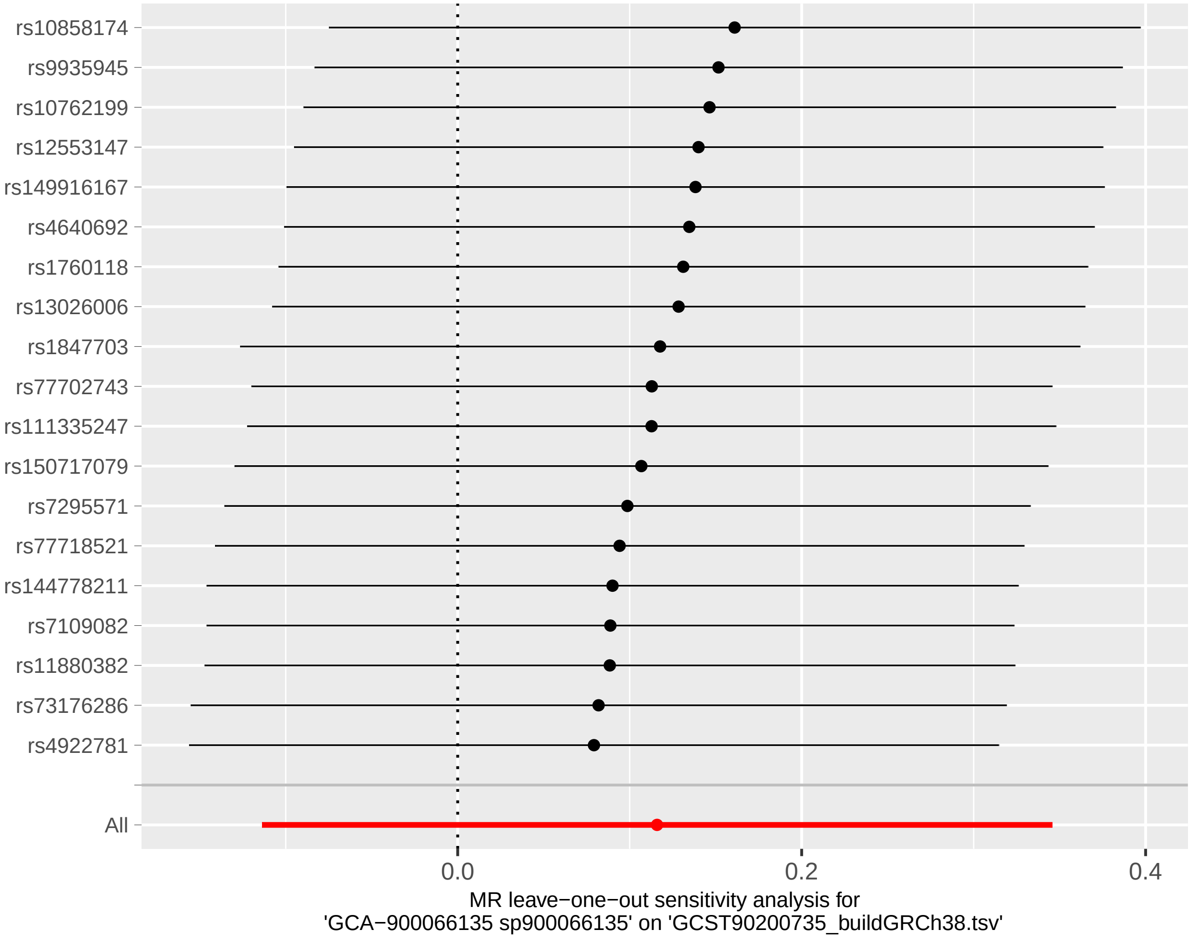

rs10858174
rs9935945
rs10762199
rs12553147
rs149916167
rs4640692
rs1760118
rs13026006
rs1847703
rs77702743
rs111335247
rs150717079
rs7295571
rs77718521
rs144778211
rs7109082
rs11880382
rs73176286
rs4922781
All
0.0
0.2
0.4
MR leave−one−out sensitivity analysis for
'GCA−900066135 sp900066135' on 'GCST90200735_buildGRCh38.tsv'

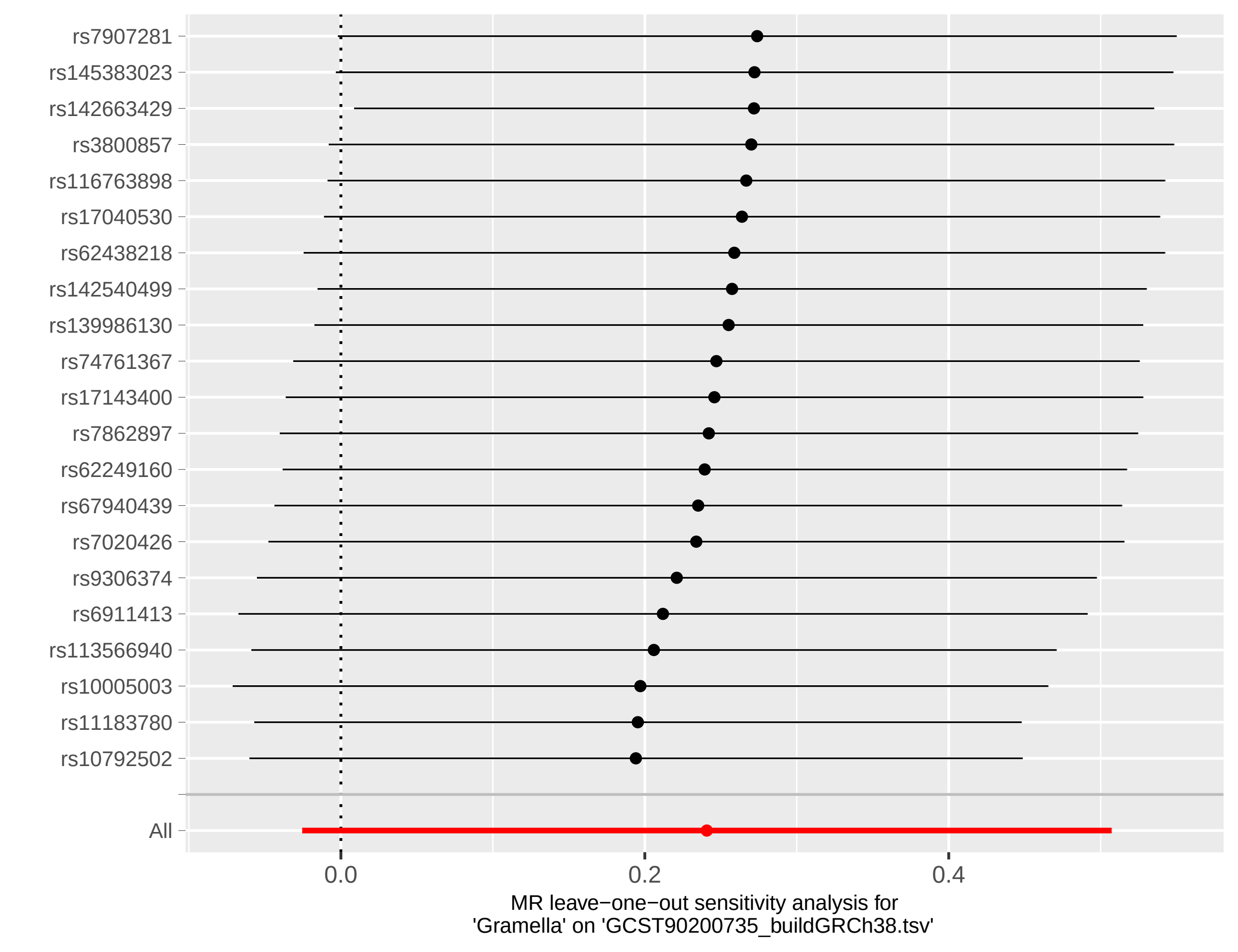

rs7907281
rs145383023
rs142663429
rs3800857
rs116763898
rs17040530
rs62438218
rs142540499
rs139986130
rs74761367
rs17143400
rs7862897
rs62249160
rs67940439
rs7020426
rs9306374
rs6911413
rs113566940
rs10005003
rs11183780
rs10792502
All
0.0
0.2
0.4
MR leave−one−out sensitivity analysis for
'Gramella' on 'GCST90200735_buildGRCh38.tsv'

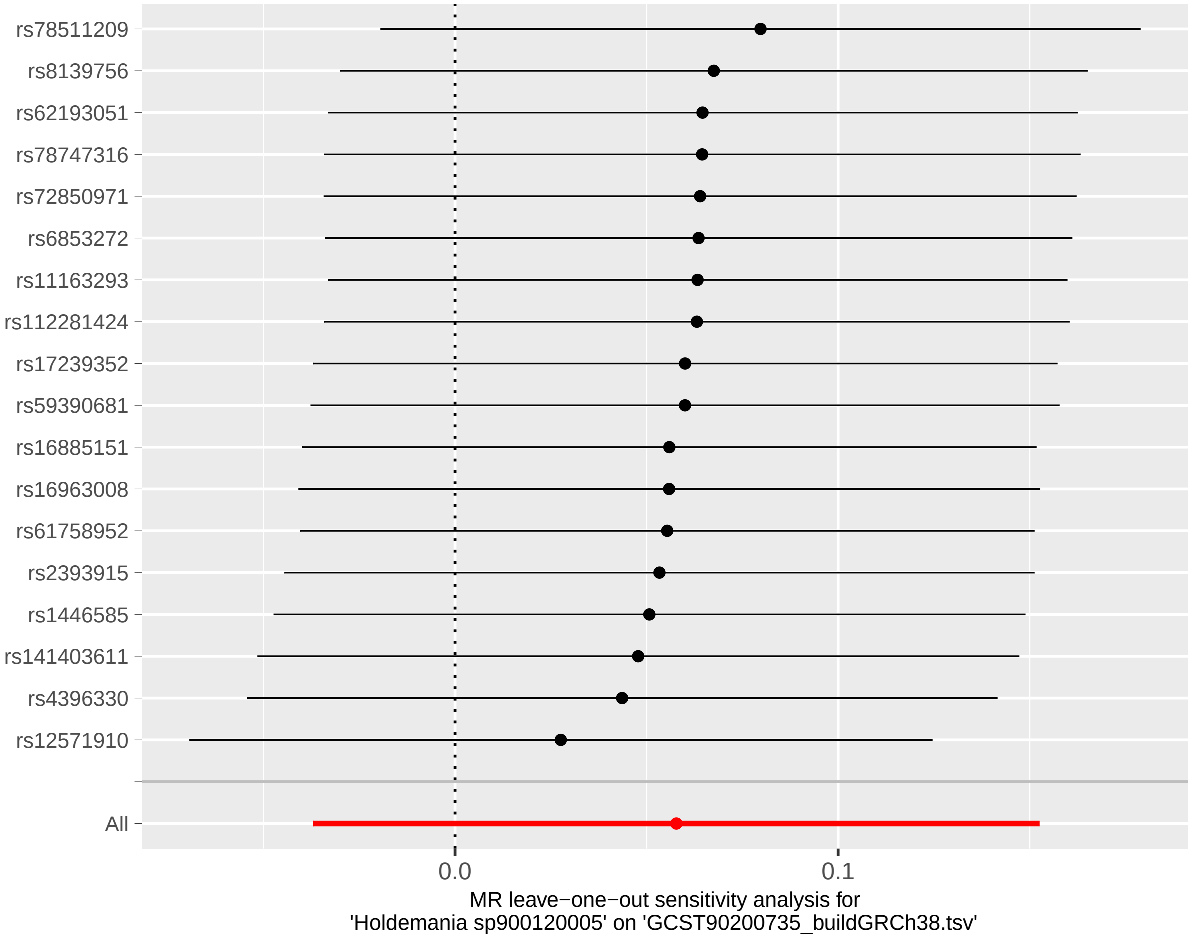

rs78511209
rs8139756
rs62193051
rs78747316
rs72850971
rs6853272
rs11163293
rs112281424
rs17239352
rs59390681
rs16885151
rs16963008
rs61758952
rs2393915
rs1446585
rs141403611
rs4396330
rs12571910
All
0.0
0.1
MR leave−one−out sensitivity analysis for
'Holdemania sp900120005' on 'GCST90200735_buildGRCh38.tsv'

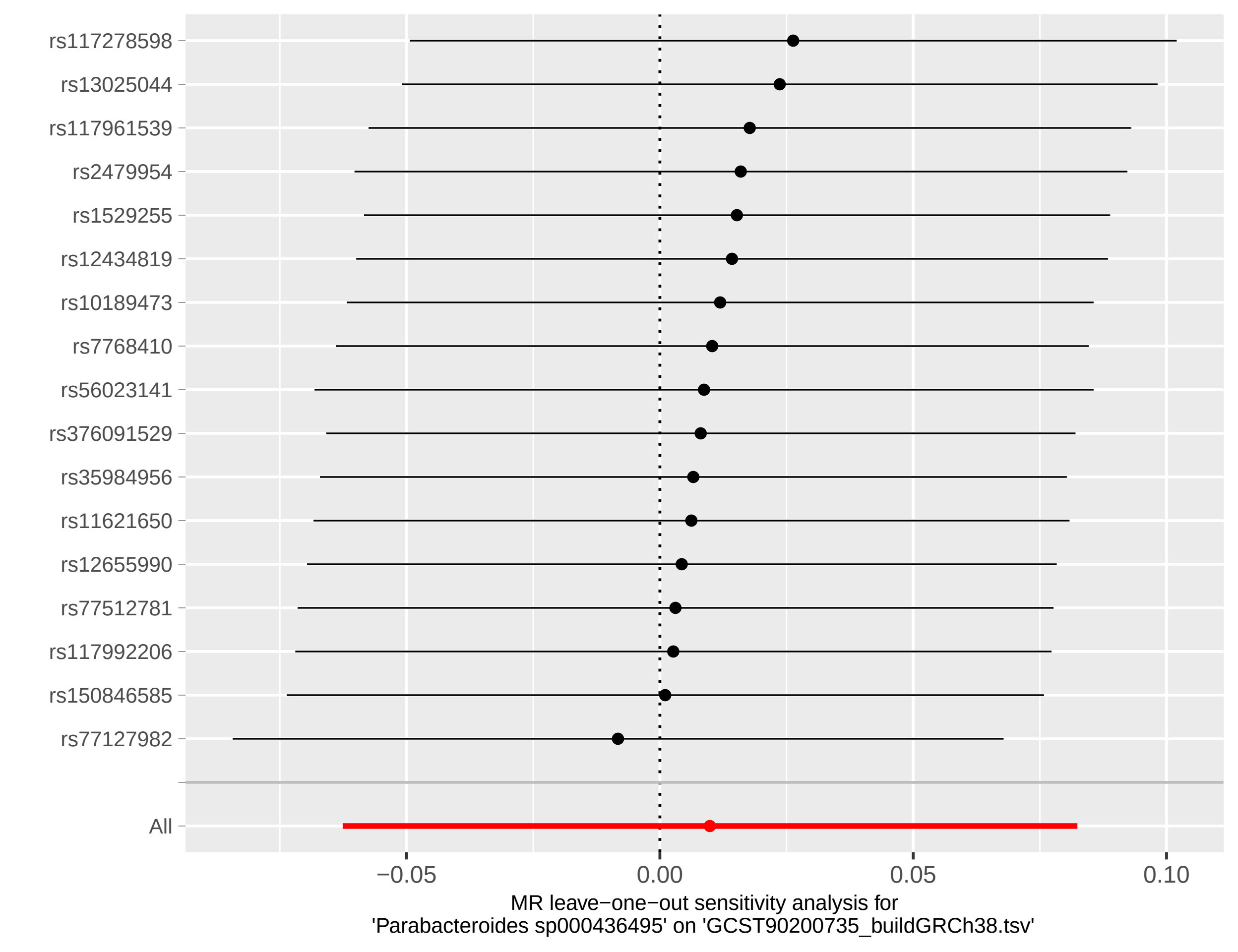

rs117278598
rs13025044
rs117961539
rs2479954
rs1529255
rs12434819
rs10189473
rs7768410
rs56023141
rs376091529
rs35984956
rs11621650
rs12655990
rs77512781
rs117992206
rs150846585
rs77127982
All
−0.05
0.00
0.05
0.10
MR leave−one−out sensitivity analysis for
'Parabacteroides sp000436495' on 'GCST90200735_buildGRCh38.tsv'

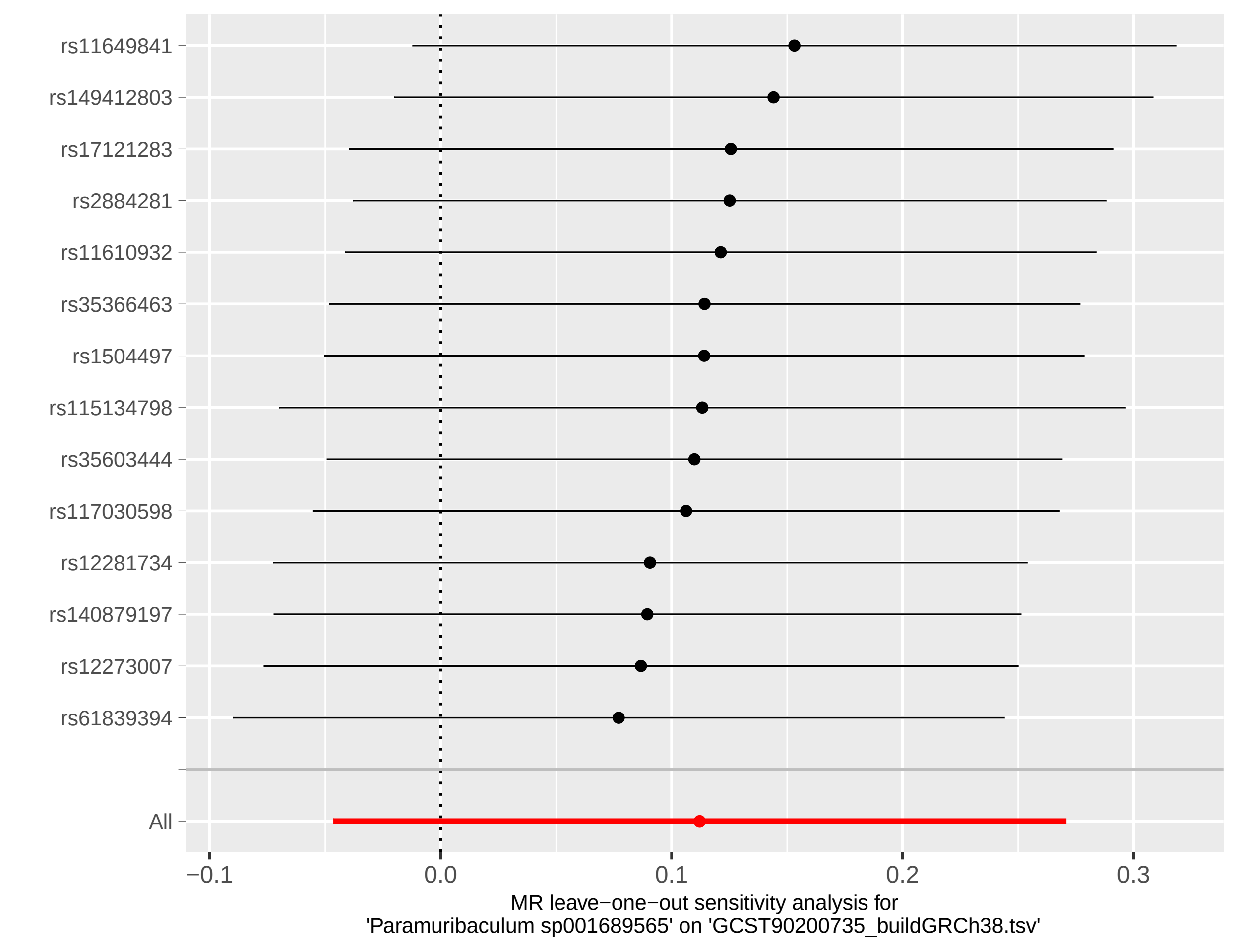

rs11649841
rs149412803
rs17121283
rs2884281
rs11610932
rs35366463
rs1504497
rs115134798
rs35603444
rs117030598
rs12281734
rs140879197
rs12273007
rs61839394
All
−0.1
0.0
0.1
0.2
0.3
MR leave−one−out sensitivity analysis for
'Paramuribaculum sp001689565' on 'GCST90200735_buildGRCh38.tsv'

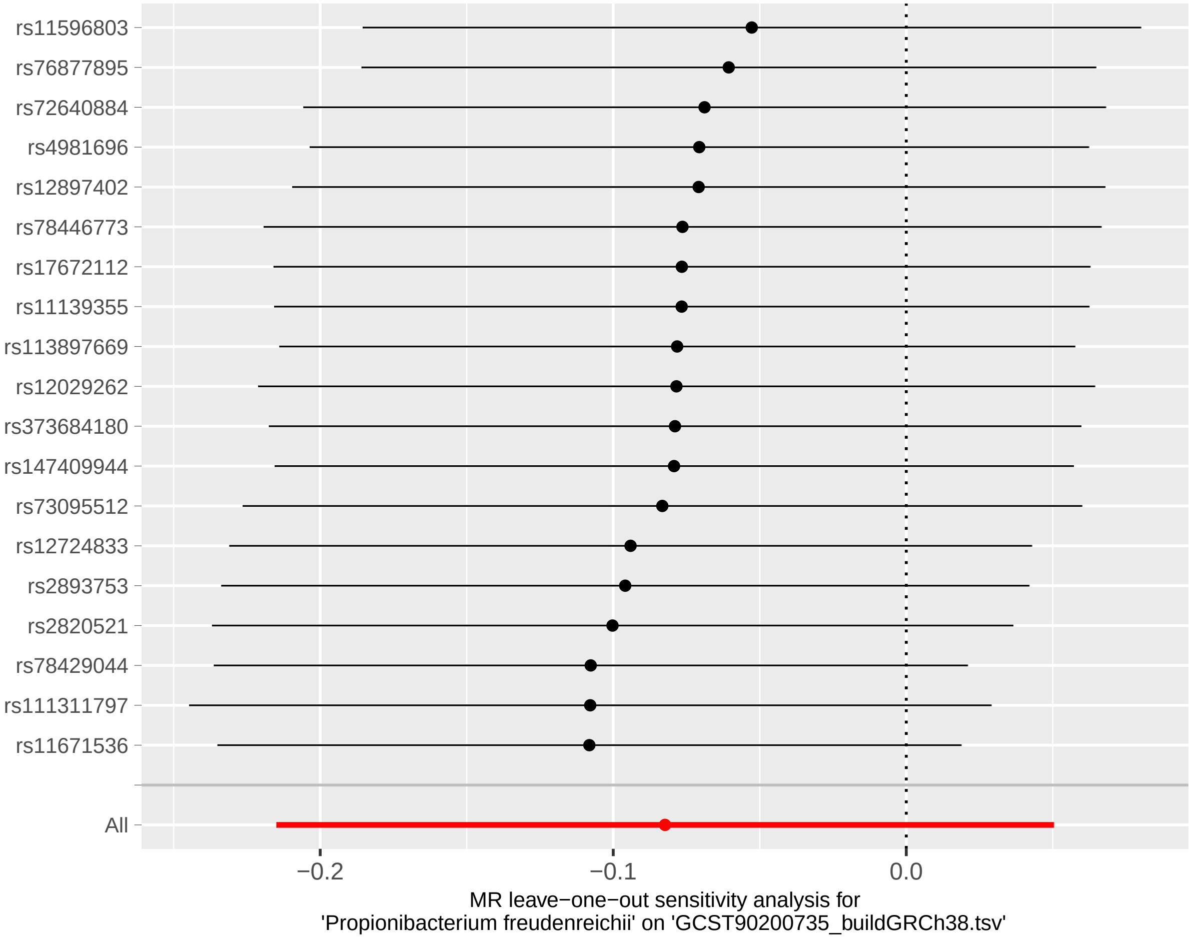
rs11596803
rs76877895
rs72640884
rs4981696
rs12897402
rs78446773
rs17672112
rs11139355
rs113897669
rs12029262
rs373684180
rs147409944
rs73095512
rs12724833
rs2893753
rs2820521
rs78429044
rs111311797
rs11671536
All
−0.2
−0.1
0.0
MR leave−one−out sensitivity analysis for
'Propionibacterium freudenreichii' on 'GCST90200735_buildGRCh38.tsv'

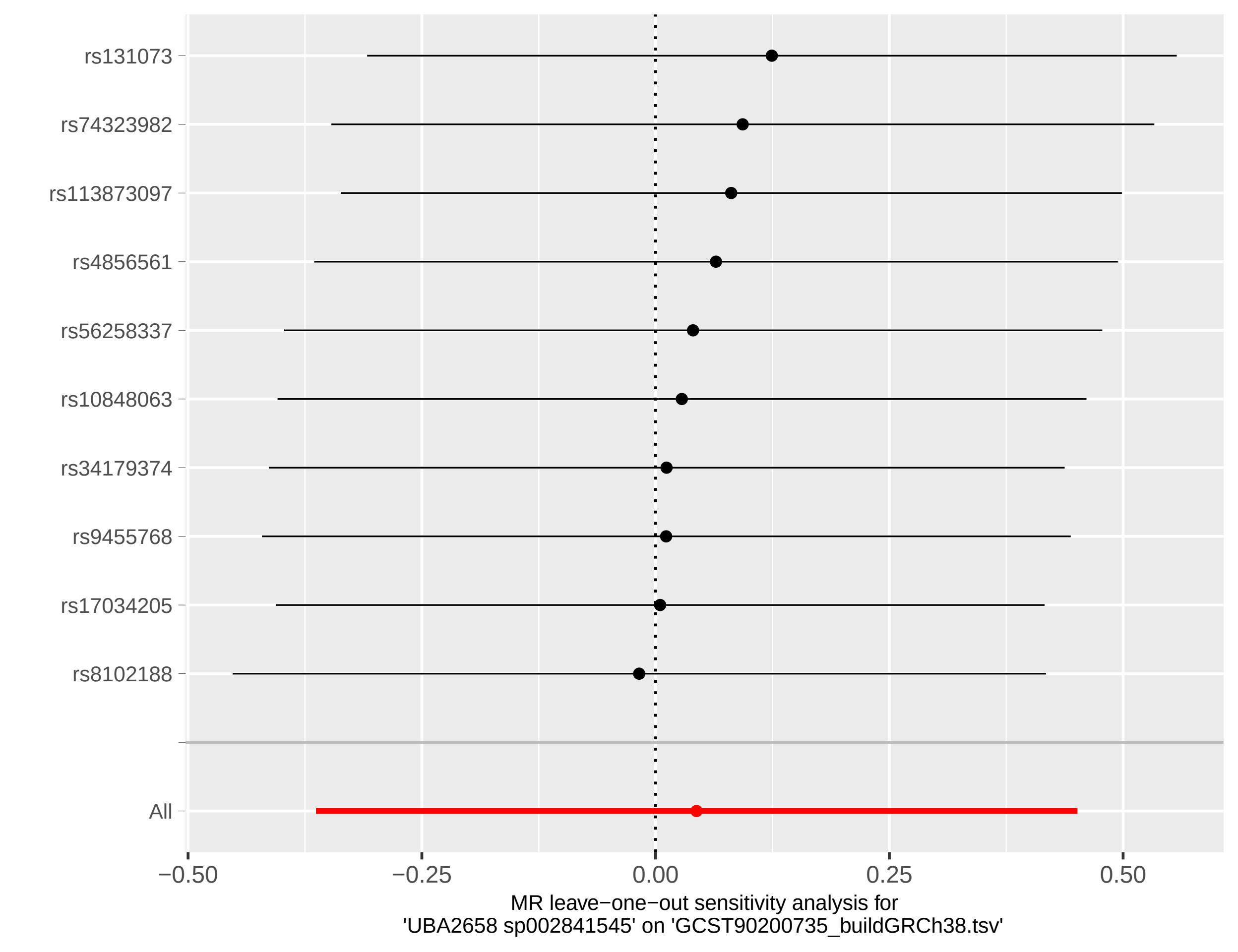
rs131073
rs74323982
rs113873097
rs4856561
rs56258337
rs10848063
rs34179374
rs9455768
rs17034205
rs8102188
All
−0.50
−0.25
0.00
0.25
0.50
MR leave−one−out sensitivity analysis for
'UBA2658 sp002841545' on 'GCST90200735_buildGRCh38.tsv'

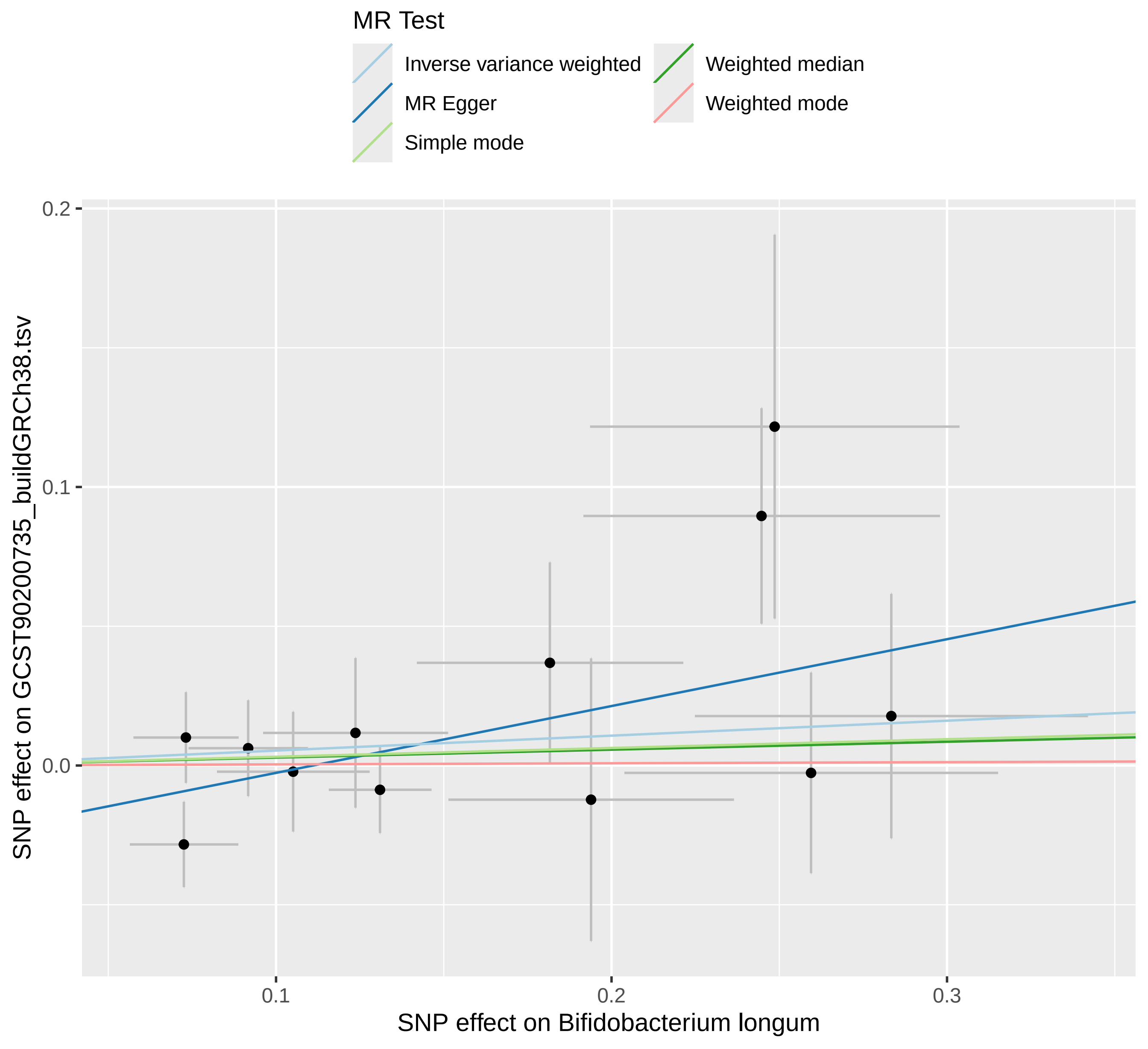
MR Test
Inverse variance weighted
MR Egger
Simple mode
Weighted median
Weighted mode
0.2
0.1
0.0
0.1
0.2
0.3
SNP effect on GCST90200735_buildGRCh38.tsv
SNP effect on Bifidobacterium longum

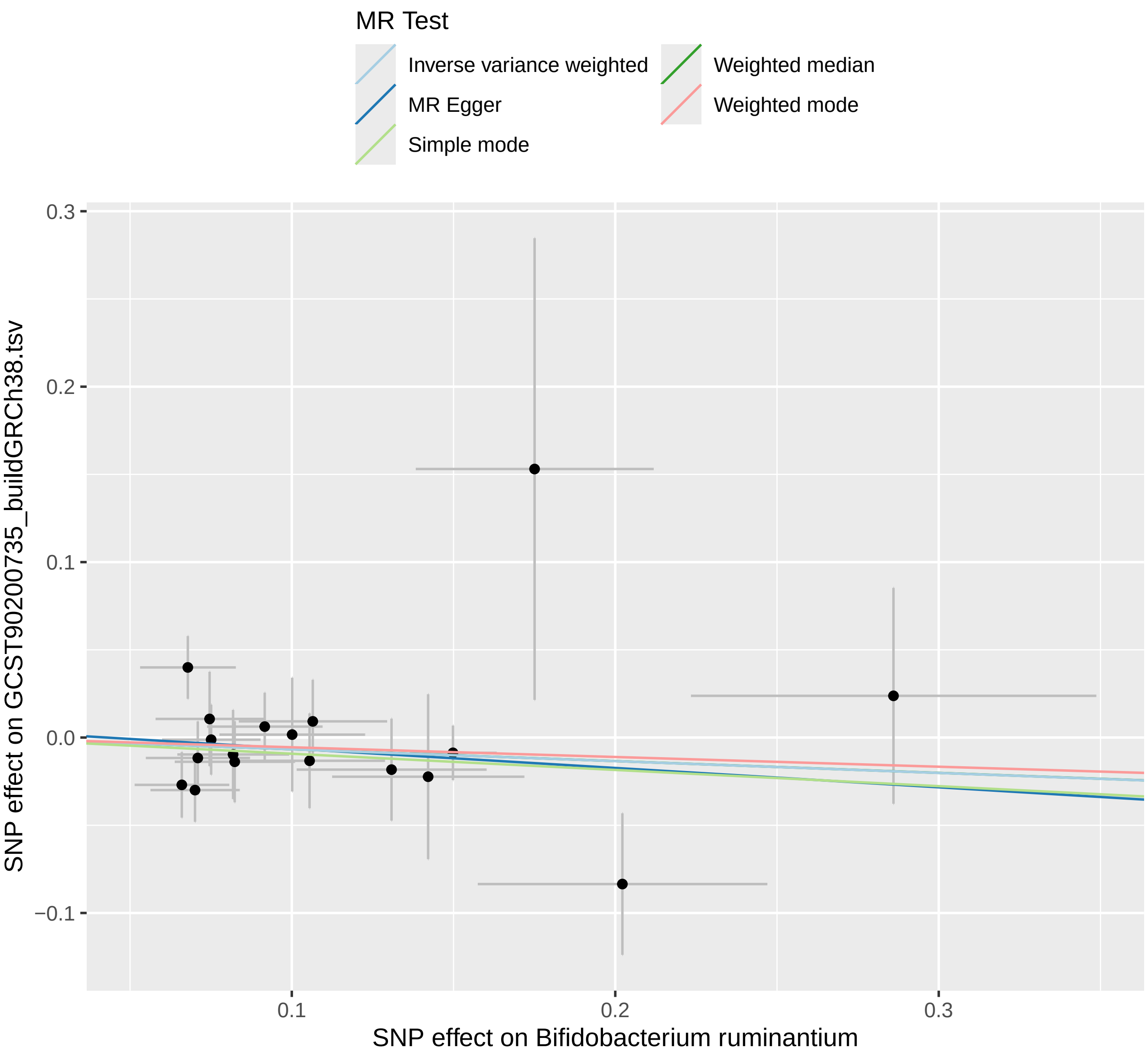

MR Test
Inverse variance weighted
MR Egger
Simple mode
Weighted median
Weighted mode
0.3
0.2
0.1
0.0
−0.1
SNP effect on GCST90200735_buildGRCh38.tsv
0.1
0.2
0.3
SNP effect on Bifidobacterium ruminantium

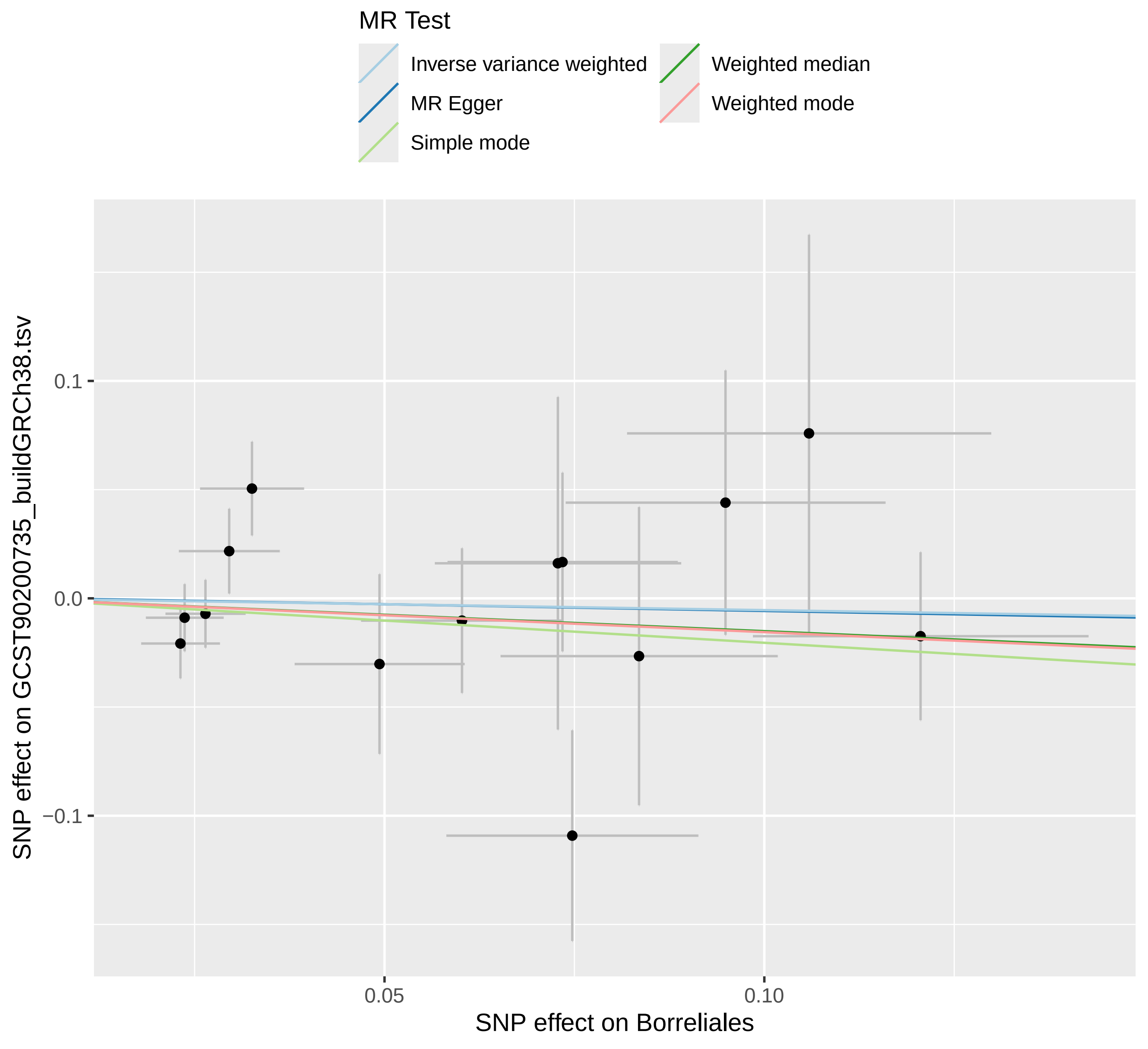

MR Test
Inverse variance weighted
MR Egger
Simple mode
Weighted median
Weighted mode
0.1
0.0
−0.1
0.05
0.10
SNP effect on GCST90200735_buildGRCh38.tsv
SNP effect on Borreliales

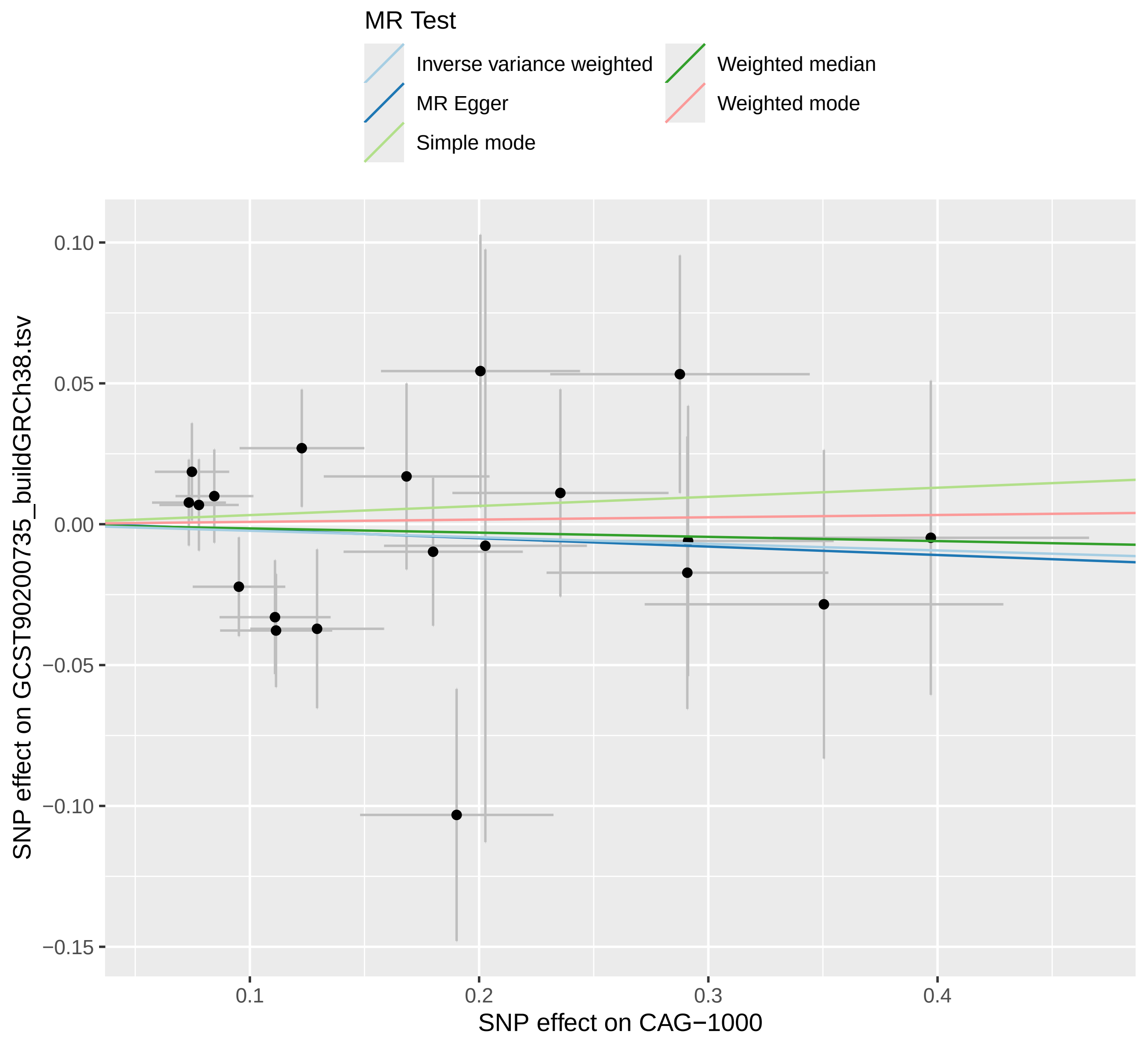
MR Test
Inverse variance weighted
MR Egger
Simple mode
Weighted median
Weighted mode
0.10
0.05
0.00
−0.05
−0.10
−0.15
SNP effect on GCST90200735_buildGRCh38.tsv
0.1
0.2
0.3
0.4
SNP effect on CAG−1000

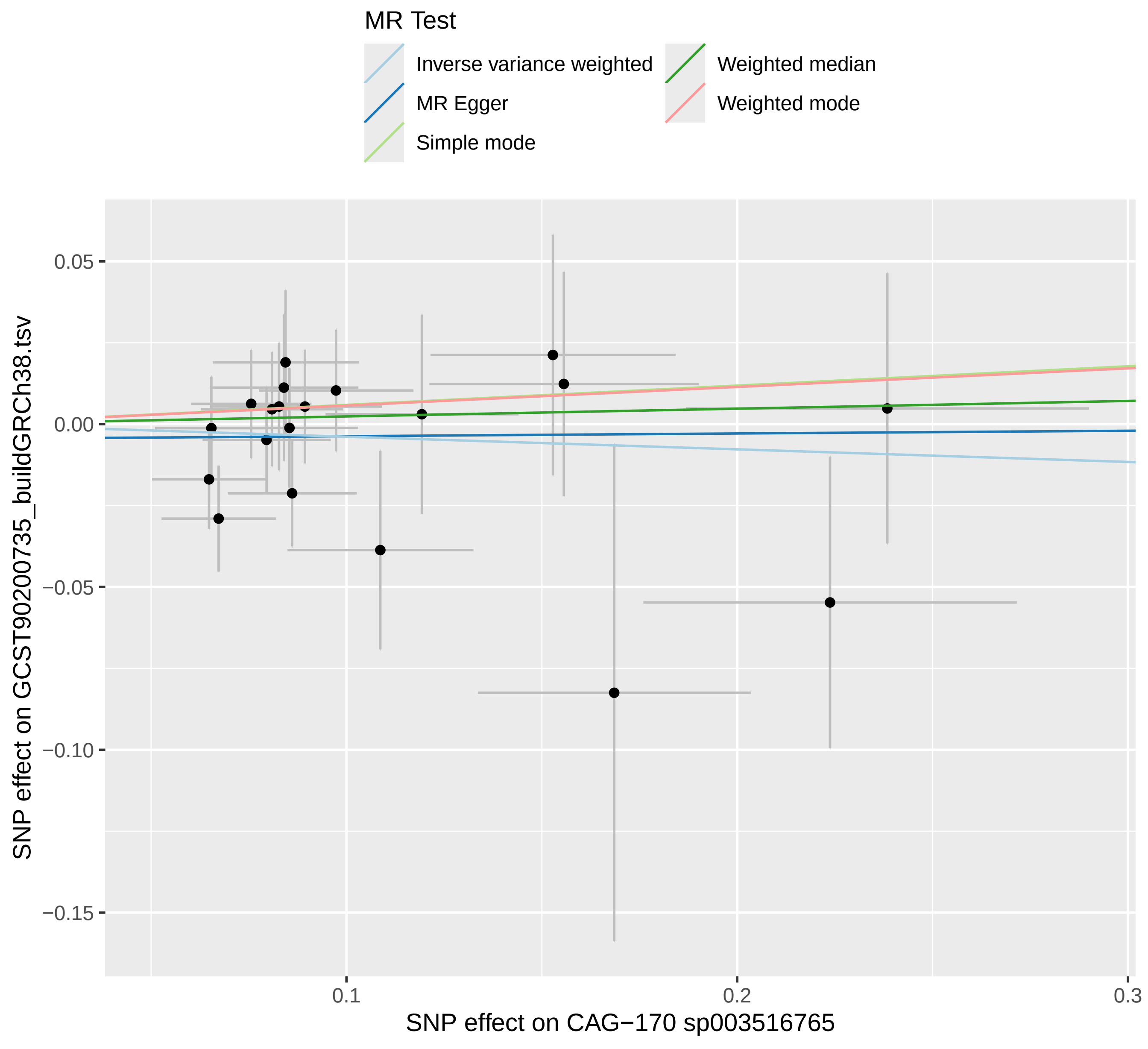

MR Test
Inverse variance weighted
MR Egger
Simple mode
Weighted median
Weighted mode
0.05
0.00
−0.05
−0.10
−0.15
0.1
0.2
0.3
SNP effect on GCST90200735_buildGRCh38.tsv
SNP effect on CAG−170 sp003516765

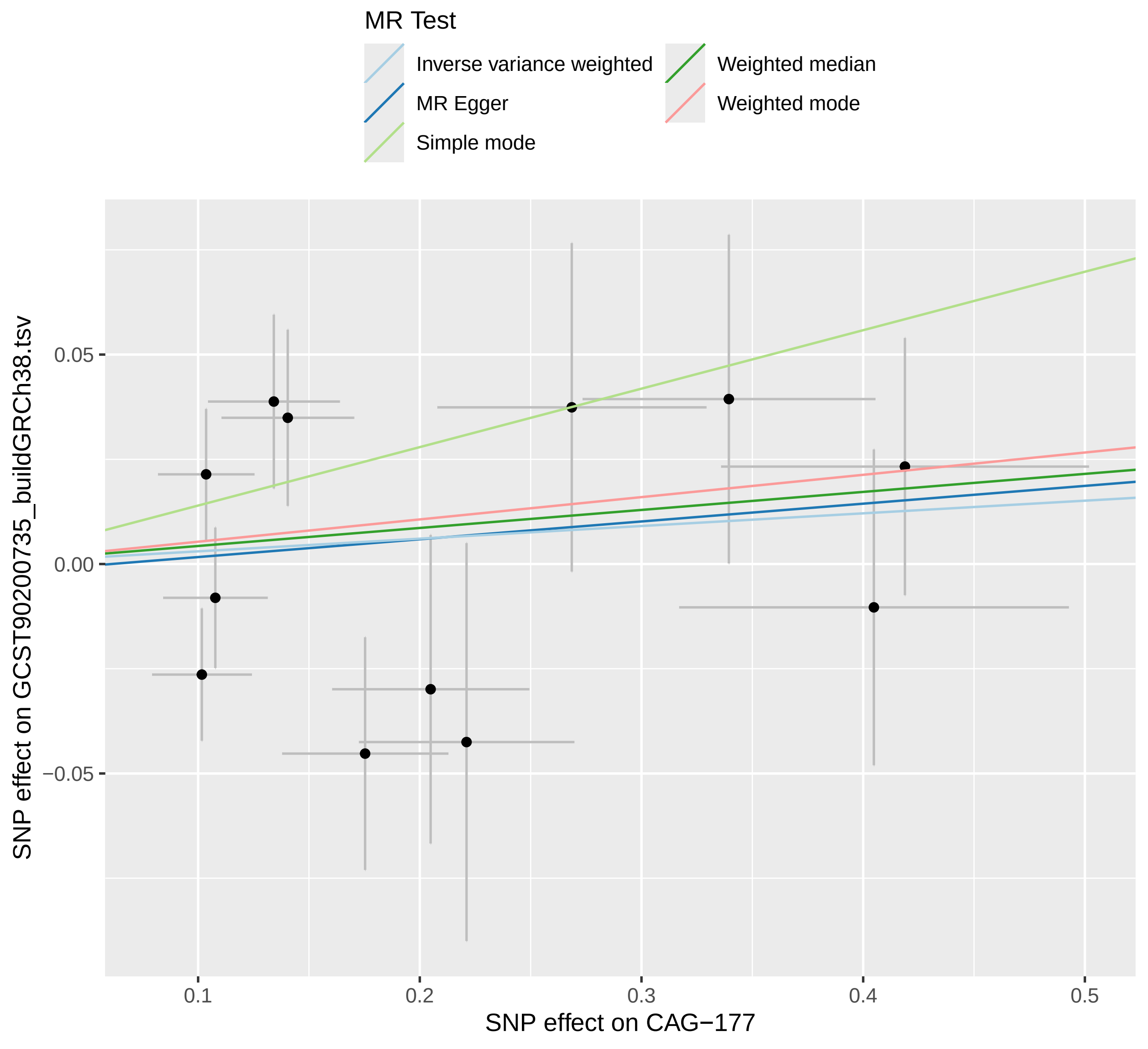
MR Test
Inverse variance weighted
MR Egger
Simple mode
Weighted median
Weighted mode
0.05
0.00
−0.05
0.1
0.2
0.3
0.4
0.5
SNP effect on CAG−177
SNP effect on GCST90200735_buildGRCh38.tsv

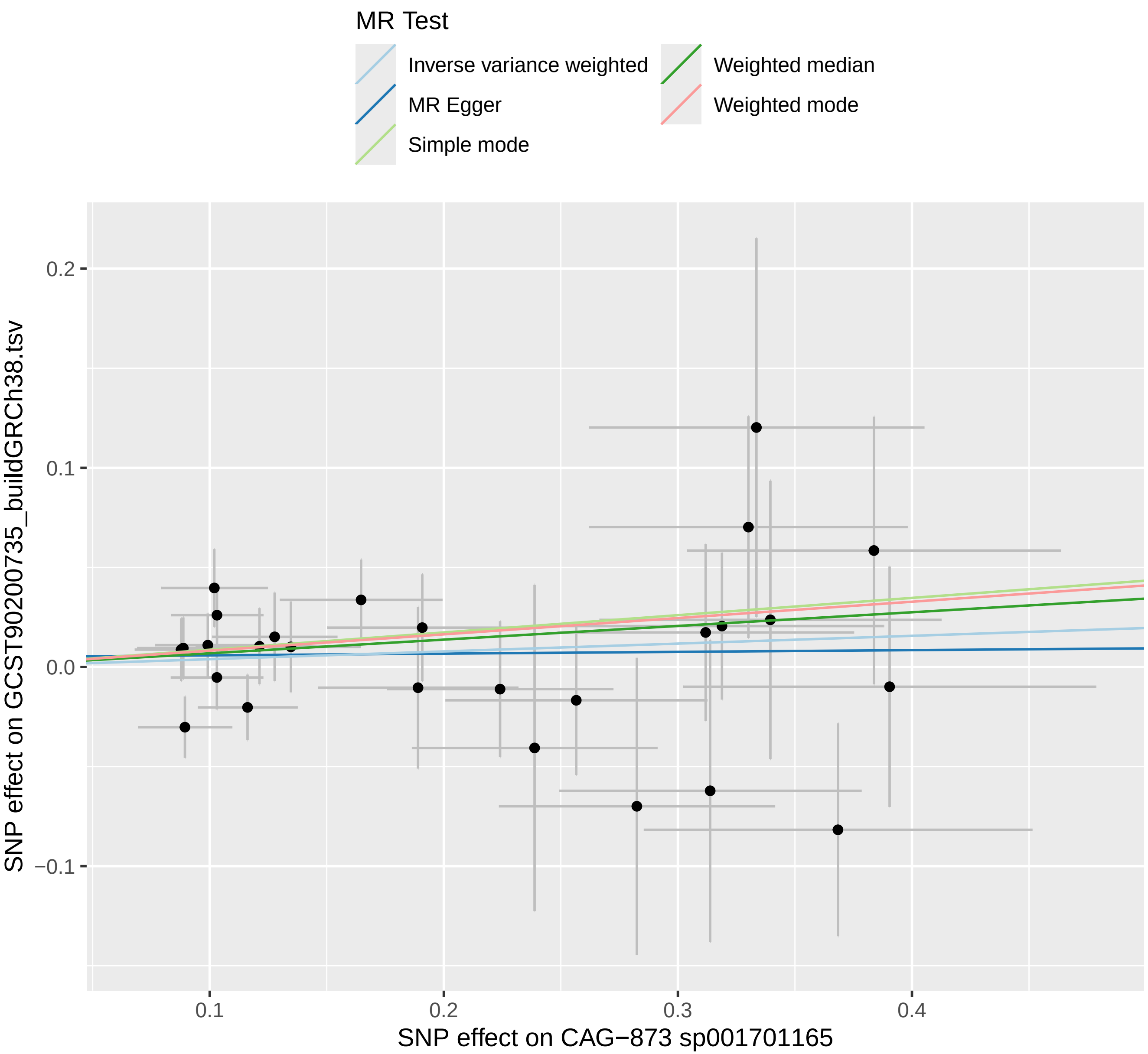

MR Test
Inverse variance weighted
MR Egger
Simple mode
Weighted median
Weighted mode
SNP effect on GCST90200735_buildGRCh38.tsv
0.2
0.1
0.0
−0.1
0.1
0.2
0.3
0.4
SNP effect on CAG−873 sp001701165

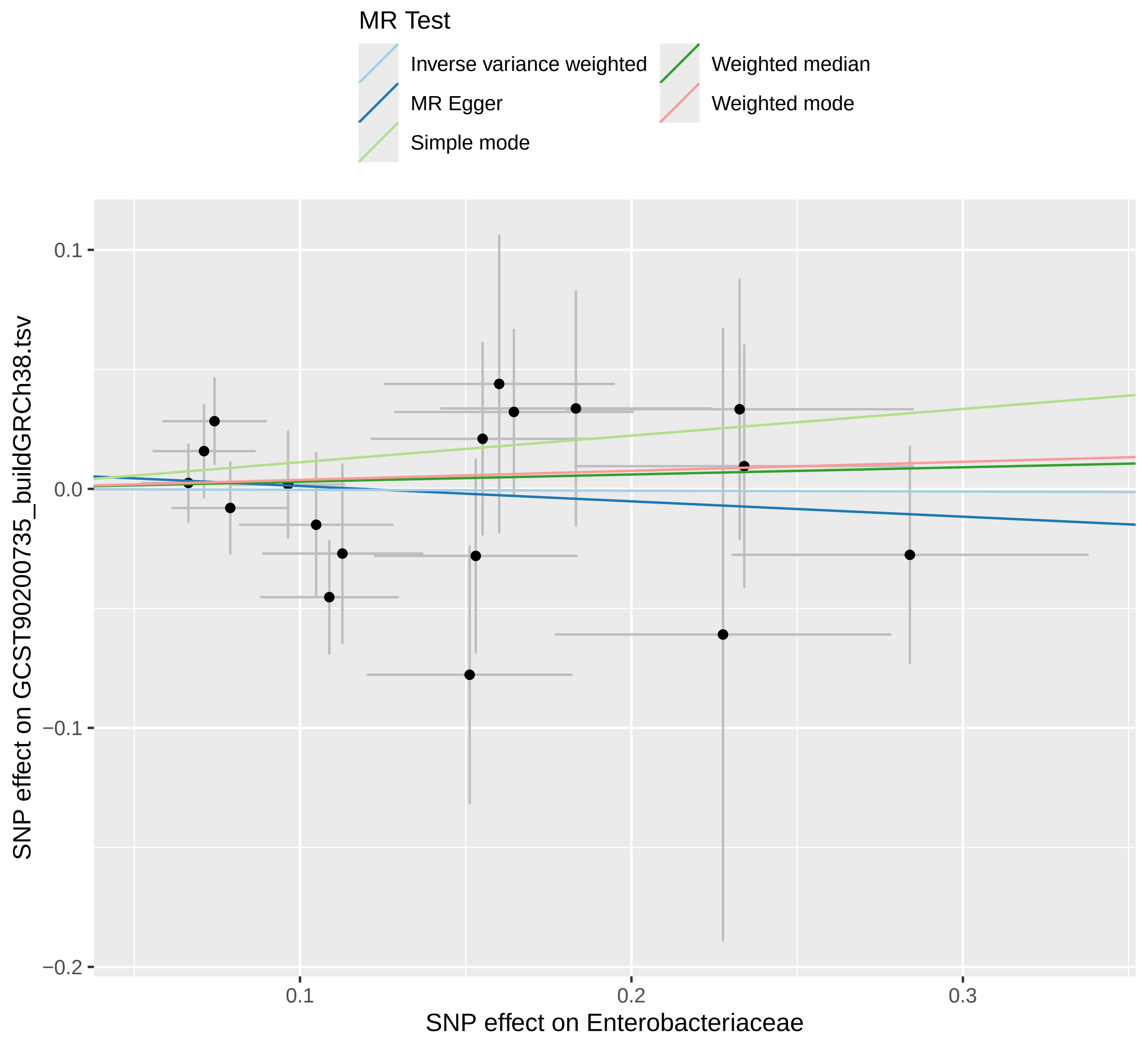
MR Test
Inverse variance weighted
MR Egger
Simple mode
Weighted median
Weighted mode
0.1
0.0
−0.1
−0.2
0.1
0.2
0.3
SNP effect on GCST90200735_buildGRCh38.tsv
SNP effect on Enterobacteriaceae

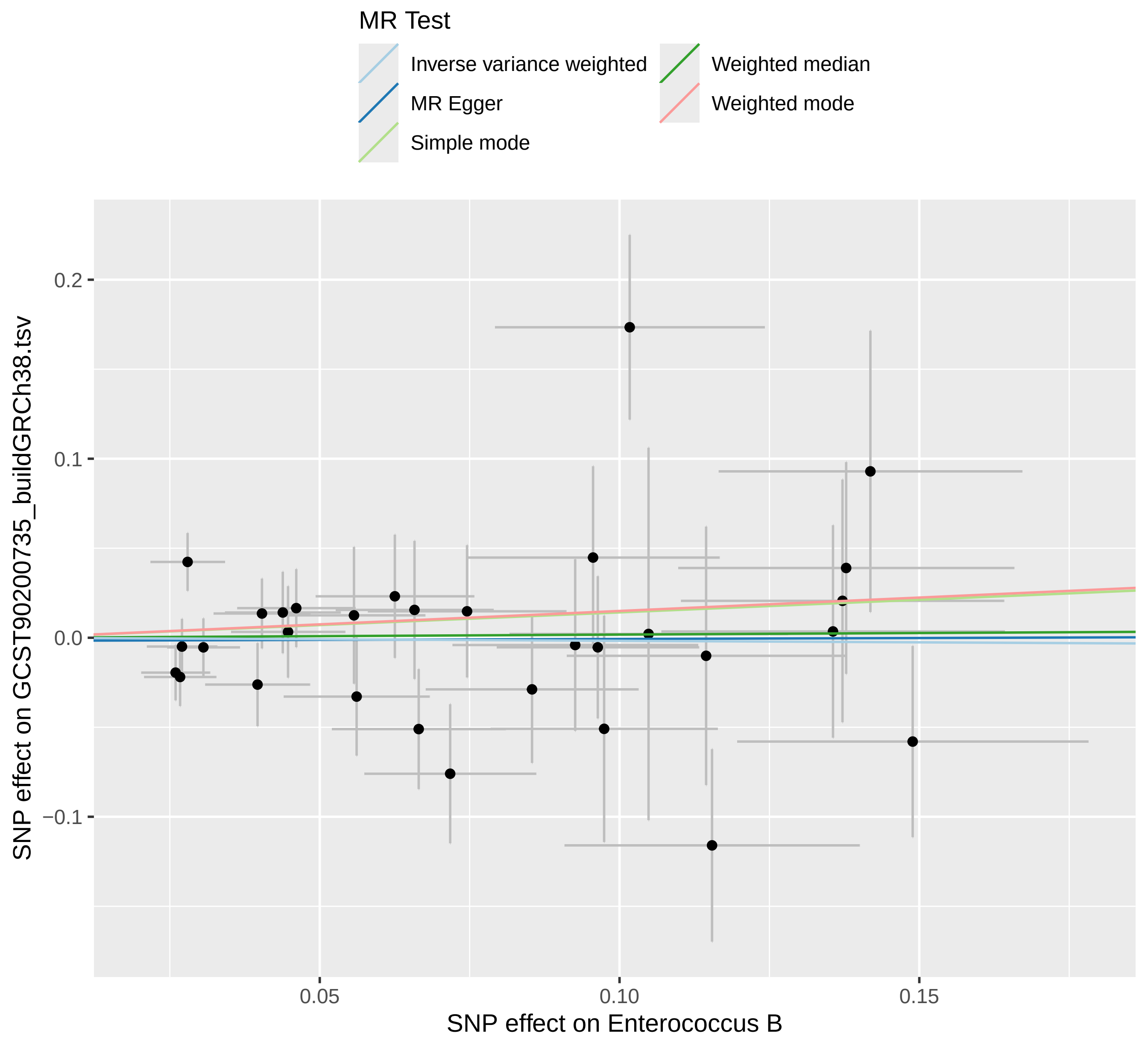

MR Test
Inverse variance weighted
MR Egger
Simple mode
Weighted median
Weighted mode
0.2
0.1
0.0
−0.1
0.05
0.10
0.15
SNP effect on GCST90200735_buildGRCh38.tsv
SNP effect on Enterococcus B

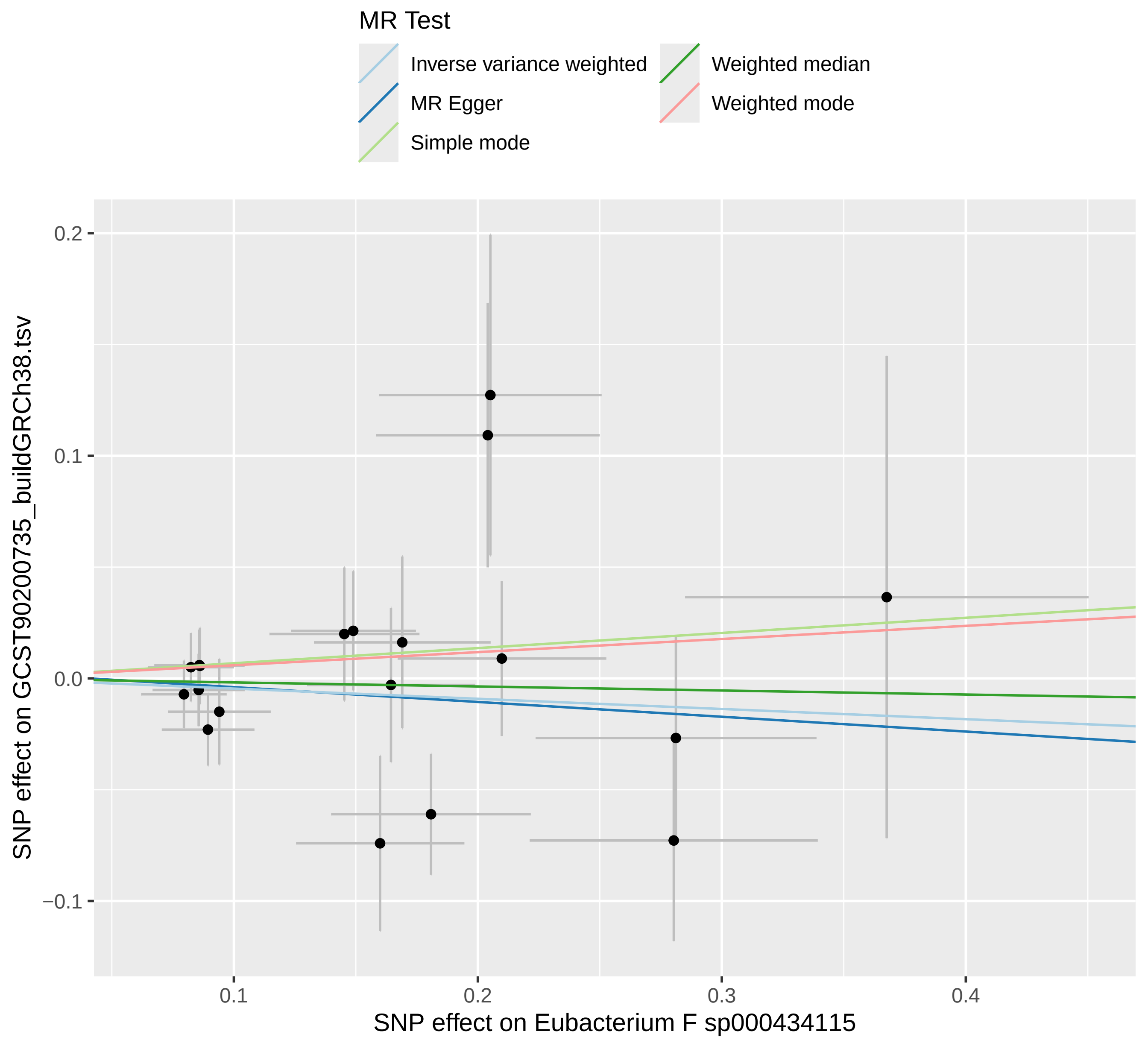

MR Test
Inverse variance weighted
MR Egger
Simple mode
Weighted median
Weighted mode
0.2
0.1
0.0
−0.1
SNP effect on GCST90200735_buildGRCh38.tsv
0.1
0.2
0.3
0.4
SNP effect on Eubacterium F sp000434115

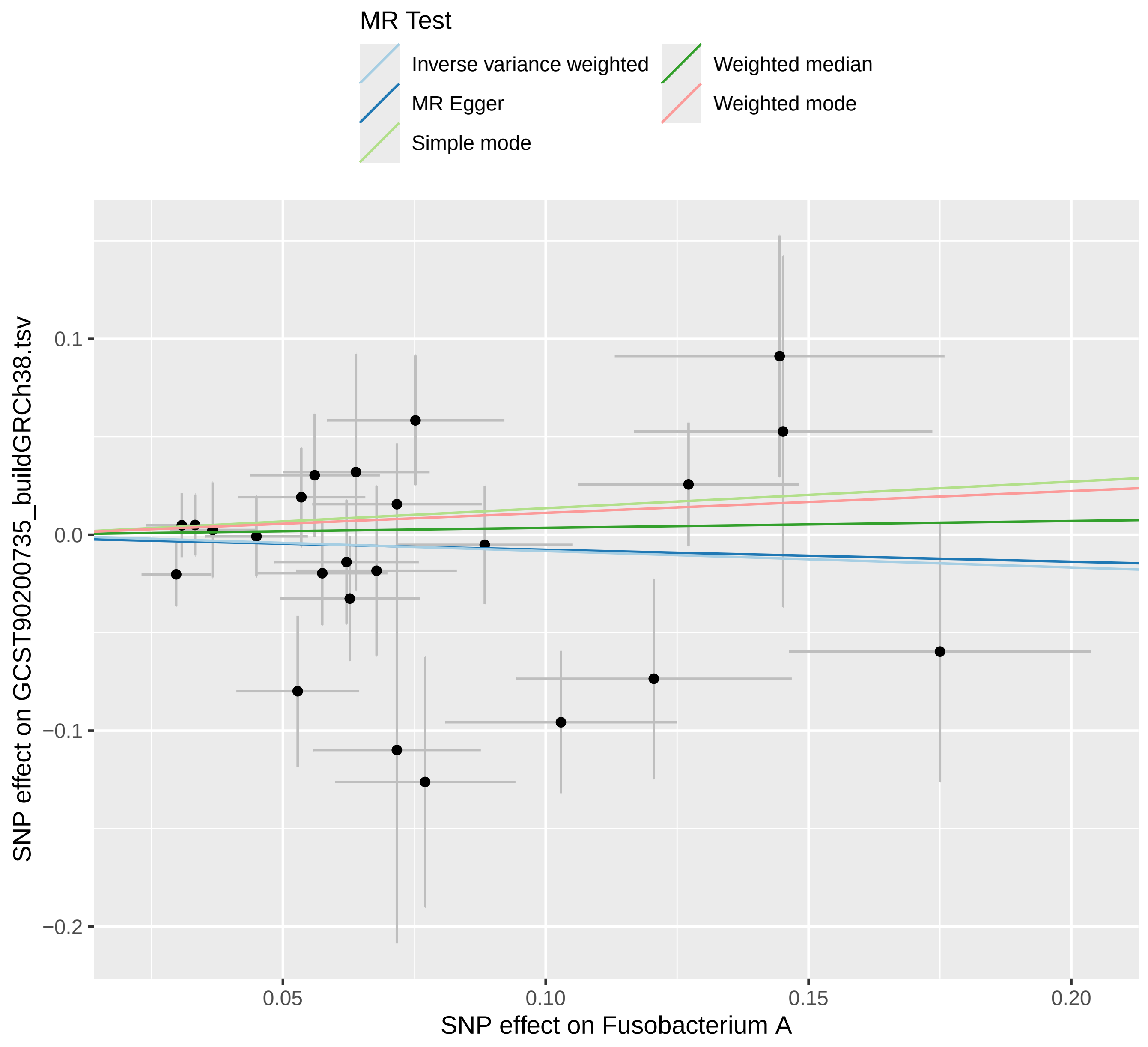
MR Test
Inverse variance weighted
MR Egger
Simple mode
Weighted median
Weighted mode
SNP effect on GCST90200735_buildGRCh38.tsv
0.1
0.0
−0.1
−0.2
0.05
0.10
0.15
0.20
SNP effect on Fusobacterium A

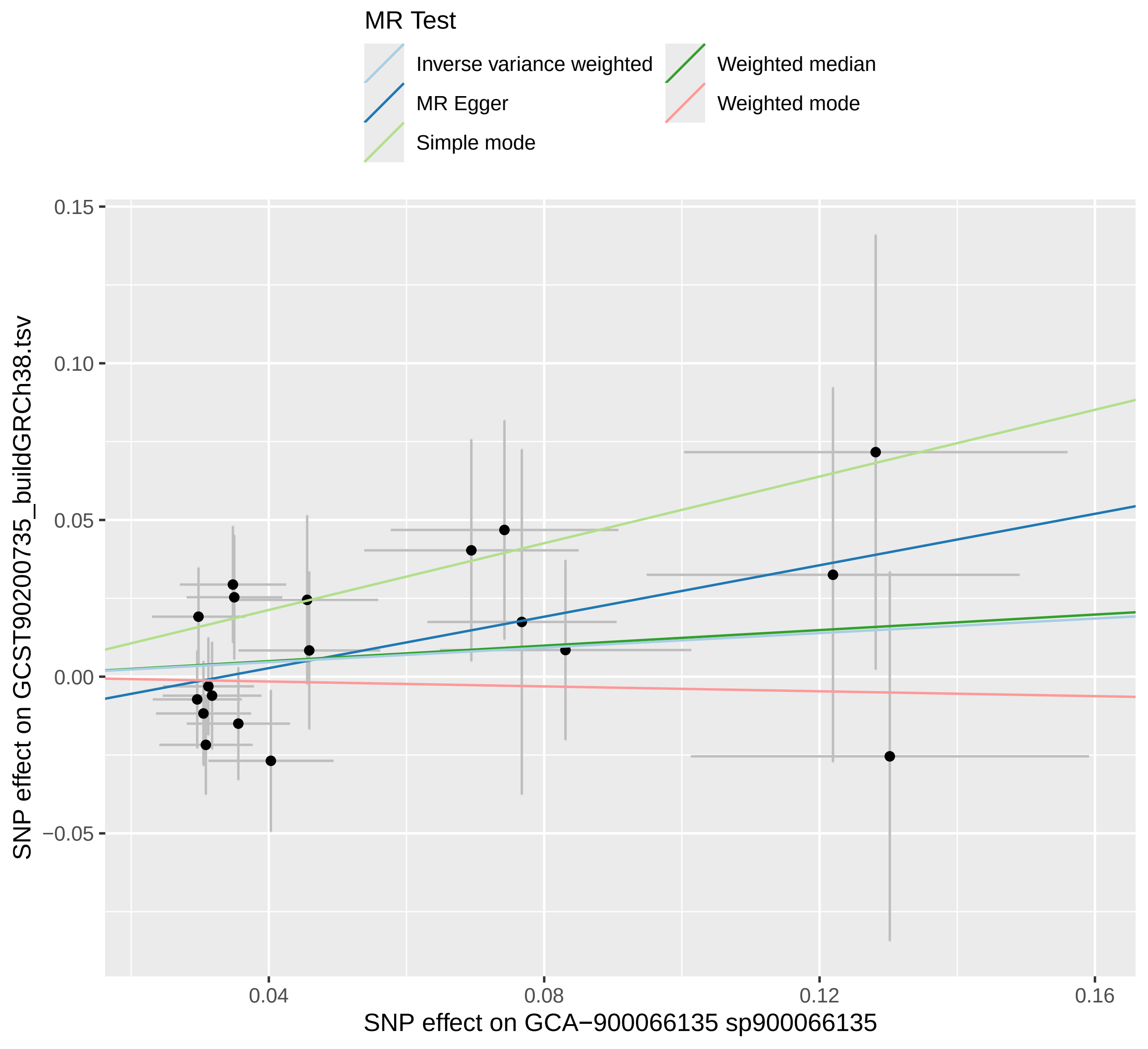
MR Test
Inverse variance weighted
MR Egger
Simple mode
Weighted median
Weighted mode
0.15
0.10
0.05
0.00
−0.05
0.04
0.08
0.12
0.16
SNP effect on GCST90200735_buildGRCh38.tsv
SNP effect on GCA−900066135 sp900066135

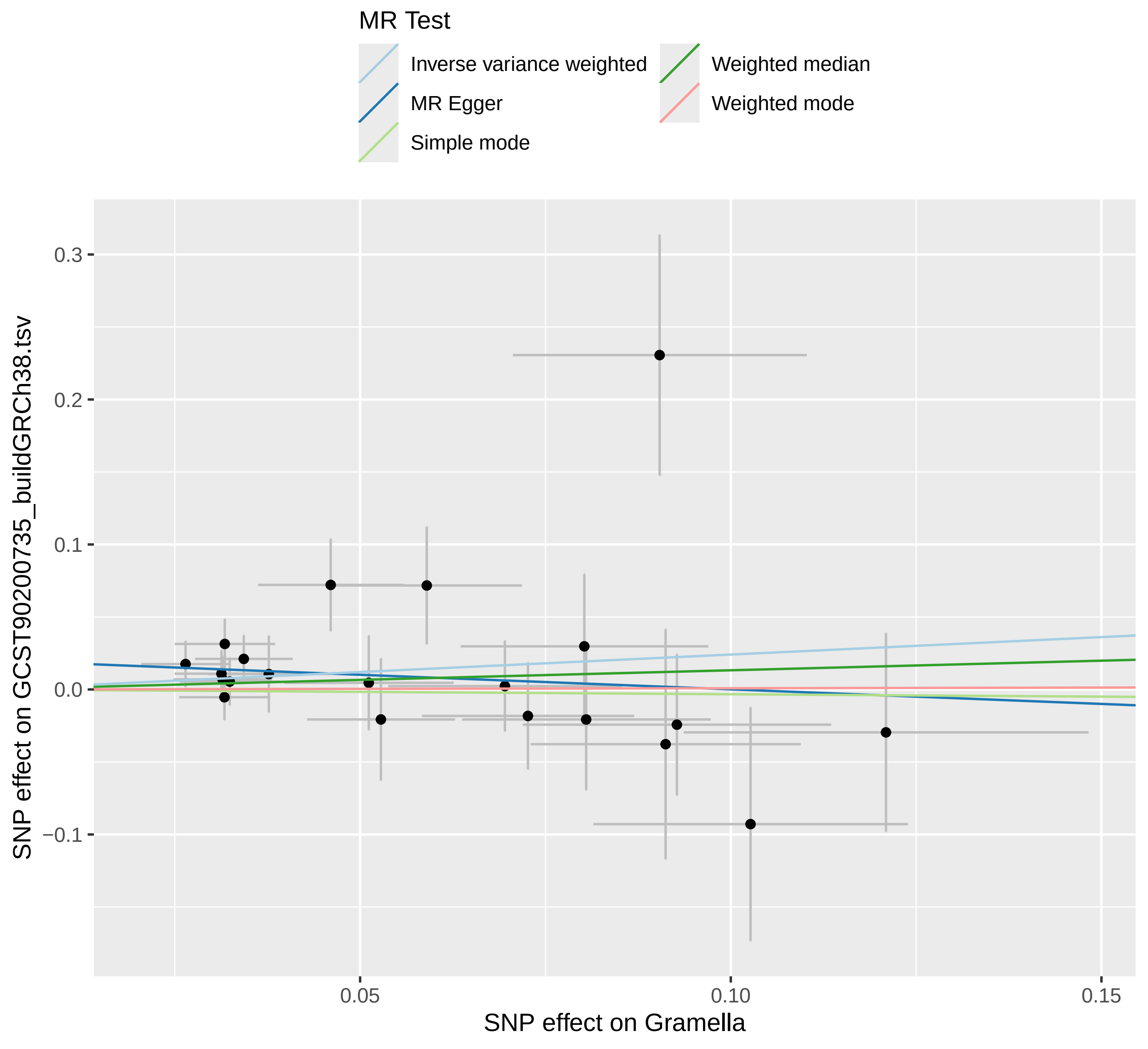
MR Test
Inverse variance weighted
Weighted median
MR Egger
Weighted mode
Simple mode
0.3
0.2
0.1
0.0
−0.1
0.05
0.10
0.15
SNP effect on GCST90200735_buildGRCh38.tsv
SNP effect on Gramella

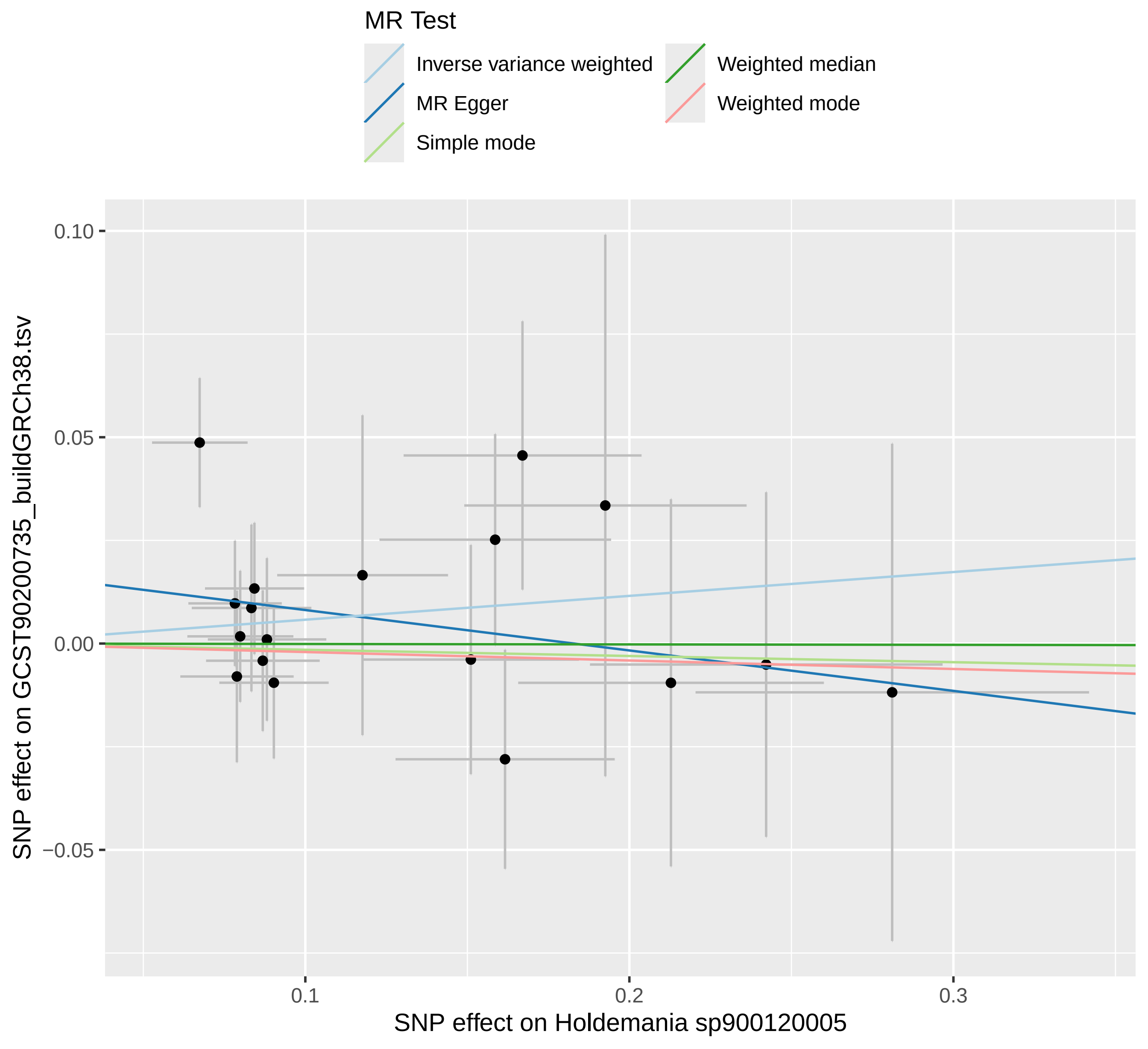
MR Test
Inverse variance weighted
MR Egger
Simple mode
Weighted median
Weighted mode
0.10
0.05
0.00
−0.05
0.1
0.2
0.3
SNP effect on GCST90200735_buildGRCh38.tsv
SNP effect on Holdemania sp900120005

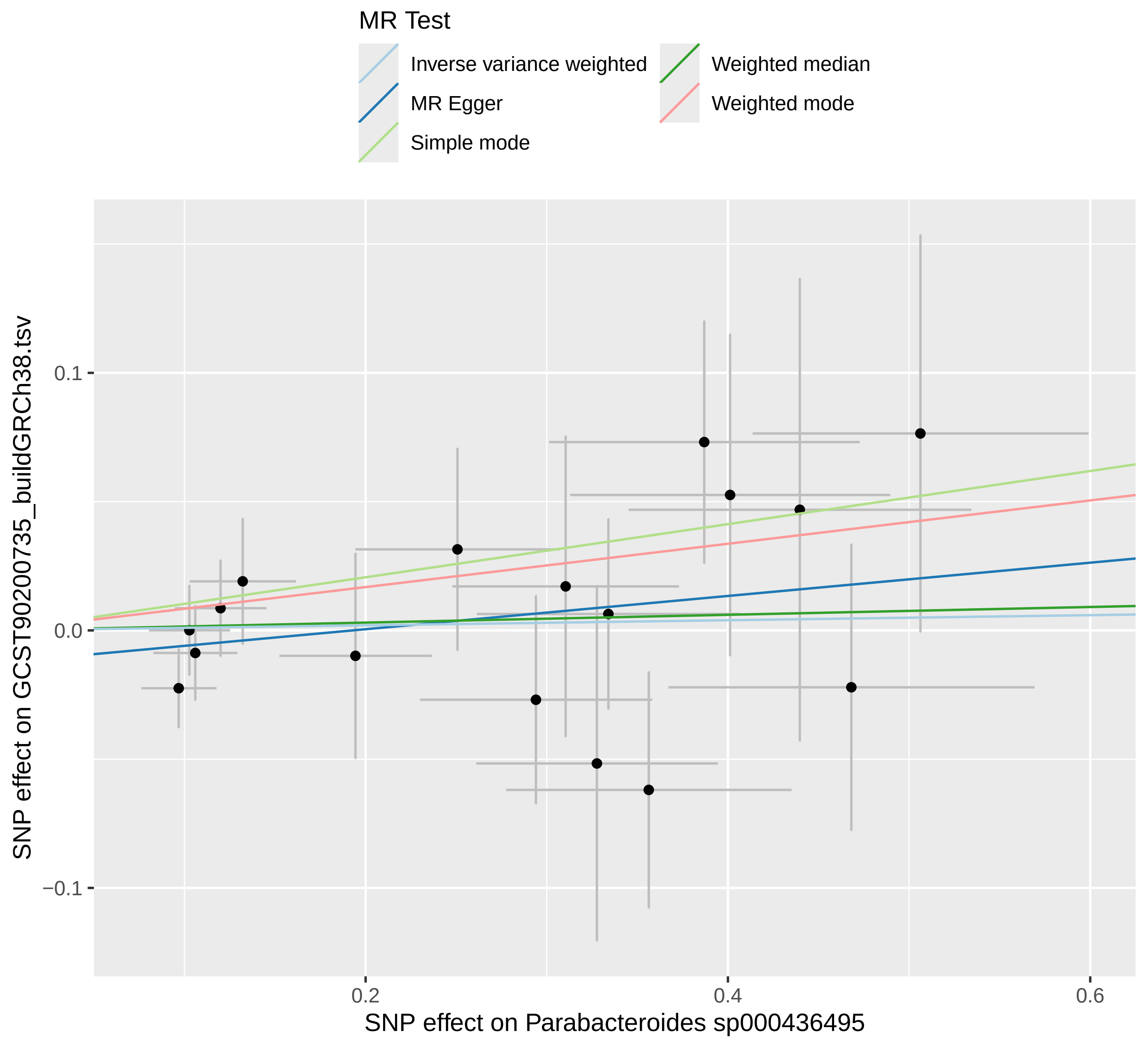

MR Test
Inverse variance weighted
MR Egger
Simple mode
Weighted median
Weighted mode
0.1
0.0
−0.1
0.2
0.4
0.6
SNP effect on GCST90200735_buildGRCh38.tsv
SNP effect on Parabacteroides sp000436495

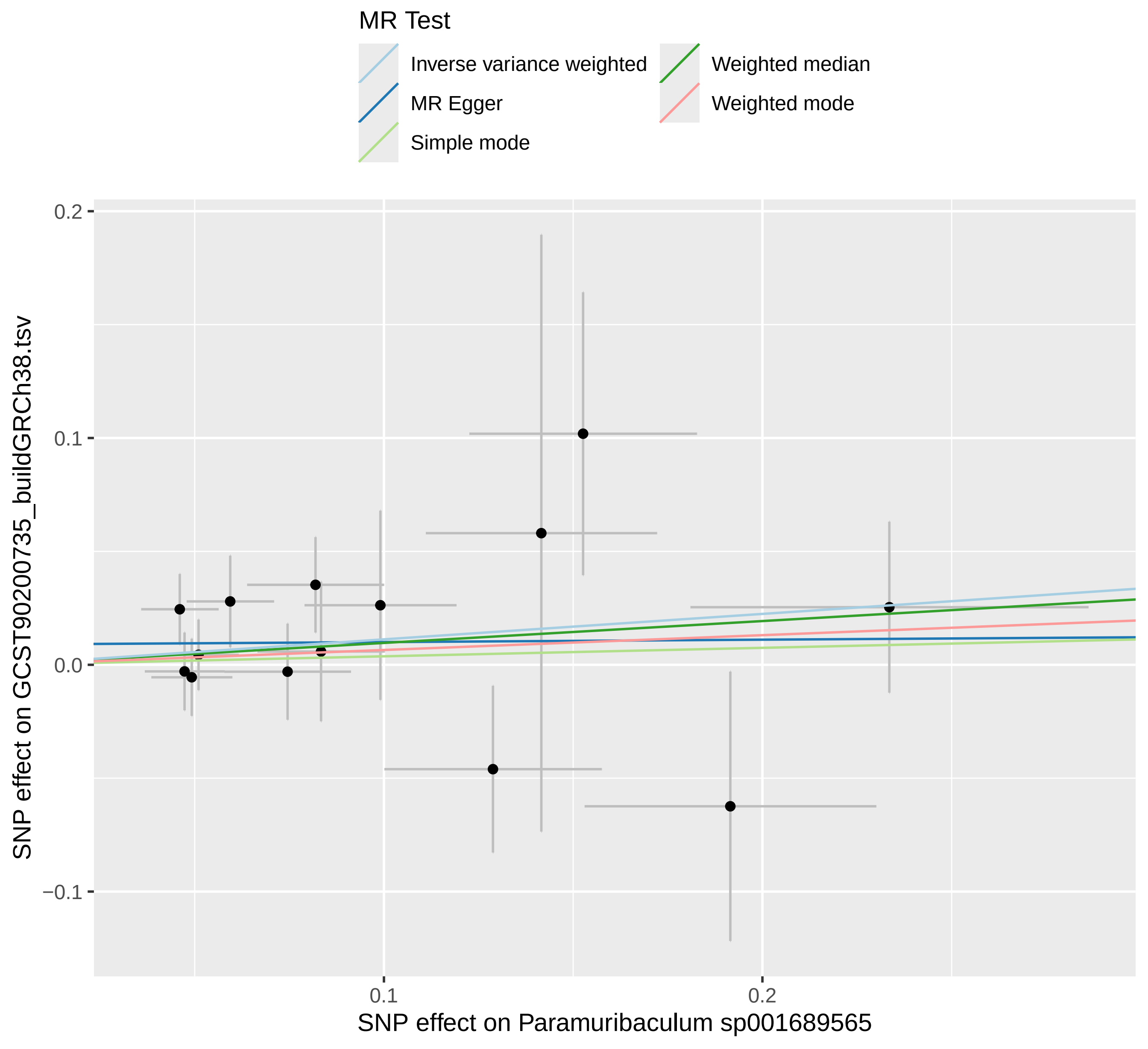
MR Test
Inverse variance weighted
MR Egger
Simple mode
Weighted median
Weighted mode
0.2
0.1
0.0
−0.1
0.1
0.2
SNP effect on GCST90200735_buildGRCh38.tsv
SNP effect on Paramuribaculum sp001689565

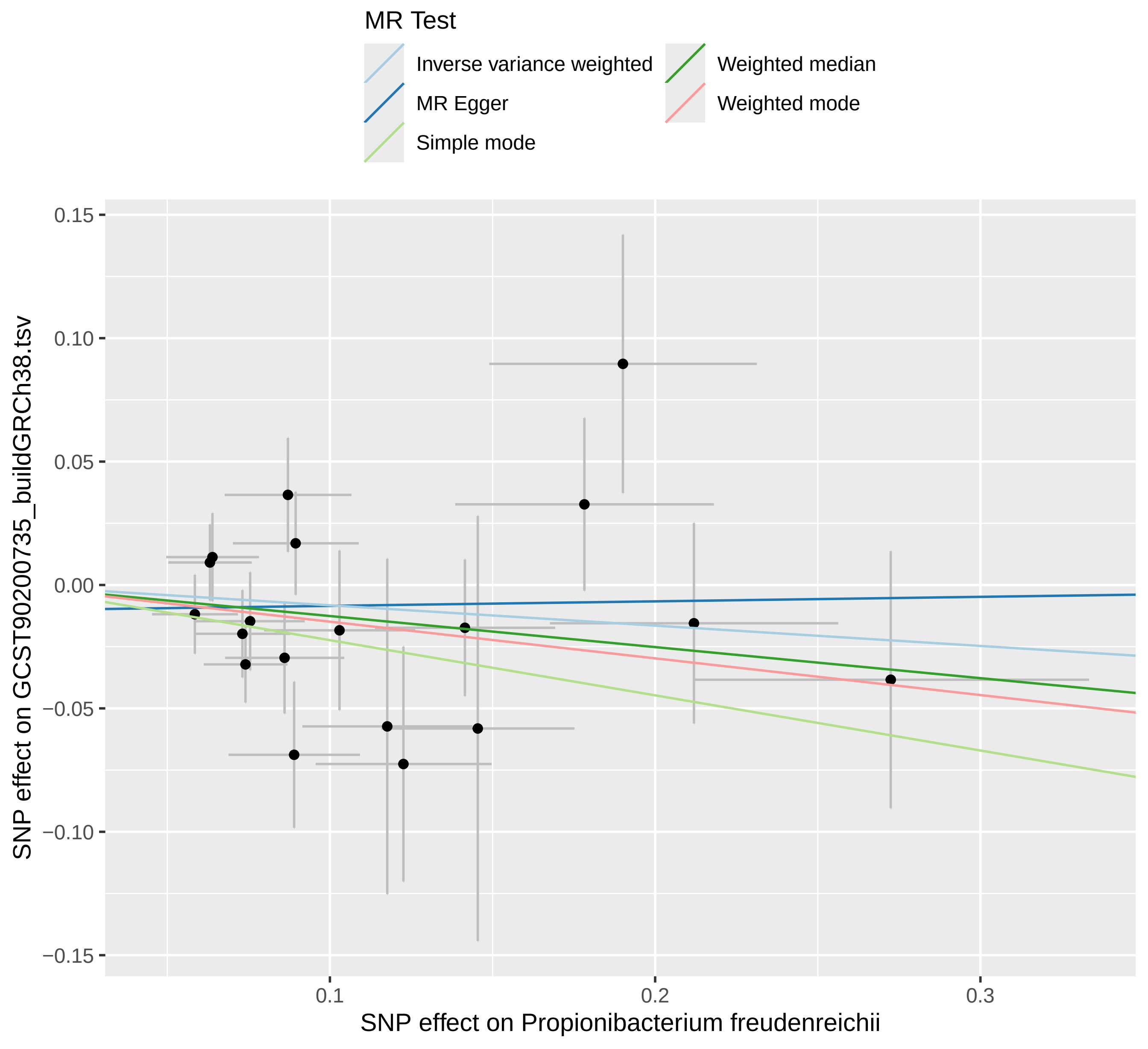

MR Test
Inverse variance weighted
MR Egger
Simple mode
Weighted median
Weighted mode
0.15
0.10
0.05
0.00
−0.05
−0.10
−0.15
0.1
0.2
0.3
SNP effect on GCST90200735_buildGRCh38.tsv
SNP effect on Propionibacterium freudenreichii

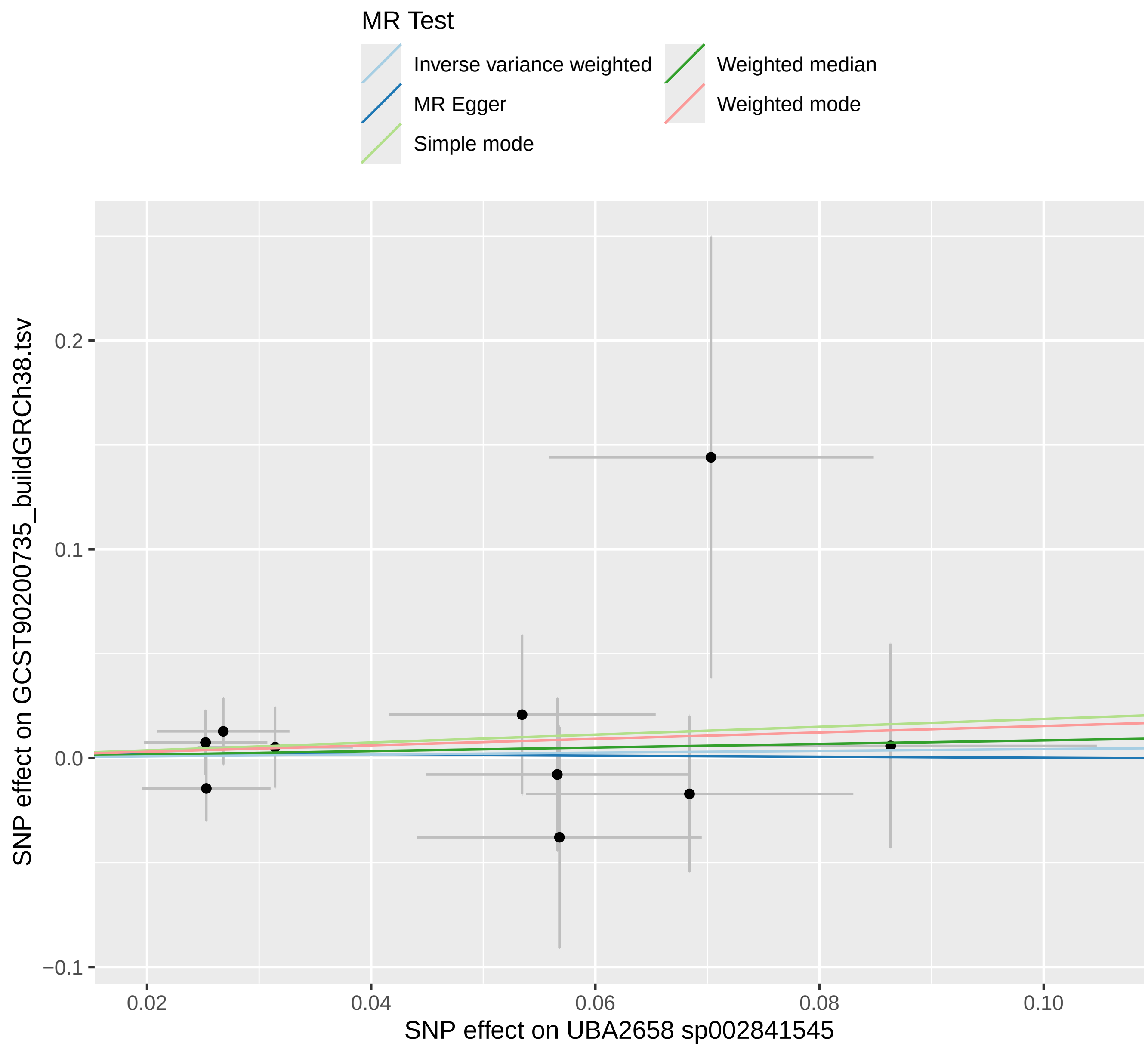

MR Test
Inverse variance weighted
MR Egger
Simple mode
Weighted median
Weighted mode
SNP effect on GCST90200735_buildGRCh38.tsv
0.2
0.1
0.0
−0.1
0.02
0.04
0.06
0.08
0.10
SNP effect on UBA2658 sp002841545

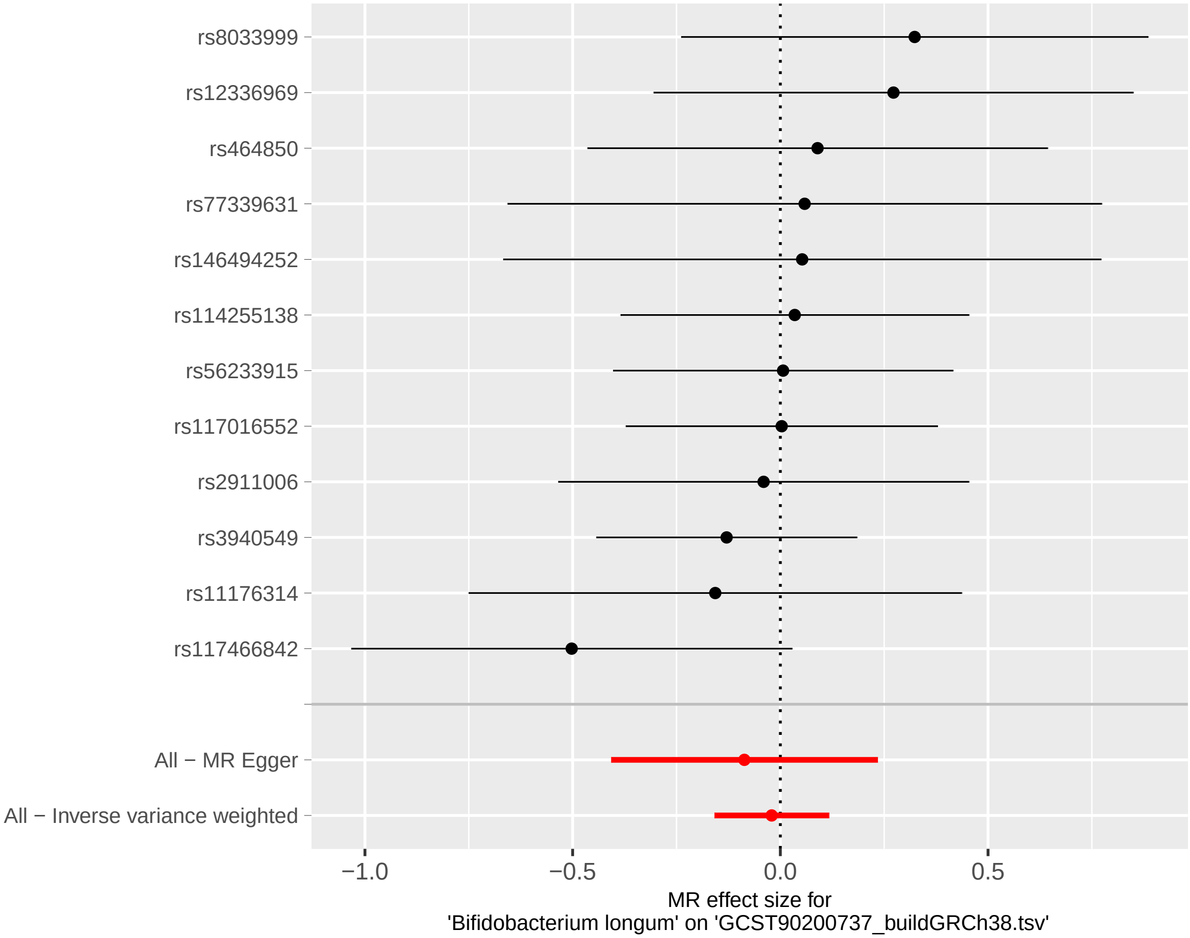
rs8033999
rs12336969
rs464850
rs77339631
rs146494252
rs114255138
rs56233915
rs117016552
rs2911006
rs3940549
rs11176314
rs117466842
All − MR Egger
All − Inverse variance weighted
−1.0
−0.5
0.0
0.5
MR effect size for
'Bifidobacterium longum' on 'GCST90200737_buildGRCh38.tsv'

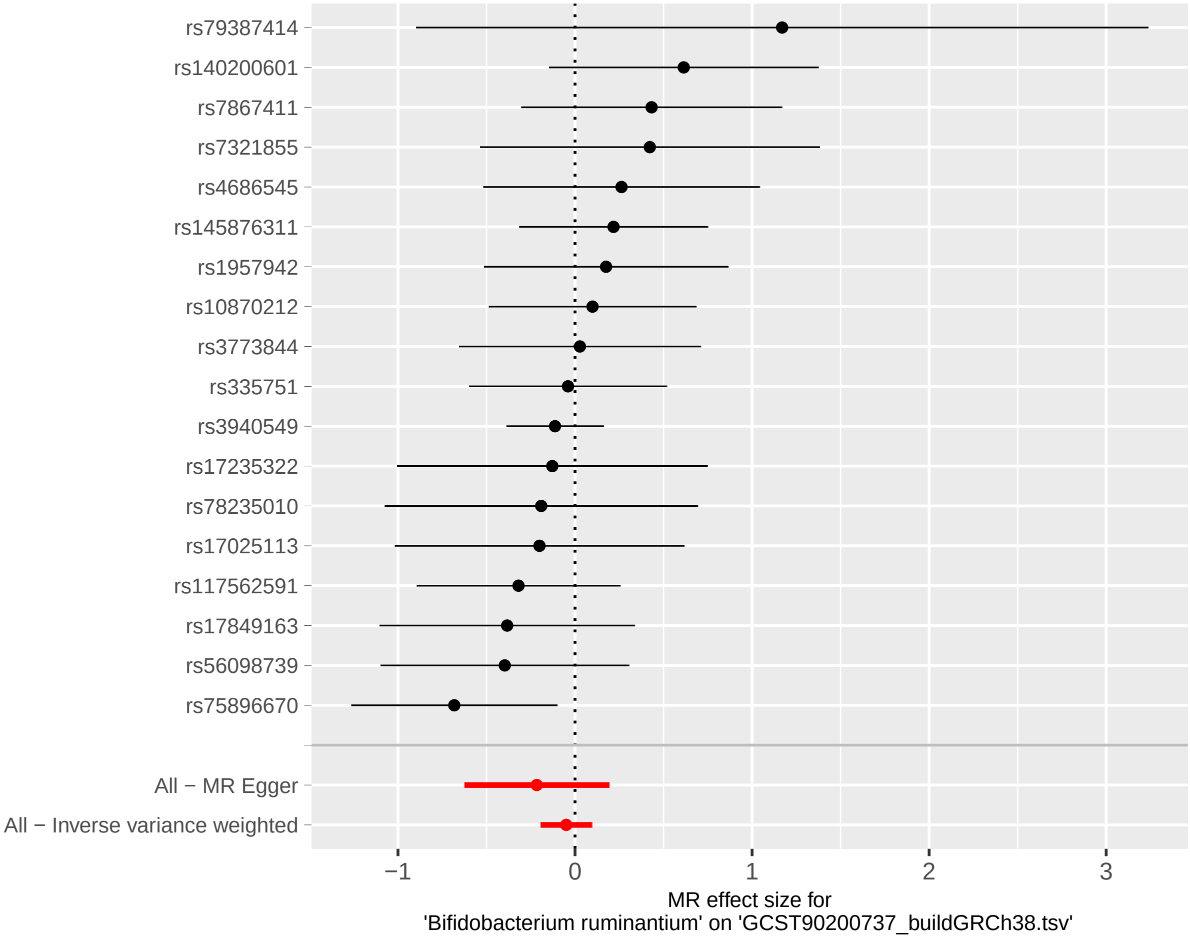
rs79387414
rs140200601
rs7867411
rs7321855
rs4686545
rs145876311
rs1957942
rs10870212
rs3773844
rs335751
rs3940549
rs17235322
rs78235010
rs17025113
rs117562591
rs17849163
rs56098739
rs75896670
All − MR Egger
All − Inverse variance weighted
−1
0
1
2
3
MR effect size for
'Bifidobacterium ruminantium' on 'GCST90200737_buildGRCh38.tsv'

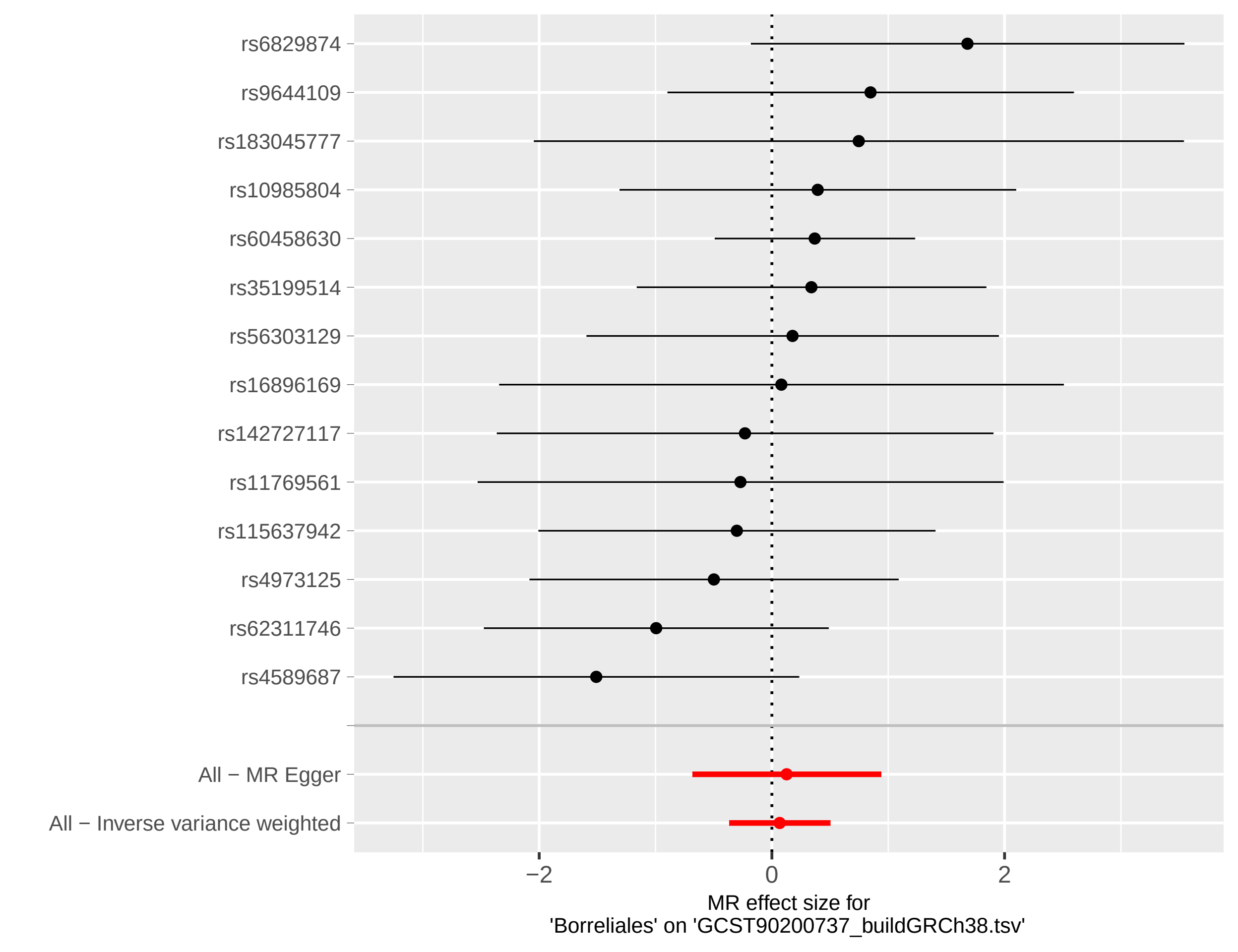

rs6829874
rs9644109
rs183045777
rs10985804
rs60458630
rs35199514
rs56303129
rs16896169
rs142727117
rs11769561
rs115637942
rs4973125
rs62311746
rs4589687
All − MR Egger
All − Inverse variance weighted
−2
0
2
MR effect size for
'Borreliales' on 'GCST90200737_buildGRCh38.tsv'

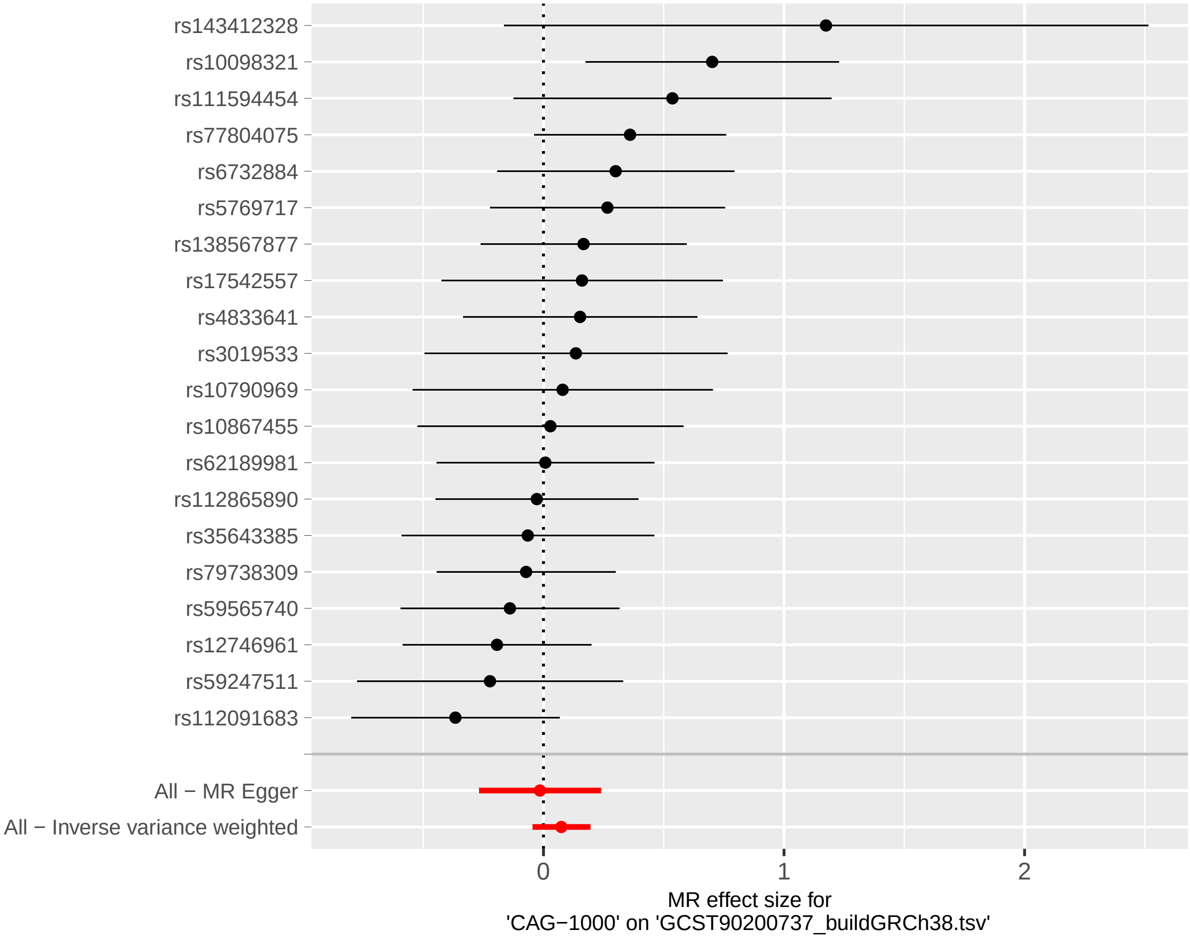
rs143412328
rs10098321
rs111594454
rs77804075
rs6732884
rs5769717
rs138567877
rs17542557
rs4833641
rs3019533
rs10790969
rs10867455
rs62189981
rs112865890
rs35643385
rs79738309
rs59565740
rs12746961
rs59247511
rs112091683
All − MR Egger
All − Inverse variance weighted
0
1
2
MR effect size for
'CAG−1000' on 'GCST90200737_buildGRCh38.tsv'

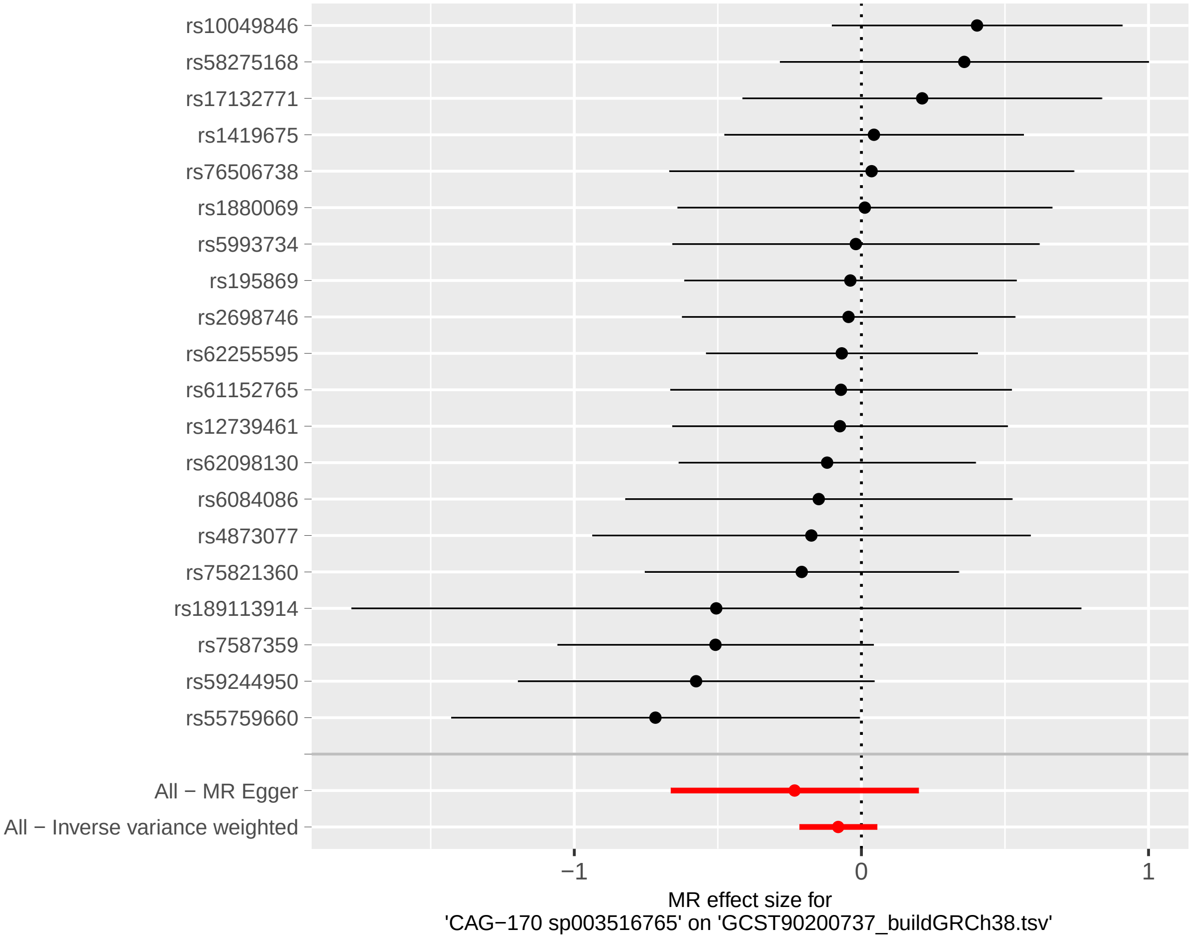
rs10049846
rs58275168
rs17132771
rs1419675
rs76506738
rs1880069
rs5993734
rs195869
rs2698746
rs62255595
rs61152765
rs12739461
rs62098130
rs6084086
rs4873077
rs75821360
rs189113914
rs7587359
rs59244950
rs55759660
All − MR Egger
All − Inverse variance weighted
−1
0
1
MR effect size for
'CAG−170 sp003516765' on 'GCST90200737_buildGRCh38.tsv'

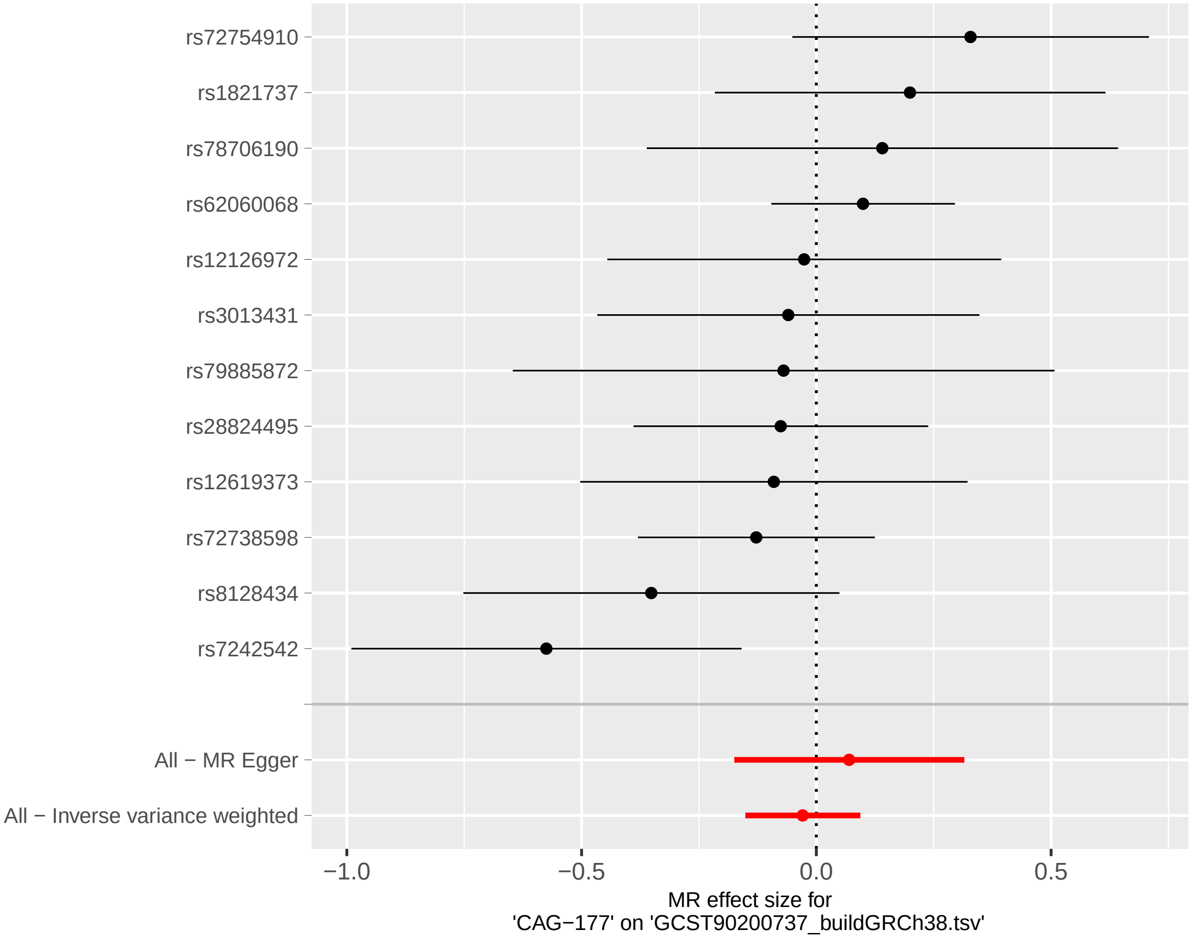
rs72754910
rs1821737
rs78706190
rs62060068
rs12126972
rs3013431
rs79885872
rs28824495
rs12619373
rs72738598
rs8128434
rs7242542
All − MR Egger
All − Inverse variance weighted
−1.0
−0.5
0.0
0.5
MR effect size for
'CAG−177' on 'GCST90200737_buildGRCh38.tsv'

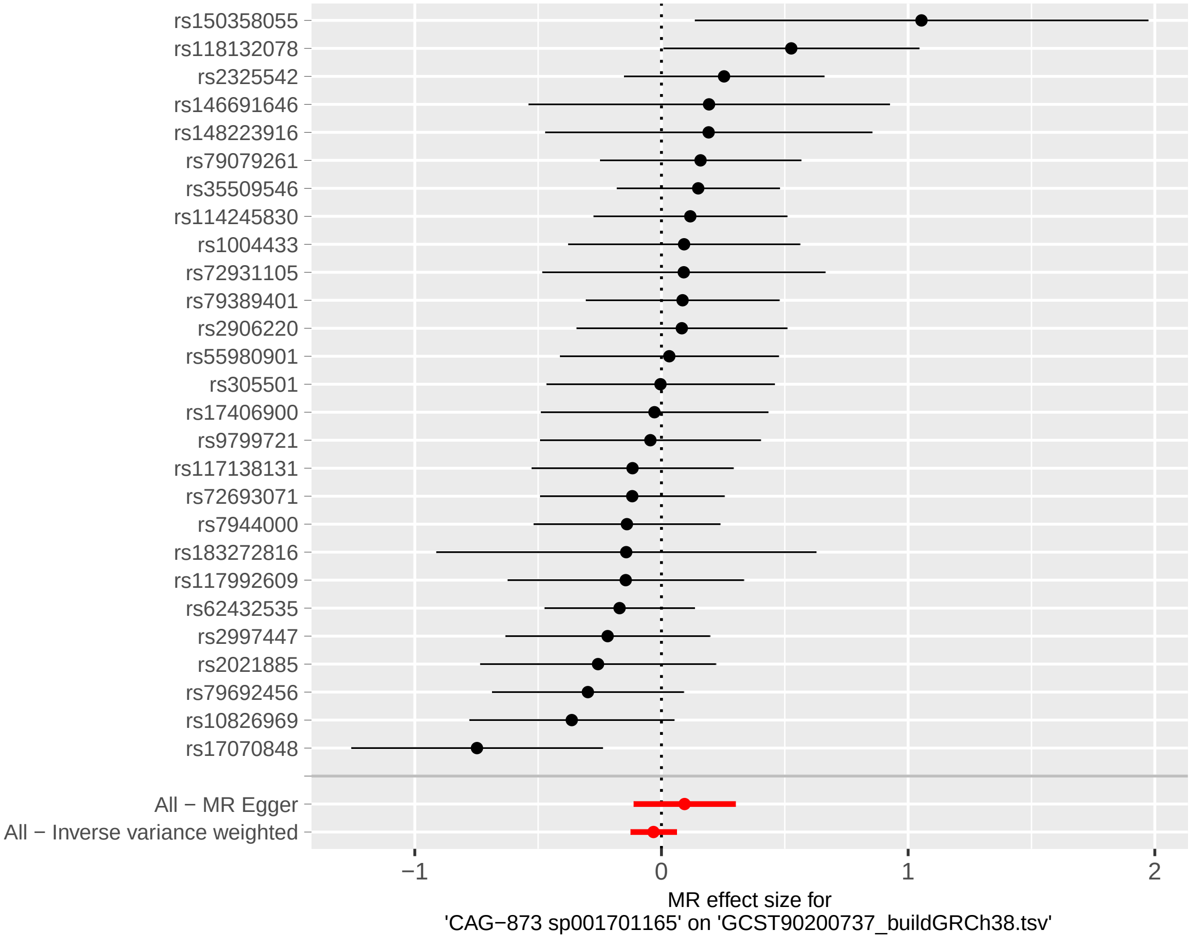
rs150358055
rs118132078
rs2325542
rs146691646
rs148223916
rs79079261
rs35509546
rs114245830
rs1004433
rs72931105
rs79389401
rs2906220
rs55980901
rs305501
rs17406900
rs9799721
rs117138131
rs72693071
rs7944000
rs183272816
rs117992609
rs62432535
rs2997447
rs2021885
rs79692456
rs10826969
rs17070848
All − MR Egger
All − Inverse variance weighted
−1
0
1
2
MR effect size for
'CAG−873 sp001701165' on 'GCST90200737_buildGRCh38.tsv'

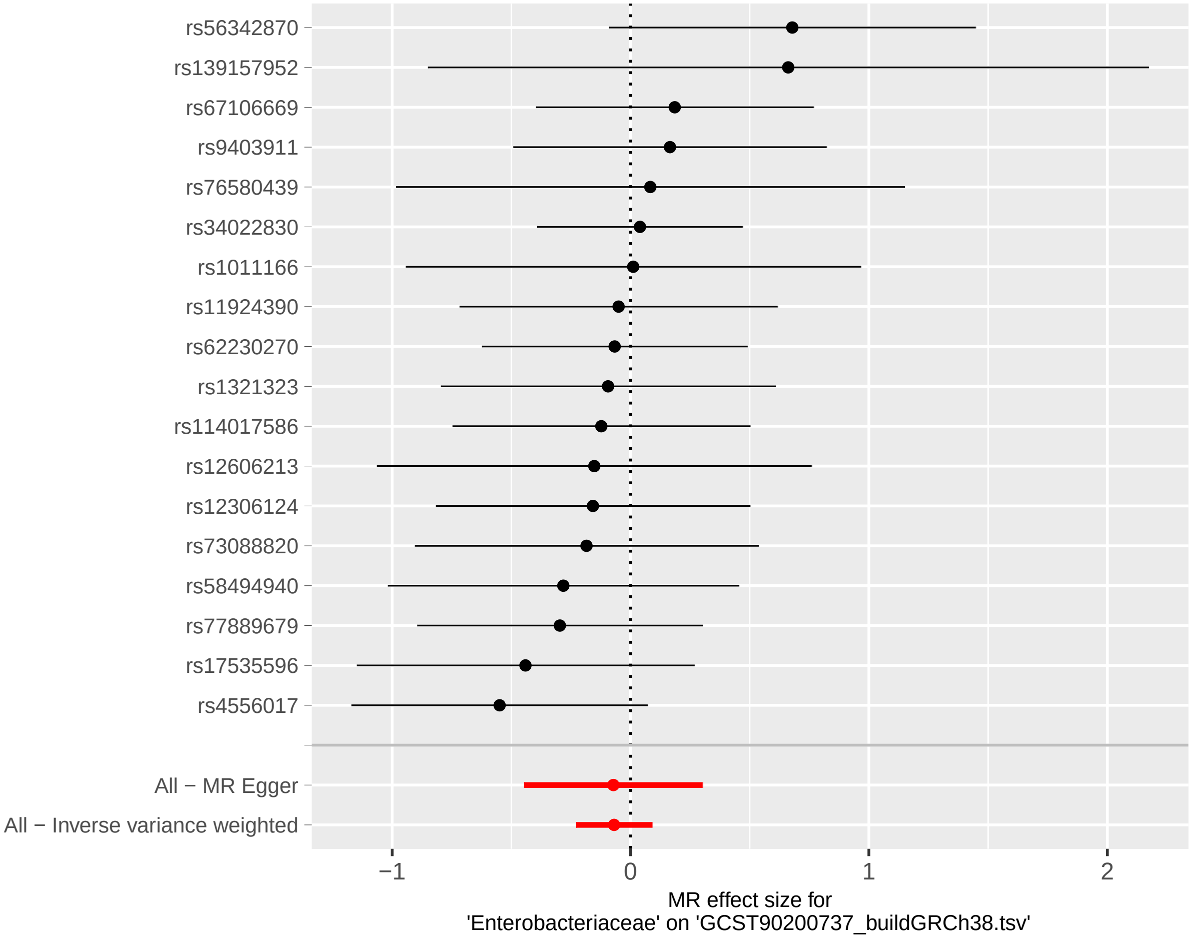
rs56342870
rs139157952
rs67106669
rs9403911
rs76580439
rs34022830
rs1011166
rs11924390
rs62230270
rs1321323
rs114017586
rs12606213
rs12306124
rs73088820
rs58494940
rs77889679
rs17535596
rs4556017
All − MR Egger
All − Inverse variance weighted
−1
0
1
2
MR effect size for
'Enterobacteriaceae' on 'GCST90200737_buildGRCh38.tsv'

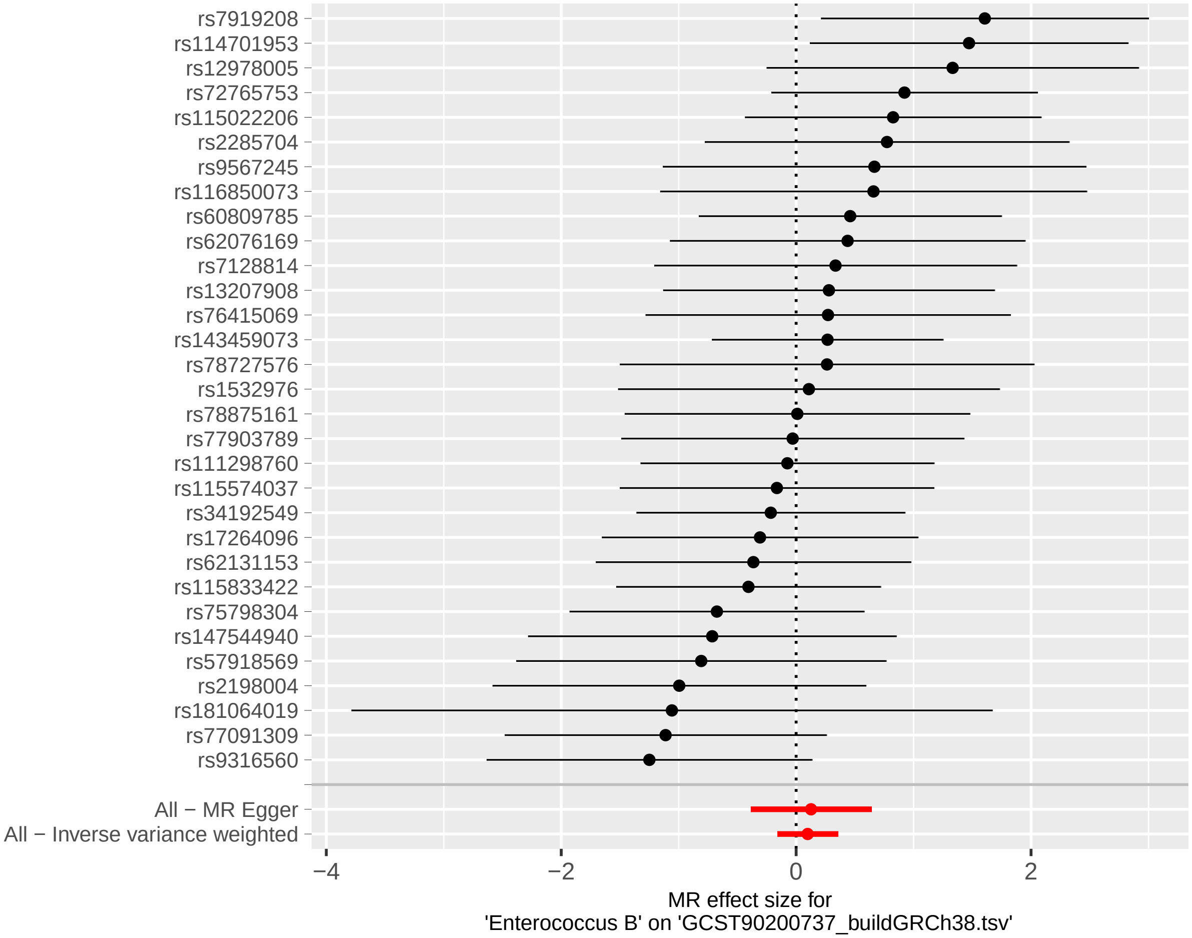
rs7919208
rs114701953
rs12978005
rs72765753
rs115022206
rs2285704
rs9567245
rs116850073
rs60809785
rs62076169
rs7128814
rs13207908
rs76415069
rs143459073
rs78727576
rs1532976
rs78875161
rs77903789
rs111298760
rs115574037
rs34192549
rs17264096
rs62131153
rs115833422
rs75798304
rs147544940
rs57918569
rs2198004
rs181064019
rs77091309
rs9316560
All − MR Egger
All − Inverse variance weighted
−4
−2
0
2
MR effect size for
'Enterococcus B' on 'GCST90200737_buildGRCh38.tsv'

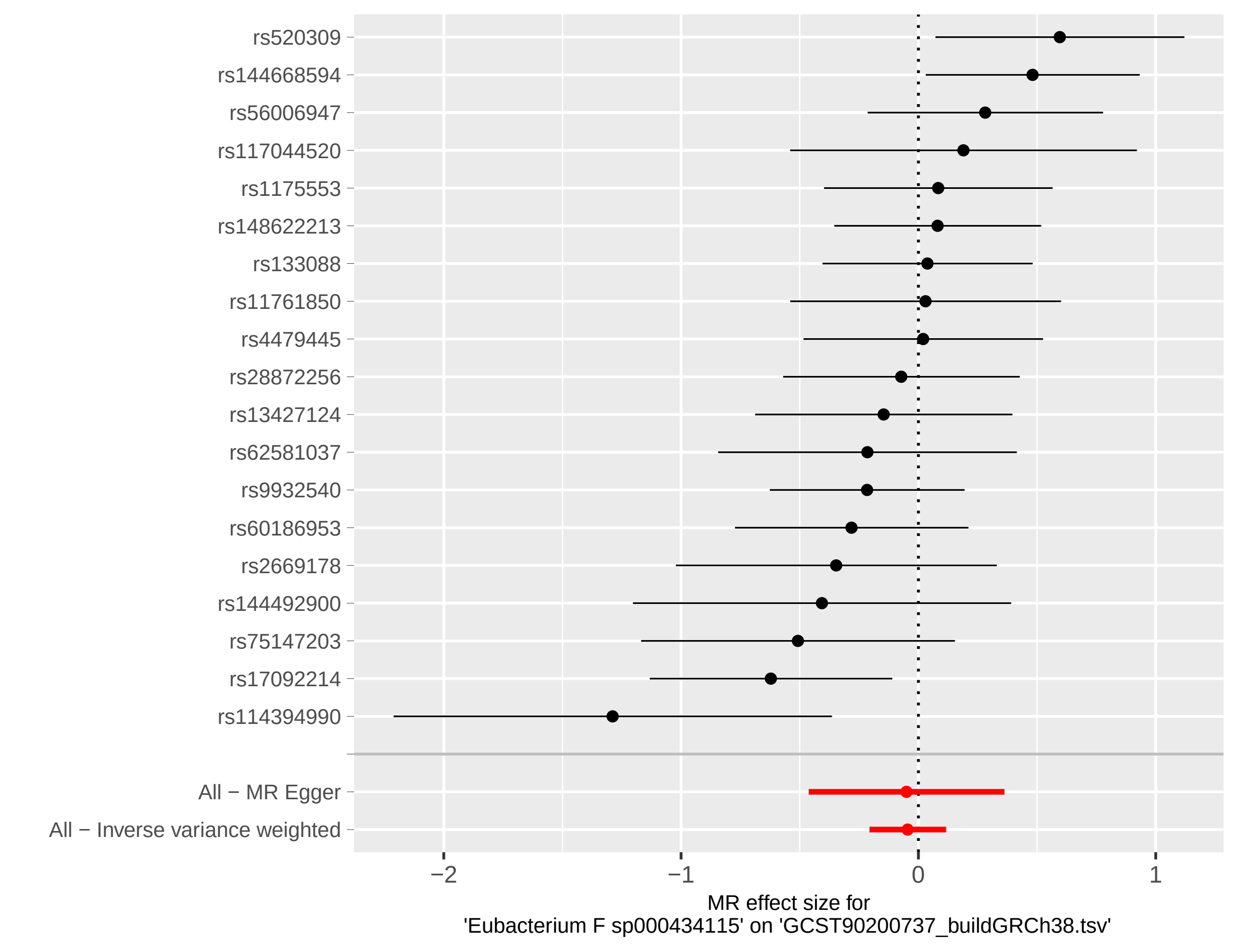
rs520309
rs144668594
rs56006947
rs117044520
rs1175553
rs148622213
rs133088
rs11761850
rs4479445
rs28872256
rs13427124
rs62581037
rs9932540
rs60186953
rs2669178
rs144492900
rs75147203
rs17092214
rs114394990
All − MR Egger
All − Inverse variance weighted
−2
−1
0
1
MR effect size for
'Eubacterium F sp000434115' on 'GCST90200737_buildGRCh38.tsv'

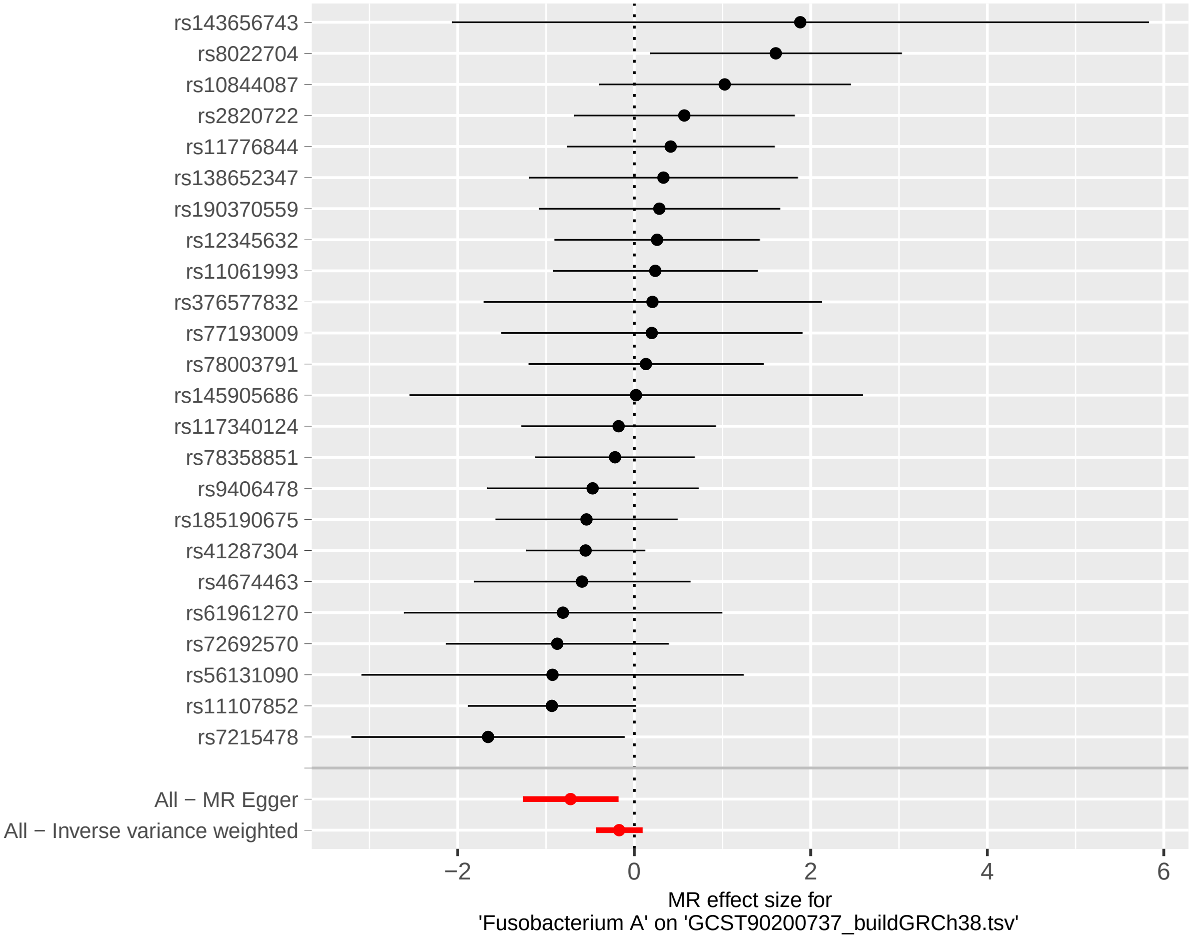

rs143656743
rs8022704
rs10844087
rs2820722
rs11776844
rs138652347
rs190370559
rs12345632
rs11061993
rs376577832
rs77193009
rs78003791
rs145905686
rs117340124
rs78358851
rs9406478
rs185190675
rs41287304
rs4674463
rs61961270
rs72692570
rs56131090
rs11107852
rs7215478
All − MR Egger
All − Inverse variance weighted
−2
0
2
4
6
MR effect size for
'Fusobacterium A' on 'GCST90200737_buildGRCh38.tsv'

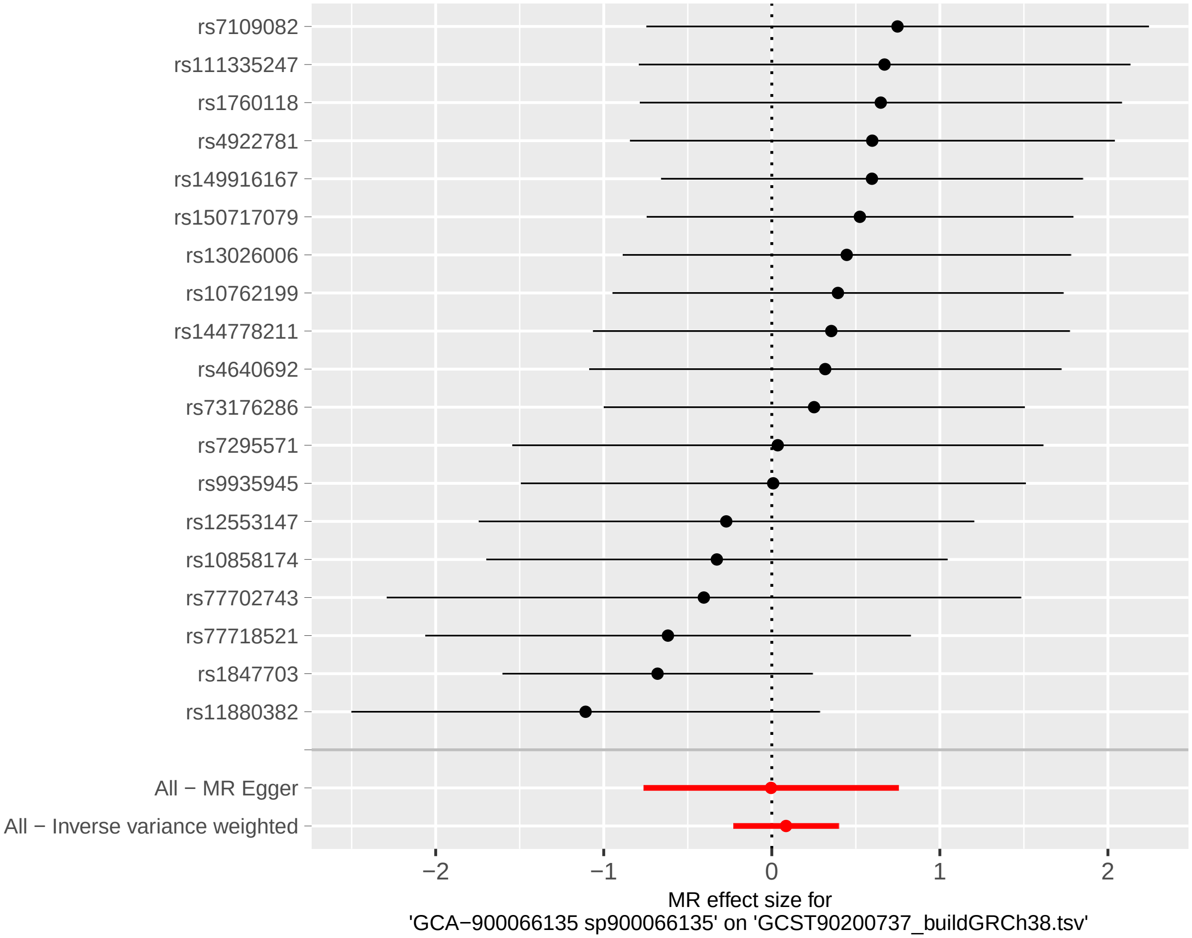
rs7109082
rs111335247
rs1760118
rs4922781
rs149916167
rs150717079
rs13026006
rs10762199
rs144778211
rs4640692
rs73176286
rs7295571
rs9935945
rs12553147
rs10858174
rs77702743
rs77718521
rs1847703
rs11880382
All − MR Egger
All − Inverse variance weighted
−2
−1
0
1
2
MR effect size for
'GCA−900066135 sp900066135' on 'GCST90200737_buildGRCh38.tsv'

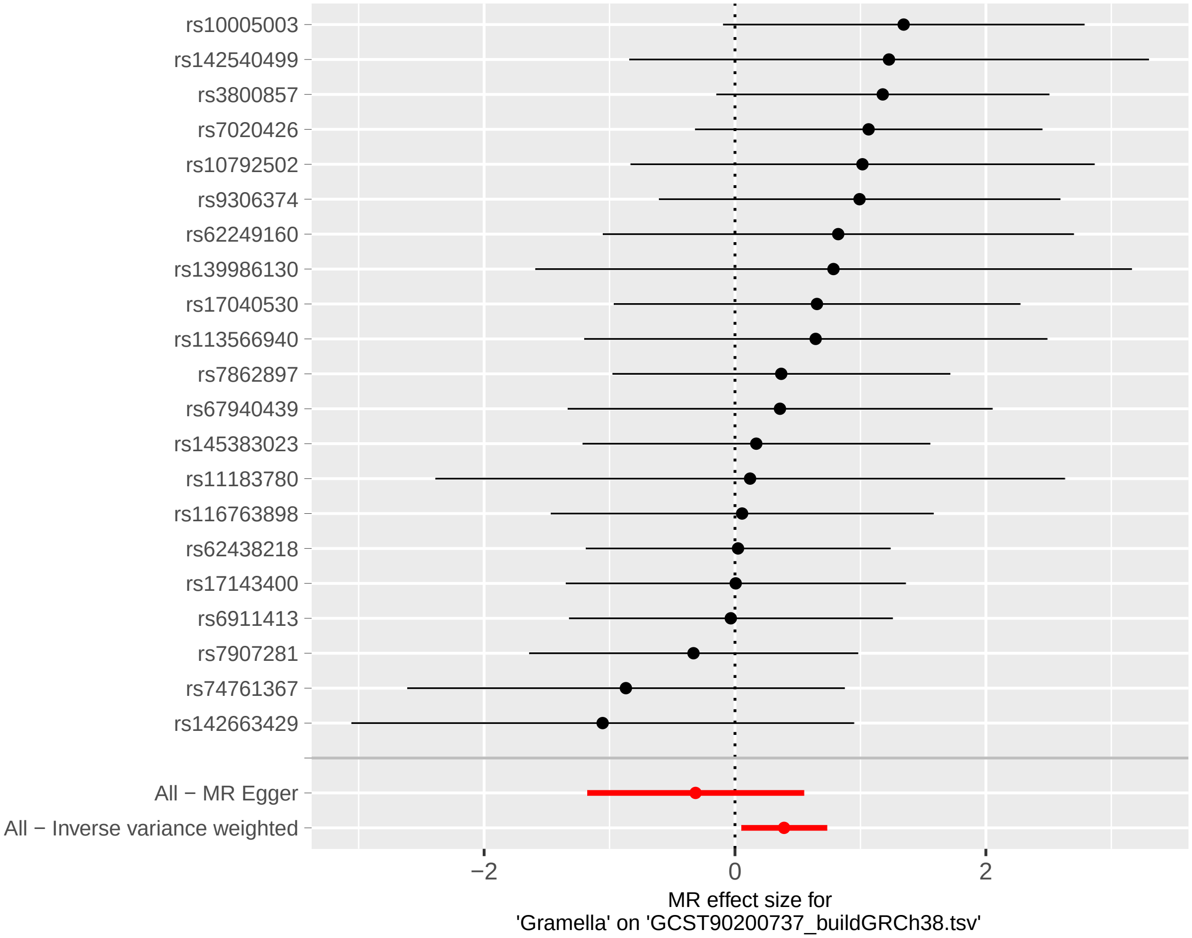
rs10005003
rs142540499
rs3800857
rs7020426
rs10792502
rs9306374
rs62249160
rs139986130
rs17040530
rs113566940
rs7862897
rs67940439
rs145383023
rs11183780
rs116763898
rs62438218
rs17143400
rs6911413
rs7907281
rs74761367
rs142663429
All − MR Egger
All − Inverse variance weighted
−2
0
2
MR effect size for
'Gramella' on 'GCST90200737_buildGRCh38.tsv'

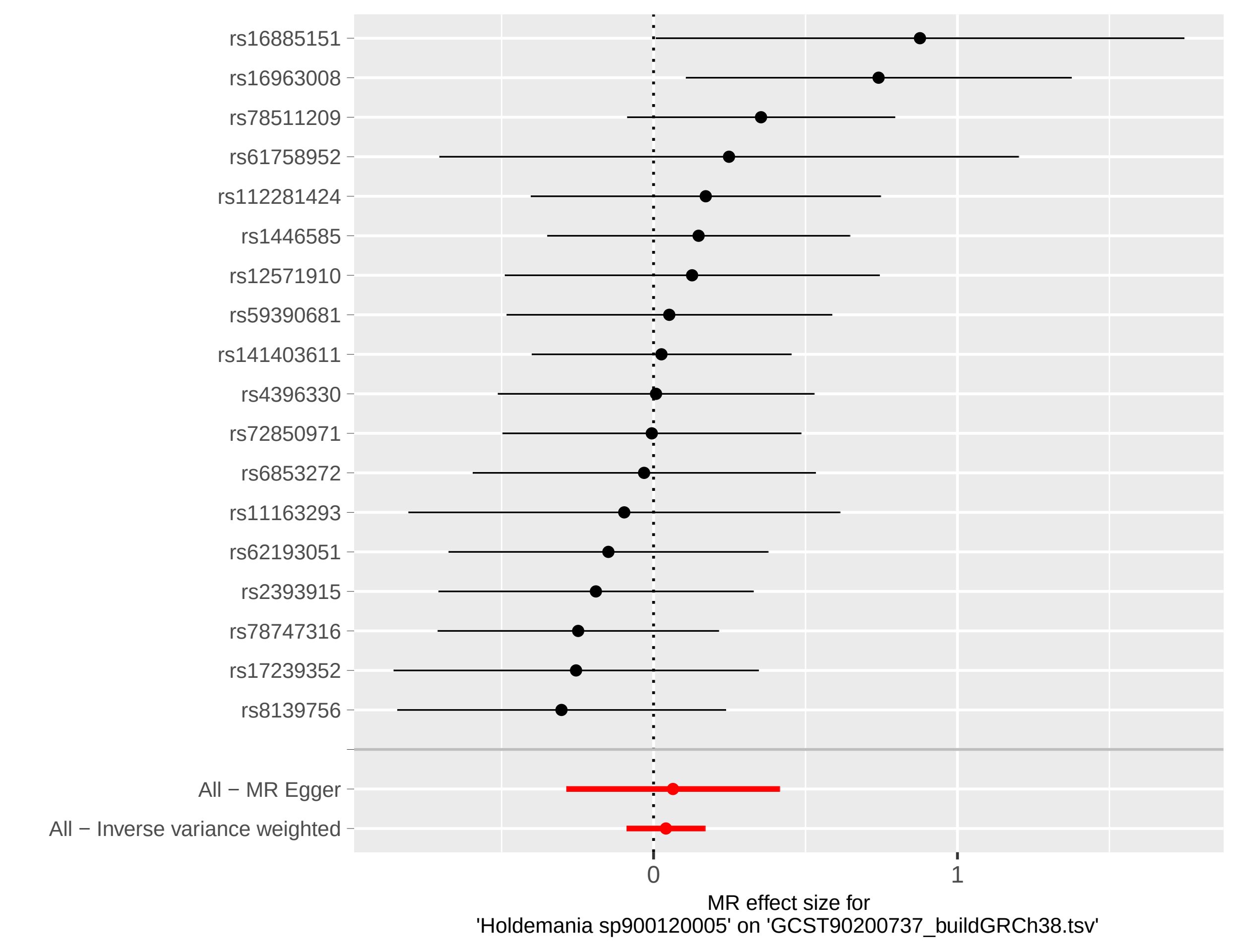
rs16885151
rs16963008
rs78511209
rs61758952
rs112281424
rs1446585
rs12571910
rs59390681
rs141403611
rs4396330
rs72850971
rs6853272
rs11163293
rs62193051
rs2393915
rs78747316
rs17239352
rs8139756
All − MR Egger
All − Inverse variance weighted
0
1
MR effect size for
'Holdemania sp900120005' on 'GCST90200737_buildGRCh38.tsv'

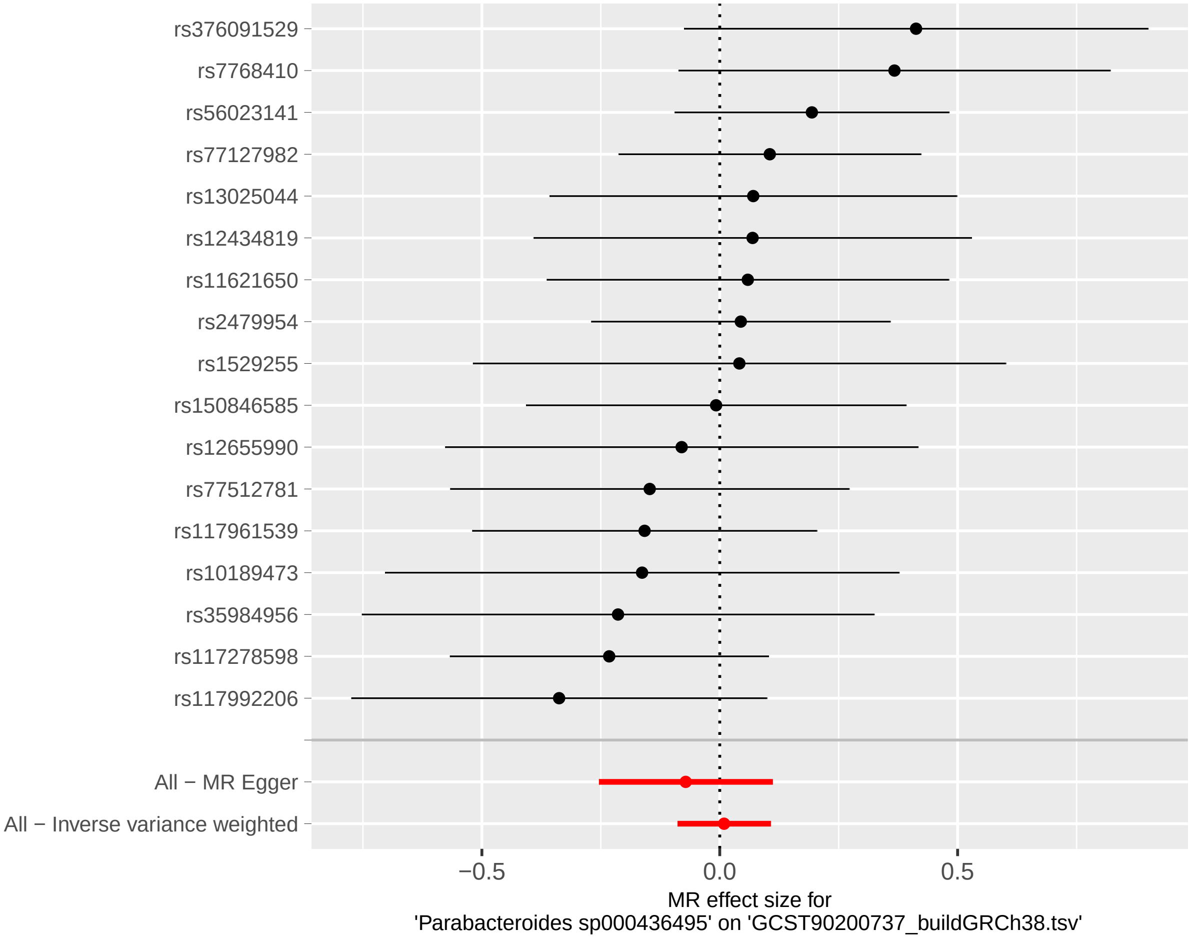
rs376091529
rs7768410
rs56023141
rs77127982
rs13025044
rs12434819
rs11621650
rs2479954
rs1529255
rs150846585
rs12655990
rs77512781
rs117961539
rs10189473
rs35984956
rs117278598
rs117992206
All − MR Egger
All − Inverse variance weighted
−0.5
0.0
0.5
MR effect size for
'Parabacteroides sp000436495' on 'GCST90200737_buildGRCh38.tsv'

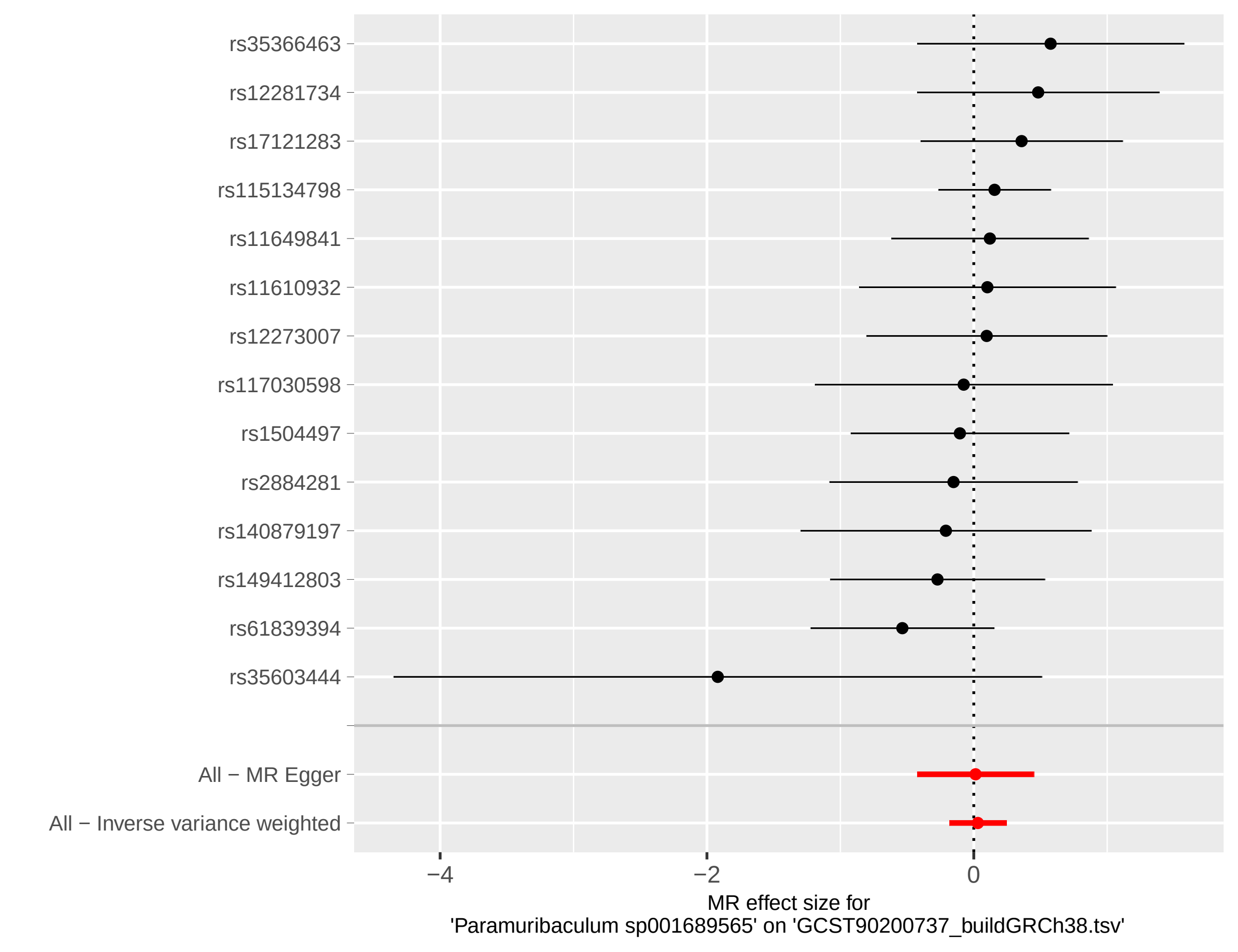
rs35366463
rs12281734
rs17121283
rs115134798
rs11649841
rs11610932
rs12273007
rs117030598
rs1504497
rs2884281
rs140879197
rs149412803
rs61839394
rs35603444
All − MR Egger
All − Inverse variance weighted
−4
−2
0
MR effect size for
'Paramuribaculum sp001689565' on 'GCST90200737_buildGRCh38.tsv'

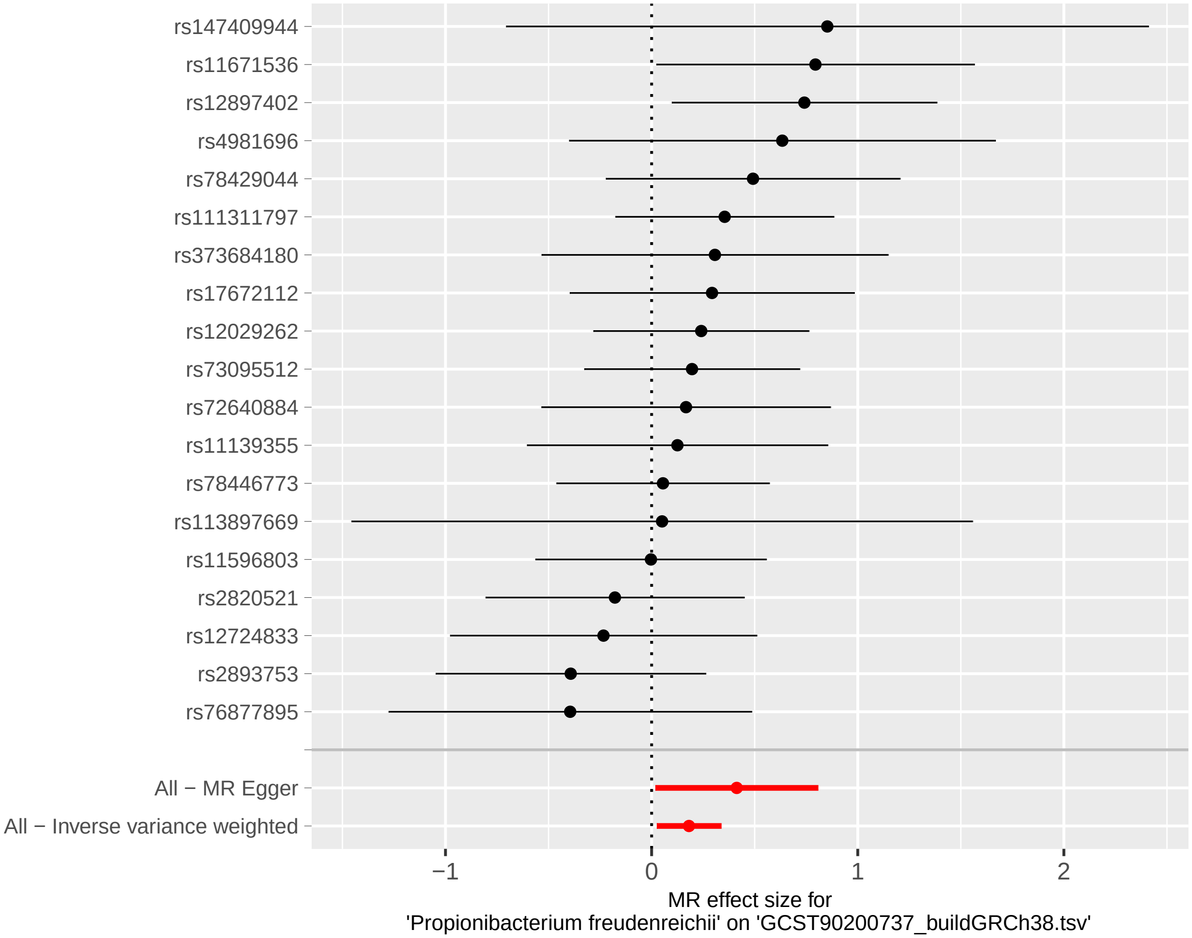
rs147409944
rs11671536
rs12897402
rs4981696
rs78429044
rs111311797
rs373684180
rs17672112
rs12029262
rs73095512
rs72640884
rs11139355
rs78446773
rs113897669
rs11596803
rs2820521
rs12724833
rs2893753
rs76877895
All − MR Egger
All − Inverse variance weighted
−1
0
1
2
MR effect size for
'Propionibacterium freudenreichii' on 'GCST90200737_buildGRCh38.tsv'

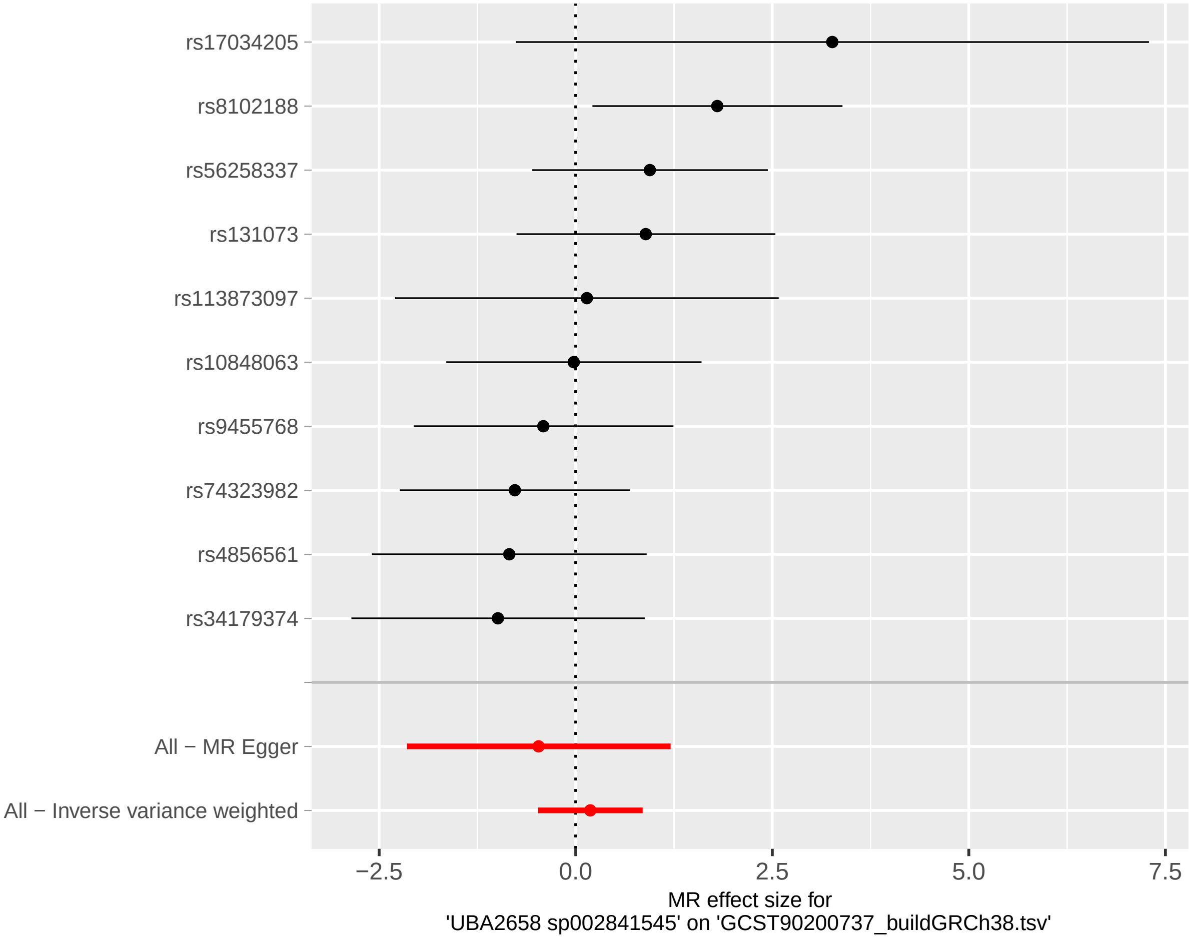

rs17034205
rs8102188
rs56258337
rs131073
rs113873097
rs10848063
rs9455768
rs74323982
rs4856561
rs34179374
All − MR Egger
All − Inverse variance weighted
−2.5
0.0
2.5
5.0
7.5
MR effect size for
'UBA2658 sp002841545' on 'GCST90200737_buildGRCh38.tsv'

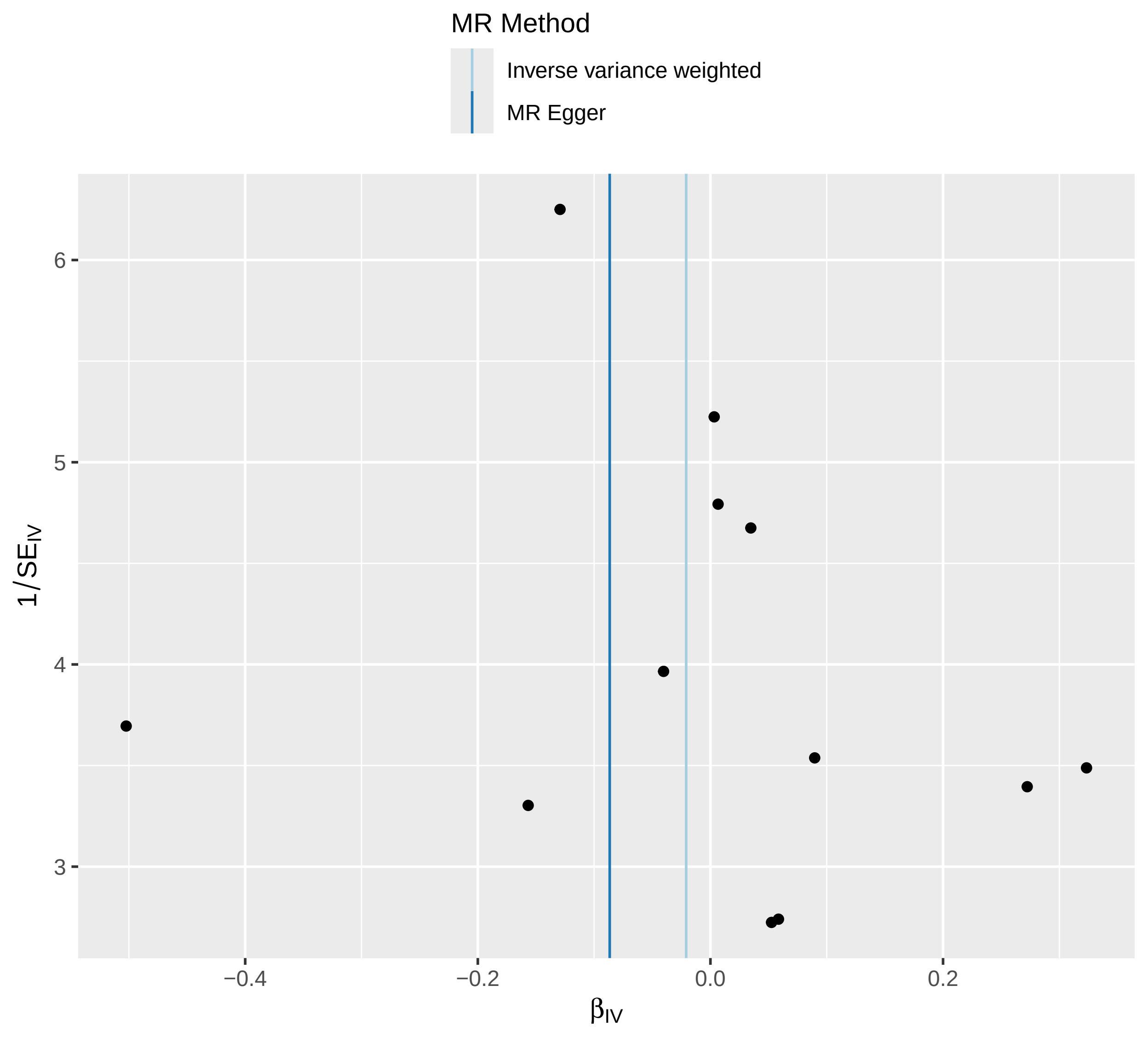
MR Method
Inverse variance weighted
MR Egger
1/SE_IV
6
5
4
3
−0.4
−0.2
0.0
0.2
β_IV

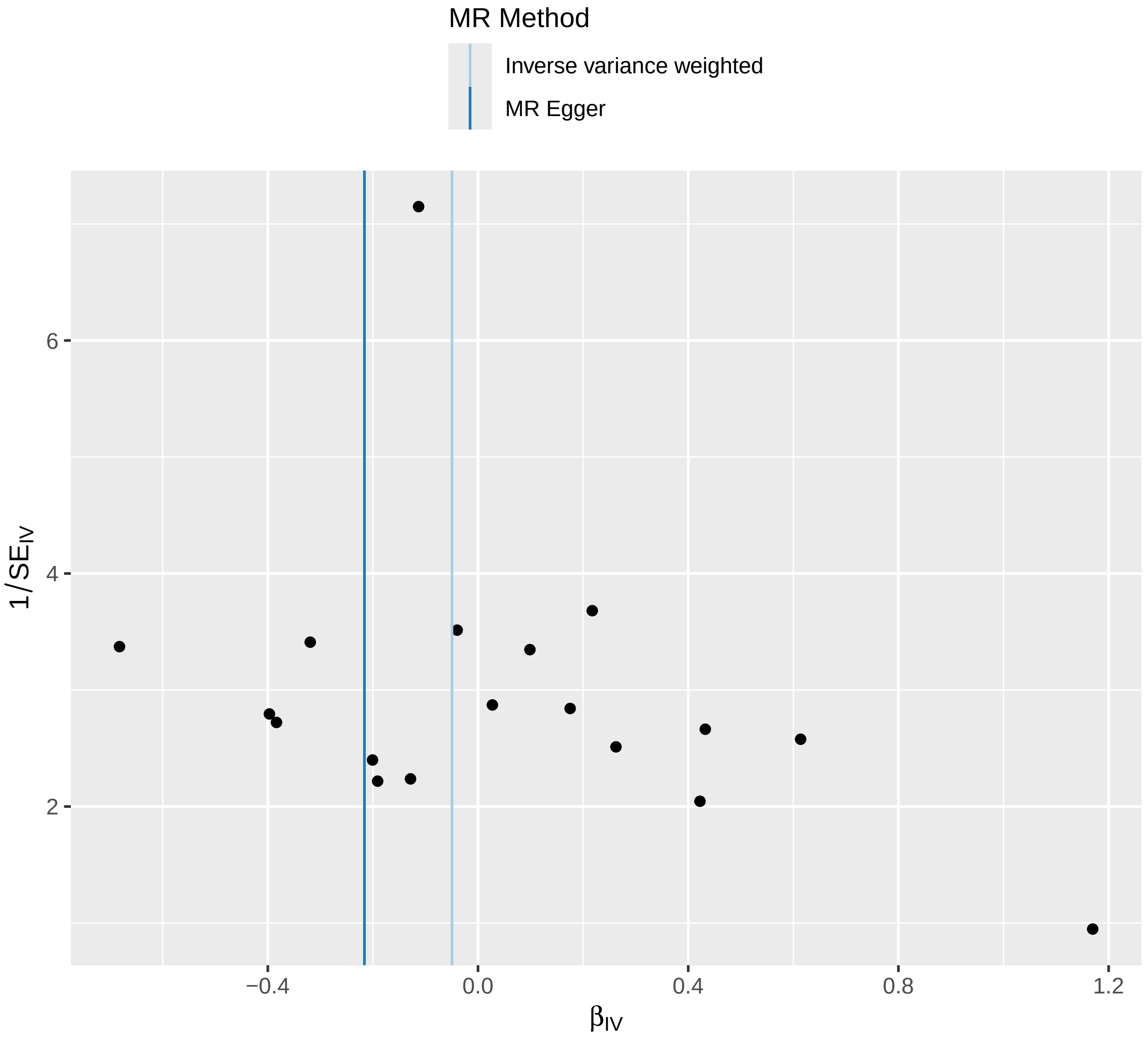
MR Method
Inverse variance weighted
MR Egger
1/SE_IV
6
4
2
−0.4
0.0
0.4
0.8
1.2
β_IV

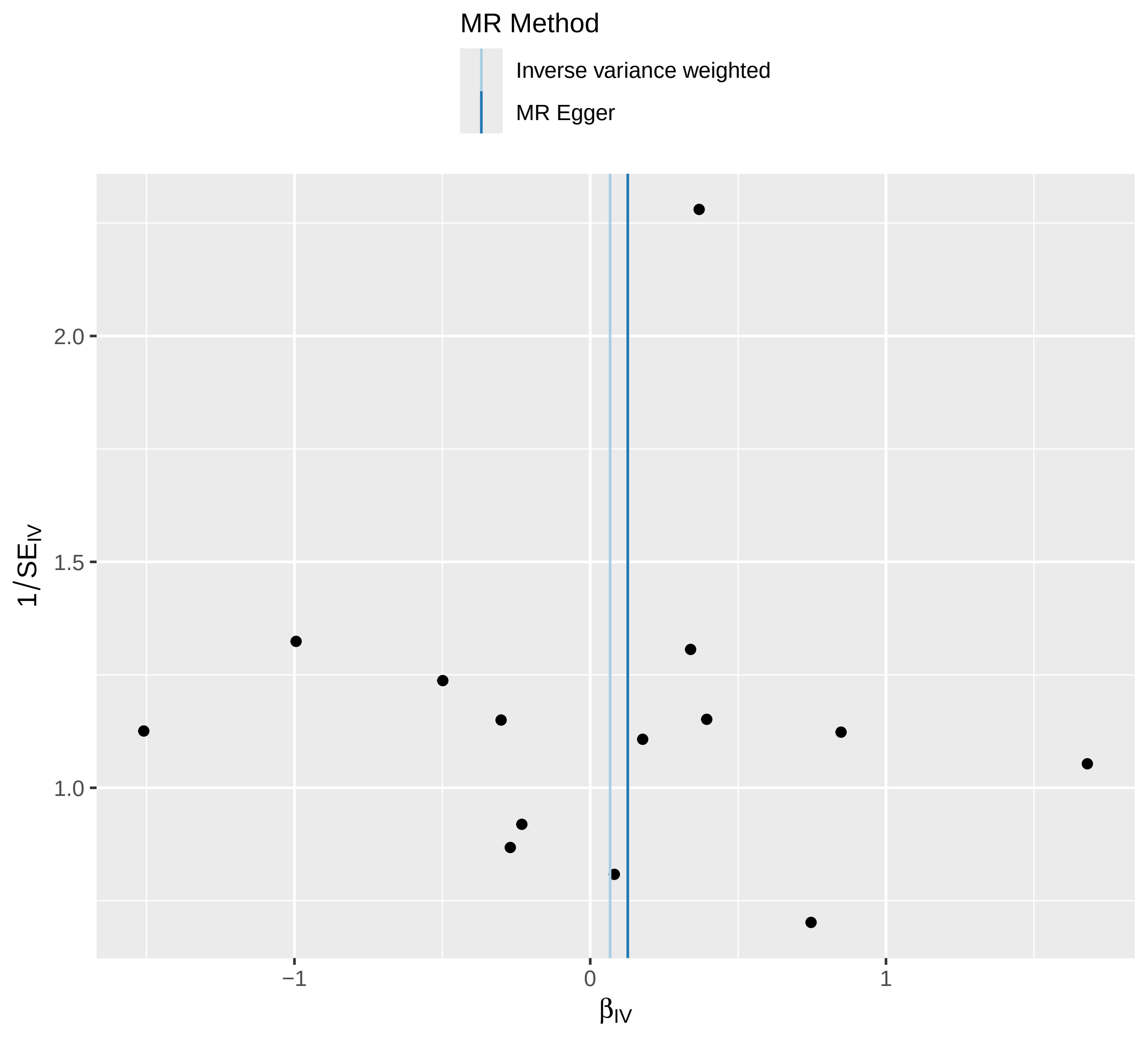
MR Method
Inverse variance weighted
MR Egger
1/SE_IV
2.0
1.5
1.0
−1
0
1
β_IV

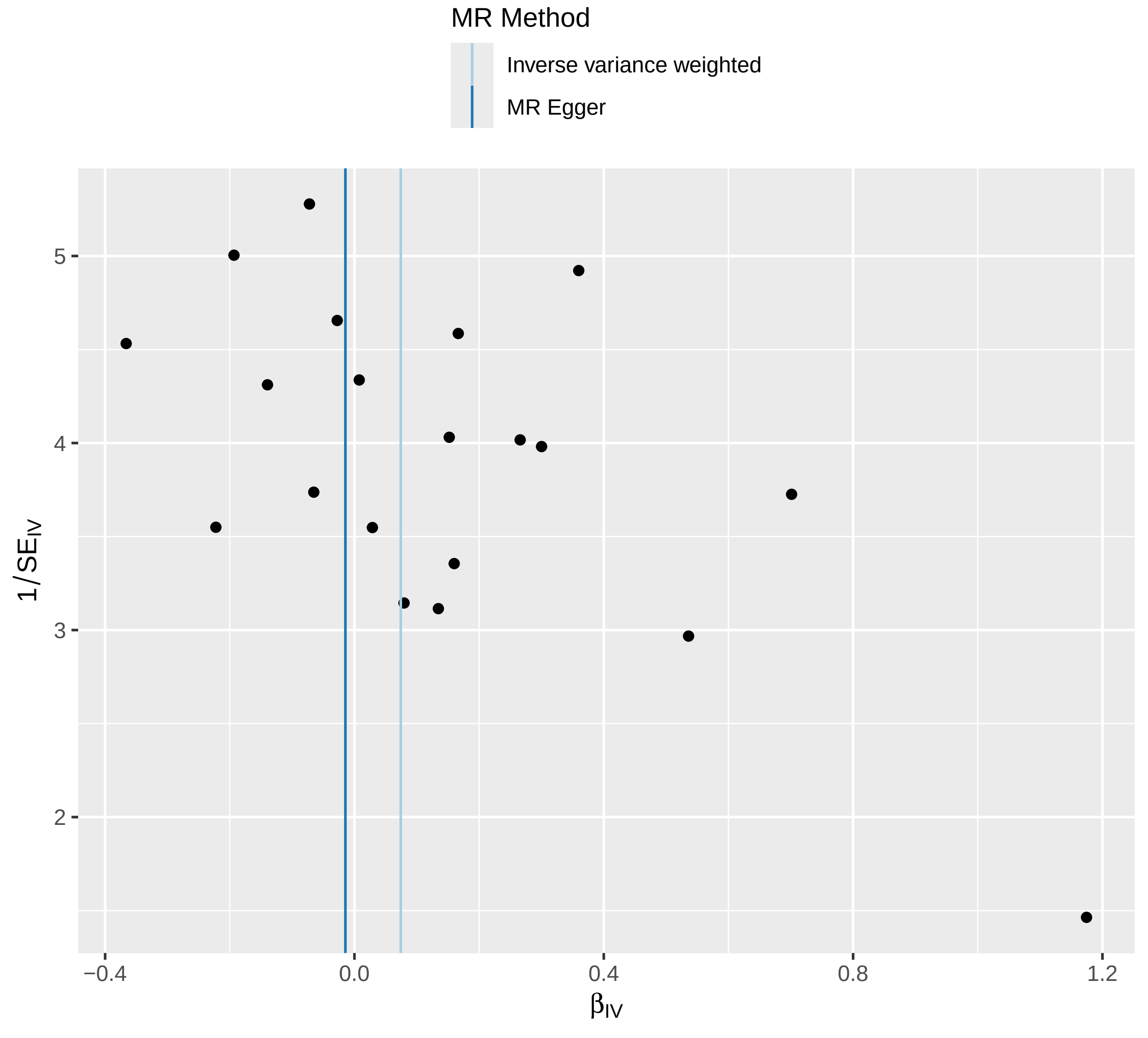

MR Method
Inverse variance weighted
MR Egger
5
4
3
2
1/SE_IV
−0.4
0.0
0.4
0.8
1.2
β_IV

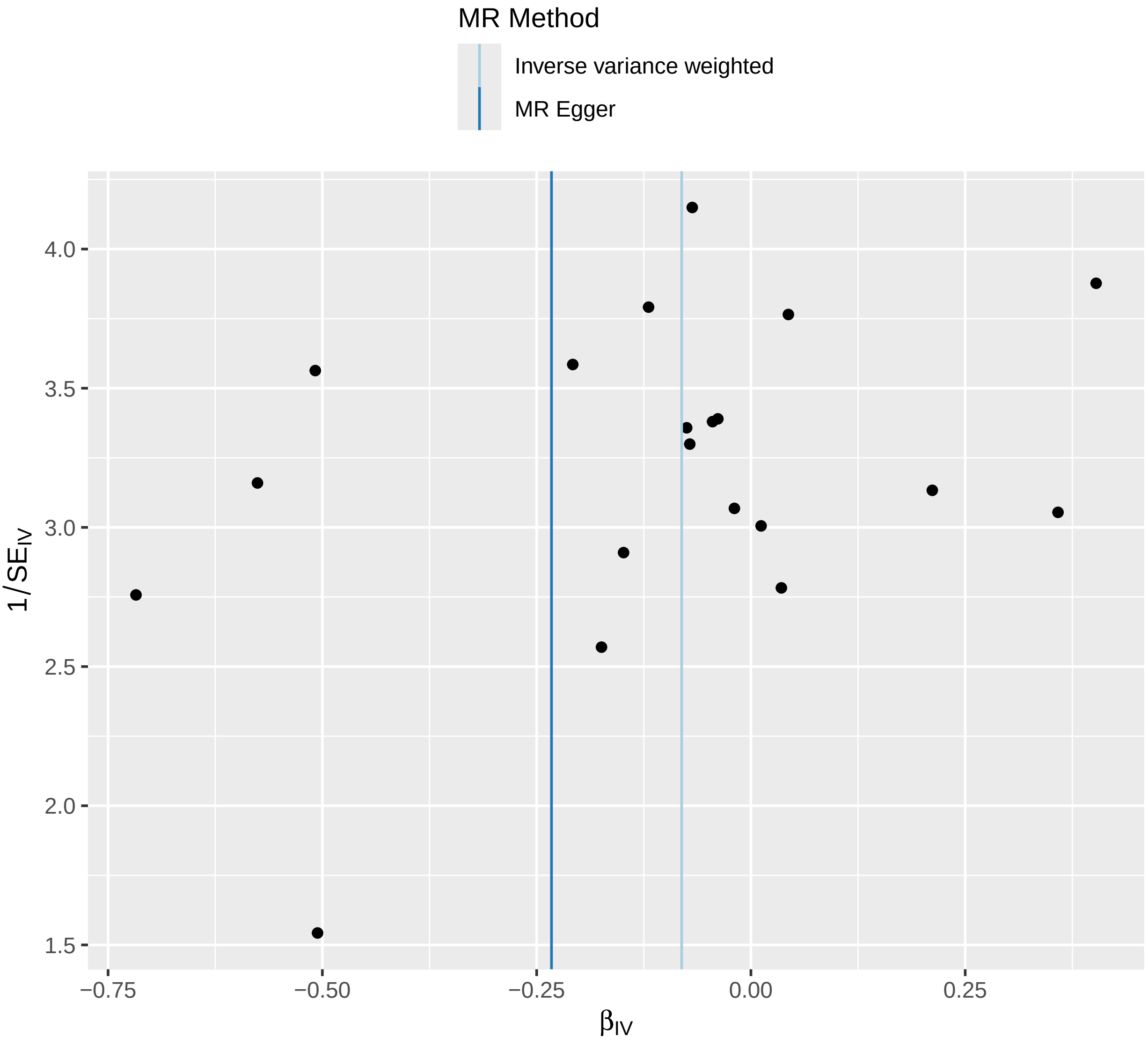
MR Method
Inverse variance weighted
MR Egger
1/SE_IV
4.0
3.5
3.0
2.5
2.0
1.5
−0.75
−0.50
−0.25
0.00
0.25
β_IV

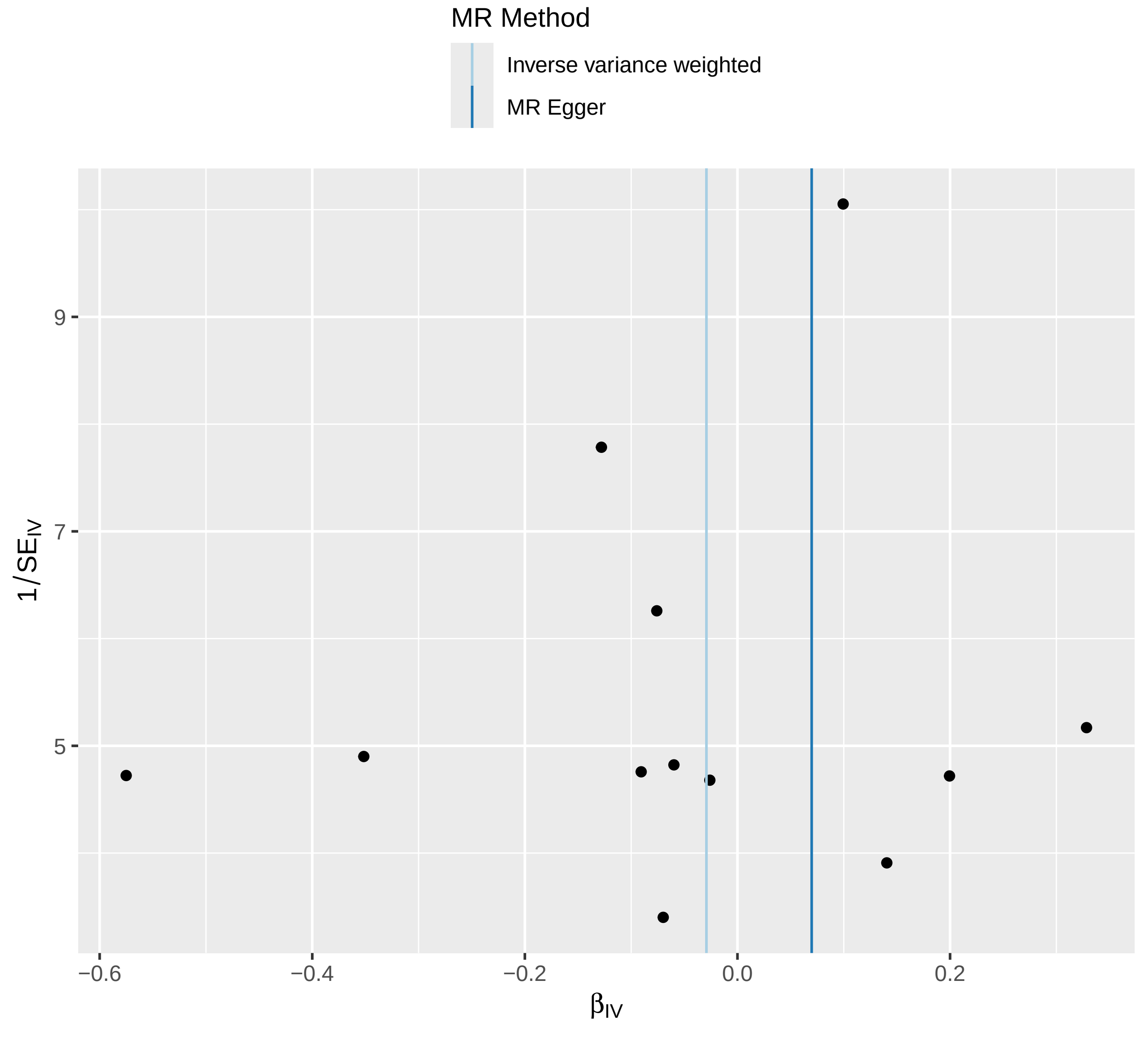

MR Method
Inverse variance weighted
MR Egger
9
7
5
1/SE_IV
−0.6
−0.4
−0.2
0.0
0.2
β_IV

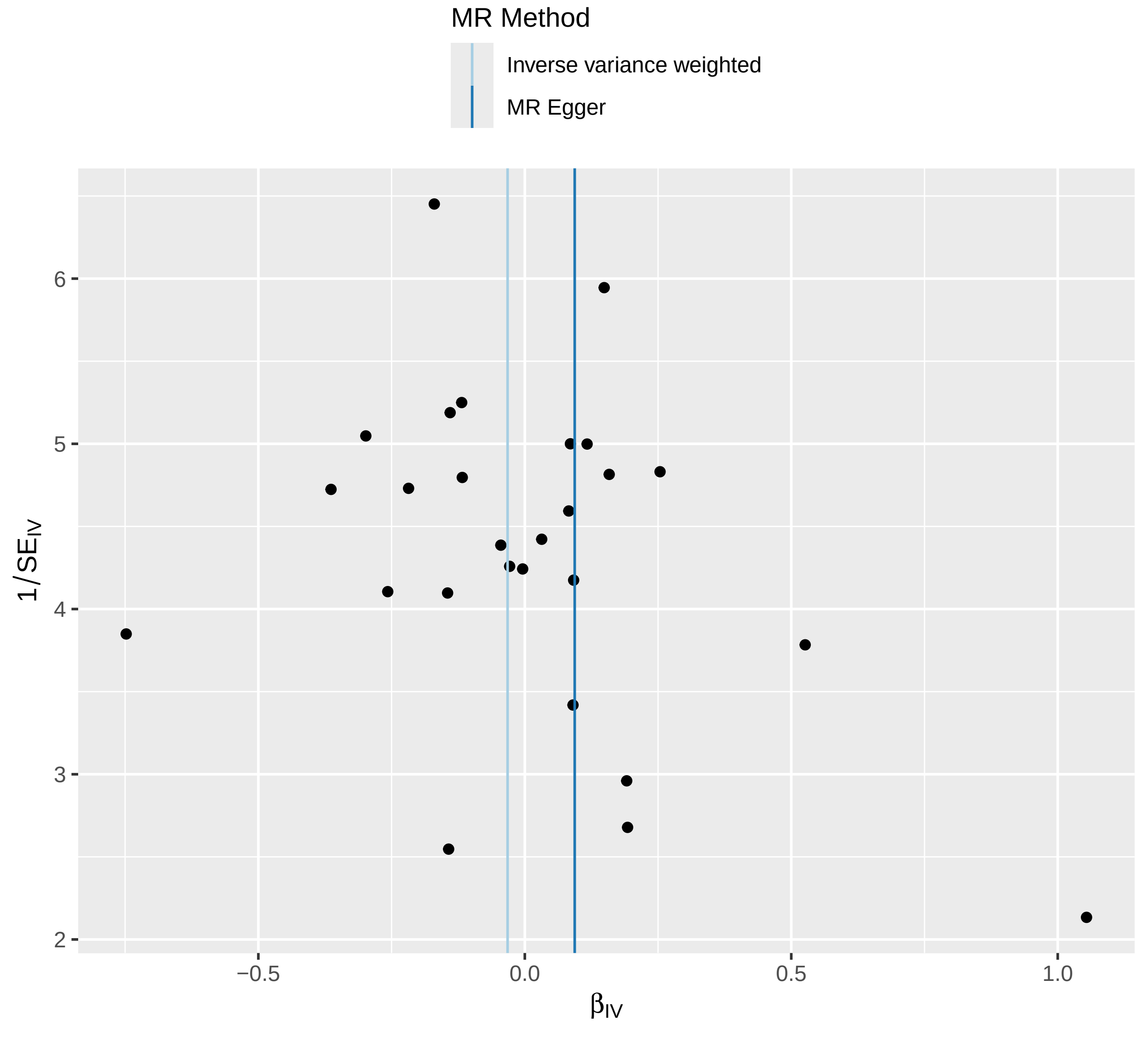
MR Method
Inverse variance weighted
MR Egger
1/SE$_{IV}$
6
5
4
3
2
−0.5
0.0
0.5
1.0
β$_{IV}$

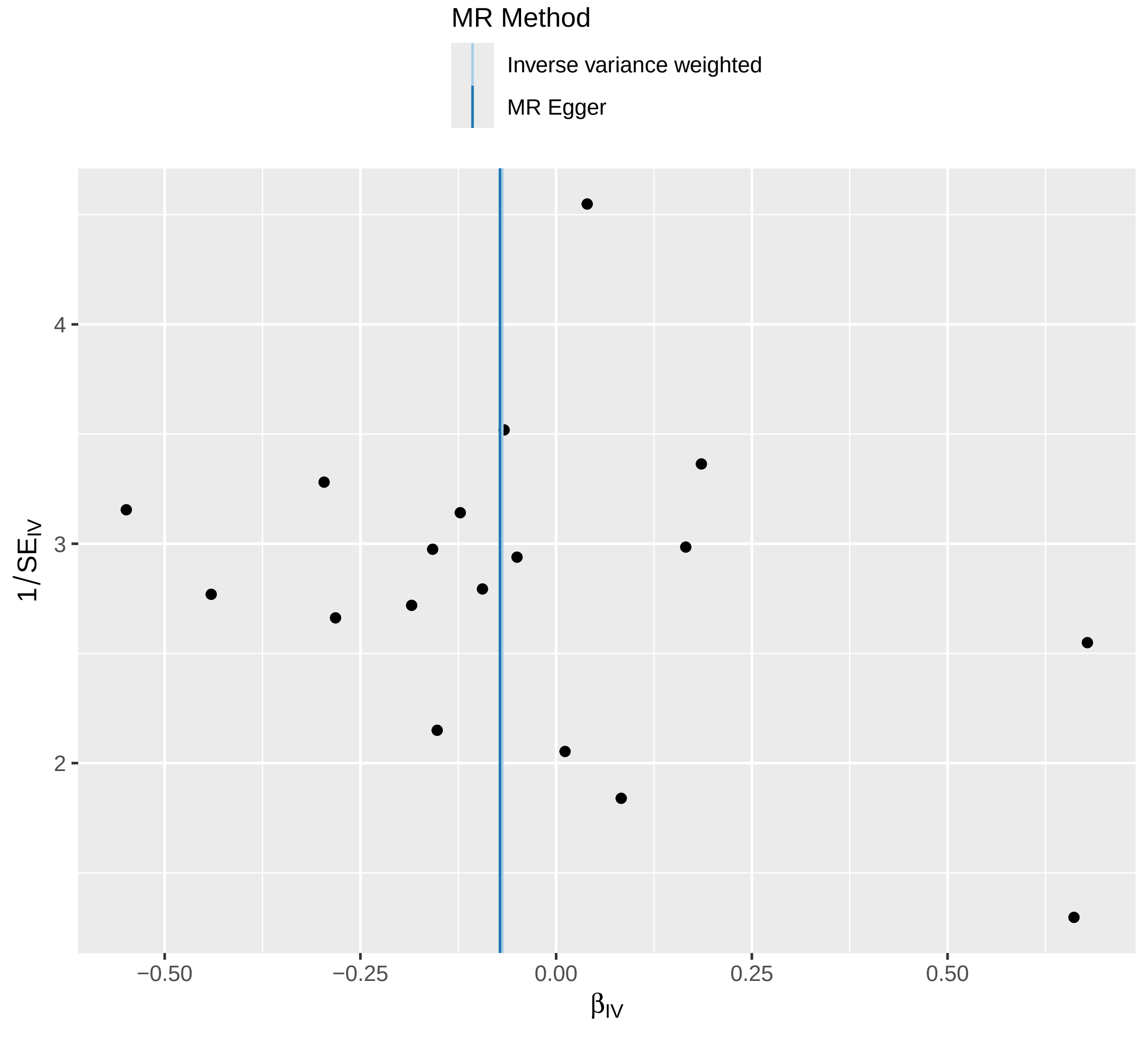

MR Method
Inverse variance weighted
MR Egger
4
3
2
$1/SE_{IV}$
−0.50
−0.25
0.00
0.25
0.50
β_{IV}

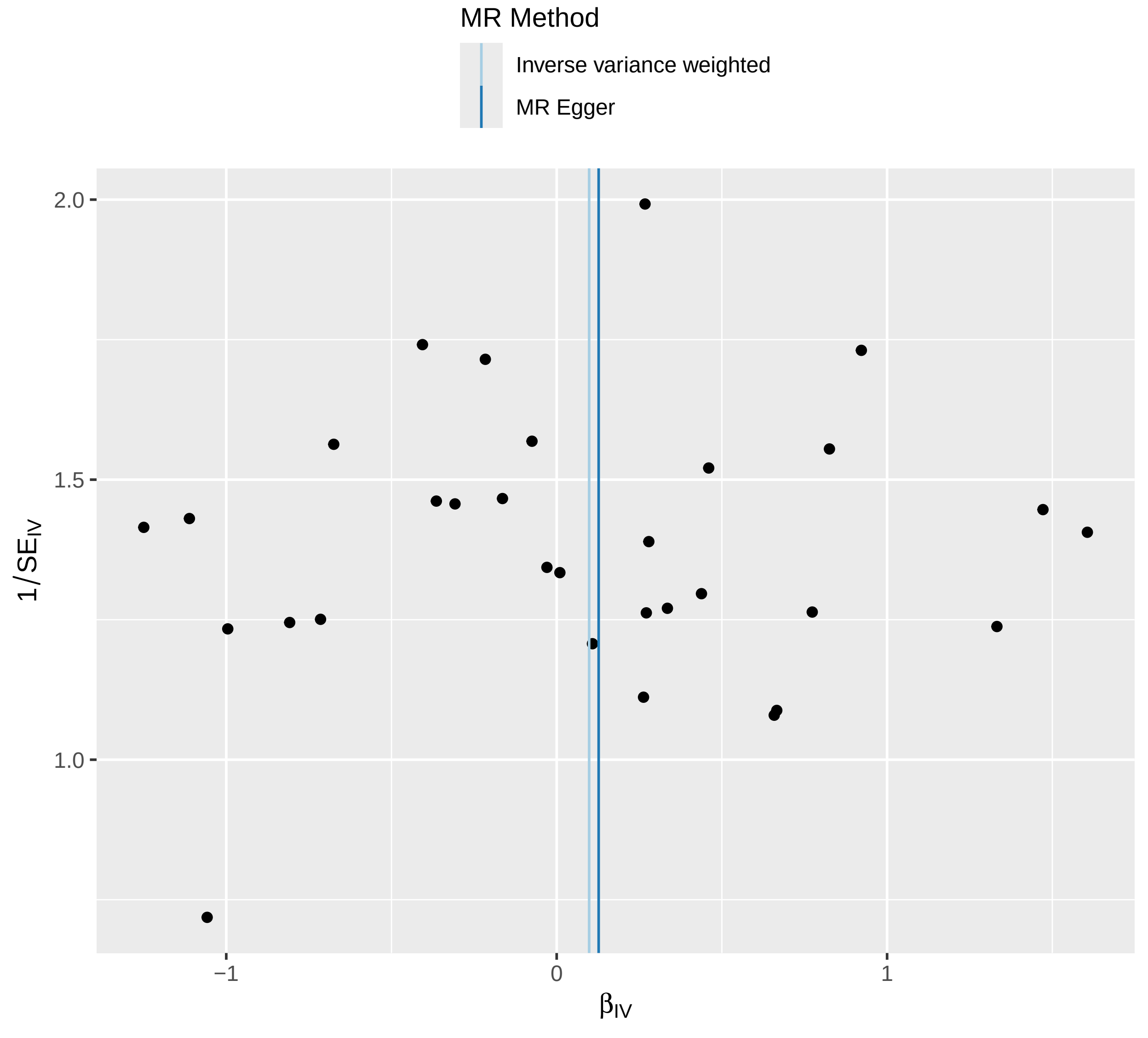

MR Method
Inverse variance weighted
MR Egger
2.0
1.5
1.0
−1
0
1
1/SE_IV
β_IV

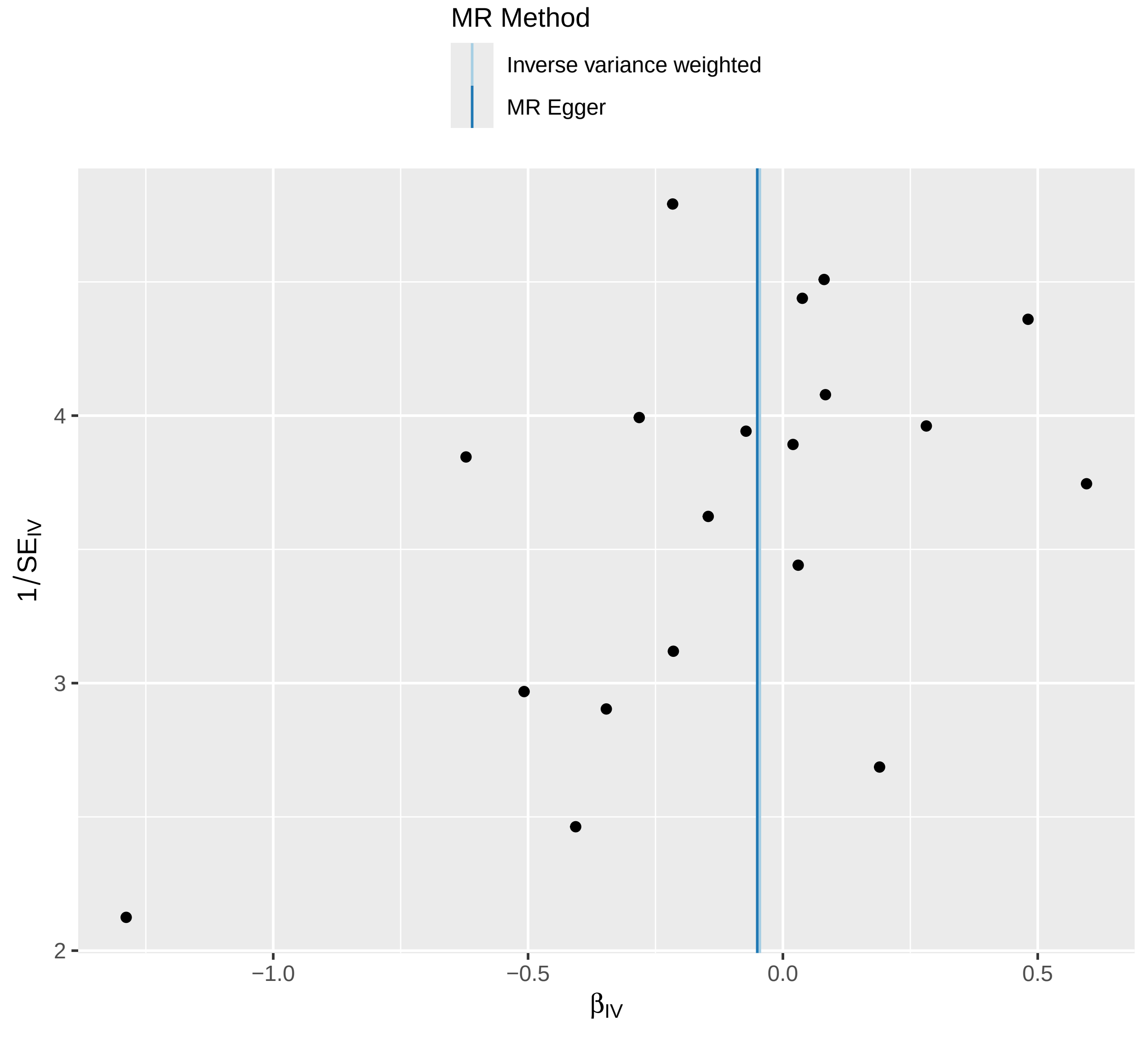

MR Method
Inverse variance weighted
MR Egger
4
3
2
1/SE_IV
−1.0
−0.5
0.0
0.5
β_IV

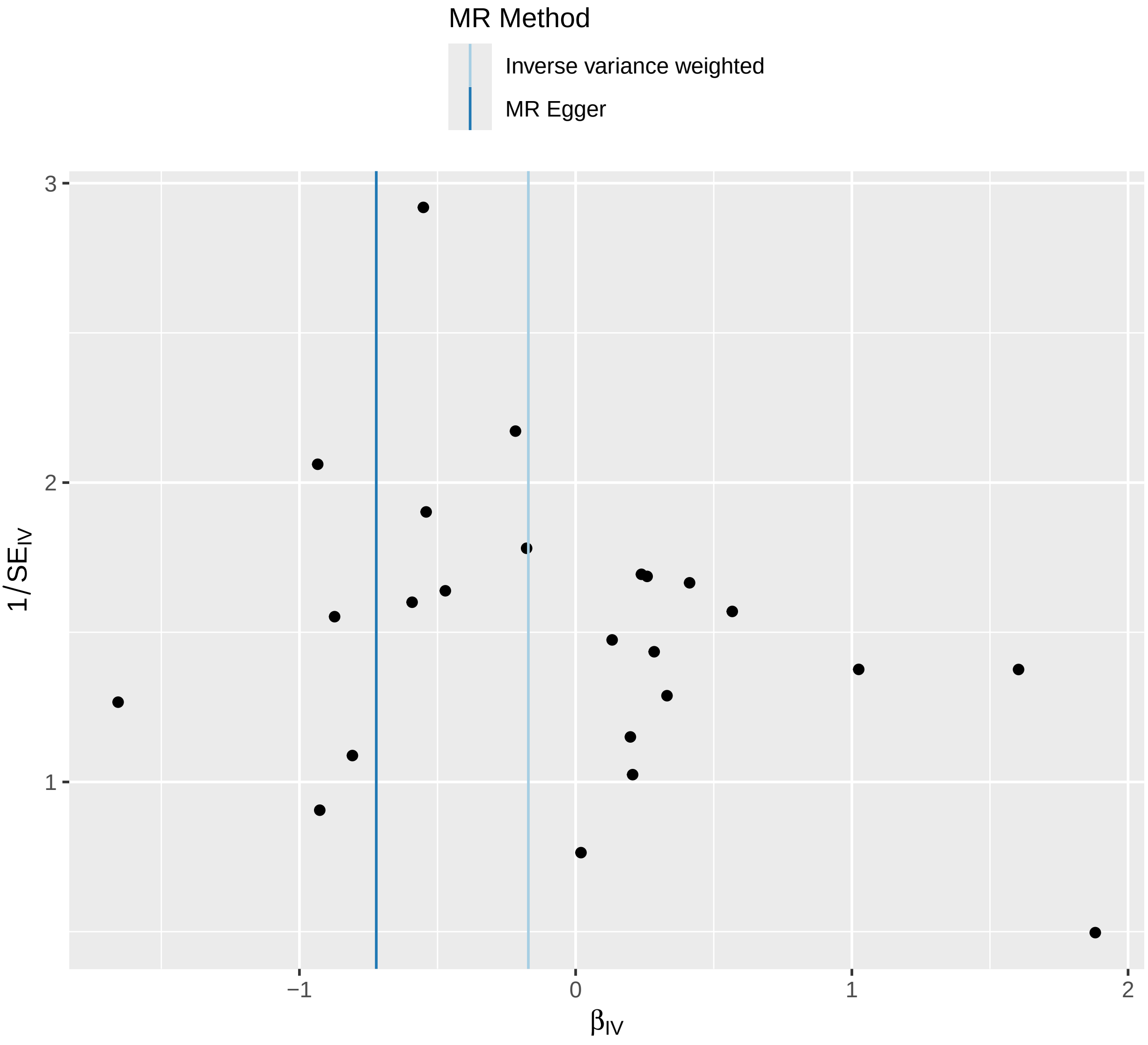
MR Method
Inverse variance weighted
MR Egger
3
2
1
1/SE$_{IV}$
−1
0
1
2
β$_{IV}$

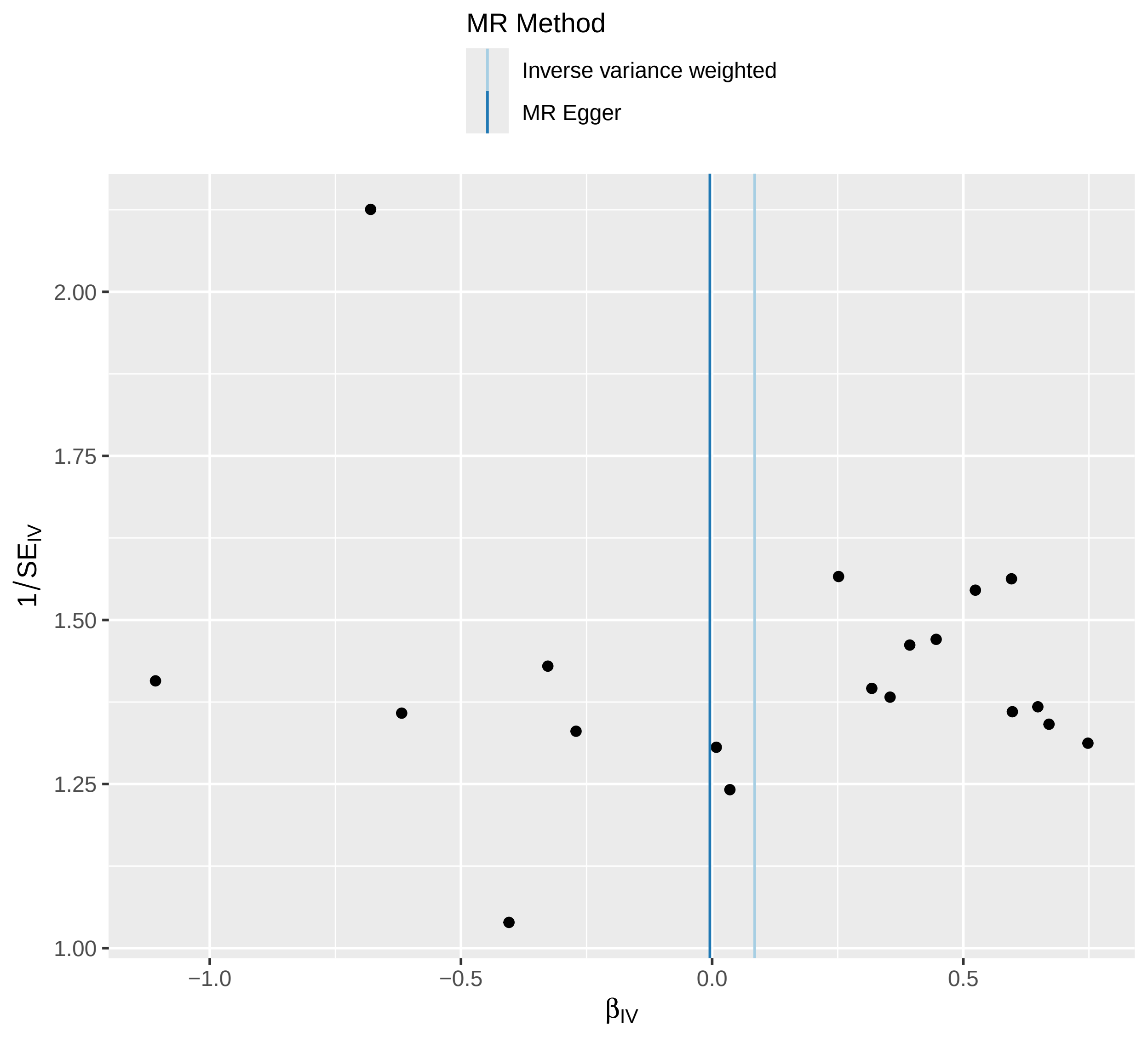
MR Method
Inverse variance weighted
MR Egger
1/SE_IV
2.00
1.75
1.50
1.25
1.00
−1.0
−0.5
0.0
0.5
β_IV

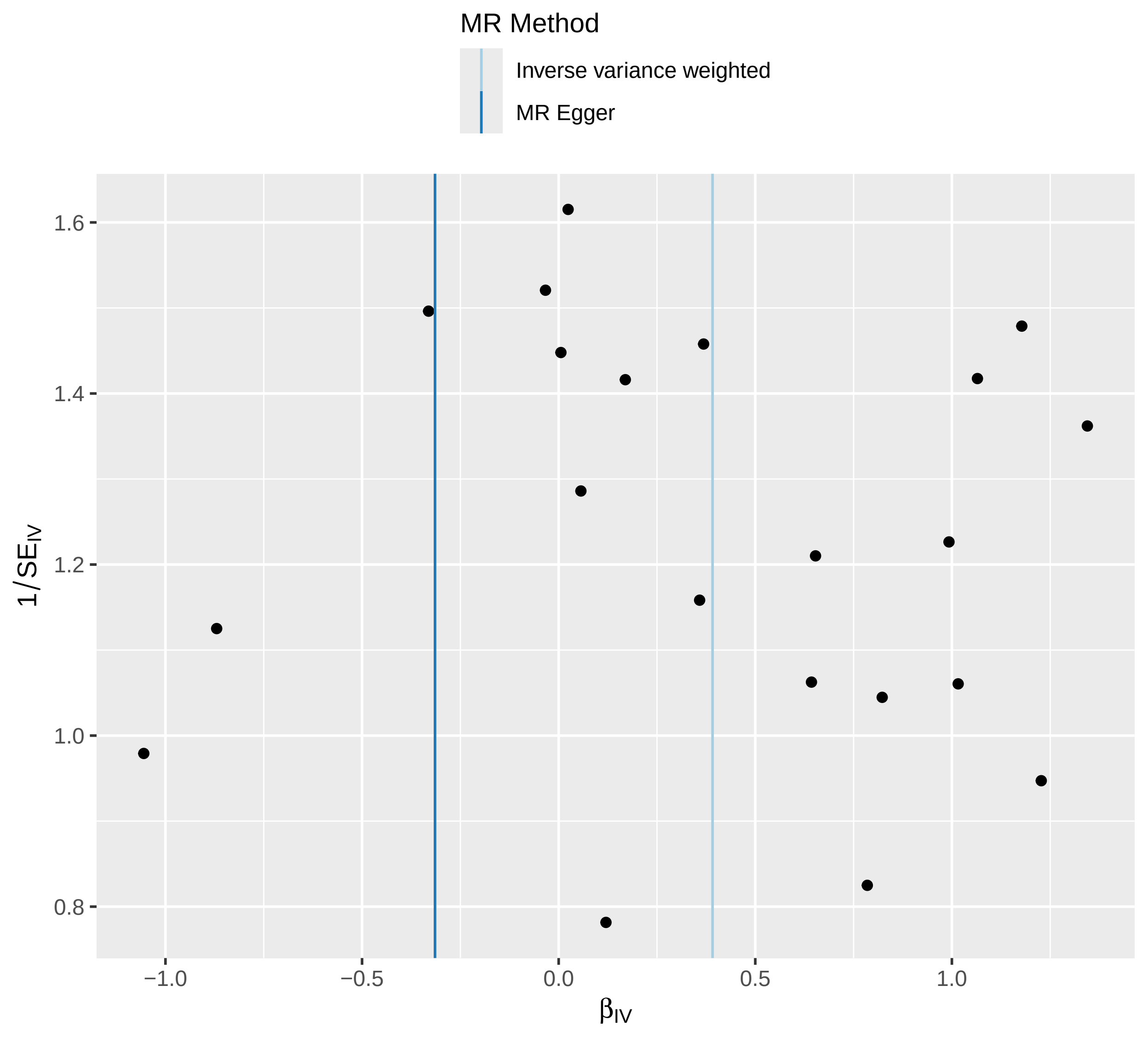

MR Method
Inverse variance weighted
MR Egger
1.6
1.4
1.2
1.0
0.8
$1/SE_{IV}$
−1.0
−0.5
0.0
0.5
1.0
β_{IV}

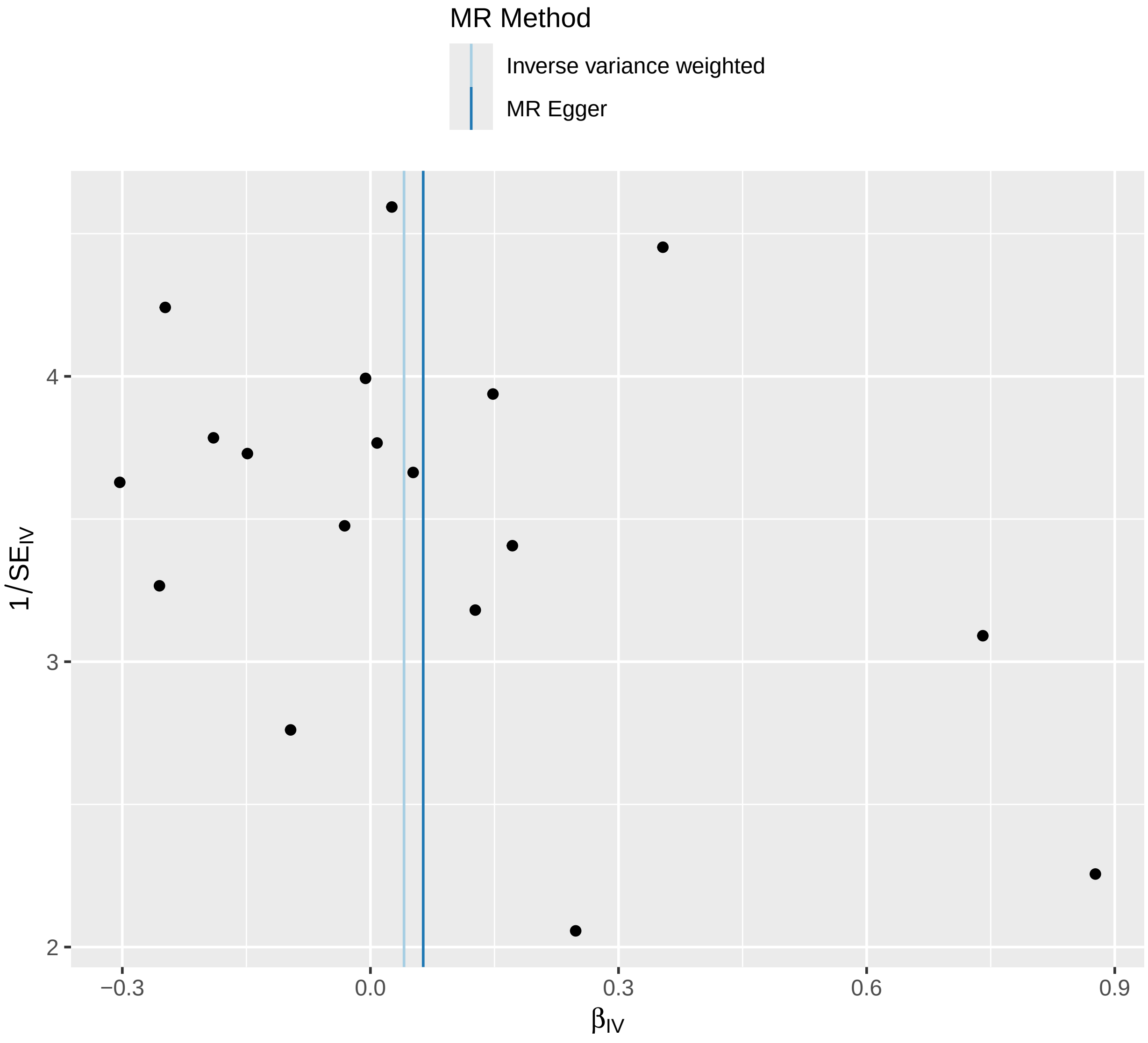

MR Method
Inverse variance weighted
MR Egger
1/SE_{IV}
4
3
2
−0.3
0.0
0.3
0.6
0.9
β_{IV}

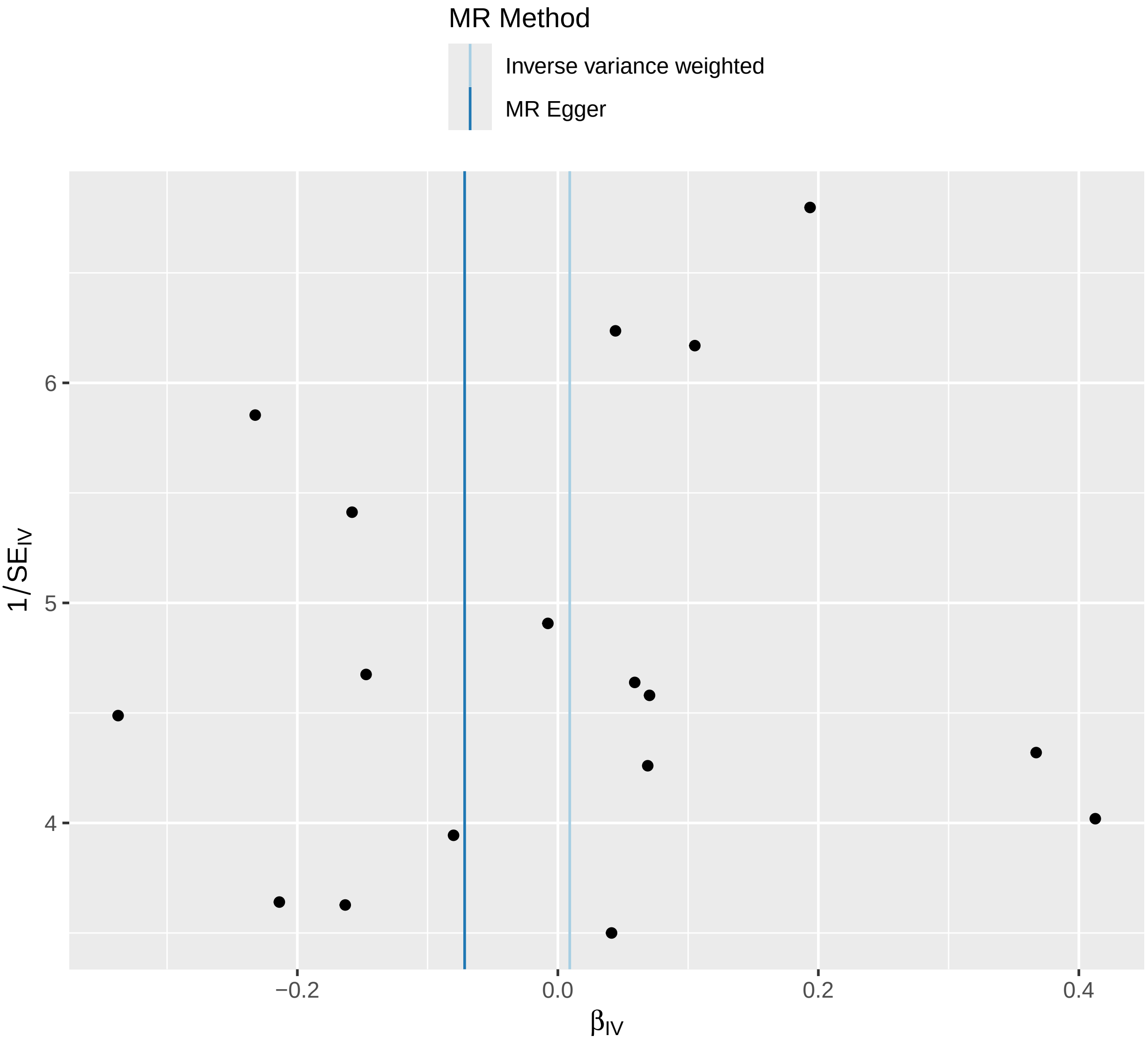
MR Method
Inverse variance weighted
MR Egger
1/SE_IV
6
5
4
−0.2
0.0
0.2
0.4
β_IV

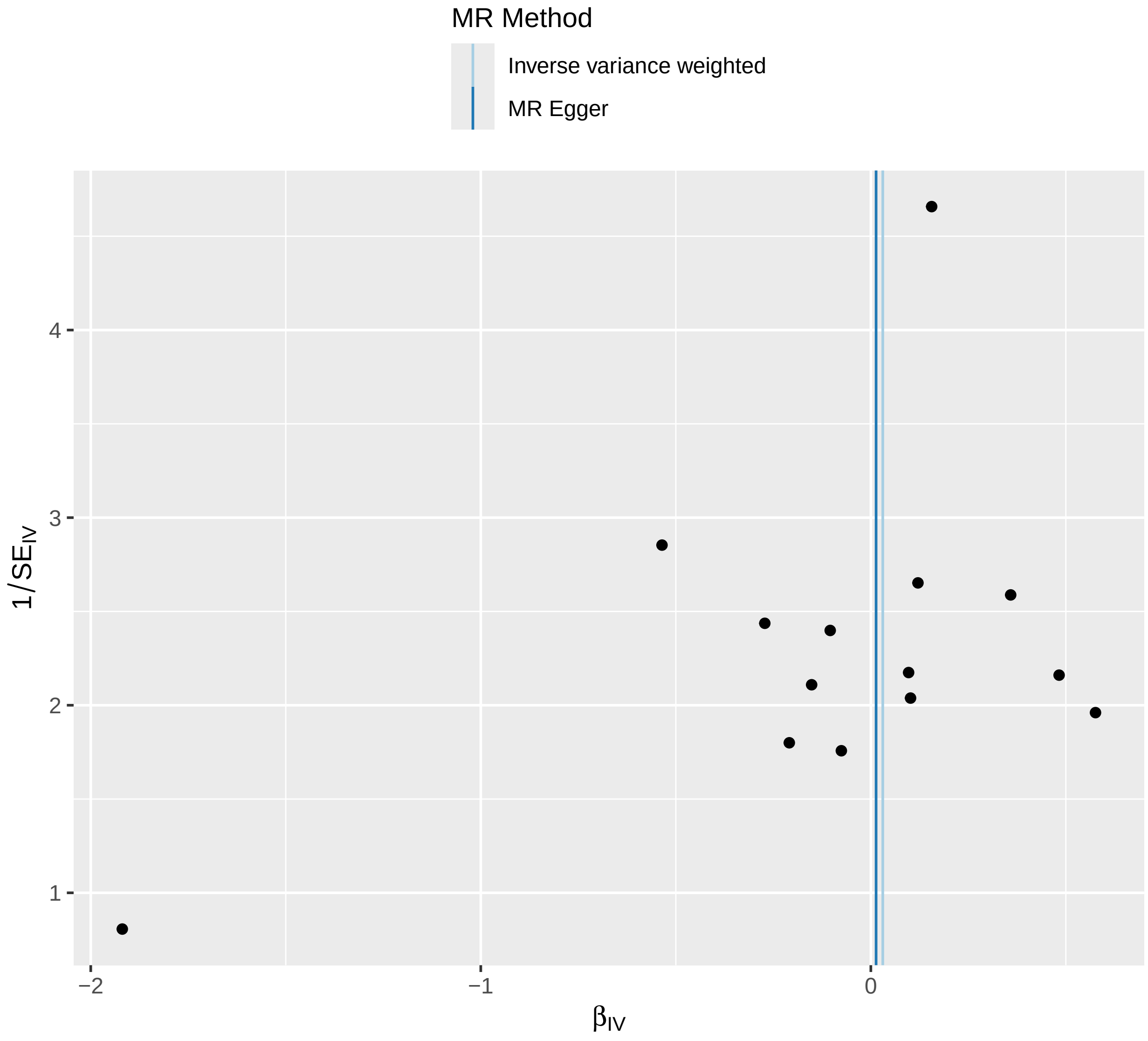
MR Method
Inverse variance weighted
MR Egger
1/SE_IV
β_IV
4
3
2
1
−2
−1
0

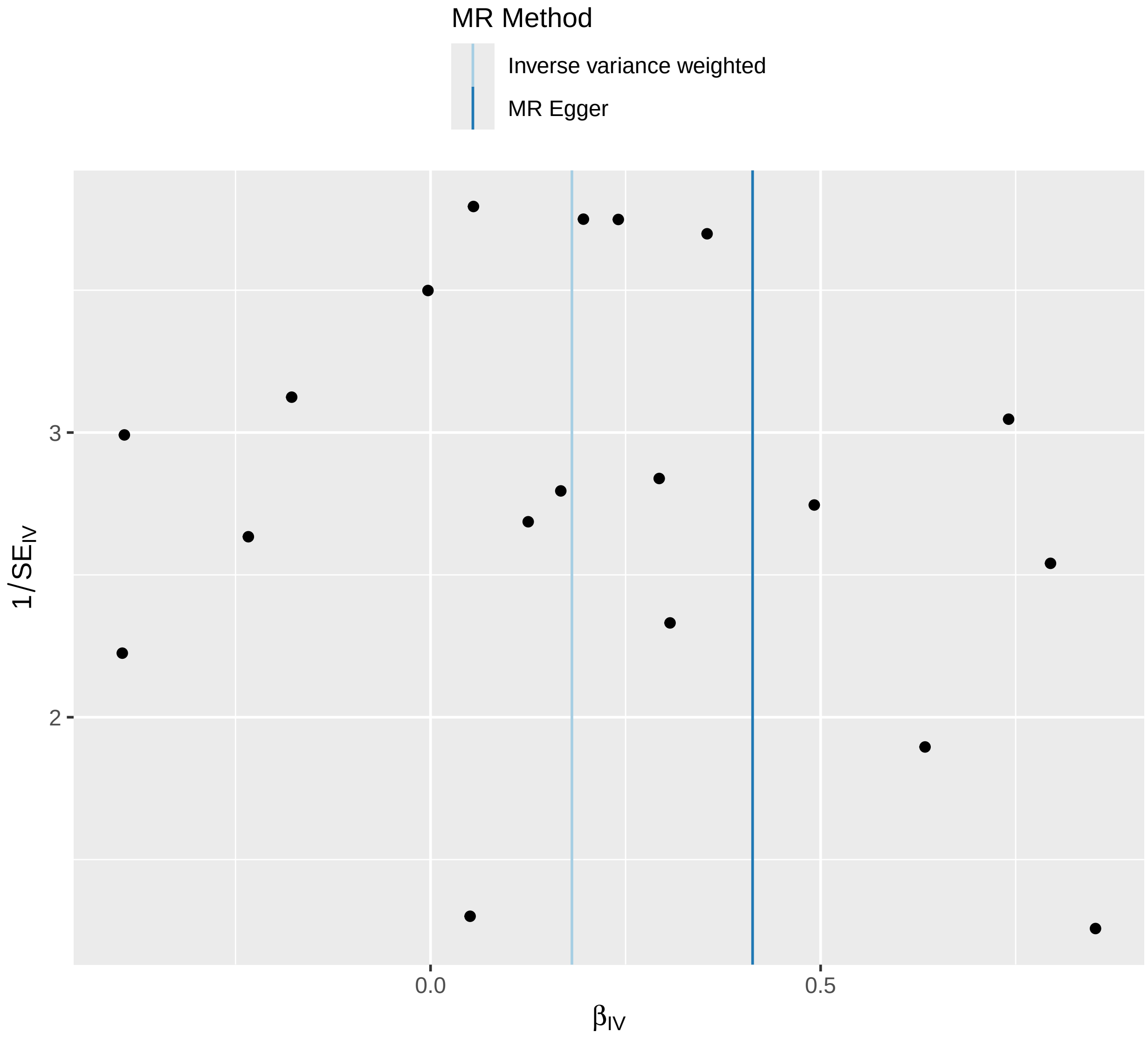
MR Method
Inverse variance weighted
MR Egger
3
2
$1/SE_{IV}$
0.0
0.5
β_{IV}

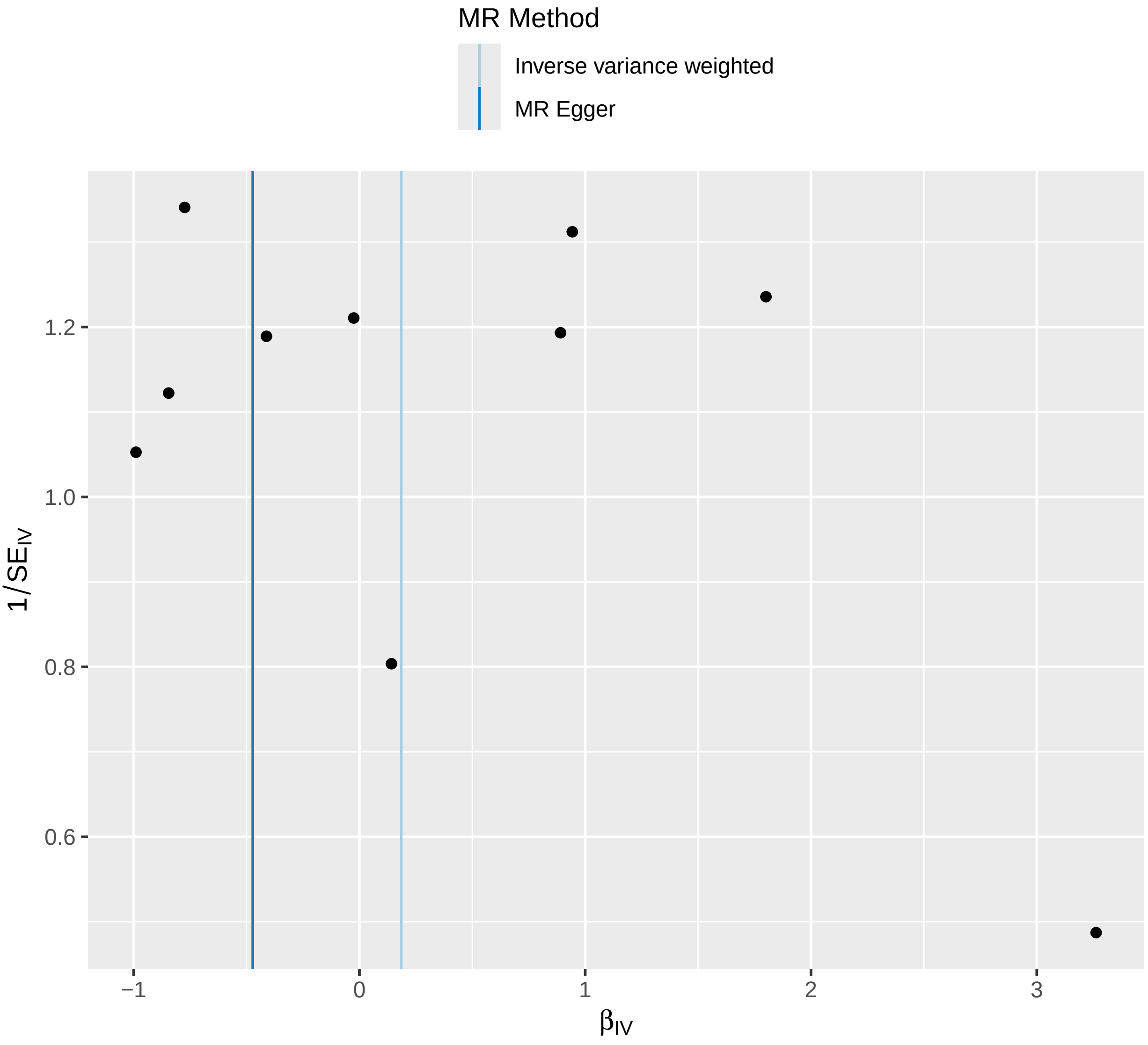
MR Method
Inverse variance weighted
MR Egger
1.2
1.0
0.8
0.6
1/SE_IV
−1
0
1
2
3
β_IV

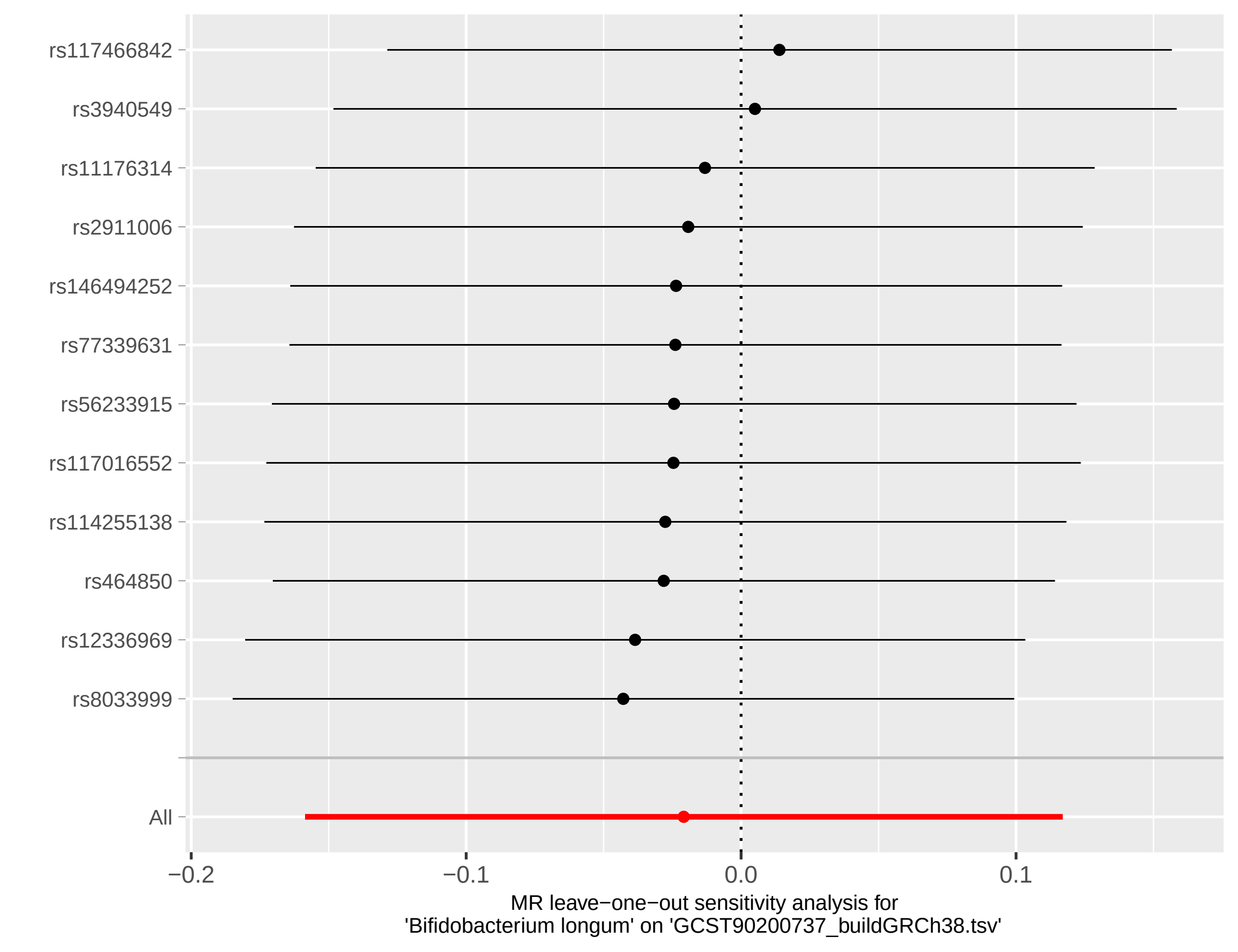
rs117466842
rs3940549
rs11176314
rs2911006
rs146494252
rs77339631
rs56233915
rs117016552
rs114255138
rs464850
rs12336969
rs8033999
All
−0.2
−0.1
0.0
0.1
MR leave−one−out sensitivity analysis for
'Bifidobacterium longum' on 'GCST90200737_buildGRCh38.tsv'

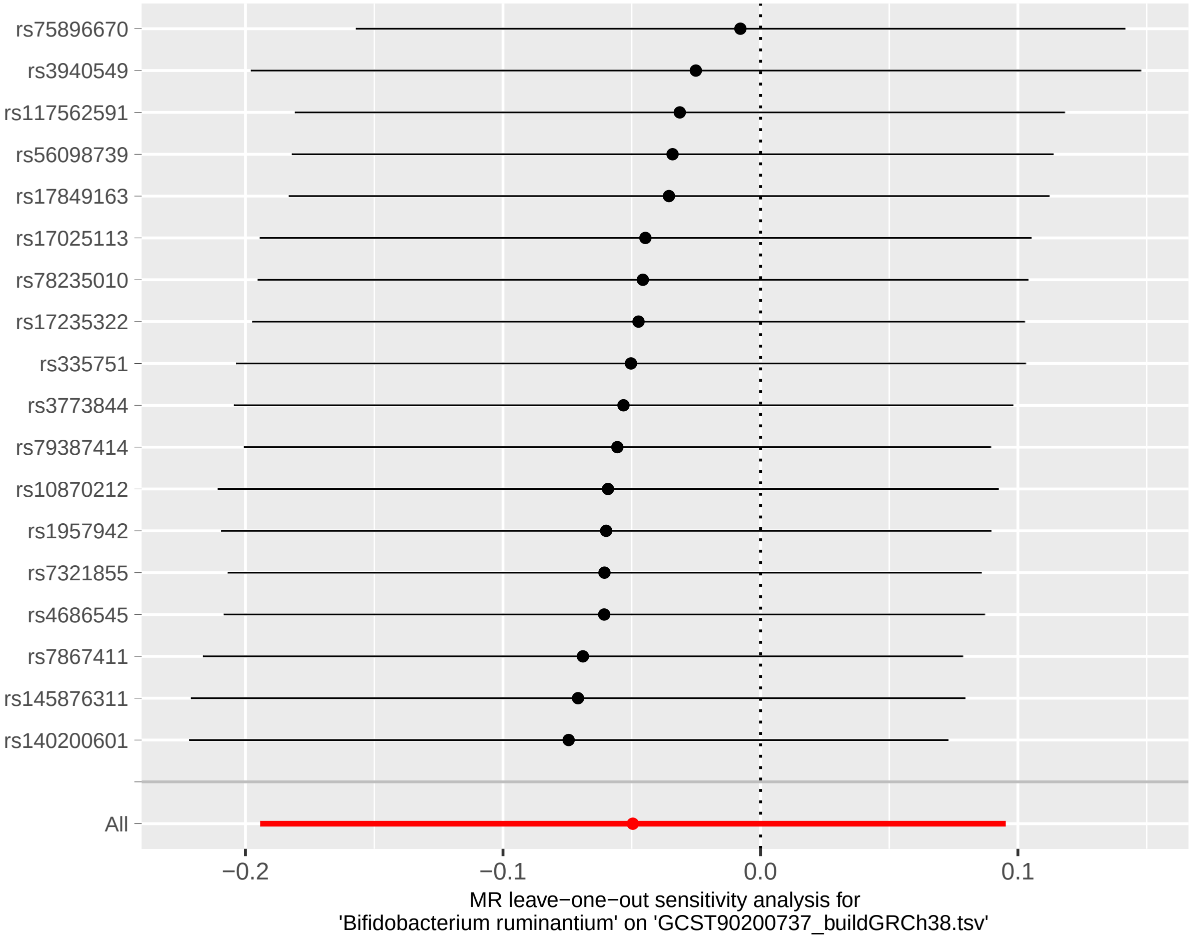

rs75896670
rs3940549
rs117562591
rs56098739
rs17849163
rs17025113
rs78235010
rs17235322
rs335751
rs3773844
rs79387414
rs10870212
rs1957942
rs7321855
rs4686545
rs7867411
rs145876311
rs140200601
All
−0.2
−0.1
0.0
0.1
MR leave−one−out sensitivity analysis for
'Bifidobacterium ruminantium' on 'GCST90200737_buildGRCh38.tsv'

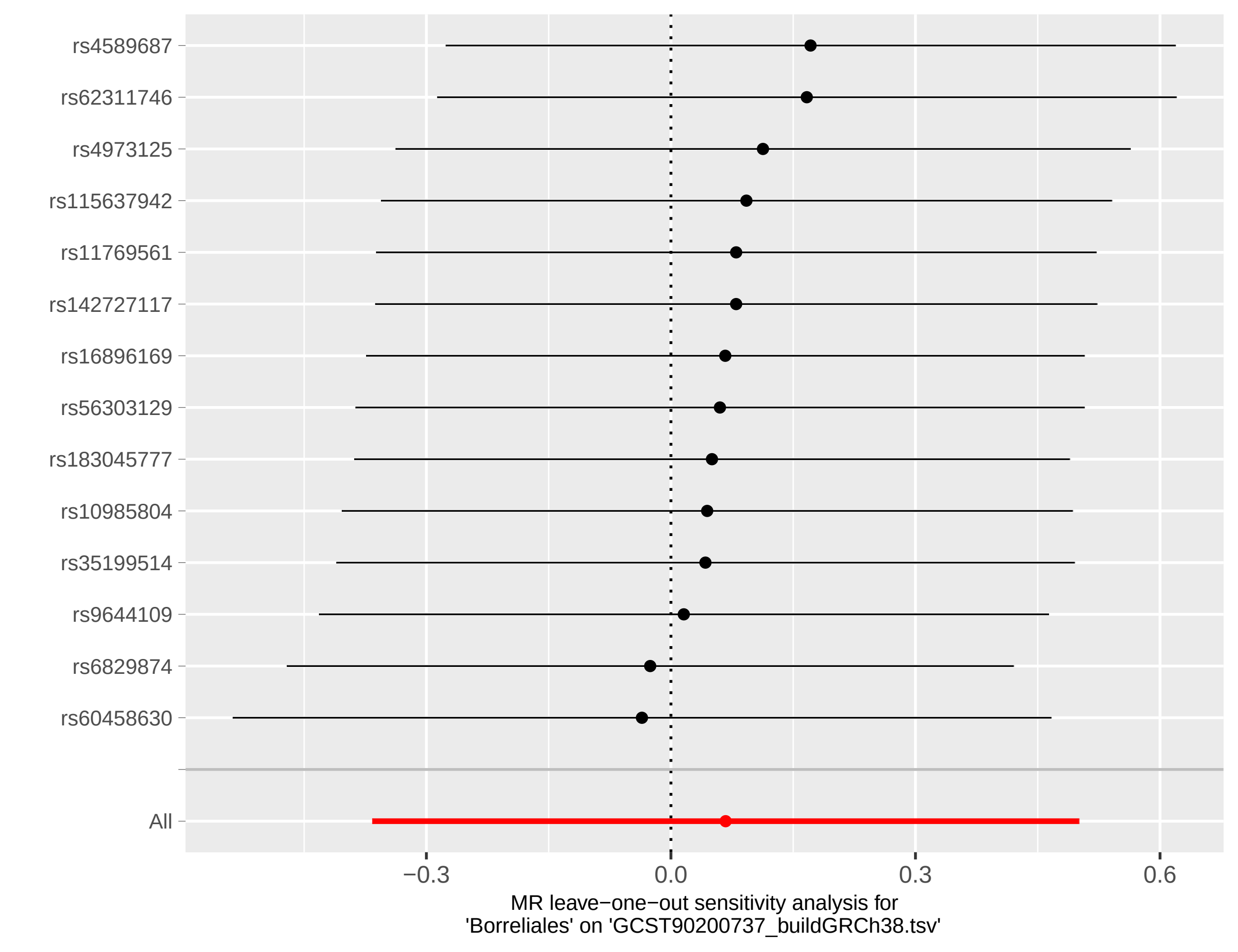
rs4589687
rs62311746
rs4973125
rs115637942
rs11769561
rs142727117
rs16896169
rs56303129
rs183045777
rs10985804
rs35199514
rs9644109
rs6829874
rs60458630
All
−0.3
0.0
0.3
0.6
MR leave−one−out sensitivity analysis for
'Borreliales' on 'GCST90200737_buildGRCh38.tsv'

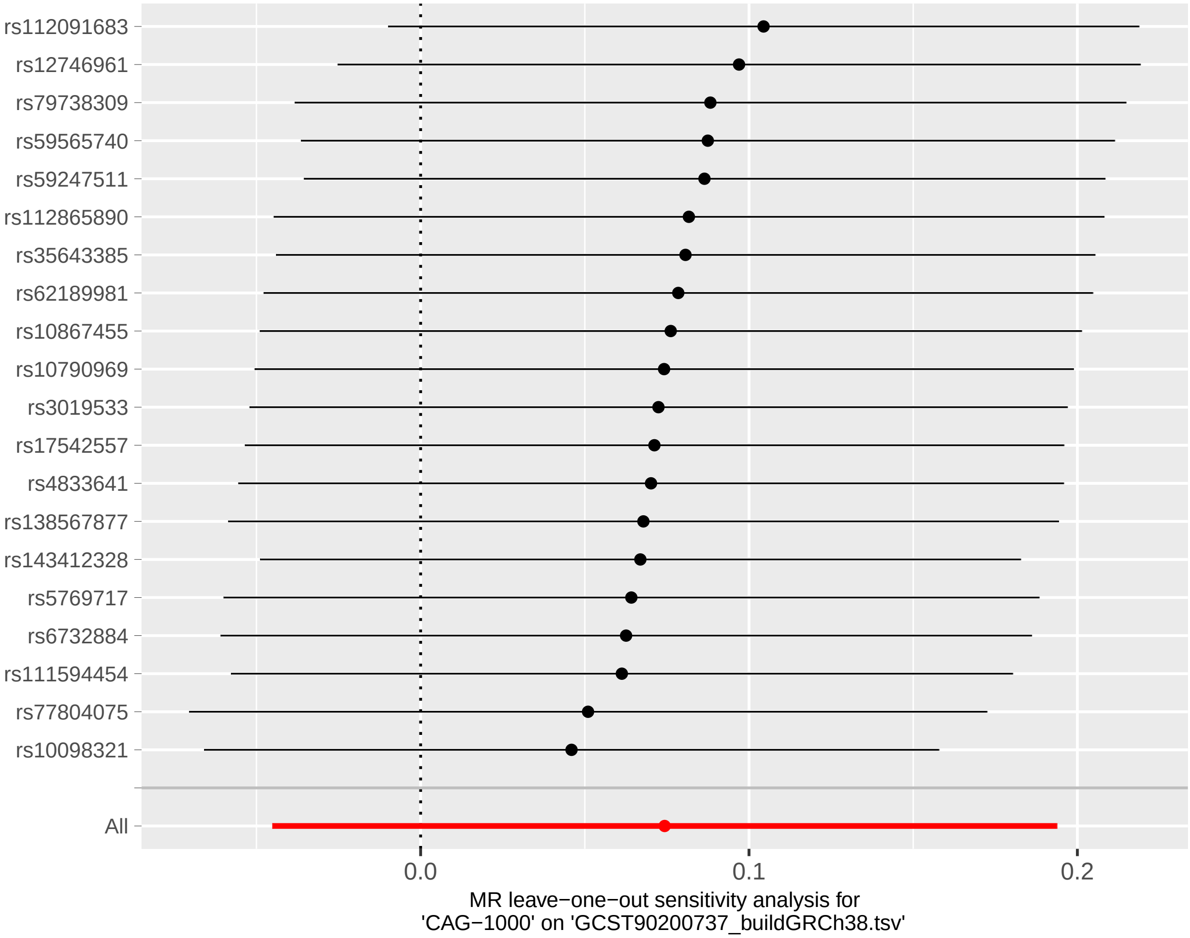

rs112091683
rs12746961
rs79738309
rs59565740
rs59247511
rs112865890
rs35643385
rs62189981
rs10867455
rs10790969
rs3019533
rs17542557
rs4833641
rs138567877
rs143412328
rs5769717
rs6732884
rs111594454
rs77804075
rs10098321
All
0.0
0.1
0.2
MR leave−one−out sensitivity analysis for
'CAG−1000' on 'GCST90200737_buildGRCh38.tsv'

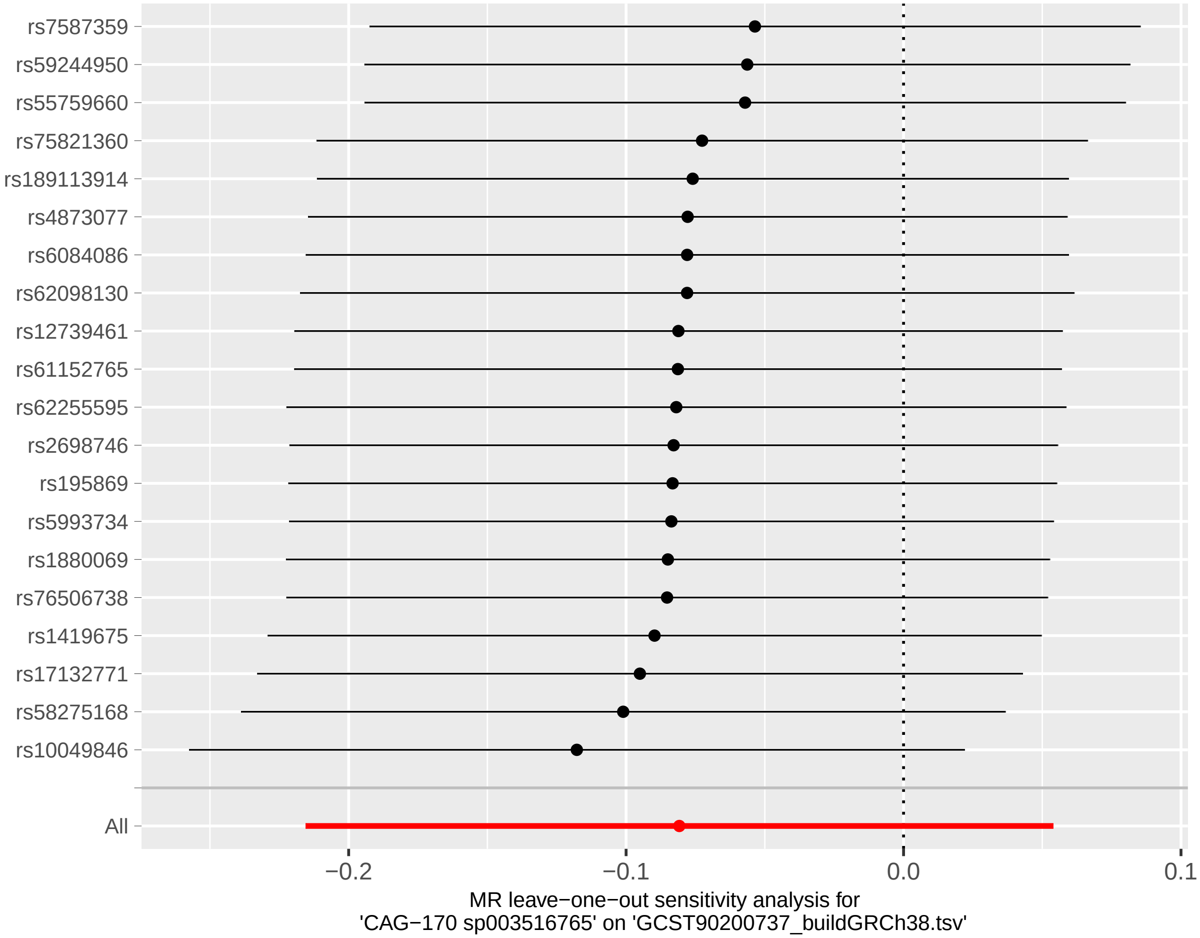
rs7587359
rs59244950
rs55759660
rs75821360
rs189113914
rs4873077
rs6084086
rs62098130
rs12739461
rs61152765
rs62255595
rs2698746
rs195869
rs5993734
rs1880069
rs76506738
rs1419675
rs17132771
rs58275168
rs10049846
All
−0.2
−0.1
0.0
0.1
MR leave−one−out sensitivity analysis for
'CAG−170 sp003516765' on 'GCST90200737_buildGRCh38.tsv'

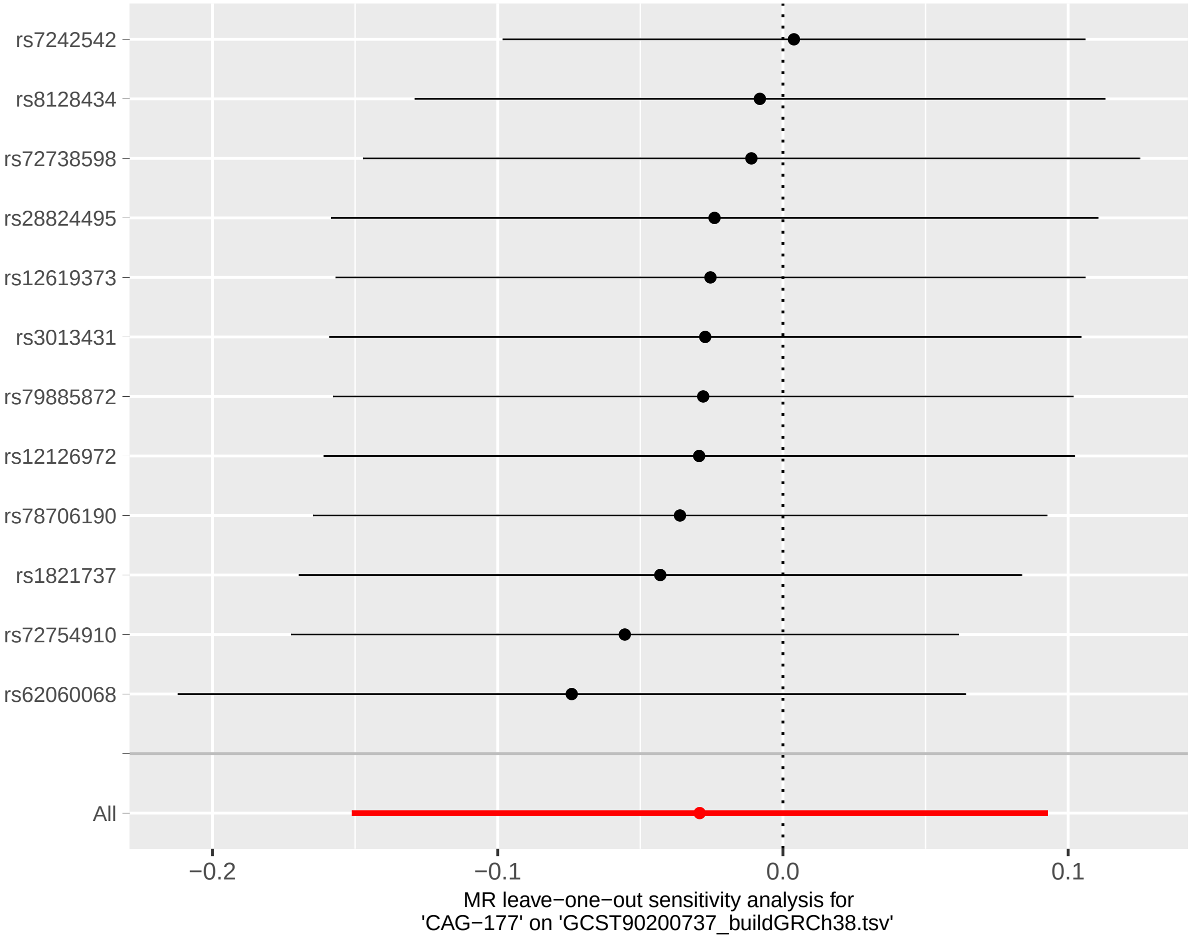

rs7242542
rs8128434
rs72738598
rs28824495
rs12619373
rs3013431
rs79885872
rs12126972
rs78706190
rs1821737
rs72754910
rs62060068
All
−0.2
−0.1
0.0
0.1
MR leave−one−out sensitivity analysis for
'CAG−177' on 'GCST90200737_buildGRCh38.tsv'

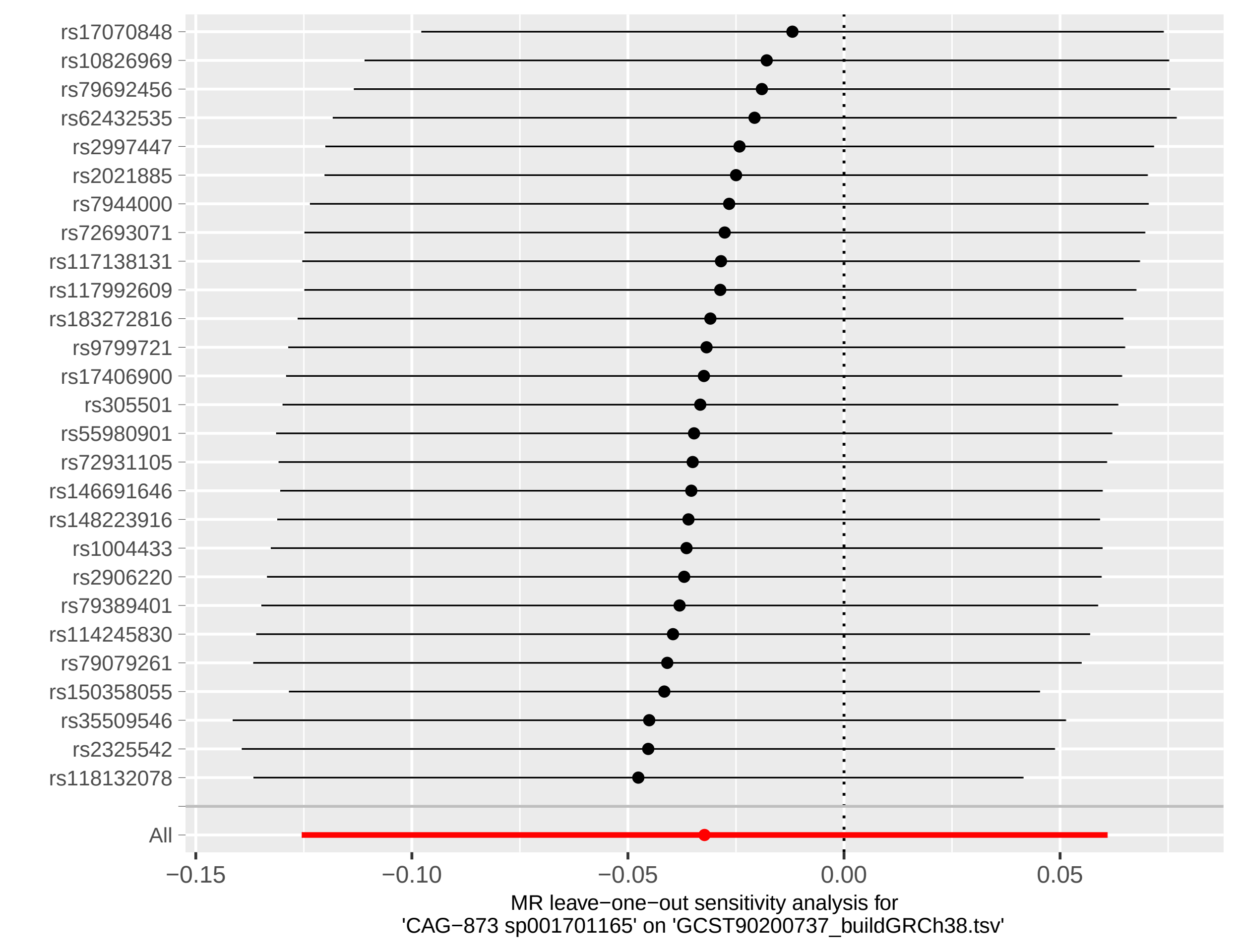

rs17070848
rs10826969
rs79692456
rs62432535
rs2997447
rs2021885
rs7944000
rs72693071
rs117138131
rs117992609
rs183272816
rs9799721
rs17406900
rs305501
rs55980901
rs72931105
rs146691646
rs148223916
rs1004433
rs2906220
rs79389401
rs114245830
rs79079261
rs150358055
rs35509546
rs2325542
rs118132078
All
−0.15
−0.10
−0.05
0.00
0.05
MR leave−one−out sensitivity analysis for
'CAG−873 sp001701165' on 'GCST90200737_buildGRCh38.tsv'

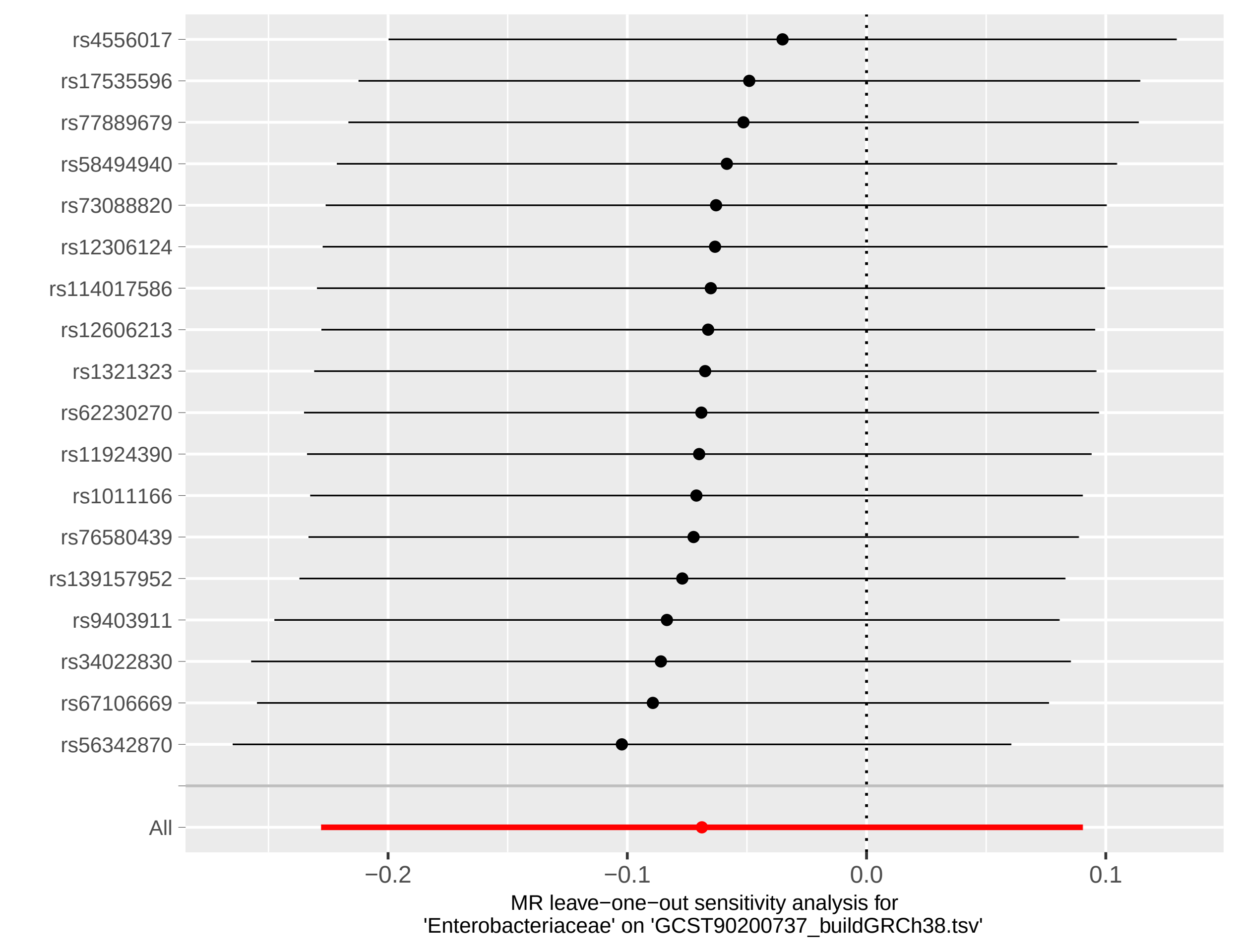
rs4556017
rs17535596
rs77889679
rs58494940
rs73088820
rs12306124
rs114017586
rs12606213
rs1321323
rs62230270
rs11924390
rs1011166
rs76580439
rs139157952
rs9403911
rs34022830
rs67106669
rs56342870
All
−0.2
−0.1
0.0
0.1
MR leave−one−out sensitivity analysis for
'Enterobacteriaceae' on 'GCST90200737_buildGRCh38.tsv'

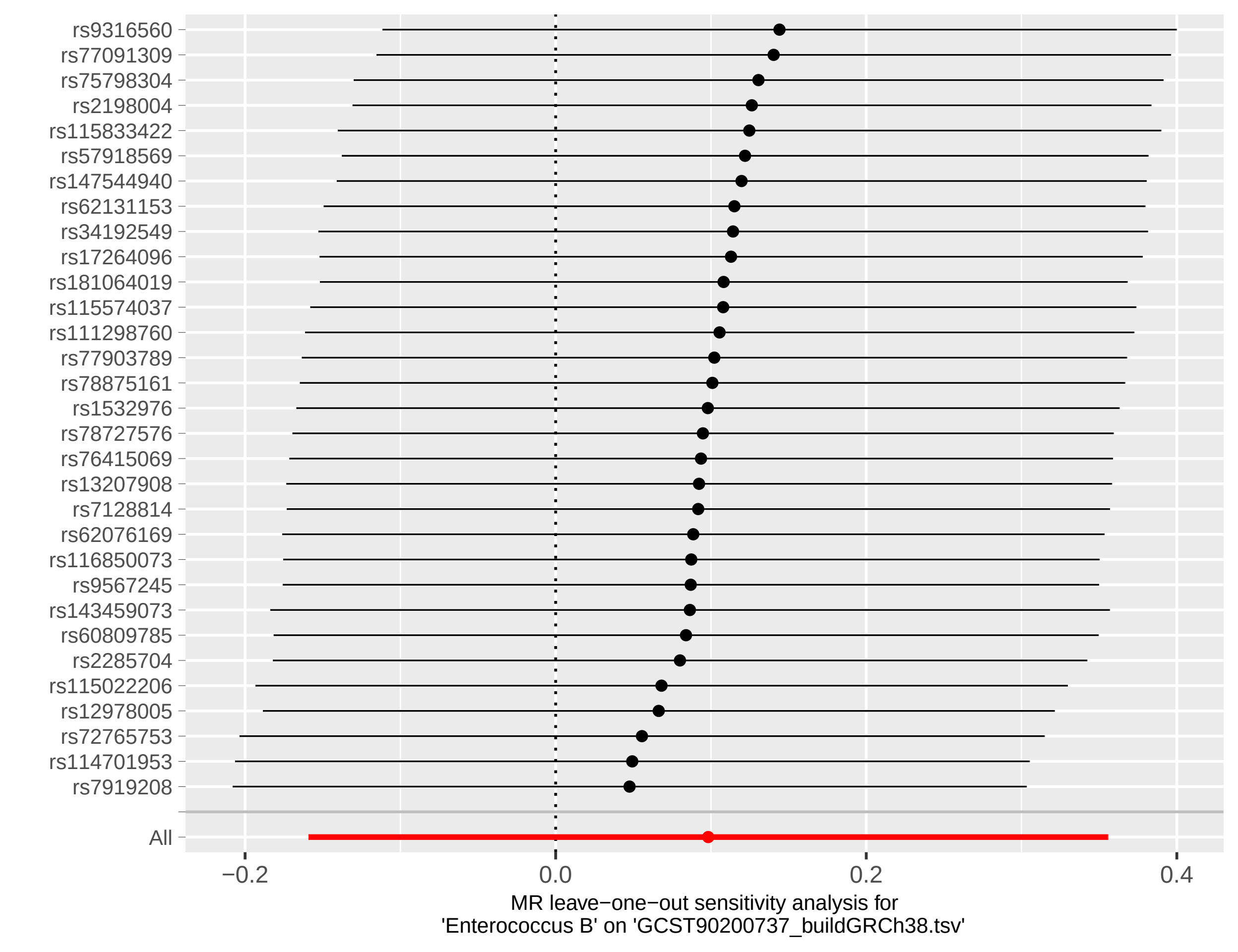
rs9316560
rs77091309
rs75798304
rs2198004
rs115833422
rs57918569
rs147544940
rs62131153
rs34192549
rs17264096
rs181064019
rs115574037
rs111298760
rs77903789
rs78875161
rs1532976
rs78727576
rs76415069
rs13207908
rs7128814
rs62076169
rs116850073
rs9567245
rs143459073
rs60809785
rs2285704
rs115022206
rs12978005
rs72765753
rs114701953
rs7919208
All
−0.2
0.0
0.2
0.4
MR leave−one−out sensitivity analysis for
'Enterococcus B' on 'GCST90200737_buildGRCh38.tsv'

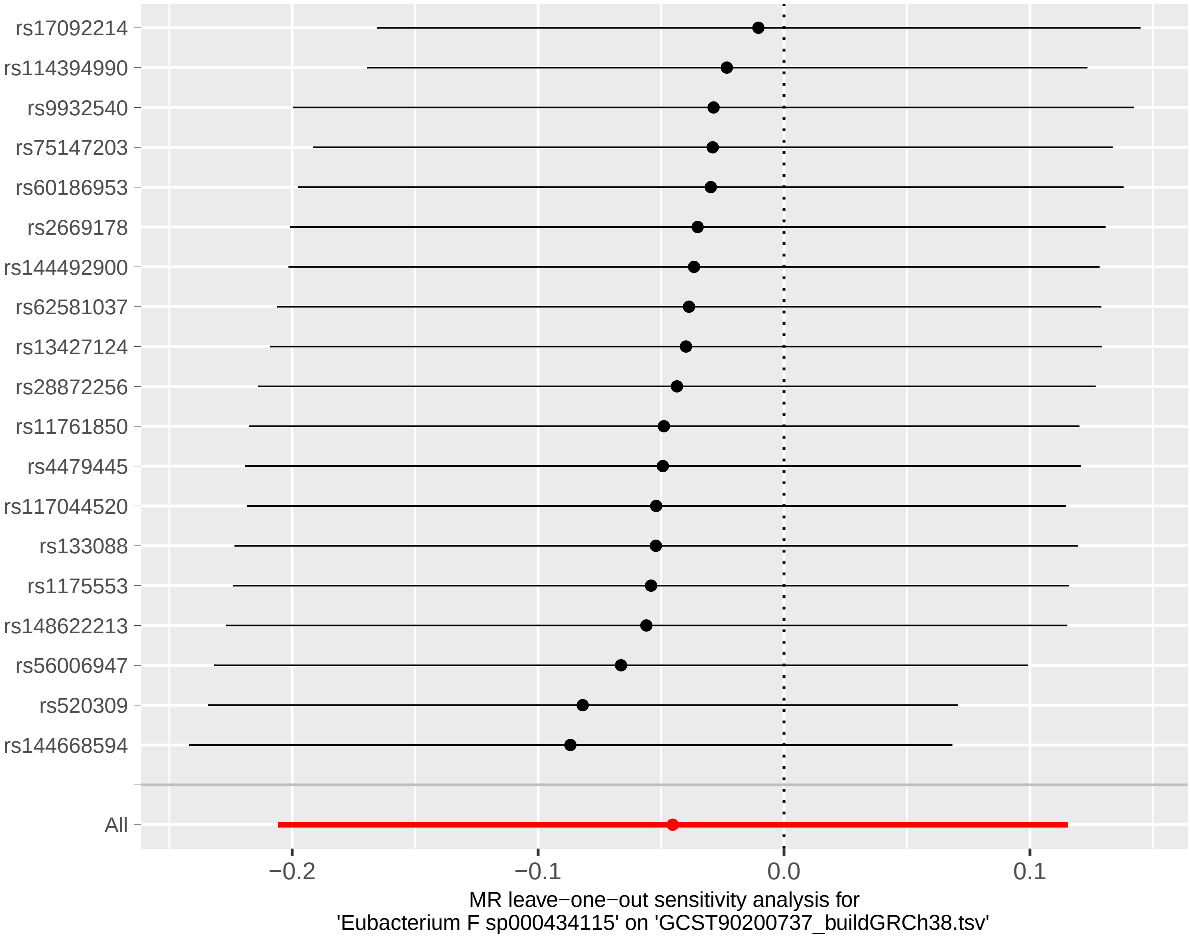

rs17092214
rs114394990
rs9932540
rs75147203
rs60186953
rs2669178
rs144492900
rs62581037
rs13427124
rs28872256
rs11761850
rs4479445
rs117044520
rs133088
rs1175553
rs148622213
rs56006947
rs520309
rs144668594
All
−0.2
−0.1
0.0
0.1
MR leave−one−out sensitivity analysis for
'Eubacterium F sp000434115' on 'GCST90200737_buildGRCh38.tsv'

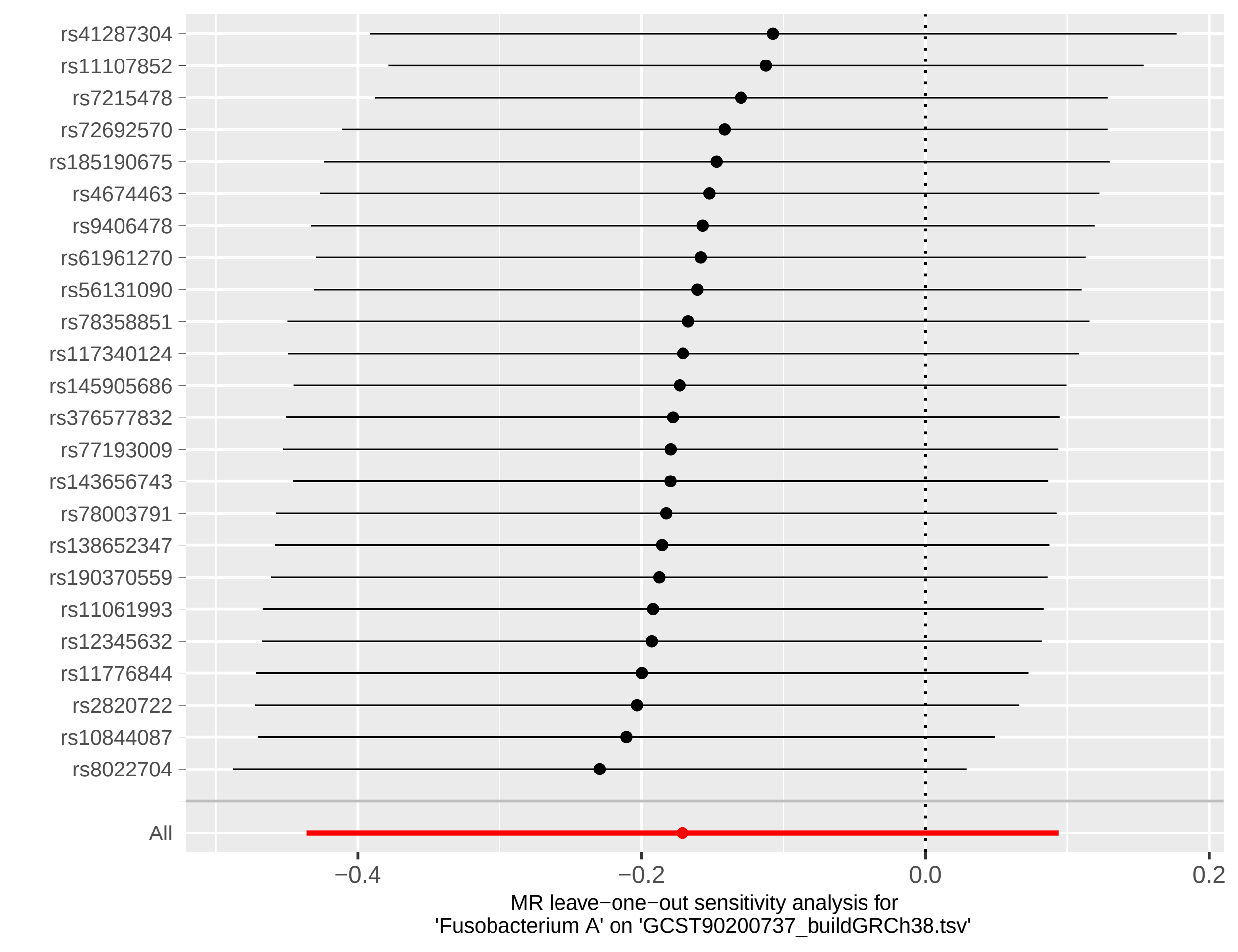
rs41287304
rs11107852
rs7215478
rs72692570
rs185190675
rs4674463
rs9406478
rs61961270
rs56131090
rs78358851
rs117340124
rs145905686
rs376577832
rs77193009
rs143656743
rs78003791
rs138652347
rs190370559
rs11061993
rs12345632
rs11776844
rs2820722
rs10844087
rs8022704
All
−0.4
−0.2
0.0
0.2
MR leave−one−out sensitivity analysis for
'Fusobacterium A' on 'GCST90200737_buildGRCh38.tsv'

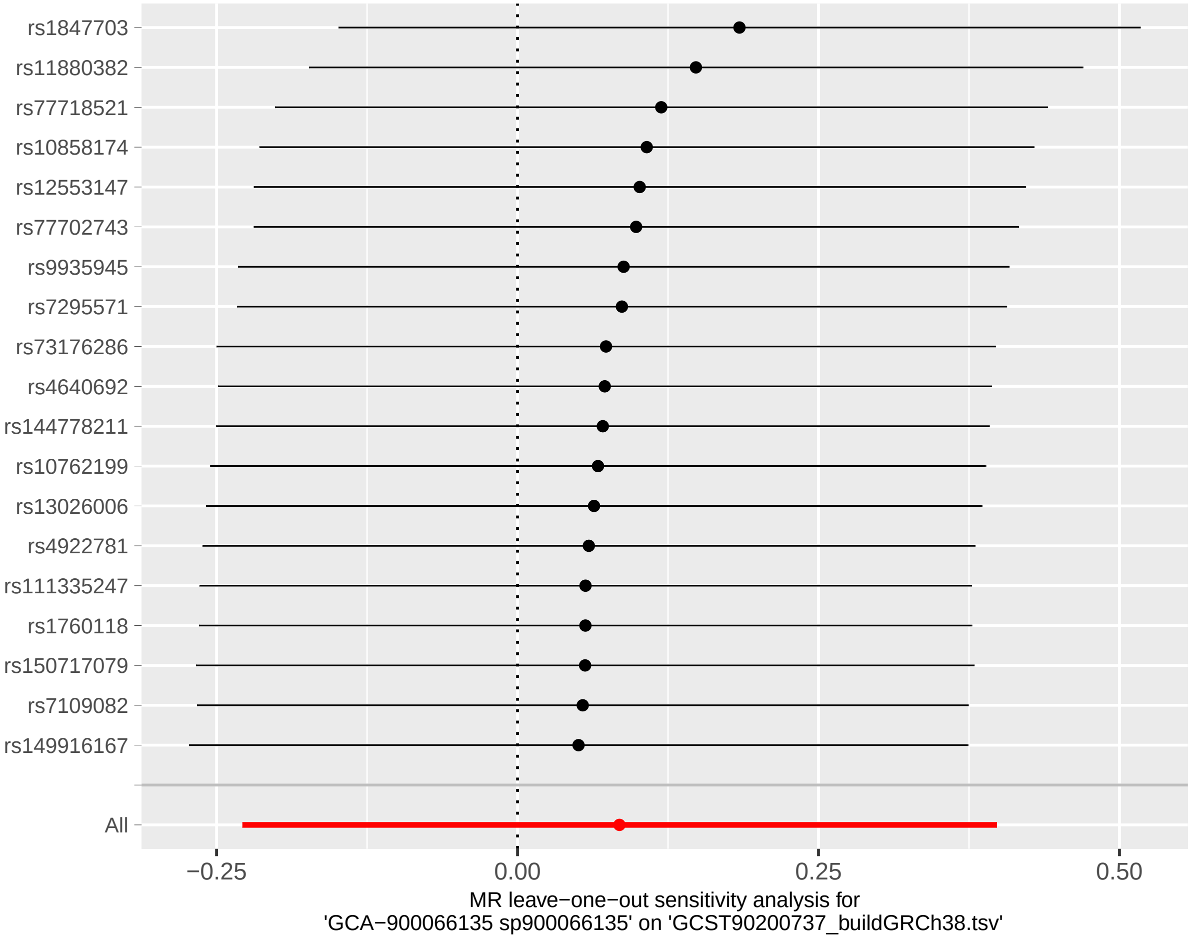
rs1847703
rs11880382
rs77718521
rs10858174
rs12553147
rs77702743
rs9935945
rs7295571
rs73176286
rs4640692
rs144778211
rs10762199
rs13026006
rs4922781
rs111335247
rs1760118
rs150717079
rs7109082
rs149916167
All
−0.25
0.00
0.25
0.50
MR leave−one−out sensitivity analysis for
'GCA−900066135 sp900066135' on 'GCST90200737_buildGRCh38.tsv'

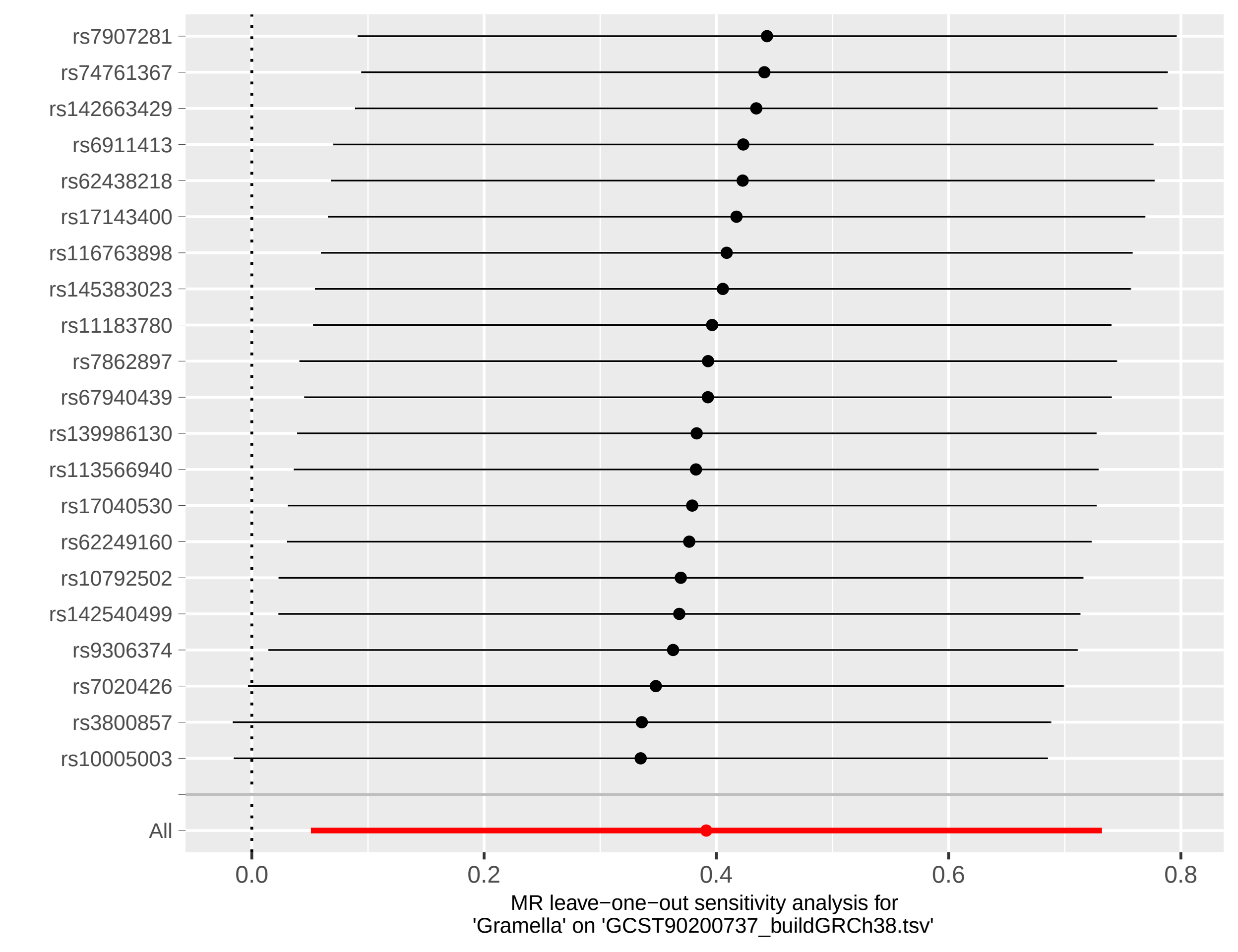

rs7907281
rs74761367
rs142663429
rs6911413
rs62438218
rs17143400
rs116763898
rs145383023
rs11183780
rs7862897
rs67940439
rs139986130
rs113566940
rs17040530
rs62249160
rs10792502
rs142540499
rs9306374
rs7020426
rs3800857
rs10005003
All
0.0
0.2
0.4
0.6
0.8
MR leave−one−out sensitivity analysis for
'Gramella' on 'GCST90200737_buildGRCh38.tsv'

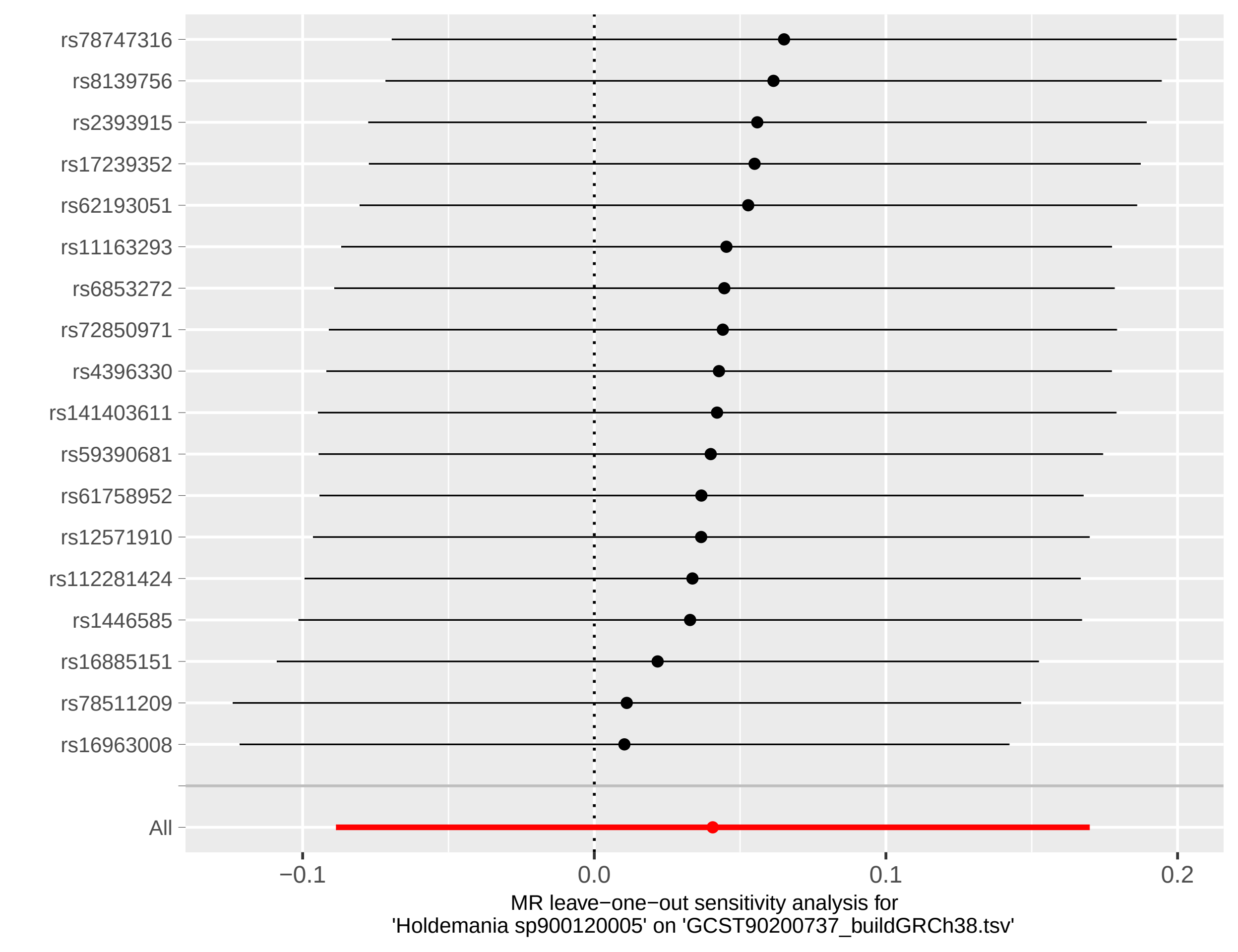
rs78747316
rs8139756
rs2393915
rs17239352
rs62193051
rs11163293
rs6853272
rs72850971
rs4396330
rs141403611
rs59390681
rs61758952
rs12571910
rs112281424
rs1446585
rs16885151
rs78511209
rs16963008
All
−0.1
0.0
0.1
0.2
MR leave−one−out sensitivity analysis for
'Holdemania sp900120005' on 'GCST90200737_buildGRCh38.tsv'

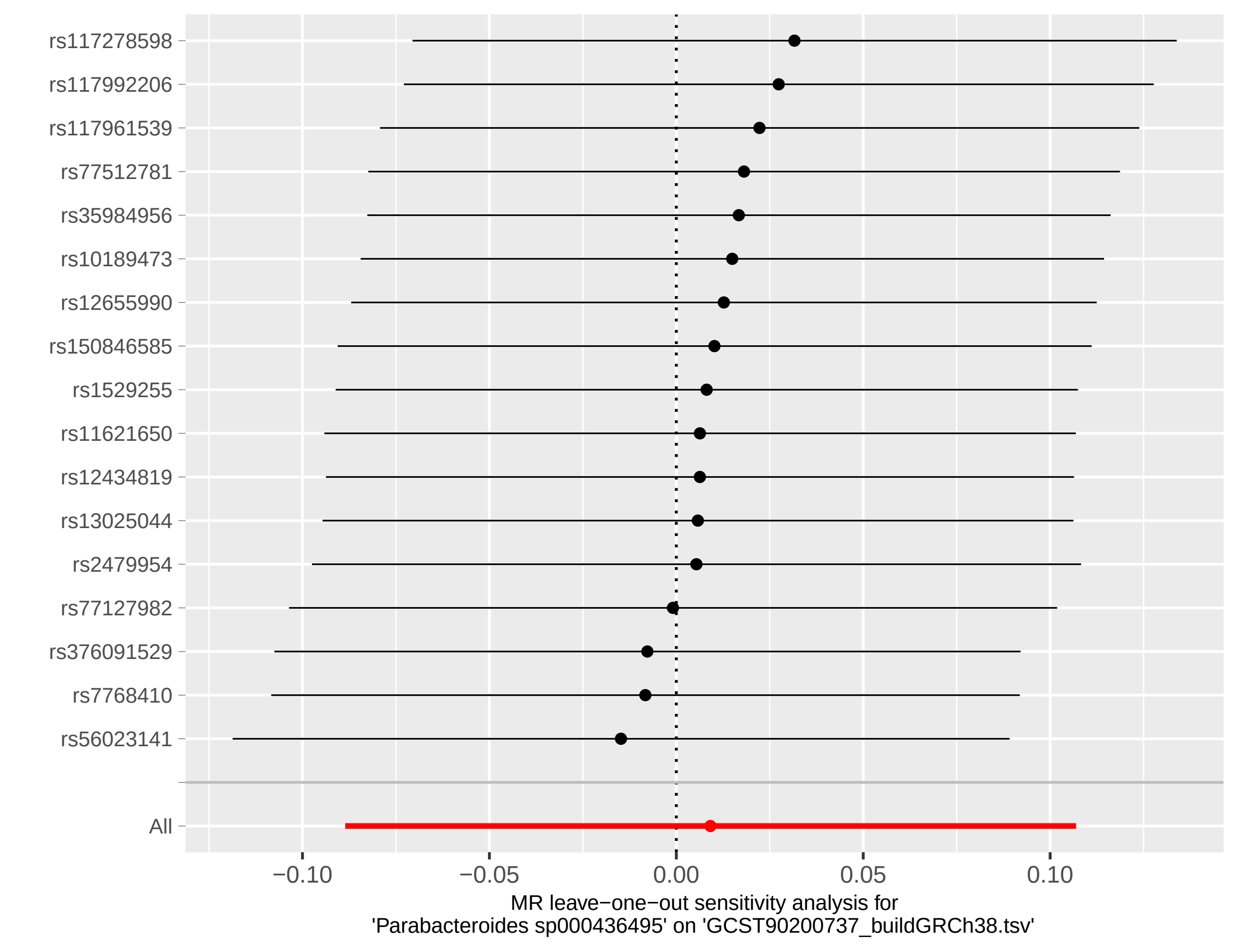
rs117278598
rs117992206
rs117961539
rs77512781
rs35984956
rs10189473
rs12655990
rs150846585
rs1529255
rs11621650
rs12434819
rs13025044
rs2479954
rs77127982
rs376091529
rs7768410
rs56023141
All
−0.10
−0.05
0.00
0.05
0.10
MR leave−one−out sensitivity analysis for
'Parabacteroides sp000436495' on 'GCST90200737_buildGRCh38.tsv'

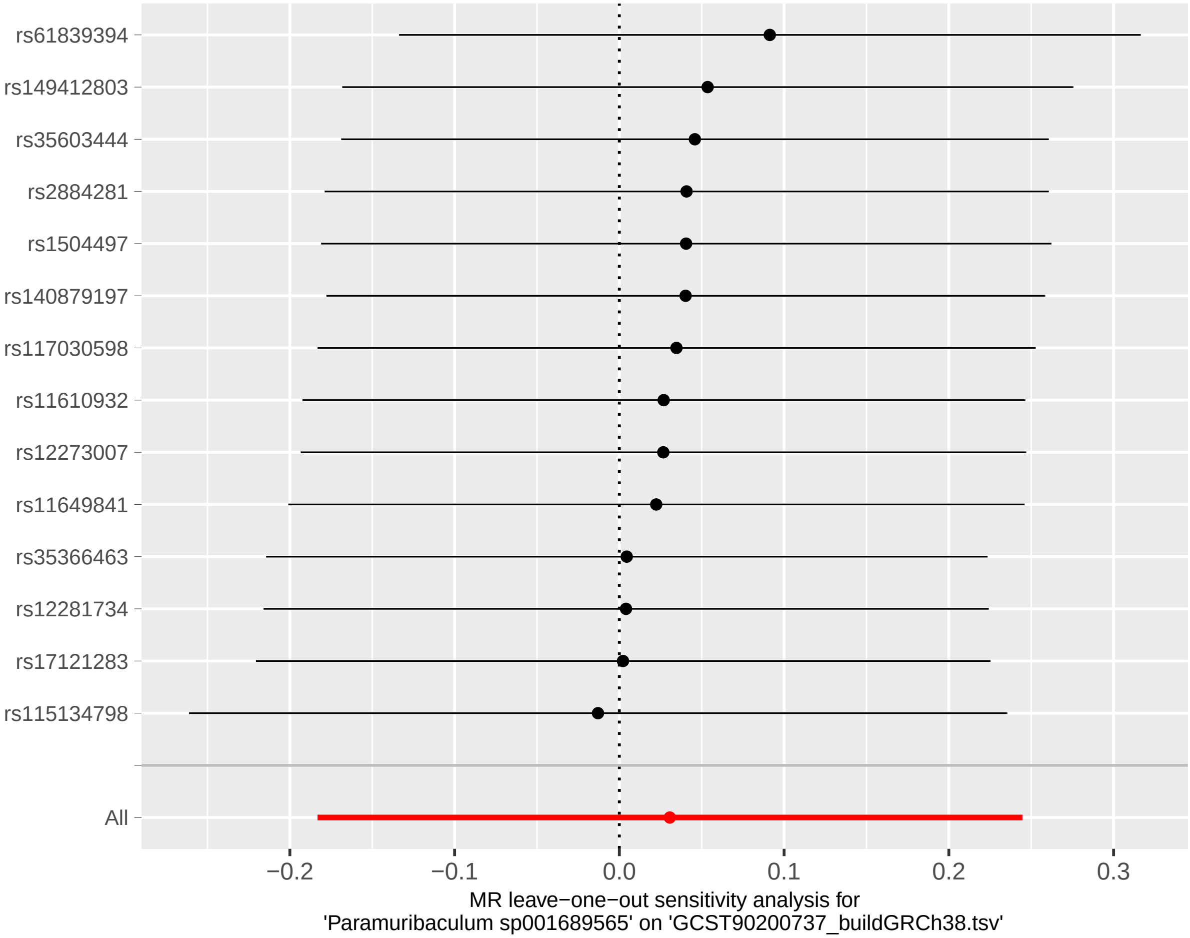

rs61839394
rs149412803
rs35603444
rs2884281
rs1504497
rs140879197
rs117030598
rs11610932
rs12273007
rs11649841
rs35366463
rs12281734
rs17121283
rs115134798
All
−0.2
−0.1
0.0
0.1
0.2
0.3
MR leave−one−out sensitivity analysis for
'Paramuribaculum sp001689565' on 'GCST90200737_buildGRCh38.tsv'

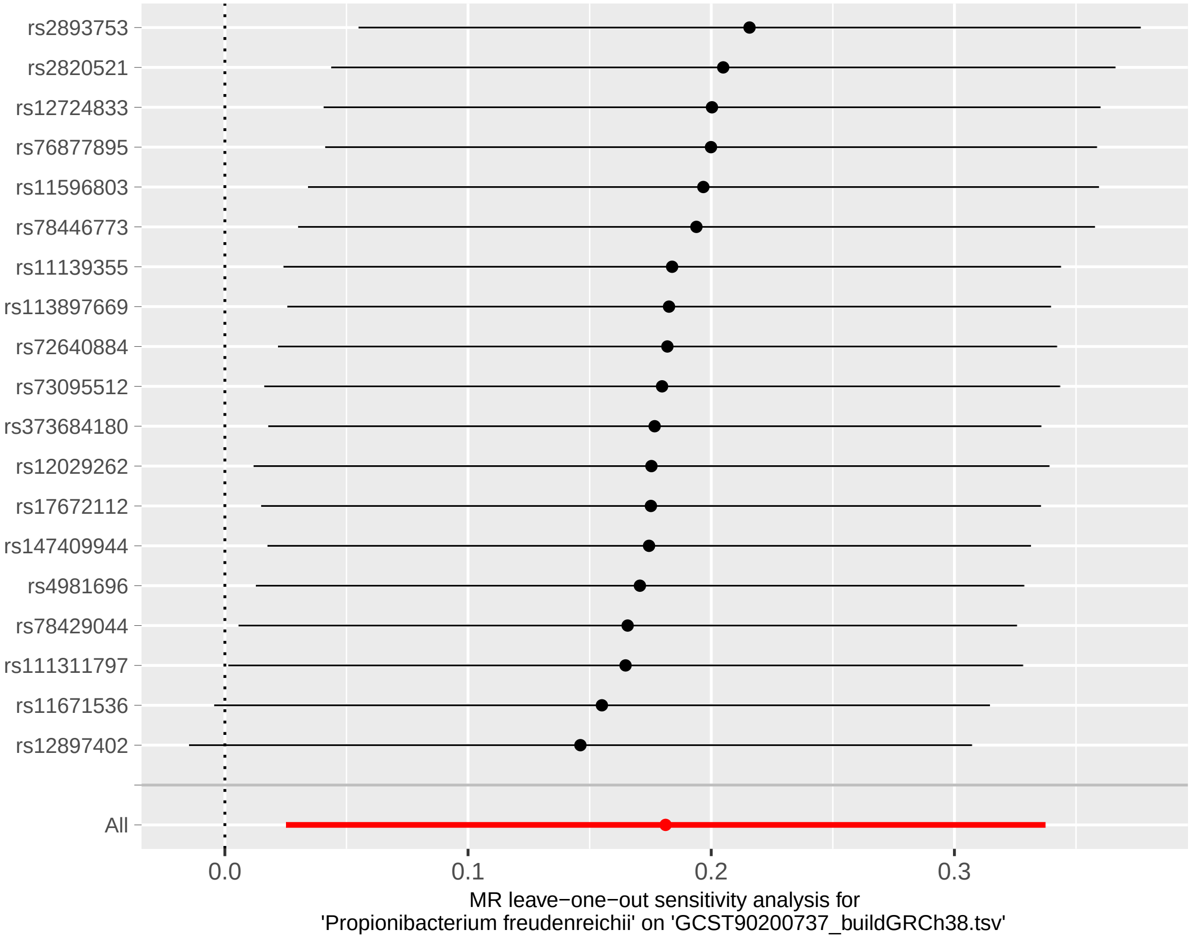

rs2893753
rs2820521
rs12724833
rs76877895
rs11596803
rs78446773
rs11139355
rs113897669
rs72640884
rs73095512
rs373684180
rs12029262
rs17672112
rs147409944
rs4981696
rs78429044
rs111311797
rs11671536
rs12897402
All
0.0
0.1
0.2
0.3
MR leave−one−out sensitivity analysis for
'Propionibacterium freudenreichii' on 'GCST90200737_buildGRCh38.tsv'

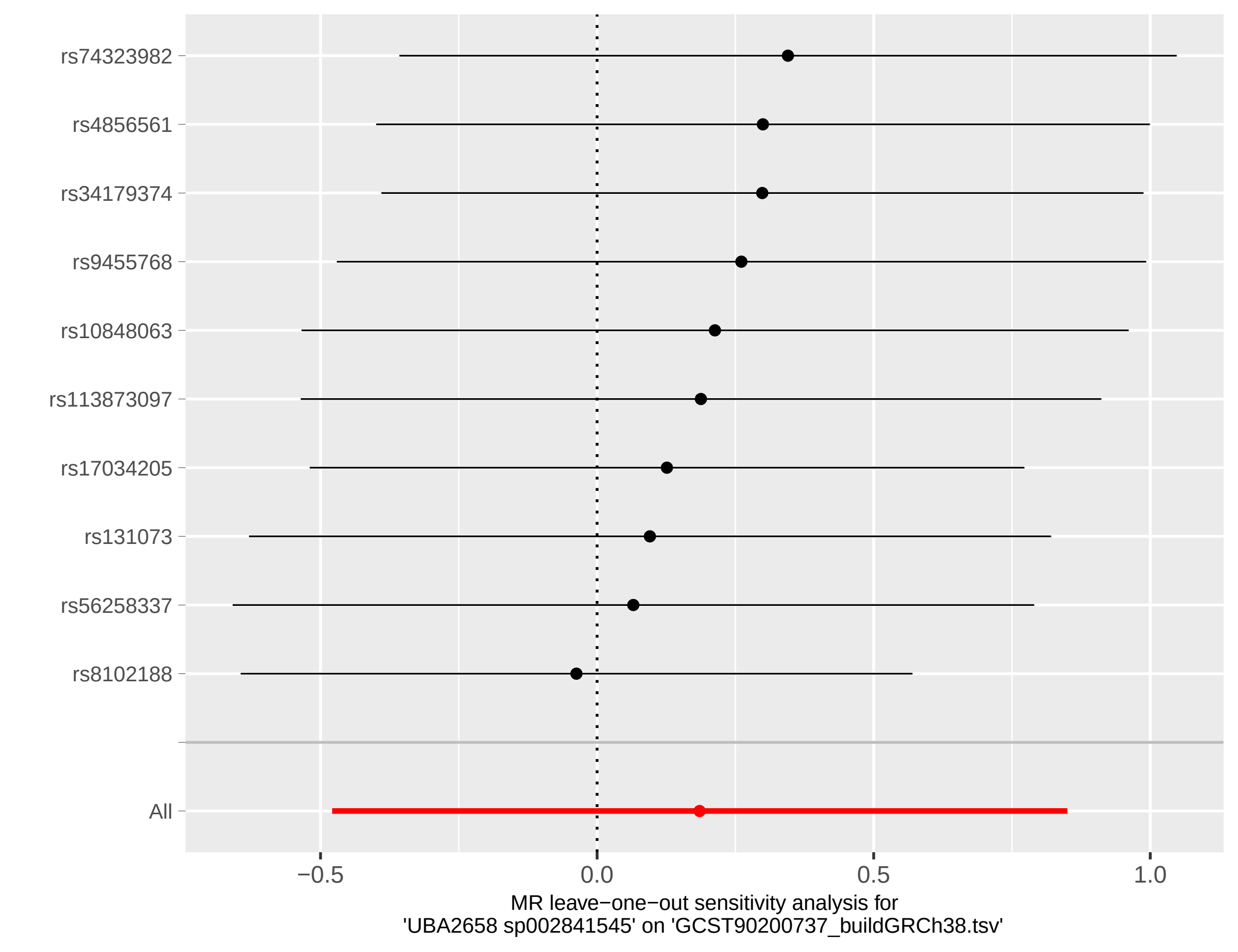

rs74323982
rs4856561
rs34179374
rs9455768
rs10848063
rs113873097
rs17034205
rs131073
rs56258337
rs8102188
All
−0.5
0.0
0.5
1.0
MR leave−one−out sensitivity analysis for
'UBA2658 sp002841545' on 'GCST90200737_buildGRCh38.tsv'

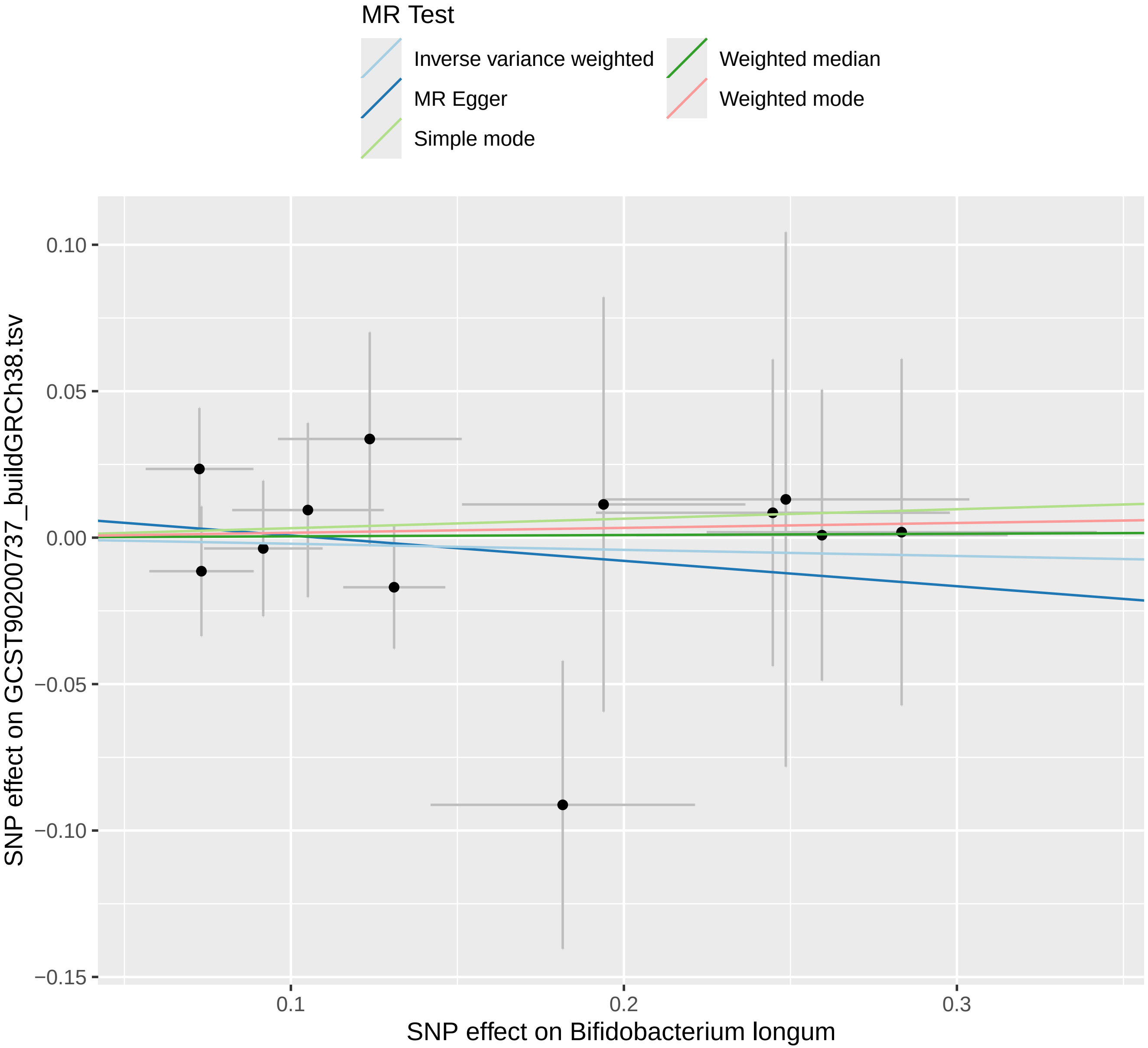
MR Test
Inverse variance weighted
MR Egger
Simple mode
Weighted median
Weighted mode
0.10
0.05
0.00
−0.05
−0.10
−0.15
SNP effect on GCST90200737_buildGRCh38.tsv
0.1
0.2
0.3
SNP effect on Bifidobacterium longum

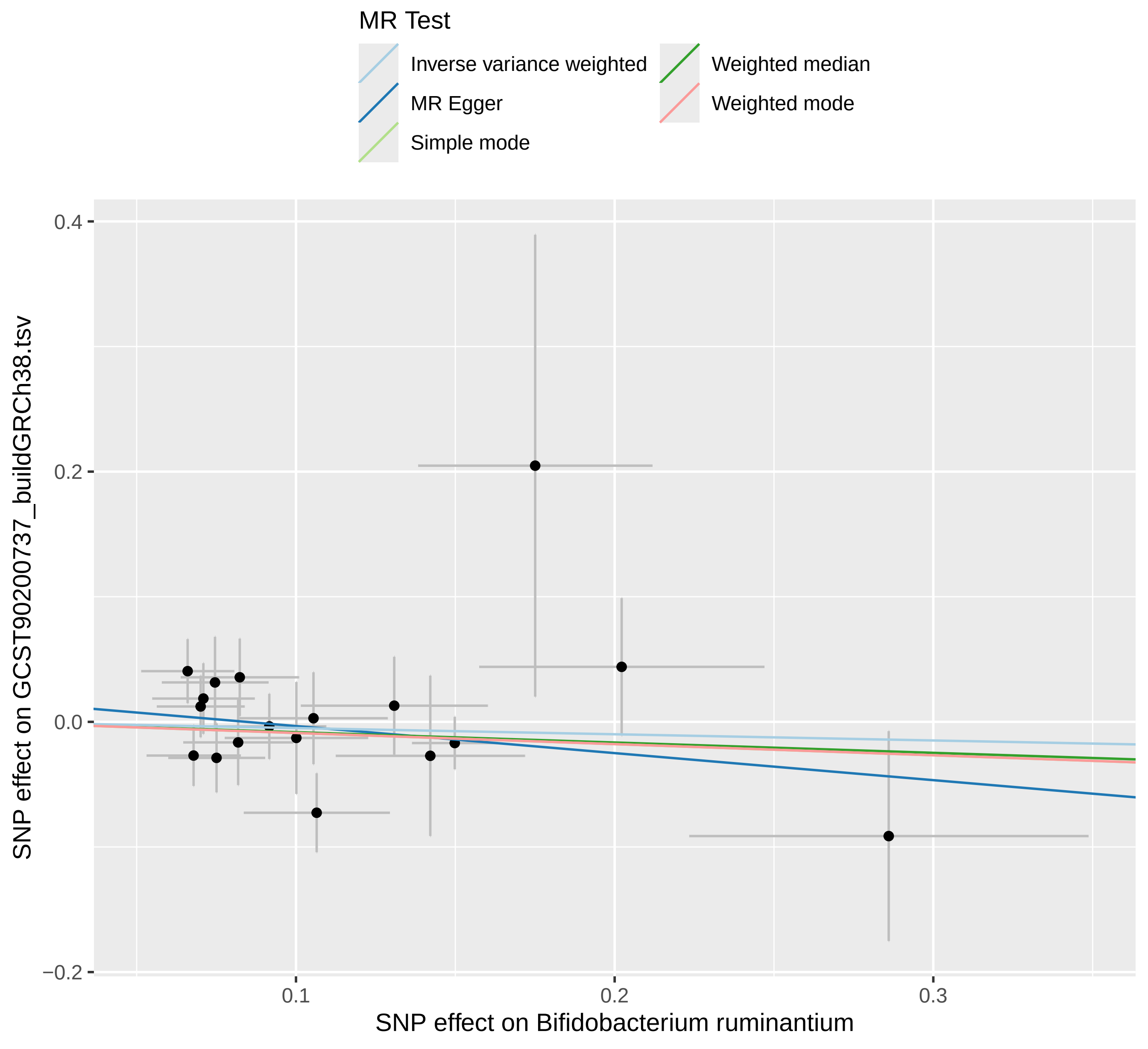
MR Test
Inverse variance weighted
MR Egger
Simple mode
Weighted median
Weighted mode
0.4
0.2
0.0
−0.2
0.1
0.2
0.3
SNP effect on GCST90200737_buildGRCh38.tsv
SNP effect on Bifidobacterium ruminantium

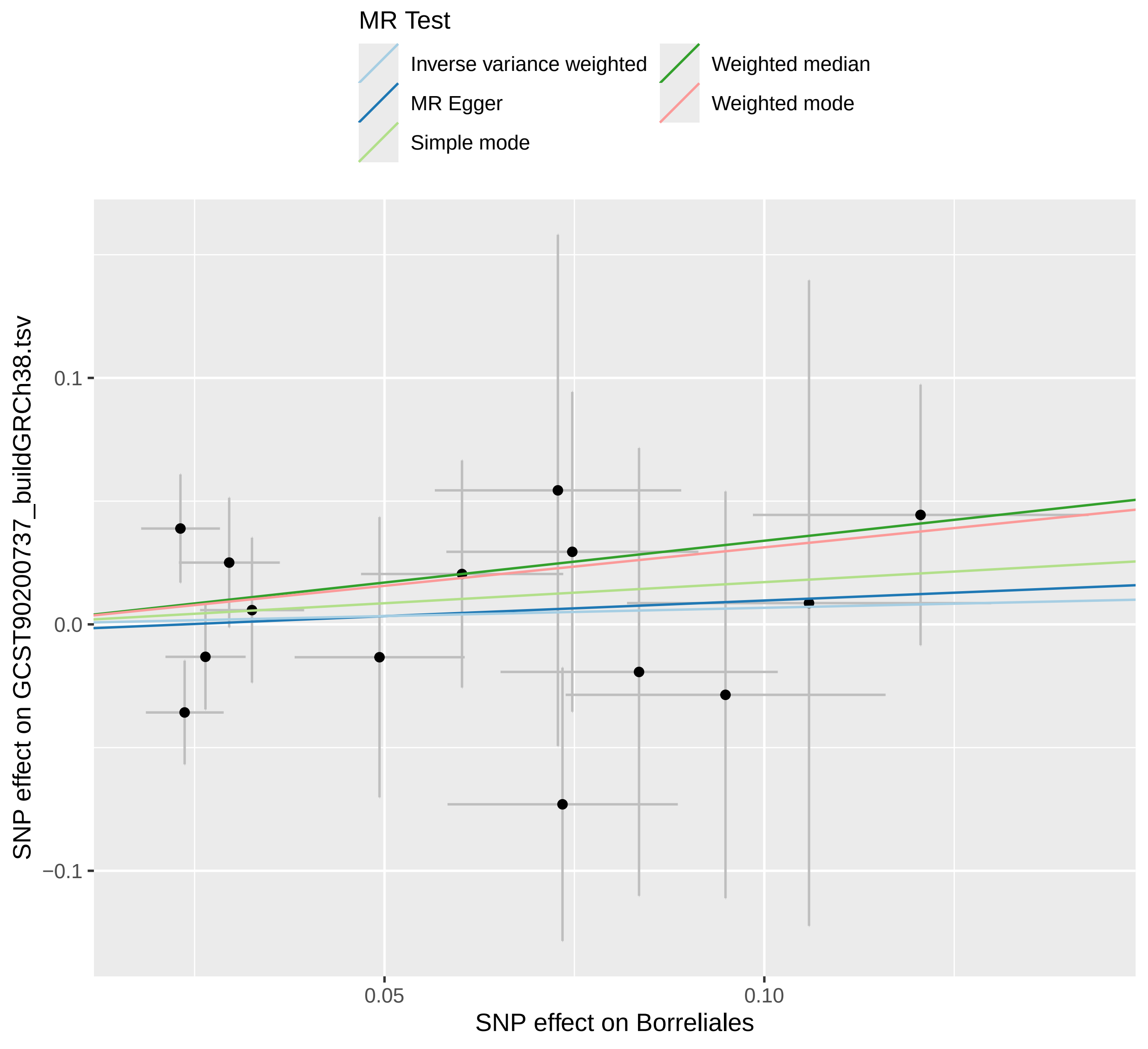

MR Test
Inverse variance weighted
MR Egger
Simple mode
Weighted median
Weighted mode
0.1
0.0
−0.1
0.05
0.10
SNP effect on GCST90200737_buildGRCh38.tsv
SNP effect on Borreliales

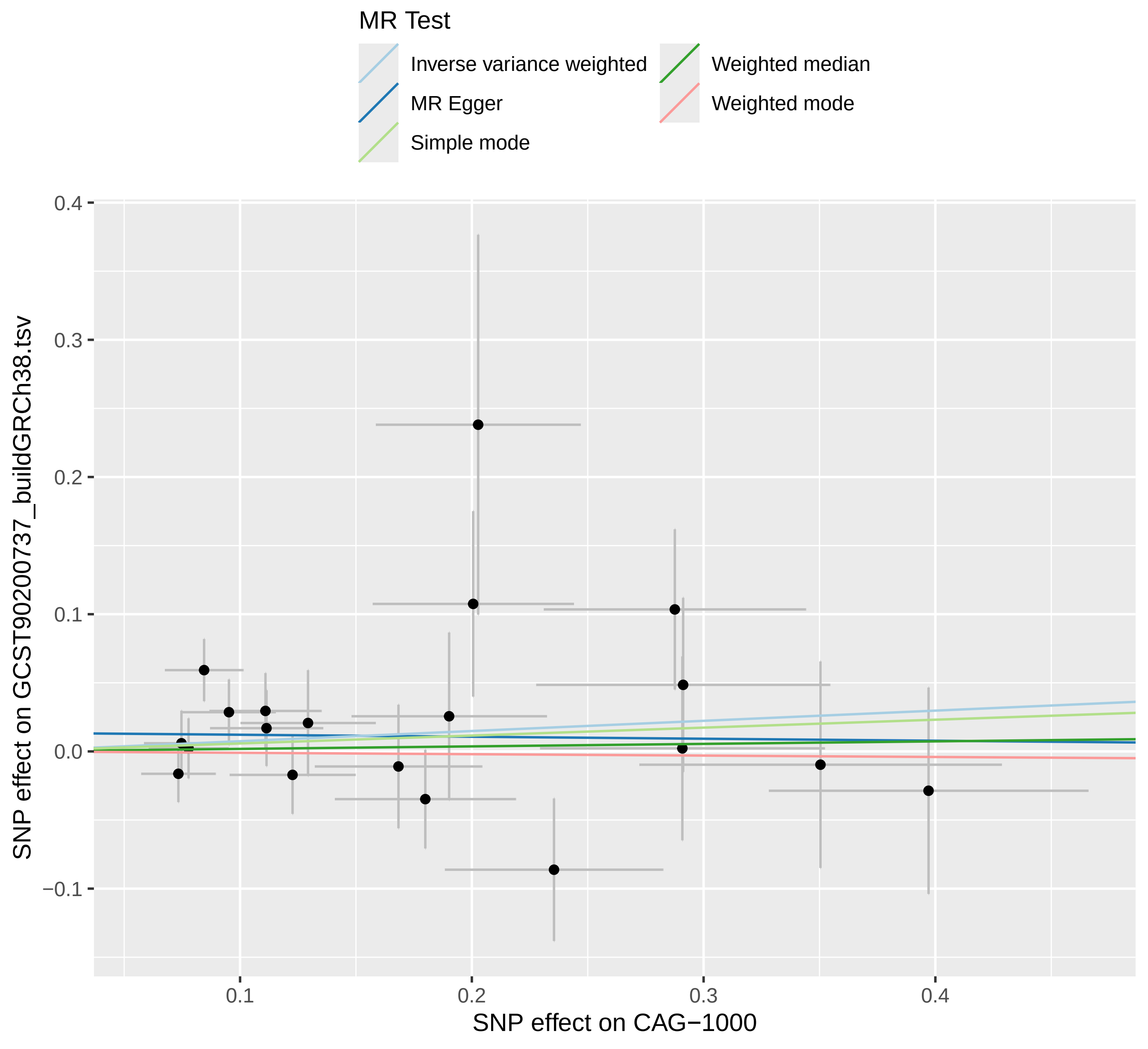

MR Test
Inverse variance weighted
MR Egger
Simple mode
Weighted median
Weighted mode
SNP effect on GCST90200737_buildGRCh38.tsv
0.4
0.3
0.2
0.1
0.0
−0.1
0.1
0.2
0.3
0.4
SNP effect on CAG−1000

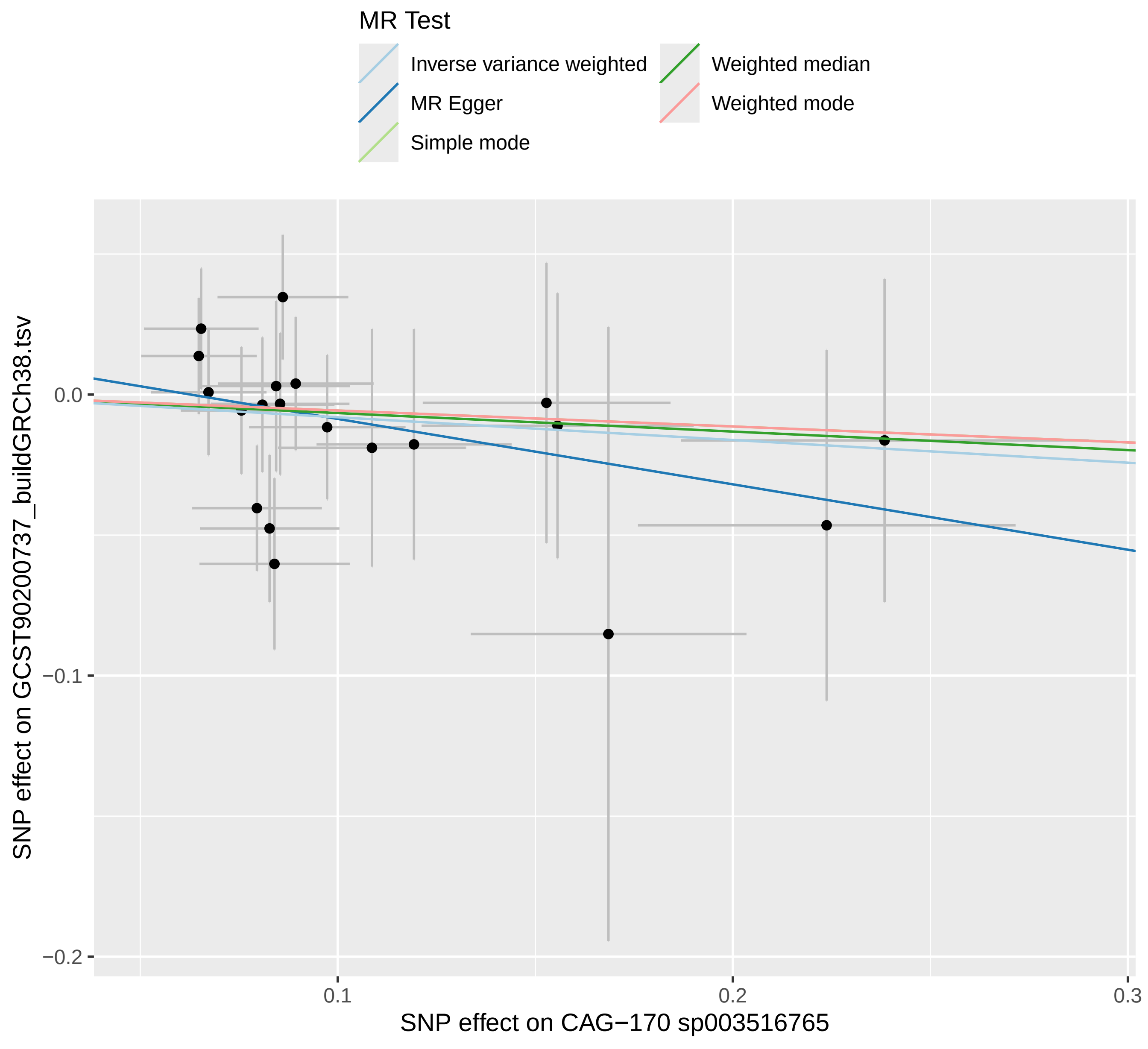

MR Test
Inverse variance weighted
MR Egger
Simple mode
Weighted median
Weighted mode
0.0
−0.1
−0.2
0.1
0.2
0.3
SNP effect on GCST90200737_buildGRCh38.tsv
SNP effect on CAG−170 sp003516765

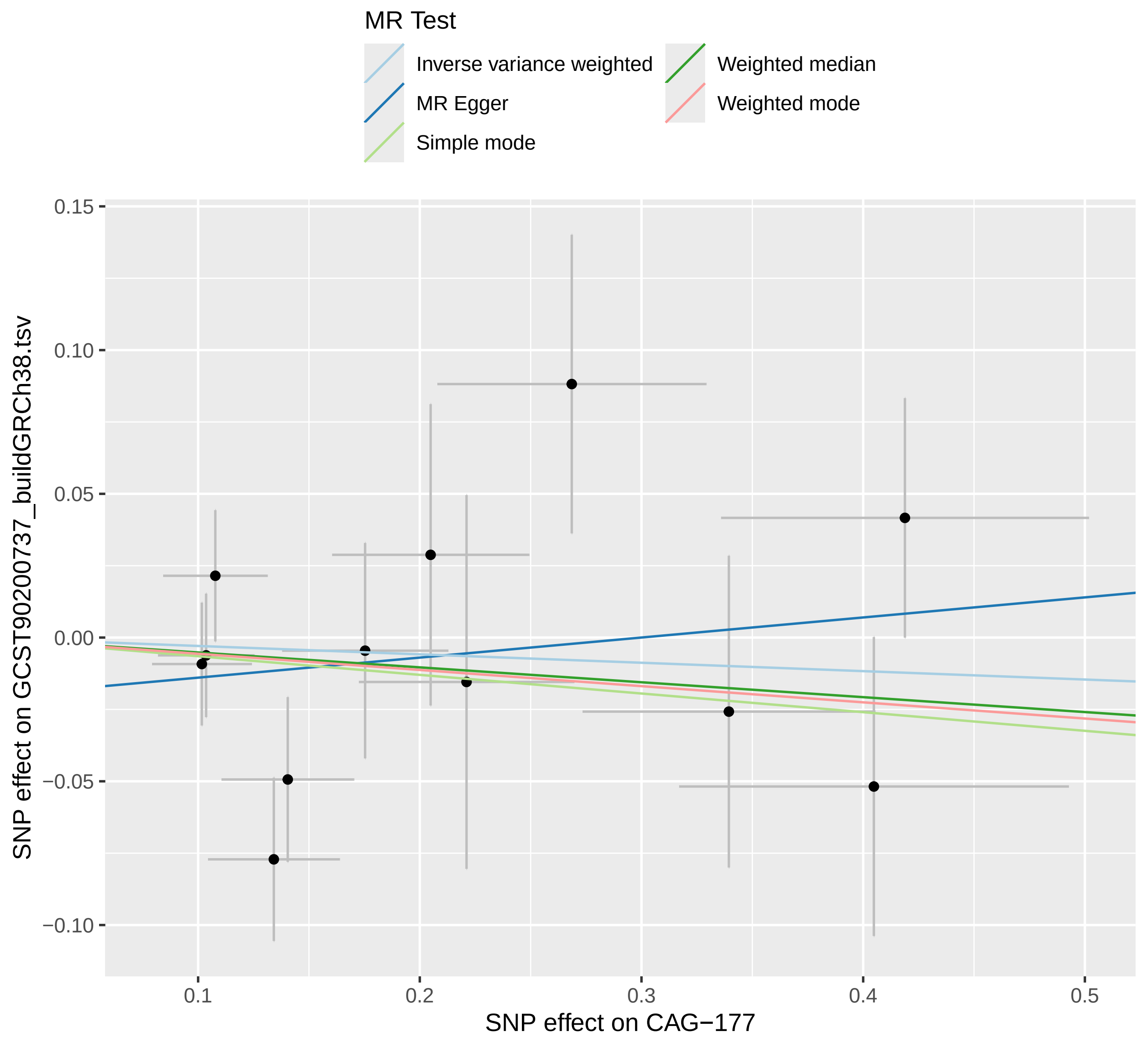
MR Test
Inverse variance weighted
MR Egger
Simple mode
Weighted median
Weighted mode
0.15
0.10
0.05
0.00
−0.05
−0.10
0.1
0.2
0.3
0.4
0.5
SNP effect on GCST90200737_buildGRCh38.tsv
SNP effect on CAG−177

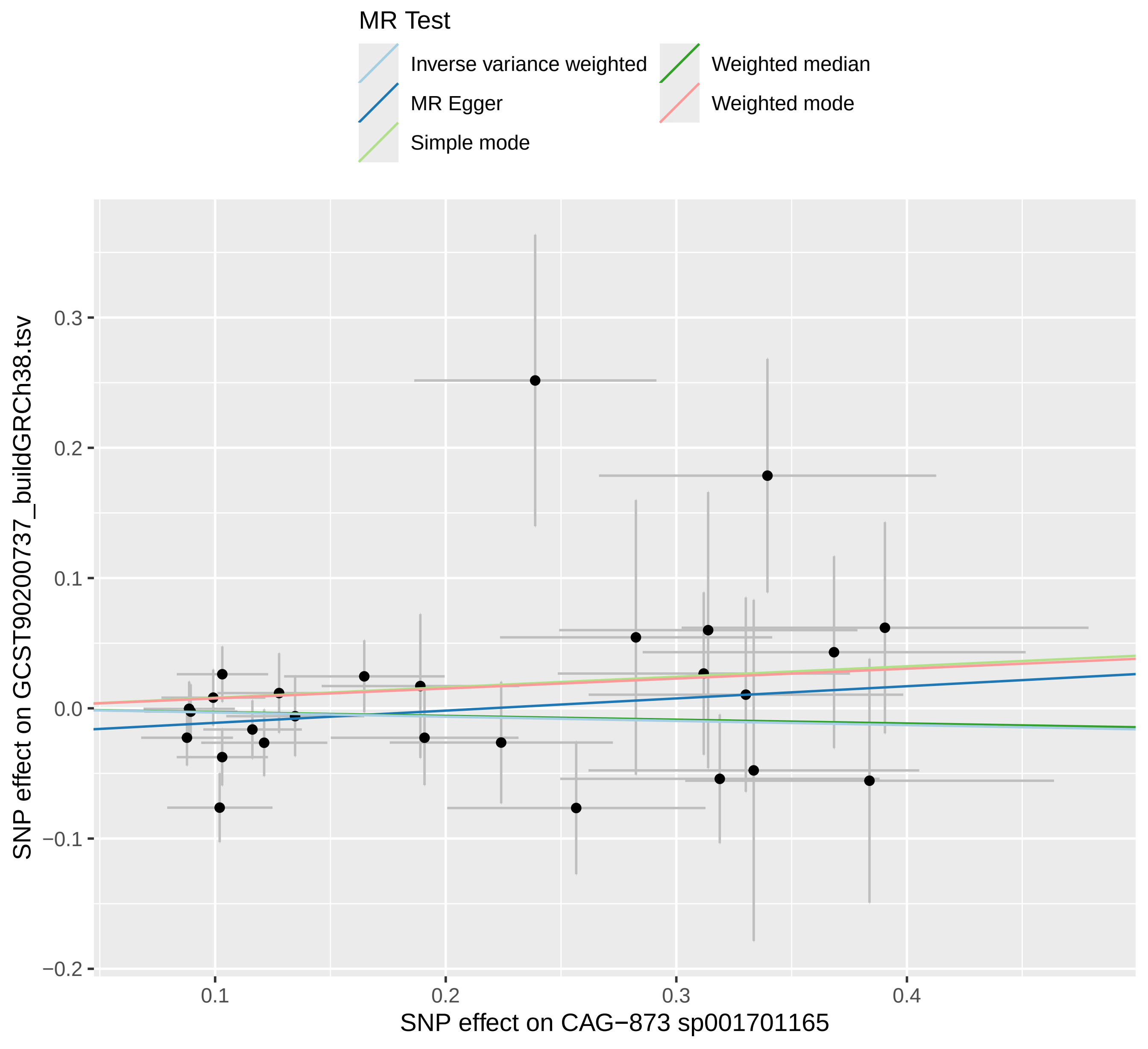
MR Test
Inverse variance weighted
MR Egger
Simple mode
Weighted median
Weighted mode
0.3
0.2
0.1
0.0
−0.1
−0.2
0.1
0.2
0.3
0.4
SNP effect on GCST90200737_buildGRCh38.tsv
SNP effect on CAG−873 sp001701165

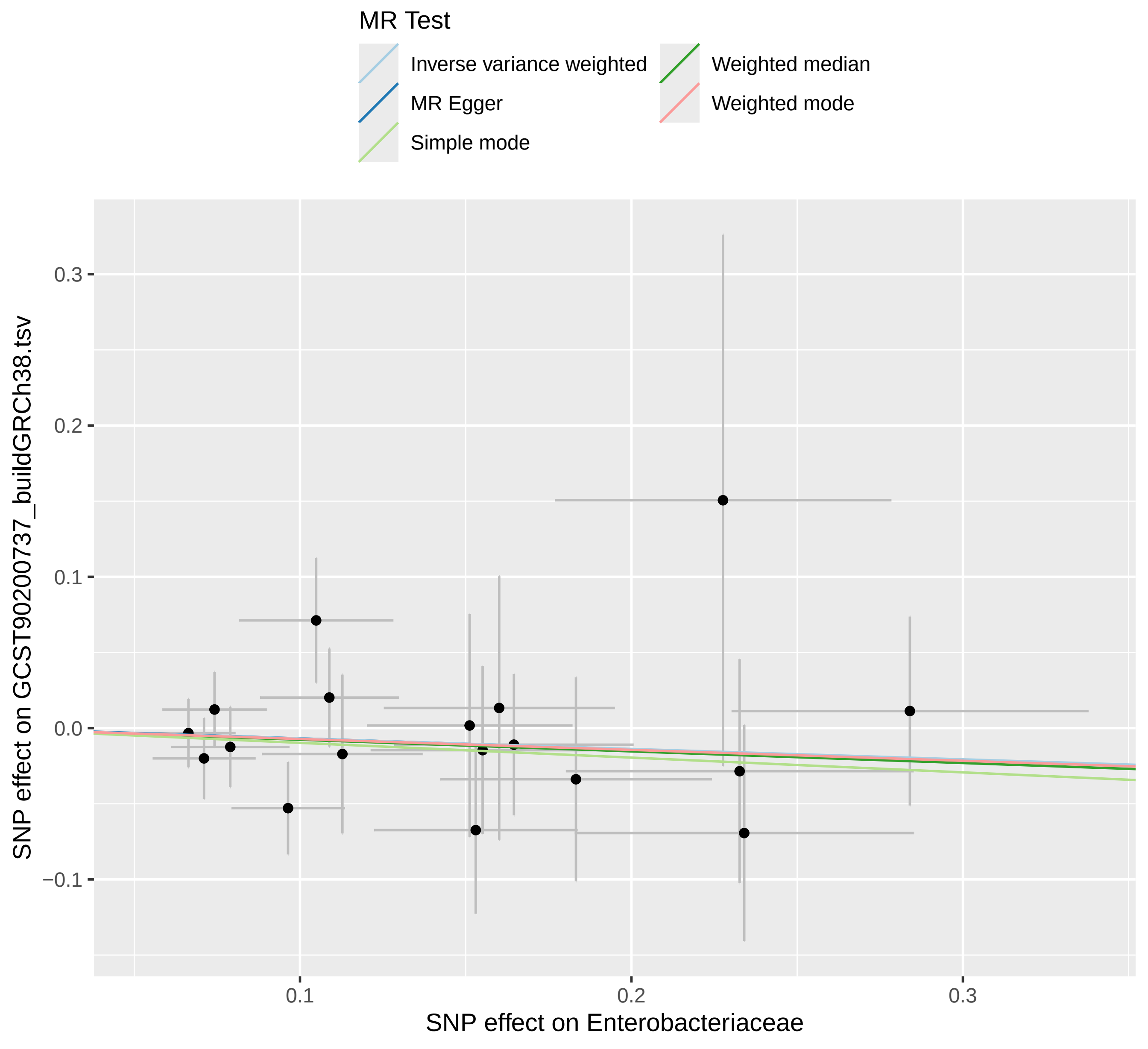

MR Test
Inverse variance weighted
MR Egger
Simple mode
Weighted median
Weighted mode
0.3
0.2
0.1
0.0
−0.1
0.1
0.2
0.3
SNP effect on GCST90200737_buildGRCh38.tsv
SNP effect on Enterobacteriaceae

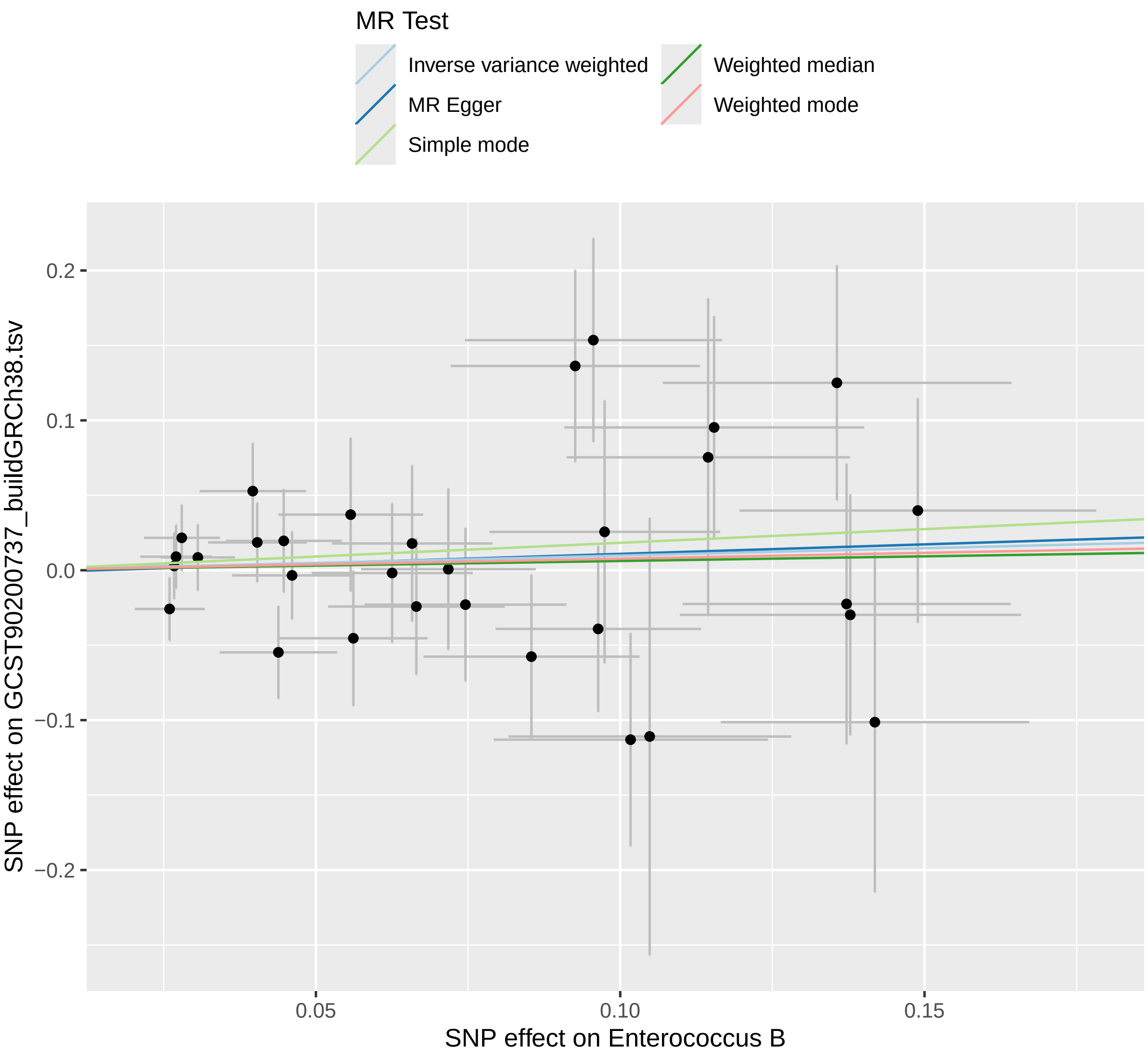
MR Test
Inverse variance weighted
MR Egger
Simple mode
Weighted median
Weighted mode
0.2
0.1
0.0
−0.1
−0.2
0.05
0.10
0.15
SNP effect on GCST90200737_buildGRCh38.tsv
SNP effect on Enterococcus B

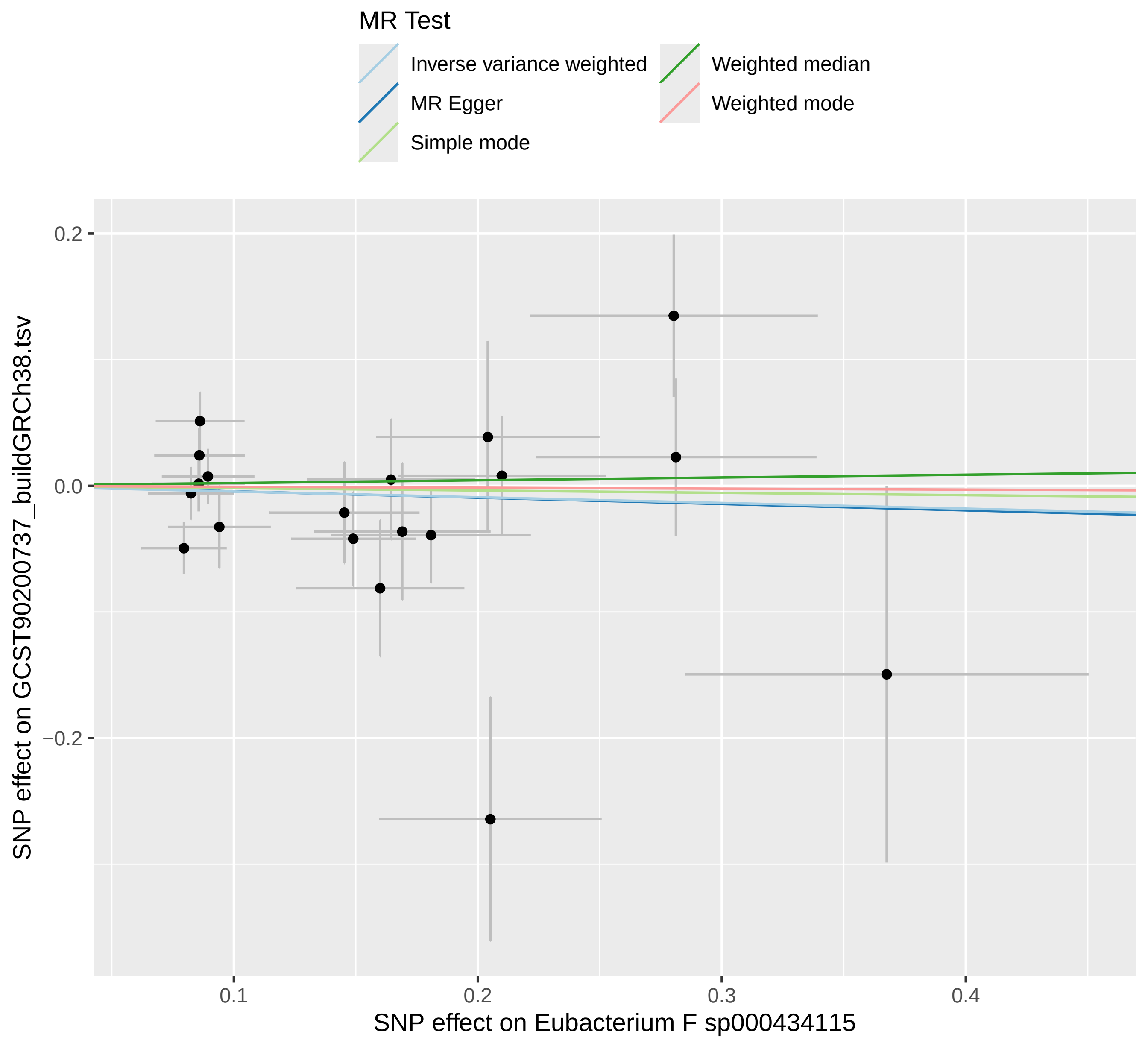
MR Test
Inverse variance weighted
MR Egger
Simple mode
Weighted median
Weighted mode
0.2
0.0
−0.2
0.1
0.2
0.3
0.4
SNP effect on GCST90200737_buildGRCh38.tsv
SNP effect on Eubacterium F sp000434115

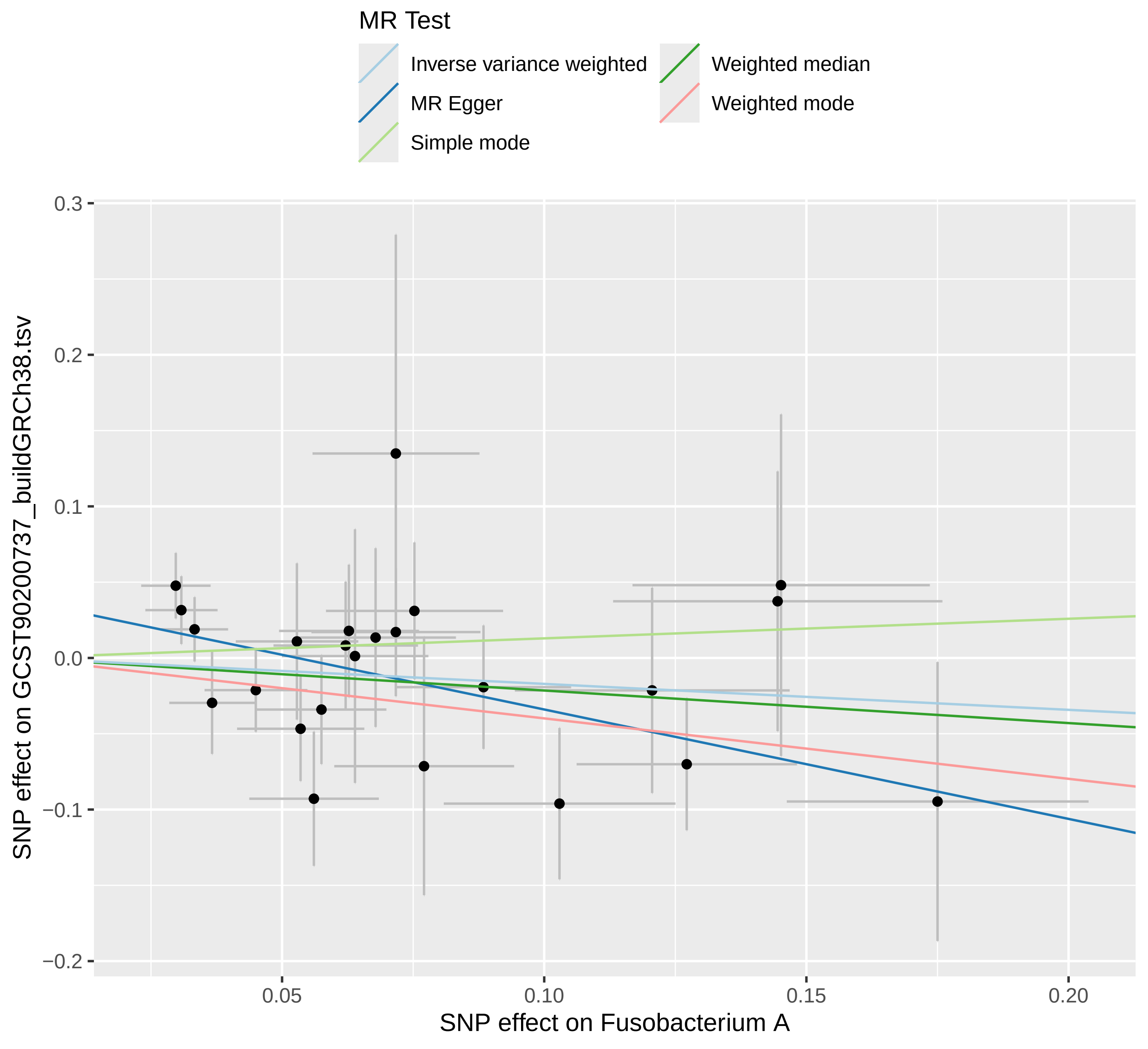
MR Test
Inverse variance weighted
MR Egger
Simple mode
Weighted median
Weighted mode
0.3
0.2
0.1
0.0
−0.1
−0.2
SNP effect on GCST90200737_buildGRCh38.tsv
0.05
0.10
0.15
0.20
SNP effect on Fusobacterium A

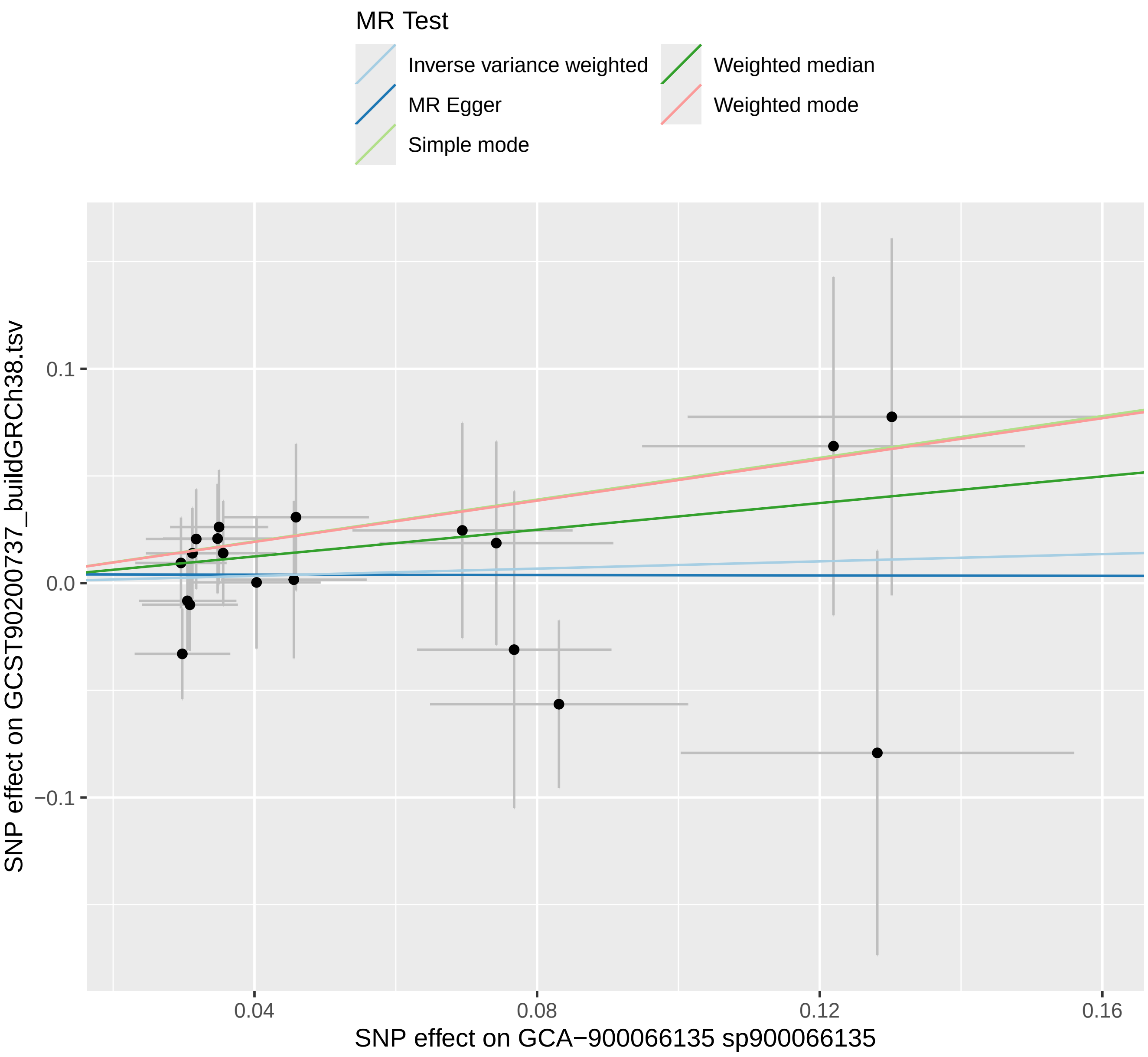

MR Test
Inverse variance weighted
MR Egger
Simple mode
Weighted median
Weighted mode
SNP effect on GCST90200737_buildGRCh38.tsv
0.1
0.0
−0.1
0.04
0.08
0.12
0.16
SNP effect on GCA−900066135 sp900066135

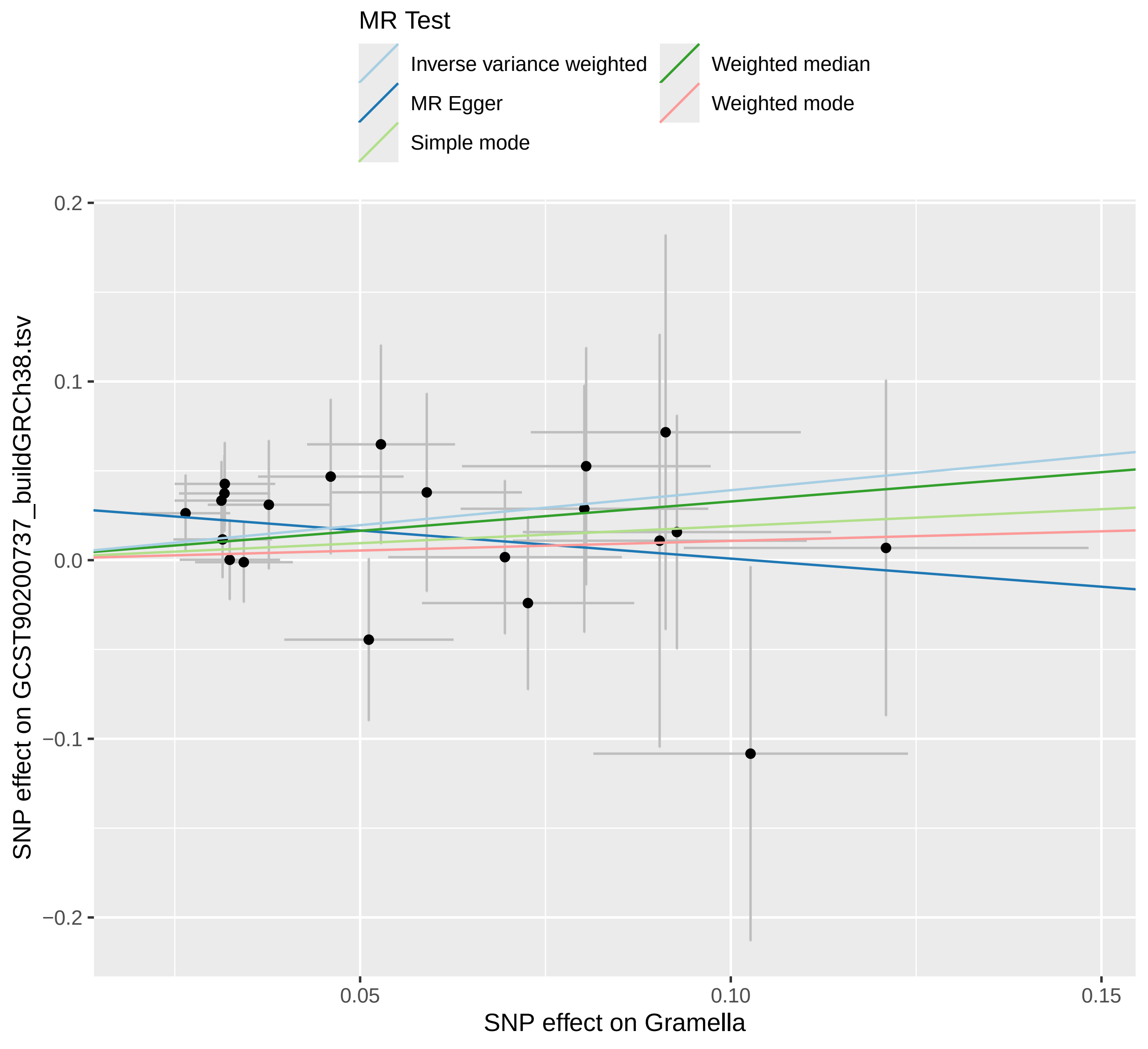
MR Test
Inverse variance weighted
MR Egger
Simple mode
Weighted median
Weighted mode
0.2
0.1
0.0
−0.1
−0.2
0.05
0.10
0.15
SNP effect on GCST90200737_buildGRCh38.tsv
SNP effect on Gramella

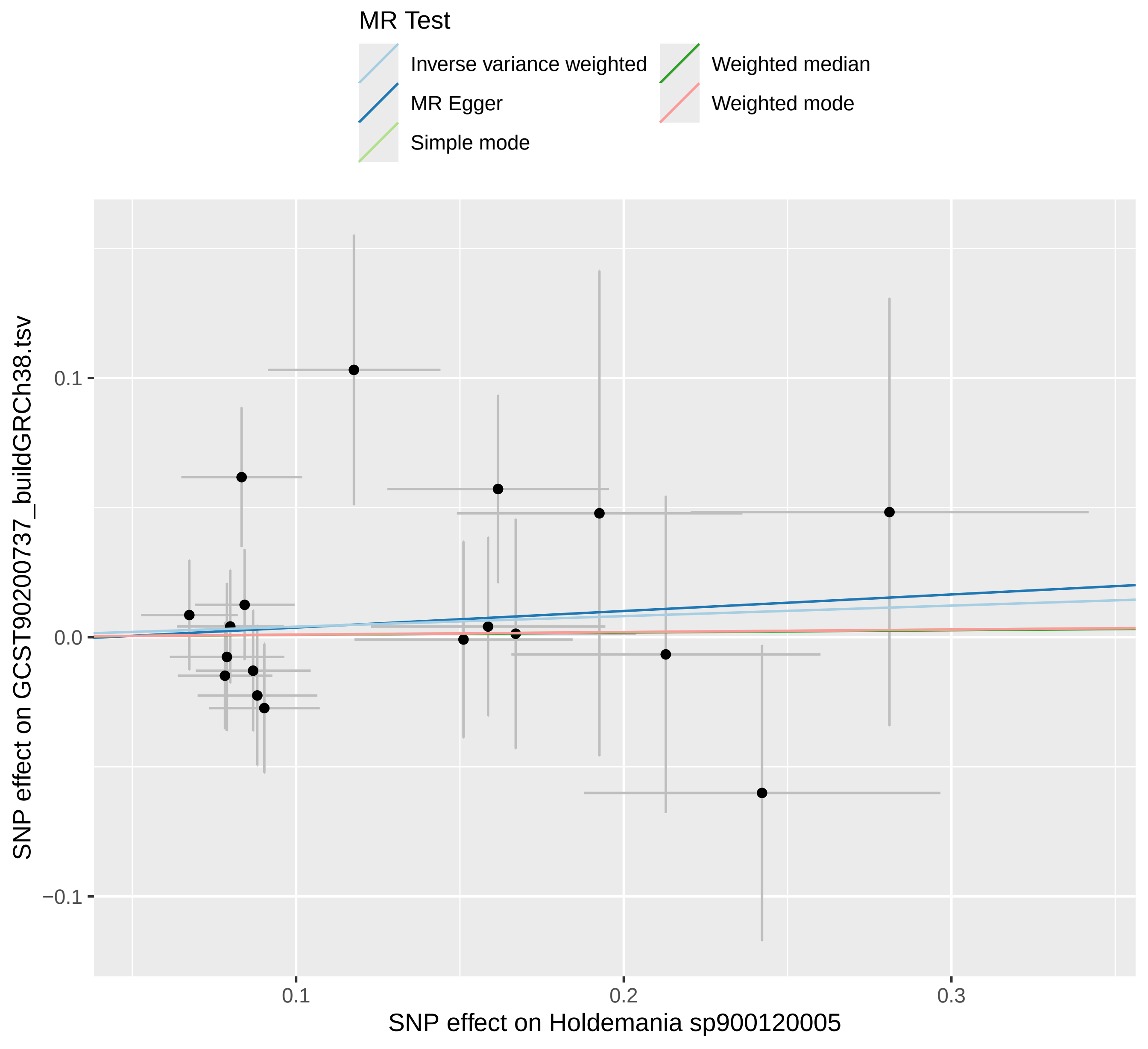

MR Test
Inverse variance weighted
MR Egger
Simple mode
Weighted median
Weighted mode
SNP effect on GCST90200737_buildGRCh38.tsv
0.1
0.0
−0.1
0.1
0.2
0.3
SNP effect on Holdemania sp900120005

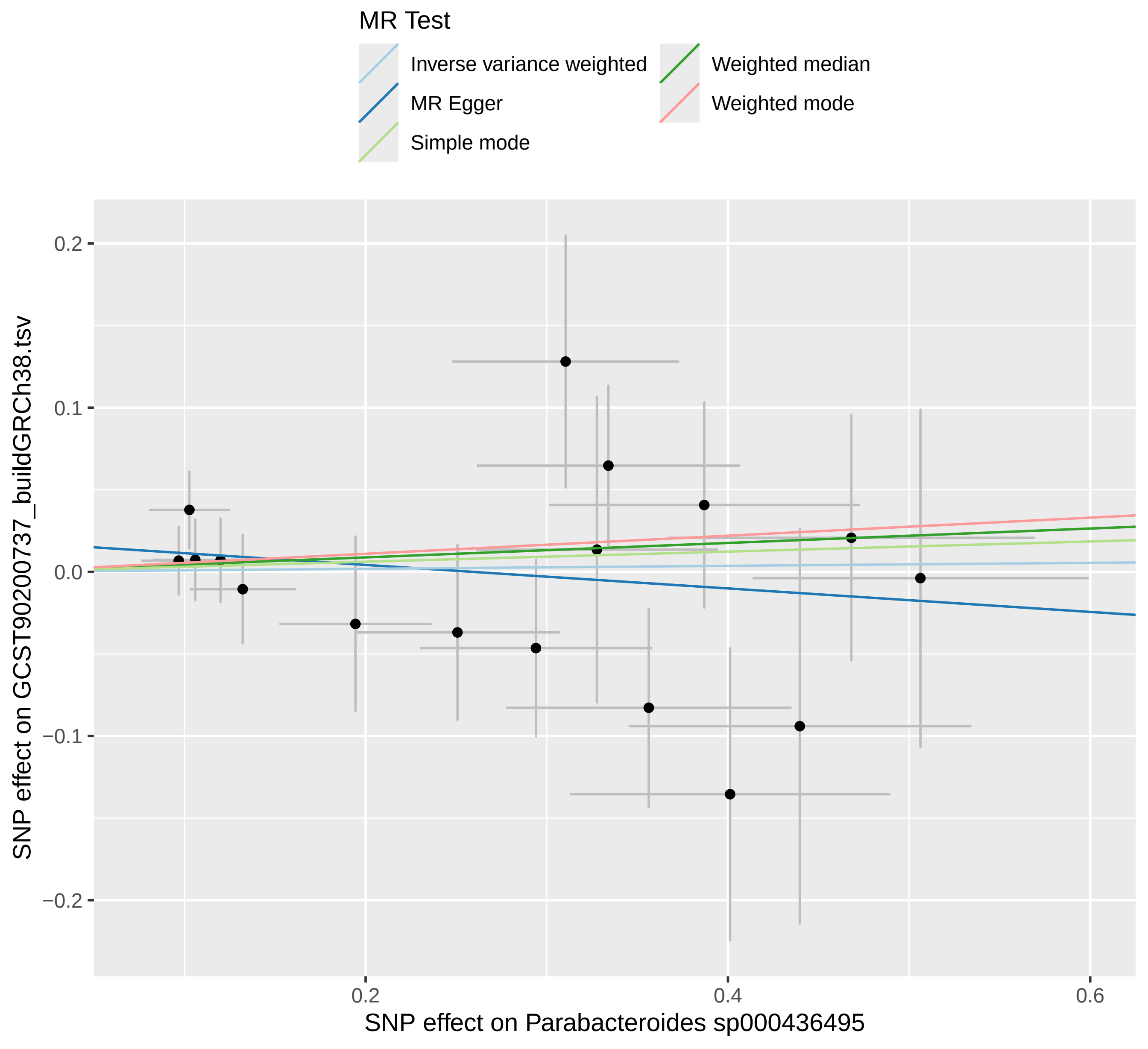

MR Test
Inverse variance weighted
MR Egger
Simple mode
Weighted median
Weighted mode
SNP effect on GCST90200737_buildGRCh38.tsv
0.2
0.1
0.0
−0.1
−0.2
0.2
0.4
0.6
SNP effect on Parabacteroides sp000436495

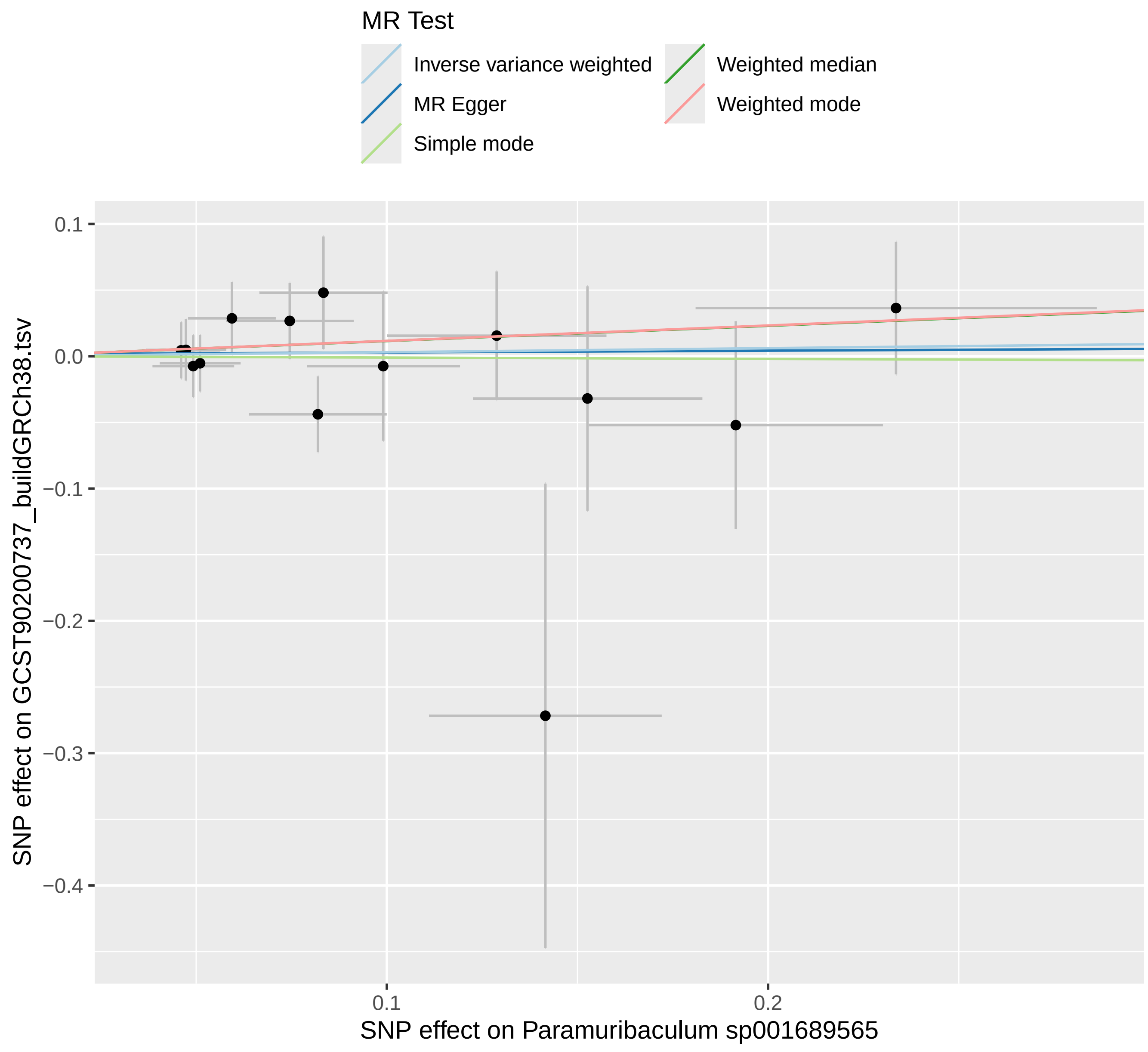
MR Test
Inverse variance weighted
MR Egger
Simple mode
Weighted median
Weighted mode
0.1
0.0
−0.1
−0.2
−0.3
−0.4
0.1
0.2
SNP effect on GCST90200737_buildGRCh38.tsv
SNP effect on Paramuribaculum sp001689565

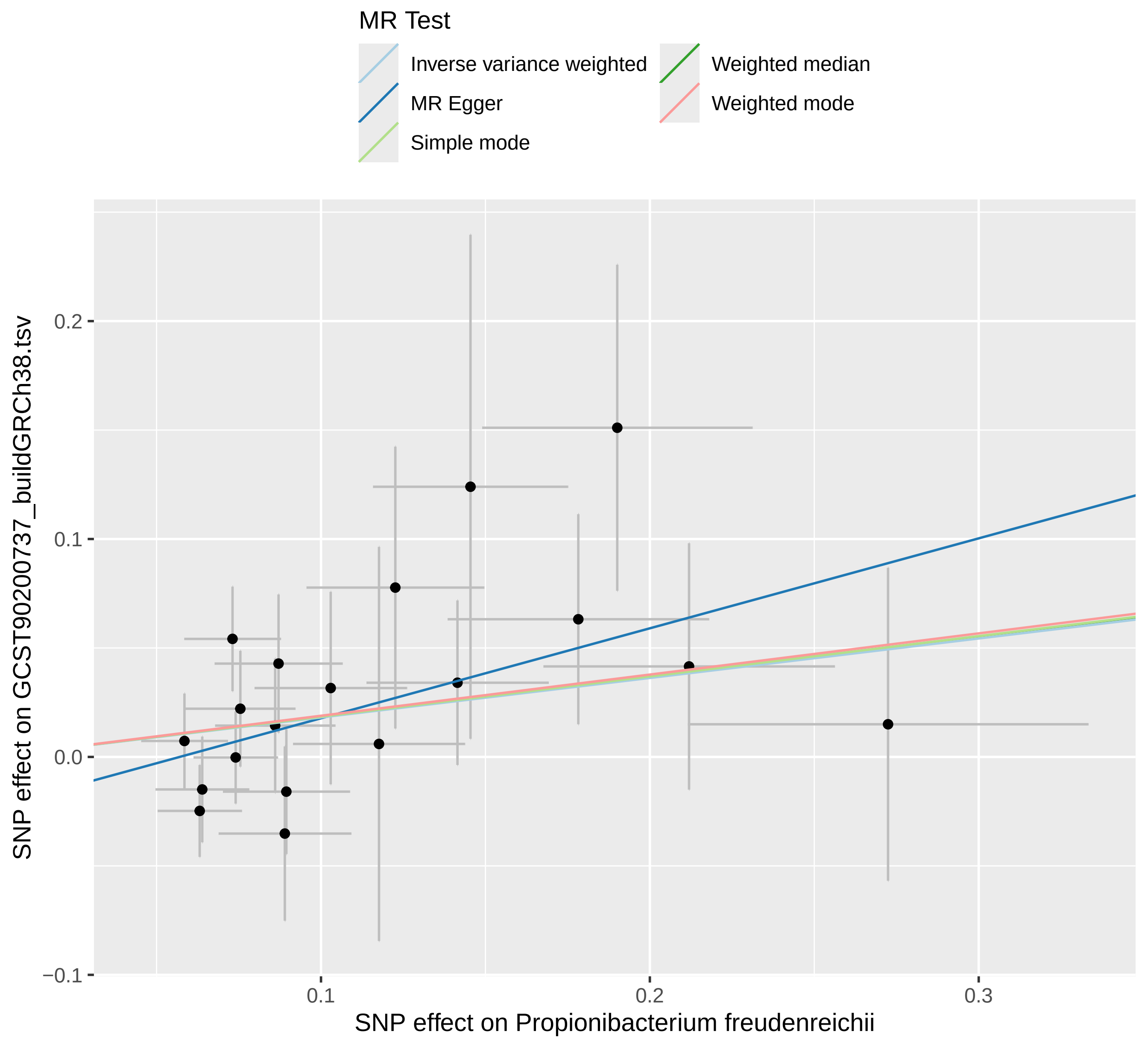

MR Test
Inverse variance weighted
MR Egger
Simple mode
Weighted median
Weighted mode
0.2
0.1
0.0
−0.1
0.1
0.2
0.3
SNP effect on GCST90200737_buildGRCh38.tsv
SNP effect on Propionibacterium freudenreichii

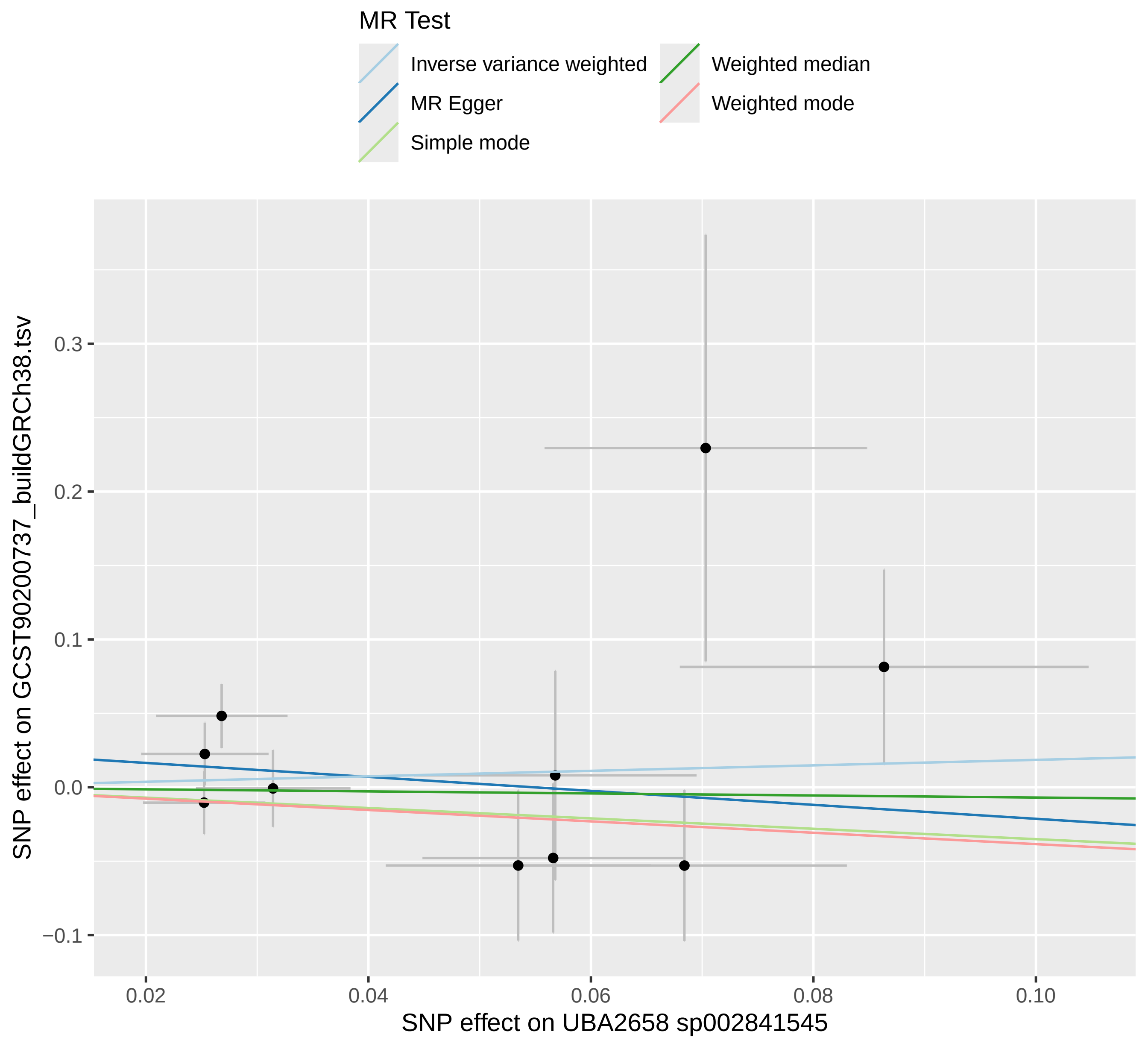
MR Test
Inverse variance weighted
MR Egger
Simple mode
Weighted median
Weighted mode
SNP effect on GCST90200737_buildGRCh38.tsv
0.3
0.2
0.1
0.0
−0.1
0.02
0.04
0.06
0.08
0.10
SNP effect on UBA2658 sp002841545

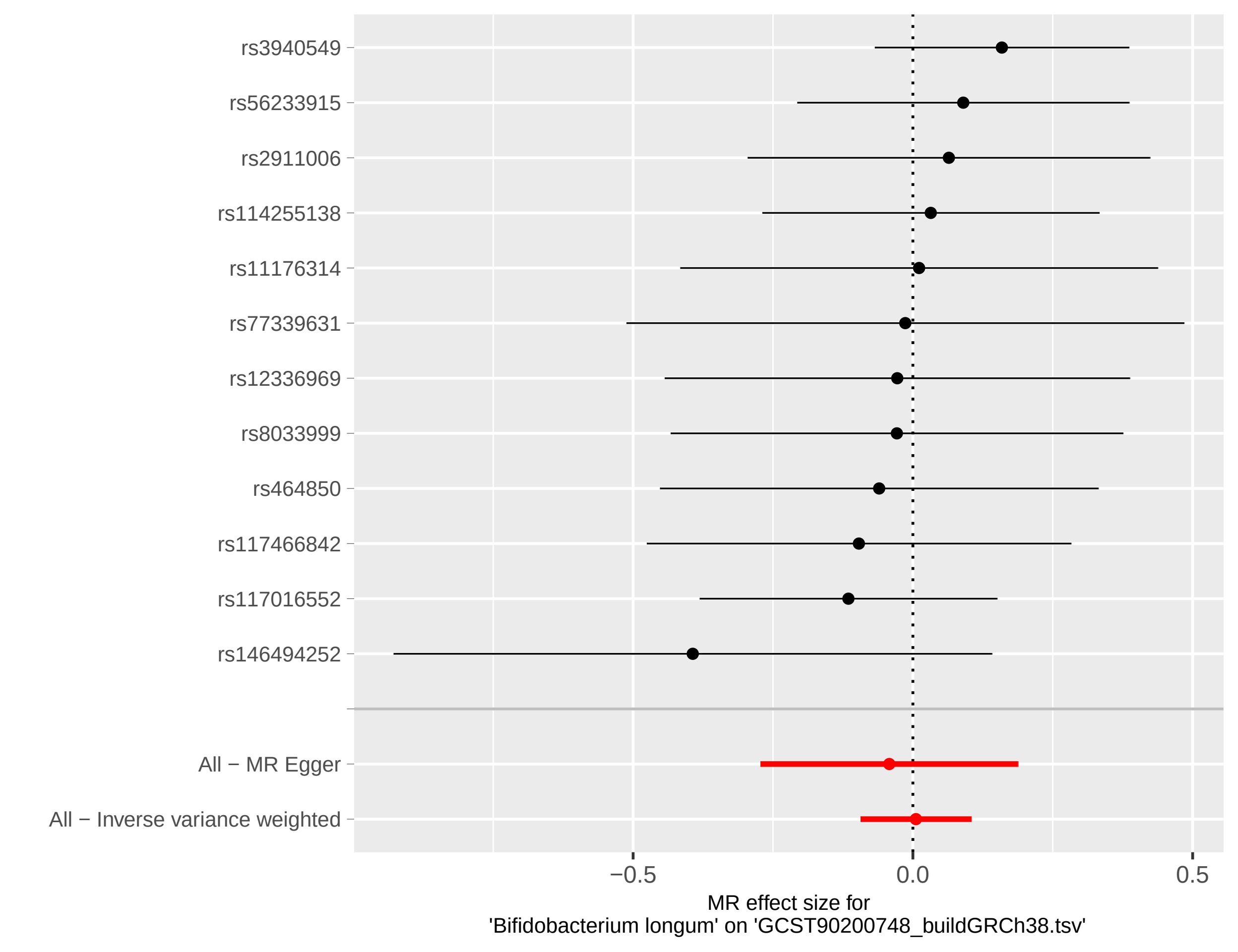
rs3940549
rs56233915
rs2911006
rs114255138
rs11176314
rs77339631
rs12336969
rs8033999
rs464850
rs117466842
rs117016552
rs146494252
All − MR Egger
All − Inverse variance weighted
−0.5
0.0
0.5
MR effect size for
'Bifidobacterium longum' on 'GCST90200748_buildGRCh38.tsv'

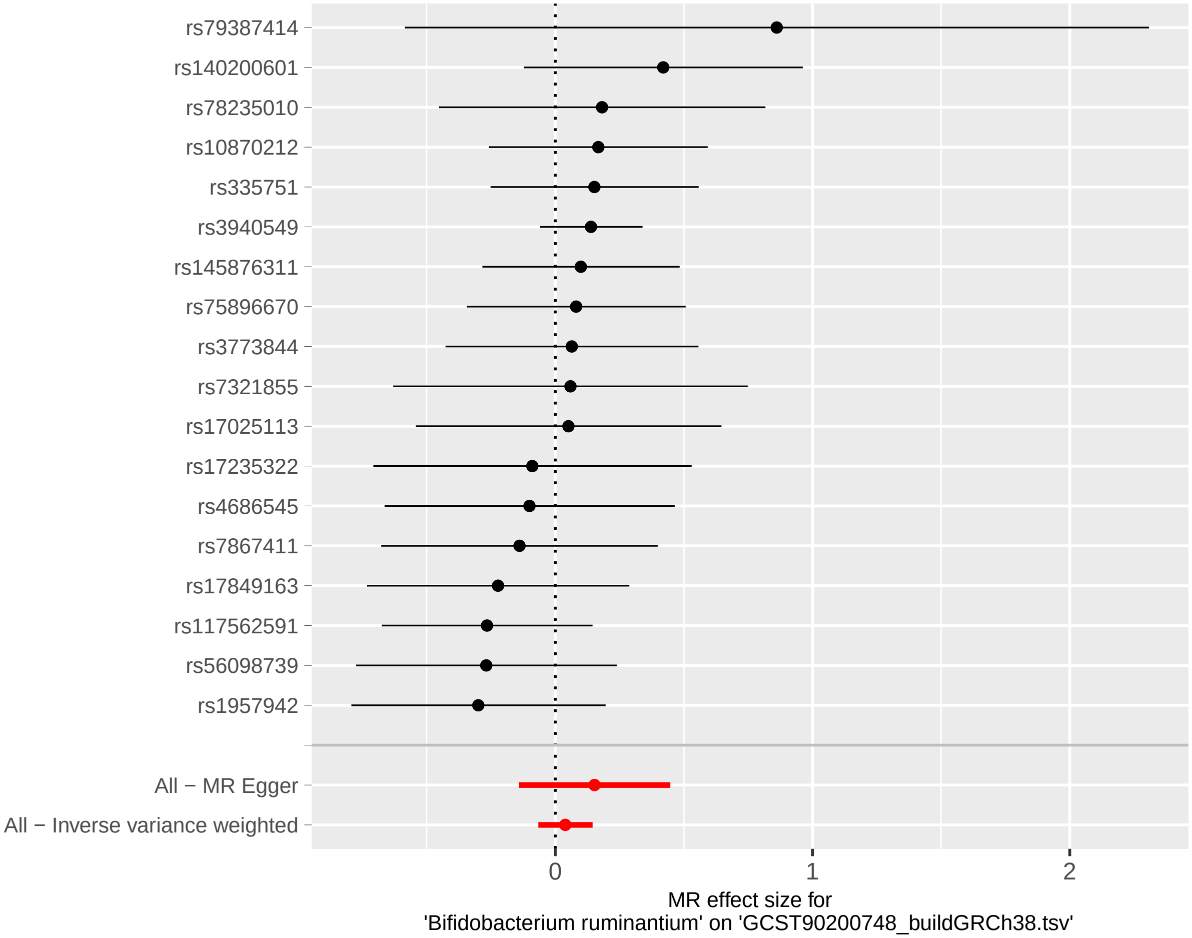

rs79387414
rs140200601
rs78235010
rs10870212
rs335751
rs3940549
rs145876311
rs75896670
rs3773844
rs7321855
rs17025113
rs17235322
rs4686545
rs7867411
rs17849163
rs117562591
rs56098739
rs1957942
All − MR Egger
All − Inverse variance weighted
0
1
2
MR effect size for
'Bifidobacterium ruminantium' on 'GCST90200748_buildGRCh38.tsv'

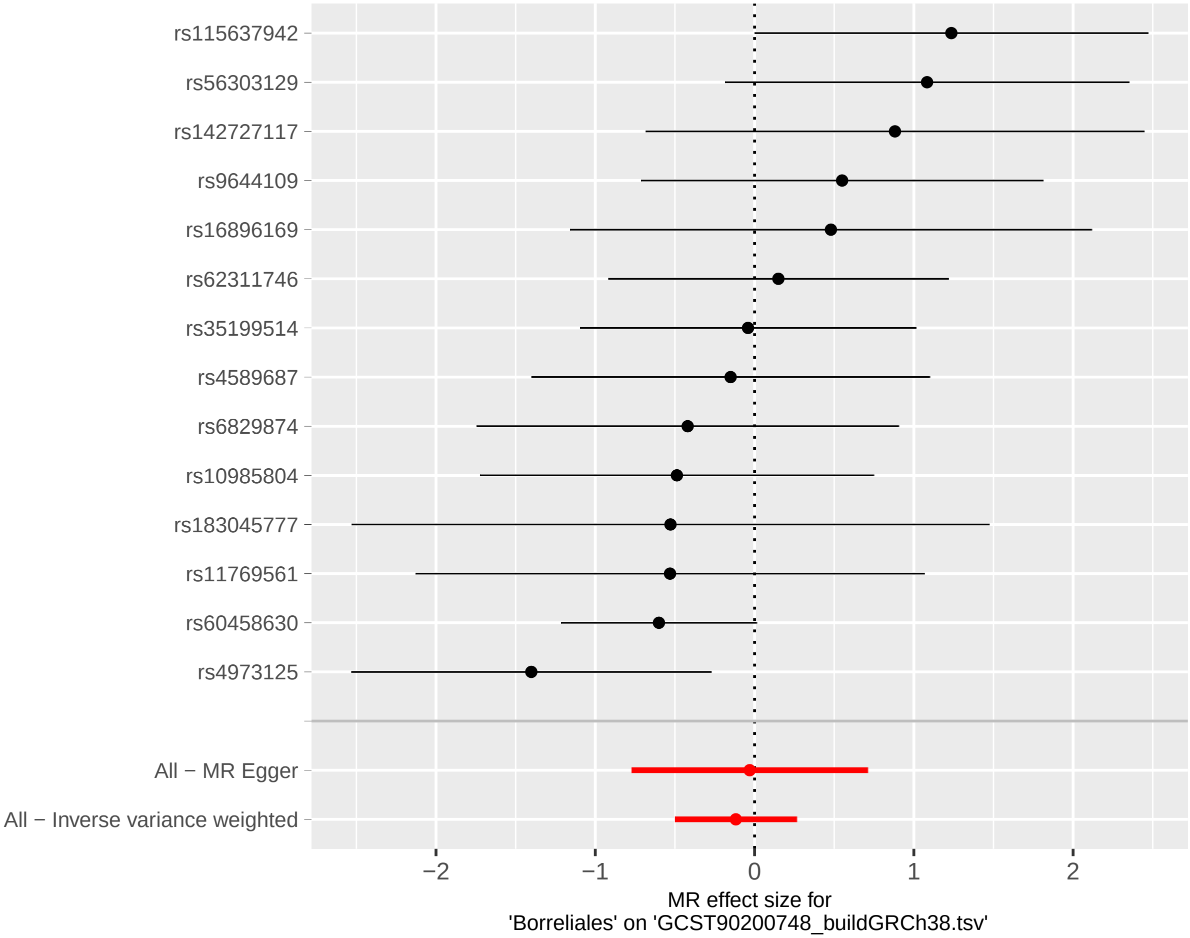
rs115637942
rs56303129
rs142727117
rs9644109
rs16896169
rs62311746
rs35199514
rs4589687
rs6829874
rs10985804
rs183045777
rs11769561
rs60458630
rs4973125
All − MR Egger
All − Inverse variance weighted
−2
−1
0
1
2
MR effect size for
'Borreliales' on 'GCST90200748_buildGRCh38.tsv'

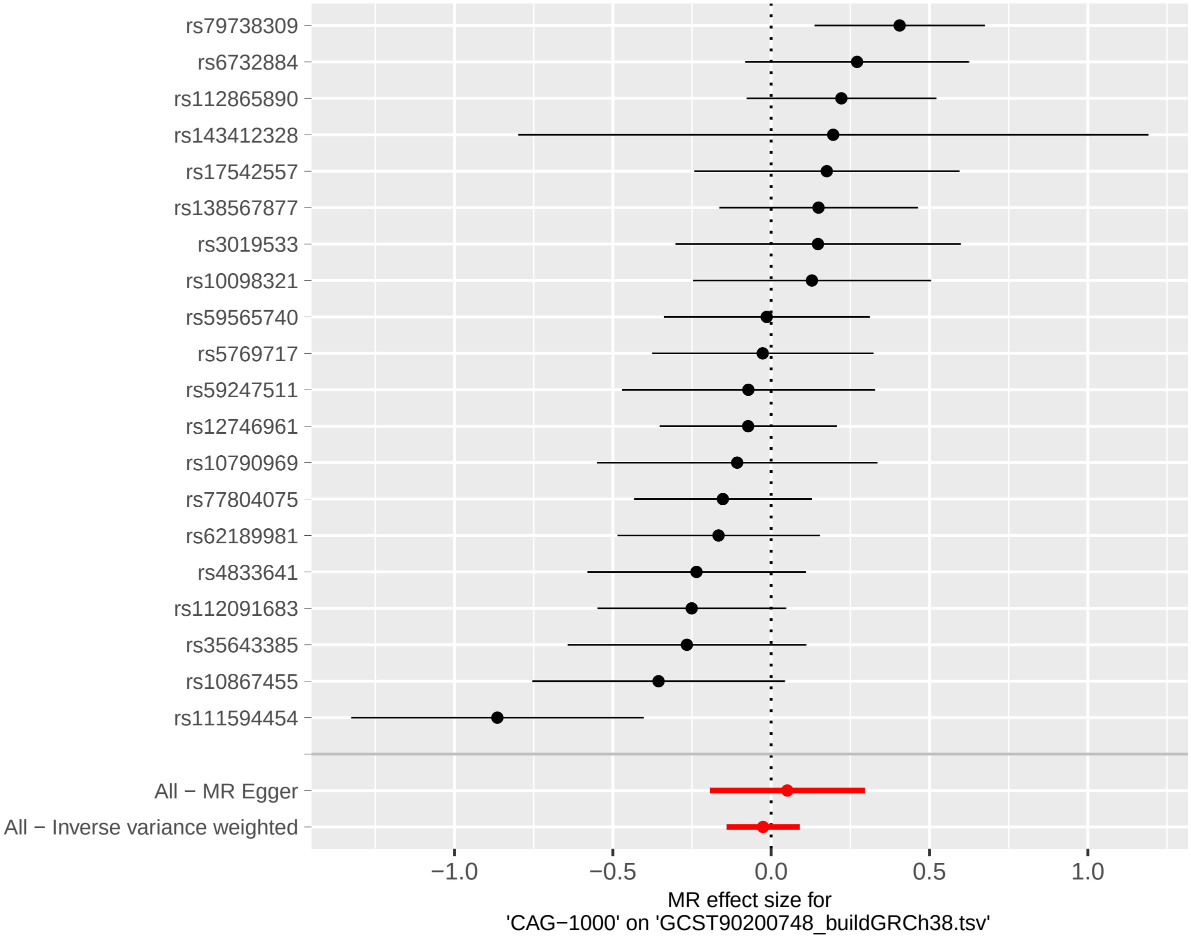

rs79738309
rs6732884
rs112865890
rs143412328
rs17542557
rs138567877
rs3019533
rs10098321
rs59565740
rs5769717
rs59247511
rs12746961
rs10790969
rs77804075
rs62189981
rs4833641
rs112091683
rs35643385
rs10867455
rs111594454
All − MR Egger
All − Inverse variance weighted
−1.0
−0.5
0.0
0.5
1.0
MR effect size for
'CAG−1000' on 'GCST90200748_buildGRCh38.tsv'

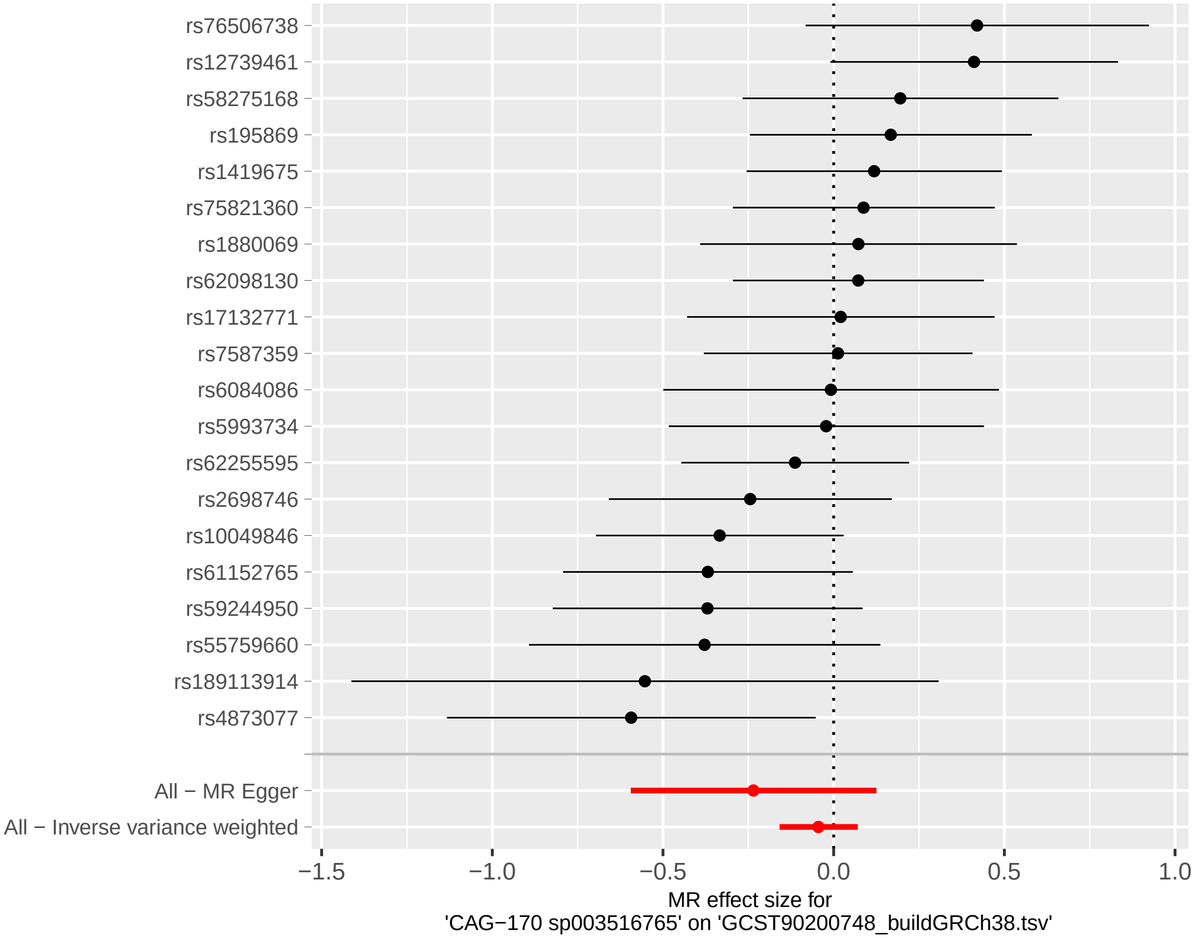
rs76506738
rs12739461
rs58275168
rs195869
rs1419675
rs75821360
rs1880069
rs62098130
rs17132771
rs7587359
rs6084086
rs5993734
rs62255595
rs2698746
rs10049846
rs61152765
rs59244950
rs55759660
rs189113914
rs4873077
All − MR Egger
All − Inverse variance weighted
−1.5
−1.0
−0.5
0.0
0.5
1.0
MR effect size for
'CAG−170 sp003516765' on 'GCST90200748_buildGRCh38.tsv'

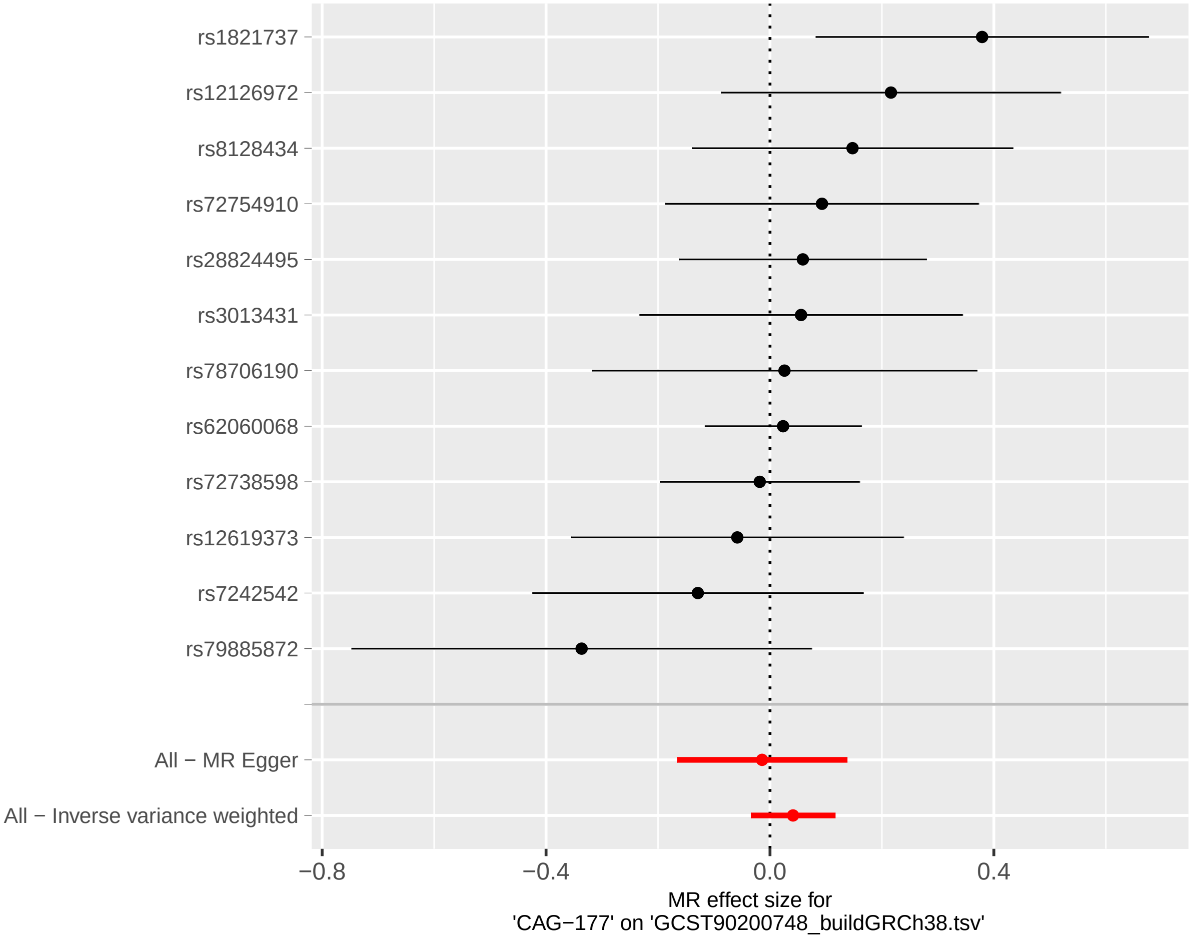
rs1821737
rs12126972
rs8128434
rs72754910
rs28824495
rs3013431
rs78706190
rs62060068
rs72738598
rs12619373
rs7242542
rs79885872
All − MR Egger
All − Inverse variance weighted
−0.8
−0.4
0.0
0.4
MR effect size for
'CAG−177' on 'GCST90200748_buildGRCh38.tsv'

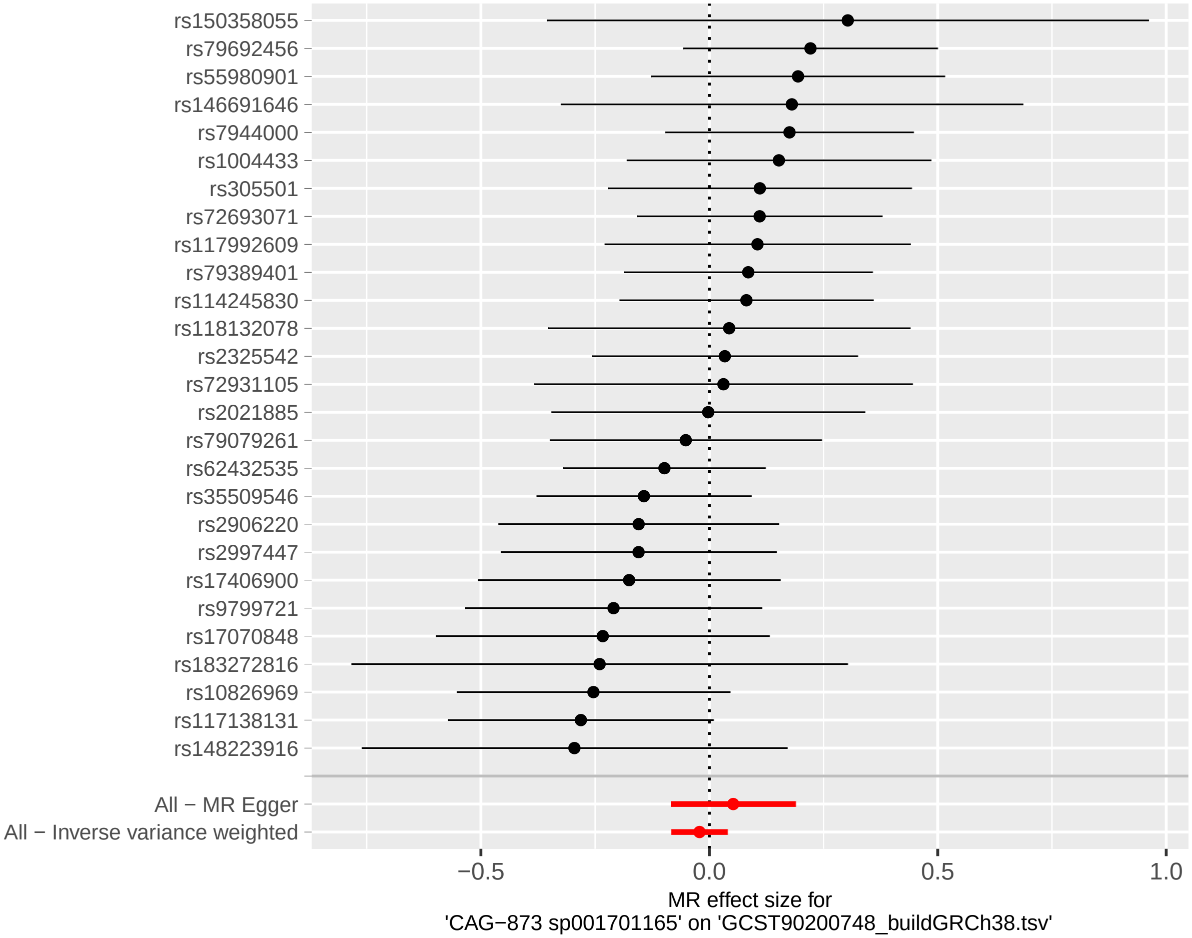
rs150358055
rs79692456
rs55980901
rs146691646
rs7944000
rs1004433
rs305501
rs72693071
rs117992609
rs79389401
rs114245830
rs118132078
rs2325542
rs72931105
rs2021885
rs79079261
rs62432535
rs35509546
rs2906220
rs2997447
rs17406900
rs9799721
rs17070848
rs183272816
rs10826969
rs117138131
rs148223916
All − MR Egger
All − Inverse variance weighted
−0.5
0.0
0.5
1.0
MR effect size for
'CAG−873 sp001701165' on 'GCST90200748_buildGRCh38.tsv'

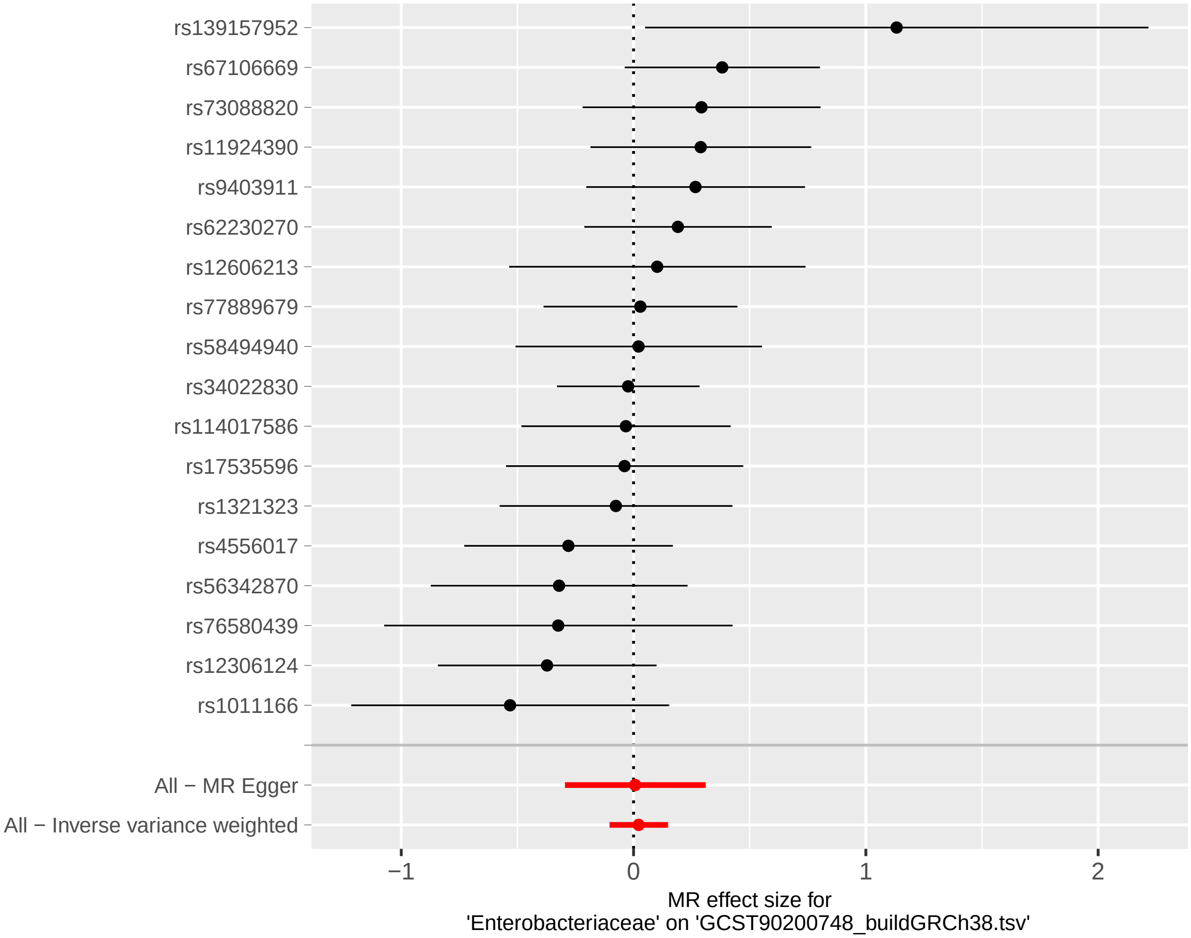

rs139157952
rs67106669
rs73088820
rs11924390
rs9403911
rs62230270
rs12606213
rs77889679
rs58494940
rs34022830
rs114017586
rs17535596
rs1321323
rs4556017
rs56342870
rs76580439
rs12306124
rs1011166
All − MR Egger
All − Inverse variance weighted
−1
0
1
2
MR effect size for
'Enterobacteriaceae' on 'GCST90200748_buildGRCh38.tsv'

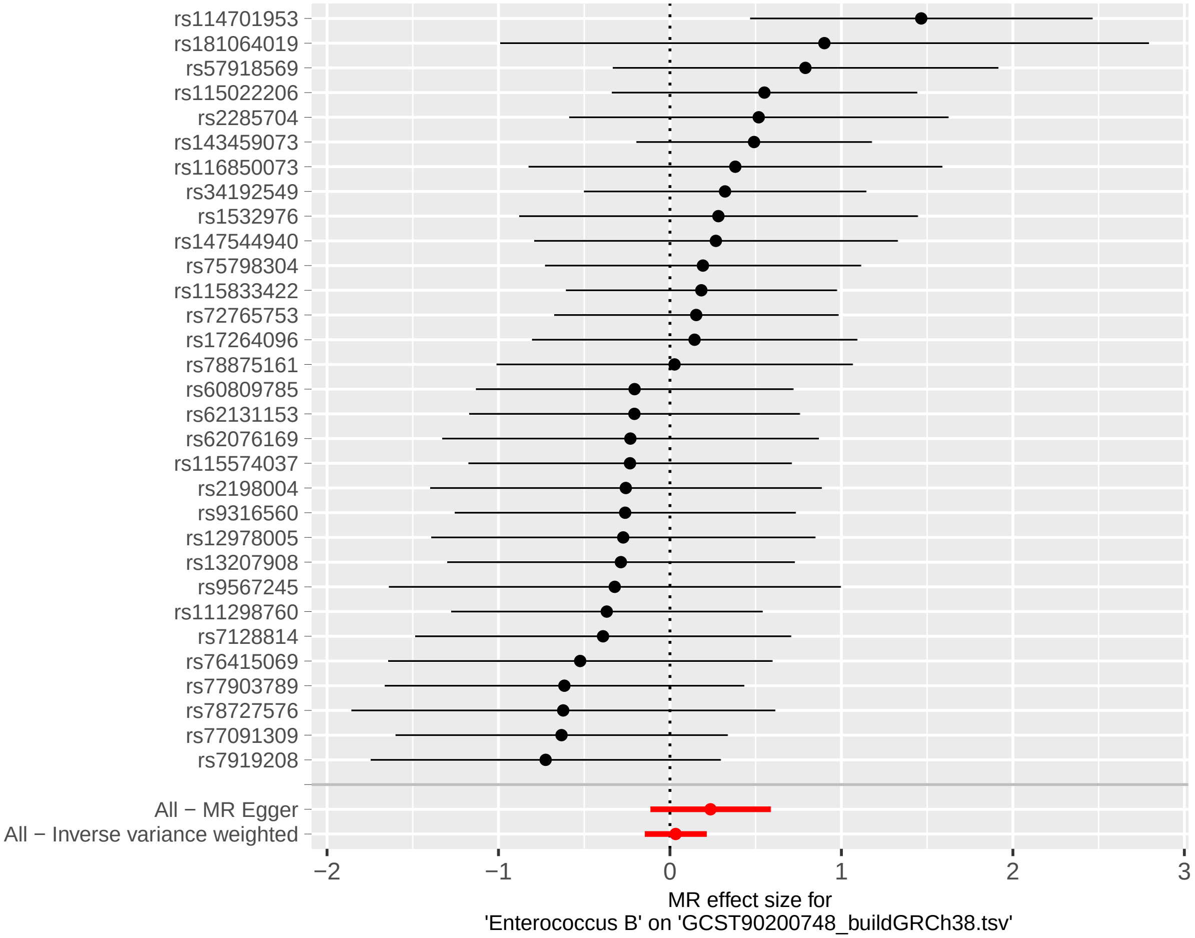
rs114701953
rs181064019
rs57918569
rs115022206
rs2285704
rs143459073
rs116850073
rs34192549
rs1532976
rs147544940
rs75798304
rs115833422
rs72765753
rs17264096
rs78875161
rs60809785
rs62131153
rs62076169
rs115574037
rs2198004
rs9316560
rs12978005
rs13207908
rs9567245
rs111298760
rs7128814
rs76415069
rs77903789
rs78727576
rs77091309
rs7919208
All − MR Egger
All − Inverse variance weighted
−2
−1
0
1
2
3
MR effect size for
'Enterococcus B' on 'GCST90200748_buildGRCh38.tsv'

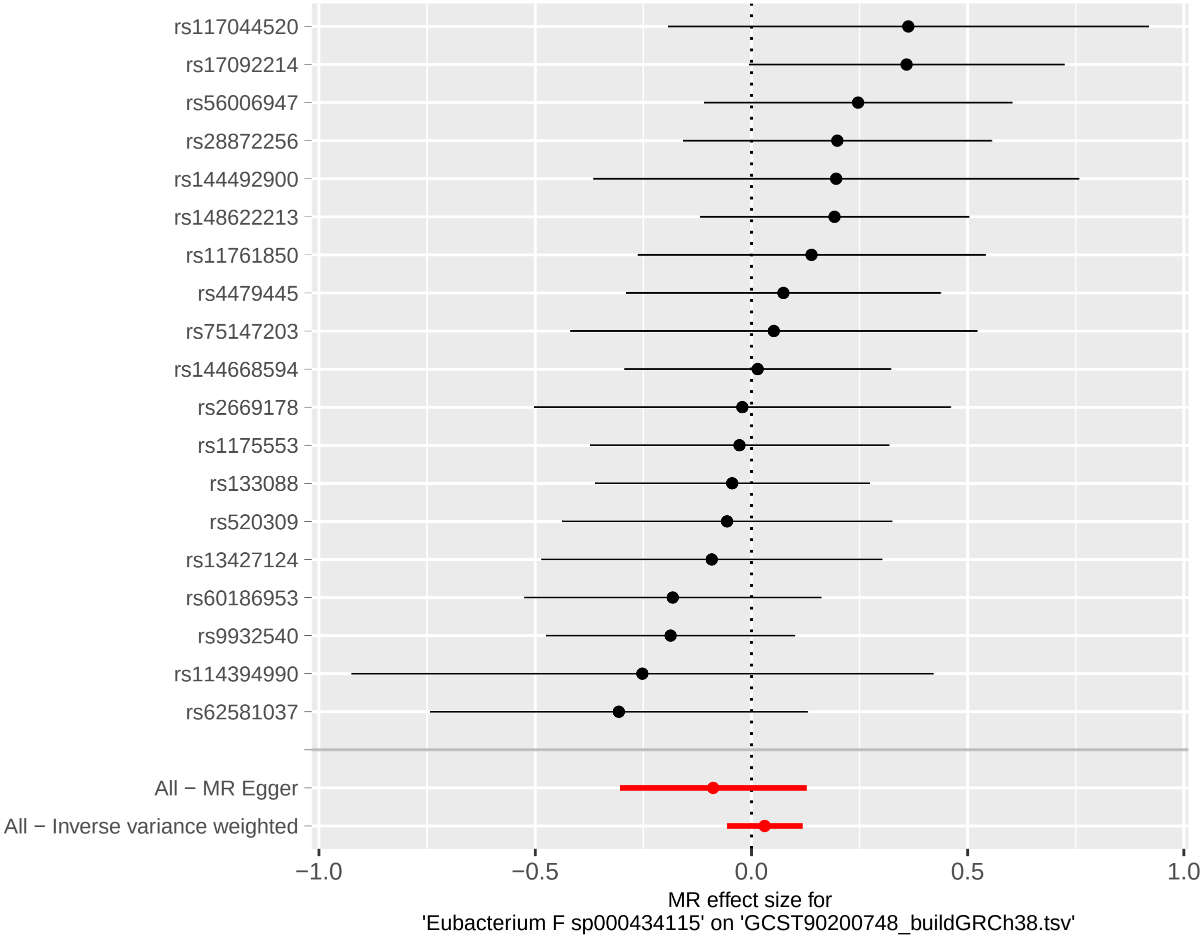
rs117044520
rs17092214
rs56006947
rs28872256
rs144492900
rs148622213
rs11761850
rs4479445
rs75147203
rs144668594
rs2669178
rs1175553
rs133088
rs520309
rs13427124
rs60186953
rs9932540
rs114394990
rs62581037
All − MR Egger
All − Inverse variance weighted
−1.0
−0.5
0.0
0.5
1.0
MR effect size for
'Eubacterium F sp000434115' on 'GCST90200748_buildGRCh38.tsv'

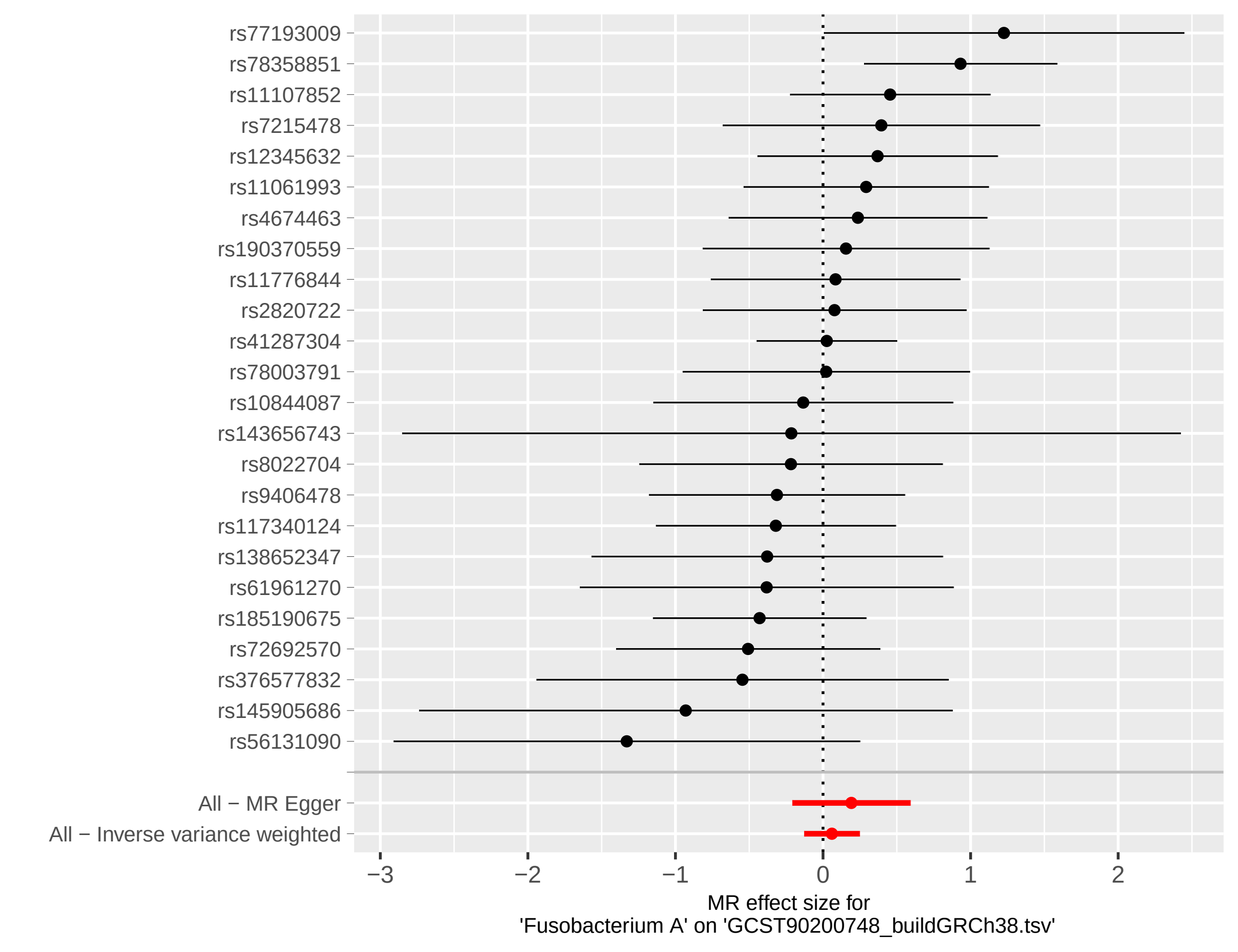

rs77193009
rs78358851
rs11107852
rs7215478
rs12345632
rs11061993
rs4674463
rs190370559
rs11776844
rs2820722
rs41287304
rs78003791
rs10844087
rs143656743
rs8022704
rs9406478
rs117340124
rs138652347
rs61961270
rs185190675
rs72692570
rs376577832
rs145905686
rs56131090
All − MR Egger
All − Inverse variance weighted
−3
−2
−1
0
1
2
MR effect size for
'Fusobacterium A' on 'GCST90200748_buildGRCh38.tsv'

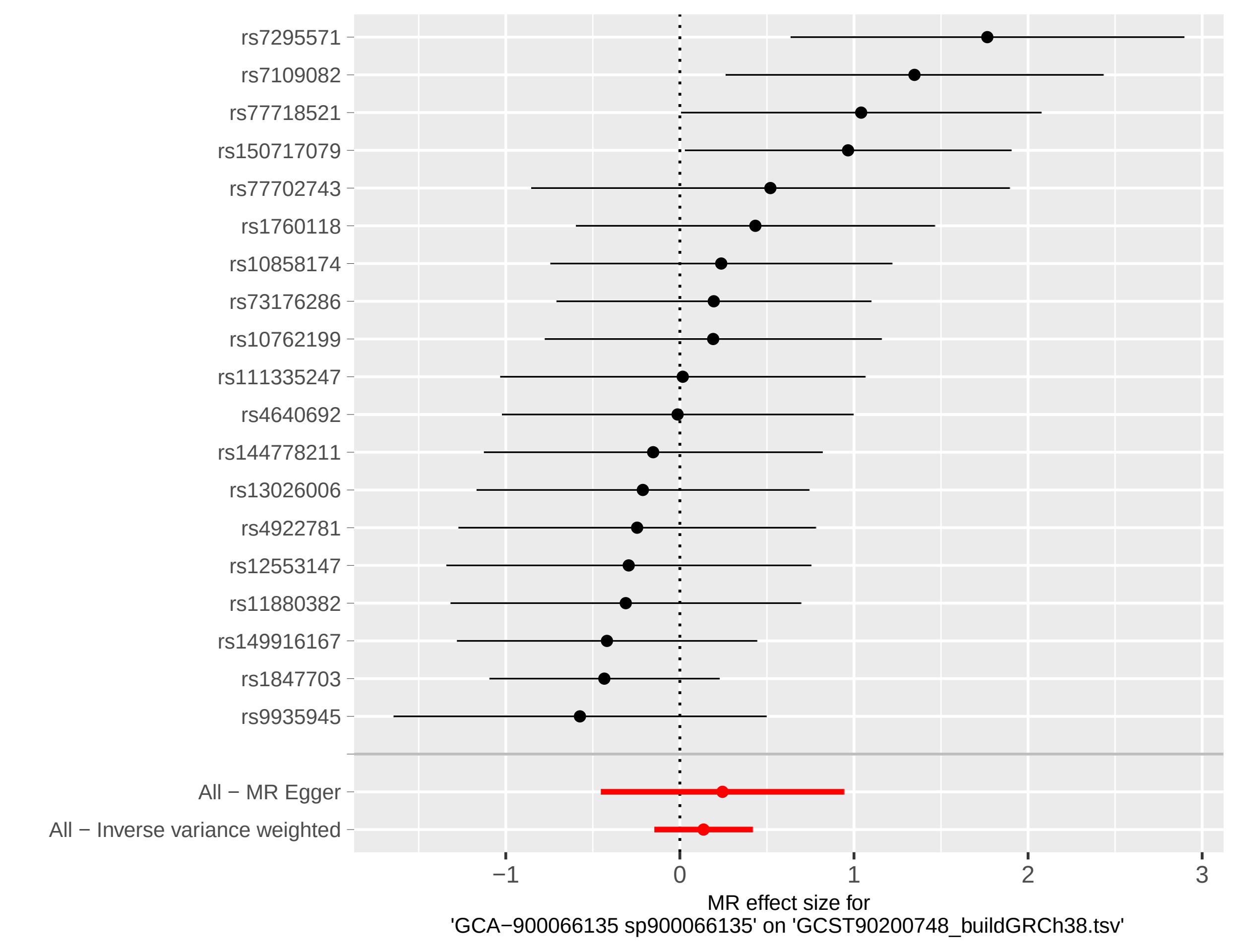

rs7295571
rs7109082
rs77718521
rs150717079
rs77702743
rs1760118
rs10858174
rs73176286
rs10762199
rs111335247
rs4640692
rs144778211
rs13026006
rs4922781
rs12553147
rs11880382
rs149916167
rs1847703
rs9935945
All − MR Egger
All − Inverse variance weighted
−1
0
1
2
3
MR effect size for
'GCA−900066135 sp900066135' on 'GCST90200748_buildGRCh38.tsv'

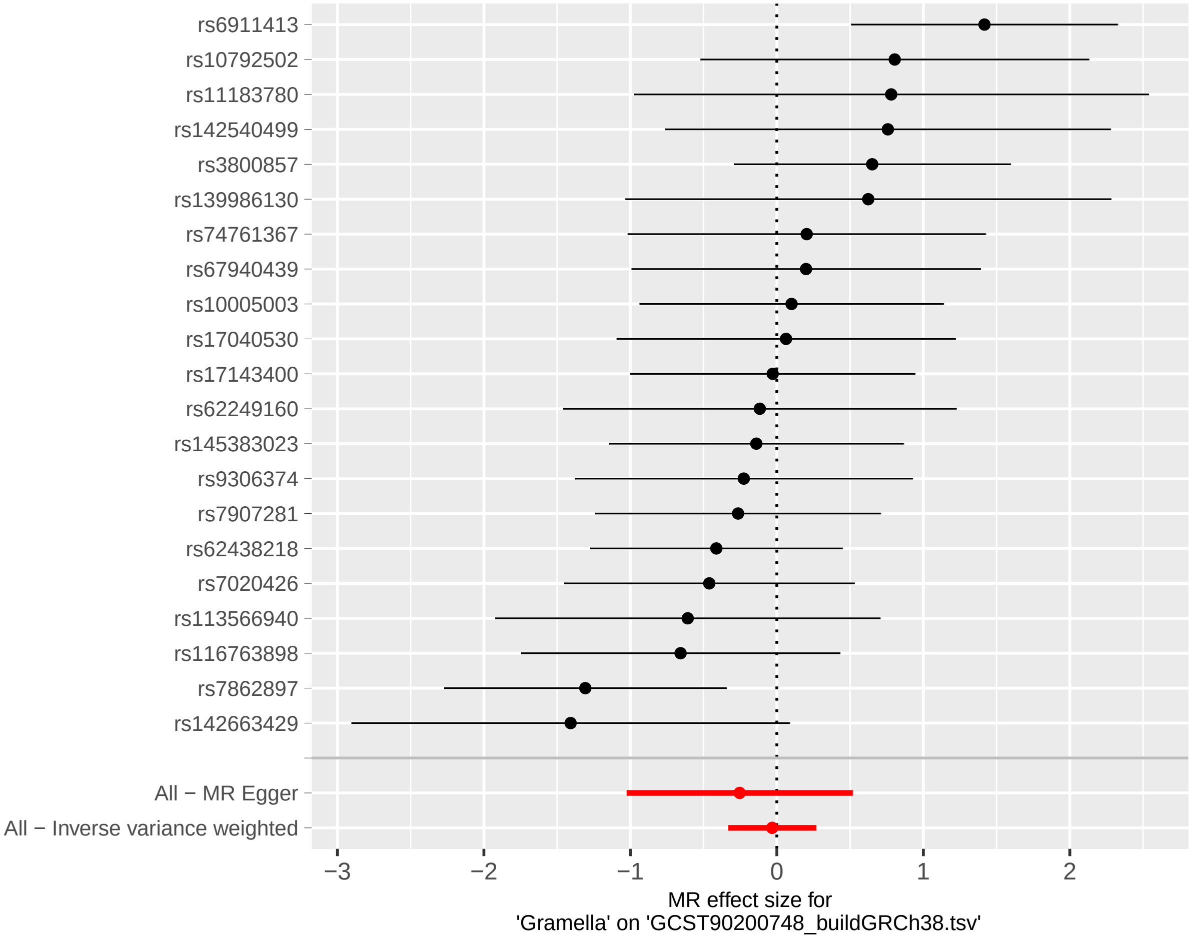
rs6911413
rs10792502
rs11183780
rs142540499
rs3800857
rs139986130
rs74761367
rs67940439
rs10005003
rs17040530
rs17143400
rs62249160
rs145383023
rs9306374
rs7907281
rs62438218
rs7020426
rs113566940
rs116763898
rs7862897
rs142663429
All − MR Egger
All − Inverse variance weighted
−3
−2
−1
0
1
2
MR effect size for
'Gramella' on 'GCST90200748_buildGRCh38.tsv'

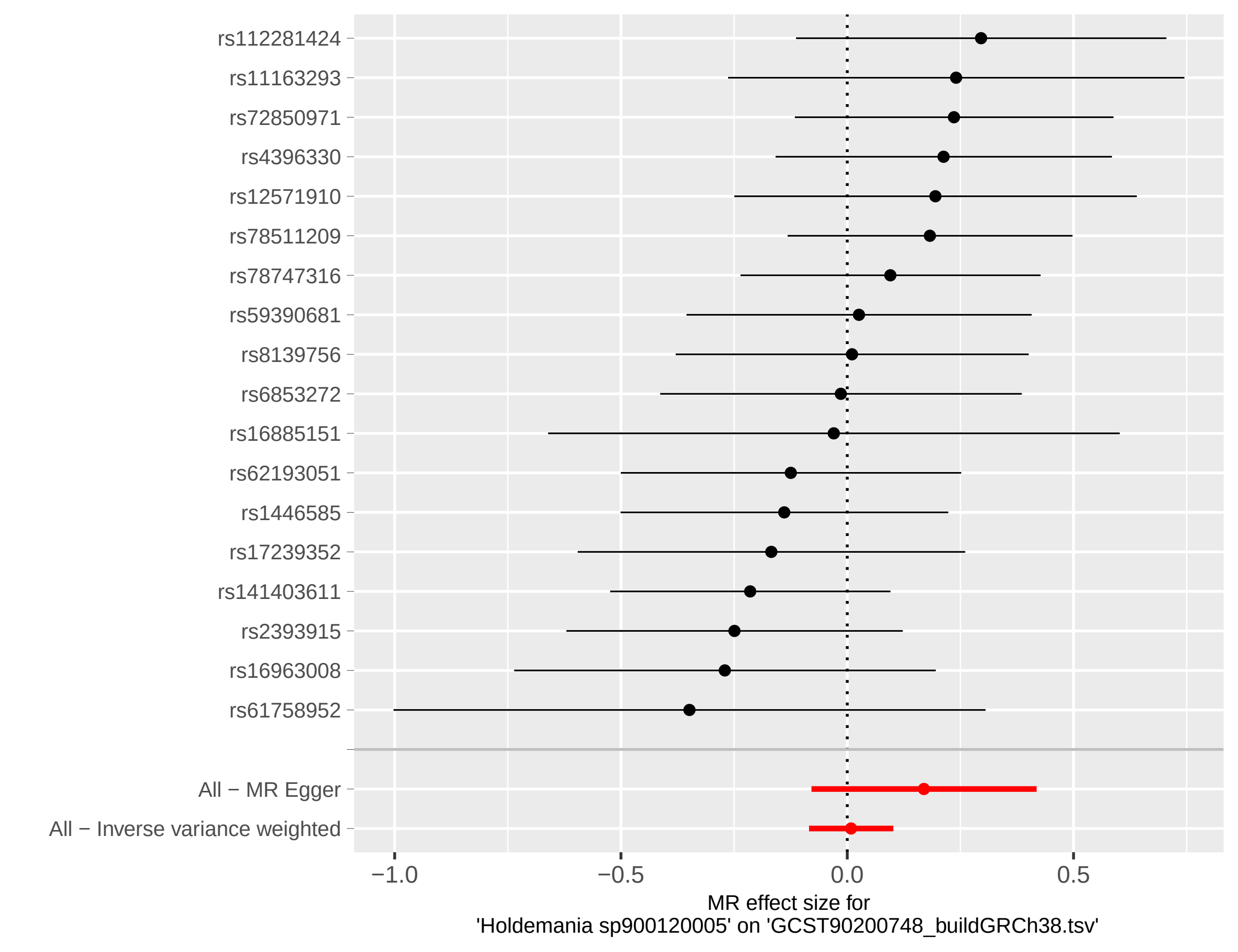

rs112281424
rs11163293
rs72850971
rs4396330
rs12571910
rs78511209
rs78747316
rs59390681
rs8139756
rs6853272
rs16885151
rs62193051
rs1446585
rs17239352
rs141403611
rs2393915
rs16963008
rs61758952
All − MR Egger
All − Inverse variance weighted
−1.0
−0.5
0.0
0.5
MR effect size for
'Holdemania sp900120005' on 'GCST90200748_buildGRCh38.tsv'

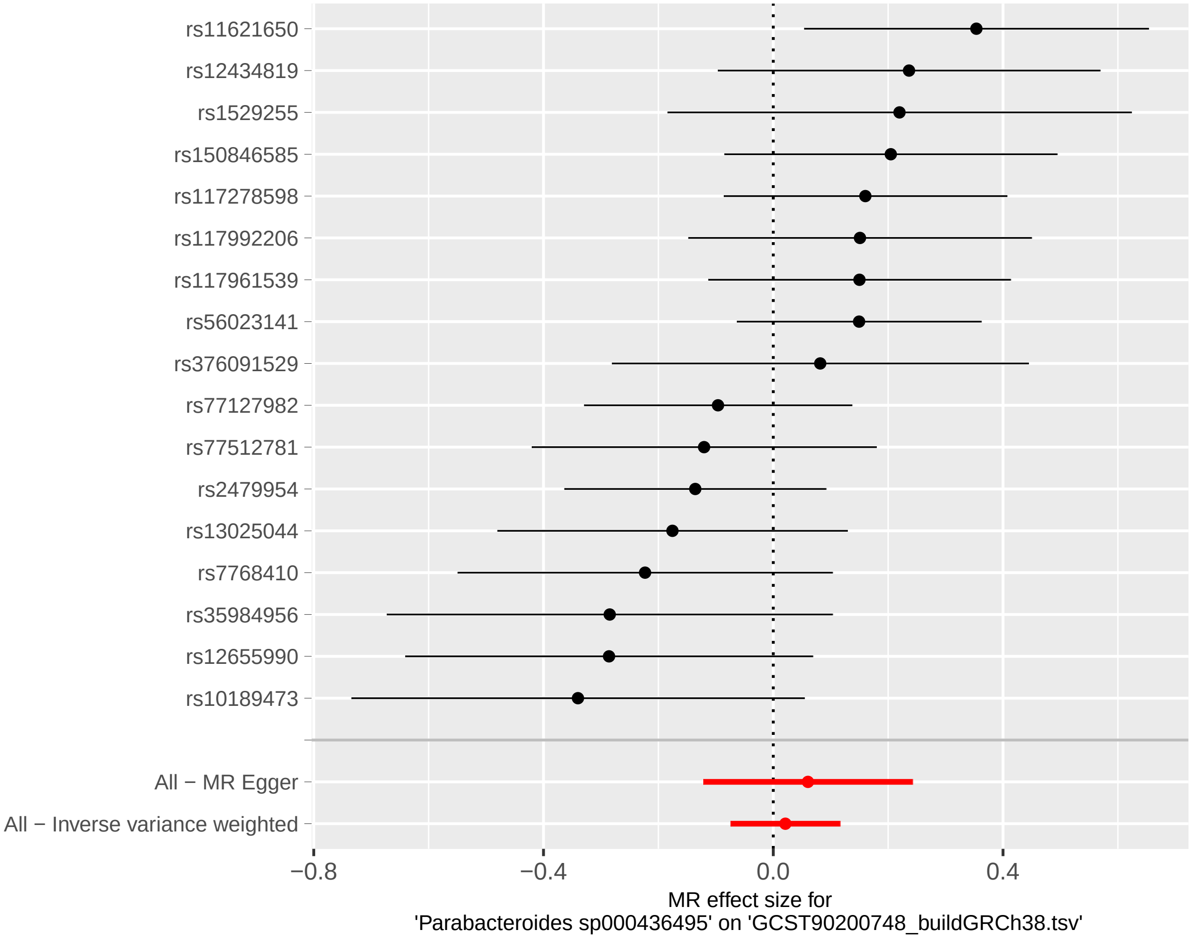
rs11621650
rs12434819
rs1529255
rs150846585
rs117278598
rs117992206
rs117961539
rs56023141
rs376091529
rs77127982
rs77512781
rs2479954
rs13025044
rs7768410
rs35984956
rs12655990
rs10189473
All − MR Egger
All − Inverse variance weighted
−0.8
−0.4
0.0
0.4
MR effect size for
'Parabacteroides sp000436495' on 'GCST90200748_buildGRCh38.tsv'

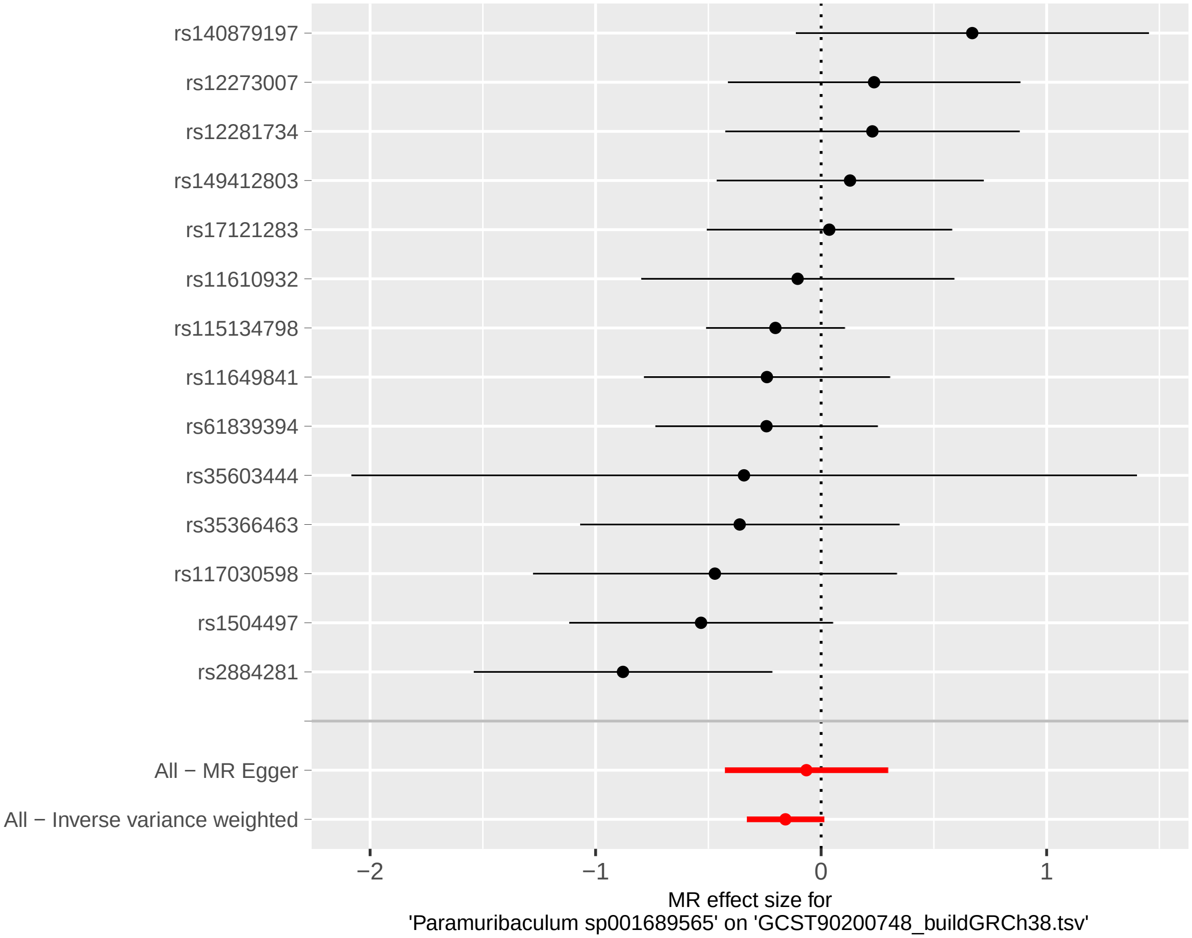

rs140879197
rs12273007
rs12281734
rs149412803
rs17121283
rs11610932
rs115134798
rs11649841
rs61839394
rs35603444
rs35366463
rs117030598
rs1504497
rs2884281
All − MR Egger
All − Inverse variance weighted
−2
−1
0
1
MR effect size for
'Paramuribaculum sp001689565' on 'GCST90200748_buildGRCh38.tsv'

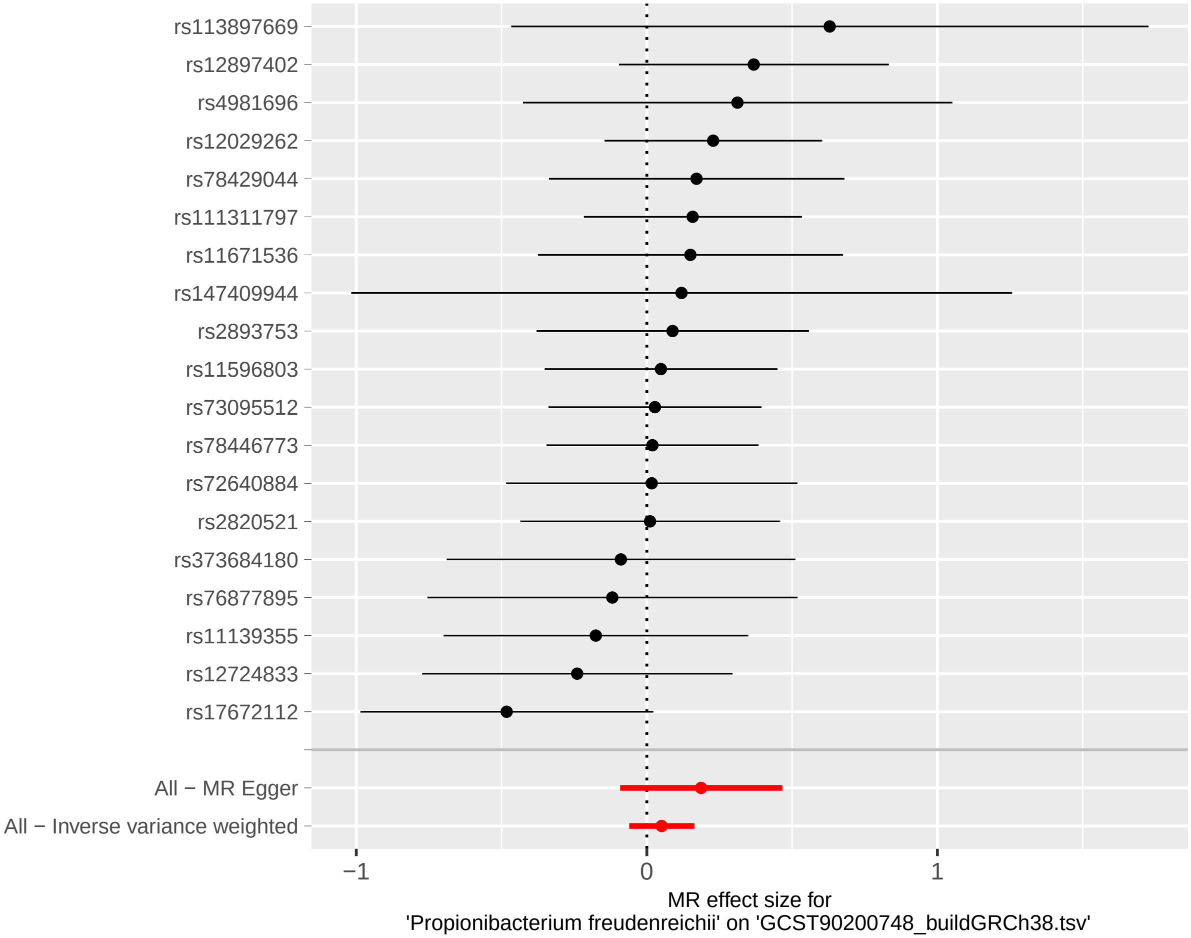
rs113897669
rs12897402
rs4981696
rs12029262
rs78429044
rs111311797
rs11671536
rs147409944
rs2893753
rs11596803
rs73095512
rs78446773
rs72640884
rs2820521
rs373684180
rs76877895
rs11139355
rs12724833
rs17672112
All − MR Egger
All − Inverse variance weighted
−1
0
1
MR effect size for
'Propionibacterium freudenreichii' on 'GCST90200748_buildGRCh38.tsv'

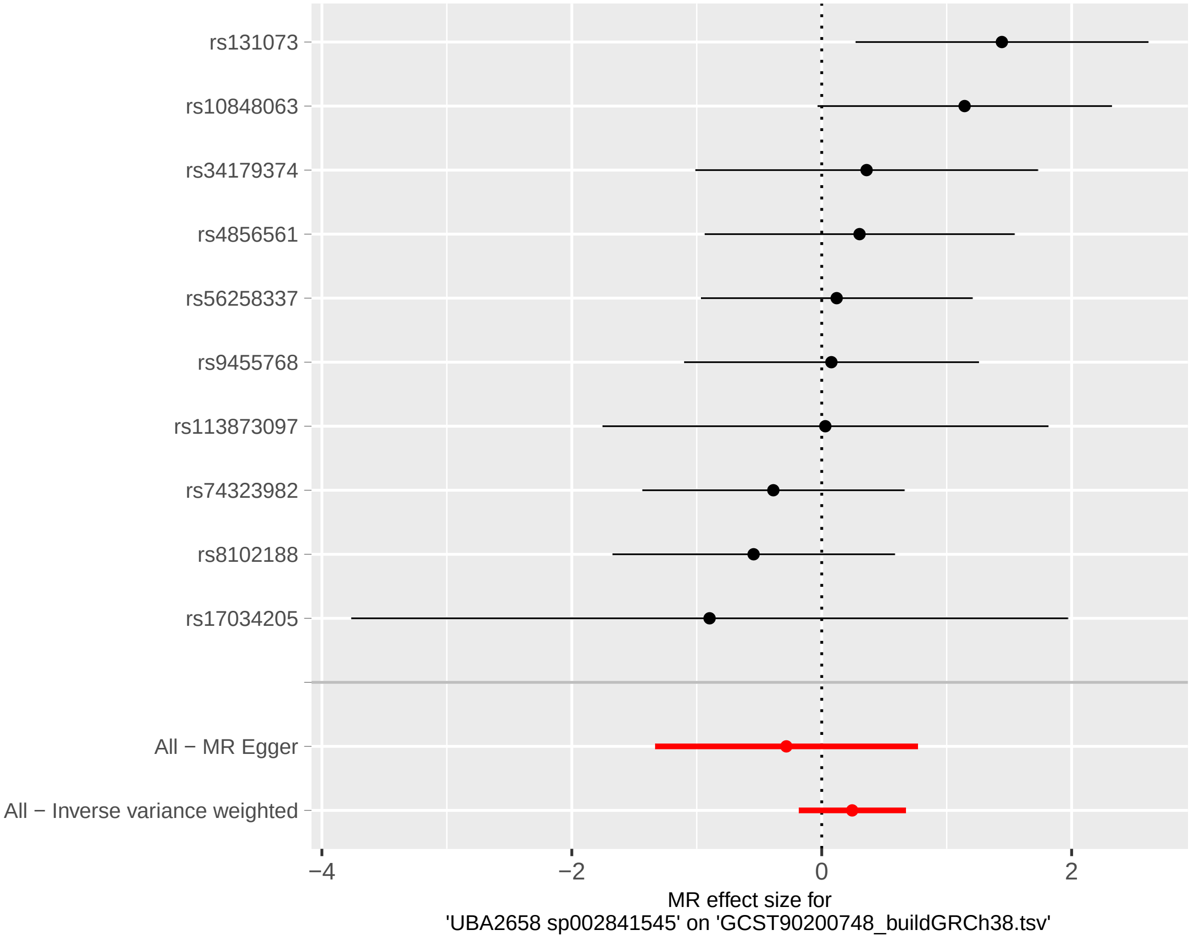

rs131073
rs10848063
rs34179374
rs4856561
rs56258337
rs9455768
rs113873097
rs74323982
rs8102188
rs17034205
All − MR Egger
All − Inverse variance weighted
−4
−2
0
2
MR effect size for
'UBA2658 sp002841545' on 'GCST90200748_buildGRCh38.tsv'

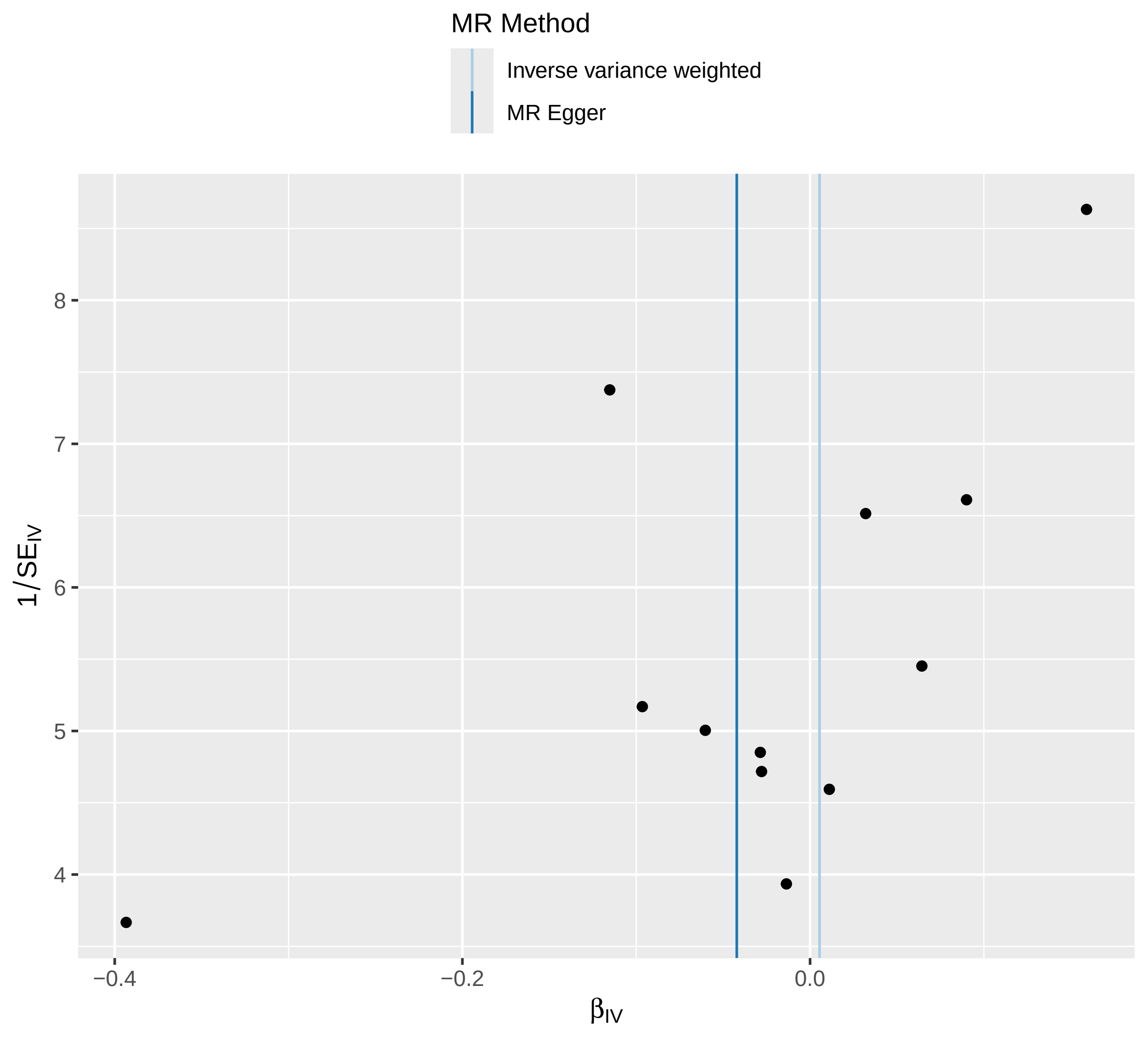
MR Method
Inverse variance weighted
MR Egger
8
7
6
5
4
1/SE_IV
−0.4
−0.2
0.0
β_IV

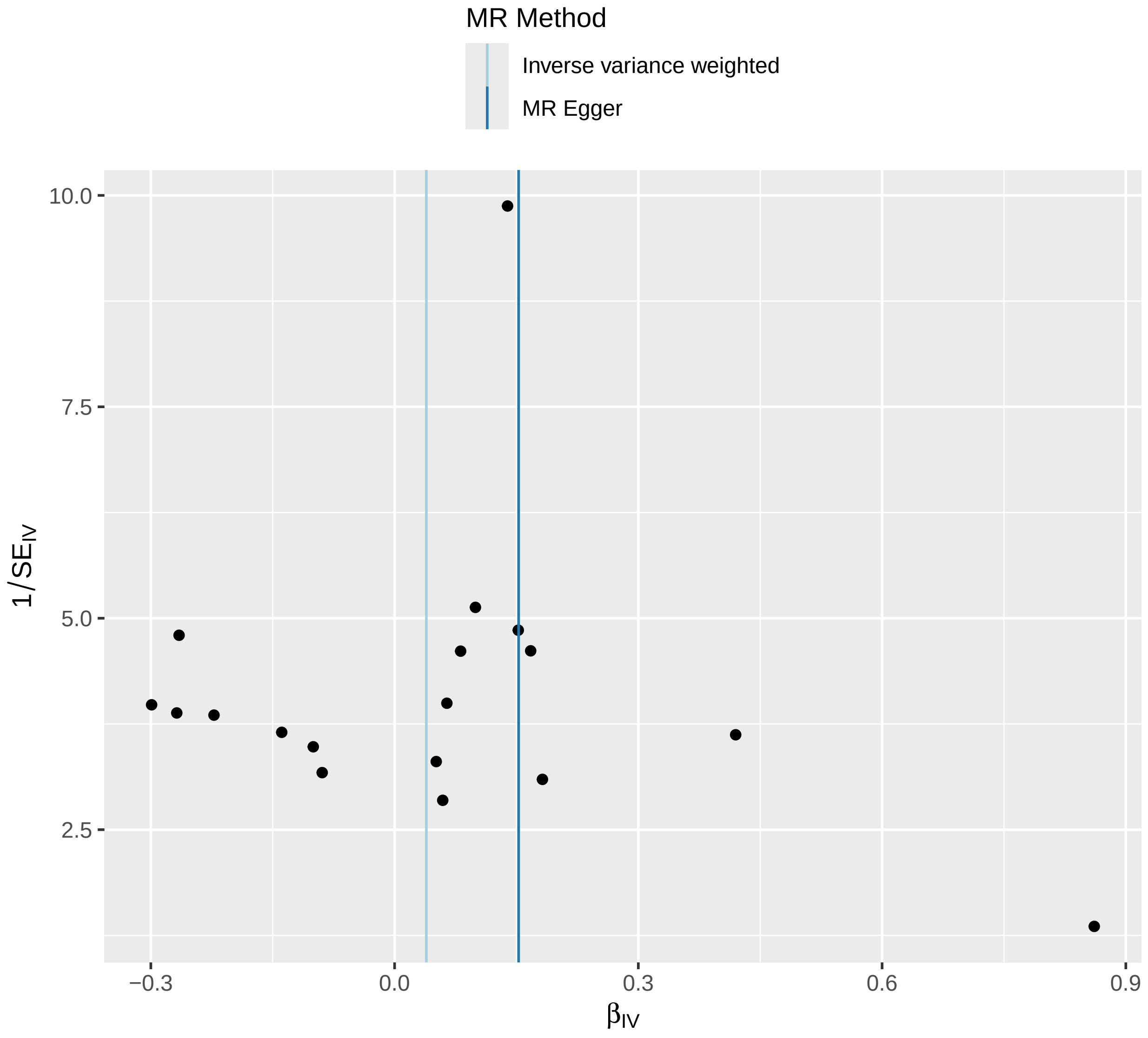

MR Method
Inverse variance weighted
MR Egger
10.0
7.5
5.0
2.5
$1/SE_{IV}$
−0.3
0.0
0.3
0.6
0.9
β_{IV}

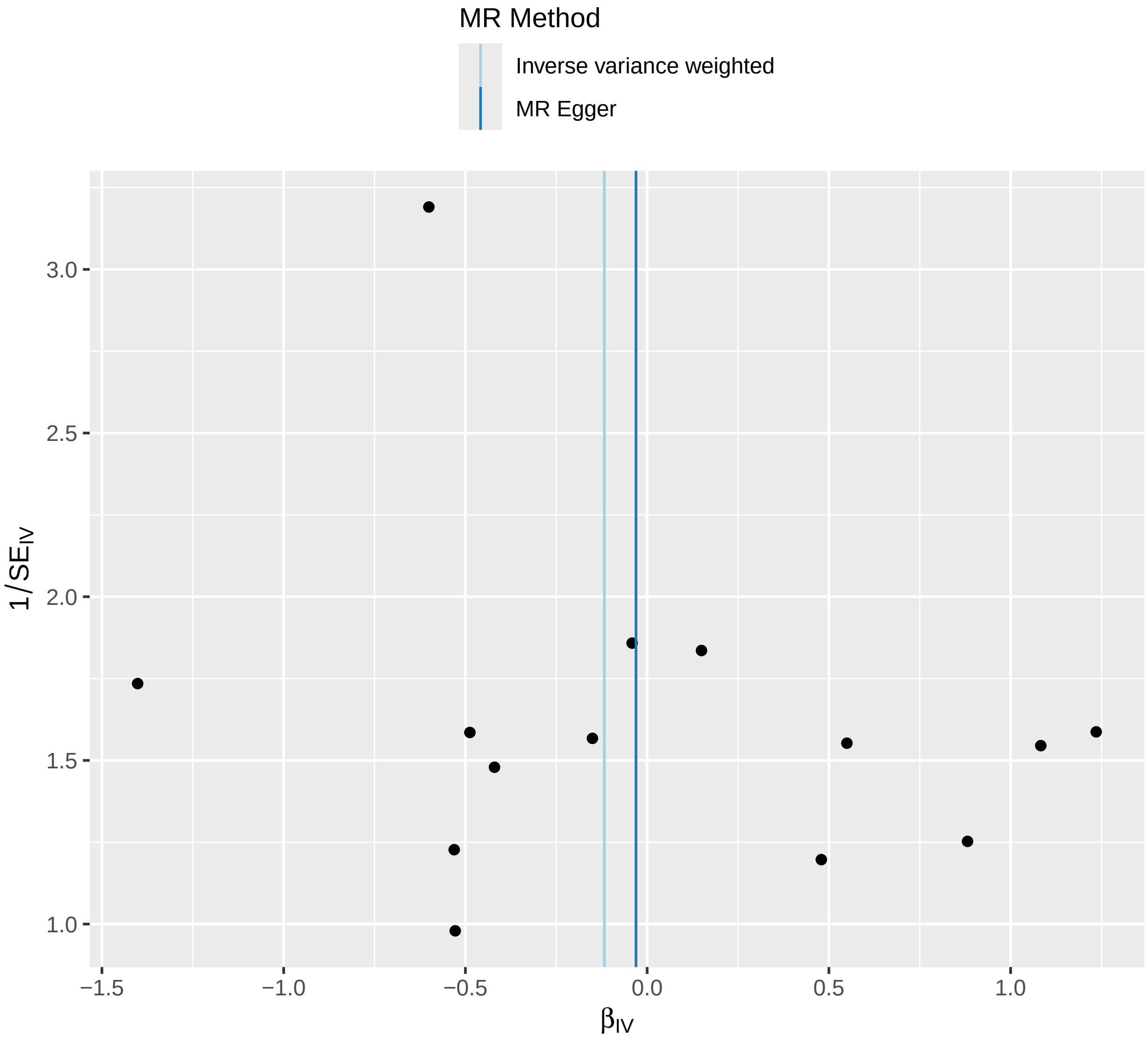
MR Method
Inverse variance weighted
MR Egger
3.0
2.5
2.0
1.5
1.0
1/SE_IV
−1.5
−1.0
−0.5
0.0
0.5
1.0
β_IV

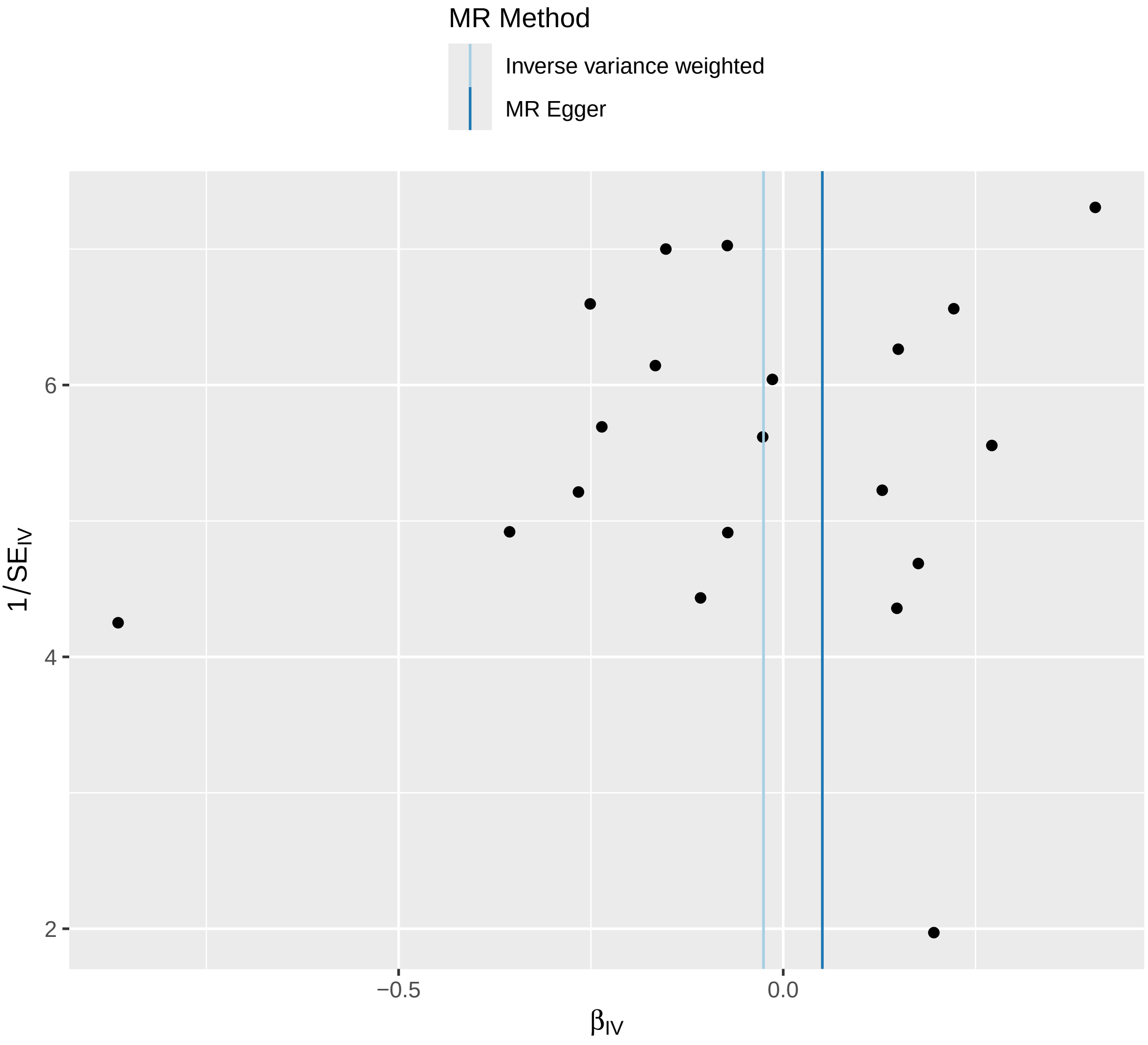
MR Method
Inverse variance weighted
MR Egger
6
4
2
−0.5
0.0
β_{IV}
$1/SE_{IV}$

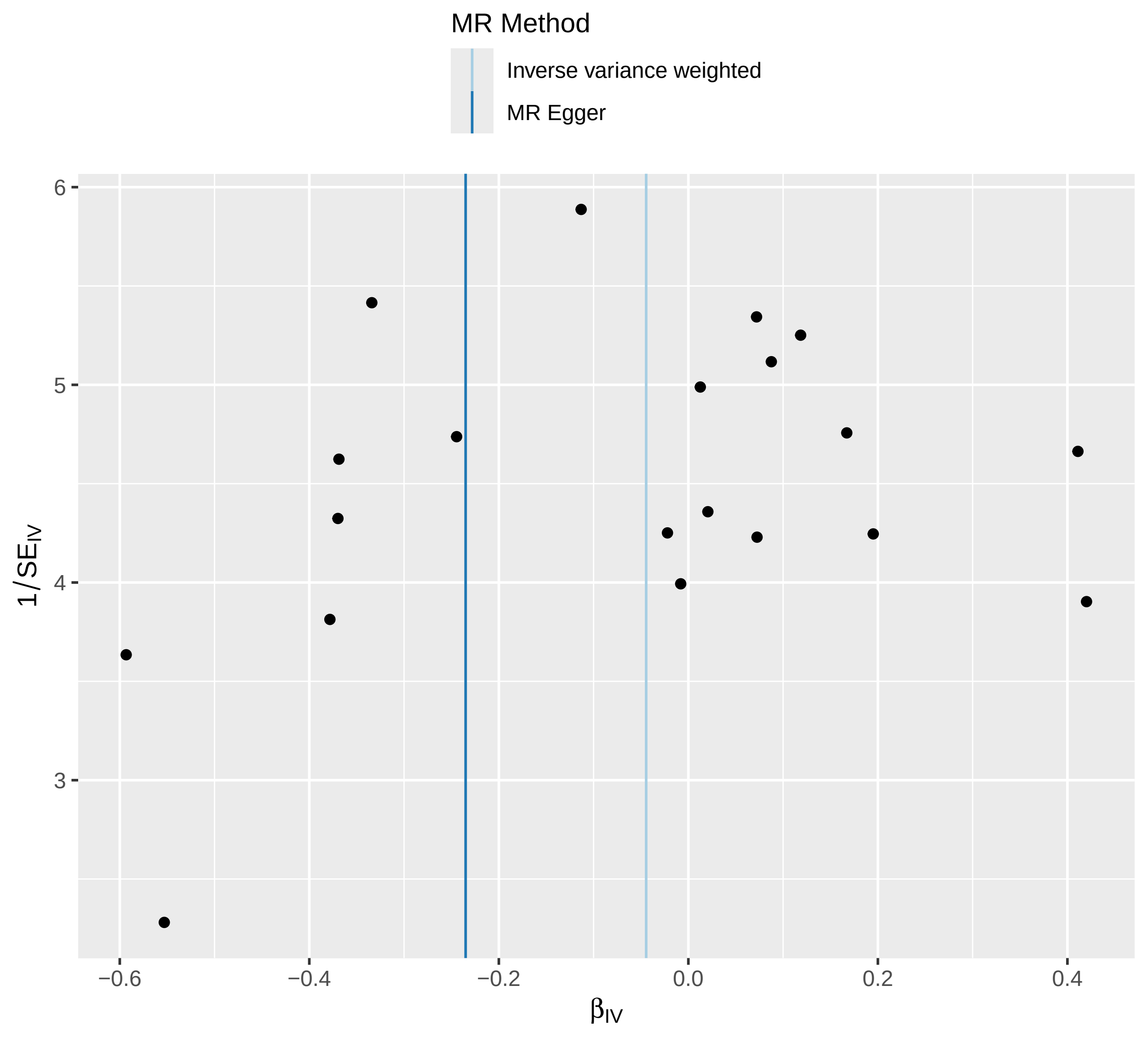
MR Method
Inverse variance weighted
MR Egger
6
5
4
3
1/SE_IV
−0.6
−0.4
−0.2
0.0
0.2
0.4
β_IV

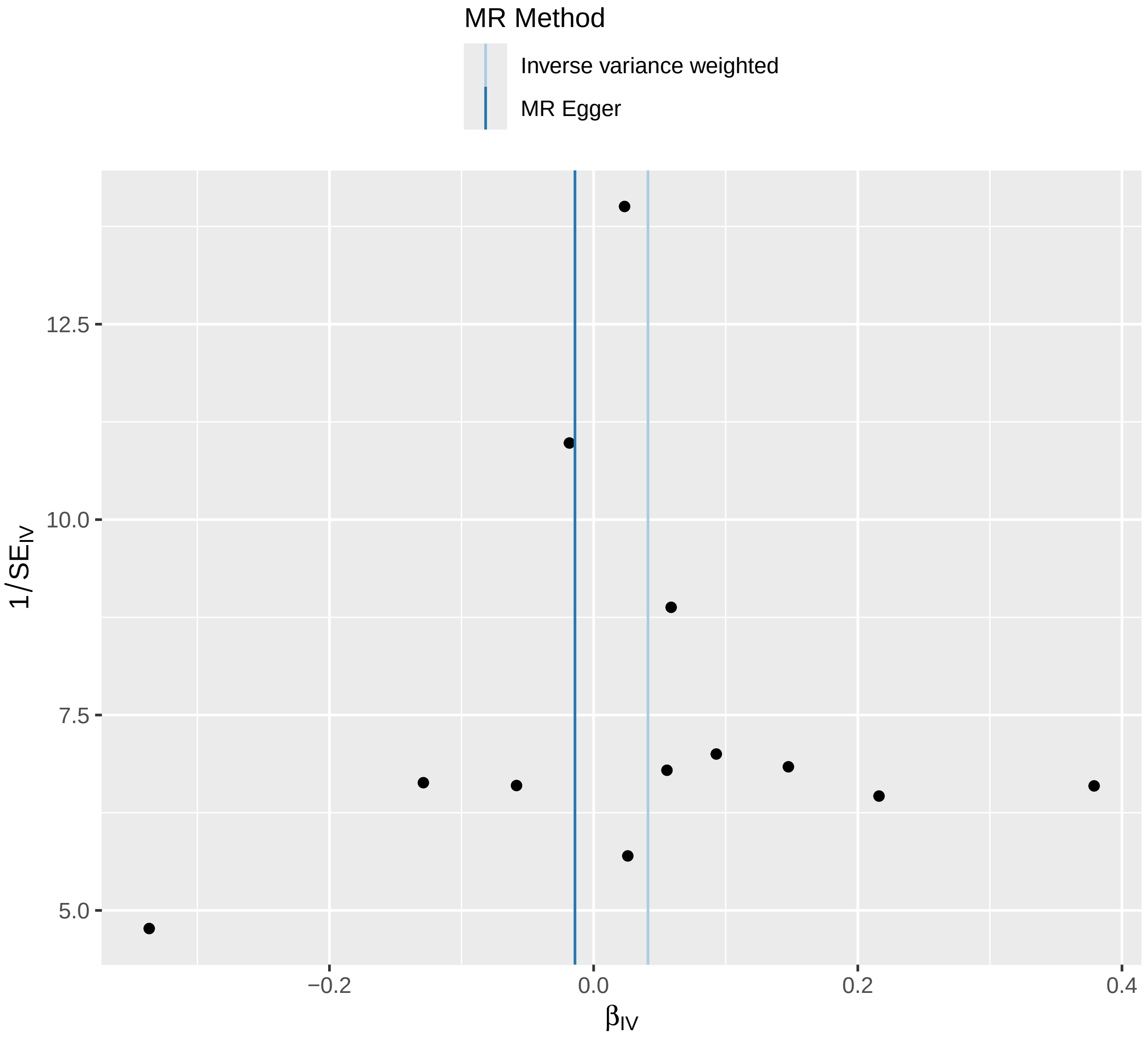

MR Method
Inverse variance weighted
MR Egger
12.5
10.0
7.5
5.0
1/SE$_{IV}$
−0.2
0.0
0.2
0.4
β$_{IV}$

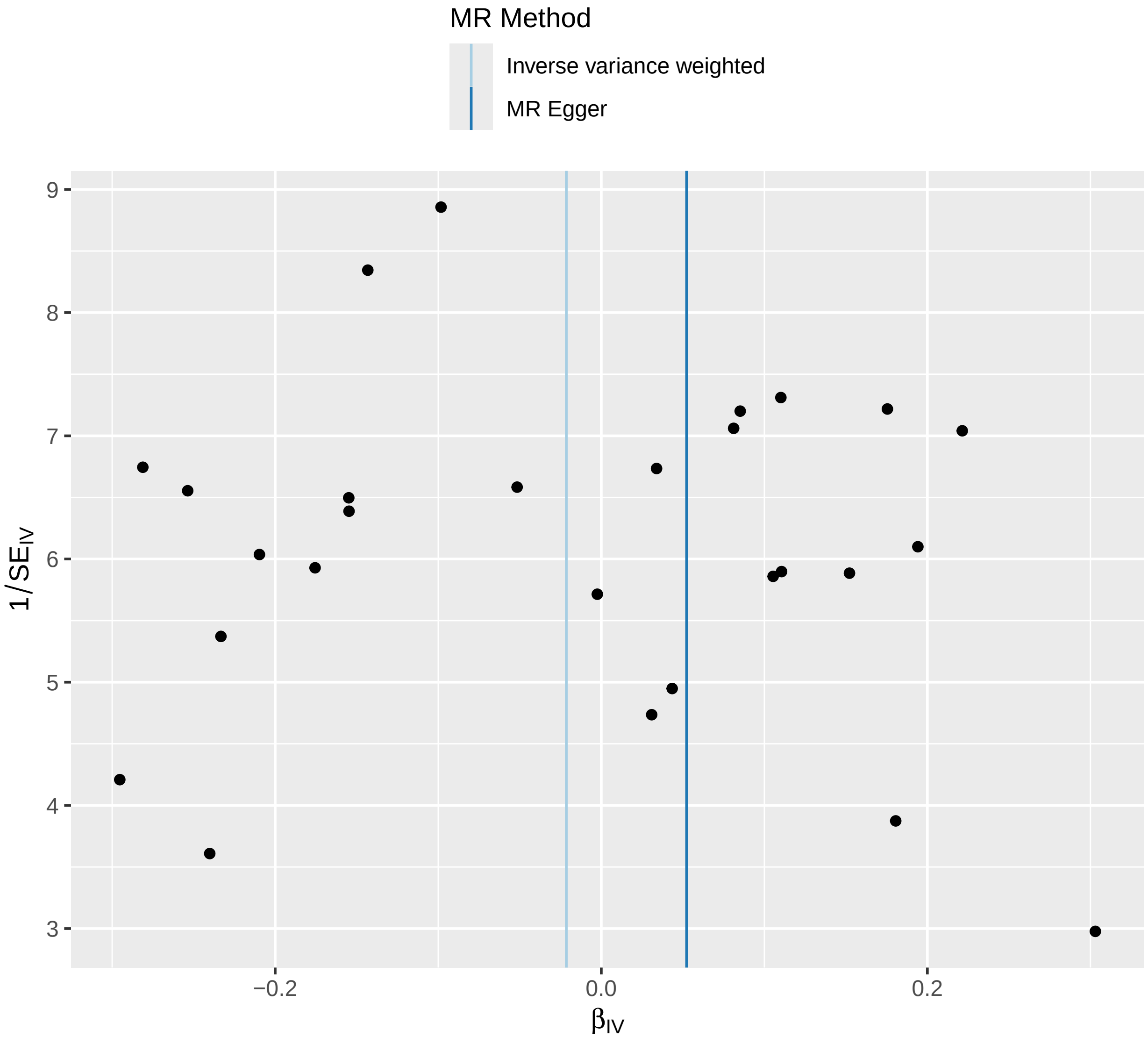

MR Method
Inverse variance weighted
MR Egger
9
8
7
6
5
4
3
1/SE_IV
−0.2
0.0
0.2
β_IV

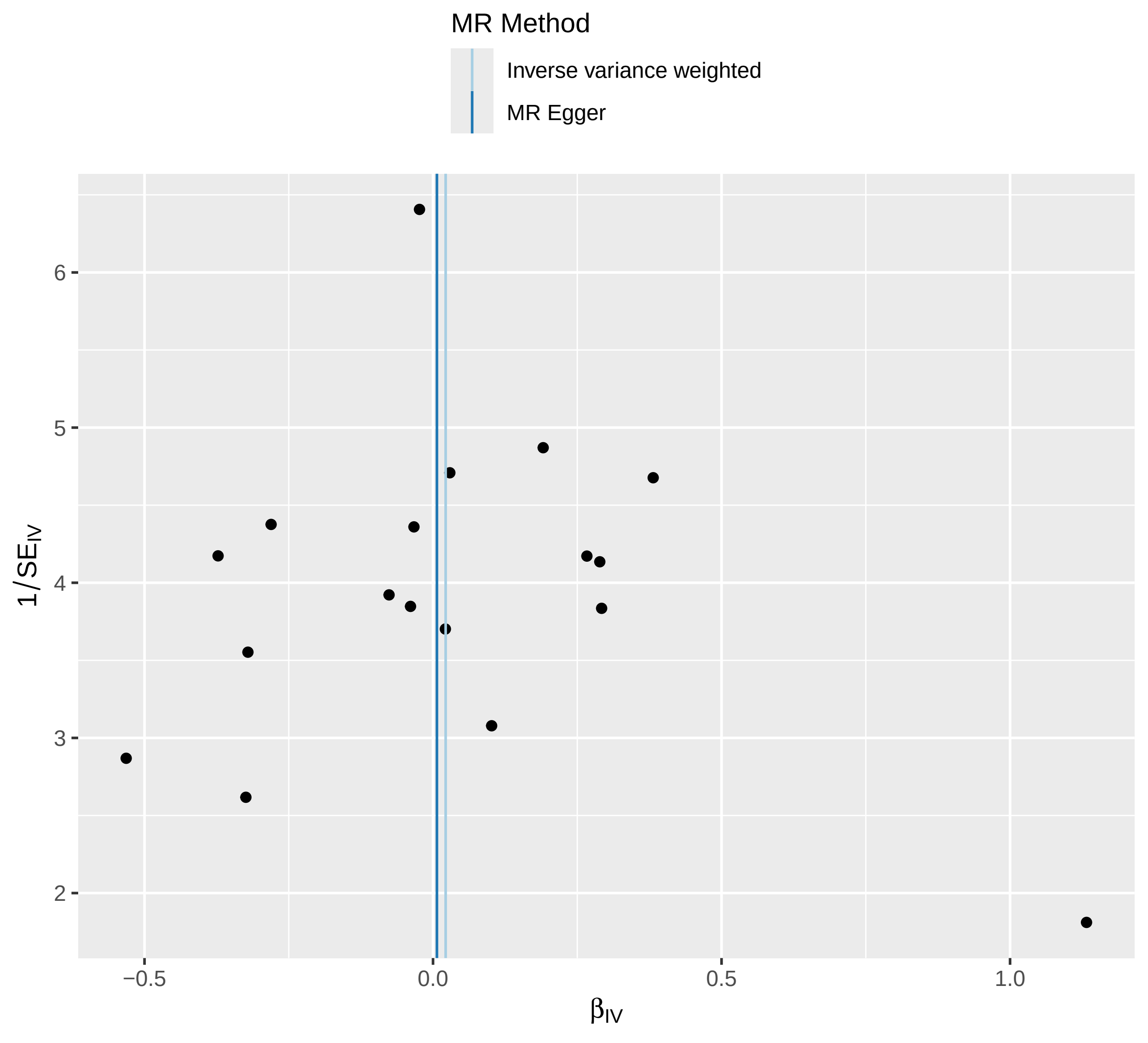
MR Method
Inverse variance weighted
MR Egger
1/SE_IV
6
5
4
3
2
−0.5
0.0
0.5
1.0
β_IV

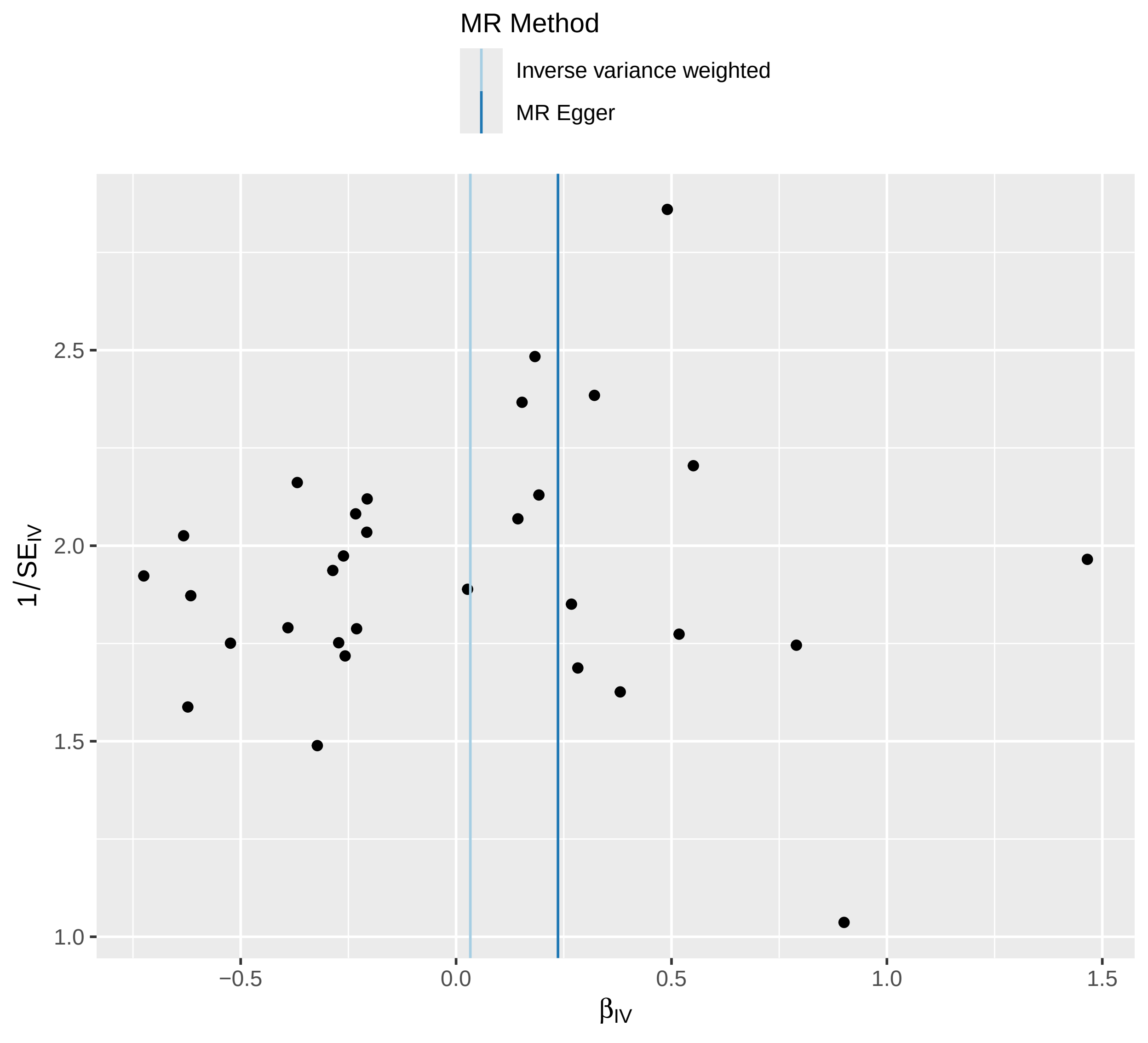

MR Method
Inverse variance weighted
MR Egger
1/SE_IV
β_IV
2.5
2.0
1.5
1.0
−0.5
0.0
0.5
1.0
1.5

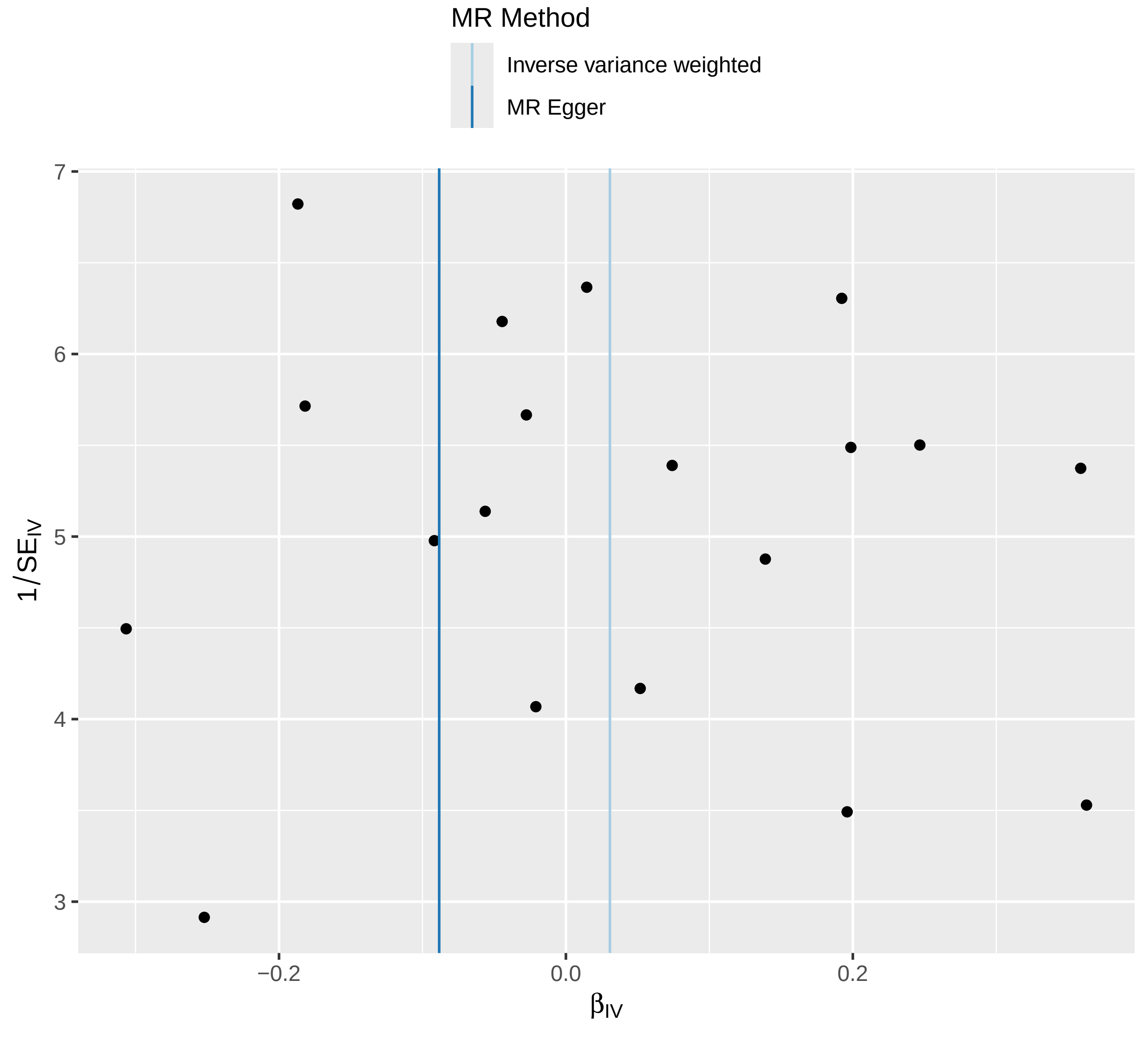

MR Method
Inverse variance weighted
MR Egger
7
6
5
4
3
1/SE_IV
−0.2
0.0
0.2
β_IV

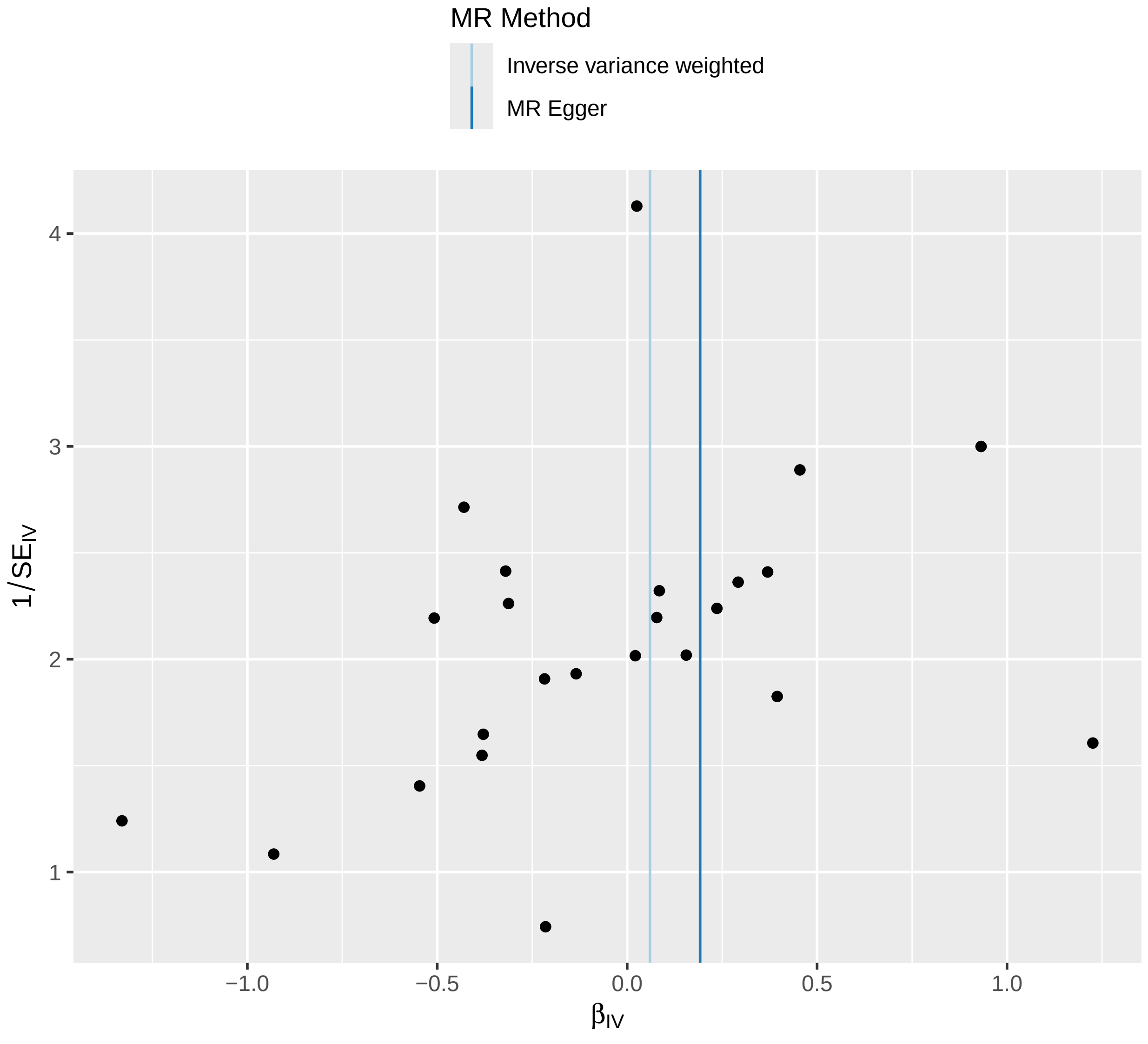
MR Method
Inverse variance weighted
MR Egger
1/SE_IV
β_IV
4
3
2
1
−1.0
−0.5
0.0
0.5
1.0

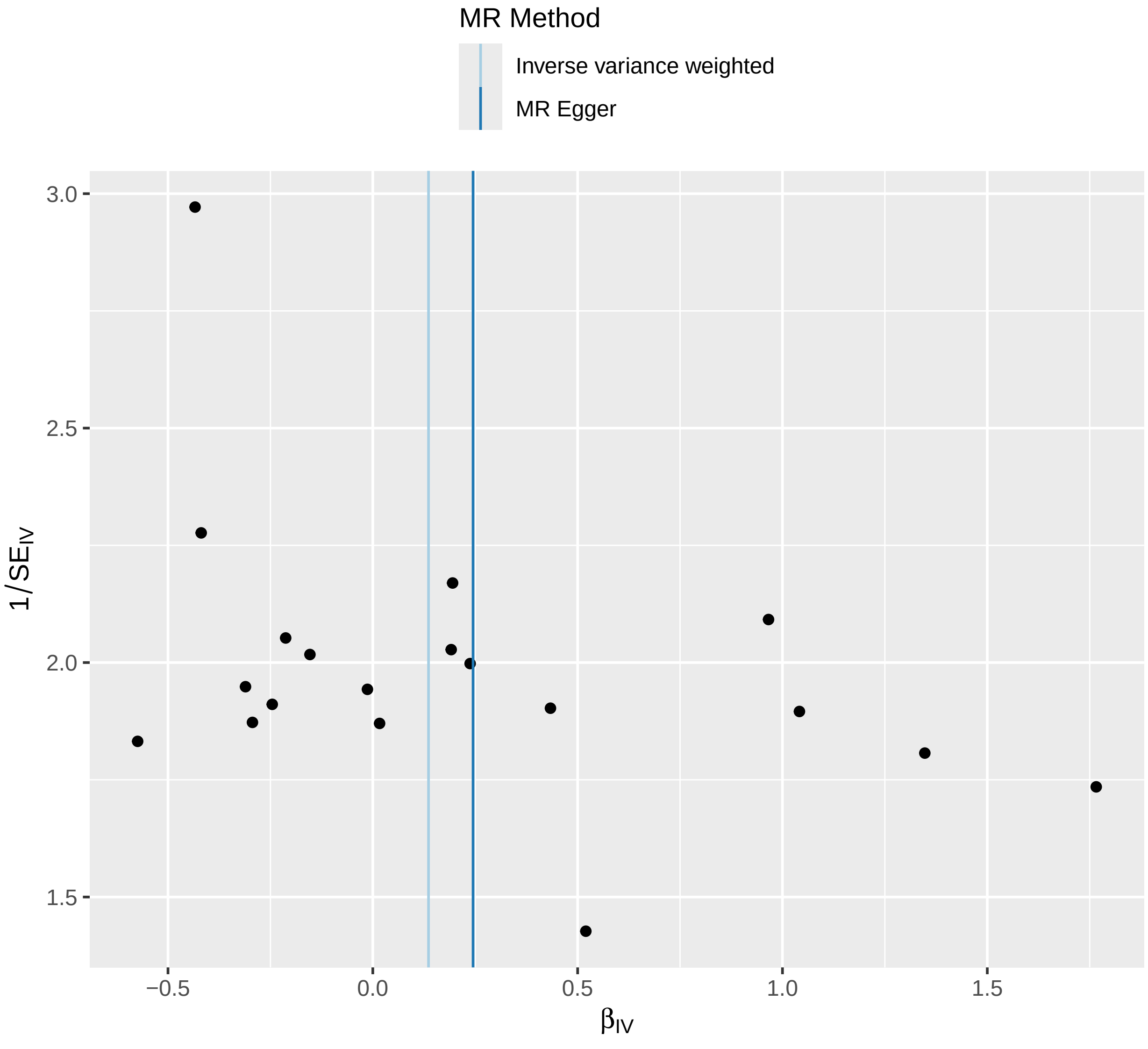
MR Method
Inverse variance weighted
MR Egger
3.0
2.5
2.0
1.5
−0.5
0.0
0.5
1.0
1.5
$1/SE_{IV}$
β_{IV}

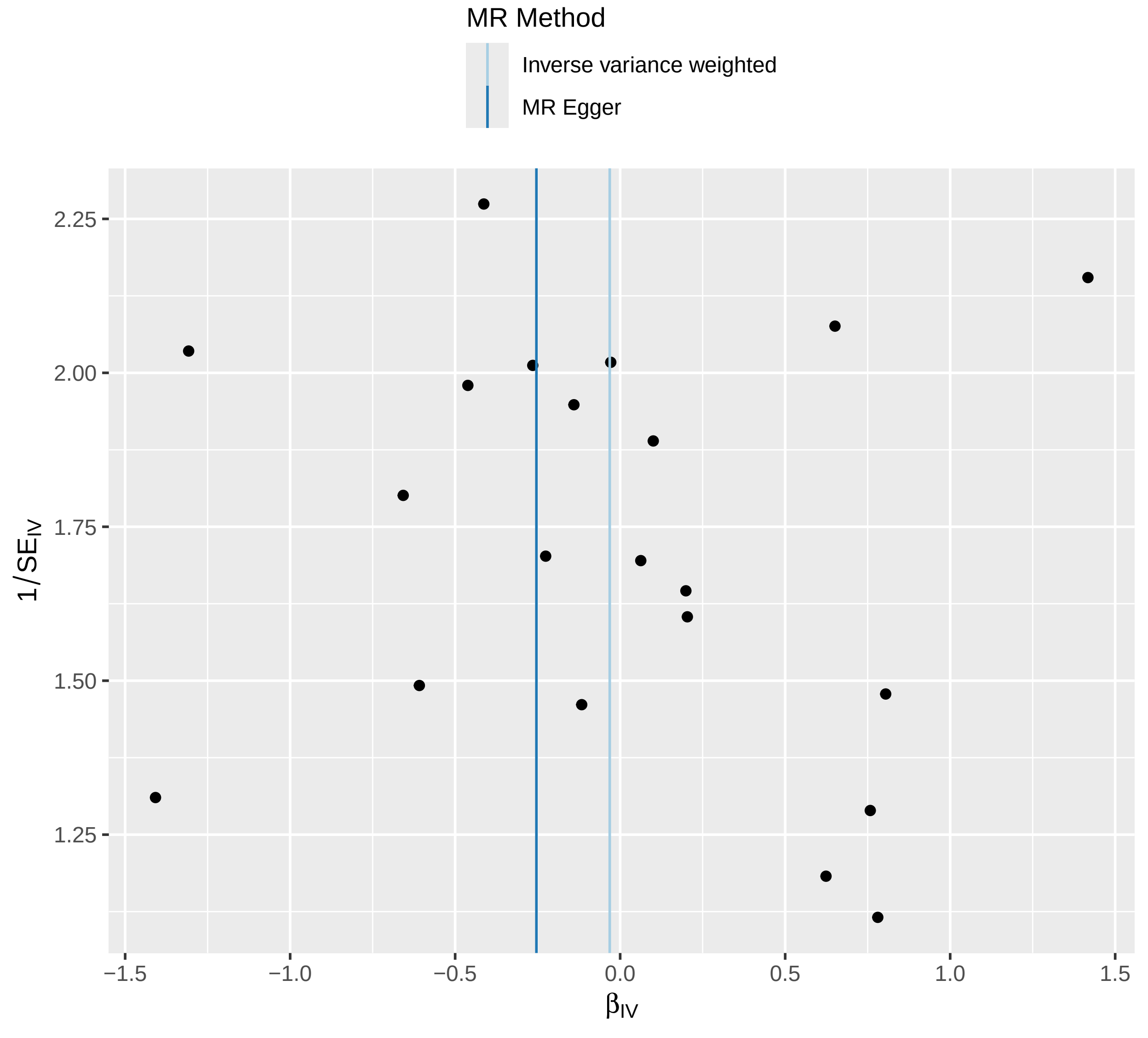
MR Method
Inverse variance weighted
MR Egger
2.25
2.00
1.75
1.50
1.25
1/SE$_{IV}$
−1.5
−1.0
−0.5
0.0
0.5
1.0
1.5
β$_{IV}$

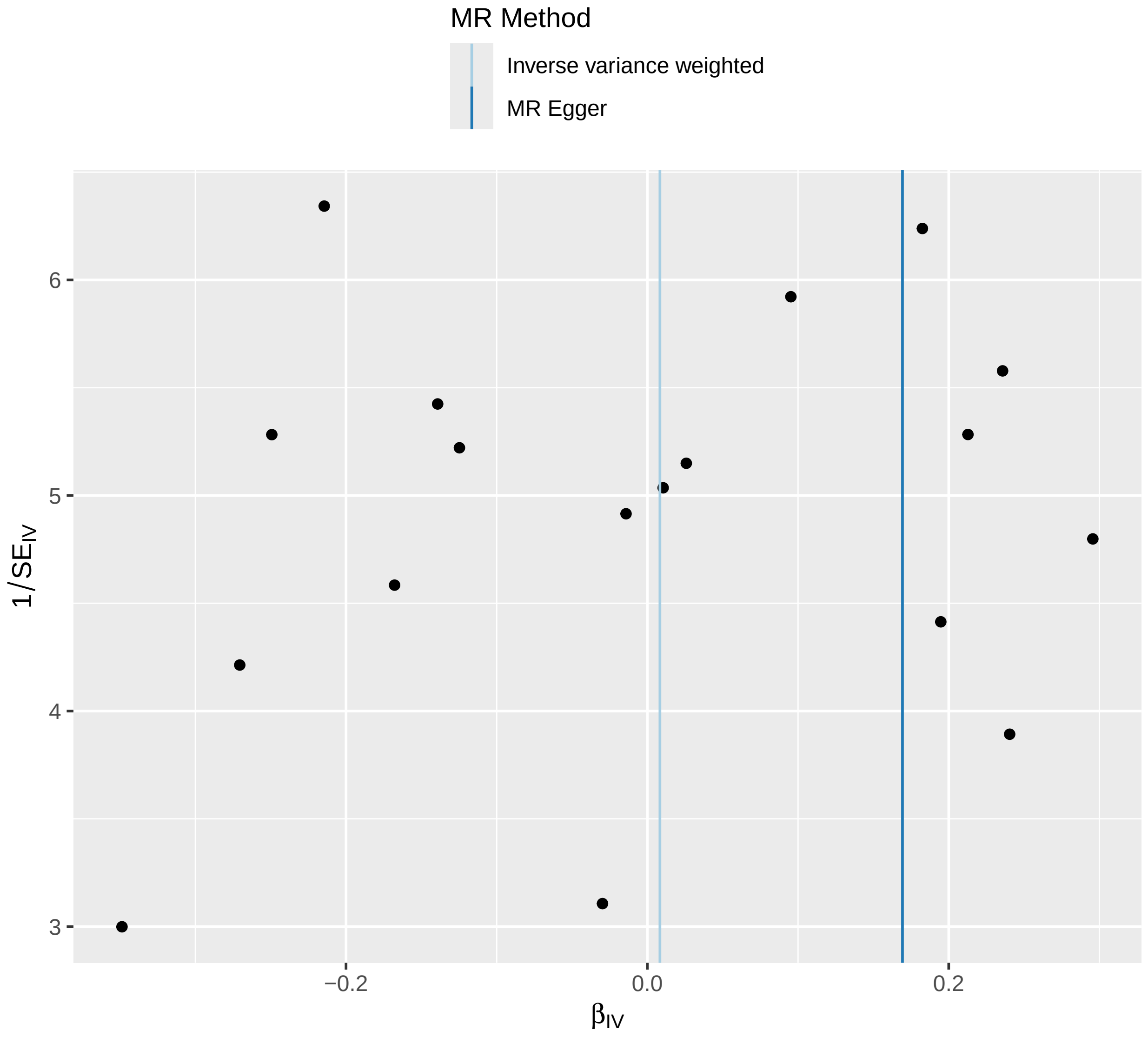
MR Method
Inverse variance weighted
MR Egger
6
5
4
3
1/SE_IV
−0.2
0.0
0.2
β_IV

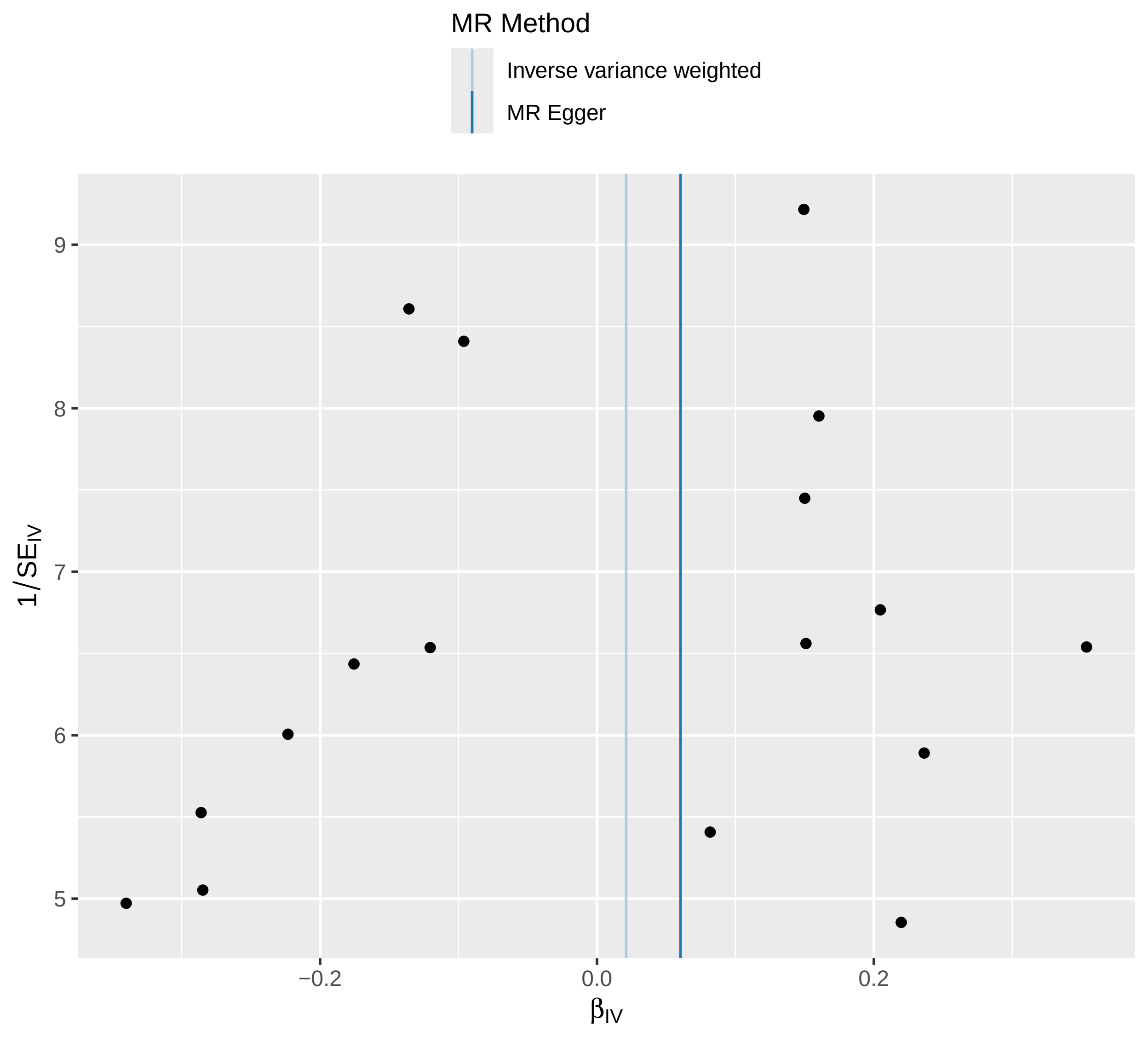

MR Method
Inverse variance weighted
MR Egger
9
8
7
6
5
1/SE$_{IV}$
−0.2
0.0
0.2
β$_{IV}$

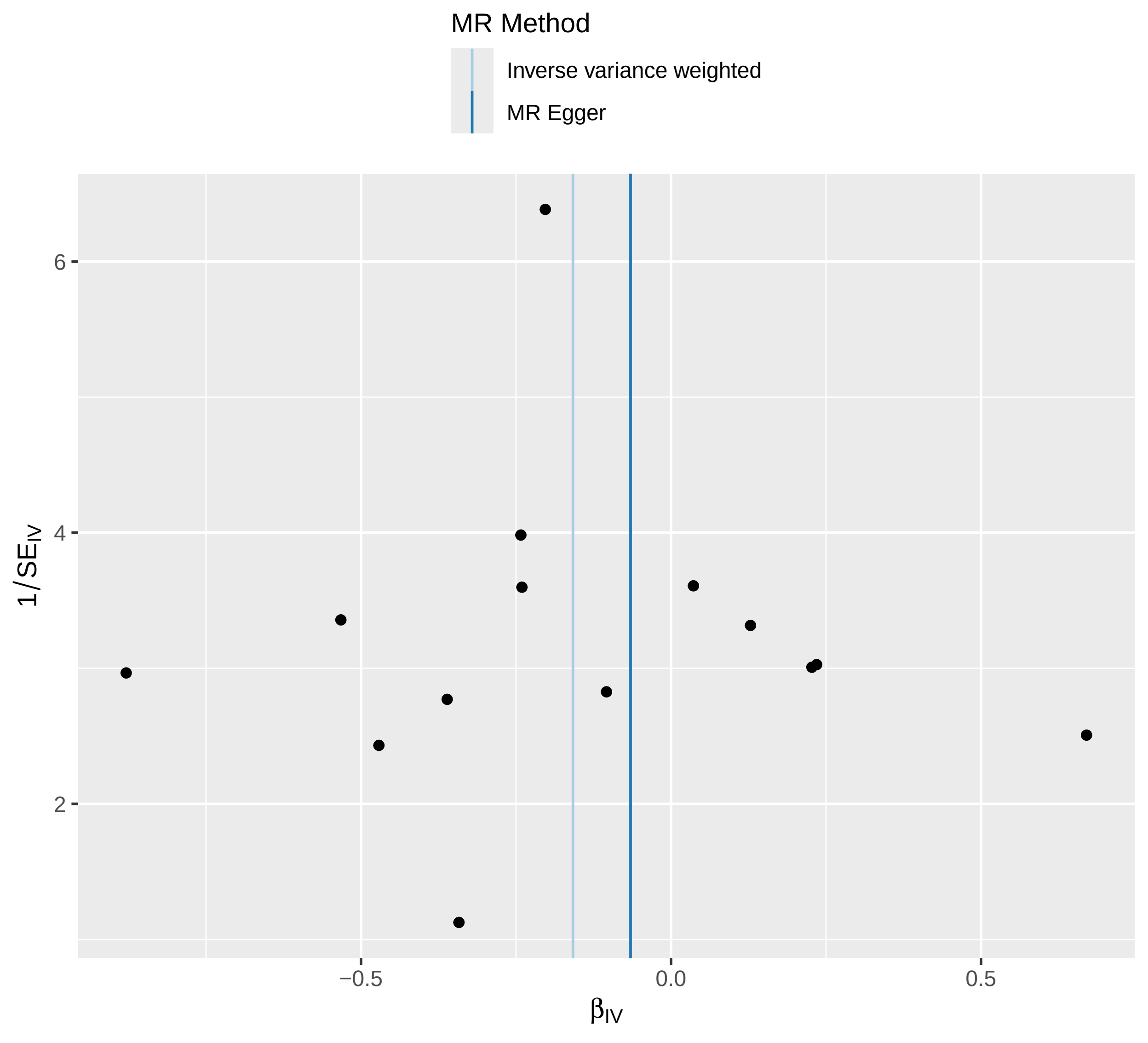

MR Method
Inverse variance weighted
MR Egger
6
4
2
1/SE$_{IV}$
−0.5
0.0
0.5
β$_{IV}$

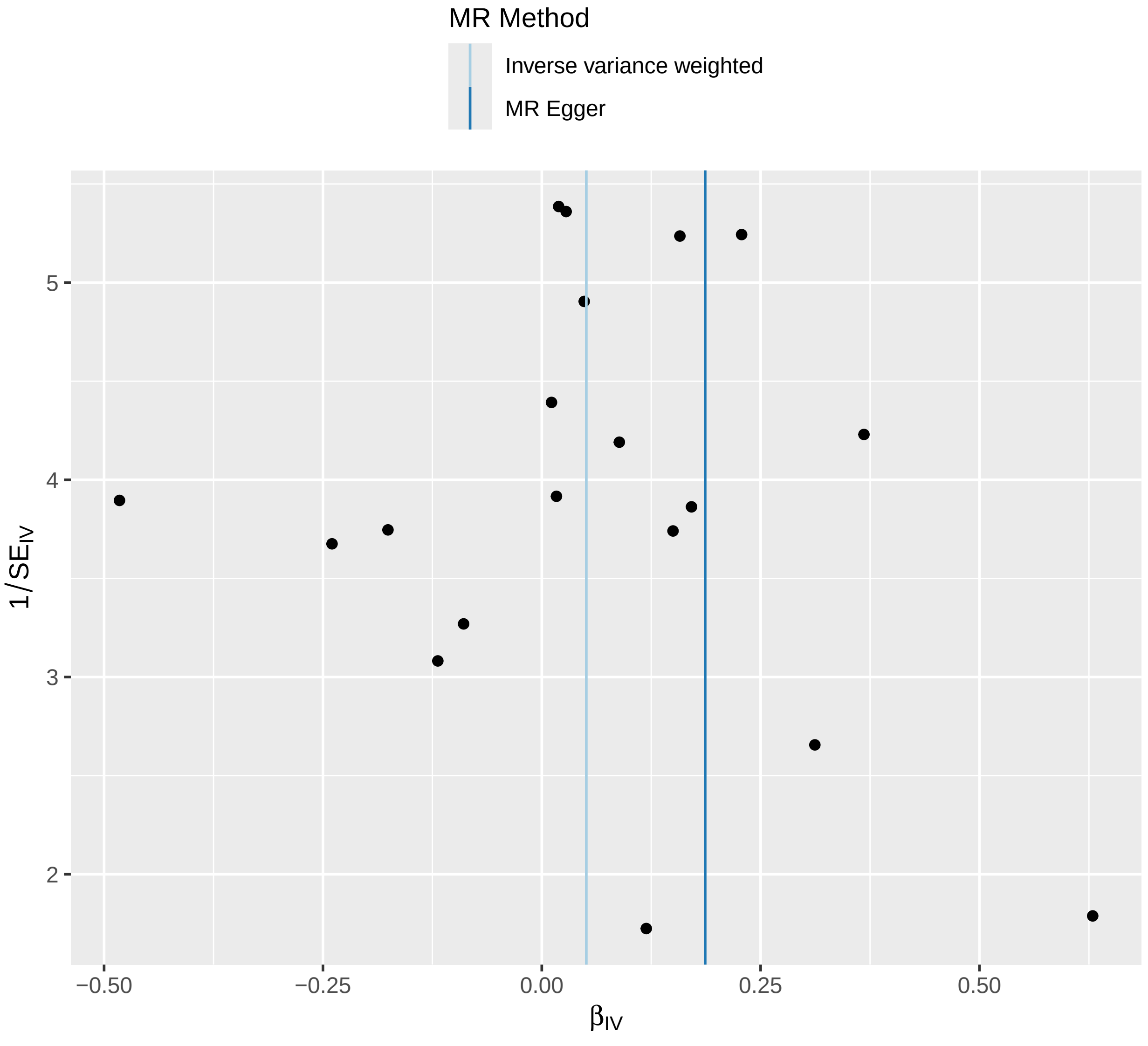

MR Method
Inverse variance weighted
MR Egger
5
4
3
2
1/SE$_{IV}$
−0.50
−0.25
0.00
0.25
0.50
β$_{IV}$

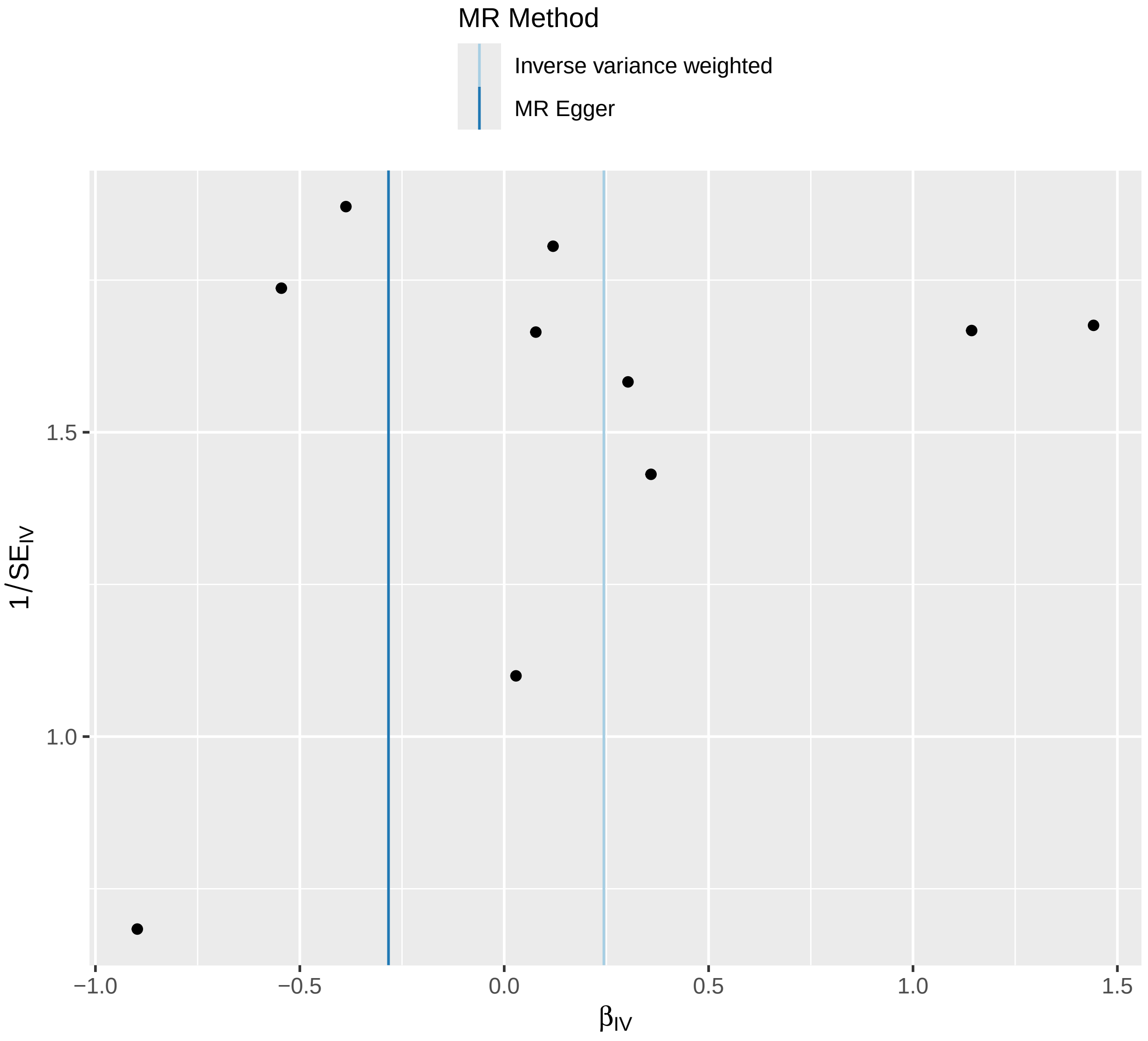

MR Method
Inverse variance weighted
MR Egger
1.5
1.0
$1/SE_{IV}$
−1.0
−0.5
0.0
0.5
1.0
1.5
β_{IV}

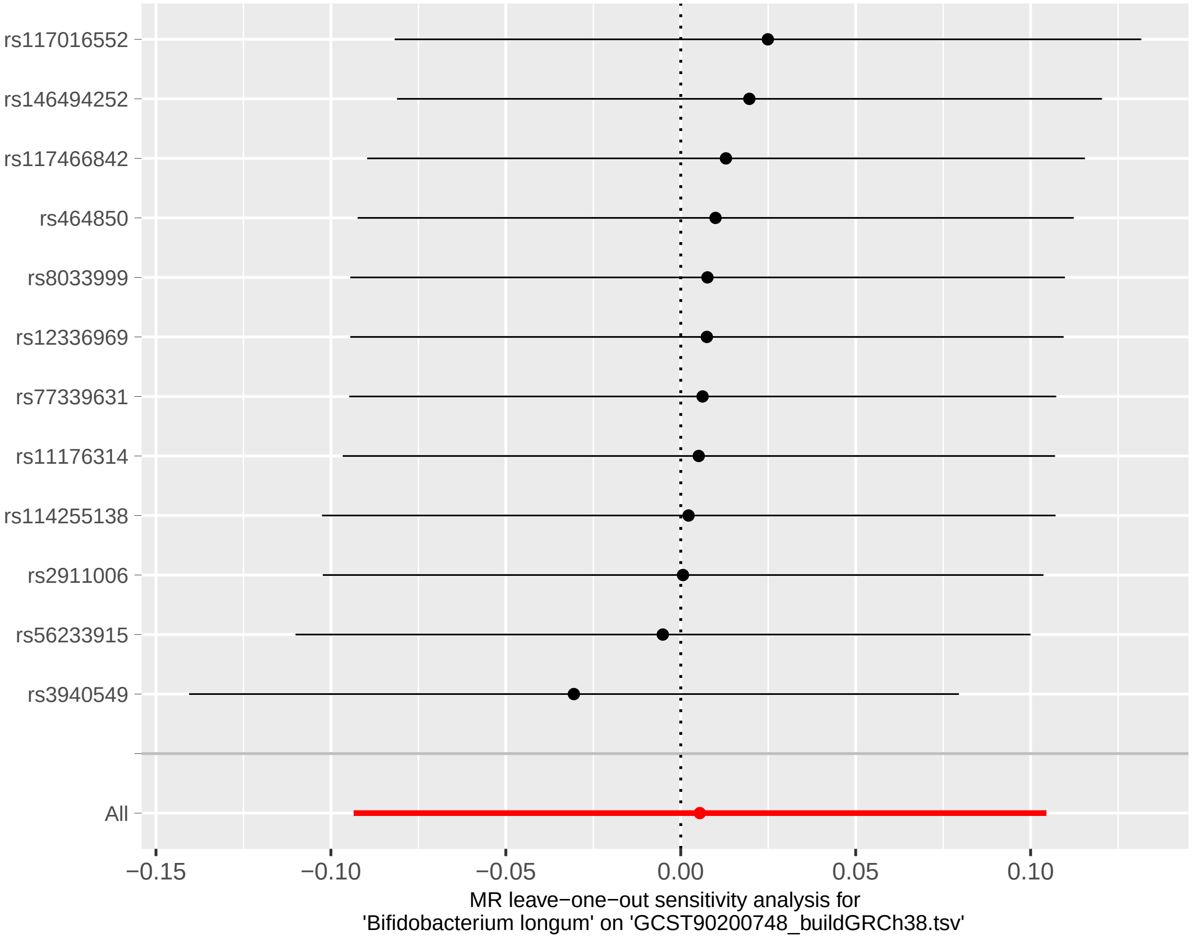
rs117016552
rs146494252
rs117466842
rs464850
rs8033999
rs12336969
rs77339631
rs11176314
rs114255138
rs2911006
rs56233915
rs3940549
All
−0.15
−0.10
−0.05
0.00
0.05
0.10
MR leave−one−out sensitivity analysis for
'Bifidobacterium longum' on 'GCST90200748_buildGRCh38.tsv'

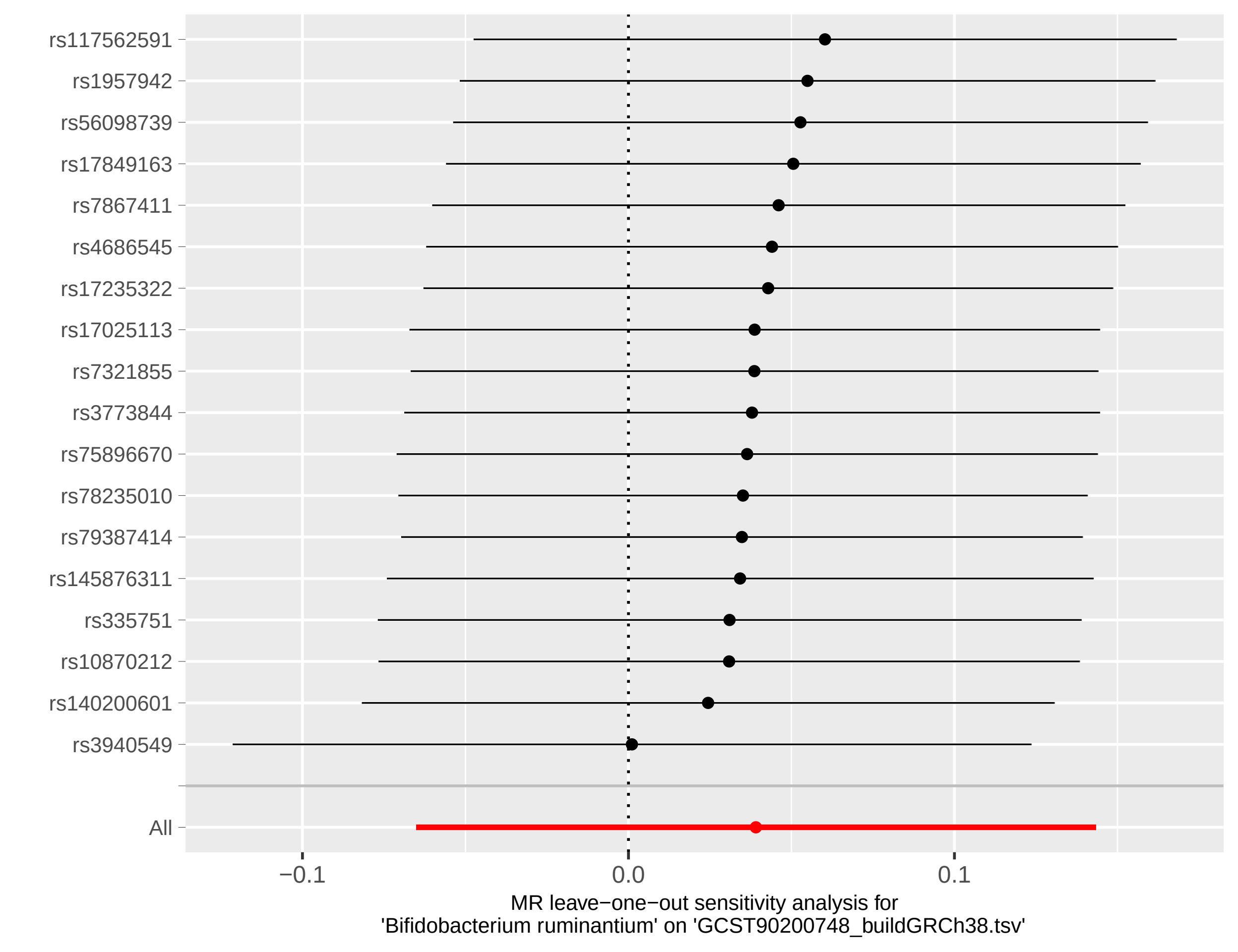
rs117562591
rs1957942
rs56098739
rs17849163
rs7867411
rs4686545
rs17235322
rs17025113
rs7321855
rs3773844
rs75896670
rs78235010
rs79387414
rs145876311
rs335751
rs10870212
rs140200601
rs3940549
All
−0.1
0.0
0.1
MR leave−one−out sensitivity analysis for
'Bifidobacterium ruminantium' on 'GCST90200748_buildGRCh38.tsv'

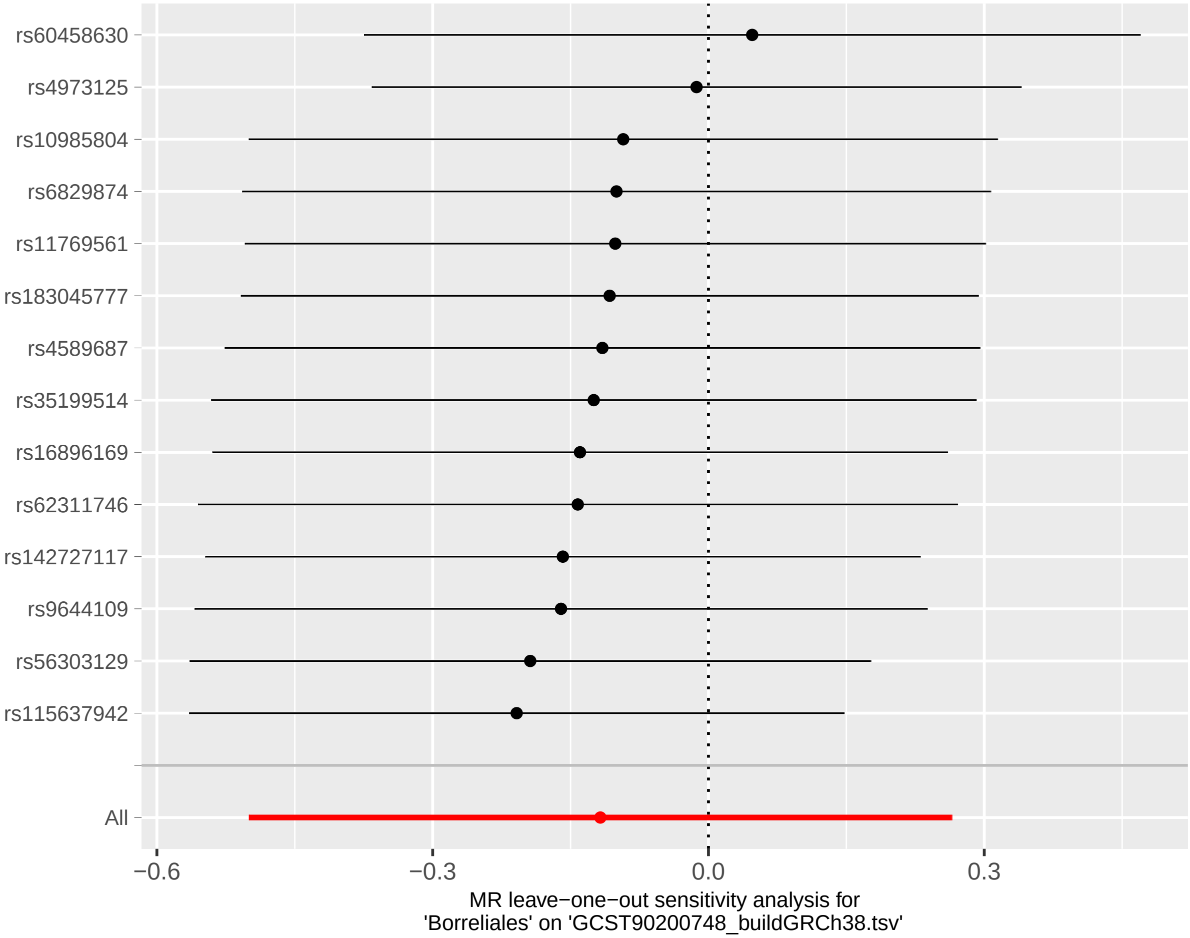
rs60458630
rs4973125
rs10985804
rs6829874
rs11769561
rs183045777
rs4589687
rs35199514
rs16896169
rs62311746
rs142727117
rs9644109
rs56303129
rs115637942
All
−0.6
−0.3
0.0
0.3
MR leave−one−out sensitivity analysis for
'Borreliales' on 'GCST90200748_buildGRCh38.tsv'

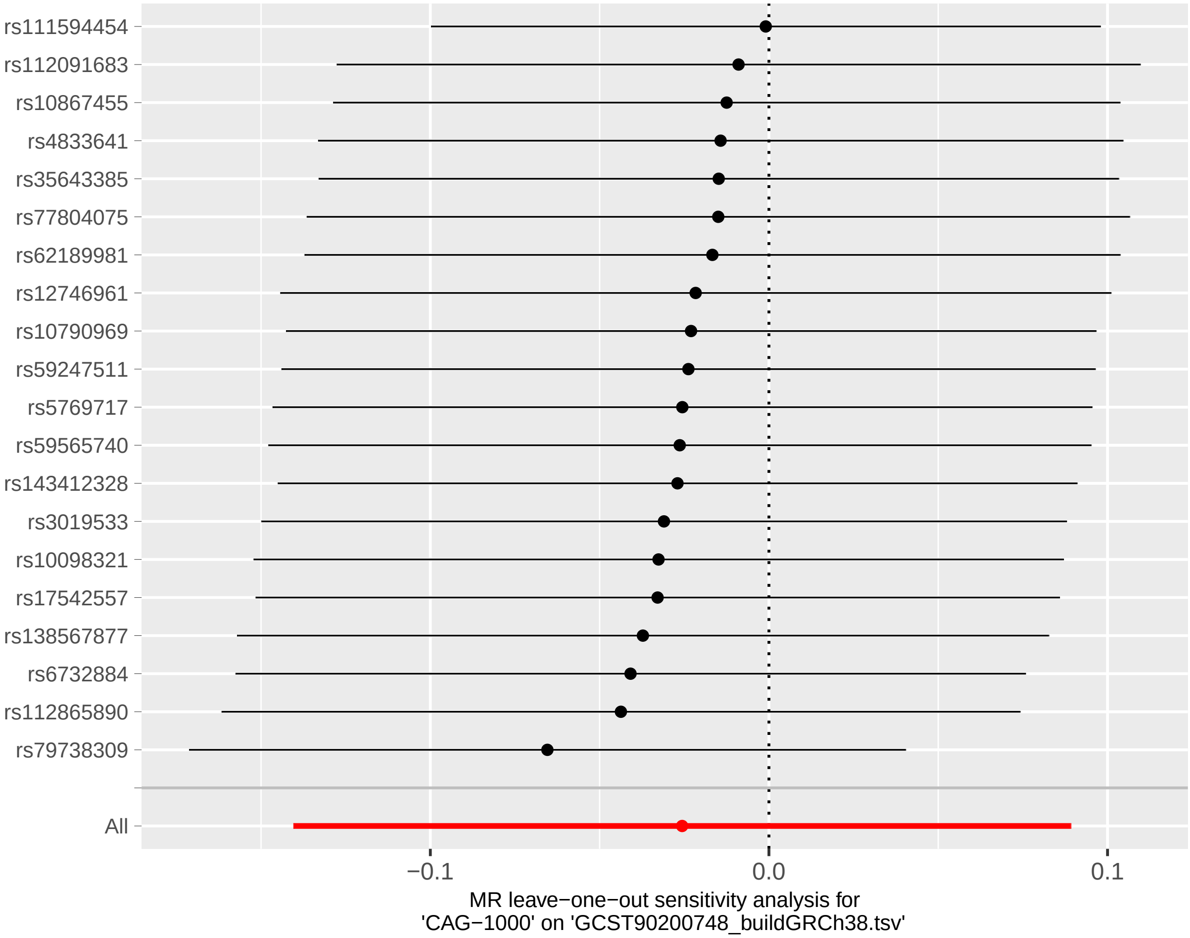

rs111594454
rs112091683
rs10867455
rs4833641
rs35643385
rs77804075
rs62189981
rs12746961
rs10790969
rs59247511
rs5769717
rs59565740
rs143412328
rs3019533
rs10098321
rs17542557
rs138567877
rs6732884
rs112865890
rs79738309
All
−0.1
0.0
0.1
MR leave−one−out sensitivity analysis for
'CAG−1000' on 'GCST90200748_buildGRCh38.tsv'

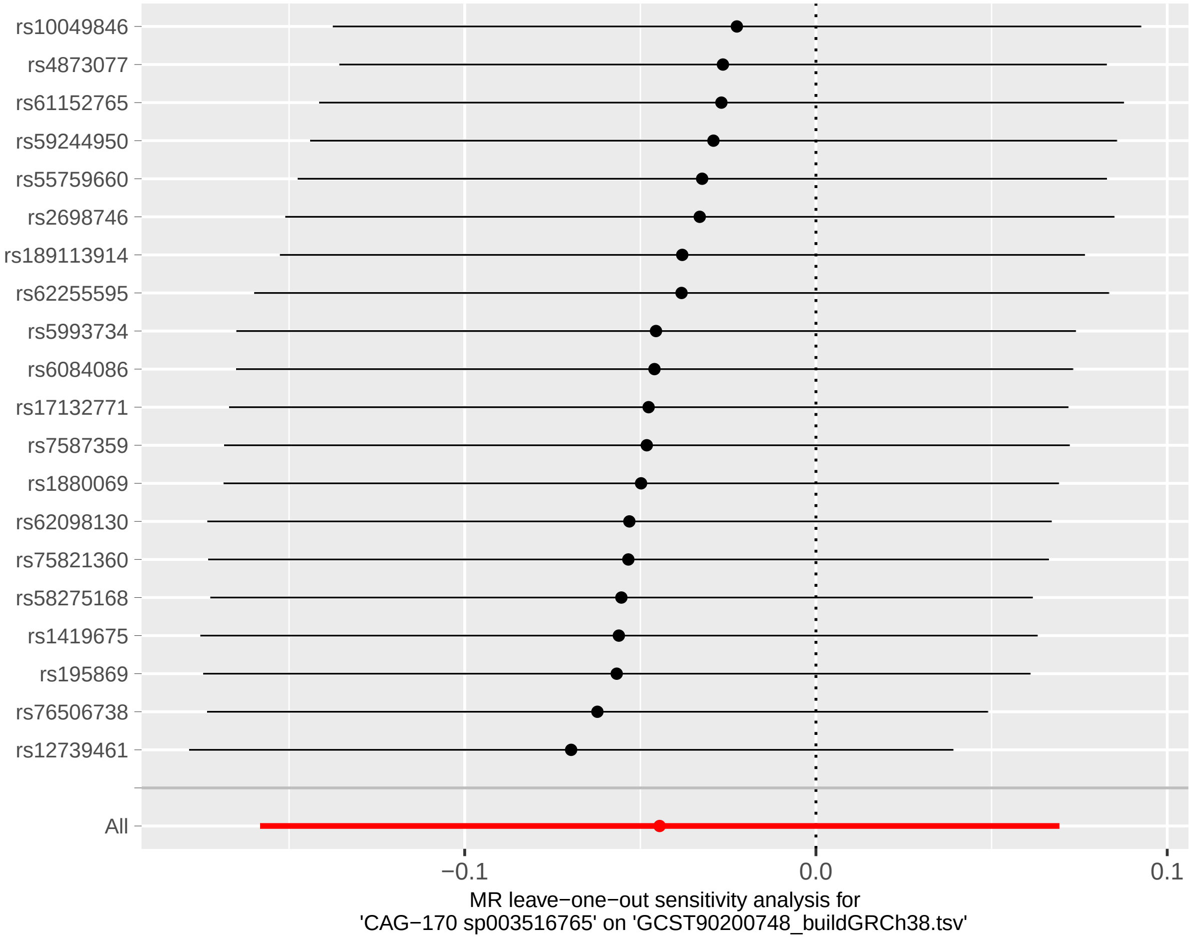
rs10049846
rs4873077
rs61152765
rs59244950
rs55759660
rs2698746
rs189113914
rs62255595
rs5993734
rs6084086
rs17132771
rs7587359
rs1880069
rs62098130
rs75821360
rs58275168
rs1419675
rs195869
rs76506738
rs12739461
All
−0.1
0.0
0.1
MR leave−one−out sensitivity analysis for
'CAG−170 sp003516765' on 'GCST90200748_buildGRCh38.tsv'

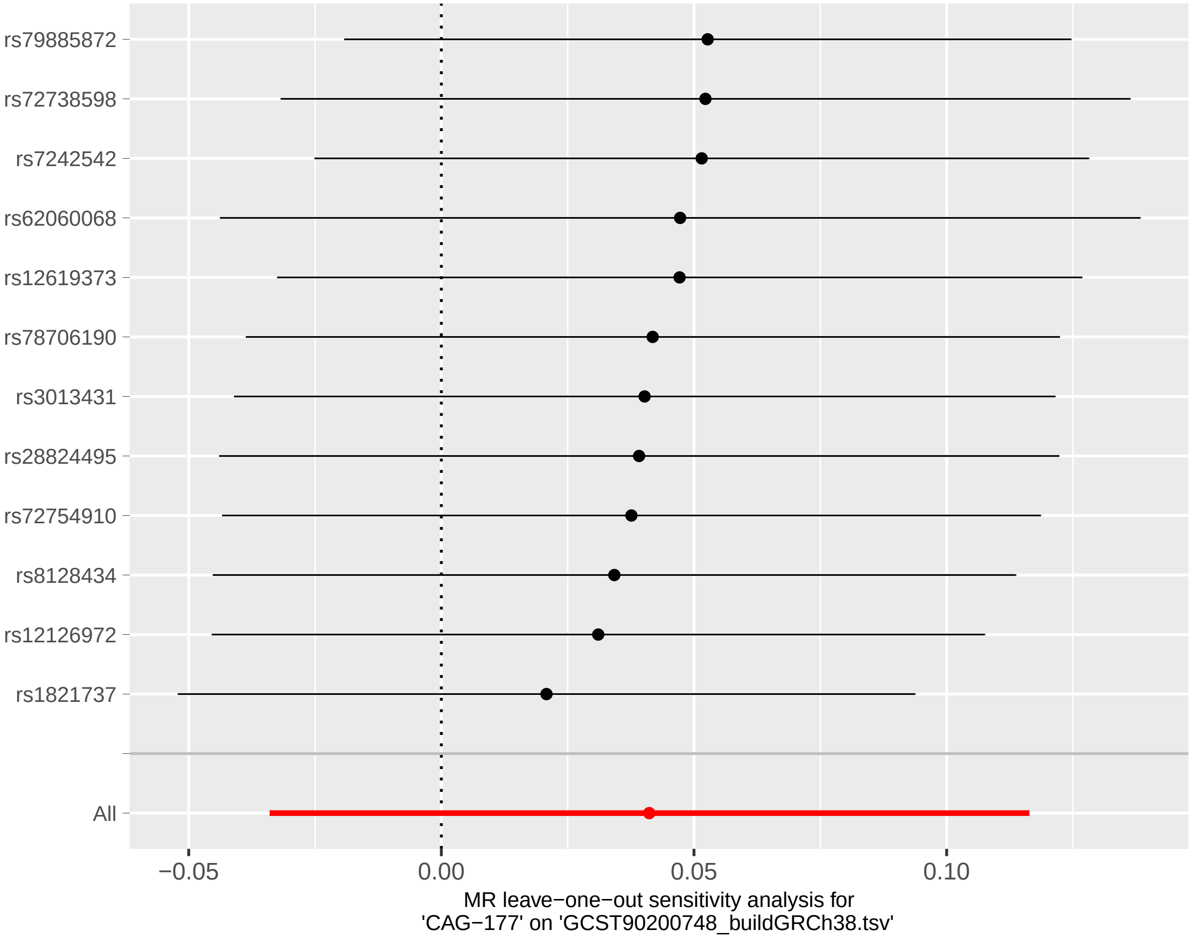
rs79885872
rs72738598
rs7242542
rs62060068
rs12619373
rs78706190
rs3013431
rs28824495
rs72754910
rs8128434
rs12126972
rs1821737
All
−0.05
0.00
0.05
0.10
MR leave−one−out sensitivity analysis for
'CAG−177' on 'GCST90200748_buildGRCh38.tsv'

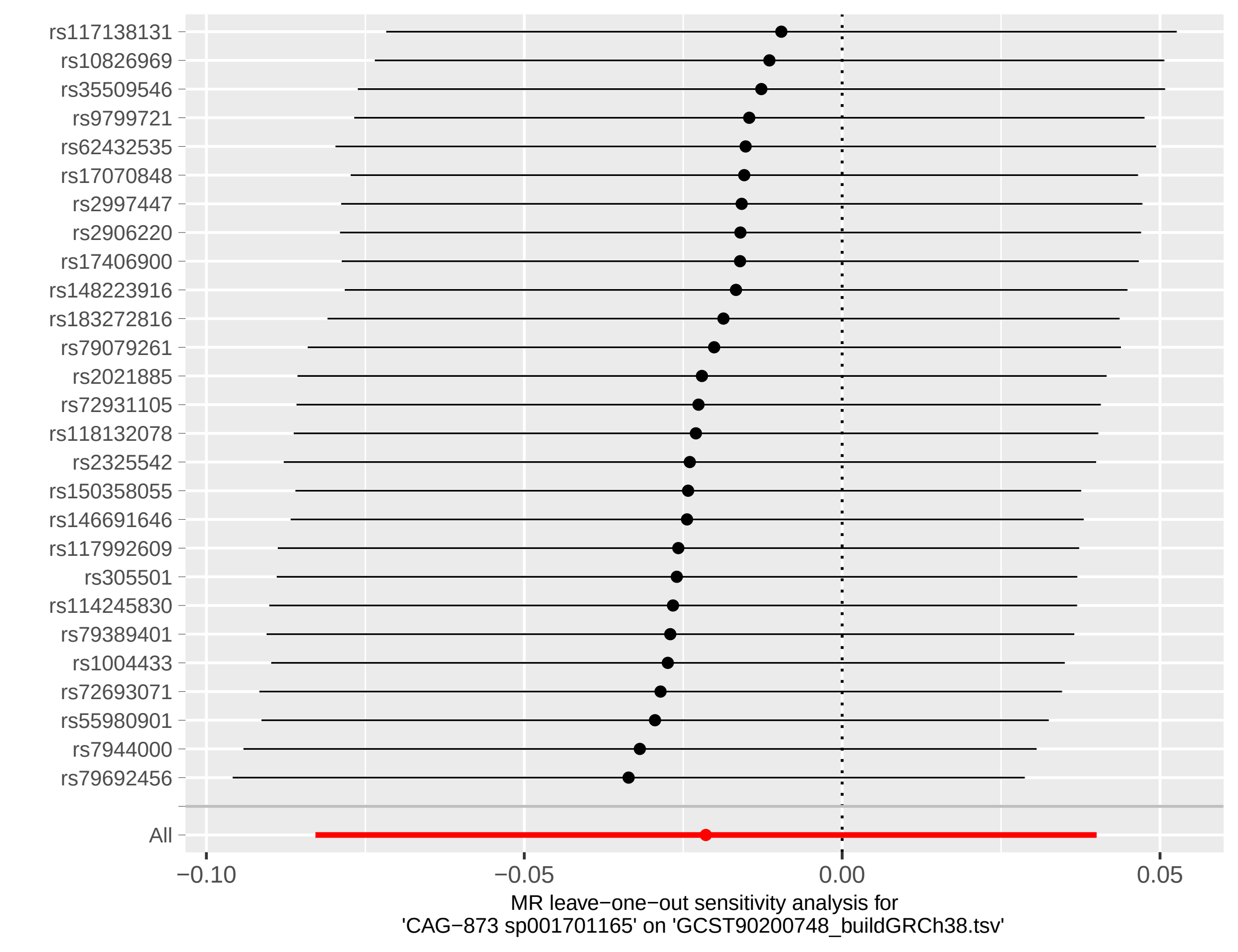

rs117138131
rs10826969
rs35509546
rs9799721
rs62432535
rs17070848
rs2997447
rs2906220
rs17406900
rs148223916
rs183272816
rs79079261
rs2021885
rs72931105
rs118132078
rs2325542
rs150358055
rs146691646
rs117992609
rs305501
rs114245830
rs79389401
rs1004433
rs72693071
rs55980901
rs7944000
rs79692456
All
−0.10
−0.05
0.00
0.05
MR leave−one−out sensitivity analysis for
'CAG−873 sp001701165' on 'GCST90200748_buildGRCh38.tsv'

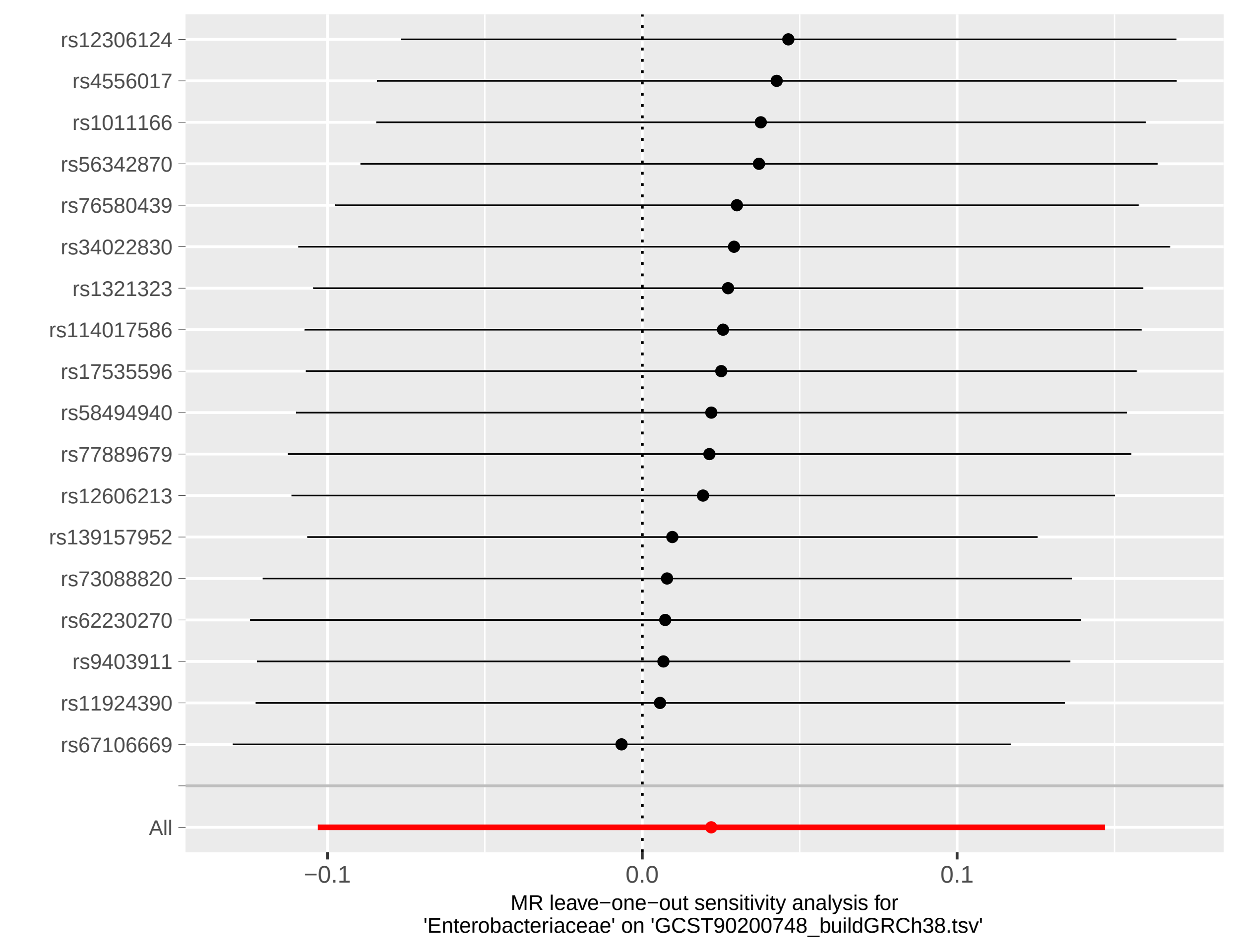
rs12306124
rs4556017
rs1011166
rs56342870
rs76580439
rs34022830
rs1321323
rs114017586
rs17535596
rs58494940
rs77889679
rs12606213
rs139157952
rs73088820
rs62230270
rs9403911
rs11924390
rs67106669
All
−0.1
0.0
0.1
MR leave−one−out sensitivity analysis for
'Enterobacteriaceae' on 'GCST90200748_buildGRCh38.tsv'

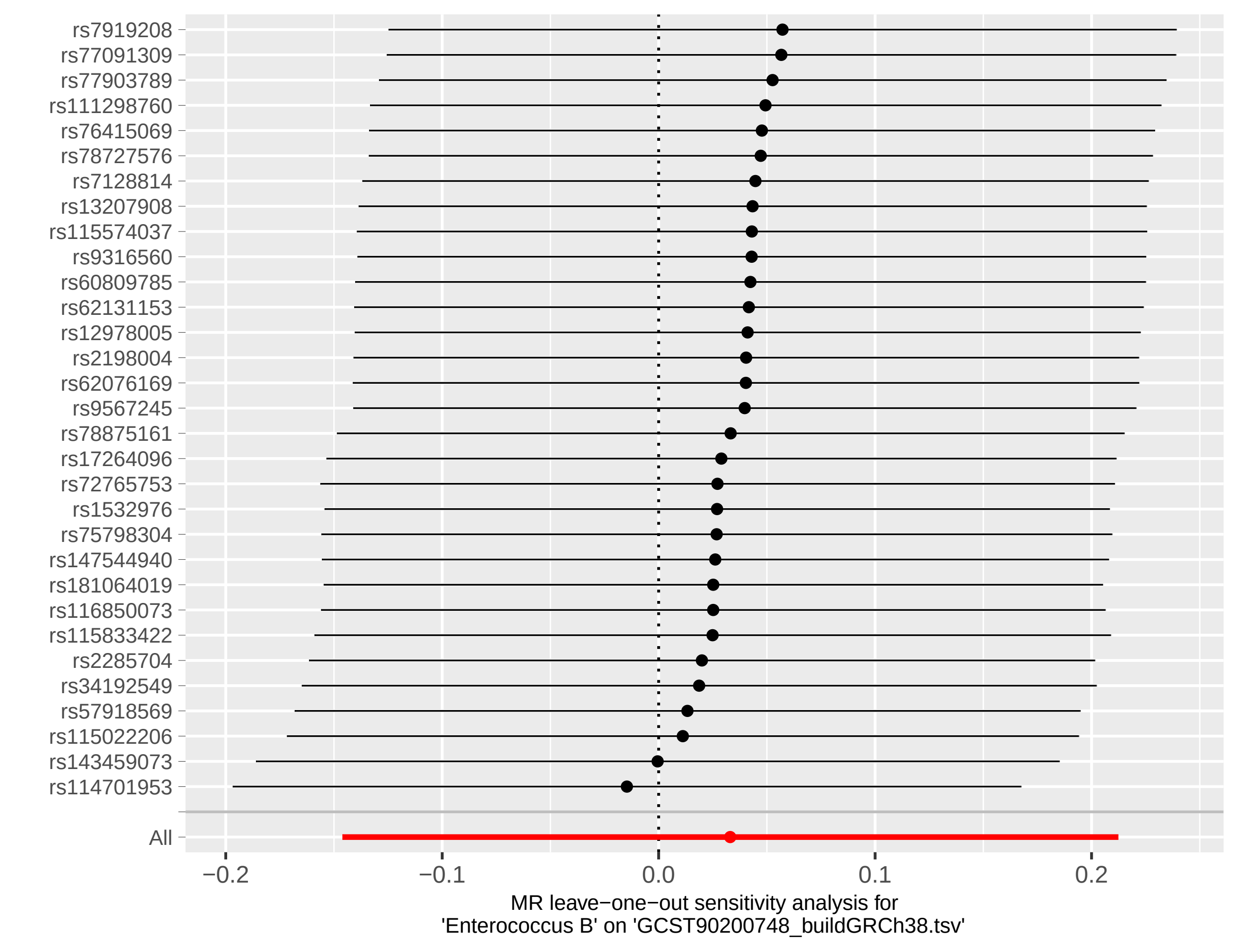
rs7919208
rs77091309
rs77903789
rs111298760
rs76415069
rs78727576
rs7128814
rs13207908
rs115574037
rs9316560
rs60809785
rs62131153
rs12978005
rs2198004
rs62076169
rs9567245
rs78875161
rs17264096
rs72765753
rs1532976
rs75798304
rs147544940
rs181064019
rs116850073
rs115833422
rs2285704
rs34192549
rs57918569
rs115022206
rs143459073
rs114701953
All
−0.2
−0.1
0.0
0.1
0.2
MR leave−one−out sensitivity analysis for
'Enterococcus B' on 'GCST90200748_buildGRCh38.tsv'

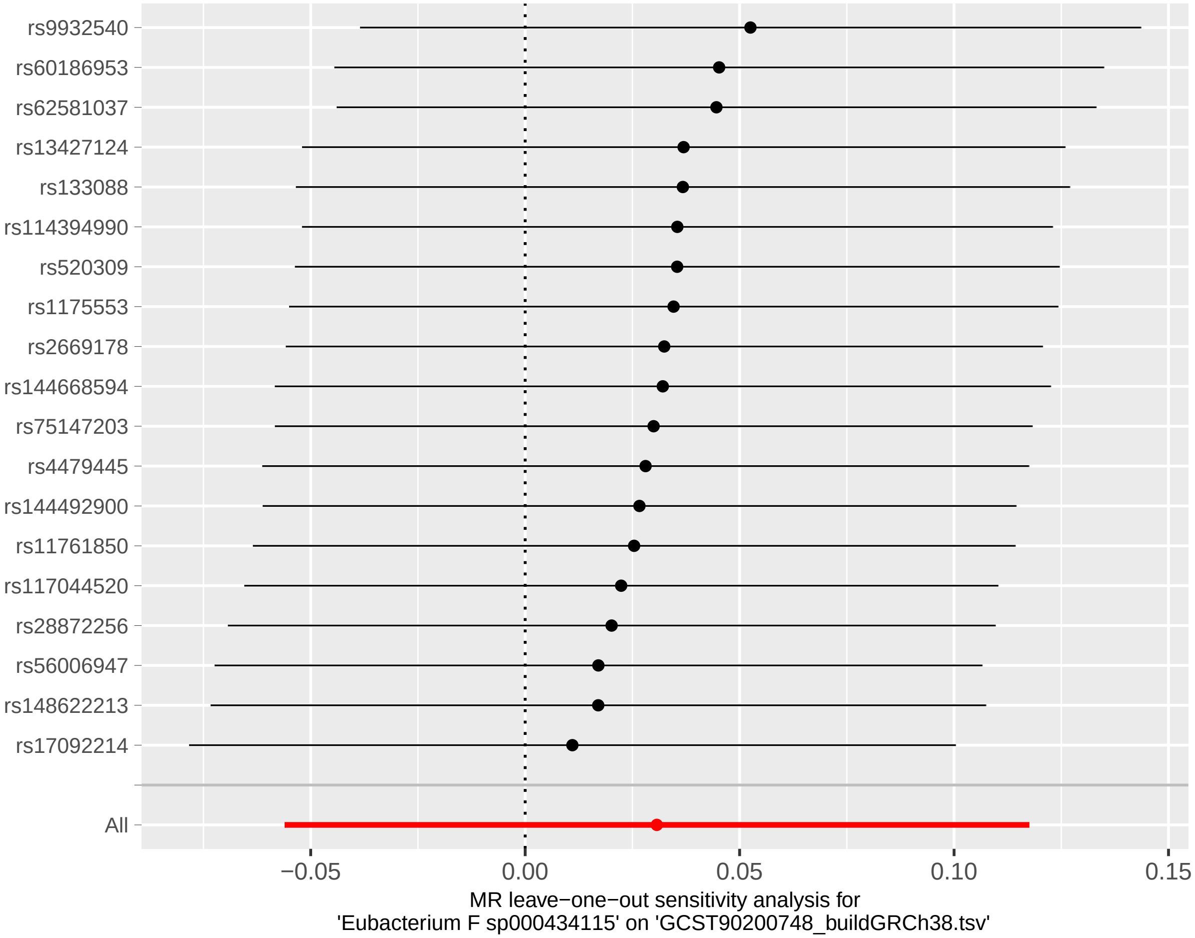
rs9932540
rs60186953
rs62581037
rs13427124
rs133088
rs114394990
rs520309
rs1175553
rs2669178
rs144668594
rs75147203
rs4479445
rs144492900
rs11761850
rs117044520
rs28872256
rs56006947
rs148622213
rs17092214
All
−0.05
0.00
0.05
0.10
0.15
MR leave−one−out sensitivity analysis for
'Eubacterium F sp000434115' on 'GCST90200748_buildGRCh38.tsv'

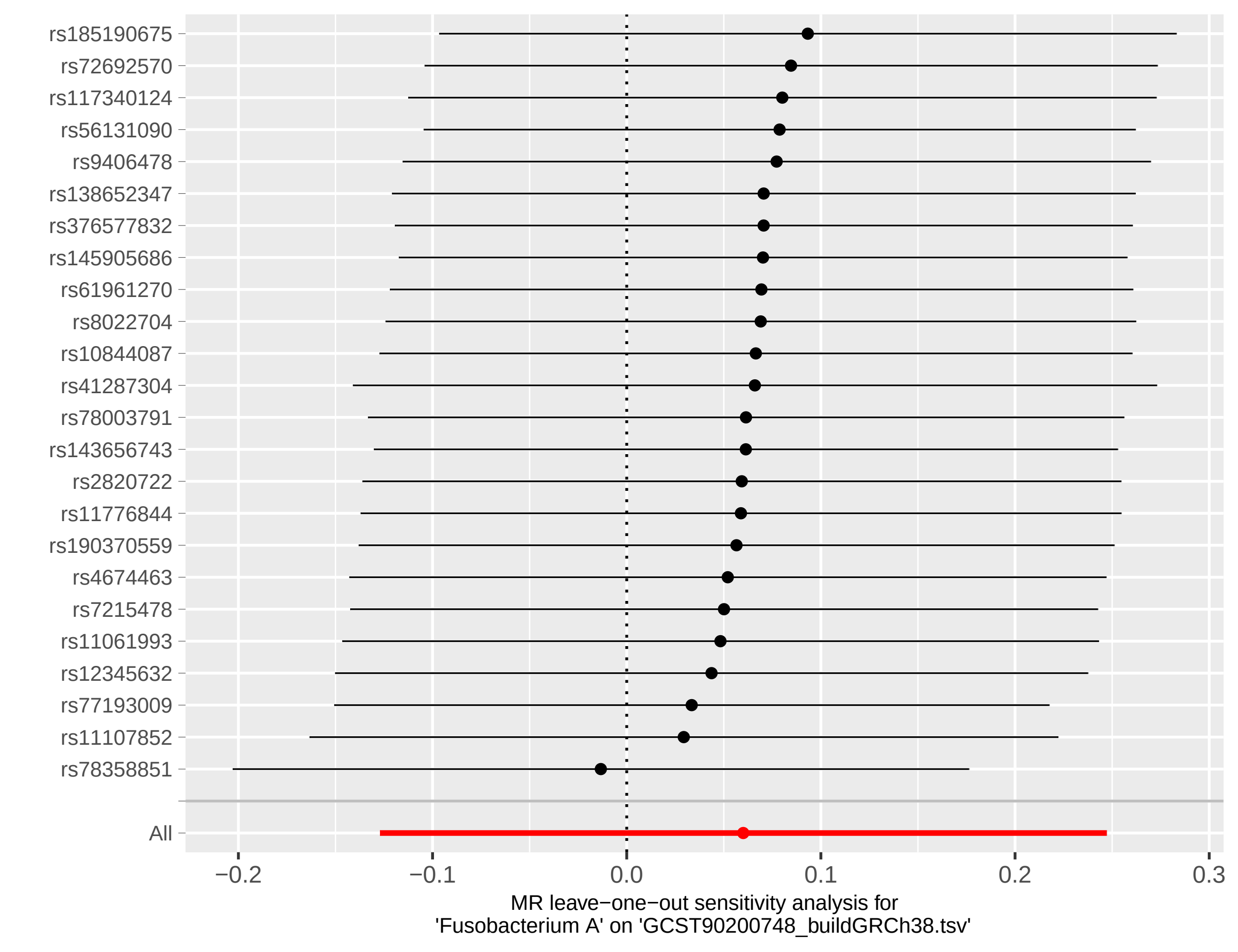

rs185190675
rs72692570
rs117340124
rs56131090
rs9406478
rs138652347
rs376577832
rs145905686
rs61961270
rs8022704
rs10844087
rs41287304
rs78003791
rs143656743
rs2820722
rs11776844
rs190370559
rs4674463
rs7215478
rs11061993
rs12345632
rs77193009
rs11107852
rs78358851
All
−0.2
−0.1
0.0
0.1
0.2
0.3
MR leave−one−out sensitivity analysis for
'Fusobacterium A' on 'GCST90200748_buildGRCh38.tsv'

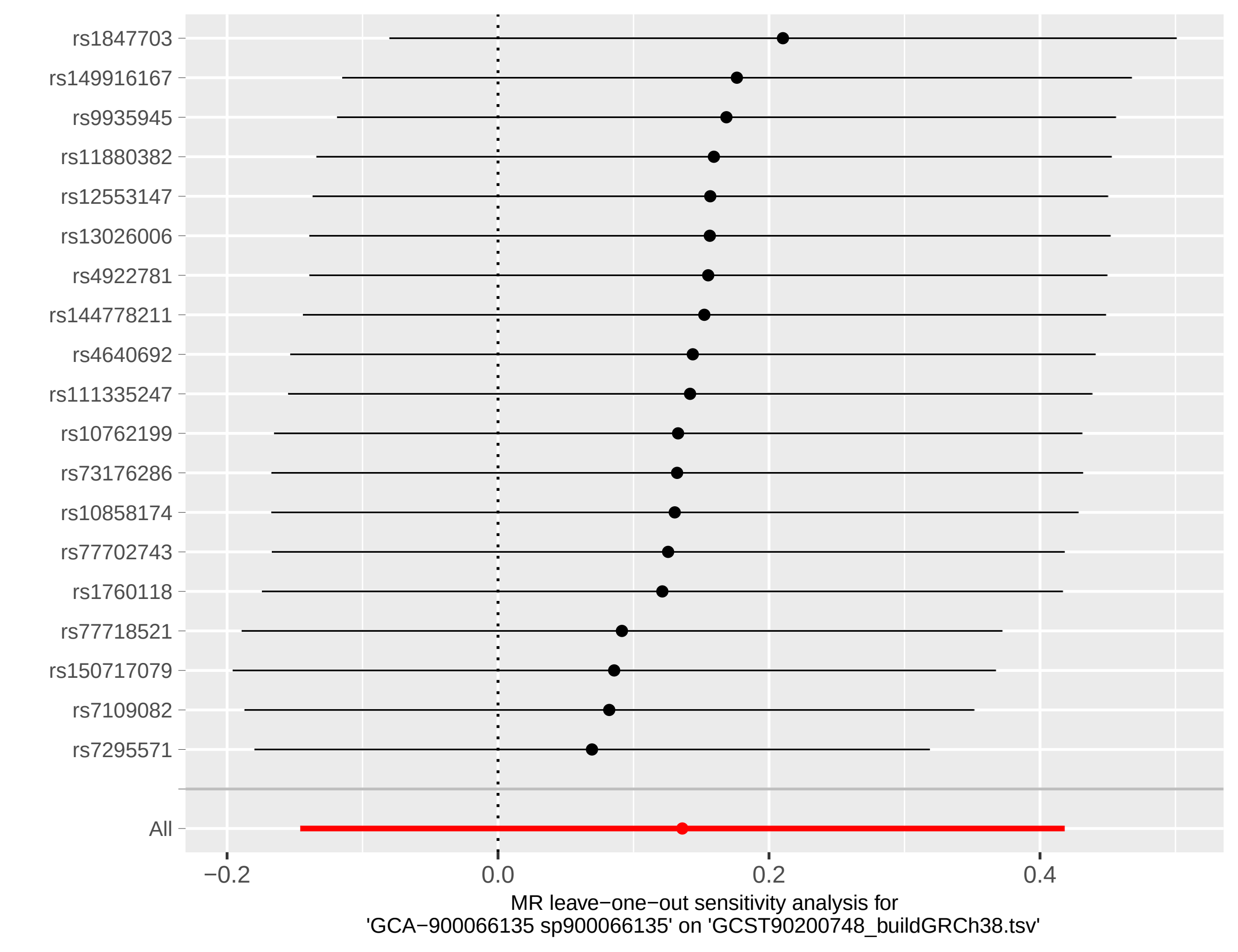

rs1847703
rs149916167
rs9935945
rs11880382
rs12553147
rs13026006
rs4922781
rs144778211
rs4640692
rs111335247
rs10762199
rs73176286
rs10858174
rs77702743
rs1760118
rs77718521
rs150717079
rs7109082
rs7295571
All
−0.2
0.0
0.2
0.4
MR leave−one−out sensitivity analysis for
'GCA−900066135 sp900066135' on 'GCST90200748_buildGRCh38.tsv'

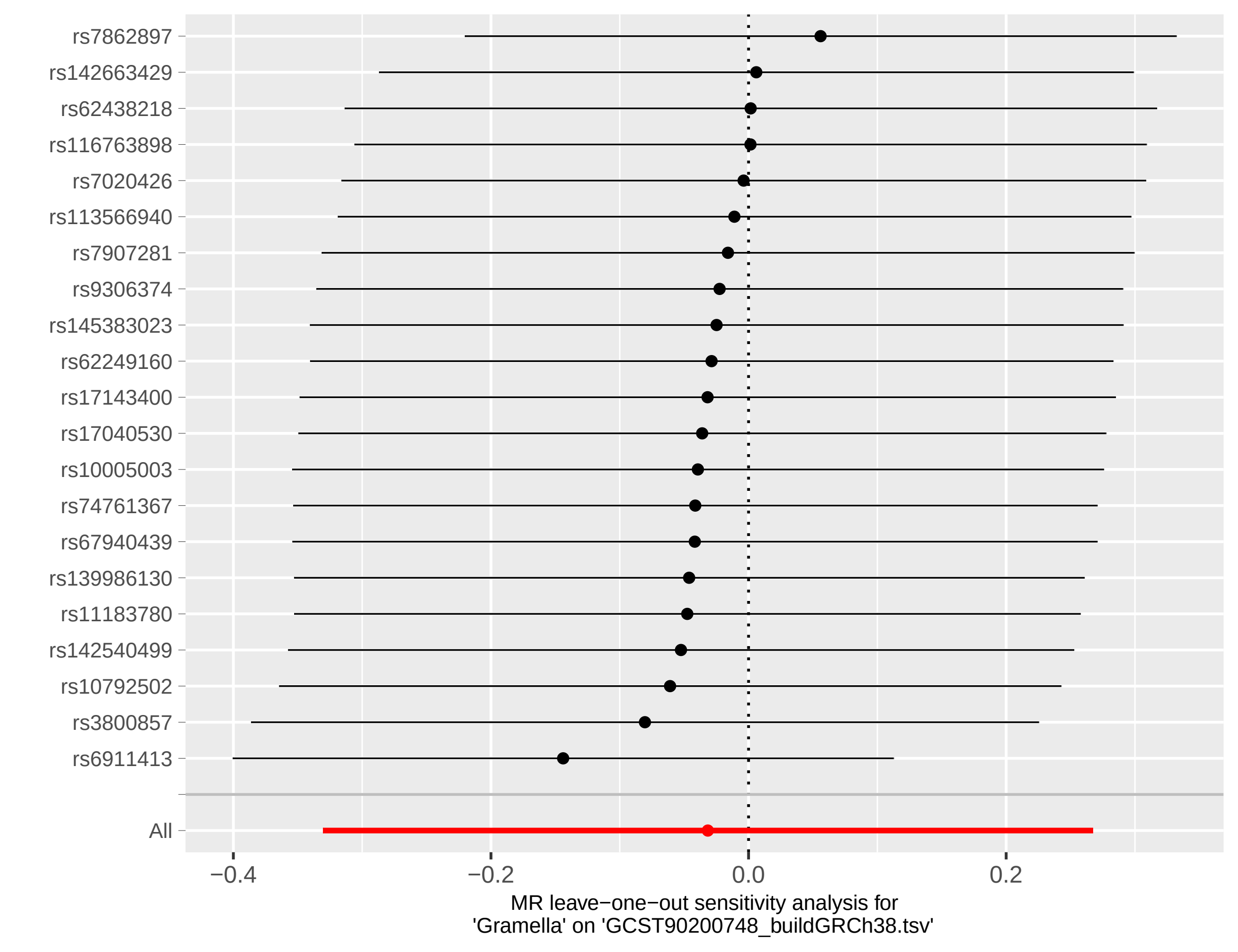

rs7862897
rs142663429
rs62438218
rs116763898
rs7020426
rs113566940
rs7907281
rs9306374
rs145383023
rs62249160
rs17143400
rs17040530
rs10005003
rs74761367
rs67940439
rs139986130
rs11183780
rs142540499
rs10792502
rs3800857
rs6911413
All
−0.4
−0.2
0.0
0.2
MR leave−one−out sensitivity analysis for
'Gramella' on 'GCST90200748_buildGRCh38.tsv'

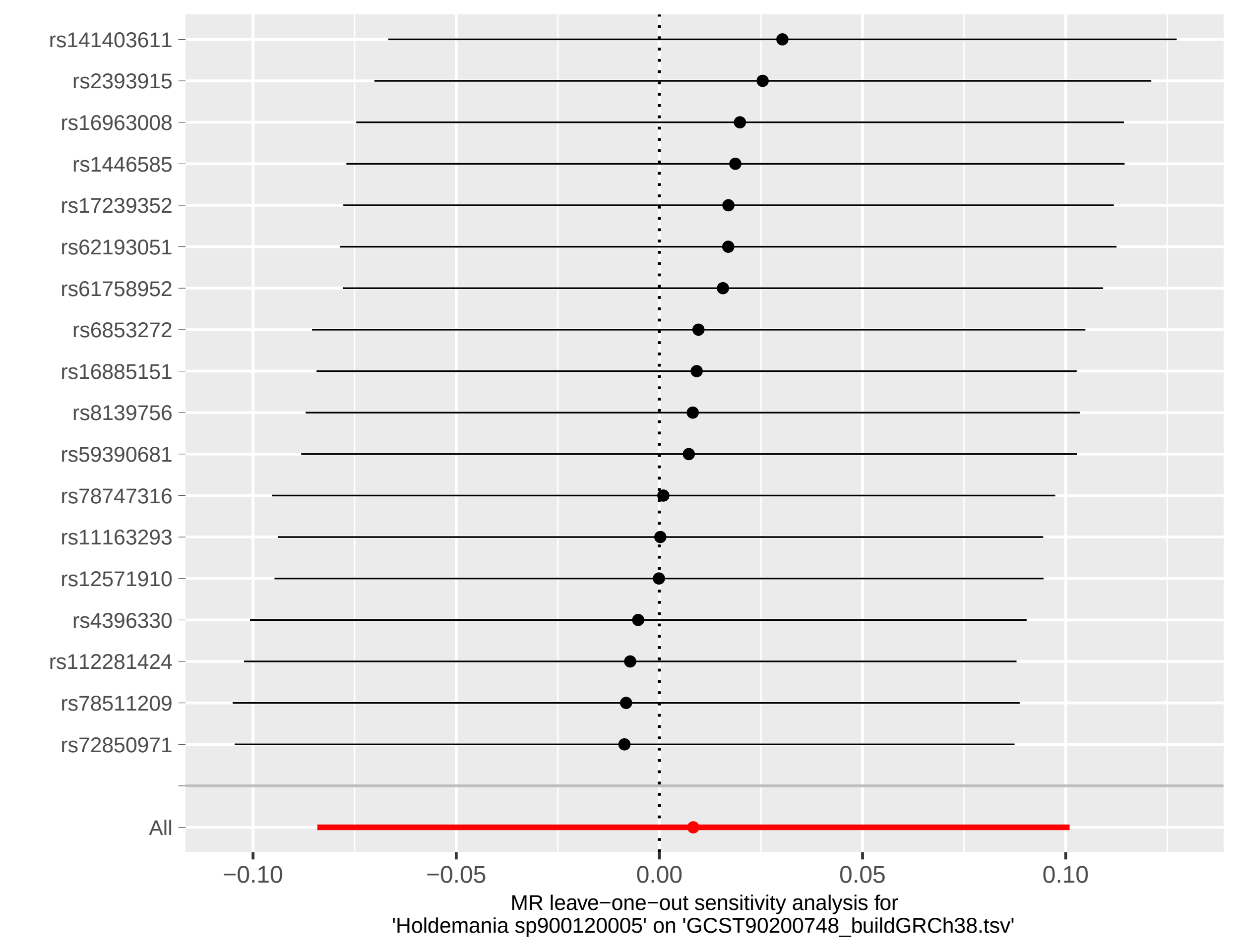

rs141403611
rs2393915
rs16963008
rs1446585
rs17239352
rs62193051
rs61758952
rs6853272
rs16885151
rs8139756
rs59390681
rs78747316
rs11163293
rs12571910
rs4396330
rs112281424
rs78511209
rs72850971
All
−0.10
−0.05
0.00
0.05
0.10
MR leave−one−out sensitivity analysis for
'Holdemania sp900120005' on 'GCST90200748_buildGRCh38.tsv'

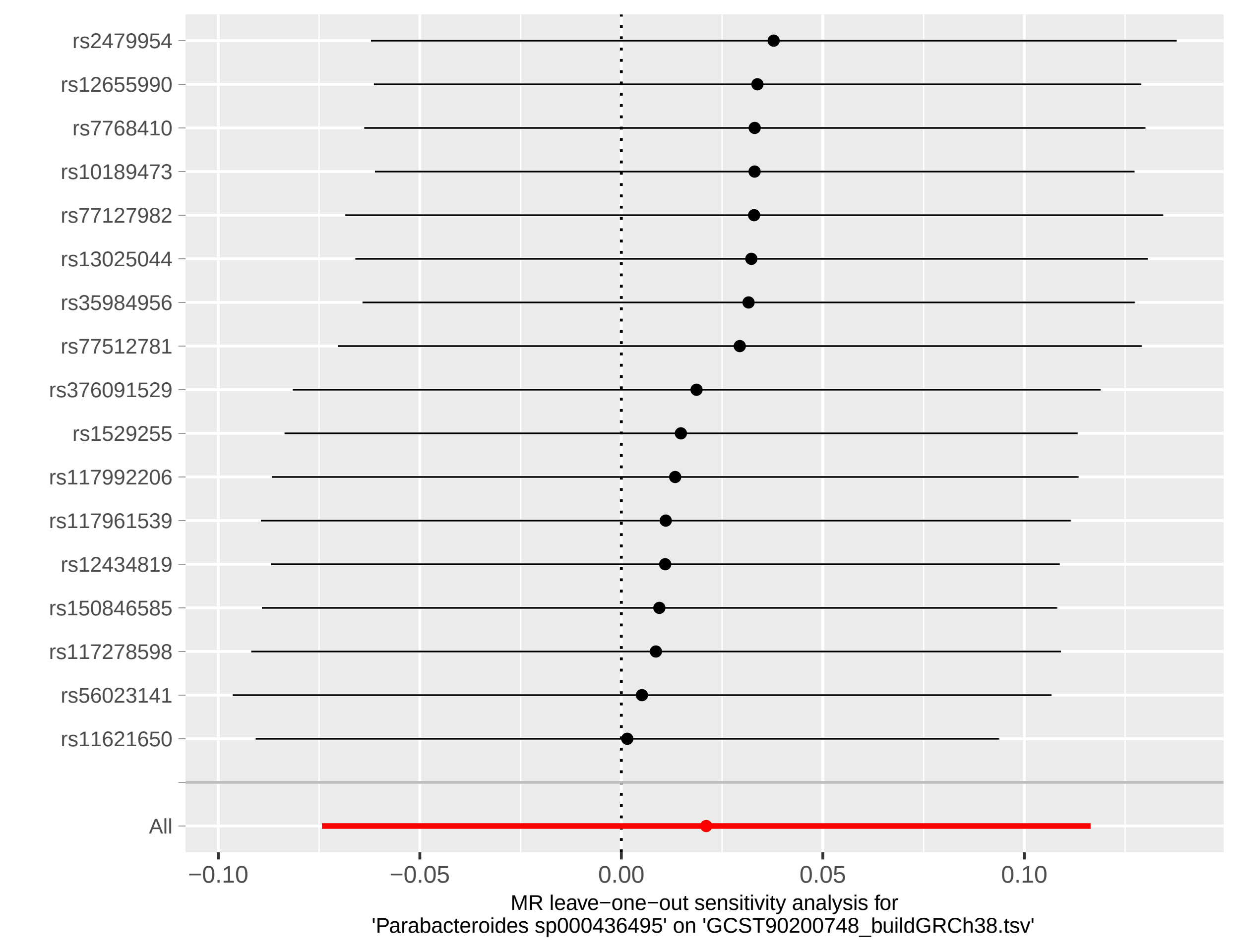

rs2479954
rs12655990
rs7768410
rs10189473
rs77127982
rs13025044
rs35984956
rs77512781
rs376091529
rs1529255
rs117992206
rs117961539
rs12434819
rs150846585
rs117278598
rs56023141
rs11621650
All
−0.10
−0.05
0.00
0.05
0.10
MR leave−one−out sensitivity analysis for
'Parabacteroides sp000436495' on 'GCST90200748_buildGRCh38.tsv'

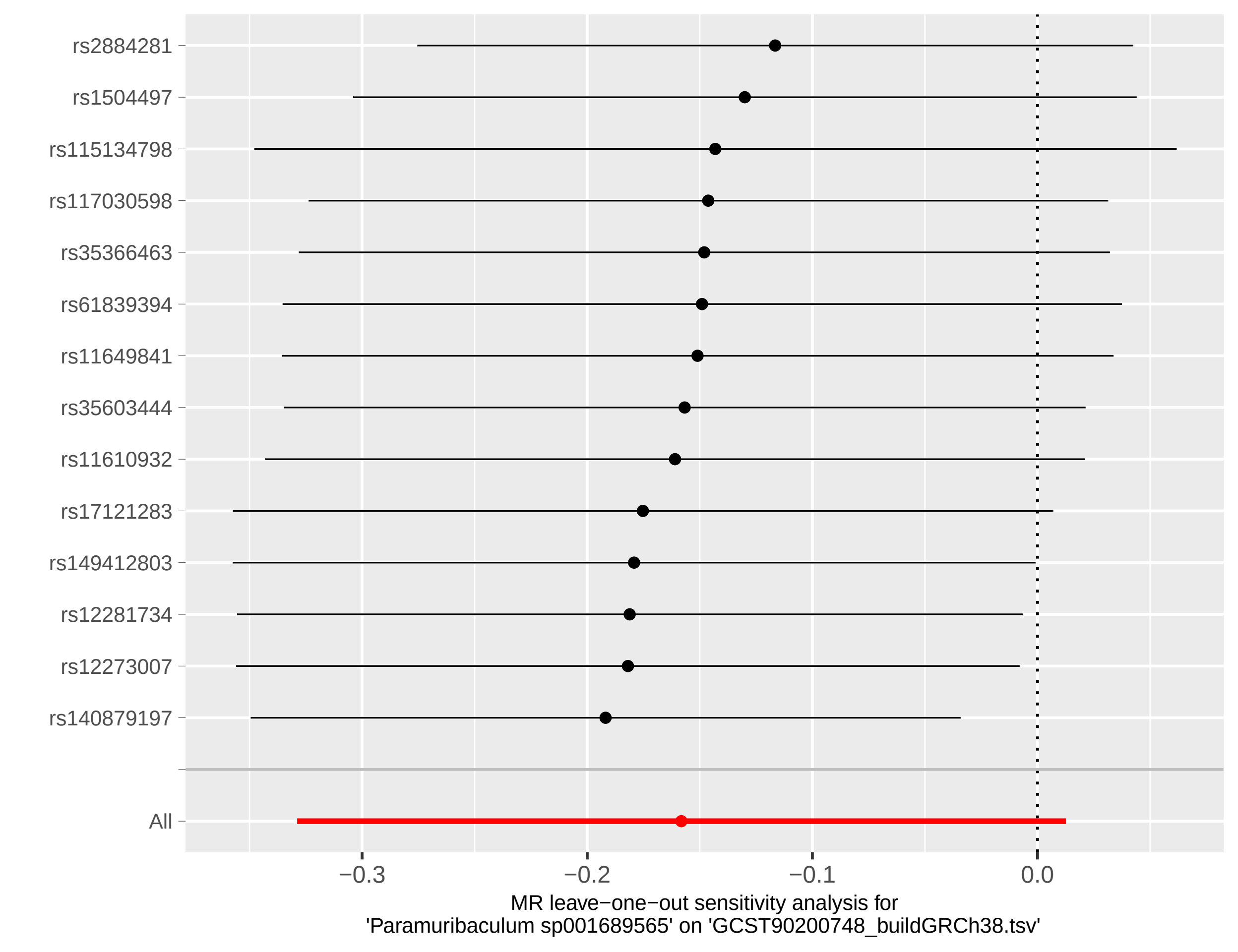
rs2884281
rs1504497
rs115134798
rs117030598
rs35366463
rs61839394
rs11649841
rs35603444
rs11610932
rs17121283
rs149412803
rs12281734
rs12273007
rs140879197
All
−0.3
−0.2
−0.1
0.0
MR leave−one−out sensitivity analysis for
'Paramuribaculum sp001689565' on 'GCST90200748_buildGRCh38.tsv'

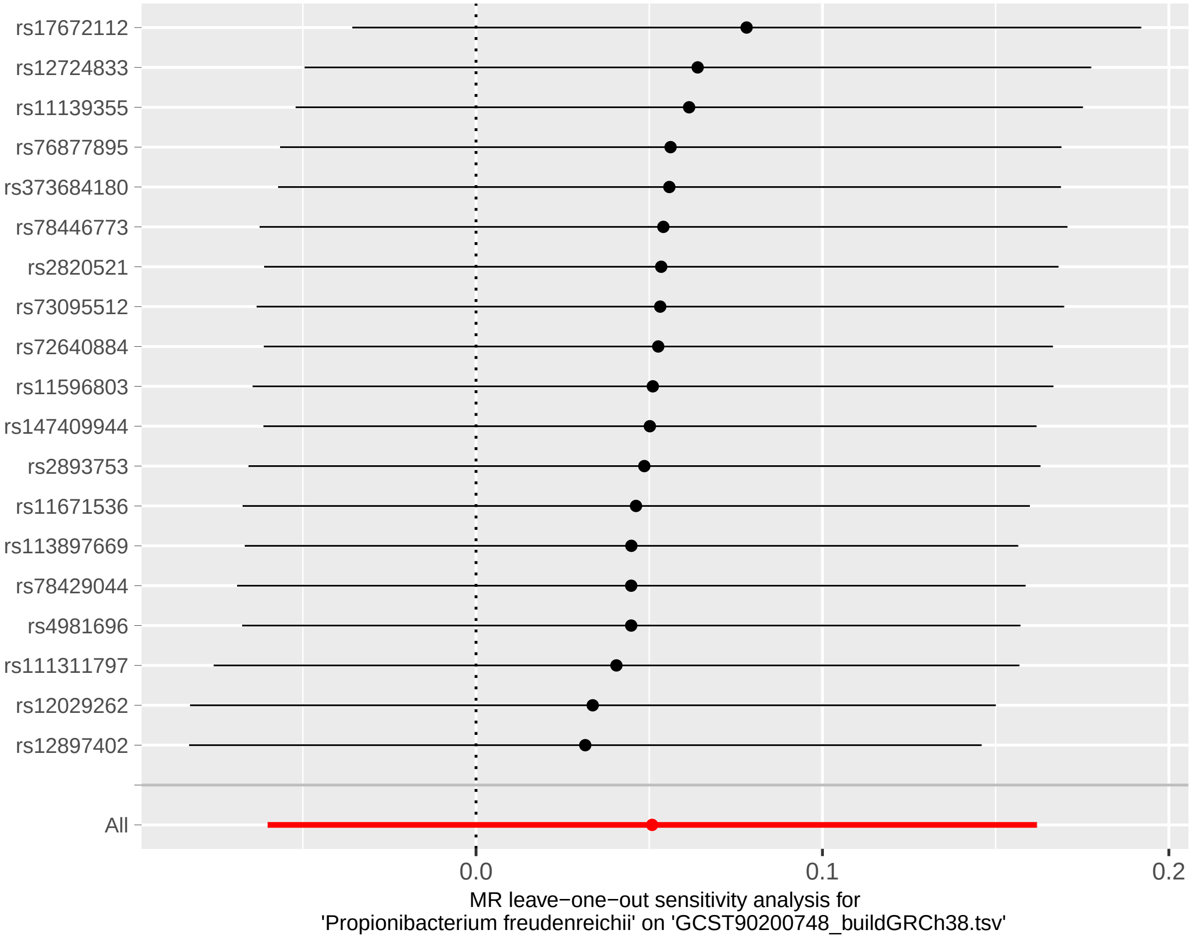
rs17672112
rs12724833
rs11139355
rs76877895
rs373684180
rs78446773
rs2820521
rs73095512
rs72640884
rs11596803
rs147409944
rs2893753
rs11671536
rs113897669
rs78429044
rs4981696
rs111311797
rs12029262
rs12897402
All
0.0
0.1
0.2
MR leave−one−out sensitivity analysis for
'Propionibacterium freudenreichii' on 'GCST90200748_buildGRCh38.tsv'

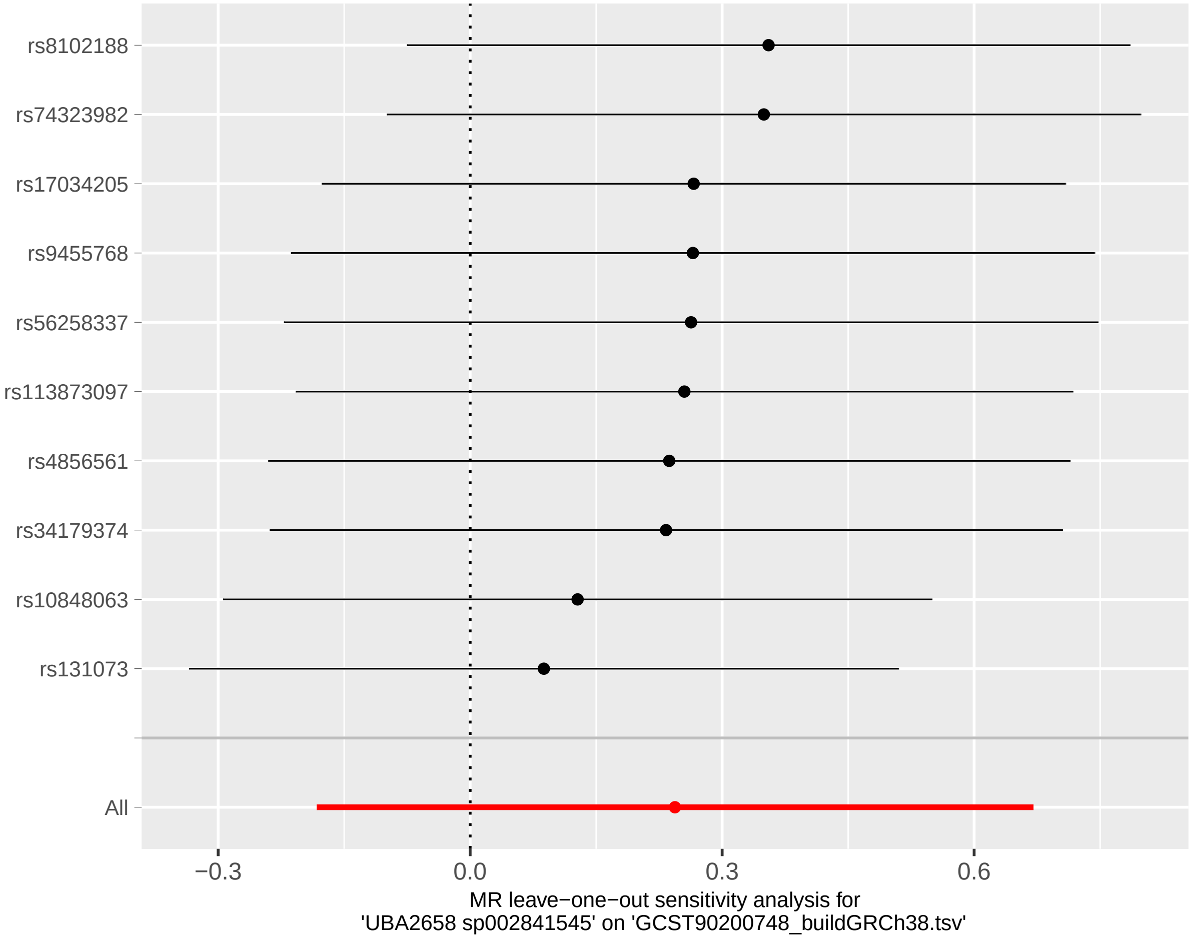
rs8102188
rs74323982
rs17034205
rs9455768
rs56258337
rs113873097
rs4856561
rs34179374
rs10848063
rs131073
All
−0.3
0.0
0.3
0.6
MR leave−one−out sensitivity analysis for
'UBA2658 sp002841545' on 'GCST90200748_buildGRCh38.tsv'

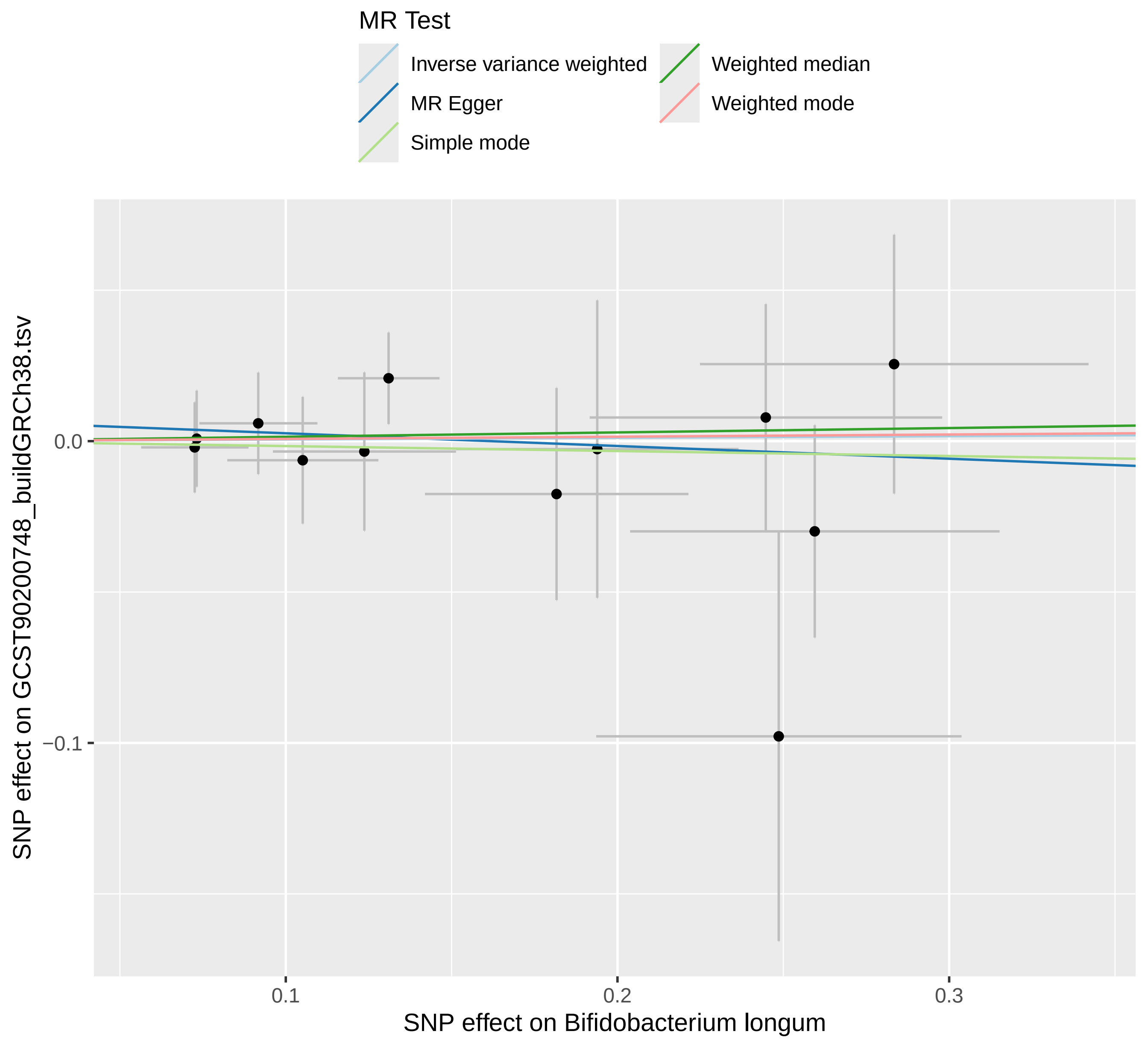

MR Test
Inverse variance weighted
MR Egger
Simple mode
Weighted median
Weighted mode
0.0
−0.1
0.1
0.2
0.3
SNP effect on GCST90200748_buildGRCh38.tsv
SNP effect on Bifidobacterium longum

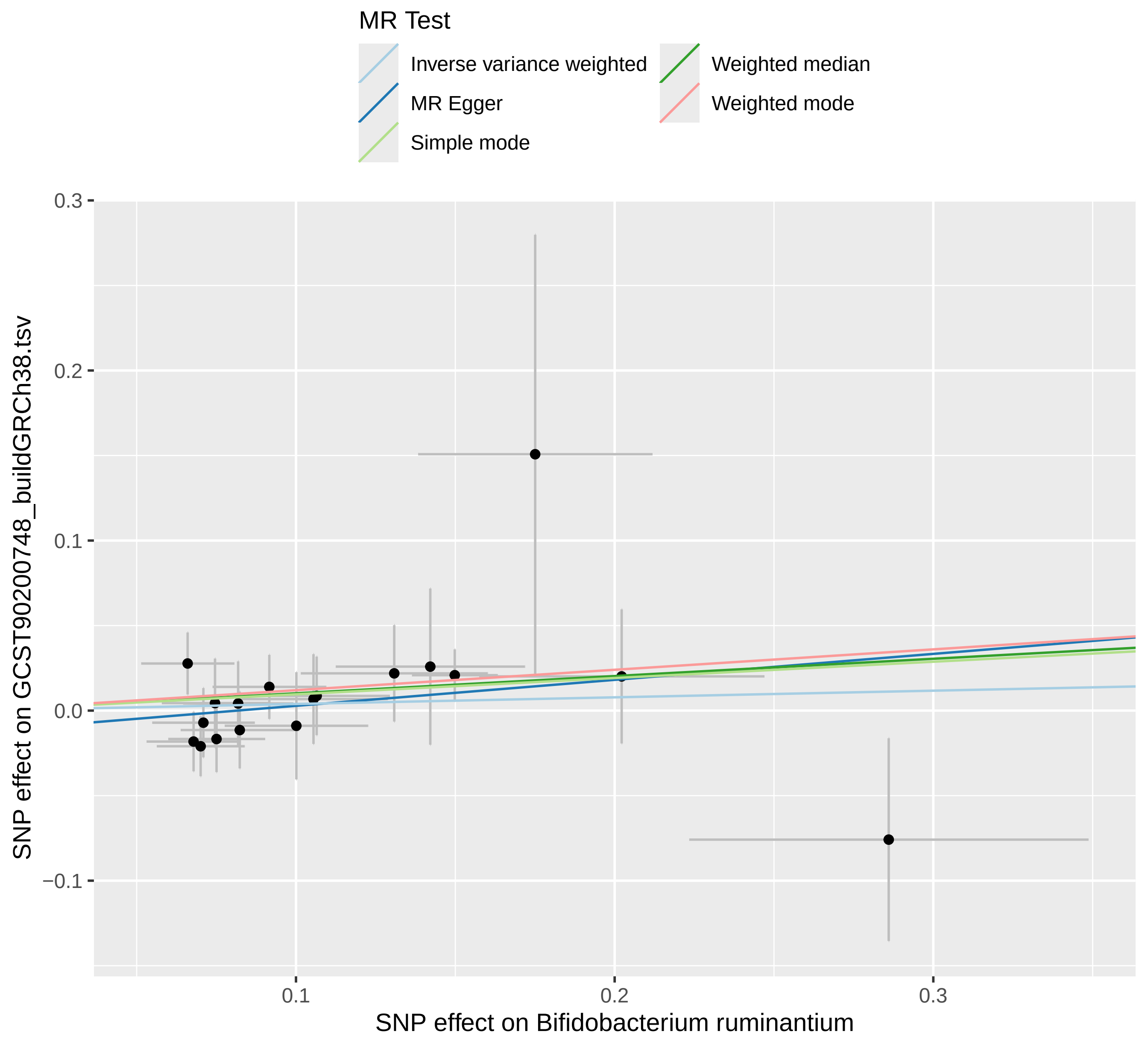
MR Test
Inverse variance weighted
MR Egger
Simple mode
Weighted median
Weighted mode
0.3
0.2
0.1
0.0
−0.1
SNP effect on GCST90200748_buildGRCh38.tsv
0.1
0.2
0.3
SNP effect on Bifidobacterium ruminantium

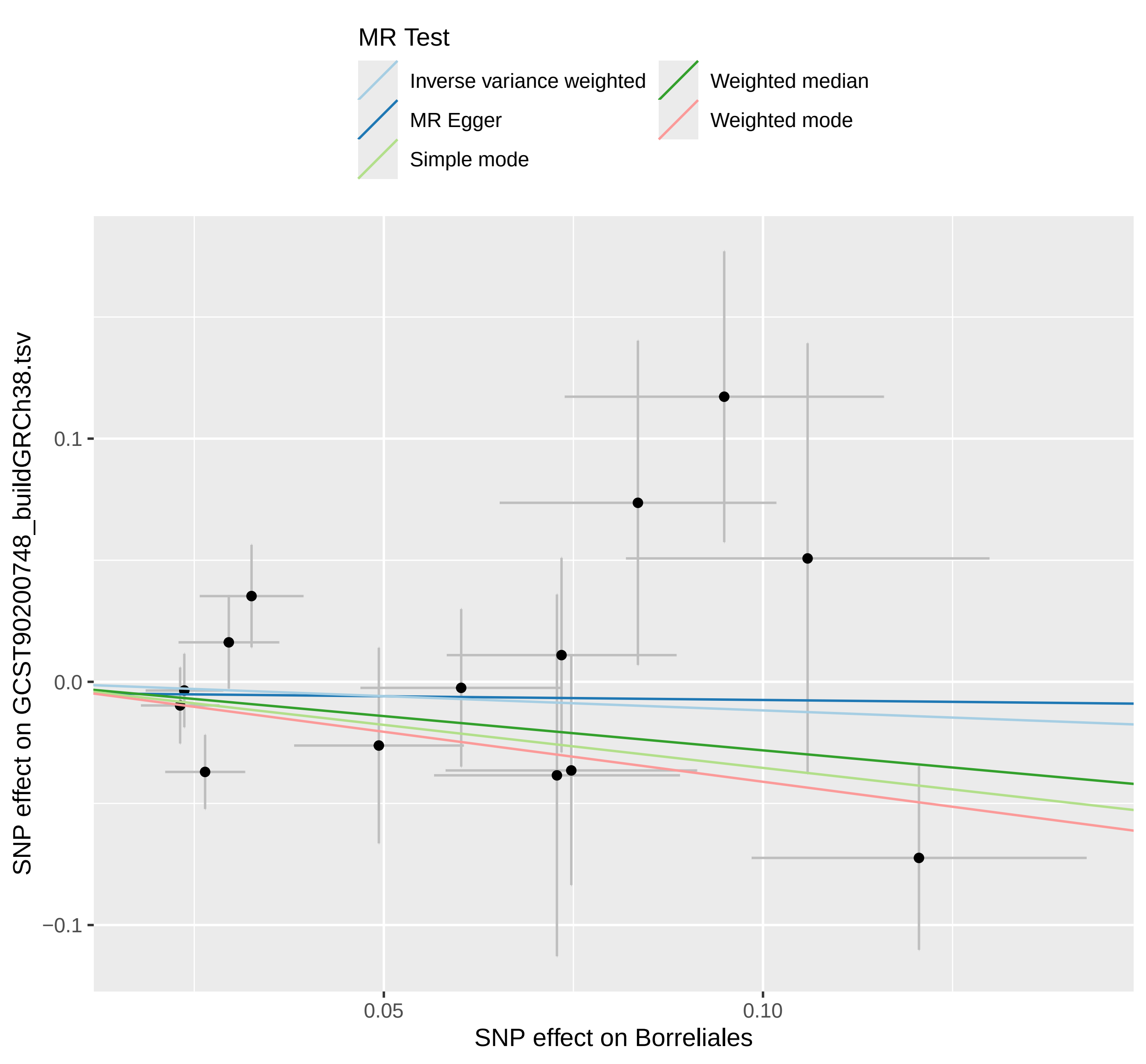
MR Test
Inverse variance weighted
MR Egger
Simple mode
Weighted median
Weighted mode
SNP effect on GCST90200748_buildGRCh38.tsv
0.1
0.0
−0.1
0.05
0.10
SNP effect on Borreliales

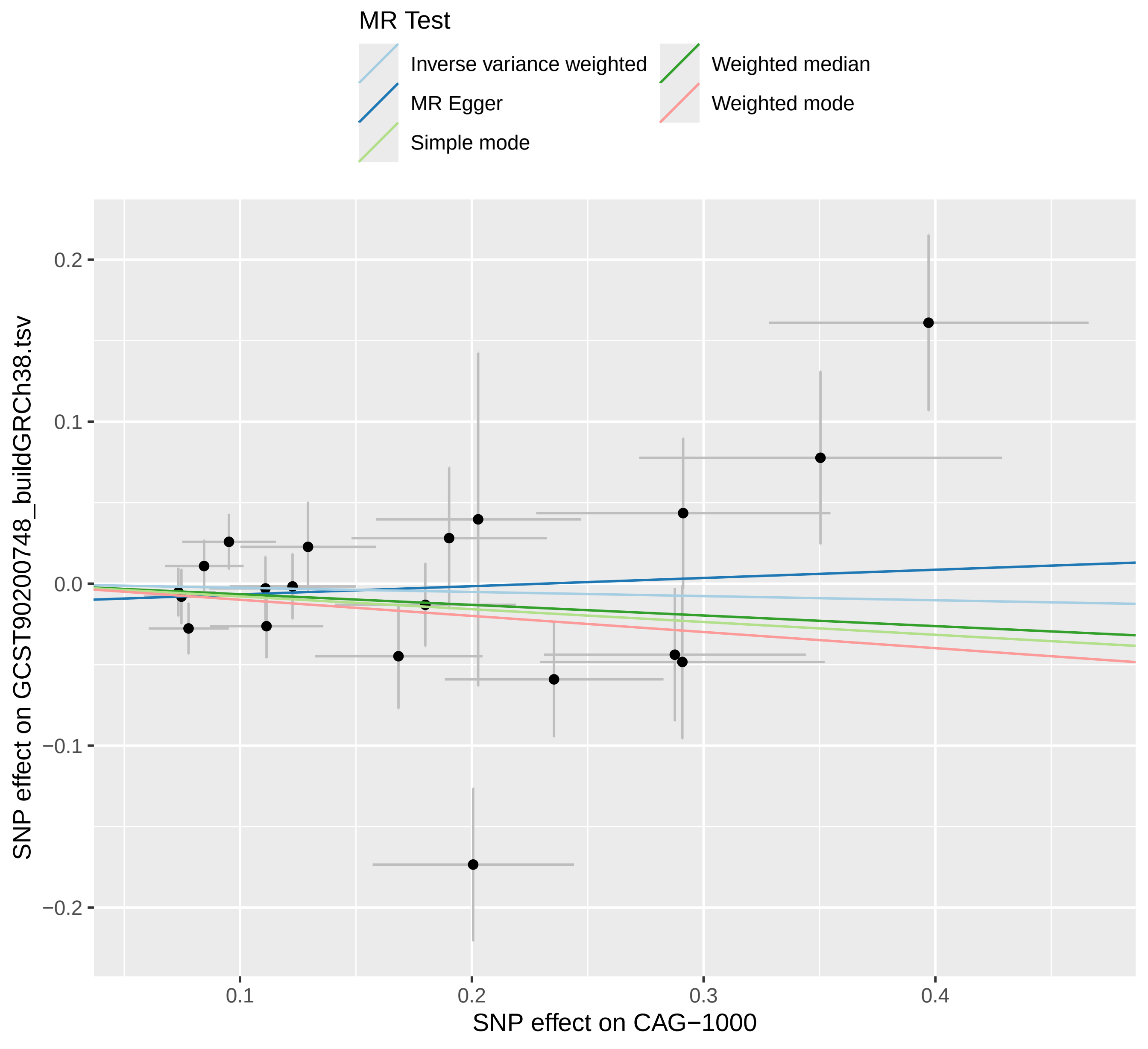
MR Test
Inverse variance weighted
MR Egger
Simple mode
Weighted median
Weighted mode
SNP effect on GCST90200748_buildGRCh38.tsv
SNP effect on CAG−1000
0.2
0.1
0.0
−0.1
−0.2
0.1
0.2
0.3
0.4

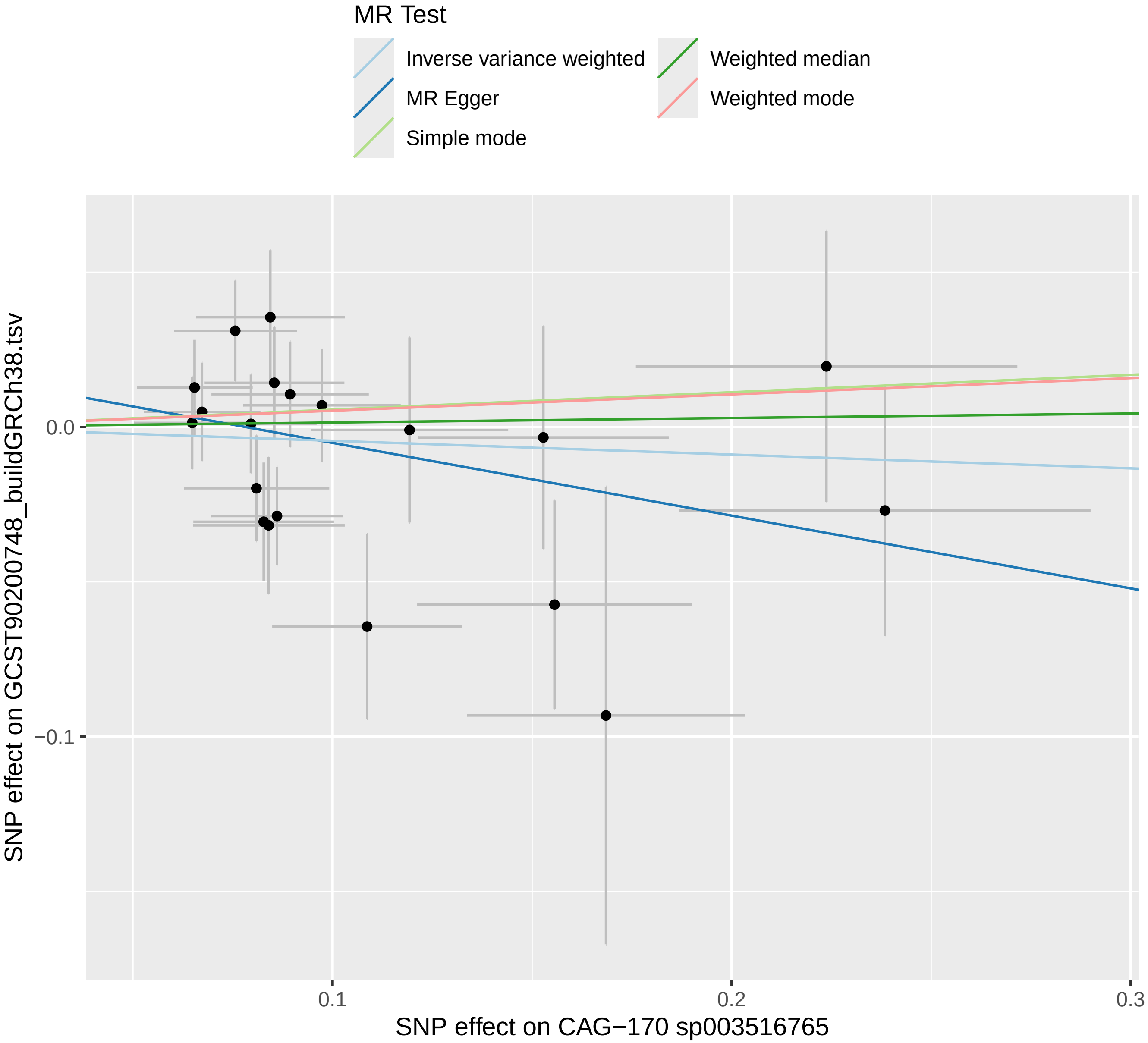
MR Test
Inverse variance weighted
MR Egger
Simple mode
Weighted median
Weighted mode
0.0
−0.1
0.1
0.2
0.3
SNP effect on GCST90200748_buildGRCh38.tsv
SNP effect on CAG−170 sp003516765

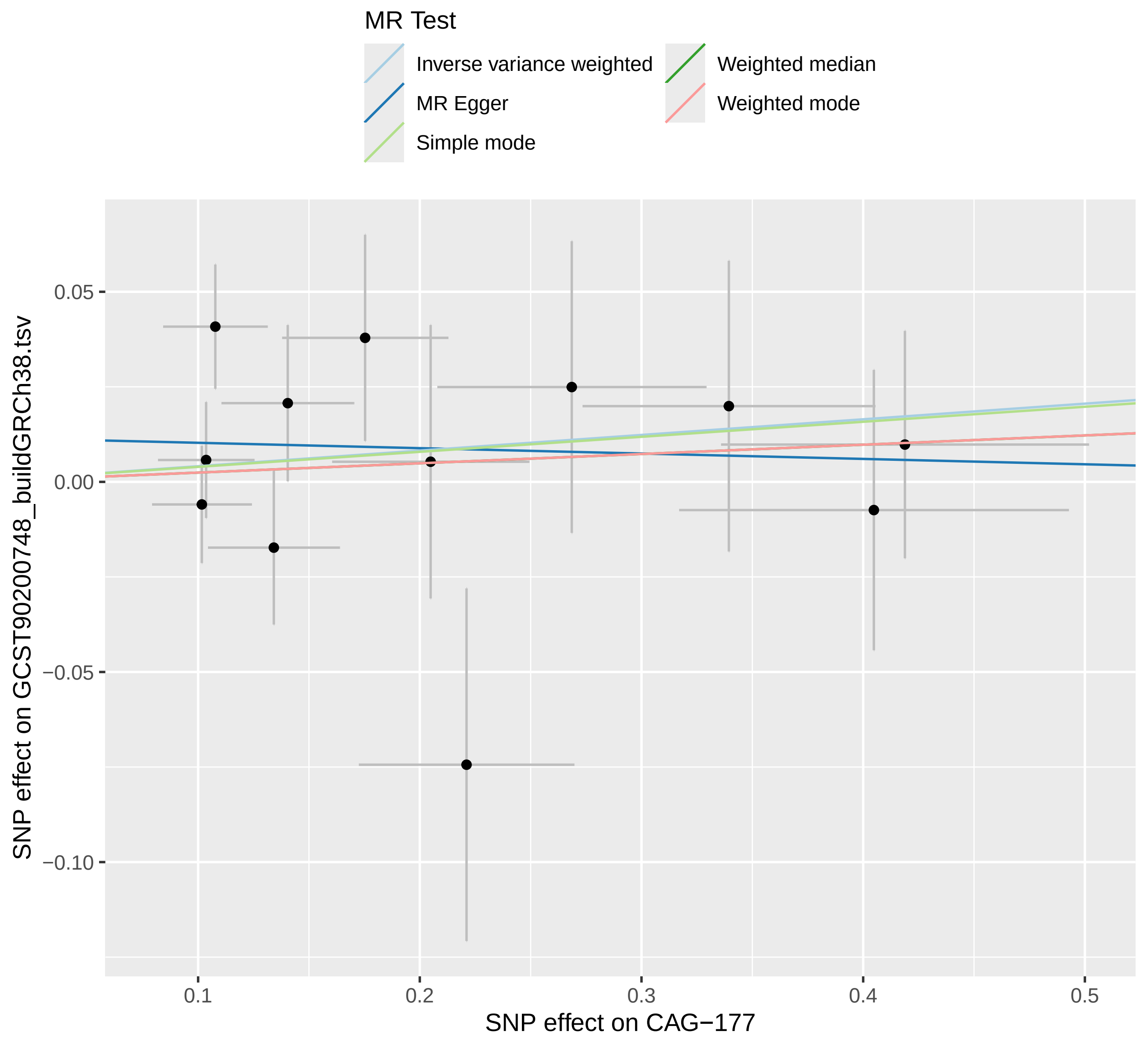

MR Test
Inverse variance weighted
MR Egger
Simple mode
Weighted median
Weighted mode
0.05
0.00
−0.05
−0.10
SNP effect on GCST90200748_buildGRCh38.tsv
0.1
0.2
0.3
0.4
0.5
SNP effect on CAG−177

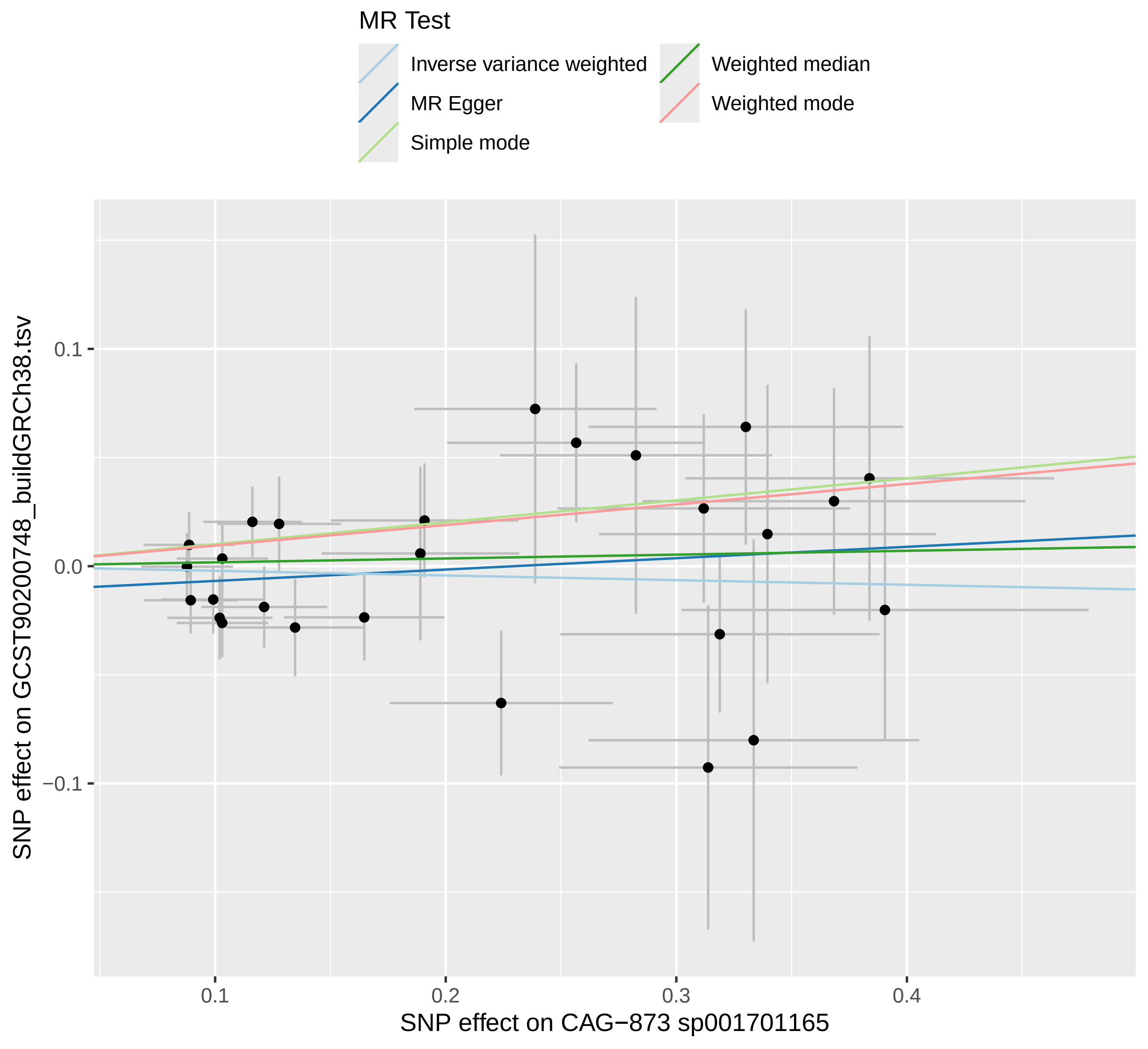

MR Test
Inverse variance weighted
MR Egger
Simple mode
Weighted median
Weighted mode
SNP effect on GCST90200748_buildGRCh38.tsv
0.1
0.0
−0.1
0.1
0.2
0.3
0.4
SNP effect on CAG−873 sp001701165

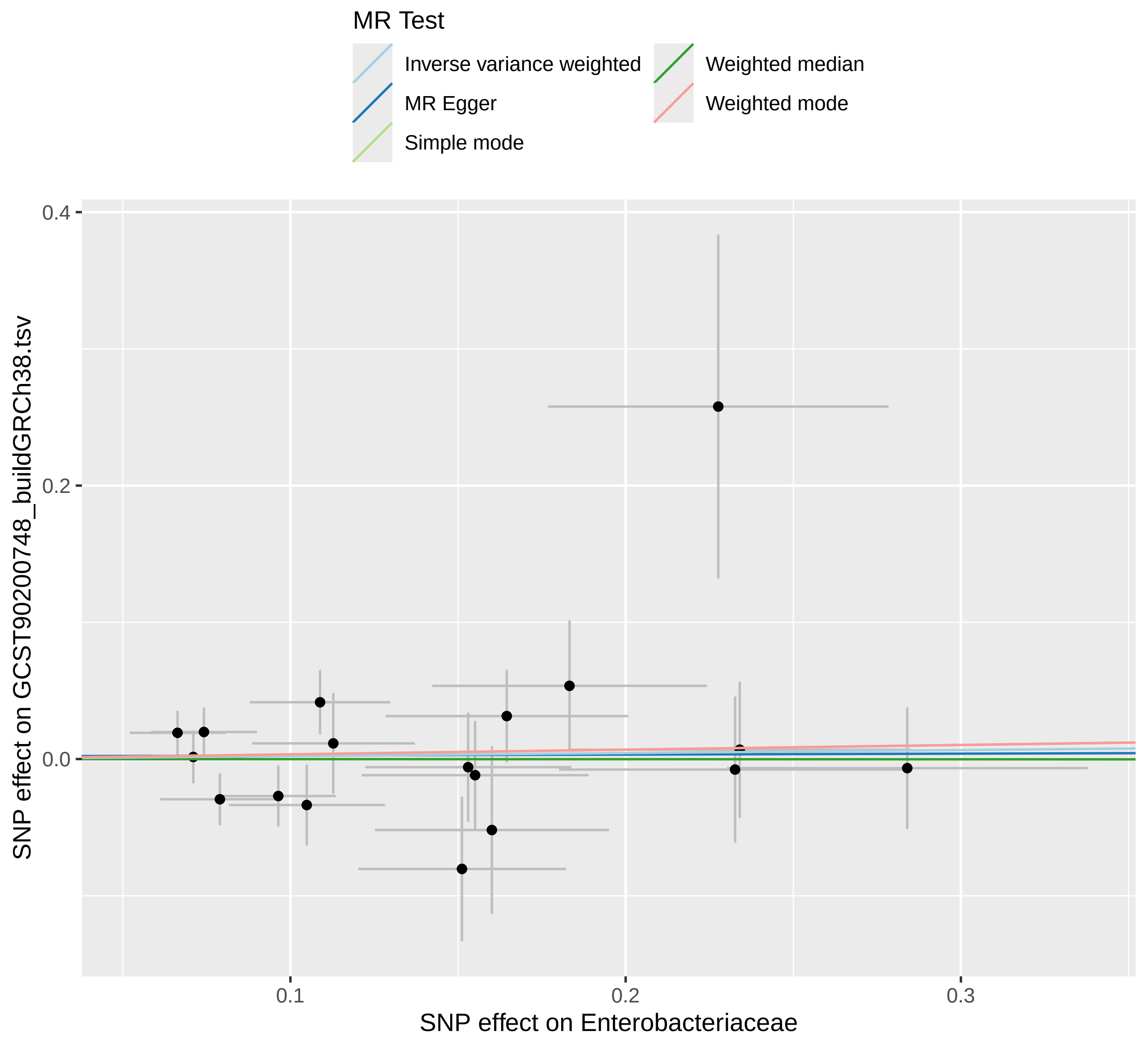

MR Test
Inverse variance weighted
MR Egger
Simple mode
Weighted median
Weighted mode
0.4
0.2
0.0
0.1
0.2
0.3
SNP effect on GCST90200748_buildGRCh38.tsv
SNP effect on Enterobacteriaceae

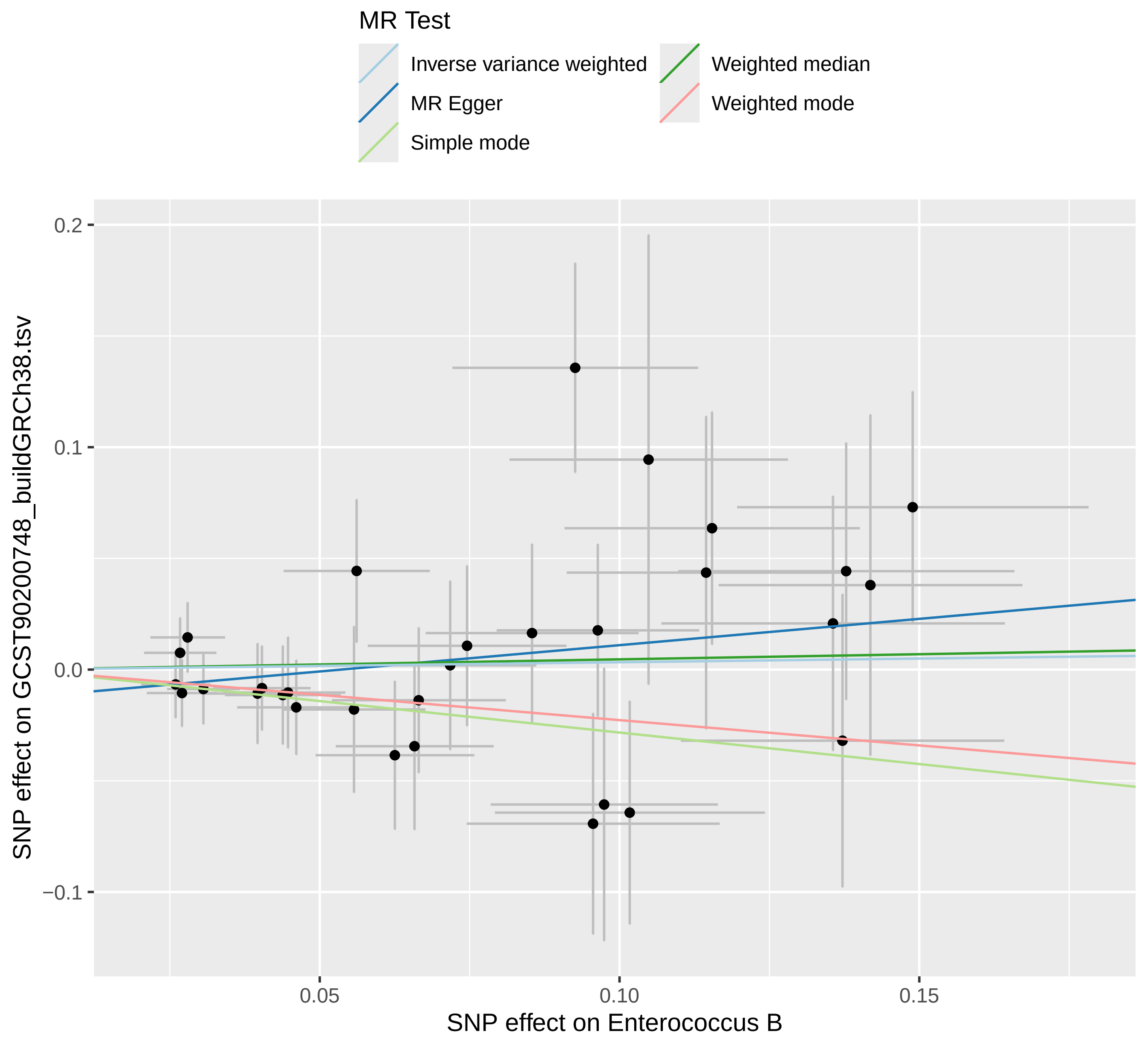
MR Test
Inverse variance weighted
MR Egger
Simple mode
Weighted median
Weighted mode
0.2
0.1
0.0
−0.1
0.05
0.10
0.15
SNP effect on GCST90200748_buildGRCh38.tsv
SNP effect on Enterococcus B

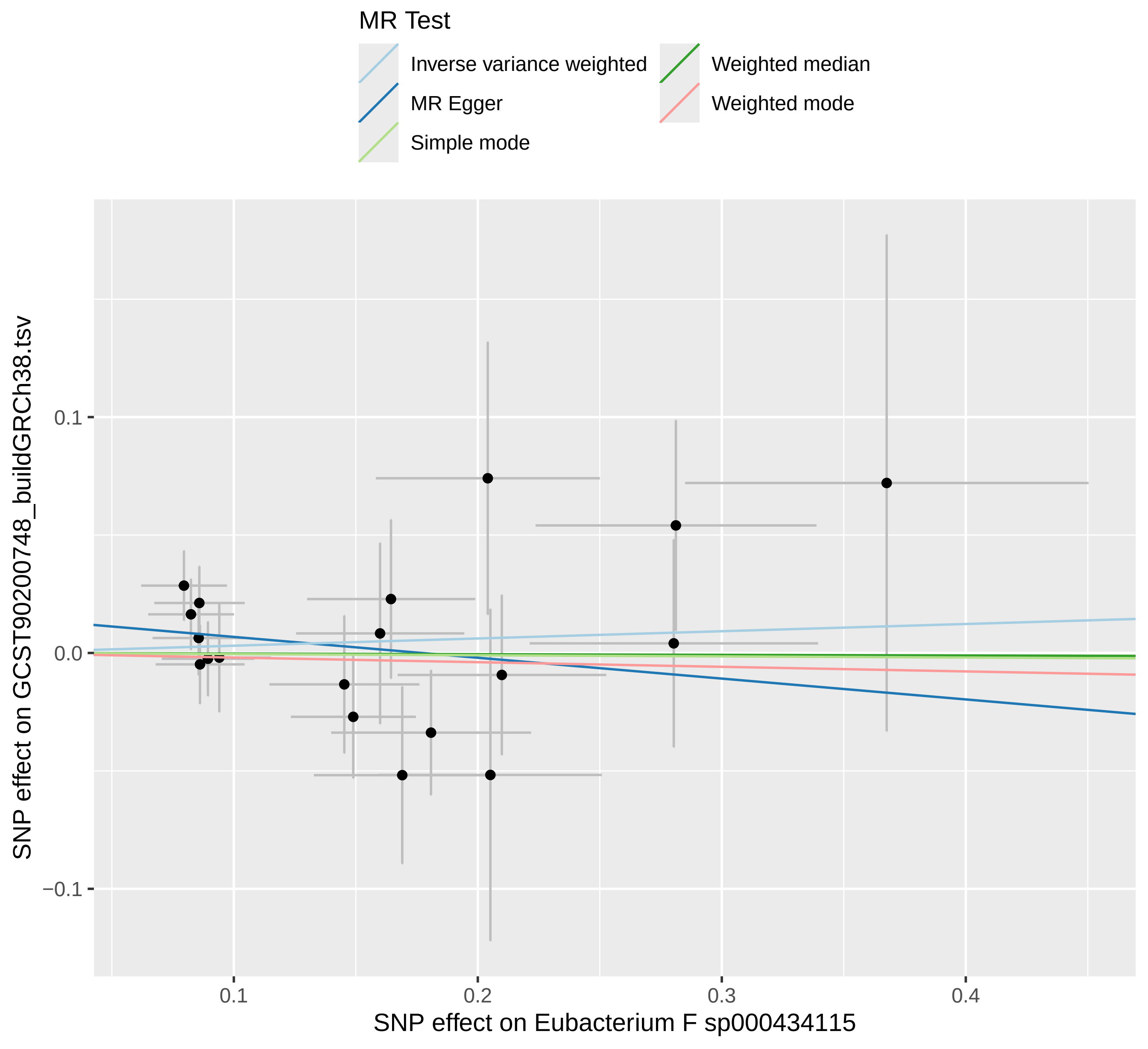

MR Test
Inverse variance weighted
MR Egger
Simple mode
Weighted median
Weighted mode
0.1
0.0
−0.1
SNP effect on GCST90200748_buildGRCh38.tsv
0.1
0.2
0.3
0.4
SNP effect on Eubacterium F sp000434115

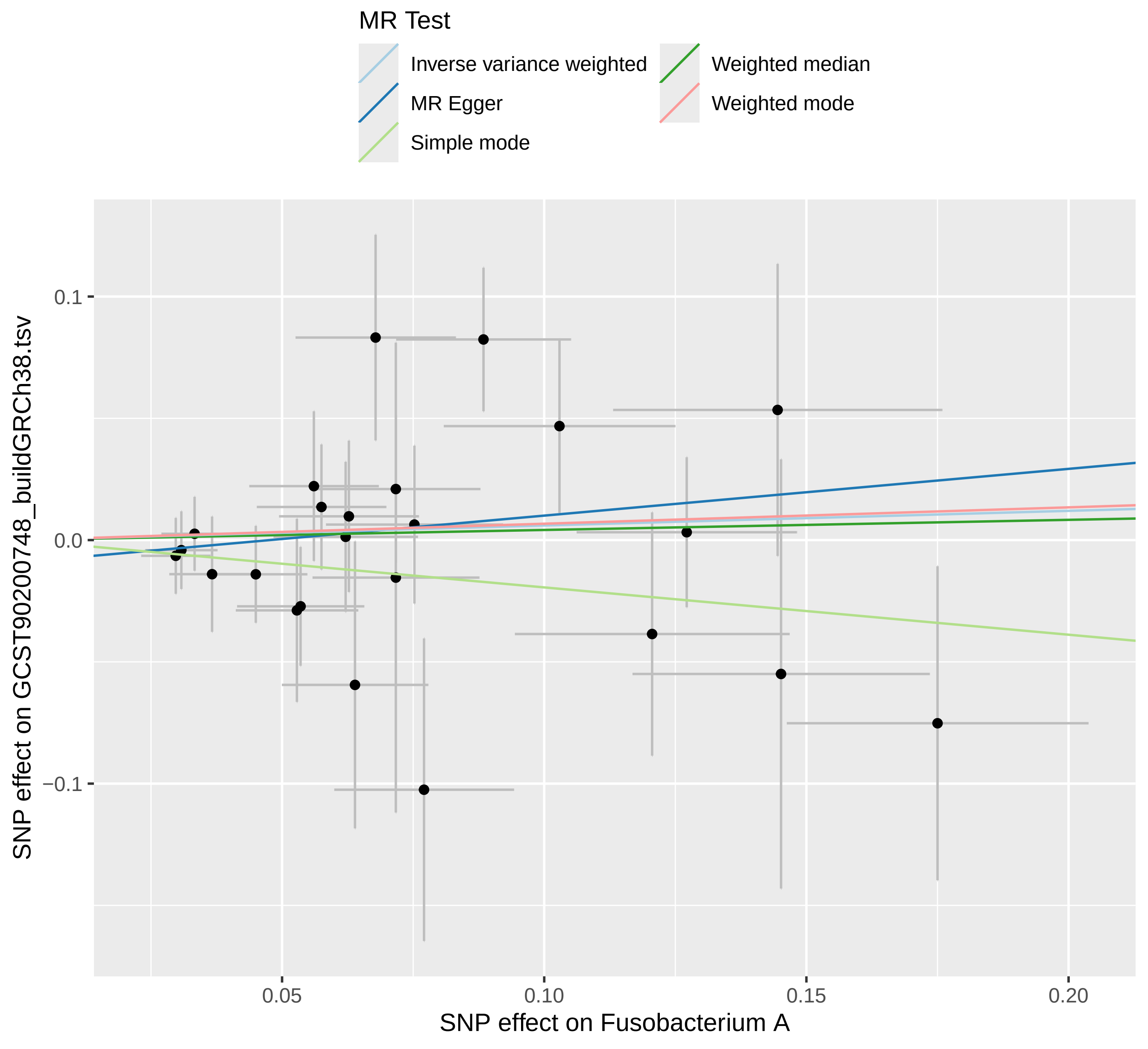
MR Test
Inverse variance weighted
MR Egger
Simple mode
Weighted median
Weighted mode
0.1
0.0
−0.1
0.05
0.10
0.15
0.20
SNP effect on GCST90200748_buildGRCh38.tsv
SNP effect on Fusobacterium A

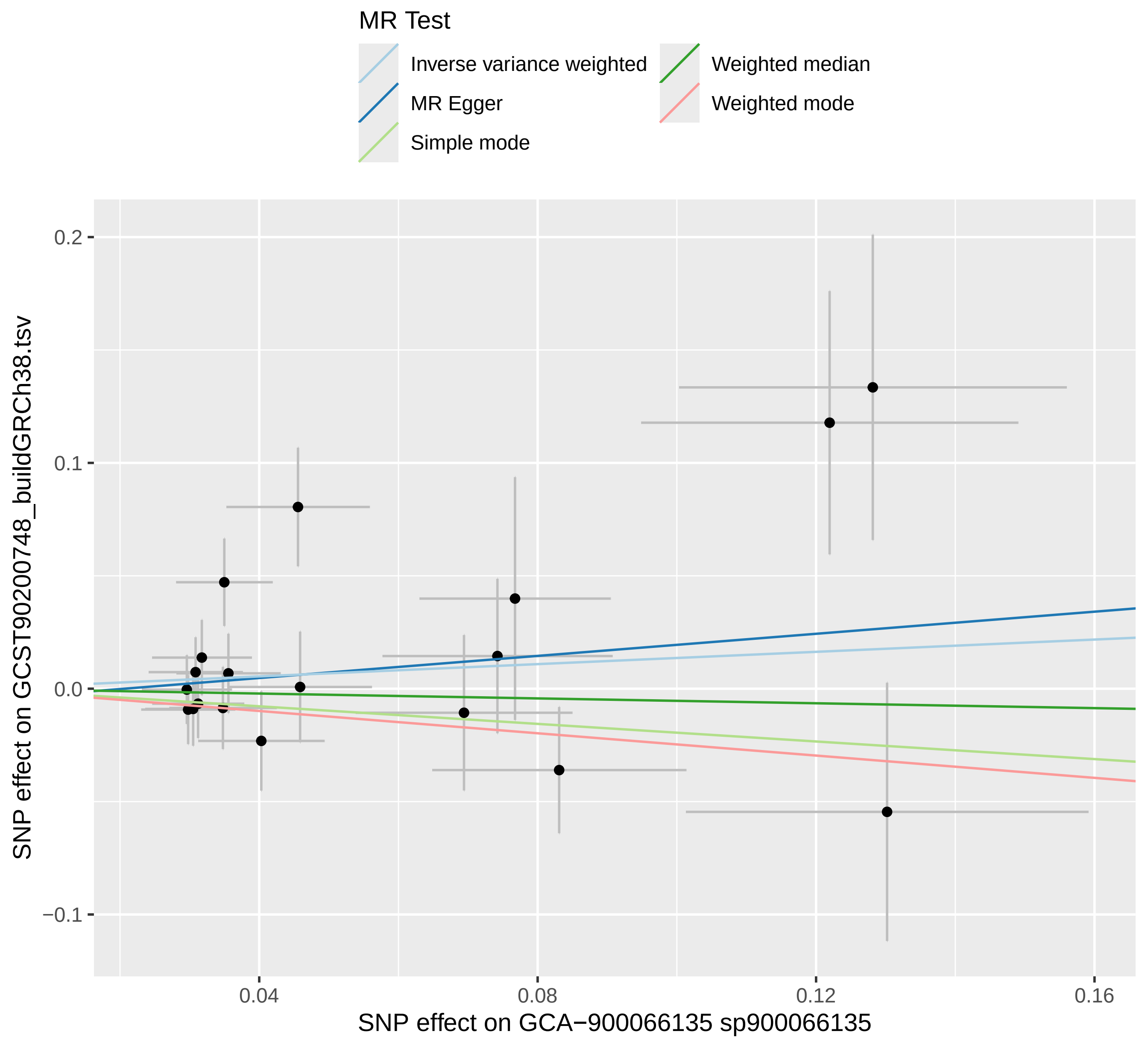

MR Test
Inverse variance weighted
MR Egger
Simple mode
Weighted median
Weighted mode
0.2
0.1
0.0
−0.1
0.04
0.08
0.12
0.16
SNP effect on GCST90200748_buildGRCh38.tsv
SNP effect on GCA−900066135 sp900066135

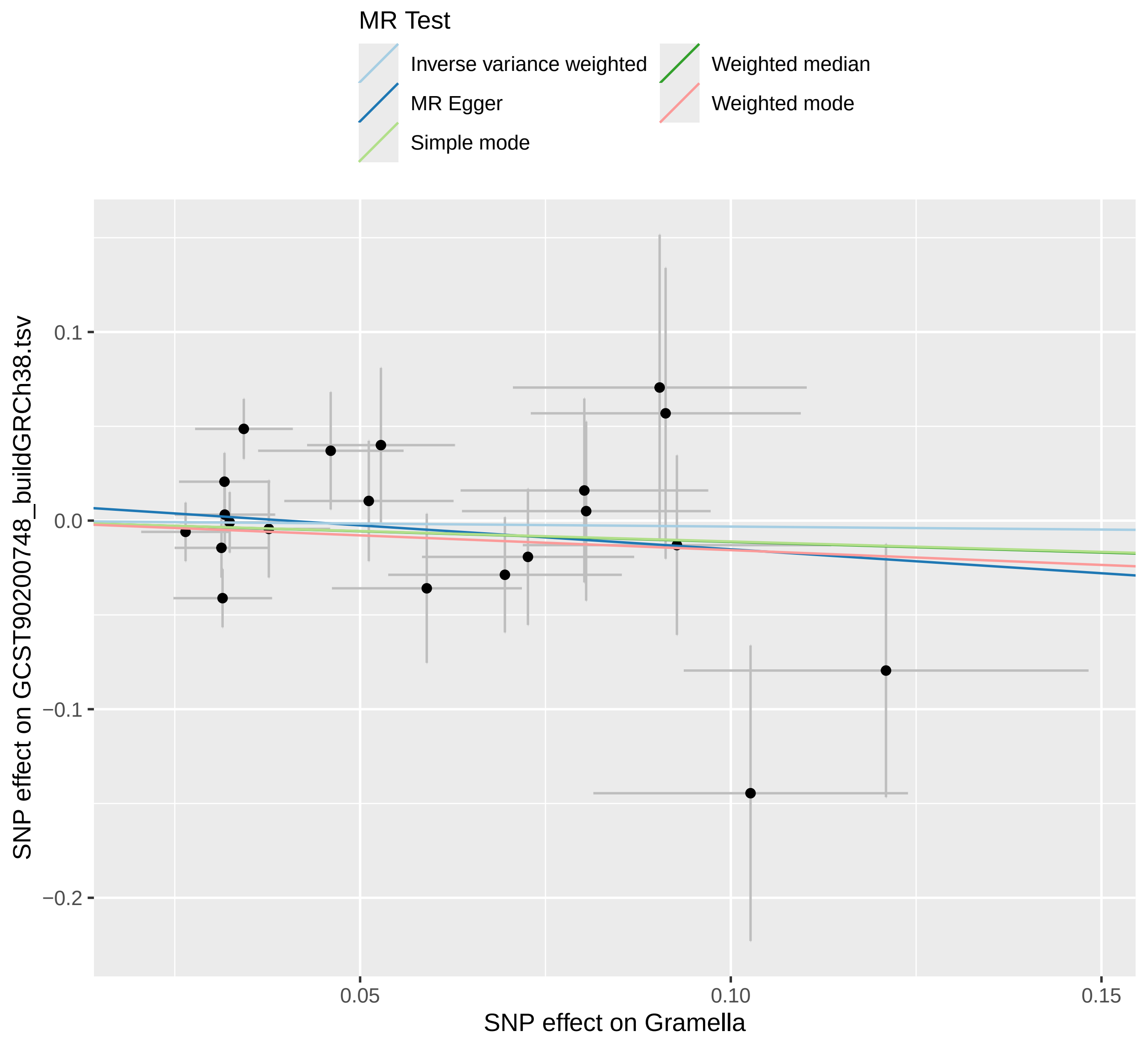
MR Test
Inverse variance weighted
MR Egger
Simple mode
Weighted median
Weighted mode
SNP effect on GCST90200748_buildGRCh38.tsv
0.1
0.0
−0.1
−0.2
0.05
0.10
0.15
SNP effect on Gramella

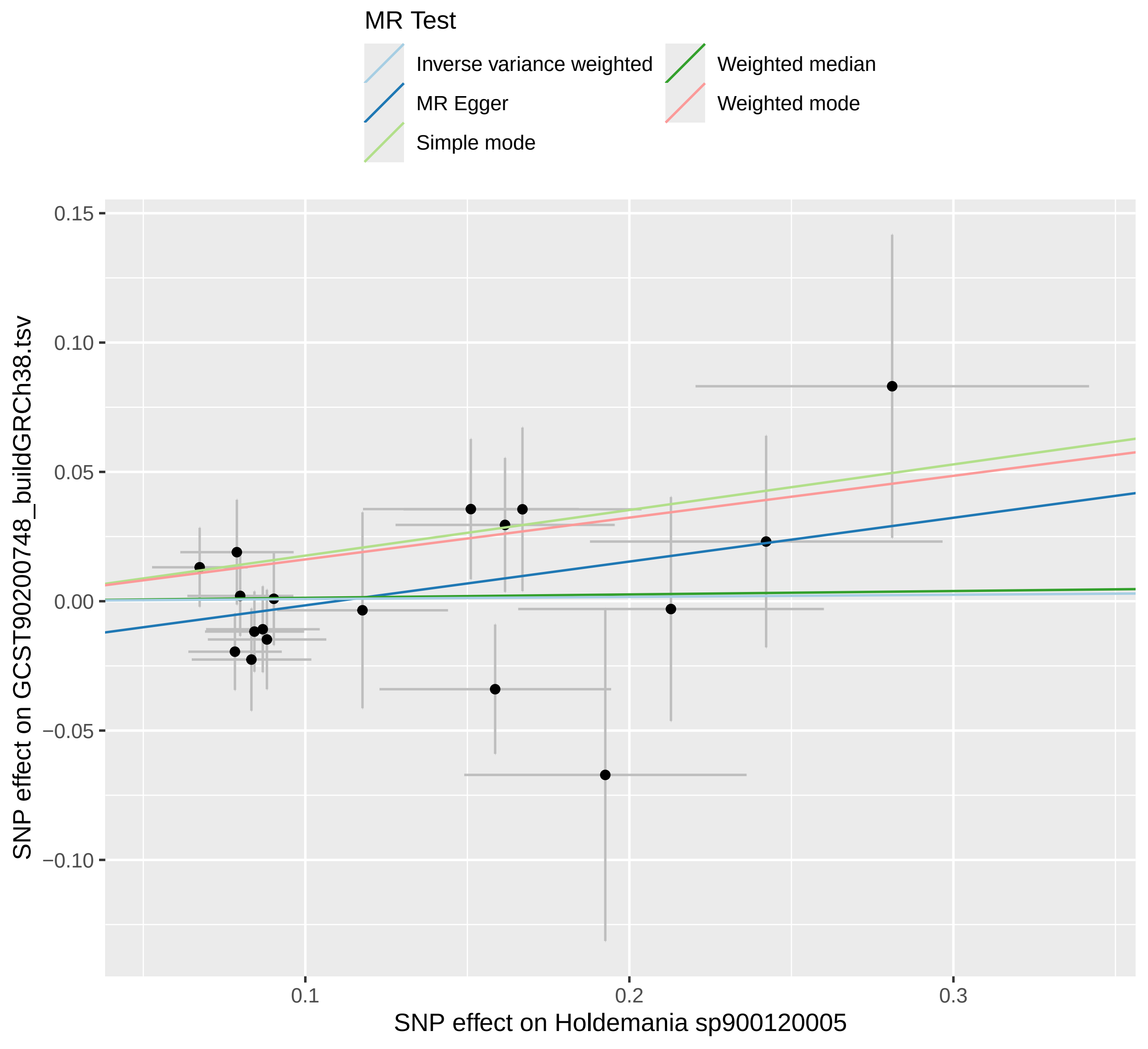
MR Test
Inverse variance weighted
MR Egger
Simple mode
Weighted median
Weighted mode
0.15
0.10
0.05
0.00
−0.05
−0.10
0.1
0.2
0.3
SNP effect on GCST90200748_buildGRCh38.tsv
SNP effect on Holdemania sp900120005

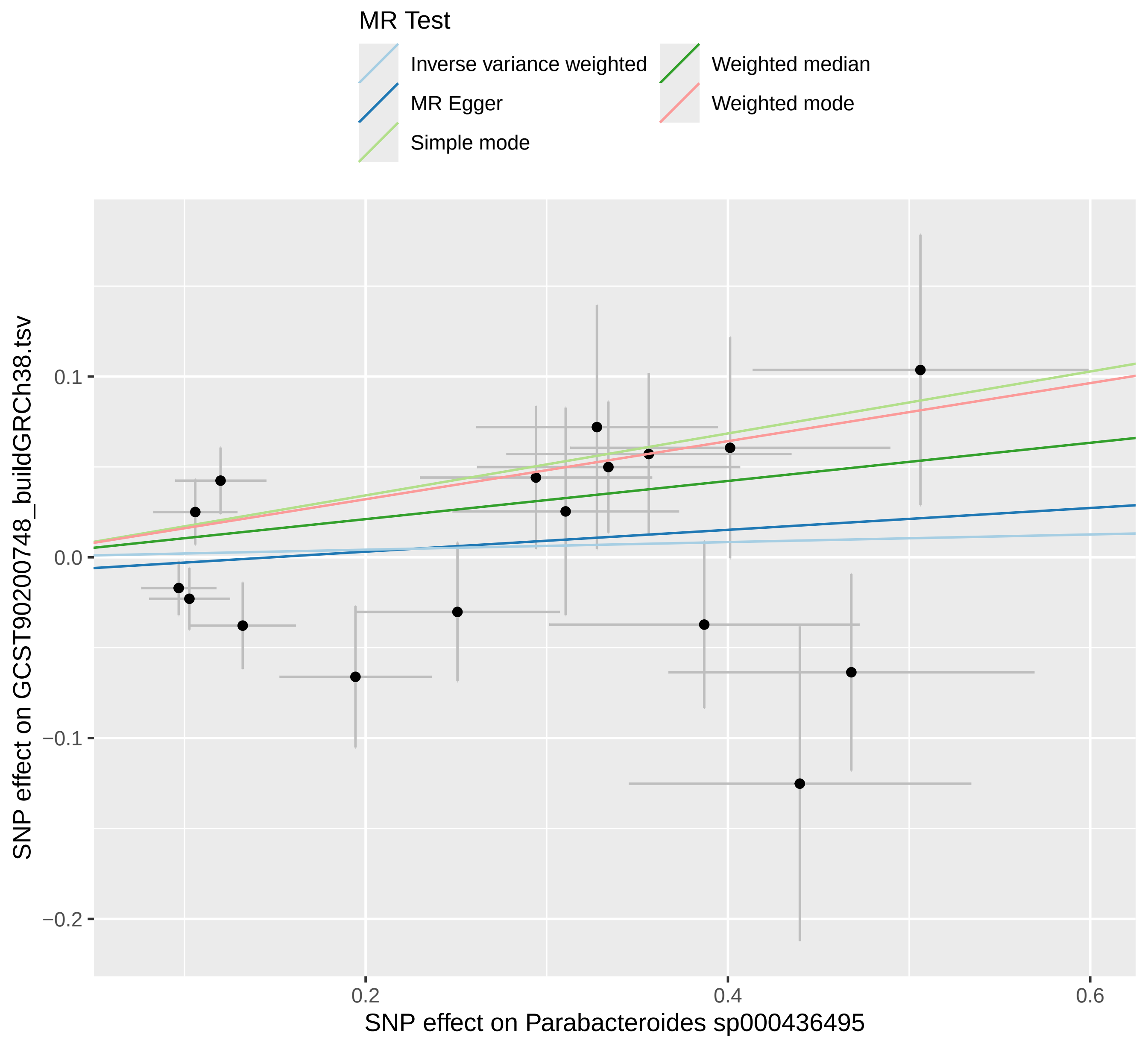

MR Test
Inverse variance weighted
MR Egger
Simple mode
Weighted median
Weighted mode
SNP effect on GCST90200748_buildGRCh38.tsv
0.1
0.0
−0.1
−0.2
0.2
0.4
0.6
SNP effect on Parabacteroides sp000436495

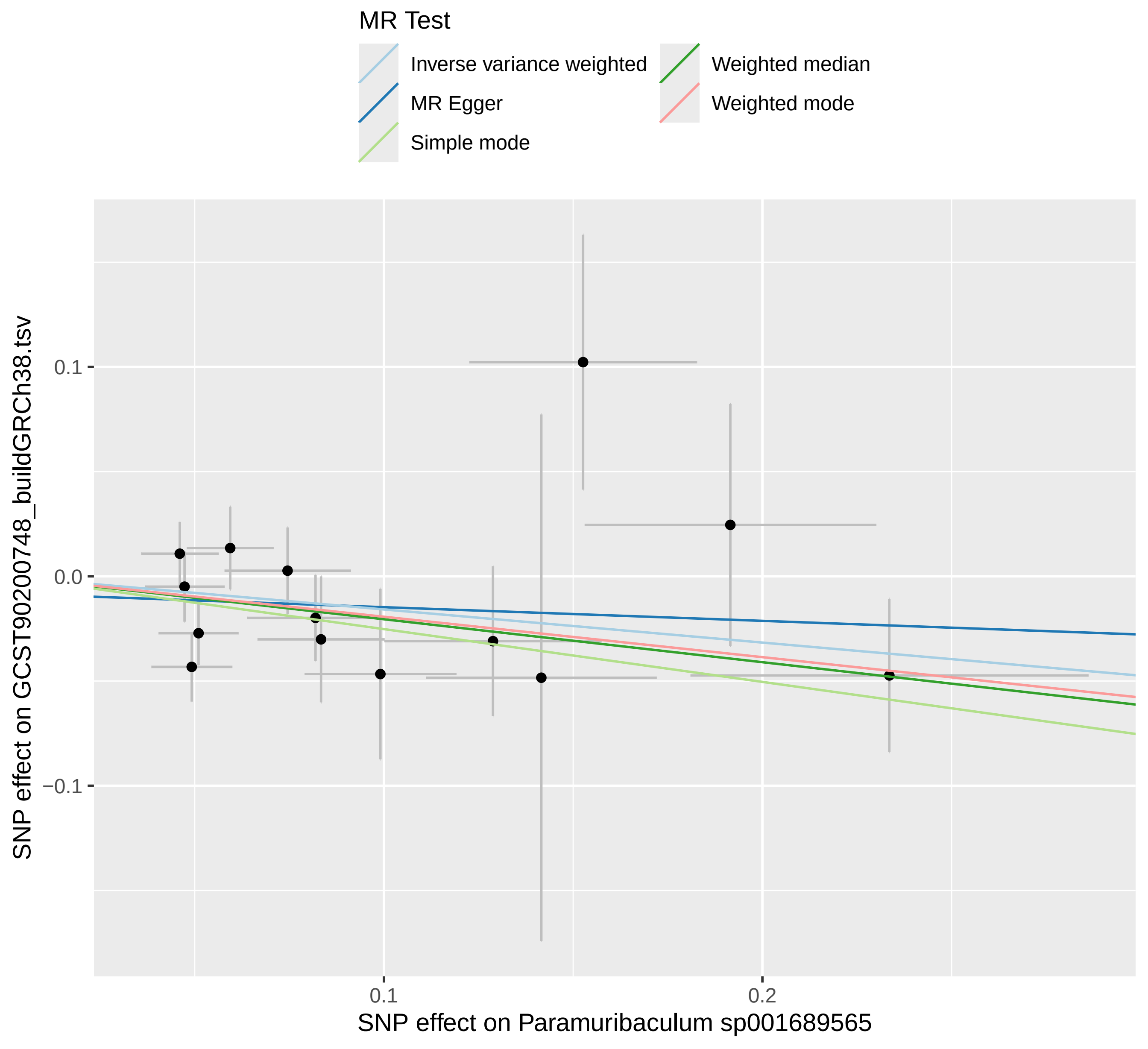
MR Test
Inverse variance weighted
MR Egger
Simple mode
Weighted median
Weighted mode
0.1
0.0
−0.1
0.1
0.2
SNP effect on GCST90200748_buildGRCh38.tsv
SNP effect on Paramuribaculum sp001689565

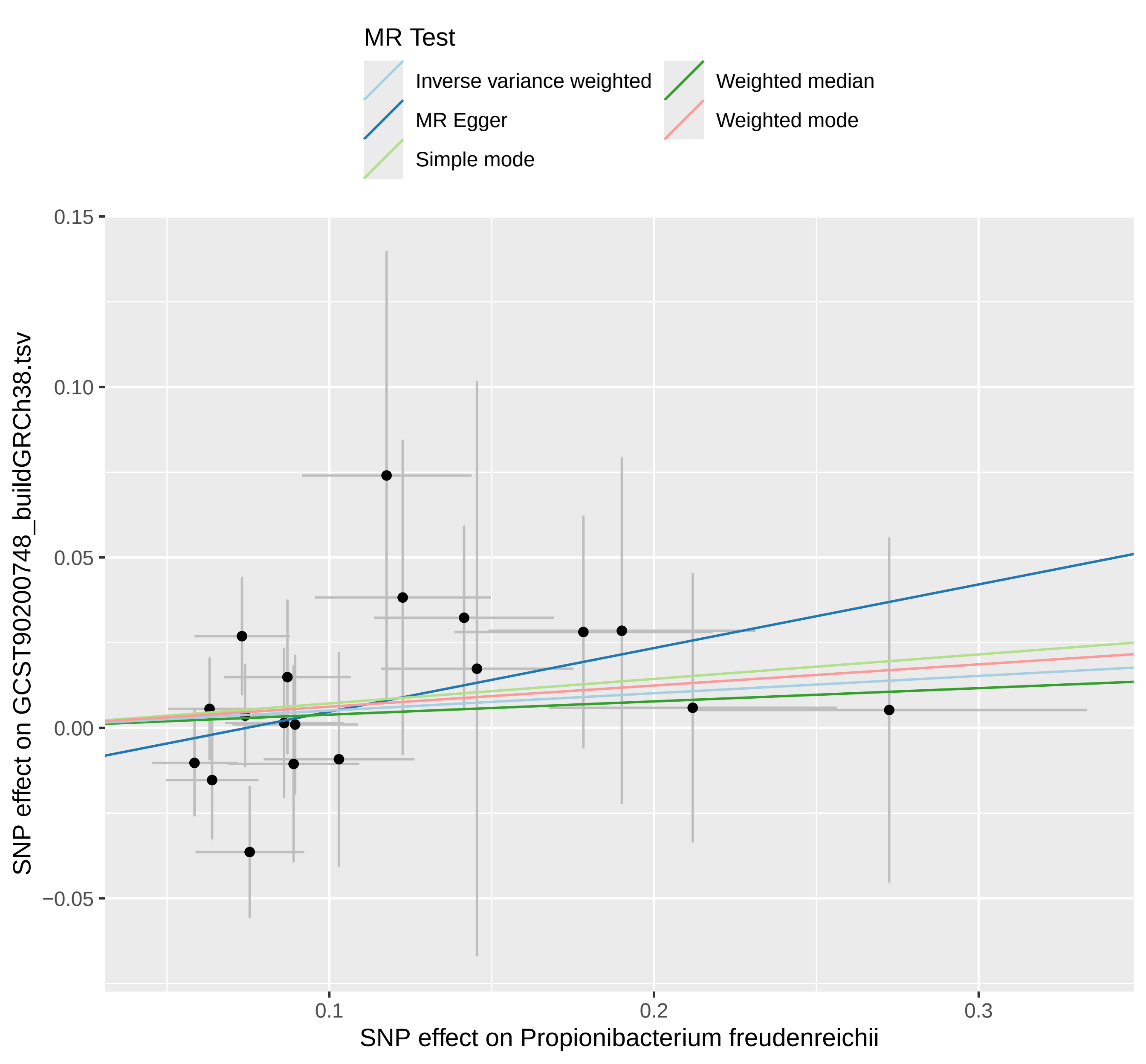
MR Test
Inverse variance weighted
MR Egger
Simple mode
Weighted median
Weighted mode
0.15
0.10
0.05
0.00
−0.05
0.1
0.2
0.3
SNP effect on GCST90200748_buildGRCh38.tsv
SNP effect on Propionibacterium freudenreichii

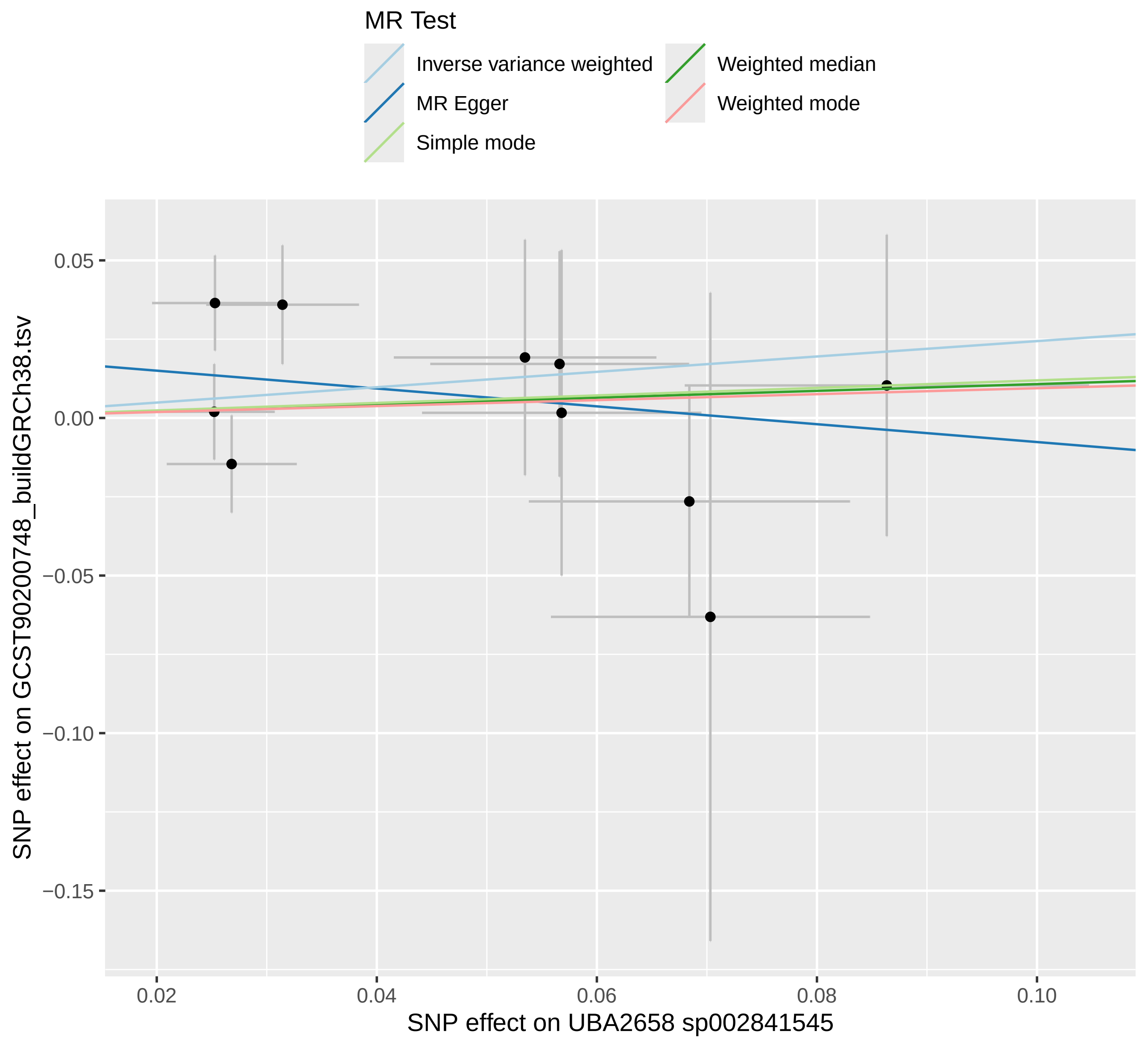
MR Test
Inverse variance weighted
MR Egger
Simple mode
Weighted median
Weighted mode
0.05
0.00
−0.05
−0.10
−0.15
SNP effect on GCST90200748_buildGRCh38.tsv
0.02
0.04
0.06
0.08
0.10
SNP effect on UBA2658 sp002841545

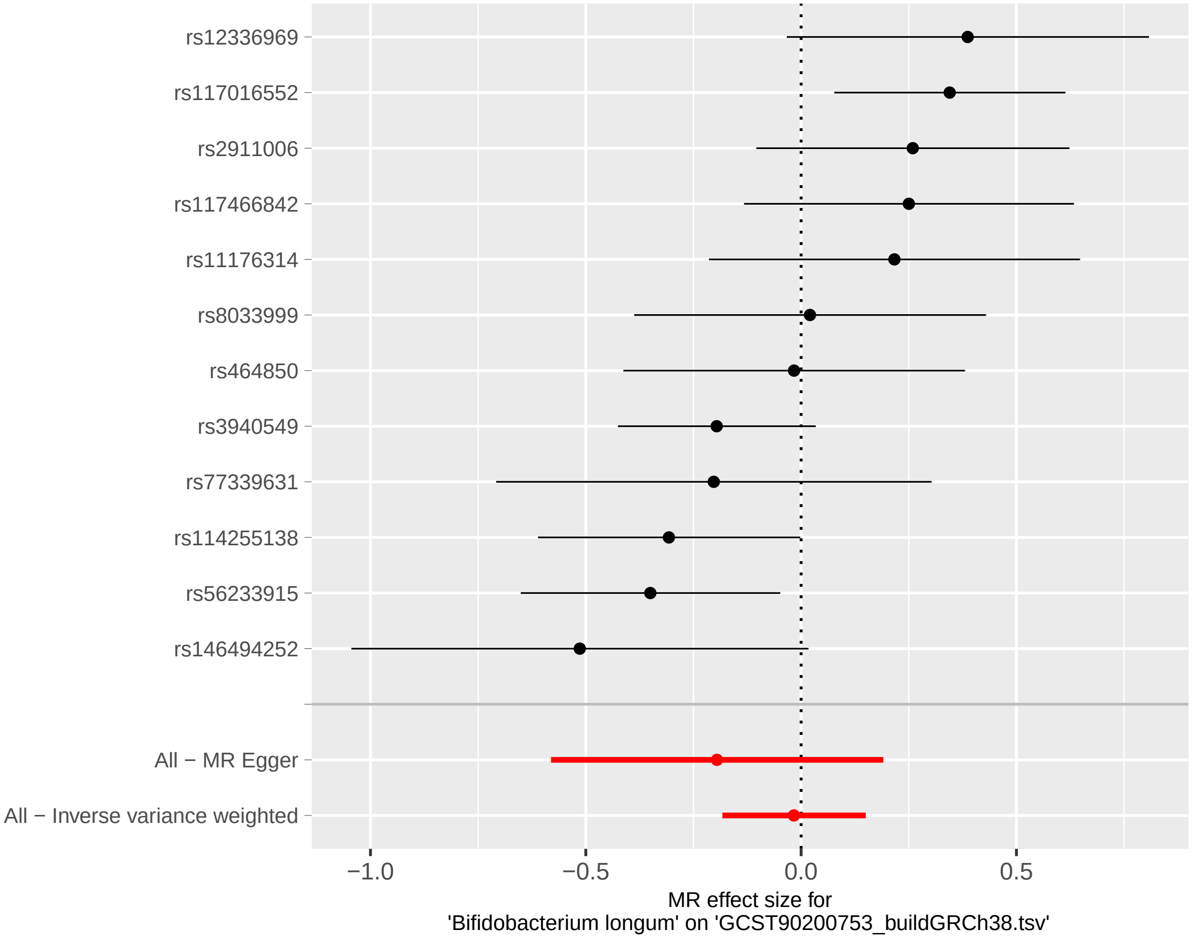
rs12336969
rs117016552
rs2911006
rs117466842
rs11176314
rs8033999
rs464850
rs3940549
rs77339631
rs114255138
rs56233915
rs146494252
All − MR Egger
All − Inverse variance weighted
−1.0
−0.5
0.0
0.5
MR effect size for
'Bifidobacterium longum' on 'GCST90200753_buildGRCh38.tsv'

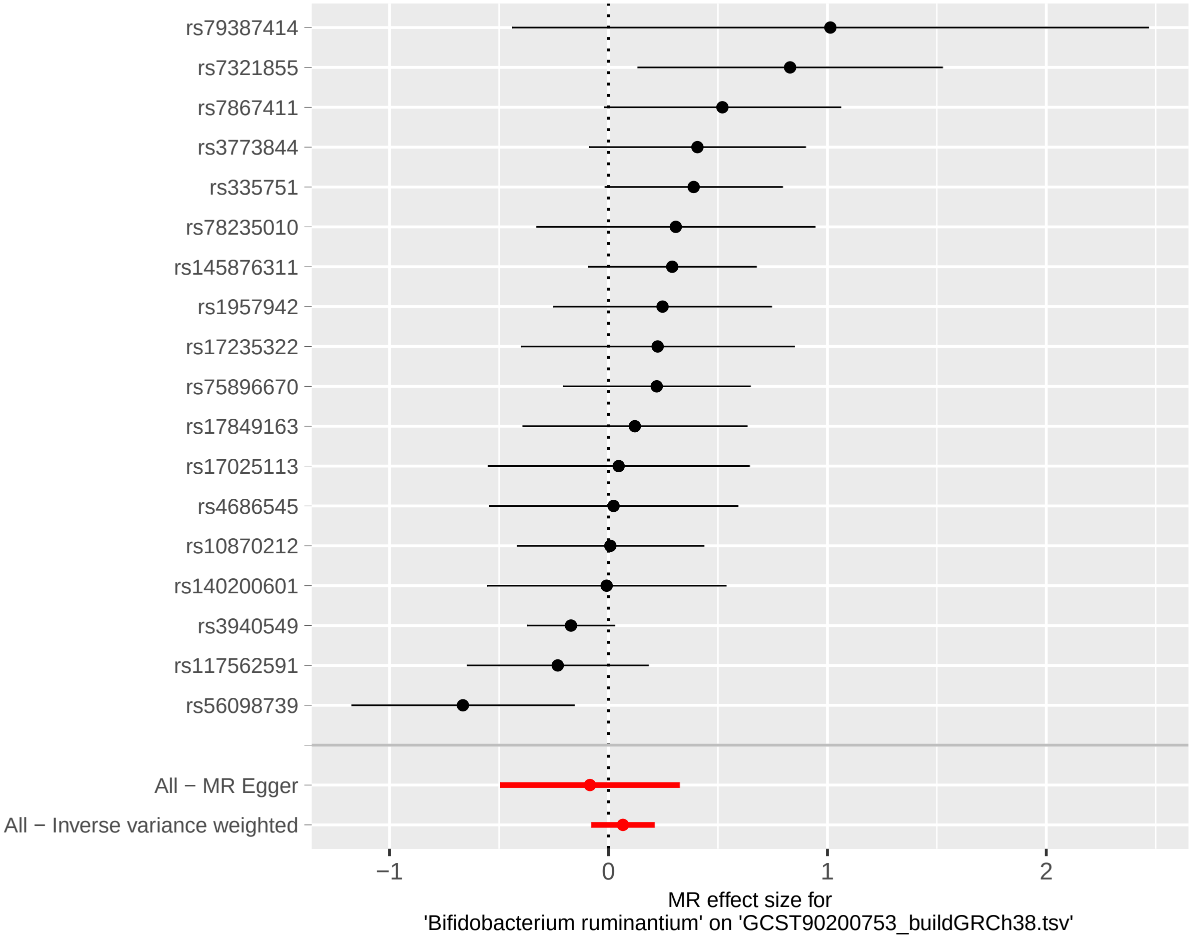
rs79387414
rs7321855
rs7867411
rs3773844
rs335751
rs78235010
rs145876311
rs1957942
rs17235322
rs75896670
rs17849163
rs17025113
rs4686545
rs10870212
rs140200601
rs3940549
rs117562591
rs56098739
All − MR Egger
All − Inverse variance weighted
−1
0
1
2
MR effect size for
'Bifidobacterium ruminantium' on 'GCST90200753_buildGRCh38.tsv'

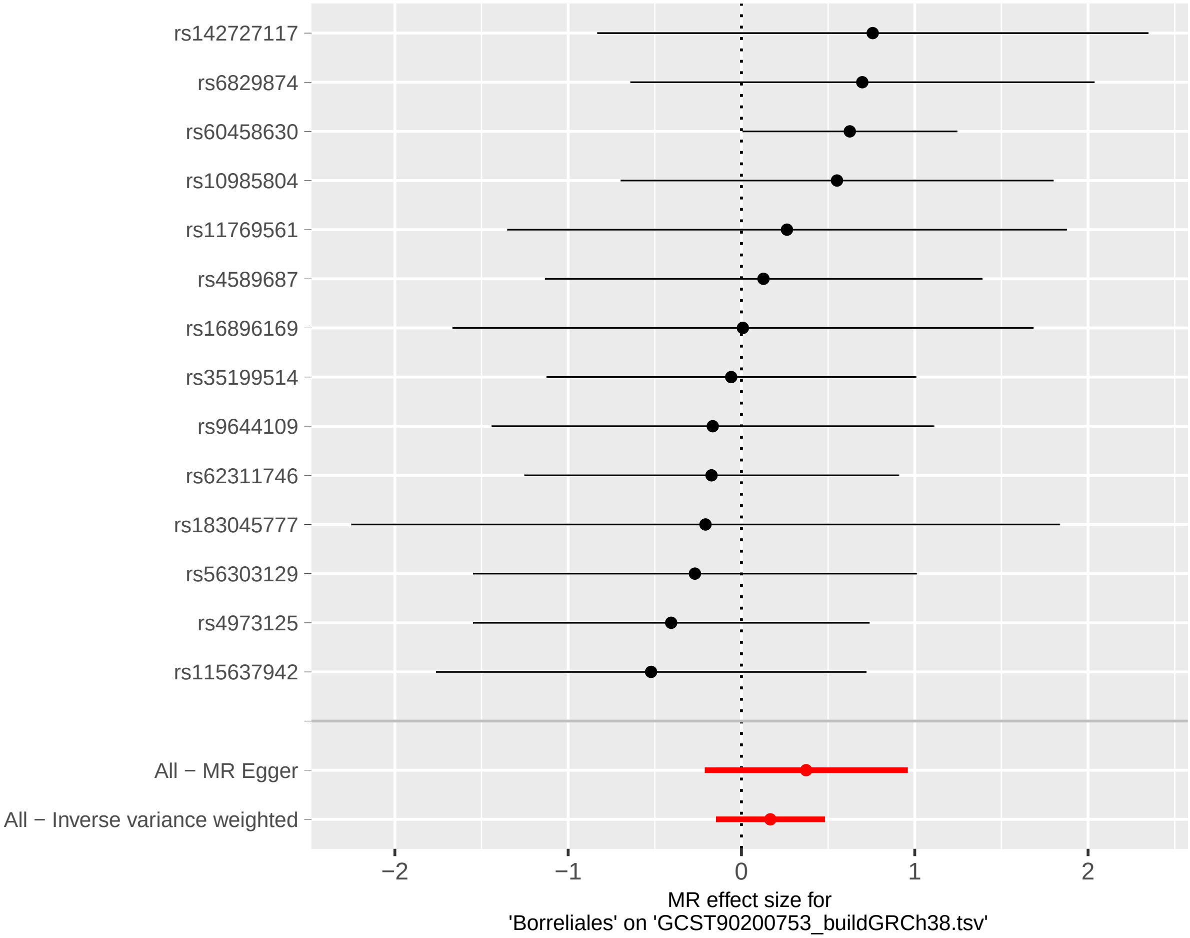

rs142727117
rs6829874
rs60458630
rs10985804
rs11769561
rs4589687
rs16896169
rs35199514
rs9644109
rs62311746
rs183045777
rs56303129
rs4973125
rs115637942
All − MR Egger
All − Inverse variance weighted
−2
−1
0
1
2
MR effect size for
'Borreliales' on 'GCST90200753_buildGRCh38.tsv'

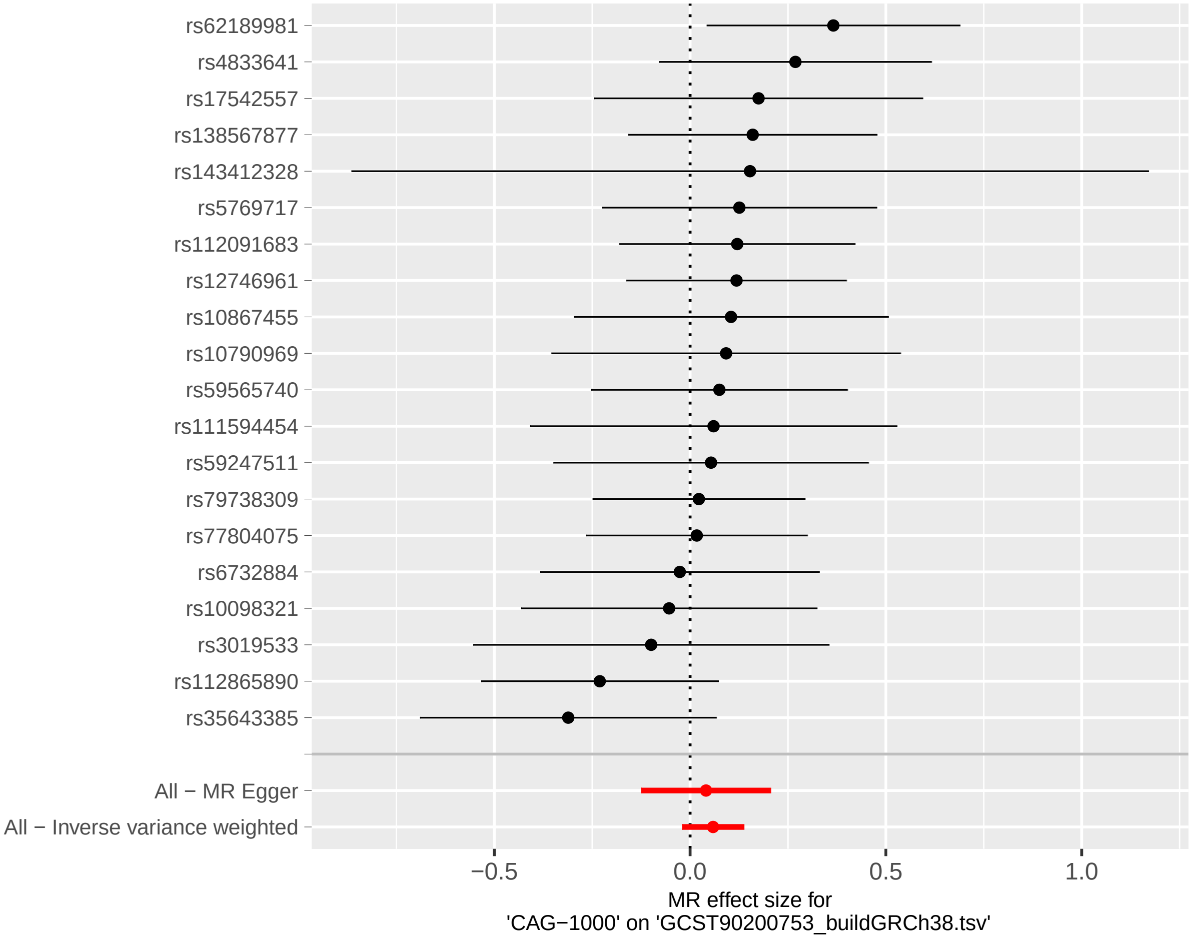

rs62189981
rs4833641
rs17542557
rs138567877
rs143412328
rs5769717
rs112091683
rs12746961
rs10867455
rs10790969
rs59565740
rs111594454
rs59247511
rs79738309
rs77804075
rs6732884
rs10098321
rs3019533
rs112865890
rs35643385
All − MR Egger
All − Inverse variance weighted
−0.5
0.0
0.5
1.0
MR effect size for
'CAG−1000' on 'GCST90200753_buildGRCh38.tsv'

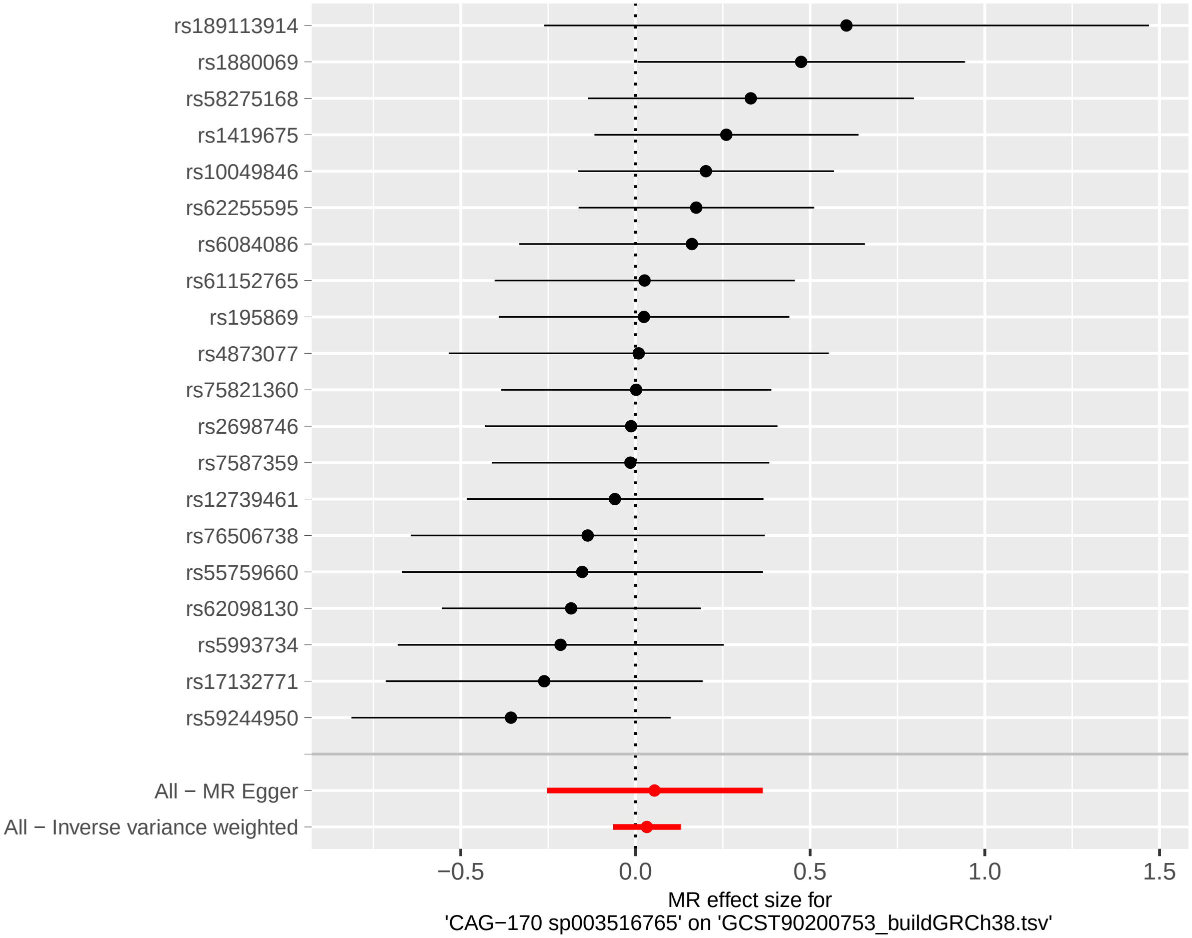
rs189113914
rs1880069
rs58275168
rs1419675
rs10049846
rs62255595
rs6084086
rs61152765
rs195869
rs4873077
rs75821360
rs2698746
rs7587359
rs12739461
rs76506738
rs55759660
rs62098130
rs5993734
rs17132771
rs59244950
All − MR Egger
All − Inverse variance weighted
−0.5
0.0
0.5
1.0
1.5
MR effect size for
'CAG−170 sp003516765' on 'GCST90200753_buildGRCh38.tsv'

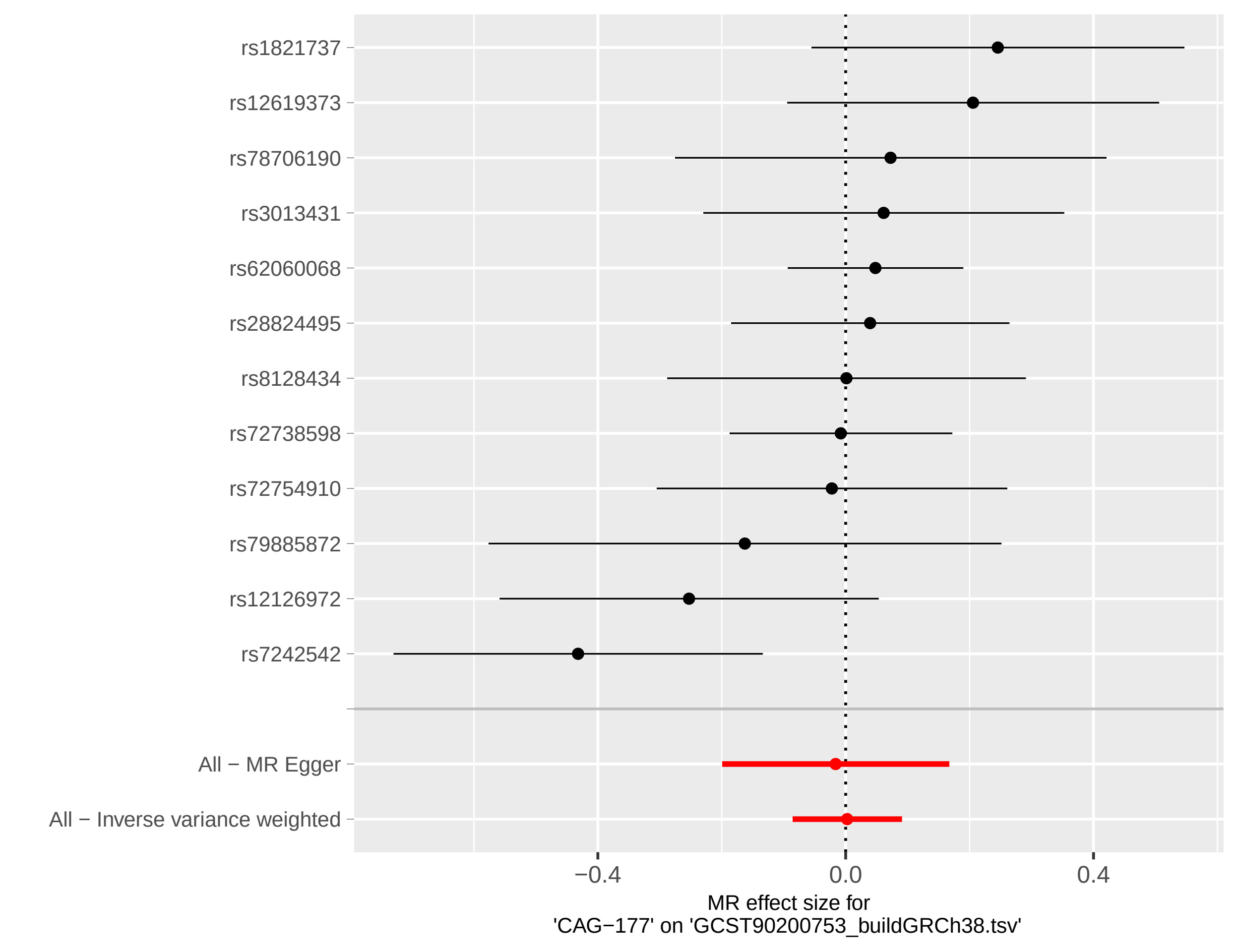

rs1821737
rs12619373
rs78706190
rs3013431
rs62060068
rs28824495
rs8128434
rs72738598
rs72754910
rs79885872
rs12126972
rs7242542
All − MR Egger
All − Inverse variance weighted
−0.4
0.0
0.4
MR effect size for
'CAG−177' on 'GCST90200753_buildGRCh38.tsv'

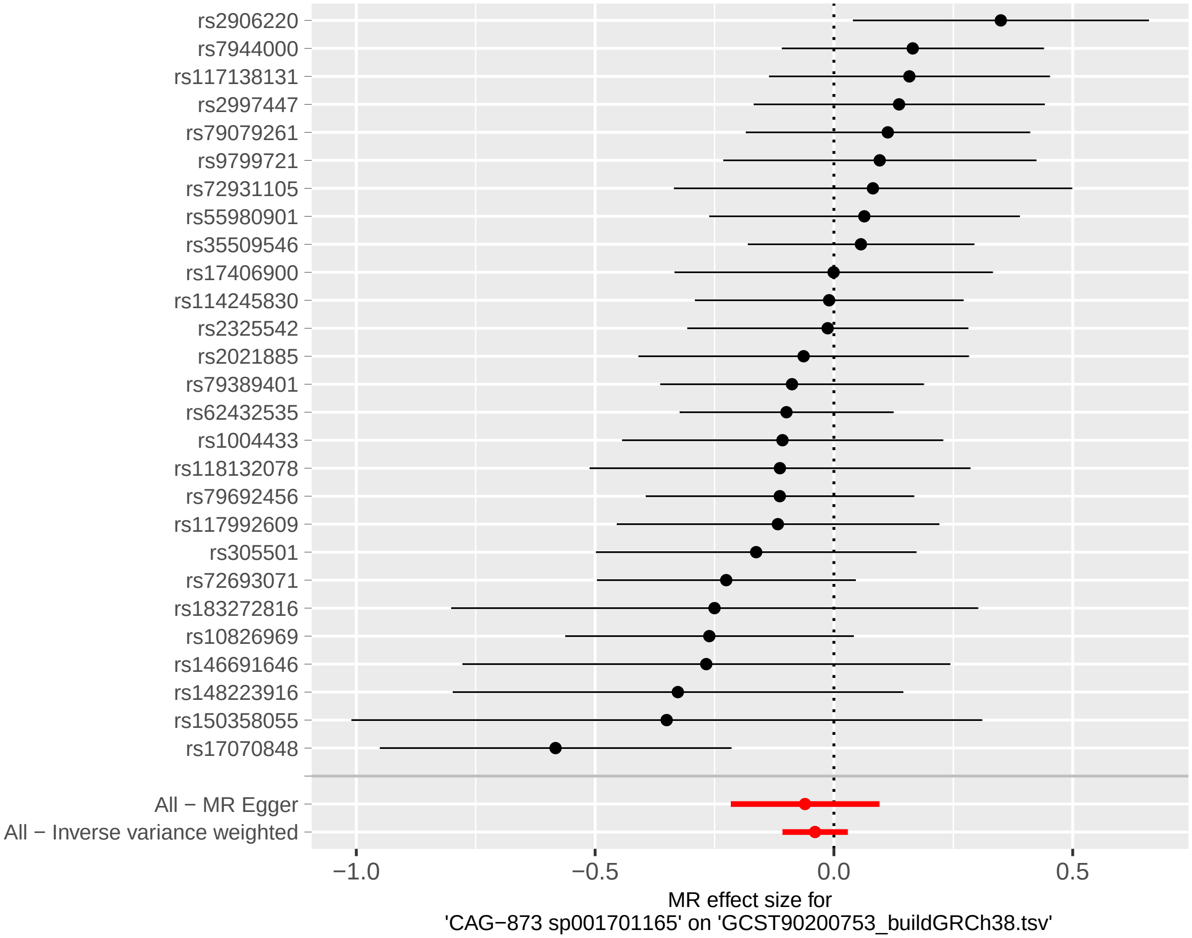
rs2906220
rs7944000
rs117138131
rs2997447
rs79079261
rs9799721
rs72931105
rs55980901
rs35509546
rs17406900
rs114245830
rs2325542
rs2021885
rs79389401
rs62432535
rs1004433
rs118132078
rs79692456
rs117992609
rs305501
rs72693071
rs183272816
rs10826969
rs146691646
rs148223916
rs150358055
rs17070848
All − MR Egger
All − Inverse variance weighted
−1.0
−0.5
0.0
0.5
MR effect size for
'CAG−873 sp001701165' on 'GCST90200753_buildGRCh38.tsv'

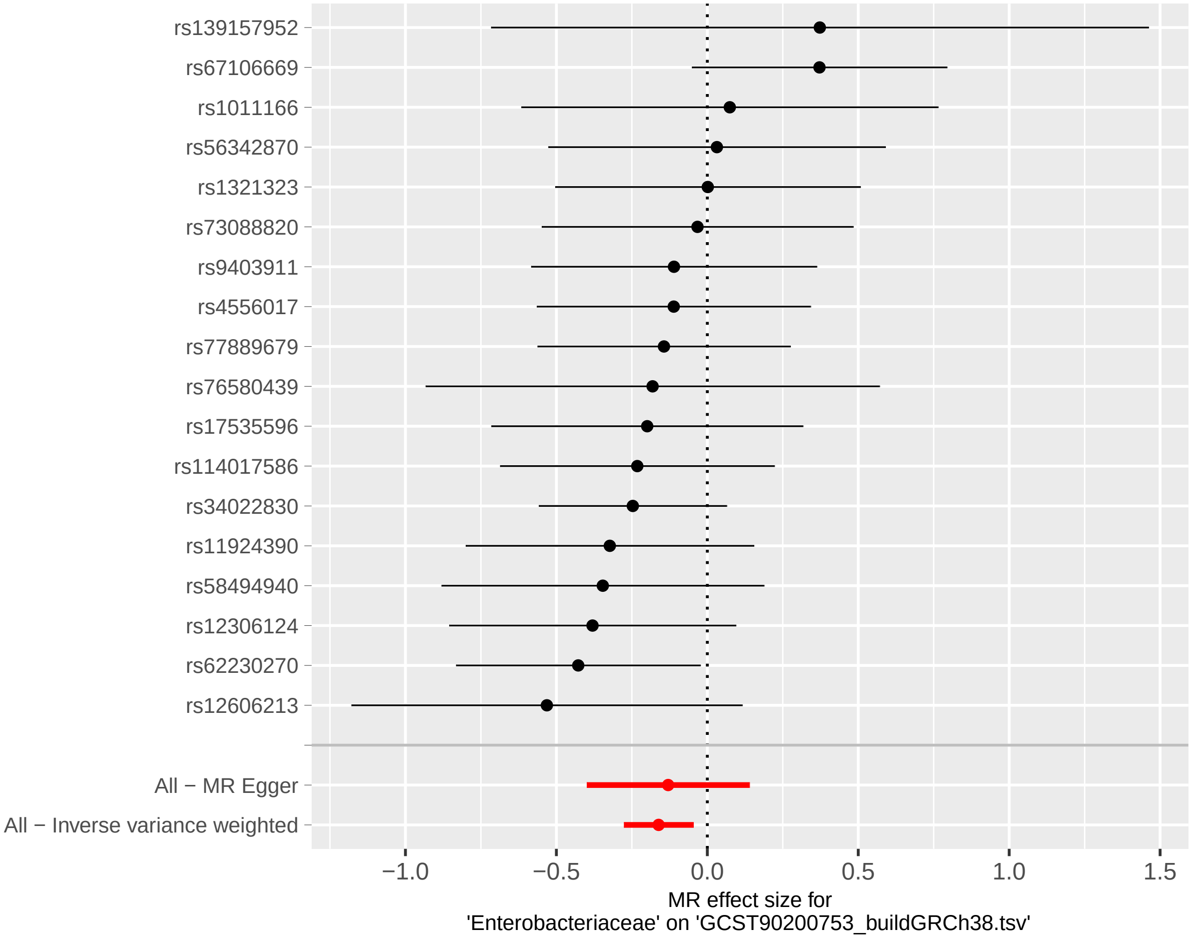
rs139157952
rs67106669
rs1011166
rs56342870
rs1321323
rs73088820
rs9403911
rs4556017
rs77889679
rs76580439
rs17535596
rs114017586
rs34022830
rs11924390
rs58494940
rs12306124
rs62230270
rs12606213
All − MR Egger
All − Inverse variance weighted
−1.0
−0.5
0.0
0.5
1.0
1.5
MR effect size for
'Enterobacteriaceae' on 'GCST90200753_buildGRCh38.tsv'

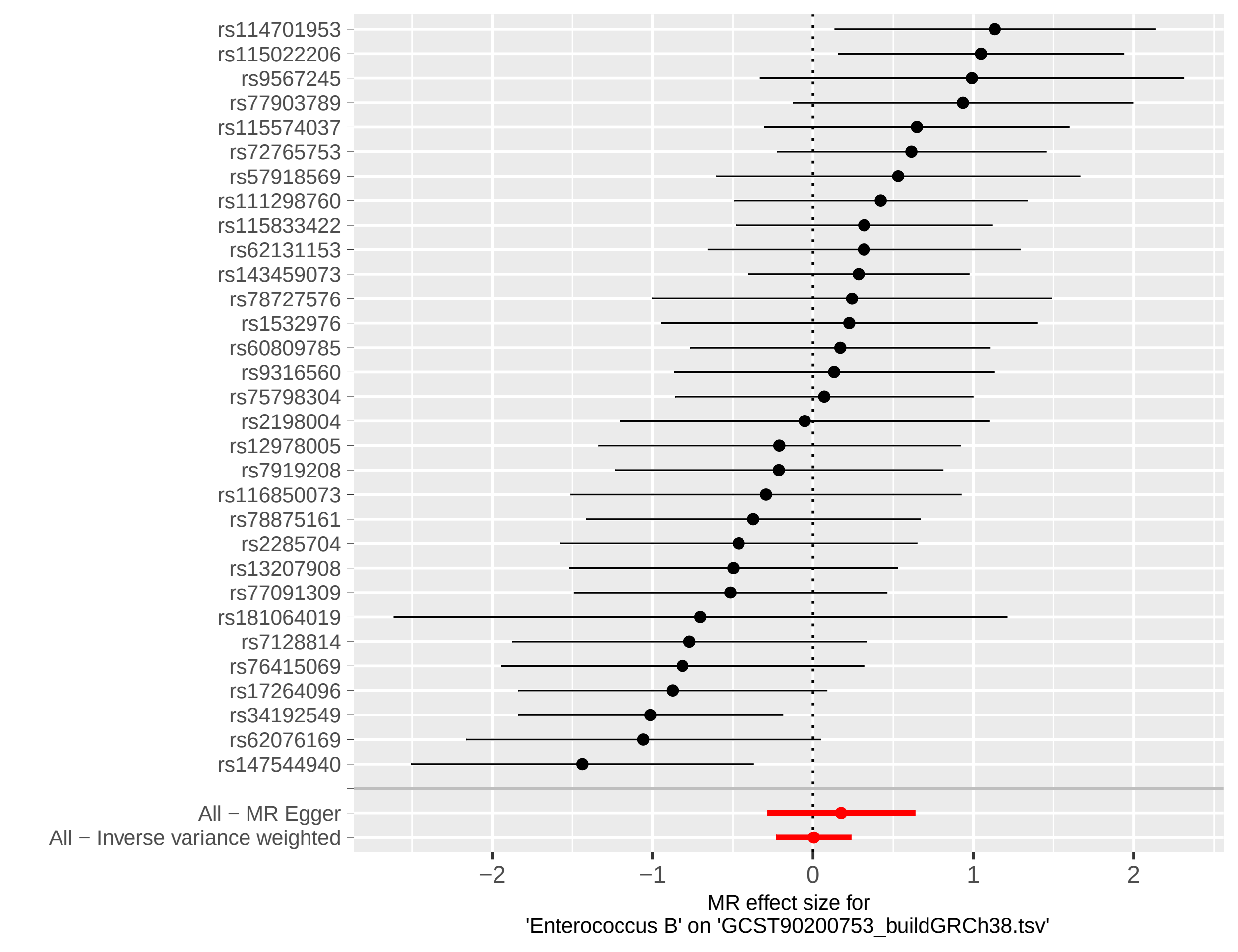
rs114701953
rs115022206
rs9567245
rs77903789
rs115574037
rs72765753
rs57918569
rs111298760
rs115833422
rs62131153
rs143459073
rs78727576
rs1532976
rs60809785
rs9316560
rs75798304
rs2198004
rs12978005
rs7919208
rs116850073
rs78875161
rs2285704
rs13207908
rs77091309
rs181064019
rs7128814
rs76415069
rs17264096
rs34192549
rs62076169
rs147544940
All − MR Egger
All − Inverse variance weighted
−2
−1
0
1
2
MR effect size for
'Enterococcus B' on 'GCST90200753_buildGRCh38.tsv'

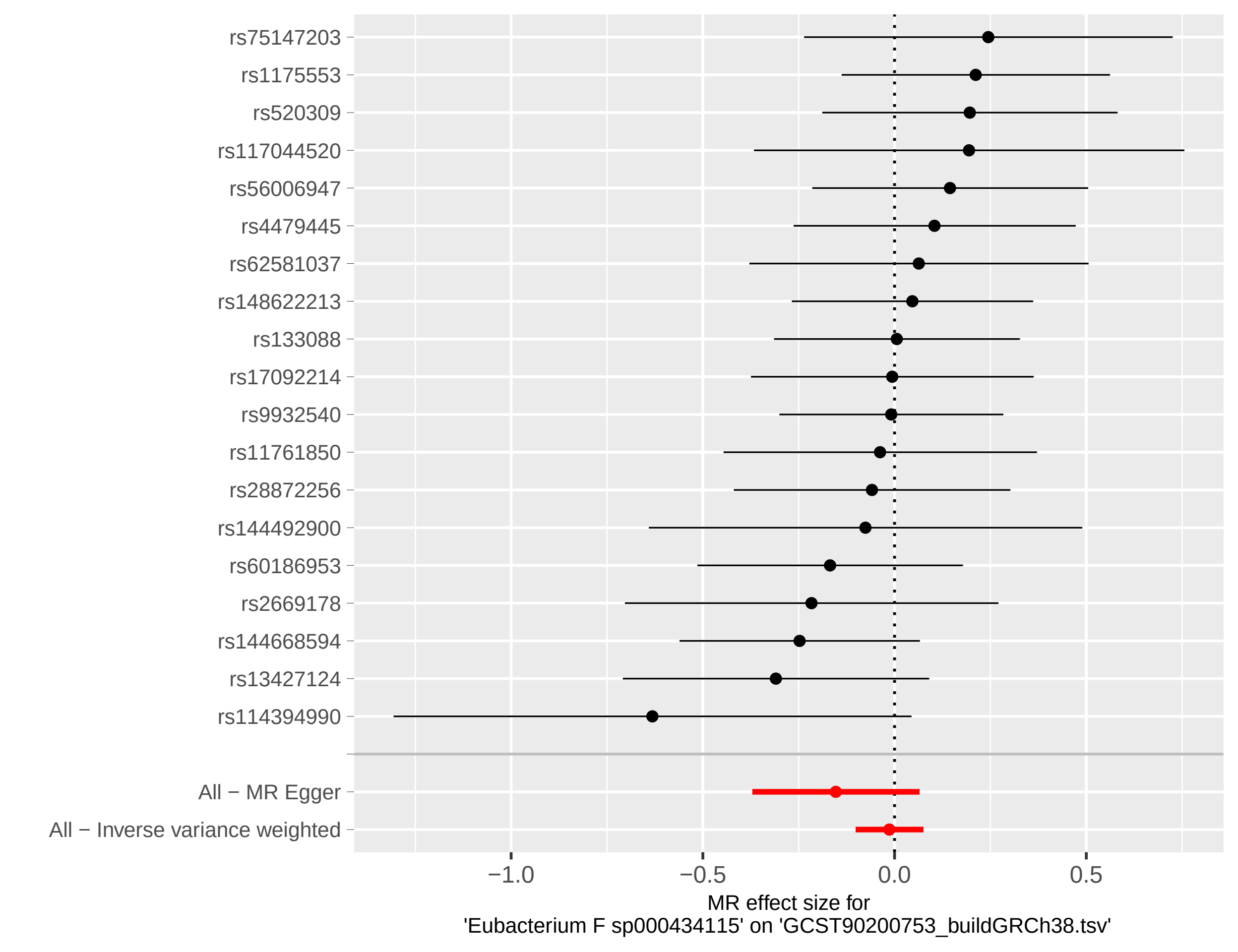
rs75147203
rs1175553
rs520309
rs117044520
rs56006947
rs4479445
rs62581037
rs148622213
rs133088
rs17092214
rs9932540
rs11761850
rs28872256
rs144492900
rs60186953
rs2669178
rs144668594
rs13427124
rs114394990
All − MR Egger
All − Inverse variance weighted
−1.0
−0.5
0.0
0.5
MR effect size for
'Eubacterium F sp000434115' on 'GCST90200753_buildGRCh38.tsv'

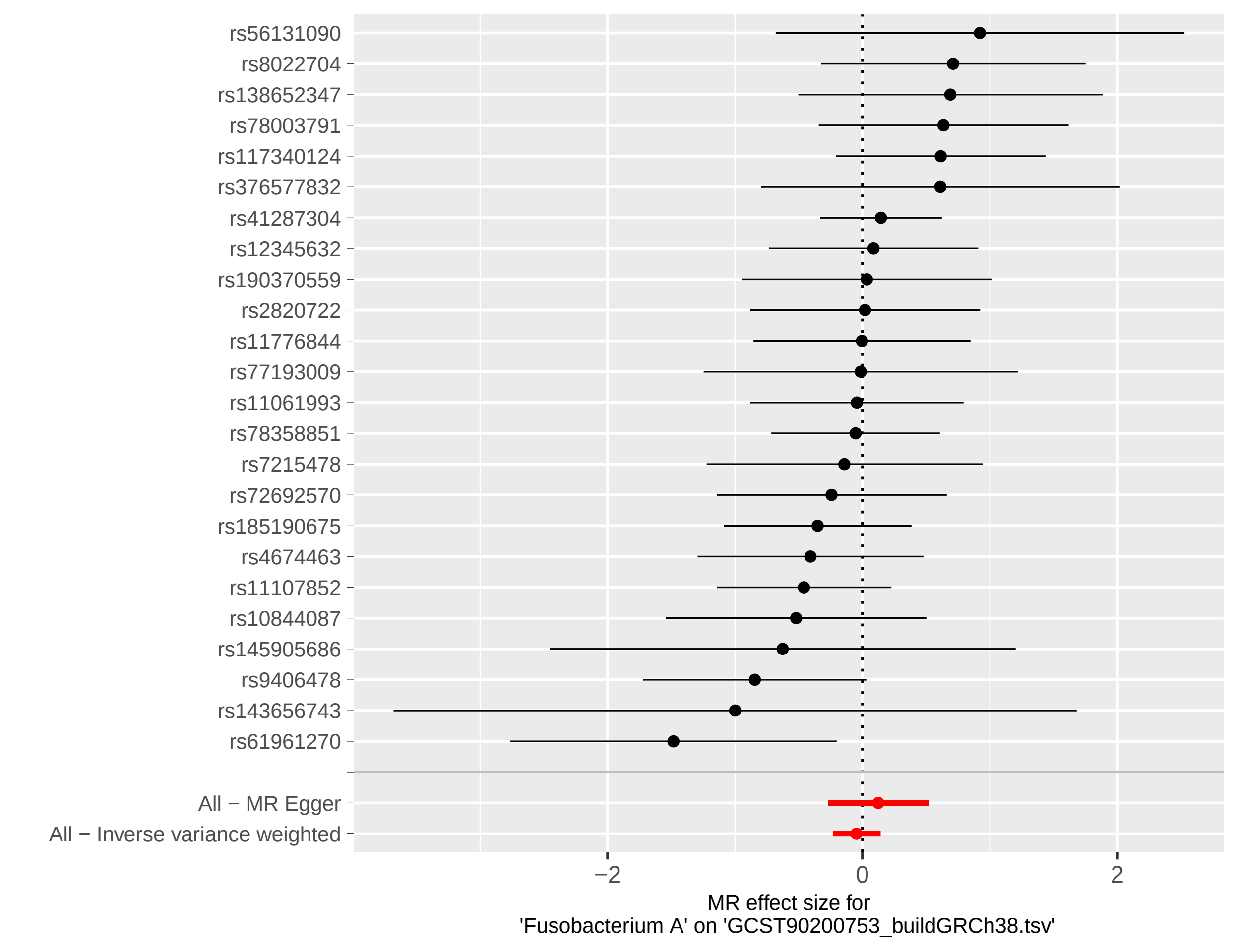

rs56131090
rs8022704
rs138652347
rs78003791
rs117340124
rs376577832
rs41287304
rs12345632
rs190370559
rs2820722
rs11776844
rs77193009
rs11061993
rs78358851
rs7215478
rs72692570
rs185190675
rs4674463
rs11107852
rs10844087
rs145905686
rs9406478
rs143656743
rs61961270
All − MR Egger
All − Inverse variance weighted
−2
0
2
MR effect size for
'Fusobacterium A' on 'GCST90200753_buildGRCh38.tsv'

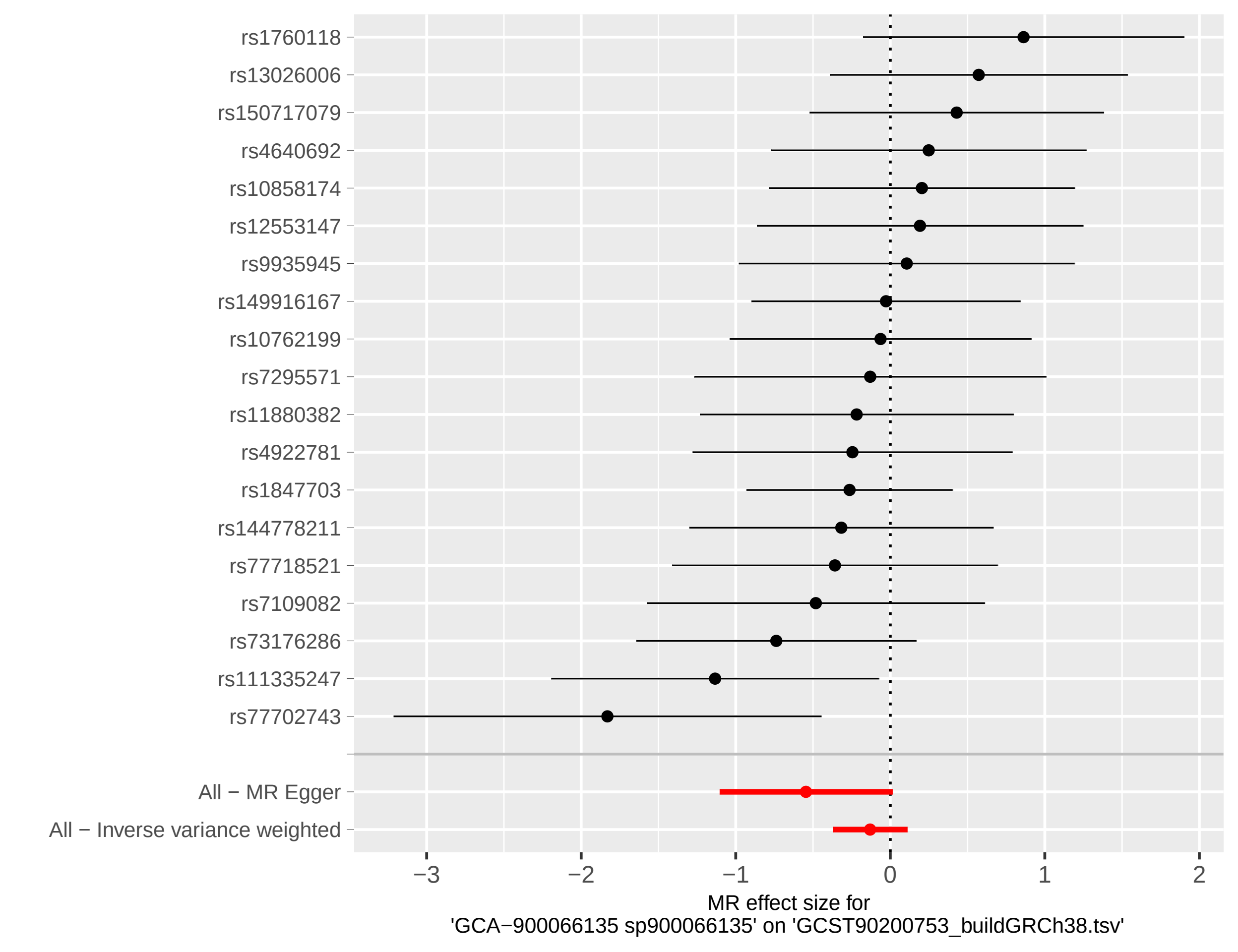

rs1760118
rs13026006
rs150717079
rs4640692
rs10858174
rs12553147
rs9935945
rs149916167
rs10762199
rs7295571
rs11880382
rs4922781
rs1847703
rs144778211
rs77718521
rs7109082
rs73176286
rs111335247
rs77702743
All − MR Egger
All − Inverse variance weighted
−3
−2
−1
0
1
2
MR effect size for
'GCA−900066135 sp900066135' on 'GCST90200753_buildGRCh38.tsv'

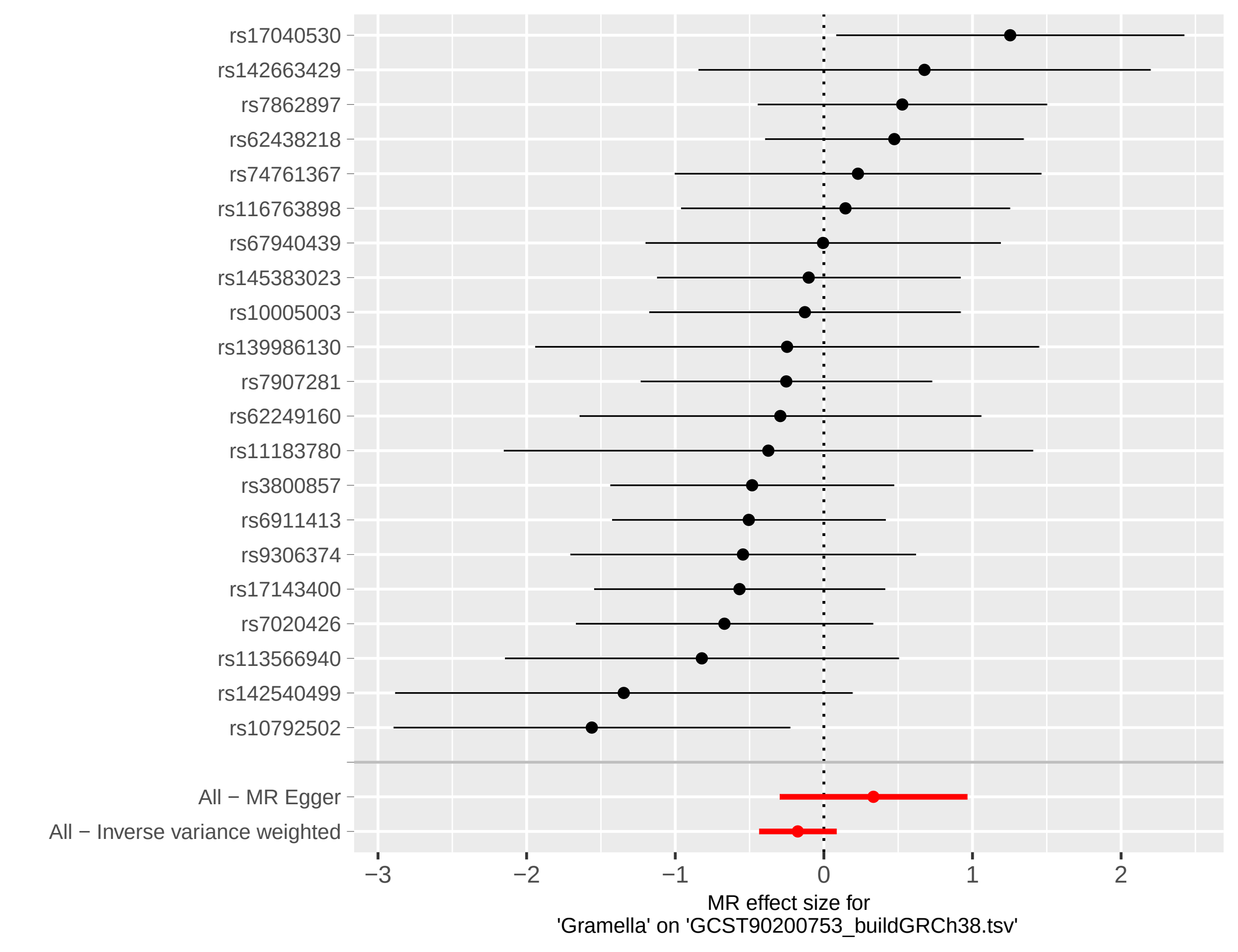
rs17040530
rs142663429
rs7862897
rs62438218
rs74761367
rs116763898
rs67940439
rs145383023
rs10005003
rs139986130
rs7907281
rs62249160
rs11183780
rs3800857
rs6911413
rs9306374
rs17143400
rs7020426
rs113566940
rs142540499
rs10792502
All − MR Egger
All − Inverse variance weighted
−3
−2
−1
0
1
2
MR effect size for
'Gramella' on 'GCST90200753_buildGRCh38.tsv'

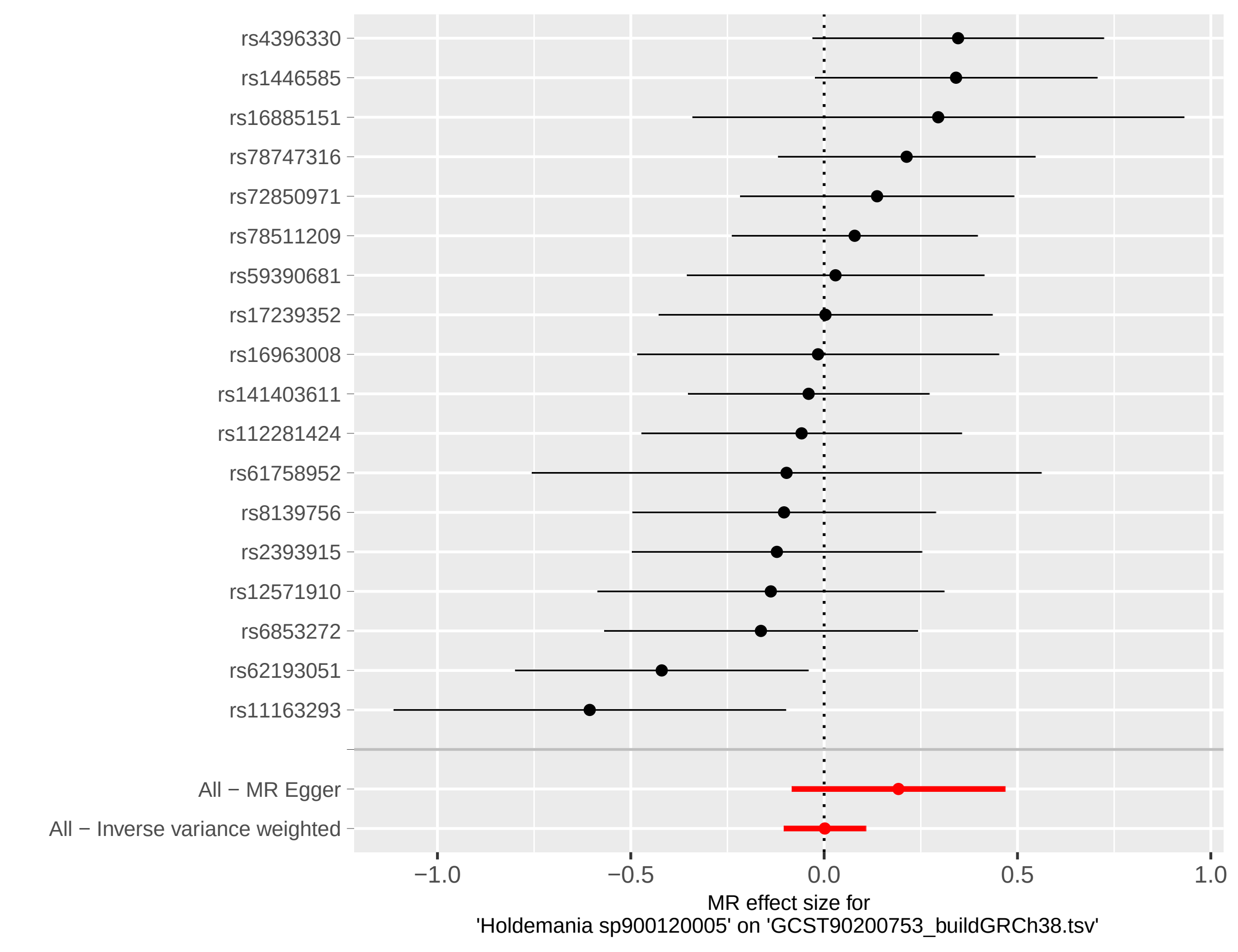
rs4396330
rs1446585
rs16885151
rs78747316
rs72850971
rs78511209
rs59390681
rs17239352
rs16963008
rs141403611
rs112281424
rs61758952
rs8139756
rs2393915
rs12571910
rs6853272
rs62193051
rs11163293
All − MR Egger
All − Inverse variance weighted
−1.0
−0.5
0.0
0.5
1.0
MR effect size for
'Holdemania sp900120005' on 'GCST90200753_buildGRCh38.tsv'

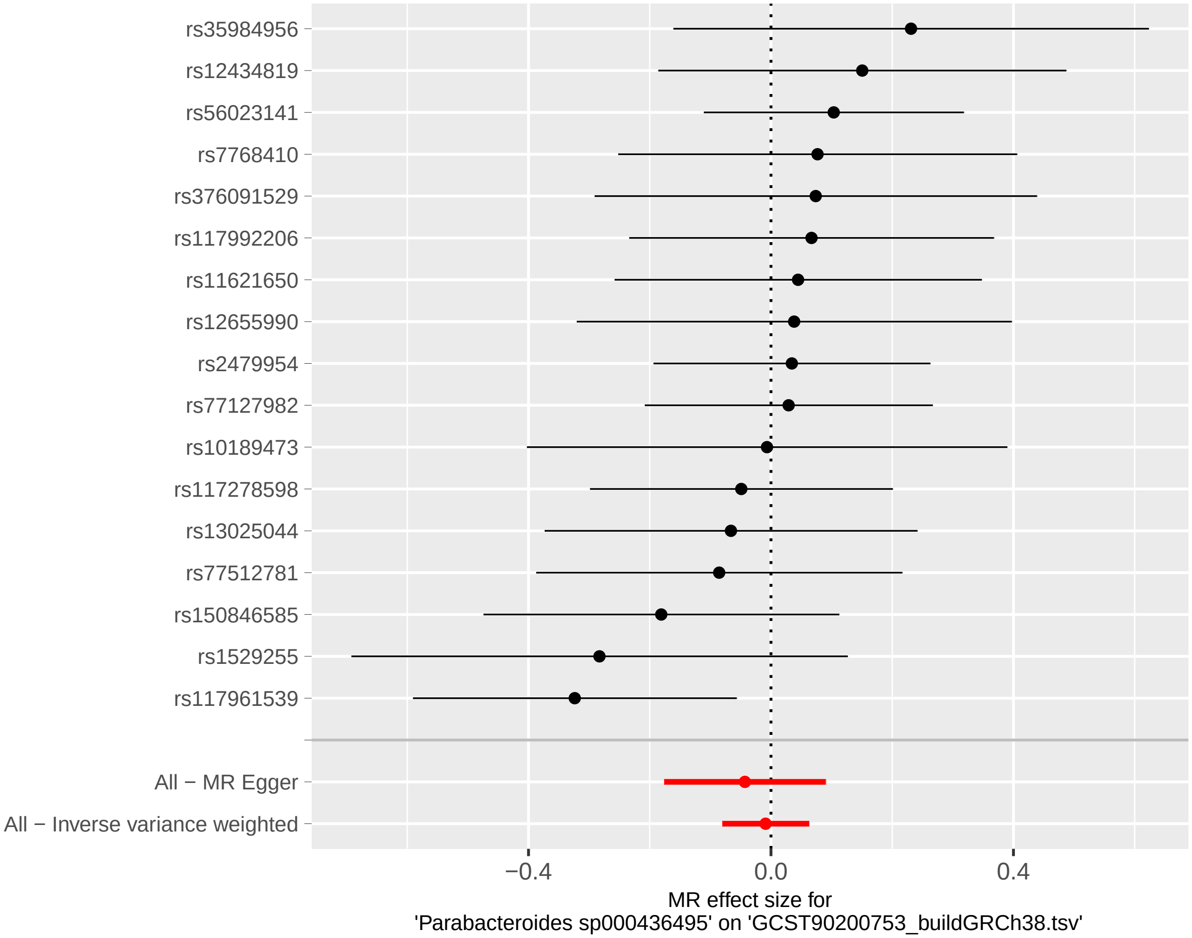

rs35984956
rs12434819
rs56023141
rs7768410
rs376091529
rs117992206
rs11621650
rs12655990
rs2479954
rs77127982
rs10189473
rs117278598
rs13025044
rs77512781
rs150846585
rs1529255
rs117961539
All − MR Egger
All − Inverse variance weighted
−0.4
0.0
0.4
MR effect size for
'Parabacteroides sp000436495' on 'GCST90200753_buildGRCh38.tsv'

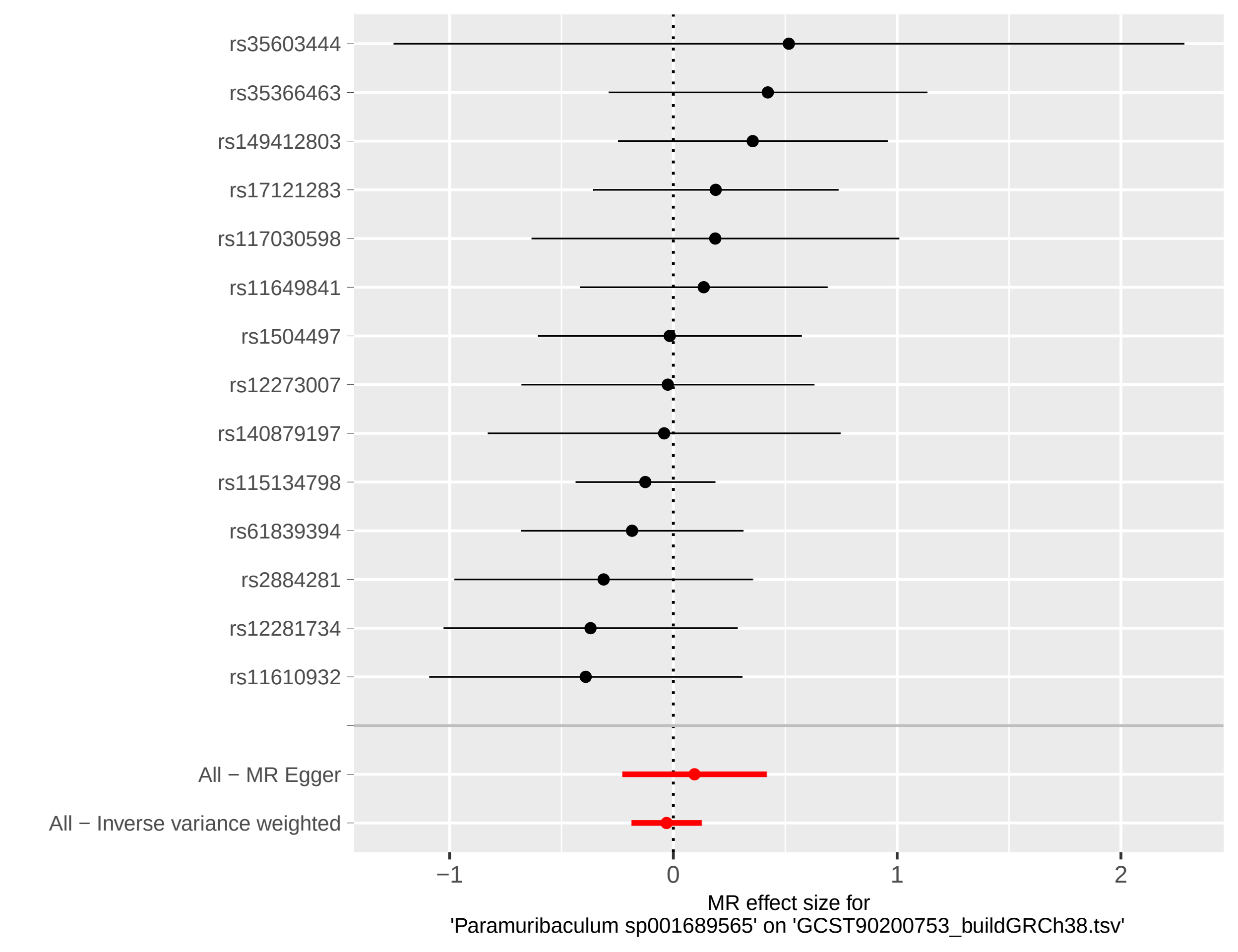
rs35603444
rs35366463
rs149412803
rs17121283
rs117030598
rs11649841
rs1504497
rs12273007
rs140879197
rs115134798
rs61839394
rs2884281
rs12281734
rs11610932
All − MR Egger
All − Inverse variance weighted
−1
0
1
2
MR effect size for
'Paramuribaculum sp001689565' on 'GCST90200753_buildGRCh38.tsv'

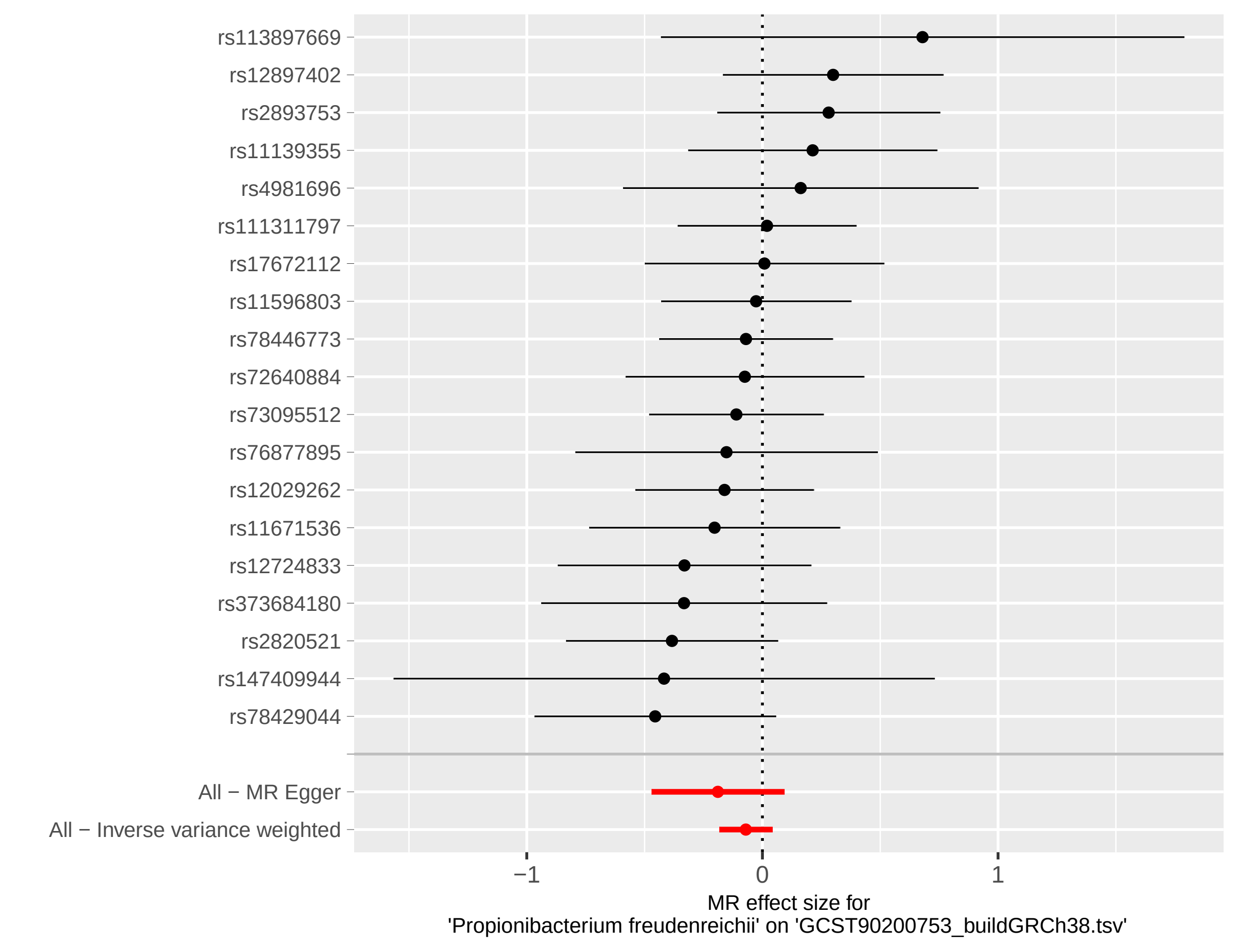
rs113897669
rs12897402
rs2893753
rs11139355
rs4981696
rs111311797
rs17672112
rs11596803
rs78446773
rs72640884
rs73095512
rs76877895
rs12029262
rs11671536
rs12724833
rs373684180
rs2820521
rs147409944
rs78429044
All − MR Egger
All − Inverse variance weighted
−1
0
1
MR effect size for
'Propionibacterium freudenreichii' on 'GCST90200753_buildGRCh38.tsv'

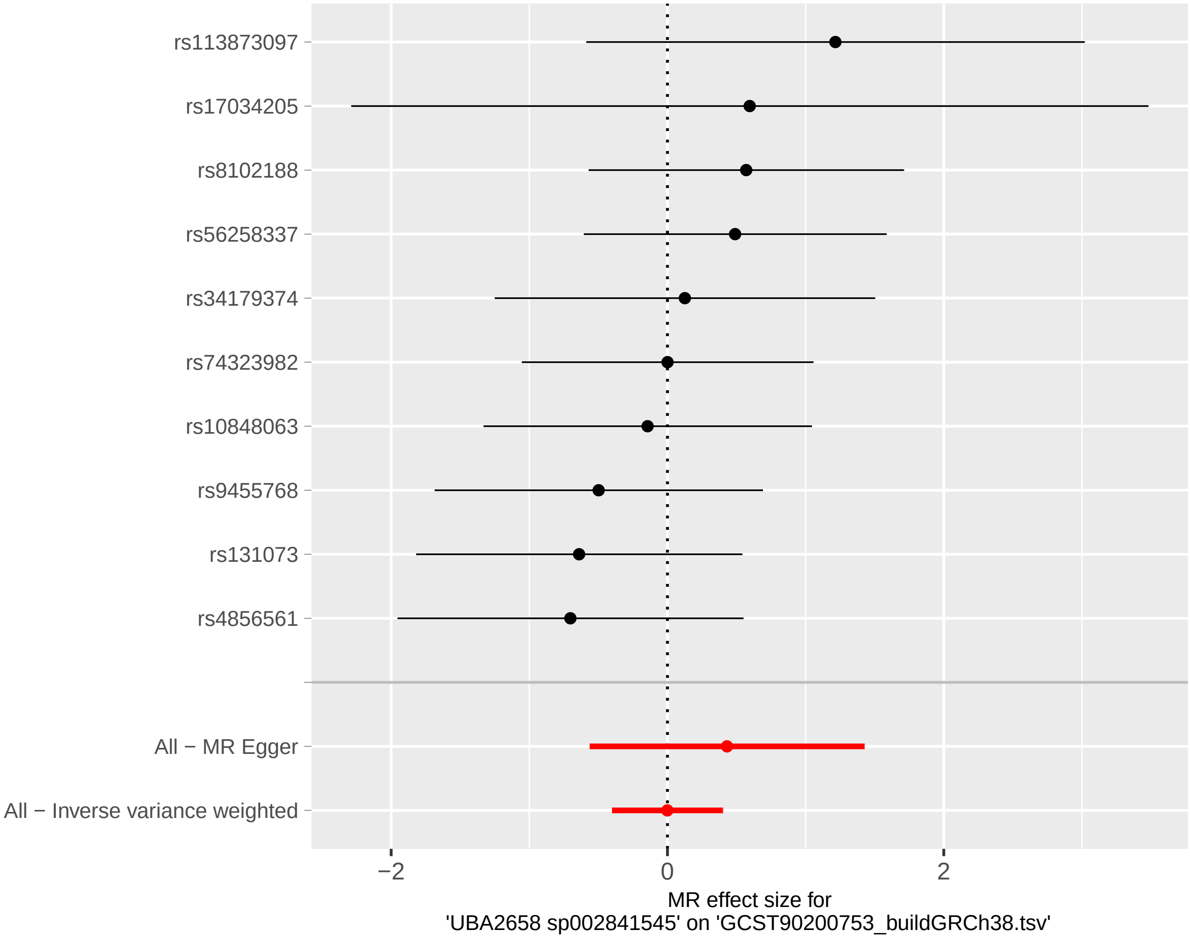
rs113873097
rs17034205
rs8102188
rs56258337
rs34179374
rs74323982
rs10848063
rs9455768
rs131073
rs4856561
All − MR Egger
All − Inverse variance weighted
−2
0
2
MR effect size for
'UBA2658 sp002841545' on 'GCST90200753_buildGRCh38.tsv'

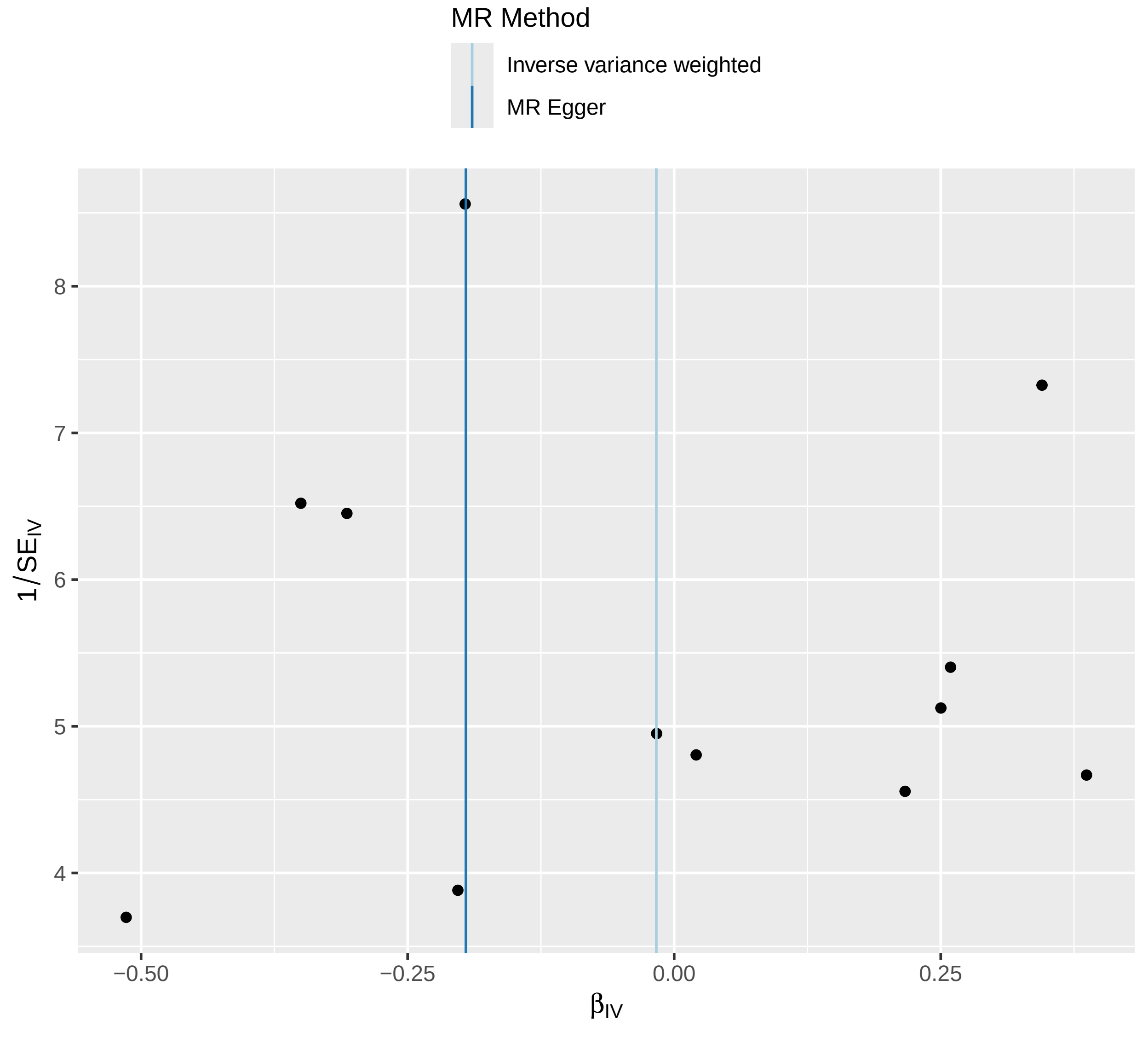
MR Method
Inverse variance weighted
MR Egger
1/SE_IV
8
7
6
5
4
−0.50
−0.25
0.00
0.25
β_IV

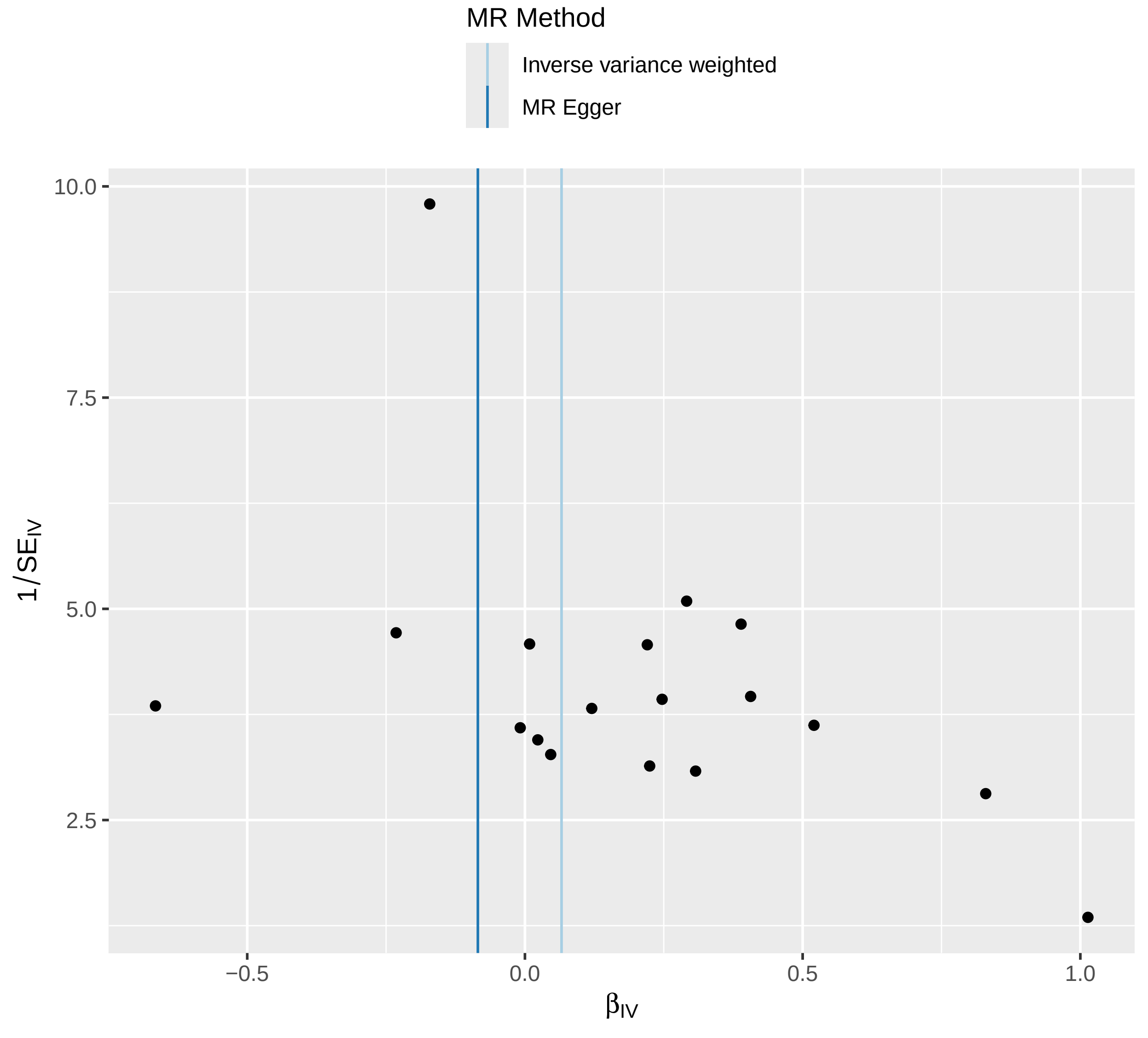

MR Method
Inverse variance weighted
MR Egger
10.0
7.5
5.0
2.5
1/SE_IV
−0.5
0.0
0.5
1.0
β_IV

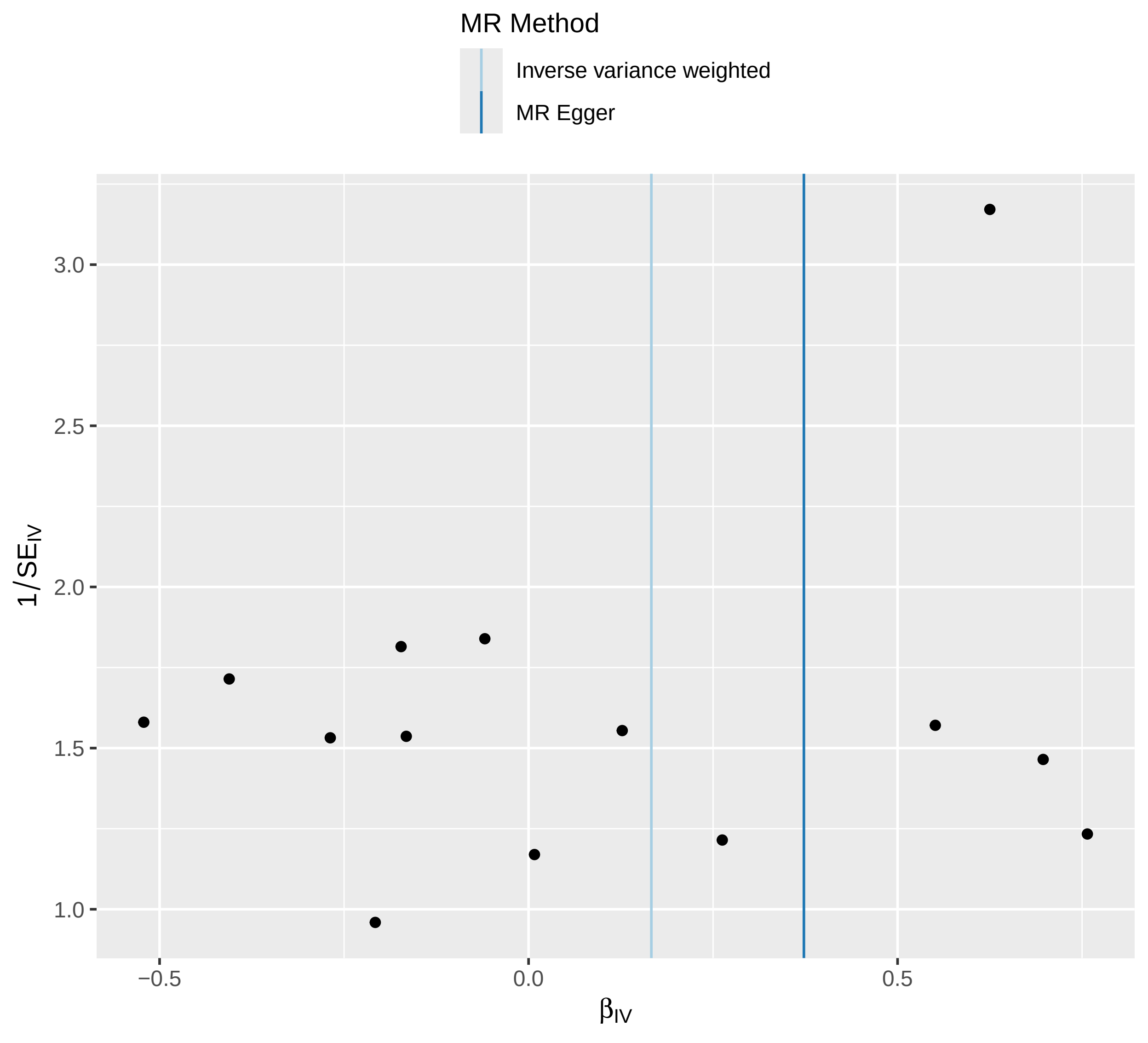
MR Method
Inverse variance weighted
MR Egger
3.0
2.5
2.0
1.5
1.0
1/SE_IV
−0.5
0.0
0.5
β_IV

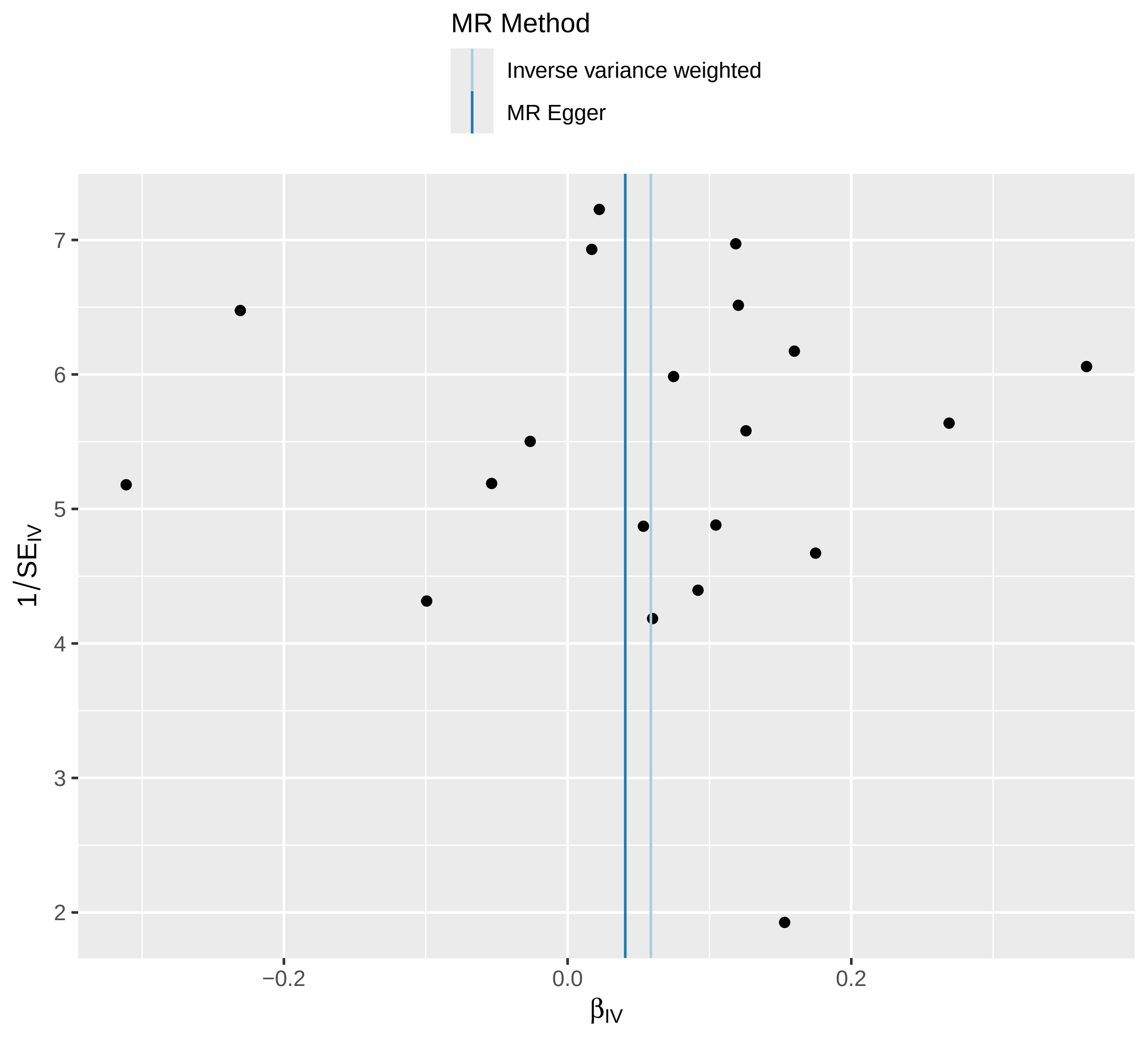

MR Method
Inverse variance weighted
MR Egger
7
6
5
4
3
2
1/SE_IV
−0.2
0.0
0.2
β_IV

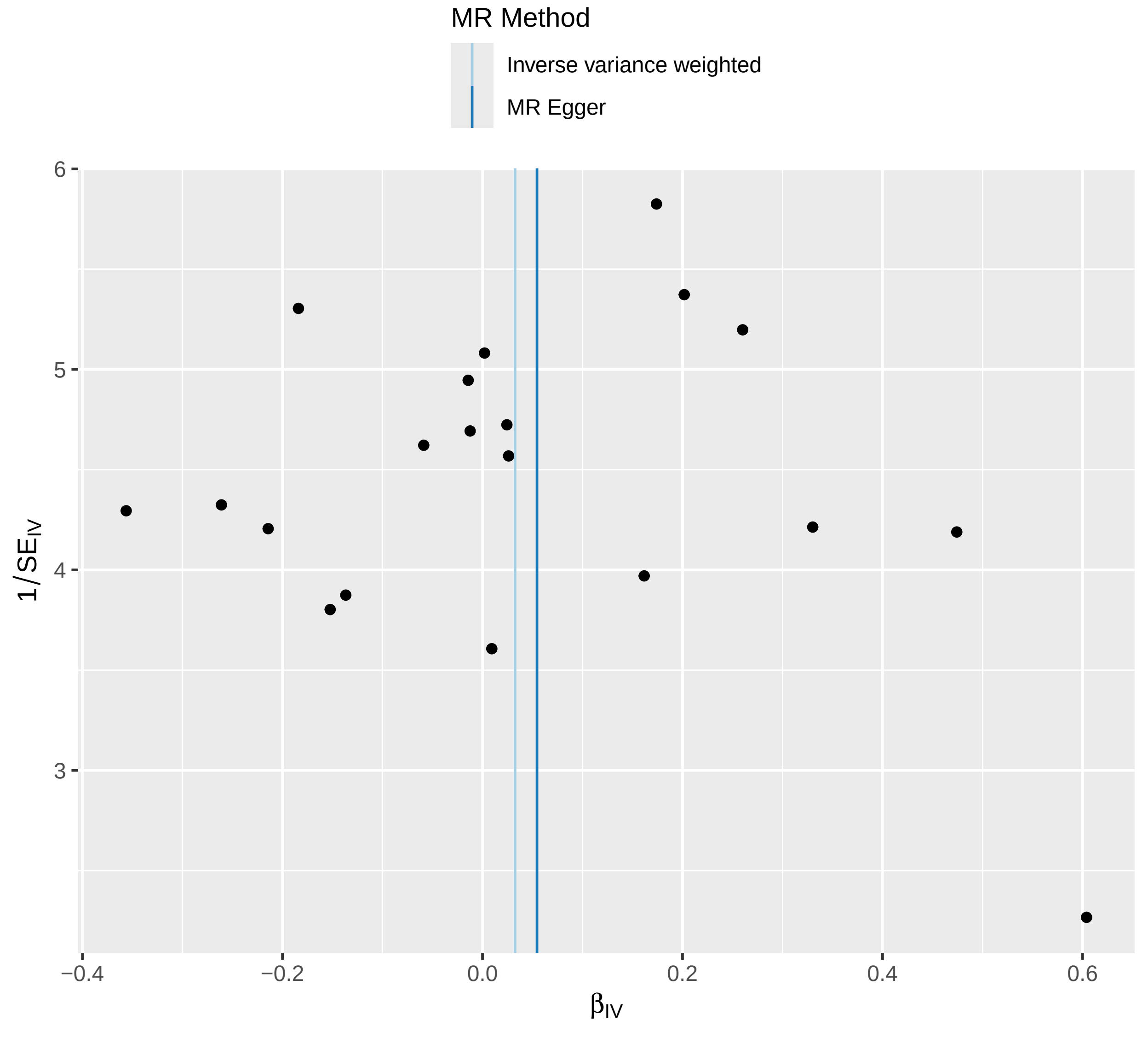
MR Method
Inverse variance weighted
MR Egger
6
5
4
3
1/SE_IV
−0.4
−0.2
0.0
0.2
0.4
0.6
β_IV

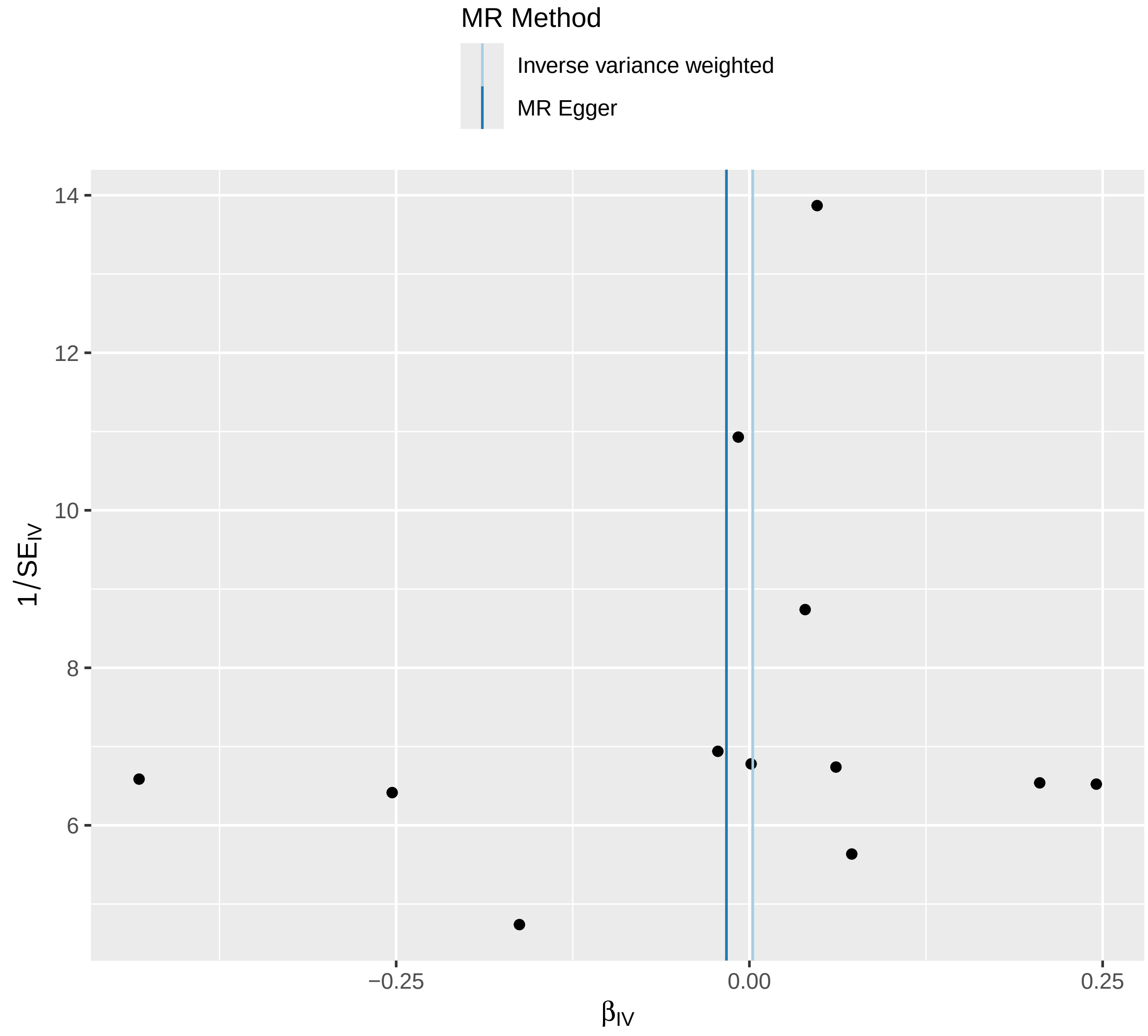
MR Method
Inverse variance weighted
MR Egger
14
12
10
8
6
1/SE_IV
−0.25
0.00
0.25
β_IV

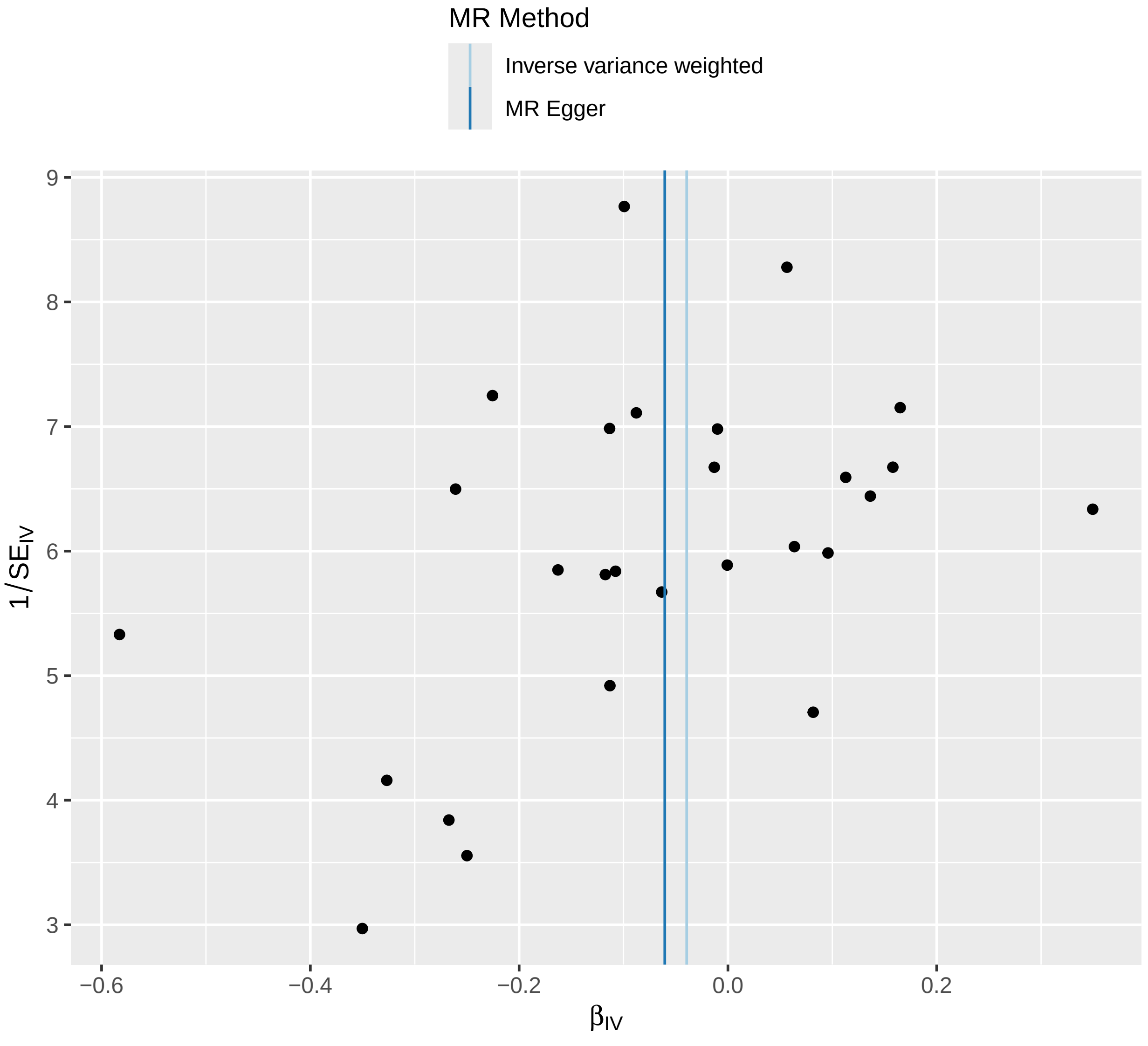
MR Method
Inverse variance weighted
MR Egger
1/SE_IV
β_IV
9
8
7
6
5
4
3
−0.6
−0.4
−0.2
0.0
0.2

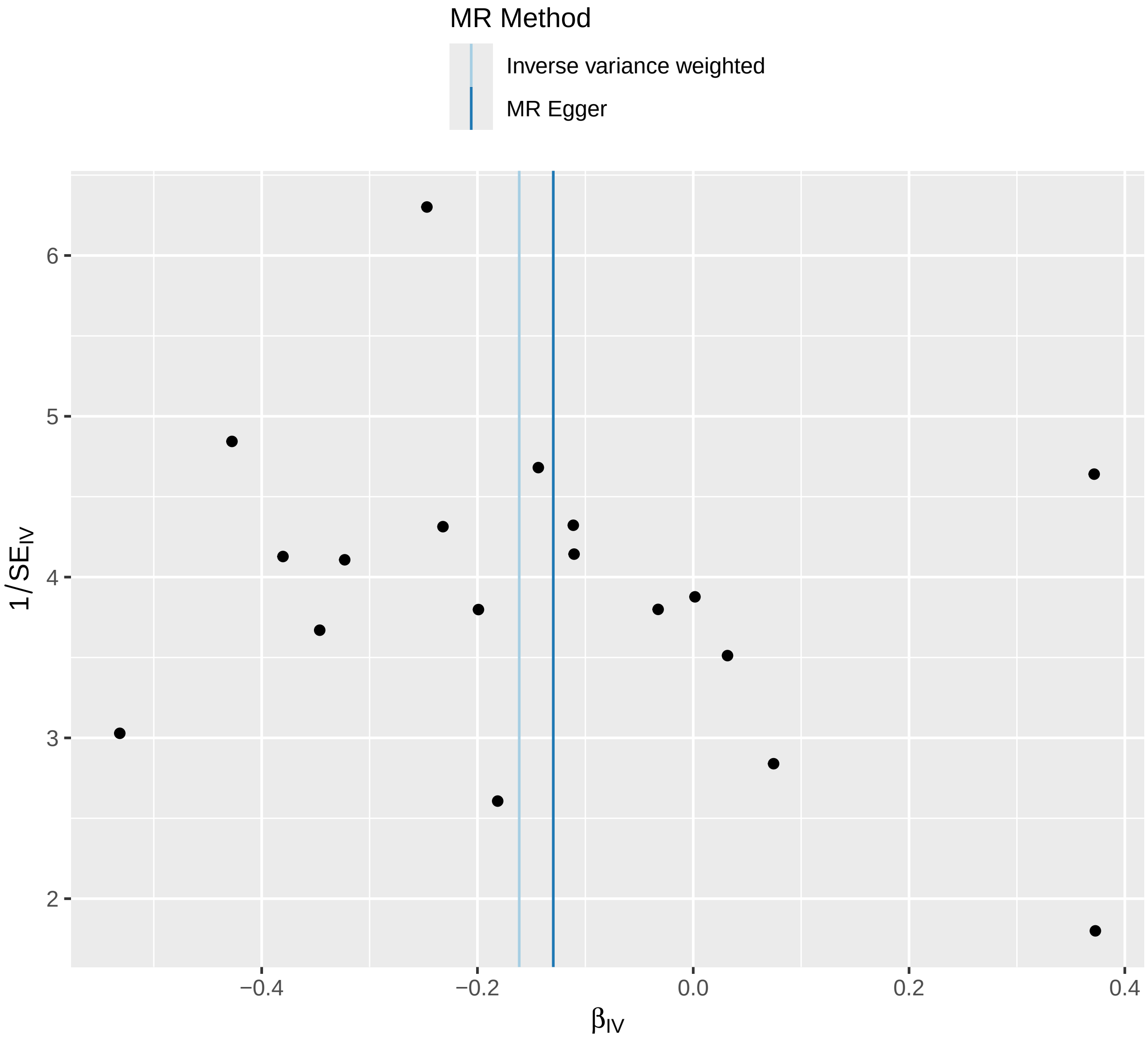

MR Method
Inverse variance weighted
MR Egger
6
5
4
3
2
1/SE_IV
−0.4
−0.2
0.0
0.2
0.4
β_IV

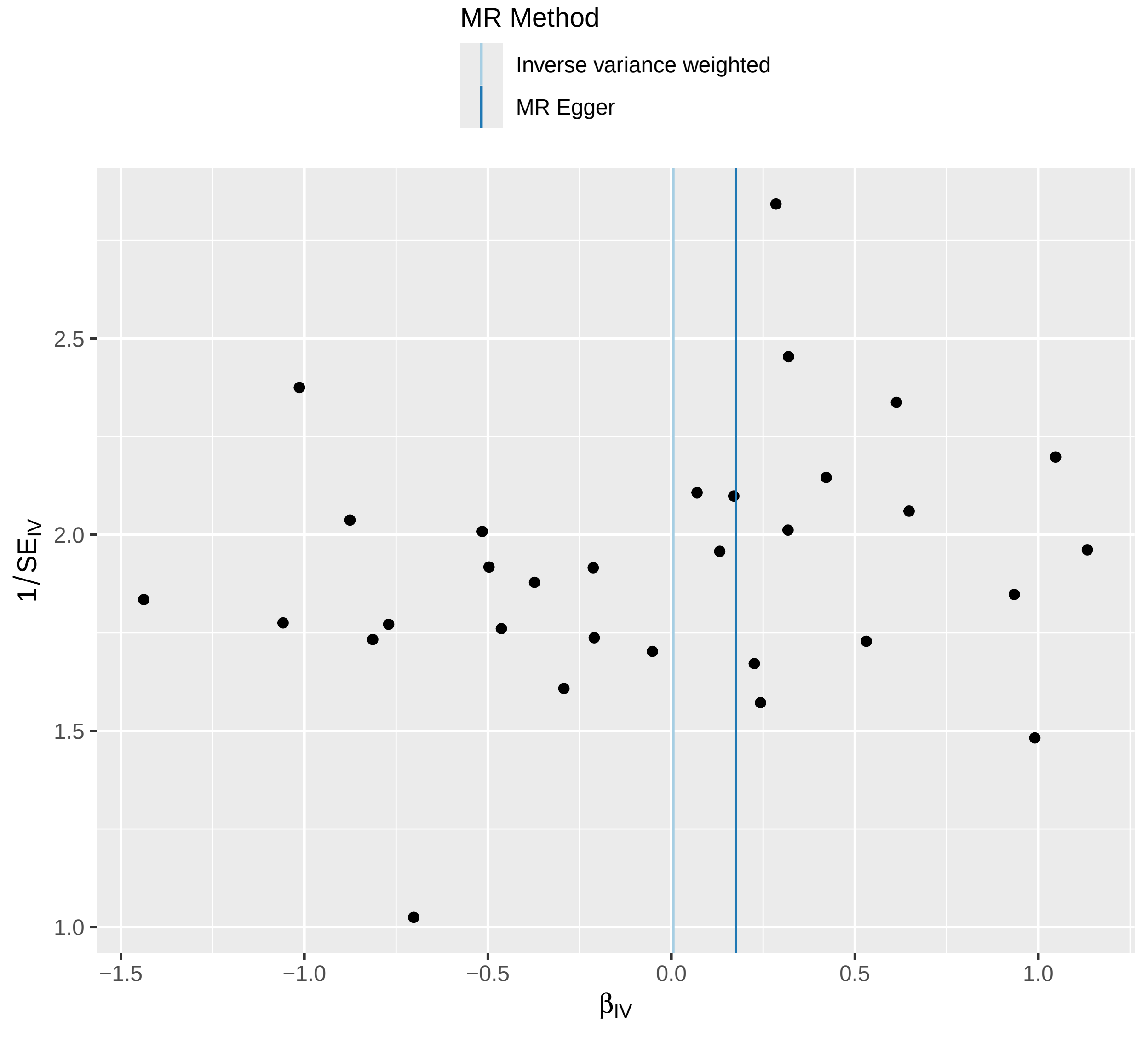

MR Method
Inverse variance weighted
MR Egger
2.5
2.0
1.5
1.0
1/SE_IV
−1.5
−1.0
−0.5
0.0
0.5
1.0
β_IV

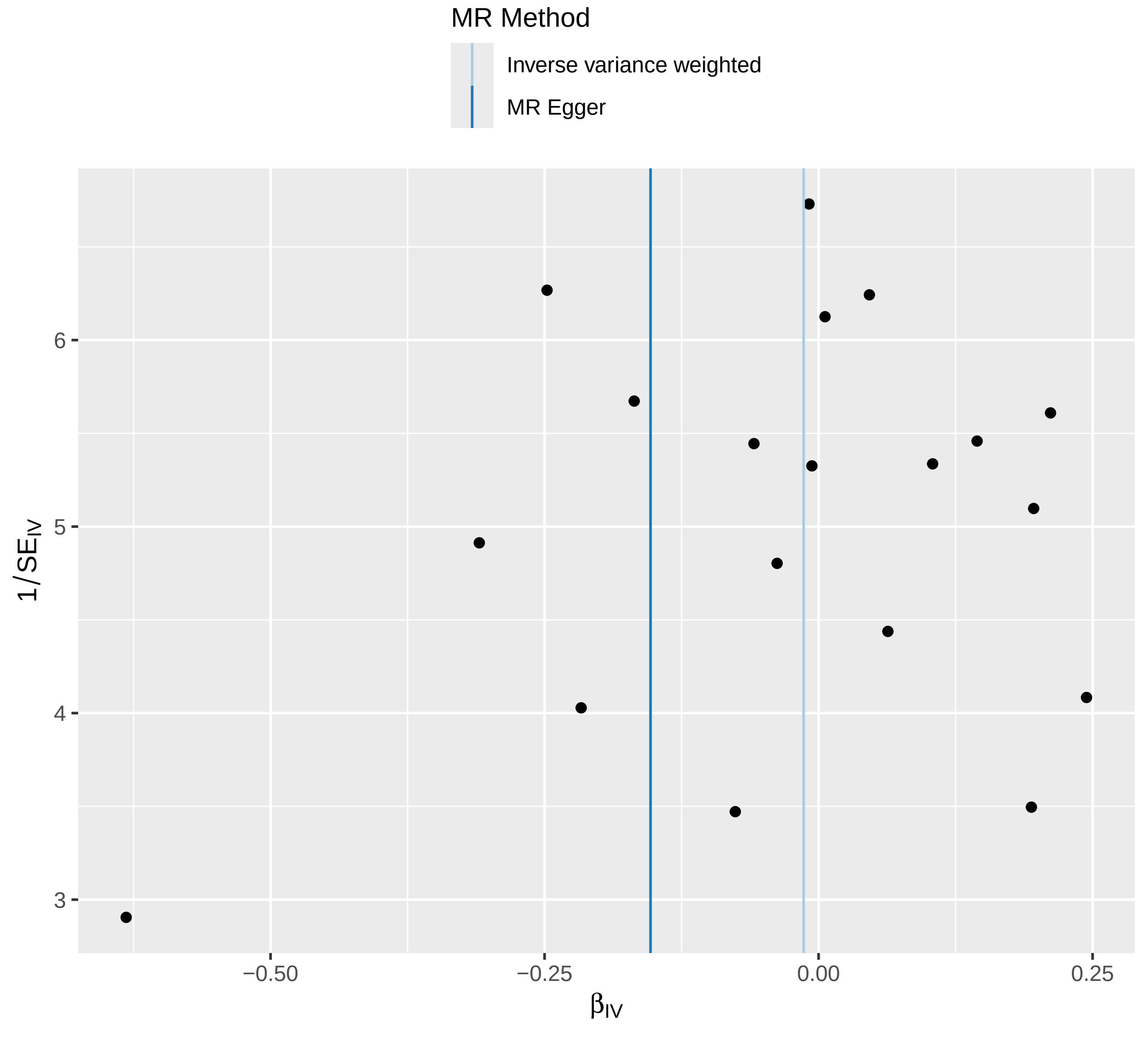
MR Method
Inverse variance weighted
MR Egger
6
5
4
3
1/SE_IV
−0.50
−0.25
0.00
0.25
β_IV

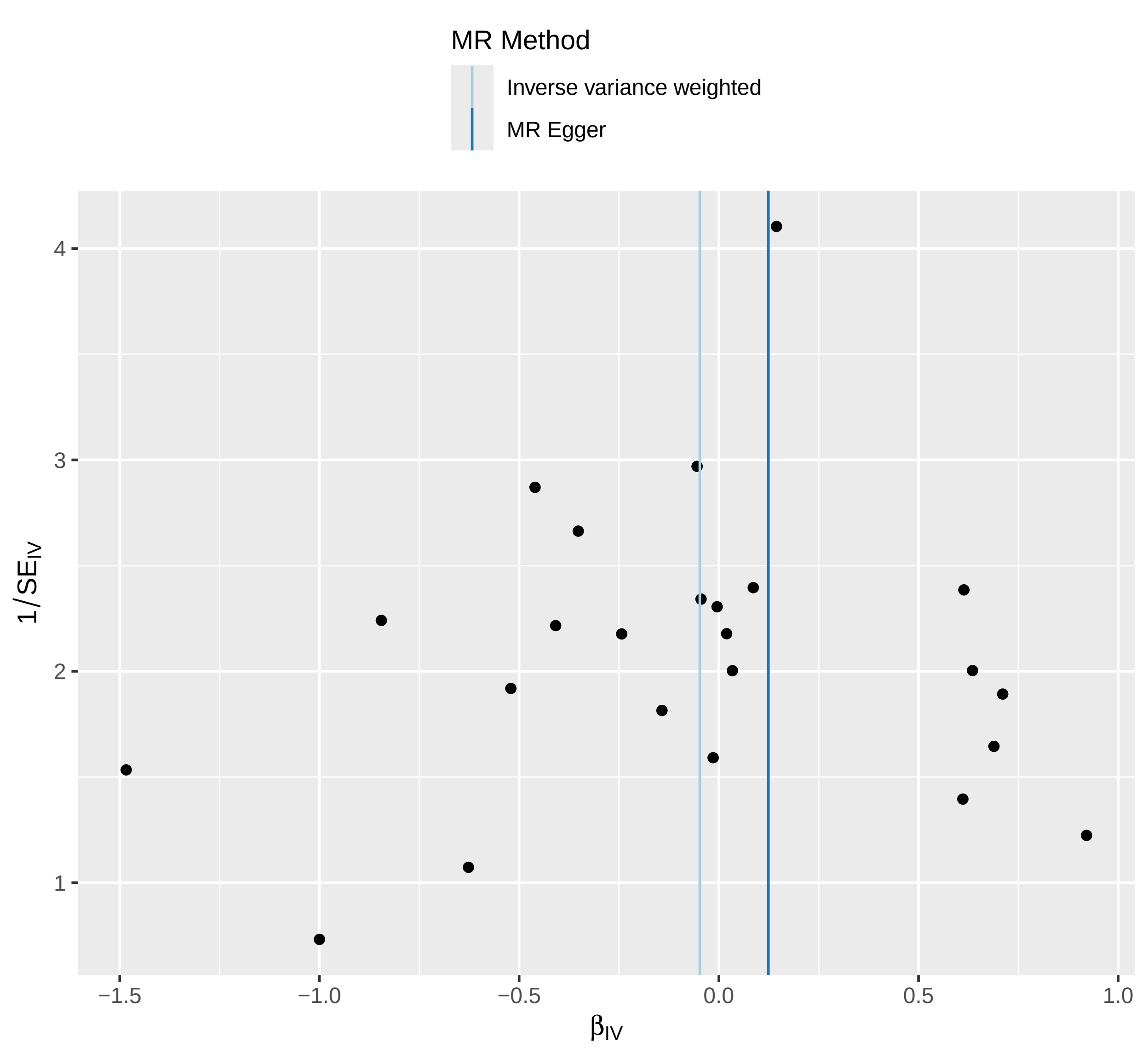
MR Method
Inverse variance weighted
MR Egger
1/SE_IV
4
3
2
1
−1.5
−1.0
−0.5
0.0
0.5
1.0
β_IV

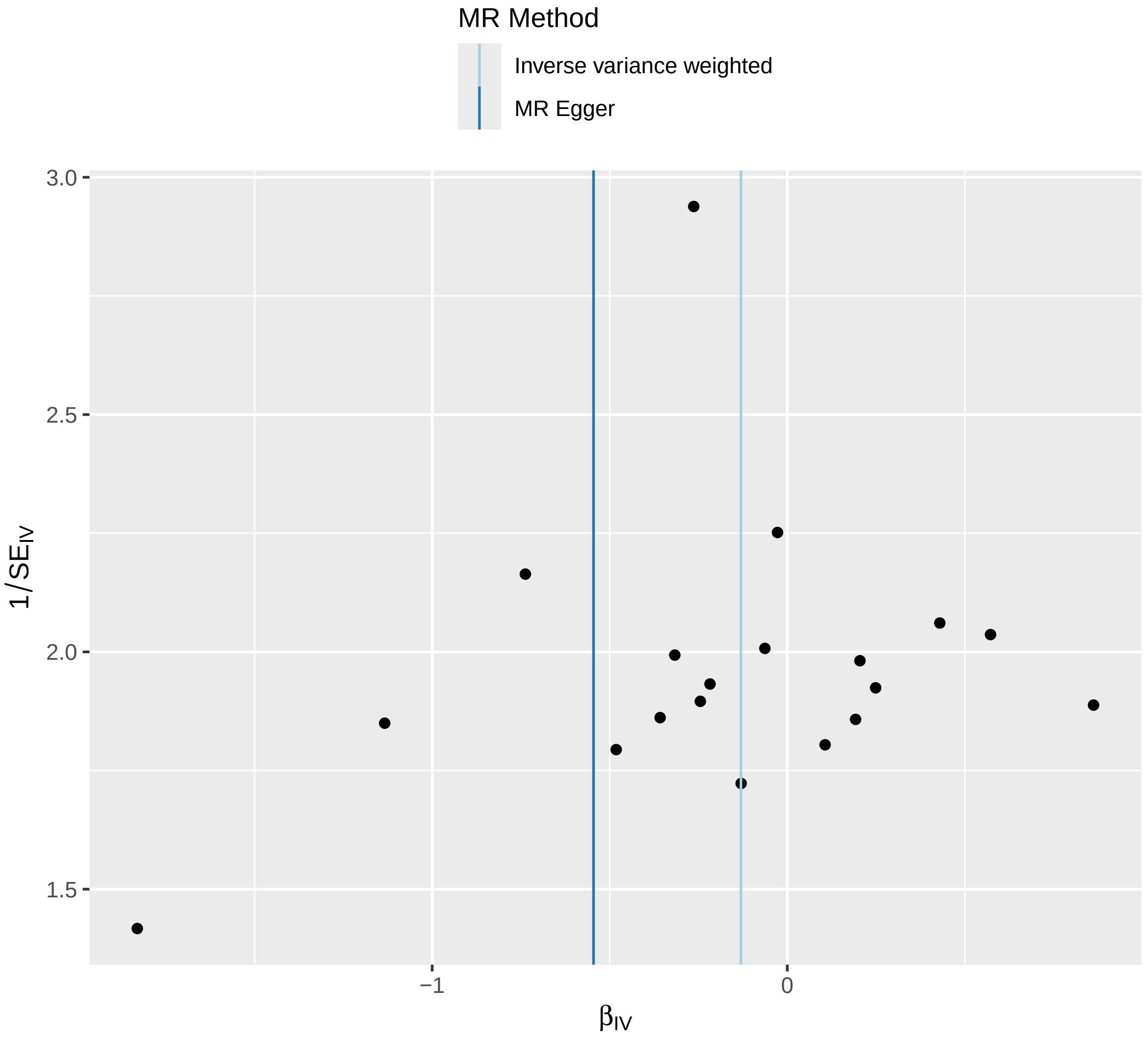
MR Method
Inverse variance weighted
MR Egger
3.0
2.5
2.0
1.5
−1
0
$1/SE_{IV}$
β_{IV}

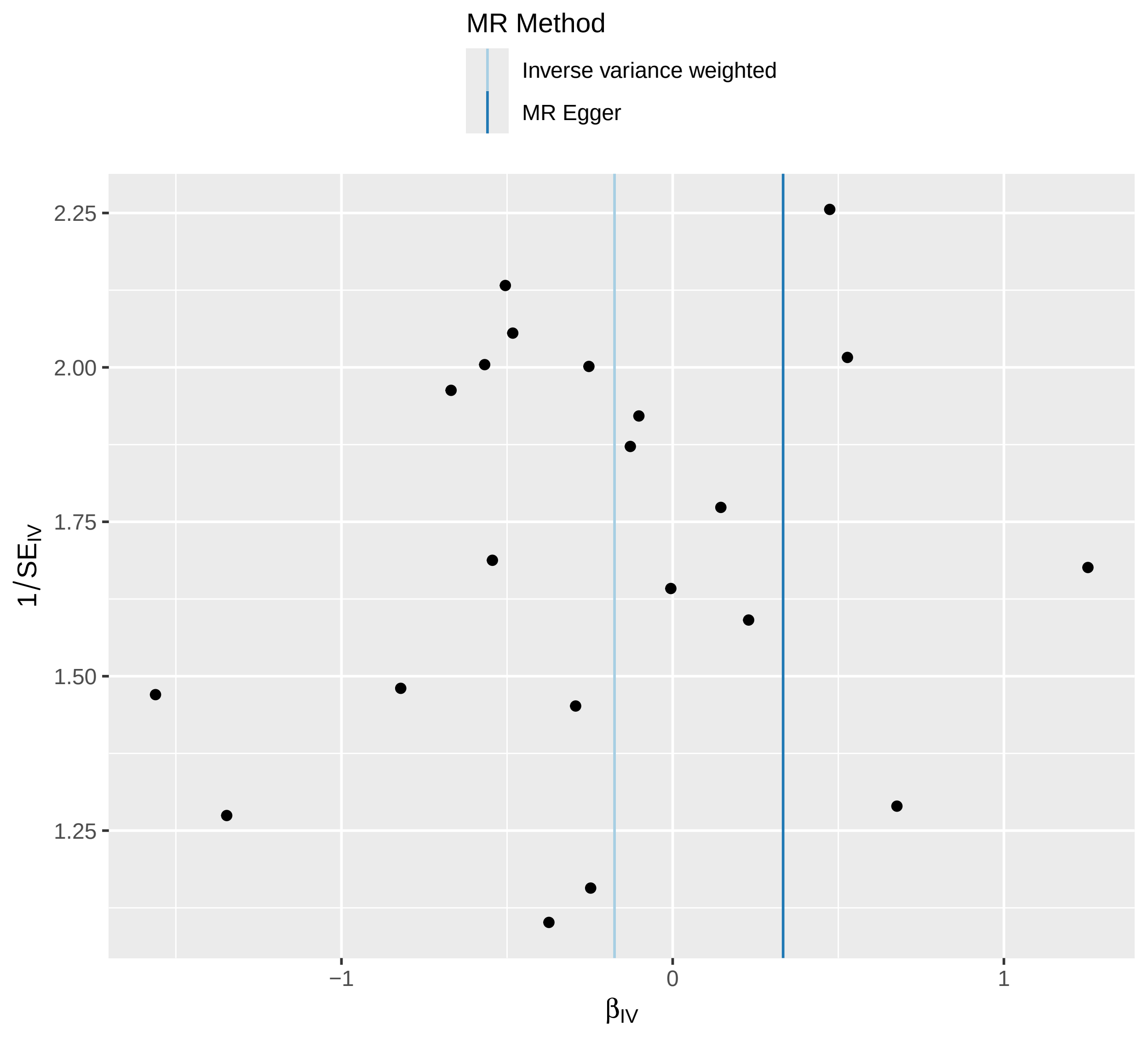

MR Method
Inverse variance weighted
MR Egger
2.25
2.00
1.75
1.50
1.25
1/SE_IV
−1
0
1
β_IV

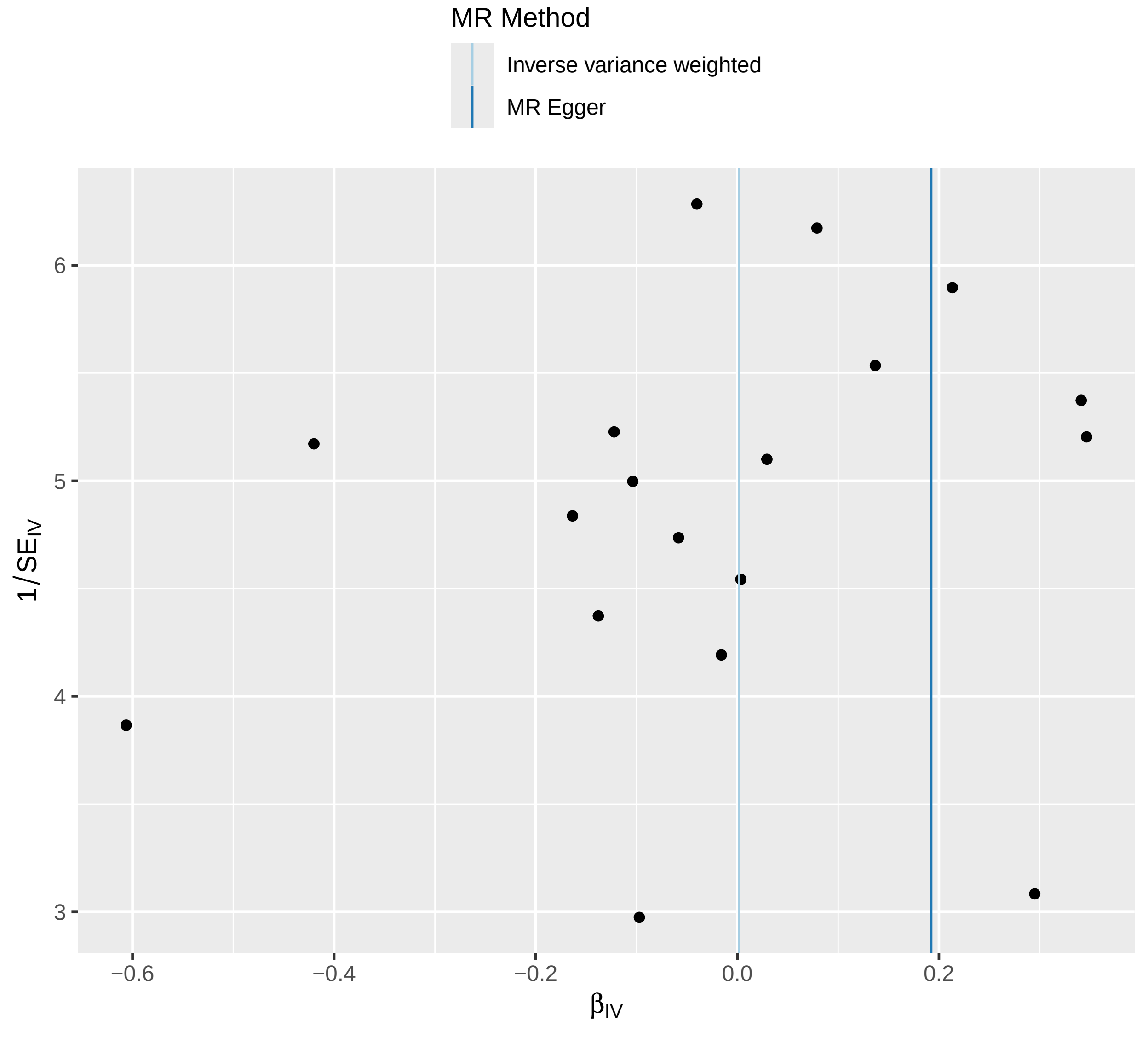
MR Method
Inverse variance weighted
MR Egger
6
5
4
3
−0.6
−0.4
−0.2
0.0
0.2
$1/SE_{IV}$
β_{IV}

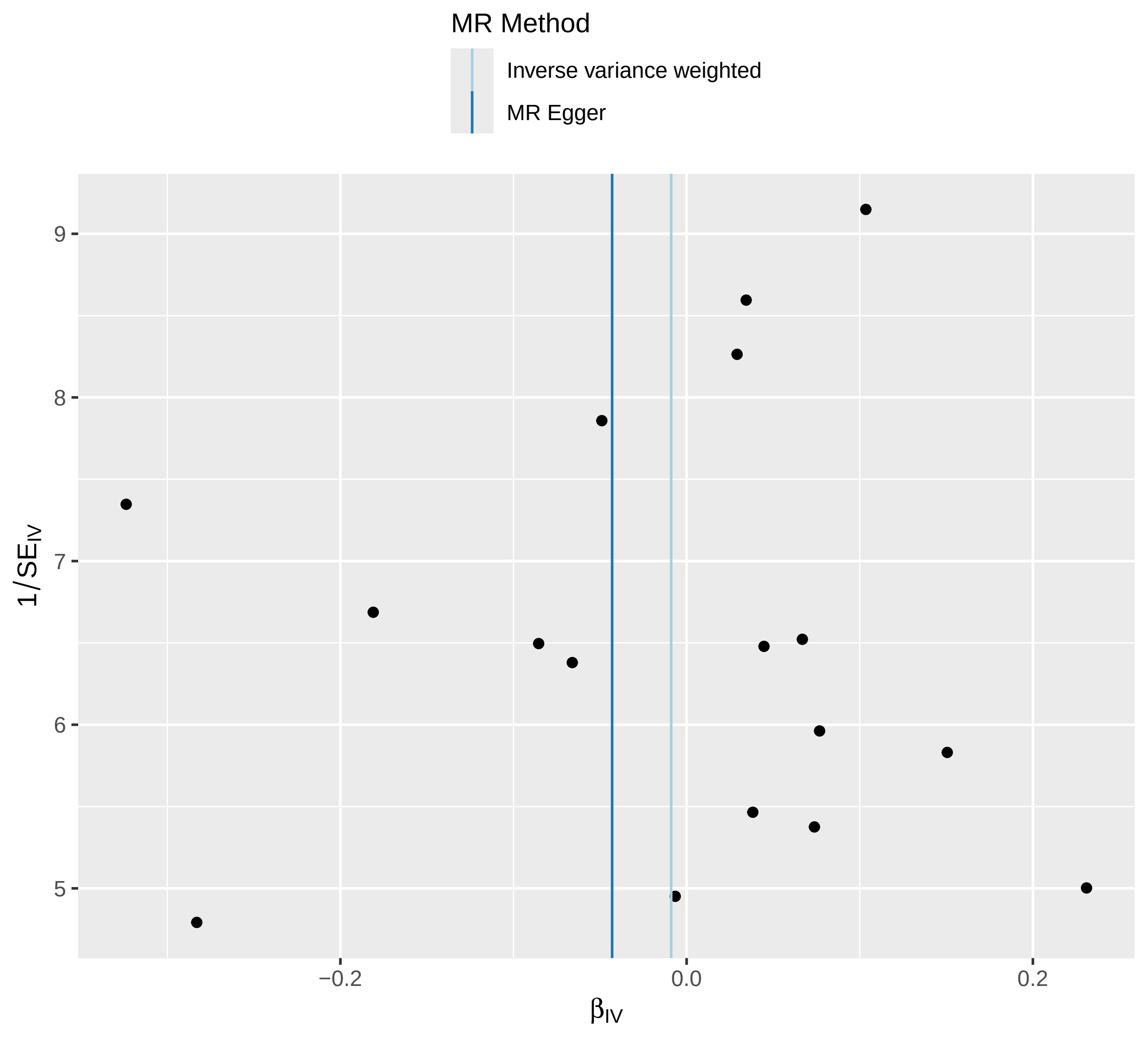
MR Method
Inverse variance weighted
MR Egger
9
8
7
6
5
$1/SE_{IV}$
−0.2
0.0
0.2
β_{IV}

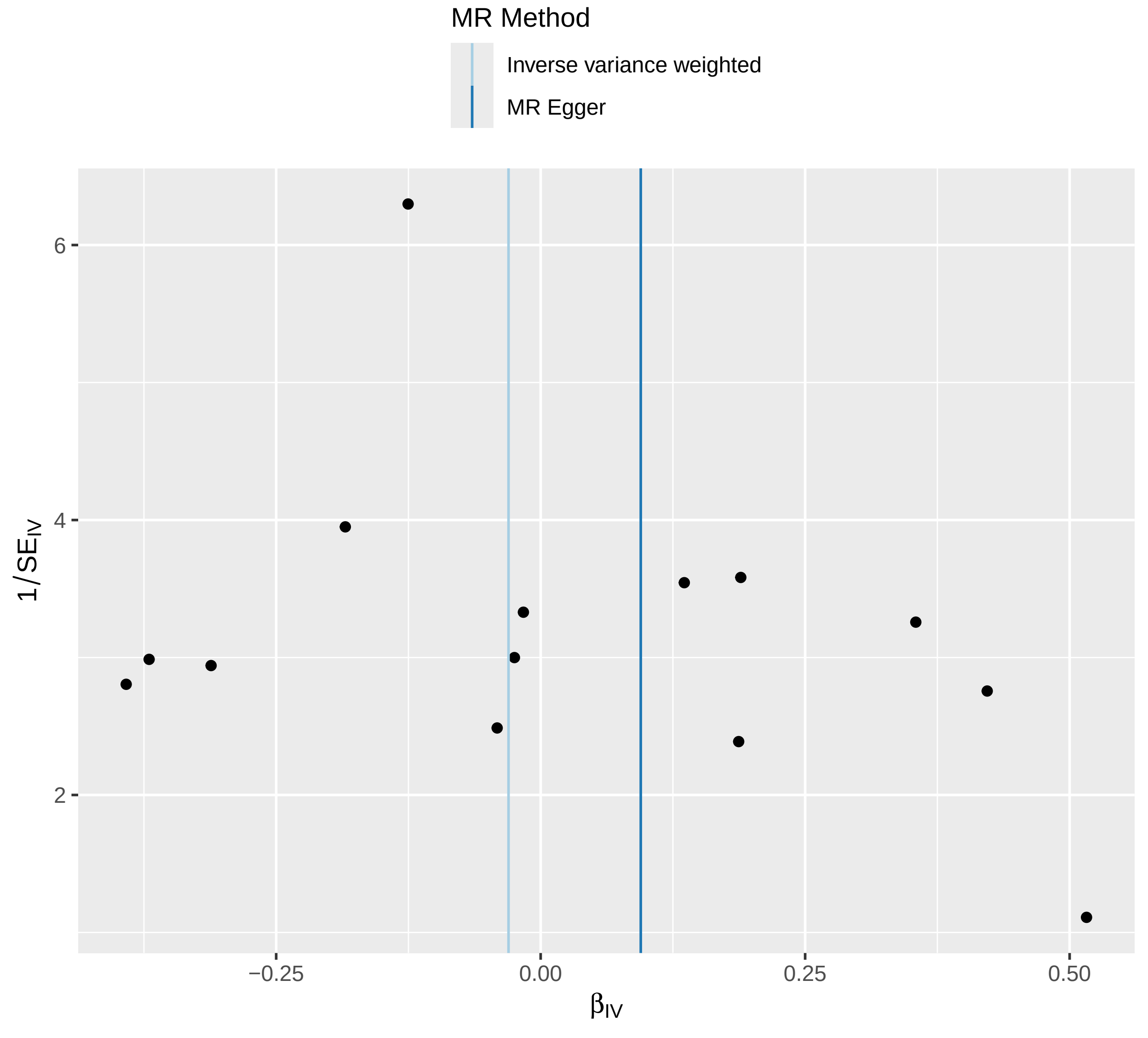
MR Method
Inverse variance weighted
MR Egger
6
4
2
$1/SE_{IV}$
−0.25
0.00
0.25
0.50
β_{IV}

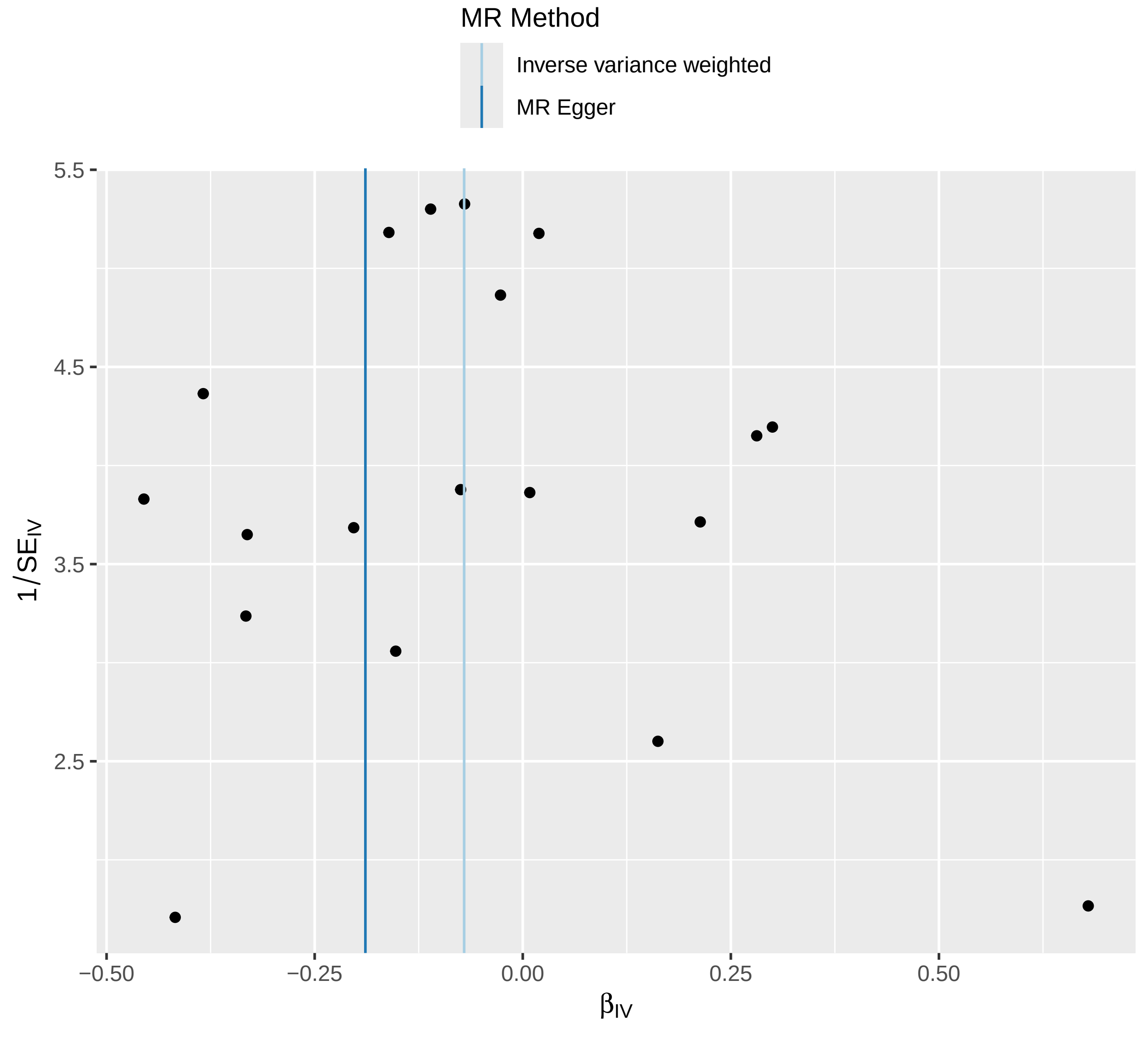

MR Method
Inverse variance weighted
MR Egger
5.5
4.5
3.5
2.5
1/SE_IV
−0.50
−0.25
0.00
0.25
0.50
β_IV

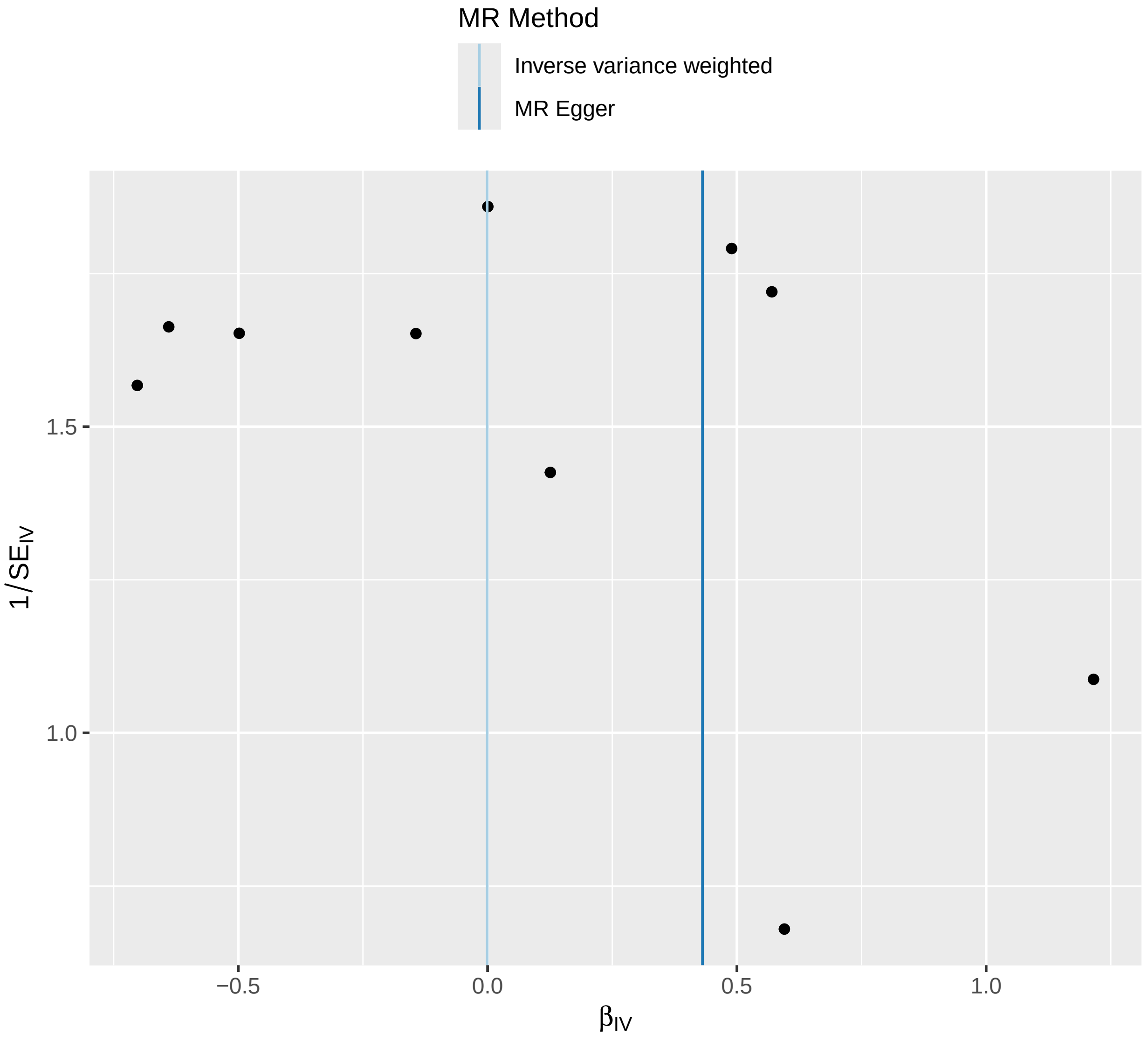

MR Method
Inverse variance weighted
MR Egger
1.5
1.0
1/SE$_{IV}$
−0.5
0.0
0.5
1.0
β$_{IV}$

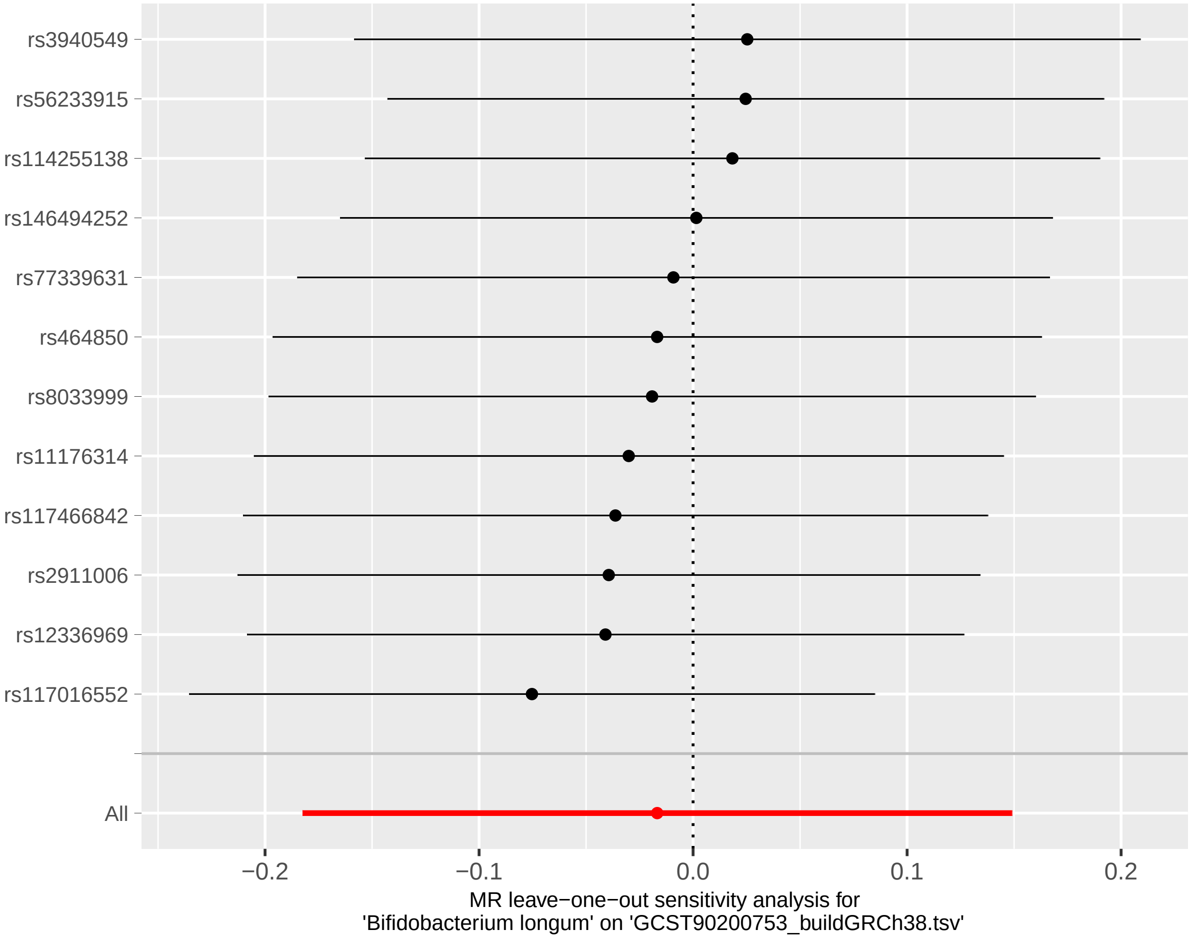
rs3940549
rs56233915
rs114255138
rs146494252
rs77339631
rs464850
rs8033999
rs11176314
rs117466842
rs2911006
rs12336969
rs117016552
All
−0.2
−0.1
0.0
0.1
0.2
MR leave−one−out sensitivity analysis for
'Bifidobacterium longum' on 'GCST90200753_buildGRCh38.tsv'

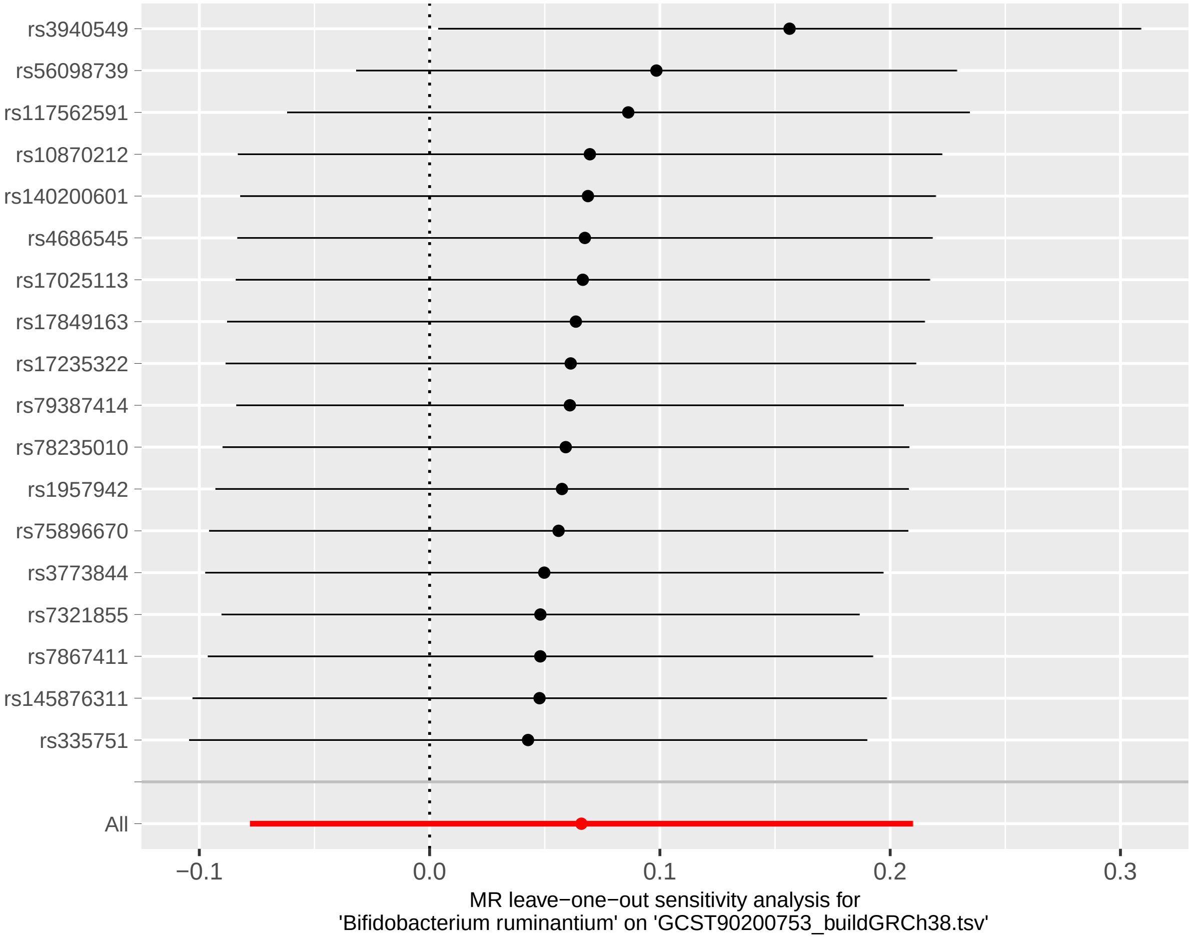

rs3940549
rs56098739
rs117562591
rs10870212
rs140200601
rs4686545
rs17025113
rs17849163
rs17235322
rs79387414
rs78235010
rs1957942
rs75896670
rs3773844
rs7321855
rs7867411
rs145876311
rs335751
All
−0.1
0.0
0.1
0.2
0.3
MR leave−one−out sensitivity analysis for
'Bifidobacterium ruminantium' on 'GCST90200753_buildGRCh38.tsv'

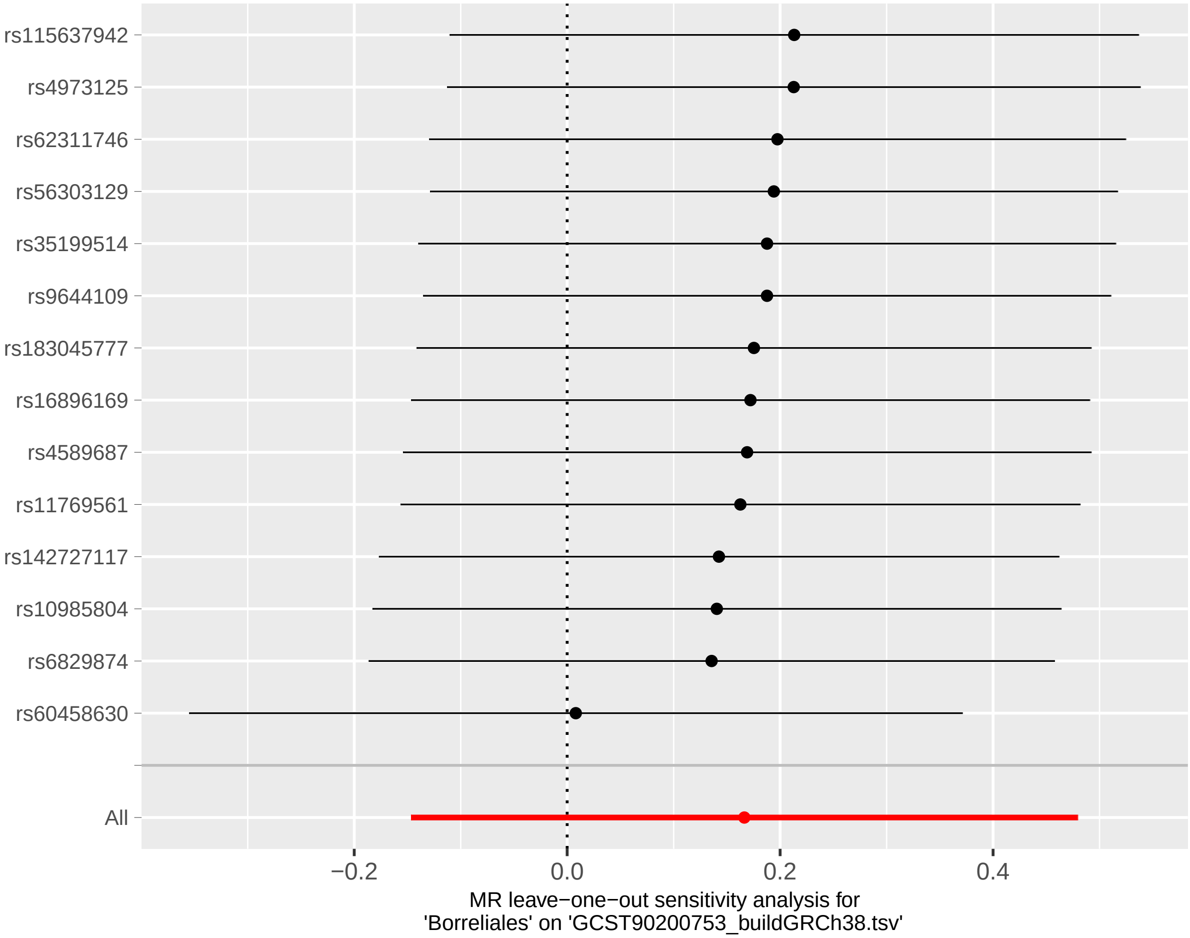
rs115637942
rs4973125
rs62311746
rs56303129
rs35199514
rs9644109
rs183045777
rs16896169
rs4589687
rs11769561
rs142727117
rs10985804
rs6829874
rs60458630
All
−0.2
0.0
0.2
0.4
MR leave−one−out sensitivity analysis for
'Borreliales' on 'GCST90200753_buildGRCh38.tsv'

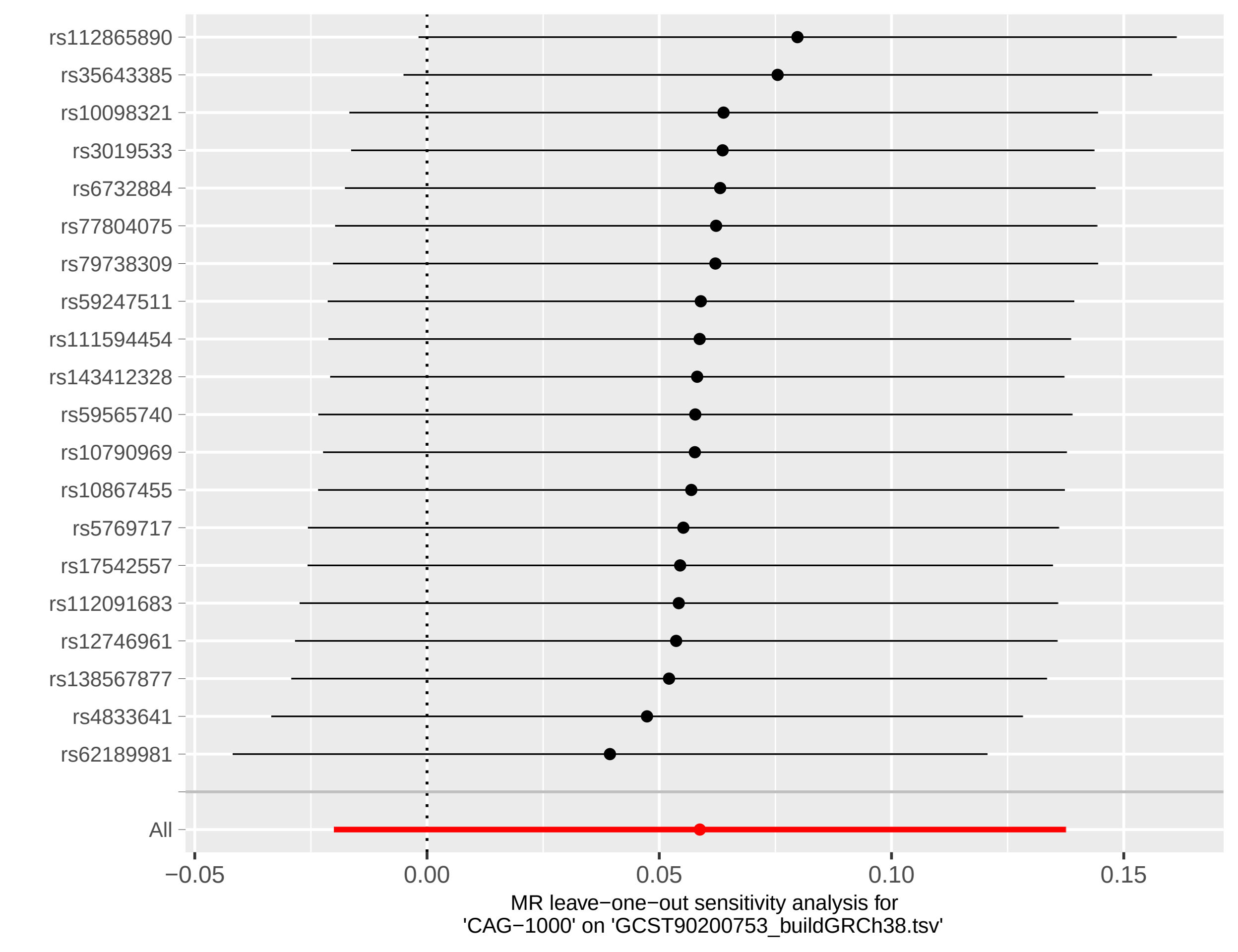
rs112865890
rs35643385
rs10098321
rs3019533
rs6732884
rs77804075
rs79738309
rs59247511
rs111594454
rs143412328
rs59565740
rs10790969
rs10867455
rs5769717
rs17542557
rs112091683
rs12746961
rs138567877
rs4833641
rs62189981
All
−0.05
0.00
0.05
0.10
0.15
MR leave−one−out sensitivity analysis for
'CAG−1000' on 'GCST90200753_buildGRCh38.tsv'

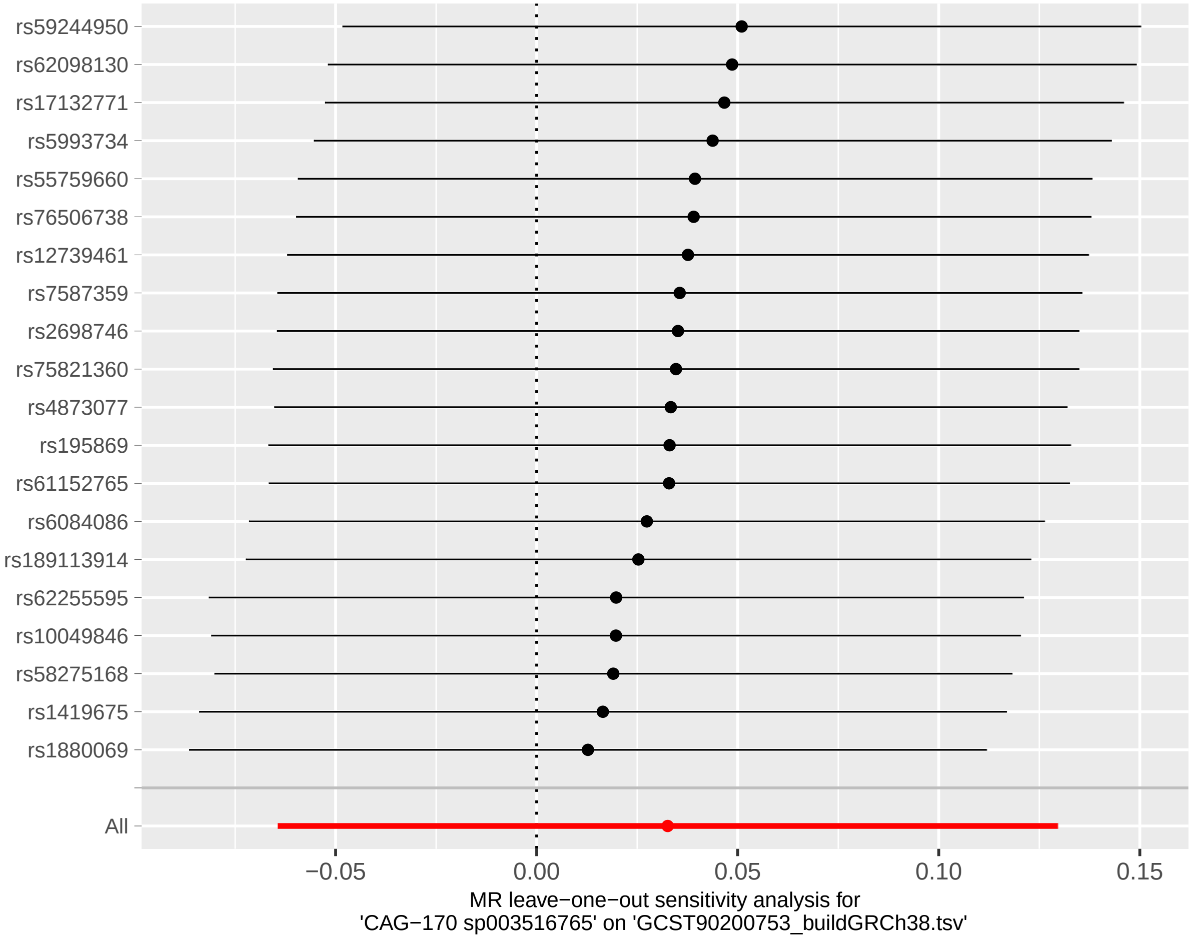
rs59244950
rs62098130
rs17132771
rs5993734
rs55759660
rs76506738
rs12739461
rs7587359
rs2698746
rs75821360
rs4873077
rs195869
rs61152765
rs6084086
rs189113914
rs62255595
rs10049846
rs58275168
rs1419675
rs1880069
All
−0.05
0.00
0.05
0.10
0.15
MR leave−one−out sensitivity analysis for
'CAG−170 sp003516765' on 'GCST90200753_buildGRCh38.tsv'

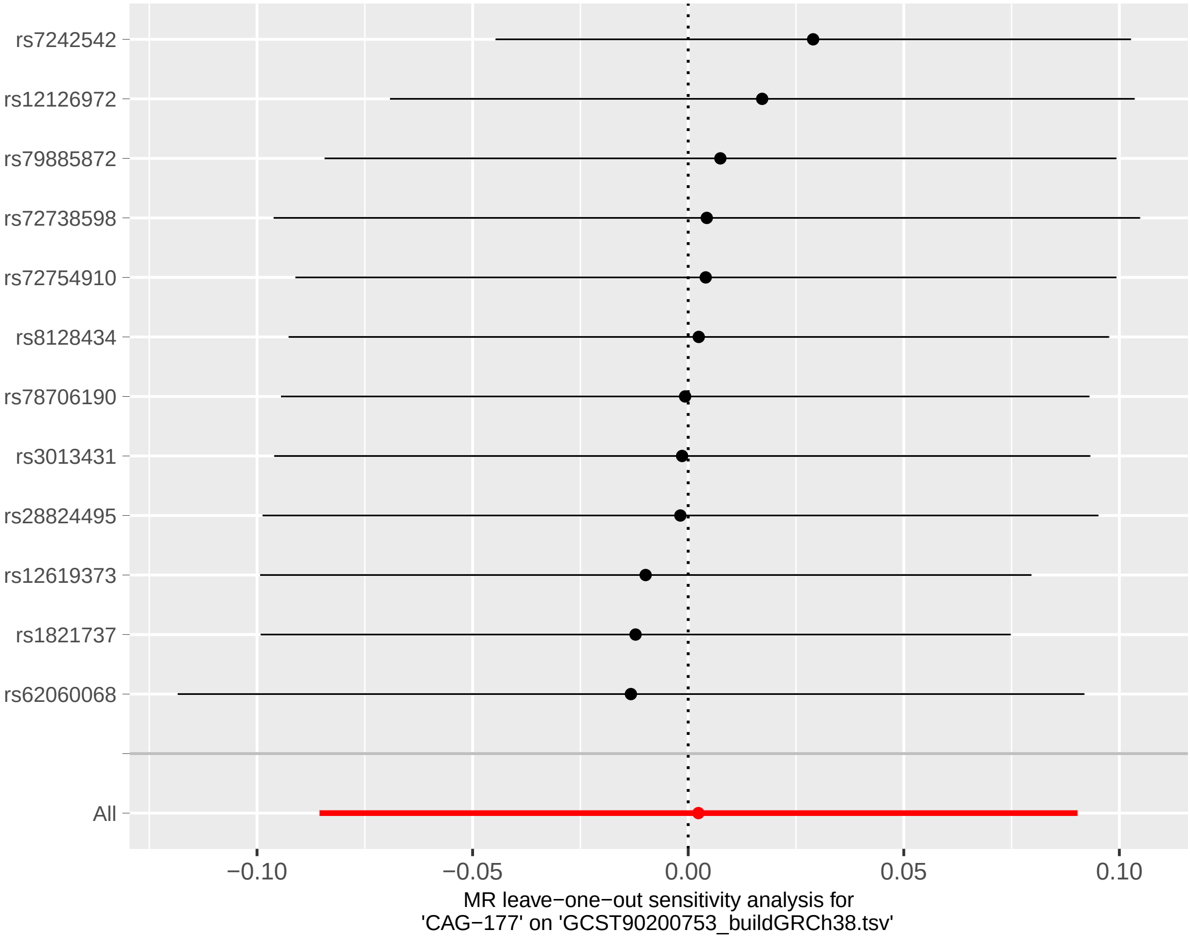
rs7242542
rs12126972
rs79885872
rs72738598
rs72754910
rs8128434
rs78706190
rs3013431
rs28824495
rs12619373
rs1821737
rs62060068
All
−0.10
−0.05
0.00
0.05
0.10
MR leave−one−out sensitivity analysis for
'CAG−177' on 'GCST90200753_buildGRCh38.tsv'

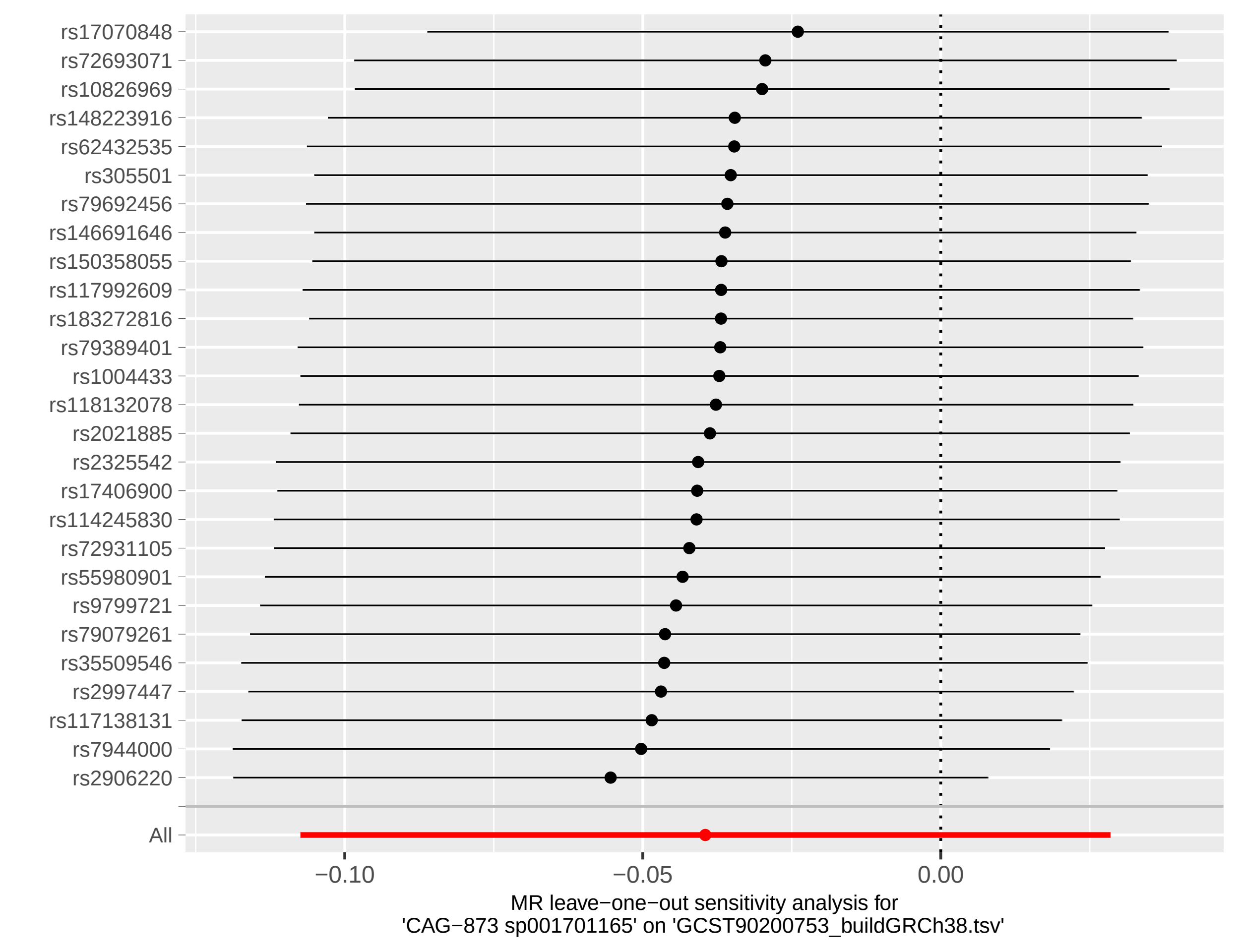
rs17070848
rs72693071
rs10826969
rs148223916
rs62432535
rs305501
rs79692456
rs146691646
rs150358055
rs117992609
rs183272816
rs79389401
rs1004433
rs118132078
rs2021885
rs2325542
rs17406900
rs114245830
rs72931105
rs55980901
rs9799721
rs79079261
rs35509546
rs2997447
rs117138131
rs7944000
rs2906220
All
−0.10
−0.05
0.00
MR leave−one−out sensitivity analysis for
'CAG−873 sp001701165' on 'GCST90200753_buildGRCh38.tsv'

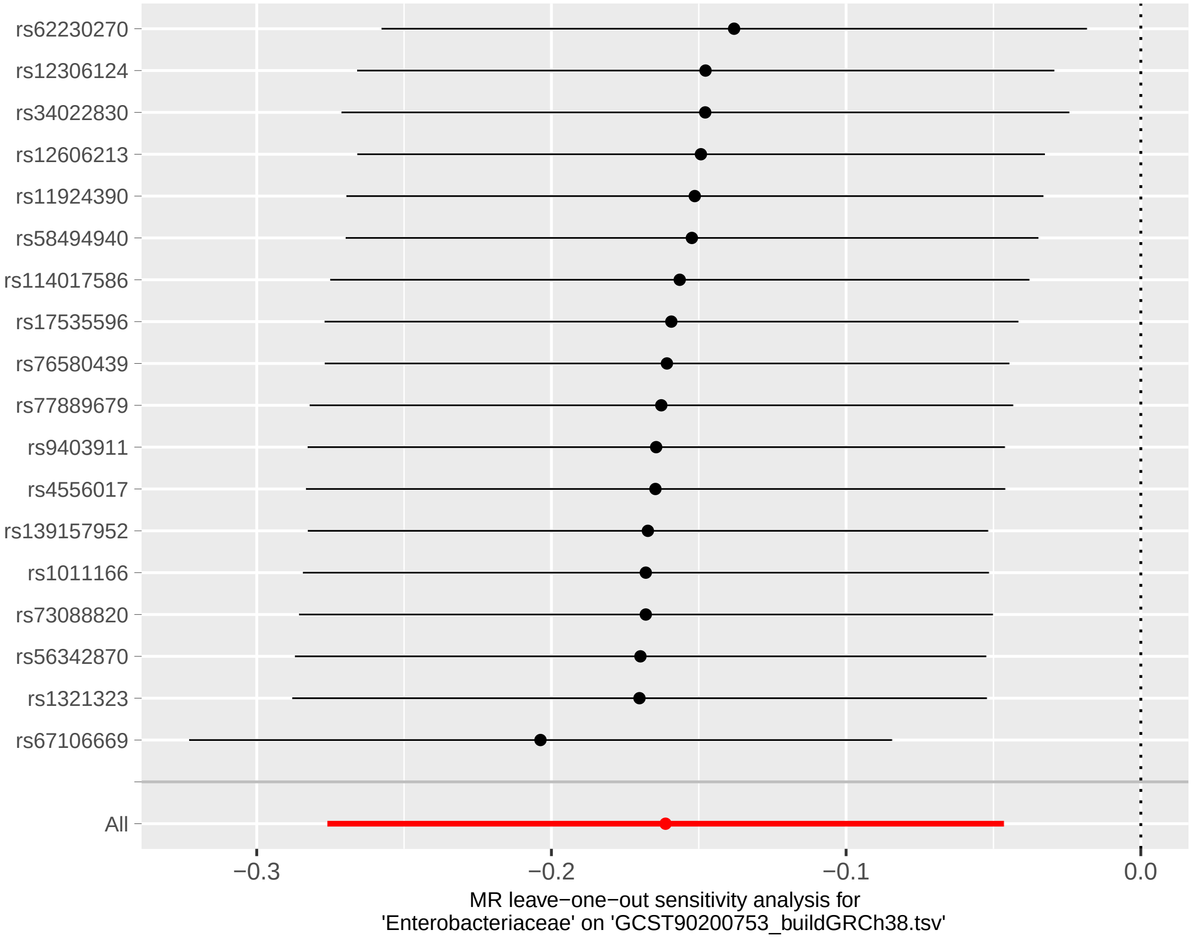

rs62230270
rs12306124
rs34022830
rs12606213
rs11924390
rs58494940
rs114017586
rs17535596
rs76580439
rs77889679
rs9403911
rs4556017
rs139157952
rs1011166
rs73088820
rs56342870
rs1321323
rs67106669
All
−0.3
−0.2
−0.1
0.0
MR leave−one−out sensitivity analysis for
'Enterobacteriaceae' on 'GCST90200753_buildGRCh38.tsv'

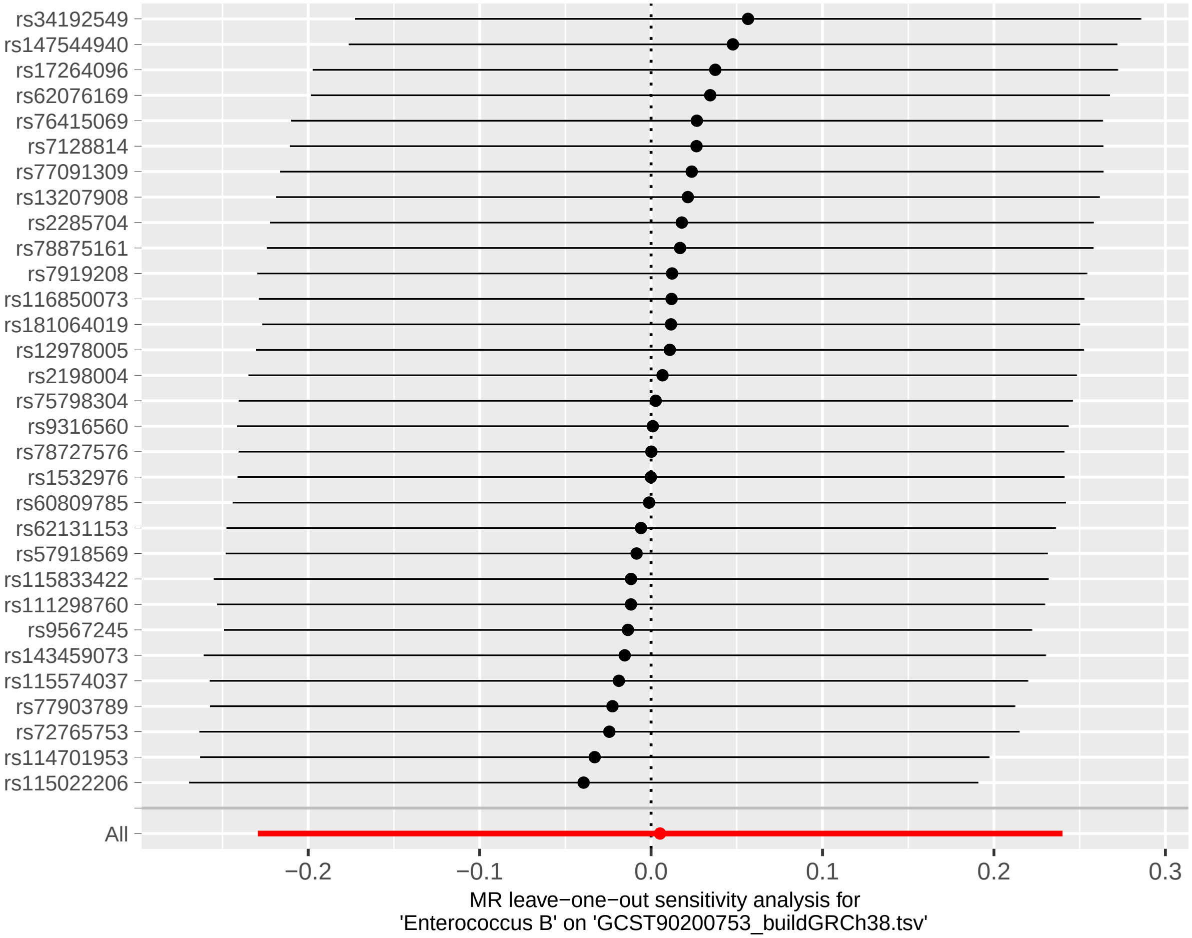
rs34192549
rs147544940
rs17264096
rs62076169
rs76415069
rs7128814
rs77091309
rs13207908
rs2285704
rs78875161
rs7919208
rs116850073
rs181064019
rs12978005
rs2198004
rs75798304
rs9316560
rs78727576
rs1532976
rs60809785
rs62131153
rs57918569
rs115833422
rs111298760
rs9567245
rs143459073
rs115574037
rs77903789
rs72765753
rs114701953
rs115022206
All
−0.2
−0.1
0.0
0.1
0.2
0.3
MR leave−one−out sensitivity analysis for
'Enterococcus B' on 'GCST90200753_buildGRCh38.tsv'

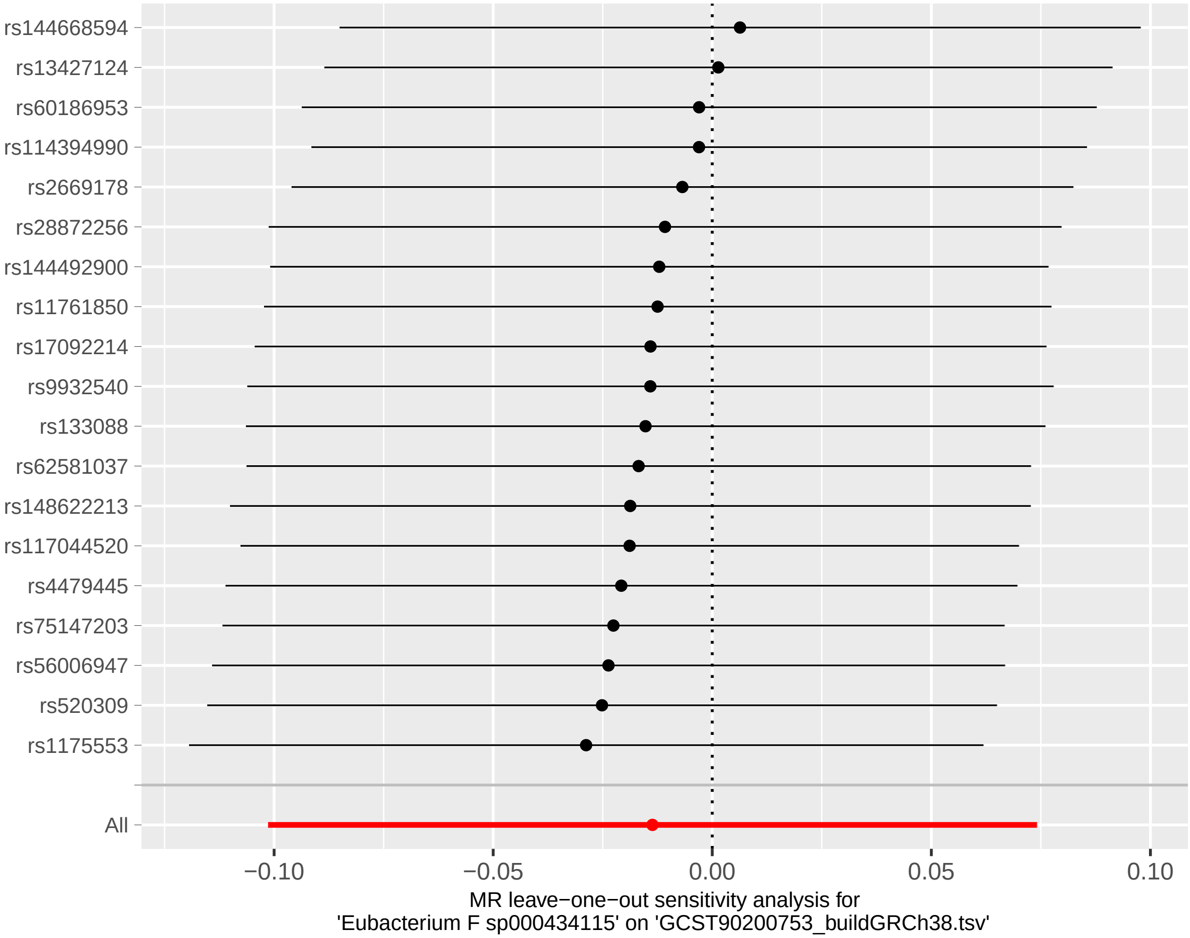
rs144668594
rs13427124
rs60186953
rs114394990
rs2669178
rs28872256
rs144492900
rs11761850
rs17092214
rs9932540
rs133088
rs62581037
rs148622213
rs117044520
rs4479445
rs75147203
rs56006947
rs520309
rs1175553
All
−0.10
−0.05
0.00
0.05
0.10
MR leave−one−out sensitivity analysis for
'Eubacterium F sp000434115' on 'GCST90200753_buildGRCh38.tsv'

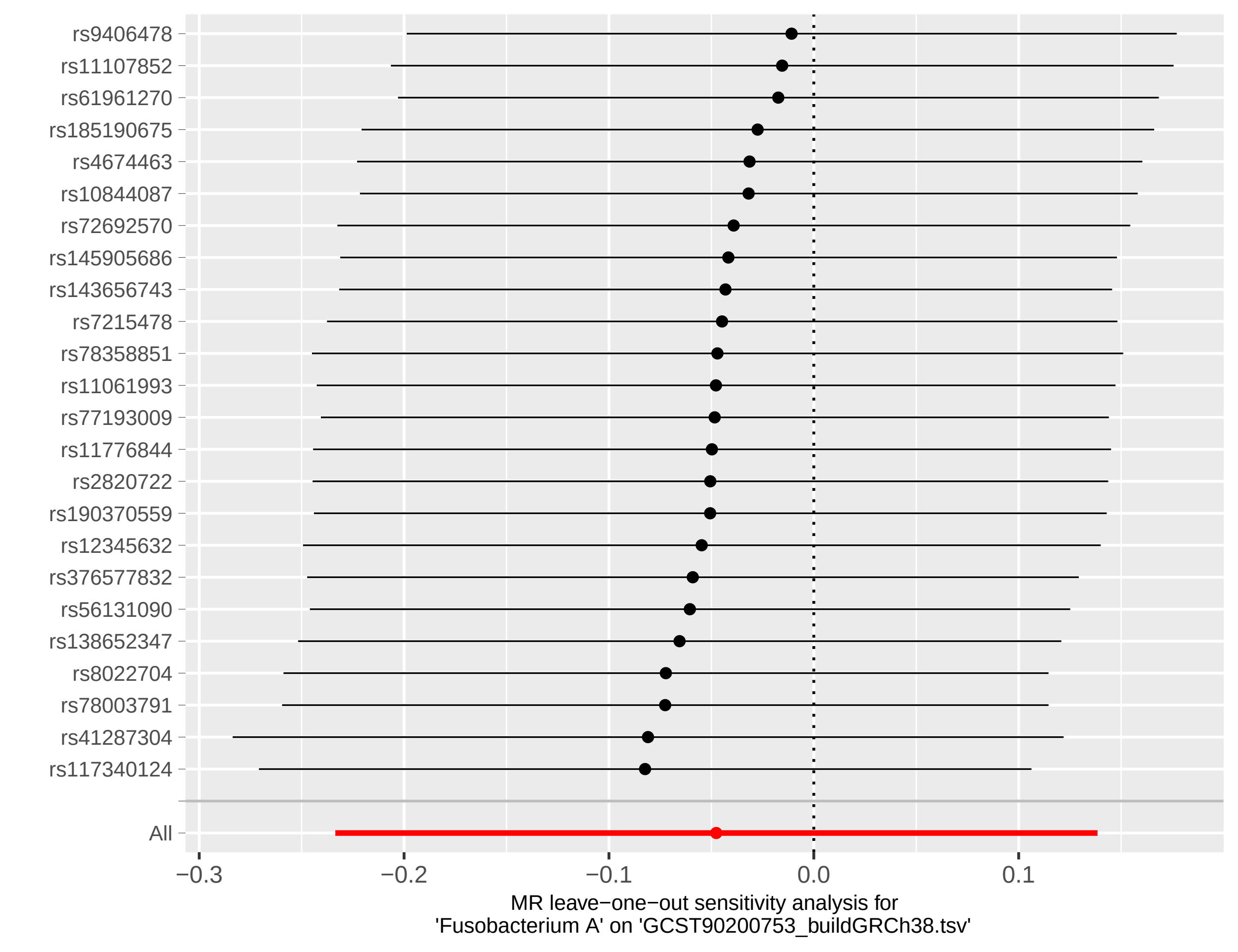
rs9406478
rs11107852
rs61961270
rs185190675
rs4674463
rs10844087
rs72692570
rs145905686
rs143656743
rs7215478
rs78358851
rs11061993
rs77193009
rs11776844
rs2820722
rs190370559
rs12345632
rs376577832
rs56131090
rs138652347
rs8022704
rs78003791
rs41287304
rs117340124
All
−0.3
−0.2
−0.1
0.0
0.1
MR leave−one−out sensitivity analysis for
'Fusobacterium A' on 'GCST90200753_buildGRCh38.tsv'

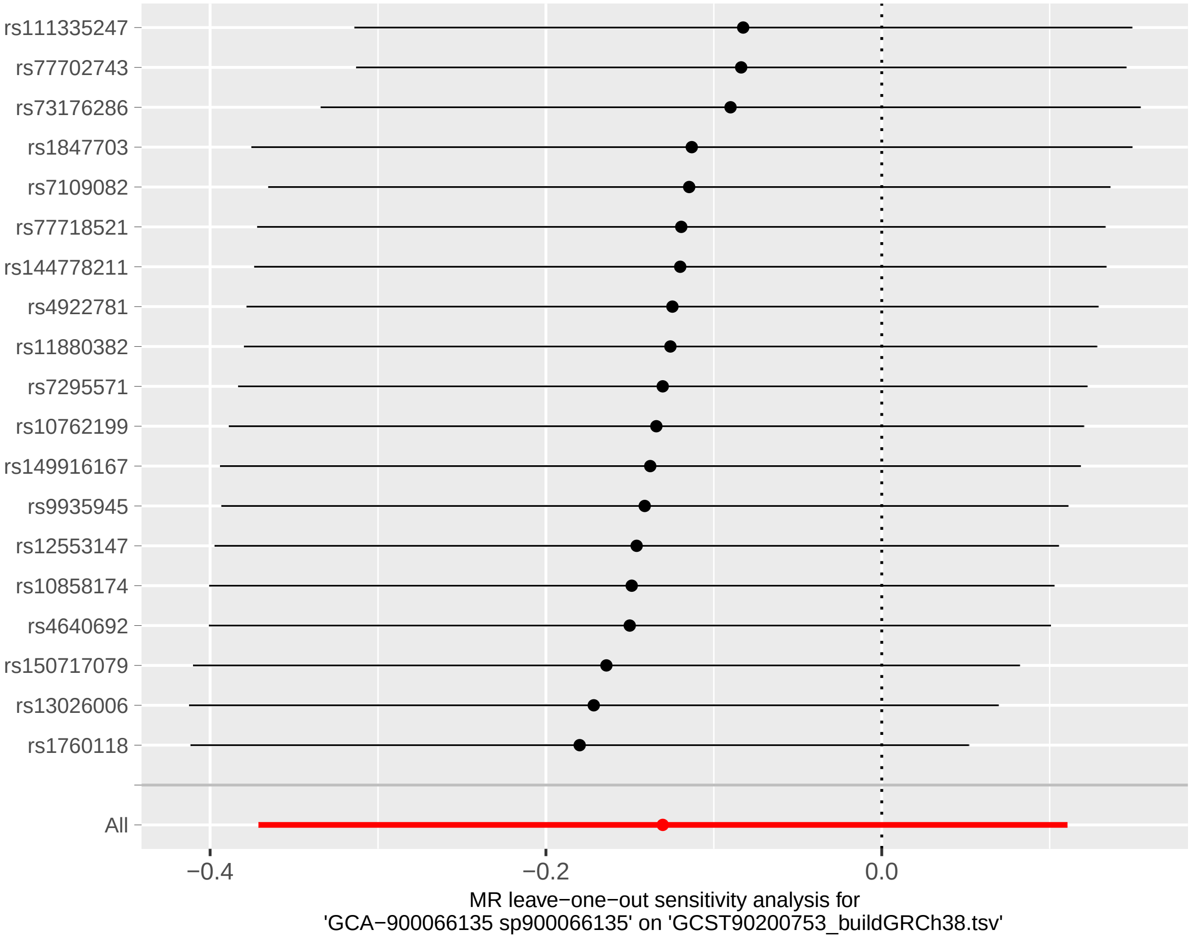
rs111335247
rs77702743
rs73176286
rs1847703
rs7109082
rs77718521
rs144778211
rs4922781
rs11880382
rs7295571
rs10762199
rs149916167
rs9935945
rs12553147
rs10858174
rs4640692
rs150717079
rs13026006
rs1760118
All
−0.4
−0.2
0.0
MR leave−one−out sensitivity analysis for
'GCA−900066135 sp900066135' on 'GCST90200753_buildGRCh38.tsv'

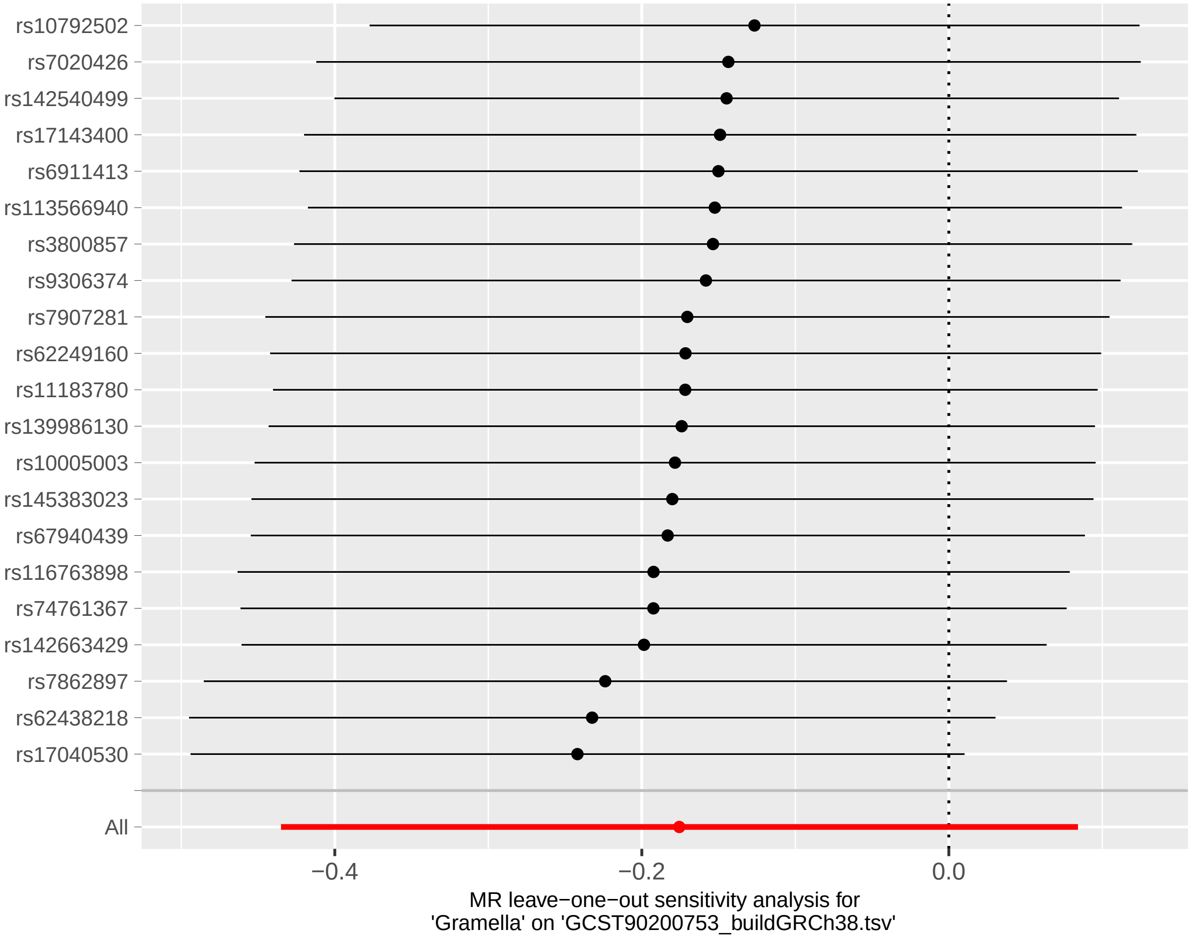

rs10792502
rs7020426
rs142540499
rs17143400
rs6911413
rs113566940
rs3800857
rs9306374
rs7907281
rs62249160
rs11183780
rs139986130
rs10005003
rs145383023
rs67940439
rs116763898
rs74761367
rs142663429
rs7862897
rs62438218
rs17040530
All
−0.4
−0.2
0.0
MR leave−one−out sensitivity analysis for
'Gramella' on 'GCST90200753_buildGRCh38.tsv'

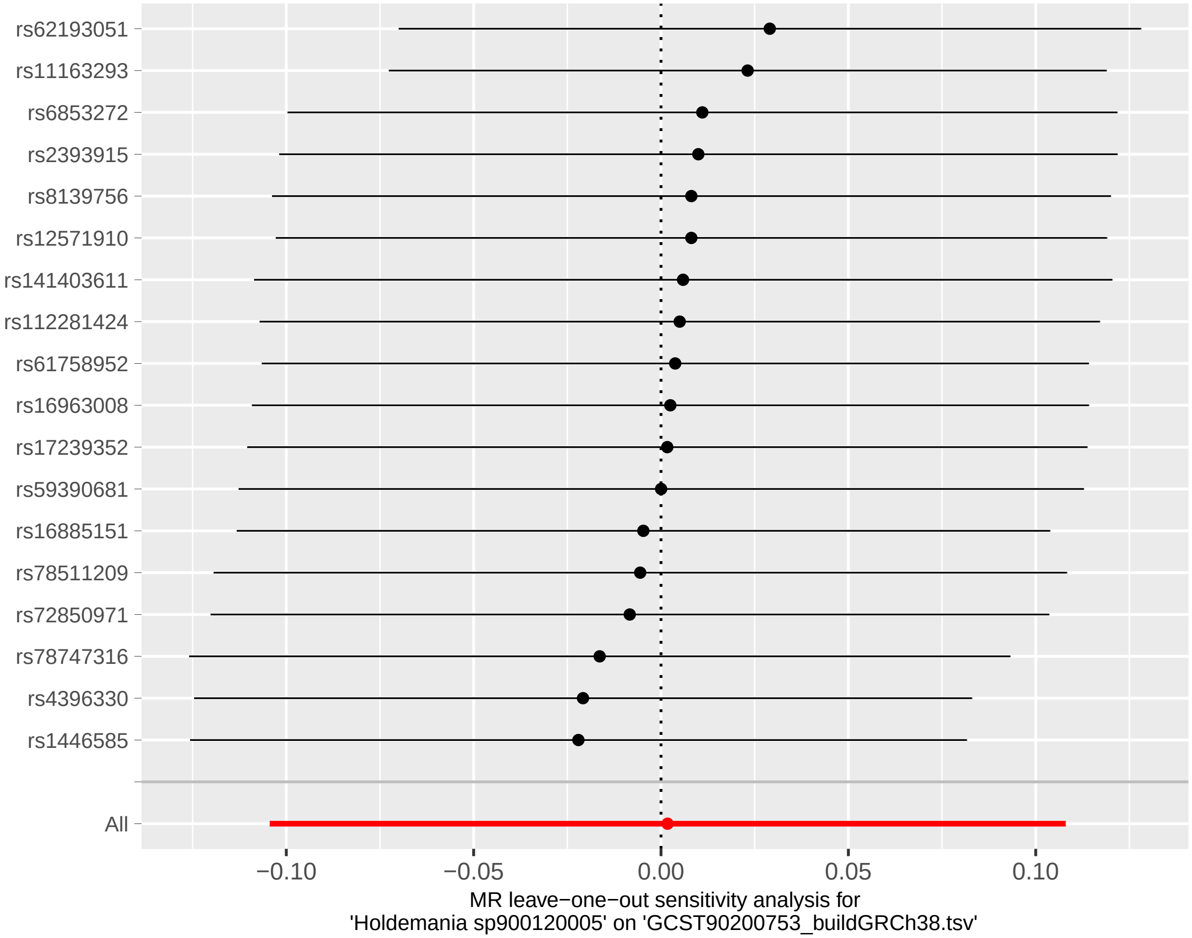

rs62193051
rs11163293
rs6853272
rs2393915
rs8139756
rs12571910
rs141403611
rs112281424
rs61758952
rs16963008
rs17239352
rs59390681
rs16885151
rs78511209
rs72850971
rs78747316
rs4396330
rs1446585
All
−0.10
−0.05
0.00
0.05
0.10
MR leave−one−out sensitivity analysis for
'Holdemania sp900120005' on 'GCST90200753_buildGRCh38.tsv'

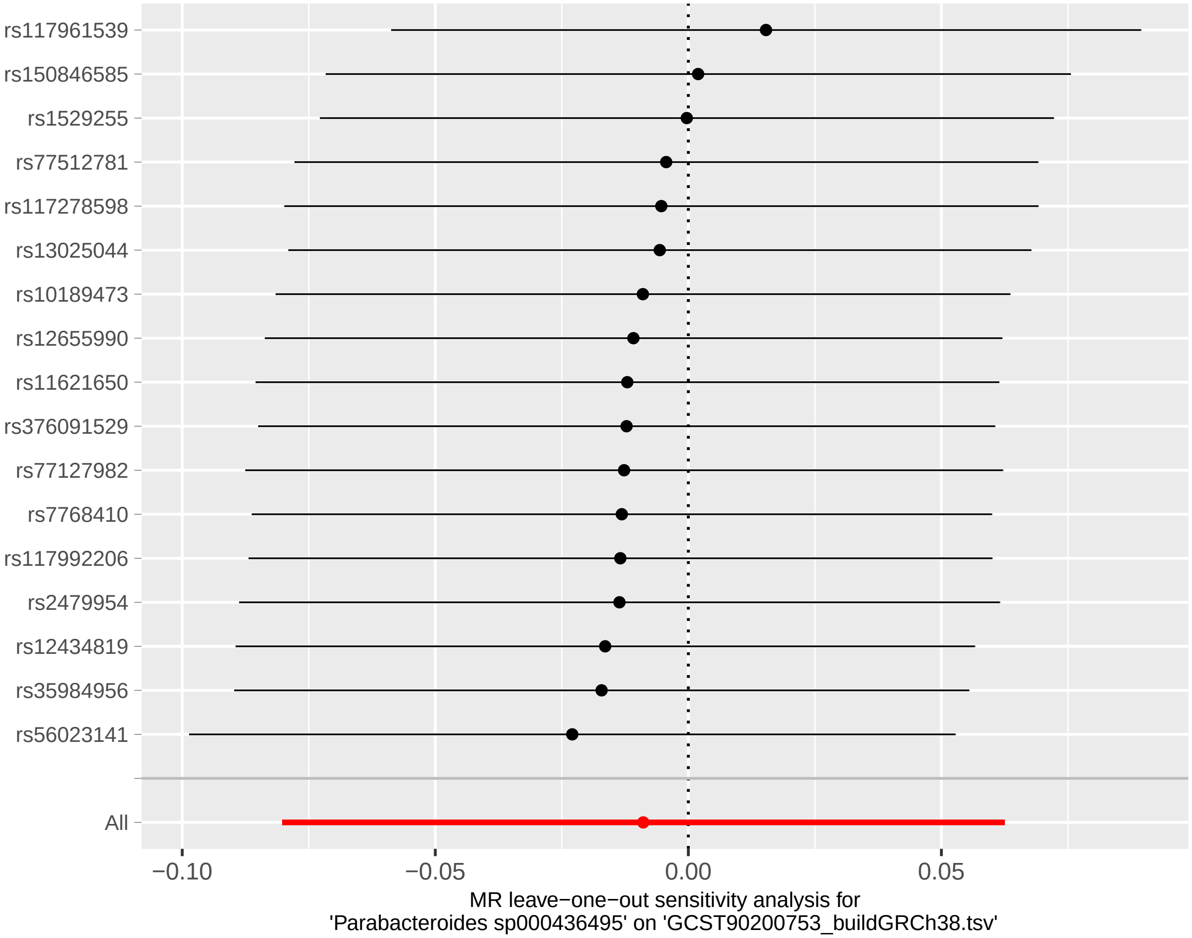
rs117961539
rs150846585
rs1529255
rs77512781
rs117278598
rs13025044
rs10189473
rs12655990
rs11621650
rs376091529
rs77127982
rs7768410
rs117992206
rs2479954
rs12434819
rs35984956
rs56023141
All
−0.10
−0.05
0.00
0.05
MR leave−one−out sensitivity analysis for
'Parabacteroides sp000436495' on 'GCST90200753_buildGRCh38.tsv'

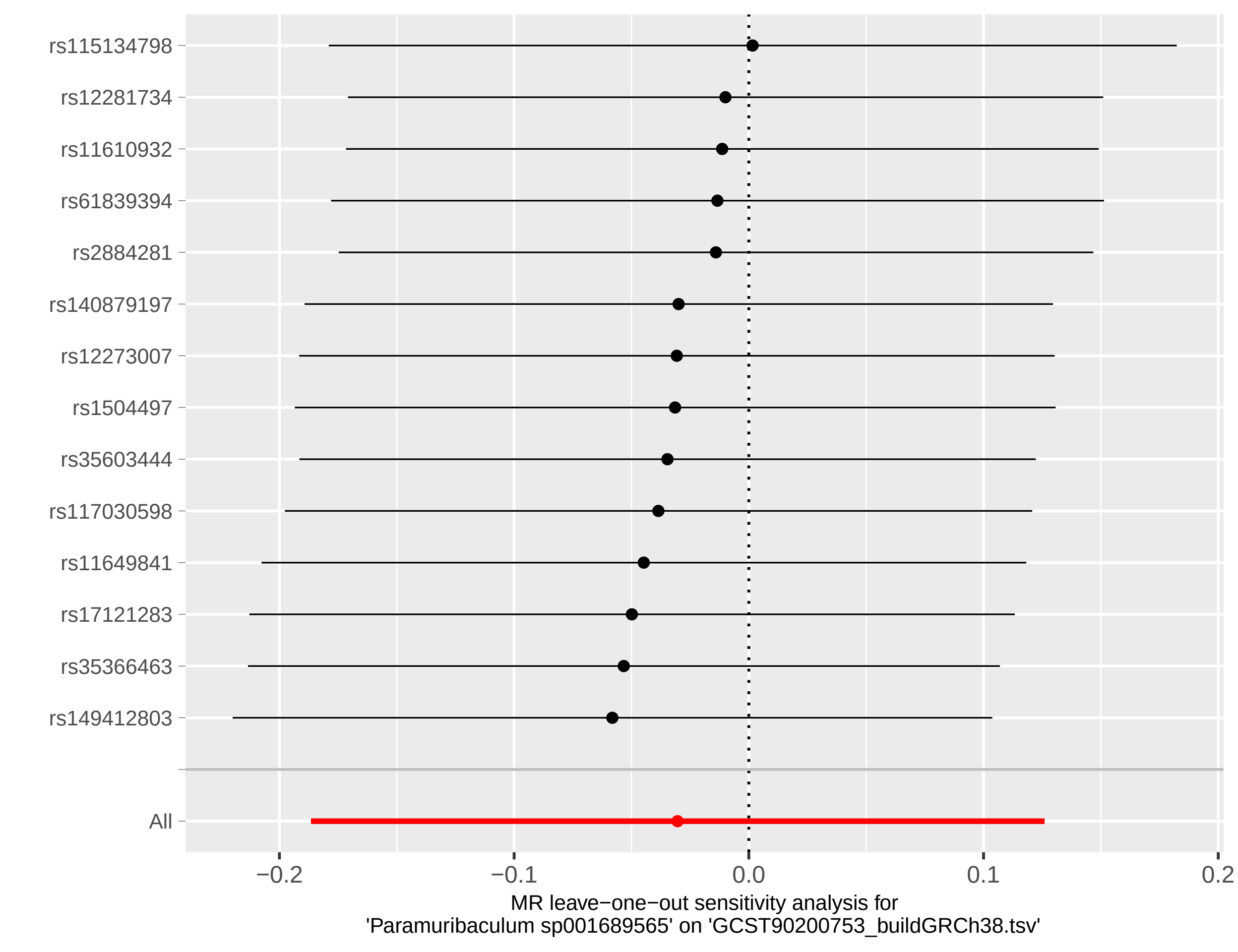
rs115134798
rs12281734
rs11610932
rs61839394
rs2884281
rs140879197
rs12273007
rs1504497
rs35603444
rs117030598
rs11649841
rs17121283
rs35366463
rs149412803
All
−0.2
−0.1
0.0
0.1
0.2
MR leave−one−out sensitivity analysis for
'Paramuribaculum sp001689565' on 'GCST90200753_buildGRCh38.tsv'

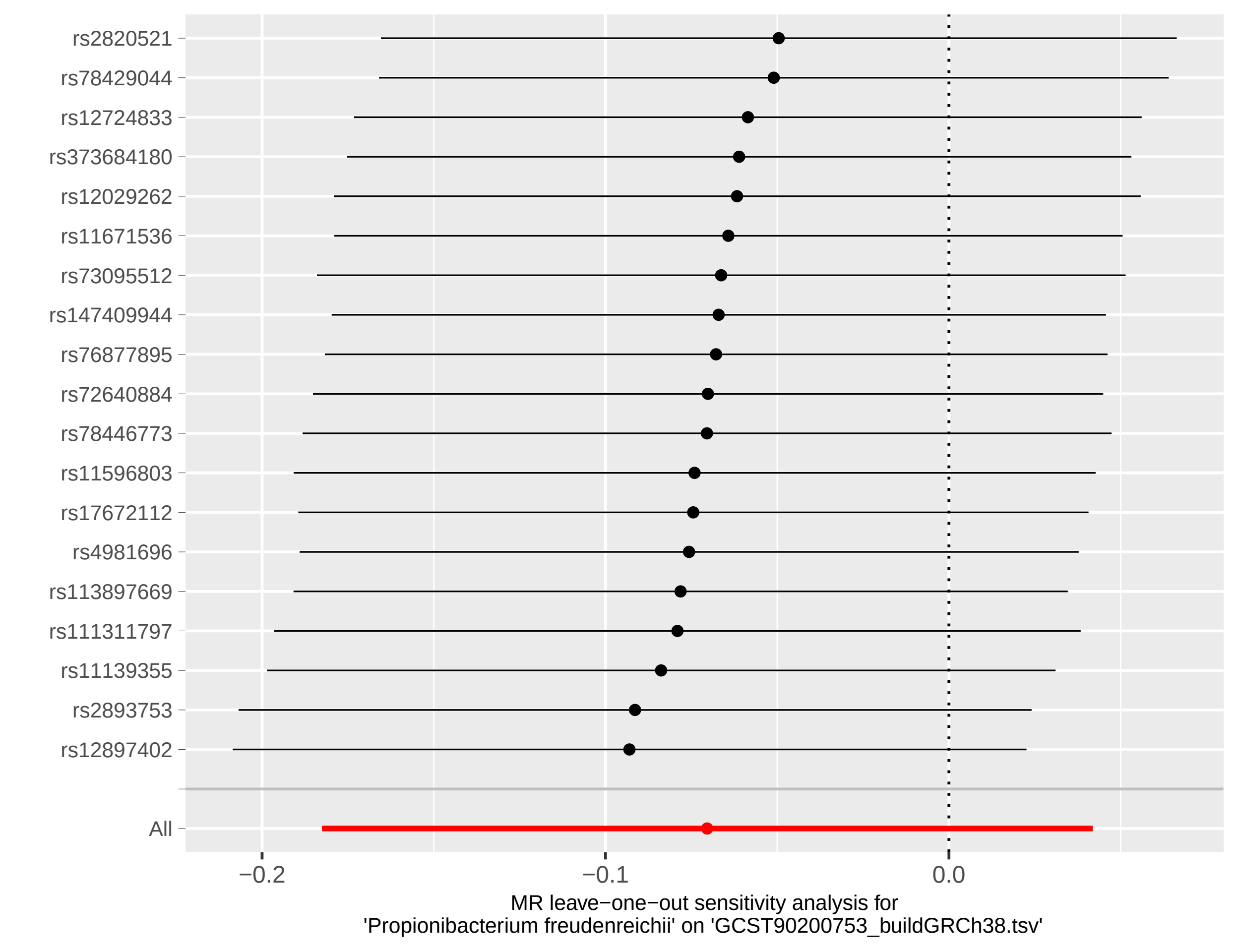

rs2820521
rs78429044
rs12724833
rs373684180
rs12029262
rs11671536
rs73095512
rs147409944
rs76877895
rs72640884
rs78446773
rs11596803
rs17672112
rs4981696
rs113897669
rs111311797
rs11139355
rs2893753
rs12897402
All
−0.2
−0.1
0.0
MR leave−one−out sensitivity analysis for
'Propionibacterium freudenreichii' on 'GCST90200753_buildGRCh38.tsv'

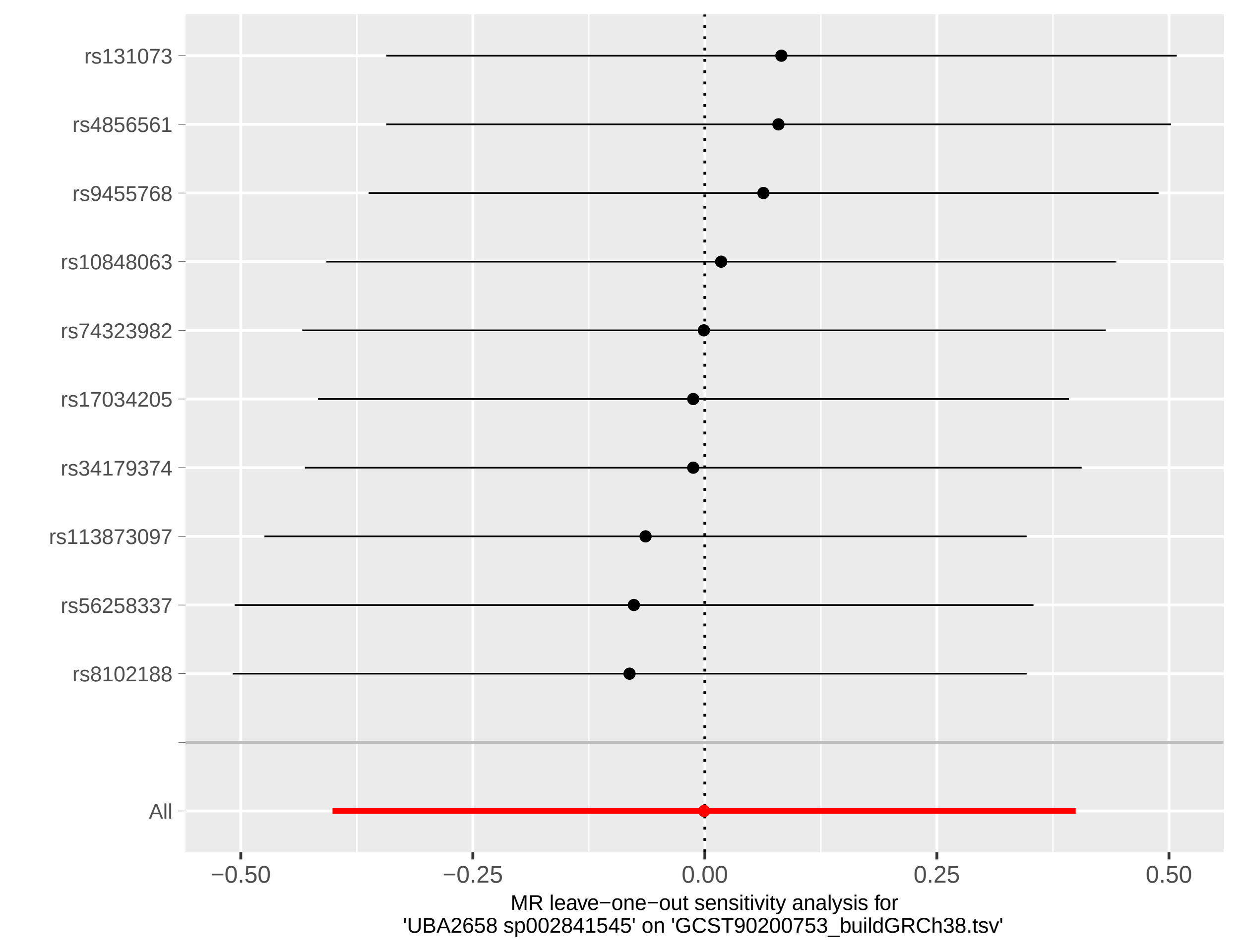

rs131073
rs4856561
rs9455768
rs10848063
rs74323982
rs17034205
rs34179374
rs113873097
rs56258337
rs8102188
All
−0.50
−0.25
0.00
0.25
0.50
MR leave−one−out sensitivity analysis for
'UBA2658 sp002841545' on 'GCST90200753_buildGRCh38.tsv'

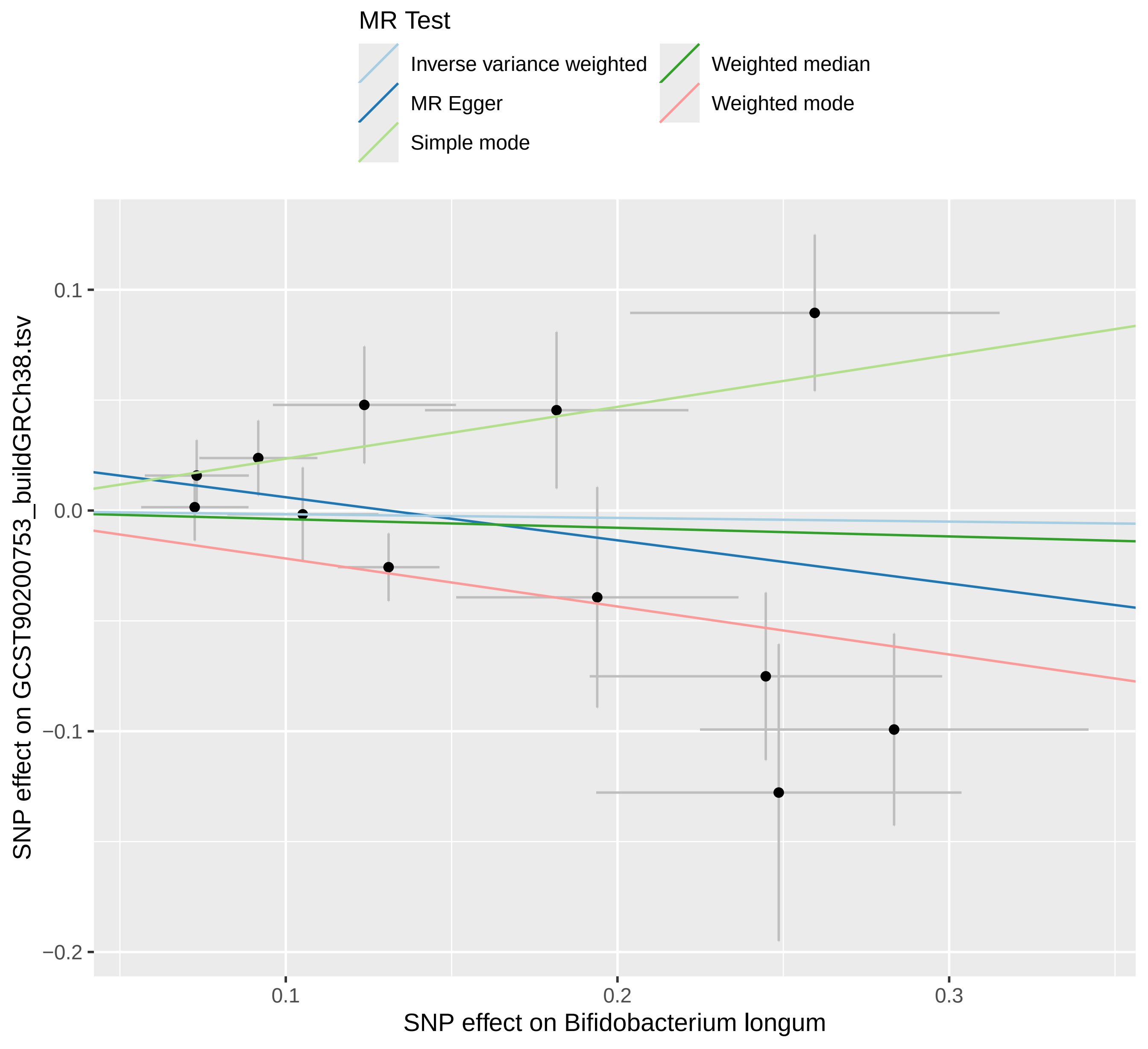
MR Test
Inverse variance weighted
MR Egger
Simple mode
Weighted median
Weighted mode
SNP effect on GCST90200753_buildGRCh38.tsv
0.1
0.0
−0.1
−0.2
0.1
0.2
0.3
SNP effect on Bifidobacterium longum

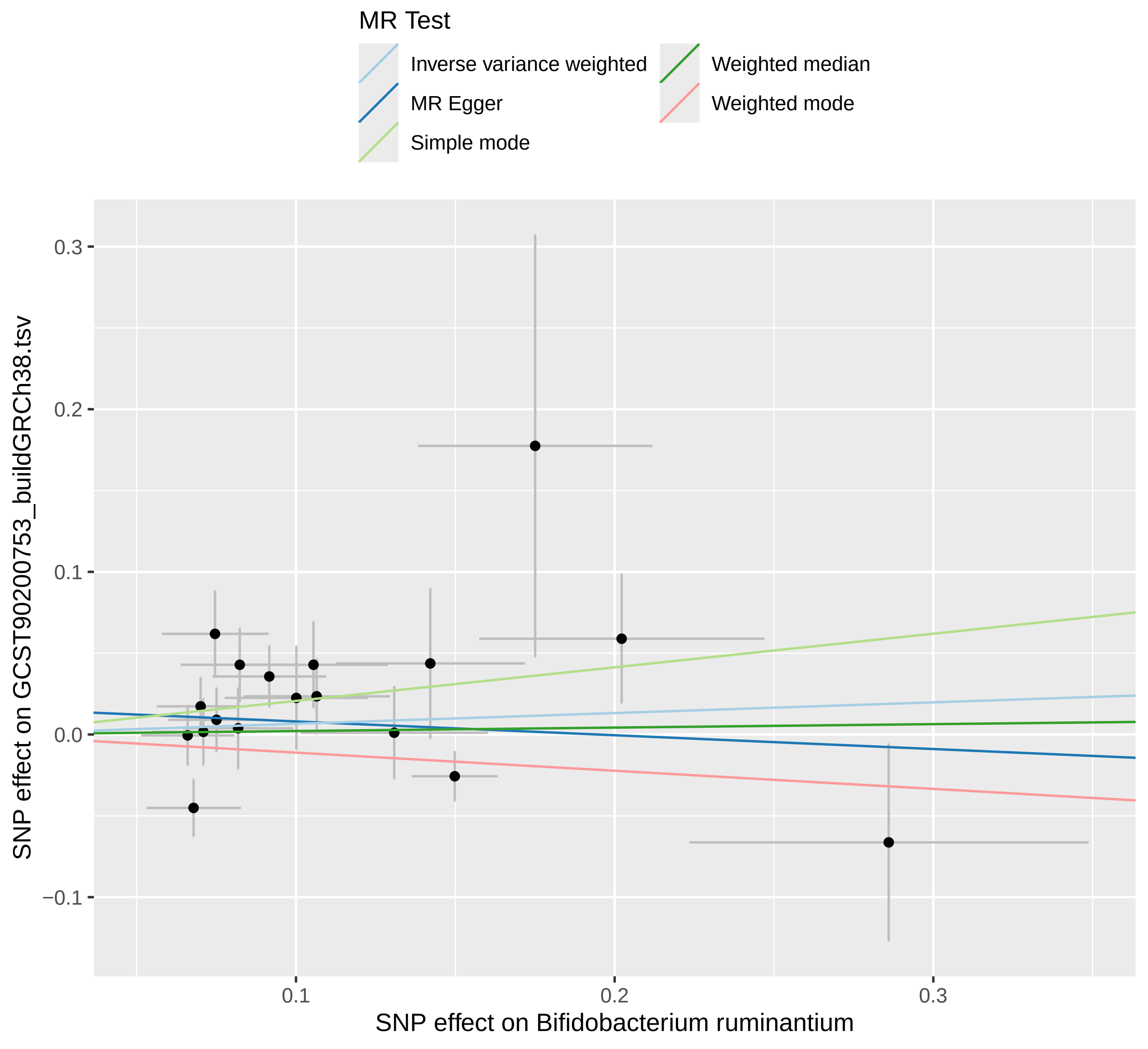
MR Test
Inverse variance weighted
MR Egger
Simple mode
Weighted median
Weighted mode
0.3
0.2
0.1
0.0
−0.1
0.1
0.2
0.3
SNP effect on GCST90200753_buildGRCh38.tsv
SNP effect on Bifidobacterium ruminantium

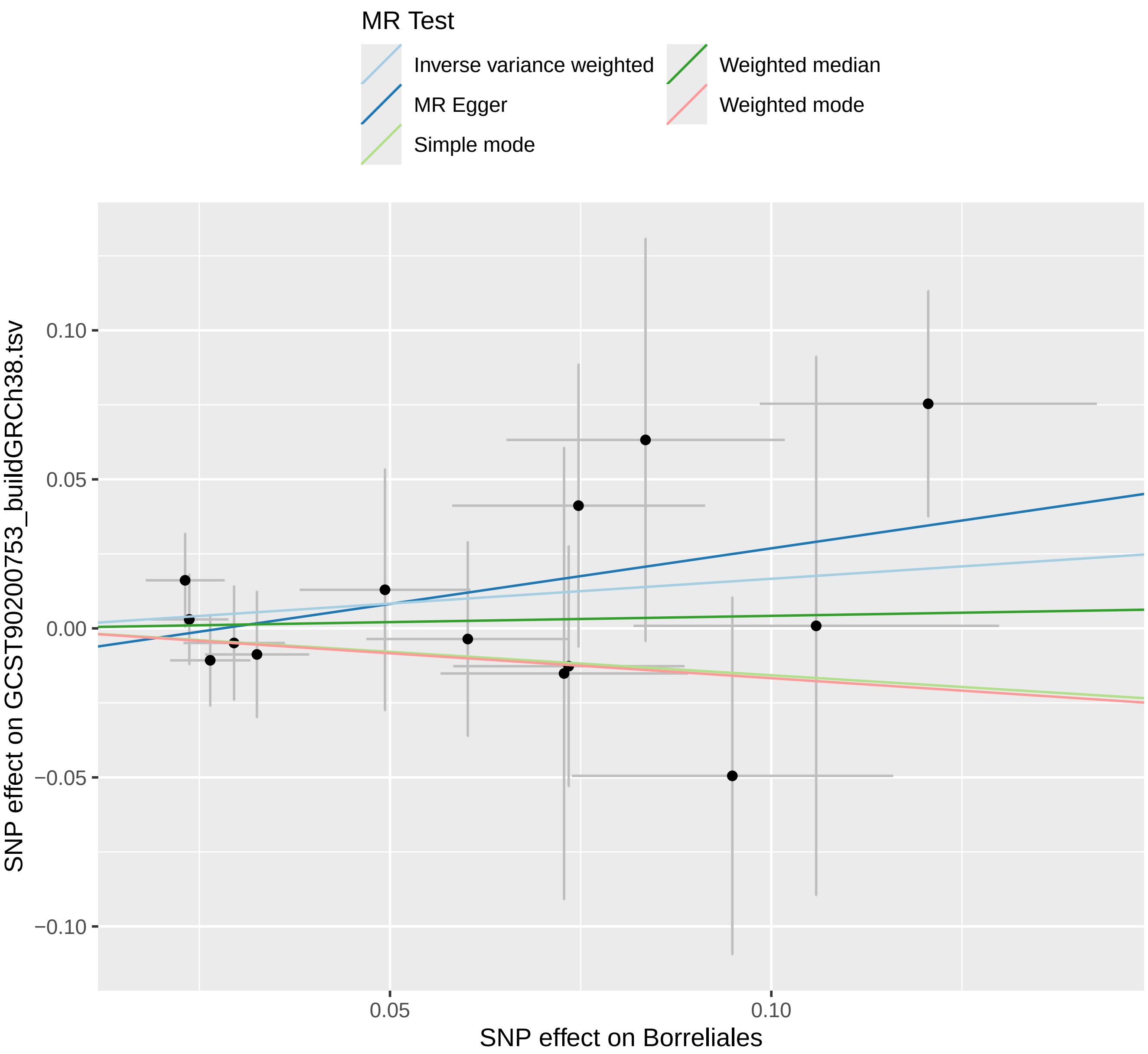
MR Test
Inverse variance weighted
MR Egger
Simple mode
Weighted median
Weighted mode
SNP effect on GCST90200753_buildGRCh38.tsv
0.10
0.05
0.00
−0.05
−0.10
0.05
0.10
SNP effect on Borreliales

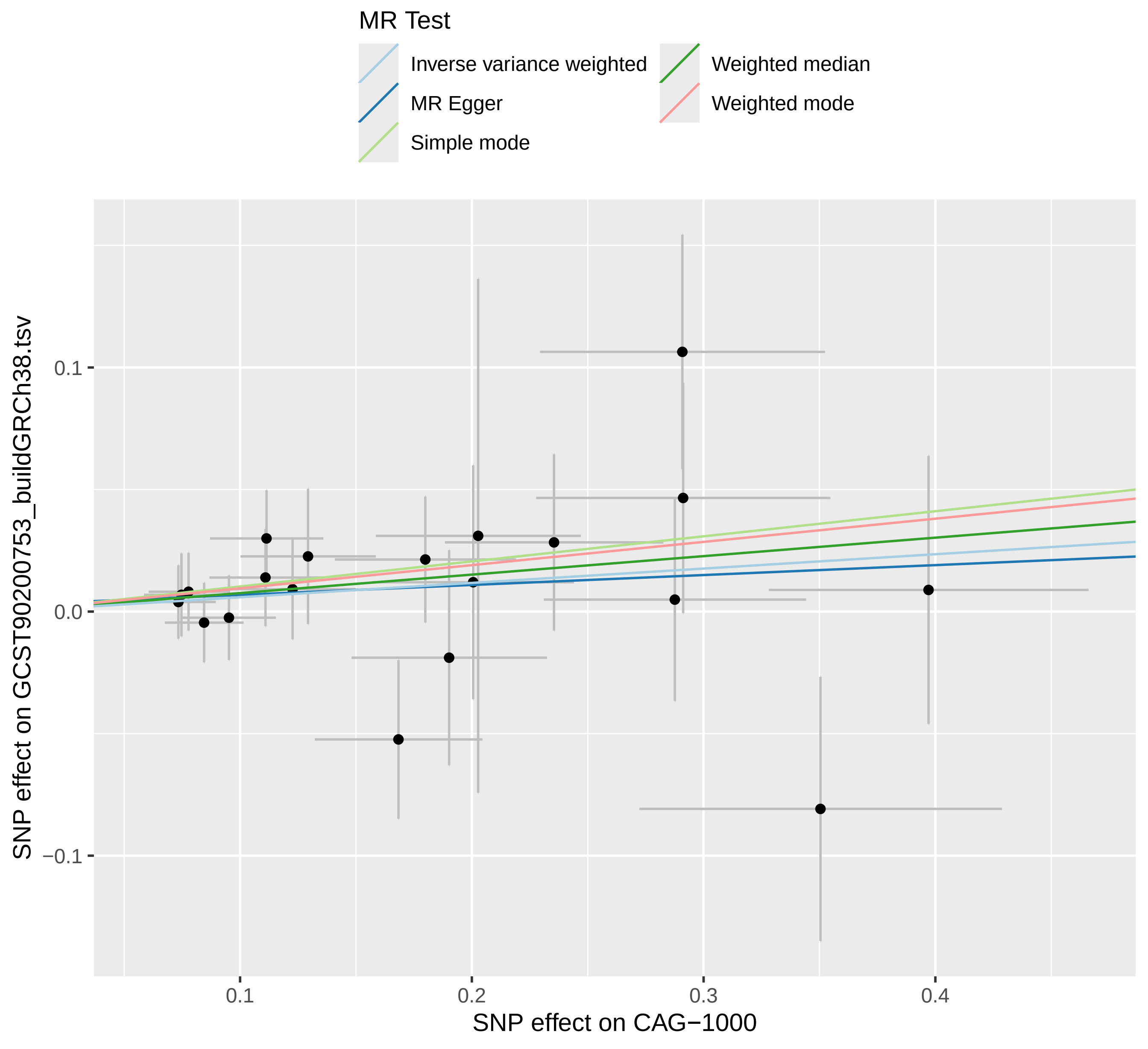
MR Test
Inverse variance weighted
MR Egger
Simple mode
Weighted median
Weighted mode
SNP effect on GCST90200753_buildGRCh38.tsv
0.1
0.0
−0.1
0.1
0.2
0.3
0.4
SNP effect on CAG−1000

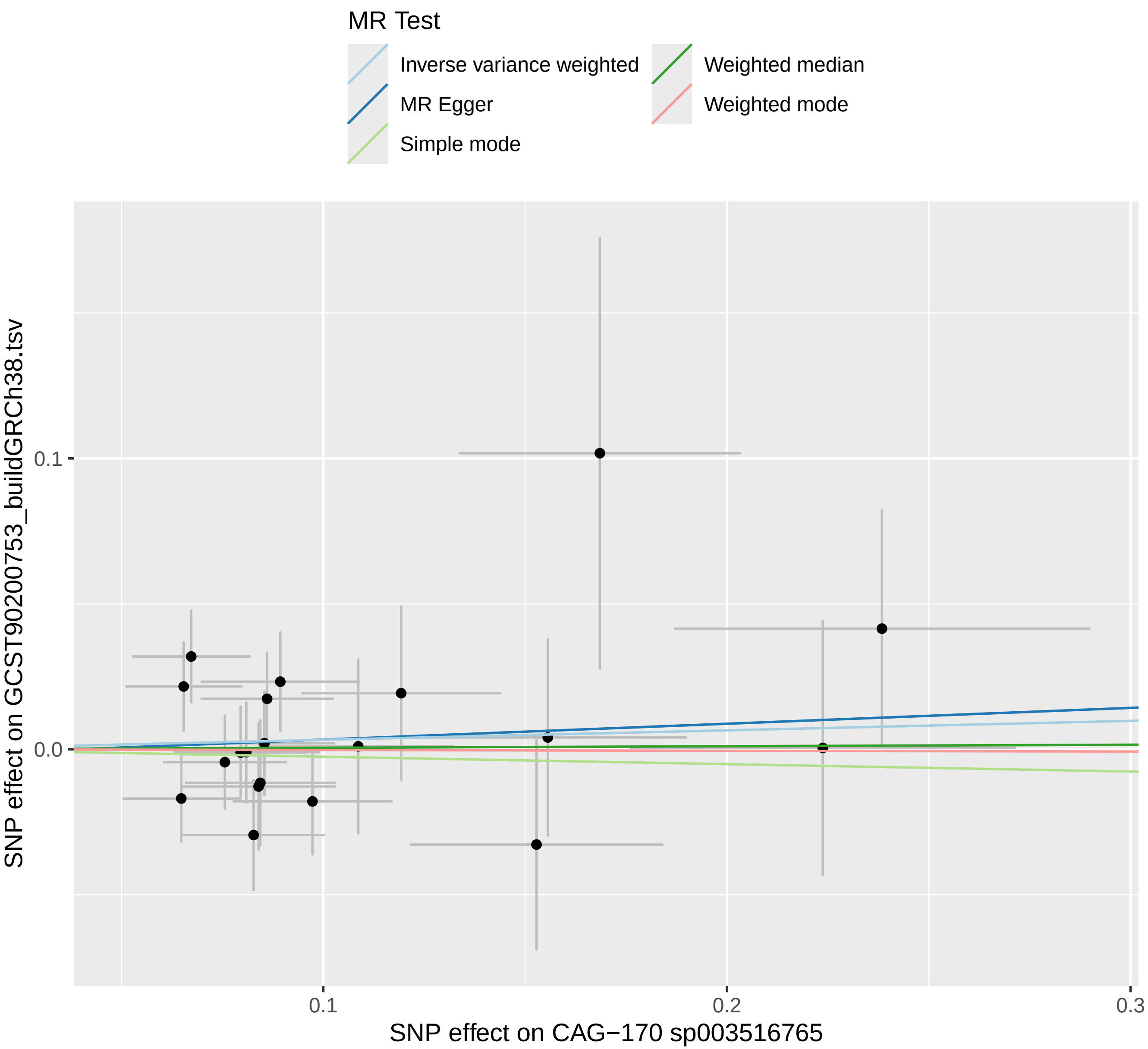
MR Test
Inverse variance weighted
MR Egger
Simple mode
Weighted median
Weighted mode
0.1
0.0
0.1
0.2
0.3
SNP effect on GCST90200753_buildGRCh38.tsv
SNP effect on CAG−170 sp003516765

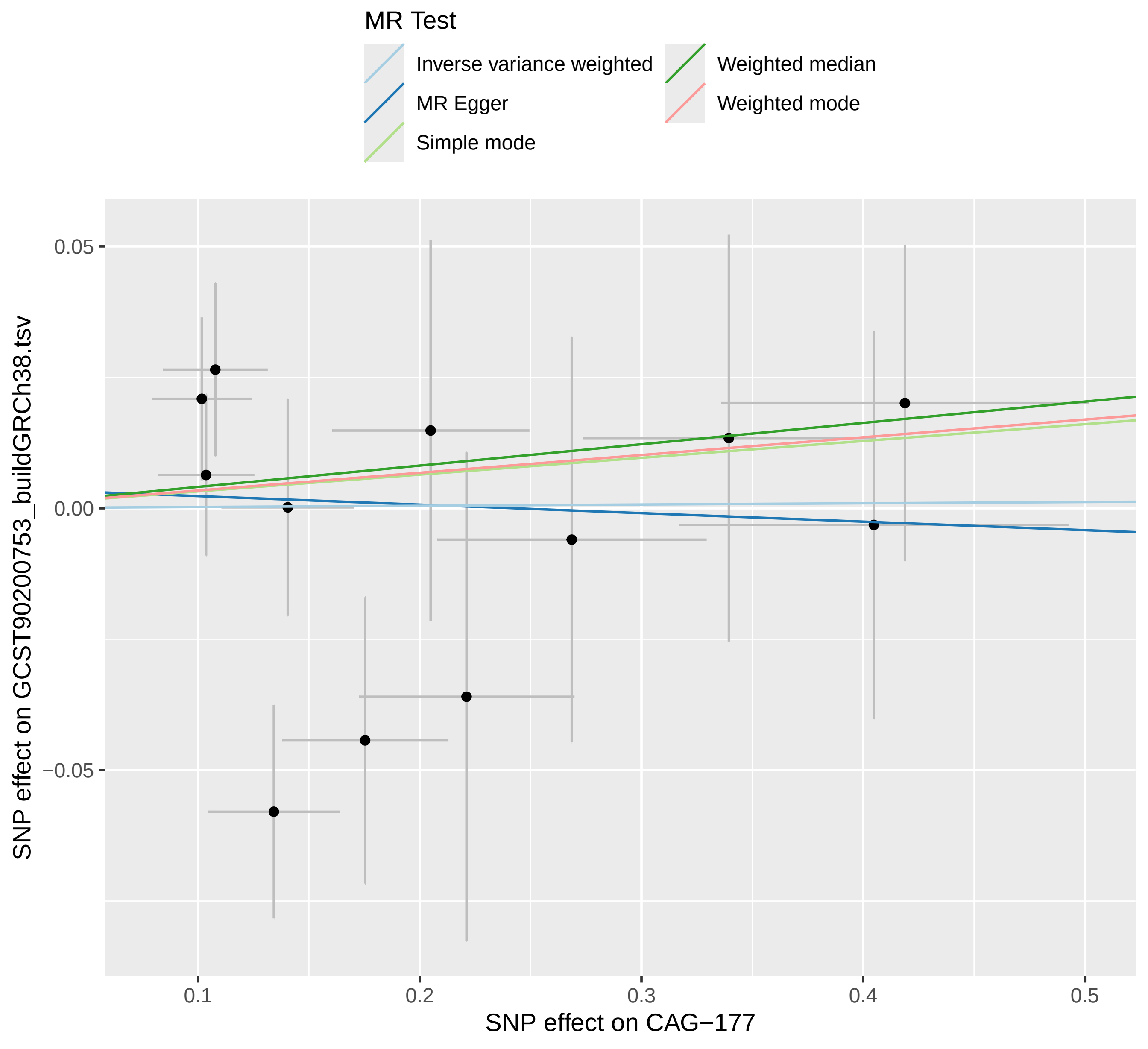
MR Test
Inverse variance weighted
MR Egger
Simple mode
Weighted median
Weighted mode
0.05
0.00
−0.05
0.1
0.2
0.3
0.4
0.5
SNP effect on GCST90200753_buildGRCh38.tsv
SNP effect on CAG−177

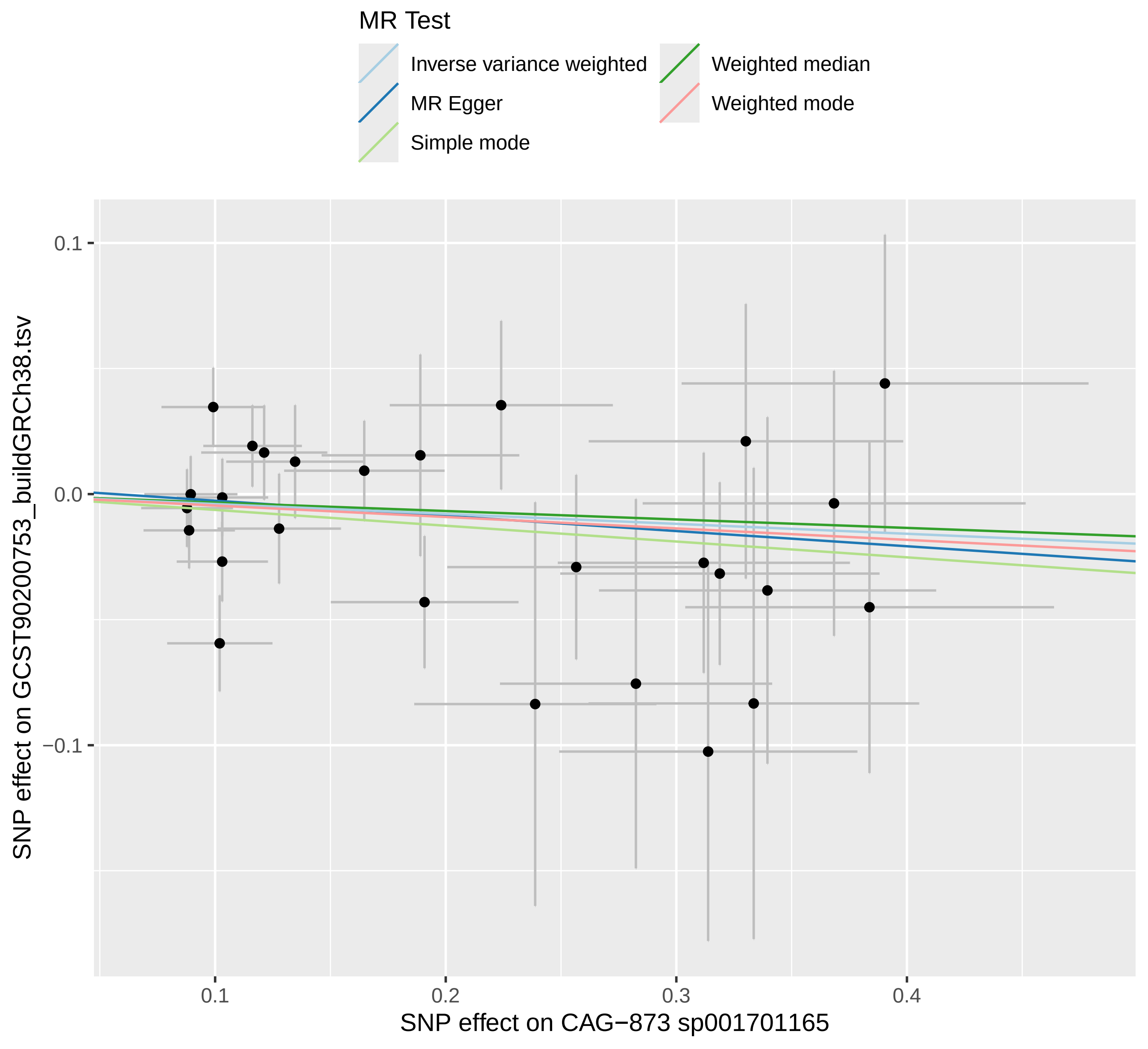

MR Test
Inverse variance weighted
MR Egger
Simple mode
Weighted median
Weighted mode
0.1
0.0
−0.1
0.1
0.2
0.3
0.4
SNP effect on GCST90200753_buildGRCh38.tsv
SNP effect on CAG−873 sp001701165

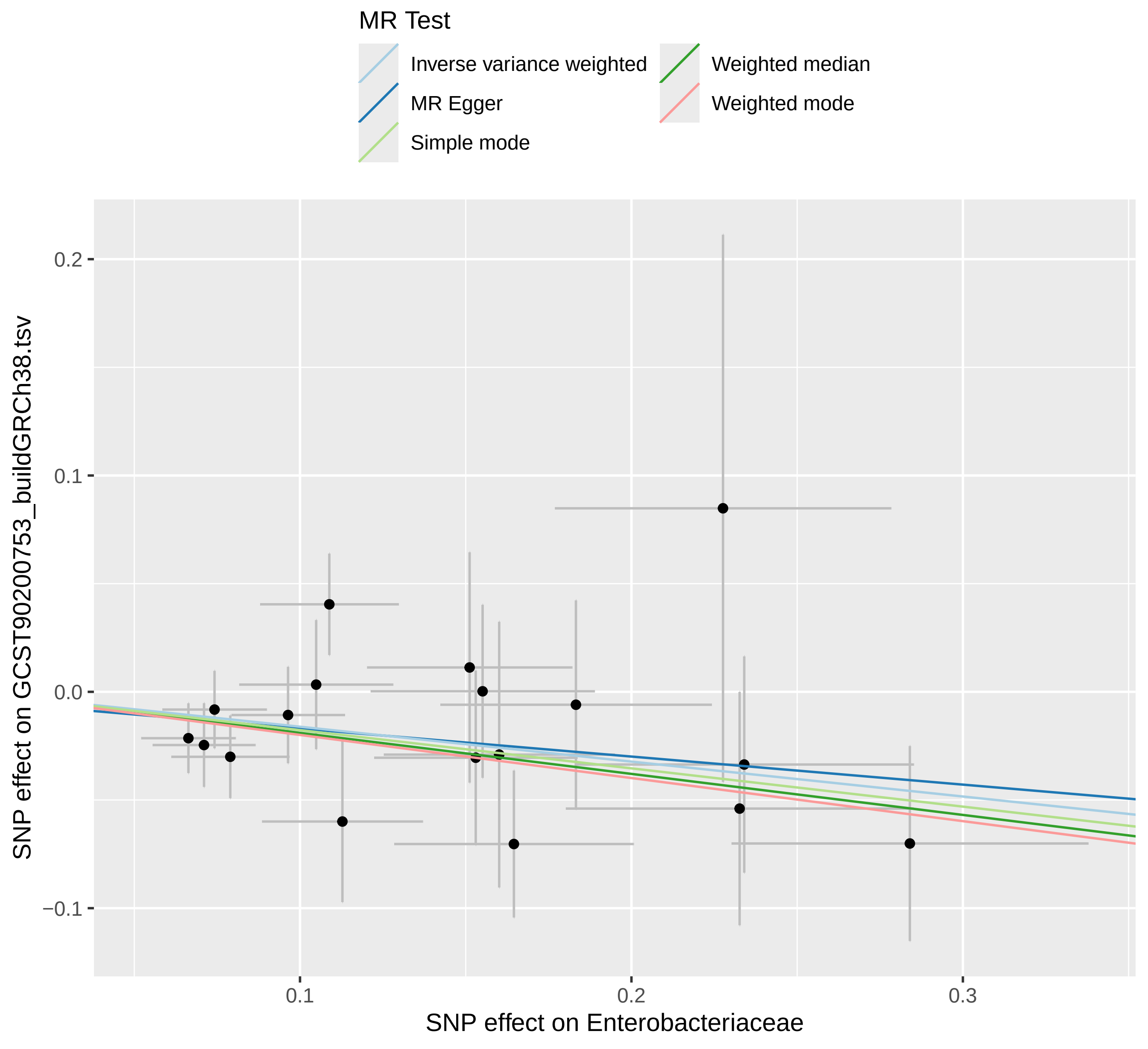
MR Test
Inverse variance weighted
MR Egger
Simple mode
Weighted median
Weighted mode
0.2
0.1
0.0
−0.1
SNP effect on GCST90200753_buildGRCh38.tsv
0.1
0.2
0.3
SNP effect on Enterobacteriaceae

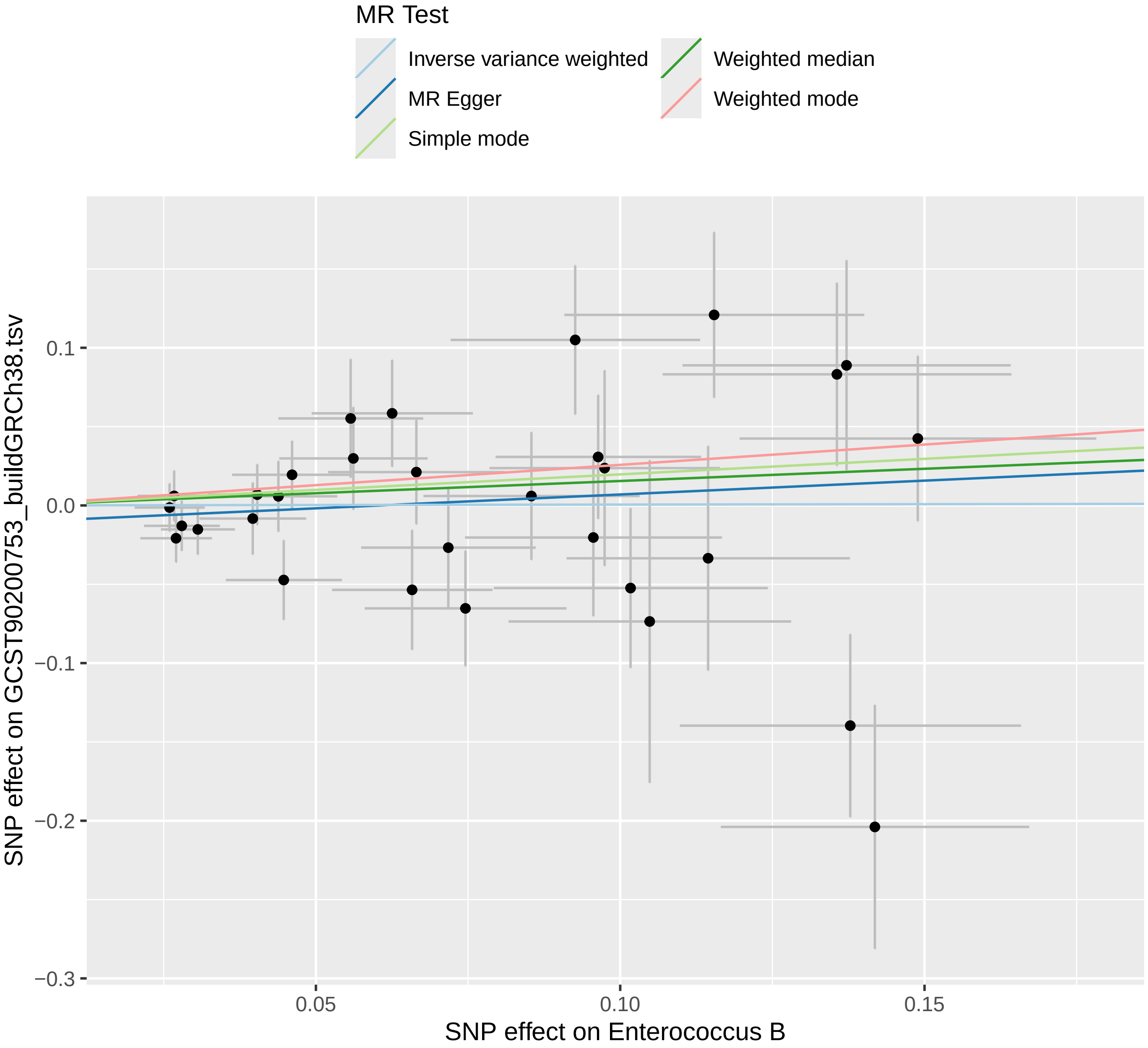
MR Test
Inverse variance weighted
MR Egger
Simple mode
Weighted median
Weighted mode
0.1
0.0
−0.1
−0.2
−0.3
0.05
0.10
0.15
SNP effect on GCST90200753_buildGRCh38.tsv
SNP effect on Enterococcus B

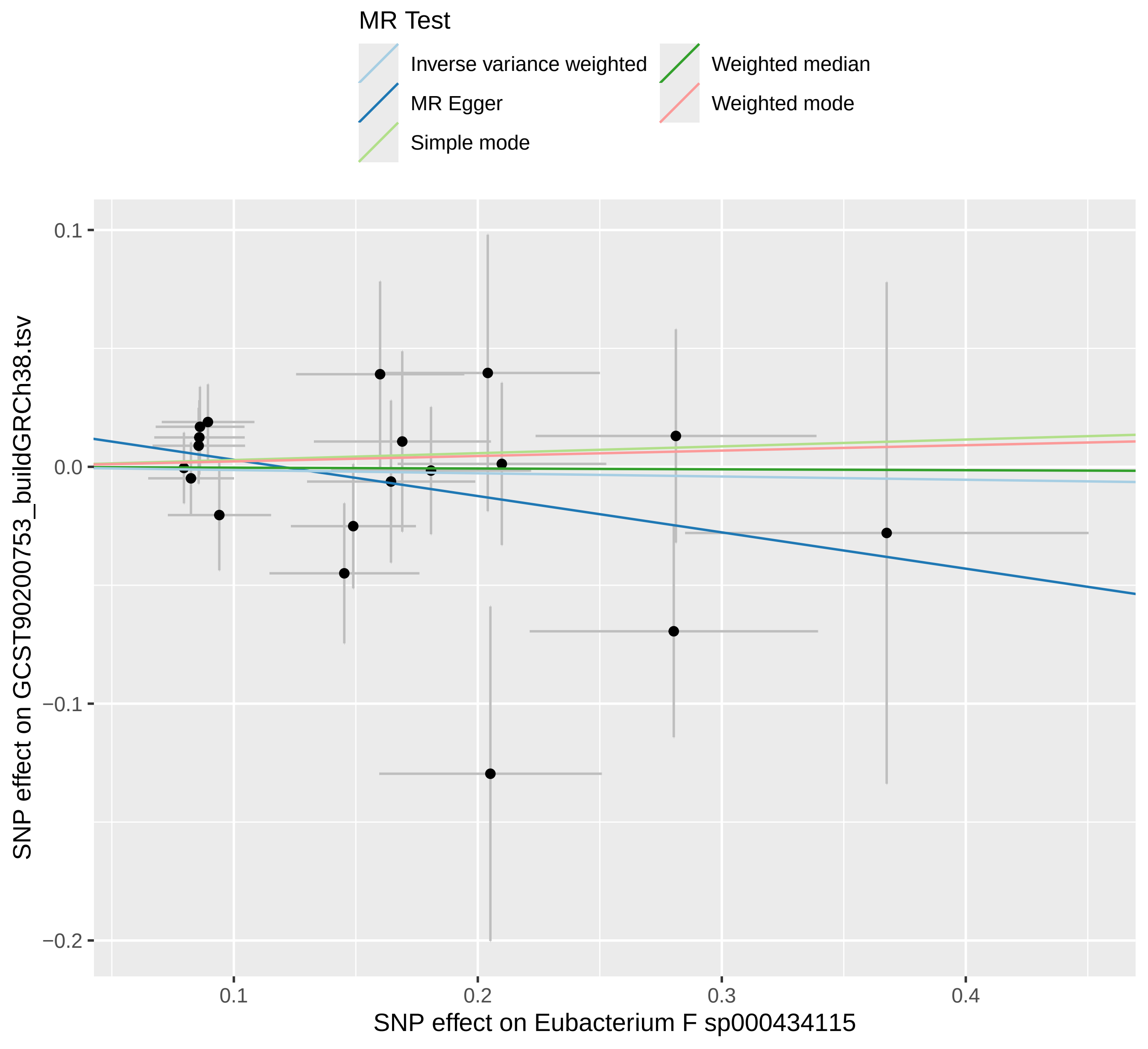
MR Test
Inverse variance weighted
MR Egger
Simple mode
Weighted median
Weighted mode
0.1
0.0
−0.1
−0.2
SNP effect on GCST90200753_buildGRCh38.tsv
0.1
0.2
0.3
0.4
SNP effect on Eubacterium F sp000434115

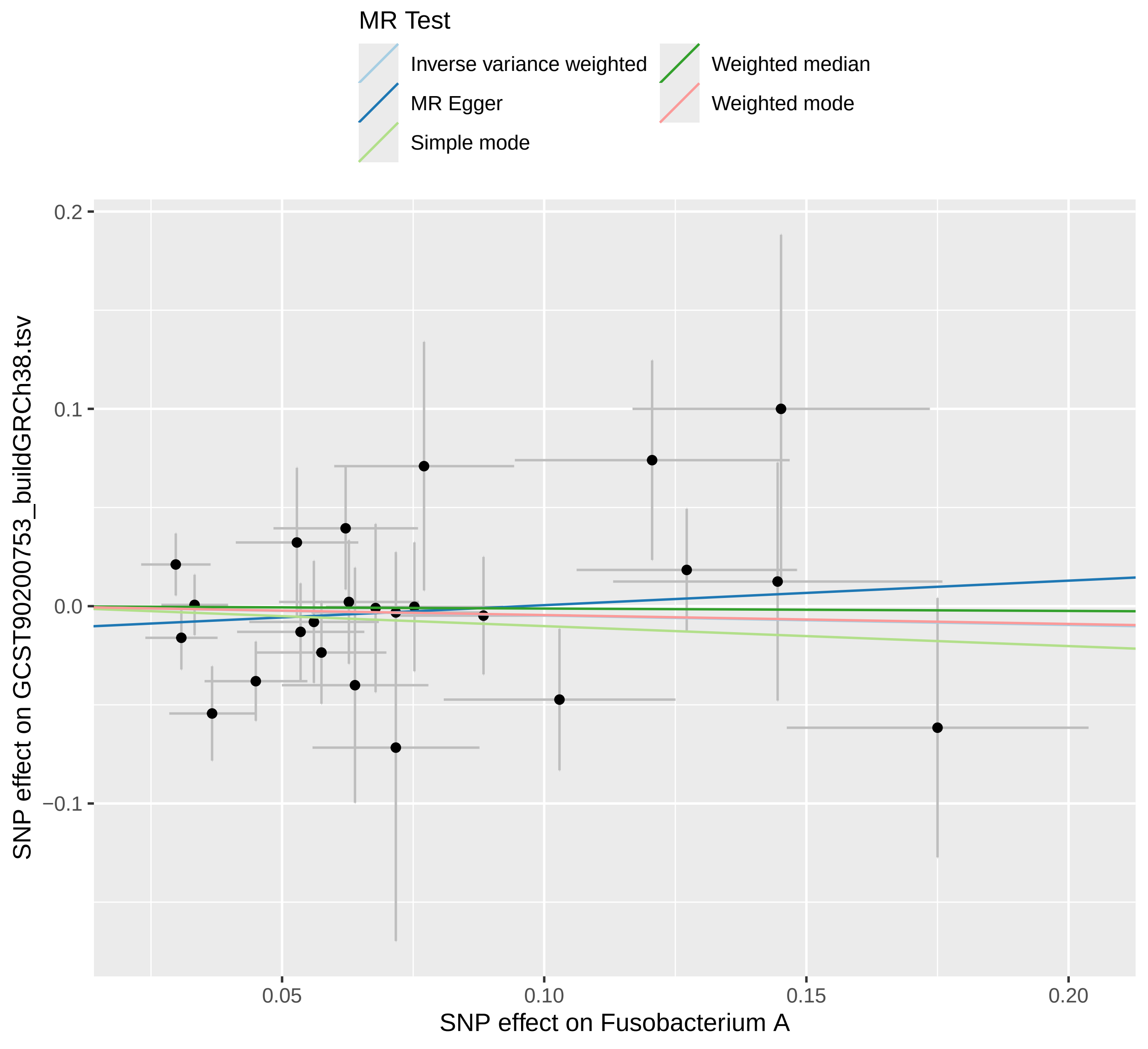

MR Test
Inverse variance weighted
MR Egger
Simple mode
Weighted median
Weighted mode
0.2
0.1
0.0
−0.1
0.05
0.10
0.15
0.20
SNP effect on GCST90200753_buildGRCh38.tsv
SNP effect on Fusobacterium A

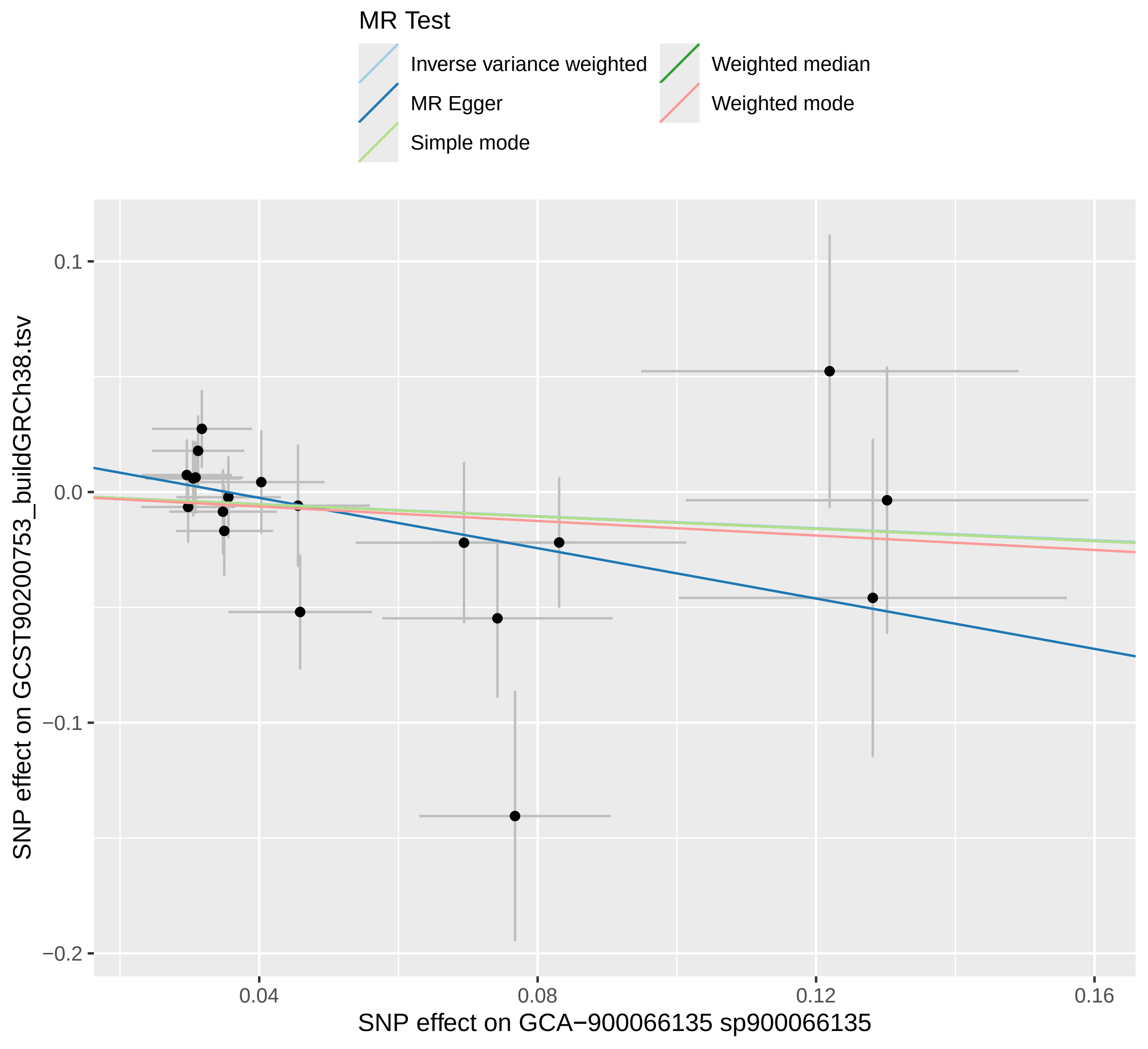
MR Test
Inverse variance weighted
MR Egger
Simple mode
Weighted median
Weighted mode
0.1
0.0
−0.1
−0.2
SNP effect on GCST90200753_buildGRCh38.tsv
0.04
0.08
0.12
0.16
SNP effect on GCA−900066135 sp900066135

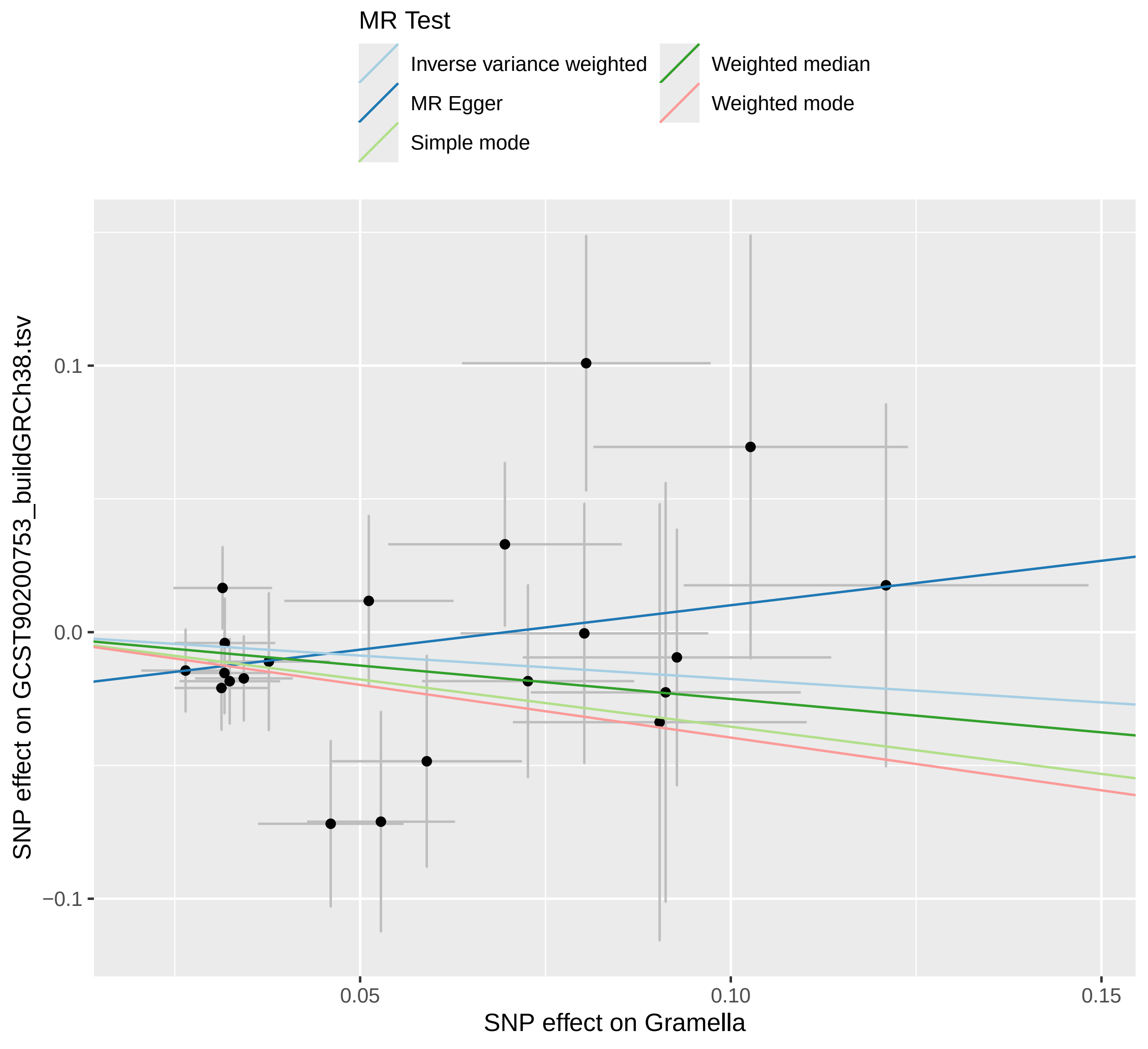

MR Test
Inverse variance weighted
MR Egger
Simple mode
Weighted median
Weighted mode
SNP effect on GCST90200753_buildGRCh38.tsv
0.1
0.0
−0.1
0.05
0.10
0.15
SNP effect on Gramella

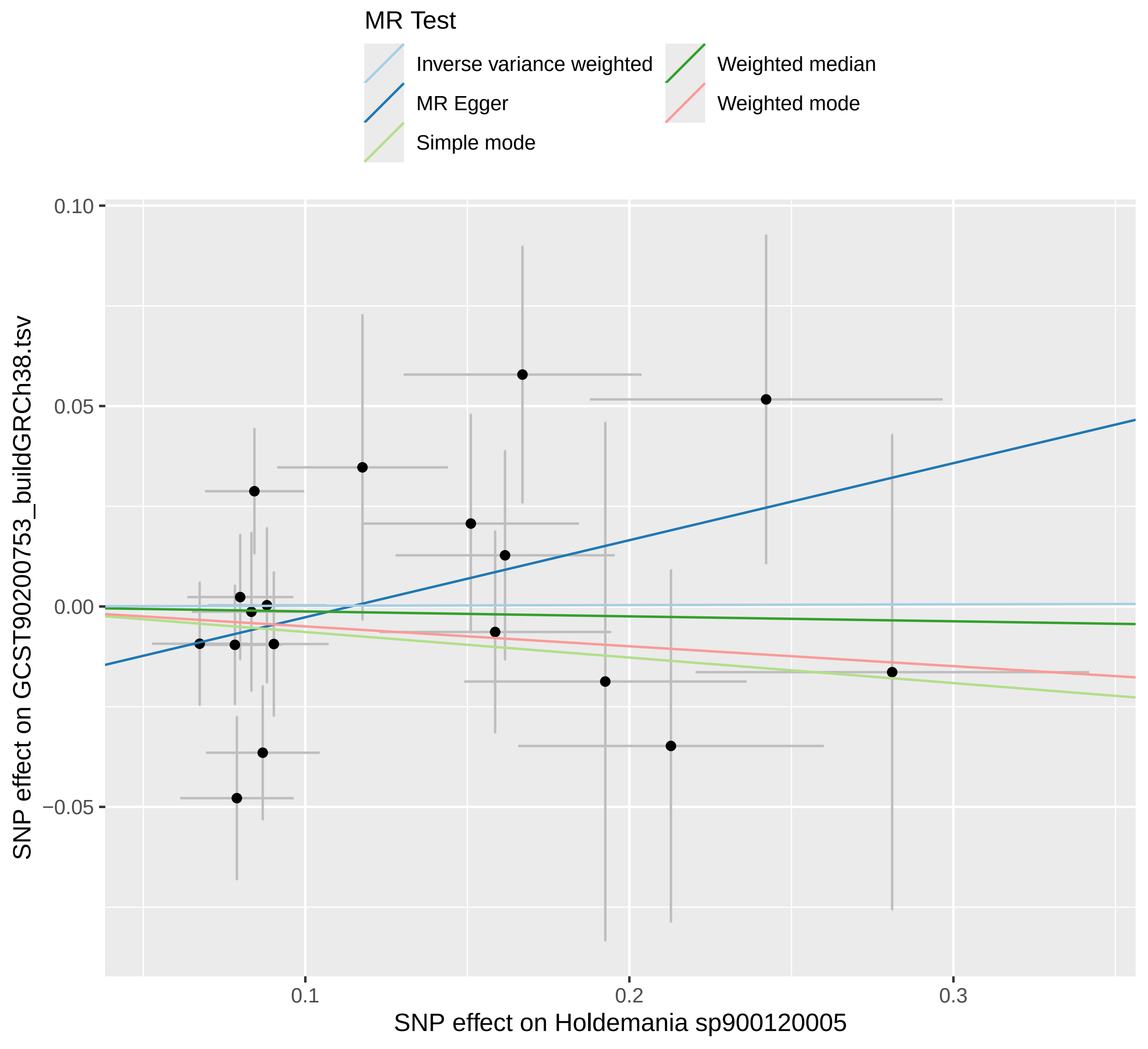

MR Test
Inverse variance weighted
MR Egger
Simple mode
Weighted median
Weighted mode
0.10
0.05
0.00
−0.05
0.1
0.2
0.3
SNP effect on GCST90200753_buildGRCh38.tsv
SNP effect on Holdemania sp900120005

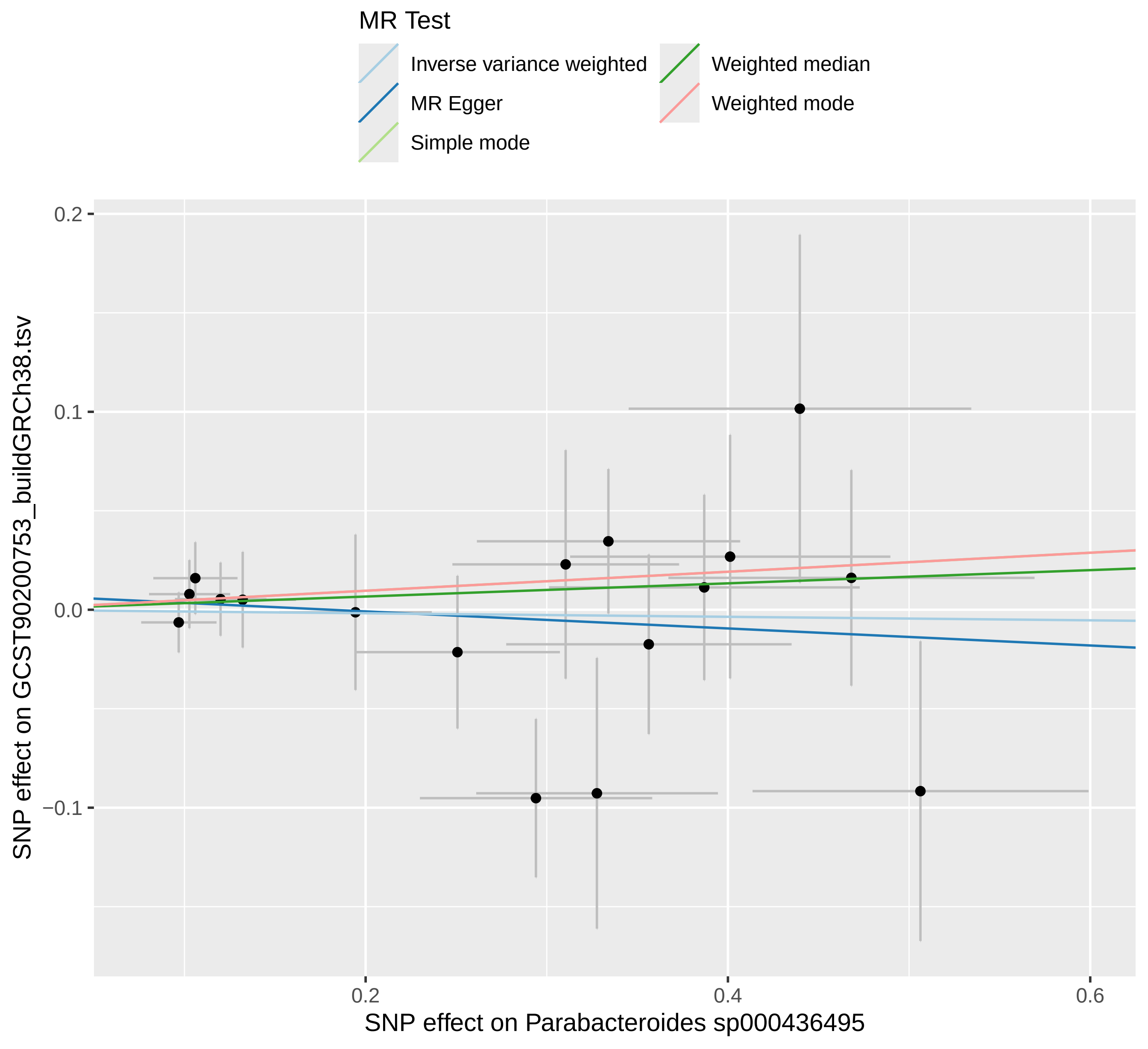

MR Test
Inverse variance weighted
MR Egger
Simple mode
Weighted median
Weighted mode
0.2
0.1
0.0
−0.1
0.2
0.4
0.6
SNP effect on GCST90200753_buildGRCh38.tsv
SNP effect on Parabacteroides sp000436495

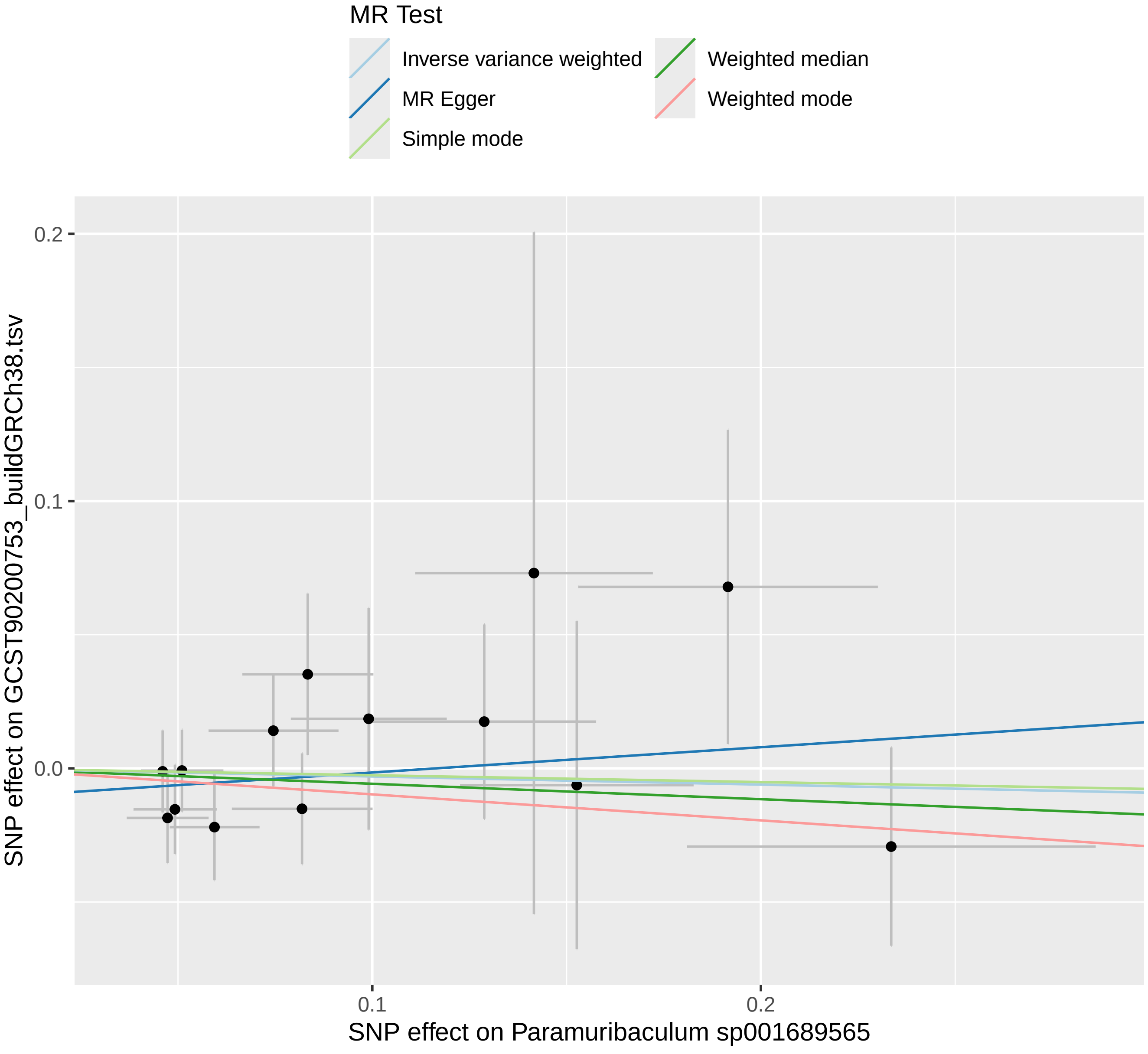
MR Test
Inverse variance weighted
MR Egger
Simple mode
Weighted median
Weighted mode
0.2
0.1
0.0
0.1
0.2
SNP effect on GCST90200753_buildGRCh38.tsv
SNP effect on Paramuribaculum sp001689565

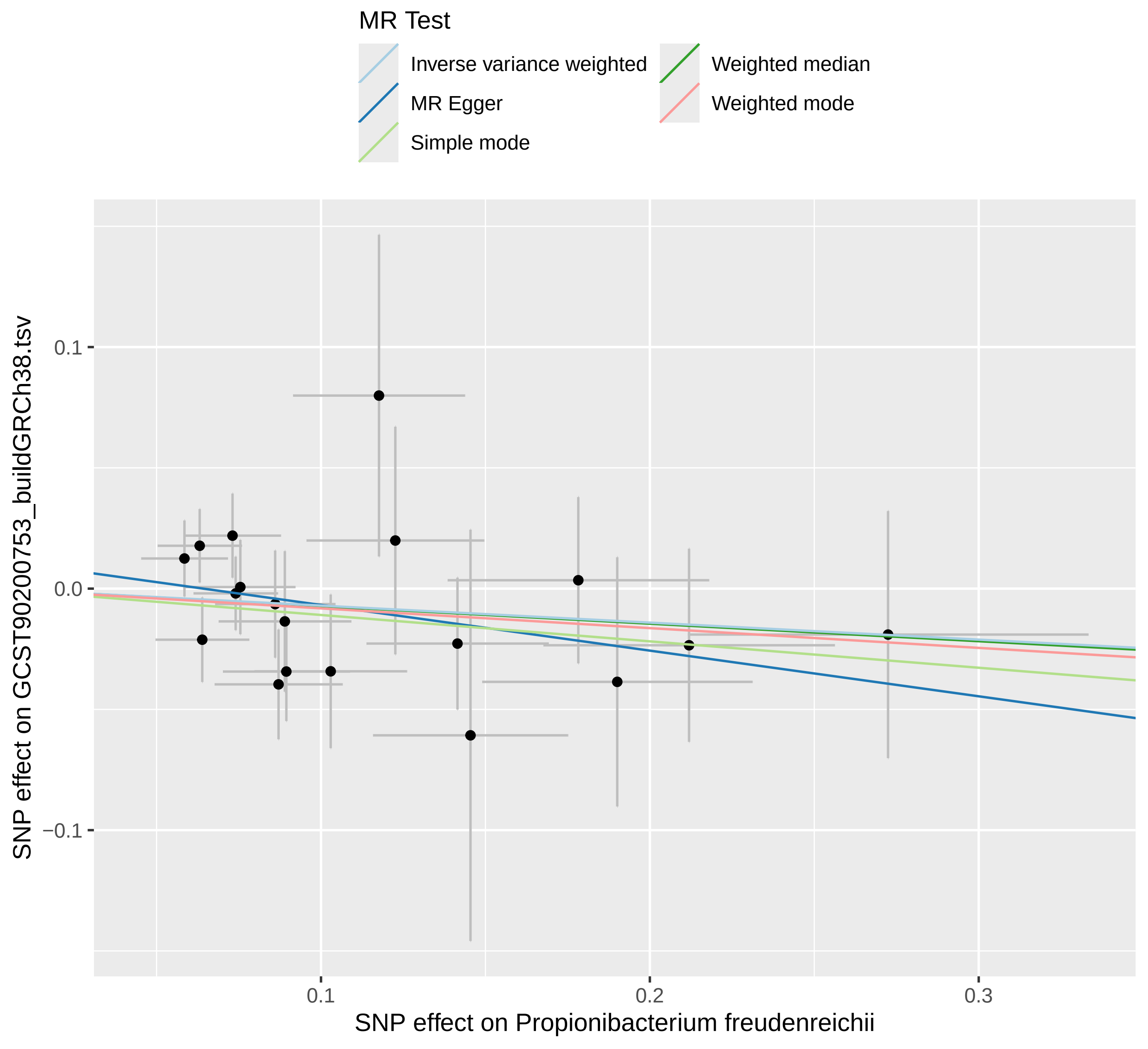

MR Test
Inverse variance weighted
MR Egger
Simple mode
Weighted median
Weighted mode
0.1
0.0
−0.1
0.1
0.2
0.3
SNP effect on GCST90200753_buildGRCh38.tsv
SNP effect on Propionibacterium freudenreichii

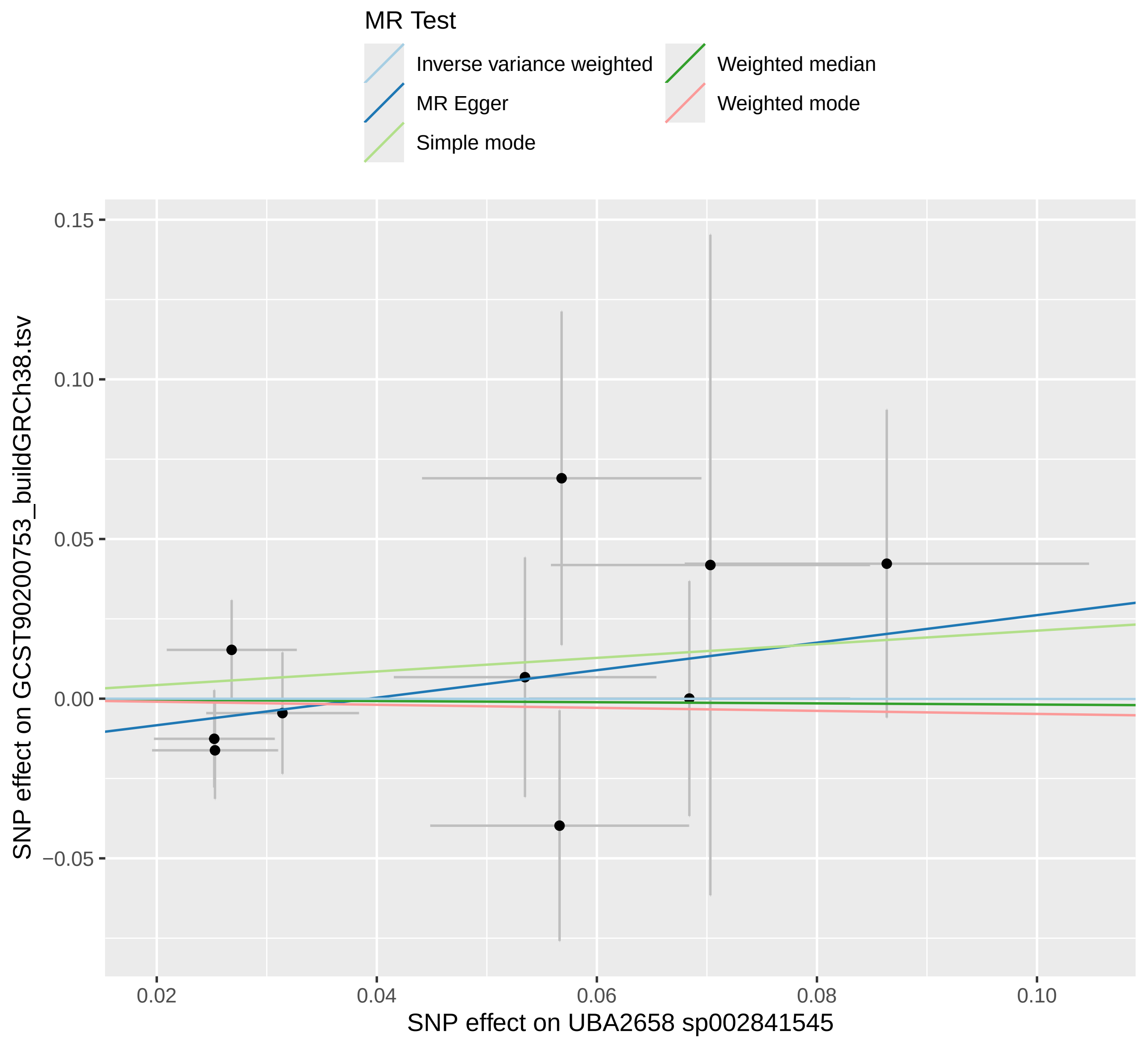
MR Test
Inverse variance weighted
MR Egger
Simple mode
Weighted median
Weighted mode
0.15
0.10
0.05
0.00
−0.05
SNP effect on GCST90200753_buildGRCh38.tsv
0.02
0.04
0.06
0.08
0.10
SNP effect on UBA2658 sp002841545

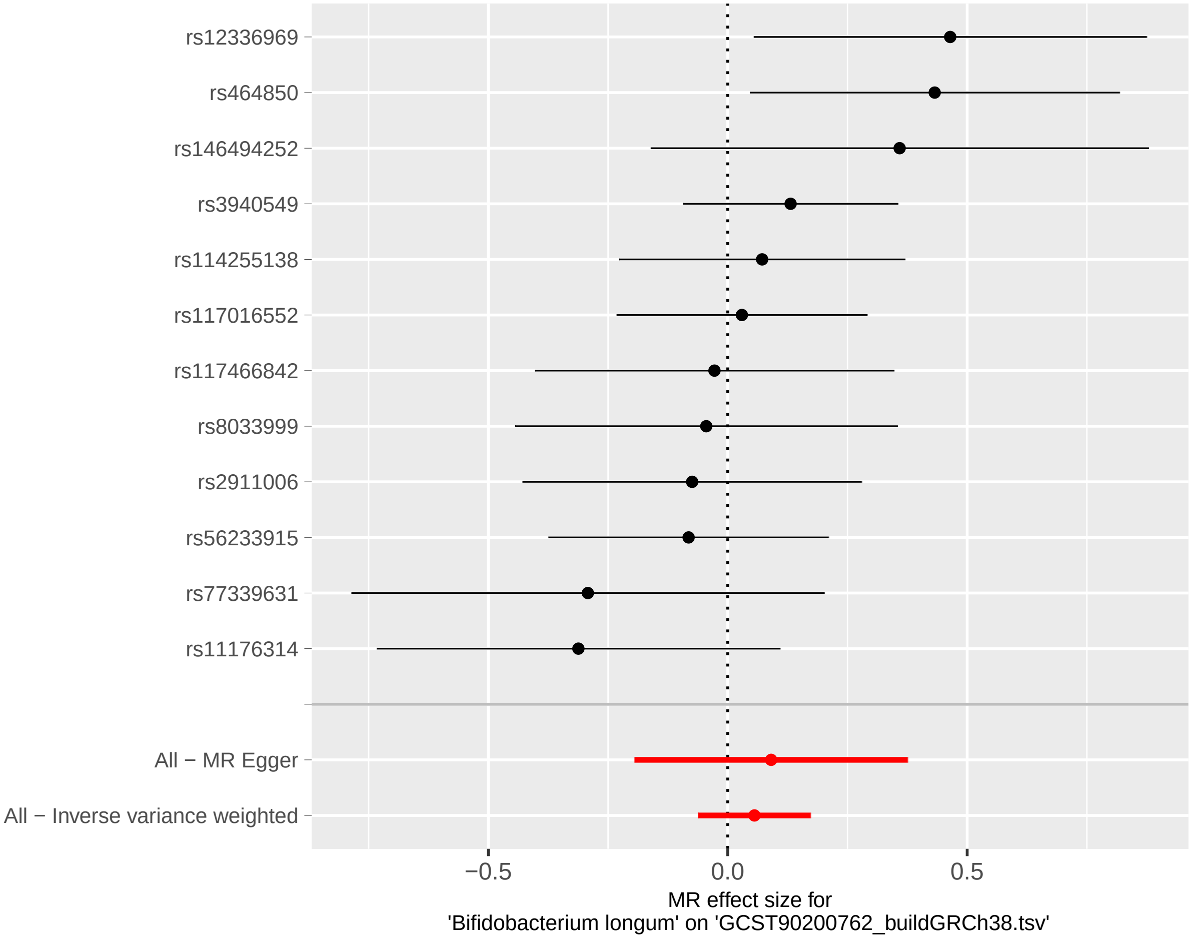
rs12336969
rs464850
rs146494252
rs3940549
rs114255138
rs117016552
rs117466842
rs8033999
rs2911006
rs56233915
rs77339631
rs11176314
All − MR Egger
All − Inverse variance weighted
−0.5
0.0
0.5
MR effect size for
'Bifidobacterium longum' on 'GCST90200762_buildGRCh38.tsv'

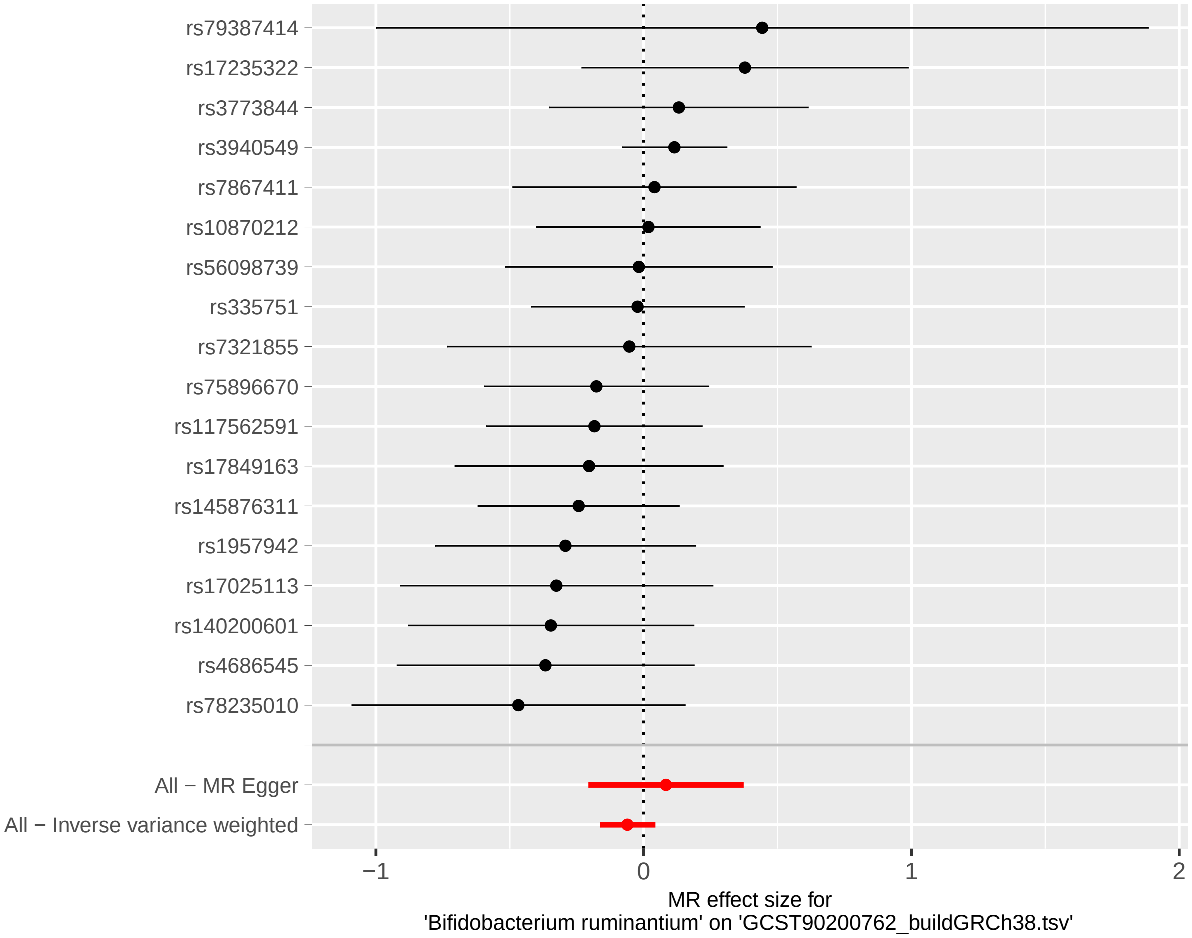

rs79387414
rs17235322
rs3773844
rs3940549
rs7867411
rs10870212
rs56098739
rs335751
rs7321855
rs75896670
rs117562591
rs17849163
rs145876311
rs1957942
rs17025113
rs140200601
rs4686545
rs78235010
All − MR Egger
All − Inverse variance weighted
−1
0
1
2
MR effect size for
'Bifidobacterium ruminantium' on 'GCST90200762_buildGRCh38.tsv'

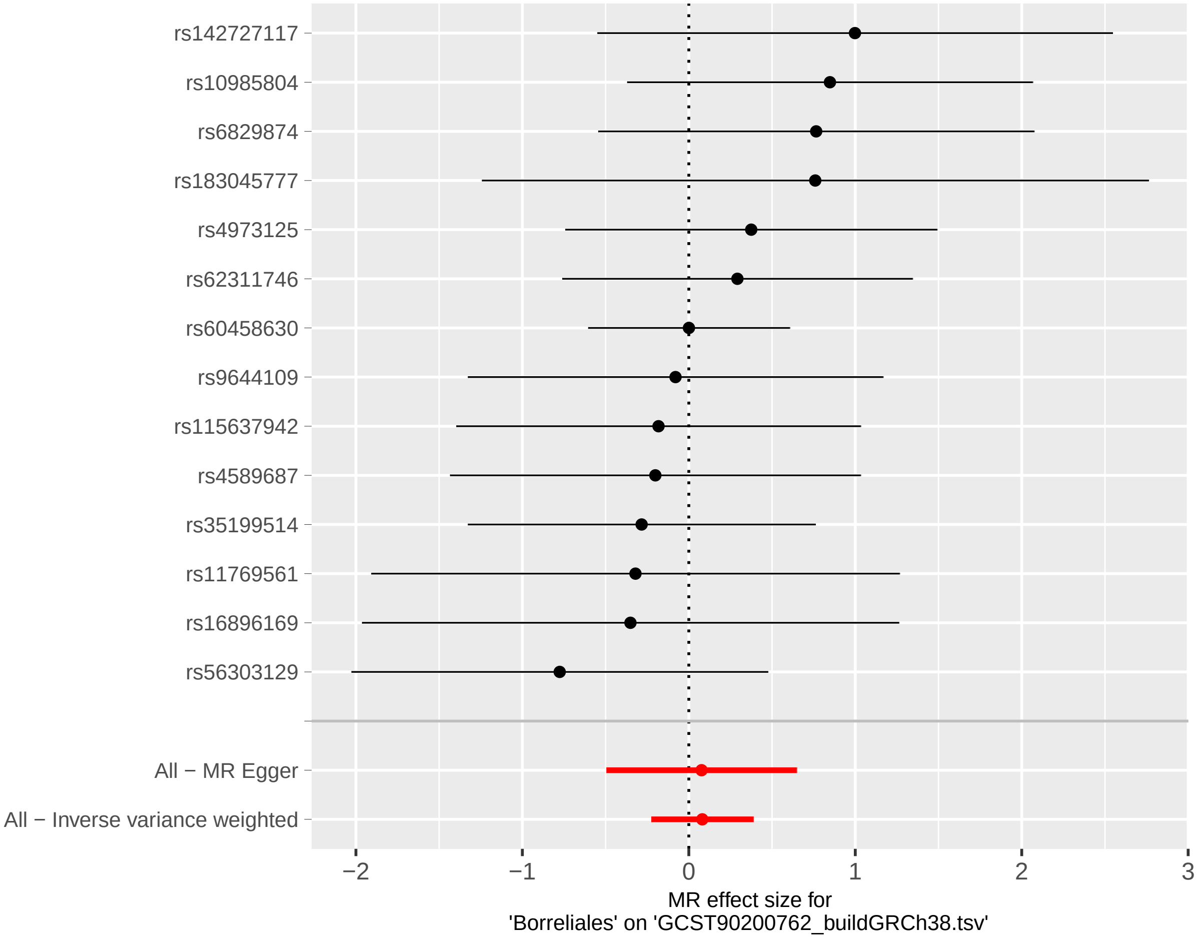
rs142727117
rs10985804
rs6829874
rs183045777
rs4973125
rs62311746
rs60458630
rs9644109
rs115637942
rs4589687
rs35199514
rs11769561
rs16896169
rs56303129
All − MR Egger
All − Inverse variance weighted
−2
−1
0
1
2
3
MR effect size for
'Borreliales' on 'GCST90200762_buildGRCh38.tsv'

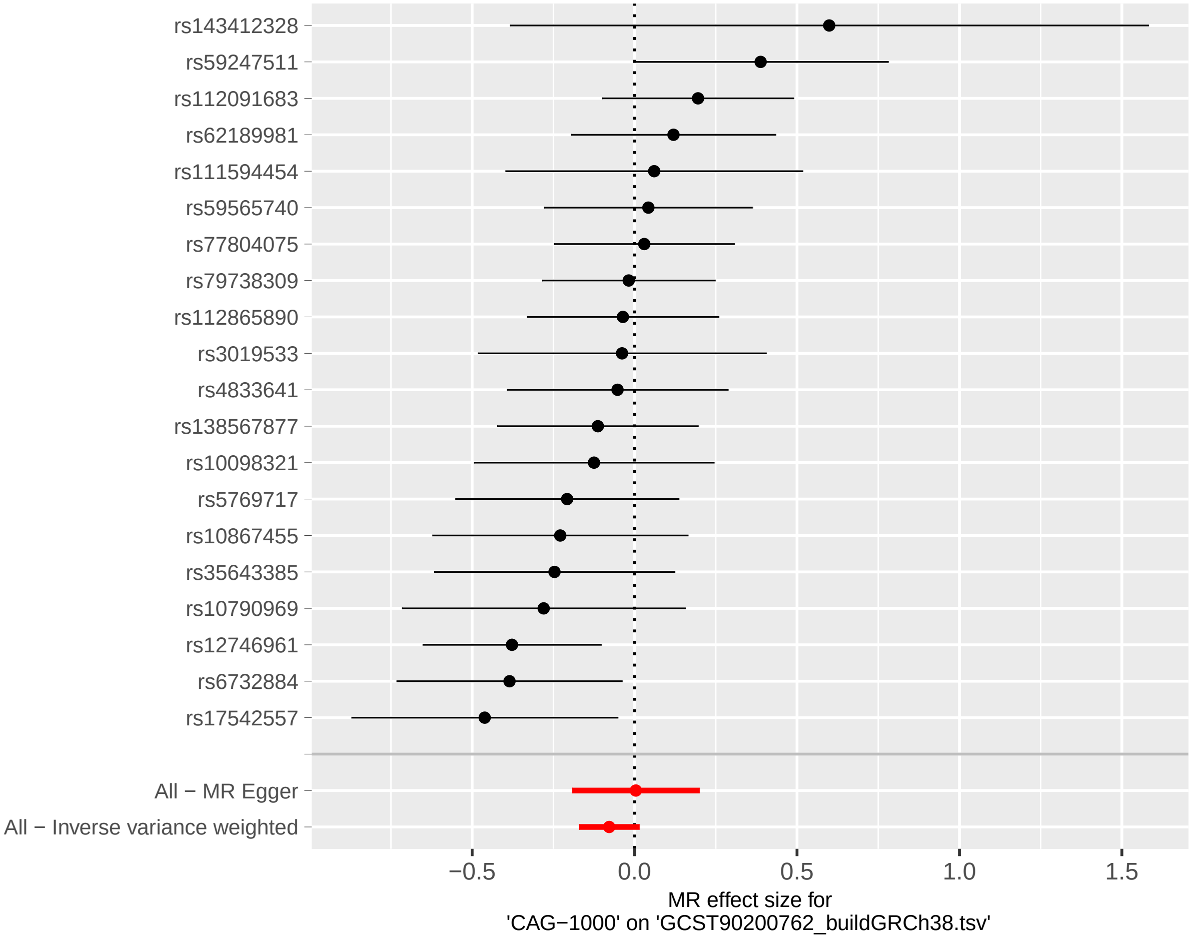
rs143412328
rs59247511
rs112091683
rs62189981
rs111594454
rs59565740
rs77804075
rs79738309
rs112865890
rs3019533
rs4833641
rs138567877
rs10098321
rs5769717
rs10867455
rs35643385
rs10790969
rs12746961
rs6732884
rs17542557
All − MR Egger
All − Inverse variance weighted
−0.5
0.0
0.5
1.0
1.5
MR effect size for
'CAG−1000' on 'GCST90200762_buildGRCh38.tsv'

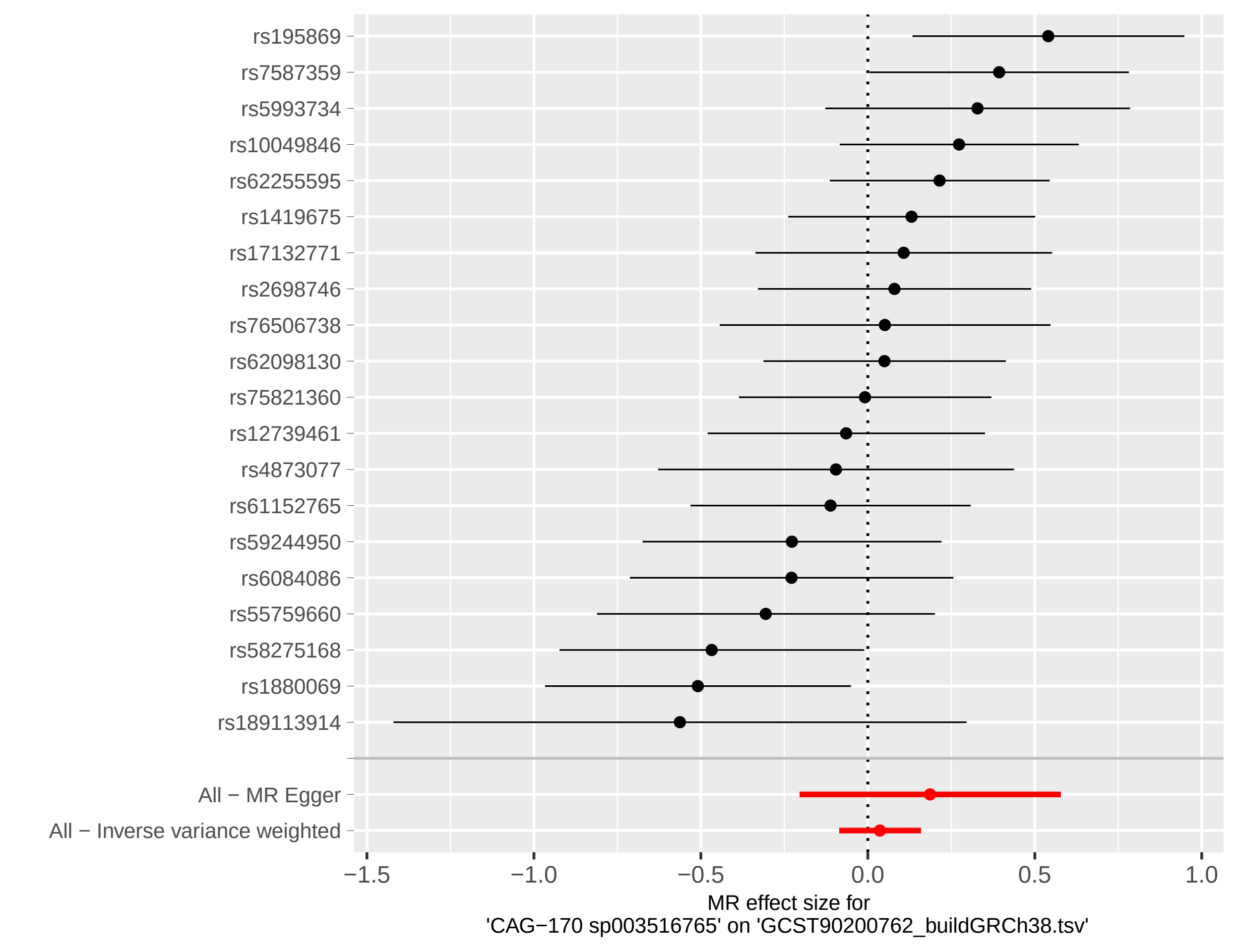

rs195869
rs7587359
rs5993734
rs10049846
rs62255595
rs1419675
rs17132771
rs2698746
rs76506738
rs62098130
rs75821360
rs12739461
rs4873077
rs61152765
rs59244950
rs6084086
rs55759660
rs58275168
rs1880069
rs189113914
All − MR Egger
All − Inverse variance weighted
−1.5
−1.0
−0.5
0.0
0.5
1.0
MR effect size for
'CAG−170 sp003516765' on 'GCST90200762_buildGRCh38.tsv'

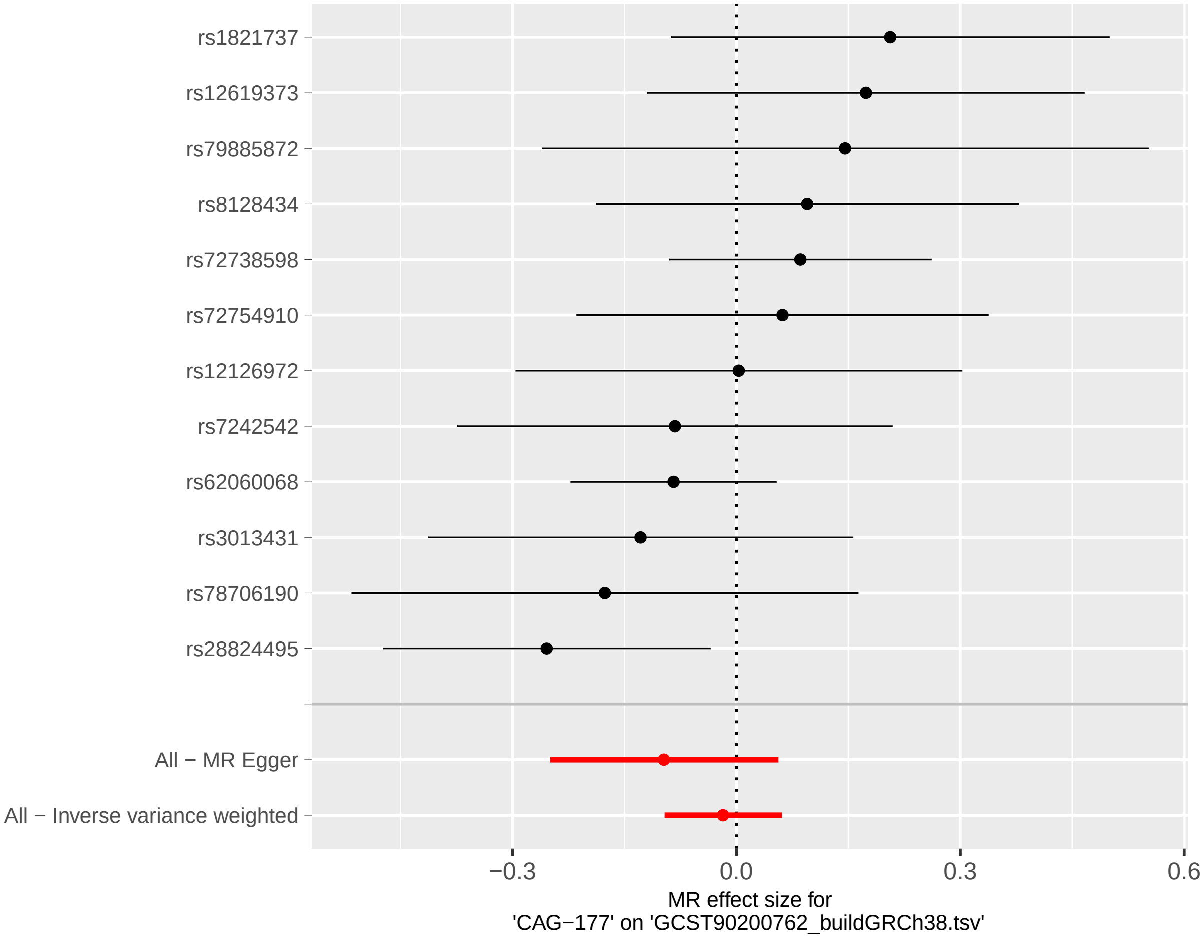

rs1821737
rs12619373
rs79885872
rs8128434
rs72738598
rs72754910
rs12126972
rs7242542
rs62060068
rs3013431
rs78706190
rs28824495
All − MR Egger
All − Inverse variance weighted
−0.3
0.0
0.3
0.6
MR effect size for
'CAG−177' on 'GCST90200762_buildGRCh38.tsv'

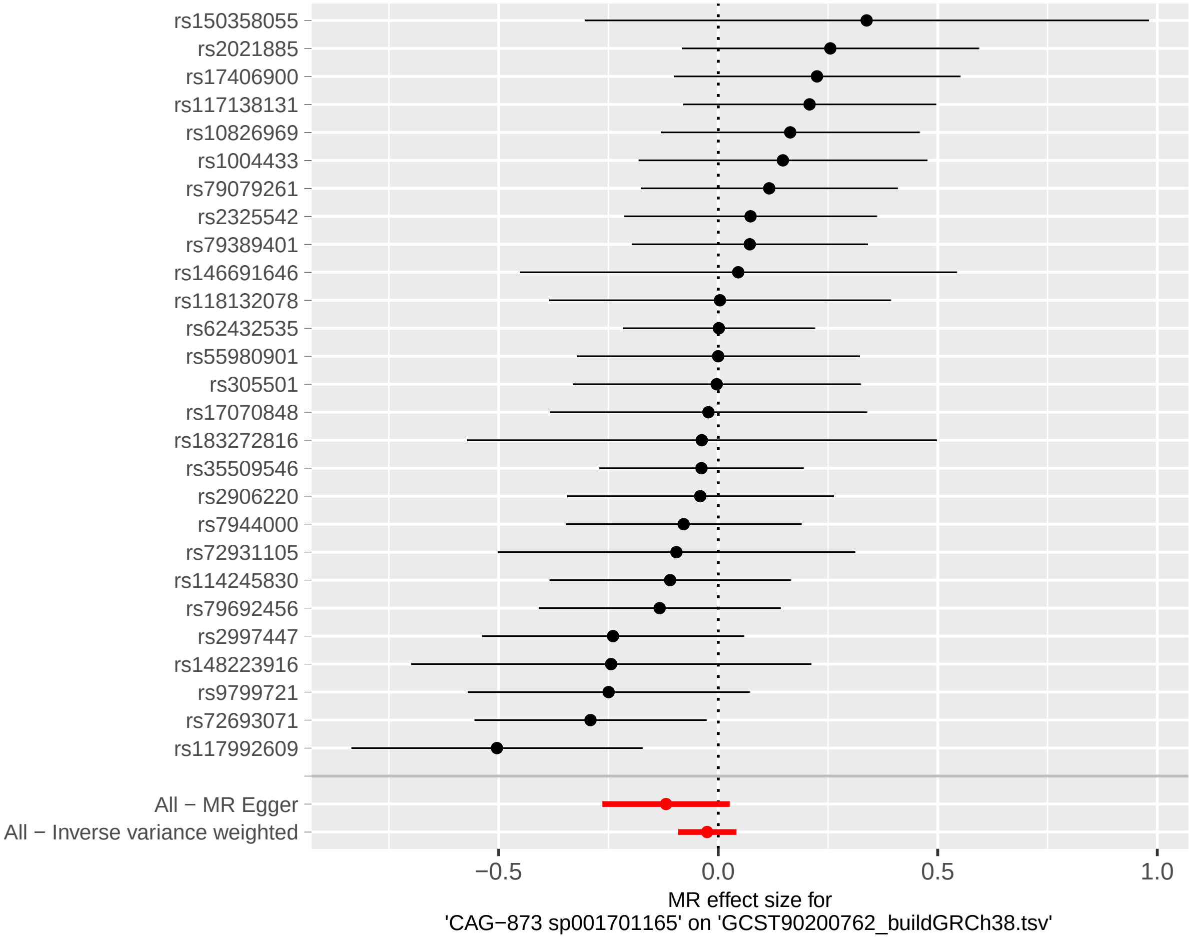
rs150358055
rs2021885
rs17406900
rs117138131
rs10826969
rs1004433
rs79079261
rs2325542
rs79389401
rs146691646
rs118132078
rs62432535
rs55980901
rs305501
rs17070848
rs183272816
rs35509546
rs2906220
rs7944000
rs72931105
rs114245830
rs79692456
rs2997447
rs148223916
rs9799721
rs72693071
rs117992609
All − MR Egger
All − Inverse variance weighted
−0.5
0.0
0.5
1.0
MR effect size for
'CAG−873 sp001701165' on 'GCST90200762_buildGRCh38.tsv'

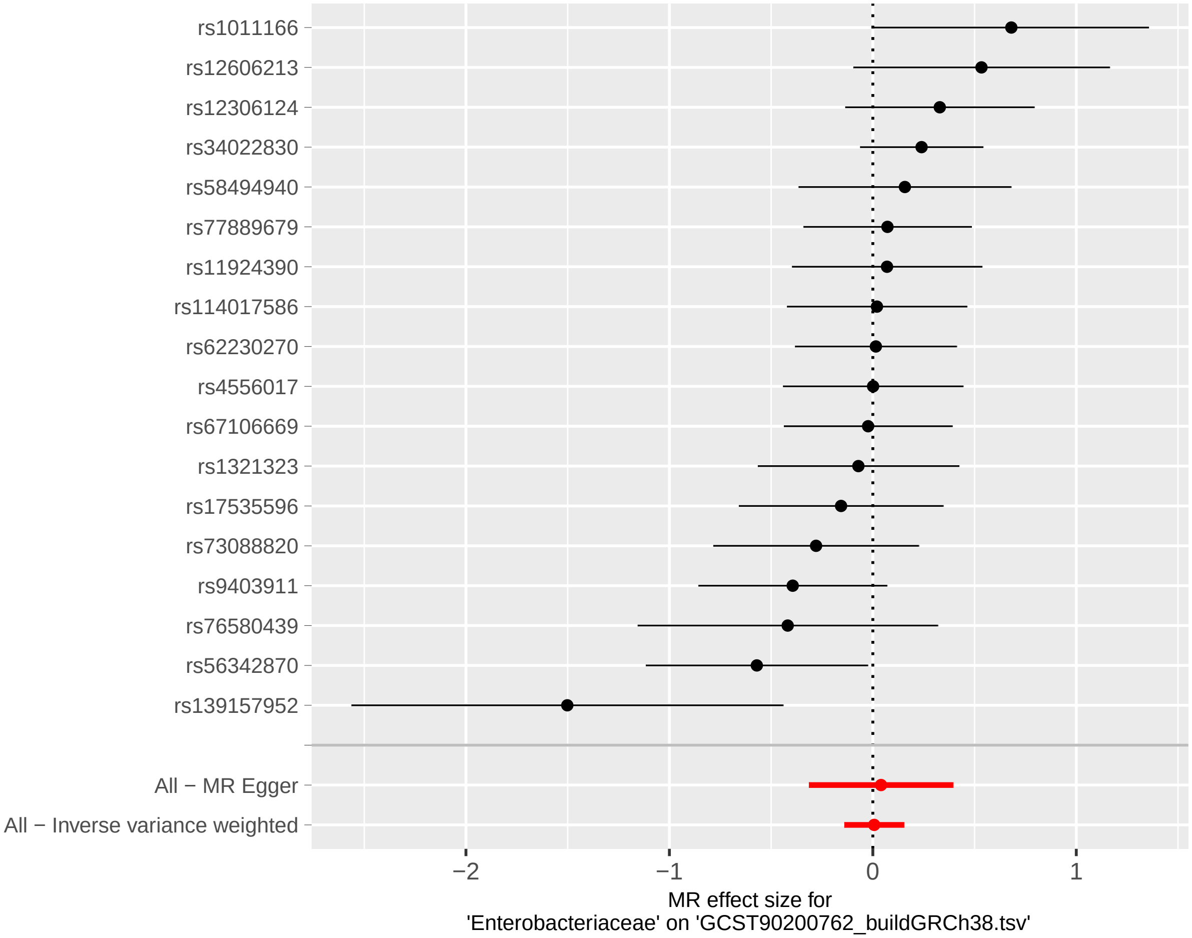

rs1011166
rs12606213
rs12306124
rs34022830
rs58494940
rs77889679
rs11924390
rs114017586
rs62230270
rs4556017
rs67106669
rs1321323
rs17535596
rs73088820
rs9403911
rs76580439
rs56342870
rs139157952
All − MR Egger
All − Inverse variance weighted
−2
−1
0
1
MR effect size for
'Enterobacteriaceae' on 'GCST90200762_buildGRCh38.tsv'

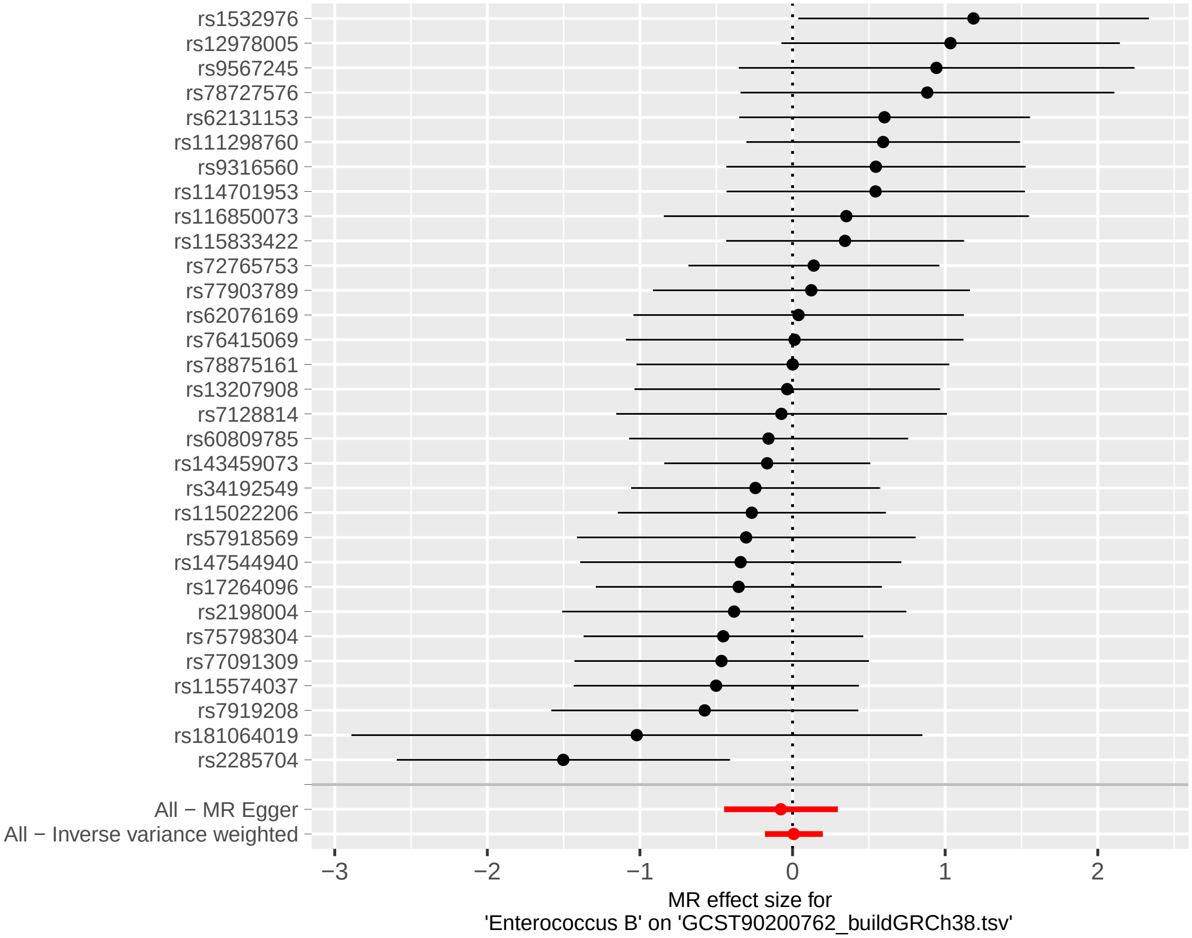

rs1532976
rs12978005
rs9567245
rs78727576
rs62131153
rs111298760
rs9316560
rs114701953
rs116850073
rs115833422
rs72765753
rs77903789
rs62076169
rs76415069
rs78875161
rs13207908
rs7128814
rs60809785
rs143459073
rs34192549
rs115022206
rs57918569
rs147544940
rs17264096
rs2198004
rs75798304
rs77091309
rs115574037
rs7919208
rs181064019
rs2285704
All − MR Egger
All − Inverse variance weighted
−3
−2
−1
0
1
2
MR effect size for
'Enterococcus B' on 'GCST90200762_buildGRCh38.tsv'

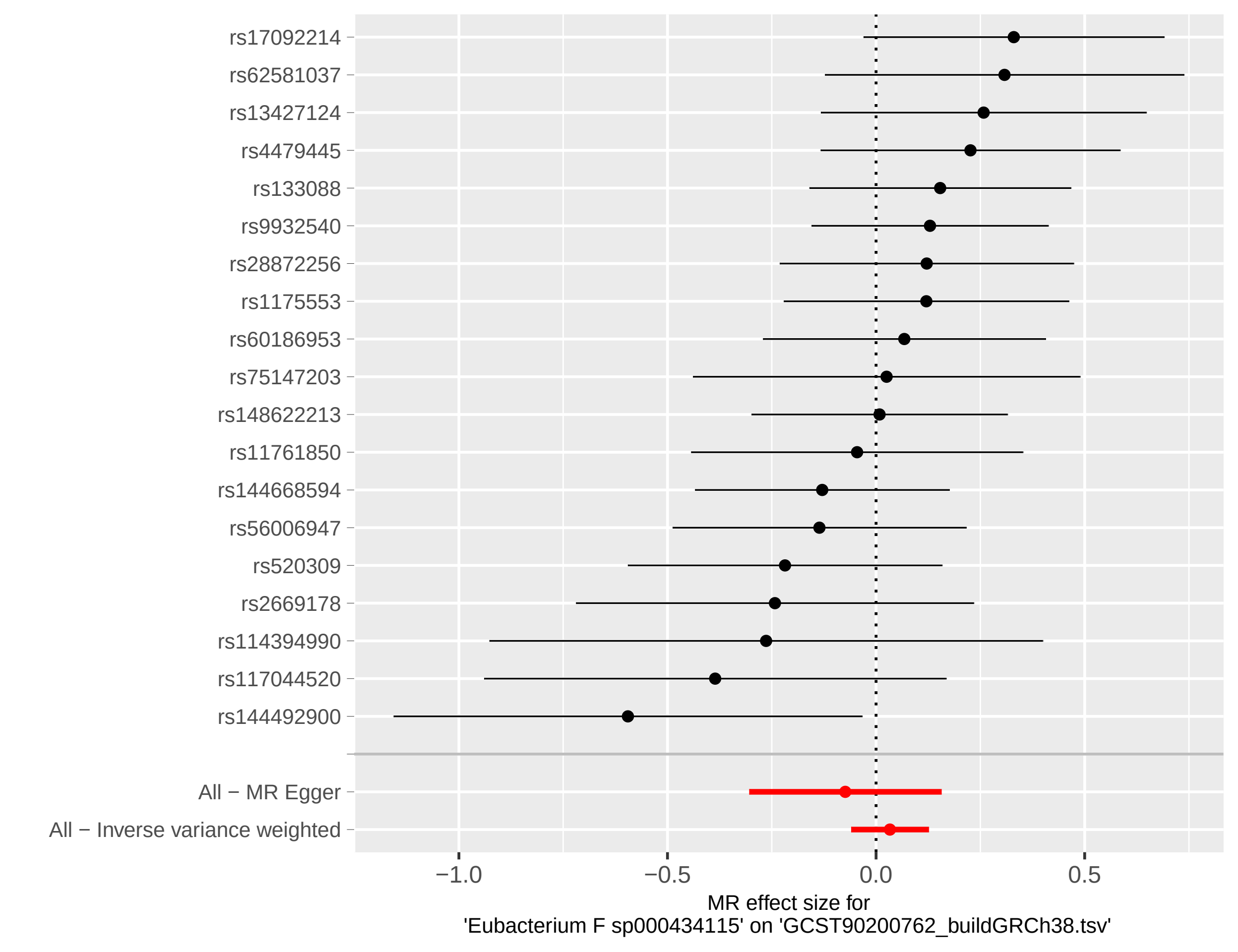

rs17092214
rs62581037
rs13427124
rs4479445
rs133088
rs9932540
rs28872256
rs1175553
rs60186953
rs75147203
rs148622213
rs11761850
rs144668594
rs56006947
rs520309
rs2669178
rs114394990
rs117044520
rs144492900
All − MR Egger
All − Inverse variance weighted
−1.0
−0.5
0.0
0.5
MR effect size for
'Eubacterium F sp000434115' on 'GCST90200762_buildGRCh38.tsv'

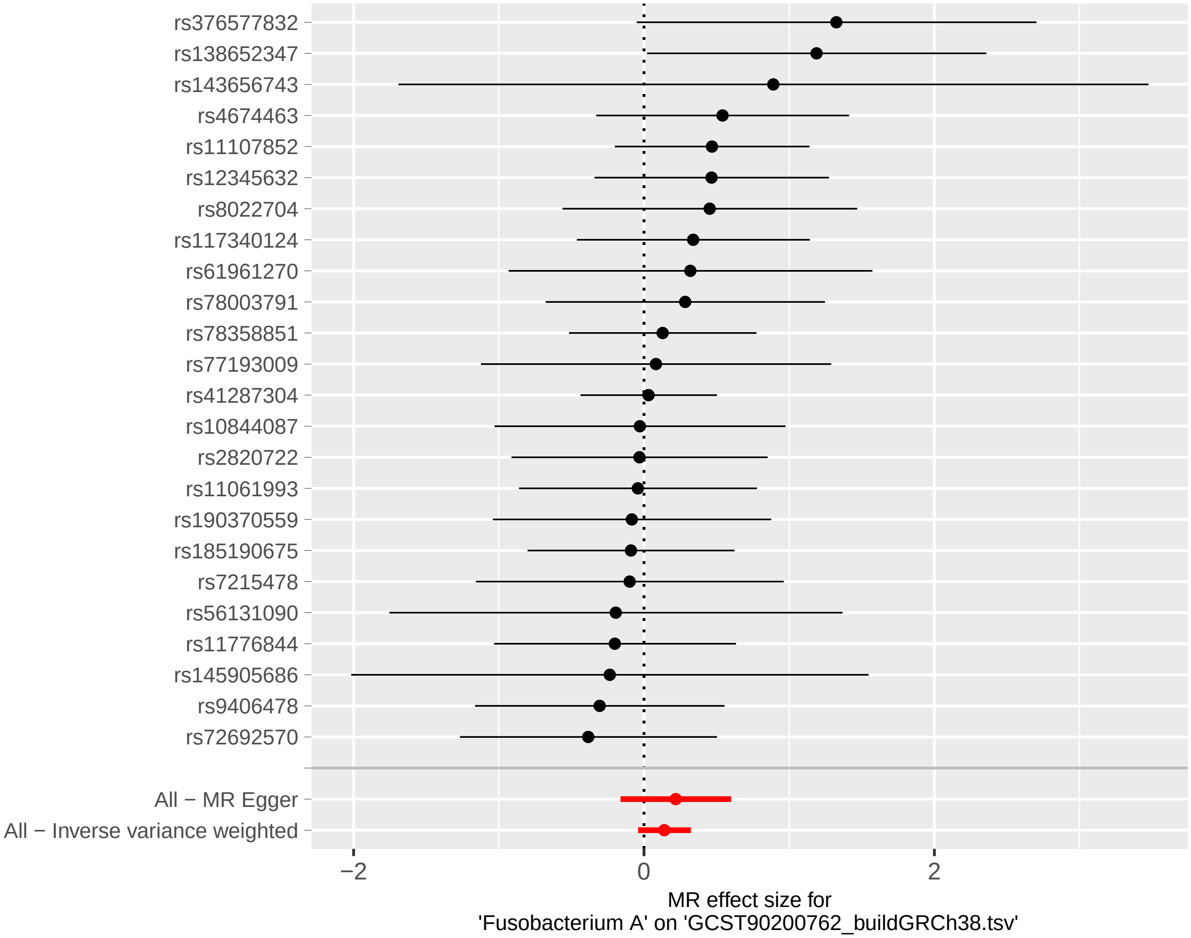
rs376577832
rs138652347
rs143656743
rs4674463
rs11107852
rs12345632
rs8022704
rs117340124
rs61961270
rs78003791
rs78358851
rs77193009
rs41287304
rs10844087
rs2820722
rs11061993
rs190370559
rs185190675
rs7215478
rs56131090
rs11776844
rs145905686
rs9406478
rs72692570
All − MR Egger
All − Inverse variance weighted
−2
0
2
MR effect size for
'Fusobacterium A' on 'GCST90200762_buildGRCh38.tsv'

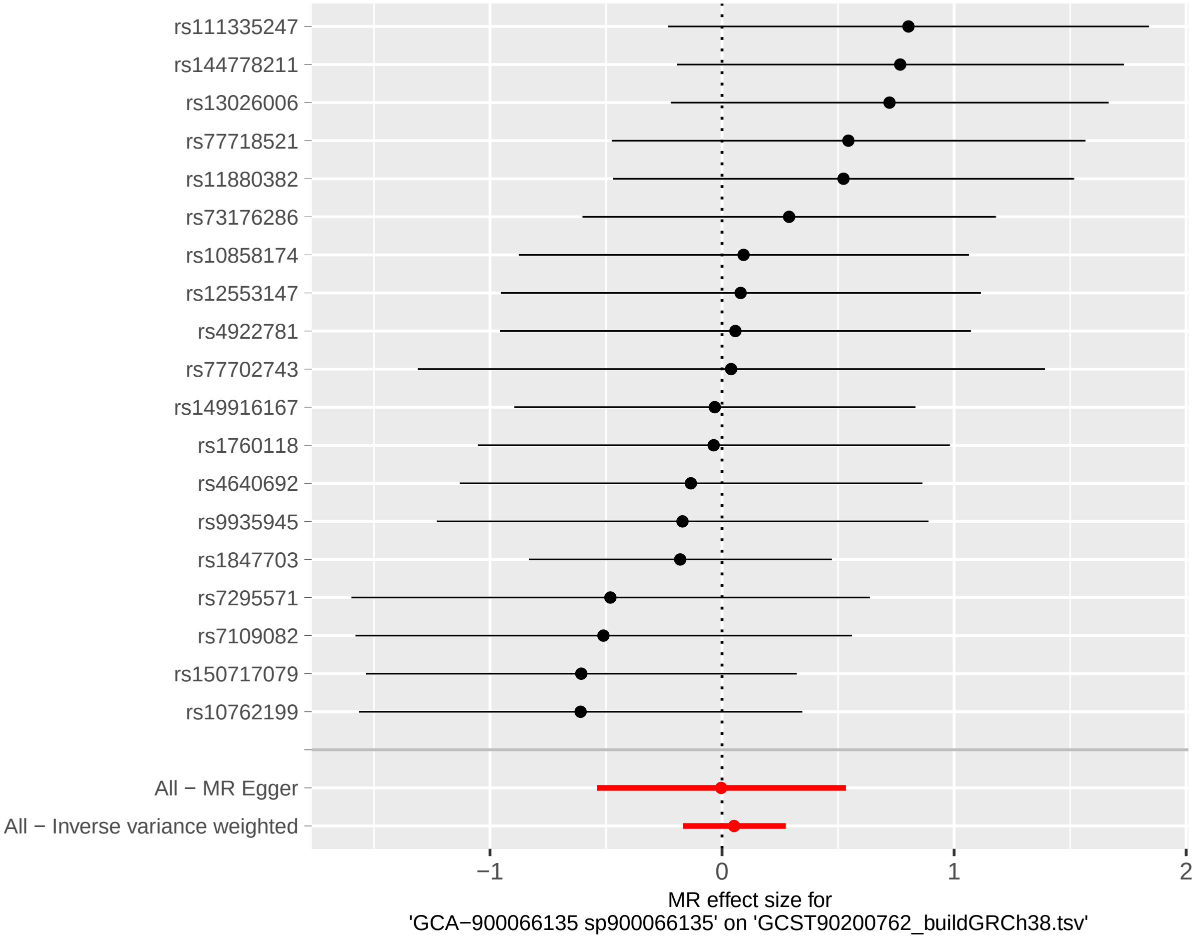

rs111335247
rs144778211
rs13026006
rs77718521
rs11880382
rs73176286
rs10858174
rs12553147
rs4922781
rs77702743
rs149916167
rs1760118
rs4640692
rs9935945
rs1847703
rs7295571
rs7109082
rs150717079
rs10762199
All − MR Egger
All − Inverse variance weighted
−1
0
1
2
MR effect size for
'GCA−900066135 sp900066135' on 'GCST90200762_buildGRCh38.tsv'

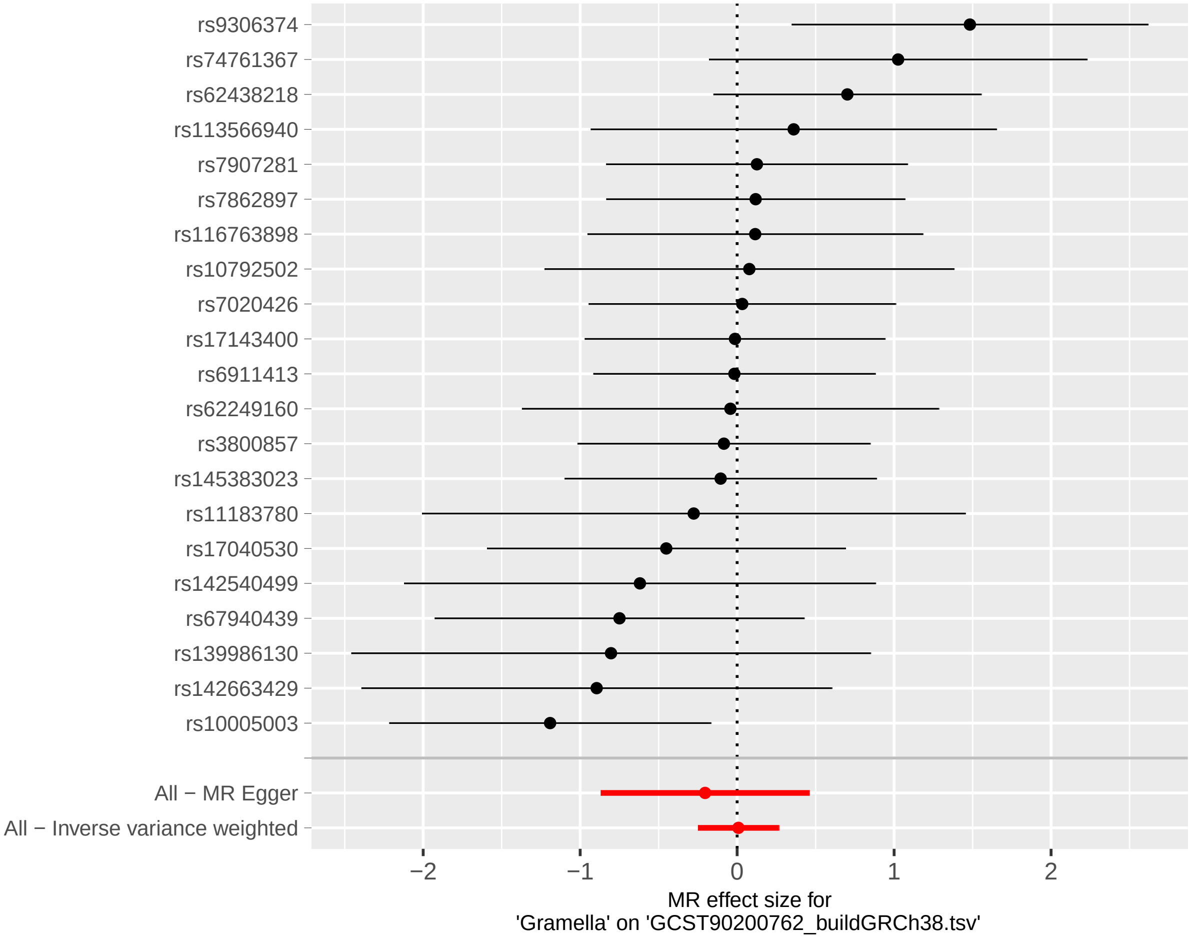
rs9306374
rs74761367
rs62438218
rs113566940
rs7907281
rs7862897
rs116763898
rs10792502
rs7020426
rs17143400
rs6911413
rs62249160
rs3800857
rs145383023
rs11183780
rs17040530
rs142540499
rs67940439
rs139986130
rs142663429
rs10005003
All − MR Egger
All − Inverse variance weighted
−2
−1
0
1
2
MR effect size for
'Gramella' on 'GCST90200762_buildGRCh38.tsv'

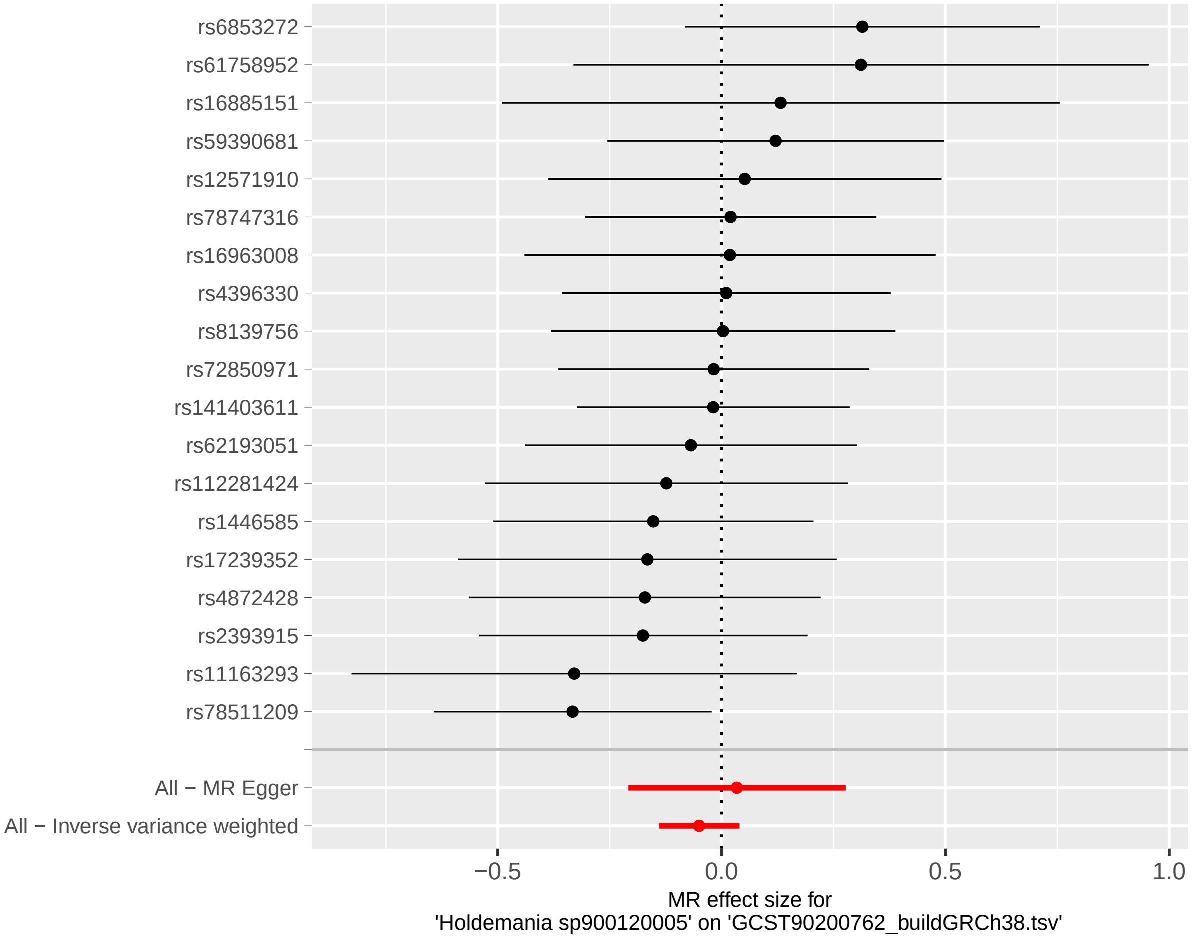
rs6853272
rs61758952
rs16885151
rs59390681
rs12571910
rs78747316
rs16963008
rs4396330
rs8139756
rs72850971
rs141403611
rs62193051
rs112281424
rs1446585
rs17239352
rs4872428
rs2393915
rs11163293
rs78511209
All − MR Egger
All − Inverse variance weighted
−0.5
0.0
0.5
1.0
MR effect size for
'Holdemania sp900120005' on 'GCST90200762_buildGRCh38.tsv'

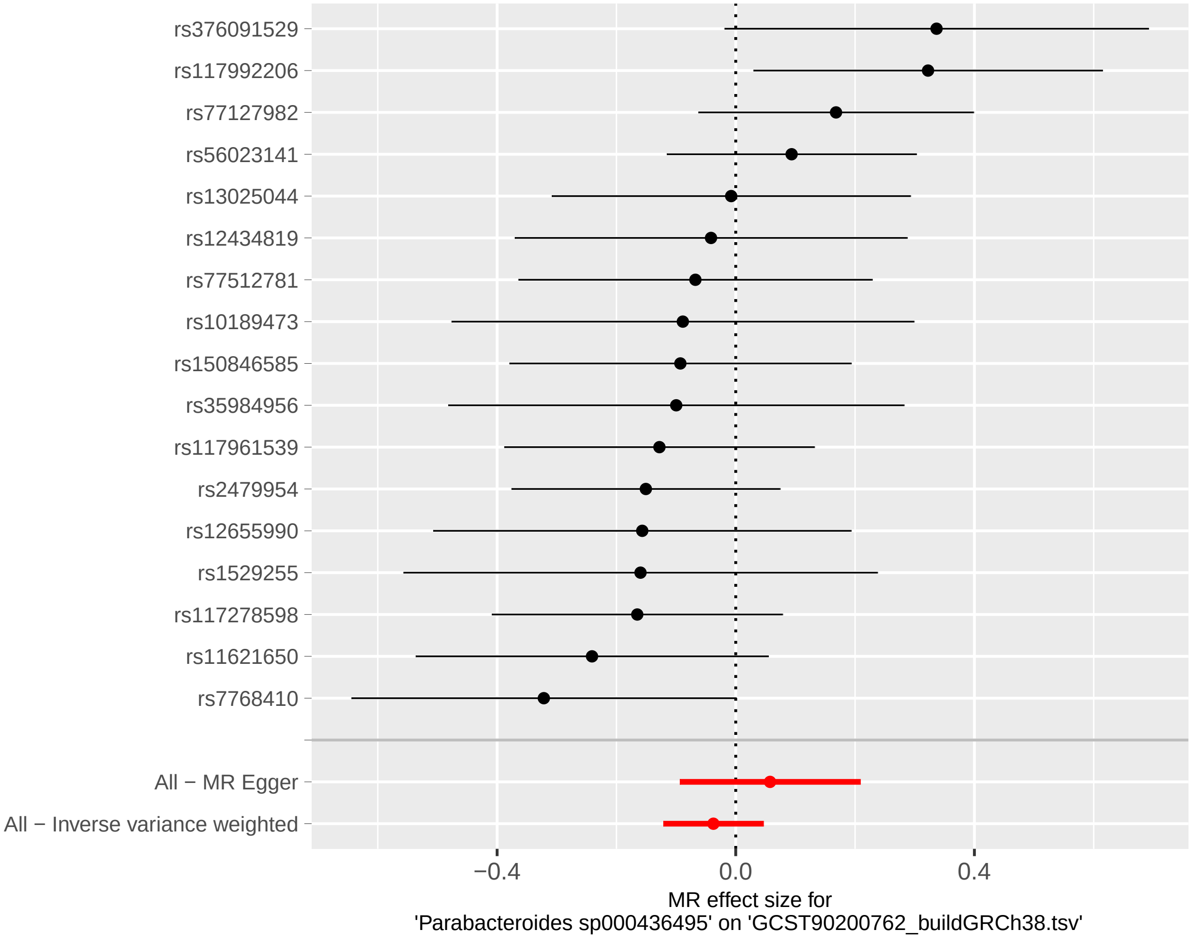

rs376091529
rs117992206
rs77127982
rs56023141
rs13025044
rs12434819
rs77512781
rs10189473
rs150846585
rs35984956
rs117961539
rs2479954
rs12655990
rs1529255
rs117278598
rs11621650
rs7768410
All − MR Egger
All − Inverse variance weighted
−0.4
0.0
0.4
MR effect size for
'Parabacteroides sp000436495' on 'GCST90200762_buildGRCh38.tsv'

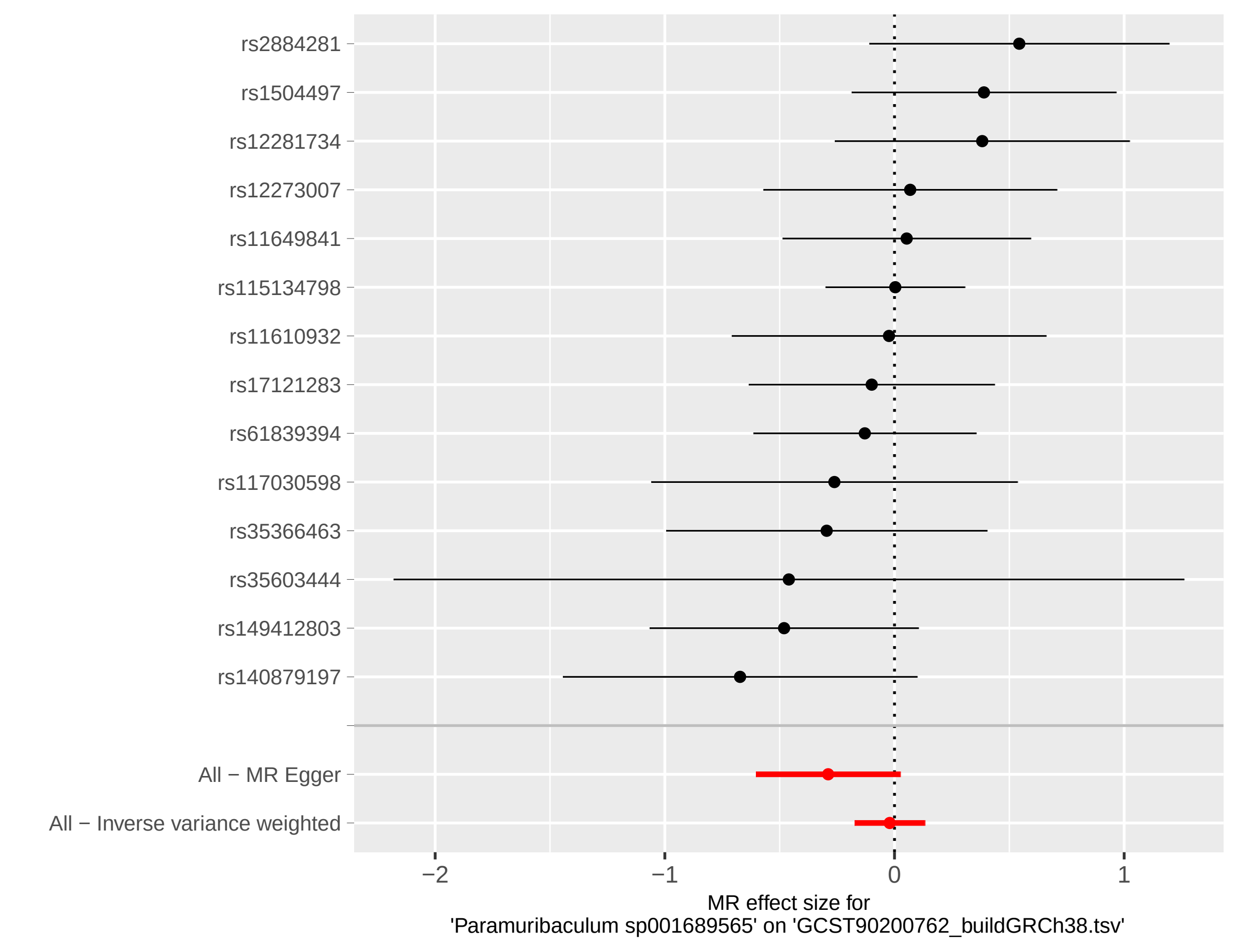

rs2884281
rs1504497
rs12281734
rs12273007
rs11649841
rs115134798
rs11610932
rs17121283
rs61839394
rs117030598
rs35366463
rs35603444
rs149412803
rs140879197
All − MR Egger
All − Inverse variance weighted
−2
−1
0
1
MR effect size for
'Paramuribaculum sp001689565' on 'GCST90200762_buildGRCh38.tsv'

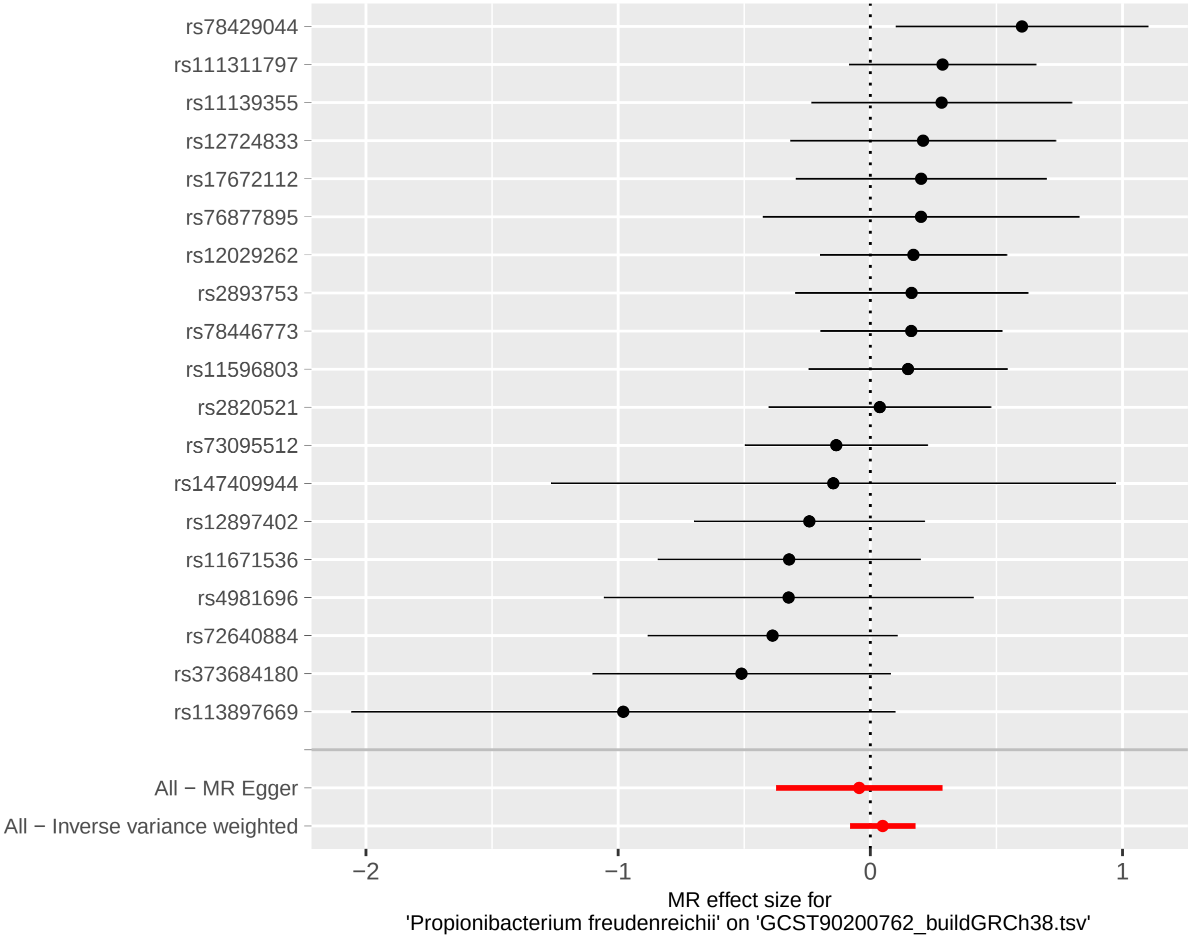
rs78429044
rs111311797
rs11139355
rs12724833
rs17672112
rs76877895
rs12029262
rs2893753
rs78446773
rs11596803
rs2820521
rs73095512
rs147409944
rs12897402
rs11671536
rs4981696
rs72640884
rs373684180
rs113897669
All − MR Egger
All − Inverse variance weighted
−2
−1
0
1
MR effect size for
'Propionibacterium freudenreichii' on 'GCST90200762_buildGRCh38.tsv'

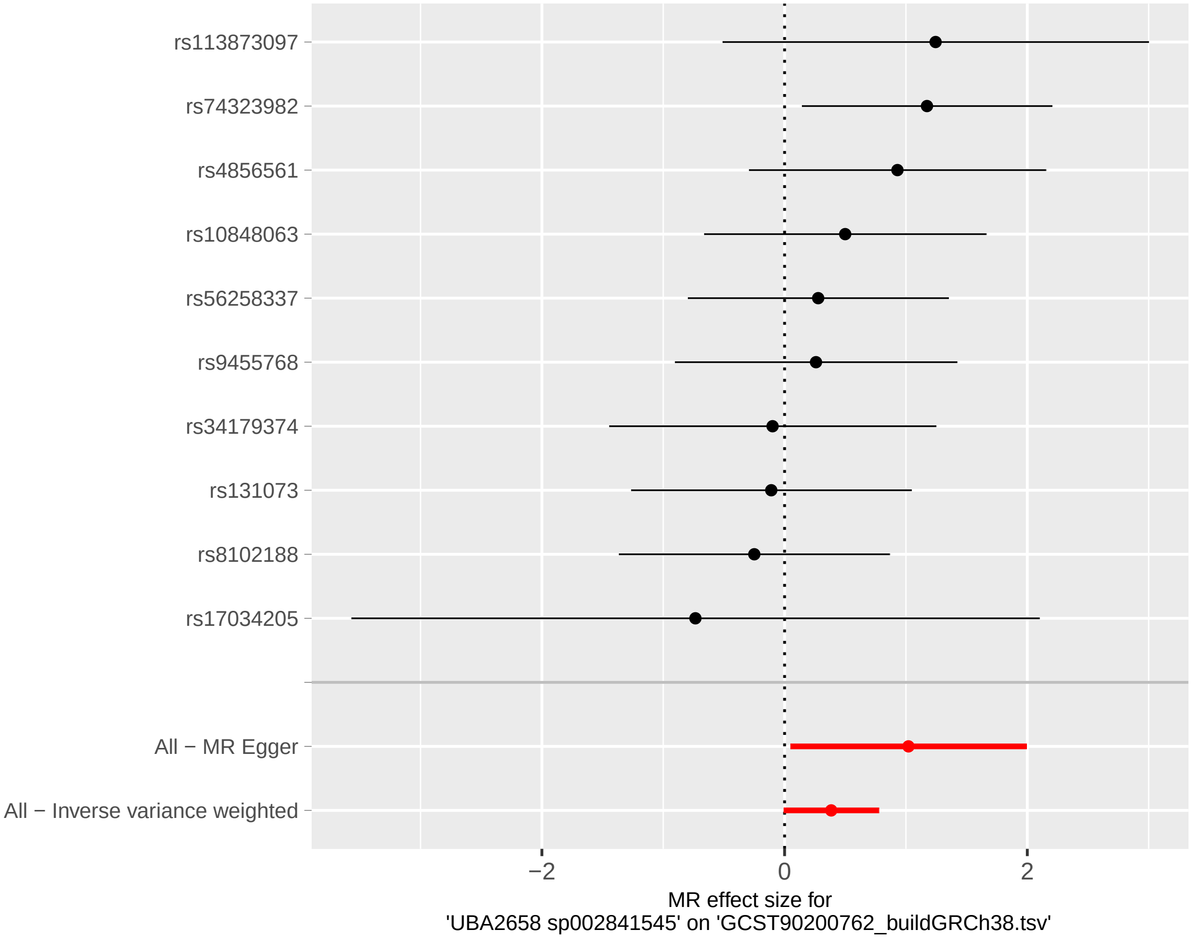

rs113873097
rs74323982
rs4856561
rs10848063
rs56258337
rs9455768
rs34179374
rs131073
rs8102188
rs17034205
All − MR Egger
All − Inverse variance weighted
−2
0
2
MR effect size for
'UBA2658 sp002841545' on 'GCST90200762_buildGRCh38.tsv'

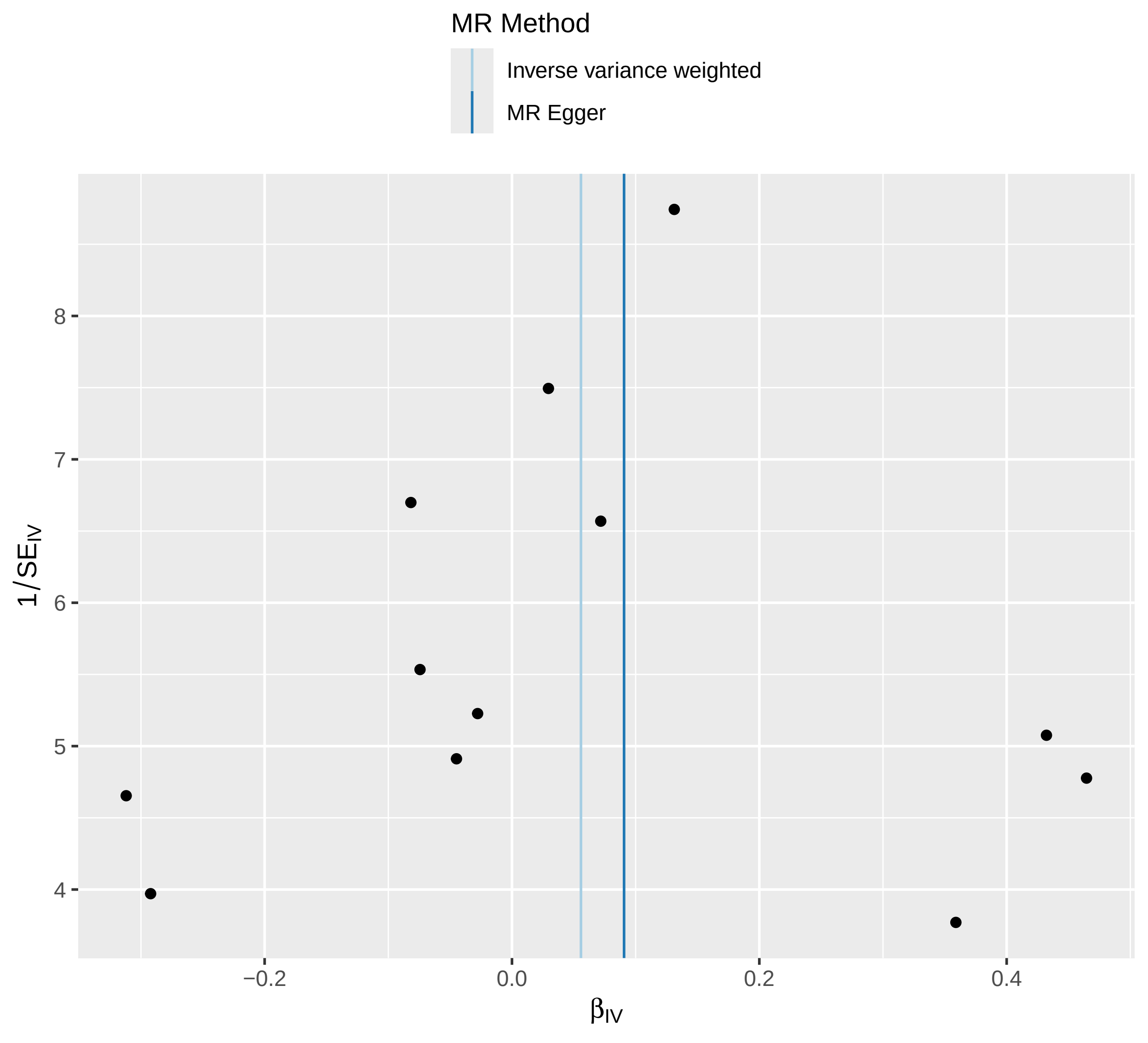
MR Method
Inverse variance weighted
MR Egger
1/SE_IV
8
7
6
5
4
−0.2
0.0
0.2
0.4
β_IV

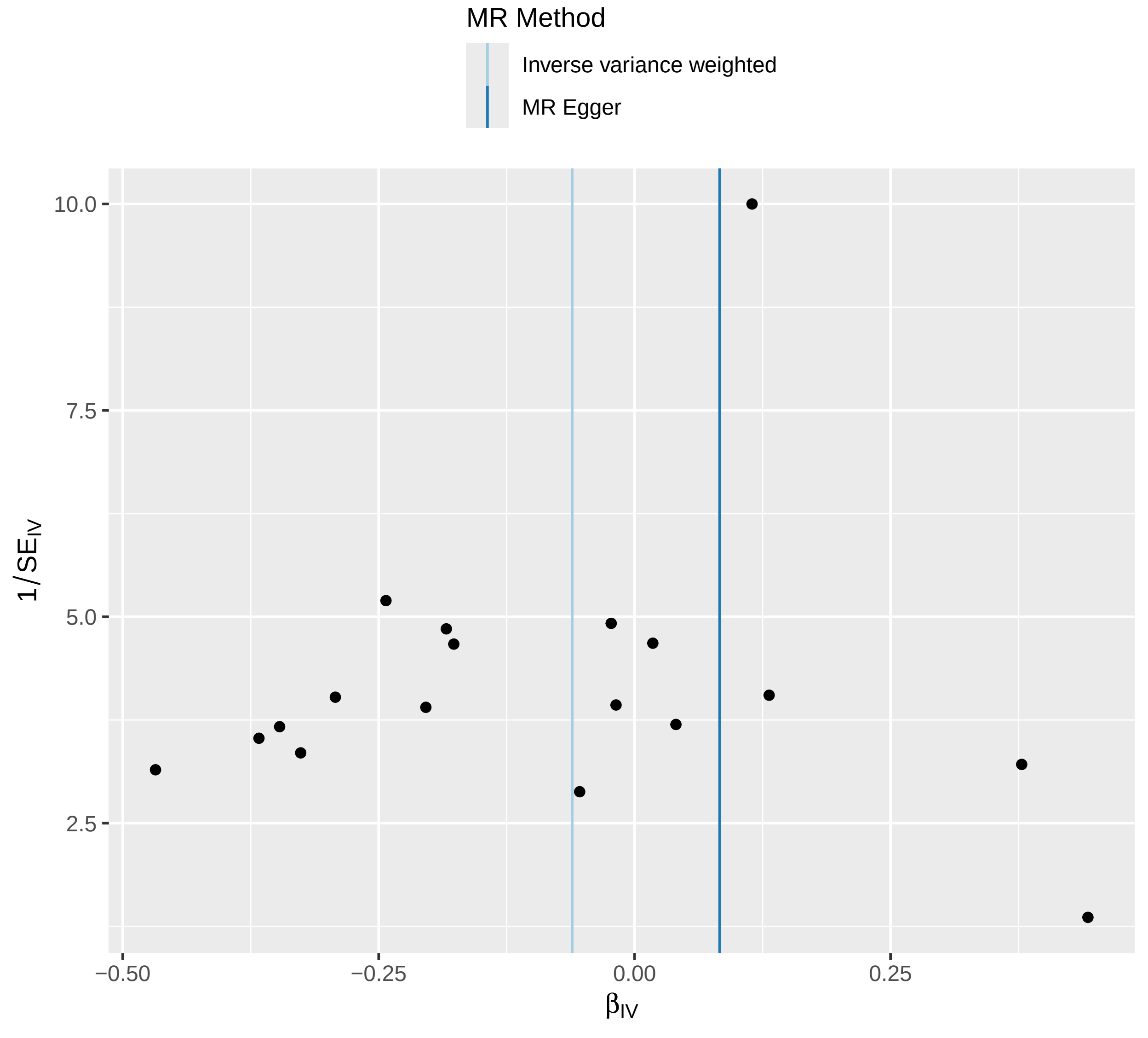

MR Method
Inverse variance weighted
MR Egger
10.0
7.5
5.0
2.5
1/SE_IV
−0.50
−0.25
0.00
0.25
β_IV

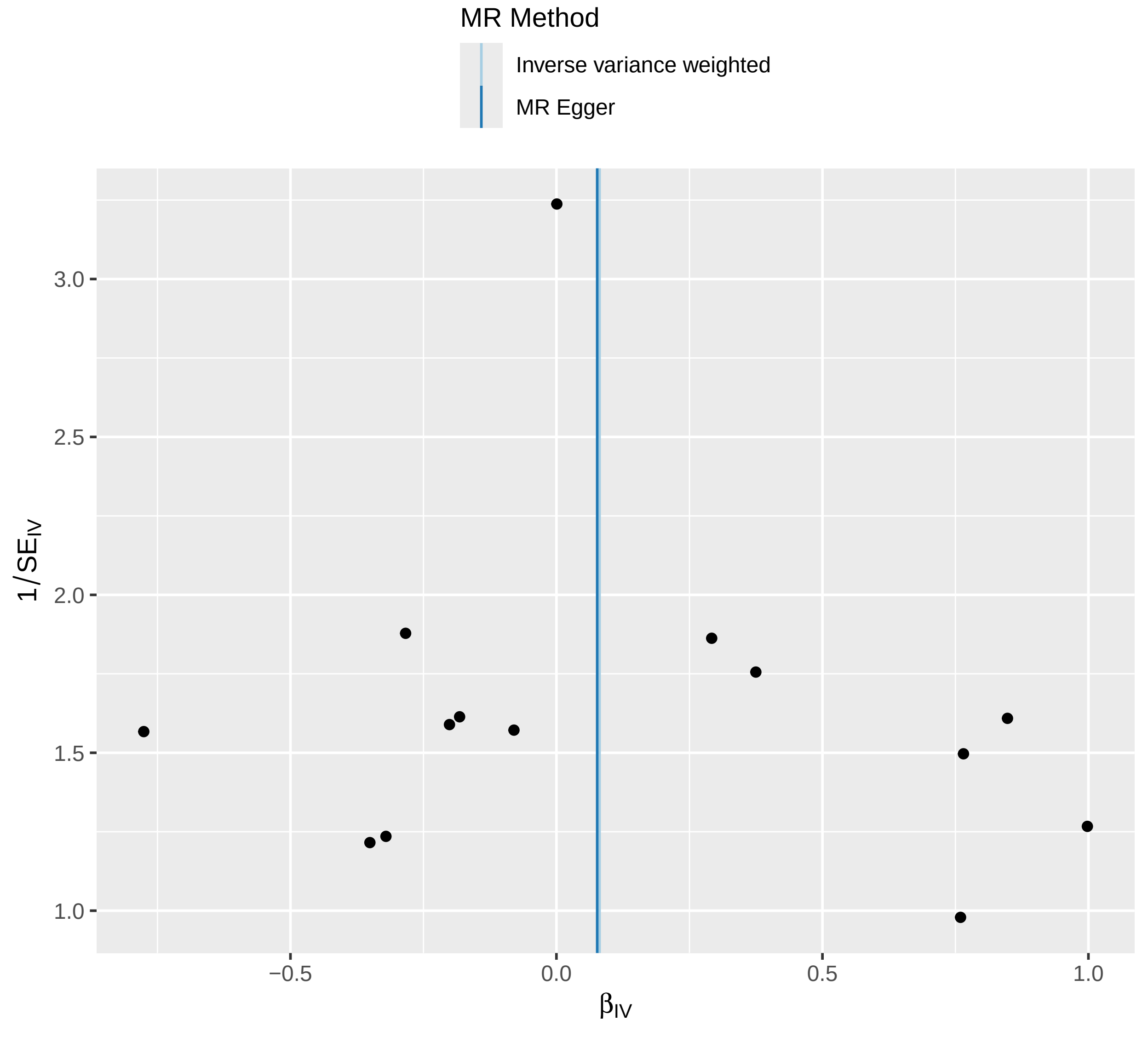
MR Method
Inverse variance weighted
MR Egger
3.0
2.5
2.0
1.5
1.0
1/SE_IV
−0.5
0.0
0.5
1.0
β_IV

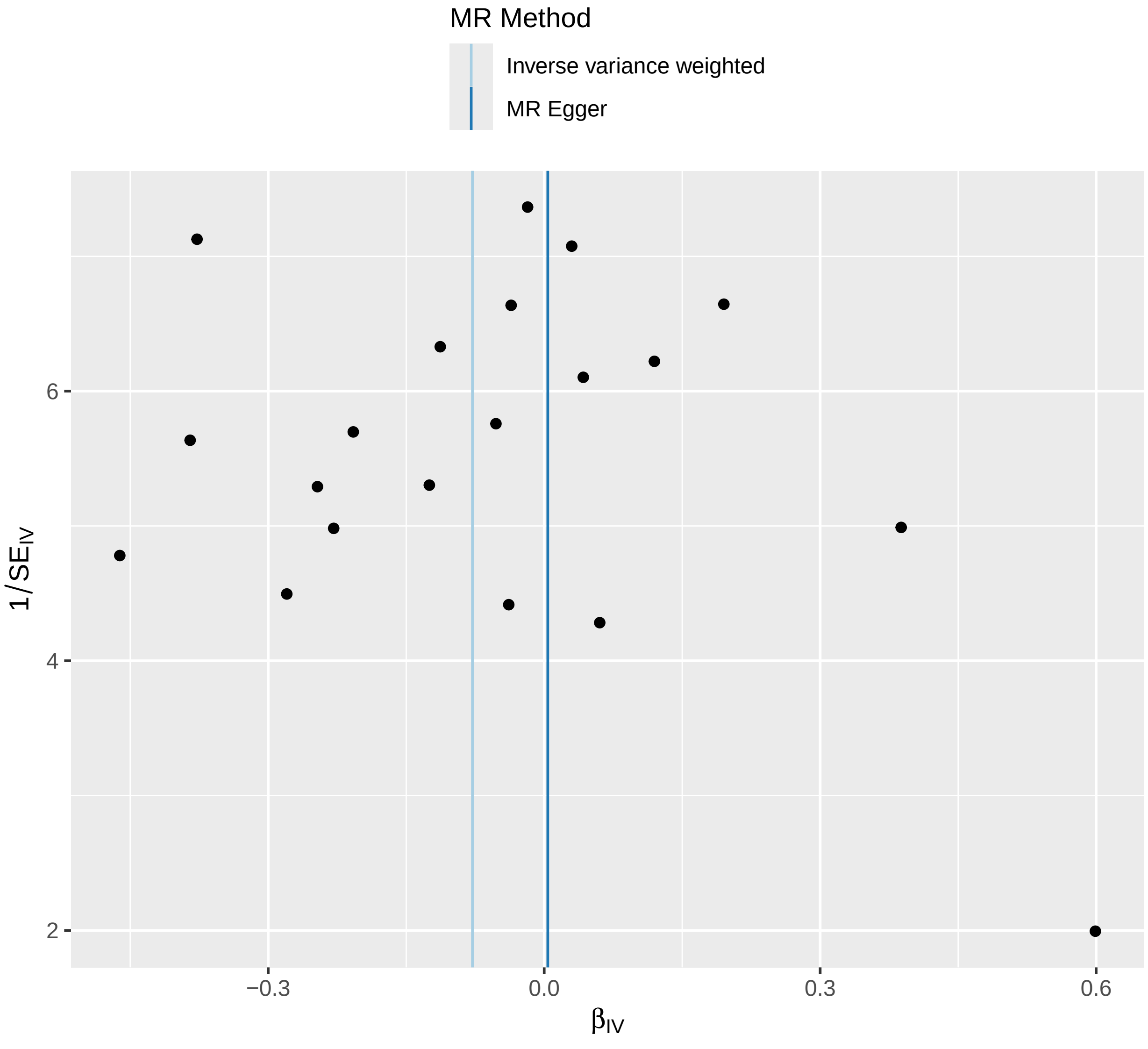
MR Method
Inverse variance weighted
MR Egger
1/SE_IV
6
4
2
−0.3
0.0
0.3
0.6
β_IV

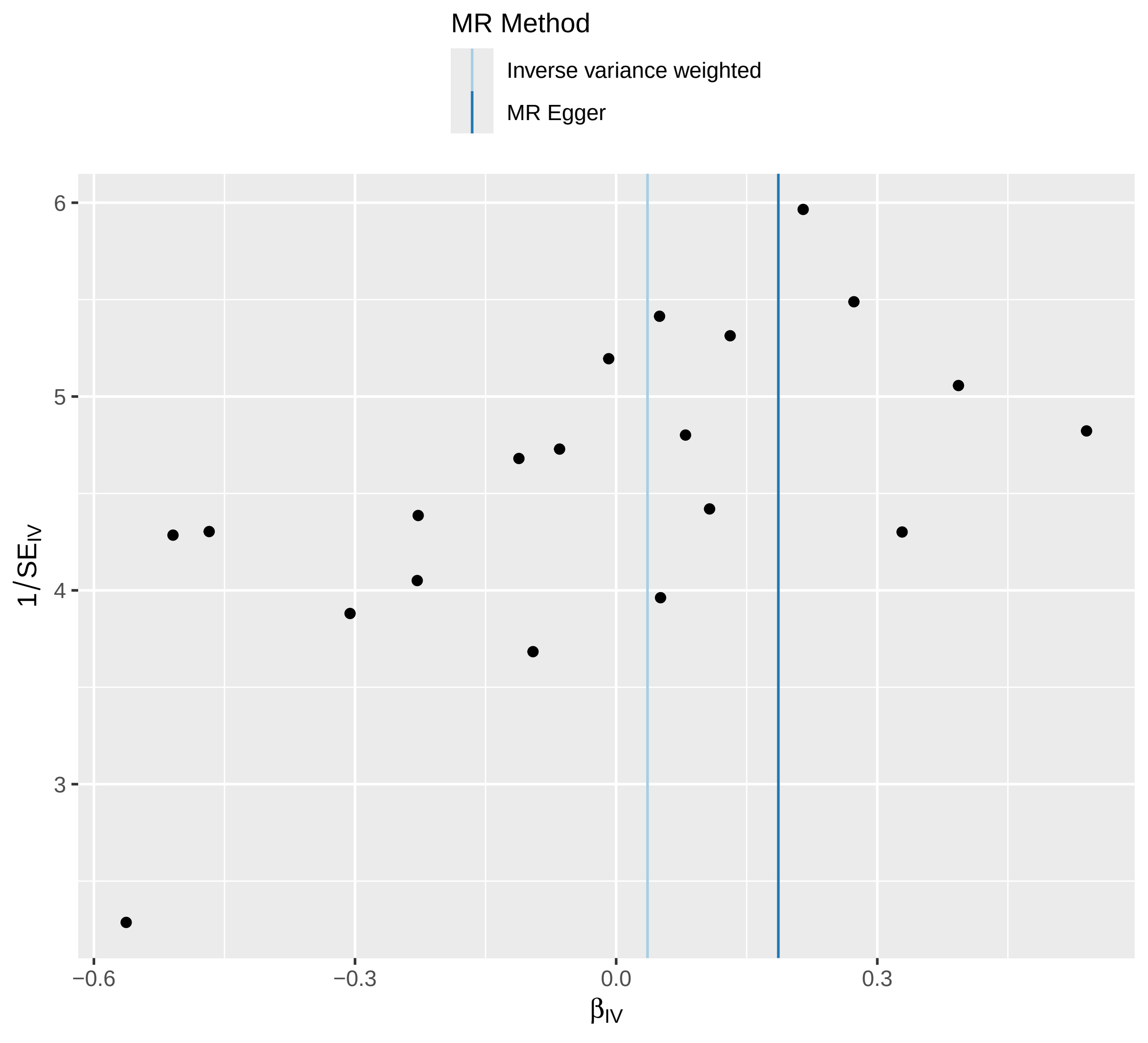
MR Method
Inverse variance weighted
MR Egger
6
5
4
3
1/SE_IV
−0.6
−0.3
0.0
0.3
β_IV

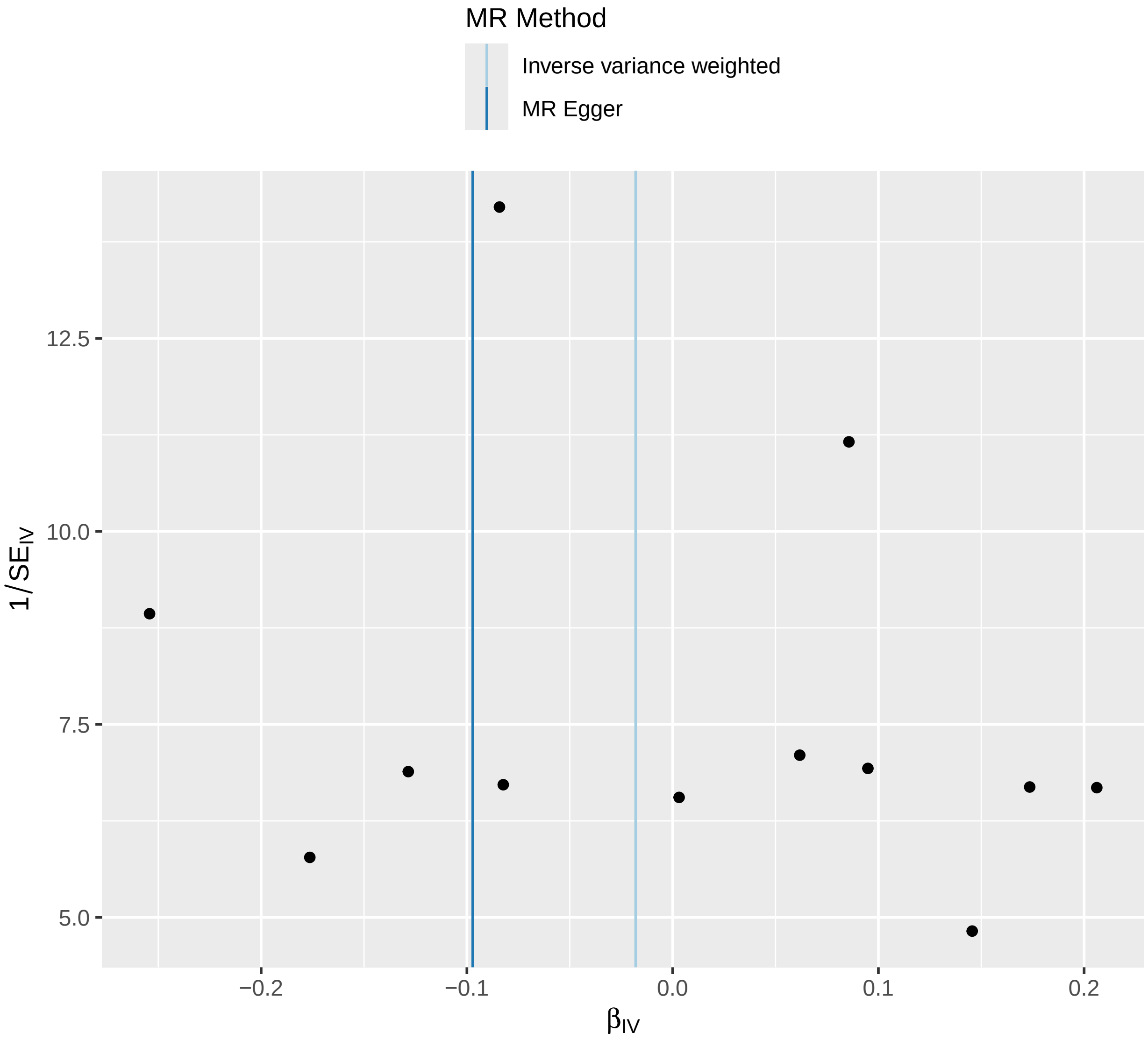
MR Method
Inverse variance weighted
MR Egger
12.5
10.0
7.5
5.0
1/SE_IV
−0.2
−0.1
0.0
0.1
0.2
β_IV

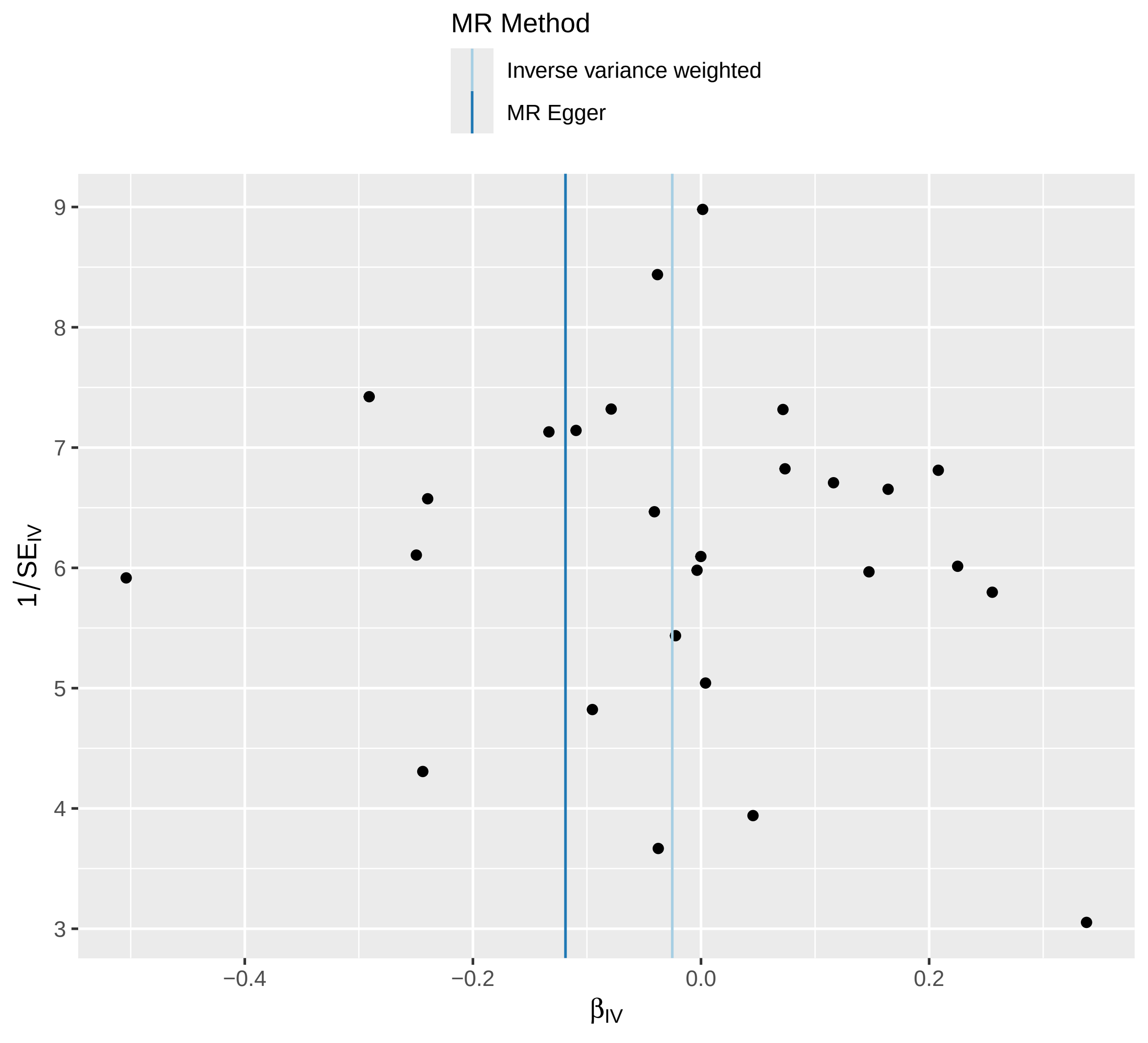
MR Method
Inverse variance weighted
MR Egger
9
8
7
6
5
4
3
1/SE_IV
−0.4
−0.2
0.0
0.2
β_IV

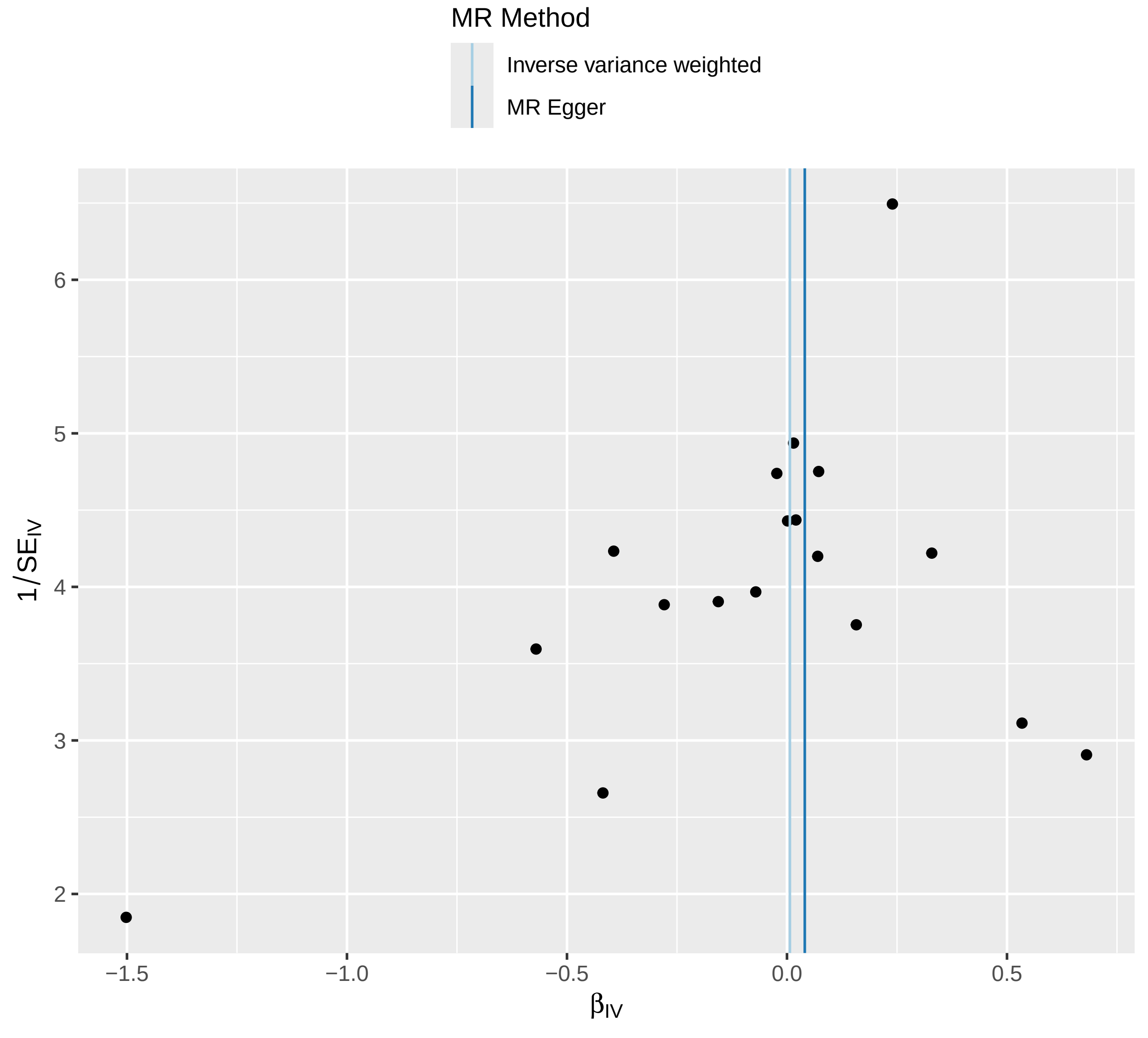
MR Method
Inverse variance weighted
MR Egger
1/SE_IV
β_IV
6
5
4
3
2
−1.5
−1.0
−0.5
0.0
0.5

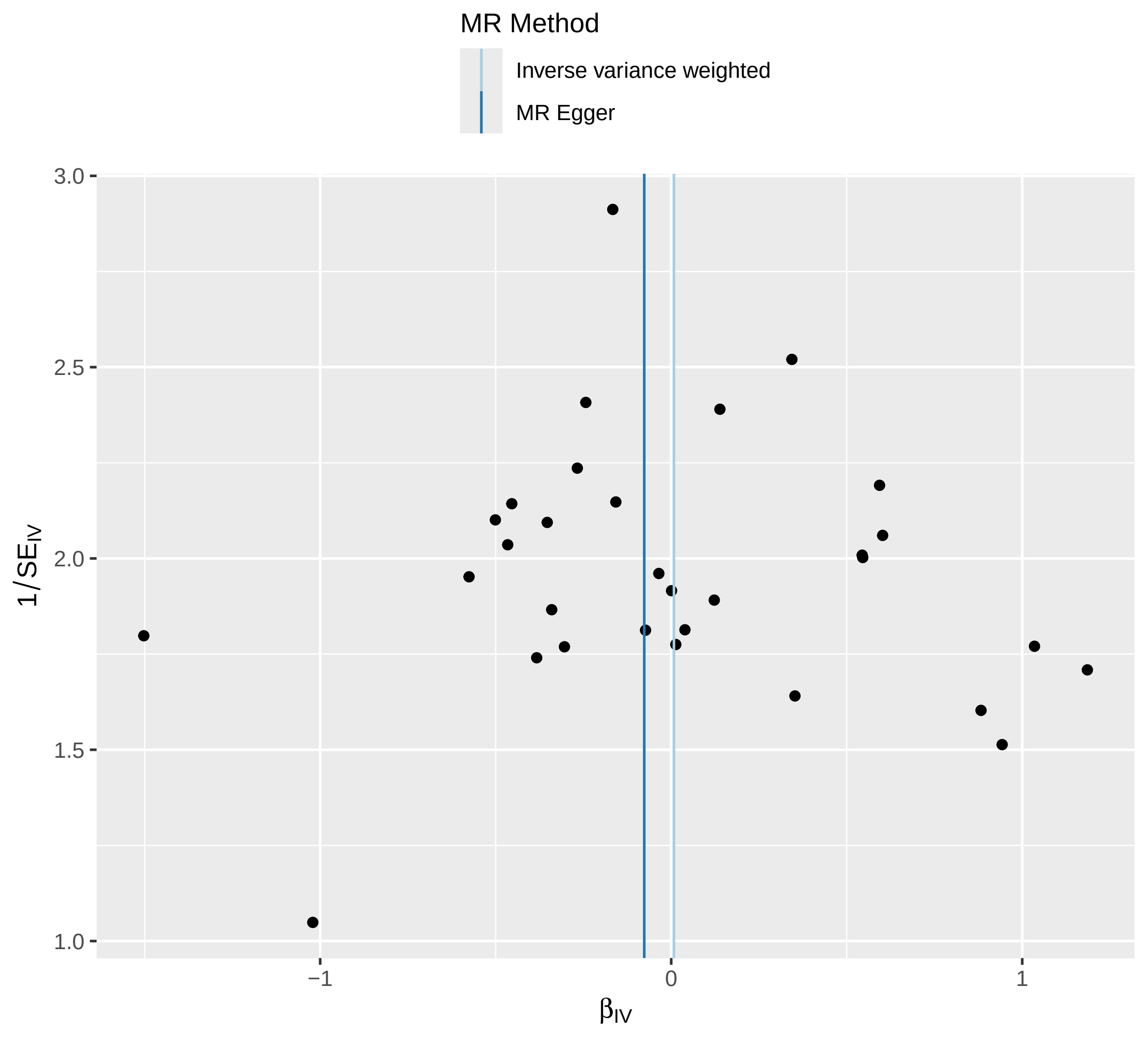
MR Method
Inverse variance weighted
MR Egger
3.0
2.5
2.0
1.5
1.0
−1
0
1
$1/SE_{IV}$
β_{IV}

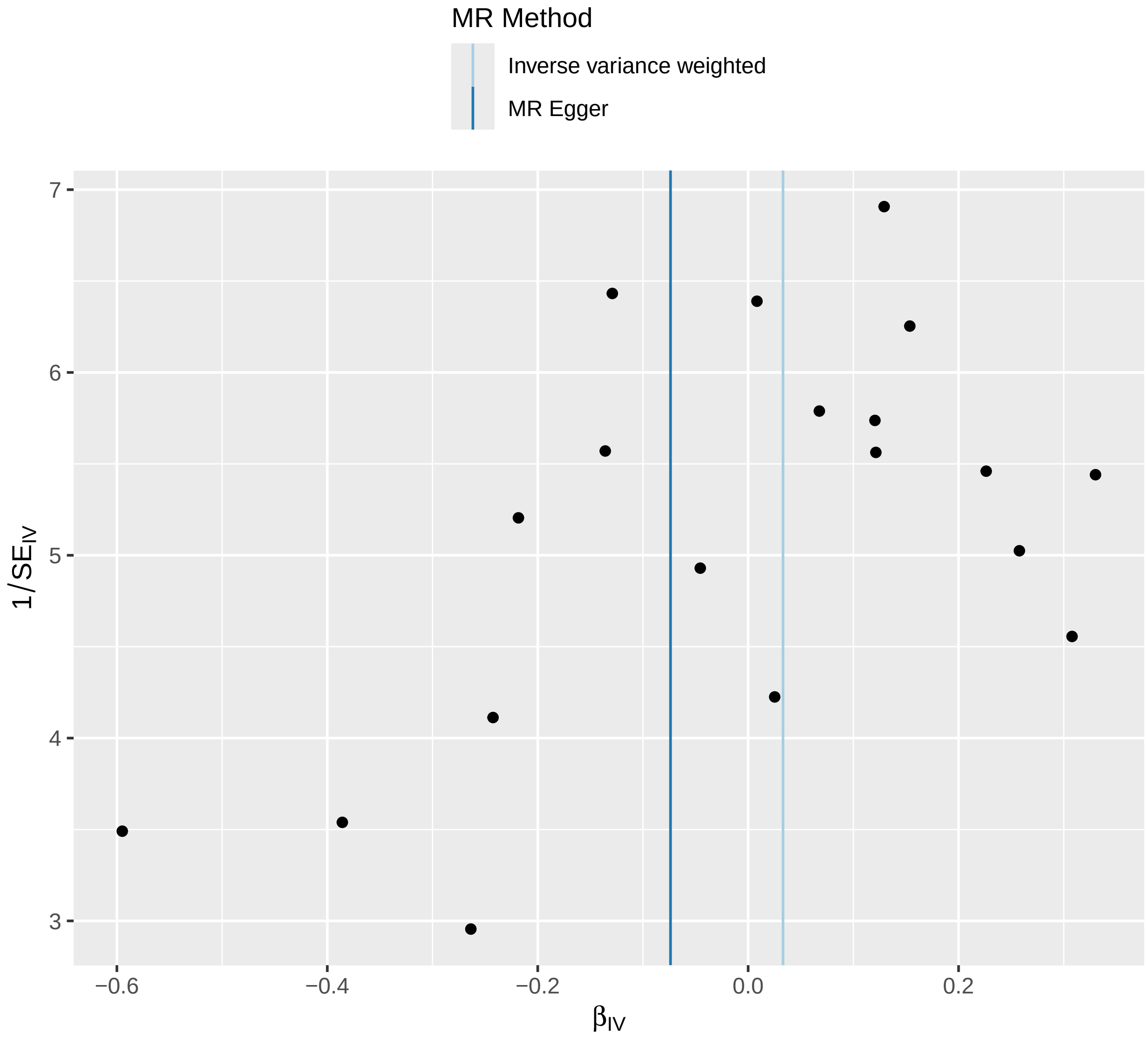
MR Method
Inverse variance weighted
MR Egger
1/SE$_{IV}$
7
6
5
4
3
−0.6
−0.4
−0.2
0.0
0.2
β$_{IV}$

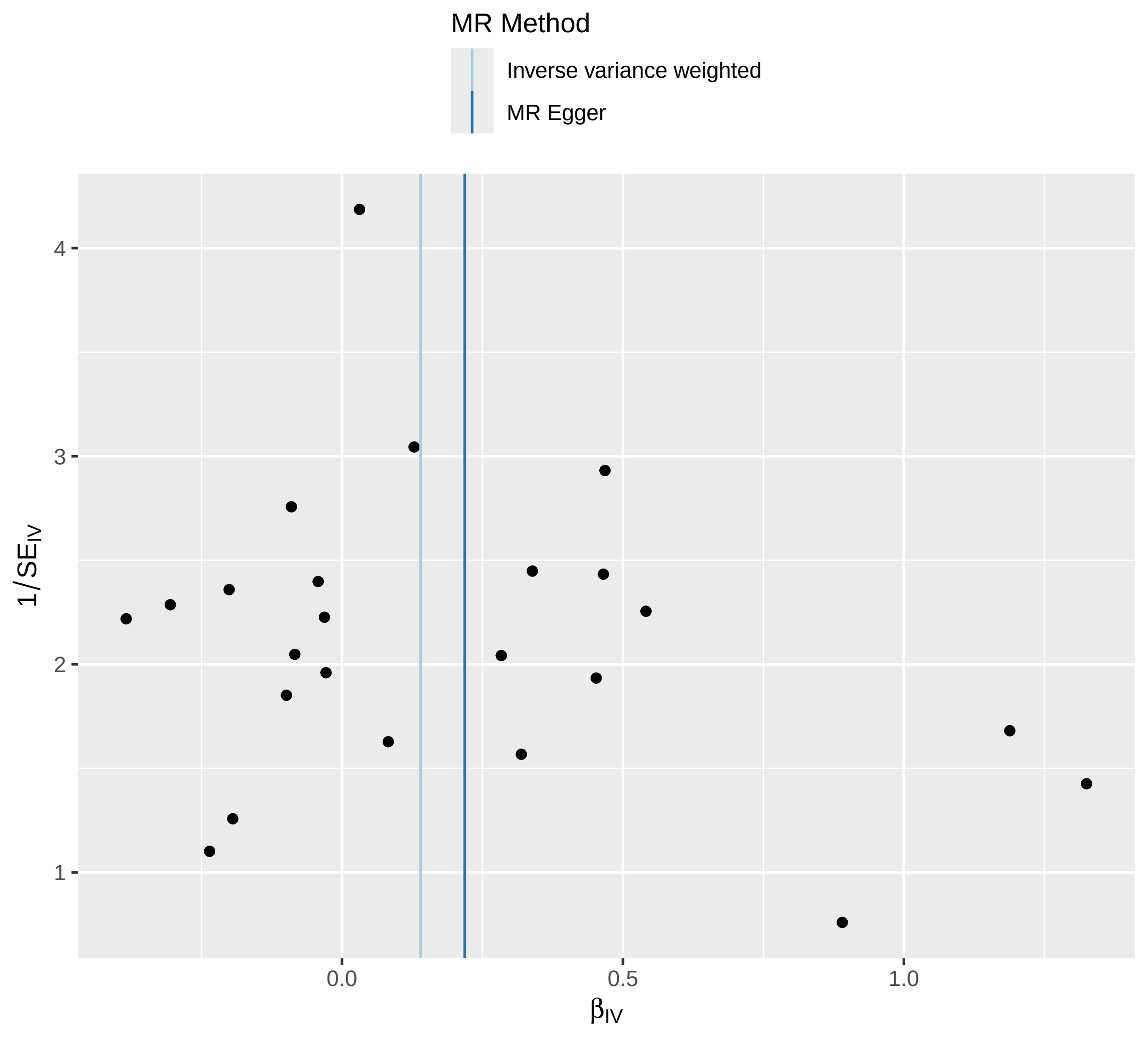

MR Method
Inverse variance weighted
MR Egger
4
3
2
1
1/SE$_{IV}$
0.0
0.5
1.0
β$_{IV}$

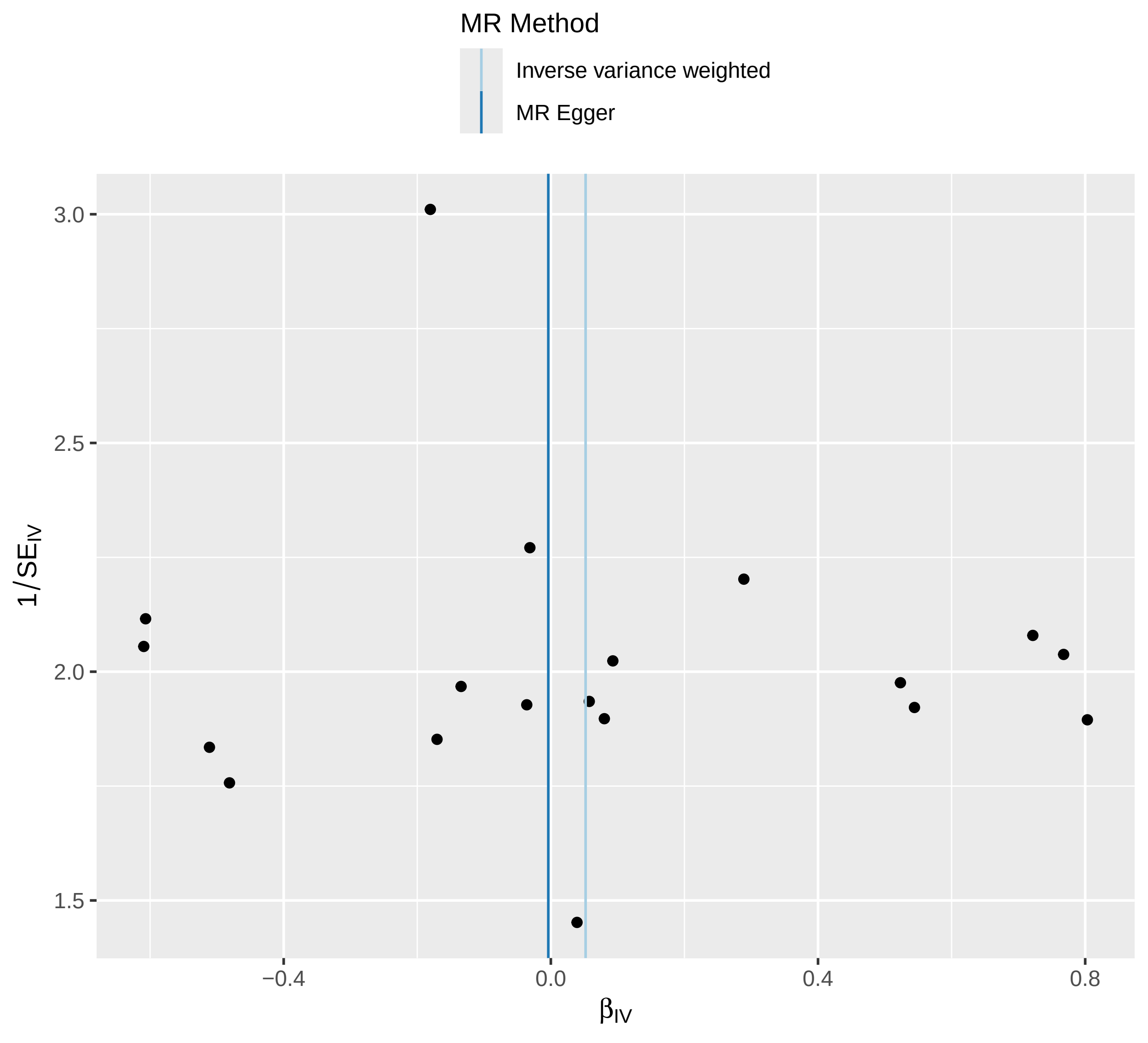
MR Method
Inverse variance weighted
MR Egger
3.0
2.5
2.0
1.5
1/SE$_{IV}$
−0.4
0.0
0.4
0.8
β$_{IV}$

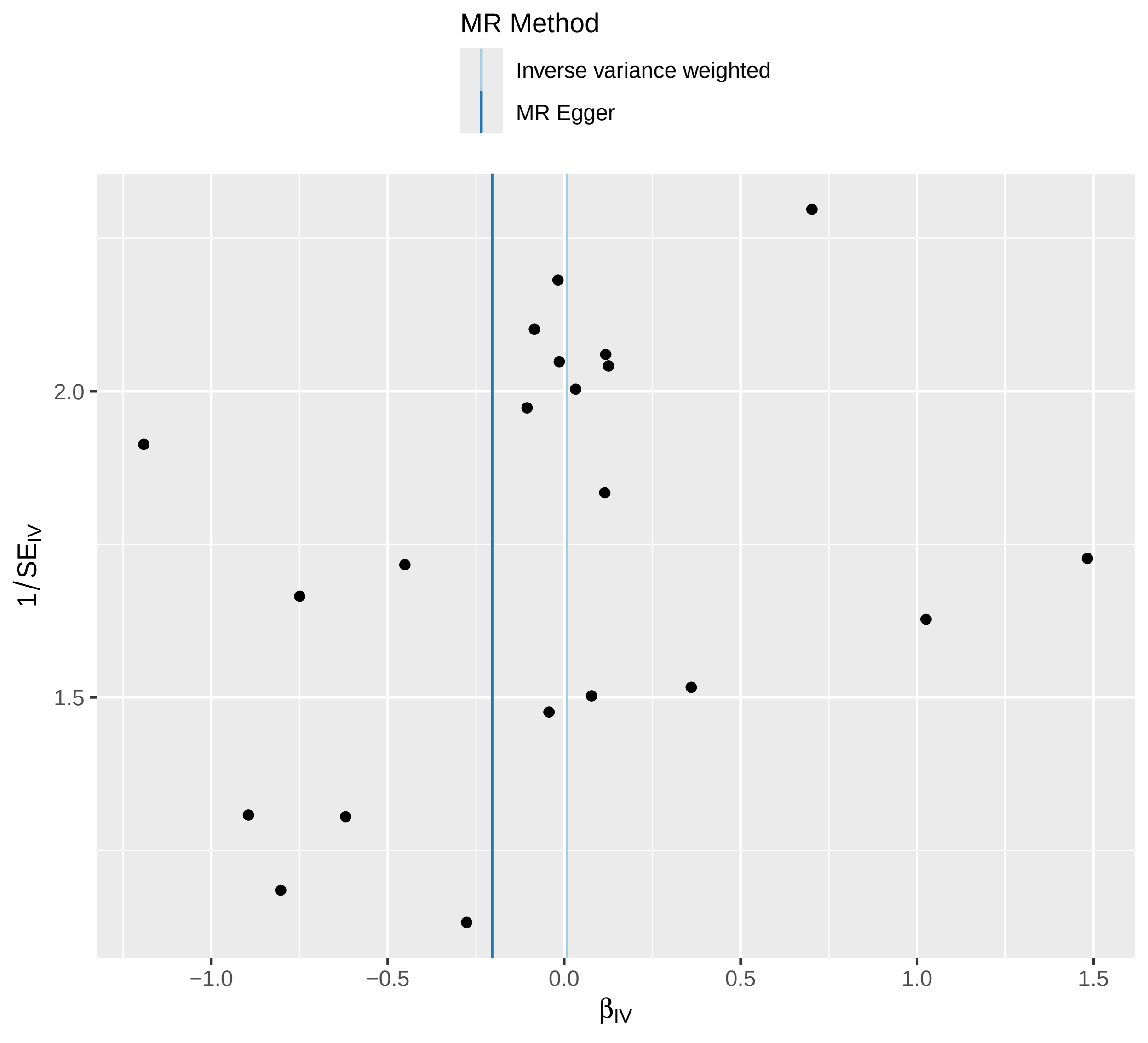

MR Method
Inverse variance weighted
MR Egger
2.0
1.5
1/SE$_{IV}$
−1.0
−0.5
0.0
0.5
1.0
1.5
β$_{IV}$

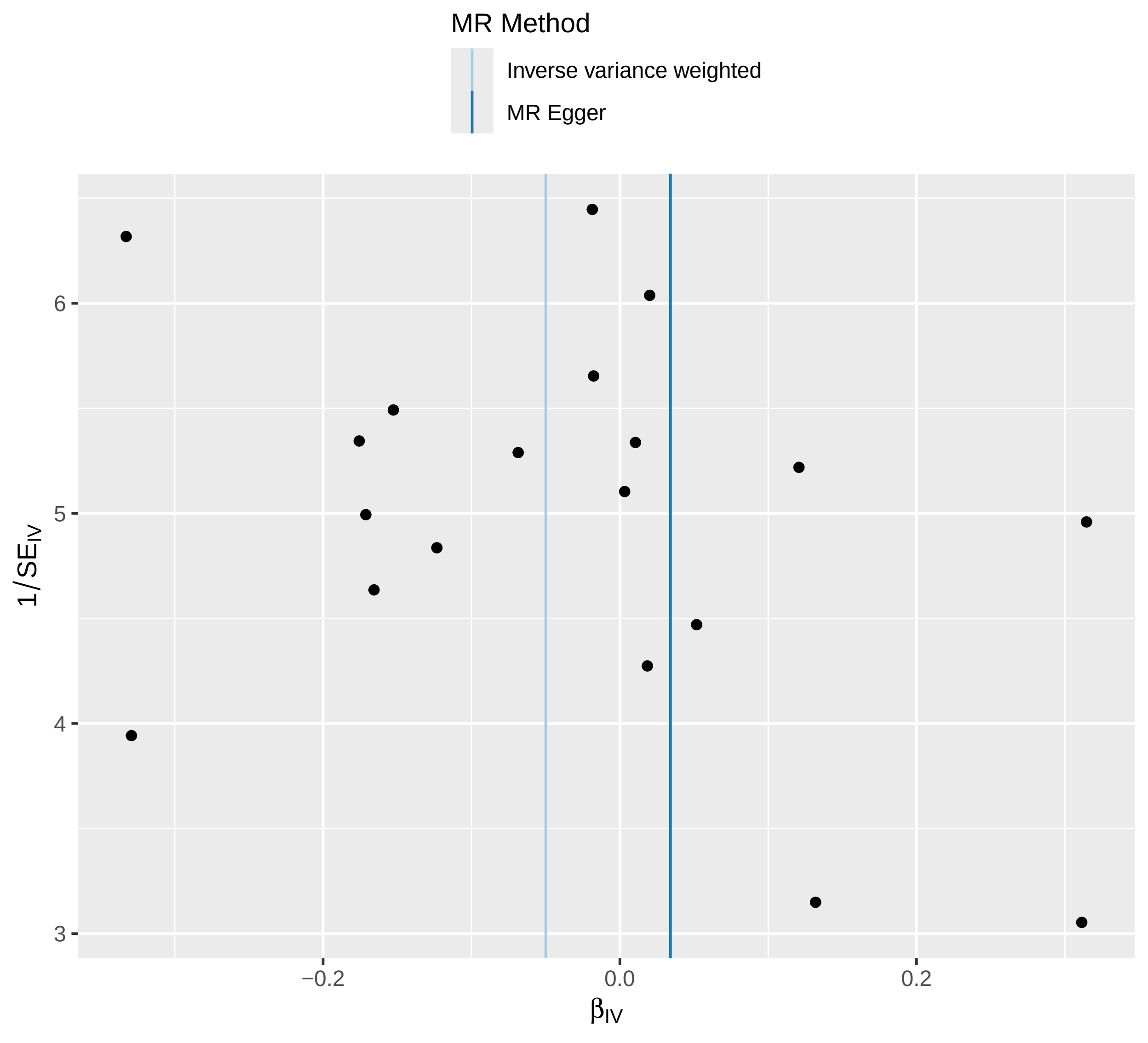

MR Method
Inverse variance weighted
MR Egger
6
5
4
3
1/SE$_{IV}$
−0.2
0.0
0.2
β$_{IV}$

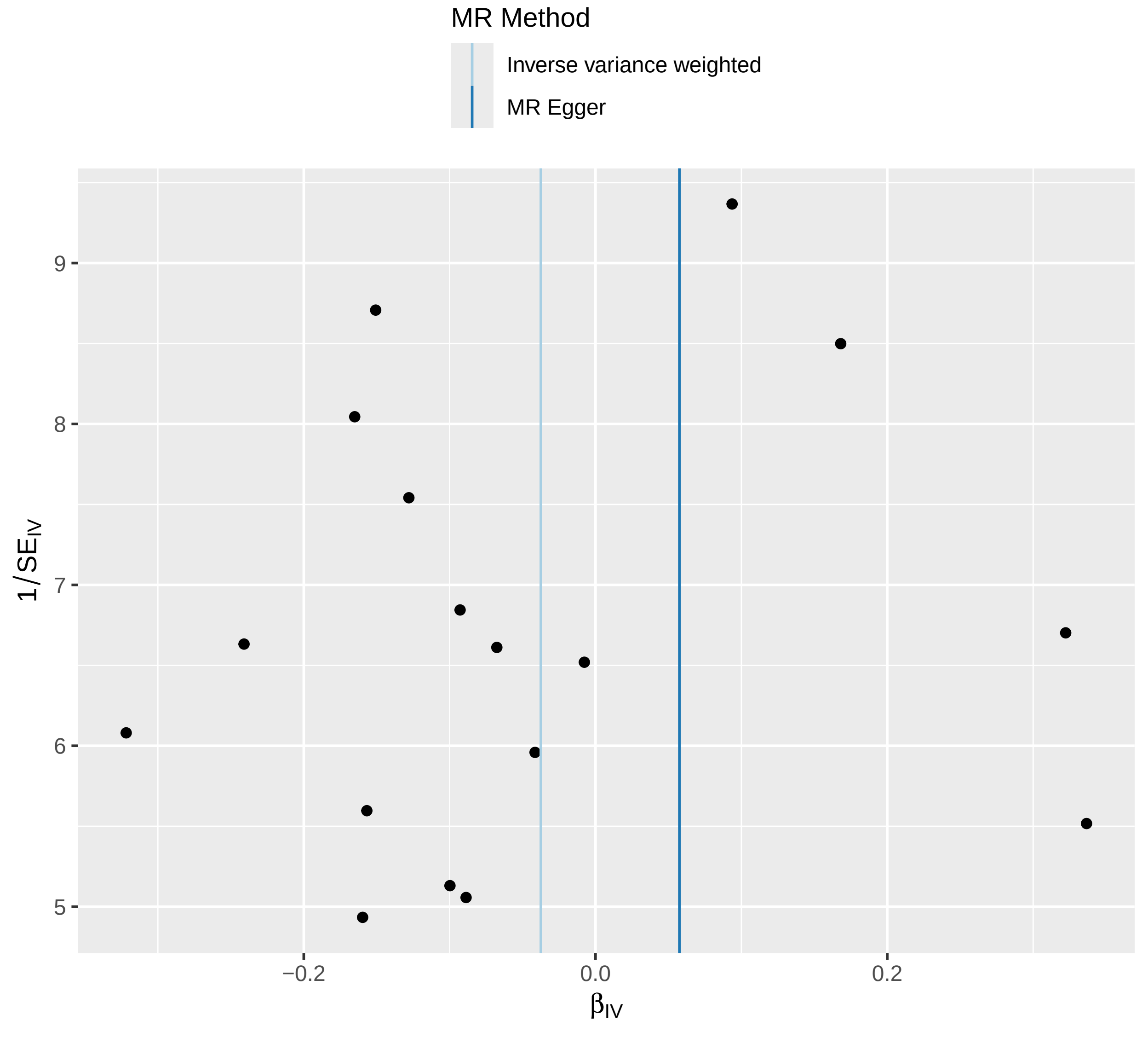
MR Method
Inverse variance weighted
MR Egger
9
8
7
6
5
1/SE_IV
−0.2
0.0
0.2
β_IV

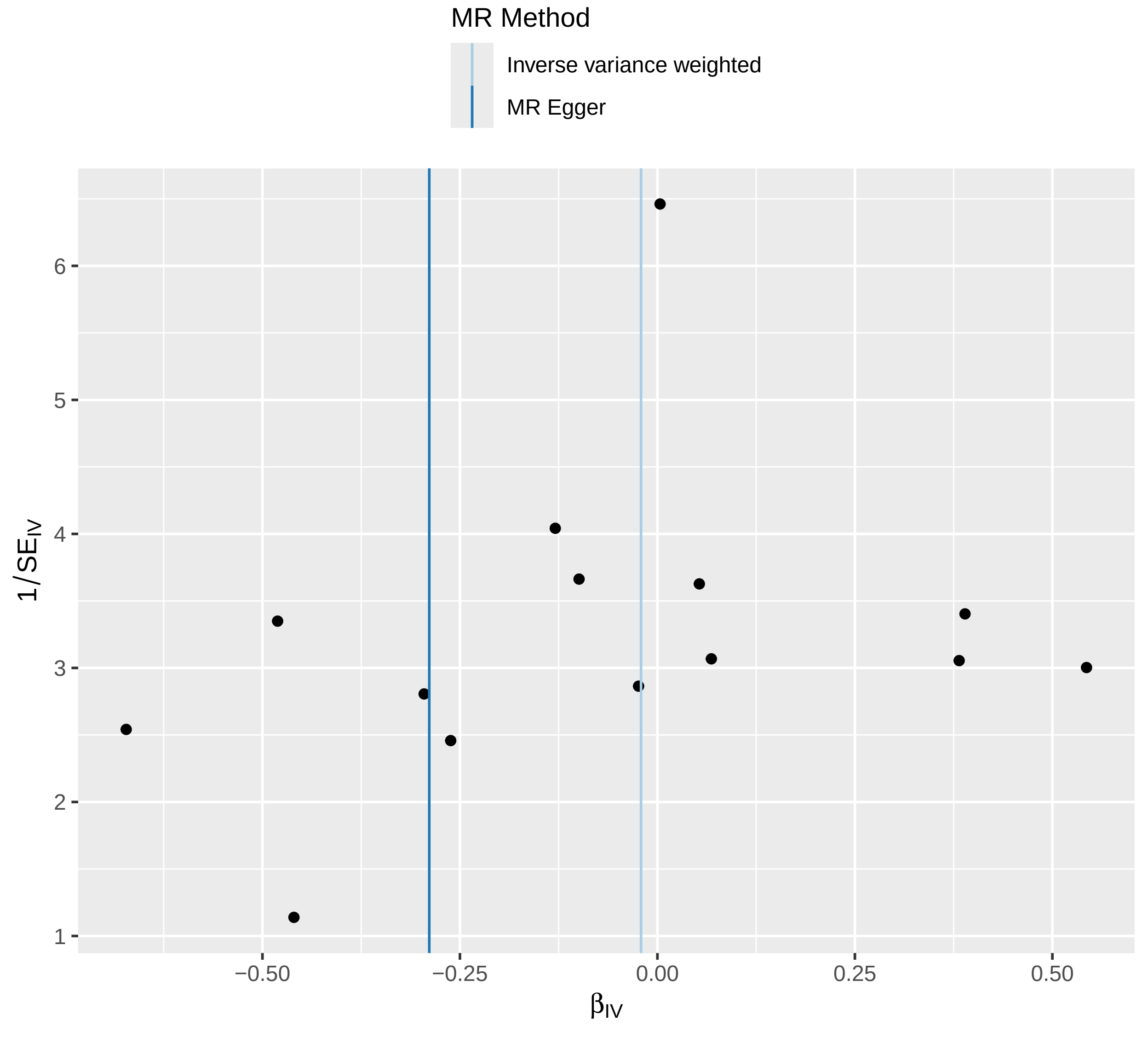

MR Method
Inverse variance weighted
MR Egger
1/SE_IV
6
5
4
3
2
1
−0.50
−0.25
0.00
0.25
0.50
β_IV

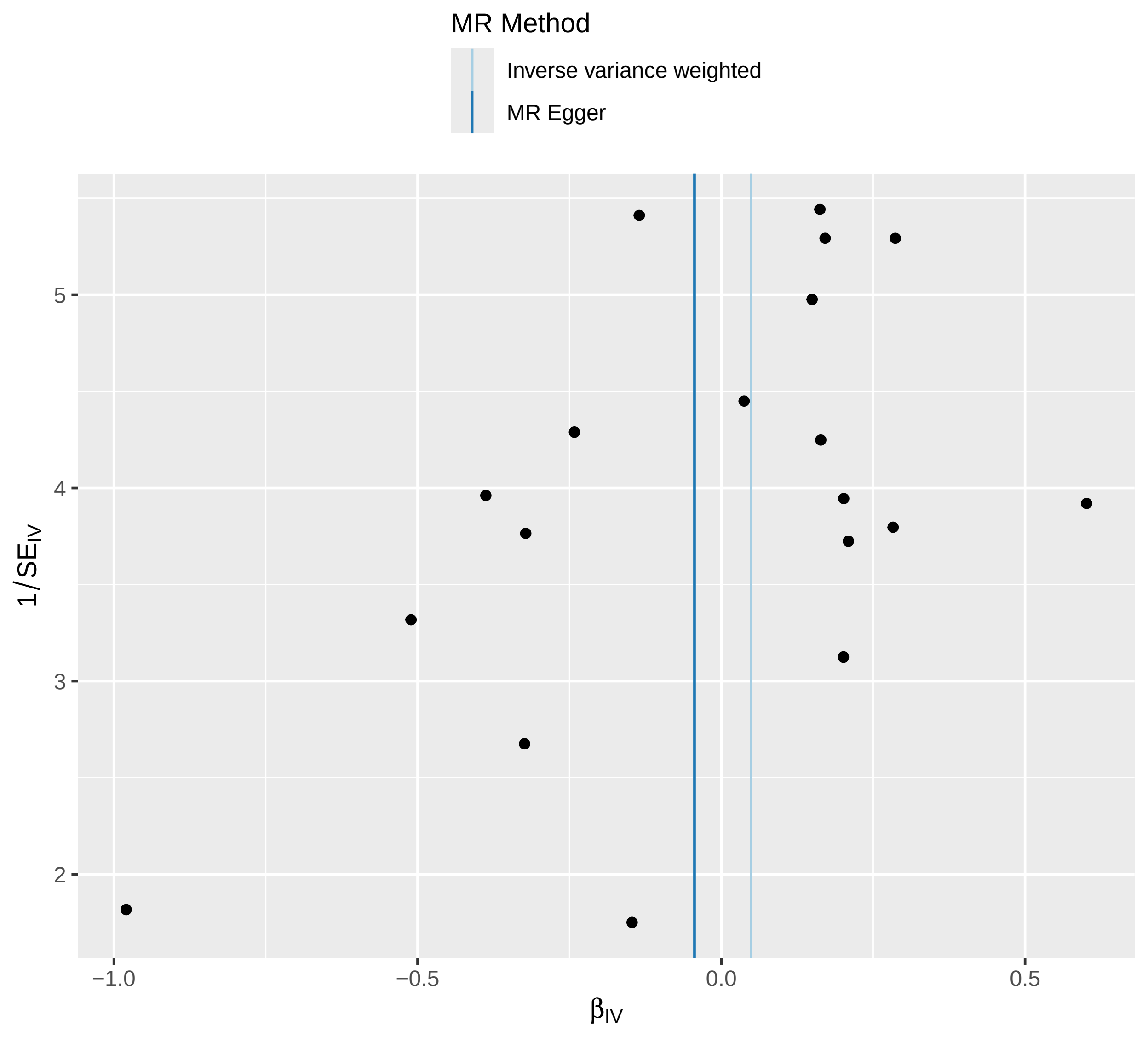

MR Method
Inverse variance weighted
MR Egger
5
4
3
2
$1/SE_{IV}$
−1.0
−0.5
0.0
0.5
β_{IV}

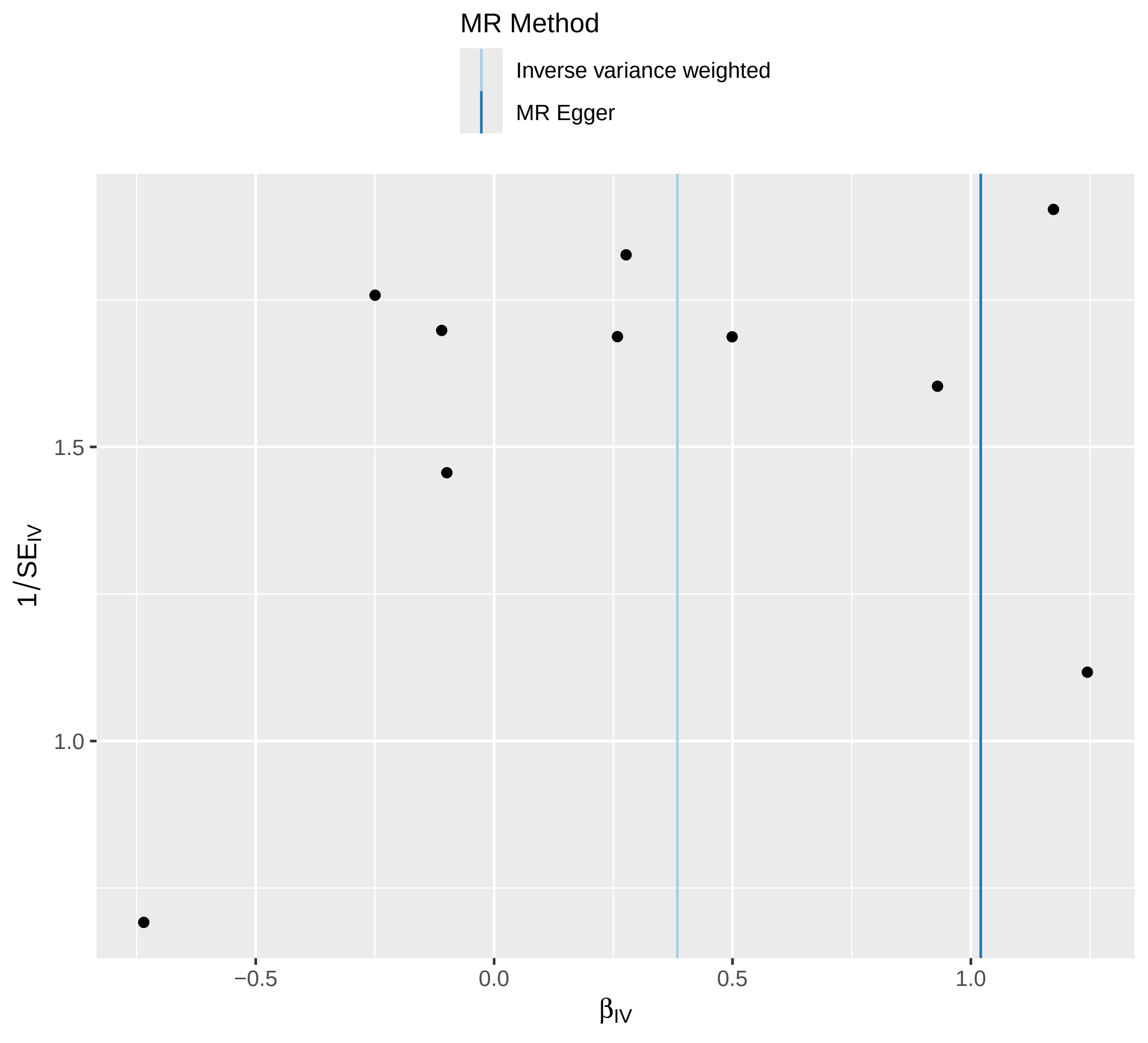
MR Method
Inverse variance weighted
MR Egger
1.5
1.0
−0.5
0.0
0.5
1.0
$1/SE_{IV}$
β_{IV}

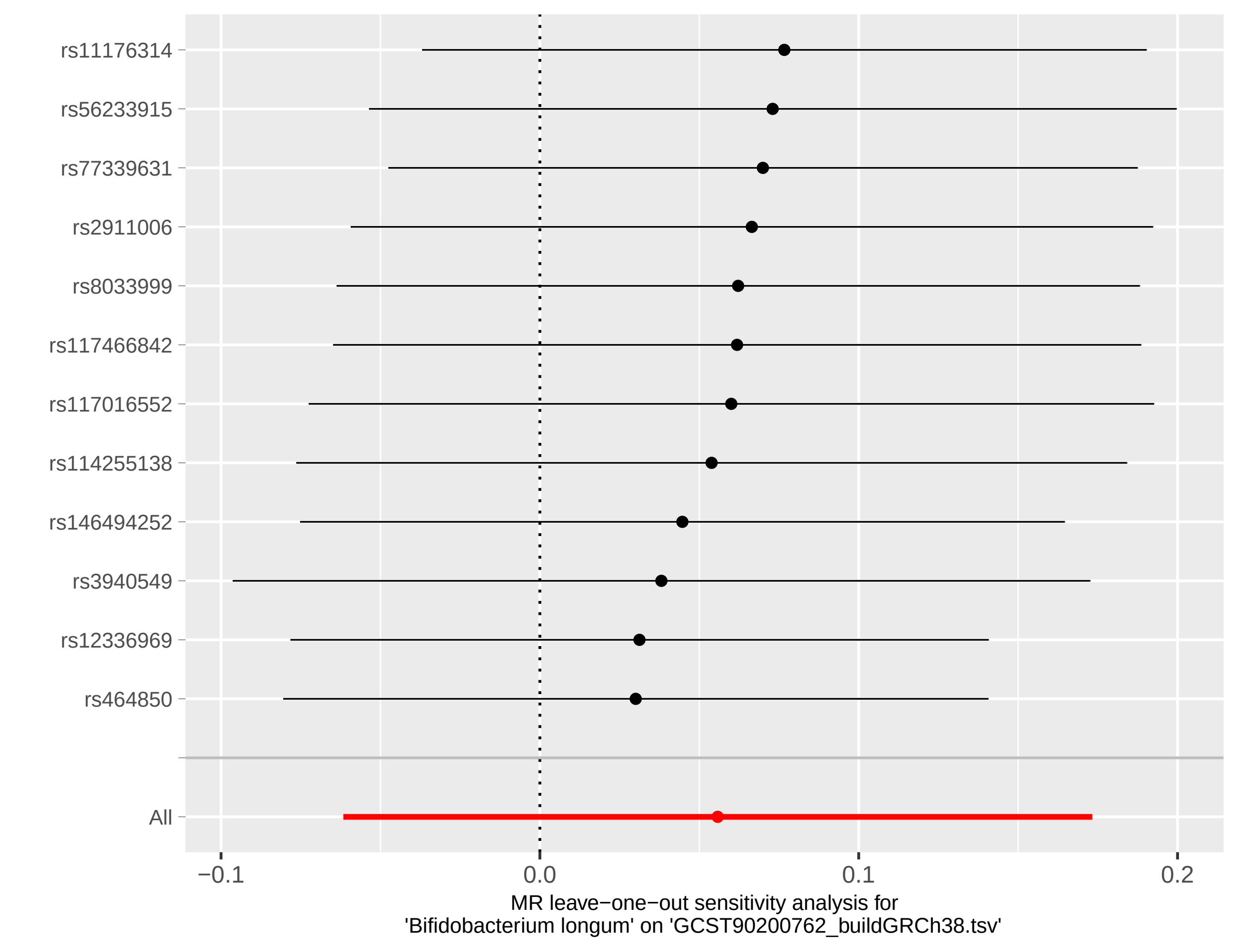

rs11176314
rs56233915
rs77339631
rs2911006
rs8033999
rs117466842
rs117016552
rs114255138
rs146494252
rs3940549
rs12336969
rs464850
All
−0.1
0.0
0.1
0.2
MR leave−one−out sensitivity analysis for
'Bifidobacterium longum' on 'GCST90200762_buildGRCh38.tsv'

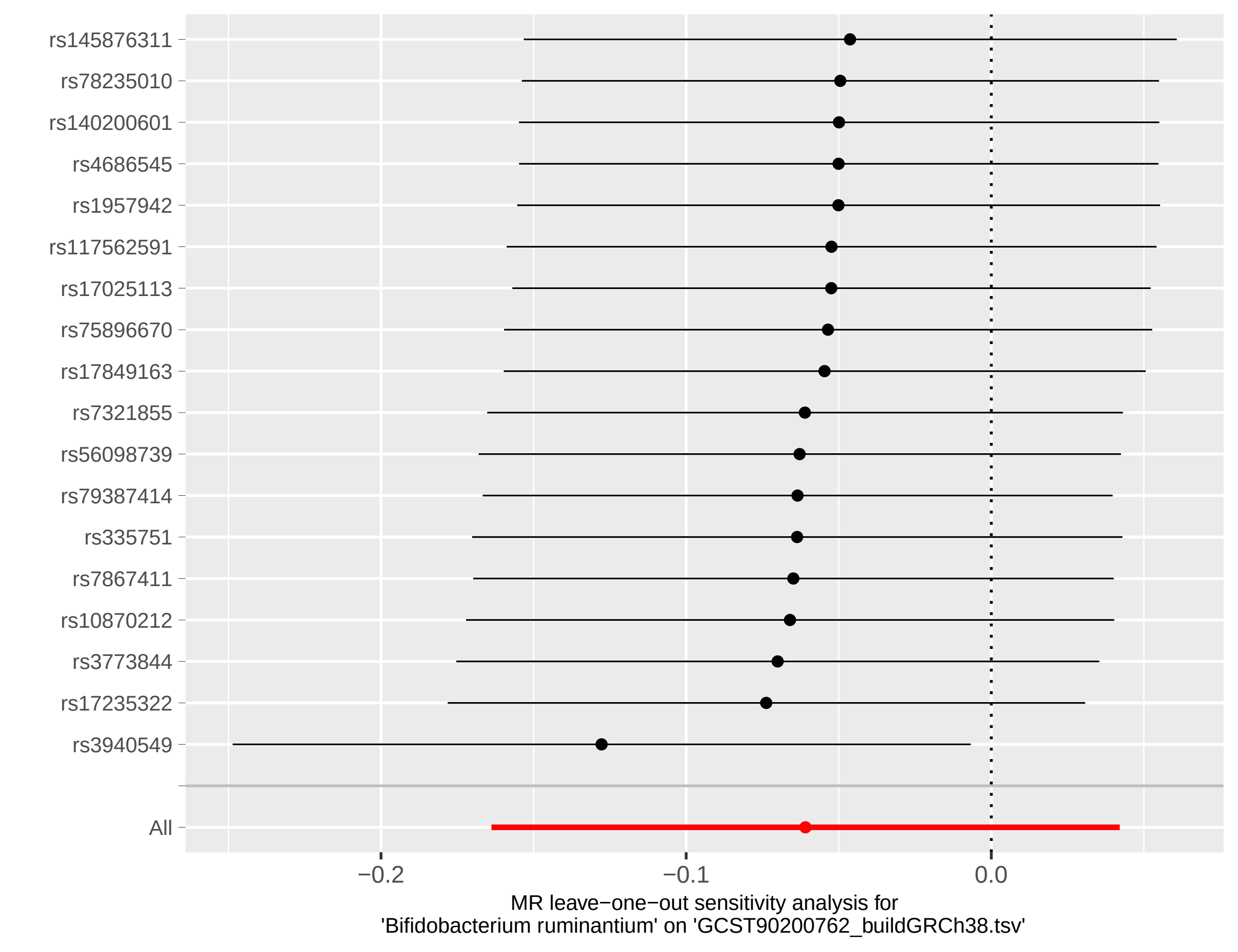
rs145876311
rs78235010
rs140200601
rs4686545
rs1957942
rs117562591
rs17025113
rs75896670
rs17849163
rs7321855
rs56098739
rs79387414
rs335751
rs7867411
rs10870212
rs3773844
rs17235322
rs3940549
All
−0.2
−0.1
0.0
MR leave−one−out sensitivity analysis for
'Bifidobacterium ruminantium' on 'GCST90200762_buildGRCh38.tsv'

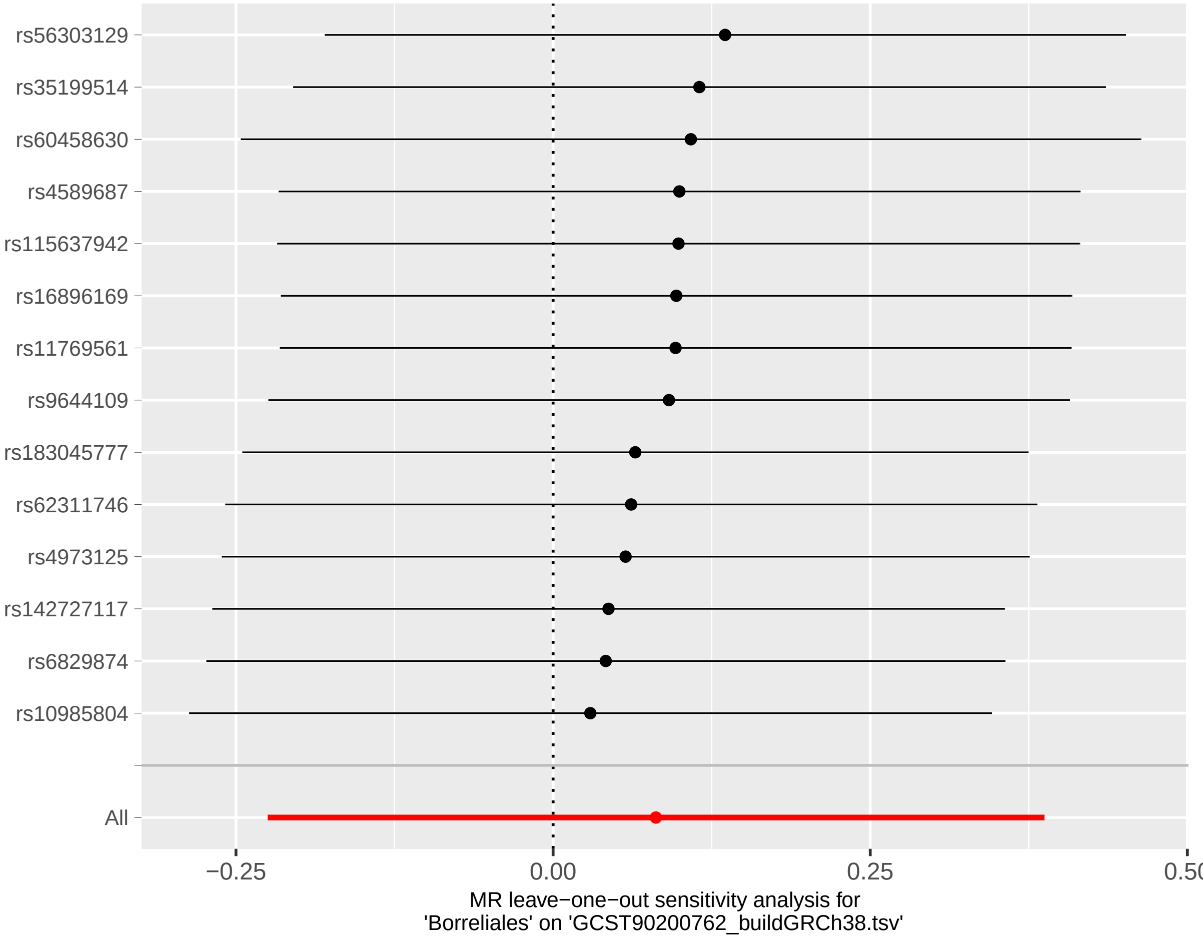
rs56303129
rs35199514
rs60458630
rs4589687
rs115637942
rs16896169
rs11769561
rs9644109
rs183045777
rs62311746
rs4973125
rs142727117
rs6829874
rs10985804
All
−0.25
0.00
0.25
0.50
MR leave−one−out sensitivity analysis for
'Borreliales' on 'GCST90200762_buildGRCh38.tsv'

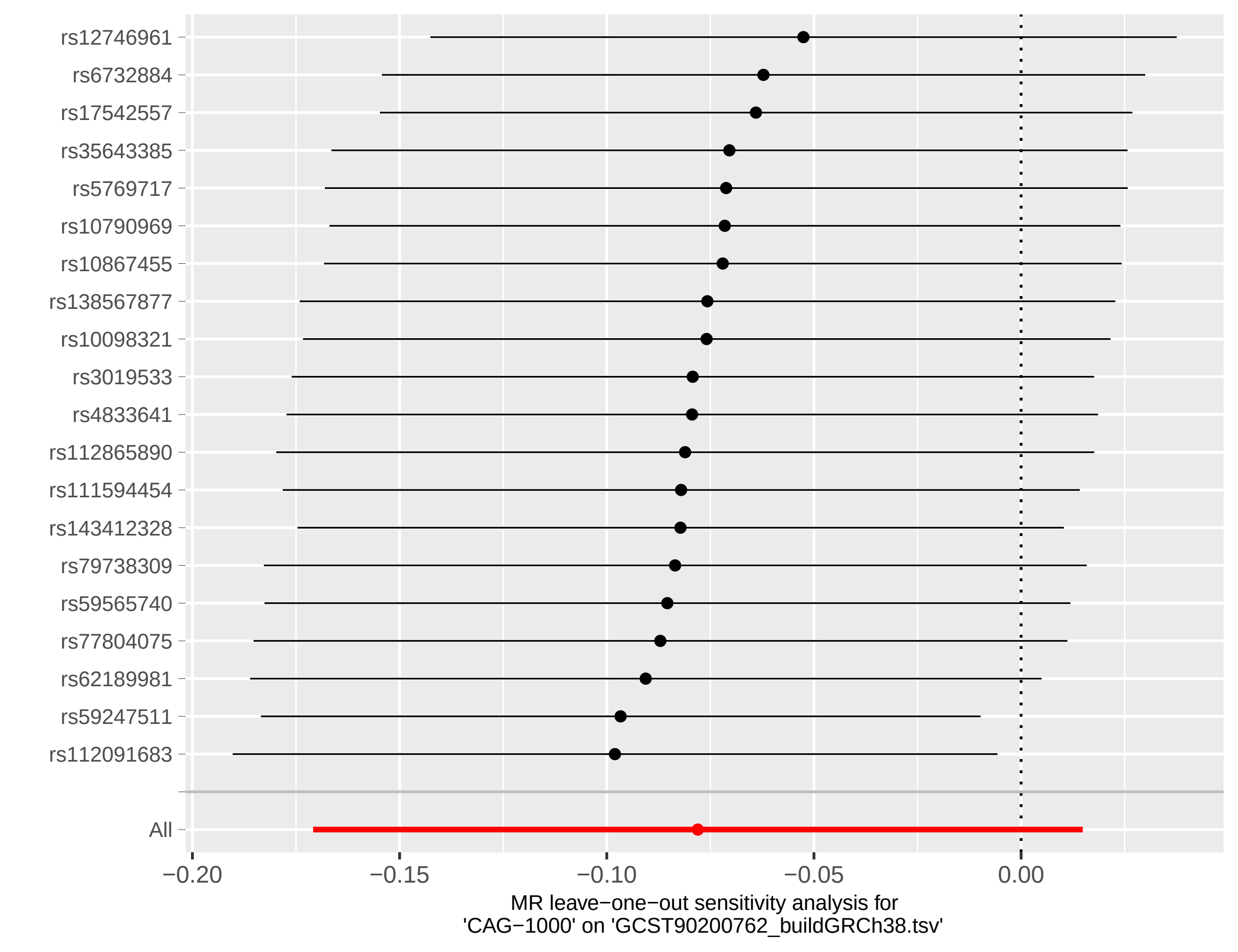

rs12746961
rs6732884
rs17542557
rs35643385
rs5769717
rs10790969
rs10867455
rs138567877
rs10098321
rs3019533
rs4833641
rs112865890
rs111594454
rs143412328
rs79738309
rs59565740
rs77804075
rs62189981
rs59247511
rs112091683
All
−0.20
−0.15
−0.10
−0.05
0.00
MR leave−one−out sensitivity analysis for
'CAG−1000' on 'GCST90200762_buildGRCh38.tsv'

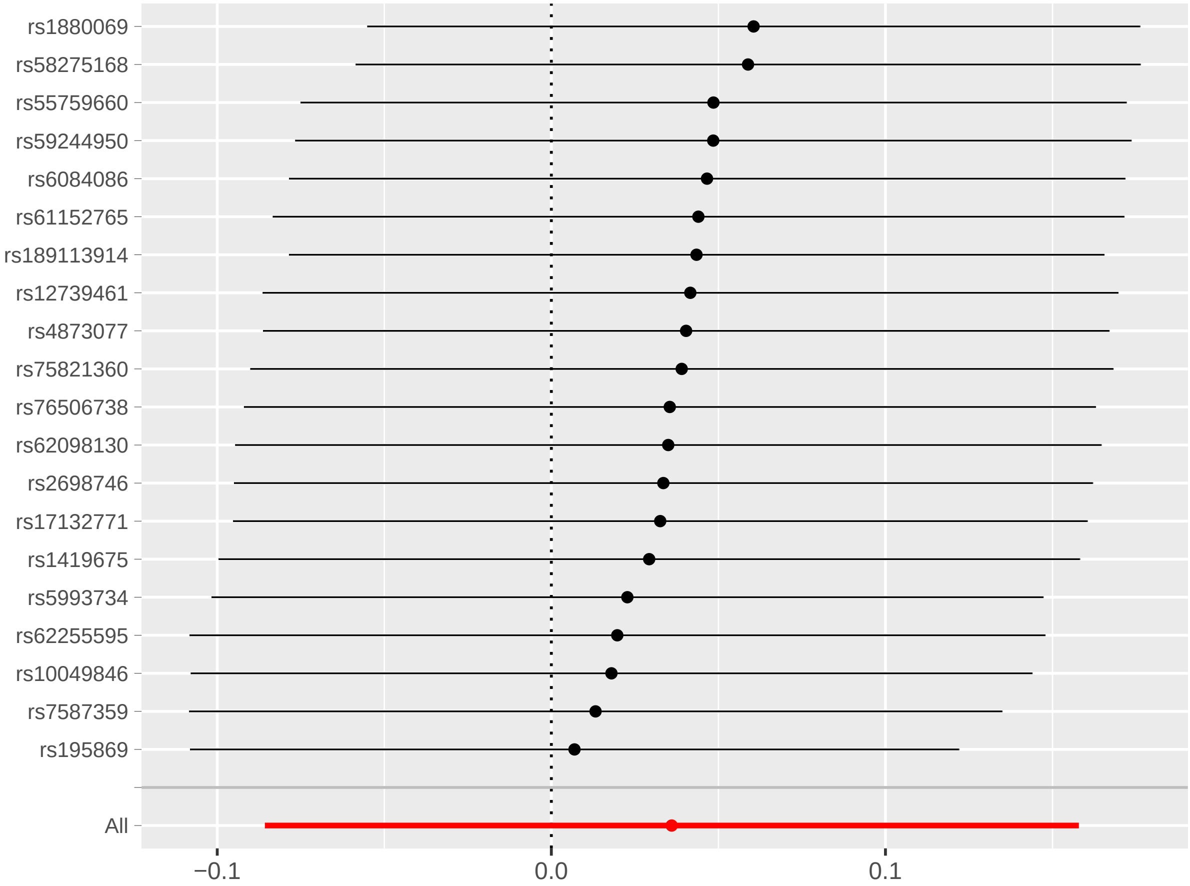

MR leave−one−out sensitivity analysis for
'CAG−170 sp003516765' on 'GCST90200762_buildGRCh38.tsv'

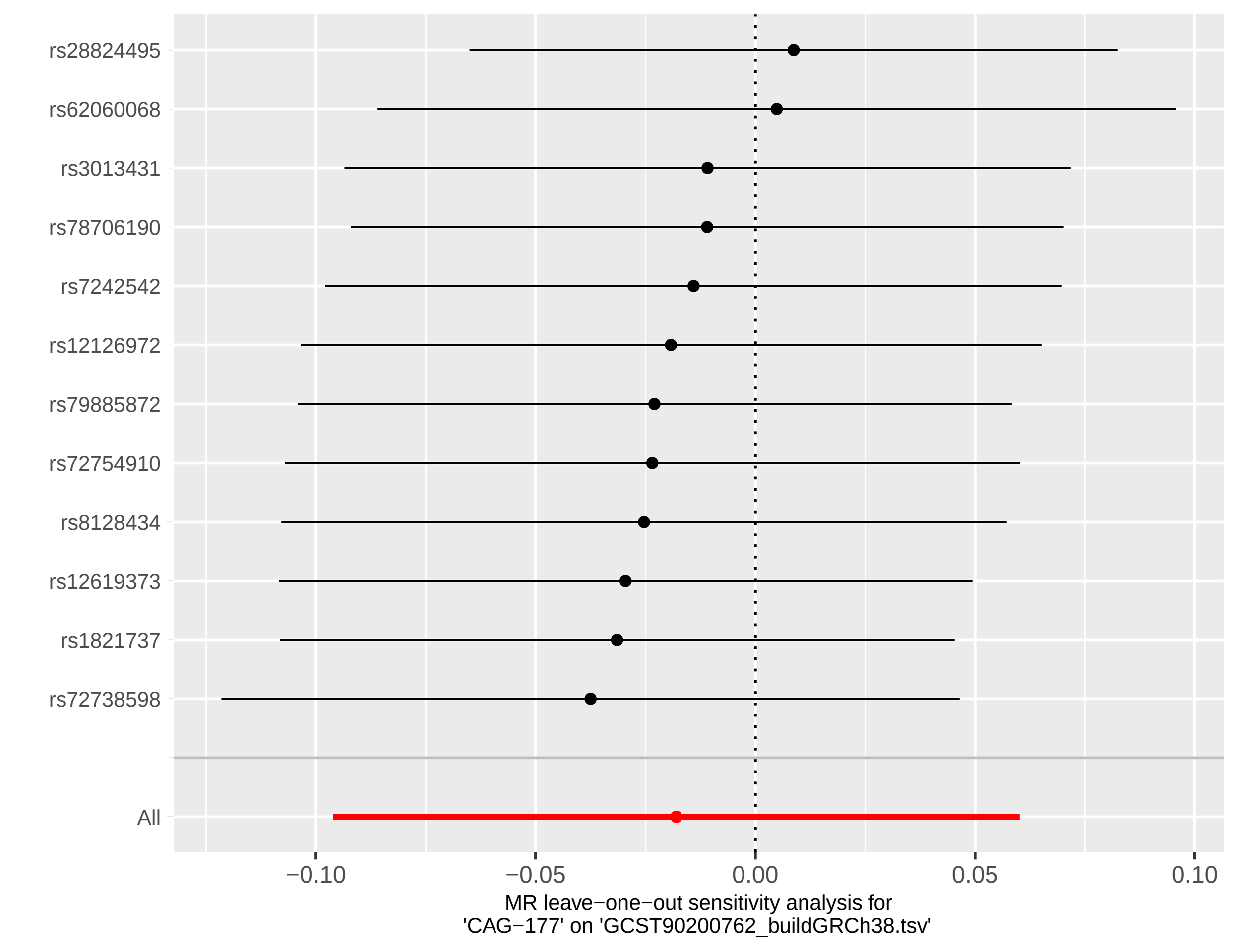

rs28824495
rs62060068
rs3013431
rs78706190
rs7242542
rs12126972
rs79885872
rs72754910
rs8128434
rs12619373
rs1821737
rs72738598
All
−0.10
−0.05
0.00
0.05
0.10
MR leave−one−out sensitivity analysis for
'CAG−177' on 'GCST90200762_buildGRCh38.tsv'

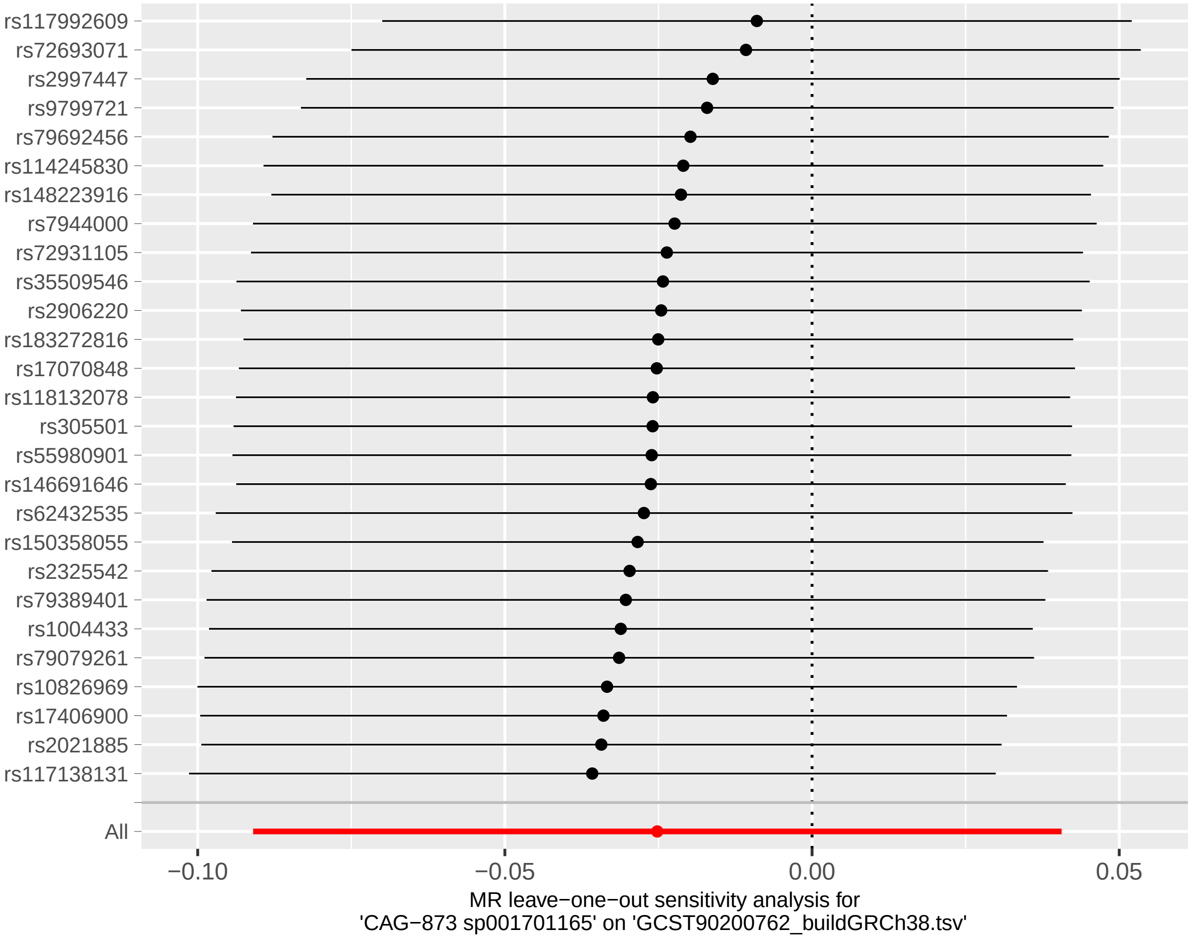

rs117992609
rs72693071
rs2997447
rs9799721
rs79692456
rs114245830
rs148223916
rs7944000
rs72931105
rs35509546
rs2906220
rs183272816
rs17070848
rs118132078
rs305501
rs55980901
rs146691646
rs62432535
rs150358055
rs2325542
rs79389401
rs1004433
rs79079261
rs10826969
rs17406900
rs2021885
rs117138131
All
−0.10
−0.05
0.00
0.05
MR leave−one−out sensitivity analysis for
'CAG−873 sp001701165' on 'GCST90200762_buildGRCh38.tsv'

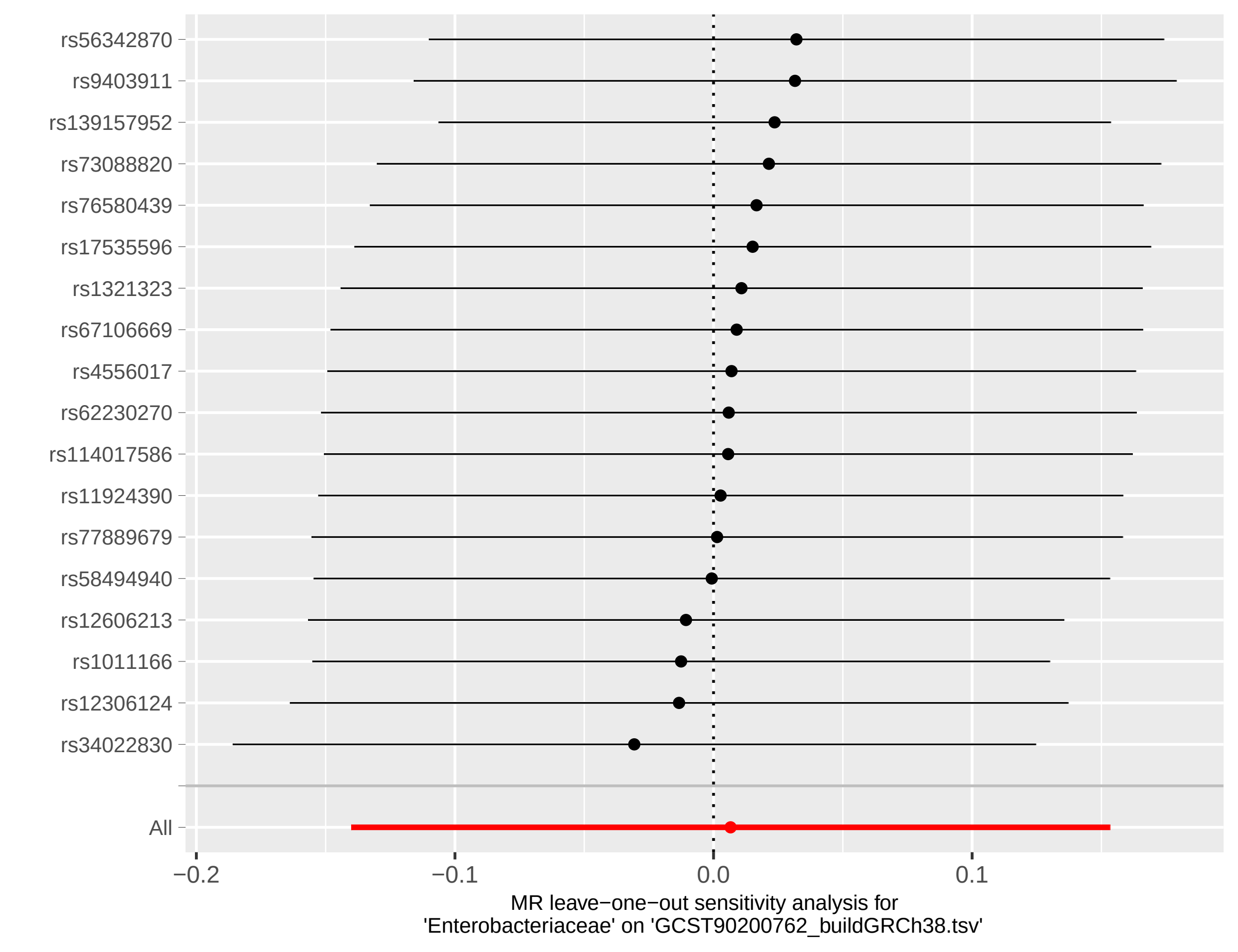
rs56342870
rs9403911
rs139157952
rs73088820
rs76580439
rs17535596
rs1321323
rs67106669
rs4556017
rs62230270
rs114017586
rs11924390
rs77889679
rs58494940
rs12606213
rs1011166
rs12306124
rs34022830
All
−0.2
−0.1
0.0
0.1
MR leave−one−out sensitivity analysis for
'Enterobacteriaceae' on 'GCST90200762_buildGRCh38.tsv'

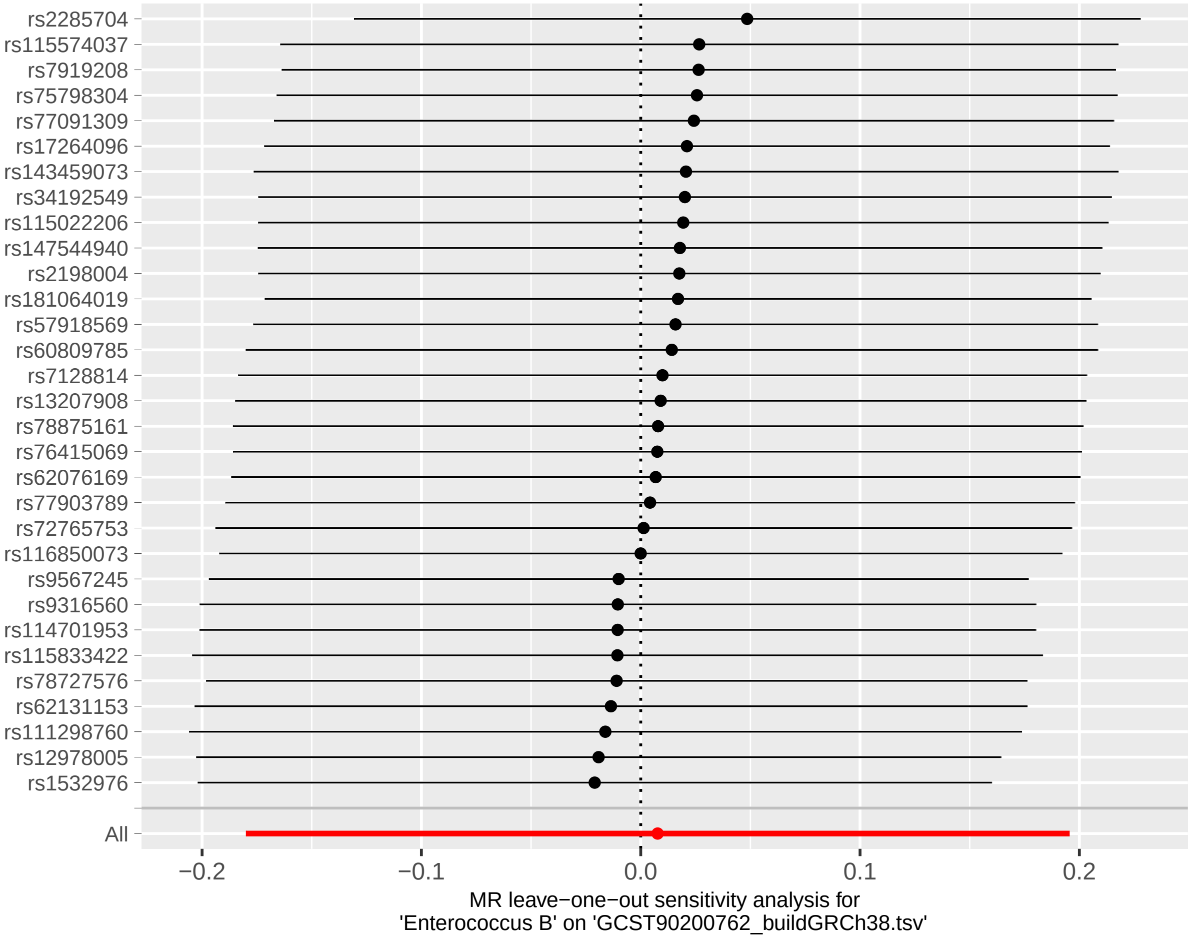
rs2285704
rs115574037
rs7919208
rs75798304
rs77091309
rs17264096
rs143459073
rs34192549
rs115022206
rs147544940
rs2198004
rs181064019
rs57918569
rs60809785
rs7128814
rs13207908
rs78875161
rs76415069
rs62076169
rs77903789
rs72765753
rs116850073
rs9567245
rs9316560
rs114701953
rs115833422
rs78727576
rs62131153
rs111298760
rs12978005
rs1532976
All
−0.2
−0.1
0.0
0.1
0.2
MR leave−one−out sensitivity analysis for
'Enterococcus B' on 'GCST90200762_buildGRCh38.tsv'

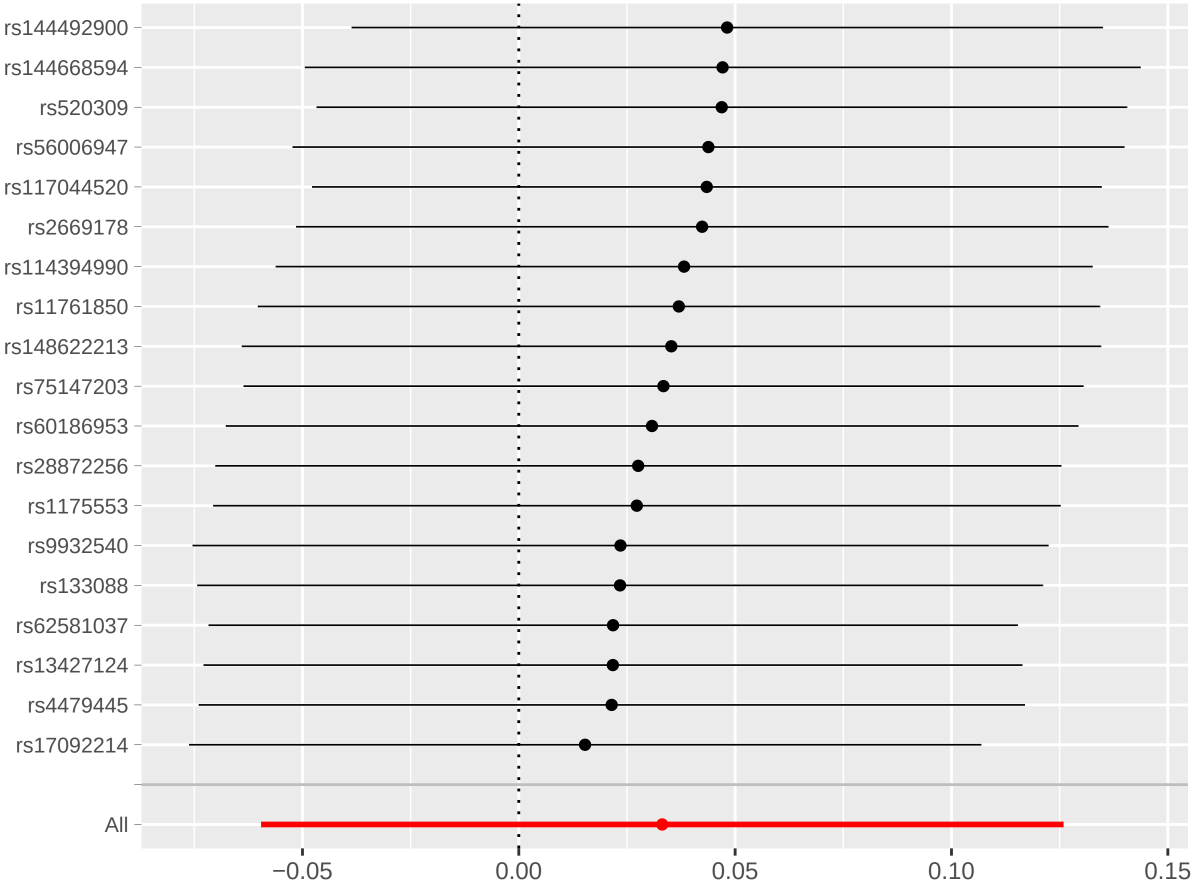

MR leave−one−out sensitivity analysis for
'Eubacterium F sp000434115' on 'GCST90200762_buildGRCh38.tsv'

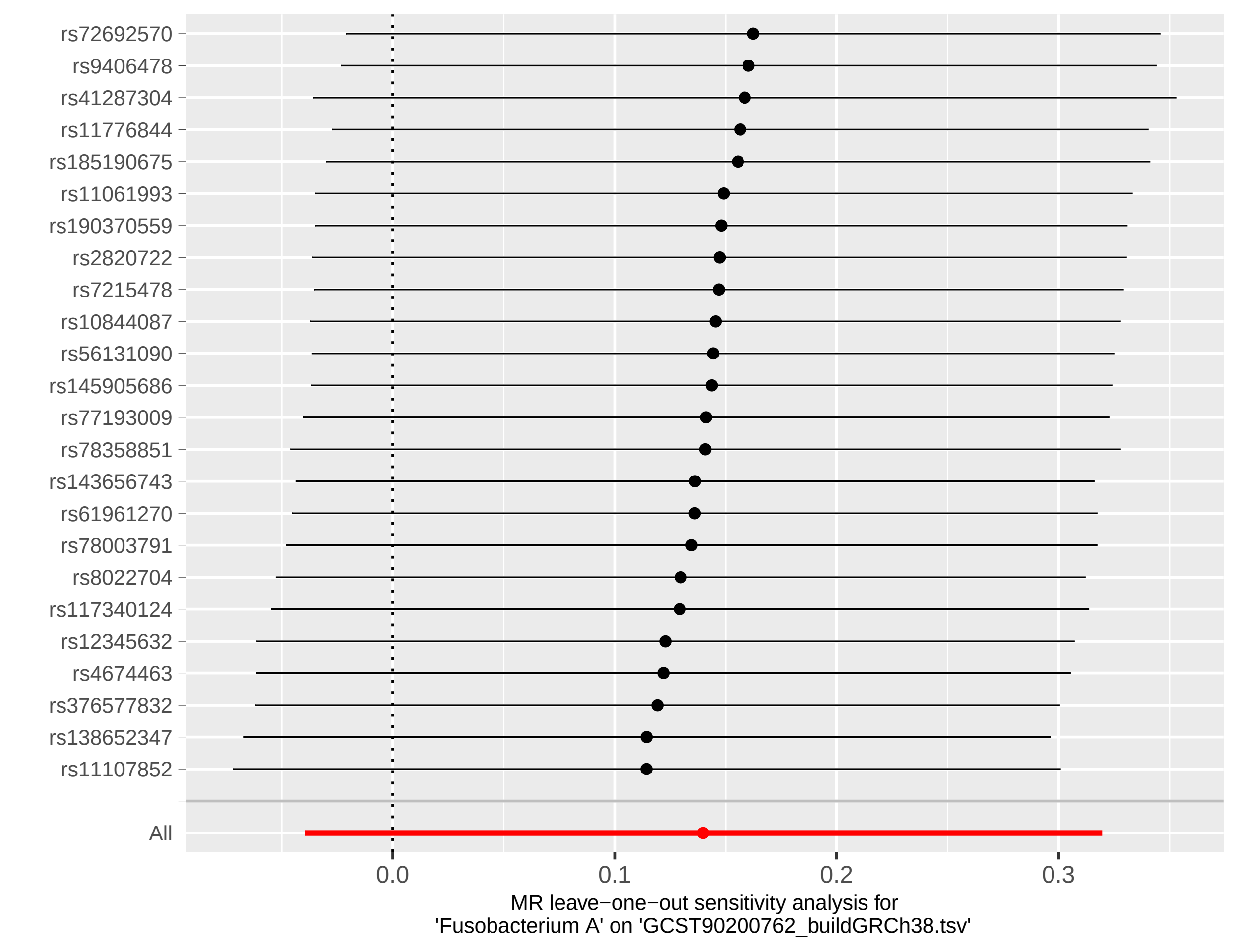
rs72692570
rs9406478
rs41287304
rs11776844
rs185190675
rs11061993
rs190370559
rs2820722
rs7215478
rs10844087
rs56131090
rs145905686
rs77193009
rs78358851
rs143656743
rs61961270
rs78003791
rs8022704
rs117340124
rs12345632
rs4674463
rs376577832
rs138652347
rs11107852
All
0.0
0.1
0.2
0.3
MR leave−one−out sensitivity analysis for
'Fusobacterium A' on 'GCST90200762_buildGRCh38.tsv'

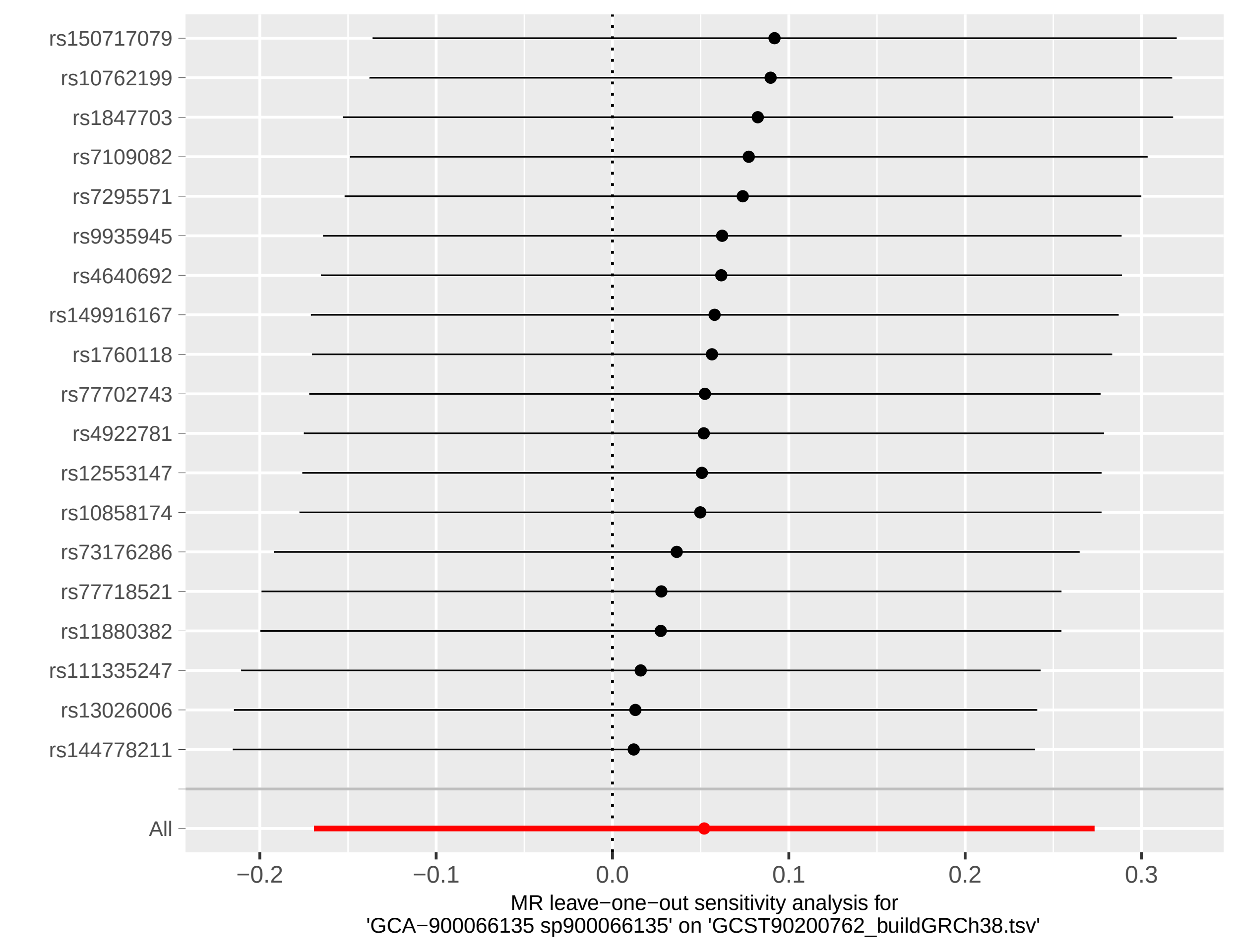
rs150717079
rs10762199
rs1847703
rs7109082
rs7295571
rs9935945
rs4640692
rs149916167
rs1760118
rs77702743
rs4922781
rs12553147
rs10858174
rs73176286
rs77718521
rs11880382
rs111335247
rs13026006
rs144778211
All
−0.2
−0.1
0.0
0.1
0.2
0.3
MR leave−one−out sensitivity analysis for
'GCA−900066135 sp900066135' on 'GCST90200762_buildGRCh38.tsv'

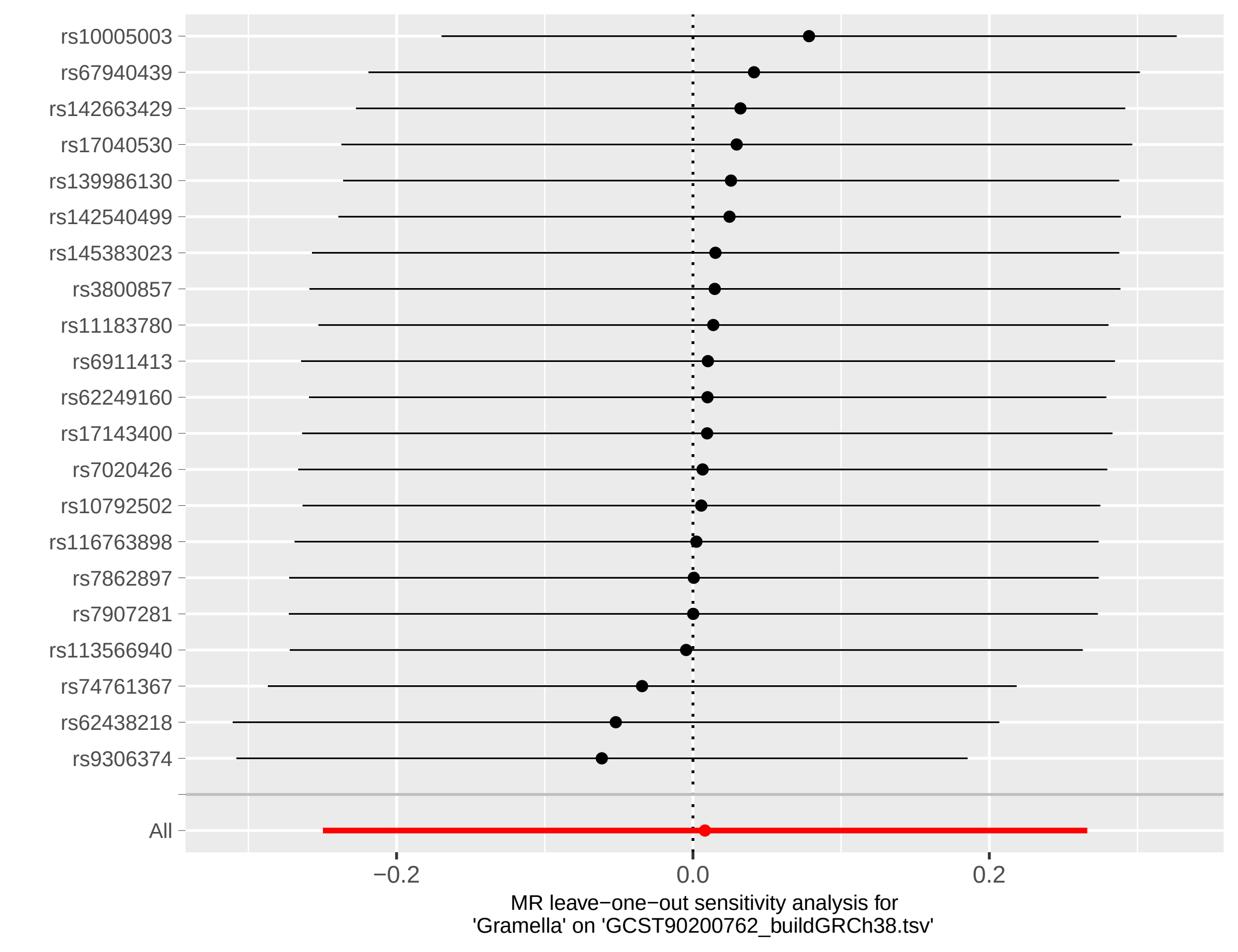

rs10005003
rs67940439
rs142663429
rs17040530
rs139986130
rs142540499
rs145383023
rs3800857
rs11183780
rs6911413
rs62249160
rs17143400
rs7020426
rs10792502
rs116763898
rs7862897
rs7907281
rs113566940
rs74761367
rs62438218
rs9306374
All
−0.2
0.0
0.2
MR leave−one−out sensitivity analysis for
'Gramella' on 'GCST90200762_buildGRCh38.tsv'

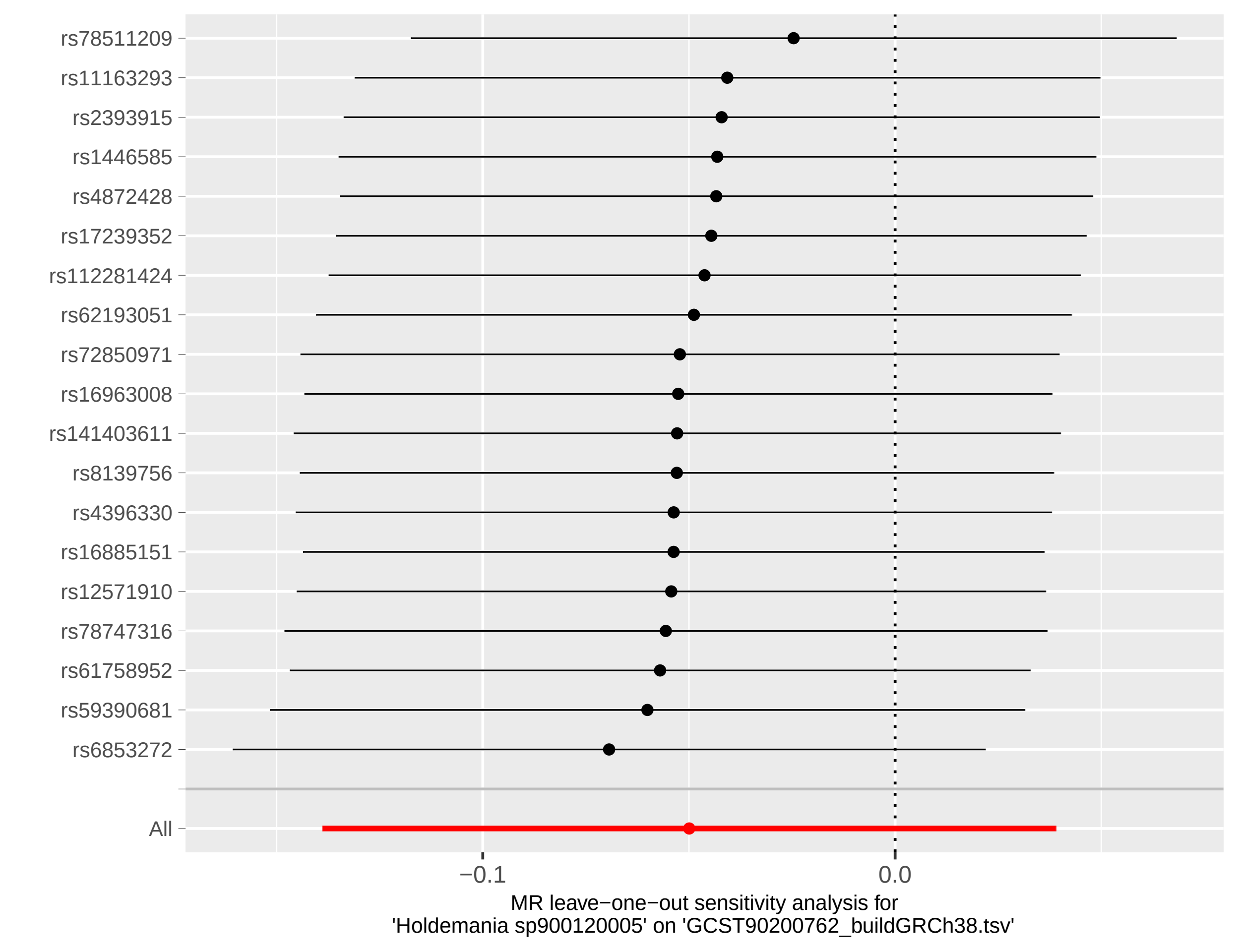

rs78511209
rs11163293
rs2393915
rs1446585
rs4872428
rs17239352
rs112281424
rs62193051
rs72850971
rs16963008
rs141403611
rs8139756
rs4396330
rs16885151
rs12571910
rs78747316
rs61758952
rs59390681
rs6853272
All
−0.1
0.0
MR leave−one−out sensitivity analysis for
'Holdemania sp900120005' on 'GCST90200762_buildGRCh38.tsv'

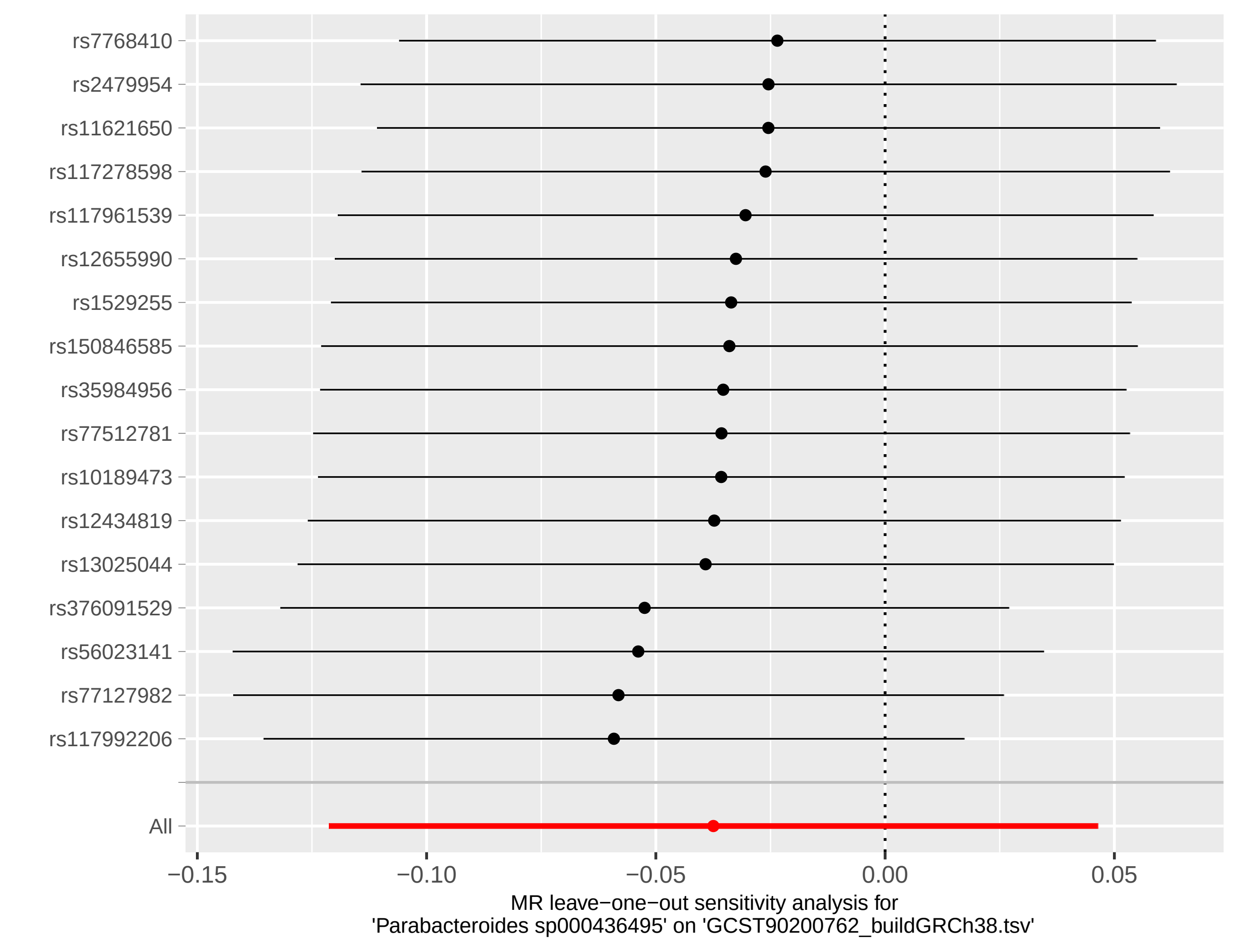
rs7768410
rs2479954
rs11621650
rs117278598
rs117961539
rs12655990
rs1529255
rs150846585
rs35984956
rs77512781
rs10189473
rs12434819
rs13025044
rs376091529
rs56023141
rs77127982
rs117992206
All
−0.15
−0.10
−0.05
0.00
0.05
MR leave−one−out sensitivity analysis for
'Parabacteroides sp000436495' on 'GCST90200762_buildGRCh38.tsv'

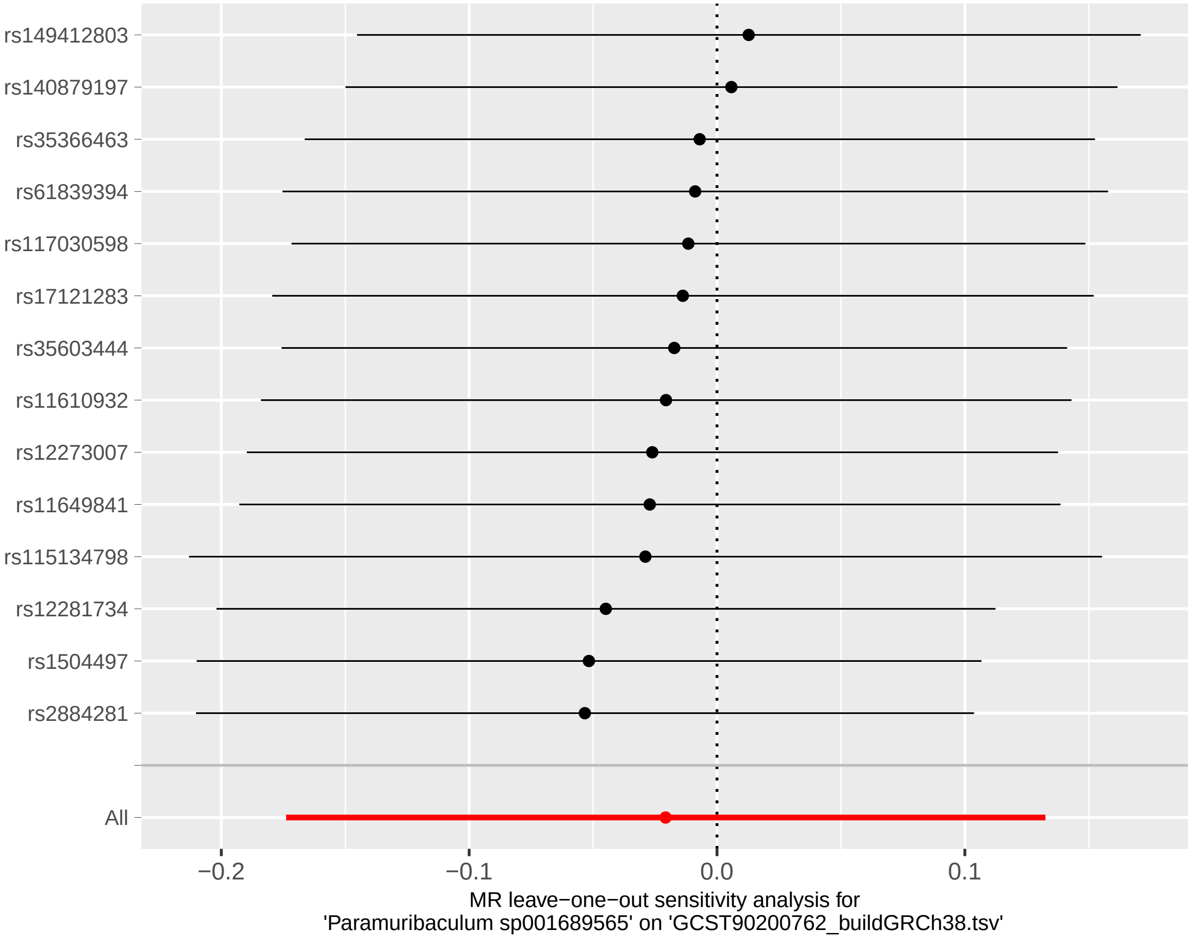
rs149412803
rs140879197
rs35366463
rs61839394
rs117030598
rs17121283
rs35603444
rs11610932
rs12273007
rs11649841
rs115134798
rs12281734
rs1504497
rs2884281
All
−0.2
−0.1
0.0
0.1
MR leave−one−out sensitivity analysis for
'Paramuribaculum sp001689565' on 'GCST90200762_buildGRCh38.tsv'

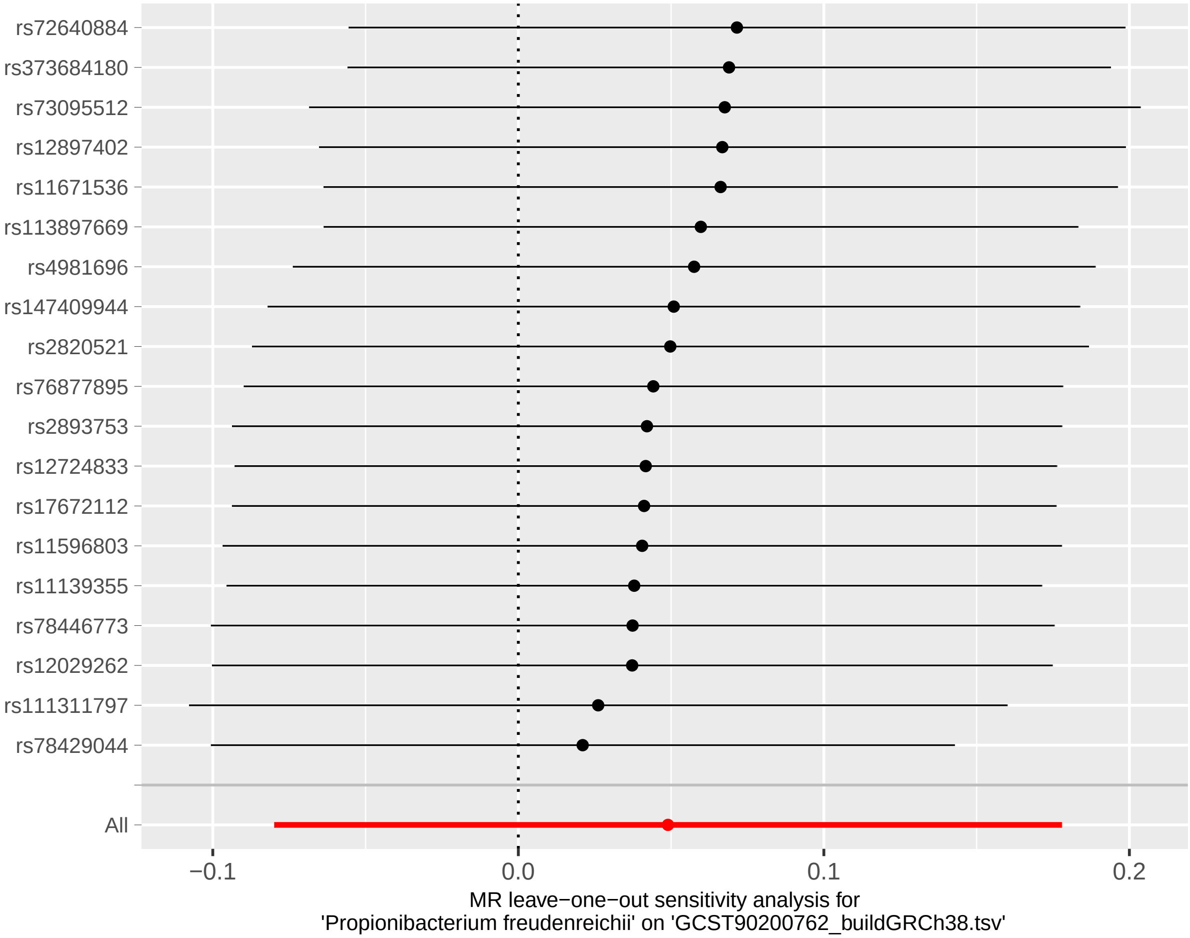

rs72640884
rs373684180
rs73095512
rs12897402
rs11671536
rs113897669
rs4981696
rs147409944
rs2820521
rs76877895
rs2893753
rs12724833
rs17672112
rs11596803
rs11139355
rs78446773
rs12029262
rs111311797
rs78429044
All
−0.1
0.0
0.1
0.2
MR leave−one−out sensitivity analysis for
'Propionibacterium freudenreichii' on 'GCST90200762_buildGRCh38.tsv'

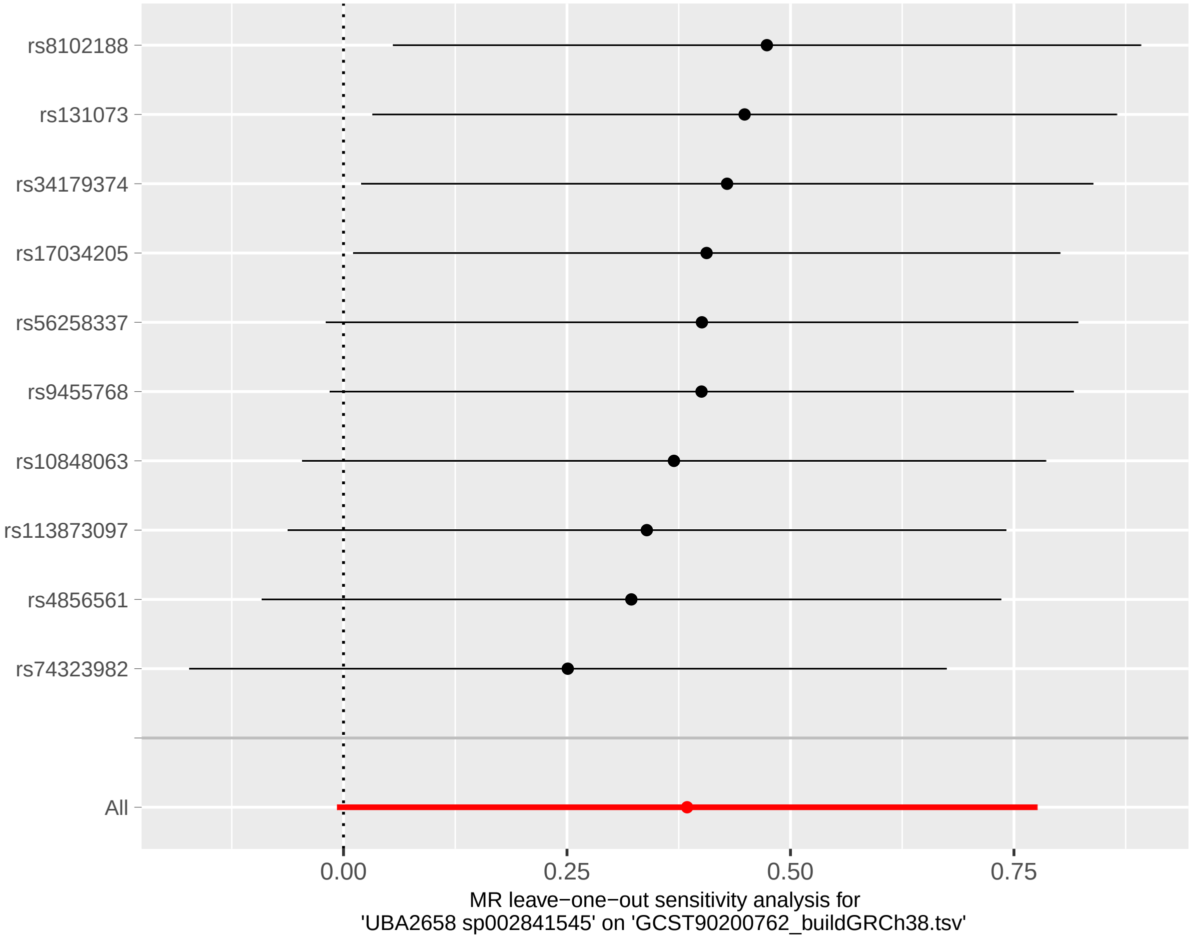
rs8102188
rs131073
rs34179374
rs17034205
rs56258337
rs9455768
rs10848063
rs113873097
rs4856561
rs74323982
All
0.00
0.25
0.50
0.75
MR leave−one−out sensitivity analysis for
'UBA2658 sp002841545' on 'GCST90200762_buildGRCh38.tsv'

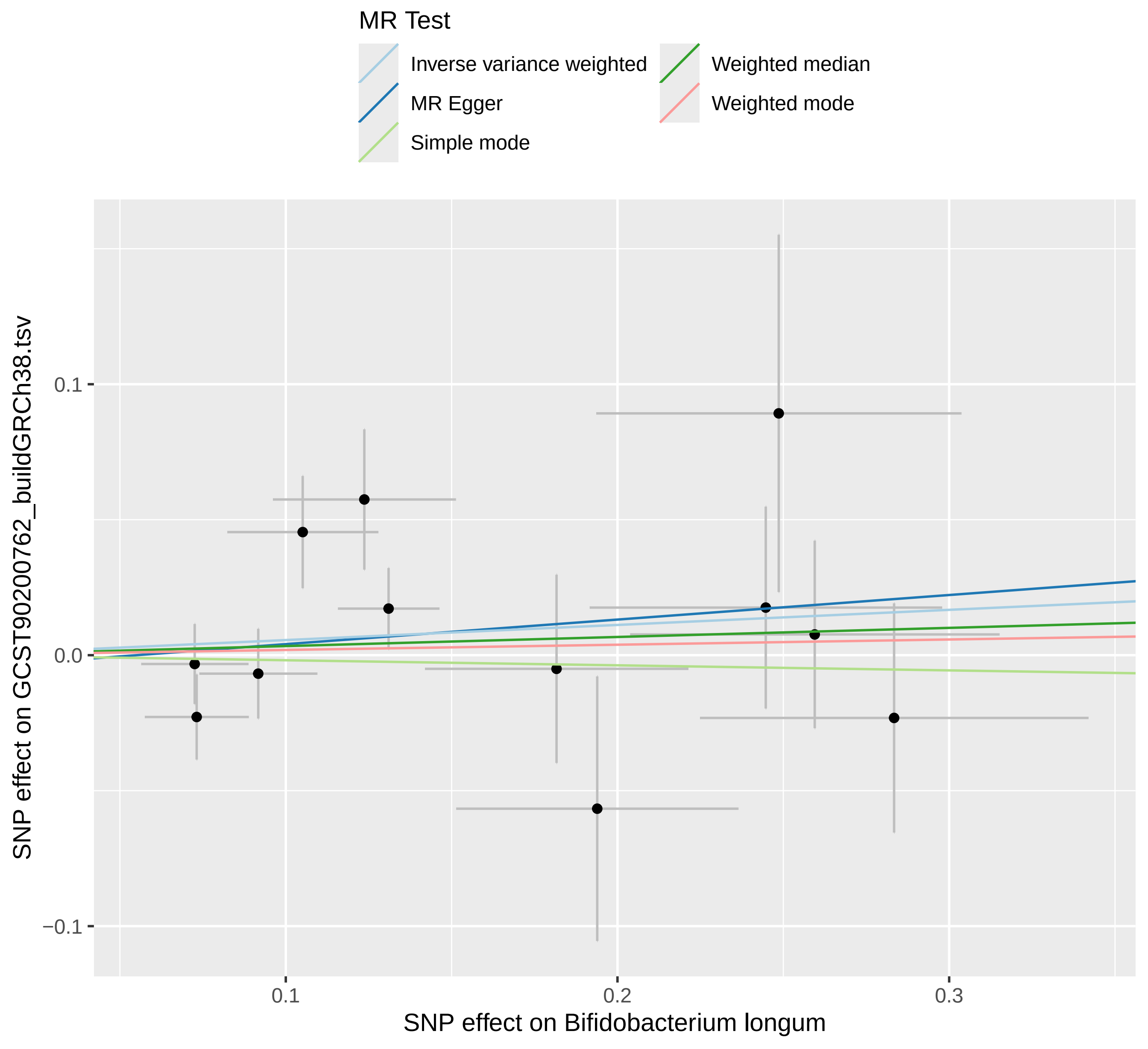
MR Test
Inverse variance weighted
MR Egger
Simple mode
Weighted median
Weighted mode
SNP effect on GCST90200762_buildGRCh38.tsv
0.1
0.0
−0.1
0.1
0.2
0.3
SNP effect on Bifidobacterium longum

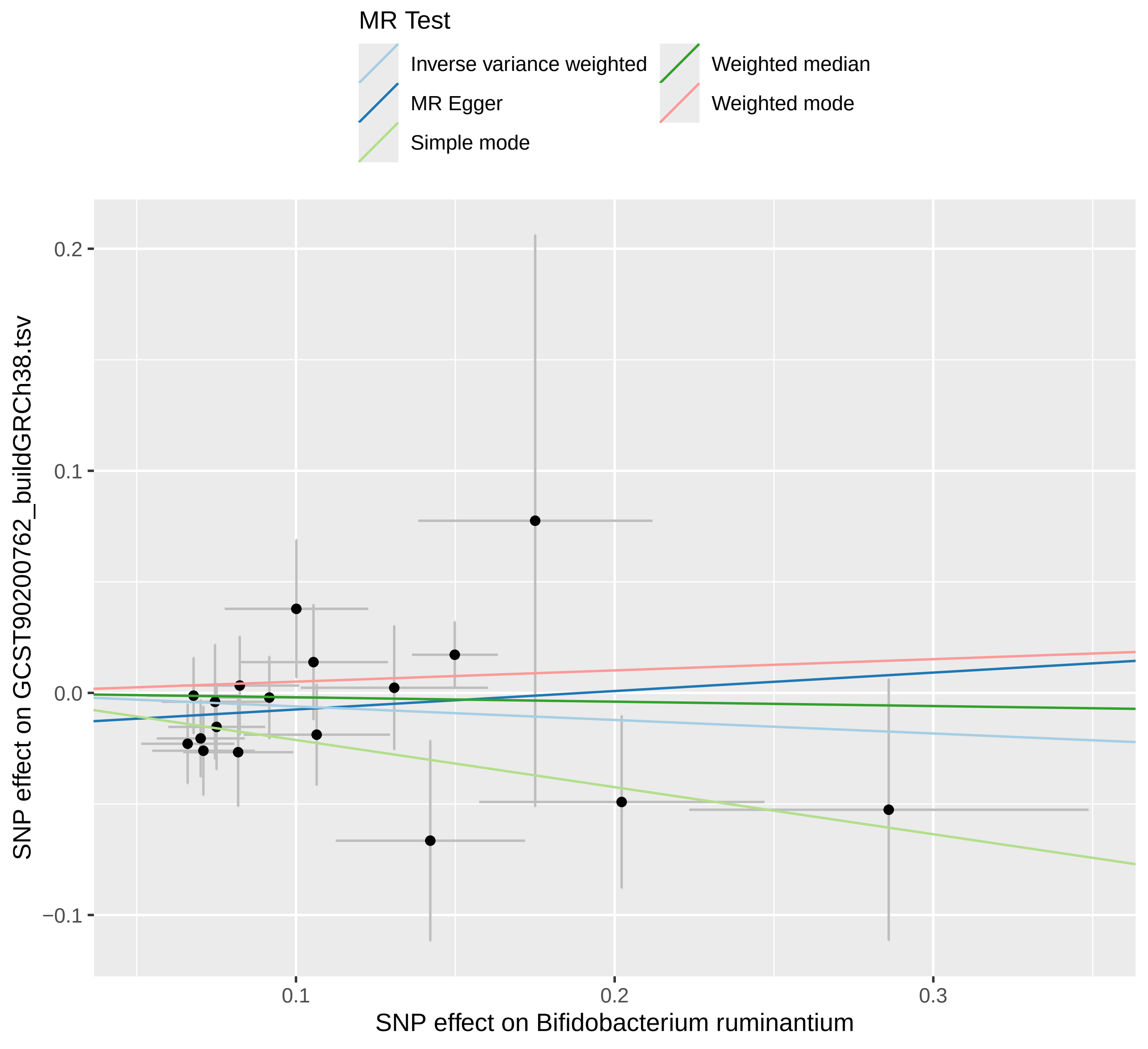
MR Test
Inverse variance weighted
MR Egger
Simple mode
Weighted median
Weighted mode
0.2
0.1
0.0
−0.1
SNP effect on GCST90200762_buildGRCh38.tsv
0.1
0.2
0.3
SNP effect on Bifidobacterium ruminantium

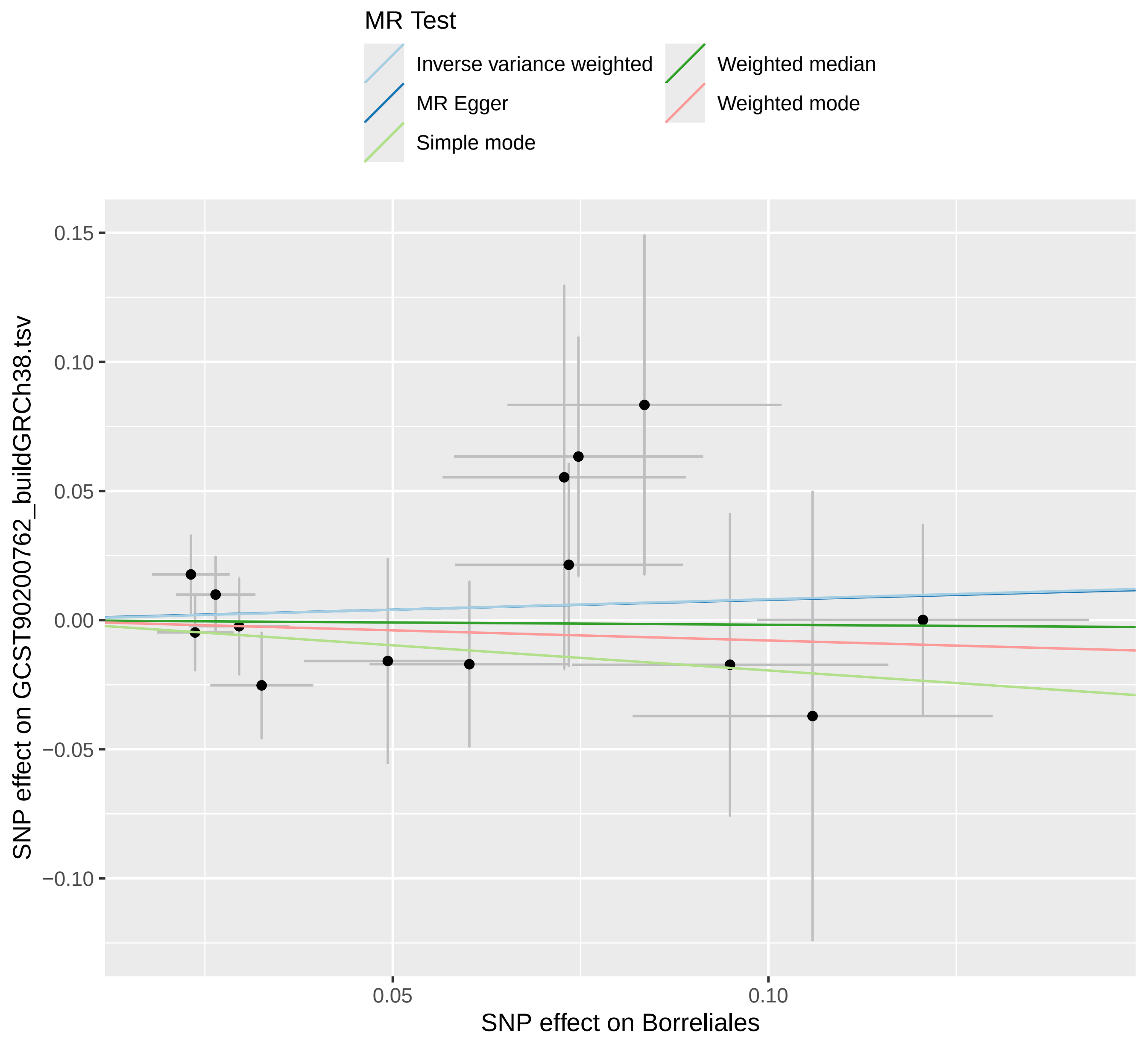

MR Test
Inverse variance weighted
MR Egger
Simple mode
Weighted median
Weighted mode
0.15
0.10
0.05
0.00
−0.05
−0.10
SNP effect on GCST90200762_buildGRCh38.tsv
0.05
0.10
SNP effect on Borreliales

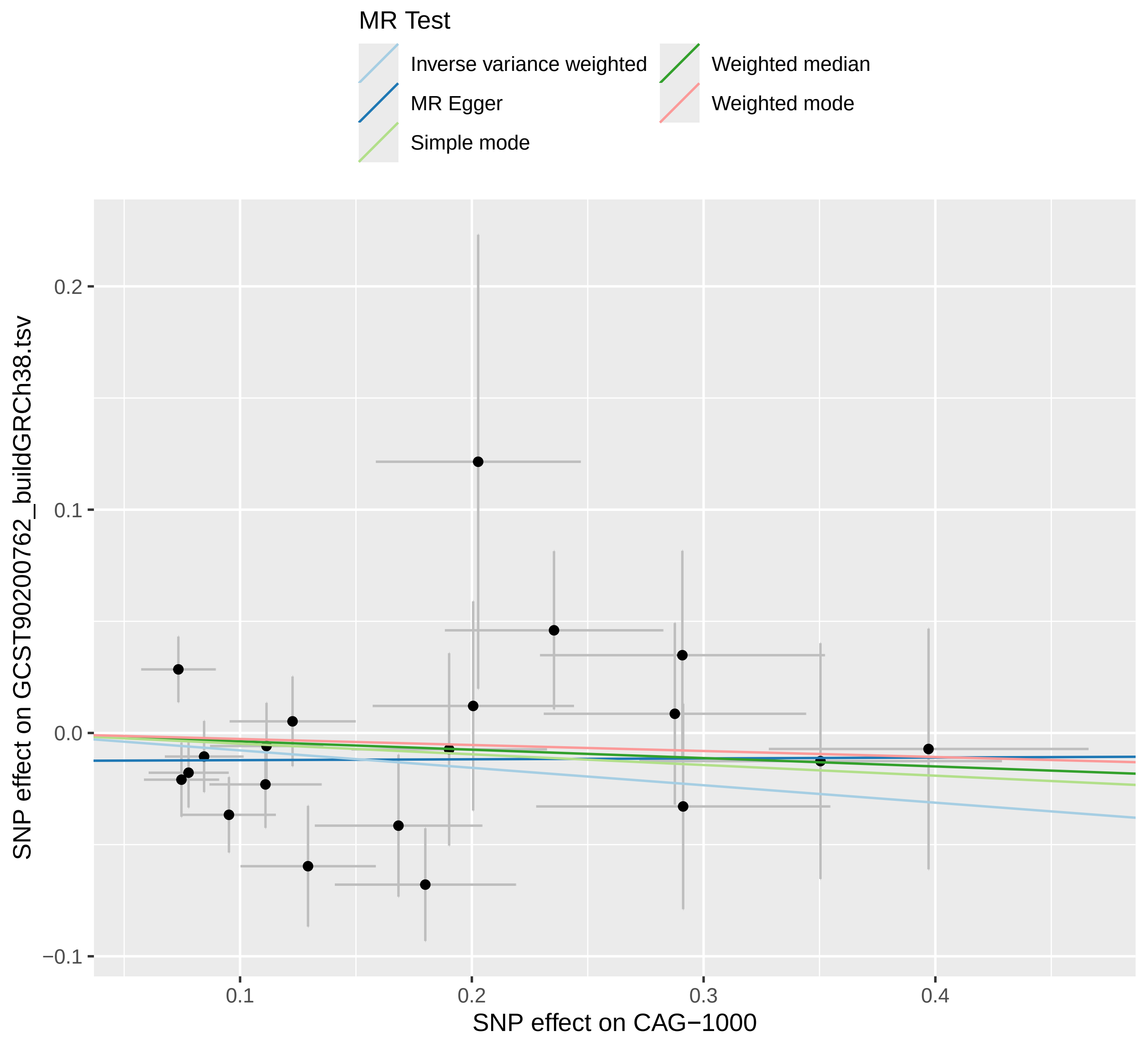

MR Test
Inverse variance weighted
MR Egger
Simple mode
Weighted median
Weighted mode
0.2
0.1
0.0
−0.1
0.1
0.2
0.3
0.4
SNP effect on GCST90200762_buildGRCh38.tsv
SNP effect on CAG−1000

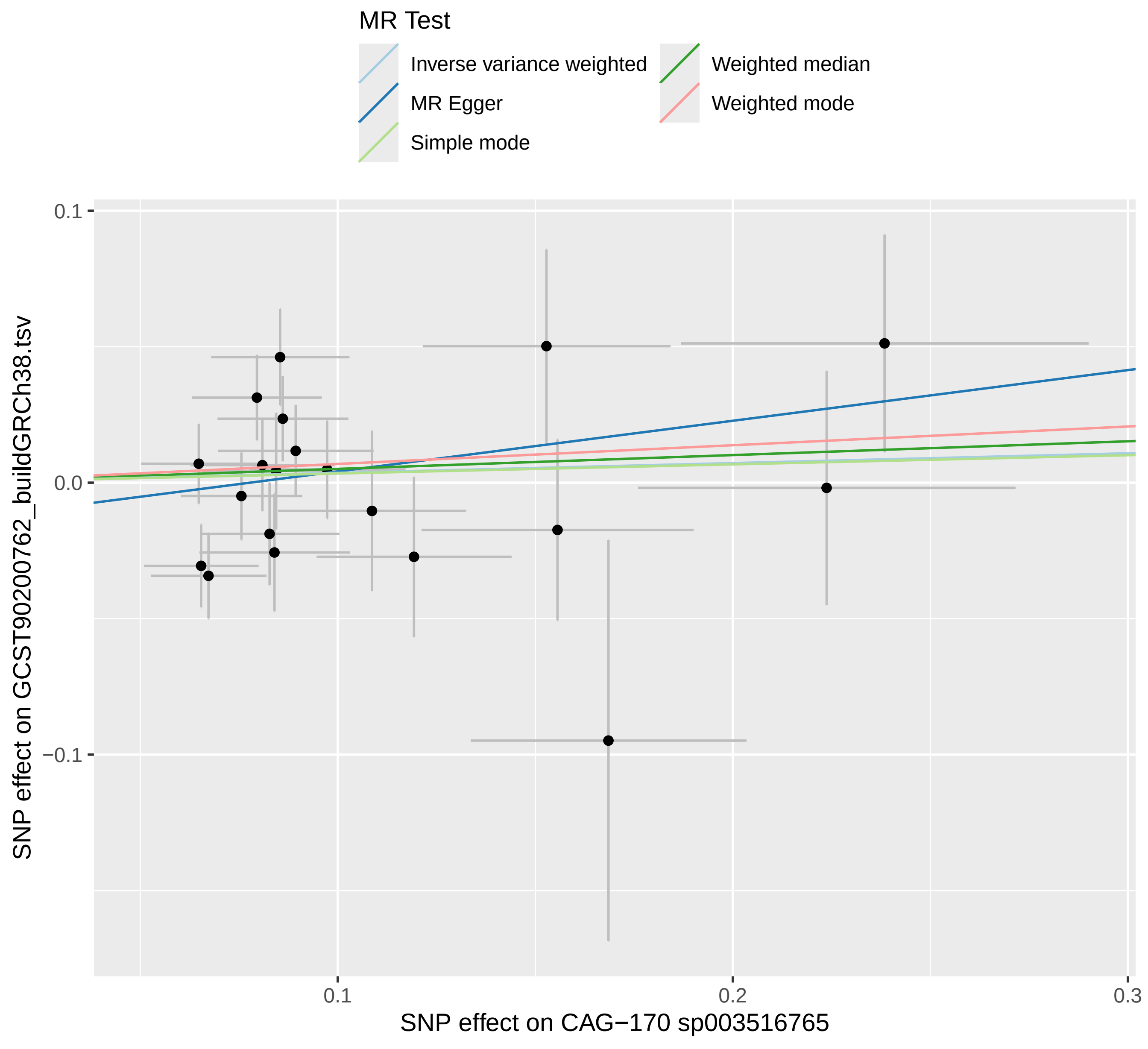
MR Test
Inverse variance weighted
MR Egger
Simple mode
Weighted median
Weighted mode
0.1
0.0
−0.1
0.1
0.2
0.3
SNP effect on GCST90200762_buildGRCh38.tsv
SNP effect on CAG−170 sp003516765

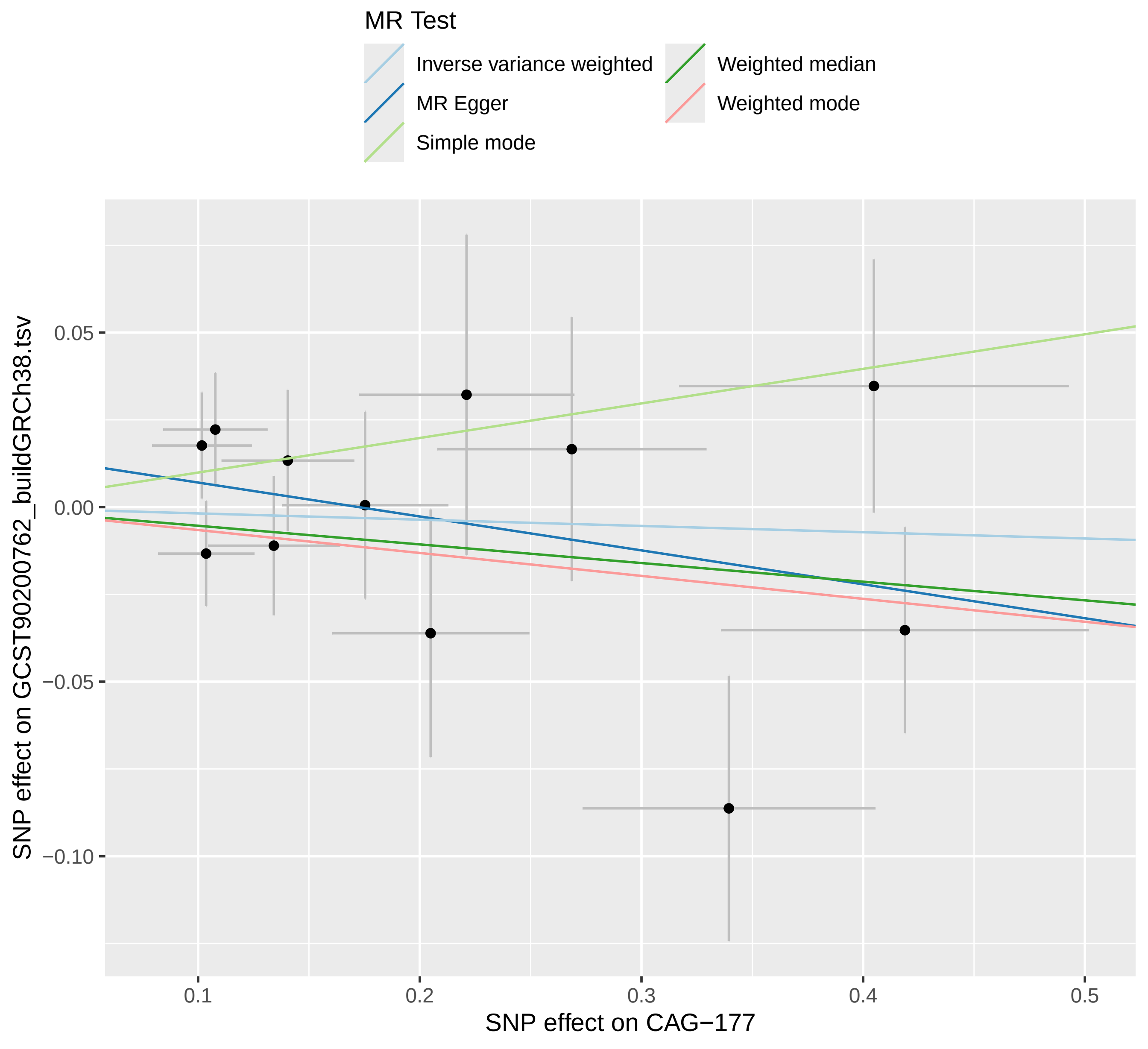
MR Test
Inverse variance weighted
MR Egger
Simple mode
Weighted median
Weighted mode
SNP effect on GCST90200762_buildGRCh38.tsv
0.05
0.00
−0.05
−0.10
0.1
0.2
0.3
0.4
0.5
SNP effect on CAG−177

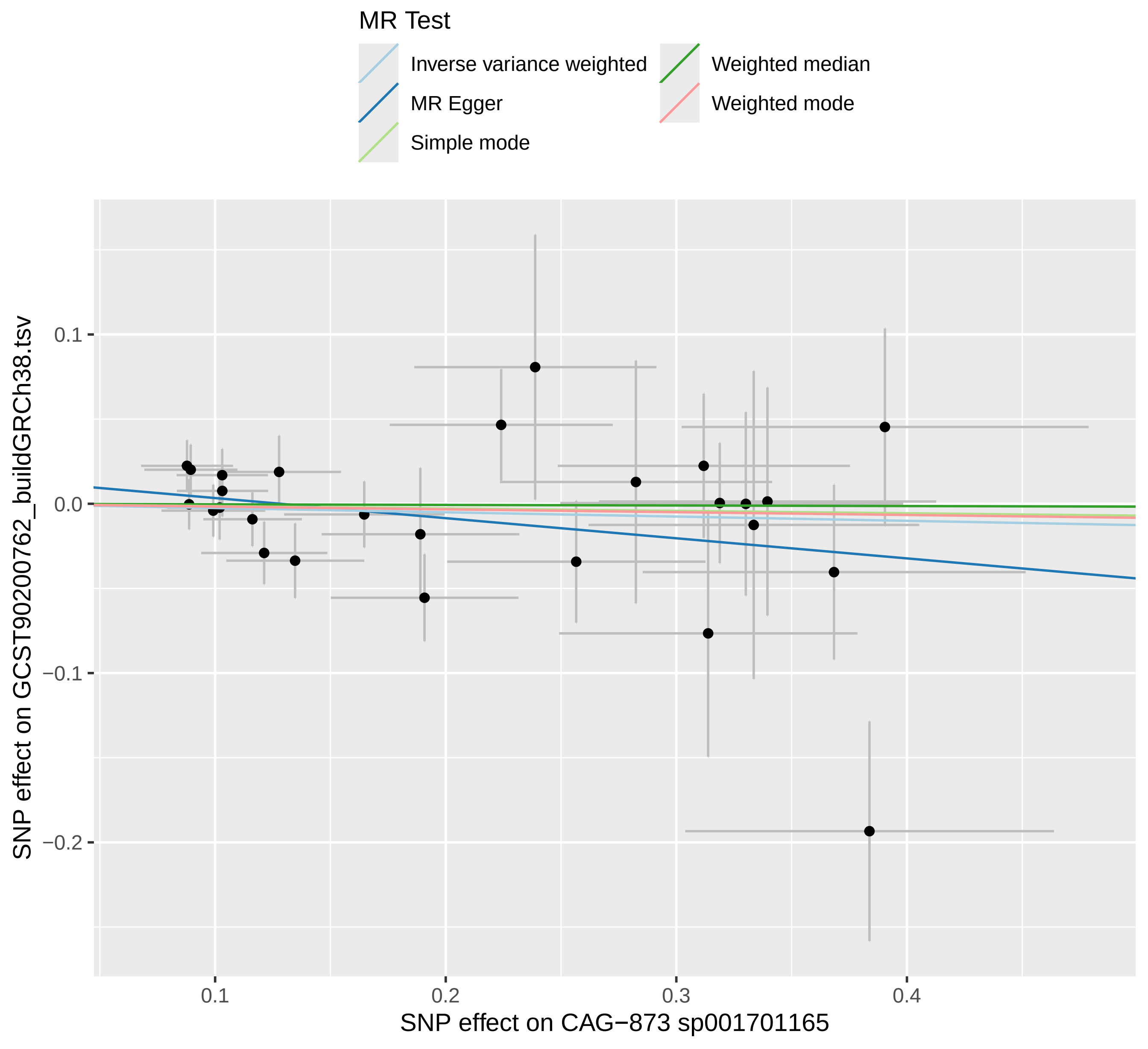

MR Test
Inverse variance weighted
MR Egger
Simple mode
Weighted median
Weighted mode
0.1
0.0
−0.1
−0.2
SNP effect on GCST90200762_buildGRCh38.tsv
0.1
0.2
0.3
0.4
SNP effect on CAG−873 sp001701165

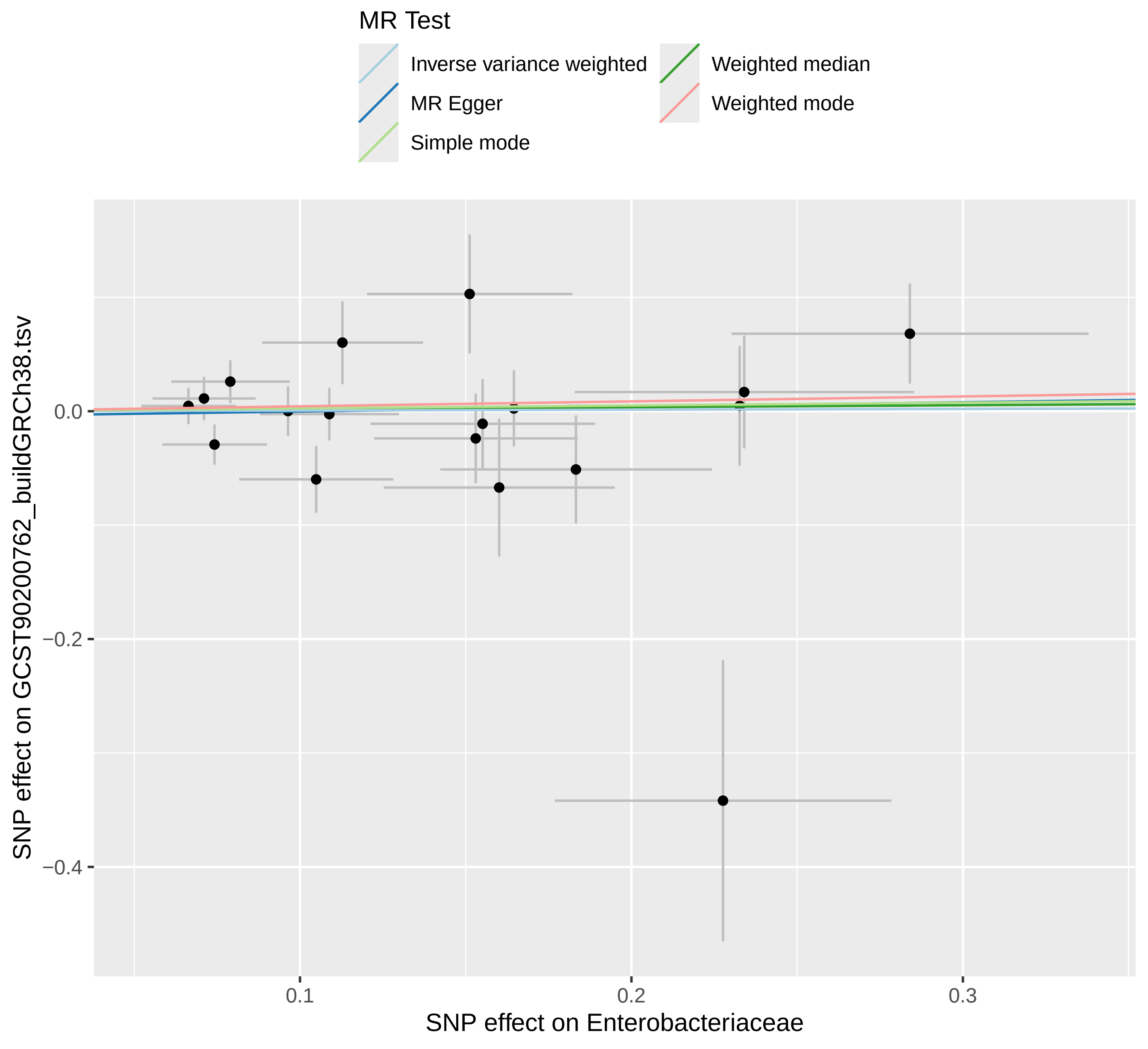

MR Test
Inverse variance weighted
MR Egger
Simple mode
Weighted median
Weighted mode
SNP effect on GCST90200762_buildGRCh38.tsv
0.0
−0.2
−0.4
0.1
0.2
0.3
SNP effect on Enterobacteriaceae

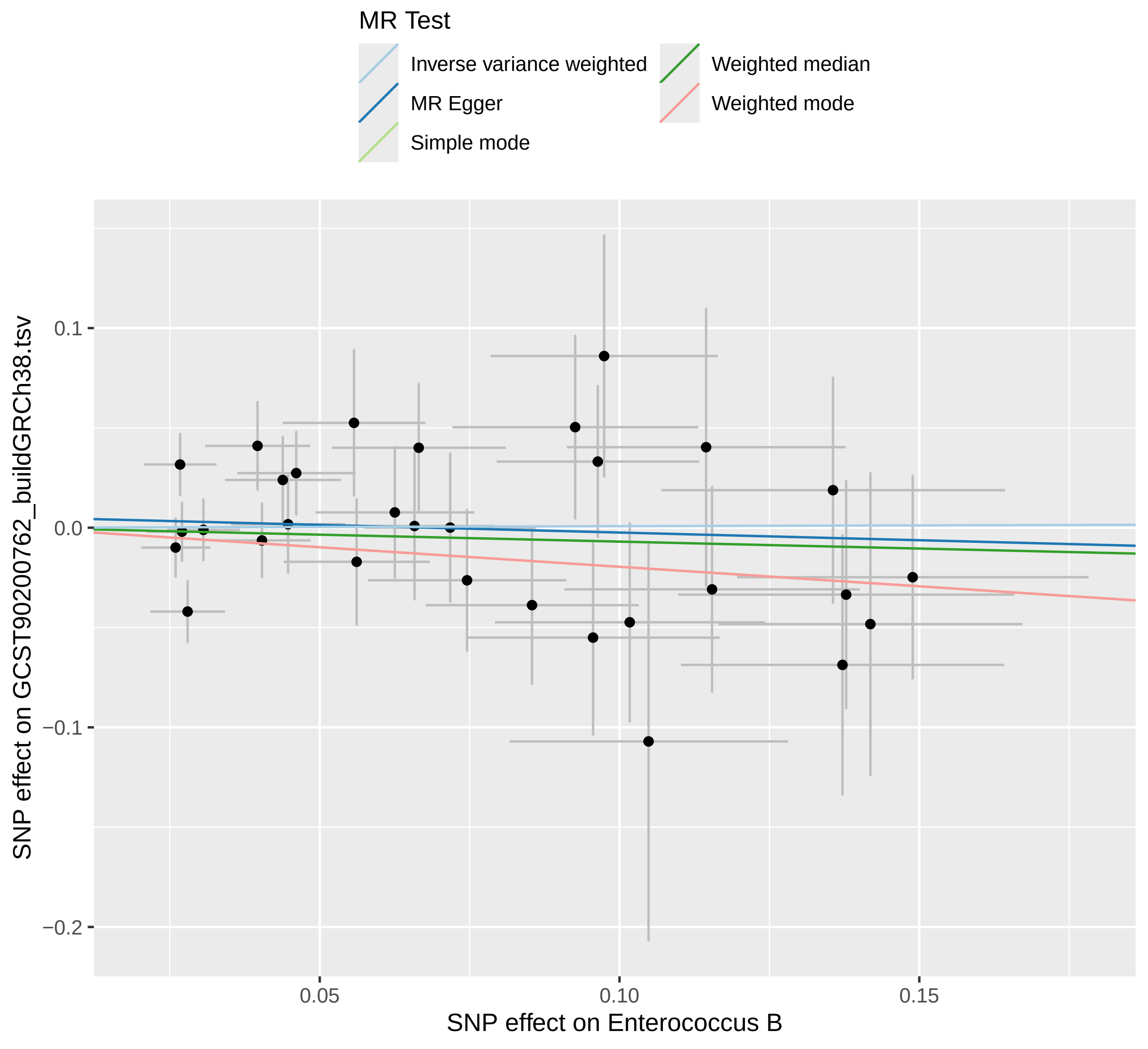
MR Test
Inverse variance weighted
MR Egger
Simple mode
Weighted median
Weighted mode
SNP effect on GCST90200762_buildGRCh38.tsv
0.1
0.0
−0.1
−0.2
0.05
0.10
0.15
SNP effect on Enterococcus B

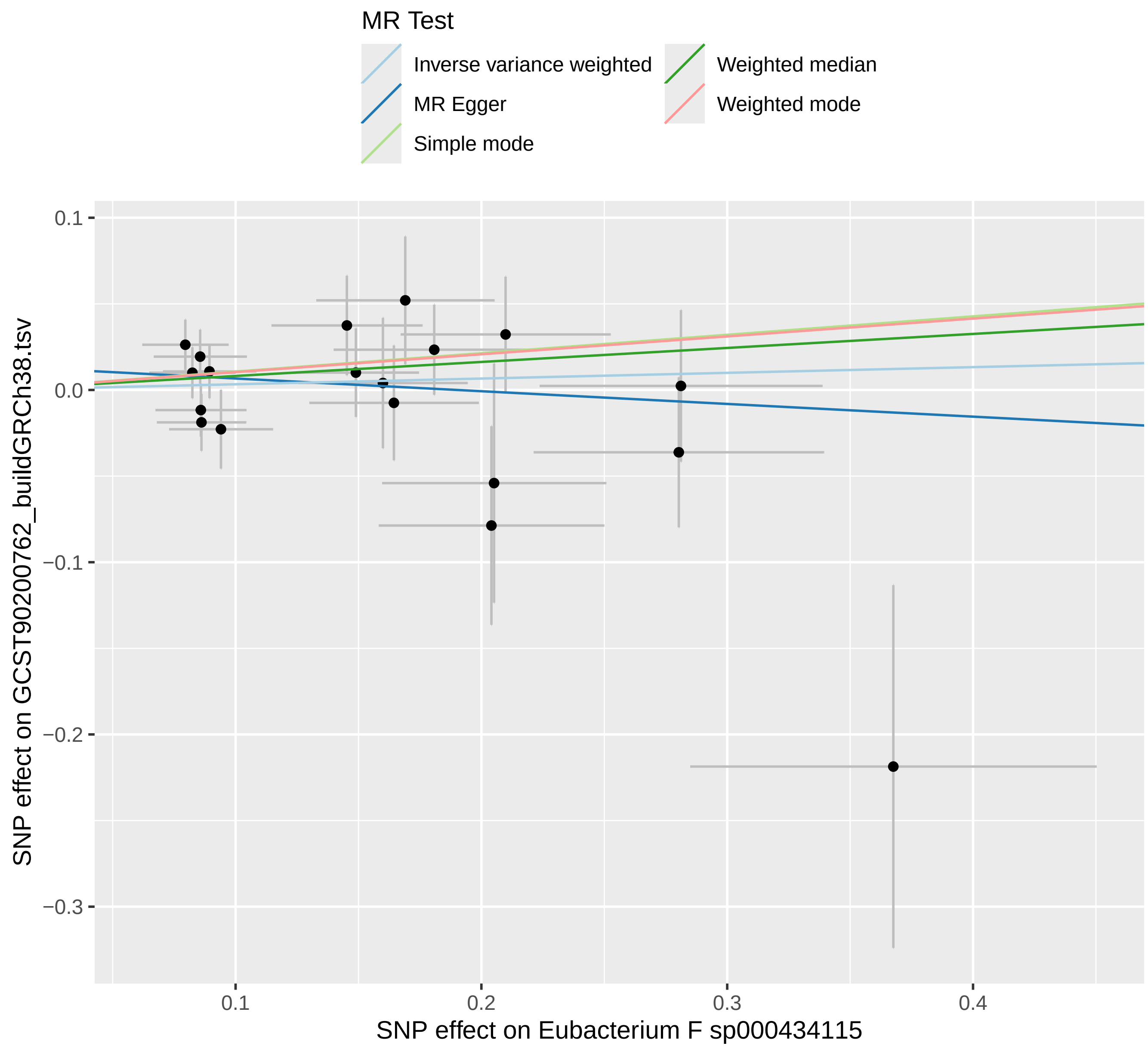

MR Test
Inverse variance weighted
MR Egger
Simple mode
Weighted median
Weighted mode
0.1
0.0
−0.1
−0.2
−0.3
SNP effect on GCST90200762_buildGRCh38.tsv
0.1
0.2
0.3
0.4
SNP effect on Eubacterium F sp000434115

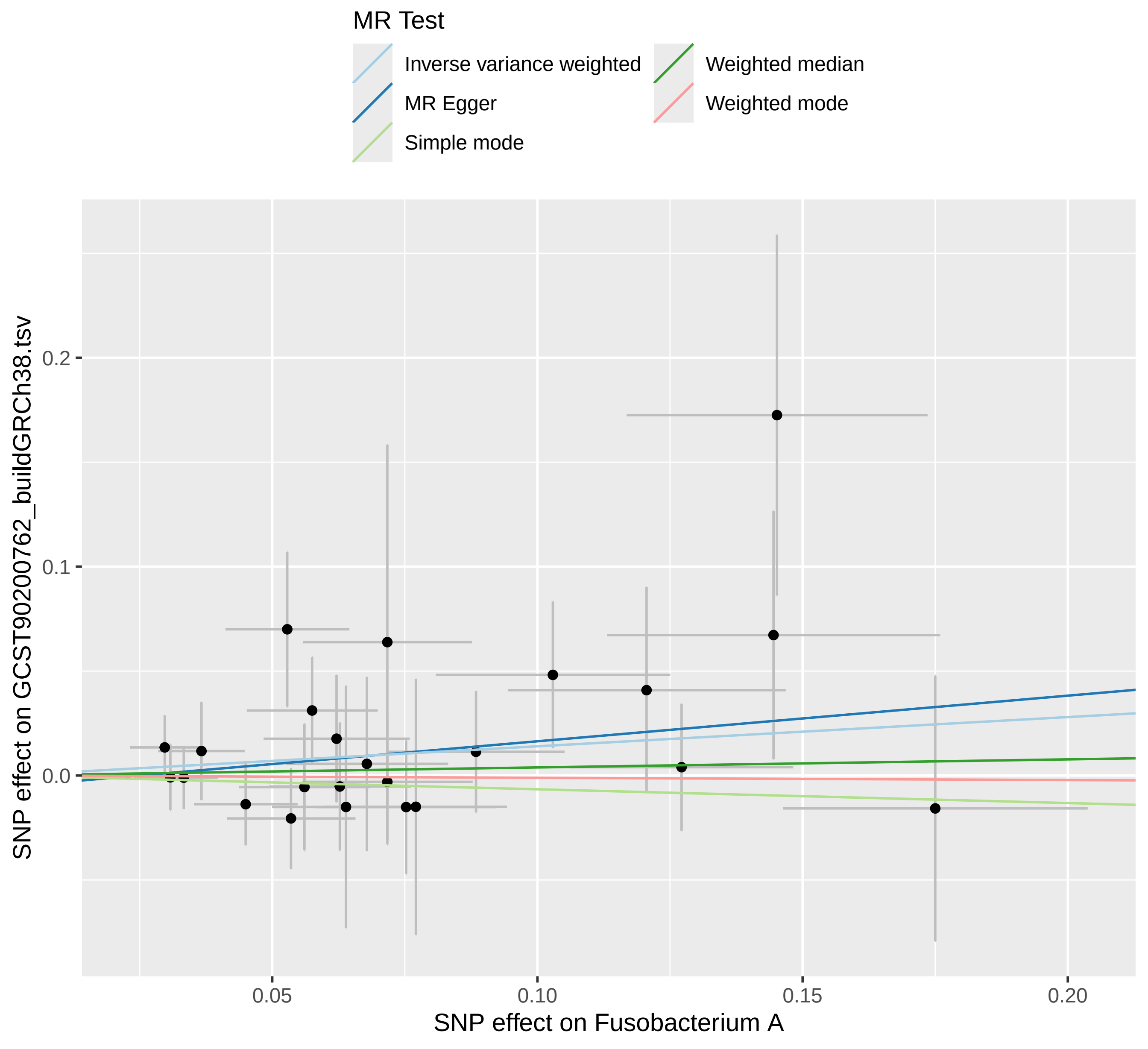
MR Test
Inverse variance weighted
MR Egger
Simple mode
Weighted median
Weighted mode
SNP effect on GCST90200762_buildGRCh38.tsv
0.2
0.1
0.0
0.05
0.10
0.15
0.20
SNP effect on Fusobacterium A

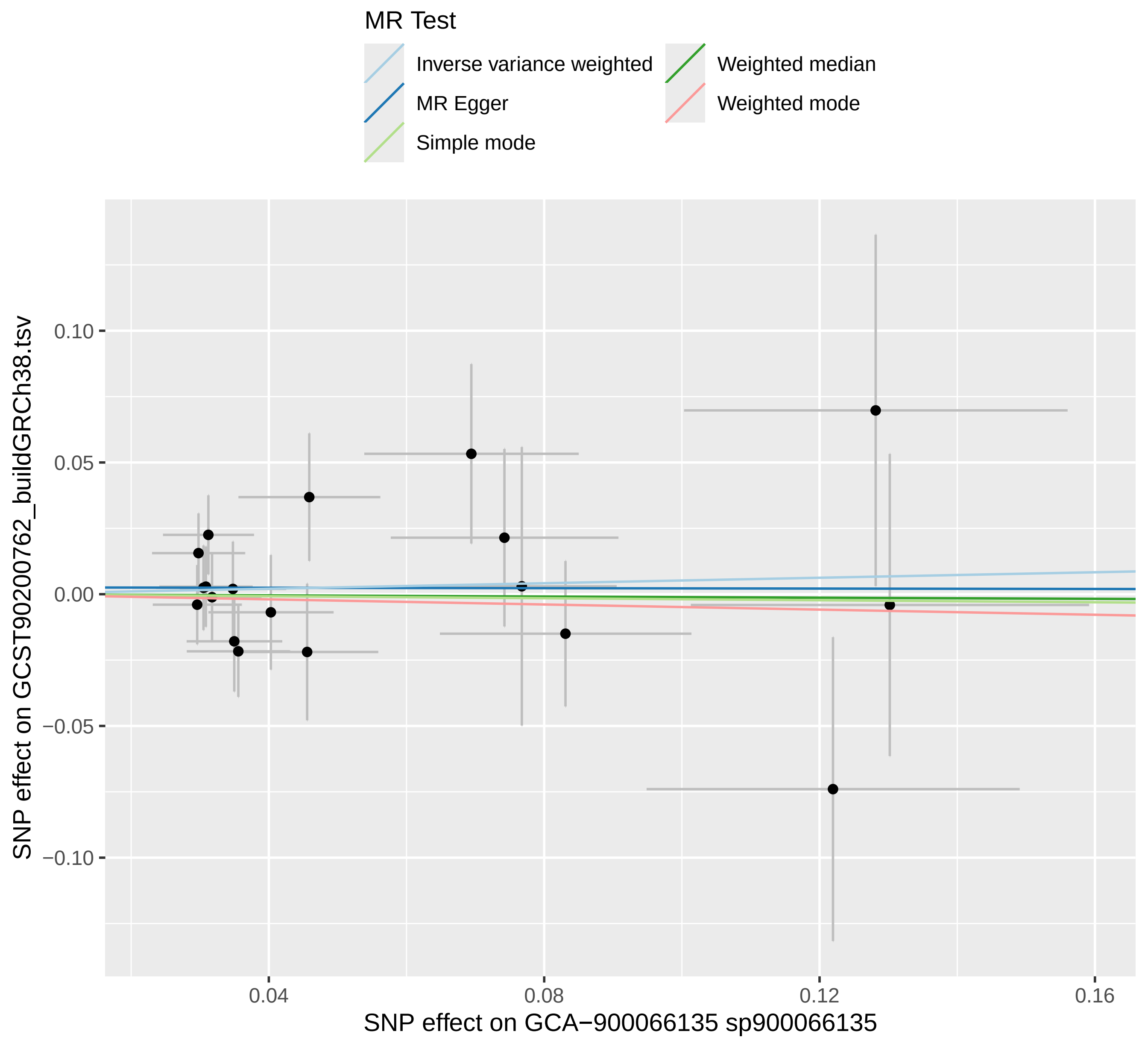

MR Test
Inverse variance weighted
MR Egger
Simple mode
Weighted median
Weighted mode
SNP effect on GCST90200762_buildGRCh38.tsv
0.10
0.05
0.00
−0.05
−0.10
0.04
0.08
0.12
0.16
SNP effect on GCA−900066135 sp900066135

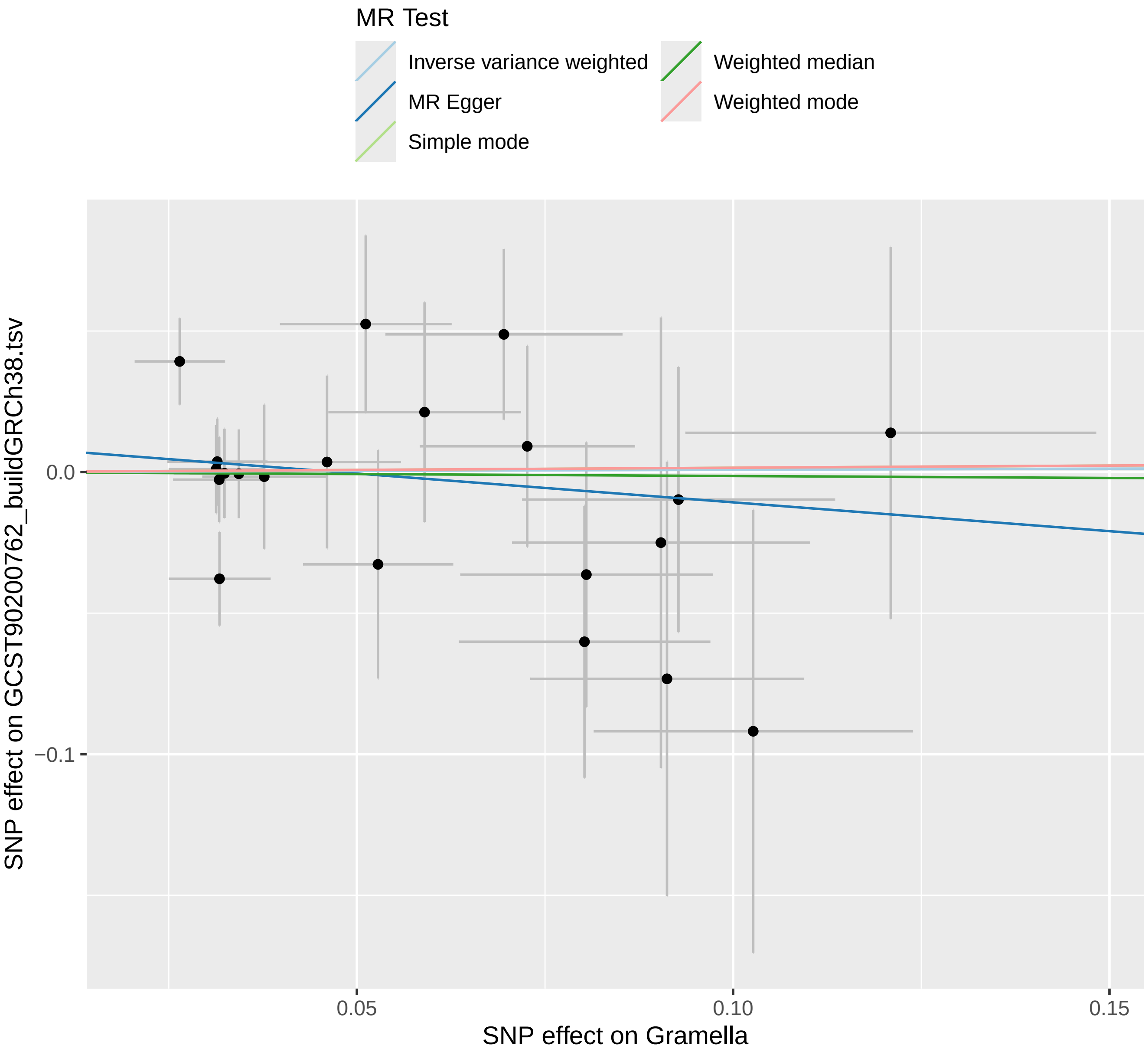
MR Test
Inverse variance weighted
MR Egger
Simple mode
Weighted median
Weighted mode
0.0
−0.1
0.05
0.10
0.15
SNP effect on GCST90200762_buildGRCh38.tsv
SNP effect on Gramella

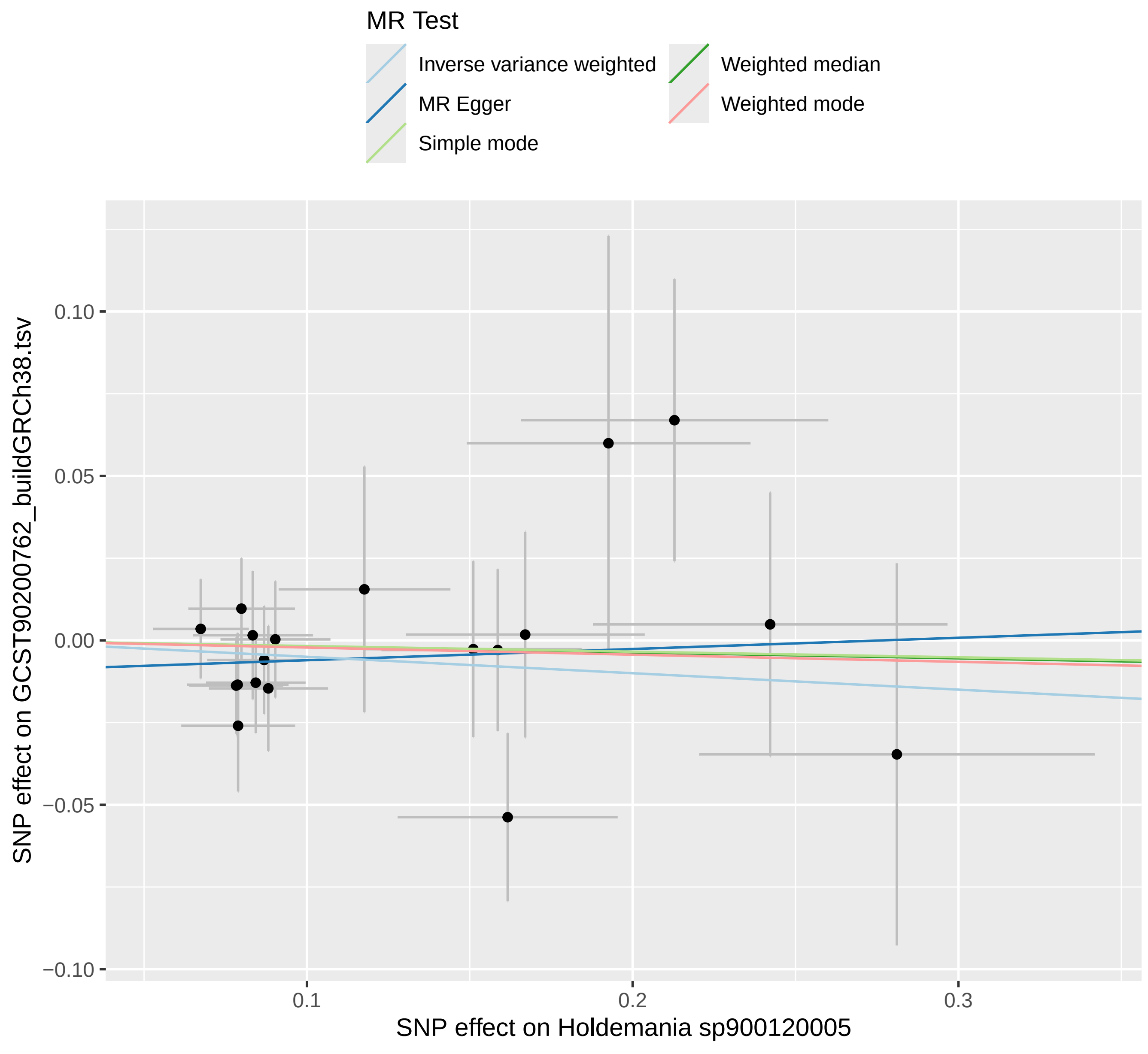
MR Test
Inverse variance weighted
MR Egger
Simple mode
Weighted median
Weighted mode
0.10
0.05
0.00
−0.05
−0.10
0.1
0.2
0.3
SNP effect on GCST90200762_buildGRCh38.tsv
SNP effect on Holdemania sp900120005

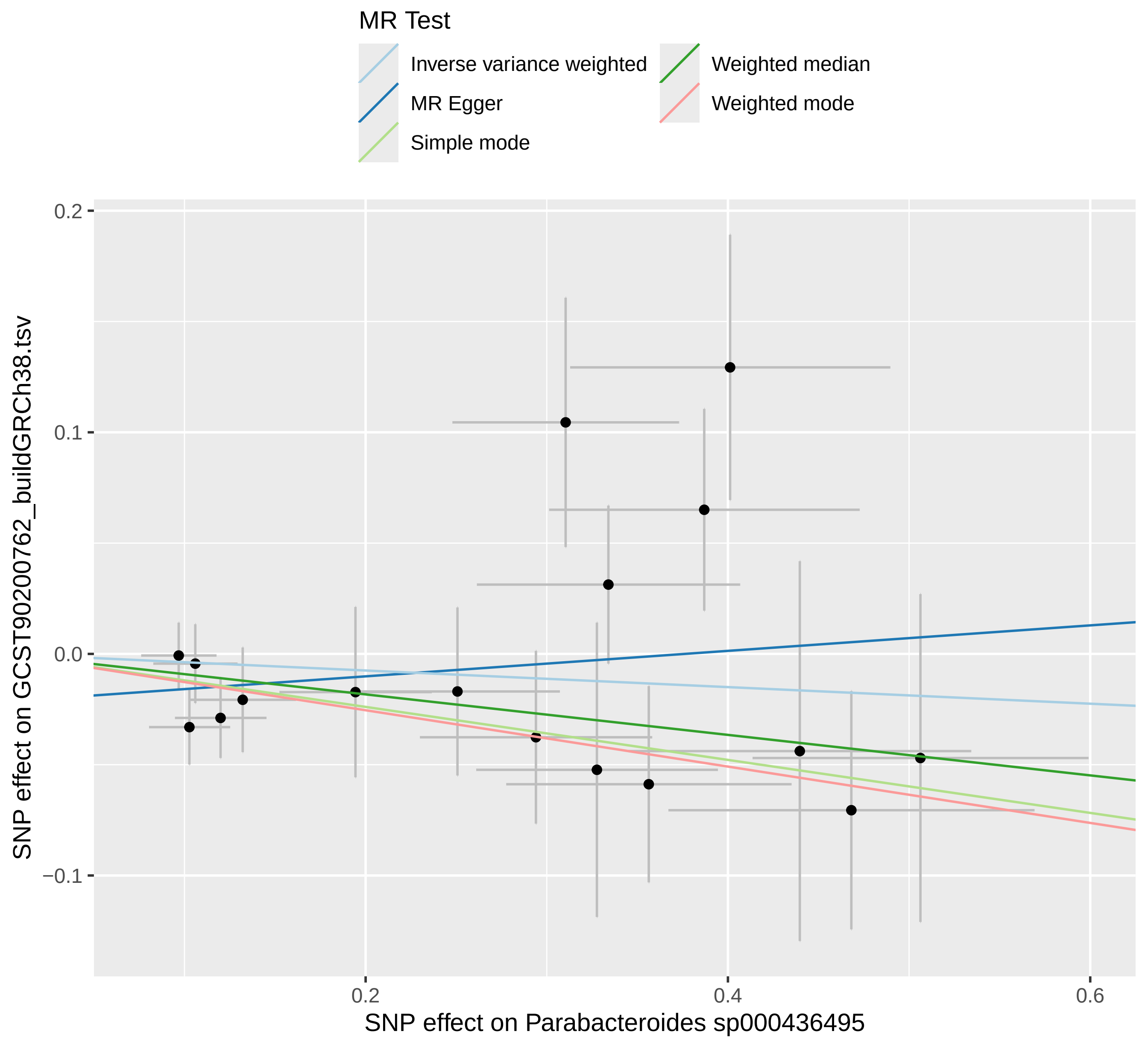
MR Test
Inverse variance weighted
MR Egger
Simple mode
Weighted median
Weighted mode
0.2
0.1
0.0
−0.1
0.2
0.4
0.6
SNP effect on GCST90200762_buildGRCh38.tsv
SNP effect on Parabacteroides sp000436495

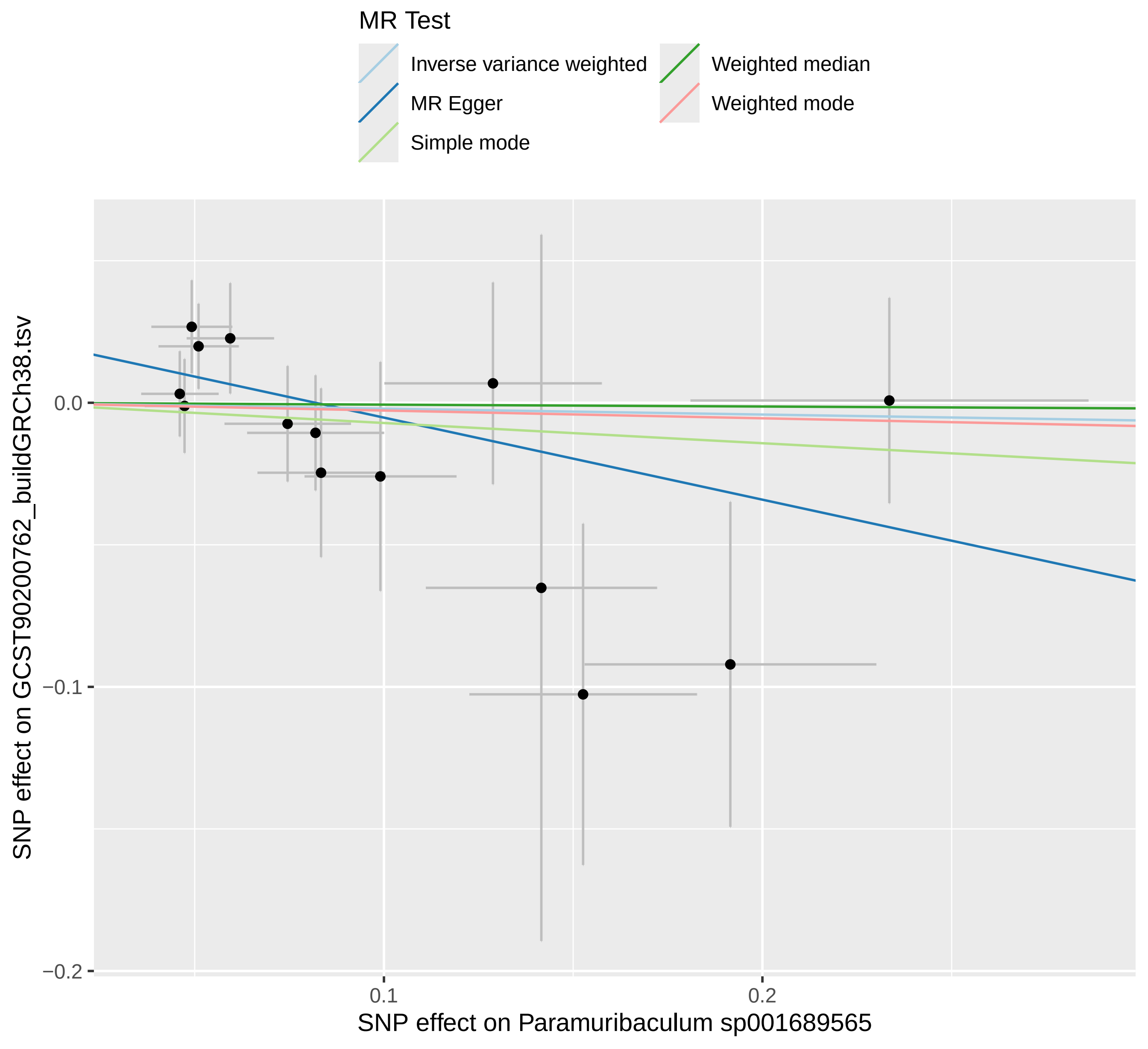

MR Test
Inverse variance weighted
MR Egger
Simple mode
Weighted median
Weighted mode
0.0
−0.1
−0.2
0.1
0.2
SNP effect on GCST90200762_buildGRCh38.tsv
SNP effect on Paramuribaculum sp001689565

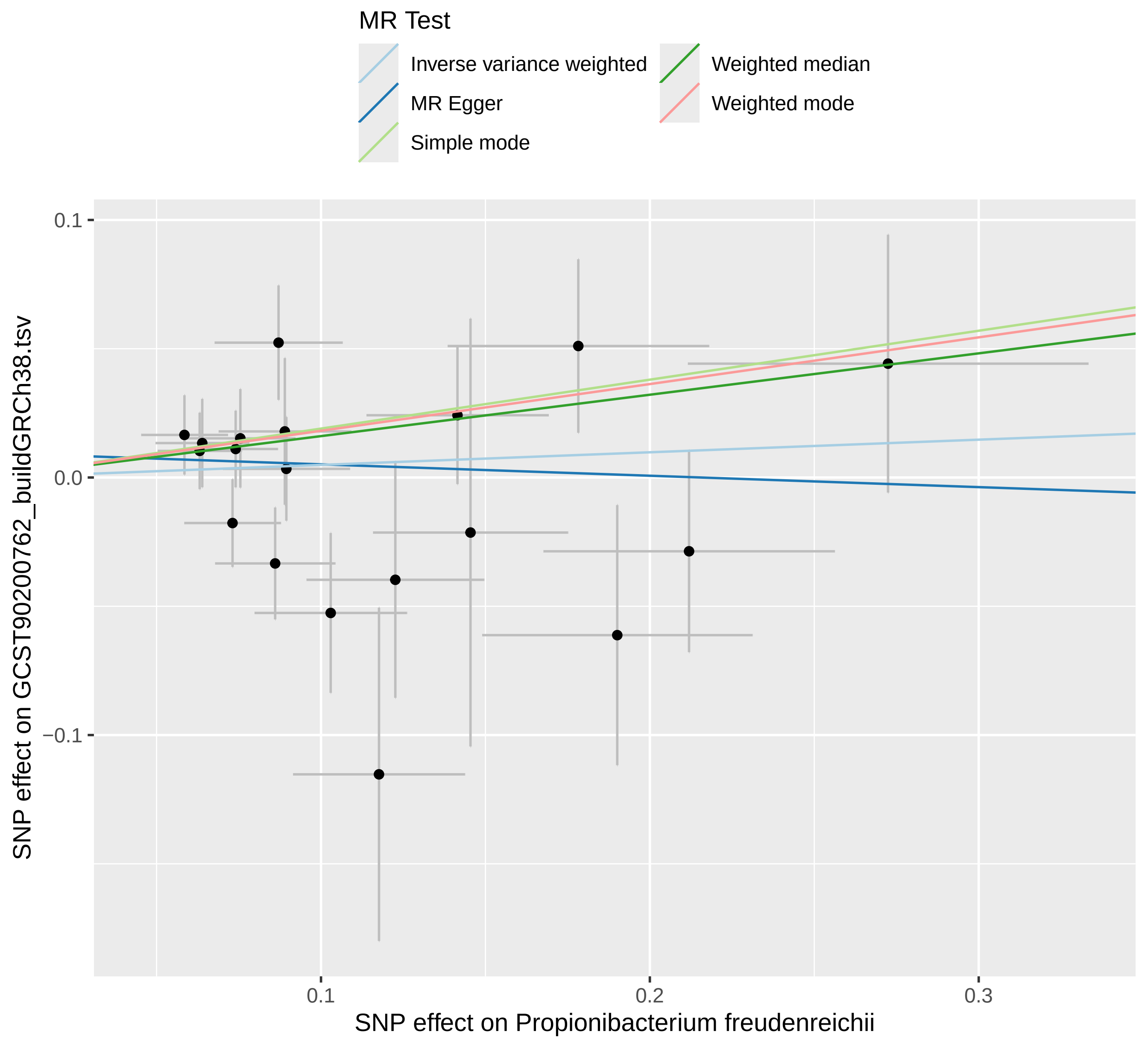

MR Test
Inverse variance weighted
MR Egger
Simple mode
Weighted median
Weighted mode
0.1
0.0
−0.1
0.1
0.2
0.3
SNP effect on GCST90200762_buildGRCh38.tsv
SNP effect on Propionibacterium freudenreichii

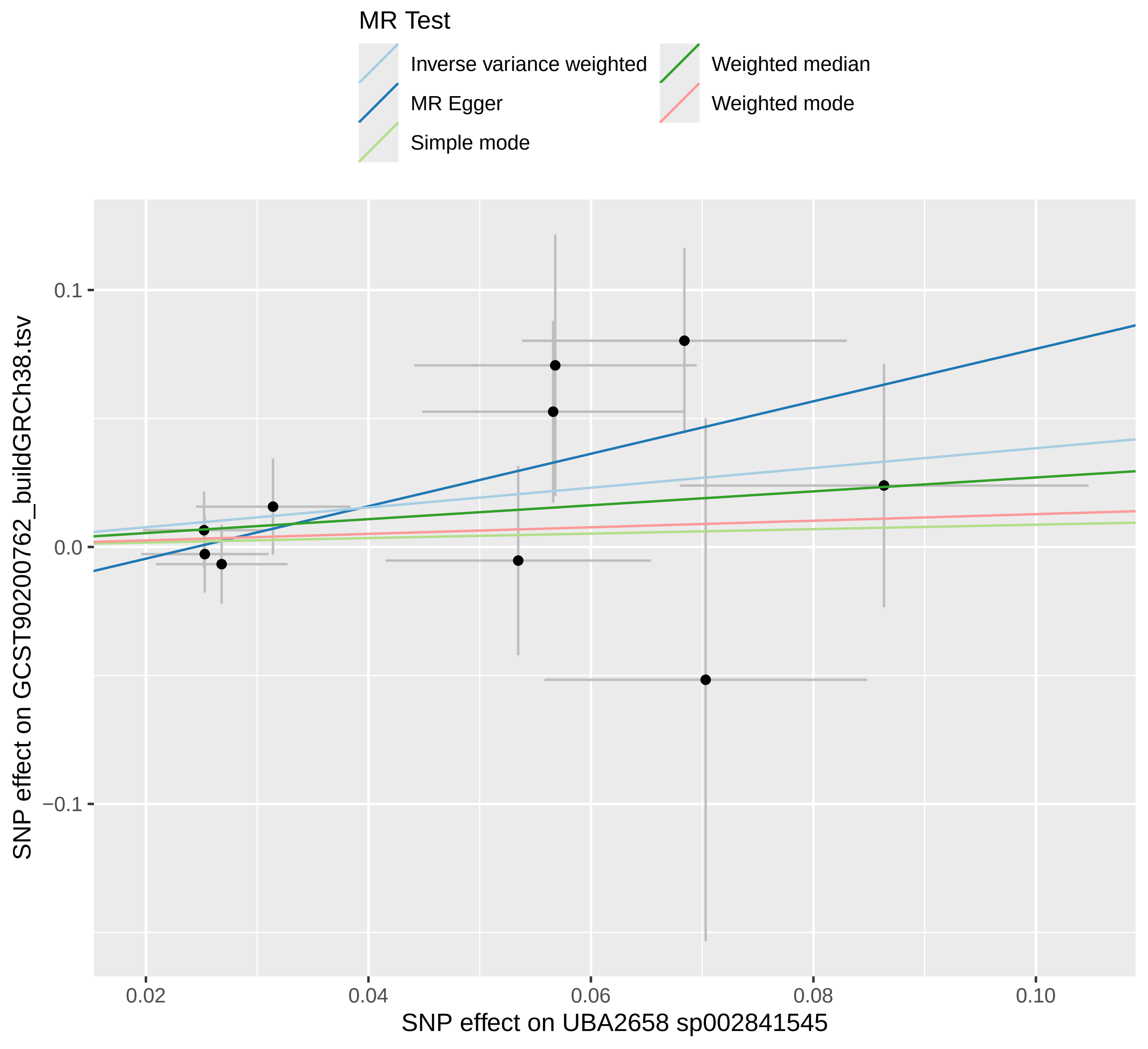
MR Test
Inverse variance weighted
MR Egger
Simple mode
Weighted median
Weighted mode
0.1
0.0
−0.1
0.02
0.04
0.06
0.08
0.10
SNP effect on GCST90200762_buildGRCh38.tsv
SNP effect on UBA2658 sp002841545

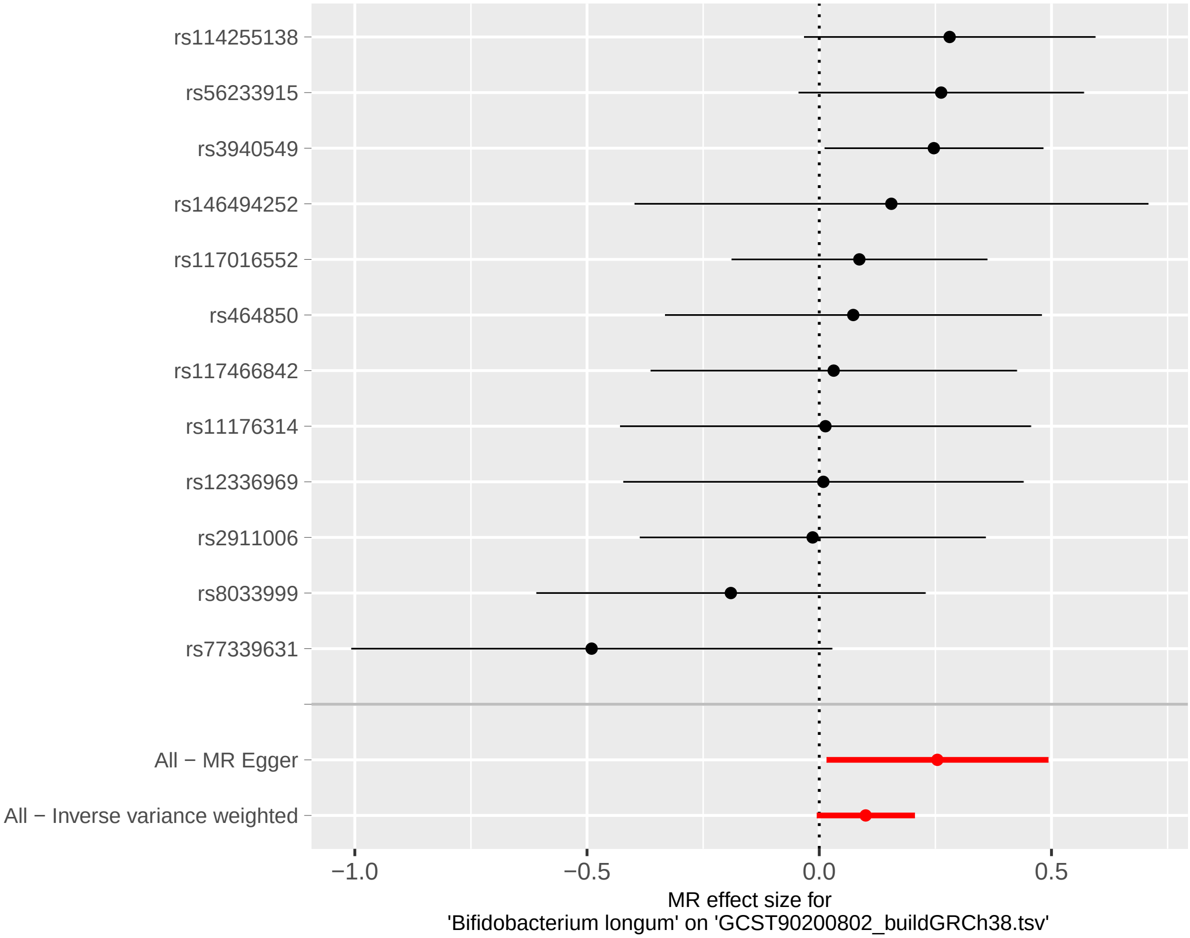
rs114255138
rs56233915
rs3940549
rs146494252
rs117016552
rs464850
rs117466842
rs11176314
rs12336969
rs2911006
rs8033999
rs77339631
All − MR Egger
All − Inverse variance weighted
−1.0
−0.5
0.0
0.5
MR effect size for
'Bifidobacterium longum' on 'GCST90200802_buildGRCh38.tsv'

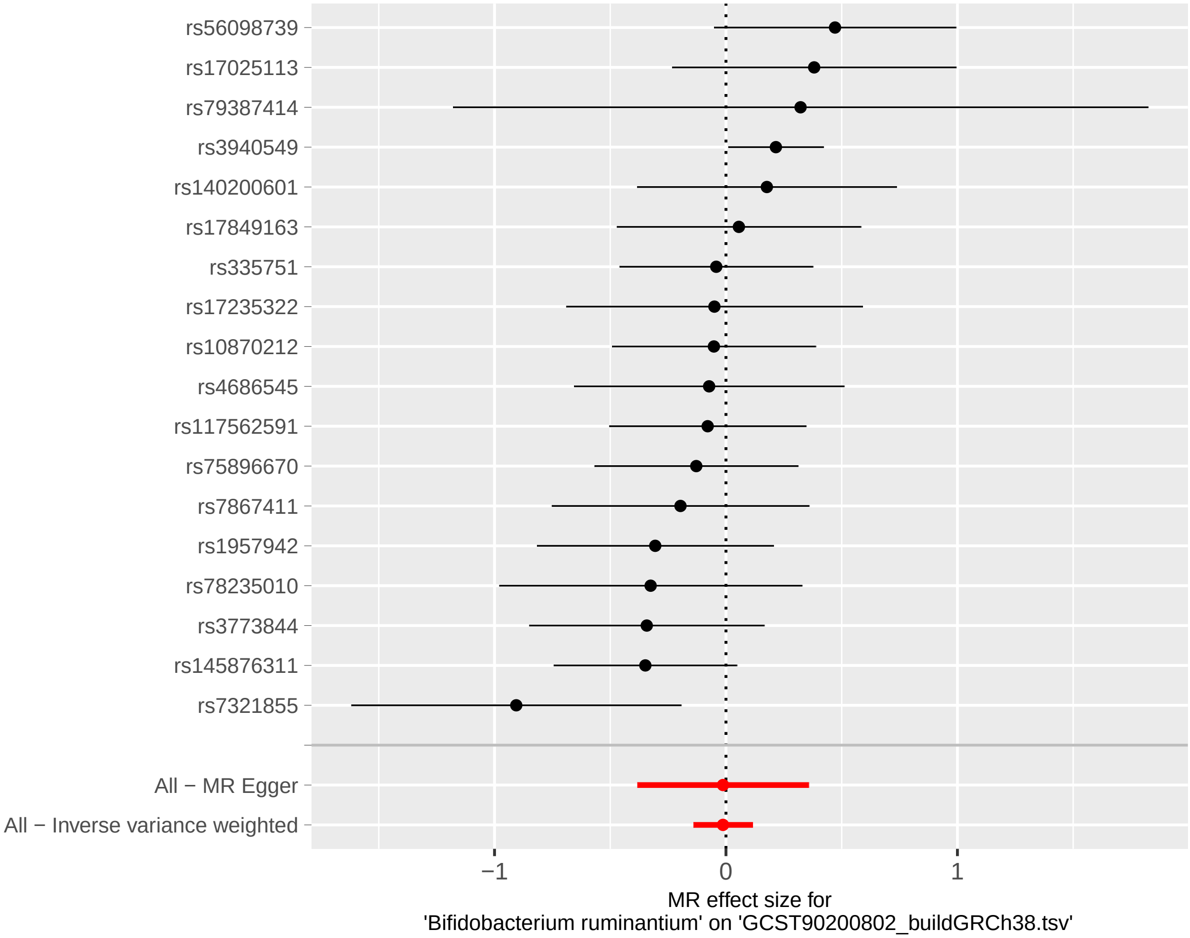
rs56098739
rs17025113
rs79387414
rs3940549
rs140200601
rs17849163
rs335751
rs17235322
rs10870212
rs4686545
rs117562591
rs75896670
rs7867411
rs1957942
rs78235010
rs3773844
rs145876311
rs7321855
All − MR Egger
All − Inverse variance weighted
−1
0
1
MR effect size for
'Bifidobacterium ruminantium' on 'GCST90200802_buildGRCh38.tsv'

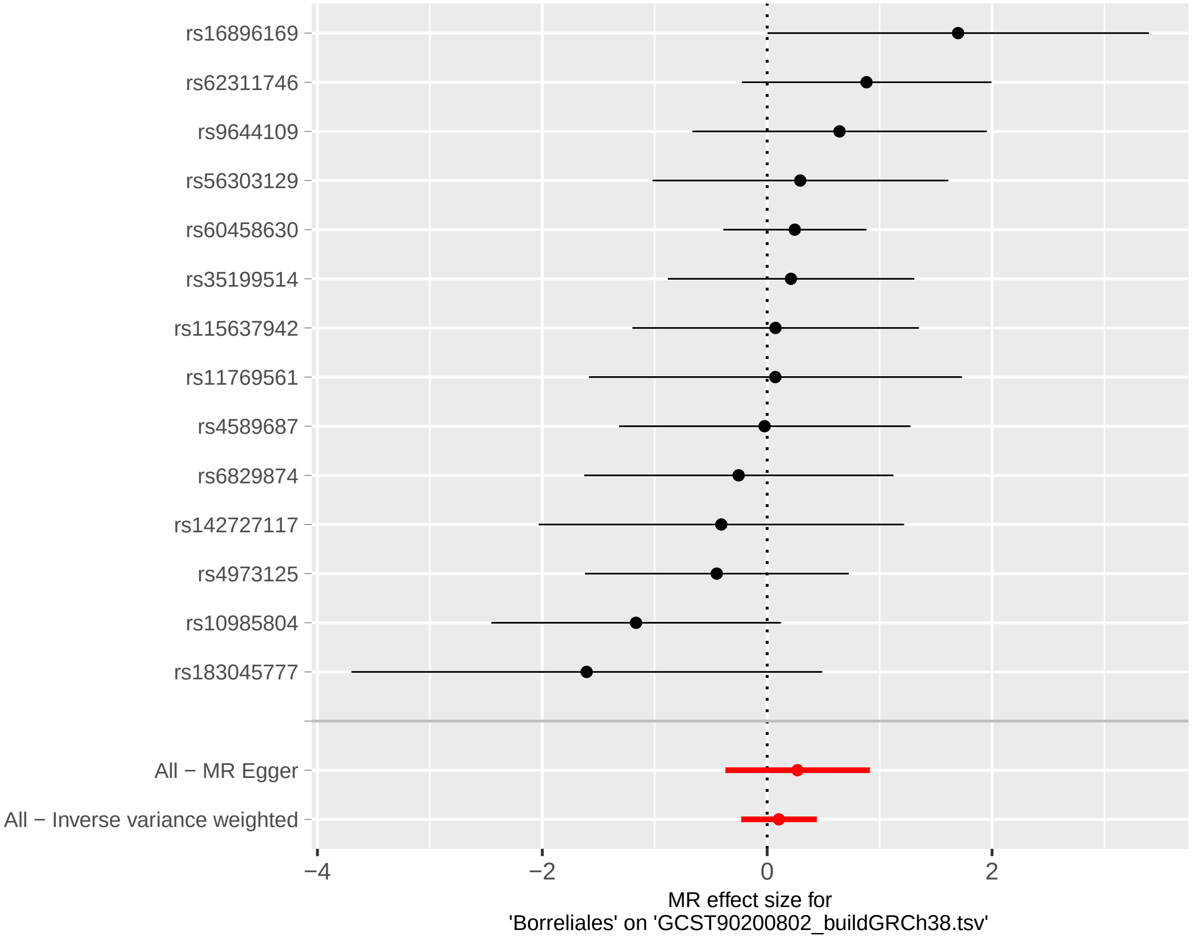

rs16896169
rs62311746
rs9644109
rs56303129
rs60458630
rs35199514
rs115637942
rs11769561
rs4589687
rs6829874
rs142727117
rs4973125
rs10985804
rs183045777
All − MR Egger
All − Inverse variance weighted
−4
−2
0
2
MR effect size for
'Borreliales' on 'GCST90200802_buildGRCh38.tsv'

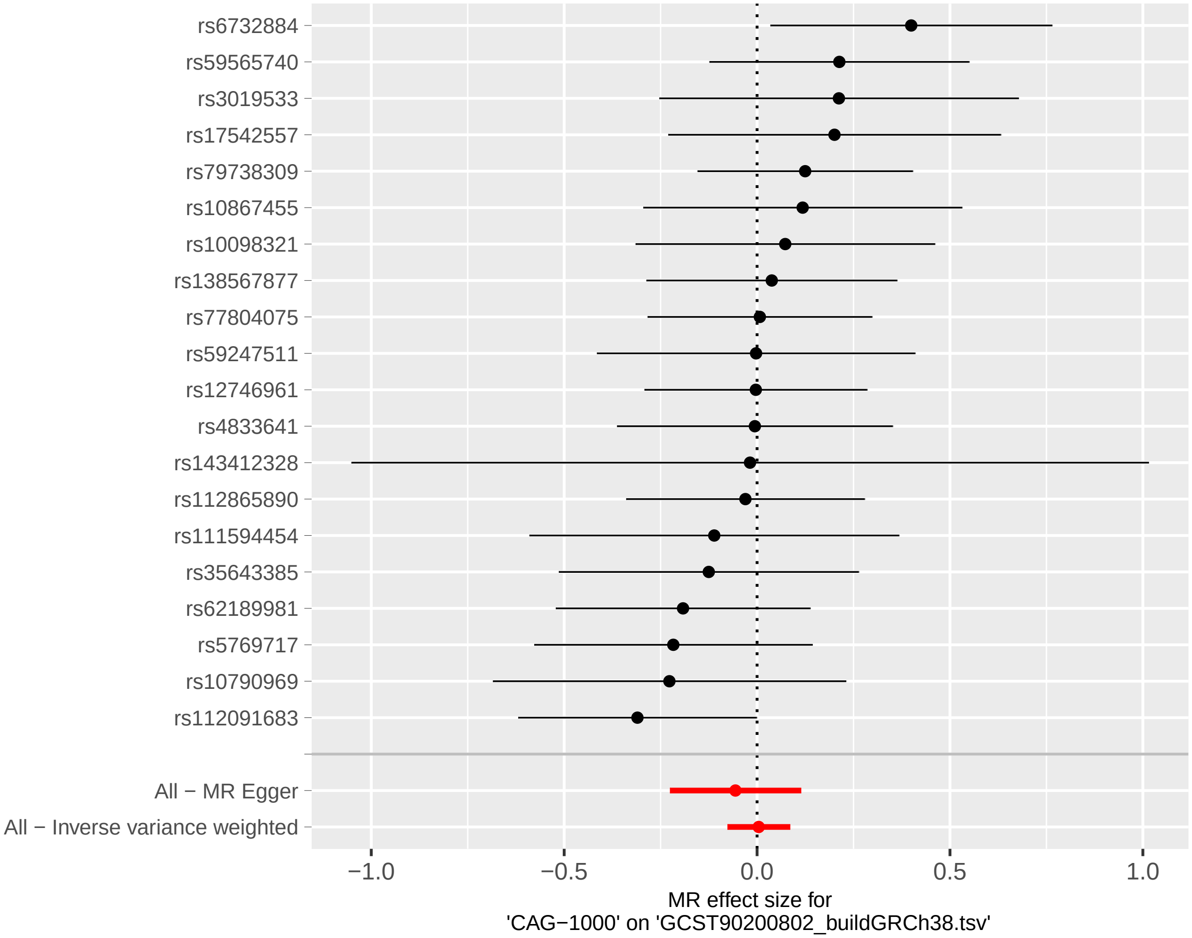
rs6732884
rs59565740
rs3019533
rs17542557
rs79738309
rs10867455
rs10098321
rs138567877
rs77804075
rs59247511
rs12746961
rs4833641
rs143412328
rs112865890
rs111594454
rs35643385
rs62189981
rs5769717
rs10790969
rs112091683
All − MR Egger
All − Inverse variance weighted
−1.0
−0.5
0.0
0.5
1.0
MR effect size for
'CAG−1000' on 'GCST90200802_buildGRCh38.tsv'

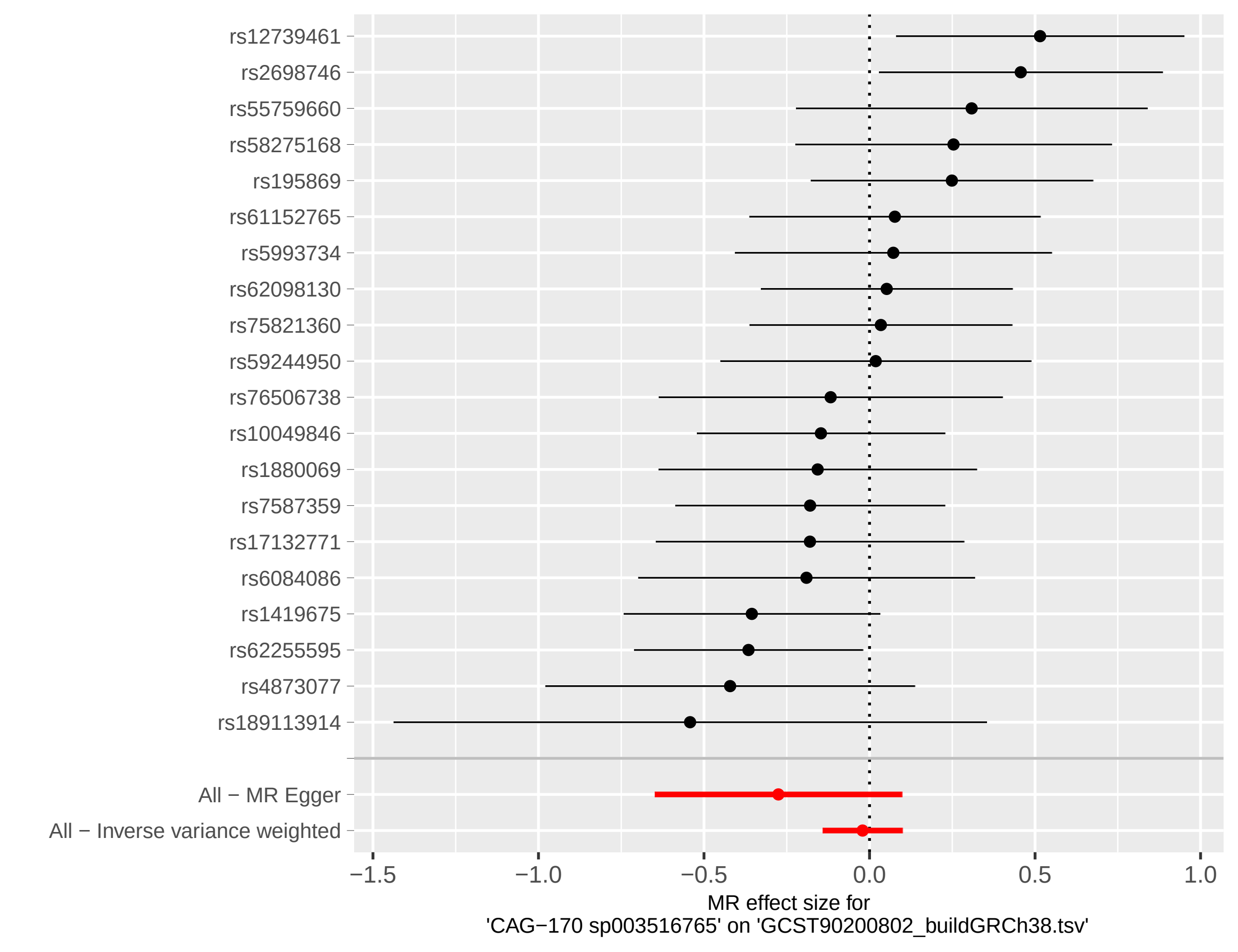
rs12739461
rs2698746
rs55759660
rs58275168
rs195869
rs61152765
rs5993734
rs62098130
rs75821360
rs59244950
rs76506738
rs10049846
rs1880069
rs7587359
rs17132771
rs6084086
rs1419675
rs62255595
rs4873077
rs189113914
All − MR Egger
All − Inverse variance weighted
−1.5
−1.0
−0.5
0.0
0.5
1.0
MR effect size for
'CAG−170 sp003516765' on 'GCST90200802_buildGRCh38.tsv'

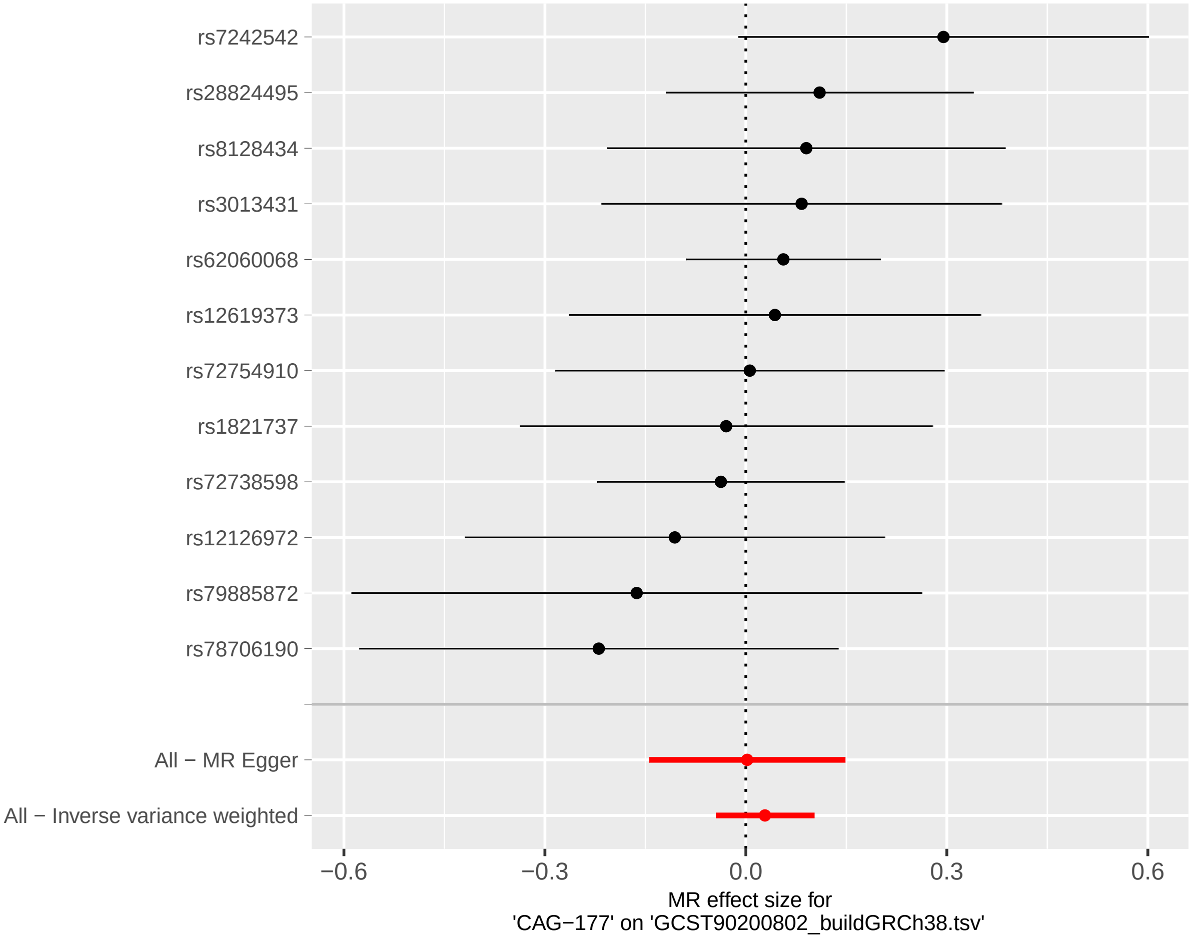

rs7242542
rs28824495
rs8128434
rs3013431
rs62060068
rs12619373
rs72754910
rs1821737
rs72738598
rs12126972
rs79885872
rs78706190
All − MR Egger
All − Inverse variance weighted
−0.6
−0.3
0.0
0.3
0.6
MR effect size for
'CAG−177' on 'GCST90200802_buildGRCh38.tsv'

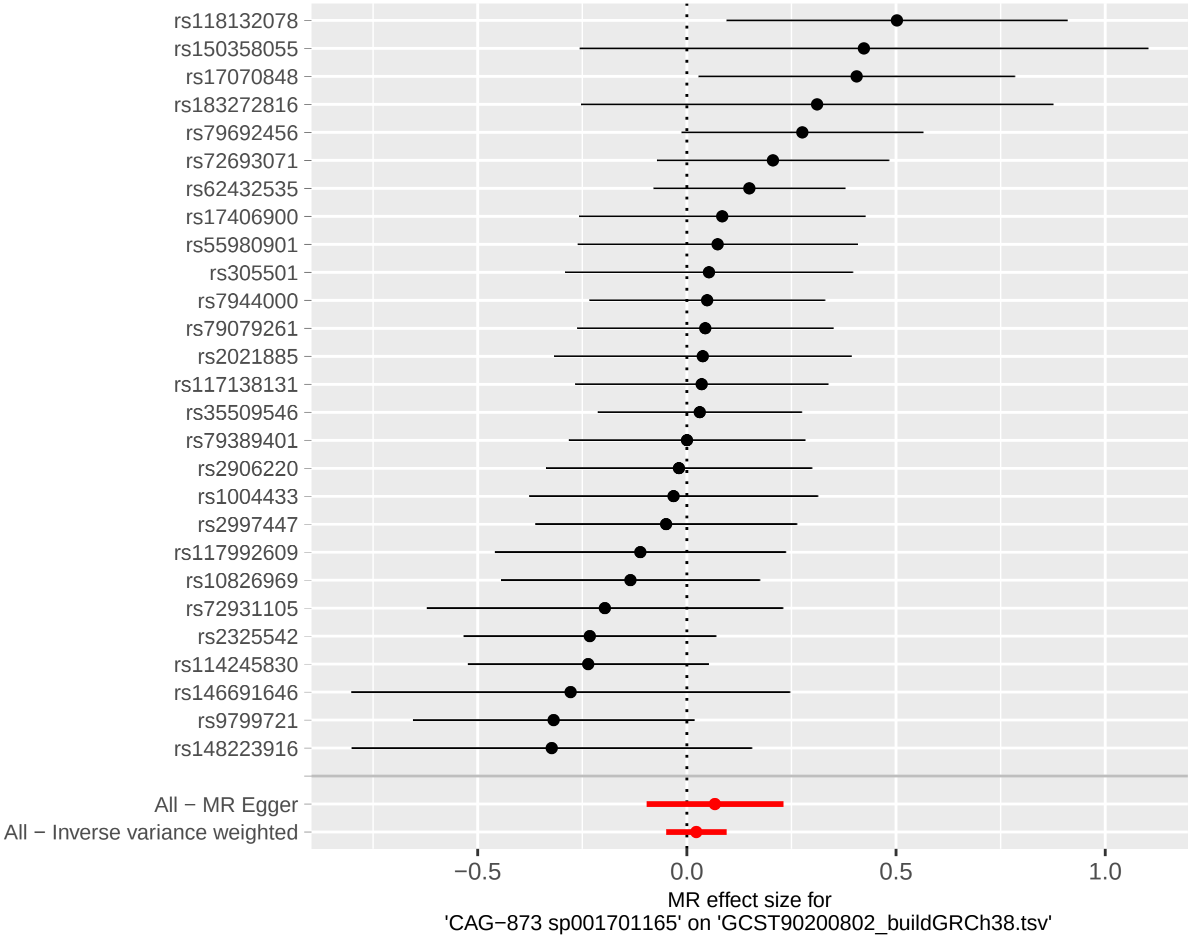

rs118132078
rs150358055
rs17070848
rs183272816
rs79692456
rs72693071
rs62432535
rs17406900
rs55980901
rs305501
rs7944000
rs79079261
rs2021885
rs117138131
rs35509546
rs79389401
rs2906220
rs1004433
rs2997447
rs117992609
rs10826969
rs72931105
rs2325542
rs114245830
rs146691646
rs9799721
rs148223916
All − MR Egger
All − Inverse variance weighted
−0.5
0.0
0.5
1.0
MR effect size for
'CAG−873 sp001701165' on 'GCST90200802_buildGRCh38.tsv'

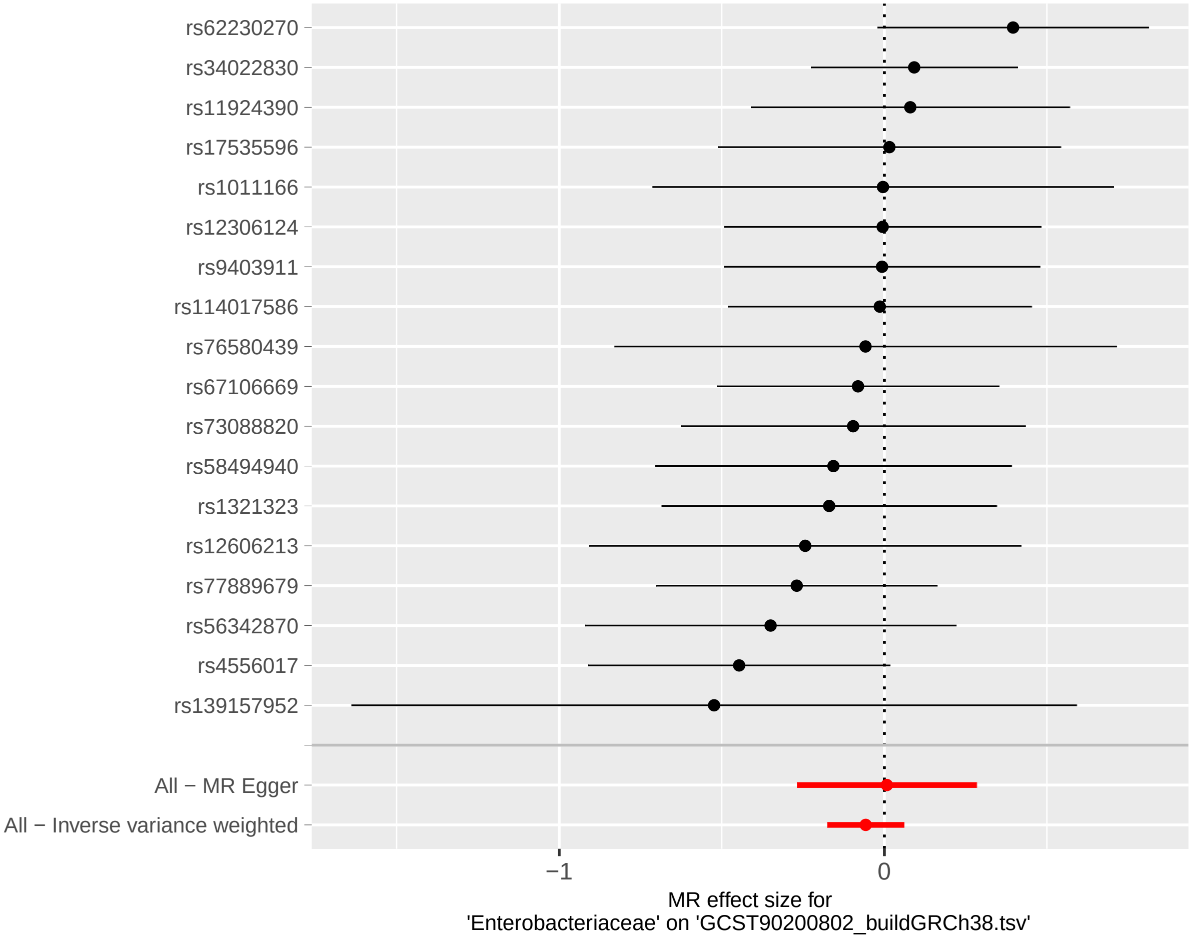
rs62230270
rs34022830
rs11924390
rs17535596
rs1011166
rs12306124
rs9403911
rs114017586
rs76580439
rs67106669
rs73088820
rs58494940
rs1321323
rs12606213
rs77889679
rs56342870
rs4556017
rs139157952
All − MR Egger
All − Inverse variance weighted
−1
0
MR effect size for
'Enterobacteriaceae' on 'GCST90200802_buildGRCh38.tsv'

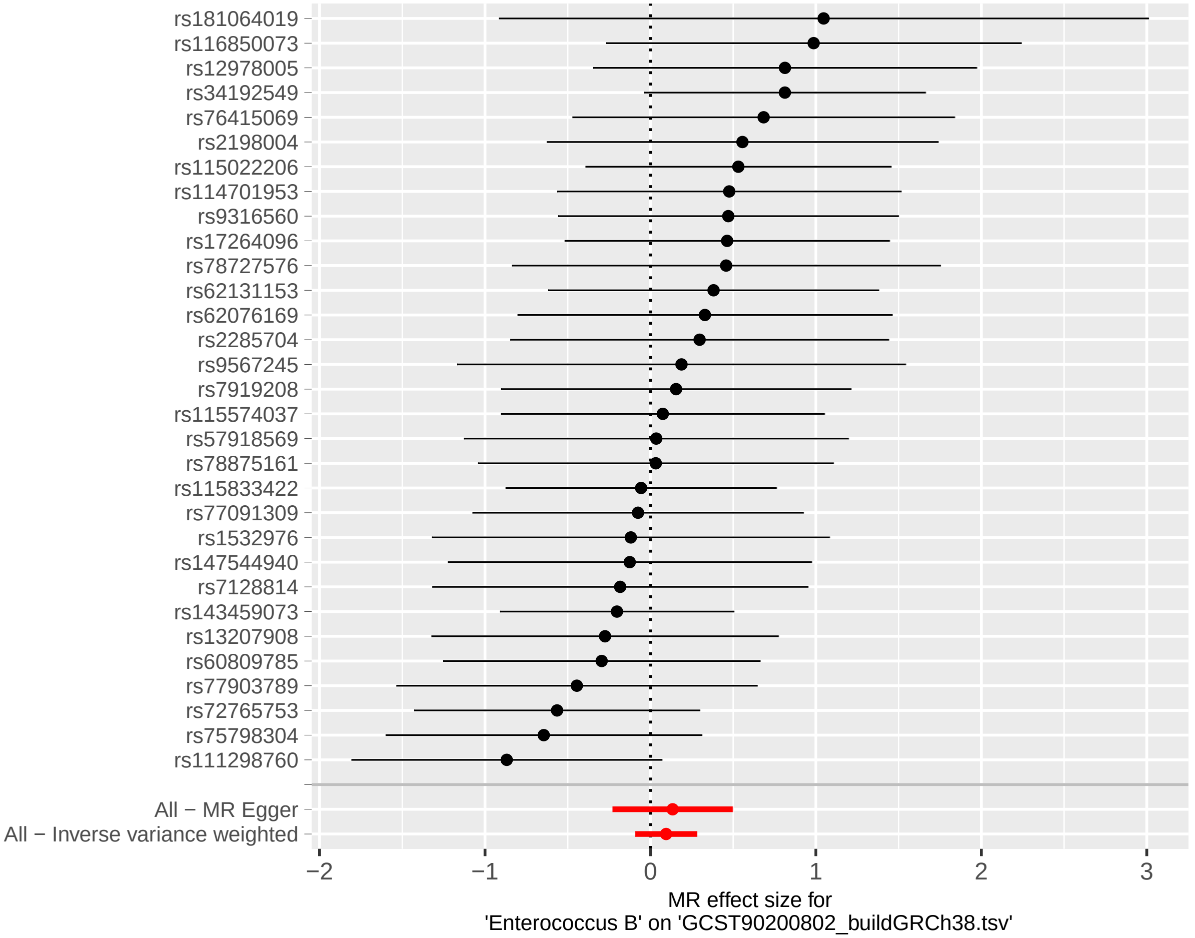
rs181064019
rs116850073
rs12978005
rs34192549
rs76415069
rs2198004
rs115022206
rs114701953
rs9316560
rs17264096
rs78727576
rs62131153
rs62076169
rs2285704
rs9567245
rs7919208
rs115574037
rs57918569
rs78875161
rs115833422
rs77091309
rs1532976
rs147544940
rs7128814
rs143459073
rs13207908
rs60809785
rs77903789
rs72765753
rs75798304
rs111298760
All − MR Egger
All − Inverse variance weighted
−2
−1
0
1
2
3
MR effect size for
'Enterococcus B' on 'GCST90200802_buildGRCh38.tsv'

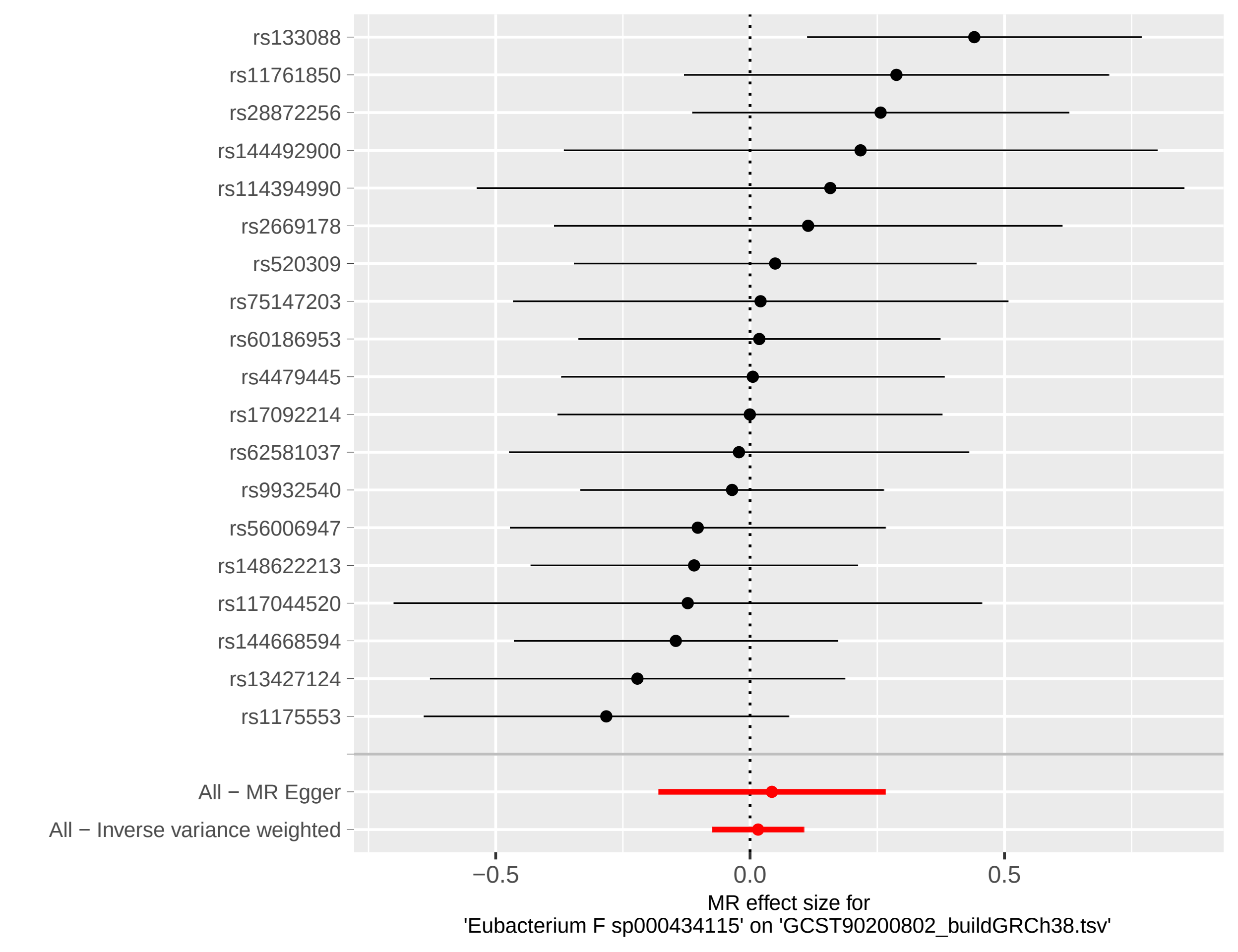
rs133088
rs11761850
rs28872256
rs144492900
rs114394990
rs2669178
rs520309
rs75147203
rs60186953
rs4479445
rs17092214
rs62581037
rs9932540
rs56006947
rs148622213
rs117044520
rs144668594
rs13427124
rs1175553
All − MR Egger
All − Inverse variance weighted
−0.5
0.0
0.5
MR effect size for
'Eubacterium F sp000434115' on 'GCST90200802_buildGRCh38.tsv'

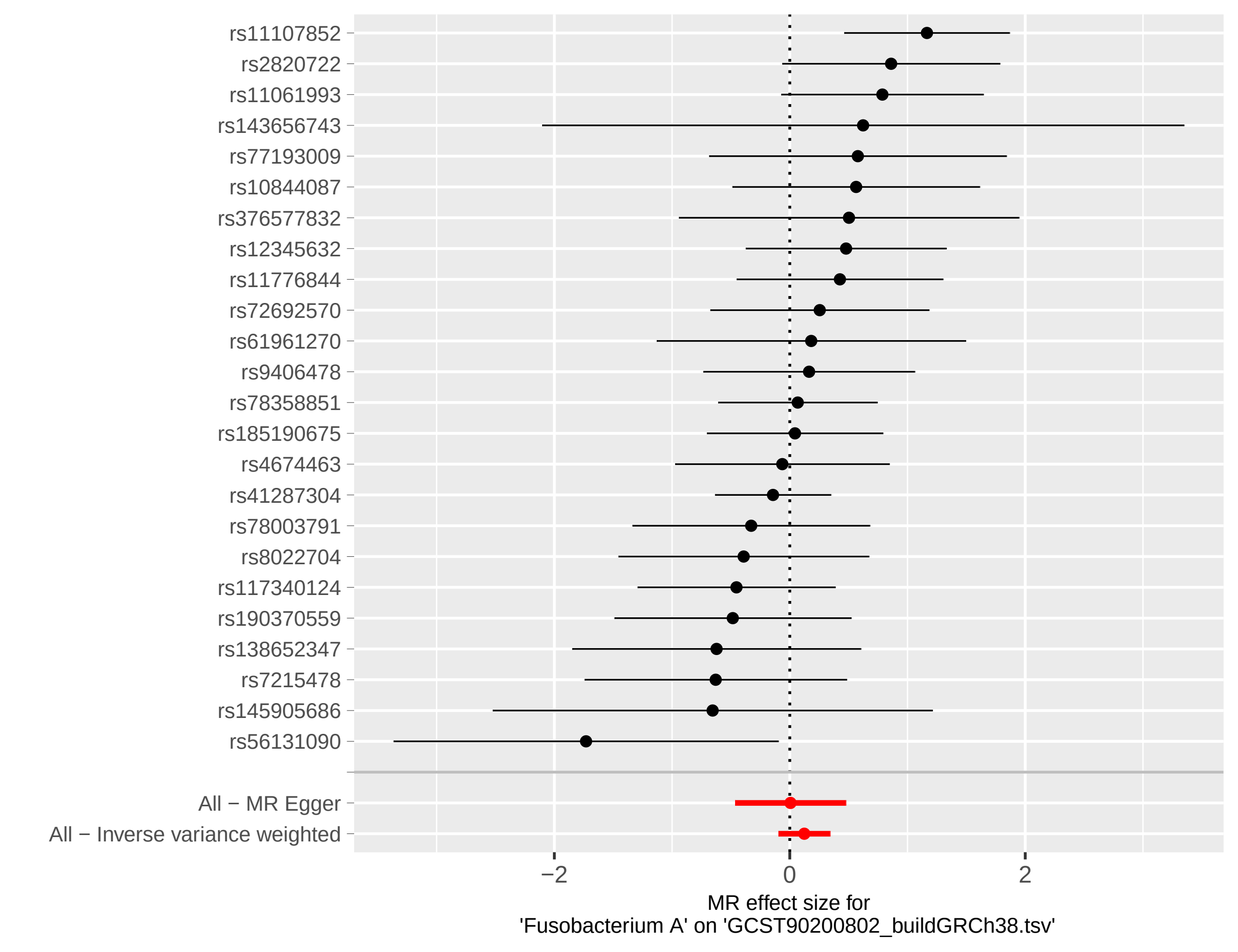
rs11107852
rs2820722
rs11061993
rs143656743
rs77193009
rs10844087
rs376577832
rs12345632
rs11776844
rs72692570
rs61961270
rs9406478
rs78358851
rs185190675
rs4674463
rs41287304
rs78003791
rs8022704
rs117340124
rs190370559
rs138652347
rs7215478
rs145905686
rs56131090
All − MR Egger
All − Inverse variance weighted
−2
0
2
MR effect size for
'Fusobacterium A' on 'GCST90200802_buildGRCh38.tsv'

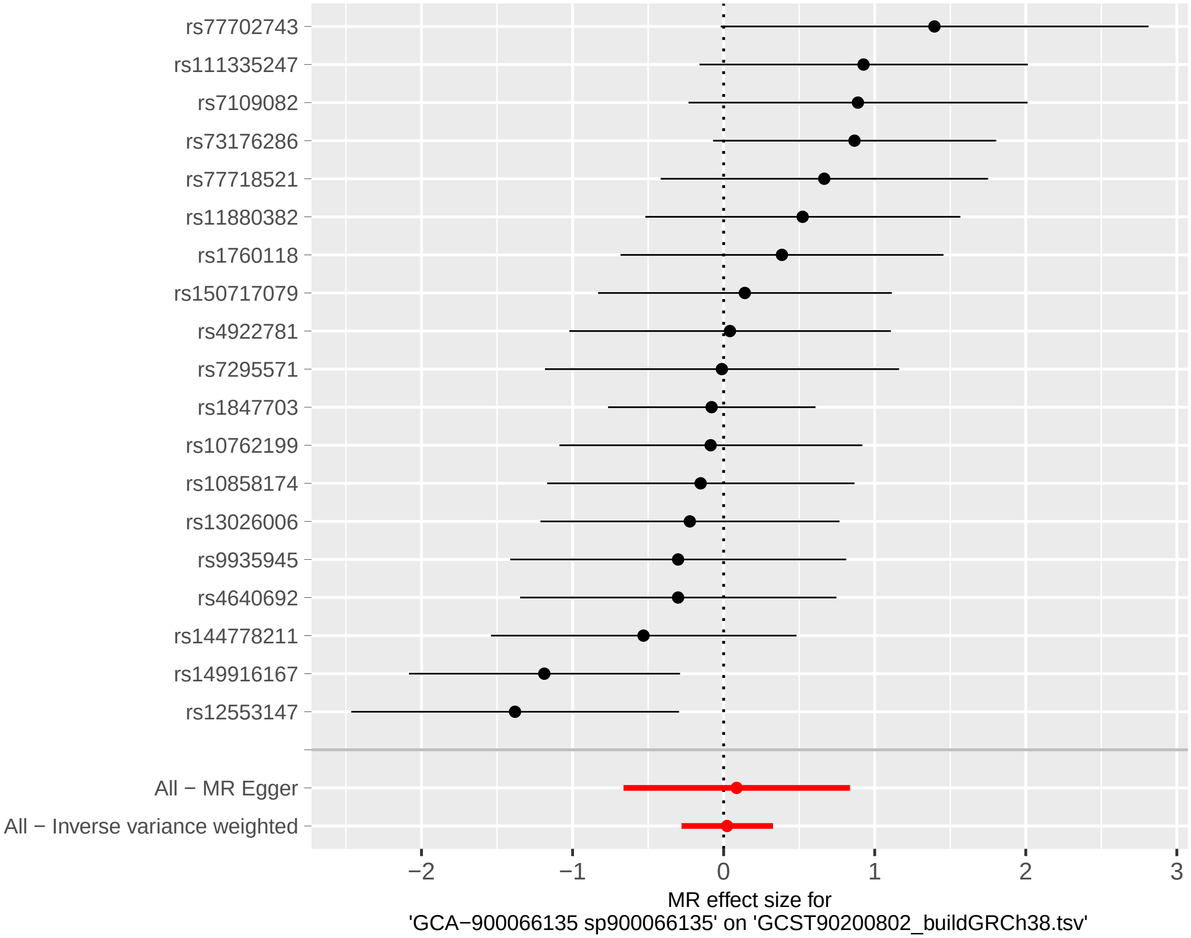
rs77702743
rs111335247
rs7109082
rs73176286
rs77718521
rs11880382
rs1760118
rs150717079
rs4922781
rs7295571
rs1847703
rs10762199
rs10858174
rs13026006
rs9935945
rs4640692
rs144778211
rs149916167
rs12553147
All − MR Egger
All − Inverse variance weighted
−2
−1
0
1
2
3
MR effect size for
'GCA−900066135 sp900066135' on 'GCST90200802_buildGRCh38.tsv'

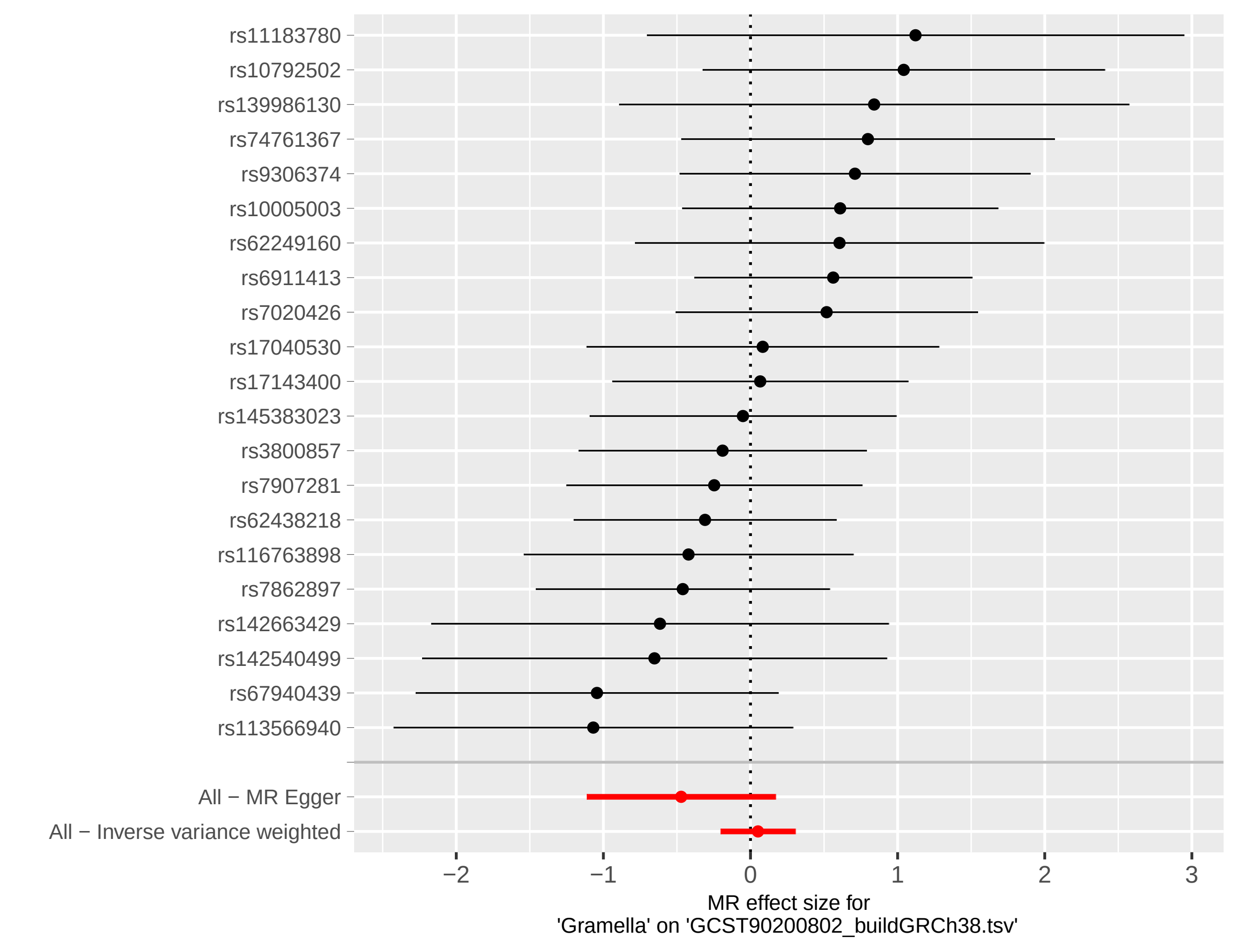

rs11183780
rs10792502
rs139986130
rs74761367
rs9306374
rs10005003
rs62249160
rs6911413
rs7020426
rs17040530
rs17143400
rs145383023
rs3800857
rs7907281
rs62438218
rs116763898
rs7862897
rs142663429
rs142540499
rs67940439
rs113566940
All − MR Egger
All − Inverse variance weighted
−2
−1
0
1
2
3
MR effect size for
'Gramella' on 'GCST90200802_buildGRCh38.tsv'

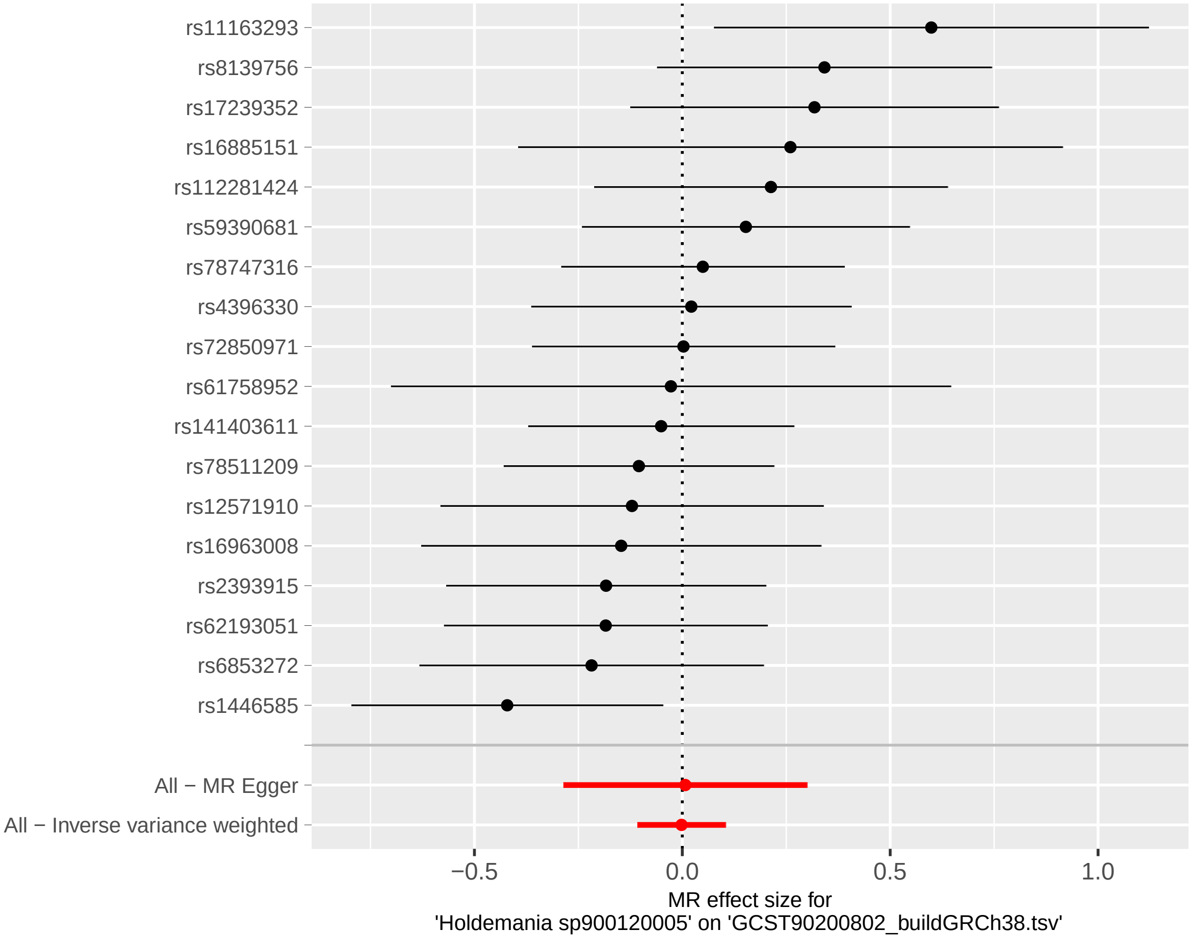
rs11163293
rs8139756
rs17239352
rs16885151
rs112281424
rs59390681
rs78747316
rs4396330
rs72850971
rs61758952
rs141403611
rs78511209
rs12571910
rs16963008
rs2393915
rs62193051
rs6853272
rs1446585
All − MR Egger
All − Inverse variance weighted
−0.5
0.0
0.5
1.0
MR effect size for
'Holdemania sp900120005' on 'GCST90200802_buildGRCh38.tsv'

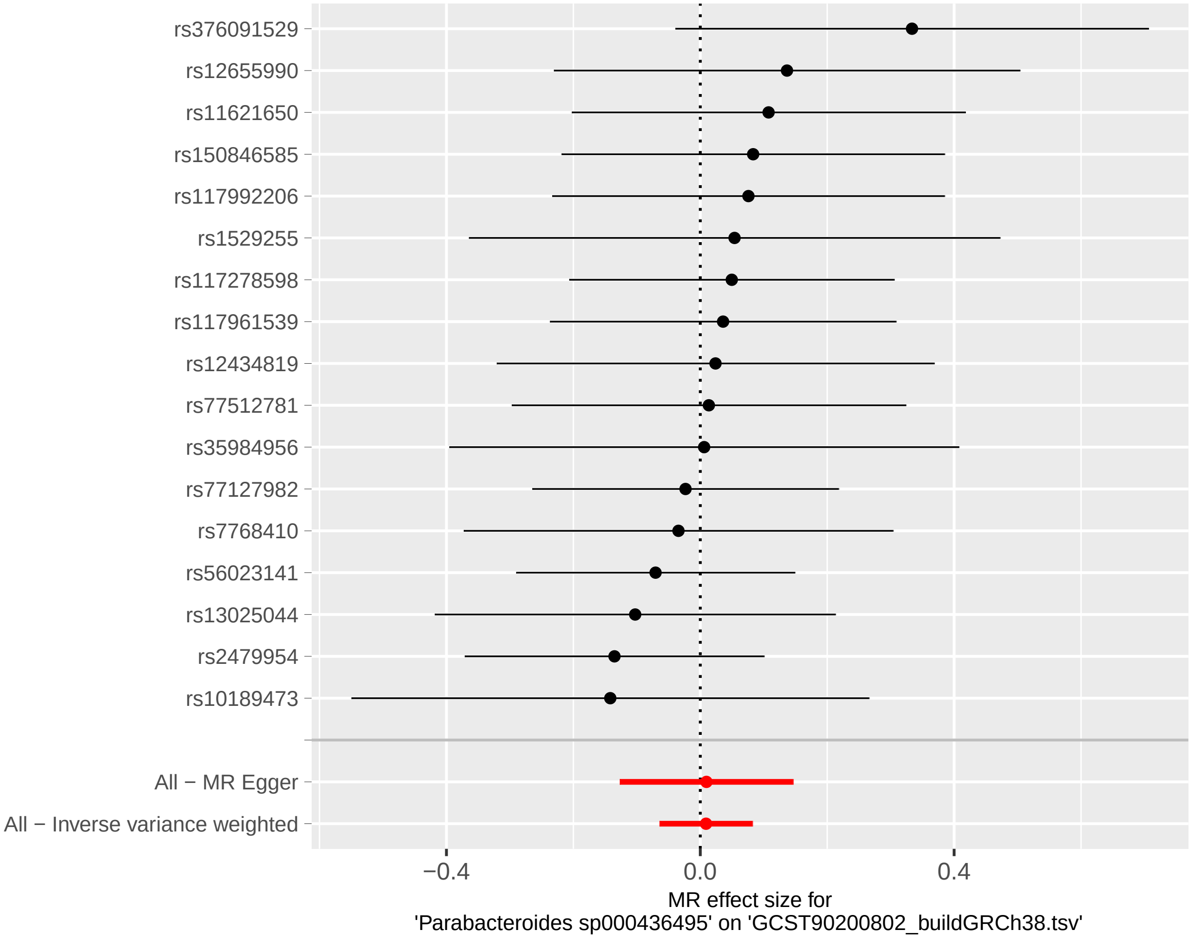
rs376091529
rs12655990
rs11621650
rs150846585
rs117992206
rs1529255
rs117278598
rs117961539
rs12434819
rs77512781
rs35984956
rs77127982
rs7768410
rs56023141
rs13025044
rs2479954
rs10189473
All − MR Egger
All − Inverse variance weighted
−0.4
0.0
0.4
MR effect size for
'Parabacteroides sp000436495' on 'GCST90200802_buildGRCh38.tsv'

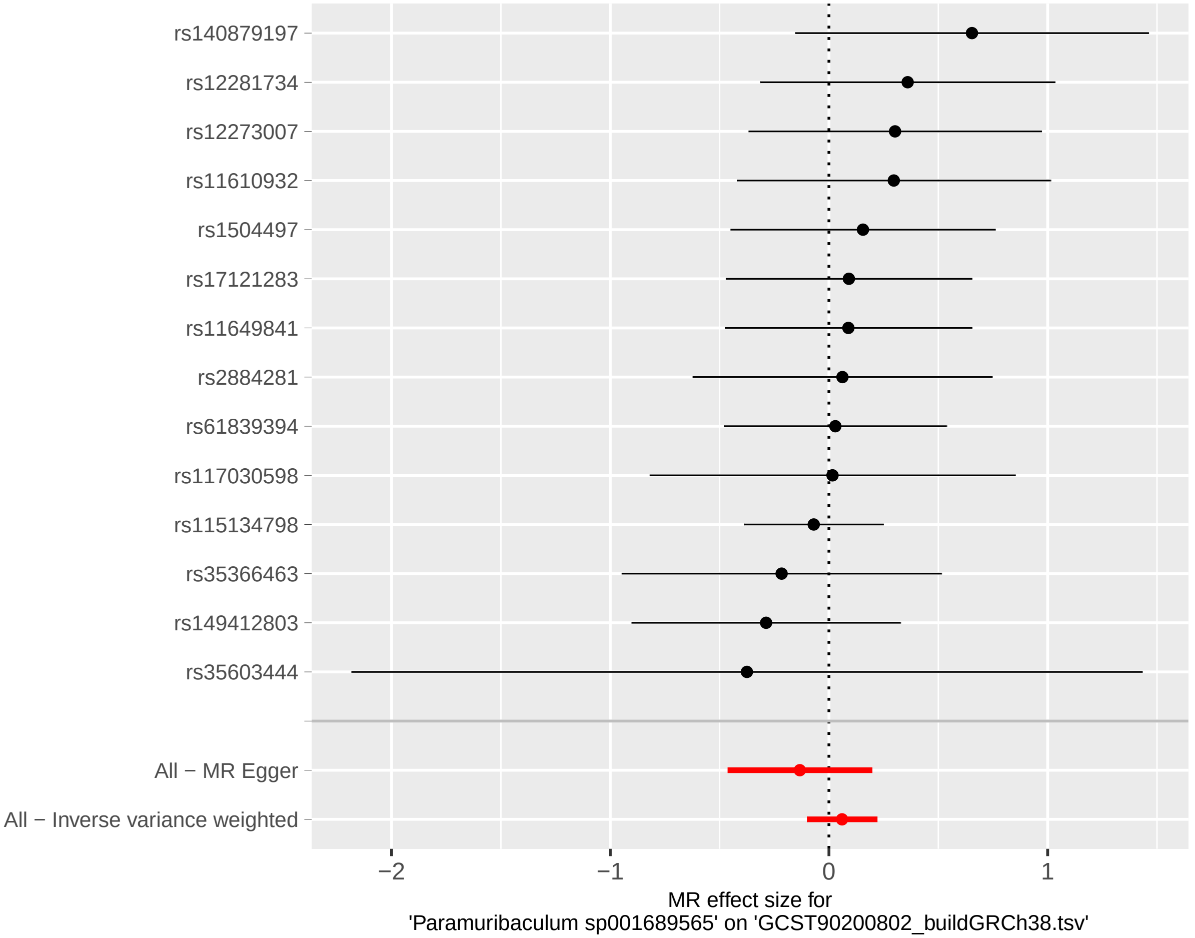
rs140879197
rs12281734
rs12273007
rs11610932
rs1504497
rs17121283
rs11649841
rs2884281
rs61839394
rs117030598
rs115134798
rs35366463
rs149412803
rs35603444
All − MR Egger
All − Inverse variance weighted
−2
−1
0
1
MR effect size for
'Paramuribaculum sp001689565' on 'GCST90200802_buildGRCh38.tsv'

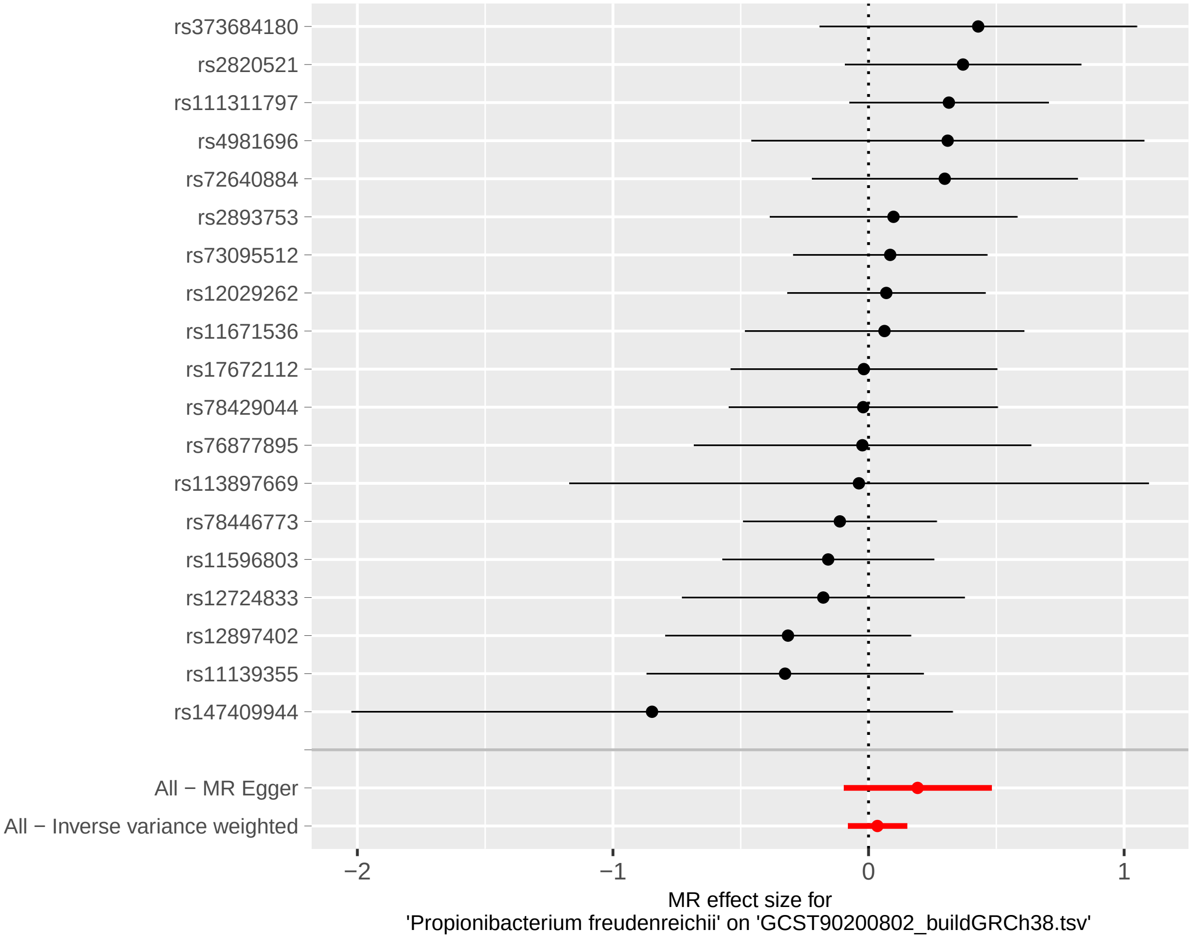
rs373684180
rs2820521
rs111311797
rs4981696
rs72640884
rs2893753
rs73095512
rs12029262
rs11671536
rs17672112
rs78429044
rs76877895
rs113897669
rs78446773
rs11596803
rs12724833
rs12897402
rs11139355
rs147409944
All − MR Egger
All − Inverse variance weighted
−2
−1
0
1
MR effect size for
'Propionibacterium freudenreichii' on 'GCST90200802_buildGRCh38.tsv'

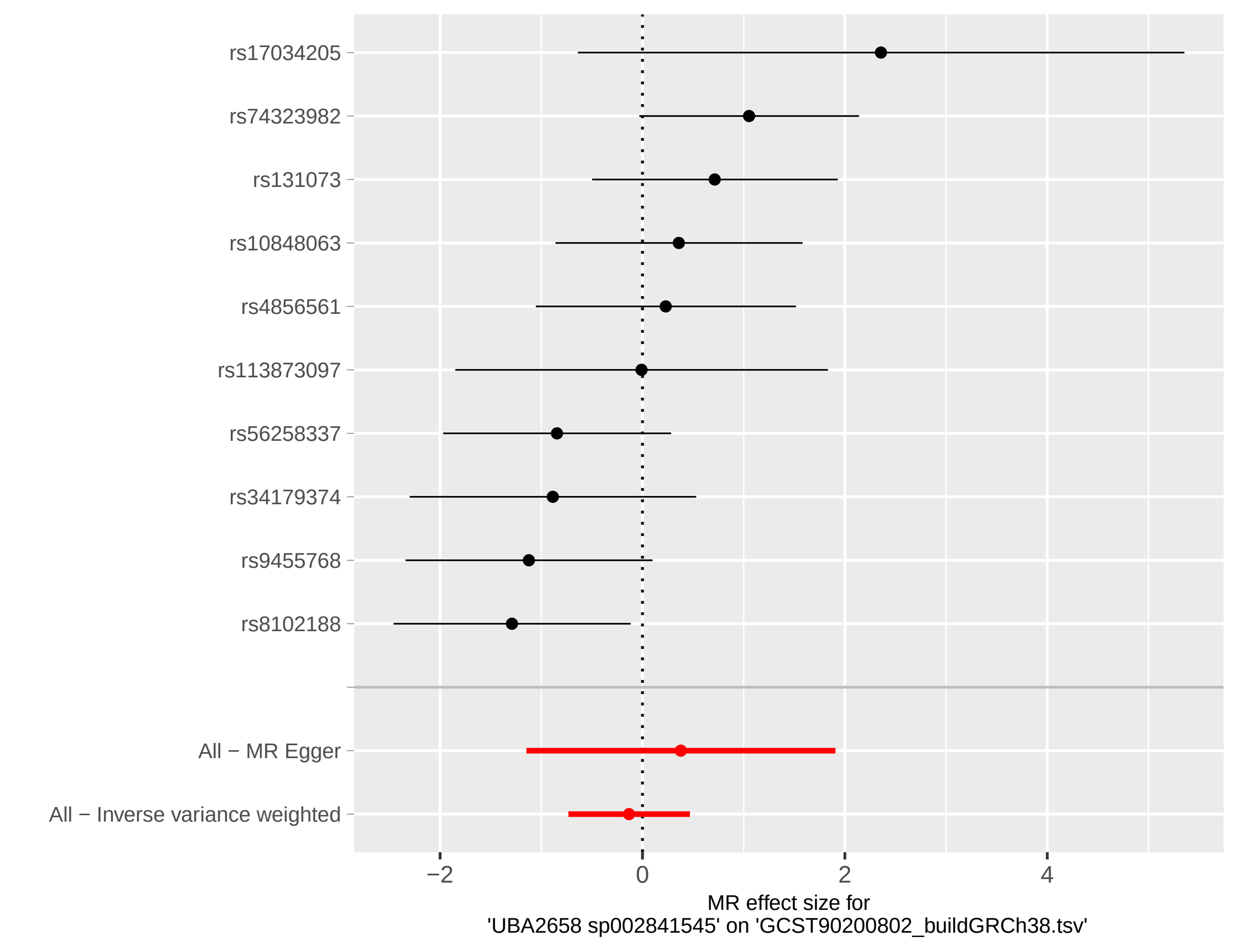

rs17034205
rs74323982
rs131073
rs10848063
rs4856561
rs113873097
rs56258337
rs34179374
rs9455768
rs8102188
All − MR Egger
All − Inverse variance weighted
−2
0
2
4
MR effect size for
'UBA2658 sp002841545' on 'GCST90200802_buildGRCh38.tsv'

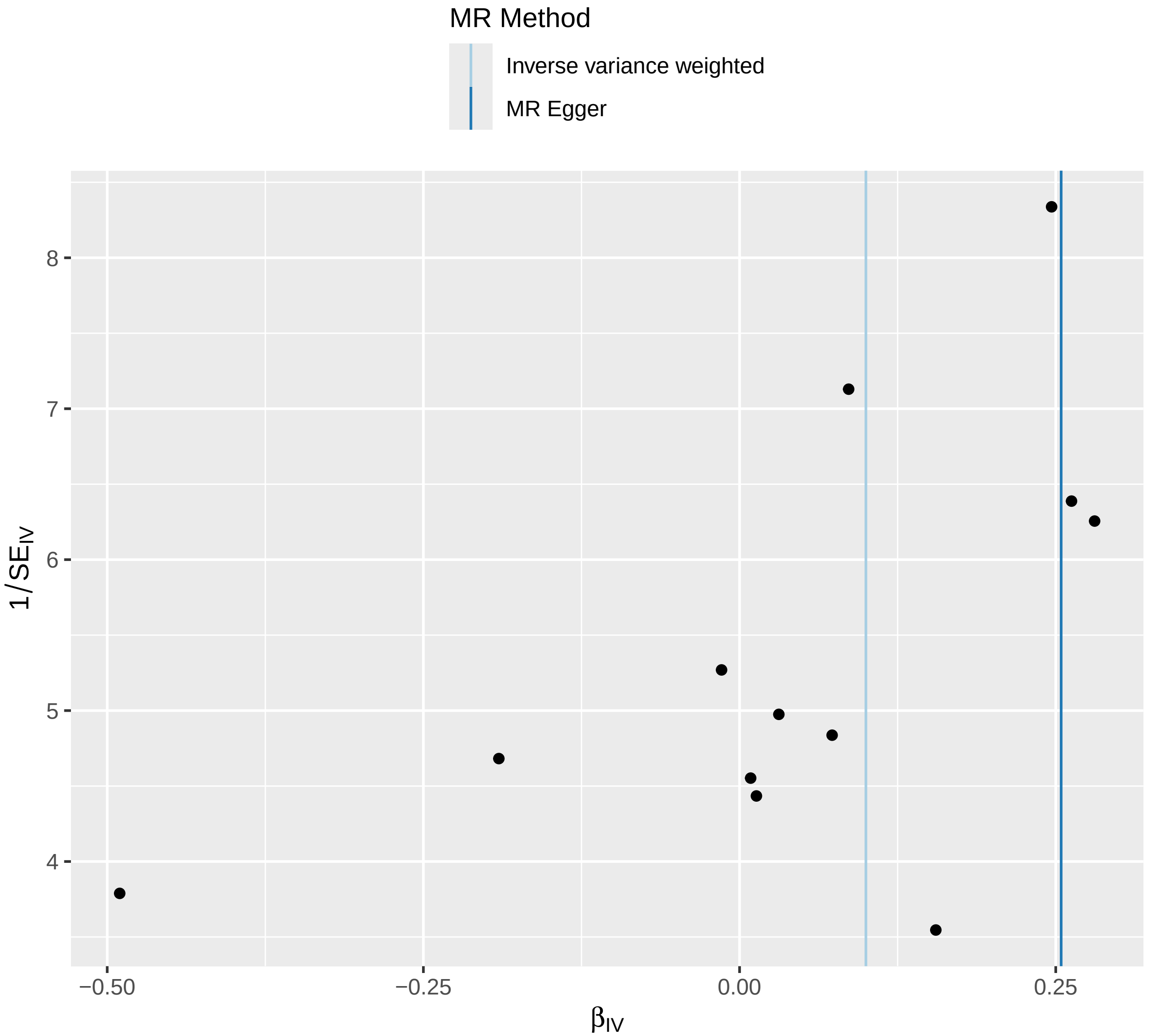
MR Method
Inverse variance weighted
MR Egger
8
7
6
5
4
−0.50
−0.25
0.00
0.25
1/SE_IV
β_IV

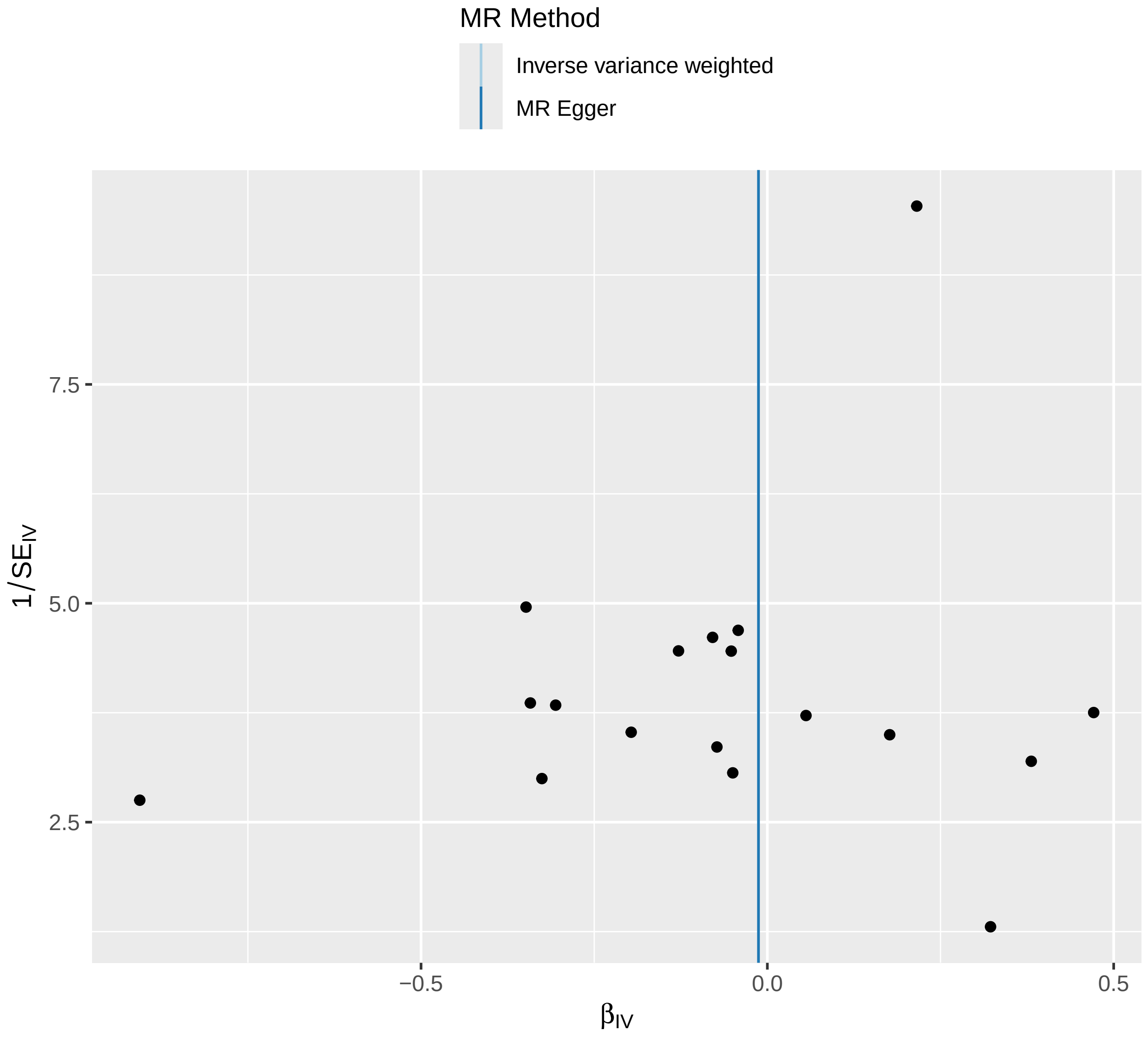
MR Method
Inverse variance weighted
MR Egger
7.5
5.0
2.5
1/SE_IV
−0.5
0.0
0.5
β_IV

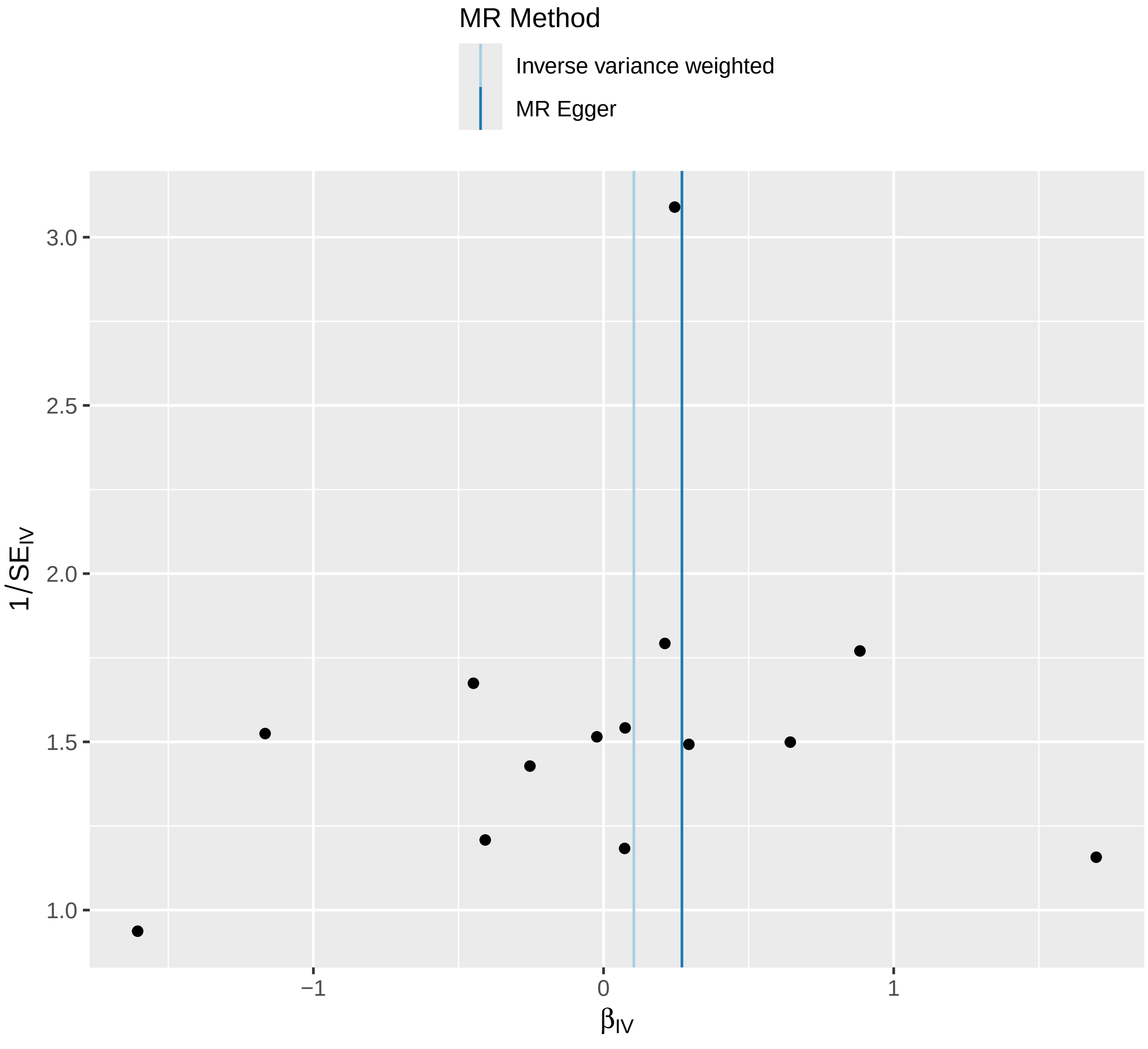
MR Method
Inverse variance weighted
MR Egger
3.0
2.5
2.0
1.5
1.0
1/SE$_{IV}$
−1
0
1
β_{IV}

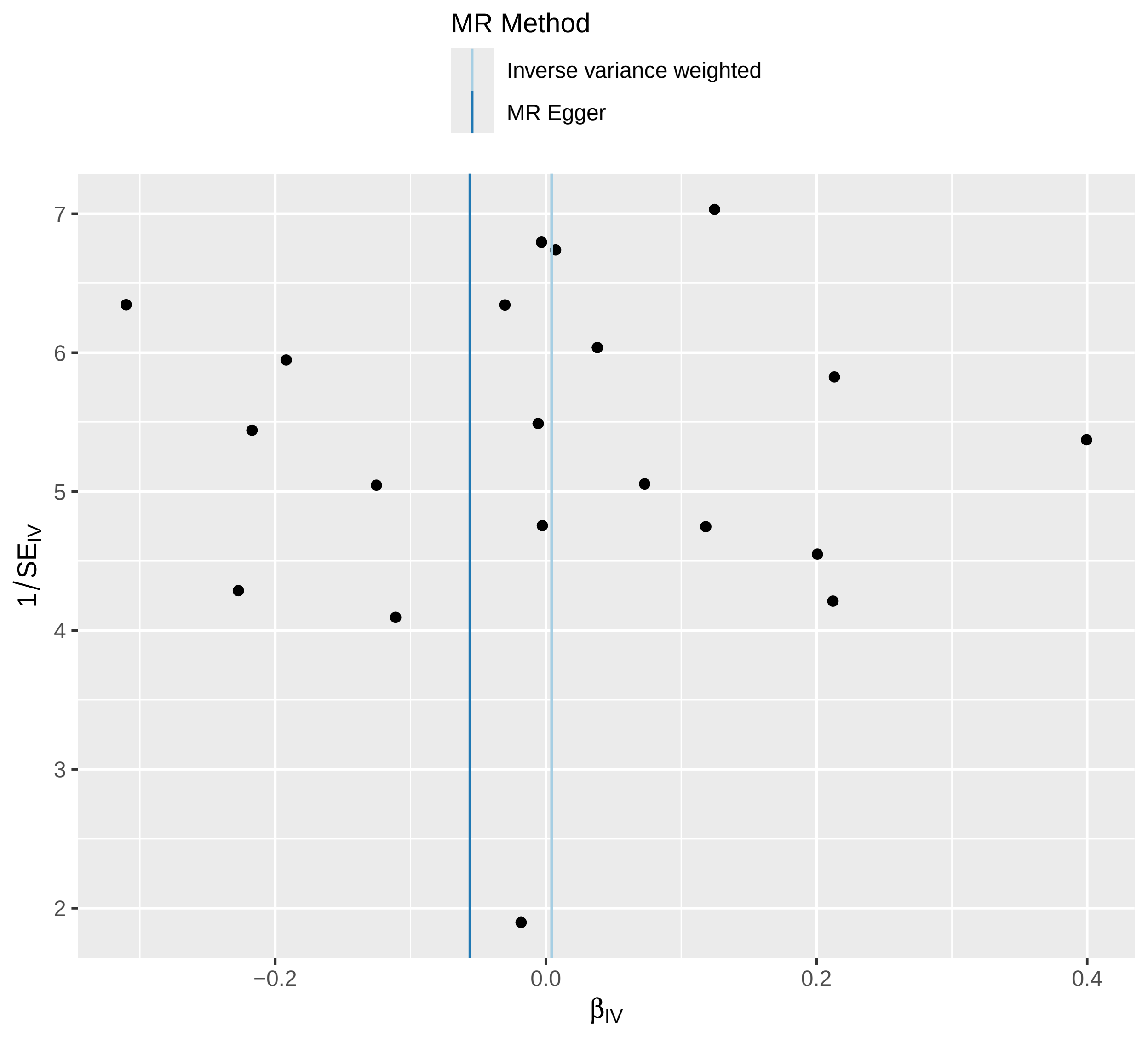
MR Method
Inverse variance weighted
MR Egger
7
6
5
4
3
2
1/SE$_{IV}$
−0.2
0.0
0.2
0.4
β$_{IV}$

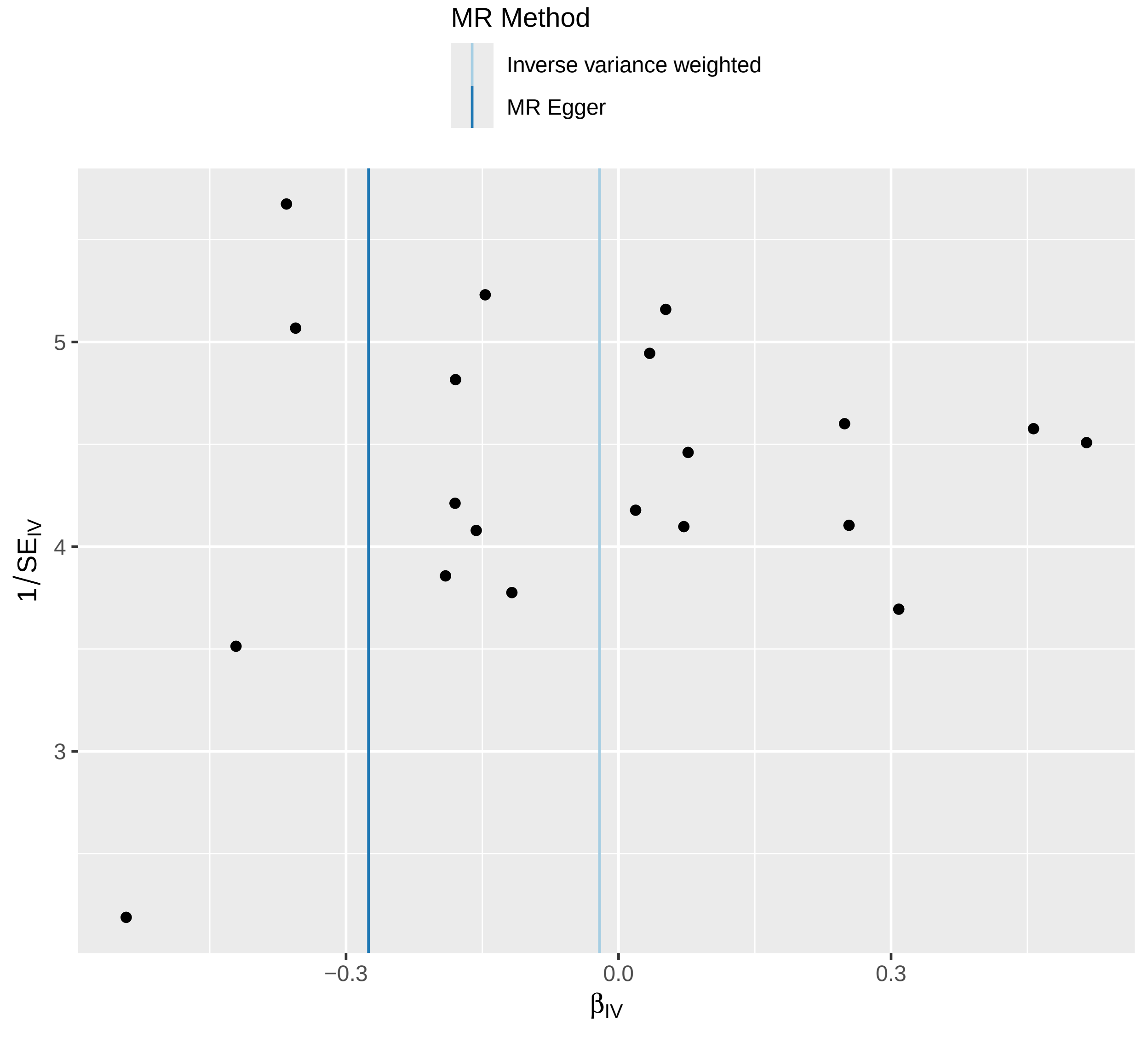
MR Method
Inverse variance weighted
MR Egger
5
4
3
1/SE$_{IV}$
−0.3
0.0
0.3
β$_{IV}$

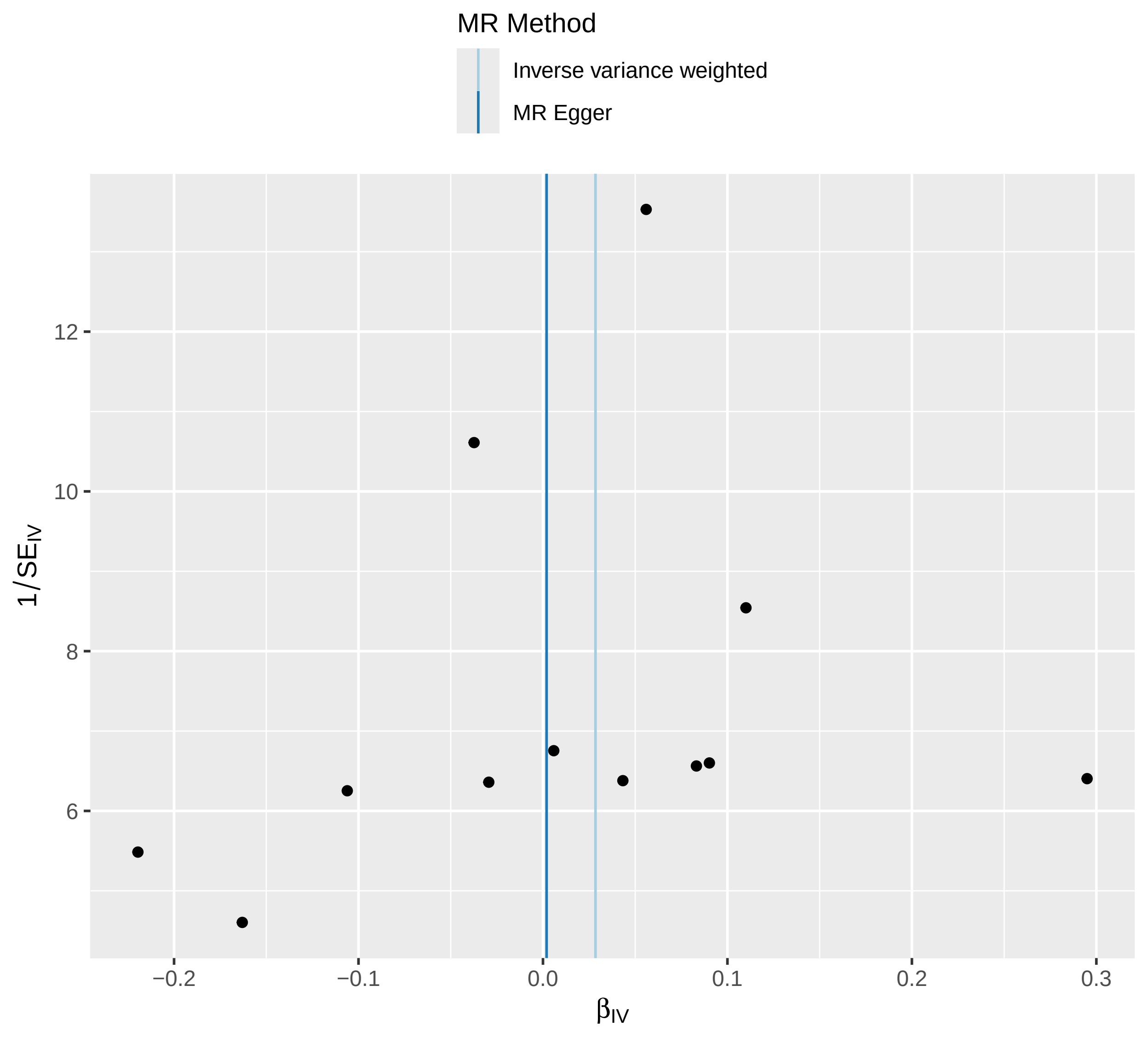
MR Method
Inverse variance weighted
MR Egger
12
10
8
6
1/SE$_{IV}$
−0.2
−0.1
0.0
0.1
0.2
0.3
β$_{IV}$

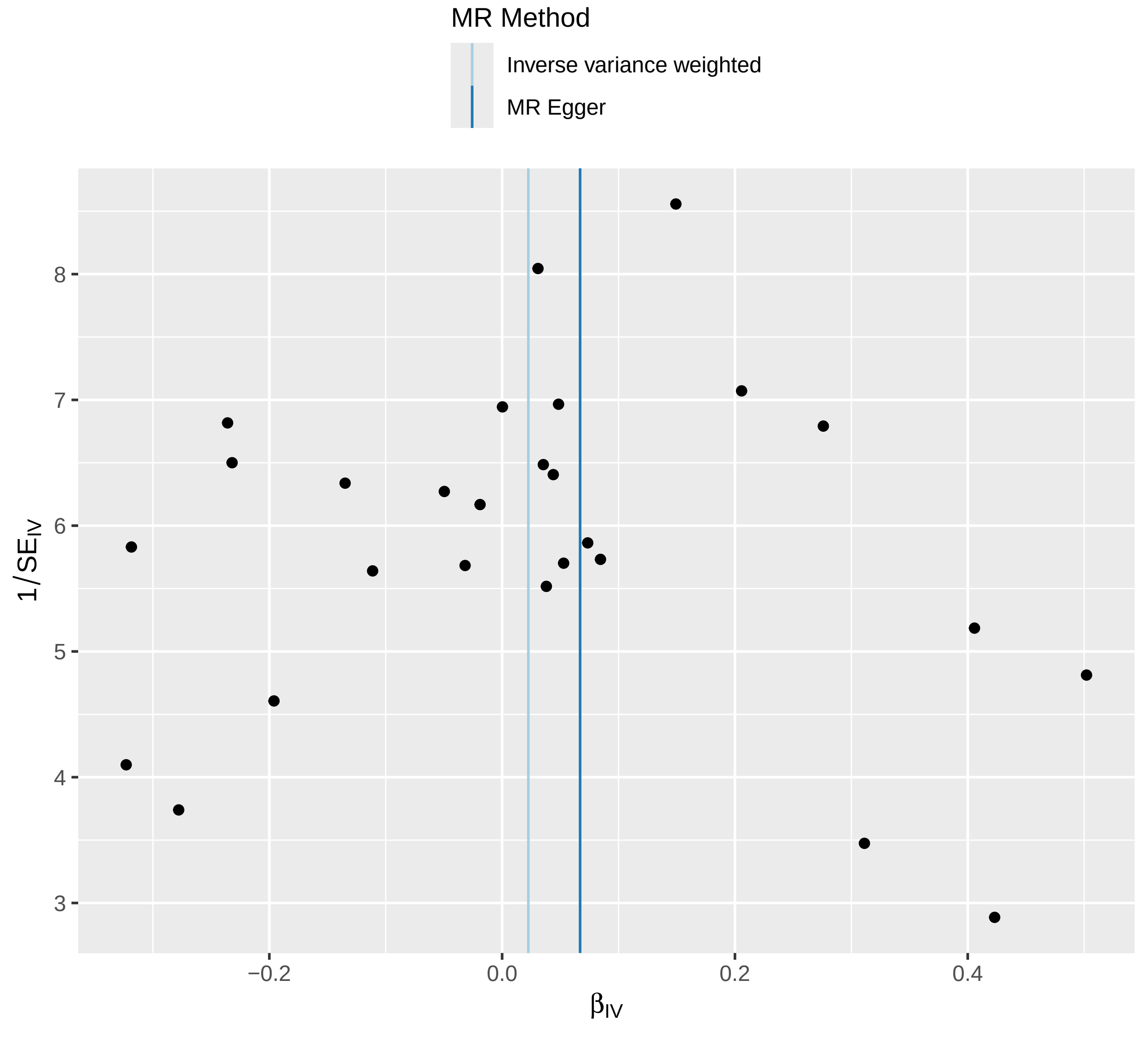

MR Method
Inverse variance weighted
MR Egger
1/SE_IV
8
7
6
5
4
3
−0.2
0.0
0.2
0.4
β_IV

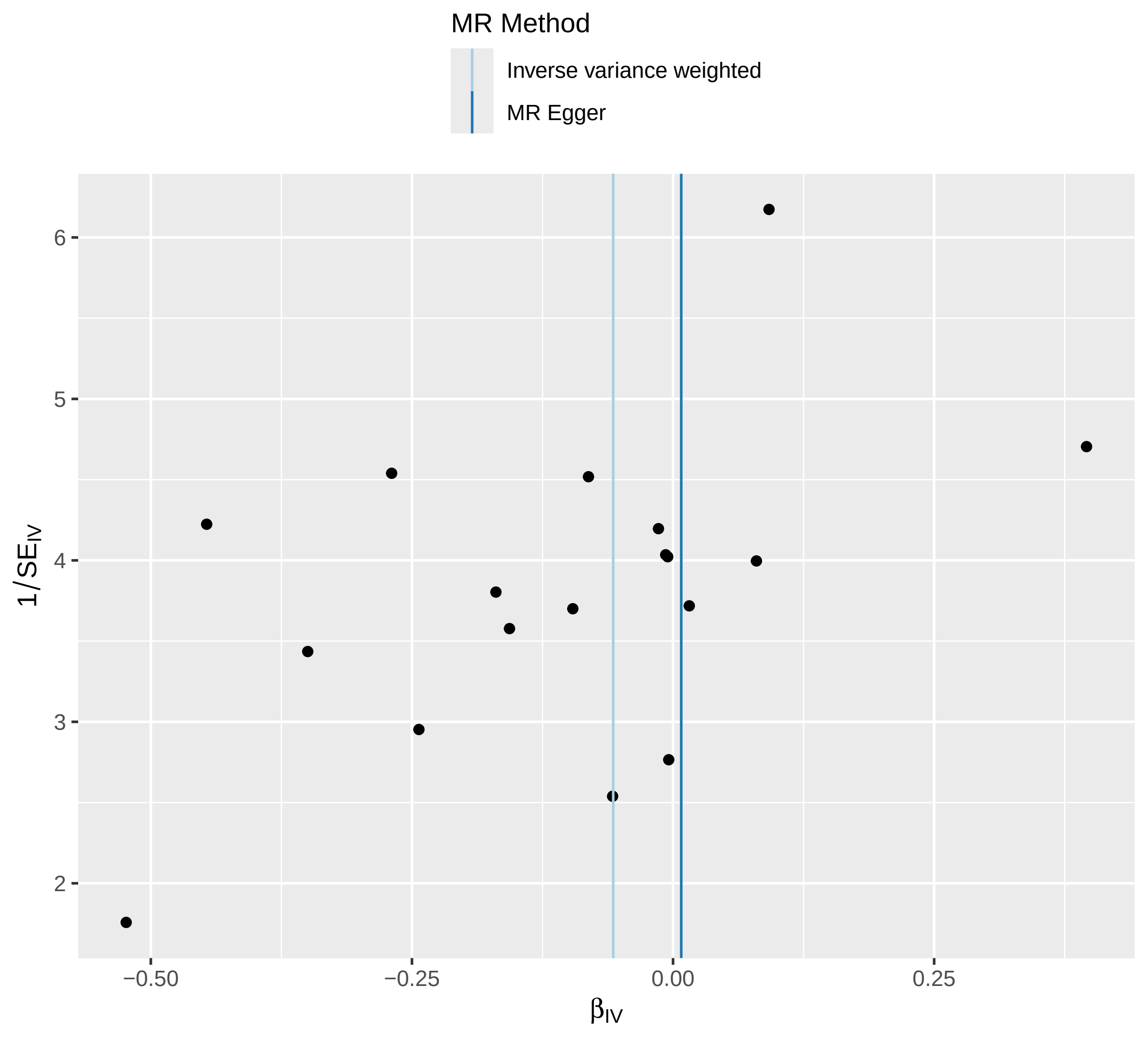
MR Method
Inverse variance weighted
MR Egger
6
5
4
3
2
1/SE_IV
−0.50
−0.25
0.00
0.25
β_IV

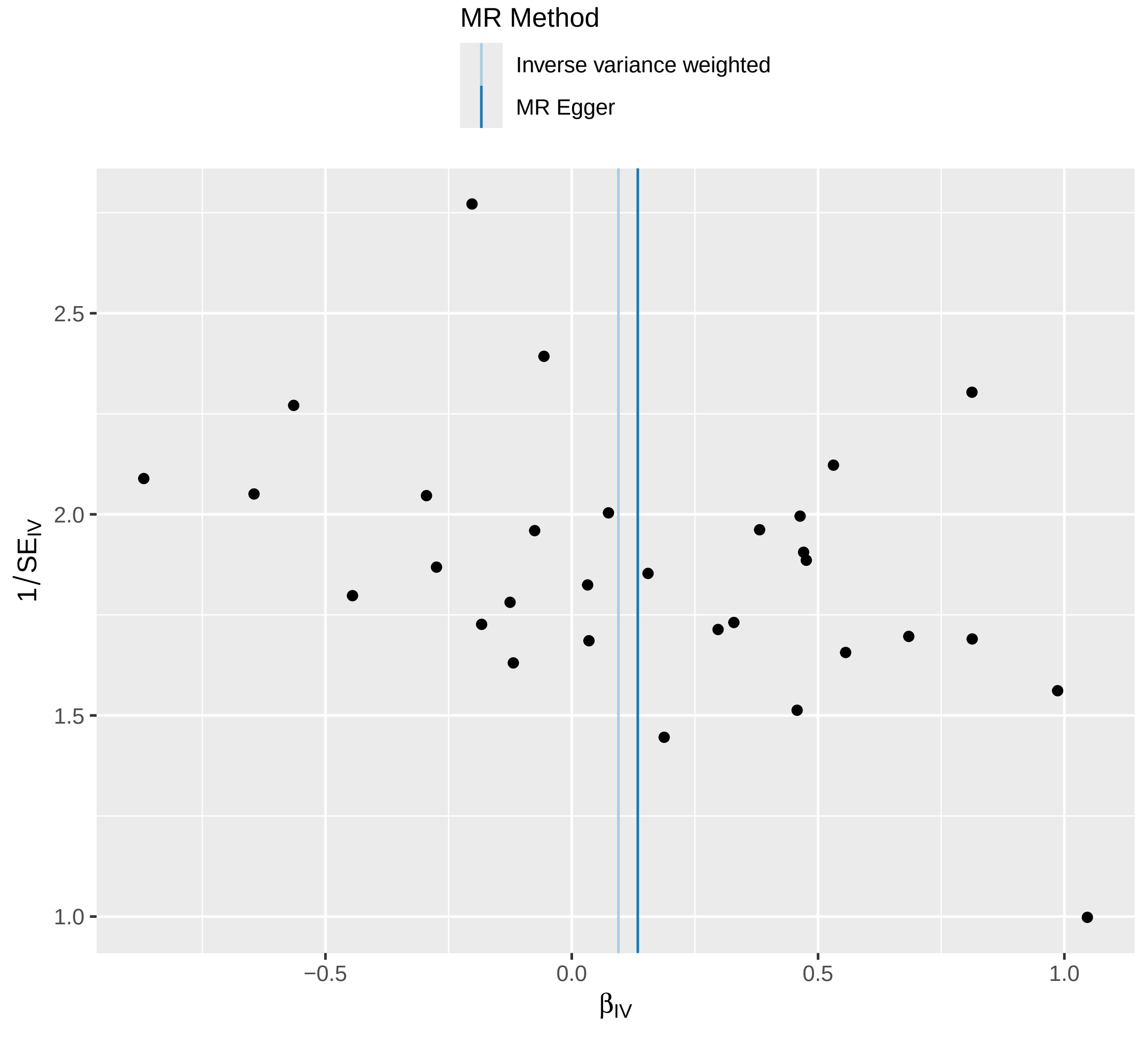

MR Method
Inverse variance weighted
MR Egger
2.5
2.0
1.5
1.0
−0.5
0.0
0.5
1.0
1/SE_IV
β_IV

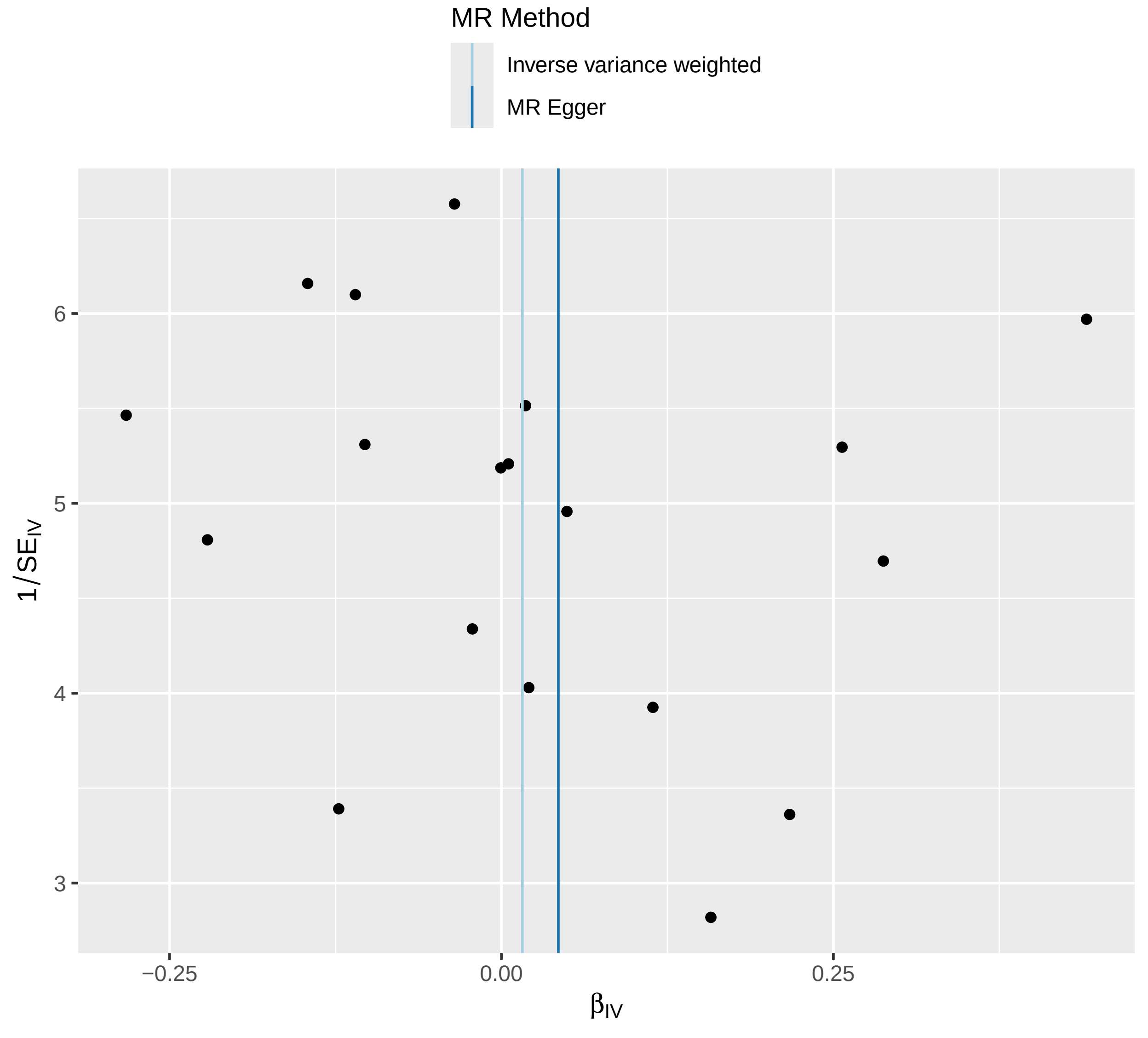

MR Method
Inverse variance weighted
MR Egger
6
5
4
3
1/SE_IV
−0.25
0.00
0.25
β_IV

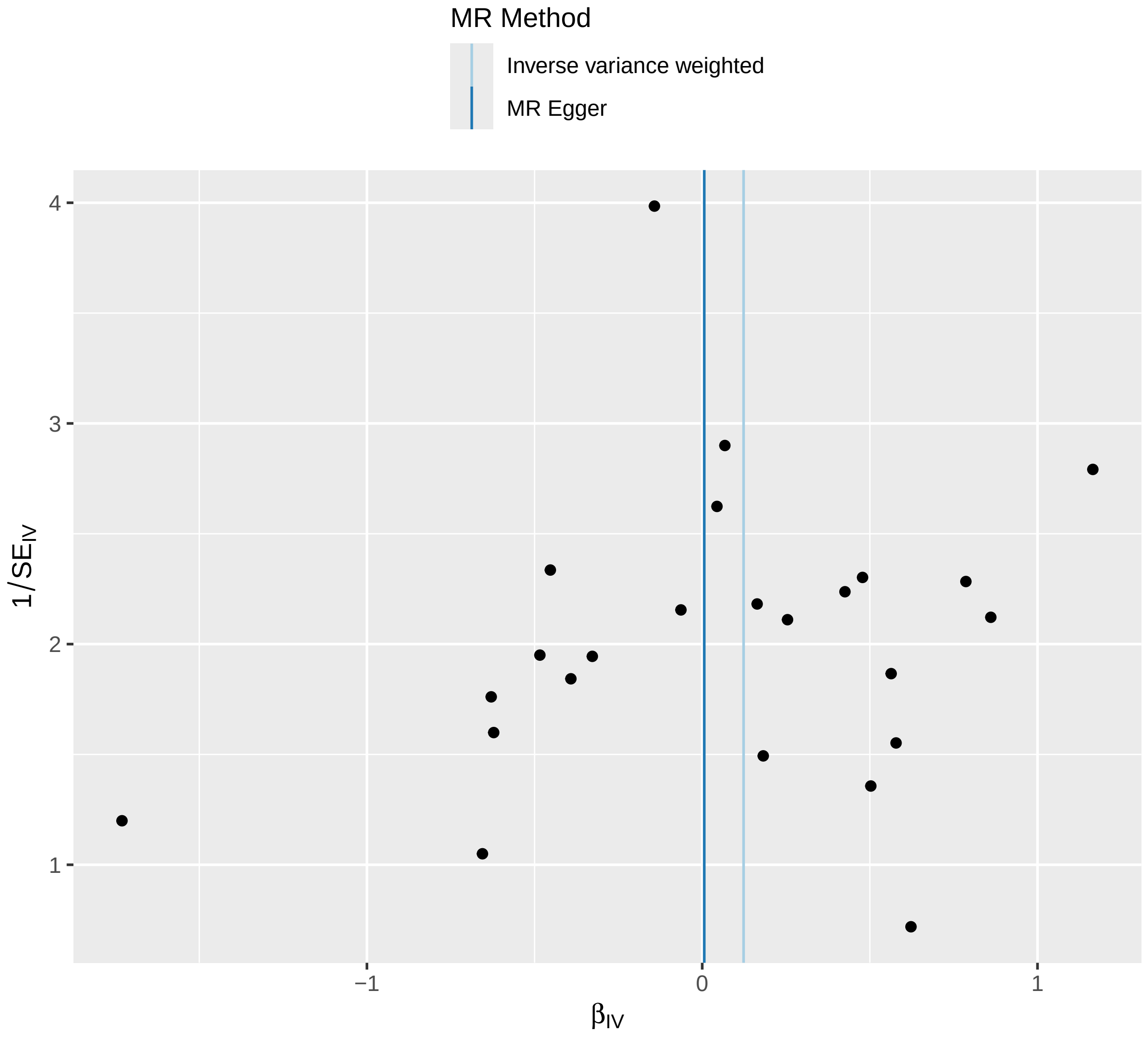
MR Method
Inverse variance weighted
MR Egger
4
3
2
1
$1/SE_{IV}$
−1
0
1
β_{IV}

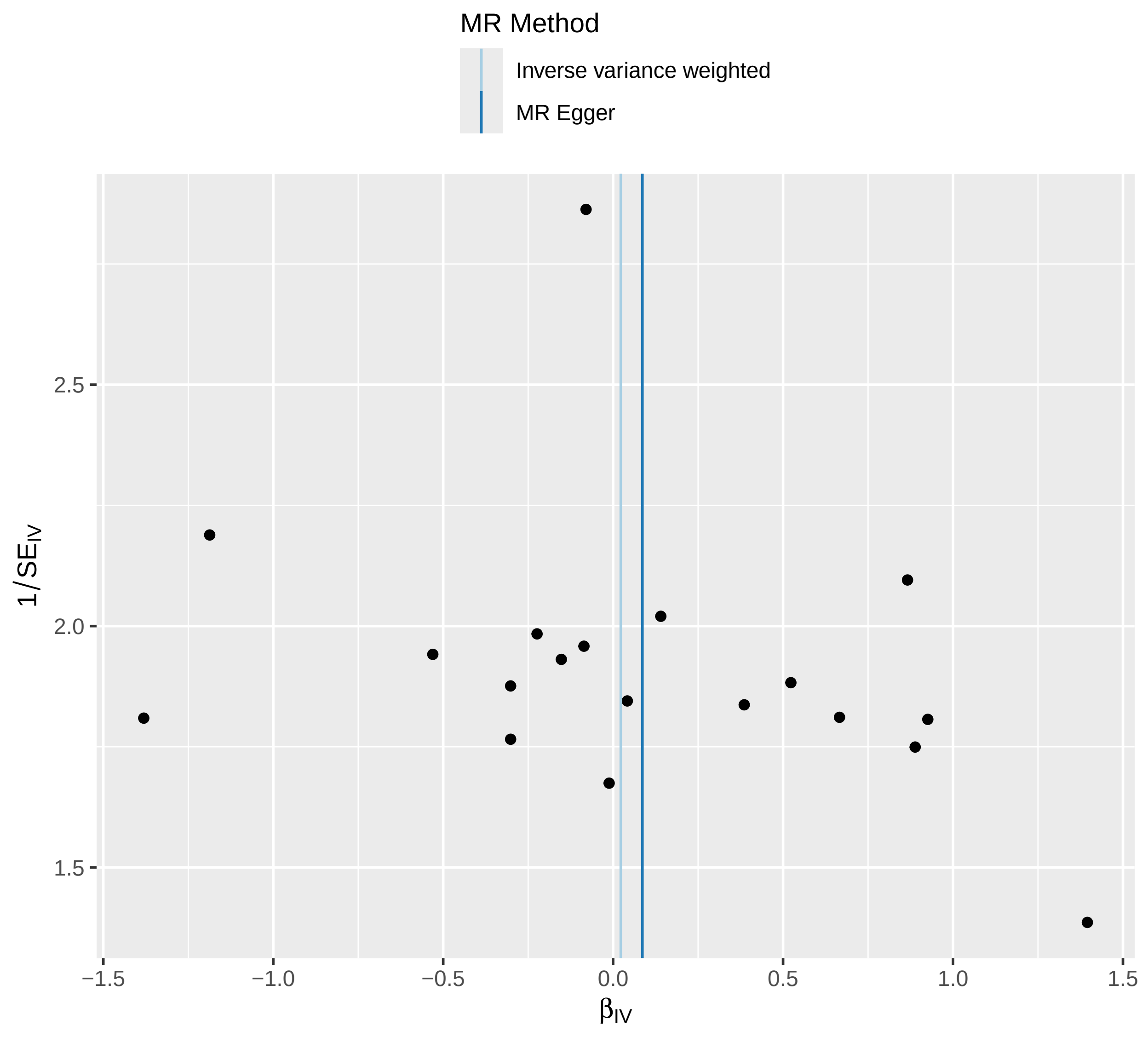

MR Method
Inverse variance weighted
MR Egger
2.5
2.0
1.5
1/SE_IV
−1.5
−1.0
−0.5
0.0
0.5
1.0
1.5
β_IV

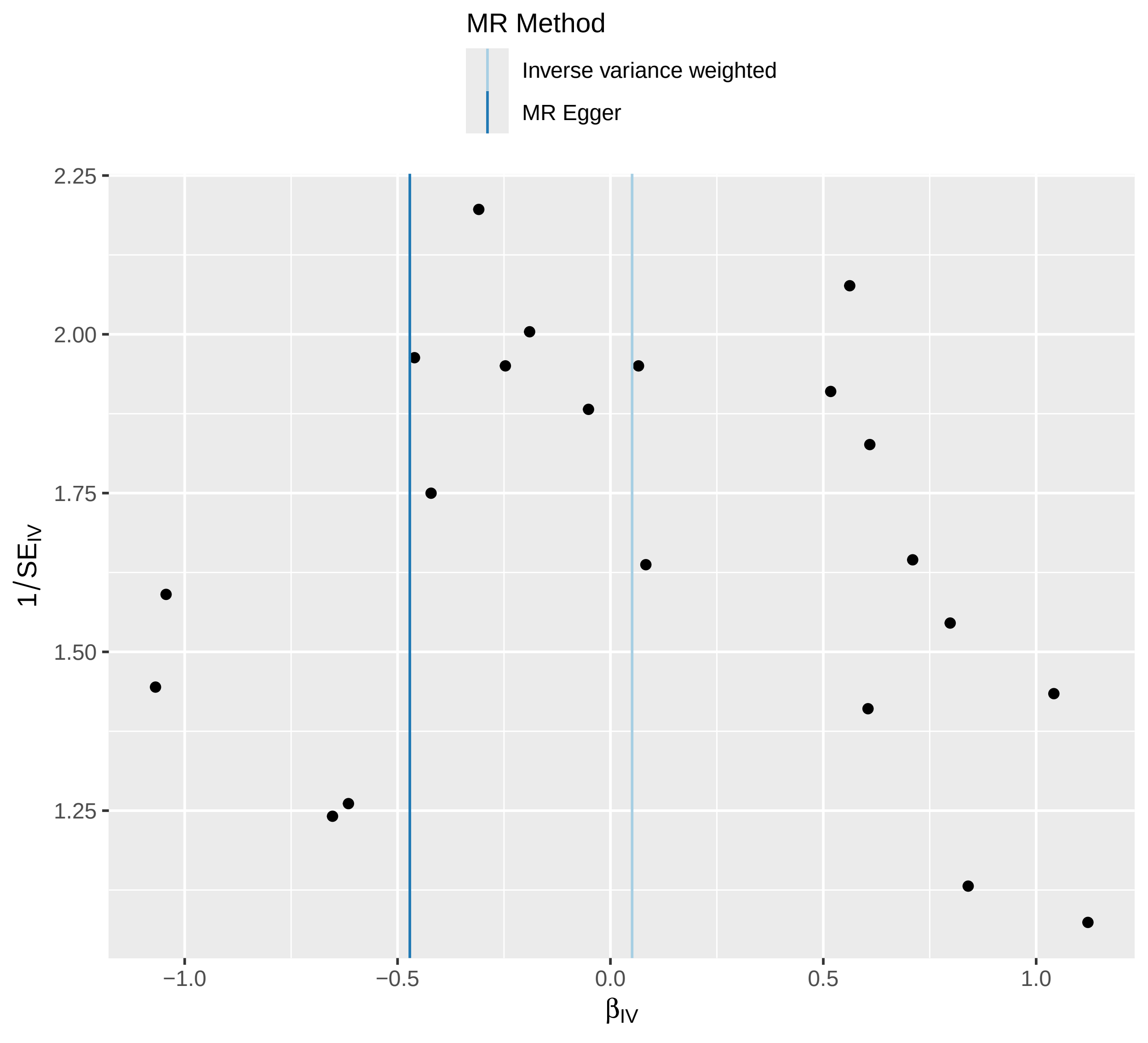
MR Method
Inverse variance weighted
MR Egger
2.25
2.00
1.75
1.50
1.25
1/SE_IV
−1.0
−0.5
0.0
0.5
1.0
β_IV

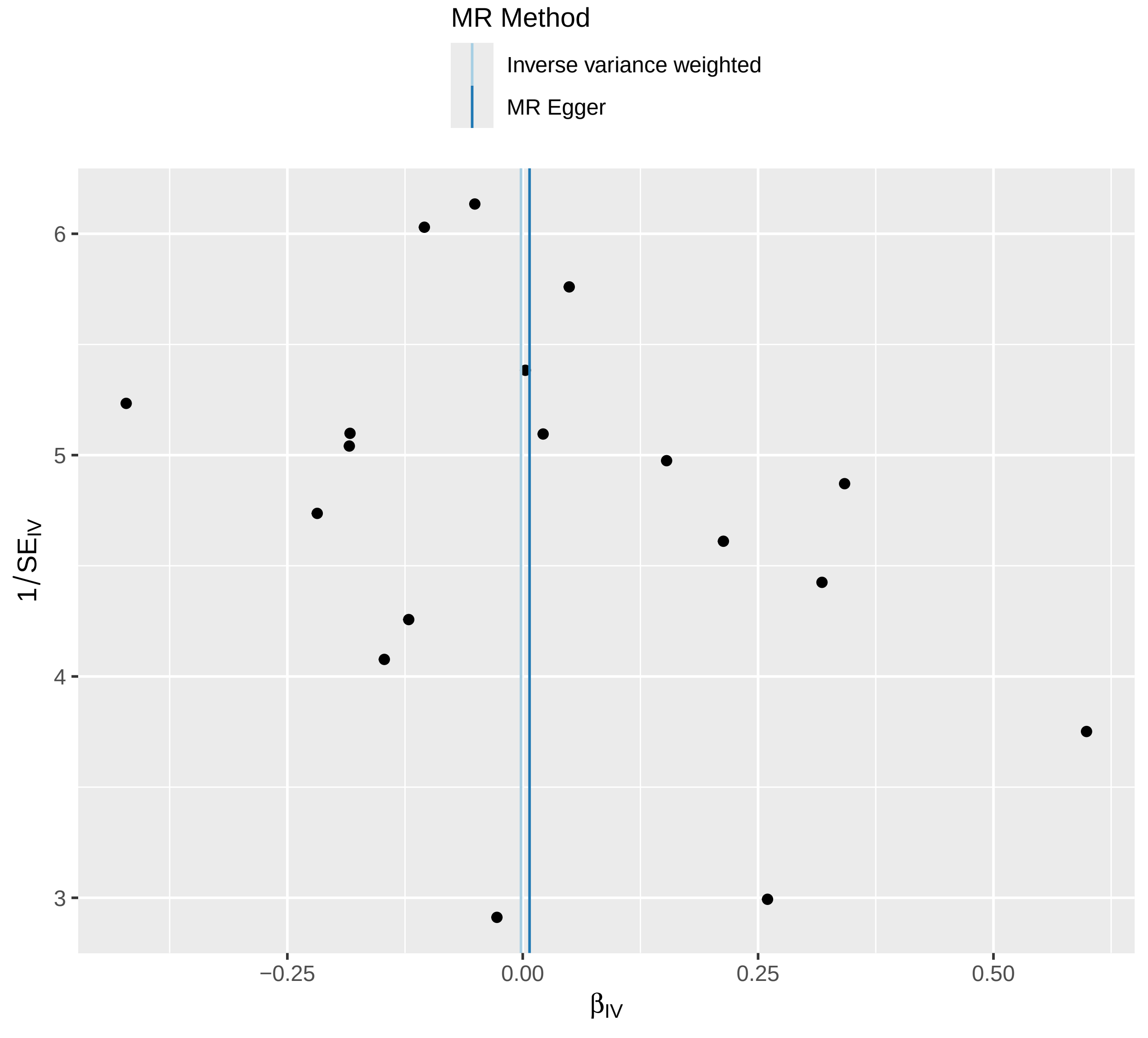
MR Method
Inverse variance weighted
MR Egger
6
5
4
3
1/SE_IV
−0.25
0.00
0.25
0.50
β_IV

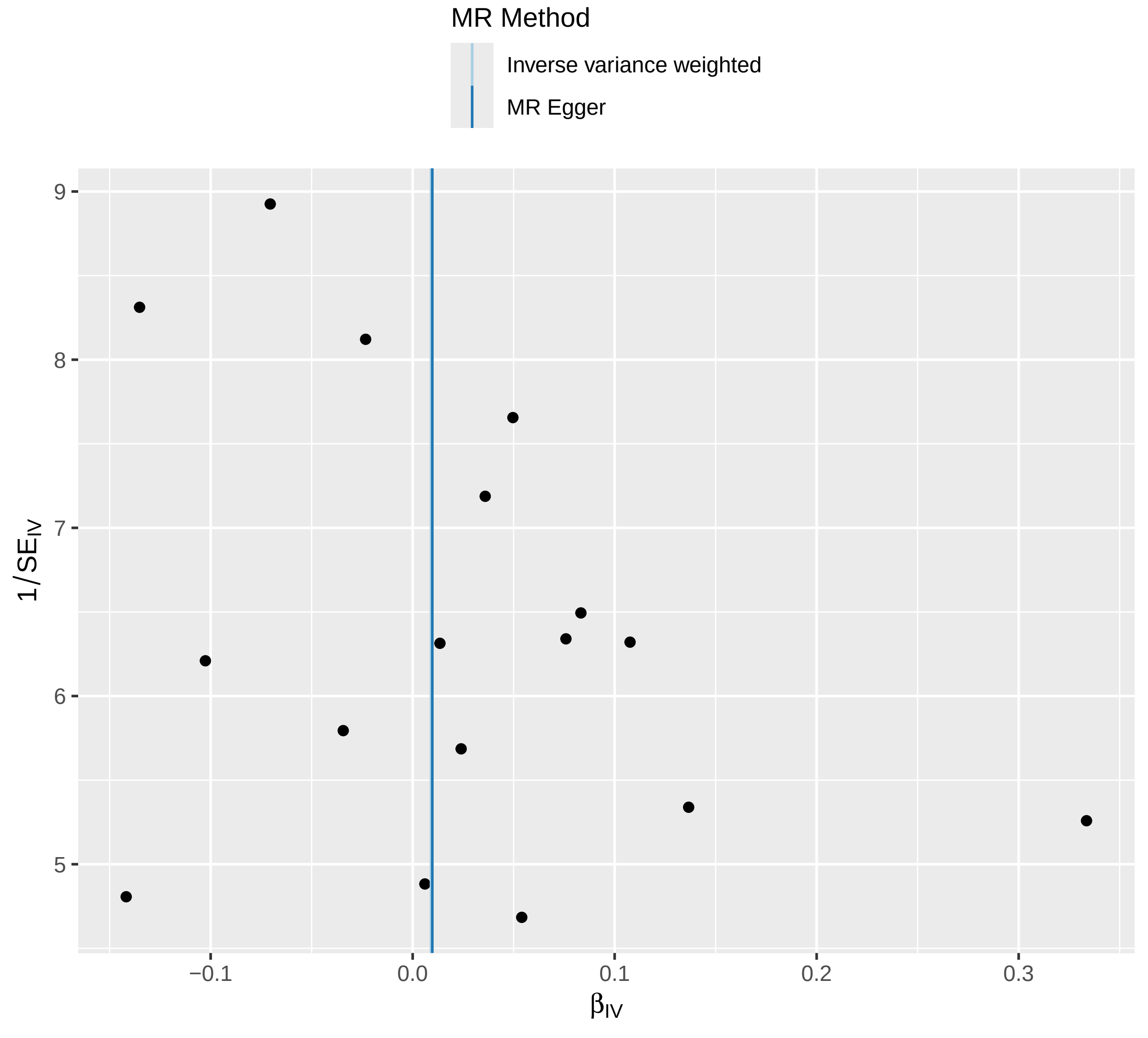
MR Method
Inverse variance weighted
MR Egger
9
8
7
6
5
$1/SE_{IV}$
−0.1
0.0
0.1
0.2
0.3
β_{IV}

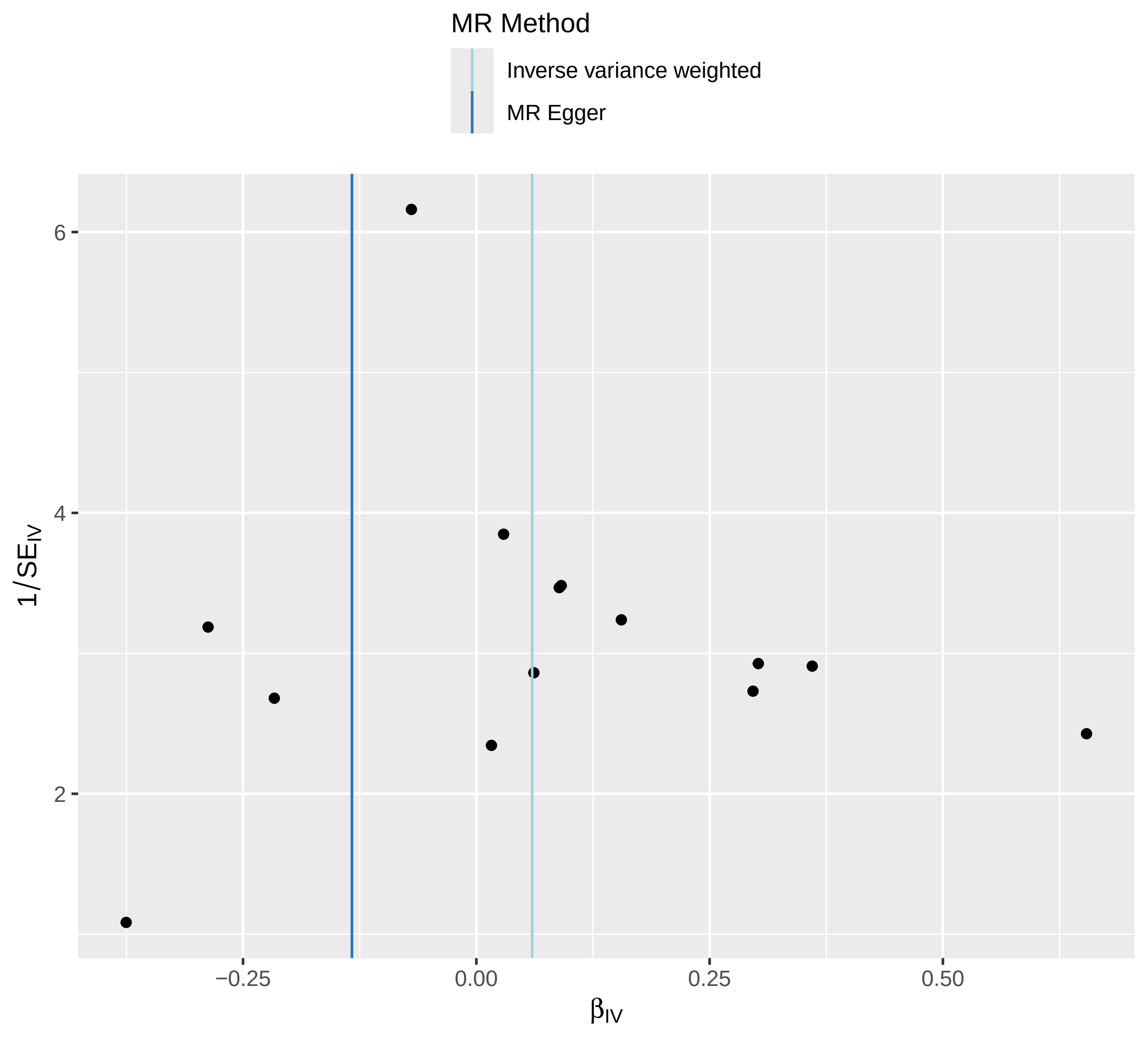
MR Method
Inverse variance weighted
MR Egger
6
4
2
1/SE_IV
−0.25
0.00
0.25
0.50
β_IV

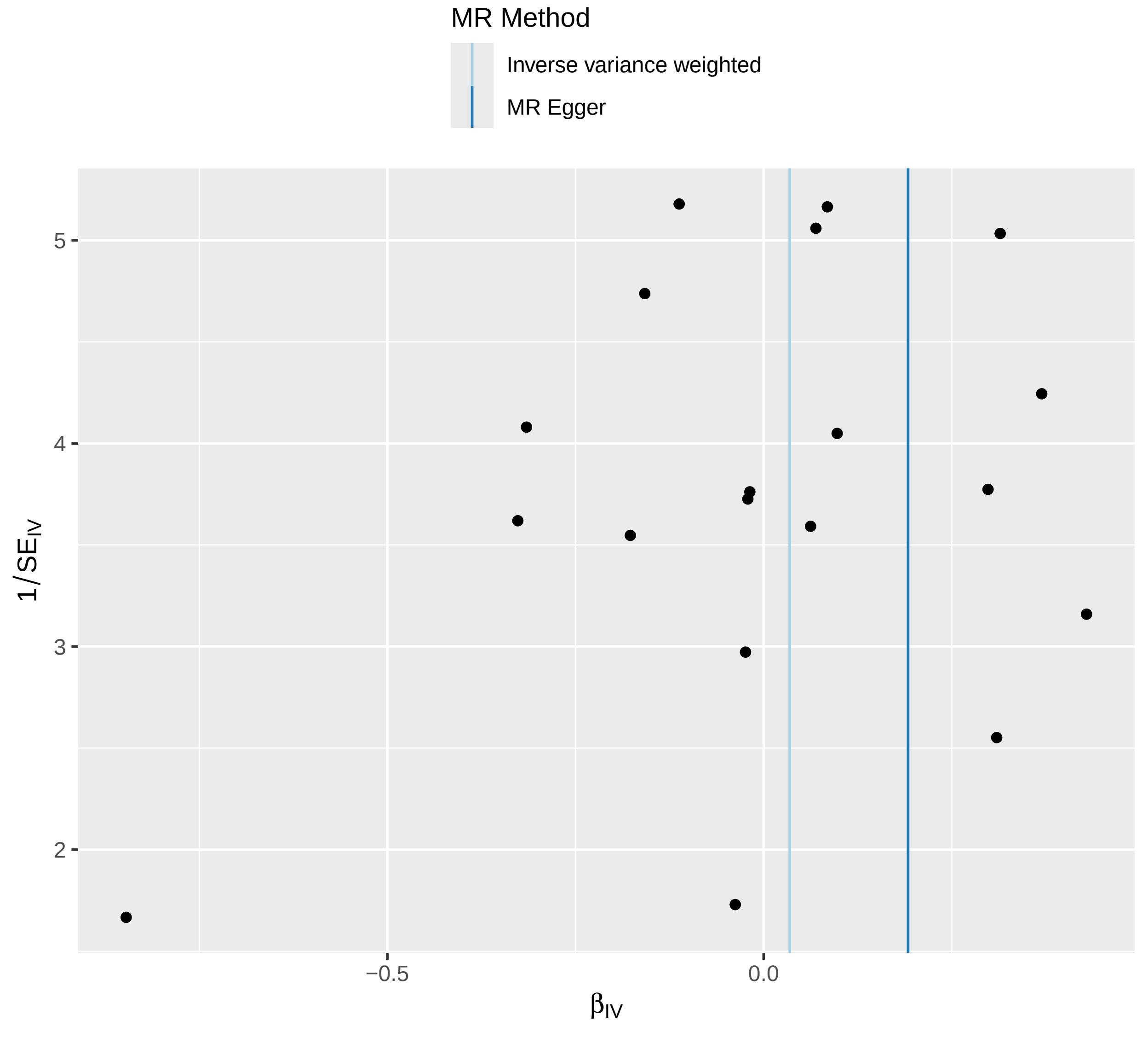

MR Method
Inverse variance weighted
MR Egger
5
4
3
2
1/SE$_{IV}$
−0.5
0.0
β$_{IV}$

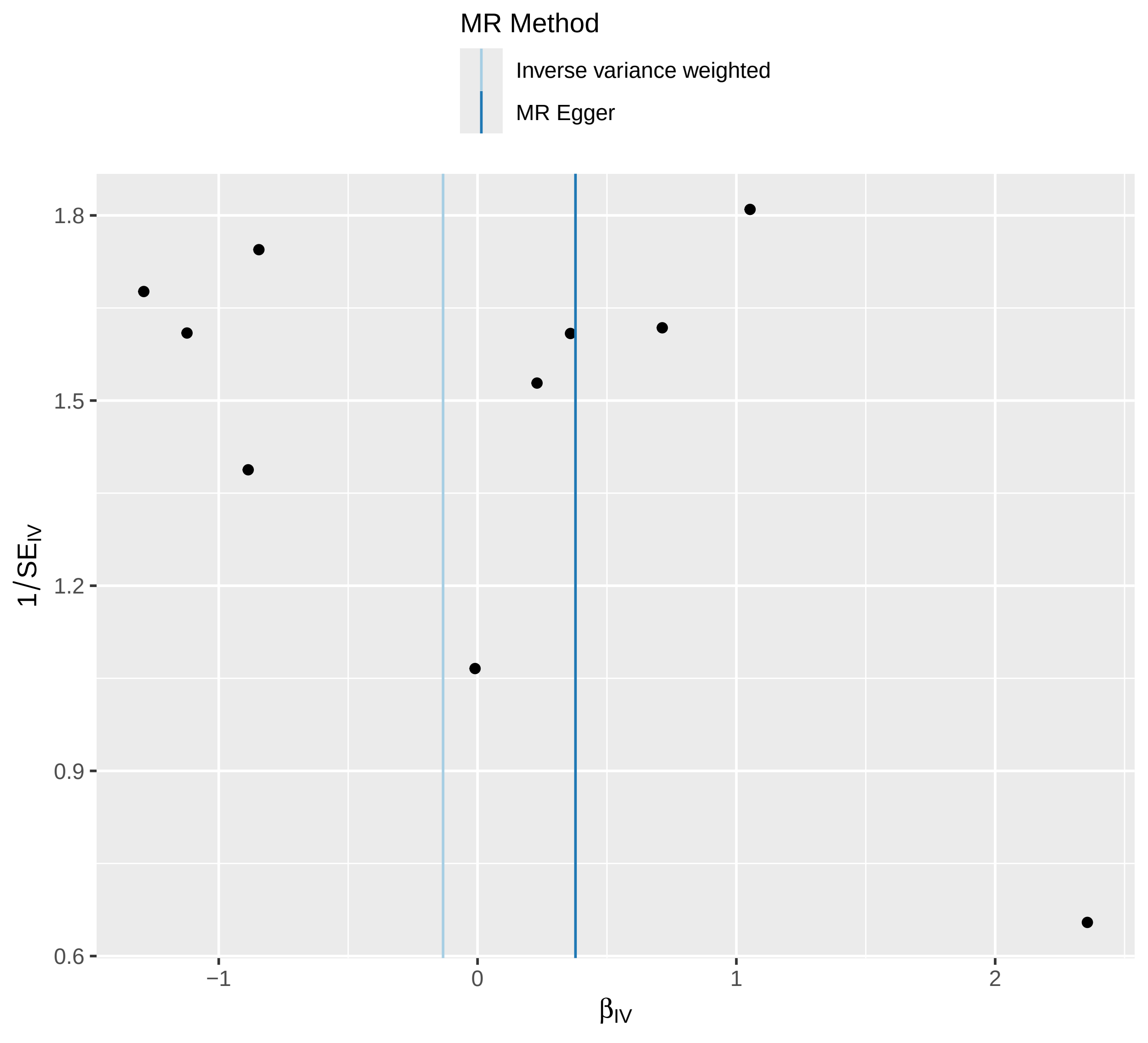
MR Method
Inverse variance weighted
MR Egger
1.8
1.5
1.2
0.9
0.6
1/SE_IV
−1
0
1
2
β_IV

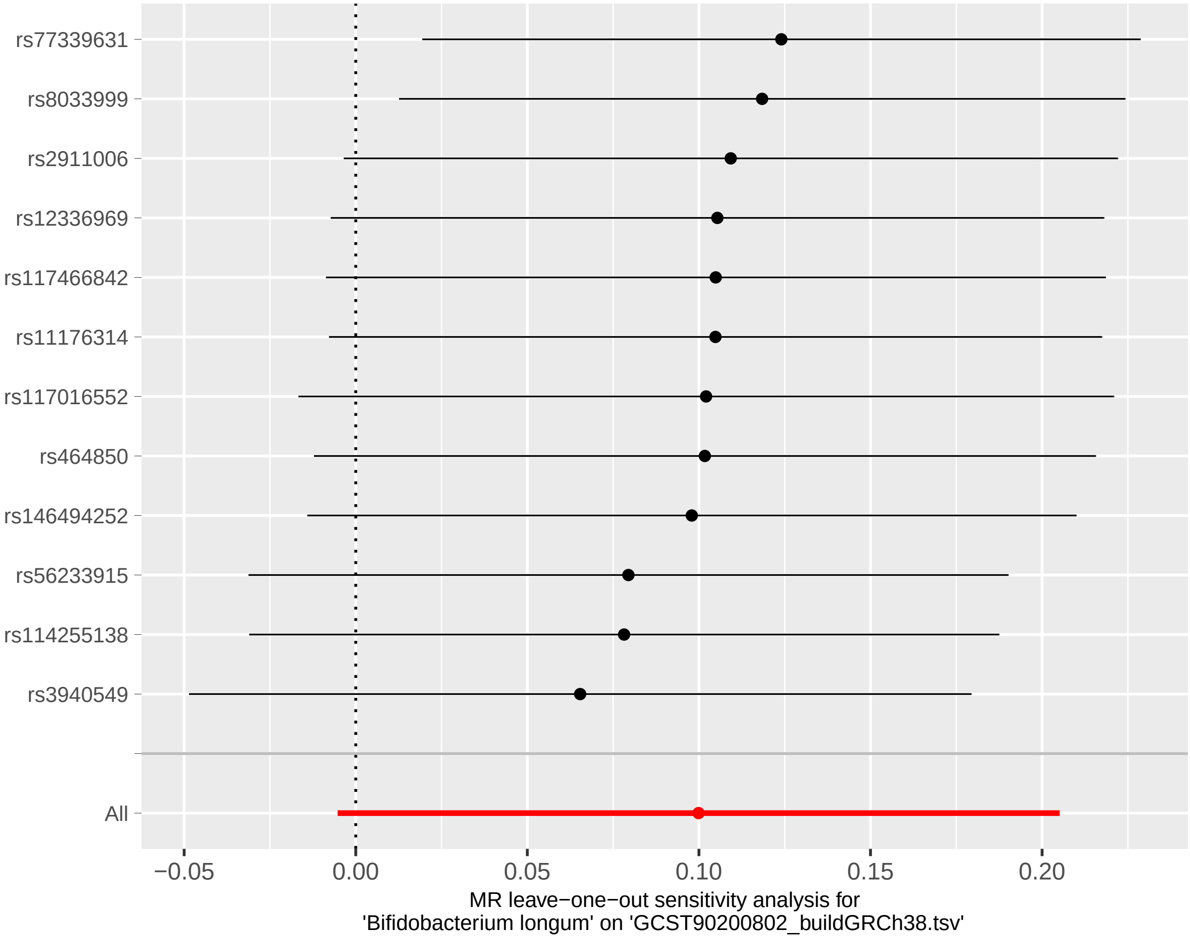

rs77339631
rs8033999
rs2911006
rs12336969
rs117466842
rs11176314
rs117016552
rs464850
rs146494252
rs56233915
rs114255138
rs3940549
All
−0.05
0.00
0.05
0.10
0.15
0.20
MR leave−one−out sensitivity analysis for
'Bifidobacterium longum' on 'GCST90200802_buildGRCh38.tsv'

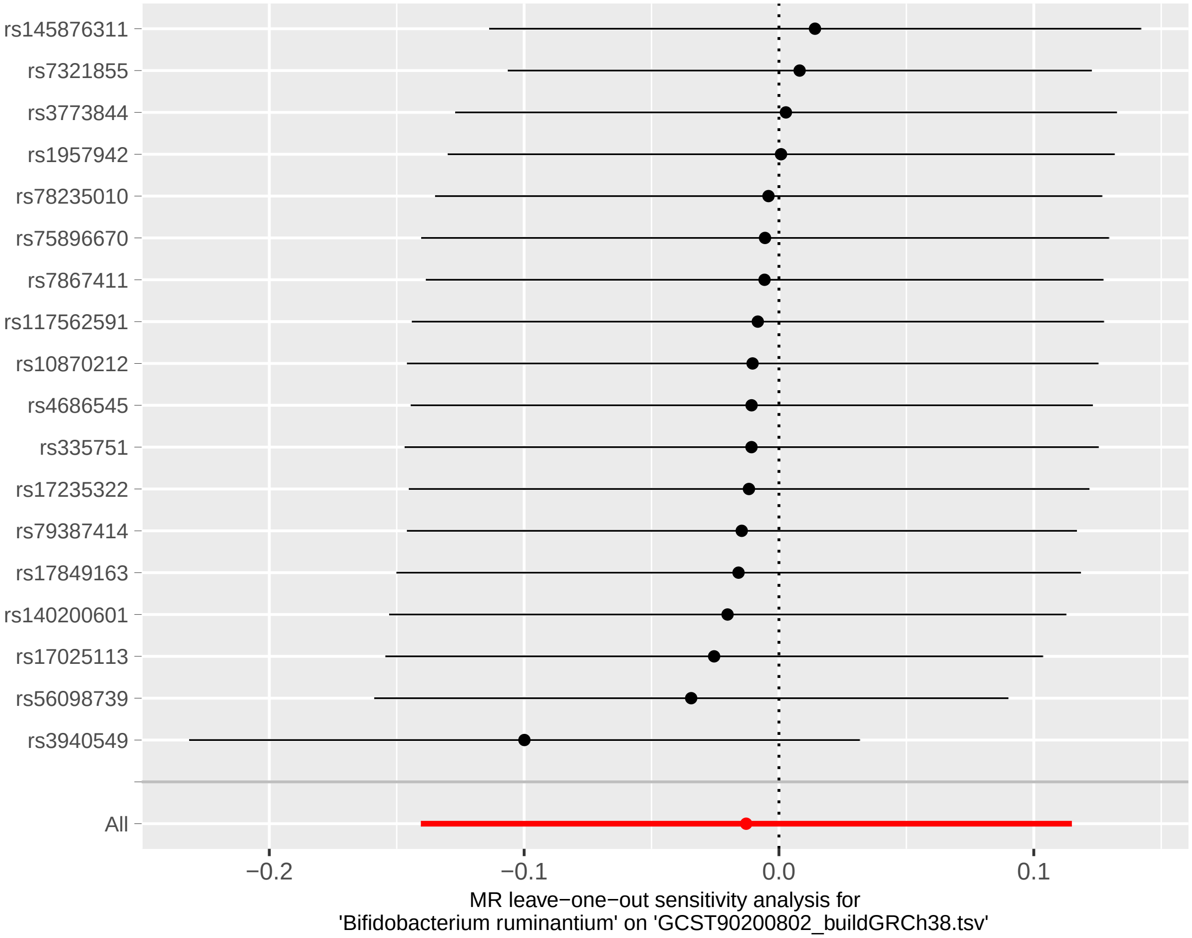

rs145876311
rs7321855
rs3773844
rs1957942
rs78235010
rs75896670
rs7867411
rs117562591
rs10870212
rs4686545
rs335751
rs17235322
rs79387414
rs17849163
rs140200601
rs17025113
rs56098739
rs3940549
All
−0.2
−0.1
0.0
0.1
MR leave−one−out sensitivity analysis for
'Bifidobacterium ruminantium' on 'GCST90200802_buildGRCh38.tsv'

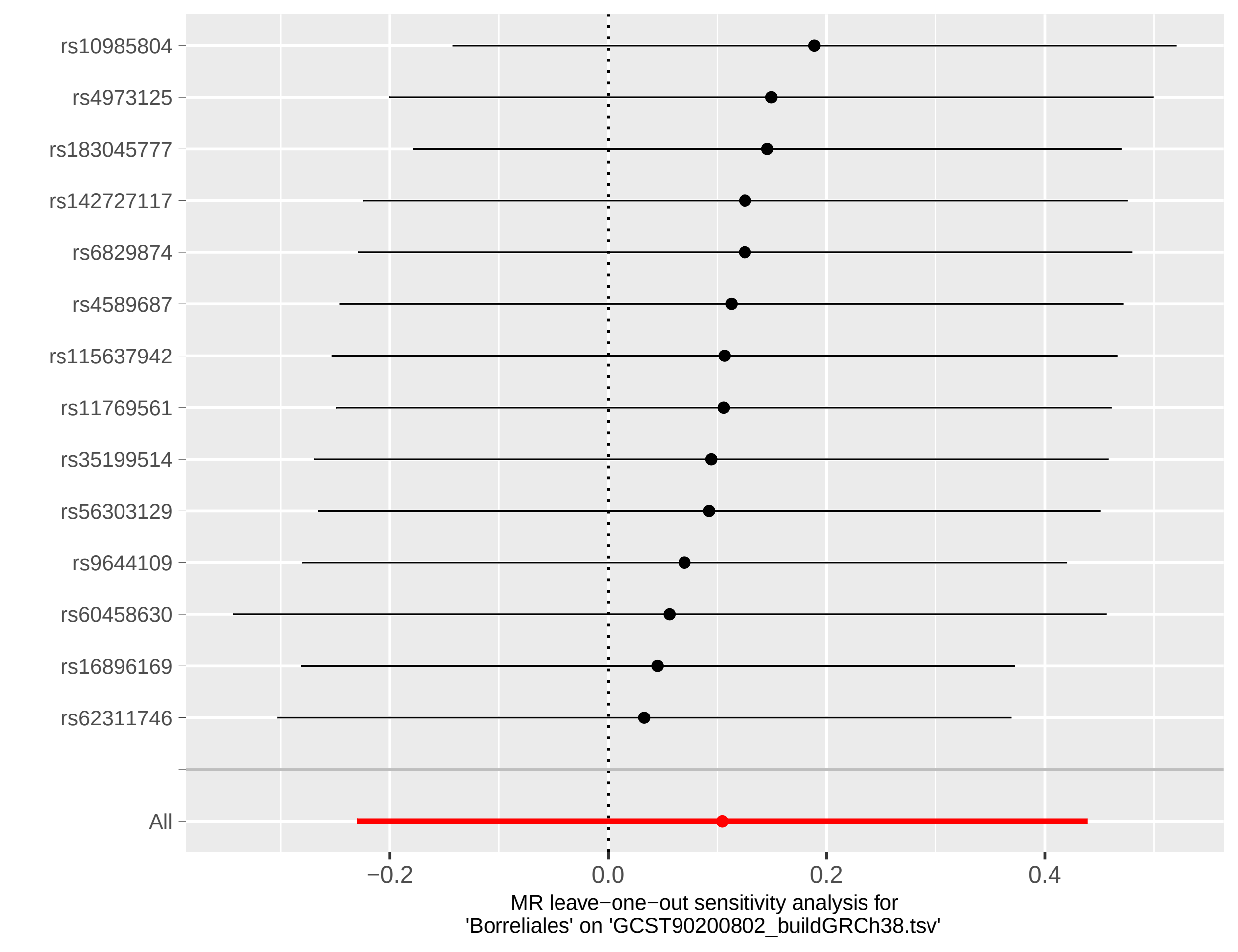
rs10985804
rs4973125
rs183045777
rs142727117
rs6829874
rs4589687
rs115637942
rs11769561
rs35199514
rs56303129
rs9644109
rs60458630
rs16896169
rs62311746
All
−0.2
0.0
0.2
0.4
MR leave−one−out sensitivity analysis for
'Borreliales' on 'GCST90200802_buildGRCh38.tsv'

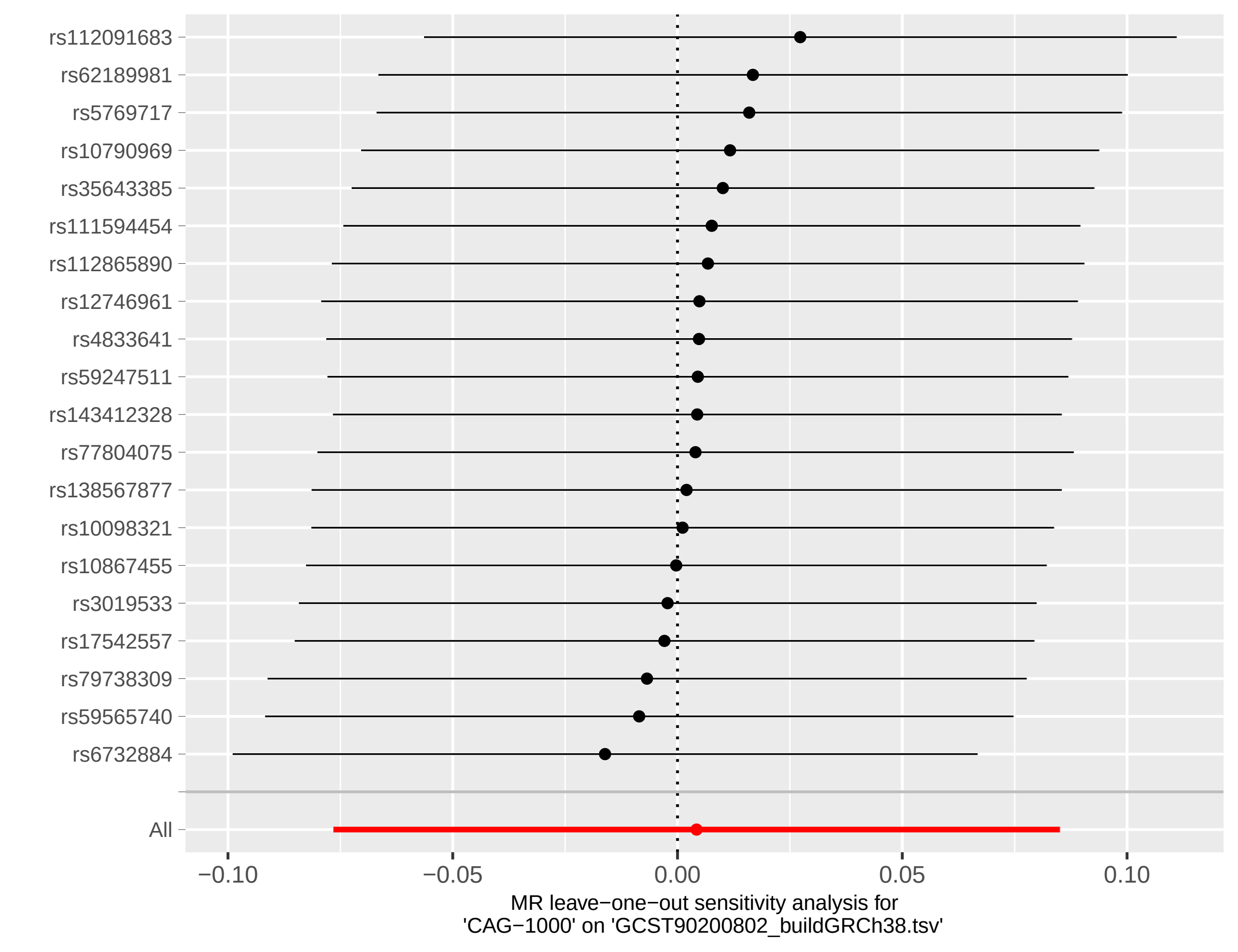
rs112091683
rs62189981
rs5769717
rs10790969
rs35643385
rs111594454
rs112865890
rs12746961
rs4833641
rs59247511
rs143412328
rs77804075
rs138567877
rs10098321
rs10867455
rs3019533
rs17542557
rs79738309
rs59565740
rs6732884
All
−0.10
−0.05
0.00
0.05
0.10
MR leave−one−out sensitivity analysis for
'CAG−1000' on 'GCST90200802_buildGRCh38.tsv'

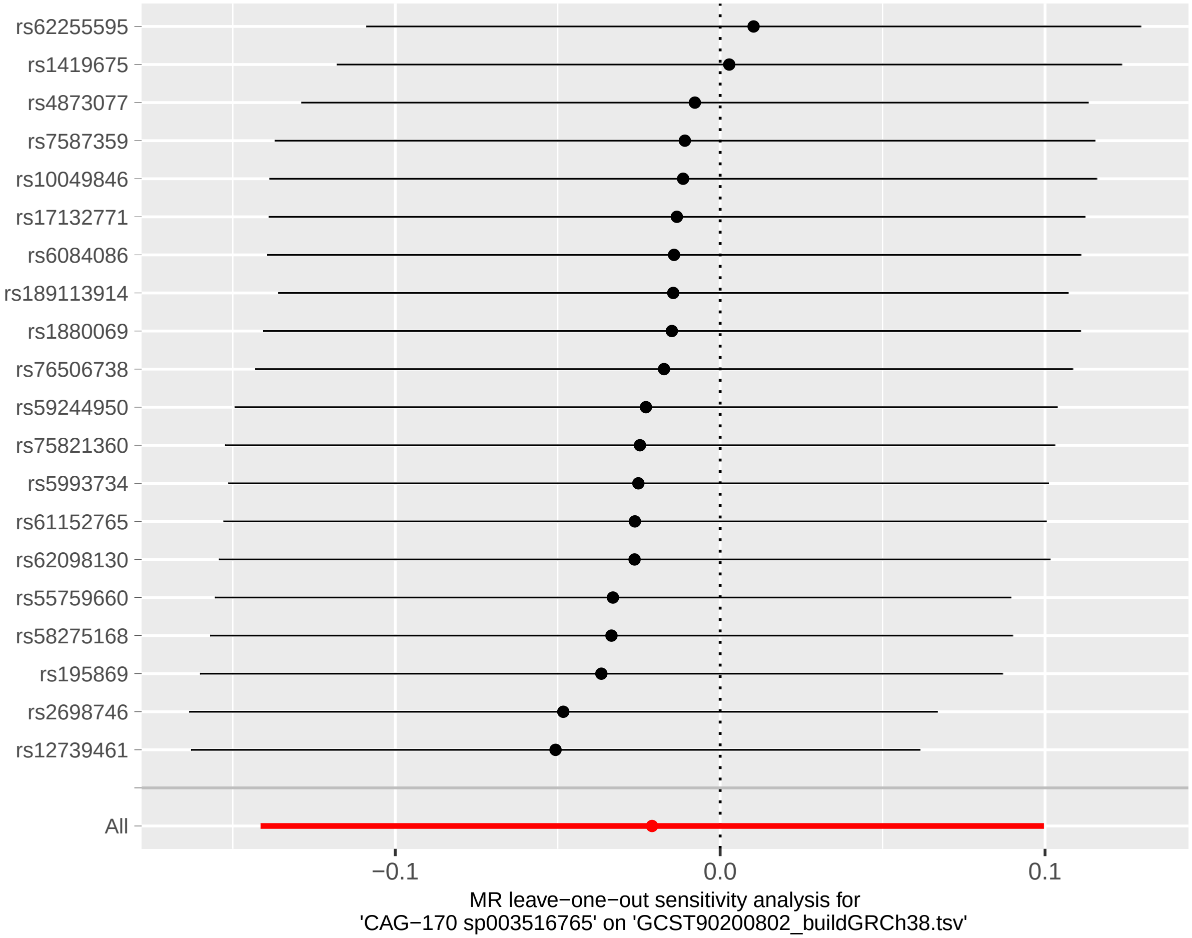

rs62255595
rs1419675
rs4873077
rs7587359
rs10049846
rs17132771
rs6084086
rs189113914
rs1880069
rs76506738
rs59244950
rs75821360
rs5993734
rs61152765
rs62098130
rs55759660
rs58275168
rs195869
rs2698746
rs12739461
All
−0.1
0.0
0.1
MR leave−one−out sensitivity analysis for
'CAG−170 sp003516765' on 'GCST90200802_buildGRCh38.tsv'

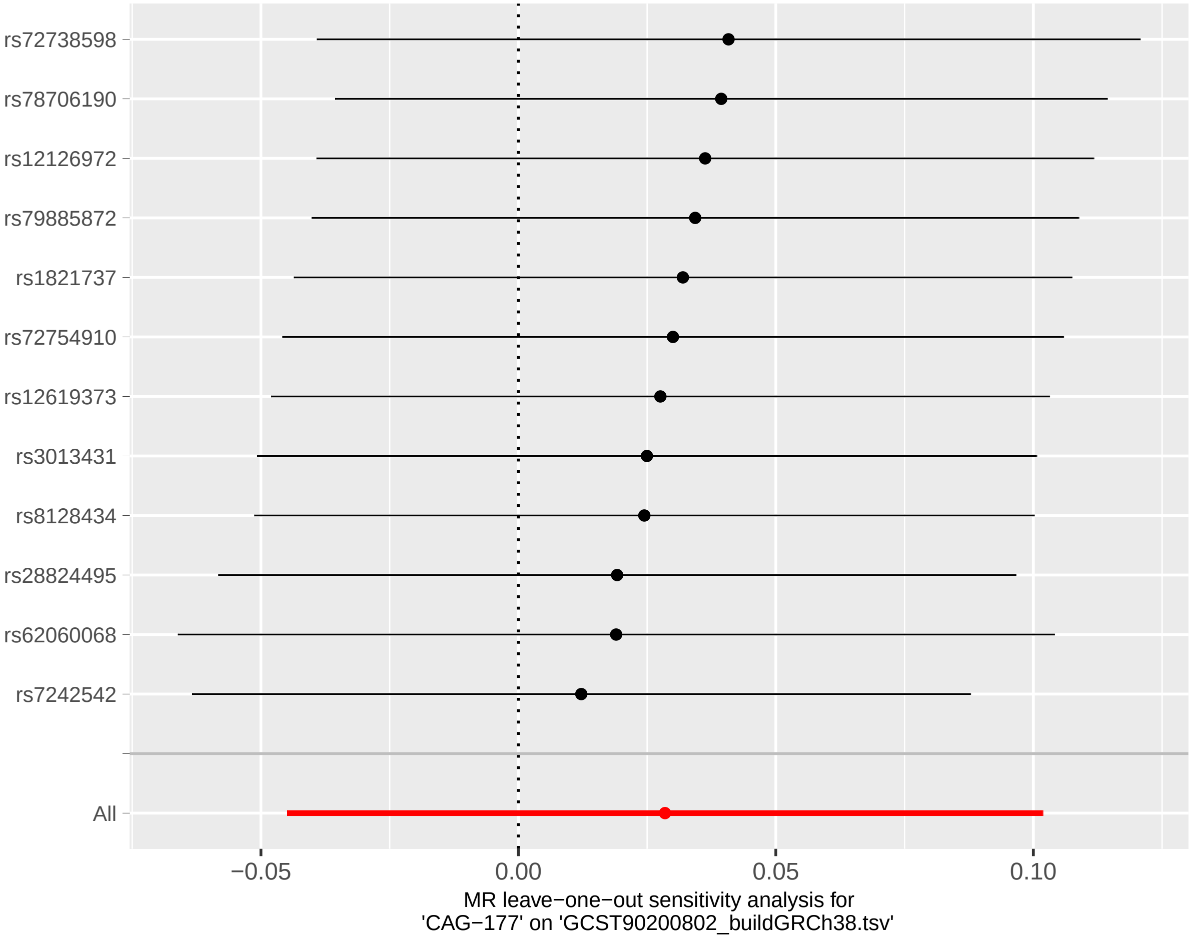
rs72738598
rs78706190
rs12126972
rs79885872
rs1821737
rs72754910
rs12619373
rs3013431
rs8128434
rs28824495
rs62060068
rs7242542
All
−0.05
0.00
0.05
0.10
MR leave−one−out sensitivity analysis for
'CAG−177' on 'GCST90200802_buildGRCh38.tsv'

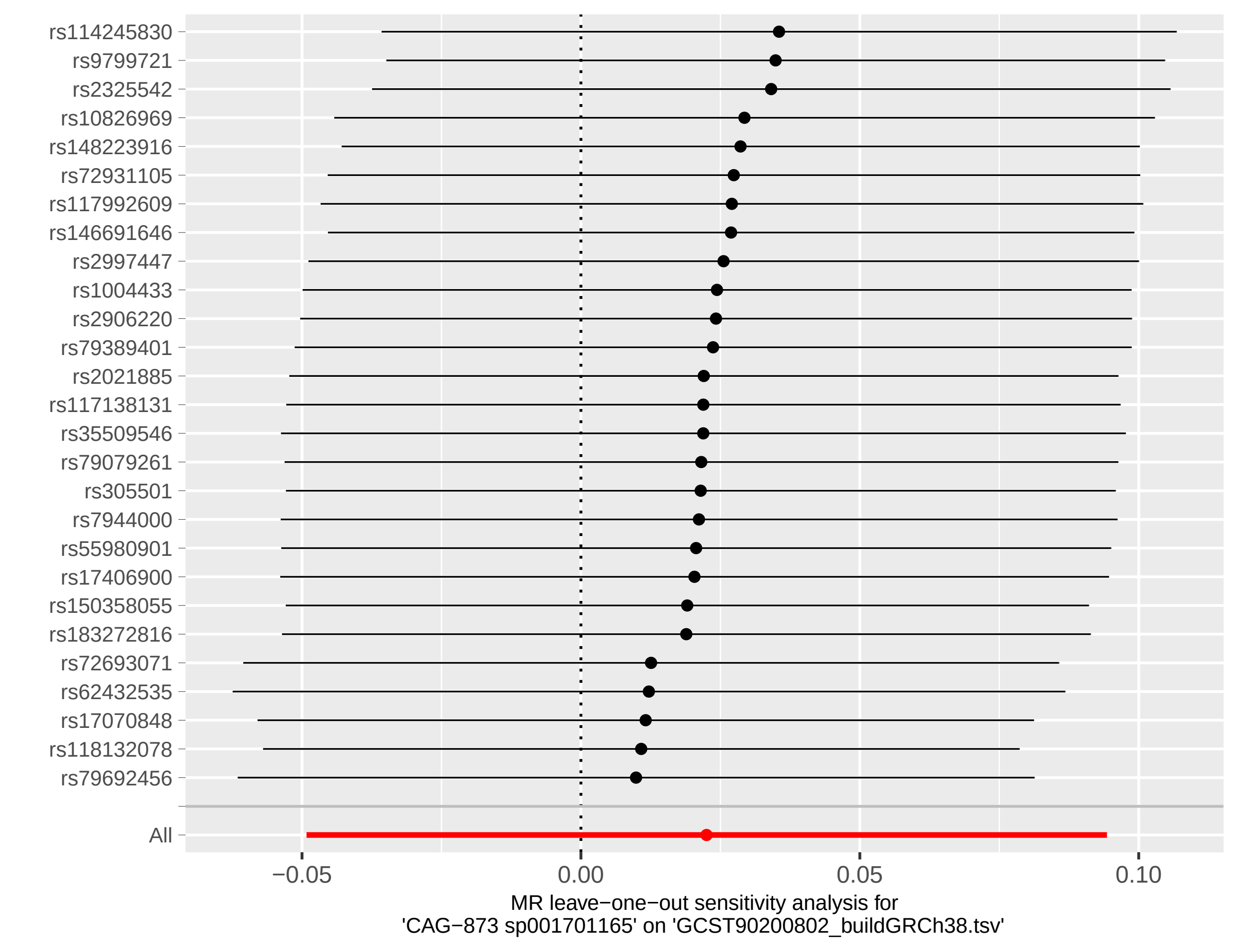

rs114245830
rs9799721
rs2325542
rs10826969
rs148223916
rs72931105
rs117992609
rs146691646
rs2997447
rs1004433
rs2906220
rs79389401
rs2021885
rs117138131
rs35509546
rs79079261
rs305501
rs7944000
rs55980901
rs17406900
rs150358055
rs183272816
rs72693071
rs62432535
rs17070848
rs118132078
rs79692456
All
−0.05
0.00
0.05
0.10
MR leave−one−out sensitivity analysis for
'CAG−873 sp001701165' on 'GCST90200802_buildGRCh38.tsv'

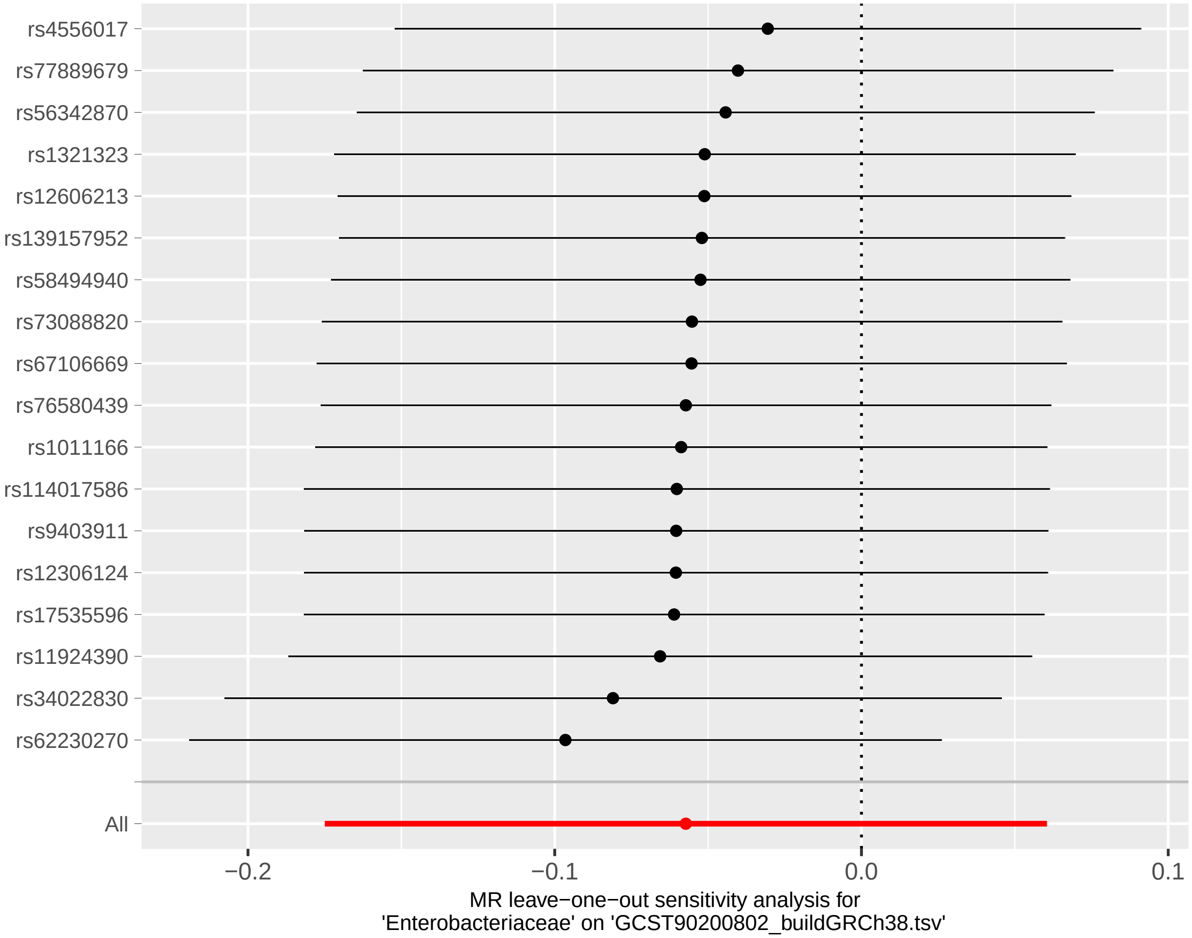

rs4556017
rs77889679
rs56342870
rs1321323
rs12606213
rs139157952
rs58494940
rs73088820
rs67106669
rs76580439
rs1011166
rs114017586
rs9403911
rs12306124
rs17535596
rs11924390
rs34022830
rs62230270
All
−0.2
−0.1
0.0
0.1
MR leave−one−out sensitivity analysis for
'Enterobacteriaceae' on 'GCST90200802_buildGRCh38.tsv'

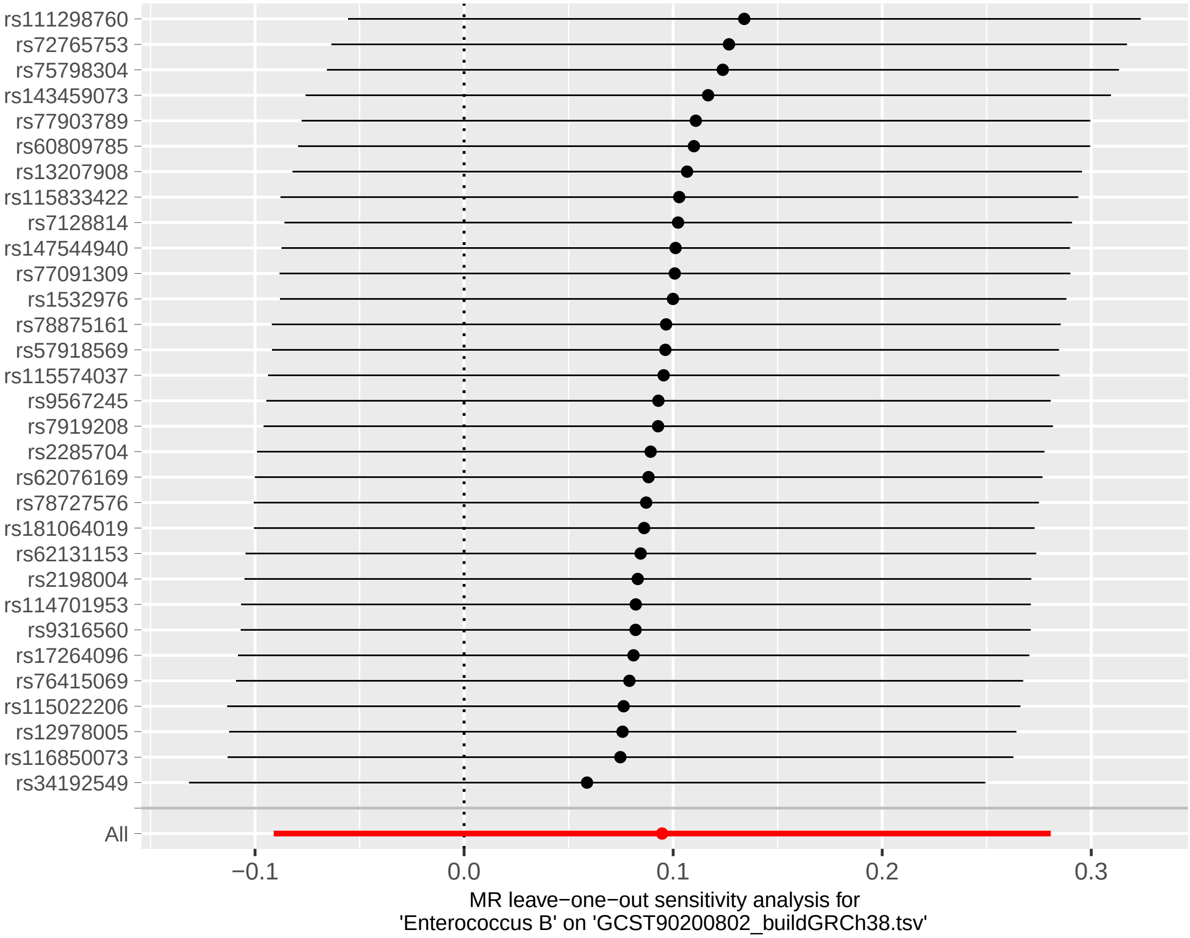
rs111298760
rs72765753
rs75798304
rs143459073
rs77903789
rs60809785
rs13207908
rs115833422
rs7128814
rs147544940
rs77091309
rs1532976
rs78875161
rs57918569
rs115574037
rs9567245
rs7919208
rs2285704
rs62076169
rs78727576
rs181064019
rs62131153
rs2198004
rs114701953
rs9316560
rs17264096
rs76415069
rs115022206
rs12978005
rs116850073
rs34192549
All
−0.1
0.0
0.1
0.2
0.3
MR leave−one−out sensitivity analysis for
'Enterococcus B' on 'GCST90200802_buildGRCh38.tsv'

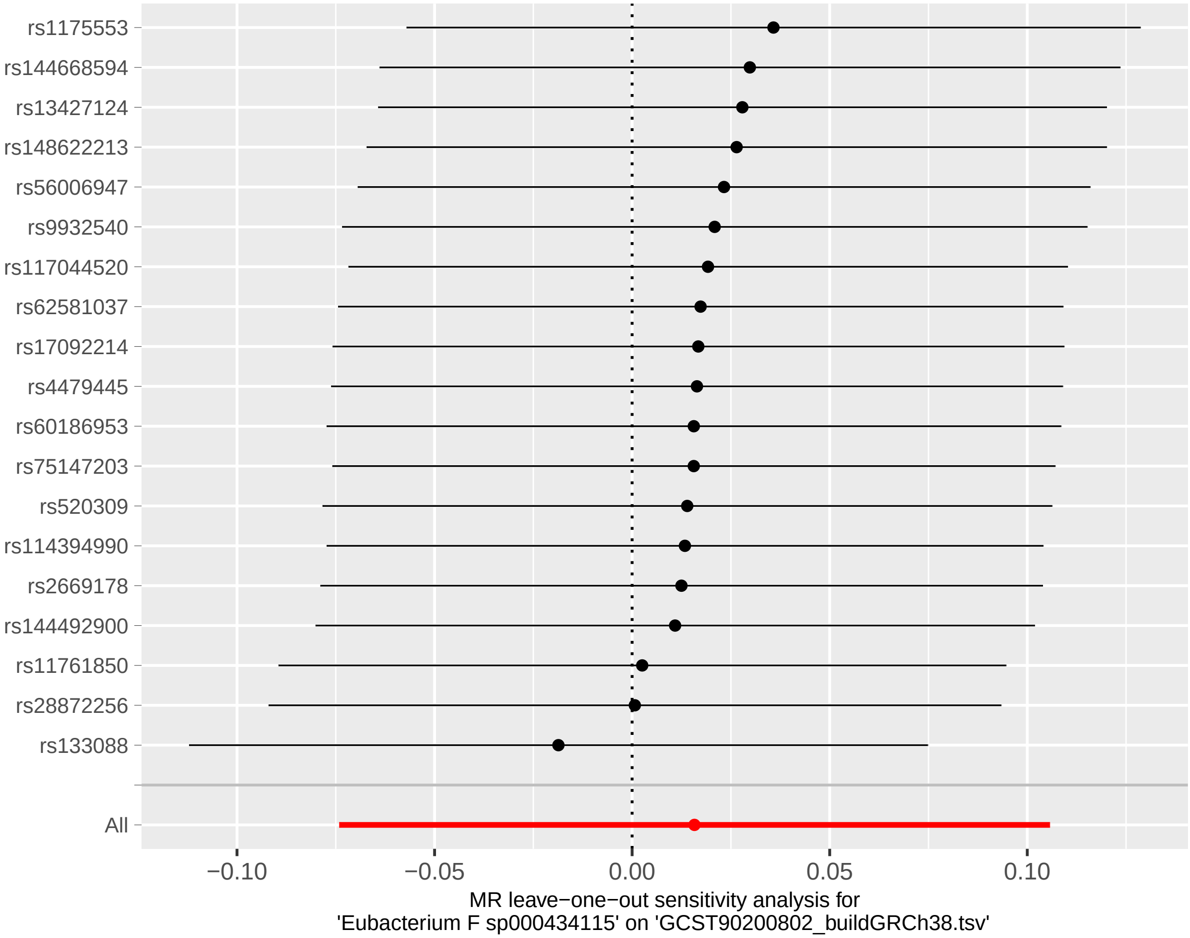
rs1175553
rs144668594
rs13427124
rs148622213
rs56006947
rs9932540
rs117044520
rs62581037
rs17092214
rs4479445
rs60186953
rs75147203
rs520309
rs114394990
rs2669178
rs144492900
rs11761850
rs28872256
rs133088
All
−0.10
−0.05
0.00
0.05
0.10
MR leave−one−out sensitivity analysis for
'Eubacterium F sp000434115' on 'GCST90200802_buildGRCh38.tsv'

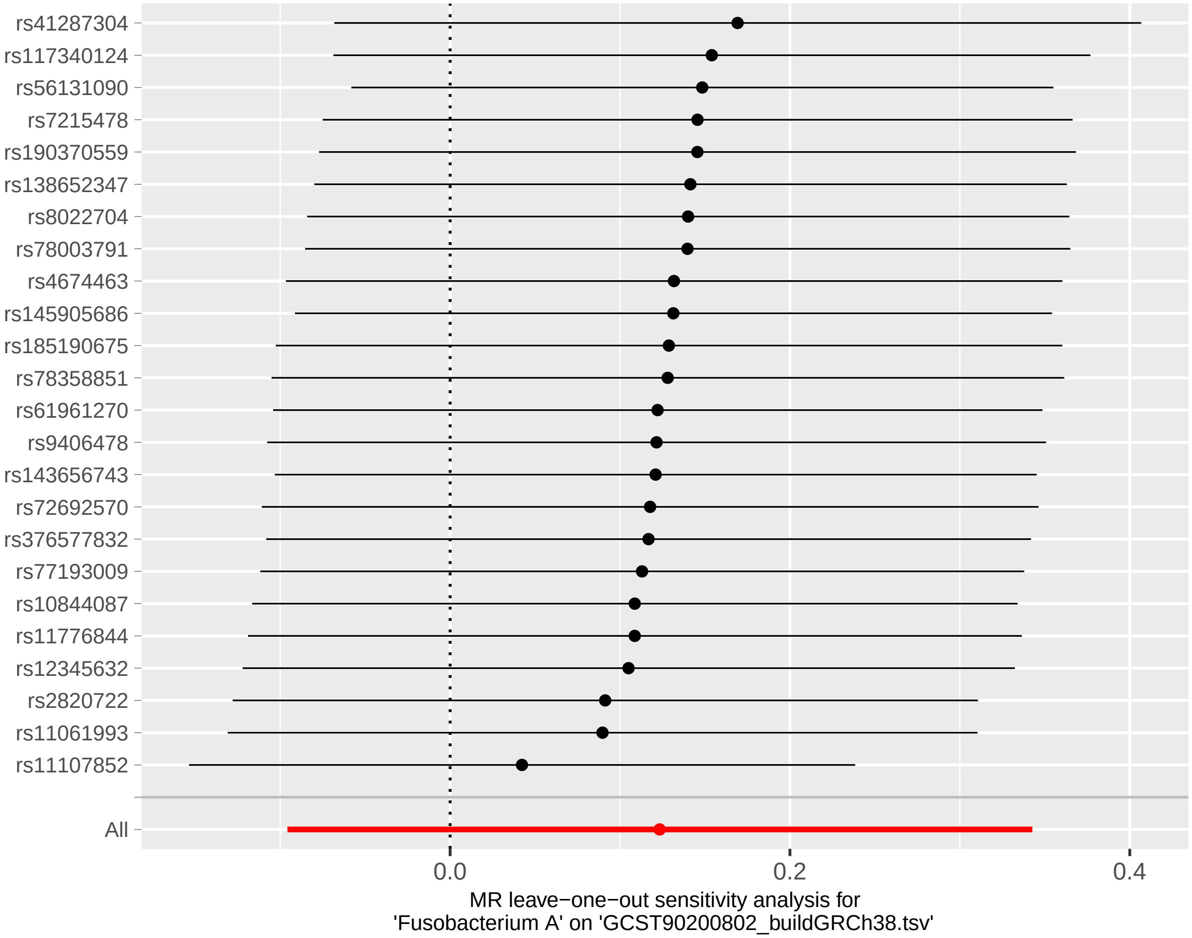

rs41287304
rs117340124
rs56131090
rs7215478
rs190370559
rs138652347
rs8022704
rs78003791
rs4674463
rs145905686
rs185190675
rs78358851
rs61961270
rs9406478
rs143656743
rs72692570
rs376577832
rs77193009
rs10844087
rs11776844
rs12345632
rs2820722
rs11061993
rs11107852
All
0.0
0.2
0.4
MR leave−one−out sensitivity analysis for
'Fusobacterium A' on 'GCST90200802_buildGRCh38.tsv'

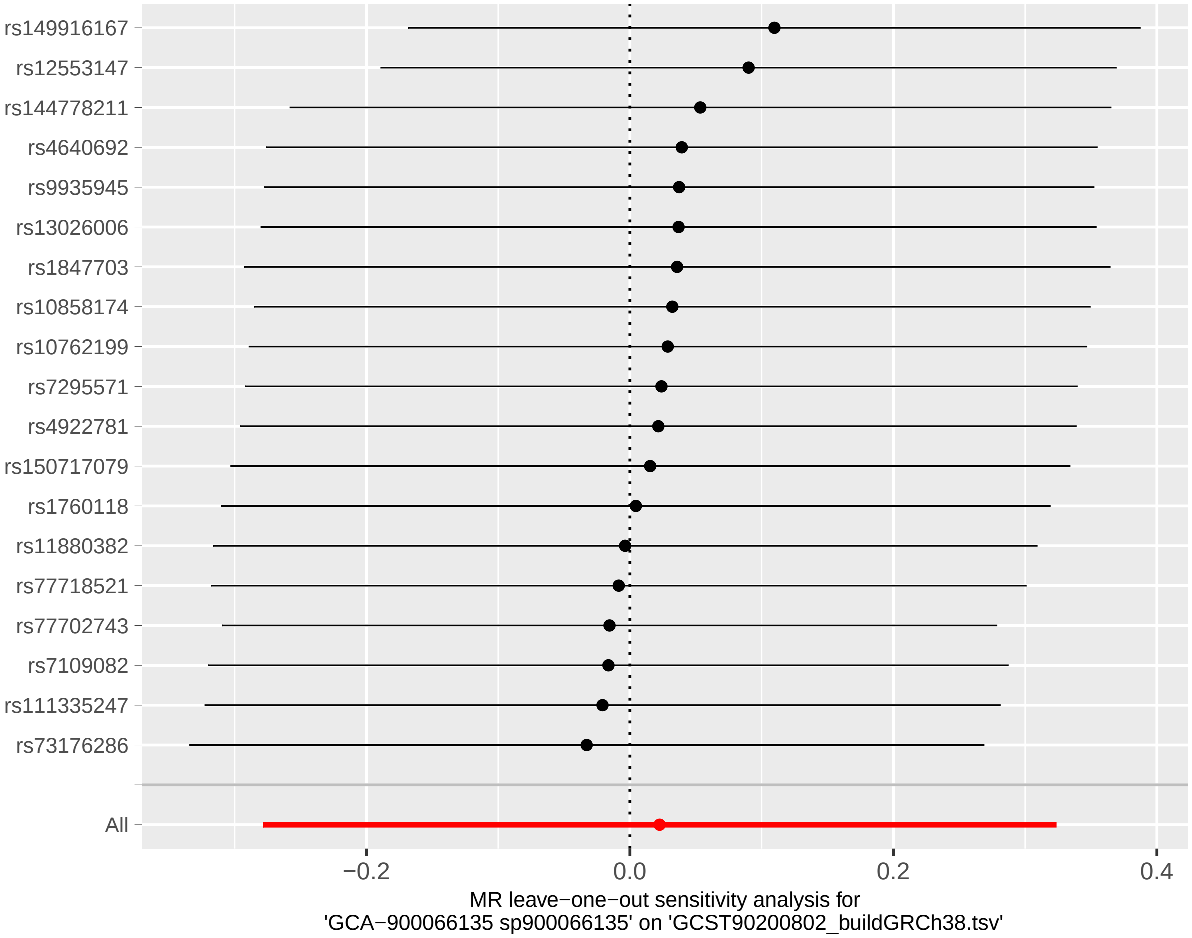
rs149916167
rs12553147
rs144778211
rs4640692
rs9935945
rs13026006
rs1847703
rs10858174
rs10762199
rs7295571
rs4922781
rs150717079
rs1760118
rs11880382
rs77718521
rs77702743
rs7109082
rs111335247
rs73176286
All
−0.2
0.0
0.2
0.4
MR leave−one−out sensitivity analysis for
'GCA−900066135 sp900066135' on 'GCST90200802_buildGRCh38.tsv'

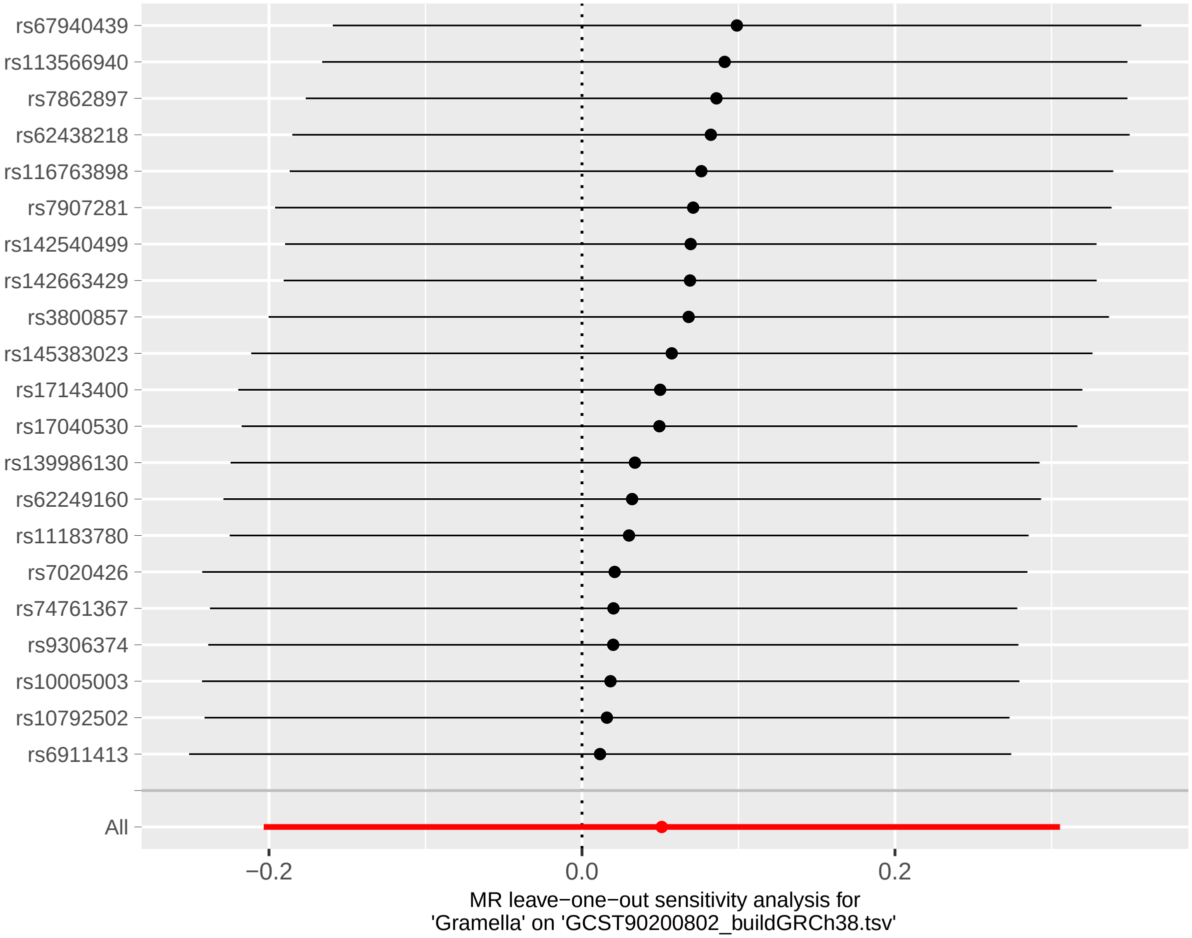

rs67940439
rs113566940
rs7862897
rs62438218
rs116763898
rs7907281
rs142540499
rs142663429
rs3800857
rs145383023
rs17143400
rs17040530
rs139986130
rs62249160
rs11183780
rs7020426
rs74761367
rs9306374
rs10005003
rs10792502
rs6911413
All
−0.2
0.0
0.2
MR leave−one−out sensitivity analysis for
'Gramella' on 'GCST90200802_buildGRCh38.tsv'

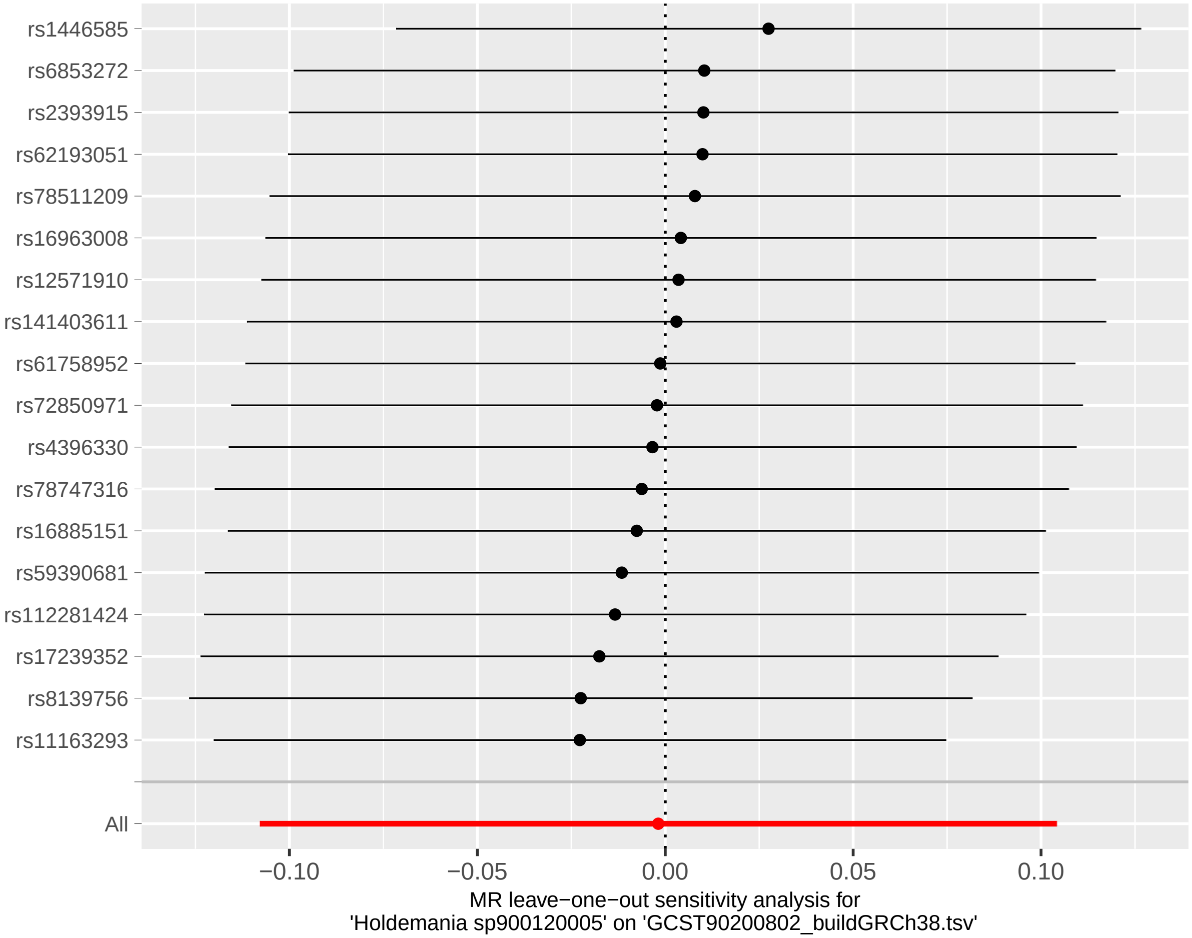
rs1446585
rs6853272
rs2393915
rs62193051
rs78511209
rs16963008
rs12571910
rs141403611
rs61758952
rs72850971
rs4396330
rs78747316
rs16885151
rs59390681
rs112281424
rs17239352
rs8139756
rs11163293
All
−0.10
−0.05
0.00
0.05
0.10
MR leave−one−out sensitivity analysis for
'Holdemania sp900120005' on 'GCST90200802_buildGRCh38.tsv'

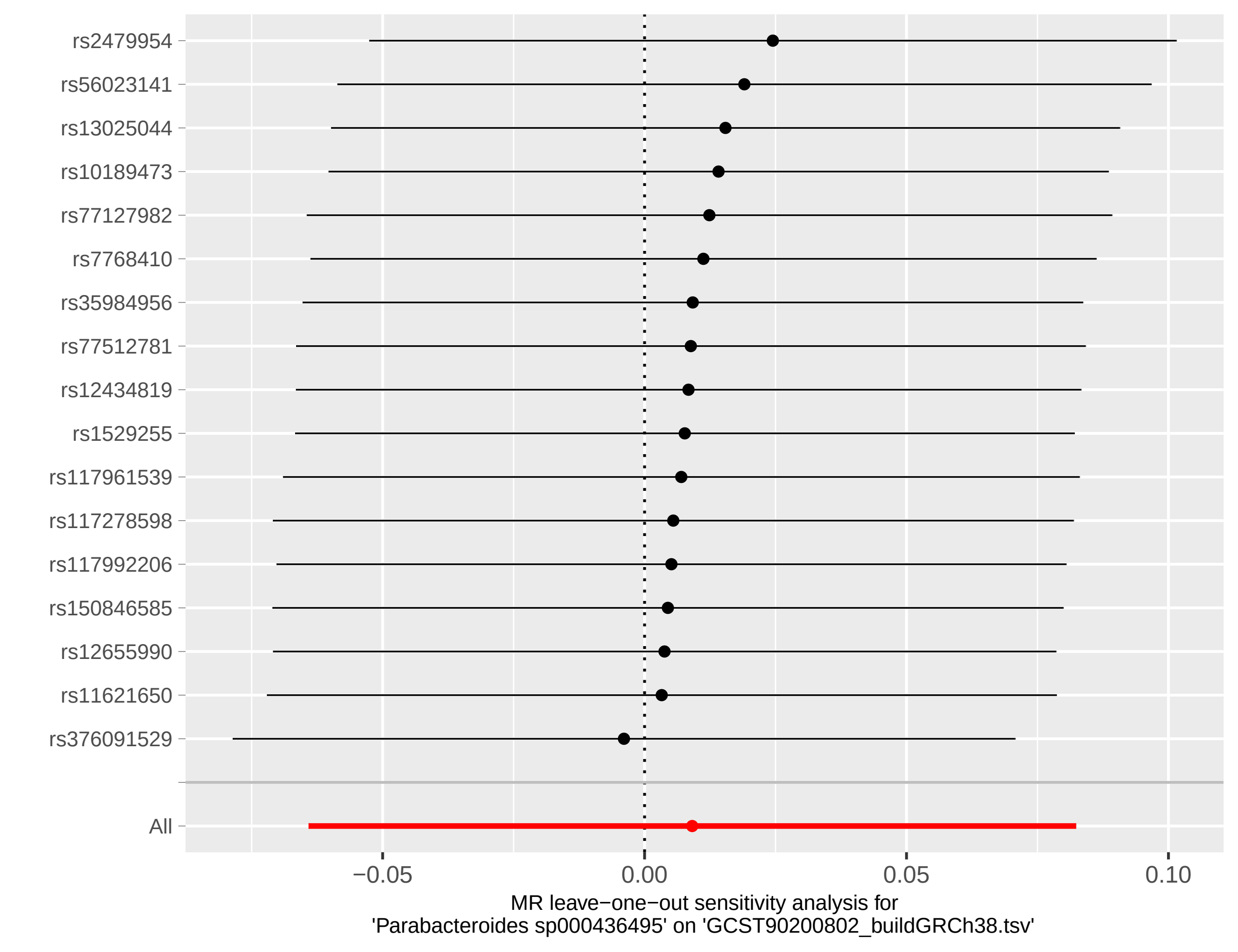

rs2479954
rs56023141
rs13025044
rs10189473
rs77127982
rs7768410
rs35984956
rs77512781
rs12434819
rs1529255
rs117961539
rs117278598
rs117992206
rs150846585
rs12655990
rs11621650
rs376091529
All
−0.05
0.00
0.05
0.10
MR leave−one−out sensitivity analysis for
'Parabacteroides sp000436495' on 'GCST90200802_buildGRCh38.tsv'

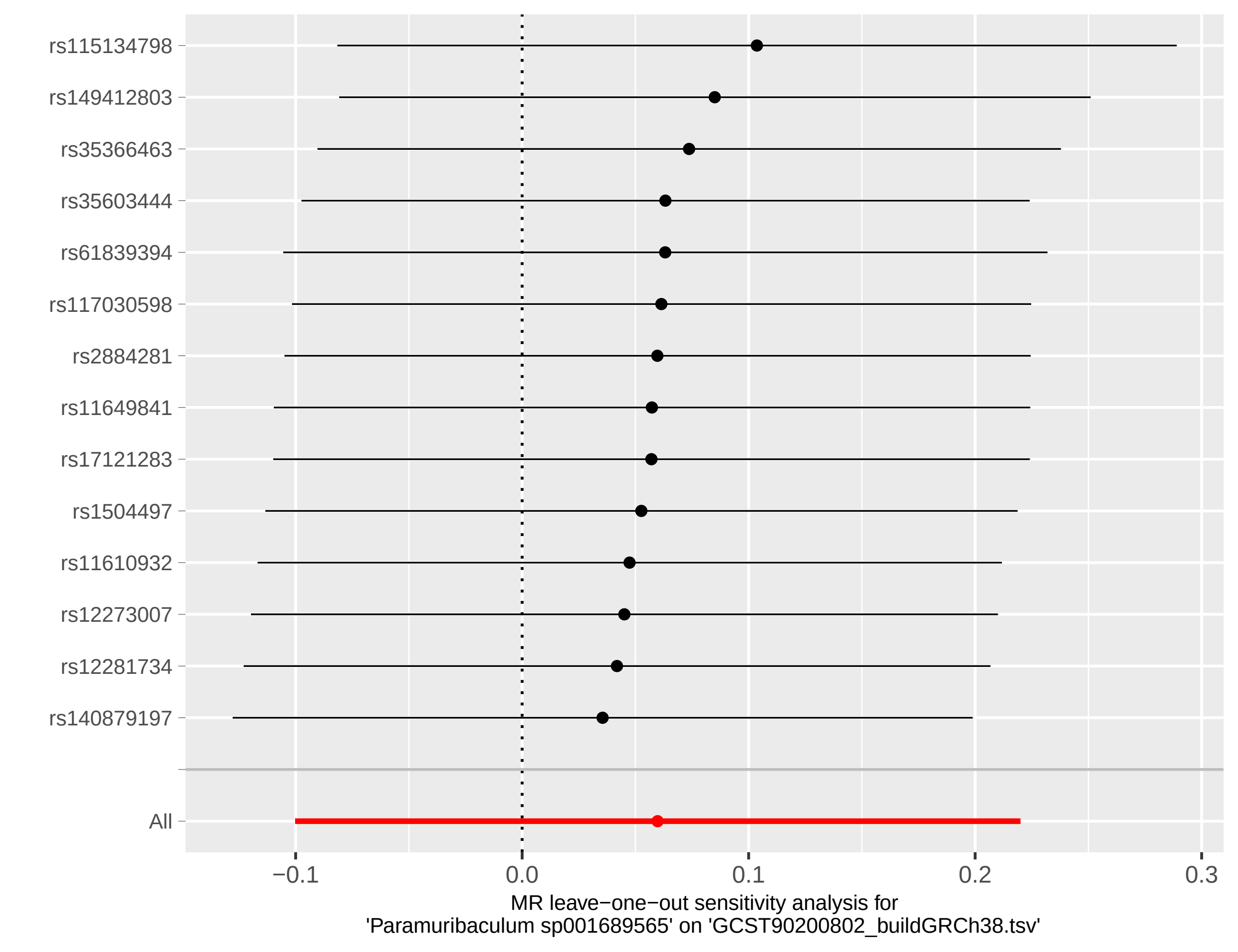
rs115134798
rs149412803
rs35366463
rs35603444
rs61839394
rs117030598
rs2884281
rs11649841
rs17121283
rs1504497
rs11610932
rs12273007
rs12281734
rs140879197
All
−0.1
0.0
0.1
0.2
0.3
MR leave−one−out sensitivity analysis for
'Paramuribaculum sp001689565' on 'GCST90200802_buildGRCh38.tsv'

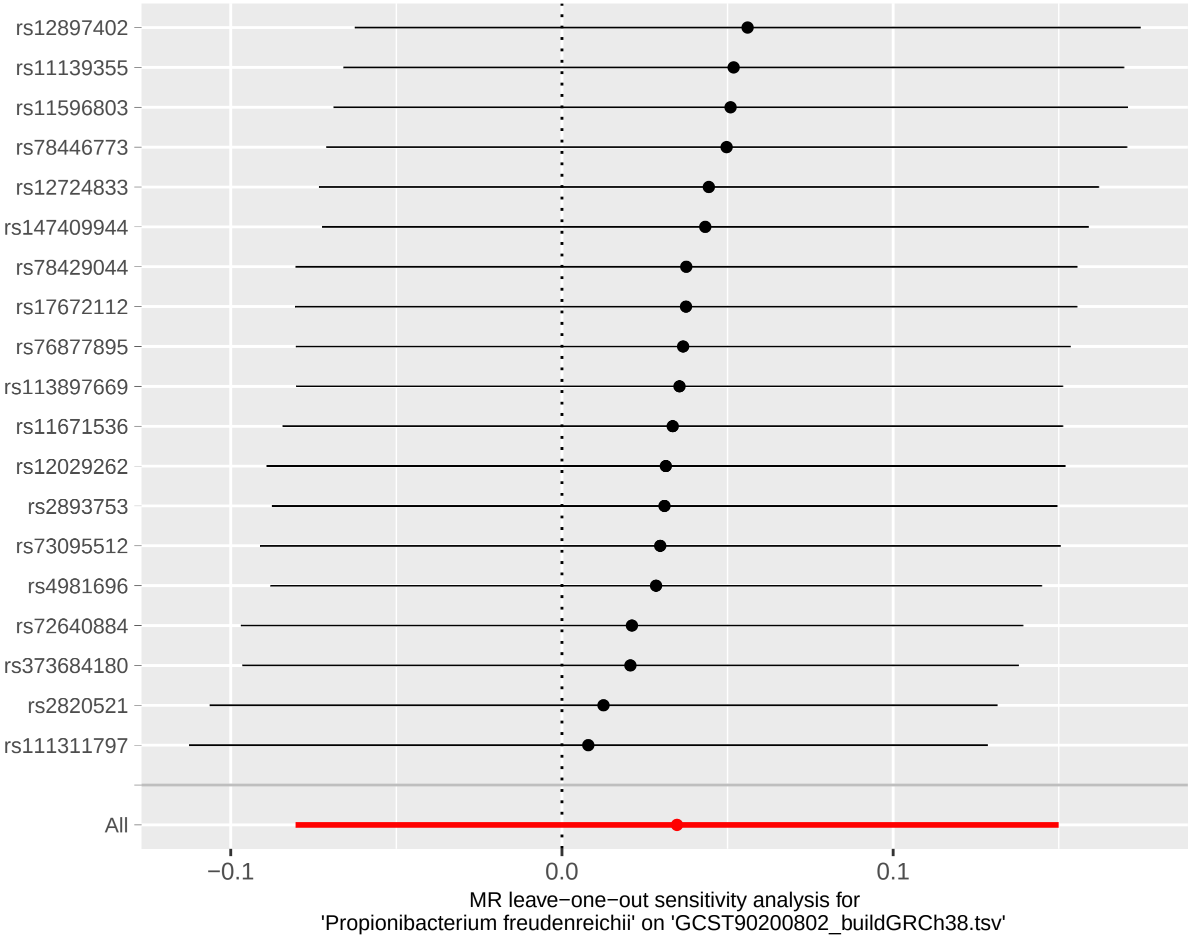

rs12897402
rs11139355
rs11596803
rs78446773
rs12724833
rs147409944
rs78429044
rs17672112
rs76877895
rs113897669
rs11671536
rs12029262
rs2893753
rs73095512
rs4981696
rs72640884
rs373684180
rs2820521
rs111311797
All
−0.1
0.0
0.1
MR leave−one−out sensitivity analysis for
'Propionibacterium freudenreichii' on 'GCST90200802_buildGRCh38.tsv'

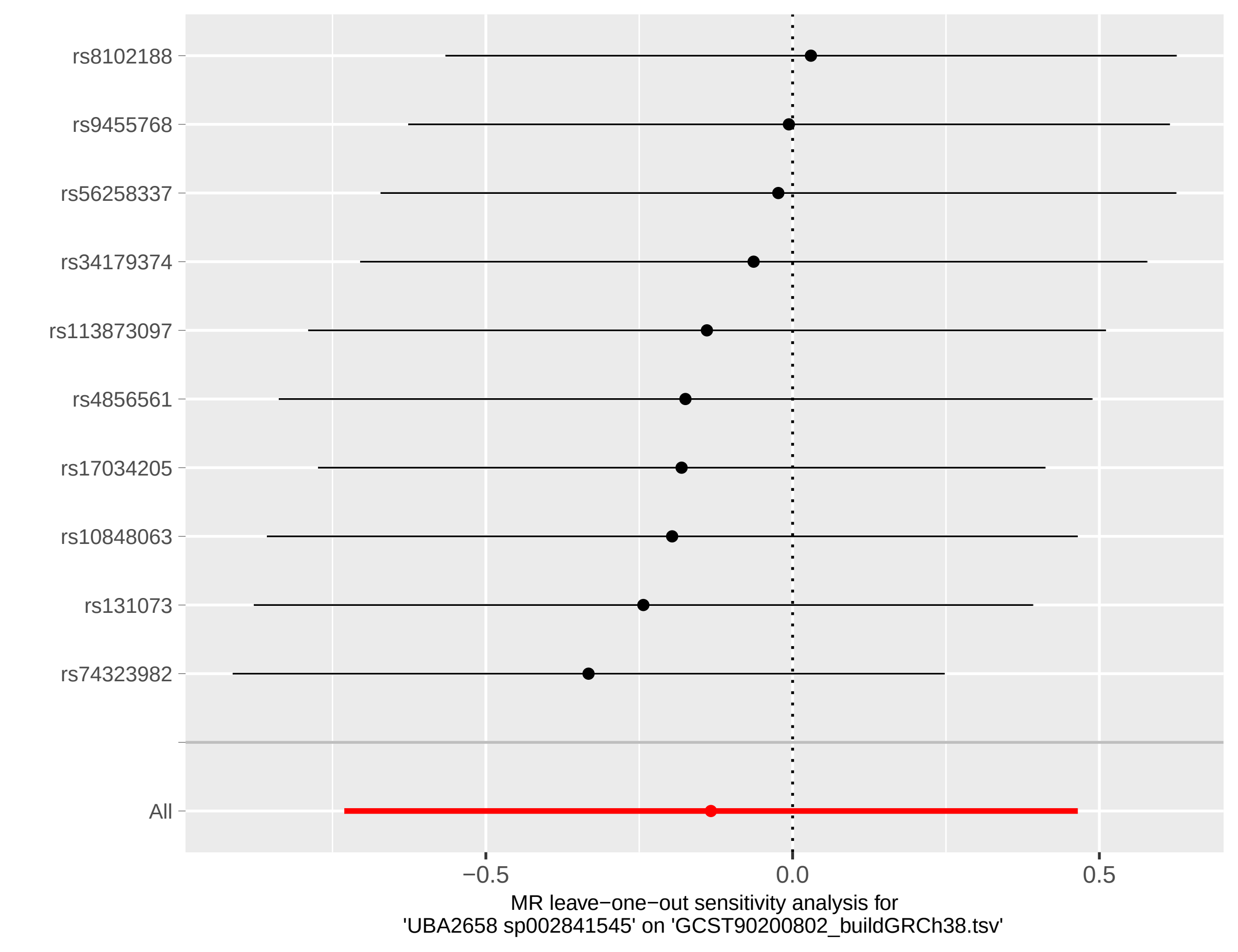
rs8102188
rs9455768
rs56258337
rs34179374
rs113873097
rs4856561
rs17034205
rs10848063
rs131073
rs74323982
All
−0.5
0.0
0.5
MR leave−one−out sensitivity analysis for
'UBA2658 sp002841545' on 'GCST90200802_buildGRCh38.tsv'

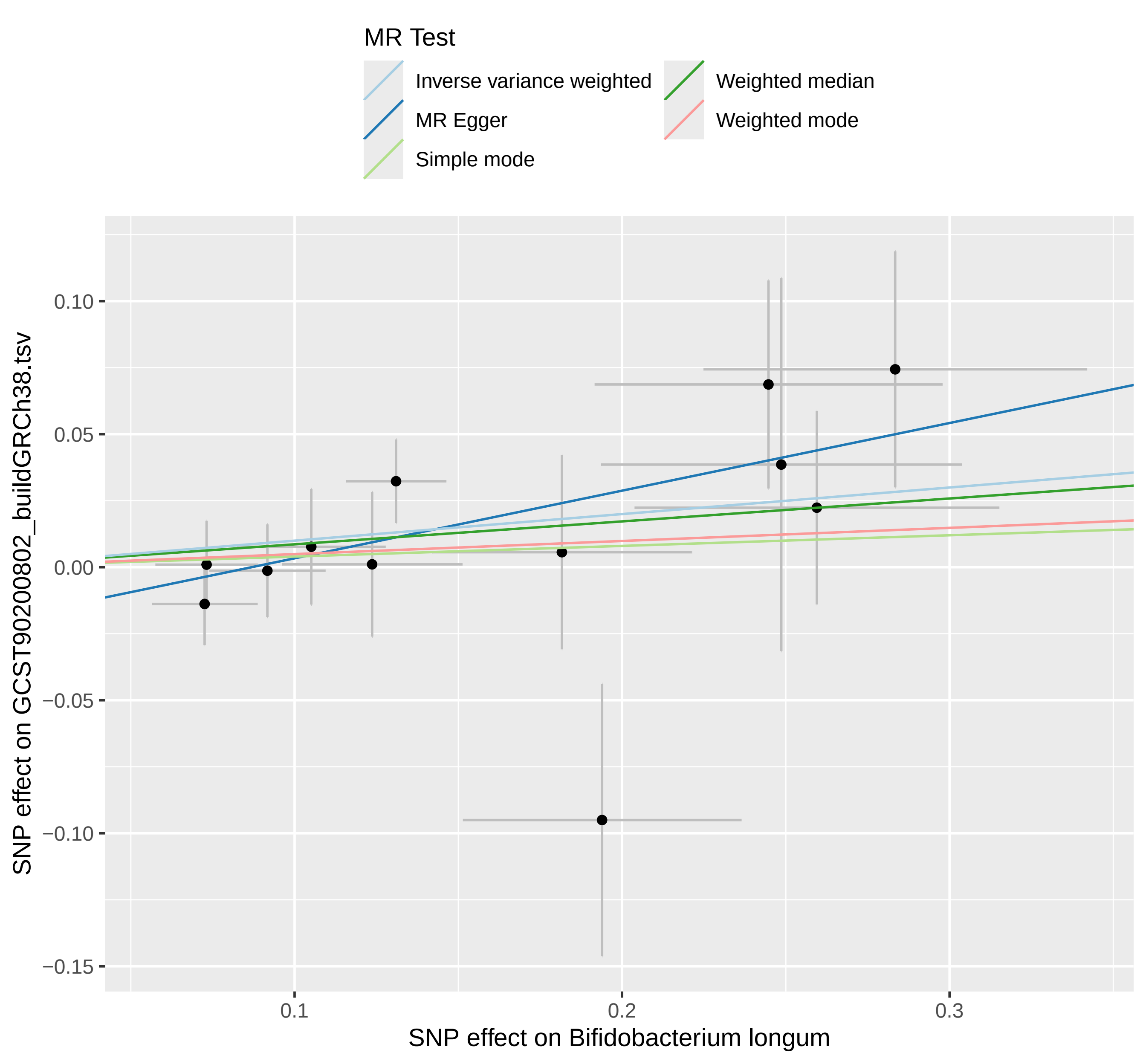

MR Test
Inverse variance weighted
MR Egger
Simple mode
Weighted median
Weighted mode
0.10
0.05
0.00
−0.05
−0.10
−0.15
SNP effect on GCST90200802_buildGRCh38.tsv
0.1
0.2
0.3
SNP effect on Bifidobacterium longum

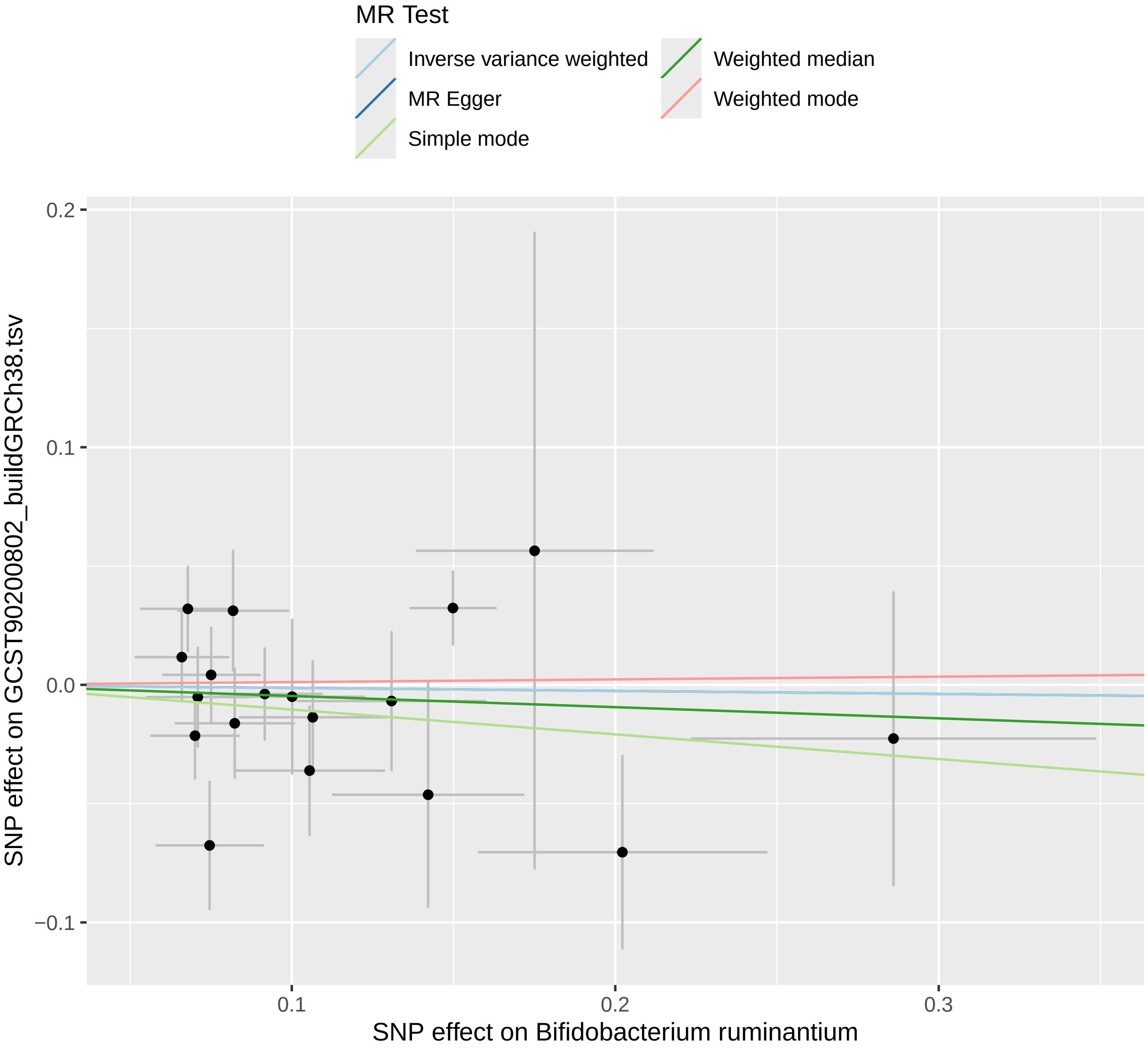
MR Test
Inverse variance weighted
MR Egger
Simple mode
Weighted median
Weighted mode
0.2
0.1
0.0
−0.1
0.1
0.2
0.3
SNP effect on GCST90200802_buildGRCh38.tsv
SNP effect on Bifidobacterium ruminantium

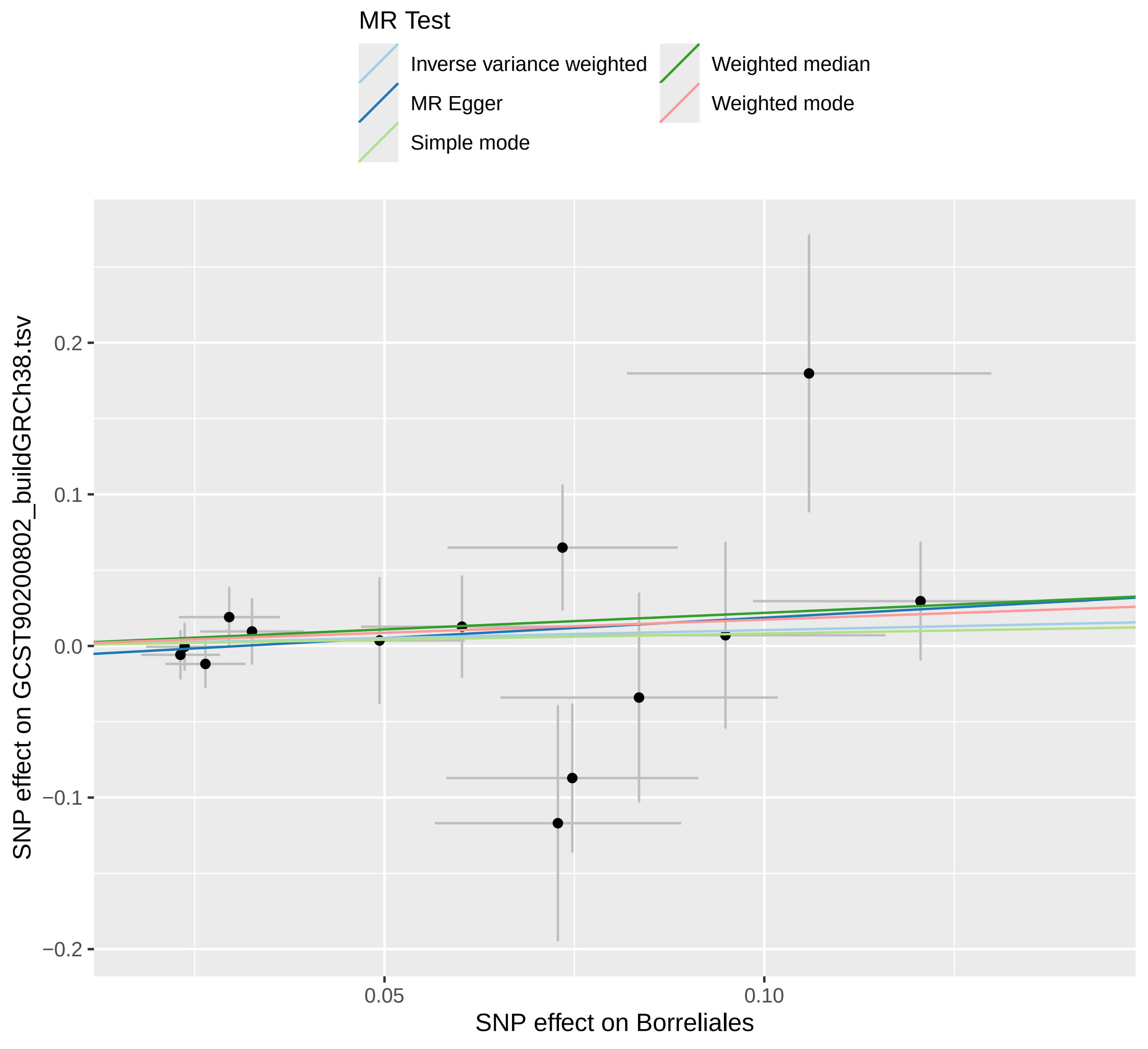
MR Test
Inverse variance weighted
MR Egger
Simple mode
Weighted median
Weighted mode
SNP effect on GCST90200802_buildGRCh38.tsv
0.2
0.1
0.0
−0.1
−0.2
0.05
0.10
SNP effect on Borreliales

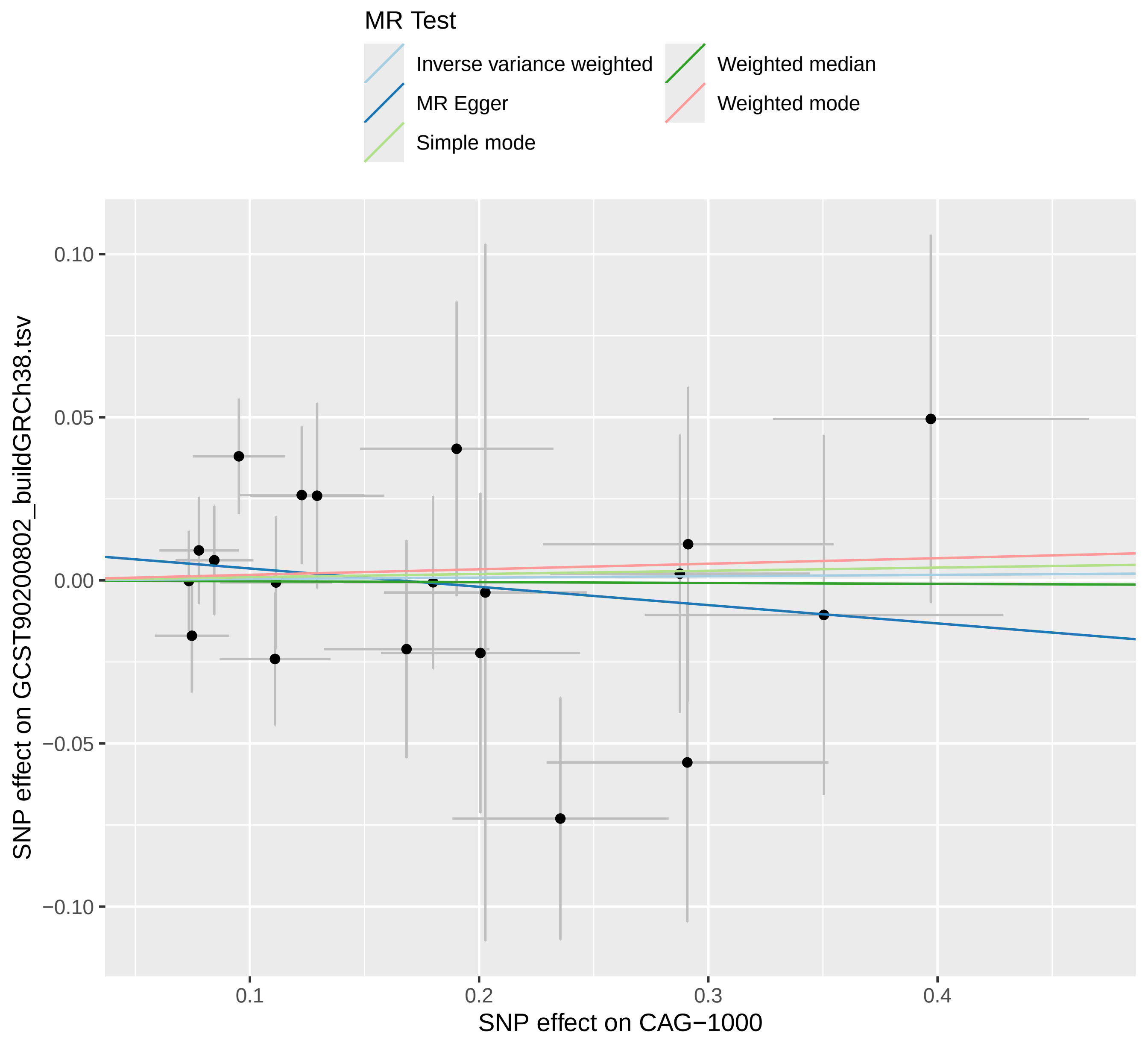

MR Test
Inverse variance weighted
MR Egger
Simple mode
Weighted median
Weighted mode
0.10
0.05
0.00
−0.05
−0.10
SNP effect on GCST90200802_buildGRCh38.tsv
0.1
0.2
0.3
0.4
SNP effect on CAG−1000

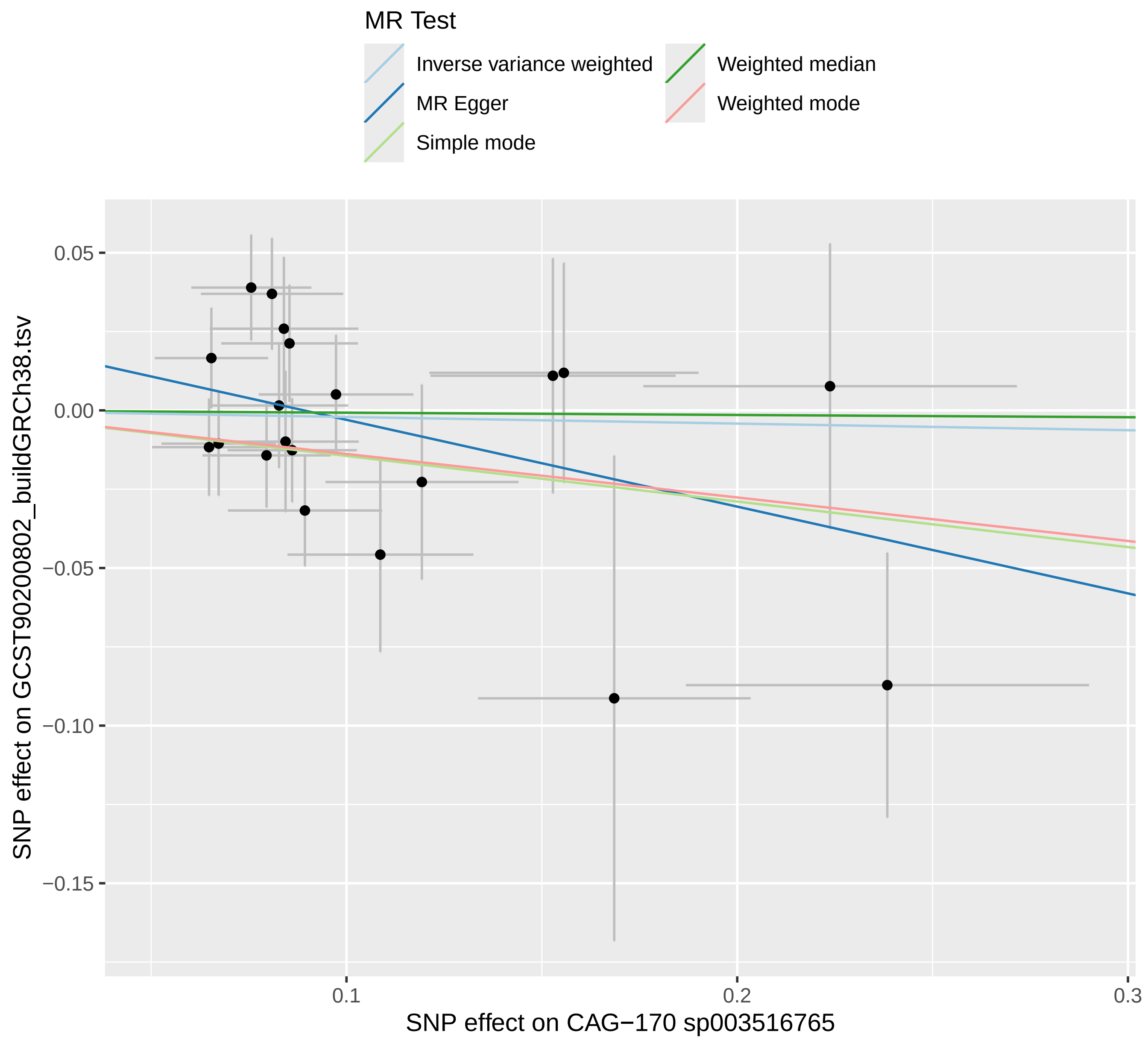

MR Test
Inverse variance weighted
MR Egger
Simple mode
Weighted median
Weighted mode
0.05
0.00
−0.05
−0.10
−0.15
0.1
0.2
0.3
SNP effect on GCST90200802_buildGRCh38.tsv
SNP effect on CAG−170 sp003516765

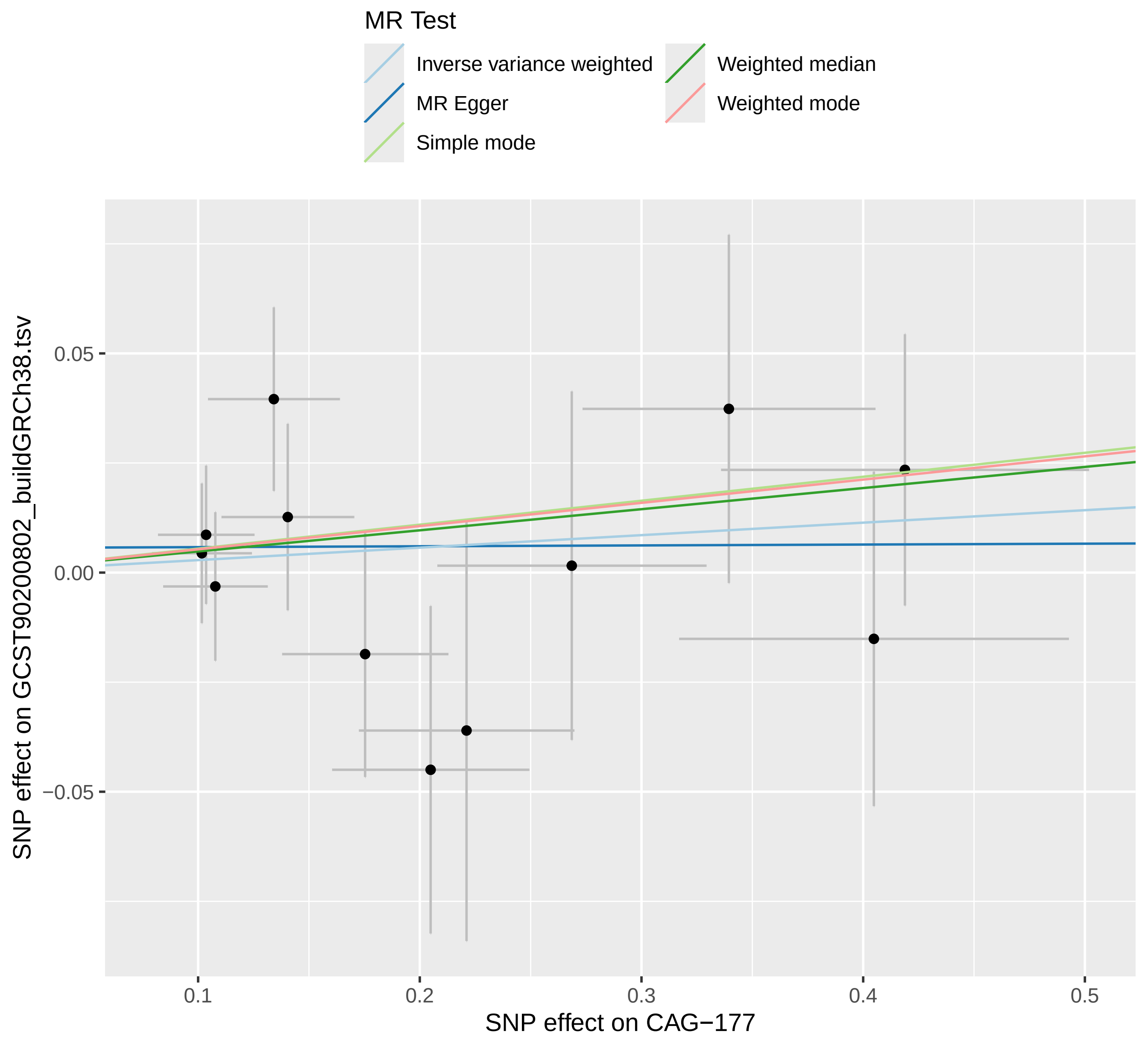

MR Test
Inverse variance weighted
MR Egger
Simple mode
Weighted median
Weighted mode
SNP effect on GCST90200802_buildGRCh38.tsv
0.05
0.00
−0.05
0.1
0.2
0.3
0.4
0.5
SNP effect on CAG−177

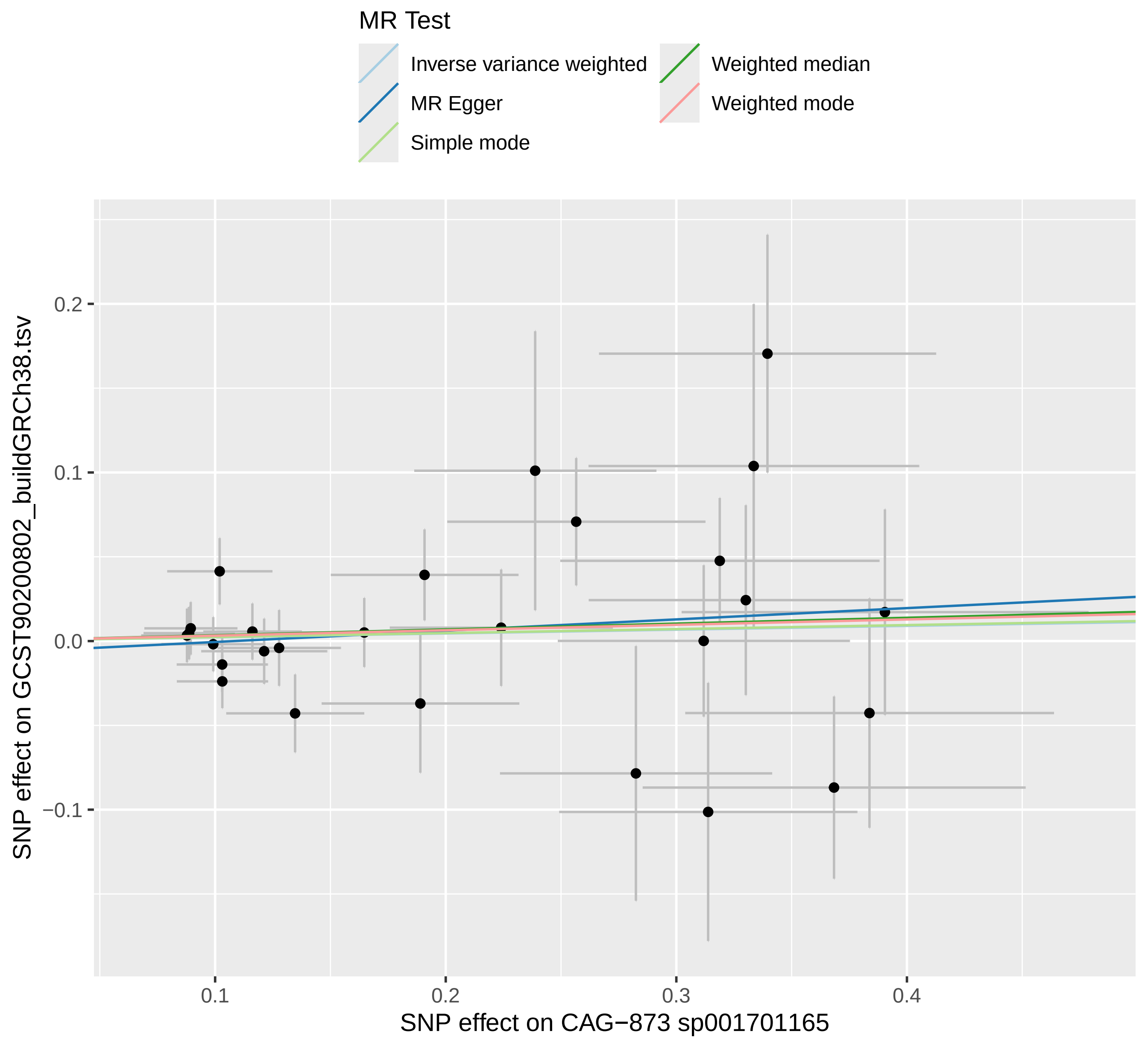
MR Test
Inverse variance weighted
MR Egger
Simple mode
Weighted median
Weighted mode
0.2
0.1
0.0
−0.1
0.1
0.2
0.3
0.4
SNP effect on GCST90200802_buildGRCh38.tsv
SNP effect on CAG−873 sp001701165

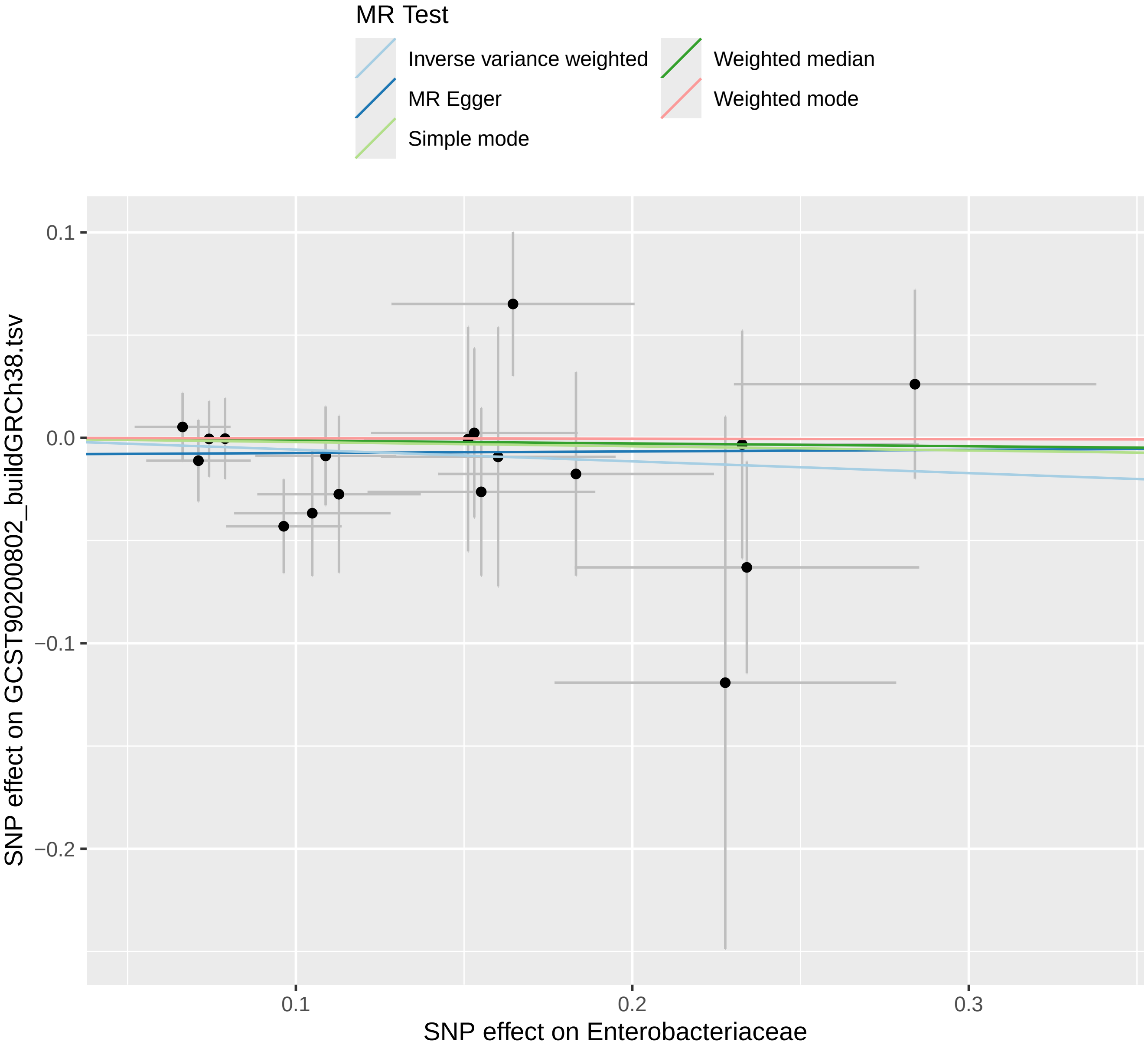

MR Test
Inverse variance weighted
MR Egger
Simple mode
Weighted median
Weighted mode
0.1
0.0
−0.1
−0.2
0.1
0.2
0.3
SNP effect on GCST90200802_buildGRCh38.tsv
SNP effect on Enterobacteriaceae

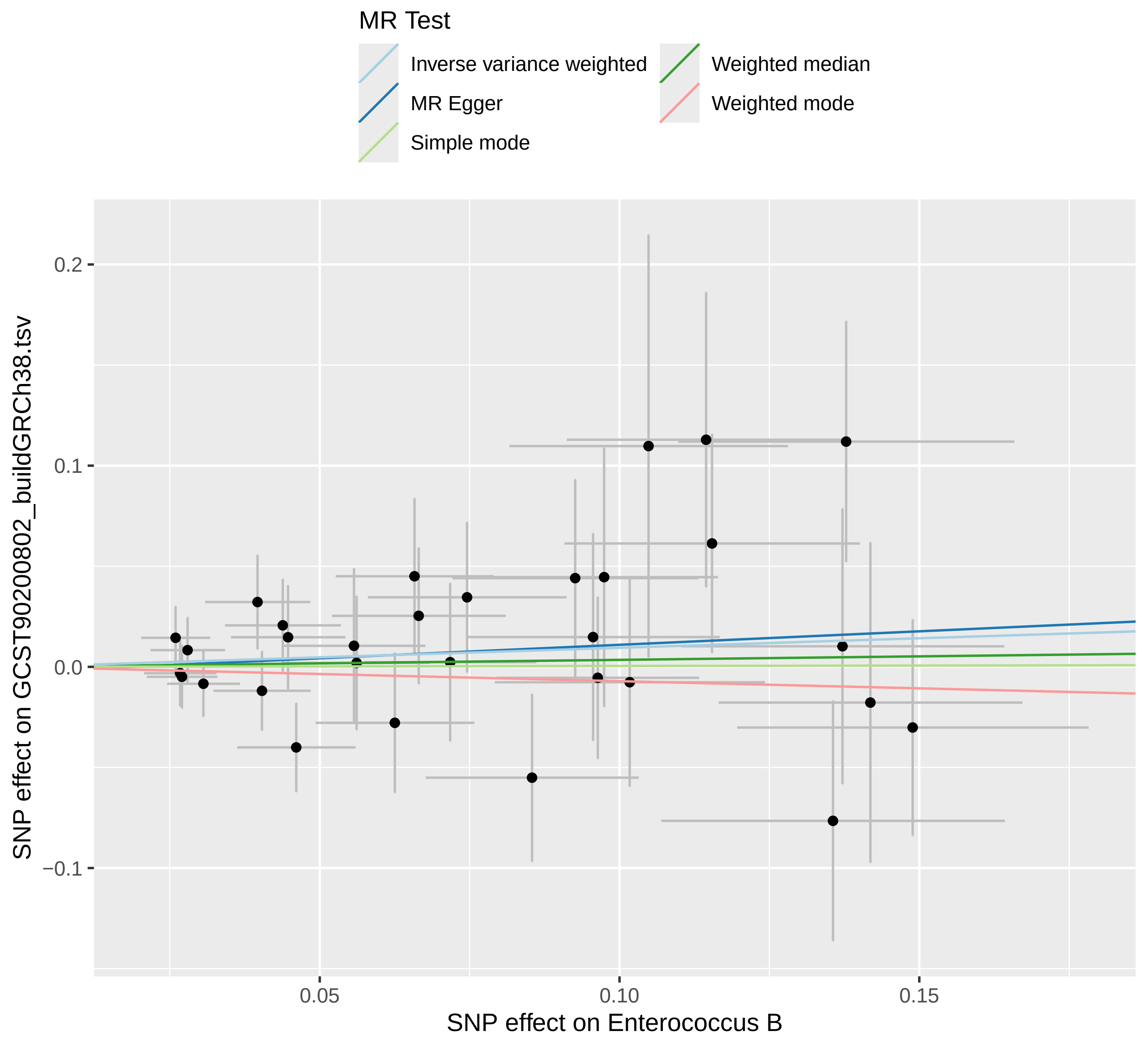
MR Test
Inverse variance weighted
MR Egger
Simple mode
Weighted median
Weighted mode
0.2
0.1
0.0
−0.1
SNP effect on GCST90200802_buildGRCh38.tsv
0.05
0.10
0.15
SNP effect on Enterococcus B

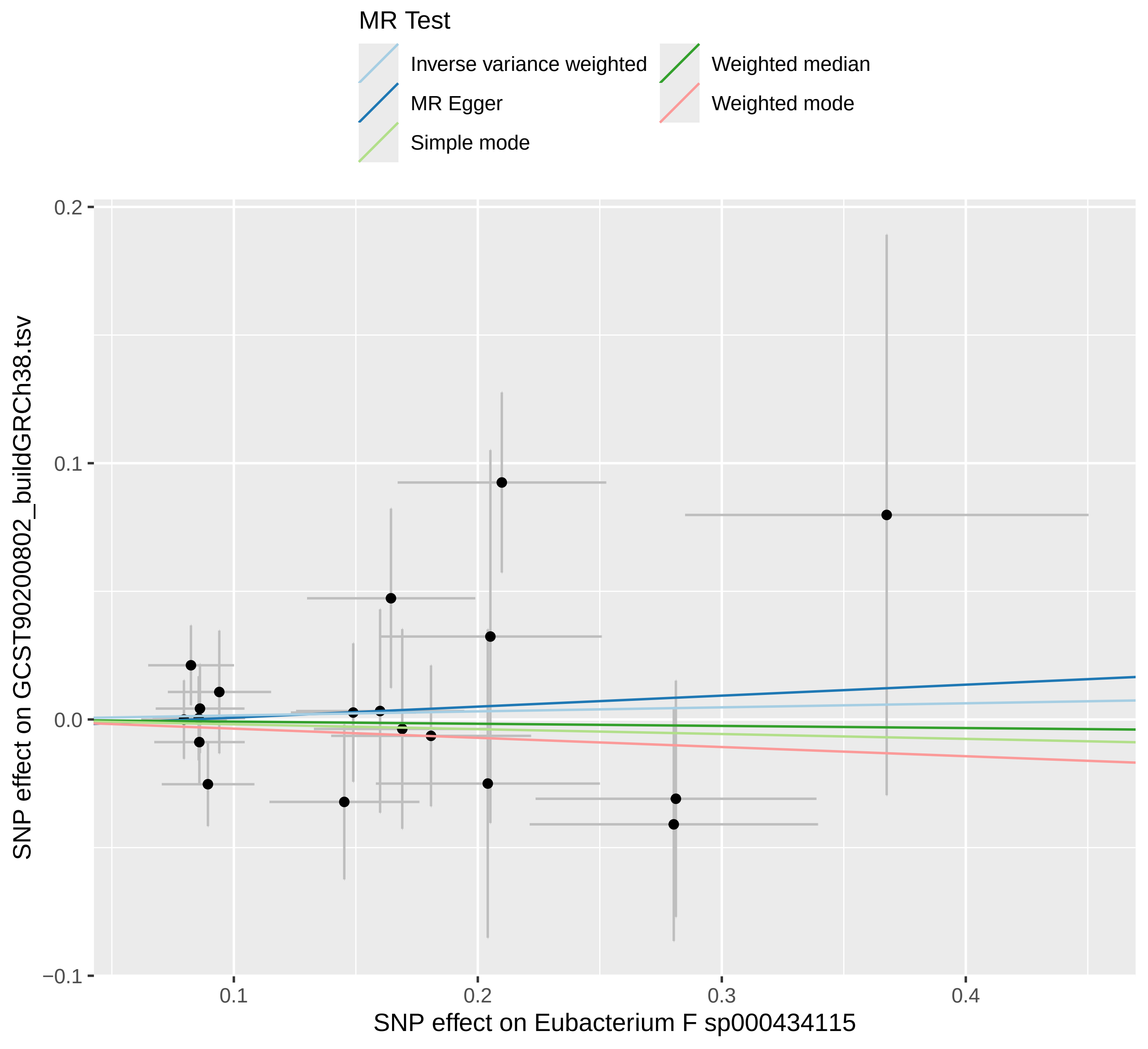

MR Test
Inverse variance weighted
MR Egger
Simple mode
Weighted median
Weighted mode
0.2
0.1
0.0
−0.1
0.1
0.2
0.3
0.4
SNP effect on GCST90200802_buildGRCh38.tsv
SNP effect on Eubacterium F sp000434115

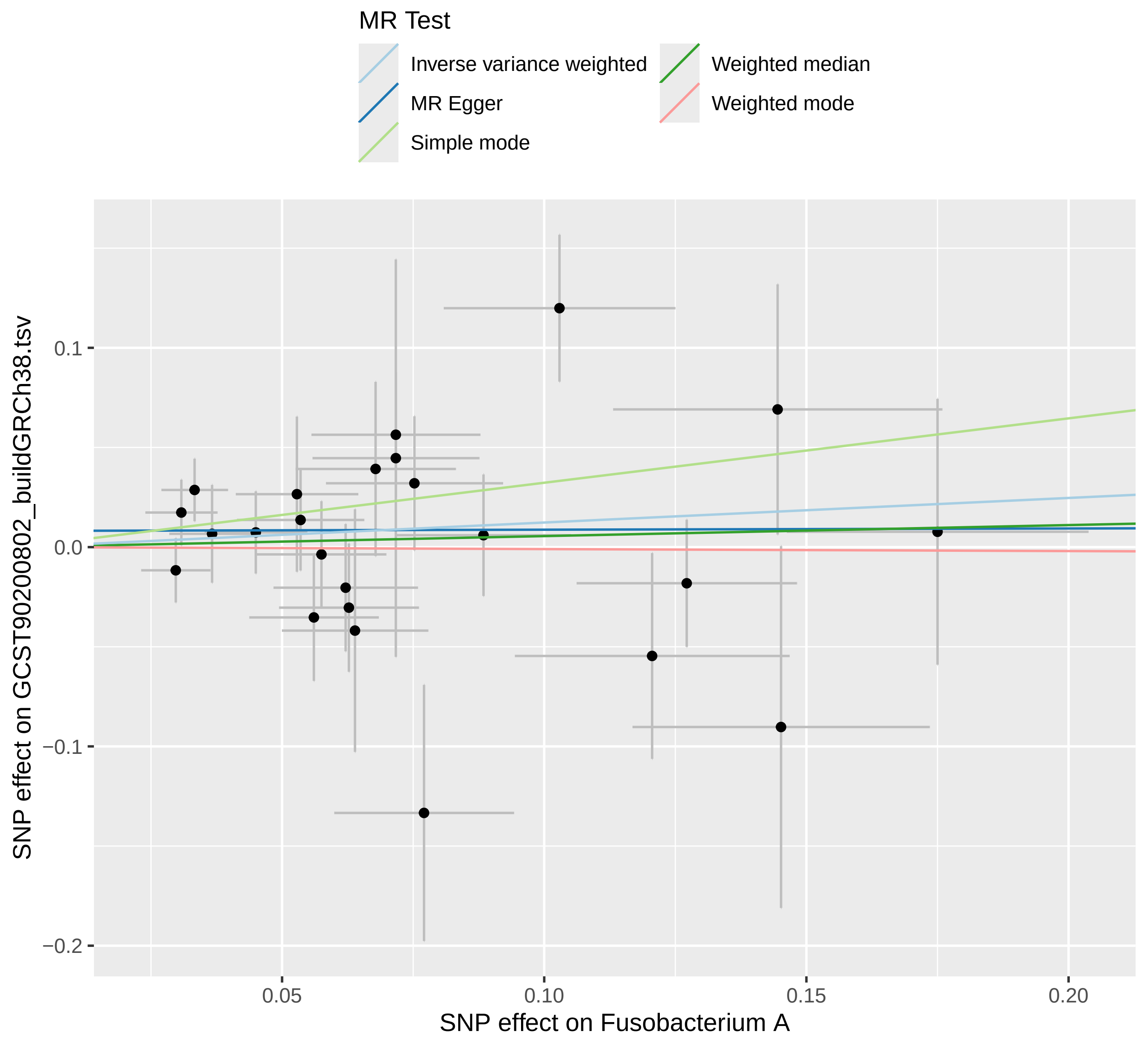
MR Test
Inverse variance weighted
MR Egger
Simple mode
Weighted median
Weighted mode
0.1
0.0
−0.1
−0.2
0.05
0.10
0.15
0.20
SNP effect on GCST90200802_buildGRCh38.tsv
SNP effect on Fusobacterium A

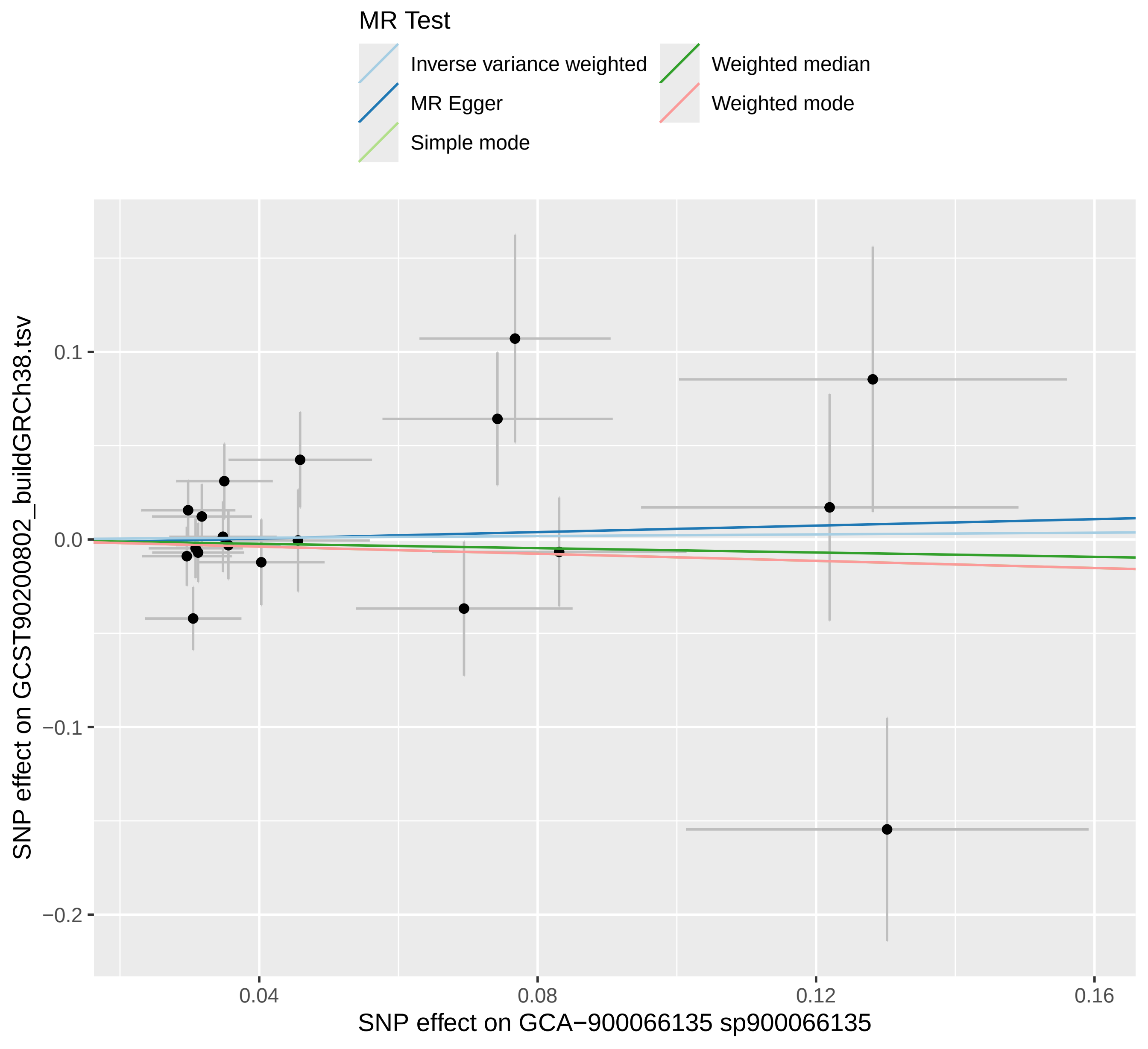
MR Test
Inverse variance weighted
MR Egger
Simple mode
Weighted median
Weighted mode
0.1
0.0
−0.1
−0.2
SNP effect on GCST90200802_buildGRCh38.tsv
0.04
0.08
0.12
0.16
SNP effect on GCA−900066135 sp900066135

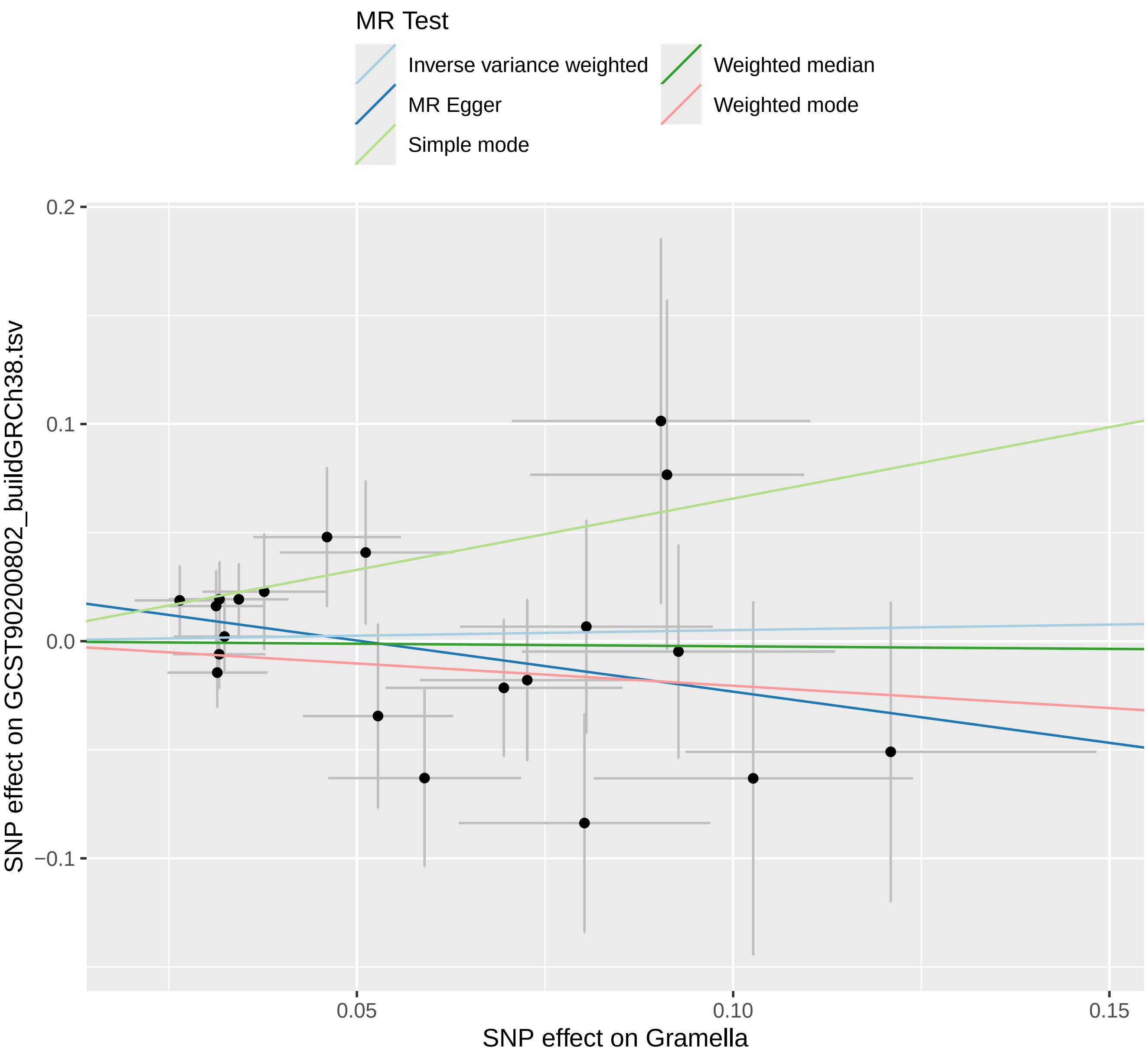
MR Test
Inverse variance weighted
MR Egger
Simple mode
Weighted median
Weighted mode
0.2
0.1
0.0
−0.1
0.05
0.10
0.15
SNP effect on GCST90200802_buildGRCh38.tsv
SNP effect on Gramella

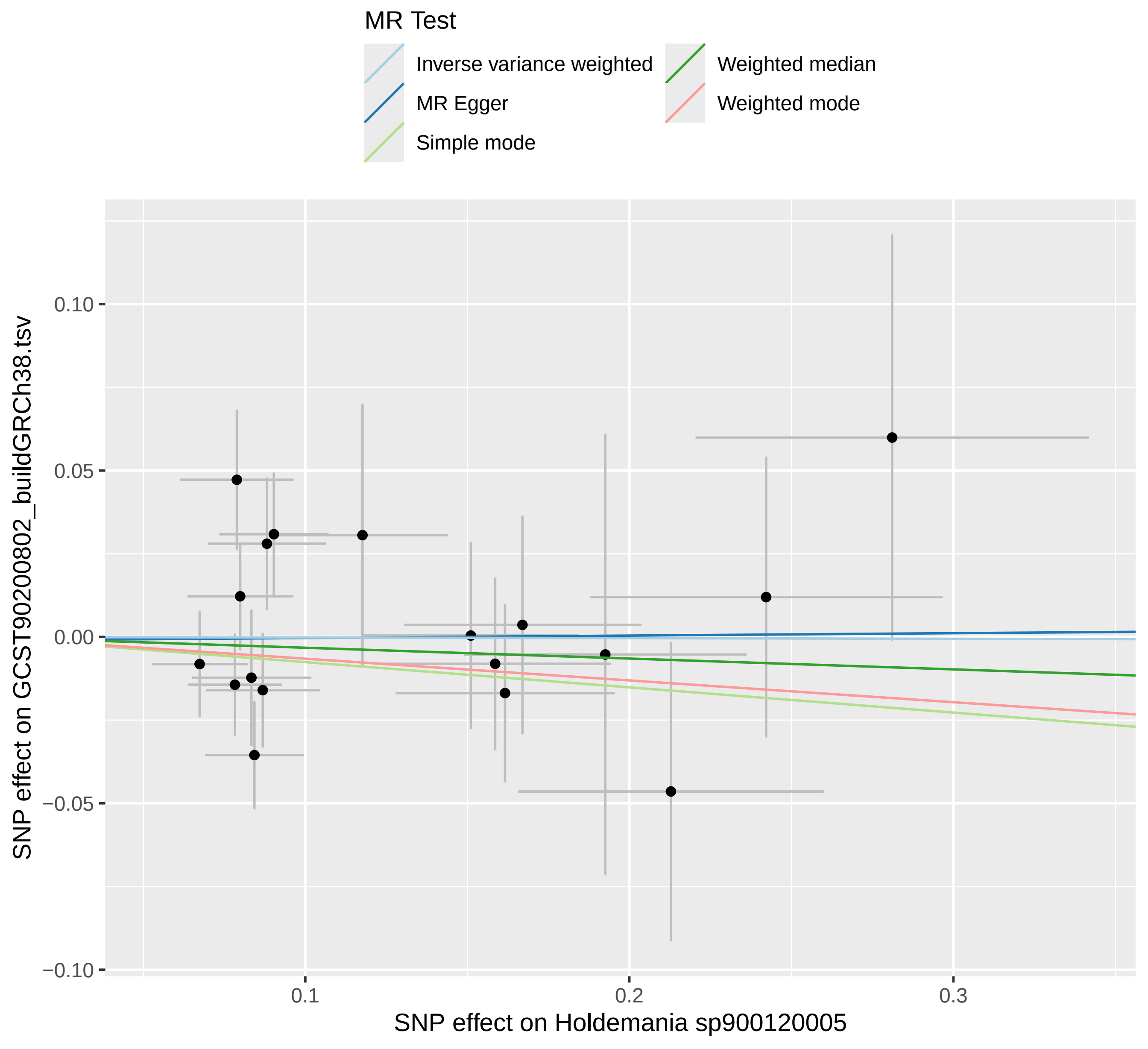
MR Test
Inverse variance weighted
MR Egger
Simple mode
Weighted median
Weighted mode
0.10
0.05
0.00
−0.05
−0.10
0.1
0.2
0.3
SNP effect on GCST90200802_buildGRCh38.tsv
SNP effect on Holdemania sp900120005

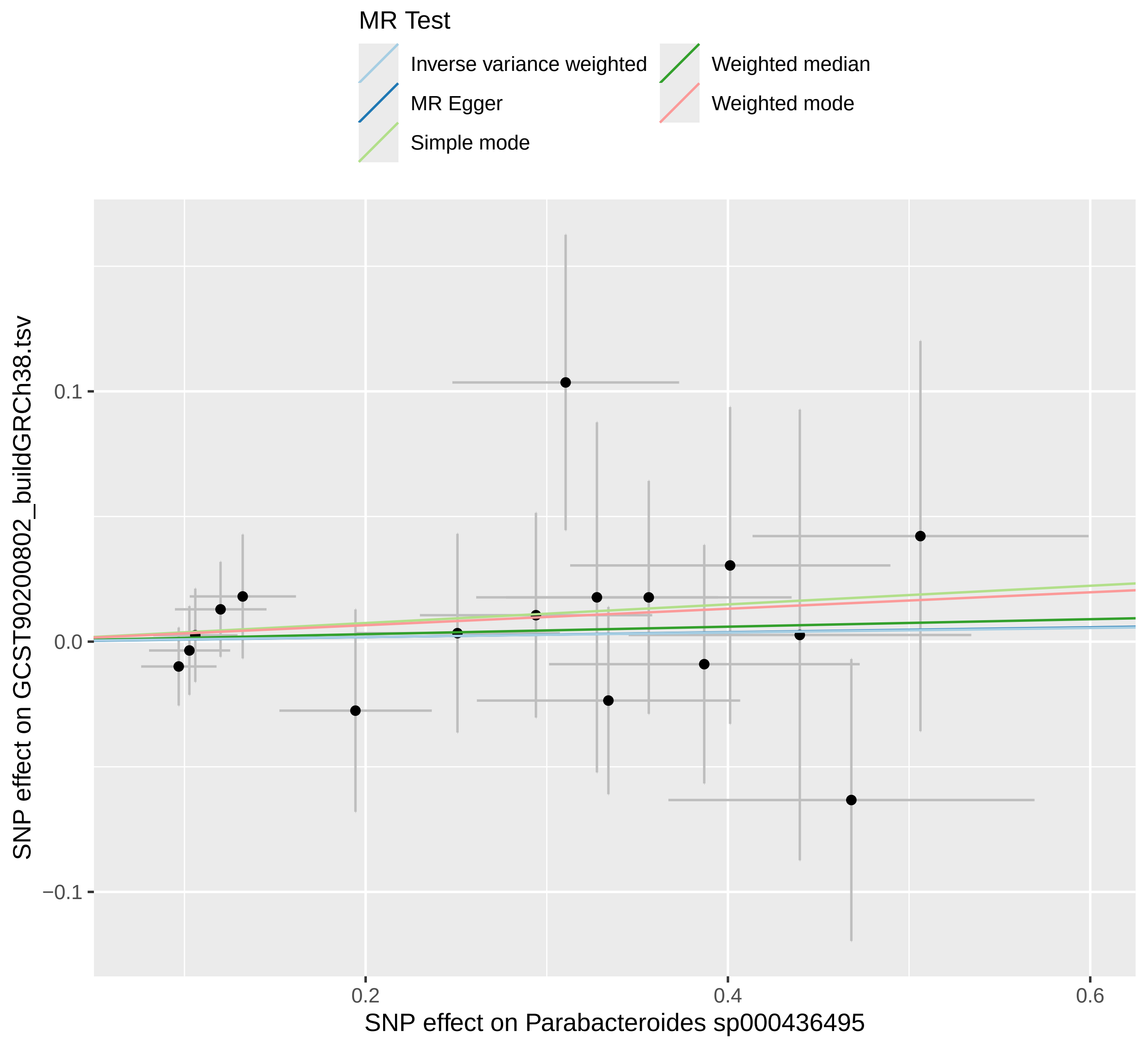

MR Test
Inverse variance weighted
MR Egger
Simple mode
Weighted median
Weighted mode
0.1
0.0
−0.1
0.2
0.4
0.6
SNP effect on GCST90200802_buildGRCh38.tsv
SNP effect on Parabacteroides sp000436495

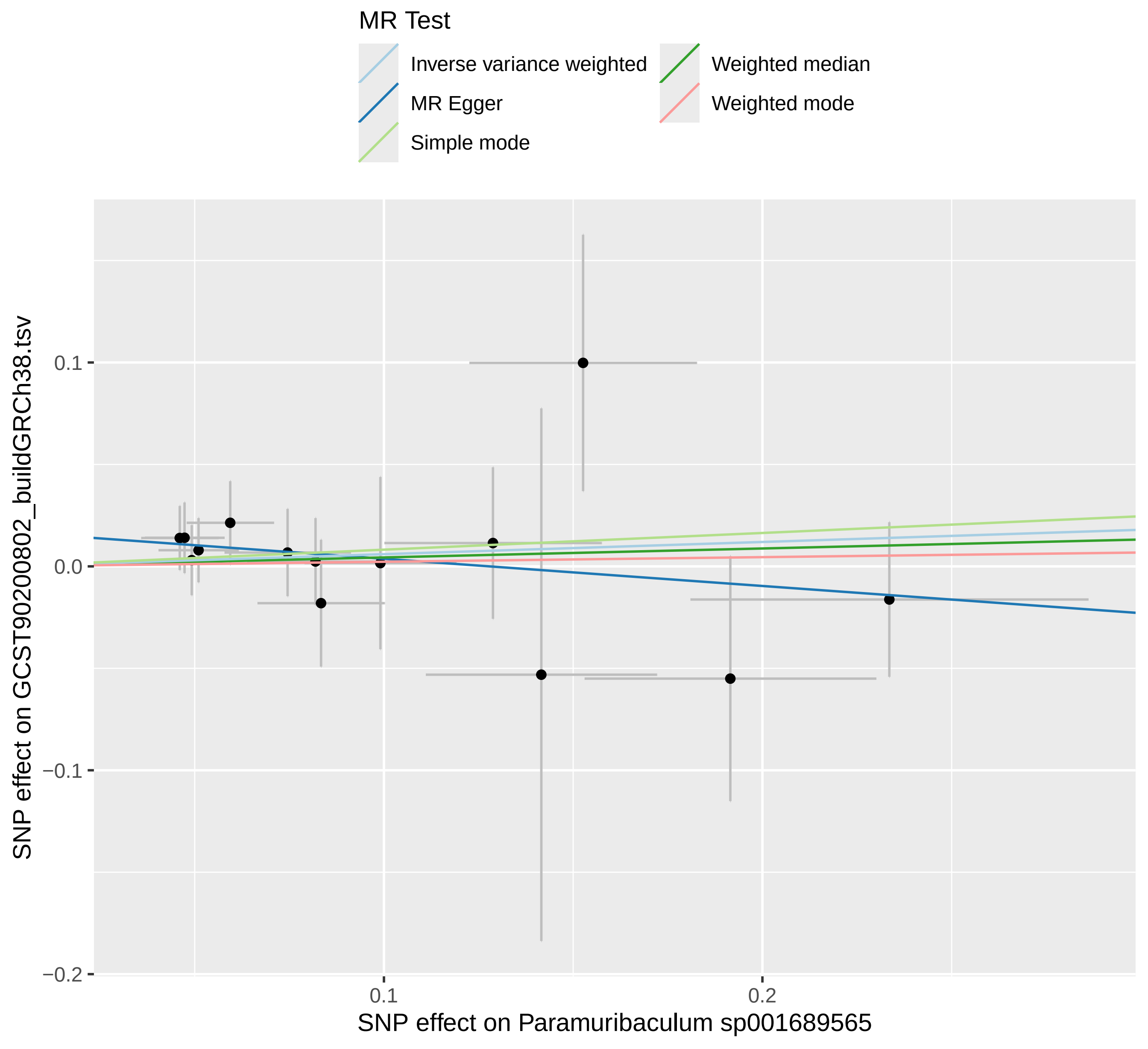
MR Test
Inverse variance weighted
MR Egger
Simple mode
Weighted median
Weighted mode
0.1
0.0
−0.1
−0.2
0.1
0.2
SNP effect on GCST90200802_buildGRCh38.tsv
SNP effect on Paramuribaculum sp001689565

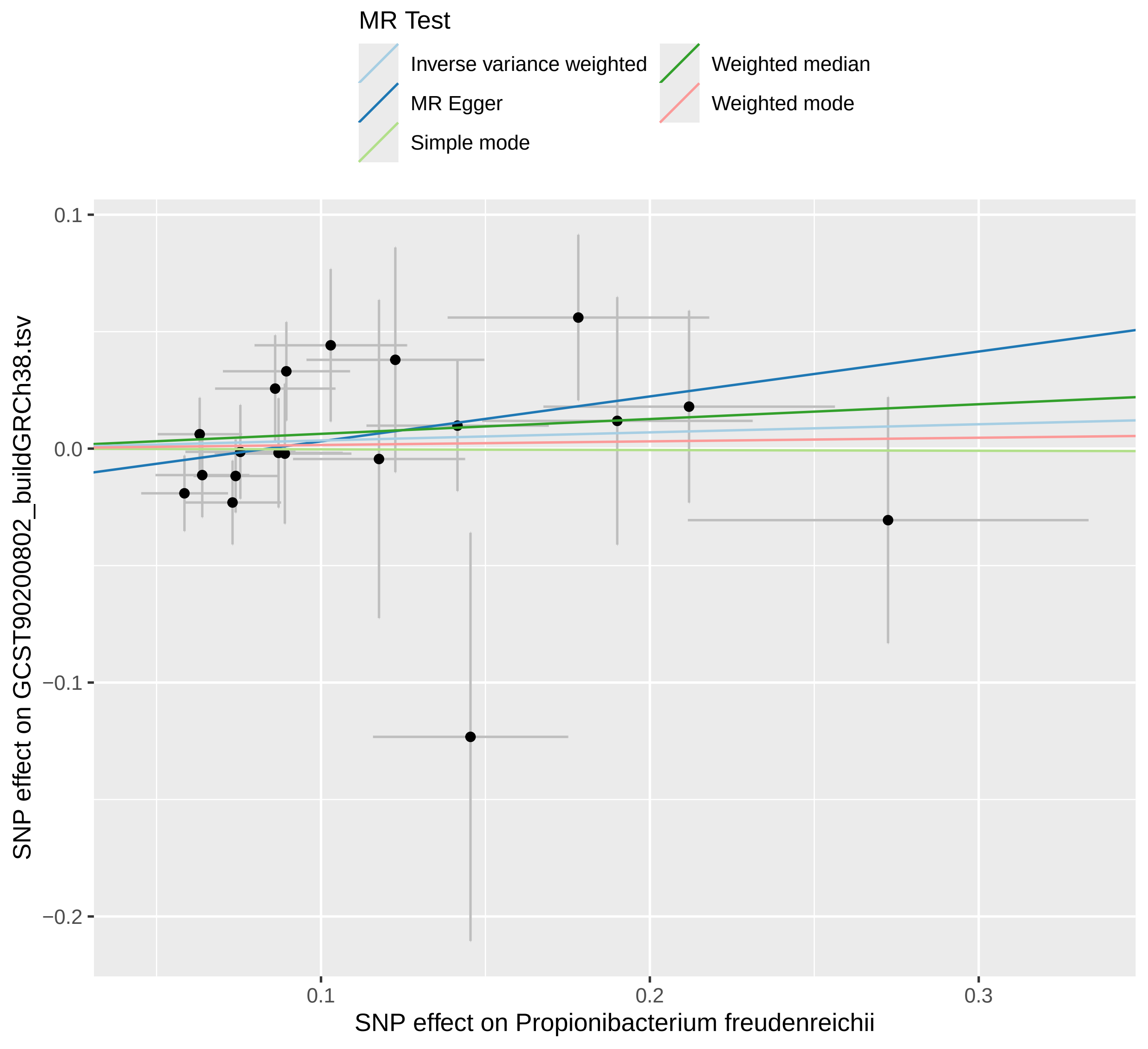
MR Test
Inverse variance weighted
MR Egger
Simple mode
Weighted median
Weighted mode
0.1
0.0
−0.1
−0.2
0.1
0.2
0.3
SNP effect on GCST90200802_buildGRCh38.tsv
SNP effect on Propionibacterium freudenreichii

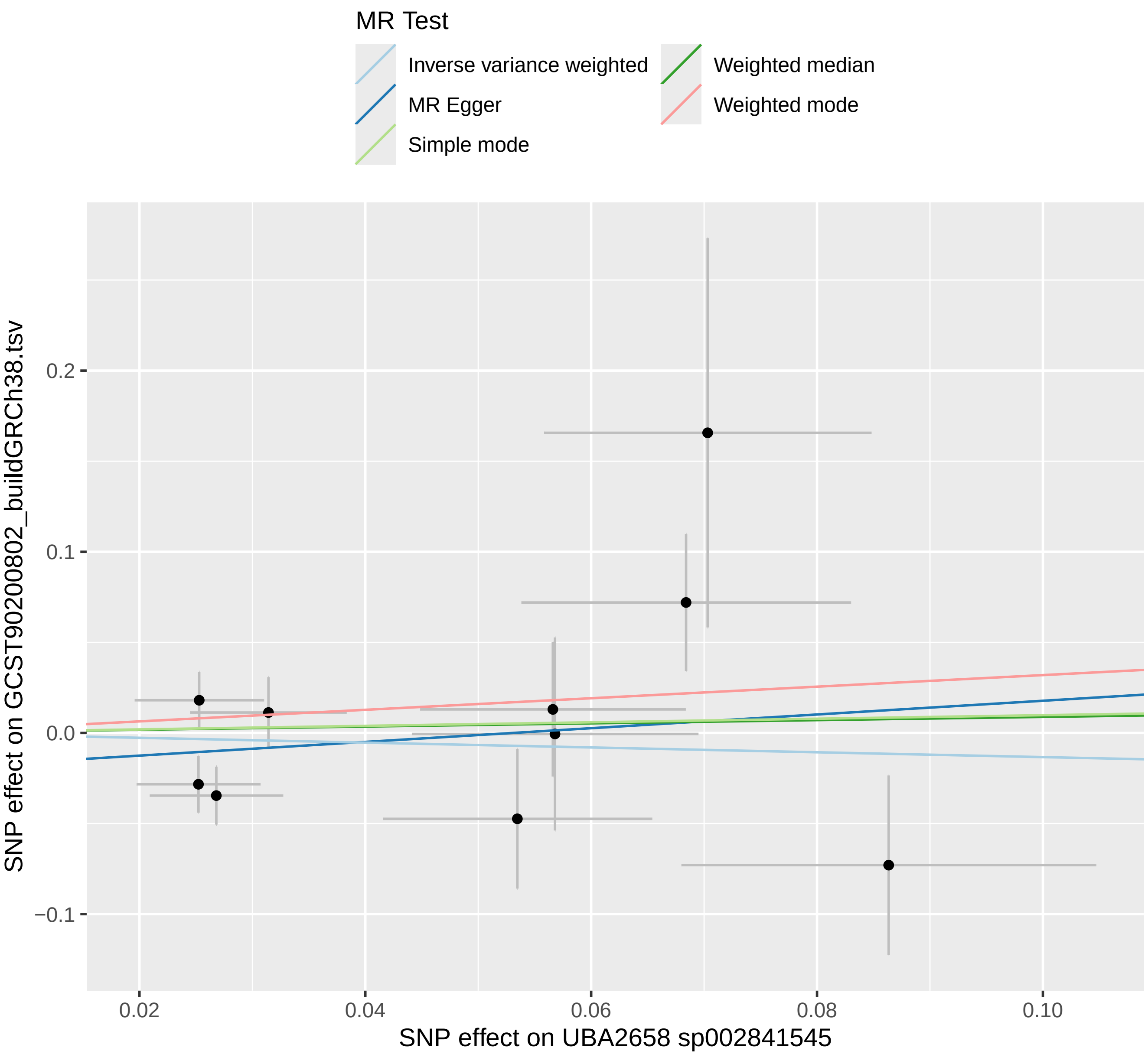
MR Test
Inverse variance weighted
MR Egger
Simple mode
Weighted median
Weighted mode
SNP effect on GCST90200802_buildGRCh38.tsv
0.2
0.1
0.0
−0.1
0.02
0.04
0.06
0.08
0.10
SNP effect on UBA2658 sp002841545

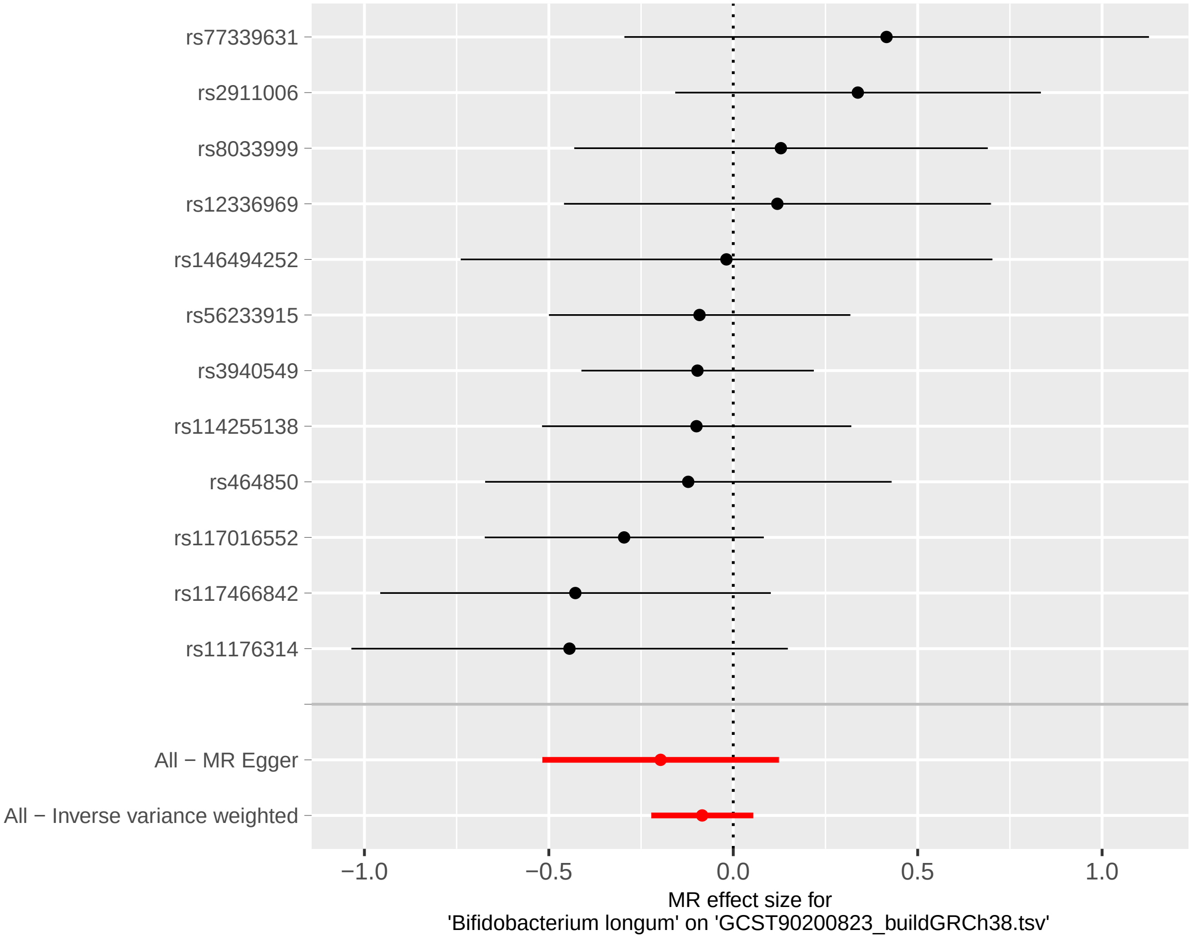

rs77339631
rs2911006
rs8033999
rs12336969
rs146494252
rs56233915
rs3940549
rs114255138
rs464850
rs117016552
rs117466842
rs11176314
All − MR Egger
All − Inverse variance weighted
−1.0
−0.5
0.0
0.5
1.0
MR effect size for
'Bifidobacterium longum' on 'GCST90200823_buildGRCh38.tsv'

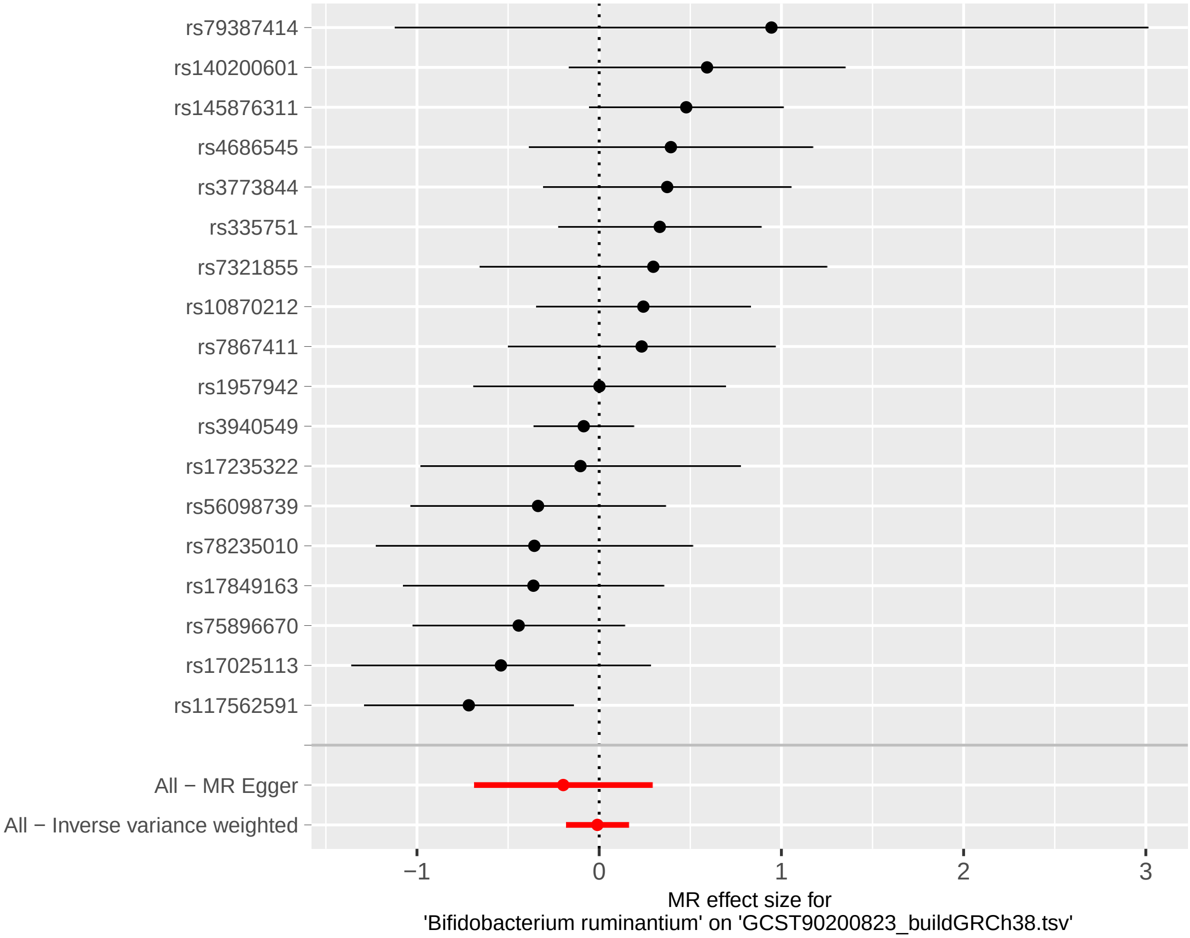
rs79387414
rs140200601
rs145876311
rs4686545
rs3773844
rs335751
rs7321855
rs10870212
rs7867411
rs1957942
rs3940549
rs17235322
rs56098739
rs78235010
rs17849163
rs75896670
rs17025113
rs117562591
All − MR Egger
All − Inverse variance weighted
−1
0
1
2
3
MR effect size for
'Bifidobacterium ruminantium' on 'GCST90200823_buildGRCh38.tsv'

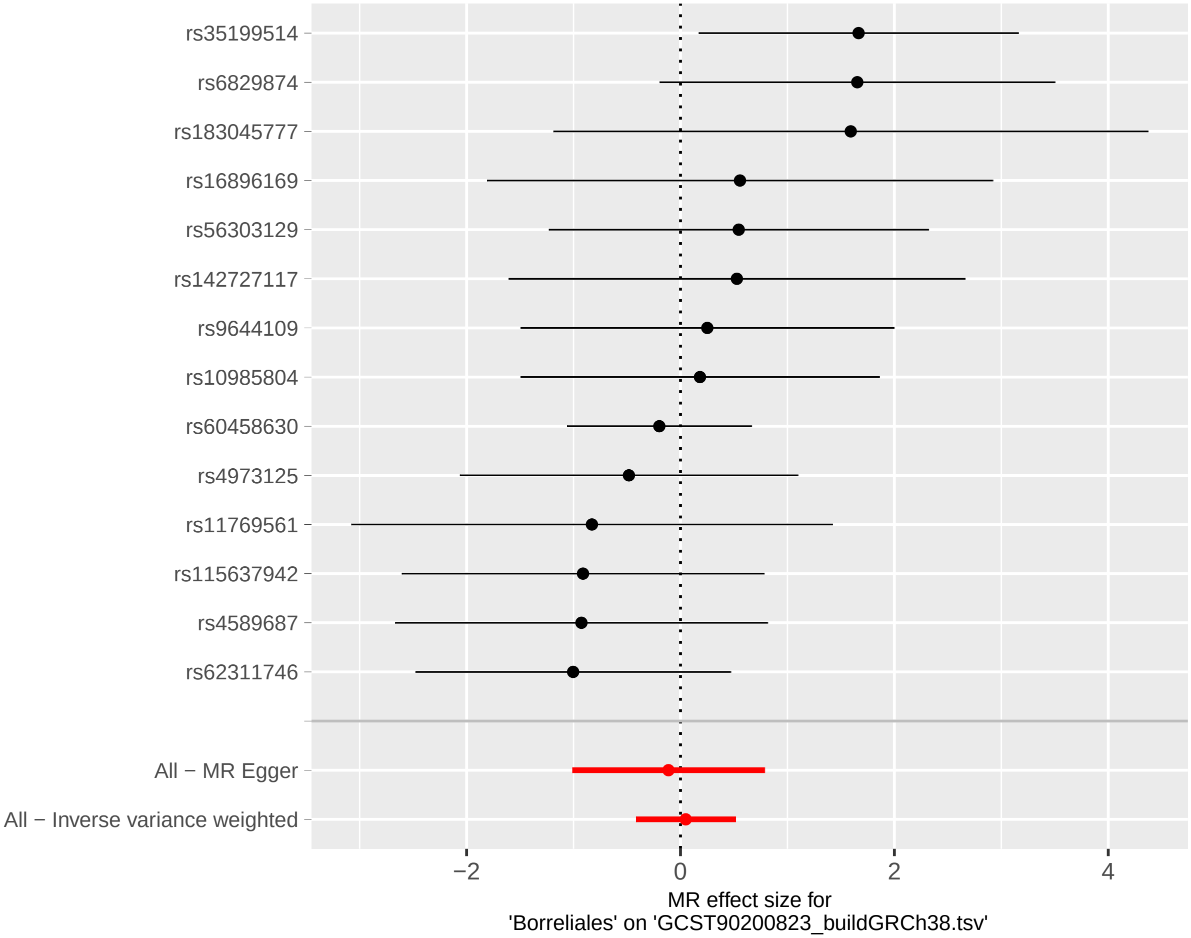
rs35199514
rs6829874
rs183045777
rs16896169
rs56303129
rs142727117
rs9644109
rs10985804
rs60458630
rs4973125
rs11769561
rs115637942
rs4589687
rs62311746
All − MR Egger
All − Inverse variance weighted
−2
0
2
4
MR effect size for
'Borreliales' on 'GCST90200823_buildGRCh38.tsv'

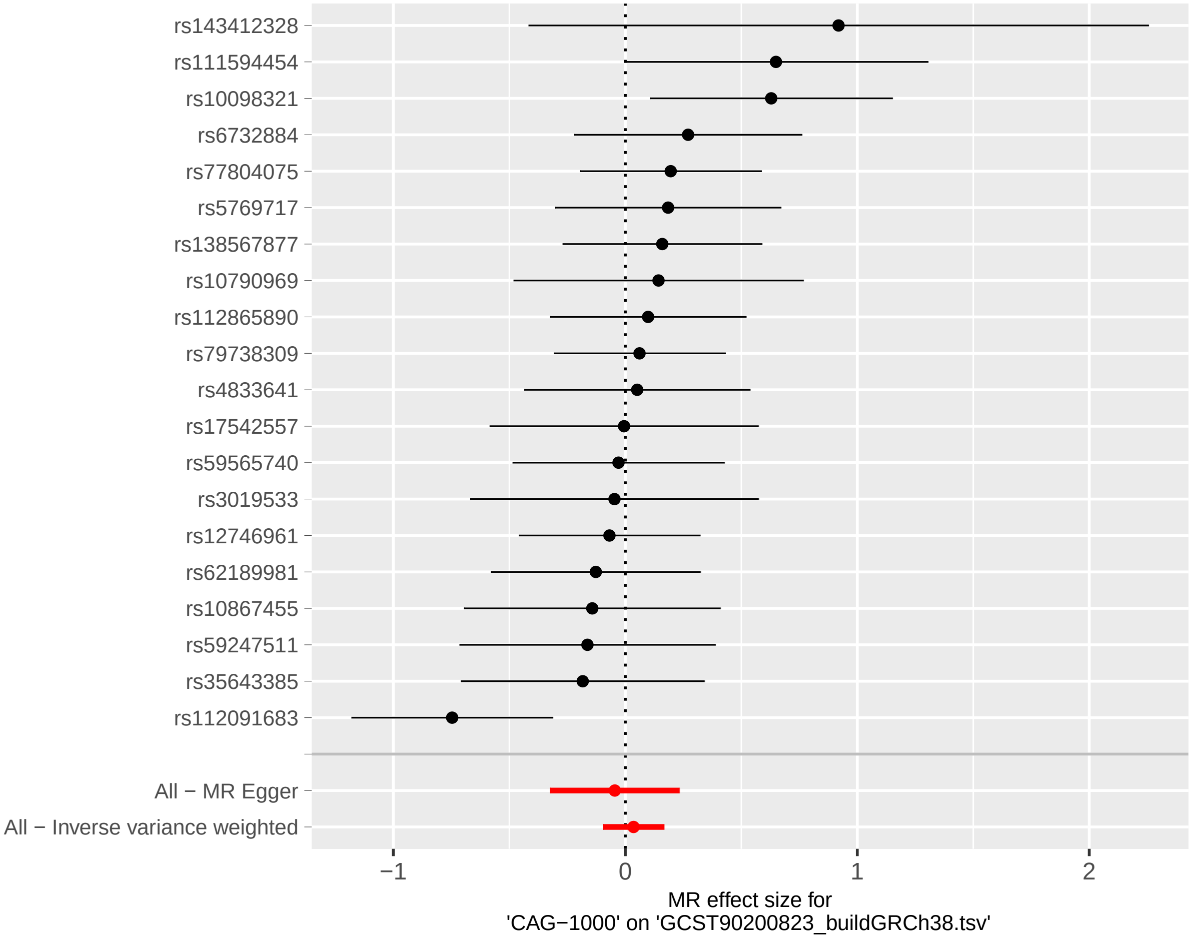
rs143412328
rs111594454
rs10098321
rs6732884
rs77804075
rs5769717
rs138567877
rs10790969
rs112865890
rs79738309
rs4833641
rs17542557
rs59565740
rs3019533
rs12746961
rs62189981
rs10867455
rs59247511
rs35643385
rs112091683
All − MR Egger
All − Inverse variance weighted
−1
0
1
2
MR effect size for
'CAG−1000' on 'GCST90200823_buildGRCh38.tsv'

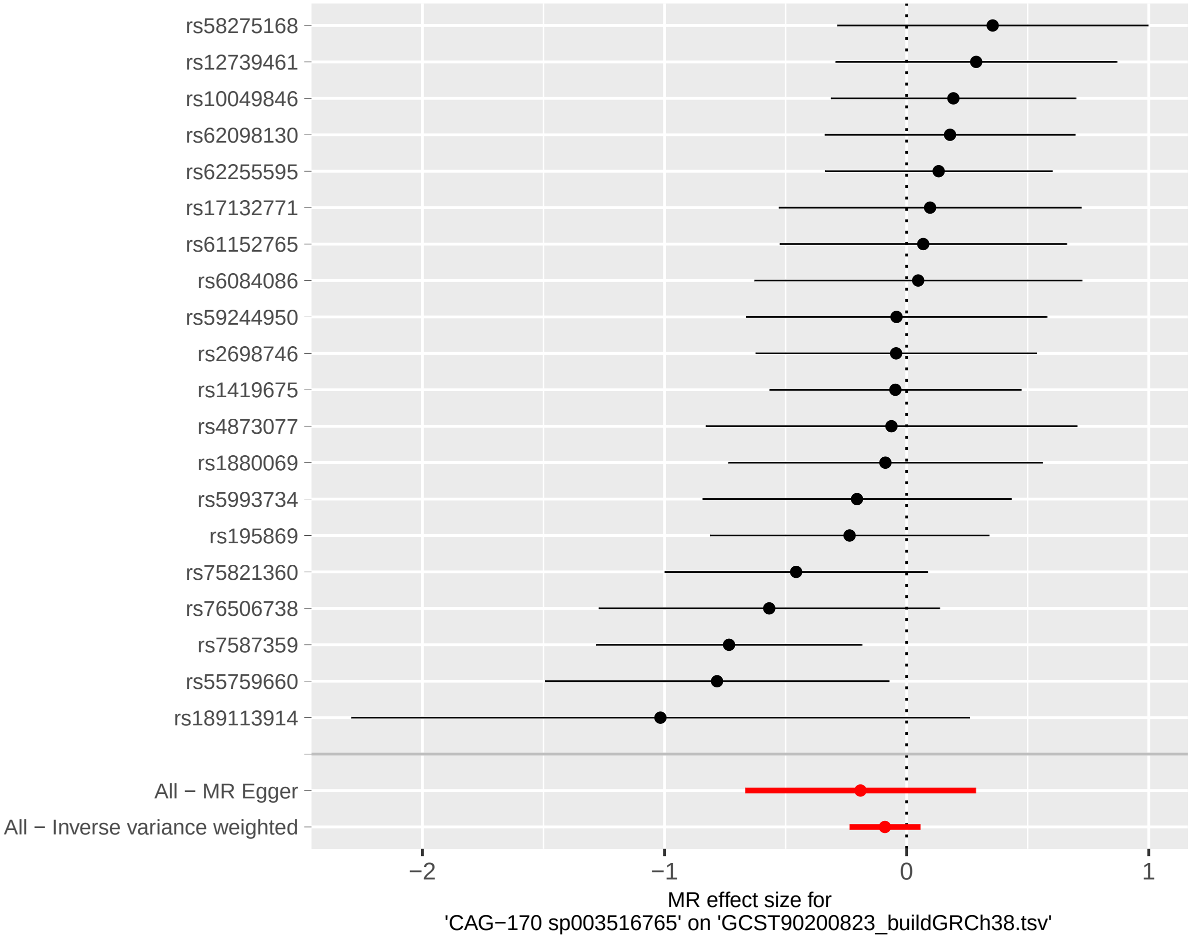
rs58275168
rs12739461
rs10049846
rs62098130
rs62255595
rs17132771
rs61152765
rs6084086
rs59244950
rs2698746
rs1419675
rs4873077
rs1880069
rs5993734
rs195869
rs75821360
rs76506738
rs7587359
rs55759660
rs189113914
All − MR Egger
All − Inverse variance weighted
−2
−1
0
1
MR effect size for
'CAG−170 sp003516765' on 'GCST90200823_buildGRCh38.tsv'

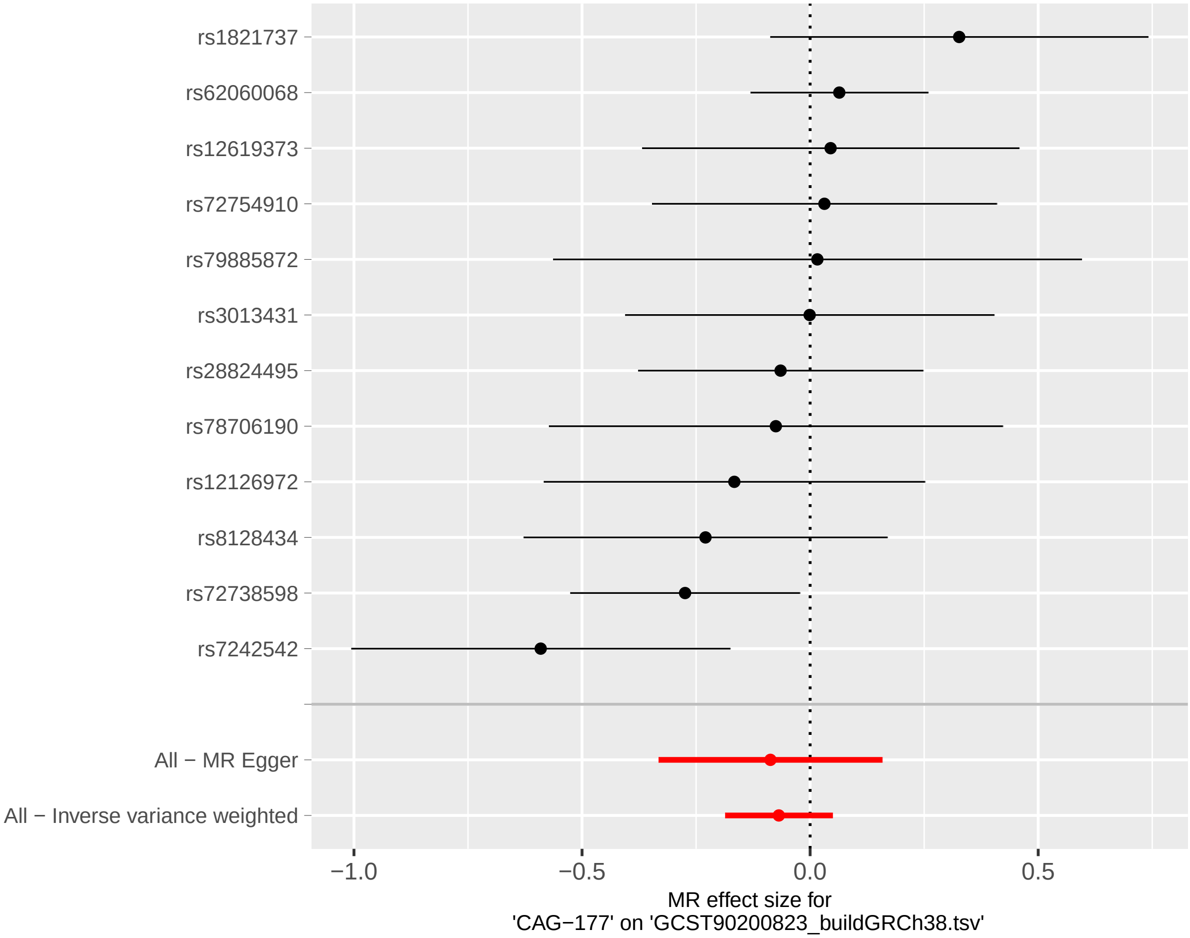
rs1821737
rs62060068
rs12619373
rs72754910
rs79885872
rs3013431
rs28824495
rs78706190
rs12126972
rs8128434
rs72738598
rs7242542
All − MR Egger
All − Inverse variance weighted
−1.0
−0.5
0.0
0.5
MR effect size for
'CAG−177' on 'GCST90200823_buildGRCh38.tsv'

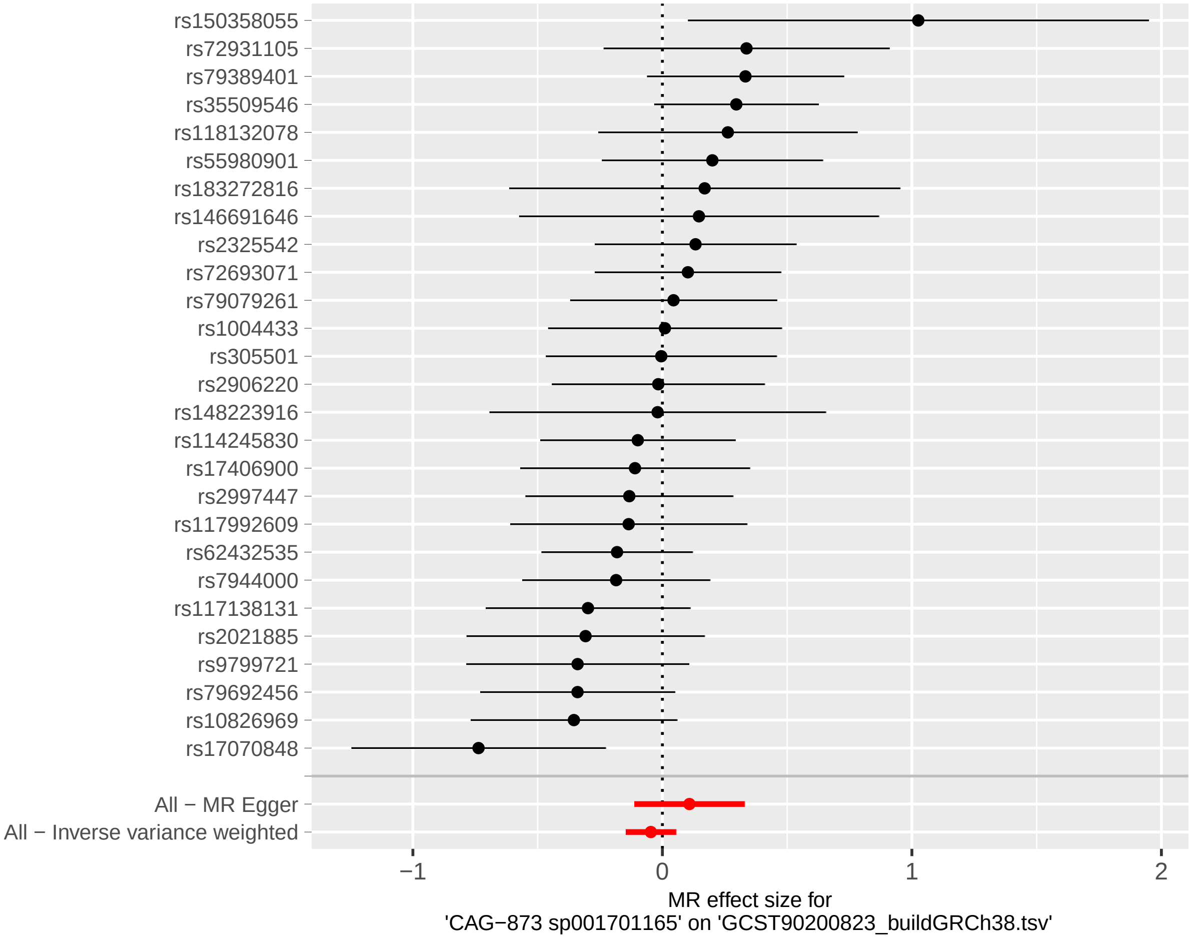

rs150358055
rs72931105
rs79389401
rs35509546
rs118132078
rs55980901
rs183272816
rs146691646
rs2325542
rs72693071
rs79079261
rs1004433
rs305501
rs2906220
rs148223916
rs114245830
rs17406900
rs2997447
rs117992609
rs62432535
rs7944000
rs117138131
rs2021885
rs9799721
rs79692456
rs10826969
rs17070848
All − MR Egger
All − Inverse variance weighted
−1
0
1
2
MR effect size for
'CAG−873 sp001701165' on 'GCST90200823_buildGRCh38.tsv'

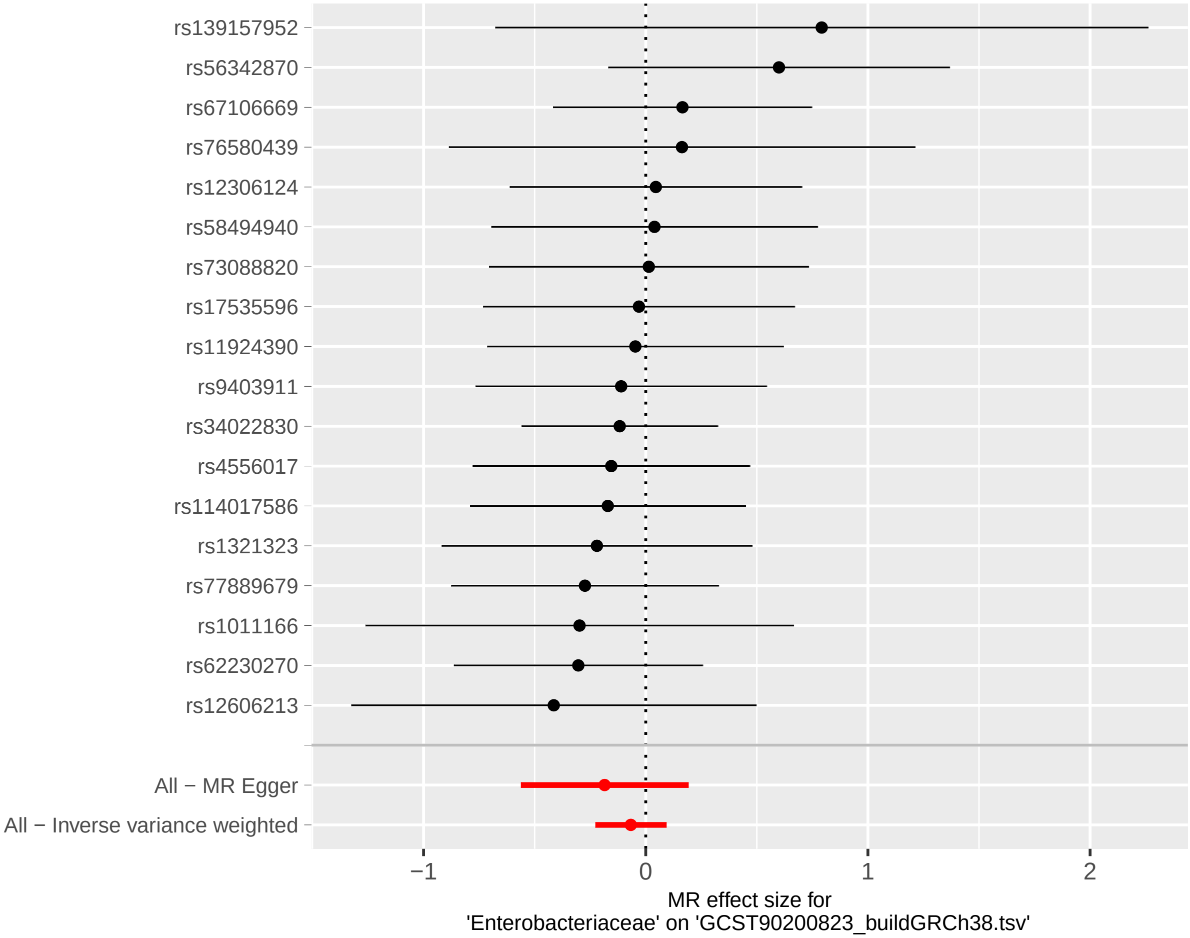
rs139157952
rs56342870
rs67106669
rs76580439
rs12306124
rs58494940
rs73088820
rs17535596
rs11924390
rs9403911
rs34022830
rs4556017
rs114017586
rs1321323
rs77889679
rs1011166
rs62230270
rs12606213
All − MR Egger
All − Inverse variance weighted
−1
0
1
2
MR effect size for
'Enterobacteriaceae' on 'GCST90200823_buildGRCh38.tsv'

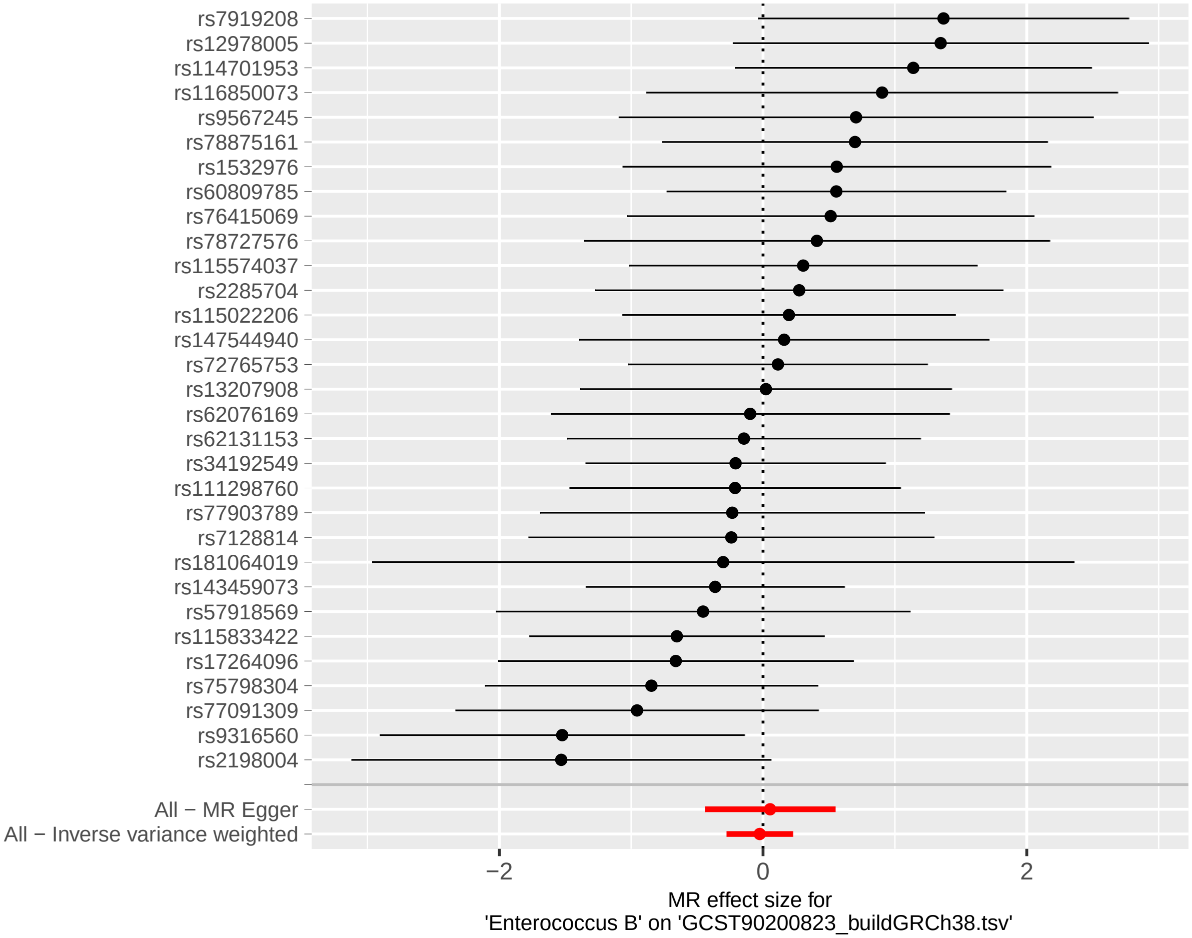
rs7919208
rs12978005
rs114701953
rs116850073
rs9567245
rs78875161
rs1532976
rs60809785
rs76415069
rs78727576
rs115574037
rs2285704
rs115022206
rs147544940
rs72765753
rs13207908
rs62076169
rs62131153
rs34192549
rs111298760
rs77903789
rs7128814
rs181064019
rs143459073
rs57918569
rs115833422
rs17264096
rs75798304
rs77091309
rs9316560
rs2198004
All − MR Egger
All − Inverse variance weighted
−2
0
2
MR effect size for
'Enterococcus B' on 'GCST90200823_buildGRCh38.tsv'

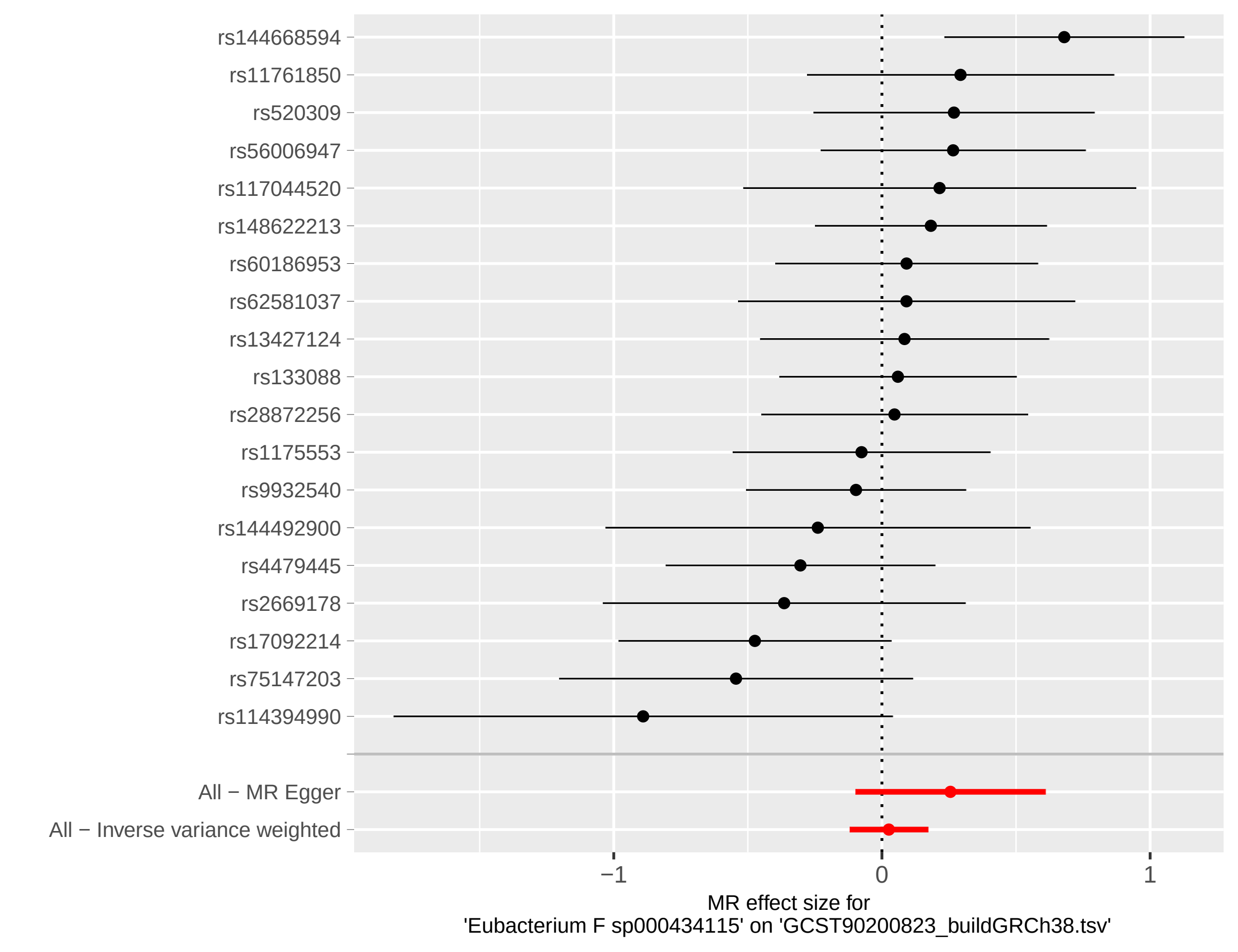
rs144668594
rs11761850
rs520309
rs56006947
rs117044520
rs148622213
rs60186953
rs62581037
rs13427124
rs133088
rs28872256
rs1175553
rs9932540
rs144492900
rs4479445
rs2669178
rs17092214
rs75147203
rs114394990
All − MR Egger
All − Inverse variance weighted
−1
0
1
MR effect size for
'Eubacterium F sp000434115' on 'GCST90200823_buildGRCh38.tsv'

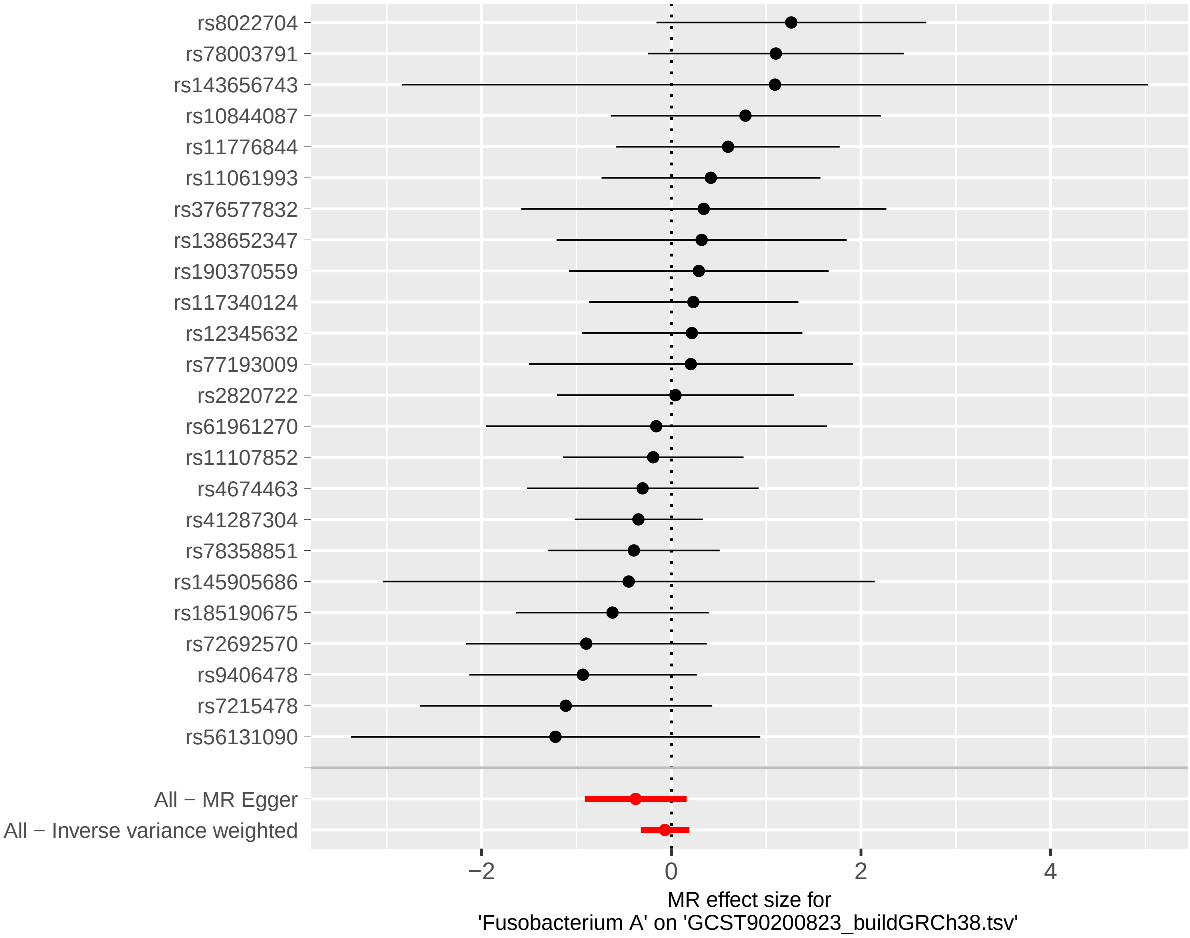

rs8022704
rs78003791
rs143656743
rs10844087
rs11776844
rs11061993
rs376577832
rs138652347
rs190370559
rs117340124
rs12345632
rs77193009
rs2820722
rs61961270
rs11107852
rs4674463
rs41287304
rs78358851
rs145905686
rs185190675
rs72692570
rs9406478
rs7215478
rs56131090
All − MR Egger
All − Inverse variance weighted
−2
0
2
4
MR effect size for
'Fusobacterium A' on 'GCST90200823_buildGRCh38.tsv'

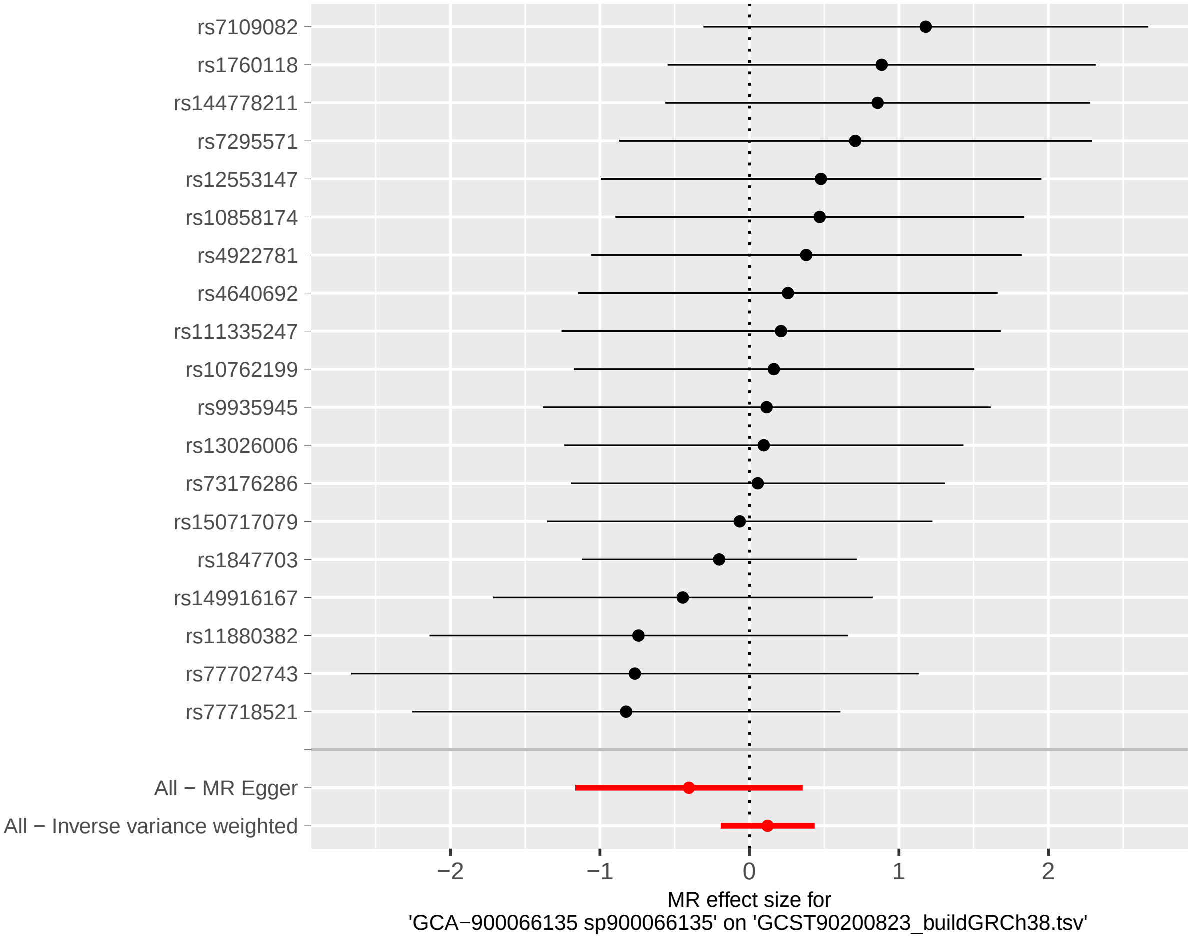

rs7109082
rs1760118
rs144778211
rs7295571
rs12553147
rs10858174
rs4922781
rs4640692
rs111335247
rs10762199
rs9935945
rs13026006
rs73176286
rs150717079
rs1847703
rs149916167
rs11880382
rs77702743
rs77718521
All − MR Egger
All − Inverse variance weighted
−2
−1
0
1
2
MR effect size for
'GCA−900066135 sp900066135' on 'GCST90200823_buildGRCh38.tsv'

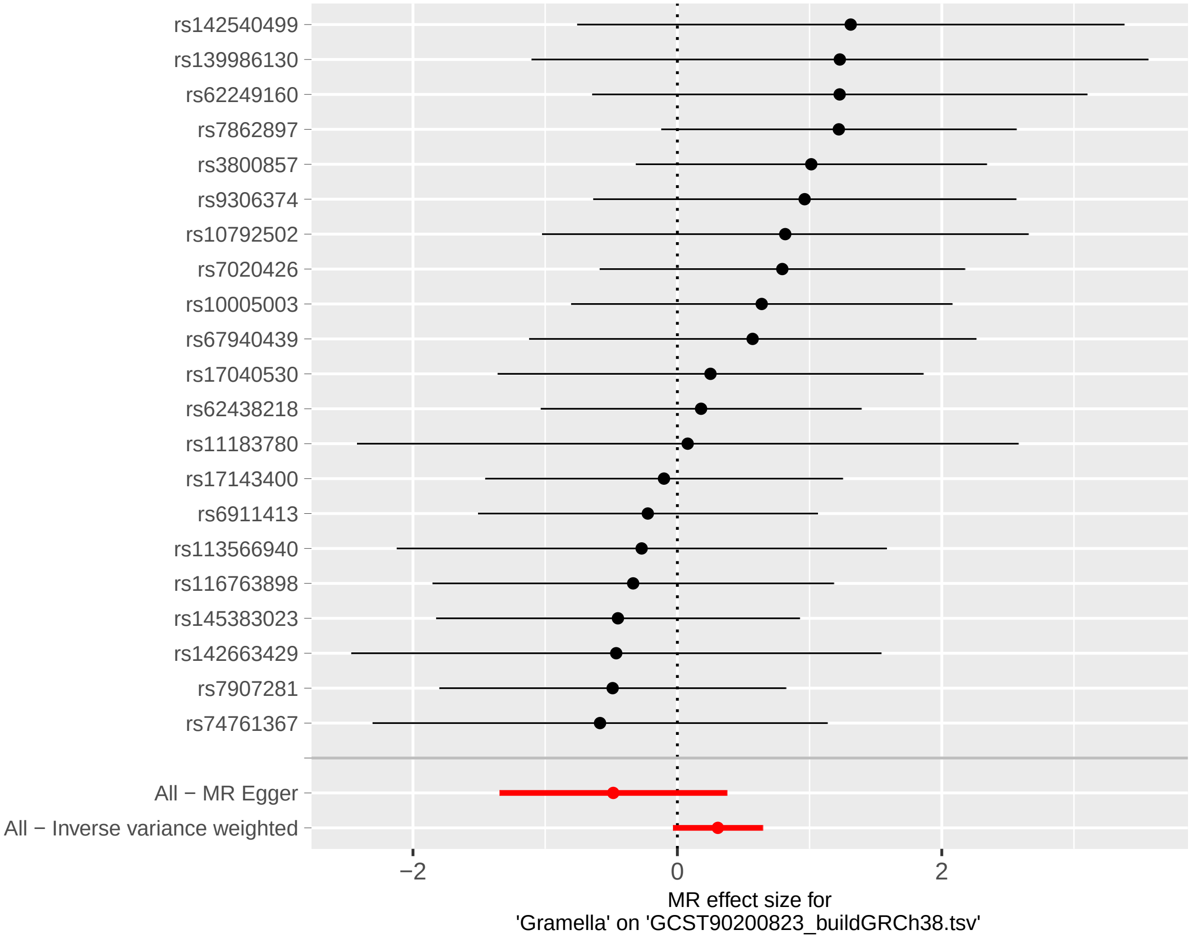
rs142540499
rs139986130
rs62249160
rs7862897
rs3800857
rs9306374
rs10792502
rs7020426
rs10005003
rs67940439
rs17040530
rs62438218
rs11183780
rs17143400
rs6911413
rs113566940
rs116763898
rs145383023
rs142663429
rs7907281
rs74761367
All − MR Egger
All − Inverse variance weighted
−2
0
2
MR effect size for
'Gramella' on 'GCST90200823_buildGRCh38.tsv'

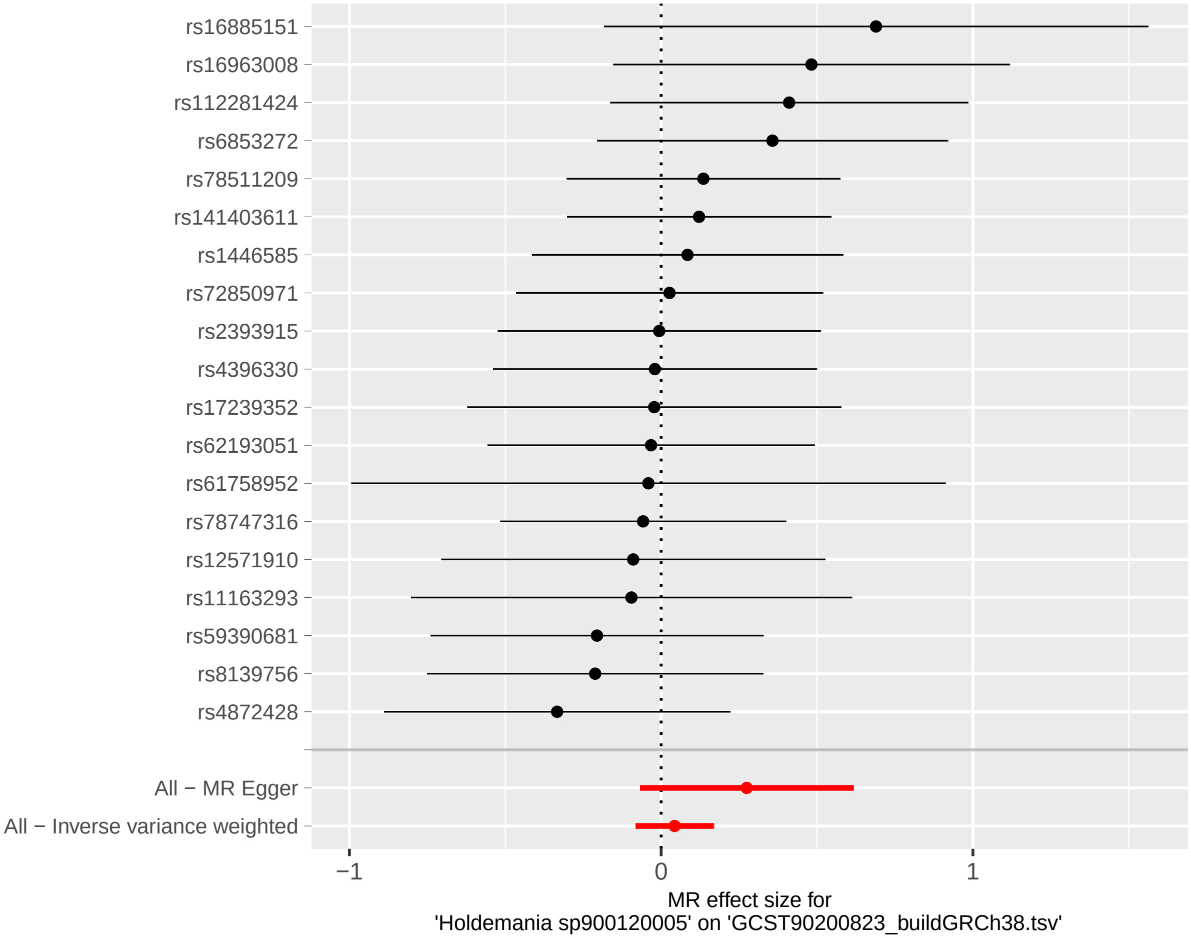

rs16885151
rs16963008
rs112281424
rs6853272
rs78511209
rs141403611
rs1446585
rs72850971
rs2393915
rs4396330
rs17239352
rs62193051
rs61758952
rs78747316
rs12571910
rs11163293
rs59390681
rs8139756
rs4872428
All − MR Egger
All − Inverse variance weighted
−1
0
1
MR effect size for
'Holdemania sp900120005' on 'GCST90200823_buildGRCh38.tsv'

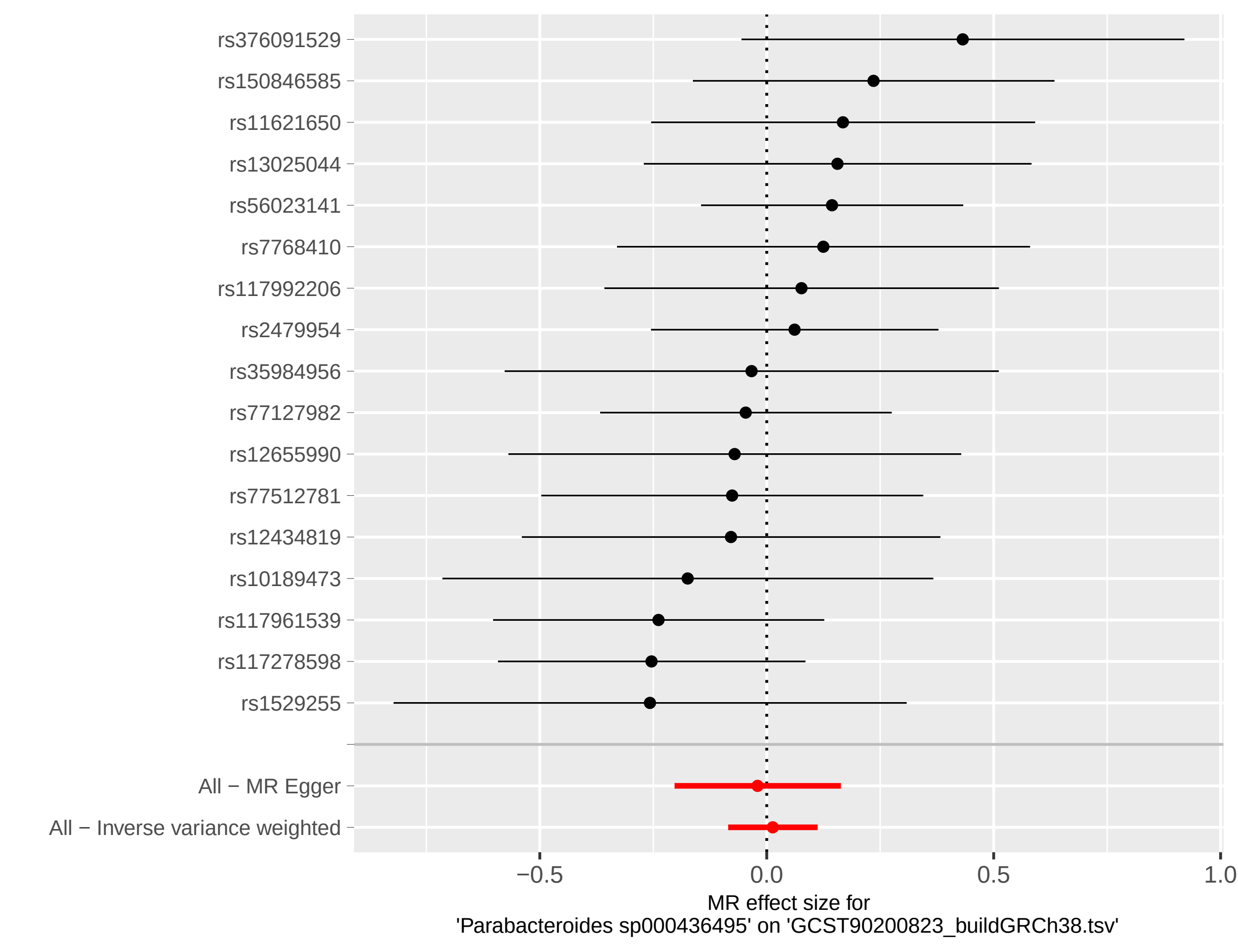

rs376091529
rs150846585
rs11621650
rs13025044
rs56023141
rs7768410
rs117992206
rs2479954
rs35984956
rs77127982
rs12655990
rs77512781
rs12434819
rs10189473
rs117961539
rs117278598
rs1529255
All − MR Egger
All − Inverse variance weighted
−0.5
0.0
0.5
1.0
MR effect size for
'Parabacteroides sp000436495' on 'GCST90200823_buildGRCh38.tsv'

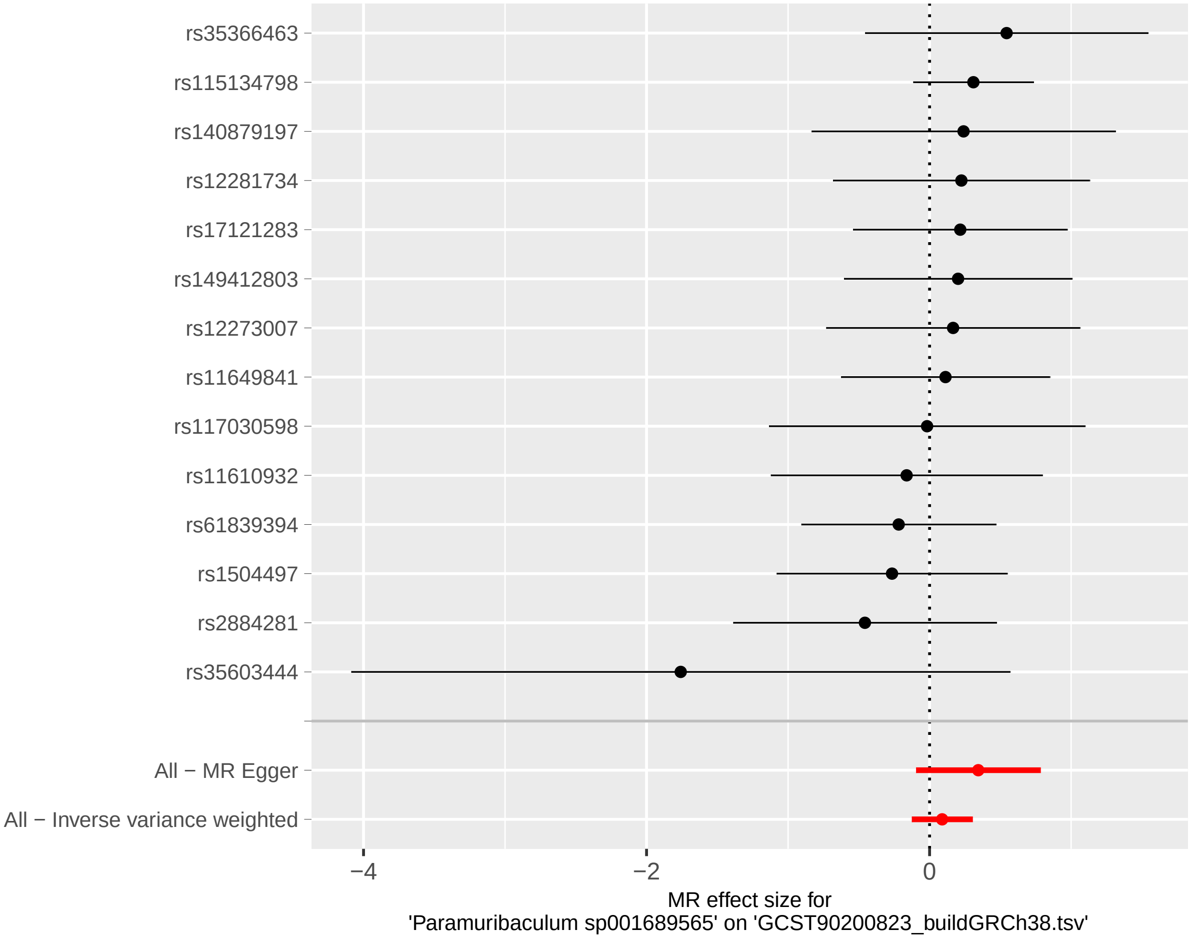
rs35366463
rs115134798
rs140879197
rs12281734
rs17121283
rs149412803
rs12273007
rs11649841
rs117030598
rs11610932
rs61839394
rs1504497
rs2884281
rs35603444
All − MR Egger
All − Inverse variance weighted
−4
−2
0
MR effect size for
'Paramuribaculum sp001689565' on 'GCST90200823_buildGRCh38.tsv'

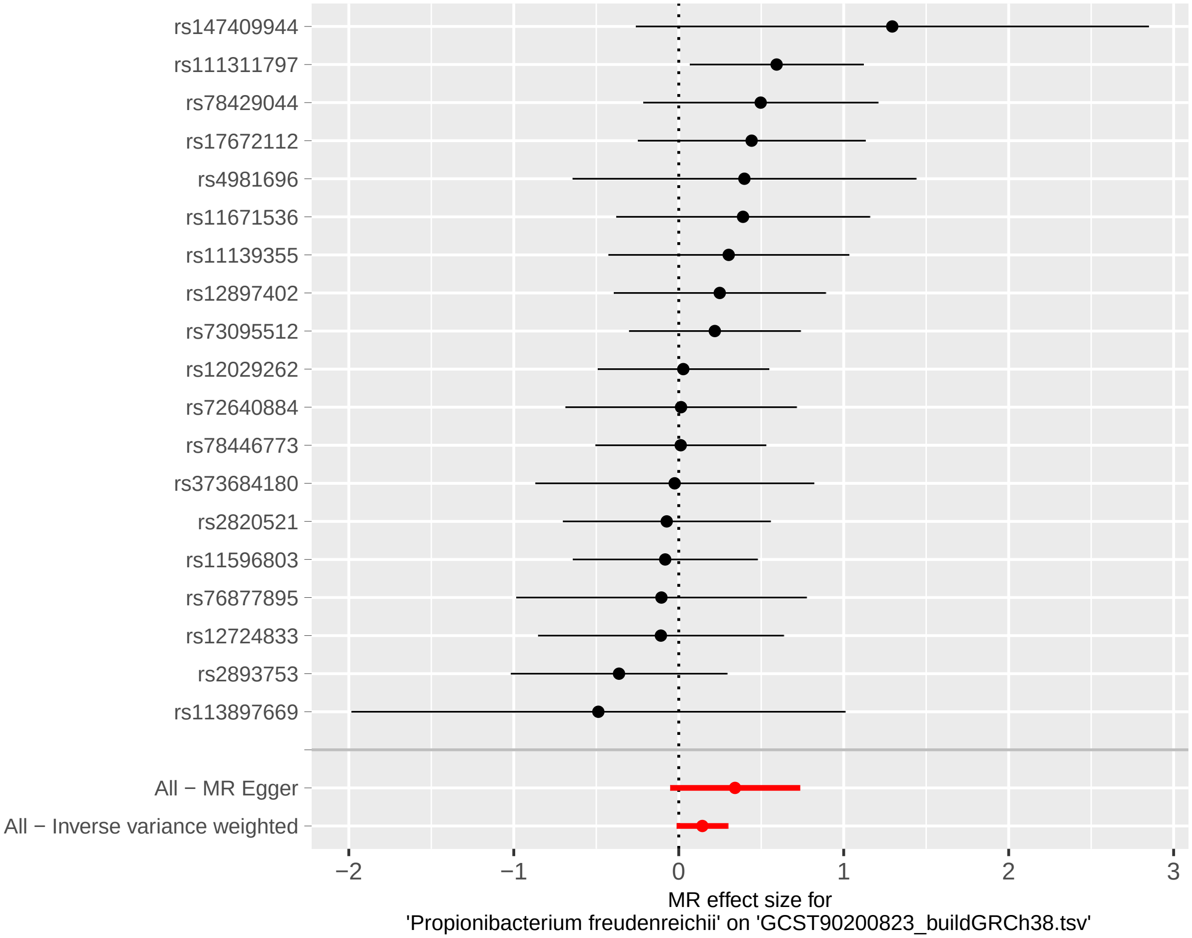

rs147409944
rs111311797
rs78429044
rs17672112
rs4981696
rs11671536
rs11139355
rs12897402
rs73095512
rs12029262
rs72640884
rs78446773
rs373684180
rs2820521
rs11596803
rs76877895
rs12724833
rs2893753
rs113897669
All − MR Egger
All − Inverse variance weighted
−2
−1
0
1
2
3
MR effect size for
'Propionibacterium freudenreichii' on 'GCST90200823_buildGRCh38.tsv'

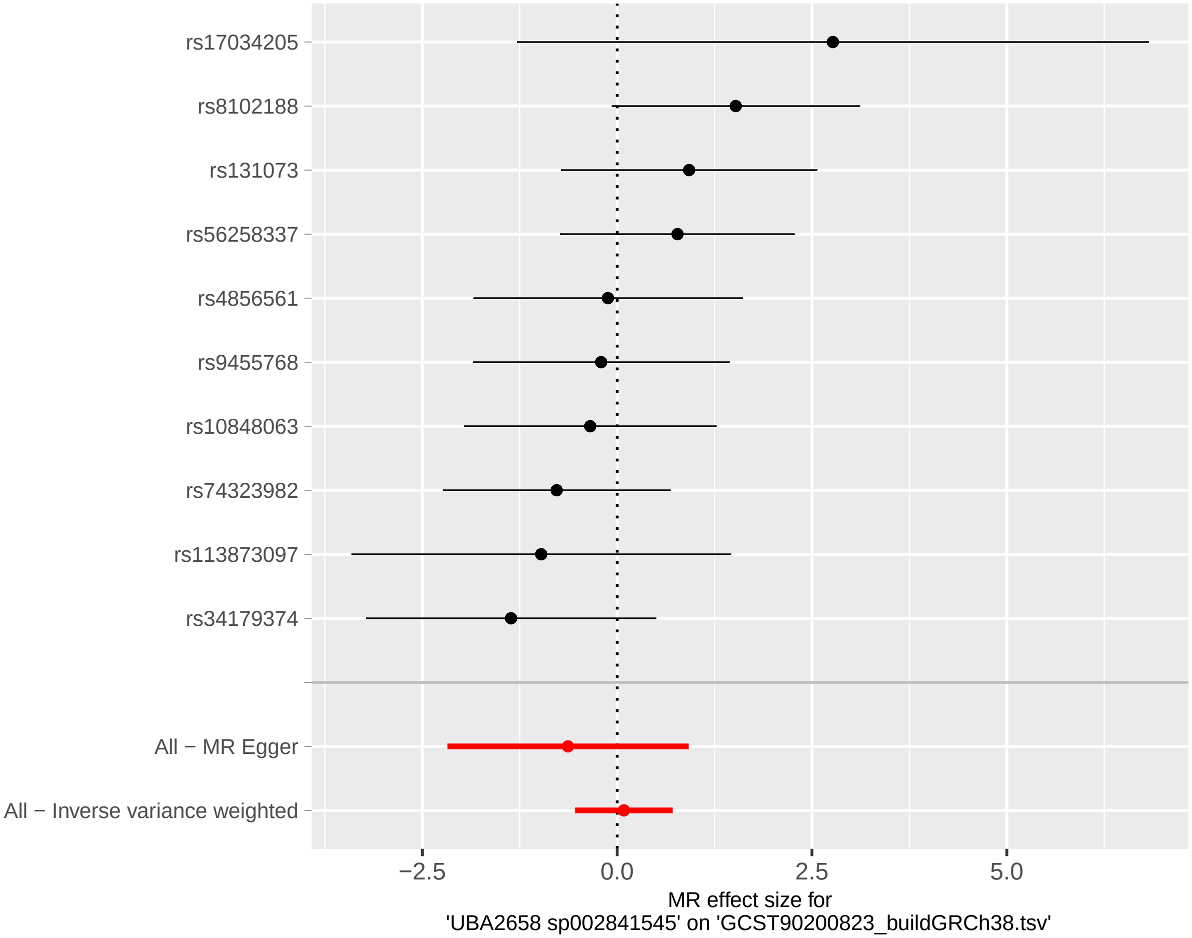

rs17034205
rs8102188
rs131073
rs56258337
rs4856561
rs9455768
rs10848063
rs74323982
rs113873097
rs34179374
All − MR Egger
All − Inverse variance weighted
−2.5
0.0
2.5
5.0
MR effect size for
'UBA2658 sp002841545' on 'GCST90200823_buildGRCh38.tsv'

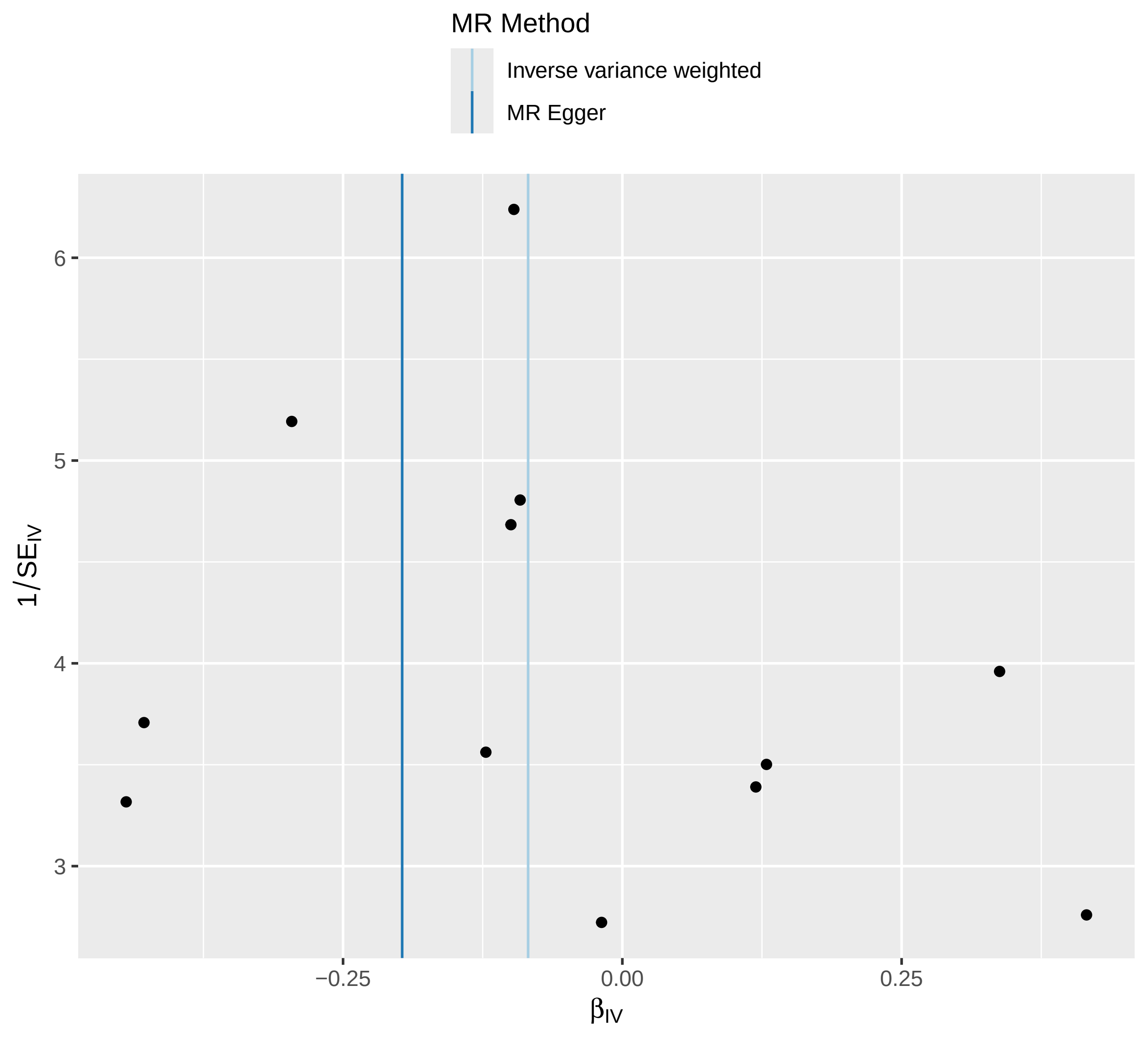
MR Method
Inverse variance weighted
MR Egger
6
5
4
3
1/SE$_{IV}$
−0.25
0.00
0.25
β$_{IV}$

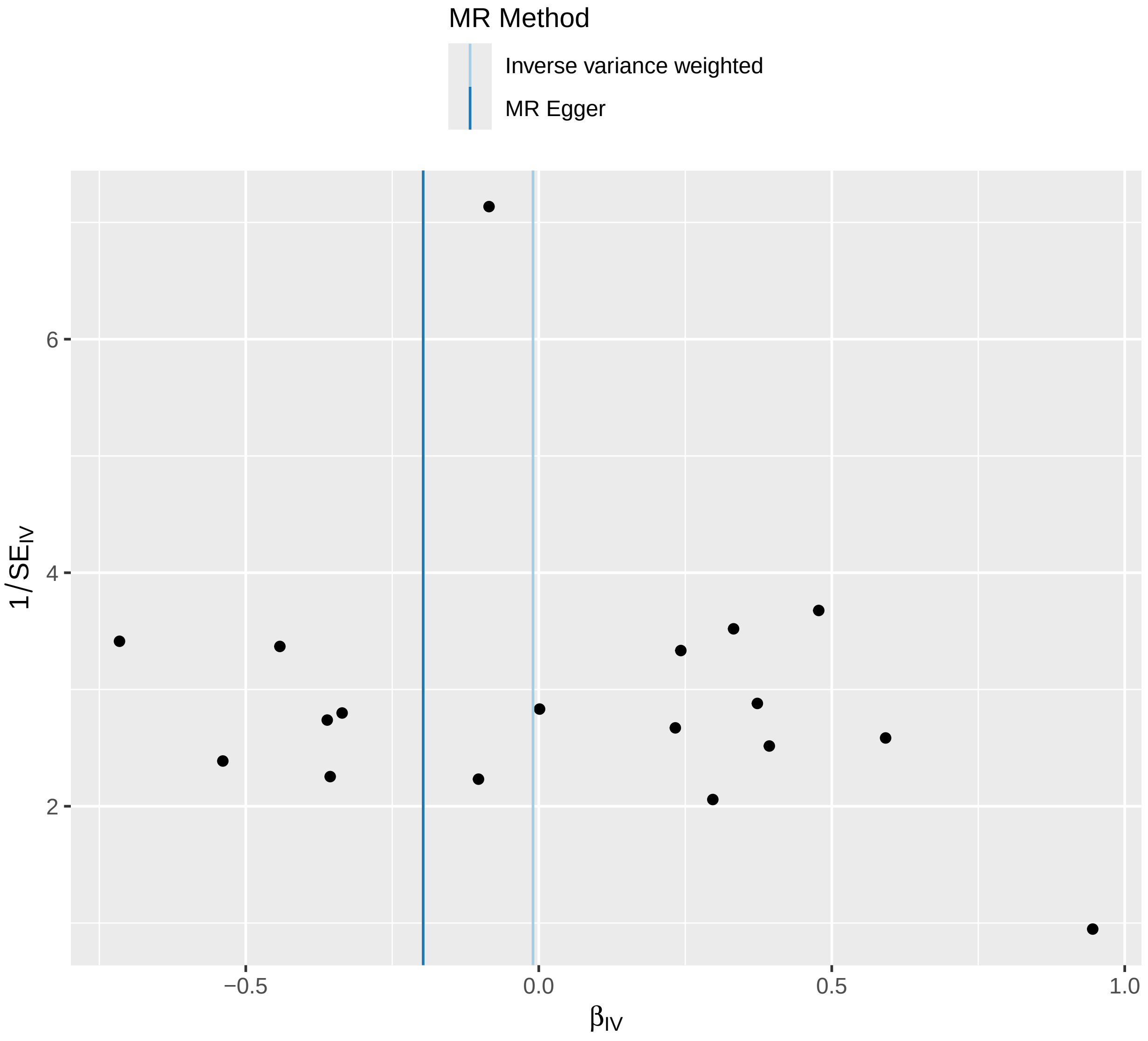

MR Method
Inverse variance weighted
MR Egger
6
4
2
1/SE$_{IV}$
−0.5
0.0
0.5
1.0
β$_{IV}$

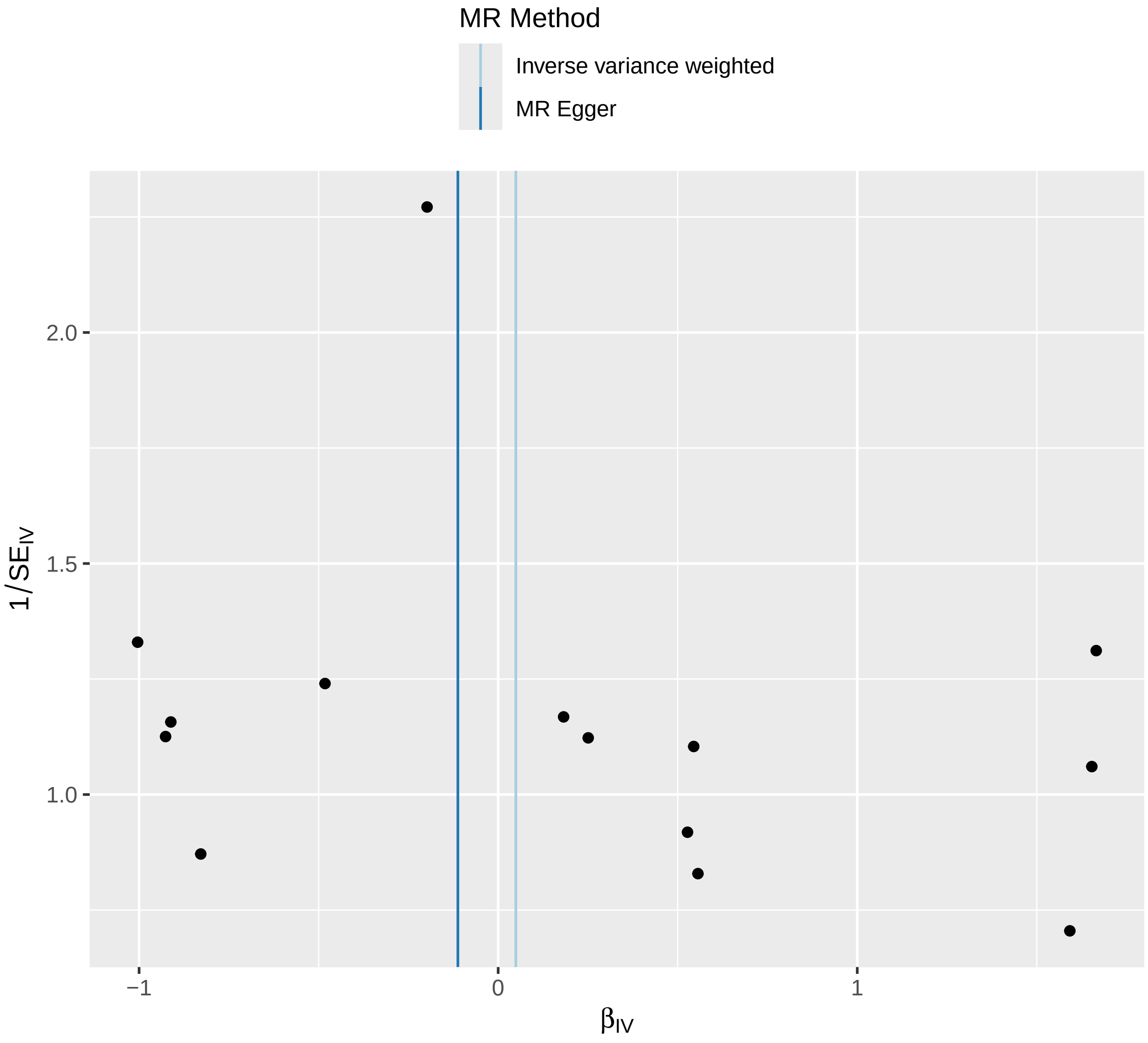

MR Method
Inverse variance weighted
MR Egger
2.0
1.5
1.0
1/SE_IV
−1
0
1
β_IV

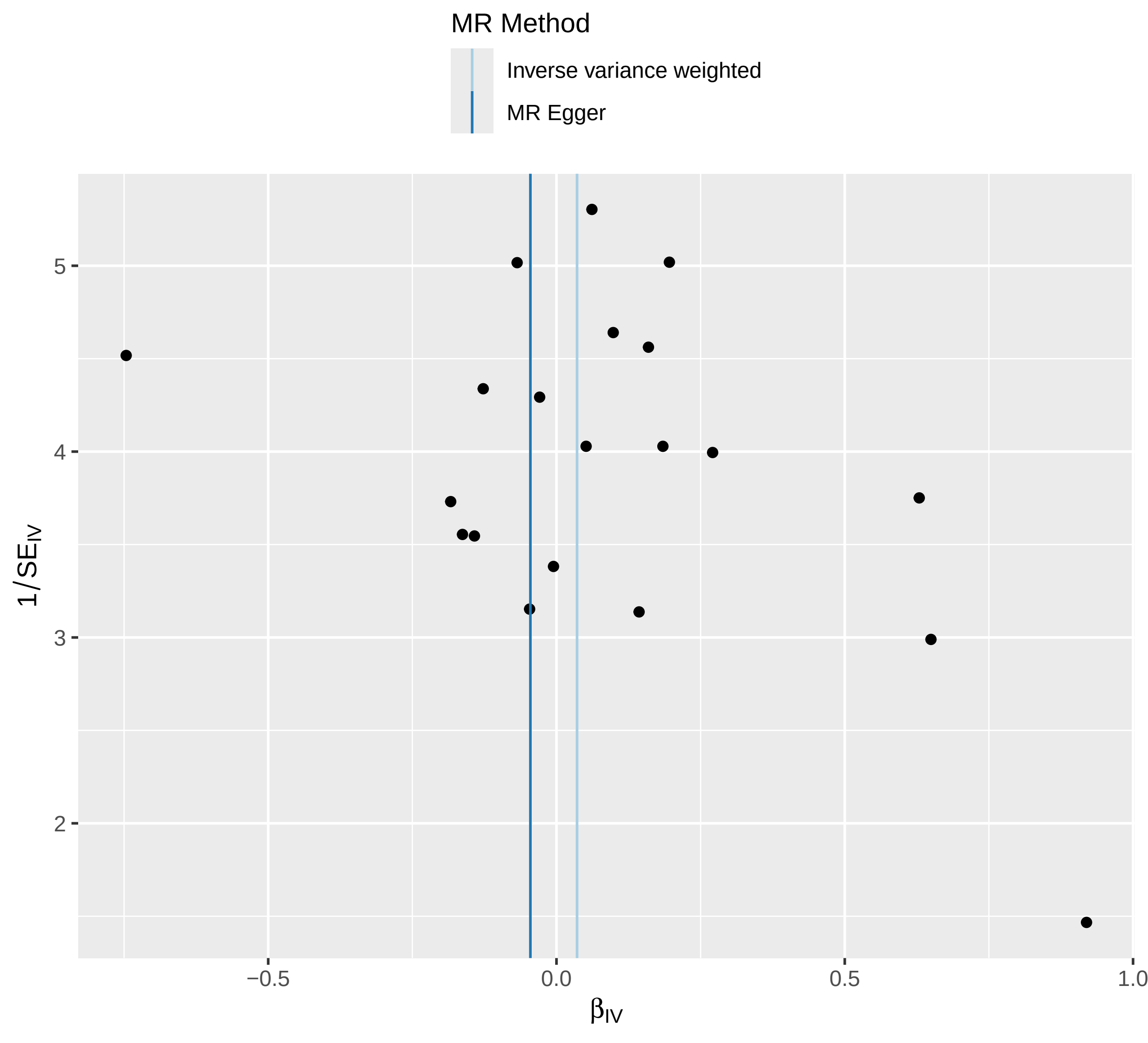
MR Method
Inverse variance weighted
MR Egger
5
4
3
2
1/SE_IV
−0.5
0.0
0.5
1.0
β_IV

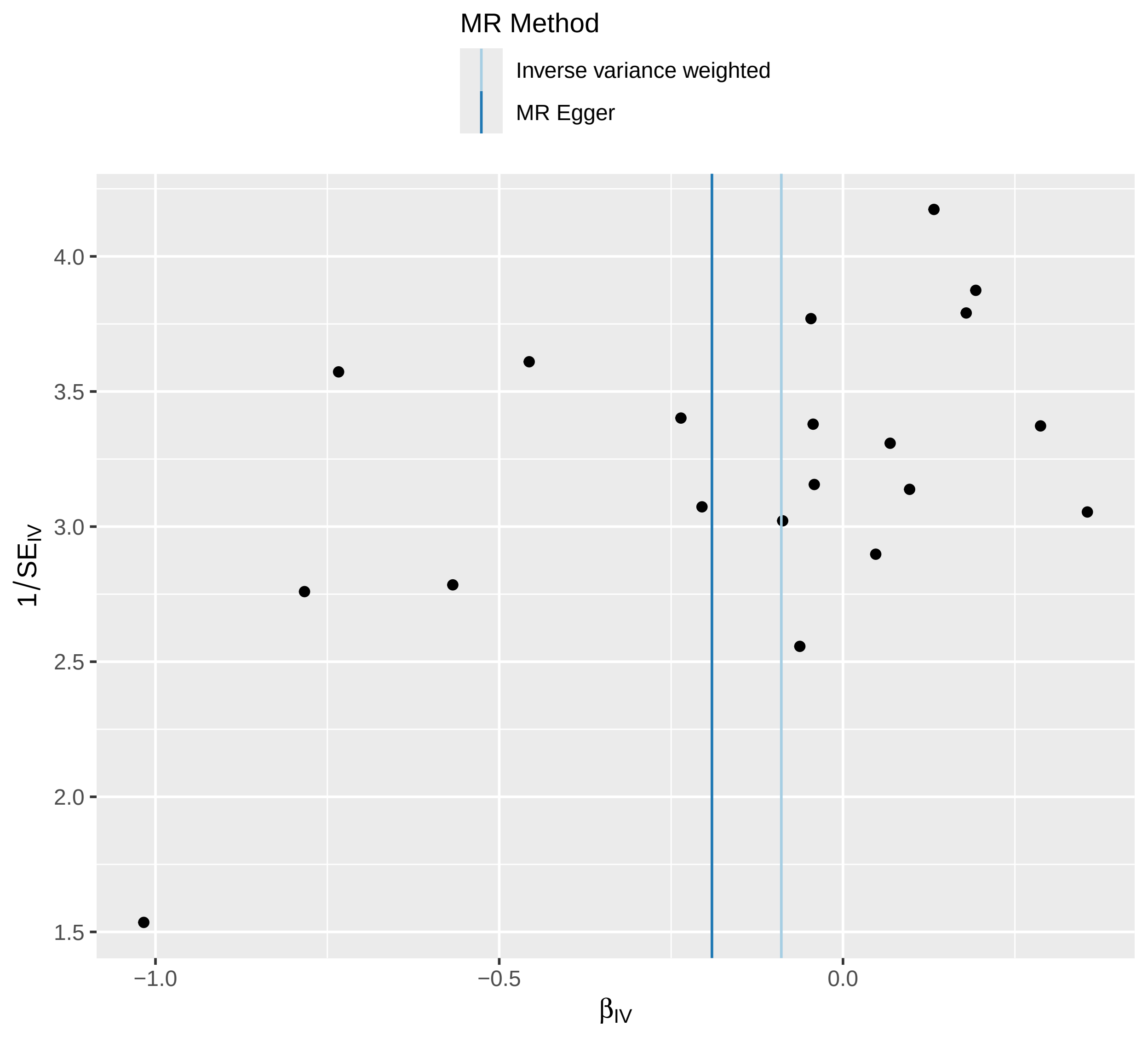

MR Method
Inverse variance weighted
MR Egger
4.0
3.5
3.0
2.5
2.0
1.5
1/SE_IV
−1.0
−0.5
0.0
β_IV

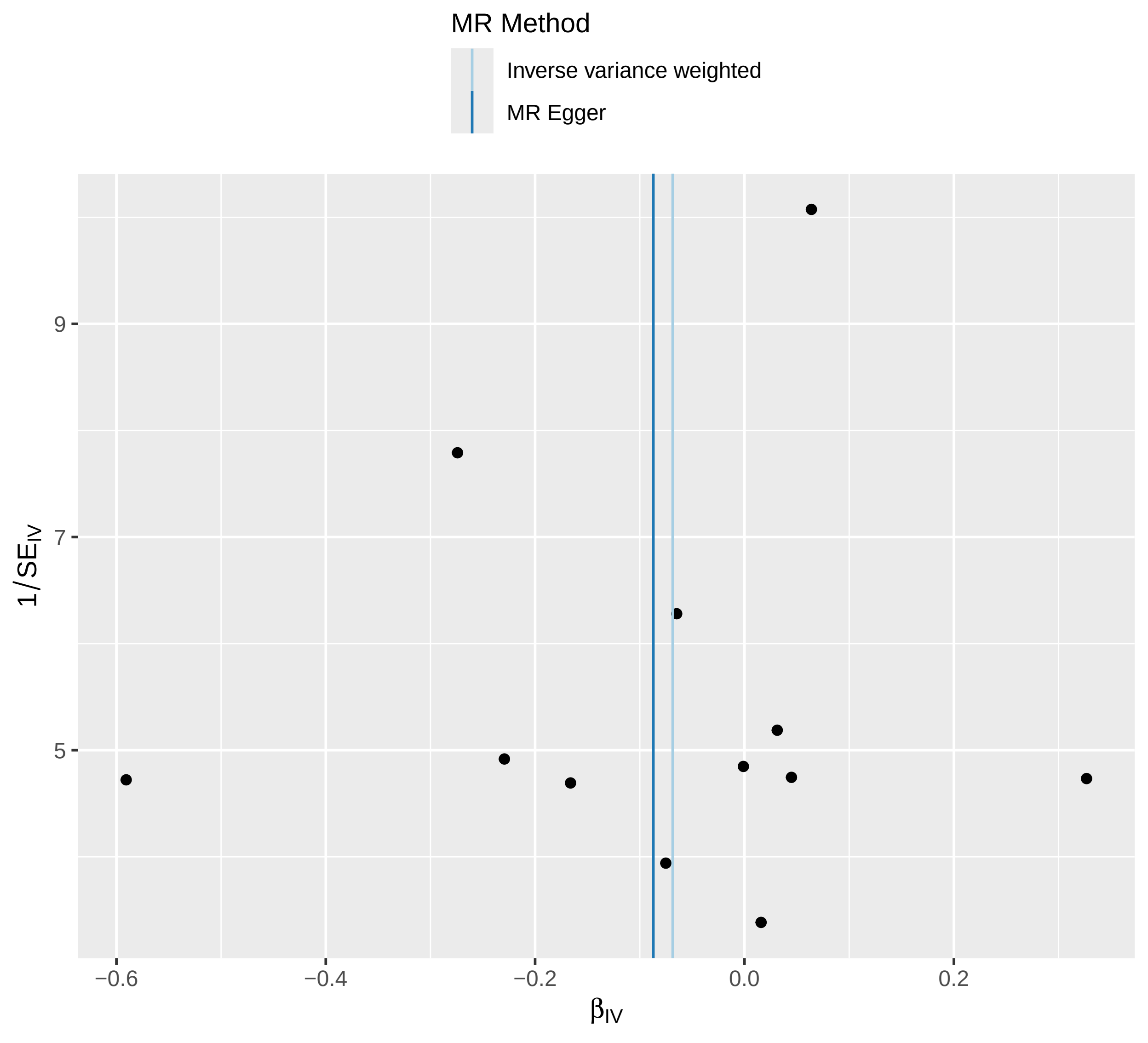
MR Method
Inverse variance weighted
MR Egger
9
7
5
−0.6
−0.4
−0.2
0.0
0.2
$1/SE_{IV}$
β_{IV}

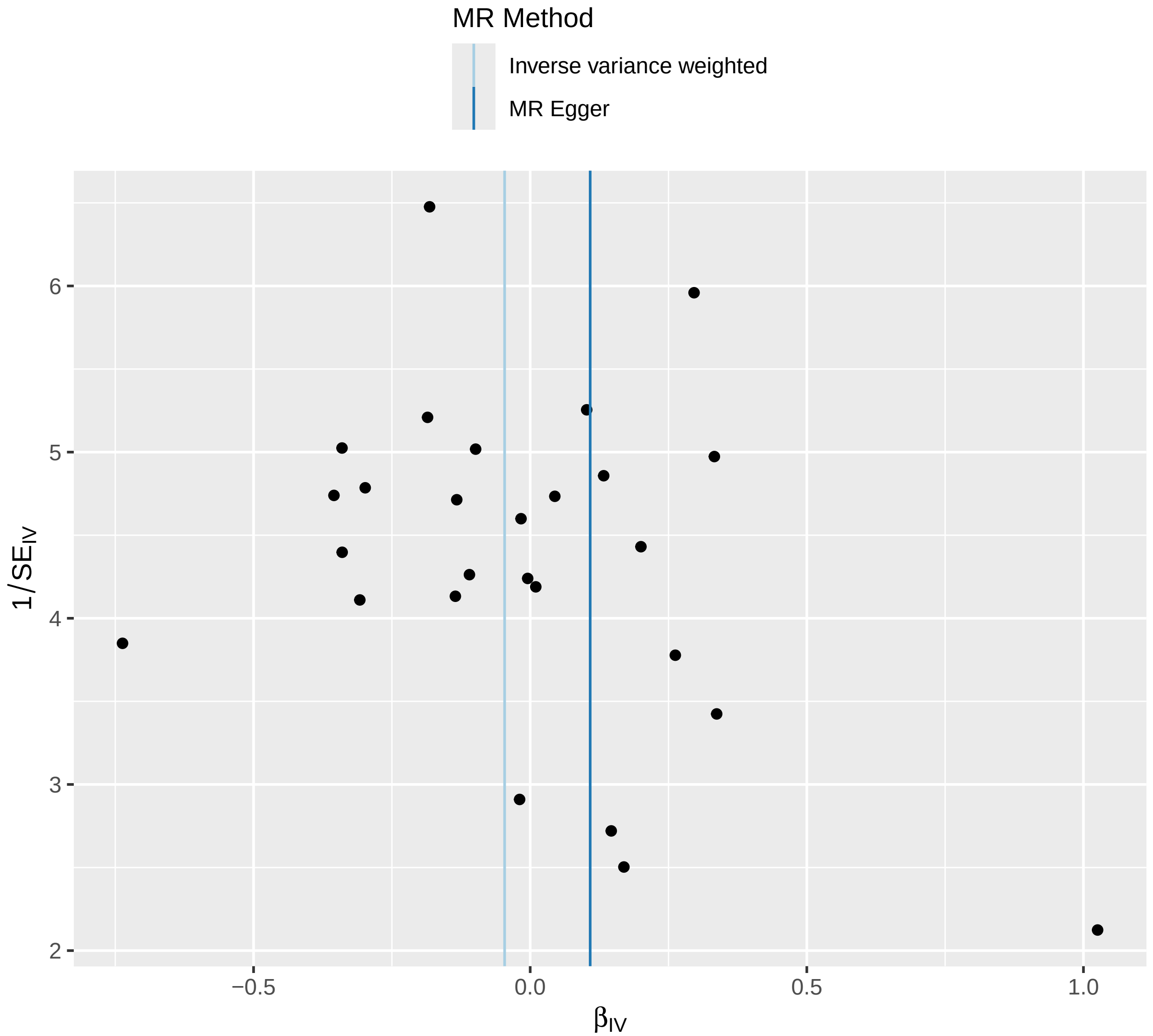
MR Method
Inverse variance weighted
MR Egger
1/SE_IV
β_IV
6
5
4
3
2
−0.5
0.0
0.5
1.0

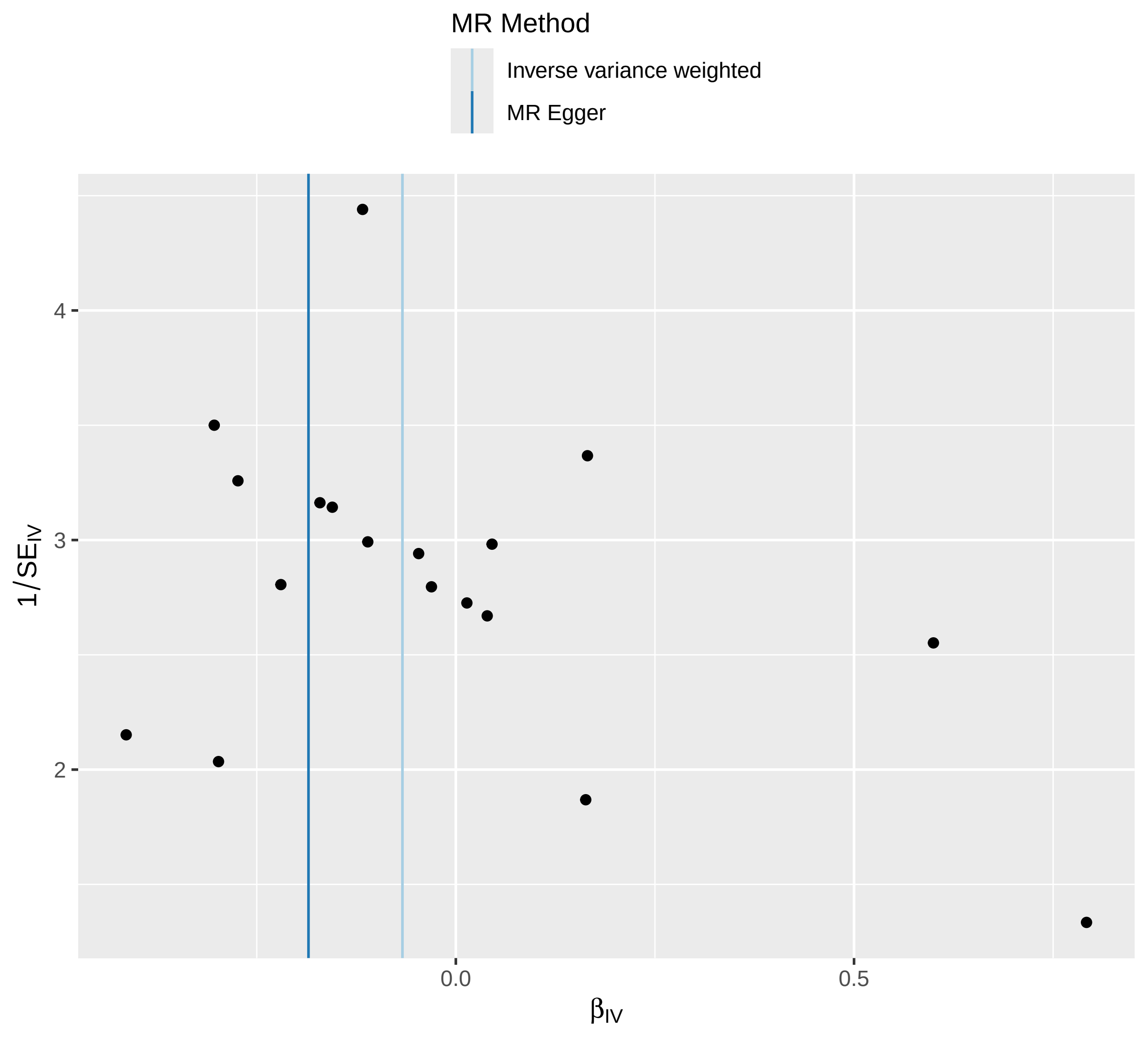
MR Method
Inverse variance weighted
MR Egger
4
3
2
$1/SE_{IV}$
0.0
0.5
β_{IV}

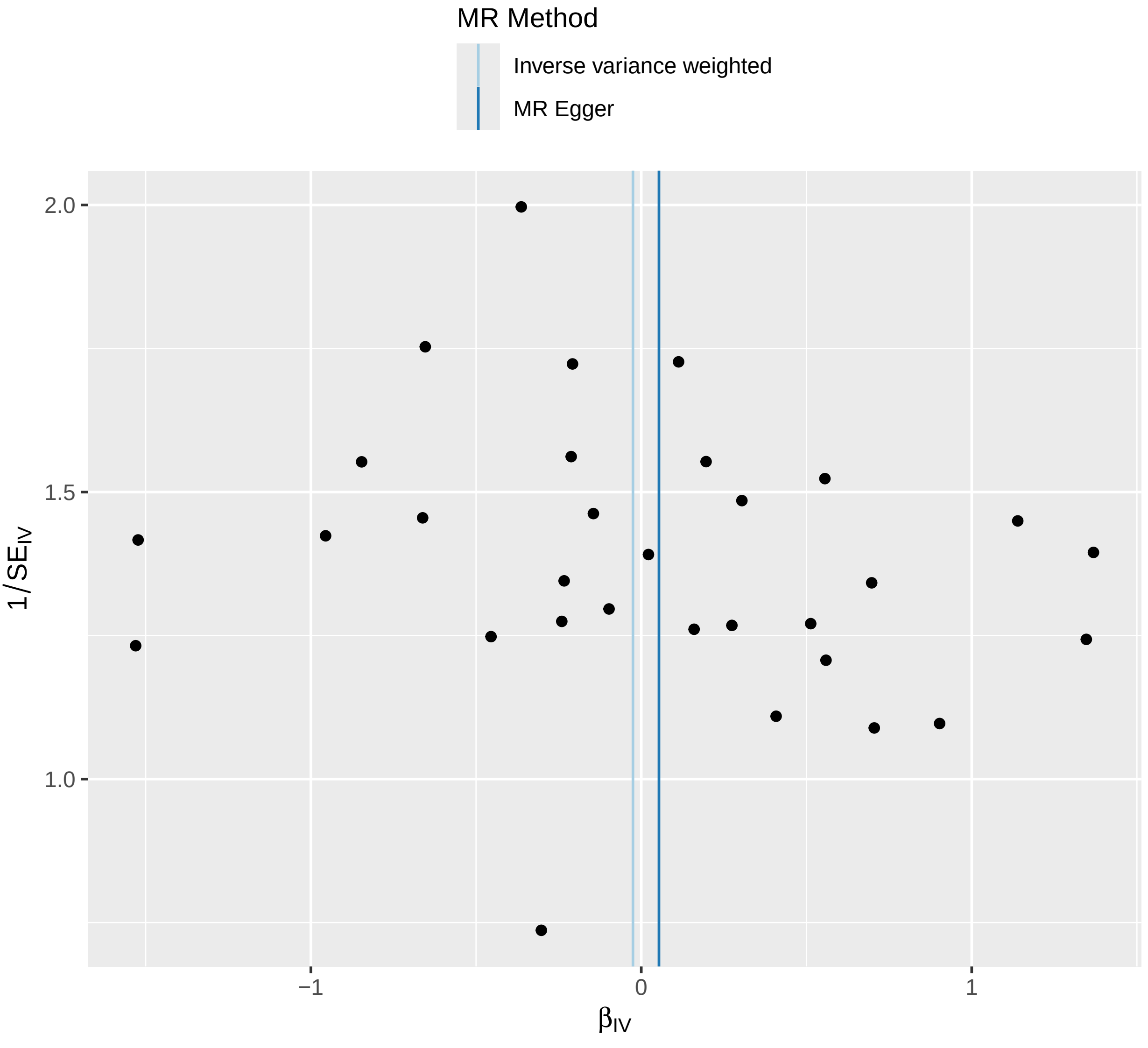

MR Method
Inverse variance weighted
MR Egger
2.0
1.5
1.0
1/SE_IV
−1
0
1
β_IV

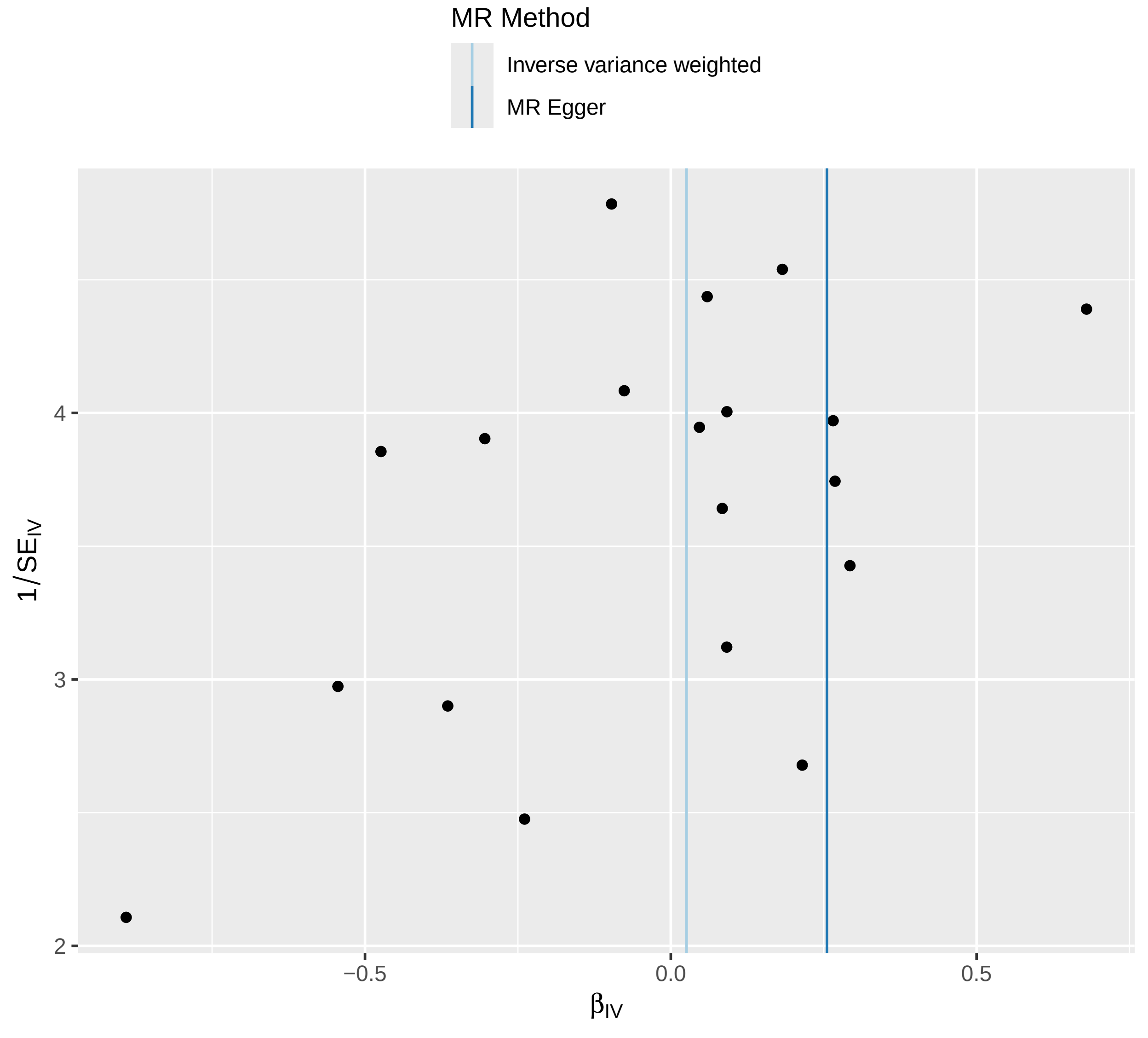

MR Method
Inverse variance weighted
MR Egger
4
3
2
1/SE$_{IV}$
−0.5
0.0
0.5
β$_{IV}$

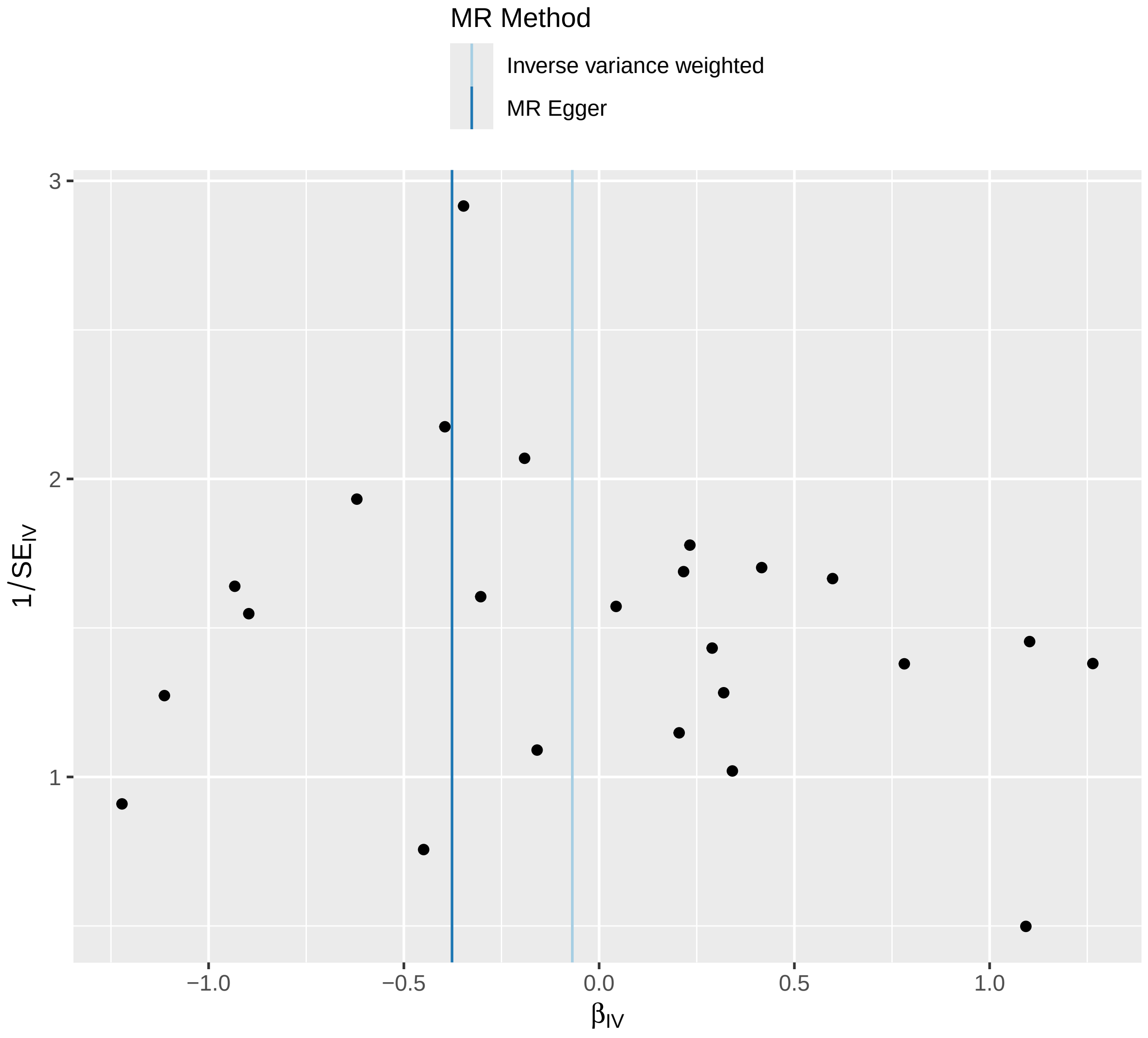
MR Method
Inverse variance weighted
MR Egger
3
2
1
$1/SE_{IV}$
−1.0
−0.5
0.0
0.5
1.0
β_{IV}

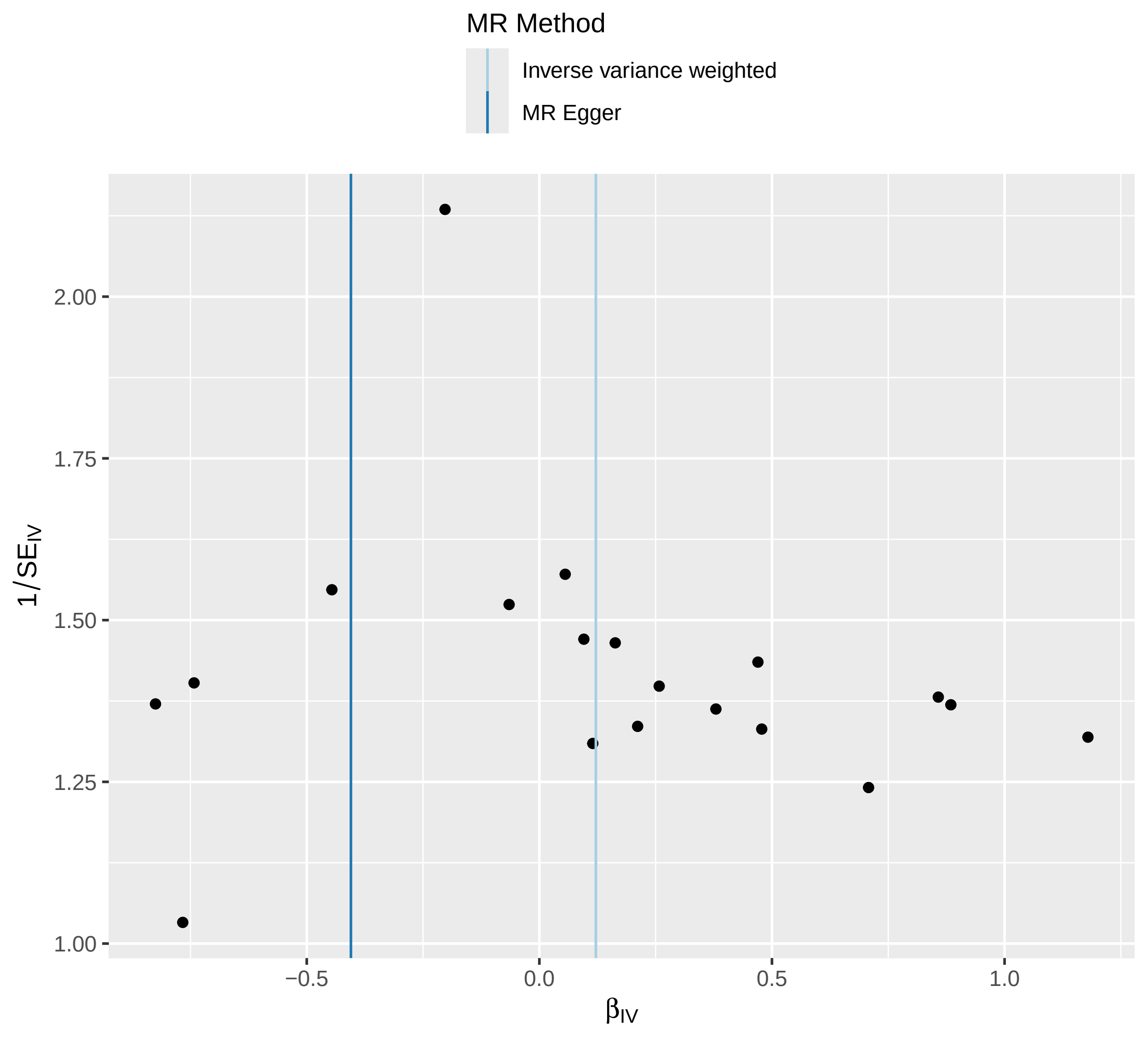
MR Method
Inverse variance weighted
MR Egger
1/SE_IV
2.00
1.75
1.50
1.25
1.00
−0.5
0.0
0.5
1.0
β_IV

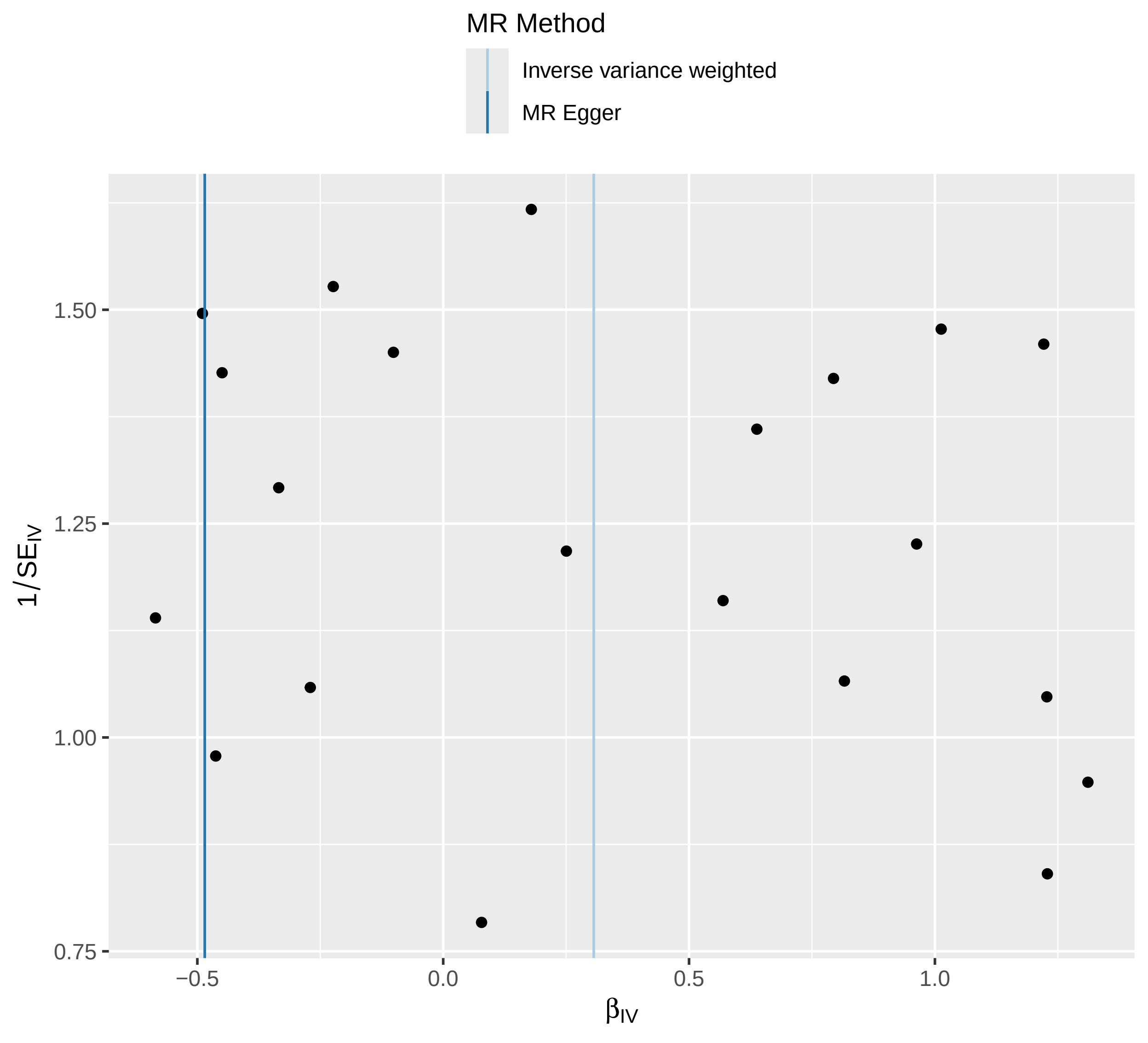
MR Method
Inverse variance weighted
MR Egger
1.50
1.25
1.00
0.75
1/SE$_{IV}$
−0.5
0.0
0.5
1.0
β$_{IV}$

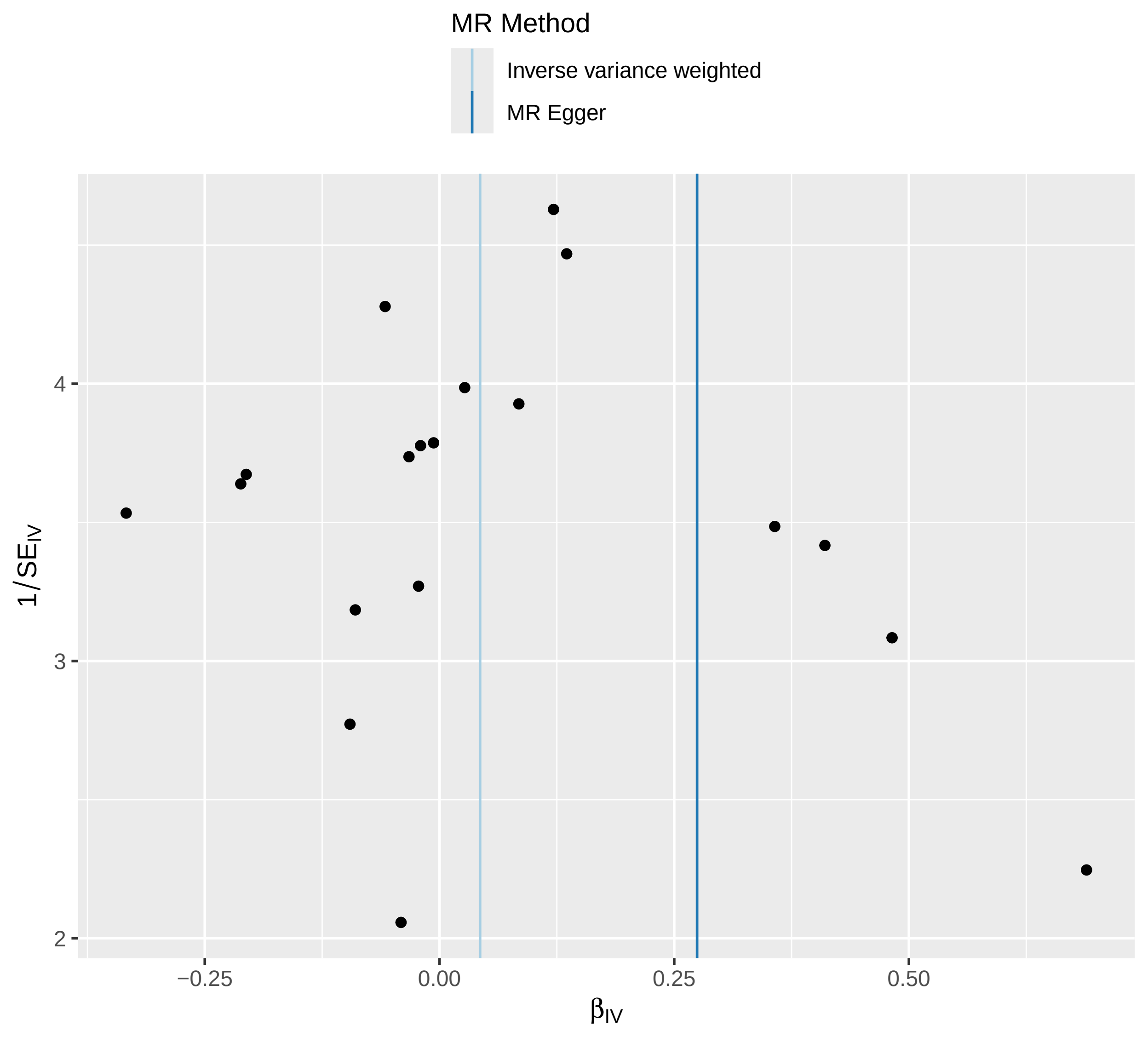

MR Method
Inverse variance weighted
MR Egger
4
3
2
−0.25
0.00
0.25
0.50
$1/SE_{IV}$
β_{IV}

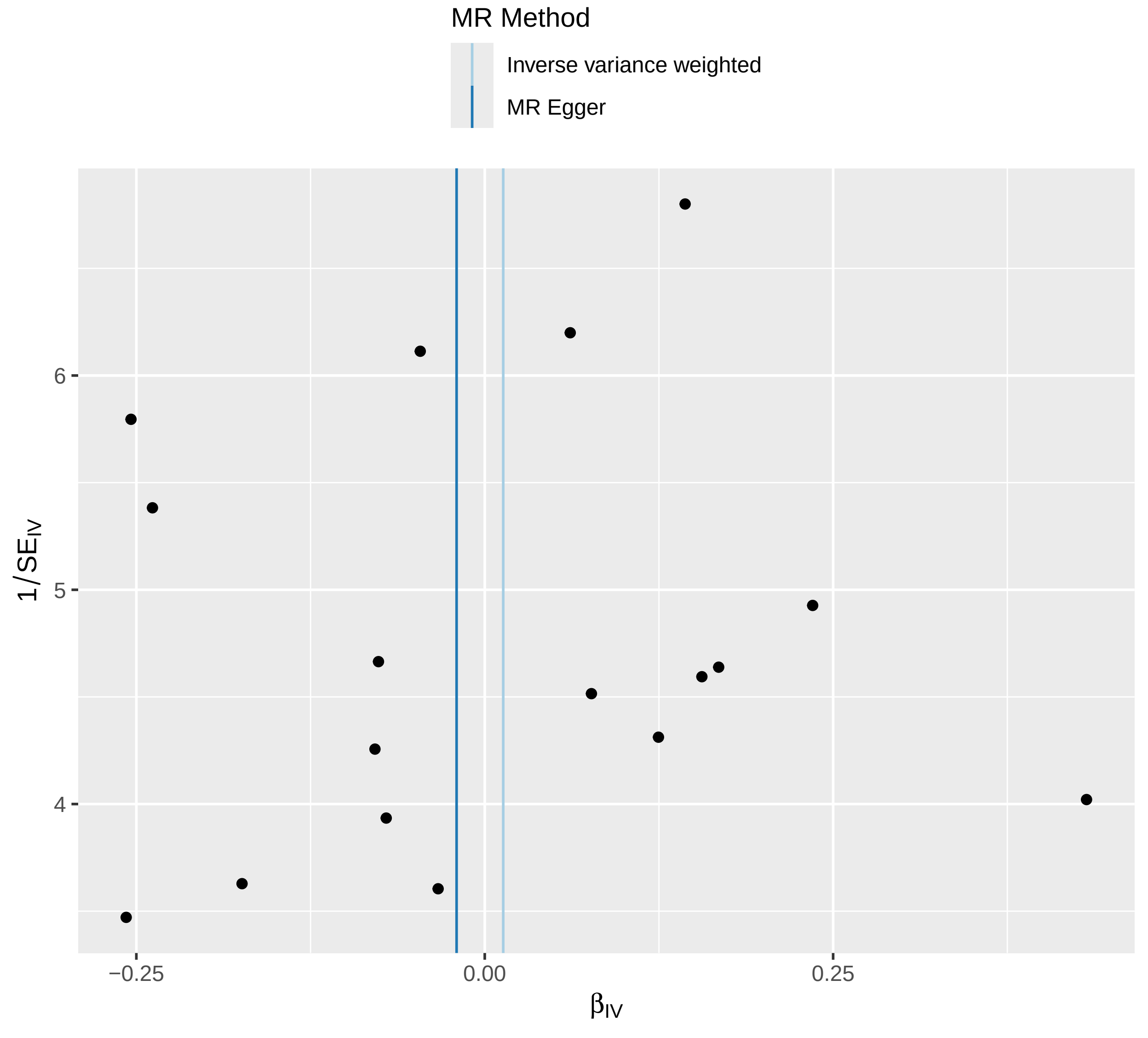

MR Method
Inverse variance weighted
MR Egger
6
5
4
1/SE_IV
−0.25
0.00
0.25
β_IV

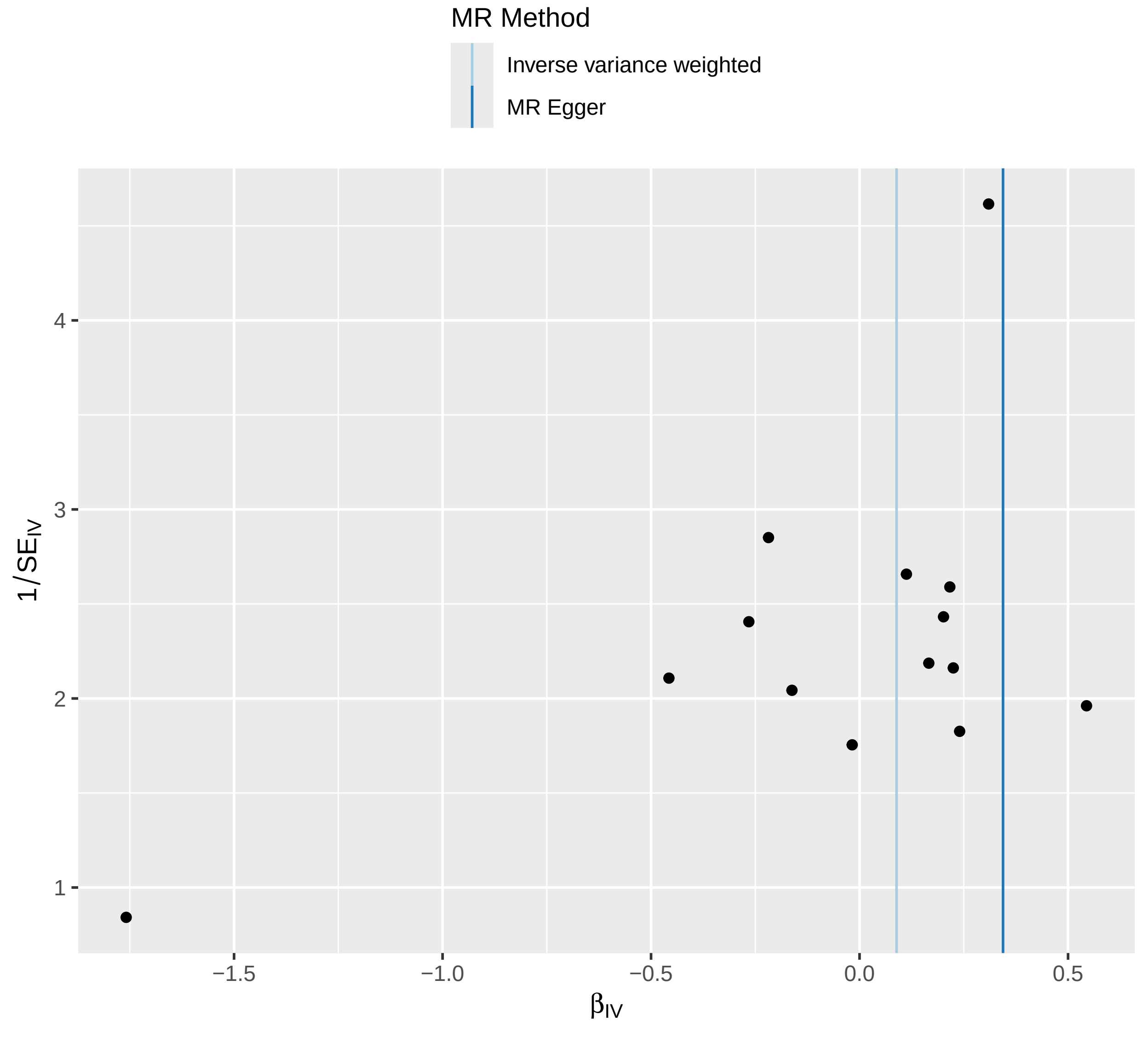

MR Method
Inverse variance weighted
MR Egger
4
3
2
1
1/SE_IV
−1.5
−1.0
−0.5
0.0
0.5
β_IV

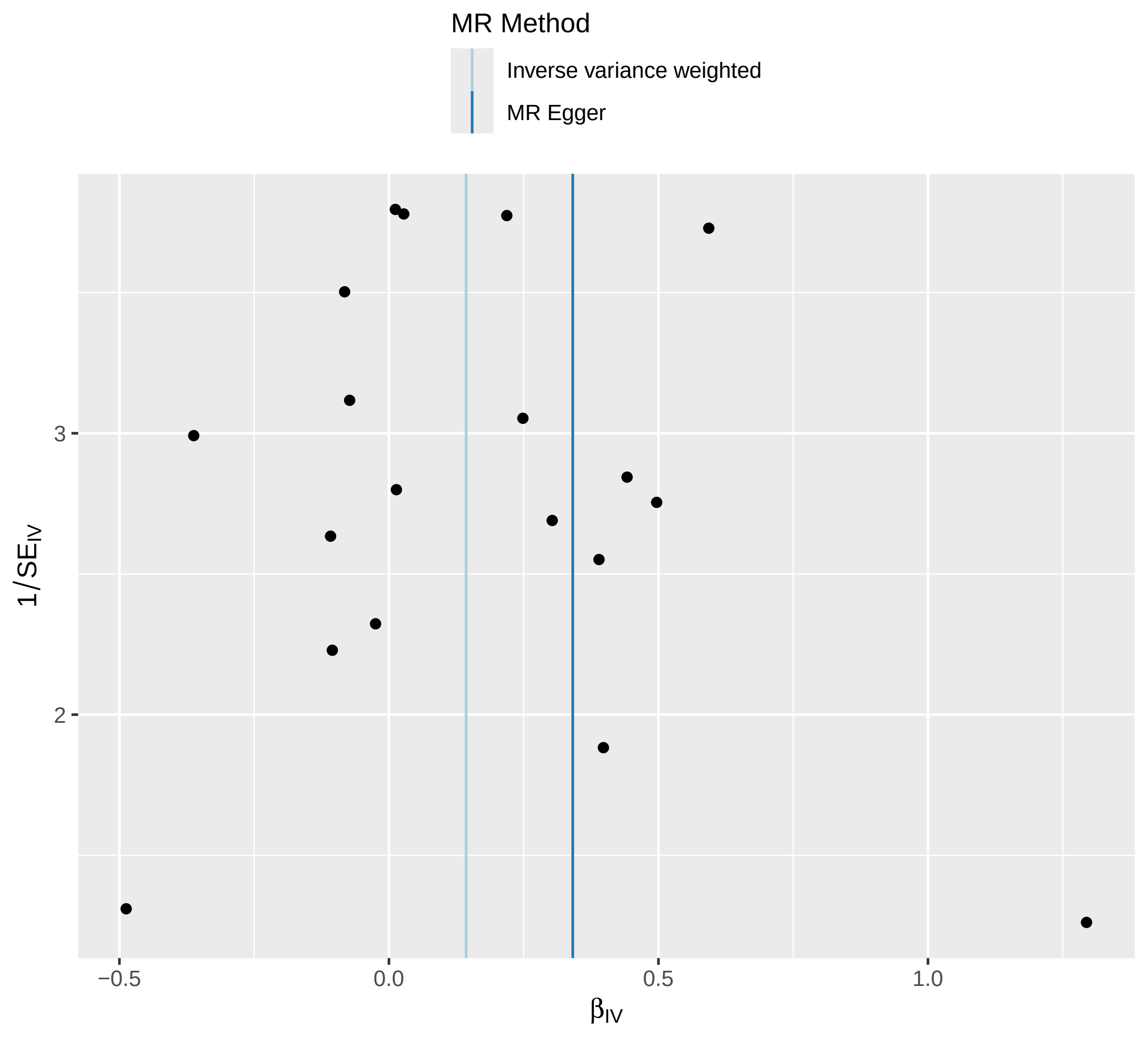

MR Method
Inverse variance weighted
MR Egger
3
2
1/SE$_{IV}$
−0.5
0.0
0.5
1.0
β$_{IV}$

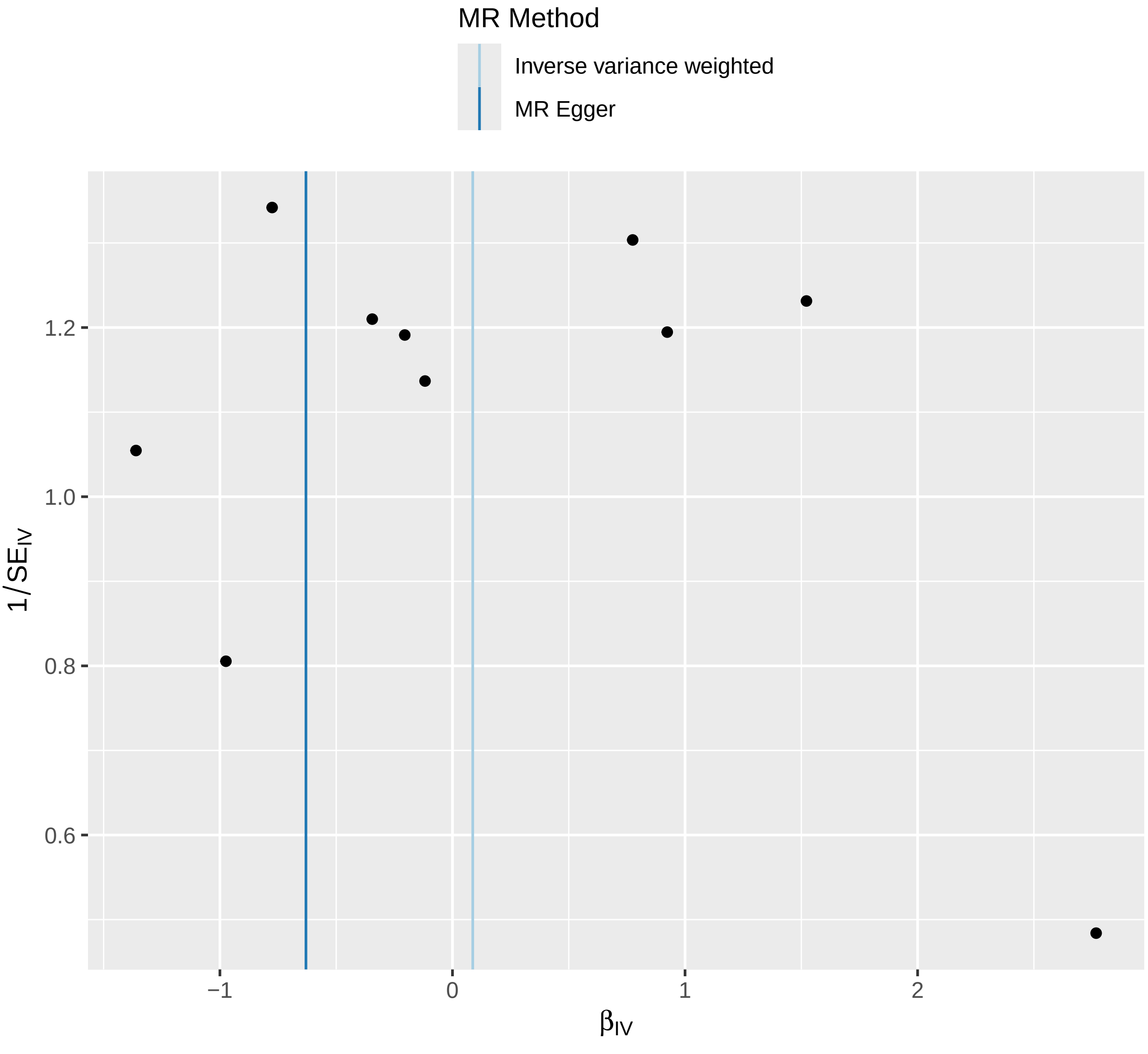
MR Method
Inverse variance weighted
MR Egger
1/SE_IV
1.2
1.0
0.8
0.6
−1
0
1
2
β_IV

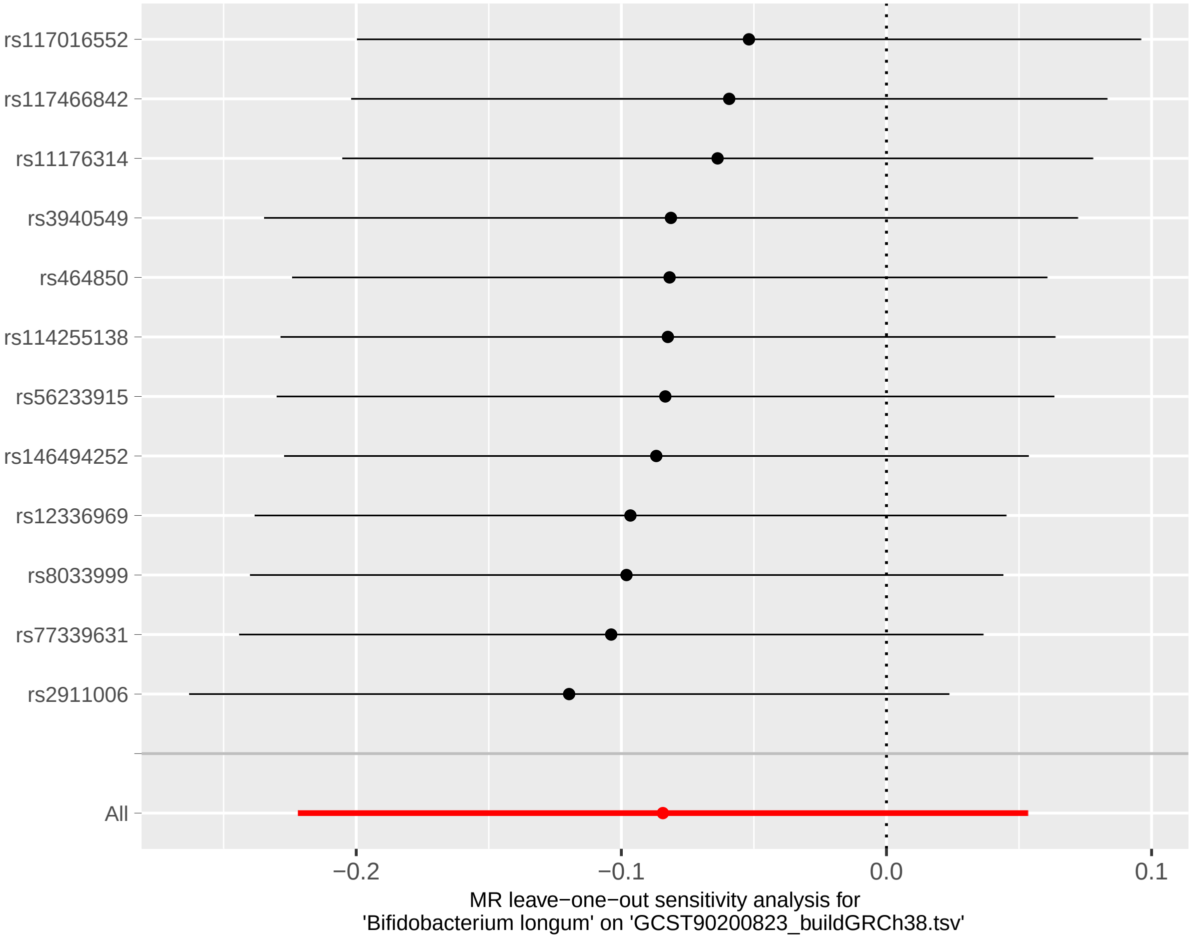

rs117016552
rs117466842
rs11176314
rs3940549
rs464850
rs114255138
rs56233915
rs146494252
rs12336969
rs8033999
rs77339631
rs2911006
All
−0.2
−0.1
0.0
0.1
MR leave−one−out sensitivity analysis for
'Bifidobacterium longum' on 'GCST90200823_buildGRCh38.tsv'

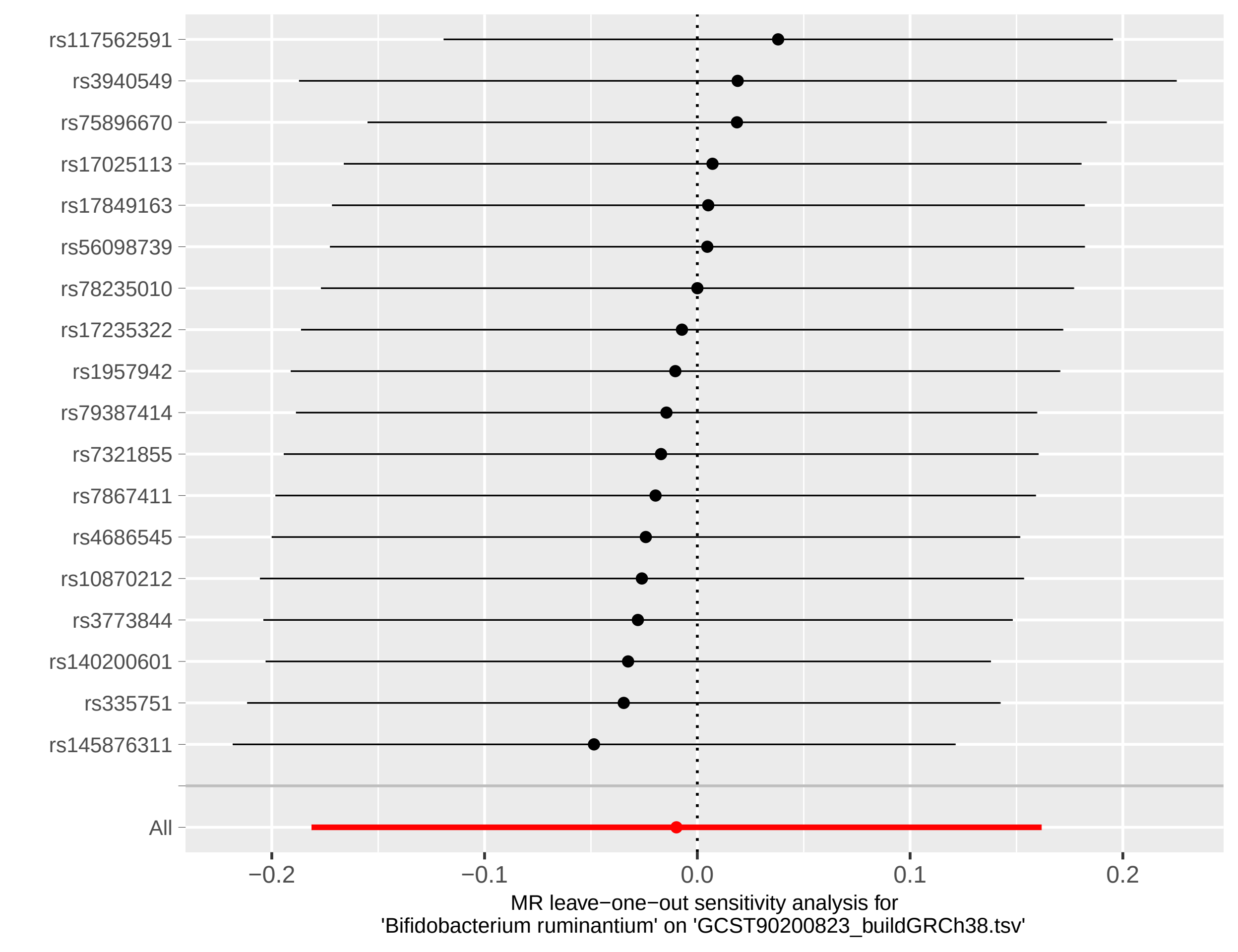
rs117562591
rs3940549
rs75896670
rs17025113
rs17849163
rs56098739
rs78235010
rs17235322
rs1957942
rs79387414
rs7321855
rs7867411
rs4686545
rs10870212
rs3773844
rs140200601
rs335751
rs145876311
All
−0.2
−0.1
0.0
0.1
0.2
MR leave−one−out sensitivity analysis for
'Bifidobacterium ruminantium' on 'GCST90200823_buildGRCh38.tsv'

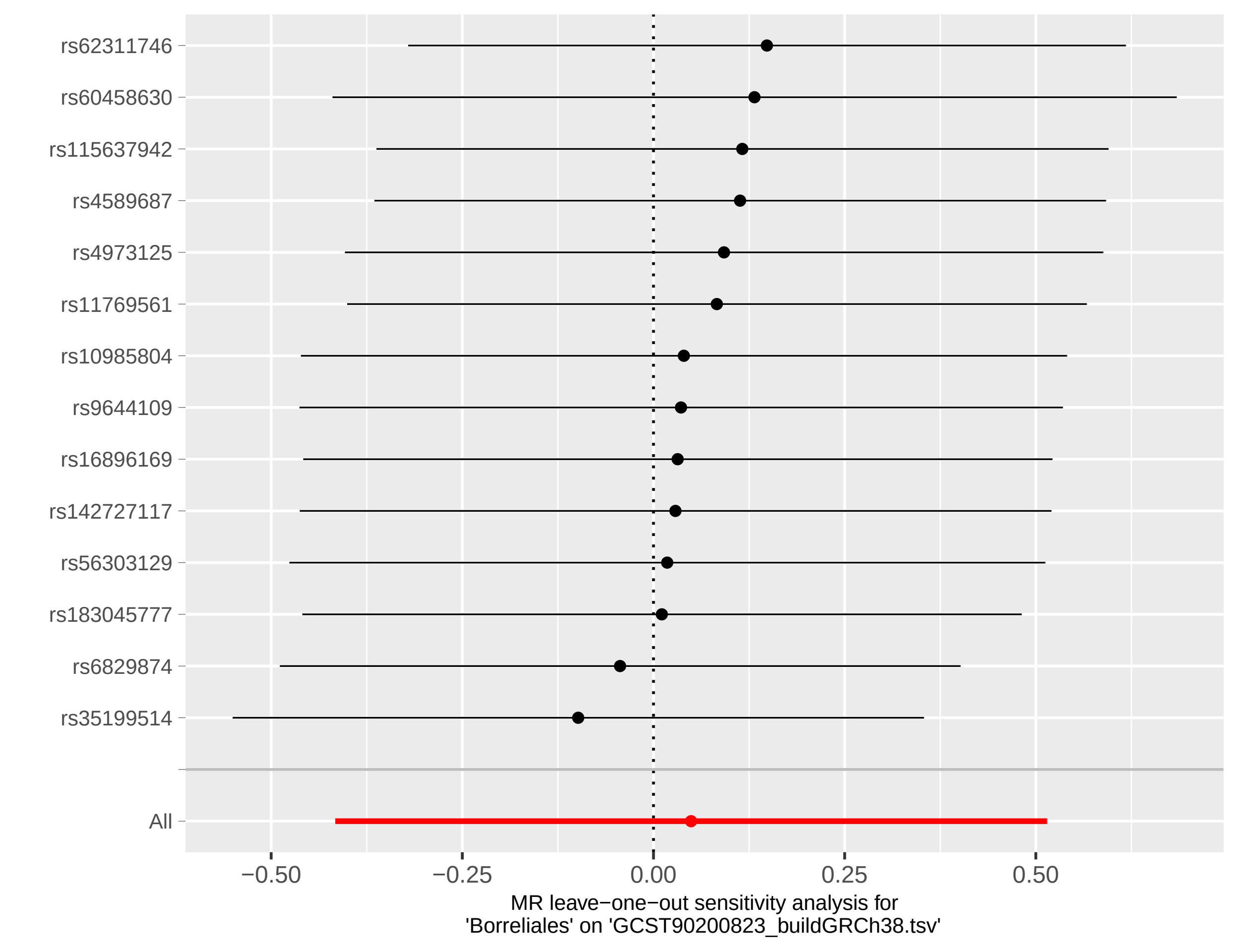

rs62311746
rs60458630
rs115637942
rs4589687
rs4973125
rs11769561
rs10985804
rs9644109
rs16896169
rs142727117
rs56303129
rs183045777
rs6829874
rs35199514
All
−0.50
−0.25
0.00
0.25
0.50
MR leave−one−out sensitivity analysis for
'Borreliales' on 'GCST90200823_buildGRCh38.tsv'

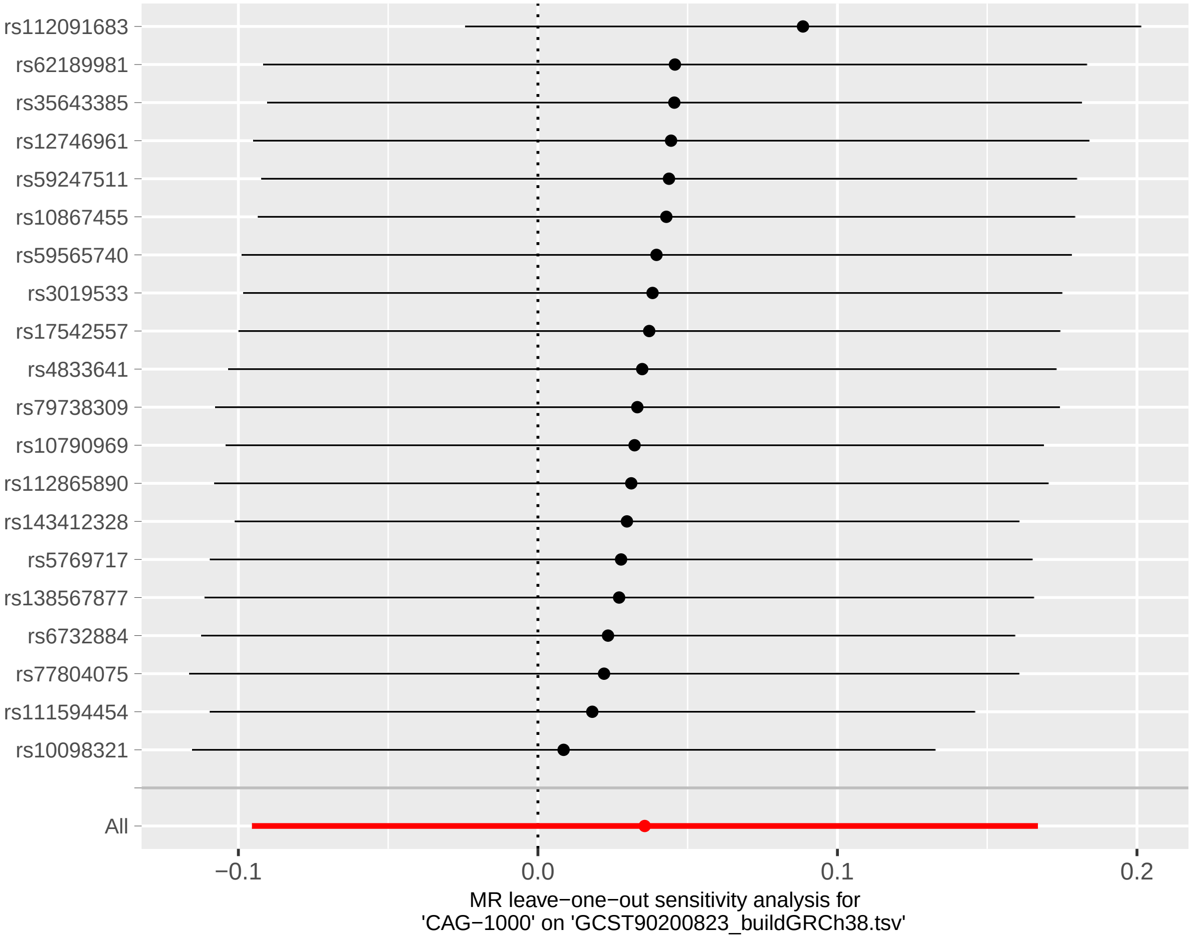
rs112091683
rs62189981
rs35643385
rs12746961
rs59247511
rs10867455
rs59565740
rs3019533
rs17542557
rs4833641
rs79738309
rs10790969
rs112865890
rs143412328
rs5769717
rs138567877
rs6732884
rs77804075
rs111594454
rs10098321
All
−0.1
0.0
0.1
0.2
MR leave−one−out sensitivity analysis for
'CAG−1000' on 'GCST90200823_buildGRCh38.tsv'

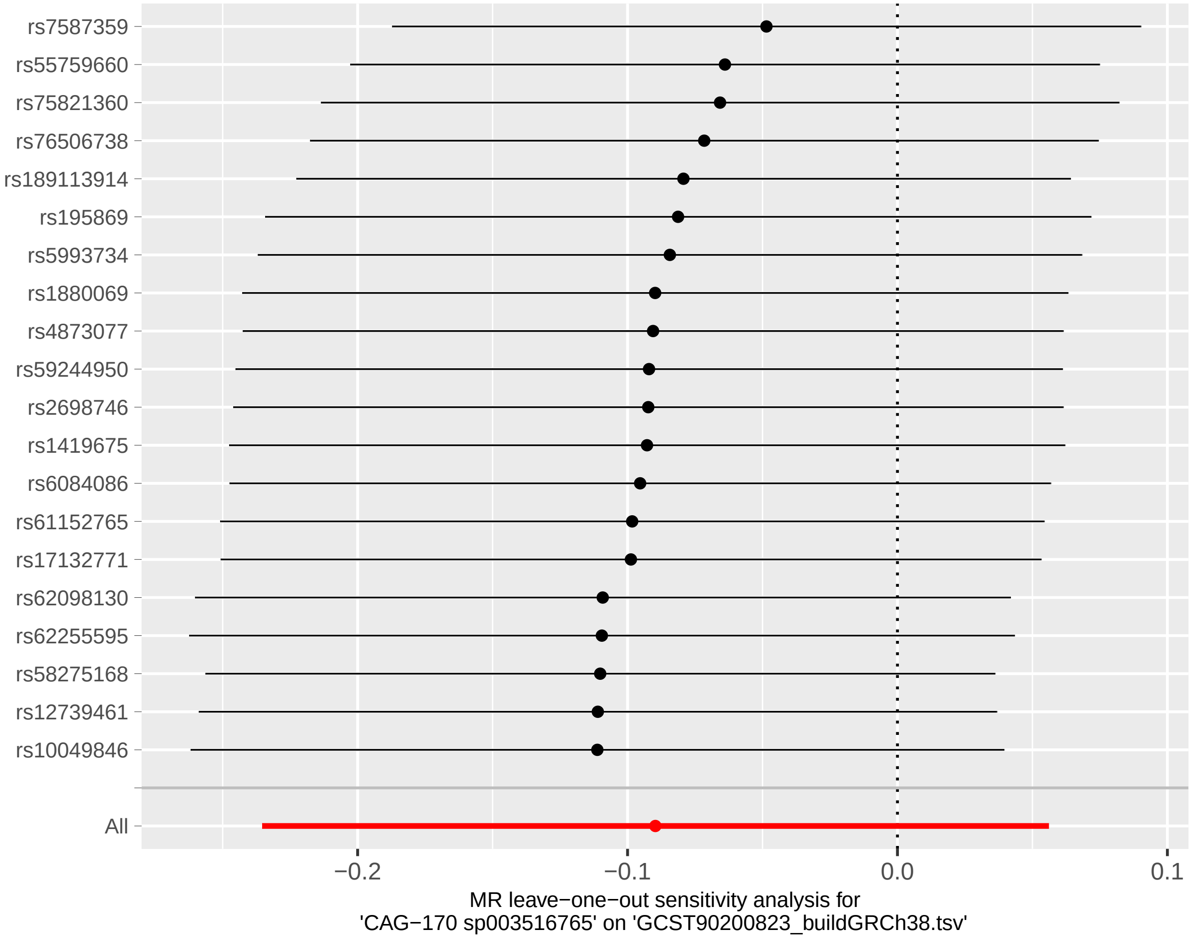
rs7587359
rs55759660
rs75821360
rs76506738
rs189113914
rs195869
rs5993734
rs1880069
rs4873077
rs59244950
rs2698746
rs1419675
rs6084086
rs61152765
rs17132771
rs62098130
rs62255595
rs58275168
rs12739461
rs10049846
All
−0.2
−0.1
0.0
0.1
MR leave−one−out sensitivity analysis for
'CAG−170 sp003516765' on 'GCST90200823_buildGRCh38.tsv'

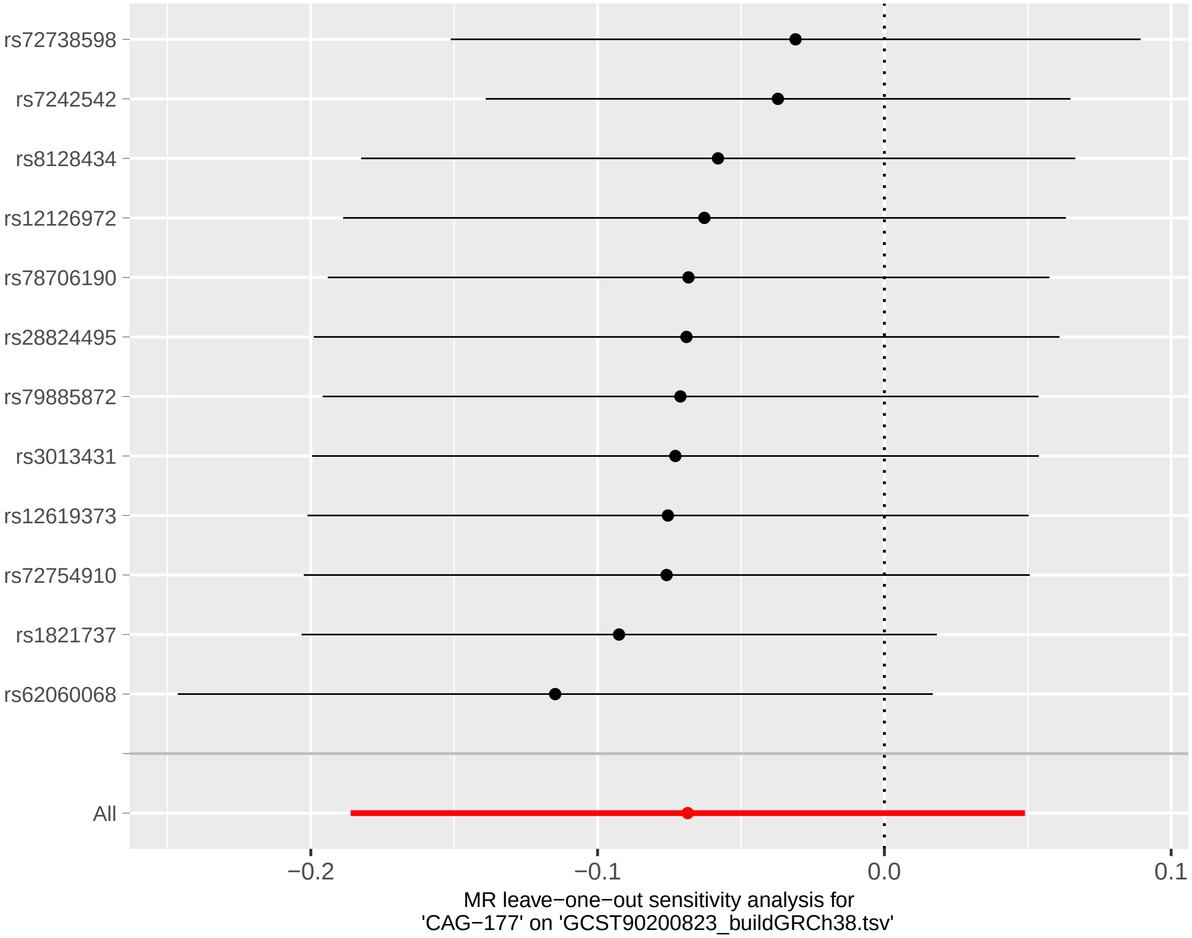
rs72738598
rs7242542
rs8128434
rs12126972
rs78706190
rs28824495
rs79885872
rs3013431
rs12619373
rs72754910
rs1821737
rs62060068
All
−0.2
−0.1
0.0
0.1
MR leave−one−out sensitivity analysis for
'CAG−177' on 'GCST90200823_buildGRCh38.tsv'

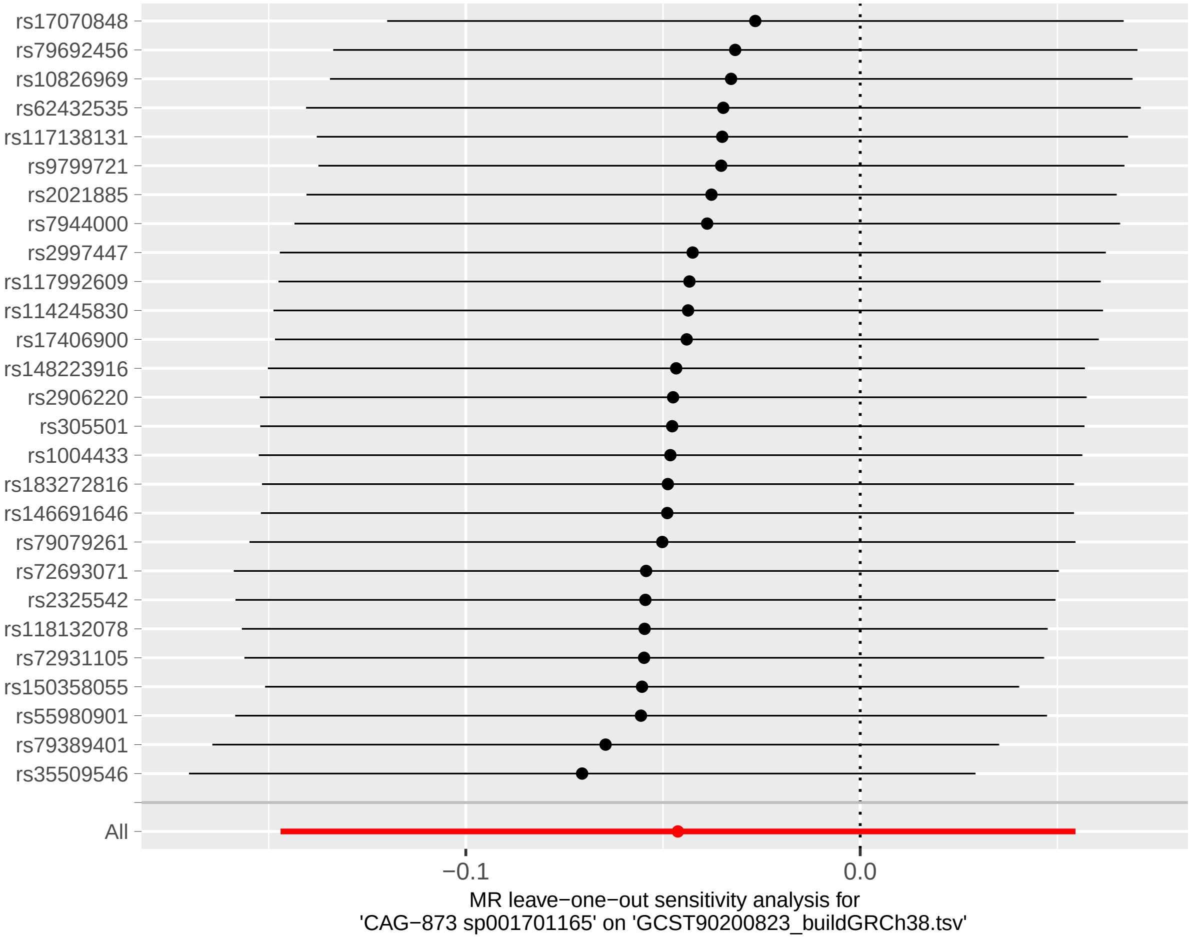

rs17070848
rs79692456
rs10826969
rs62432535
rs117138131
rs9799721
rs2021885
rs7944000
rs2997447
rs117992609
rs114245830
rs17406900
rs148223916
rs2906220
rs305501
rs1004433
rs183272816
rs146691646
rs79079261
rs72693071
rs2325542
rs118132078
rs72931105
rs150358055
rs55980901
rs79389401
rs35509546
All
−0.1
0.0
MR leave−one−out sensitivity analysis for
'CAG−873 sp001701165' on 'GCST90200823_buildGRCh38.tsv'

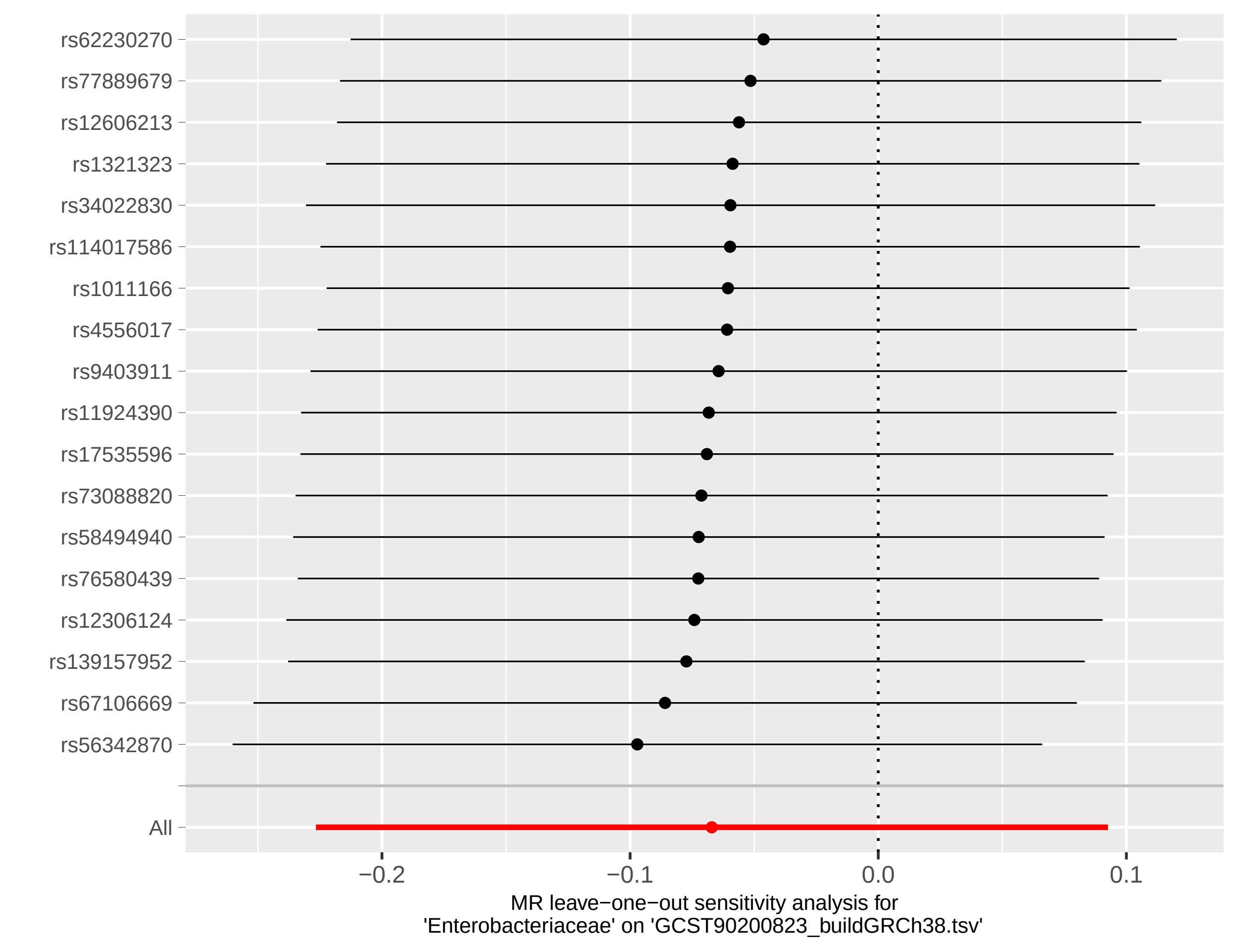
rs62230270
rs77889679
rs12606213
rs1321323
rs34022830
rs114017586
rs1011166
rs4556017
rs9403911
rs11924390
rs17535596
rs73088820
rs58494940
rs76580439
rs12306124
rs139157952
rs67106669
rs56342870
All
−0.2
−0.1
0.0
0.1
MR leave−one−out sensitivity analysis for
'Enterobacteriaceae' on 'GCST90200823_buildGRCh38.tsv'

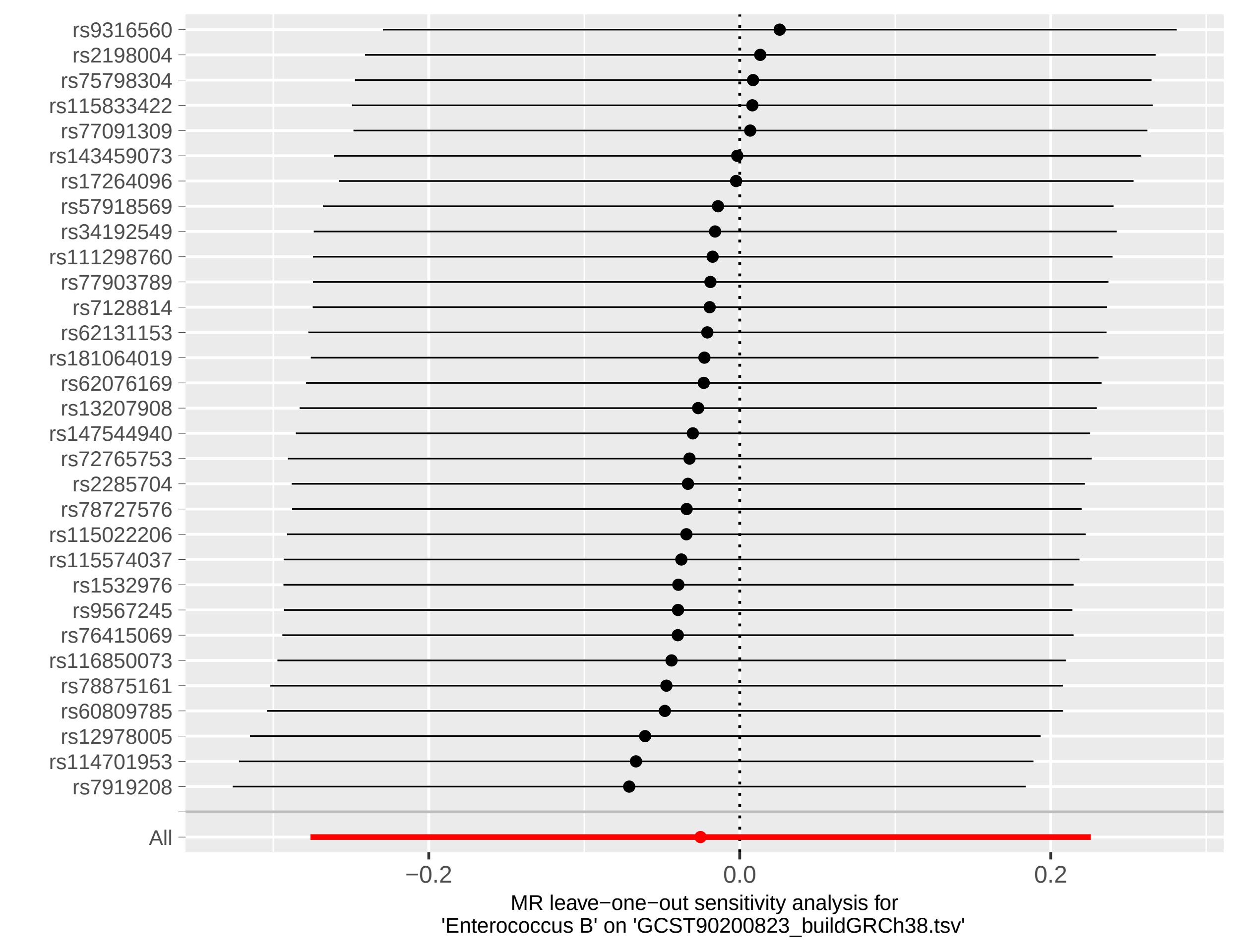
rs9316560
rs2198004
rs75798304
rs115833422
rs77091309
rs143459073
rs17264096
rs57918569
rs34192549
rs111298760
rs77903789
rs7128814
rs62131153
rs181064019
rs62076169
rs13207908
rs147544940
rs72765753
rs2285704
rs78727576
rs115022206
rs115574037
rs1532976
rs9567245
rs76415069
rs116850073
rs78875161
rs60809785
rs12978005
rs114701953
rs7919208
All
−0.2
0.0
0.2
MR leave−one−out sensitivity analysis for
'Enterococcus B' on 'GCST90200823_buildGRCh38.tsv'

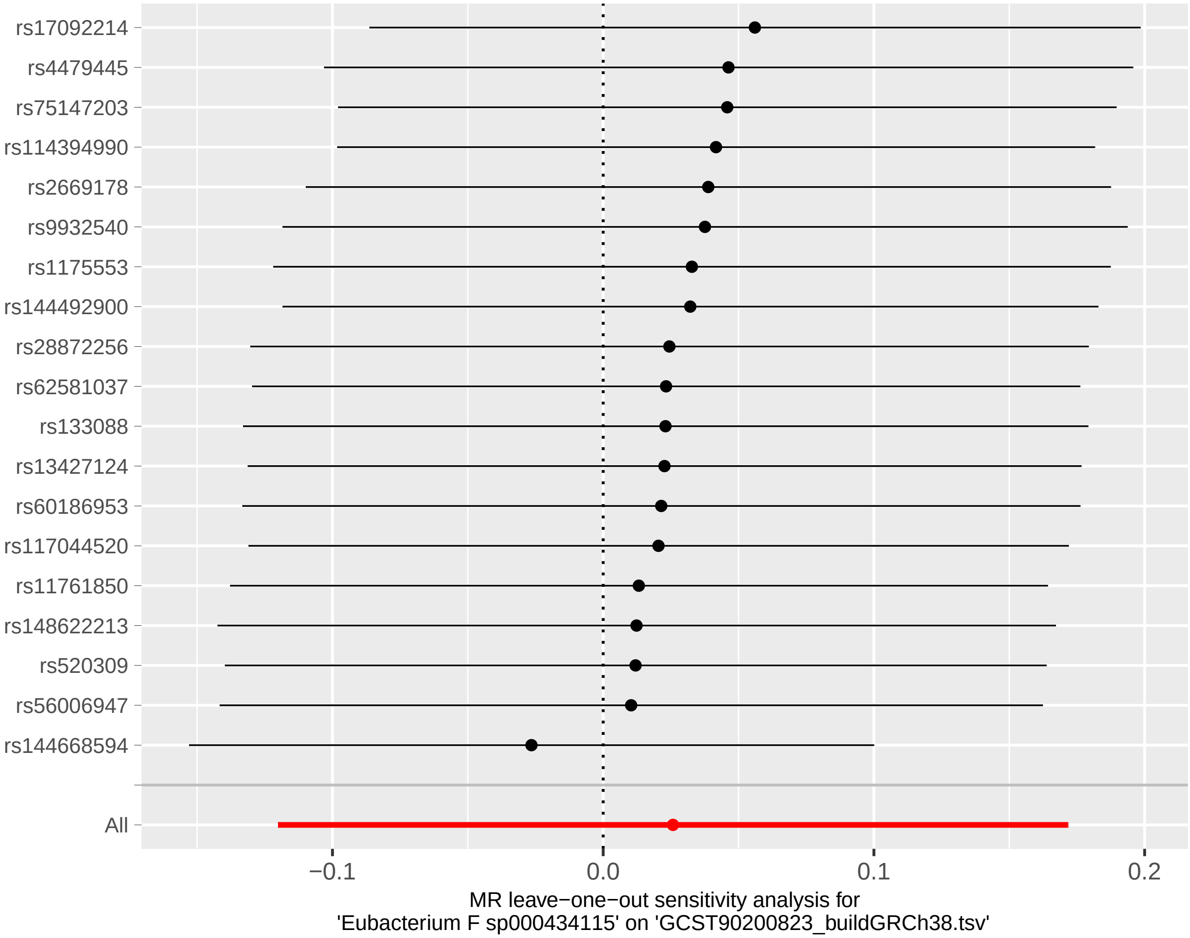

rs17092214
rs4479445
rs75147203
rs114394990
rs2669178
rs9932540
rs1175553
rs144492900
rs28872256
rs62581037
rs133088
rs13427124
rs60186953
rs117044520
rs11761850
rs148622213
rs520309
rs56006947
rs144668594
All
−0.1
0.0
0.1
0.2
MR leave−one−out sensitivity analysis for
'Eubacterium F sp000434115' on 'GCST90200823_buildGRCh38.tsv'

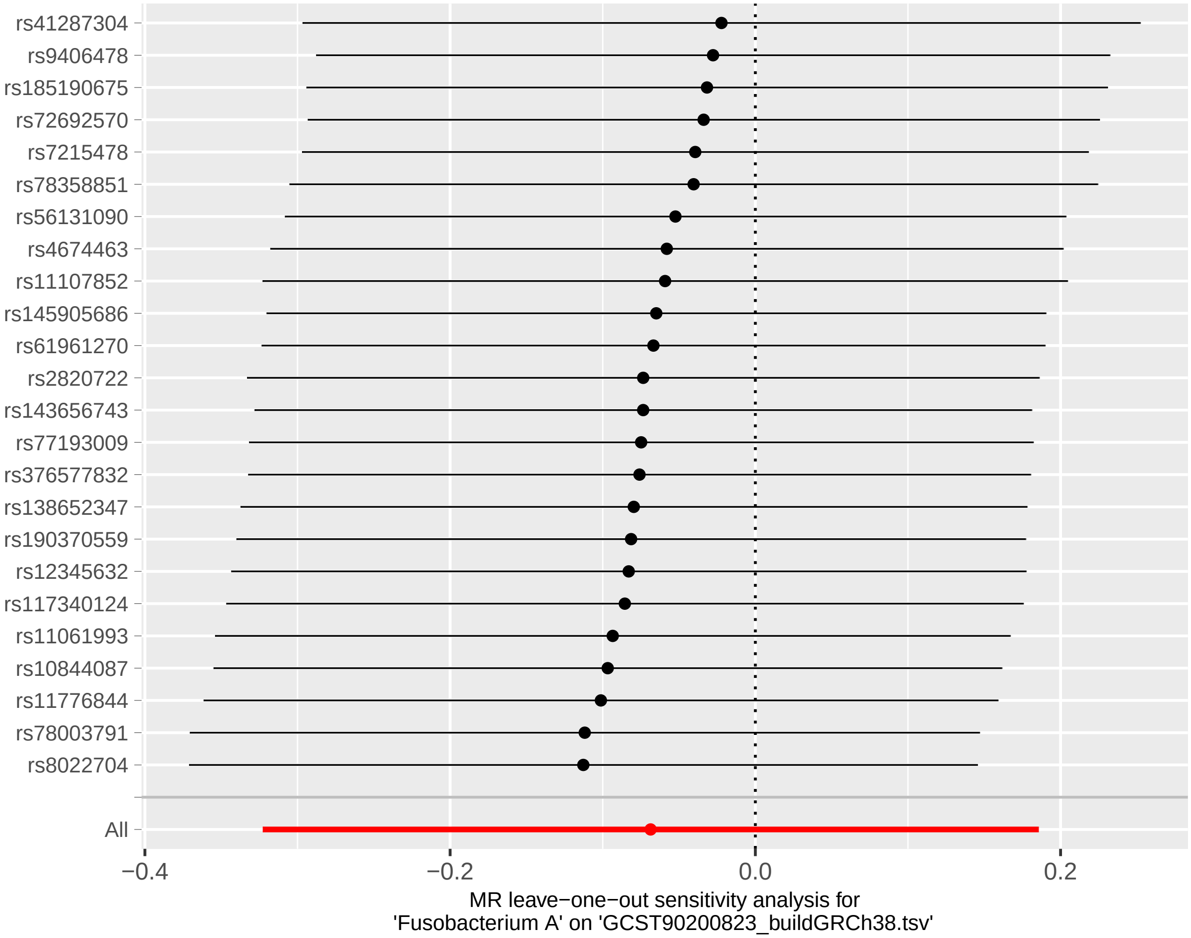
rs41287304
rs9406478
rs185190675
rs72692570
rs7215478
rs78358851
rs56131090
rs4674463
rs11107852
rs145905686
rs61961270
rs2820722
rs143656743
rs77193009
rs376577832
rs138652347
rs190370559
rs12345632
rs117340124
rs11061993
rs10844087
rs11776844
rs78003791
rs8022704
All
−0.4
−0.2
0.0
0.2
MR leave−one−out sensitivity analysis for
'Fusobacterium A' on 'GCST90200823_buildGRCh38.tsv'

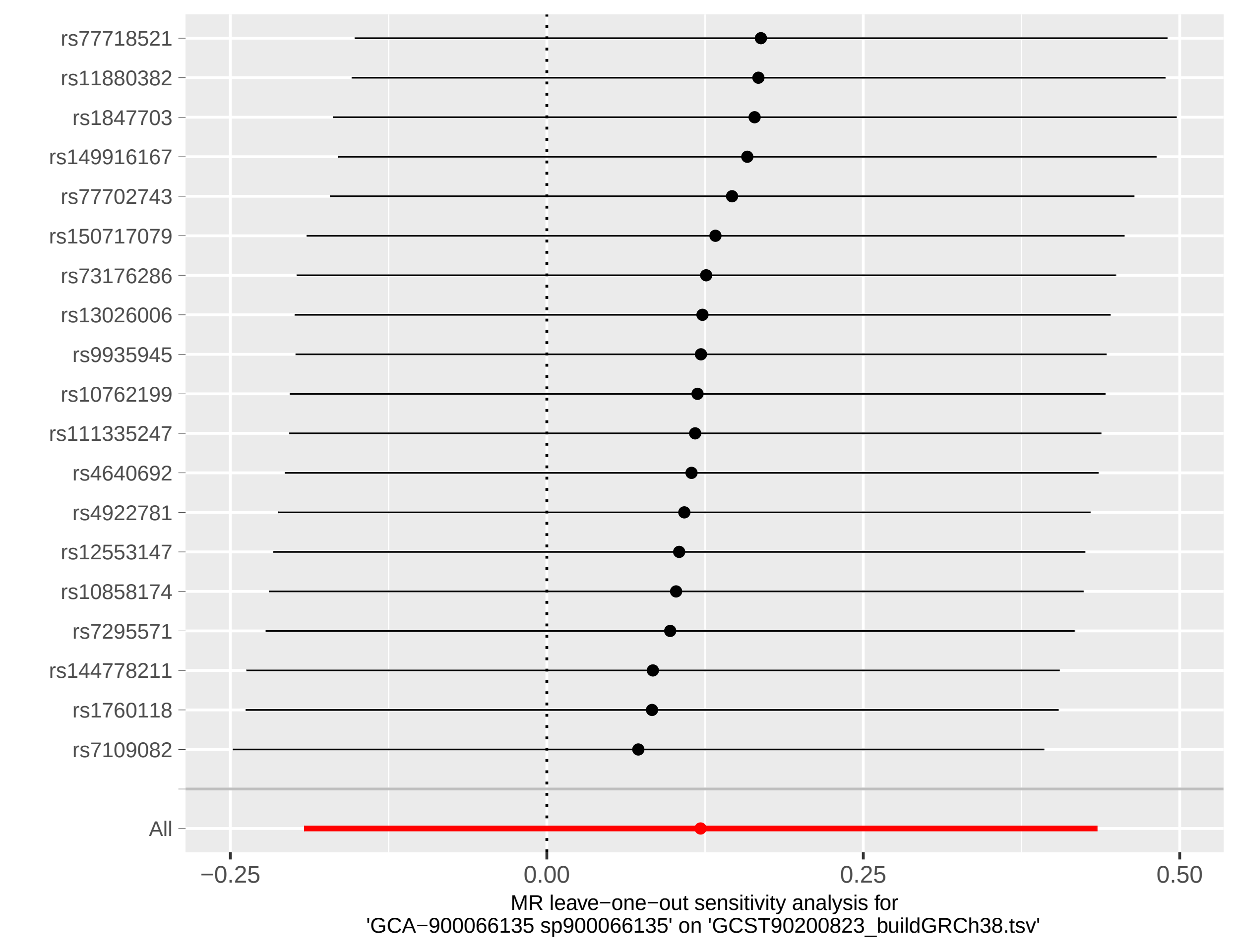
rs77718521
rs11880382
rs1847703
rs149916167
rs77702743
rs150717079
rs73176286
rs13026006
rs9935945
rs10762199
rs111335247
rs4640692
rs4922781
rs12553147
rs10858174
rs7295571
rs144778211
rs1760118
rs7109082
All
−0.25
0.00
0.25
0.50
MR leave−one−out sensitivity analysis for
'GCA−900066135 sp900066135' on 'GCST90200823_buildGRCh38.tsv'

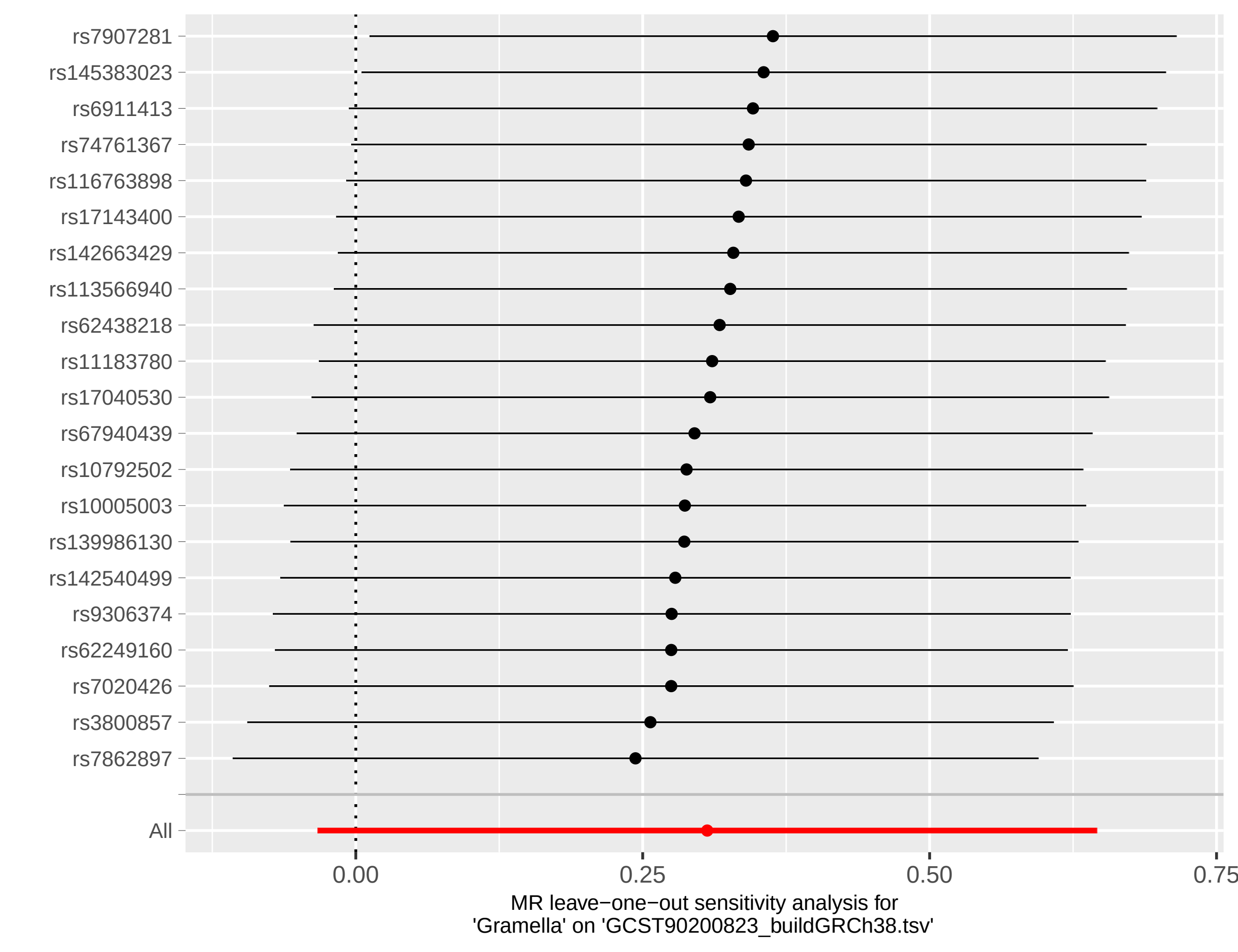
rs7907281
rs145383023
rs6911413
rs74761367
rs116763898
rs17143400
rs142663429
rs113566940
rs62438218
rs11183780
rs17040530
rs67940439
rs10792502
rs10005003
rs139986130
rs142540499
rs9306374
rs62249160
rs7020426
rs3800857
rs7862897
All
0.00
0.25
0.50
0.75
MR leave−one−out sensitivity analysis for
'Gramella' on 'GCST90200823_buildGRCh38.tsv'

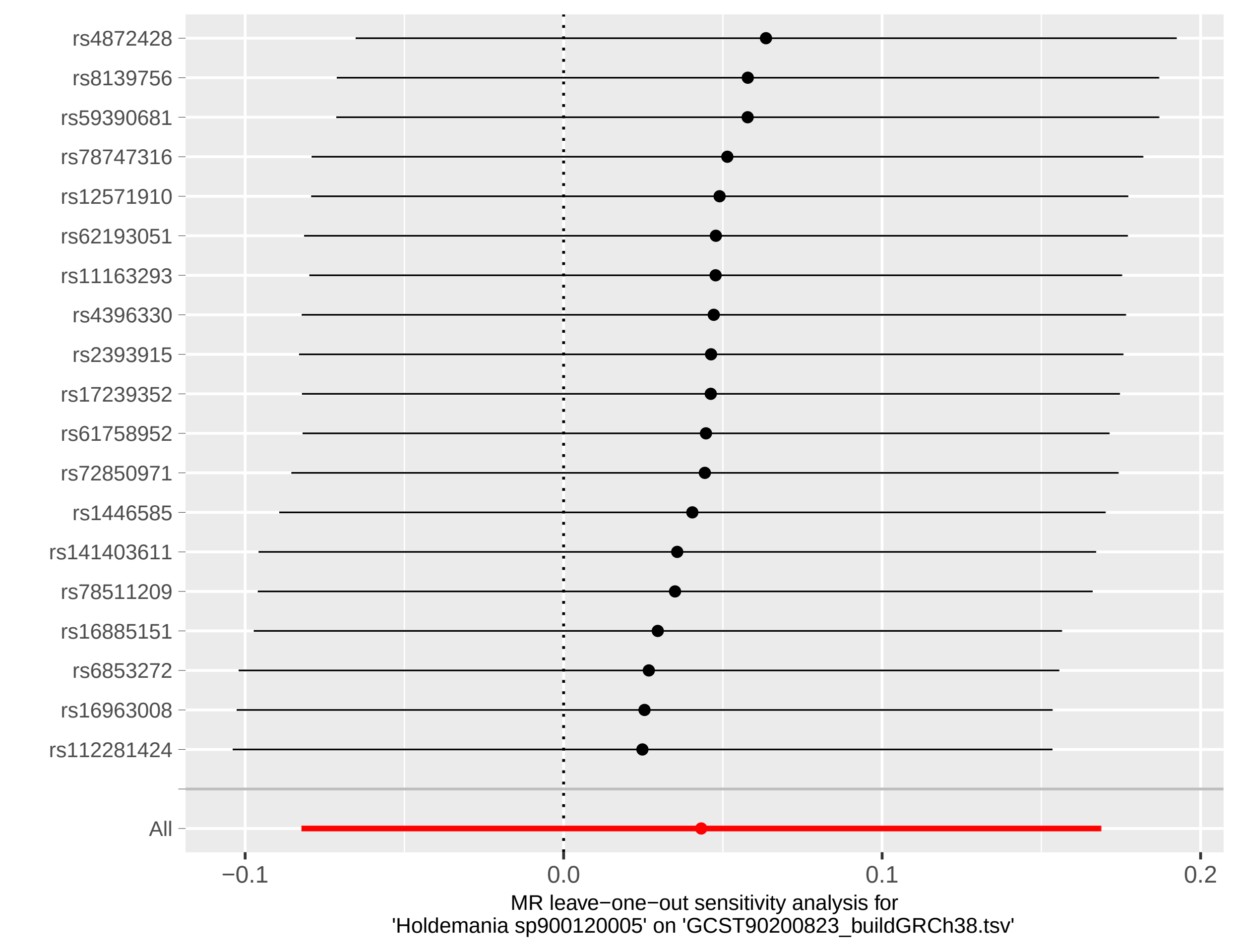
rs4872428
rs8139756
rs59390681
rs78747316
rs12571910
rs62193051
rs11163293
rs4396330
rs2393915
rs17239352
rs61758952
rs72850971
rs1446585
rs141403611
rs78511209
rs16885151
rs6853272
rs16963008
rs112281424
All
−0.1
0.0
0.1
0.2
MR leave−one−out sensitivity analysis for
'Holdemania sp900120005' on 'GCST90200823_buildGRCh38.tsv'

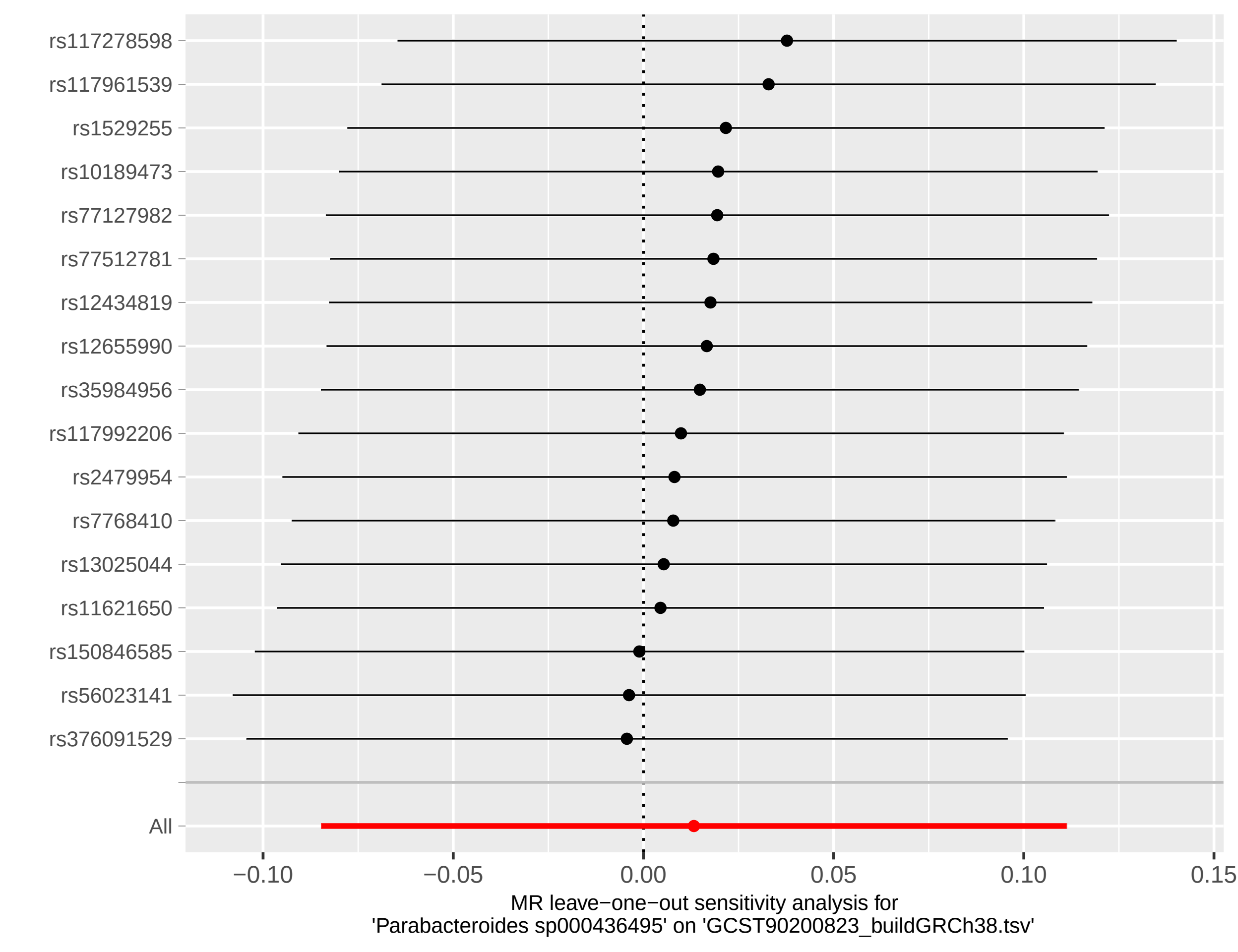
rs117278598
rs117961539
rs1529255
rs10189473
rs77127982
rs77512781
rs12434819
rs12655990
rs35984956
rs117992206
rs2479954
rs7768410
rs13025044
rs11621650
rs150846585
rs56023141
rs376091529
All
−0.10
−0.05
0.00
0.05
0.10
0.15
MR leave−one−out sensitivity analysis for
'Parabacteroides sp000436495' on 'GCST90200823_buildGRCh38.tsv'

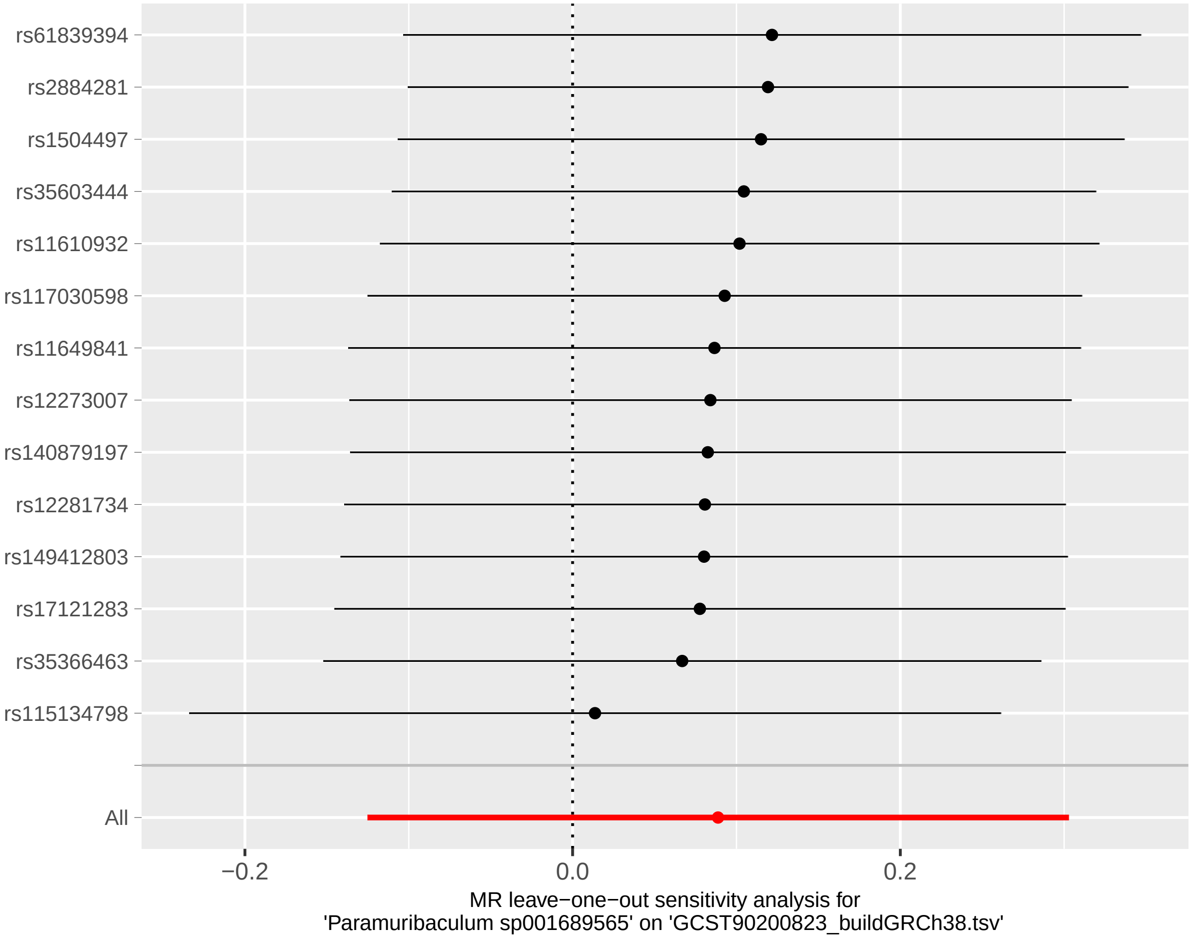

rs61839394
rs2884281
rs1504497
rs35603444
rs11610932
rs117030598
rs11649841
rs12273007
rs140879197
rs12281734
rs149412803
rs17121283
rs35366463
rs115134798
All
−0.2
0.0
0.2
MR leave−one−out sensitivity analysis for
'Paramuribaculum sp001689565' on 'GCST90200823_buildGRCh38.tsv'

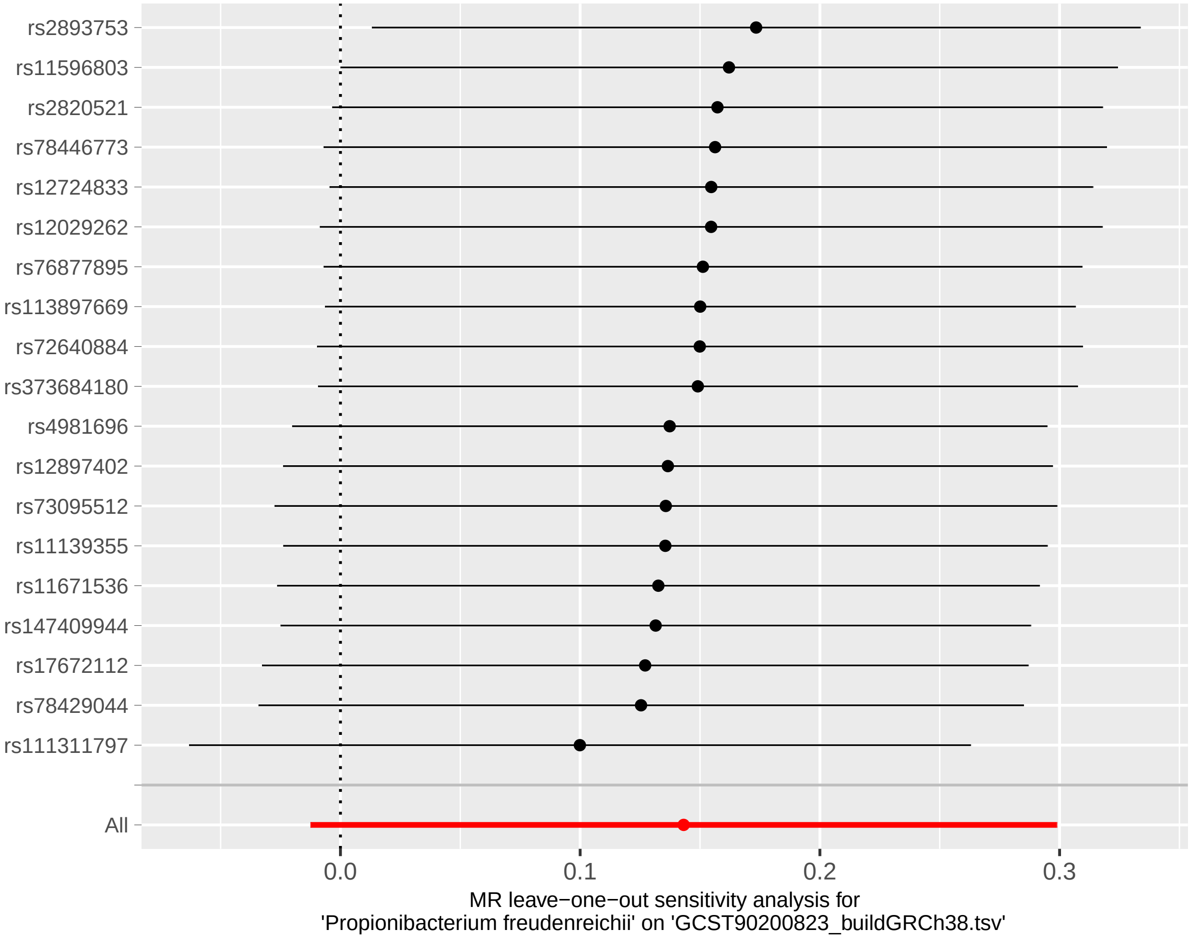
rs2893753
rs11596803
rs2820521
rs78446773
rs12724833
rs12029262
rs76877895
rs113897669
rs72640884
rs373684180
rs4981696
rs12897402
rs73095512
rs11139355
rs11671536
rs147409944
rs17672112
rs78429044
rs111311797
All
0.0
0.1
0.2
0.3
MR leave−one−out sensitivity analysis for
'Propionibacterium freudenreichii' on 'GCST90200823_buildGRCh38.tsv'

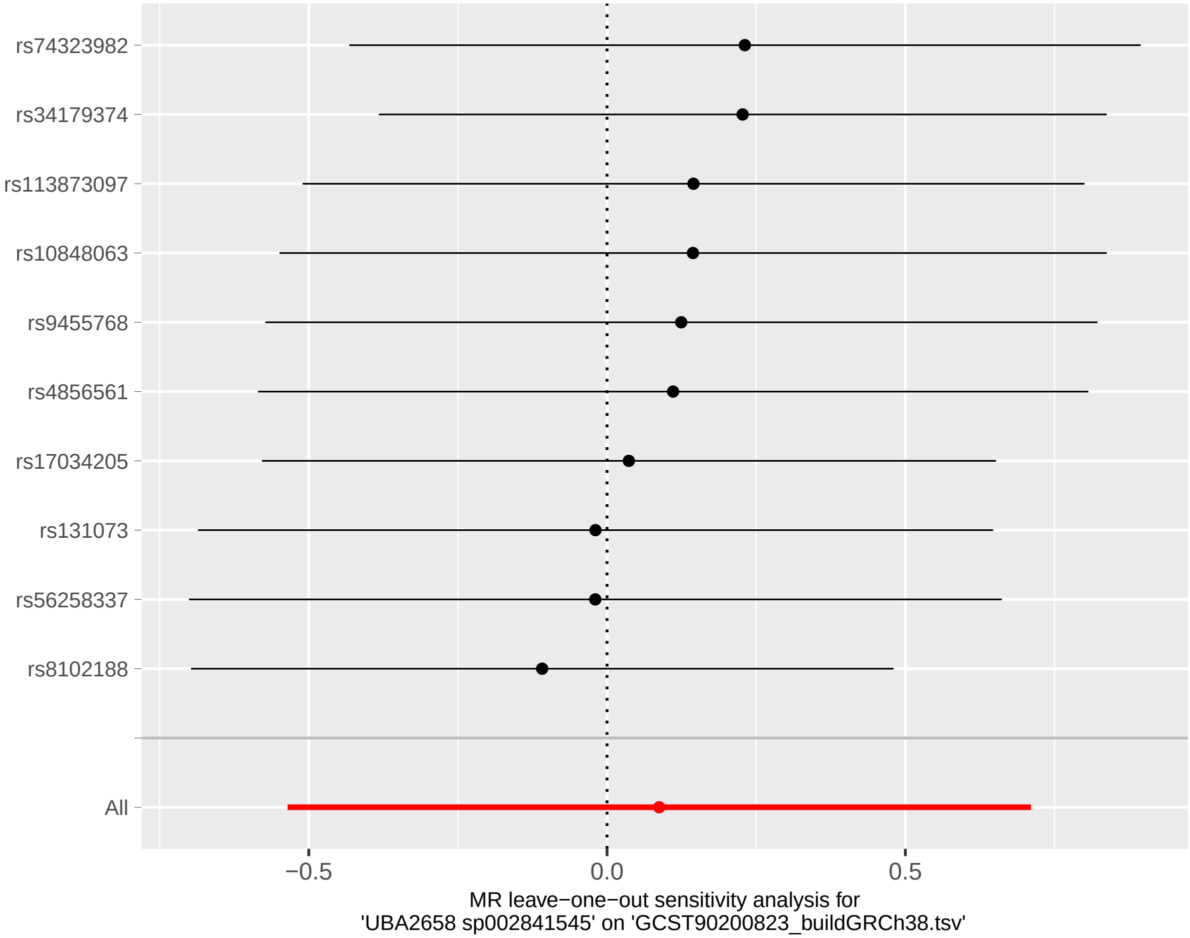
rs74323982
rs34179374
rs113873097
rs10848063
rs9455768
rs4856561
rs17034205
rs131073
rs56258337
rs8102188
All
−0.5
0.0
0.5
MR leave−one−out sensitivity analysis for
'UBA2658 sp002841545' on 'GCST90200823_buildGRCh38.tsv'

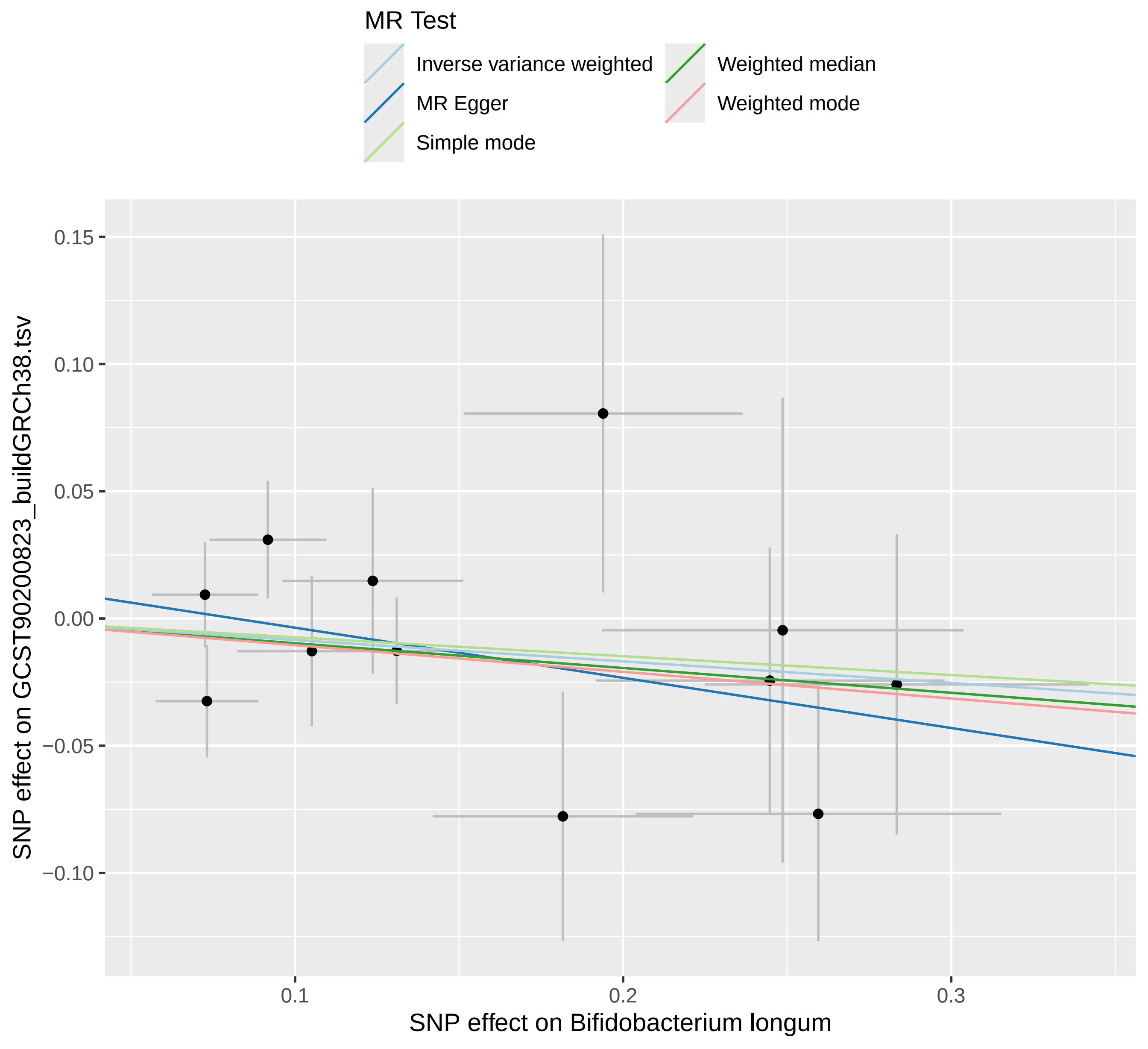
MR Test
Inverse variance weighted
MR Egger
Simple mode
Weighted median
Weighted mode
0.15
0.10
0.05
0.00
−0.05
−0.10
0.1
0.2
0.3
SNP effect on GCST90200823_buildGRCh38.tsv
SNP effect on Bifidobacterium longum

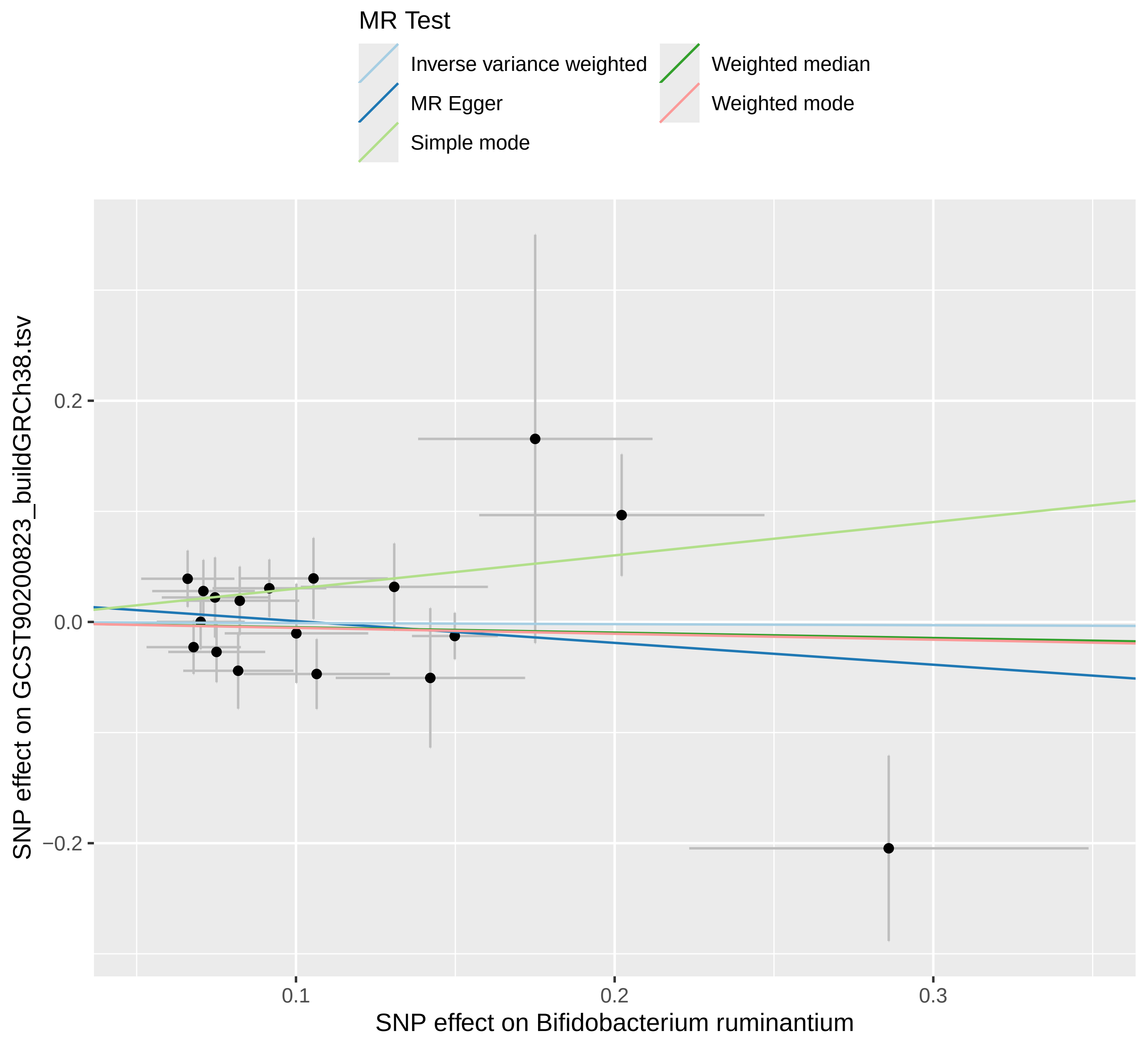
MR Test
Inverse variance weighted
MR Egger
Simple mode
Weighted median
Weighted mode
0.2
0.0
−0.2
0.1
0.2
0.3
SNP effect on GCST90200823_buildGRCh38.tsv
SNP effect on Bifidobacterium ruminantium

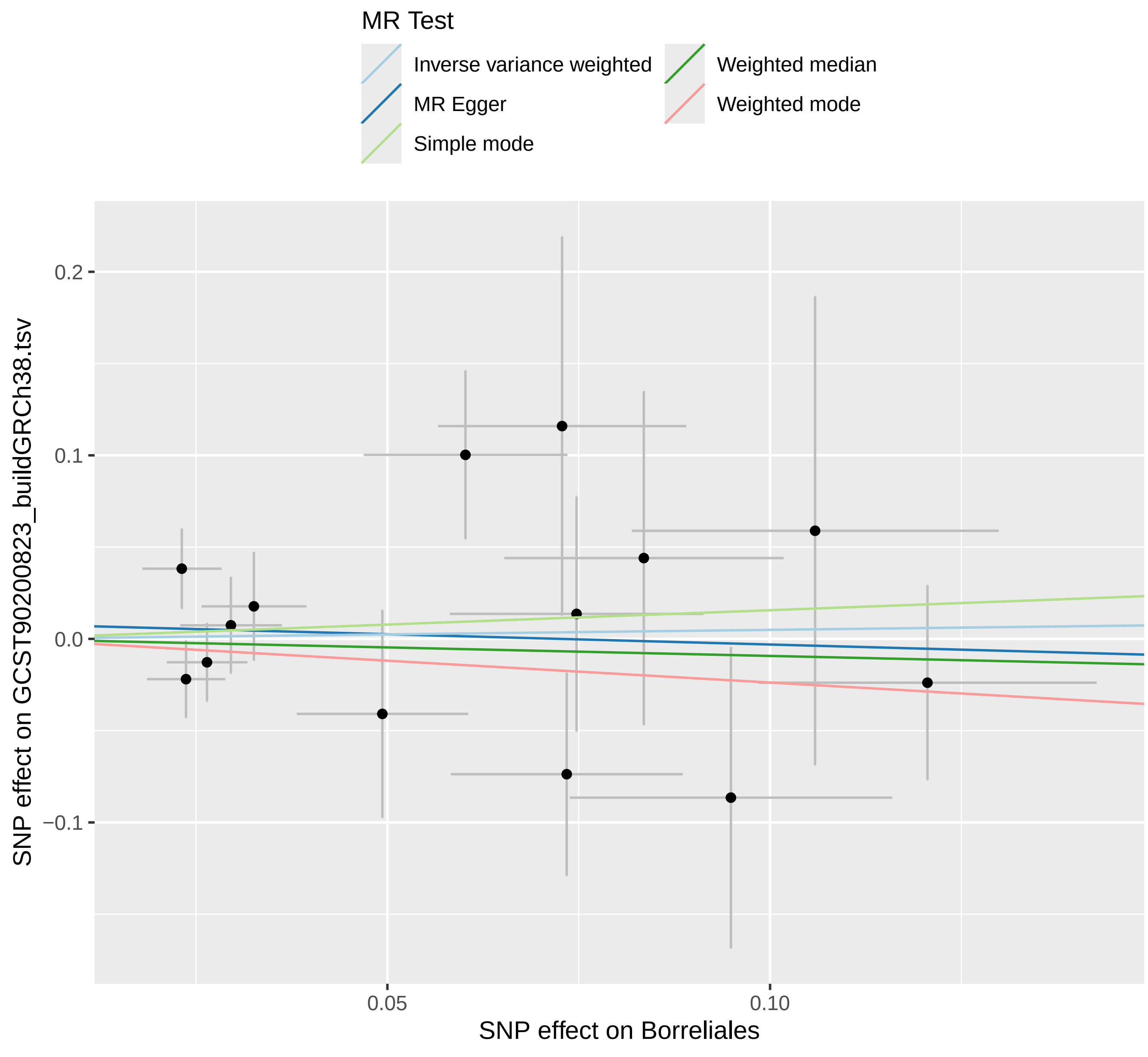
MR Test
Inverse variance weighted
MR Egger
Simple mode
Weighted median
Weighted mode
0.2
0.1
0.0
−0.1
0.05
0.10
SNP effect on GCST90200823_buildGRCh38.tsv
SNP effect on Borreliales

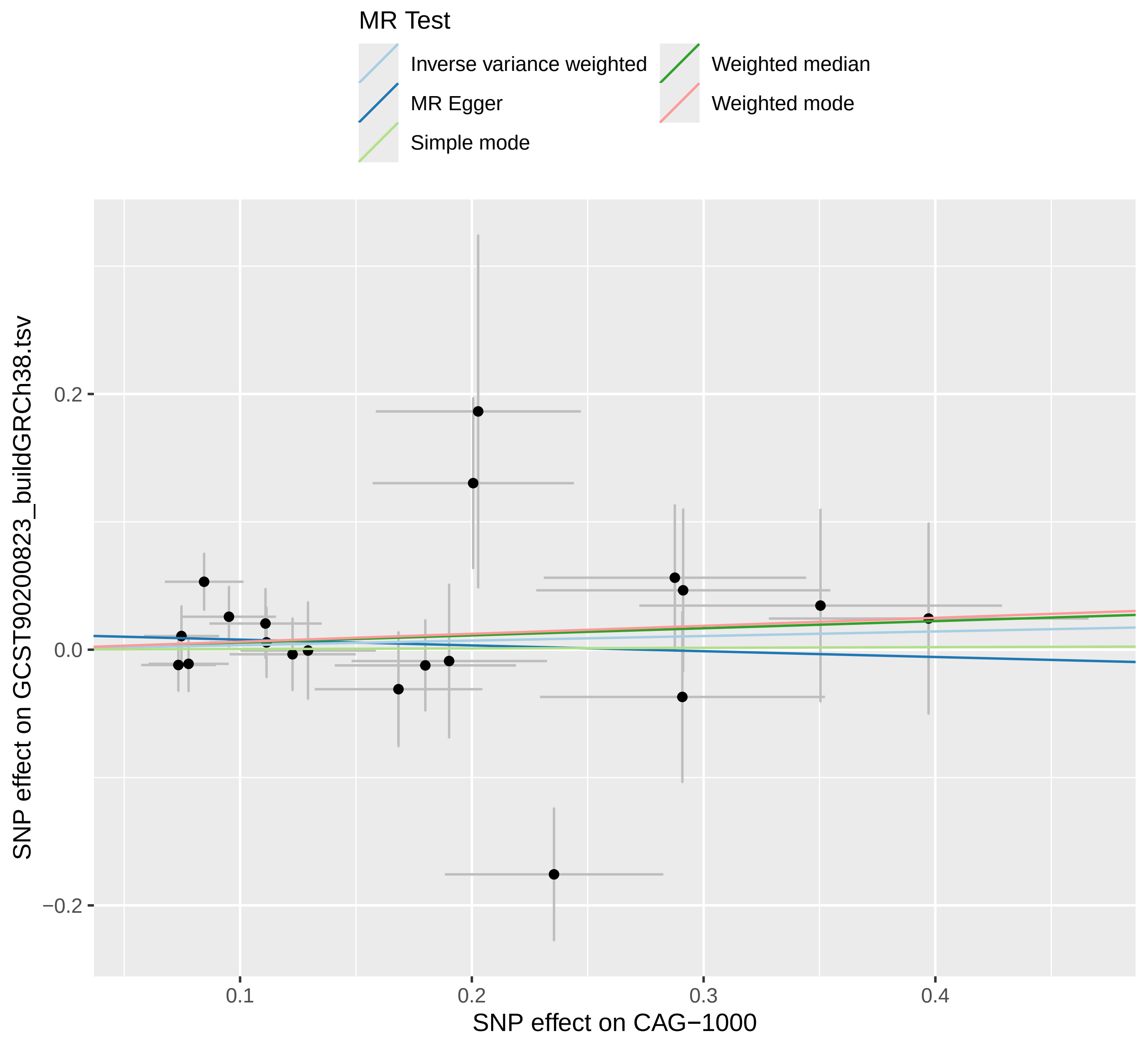

MR Test
Inverse variance weighted
MR Egger
Simple mode
Weighted median
Weighted mode
SNP effect on GCST90200823_buildGRCh38.tsv
0.2
0.0
−0.2
0.1
0.2
0.3
0.4
SNP effect on CAG−1000

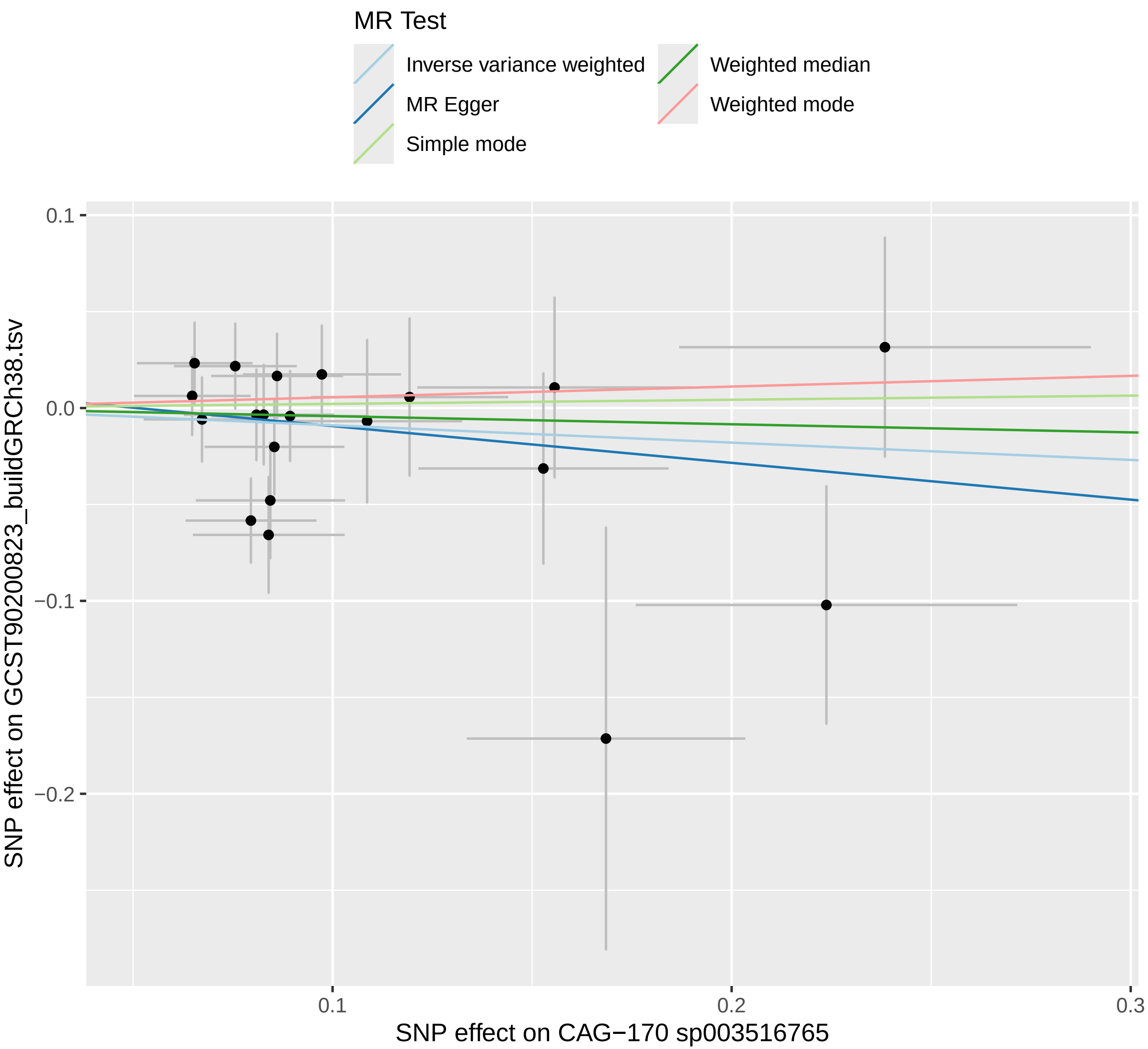

MR Test
Inverse variance weighted
MR Egger
Simple mode
Weighted median
Weighted mode
0.1
0.0
−0.1
−0.2
0.1
0.2
0.3
SNP effect on GCST90200823_buildGRCh38.tsv
SNP effect on CAG−170 sp003516765

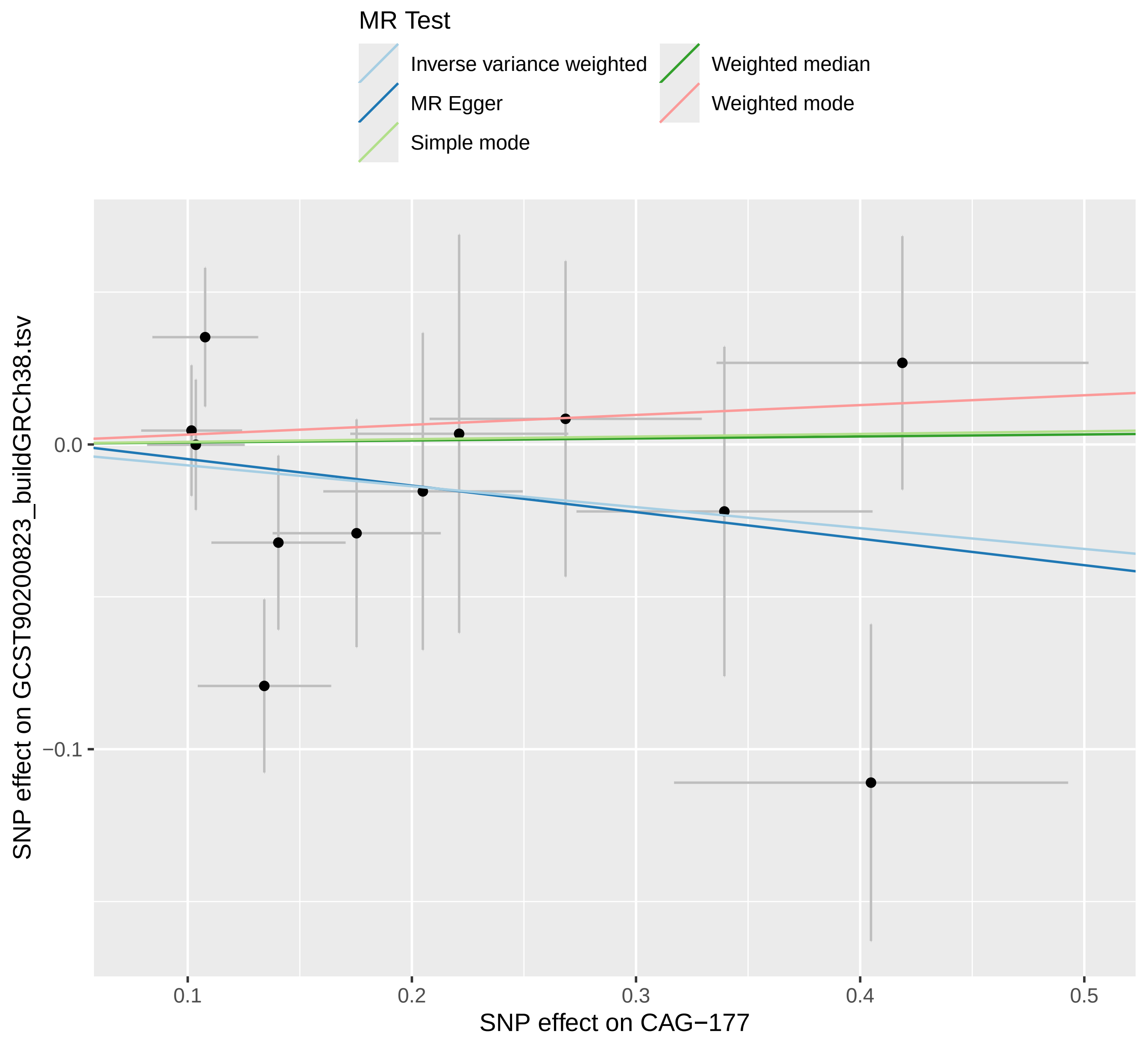

MR Test
Inverse variance weighted
MR Egger
Simple mode
Weighted median
Weighted mode
SNP effect on GCST90200823_buildGRCh38.tsv
0.0
−0.1
0.1
0.2
0.3
0.4
0.5
SNP effect on CAG−177

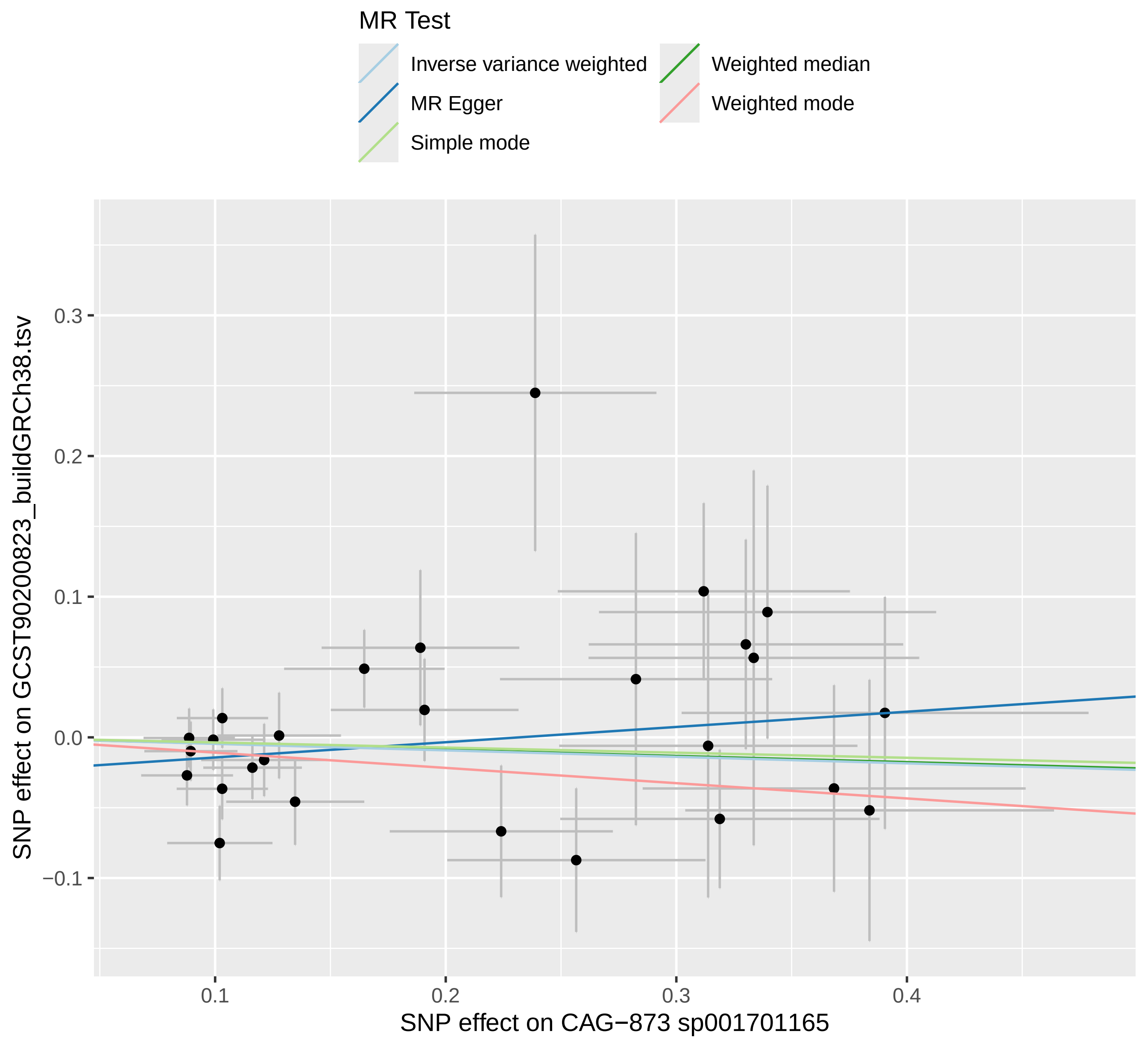
MR Test
Inverse variance weighted
MR Egger
Simple mode
Weighted median
Weighted mode
0.3
0.2
0.1
0.0
−0.1
SNP effect on GCST90200823_buildGRCh38.tsv
0.1
0.2
0.3
0.4
SNP effect on CAG−873 sp001701165

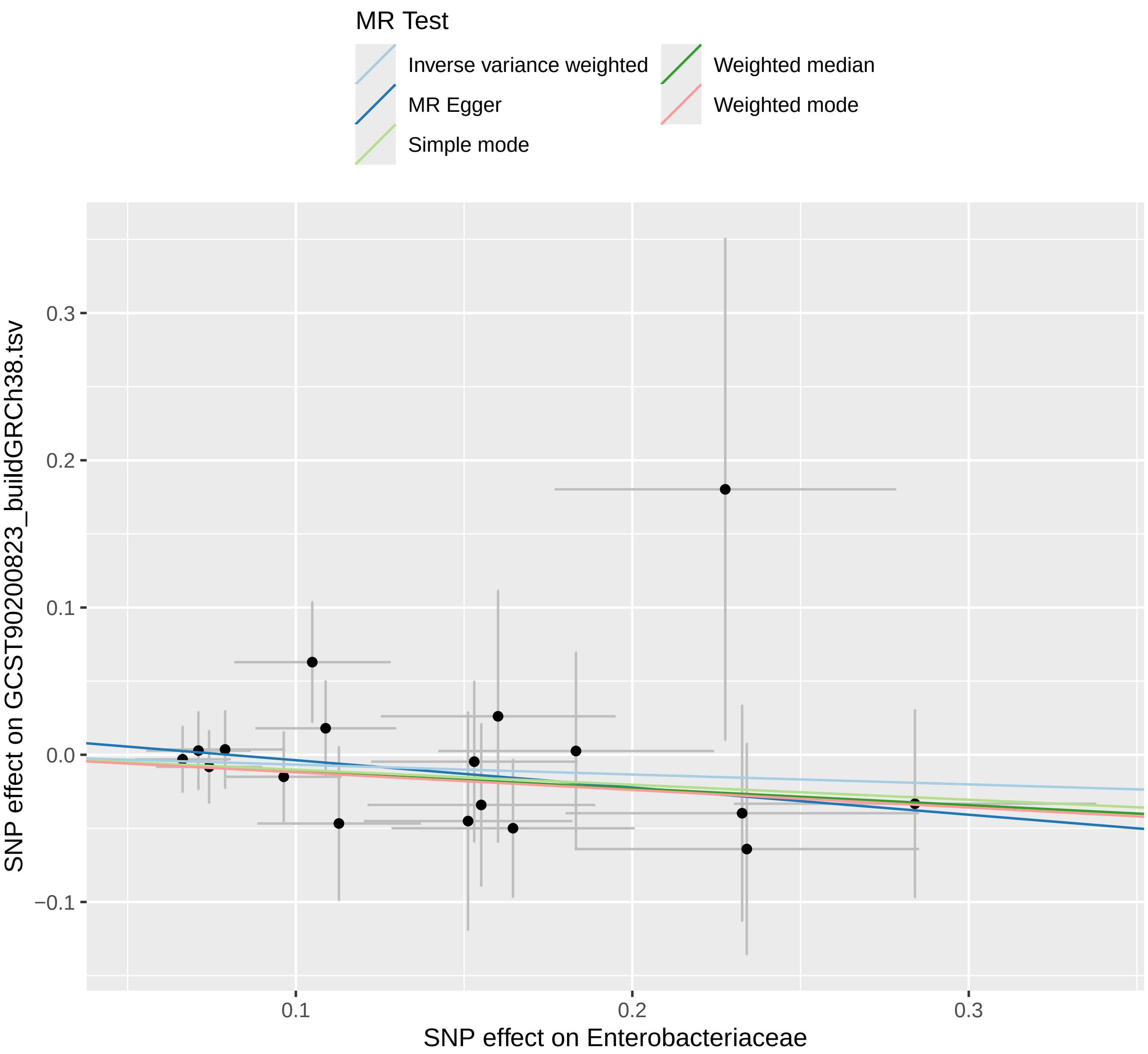

MR Test
Inverse variance weighted
MR Egger
Simple mode
Weighted median
Weighted mode
0.3
0.2
0.1
0.0
−0.1
SNP effect on GCST90200823_buildGRCh38.tsv
0.1
0.2
0.3
SNP effect on Enterobacteriaceae

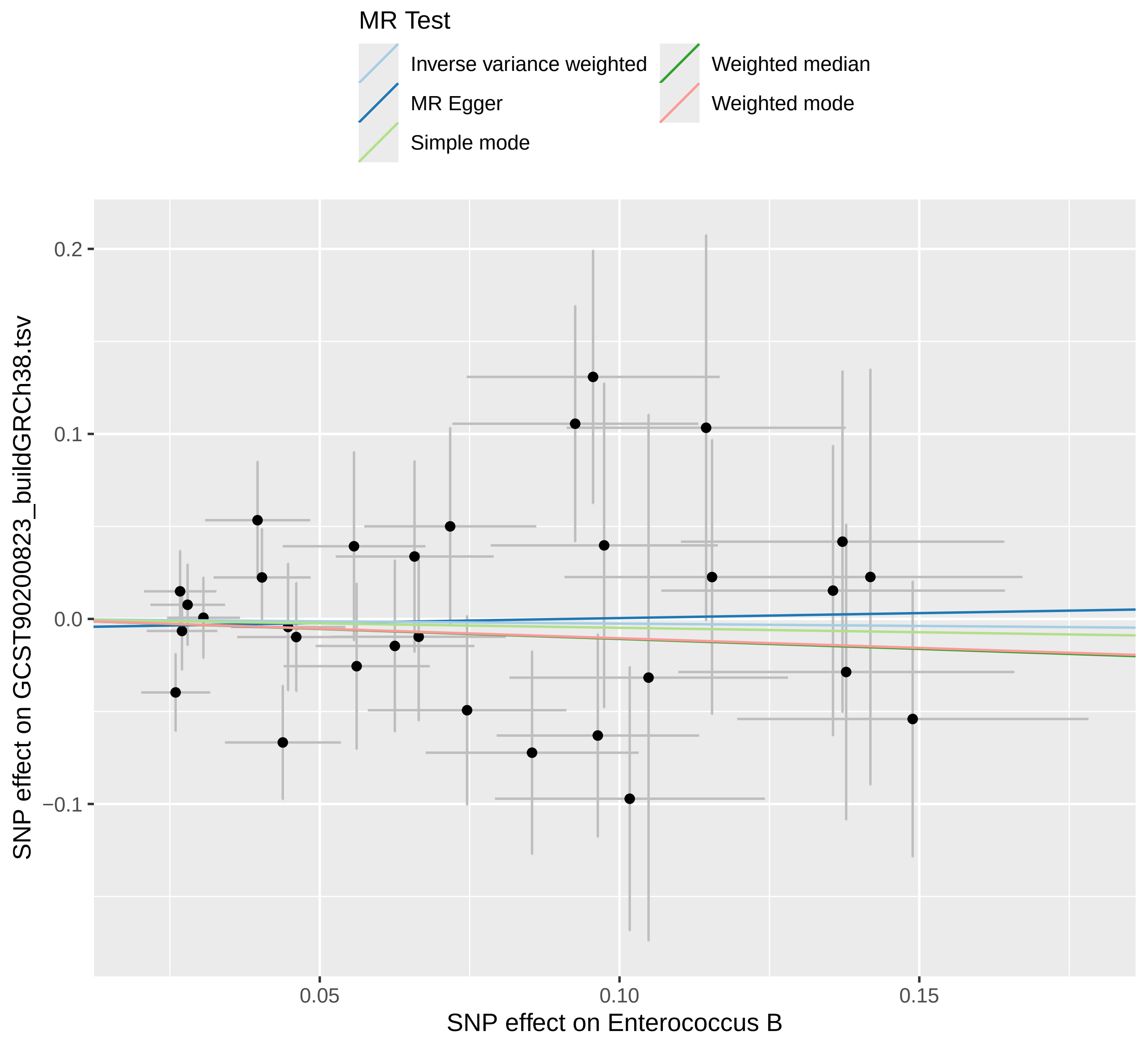
MR Test
Inverse variance weighted
MR Egger
Simple mode
Weighted median
Weighted mode
SNP effect on GCST90200823_buildGRCh38.tsv
0.2
0.1
0.0
−0.1
0.05
0.10
0.15
SNP effect on Enterococcus B

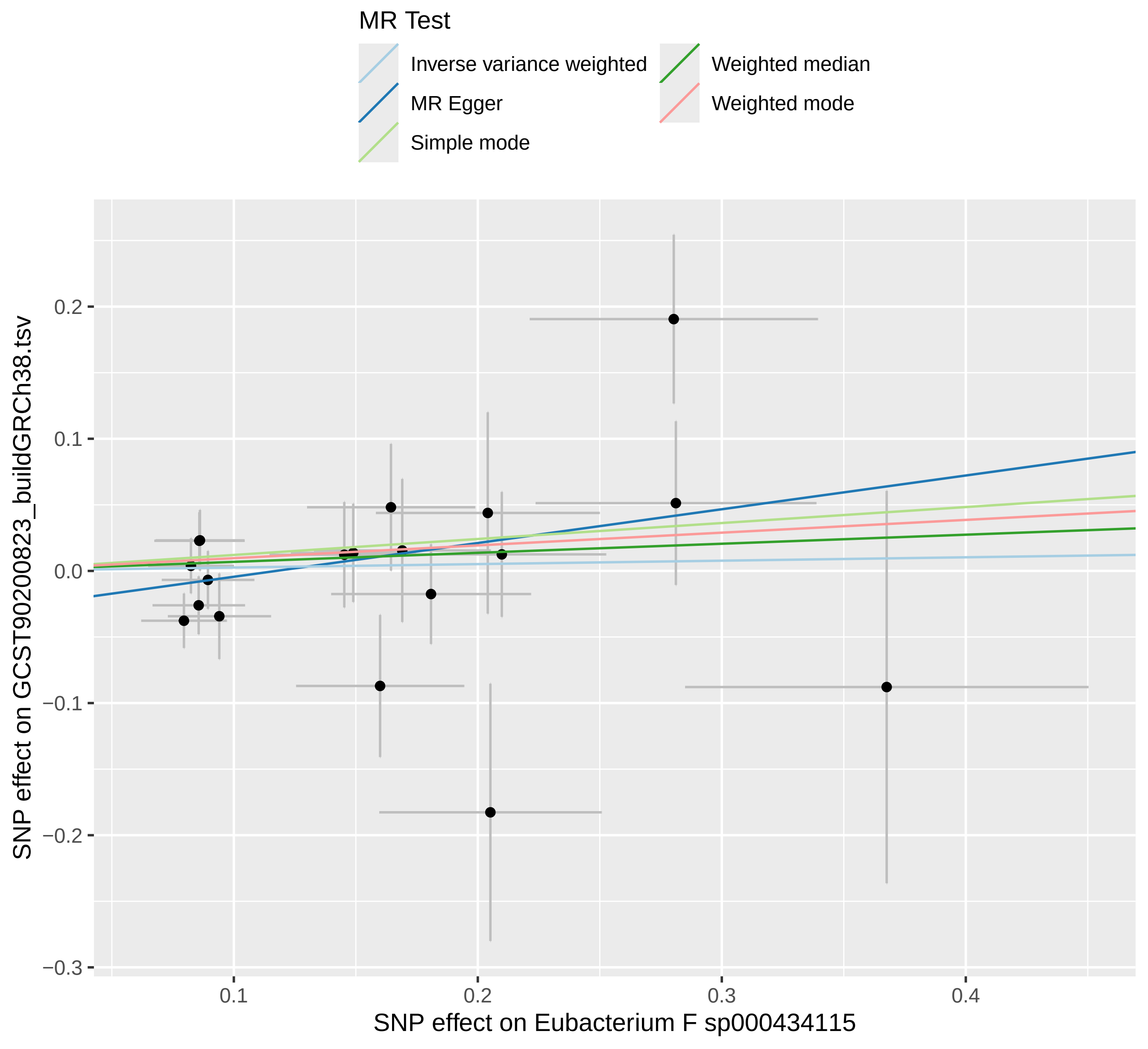
MR Test
Inverse variance weighted
MR Egger
Simple mode
Weighted median
Weighted mode
SNP effect on GCST90200823_buildGRCh38.tsv
0.2
0.1
0.0
−0.1
−0.2
−0.3
0.1
0.2
0.3
0.4
SNP effect on Eubacterium F sp000434115

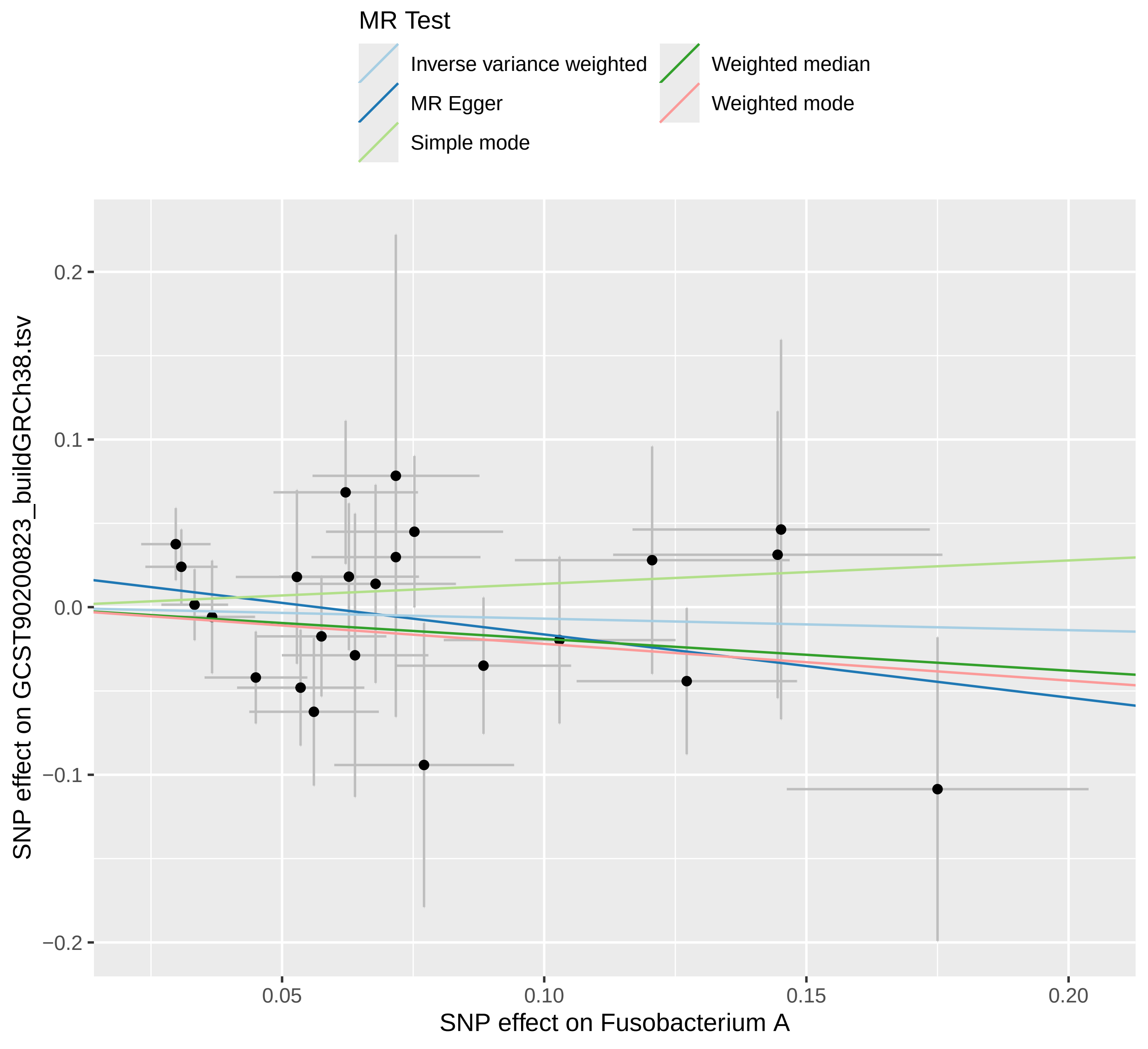
MR Test
Inverse variance weighted
MR Egger
Simple mode
Weighted median
Weighted mode
SNP effect on GCST90200823_buildGRCh38.tsv
0.2
0.1
0.0
−0.1
−0.2
0.05
0.10
0.15
0.20
SNP effect on Fusobacterium A

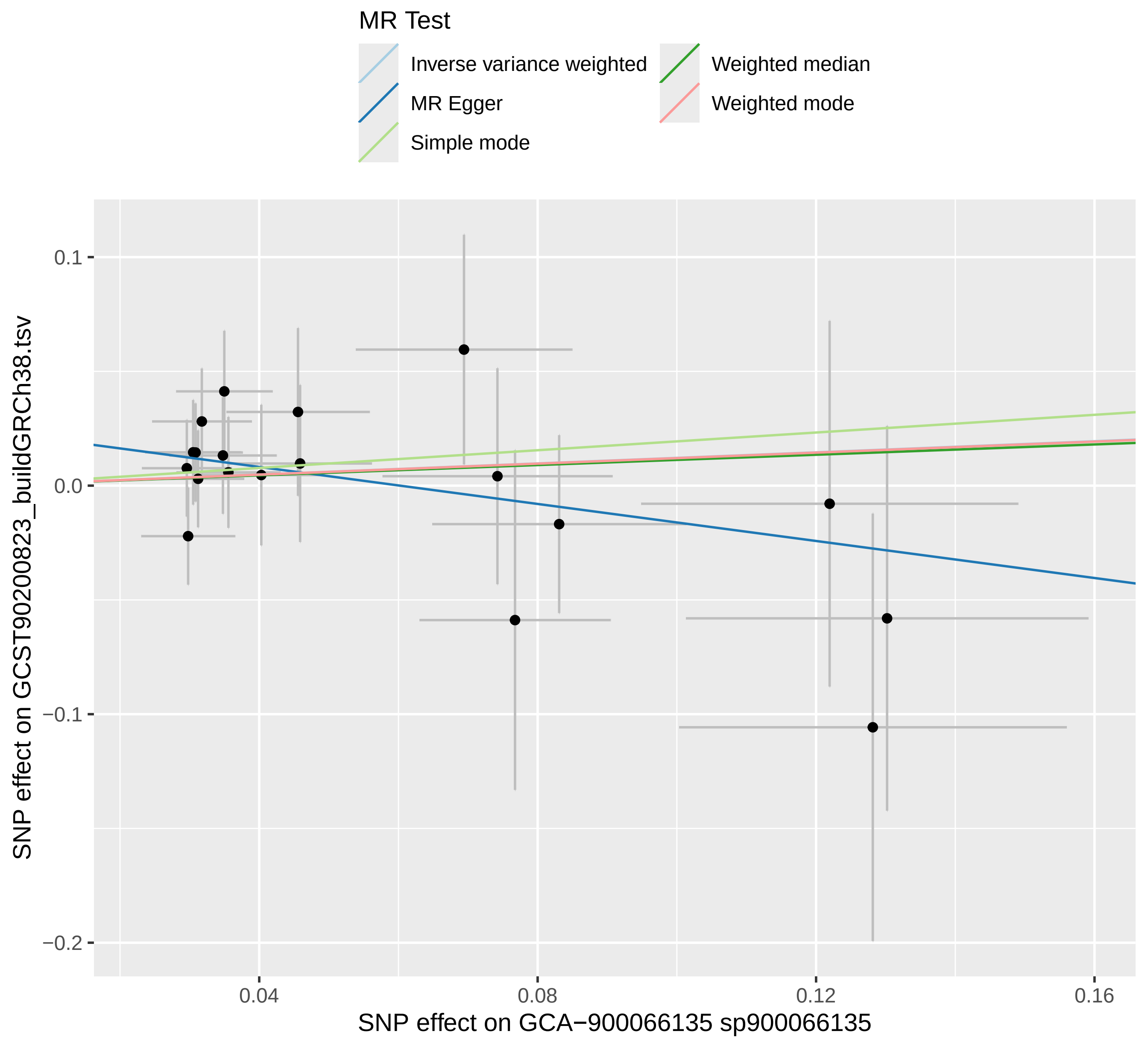
MR Test
Inverse variance weighted
MR Egger
Simple mode
Weighted median
Weighted mode
0.1
0.0
−0.1
−0.2
SNP effect on GCST90200823_buildGRCh38.tsv
0.04
0.08
0.12
0.16
SNP effect on GCA−900066135 sp900066135

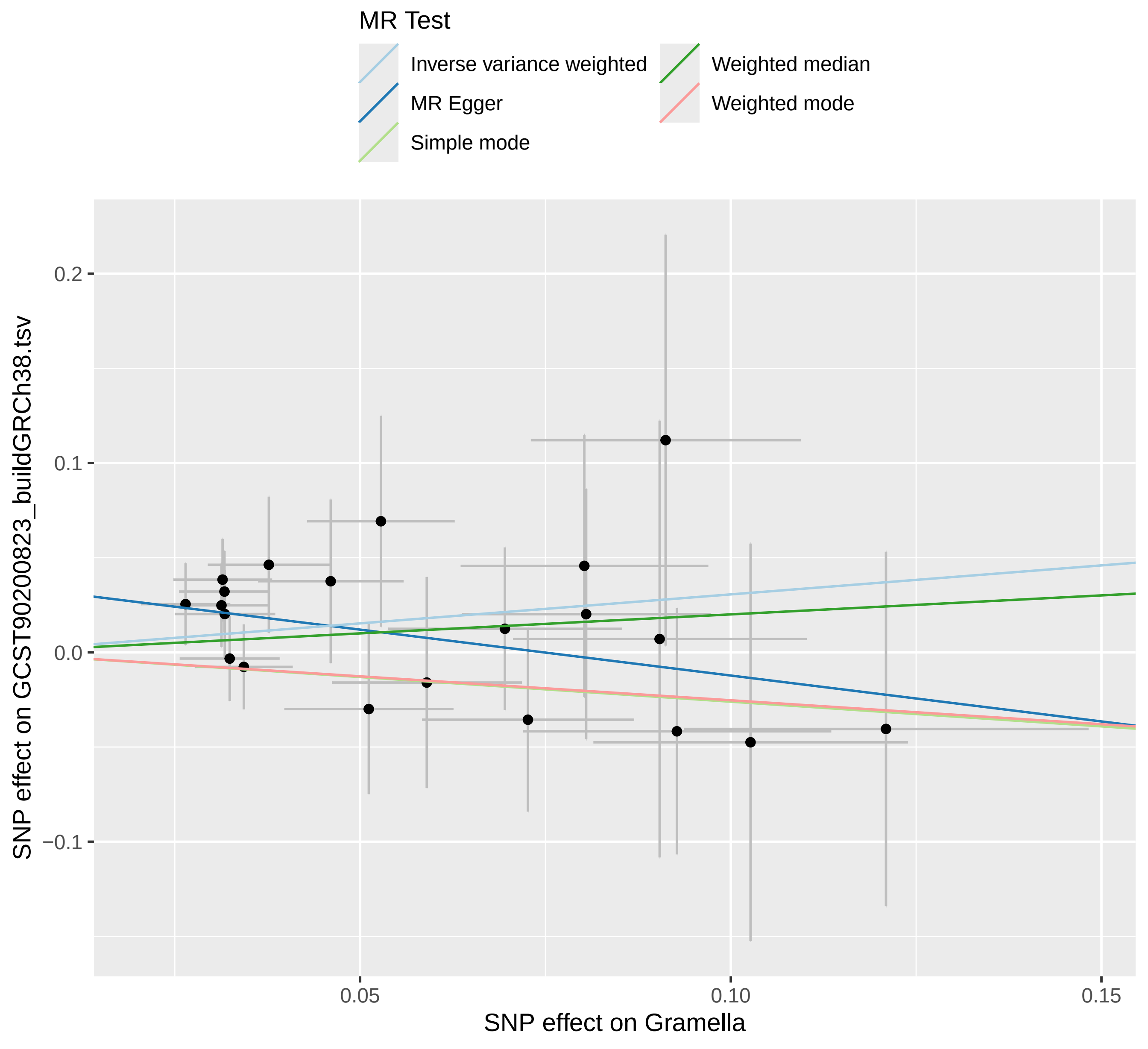
MR Test
Inverse variance weighted
MR Egger
Simple mode
Weighted median
Weighted mode
0.2
0.1
0.0
−0.1
0.05
0.10
0.15
SNP effect on GCST90200823_buildGRCh38.tsv
SNP effect on Gramella

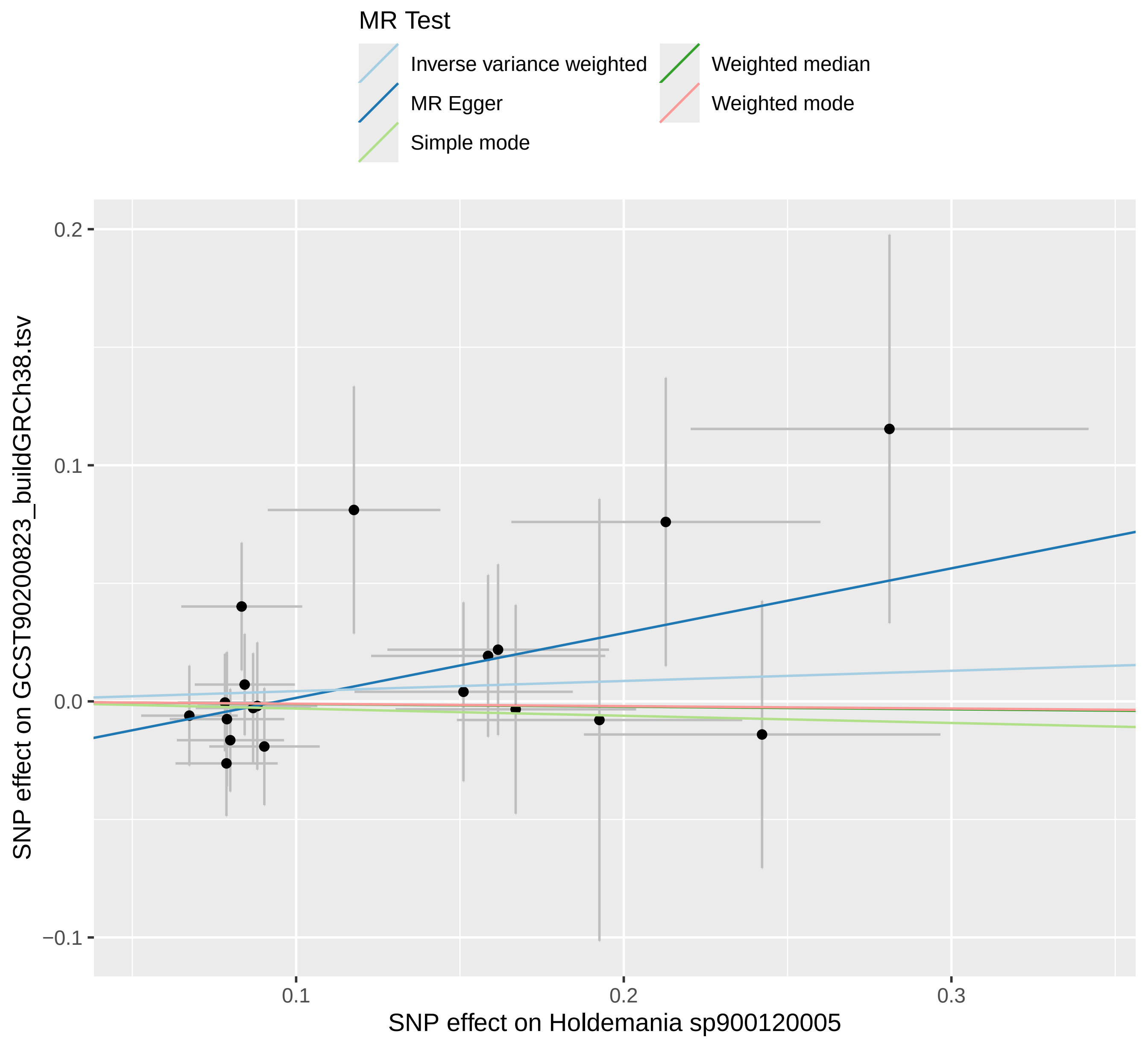
MR Test
Inverse variance weighted
MR Egger
Simple mode
Weighted median
Weighted mode
0.2
0.1
0.0
−0.1
0.1
0.2
0.3
SNP effect on GCST90200823_buildGRCh38.tsv
SNP effect on Holdemania sp900120005

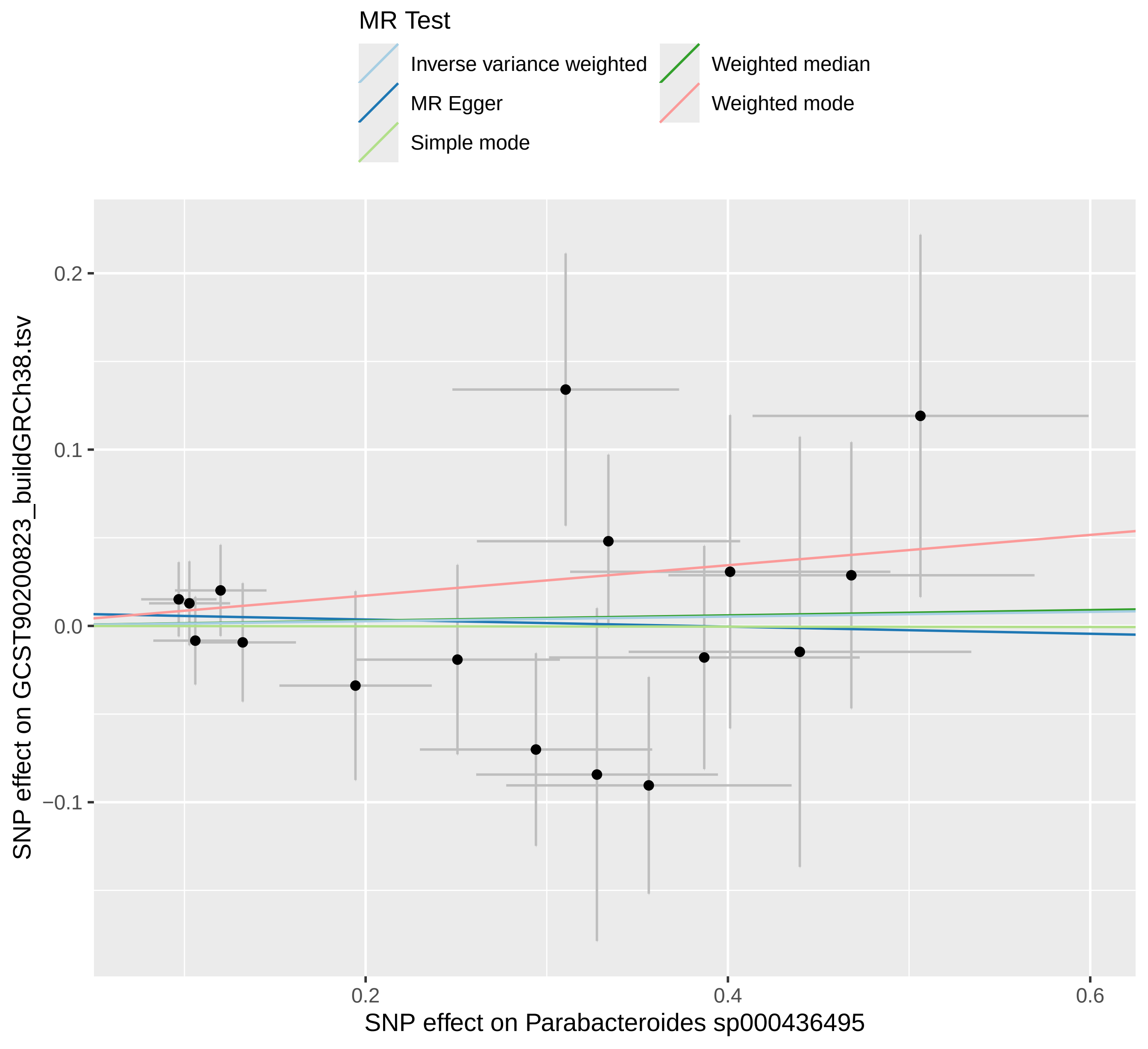

MR Test
Inverse variance weighted
MR Egger
Simple mode
Weighted median
Weighted mode
0.2
0.1
0.0
−0.1
0.2
0.4
0.6
SNP effect on GCST90200823_buildGRCh38.tsv
SNP effect on Parabacteroides sp000436495

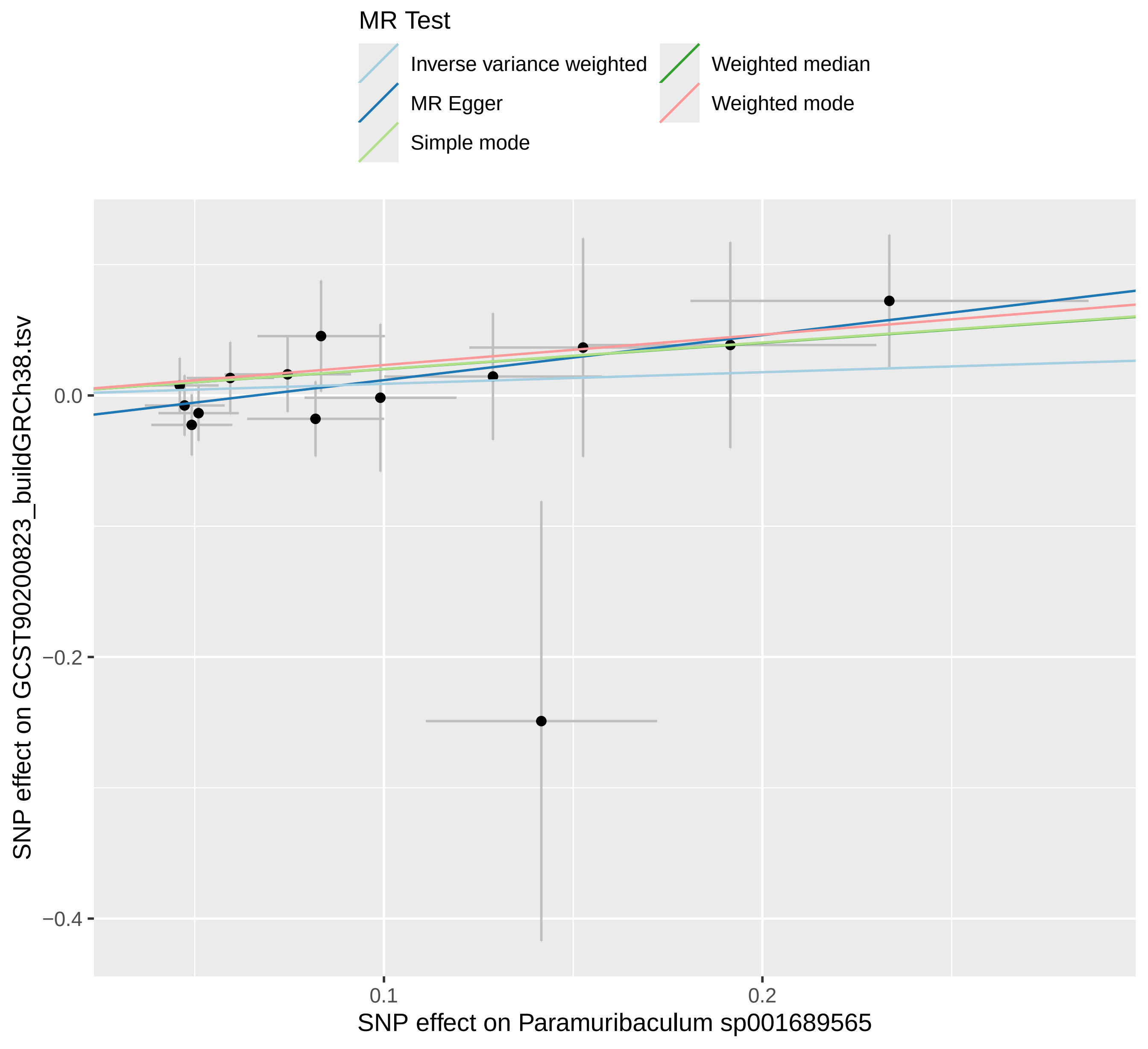
MR Test
Inverse variance weighted
MR Egger
Simple mode
Weighted median
Weighted mode
SNP effect on GCST90200823_buildGRCh38.tsv
0.0
−0.2
−0.4
0.1
0.2
SNP effect on Paramuribaculum sp001689565

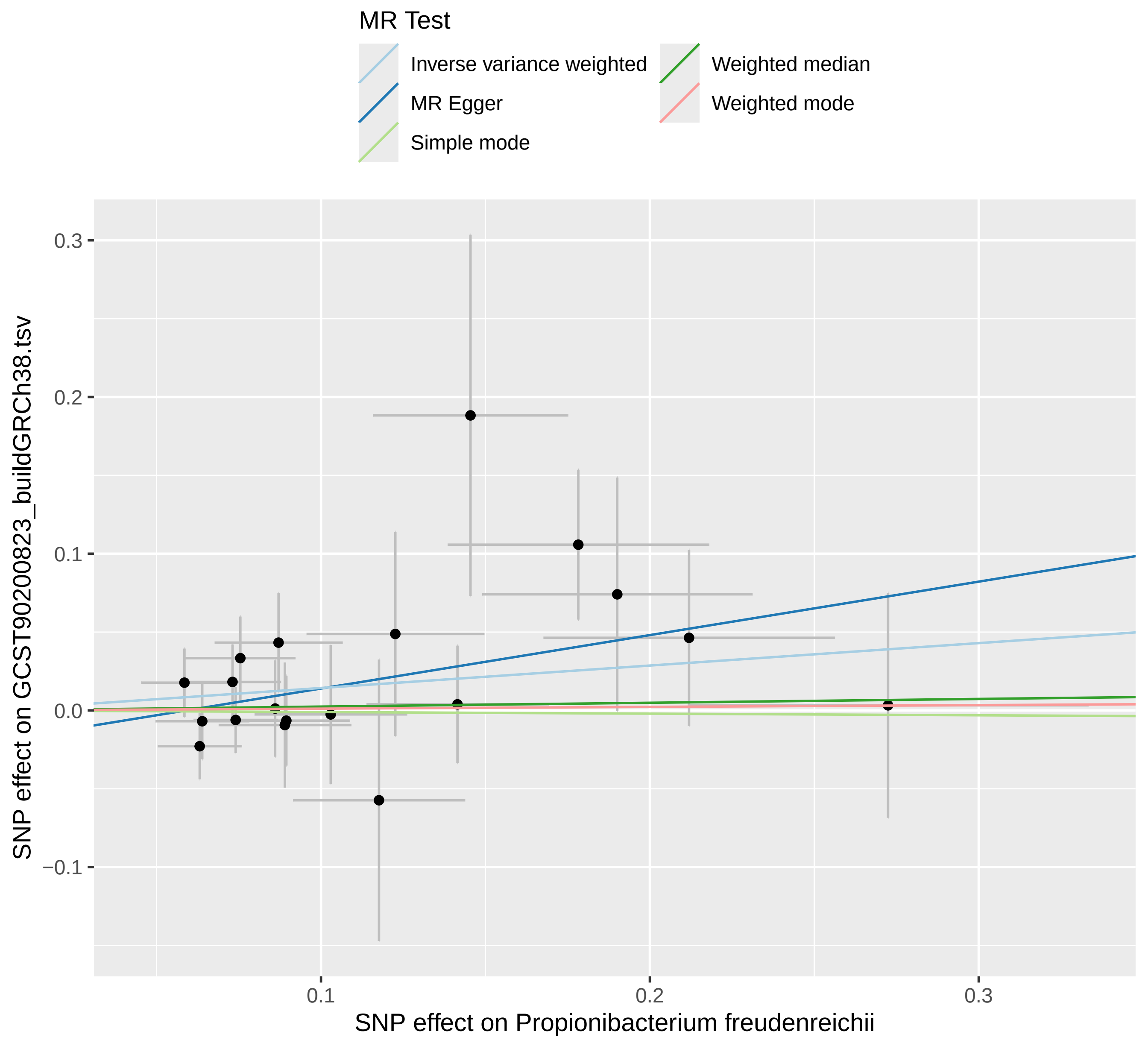
MR Test
Inverse variance weighted
MR Egger
Simple mode
Weighted median
Weighted mode
0.3
0.2
0.1
0.0
−0.1
0.1
0.2
0.3
SNP effect on GCST90200823_buildGRCh38.tsv
SNP effect on Propionibacterium freudenreichii

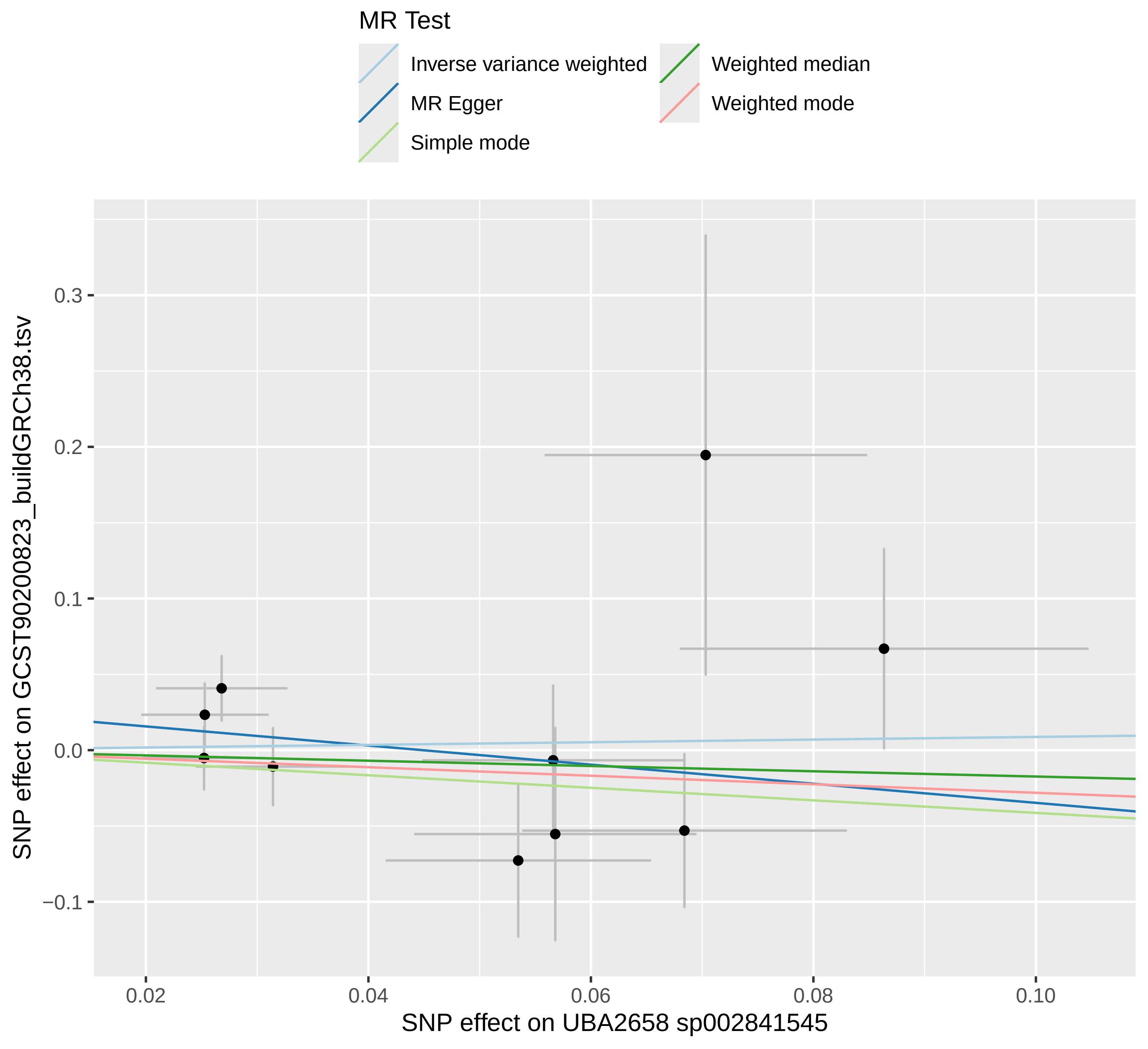

MR Test
Inverse variance weighted
MR Egger
Simple mode
Weighted median
Weighted mode
0.3
0.2
0.1
0.0
−0.1
0.02
0.04
0.06
0.08
0.10
SNP effect on GCST90200823_buildGRCh38.tsv
SNP effect on UBA2658 sp002841545

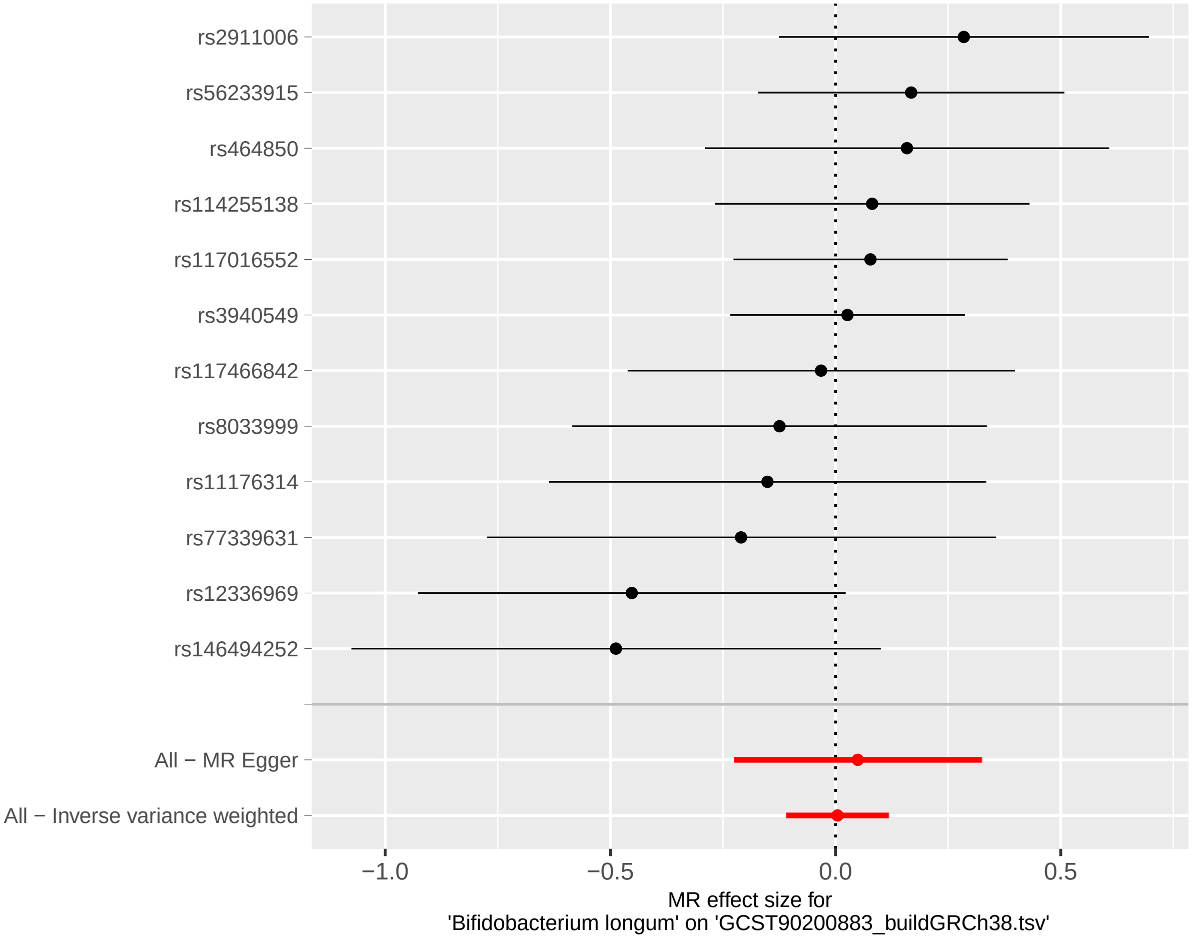

rs2911006
rs56233915
rs464850
rs114255138
rs117016552
rs3940549
rs117466842
rs8033999
rs11176314
rs77339631
rs12336969
rs146494252
All − MR Egger
All − Inverse variance weighted
−1.0
−0.5
0.0
0.5
MR effect size for
'Bifidobacterium longum' on 'GCST90200883_buildGRCh38.tsv'

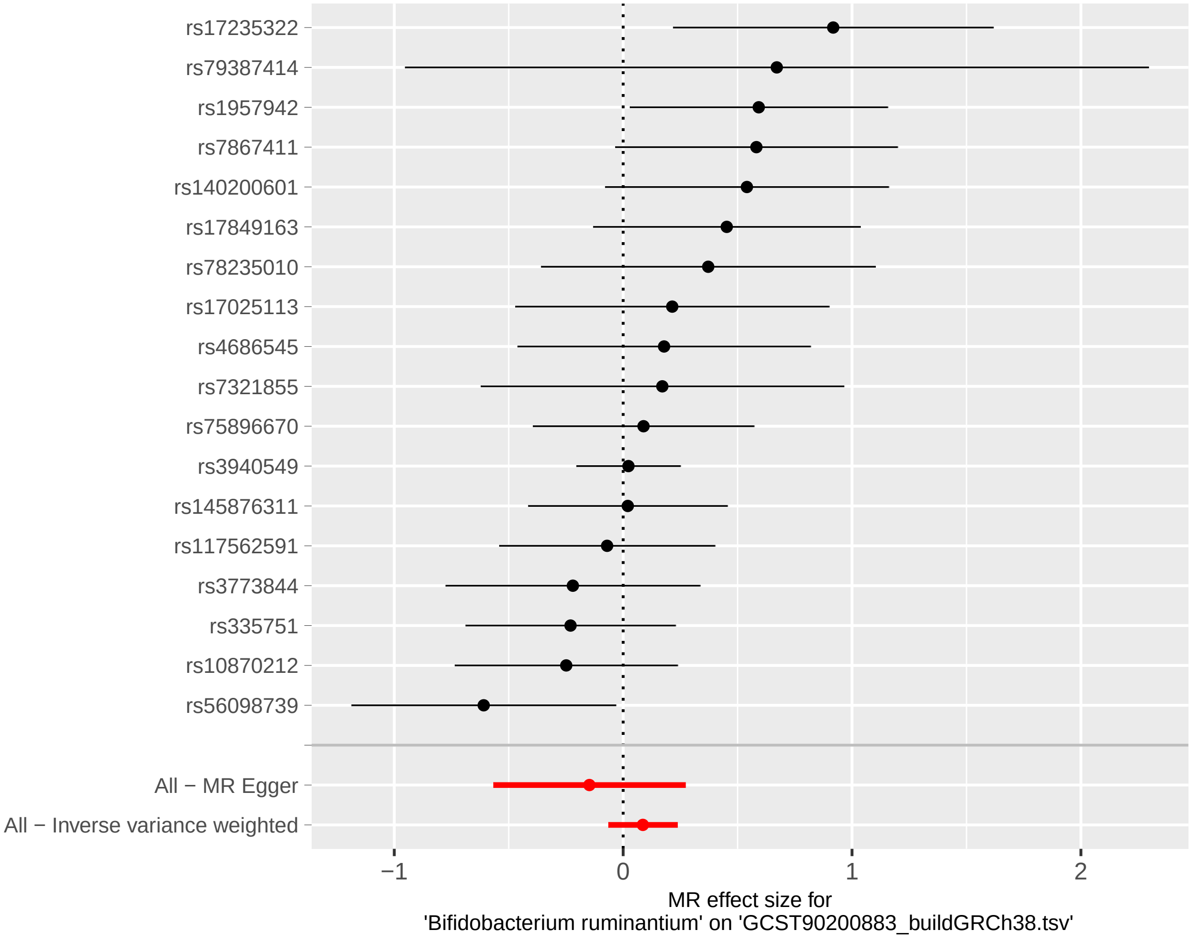
rs17235322
rs79387414
rs1957942
rs7867411
rs140200601
rs17849163
rs78235010
rs17025113
rs4686545
rs7321855
rs75896670
rs3940549
rs145876311
rs117562591
rs3773844
rs335751
rs10870212
rs56098739
All − MR Egger
All − Inverse variance weighted
−1
0
1
2
MR effect size for
'Bifidobacterium ruminantium' on 'GCST90200883_buildGRCh38.tsv'

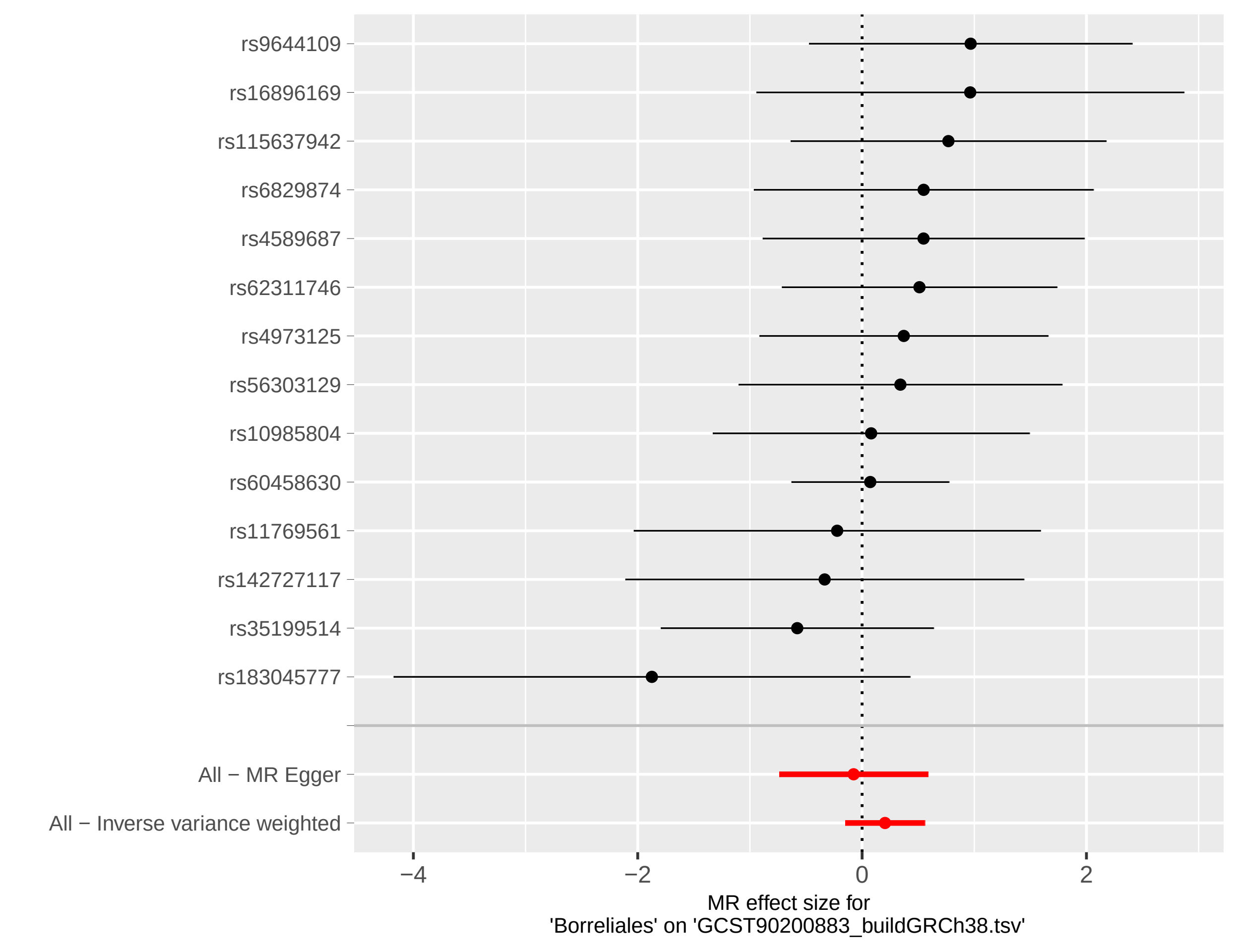
rs9644109
rs16896169
rs115637942
rs6829874
rs4589687
rs62311746
rs4973125
rs56303129
rs10985804
rs60458630
rs11769561
rs142727117
rs35199514
rs183045777
All − MR Egger
All − Inverse variance weighted
−4
−2
0
2
MR effect size for
'Borreliales' on 'GCST90200883_buildGRCh38.tsv'

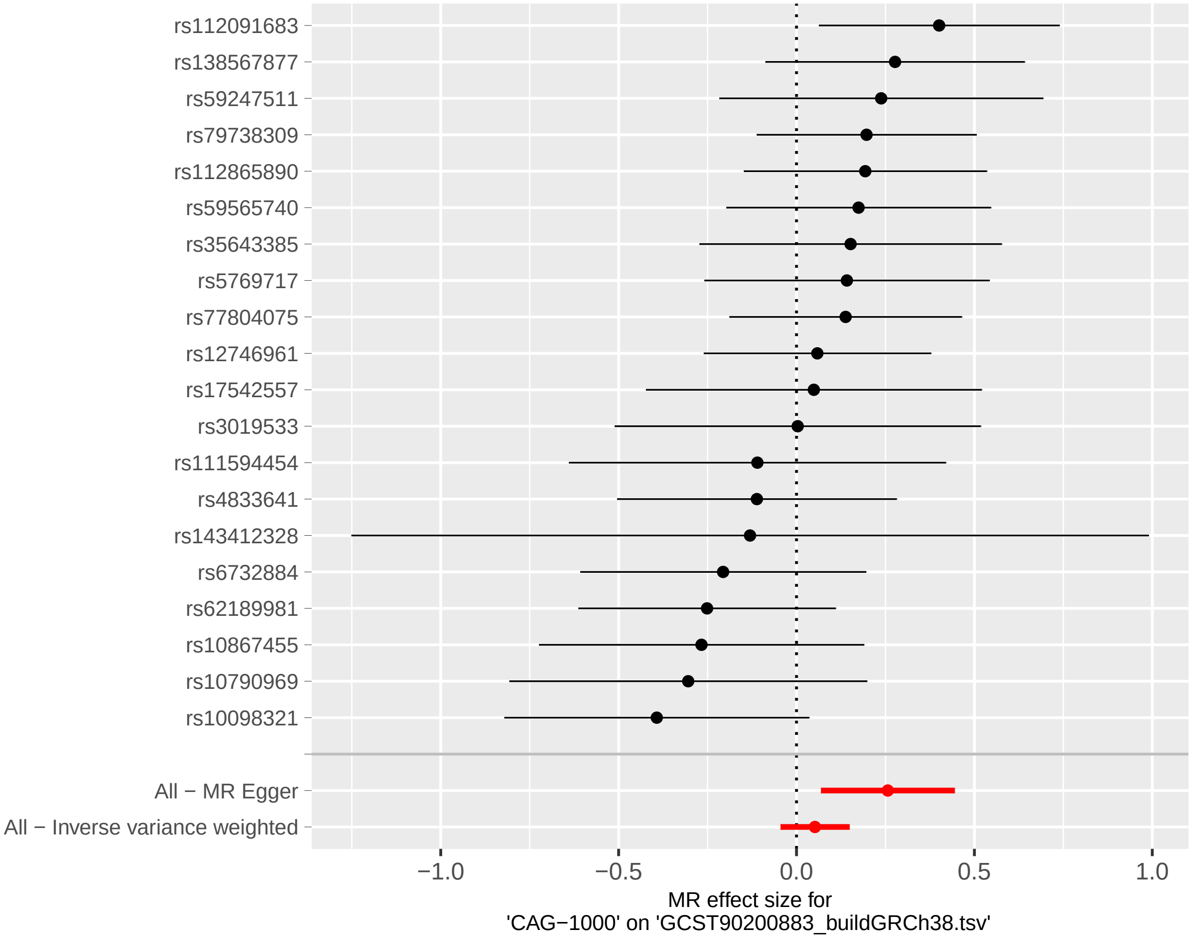
rs112091683
rs138567877
rs59247511
rs79738309
rs112865890
rs59565740
rs35643385
rs5769717
rs77804075
rs12746961
rs17542557
rs3019533
rs111594454
rs4833641
rs143412328
rs6732884
rs62189981
rs10867455
rs10790969
rs10098321
All − MR Egger
All − Inverse variance weighted
−1.0
−0.5
0.0
0.5
1.0
MR effect size for
'CAG−1000' on 'GCST90200883_buildGRCh38.tsv'

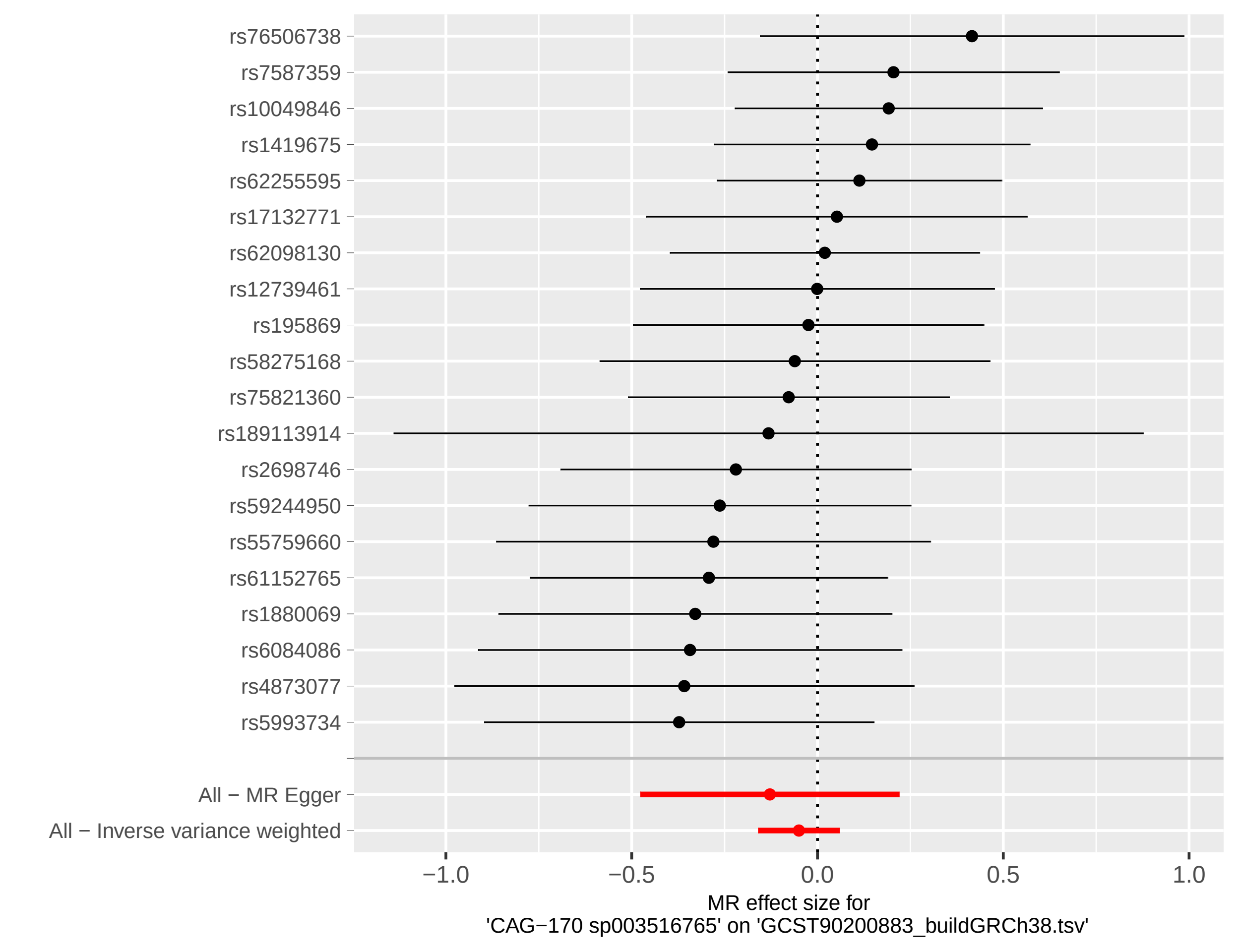

rs76506738
rs7587359
rs10049846
rs1419675
rs62255595
rs17132771
rs62098130
rs12739461
rs195869
rs58275168
rs75821360
rs189113914
rs2698746
rs59244950
rs55759660
rs61152765
rs1880069
rs6084086
rs4873077
rs5993734
All − MR Egger
All − Inverse variance weighted
−1.0
−0.5
0.0
0.5
1.0
MR effect size for
'CAG−170 sp003516765' on 'GCST90200883_buildGRCh38.tsv'

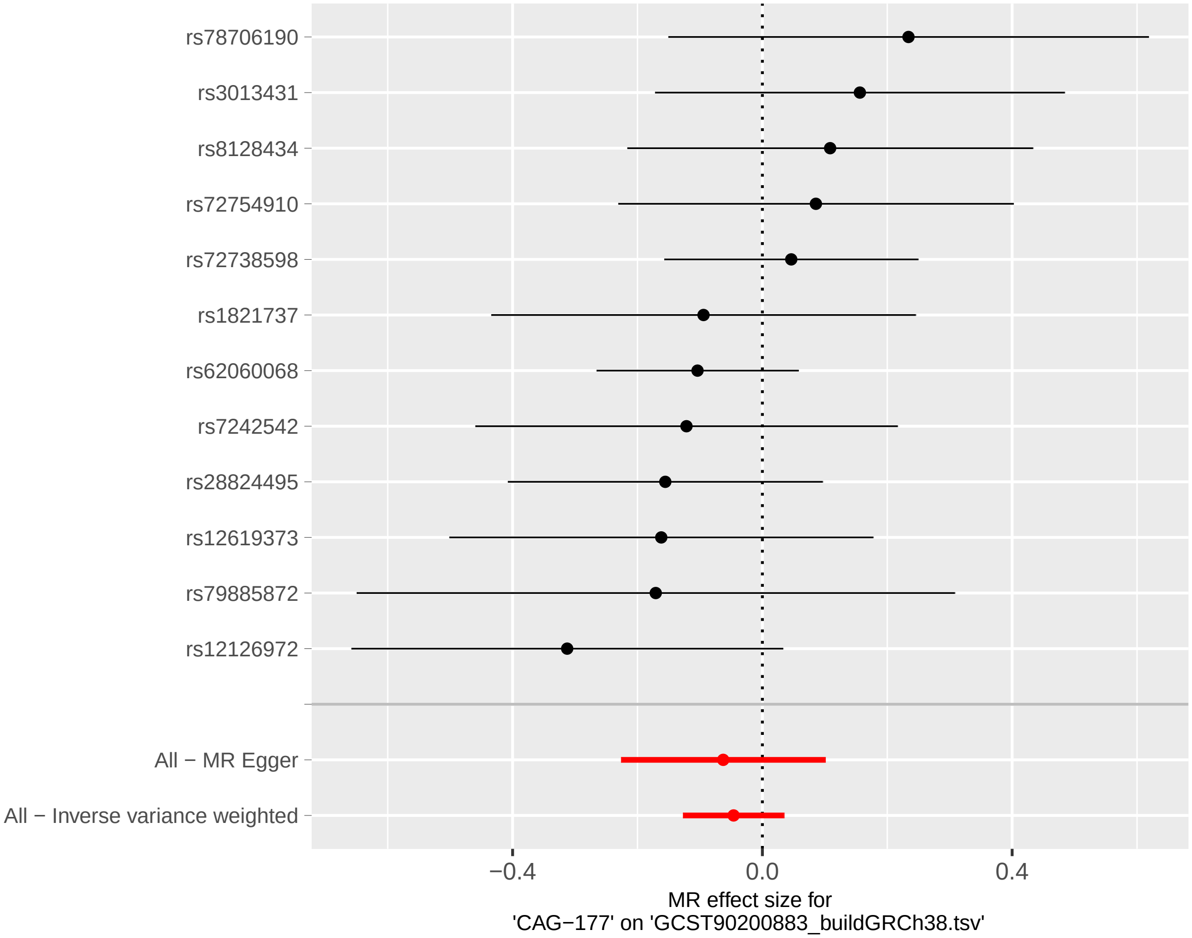
rs78706190
rs3013431
rs8128434
rs72754910
rs72738598
rs1821737
rs62060068
rs7242542
rs28824495
rs12619373
rs79885872
rs12126972
All − MR Egger
All − Inverse variance weighted
−0.4
0.0
0.4
MR effect size for
'CAG−177' on 'GCST90200883_buildGRCh38.tsv'

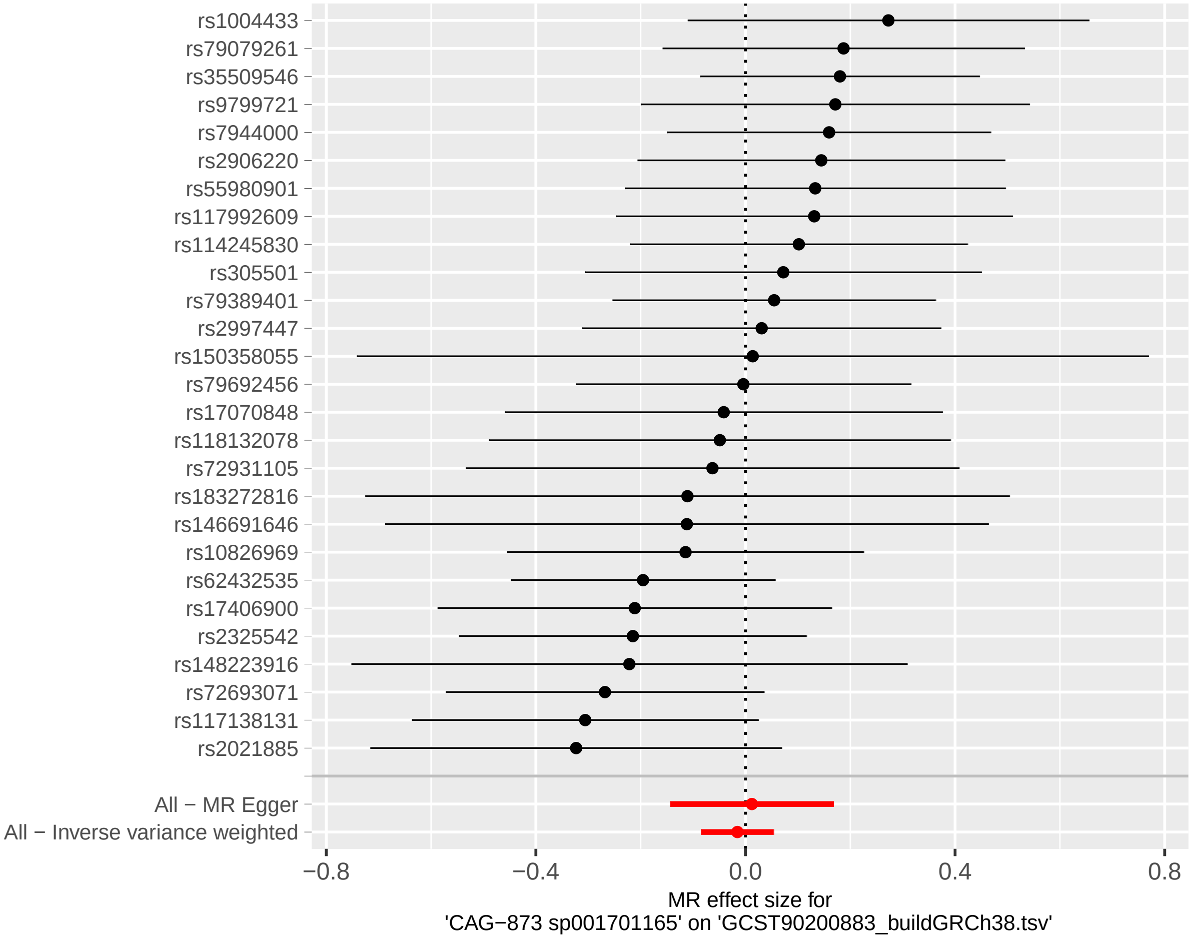

rs1004433
rs79079261
rs35509546
rs9799721
rs7944000
rs2906220
rs55980901
rs117992609
rs114245830
rs305501
rs79389401
rs2997447
rs150358055
rs79692456
rs17070848
rs118132078
rs72931105
rs183272816
rs146691646
rs10826969
rs62432535
rs17406900
rs2325542
rs148223916
rs72693071
rs117138131
rs2021885
All − MR Egger
All − Inverse variance weighted
−0.8
−0.4
0.0
0.4
0.8
MR effect size for
'CAG−873 sp001701165' on 'GCST90200883_buildGRCh38.tsv'

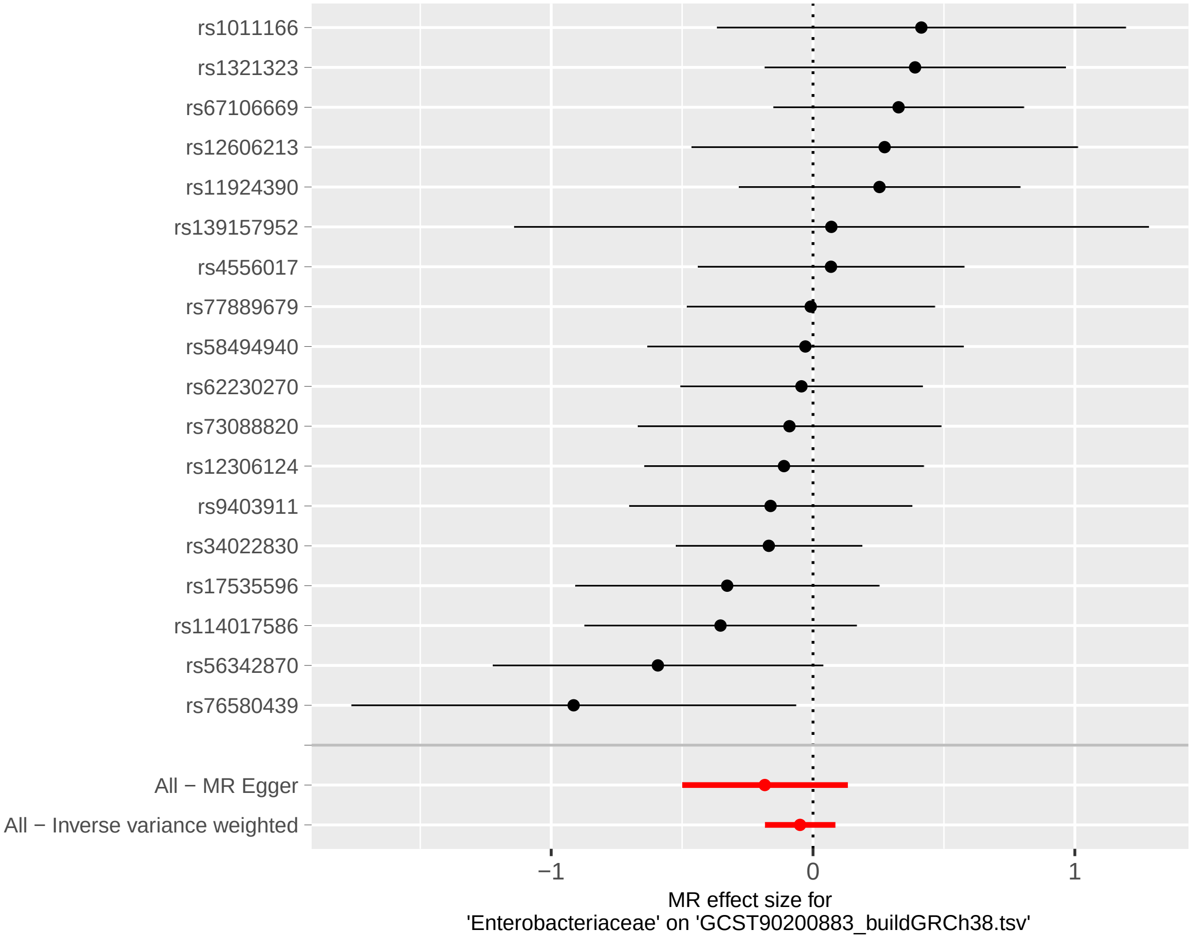
rs1011166
rs1321323
rs67106669
rs12606213
rs11924390
rs139157952
rs4556017
rs77889679
rs58494940
rs62230270
rs73088820
rs12306124
rs9403911
rs34022830
rs17535596
rs114017586
rs56342870
rs76580439
All − MR Egger
All − Inverse variance weighted
−1
0
1
MR effect size for
'Enterobacteriaceae' on 'GCST90200883_buildGRCh38.tsv'

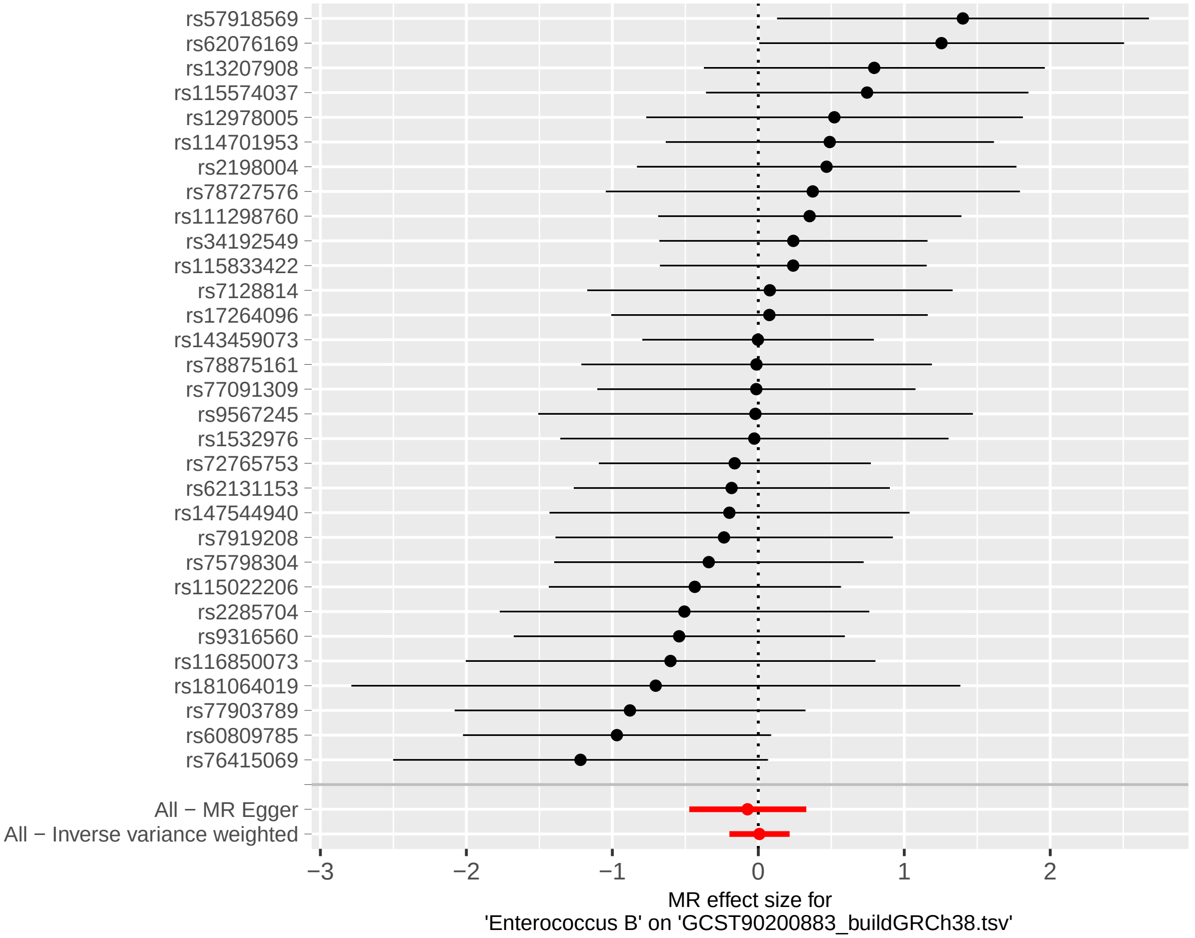
rs57918569
rs62076169
rs13207908
rs115574037
rs12978005
rs114701953
rs2198004
rs78727576
rs111298760
rs34192549
rs115833422
rs7128814
rs17264096
rs143459073
rs78875161
rs77091309
rs9567245
rs1532976
rs72765753
rs62131153
rs147544940
rs7919208
rs75798304
rs115022206
rs2285704
rs9316560
rs116850073
rs181064019
rs77903789
rs60809785
rs76415069
All − MR Egger
All − Inverse variance weighted
−3
−2
−1
0
1
2
MR effect size for
'Enterococcus B' on 'GCST90200883_buildGRCh38.tsv'

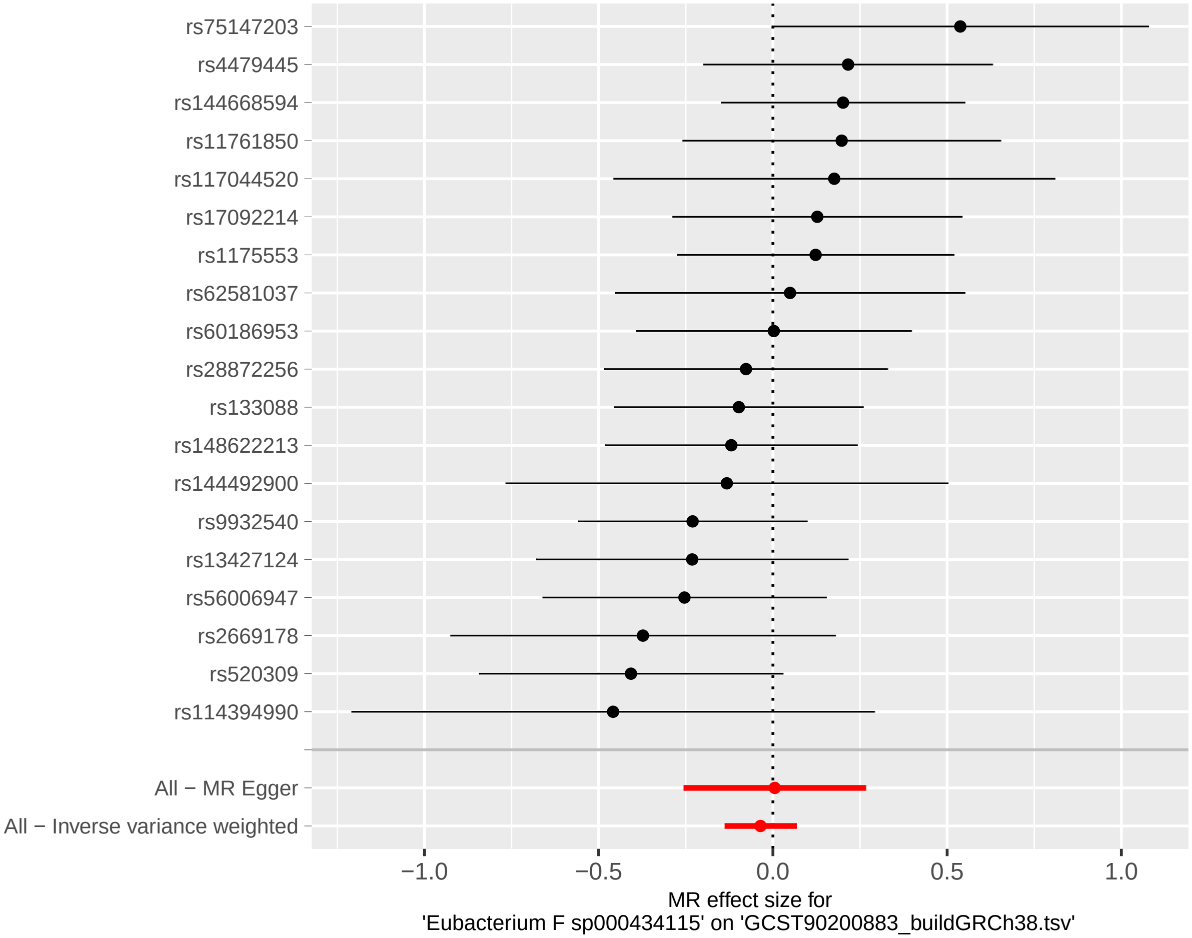

rs75147203
rs4479445
rs144668594
rs11761850
rs117044520
rs17092214
rs1175553
rs62581037
rs60186953
rs28872256
rs133088
rs148622213
rs144492900
rs9932540
rs13427124
rs56006947
rs2669178
rs520309
rs114394990
All − MR Egger
All − Inverse variance weighted
−1.0
−0.5
0.0
0.5
1.0
MR effect size for
'Eubacterium F sp000434115' on 'GCST90200883_buildGRCh38.tsv'

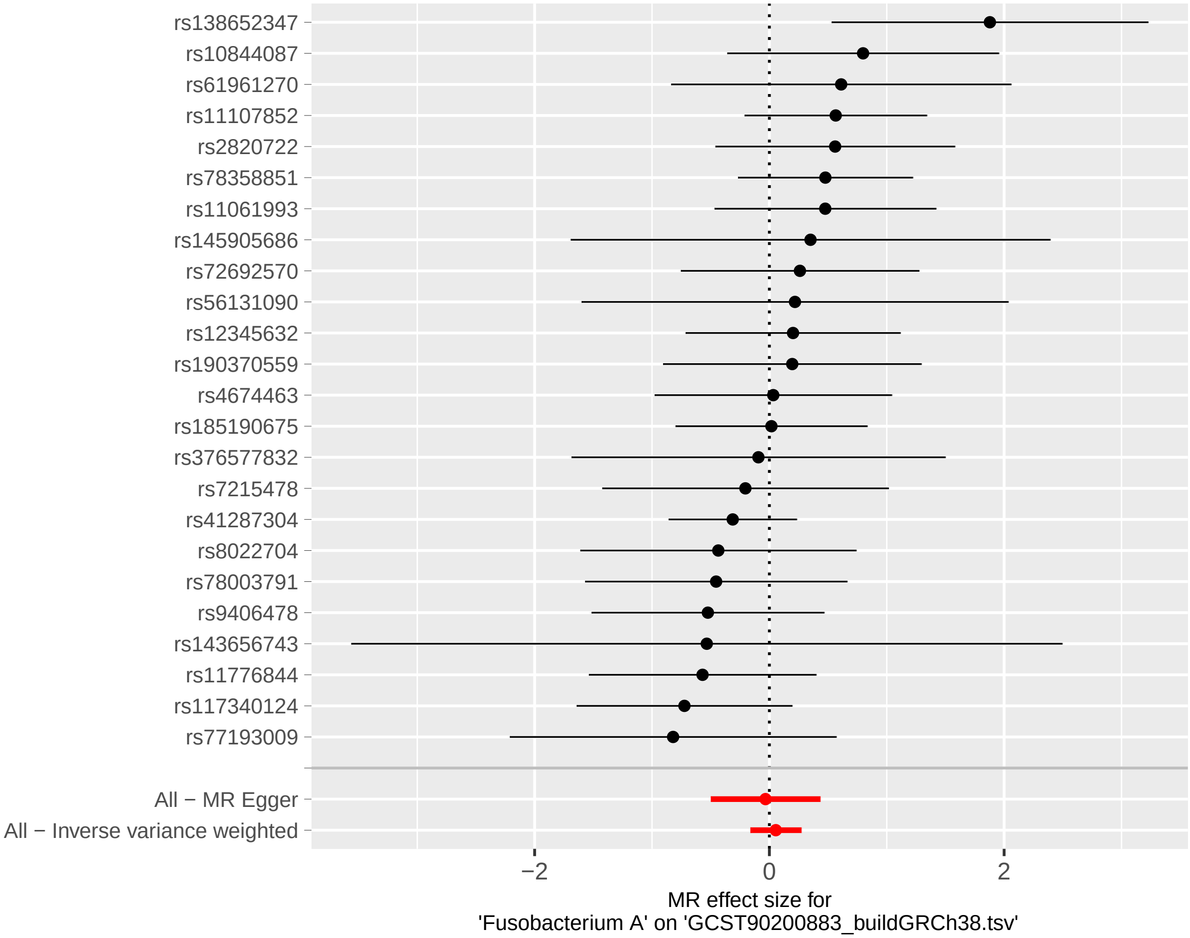

rs138652347
rs10844087
rs61961270
rs11107852
rs2820722
rs78358851
rs11061993
rs145905686
rs72692570
rs56131090
rs12345632
rs190370559
rs4674463
rs185190675
rs376577832
rs7215478
rs41287304
rs8022704
rs78003791
rs9406478
rs143656743
rs11776844
rs117340124
rs77193009
All − MR Egger
All − Inverse variance weighted
−2
0
2
MR effect size for
'Fusobacterium A' on 'GCST90200883_buildGRCh38.tsv'

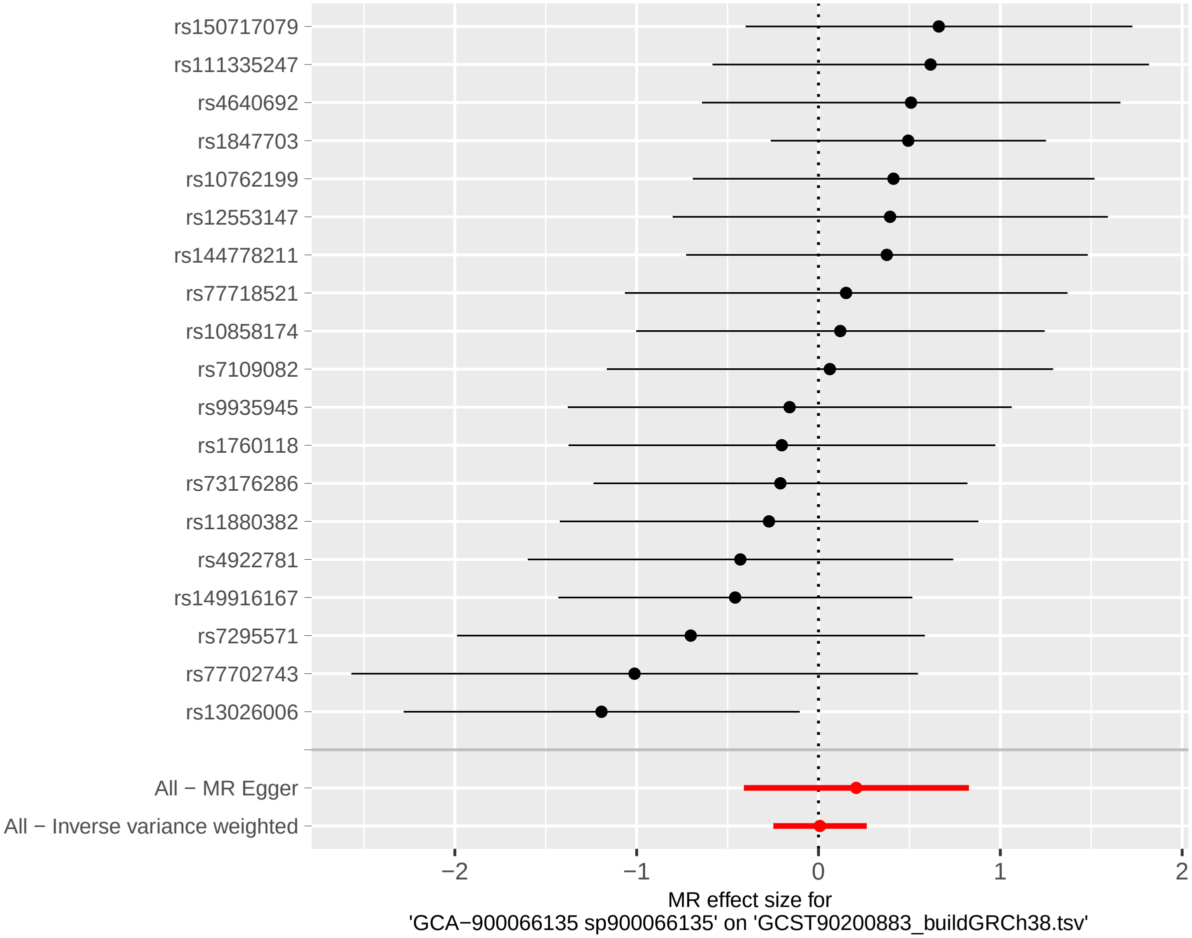
rs150717079
rs111335247
rs4640692
rs1847703
rs10762199
rs12553147
rs144778211
rs77718521
rs10858174
rs7109082
rs9935945
rs1760118
rs73176286
rs11880382
rs4922781
rs149916167
rs7295571
rs77702743
rs13026006
All − MR Egger
All − Inverse variance weighted
−2
−1
0
1
2
MR effect size for
'GCA−900066135 sp900066135' on 'GCST90200883_buildGRCh38.tsv'

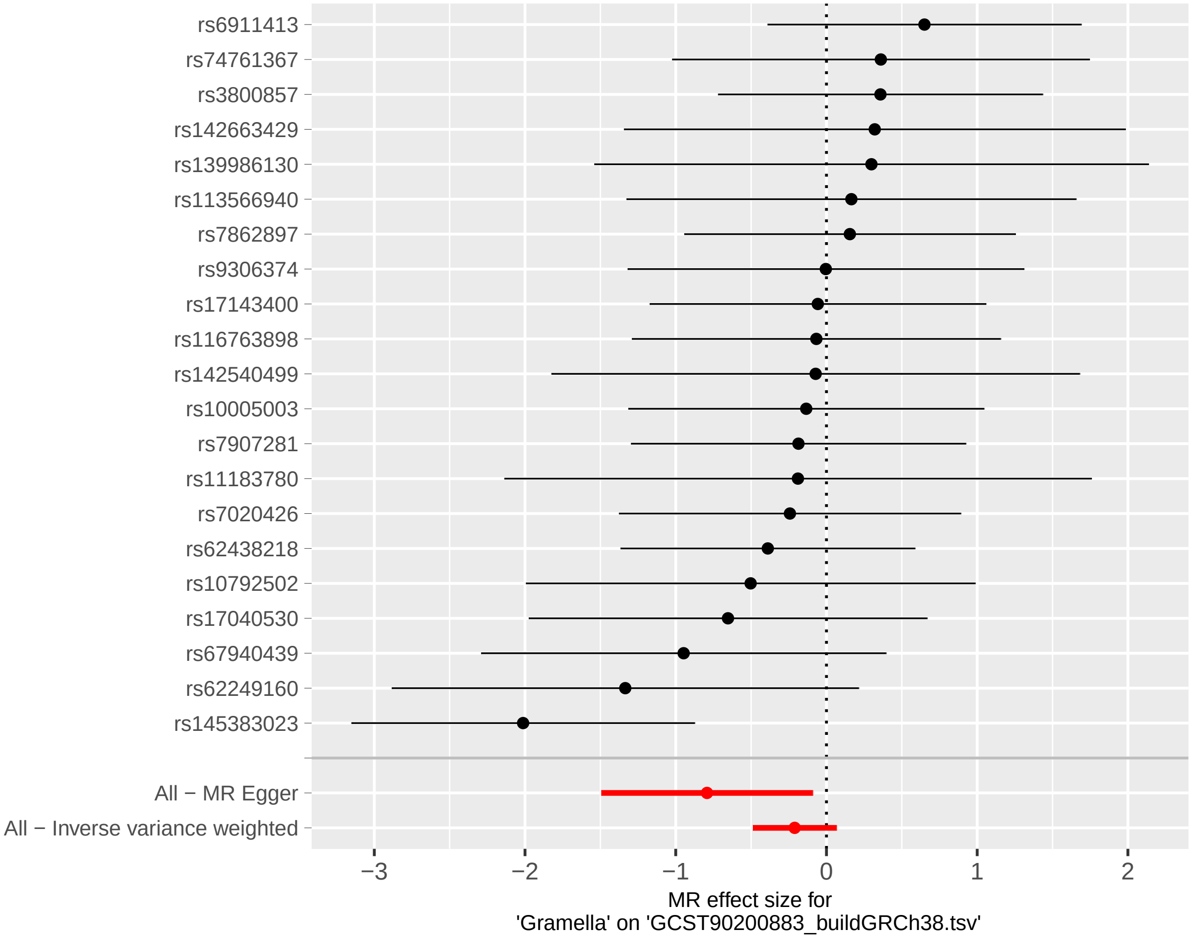
rs6911413
rs74761367
rs3800857
rs142663429
rs139986130
rs113566940
rs7862897
rs9306374
rs17143400
rs116763898
rs142540499
rs10005003
rs7907281
rs11183780
rs7020426
rs62438218
rs10792502
rs17040530
rs67940439
rs62249160
rs145383023
All − MR Egger
All − Inverse variance weighted
−3
−2
−1
0
1
2
MR effect size for
'Gramella' on 'GCST90200883_buildGRCh38.tsv'

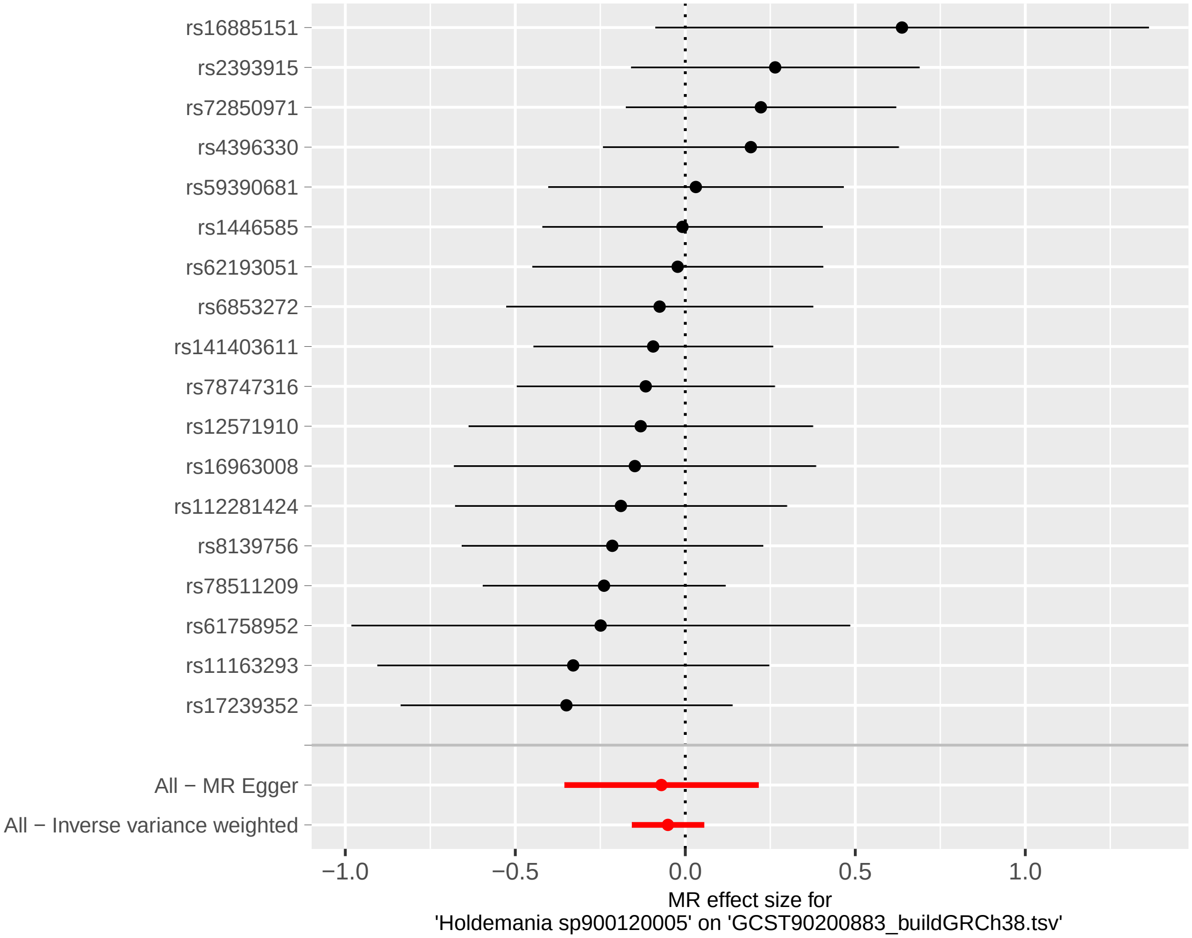
rs16885151
rs2393915
rs72850971
rs4396330
rs59390681
rs1446585
rs62193051
rs6853272
rs141403611
rs78747316
rs12571910
rs16963008
rs112281424
rs8139756
rs78511209
rs61758952
rs11163293
rs17239352
All − MR Egger
All − Inverse variance weighted
−1.0
−0.5
0.0
0.5
1.0
MR effect size for
'Holdemania sp900120005' on 'GCST90200883_buildGRCh38.tsv'

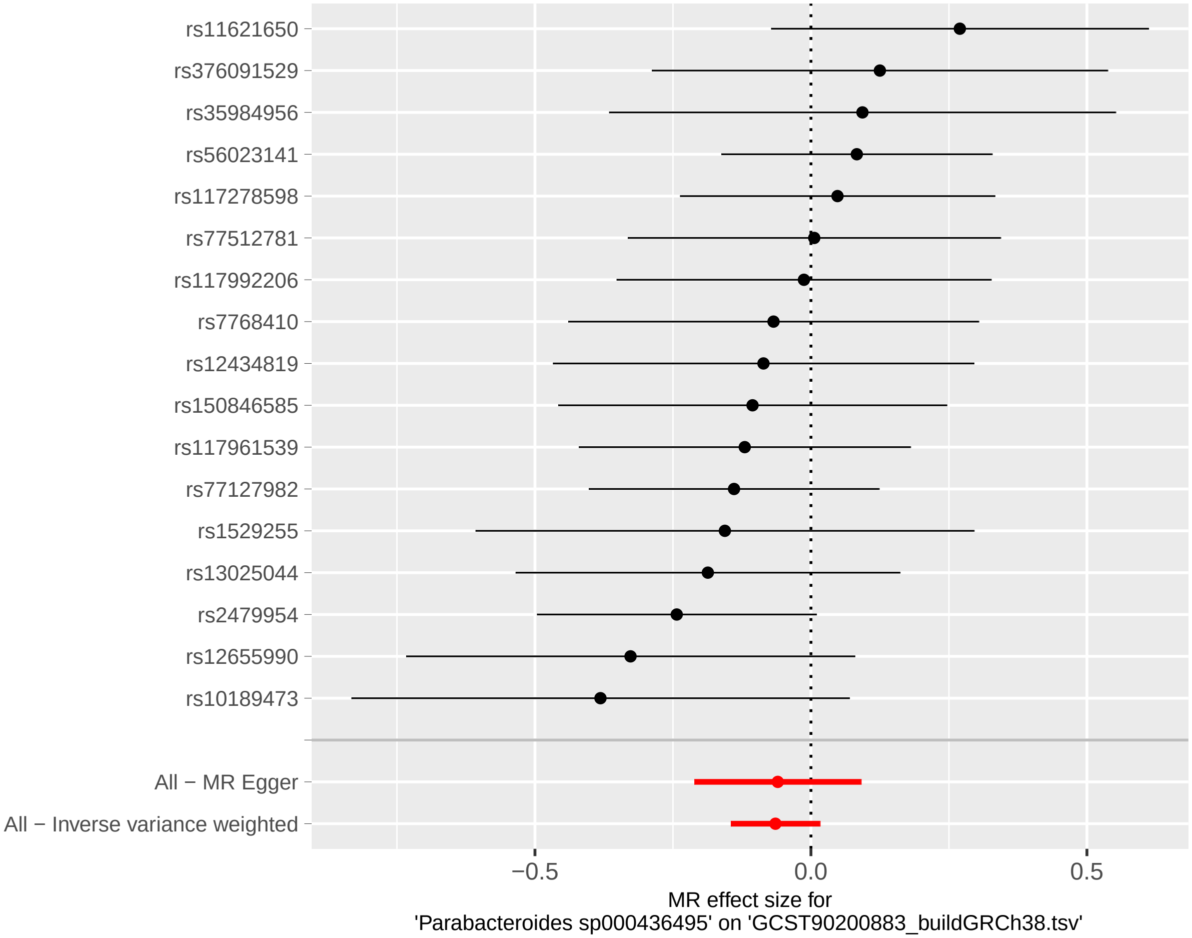

rs11621650
rs376091529
rs35984956
rs56023141
rs117278598
rs77512781
rs117992206
rs7768410
rs12434819
rs150846585
rs117961539
rs77127982
rs1529255
rs13025044
rs2479954
rs12655990
rs10189473
All − MR Egger
All − Inverse variance weighted
−0.5
0.0
0.5
MR effect size for
'Parabacteroides sp000436495' on 'GCST90200883_buildGRCh38.tsv'

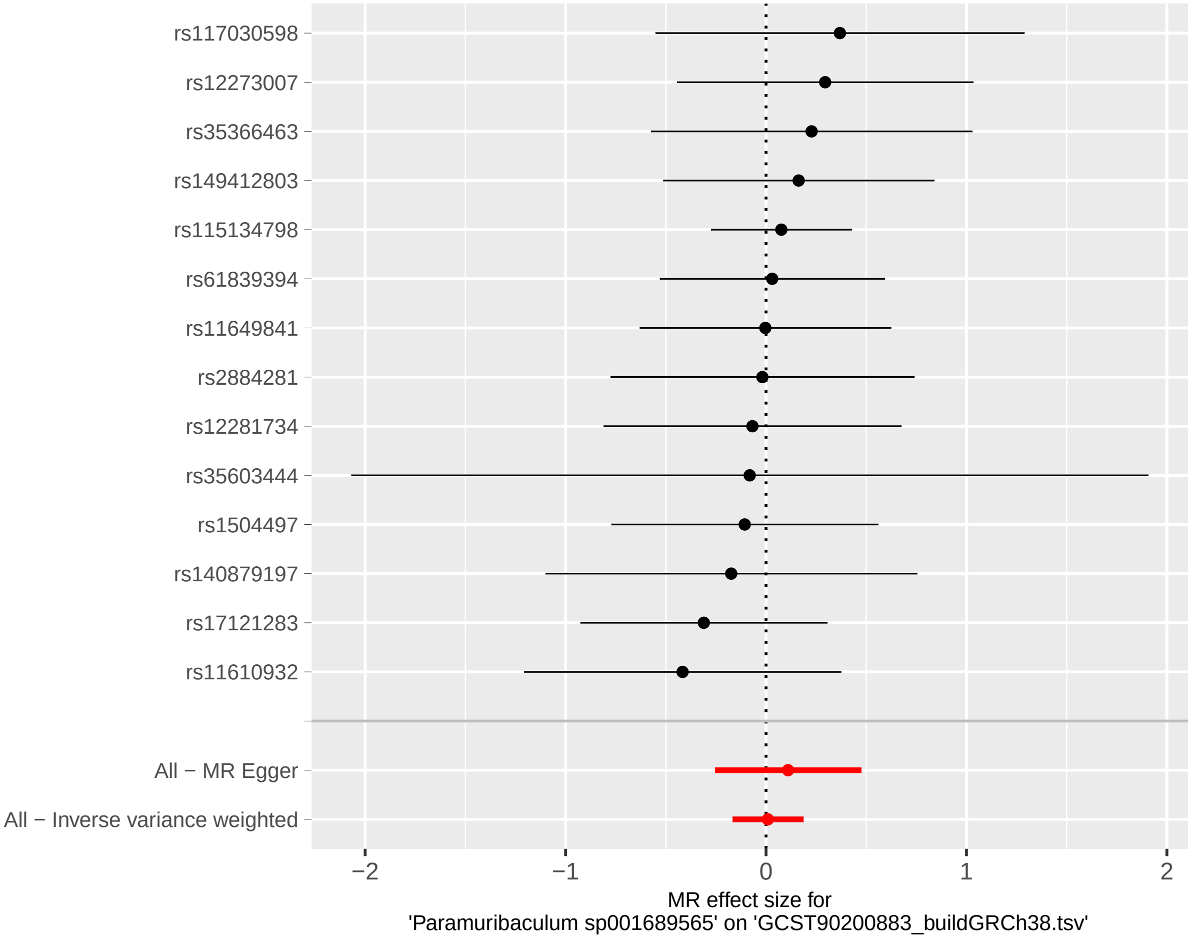

rs117030598
rs12273007
rs35366463
rs149412803
rs115134798
rs61839394
rs11649841
rs2884281
rs12281734
rs35603444
rs1504497
rs140879197
rs17121283
rs11610932
All − MR Egger
All − Inverse variance weighted
−2
−1
0
1
2
MR effect size for
'Paramuribaculum sp001689565' on 'GCST90200883_buildGRCh38.tsv'

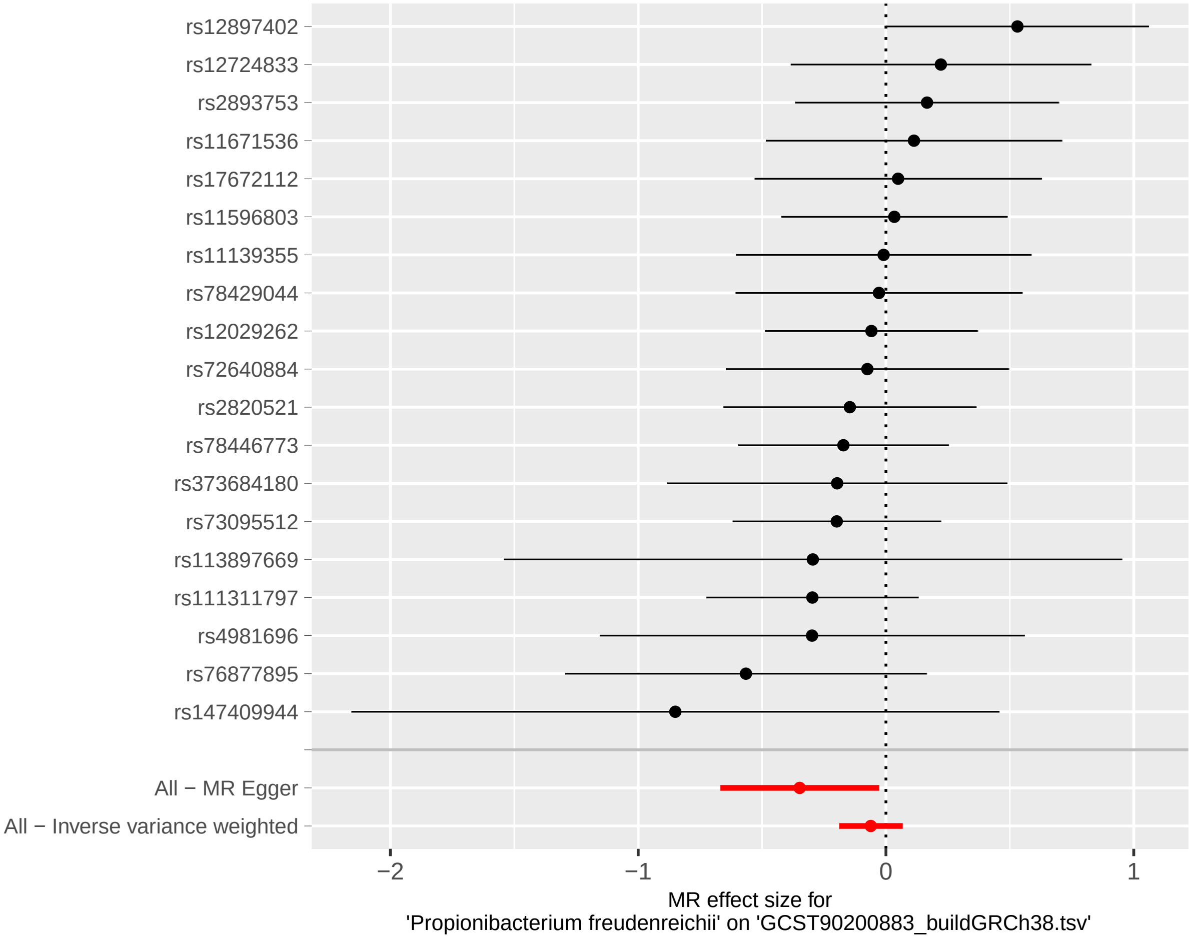

rs12897402
rs12724833
rs2893753
rs11671536
rs17672112
rs11596803
rs11139355
rs78429044
rs12029262
rs72640884
rs2820521
rs78446773
rs373684180
rs73095512
rs113897669
rs111311797
rs4981696
rs76877895
rs147409944
All − MR Egger
All − Inverse variance weighted
−2
−1
0
1
MR effect size for
'Propionibacterium freudenreichii' on 'GCST90200883_buildGRCh38.tsv'

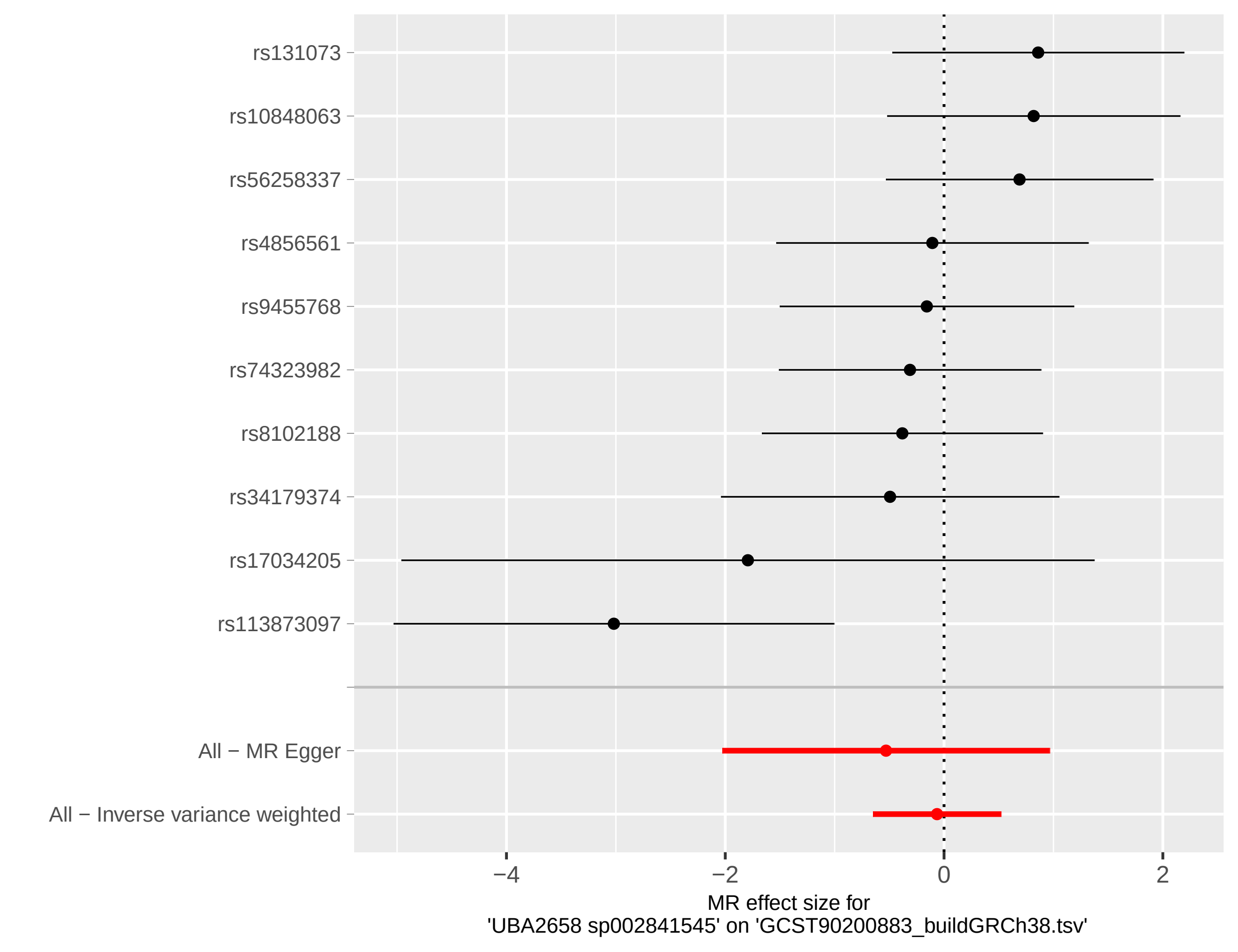

rs131073
rs10848063
rs56258337
rs4856561
rs9455768
rs74323982
rs8102188
rs34179374
rs17034205
rs113873097
All − MR Egger
All − Inverse variance weighted
−4
−2
0
2
MR effect size for
'UBA2658 sp002841545' on 'GCST90200883_buildGRCh38.tsv'

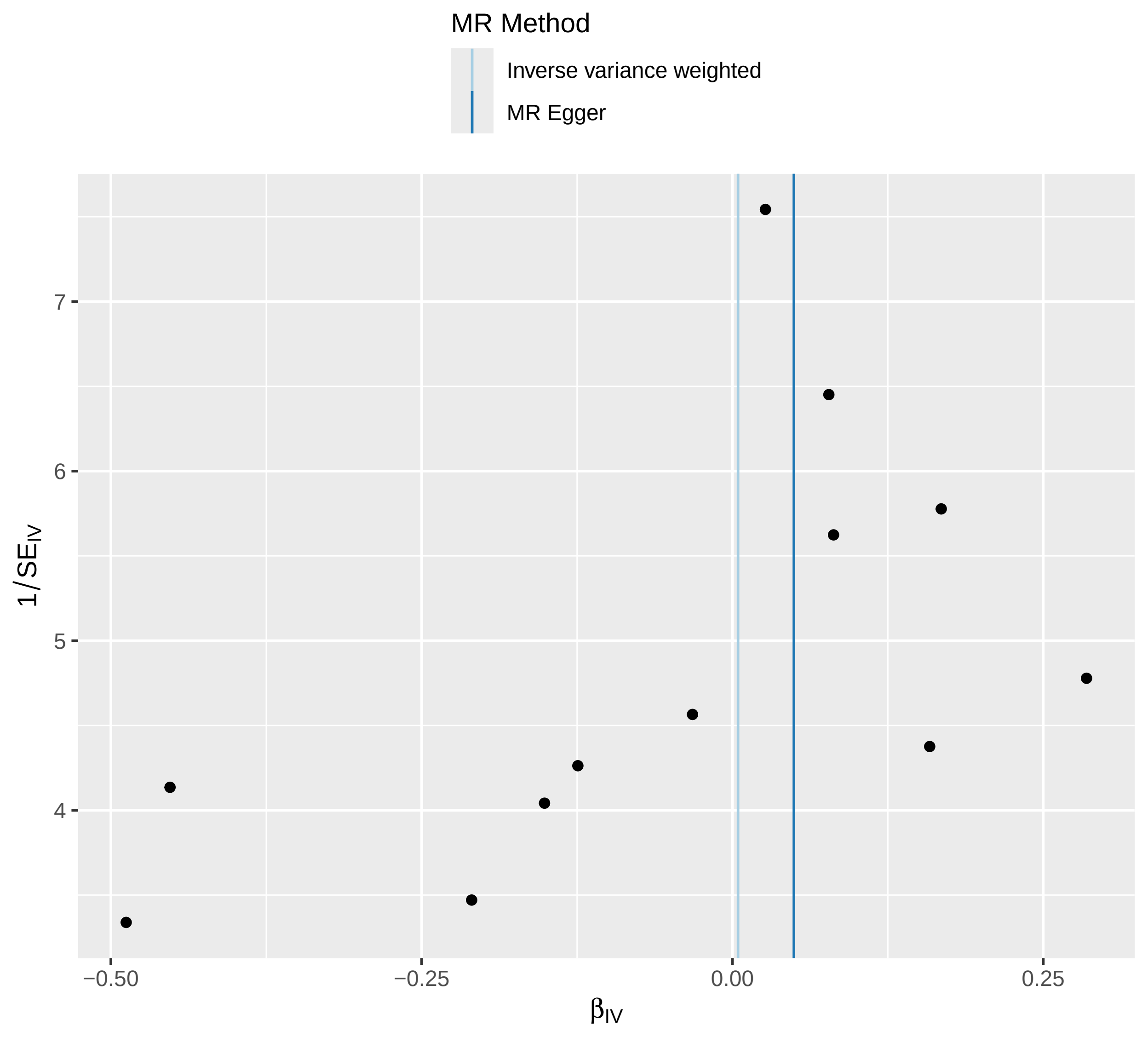

MR Method
Inverse variance weighted
MR Egger
7
6
5
4
1/SE$_{IV}$
−0.50
−0.25
0.00
0.25
β$_{IV}$

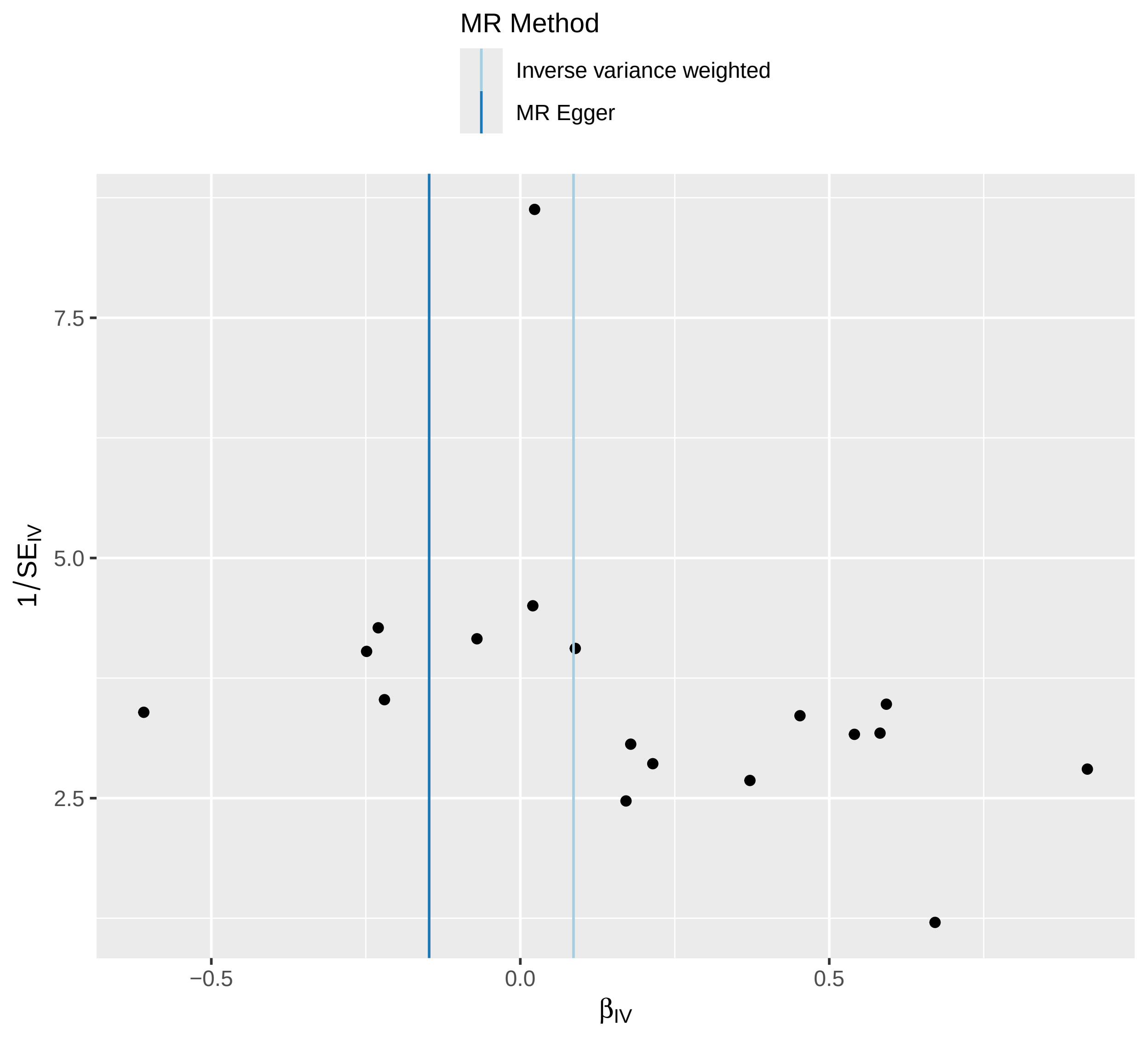
MR Method
Inverse variance weighted
MR Egger
7.5
5.0
2.5
$1/SE_{IV}$
−0.5
0.0
0.5
β_{IV}

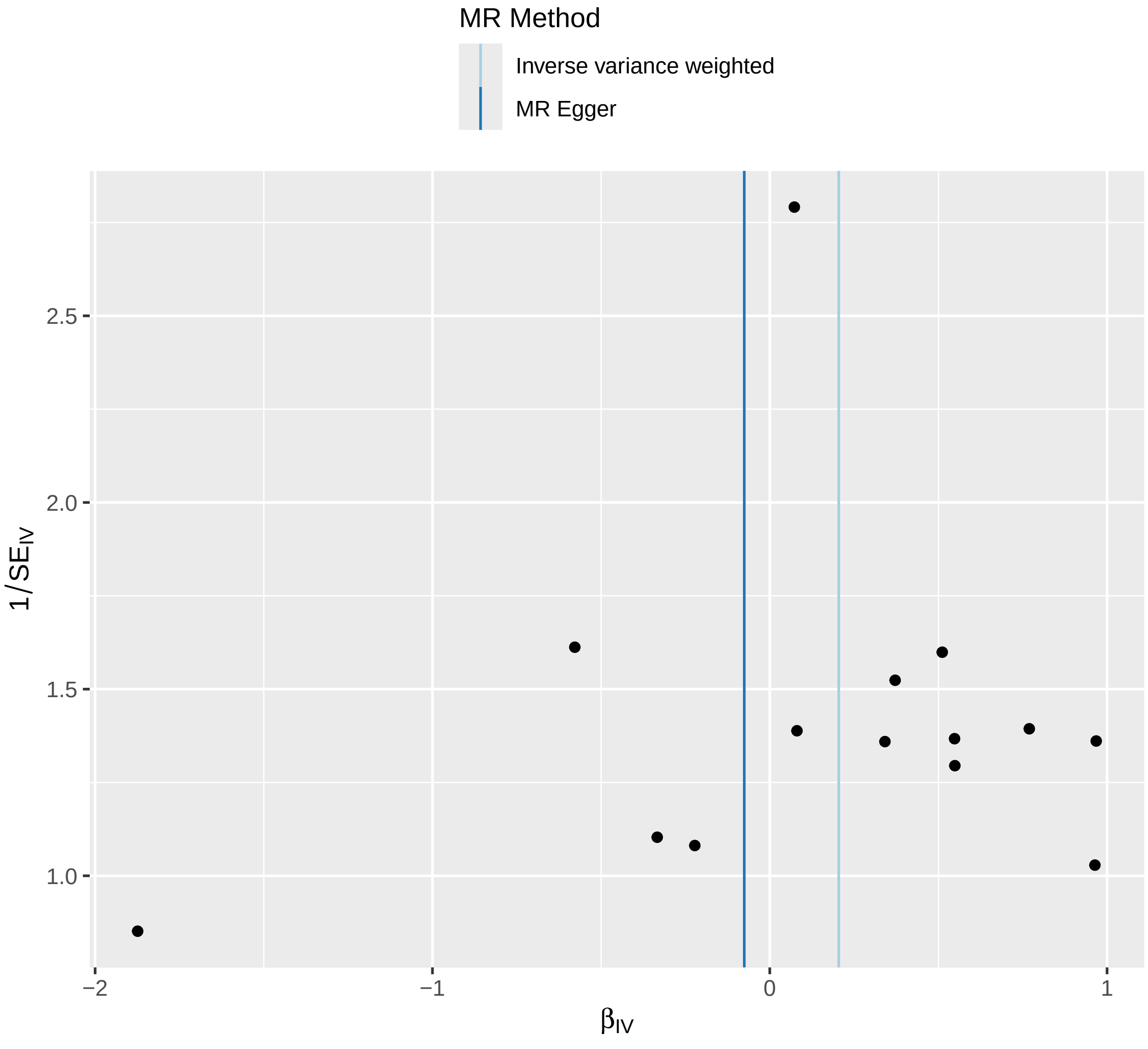
MR Method
Inverse variance weighted
MR Egger
2.5
2.0
1.5
1.0
$1/SE_{IV}$
−2
−1
0
1
β_{IV}

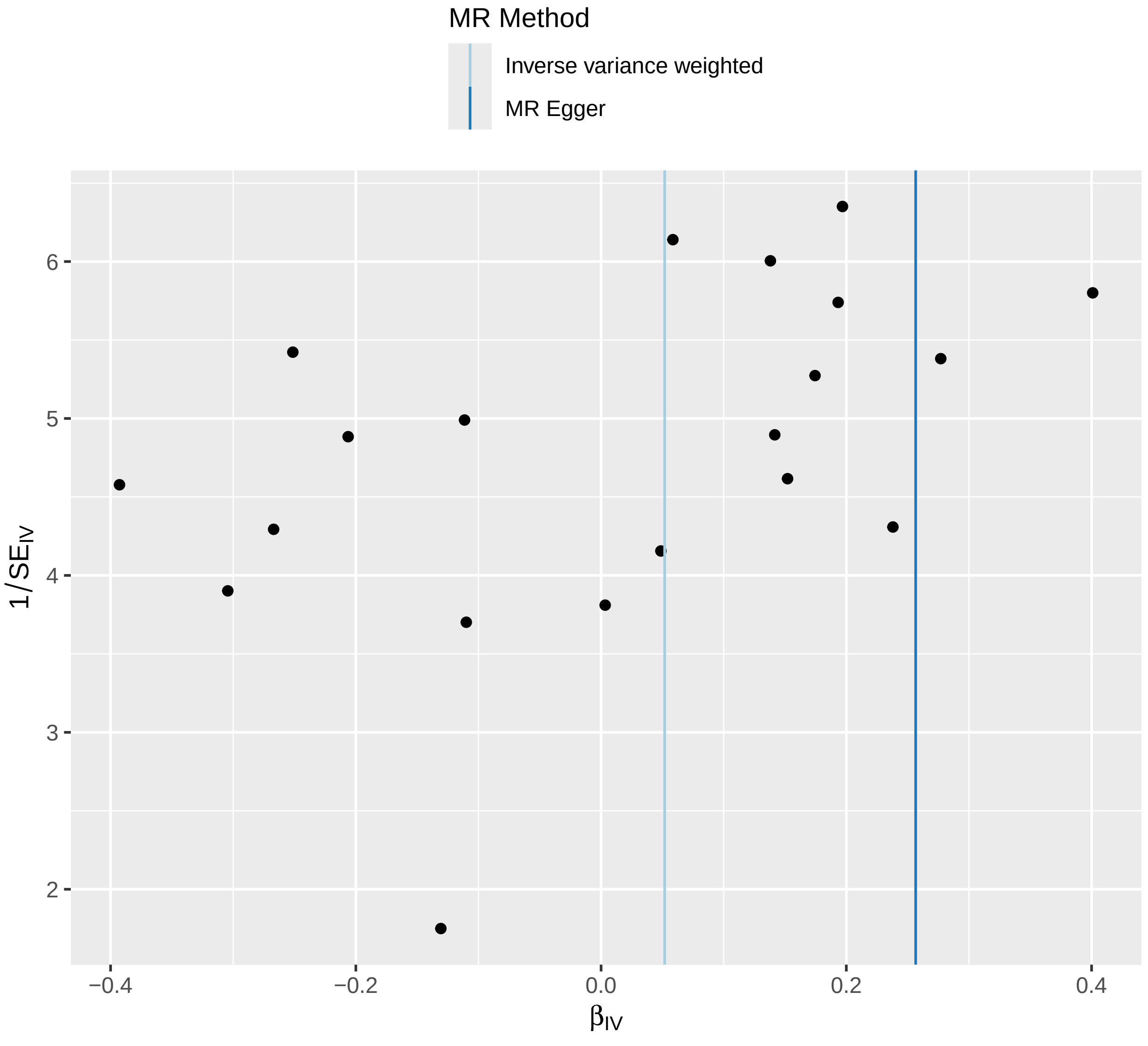

MR Method
Inverse variance weighted
MR Egger
1/SE_IV
6
5
4
3
2
β_IV
−0.4
−0.2
0.0
0.2
0.4

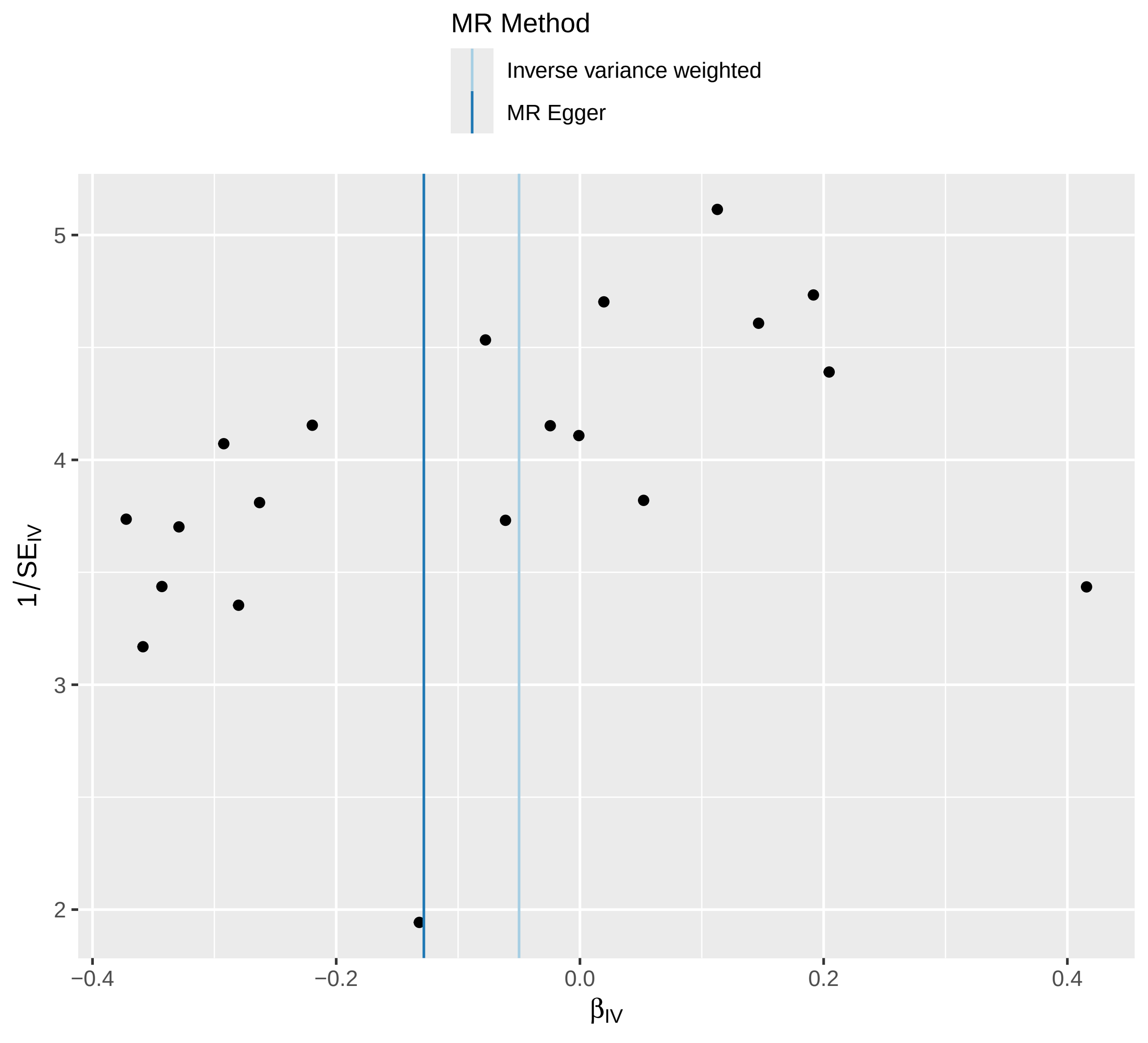
MR Method
Inverse variance weighted
MR Egger
1/SE_IV
5
4
3
2
−0.4
−0.2
0.0
0.2
0.4
β_IV

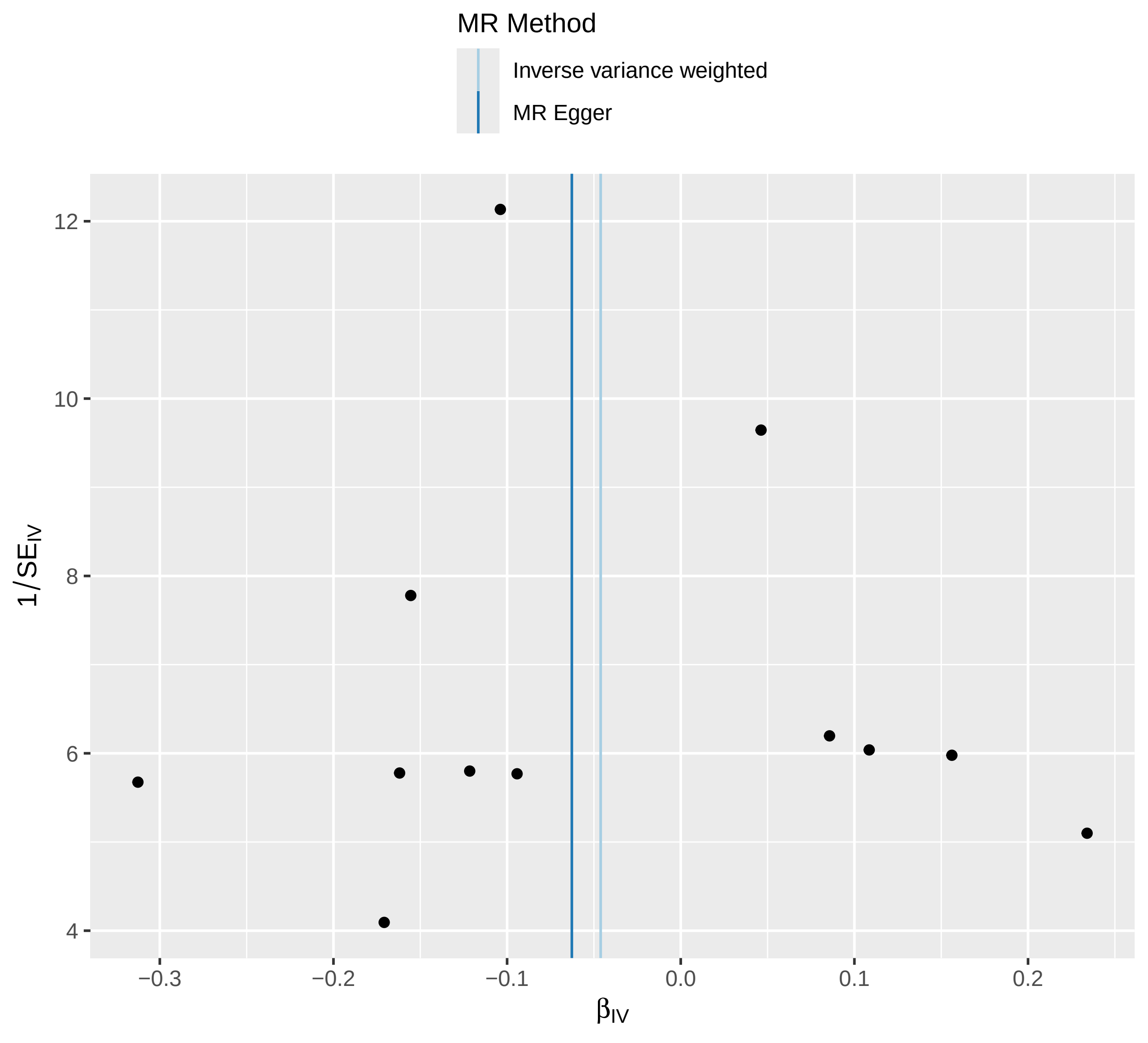

MR Method
Inverse variance weighted
MR Egger
12
10
8
6
4
1/SE_IV
−0.3
−0.2
−0.1
0.0
0.1
0.2
β_IV

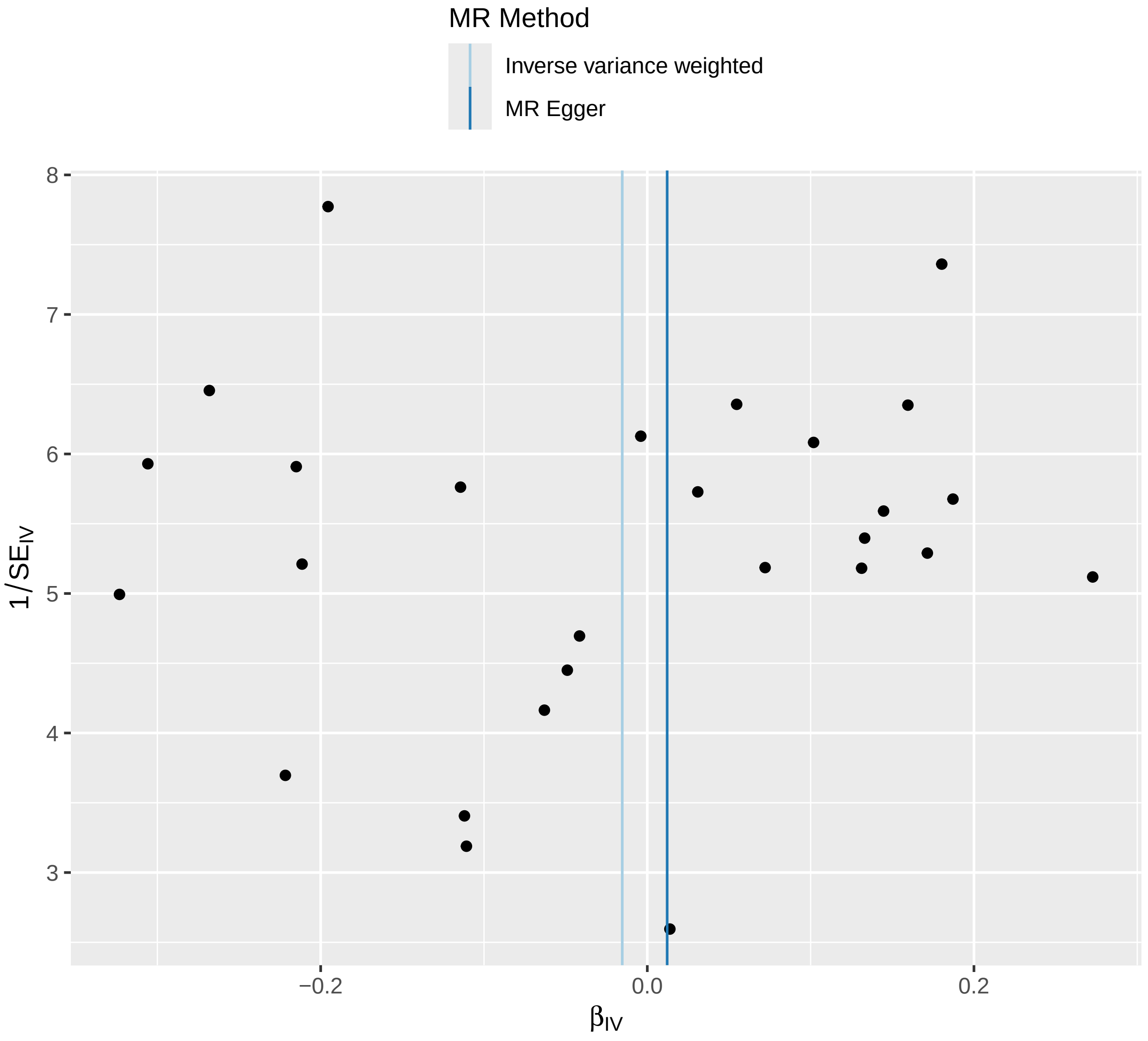

MR Method
Inverse variance weighted
MR Egger
8
7
6
5
4
3
1/SE$_{IV}$
−0.2
0.0
0.2
β$_{IV}$

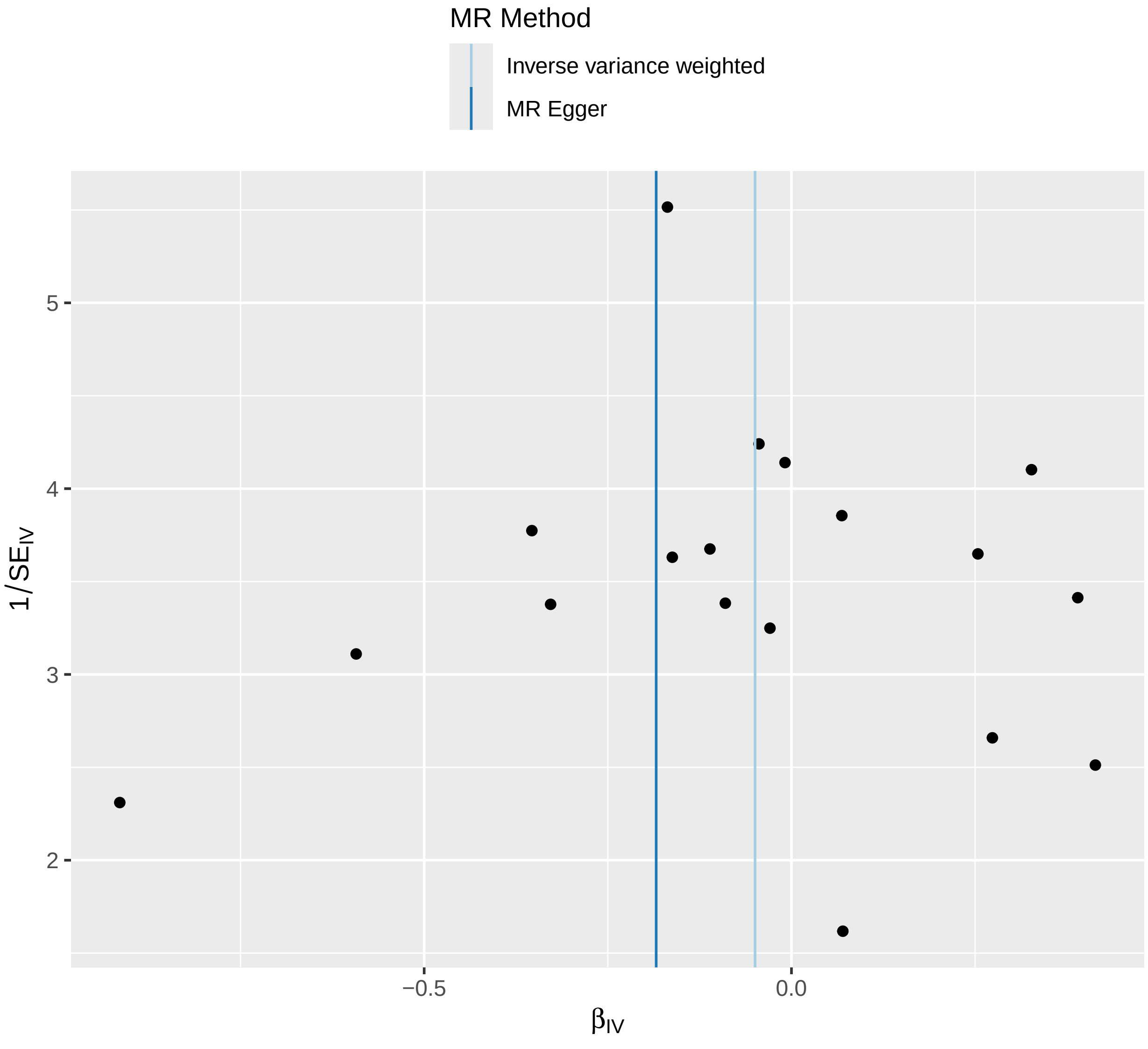
MR Method
Inverse variance weighted
MR Egger
5
4
3
2
$1/SE_{IV}$
−0.5
0.0
β_{IV}

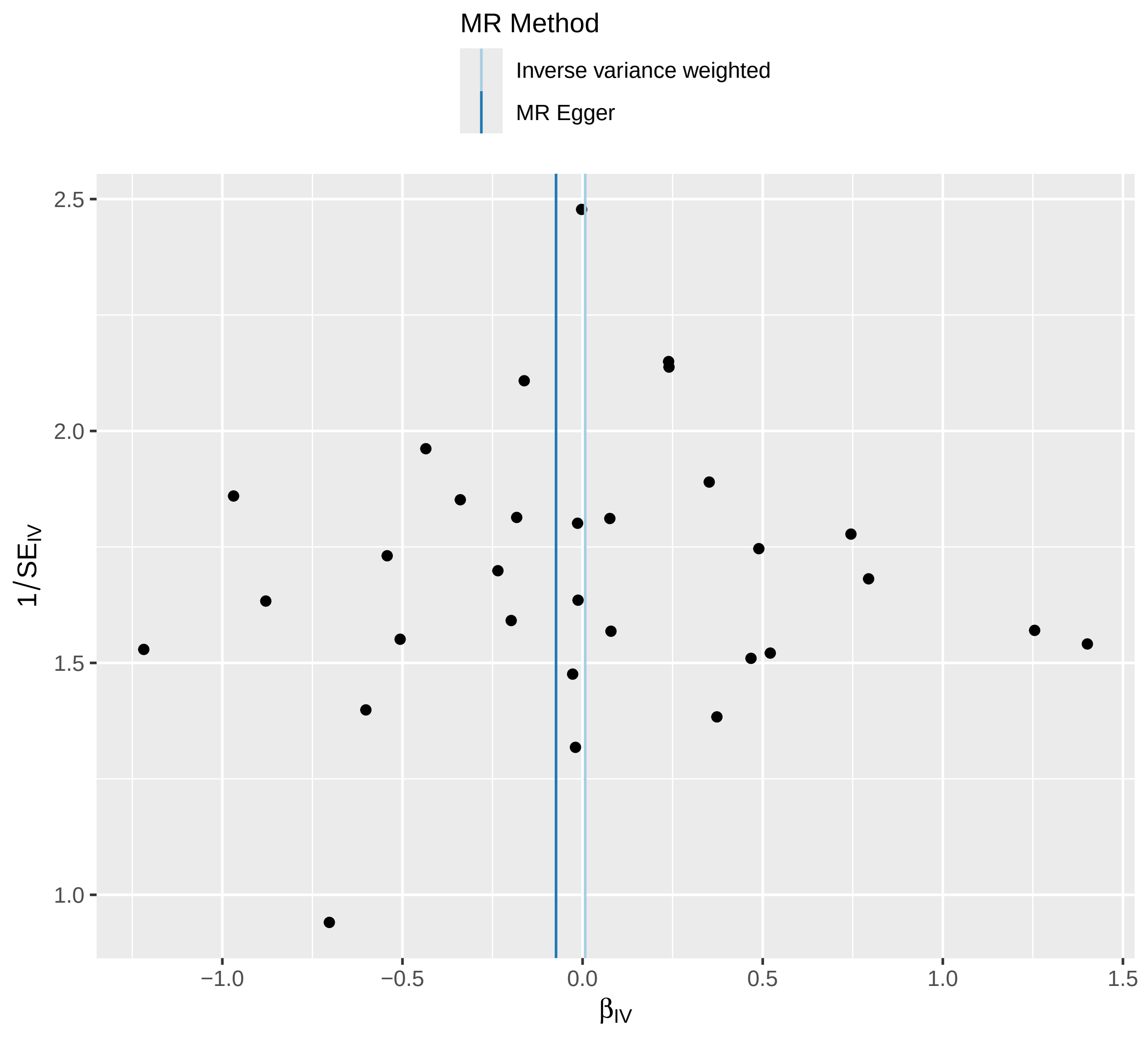

MR Method
Inverse variance weighted
MR Egger
2.5
2.0
1.5
1.0
1/SE$_{IV}$
−1.0
−0.5
0.0
0.5
1.0
1.5
β$_{IV}$

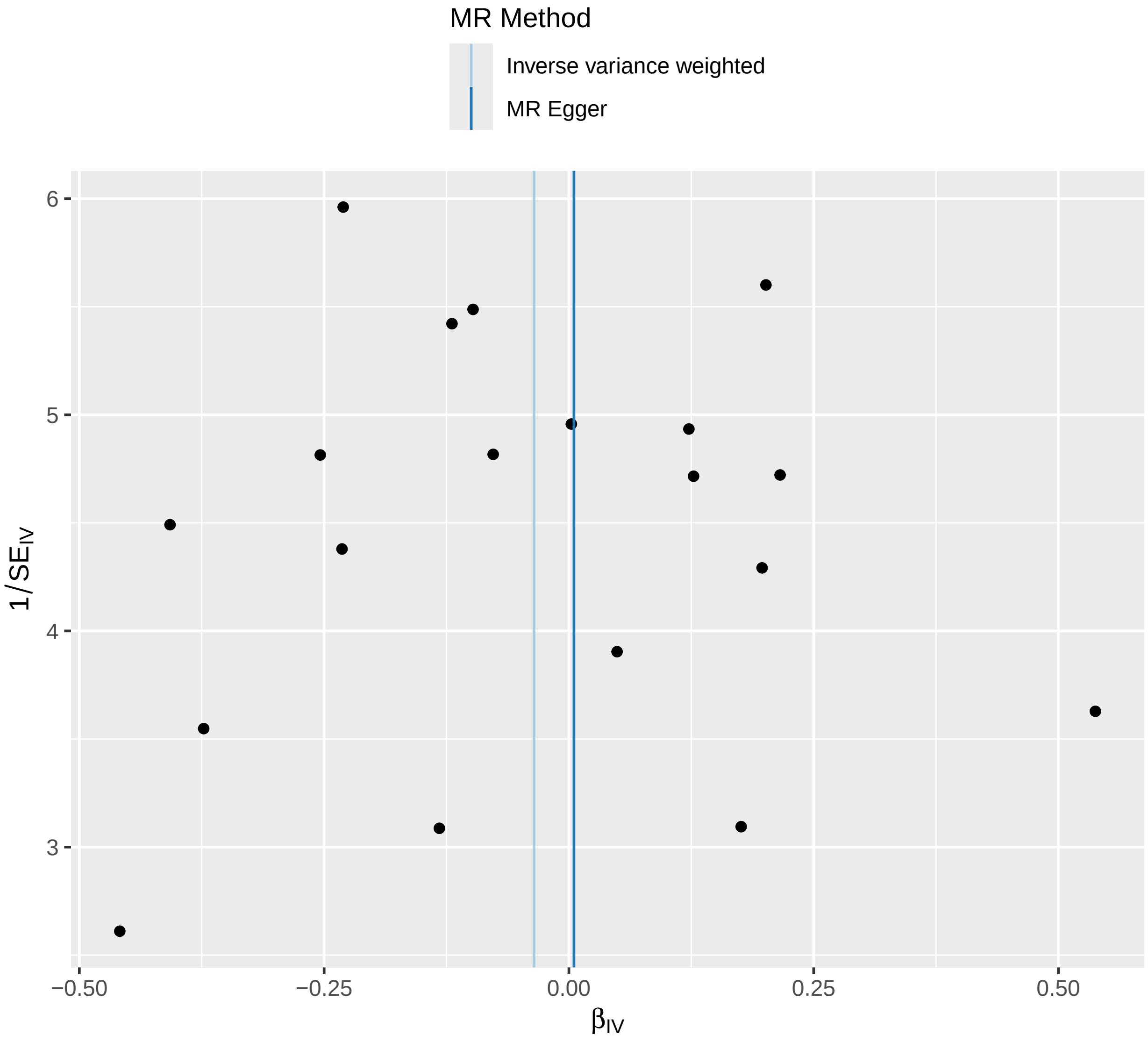

MR Method
Inverse variance weighted
MR Egger
6
5
4
3
$1/SE_{IV}$
−0.50
−0.25
0.00
0.25
0.50
β_{IV}

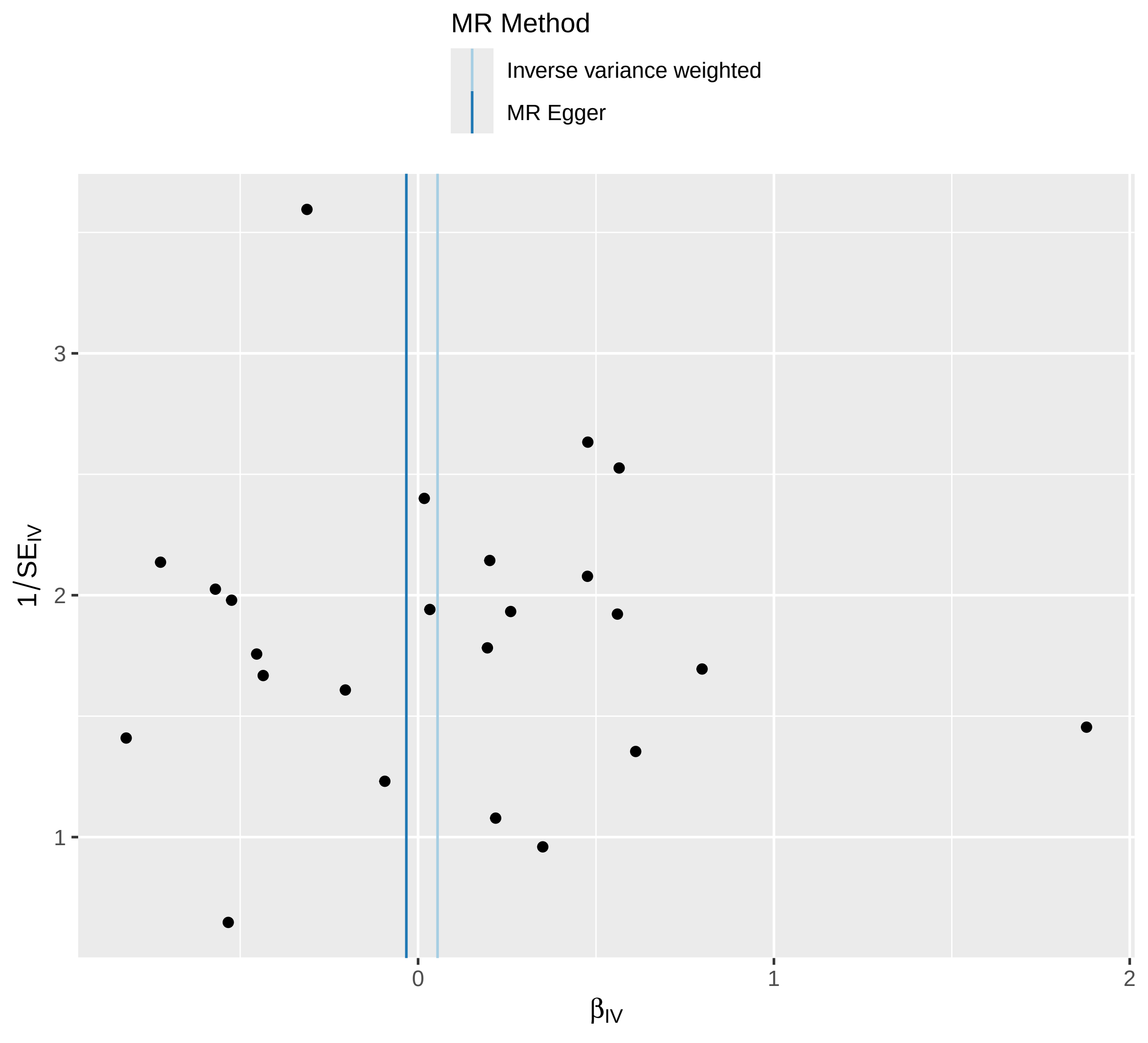

MR Method
Inverse variance weighted
MR Egger
3
2
1
1/SE$_{IV}$
0
1
2
β$_{IV}$

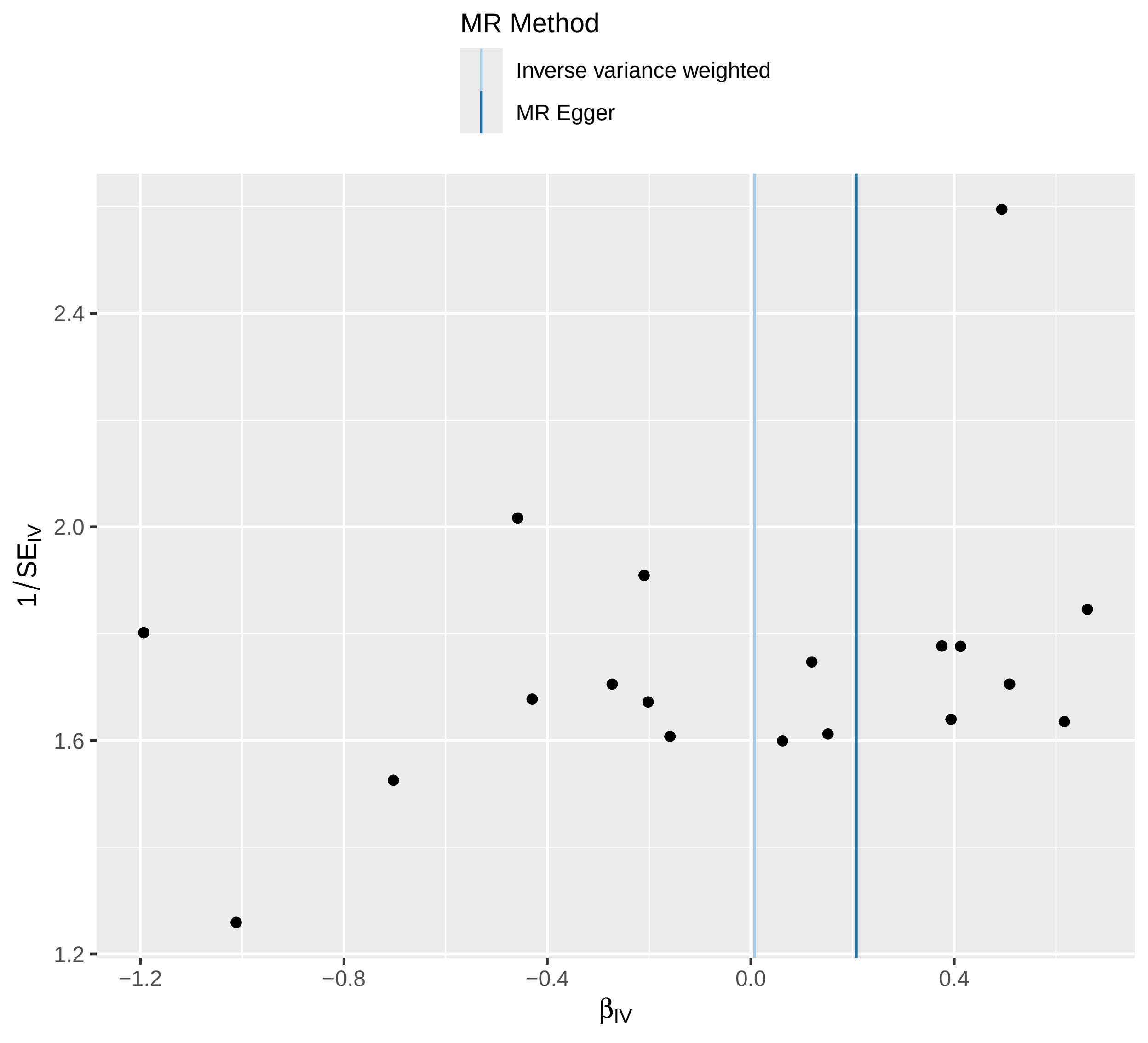
MR Method
Inverse variance weighted
MR Egger
2.4
2.0
1.6
1.2
1/SE$_{IV}$
−1.2
−0.8
−0.4
0.0
0.4
β_{IV}

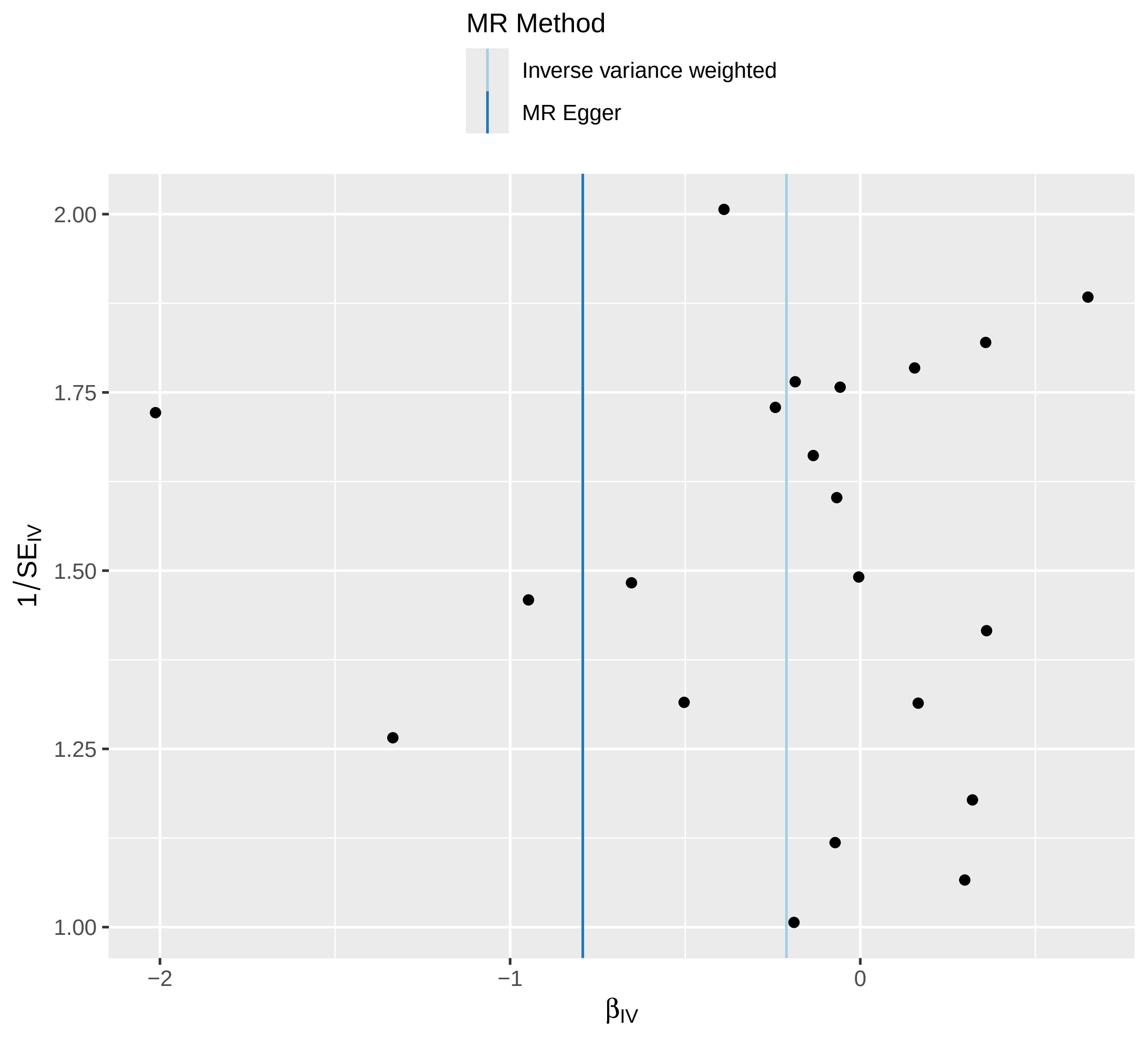

MR Method
Inverse variance weighted
MR Egger
2.00
1.75
1.50
1.25
1.00
$1/SE_{IV}$
−2
−1
0
β_{IV}

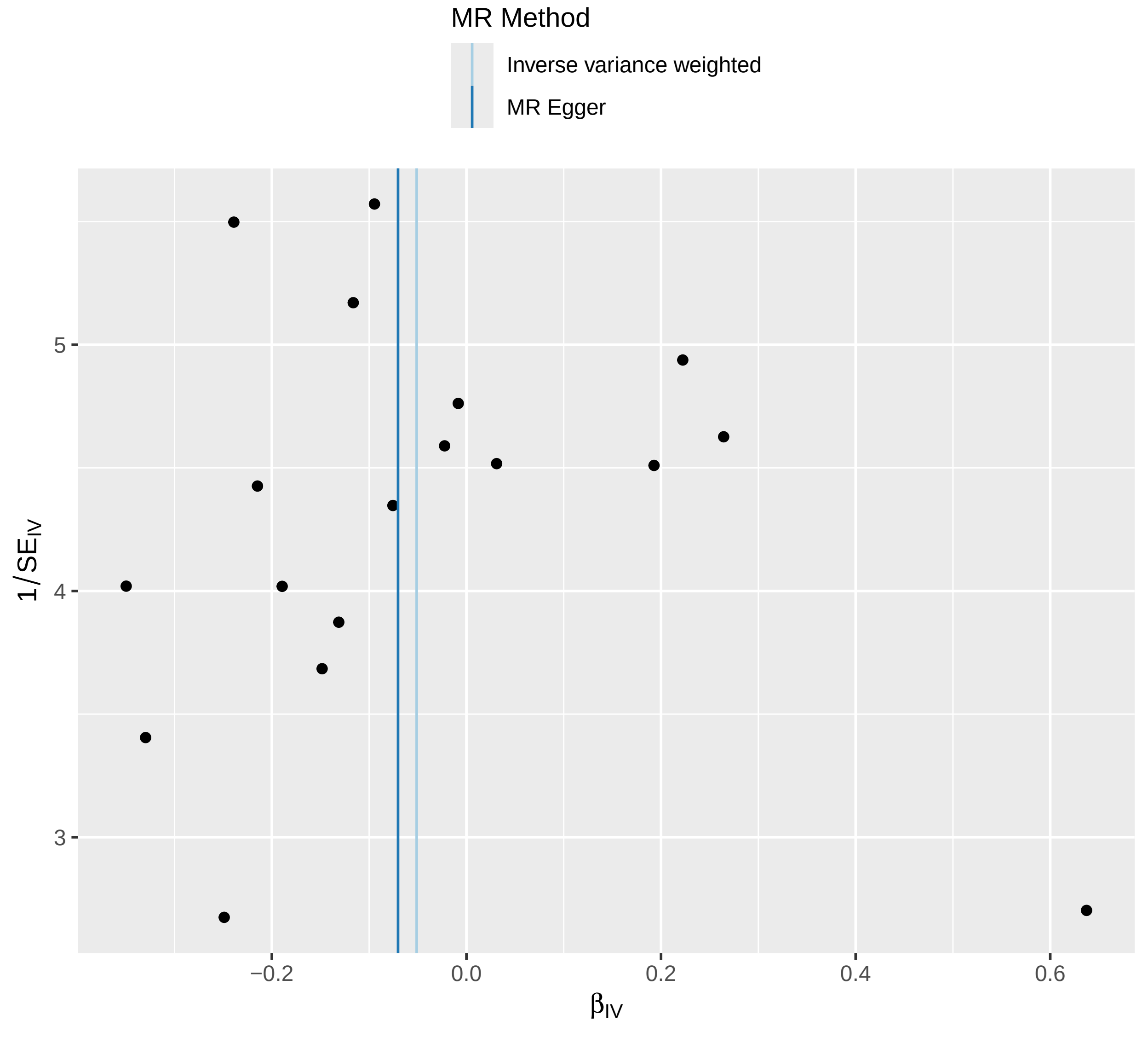

MR Method
Inverse variance weighted
MR Egger
5
4
3
1/SE_IV
−0.2
0.0
0.2
0.4
0.6
β_IV

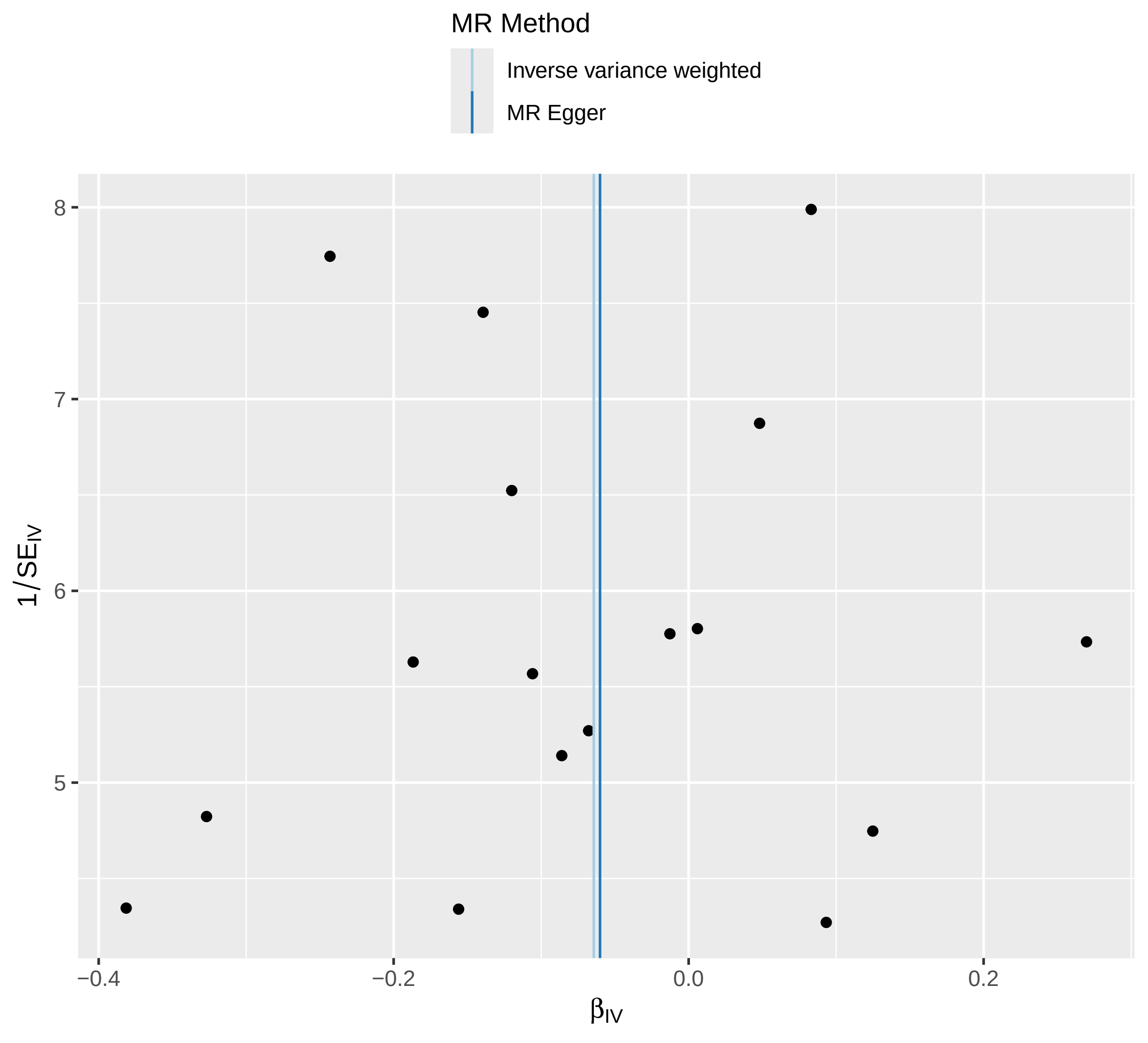

MR Method
Inverse variance weighted
MR Egger
8
7
6
5
1/SE$_{IV}$
−0.4
−0.2
0.0
0.2
β$_{IV}$

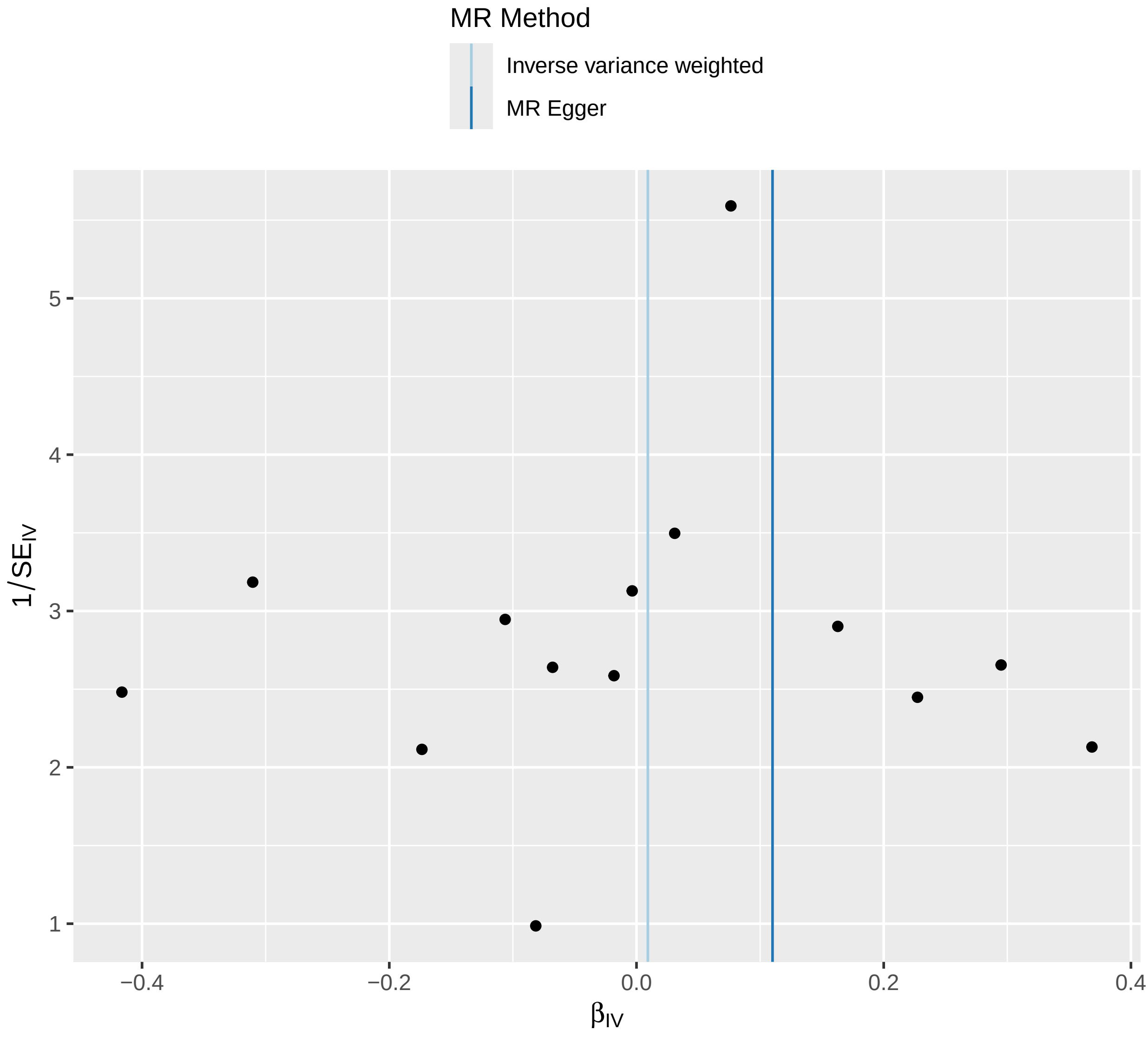

MR Method
Inverse variance weighted
MR Egger
1/SE$_{IV}$
5
4
3
2
1
−0.4
−0.2
0.0
0.2
0.4
β$_{IV}$

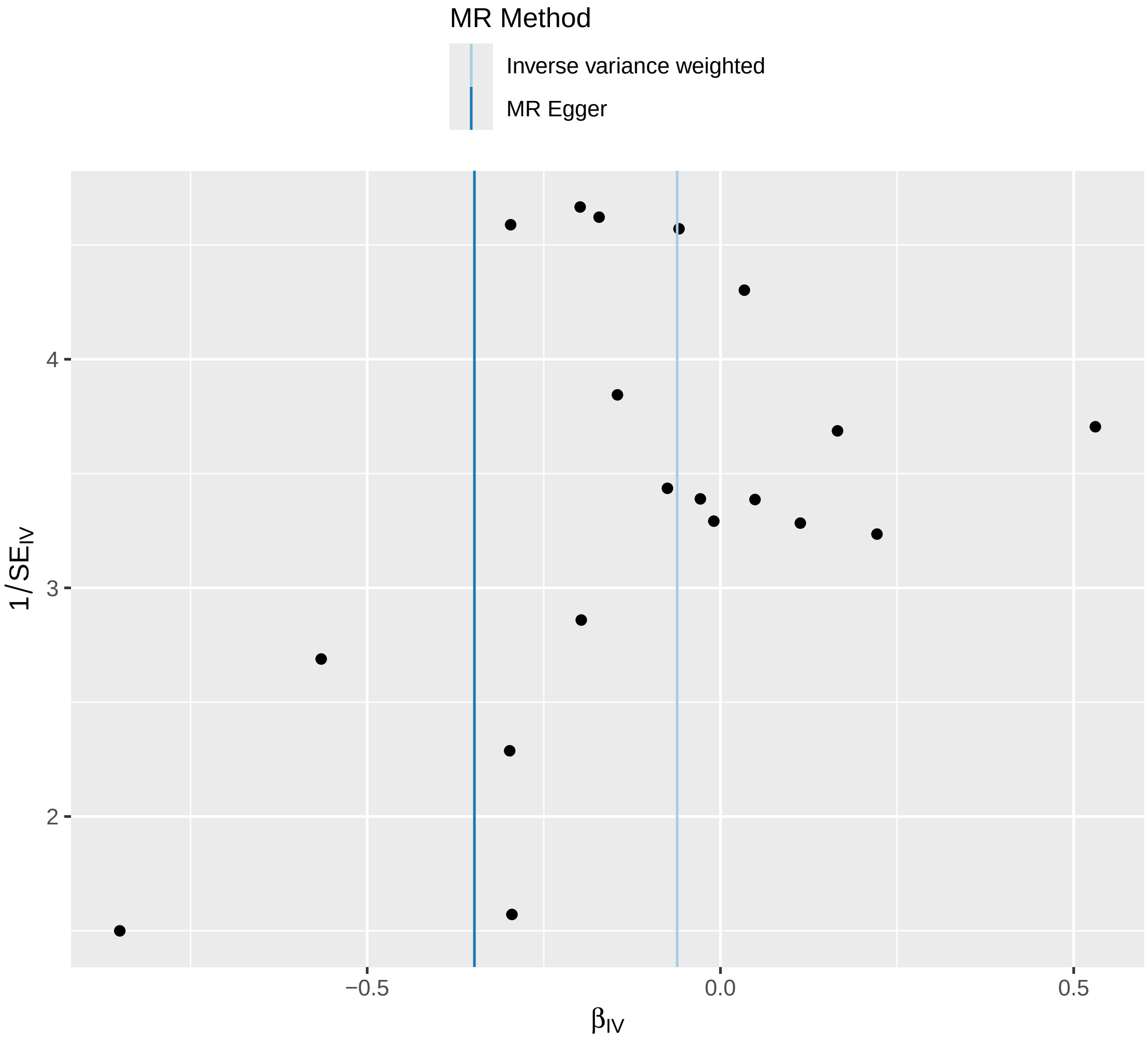
MR Method
Inverse variance weighted
MR Egger
4
3
2
1/SE_IV
−0.5
0.0
0.5
β_IV

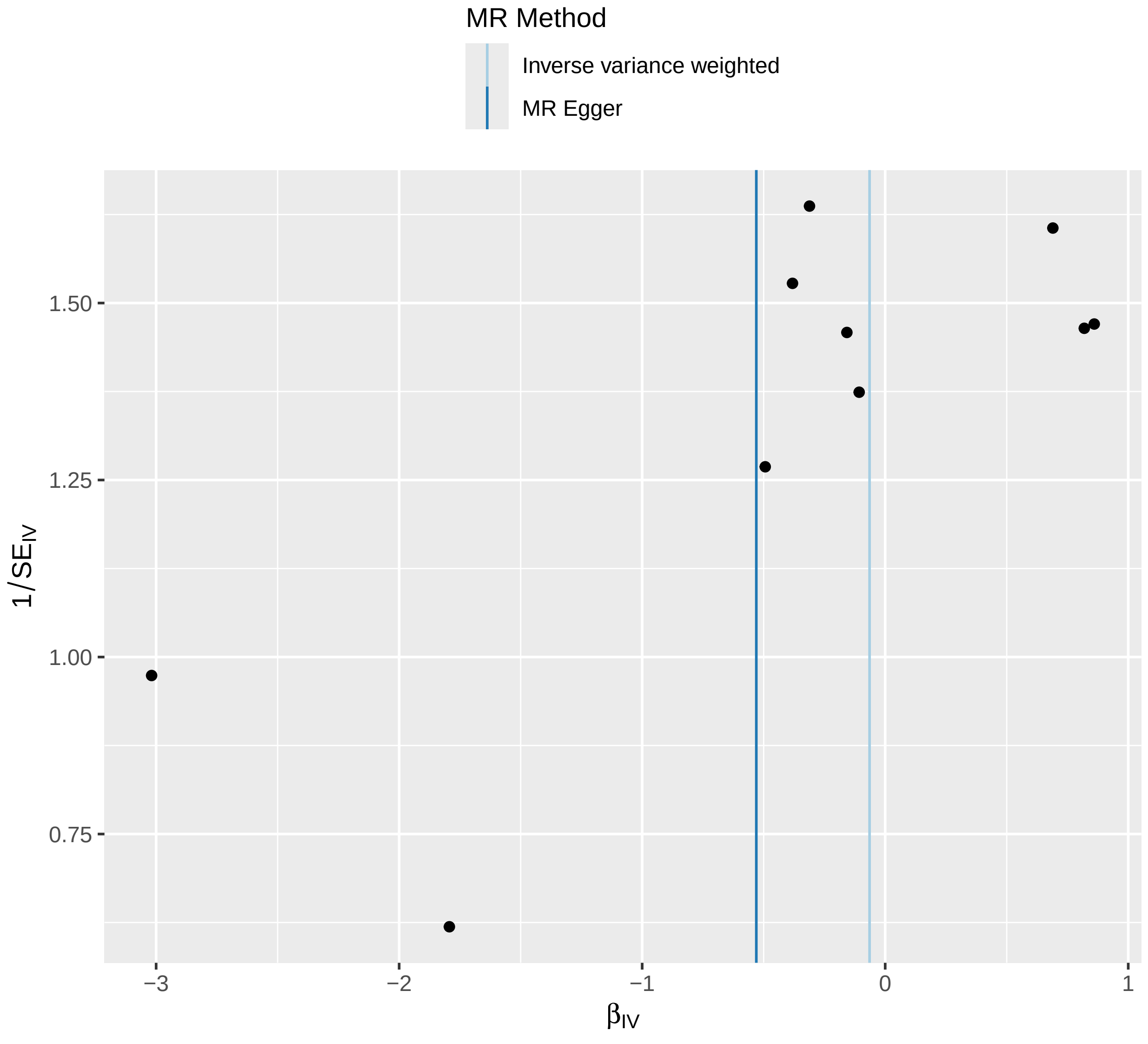
MR Method
Inverse variance weighted
MR Egger
1.50
1.25
1.00
0.75
1/SE$_{IV}$
−3
−2
−1
0
1
β$_{IV}$

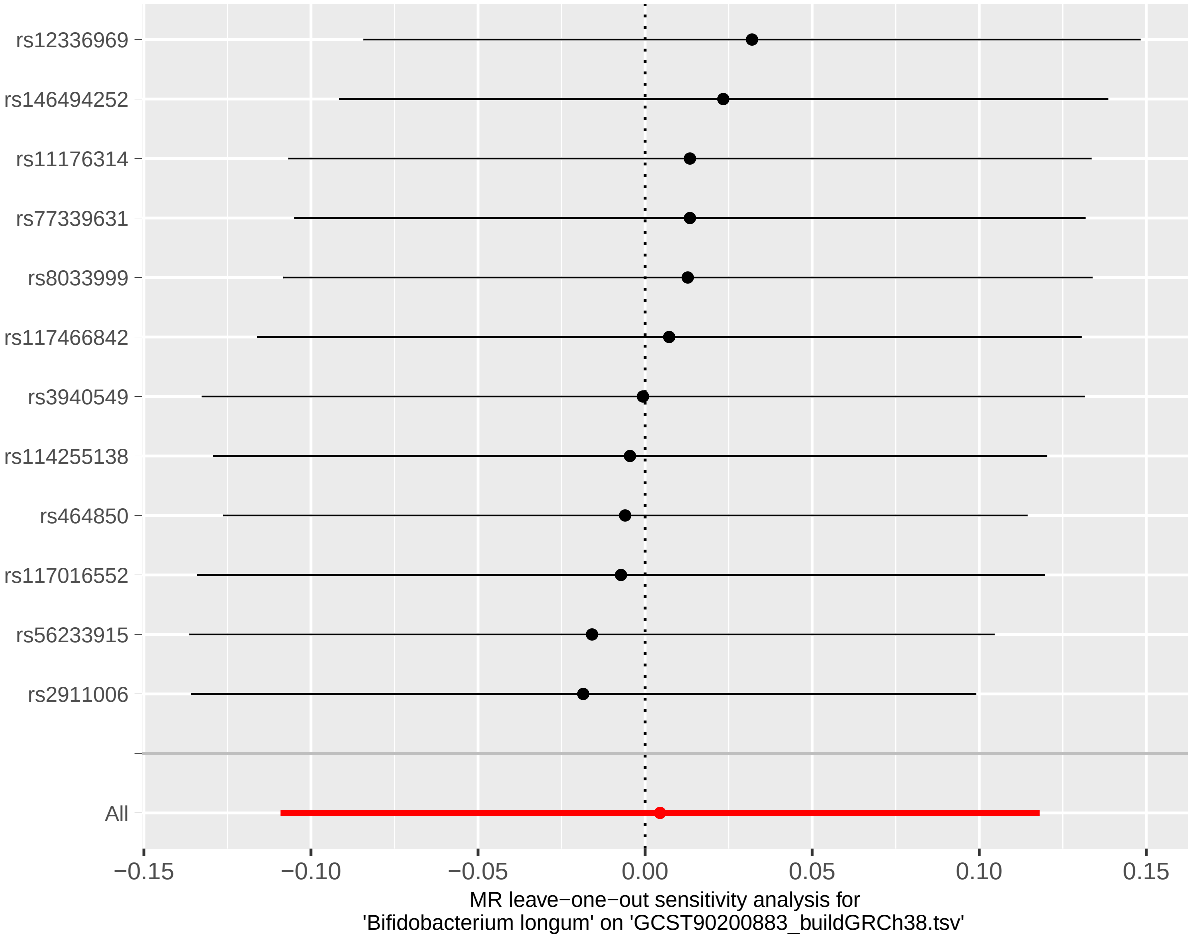
rs12336969
rs146494252
rs11176314
rs77339631
rs8033999
rs117466842
rs3940549
rs114255138
rs464850
rs117016552
rs56233915
rs2911006
All
−0.15
−0.10
−0.05
0.00
0.05
0.10
0.15
MR leave−one−out sensitivity analysis for
'Bifidobacterium longum' on 'GCST90200883_buildGRCh38.tsv'

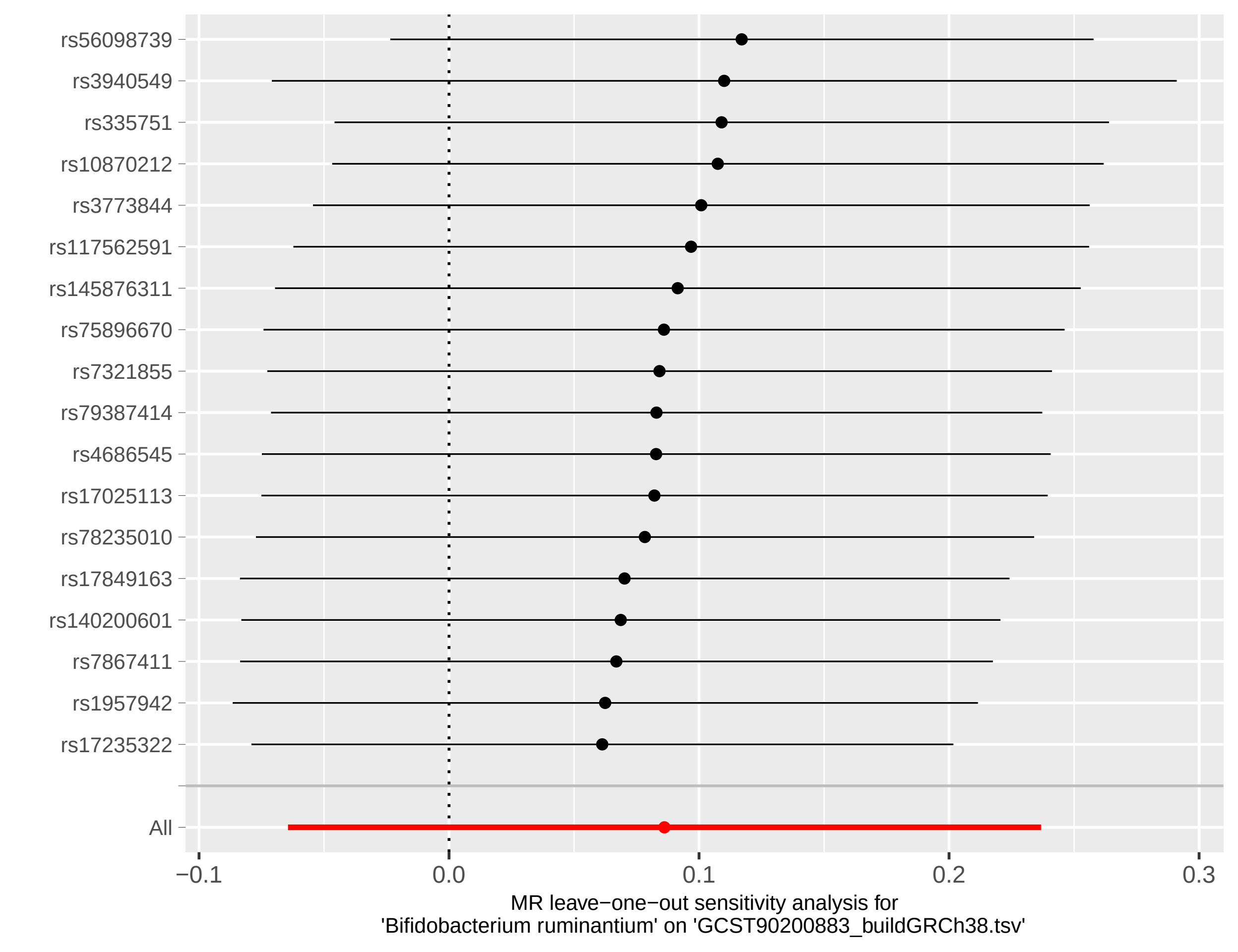
rs56098739
rs3940549
rs335751
rs10870212
rs3773844
rs117562591
rs145876311
rs75896670
rs7321855
rs79387414
rs4686545
rs17025113
rs78235010
rs17849163
rs140200601
rs7867411
rs1957942
rs17235322
All
−0.1
0.0
0.1
0.2
0.3
MR leave−one−out sensitivity analysis for
'Bifidobacterium ruminantium' on 'GCST90200883_buildGRCh38.tsv'

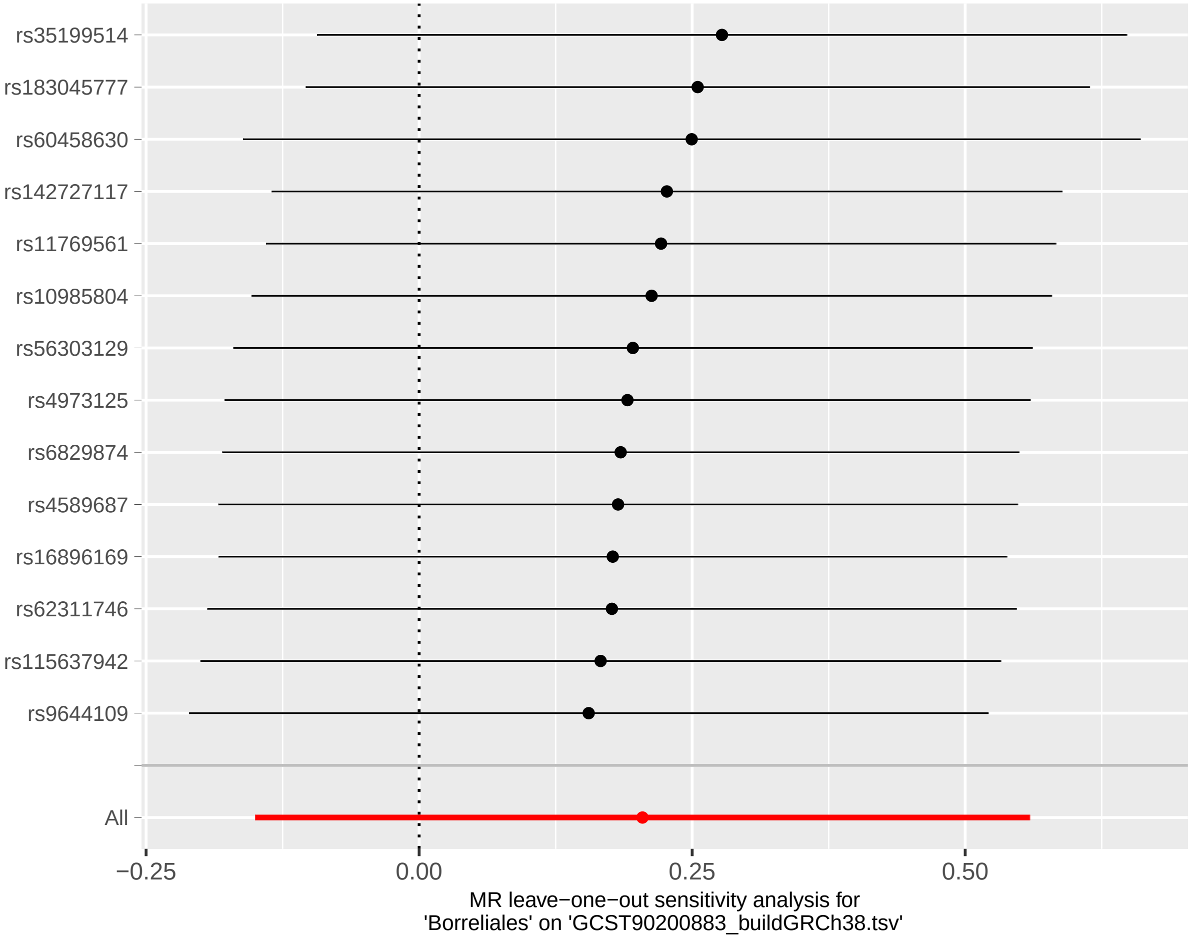

rs35199514
rs183045777
rs60458630
rs142727117
rs11769561
rs10985804
rs56303129
rs4973125
rs6829874
rs4589687
rs16896169
rs62311746
rs115637942
rs9644109
All
−0.25
0.00
0.25
0.50
MR leave−one−out sensitivity analysis for
'Borreliales' on 'GCST90200883_buildGRCh38.tsv'

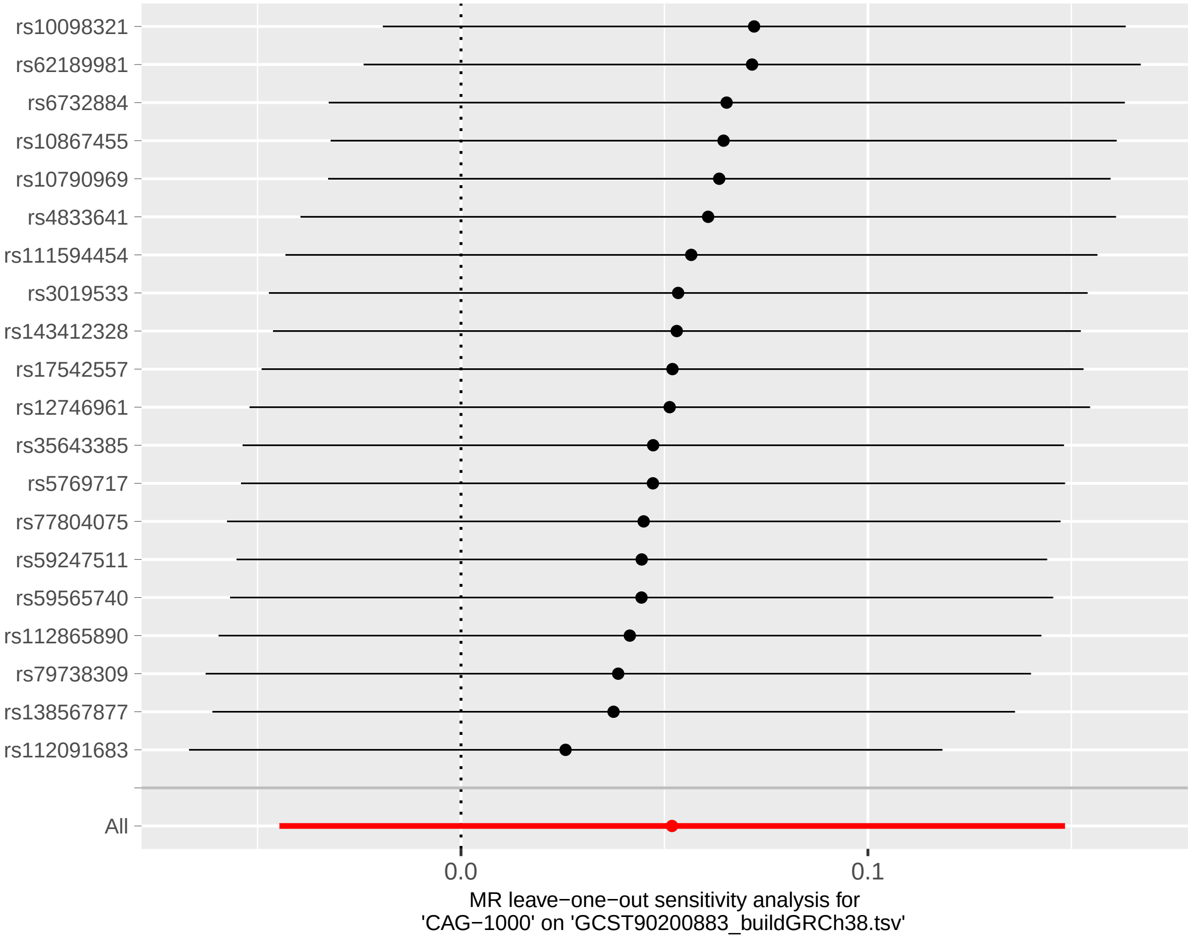

rs10098321
rs62189981
rs6732884
rs10867455
rs10790969
rs4833641
rs111594454
rs3019533
rs143412328
rs17542557
rs12746961
rs35643385
rs5769717
rs77804075
rs59247511
rs59565740
rs112865890
rs79738309
rs138567877
rs112091683
All
0.0
0.1
MR leave−one−out sensitivity analysis for
'CAG−1000' on 'GCST90200883_buildGRCh38.tsv'

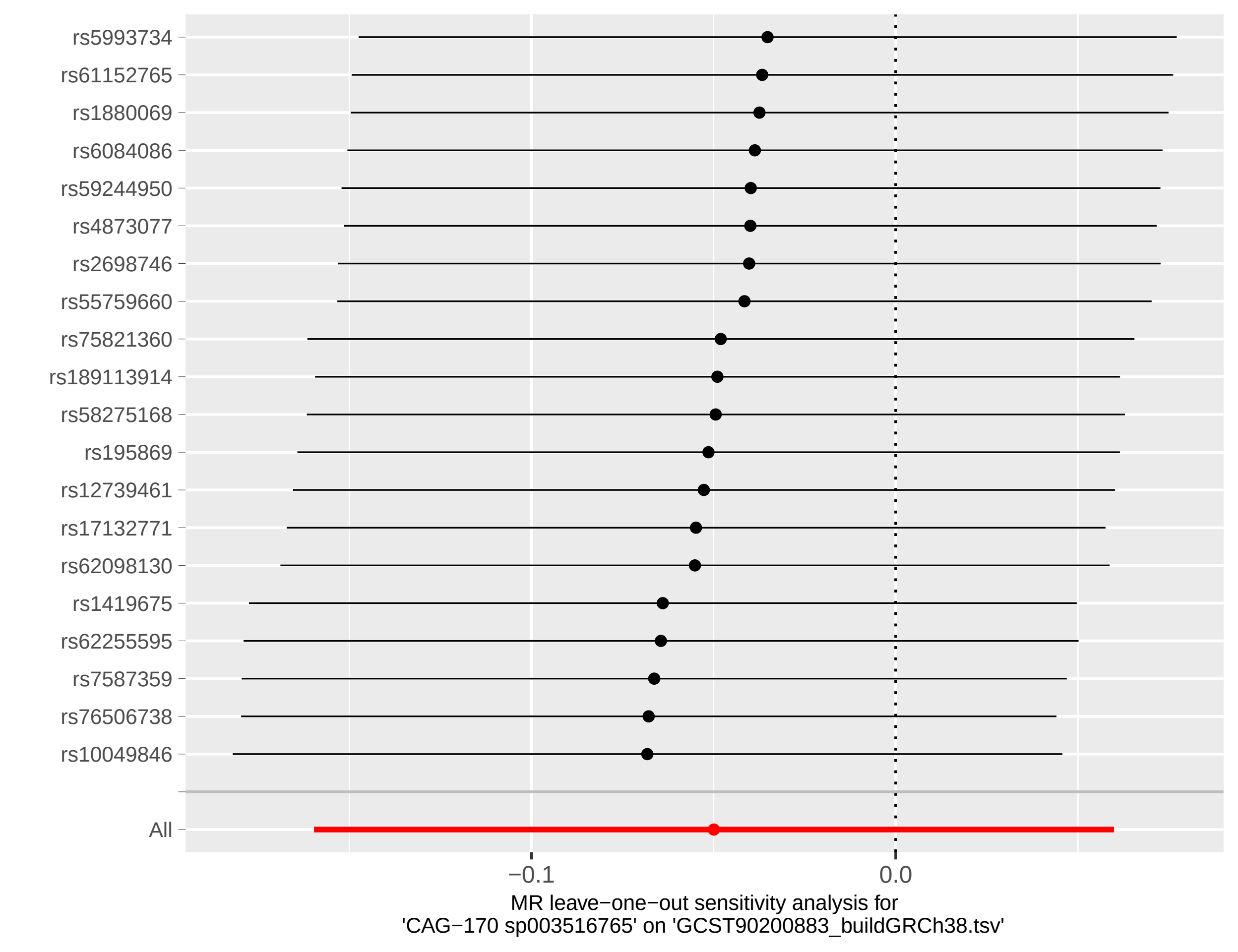
rs5993734
rs61152765
rs1880069
rs6084086
rs59244950
rs4873077
rs2698746
rs55759660
rs75821360
rs189113914
rs58275168
rs195869
rs12739461
rs17132771
rs62098130
rs1419675
rs62255595
rs7587359
rs76506738
rs10049846
All
−0.1
0.0
MR leave−one−out sensitivity analysis for
'CAG−170 sp003516765' on 'GCST90200883_buildGRCh38.tsv'

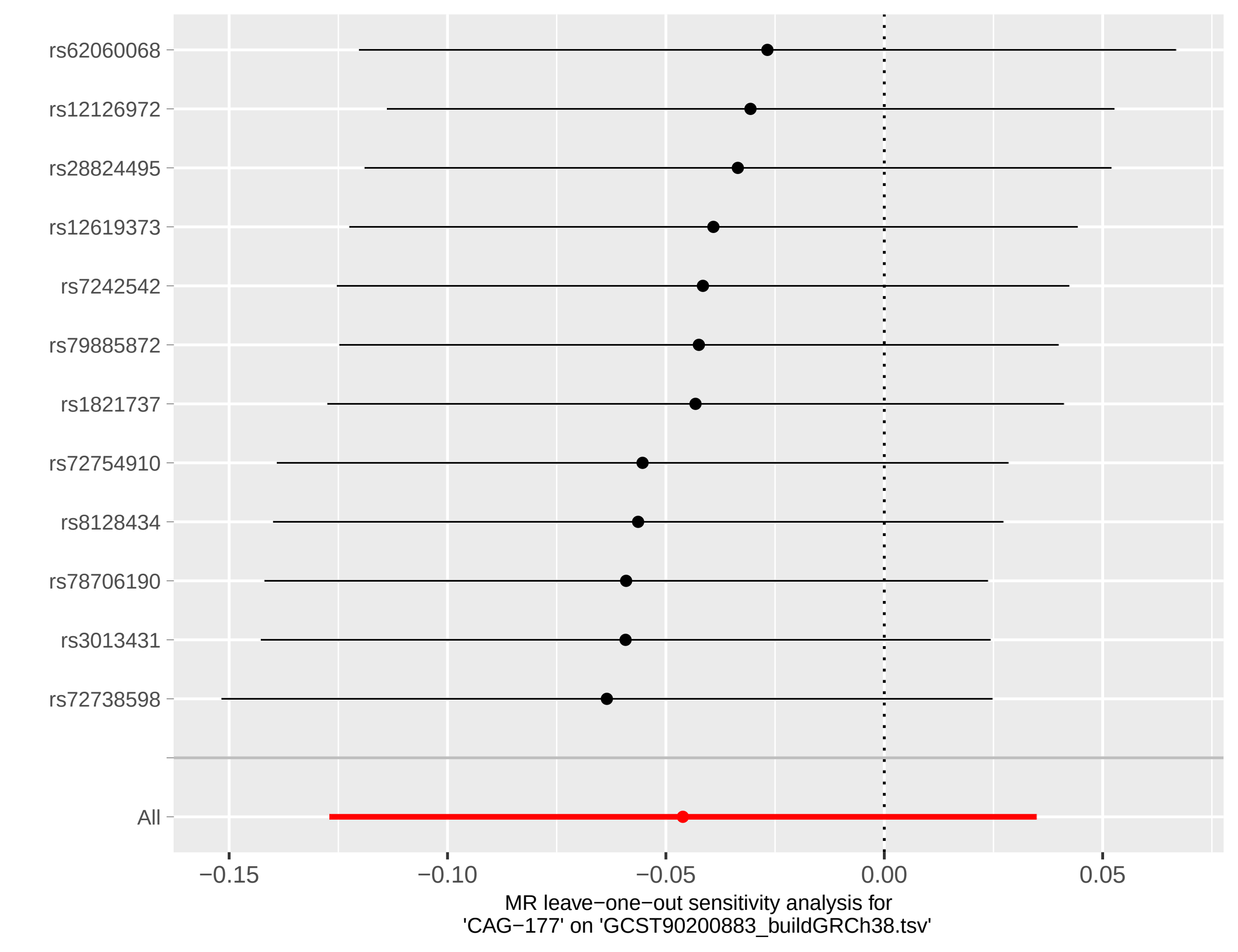
rs62060068
rs12126972
rs28824495
rs12619373
rs7242542
rs79885872
rs1821737
rs72754910
rs8128434
rs78706190
rs3013431
rs72738598
All
−0.15
−0.10
−0.05
0.00
0.05
MR leave−one−out sensitivity analysis for
'CAG−177' on 'GCST90200883_buildGRCh38.tsv'

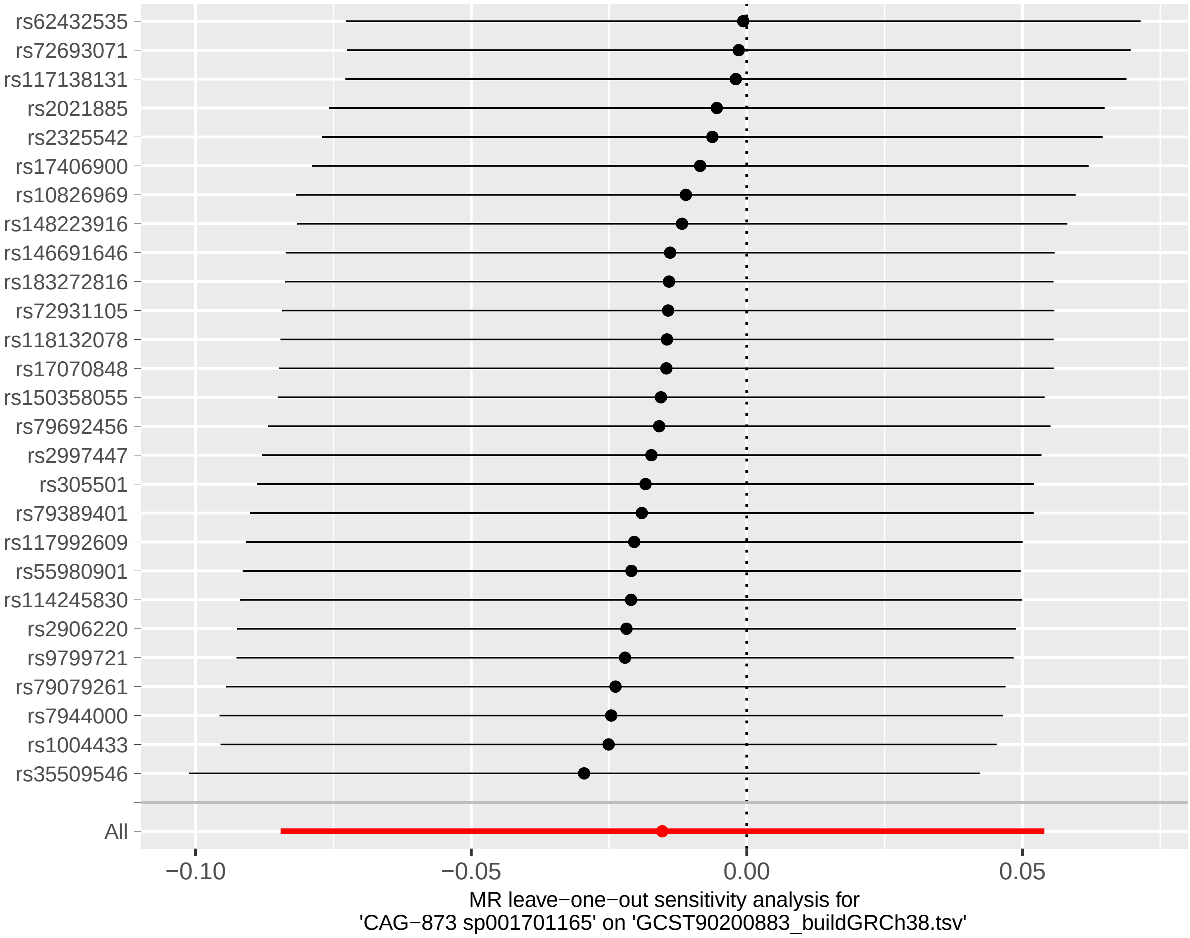
rs62432535
rs72693071
rs117138131
rs2021885
rs2325542
rs17406900
rs10826969
rs148223916
rs146691646
rs183272816
rs72931105
rs118132078
rs17070848
rs150358055
rs79692456
rs2997447
rs305501
rs79389401
rs117992609
rs55980901
rs114245830
rs2906220
rs9799721
rs79079261
rs7944000
rs1004433
rs35509546
All
−0.10
−0.05
0.00
0.05
MR leave−one−out sensitivity analysis for
'CAG−873 sp001701165' on 'GCST90200883_buildGRCh38.tsv'

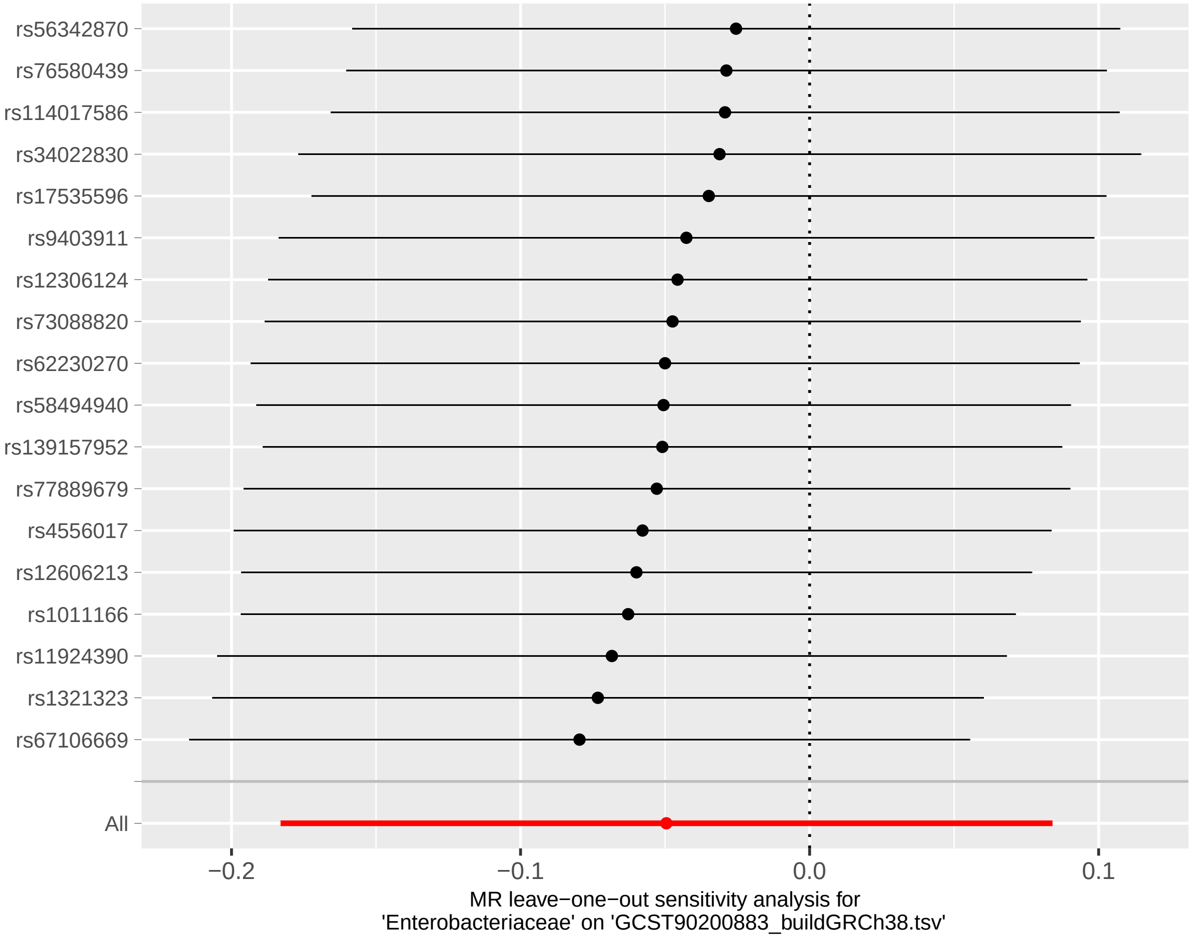

rs56342870
rs76580439
rs114017586
rs34022830
rs17535596
rs9403911
rs12306124
rs73088820
rs62230270
rs58494940
rs139157952
rs77889679
rs4556017
rs12606213
rs1011166
rs11924390
rs1321323
rs67106669
All
−0.2
−0.1
0.0
0.1
MR leave−one−out sensitivity analysis for
'Enterobacteriaceae' on 'GCST90200883_buildGRCh38.tsv'

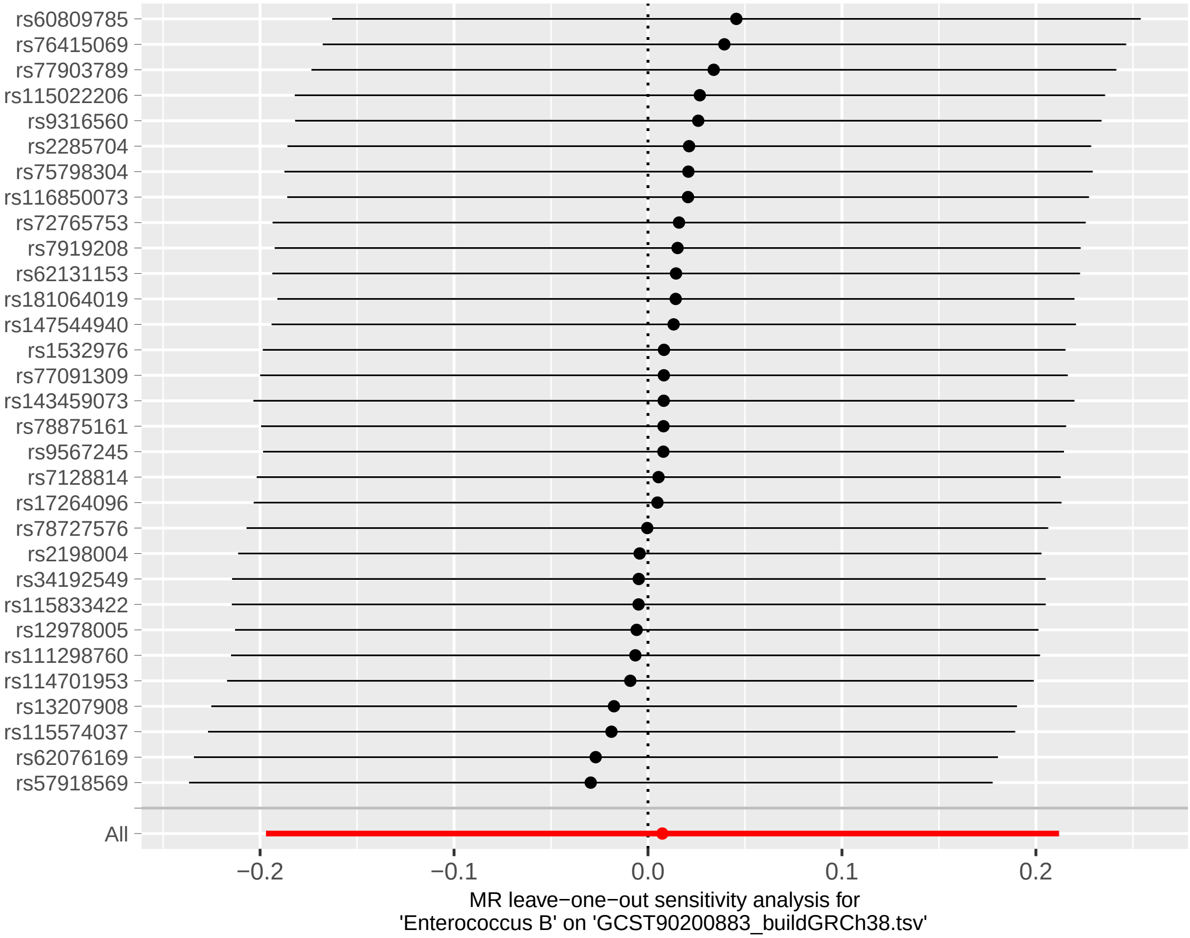
rs60809785
rs76415069
rs77903789
rs115022206
rs9316560
rs2285704
rs75798304
rs116850073
rs72765753
rs7919208
rs62131153
rs181064019
rs147544940
rs1532976
rs77091309
rs143459073
rs78875161
rs9567245
rs7128814
rs17264096
rs78727576
rs2198004
rs34192549
rs115833422
rs12978005
rs111298760
rs114701953
rs13207908
rs115574037
rs62076169
rs57918569
All
−0.2
−0.1
0.0
0.1
0.2
MR leave−one−out sensitivity analysis for
'Enterococcus B' on 'GCST90200883_buildGRCh38.tsv'

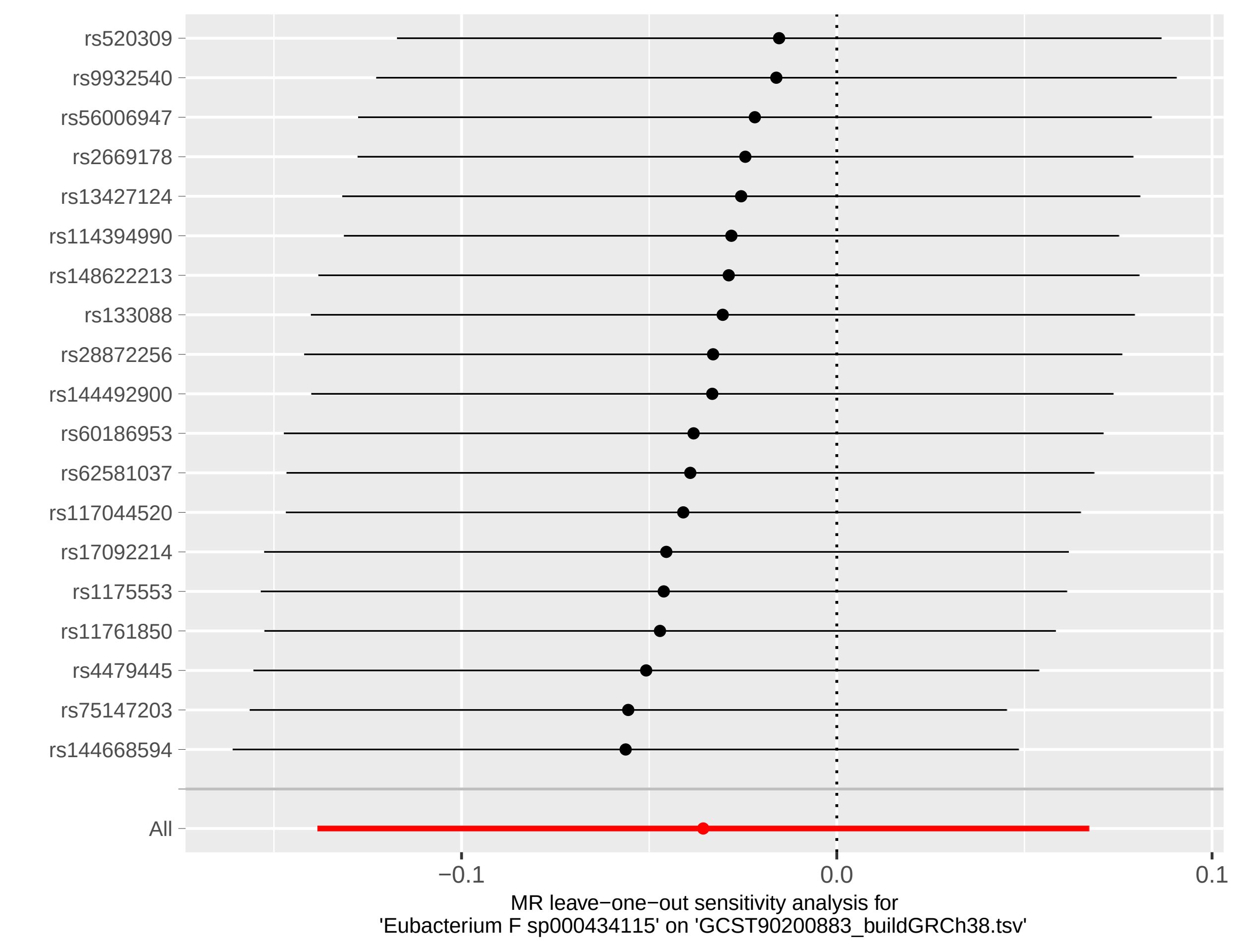
rs520309
rs9932540
rs56006947
rs2669178
rs13427124
rs114394990
rs148622213
rs133088
rs28872256
rs144492900
rs60186953
rs62581037
rs117044520
rs17092214
rs1175553
rs11761850
rs4479445
rs75147203
rs144668594
All
−0.1
0.0
0.1
MR leave−one−out sensitivity analysis for
'Eubacterium F sp000434115' on 'GCST90200883_buildGRCh38.tsv'

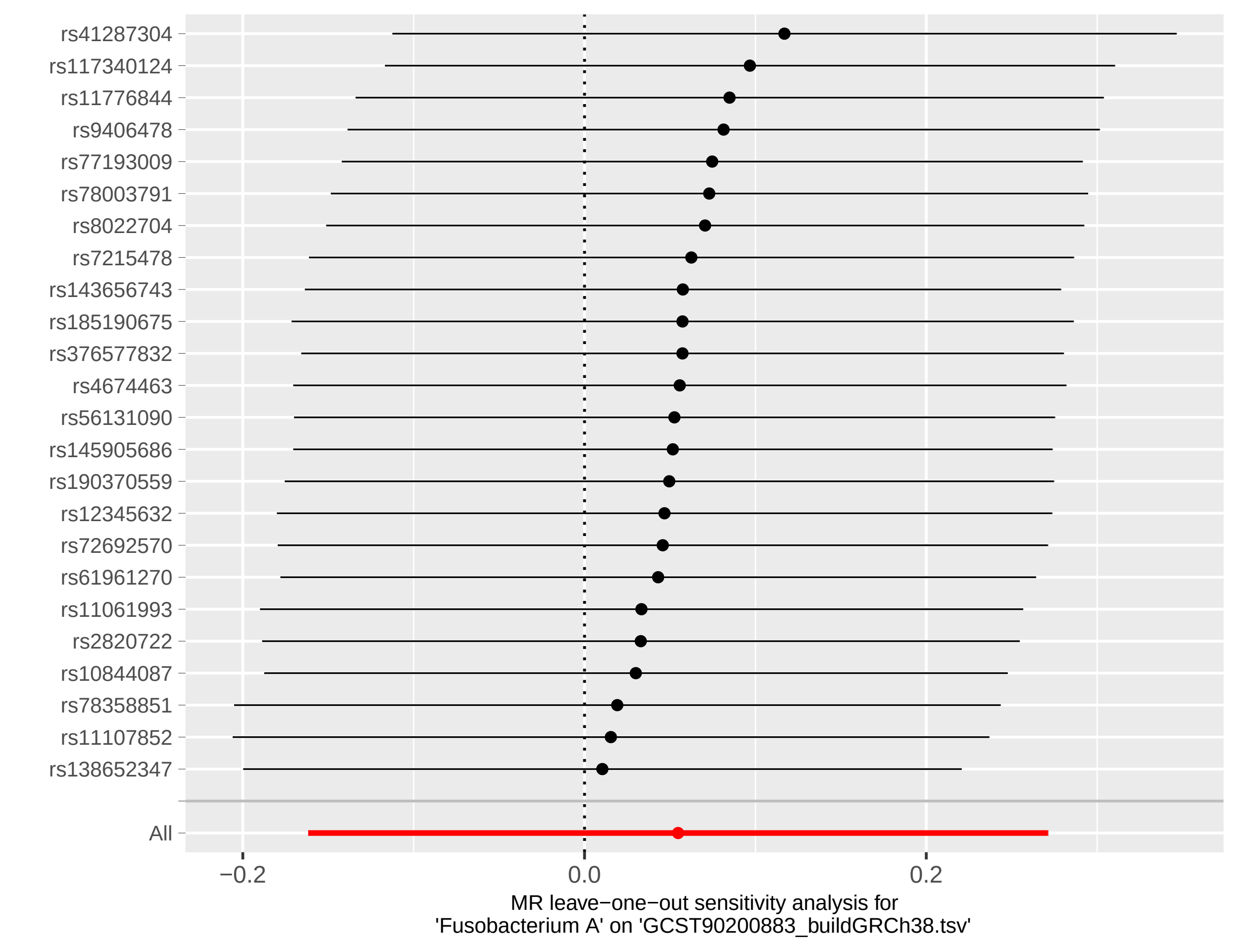
rs41287304
rs117340124
rs11776844
rs9406478
rs77193009
rs78003791
rs8022704
rs7215478
rs143656743
rs185190675
rs376577832
rs4674463
rs56131090
rs145905686
rs190370559
rs12345632
rs72692570
rs61961270
rs11061993
rs2820722
rs10844087
rs78358851
rs11107852
rs138652347
All
−0.2
0.0
0.2
MR leave−one−out sensitivity analysis for
'Fusobacterium A' on 'GCST90200883_buildGRCh38.tsv'

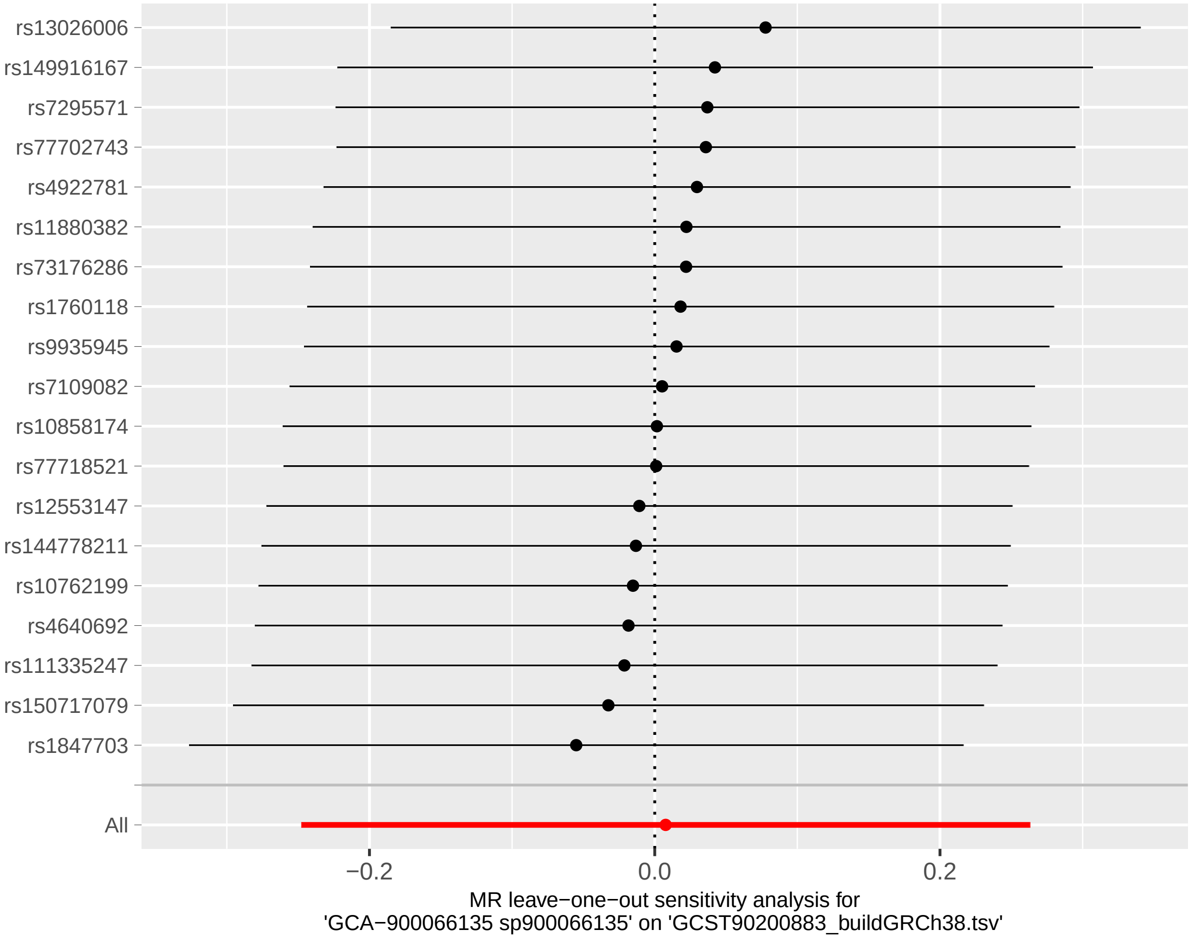
rs13026006
rs149916167
rs7295571
rs77702743
rs4922781
rs11880382
rs73176286
rs1760118
rs9935945
rs7109082
rs10858174
rs77718521
rs12553147
rs144778211
rs10762199
rs4640692
rs111335247
rs150717079
rs1847703
All
−0.2
0.0
0.2
MR leave−one−out sensitivity analysis for
'GCA−900066135 sp900066135' on 'GCST90200883_buildGRCh38.tsv'

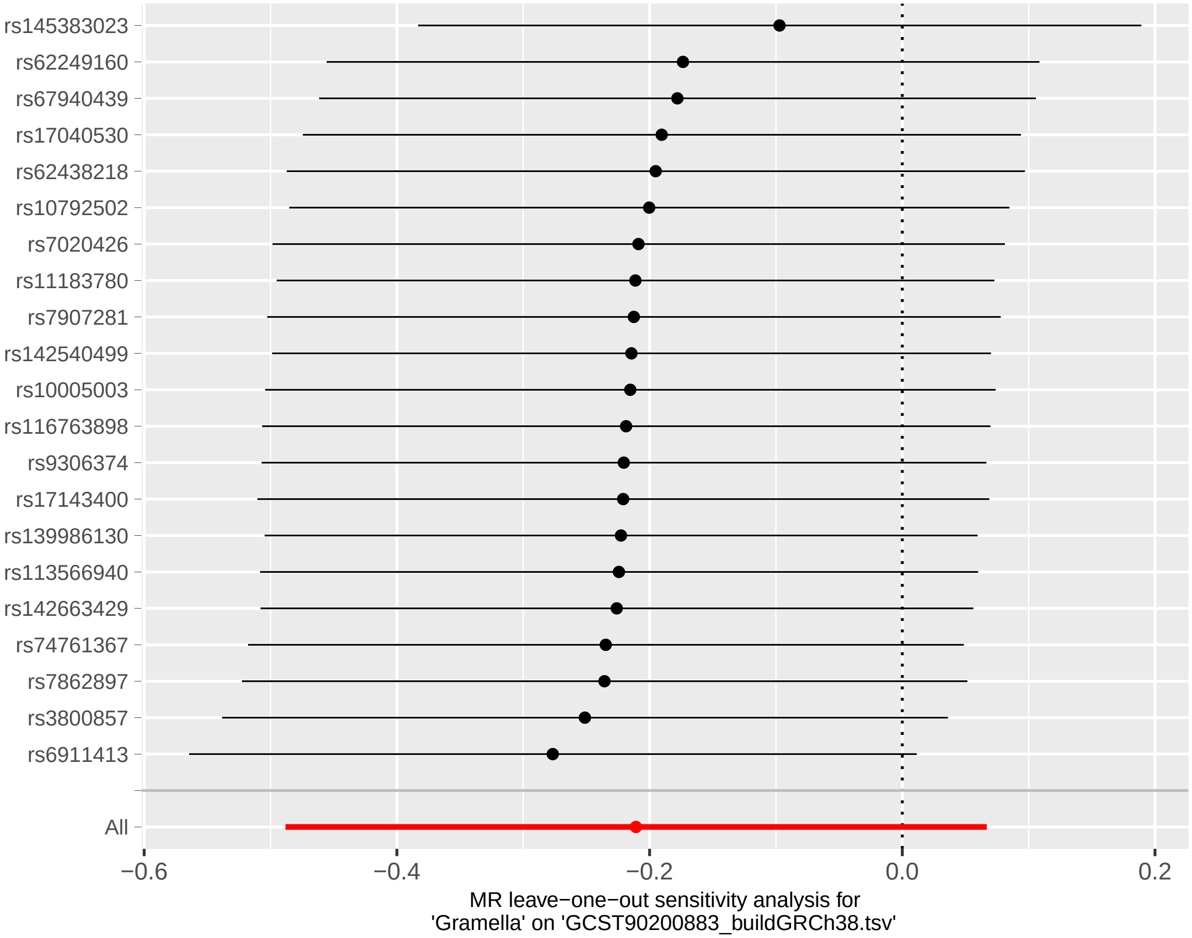

rs145383023
rs62249160
rs67940439
rs17040530
rs62438218
rs10792502
rs7020426
rs11183780
rs7907281
rs142540499
rs10005003
rs116763898
rs9306374
rs17143400
rs139986130
rs113566940
rs142663429
rs74761367
rs7862897
rs3800857
rs6911413
All
−0.6
−0.4
−0.2
0.0
0.2
MR leave−one−out sensitivity analysis for
'Gramella' on 'GCST90200883_buildGRCh38.tsv'

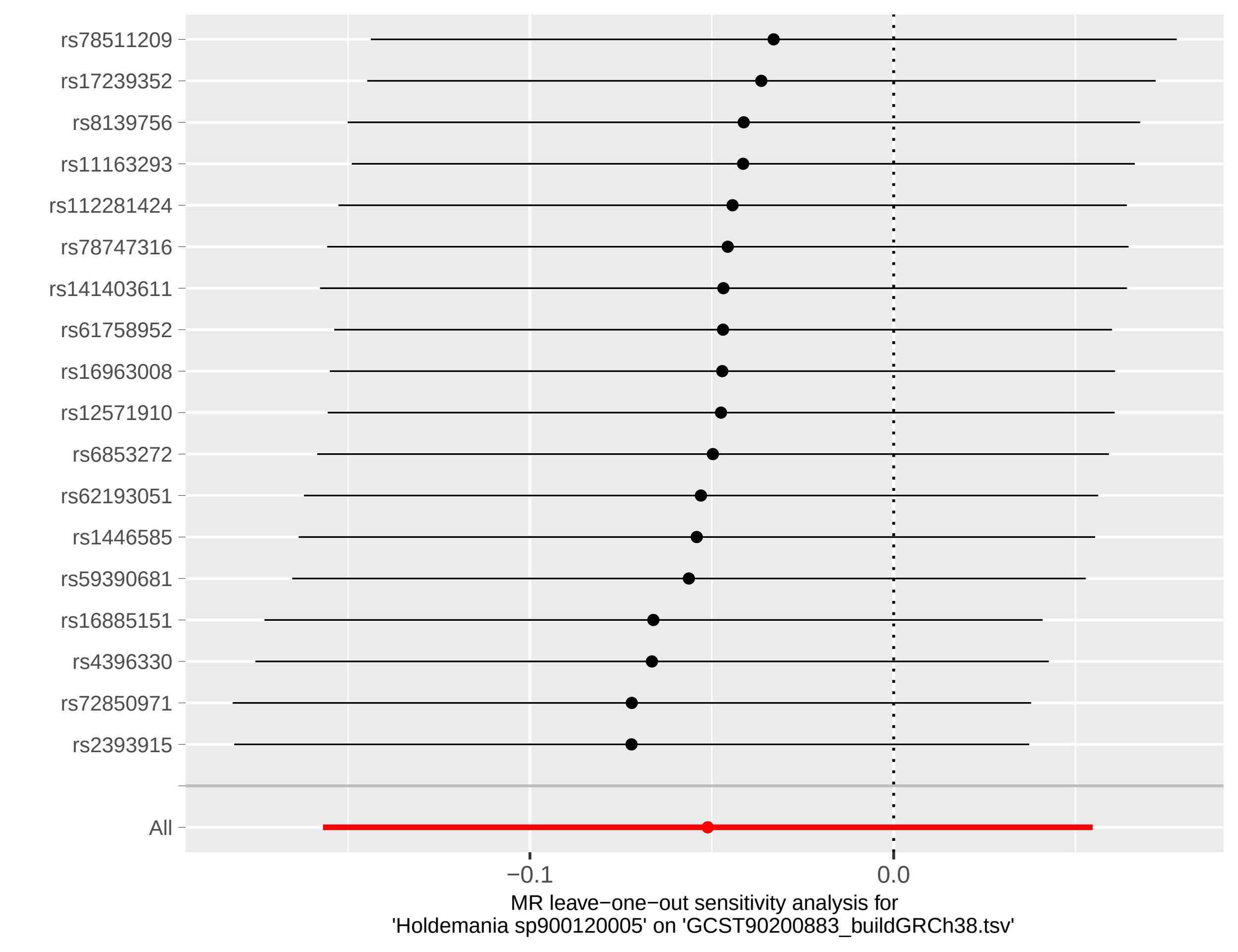
rs78511209
rs17239352
rs8139756
rs11163293
rs112281424
rs78747316
rs141403611
rs61758952
rs16963008
rs12571910
rs6853272
rs62193051
rs1446585
rs59390681
rs16885151
rs4396330
rs72850971
rs2393915
All
−0.1
0.0
MR leave−one−out sensitivity analysis for
'Holdemania sp900120005' on 'GCST90200883_buildGRCh38.tsv'

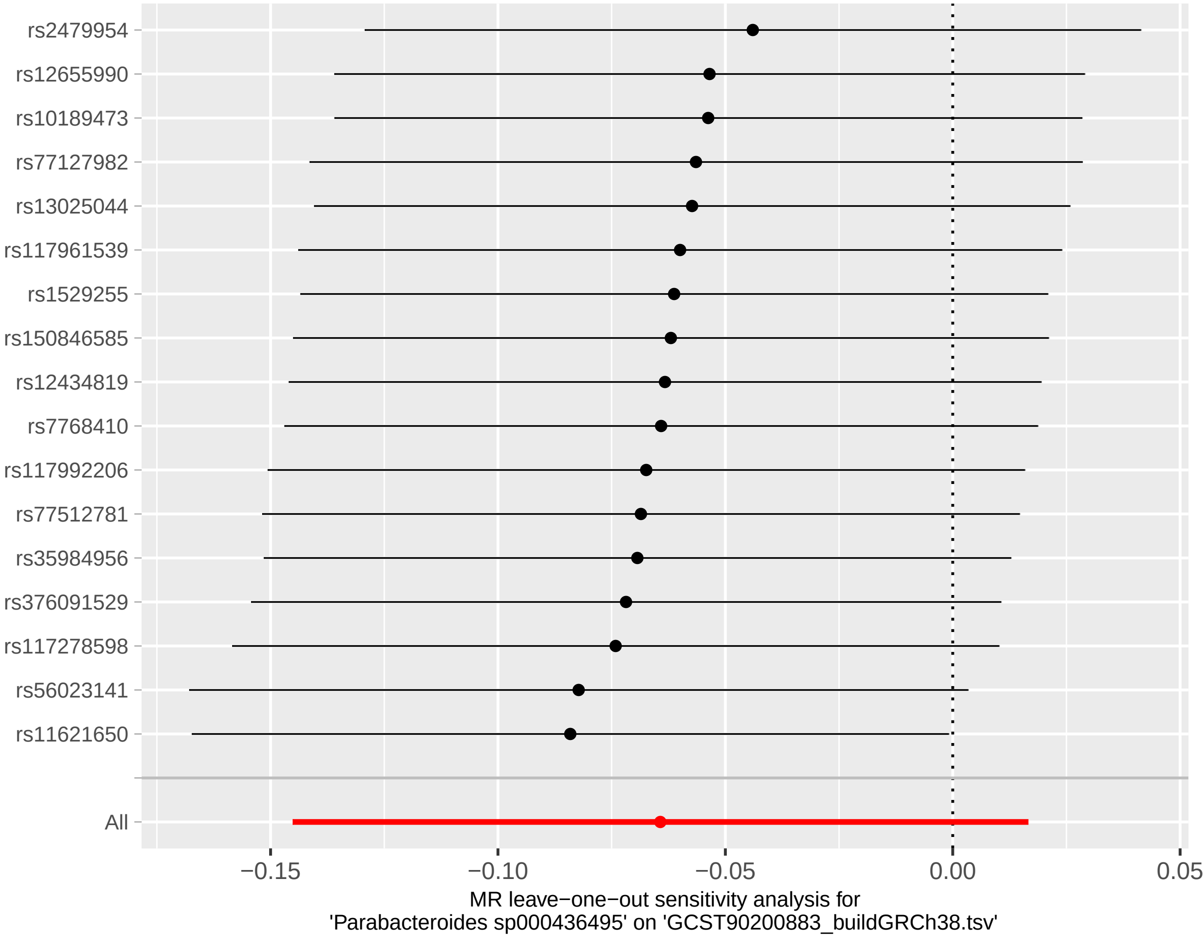

rs2479954
rs12655990
rs10189473
rs77127982
rs13025044
rs117961539
rs1529255
rs150846585
rs12434819
rs7768410
rs117992206
rs77512781
rs35984956
rs376091529
rs117278598
rs56023141
rs11621650
All
−0.15
−0.10
−0.05
0.00
0.05
MR leave−one−out sensitivity analysis for
'Parabacteroides sp000436495' on 'GCST90200883_buildGRCh38.tsv'

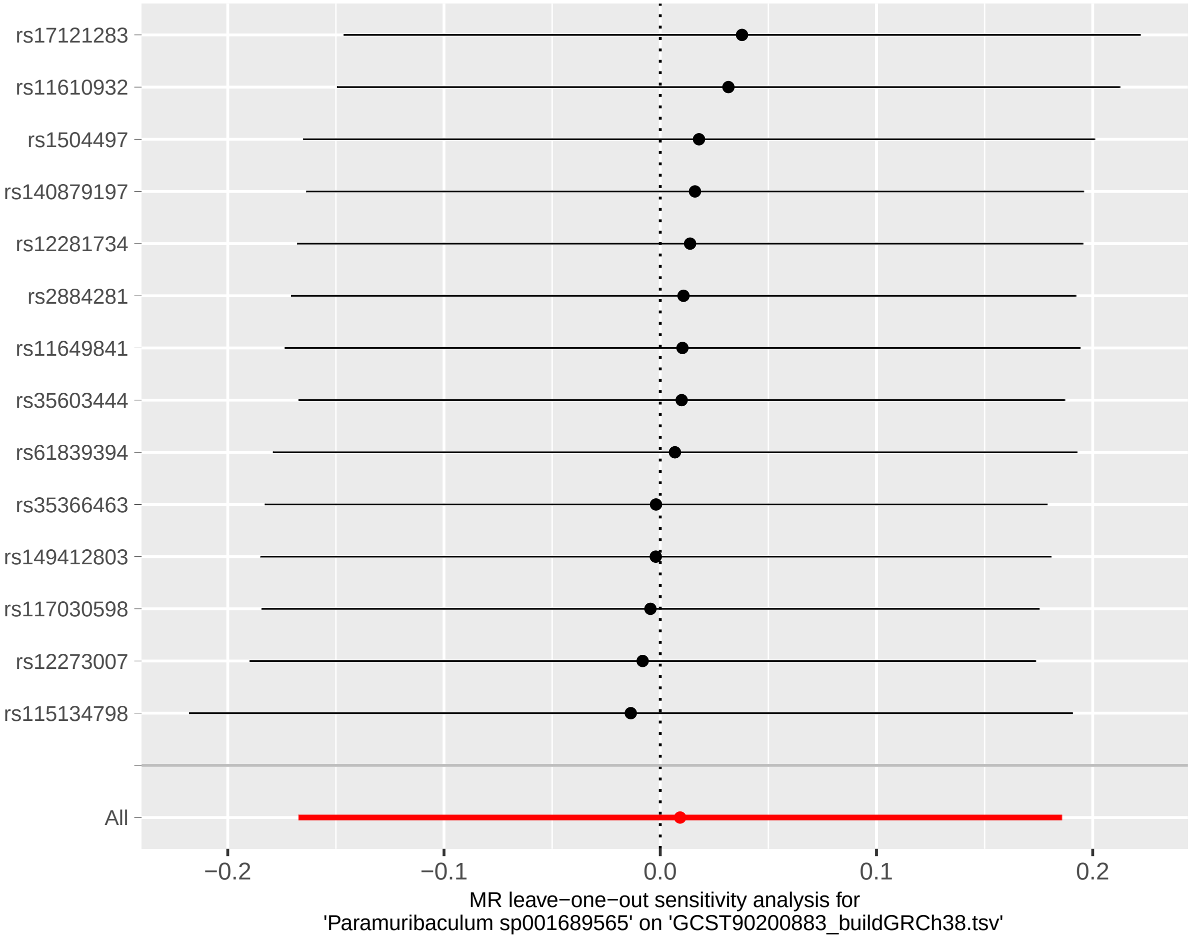

rs17121283
rs11610932
rs1504497
rs140879197
rs12281734
rs2884281
rs11649841
rs35603444
rs61839394
rs35366463
rs149412803
rs117030598
rs12273007
rs115134798
All
−0.2
−0.1
0.0
0.1
0.2
MR leave−one−out sensitivity analysis for
'Paramuribaculum sp001689565' on 'GCST90200883_buildGRCh38.tsv'

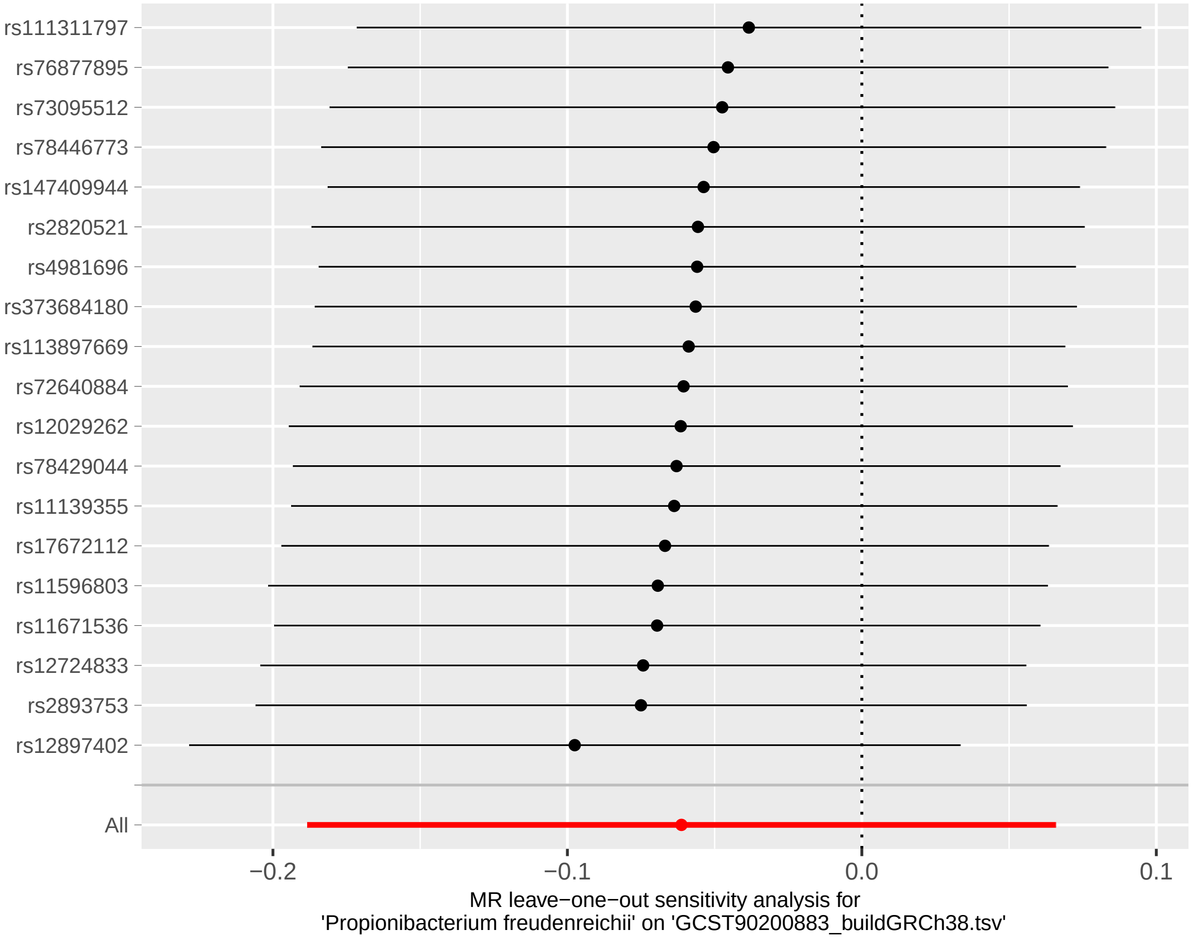

rs111311797
rs76877895
rs73095512
rs78446773
rs147409944
rs2820521
rs4981696
rs373684180
rs113897669
rs72640884
rs12029262
rs78429044
rs11139355
rs17672112
rs11596803
rs11671536
rs12724833
rs2893753
rs12897402
All
−0.2
−0.1
0.0
0.1
MR leave−one−out sensitivity analysis for
'Propionibacterium freudenreichii' on 'GCST90200883_buildGRCh38.tsv'

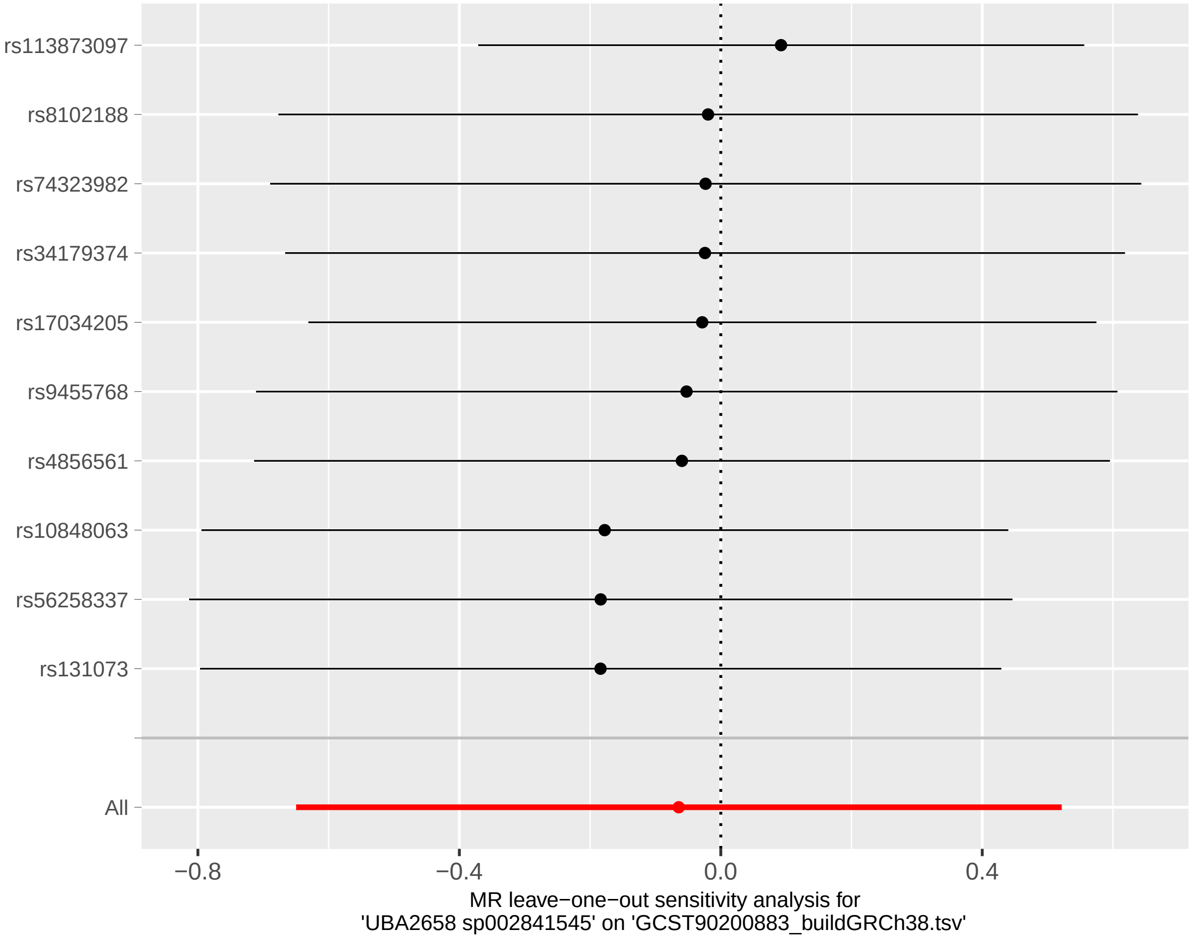
rs113873097
rs8102188
rs74323982
rs34179374
rs17034205
rs9455768
rs4856561
rs10848063
rs56258337
rs131073
All
−0.8
−0.4
0.0
0.4
MR leave−one−out sensitivity analysis for
'UBA2658 sp002841545' on 'GCST90200883_buildGRCh38.tsv'

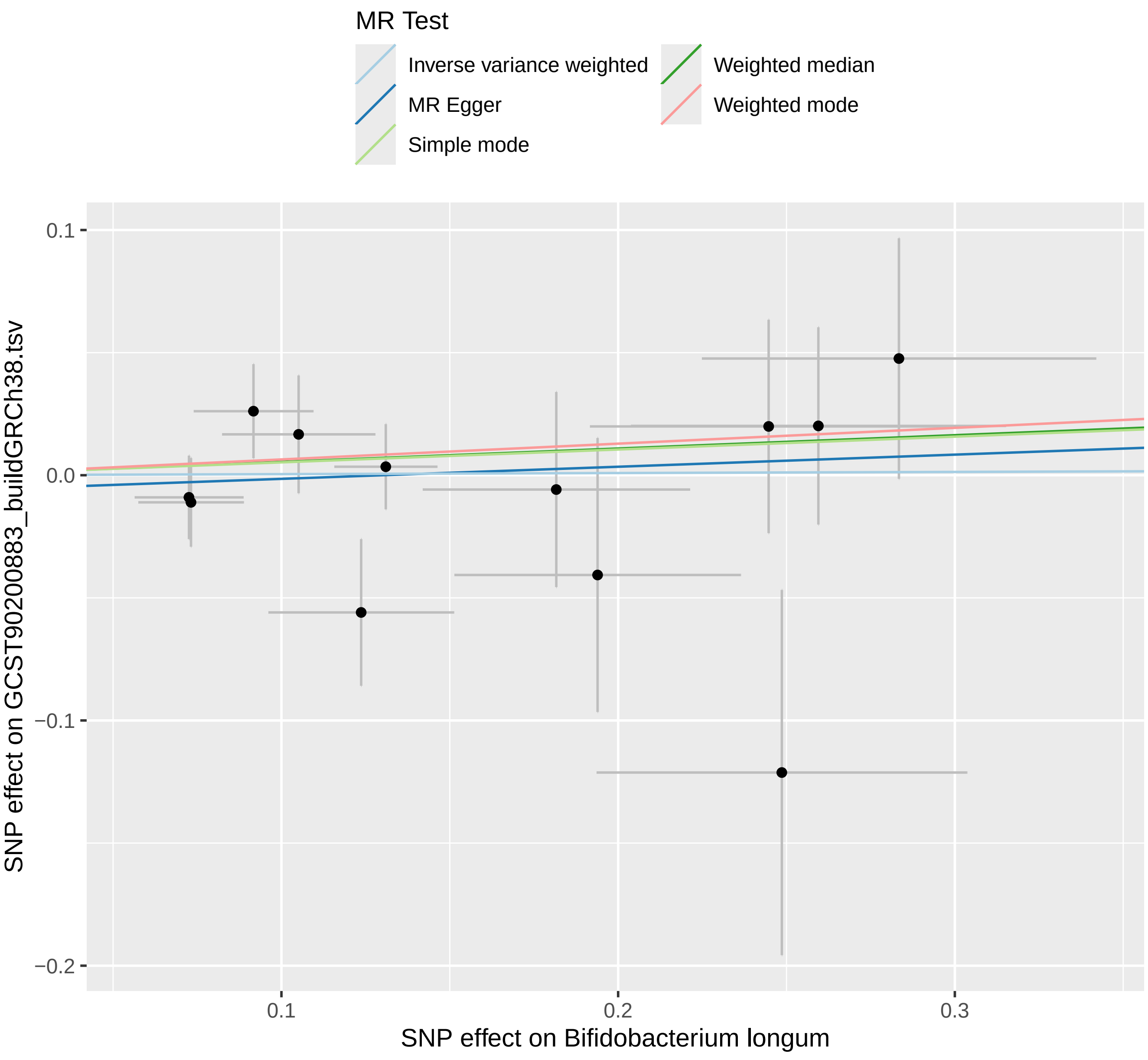
MR Test
Inverse variance weighted
MR Egger
Simple mode
Weighted median
Weighted mode
0.1
0.0
−0.1
−0.2
0.1
0.2
0.3
SNP effect on GCST90200883_buildGRCh38.tsv
SNP effect on Bifidobacterium longum

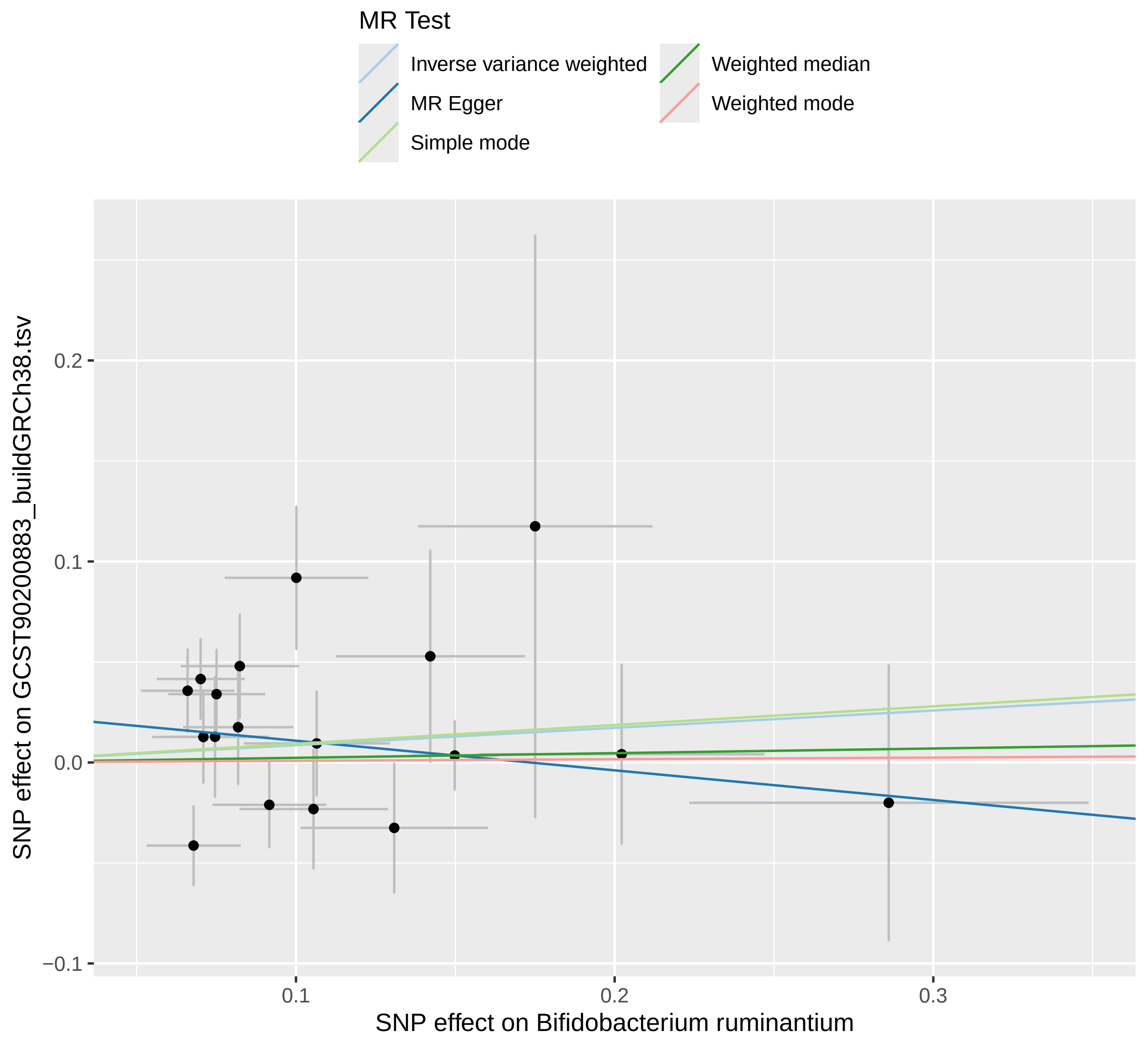
MR Test
Inverse variance weighted
MR Egger
Simple mode
Weighted median
Weighted mode
SNP effect on GCST90200883_buildGRCh38.tsv
0.2
0.1
0.0
−0.1
0.1
0.2
0.3
SNP effect on Bifidobacterium ruminantium

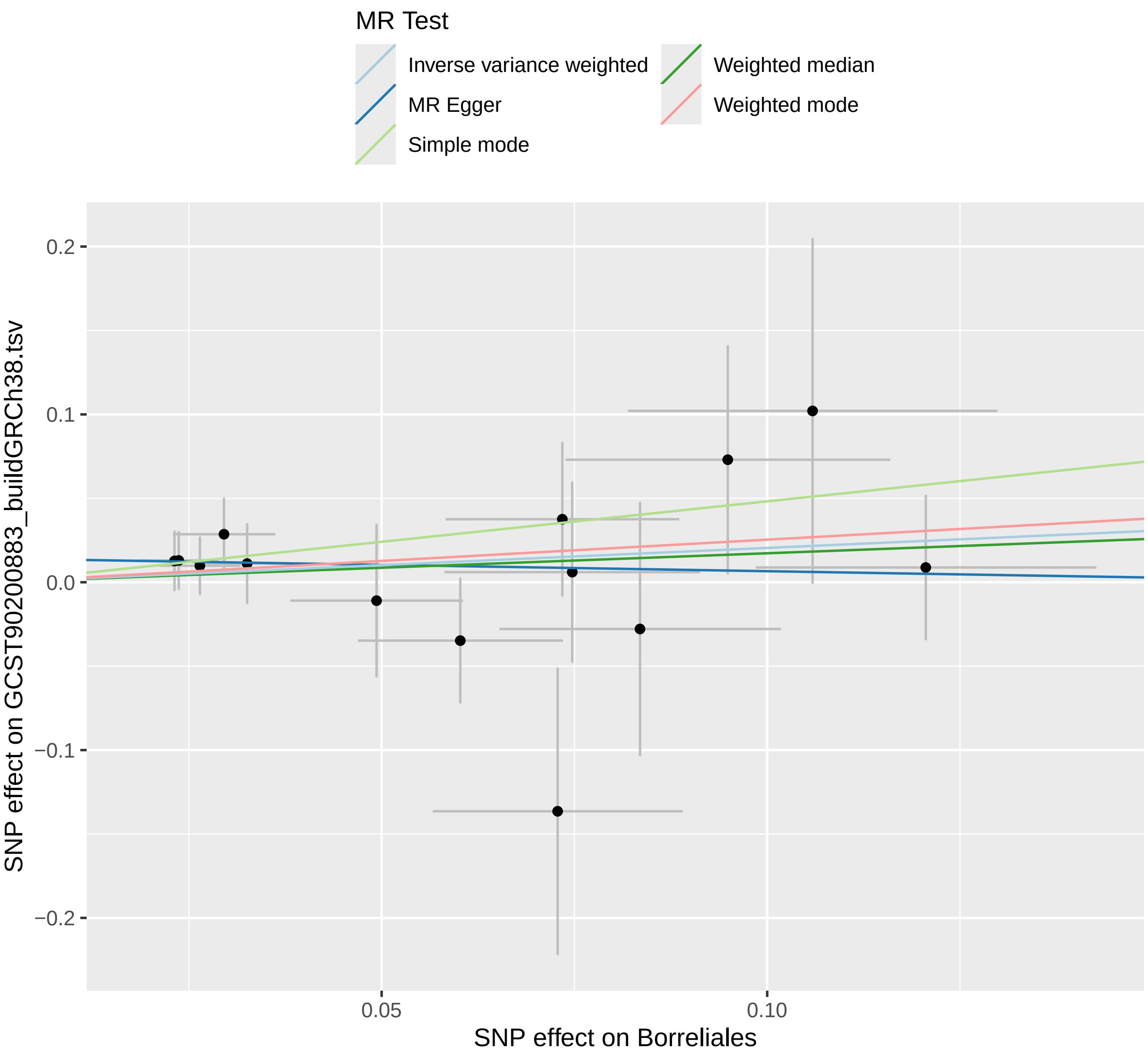
MR Test
Inverse variance weighted
MR Egger
Simple mode
Weighted median
Weighted mode
0.2
0.1
0.0
−0.1
−0.2
0.05
0.10
SNP effect on GCST90200883_buildGRCh38.tsv
SNP effect on Borreliales

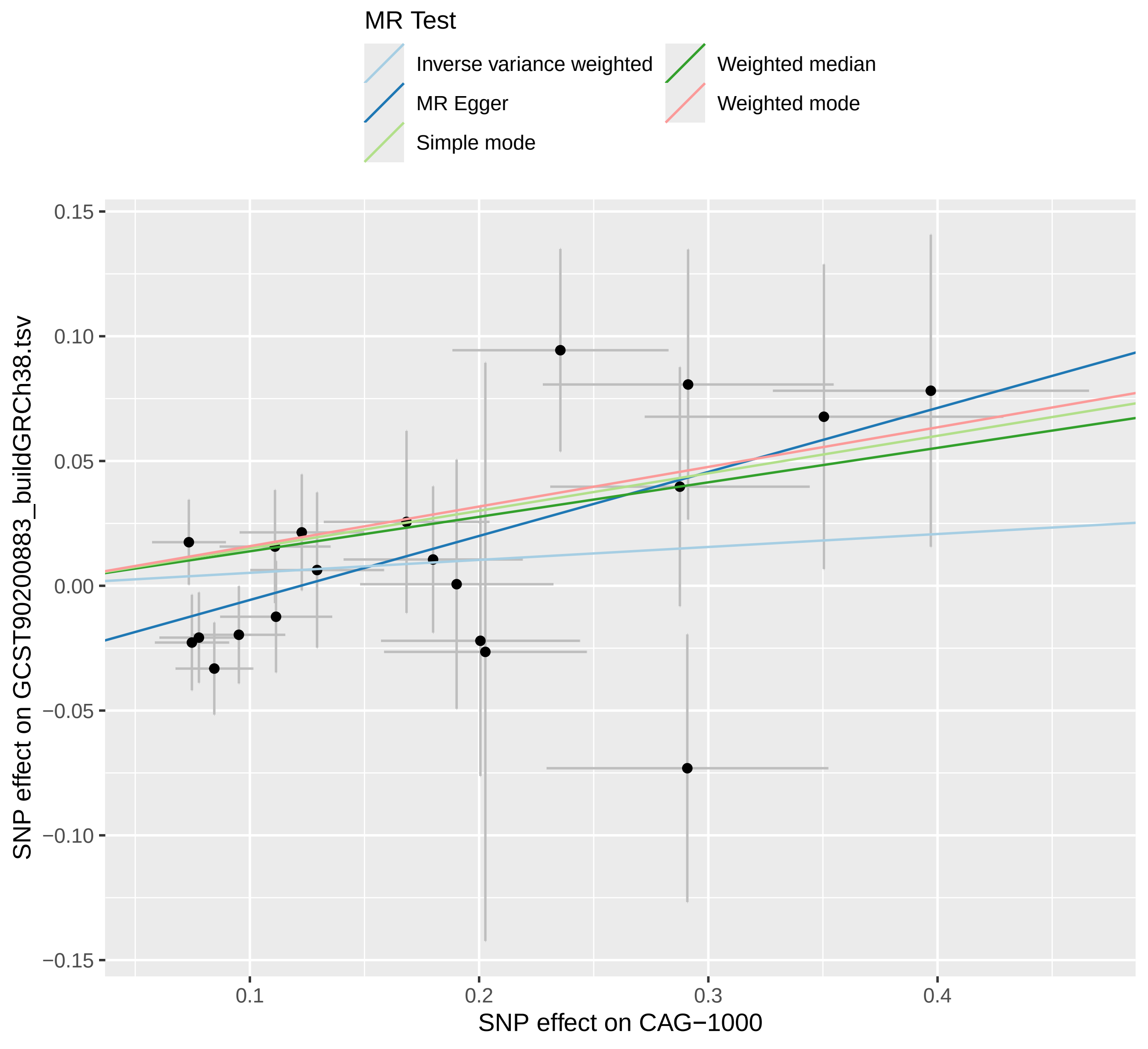
MR Test
Inverse variance weighted
MR Egger
Simple mode
Weighted median
Weighted mode
0.15
0.10
0.05
0.00
−0.05
−0.10
−0.15
SNP effect on GCST90200883_buildGRCh38.tsv
0.1
0.2
0.3
0.4
SNP effect on CAG−1000

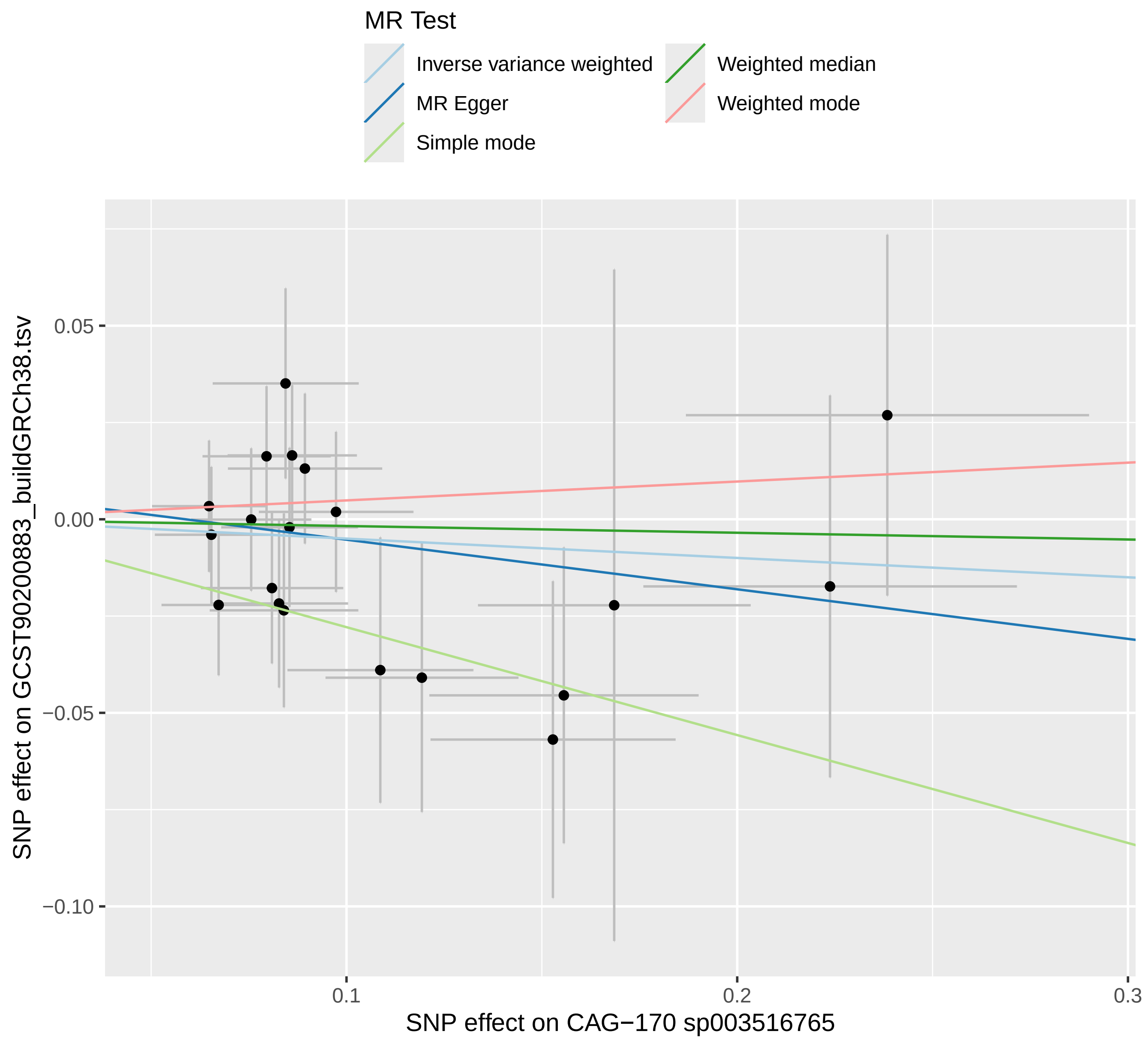
MR Test
Inverse variance weighted
MR Egger
Simple mode
Weighted median
Weighted mode
0.05
0.00
−0.05
−0.10
0.1
0.2
0.3
SNP effect on GCST90200883_buildGRCh38.tsv
SNP effect on CAG−170 sp003516765

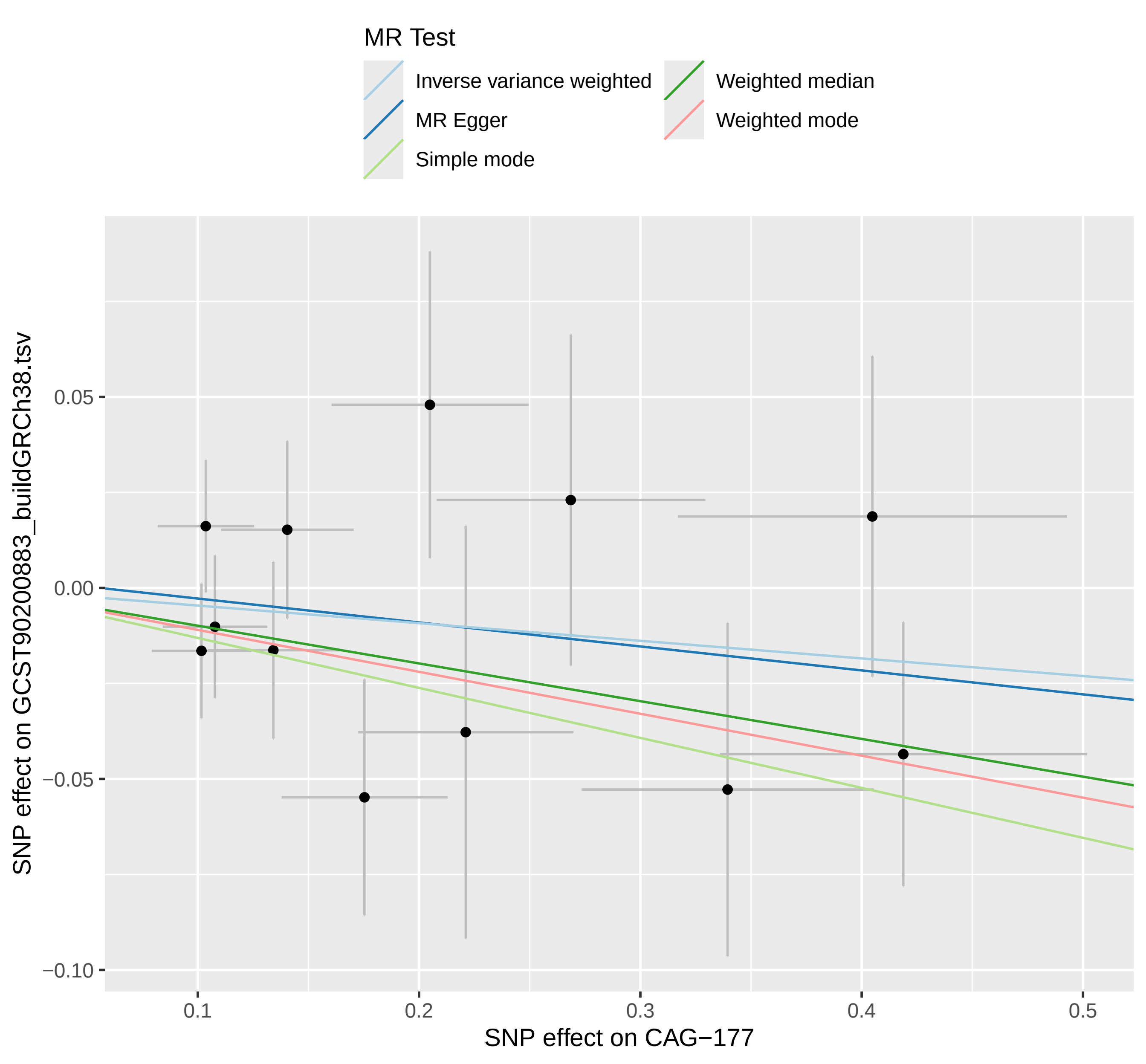
MR Test
Inverse variance weighted
MR Egger
Simple mode
Weighted median
Weighted mode
0.05
0.00
−0.05
−0.10
0.1
0.2
0.3
0.4
0.5
SNP effect on GCST90200883_buildGRCh38.tsv
SNP effect on CAG−177

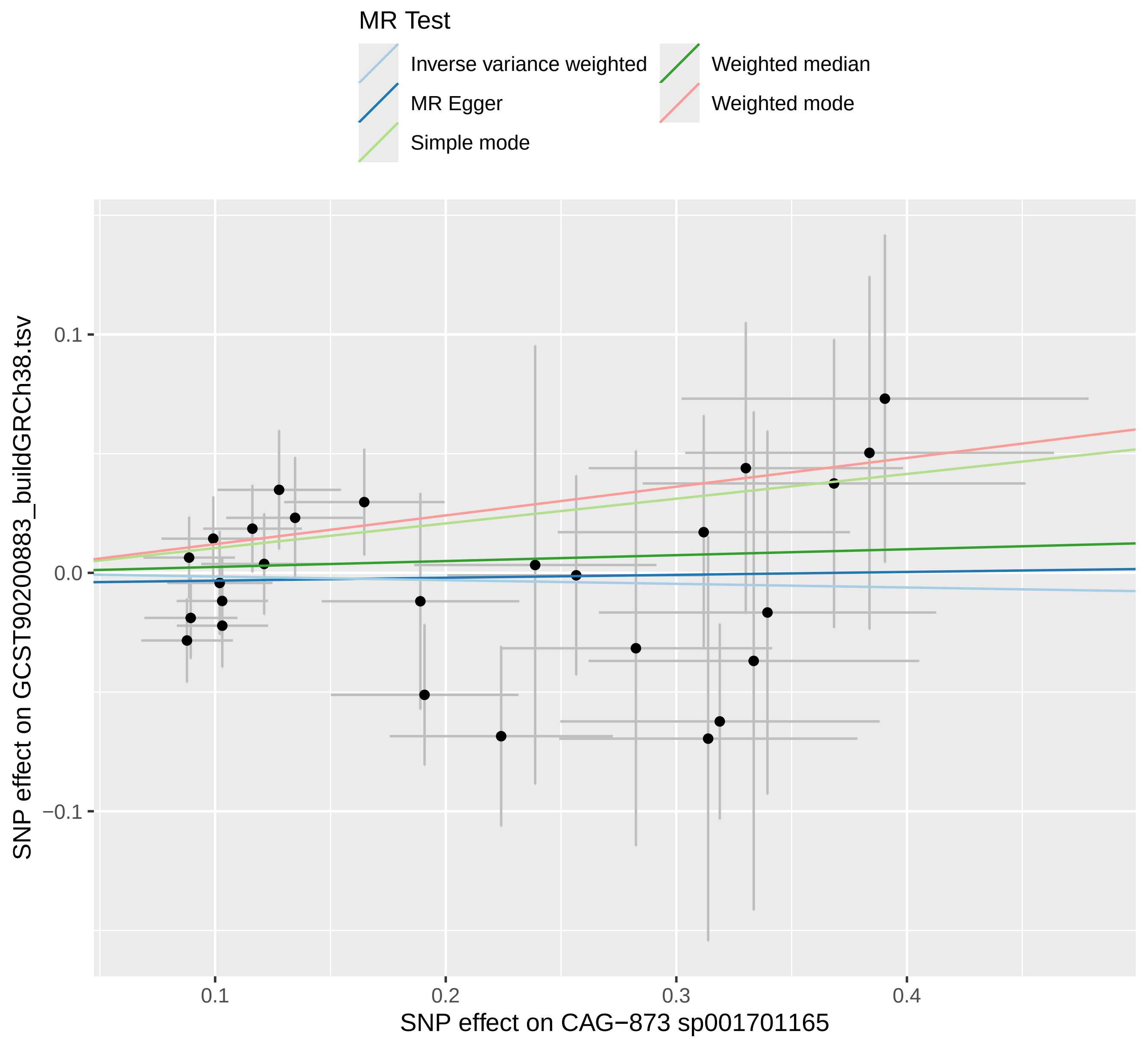

MR Test
Inverse variance weighted
MR Egger
Simple mode
Weighted median
Weighted mode
SNP effect on GCST90200883_buildGRCh38.tsv
0.1
0.0
−0.1
0.1
0.2
0.3
0.4
SNP effect on CAG−873 sp001701165

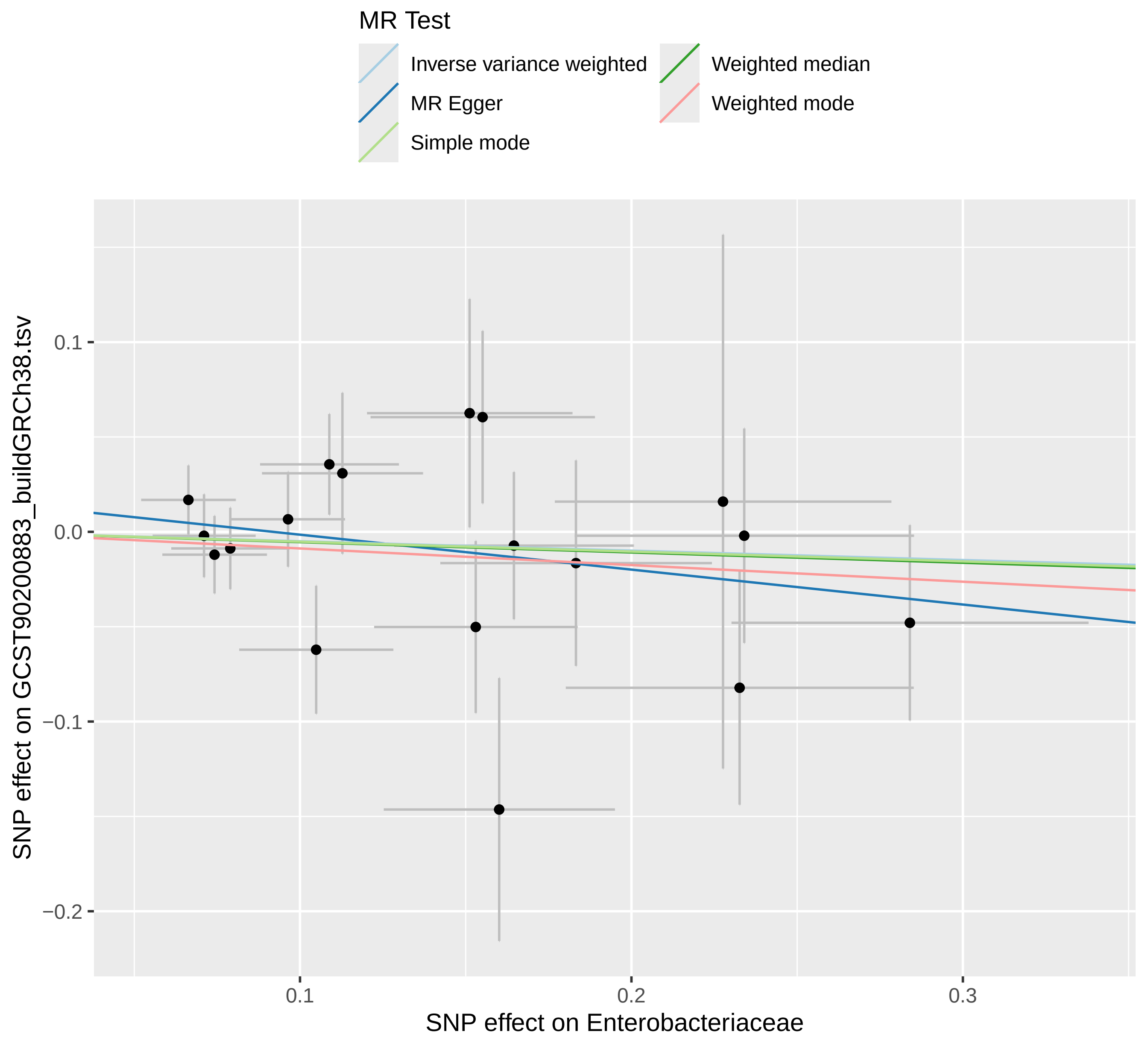
MR Test
Inverse variance weighted
MR Egger
Simple mode
Weighted median
Weighted mode
SNP effect on GCST90200883_buildGRCh38.tsv
0.1
0.0
−0.1
−0.2
0.1
0.2
0.3
SNP effect on Enterobacteriaceae

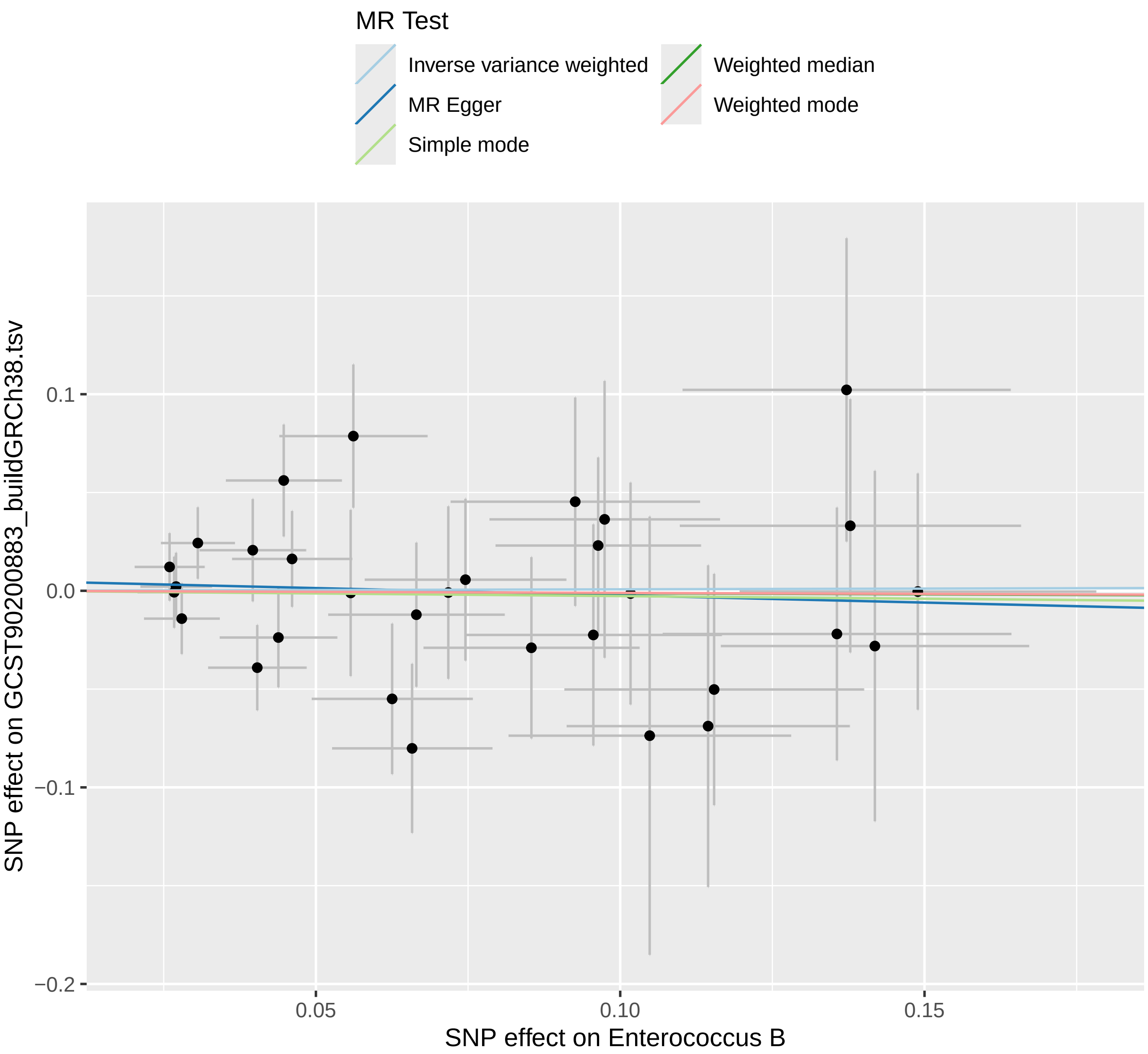
MR Test
Inverse variance weighted
MR Egger
Simple mode
Weighted median
Weighted mode
SNP effect on GCST90200883_buildGRCh38.tsv
0.1
0.0
−0.1
−0.2
0.05
0.10
0.15
SNP effect on Enterococcus B

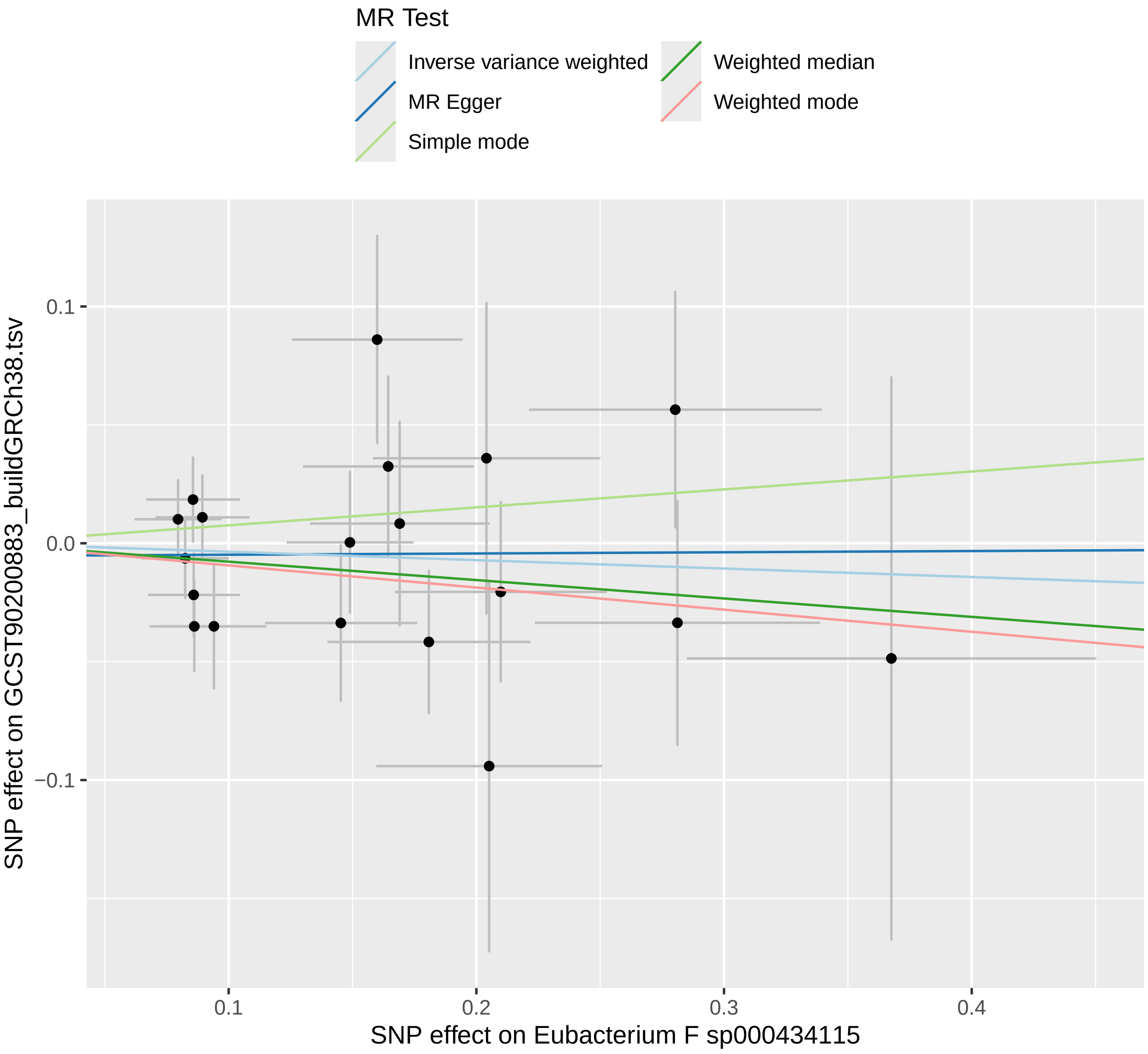

MR Test
Inverse variance weighted
MR Egger
Simple mode
Weighted median
Weighted mode
0.1
0.0
−0.1
0.1
0.2
0.3
0.4
SNP effect on GCST90200883_buildGRCh38.tsv
SNP effect on Eubacterium F sp000434115

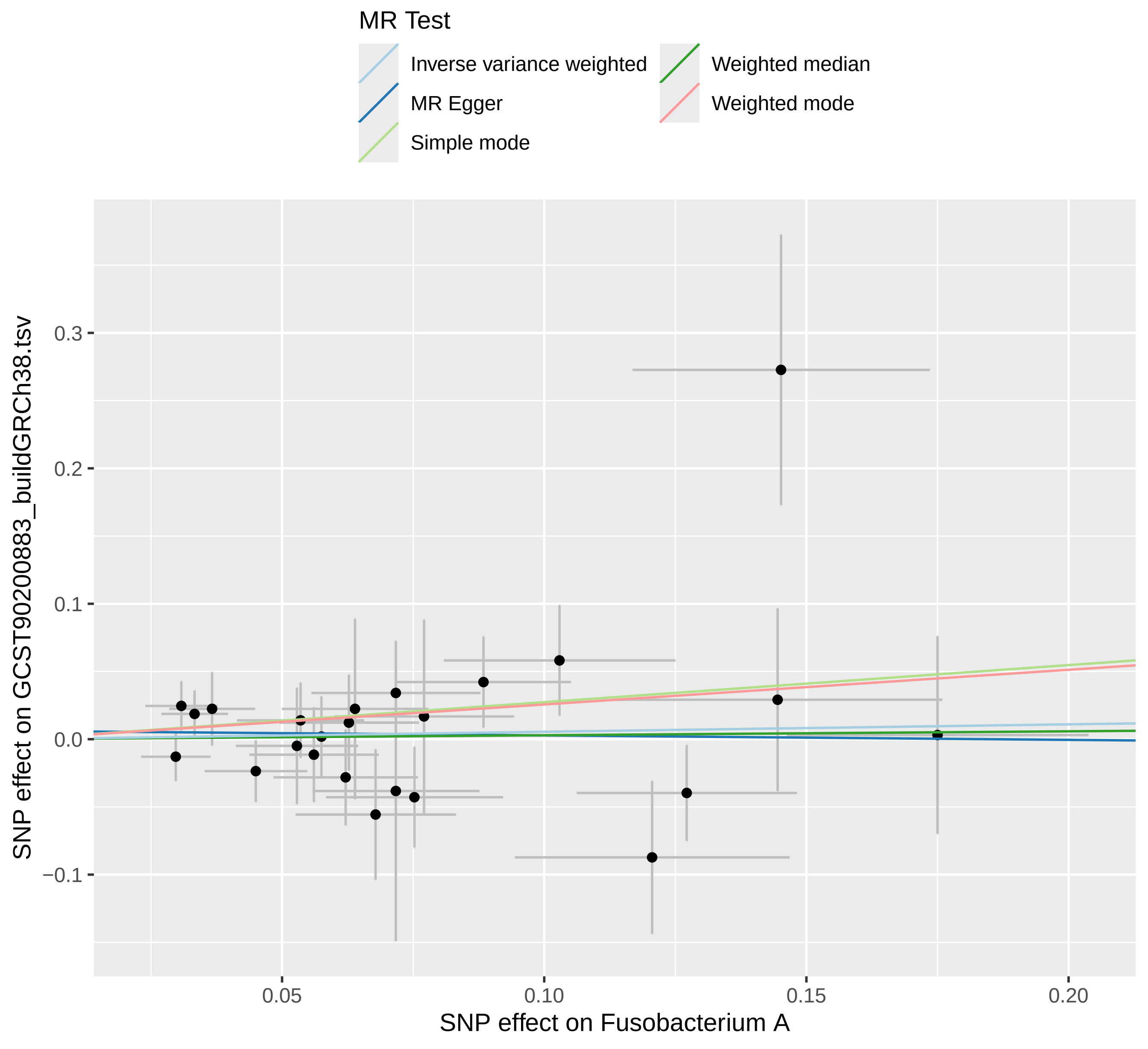
MR Test
Inverse variance weighted
MR Egger
Simple mode
Weighted median
Weighted mode
SNP effect on GCST90200883_buildGRCh38.tsv
0.3
0.2
0.1
0.0
−0.1
0.05
0.10
0.15
0.20
SNP effect on Fusobacterium A

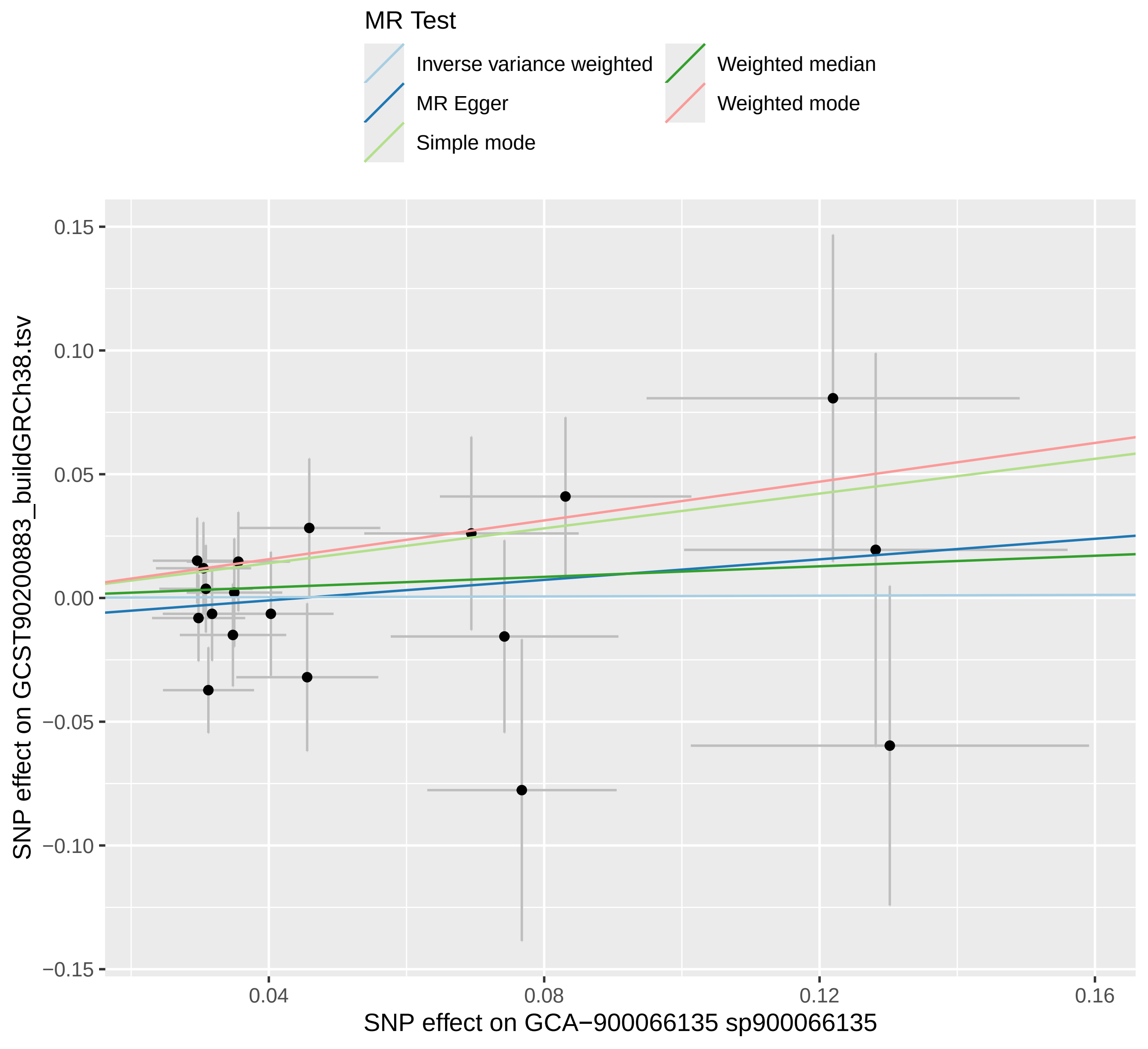
MR Test
Inverse variance weighted
MR Egger
Simple mode
Weighted median
Weighted mode
0.15
0.10
0.05
0.00
−0.05
−0.10
−0.15
0.04
0.08
0.12
0.16
SNP effect on GCA−900066135 sp900066135
SNP effect on GCST90200883_buildGRCh38.tsv

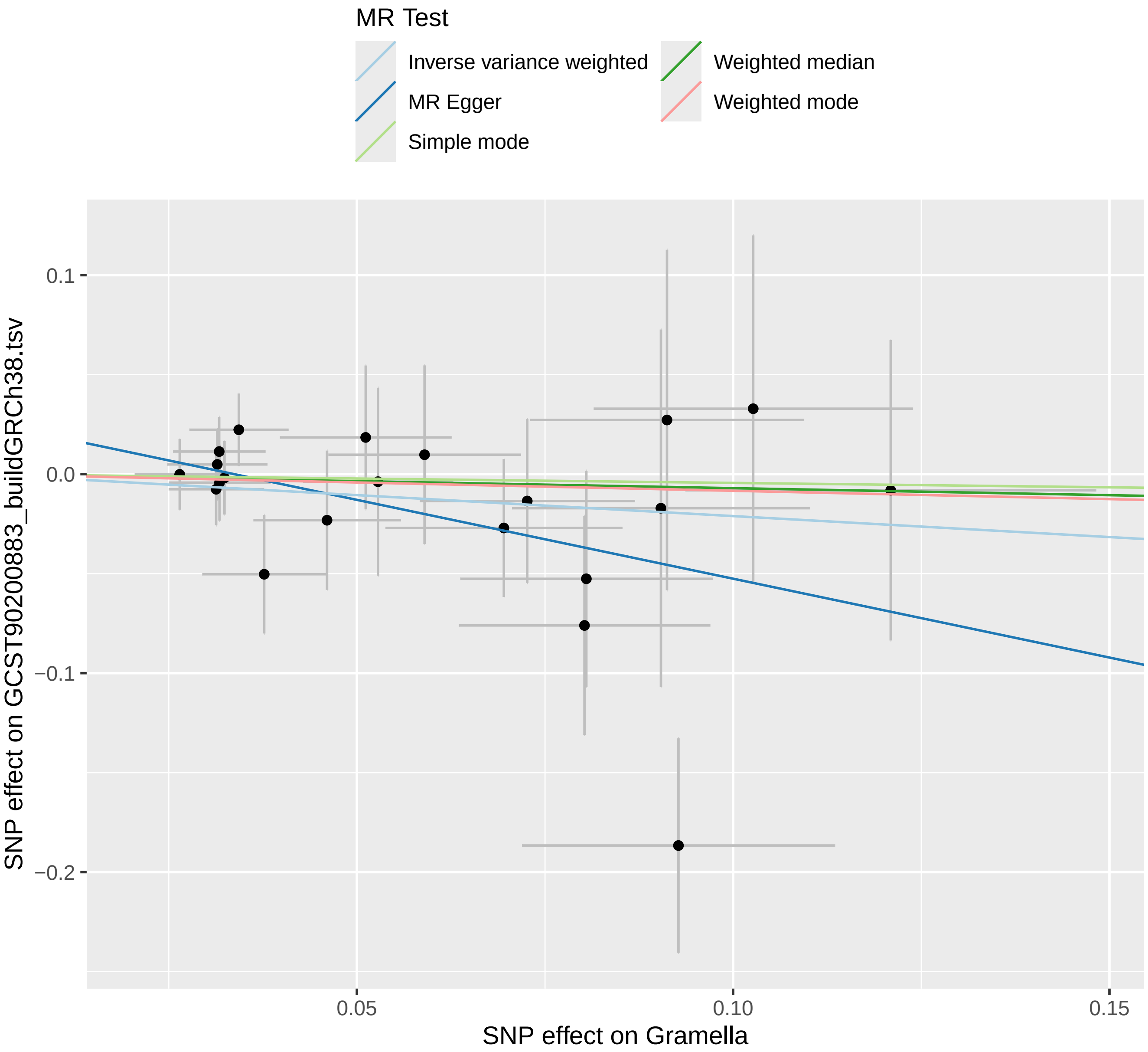
MR Test
Inverse variance weighted
MR Egger
Simple mode
Weighted median
Weighted mode
0.1
0.0
−0.1
−0.2
SNP effect on GCST90200883_buildGRCh38.tsv
0.05
0.10
0.15
SNP effect on Gramella

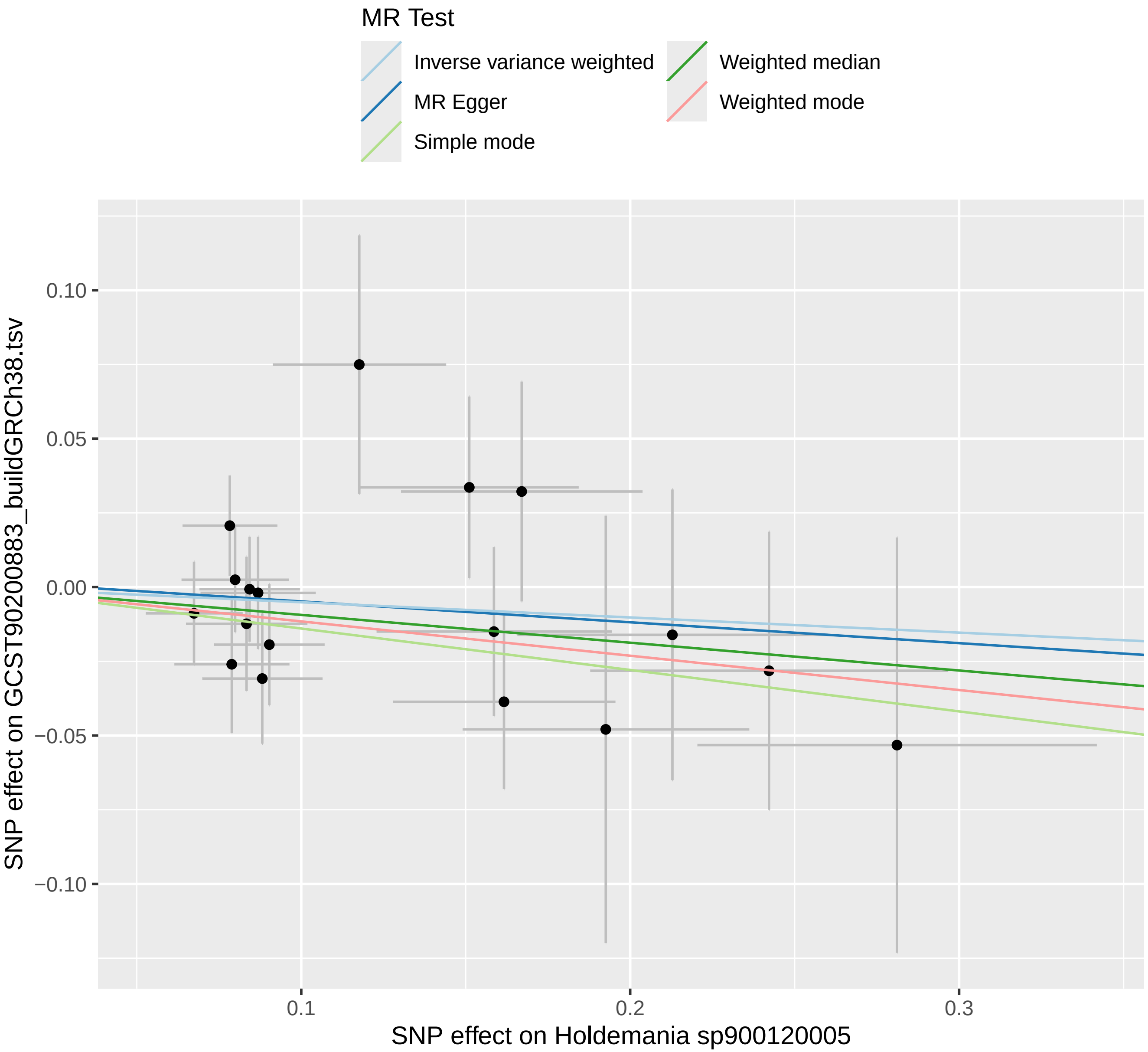
MR Test
Inverse variance weighted
MR Egger
Simple mode
Weighted median
Weighted mode
0.10
0.05
0.00
−0.05
−0.10
SNP effect on GCST90200883_buildGRCh38.tsv
0.1
0.2
0.3
SNP effect on Holdemania sp900120005

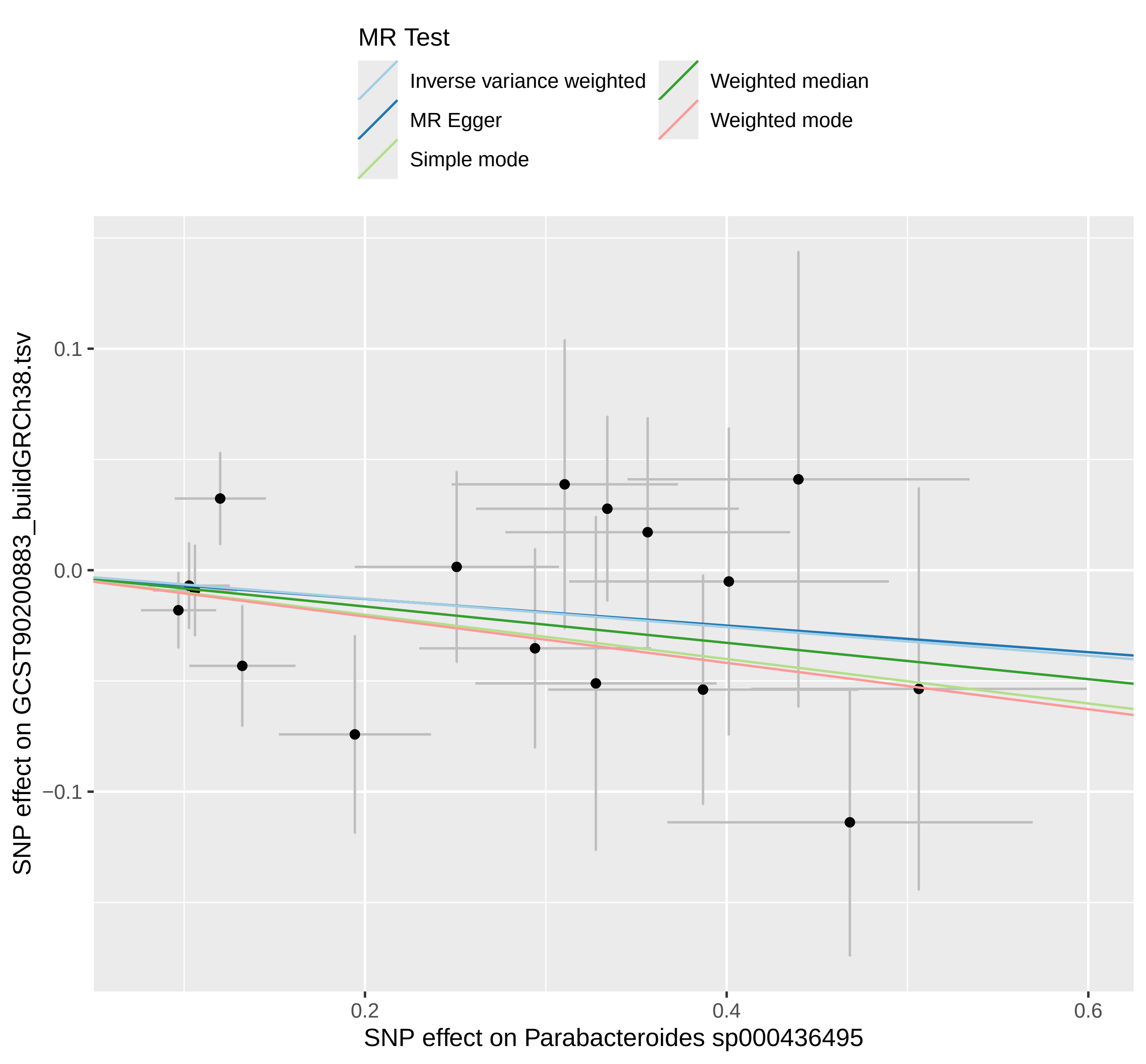
MR Test
Inverse variance weighted
MR Egger
Simple mode
Weighted median
Weighted mode
0.1
0.0
−0.1
0.2
0.4
0.6
SNP effect on GCST90200883_buildGRCh38.tsv
SNP effect on Parabacteroides sp000436495

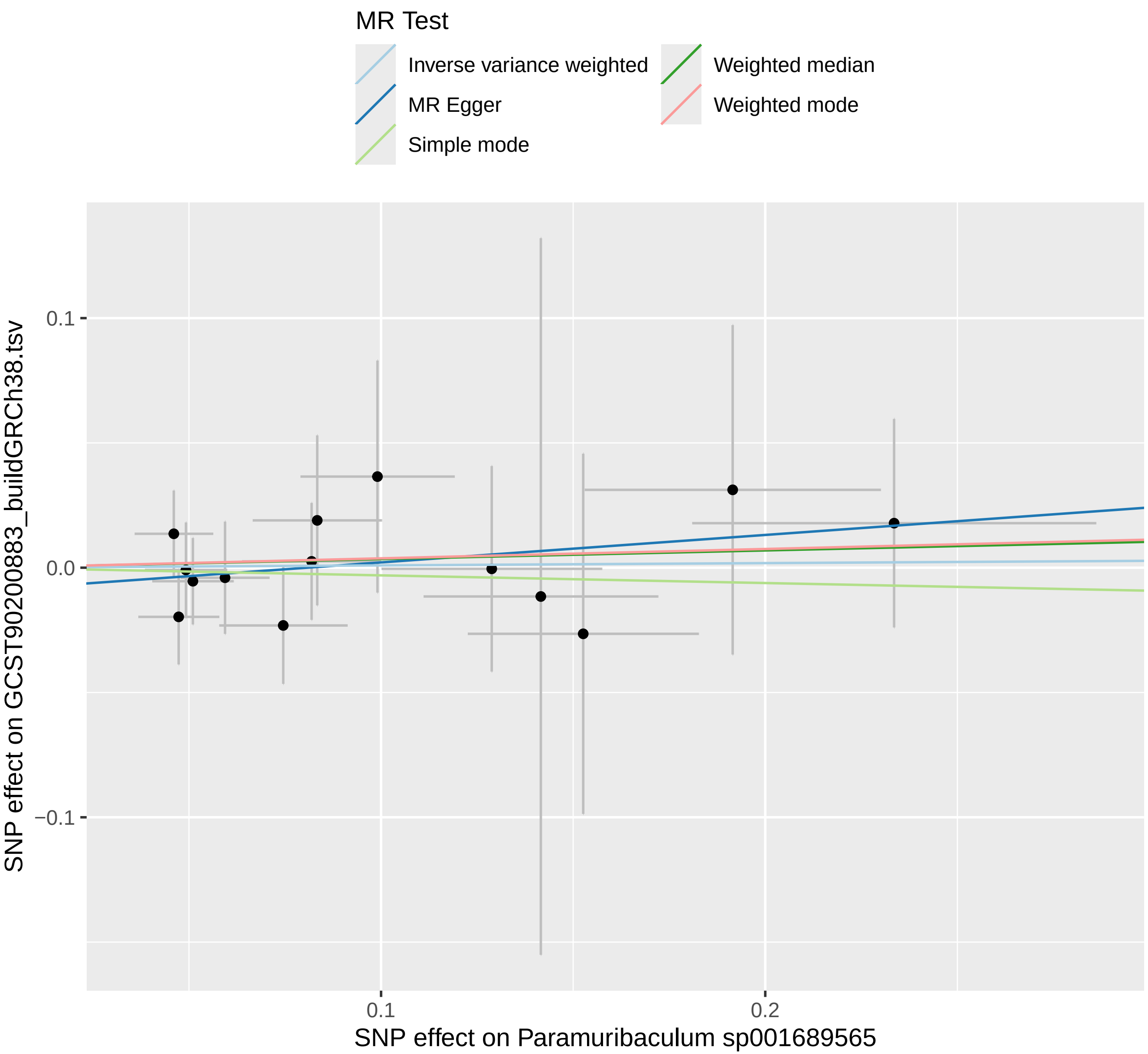
MR Test
Inverse variance weighted
MR Egger
Simple mode
Weighted median
Weighted mode
0.1
0.0
−0.1
0.1
0.2
SNP effect on GCST90200883_buildGRCh38.tsv
SNP effect on Paramuribaculum sp001689565

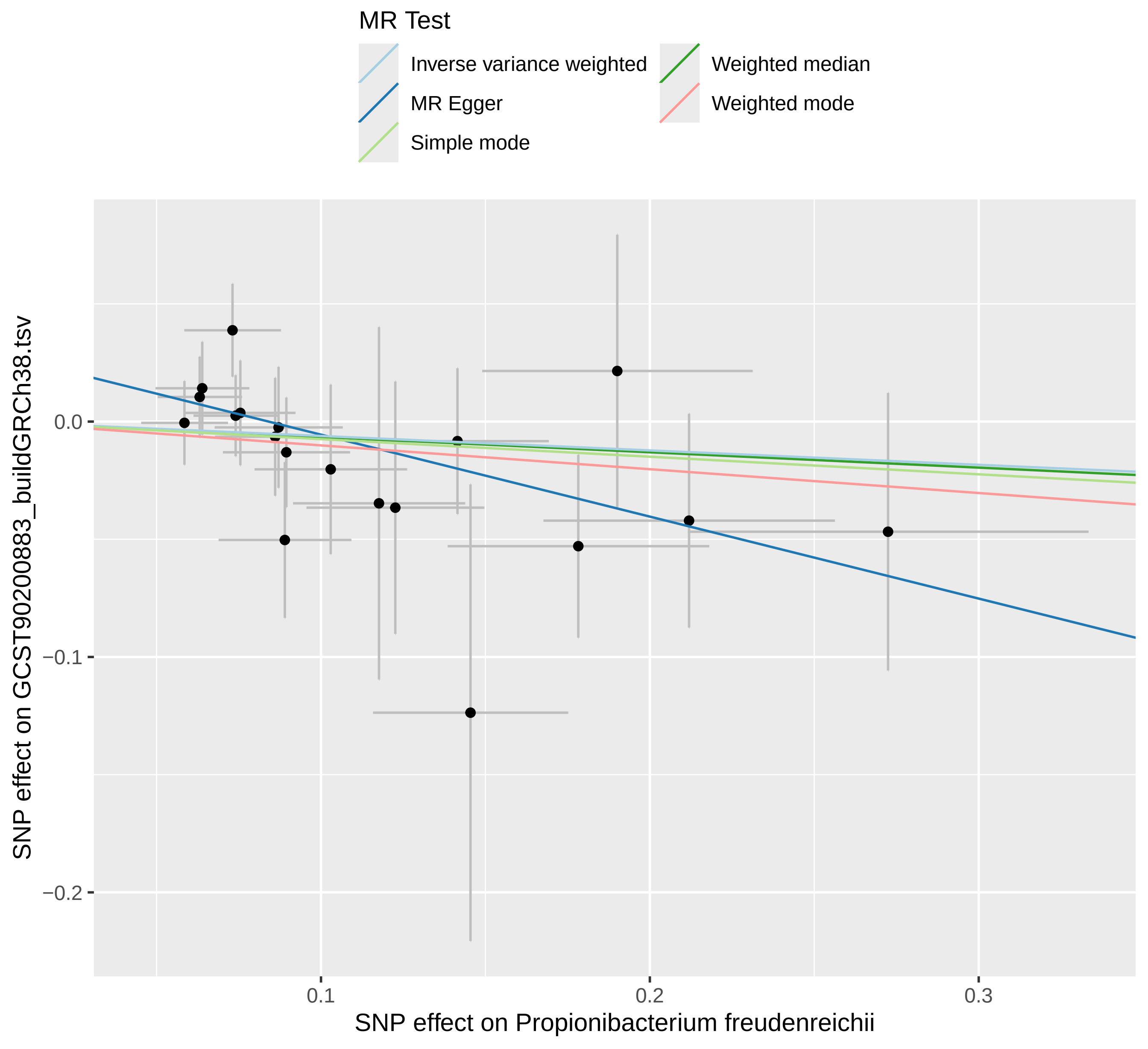
MR Test
Inverse variance weighted
MR Egger
Simple mode
Weighted median
Weighted mode
SNP effect on GCST90200883_buildGRCh38.tsv
0.0
−0.1
−0.2
0.1
0.2
0.3
SNP effect on Propionibacterium freudenreichii

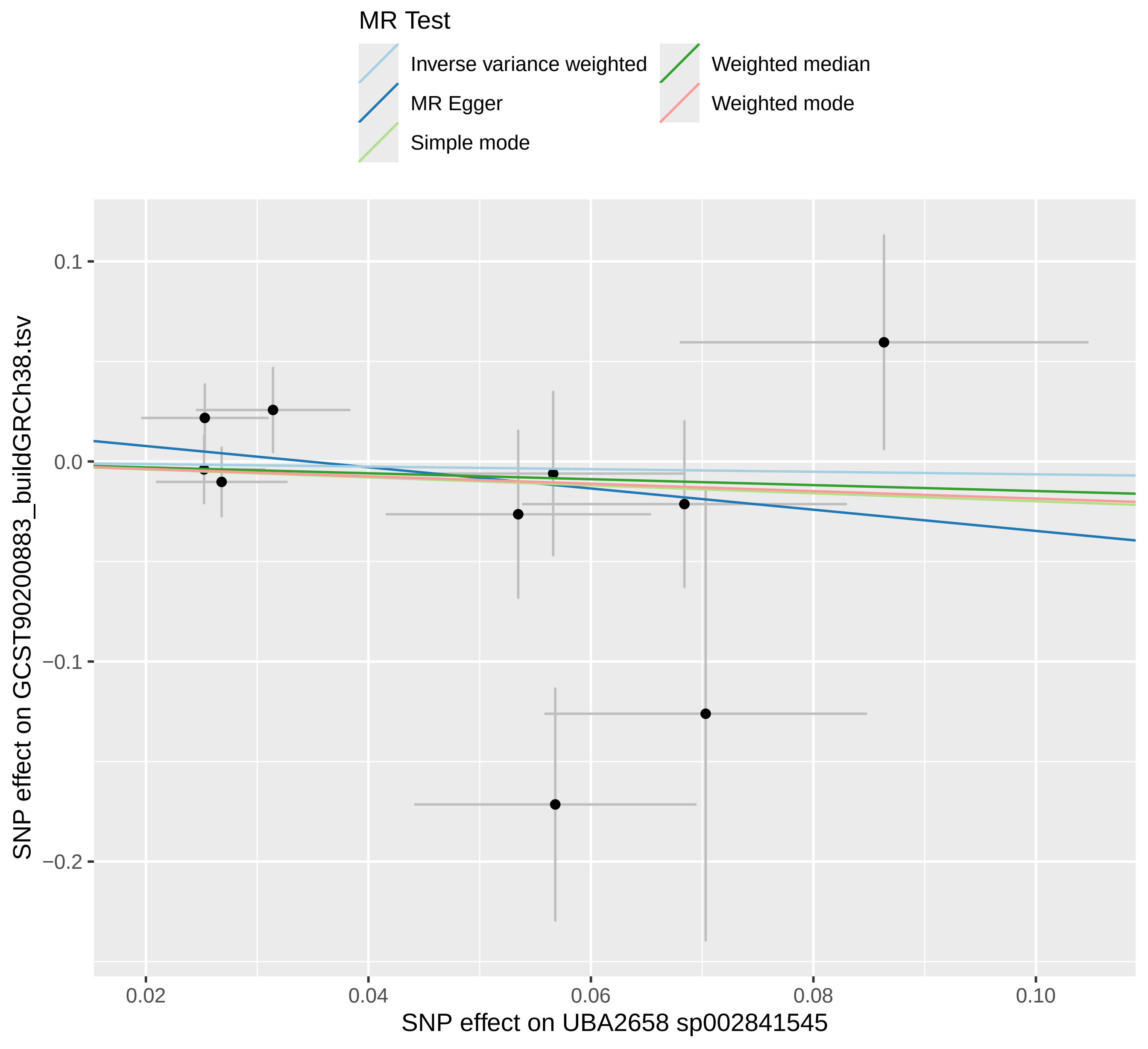
MR Test
Inverse variance weighted
MR Egger
Simple mode
Weighted median
Weighted mode
0.1
0.0
−0.1
−0.2
SNP effect on GCST90200883_buildGRCh38.tsv
0.02
0.04
0.06
0.08
0.10
SNP effect on UBA2658 sp002841545

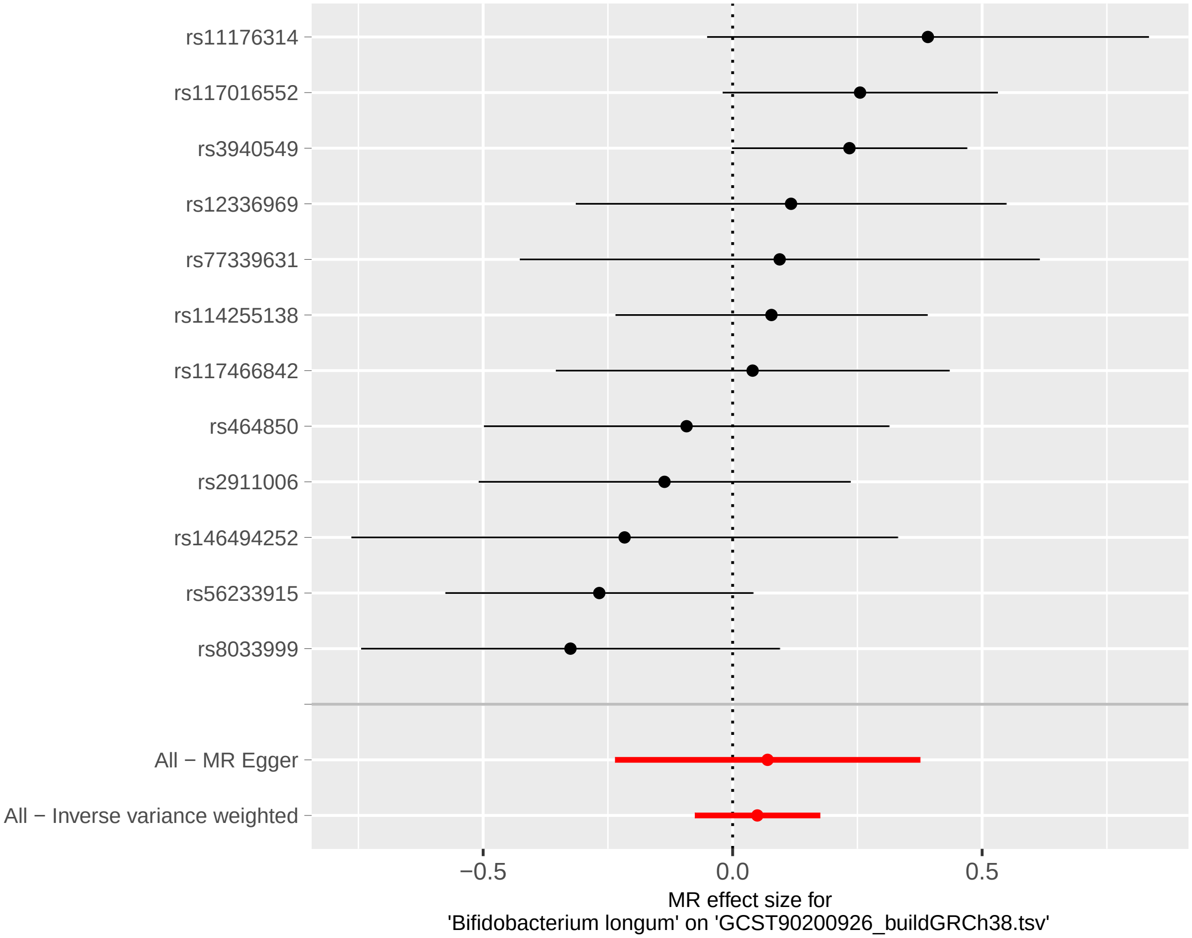

rs11176314
rs117016552
rs3940549
rs12336969
rs77339631
rs114255138
rs117466842
rs464850
rs2911006
rs146494252
rs56233915
rs8033999
All − MR Egger
All − Inverse variance weighted
−0.5
0.0
0.5
MR effect size for
'Bifidobacterium longum' on 'GCST90200926_buildGRCh38.tsv'

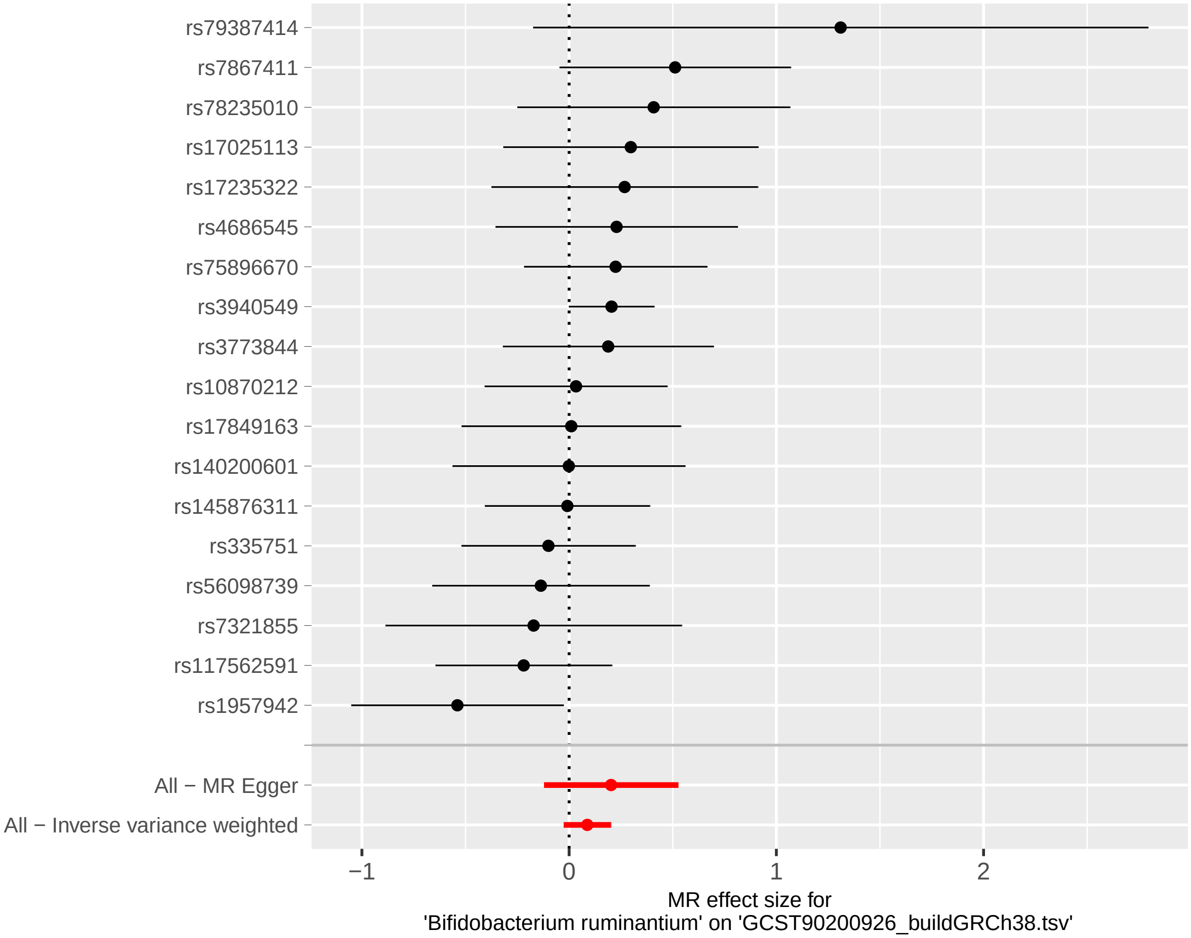

rs79387414
rs7867411
rs78235010
rs17025113
rs17235322
rs4686545
rs75896670
rs3940549
rs3773844
rs10870212
rs17849163
rs140200601
rs145876311
rs335751
rs56098739
rs7321855
rs117562591
rs1957942
All − MR Egger
All − Inverse variance weighted
−1
0
1
2
MR effect size for
'Bifidobacterium ruminantium' on 'GCST90200926_buildGRCh38.tsv'

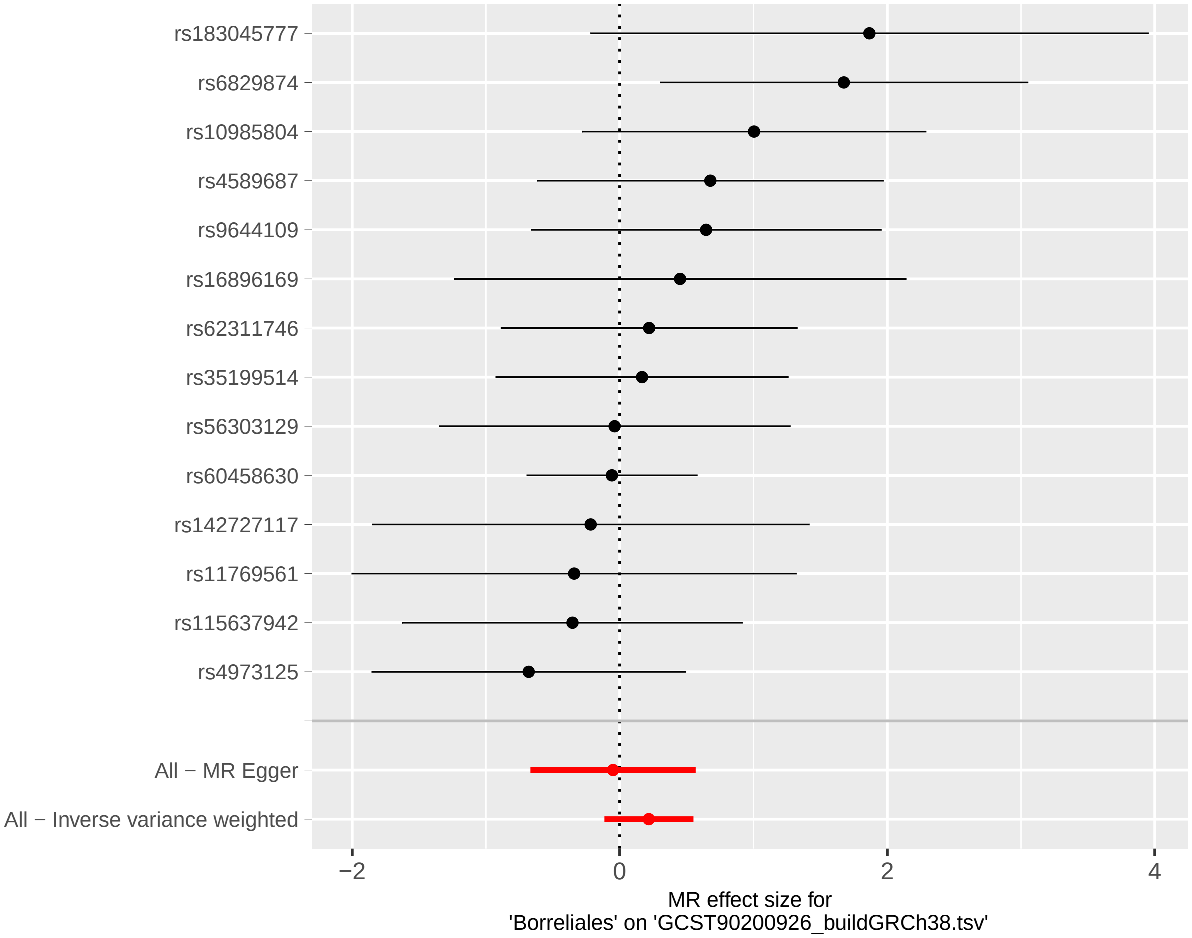
rs183045777
rs6829874
rs10985804
rs4589687
rs9644109
rs16896169
rs62311746
rs35199514
rs56303129
rs60458630
rs142727117
rs11769561
rs115637942
rs4973125
All − MR Egger
All − Inverse variance weighted
−2
0
2
4
MR effect size for
'Borreliales' on 'GCST90200926_buildGRCh38.tsv'

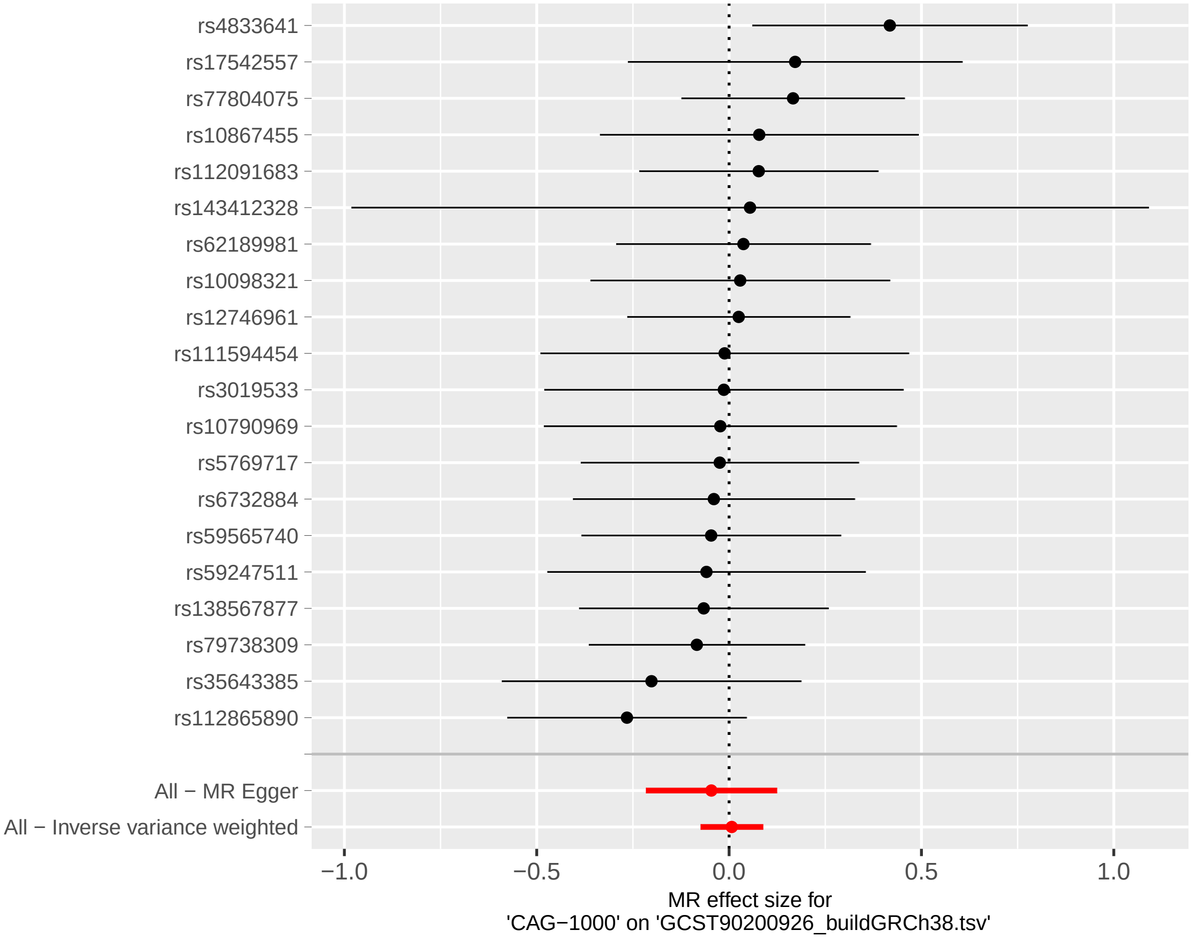
rs4833641
rs17542557
rs77804075
rs10867455
rs112091683
rs143412328
rs62189981
rs10098321
rs12746961
rs111594454
rs3019533
rs10790969
rs5769717
rs6732884
rs59565740
rs59247511
rs138567877
rs79738309
rs35643385
rs112865890
All − MR Egger
All − Inverse variance weighted
−1.0
−0.5
0.0
0.5
1.0
MR effect size for
'CAG−1000' on 'GCST90200926_buildGRCh38.tsv'

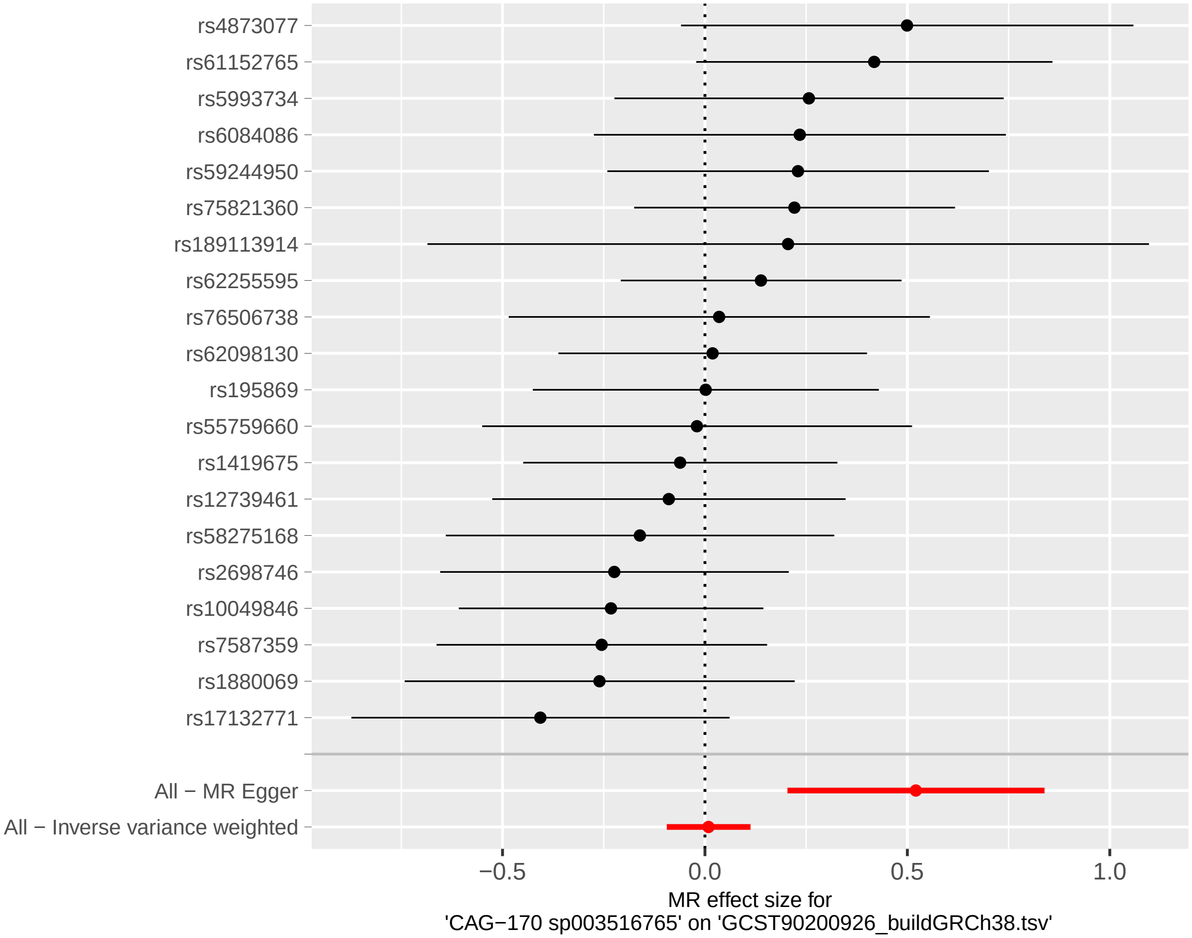

rs4873077
rs61152765
rs5993734
rs6084086
rs59244950
rs75821360
rs189113914
rs62255595
rs76506738
rs62098130
rs195869
rs55759660
rs1419675
rs12739461
rs58275168
rs2698746
rs10049846
rs7587359
rs1880069
rs17132771
All − MR Egger
All − Inverse variance weighted
−0.5
0.0
0.5
1.0
MR effect size for
'CAG−170 sp003516765' on 'GCST90200926_buildGRCh38.tsv'

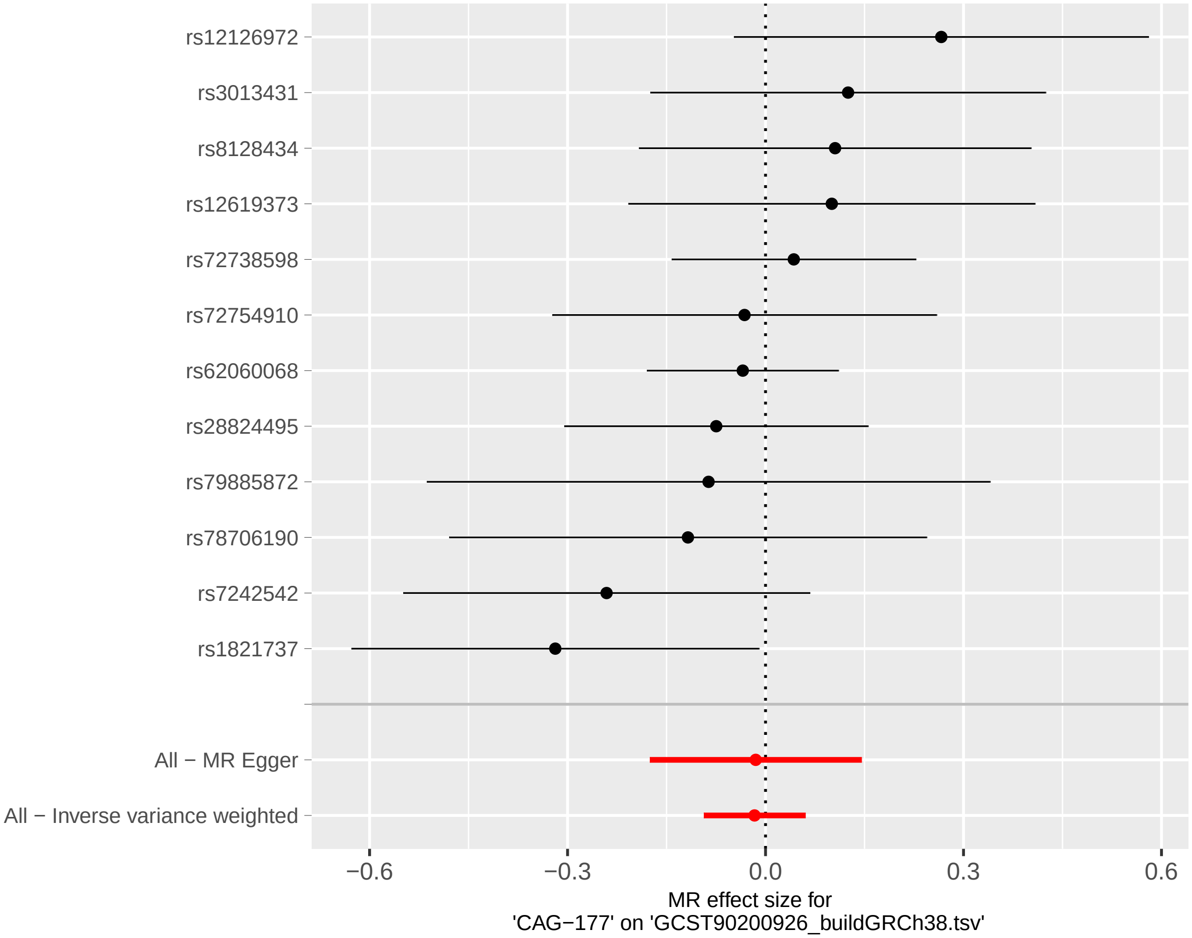

rs12126972
rs3013431
rs8128434
rs12619373
rs72738598
rs72754910
rs62060068
rs28824495
rs79885872
rs78706190
rs7242542
rs1821737
All − MR Egger
All − Inverse variance weighted
−0.6
−0.3
0.0
0.3
0.6
MR effect size for
'CAG−177' on 'GCST90200926_buildGRCh38.tsv'

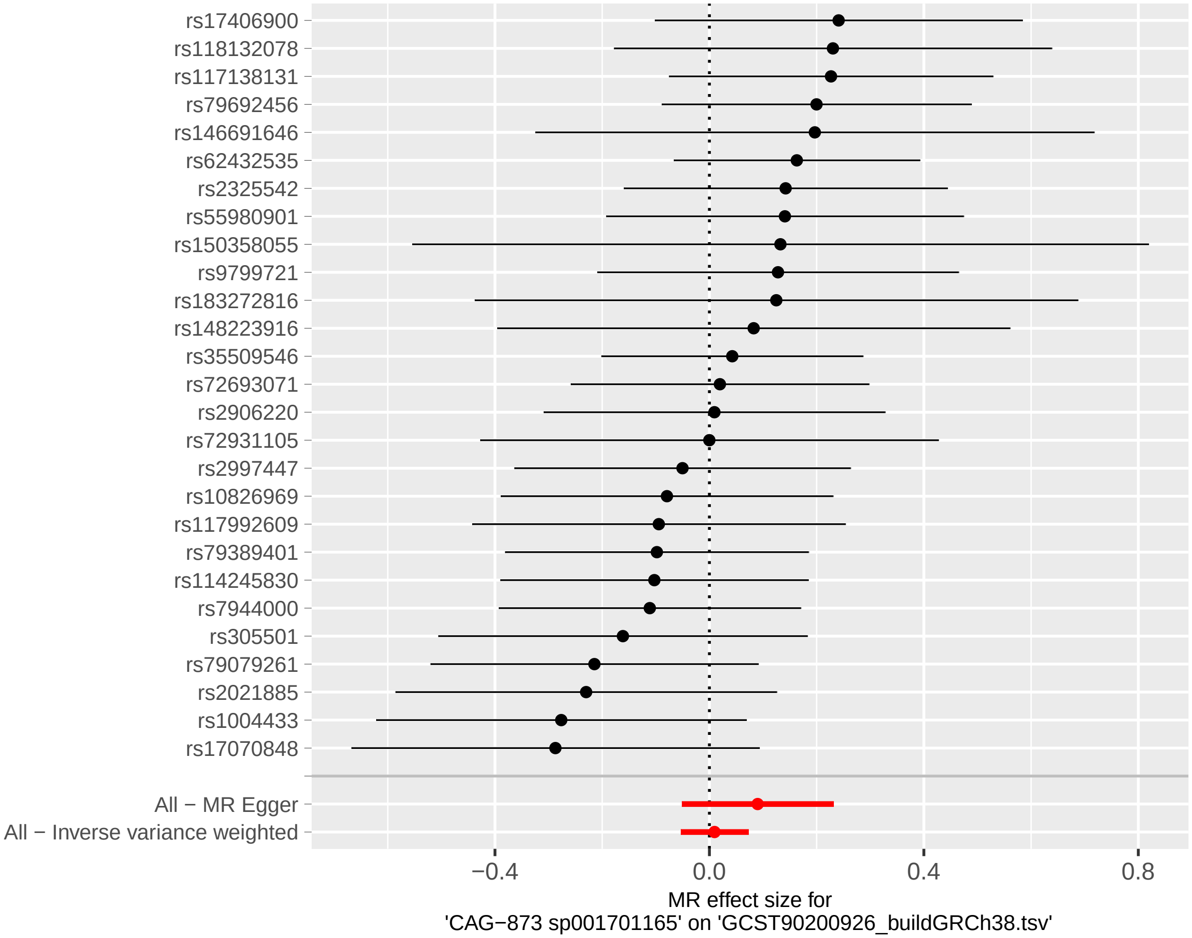

rs17406900
rs118132078
rs117138131
rs79692456
rs146691646
rs62432535
rs2325542
rs55980901
rs150358055
rs9799721
rs183272816
rs148223916
rs35509546
rs72693071
rs2906220
rs72931105
rs2997447
rs10826969
rs117992609
rs79389401
rs114245830
rs7944000
rs305501
rs79079261
rs2021885
rs1004433
rs17070848
All − MR Egger
All − Inverse variance weighted
−0.4
0.0
0.4
0.8
MR effect size for
'CAG−873 sp001701165' on 'GCST90200926_buildGRCh38.tsv'

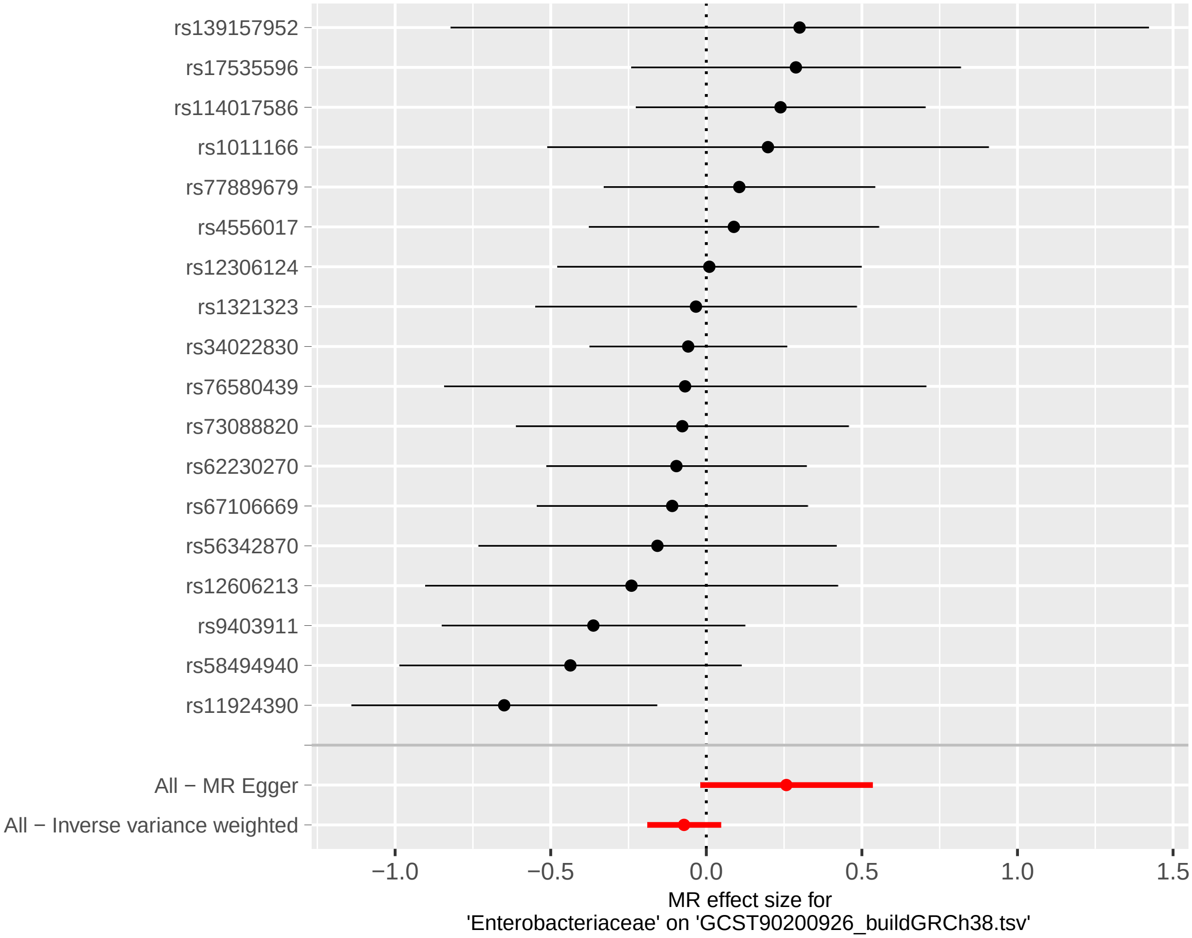

rs139157952
rs17535596
rs114017586
rs1011166
rs77889679
rs4556017
rs12306124
rs1321323
rs34022830
rs76580439
rs73088820
rs62230270
rs67106669
rs56342870
rs12606213
rs9403911
rs58494940
rs11924390
All − MR Egger
All − Inverse variance weighted
−1.0
−0.5
0.0
0.5
1.0
1.5
MR effect size for
'Enterobacteriaceae' on 'GCST90200926_buildGRCh38.tsv'

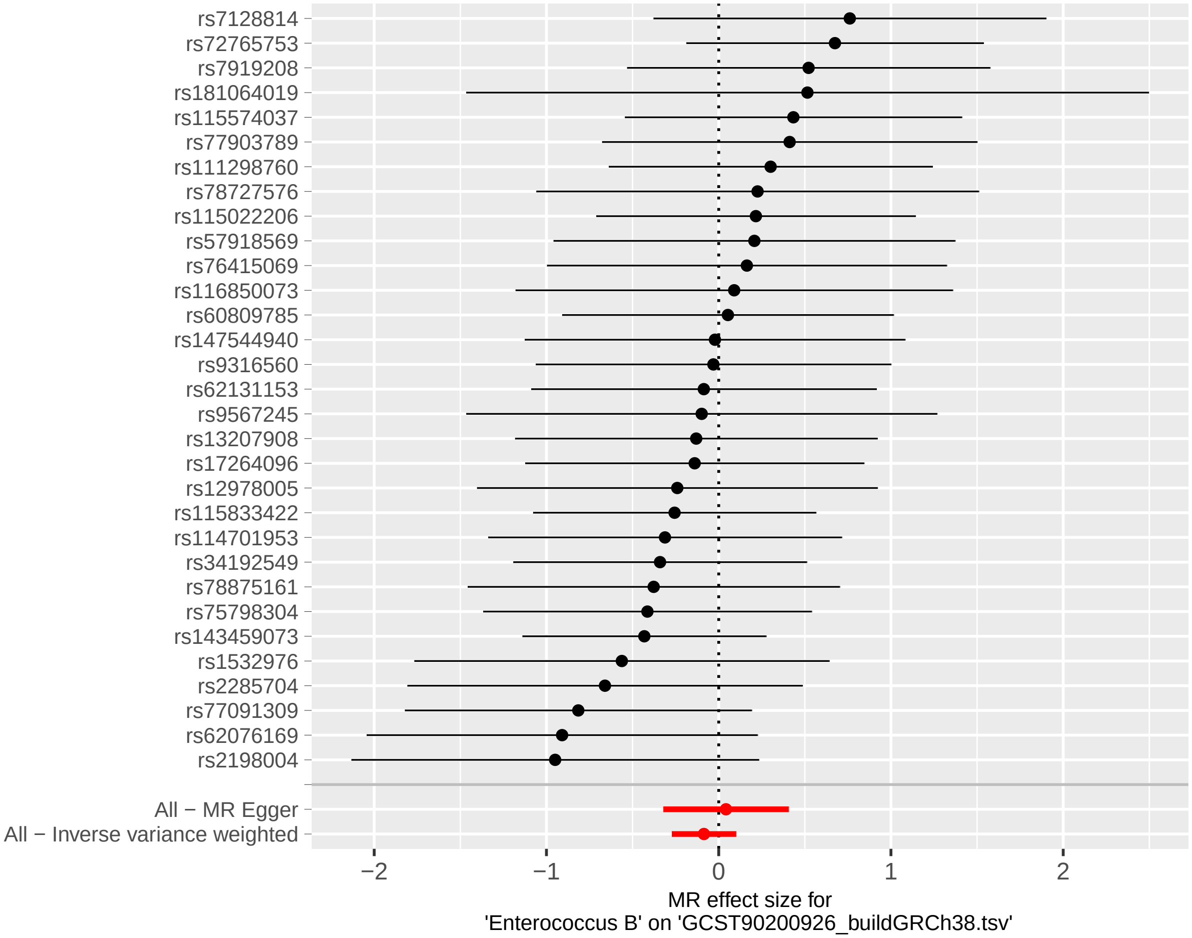
rs7128814
rs72765753
rs7919208
rs181064019
rs115574037
rs77903789
rs111298760
rs78727576
rs115022206
rs57918569
rs76415069
rs116850073
rs60809785
rs147544940
rs9316560
rs62131153
rs9567245
rs13207908
rs17264096
rs12978005
rs115833422
rs114701953
rs34192549
rs78875161
rs75798304
rs143459073
rs1532976
rs2285704
rs77091309
rs62076169
rs2198004
All − MR Egger
All − Inverse variance weighted
−2
−1
0
1
2
MR effect size for
'Enterococcus B' on 'GCST90200926_buildGRCh38.tsv'

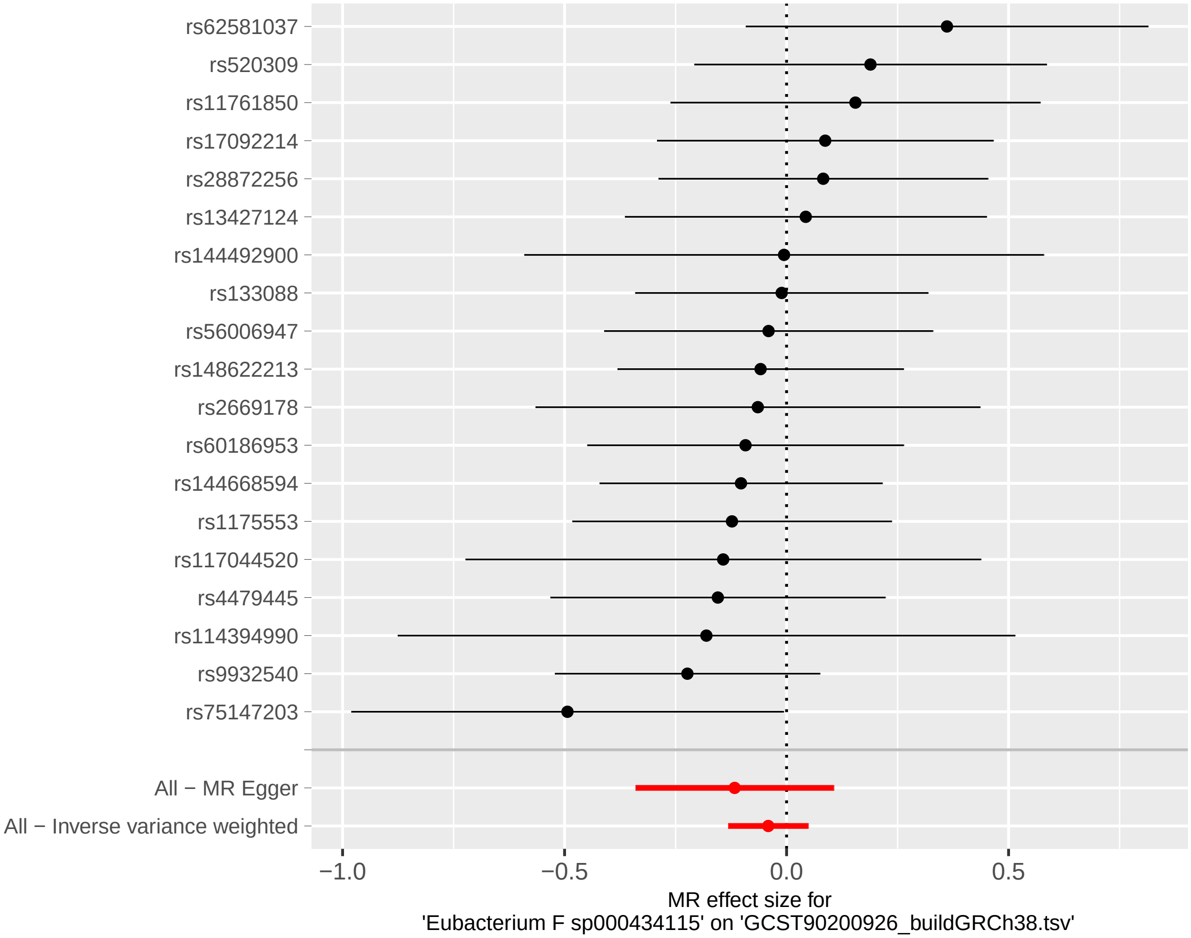
rs62581037
rs520309
rs11761850
rs17092214
rs28872256
rs13427124
rs144492900
rs133088
rs56006947
rs148622213
rs2669178
rs60186953
rs144668594
rs1175553
rs117044520
rs4479445
rs114394990
rs9932540
rs75147203
All − MR Egger
All − Inverse variance weighted
−1.0
−0.5
0.0
0.5
MR effect size for
'Eubacterium F sp000434115' on 'GCST90200926_buildGRCh38.tsv'

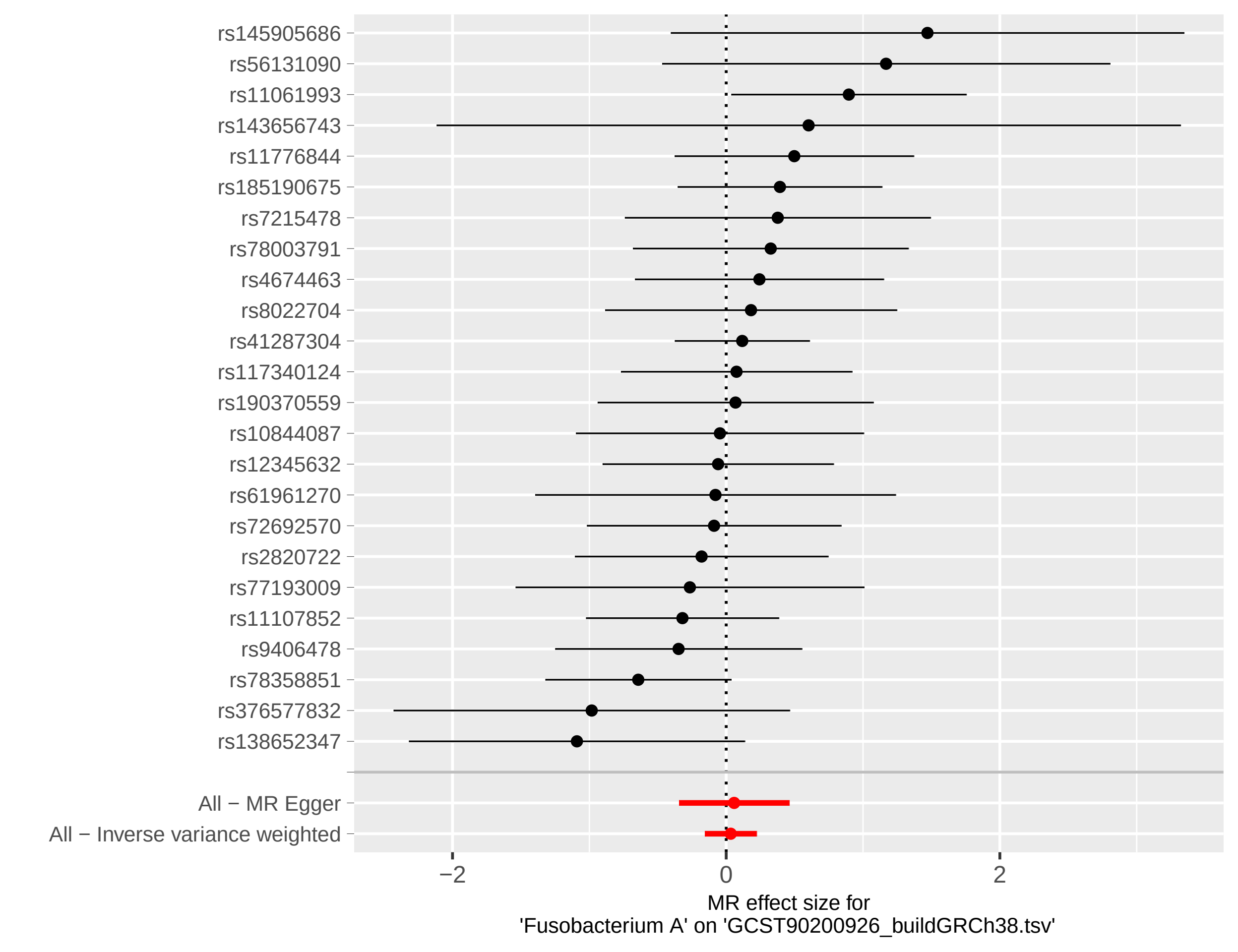

rs145905686
rs56131090
rs11061993
rs143656743
rs11776844
rs185190675
rs7215478
rs78003791
rs4674463
rs8022704
rs41287304
rs117340124
rs190370559
rs10844087
rs12345632
rs61961270
rs72692570
rs2820722
rs77193009
rs11107852
rs9406478
rs78358851
rs376577832
rs138652347
All − MR Egger
All − Inverse variance weighted
−2
0
2
MR effect size for
'Fusobacterium A' on 'GCST90200926_buildGRCh38.tsv'

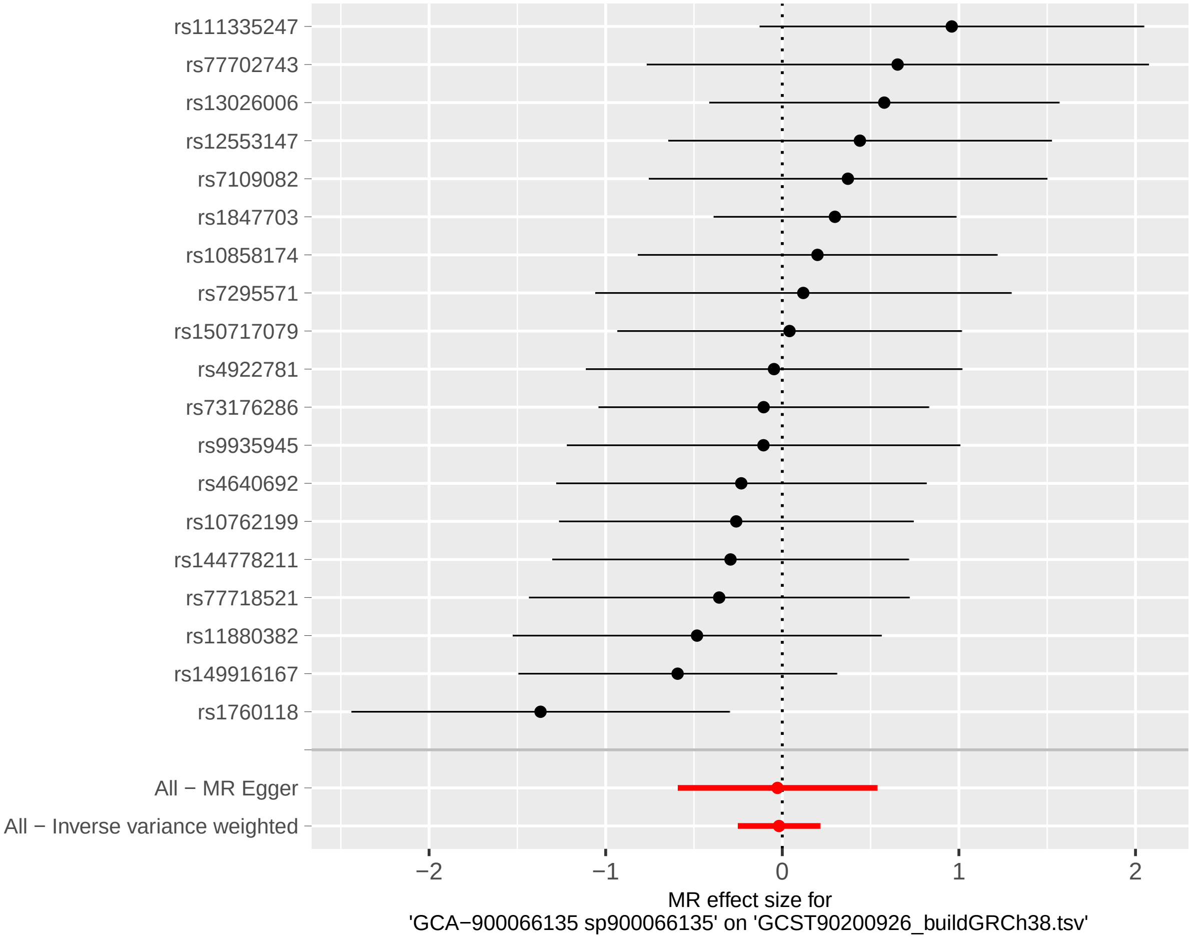

rs111335247
rs77702743
rs13026006
rs12553147
rs7109082
rs1847703
rs10858174
rs7295571
rs150717079
rs4922781
rs73176286
rs9935945
rs4640692
rs10762199
rs144778211
rs77718521
rs11880382
rs149916167
rs1760118
All − MR Egger
All − Inverse variance weighted
−2
−1
0
1
2
MR effect size for
'GCA−900066135 sp900066135' on 'GCST90200926_buildGRCh38.tsv'

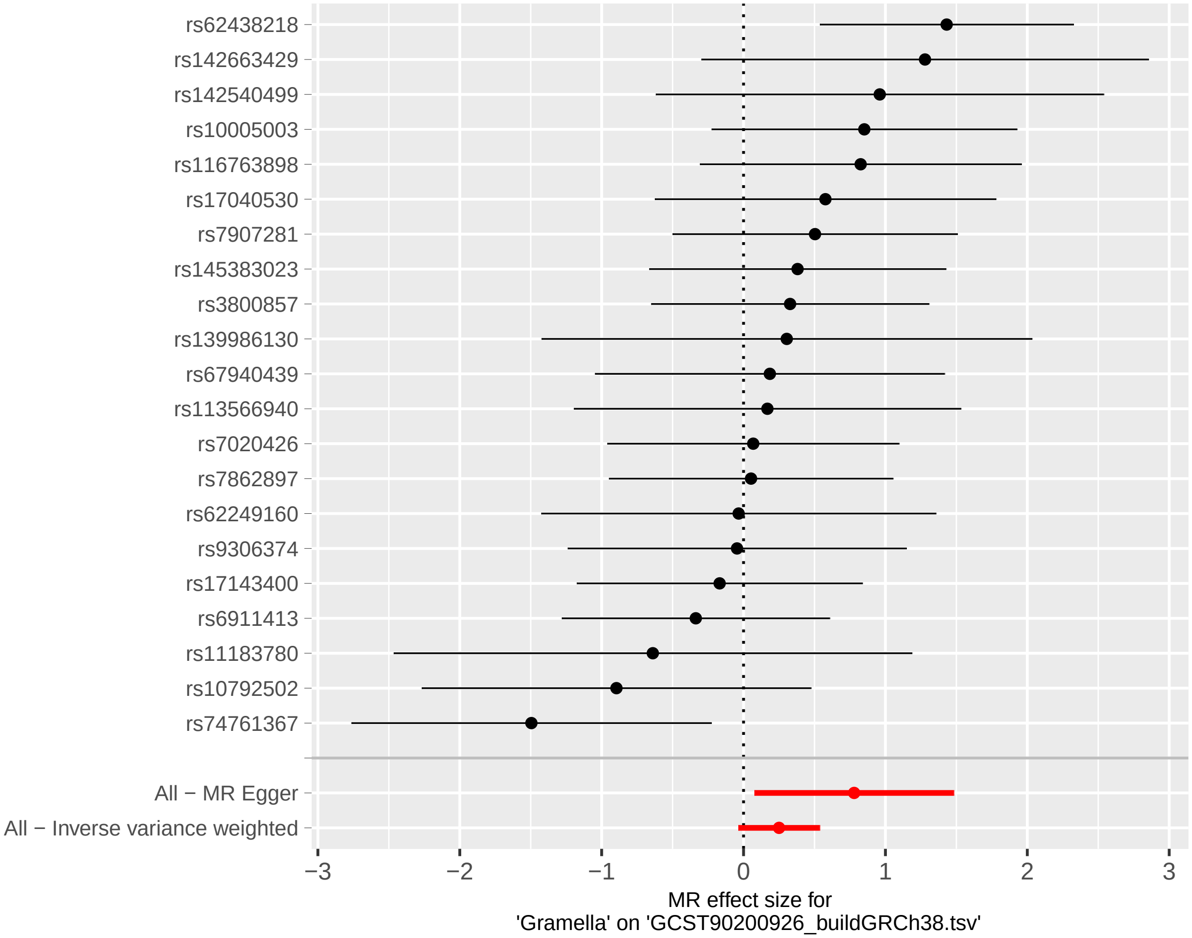

rs62438218
rs142663429
rs142540499
rs10005003
rs116763898
rs17040530
rs7907281
rs145383023
rs3800857
rs139986130
rs67940439
rs113566940
rs7020426
rs7862897
rs62249160
rs9306374
rs17143400
rs6911413
rs11183780
rs10792502
rs74761367
All − MR Egger
All − Inverse variance weighted
−3
−2
−1
0
1
2
3
MR effect size for
'Gramella' on 'GCST90200926_buildGRCh38.tsv'

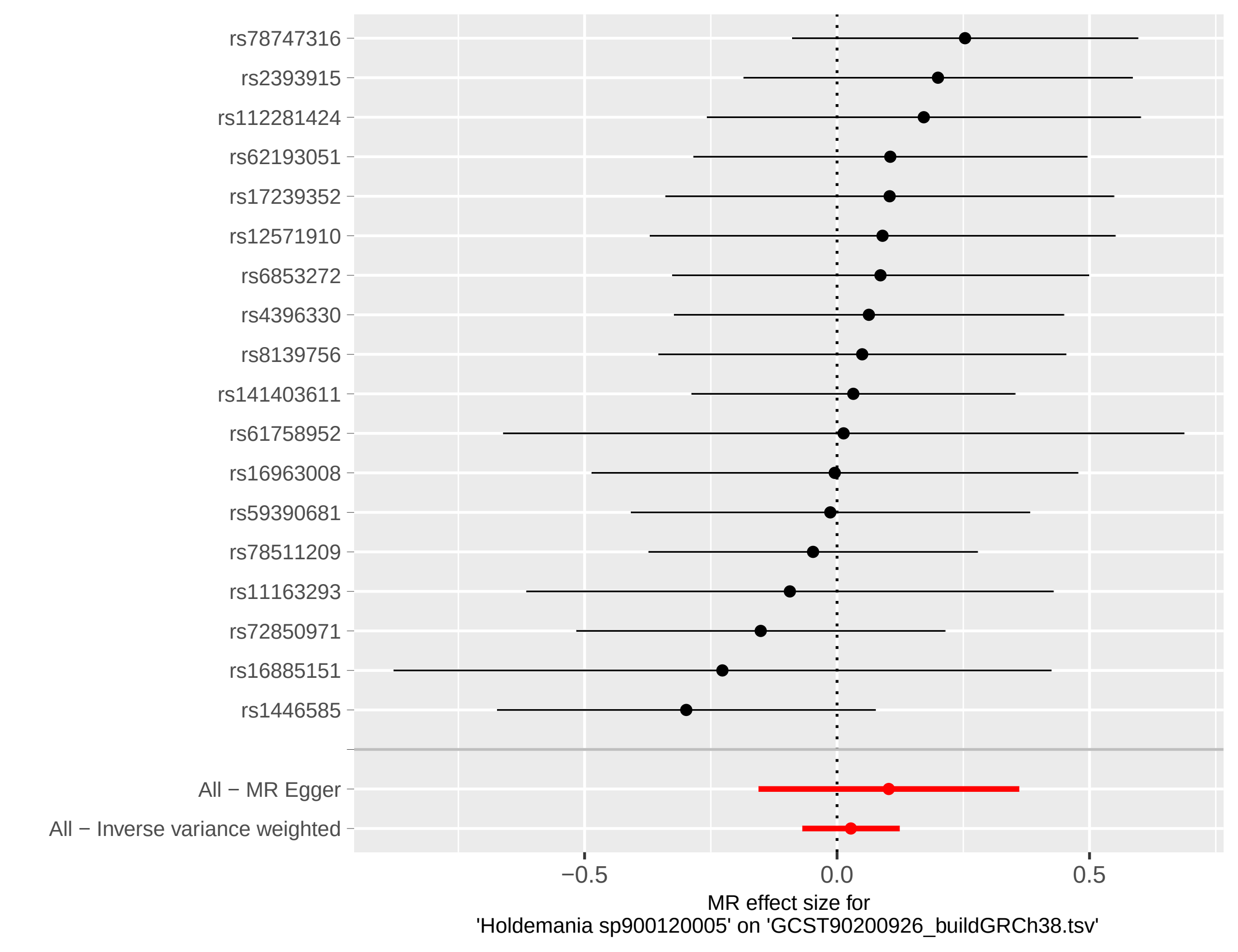
rs78747316
rs2393915
rs112281424
rs62193051
rs17239352
rs12571910
rs6853272
rs4396330
rs8139756
rs141403611
rs61758952
rs16963008
rs59390681
rs78511209
rs11163293
rs72850971
rs16885151
rs1446585
All − MR Egger
All − Inverse variance weighted
−0.5
0.0
0.5
MR effect size for
'Holdemania sp900120005' on 'GCST90200926_buildGRCh38.tsv'

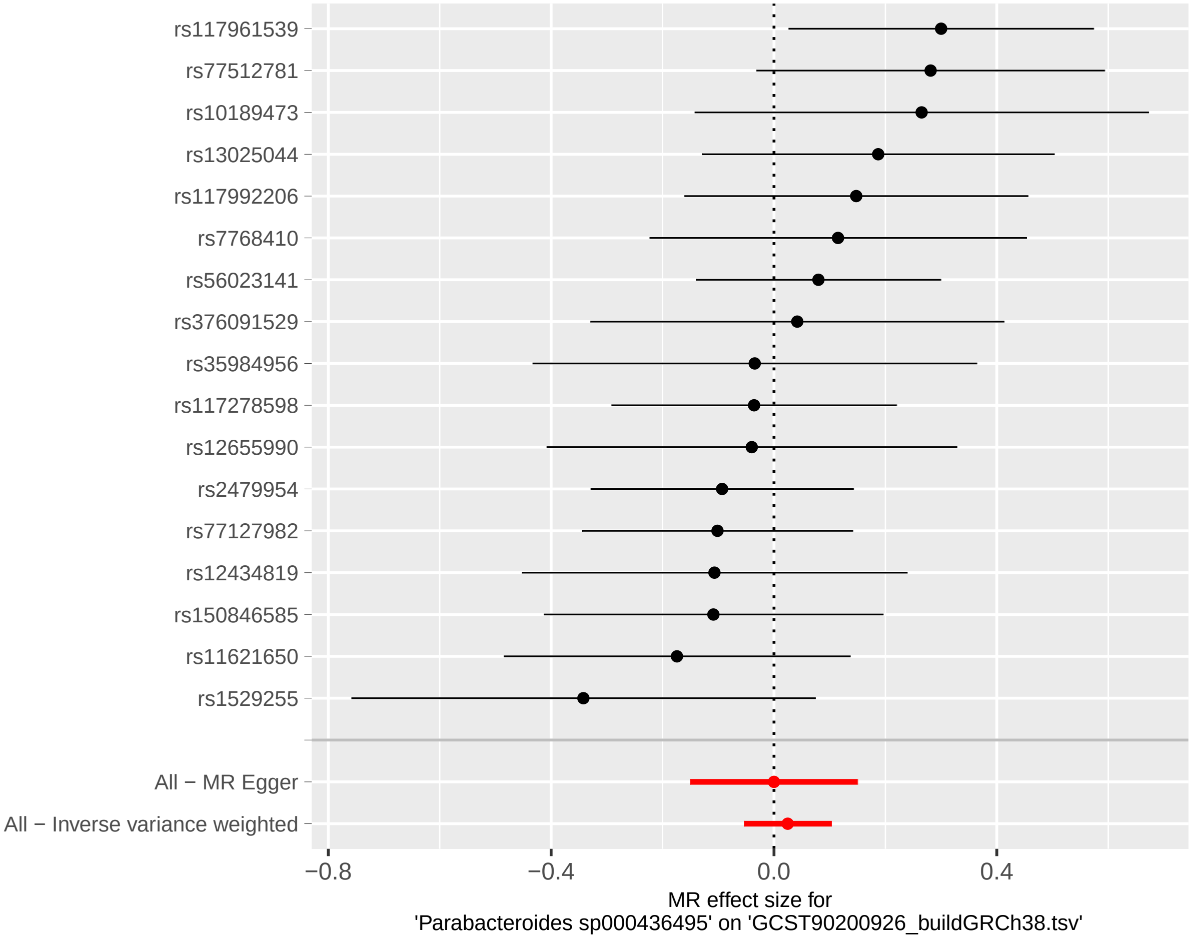
rs117961539
rs77512781
rs10189473
rs13025044
rs117992206
rs7768410
rs56023141
rs376091529
rs35984956
rs117278598
rs12655990
rs2479954
rs77127982
rs12434819
rs150846585
rs11621650
rs1529255
All − MR Egger
All − Inverse variance weighted
−0.8
−0.4
0.0
0.4
MR effect size for
'Parabacteroides sp000436495' on 'GCST90200926_buildGRCh38.tsv'

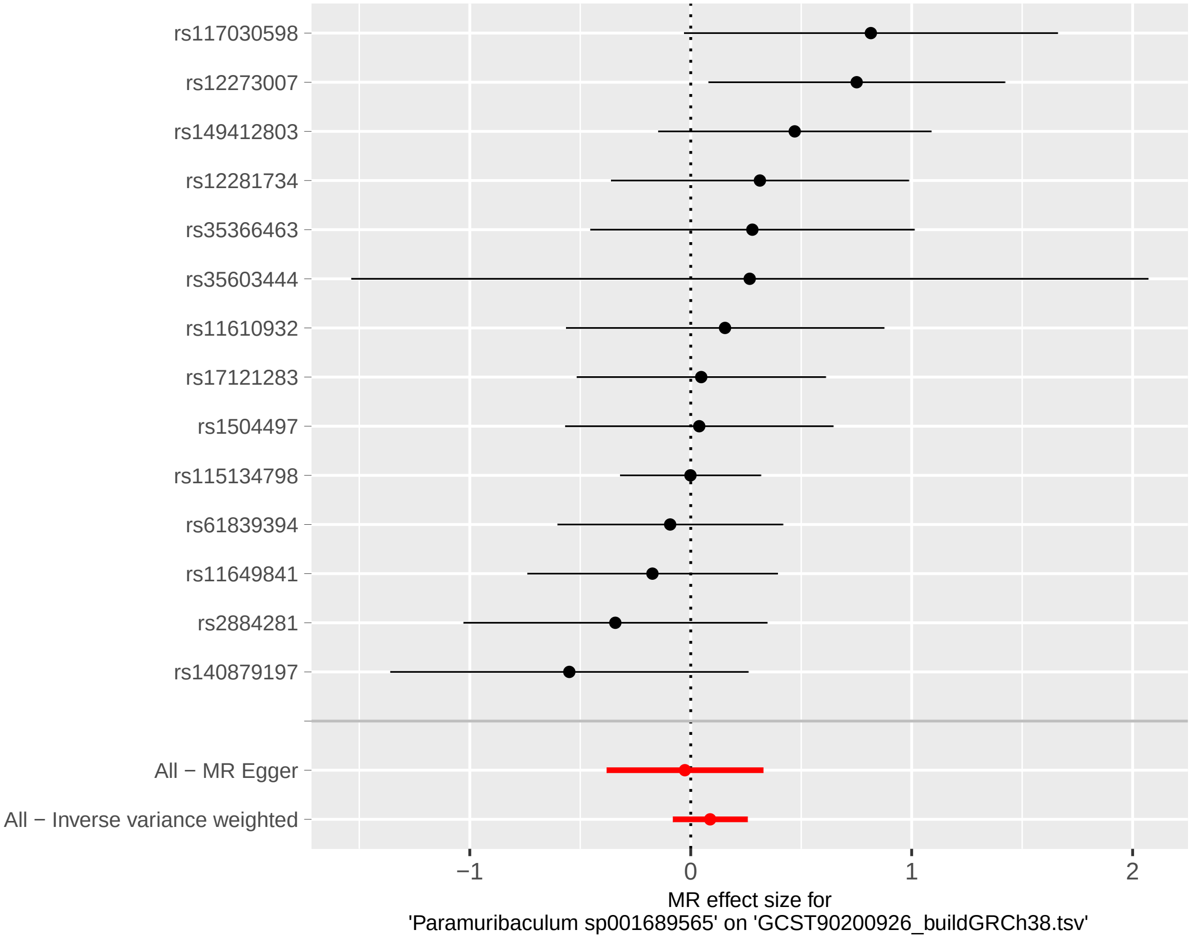

rs117030598
rs12273007
rs149412803
rs12281734
rs35366463
rs35603444
rs11610932
rs17121283
rs1504497
rs115134798
rs61839394
rs11649841
rs2884281
rs140879197
All − MR Egger
All − Inverse variance weighted
−1
0
1
2
MR effect size for
'Paramuribaculum sp001689565' on 'GCST90200926_buildGRCh38.tsv'

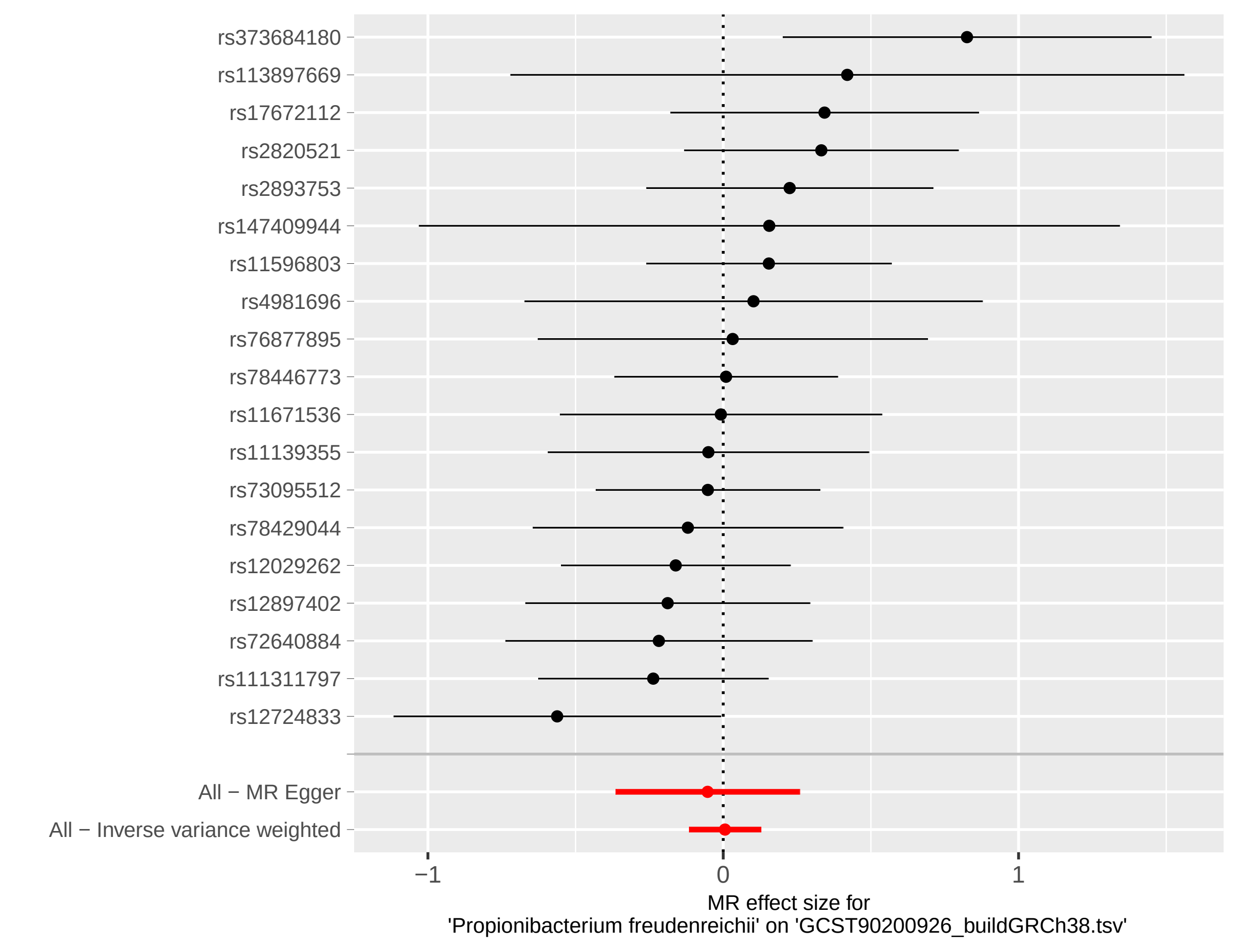
rs373684180
rs113897669
rs17672112
rs2820521
rs2893753
rs147409944
rs11596803
rs4981696
rs76877895
rs78446773
rs11671536
rs11139355
rs73095512
rs78429044
rs12029262
rs12897402
rs72640884
rs111311797
rs12724833
All − MR Egger
All − Inverse variance weighted
−1
0
1
MR effect size for
'Propionibacterium freudenreichii' on 'GCST90200926_buildGRCh38.tsv'

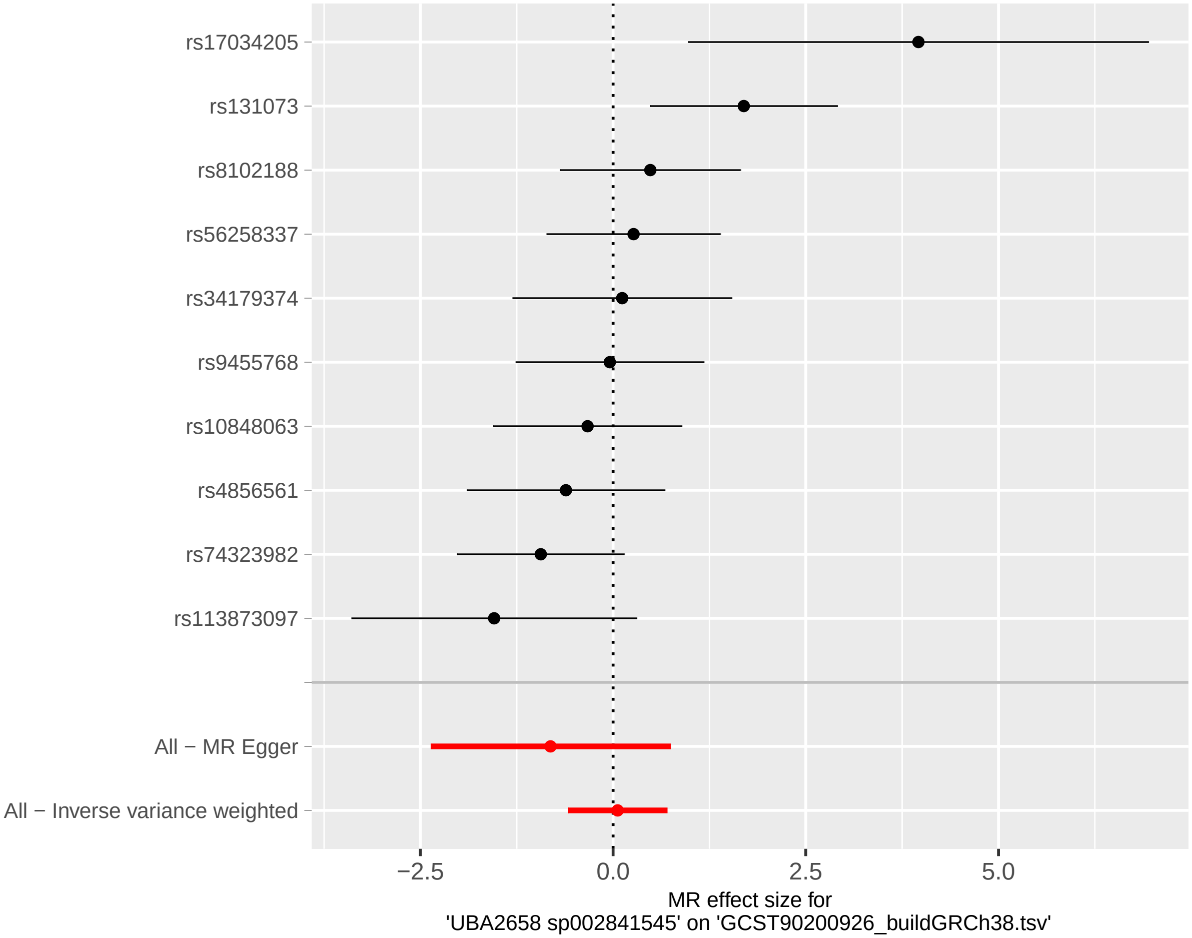

rs17034205
rs131073
rs8102188
rs56258337
rs34179374
rs9455768
rs10848063
rs4856561
rs74323982
rs113873097
All − MR Egger
All − Inverse variance weighted
−2.5
0.0
2.5
5.0
MR effect size for
'UBA2658 sp002841545' on 'GCST90200926_buildGRCh38.tsv'

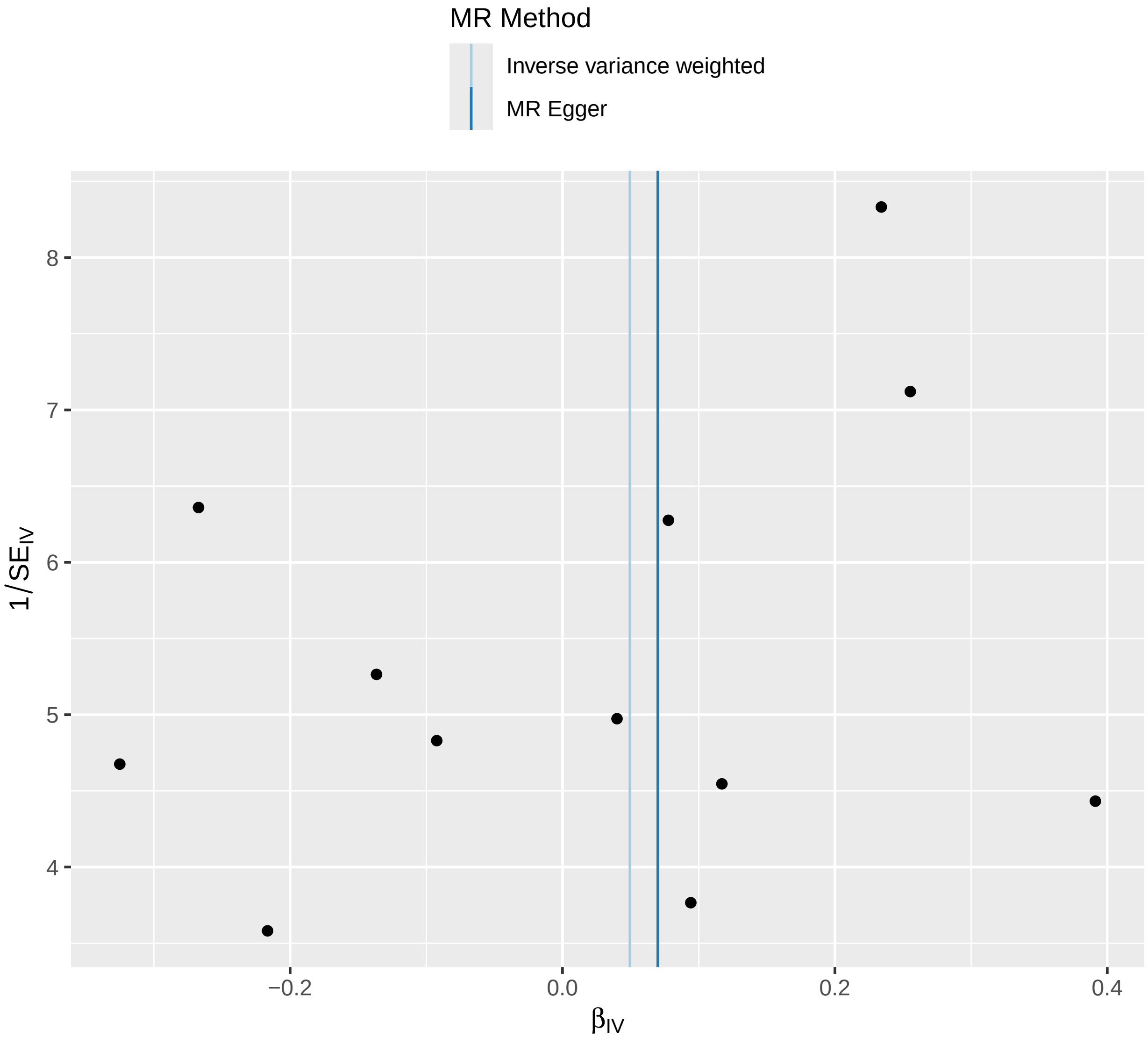
MR Method
Inverse variance weighted
MR Egger
8
7
6
5
4
1/SE_IV
−0.2
0.0
0.2
0.4
β_IV

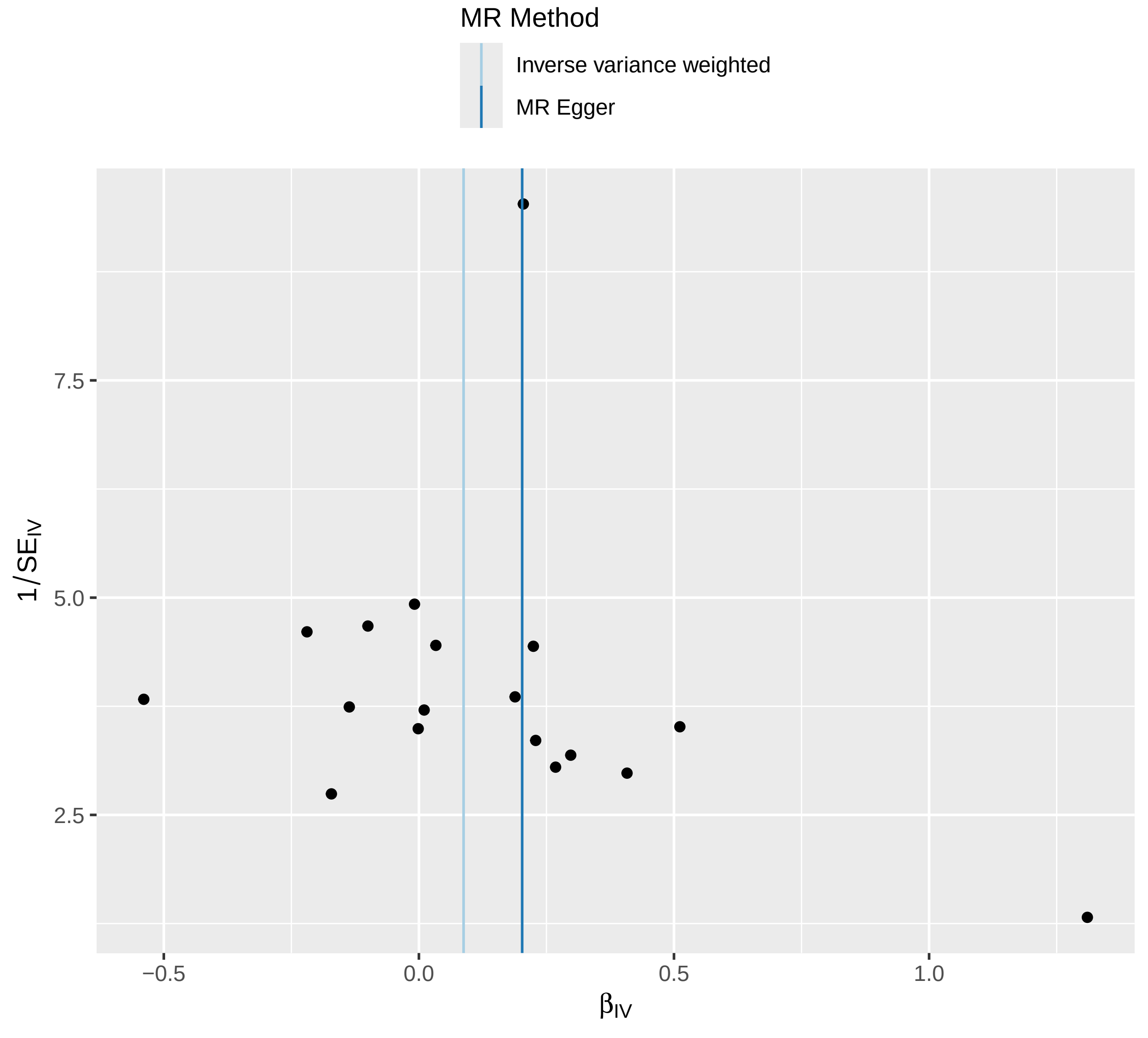
MR Method
Inverse variance weighted
MR Egger
7.5
5.0
2.5
−0.5
0.0
0.5
1.0
$1/SE_{IV}$
β_{IV}

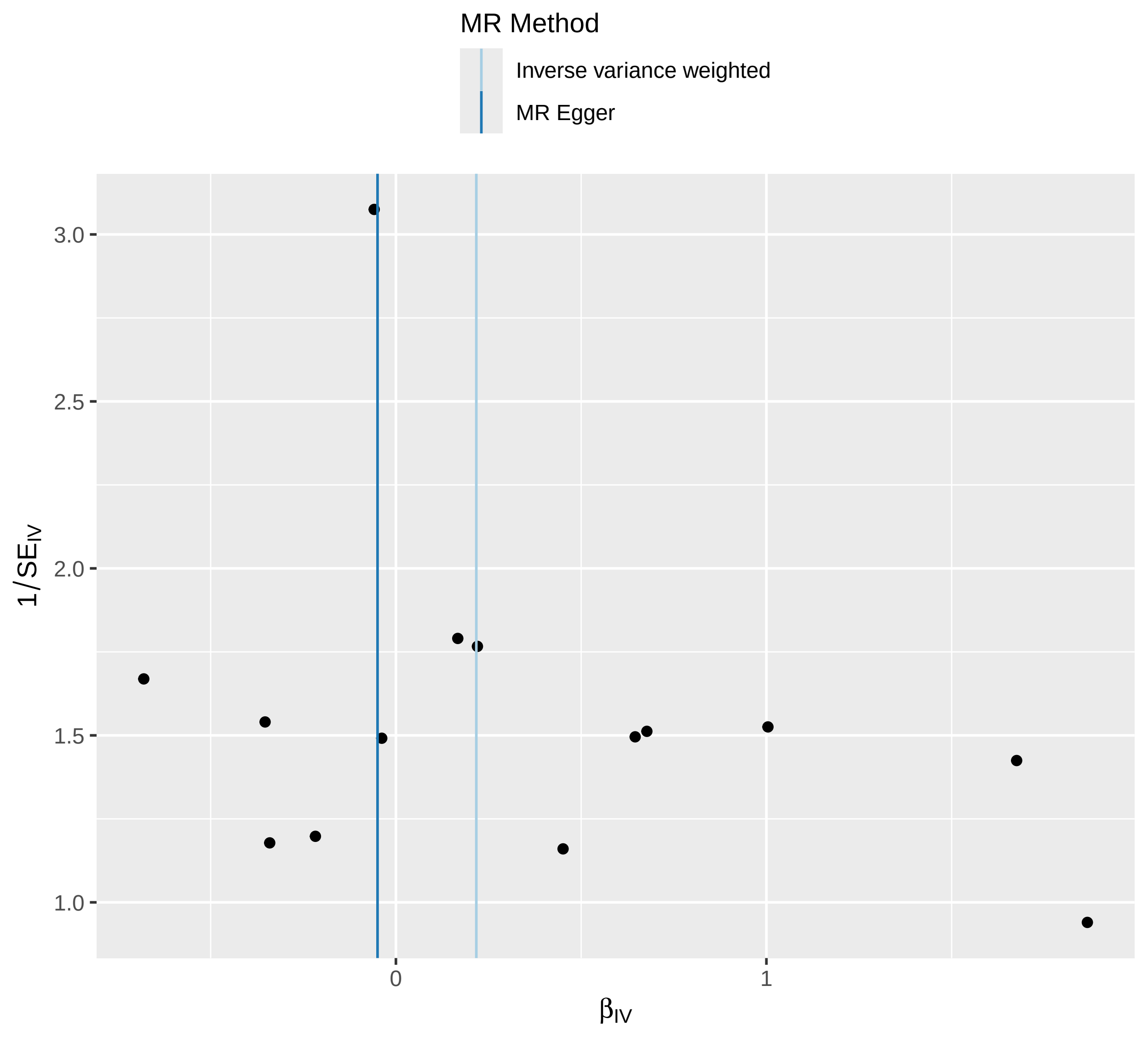
MR Method
Inverse variance weighted
MR Egger
3.0
2.5
2.0
1.5
1.0
$1/SE_{IV}$
0
1
β_{IV}

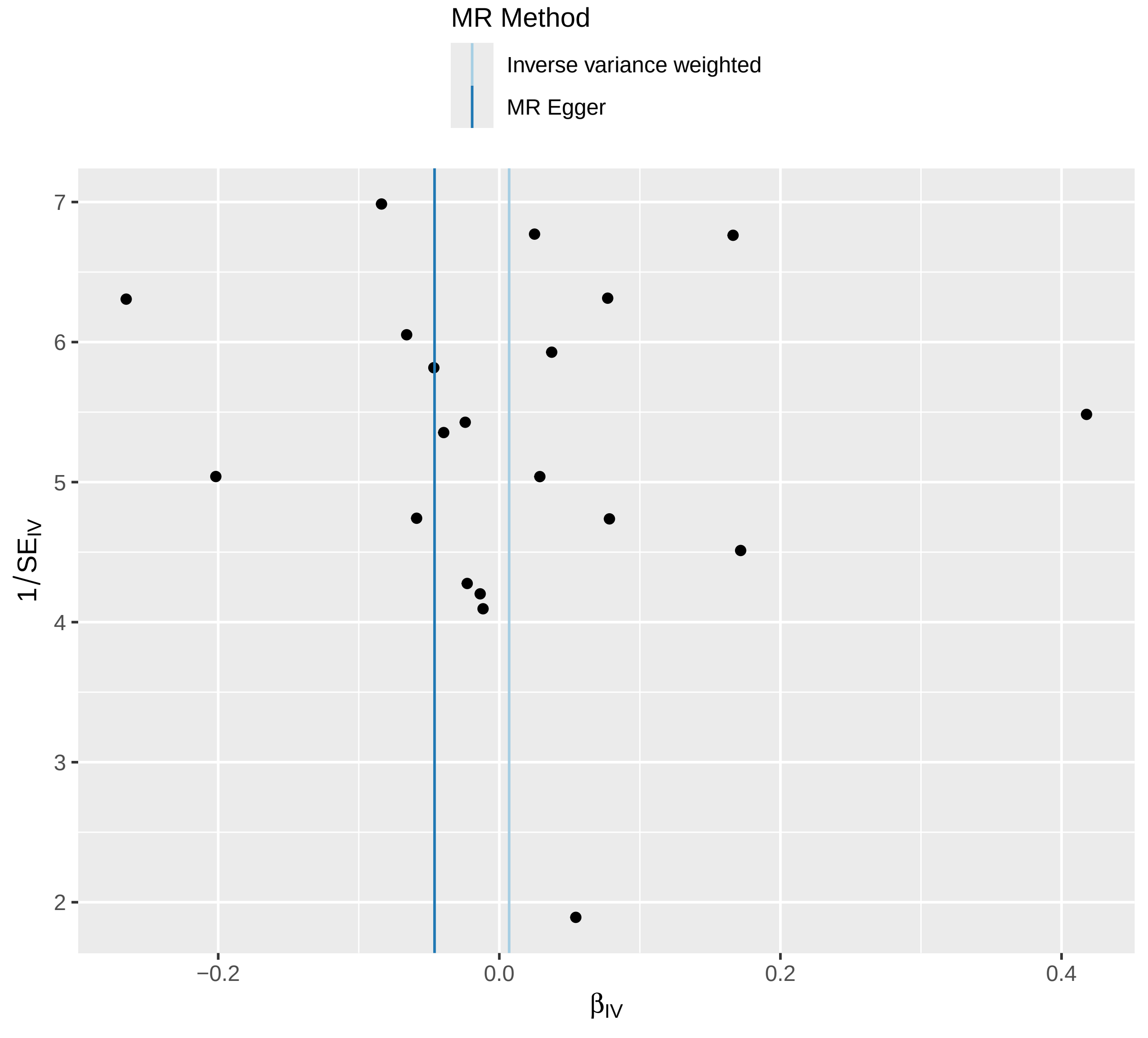
MR Method
Inverse variance weighted
MR Egger
1/SE_IV
β_IV
7
6
5
4
3
2
−0.2
0.0
0.2
0.4

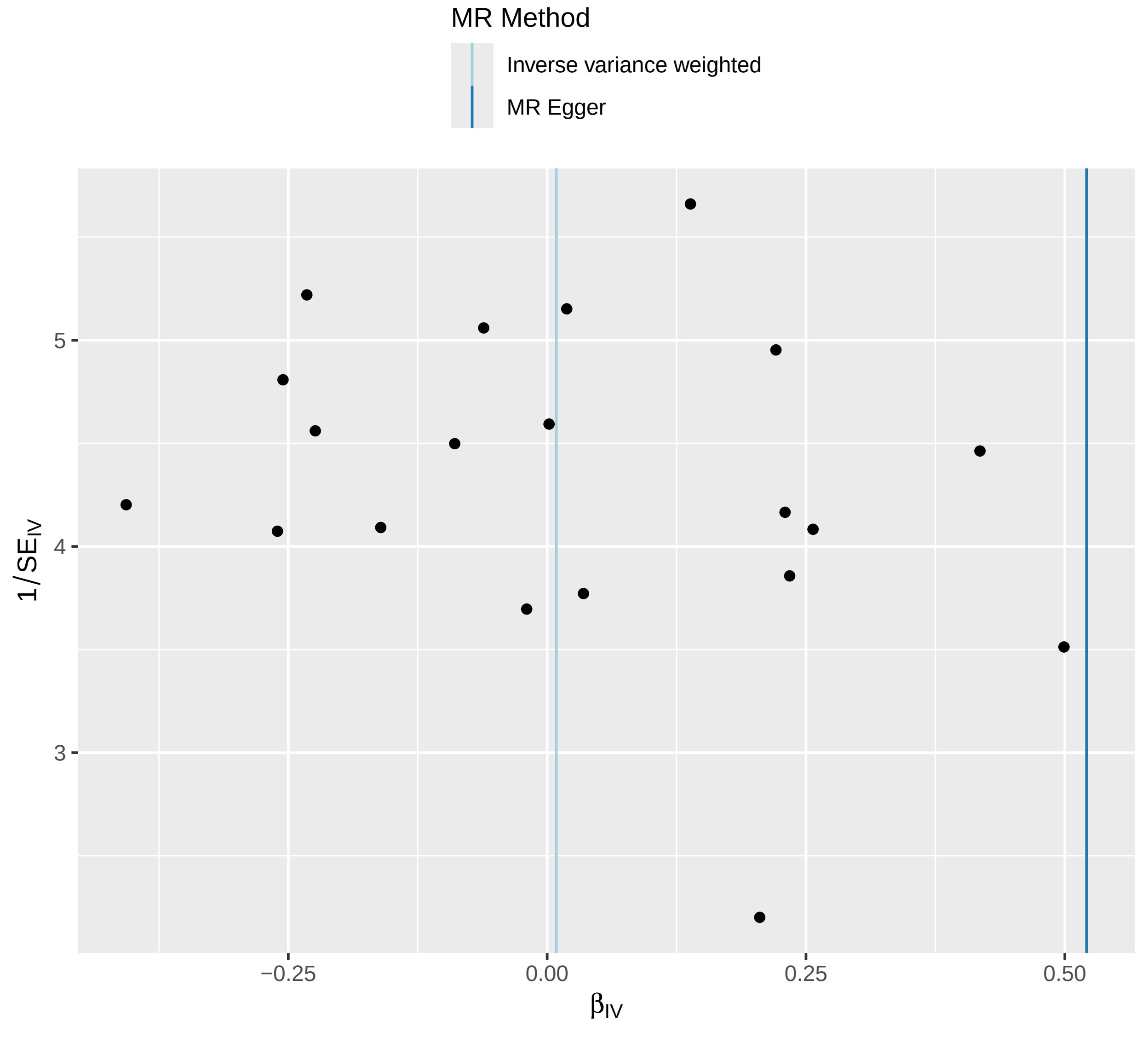
MR Method
Inverse variance weighted
MR Egger
5
4
3
1/SE_IV
−0.25
0.00
0.25
0.50
β_IV

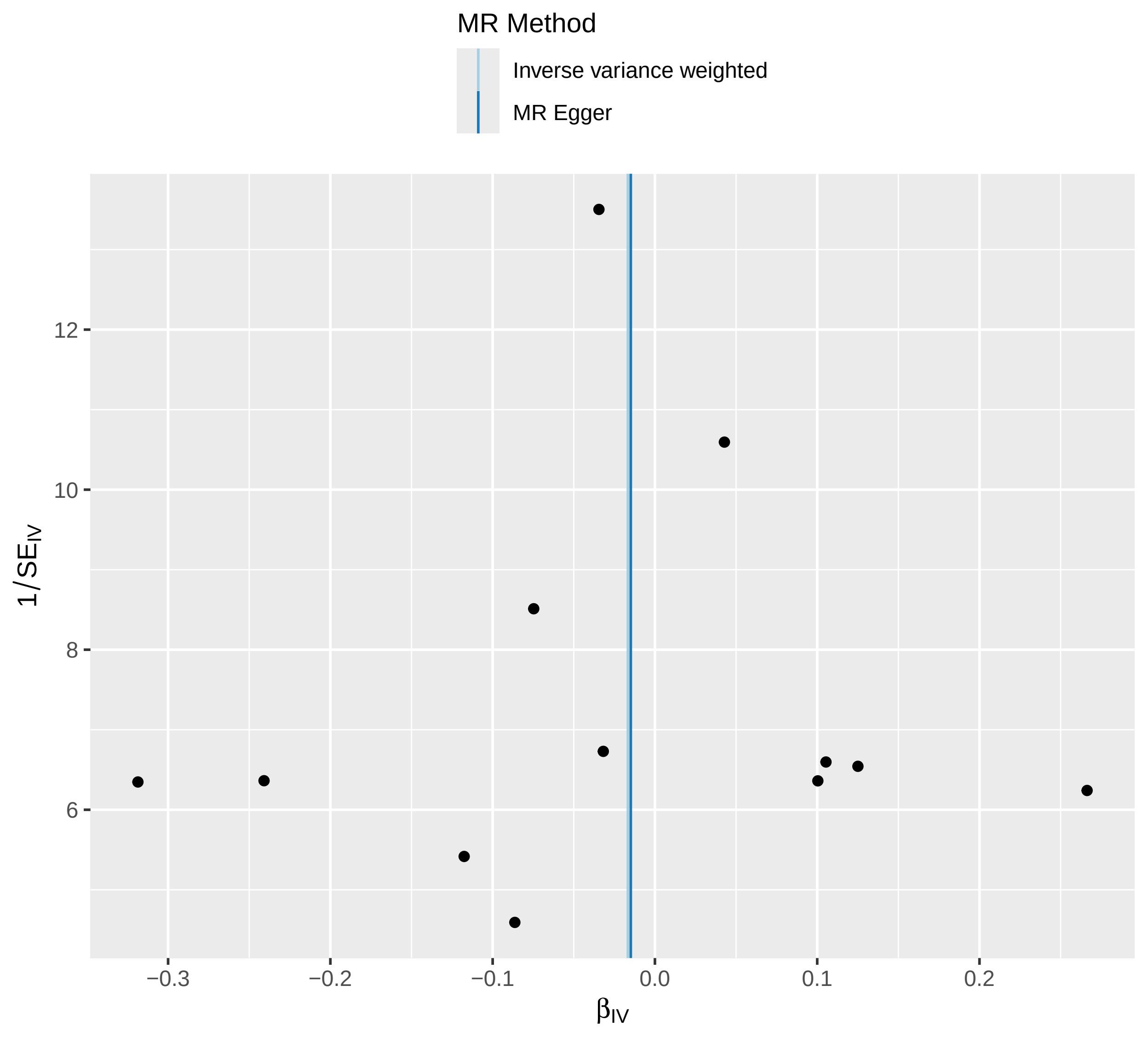
MR Method
Inverse variance weighted
MR Egger
12
10
8
6
1/SE_IV
−0.3
−0.2
−0.1
0.0
0.1
0.2
β_IV

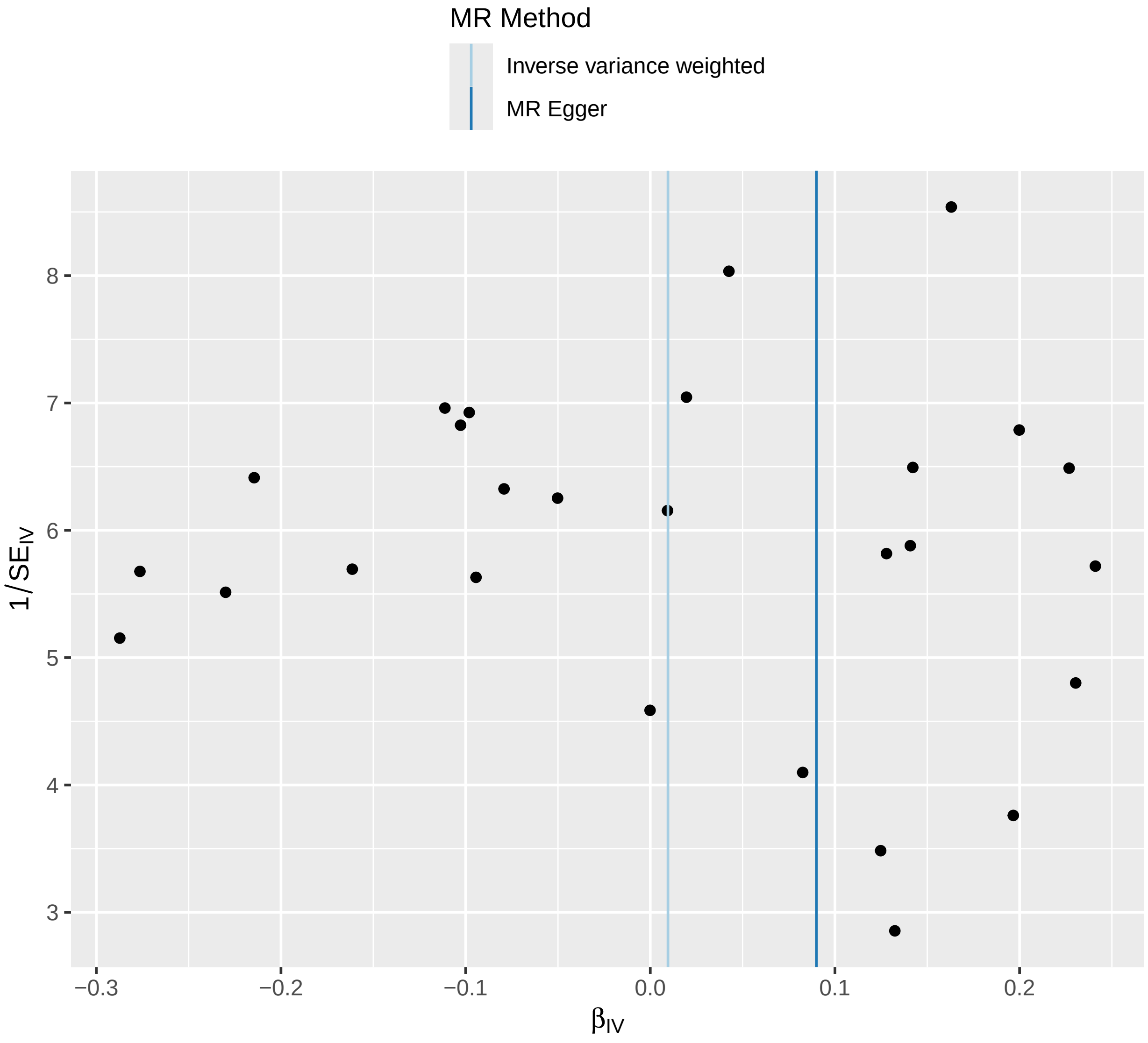

MR Method
Inverse variance weighted
MR Egger
8
7
6
5
4
3
1/SE$_{IV}$
−0.3
−0.2
−0.1
0.0
0.1
0.2
β$_{IV}$

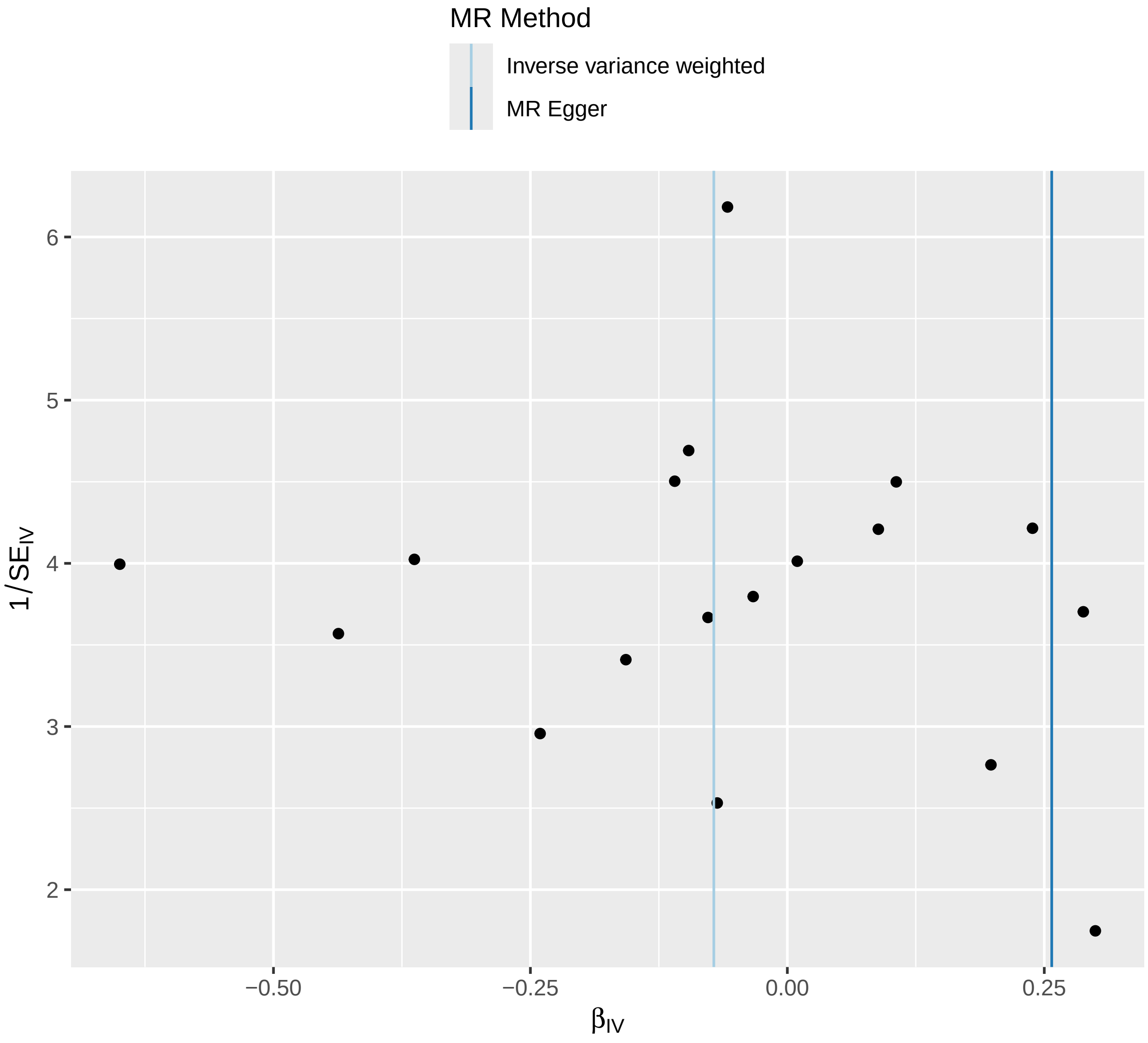
MR Method
Inverse variance weighted
MR Egger
6
5
4
3
2
1/SE_IV
−0.50
−0.25
0.00
0.25
β_IV

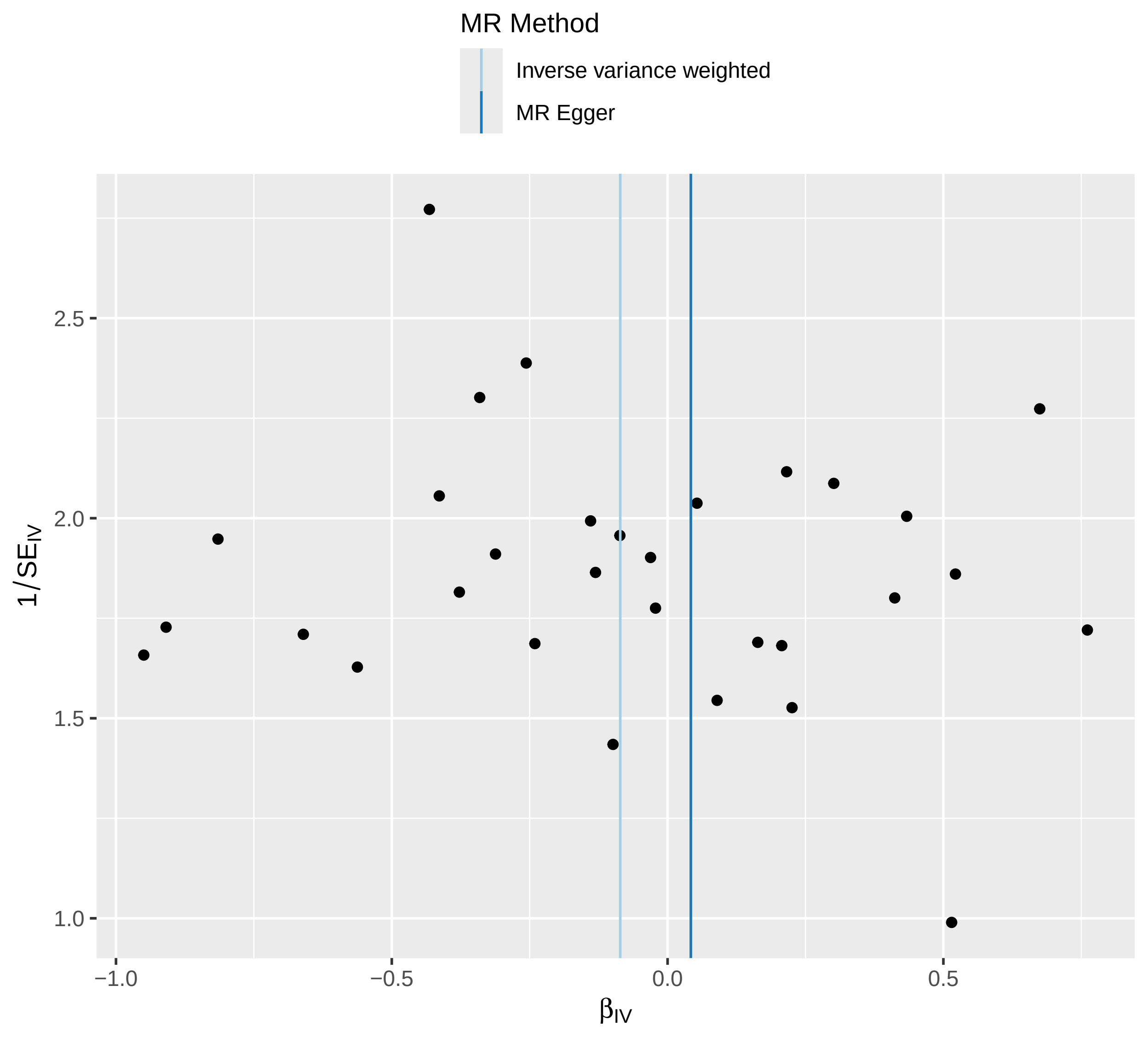

MR Method
Inverse variance weighted
MR Egger
2.5
2.0
1.5
1.0
$1/SE_{IV}$
−1.0
−0.5
0.0
0.5
β_{IV}

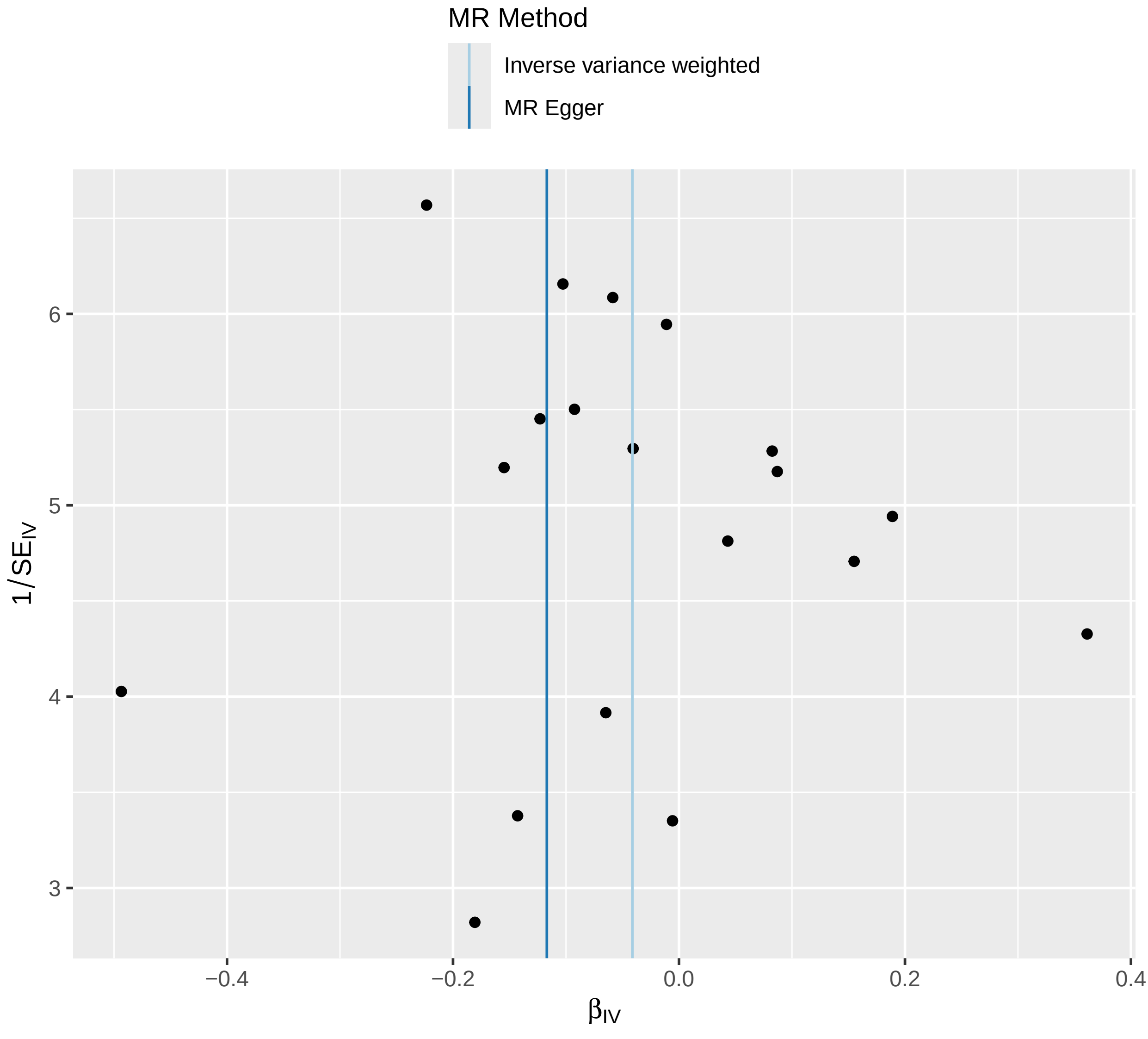

MR Method
Inverse variance weighted
MR Egger
1/SE$_{IV}$
6
5
4
3
−0.4
−0.2
0.0
0.2
0.4
β$_{IV}$

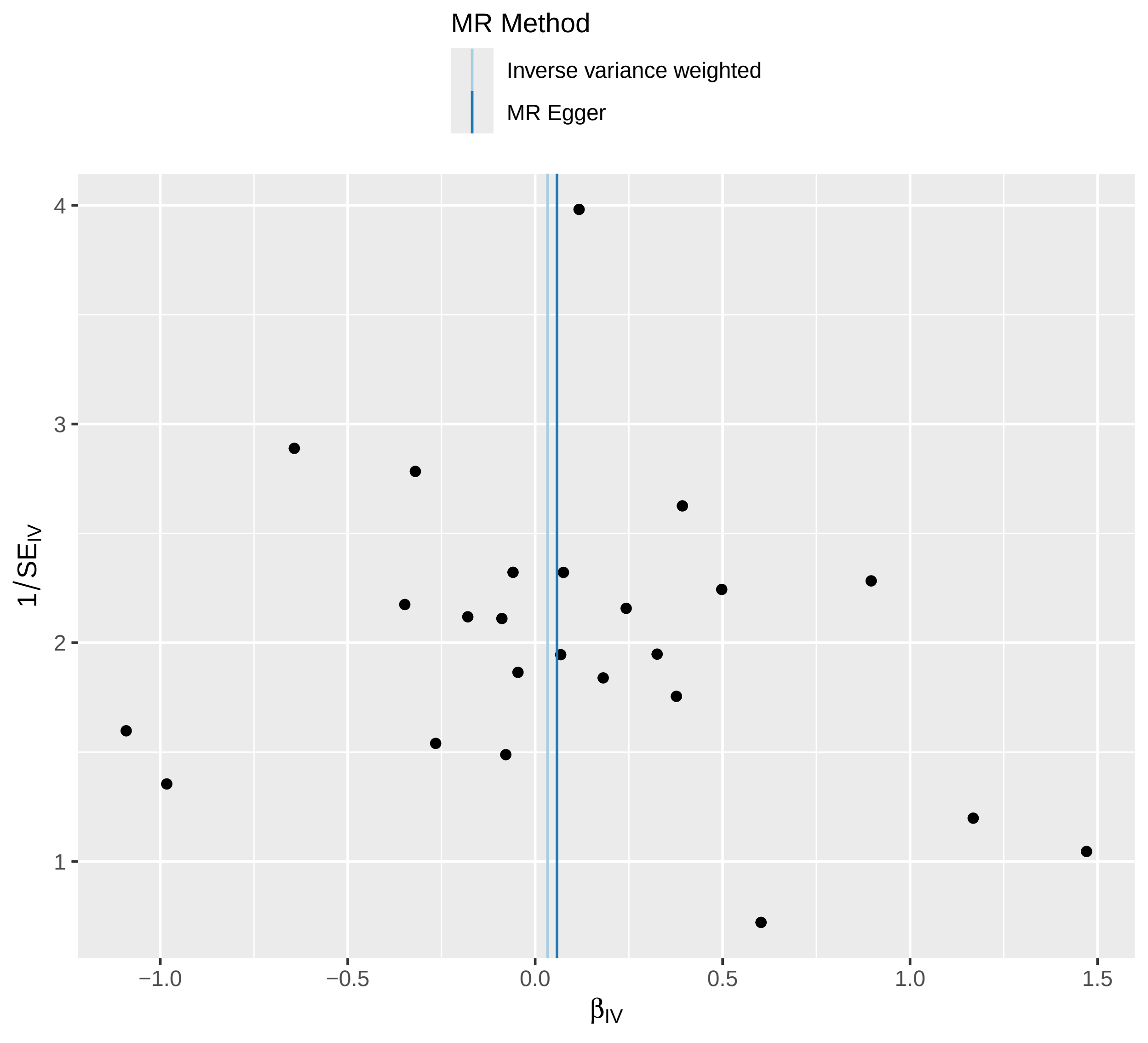
MR Method
Inverse variance weighted
MR Egger
4
3
2
1
1/SE_IV
−1.0
−0.5
0.0
0.5
1.0
1.5
β_IV

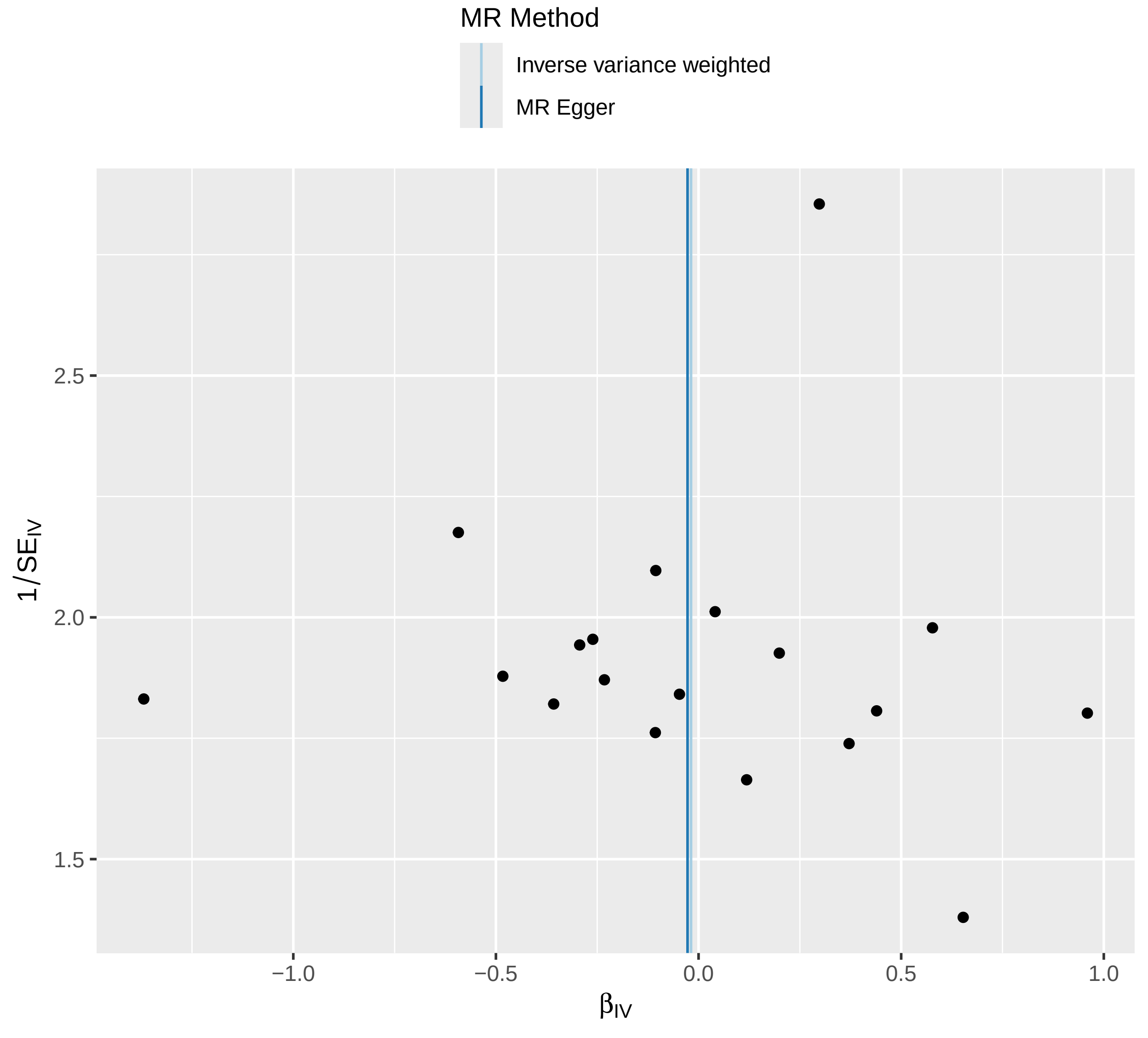

MR Method
Inverse variance weighted
MR Egger
2.5
2.0
1.5
1/SE_IV
−1.0
−0.5
0.0
0.5
1.0
β_IV

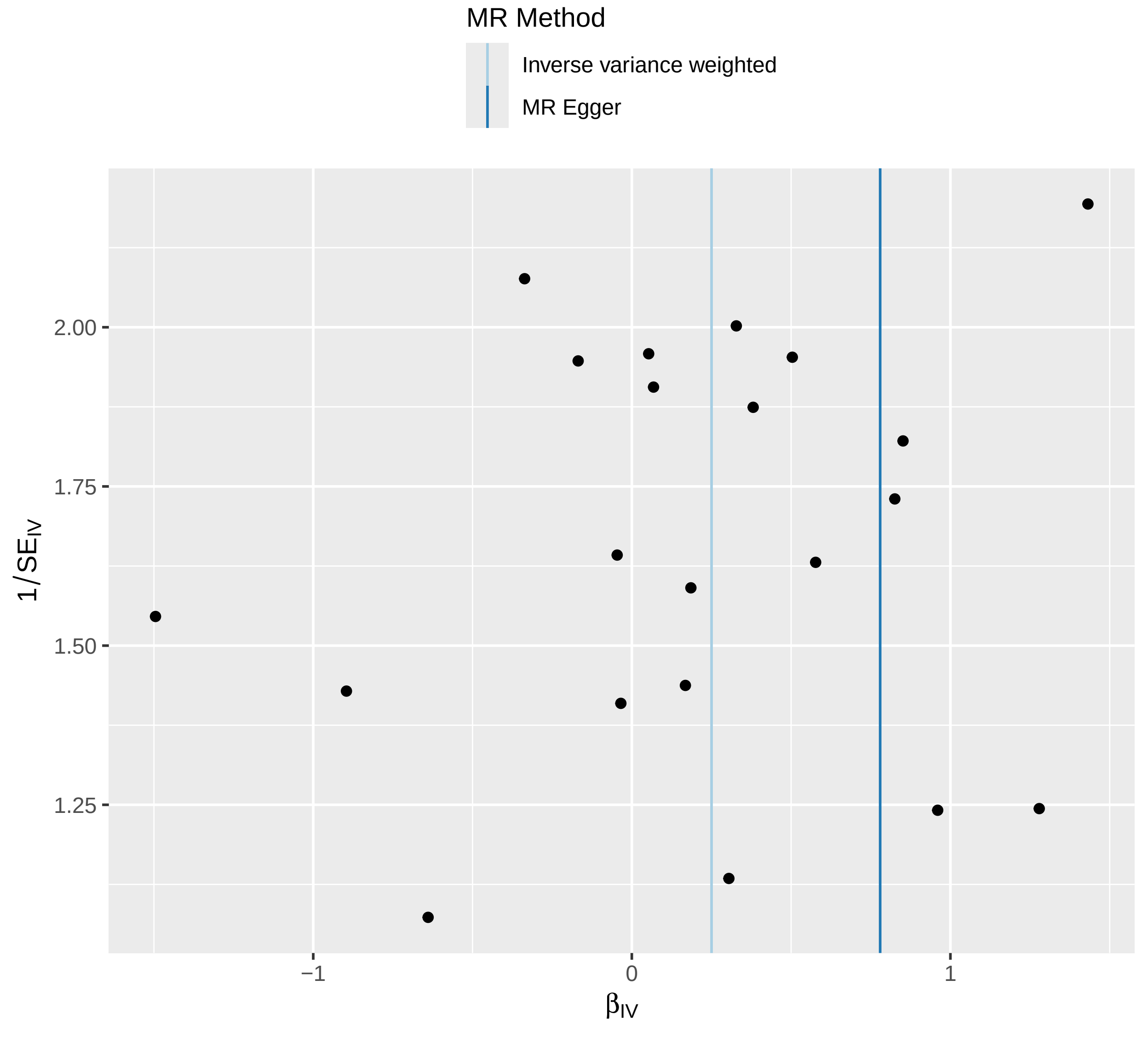
MR Method
Inverse variance weighted
MR Egger
2.00
1.75
1.50
1.25
$1/SE_{IV}$
−1
0
1
β_{IV}

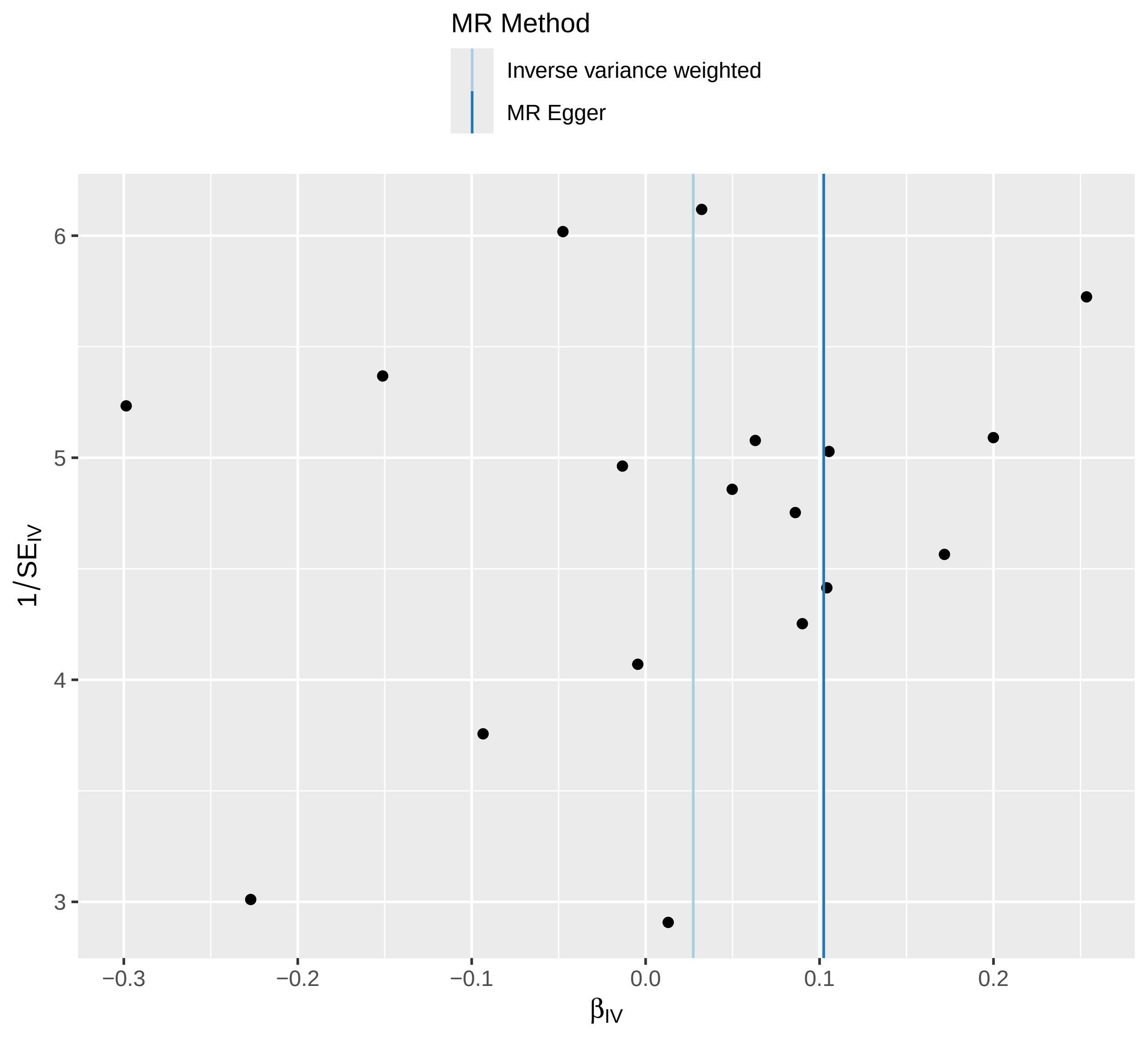
MR Method
Inverse variance weighted
MR Egger
1/SE_IV
6
5
4
3
−0.3
−0.2
−0.1
0.0
0.1
0.2
β_IV

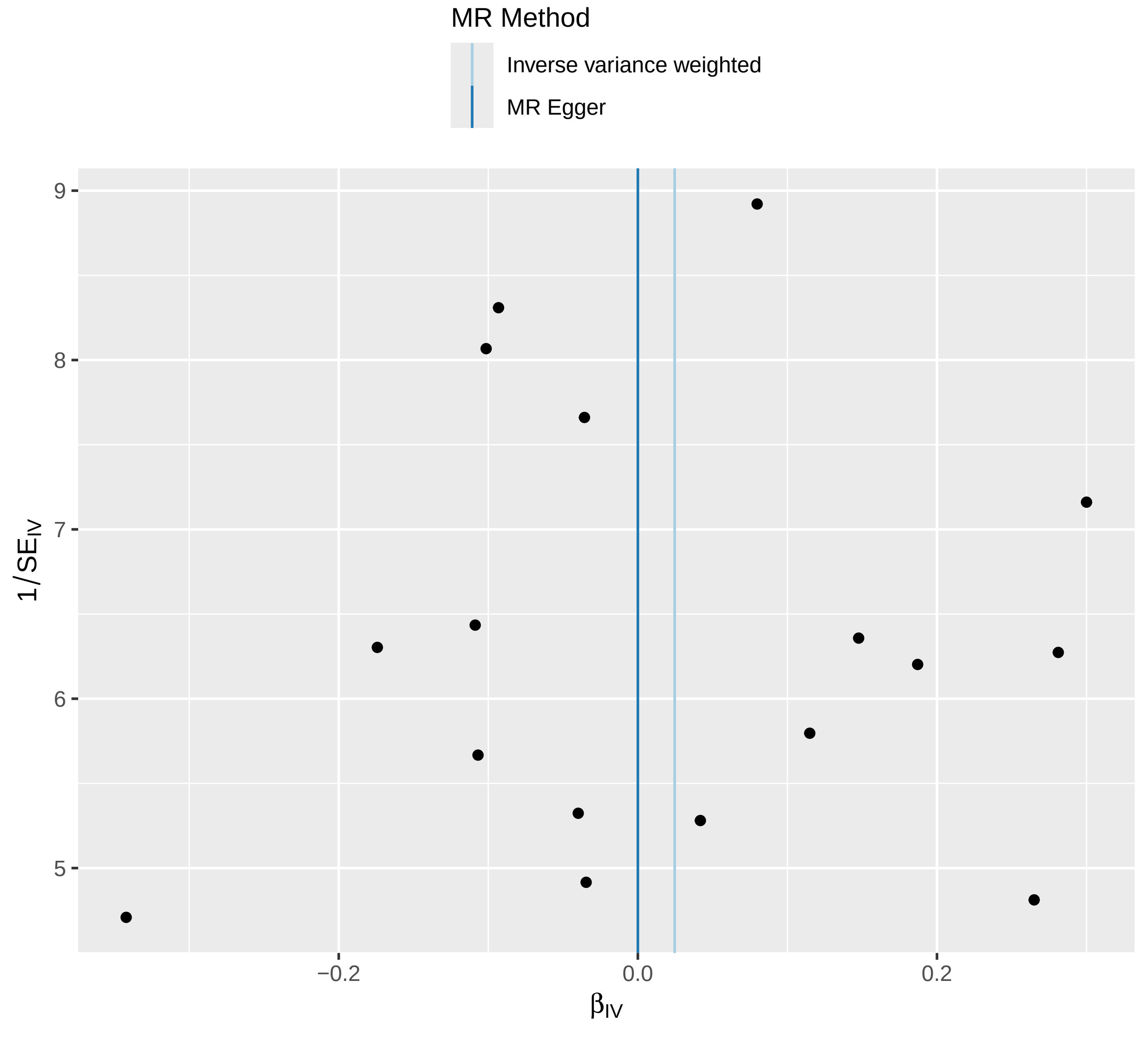

MR Method
Inverse variance weighted
MR Egger
9
8
7
6
5
1/SE$_{IV}$
−0.2
0.0
0.2
β$_{IV}$

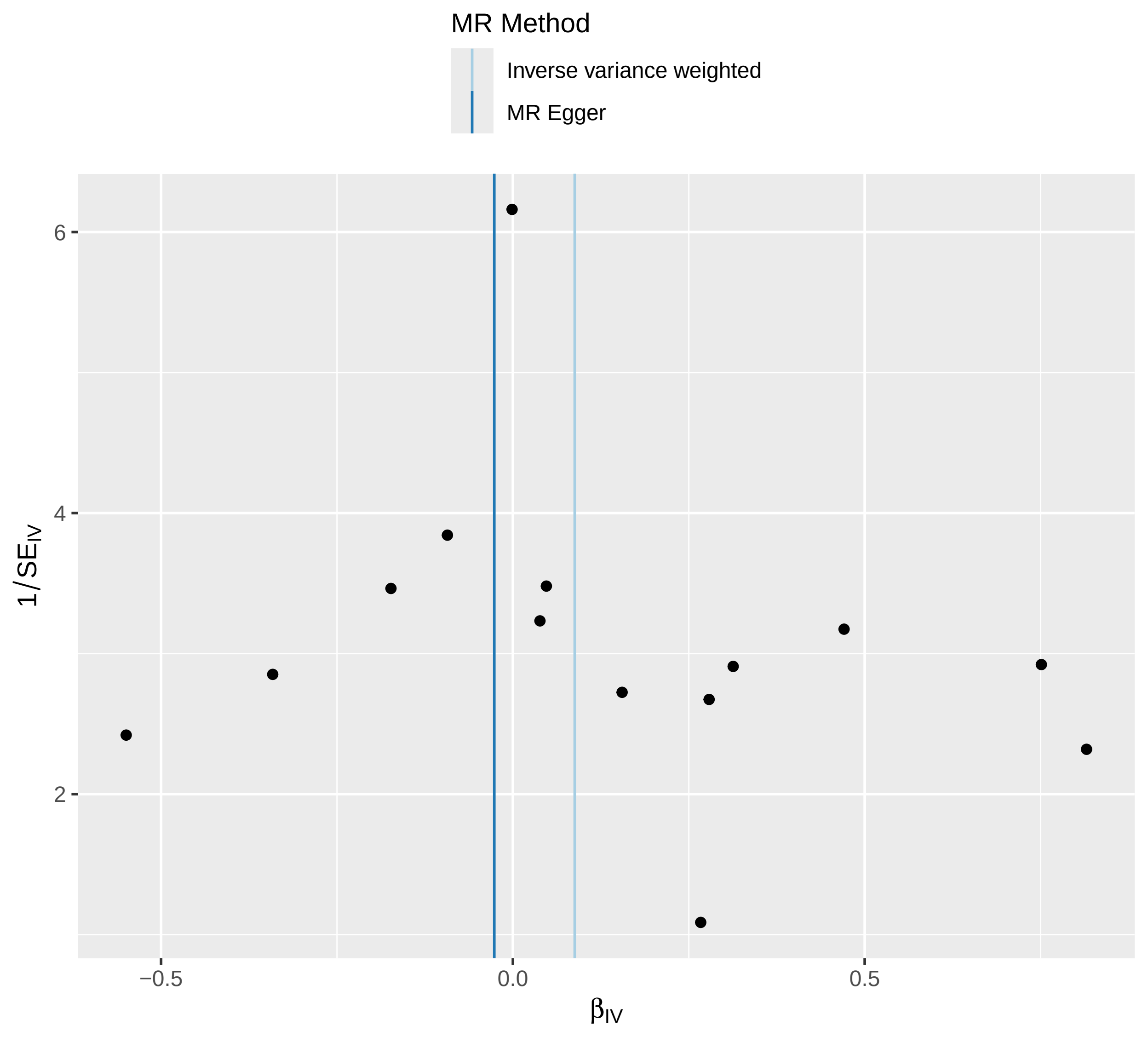
MR Method
Inverse variance weighted
MR Egger
6
4
2
1/SE_IV
−0.5
0.0
0.5
β_IV

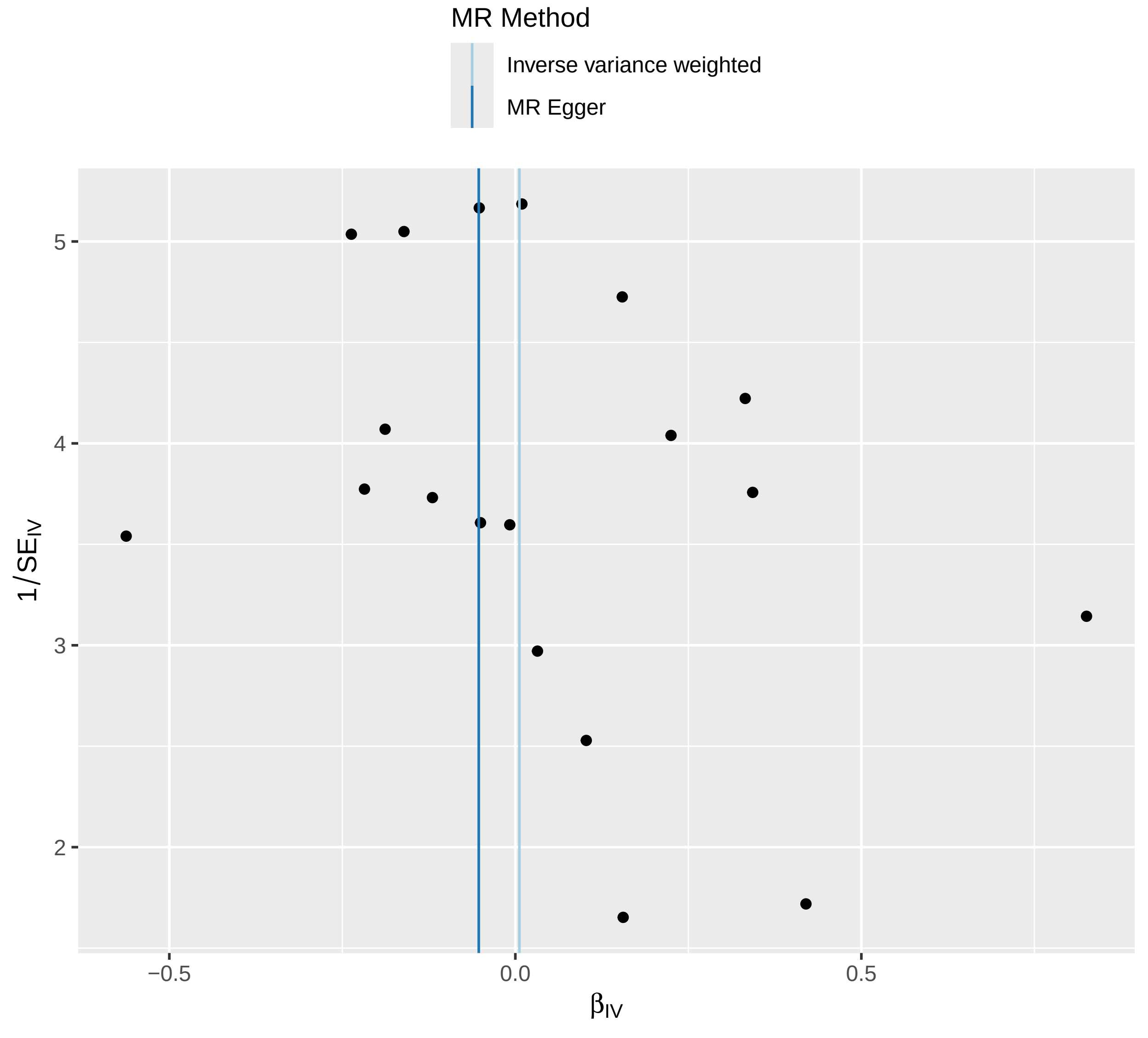
MR Method
Inverse variance weighted
MR Egger
5
4
3
2
−0.5
0.0
0.5
$1/SE_{IV}$
β_{IV}

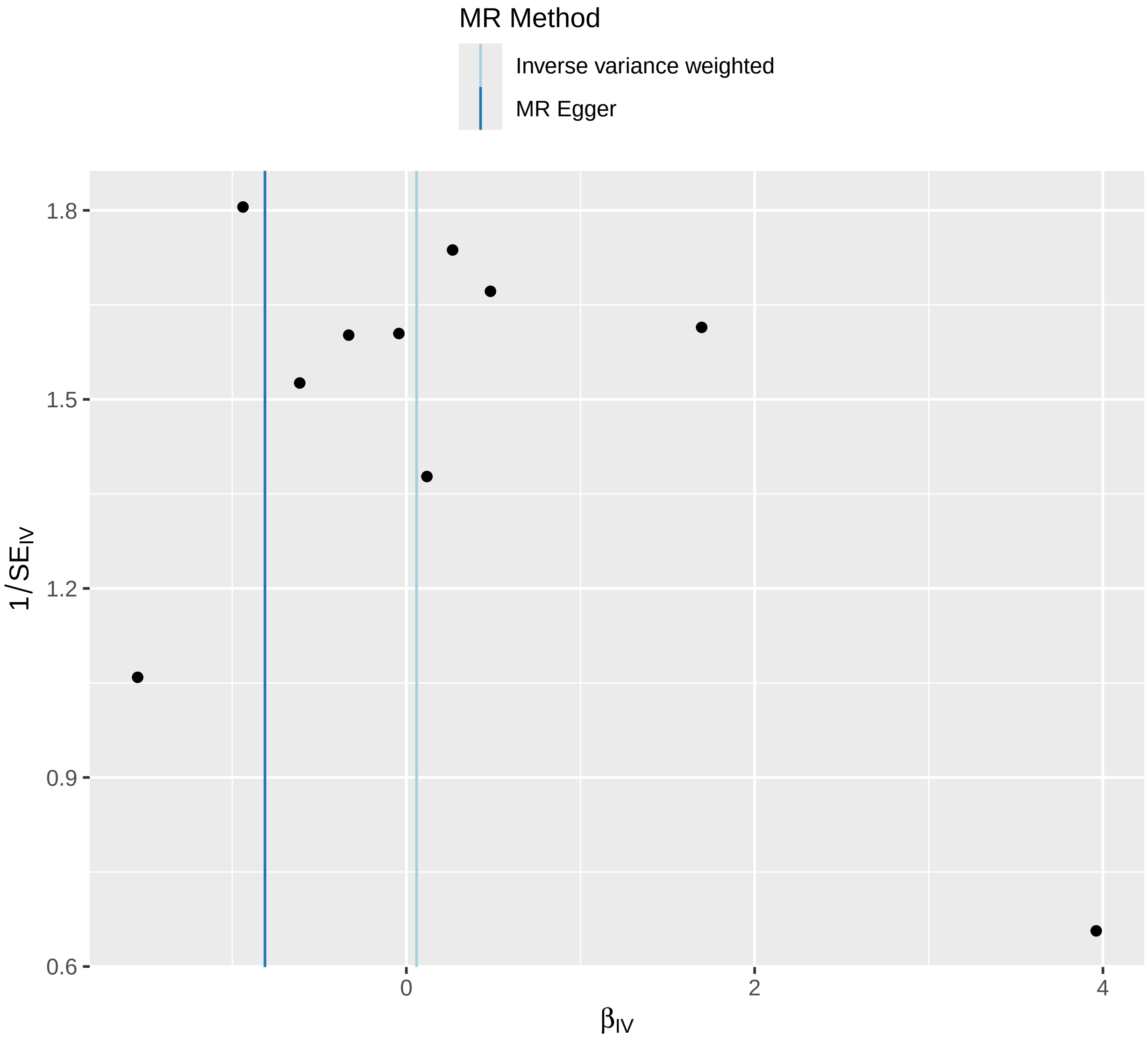
MR Method
Inverse variance weighted
MR Egger
1.8
1.5
1.2
0.9
0.6
1/SE_IV
0
2
4
β_IV

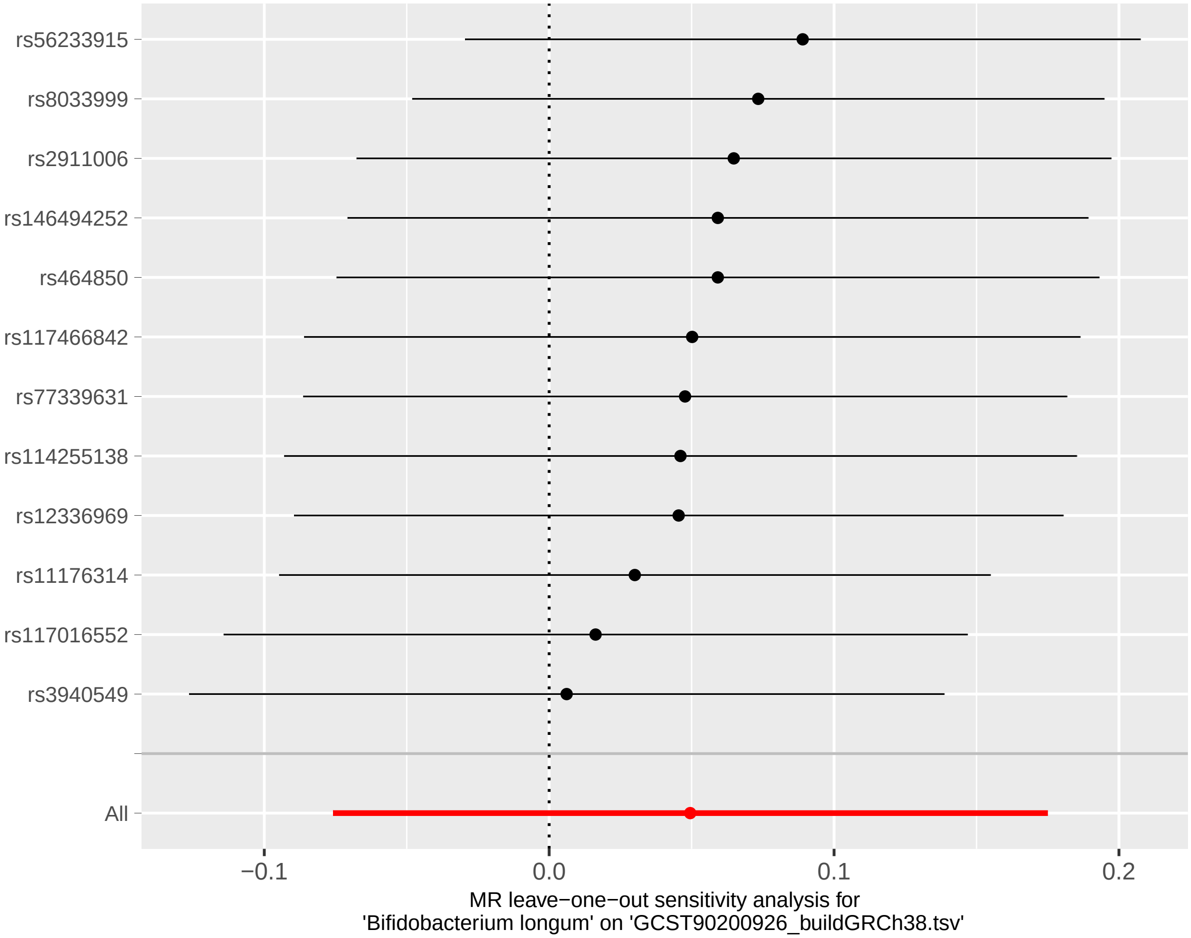

rs56233915
rs8033999
rs2911006
rs146494252
rs464850
rs117466842
rs77339631
rs114255138
rs12336969
rs11176314
rs117016552
rs3940549
All
−0.1
0.0
0.1
0.2
MR leave−one−out sensitivity analysis for
'Bifidobacterium longum' on 'GCST90200926_buildGRCh38.tsv'

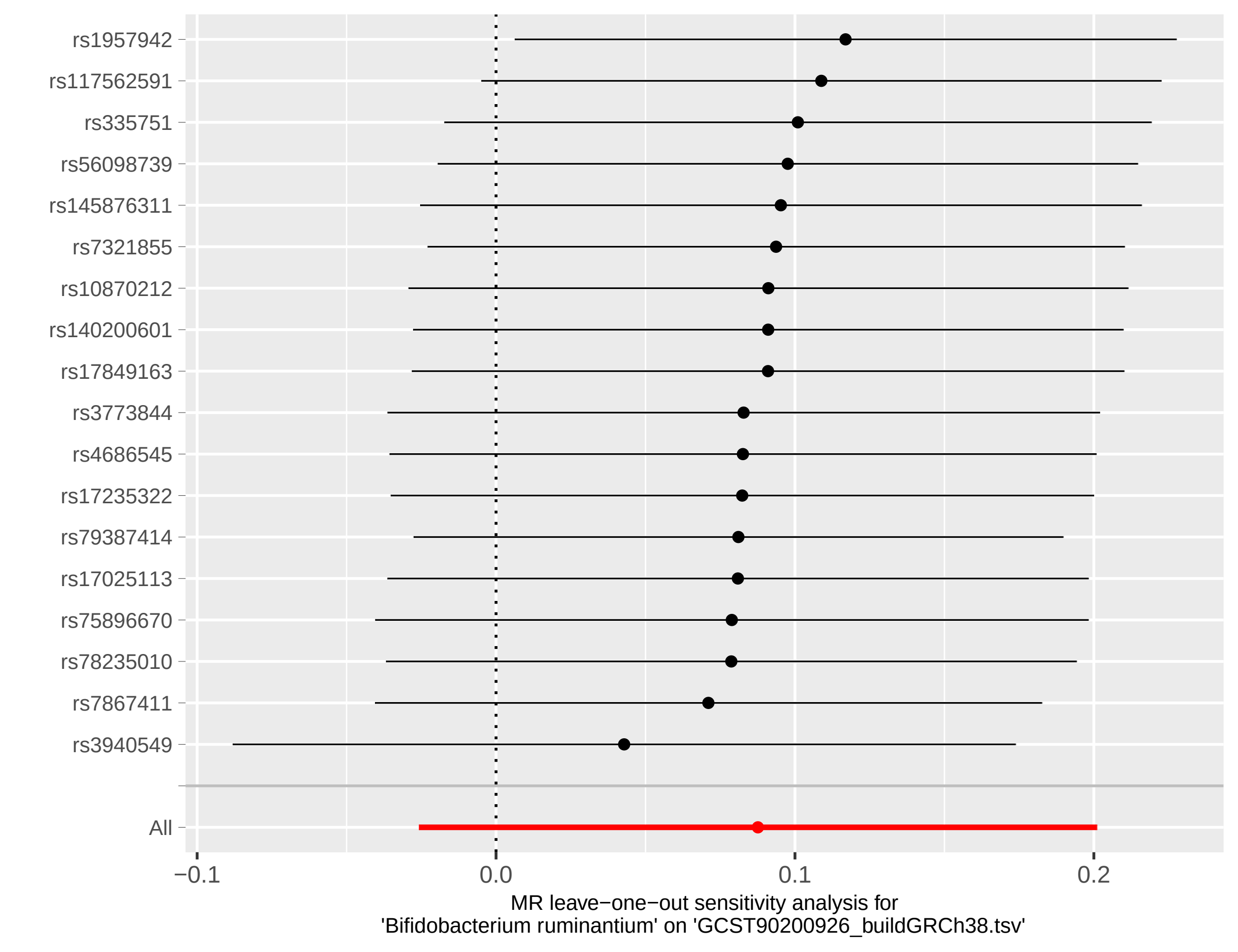
rs1957942
rs117562591
rs335751
rs56098739
rs145876311
rs7321855
rs10870212
rs140200601
rs17849163
rs3773844
rs4686545
rs17235322
rs79387414
rs17025113
rs75896670
rs78235010
rs7867411
rs3940549
All
−0.1
0.0
0.1
0.2
MR leave−one−out sensitivity analysis for
'Bifidobacterium ruminantium' on 'GCST90200926_buildGRCh38.tsv'

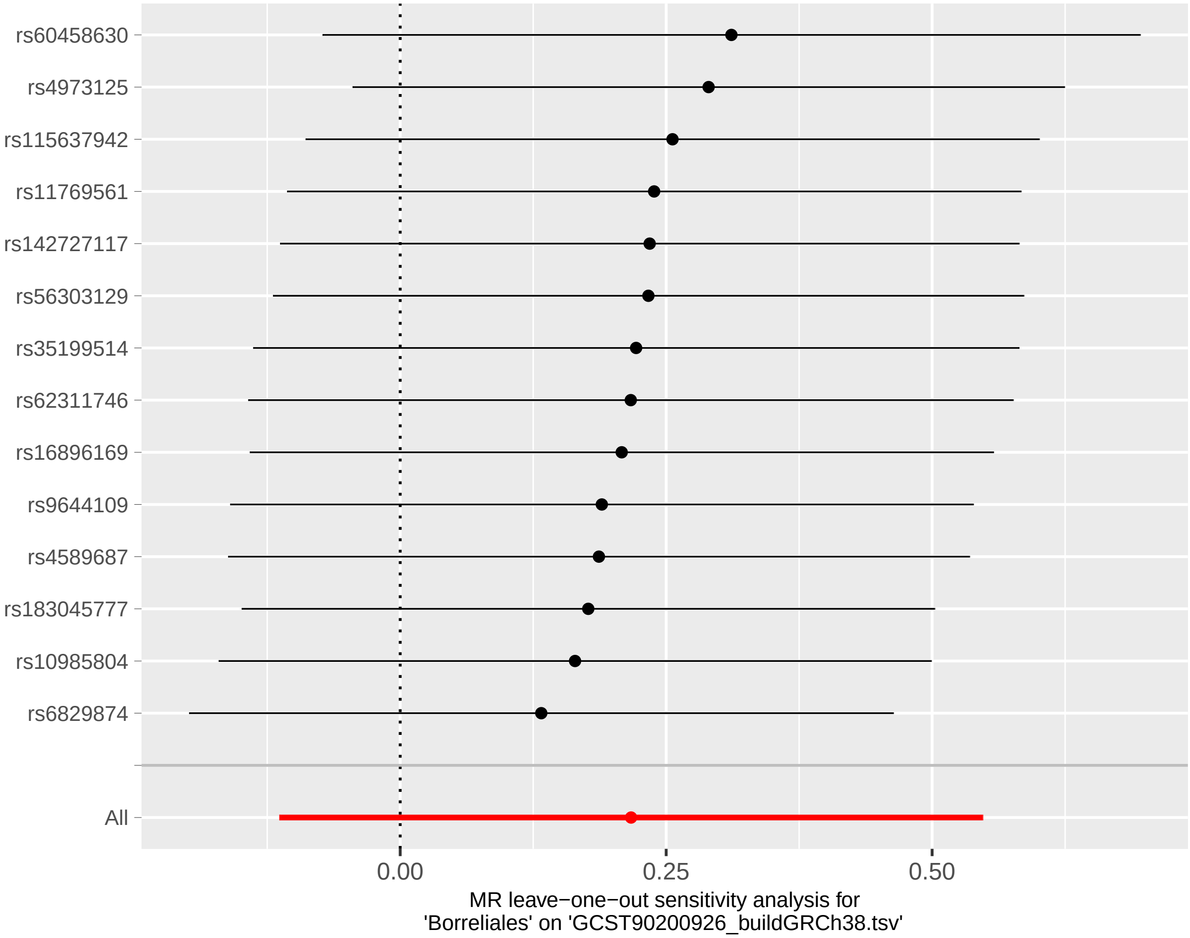
rs60458630
rs4973125
rs115637942
rs11769561
rs142727117
rs56303129
rs35199514
rs62311746
rs16896169
rs9644109
rs4589687
rs183045777
rs10985804
rs6829874
All
0.00
0.25
0.50
MR leave−one−out sensitivity analysis for
'Borreliales' on 'GCST90200926_buildGRCh38.tsv'

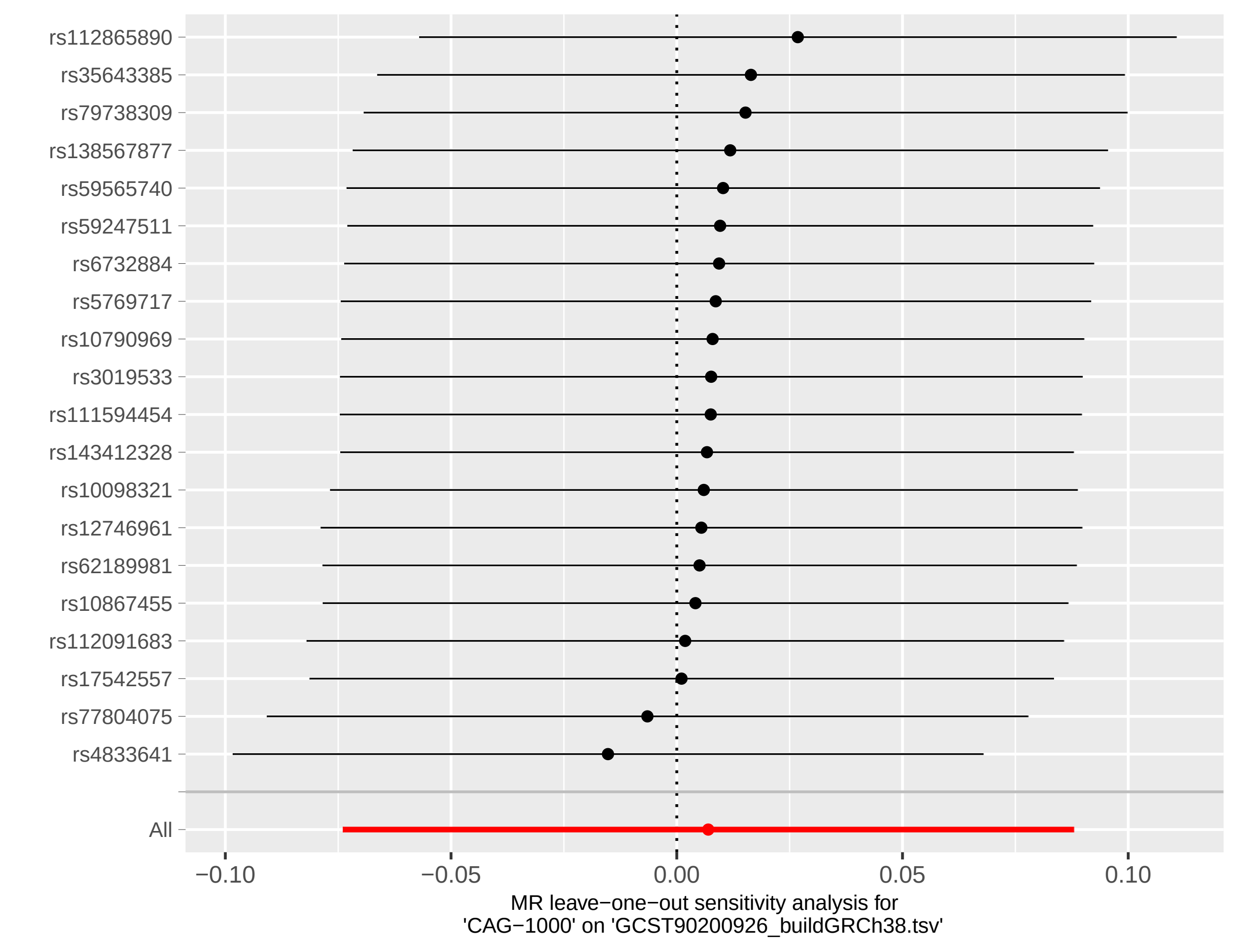
rs112865890
rs35643385
rs79738309
rs138567877
rs59565740
rs59247511
rs6732884
rs5769717
rs10790969
rs3019533
rs111594454
rs143412328
rs10098321
rs12746961
rs62189981
rs10867455
rs112091683
rs17542557
rs77804075
rs4833641
All
−0.10
−0.05
0.00
0.05
0.10
MR leave−one−out sensitivity analysis for
'CAG−1000' on 'GCST90200926_buildGRCh38.tsv'

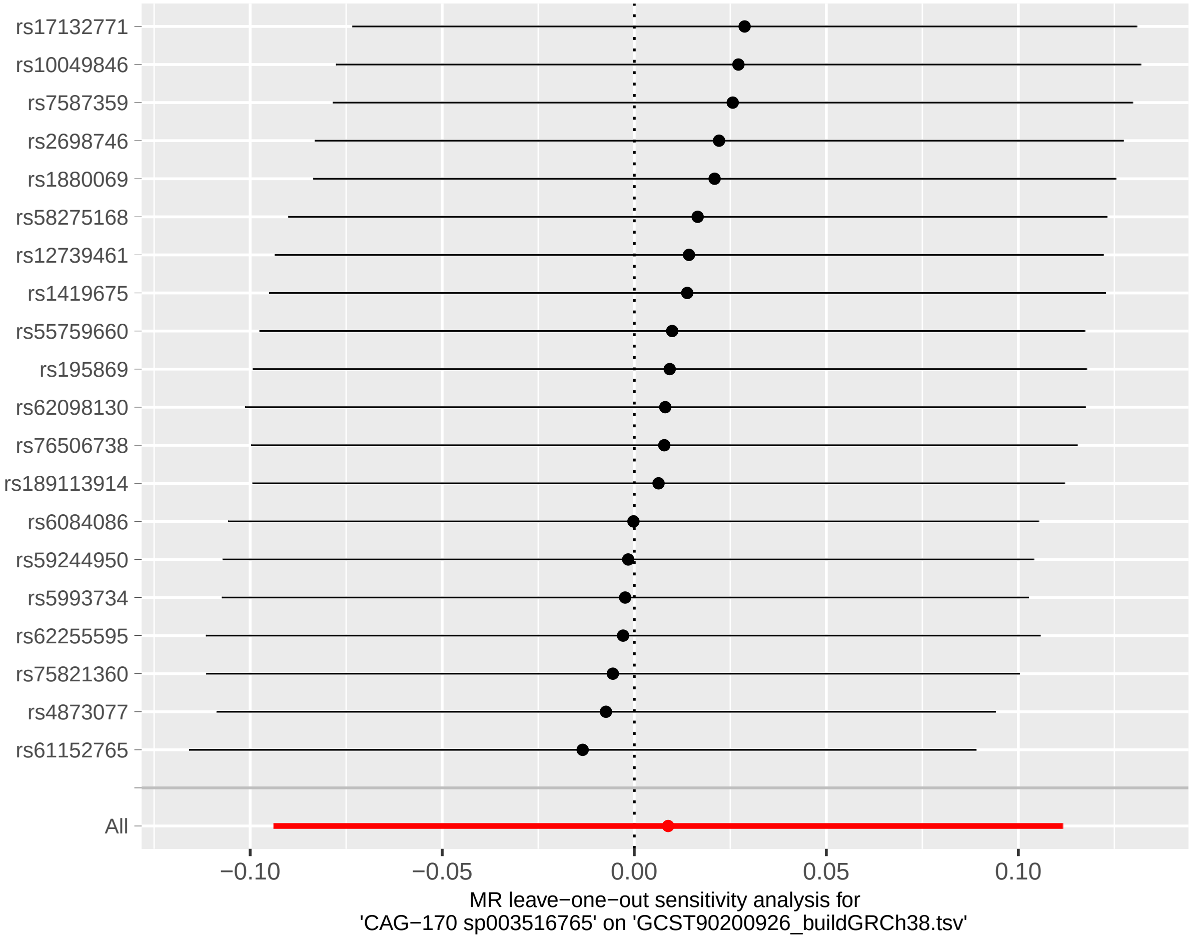
rs17132771
rs10049846
rs7587359
rs2698746
rs1880069
rs58275168
rs12739461
rs1419675
rs55759660
rs195869
rs62098130
rs76506738
rs189113914
rs6084086
rs59244950
rs5993734
rs62255595
rs75821360
rs4873077
rs61152765
All
−0.10
−0.05
0.00
0.05
0.10
MR leave−one−out sensitivity analysis for
'CAG−170 sp003516765' on 'GCST90200926_buildGRCh38.tsv'

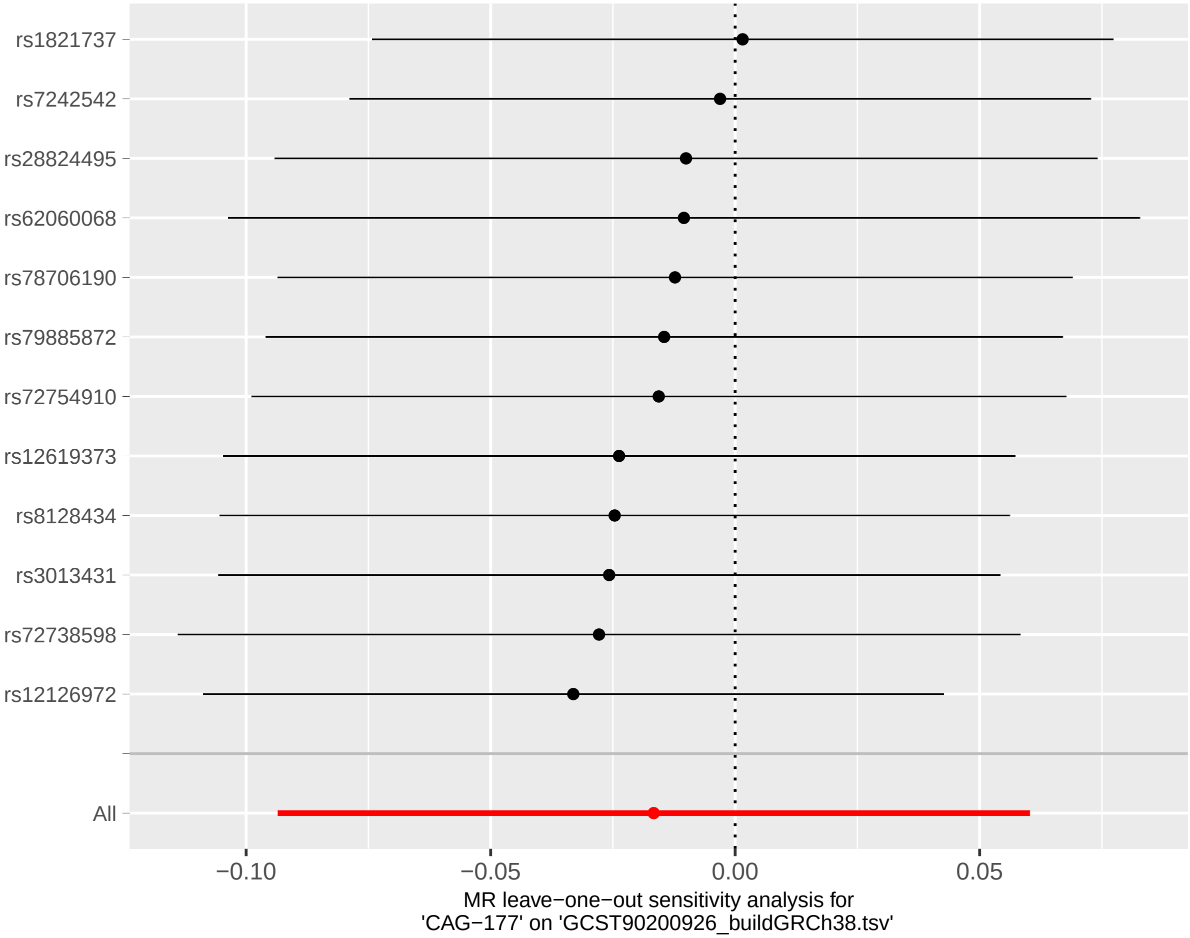

rs1821737
rs7242542
rs28824495
rs62060068
rs78706190
rs79885872
rs72754910
rs12619373
rs8128434
rs3013431
rs72738598
rs12126972
All
−0.10
−0.05
0.00
0.05
MR leave−one−out sensitivity analysis for
'CAG−177' on 'GCST90200926_buildGRCh38.tsv'

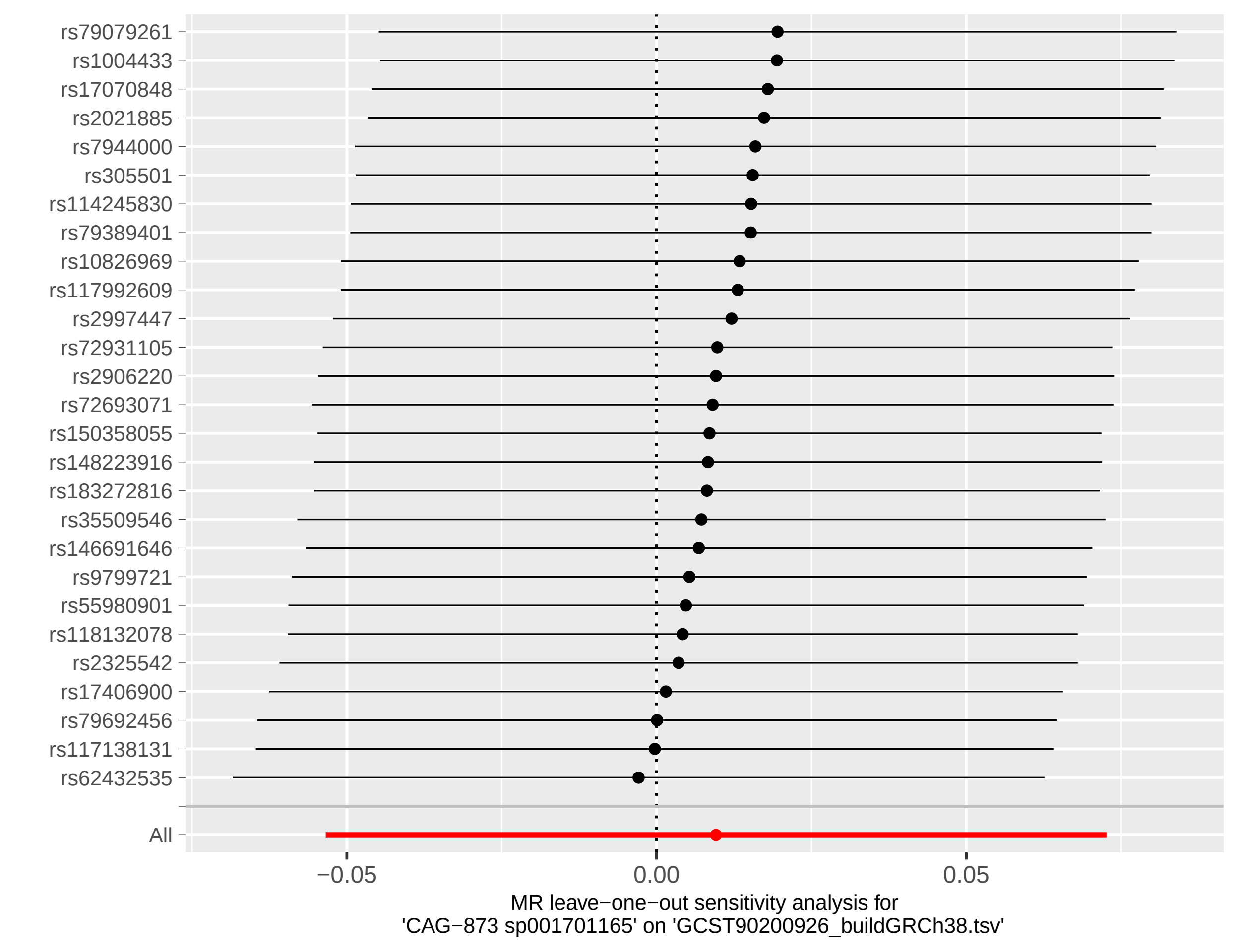

rs79079261
rs1004433
rs17070848
rs2021885
rs7944000
rs305501
rs114245830
rs79389401
rs10826969
rs117992609
rs2997447
rs72931105
rs2906220
rs72693071
rs150358055
rs148223916
rs183272816
rs35509546
rs146691646
rs9799721
rs55980901
rs118132078
rs2325542
rs17406900
rs79692456
rs117138131
rs62432535
All
−0.05
0.00
0.05
MR leave−one−out sensitivity analysis for
'CAG−873 sp001701165' on 'GCST90200926_buildGRCh38.tsv'

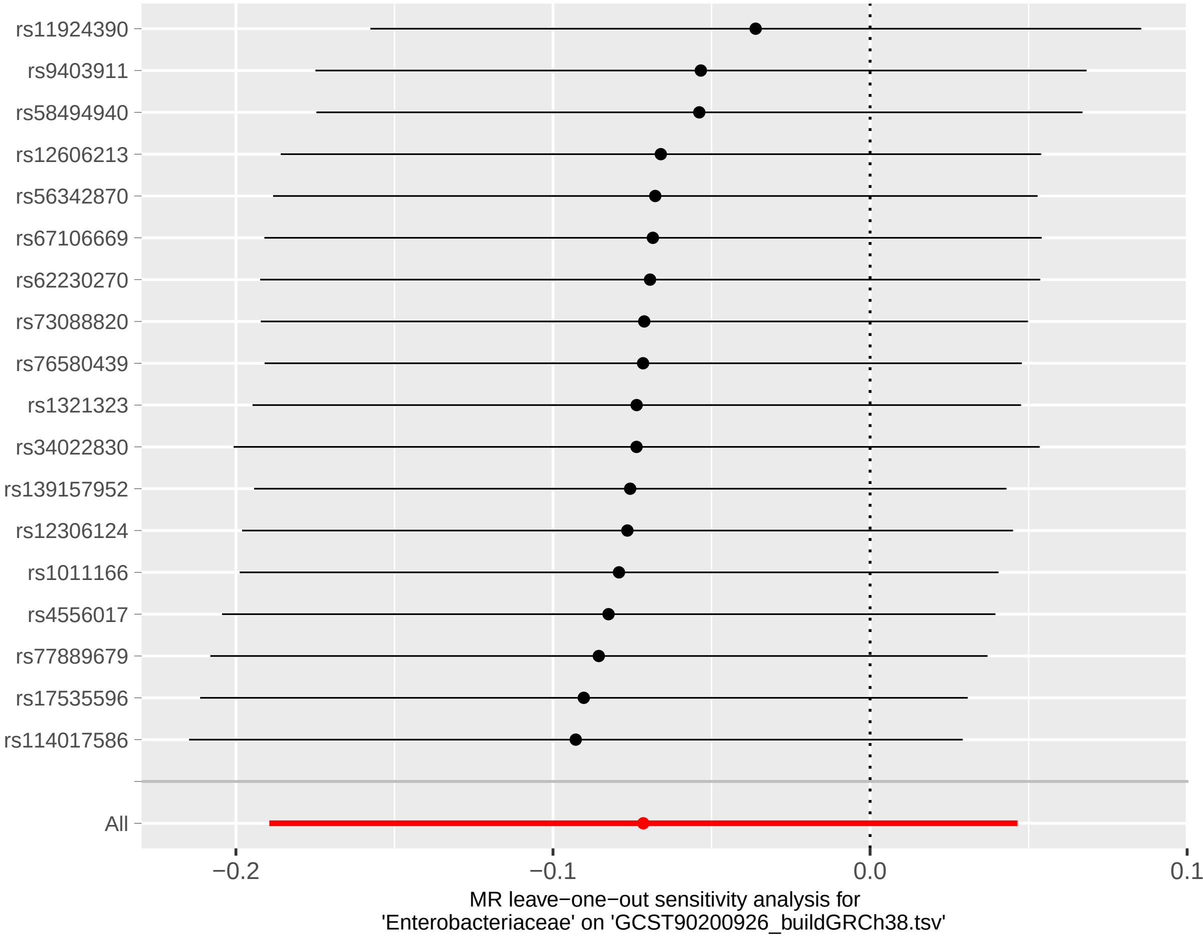
rs11924390
rs9403911
rs58494940
rs12606213
rs56342870
rs67106669
rs62230270
rs73088820
rs76580439
rs1321323
rs34022830
rs139157952
rs12306124
rs1011166
rs4556017
rs77889679
rs17535596
rs114017586
All
−0.2
−0.1
0.0
0.1
MR leave−one−out sensitivity analysis for
'Enterobacteriaceae' on 'GCST90200926_buildGRCh38.tsv'

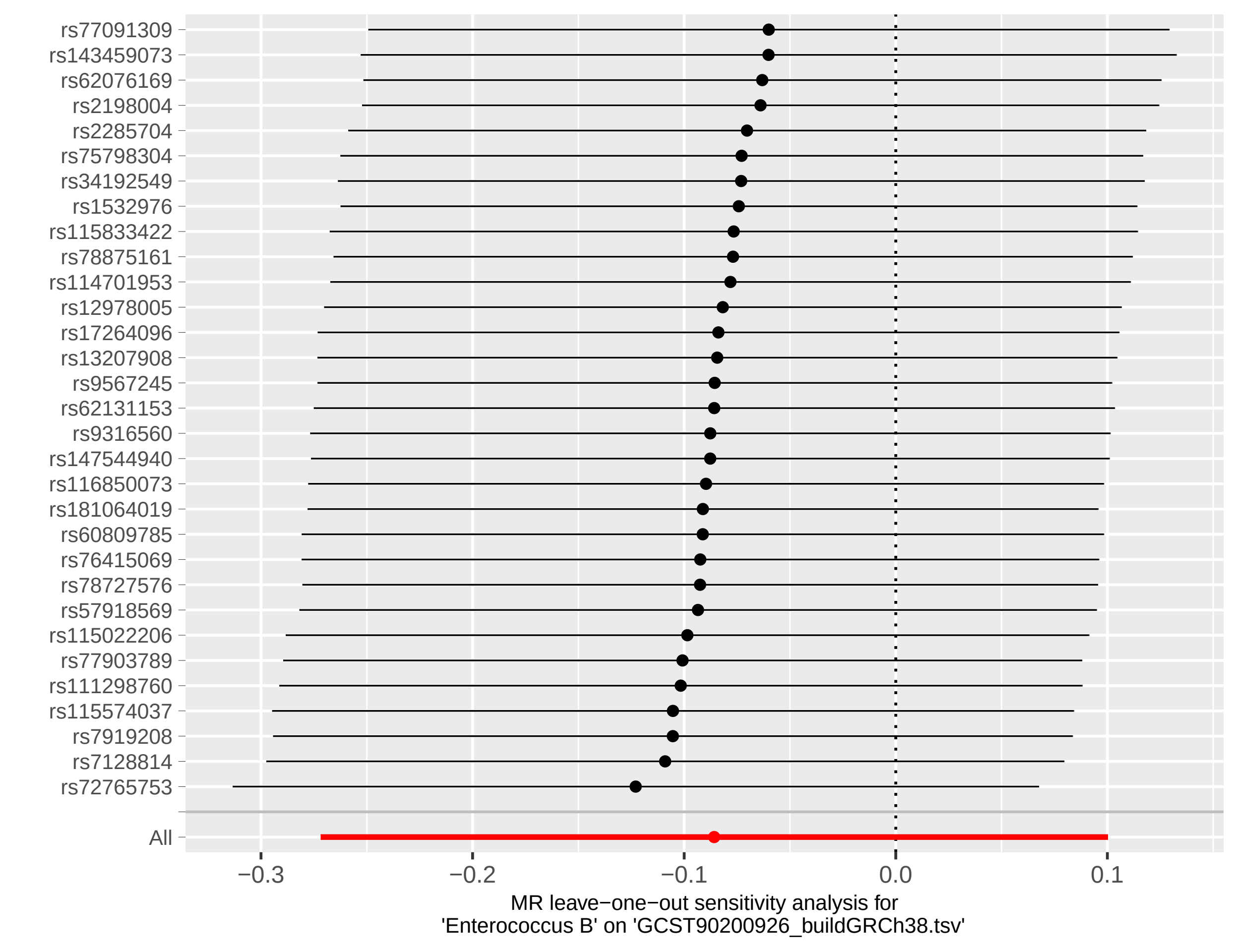

rs77091309
rs143459073
rs62076169
rs2198004
rs2285704
rs75798304
rs34192549
rs1532976
rs115833422
rs78875161
rs114701953
rs12978005
rs17264096
rs13207908
rs9567245
rs62131153
rs9316560
rs147544940
rs116850073
rs181064019
rs60809785
rs76415069
rs78727576
rs57918569
rs115022206
rs77903789
rs111298760
rs115574037
rs7919208
rs7128814
rs72765753
All
−0.3
−0.2
−0.1
0.0
0.1
MR leave−one−out sensitivity analysis for
'Enterococcus B' on 'GCST90200926_buildGRCh38.tsv'

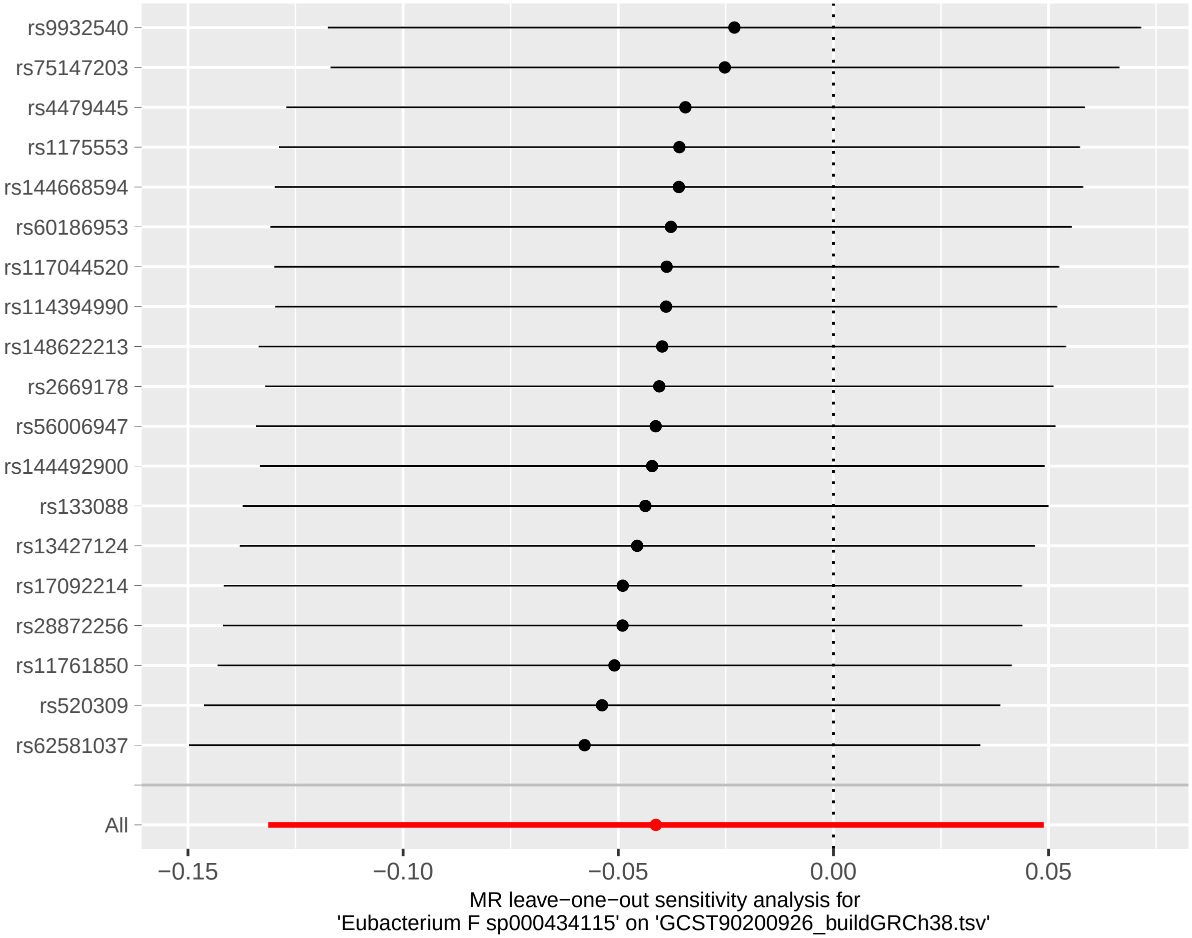
rs9932540
rs75147203
rs4479445
rs1175553
rs144668594
rs60186953
rs117044520
rs114394990
rs148622213
rs2669178
rs56006947
rs144492900
rs133088
rs13427124
rs17092214
rs28872256
rs11761850
rs520309
rs62581037
All
−0.15
−0.10
−0.05
0.00
0.05
MR leave−one−out sensitivity analysis for
'Eubacterium F sp000434115' on 'GCST90200926_buildGRCh38.tsv'

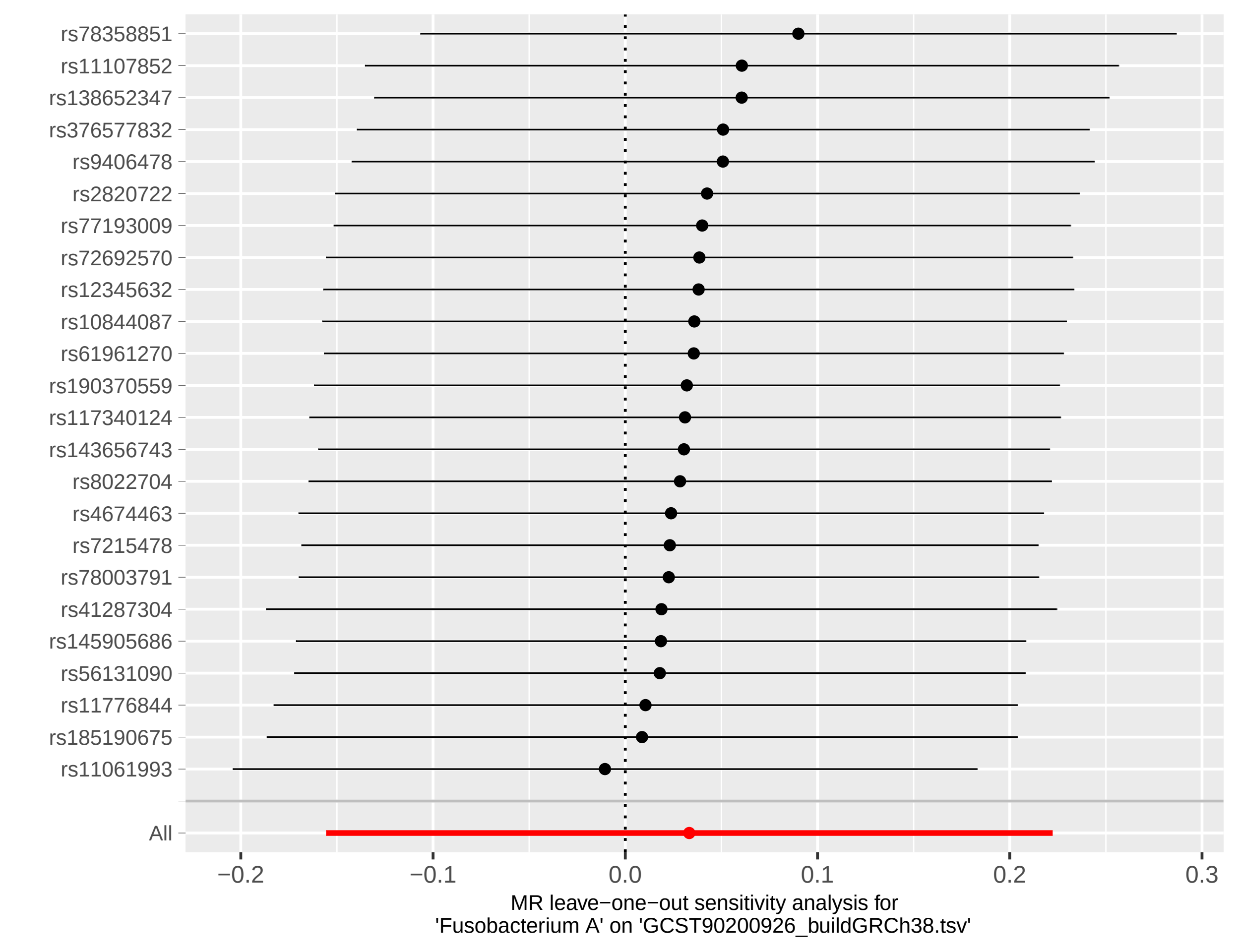
rs78358851
rs11107852
rs138652347
rs376577832
rs9406478
rs2820722
rs77193009
rs72692570
rs12345632
rs10844087
rs61961270
rs190370559
rs117340124
rs143656743
rs8022704
rs4674463
rs7215478
rs78003791
rs41287304
rs145905686
rs56131090
rs11776844
rs185190675
rs11061993
All
−0.2
−0.1
0.0
0.1
0.2
0.3
MR leave−one−out sensitivity analysis for
'Fusobacterium A' on 'GCST90200926_buildGRCh38.tsv'

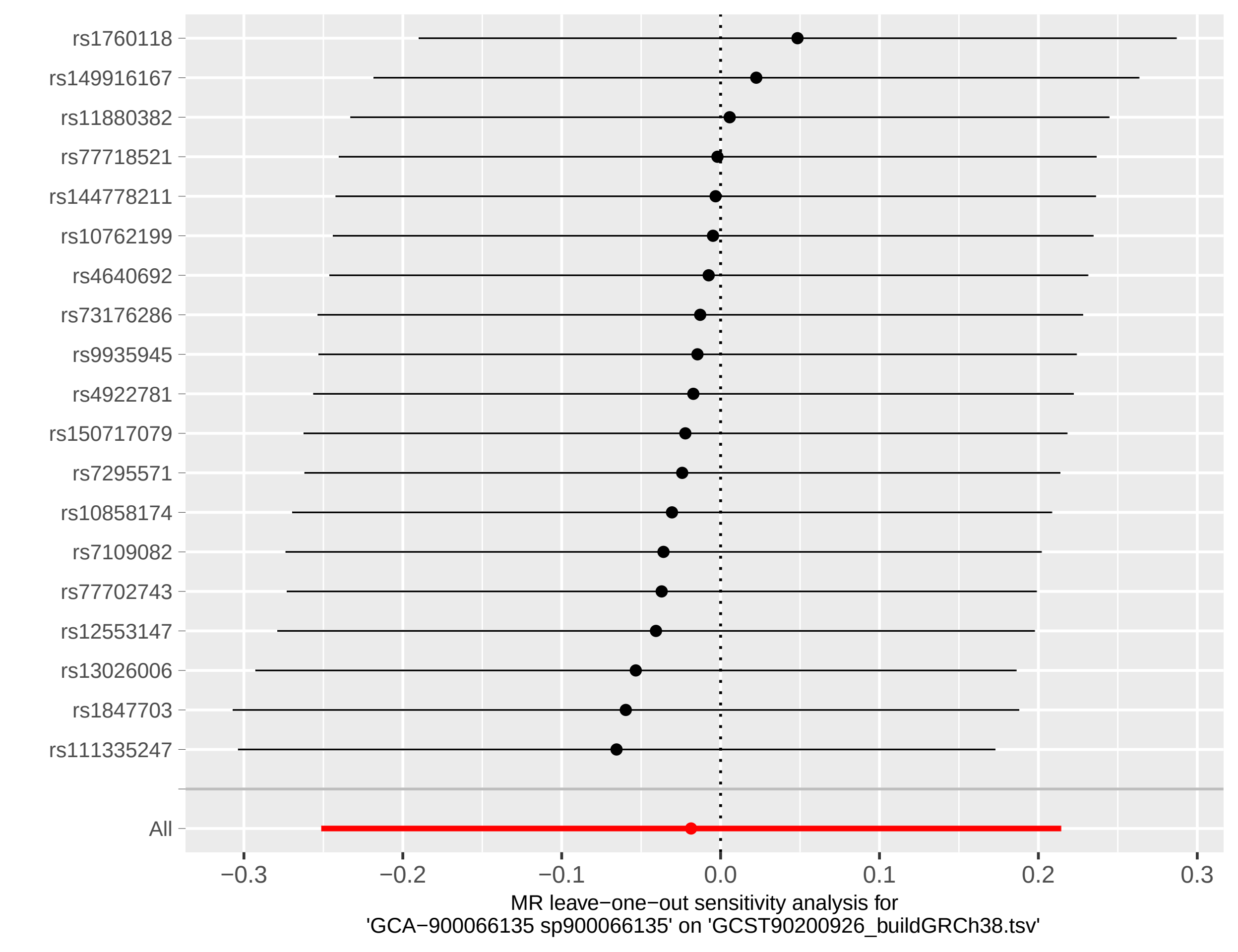

rs1760118
rs149916167
rs11880382
rs77718521
rs144778211
rs10762199
rs4640692
rs73176286
rs9935945
rs4922781
rs150717079
rs7295571
rs10858174
rs7109082
rs77702743
rs12553147
rs13026006
rs1847703
rs111335247
All
−0.3
−0.2
−0.1
0.0
0.1
0.2
0.3
MR leave−one−out sensitivity analysis for
'GCA−900066135 sp900066135' on 'GCST90200926_buildGRCh38.tsv'

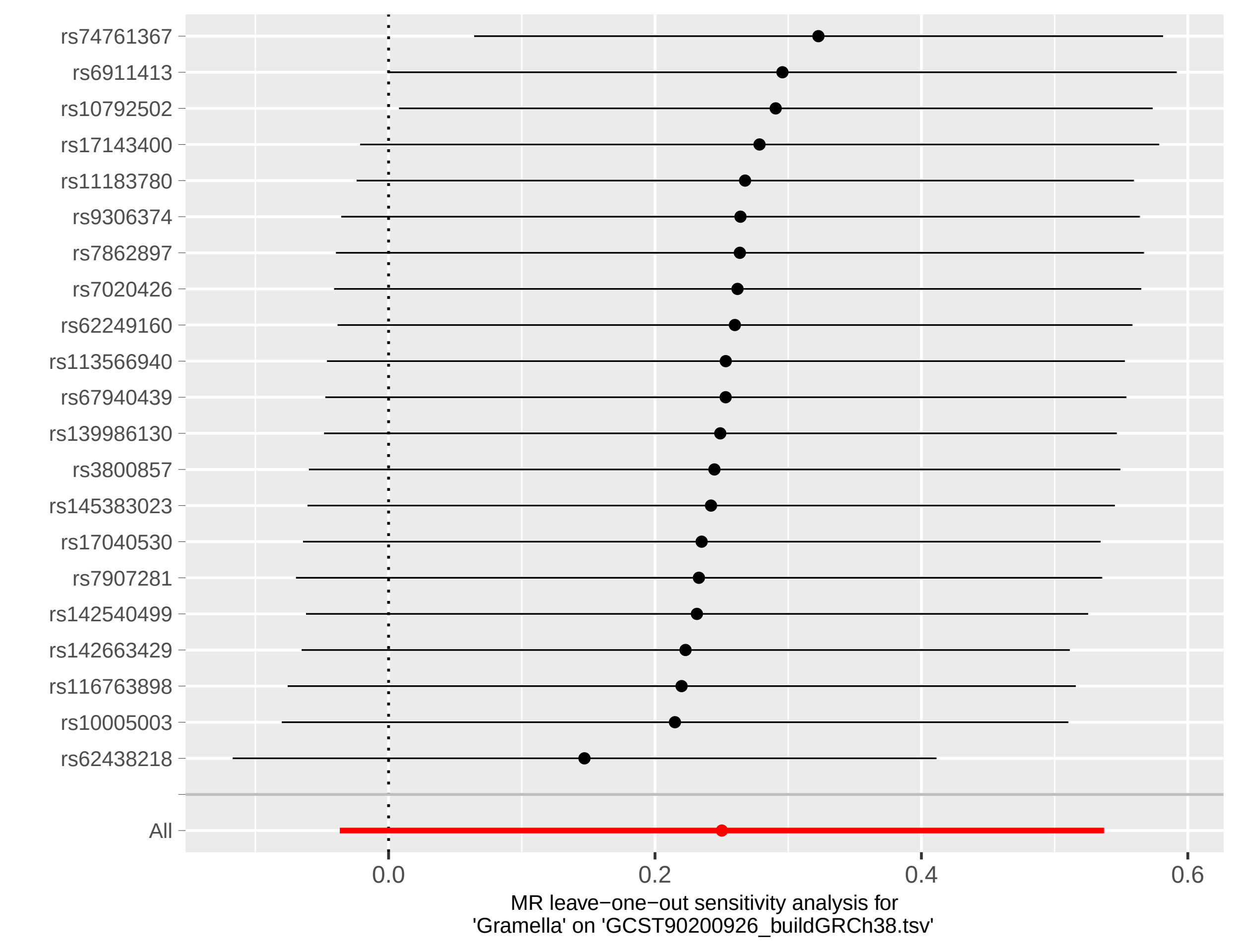
rs74761367
rs6911413
rs10792502
rs17143400
rs11183780
rs9306374
rs7862897
rs7020426
rs62249160
rs113566940
rs67940439
rs139986130
rs3800857
rs145383023
rs17040530
rs7907281
rs142540499
rs142663429
rs116763898
rs10005003
rs62438218
All
0.0
0.2
0.4
0.6
MR leave−one−out sensitivity analysis for
'Gramella' on 'GCST90200926_buildGRCh38.tsv'

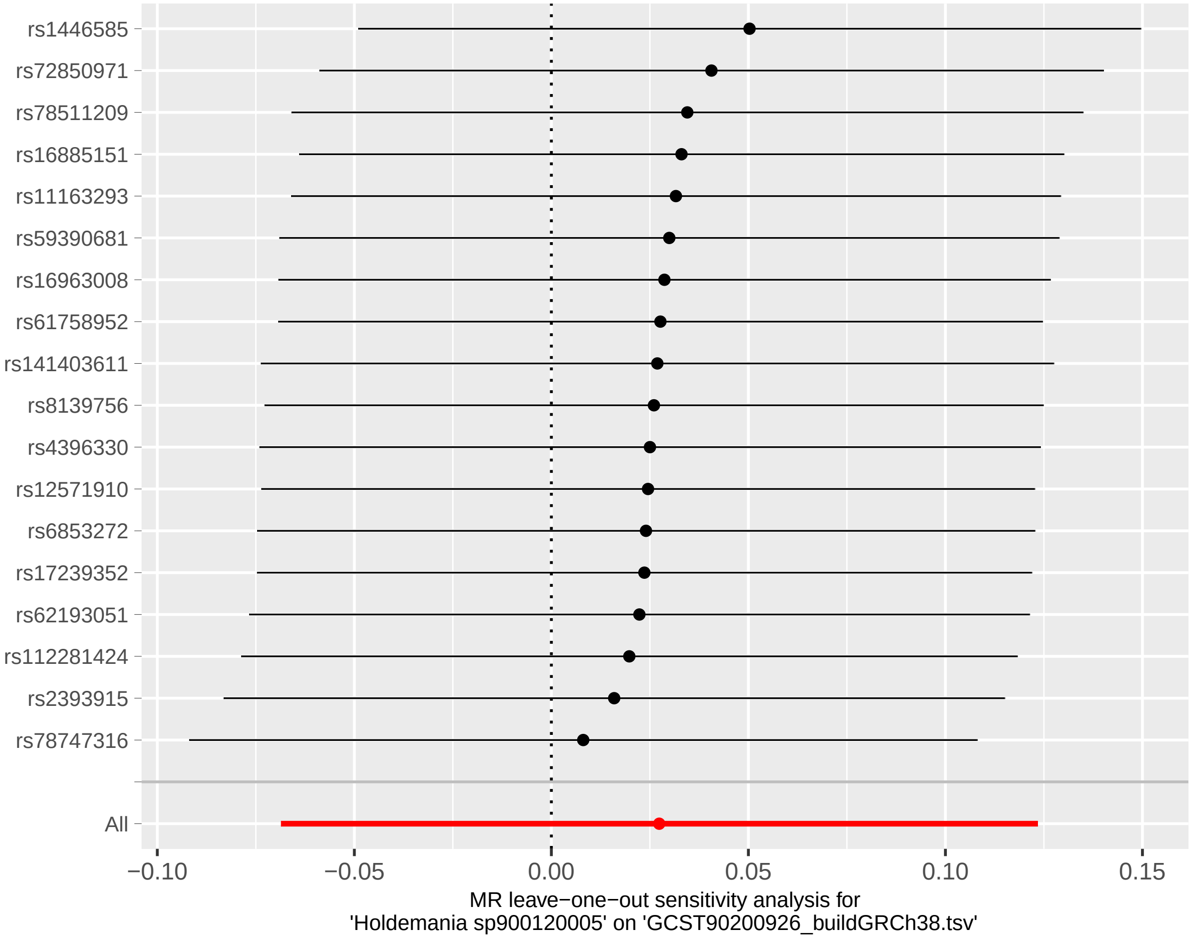
rs1446585
rs72850971
rs78511209
rs16885151
rs11163293
rs59390681
rs16963008
rs61758952
rs141403611
rs8139756
rs4396330
rs12571910
rs6853272
rs17239352
rs62193051
rs112281424
rs2393915
rs78747316
All
−0.10
−0.05
0.00
0.05
0.10
0.15
MR leave−one−out sensitivity analysis for
'Holdemania sp900120005' on 'GCST90200926_buildGRCh38.tsv'

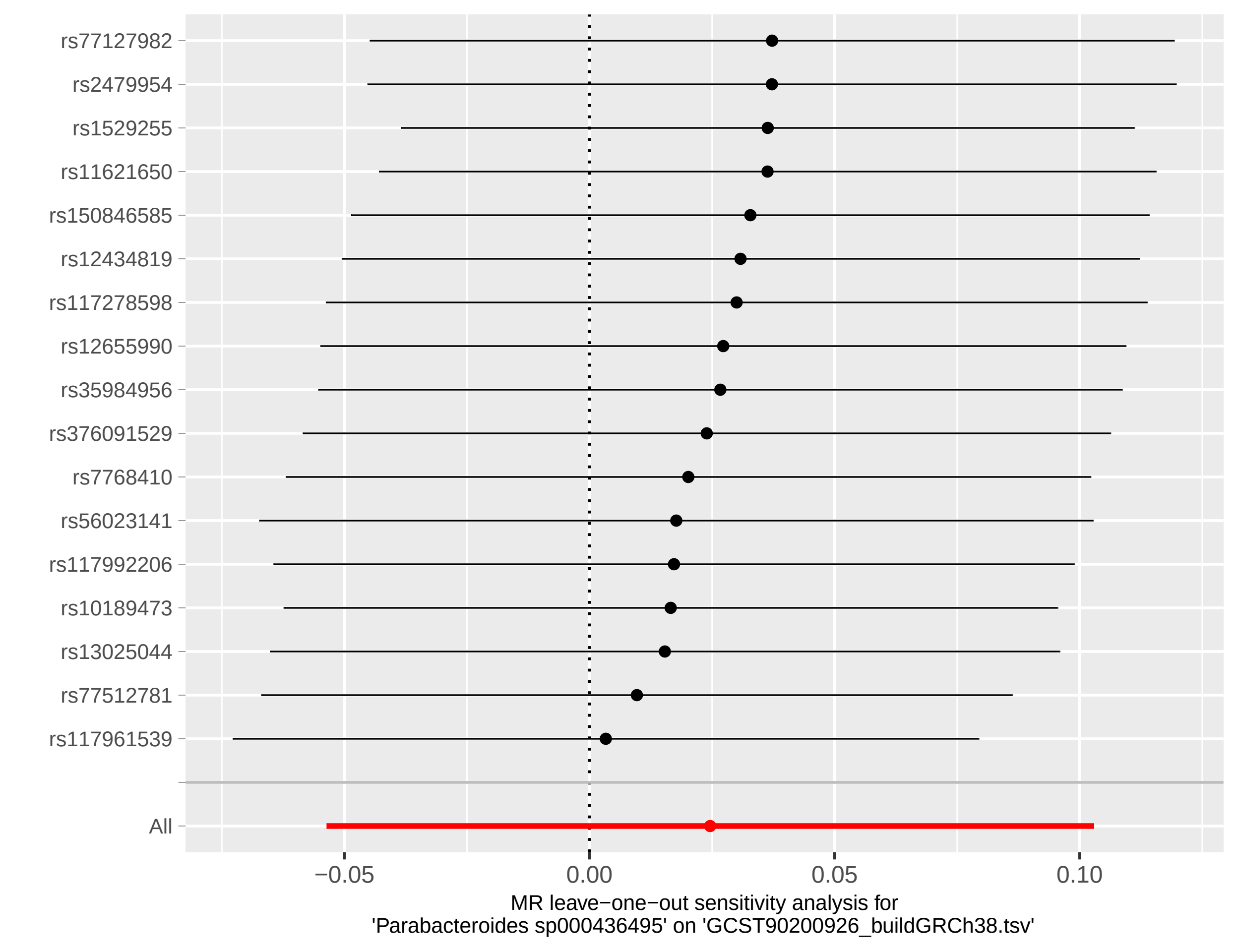
rs77127982
rs2479954
rs1529255
rs11621650
rs150846585
rs12434819
rs117278598
rs12655990
rs35984956
rs376091529
rs7768410
rs56023141
rs117992206
rs10189473
rs13025044
rs77512781
rs117961539
All
−0.05
0.00
0.05
0.10
MR leave−one−out sensitivity analysis for
'Parabacteroides sp000436495' on 'GCST90200926_buildGRCh38.tsv'

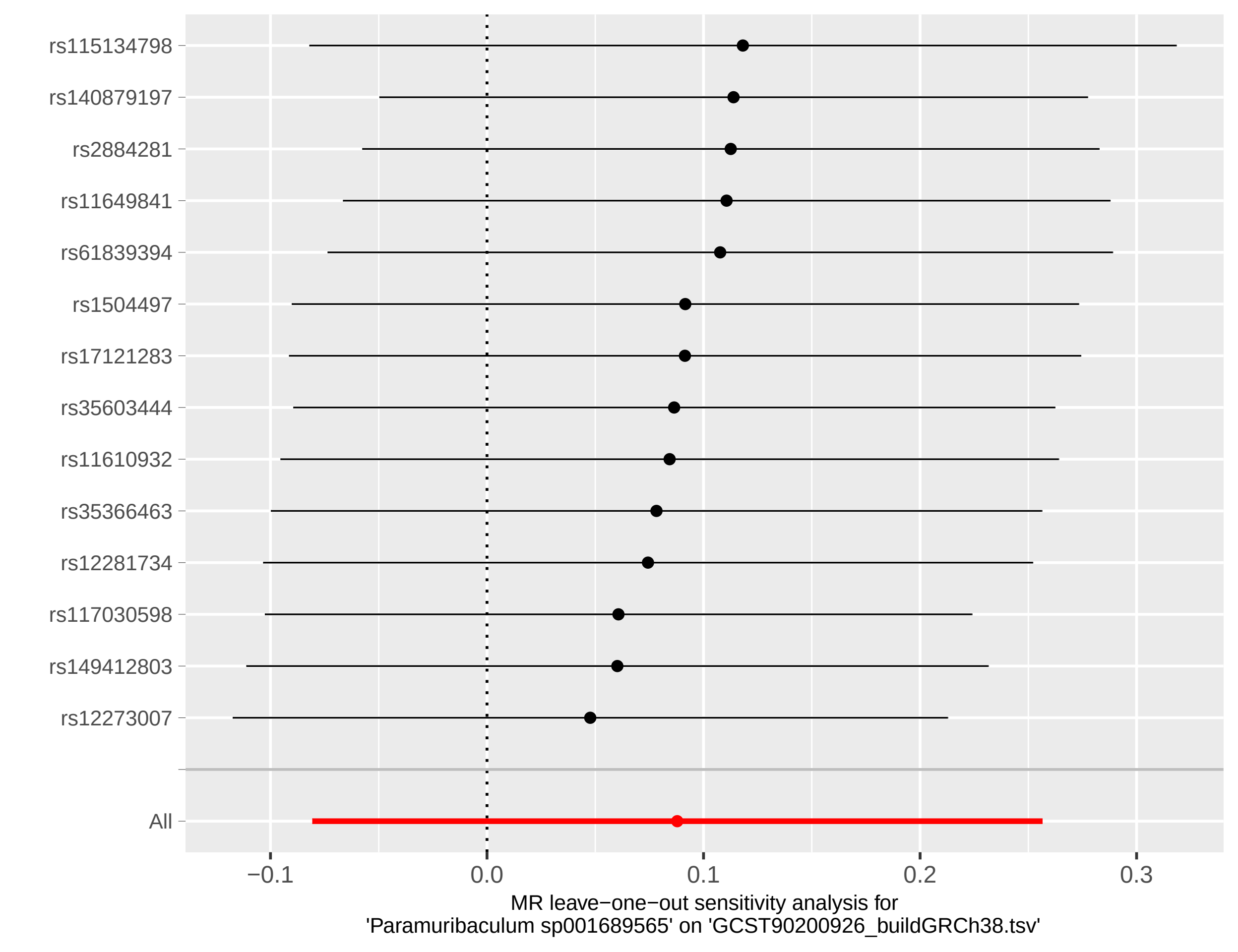
rs115134798
rs140879197
rs2884281
rs11649841
rs61839394
rs1504497
rs17121283
rs35603444
rs11610932
rs35366463
rs12281734
rs117030598
rs149412803
rs12273007
All
−0.1
0.0
0.1
0.2
0.3
MR leave−one−out sensitivity analysis for
'Paramuribaculum sp001689565' on 'GCST90200926_buildGRCh38.tsv'

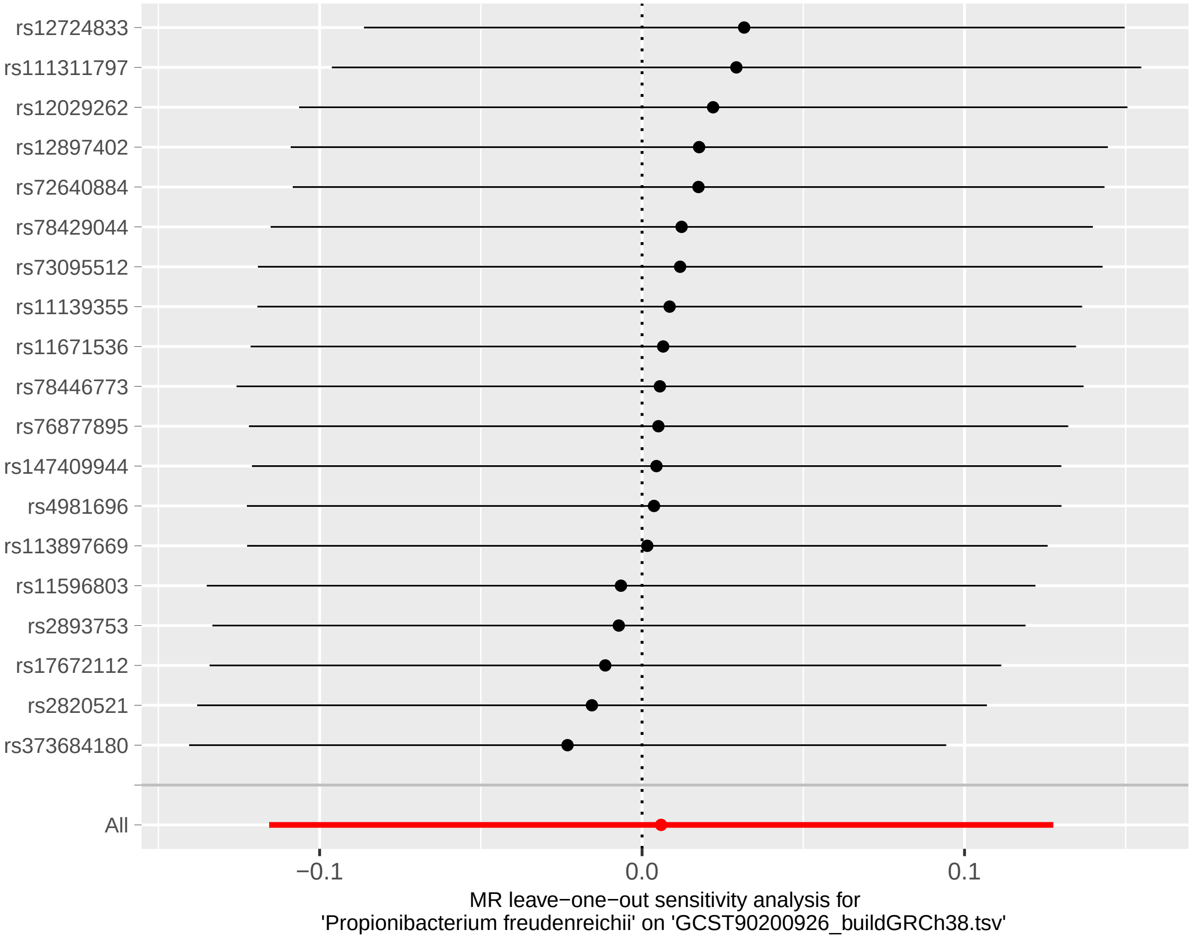

rs12724833
rs111311797
rs12029262
rs12897402
rs72640884
rs78429044
rs73095512
rs11139355
rs11671536
rs78446773
rs76877895
rs147409944
rs4981696
rs113897669
rs11596803
rs2893753
rs17672112
rs2820521
rs373684180
All
−0.1
0.0
0.1
MR leave−one−out sensitivity analysis for
'Propionibacterium freudenreichii' on 'GCST90200926_buildGRCh38.tsv'

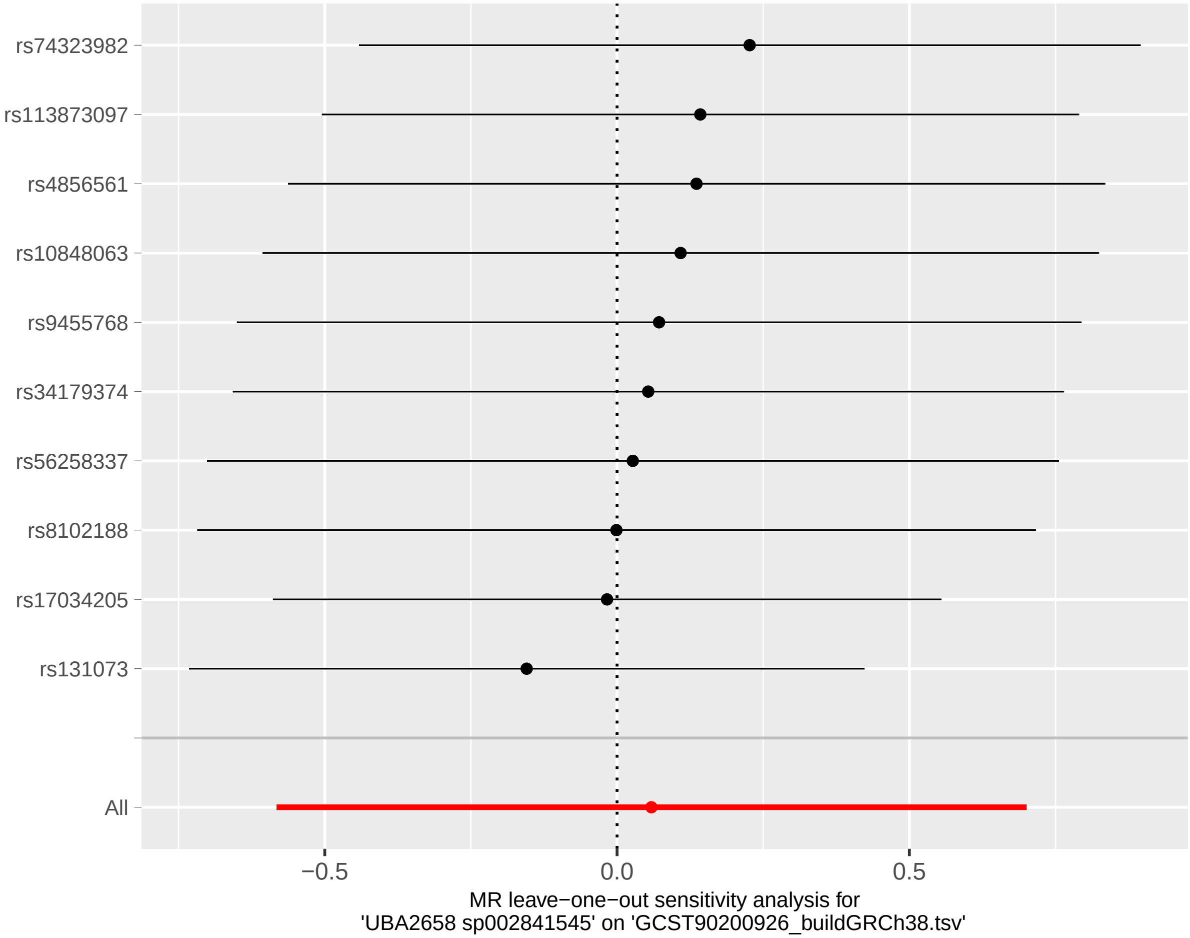
rs74323982
rs113873097
rs4856561
rs10848063
rs9455768
rs34179374
rs56258337
rs8102188
rs17034205
rs131073
All
−0.5
0.0
0.5
MR leave−one−out sensitivity analysis for
'UBA2658 sp002841545' on 'GCST90200926_buildGRCh38.tsv'

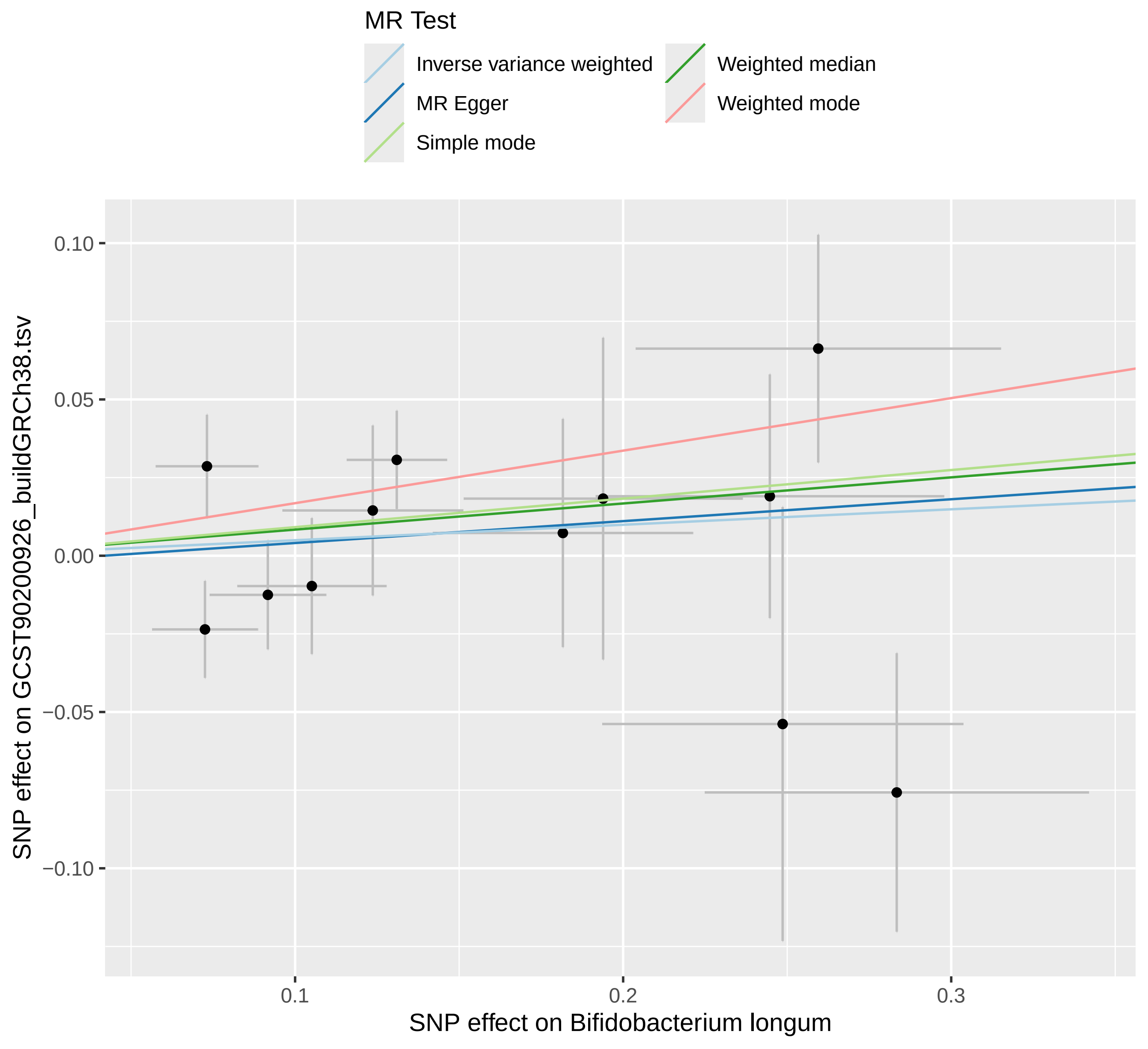

MR Test
Inverse variance weighted
MR Egger
Simple mode
Weighted median
Weighted mode
0.10
0.05
0.00
−0.05
−0.10
SNP effect on GCST90200926_buildGRCh38.tsv
0.1
0.2
0.3
SNP effect on Bifidobacterium longum

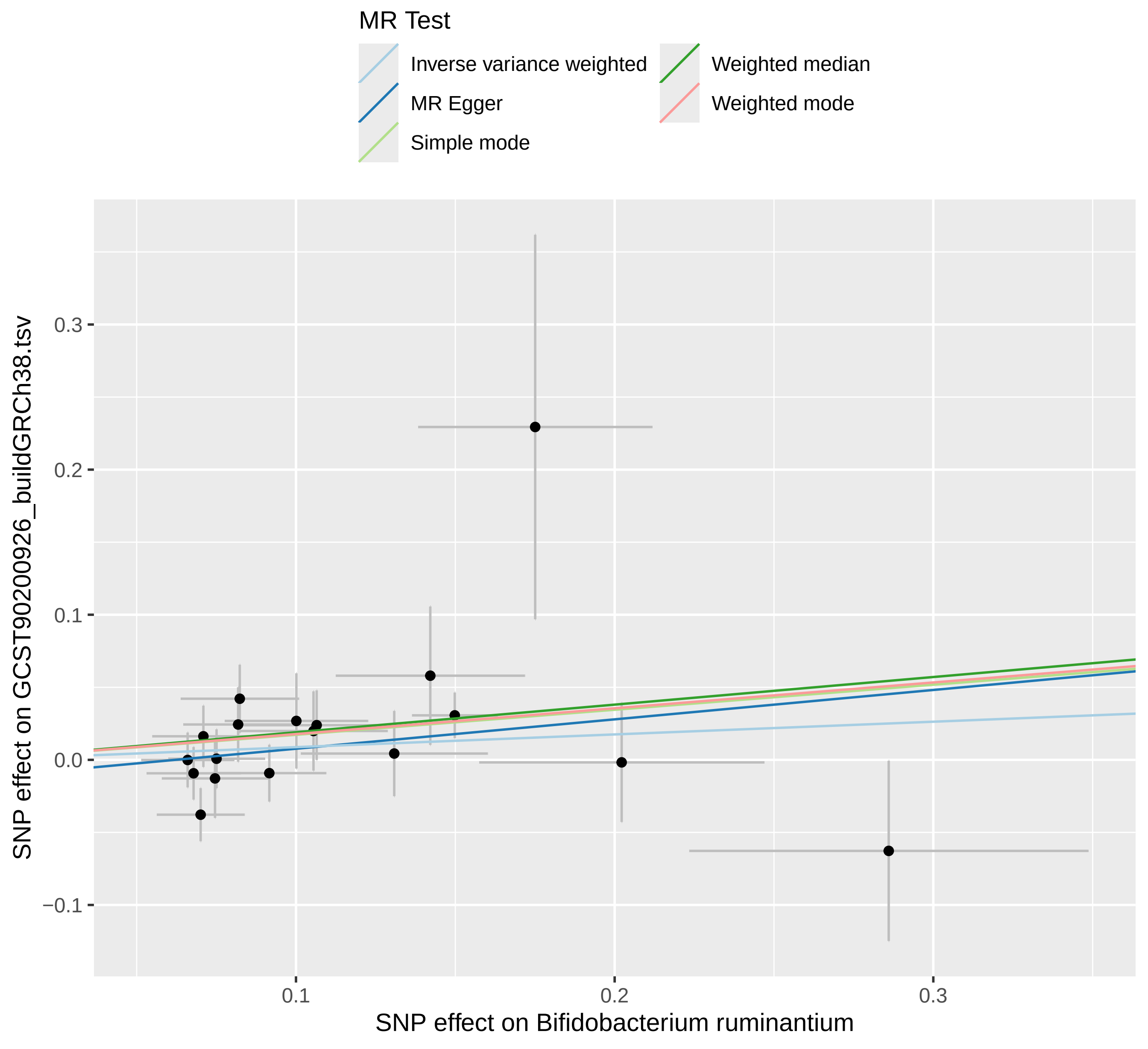
MR Test
Inverse variance weighted
MR Egger
Simple mode
Weighted median
Weighted mode
SNP effect on GCST90200926_buildGRCh38.tsv
0.3
0.2
0.1
0.0
−0.1
0.1
0.2
0.3
SNP effect on Bifidobacterium ruminantium

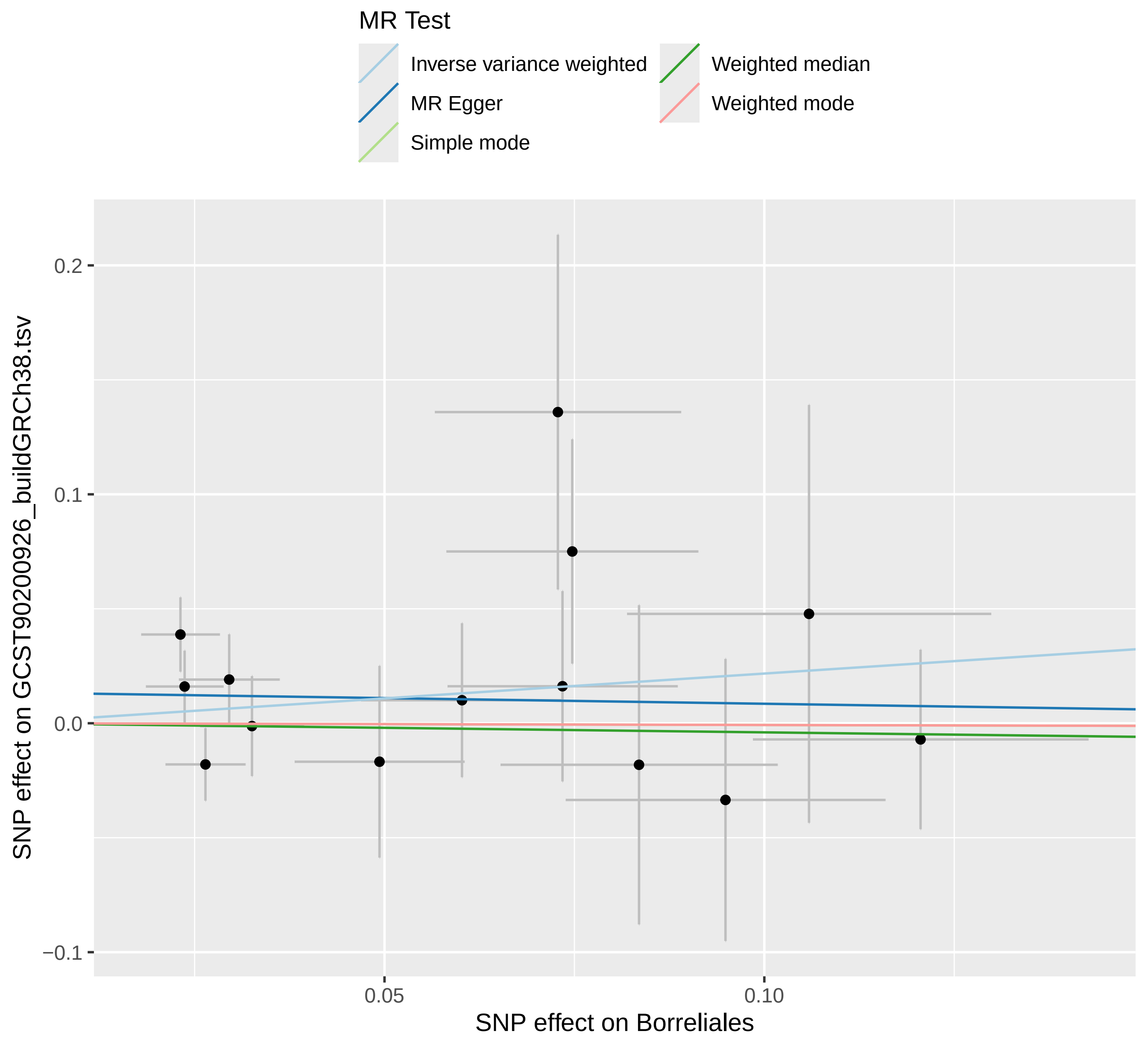

MR Test
Inverse variance weighted
MR Egger
Simple mode
Weighted median
Weighted mode
0.2
0.1
0.0
−0.1
0.05
0.10
SNP effect on GCST90200926_buildGRCh38.tsv
SNP effect on Borreliales

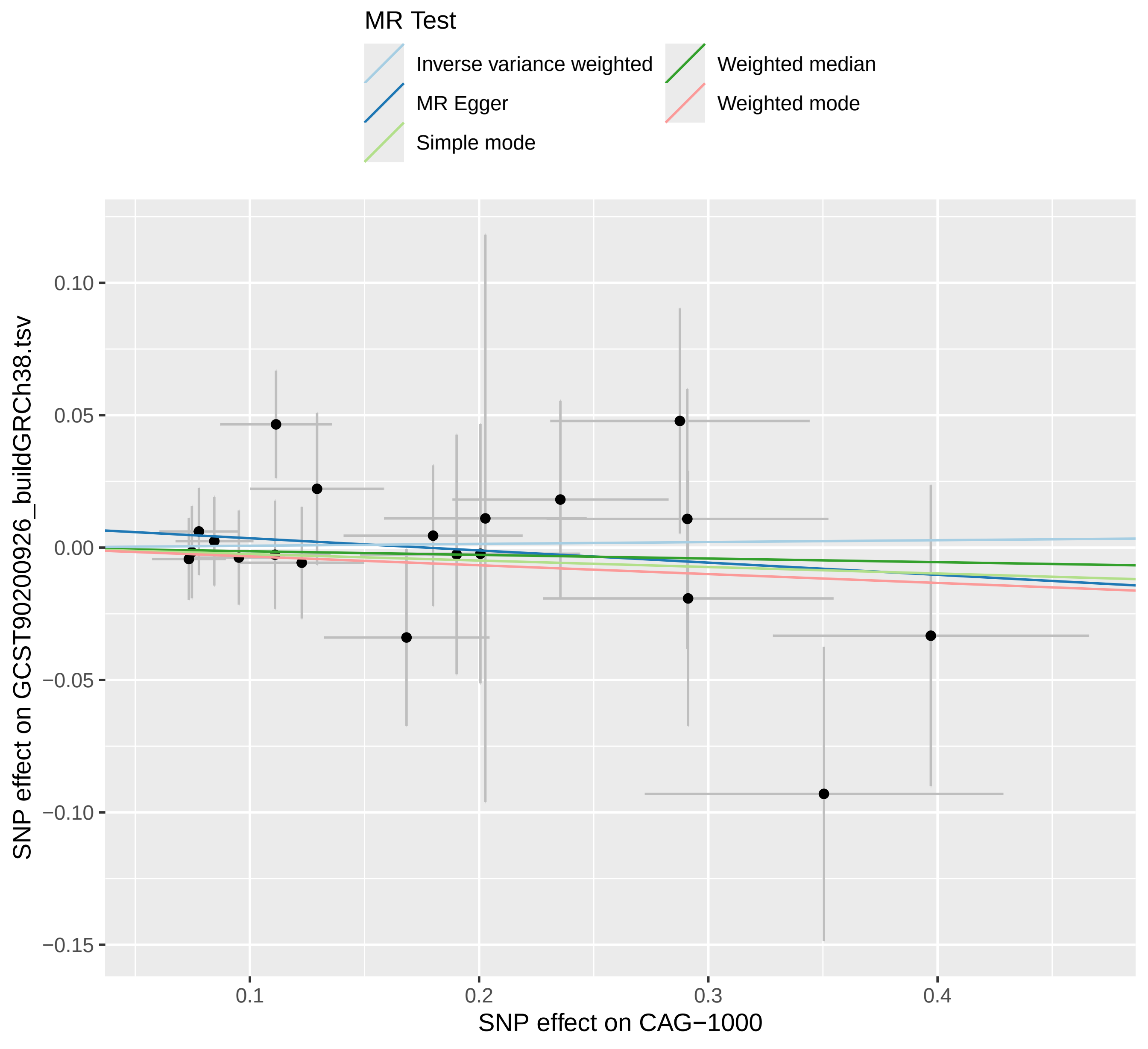
MR Test
Inverse variance weighted
MR Egger
Simple mode
Weighted median
Weighted mode
SNP effect on GCST90200926_buildGRCh38.tsv
0.10
0.05
0.00
−0.05
−0.10
−0.15
0.1
0.2
0.3
0.4
SNP effect on CAG−1000

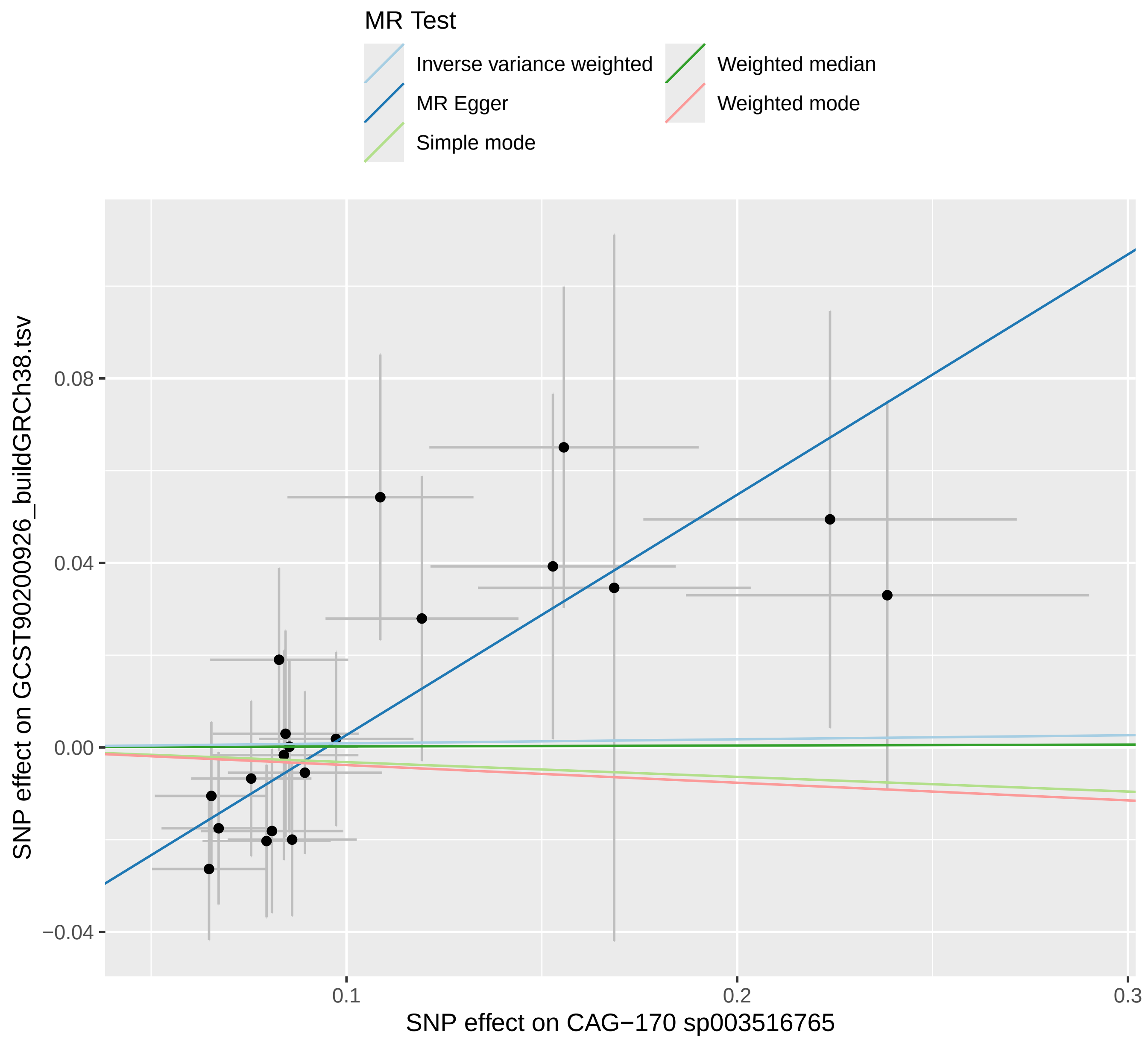
MR Test
Inverse variance weighted
MR Egger
Simple mode
Weighted median
Weighted mode
0.08
0.04
0.00
−0.04
0.1
0.2
0.3
SNP effect on GCST90200926_buildGRCh38.tsv
SNP effect on CAG−170 sp003516765

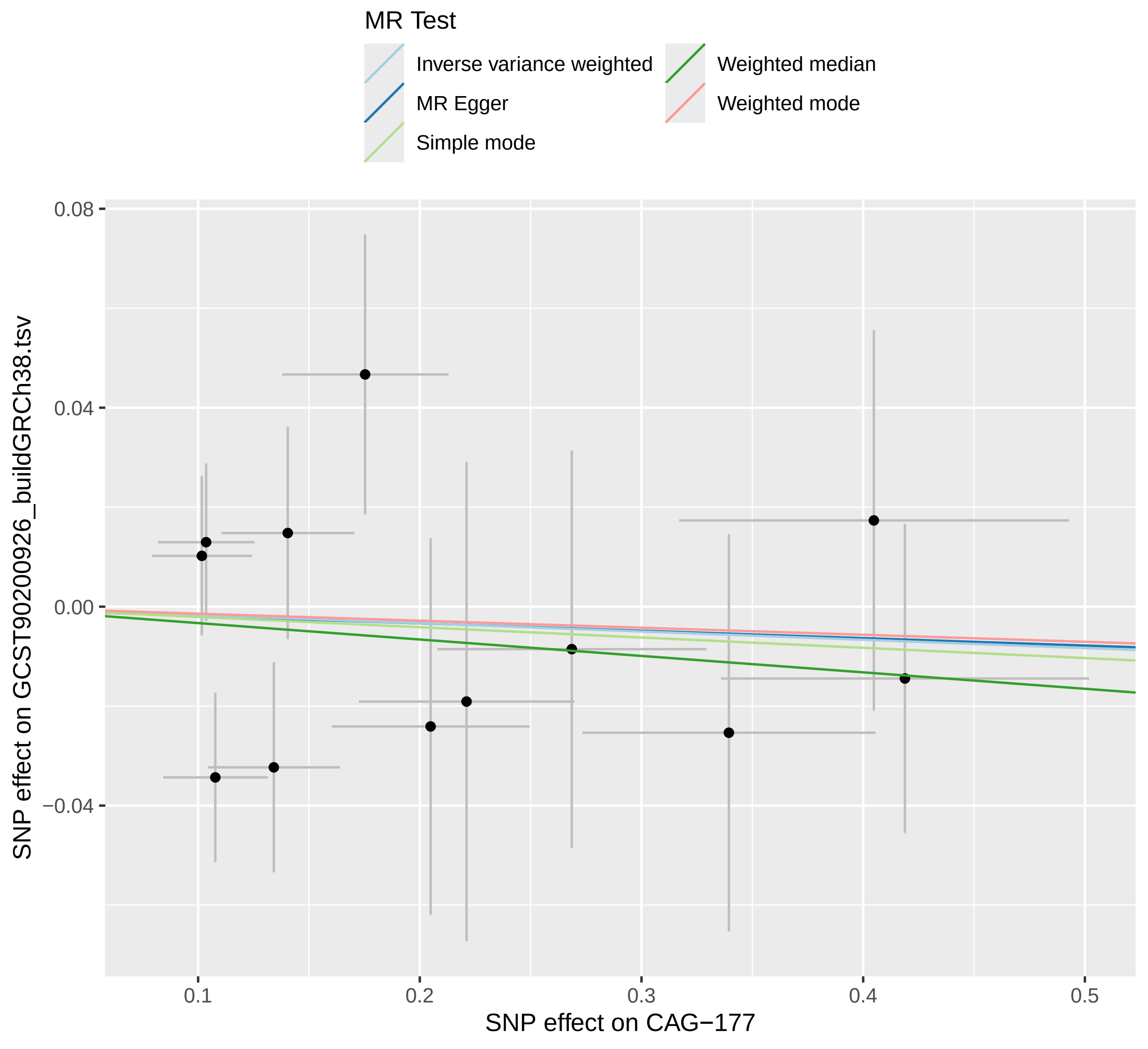
MR Test
Inverse variance weighted
MR Egger
Simple mode
Weighted median
Weighted mode
0.08
0.04
0.00
−0.04
0.1
0.2
0.3
0.4
0.5
SNP effect on GCST90200926_buildGRCh38.tsv
SNP effect on CAG−177

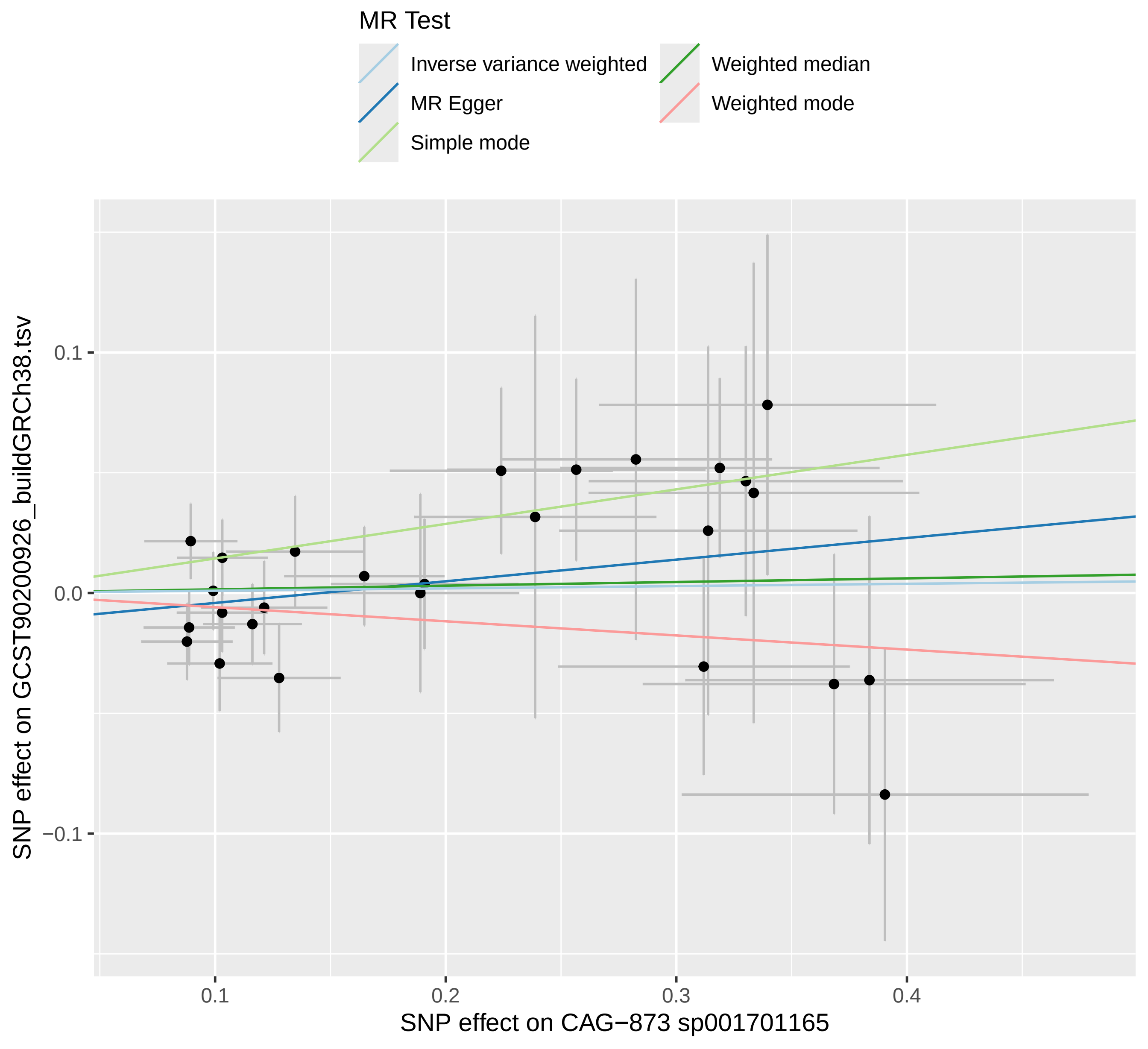
MR Test
Inverse variance weighted
MR Egger
Simple mode
Weighted median
Weighted mode
SNP effect on GCST90200926_buildGRCh38.tsv
0.1
0.0
−0.1
0.1
0.2
0.3
0.4
SNP effect on CAG−873 sp001701165

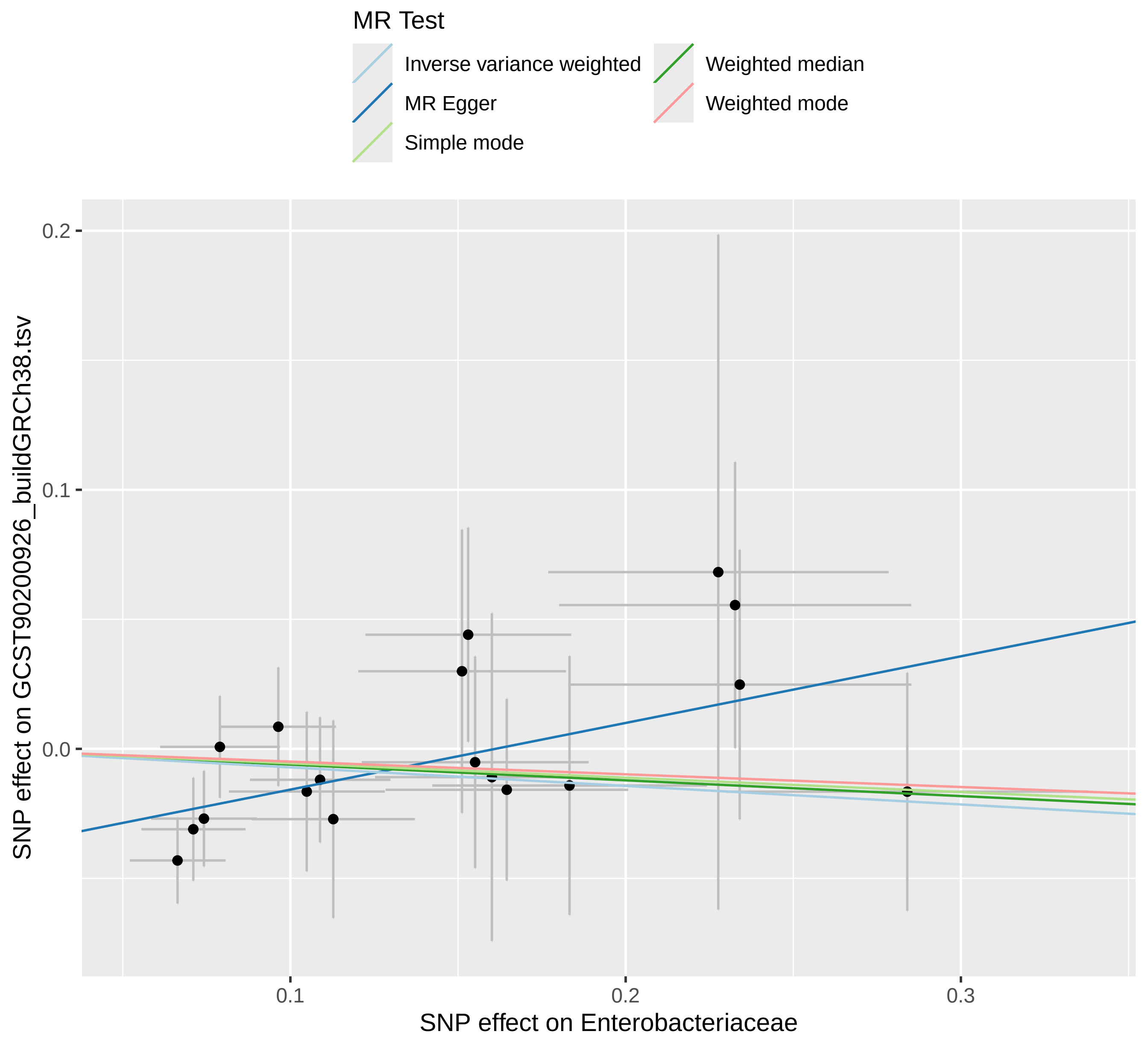

MR Test
Inverse variance weighted
MR Egger
Simple mode
Weighted median
Weighted mode
0.2
0.1
0.0
0.1
0.2
0.3
SNP effect on GCST90200926_buildGRCh38.tsv
SNP effect on Enterobacteriaceae

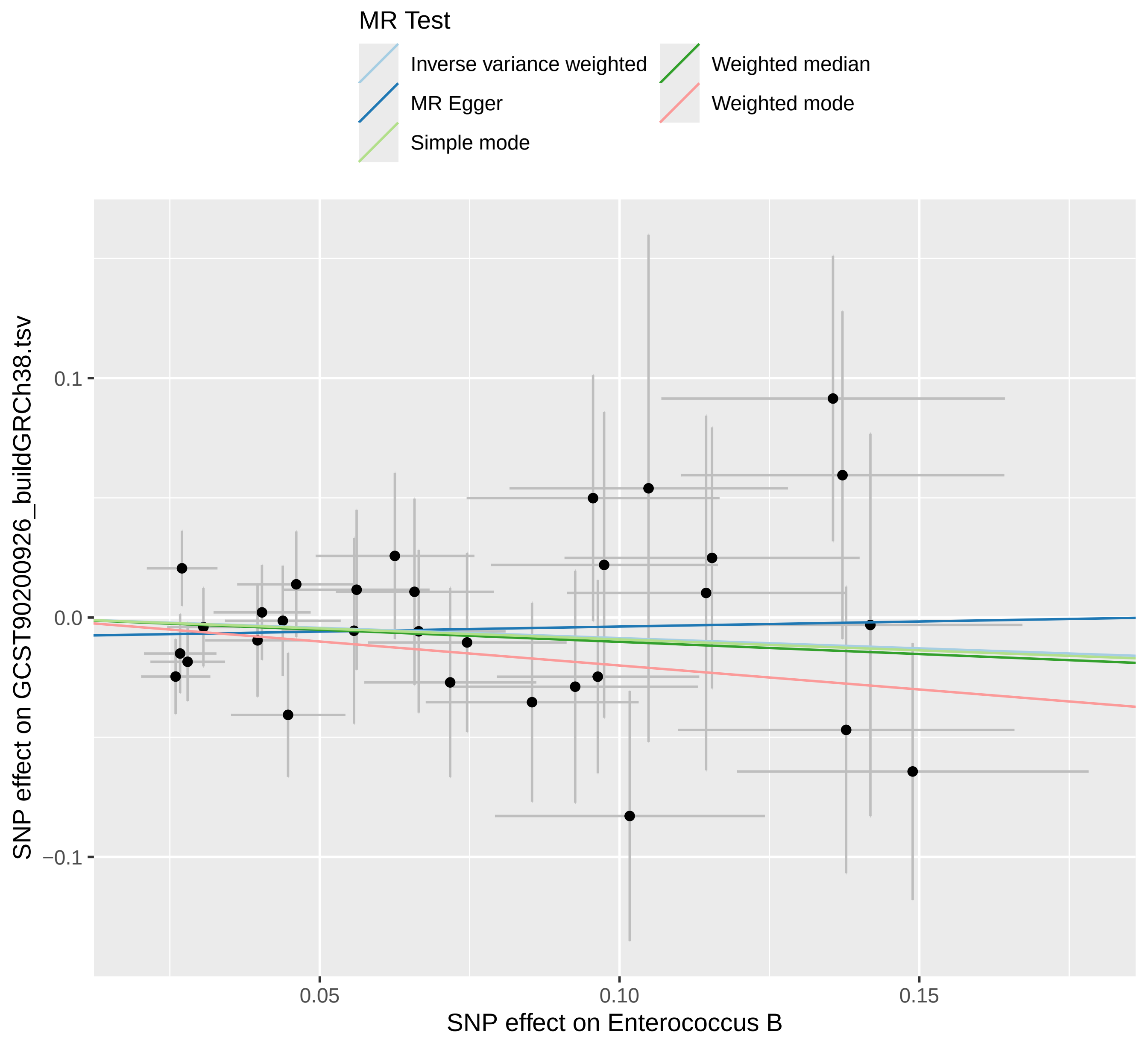

MR Test
Inverse variance weighted
MR Egger
Simple mode
Weighted median
Weighted mode
SNP effect on GCST90200926_buildGRCh38.tsv
0.1
0.0
−0.1
0.05
0.10
0.15
SNP effect on Enterococcus B

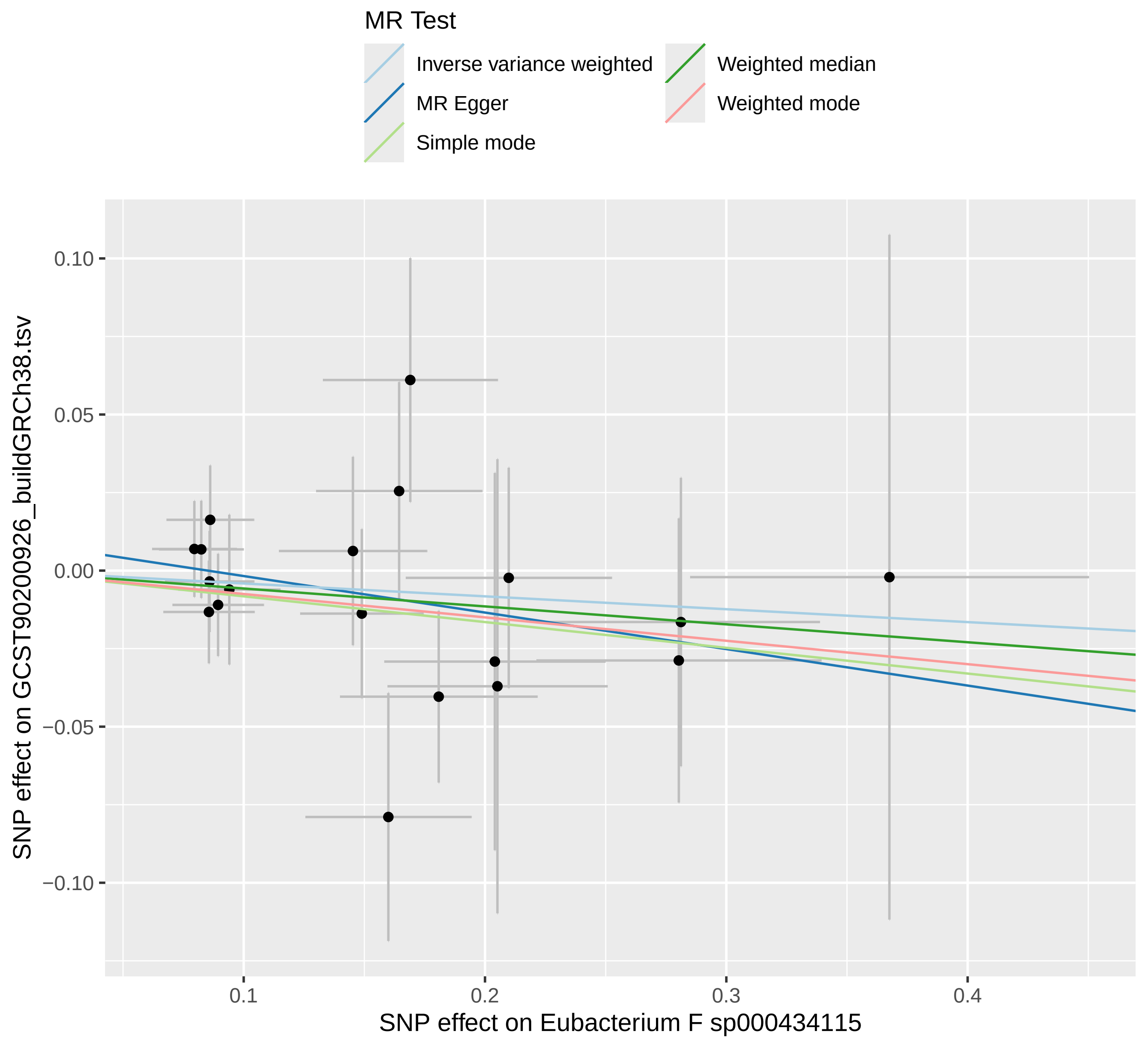
MR Test
Inverse variance weighted
MR Egger
Simple mode
Weighted median
Weighted mode
0.10
0.05
0.00
−0.05
−0.10
SNP effect on GCST90200926_buildGRCh38.tsv
0.1
0.2
0.3
0.4
SNP effect on Eubacterium F sp000434115

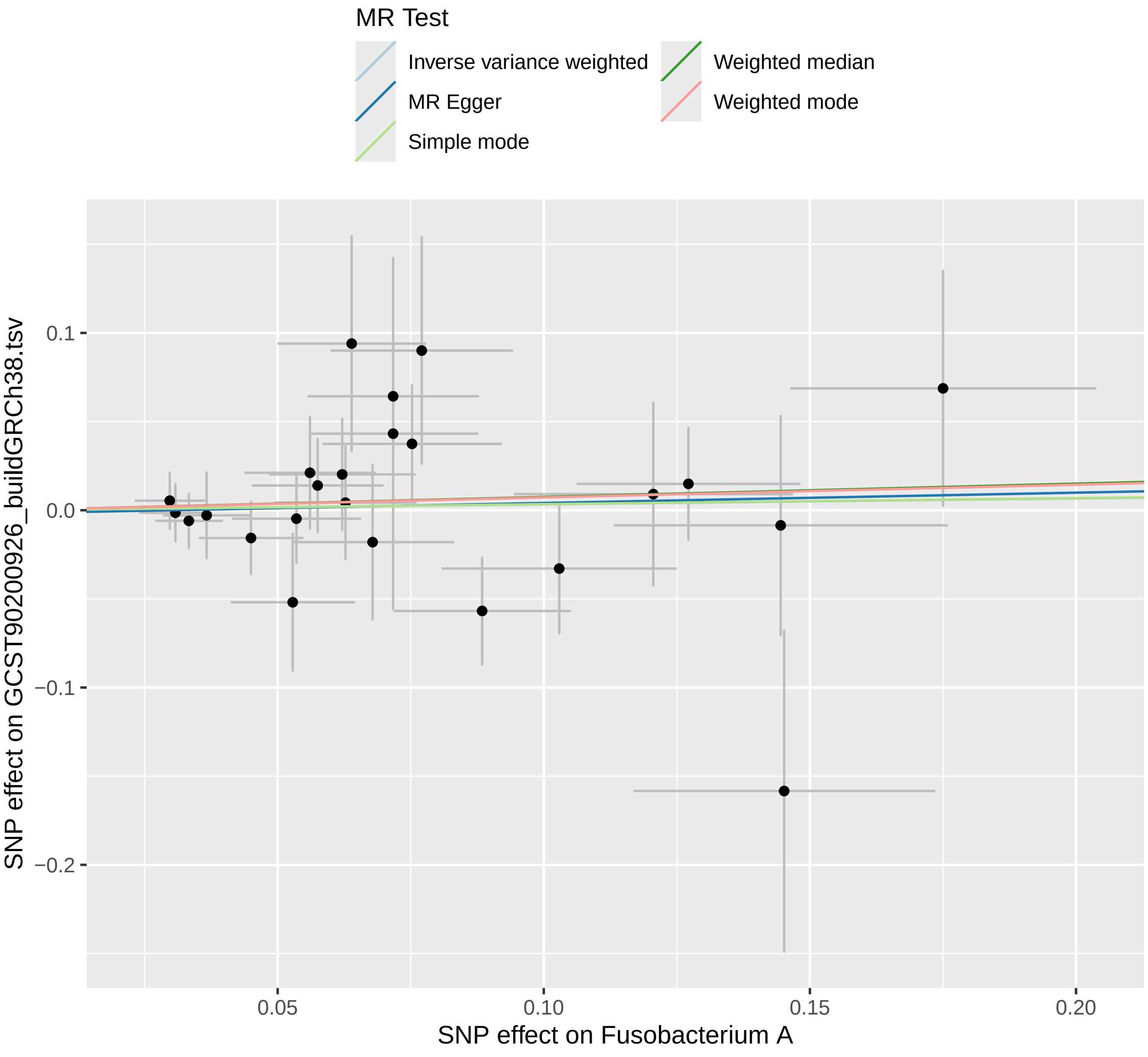
MR Test
Inverse variance weighted
MR Egger
Simple mode
Weighted median
Weighted mode
SNP effect on GCST90200926_buildGRCh38.tsv
0.1
0.0
−0.1
−0.2
0.05
0.10
0.15
0.20
SNP effect on Fusobacterium A

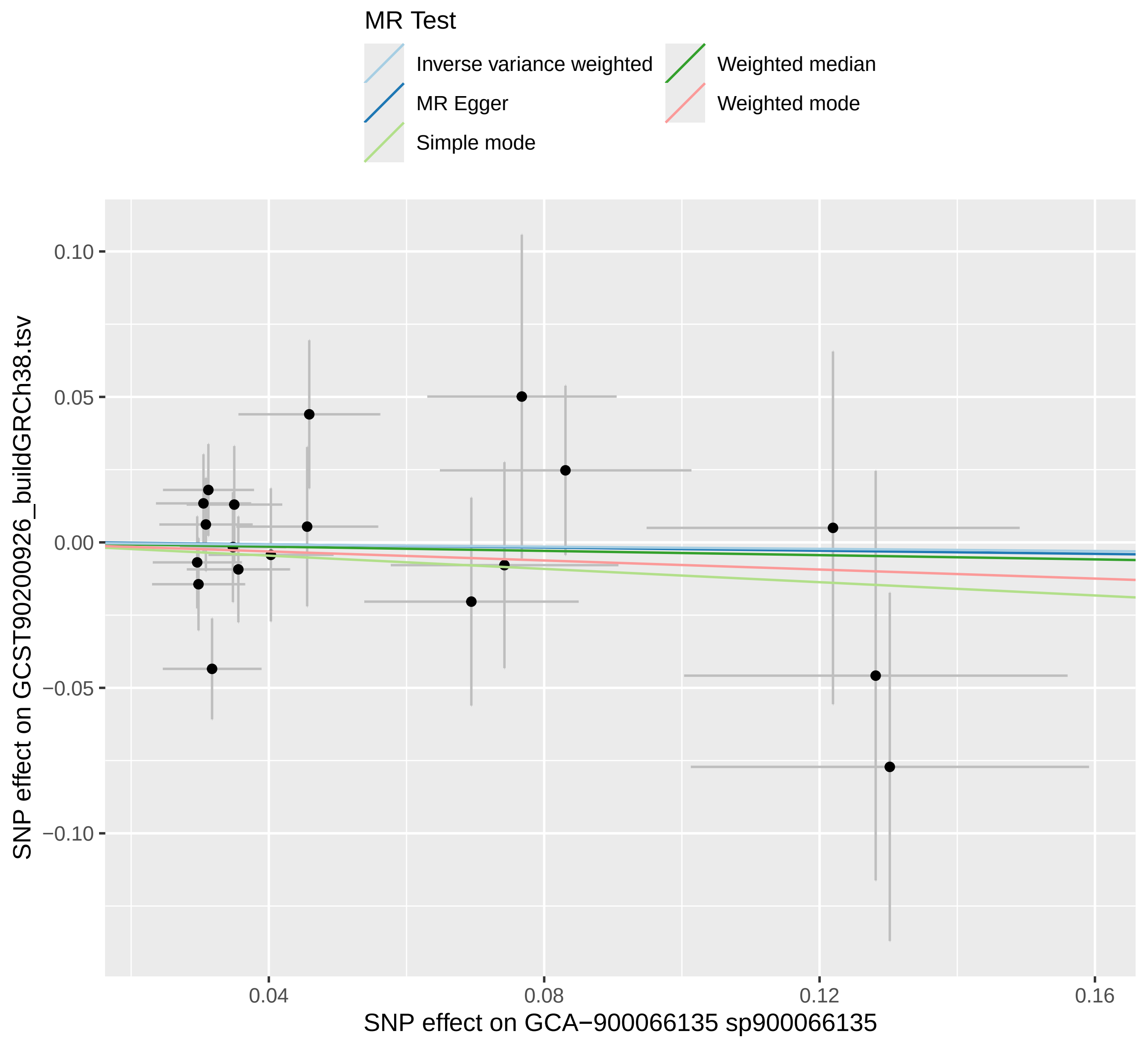
MR Test
Inverse variance weighted
MR Egger
Simple mode
Weighted median
Weighted mode
0.10
0.05
0.00
−0.05
−0.10
0.04
0.08
0.12
0.16
SNP effect on GCST90200926_buildGRCh38.tsv
SNP effect on GCA−900066135 sp900066135

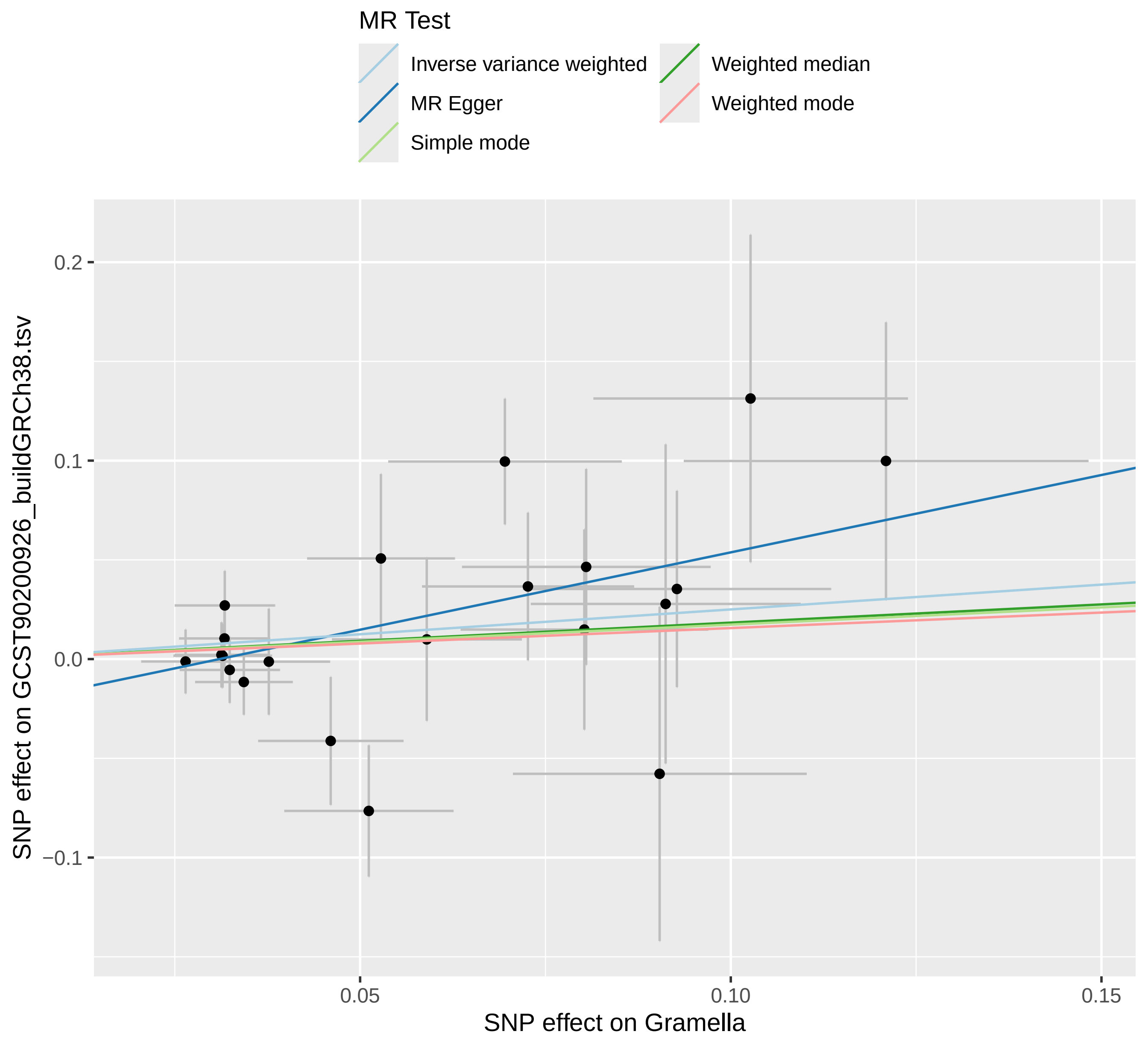

MR Test
Inverse variance weighted
MR Egger
Simple mode
Weighted median
Weighted mode
SNP effect on GCST90200926_buildGRCh38.tsv
0.2
0.1
0.0
−0.1
0.05
0.10
0.15
SNP effect on Gramella

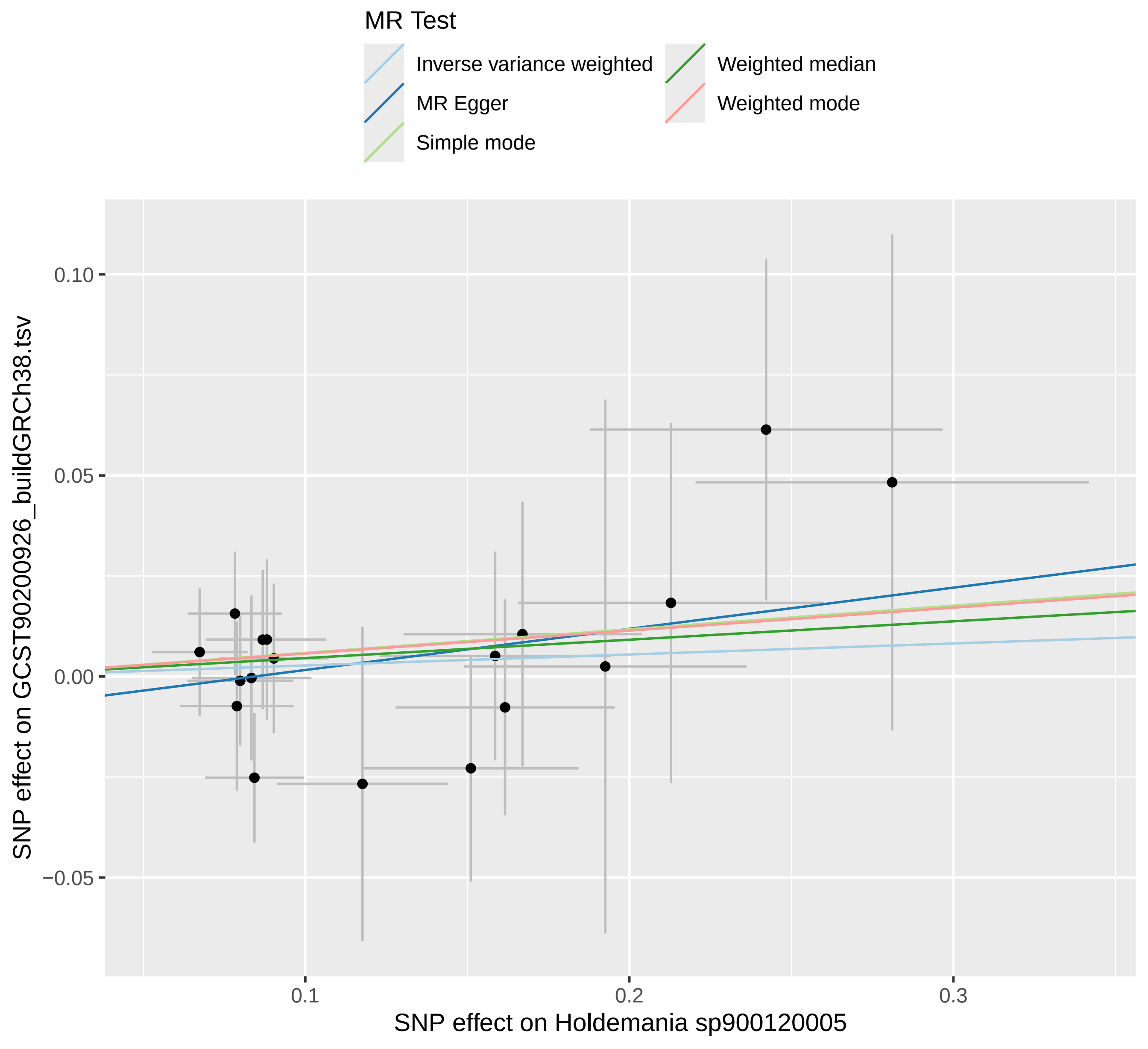

MR Test
Inverse variance weighted
MR Egger
Simple mode
Weighted median
Weighted mode
0.10
0.05
0.00
−0.05
0.1
0.2
0.3
SNP effect on GCST90200926_buildGRCh38.tsv
SNP effect on Holdemania sp900120005

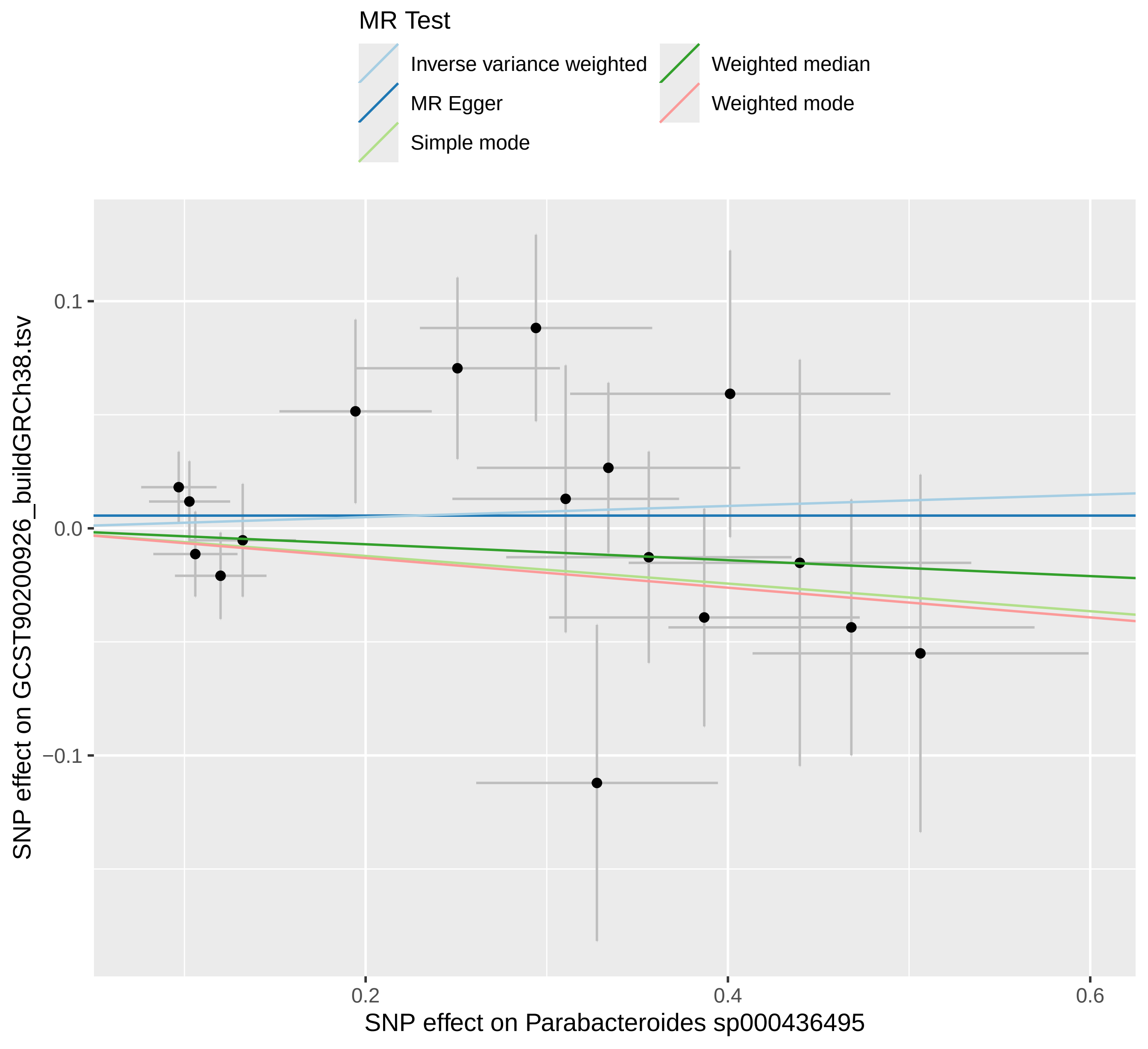

MR Test
Inverse variance weighted
MR Egger
Simple mode
Weighted median
Weighted mode
0.1
0.0
−0.1
0.2
0.4
0.6
SNP effect on GCST90200926_buildGRCh38.tsv
SNP effect on Parabacteroides sp000436495

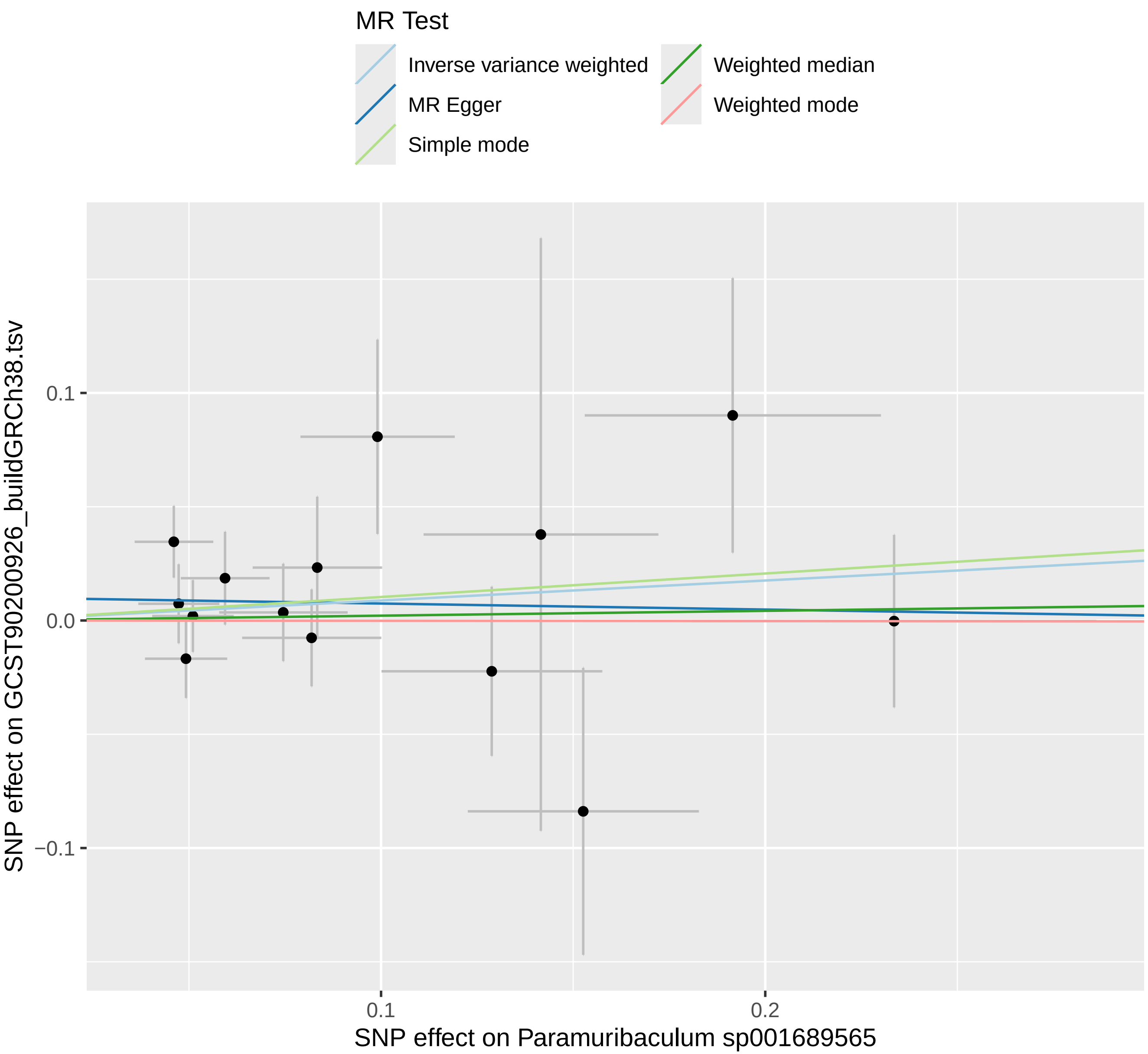
MR Test
Inverse variance weighted
MR Egger
Simple mode
Weighted median
Weighted mode
0.1
0.0
−0.1
0.1
0.2
SNP effect on GCST90200926_buildGRCh38.tsv
SNP effect on Paramuribaculum sp001689565

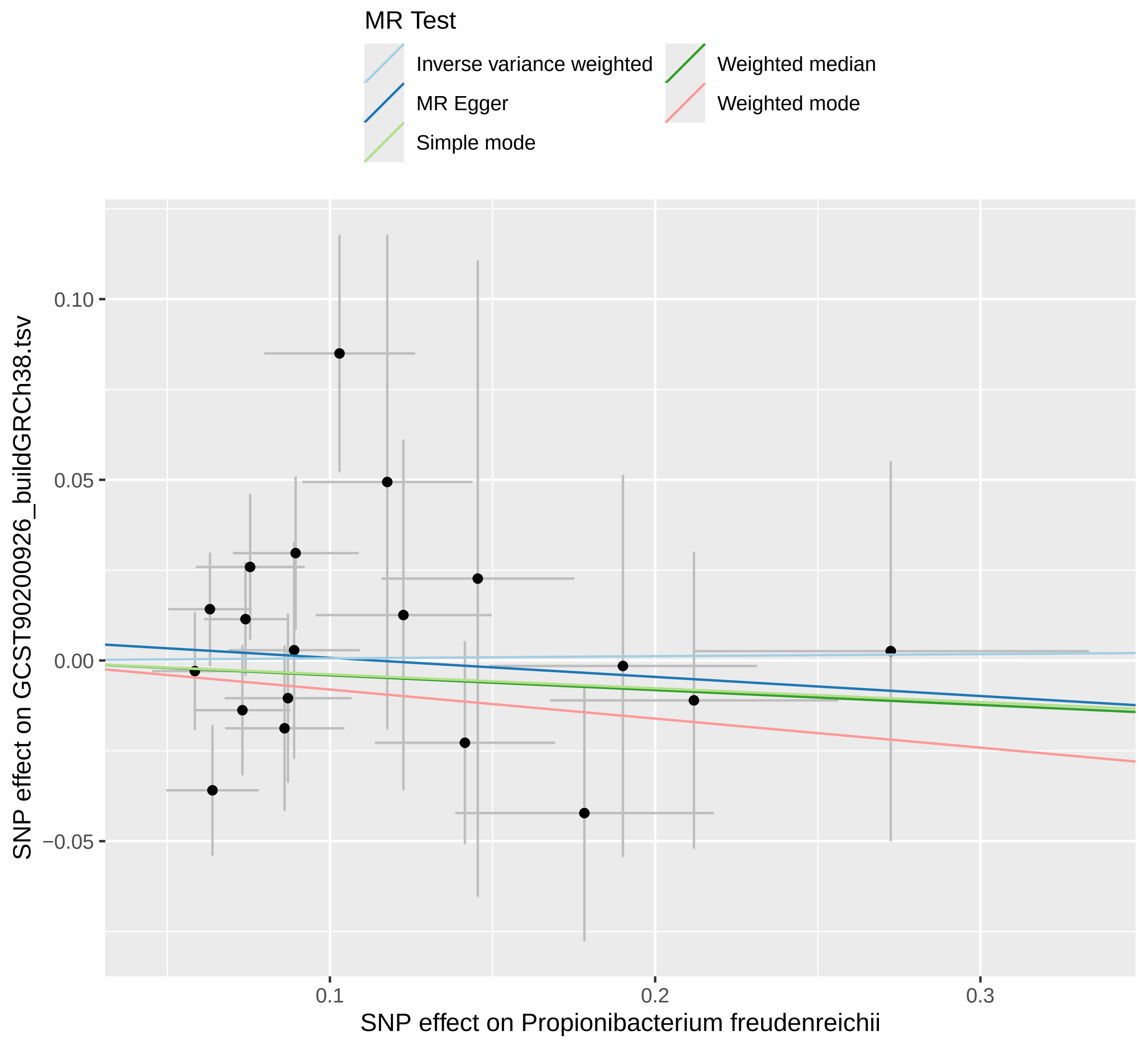
MR Test
Inverse variance weighted
MR Egger
Simple mode
Weighted median
Weighted mode
0.10
0.05
0.00
−0.05
0.1
0.2
0.3
SNP effect on GCST90200926_buildGRCh38.tsv
SNP effect on Propionibacterium freudenreichii

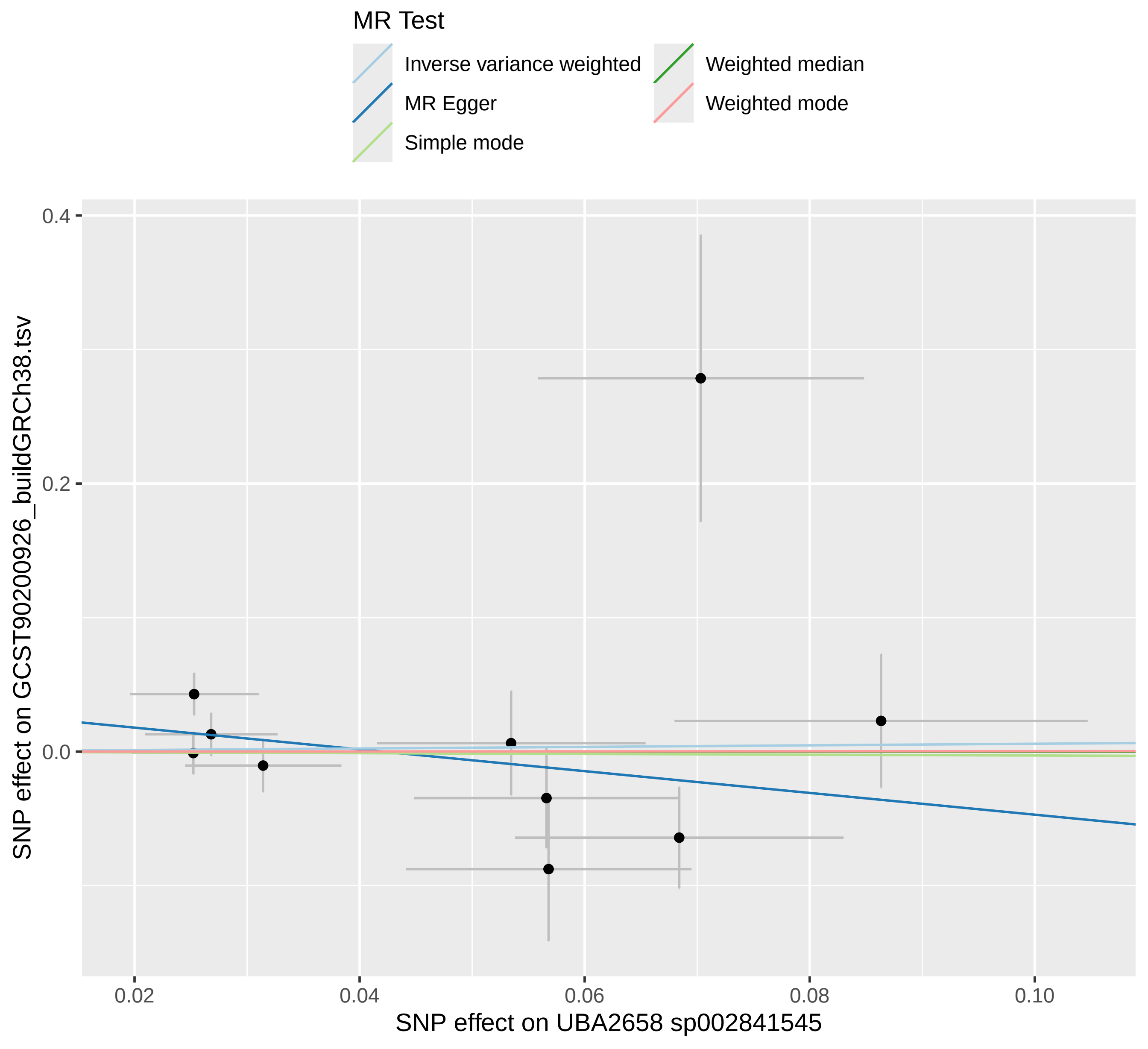
MR Test
Inverse variance weighted
MR Egger
Simple mode
Weighted median
Weighted mode
0.4
0.2
0.0
0.02
0.04
0.06
0.08
0.10
SNP effect on GCST90200926_buildGRCh38.tsv
SNP effect on UBA2658 sp002841545

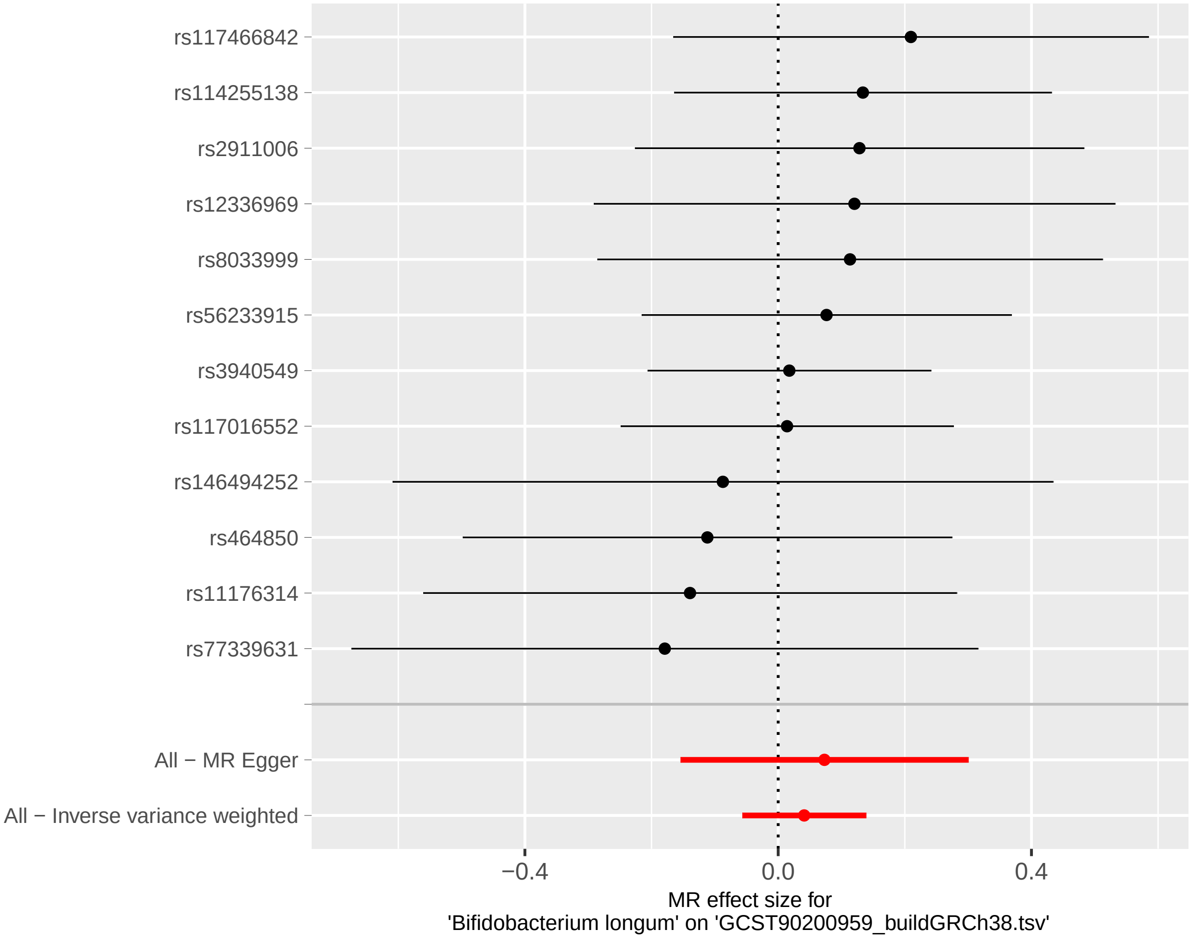
rs117466842
rs114255138
rs2911006
rs12336969
rs8033999
rs56233915
rs3940549
rs117016552
rs146494252
rs464850
rs11176314
rs77339631
All − MR Egger
All − Inverse variance weighted
−0.4
0.0
0.4
MR effect size for
'Bifidobacterium longum' on 'GCST90200959_buildGRCh38.tsv'

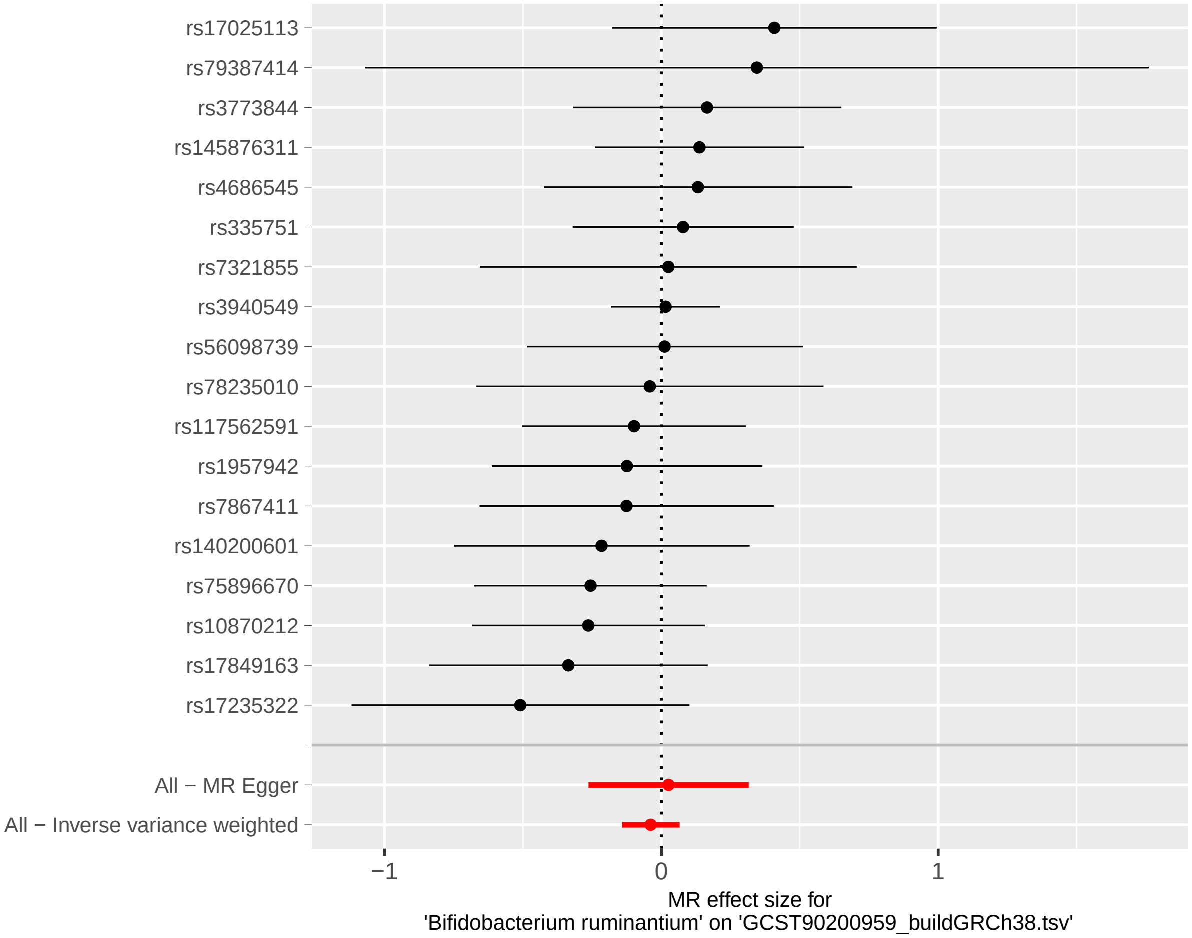
rs17025113
rs79387414
rs3773844
rs145876311
rs4686545
rs335751
rs7321855
rs3940549
rs56098739
rs78235010
rs117562591
rs1957942
rs7867411
rs140200601
rs75896670
rs10870212
rs17849163
rs17235322
All − MR Egger
All − Inverse variance weighted
−1
0
1
MR effect size for
'Bifidobacterium ruminantium' on 'GCST90200959_buildGRCh38.tsv'

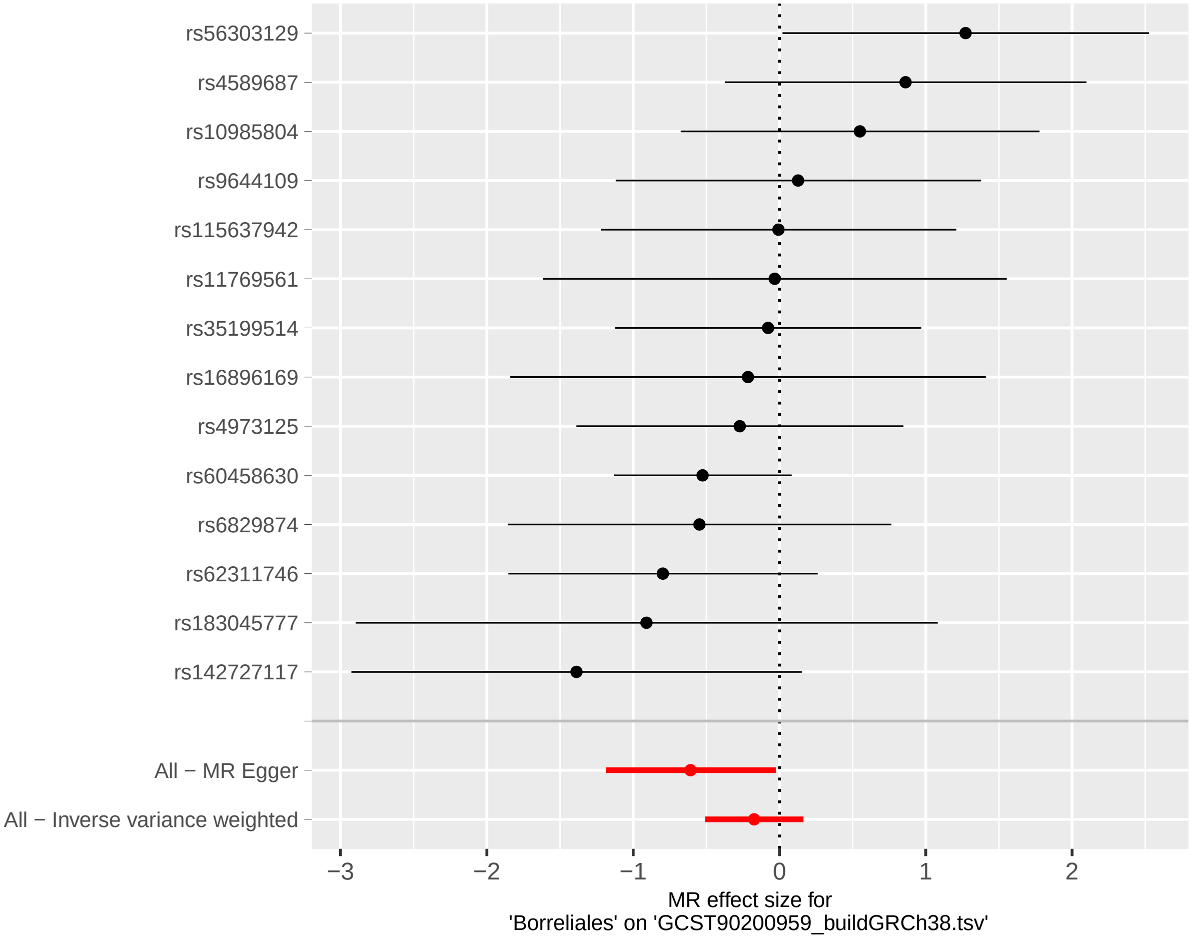

rs56303129
rs4589687
rs10985804
rs9644109
rs115637942
rs11769561
rs35199514
rs16896169
rs4973125
rs60458630
rs6829874
rs62311746
rs183045777
rs142727117
All − MR Egger
All − Inverse variance weighted
−3
−2
−1
0
1
2
MR effect size for
'Borreliales' on 'GCST90200959_buildGRCh38.tsv'

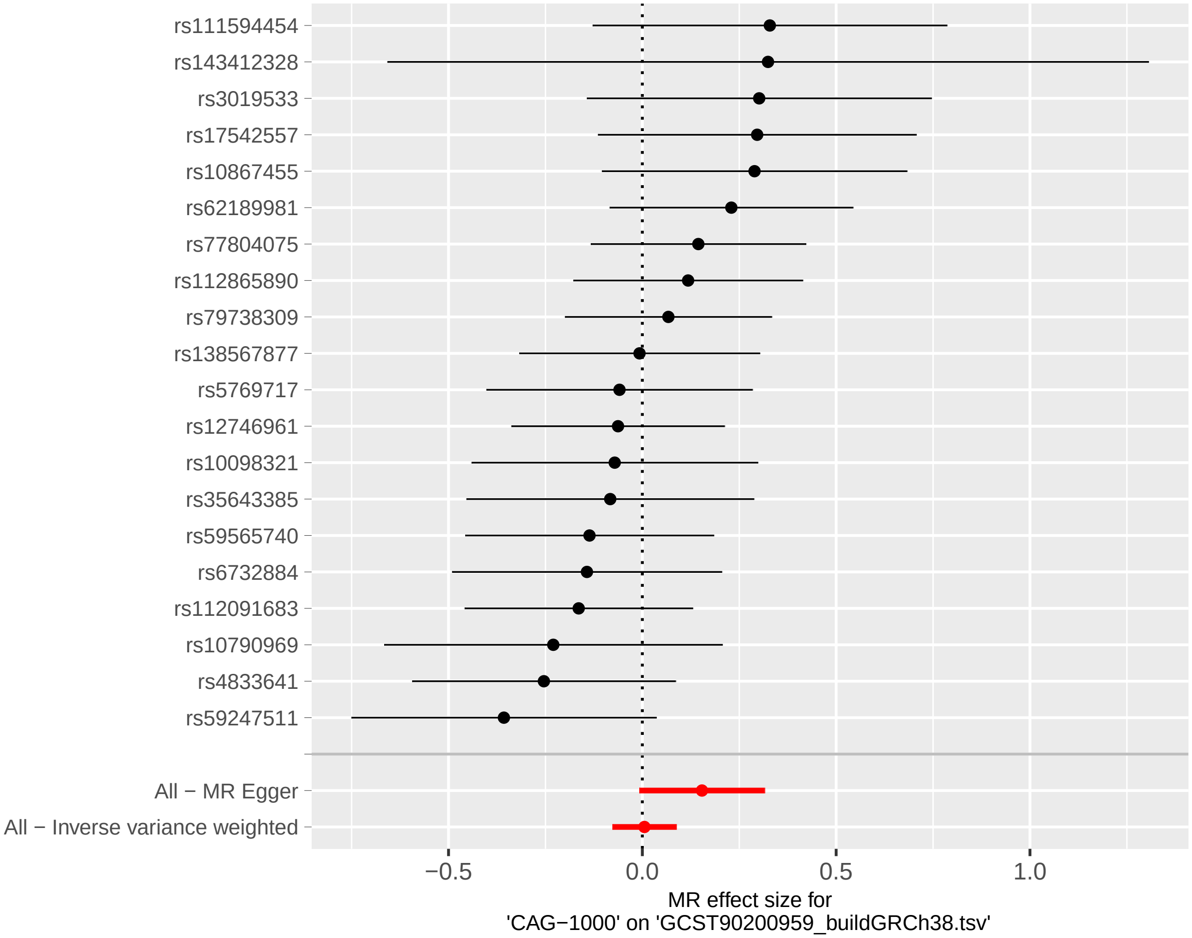
rs111594454
rs143412328
rs3019533
rs17542557
rs10867455
rs62189981
rs77804075
rs112865890
rs79738309
rs138567877
rs5769717
rs12746961
rs10098321
rs35643385
rs59565740
rs6732884
rs112091683
rs10790969
rs4833641
rs59247511
All − MR Egger
All − Inverse variance weighted
−0.5
0.0
0.5
1.0
MR effect size for
'CAG−1000' on 'GCST90200959_buildGRCh38.tsv'

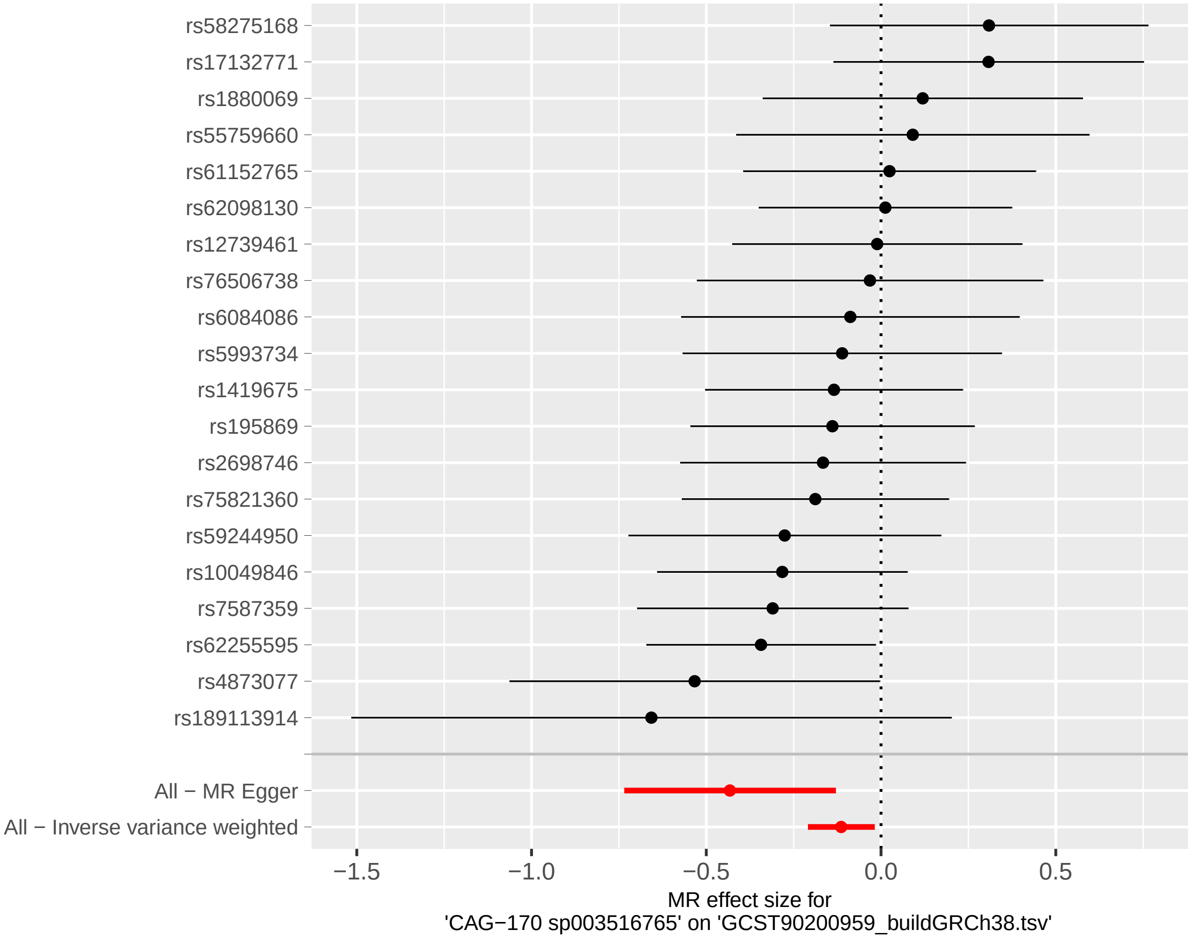
rs58275168
rs17132771
rs1880069
rs55759660
rs61152765
rs62098130
rs12739461
rs76506738
rs6084086
rs5993734
rs1419675
rs195869
rs2698746
rs75821360
rs59244950
rs10049846
rs7587359
rs62255595
rs4873077
rs189113914
All − MR Egger
All − Inverse variance weighted
−1.5
−1.0
−0.5
0.0
0.5
MR effect size for
'CAG−170 sp003516765' on 'GCST90200959_buildGRCh38.tsv'

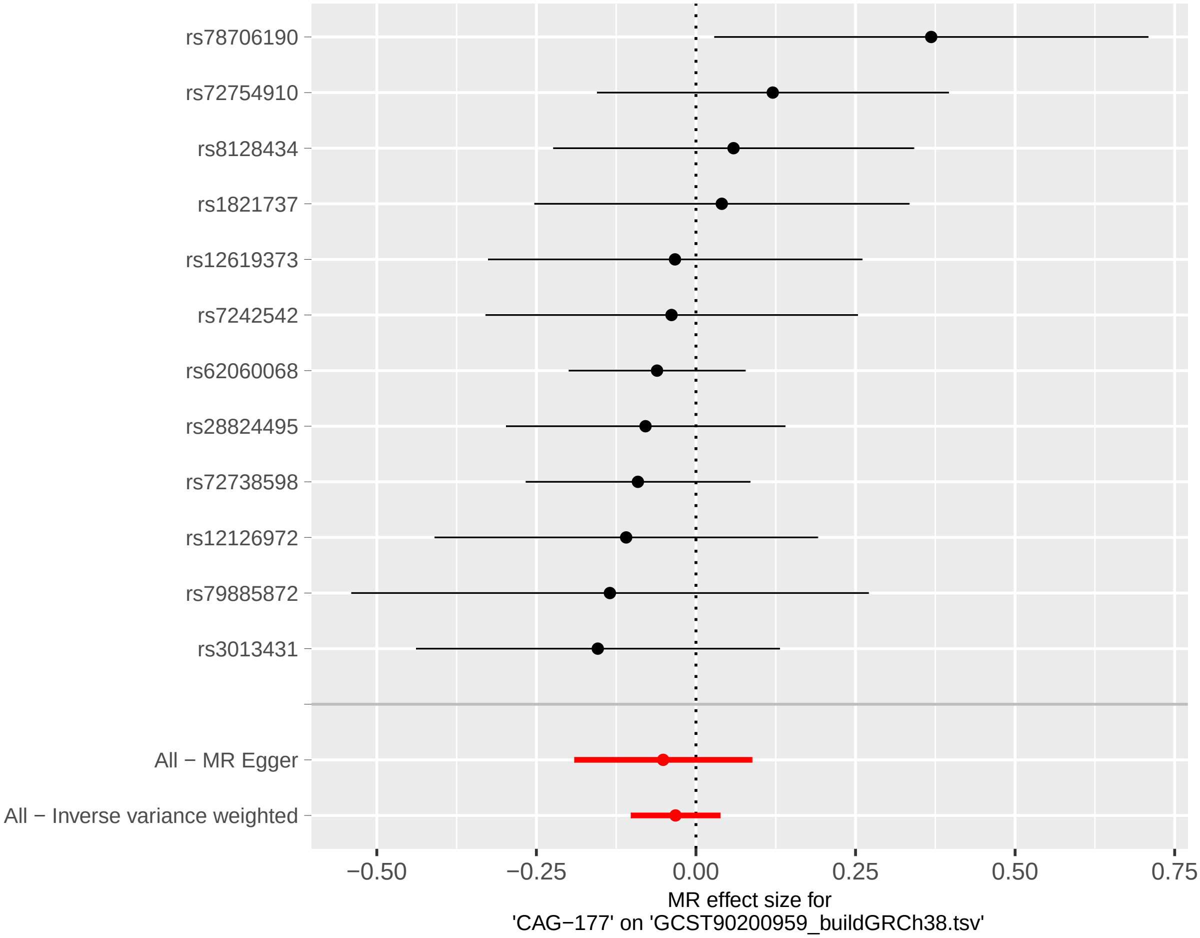

rs78706190
rs72754910
rs8128434
rs1821737
rs12619373
rs7242542
rs62060068
rs28824495
rs72738598
rs12126972
rs79885872
rs3013431
All − MR Egger
All − Inverse variance weighted
−0.50
−0.25
0.00
0.25
0.50
0.75
MR effect size for
'CAG−177' on 'GCST90200959_buildGRCh38.tsv'

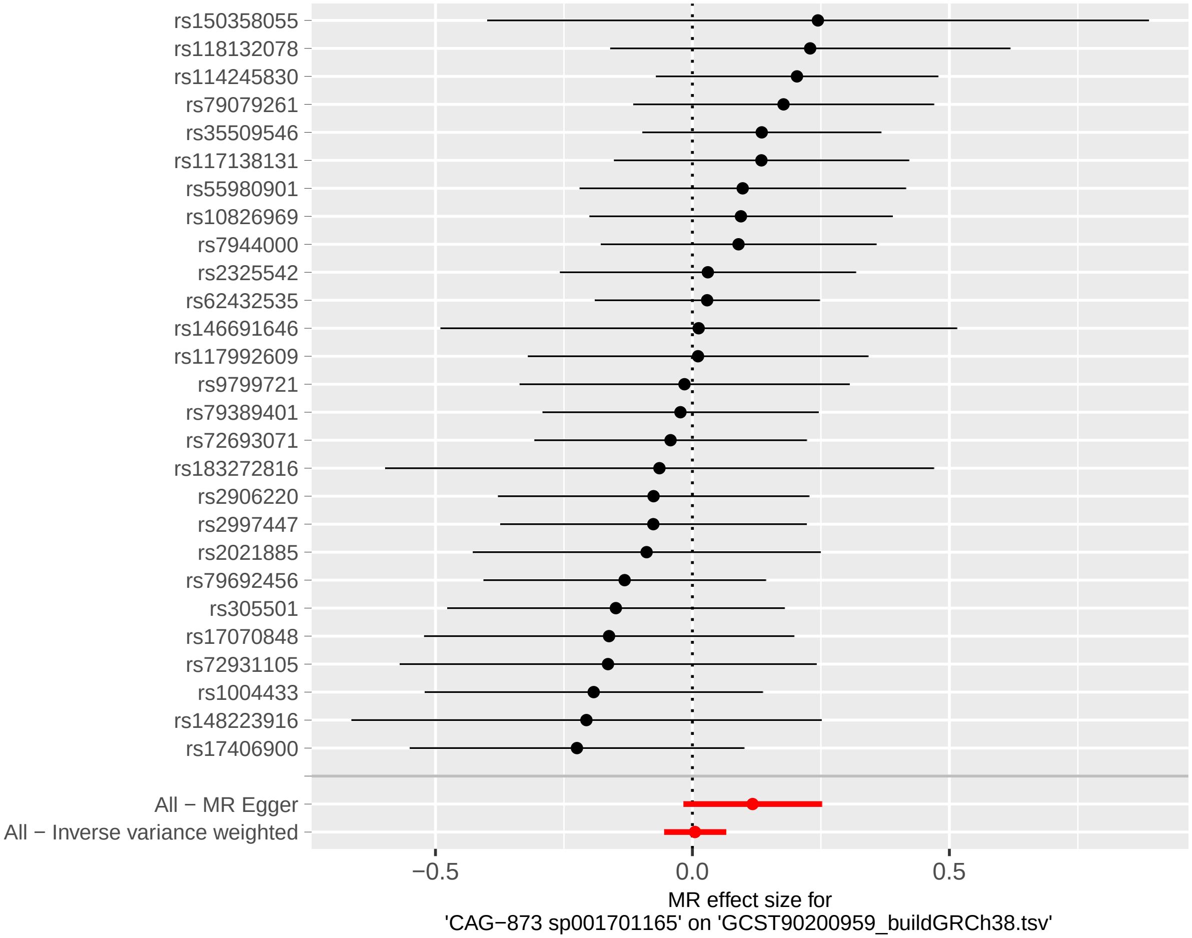
rs150358055
rs118132078
rs114245830
rs79079261
rs35509546
rs117138131
rs55980901
rs10826969
rs7944000
rs2325542
rs62432535
rs146691646
rs117992609
rs9799721
rs79389401
rs72693071
rs183272816
rs2906220
rs2997447
rs2021885
rs79692456
rs305501
rs17070848
rs72931105
rs1004433
rs148223916
rs17406900
All − MR Egger
All − Inverse variance weighted
−0.5
0.0
0.5
MR effect size for
'CAG−873 sp001701165' on 'GCST90200959_buildGRCh38.tsv'

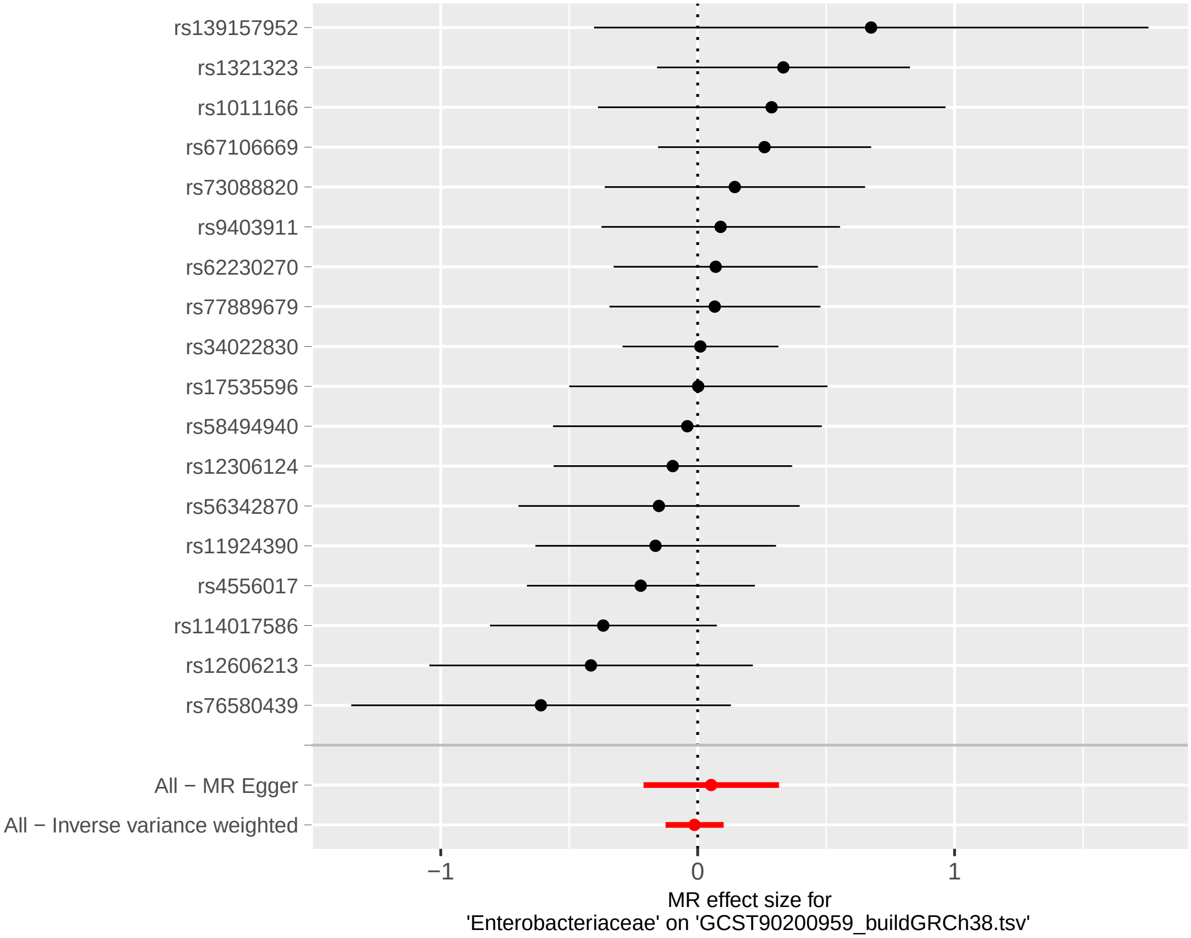
rs139157952
rs1321323
rs1011166
rs67106669
rs73088820
rs9403911
rs62230270
rs77889679
rs34022830
rs17535596
rs58494940
rs12306124
rs56342870
rs11924390
rs4556017
rs114017586
rs12606213
rs76580439
All − MR Egger
All − Inverse variance weighted
−1
0
1
MR effect size for
'Enterobacteriaceae' on 'GCST90200959_buildGRCh38.tsv'

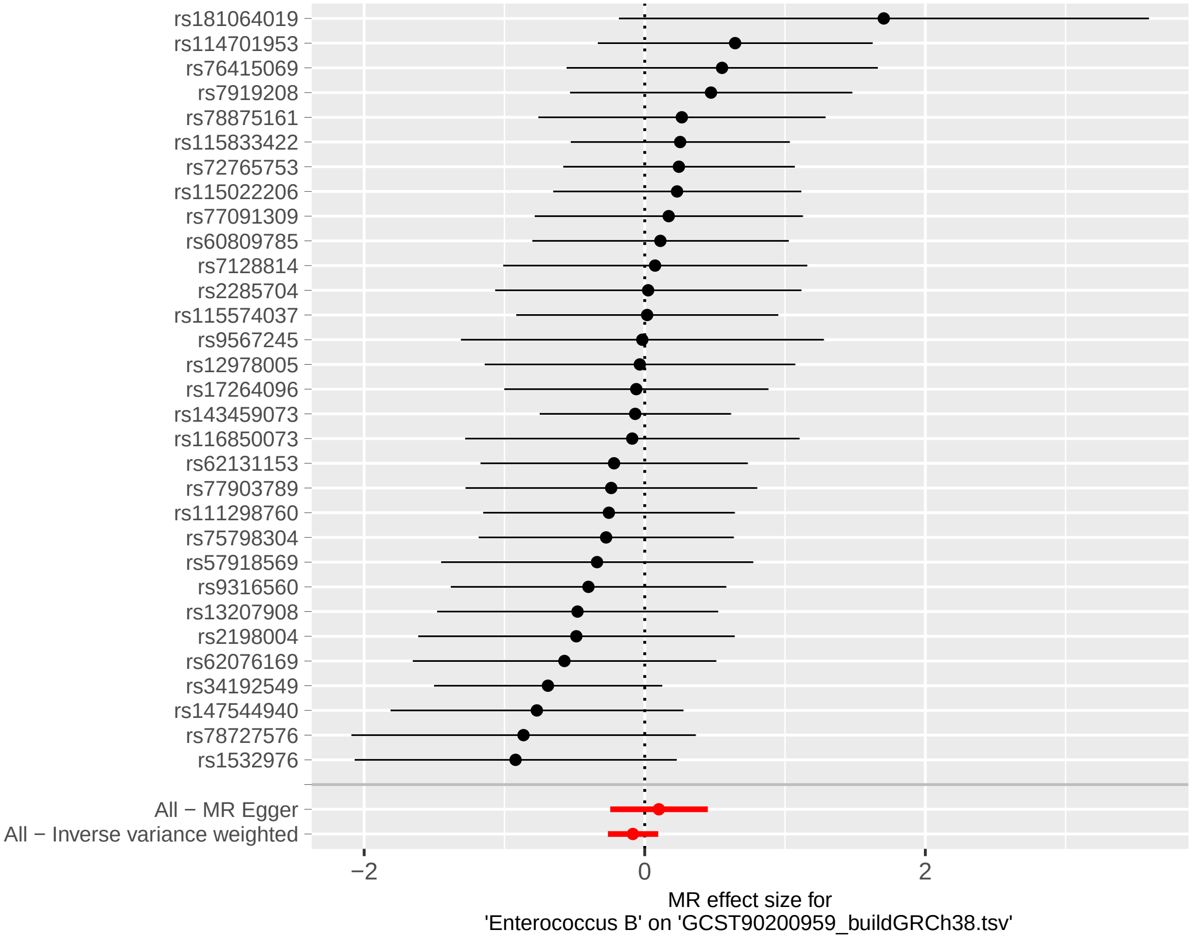

rs181064019
rs114701953
rs76415069
rs7919208
rs78875161
rs115833422
rs72765753
rs115022206
rs77091309
rs60809785
rs7128814
rs2285704
rs115574037
rs9567245
rs12978005
rs17264096
rs143459073
rs116850073
rs62131153
rs77903789
rs111298760
rs75798304
rs57918569
rs9316560
rs13207908
rs2198004
rs62076169
rs34192549
rs147544940
rs78727576
rs1532976
All − MR Egger
All − Inverse variance weighted
−2
0
2
MR effect size for
'Enterococcus B' on 'GCST90200959_buildGRCh38.tsv'

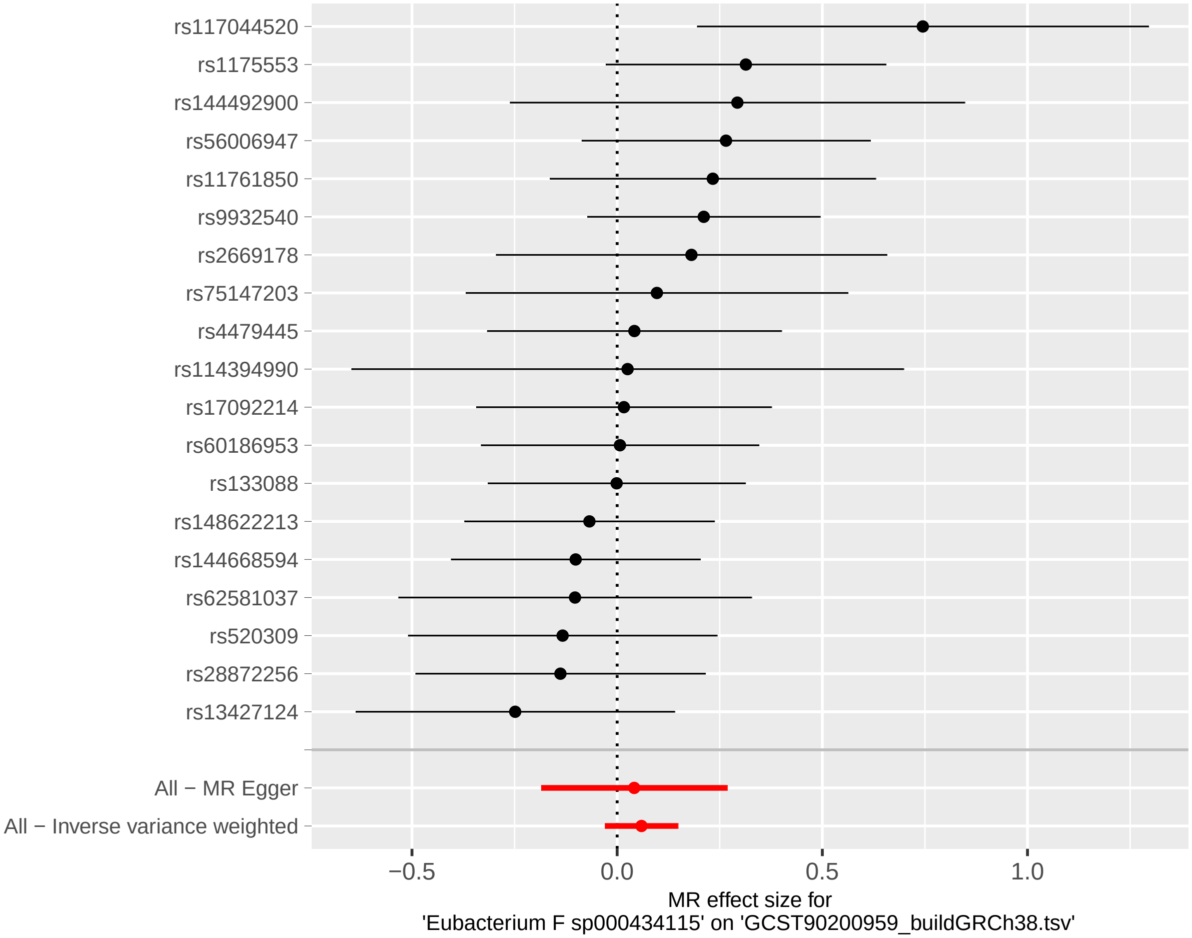
rs117044520
rs1175553
rs144492900
rs56006947
rs11761850
rs9932540
rs2669178
rs75147203
rs4479445
rs114394990
rs17092214
rs60186953
rs133088
rs148622213
rs144668594
rs62581037
rs520309
rs28872256
rs13427124
All − MR Egger
All − Inverse variance weighted
−0.5
0.0
0.5
1.0
MR effect size for
'Eubacterium F sp000434115' on 'GCST90200959_buildGRCh38.tsv'

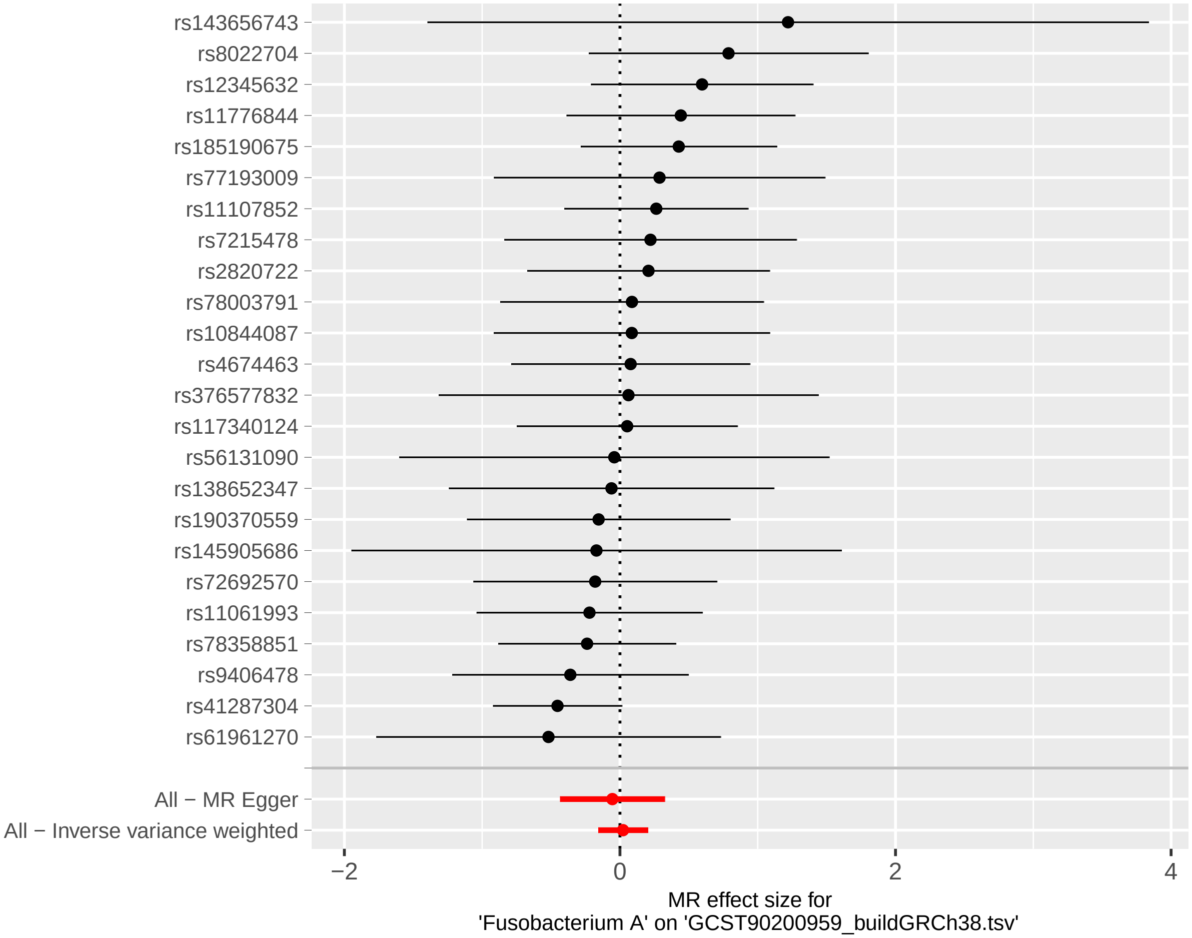
rs143656743
rs8022704
rs12345632
rs11776844
rs185190675
rs77193009
rs11107852
rs7215478
rs2820722
rs78003791
rs10844087
rs4674463
rs376577832
rs117340124
rs56131090
rs138652347
rs190370559
rs145905686
rs72692570
rs11061993
rs78358851
rs9406478
rs41287304
rs61961270
All − MR Egger
All − Inverse variance weighted
−2
0
2
4
MR effect size for
'Fusobacterium A' on 'GCST90200959_buildGRCh38.tsv'

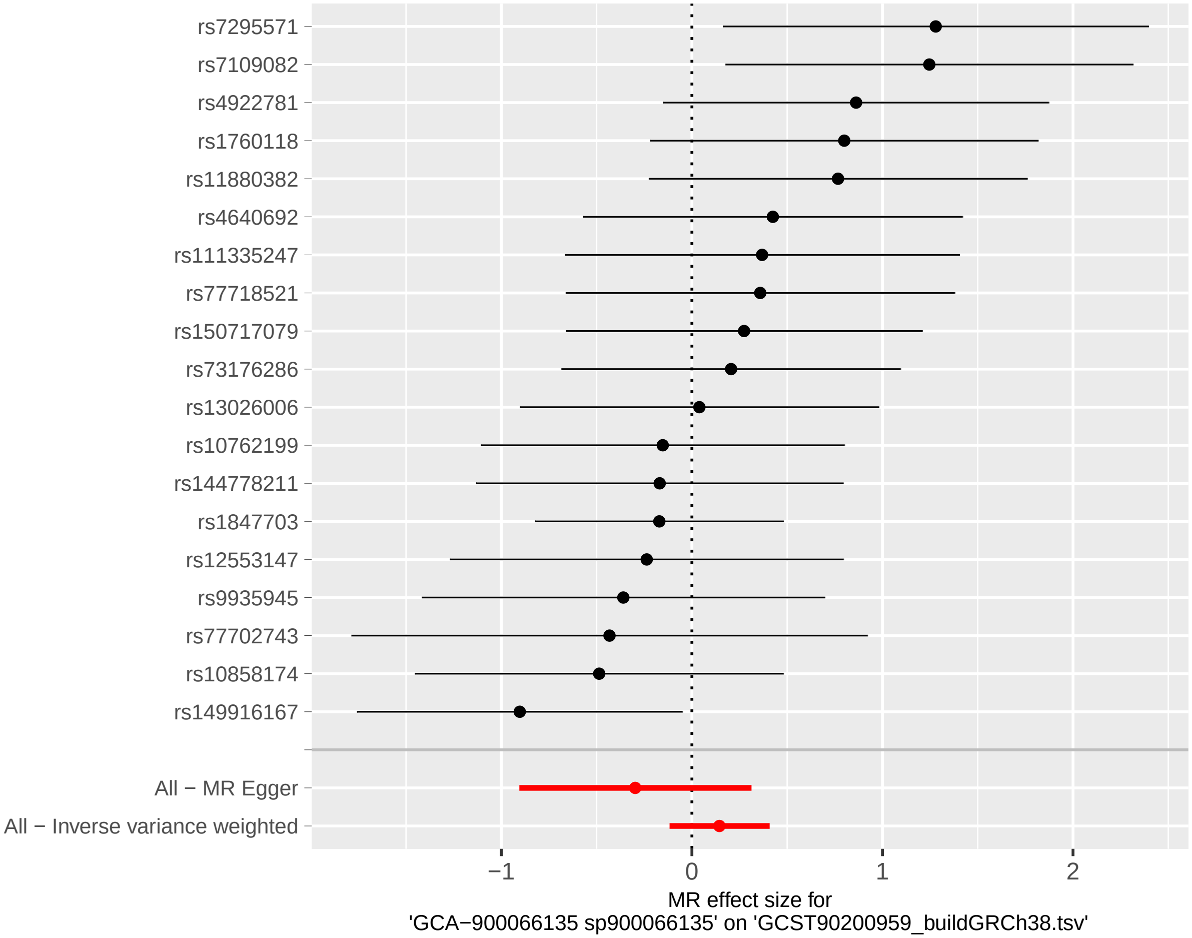
rs7295571
rs7109082
rs4922781
rs1760118
rs11880382
rs4640692
rs111335247
rs77718521
rs150717079
rs73176286
rs13026006
rs10762199
rs144778211
rs1847703
rs12553147
rs9935945
rs77702743
rs10858174
rs149916167
All − MR Egger
All − Inverse variance weighted
−1
0
1
2
MR effect size for
'GCA−900066135 sp900066135' on 'GCST90200959_buildGRCh38.tsv'

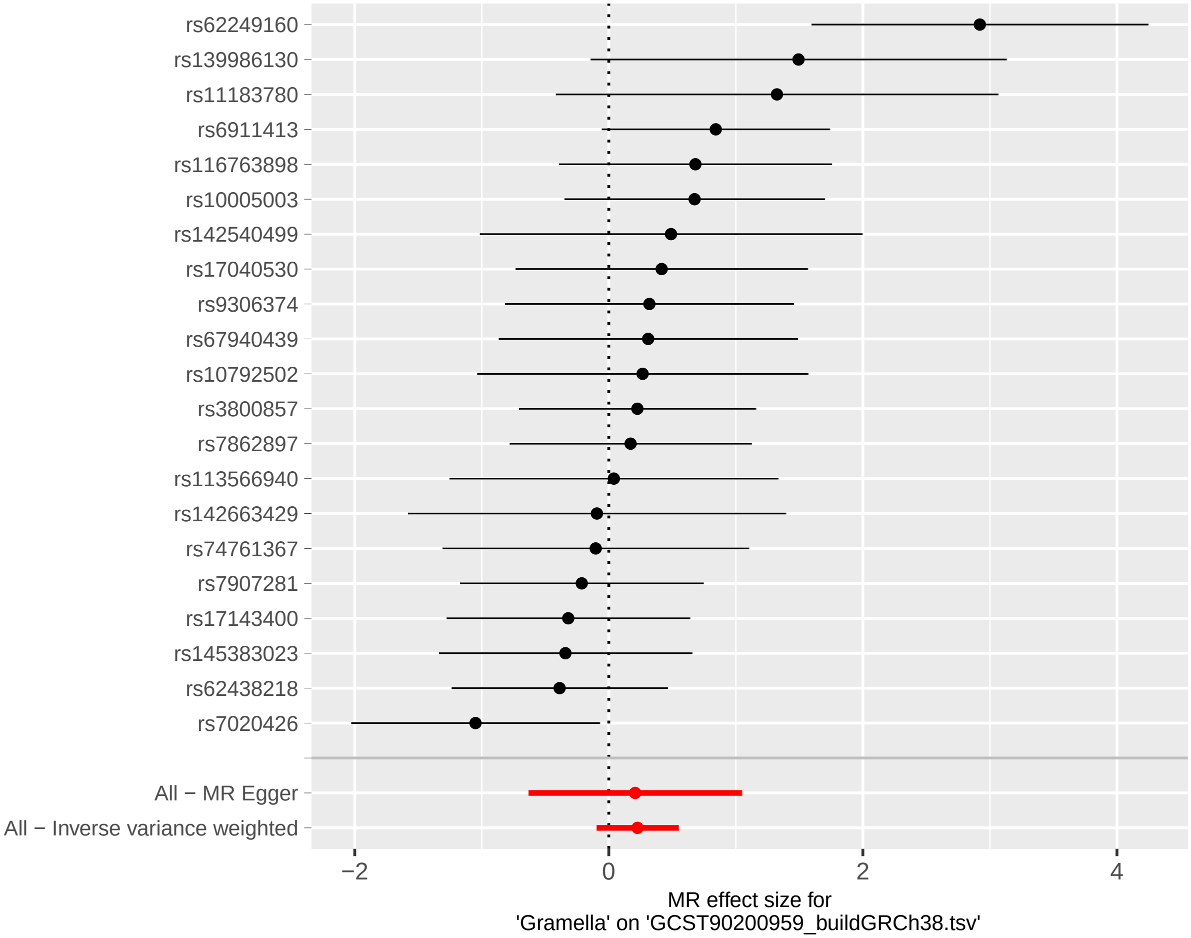
rs62249160
rs139986130
rs11183780
rs6911413
rs116763898
rs10005003
rs142540499
rs17040530
rs9306374
rs67940439
rs10792502
rs3800857
rs7862897
rs113566940
rs142663429
rs74761367
rs7907281
rs17143400
rs145383023
rs62438218
rs7020426
All − MR Egger
All − Inverse variance weighted
−2
0
2
4
MR effect size for
'Gramella' on 'GCST90200959_buildGRCh38.tsv'

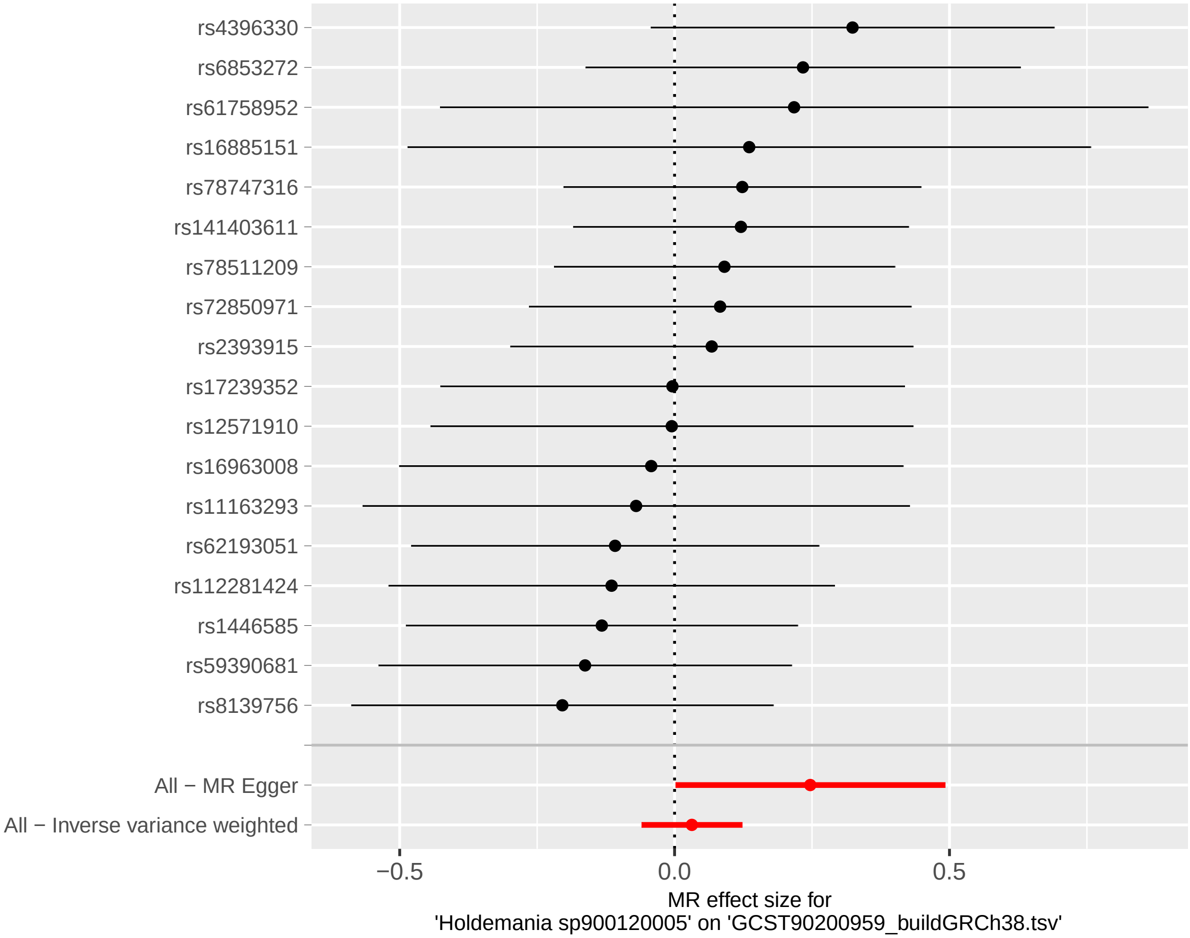
rs4396330
rs6853272
rs61758952
rs16885151
rs78747316
rs141403611
rs78511209
rs72850971
rs2393915
rs17239352
rs12571910
rs16963008
rs11163293
rs62193051
rs112281424
rs1446585
rs59390681
rs8139756
All − MR Egger
All − Inverse variance weighted
−0.5
0.0
0.5
MR effect size for
'Holdemania sp900120005' on 'GCST90200959_buildGRCh38.tsv'

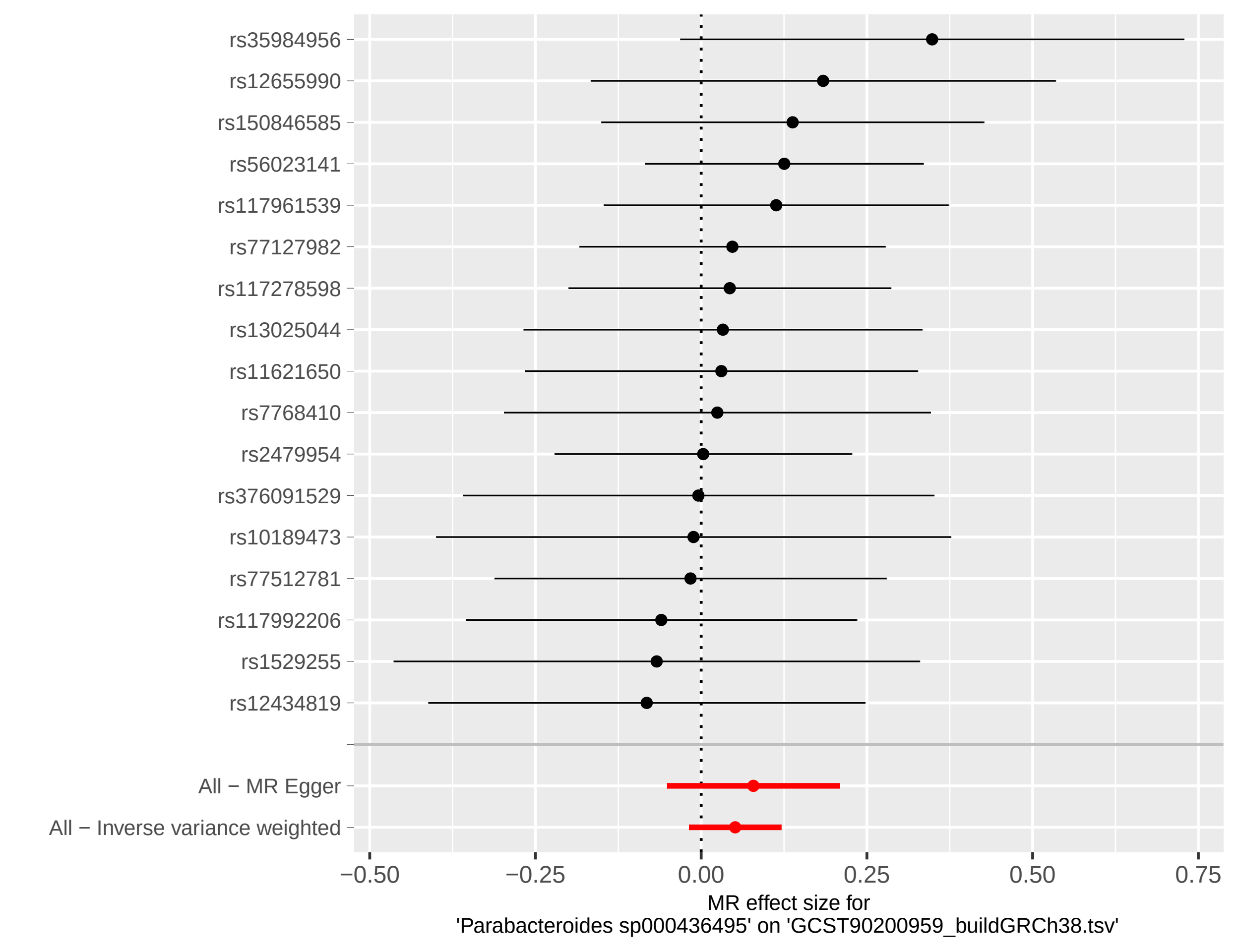
rs35984956
rs12655990
rs150846585
rs56023141
rs117961539
rs77127982
rs117278598
rs13025044
rs11621650
rs7768410
rs2479954
rs376091529
rs10189473
rs77512781
rs117992206
rs1529255
rs12434819
All − MR Egger
All − Inverse variance weighted
−0.50
−0.25
0.00
0.25
0.50
0.75
MR effect size for
'Parabacteroides sp000436495' on 'GCST90200959_buildGRCh38.tsv'

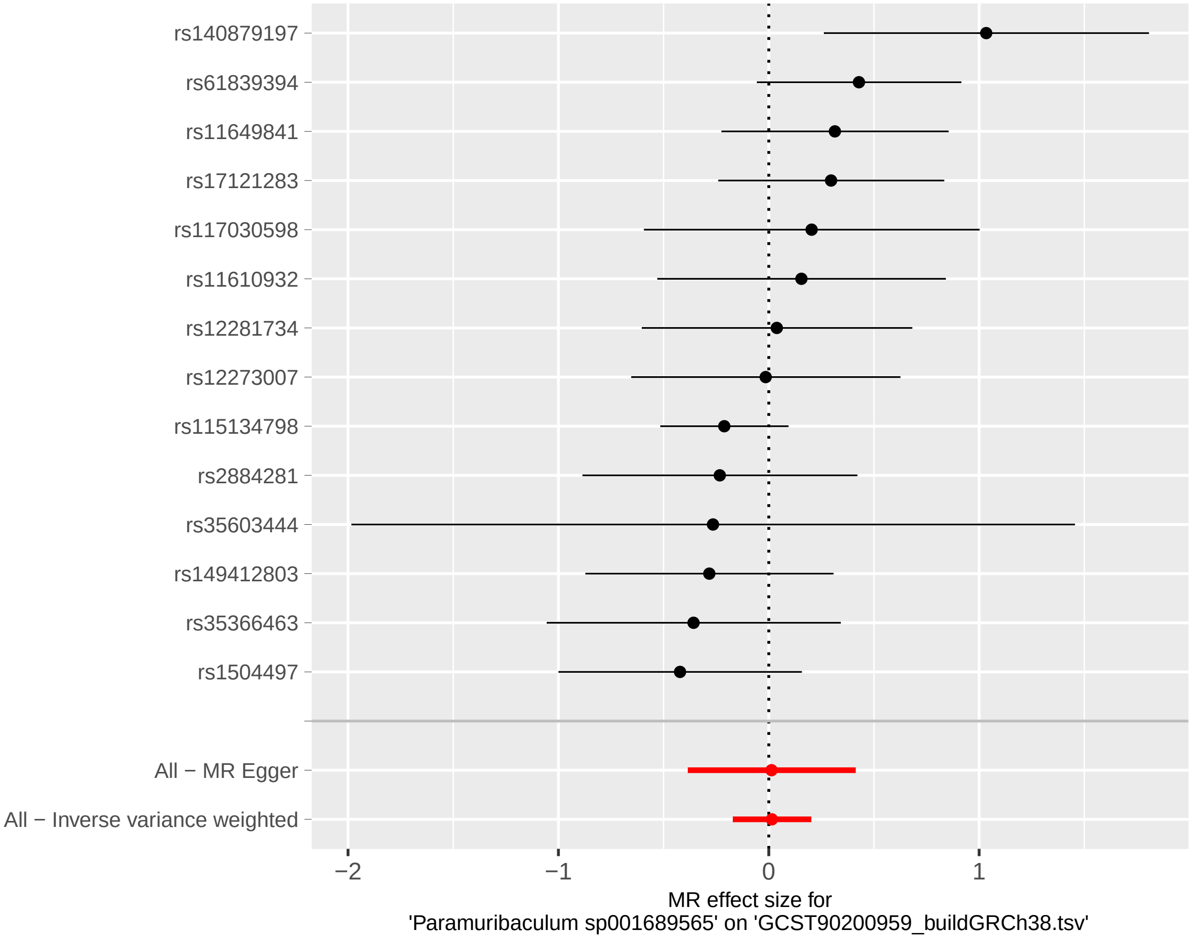
rs140879197
rs61839394
rs11649841
rs17121283
rs117030598
rs11610932
rs12281734
rs12273007
rs115134798
rs2884281
rs35603444
rs149412803
rs35366463
rs1504497
All − MR Egger
All − Inverse variance weighted
−2
−1
0
1
MR effect size for
'Paramuribaculum sp001689565' on 'GCST90200959_buildGRCh38.tsv'

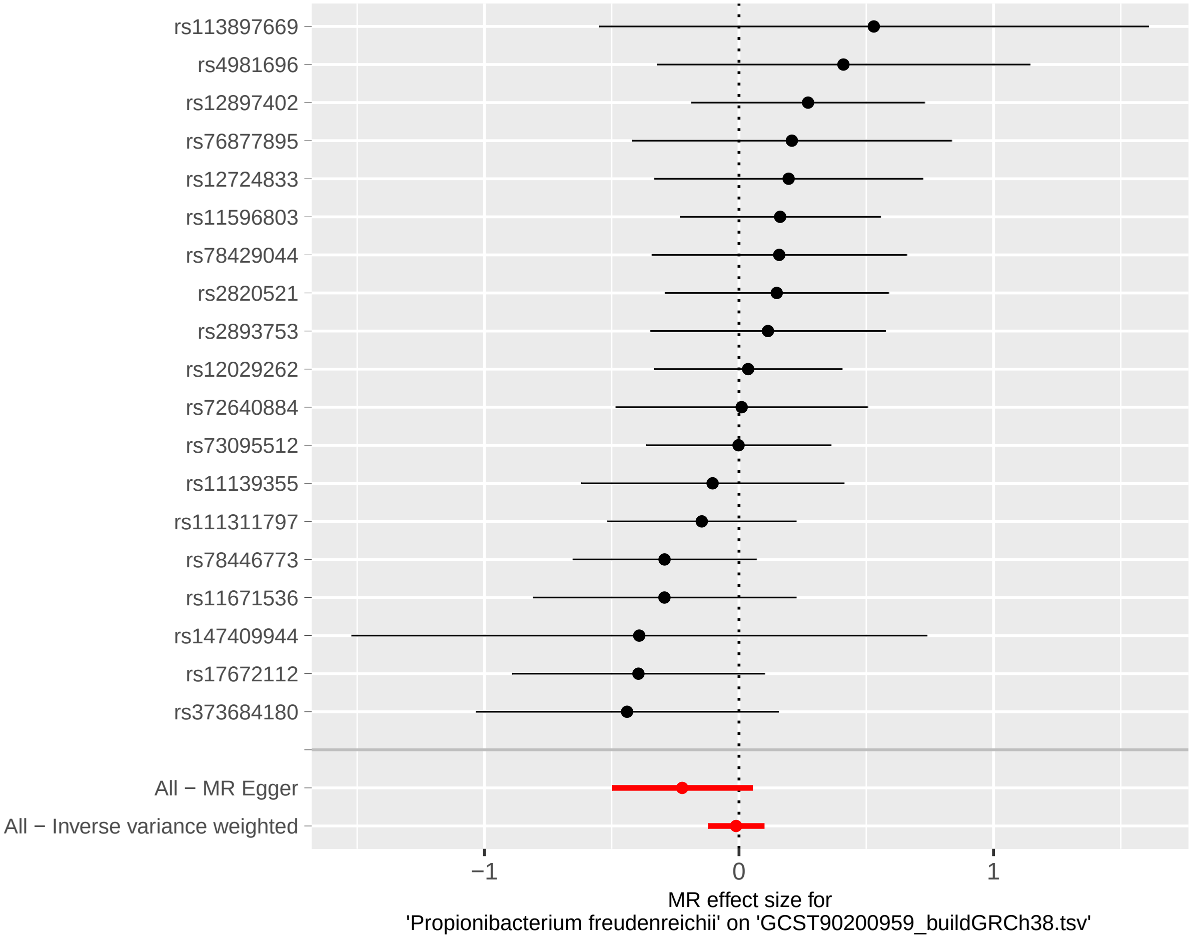
rs113897669
rs4981696
rs12897402
rs76877895
rs12724833
rs11596803
rs78429044
rs2820521
rs2893753
rs12029262
rs72640884
rs73095512
rs11139355
rs111311797
rs78446773
rs11671536
rs147409944
rs17672112
rs373684180
All − MR Egger
All − Inverse variance weighted
−1
0
1
MR effect size for
'Propionibacterium freudenreichii' on 'GCST90200959_buildGRCh38.tsv'

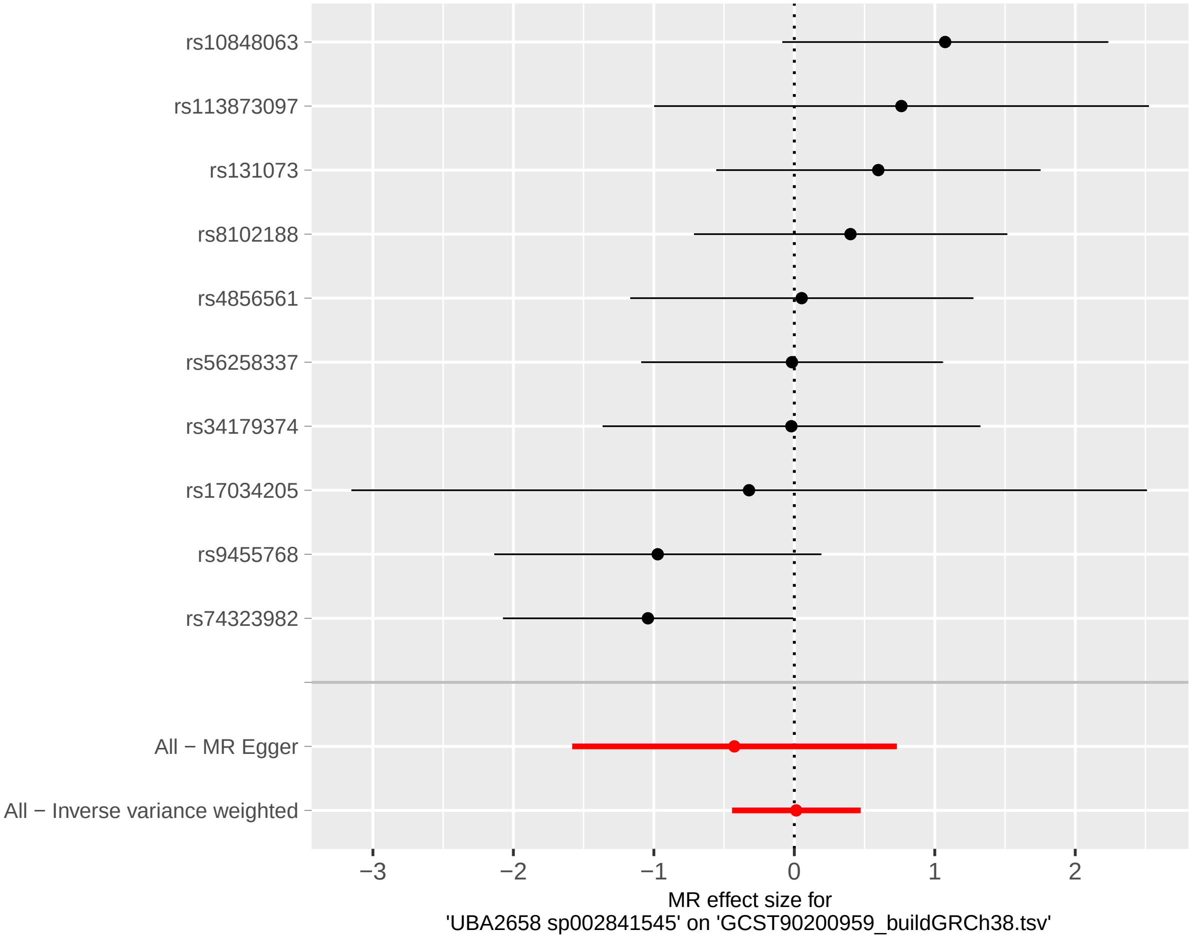
rs10848063
rs113873097
rs131073
rs8102188
rs4856561
rs56258337
rs34179374
rs17034205
rs9455768
rs74323982
All − MR Egger
All − Inverse variance weighted
−3
−2
−1
0
1
2
MR effect size for
'UBA2658 sp002841545' on 'GCST90200959_buildGRCh38.tsv'

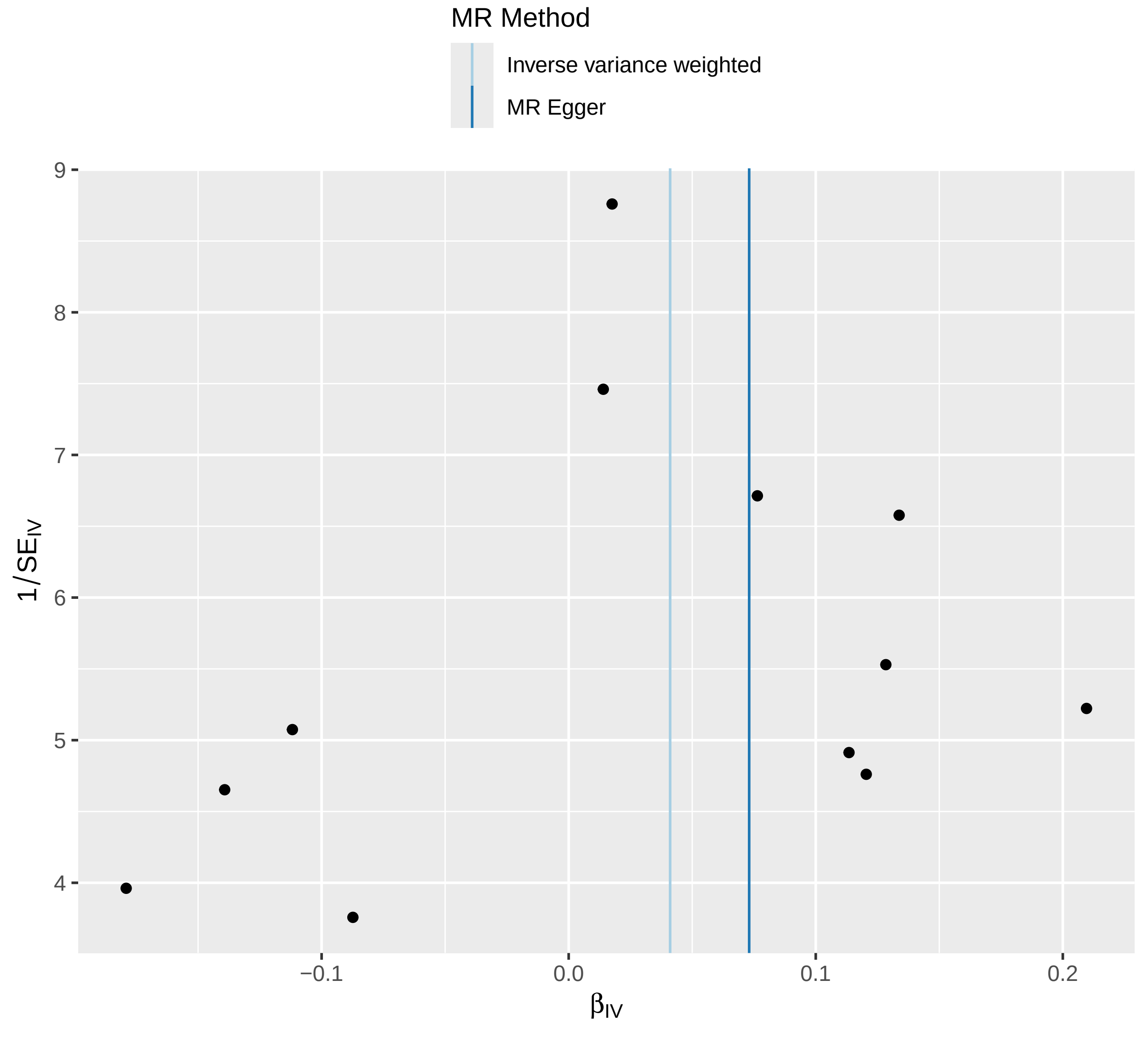
MR Method
Inverse variance weighted
MR Egger
9
8
7
6
5
4
1/SE_IV
−0.1
0.0
0.1
0.2
β_IV

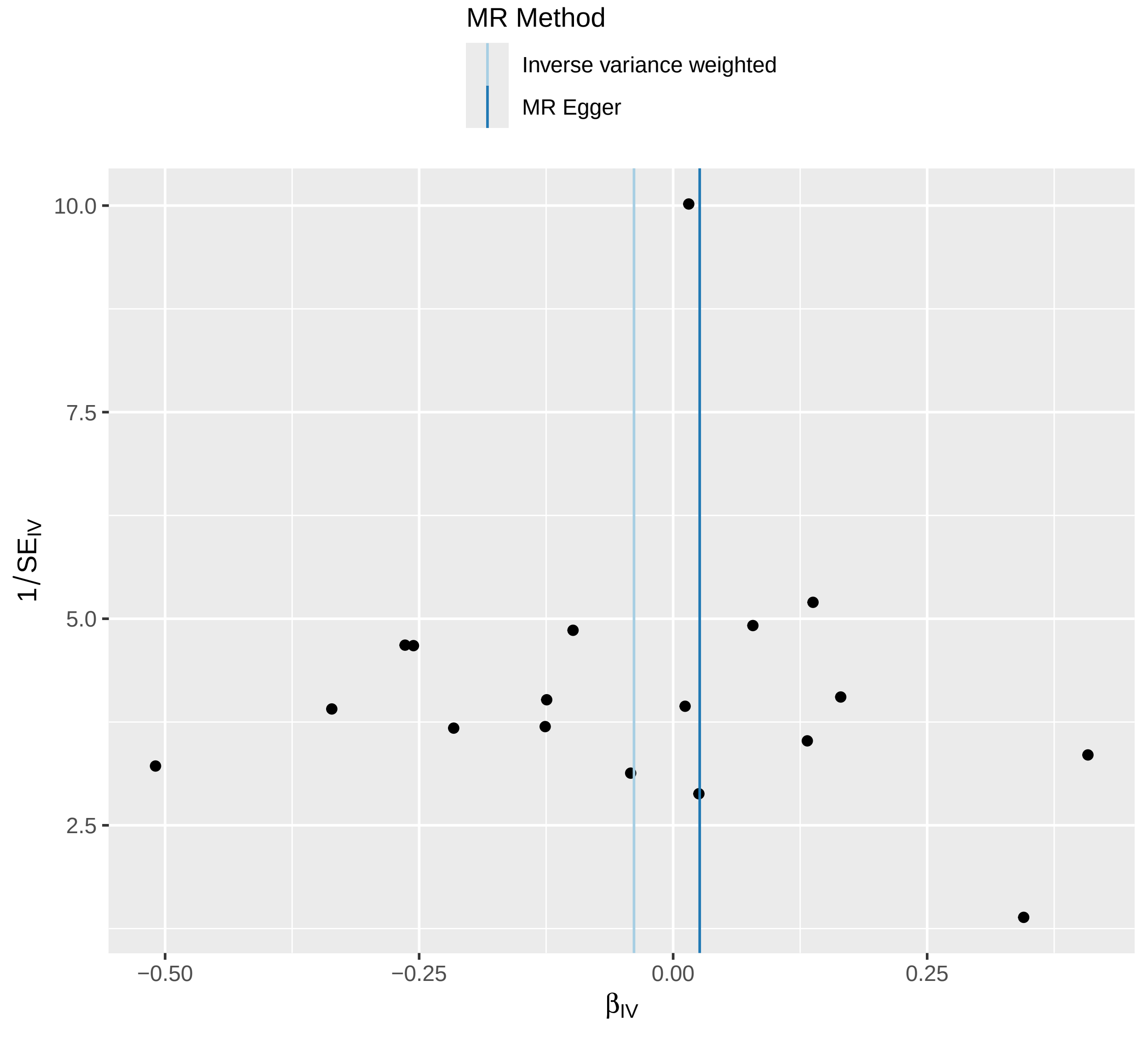
MR Method
Inverse variance weighted
MR Egger
10.0
7.5
5.0
2.5
1/SE_IV
−0.50
−0.25
0.00
0.25
β_IV

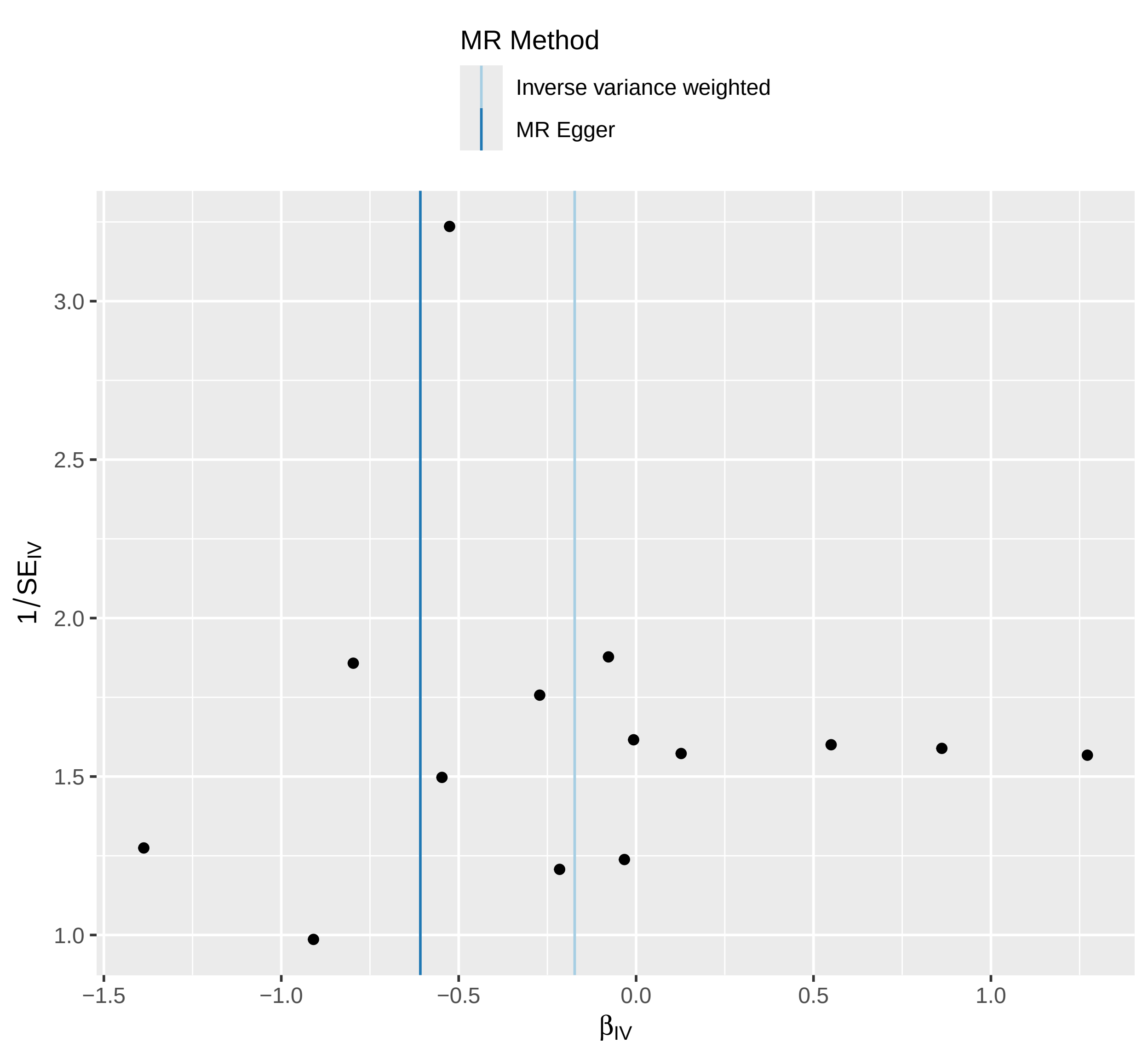

MR Method
Inverse variance weighted
MR Egger
3.0
2.5
2.0
1.5
1.0
$1/SE_{IV}$
−1.5
−1.0
−0.5
0.0
0.5
1.0
β_{IV}

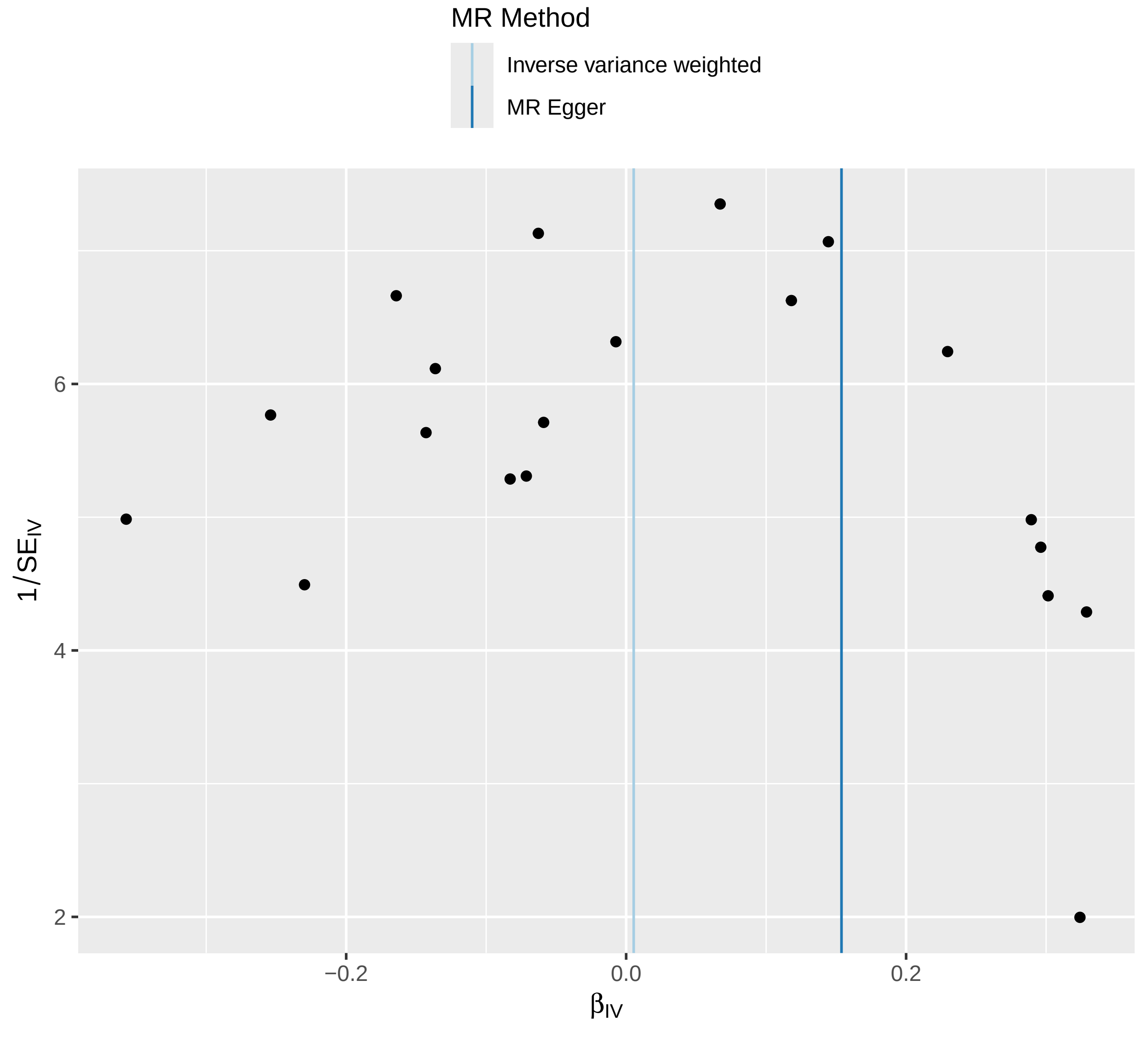
MR Method
Inverse variance weighted
MR Egger
1/SE_IV
6
4
2
−0.2
0.0
0.2
β_IV

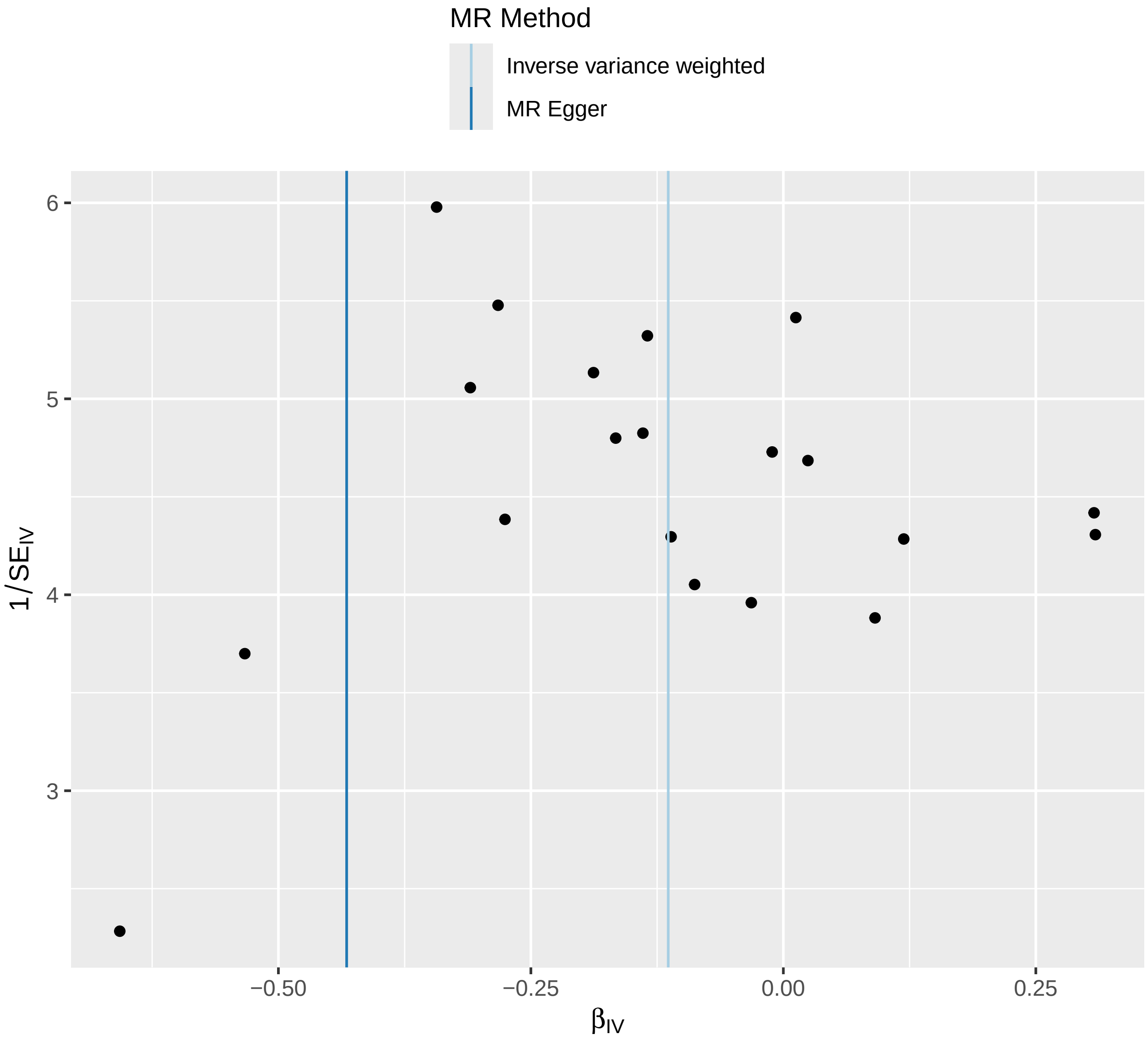
MR Method
Inverse variance weighted
MR Egger
6
5
4
3
1/SE$_{IV}$
−0.50
−0.25
0.00
0.25
β$_{IV}$

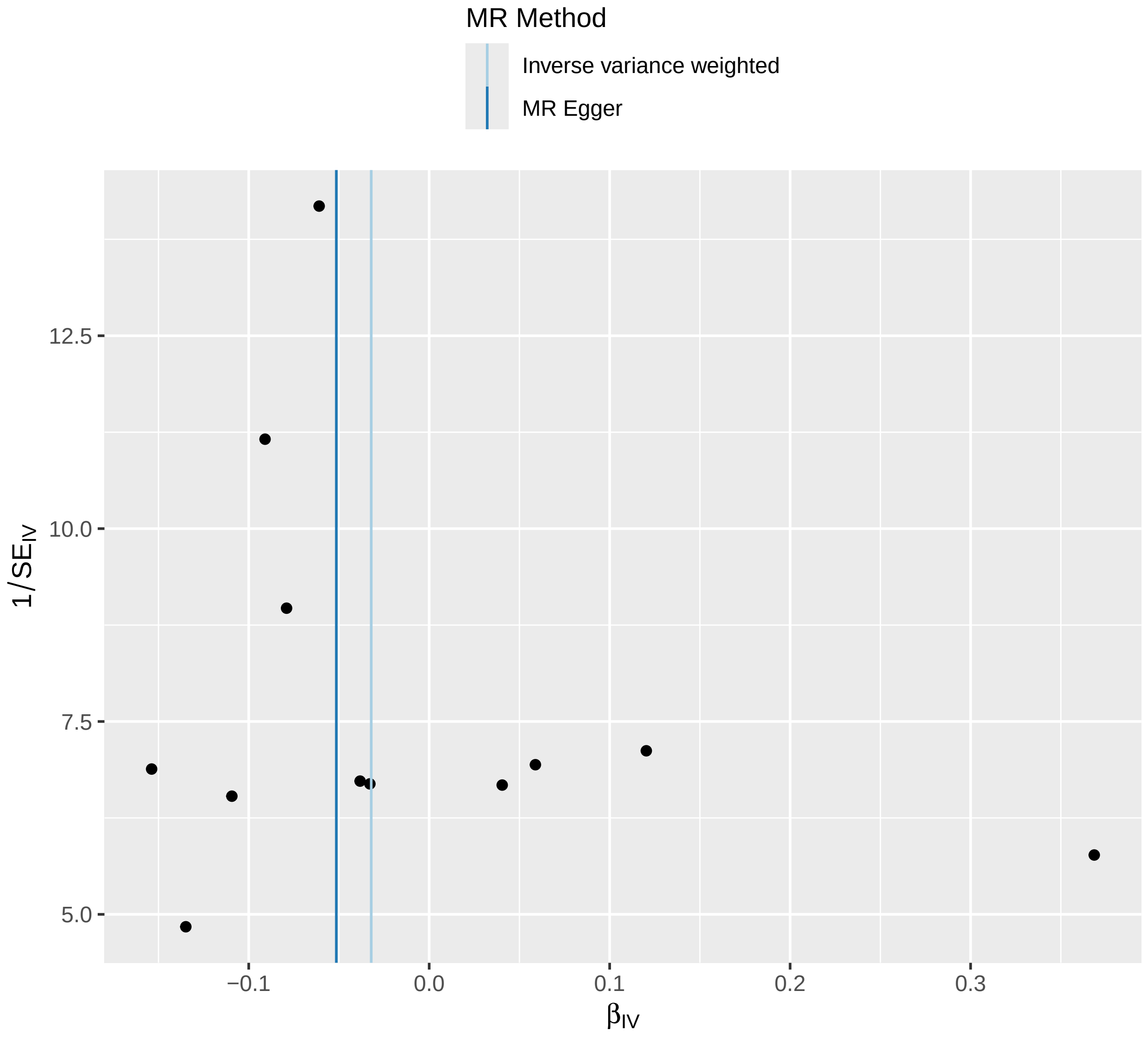
MR Method
Inverse variance weighted
MR Egger
12.5
10.0
7.5
5.0
1/SE_IV
−0.1
0.0
0.1
0.2
0.3
β_IV

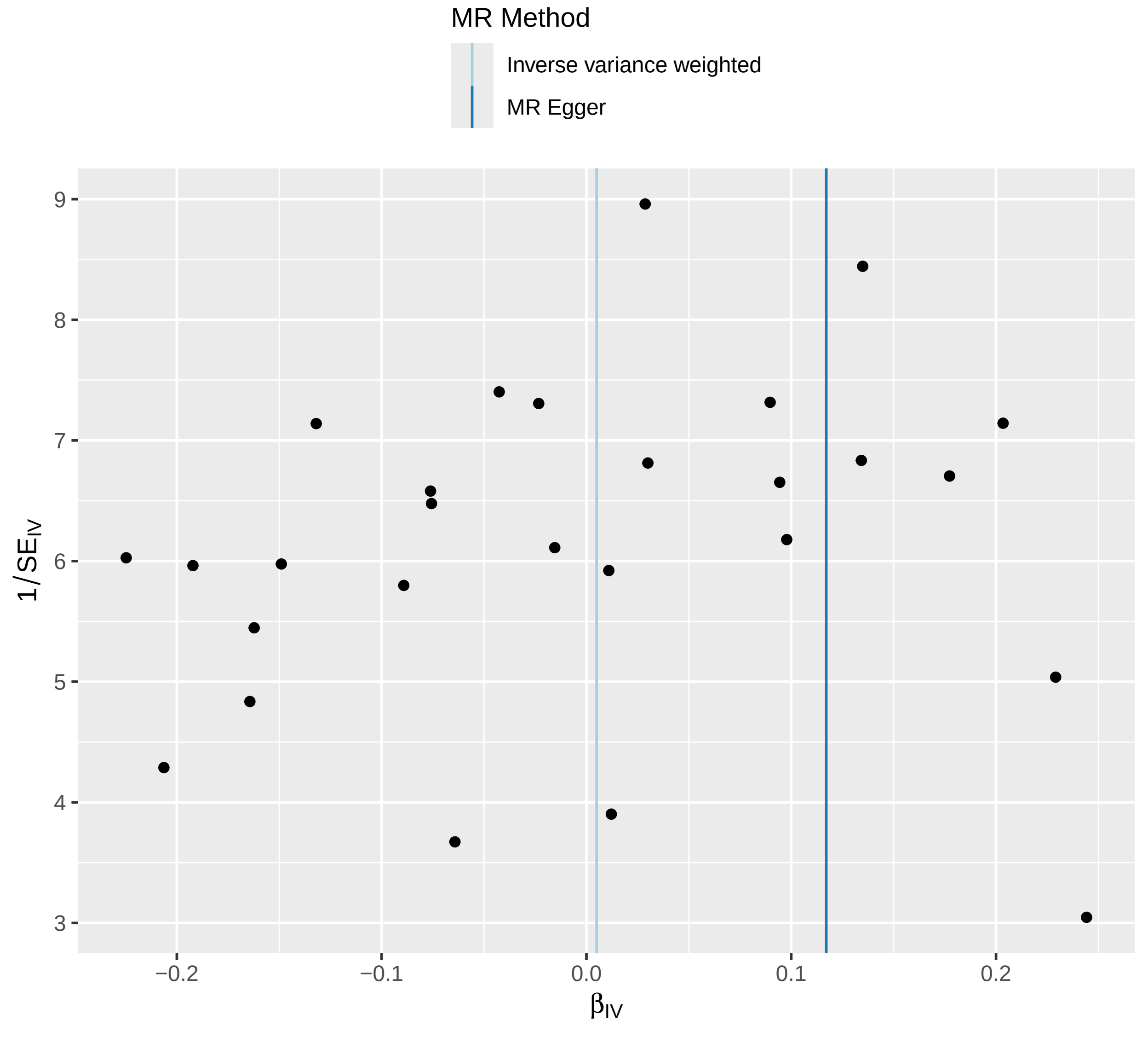
MR Method
Inverse variance weighted
MR Egger
9
8
7
6
5
4
3
1/SE_IV
−0.2
−0.1
0.0
0.1
0.2
β_IV

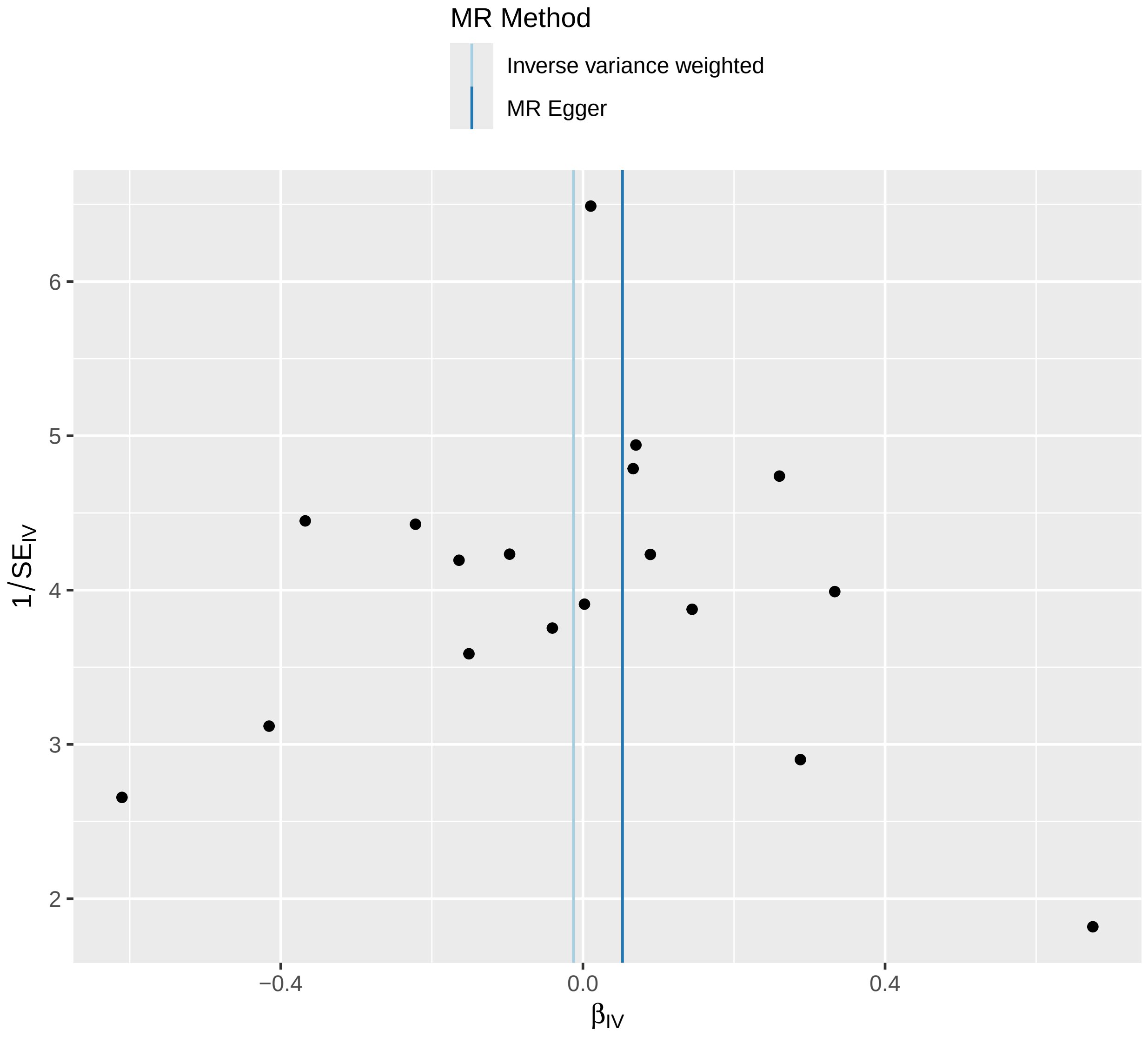

MR Method
Inverse variance weighted
MR Egger
6
5
4
3
2
1/SE_IV
−0.4
0.0
0.4
β_IV

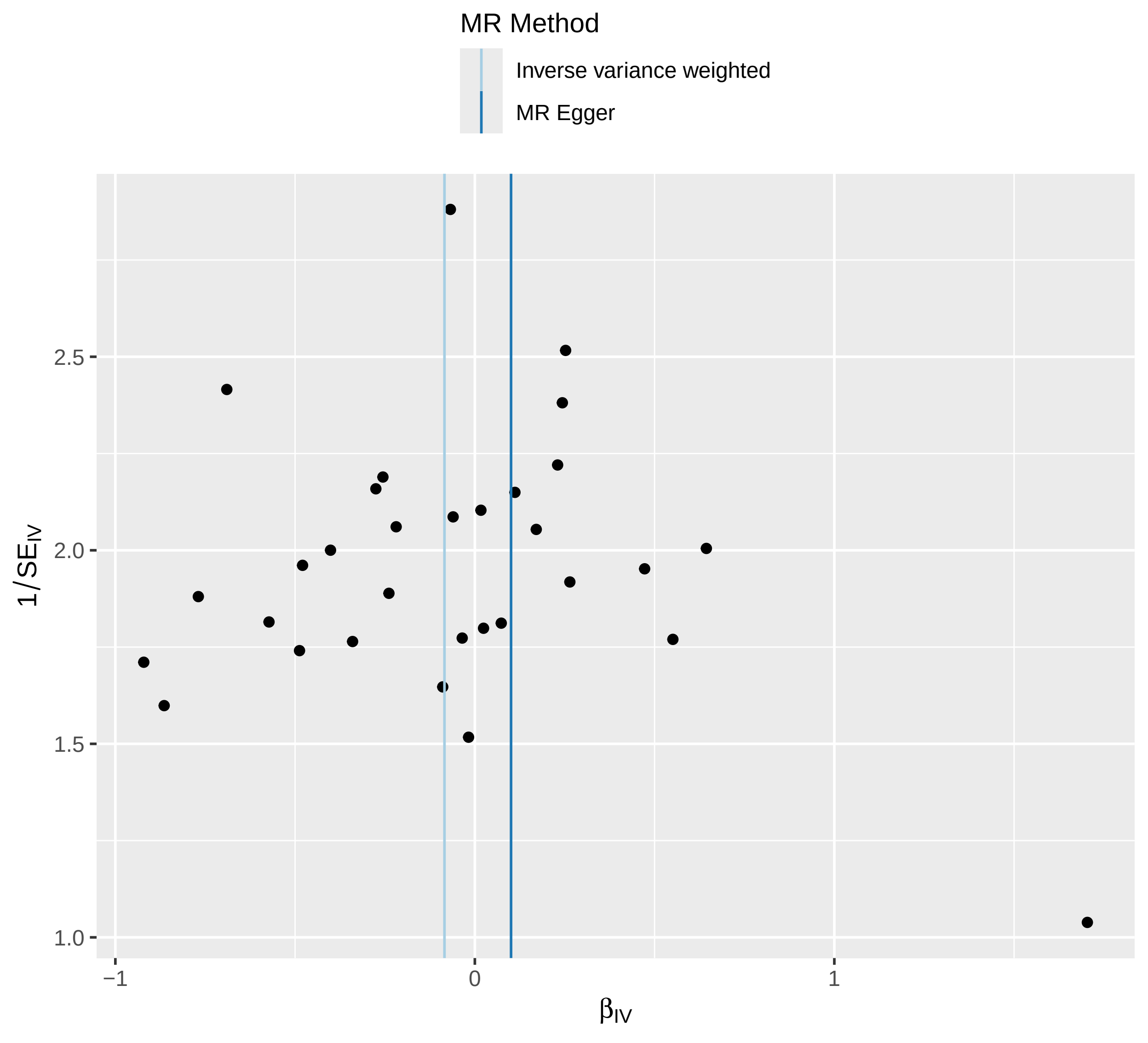

MR Method
Inverse variance weighted
MR Egger
2.5
2.0
1.5
1.0
1/SE_IV
−1
0
1
β_IV

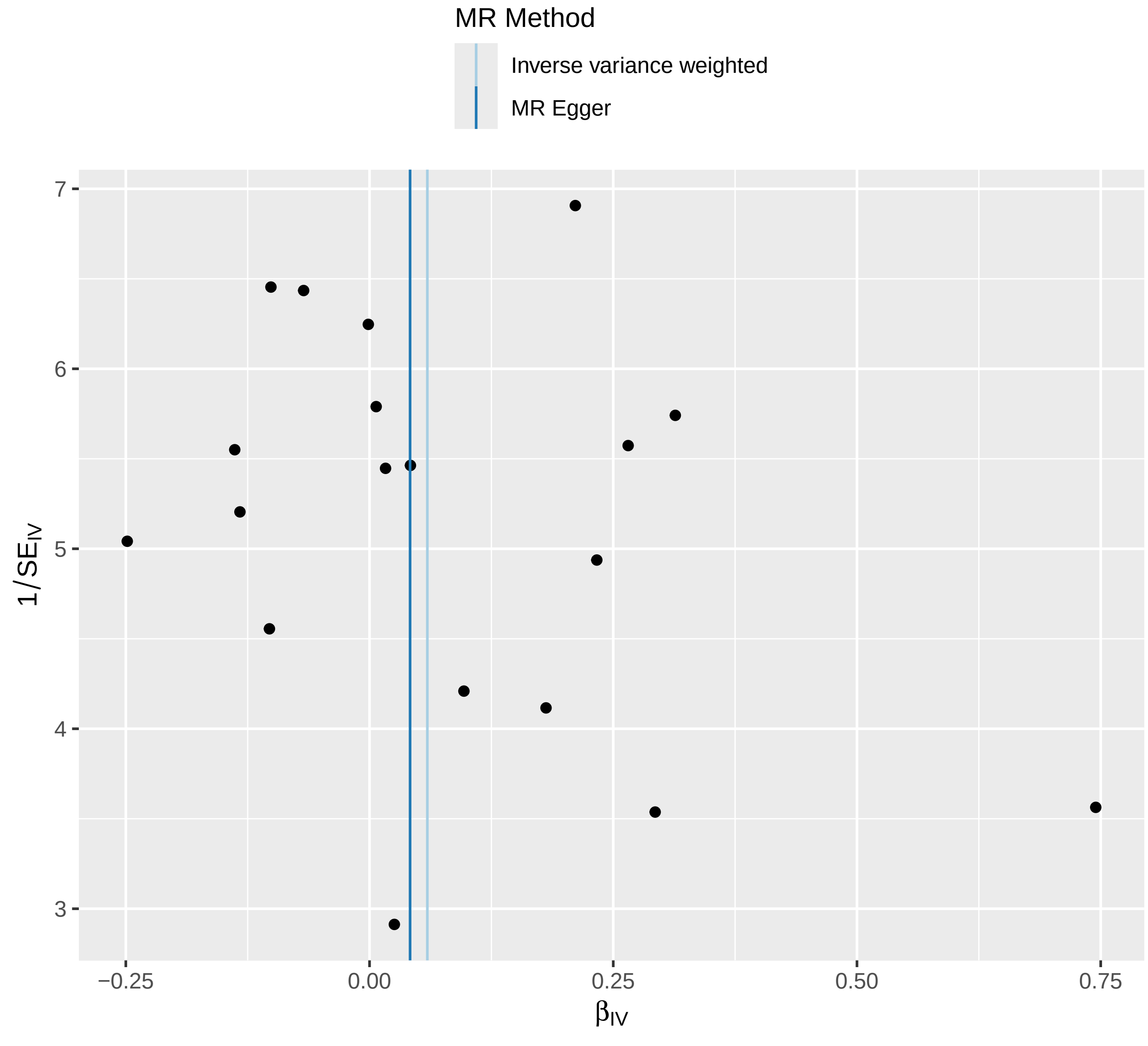
MR Method
Inverse variance weighted
MR Egger
7
6
5
4
3
1/SE_IV
−0.25
0.00
0.25
0.50
0.75
β_IV

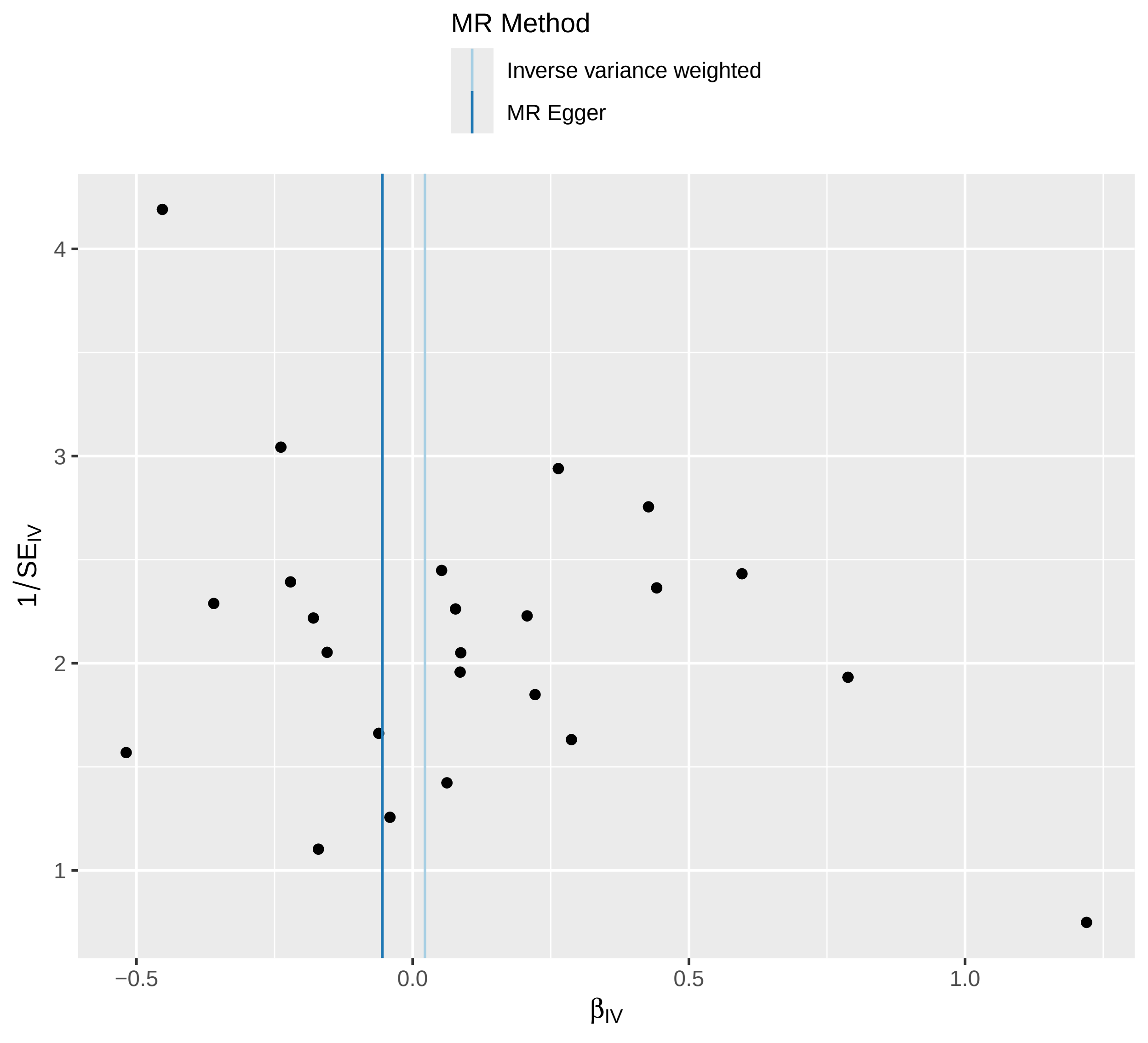

MR Method
Inverse variance weighted
MR Egger
4
3
2
1
1/SE_IV
−0.5
0.0
0.5
1.0
β_IV

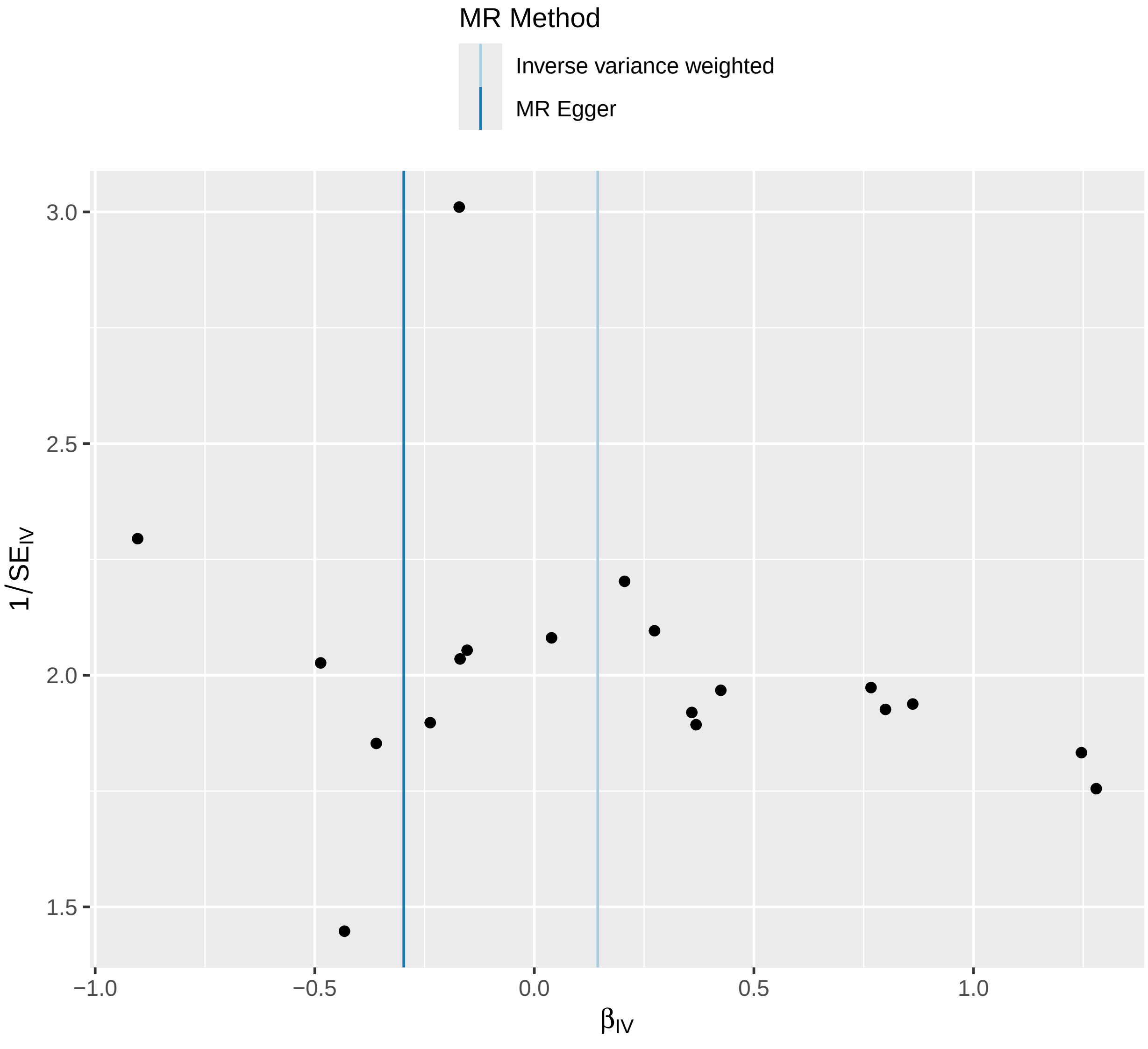
MR Method
Inverse variance weighted
MR Egger
3.0
2.5
2.0
1.5
$1/SE_{IV}$
−1.0
−0.5
0.0
0.5
1.0
β_{IV}

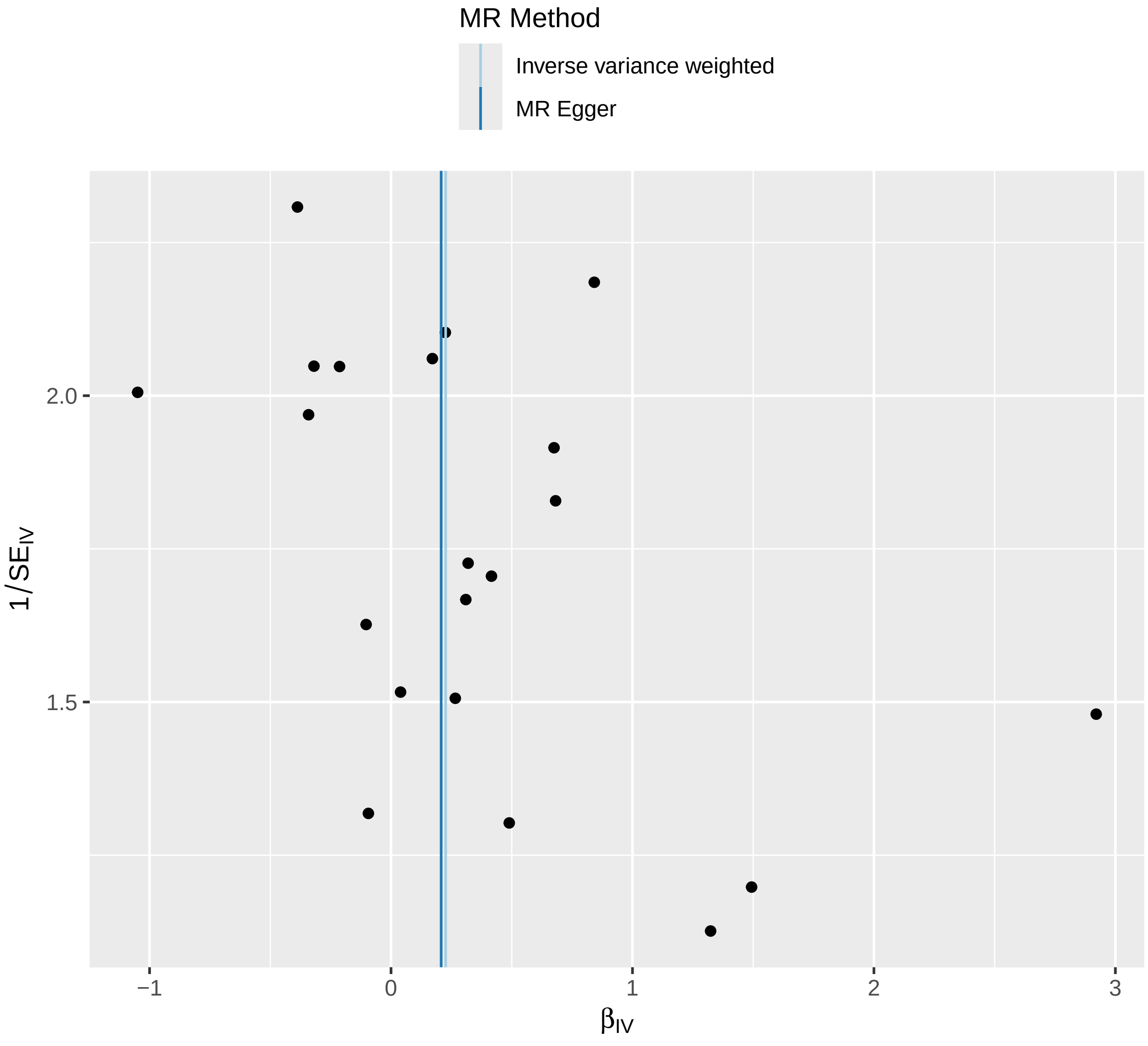
MR Method
Inverse variance weighted
MR Egger
2.0
1.5
$1/SE_{IV}$
−1
0
1
2
3
β_{IV}

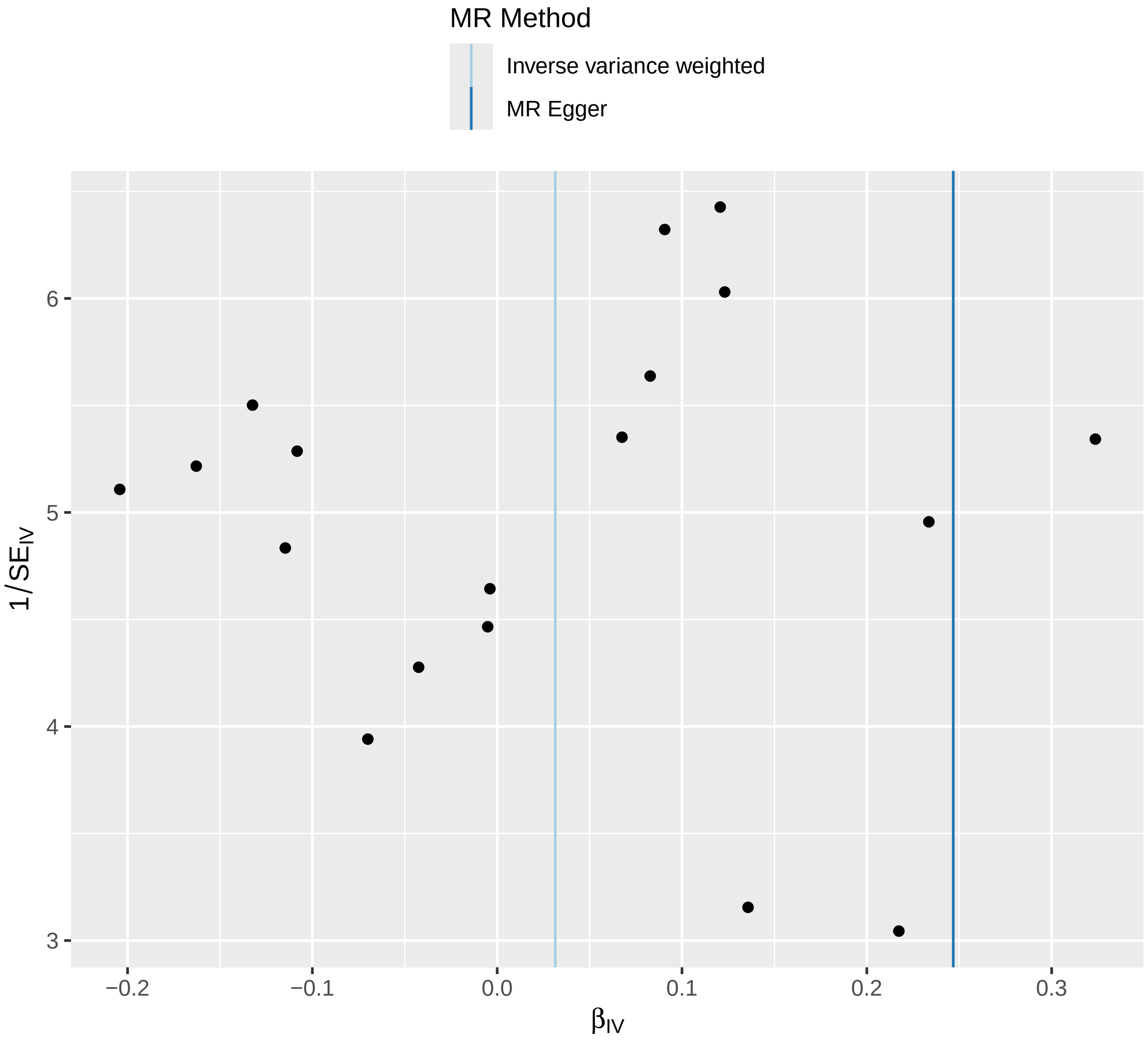

MR Method
Inverse variance weighted
MR Egger
6
5
4
3
1/SE$_{IV}$
−0.2
−0.1
0.0
0.1
0.2
0.3
β$_{IV}$

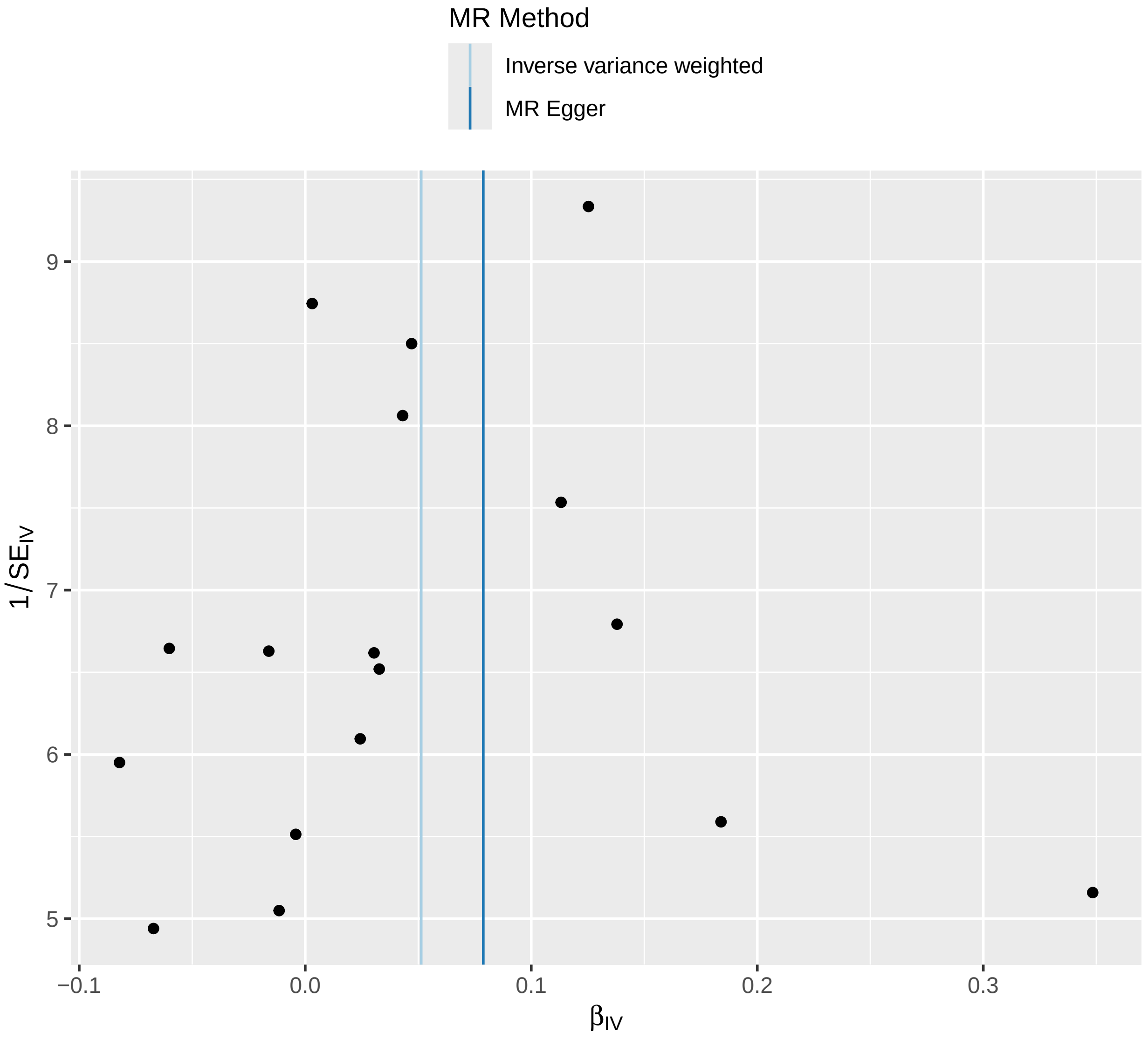
MR Method
Inverse variance weighted
MR Egger
1/SE$_{IV}$
9
8
7
6
5
−0.1
0.0
0.1
0.2
0.3
β$_{IV}$

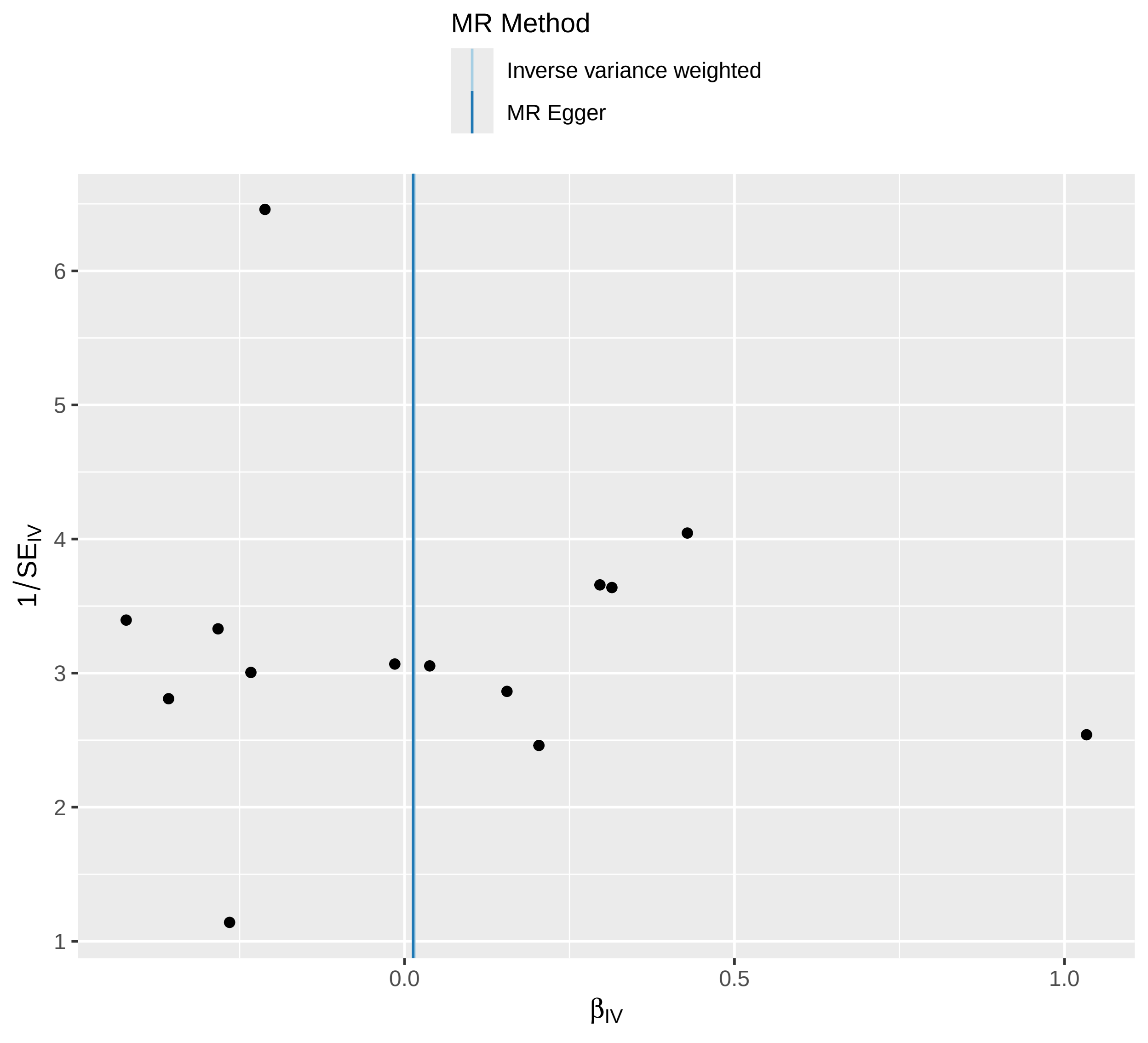
MR Method
Inverse variance weighted
MR Egger
1/SE_IV
β_IV
6
5
4
3
2
1
0.0
0.5
1.0

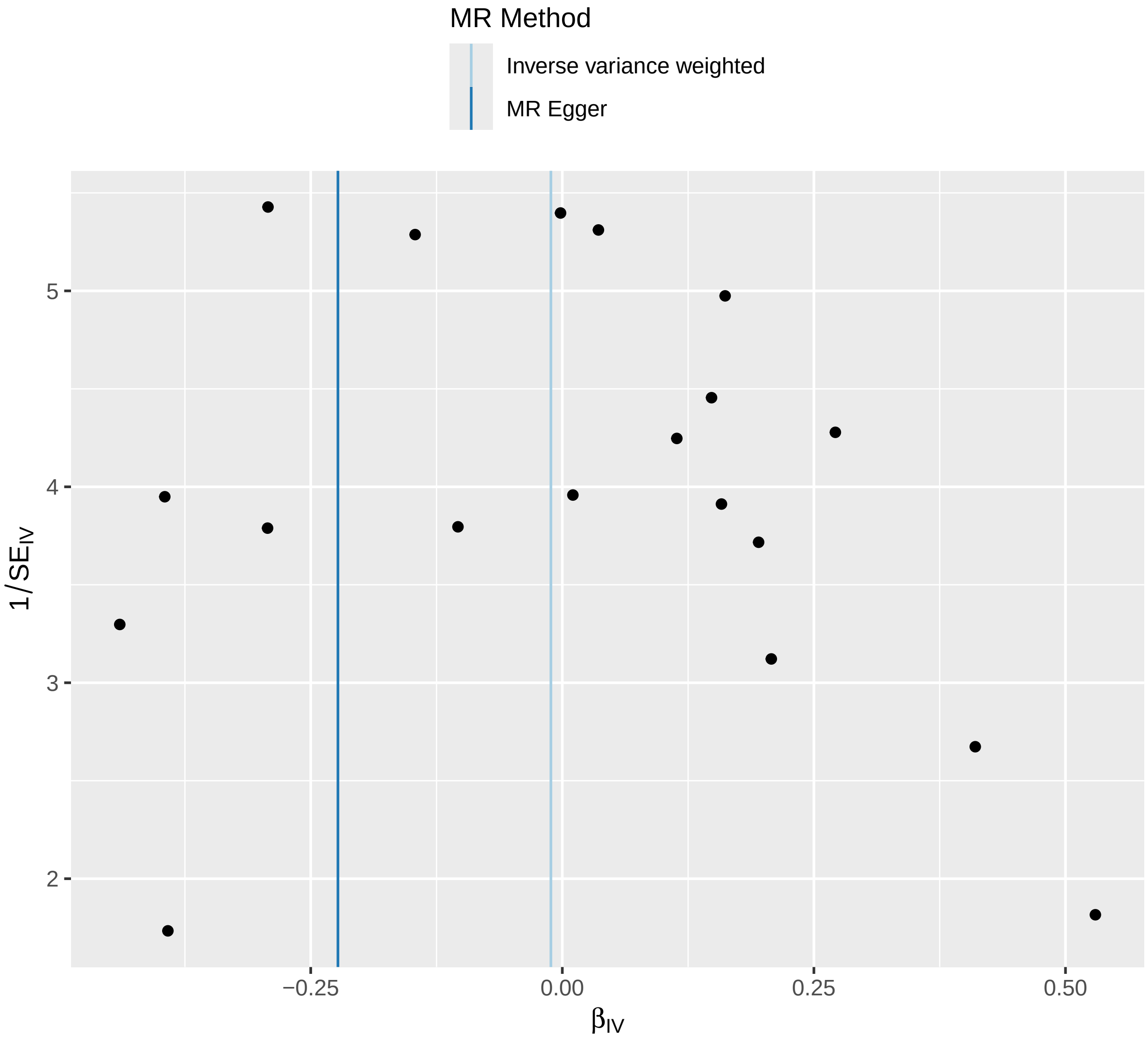
MR Method
Inverse variance weighted
MR Egger
5
4
3
2
1/SE$_{IV}$
−0.25
0.00
0.25
0.50
β$_{IV}$

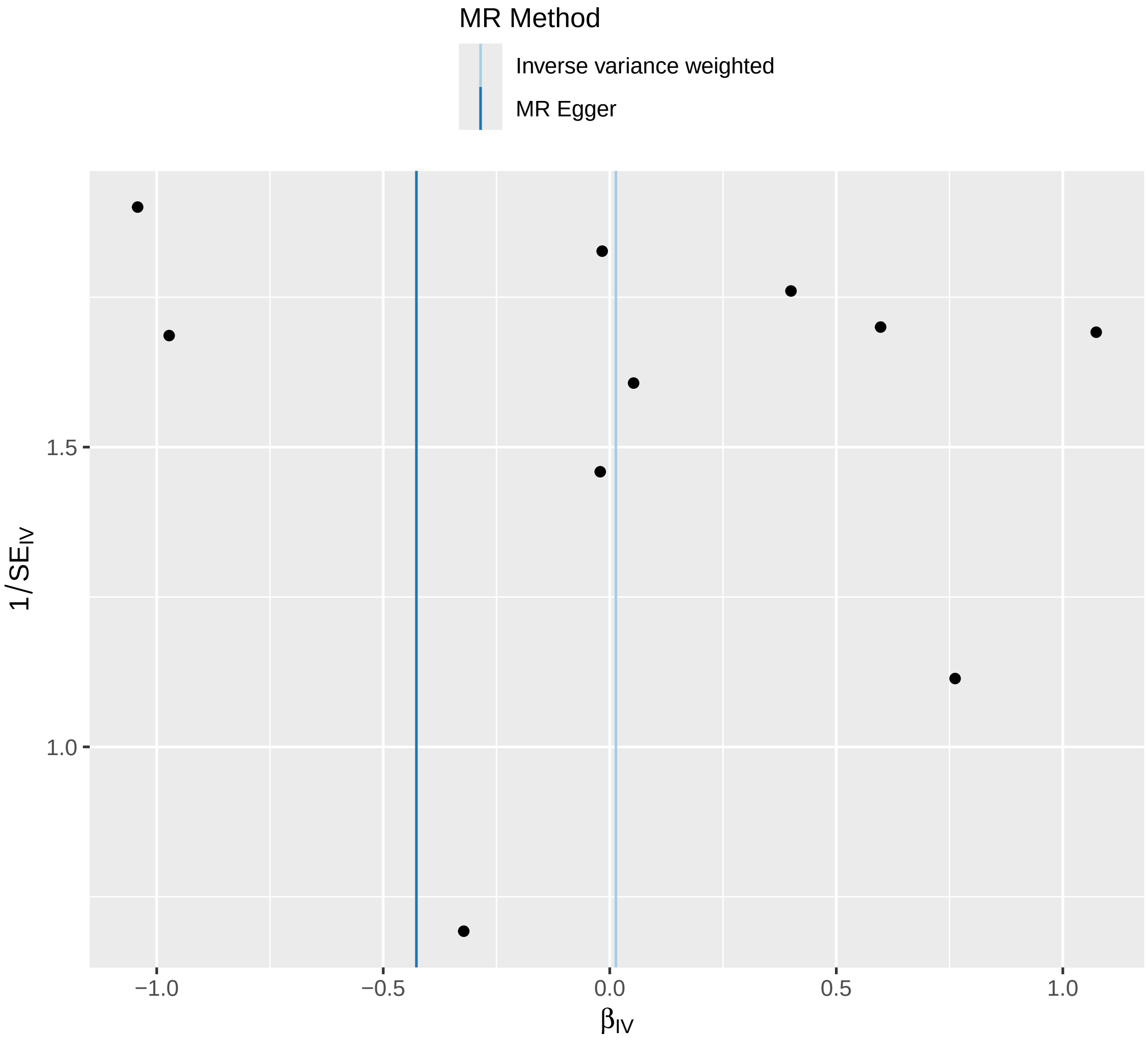
MR Method
Inverse variance weighted
MR Egger
1.5
1.0
−1.0
−0.5
0.0
0.5
1.0
$1/SE_{IV}$
β_{IV}

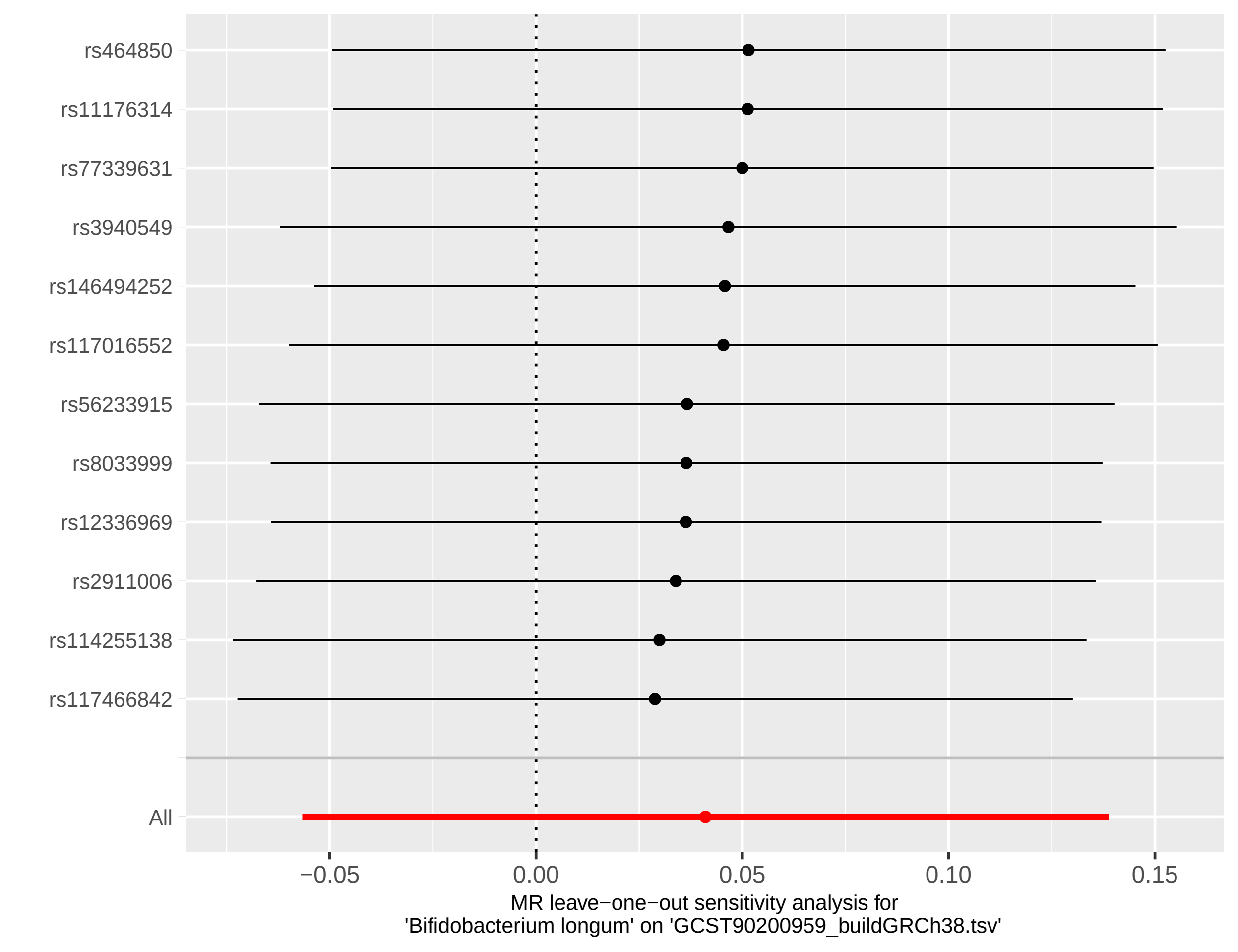

rs464850
rs11176314
rs77339631
rs3940549
rs146494252
rs117016552
rs56233915
rs8033999
rs12336969
rs2911006
rs114255138
rs117466842
All
−0.05
0.00
0.05
0.10
0.15
MR leave−one−out sensitivity analysis for
'Bifidobacterium longum' on 'GCST90200959_buildGRCh38.tsv'

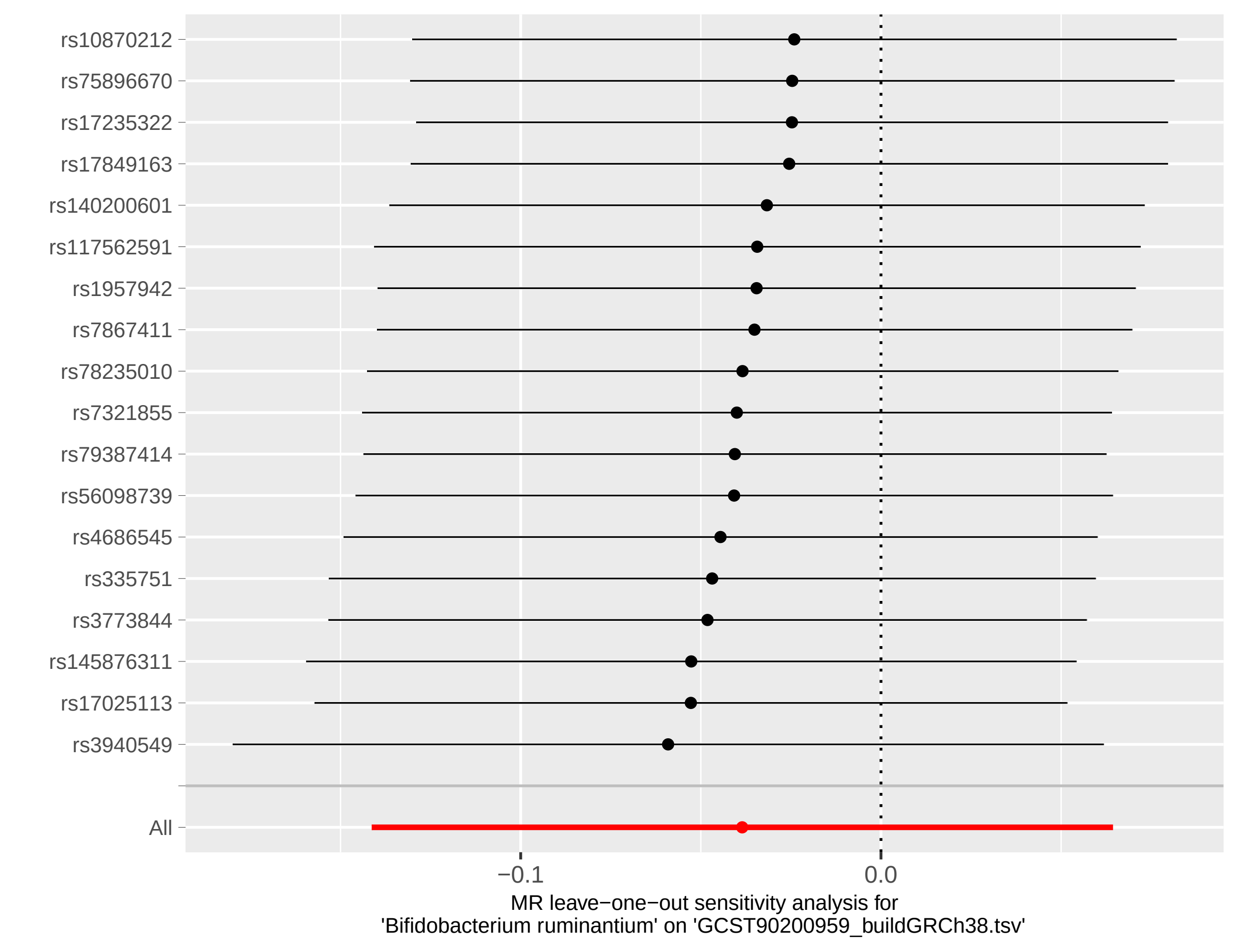

rs10870212
rs75896670
rs17235322
rs17849163
rs140200601
rs117562591
rs1957942
rs7867411
rs78235010
rs7321855
rs79387414
rs56098739
rs4686545
rs335751
rs3773844
rs145876311
rs17025113
rs3940549
All
−0.1
0.0
MR leave−one−out sensitivity analysis for
'Bifidobacterium ruminantium' on 'GCST90200959_buildGRCh38.tsv'

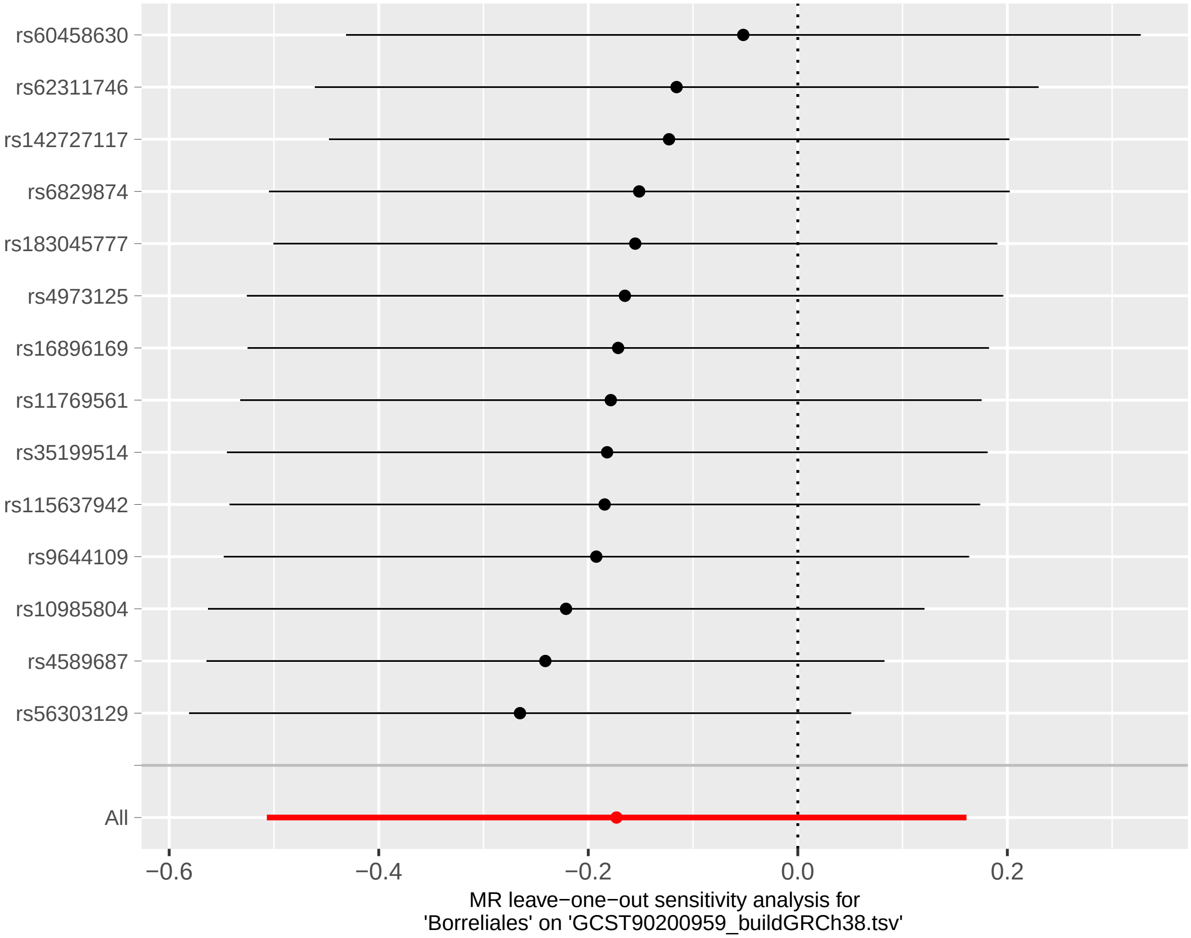
rs60458630
rs62311746
rs142727117
rs6829874
rs183045777
rs4973125
rs16896169
rs11769561
rs35199514
rs115637942
rs9644109
rs10985804
rs4589687
rs56303129
All
−0.6
−0.4
−0.2
0.0
0.2
MR leave−one−out sensitivity analysis for
'Borreliales' on 'GCST90200959_buildGRCh38.tsv'

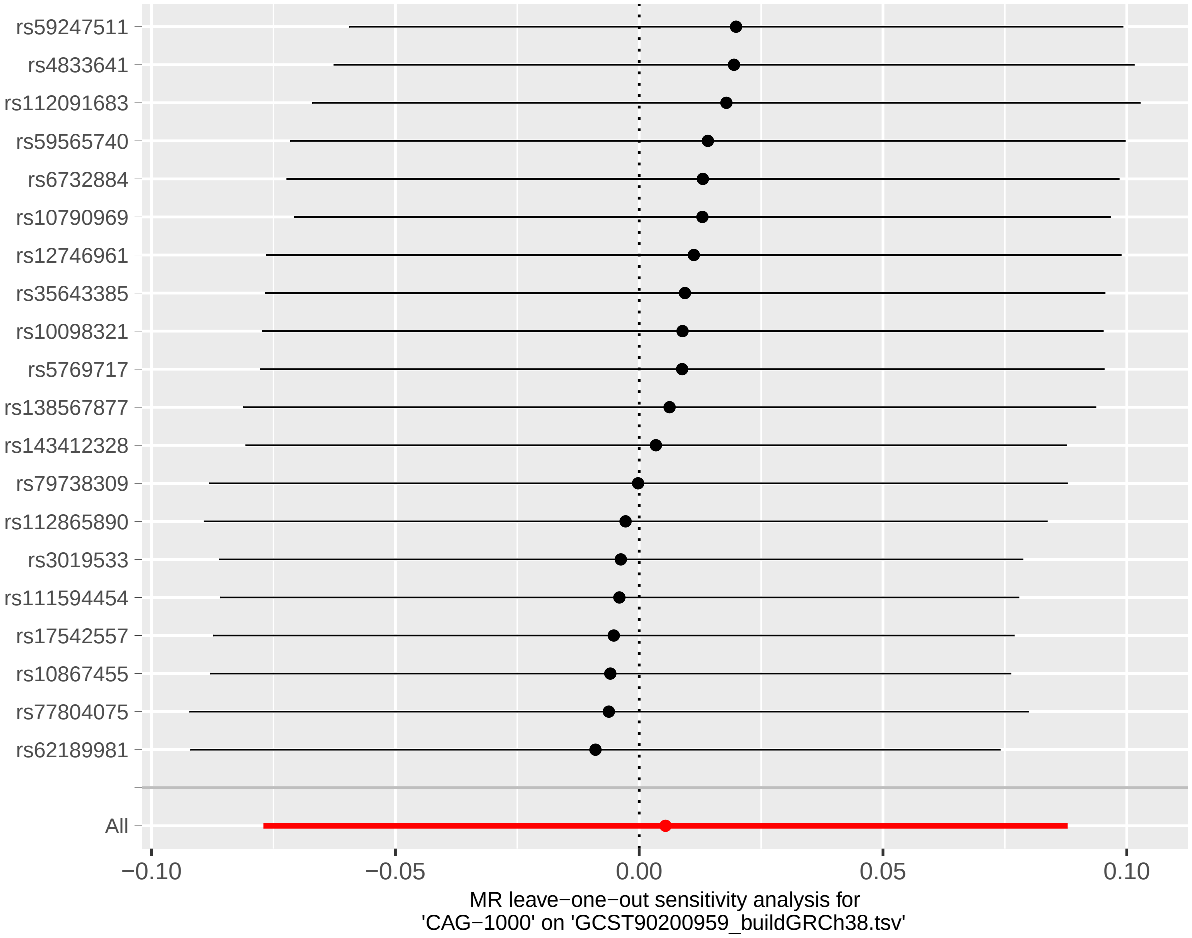

rs59247511
rs4833641
rs112091683
rs59565740
rs6732884
rs10790969
rs12746961
rs35643385
rs10098321
rs5769717
rs138567877
rs143412328
rs79738309
rs112865890
rs3019533
rs111594454
rs17542557
rs10867455
rs77804075
rs62189981
All
−0.10
−0.05
0.00
0.05
0.10
MR leave−one−out sensitivity analysis for
'CAG−1000' on 'GCST90200959_buildGRCh38.tsv'

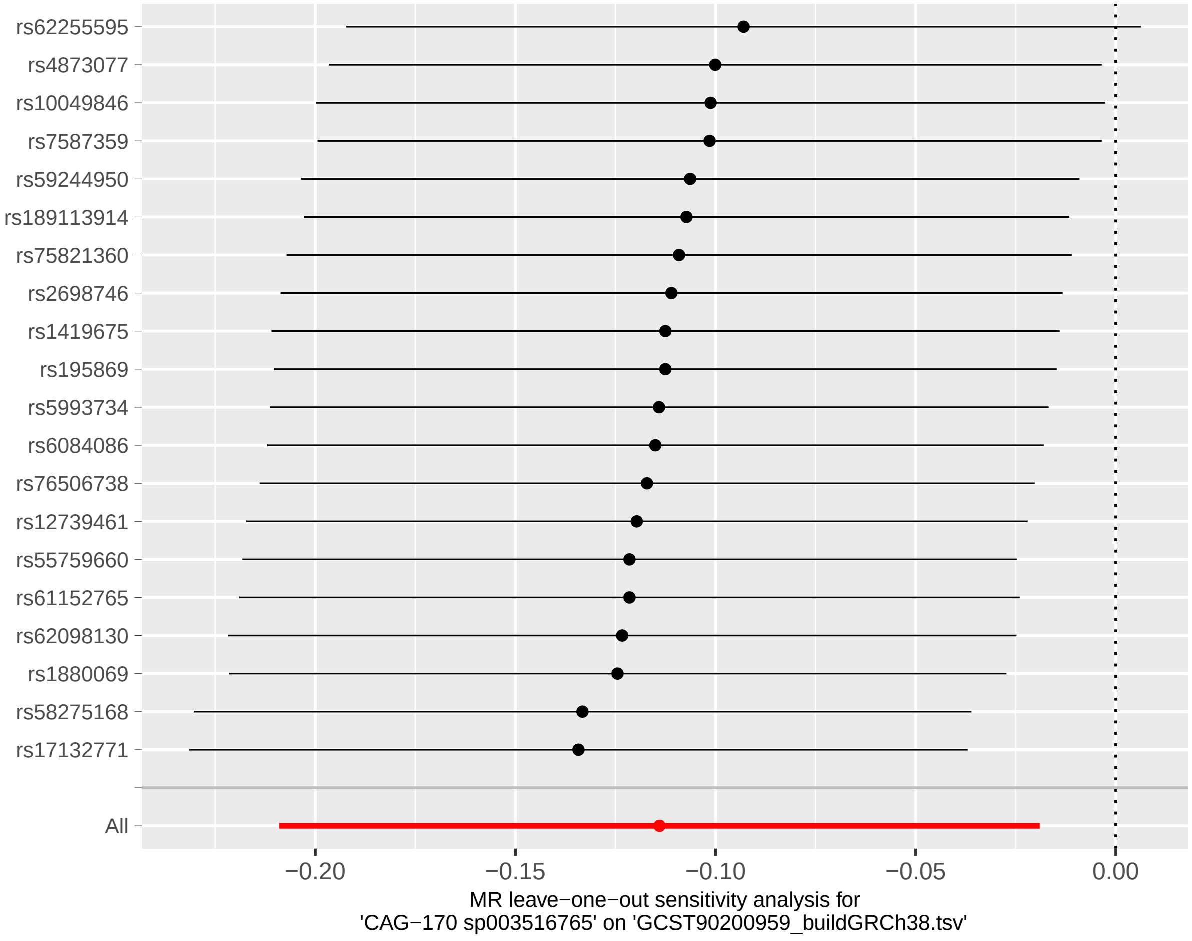
rs62255595
rs4873077
rs10049846
rs7587359
rs59244950
rs189113914
rs75821360
rs2698746
rs1419675
rs195869
rs5993734
rs6084086
rs76506738
rs12739461
rs55759660
rs61152765
rs62098130
rs1880069
rs58275168
rs17132771
All
−0.20
−0.15
−0.10
−0.05
0.00
MR leave−one−out sensitivity analysis for
'CAG−170 sp003516765' on 'GCST90200959_buildGRCh38.tsv'

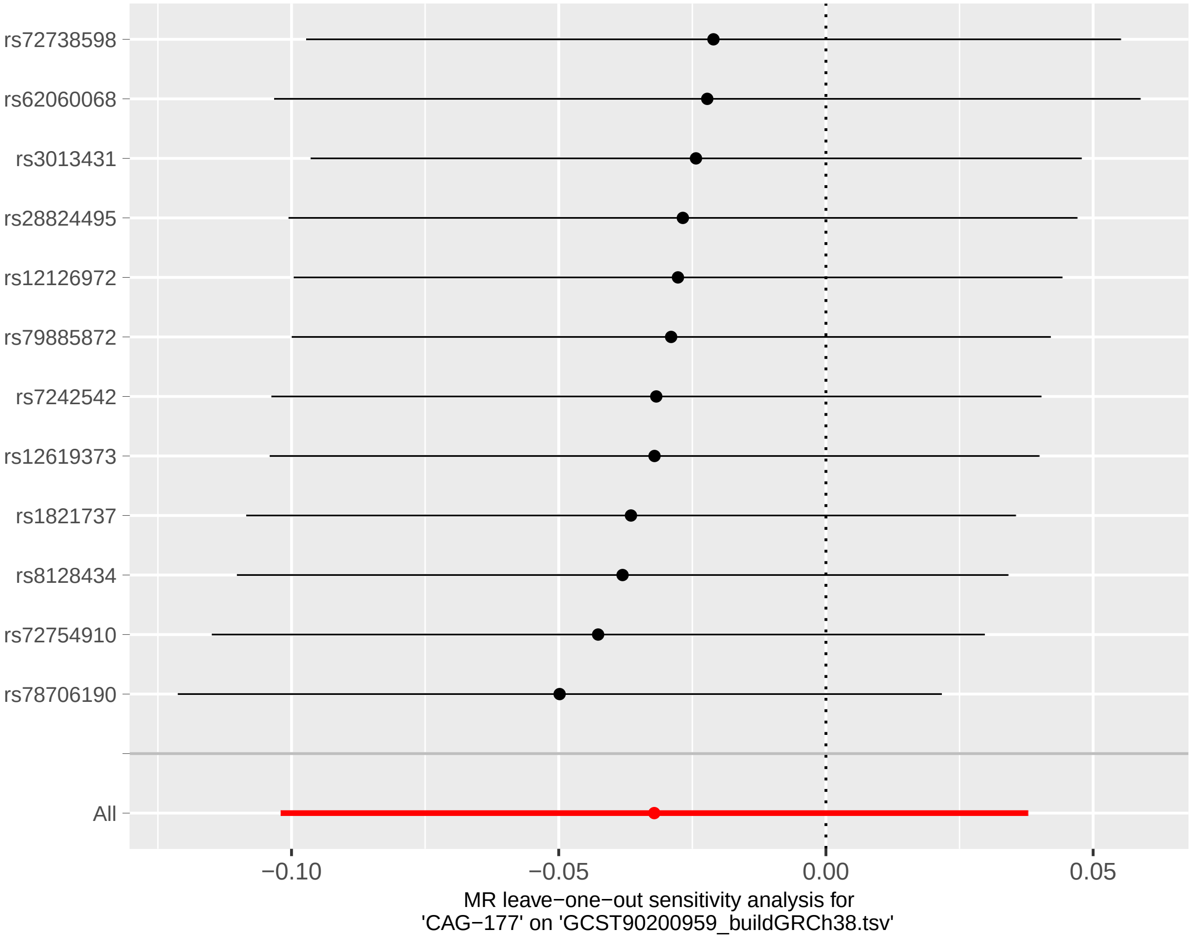

rs72738598
rs62060068
rs3013431
rs28824495
rs12126972
rs79885872
rs7242542
rs12619373
rs1821737
rs8128434
rs72754910
rs78706190
All
−0.10
−0.05
0.00
0.05
MR leave−one−out sensitivity analysis for
'CAG−177' on 'GCST90200959_buildGRCh38.tsv'

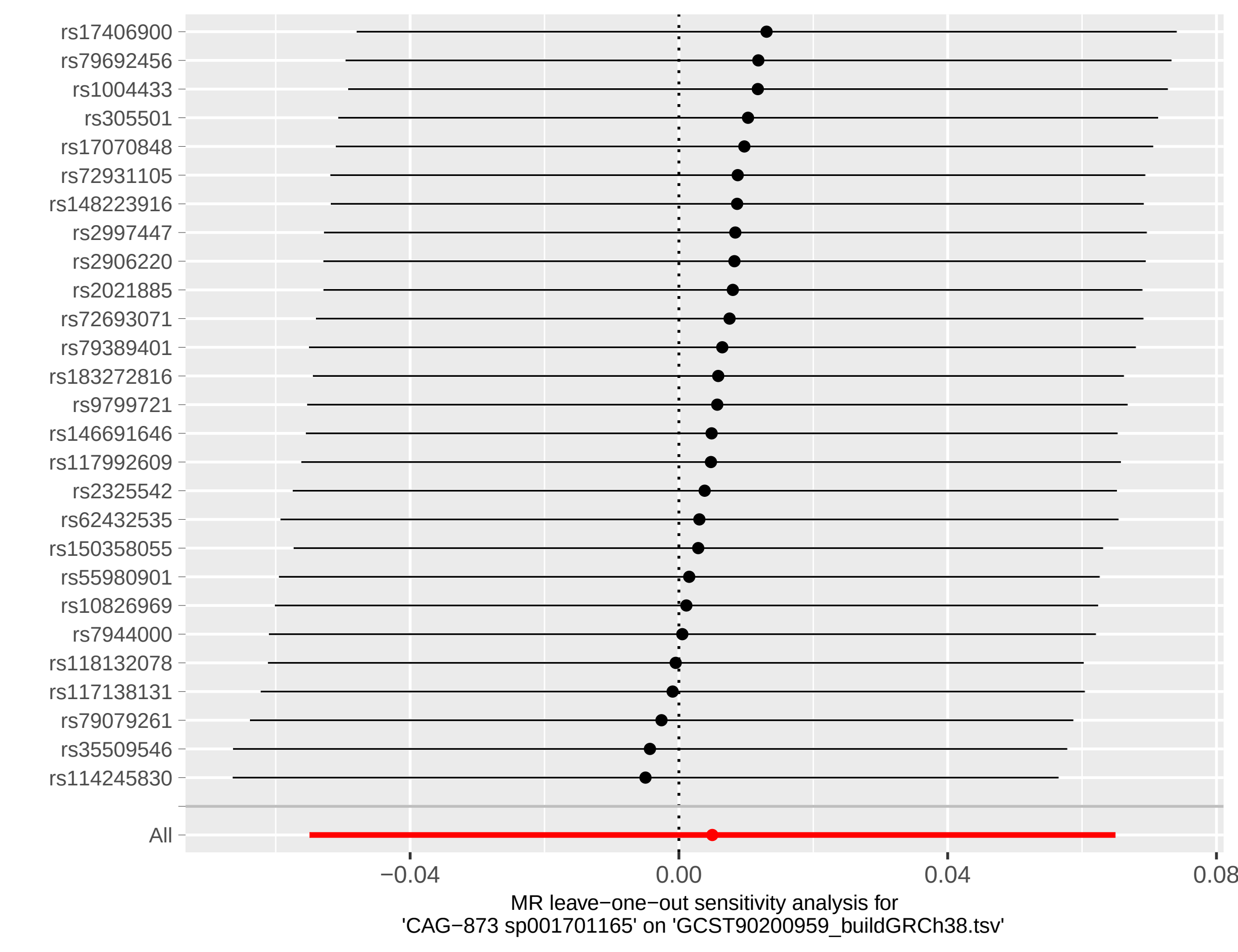
rs17406900
rs79692456
rs1004433
rs305501
rs17070848
rs72931105
rs148223916
rs2997447
rs2906220
rs2021885
rs72693071
rs79389401
rs183272816
rs9799721
rs146691646
rs117992609
rs2325542
rs62432535
rs150358055
rs55980901
rs10826969
rs7944000
rs118132078
rs117138131
rs79079261
rs35509546
rs114245830
All
−0.04
0.00
0.04
0.08
MR leave−one−out sensitivity analysis for
'CAG−873 sp001701165' on 'GCST90200959_buildGRCh38.tsv'

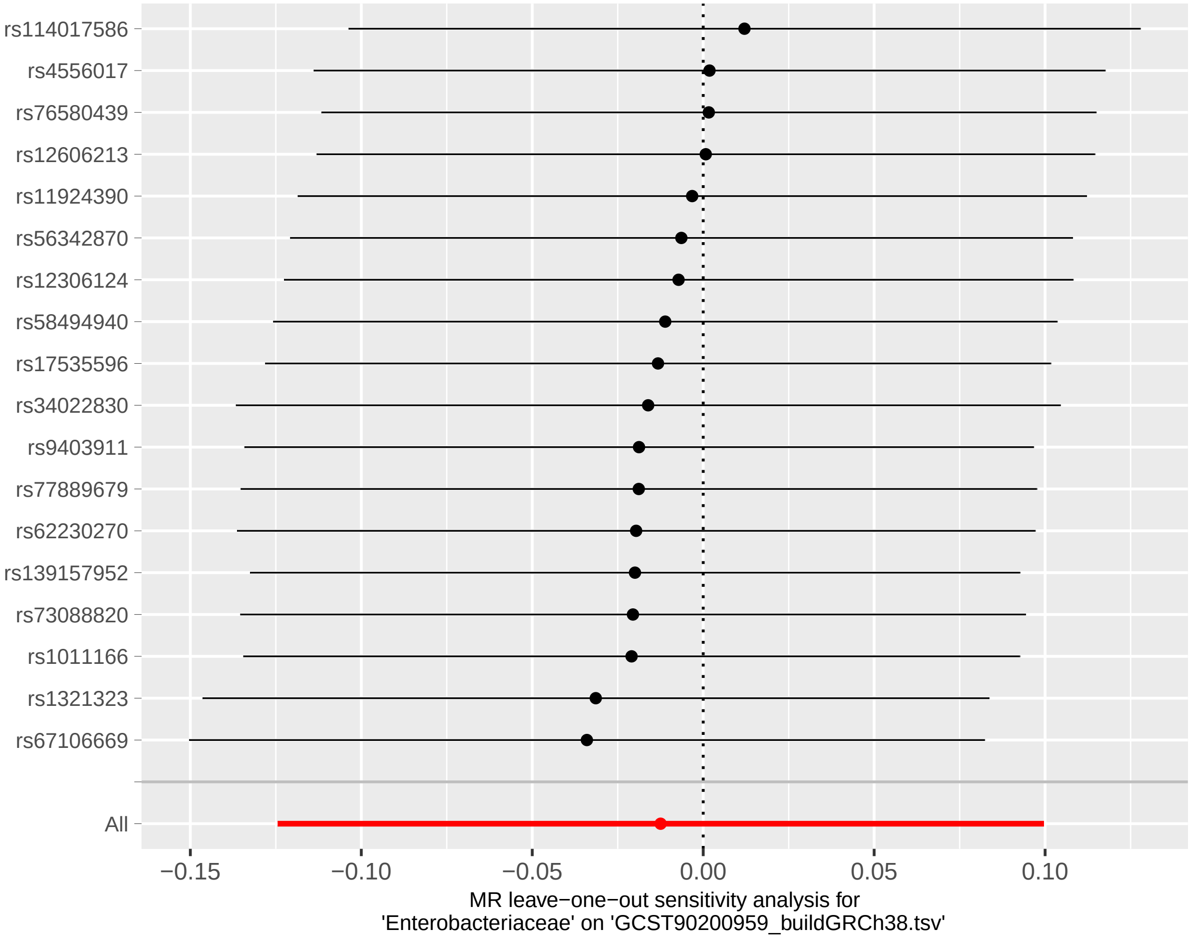

rs114017586
rs4556017
rs76580439
rs12606213
rs11924390
rs56342870
rs12306124
rs58494940
rs17535596
rs34022830
rs9403911
rs77889679
rs62230270
rs139157952
rs73088820
rs1011166
rs1321323
rs67106669
All
−0.15
−0.10
−0.05
0.00
0.05
0.10
MR leave−one−out sensitivity analysis for
'Enterobacteriaceae' on 'GCST90200959_buildGRCh38.tsv'

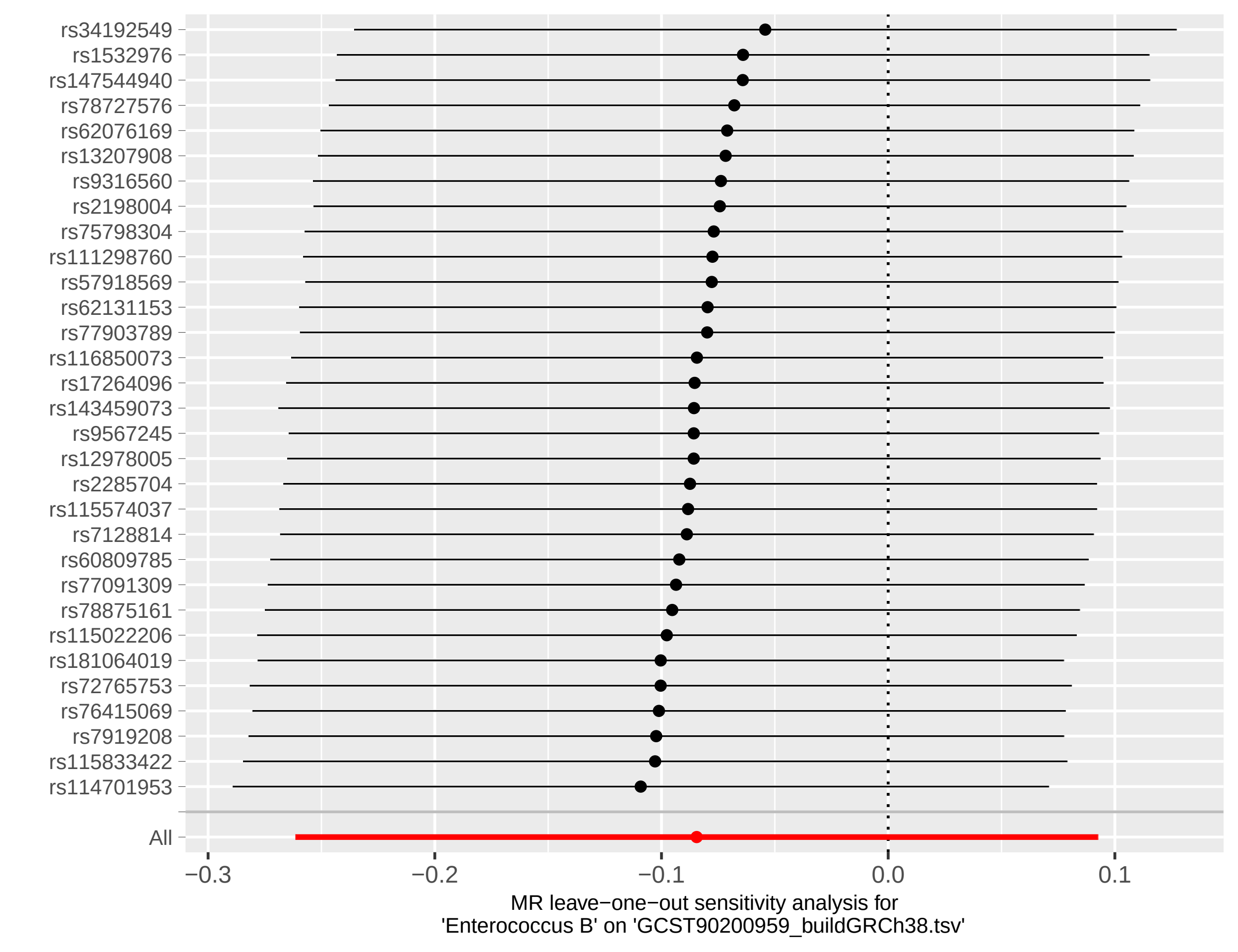
rs34192549
rs1532976
rs147544940
rs78727576
rs62076169
rs13207908
rs9316560
rs2198004
rs75798304
rs111298760
rs57918569
rs62131153
rs77903789
rs116850073
rs17264096
rs143459073
rs9567245
rs12978005
rs2285704
rs115574037
rs7128814
rs60809785
rs77091309
rs78875161
rs115022206
rs181064019
rs72765753
rs76415069
rs7919208
rs115833422
rs114701953
All
−0.3
−0.2
−0.1
0.0
0.1
MR leave−one−out sensitivity analysis for
'Enterococcus B' on 'GCST90200959_buildGRCh38.tsv'

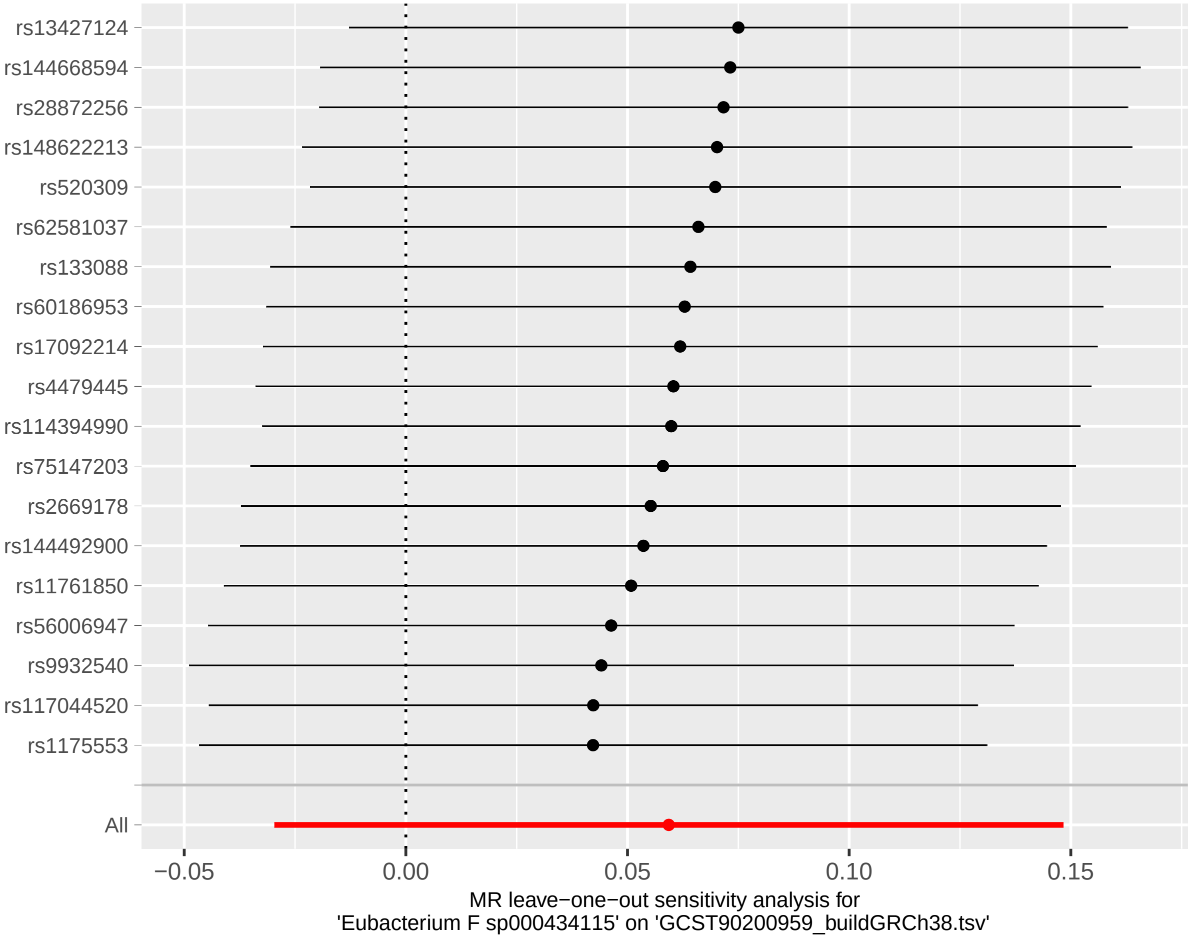

rs13427124
rs144668594
rs28872256
rs148622213
rs520309
rs62581037
rs133088
rs60186953
rs17092214
rs4479445
rs114394990
rs75147203
rs2669178
rs144492900
rs11761850
rs56006947
rs9932540
rs117044520
rs1175553
All
−0.05
0.00
0.05
0.10
0.15
MR leave−one−out sensitivity analysis for
'Eubacterium F sp000434115' on 'GCST90200959_buildGRCh38.tsv'

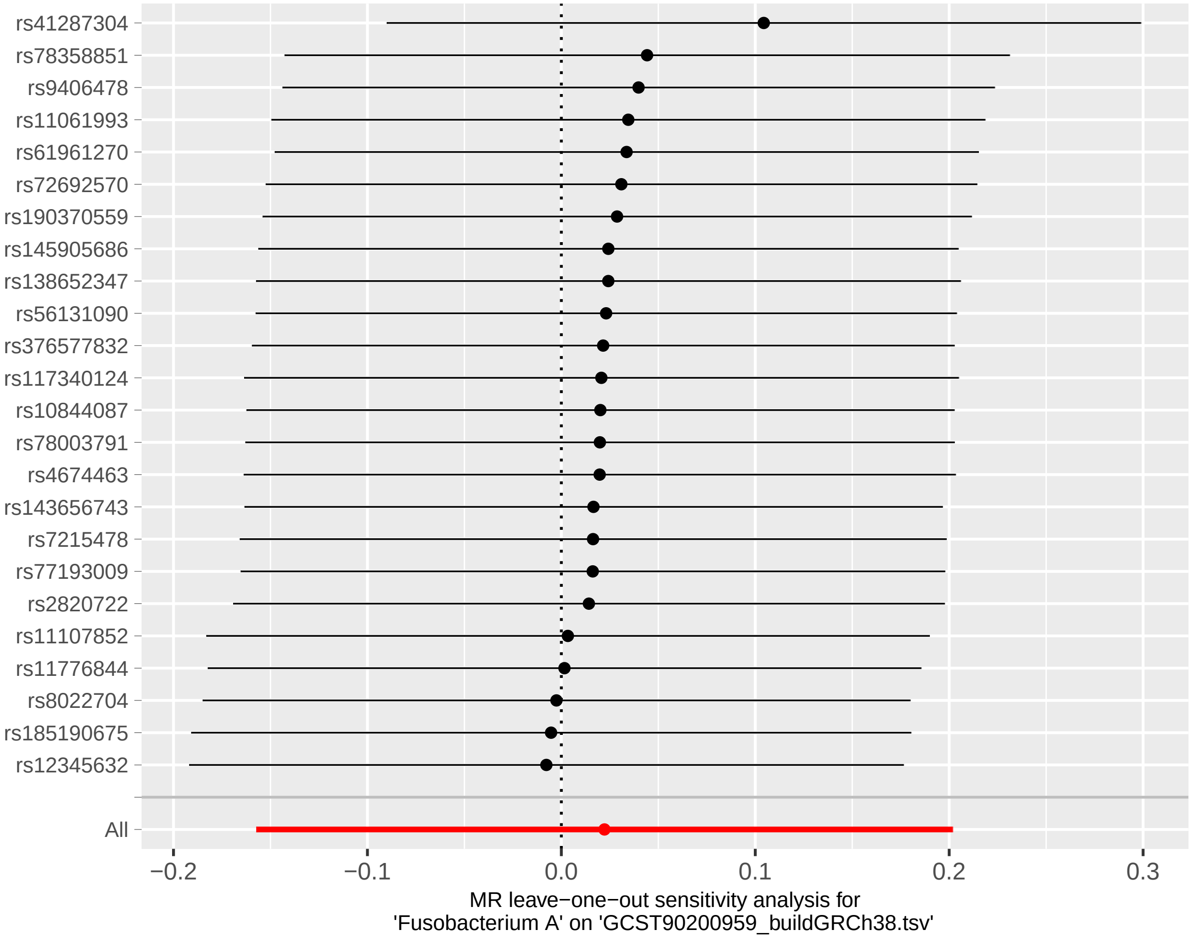
rs41287304
rs78358851
rs9406478
rs11061993
rs61961270
rs72692570
rs190370559
rs145905686
rs138652347
rs56131090
rs376577832
rs117340124
rs10844087
rs78003791
rs4674463
rs143656743
rs7215478
rs77193009
rs2820722
rs11107852
rs11776844
rs8022704
rs185190675
rs12345632
All
−0.2
−0.1
0.0
0.1
0.2
0.3
MR leave−one−out sensitivity analysis for
'Fusobacterium A' on 'GCST90200959_buildGRCh38.tsv'

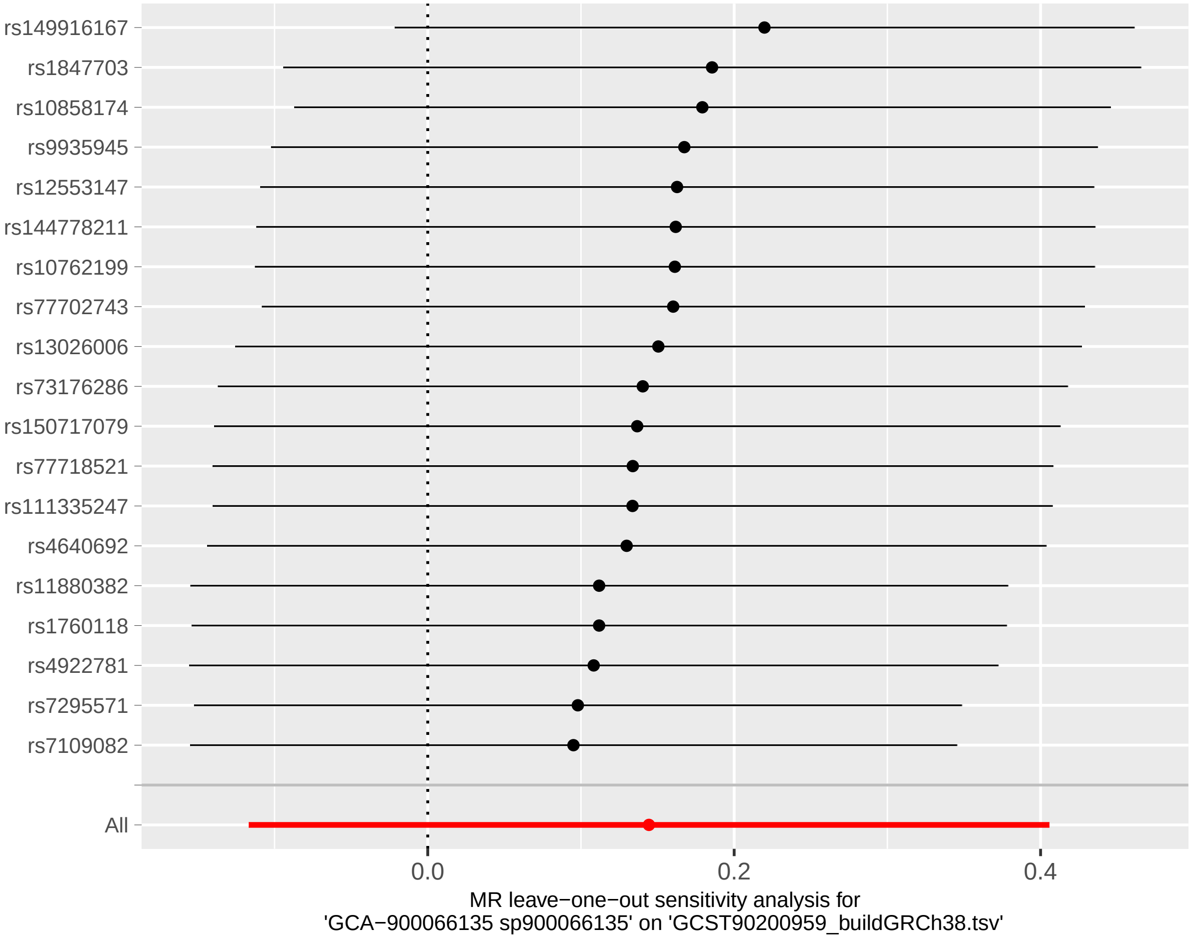
rs149916167
rs1847703
rs10858174
rs9935945
rs12553147
rs144778211
rs10762199
rs77702743
rs13026006
rs73176286
rs150717079
rs77718521
rs111335247
rs4640692
rs11880382
rs1760118
rs4922781
rs7295571
rs7109082
All
0.0
0.2
0.4
MR leave−one−out sensitivity analysis for
'GCA−900066135 sp900066135' on 'GCST90200959_buildGRCh38.tsv'

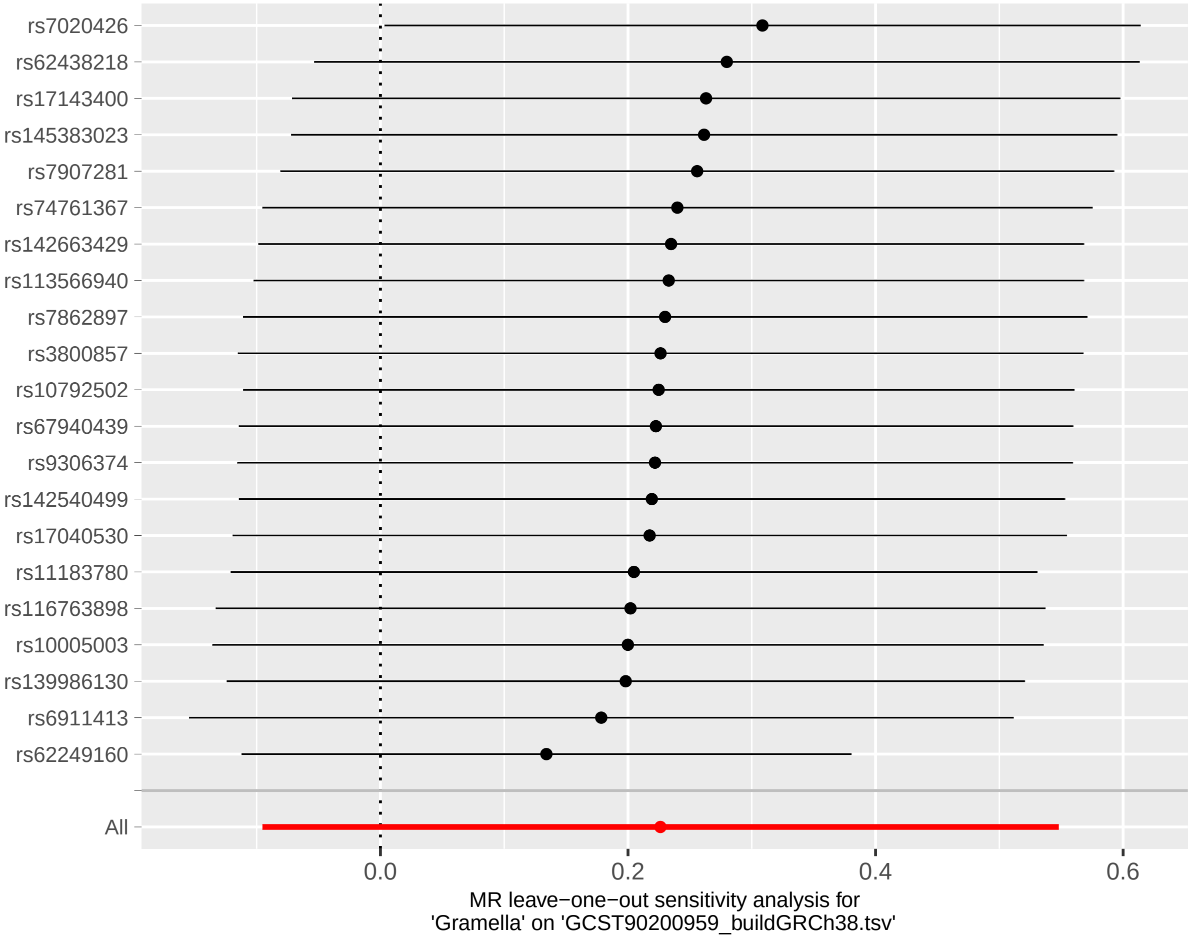
rs7020426
rs62438218
rs17143400
rs145383023
rs7907281
rs74761367
rs142663429
rs113566940
rs7862897
rs3800857
rs10792502
rs67940439
rs9306374
rs142540499
rs17040530
rs11183780
rs116763898
rs10005003
rs139986130
rs6911413
rs62249160
All
0.0
0.2
0.4
0.6
MR leave−one−out sensitivity analysis for
'Gramella' on 'GCST90200959_buildGRCh38.tsv'

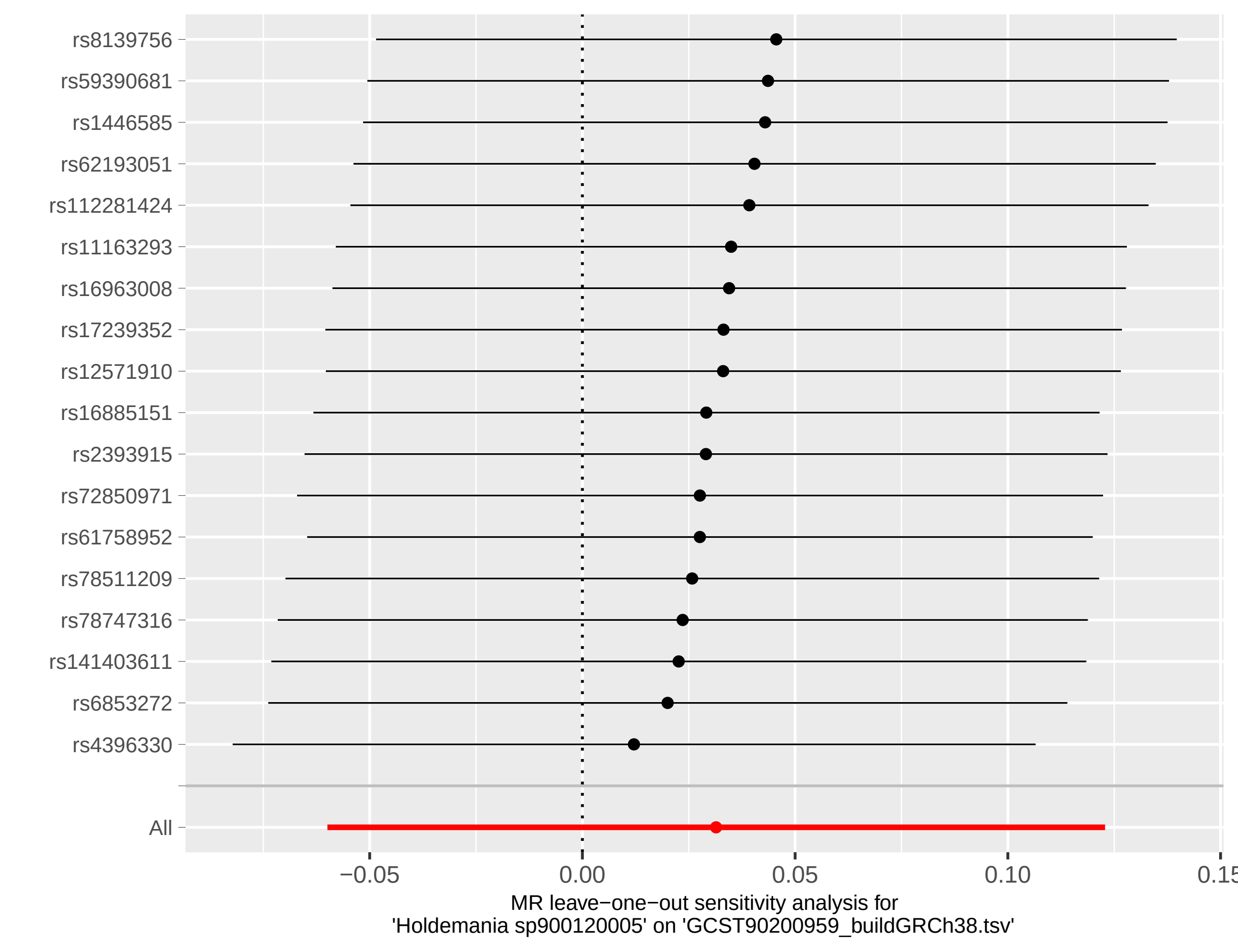

rs8139756
rs59390681
rs1446585
rs62193051
rs112281424
rs11163293
rs16963008
rs17239352
rs12571910
rs16885151
rs2393915
rs72850971
rs61758952
rs78511209
rs78747316
rs141403611
rs6853272
rs4396330
All
−0.05
0.00
0.05
0.10
0.15
MR leave−one−out sensitivity analysis for
'Holdemania sp900120005' on 'GCST90200959_buildGRCh38.tsv'

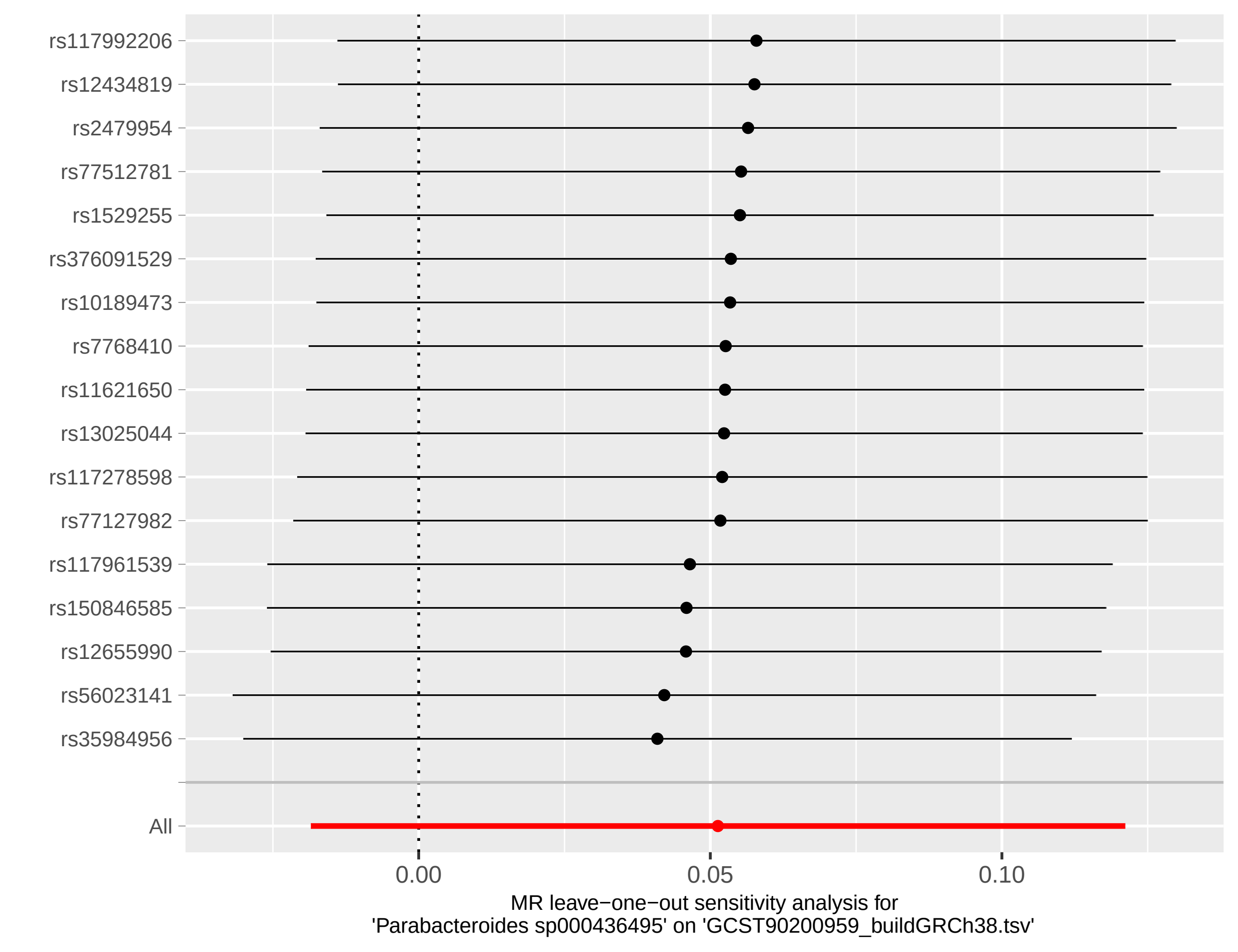
rs117992206
rs12434819
rs2479954
rs77512781
rs1529255
rs376091529
rs10189473
rs7768410
rs11621650
rs13025044
rs117278598
rs77127982
rs117961539
rs150846585
rs12655990
rs56023141
rs35984956
All
0.00
0.05
0.10
MR leave−one−out sensitivity analysis for
'Parabacteroides sp000436495' on 'GCST90200959_buildGRCh38.tsv'

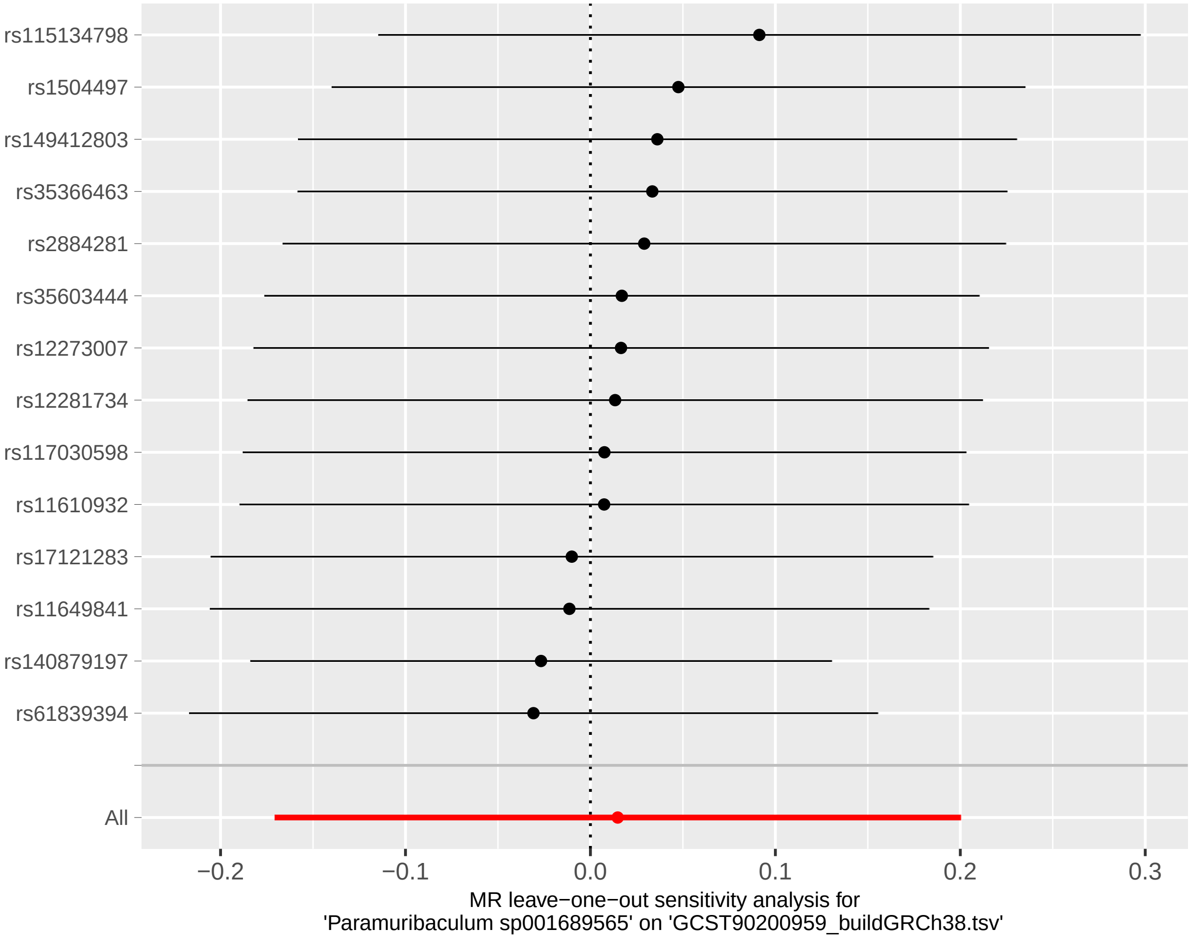
rs115134798
rs1504497
rs149412803
rs35366463
rs2884281
rs35603444
rs12273007
rs12281734
rs117030598
rs11610932
rs17121283
rs11649841
rs140879197
rs61839394
All
−0.2
−0.1
0.0
0.1
0.2
0.3
MR leave−one−out sensitivity analysis for
'Paramuribaculum sp001689565' on 'GCST90200959_buildGRCh38.tsv'

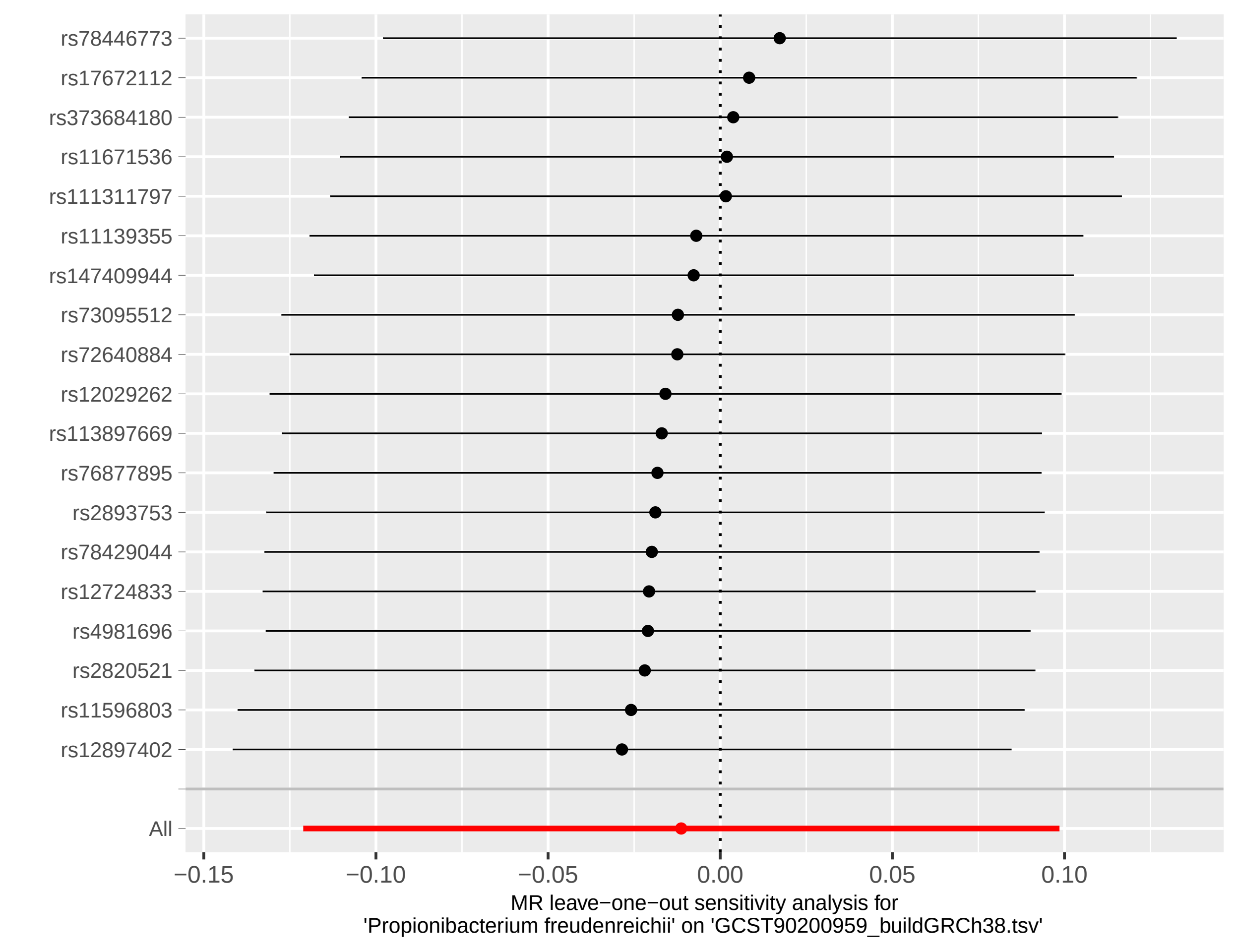
rs78446773
rs17672112
rs373684180
rs11671536
rs111311797
rs11139355
rs147409944
rs73095512
rs72640884
rs12029262
rs113897669
rs76877895
rs2893753
rs78429044
rs12724833
rs4981696
rs2820521
rs11596803
rs12897402
All
−0.15
−0.10
−0.05
0.00
0.05
0.10
MR leave−one−out sensitivity analysis for
'Propionibacterium freudenreichii' on 'GCST90200959_buildGRCh38.tsv'

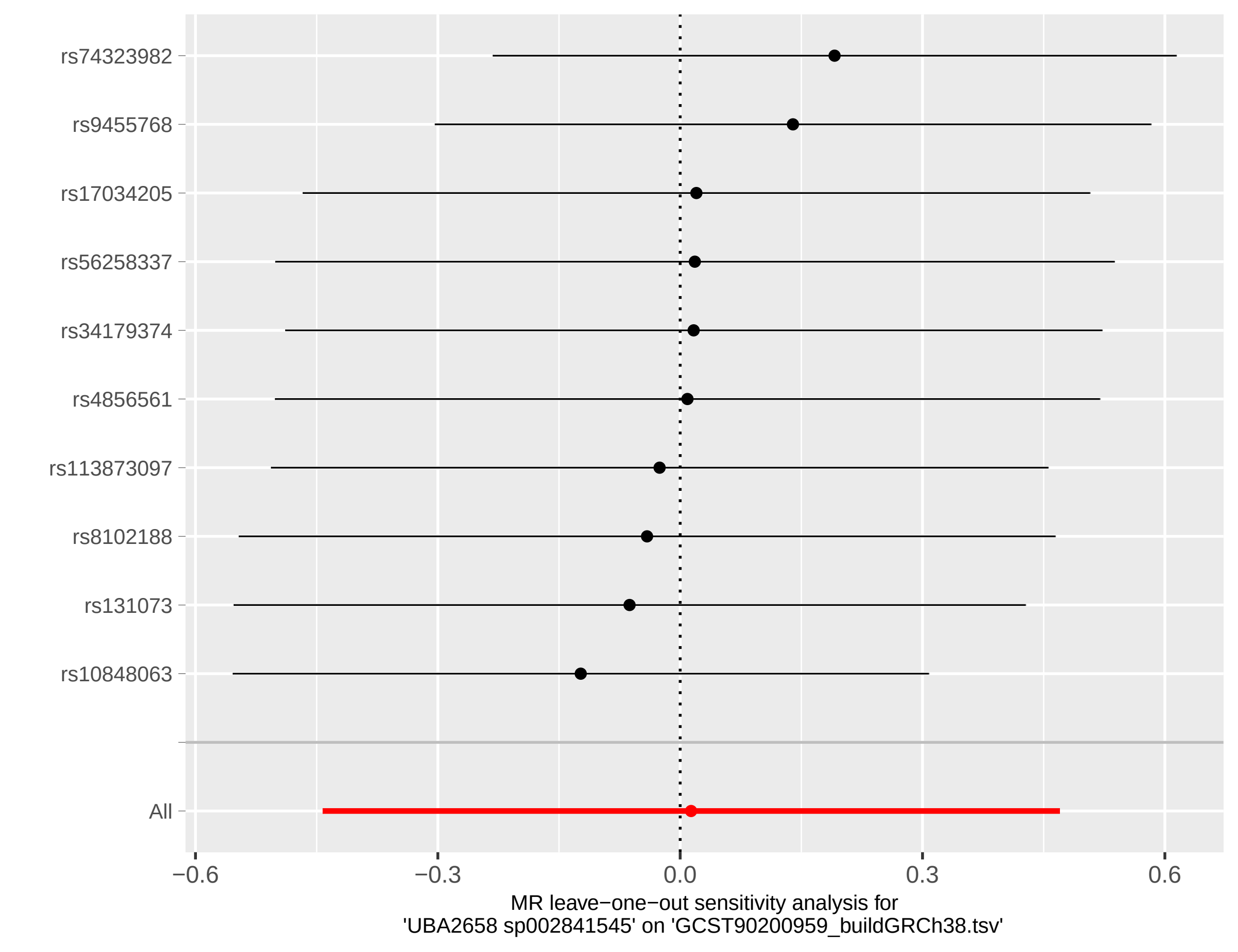

rs74323982
rs9455768
rs17034205
rs56258337
rs34179374
rs4856561
rs113873097
rs8102188
rs131073
rs10848063
All
−0.6
−0.3
0.0
0.3
0.6
MR leave−one−out sensitivity analysis for
'UBA2658 sp002841545' on 'GCST90200959_buildGRCh38.tsv'

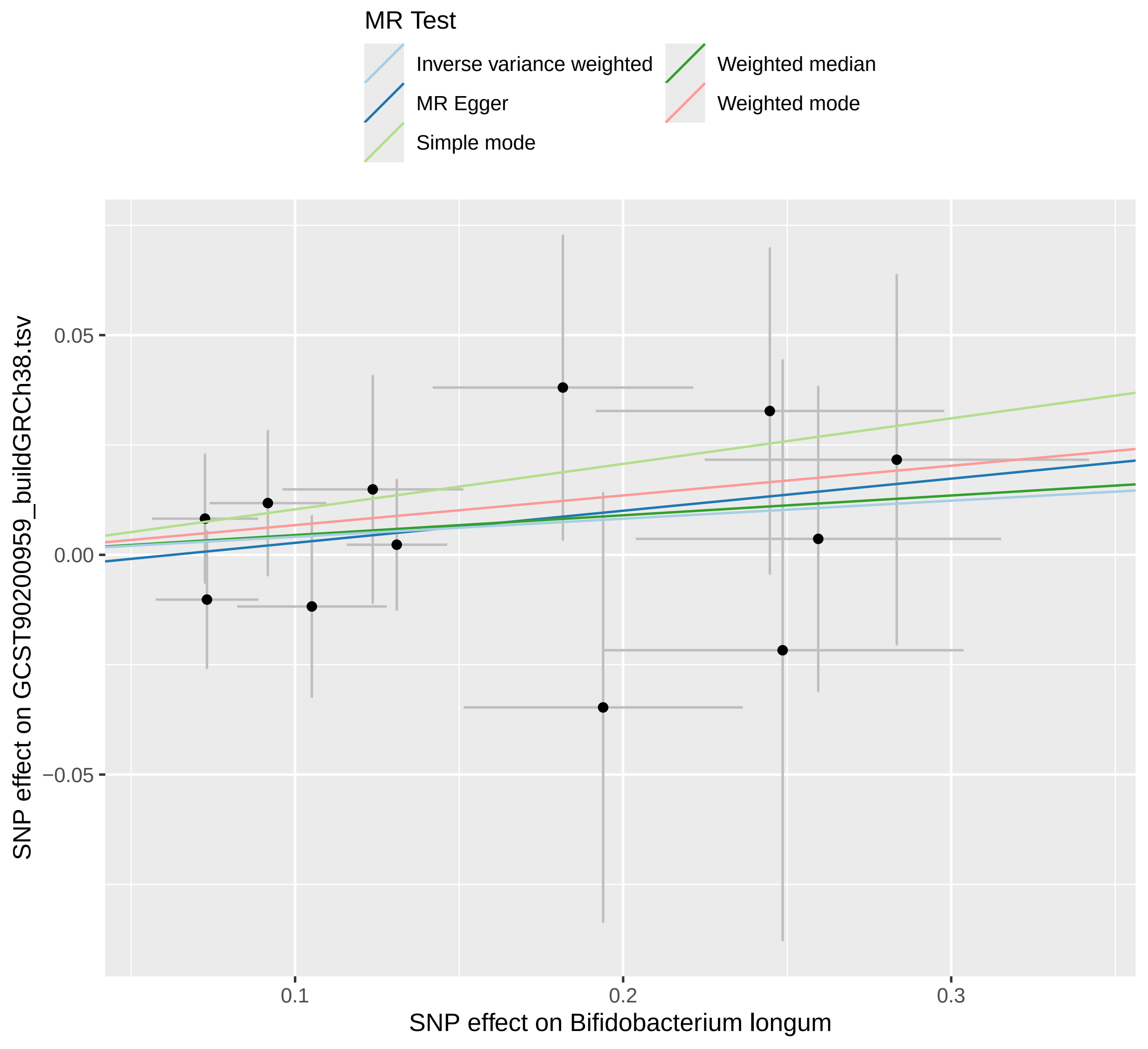

MR Test
Inverse variance weighted
MR Egger
Simple mode
Weighted median
Weighted mode
SNP effect on GCST90200959_buildGRCh38.tsv
0.05
0.00
−0.05
0.1
0.2
0.3
SNP effect on Bifidobacterium longum

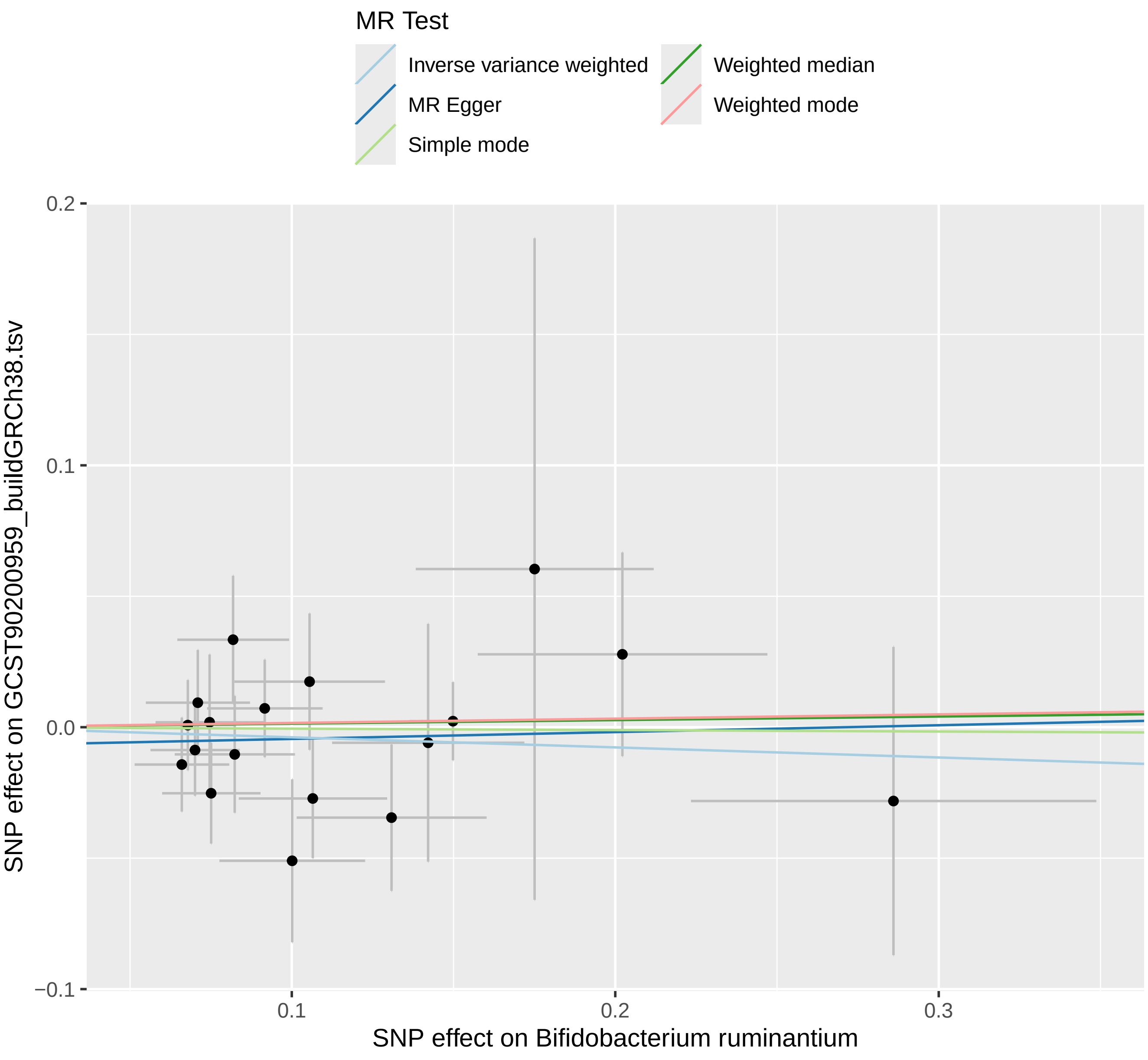

MR Test
Inverse variance weighted
MR Egger
Simple mode
Weighted median
Weighted mode
0.2
0.1
0.0
−0.1
0.1
0.2
0.3
SNP effect on GCST90200959_buildGRCh38.tsv
SNP effect on Bifidobacterium ruminantium

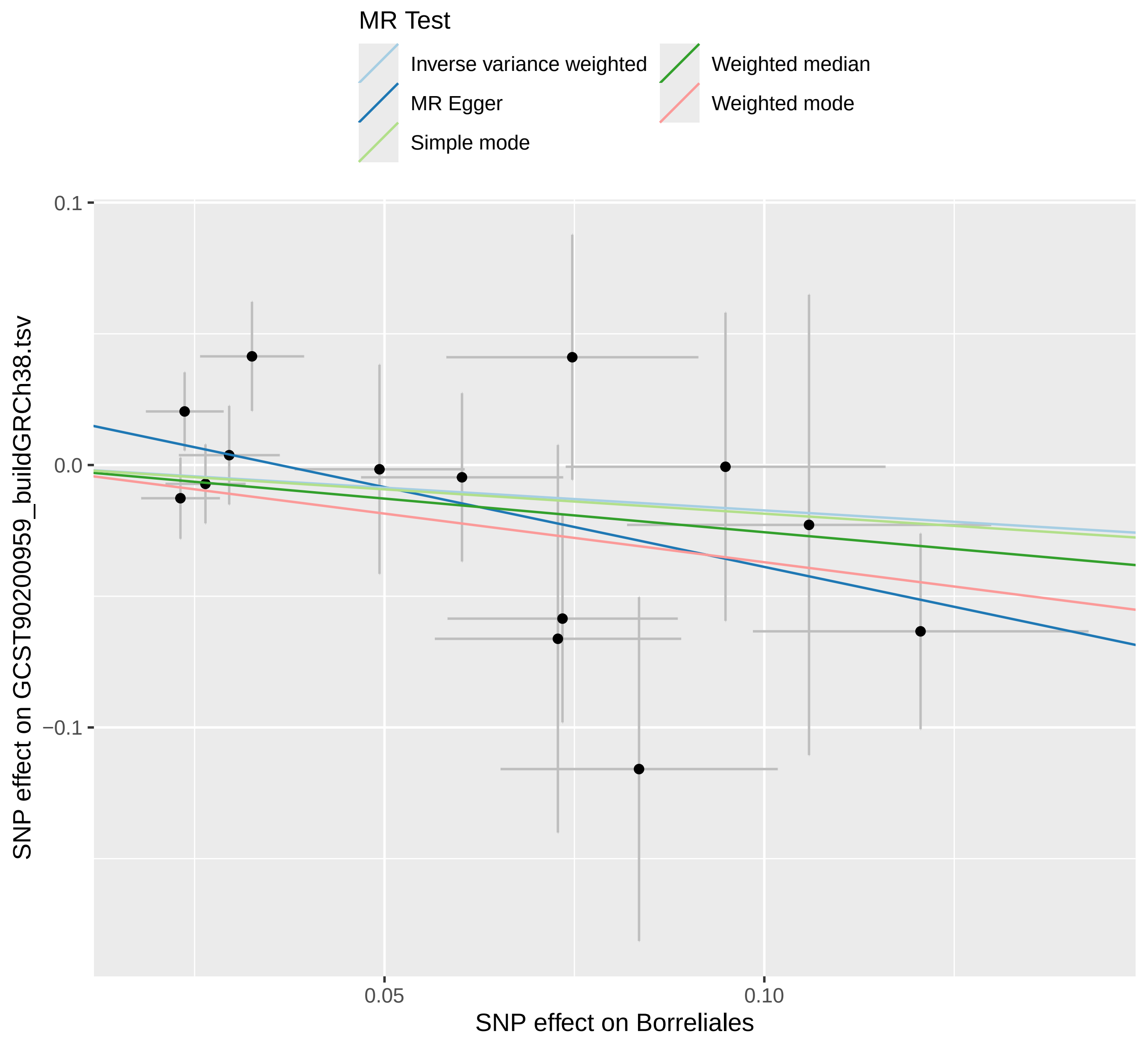
MR Test
Inverse variance weighted
MR Egger
Simple mode
Weighted median
Weighted mode
0.1
0.0
−0.1
0.05
0.10
SNP effect on Borreliales
SNP effect on GCST90200959_buildGRCh38.tsv

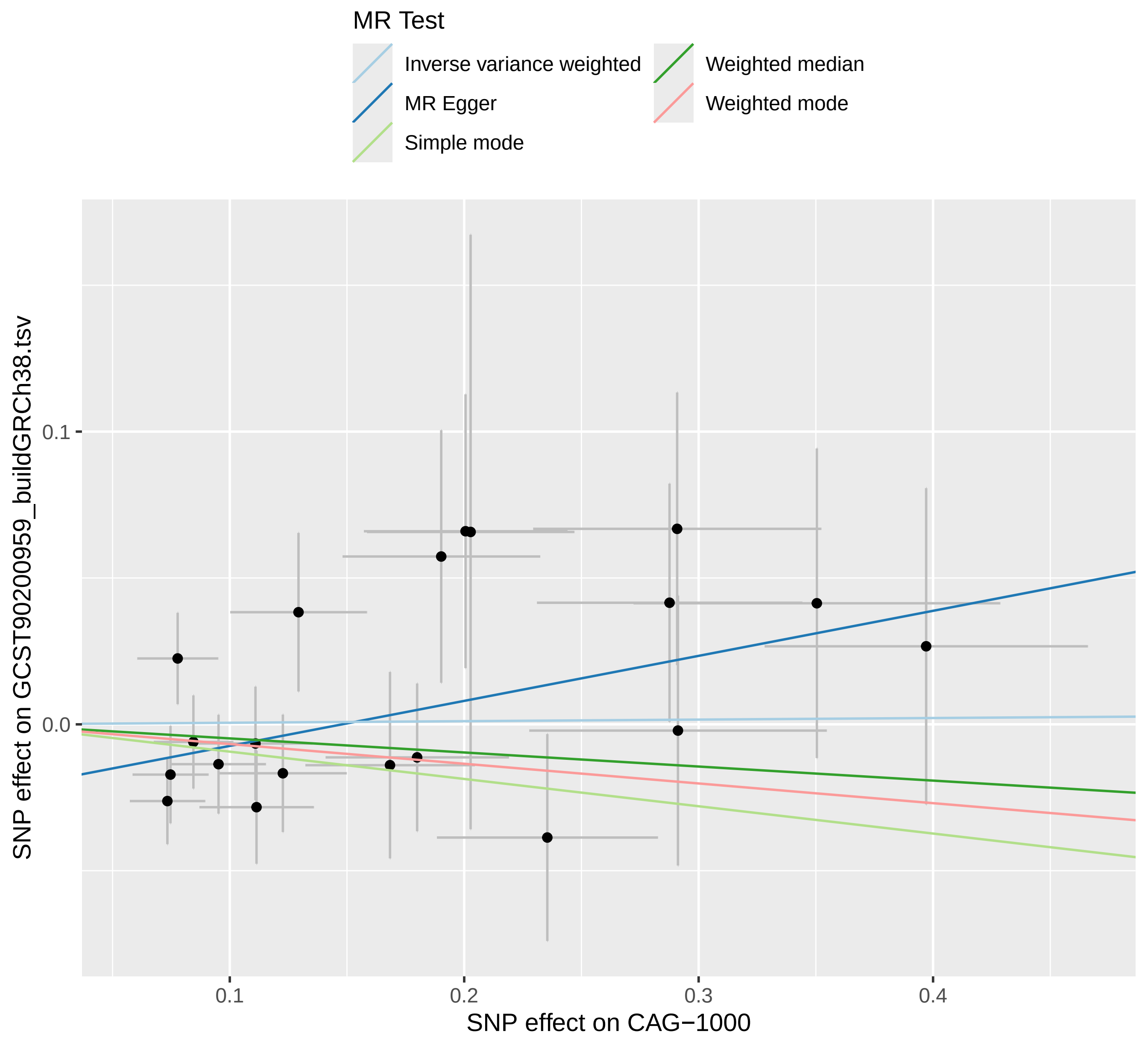

MR Test
Inverse variance weighted
MR Egger
Simple mode
Weighted median
Weighted mode
0.1
0.0
0.1
0.2
0.3
0.4
SNP effect on GCST90200959_buildGRCh38.tsv
SNP effect on CAG−1000

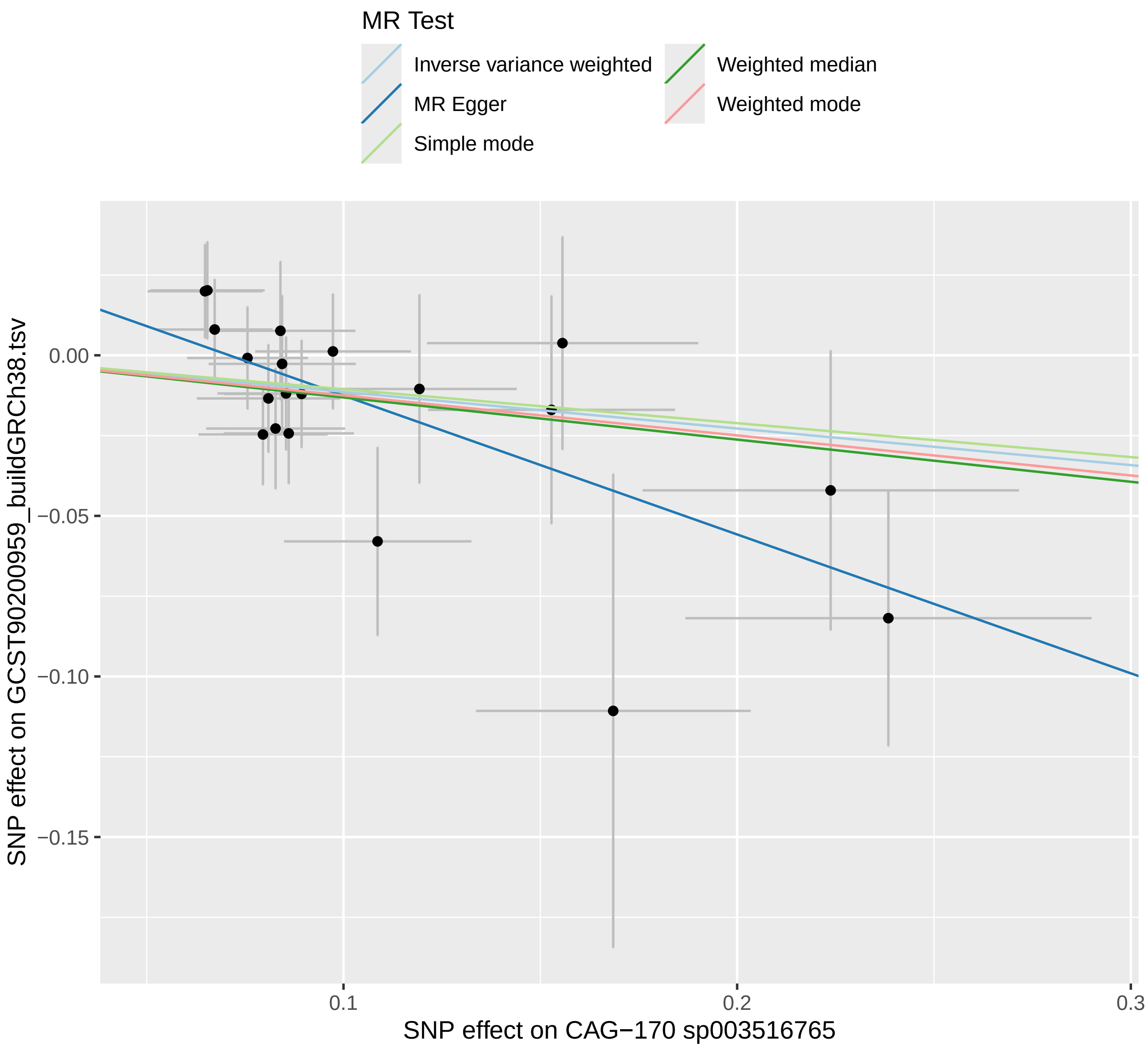
MR Test
Inverse variance weighted
MR Egger
Simple mode
Weighted median
Weighted mode
SNP effect on GCST90200959_buildGRCh38.tsv
0.00
−0.05
−0.10
−0.15
0.1
0.2
0.3
SNP effect on CAG−170 sp003516765

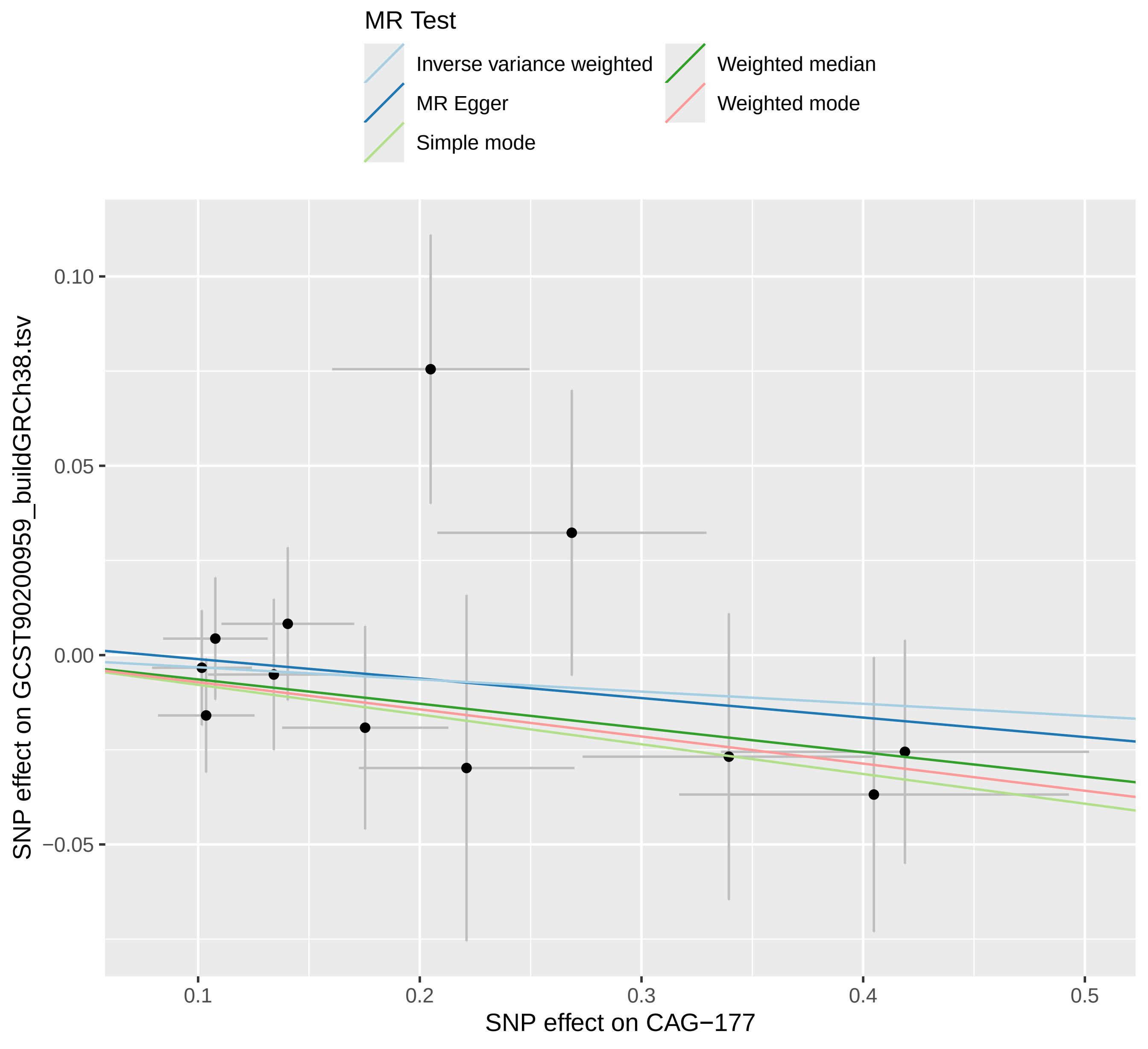
MR Test
Inverse variance weighted
MR Egger
Simple mode
Weighted median
Weighted mode
0.10
0.05
0.00
−0.05
SNP effect on GCST90200959_buildGRCh38.tsv
0.1
0.2
0.3
0.4
0.5
SNP effect on CAG−177

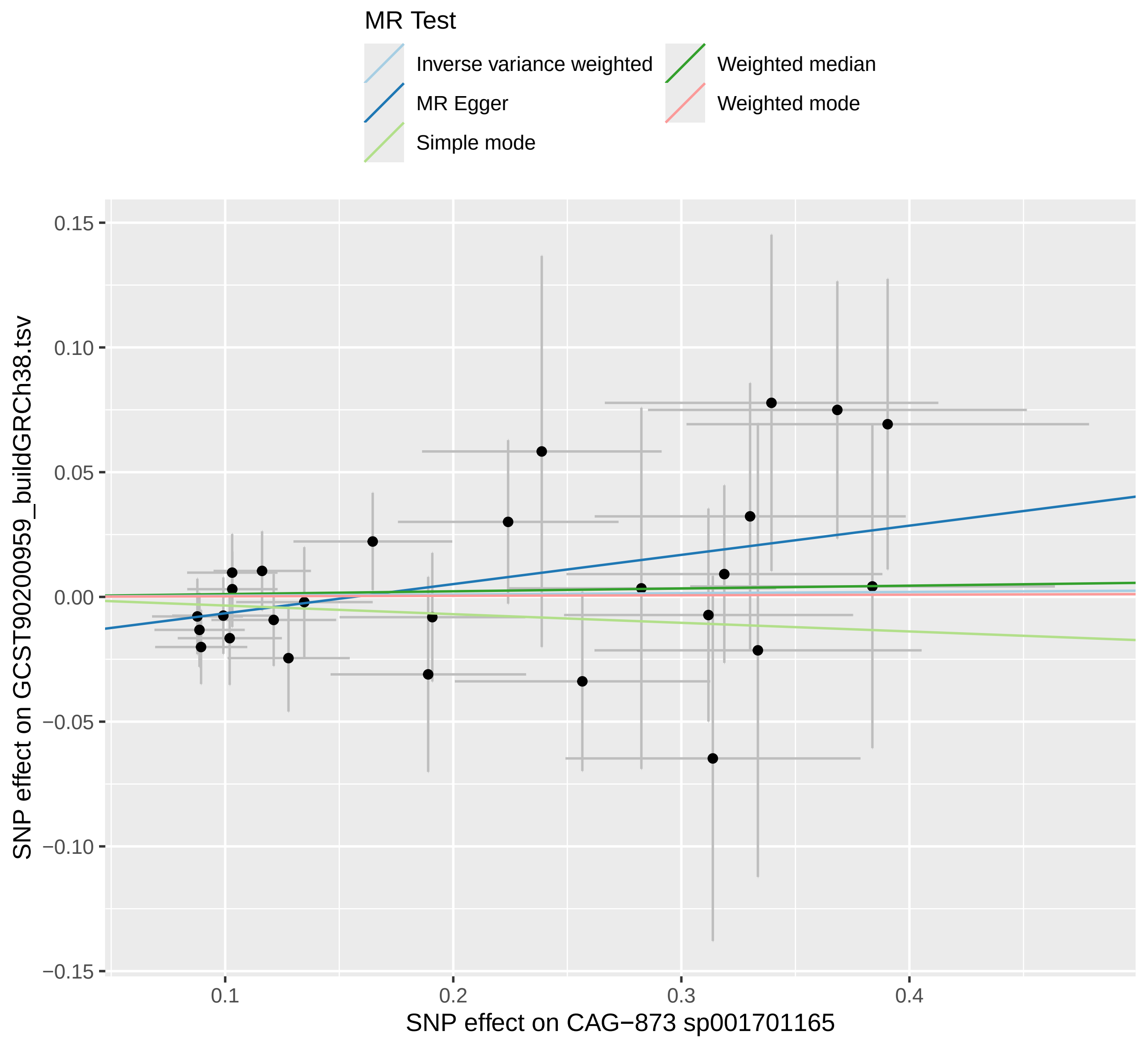
MR Test
Inverse variance weighted
MR Egger
Simple mode
Weighted median
Weighted mode
0.15
0.10
0.05
0.00
−0.05
−0.10
−0.15
0.1
0.2
0.3
0.4
SNP effect on GCST90200959_buildGRCh38.tsv
SNP effect on CAG−873 sp001701165

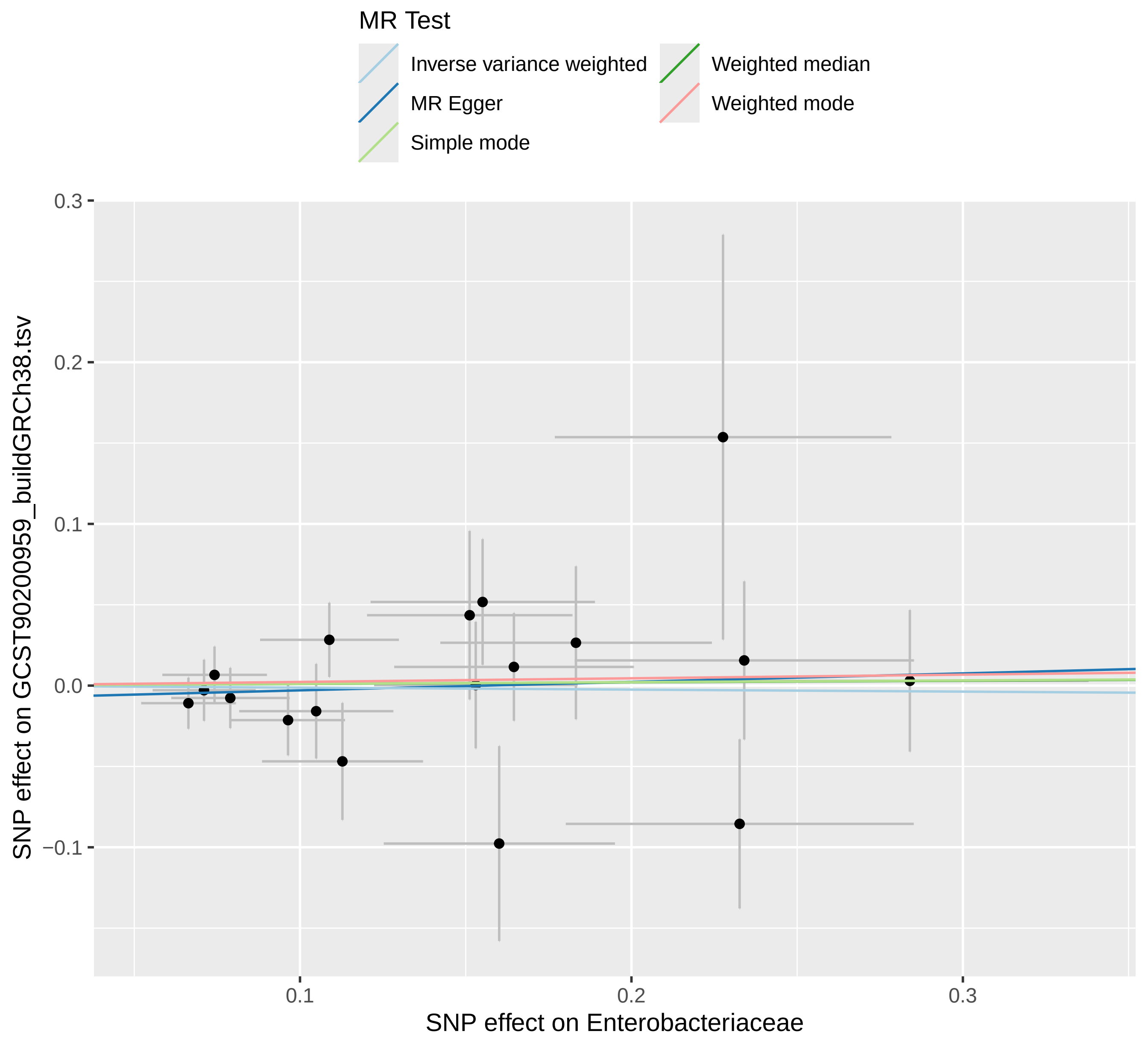
MR Test
Inverse variance weighted
MR Egger
Simple mode
Weighted median
Weighted mode
0.3
0.2
0.1
0.0
−0.1
SNP effect on GCST90200959_buildGRCh38.tsv
0.1
0.2
0.3
SNP effect on Enterobacteriaceae

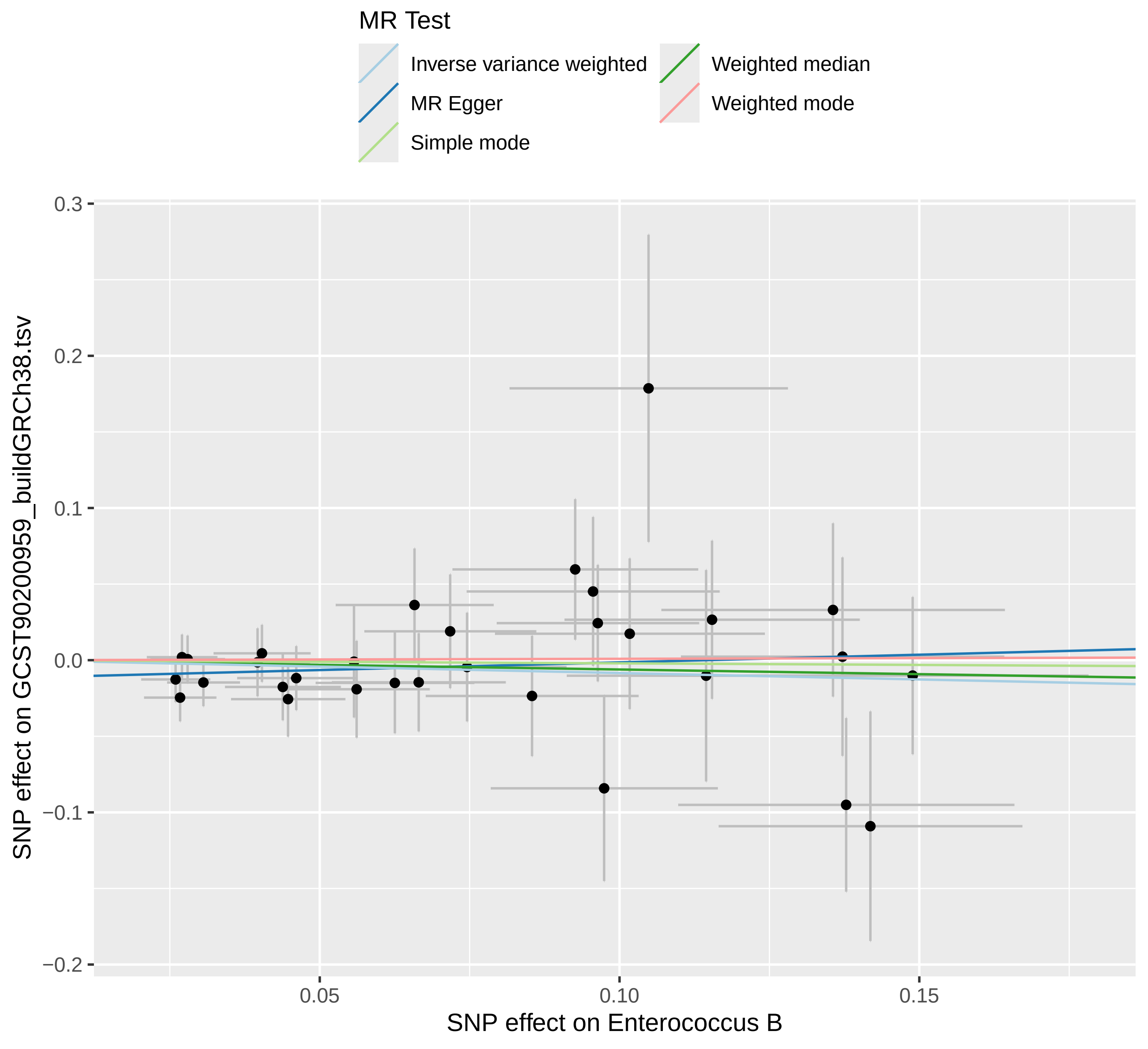

MR Test
Inverse variance weighted
MR Egger
Simple mode
Weighted median
Weighted mode
0.3
0.2
0.1
0.0
−0.1
−0.2
0.05
0.10
0.15
SNP effect on GCST90200959_buildGRCh38.tsv
SNP effect on Enterococcus B

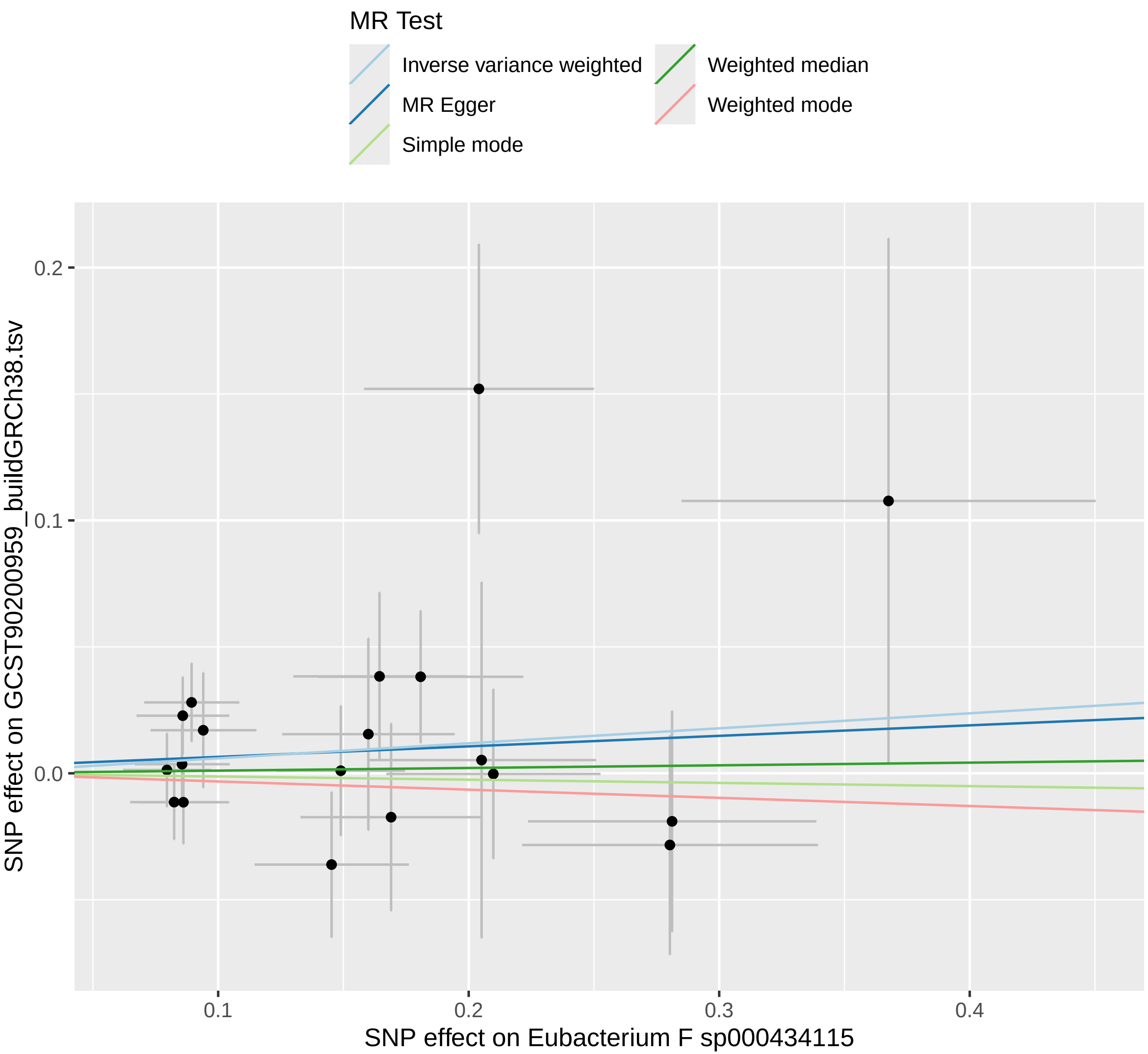
MR Test
Inverse variance weighted
MR Egger
Simple mode
Weighted median
Weighted mode
0.2
0.1
0.0
0.1
0.2
0.3
0.4
SNP effect on GCST90200959_buildGRCh38.tsv
SNP effect on Eubacterium F sp000434115

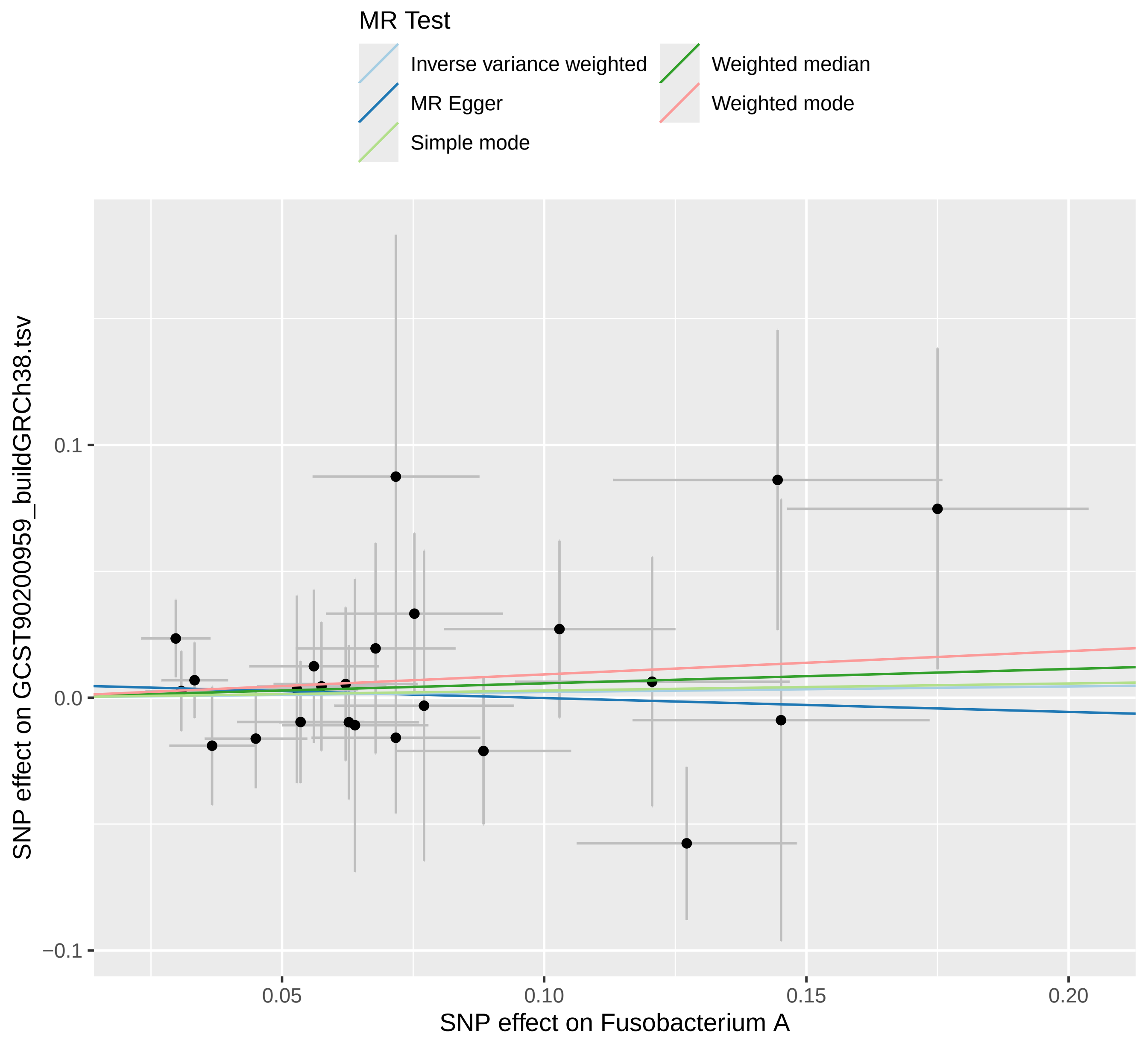

MR Test
Inverse variance weighted
MR Egger
Simple mode
Weighted median
Weighted mode
SNP effect on GCST90200959_buildGRCh38.tsv
0.1
0.0
−0.1
0.05
0.10
0.15
0.20
SNP effect on Fusobacterium A

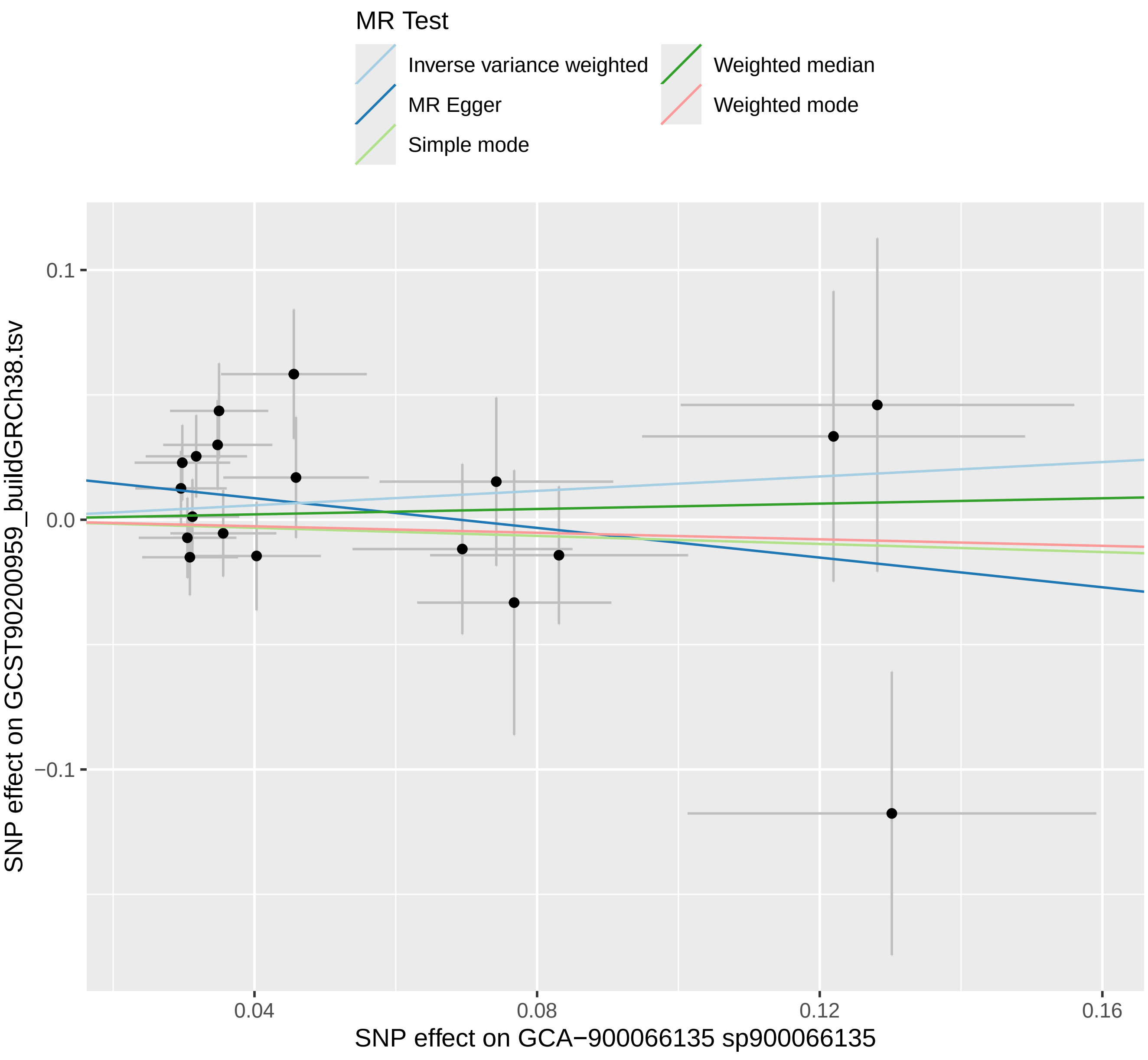
MR Test
Inverse variance weighted
MR Egger
Simple mode
Weighted median
Weighted mode
0.1
0.0
−0.1
0.04
0.08
0.12
0.16
SNP effect on GCST90200959_buildGRCh38.tsv
SNP effect on GCA−900066135 sp900066135

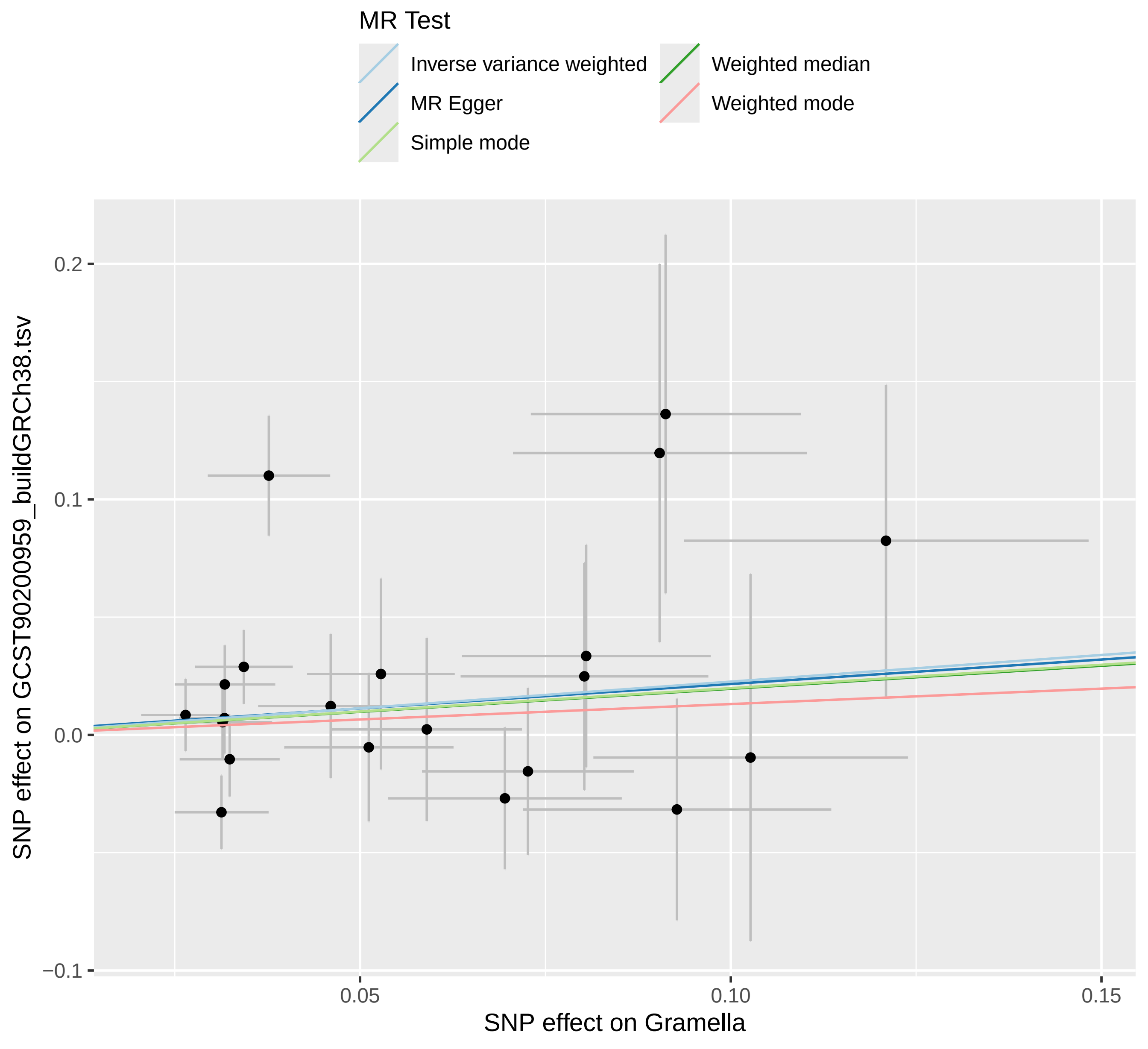
MR Test
Inverse variance weighted
MR Egger
Simple mode
Weighted median
Weighted mode
0.2
0.1
0.0
−0.1
0.05
0.10
0.15
SNP effect on GCST90200959_buildGRCh38.tsv
SNP effect on Gramella

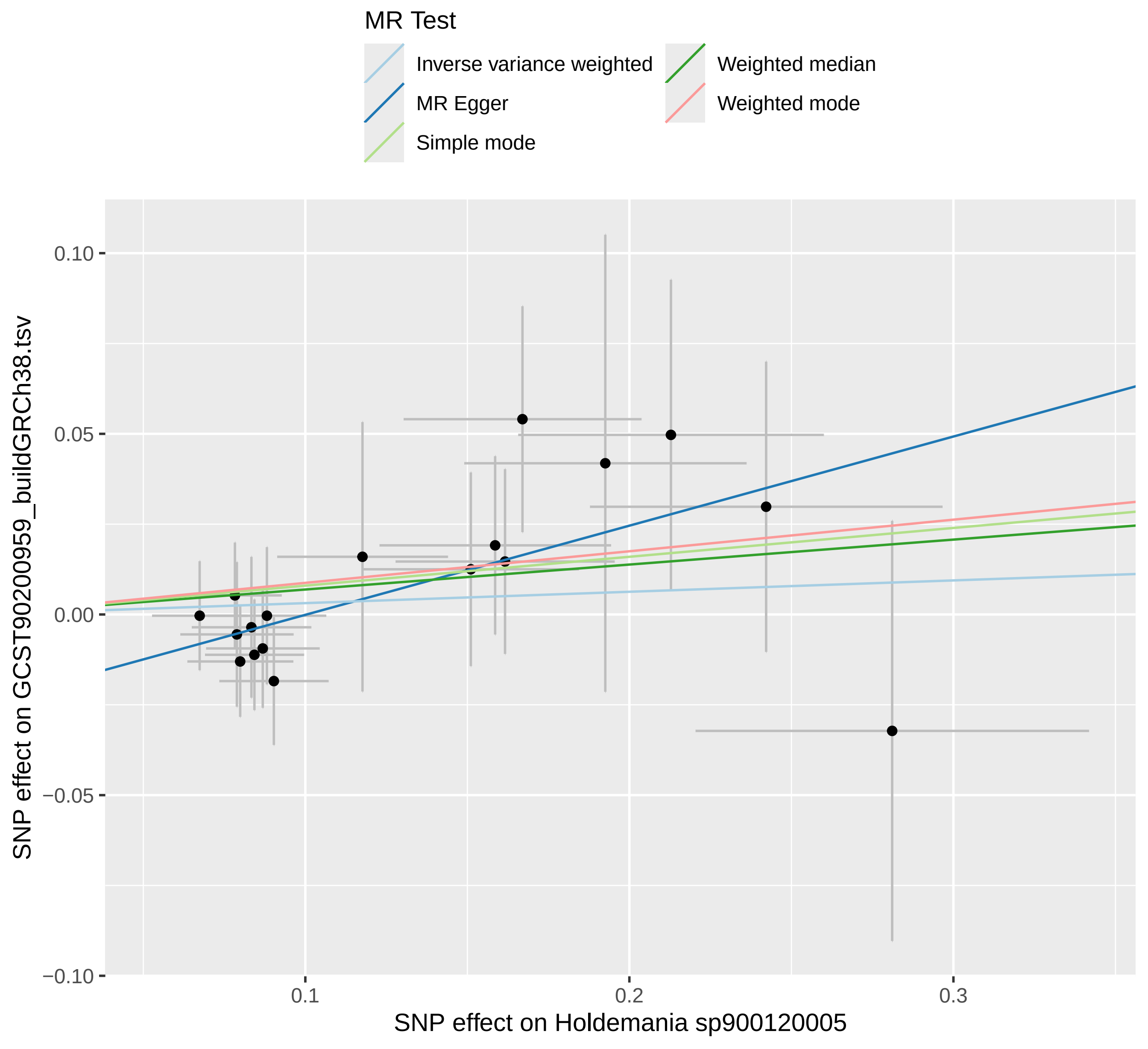

MR Test
Inverse variance weighted
MR Egger
Simple mode
Weighted median
Weighted mode
0.10
0.05
0.00
−0.05
−0.10
0.1
0.2
0.3
SNP effect on GCST90200959_buildGRCh38.tsv
SNP effect on Holdemania sp900120005

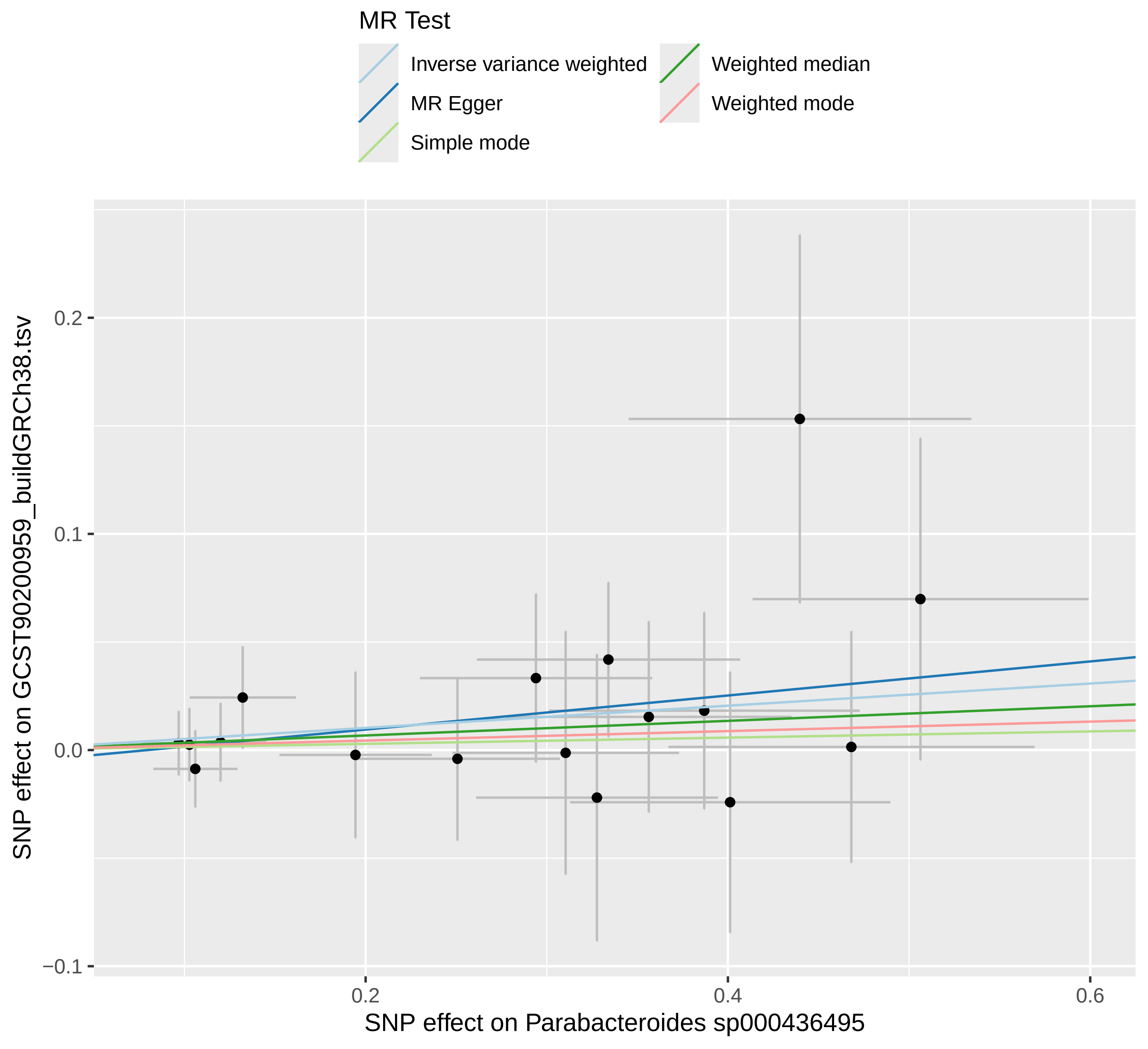
MR Test
Inverse variance weighted
MR Egger
Simple mode
Weighted median
Weighted mode
SNP effect on GCST90200959_buildGRCh38.tsv
0.2
0.1
0.0
−0.1
0.2
0.4
0.6
SNP effect on Parabacteroides sp000436495

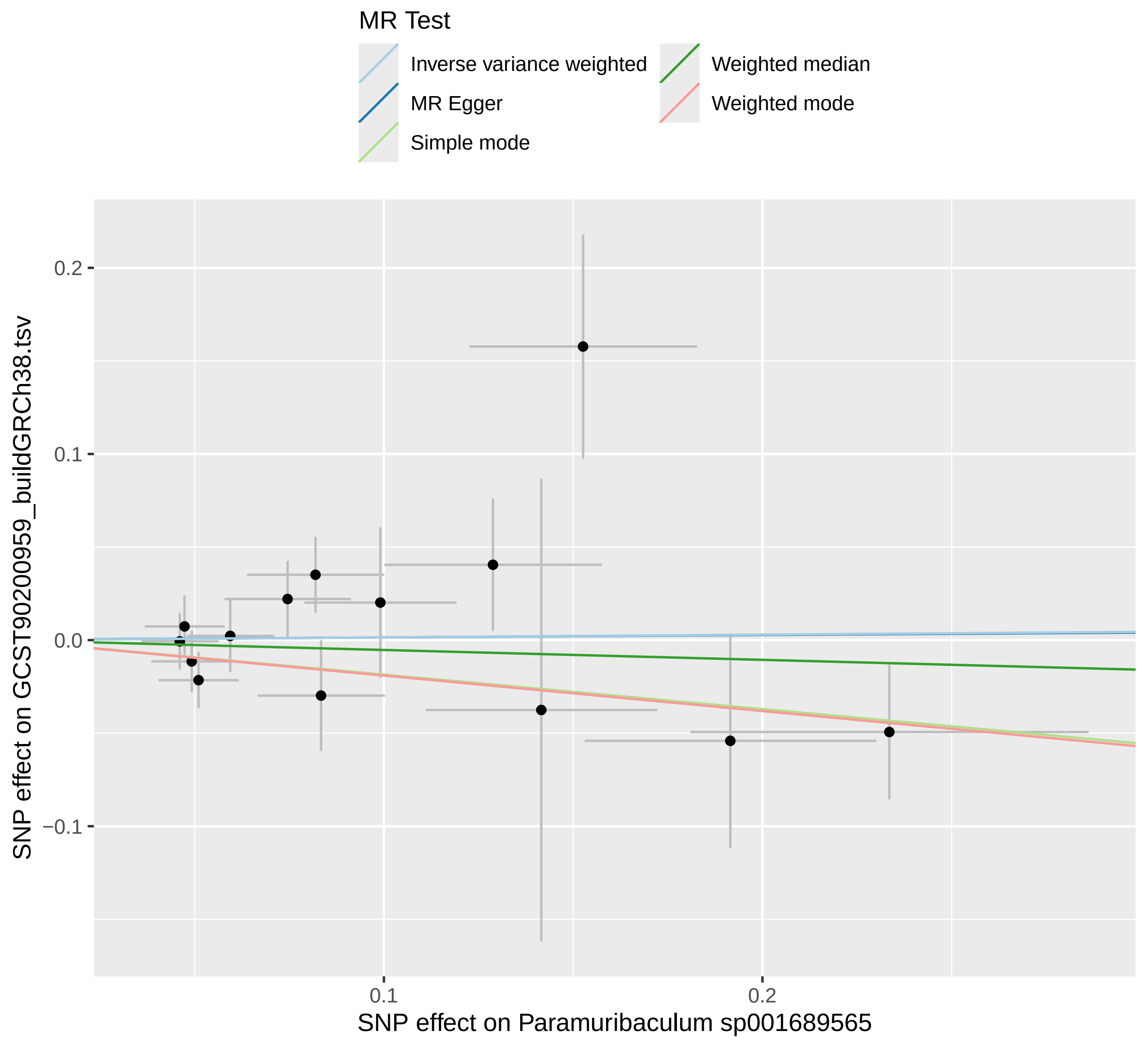
MR Test
Inverse variance weighted
MR Egger
Simple mode
Weighted median
Weighted mode
0.2
0.1
0.0
−0.1
0.1
0.2
SNP effect on GCST90200959_buildGRCh38.tsv
SNP effect on Paramuribaculum sp001689565

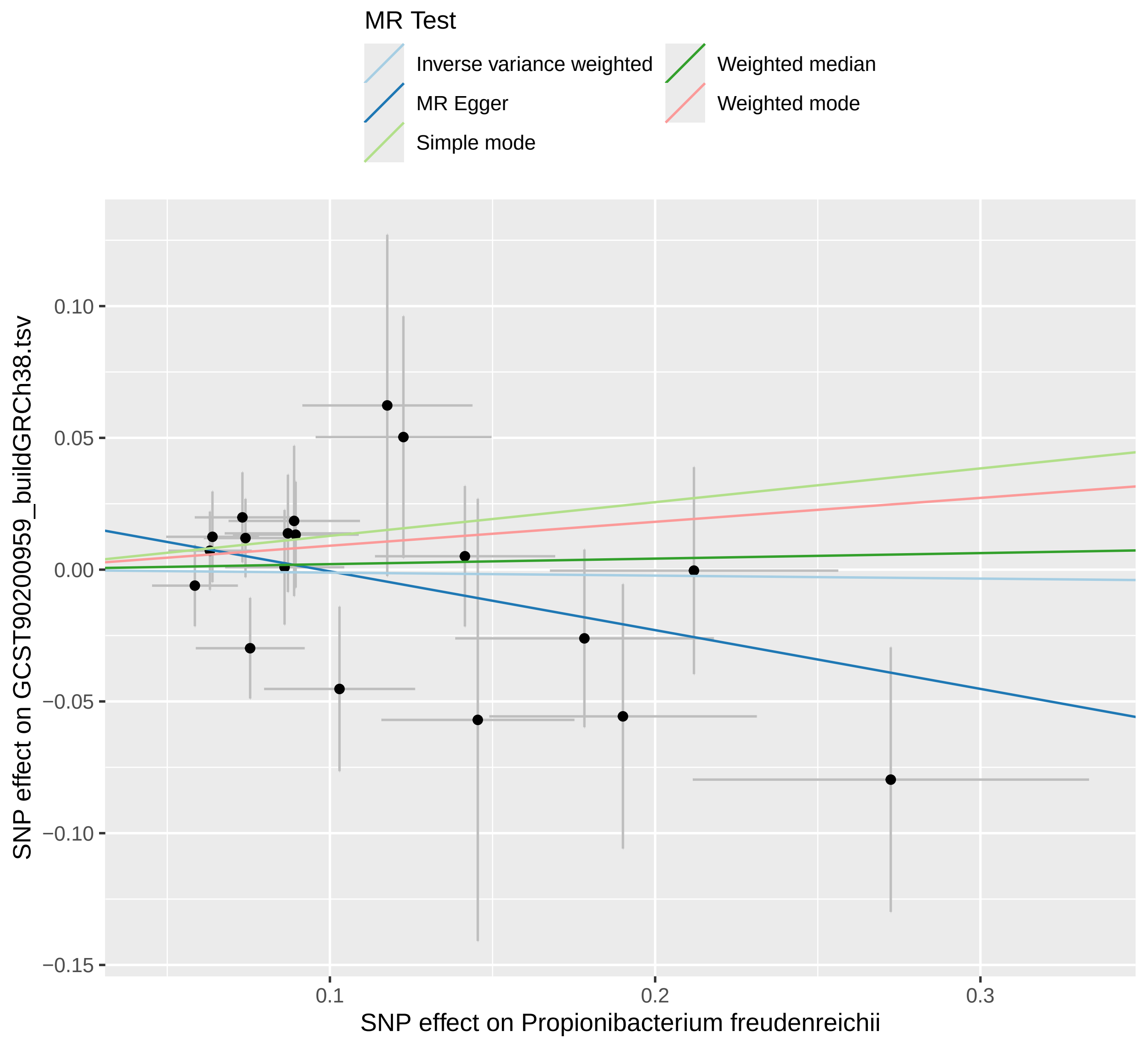
MR Test
Inverse variance weighted
MR Egger
Simple mode
Weighted median
Weighted mode
0.10
0.05
0.00
−0.05
−0.10
−0.15
0.1
0.2
0.3
SNP effect on GCST90200959_buildGRCh38.tsv
SNP effect on Propionibacterium freudenreichii

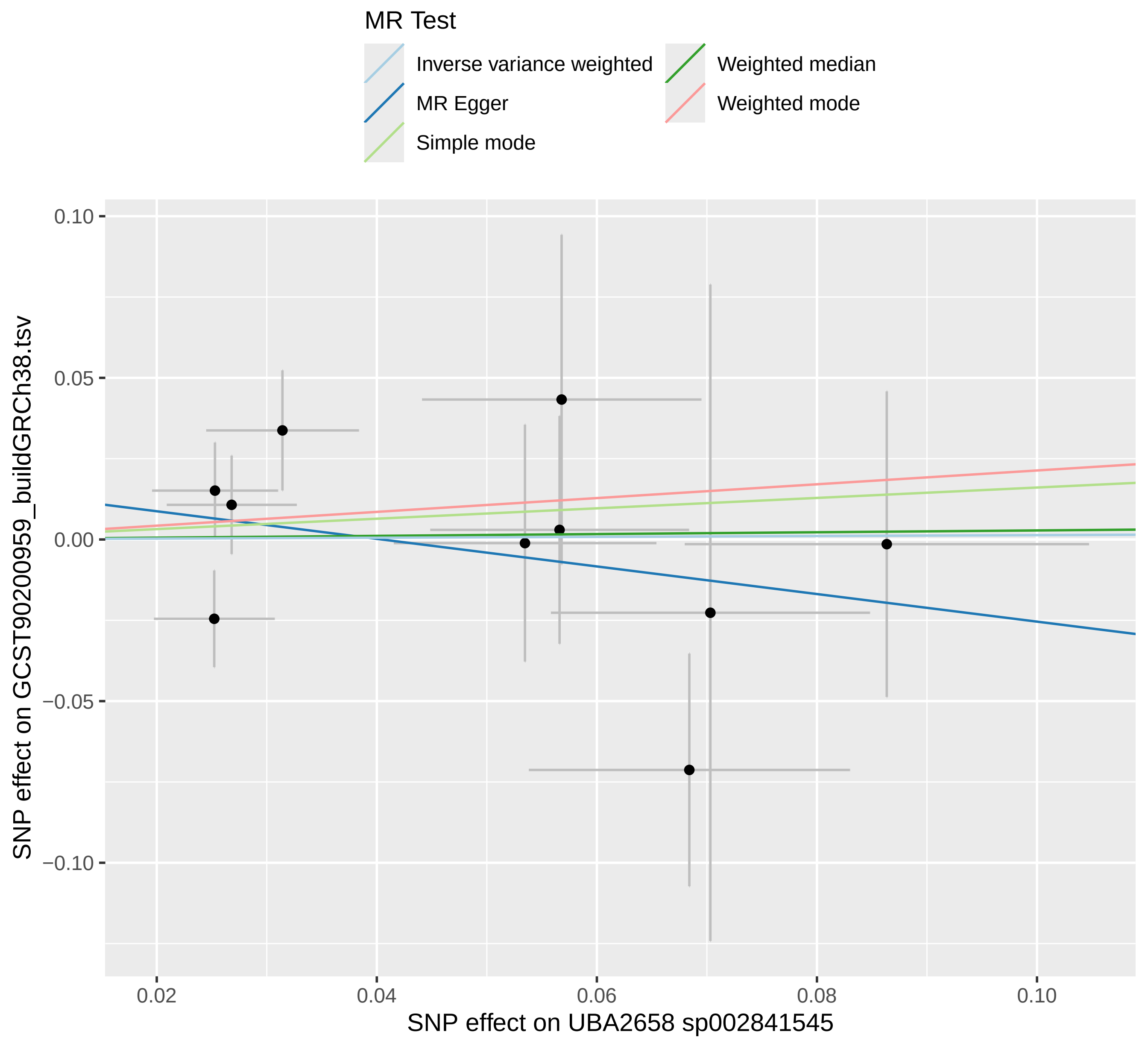
MR Test
Inverse variance weighted
MR Egger
Simple mode
Weighted median
Weighted mode
0.10
0.05
0.00
−0.05
−0.10
0.02
0.04
0.06
0.08
0.10
SNP effect on GCST90200959_buildGRCh38.tsv
SNP effect on UBA2658 sp002841545
